# Serum AGP-1-Le^x^ Glycoforms Report on Survivorship of Patients with Septic Shock Upon Admission to Intensive Care Unit

**DOI:** 10.1016/j.mcpro.2025.101470

**Published:** 2025-11-17

**Authors:** The Huong Chau, Sayantani Chatterjee, Liam Caulfield, Anastasia Chernykh, Mathew Traini, Joshua Fehring, Heeyoun Hwang, Rebeca Kawahara, Emily J. Meyer, David J. Torpy, Morten Thaysen-Andersen

**Affiliations:** 1 School of Natural Sciences, Macquarie University, Sydney, New South Wales, Australia; 2 Institute for Glyco-Core Research, Nagoya University, Nagoya, Aichi, Japan; 3 Department of Biochemistry and Molecular Biology & Biomedicine Discovery Institute, Monash University, Melbourne, Australia; 4 Digital OMICs Research Center, Korea Basic Science Institute, Cheongju, Republic of Korea; 5 Bio-Analytical School, University of Science and Technology, Daejeon, Republic of Korea; 6 Department of Medicine, University of Adelaide, Adelaide, South Australia, Australia; 7 Endocrine and Metabolic Unit, Royal Adelaide Hospital, Adelaide, South Australia, Australia

**Keywords:** alpha-1-acid-glycoprotein, glycomics, glycoproteomics, Lewis x, septic shock, serum *N*-glycosylation, survivorship

## Abstract

Septic shock, the excessive immune response to pathogen infection, accounts globally for ∼20% of all deaths. Current methods to establish disease severity are unacceptably slow, unspecific, and insensitive, hindering timely and effective treatment. Aiming to establish easy-to-measure glyco-signatures that may identify the most critically unwell patients, we applied comparative glycomics and glycoproteomics to sera longitudinally collected from septic shock survivors (n = 29) and nonsurvivors (n = 8). Glycomics of all 134 serum samples (sampled daily until recovery/death) revealed significant *N*-glycome dynamics across both patient groups. Unsupervised clustering of the serum *N*-glycome measured upon intensive care unit (ICU) admission (day 1) indicated survivorship-specific glyco-signatures. We therefore employed machine learning to train a random forest model using the serum *N*-glycome data. The model accurately classified survivorship outcomes of 35 of 37 patients (accuracy 94.6%) and correctly predicted 29 of 29 survivors (specificity 100%) and six of eight nonsurvivors (sensitivity 75%). Interrogation of the serum *N*-glycome data revealed that Lewis x (Le^x^)-type *N*-glycans are elevated in nonsurvivors relative to survivors at ICU admission, a finding recapitulated by glycoproteomics. Among the 58 other Le^x^-containing serum glycoproteins that were strongly associated with acute phase response and stress pathways, alpha-1-acid-glycoprotein (AGP-1) was identified as a principal carrier of Le^x^ glycoepitopes with a potential to stratify septic shock survivors from nonsurvivors (AUC 0.90). This study lays a foundation for risk stratification of septic shock patients by uncovering easy-to-assay AGP-1-Le^x^ glycoforms that identify individuals experiencing poor survival outcomes already upon ICU admission, with the potential to translate to early individualized clinical care at the bedside.

## Highlights

• Deep and temporal *N*-glycoproteome profiling of septic shock sera using multi-omics.
• Glycomics-trained ML model identifies septic shock survivors from nonsurvivors.
• Lewis x (Le^x^)-type glycoepitopes are raised early in fatal septic shock.
• α-1-acid glycoprotein-Le^x^ glycoforms stratify patient outcome upon ICU admission.

## Graphical Abstract

**Figure:**
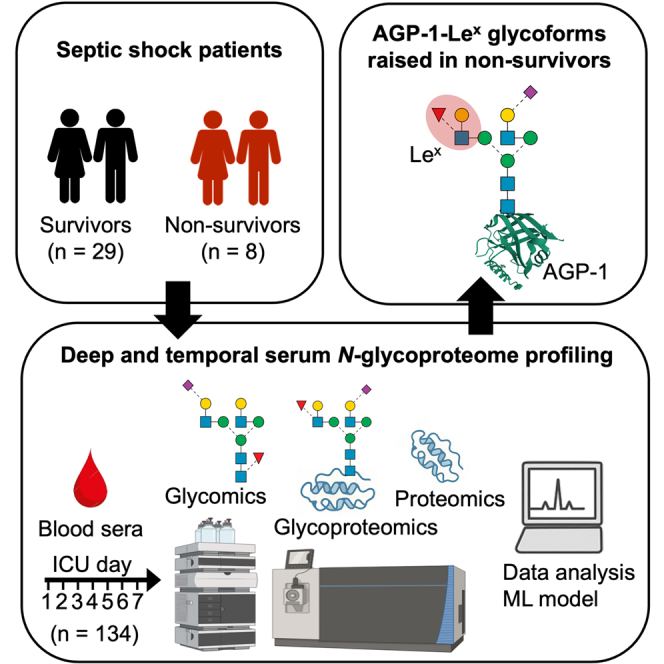
Deep and temporal glycoproteome profiling of septic shock sera using multi-omics and ML approaches reveals that α-1-acid glycoprotein-Le^x^ glycoforms stratify patient outcome upon ICU admission.

## In Brief

Septic shock lacks rapid prognostic markers. To tackle this clinical gap, this study performed comprehensive glycoproteome profiling of sera longitudinally collected from septic shock survivors and nonsurvivors using glycomics and glycoproteomics. Enabled by machine learning and deep data analysis, Lewis x glycoepitopes carried by alpha-1-acid glycoprotein were found to be elevated in fatal septic shock relative to disease survivors upon ICU admission. Our work indicates that specific glycoforms have prognostic value thereby supporting the precision management of septic shock patients.

Sepsis is the exaggerated immune response to pathogen infection and often escalates into life-threatening septic shock. These serious conditions pose a major health concern accounting globally for around 20% of all reported deaths costing annually a staggering $30 billion (USD) worldwide (1, 2, 3, 4, 5).

Despite the unacceptable burden of sepsis and septic shock on the health care systems, advances in early diagnosis, risk stratification, and tailored therapeutic interventions have been modest over the past two decades (6). A key challenge in the clinical management of septic shock is the lack of reliable biomarkers that can rapidly and accurately provide insight into the disease trajectory of individual patients. Current diagnostic approaches, including culture-based identification of pathogens and nonspecific markers of inflammation, are often too slow or insensitive to support the timely decision-making required for such acute health conditions (7). Moreover, existing prognostic tools lack the specificity needed to guide personalized treatment pathways, particularly within the critical first 24 h of admission to the intensive care unit (ICU) when intervention is likely to benefit patients the most (8). Despite the severity of sepsis and septic shock, there have been limited advances in patient management leaving clinicians with few, often imprecise, therapeutic, and prognostic tools (9). Sepsis is a complex and still poorly understood syndrome governed by a dynamic interplay of competing pro- and anti-inflammatory processes that ultimately shape patient outcomes (10).

To address the critical lack of diagnostic and prognostic tools, novel biomarker strategies that can capture dynamic aspects of the host response to infection and that accurately reflect the health status of patients are urgently needed. In this context, glycosylation, the covalent attachment of complex sugar moieties (glycans) to proteins, has emerged as a promising yet still underutilized source of biomarker information (11, 12). Glycosylation is a prevalent modification of proteins that is widely recognized to undergo considerable structural changes with altered physiology and across a wide spectrum of disease conditions (13, 14, 15) including those accompanying sepsis and septic shock events (16, 17, 18, 19, 20, 21). Glycoproteins present in blood serum, such as alpha-1-acid glycoprotein (AGP-1) and immunoglobulins, are key mediators of the acute phase response and are subject to glycan remodeling in response to systemic inflammation triggered by pathogenic insults (22, 23, 24, 25).

Serum, a readily accessible clinical biospecimen, is rich in glycoproteins and reflects the systemic physiology of patients (26). Importantly, serum is routinely collected from critically ill individuals in ICU settings from central venous catheters, making glycan-based diagnostics compatible with existing clinical routines. Advances in high-resolution mass spectrometry (MS) and data analytics now enable comprehensive profiling of serum glycosylation with high sensitivity (low microliter serum input), precision, and throughput (27, 28, 29, 30, 31). Notably, recent improvements in glycomics and glycoproteomics techniques have opened opportunities to map glycosylation in complex biological samples such as serum providing system-wide information of the fine structural details of entire populations of glycans in a sample as well as their protein carriers and specific polypeptide attachment sites (32). Such comprehensive knowledge of the glycoproteome can be critical to understand the molecular mechanisms of and establish markers for complex diseases such as septic shock (33, 34, 35, 36, 37, 38).

Previous work by our group has shown that serum *N*-glycosylation is significantly altered in modestly ill patients with bacteremia, a preseptic shock condition defined as the presence of pathogenic bacteria in the normally sterile blood stream (19). In that study, we found that the serum *N*-glycome holds a promise to stratify preseptic shock individuals based on the infecting bacterial pathogen. Other researchers used glycoproteomics to identify a panel of *N*-glycopeptides that separated bacteremic patients from febrile controls with negative blood culture reports (39) and that associated with sepsis patient outcome (40). Expanding on these findings, we recently investigated severely ill septic shock patients infected by different infectious agents, and found prominent pathogen class-specific serum *N*-glycome alterations in particular for the subset of individuals experiencing candidemia, serious fungal infections of the bloodstream caused by *Candida* spp. (41). These studies highlight the potential of glycan-based profiling to identify the disease-causing pathogen(s) and reveal pathogen-specific host responses that are mirrored in the serum *N*-glycome. However, the prognostic utility of serum glycosylation, particularly its capacity, early in the disease, to predict disease trajectory and survivorship of critically ill patients with septic shock, remains largely unexplored.

Based on this rationale, we hypothesized that the serum *N*-glycoproteome may provide prognostic insight into disease severity and survivorship outcome of patients with septic shock. Our multi-omics study contributes to the growing body of evidence supporting the clinical utility of serum glycoprofiling in infectious diseases. The work addresses a significant unmet need in the precision management of individuals experiencing septic shock.

## Experimental Procedures

### Serum Collection

Sera from septic shock patients were obtained from whole blood (1–10 ml/drawing) collected with informed consent at the Royal Adelaide Hospital (RAH), Adelaide, Australia. Briefly, blood was drawn from the study participant’s central lines and transferred into VACUETTE Serum Clot Activator tubes (PREMIUM 5 ml tubes, Greiner Bio-One). Tubes were centrifuged at 3200 rpm for 15 min at 4 °C. Sera were aliquoted into 1 ml Eppendorf safe lock tubes and stored without any additives at −80 °C until further handling and analysis. Samples were processed and frozen within 30 min of blood draw and were subjected to a maximum of two freeze/thaw cycles over the entire study.

Sera were collected from 37 patients clinically diagnosed with septic shock (mean baseline sequential organ failure assessment (SOFA) score of 11 [range 4–19] and acute physiology and chronic health (APACHE) score of 23 [range 9–41]) immediately upon admission to the ICU at RAH (ICU day 1) and then daily until recovery (ICU discharge) or ICU death, see Supplementary Table S1 for details of the patient cohort and key patient metadata including all blood transfusion records. In line with the 2012 Surviving Sepsis international consensus (42), all patients received guideline-directed core therapies, including intravenous fluids, noradrenaline infusions, and broad-spectrum antibiotics as the mainstay of care. Noradrenaline infusions were given universally to all participants under the same study protocol to ensure best clinical practice, standardization of care, and safeguard against any study-related risk of harm to the participants. Additional interventions such as intubation and ventilation, renal replacement therapy, parenteral nutrition, and blood product transfusion were instituted on an individualized basis according to clinical need as per the treating intensivist.

From this cohort, eight patients died within ICU care from whom a total of 40 serum samples were longitudinally collected (average five samples/patient), while 29 patients recovered and were discharged from the ICU from whom 94 serum samples were longitudinally collected (average four samples/patient). Ethics approval for the collection and biochemical analysis of the septic shock sera was granted by the RAH Human Research Ethics Committee (HREC/16/RAH/29). This study abides by the Declaration of Helsinki principles.

### Protein Handling

The protein concentrations of the neat serum samples were determined using a bicinchoninic acid assay (Thermo Fisher Scientific), following the manufacturer’s protocol and using a bovine serum albumin standard curve. Samples were diluted with triethylammonium bicarbonate (TEAB) to a final concentration of 1.5 μg/μl serum protein in 50 mM TEAB. Samples were reduced with 10 mM dithiothreitol (DTT) for 45 min at 56 °C, alkylated with 30 mM aqueous iodoacetamide (both final concentration) for 30 min in the dark and quenched with excess DTT. The serum protein extracts were used for both comparative glycomics and comparative glyco/proteomics experiments as described below.

### *N*-Glycomics Sample Preparation

Glycomics sample preparation of serum protein extracts followed a well-established method (33, 34, 43). For each sample, 50 μg of reduced and alkylated protein was spotted onto an activated 0.45 μm polyvinylidene fluoride membrane (Merck-Millipore), dried, stained with direct blue, and excised. The excised spots were transferred to separate wells in a flat bottom polypropylene 96-well plate (Corning Life Sciences), blocked with 1% (w/v) polyvinylpyrrolidone in 50% (v/v) aqueous methanol, and washed with ultra-pure water from a MilliQ source (used throughout the protocol).

The *N*-glycans were exhaustively released using 0.5 U/μl *Elizabethkingia miricola* peptide:*N*-glycosidase F (PNGase F) recombinantly expressed in *Escherichia coli* (Promega) per well and incubated for 16 h at 37 °C. The released *N*-glycans were transferred into fresh tubes and hydroxylated by the addition of 100 mM aqueous ammonium acetate, pH 5 for 1 h at 20 °C. The glycans were reduced using 1 M sodium borohydride in 50 mM aqueous potassium hydroxide for 3 h at 50 °C. The reduction reaction was quenched using glacial acetic acid.

Dual desalting of the reduced *N*-glycans was performed using firstly strong cation exchange resin (AG 50W-X8 Resin, Bio-Rad), followed by porous graphitized carbon (PGC) resin custom packed as microcolumns on top of C18 discs (Merck-Millipore) in P10 solid-phase extraction (SPE) formats. Following microcolumn equilibration and sample loading and washing, the *N*-glycans were eluted from the PGC-C18-SPE microcolumns using 0.1% trifluoroacetic acid (TFA)/50% acetonitrile (ACN)/49.9% water (v/v/v), dried and resuspended in 20 μl water. Samples were spun at 14,000*g* for 10 min at 4 °C and the clear supernatant fractions were carefully transferred to high recovery glass vials (Thermo Fisher Scientific) to avoid debris and particulates in the liquid chromatography-tandem mass spectrometry (LC-MS/MS) injection vials.

### *N*-Glycan Profiling

The *N*-glycans were profiled using a well-established PGC-LC-MS/MS method (19, 41, 44, 45). In brief, the *N*-glycan samples were injected on a HyperCarb KAPPA PGC-LC column (particle/pore size, 3 μm/250 Å; column length, 30 mm; inner diameter, 1 mm, Thermo Hypersil, Runcorn, UK) heated to 50 °C. To avoid data acquisition batch effects and injection sequence bias, the 134 *N*-glycan samples were injected on the LC-MS/MS instrument in a randomized order considering both survivorship status and ICU time points. The *N*-glycans were separated over the following 60 min gradient of solvent B (70% ACN in 10 mM ammonium bicarbonate) in solvent A (10 mM aqueous ammonium bicarbonate). The gradient parameters were: 0 to 3 min - 0% B, 4 min - 14% B, 40 min - 40% B, 48 min - 56% B, 50 to 54 min - 100% B, 56 to 60 min - 0% B. The gradient was delivered by a 1260 Infinity Capillary HPLC system (Agilent) operating with a constant flow rate of 20 μl/min. The separated *N*-glycans were introduced directly into the mass spectrometer, ionized using electrospray ionization and detected in negative ion polarity mode using a linear trap quadrupole Velos Pro ion trap mass spectrometer (Thermo Fisher Scientific). The acquisition settings included a full MS1 scan within the acquisition range of *m/z* 570–2,000, resolution of *m/z* 0.25 full width half maximum and a source voltage of +3.2 kV. The automatic gain control (AGC) for the MS1 scans was set to 5 × 10^4^ with a maximum accumulation time of 50 ms. For the MS/MS events, the resolution was set to *m/z* 0.25 full width half maximum, the AGC was 2 × 10^4^ and the maximum accumulation time was 300 ms. Data-dependent acquisition was enabled for all samples. The five most abundant precursors in each MS1 full scan were selected for fragmentation using resonance activation (ion trap) collision-induced dissociation (CID) at a normalized collision energy (NCE) of 33%. Dynamic exclusion of precursors was inactivated. All MS1 and MS/MS data were acquired in profile mode. The mass accuracy of the precursor and product ions was typically better than 0.2 Da. The LC-MS/MS instrument was tuned and calibrated, and its performance bench marked using well-characterized bovine fetuin *N*-glycan standards analyzed at the same time as the samples of interest.

### *N*-Glycomics Data Analysis

The generated LC-MS/MS glycomics raw data files were browsed, interrogated, and manually annotated using Xcalibur v2.2 (Thermo Fisher Scientific), GlycoMod (46) and GlycoWorkBench v2.1 (47) as previously described (19). Briefly, the *N*-glycans were identified based on the match between the observed and theoretical monoisotopic precursor mass and the MS/MS fragmentation pattern generated *in silico* using GlycoWorkBench, and the expected relative and absolute PGC-LC retention time of each glycan isomer. The relative abundances of the confidently identified *N*-glycans were determined from area under the-curve (AUC) measurements based on extracted ion chromatograms performed for all relevant charge states of the monoisotopic precursor *m/z* using RawMeat v2.1 (Vast Scientific) and Skyline (64 bit) v24.1 (48, 49).

A machine learning (ML) model was established to assess whether the serum *N*-glycome profiles measured upon ICU admission (day 1) could predict survivorship outcomes of the septic shock patients. Specifically, a supervised random forest model was established using *N*-glycomics data acquired from the longitudinally collected serum samples. To ensure independence of the training and testing data and to maximize the clinical relevance, the model was trained exclusively on serum *N*-glycome data collected from ICU day ≥2 and tested using ICU day 1 data. All ML modeling, including data preprocessing, feature selection, model training, and evaluation, was performed in R using in-house custom scripts. Nontransformed glycome data comprising glycan relative abundance data were used as input into the ML model. As per conventions in biomarker studies (50), accuracy, sensitivity, and specificity were used to assess the performance of the model in terms of predicting patient survivorship. For this purpose, the eight nonsurvivor cases were defined as positives and the 29 survivors as negatives. The model performance was established based on the true positive (TP, nonsurvivors correctly identified out of all nonsurvivors), true negative (TN, survivors correctly identified out of all survivors), false positive (FP, survivors incorrectly identified as nonsurvivors) and false negative (FN, nonsurvivors incorrectly identified as survivors). Performance was measured using the following metrics: Accuracy: (TP + TN)/(TP + TN + FP + FN) (overall proportion of survivorship/nonsurvivorship status correctly identified), sensitivity: TP/(TP + FN) (proportion of nonsurvivorship status in actual nonsurvivors correctly identified) and specificity: TN/(TN + FP) (proportion of survivorship in actual survivors correctly identified).

### *N*-Glycoproteomics and Proteomics Sample Preparation

For the glyco/proteomics analyses, the serum protein extracts from ICU day 1 (37 samples) were exhaustively digested using sequencing-grade modified porcine trypsin (Promega) at a 1:50 ratio (enzyme:substrate, w/w) at 37 °C for 17 h. The digestion was stopped by acidification with 1% (v/v) TFA (final concentration). Serum peptides were desalted using Oligo R3-C18-SPE clean up as previously described (33, 34). The desalted peptides were split into (1) a minor fraction (10%) used for proteome analysis by direct LC-MS/MS detection without glycopeptide enrichment (used to establish relative protein levels across patient groups) and (2) a major fraction (90%) used for glycoproteome analysis by LC-MS/MS detection after glycopeptide enrichment (used to establish the relative *N*-glycan distribution at each site or protein across patient groups). For (1), the fraction was dried and resuspended in 20 μl 0.1% (v/v) formic acid in preparation for direct LC-MS/MS analysis. For (2), the fraction was dried and resuspended in 50 μl 0.1% (v/v) TFA in 80% (v/v) ACN in preparation for glycopeptide enrichment.

Glycopeptides were enriched utilizing an established hydrophilic interaction liquid chromatography (HILIC)-SPE based enrichment method (51). Custom-made HILIC-C8-SPE microcolumns were prepared by inserting 1 mm Empore discs (Octyl C8 47 mm Extraction DISK 66882-U, Thermo Fisher Scientific) into P10 pipette tips (Eppendorf) onto which microcolumns of zwitterionic HILIC resin (ZIC-HILIC, 10 μm particle size, 200 Å pore size, kindly donated by Merck KGaA) were packed (33, 34). The HILIC-C8-SPE microcolumns were conditioned after which the serum peptides were gently loaded and reloaded onto the microcolumns. Following thorough washing, glycopeptides were eluted sequentially using 50 μl 0.1% (v/v) TFA, 50 μl 25 mM aqueous NH_4_HCO_3_ and finally 50 μl 50% (v/v) ACN. The eluted glycopeptide fractions were combined, dried and resuspended in 20 μl 0.1% (v/v) formic acid for LC-MS/MS analysis.

### LC-MS/MS-Based Glyco/proteomics

Unenriched serum peptides were analyzed in a single batch on a Q-Exactive HF-X Hybrid Quadrupole-Orbitrap mass spectrometer (Thermo Fisher Scientific) operated in positive ion polarity mode. To avoid data acquisition batch effects and injection sequence bias, samples were injected on the LC-MS/MS instrument in a randomized order. Peptides were loaded on an Acclaim PepMap C18 reversed phase HPLC trap column (5 mm length × 300 μm inner diameter) and were separated on a nanoLC column (30 cm length x 75 μm inner diameter) packed in-house with Solidcore Halo 2.7 μm 150 Å ES-C18 resin (Advanced Material Technology) operated at 45 °C by an UltiMate NCS-3500RS HPLC system (Thermo Fisher Scientific). The HPLC produced a constant flow rate of 300 nl/min using a binary mobile phase system comprising 0.1% (v/v) formic acid (solvent A) and 0.1% (v/v) formic acid in 99% (v/v) ACN (solvent B). Following an initial equilibration for 5 min using solvent A, peptides were separated over a multi-step linear gradient ramping from 3.2 to 36% solvent B over 56 min followed by 36 to 95% solvent B over 2 min and an 8 min wash at 95% solvent B for a total run time of 75 min per sample. MS1 full scans (*m/z* 400–2000) were acquired with a resolution of 60,000 and a normalized AGC target of 3 x 10^6^, a maximum injection time of 100 ms and a 1 s cycle time. Data-dependent acquisition was used to select the 10 most abundant precursors in each MS1 scan for MS/MS, which were performed using higher-energy collisional dissociation (HCD) at 27.5% NCE. The precursor isolation window was 1.4 Th. MS/MS data were acquired with a resolution of 15,000, AGC target of 1 x 10^5^ and a maximum injection time of 100 ms. Selected precursors were dynamically excluded for 15 s. Unassigned, singly and highly charged precursors (z > 7) were excluded for MS/MS.

Enriched serum glycopeptides were analyzed in a single batch on a Orbitrap Exploris 240 mass spectrometer (Thermo Fisher Scientific) operated in positive ion polarity mode. These samples were also injected in a randomized order on the LC-MS/MS instrument. Glycopeptides were loaded on an Aurora Ultimate nanoLC column (25 cm length × 75 μm inner diameter, 1.7 μm particle size) operated at 60 °C by a Vanquish Neo UHPLC System (Thermo Fisher Scientific). The HPLC provided a constant flow rate of 300 nl/min using the same solvent A and B as above. Peptides were separated over a multi-step linear gradient ramping from 3 to 35% solvent B over 90 min, 35 to 50% solvent B over 8 min followed by 50 to 90% solvent B over 2 min and a 10 min wash at 95% solvent B for a total run time of 110 min per sample. MS1 full scans (*m/z* 600–2,000) were acquired with a resolution of 120,000 using a standard AGC target with 100 ms maximum accumulation time. Data-dependent acquisition with a 3 s cycle time was used to select the 10 most abundant precursors in each MS1 scan for HCD-MS/MS at 30% NCE. Fragment spectra were acquired within the Orbitrap with an isolation window of 1.4 Th and resolution of 15,000. The AGC target was set to 2 x 10^5^ and maximum accumulation time was 250 ms with a dynamic exclusion window of 20 s after a single round of isolation and fragmentation of any given precursor. Unassigned, singly charged and highly charged precursors (z > 7) were excluded for MS/MS.

### Glyco/proteomics Data Analysis

The raw glyco/proteomics LC-MS/MS datasets were browsed and manually inspected using Xcalibur v2.2 or FreeStyle v1.8 (Thermo Fisher Scientific). Proteomics and glycoproteomics data from the unenriched and enriched peptide samples were analyzed separately to identify and quantify the nonmodified and glycosylated peptides and their source proteins, respectively.

The glycoproteomics data (HCD-MS/MS) were searched using both Byonic v4.5.2 (Protein Metrics) and the Integrated GlycoProteome Analyzer (I-GPA) search engine (52). For the Byonic analyses, searches were performed employing a protein database comprising a reviewed UniProtKB *Homo sapiens* database (downloaded November 2021, 20,360 entries) alongside a glycan search space of 287 mammalian *N-*glycans without sodium adducts, see Supplementary Table S2*A*. The precursor and product tolerance thresholds were 10 ppm and 20 ppm, respectively. Semispecific tryptic (RK) searches were performed with N-ragged cleavage sites allowing two missed cleavages. Methionine oxidation (+15.994 Da @ M) was considered a common variable modification while cysteine carbamidomethylation (+57.021 Da @ C) was considered a fixed modification. A maximum of two common and one rare (glycan) modification was permitted for each peptide candidate. In addition to this untargeted (“broad”) search, a targeted (“narrow”) search was performed using Byonic, which targeted only those MS/MS spectra that contained a diagnostic ion for Lewis fucosylation at *m/z* 512.20 (representing Hex-(Fuc)-HexNAc [H+]). The narrow search employed an *m/z* tolerance of 0.02 Th and a rank cutoff of 50 against the target fragment ions, but otherwise used identical search settings as the broad Byonic searches. The narrow search was included to selectively identify peptides carrying Lewis-type *N*-glycans. The outputs from all Byonic searches were filtered to <1% false discovery rate (FDR) at the protein level and 0% at the peptide level using a decoy database and only confidently identified glycopeptides (PEP 2D < 0.001) were considered. For I-GPA searches, the LC-MS/MS raw files were converted into mzML files using MSConvert (v3.0.21193-ccb3e0136; https://proteowizard.sourceforge.io/) with peak picking (vendor MS1) and default options. MS1 and MS/MS data were extracted from the mzML files with an in-house program coded by Python v3.8. I-GPA (v2.0) searches were then performed to classify the fucosylation type (core or Lewis) using an artificial neural network model generated with Tensorflow (v2.0) (52). The detailed parameters for the identification of *N*-glycopeptides were as following: noise peak thresholds of 50.0 and 2.0 for MS1 and MS/MS, respectively; precursor mass tolerance of ±0.05 Da; MS/MS tolerance of ±0.02 Da for HCD; I-GPA used a glycan search space of 226 *N*-glycan compositions, see Supplementary Table S2*B*. The cutoff threshold of M−, and S− score was 1.2, and 98.0, respectively, corresponding to less than 1.0% of the estimated FDR. For all Byonic and I-GPA outputs, the relative abundances of the identified *N-*glycopeptides or select subsets thereof were determined using a spectral counting approach considering the glycopeptide-to-spectrum matches (glycoPSMs).

For protein identification and quantification, raw files (unenriched fractions) were imported into MaxQuant v2.4.13.0. The Andromeda search engine was used to search the HCD-MS/MS data against the reviewed UniProtKB *H*. *sapiens* database (downloaded November 2021, 20,360 entries) with a precursor and product ion mass tolerance of 4.5 ppm and 20 ppm, respectively, and employing a trypsin-specific (RK) search allowing two missed cleavages. Carbamidomethylation of cysteine (+57.021 Da) was set as a fixed modification. Oxidation of methionine (+15.994 Da) and protein *N*-terminal acetylation (+42.010 Da) were selected as variable modifications. All identifications were filtered to <1% FDR at the protein and peptide level using a conventional decoy approach. For label-free AUC-based quantification, the “match between runs” feature of MaxQuant was enabled with a 0.7 min match time window and 20 min alignment time window. Label-free AUC-based quantification was performed, and protein abundance was calculated based on the normalized protein intensity (53).

Gene ontology (GO) analysis probing associations to biological processes was performed using the GO Enrichment Analysis tool (54, 55), with the protein carriers of Lewis fucosylation as input and using the human proteome as the reference set. GO terms with *p* < 0.01 were considered significantly enriched.

### Diagnostic Ion Profiling

To quantify key diagnostic fragment ions for Lewis fucosylation in the glycoproteomics LC-MS/MS data (i.e. *m/z* 512.20 and *m/z* 803.29 arising from Lewis and sialyl Lewis, respectively) and in the glycomics LC-MS/MS data (*m/z* 348.2 for Lewis a [Le^a^] and *m/z* 364.2 for Lewis x [Le^x^]) from ICU day 1, we employed (1) a custom Python script and (2) the GlyCounter tool (https://github.com/riley-research/GlyCounter) (56).

(i) For the custom script, raw LC-MS/MS files were converted to .mgf file format using MSConvert (v3.0.21188) with peak picking set to vendor msLevel = 1- and activation set to HCD (57). A Python script (v3.9.2, github.com/jfeh0001/glyco_Y0) was used to scan for the presence of *m/z* 512.20 and *m/z* 803.29 fragment ions in the .mgf files counting peaks with intensity >1% of the base peak in each fragment spectrum within ±0.01 Th of target *m/z* values. The script was also used to scan for the presence of *m/z* 348.2 and *m/z* 364.2 fragment ions in the glycomics data counting peaks with intensity >1% of the base peak in each fragment spectrum and increasing the *m/z* window to ±0.20 Th of target *m/z* values.
(ii) For the GlyCounter approach, the glycoproteomics raw files were scanned directly for the presence of *m/z* 512.20 and *m/z* 803.29 fragment ions using the settings: Tolerance = 10 ppm, signal-to-noise requirement = 3, and intensity threshold = 1000. HCD-MS/MS-specific scan settings: Must be within N most intense peaks = 50, HCD TIC fraction = 0.20, oxonium count requirement = 0.

### Experimental Design and Statistical Rationale

The discovery phase of the study was performed using serum longitudinally collected from a cohort of septic shock survivors (n = 29) and nonsurvivors (n = 8), which provides sufficient statistical power to detect glycosylation changes and associations to survivorship outcome from glyco(proteo)mics experiments. The study did not aim to elucidate causal relationships between serum glycosylation and septic shock mortality.

The glycomics and glycoproteomics data were subjected to statistical tests performed without data transformation and were visualized in various ways with or without transformation of the relative abundance data. Mann-Whitney U tests were performed to compare individual glycans/glycopeptides/glycoproteins or groups of glycosylation features between the two patient groups (unpaired data points). In contrast, Wilcoxon tests were applied when comparing paired data points from each patient. These nonparametric statistical tests were appropriate for this study as the distribution of the -omics data was not normal and since the two patient groups featured unequal size. Two-tailed tests were used for the discovery (hypothesis-free) part of the study, while one-tailed tests were used for the targeted (hypothesis-driven) statistical analyses later in the study as detailed in the respective figure legends. For all tests, *p* < 0.05 was chosen as the confidence threshold.

Heat-maps and hierarchical clustering analyses were performed with Perseus v1.6.7 using Euclidean distance with average linkages (58). The relative abundance values of the glycans were used as input data for these analyses after log2 transformation. Receiver operating characteristic (ROC) curves of both univariate and multivariate analyses were performed using linear support vector machines classification methods. The confidence threshold was AUC >0.75 with 1.00 representing perfect separation between the two patient groups. For volcano plot and ROC curves (performed using MetaboAnalyst (59)), untransformed or log2 transformed relative abundance values of the glycans/glycopeptides from each patient group were used as input. If not mentioned otherwise, data have been plotted as mean (average) and error bars indicate SD. Biological replicates and statistical significance have been stated directly in figures or in the accompanying figure legends.

## Results

### Leveraging Multi-omics to Map the Serum *N*-Glycoproteome Across Septic Shock Events

**Fig. 1:**
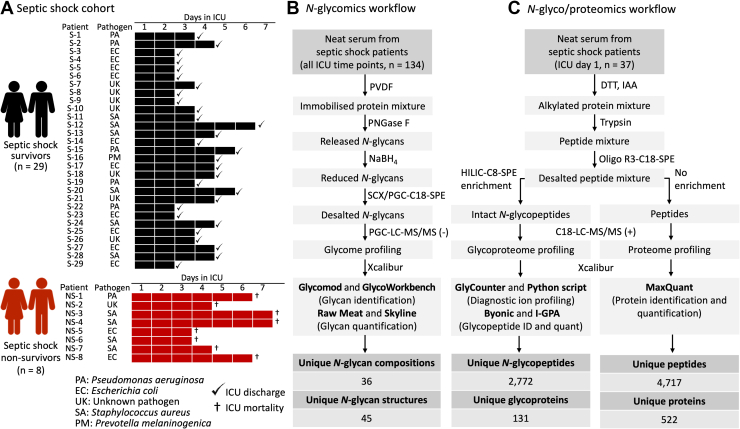
**Multi-omics temporal profiling of the serum *N*-glycoproteome during a septic shock event**. *A*, overview of the septic shock cohort comprising both patients who recovered (survivors, n = 29, labeled S-1 to S-29, in *black*) and patients who died (nonsurvivors, n = 8, NS-1 to NS-8, in *red*) from the disease. Serum was collected daily from ICU admission (day 1) until ICU discharge (√) or death (†) as indicated for each patient. A variety of disease-causing pathogens were identified across the patient cohort, see key. See Supplementary Table S1 for additional metadata (age, gender, body mass index, baseline APACHE and SOFA scores, and blood transfusion records) for the septic shock cohort. Experimental approach for the comparative glycomics (*B*) and comparative glyco/proteomics (*C*) methods applied to septic shock sera. The number of unique glycans, glyco/peptides and glyco/proteins identified in the septic shock sera are indicated. APACHE, acute physiology and chronic health evaluation; ICU, intensive care unit; I-GPA, Integrated GlycoProteome Analyzer; LC-MS/MS, liquid chromatography-tandem mass spectrometry; PVDF, polyvinylidene fluoride; SOFA, sequential organ failure assessment.

The goal of this study was to establish clinically informative glyco-signatures that may identify and thus guide the management of the most severely affected individuals with septic shock. For this, we took a multi-omics approach to comprehensively survey the *N*-glycoproteome of serum collected from a cohort of patients clinically diagnosed with septic shock who either recovered (n = 29 survivors) or died (n = 8 nonsurvivors) from the condition, Figure 1*A*. Importantly, serum was collected daily from both the survivors (from ICU admission to discharge, 2–6 samples per patient, in total n = 94 samples) and from the nonsurvivors (from ICU admission to death, 3–7 samples per patient, in total n = 40 samples). For most of the septic shock patients, different bacteria were identified as the disease-causing pathogens including *Pseudomonas aeruginosa* (n = 6), *Escherichia* *coli* (n = 12), *Staphylococcus aureus* (n = 10), *Prevotella melaninogenica* (n = 1) while no infecting pathogen was confidently identified for a subset of the septic shock patients (n = 8, unknown pathogen). The patient cohort included both males and females and spanned a large range in terms of age (40–86 years old) and body mass index (17.9–62.3 kg/m^2^), Supplementary Table S1. Although the two patient groups were appropriately gender and age matched, the patients formed a relatively heterogenous cohort in terms of disease severity upon ICU admission and treatments received reflecting the makeup of the individuals affected by this disease.

Enabled by our glycomics-guided glycoproteomics technology (33, 34), we performed comparative glycomics, glycoproteomics, and proteomics of the septic shock serum cohort, Figure 1*B*–*C*. Glycomics, involving the detailed structural characterization of liberated *N*-glycans from all ICU sampling points (134 serum samples) using PGC-LC-MS/MS, provided the relative abundance of a total 45 *N*-glycan structures (including isomers) across 36 different glycan compositions within the septic shock serum *N*-glycome (detailed further below), see Supplementary Data S1 for all annotated glycomics spectral data. To obtain important complementary information, the serum *N*-glycoproteome of the clinically valuable ICU day 1 sampling point (37 serum samples) was surveyed using both proteomics and glycoproteomics involving reversed phased-LC-MS/MS of serum peptide mixtures that were either analyzed directly or after enrichment for intact glycopeptides, respectively. The glycoproteomics experiments identified a total of 2772 unique (nonredundant) *N*-glycopeptides originating from 131 serum glycoproteins, see Supplementary Data S2 and deposited Byonic viewer files for all annotated glycopeptide spectral data. Separately, the proteomics experiments, carried out to compare the relative protein levels across the two patient groups, identified 4717 unique peptides derived from 522 serum proteins. Expanding on existing literature (17, 39, 40, 60, 61), these integrated multi-omics analyses have provided, to the best of our knowledge, to date, the most comprehensive map of the serum *N*-glycoproteome across the septic shock disease course.

### Surveying *N*-Glycome Dynamics in Septic Shock Reveals Signatures with Stratification Potential

**Fig. 2:**
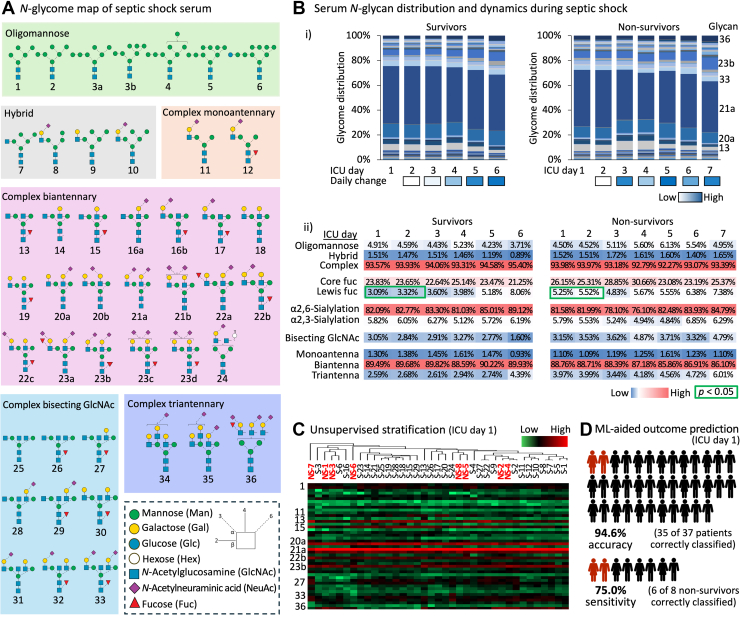
***N*-glycan profiling of septic shock sera reveals signatures with stratification potential**. *A*, map of *N*-glycan structures identified in septic shock sera. Glycan cartoons are drawn using the latest SNFG nomenclature (88), see insert for key. *B*, (i) distribution of serum *N*-glycans from survivors (*left*, average of n = 29) and nonsurvivors (*right*, average of n = 8) of each ICU sampling points during the septic shock event. Only dominant glycan structures are labeled for simplicity. See Supplementary Table S3 for all tabulated *N*-glycome data. The relative day-to-day change in the serum *N*-glycome (average across all patients) is indicated by heat map colors ranging from low (*white*) to high (*blue*) day-to-day fluctuations, see key. (ii) longitudinal plot of the relative level of discrete *N*-glycan features in sera from septic shock survivors (*left*, average of n = 29) and nonsurvivors (*right*, average of n = 8). Values are accompanied by heat map colors ranging from low (*blue*) to medium (*white*) to high (*red*) relative abundance levels. Unpaired two-tailed Mann-Whitney U tests were used to compare glycan features across survivors and nonsurvivors for each ICU time point; *green box* indicates statistical significance (*p* < 0.05). *C*, partial stratification of septic shock nonsurvivors (NS, bolded in *red*) from survivors (S, in *black*) using unsupervised hierarchical clustering on the entire serum *N*-glycome data from ICU day 1. The *N*-glycome data were visualized with heat map colors ranging from low (*green*) to medium (*black*) to high (*red*) relative abundance, see key. *D*, ML-guided prediction of survivorship outcome of septic shock patients based on the entire serum *N*-glycome. A random forest model was trained using all serum *N*-glycome data from all ICU time points except ICU day 1 and the performance of the prediction model was tested using the clinically strategic ICU day 1 data. ICU, intensive care unit; ML, machine learning; SNFG, Symbol Nomenclature For Glycans.

The 45 *N*-glycan structures that were identified by the glycomics profiling of septic shock sera featured, as expected from established serum *N*-glycome literature (19, 41, 62), primarily complex-type *N*-glycans spanning predominantly bi- and tri-antennary *N*-glycans and less abundant mono-antennary and bisecting GlcNAcylated *N*-glycans, Figure 2*A*. Also aligning with expectations, oligomannosidic- and hybrid-type *N*-glycans formed relatively minor subsets of the septic shock serum *N*-glycome, see Supplementary Table S3 for all tabulated *N*-glycome data.

Although protein *N*-glycosylation is considered relatively stable in serum with limited intrapersonal and interpersonal variations even under pathophysiological conditions (23, 24, 63), longitudinal plots of the *N*-glycan distribution revealed considerable dynamics of the serum *N*-glycome over the course of a septic shock event, Figure 2*B*i. For the septic shock survivors, the day-to-day *N*-glycome fluctuations increased with the recovery trajectory and was most pronounced around the time of discharge from the ICU whereas for the nonsurvivors, profound fluctuations in the *N*-glycome were observed on ICU days 3 to 5 mirroring the progression of sepsis shock to ICU death. However, when assessing the day-to-day fluctuations at the *N*-glycan feature level, the sera appeared relatively stable throughout the septic shock event for both survivors and nonsurvivors, Figure 2*B*ii. No statistically significant differences in *N*-glycan type distribution (e.g., oligomannose, 3.7%–6.1%), core fucosylation (21.2%–30.7%), sialylation i.e., α2,3- (4.8%–6.9%) and α2,6-sialylation (76.1%–89.1%) and glycan branching (e.g., biantennary, 86.1%–90.2%) were observed within or between the patient groups while in ICU care. As an important exception to these trends, the outer arm (antennary, hereafter referred consistently to as Lewis) fucosylation was found to be significantly raised in nonsurvivor sera on ICU day 1 and day 2 (5.25%–5.52%) relative to levels in survivor sera (3.09%–3.32%, *p* < 0.05). This survivorship-associated glycan feature is explored in greater detail below.

Taking firstly an unsupervised approach, we used unsupervised hierarchical clustering to explore if the *N*-glycome in serum collected upon ICU admission (ICU day 1) could discriminate septic shock survivors from nonsurvivors, Figure 2*C*. Although no clean separation was achieved between the two patient groups, the clustering analysis partially grouped the septic shock nonsurvivors indicating some stratification potential of the serum *N*-glycome already upon hospital admission. Given our access to a relatively large collection of glycomics data, we therefore sought to establish a glycome-based prediction model using an ML approach employing all ICU time points except for ICU day 1 data to train a random forest model. The serum *N*-glycome data from ICU day 1 were separately used to test the predictive performance of the model. Encouragingly, the ML-guided model showed very high accuracy (94.6%) by correctly predicting the survivorship outcome of 35 of the 37 septic shock patients, Figure 2*D*. Moreover, the model showed perfect specificity (100%) by correctly identifying all 29 septic shock survivors and featured high sensitivity (75.0%) by correctly classifying six of the eight nonsurvivors. Upon inspection of the ML-guided prediction model, glycan 22a and glycan 23a (both carrying Lewis fucosylation, see below), were found to be the main contributors to the high performance of the ML model.

### Le^x^-type *N*-Glycans Stratify Nonsurvivors From Survivors Early in Septic Shock

**Fig. 3:**
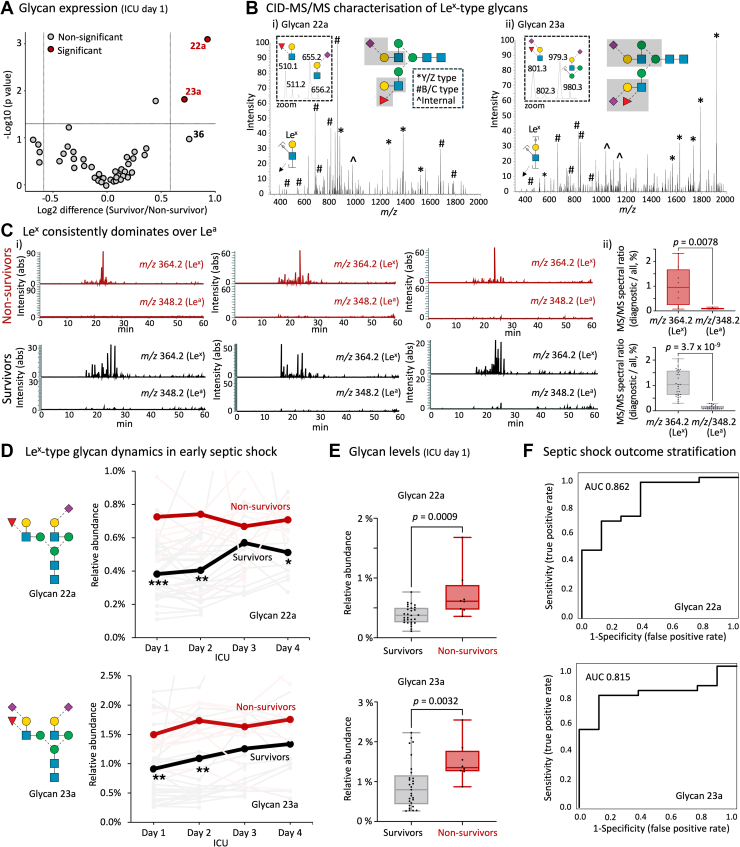
**Le^x^-type *N*-glycans stratify nonsurvivors from survivors early in sepsis shock**. *A*, comparison of the relative abundance of individual serum *N*-glycans from survivors (n = 29) and nonsurvivors (n = 8). *N*-glycans that differ between patient groups (*p* < 0.05, glycan 22a and glycan 23a) are in *red*; unpaired Wilcoxon tests, two-tailed. *B*, CID-MS/MS-based characterization of (i) glycan 22a and (ii) glycan 23a. Spectral features have been annotated (see insert for key to fragment ion types) with a focus on the terminal fragments including Lewis-specific fragment ions. See Supplementary Data S1 for fully annotated MS/MS spectra of all *N*-glycans identified in septic shock sera. *C*, (i) XICs tracking of key diagnostic fragment ions (*m/z* 364.2 for Le^x^ and *m/z* 348.2 for Le^a^ (66, 67)) in glycomics data from three representative septic shock nonsurvivors (*top*) and survivors (*bottom*). (ii) Quantitation of Le^x^ and Le^a^-specific ions across the entire patient cohort, see also Supplementary Table S4. Paired Wilcoxon tests, two-tailed. *D*, Longitudinal plots of glycan 22a (*top*) and glycan 23a (*bottom*) in sera from survivors (*black trace*, averages of 29 patients) and nonsurvivors (*red trace*, averages of eight patients) in the early stages of septic shock (ICU day 1–4). Patient-specific traces are shown in faint *gray* (survivors) and faint *red* (nonsurvivors), see Supplementary Figure S1 for full view of patient-specific plots of glycan 22a and glycan 23a. Glycan levels were compared between patient groups at each ICU time point using unpaired Mann–Whitney U tests, one-tailed, ∗*p* < 0.05, ∗∗*p* < 0.01, ∗∗∗*p* < 0.001. *E*, Boxplots showing relative level of glycan 22a (*top*) and glycan 23a (*bottom*) in sera collected on ICU day 1 from survivors (n = 29) and nonsurvivors (n = 8). Unpaired Mann–Whitney U tests, one-tailed. *F*, ROC plots showing the ability of glycan 22a (*top*) and glycan 23a (*bottom*) to predict the survivorship outcome of septic shock patients when measured on ICU day 1 serum. AUC, area under the curve; CID-MS/MS, collision-induced dissociation tandem mass spectrometry; ICU, intensive care unit; Le^x^, Lewis x; MS/MS, tandem mass spectrometry; ROC, receiver operating characteristic.

Comparative analyses of the relative abundances of the individual *N*-glycan structures identified in septic shock sera revealed that glycan 22a and glycan 23a were significantly elevated in nonsurvivors relative to survivors at ICU day 1, Figure 3*A*. Although glycan 36 also appeared elevated in nonsurvivor compared to survivor sera, this structure did not reach statistical significance. Notably, no *N*-glycan structures appeared significantly reduced in nonsurvivors compared to survivors suggesting that the relative elevation of glycan 22a and 23a was not a result of a considerable reduction of other specific structures in the serum *N*-glycome.

Detailed collision-induced dissociation tandem mass spectrometry (CID-MS/MS)-based characterization was used to elucidate the fine structural details of the observed serum *N*-glycans including glycan 22a and glycan 23a, Figure 3*B*. A common feature of glycan 22a and glycan 23a (and of the slightly elevated glycan 36), was the Lewis-type fucosylation on the 3′ arm of the biantennary complex-type *N*-glycan structures appearing as a Lewis epitope on glycan 22a (i) and a sialyl Lewis epitope on glycan 23a (ii) (as well as on glycan 36). In line with robust literature on septic shock serum (20, 64, 65), our detailed PGC-LC-MS/MS-based glycomics data indicated that the glycoepitopes were Lewis x (Le^x^) rather than Le^a^ type. This was supported by the consistent dominance of the Le^x^-specific diagnostic ion (*m/z* 364.2) over the Le^a^-specific fragment ion (*m/z* 348.2) across the entire cohort (*p* < 0.01), Figure 3*C* and Supplementary Table S4 (66, 67, 68, 69). In addition to the Lewis fucosylation, all three biosynthetically related structures (glycans 22a, 23a, 36) featured a sialyl LacNAc on the 6′ arm and did not carry core fucosylation.

Mapping the temporal expression of glycan 22a and glycan 23a across the initial stages of a septic shock event (ICU day 1–4) illustrated that these two glycans were significantly overrepresented in nonsurvivor relative to survivor sera collected on ICU day 1 (glycan 22a: *p* = 0.0009, glycan 23a: *p* = 0.0032) and on ICU day 2 (glycan 22a: *p* = 0.0016, glycan 23a: *p* = 0.0060), Figure 3*D*. Resulting from a gradual increase of these two glycan isomers within the survivor group, similar levels of glycan 22a and glycan 23a were generally detected at ICU days 3 and 4 for the two patient groups (*p* ≥ 0.05). Excitingly, the biggest difference between nonsurvivors and survivors was observed on the clinically important ICU day 1 for both glycan 22a (*p* = 0.0009) and glycan 23a (*p* = 0.0032) when intervention options are most beneficial, Figure 3*E*. Accordingly, targeted ROC analyses demonstrated a potential of both glycan 22a (AUC: 0.862) and glycan 23a (AUC: 0.815) to predict septic shock survivorship outcome, Figure 3*F*.

### AGP-1-Le^x^ Glycoforms Separate Septic Shock Nonsurvivors From Survivors on ICU Day 1

Prompted by the glycomics data indicating a stratification potential of Le^x^-type *N*-glycans in serum from early stage septic shock patients, we performed comparative glycoproteomics of serum collected on ICU day 1 in attempts to quantitatively support this interesting observation and determine the protein carrier(s) of this outcome-associated glycosylation feature.

**Fig. 4:**
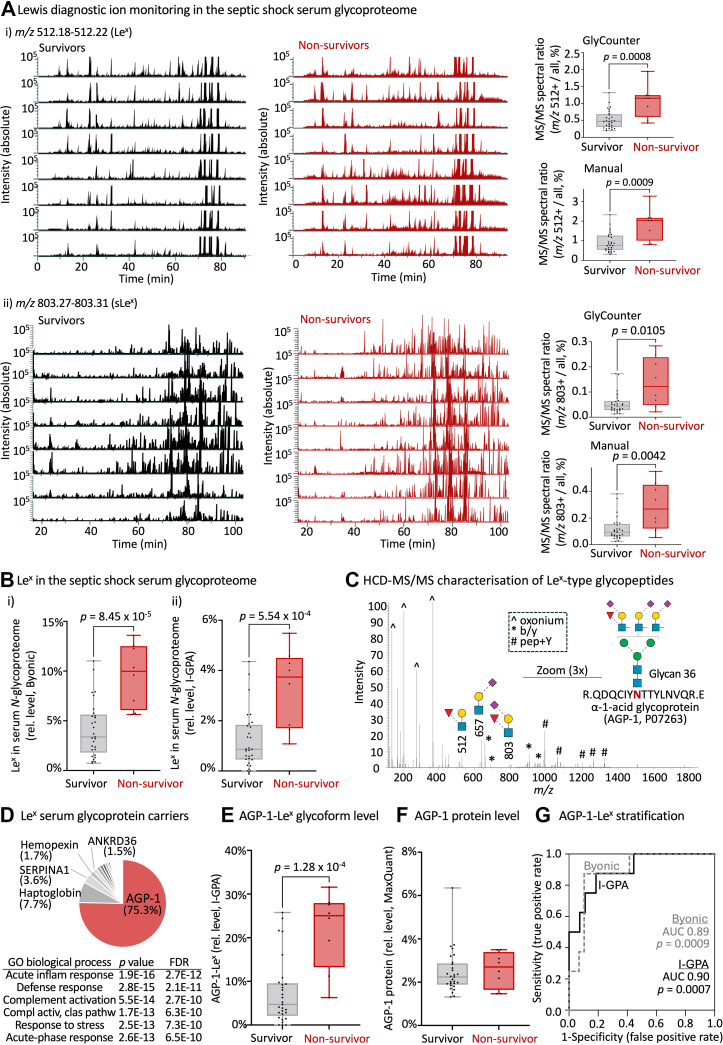
**AGP-1-Le^x^ glycoforms separate septic shock nonsurvivors from survivors upon hospital admission**. *A*, (i-ii) *Left*: stacked XICs tracking key Lewis diagnostic ions (*m/z* 512.18–512.22 and *m/z* 803.27–0.803.31 corresponding to Le^x^ and sLe^x^, respectively) within the glycopeptide enriched glycoproteomics data of eight septic shock survivors (*black traces*, *left*) and all eight nonsurvivors (*red traces*, *right*). *Right*: quantification of the relative level of Lewis fragments across the entire patient cohort as measured by the ratio of MS/MS spectra featuring the diagnostic ions using both GlyCounter (56) and an in-house Python script (manual) across all septic shock survivors (n = 29) and nonsurvivors (n = 8), see also Supplementary Table S5. Unpaired Mann-Whitney U tests, one-tailed. *B*, Global level of Le^x^ in the serum *N*-glycoproteome of septic shock survivors (n = 29) and nonsurvivors (n = 8) as determined using (i) Byonic and (ii) I-GPA search engines on the glycoproteomics data from ICU day 1 serum. See Supplementary Table S6-S7 and Supplementary Table S9 for all tabulated Byonic and I-GPA glycoproteome data, respectively, and Supplementary Table S8 for relative Le^x^ levels. Unpaired Mann-Whitney U tests, one-tailed. *C*, HCD-MS/MS-based characterization of a representative Le^x^-containing glycopeptide derived from AGP-1. Spectral features have been annotated (see insert for key to fragment ion types) with a focus on key terminal fragments including Lewis-specific fragment ions. An *m/z* region was magnified to visualize b/y peptide ions. *D*, Distribution of Le^x^ glycoproteins in septic shock serum. The five most abundant Le^x^-containing proteins including AGP-1 (in *red*) have been labeled (see Supplementary Table S10 for all identified Le^x^ glycoproteins). Pathway enrichment analysis showing the six most enriched pathways related to the Le^x^ glycoproteins identified in septic shock serum. *E*, Relative level of AGP-1-Le^x^ glycoforms in sera from septic shock survivors (n = 29) and nonsurvivors (n = 8) as determined by I-GPA using glycoproteomics data from ICU day 1 serum. Unpaired Mann–Whitney U tests, one-tailed. Observation was supported by Byonic data, see Supplementary Table S11 for AGP-1-Le^x^ data. *F*, Relative level of AGP-1 protein in sera from septic shock survivors (n = 29) and nonsurvivors (n = 8) as determined by MaxQuant on proteomics (unenriched) data from ICU day 1 serum, see Supplementary Table S12 for all tabulated proteome data and Supplementary Table S13 for AGP-1 protein level data. Unpaired Mann-Whitney U tests, one-tailed. *G*, ROC plots using both I-GPA (*black line*) and Byonic (*gray*, *broken line*) glycoproteomics data showing the ability of AGP-1-Le^x^ glycoforms to accurately predict the survivorship outcome of septic shock patients when measured on ICU day 1 serum. AGP-1, alpha-1-acid-glycoprotein; HCD-MS/MS, higher-energy collisional dissociation tandem mass spectrometry; ICU, intensive care unit; I-GPA, Integrated GlycoProteome Analyzer; Le^x^, Lewis x; ROC, receiver operating characteristic.

We firstly performed an initial high-level XIC-based monitoring of Lewis-specific fragment ions (*m/z* 512.20 and *m/z* 803.29, representing Hex-(Fuc)-HexNAc [H+] and NeuAc-Hex-(Fuc)-HexNAc [H+], respectively), across the glycoproteomics LC-MS/MS datasets (68, 69). We interpreted these ions as fragments of (s)Le^x^ glycopeptides due to the absence of detectable (s)Le^a^ glycoepitopes (see above). The diagnostic ion profiling indicated visibly stronger signal traces in septic shock nonsurvivor relative to survivor samples, Figure 4, *A–B*. Quantifying these diagnostic ions at the MS/MS spectral level using the GlyCounter tool (56) and our own in-house Python script indeed showed a significantly higher level of both Le^x^- and sLe^x^-glycoepitopes for septic shock nonsurvivors compared to survivors (all *p* < 0.05), Supplementary Table S5. The Le^x^ diagnostic ion (*m/z* 512.20) was consistently stronger than the sLe^x^ diagnostic ion (*m/z* 803.29) in the glycoproteomics fragment spectra likely due to the charge difference and the propensity of sLe^x^ fragments to decompose to Le^x^ ions. This prompted us to focus on Le^x^ glycopeptides in the subsequent analyses.

We then mined the glycoproteomics data using two complementary search engines, Byonic and I-GPA, with a focus on comparing Le^x^ glycopeptides between patient groups. On one hand, a two-step Byonic-based approach was used involving, firstly, an untargeted (broad) search against all HCD-MS/MS spectra (see Supplementary Table S6 for search output), and, secondly, a targeted (narrow) search directed against only those HCD-MS/MS spectra that contained prominent Le^x^ diagnostic ions (Supplementary Table S7). This approach identified a total of 6385 Le^x^-glycopeptides across the patient cohort (average ∼173 glycoPSMs/sample). Importantly, the data confirmed that Le^x^-glycopeptides were raised in septic shock nonsurvivors compared to survivors already upon ICU admission (*p* = 8.45 x 10^-5^), Figure 4*B*i and Supplementary Table S8*A*. On the other hand, we also employed the I-GPA search engine that has been trained to distinguish Lewis fucosylated glycopeptides from glycopeptides carrying core fucosylation (52). Although the I-GPA-based search identified comparably less Le^x^-glycopeptides (1019 across the patient cohort) due to a more refined ML-guided annotation of the fucose position, this alternative search engine reassuringly validated that Le^x^-glycopeptides were elevated in nonsurvivors compared to survivors (*p* = 5.54 x 10^-4^), Figure 4*B*ii and Supplementary Table S8*B* (see Supplementary Table S9 for search output). HCD-MS/MS-based characterization of the peptides carrying Le^x^-glycoforms not only revealed the protein carrier identities (in this representative example, AGP-1) but also confirmed the structure of the conjugated glycans (in this case glycan 36) and the terminal glycoepitopes (Lewis fucosylation i.e., *m/z* 512 and *m/z* 803 as well as sialyl LacNAc i.e., *m/z* 657), Figure 4*C*.

The Byonic-annotated *N*-glycoproteome data were then interrogated to determine the set of proteins carrying Lewis fucosylation in septic shock serum. In total 59 different serum Le^x^-type glycoproteins were identified in septic shock serum including most prominently AGP-1 (75.3%) while haptoglobin (7.7%), alpha-1-anti-trypsin (SERPINA1, 3.6%), hemopexin (1.7%), and ankyrin repeat domain-containing protein 36A (ANKRD36, 1.5%) among many other serum proteins were of lower abundances, Figure 4*D* and Supplementary Table S10. In support, I-GPA also identified AGP-1 as the principal protein carrier of Le^x^ glycoepitopes (∼75%). Interestingly, pathway enrichment analyses using GO of all 59 Le^x^-containing glycoproteins identified in septic shock sera against a human proteome reference set revealed strong overrepresentation of pathways known to be associated with septic shock including, for example, acute inflammatory response (*p* = 1.9 x 10^-16^, FDR 2.7 x 10^-12^), complement activation (*p* = 5.5 x 10^-14^, FDR 2.7 x 10^-10^), response to stress (*p* = 2.5 x 10^-13^, FDR 7.3 x 10^-10^), and acute-phase response (*p* = 2.6 x 10^-13^, FDR 6.5 x 10^-10^).

Given the profound dominance of AGP-1 among the other less abundant Le^x^ protein carriers, we used the Byonic and I-GPA search outputs to explore the relative level of AGP-1-Le^x^ glycoforms in the two patient groups. Based on the I-GPA search output, AGP-1-Le^x^ glycoforms were found to be significantly elevated in septic shock nonsurvivors relative to the survivor group (*p* = 1.28 x 10^-4^), Figure 4*E* and Supplementary Table S11*A*. This finding was supported by the Byonic search output demonstrating a similar elevation (*p* = 1.90 x 10^-4^, see Supplementary Table S11*B* for data) confirming that AGP-1-Le^x^ glycoforms are raised in septic shock nonsurvivors.

As AGP-1, similar to many other serum proteins carrying Le^x^ glycoforms, is known to be a positive acute phase protein and thereby raised in circulation as a result of systemic infection, we sought to establish the relative AGP-1 protein levels in septic shock survivor and nonsurvivor sera. As measured using the quantitative proteomics data (see Supplementary Table S12 for all tabulated proteome data), the AGP-1 level was found to be similar in nonsurvivors and survivors upon hospital admission (*p* ≥ 0.05) suggesting a glycosylation- rather than a protein-based regulation underpinning the molecular differences observed between the patient groups, Figure 4*F* and Supplementary Table S13. Excitingly, ROC analyses demonstrated that AGP-1-Le^x^ glycoforms accurately predicted survivorship outcome in septic shock patients from serum collected upon ICU admission (day 1) (I-GPA data: AUC: 0.90, *p* = 0.0007 and Byonic data: AUC: 0.89, *p* = 0.0009), Figure 4*G*.

## Discussion

Septic shock, the unbalanced and often exaggerated immune response to infection, remains a major cause of global mortality, yet tools for accurate patient risk stratification and prediction of disease trajectory are still unavailable to ICU clinicians. The lack of efficient prognostic tools contributes to the unacceptably high mortality and disease burden of septic shock. To address this significant clinical gap, we here applied an integrated glycomics and glycoproteomics method to sera from a cohort of septic shock survivors and nonsurvivors, collected daily within the ICU until recovery or death. These efforts enabled us to generate a comprehensive temporal profile of the serum *N*-glycoproteome across the septic shock disease course.

Our -omics investigations revealed dynamic changes in the serum *N*-glycome and identified distinct glycosylation patterns in septic shock survivors and nonsurvivors as early as upon hospital admission (ICU day 1). Using an ML-guided approach, a random forest model trained on the serum *N*-glycome data correctly predicted the survival outcome of all 29 survivors and six of the eight nonsurvivors using ICU day 1 data. Le^x^-type glycoepitopes, predominantly conjugated to serum AGP-1, were significantly elevated in septic shock nonsurvivors and AGP-1-Le^x^ glycoforms were a strong predictor of poor outcome upon ICU admission.

In line with our findings, an early study used simple lectin and antibody-based electrophoretic methods to suggest that Le^x^-containing AGP-1 glycoforms, rather than the AGP-1 protein level itself, is raised in septic shock nonsurvivors relative to survivors (64). Our multi-omics approach not only validates these early findings, but also adds important biochemical details (structural depth and robust quantitation) and offers additional insights into the dynamics of the serum *N*-glyco(proteo)me across the septic shock event. From a temporal perspective, the most prominent glycosylation differences between the two patient groups were observed upon ICU admission suggesting that Le^x^-type glycoepitopes can be harnessed for prediction of disease trajectory and to guide clinical decision-making in the context of septic shock.

Lewis antigens have been previously associated with leukocyte adhesion, selectin binding, and immunosuppressive signaling in cancer (70, 71, 72) and inflammation (73), but their use as prognostic markers in acute critical illness remains underexplored. The functional implications of aberrant AGP-1 glycosylation in inflammation and details of how serum Le^x^ glycoforms may mechanistically link to poor septic shock outcome are yet unknown. However, given the considerable affinity for endothelial receptors such as E-selectin and P-selectin, AGP-1-Le^x^ glycoforms have been suggested to interfere with leukocyte extravasation by competing for binding to these adhesion molecules providing possible clues to functional involvement (73, 74, 75). Conceivably, raised levels of serum AGP-1-Le^x^ glycoforms in fatal septic shock may therefore severely impact leukocyte trafficking impairing their efficient homing to site(s) of inflammation, in turn, potentially impeding their pathogen-fighting and inflammation-resolving functions and altering interactions to other critical blood cells, for example, to platelets that abundantly express Le^x^-recognizing P-selectins. As such, several underlying mechanisms and molecular effector functions of AGP-1-Le^x^ glycoforms may potentially contribute to the poor outcome in septic shock. However, we stress that the aim of this biomarker-centric study was to establish robust associations between serum *N*-glycosylation and septic shock survivorship. The study was neither designed to reveal any molecular mechanisms driving septic shock nor establish causal links between AGP-1-Le^x^ and septic shock outcome, which therefore require further investigation.

In addition to the *N*-glycans reported in this work, *O*-glycans also carry Lewis motifs (76); it remains to be investigated whether AGP-1 or other serum glycoproteins carry *O*-linked Le^x^-type glycans in septic shock sera and if these also associate with poor patient outcome.

Although we and others did not observe any protein-level differences in serum AGP-1 between septic shock survivors and nonsurvivors, this positive acute phase protein is known to be substantially raised in all septic shock sufferers (∼1.6–1.7 mg/ml) relative to healthy donors (∼0.8 mg/ml) (64). The additional AGP-1 protein in septic shock sera is predictably from hepatic origins, however, since neutrophils are also known to produce, store and upon activation release AGP-1 displaying glycosylation features that are different to those carried by liver-derived AGP-1 (77), future efforts are required to determine if septic shock survivors and nonsurvivors carry AGP-1 from different tissue origins as this may explain the different glycosylation patterns observed across the two patient groups. In line with our observation of AGP-1-Le^x^ glycoforms in septic shock sera, Heck et al., have reported over-fucosylation of other acute phase proteins of hepatic origin in septic shock conditions including serum fetuin/α-2-HS-glycoprotein (61) and alpha-1-antichymotrypsin (17).

Biosynthetically, Lewis x-type fucosylation is mediated by a group of fucosyltransferases such as FUT3, FUT4, FUT5, FUT6, FUT7, and FUT9 (78, 79), which may conceivably be differentially regulated in septic shock survivors and nonsurvivors due to differences in cytokine signaling, epigenetics, or hepatic (dys)function. If Le^x^ glycoepitopes are found to be functionally involved in septic shock processes and thus impacting or even driving patient outcome, these enzymes may be potential targets for therapeutic intervention strategies against septic shock.

Reflecting both the considerable technical challenge of performing large multi-omics studies as well as the logistic challenge of recruiting critically ill septic shock individuals for daily serum sampling within the ICU, a limitation of this study was the modest cohort size and the substantial patient heterogeneity, including differences in age, gender, body mass index, infection source, treatment regimens, and comorbidities. Given the emerging links between the serum *N*-glycome and age, gender and other physiological conditions (80, 81), these various factors may influence the glycosylation in septic shock serum independently of clinical trajectory, and could confound associations if not adequately controlled. Although our cohorts spanned considerable heterogeneity, we ensured that the two patient groups were matched for both age and gender thereby mitigating any bias resulting from these two critical factors.

Another complicating factor when exploring blood markers for septic shock is the fact that patients routinely receive blood products (i.e., platelets, red cells, fresh frozen plasma [FFP], albumin), which may interfere with molecular and cellular biomarker readings. Blood transfusion was indeed part of standard care in this single-center ICU study, with 72% of patients receiving at least one blood product during the study period (see Supplementary Table S1 for details). Importantly, most of the transfusion volume (74%) comprised infusions of albumin, a colloid volume expander, which due to the lack of *N*-glycosylation is not expected to interfere with the endogenous serum *N*-glycoproteome investigated herein. In contrast, FFP, another blood product used for septic shock patient management, contains *N*-glycoproteins that could potentially skew measurements of the endogenous serum *N*-glycoproteome if administered in significant quantities. However, the fact that only 7.125 ml FFP was administered across the entire 37 patient-cohort, constituting a neglectable proportion of the endogenous blood plasma (7,125 ml FFP/(37 patients x 4,625 ml plasma estimated per patient) = 4.16%) and that similar volumes of FFP were administered to both survivors (11 bags) and nonsurvivors (8 bags) prior to ICU admission, it is unlikely that FFP administration accounts for the prominent glycomics differences observed between survivors and nonsurvivors already at ICU day 1. Nevertheless, as with other treatments, transfusion remains a potential confounder that must be carefully considered. Although this single ICU study ensured uniform treatment practices (42), future multisite studies are required to evaluate if changes in serum fucosylation are generalizable across multiple ICUs featuring diverse treatment practices.

Sepsis remains a highly complex and heterogeneous syndrome, and to fully establish the diagnostic and prognostic utility of glycan-based biomarkers, future studies will require larger, prospectively stratified cohorts. Our recent findings demonstrating that the infecting pathogen class impacts the host serum *N*-glycome profile in both preseptic shock (19) and septic shock conditions (41), demand the inclusion of diverse patient groups featuring both bacterial, viral and fungal infections as well as varying disease severities. Furthermore, longitudinal monitoring of patients from the early stages of sepsis through progression to septic shock within the ICU and extending through to either death or recovery into the post-ICU period, will serve to validate the survivorship outcome-specific glycan signatures observed herein and determine their temporal dynamics relative to each patient’s baseline. Sizeable patient cohorts will also enable more thorough training and thus stronger predictive power of the ML model employed in this study as recently demonstrated in an ML-based prediction of septic shock using serum metabolites from 124 individuals (82). In addition, expanding the analytical scope to other glycan classes, such as *O*-glycans, glycosphingolipids and glycosaminoglycans, may provide further insight into the host response and potentially reveal other outcome marker candidates.

From a translational perspective, the ability of Le^x^-type glycoepitopes as biologically relevant glycan motifs to report on septic shock patient outcome is interesting particularly given the current lack of prognostic tools available to ICU clinicians. The fact these functional glycoepitopes can be analytically measured by relatively unsophisticated methods (antibodies, lectins) directly in crude matrices including serum highlights their potential as dynamic biomarker candidates for risk stratification of patients with septic shock. Offering even more predictive performance, the protein-specific AGP-1-Le^x^ glycoforms may represent a viable target for point-of-care risk stratification using combined antibody- and lectin-based readouts in array formats (83, 84) or using LC-MS/MS methods (24, 81, 85) from a few microliters of neat serum or whole blood from septic shock sufferers. Such approaches could enable rapid, minimally invasive patient outcome prediction in the ICU, supporting more personalized and timely management of septic shock ultimately improving patient survival.

## Data Availability

Glycomics PGC-LC-MS/MS raw data were deposited to GlycoPOST (86), accession number GPST000599, URL: https://glycopost.glycosmos.org/preview/177519572068520a17e26ef, password: 4352). See Supplementary Data S1 for annotated glycomics spectral data. The glycoproteomics and proteomics C18-LC-MS/MS raw data and Byonic Viewer search files (broad and narrow 512-focused) were deposited to the ProteomeXchange Consortium (http://proteomecentral.proteomexhange.org) via the PRIDE partner repository (87), accession number PXD065024. See Supplementary Data S2 and the deposited Byonic Viewer search files for annotated glycopeptide spectral data from I-GPA and Byonic, respectively.

## Supplemental Data

This article contains supplemental data.

## Conflict of Interest

The authors declare no competing interests.
