# Supplementary material for: Serum AGP-1-Lex Glycoforms Report on Survivorship of Patients with Septic Shock Upon Admission to Intensive Care Unit
Source: Mol Cell Proteomics. 2025 Nov 17;25(1):101470. doi: 10.1016/j.mcpro.2025.101470 (PMC12794580; doi:10.1016/j.mcpro.2025.101470)
Supplement: Supplementary Data 1 [file mmc1.pdf]

Supplementary Data S1

for

Serum AGP-1-Le^x glycoforms report on survivorship of patients with septic shock upon admission to intensive care unit

The Huong Chau[1,2^], Sayantani Chatterjee[1^], Liam Caulfield[1], Anastasia Chernykh[1], Mathew Traini[1], Joshua Fehring[3], Heeyoun Hwang[4,5], Rebeca Kawahara[1,2], Emily J. Meyer[6,7], David J. Torpy[6,7], Morten Thaysen-Andersen[1,2*]

1. School of Natural Sciences, Macquarie University, Sydney, NSW, Australia
2. Institute for Glyco-core Research, Nagoya University, Nagoya, Aichi, Japan
3. Department of Biochemistry and Molecular Biology & Biomedicine Discovery Institute, Monash University, Australia
4. Digital OMICs Research Center, Korea Basic Science Institute, Cheongju, Republic of Korea
5. Bio-Analytical School, University of Science and Technology, Daejeon, Republic of Korea
6. Department of Medicine, University of Adelaide, Adelaide, South Australia, Australia
7. Endocrine and Metabolic Unit, Royal Adelaide Hospital, Adelaide, South Australia, Australia

^Contributed equally

***Corresponding author**:
A/Prof Morten Thaysen-Andersen, PhD
School of Natural Sciences
Macquarie University, NSW-2109, Sydney, Australia
Ph: +61 2 9850 7487
Email: morten.andersen@mq.edu.au

Annotation and Fragmentation Key

Mannose (Man) (162.0528 Da)

Galactose (Gal) (162.0528 Da)

N-Acetylglucosamine (GlcNAc) (203.0794 Da)

N-Acetylneuraminic acid (NeuAc) (291.0954 Da)

Fucose (Fuc) (146.0579 Da)

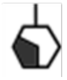 Cross-ring fragment (unspecified)

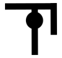 Indicates mostly Y ions (includes oxygen of glyosidic linkage)

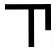 Indicates mostly Z ions (excludes oxygen of glyosidic linkage)

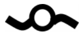 Reduced reducing end

N-glycans identified from sera of septic shock survivors and non-survivors

Glycan #1

Observed *m/z* 617.22 (2-), RT: ~22.3 min

[M-H]$^-$ 1235.44 Da

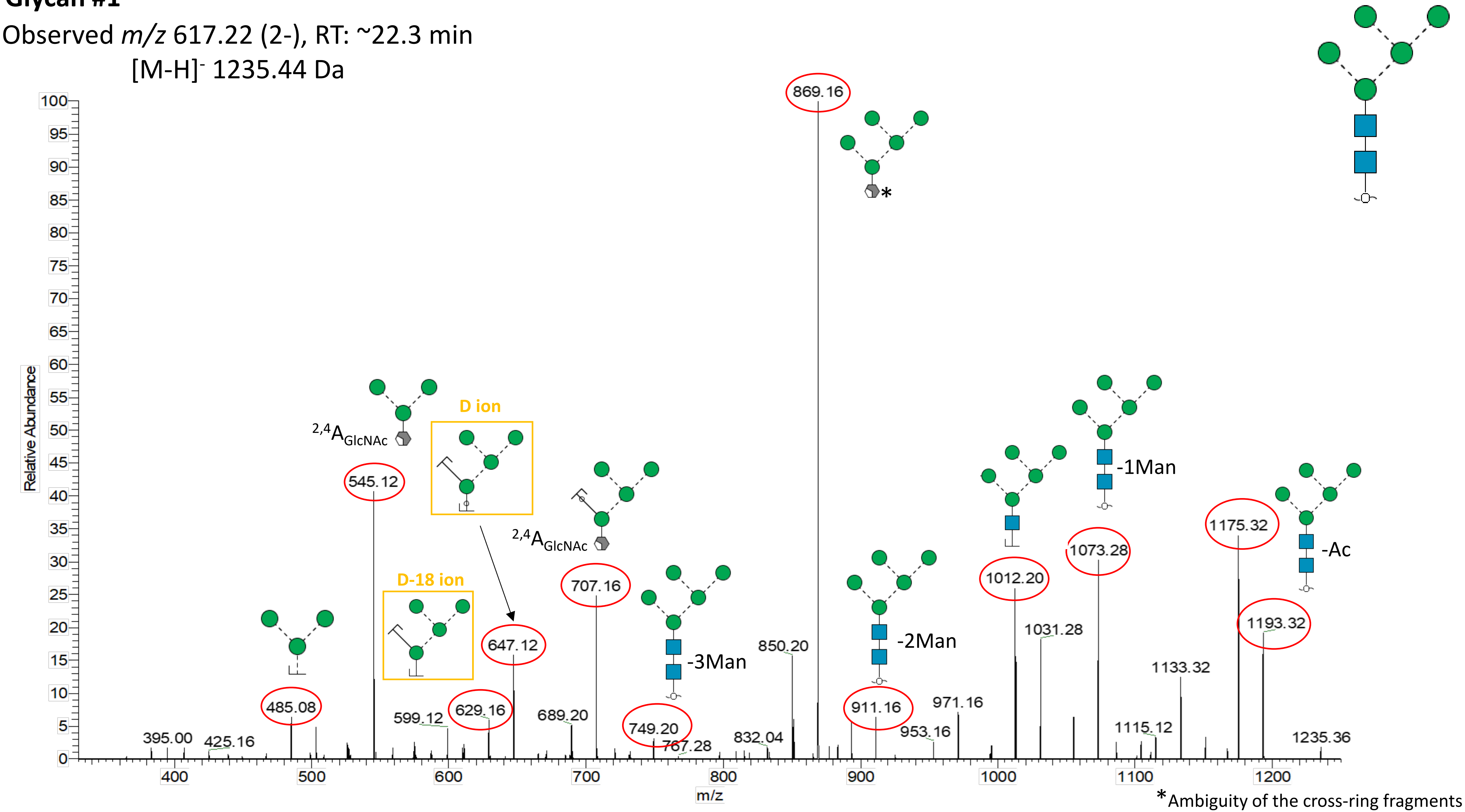

*Ambiguity of the cross-ring fragments

Glycan #2

Observed *m/z* 698.36 (2-), RT: ~19.0 min

$[M\text{-}H]^-$ 1397.49 Da

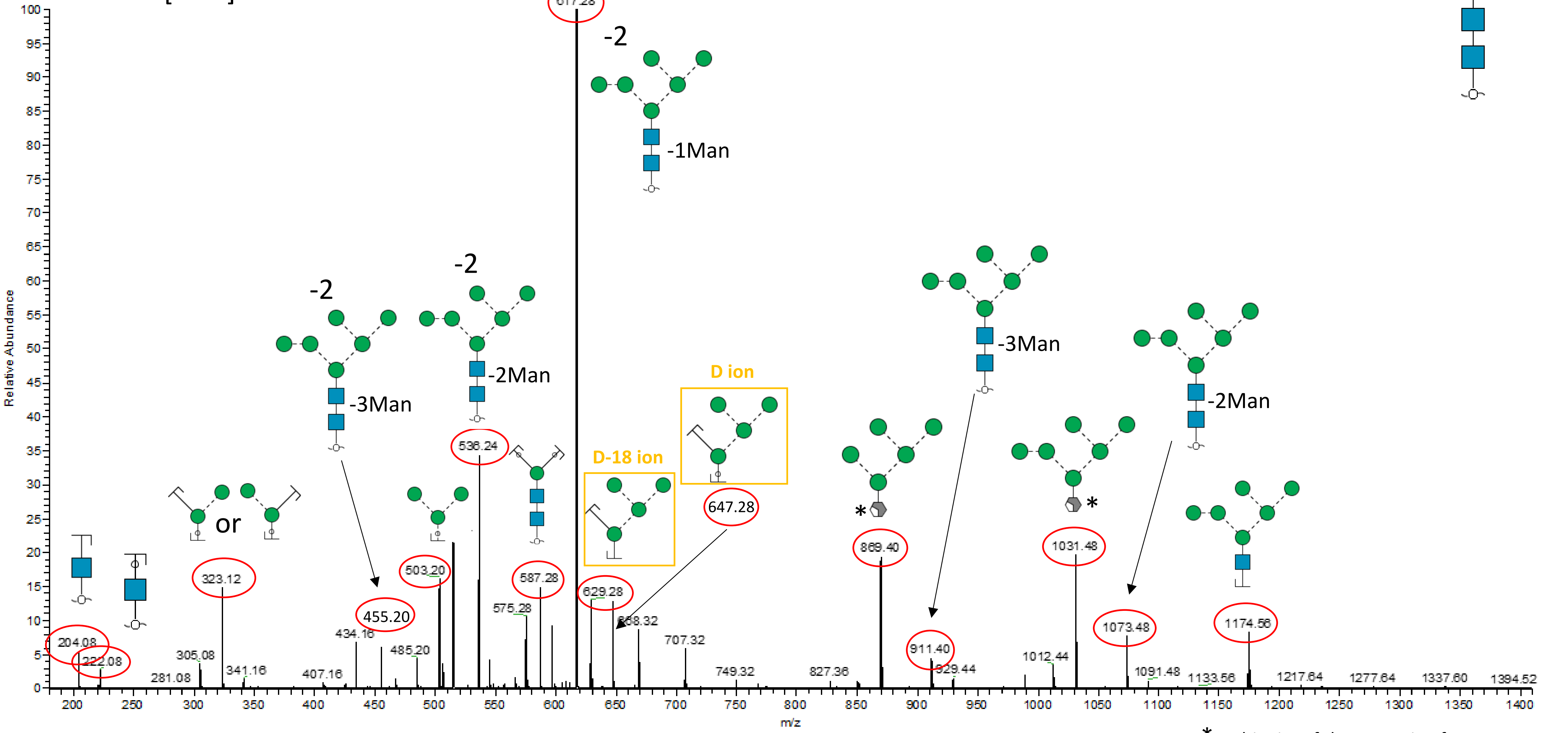

*Ambiguity of the cross-ring fragments

Glycan #3a

Observed *m/z* 779.38 (2-), RT: ~18.6 min

$[M-H]^-$ 1559.54 Da

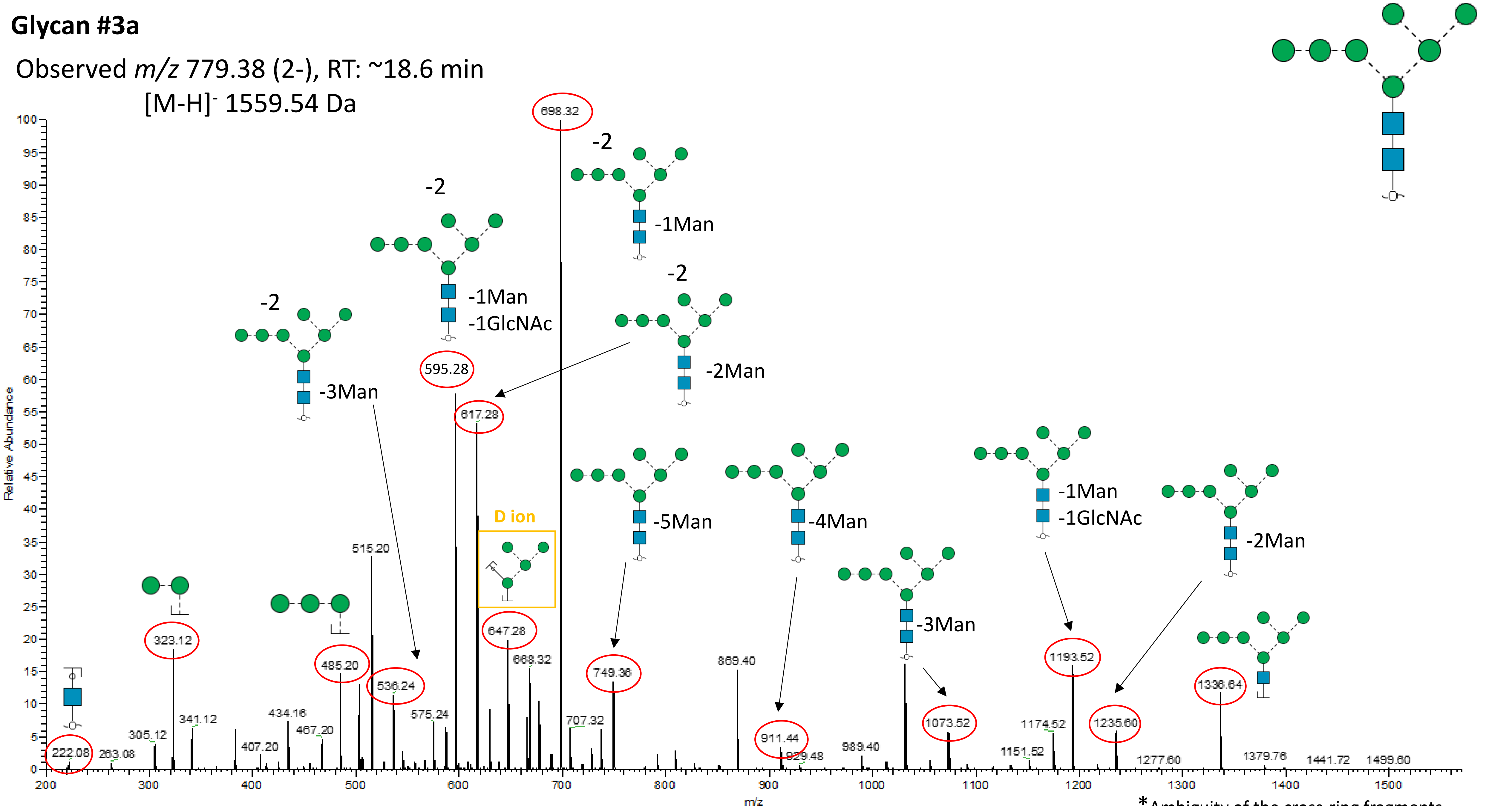

*Ambiguity of the cross-ring fragments

Glycan #3b

Observed *m/z* 779.38 (2-), RT: ~19.3 min

[M-H]$^-$ 1559.54 Da

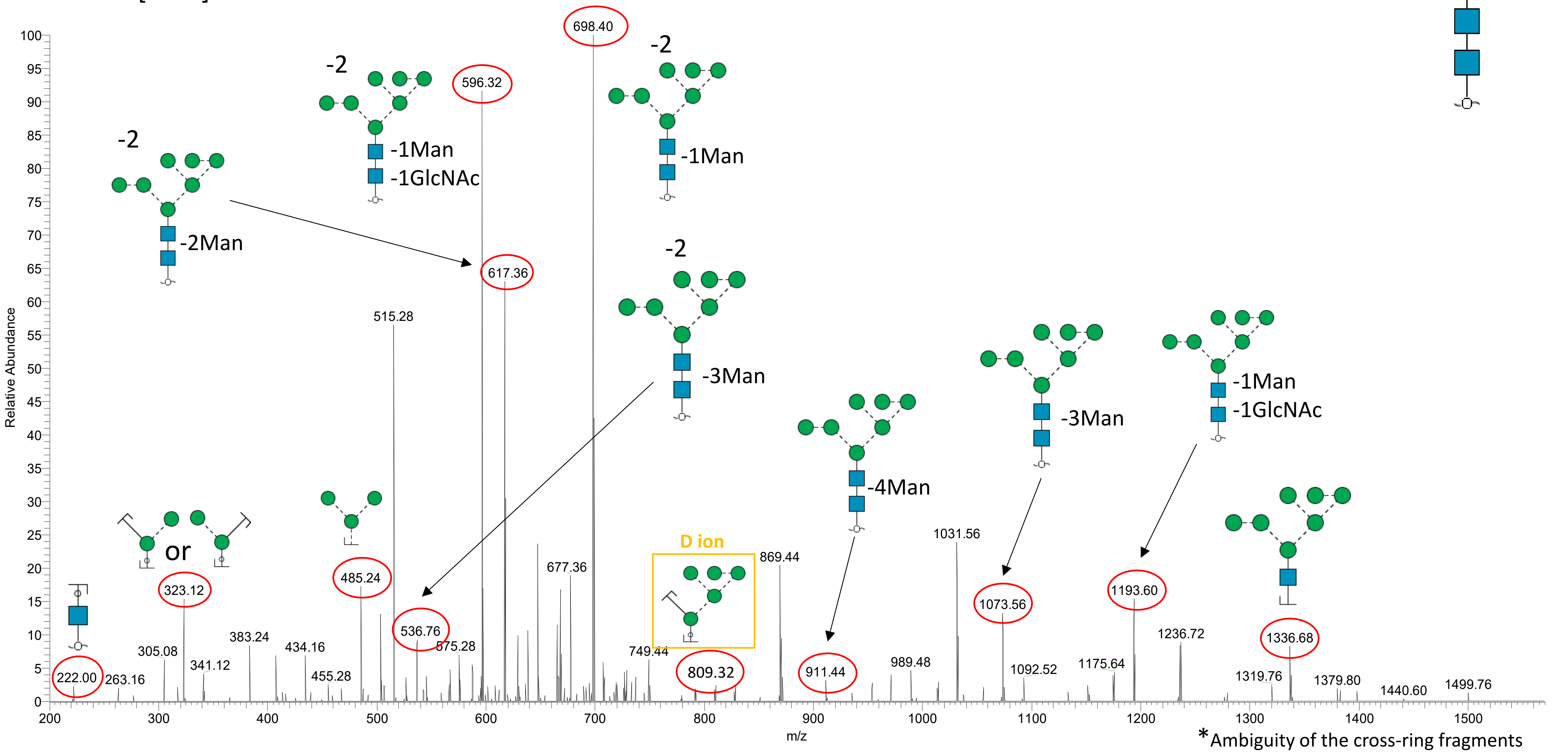

*Ambiguity of the cross-ring fragments

Glycan #4

Observed *m/z* 860.29 (2-), RT: ~18.5 min

[M-H]$^-$ 1721.60 Da

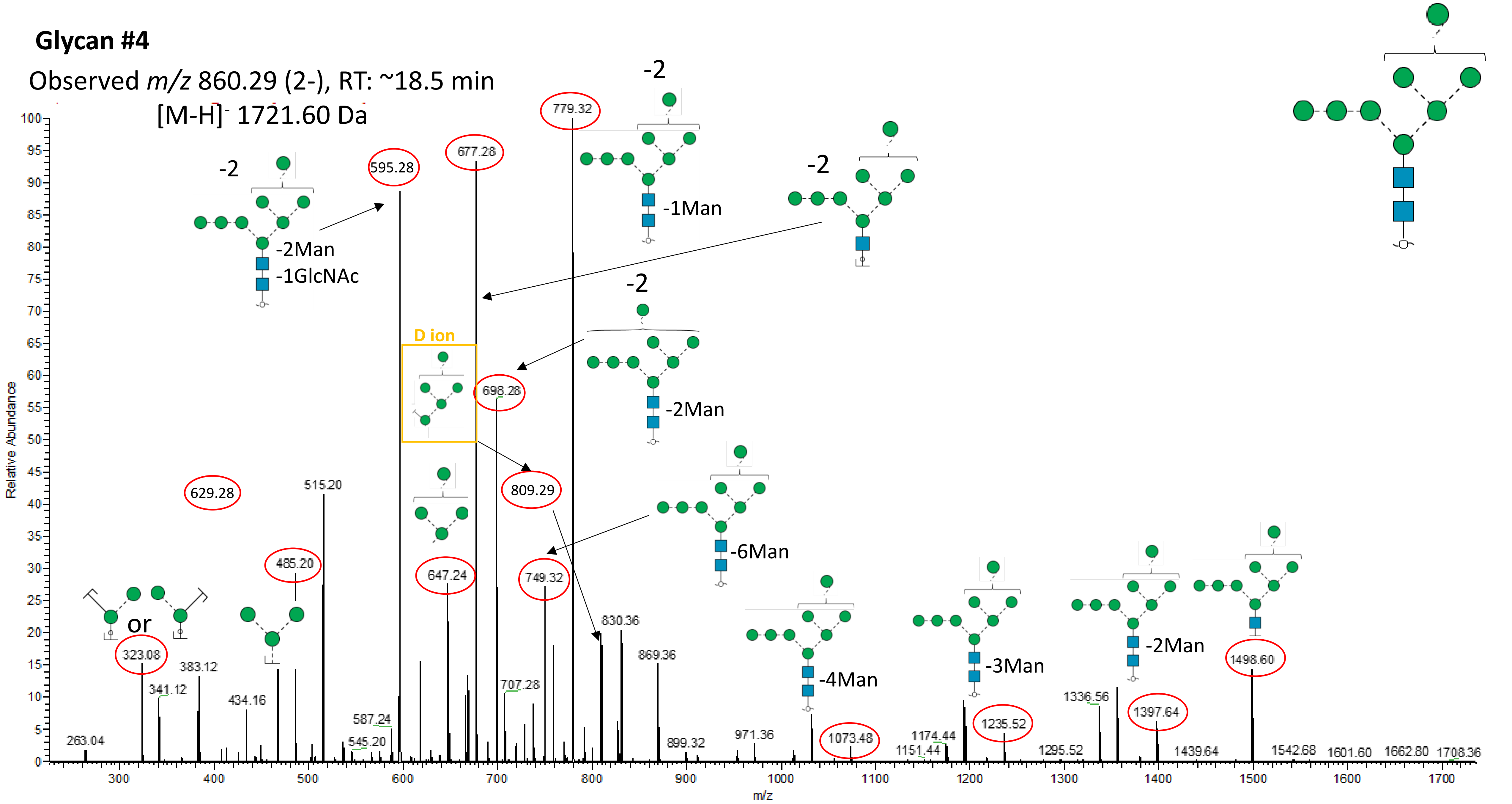

*Ambiguity of the cross-ring fragments

Glycan #5

Observed *m/z* 941.32 (2-), RT: ~18.6 min

$[M-H]^-$ 1883.65 Da

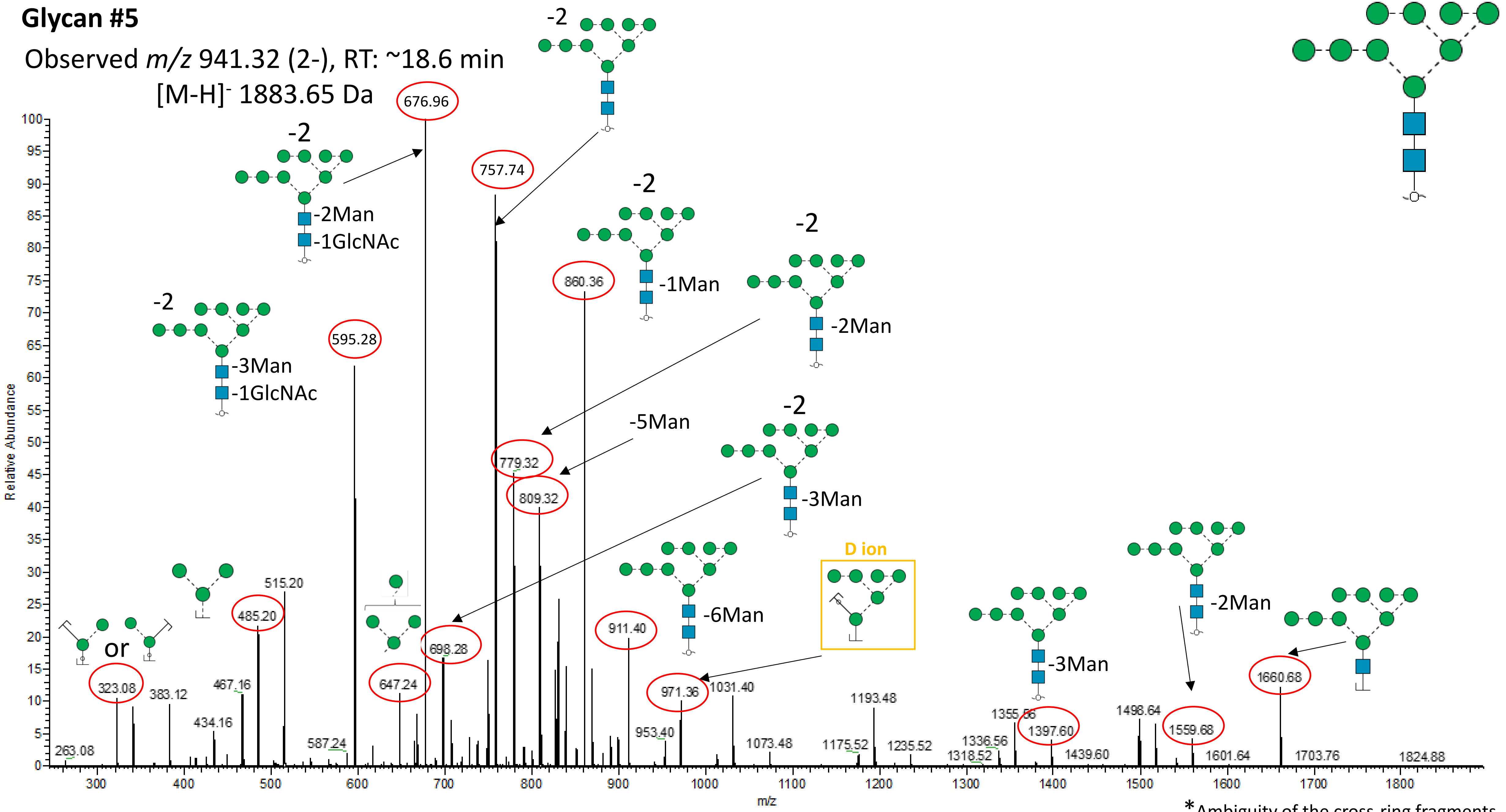

*Ambiguity of the cross-ring fragments

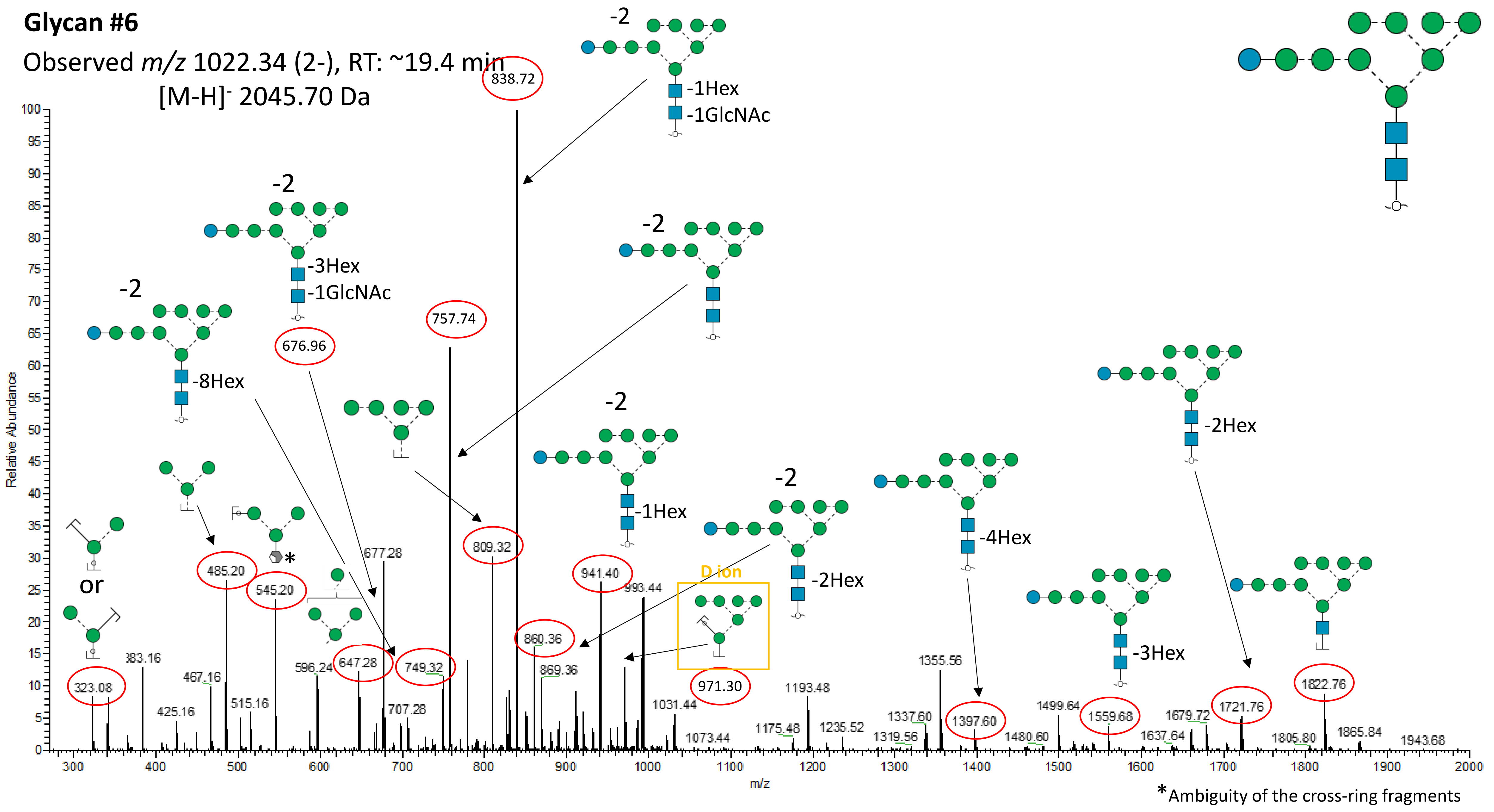
Glycan #6
Observed m/z 1022.34 (2-), RT: ~19.4 min
[M-H]- 2045.70 Da
838.72
-2
-1Hex
-1GlcNAc
-3Hex
-8Hex
-1Hex
-2Hex
-4Hex
-3Hex
D ion
757.74
676.96
677.28
809.32
941.40
993.44
485.20
545.20
860.36
869.36
971.30
323.08
383.16
425.16
467.16
515.16
596.24
647.28
707.28
749.32
1031.44
1073.44
1175.48
1193.48
1235.52
1319.56
1337.60
1355.56
1397.60
1480.60
1499.64
1559.68
1637.64
1679.72
1721.76
1805.80
1822.76
1865.84
1943.68
or
*
Relative Abundance
m/z
*Ambiguity of the cross-ring fragments

Glycan #7

Observed *m/z* 718.75 (2-), RT: ~19.8 min

$[M\text{-}H]^-$ 1438.52 Da

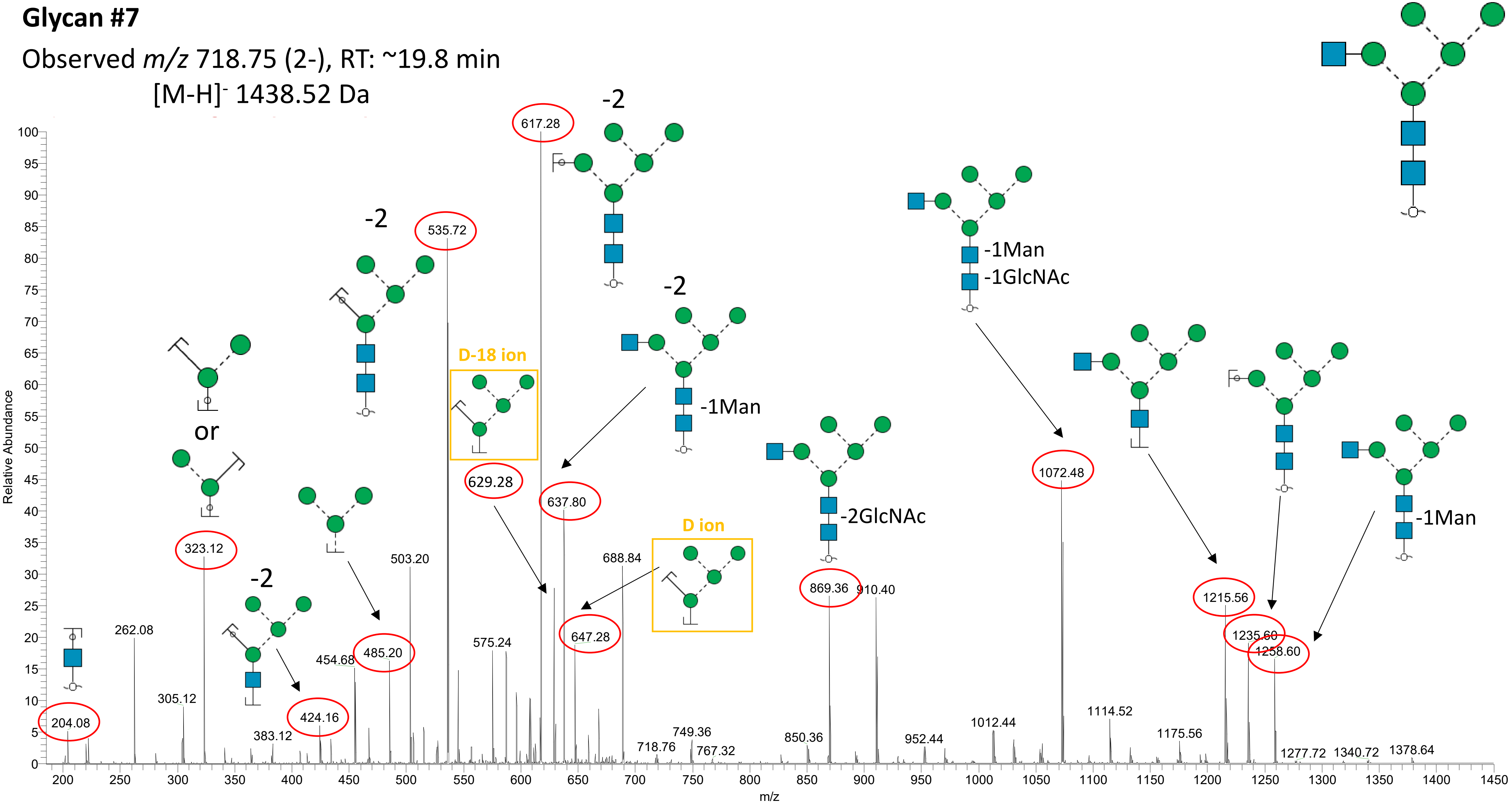

*Ambiguity in the cross-ring fragments

Glycan #8

Observed *m/z* 864.30 (2-), RT: ~20.4 min

[M-H]- 1729.61 Da

Note: Based on PGC-LC elution pattern, this glycan is annotated as the α2,6-sialyl linkage isomer.

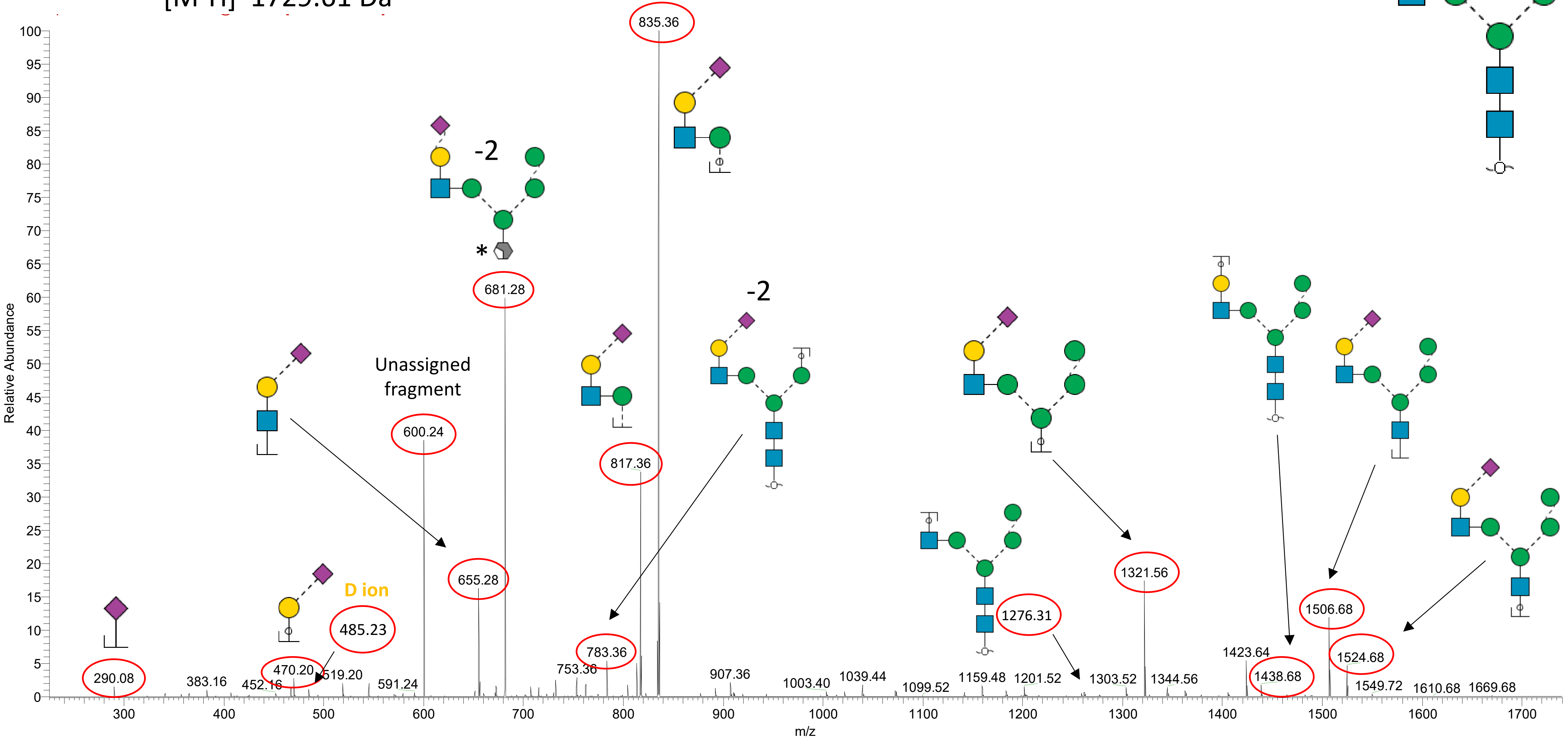

*Ambiguity in the cross-ring fragments

Glycan #9

Observed *m/z* 799.78 (2-), RT: ~20.8 min

[M-H]⁻ 1600.57 Da

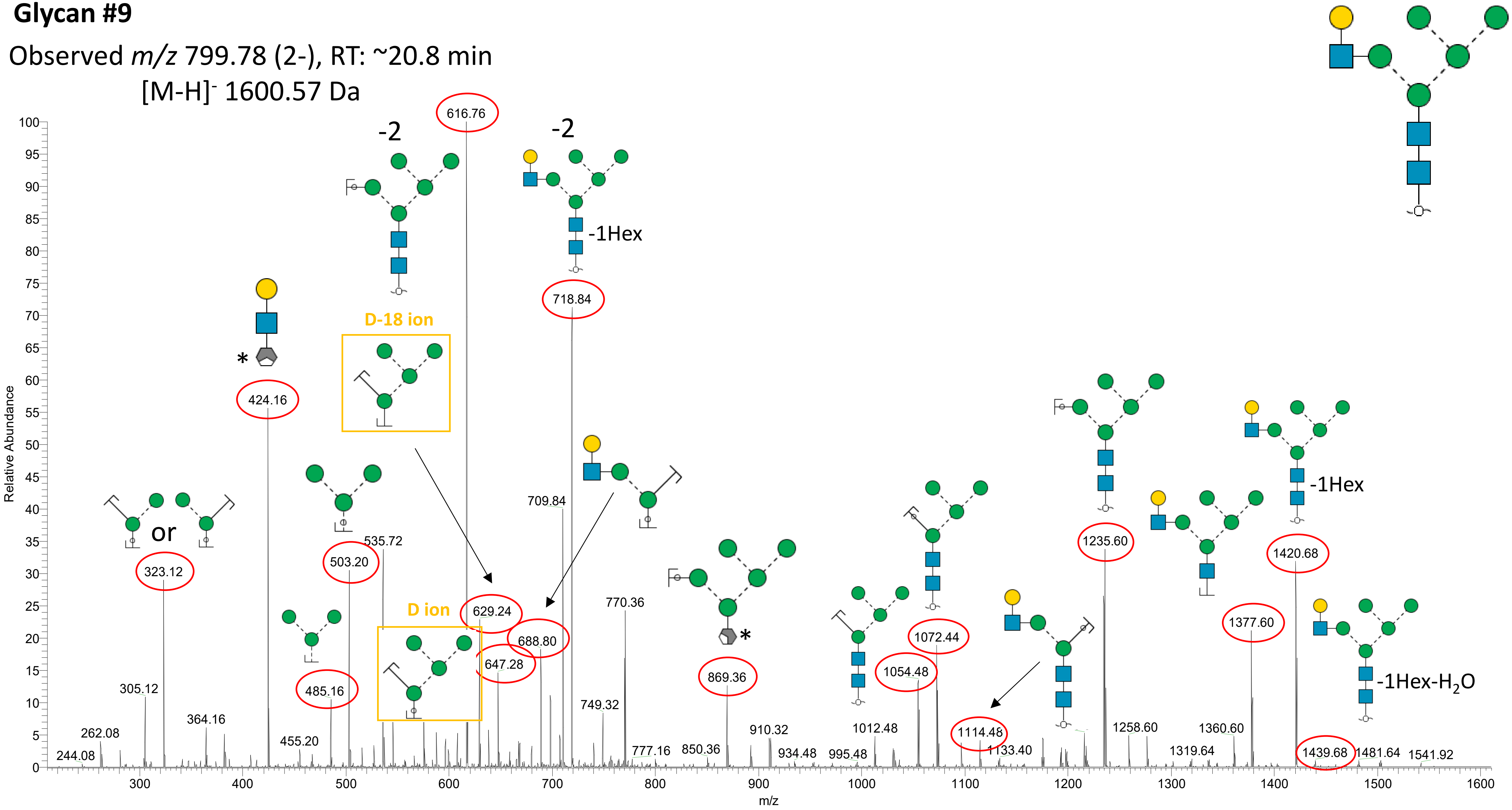

*Ambiguity in the cross-ring fragments

Glycan #10

Observed *m/z* 945.33 (2-), RT: ~20.8 min

[M-H]- 1891.66 Da

Note: Based on PGC-LC elution pattern, this glycan is annotated as the α2,6-sialyl linkage isomer.

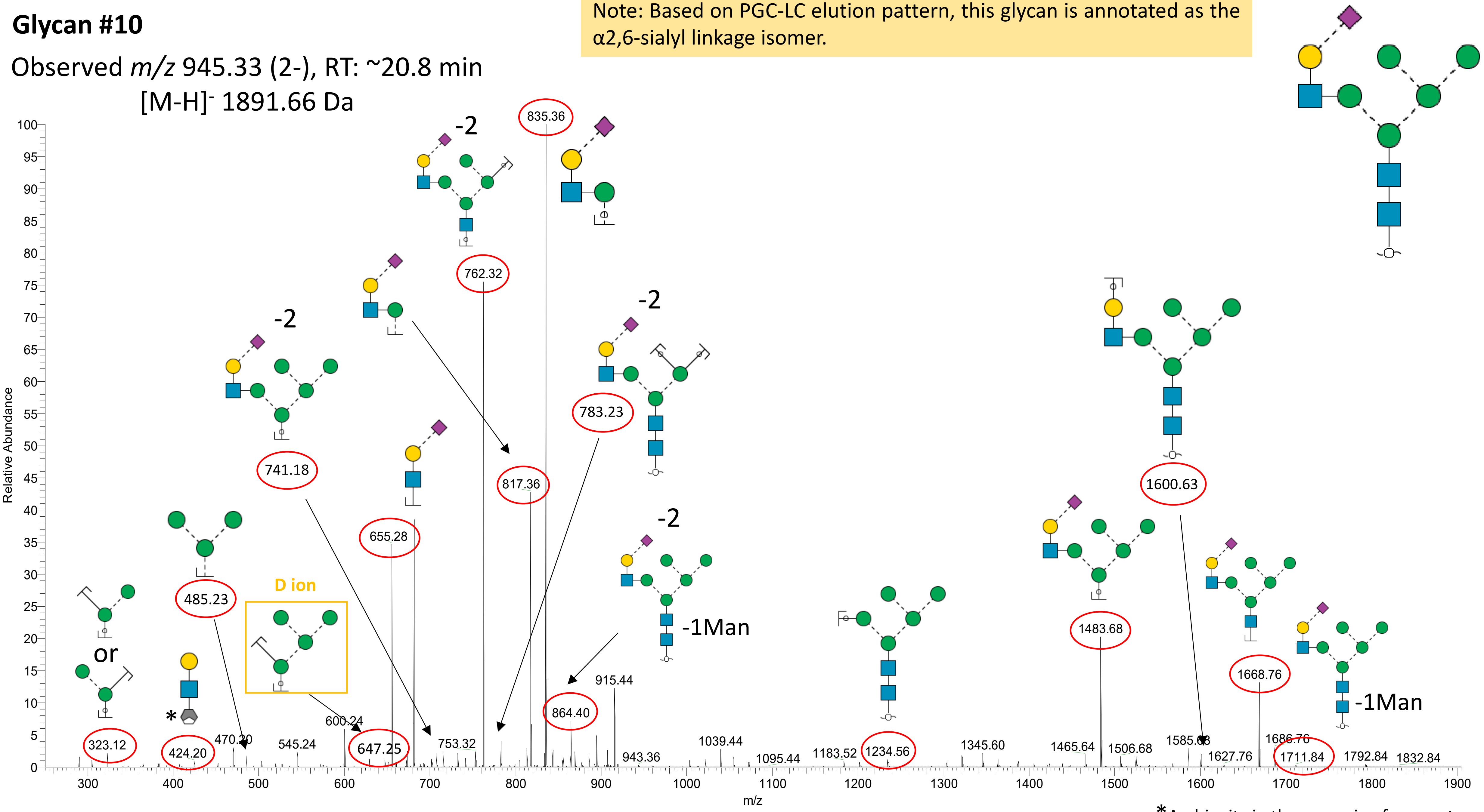

*Ambiguity in the cross-ring fragments

Glycan #11

Observed *m/z* 783.28 (2-), RT: ~21.2 min

$[M-H]^-$ 1567.56 Da

Note: From biosynthetic pathway and the lack of D and/or D-18 ions, monoantennary sialylation is predicted to occupy the α-1,3 arm. Based on early PGC-LC elution, this glycan is annotated as the α2,6-sialyl linkage isomer.

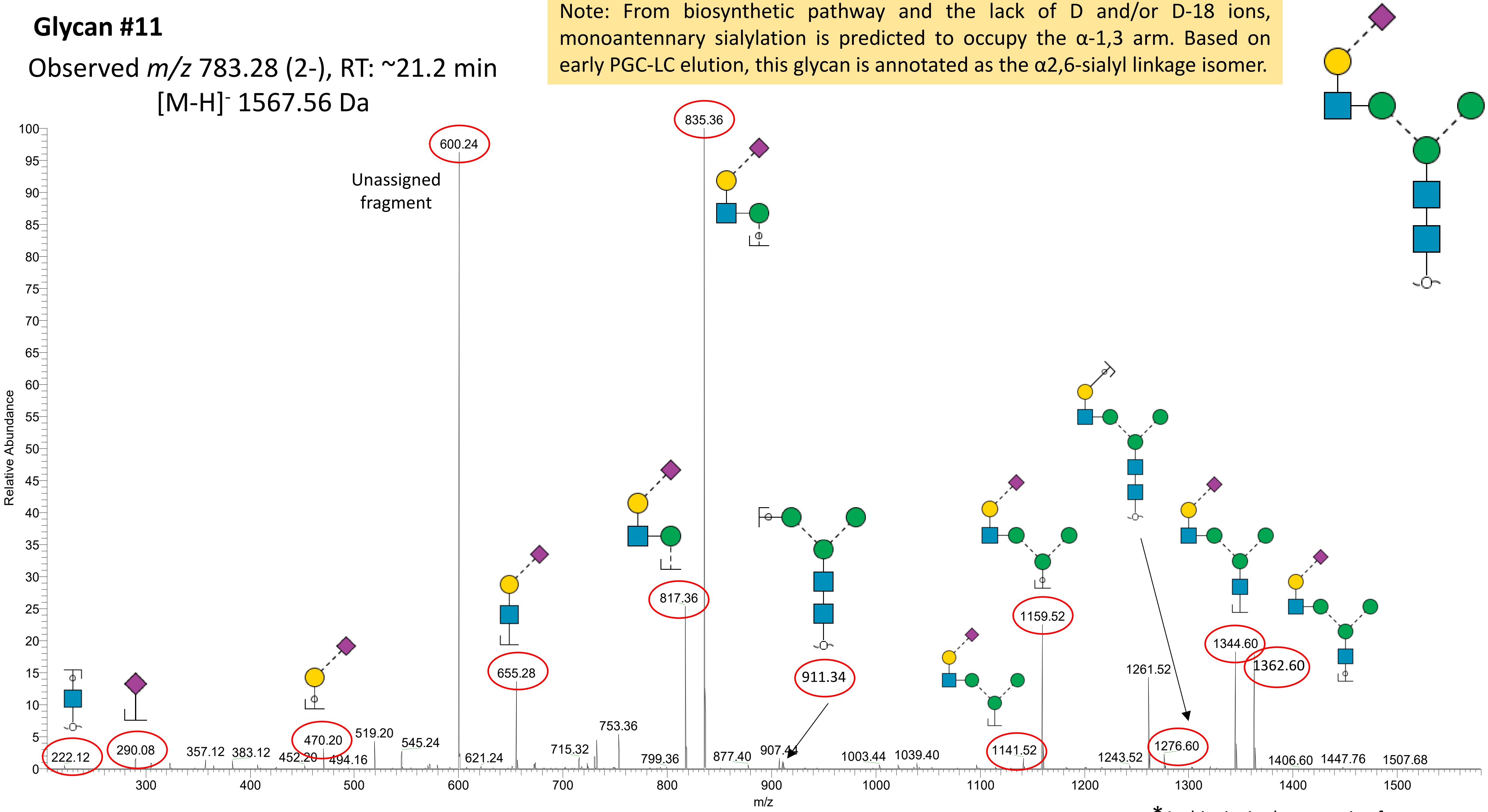

*Ambiguity in the cross-ring fragments

Glycan #12

Observed *m/z* 856.3 (1-), RT: ~22.5 min

$[M-H]^-$ 1713.62 Da

Note: From biosynthetic pathway and the lack of D and/or D-18 ions, monoantennary sialylation is predicted to occupy the α-1,3 arm. Based on early PGC-LC elution, this glycan is annotated as the α2,6-sialyl linkage isomer.

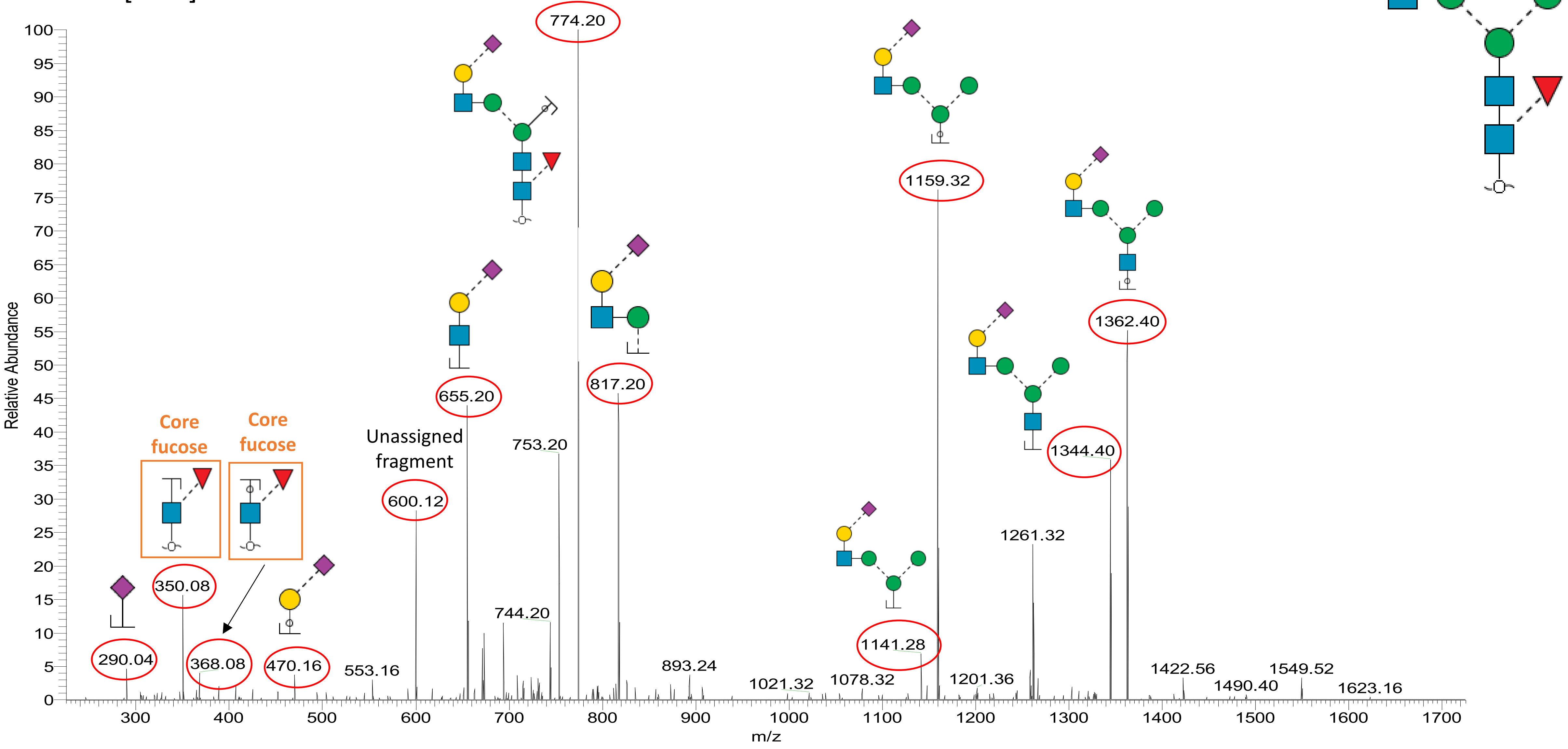

*Ambiguity of the cross-ring fragments

Glycan #13

Observed *m/z* 731.28 (2-), RT: ~22.6 min

$[M\text{-}H]^-$ 1463.55 Da

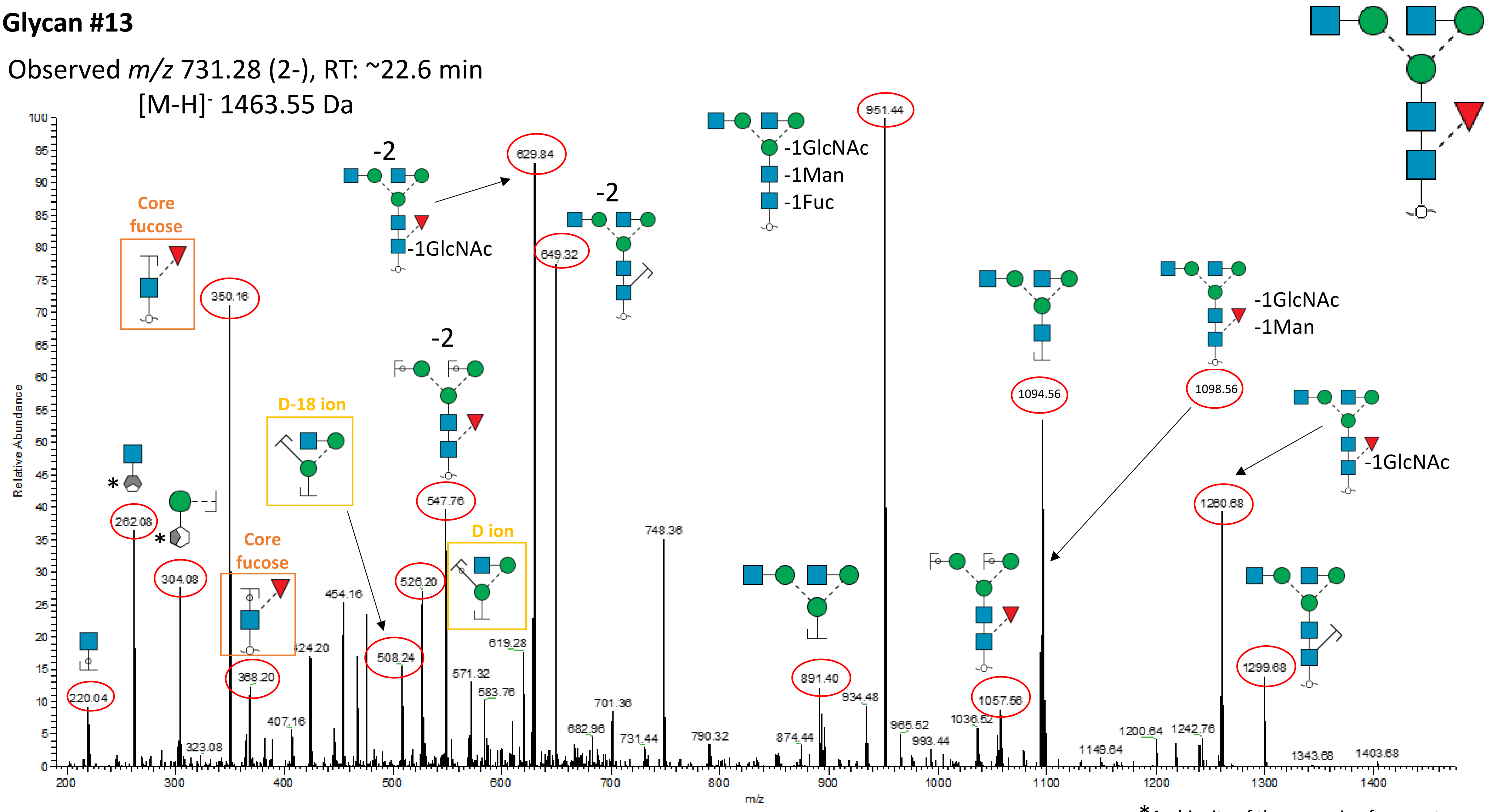

*Ambiguity of the cross-ring fragments

Glycan #14

Observed *m/z* 739.22 (2-), RT: ~22.3 min

$[M-H]^-$ 1479.54 Da

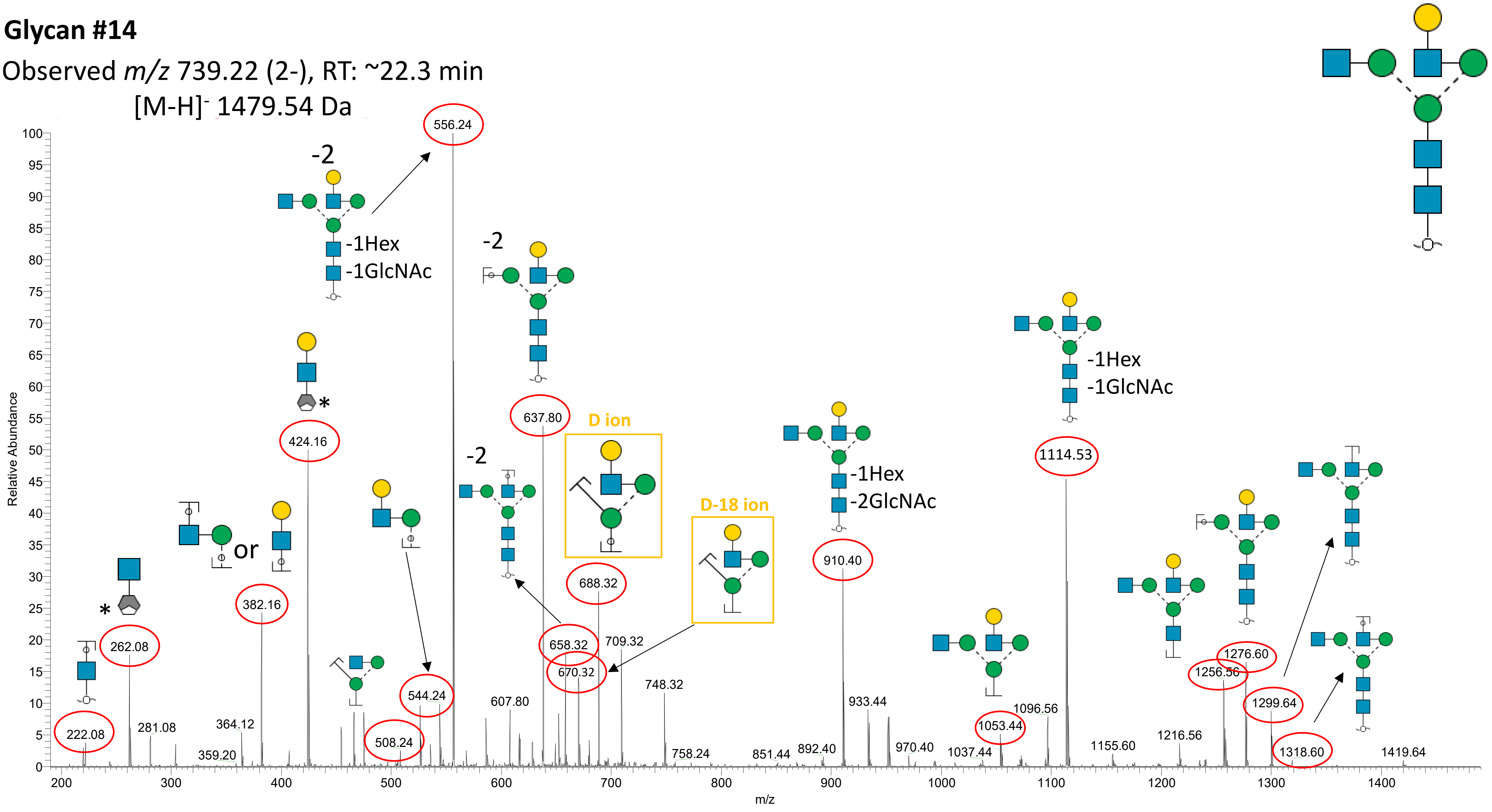

*Ambiguity of the cross-ring fragments

Glycan #15

Observed *m/z* 812.29 (2-), RT: ~24.2 min

[M-H]- 1625.60 Da

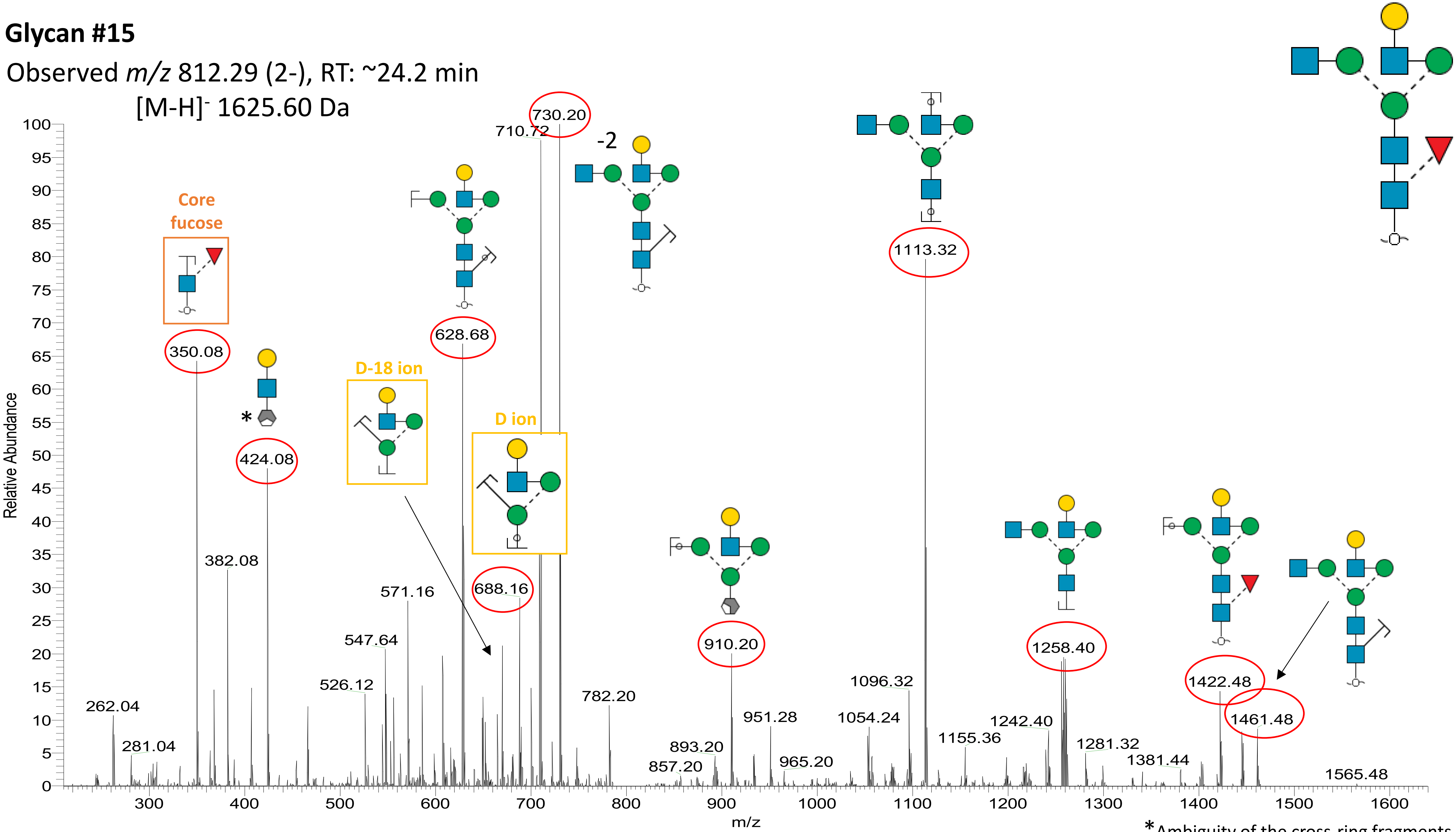

*Ambiguity of the cross-ring fragments

Glycan #16a

Observed *m/z* 884.81 (2-), RT: ~23.9 min

[M-H]$^-$ 1770.64 Da

Note: Based on PGC-LC elution pattern, this glycan is annotated as the α2,6-sialyl linkage isomer.

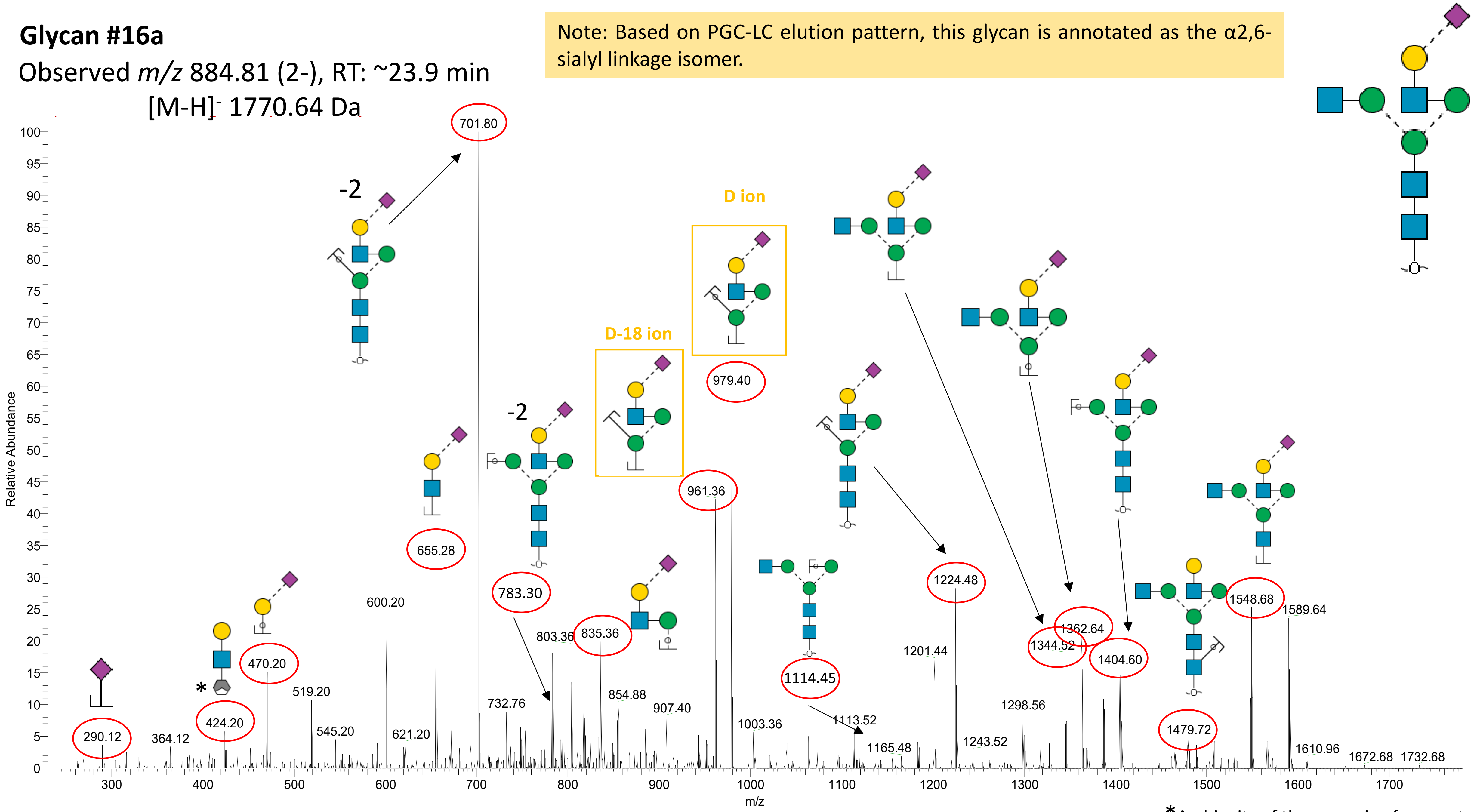

*Ambiguity of the cross-ring fragments

Glycan #16b

Observed *m/z* 884.8 (2-), RT: ~25.2 min

[M-H]⁻ 1625.60 Da

Note: Based on PGC-LC elution pattern, this glycan is annotated as the α2,6-sialyl linkage isomer.

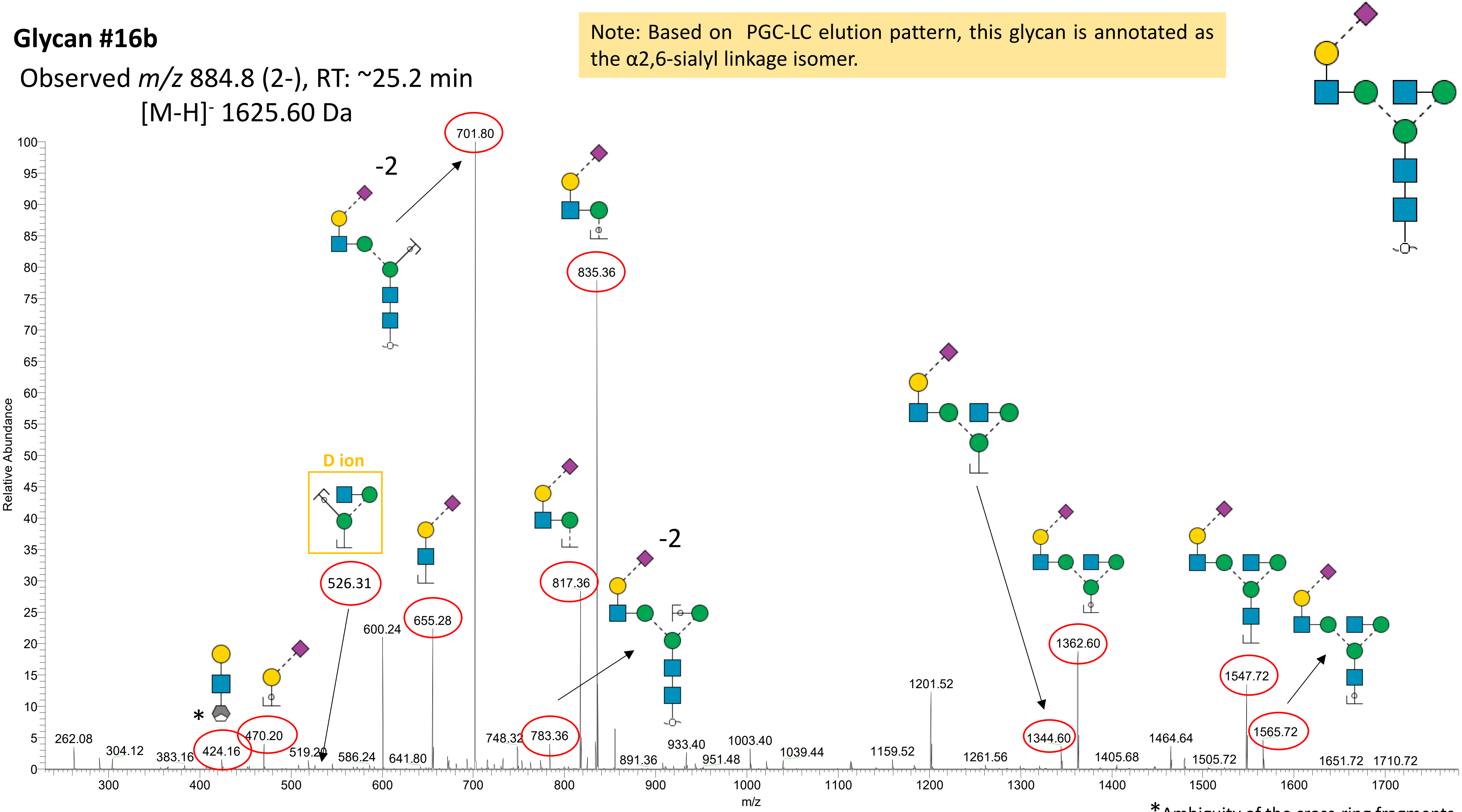

*Ambiguity of the cross-ring fragments

Glycan #17

Observed *m/z* 957.85 (2-), RT: ~29.3 min

[M-H]$^-$ 1916.70 Da

Note: Based on PGC-LC elution pattern, this glycan is annotated as the α2,6-sialyl linkage isomer.

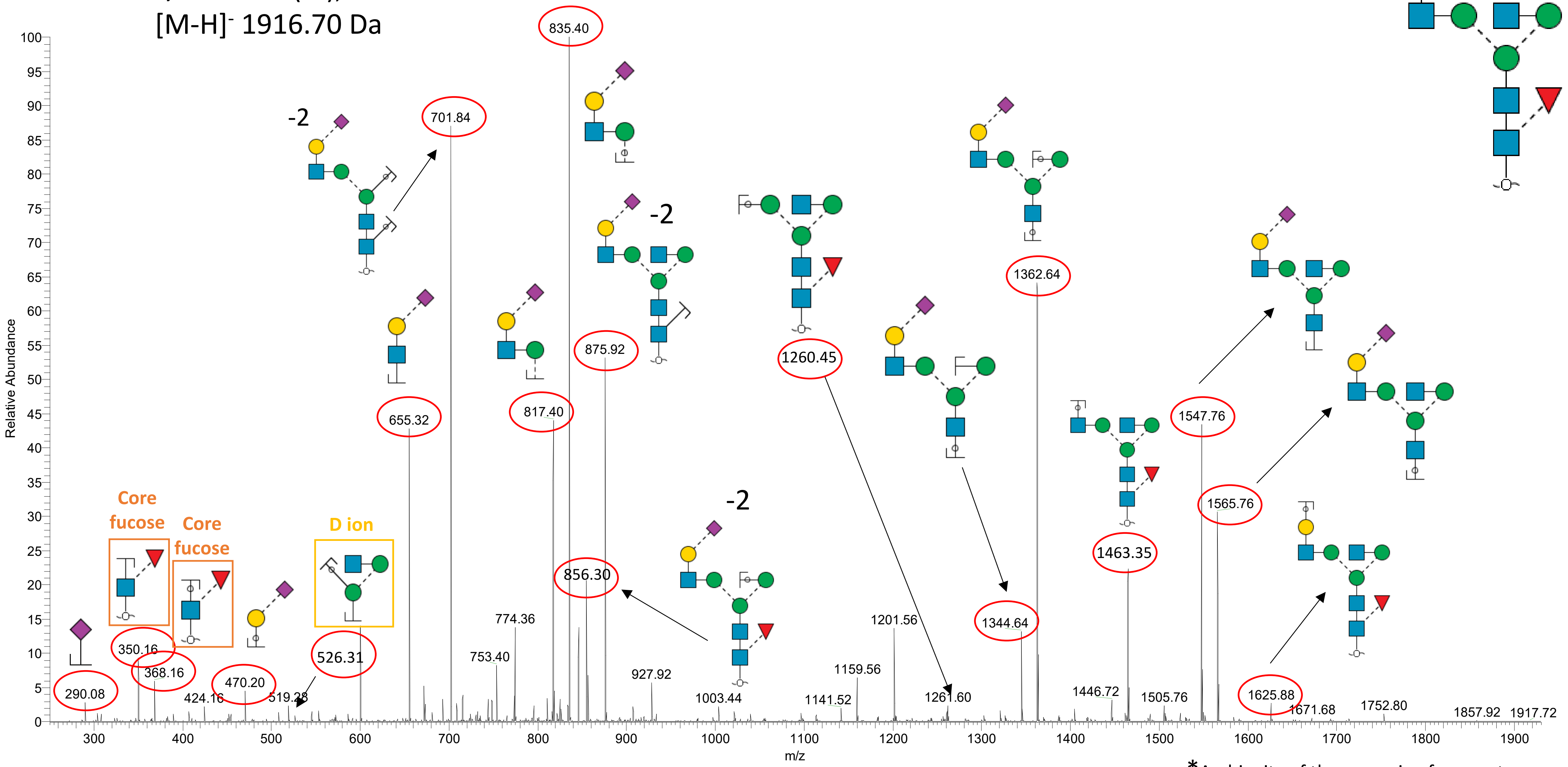

*Ambiguity of the cross-ring fragments

Glycan #18

Observed *m/z* 820.29 (2-), RT: ~25.6 min

[M-H]- 1641.60 Da

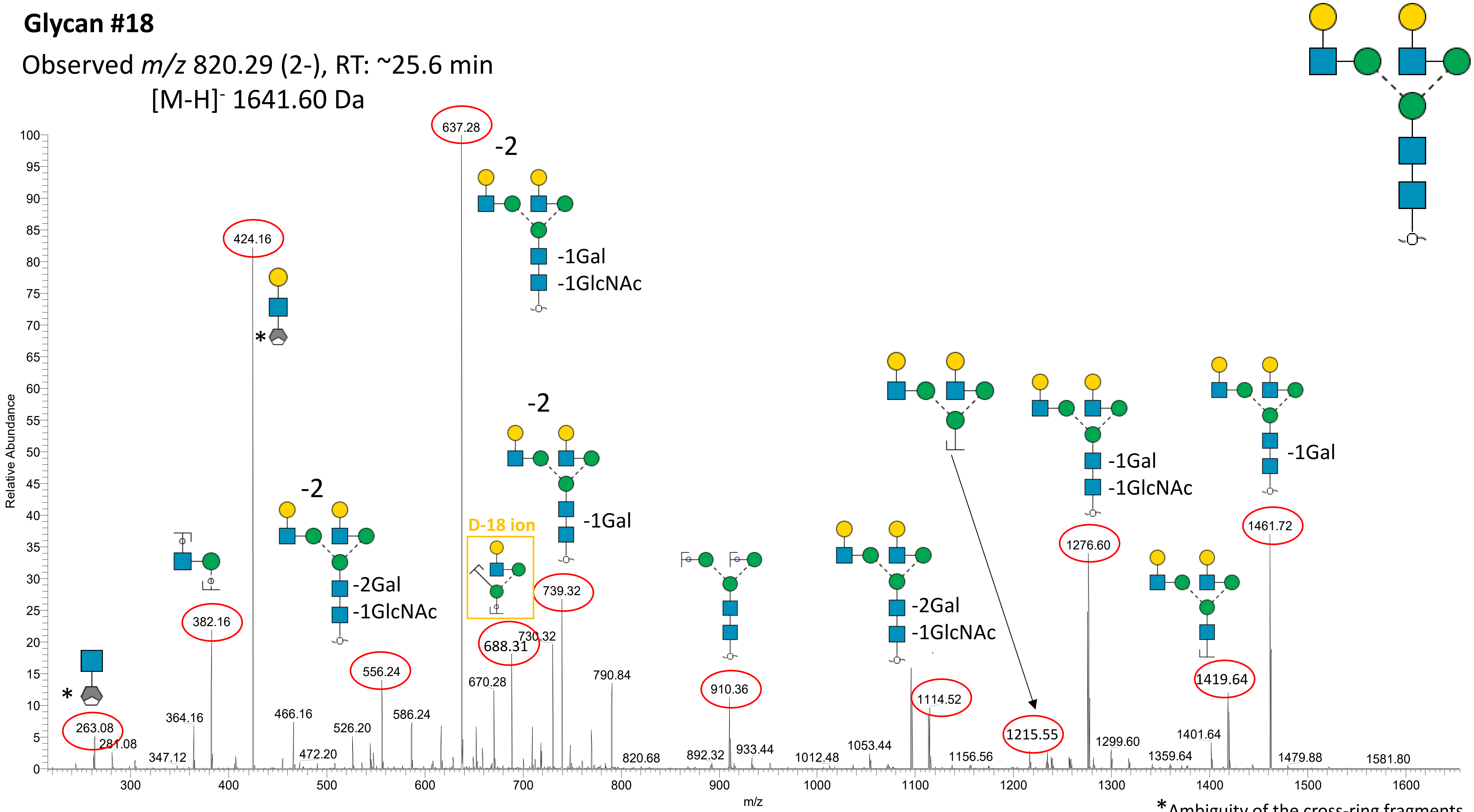

*Ambiguity of the cross-ring fragments

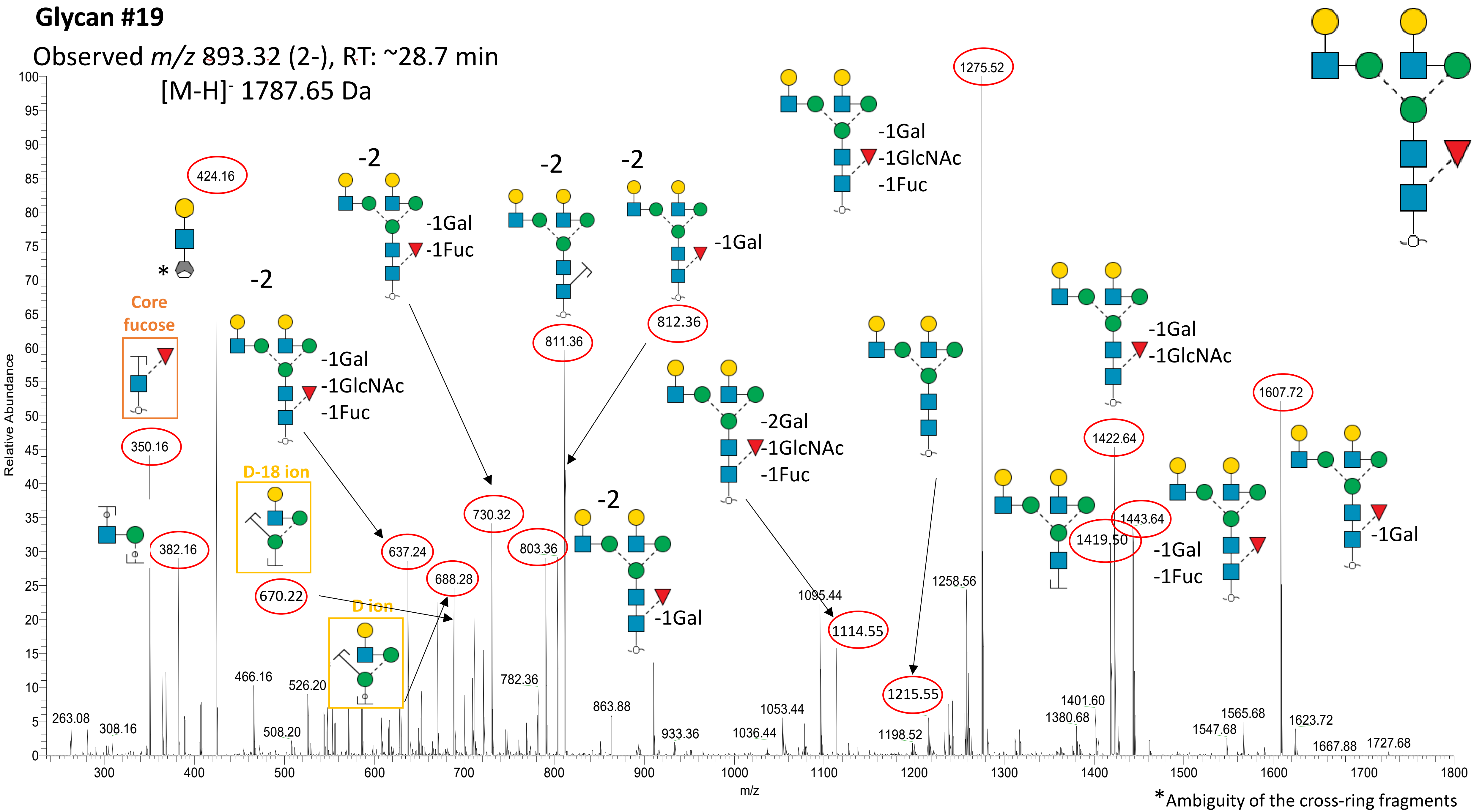
Glycan #19
Observed m/z 893.32 (2-), RT: ~28.7 min
[M-H]- 1787.65 Da
Relative Abundance
m/z
Core fucose
D-18 ion
D ion
-2
-1Gal
-1GlcNAc
-1Fuc
-2Gal
424.16
350.16
382.16
637.24
670.22
688.28
730.32
803.36
811.36
812.36
1095.44
1114.55
1215.55
1258.56
1275.52
1419.50
1422.64
1443.64
1607.72
263.08
308.16
466.16
508.20
526.20
782.36
863.88
933.36
1036.44
1053.44
1198.52
1380.68
1401.60
1547.68
1565.68
1623.72
1667.88
1727.68
*Ambiguity of the cross-ring fragments

Glycan #20a

Observed *m/z* 965.84 (2-), RT: ~23.2 min

$[M-H]^-$ 1932.69 Da

Note: Based on PGC-LC elution pattern, this glycan is annotated as the α2,6-sialyl linkage isomer.

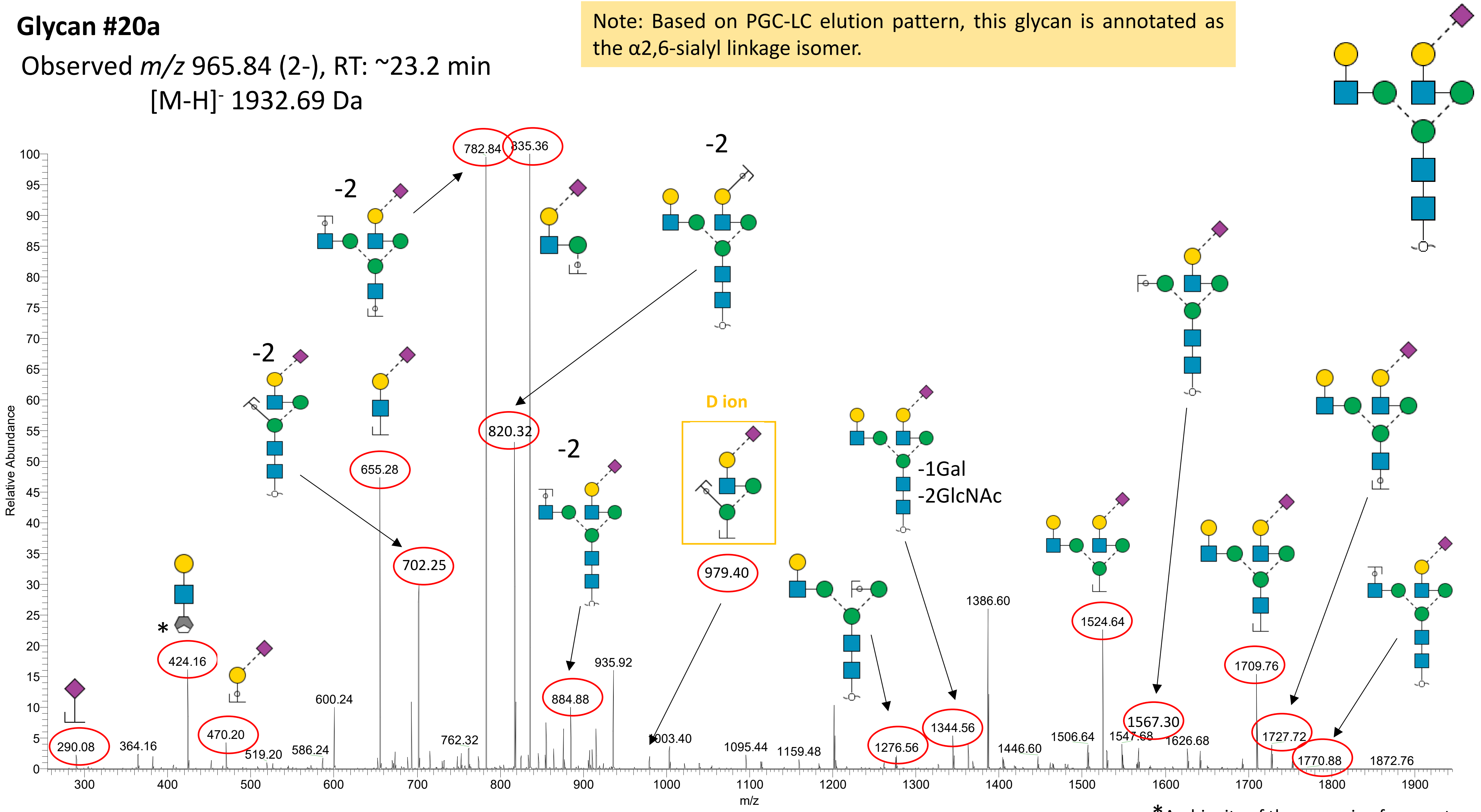

*Ambiguity of the cross-ring fragments

Glycan #20b
Observed *m/z* 965.84 (2-), RT: ~27.8 min
[M-H]⁻ 1932.69 Da

Note: Based on PGC-LC elution pattern, this glycan is annotated as the α2,3-sialyl linkage isomer.

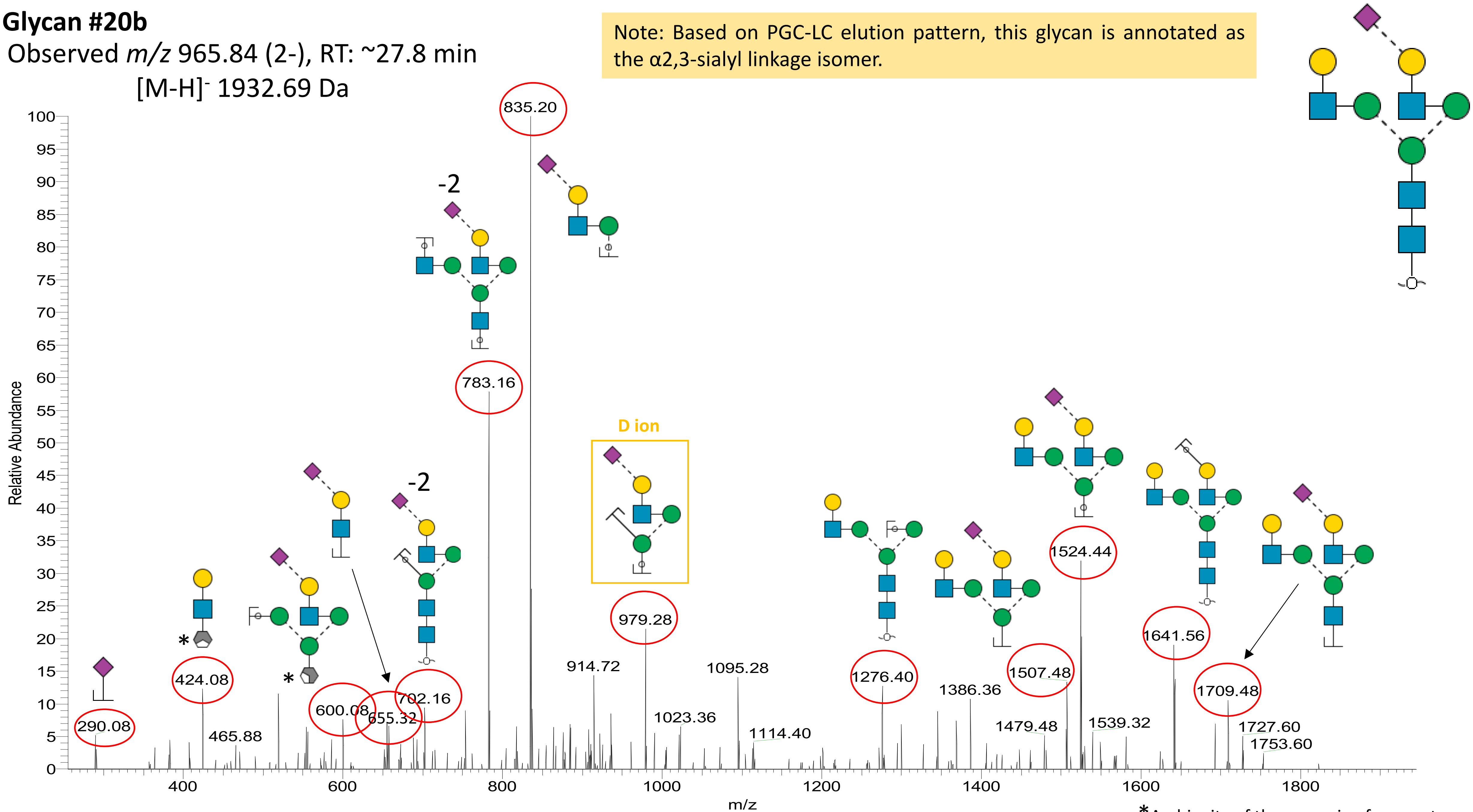

*Ambiguity of the cross-ring fragments

Glycan #21a

Observed *m/z* 1111.39 (2-), RT: ~22.2 min

$[M-H]^-$ 2223.79 Da

Note: Based on PGC-LC elution pattern, this glycan is annotated as the α2,6-α2,6-sialyl linkage isomer.

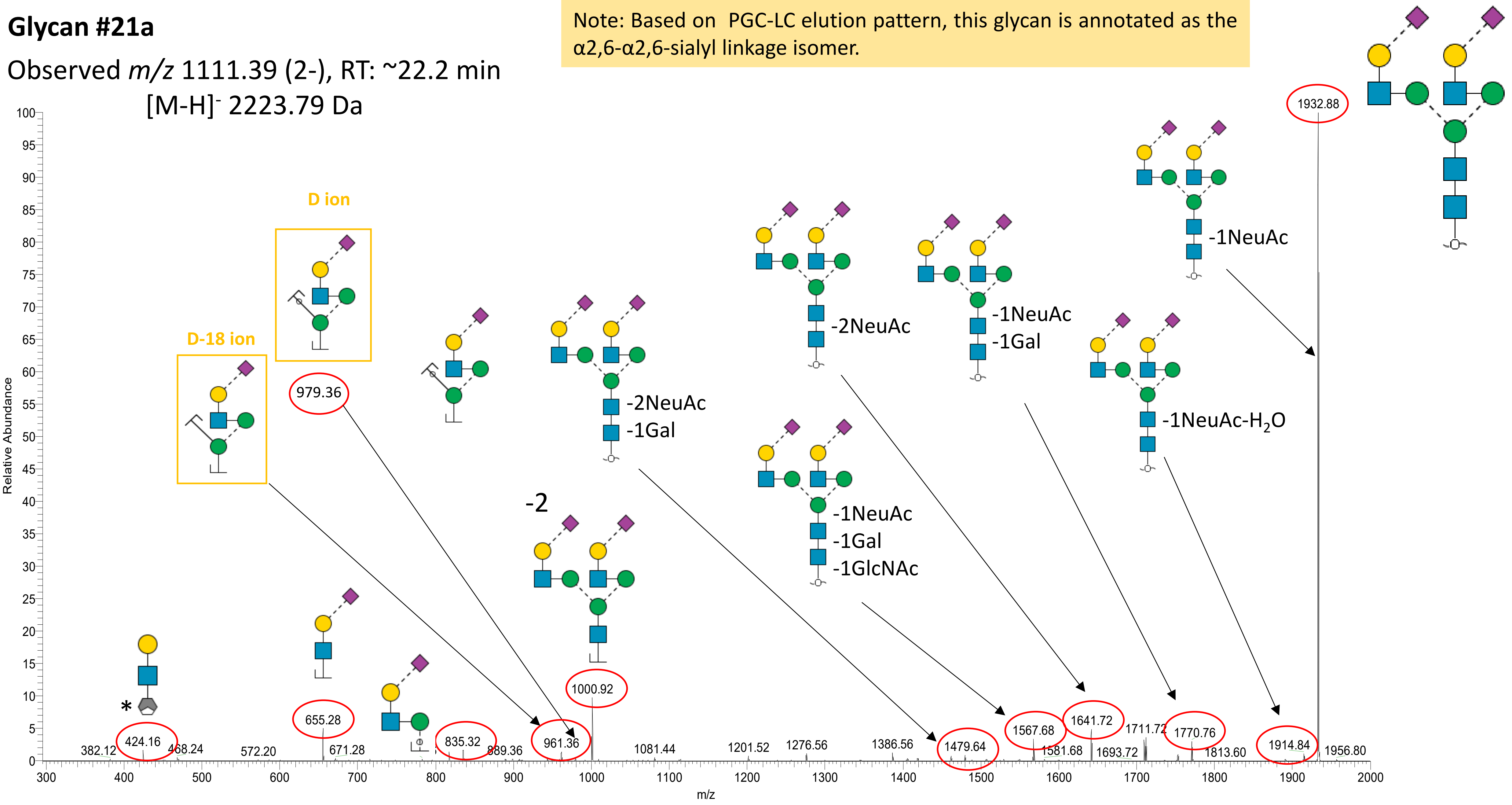

*Ambiguity of the cross-ring fragments

Glycan #21b

Observed *m/z* 1111.39 (2-), RT: ~28.6 min

[M-H]$^-$ 2223.79 Da

Note: Based on PGC-LC elution pattern, this glycan is annotated as the α2,6-α2,3-sialyl linkage isomer. The antenna positions of the linkage-specific sialic acid residues cannot be determined.

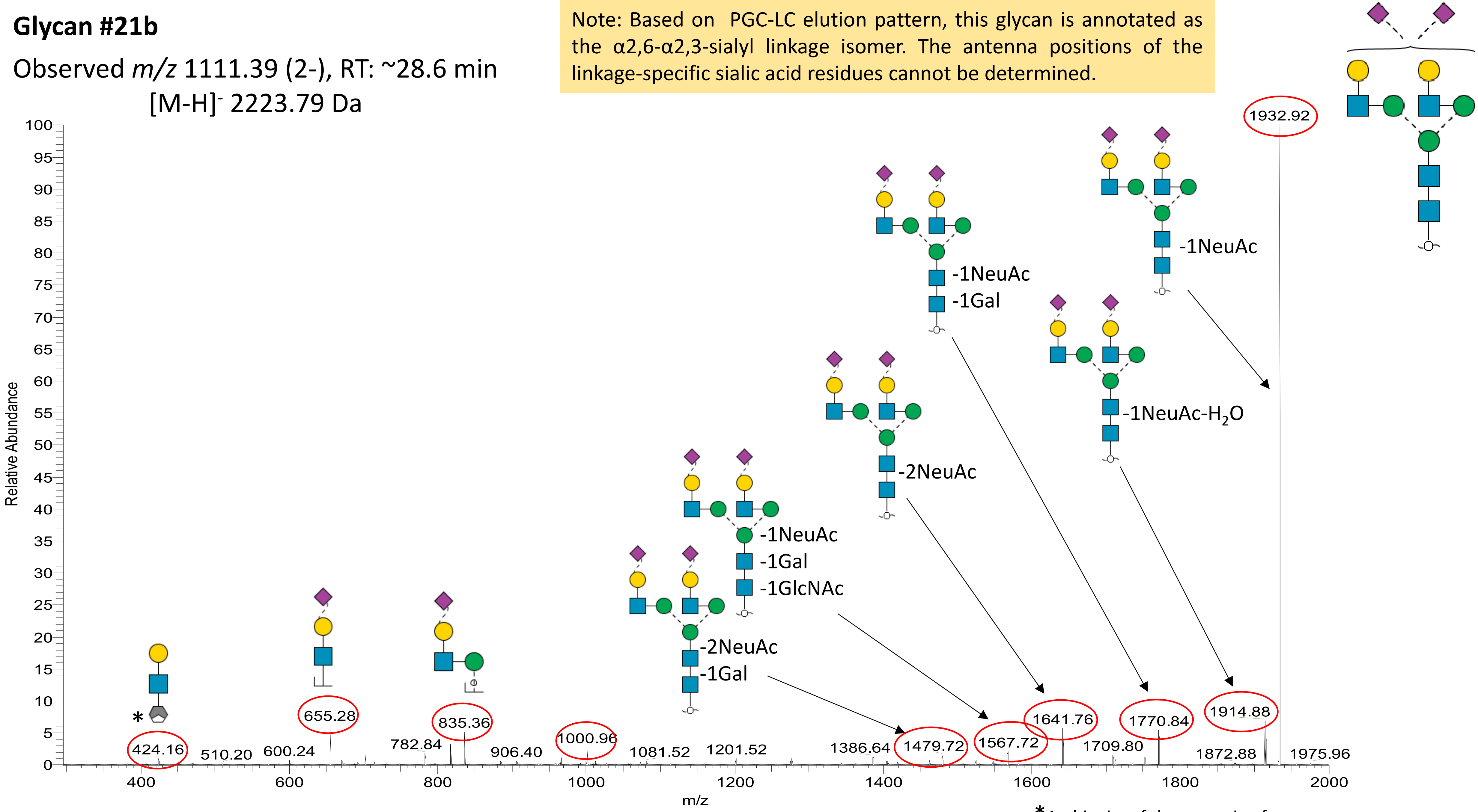

*Ambiguity of the cross-ring fragments

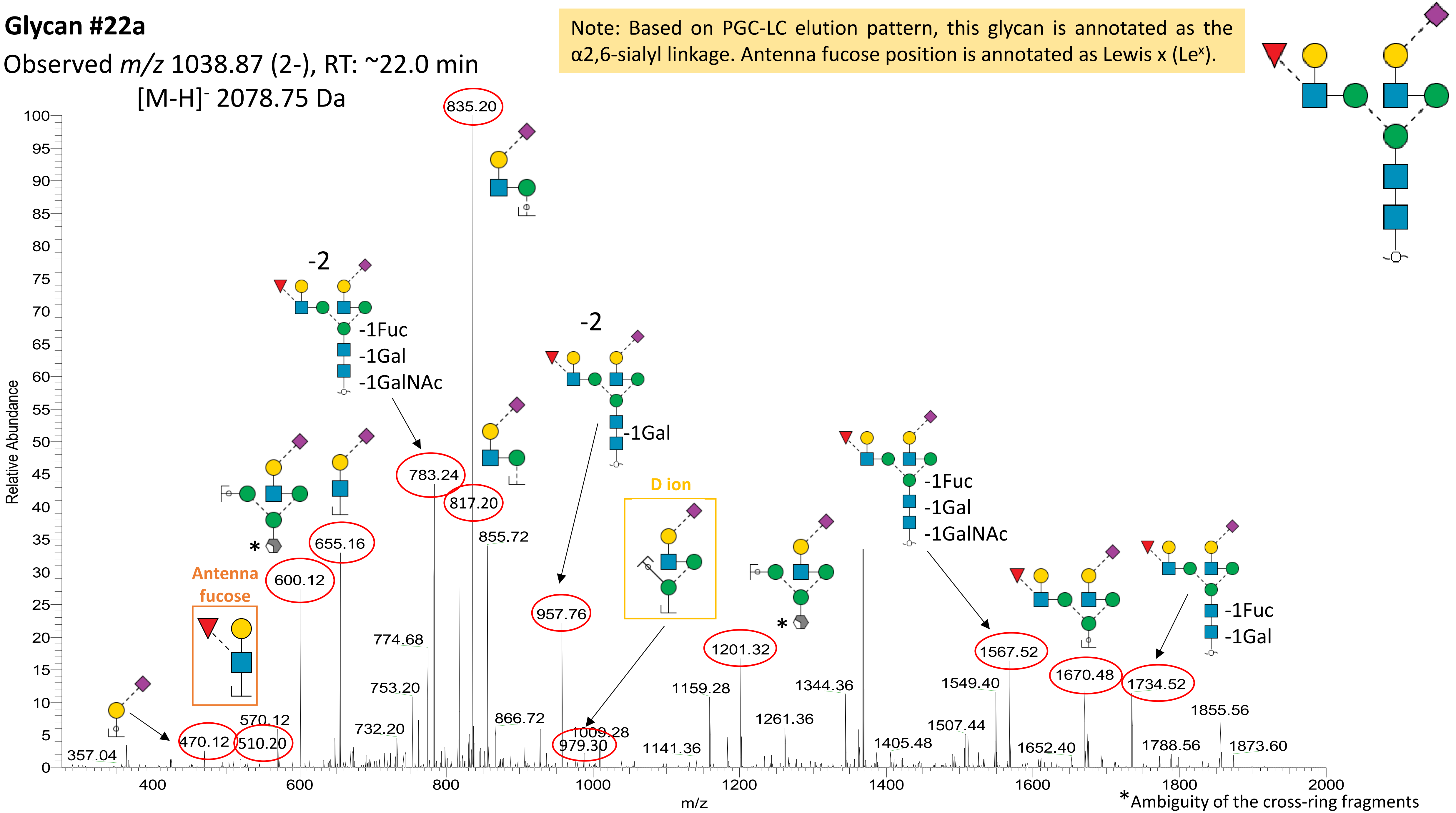
Glycan #22a
Observed m/z 1038.87 (2-), RT: ~22.0 min
[M-H]- 2078.75 Da
Note: Based on PGC-LC elution pattern, this glycan is annotated as the α2,6-sialyl linkage. Antenna fucose position is annotated as Lewis x (Le^x).
Relative Abundance
m/z
835.20
-2
-1Fuc
-1Gal
-1GalNAc
-2
-1Gal
783.24
817.20
855.72
655.16
600.12
957.76
D ion
Antenna fucose
774.68
753.20
732.20
866.72
1009.28
979.30
570.12
470.12
510.20
357.04
1141.36
1159.28
1201.32
1261.36
1344.36
1405.48
1507.44
1549.40
1567.52
1652.40
1670.48
1734.52
1788.56
1855.56
1873.60
-1Fuc
-1Gal
-1GalNAc
-1Fuc
-1Gal
*
*
*Ambiguity of the cross-ring fragments

Glycan #22b

Observed *m/z* 1038.87 (2-), RT: ~26.2 min

[M-H]⁻ 2078.75 Da

Note: Based on PGC-LC elution pattern, this glycan is annotated as the α2,6-sialyl linkage isomer.

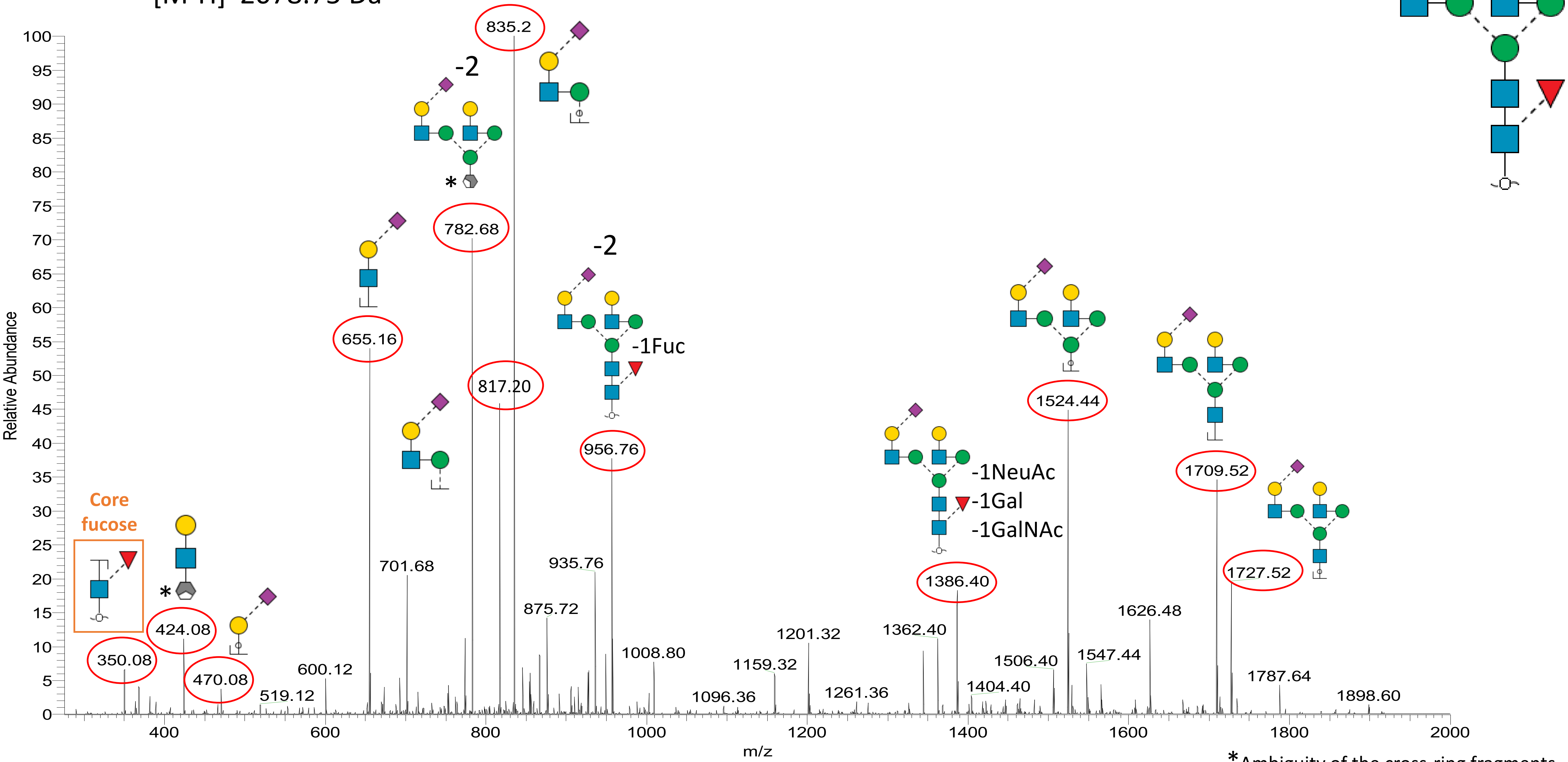

*Ambiguity of the cross-ring fragments

Glycan #22c

Observed *m/z* 1038.87 (2-), RT: ~30.8 min

[M-H]$^-$ 2078.75 Da

Note: Based on PGC-LC elution pattern, this glycan is annotated as the α2,3-sialyl linkage isomer.

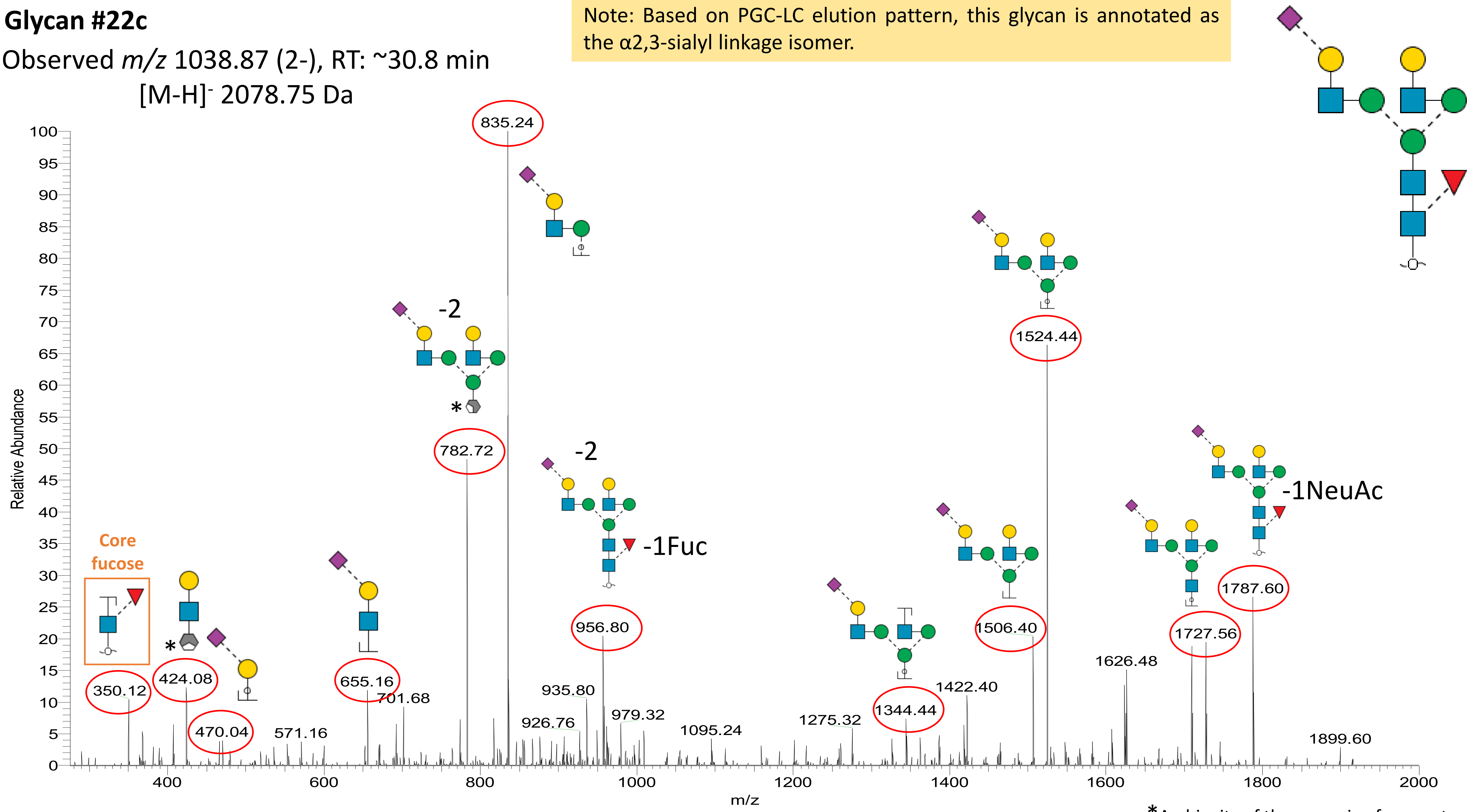

*Ambiguity of the cross-ring fragments

Glycan #23a

Observed *m/z* 1184.42 (2-), RT: ~25.1 min

$[M-H]^-$ 2369.84 Da

Note: Based on PGC-LC elution pattern, this glycan is annotated as the α2,6-sialyl linkage. Antenna fucose position is annotated as sialyl (α2,3-) Lewis x (sLe^x).

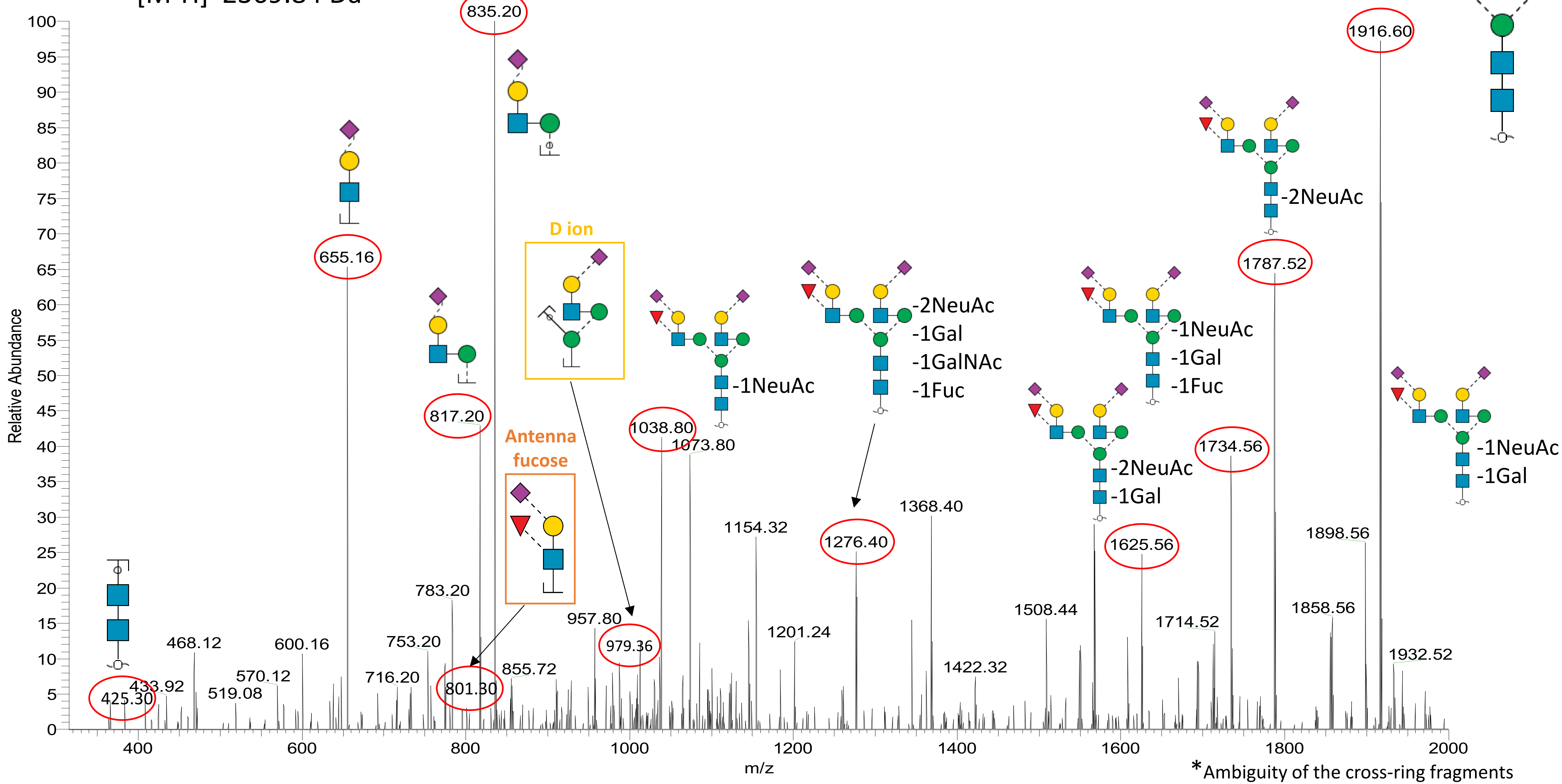

*Ambiguity of the cross-ring fragments

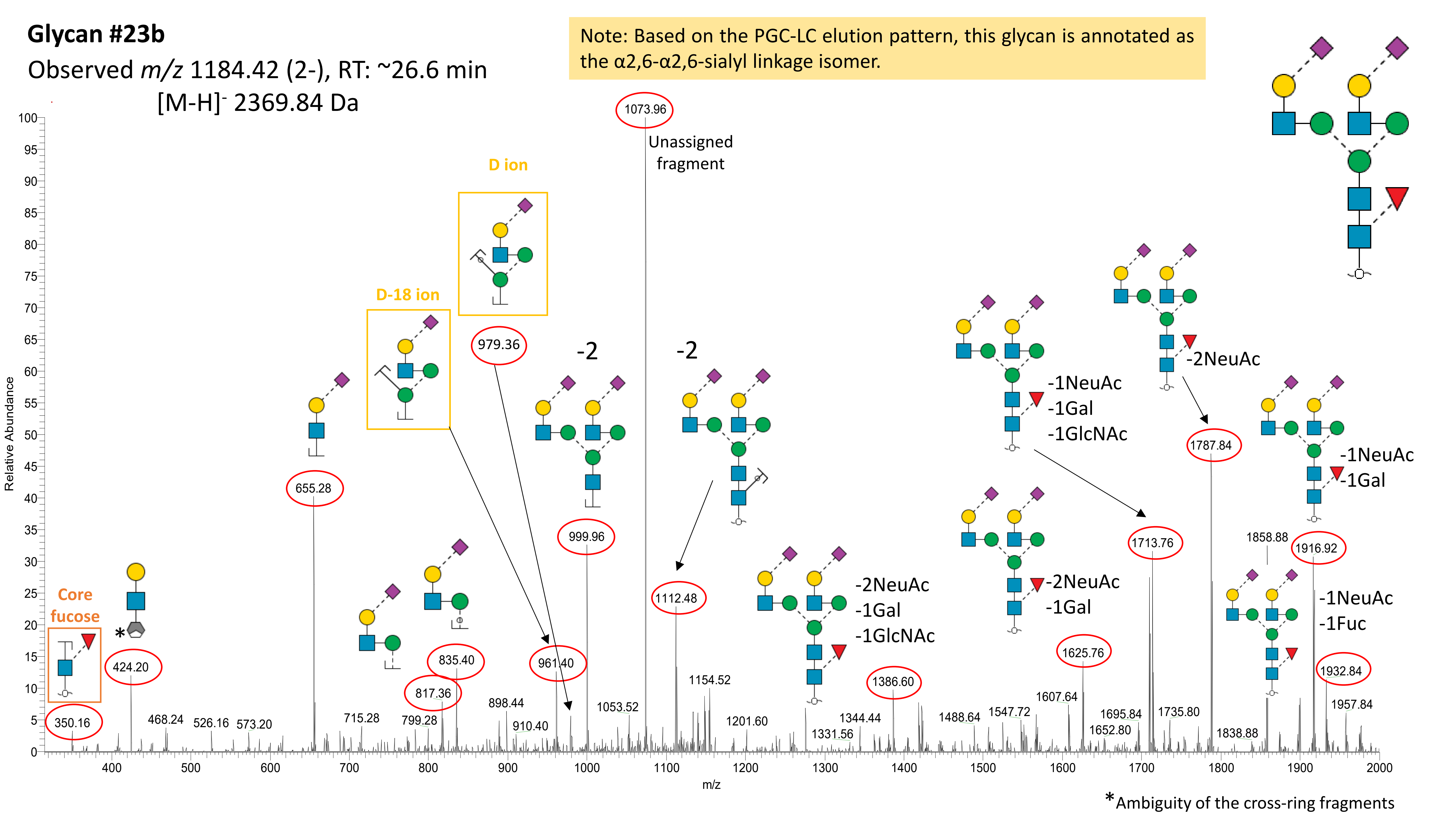

Glycan #23b
Observed m/z 1184.42 (2-), RT: ~26.6 min
[M-H]- 2369.84 Da
Note: Based on the PGC-LC elution pattern, this glycan is annotated as the α2,6-α2,6-sialyl linkage isomer.
Relative Abundance
m/z
1073.96
Unassigned fragment
D ion
D-18 ion
979.36
-2
-2
655.28
999.96
1112.48
-1NeuAc
-1Gal
-1GlcNAc
-2NeuAc
1787.84
-1NeuAc
-1Gal
1713.76
1858.88
1916.92
Core fucose
424.20
350.16
835.40
817.36
961.40
-2NeuAc
-1Gal
-1GlcNAc
-2NeuAc
-1Gal
-1NeuAc
-1Fuc
1154.52
1386.60
1625.76
1932.84
468.24
526.16
573.20
715.28
799.28
898.44
910.40
1053.52
1201.60
1331.56
1344.44
1488.64
1547.72
1607.64
1652.80
1695.84
1735.80
1838.88
1957.84
*Ambiguity of the cross-ring fragments

Glycan #23c

Observed *m/z* 1184.42 (2-), RT: ~31.2 min

$[M-H]^-$ 2369.84 Da

Note: Based on the PGC-LC elution pattern, this glycan is annotated as the α2,6-α2,3-sialyl linkage isomer. The antenna positions of the linkage-specific sialic acid residues cannot be determined.

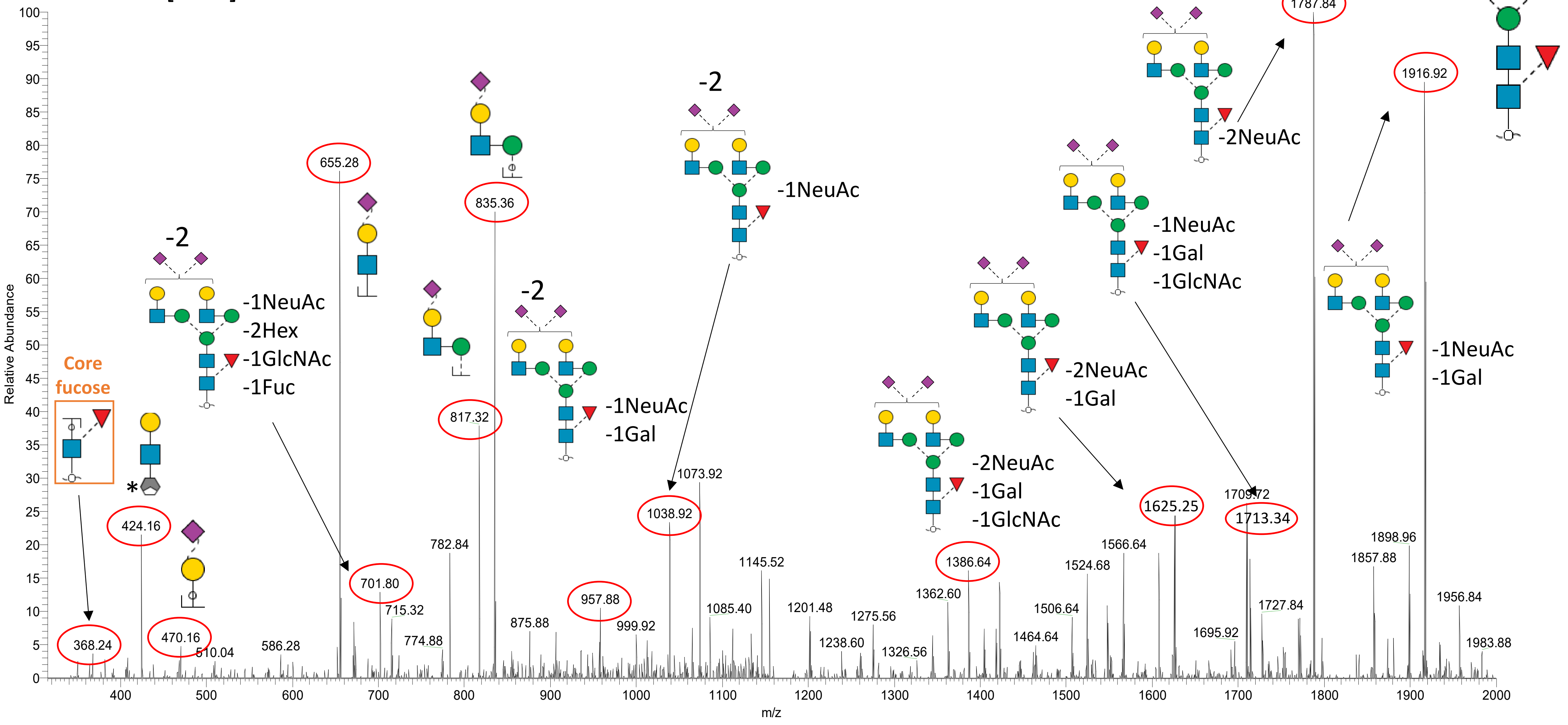

*Ambiguity of the cross-ring fragments

Glycan #23d

Observed *m/z* 1184.42 (2-), RT: ~35.2 min

$[M\text{-}H]^-$ 2369.84 Da

Note: Based on the PGC-LC elution pattern, this glycan is annotated as the α2,3-α2,6-sialyl linkage isomer. The antenna positions of the linkage-specific sialic acid residues cannot be determined.

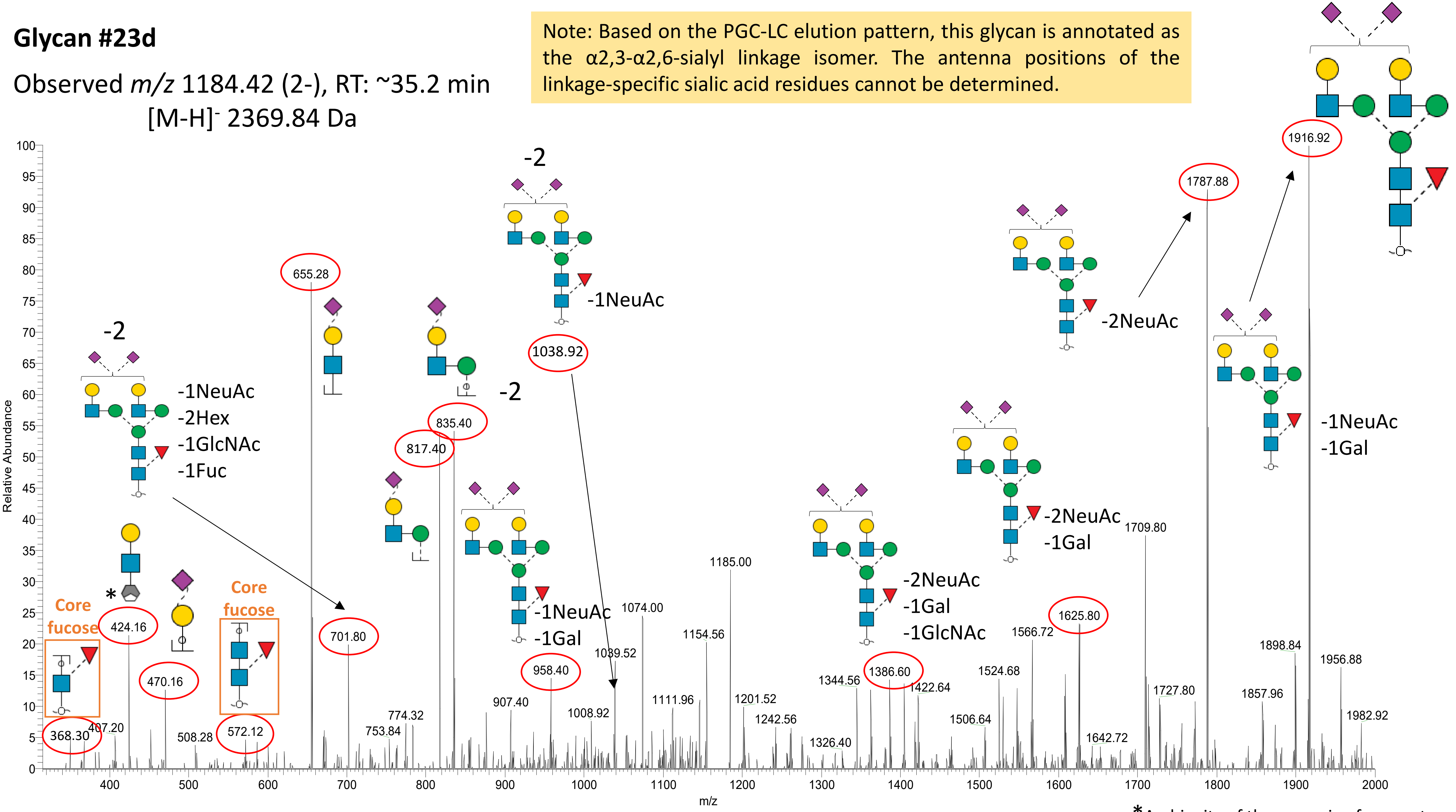

*Ambiguity of the cross-ring fragments

Glycan #24

Observed *m/z* 1046.9 (2-), RT: ~24.3 min

[M-H]- 2094.74 Da

Note: Based on the PGC-LC elution pattern, this glycan is annotated as the α2,6-sialyl linkage isomer. Due to lack of MS2 evidence, the structure has been left undetermined.

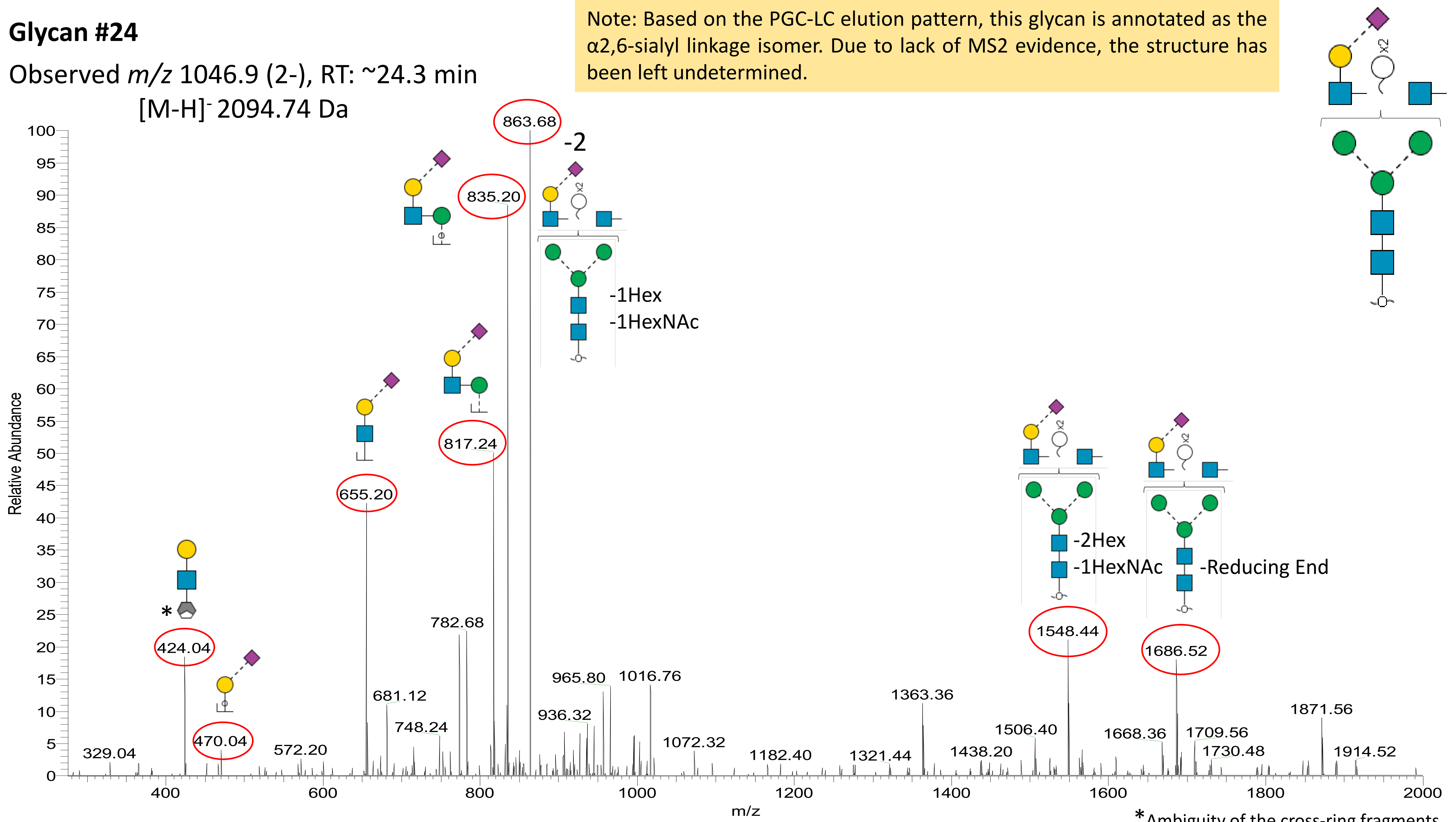

*Ambiguity of the cross-ring fragments

Glycan #25

Observed *m/z* 759.78 (2-), RT: ~16.8 min

$[M-H]^-$ 1520.57 Da

Note: Based on the very early PGC-LC elution time and the abundant D-ion, this structure is assigned as a bisecting GlcNAc.

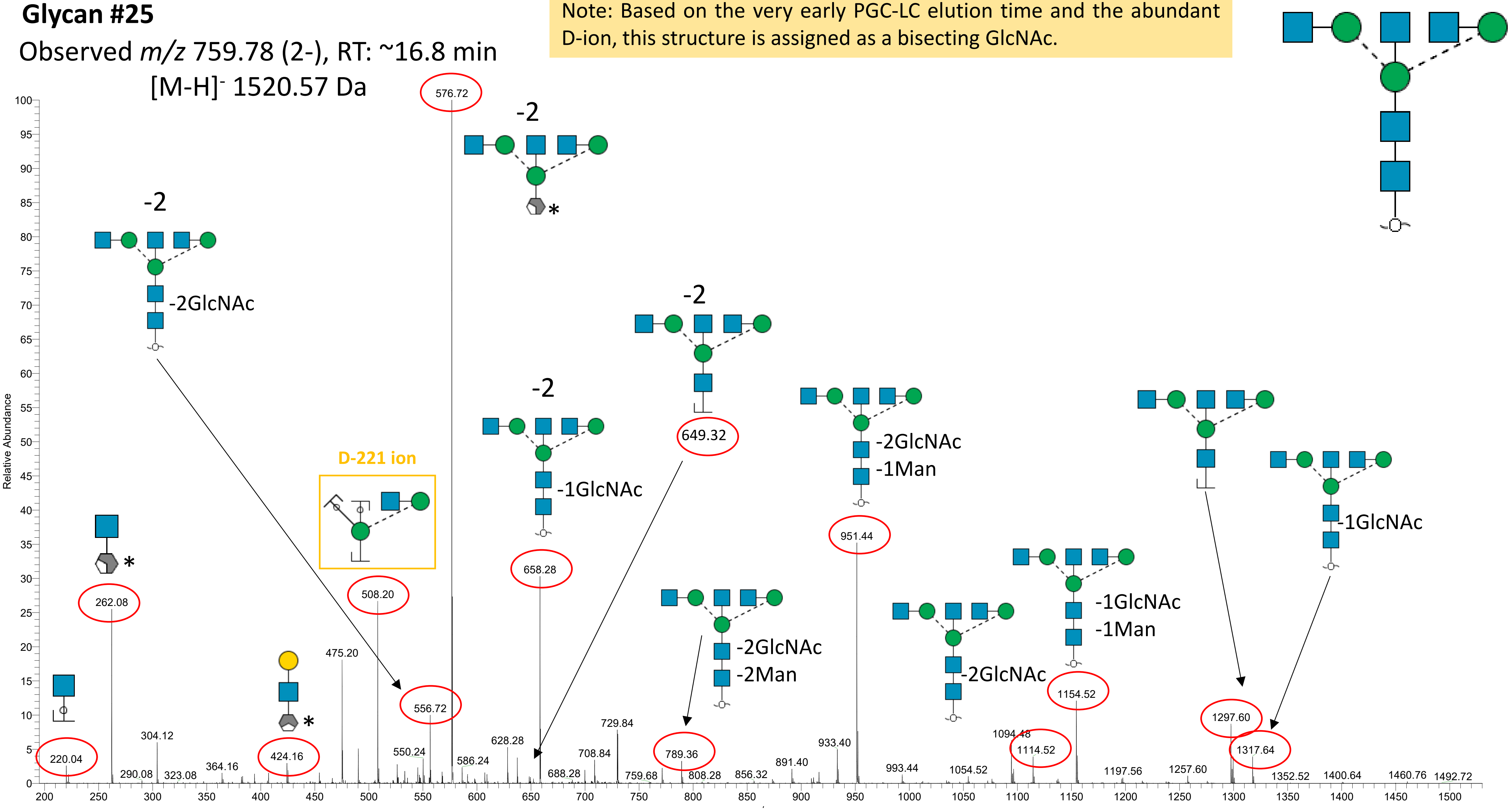

Ambiguity of the cross-ring fragments

Glycan #26

Observed *m/z* 832.81 (2-), RT: ~18.7 min

$[M-H]^-$ 1666.63 Da

Note: Based on the very early PGC-LC elution time and the abundant D-ion, this structure is assigned as a bisecting GlcNAc.

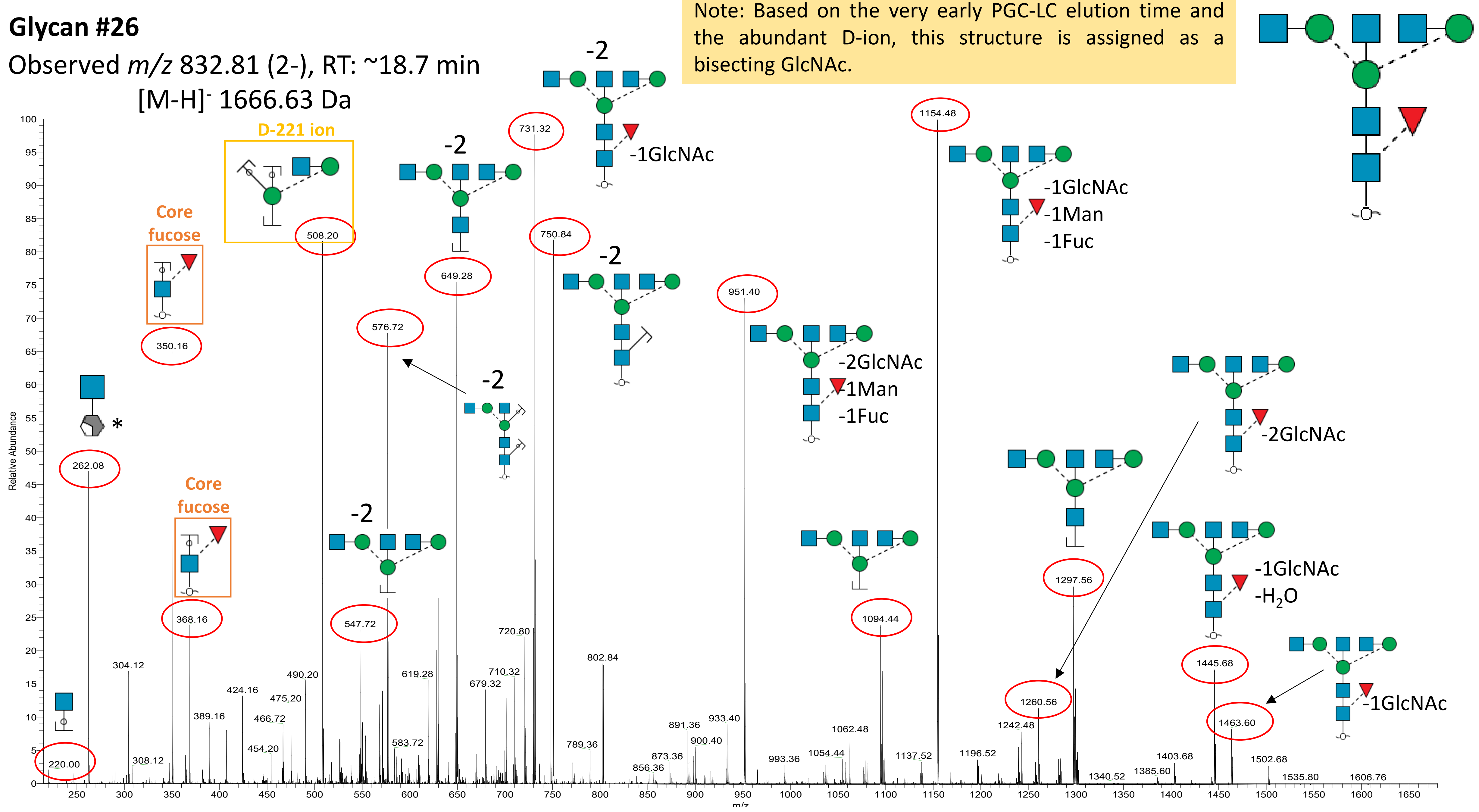

*Ambiguity of the cross-ring fragments

Glycan #27

Observed *m/z* 913.88 (2-), RT: ~19.6 min

$[M-H]^-$ 1828.68 Da

Note: Based on the very early PGC-LC elution time and the abundant D-ion, this structure is assigned as a bisecting GlcNAc.

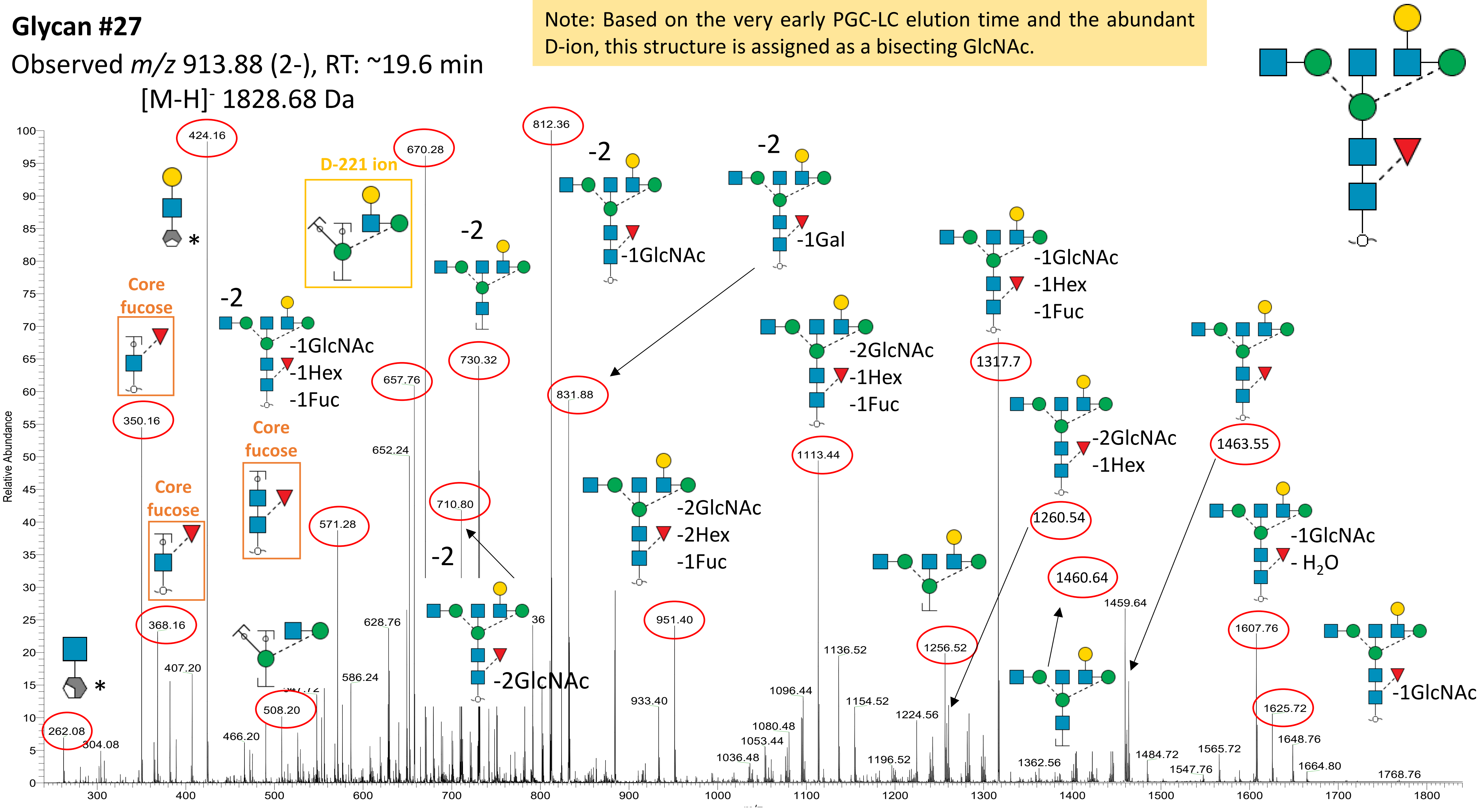

* Ambiguity of the cross-ring fragments

Glycan #28

Observed *m/z* 986.39 (2-), RT: ~18.1 min

[M-H]⁻ 1973.72 Da

Note: Based on the very early PGC-LC elution time and the D-221 ion, this structure is assigned as a bisecting GlcNAc. Based on PGC-LC elution pattern, this glycan is annotated as the α2,6-sialyl linkage isomer.

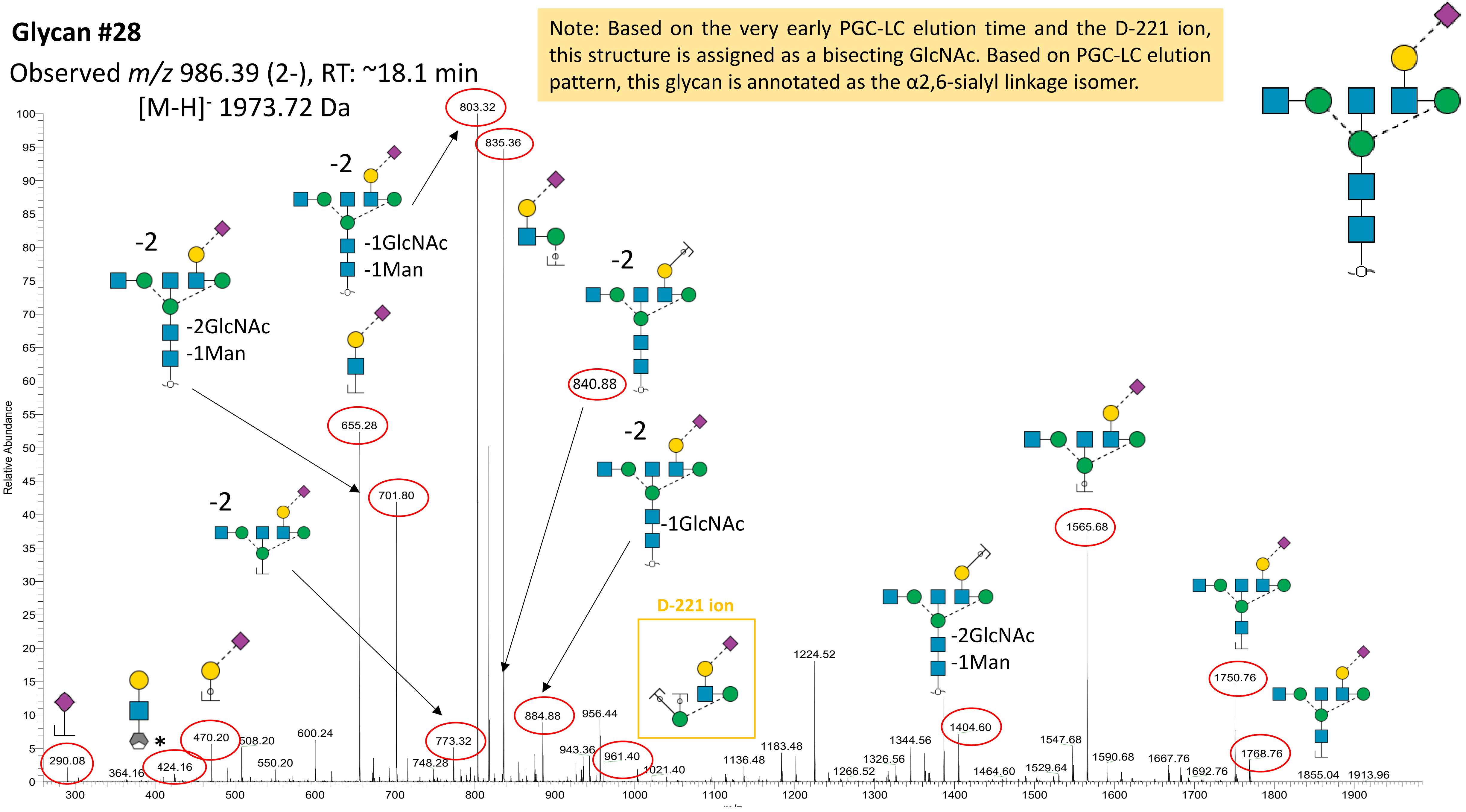

Ambiguity of the cross-ring fragments

Glycan #29

Observed *m/z* 1059.44 (2-), RT: ~20.2 min

[M-H]⁻ 2119.78 Da

Note: Based on the very early PGC-LC elution time and the abundant D-221 ion, this structure is assigned as a bisecting GlcNAc. Based on PGC-LC elution pattern, this glycan is annotated as the α2,6-sialyl linkage isomer.

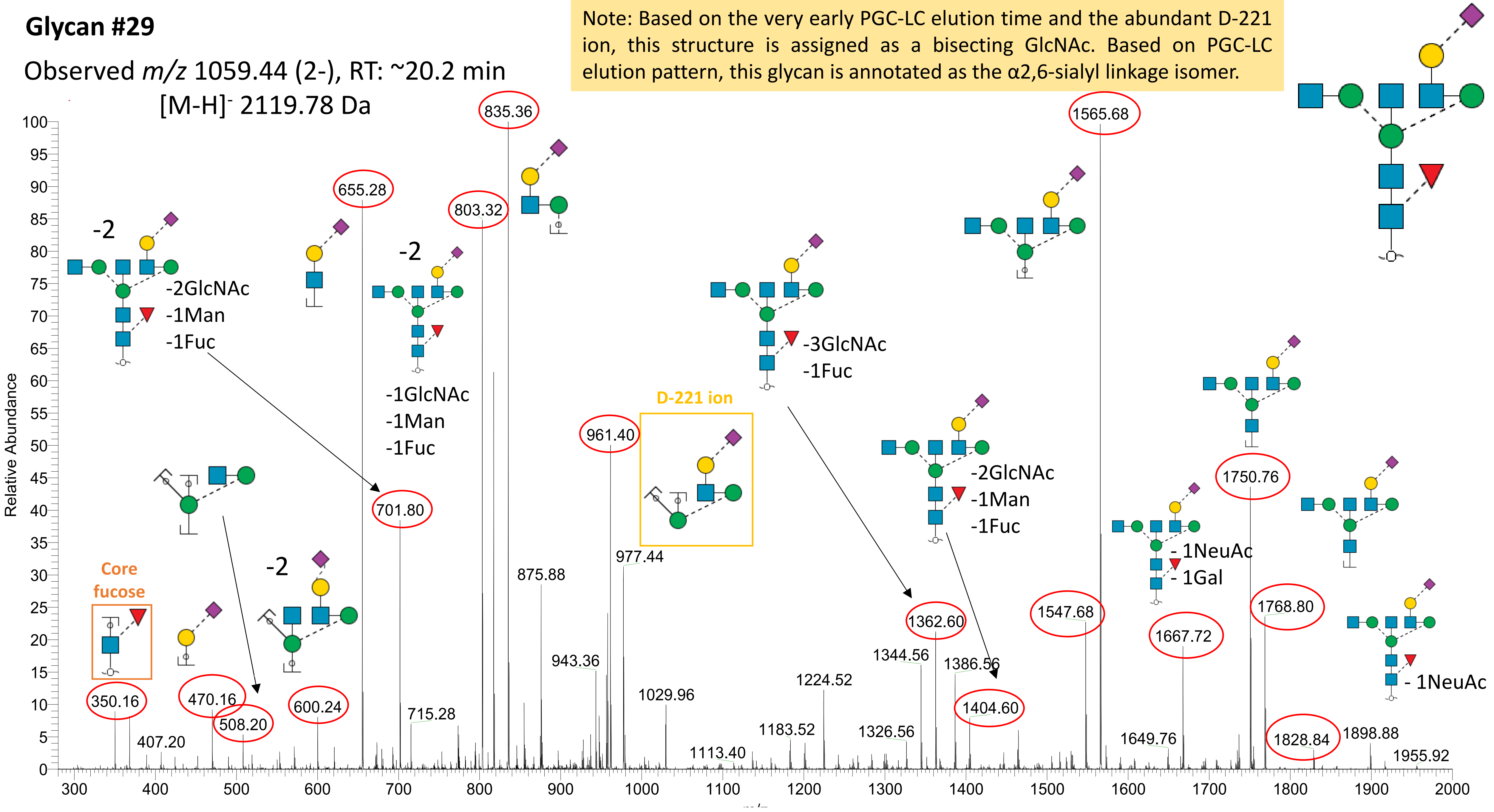

Glycan #30

Observed *m/z* 994.91 (2-), RT: ~20.3 min

$[M-H]^-$ 1990.73 Da

Note: Based on the very early PGC-LC elution time and the abundant D-221 ion, this structure is assigned as a bisecting GlcNAc.

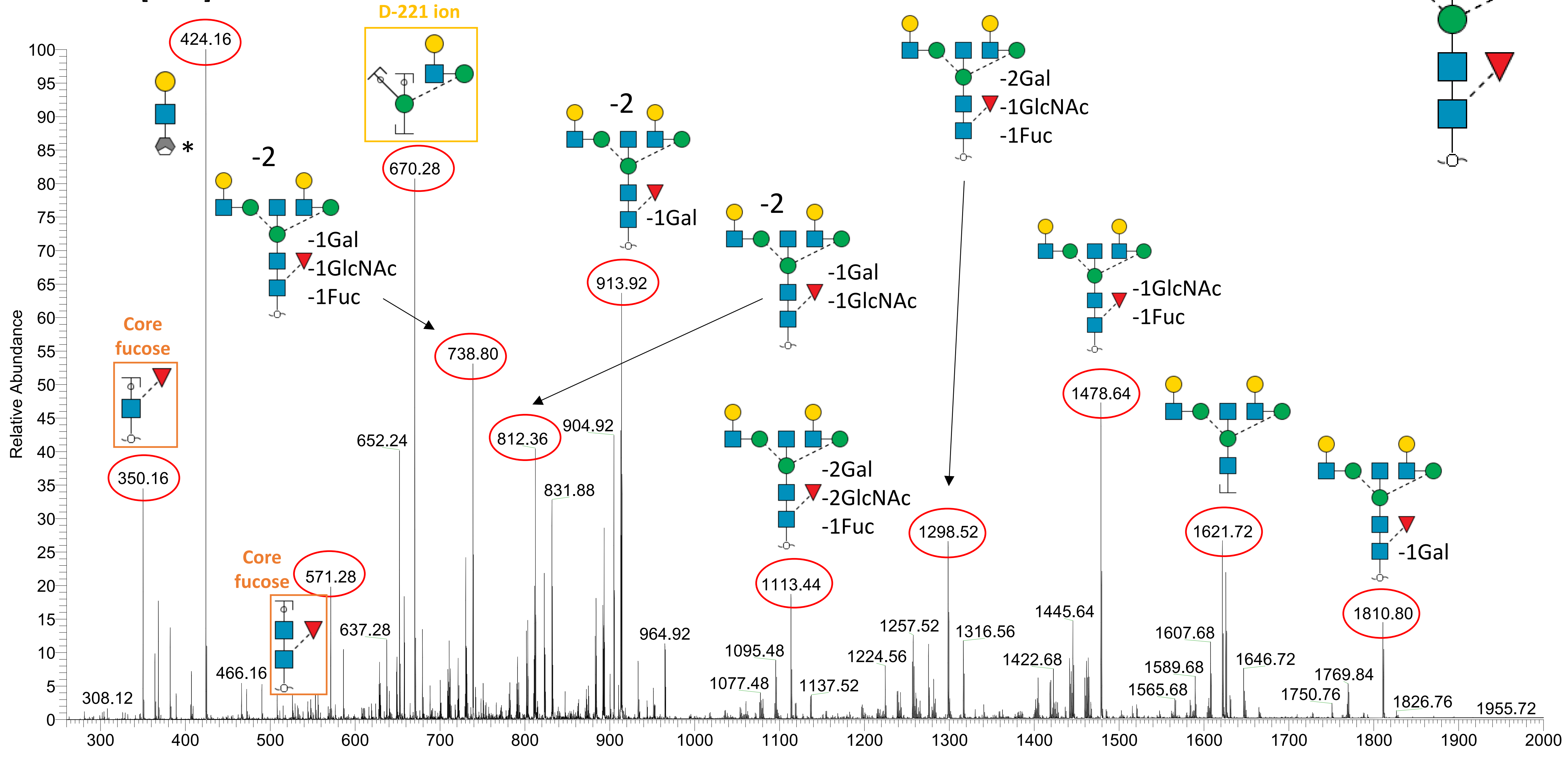

Glycan #31

Observed *m/z* 1067.43 (2-), RT: ~20.5 min

[M-H]- 2135.77 Da

Note: Based on the very early PGC-LC elution time and the D-221 ion, this structure is assigned as a bisecting GlcNAc with sialylation, predicted to occupy the α-1,3 arm. Based on PGC-LC elution pattern, this glycan is annotated as the α2,6-sialyl linkage isomer.

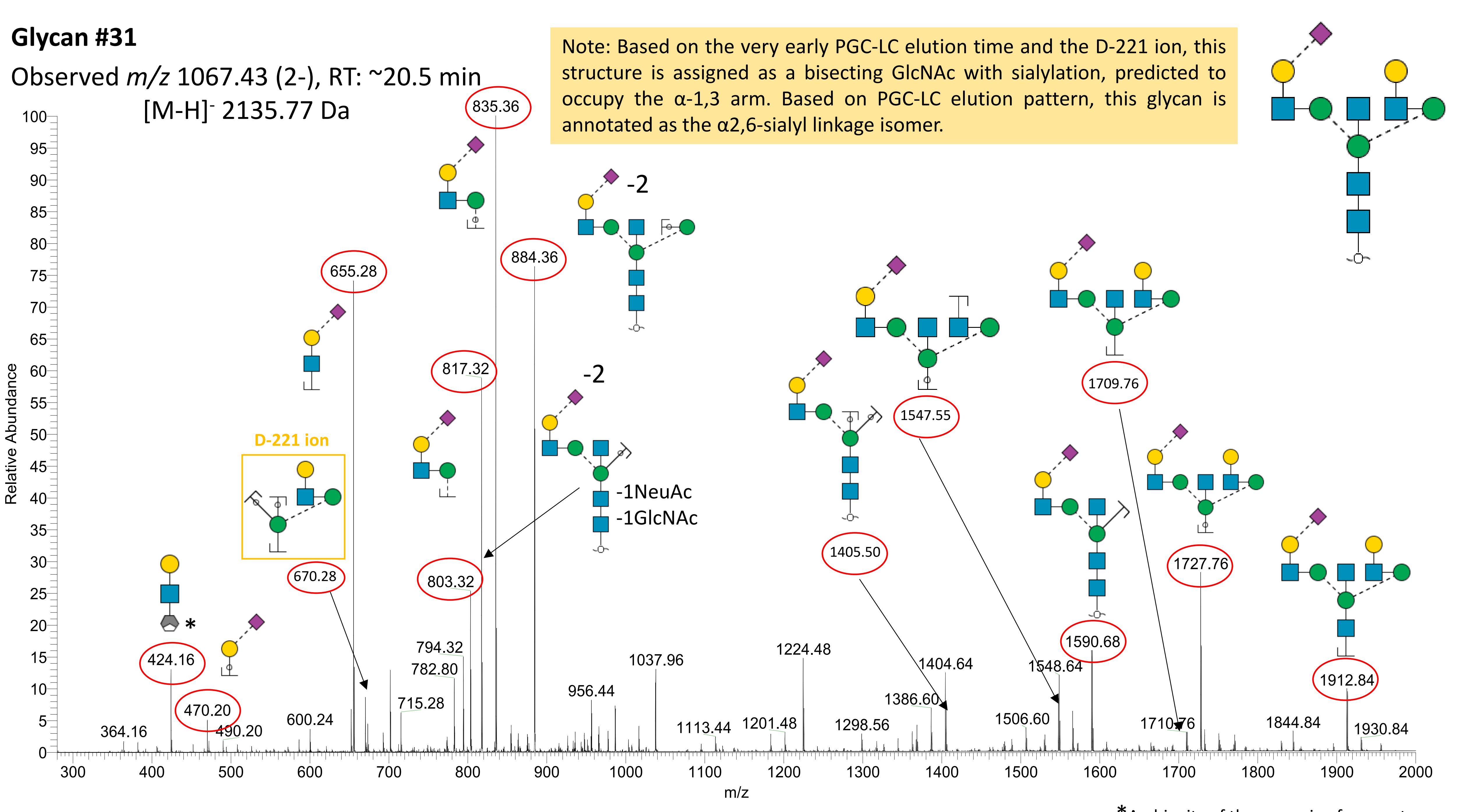

*Ambiguity of the cross-ring fragments

Glycan #32

Observed *m/z* 1140.41 (2-), RT: ~23.7 min

[M-H]$^-$ 2135.77 Da

Note: Based on the very early PGC-LC elution time and the D-221 ion, this structure is assigned as a bisecting GlcNAc with sialylation, predicted to occupy the α-1,3 arm. Based on PGC-LC elution pattern, this glycan is annotated as the α2,6-sialyl linkage isomer.

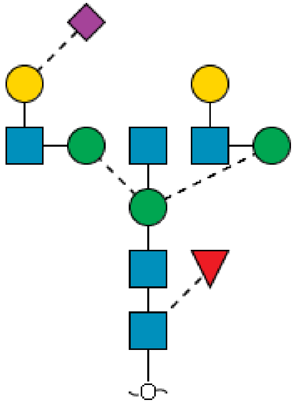

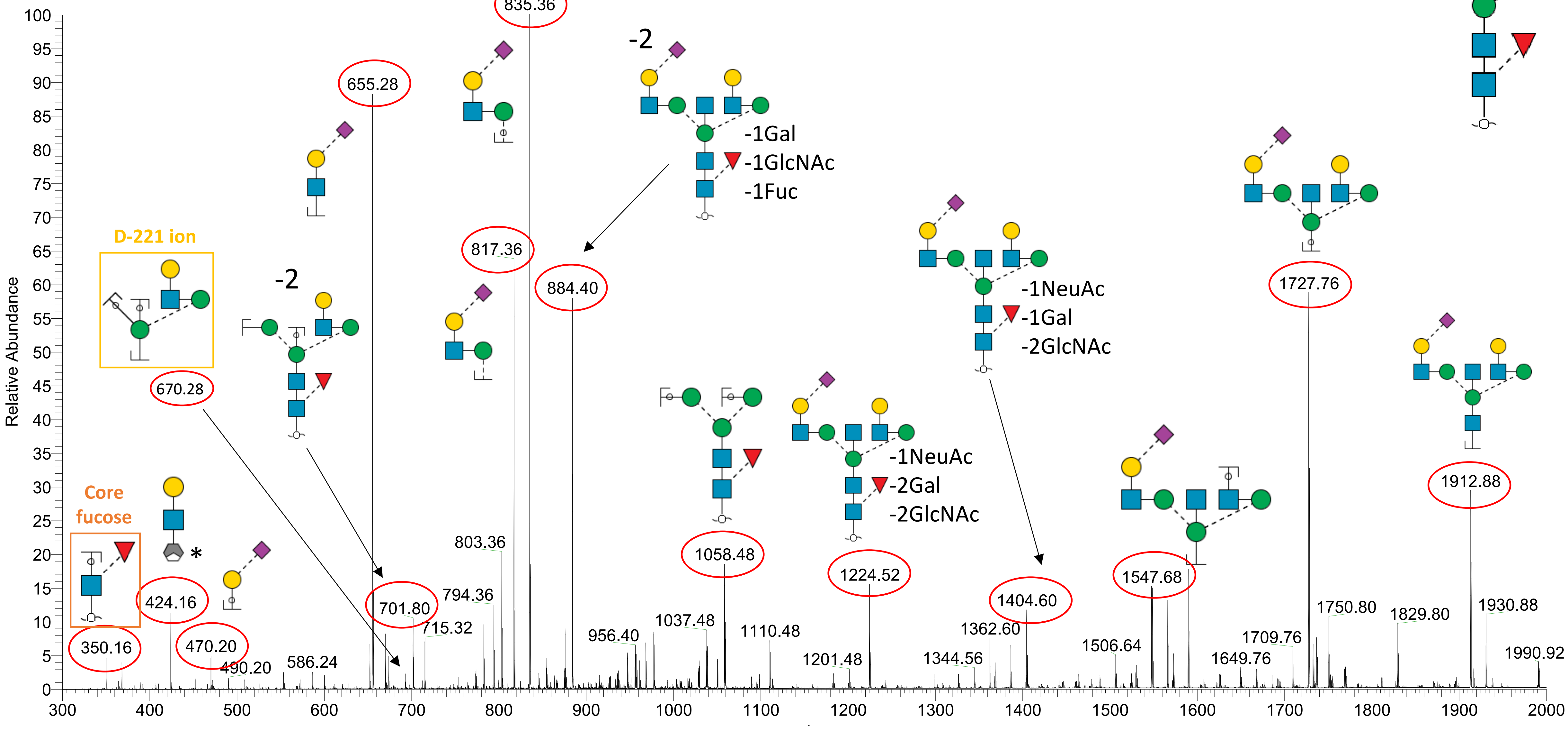

Glycan #33

Observed *m/z* 1285.96 (2-), RT: ~25.1 min

$[M-H]^-$ 2572.92 Da

Note: Based on the very early PGC-LC elution time and the D-221 ion, this structure is assigned as a bisecting GlcNAc. Based on PGC-LC elution pattern, this glycan is annotated as the α2,6-α2,6-sialyl linkage isomer.

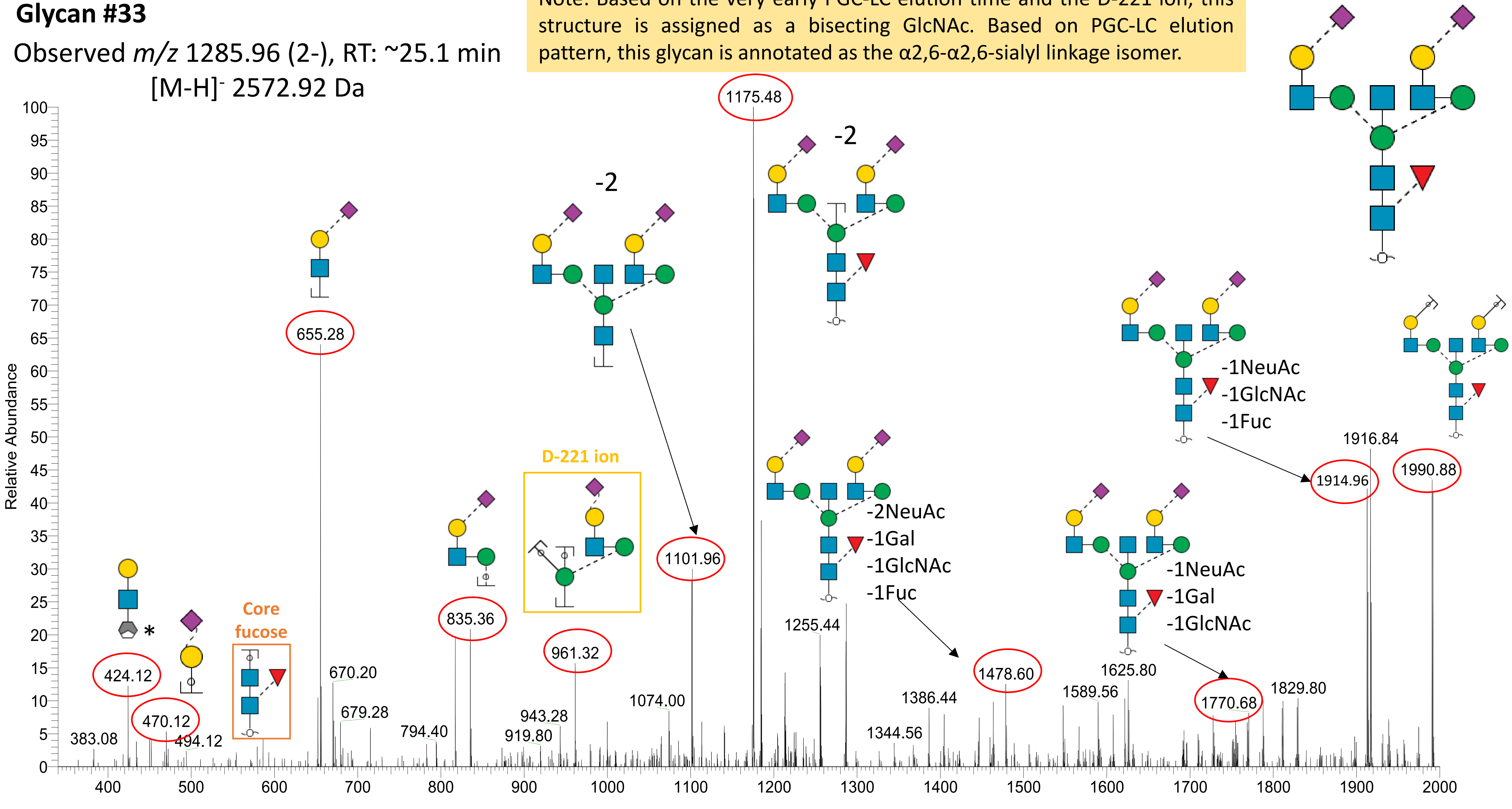

Glycan #34

Observed *m/z* 1148.41 (2-), RT: ~24.5 min
[M-H]$^-$ 2297.82 Da

Note: Based on PGC-LC elution pattern, this glycan is annotated as the α2,6-sialyl linkage isomer but the exact position cannot be determined. No D ion observed to allocate antennary branching and sialic acid.

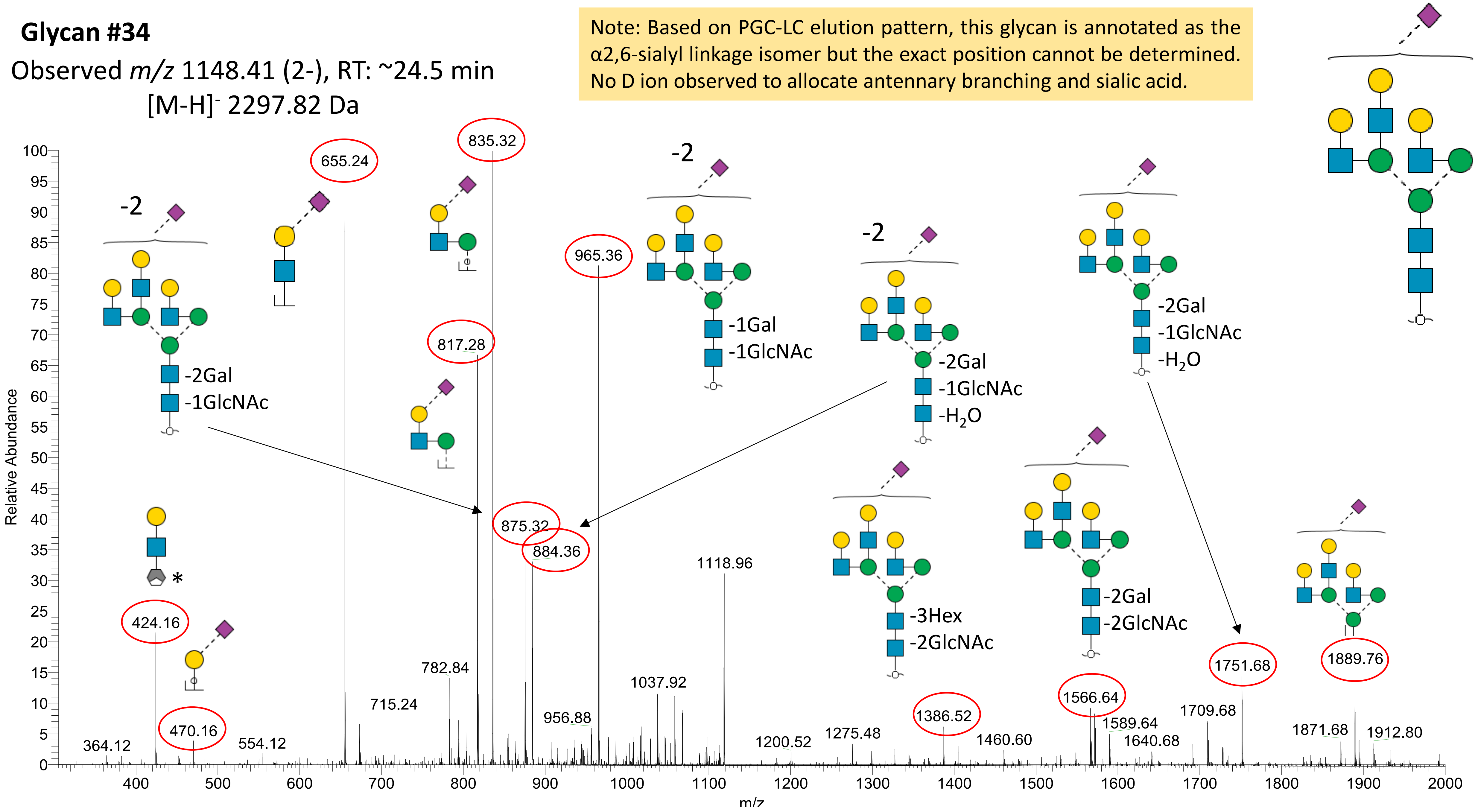

* Ambiguity of the cross-ring fragments

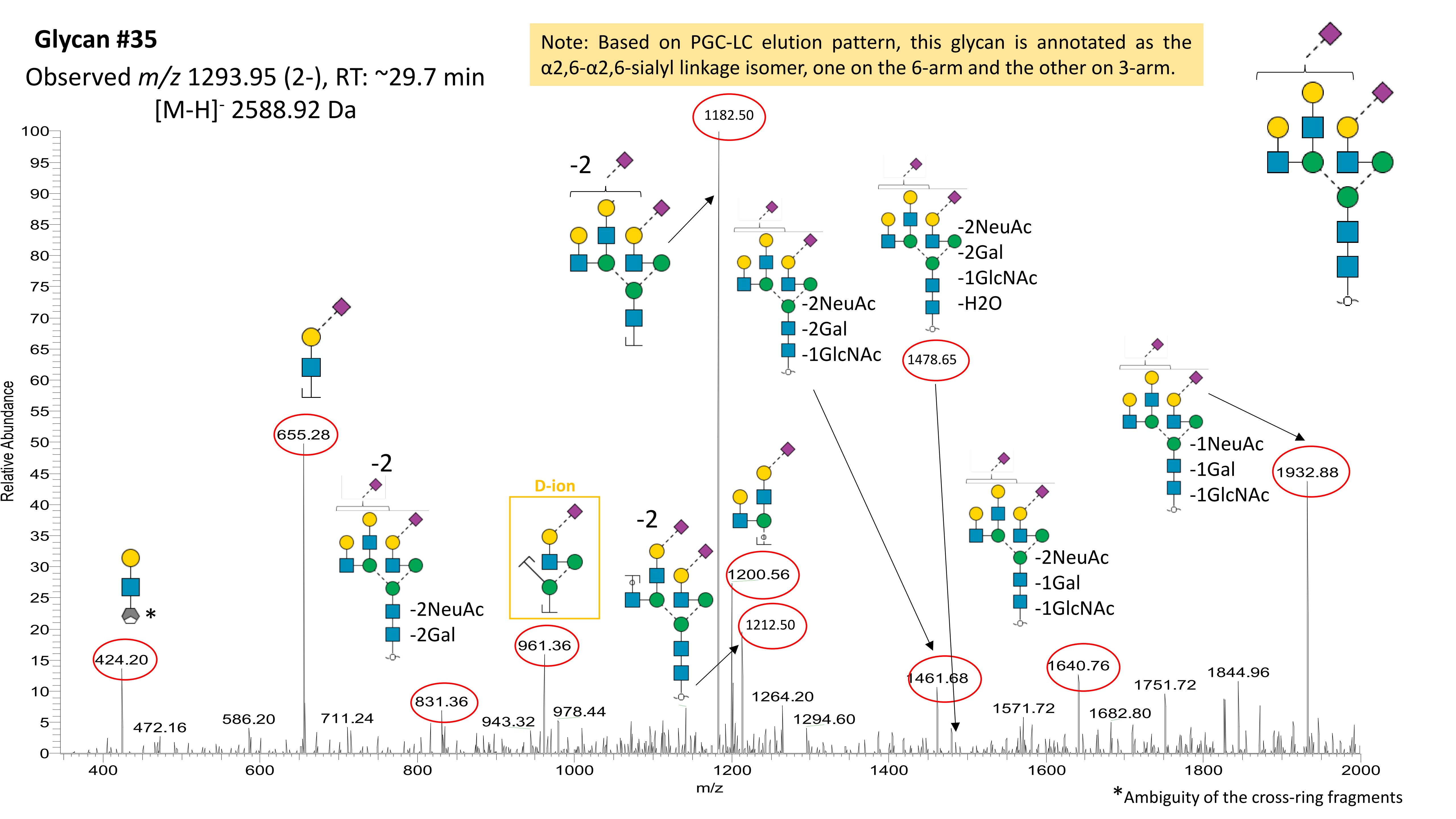

Glycan #35
Observed m/z 1293.95 (2-), RT: ~29.7 min
[M-H]- 2588.92 Da
Note: Based on PGC-LC elution pattern, this glycan is annotated as the α2,6-α2,6-sialyl linkage isomer, one on the 6-arm and the other on 3-arm.
Relative Abundance
m/z
424.20
472.16
586.20
655.28
711.24
831.36
943.32
961.36
978.44
1182.50
1200.56
1212.50
1264.20
1294.60
1461.68
1478.65
1571.72
1640.76
1682.80
1751.72
1844.96
1932.88
-2
-2
-2
D-ion
-2NeuAc
-2Gal
-2NeuAc
-2Gal
-1GlcNAc
-2NeuAc
-2Gal
-1GlcNAc
-H2O
-2NeuAc
-1Gal
-1GlcNAc
-1NeuAc
-1Gal
-1GlcNAc
*Ambiguity of the cross-ring fragments

Glycan #36

Observed *m/z* 1008.0 (3-), RT: ~32.3 min

$[M-H]^-$ 3026.06 Da, $[M-2H]^{2-}$ 1512.5 Da

Note: Based on PGC-LC elution pattern, this glycan is annotated as the α2,6-α2,6-sialyl linkage isomer but the antennary branching cannot be determined. Antenna fucose position is annotated as sialyl (α2,3-) Lewis x (sLe^x).

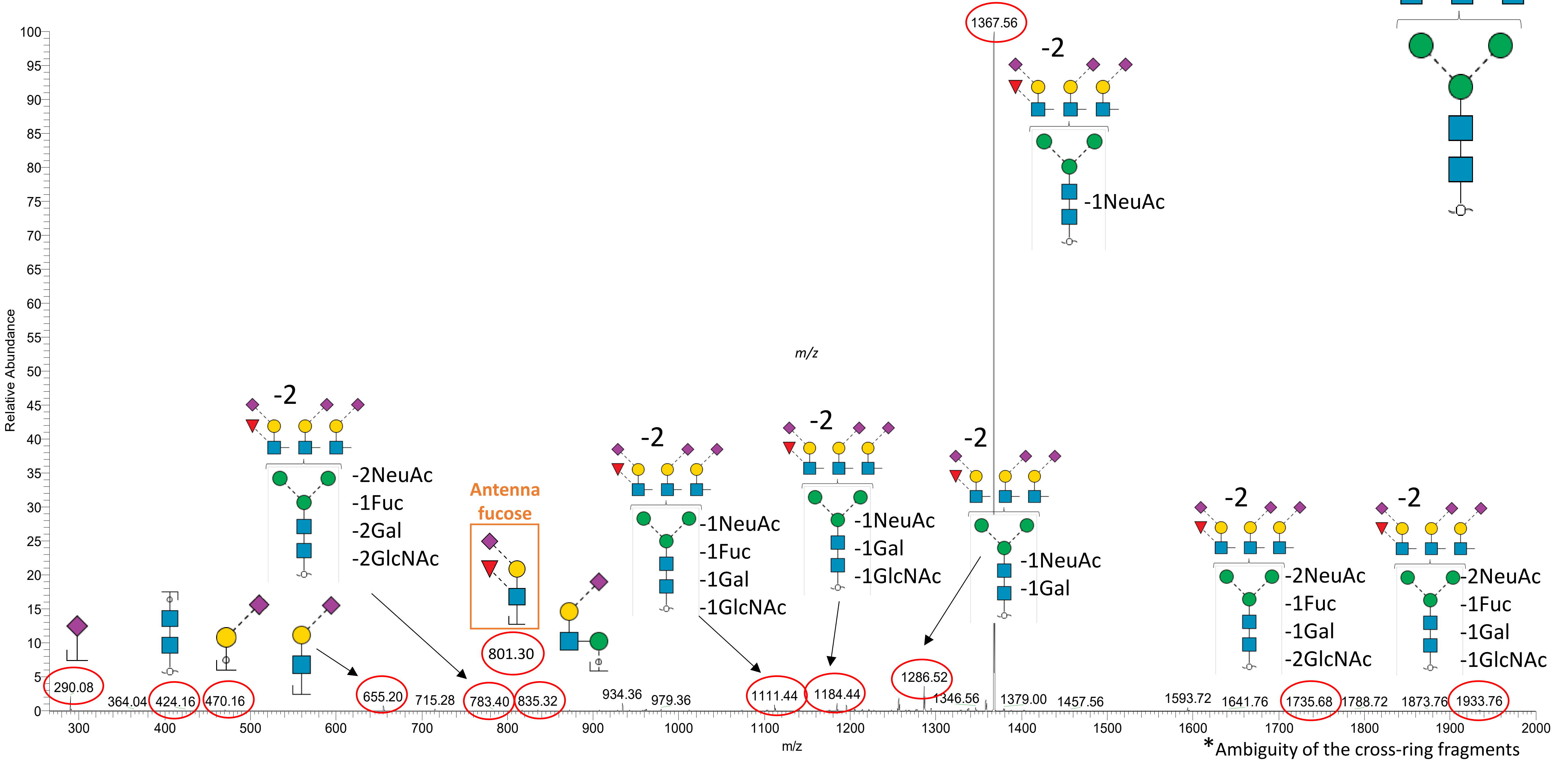

*Ambiguity of the cross-ring fragments
