# Supplementary material for: Serum AGP-1-Lex Glycoforms Report on Survivorship of Patients with Septic Shock Upon Admission to Intensive Care Unit
Source: Mol Cell Proteomics. 2025 Nov 17;25(1):101470. doi: 10.1016/j.mcpro.2025.101470 (PMC12794580; doi:10.1016/j.mcpro.2025.101470)
Supplement: Supplementary Figure S1 [file mmc3.pdf]

Supplementary Figures

for

Serum AGP-1-Le^x glycoforms report on survivorship of patients with septic shock upon admission to intensive care unit

The Huong Chau[1,2^], Sayantani Chatterjee[1^], Liam Caulfield[1], Anastasia Chernykh[1], Mathew Traini[1], Joshua Fehring[3], Heeyoun Hwang[4,5], Rebeca Kawahara[1,2], Emily J. Meyer[6,7], David J. Torpy[6,7], Morten Thaysen-Andersen[1,2]*

1. School of Natural Sciences, Macquarie University, Sydney, NSW, Australia
2. Institute for Glyco-core Research, Nagoya University, Nagoya, Aichi, Japan
3. Department of Biochemistry and Molecular Biology & Biomedicine Discovery Institute, Monash University, Australia
4. Digital OMICs Research Center, Korea Basic Science Institute, Cheongju, Republic of Korea
5. Bio-Analytical School, University of Science and Technology, Daejeon, Republic of Korea
6. Department of Medicine, University of Adelaide, Adelaide, South Australia, Australia
7. Endocrine and Metabolic Unit, Royal Adelaide Hospital, Adelaide, South Australia, Australia

^Contributed equally

Running title: Serum AGP-1-Le^x predicts septic shock survivorship

*Corresponding author:

Associate Professor Morten Thaysen-Andersen

School of Natural Sciences

Macquarie University

New South Wales 2109, Macquarie Park

Sydney, Australia

Phone: +61 2 9850 7487

Email: morten.andersen@mq.edu.au

Supplementary Figure S1

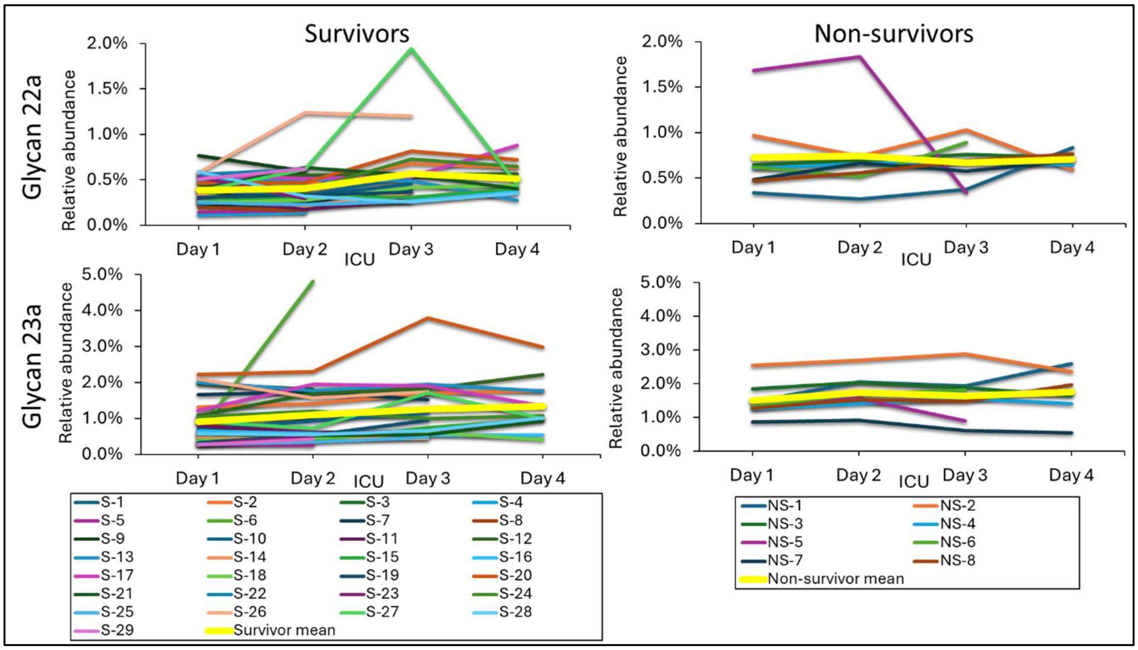

Supplementary Figure S1. Longitudinal patient-specific levels of the Lewis-containing glycan 22a (top) and glycan 23a (bottom) in septic shock survivors (left) and non-survivors (right) as determined by comparative glycomics of the early disease course (ICU day 1-4). See **Supplementary Table S3** for all tabulated glycomics data.
