# Supplementary material for: Serum AGP-1-Lex Glycoforms Report on Survivorship of Patients with Septic Shock Upon Admission to Intensive Care Unit
Source: Mol Cell Proteomics. 2025 Nov 17;25(1):101470. doi: 10.1016/j.mcpro.2025.101470 (PMC12794580; doi:10.1016/j.mcpro.2025.101470)
Supplement: Supplementary Data 2 [file mmc2.pdf]

Supplementary Data S2

for

Serum AGP-1-Le^x glycoforms report on survivorship of patients with septic shock upon admission to intensive care unit

The Huong Chau[1,2^], Sayantani Chatterjee[1^], Liam Caulfield[1], Anastasia Chernykh[1], Mathew Traini[1], Joshua Fehring[3], Heeyoun Hwang[4,5], Rebeca Kawahara[1,2], Emily J. Meyer[6,7], David J. Torpy[6,7], Morten Thaysen-Andersen[1,2*]

Page 2 – page 1020: Le^x-glycopeptides identified by I-GPA in septic shock sera

Page 1021 – page 60147: All glycopeptides identified by I-GPA in septic shock sera

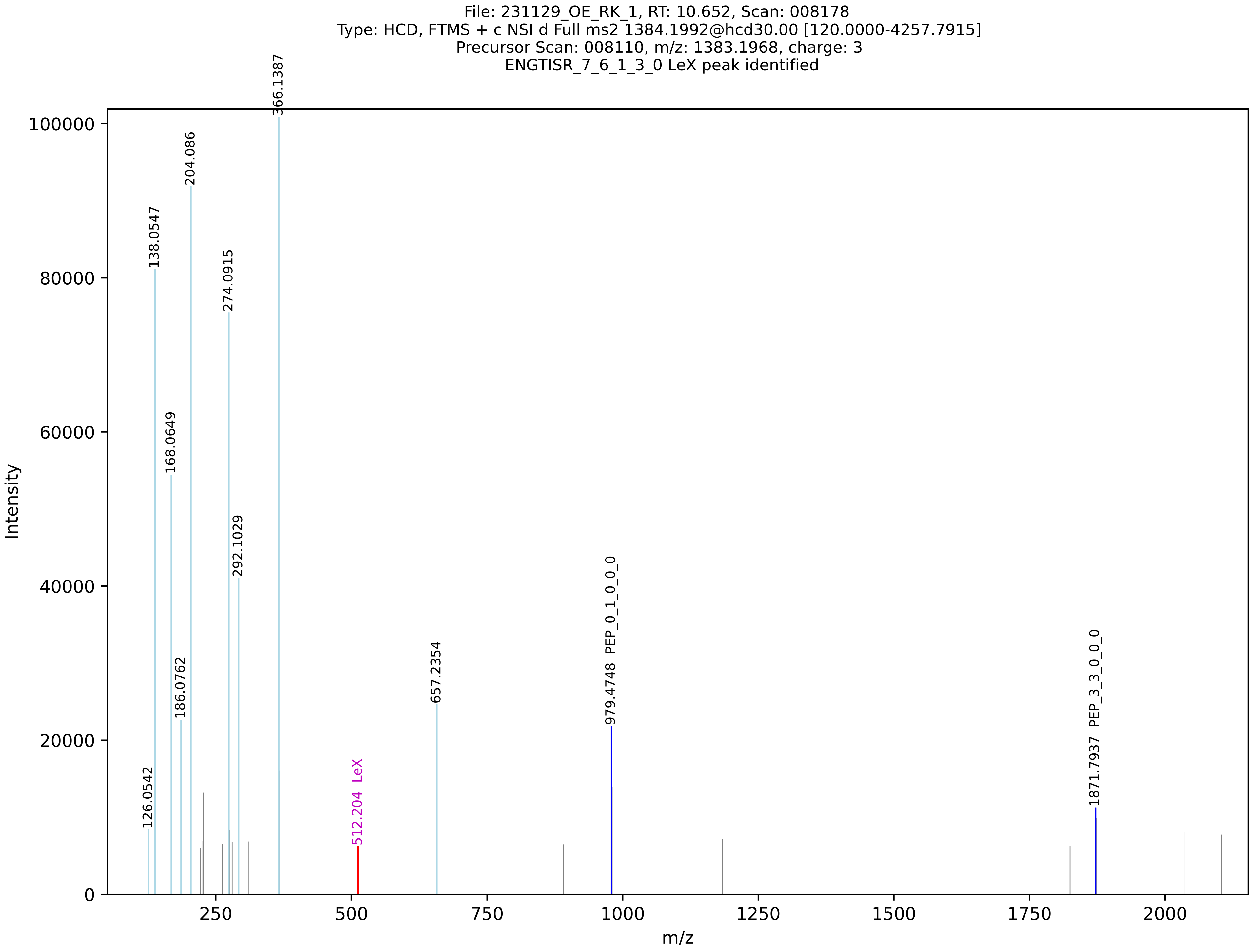

File: 231129_OE_RK_1, RT: 10.652, Scan: 008178
Type: HCD, FTMS + c NSI d Full ms2 1384.1992@hcd30.00 [120.0000-4257.7915]
Precursor Scan: 008110, m/z: 1383.1968, charge: 3
ENGTISR_7_6_1_3_0 LeX peak identified
Intensity
m/z
366.1387
204.086
138.0547
274.0915
168.0649
292.1029
186.0762
126.0542
512.204 LeX
657.2354
979.4748 PEP_0_1_0_0_0
1871.7937 PEP_3_3_0_0_0

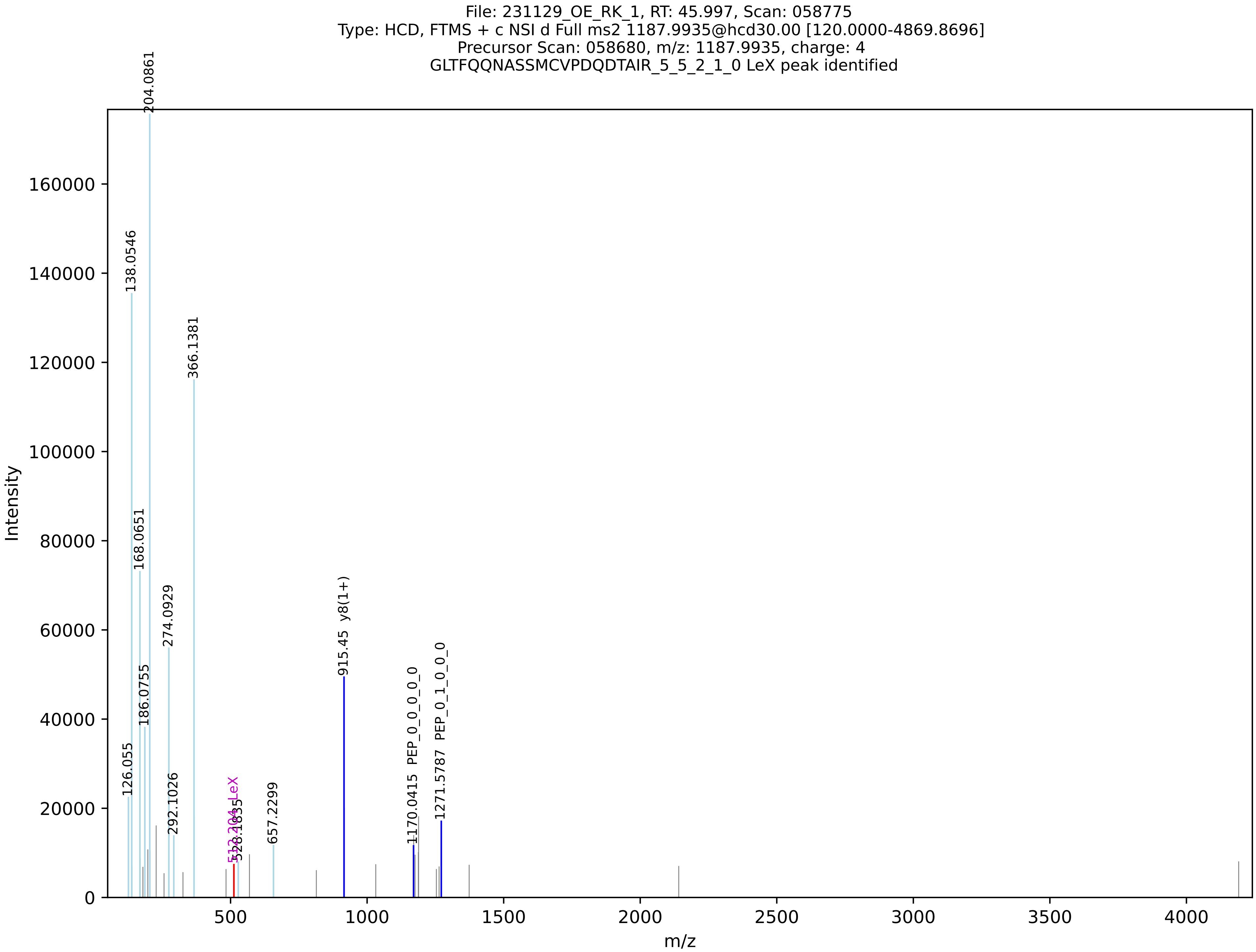
File: 231129_OE_RK_1, RT: 45.997, Scan: 058775
Type: HCD, FTMS + c NSI d Full ms2 1187.9935@hcd30.00 [120.0000-4869.8696]
Precursor Scan: 058680, m/z: 1187.9935, charge: 4
GLTFQQNASSMCVPDQDTAIR_5_5_2_1_0 LeX peak identified
204.0861
138.0546
366.1381
168.0651
274.0929
186.0755
126.055
292.1026
512.204 LeX
528.1835
657.2299
915.45 y8(1+)
1170.0415 PEP_0_0_0_0_0
1271.5787 PEP_0_1_0_0_0
Intensity
m/z
0
20000
40000
60000
80000
100000
120000
140000
160000
500
1000
1500
2000
2500
3000
3500
4000

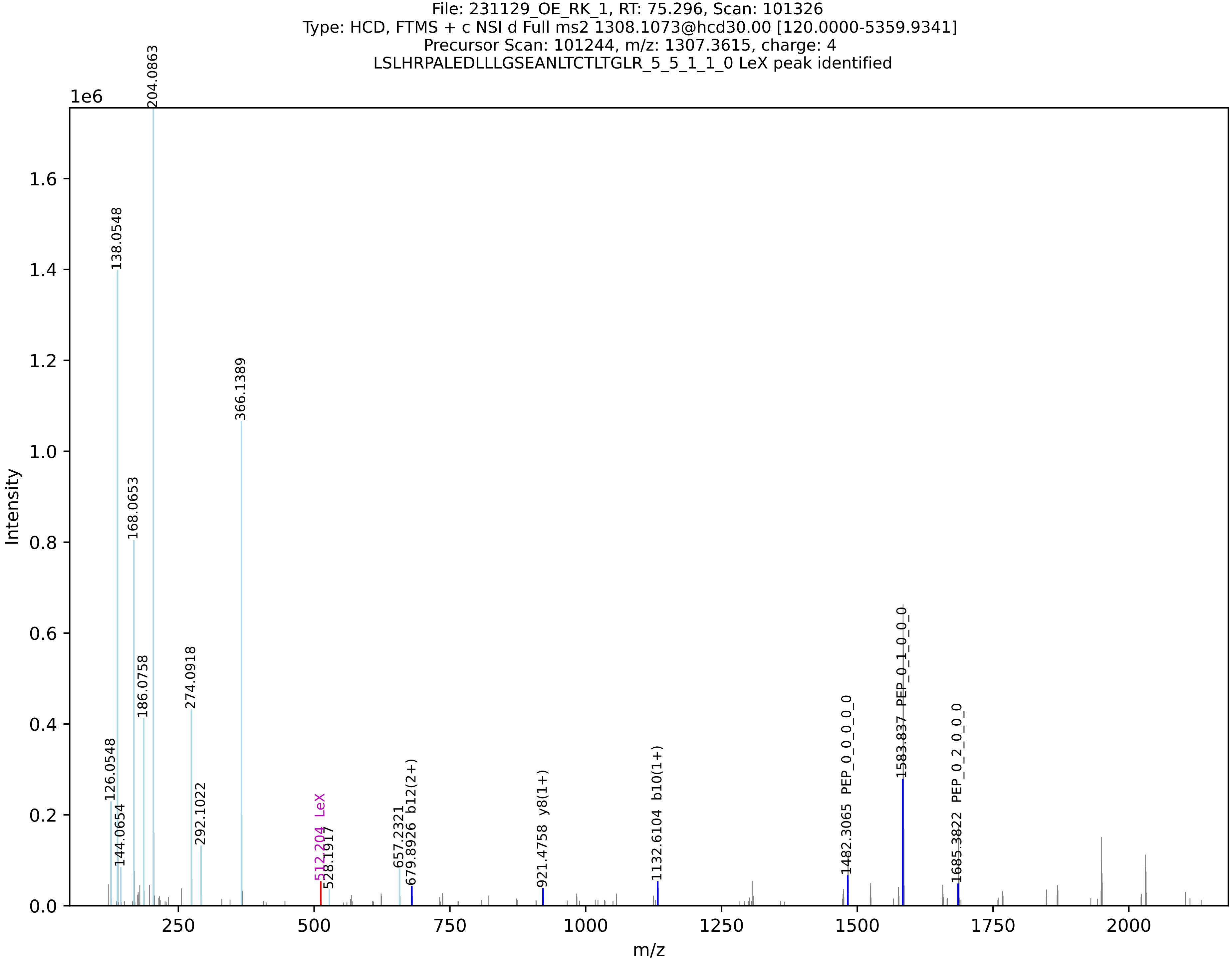
File: 231129_OE_RK_1, RT: 75.296, Scan: 101326
Type: HCD, FTMS + c NSI d Full ms2 1308.1073@hcd30.00 [120.0000-5359.9341]
Precursor Scan: 101244, m/z: 1307.3615, charge: 4
LSLHRPALEDLLLGSEANLTCTLTGLR_5_5_1_1_0 LeX peak identified
1e6
Intensity
m/z
204.0863
138.0548
366.1389
168.0653
186.0758
274.0918
126.0548
144.0654
292.1022
512.204 LeX
528.1917
657.2321
679.8926 b12(2+)
921.4758 y8(1+)
1132.6104 b10(1+)
1482.3065 PEP_0_0_0_0_0
1583.837 PEP_0_1_0_0_0
1685.3822 PEP_0_2_0_0_0

File: 231129_OE_RK_2_6ul, RT: 43.28, Scan: 054200
Type: HCD, FTMS + c NSI d Full ms2 1667.0028@hcd30.00 [120.0000-5123.1704]
Precursor Scan: 054118, m/z: 1666.3346, charge: 3
QDQCIYNTTYLNVQR_7_6_1_2_0 LeX peak identified

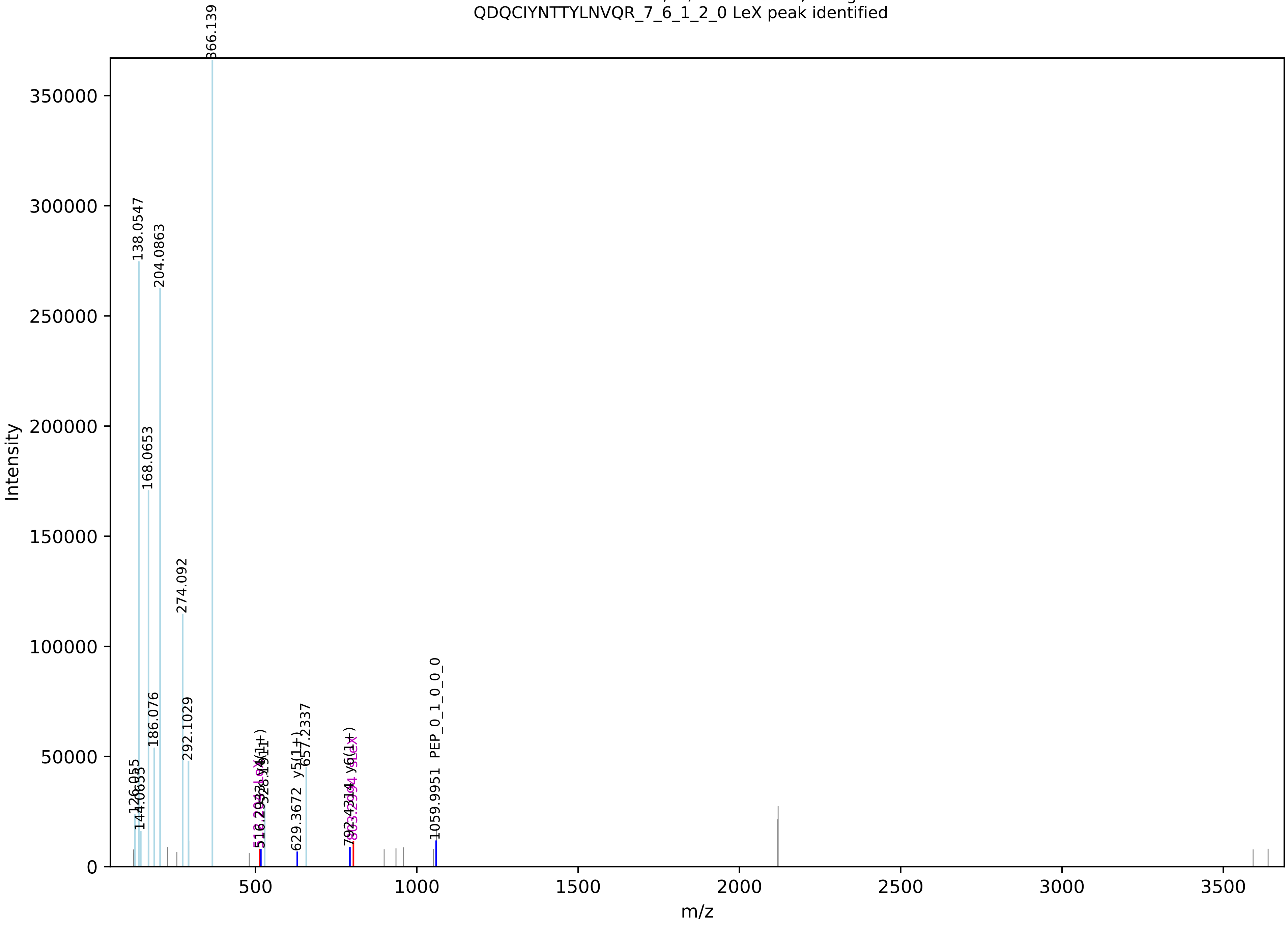

File: 231129_OE_RK_2_6ul, RT: 43.379, Scan: 054342
Type: HCD, FTMS + c NSI d Full ms2 1158.9707@hcd30.00 [120.0000-4751.4565]
Precursor Scan: 054266, m/z: 1158.7225, charge: 4
QDQCIYNTTYLNVQR_6_5_1_2_0 LeX peak identified

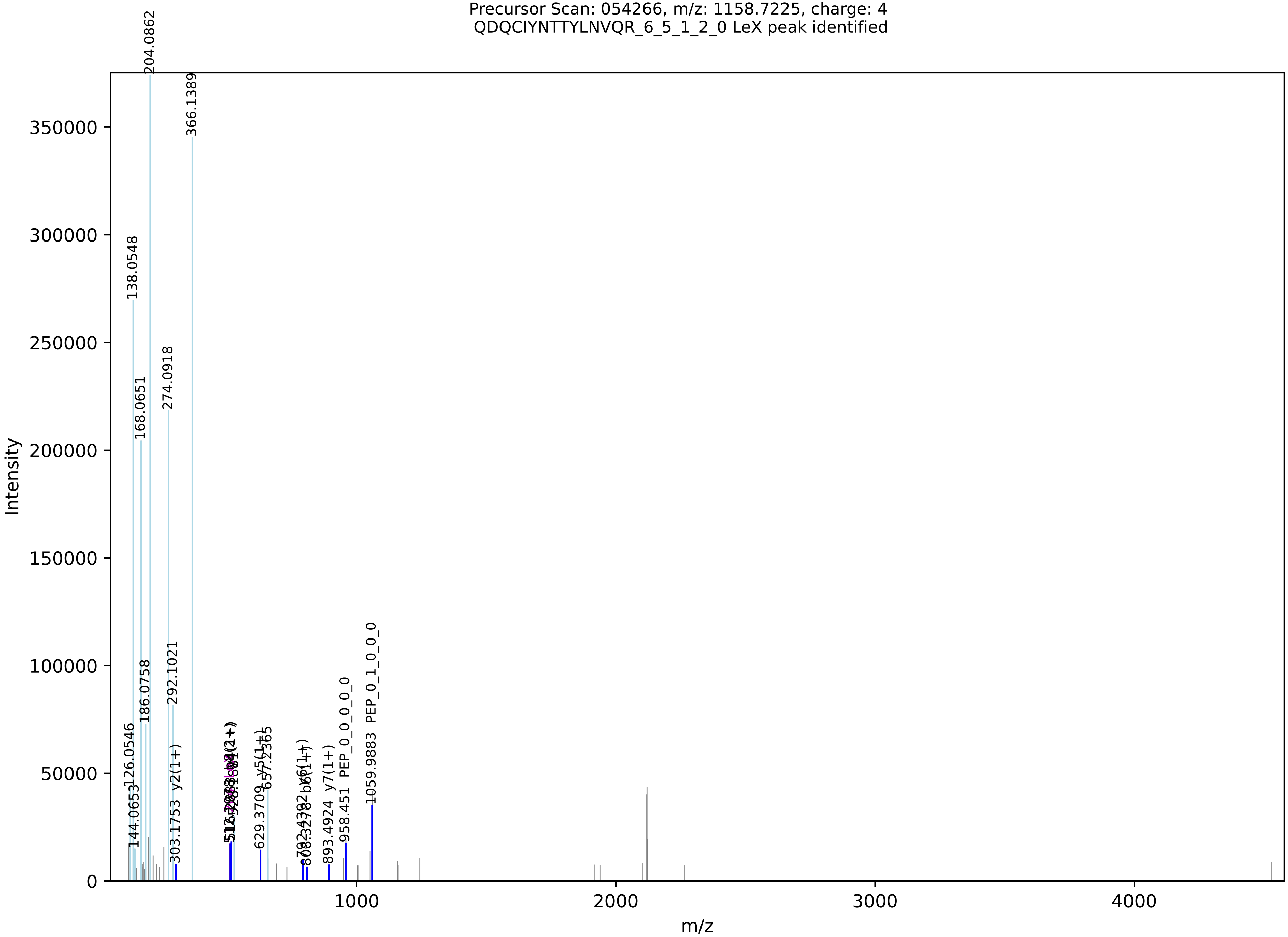

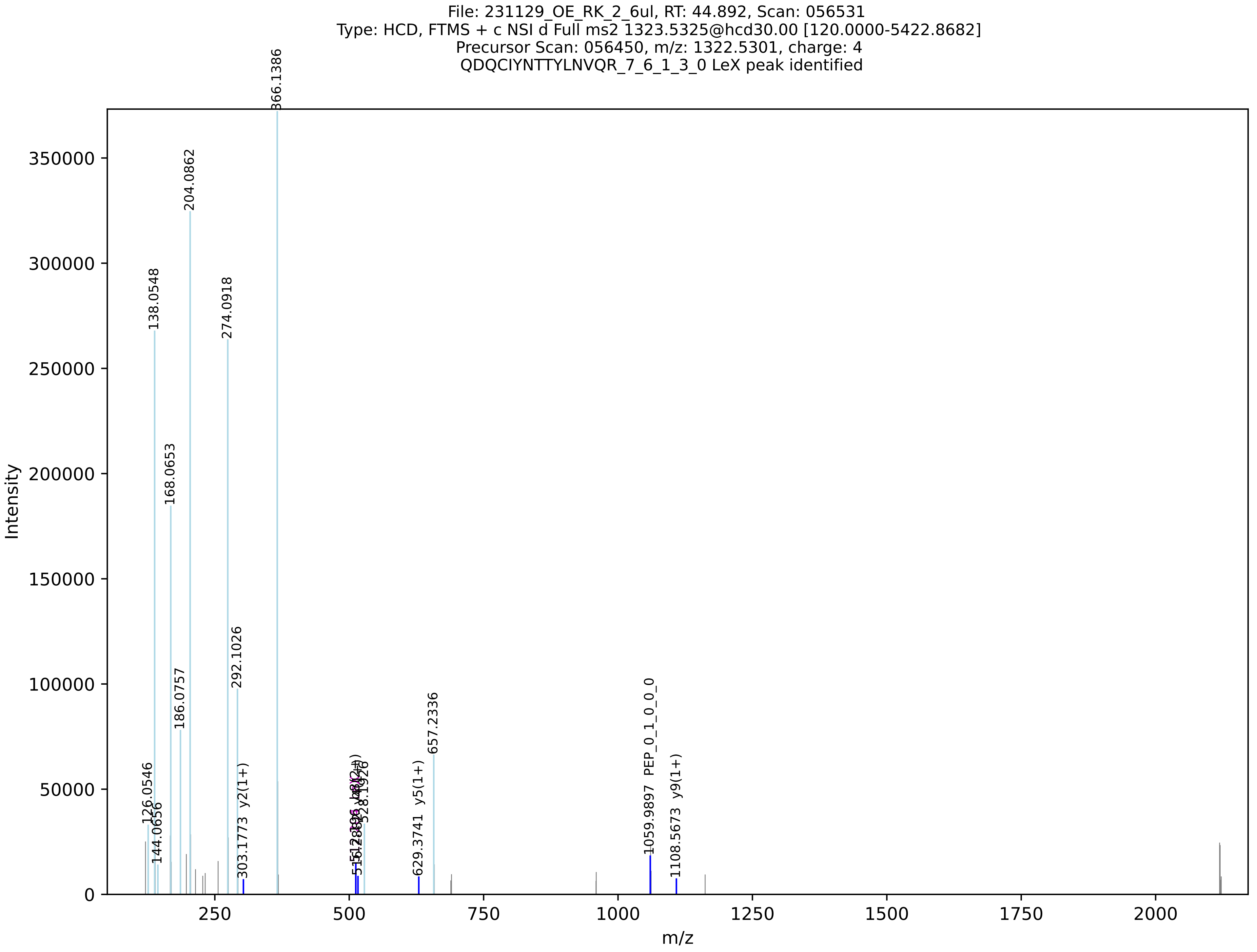
File: 231129_OE_RK_2_6ul, RT: 44.892, Scan: 056531
Type: HCD, FTMS + c NSI d Full ms2 1323.5325@hcd30.00 [120.0000-5422.8682]
Precursor Scan: 056450, m/z: 1322.5301, charge: 4
QDQCIYNTTYLNVQR_7_6_1_3_0 LeX peak identified
Intensity
m/z
0
50000
100000
150000
200000
250000
300000
350000
250
500
750
1000
1250
1500
1750
2000
366.1386
204.0862
138.0548
274.0918
168.0653
292.1026
186.0757
126.0546
144.0656
303.1773 y2(1+)
629.3741 y5(1+)
657.2336
1059.9897 PEP_0_1_0_0_0
1108.5673 y9(1+)

File: 231129_OE_RK_2_6ul, RT: 45.504, Scan: 057424
Type: HCD, FTMS + c NSI d Full ms2 1323.2760@hcd30.00 [120.0000-5421.8223]
Precursor Scan: 057345, m/z: 1322.7742, charge: 4
QDQCIYNTTYLNVQR_7_6_1_3_0 LeX peak identified

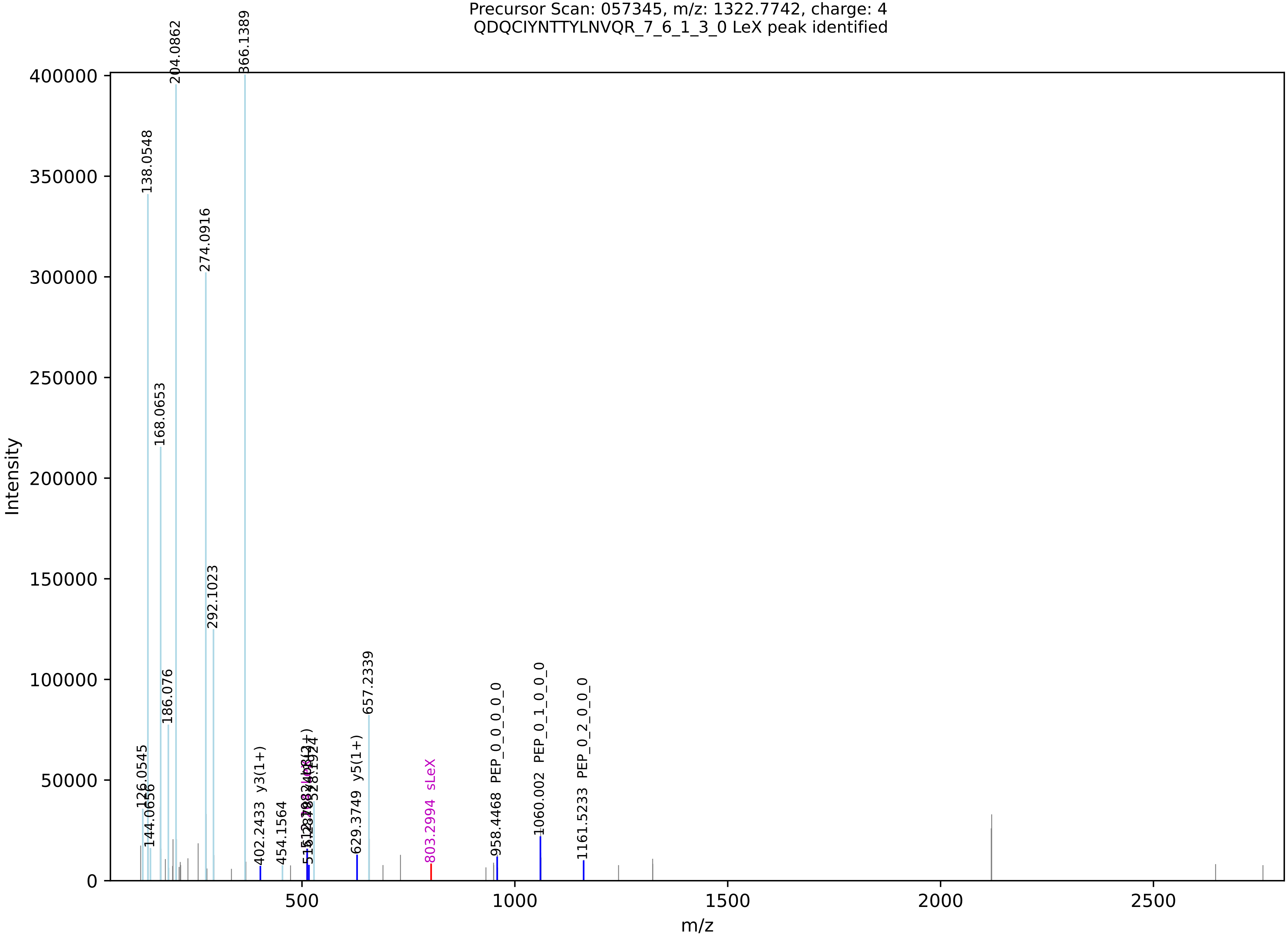

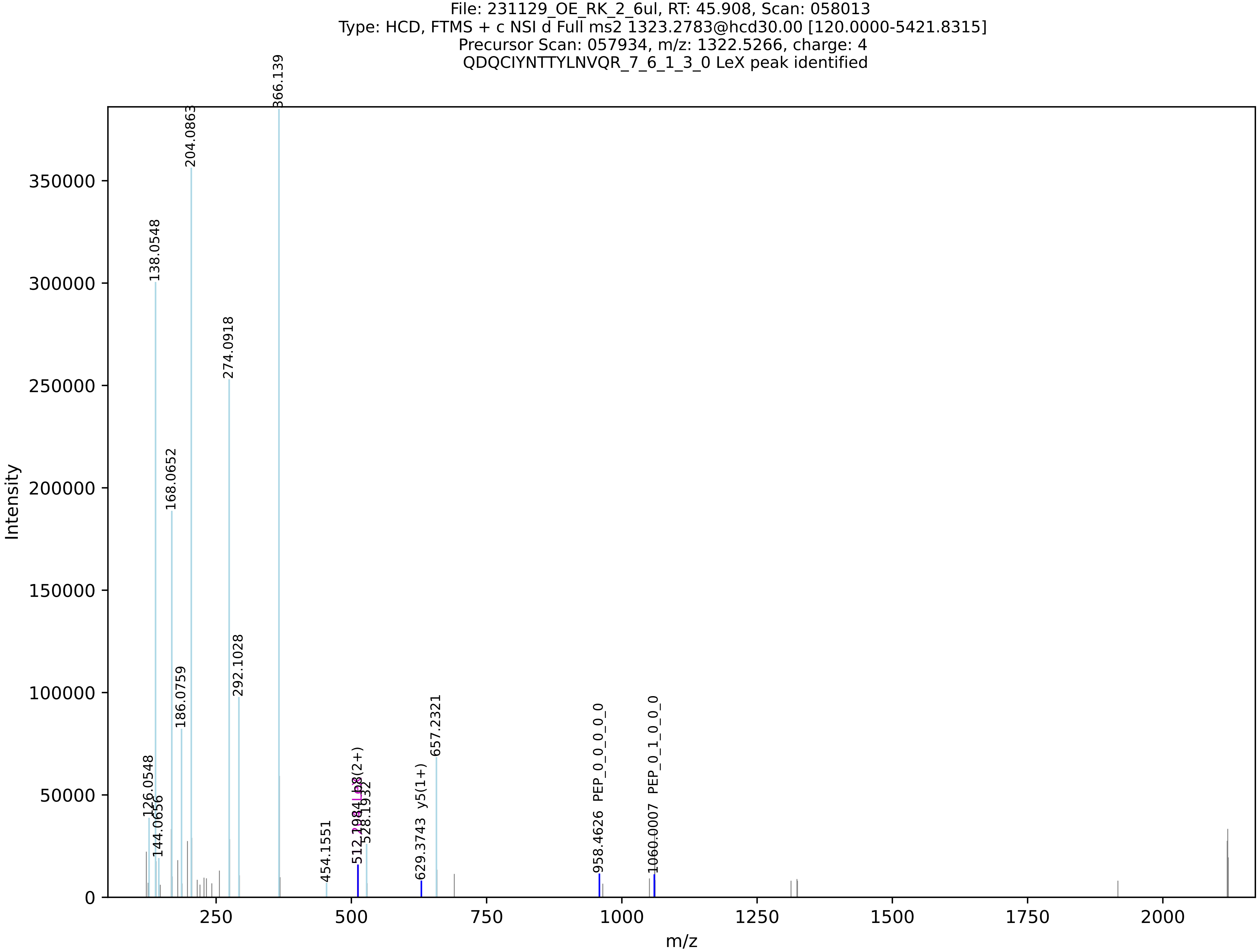

File: 231129_OE_RK_2_6ul, RT: 45.908, Scan: 058013
Type: HCD, FTMS + c NSI d Full ms2 1323.2783@hcd30.00 [120.0000-5421.8315]
Precursor Scan: 057934, m/z: 1322.5266, charge: 4
QDQCIYNTTYLNVQR_7_6_1_3_0 LeX peak identified
Intensity
m/z
366.139
204.0863
138.0548
274.0918
168.0652
292.1028
186.0759
126.0548
144.0656
454.1551
512.1984 b8(2+)
528.1932
629.3743 y5(1+)
657.2321
958.4626 PEP_0_0_0_0_0
1060.0007 PEP_0_1_0_0_0

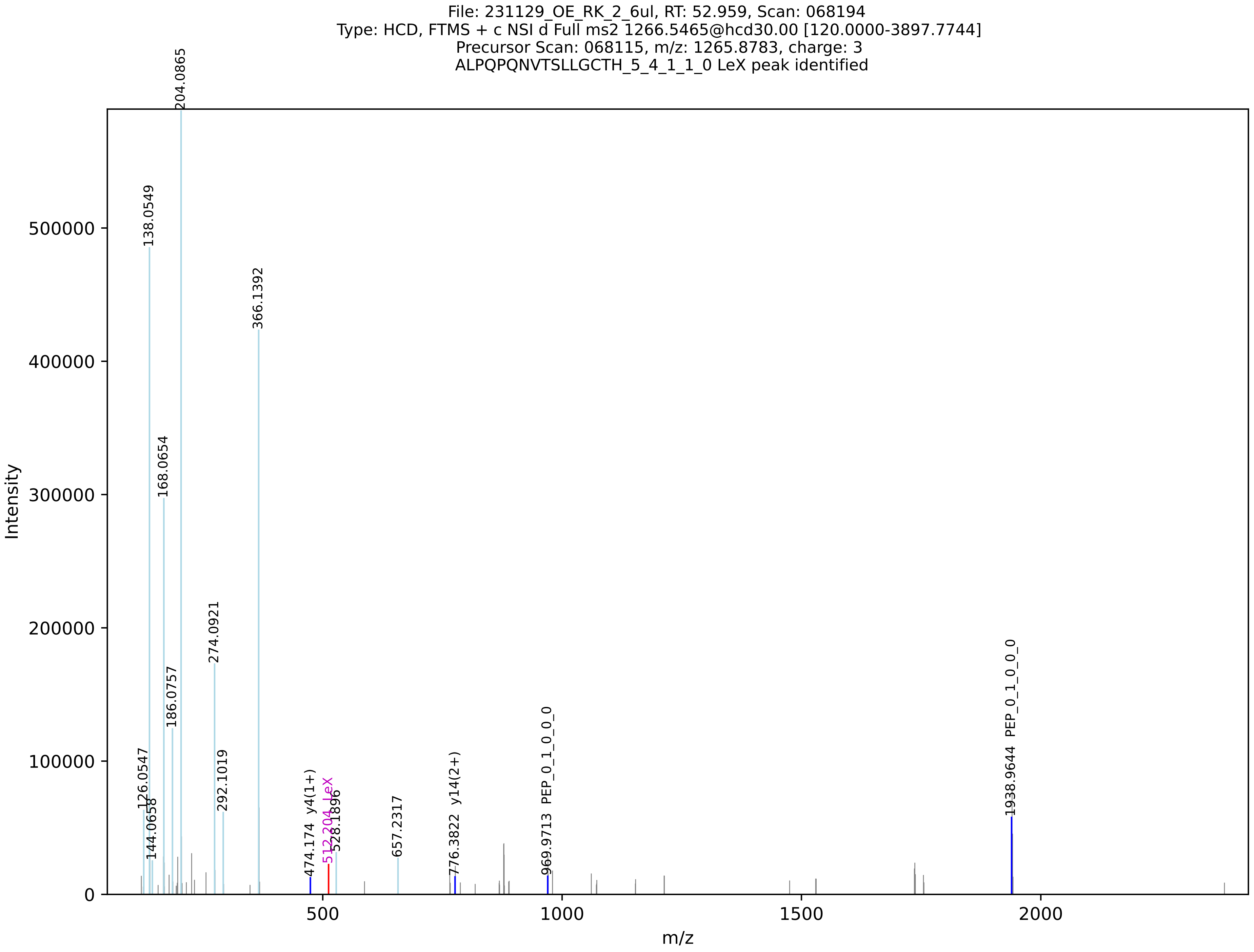
File: 231129_OE_RK_2_6ul, RT: 52.959, Scan: 068194
Type: HCD, FTMS + c NSI d Full ms2 1266.5465@hcd30.00 [120.0000-3897.7744]
Precursor Scan: 068115, m/z: 1265.8783, charge: 3
ALPQPQNVTSLLGCTH_5_4_1_1_0 LeX peak identified
Intensity
m/z
204.0865
138.0549
366.1392
168.0654
274.0921
186.0757
126.0547
144.0658
292.1019
474.174 y4(1+)
512.204_LeX
528.1896
657.2317
776.3822 y14(2+)
969.9713 PEP_0_1_0_0_0
1938.9644 PEP_0_1_0_0_0
0
100000
200000
300000
400000
500000
500
1000
1500
2000

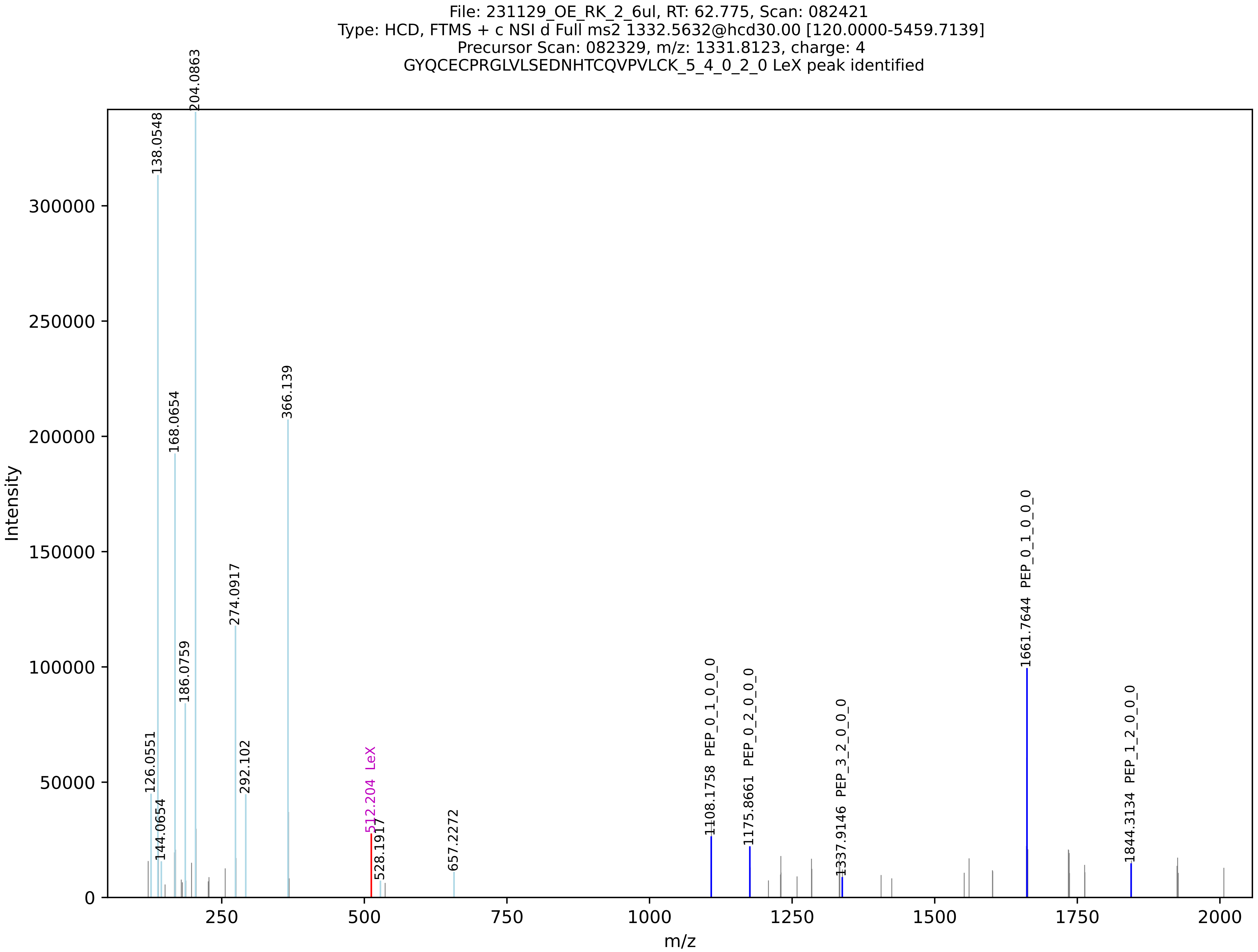
File: 231129_OE_RK_2_6ul, RT: 62.775, Scan: 082421
Type: HCD, FTMS + c NSI d Full ms2 1332.5632@hcd30.00 [120.0000-5459.7139]
Precursor Scan: 082329, m/z: 1331.8123, charge: 4
GYQCECPRGLVLSEDNHTCQVPVLCK_5_4_0_2_0 LeX peak identified
204.0863
138.0548
168.0654
366.139
274.0917
186.0759
126.0551
292.102
144.0654
512.204 LeX
528.1917
657.2272
1108.1758 PEP_0_1_0_0_0
1175.8661 PEP_0_2_0_0_0
1337.9146 PEP_3_2_0_0_0
1661.7644 PEP_0_1_0_0_0
1844.3134 PEP_1_2_0_0_0
Intensity
0
50000
100000
150000
200000
250000
300000
250
500
750
1000
1250
1500
1750
2000
m/z

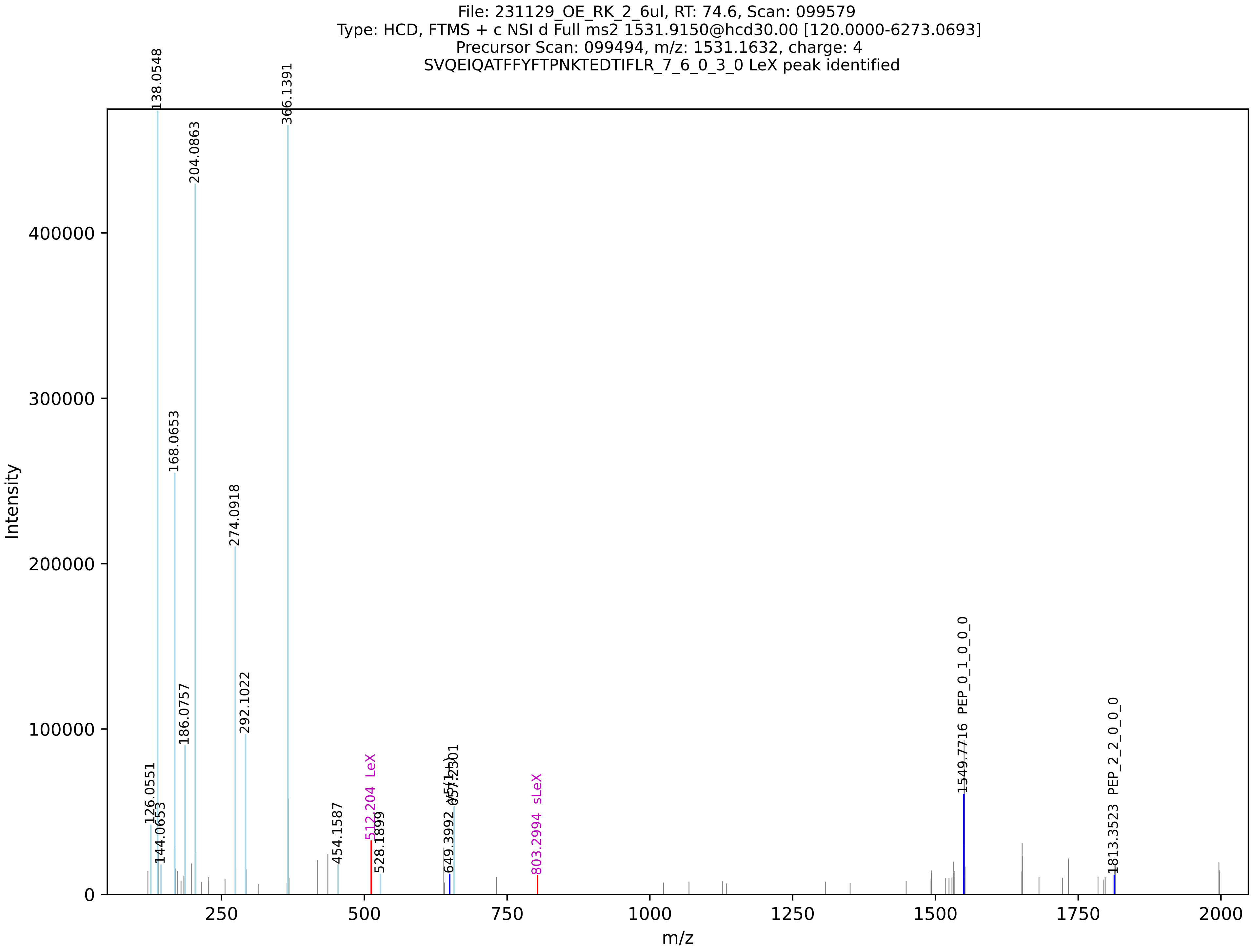

File: 231129_OE_RK_2_6ul, RT: 74.6, Scan: 099579
Type: HCD, FTMS + c NSI d Full ms2 1531.9150@hcd30.00 [120.0000-6273.0693]
Precursor Scan: 099494, m/z: 1531.1632, charge: 4
SVQEIQATFFYFTPNKTEDTIFLR_7_6_0_3_0 LeX peak identified
138.0548
366.1391
204.0863
168.0653
274.0918
292.1022
186.0757
126.0551
144.0653
454.1587
512.204 LeX
528.1899
649.3992 y5(1+)
657.2301
803.2994 sLeX
1549.7716 PEP_0_1_0_0_0
1813.3523 PEP_2_2_0_0_0
Intensity
0
100000
200000
300000
400000
250
500
750
1000
1250
1500
1750
2000
m/z

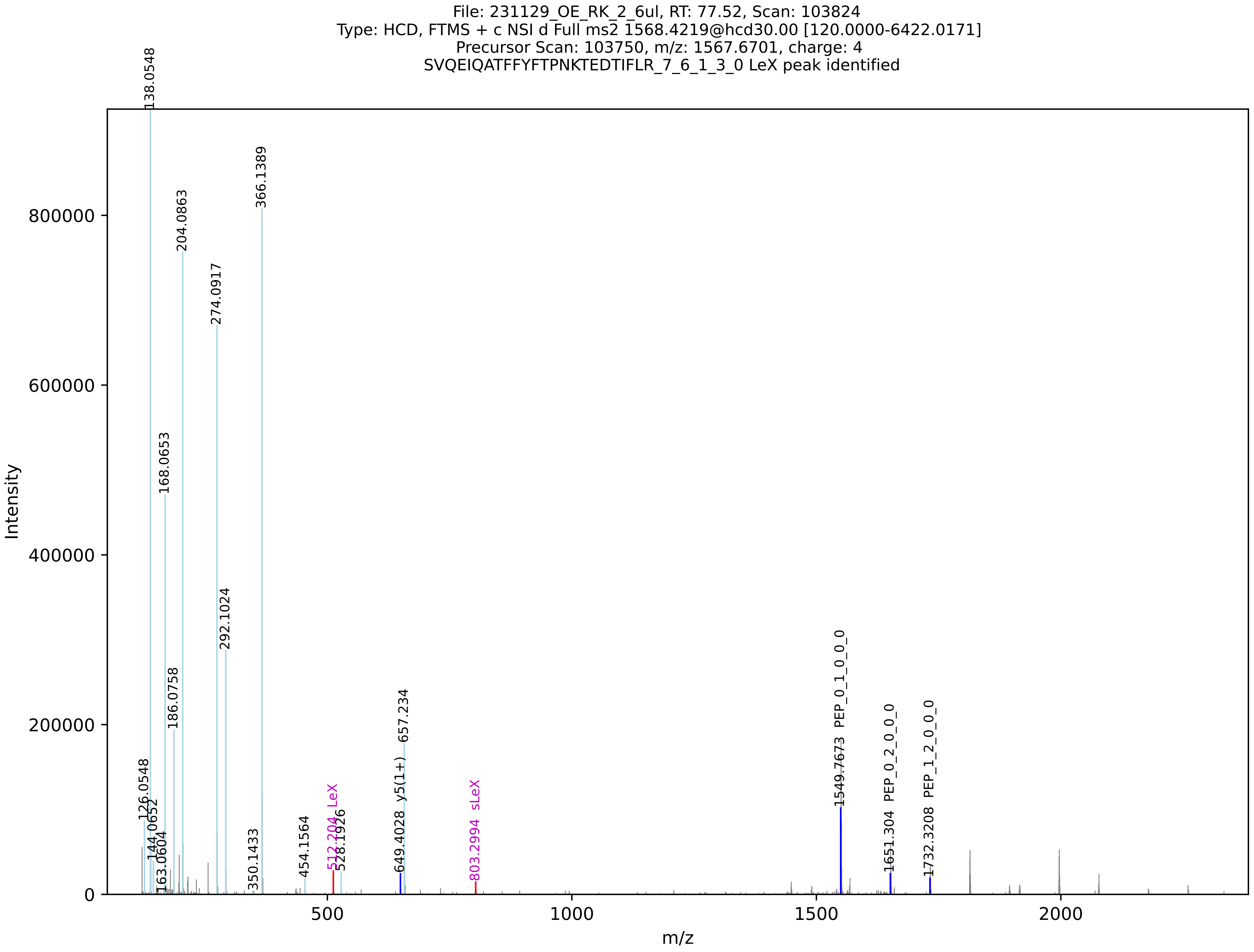
File: 231129_OE_RK_2_6ul, RT: 77.52, Scan: 103824
Type: HCD, FTMS + c NSI d Full ms2 1568.4219@hcd30.00 [120.0000-6422.0171]
Precursor Scan: 103750, m/z: 1567.6701, charge: 4
SVQEIQATFFYFTPNKTEDTIFLR_7_6_1_3_0 LeX peak identified
Intensity
m/z
138.0548
366.1389
204.0863
274.0917
168.0653
292.1024
186.0758
126.0548
144.0652
163.0604
350.1433
454.1564
512.204 LeX
528.1926
649.4028 y5(1+)
657.234
803.2994 sLeX
1549.7673 PEP_0_1_0_0_0
1651.304 PEP_0_2_0_0_0
1732.3208 PEP_1_2_0_0_0
0
200000
400000
600000
800000
500
1000
1500
2000

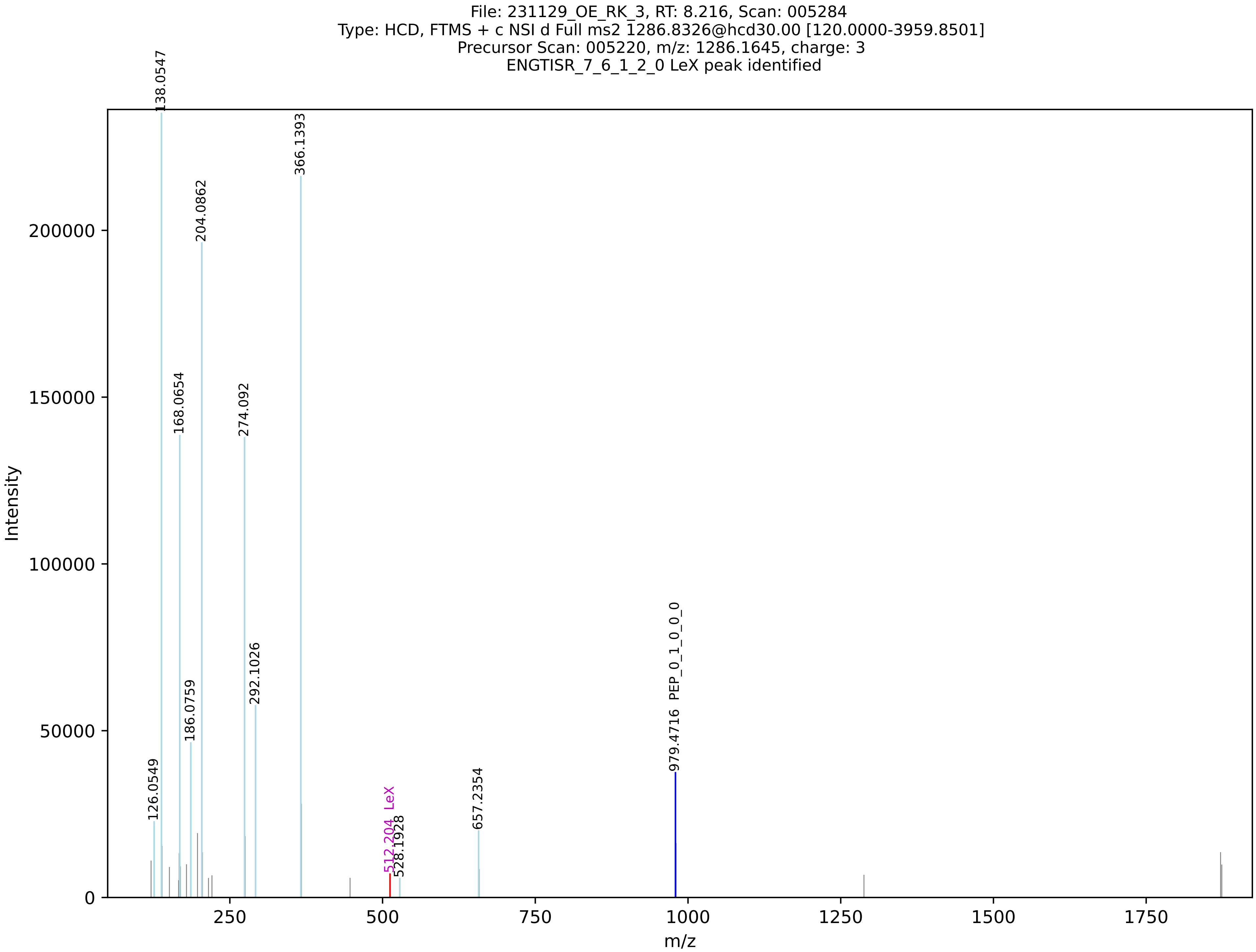
File: 231129_OE_RK_3, RT: 8.216, Scan: 005284
Type: HCD, FTMS + c NSI d Full ms2 1286.8326@hcd30.00 [120.0000-3959.8501]
Precursor Scan: 005220, m/z: 1286.1645, charge: 3
ENGTISR_7_6_1_2_0 LeX peak identified
138.0547
366.1393
204.0862
168.0654
274.092
292.1026
186.0759
126.0549
512.204 LeX
528.1928
657.2354
979.4716 PEP_0_1_0_0_0
Intensity
m/z
0
50000
100000
150000
200000
250
500
750
1000
1250
1500
1750

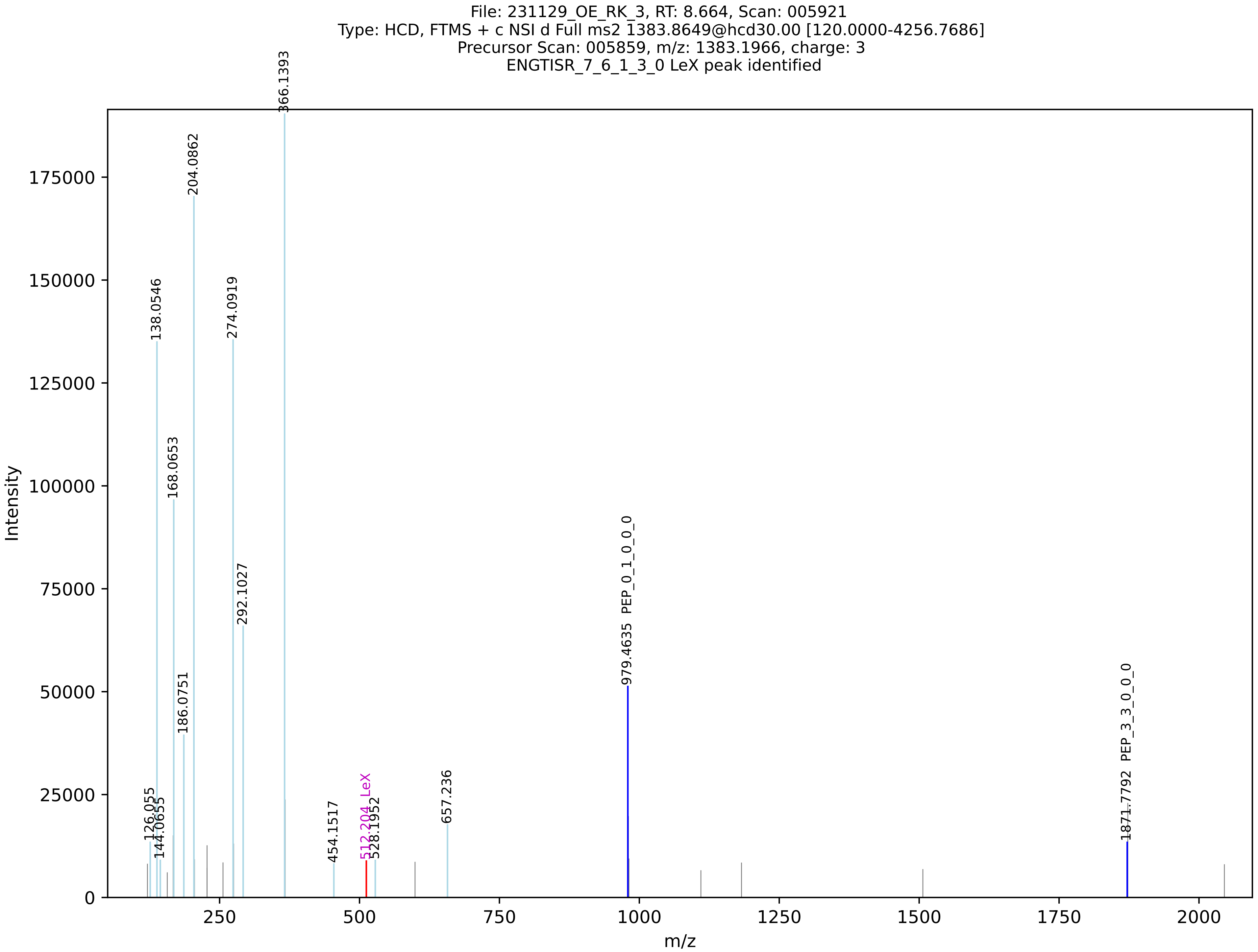
File: 231129_OE_RK_3, RT: 8.664, Scan: 005921
Type: HCD, FTMS + c NSI d Full ms2 1383.8649@hcd30.00 [120.0000-4256.7686]
Precursor Scan: 005859, m/z: 1383.1966, charge: 3
ENGTISR_7_6_1_3_0 LeX peak identified
Intensity
m/z
366.1393
204.0862
138.0546
274.0919
168.0653
292.1027
186.0751
126.055
144.0655
454.1517
512.204_LeX
528.1952
657.236
979.4635 PEP_0_1_0_0_0_
1871.7792 PEP_3_3_0_0_0_

File: 231129_OE_RK_3, RT: 10.602, Scan: 008694
Type: HCD, FTMS + c NSI d Full ms2 1384.2001@hcd30.00 [120.0000-4257.7939]
Precursor Scan: 008634, m/z: 1383.1977, charge: 3
ENGTISR_7_6_1_3_0 LeX peak identified

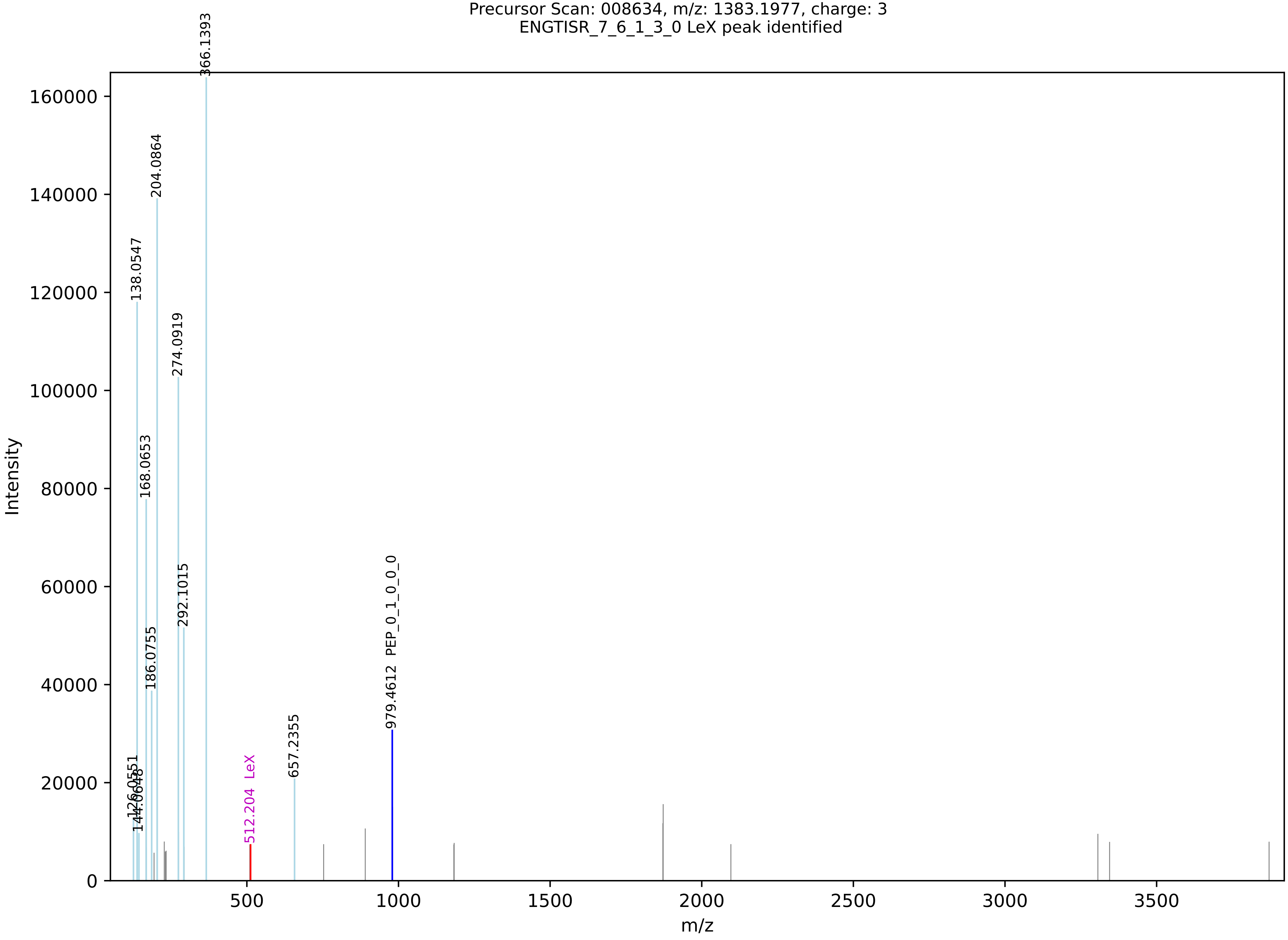

File: 231129_OE_RK_3, RT: 44.428, Scan: 057181
Type: HCD, FTMS + c NSI d Full ms2 1159.2211@hcd30.00 [120.0000-4752.4780]
Precursor Scan: 057095, m/z: 1158.7199, charge: 4
QDQCIYNTTYLNVQR_6_5_1_2_0 LeX peak identified

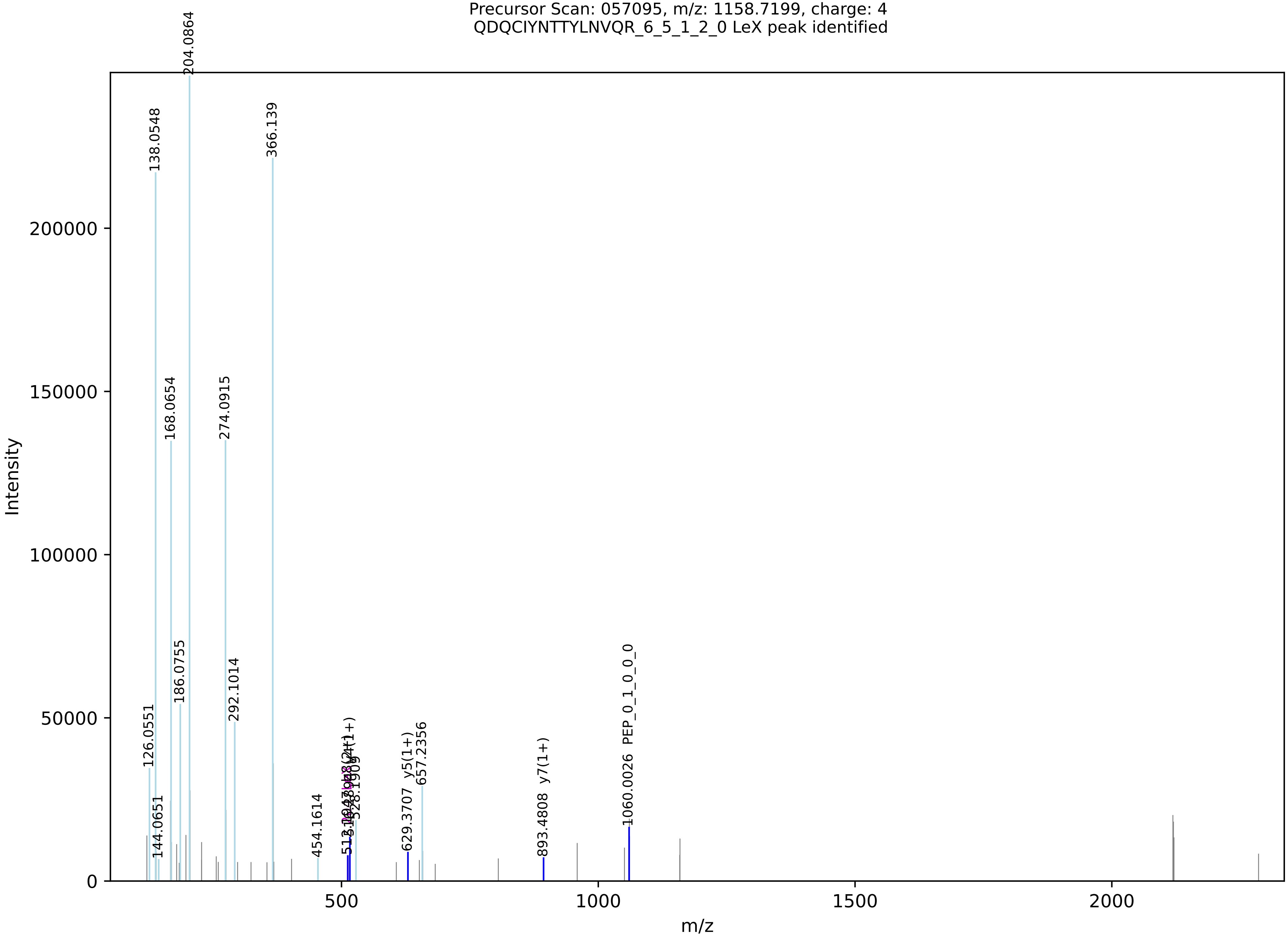

File: 231129_OE_RK_3, RT: 46.338, Scan: 059950
Type: HCD, FTMS + c NSI d Full ms2 1151.9799@hcd30.00 [120.0000-4722.9341]
Precursor Scan: 059873, m/z: 1151.2278, charge: 4
GLTFQQNASSMCVPDQDTAIR_5_5_1_1_0 LeX peak identified

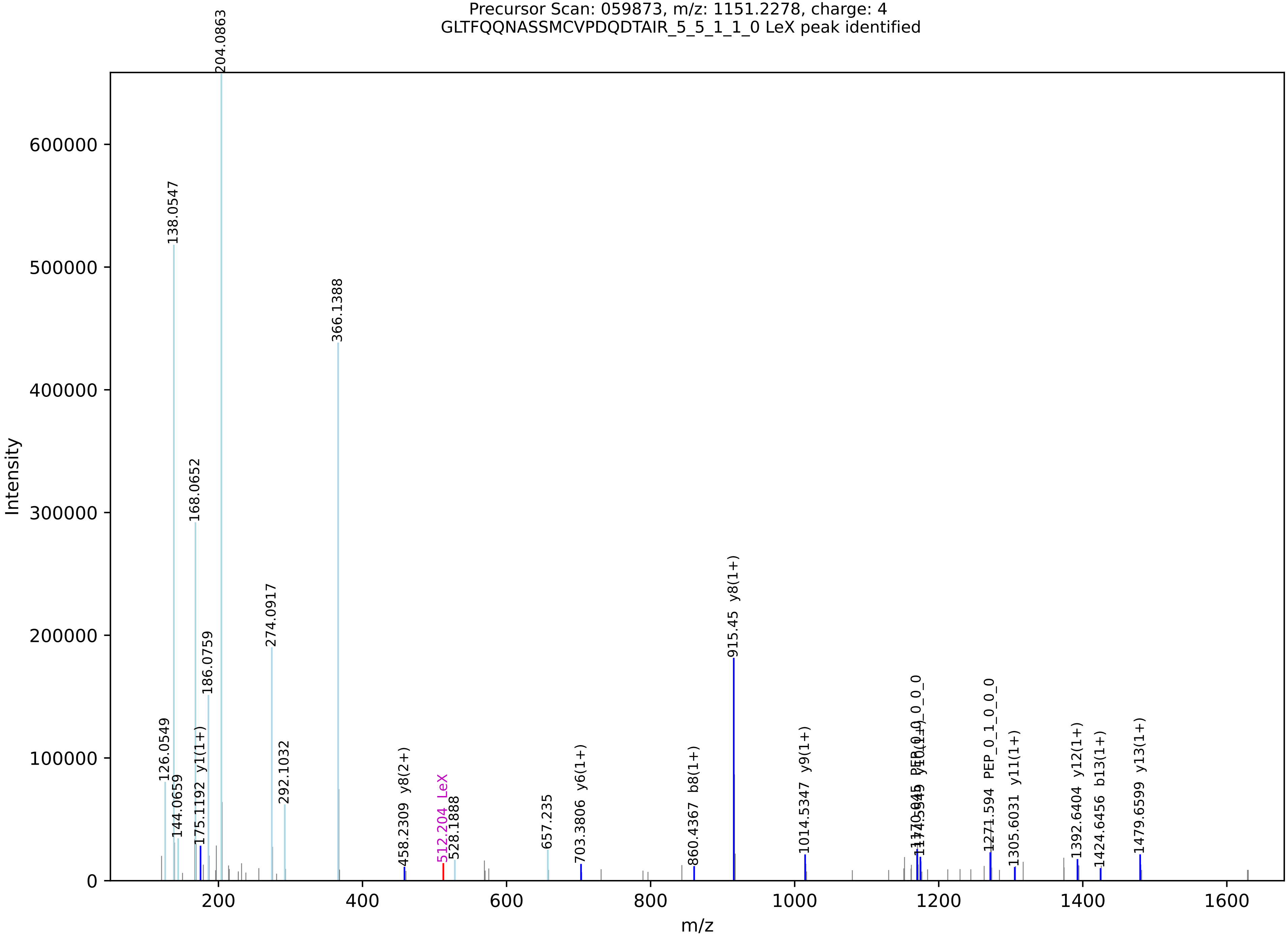

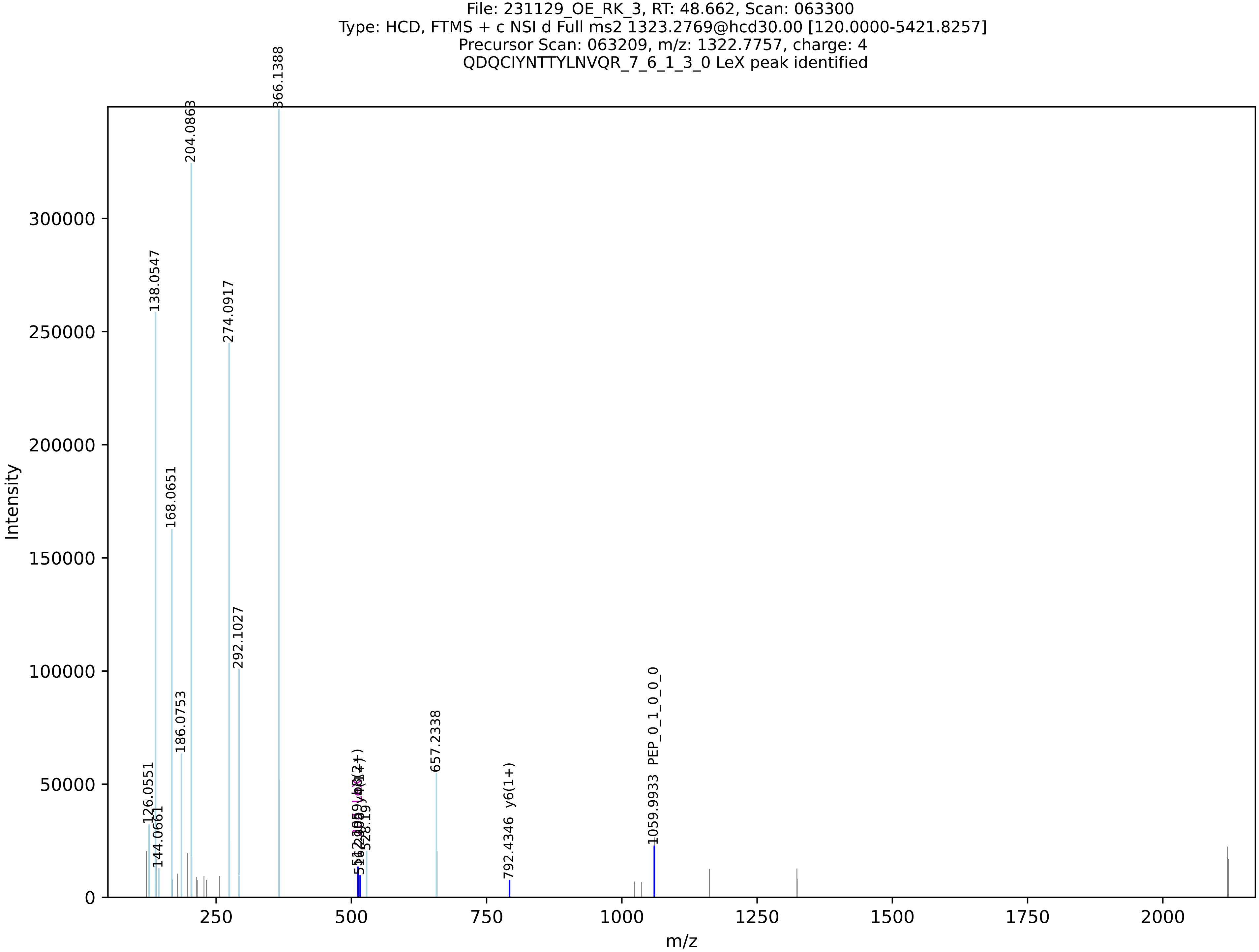
File: 231129_OE_RK_3, RT: 48.662, Scan: 063300
Type: HCD, FTMS + c NSI d Full ms2 1323.2769@hcd30.00 [120.0000-5421.8257]
Precursor Scan: 063209, m/z: 1322.7757, charge: 4
QDQCIYNTTYLNVQR_7_6_1_3_0 LeX peak identified
366.1388
204.0863
138.0547
274.0917
168.0651
292.1027
186.0753
126.0551
144.0661
657.2338
792.4346 y6(1+)
1059.9933 PEP_0_1_0_0_0_
Intensity
300000
250000
200000
150000
100000
50000
0
250
500
750
1000
1250
1500
1750
2000
m/z

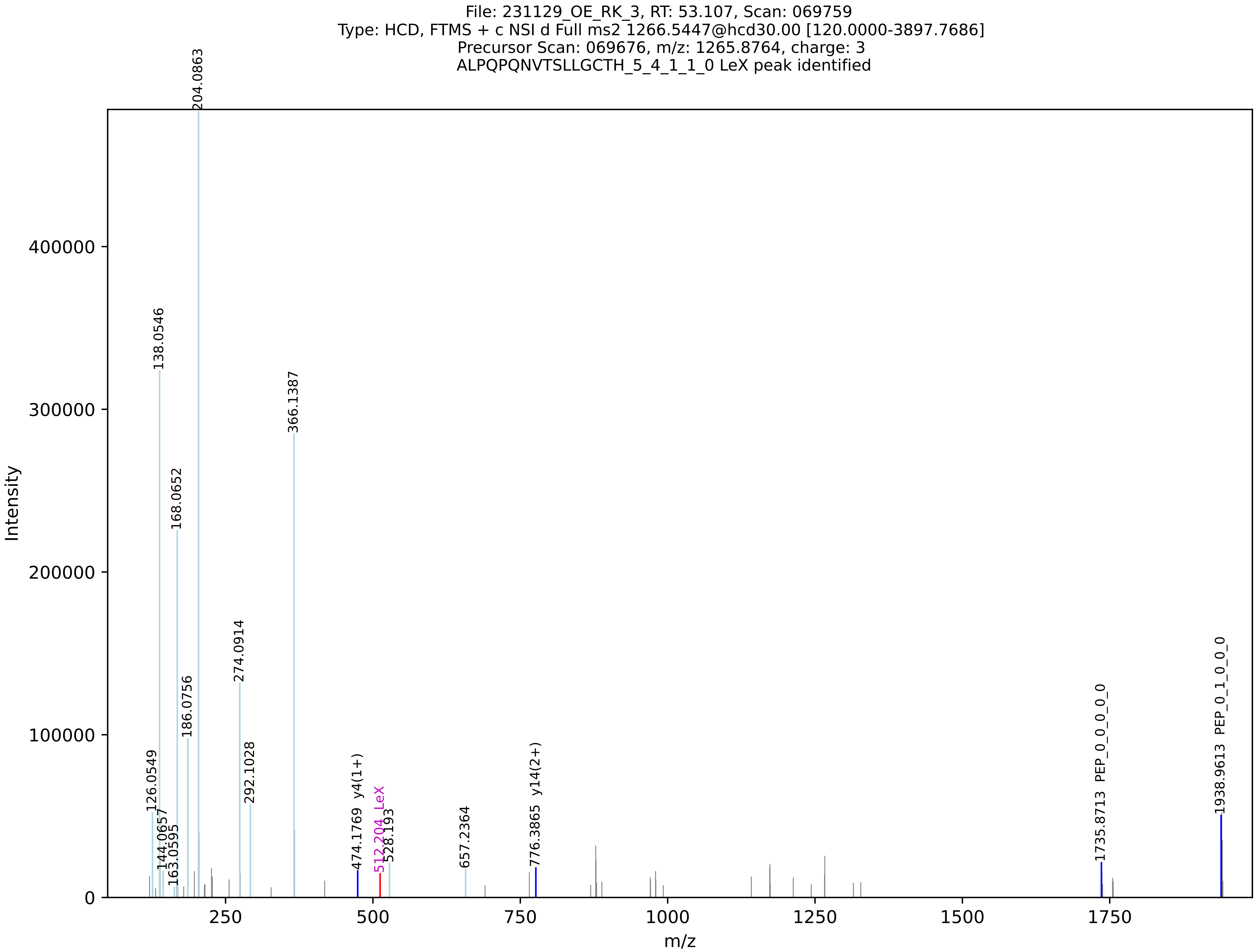

File: 231129_OE_RK_3, RT: 53.107, Scan: 069759
Type: HCD, FTMS + c NSI d Full ms2 1266.5447@hcd30.00 [120.0000-3897.7686]
Precursor Scan: 069676, m/z: 1265.8764, charge: 3
ALPQPQNVTSLLGCTH_5_4_1_1_0 LeX peak identified
Intensity
m/z
204.0863
138.0546
366.1387
168.0652
274.0914
186.0756
292.1028
126.0549
144.0657
163.0595
474.1769 y4(1+)
512.204_LeX
528.193
657.2364
776.3865 y14(2+)
1735.8713 PEP_0_0_0_0_0
1938.9613 PEP_0_1_0_0_0

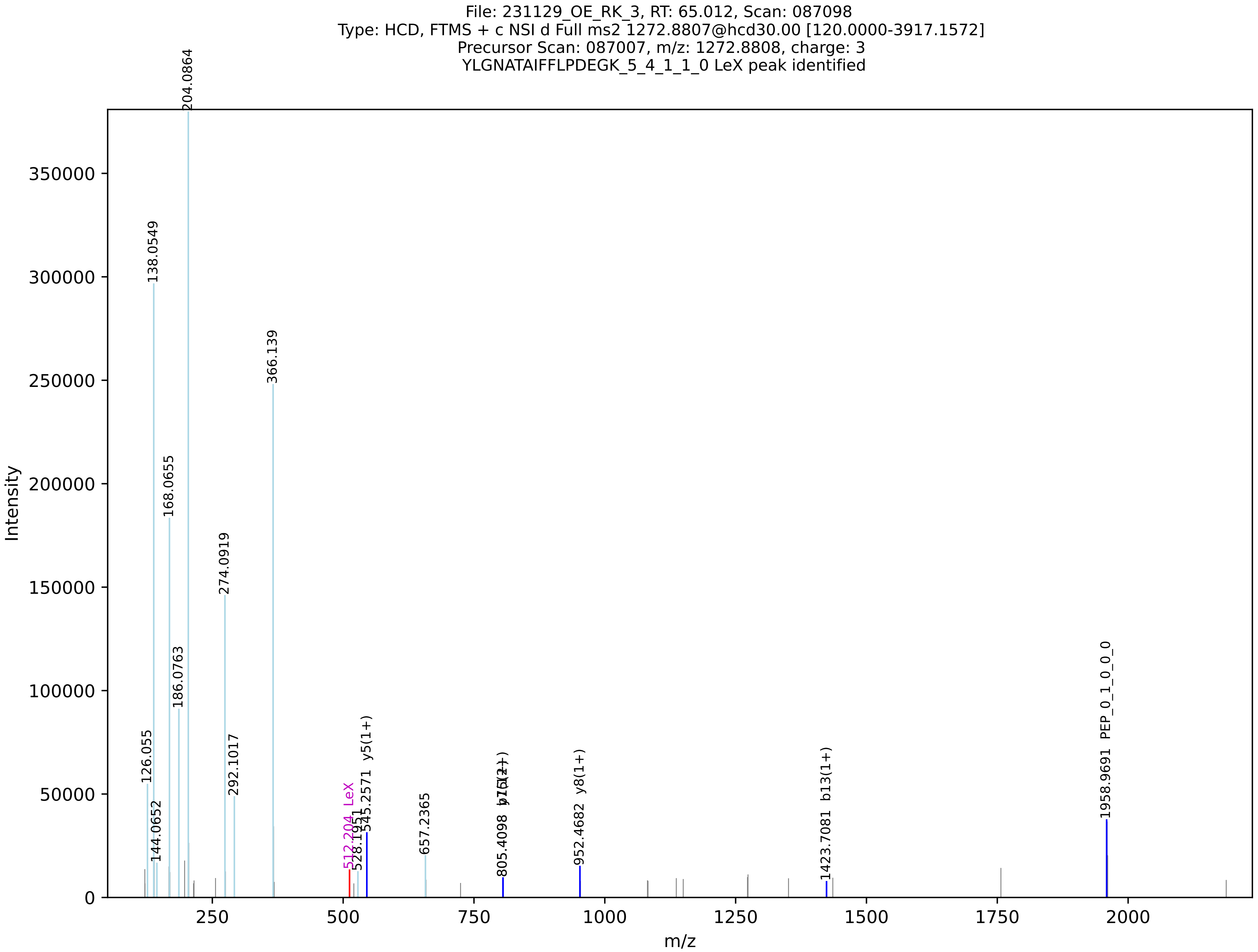
File: 231129_OE_RK_3, RT: 65.012, Scan: 087098
Type: HCD, FTMS + c NSI d Full ms2 1272.8807@hcd30.00 [120.0000-3917.1572]
Precursor Scan: 087007, m/z: 1272.8808, charge: 3
YLGNATAIFFLPDEGK_5_4_1_1_0 LeX peak identified
Intensity
0
50000
100000
150000
200000
250000
300000
350000
m/z
250
500
750
1000
1250
1500
1750
2000
204.0864
138.0549
366.139
168.0655
274.0919
186.0763
126.055
292.1017
144.0652
512.204 LeX
528.1951
545.2571 y5(1+)
657.2365
805.4098
952.4682 y8(1+)
1423.7081 b13(1+)
1958.9691 PEP_0_1_0_0_0_

File: 231129_OE_RK_3, RT: 65.268, Scan: 087472
Type: HCD, FTMS + c NSI d Full ms2 1273.5518@hcd30.00 [120.0000-3919.2102]
Precursor Scan: 087371, m/z: 1272.5494, charge: 3
YLGNATAIFFLPDEGK_5_4_1_1_0 LeX peak identified

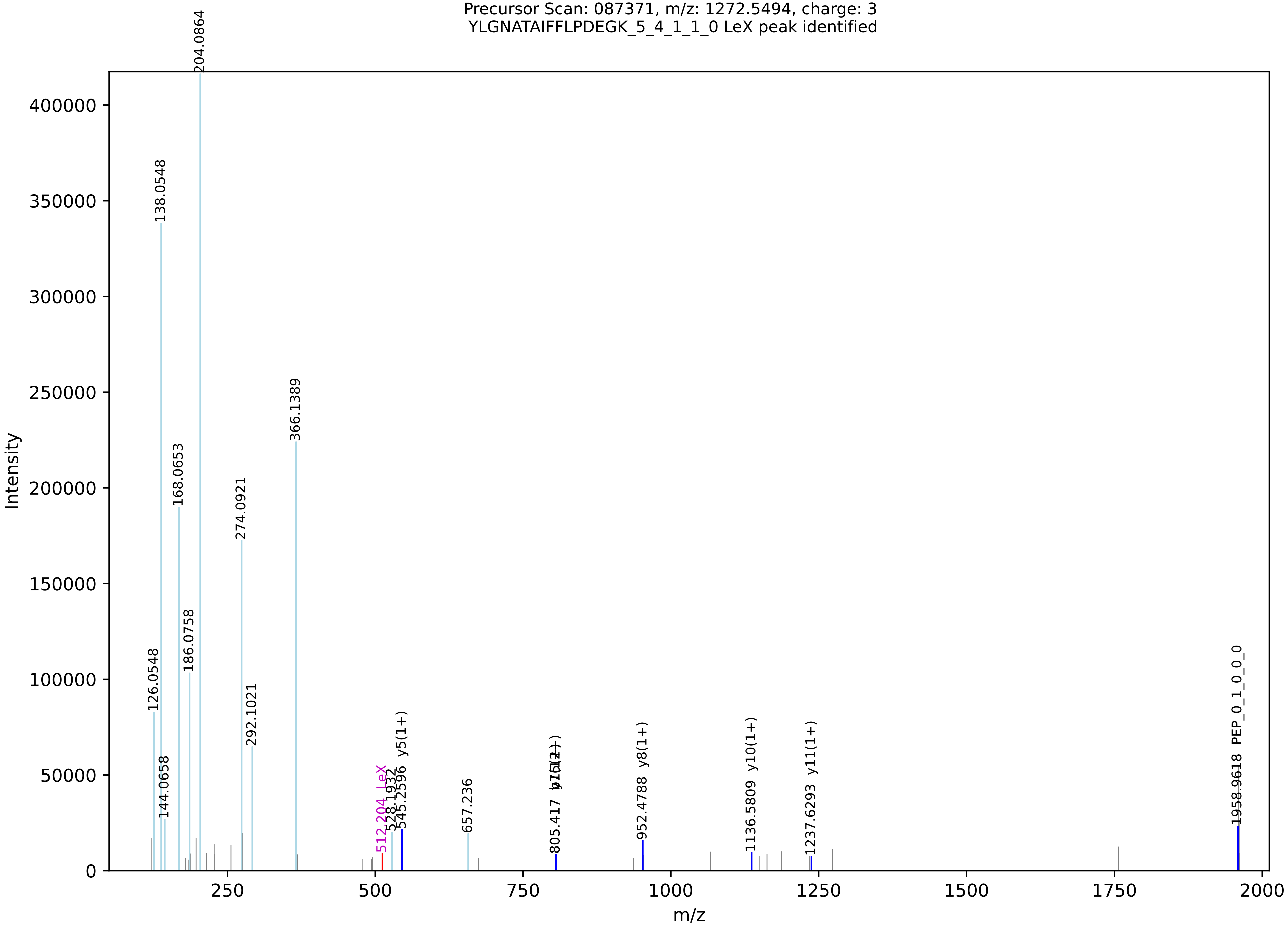

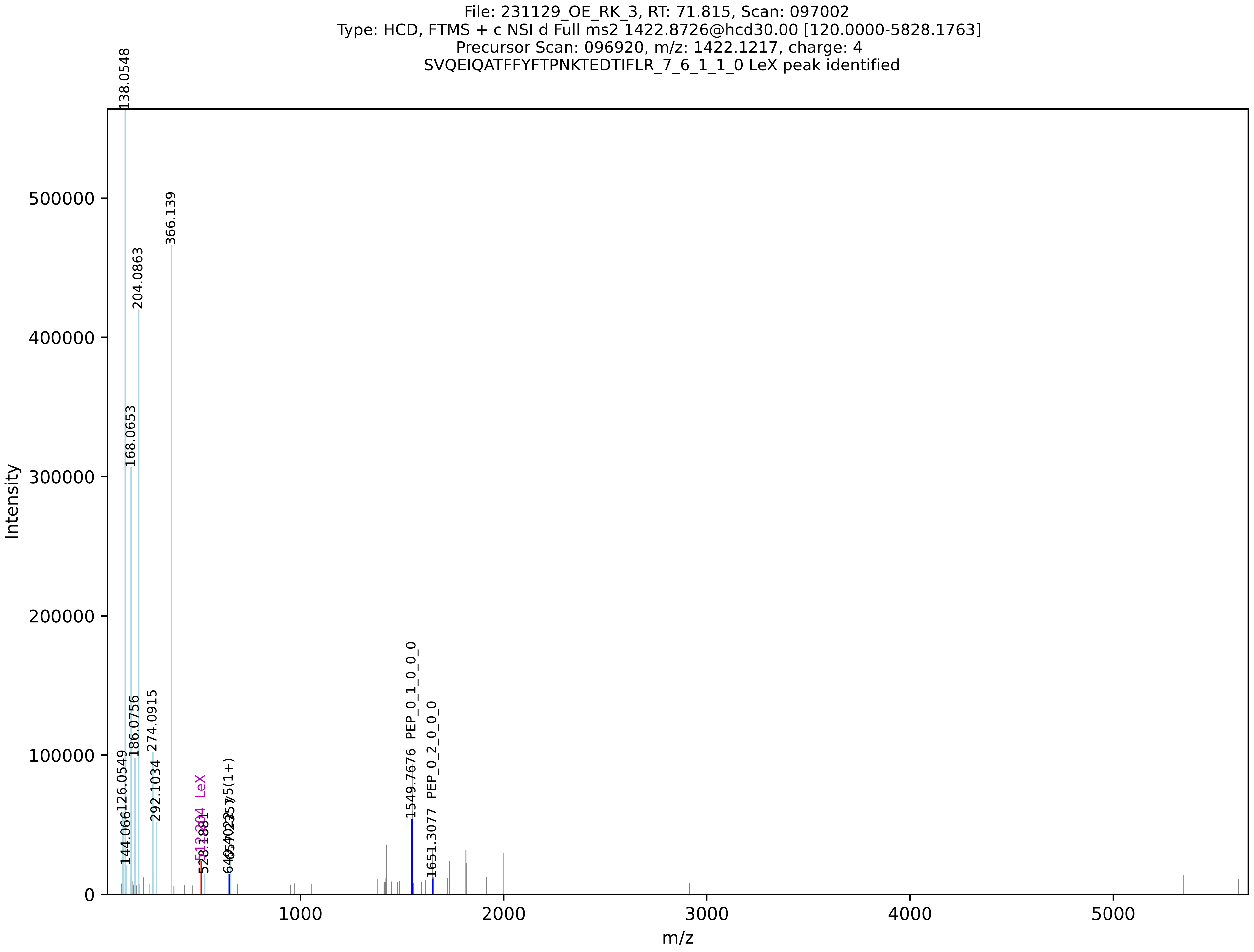
File: 231129_OE_RK_3, RT: 71.815, Scan: 097002
Type: HCD, FTMS + c NSI d Full ms2 1422.8726@hcd30.00 [120.0000-5828.1763]
Precursor Scan: 096920, m/z: 1422.1217, charge: 4
SVQEIQATFFYFTPNKTEDTIFLR_7_6_1_1_0 LeX peak identified
138.0548
366.139
204.0863
168.0653
186.0756
274.0915
126.0549
292.1034
144.066
LeX
528.1881
y5(1+)
1549.7676 PEP_0_1_0_0_0
1651.3077 PEP_0_2_0_0_0
Intensity
m/z
0
100000
200000
300000
400000
500000
1000
2000
3000
4000
5000

File: 231129_OE_RK_3, RT: 75.09, Scan: 101768
Type: HCD, FTMS + c NSI d Full ms2 1404.6152@hcd30.00 [120.0000-5753.6860]
Precursor Scan: 101692, m/z: 1403.6128, charge: 4
SVQEIQATFFYFTPNKTEDTIFLR_6_5_1_2_0 LeX peak identified

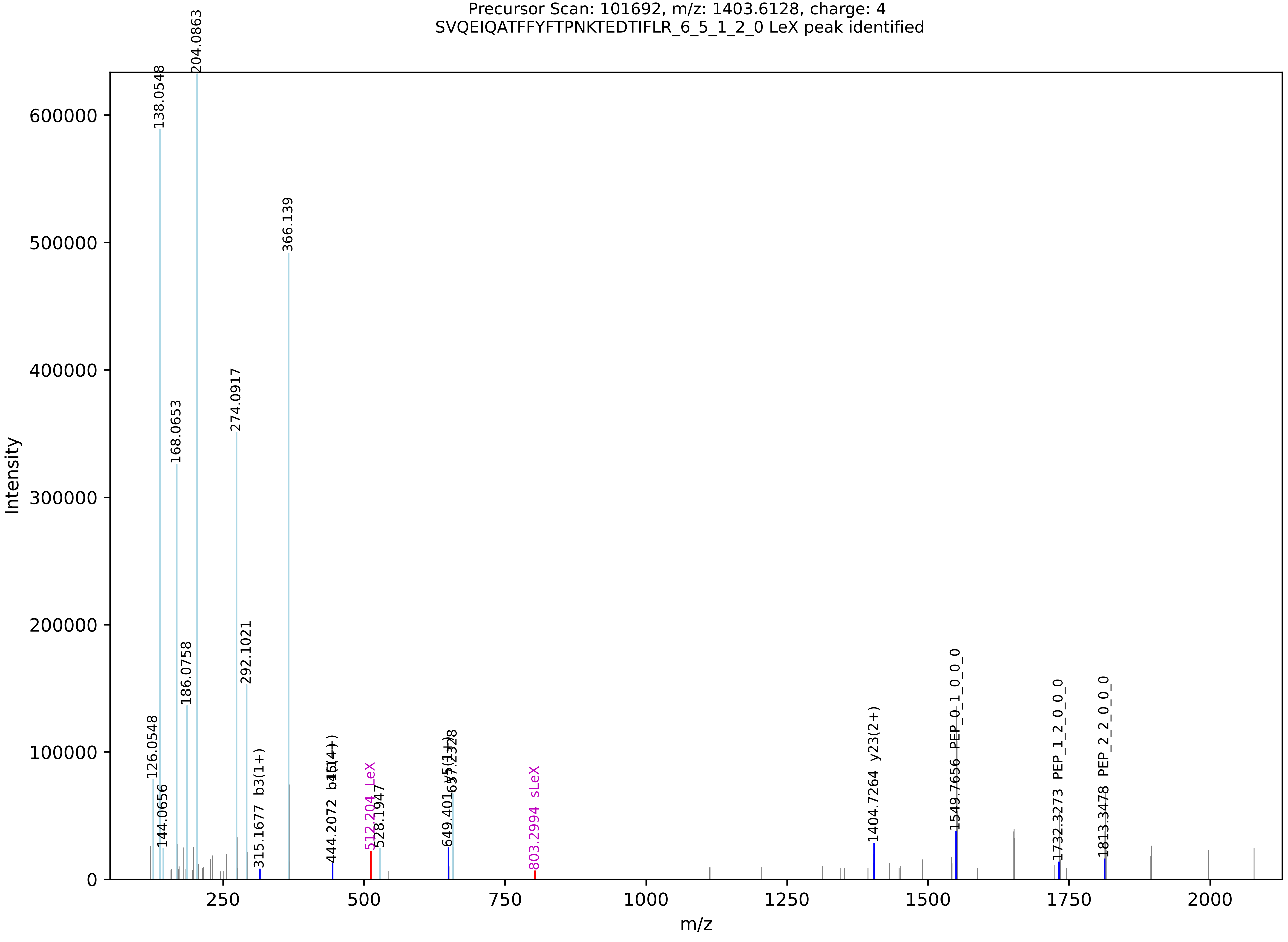

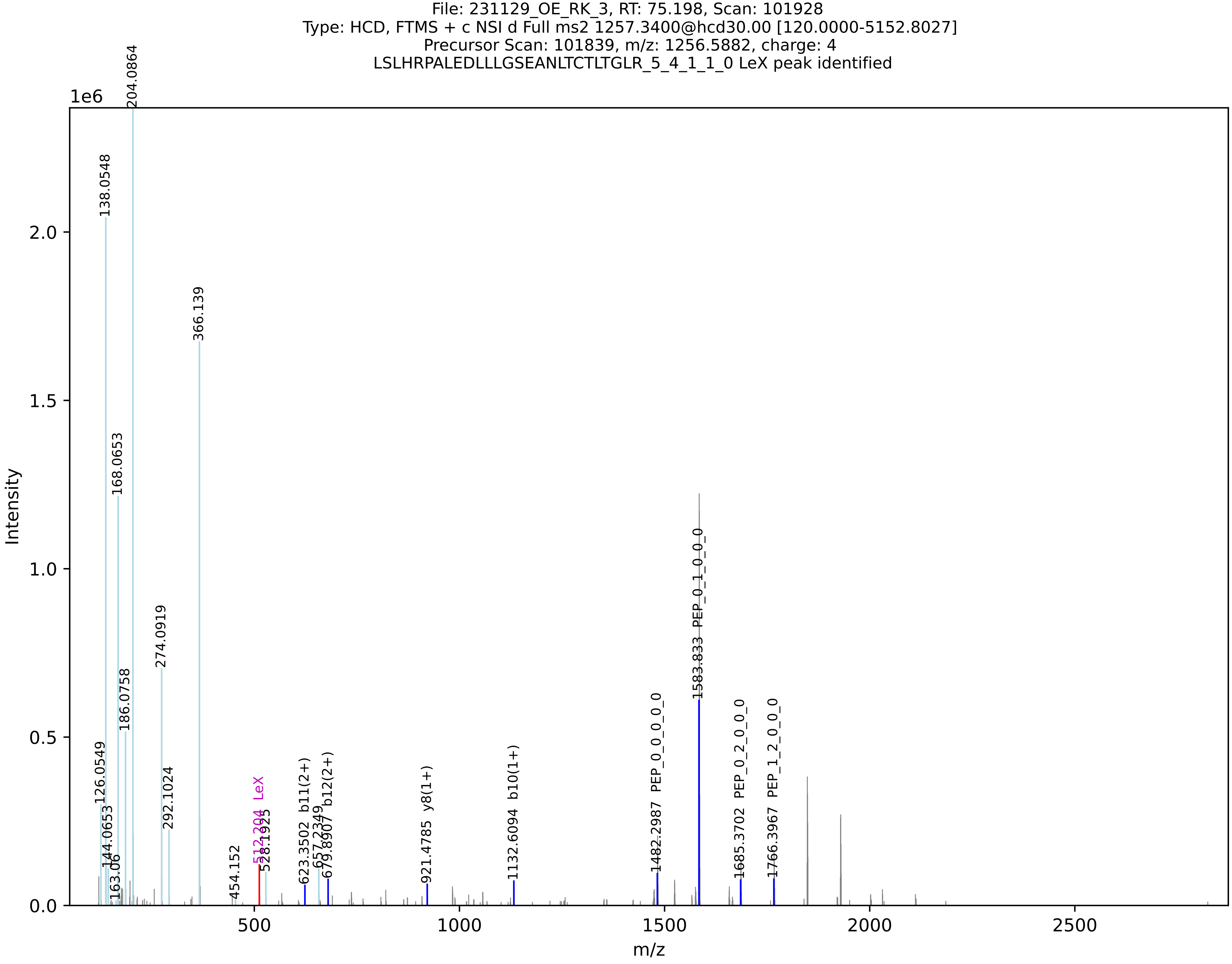

File: 231129_OE_RK_3, RT: 75.198, Scan: 101928
Type: HCD, FTMS + c NSI d Full ms2 1257.3400@hcd30.00 [120.0000-5152.8027]
Precursor Scan: 101839, m/z: 1256.5882, charge: 4
LSLHRPALEDLLLGSEANLTCTLTGLR_5_4_1_1_0 LeX peak identified
1e6
Intensity
m/z
204.0864
138.0548
366.139
168.0653
274.0919
186.0758
126.0549
292.1024
144.0653
163.06
454.152
512.204 LeX
528.1925
623.3502 b11(2+)
657.2349
679.8907 b12(2+)
921.4785 y8(1+)
1132.6094 b10(1+)
1482.2987 PEP_0_0_0_0_0
1583.833 PEP_0_1_0_0_0
1685.3702 PEP_0_2_0_0_0
1766.3967 PEP_1_2_0_0_0

File: 231129_OE_RK_3, RT: 79.446, Scan: 108122
Type: HCD, FTMS + c NSI d Full ms2 1568.6746@hcd30.00 [120.0000-6423.0483]
Precursor Scan: 108039, m/z: 1567.6722, charge: 4
SVQEIQATFFYFTPNKTEDTIFLR_9_8_0_1_0 LeX peak identified

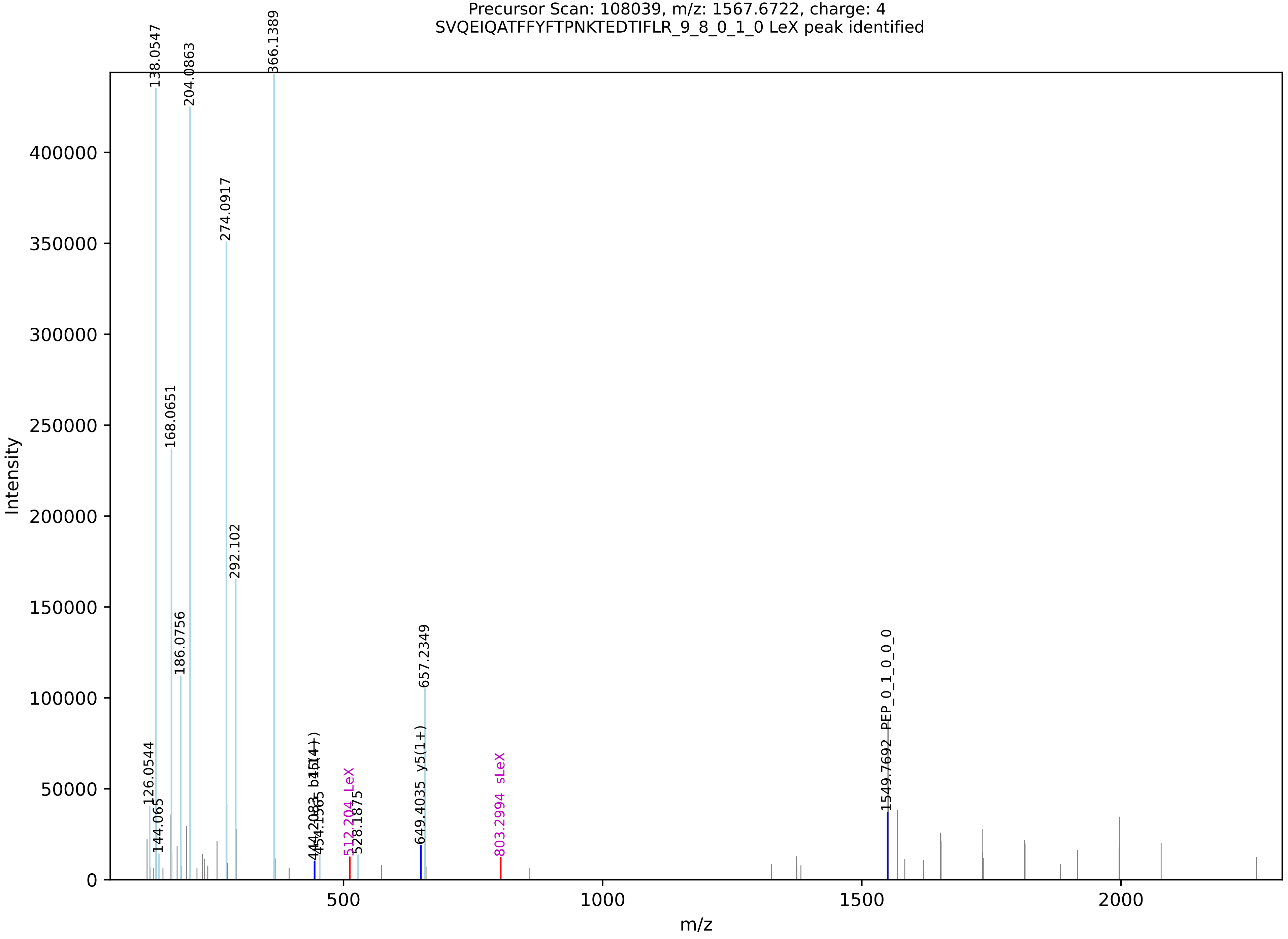

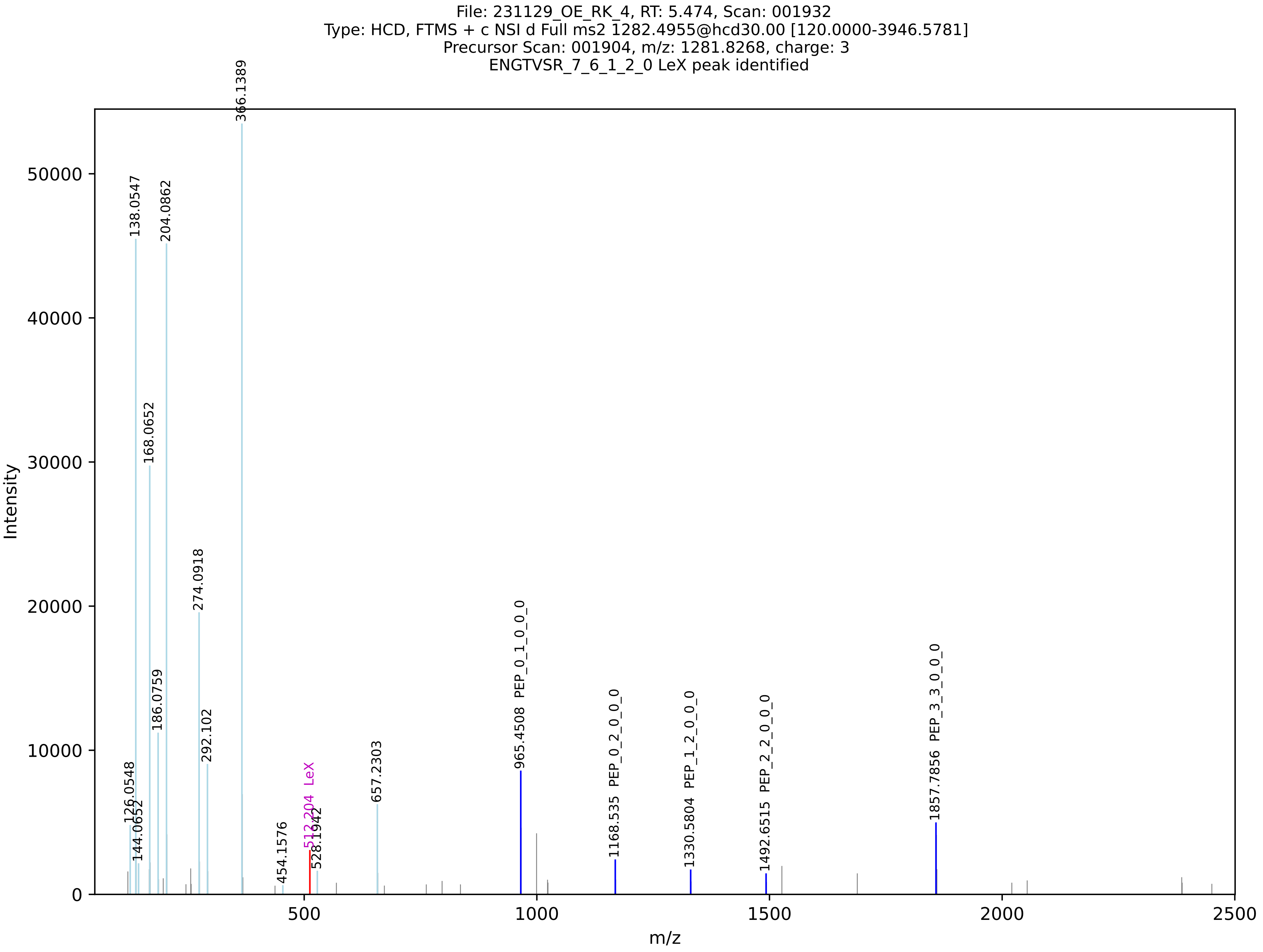
File: 231129_OE_RK_4, RT: 5.474, Scan: 001932
Type: HCD, FTMS + c NSI d Full ms2 1282.4955@hcd30.00 [120.0000-3946.5781]
Precursor Scan: 001904, m/z: 1281.8268, charge: 3
ENGTVSR_7_6_1_2_0 LeX peak identified
366.1389
138.0547
204.0862
168.0652
274.0918
186.0759
292.102
126.0548
144.0652
454.1576
512.204 LeX
528.1942
657.2303
965.4508 PEP_0_1_0_0_0
1168.535 PEP_0_2_0_0_0
1330.5804 PEP_1_2_0_0_0
1492.6515 PEP_2_2_0_0_0
1857.7856 PEP_3_3_0_0_0
Intensity
m/z
0
10000
20000
30000
40000
50000
500
1000
1500
2000
2500

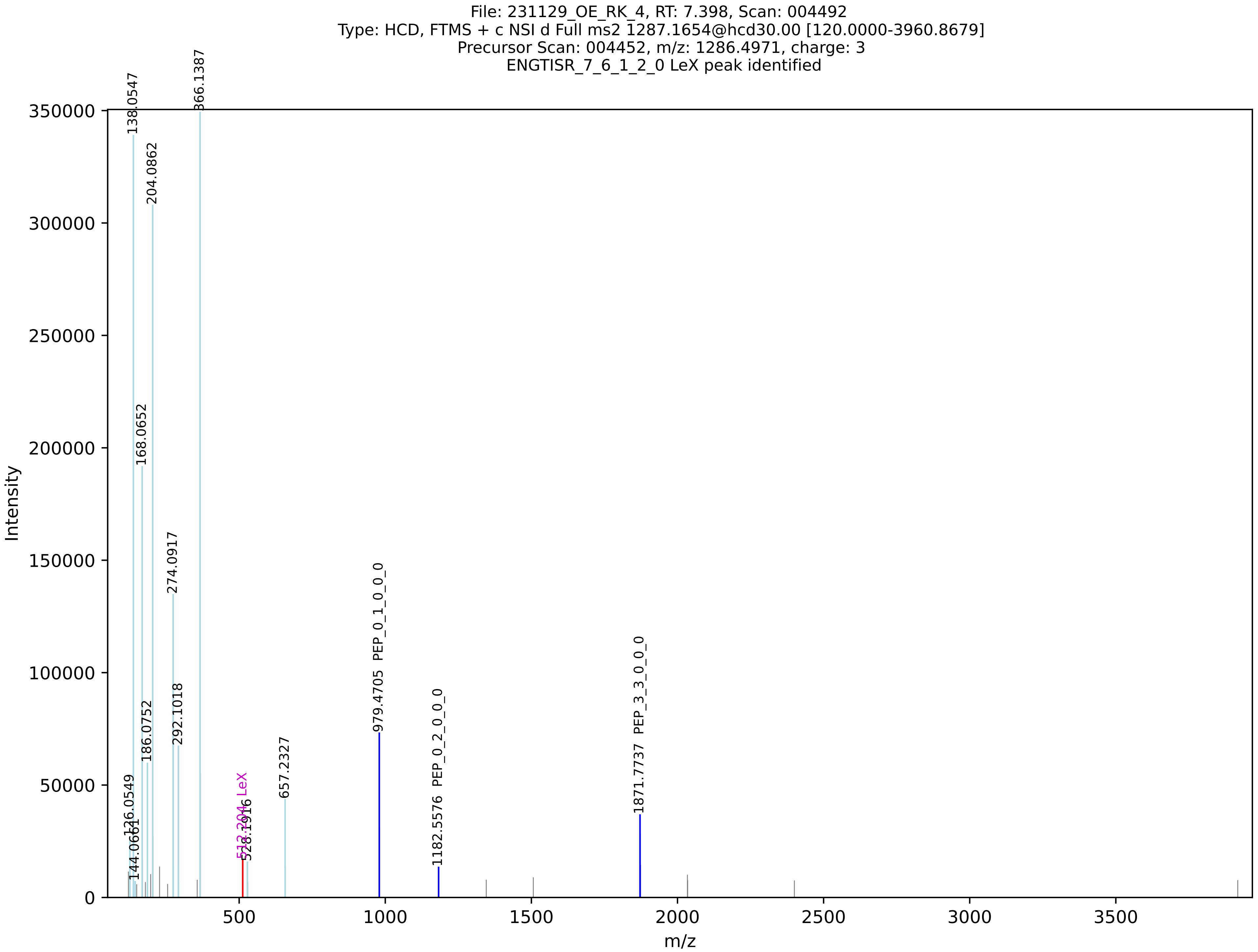
File: 231129_OE_RK_4, RT: 7.398, Scan: 004492
Type: HCD, FTMS + c NSI d Full ms2 1287.1654@hcd30.00 [120.0000-3960.8679]
Precursor Scan: 004452, m/z: 1286.4971, charge: 3
ENGTISR_7_6_1_2_0 LeX peak identified
Intensity
m/z
138.0547
366.1387
204.0862
168.0652
274.0917
186.0752
292.1018
126.0549
144.0661
512.204 LeX
528.1916
657.2327
979.4705 PEP_0_1_0_0_0
1182.5576 PEP_0_2_0_0_0
1871.7737 PEP_3_3_0_0_0

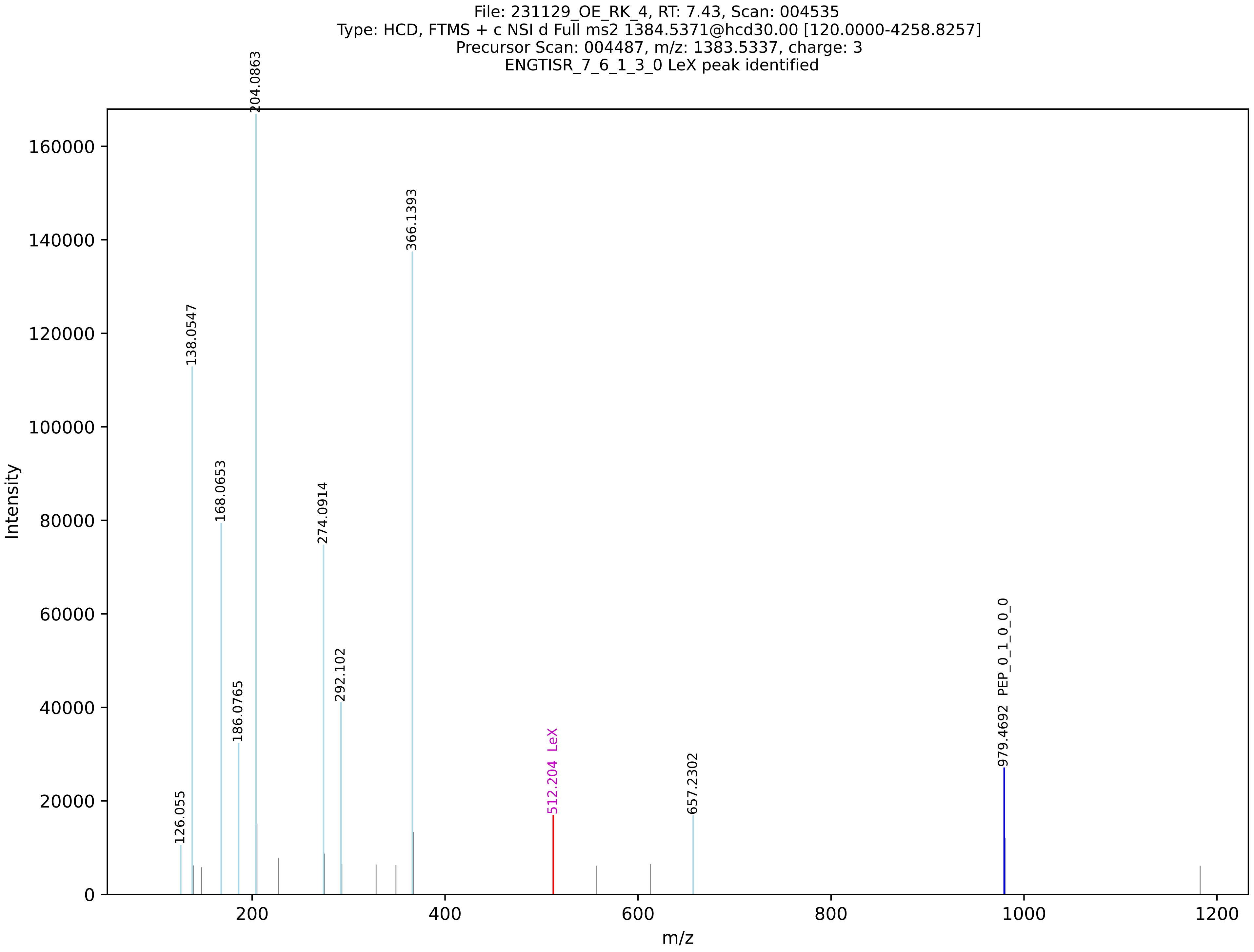
File: 231129_OE_RK_4, RT: 7.43, Scan: 004535
Type: HCD, FTMS + c NSI d Full ms2 1384.5371@hcd30.00 [120.0000-4258.8257]
Precursor Scan: 004487, m/z: 1383.5337, charge: 3
ENGTISR_7_6_1_3_0 LeX peak identified
Intensity
m/z
204.0863
366.1393
138.0547
168.0653
274.0914
292.102
186.0765
126.055
512.204 LeX
657.2302
979.4692 PEP_0_1_0_0_
0
20000
40000
60000
80000
100000
120000
140000
160000
200
400
600
800
1000
1200

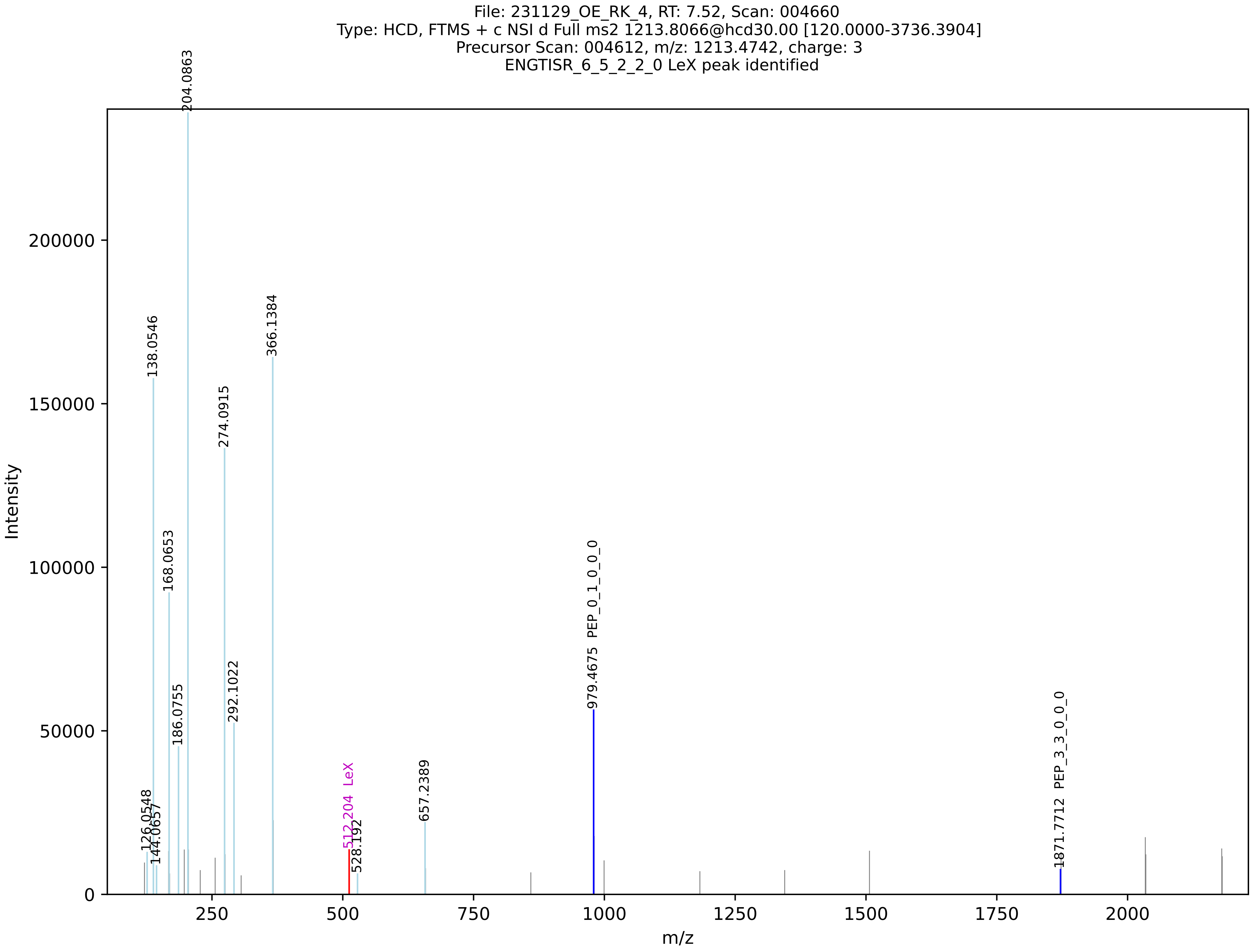

File: 231129_OE_RK_4, RT: 7.52, Scan: 004660
Type: HCD, FTMS + c NSI d Full ms2 1213.8066@hcd30.00 [120.0000-3736.3904]
Precursor Scan: 004612, m/z: 1213.4742, charge: 3
ENGTISR_6_5_2_2_0 LeX peak identified
Intensity
m/z
204.0863
138.0546
366.1384
274.0915
168.0653
186.0755
292.1022
126.0548
144.0657
512.204 LeX
528.192
657.2389
979.4675 PEP_0_1_0_0_0
1871.7712 PEP_3_3_0_0_0

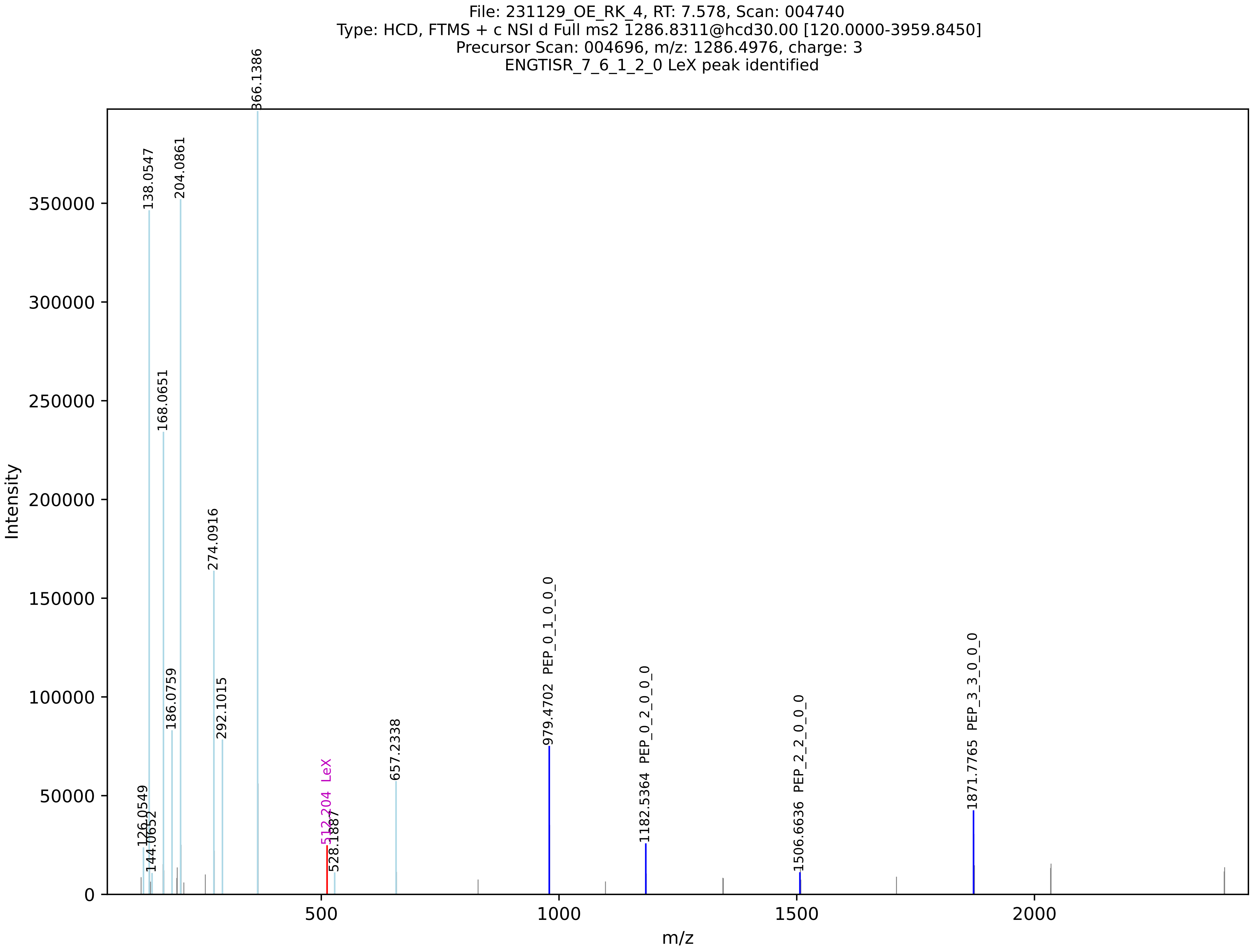

File: 231129_OE_RK_4, RT: 7.578, Scan: 004740
Type: HCD, FTMS + c NSI d Full ms2 1286.8311@hcd30.00 [120.0000-3959.8450]
Precursor Scan: 004696, m/z: 1286.4976, charge: 3
ENGTISR_7_6_1_2_0 LeX peak identified
366.1386
138.0547
204.0861
168.0651
274.0916
186.0759
292.1015
126.0549
144.0652
512.204 LeX
528.1887
657.2338
979.4702 PEP_0_1_0_0_0
1182.5364 PEP_0_2_0_0_0
1506.6636 PEP_2_2_0_0_0
1871.7765 PEP_3_3_0_0_0
Intensity
m/z
0
50000
100000
150000
200000
250000
300000
350000
500
1000
1500
2000

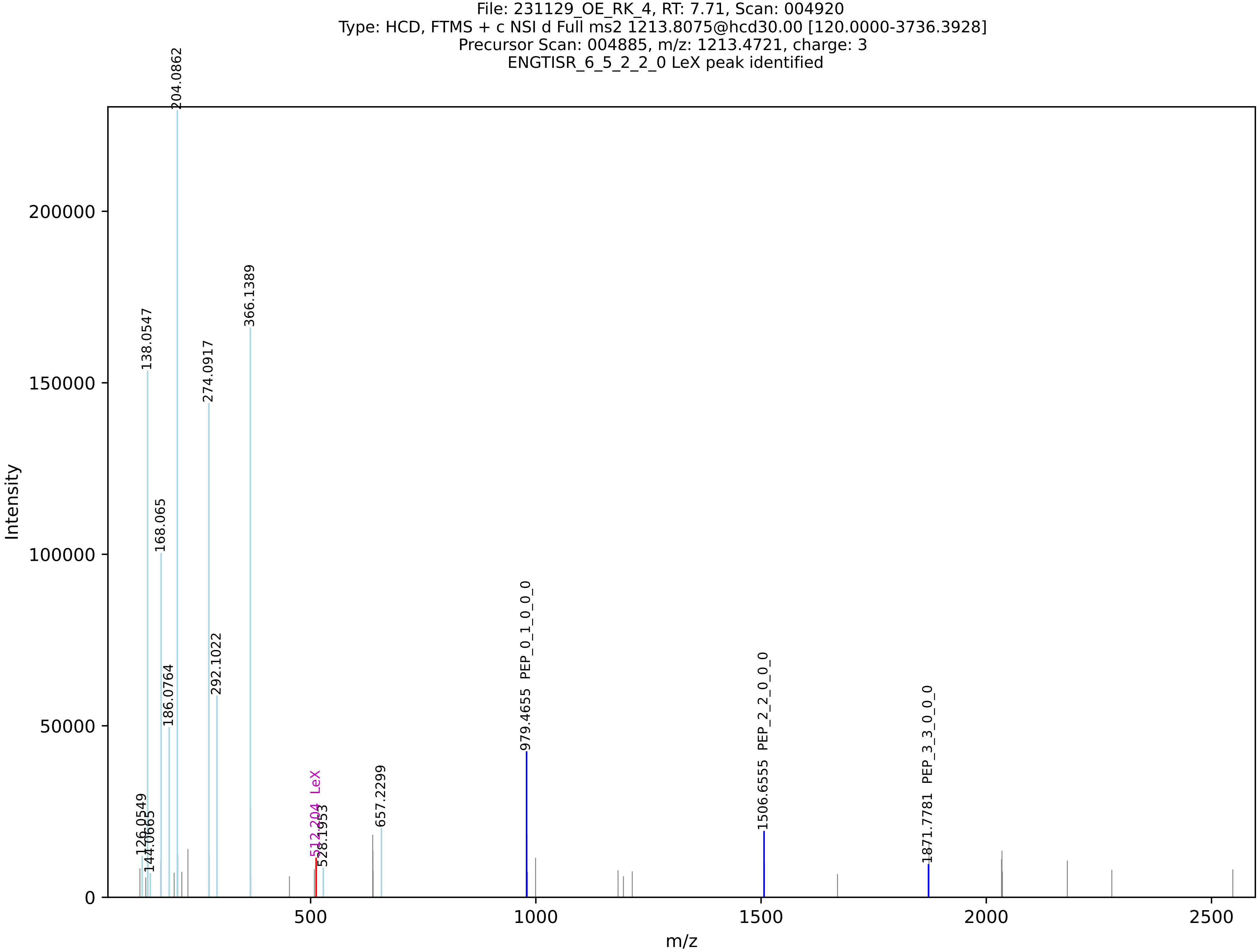
File: 231129_OE_RK_4, RT: 7.71, Scan: 004920
Type: HCD, FTMS + c NSI d Full ms2 1213.8075@hcd30.00 [120.0000-3736.3928]
Precursor Scan: 004885, m/z: 1213.4721, charge: 3
ENGTISR_6_5_2_2_0 LeX peak identified
Intensity
m/z
204.0862
366.1389
138.0547
274.0917
168.065
292.1022
186.0764
126.0549
144.0665
512.204 LeX
528.1953
657.2299
979.4655 PEP_0_1_0_0_0
1506.6555 PEP_2_2_0_0_0
1871.7781 PEP_3_3_0_0_0
0
50000
100000
150000
200000
500
1000
1500
2000
2500

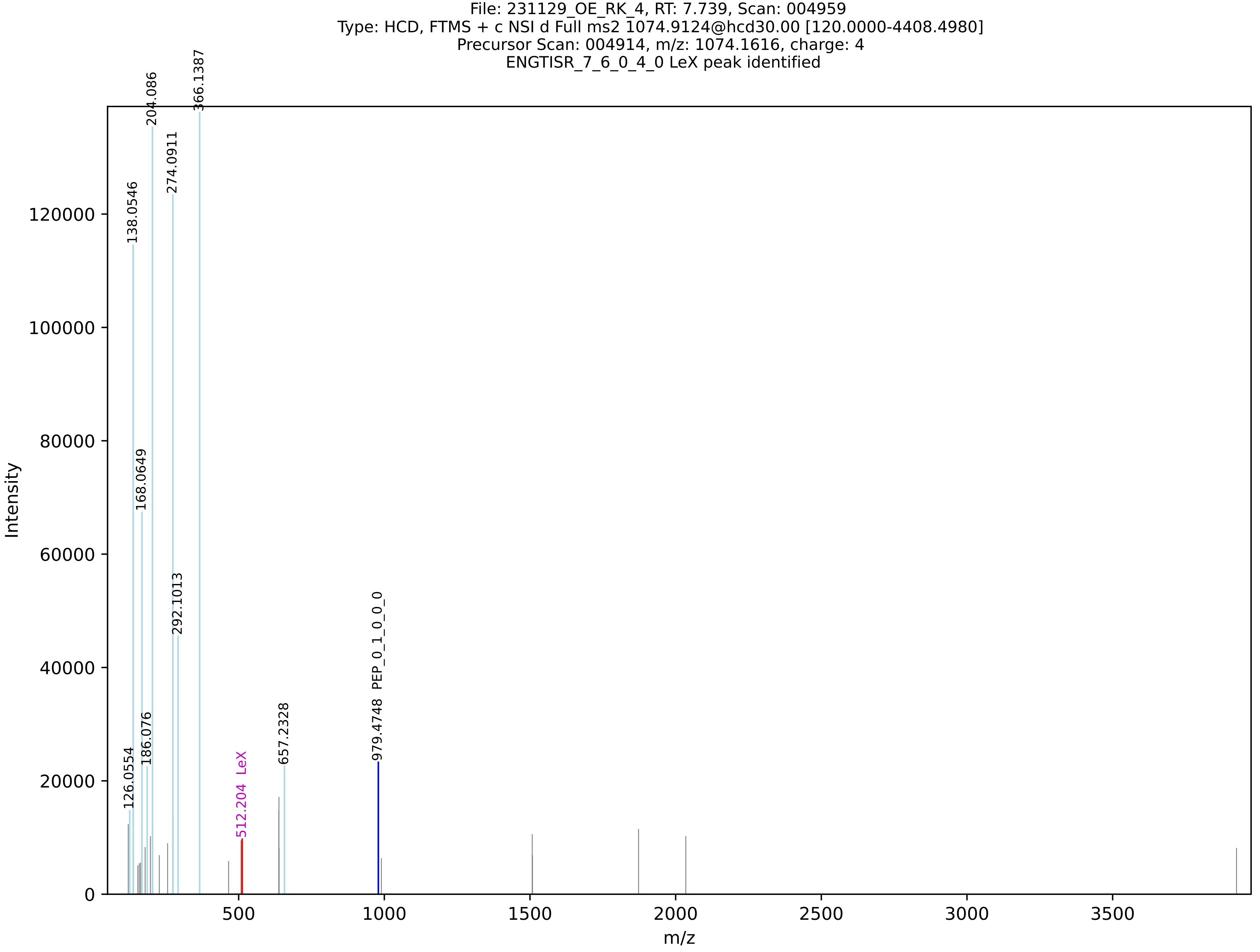
File: 231129_OE_RK_4, RT: 7.739, Scan: 004959
Type: HCD, FTMS + c NSI d Full ms2 1074.9124@hcd30.00 [120.0000-4408.4980]
Precursor Scan: 004914, m/z: 1074.1616, charge: 4
ENGTISR_7_6_0_4_0 LeX peak identified
Intensity
m/z
366.1387
204.086
274.0911
138.0546
168.0649
292.1013
979.4748 PEP_0_1_0_0_0_
657.2328
186.076
126.0554
512.204 LeX
0
20000
40000
60000
80000
100000
120000
500
1000
1500
2000
2500
3000
3500

File: 231129_OE_RK_4, RT: 7.926, Scan: 005225
Type: HCD, FTMS + c NSI d Full ms2 1074.6630@hcd30.00 [120.0000-4407.4810]
Precursor Scan: 005152, m/z: 1074.4133, charge: 4
ENGTISR_7_6_2_3_0 LeX peak identified

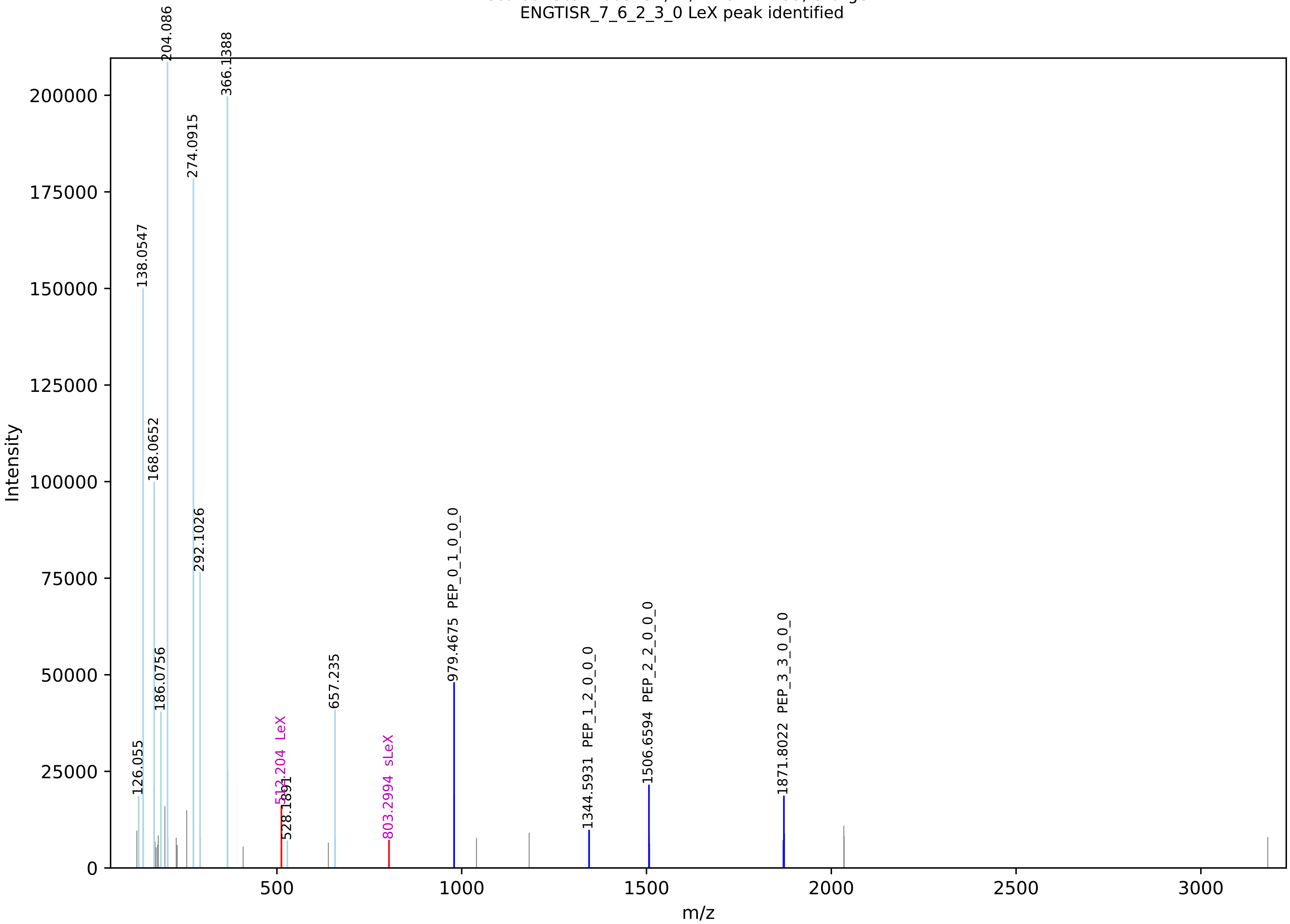

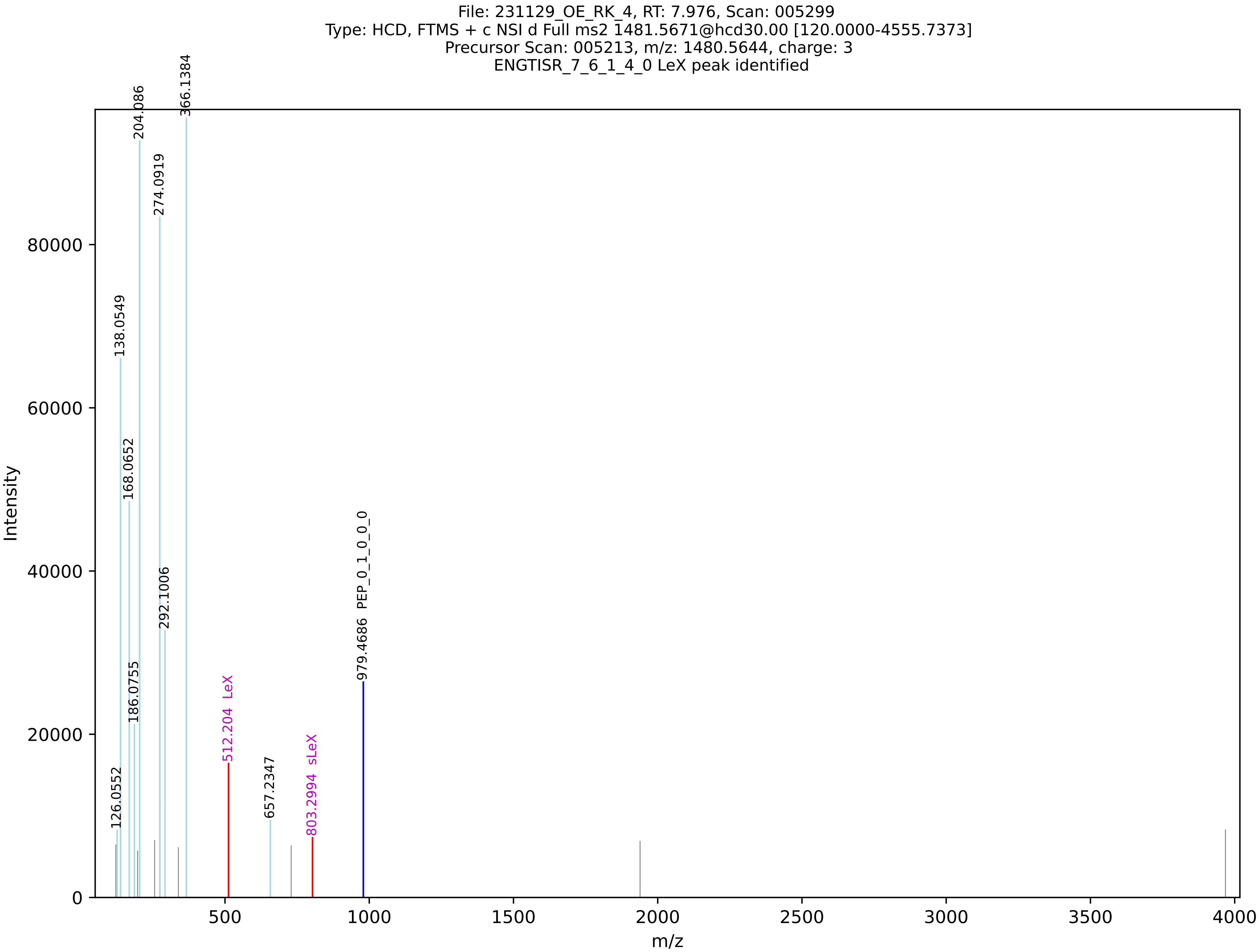
File: 231129_OE_RK_4, RT: 7.976, Scan: 005299
Type: HCD, FTMS + c NSI d Full ms2 1481.5671@hcd30.00 [120.0000-4555.7373]
Precursor Scan: 005213, m/z: 1480.5644, charge: 3
ENGTISR_7_6_1_4_0 LeX peak identified
Intensity
m/z
0
20000
40000
60000
80000
500
1000
1500
2000
2500
3000
3500
4000
366.1384
204.086
274.0919
138.0549
168.0652
292.1006
186.0755
126.0552
512.204 LeX
657.2347
803.2994 sLeX
979.4686 PEP_0_1_0_0_0

File: 231129_OE_RK_4, RT: 7.979, Scan: 005304
Type: HCD, FTMS + c NSI d Full ms2 1170.7942@hcd30.00 [120.0000-3604.7722]
Precursor Scan: 005213, m/z: 1170.1259, charge: 3
ENGTISR_7_5_2_1_0 LeX peak identified

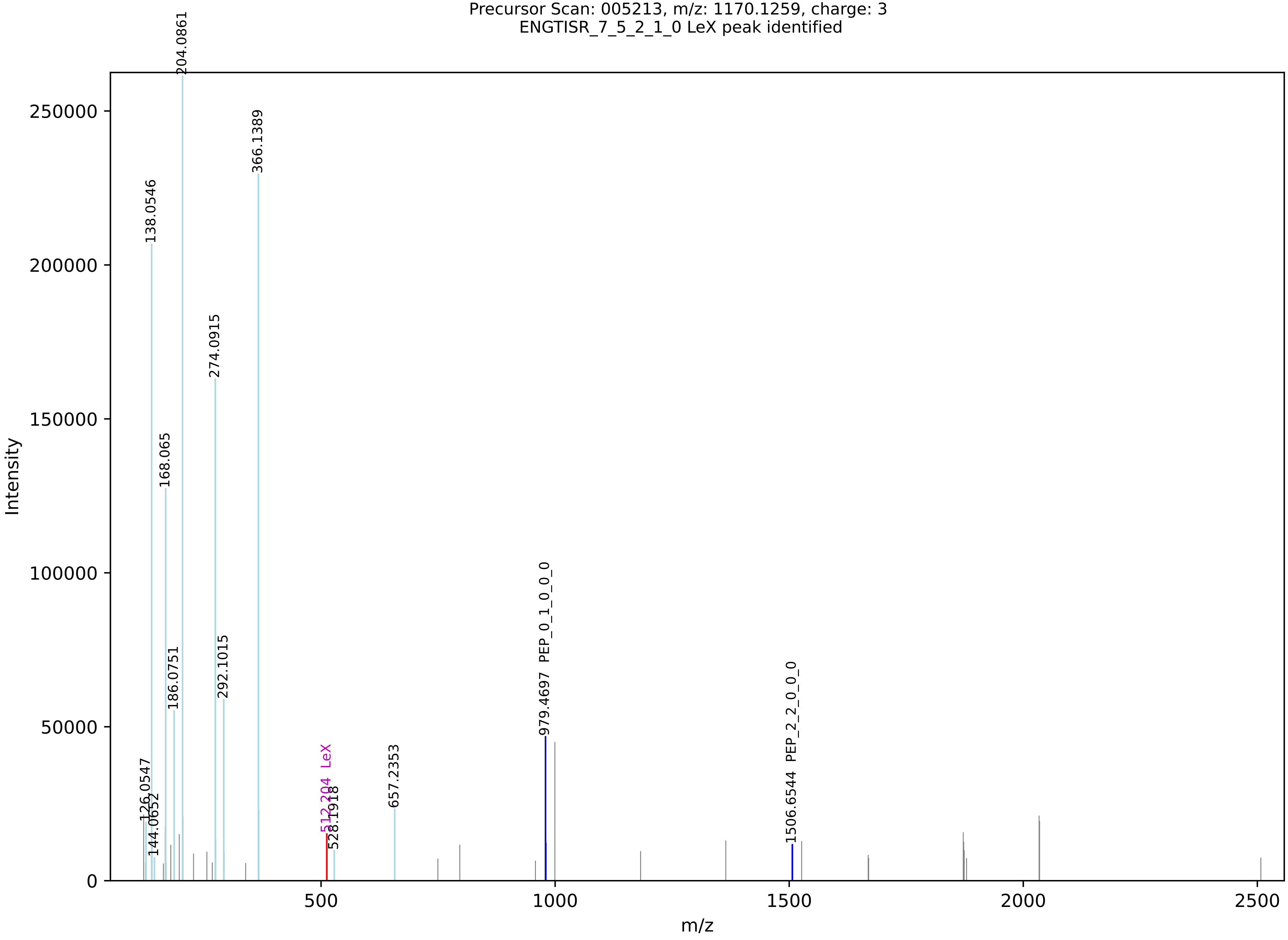

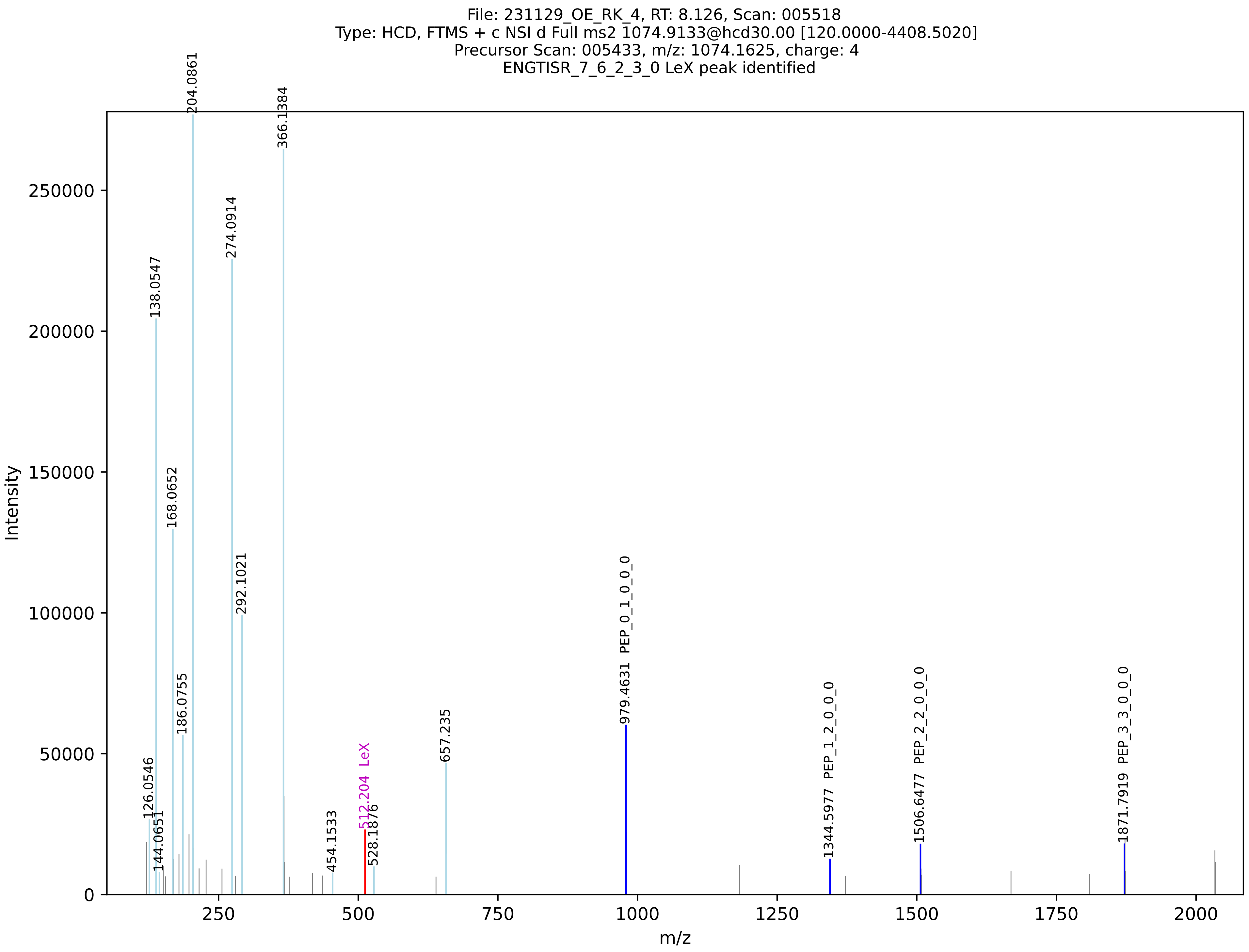
File: 231129_OE_RK_4, RT: 8.126, Scan: 005518
Type: HCD, FTMS + c NSI d Full ms2 1074.9133@hcd30.00 [120.0000-4408.5020]
Precursor Scan: 005433, m/z: 1074.1625, charge: 4
ENGTISR_7_6_2_3_0 LeX peak identified
Intensity
m/z
204.0861
366.1384
274.0914
138.0547
168.0652
292.1021
186.0755
126.0546
144.0651
454.1533
512.204 LeX
528.1876
657.235
979.4631 PEP_0_1_0_0_0
1344.5977 PEP_1_2_0_0_0
1506.6477 PEP_2_2_0_0_0
1871.7919 PEP_3_3_0_0_0

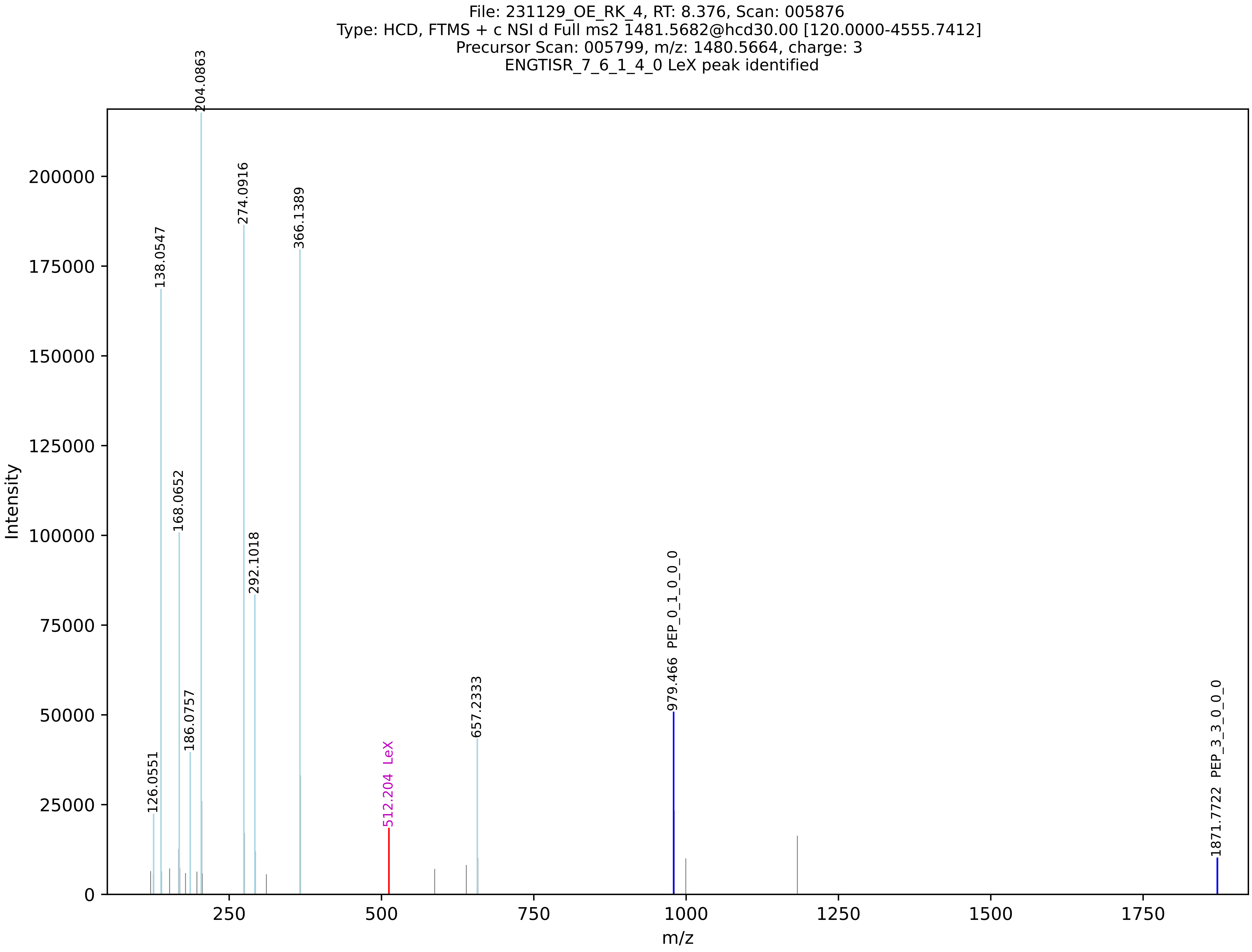
File: 231129_OE_RK_4, RT: 8.376, Scan: 005876
Type: HCD, FTMS + c NSI d Full ms2 1481.5682@hcd30.00 [120.0000-4555.7412]
Precursor Scan: 005799, m/z: 1480.5664, charge: 3
ENGTISR_7_6_1_4_0 LeX peak identified
Intensity
m/z
204.0863
274.0916
366.1389
138.0547
168.0652
292.1018
186.0757
126.0551
512.204 LeX
657.2333
979.466 PEP_0_1_0_0_0
1871.7722 PEP_3_3_0_0_0

File: 231129_OE_RK_4, RT: 8.978, Scan: 006743
Type: HCD, FTMS + c NSI d Full ms2 1147.6897@hcd30.00 [120.0000-4705.4302]
Precursor Scan: 006670, m/z: 1147.1881, charge: 4
ENGTISR_7_6_2_4_0 LeX peak identified

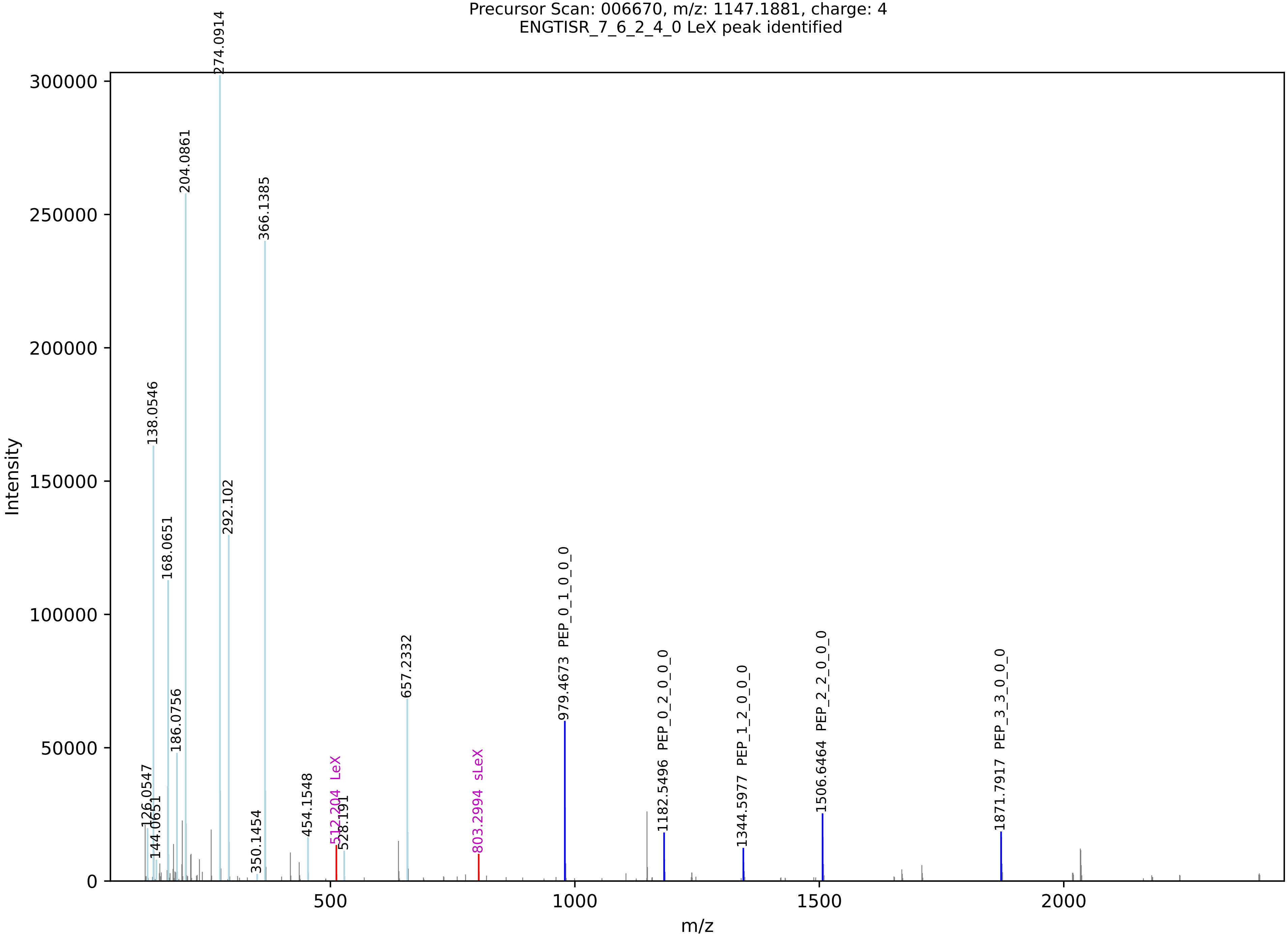

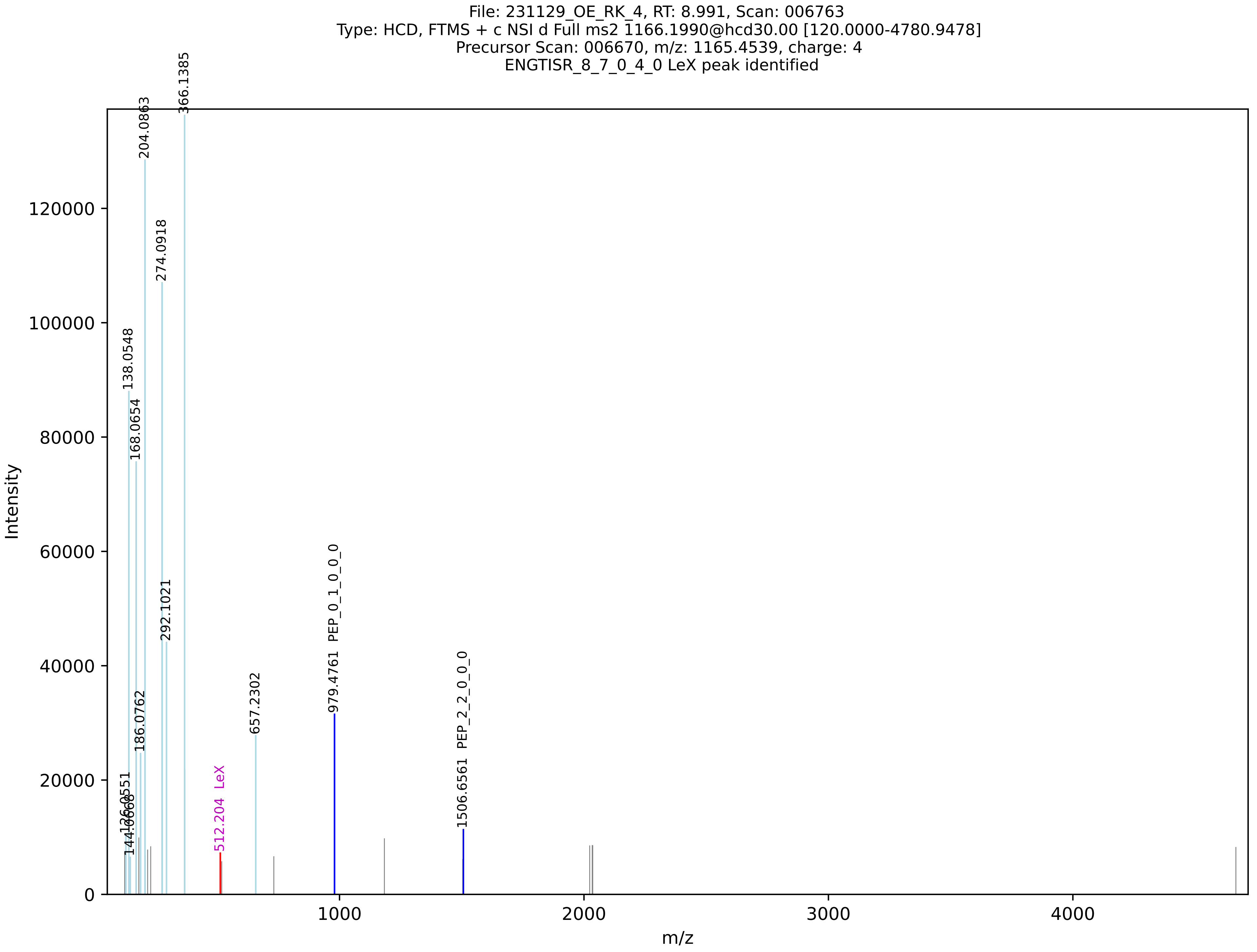

File: 231129_OE_RK_4, RT: 8.991, Scan: 006763
Type: HCD, FTMS + c NSI d Full ms2 1166.1990@hcd30.00 [120.0000-4780.9478]
Precursor Scan: 006670, m/z: 1165.4539, charge: 4
ENGTISR_8_7_0_4_0 LeX peak identified
366.1385
204.0863
274.0918
138.0548
168.0654
292.1021
186.0762
126.0551
144.0668
512.204 LeX
657.2302
979.4761 PEP_0_1_0_0_0
1506.6561 PEP_2_2_0_0_0
Intensity
m/z
0
20000
40000
60000
80000
100000
120000
1000
2000
3000
4000

File: 231129_OE_RK_4, RT: 9.08, Scan: 006892
Type: HCD, FTMS + c NSI d Full ms2 1432.5494@hcd30.00 [120.0000-4405.7432]
Precursor Scan: 006816, m/z: 1431.8812, charge: 3
ENGTISR_7_6_2_3_0 LeX peak identified

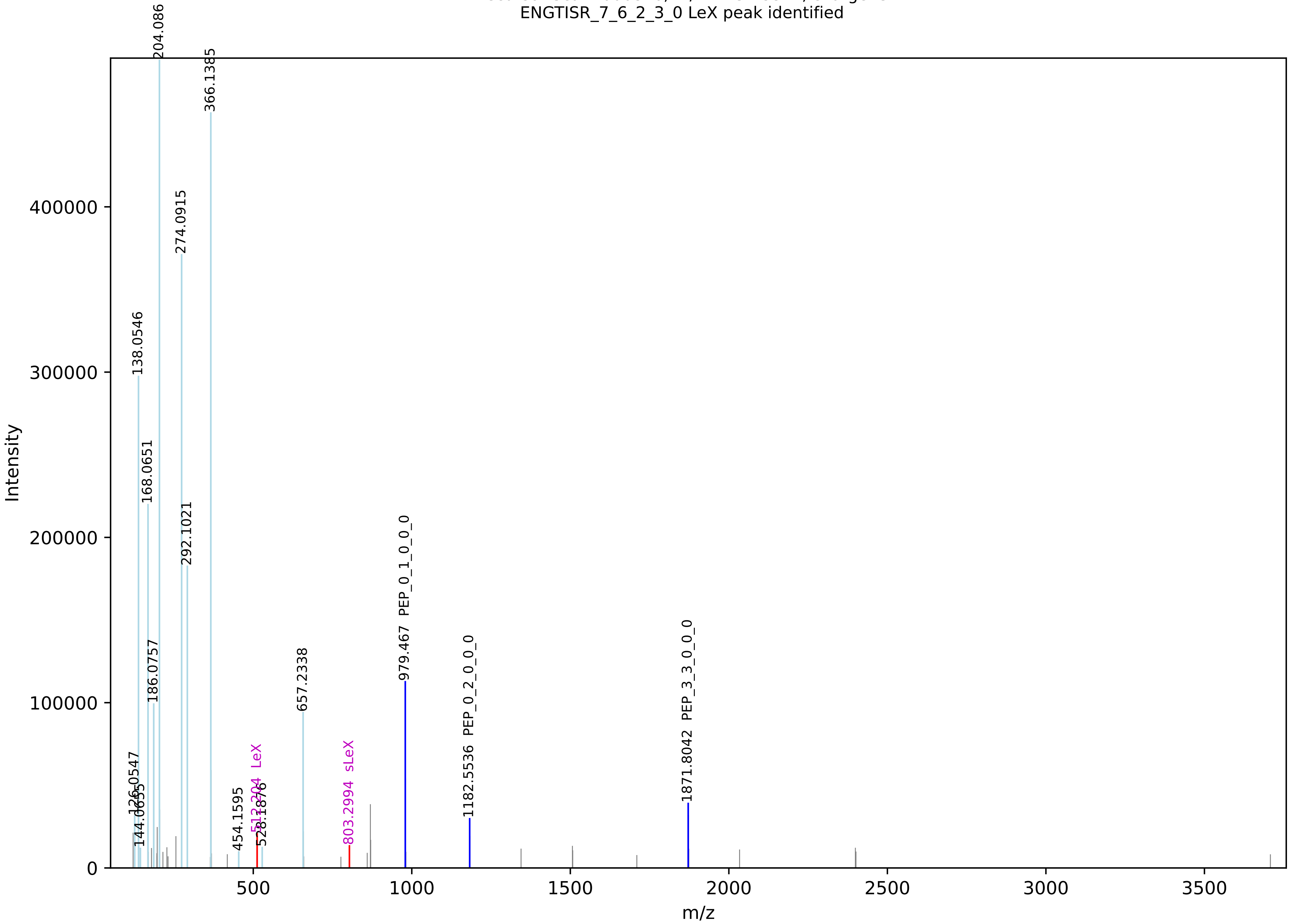

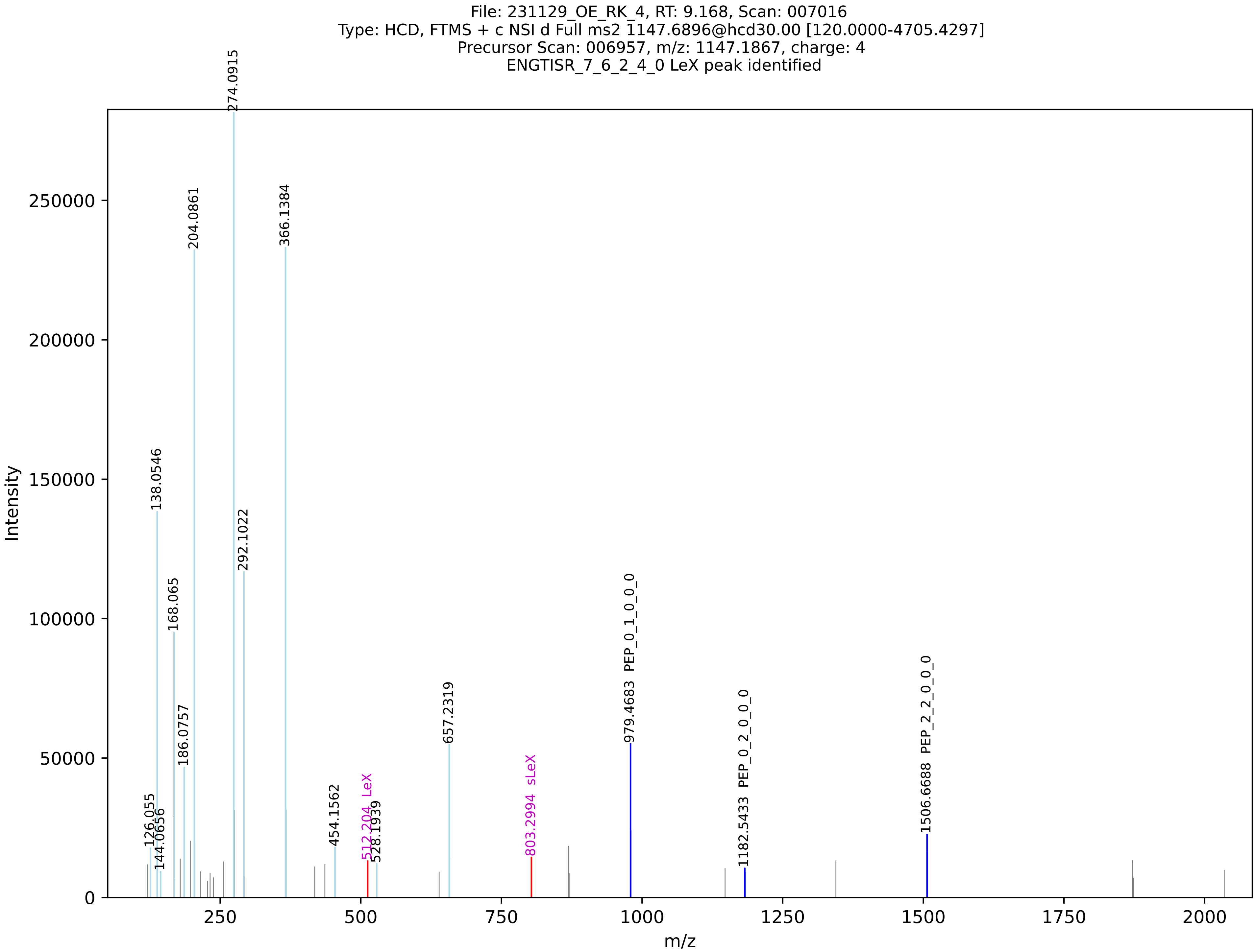
File: 231129_OE_RK_4, RT: 9.168, Scan: 007016
Type: HCD, FTMS + c NSI d Full ms2 1147.6896@hcd30.00 [120.0000-4705.4297]
Precursor Scan: 006957, m/z: 1147.1867, charge: 4
ENGTISR_7_6_2_4_0 LeX peak identified
274.0915
204.0861
366.1384
138.0546
292.1022
168.065
186.0757
126.055
144.0656
454.1562
512.204 LeX
528.1939
657.2319
803.2994 sLeX
979.4683 PEP_0_1_0_0_0_
1182.5433 PEP_0_2_0_0_0
1506.6688 PEP_2_2_0_0_0
Intensity
m/z
0
50000
100000
150000
200000
250000
250
500
750
1000
1250
1500
1750
2000

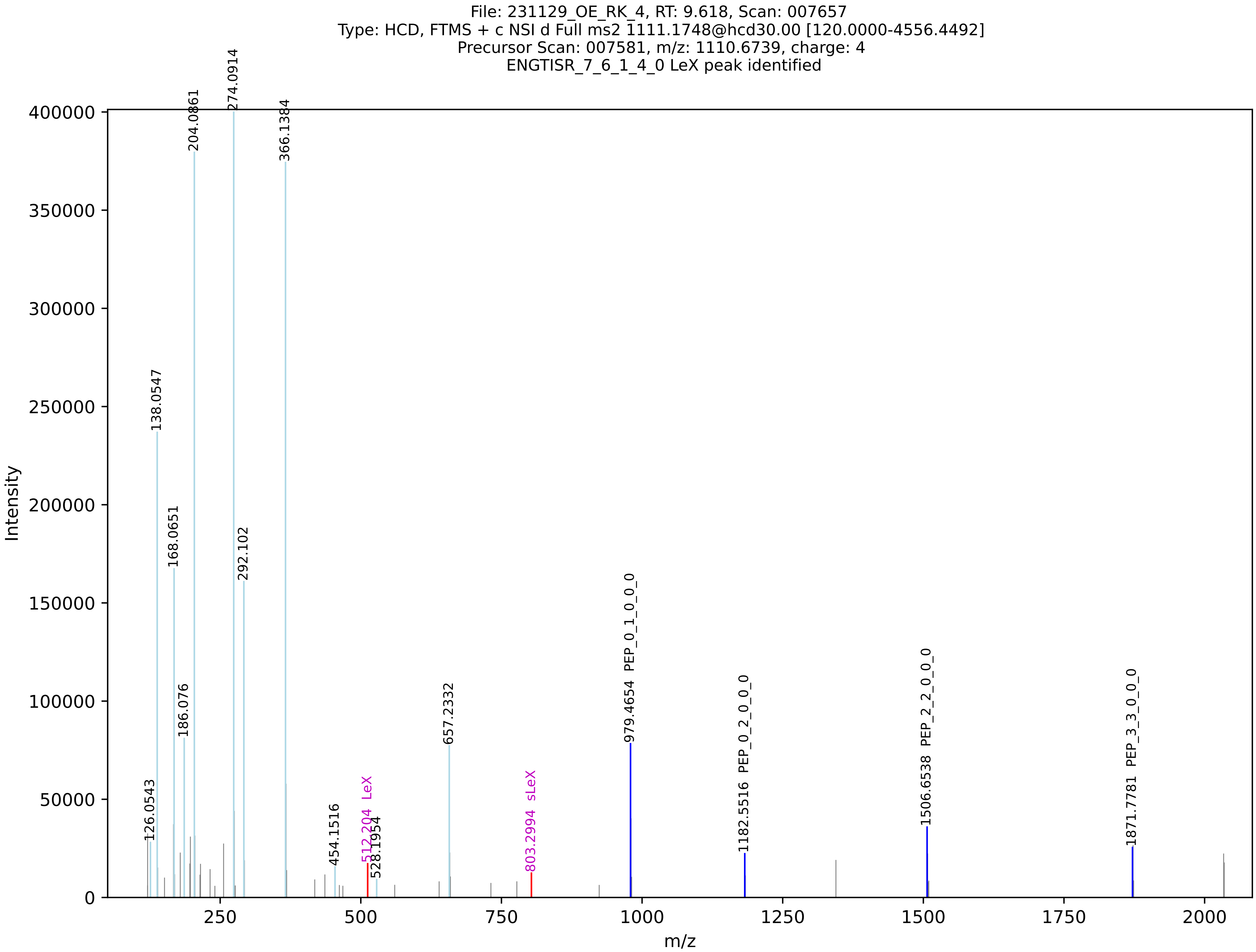
File: 231129_OE_RK_4, RT: 9.618, Scan: 007657
Type: HCD, FTMS + c NSI d Full ms2 1111.1748@hcd30.00 [120.0000-4556.4492]
Precursor Scan: 007581, m/z: 1110.6739, charge: 4
ENGTISR_7_6_1_4_0 LeX peak identified
Intensity
m/z
126.0543
138.0547
168.0651
186.076
204.0861
274.0914
292.102
366.1384
454.1516
512.204 LeX
528.1954
657.2332
803.2994 sLeX
979.4654 PEP_0_1_0_0_0_
1182.5516 PEP_0_2_0_0_0
1506.6538 PEP_2_2_0_0_0
1871.7781 PEP_3_3_0_0_0

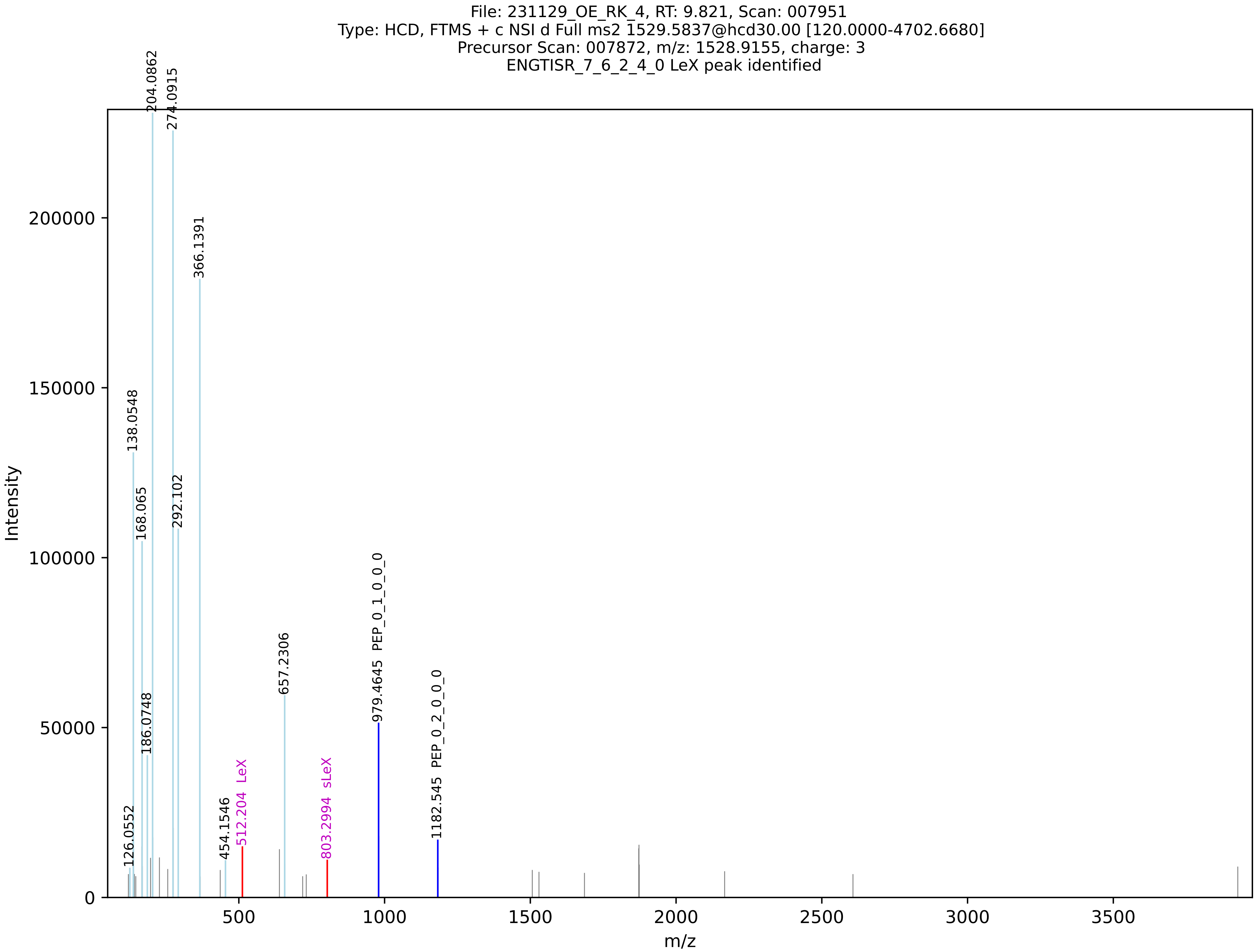

File: 231129_OE_RK_4, RT: 9.821, Scan: 007951
Type: HCD, FTMS + c NSI d Full ms2 1529.5837@hcd30.00 [120.0000-4702.6680]
Precursor Scan: 007872, m/z: 1528.9155, charge: 3
ENGTISR_7_6_2_4_0 LeX peak identified
Intensity
m/z
204.0862
274.0915
366.1391
138.0548
168.065
292.102
186.0748
126.0552
454.1546
512.204 LeX
657.2306
803.2994 sLeX
979.4645 PEP_0_1_0_0_0_
1182.545 PEP_0_2_0_0_0

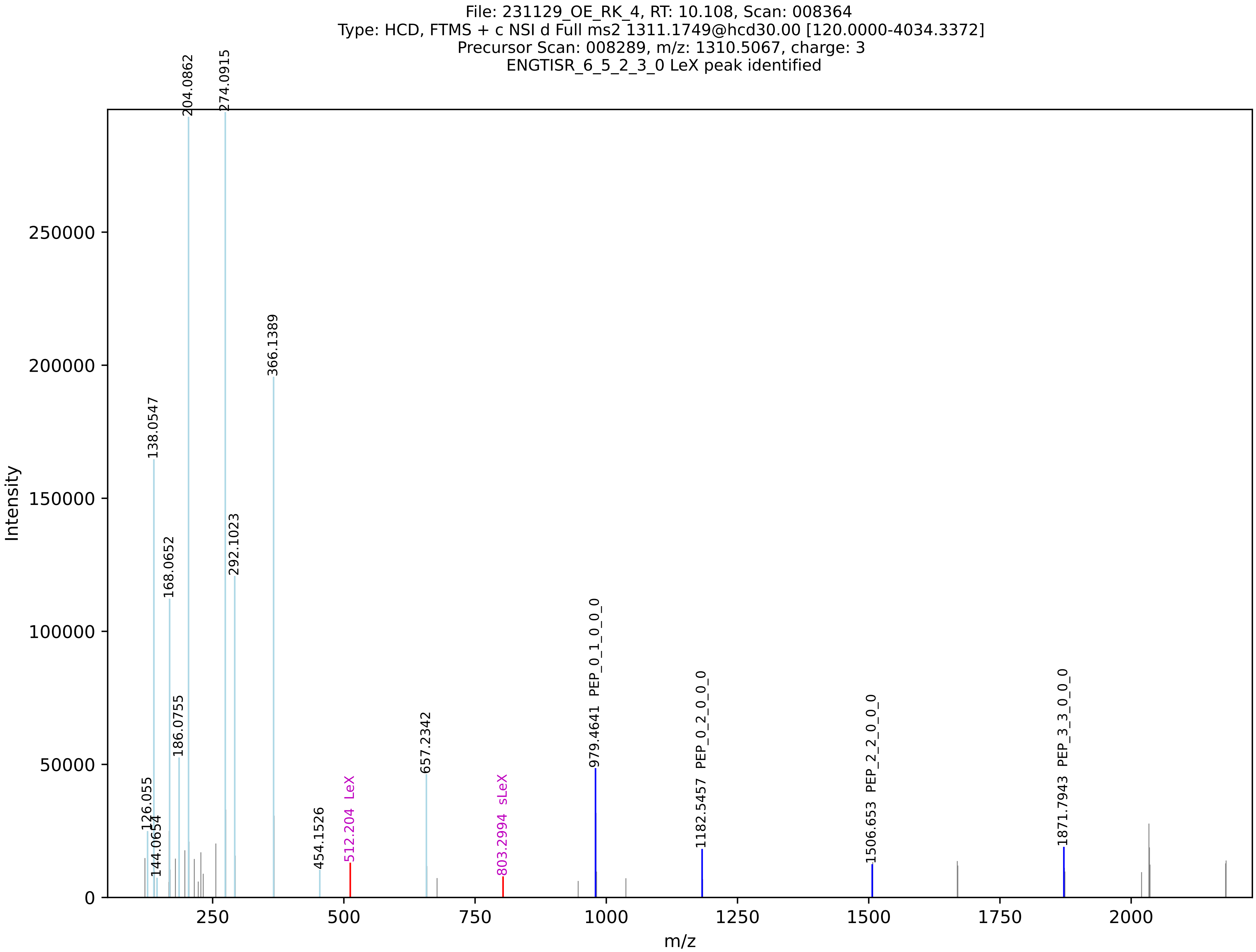
File: 231129_OE_RK_4, RT: 10.108, Scan: 008364
Type: HCD, FTMS + c NSI d Full ms2 1311.1749@hcd30.00 [120.0000-4034.3372]
Precursor Scan: 008289, m/z: 1310.5067, charge: 3
ENGTISR_6_5_2_3_0 LeX peak identified
Intensity
m/z
204.0862
274.0915
366.1389
138.0547
292.1023
168.0652
186.0755
126.055
144.0654
454.1526
512.204 LeX
657.2342
803.2994 sLeX
979.4641 PEP_0_1_0_0_0
1182.5457 PEP_0_2_0_0_0
1506.653 PEP_2_2_0_0_0
1871.7943 PEP_3_3_0_0_0

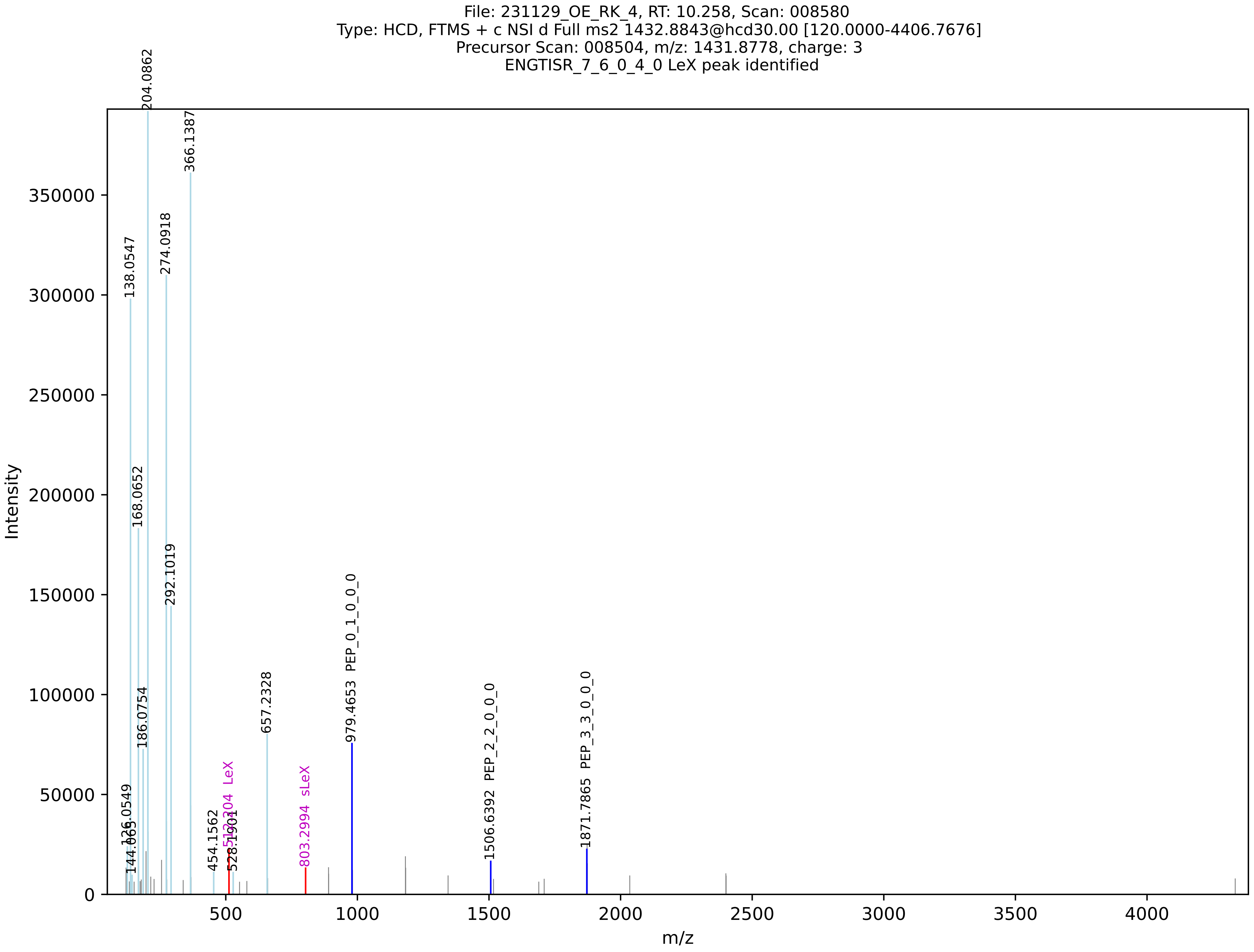
File: 231129_OE_RK_4, RT: 10.258, Scan: 008580
Type: HCD, FTMS + c NSI d Full ms2 1432.8843@hcd30.00 [120.0000-4406.7676]
Precursor Scan: 008504, m/z: 1431.8778, charge: 3
ENGTISR_7_6_0_4_0 LeX peak identified
Intensity
m/z
0
50000
100000
150000
200000
250000
300000
350000
500
1000
1500
2000
2500
3000
3500
4000
204.0862
366.1387
274.0918
138.0547
168.0652
292.1019
186.0754
126.0549
144.065
454.1562
LeX
528.1901
657.2328
803.2994 sLeX
979.4653 PEP_0_1_0_0_0
1506.6392 PEP_2_2_0_0_0
1871.7865 PEP_3_3_0_0_0

File: 231129_OE_RK_4, RT: 10.502, Scan: 008931
Type: HCD, FTMS + c NSI d Full ms2 1384.1980@hcd30.00 [120.0000-4257.7881]
Precursor Scan: 008856, m/z: 1383.5298, charge: 3
ENGTISR_7_6_1_3_0 LeX peak identified

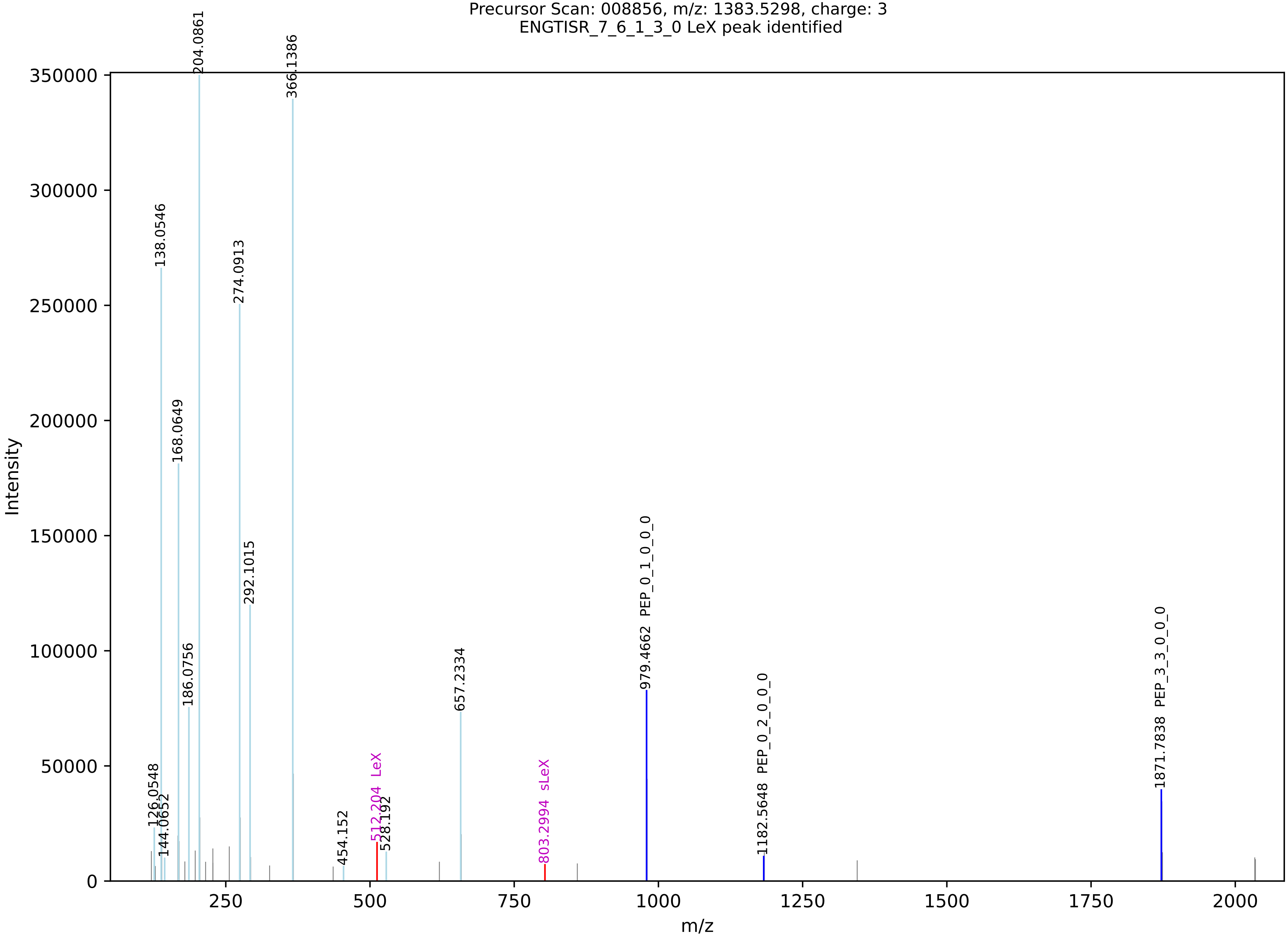

File: 231129_OE_RK_4, RT: 10.602, Scan: 009077
Type: HCD, FTMS + c NSI d Full ms2 1147.6896@hcd30.00 [120.0000-4705.4292]
Precursor Scan: 009000, m/z: 1147.1881, charge: 4
ENGTISR_7_6_2_4_0 LeX peak identified

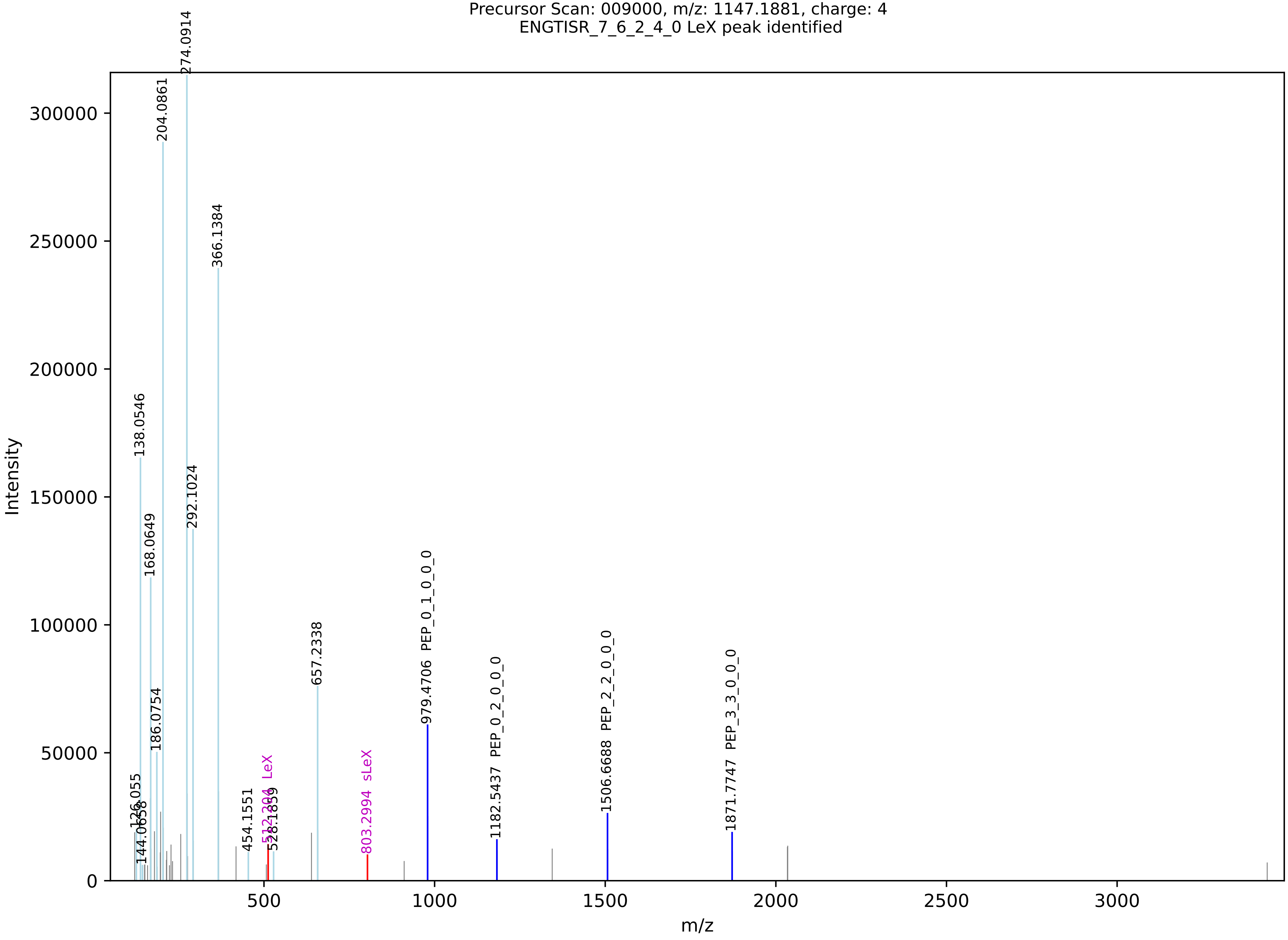

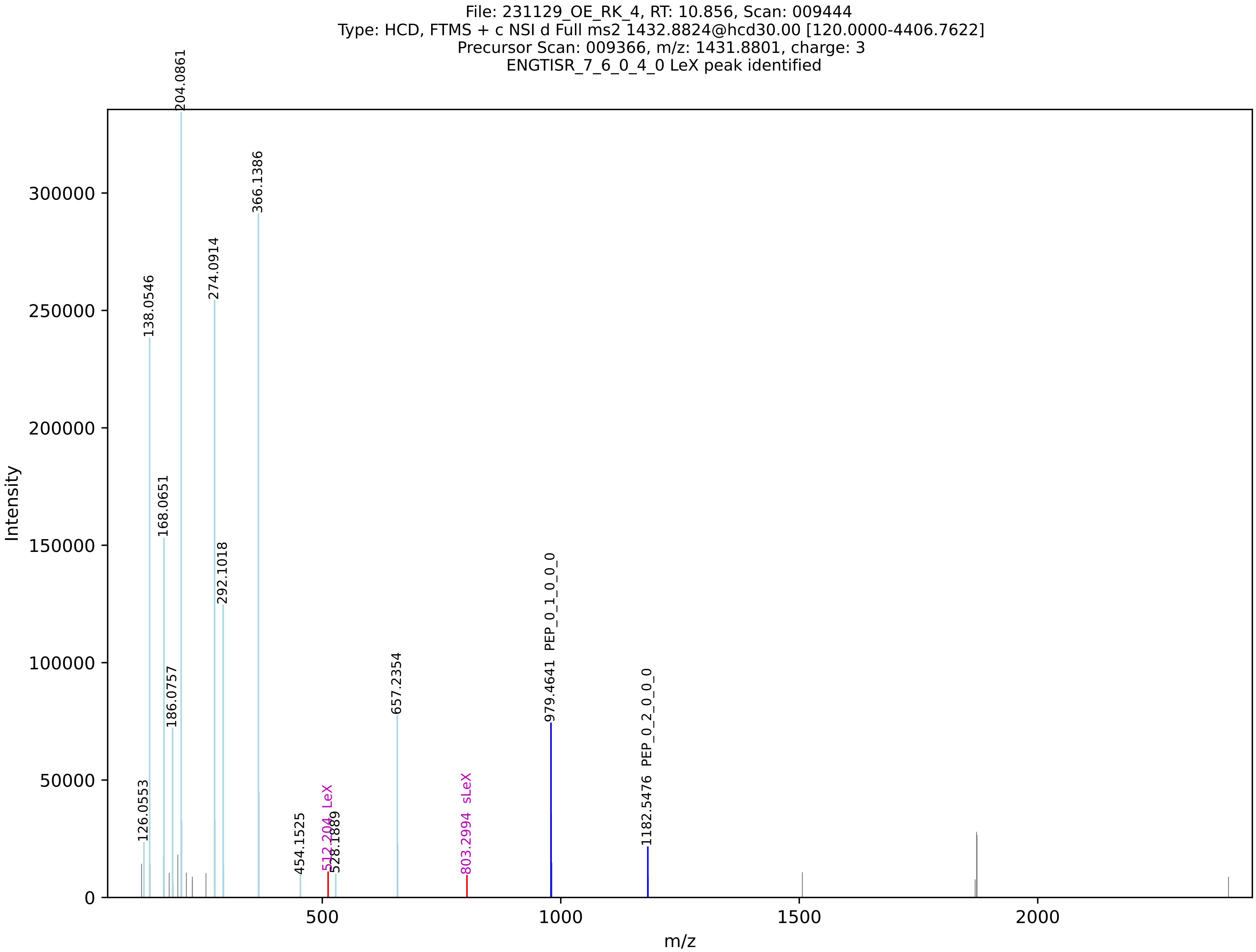

File: 231129_OE_RK_4, RT: 10.856, Scan: 009444
Type: HCD, FTMS + c NSI d Full ms2 1432.8824@hcd30.00 [120.0000-4406.7622]
Precursor Scan: 009366, m/z: 1431.8801, charge: 3
ENGTISR_7_6_0_4_0 LeX peak identified
Intensity
m/z
204.0861
366.1386
274.0914
138.0546
168.0651
292.1018
186.0757
126.0553
454.1525
512.204 LeX
528.1889
657.2354
803.2994 sLeX
979.4641 PEP_0_1_0_0_0
1182.5476 PEP_0_2_0_0_0

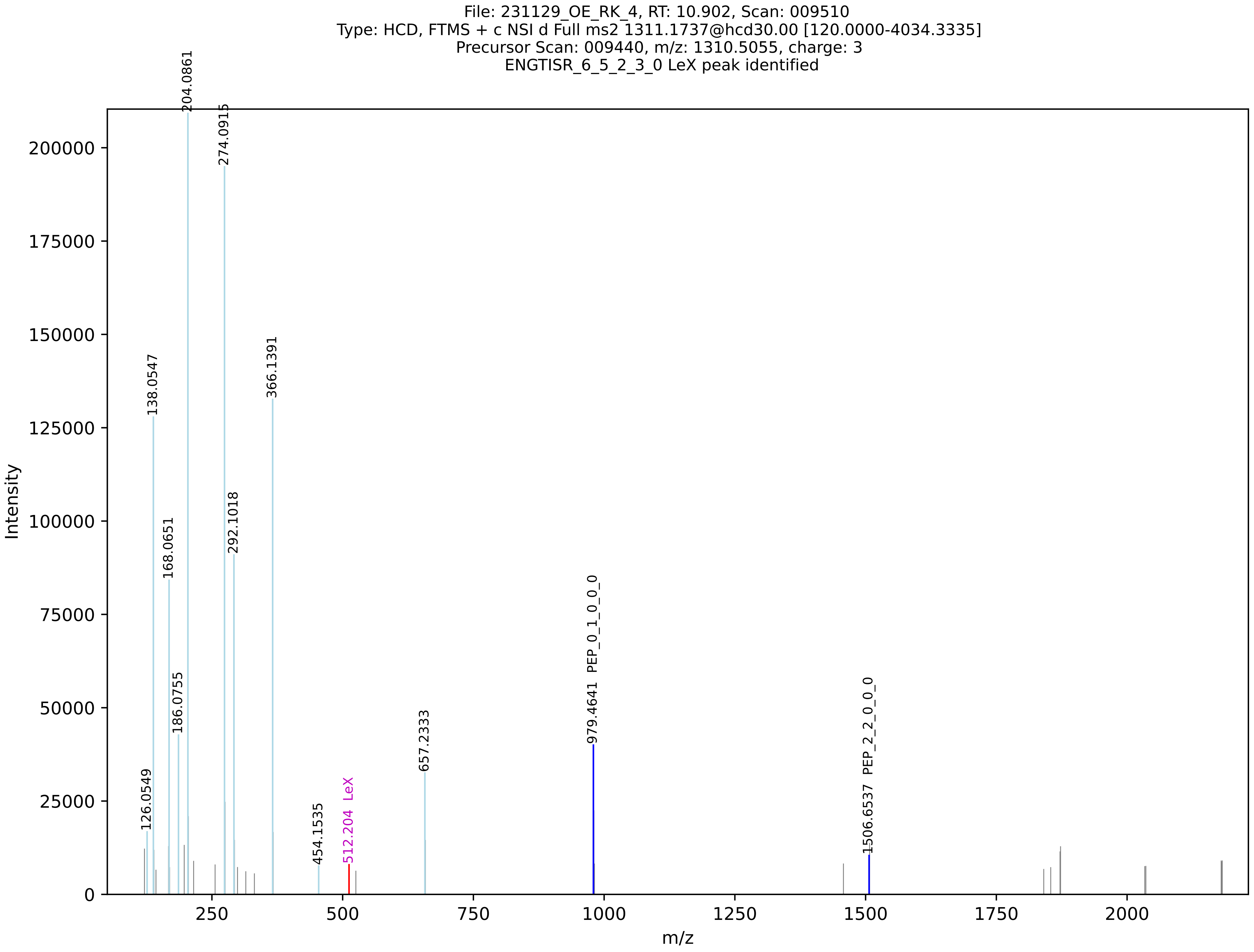

File: 231129_OE_RK_4, RT: 10.902, Scan: 009510
Type: HCD, FTMS + c NSI d Full ms2 1311.1737@hcd30.00 [120.0000-4034.3335]
Precursor Scan: 009440, m/z: 1310.5055, charge: 3
ENGTISR_6_5_2_3_0 LeX peak identified
Intensity
m/z
0
25000
50000
75000
100000
125000
150000
175000
200000
250
500
750
1000
1250
1500
1750
2000
204.0861
274.0915
138.0547
366.1391
292.1018
168.0651
186.0755
126.0549
454.1535
512.204 LeX
657.2333
979.4641 PEP_0_1_0_0_0_
1506.6537 PEP_2_2_0_0_0_

File: 231129_OE_RK_4, RT: 11.437, Scan: 010285
Type: HCD, FTMS + c NSI d Full ms2 1529.5824@hcd30.00 [120.0000-4702.6641]
Precursor Scan: 010206, m/z: 1529.249, charge: 3
ENGTISR_7_6_2_4_0 LeX peak identified

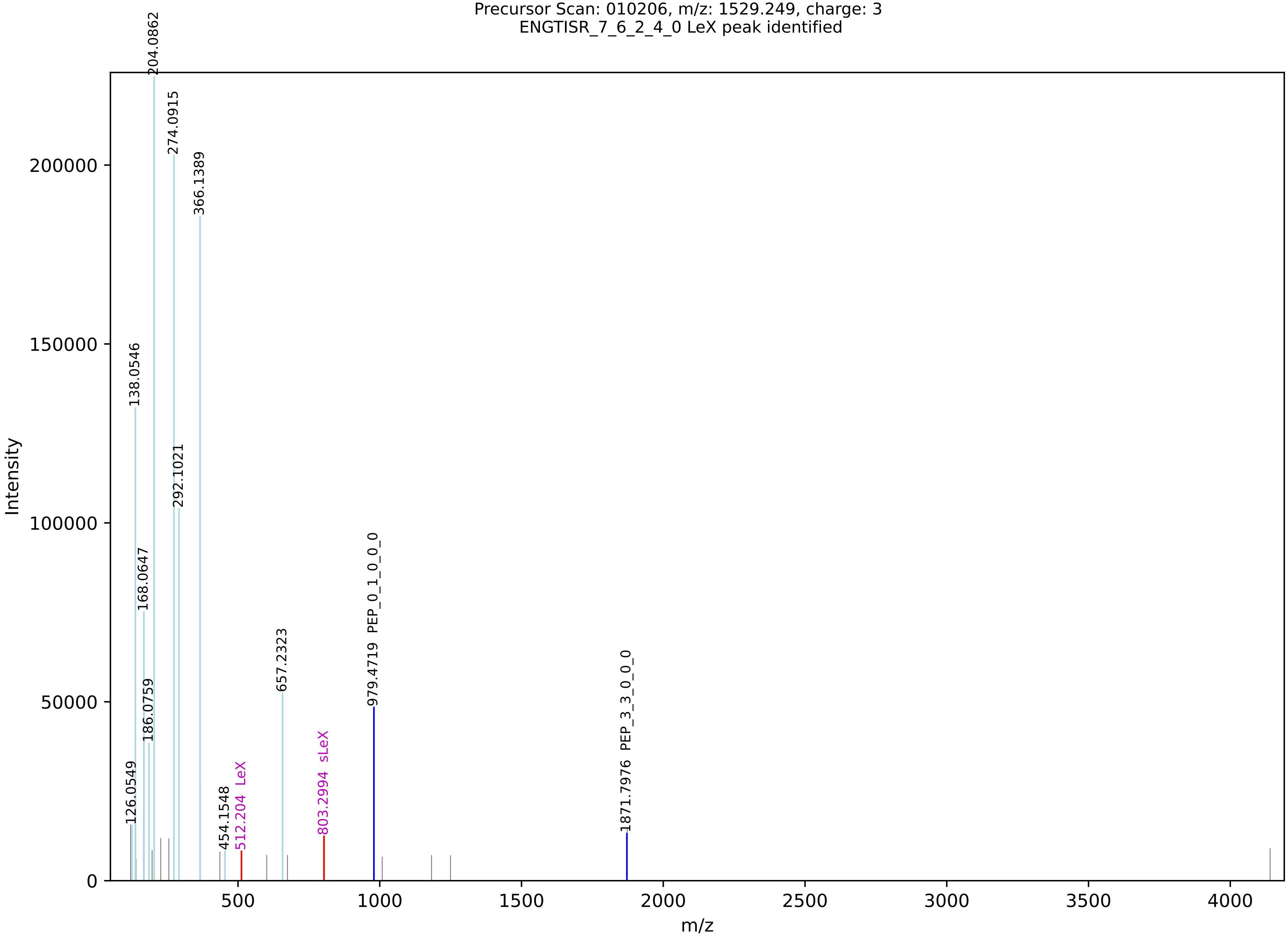

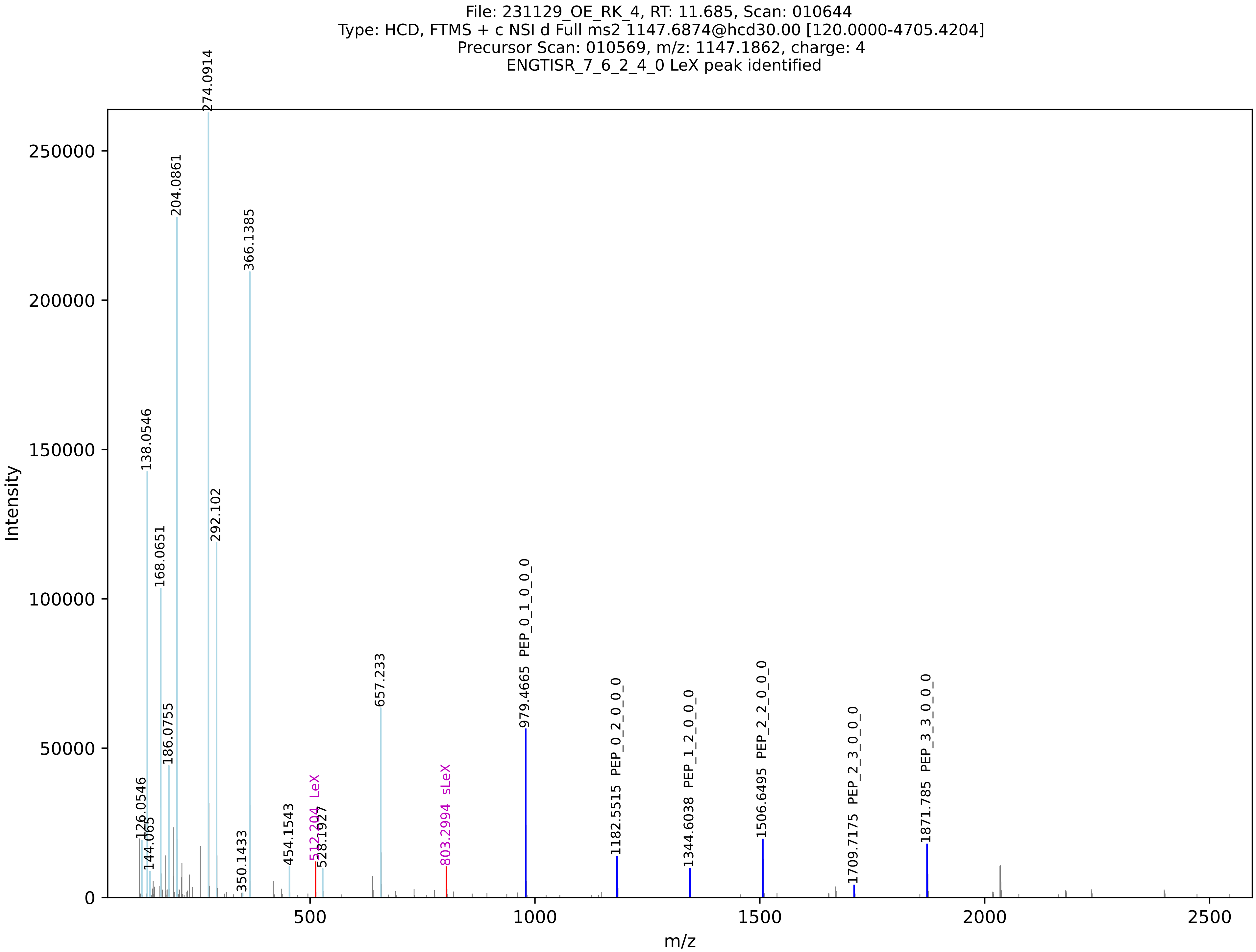
File: 231129_OE_RK_4, RT: 11.685, Scan: 010644
Type: HCD, FTMS + c NSI d Full ms2 1147.6874@hcd30.00 [120.0000-4705.4204]
Precursor Scan: 010569, m/z: 1147.1862, charge: 4
ENGTISR_7_6_2_4_0 LeX peak identified
Intensity
m/z
274.0914
204.0861
366.1385
138.0546
292.102
168.0651
186.0755
126.0546
144.065
350.1433
454.1543
512.204 LeX
528.1927
657.233
803.2994 sLeX
979.4665 PEP_0_1_0_0_0_
1182.5515 PEP_0_2_0_0_0_
1344.6038 PEP_1_2_0_0_0_
1506.6495 PEP_2_2_0_0_0_
1709.7175 PEP_2_3_0_0_0_
1871.785 PEP_3_3_0_0_0_
0
50000
100000
150000
200000
250000
500
1000
1500
2000
2500

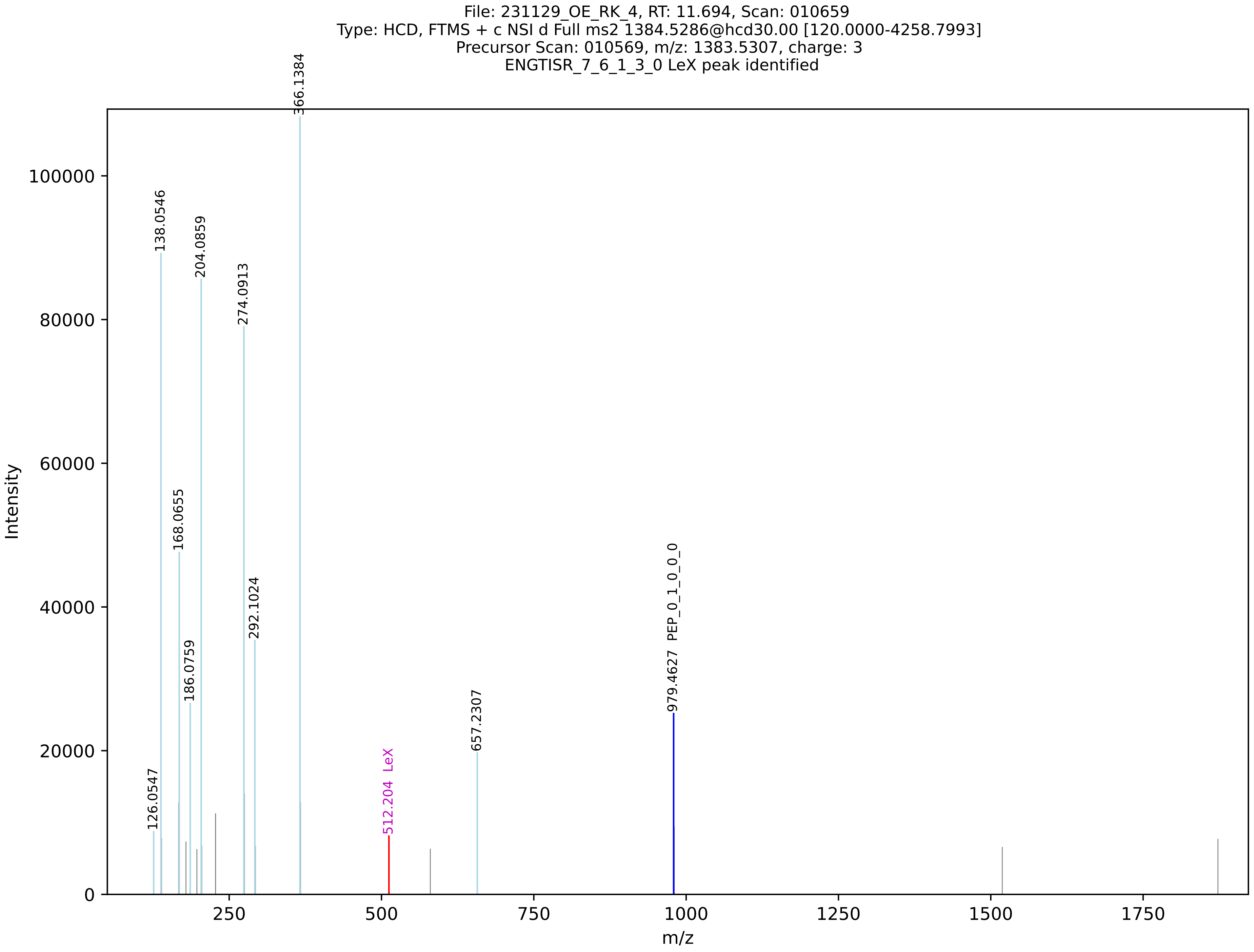

File: 231129_OE_RK_4, RT: 11.694, Scan: 010659
Type: HCD, FTMS + c NSI d Full ms2 1384.5286@hcd30.00 [120.0000-4258.7993]
Precursor Scan: 010569, m/z: 1383.5307, charge: 3
ENGTISR_7_6_1_3_0 LeX peak identified
366.1384
138.0546
204.0859
274.0913
168.0655
292.1024
186.0759
126.0547
512.204 LeX
657.2307
979.4627 PEP_0_1_0_0_0
Intensity
m/z
0
20000
40000
60000
80000
100000
250
500
750
1000
1250
1500
1750

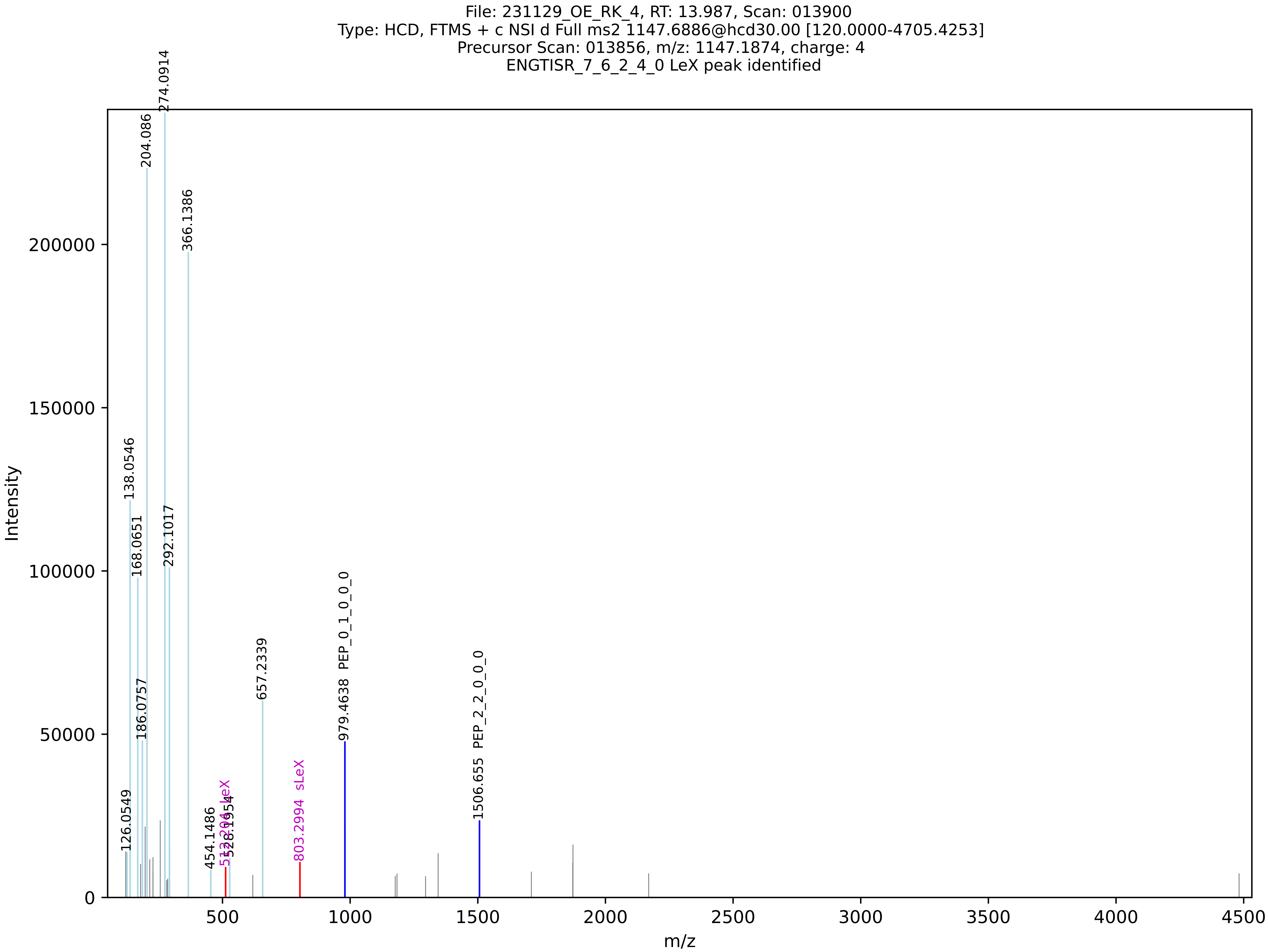
File: 231129_OE_RK_4, RT: 13.987, Scan: 013900
Type: HCD, FTMS + c NSI d Full ms2 1147.6886@hcd30.00 [120.0000-4705.4253]
Precursor Scan: 013856, m/z: 1147.1874, charge: 4
ENGTISR_7_6_2_4_0 LeX peak identified
Intensity
m/z
274.0914
204.086
366.1386
138.0546
168.0651
292.1017
186.0757
126.0549
454.1486
528.1954
657.2339
803.2994 sLeX
979.4638 PEP_0_1_0_0_0_
1506.655 PEP_2_2_0_0_0_
0
50000
100000
150000
200000
500
1000
1500
2000
2500
3000
3500
4000
4500

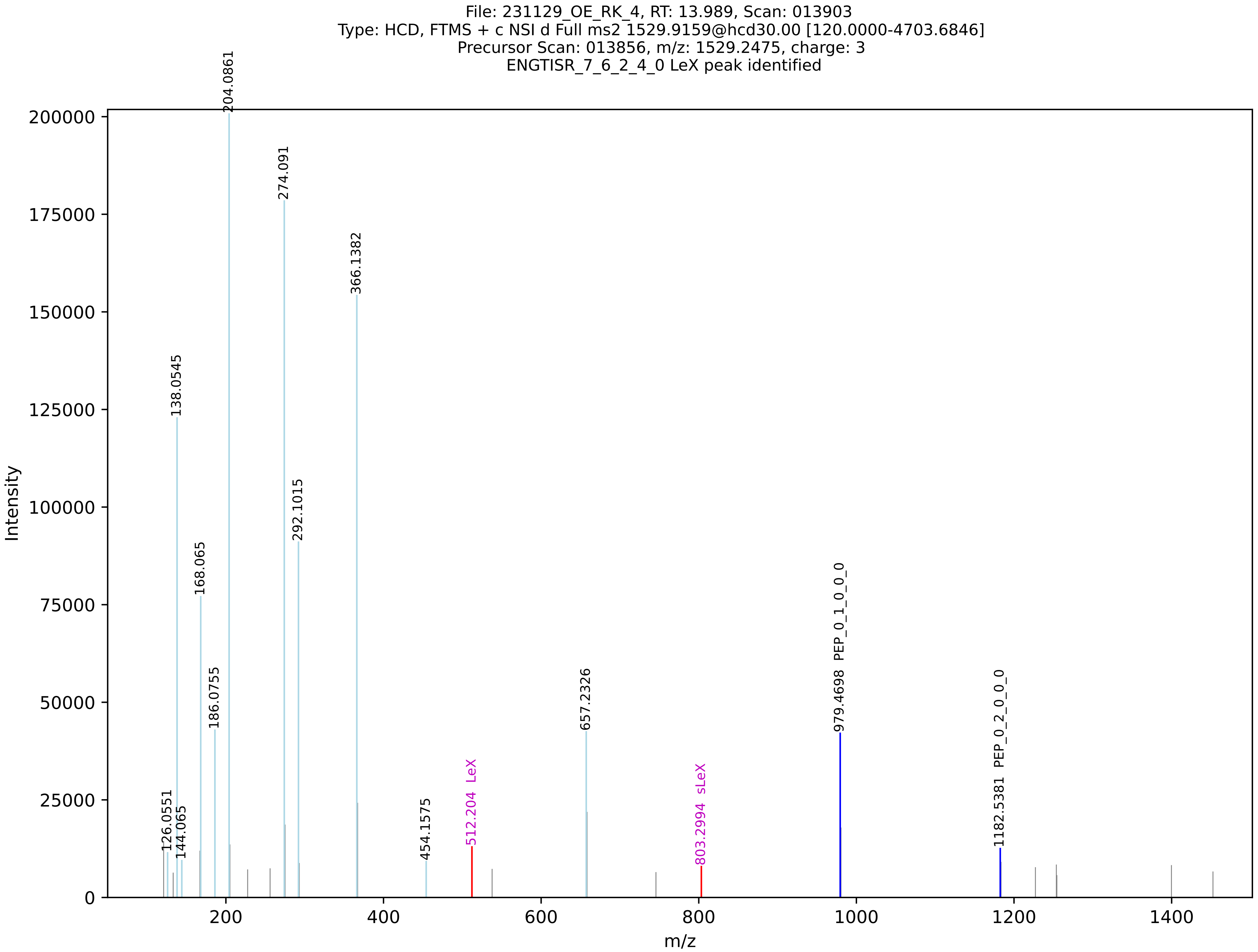

File: 231129_OE_RK_4, RT: 13.989, Scan: 013903
Type: HCD, FTMS + c NSI d Full ms2 1529.9159@hcd30.00 [120.0000-4703.6846]
Precursor Scan: 013856, m/z: 1529.2475, charge: 3
ENGTISR_7_6_2_4_0 LeX peak identified
Intensity
m/z
204.0861
274.091
366.1382
138.0545
292.1015
168.065
186.0755
126.0551
144.065
454.1575
512.204 LeX
657.2326
803.2994 sLeX
979.4698 PEP_0_1_0_0_0
1182.5381 PEP_0_2_0_0_0

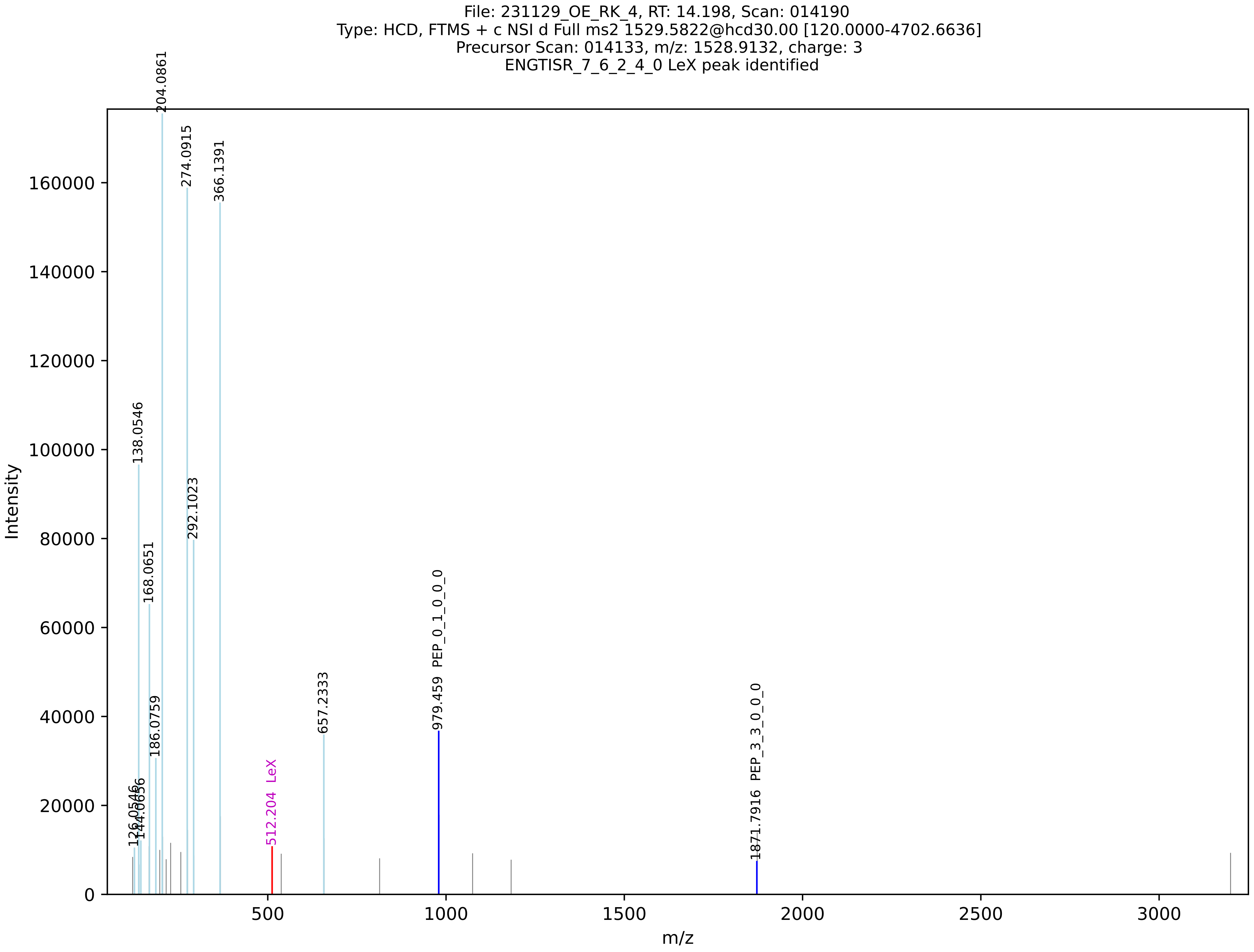
File: 231129_OE_RK_4, RT: 14.198, Scan: 014190
Type: HCD, FTMS + c NSI d Full ms2 1529.5822@hcd30.00 [120.0000-4702.6636]
Precursor Scan: 014133, m/z: 1528.9132, charge: 3
ENGTISR_7_6_2_4_0 LeX peak identified
204.0861
274.0915
366.1391
138.0546
292.1023
168.0651
186.0759
126.0546
144.0656
512.204 LeX
657.2333
979.459 PEP_0_1_0_0_0
1871.7916 PEP_3_3_0_0_0
Intensity
m/z
0
20000
40000
60000
80000
100000
120000
140000
160000
500
1000
1500
2000
2500
3000

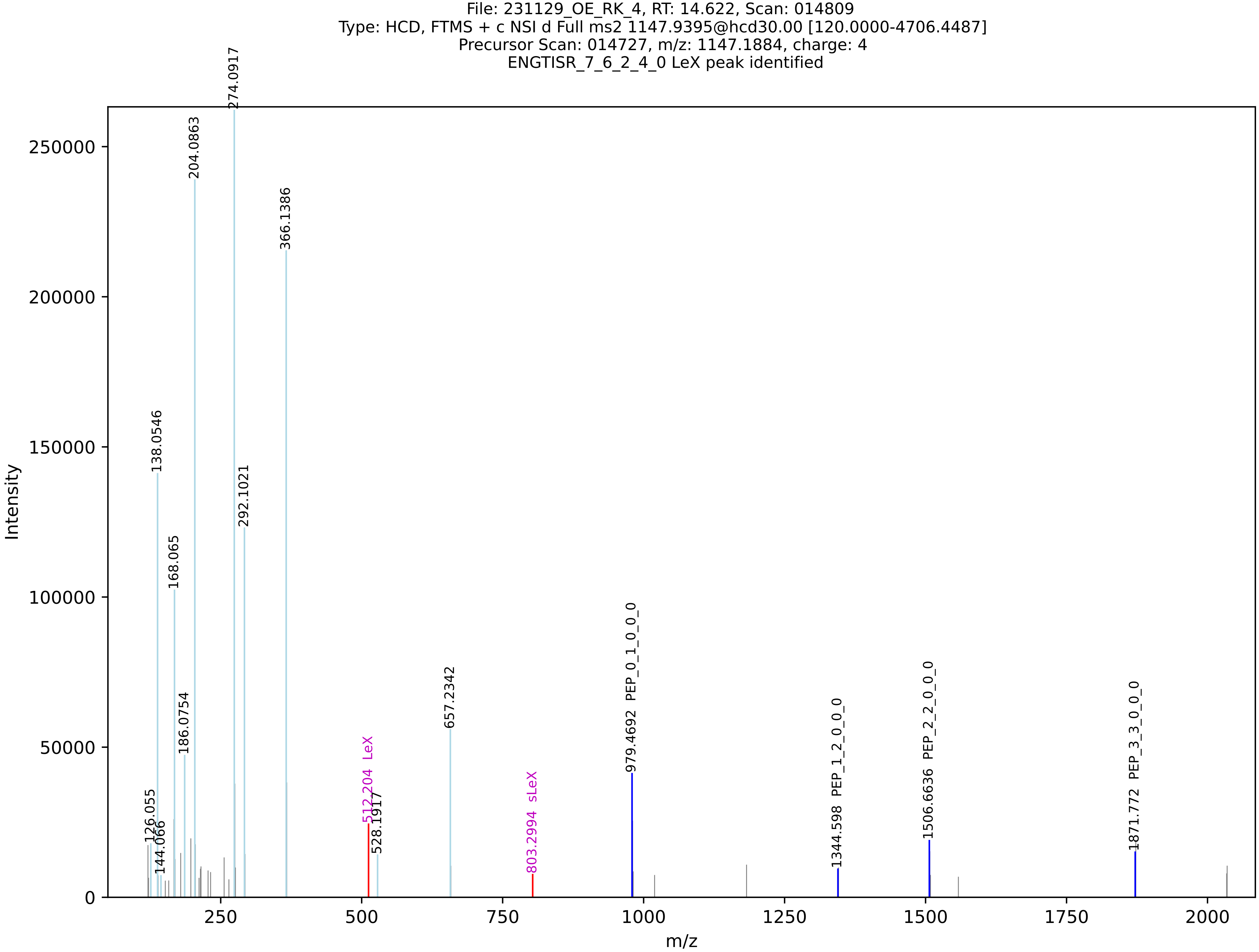

File: 231129_OE_RK_4, RT: 14.622, Scan: 014809
Type: HCD, FTMS + c NSI d Full ms2 1147.9395@hcd30.00 [120.0000-4706.4487]
Precursor Scan: 014727, m/z: 1147.1884, charge: 4
ENGTISR_7_6_2_4_0 LeX peak identified
Intensity
m/z
274.0917
204.0863
366.1386
138.0546
292.1021
168.065
186.0754
126.055
144.066
512.204 LeX
528.1917
657.2342
803.2994 sLeX
979.4692 PEP_0_1_0_0_0
1344.598 PEP_1_2_0_0_0
1506.6636 PEP_2_2_0_0_0
1871.772 PEP_3_3_0_0_0

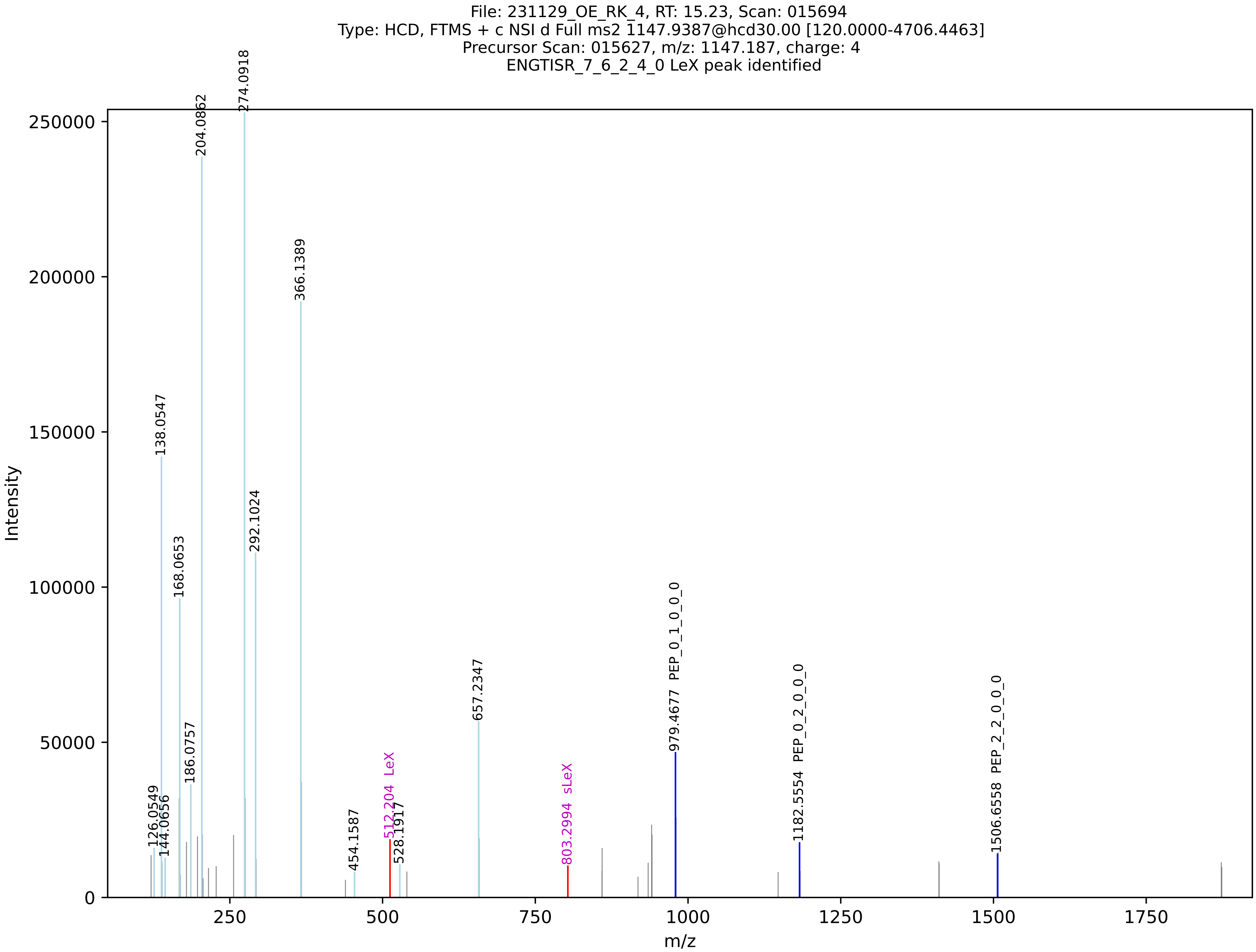
File: 231129_OE_RK_4, RT: 15.23, Scan: 015694
Type: HCD, FTMS + c NSI d Full ms2 1147.9387@hcd30.00 [120.0000-4706.4463]
Precursor Scan: 015627, m/z: 1147.187, charge: 4
ENGTISR_7_6_2_4_0 LeX peak identified
Intensity
m/z
274.0918
204.0862
366.1389
138.0547
292.1024
168.0653
186.0757
126.0549
144.0656
454.1587
512.204 LeX
528.1917
657.2347
803.2994 sLeX
979.4677 PEP_0_1_0_0_0
1182.5554 PEP_0_2_0_0_0
1506.6558 PEP_2_2_0_0_0
0
50000
100000
150000
200000
250000
250
500
750
1000
1250
1500
1750

File: 231129_OE_RK_4, RT: 15.316, Scan: 015818
Type: HCD, FTMS + c NSI d Full ms2 1529.9166@hcd30.00 [120.0000-4703.6870]
Precursor Scan: 015758, m/z: 1529.2453, charge: 3
ENGTISR_7_6_2_4_0 LeX peak identified

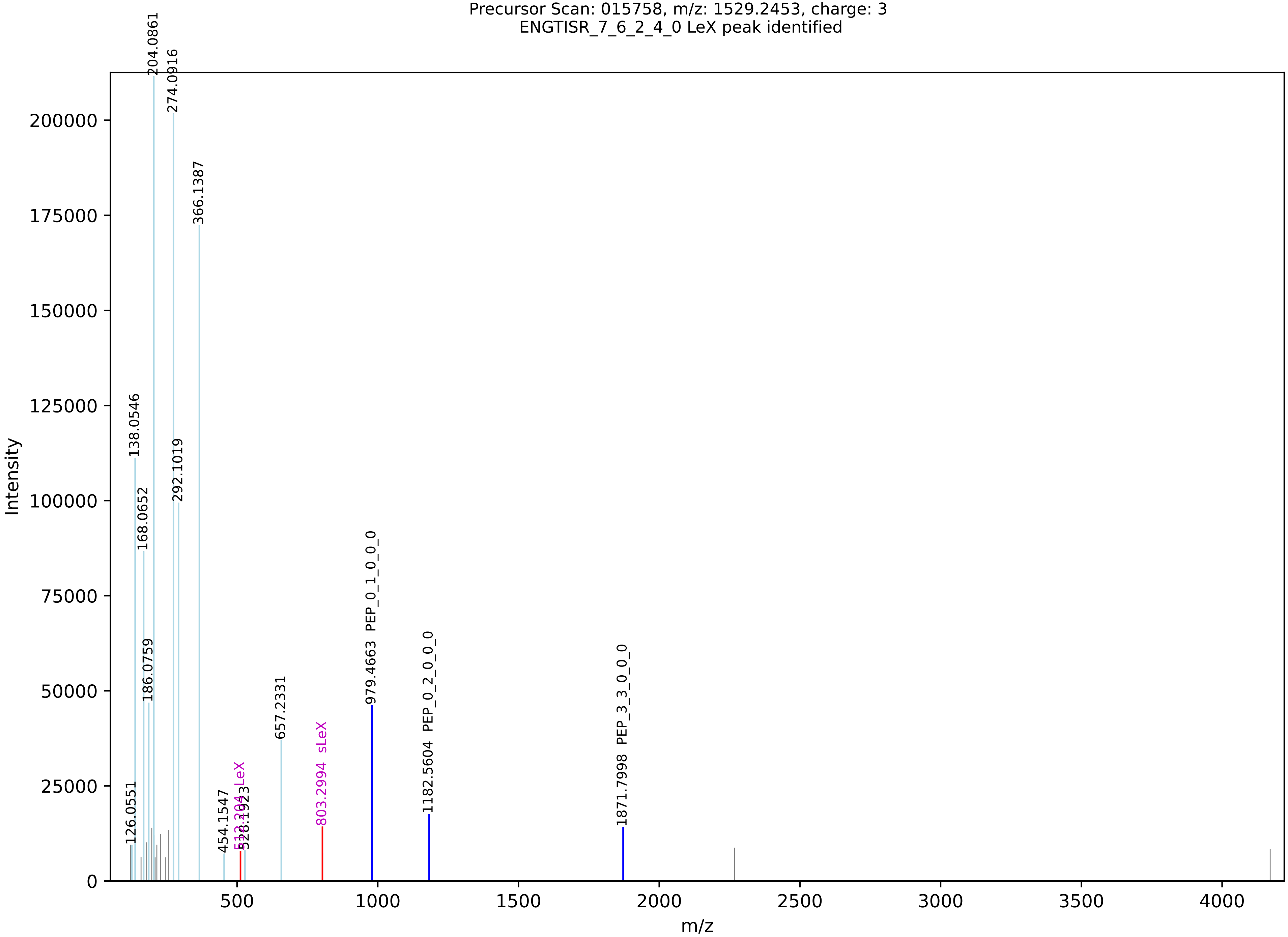

File: 231129_OE_RK_4, RT: 15.615, Scan: 016250
Type: HCD, FTMS + c NSI d Full ms2 1147.6897@hcd30.00 [120.0000-4705.4302]
Precursor Scan: 016196, m/z: 1147.1882, charge: 4
ENGTISR_7_6_2_4_0 LeX peak identified

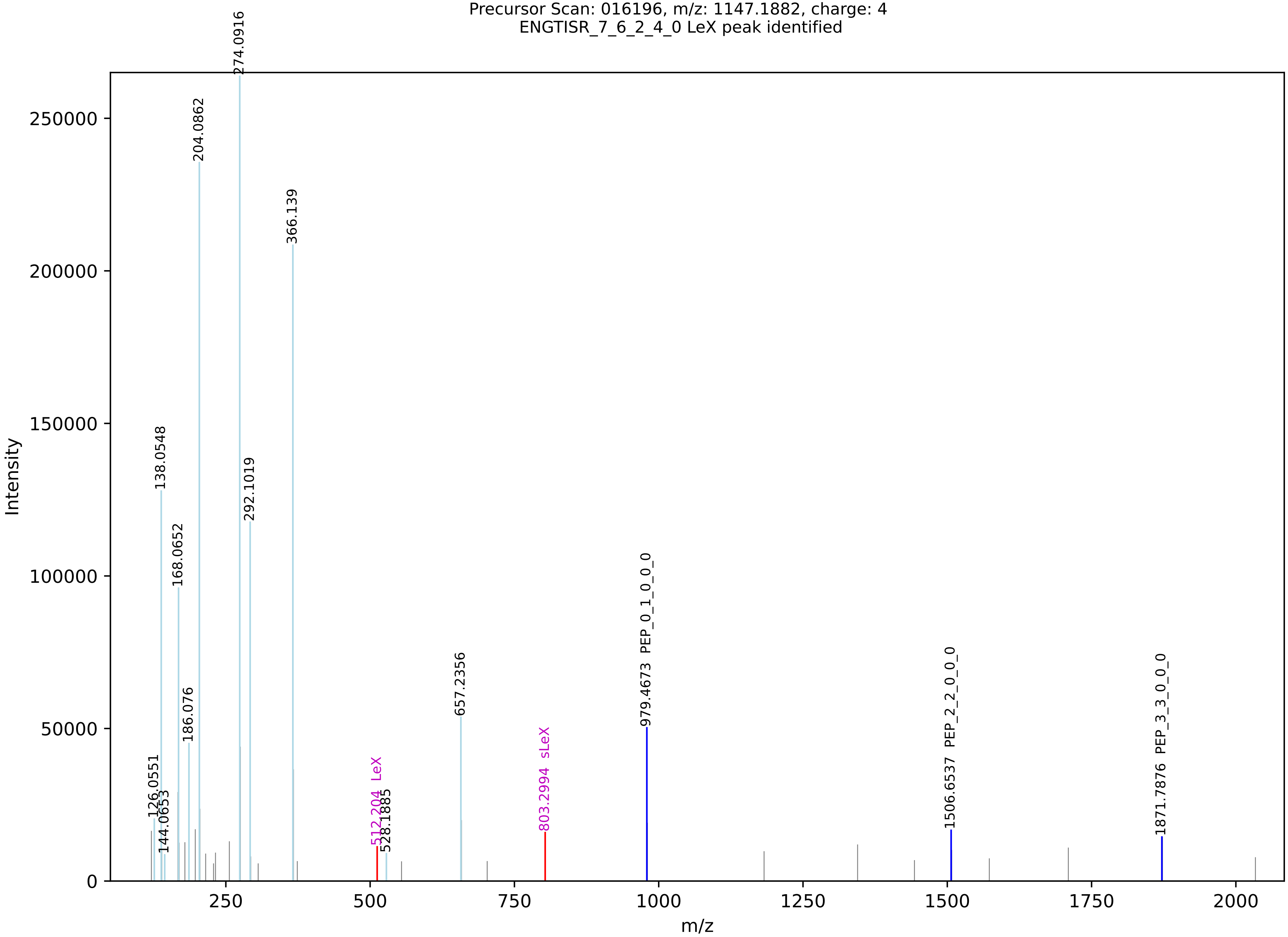

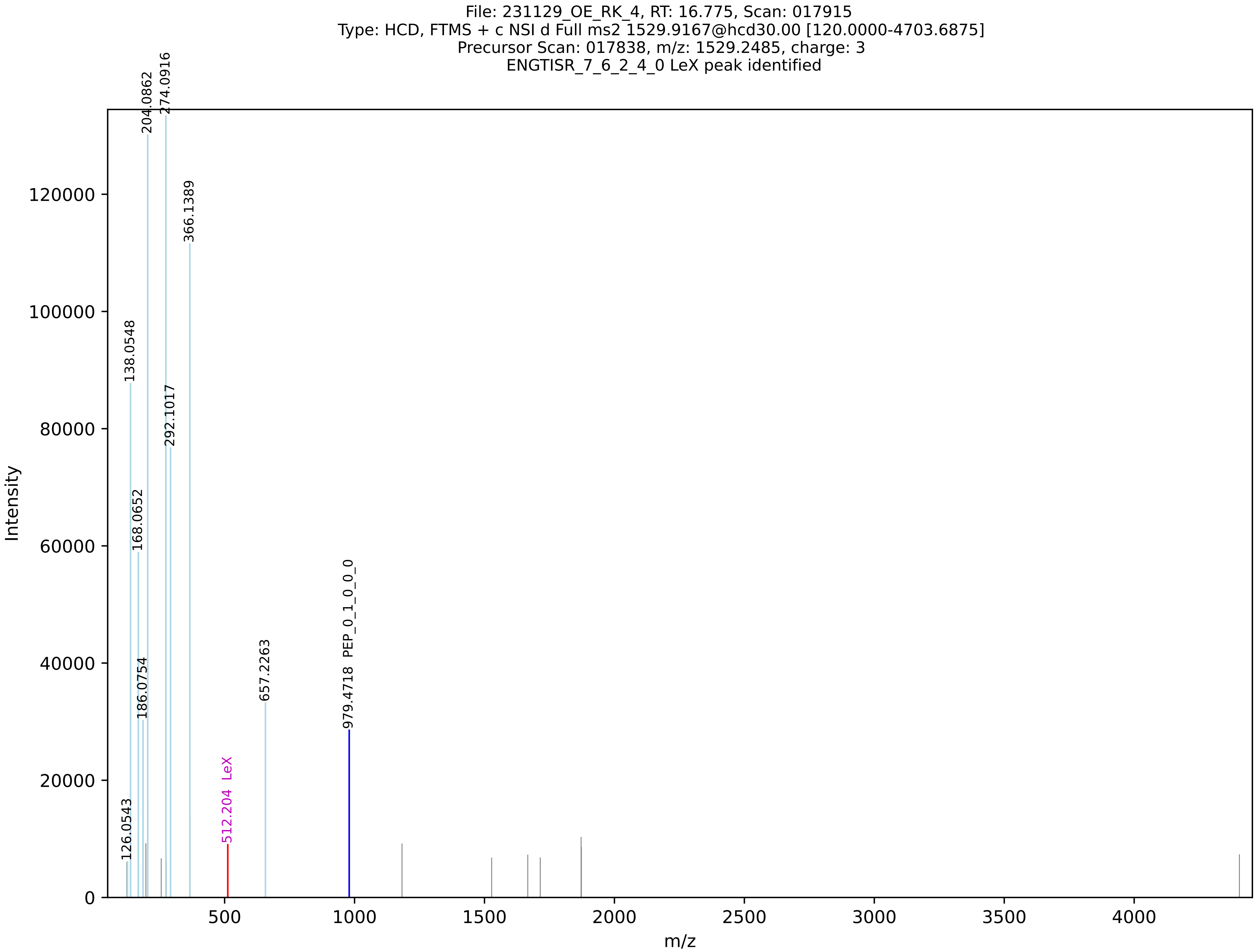
File: 231129_OE_RK_4, RT: 16.775, Scan: 017915
Type: HCD, FTMS + c NSI d Full ms2 1529.9167@hcd30.00 [120.0000-4703.6875]
Precursor Scan: 017838, m/z: 1529.2485, charge: 3
ENGTISR_7_6_2_4_0 LeX peak identified
Intensity
m/z
204.0862
274.0916
366.1389
138.0548
292.1017
168.0652
186.0754
657.2263
979.4718 PEP_0_1_0_0_0
512.204 LeX
126.0543
0
20000
40000
60000
80000
100000
120000
500
1000
1500
2000
2500
3000
3500
4000

File: 231129_OE_RK_4, RT: 16.974, Scan: 018203
Type: HCD, FTMS + c NSI d Full ms2 1481.2294@hcd30.00 [120.0000-4554.7036]
Precursor Scan: 018130, m/z: 1480.56, charge: 3
ENGTISR_7_6_1_4_0 LeX peak identified

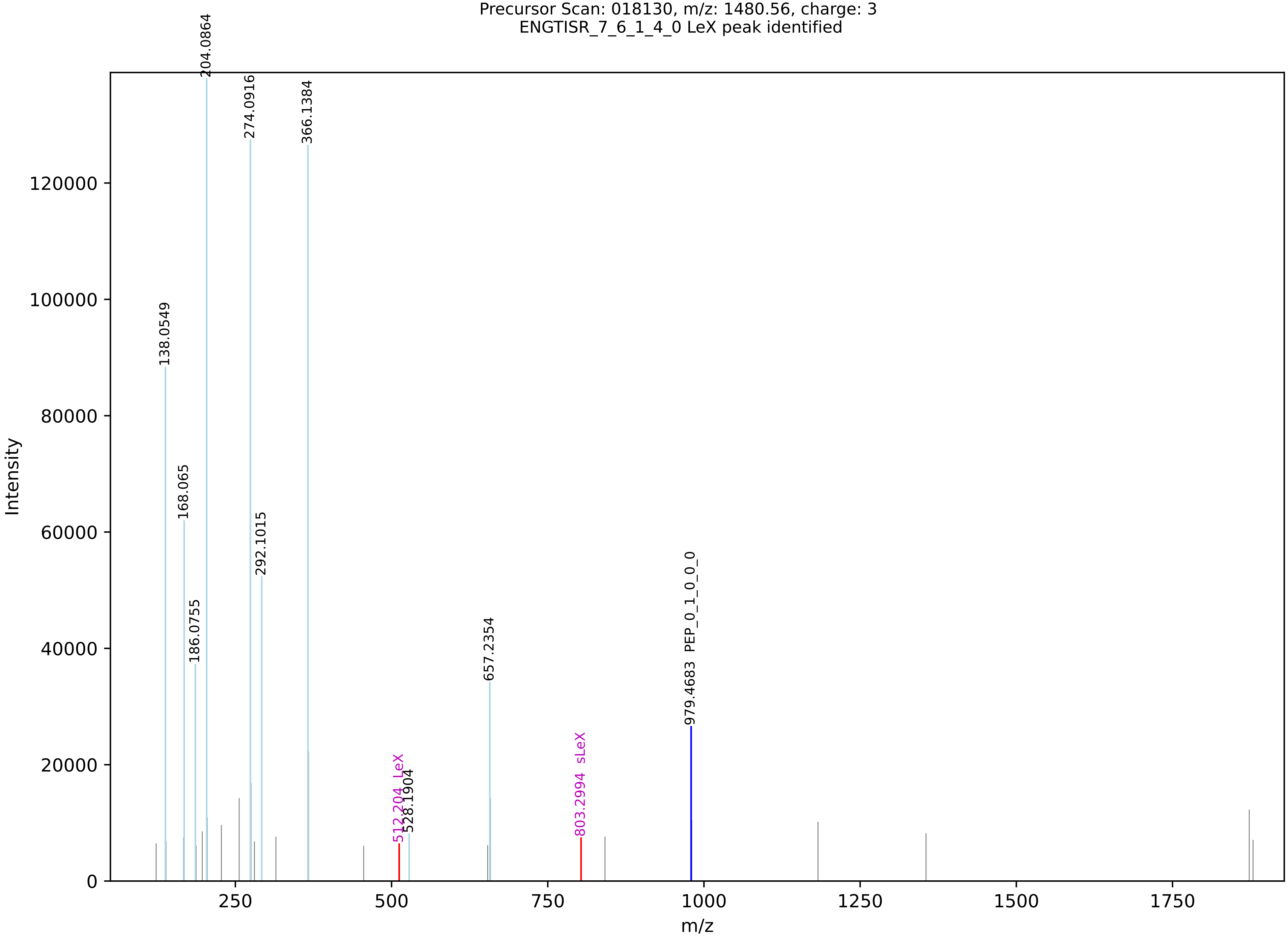

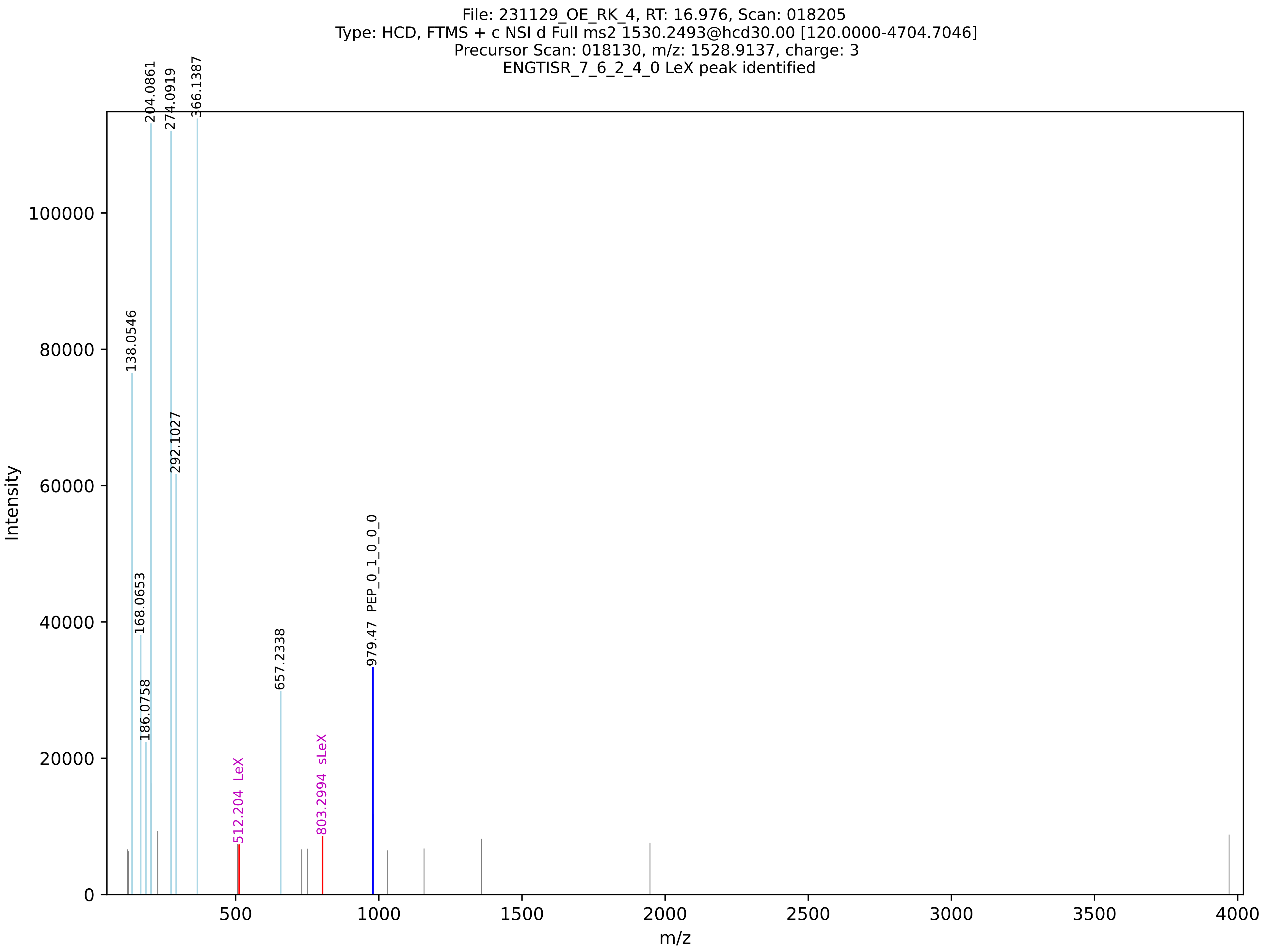
File: 231129_OE_RK_4, RT: 16.976, Scan: 018205
Type: HCD, FTMS + c NSI d Full ms2 1530.2493@hcd30.00 [120.0000-4704.7046]
Precursor Scan: 018130, m/z: 1528.9137, charge: 3
ENGTISR_7_6_2_4_0 LeX peak identified
Intensity
m/z
204.0861
274.0919
366.1387
138.0546
292.1027
168.0653
186.0758
657.2338
979.47 PEP_0_1_0_0_0
512.204 LeX
803.2994 sLeX
0
20000
40000
60000
80000
100000
500
1000
1500
2000
2500
3000
3500
4000

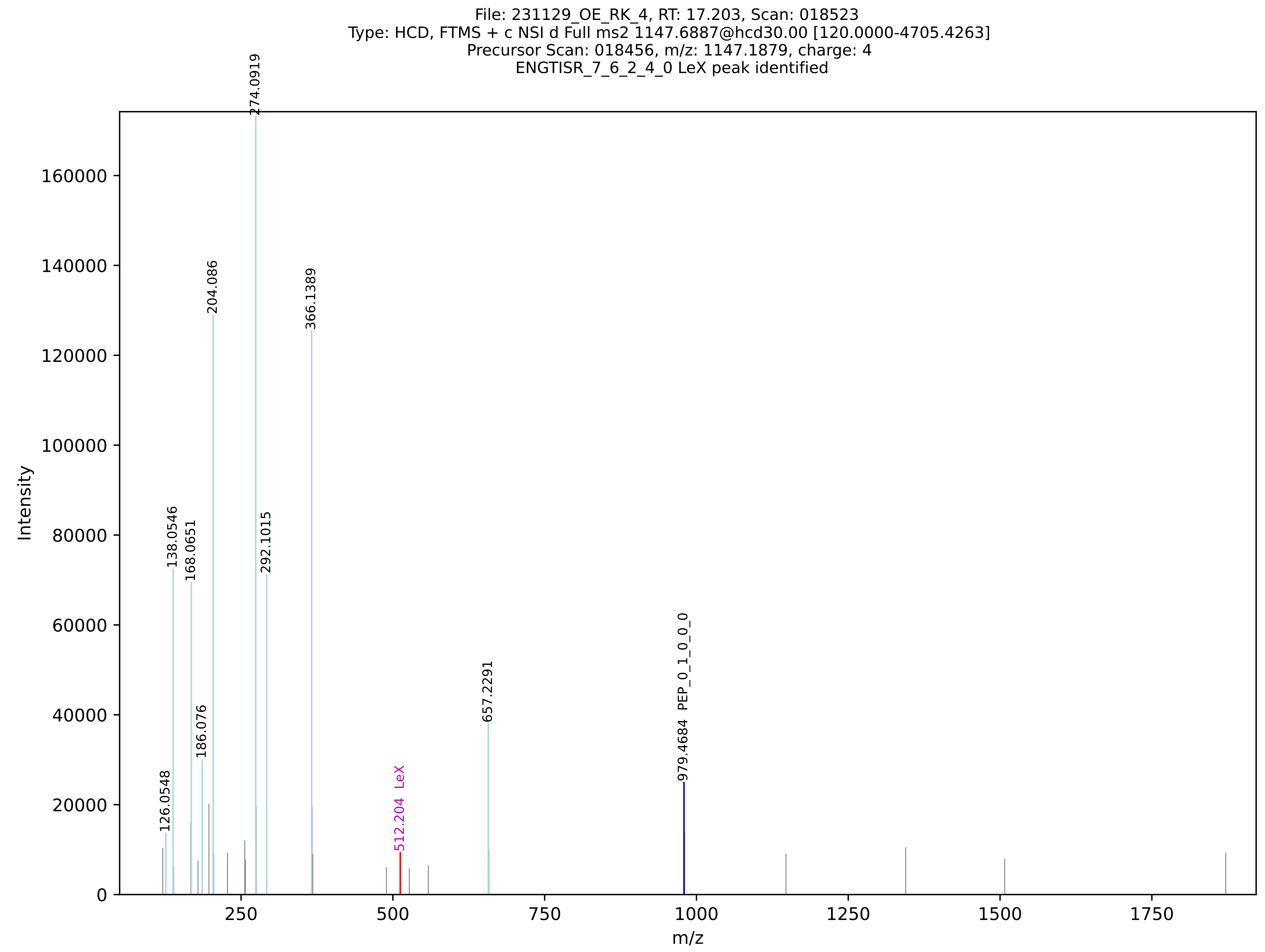
File: 231129_OE_RK_4, RT: 17.203, Scan: 018523
Type: HCD, FTMS + c NSI d Full ms2 1147.6887@hcd30.00 [120.0000-4705.4263]
Precursor Scan: 018456, m/z: 1147.1879, charge: 4
ENGTISR_7_6_2_4_0 LeX peak identified
Intensity
m/z
274.0919
204.086
366.1389
138.0546
168.0651
292.1015
186.076
126.0548
512.204 LeX
657.2291
979.4684 PEP_0_1_0_0_0

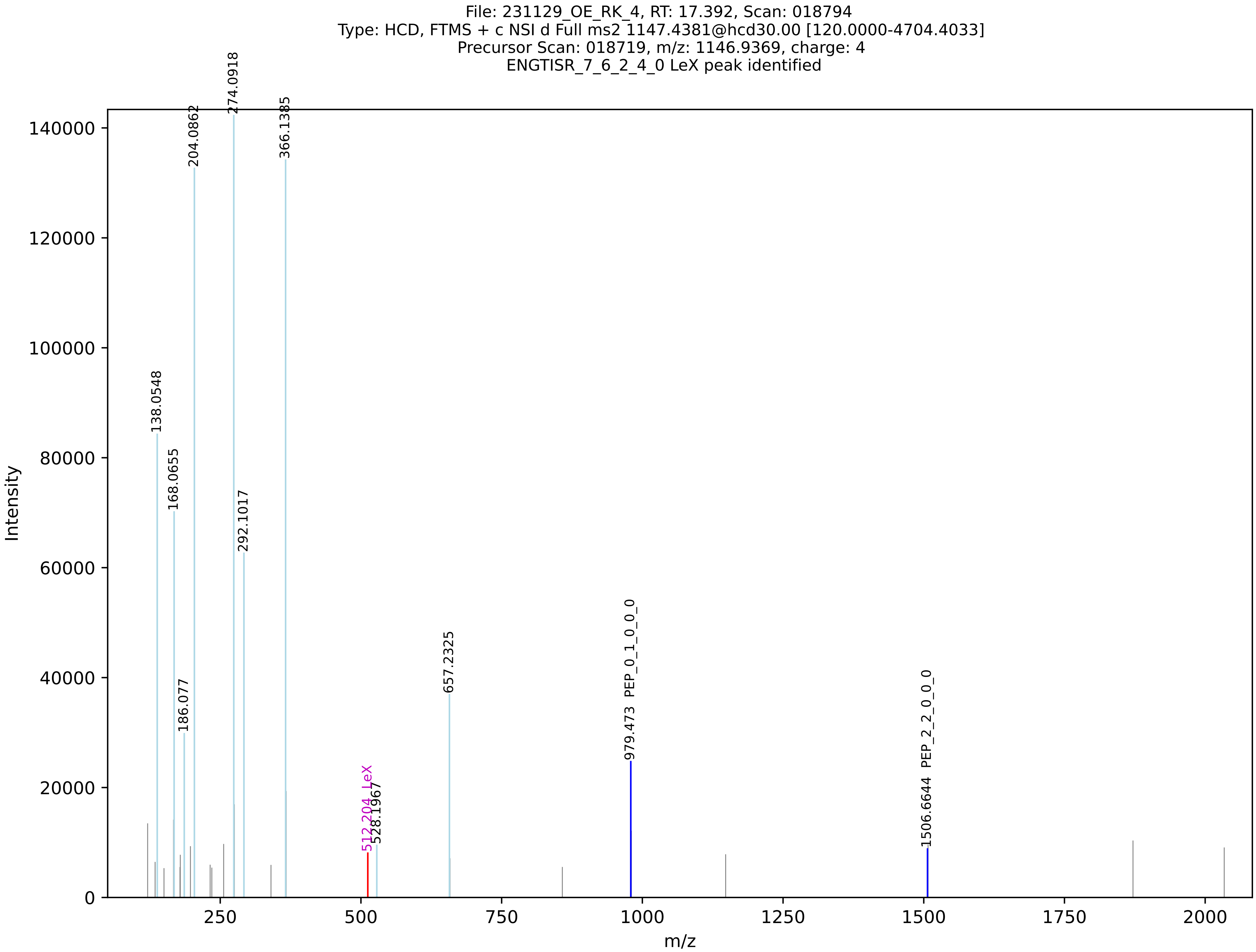
File: 231129_OE_RK_4, RT: 17.392, Scan: 018794
Type: HCD, FTMS + c NSI d Full ms2 1147.4381@hcd30.00 [120.0000-4704.4033]
Precursor Scan: 018719, m/z: 1146.9369, charge: 4
ENGTISR_7_6_2_4_0 LeX peak identified
Intensity
m/z
274.0918
366.1385
204.0862
138.0548
168.0655
292.1017
186.077
657.2325
512.204_LeX
528.1967
979.473 PEP_0_1_0_0_0
1506.6644 PEP_2_2_0_0_0
0
20000
40000
60000
80000
100000
120000
140000
250
500
750
1000
1250
1500
1750
2000

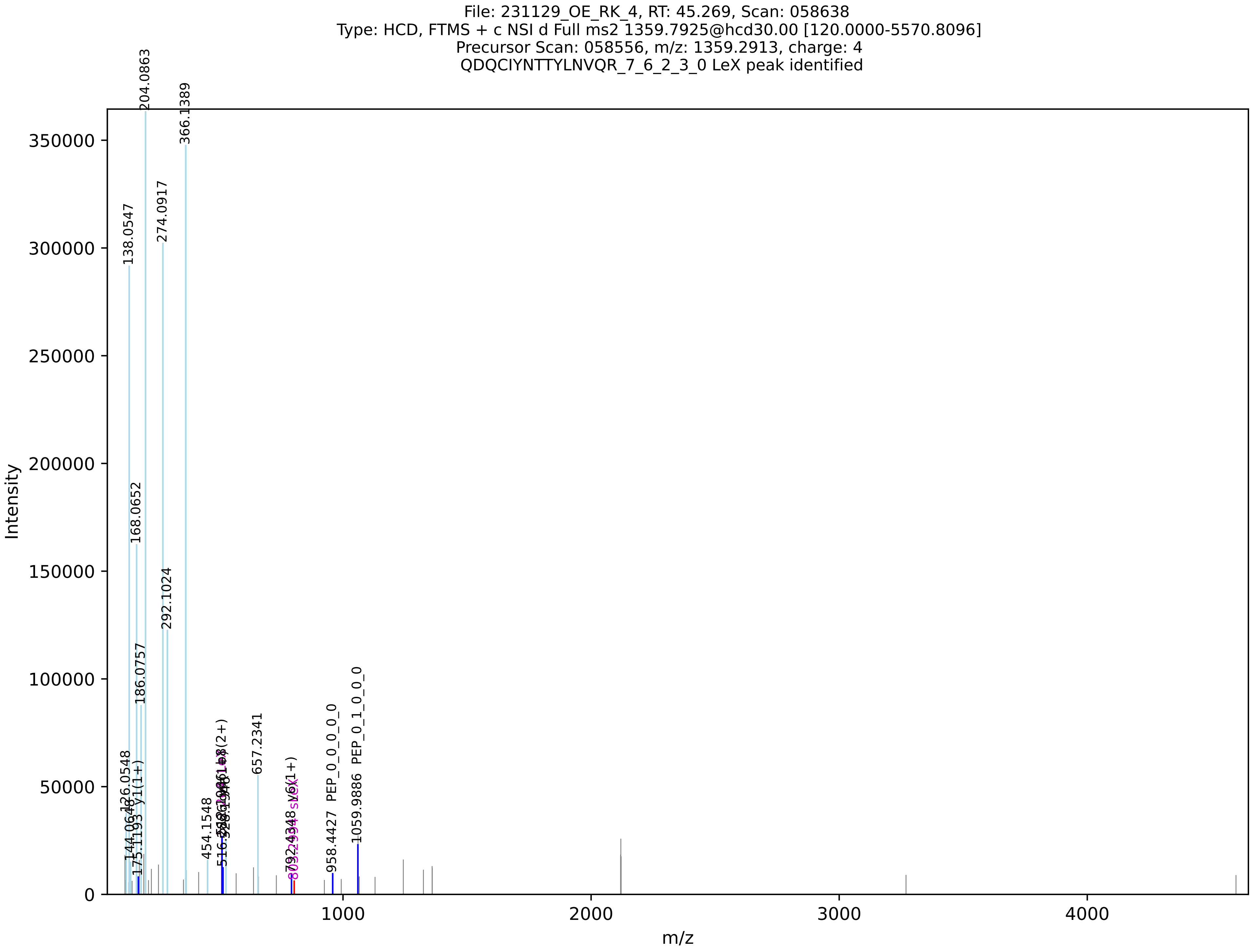
File: 231129_OE_RK_4, RT: 45.269, Scan: 058638
Type: HCD, FTMS + c NSI d Full ms2 1359.7925@hcd30.00 [120.0000-5570.8096]
Precursor Scan: 058556, m/z: 1359.2913, charge: 4
QDQCIYNTTYLNVQR_7_6_2_3_0 LeX peak identified
Intensity
m/z
204.0863
366.1389
274.0917
138.0547
168.0652
292.1024
186.0757
126.0548
144.0648
175.1193 y1(1+)
454.1548
657.2341
y6(1+)
958.4427 PEP_0_0_0_0_0
1059.9886 PEP_0_1_0_0_0
0
50000
100000
150000
200000
250000
300000
350000
1000
2000
3000
4000

File: 231129_OE_RK_4, RT: 46.277, Scan: 060103
Type: HCD, FTMS + c NSI d Full ms2 1359.7920@hcd30.00 [120.0000-5570.8076]
Precursor Scan: 060027, m/z: 1359.0403, charge: 4
QDQCIYNTTYLNVQR_9_8_1_1_0 LeX peak identified

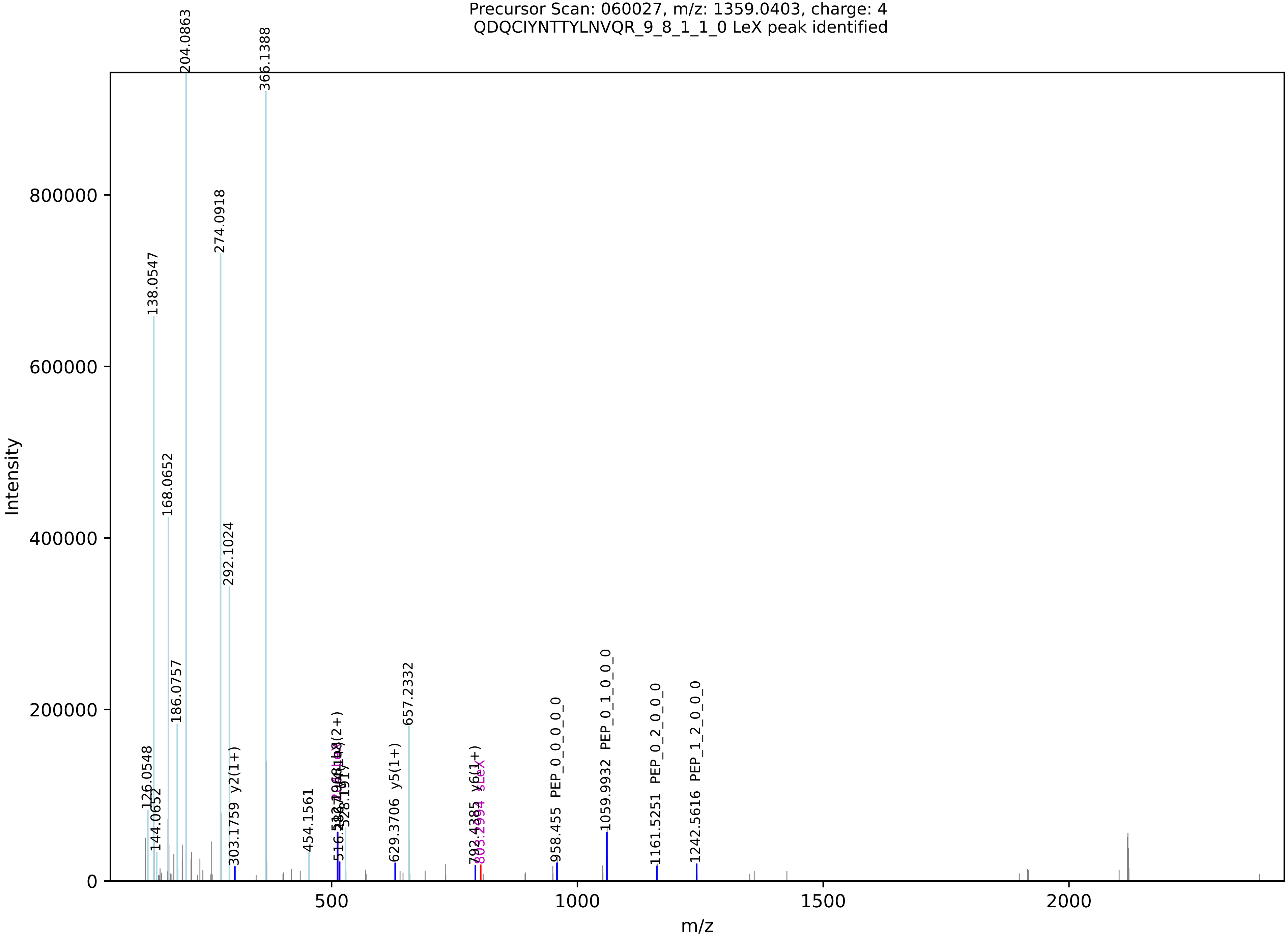

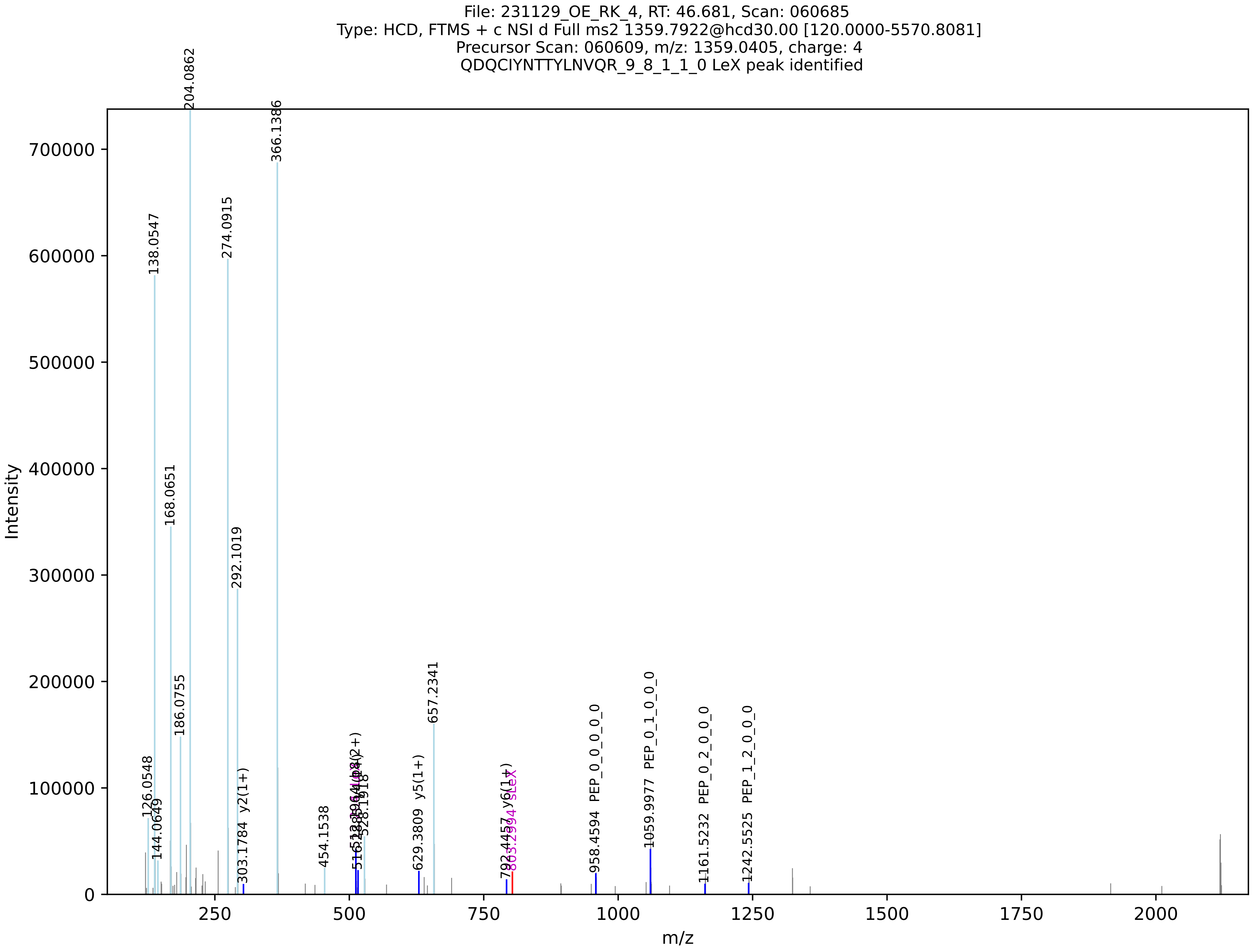

File: 231129_OE_RK_4, RT: 46.681, Scan: 060685
Type: HCD, FTMS + c NSI d Full ms2 1359.7922@hcd30.00 [120.0000-5570.8081]
Precursor Scan: 060609, m/z: 1359.0405, charge: 4
QDQCIYNTTYLNVQR_9_8_1_1_0 LeX peak identified
Intensity
m/z
204.0862
366.1386
274.0915
138.0547
168.0651
292.1019
186.0755
657.2341
126.0548
144.0649
303.1784 y2(1+)
454.1538
528.1918
629.3809 y5(1+)
792.4457 y6(1+)
803.2994 sLeX
958.4594 PEP_0_0_0_0_0
1059.9977 PEP_0_1_0_0_0
1161.5232 PEP_0_2_0_0_0
1242.5525 PEP_1_2_0_0_0

File: 231129_OE_RK_4, RT: 46.686, Scan: 060693
Type: HCD, FTMS + c NSI d Full ms2 1396.3062@hcd30.00 [120.0000-5719.7852]
Precursor Scan: 060609, m/z: 1395.5544, charge: 4
QDQCIYNTTYLNVQR_7_6_1_4_0 LeX peak identified

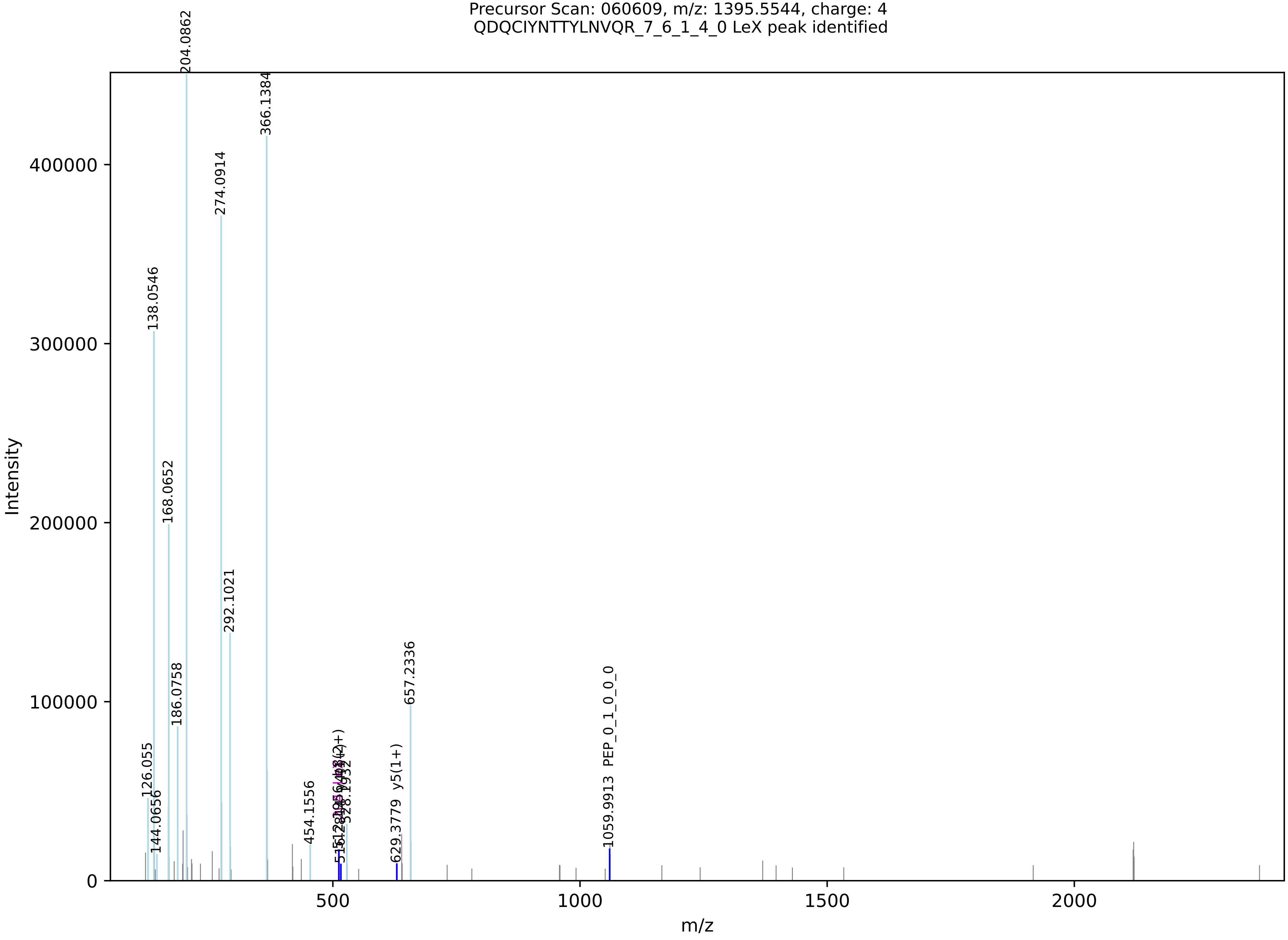

File: 231129_OE_RK_4, RT: 46.935, Scan: 061054
Type: HCD, FTMS + c NSI d Full ms2 1396.3033@hcd30.00 [120.0000-5719.7734]
Precursor Scan: 060974, m/z: 1395.5515, charge: 4
QDQCIYNTTYLNVQR_7_6_1_4_0 LeX peak identified

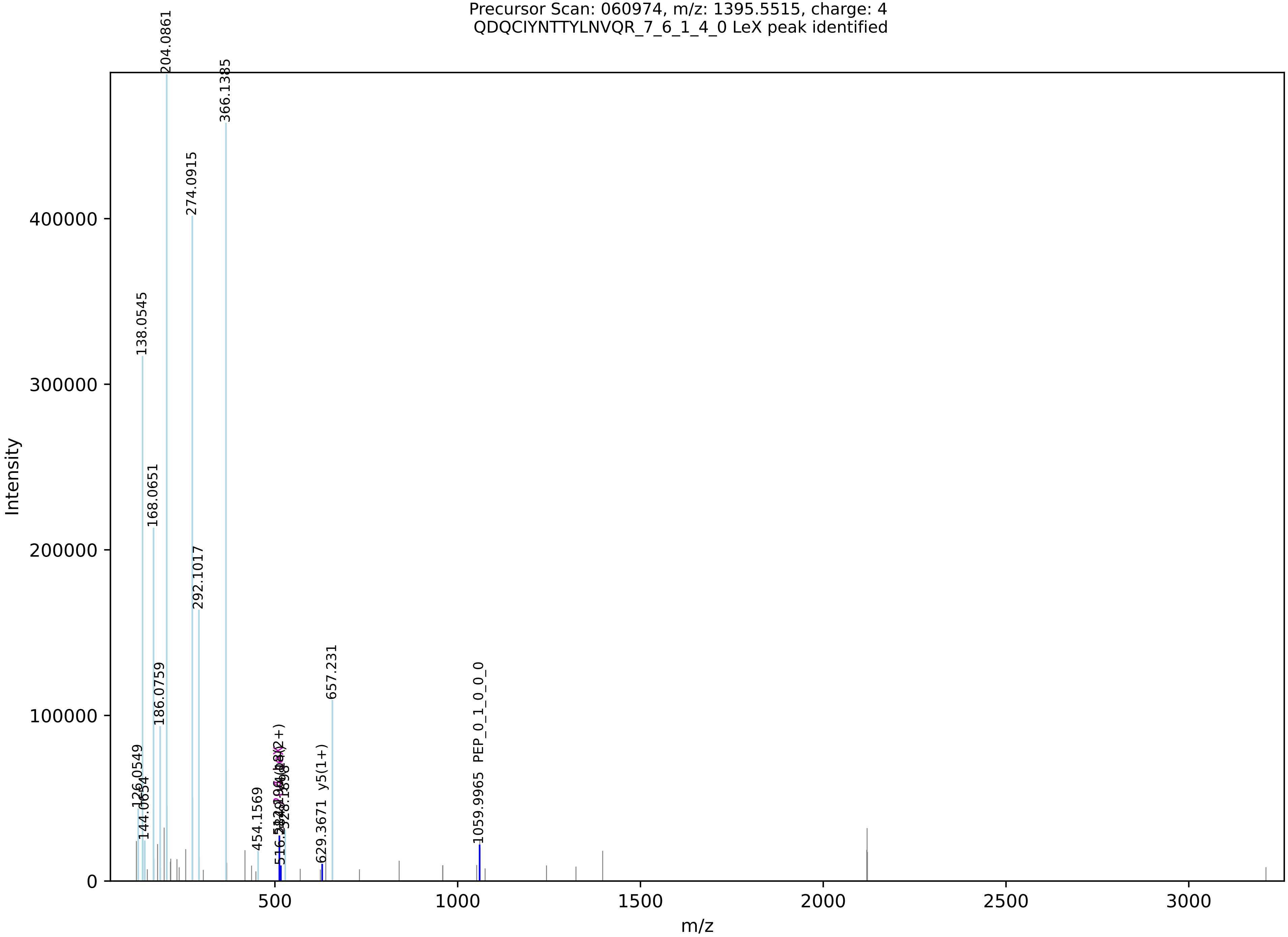

File: 231129_OE_RK_4, RT: 47.361, Scan: 061677
Type: HCD, FTMS + c NSI d Full ms2 1763.6969@hcd30.00 [120.0000-5419.0547]
Precursor Scan: 061589, m/z: 1763.3633, charge: 3
QDQCIYNTTYLNVQR_9_8_0_1_0 LeX peak identified

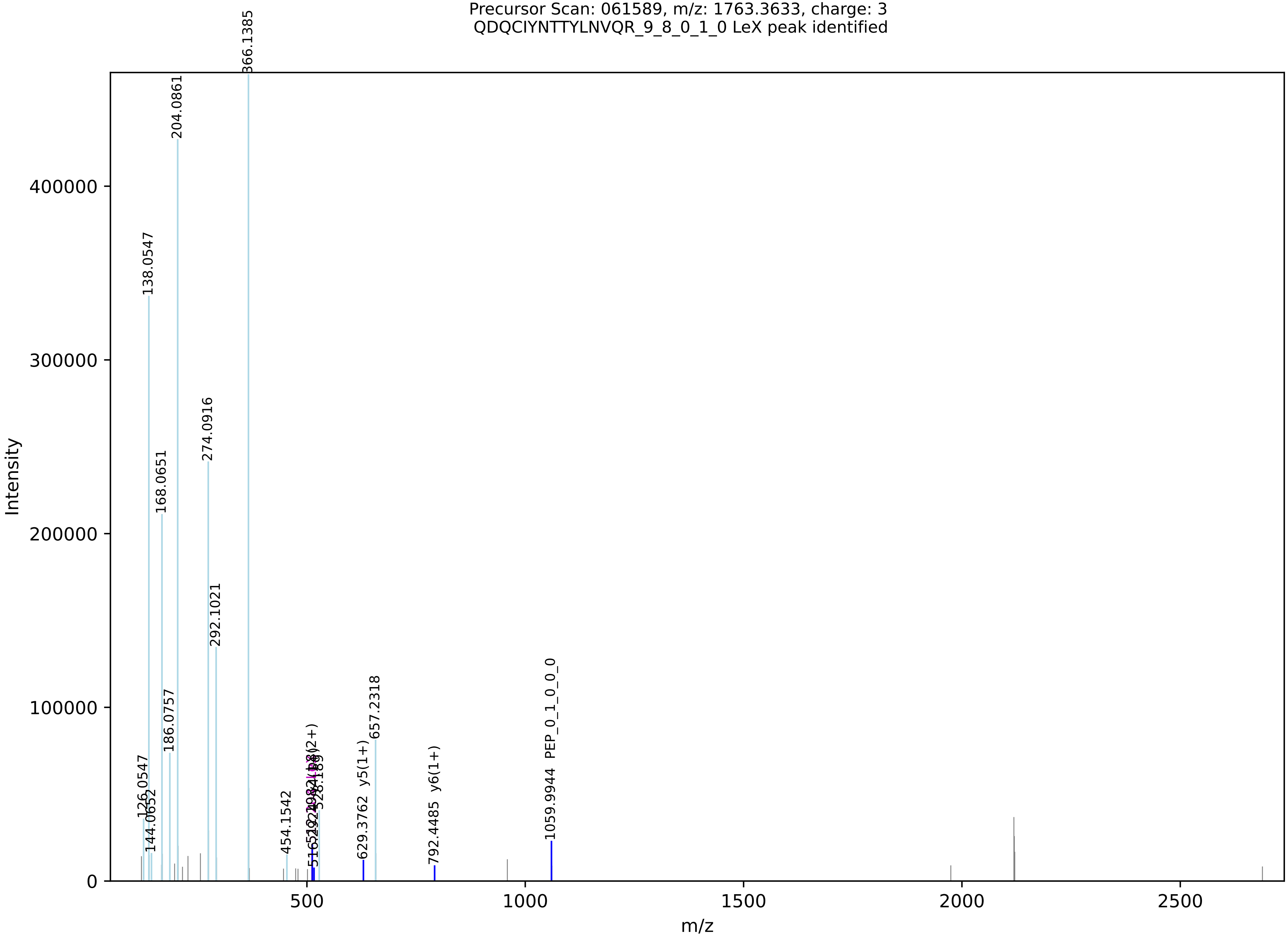

File: 231129_OE_RK_4, RT: 47.759, Scan: 062255
Type: HCD, FTMS + c NSI d Full ms2 1323.5265@hcd30.00 [120.0000-5422.8438]
Precursor Scan: 062180, m/z: 1322.7747, charge: 4
QDQCIYNTTYLNVQR_7_6_1_3_0 LeX peak identified

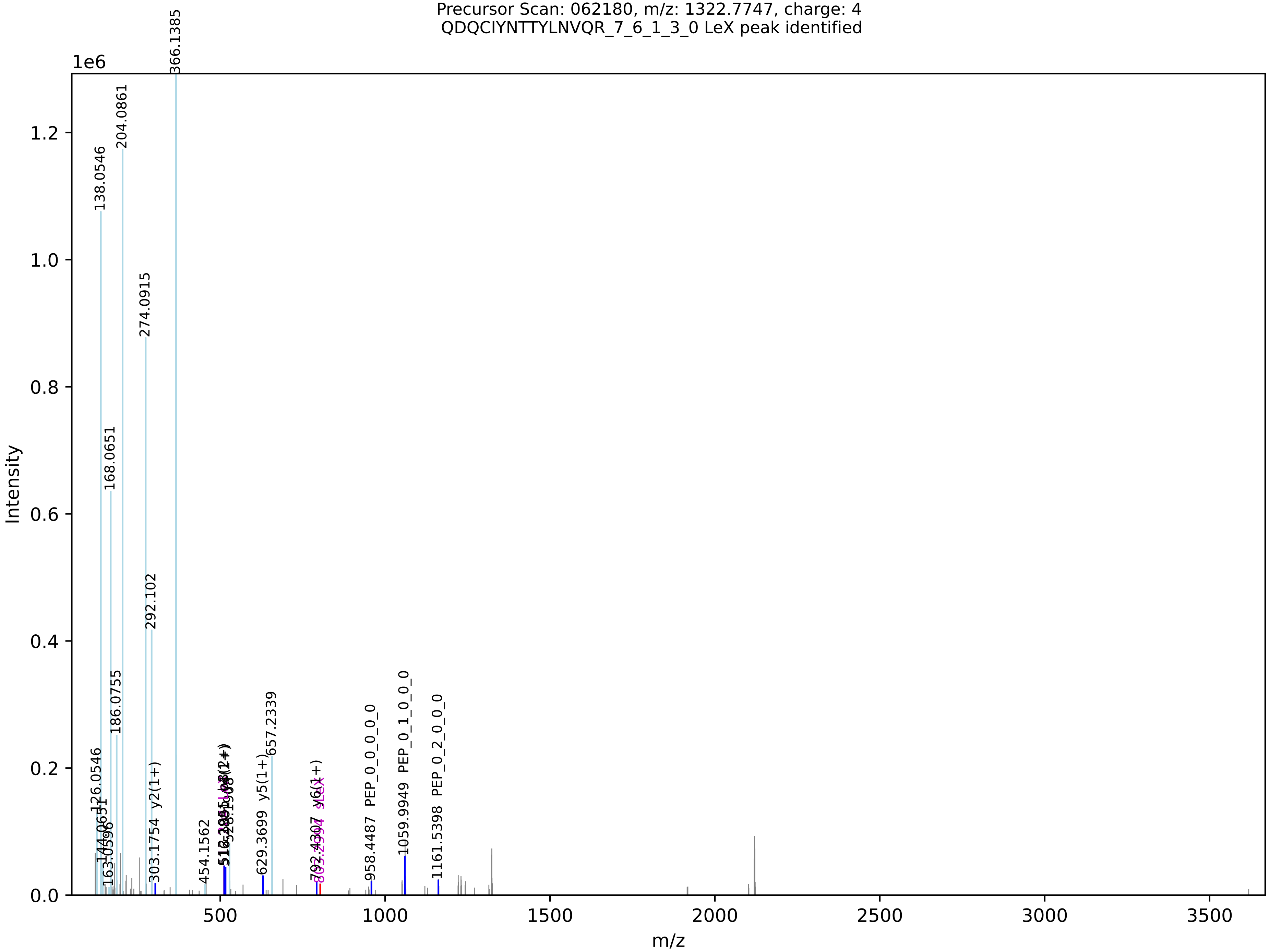

File: 231129_OE_RK_4, RT: 48.147, Scan: 062817
Type: HCD, FTMS + c NSI d Full ms2 1764.3629@hcd30.00 [120.0000-5421.0928]
Precursor Scan: 062759, m/z: 1763.3606, charge: 3
QDQCIYNTTYLNVQR_7_6_1_3_0 LeX peak identified

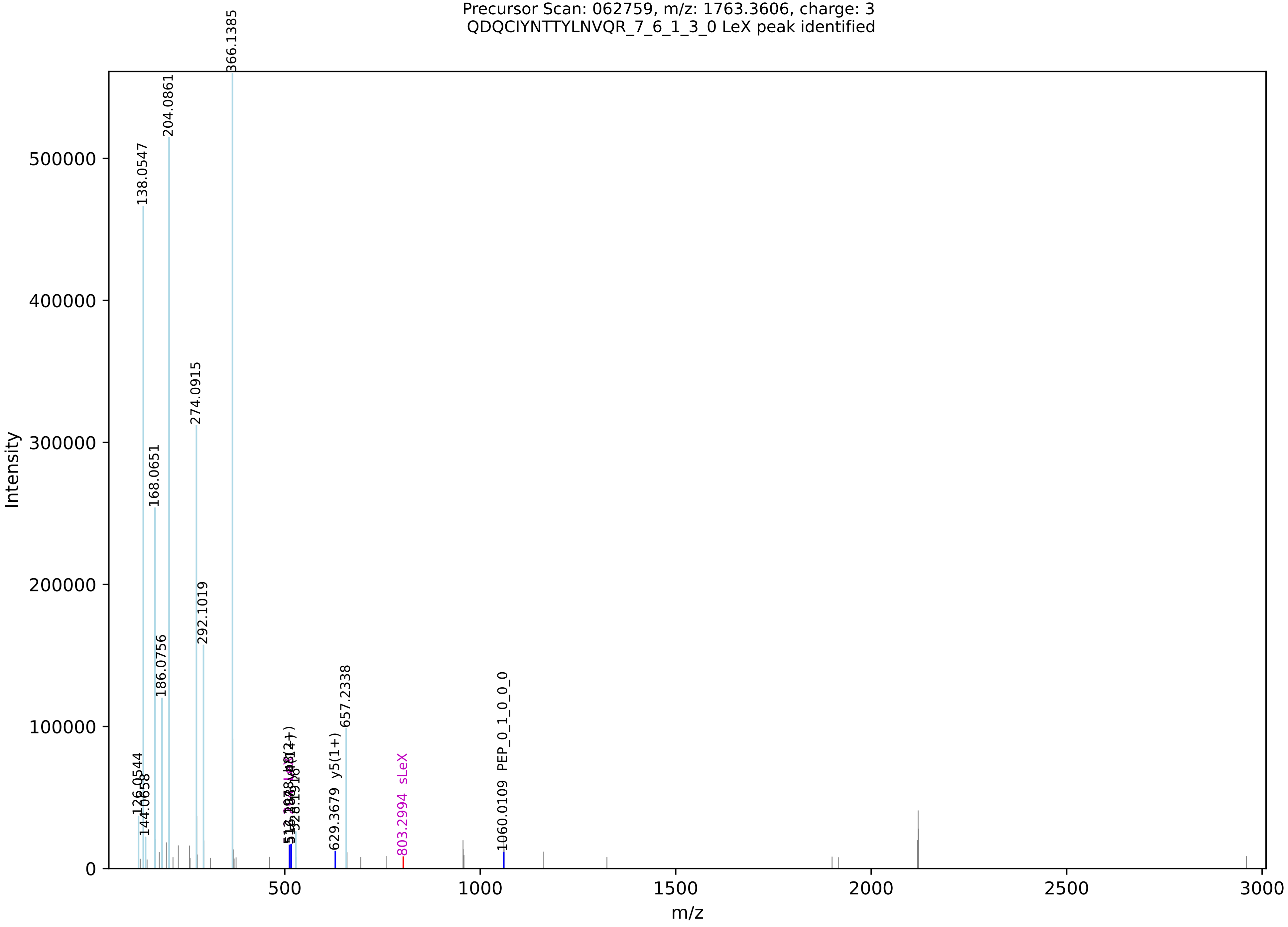

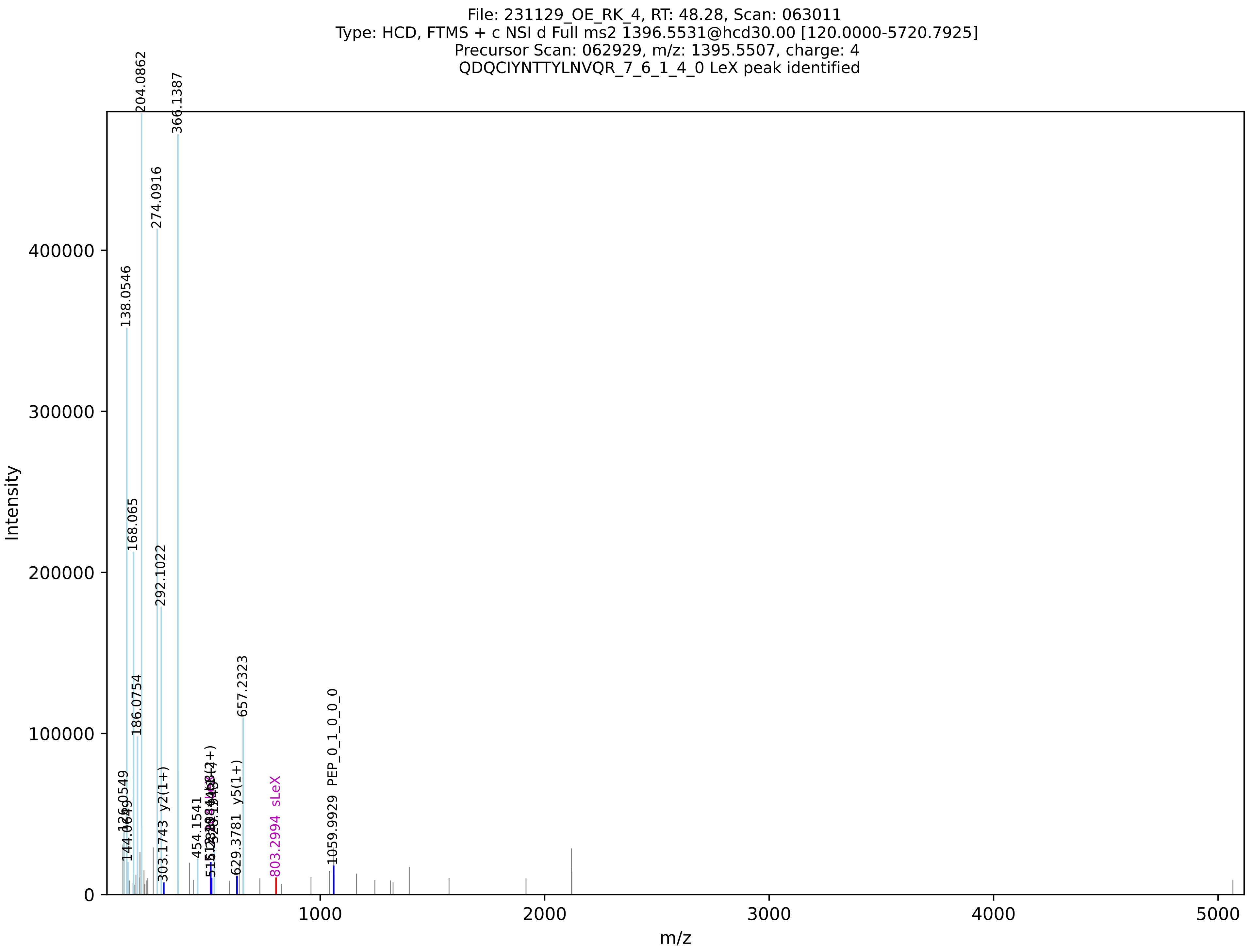

File: 231129_OE_RK_4, RT: 48.28, Scan: 063011
Type: HCD, FTMS + c NSI d Full ms2 1396.5531@hcd30.00 [120.0000-5720.7925]
Precursor Scan: 062929, m/z: 1395.5507, charge: 4
QDQCIYNTTYLNVQR_7_6_1_4_0 LeX peak identified
204.0862
366.1387
274.0916
138.0546
168.065
292.1022
186.0754
657.2323
PEP_0_1_0_0_0
1059.9929
803.2994 sLeX
629.3781 y5(1+)
303.1743 y2(1+)
454.1541
126.0549
144.0649
Intensity
m/z
0
100000
200000
300000
400000
1000
2000
3000
4000
5000

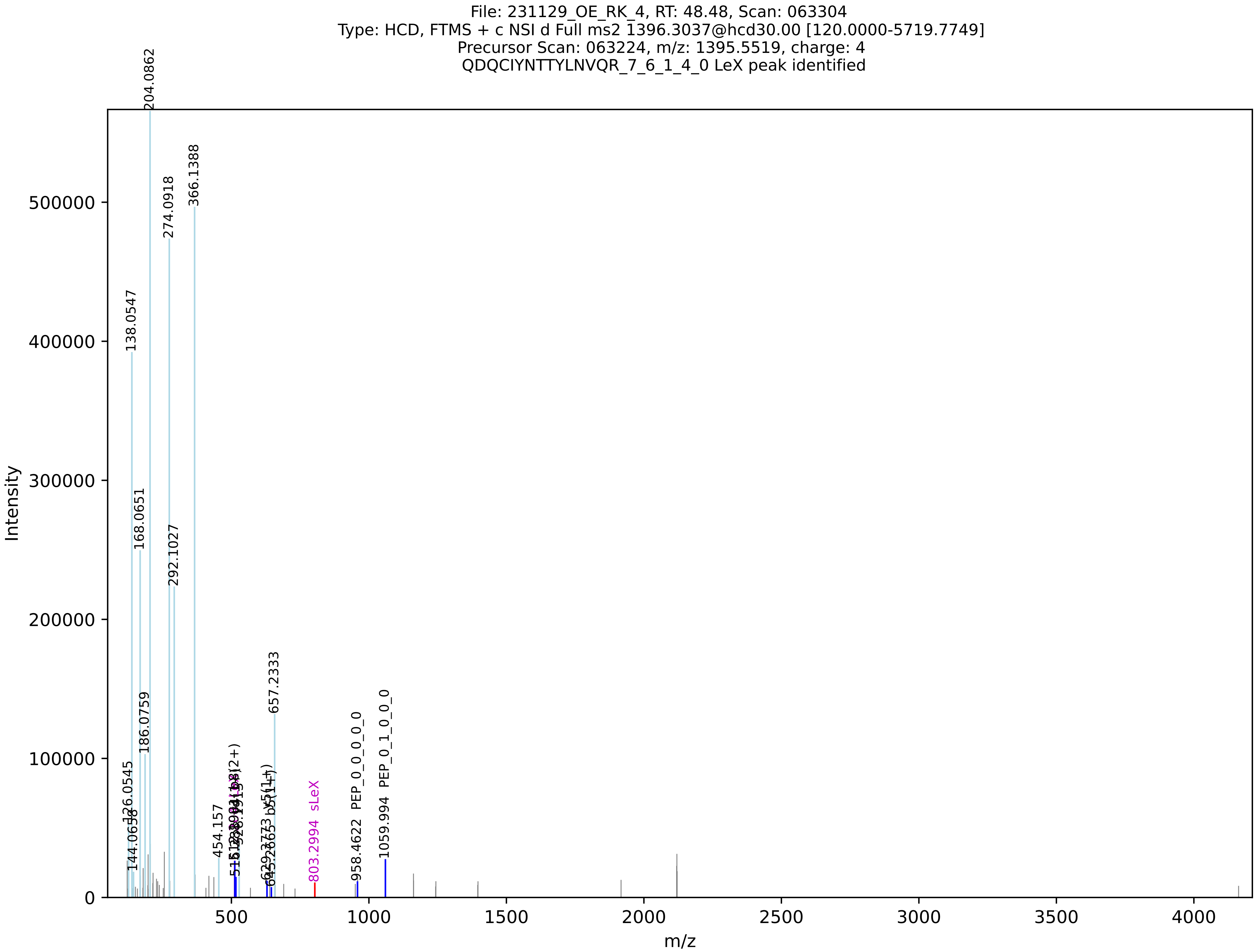
File: 231129_OE_RK_4, RT: 48.48, Scan: 063304
Type: HCD, FTMS + c NSI d Full ms2 1396.3037@hcd30.00 [120.0000-5719.7749]
Precursor Scan: 063224, m/z: 1395.5519, charge: 4
QDQCIYNTTYLNVQR_7_6_1_4_0 LeX peak identified
Intensity
m/z
204.0862
366.1388
274.0918
138.0547
168.0651
292.1027
186.0759
657.2333
126.0545
144.0658
454.157
803.2994 sLeX
958.4622 PEP_0_0_0_0_0
1059.994 PEP_0_1_0_0_0
0
100000
200000
300000
400000
500000
500
1000
1500
2000
2500
3000
3500
4000

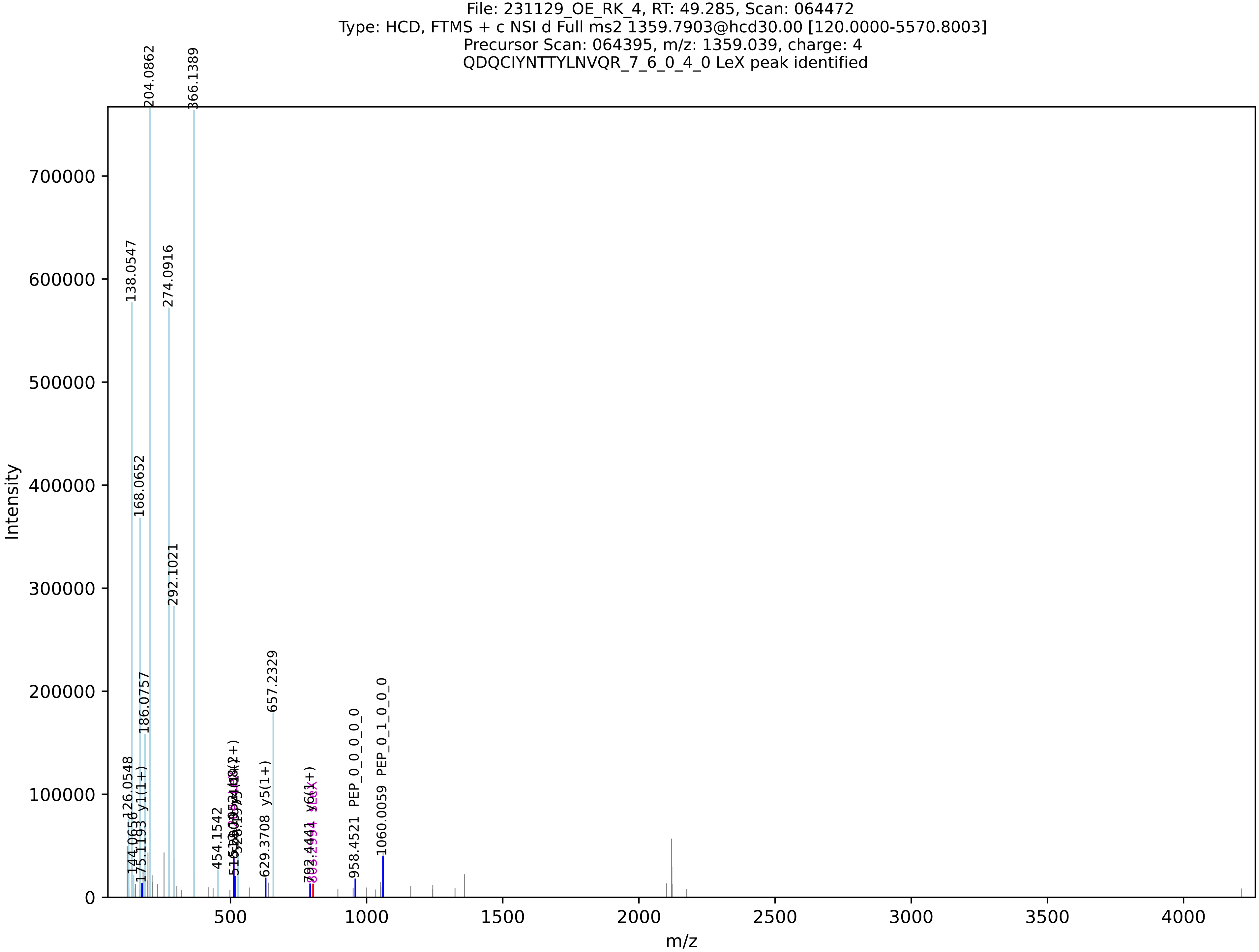
File: 231129_OE_RK_4, RT: 49.285, Scan: 064472
Type: HCD, FTMS + c NSI d Full ms2 1359.7903@hcd30.00 [120.0000-5570.8003]
Precursor Scan: 064395, m/z: 1359.039, charge: 4
QDQCIYNTTYLNVQR_7_6_0_4_0 LeX peak identified
Intensity
m/z
204.0862
366.1389
138.0547
274.0916
168.0652
292.1021
186.0757
657.2329
126.0548
y1(1+)
175.1193
454.1542
629.3708
y5(1+)
958.4521
PEP_0_0_0_0_0_
1060.0059
PEP_0_1_0_0_0_
0
100000
200000
300000
400000
500000
600000
700000
500
1000
1500
2000
2500
3000
3500
4000

File: 231129_OE_RK_4, RT: 49.339, Scan: 064551
Type: HCD, FTMS + c NSI d Full ms2 1396.5536@hcd30.00 [120.0000-5720.7944]
Precursor Scan: 064469, m/z: 1395.5512, charge: 4
QDQCIYNTTYLNVQR_7_6_1_4_0 LeX peak identified

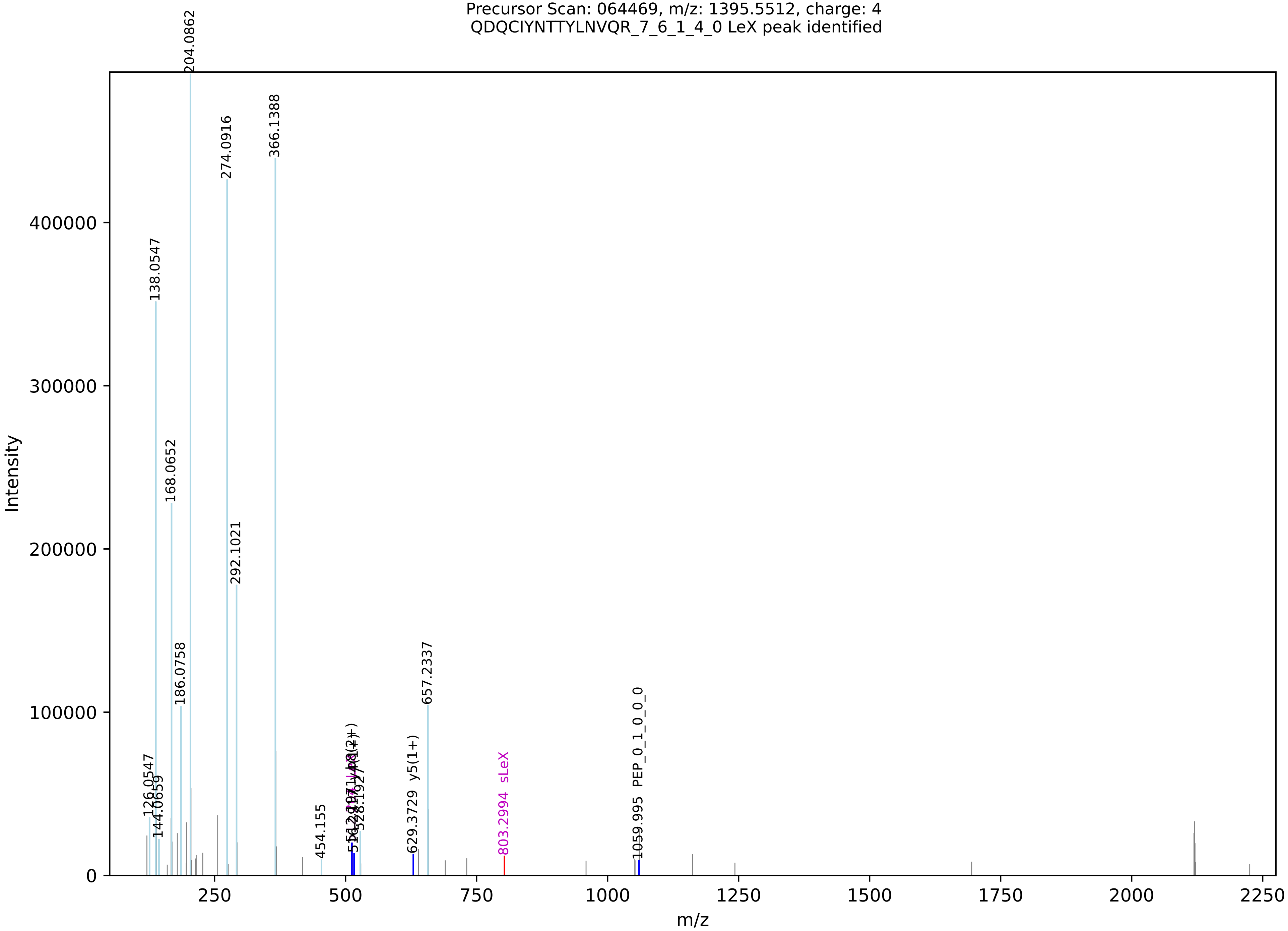

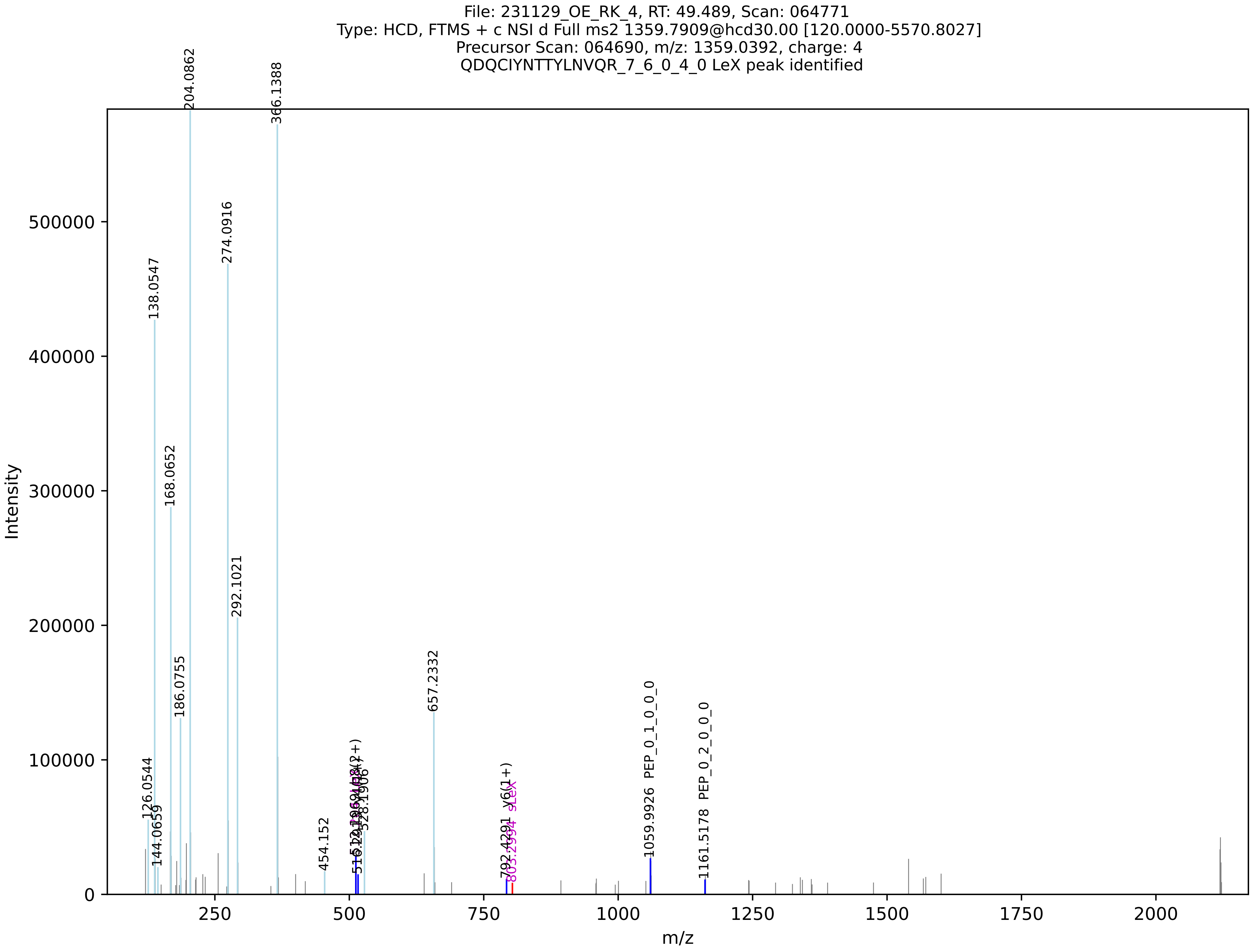
File: 231129_OE_RK_4, RT: 49.489, Scan: 064771
Type: HCD, FTMS + c NSI d Full ms2 1359.7909@hcd30.00 [120.0000-5570.8027]
Precursor Scan: 064690, m/z: 1359.0392, charge: 4
QDQCIYNTTYLNVQR_7_6_0_4_0 LeX peak identified
Intensity
m/z
0
100000
200000
300000
400000
500000
250
500
750
1000
1250
1500
1750
2000
204.0862
366.1388
274.0916
138.0547
168.0652
292.1021
186.0755
657.2332
126.0544
144.0659
454.152
528.1906
792.4291 y6(1+)
803.2994 sLeX
1059.9926 PEP_0_1_0_0_0_
1161.5178 PEP_0_2_0_0_0_

File: 231129_OE_RK_4, RT: 49.542, Scan: 064845
Type: HCD, FTMS + c NSI d Full ms2 1396.3041@hcd30.00 [120.0000-5719.7764]
Precursor Scan: 064764, m/z: 1395.5523, charge: 4
QDQCIYNTTYLNVQR_7_6_1_4_0 LeX peak identified

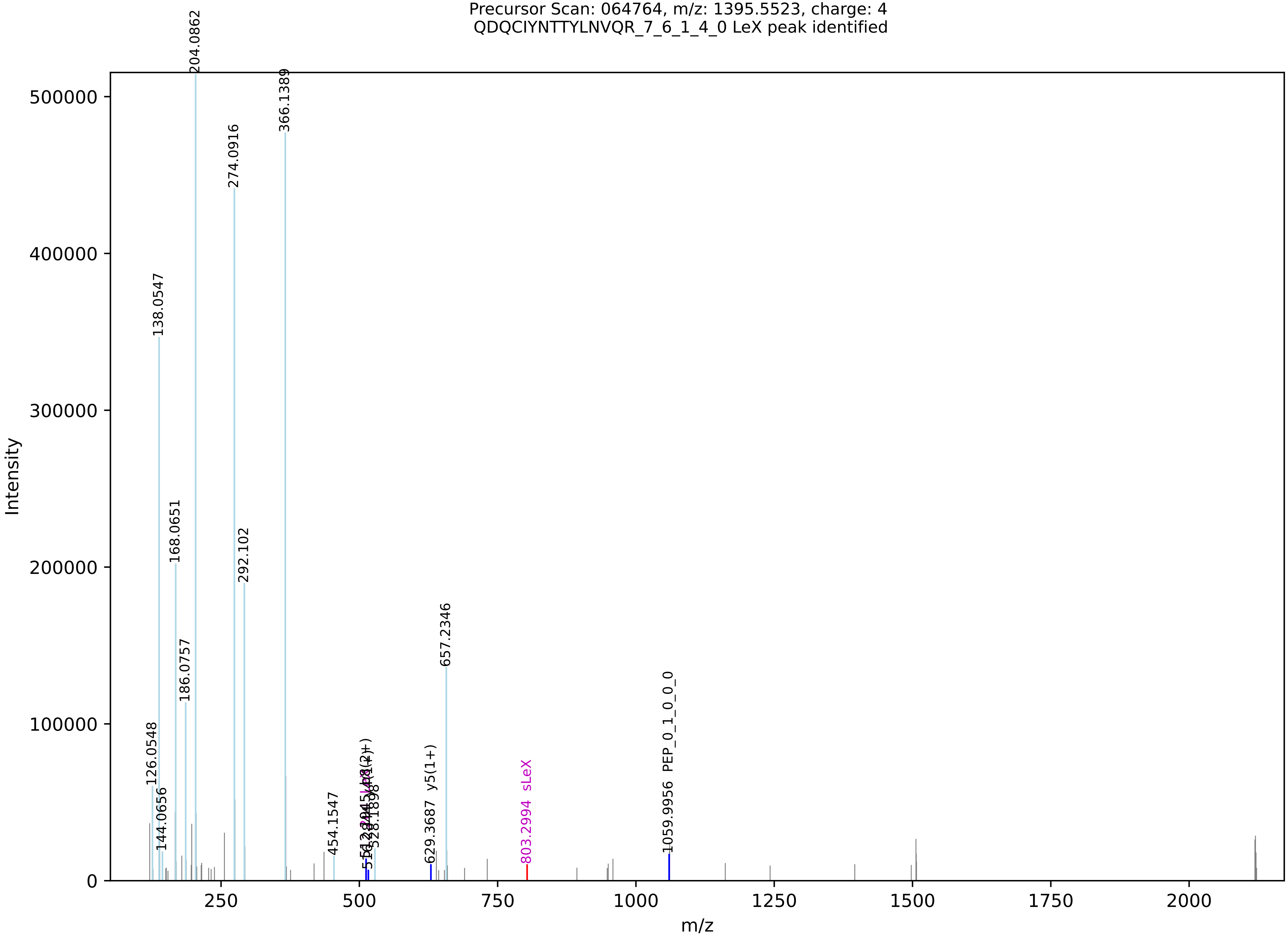

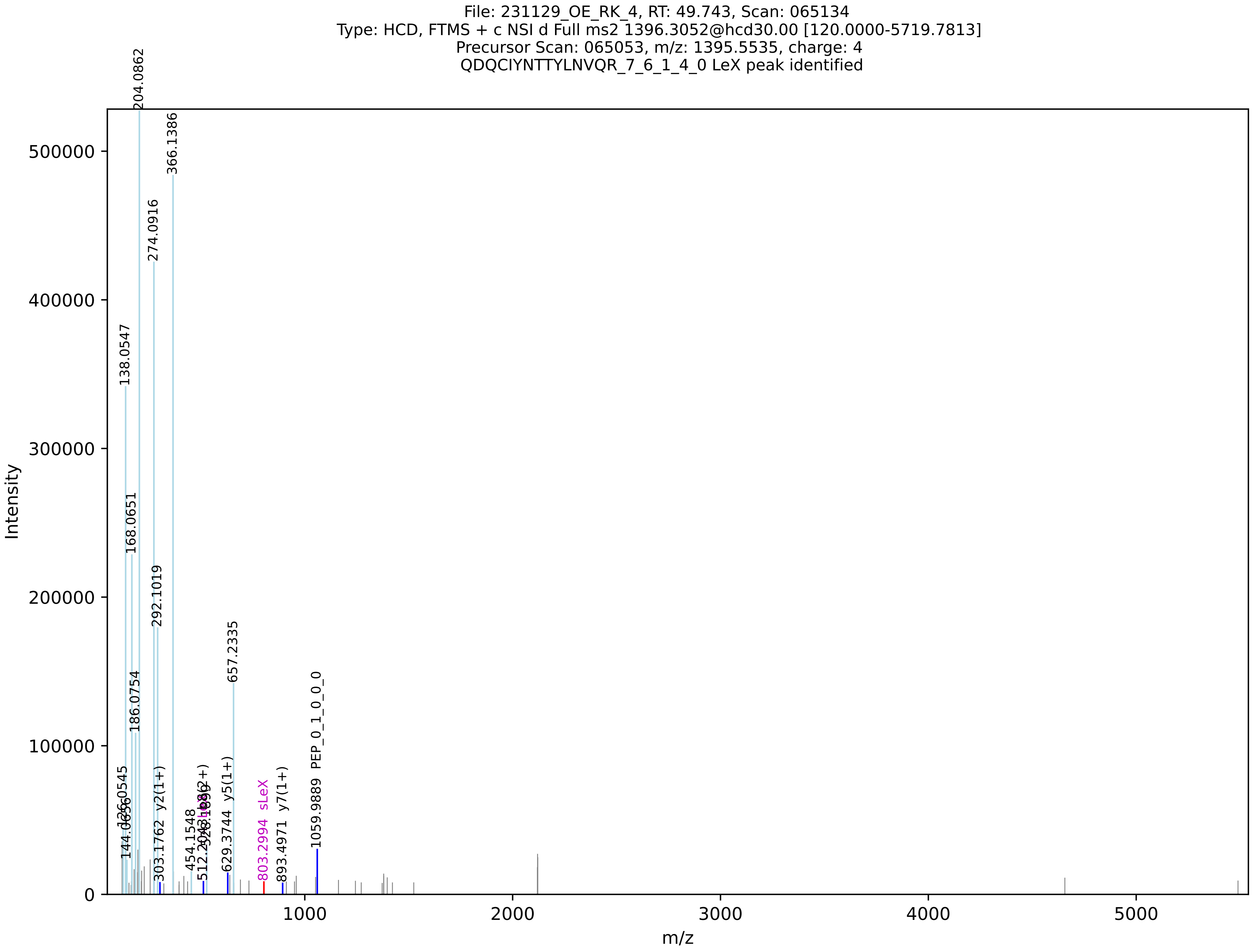
File: 231129_OE_RK_4, RT: 49.743, Scan: 065134
Type: HCD, FTMS + c NSI d Full ms2 1396.3052@hcd30.00 [120.0000-5719.7813]
Precursor Scan: 065053, m/z: 1395.5535, charge: 4
QDQCIYNTTYLNVQR_7_6_1_4_0 LeX peak identified
204.0862
366.1386
274.0916
138.0547
168.0651
292.1019
657.2335
186.0754
PEP_0_1_0_0_0
126.0545
144.0656
y2(1+)
303.1762
454.1548
y5(1+)
629.3744
sLeX
803.2994
y7(1+)
893.4971
1059.9889
Intensity
m/z
0
100000
200000
300000
400000
500000
1000
2000
3000
4000
5000

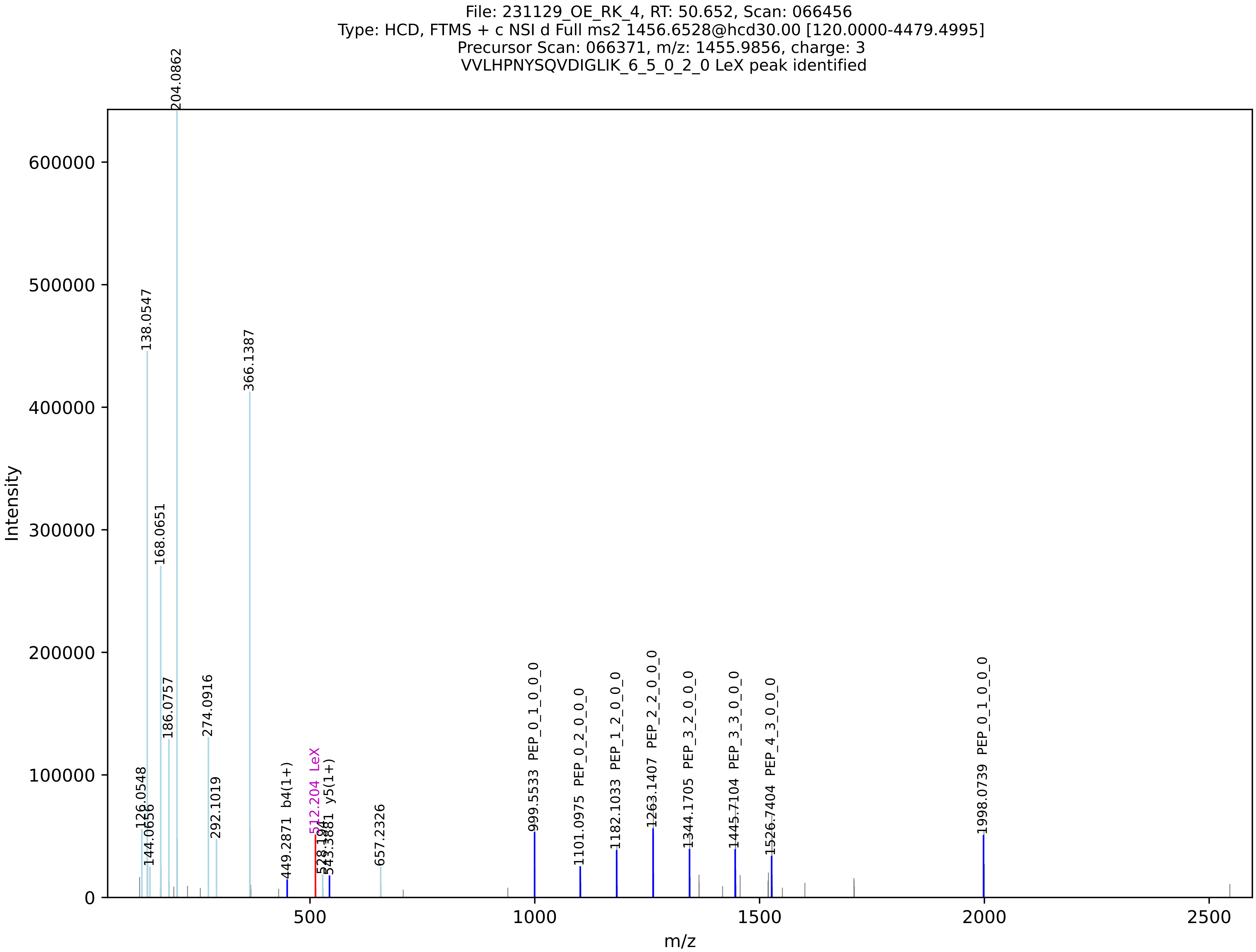
File: 231129_OE_RK_4, RT: 50.652, Scan: 066456
Type: HCD, FTMS + c NSI d Full ms2 1456.6528@hcd30.00 [120.0000-4479.4995]
Precursor Scan: 066371, m/z: 1455.9856, charge: 3
VVLHPNYSQVDIGLIK_6_5_0_2_0 LeX peak identified
Intensity
m/z
204.0862
138.0547
366.1387
168.0651
186.0757
274.0916
126.0548
144.0656
292.1019
449.2871 b4(1+)
512.204 LeX
543.3881 y5(1+)
657.2326
999.5533 PEP_0_1_0_0_0
1101.0975 PEP_0_2_0_0_0
1182.1033 PEP_1_2_0_0_0
1263.1407 PEP_2_2_0_0_0
1344.1705 PEP_3_2_0_0_0
1445.7104 PEP_3_3_0_0_0
1526.7404 PEP_4_3_0_0_0
1998.0739 PEP_0_1_0_0_0

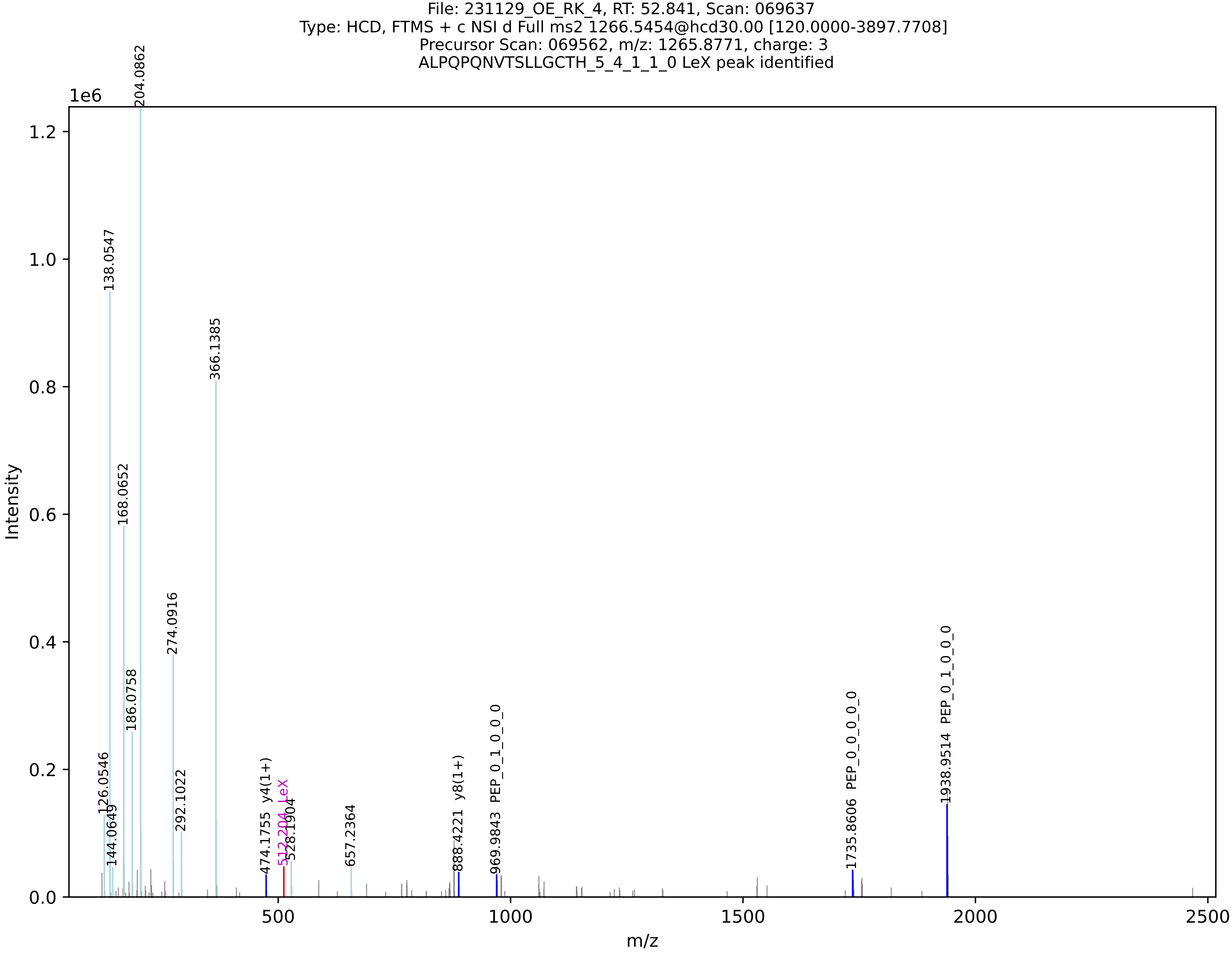
File: 231129_OE_RK_4, RT: 52.841, Scan: 069637
Type: HCD, FTMS + c NSI d Full ms2 1266.5454@hcd30.00 [120.0000-3897.7708]
Precursor Scan: 069562, m/z: 1265.8771, charge: 3
ALPQPQNVTSLLGCTH_5_4_1_1_0 LeX peak identified
1e6
Intensity
m/z
204.0862
138.0547
366.1385
168.0652
274.0916
186.0758
292.1022
126.0546
144.0649
474.1755 y4(1+)
512.204 LeX
528.1904
657.2364
888.4221 y8(1+)
969.9843 PEP_0_1_0_0_0
1735.8606 PEP_0_0_0_0_0
1938.9514 PEP_0_1_0_0_0

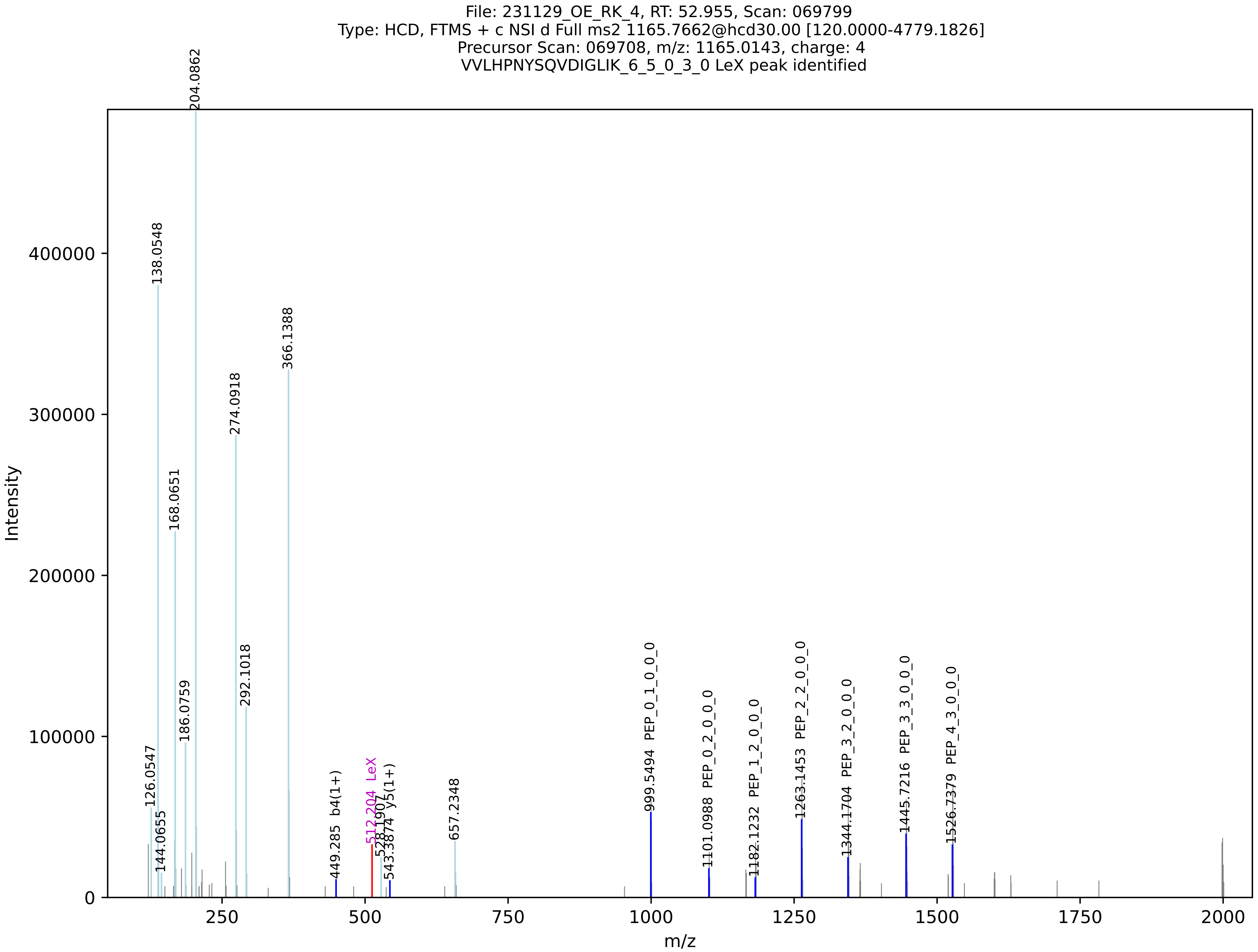
File: 231129_OE_RK_4, RT: 52.955, Scan: 069799
Type: HCD, FTMS + c NSI d Full ms2 1165.7662@hcd30.00 [120.0000-4779.1826]
Precursor Scan: 069708, m/z: 1165.0143, charge: 4
VVLHPNYSQVDIGLIK_6_5_0_3_0 LeX peak identified
Intensity
m/z
204.0862
138.0548
168.0651
186.0759
126.0547
144.0655
274.0918
292.1018
366.1388
449.285 b4(1+)
512.204 LeX
528.1907
543.3874 y5(1+)
657.2348
999.5494 PEP_0_1_0_0_0
1101.0988 PEP_0_2_0_0_0
1182.1232 PEP_1_2_0_0_0
1263.1453 PEP_2_2_0_0_0
1344.1704 PEP_3_2_0_0_0
1445.7216 PEP_3_3_0_0_0
1526.7379 PEP_4_3_0_0_0

File: 231129_OE_RK_4, RT: 53.045, Scan: 069929
Type: HCD, FTMS + c NSI d Full ms2 1266.5438@hcd30.00 [120.0000-3897.7664]
Precursor Scan: 069850, m/z: 1265.8756, charge: 3
ALPQPQNVTSLLGCTH_5_4_1_1_0 LeX peak identified

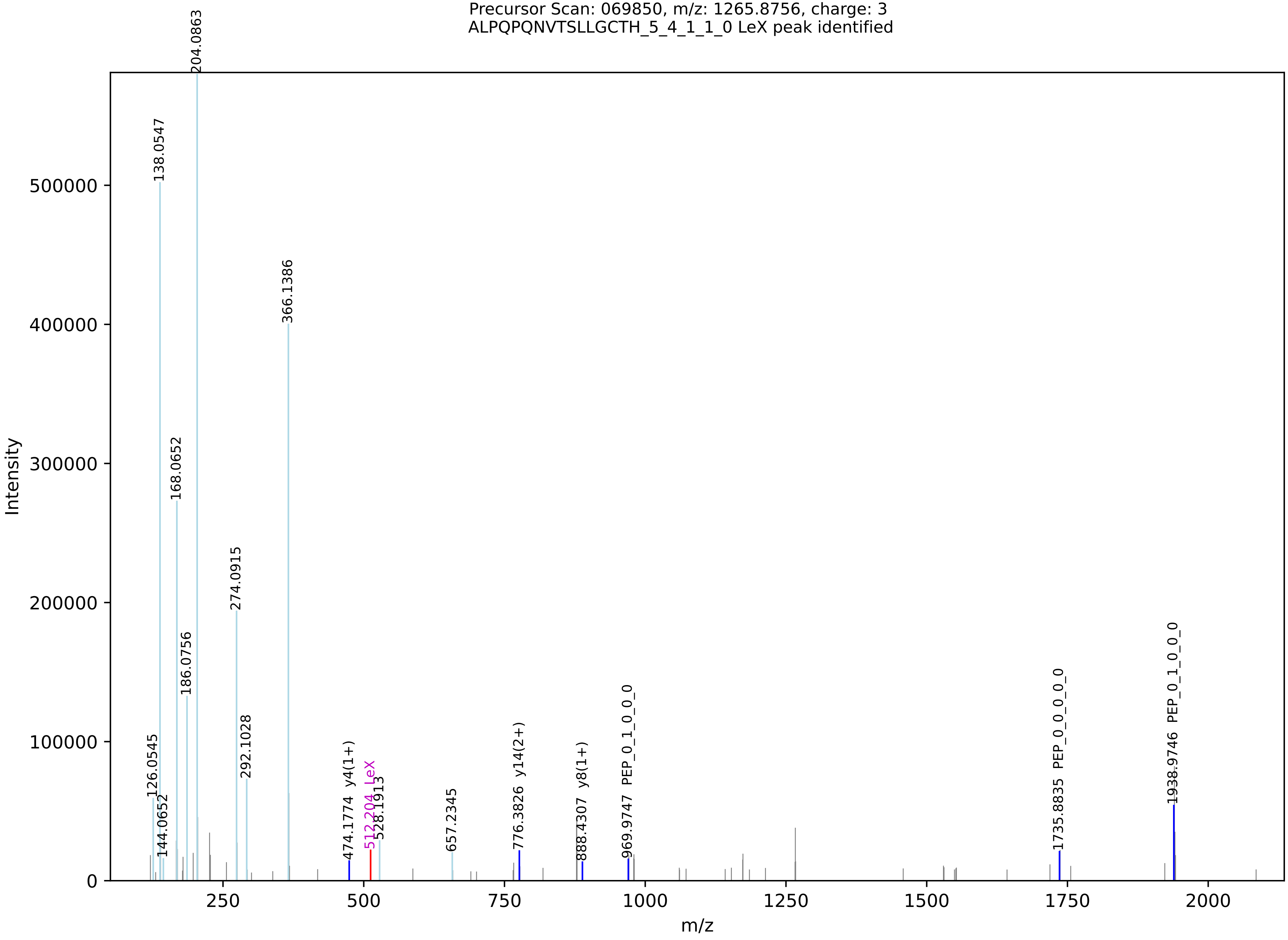

File: 231129_OE_RK_4, RT: 53.203, Scan: 070159
Type: HCD, FTMS + c NSI d Full ms2 1396.0516@hcd30.00 [120.0000-5718.7471]
Precursor Scan: 070072, m/z: 1395.5485, charge: 4
QDQCIYNTTYLNVQR_7_6_1_4_0 LeX peak identified

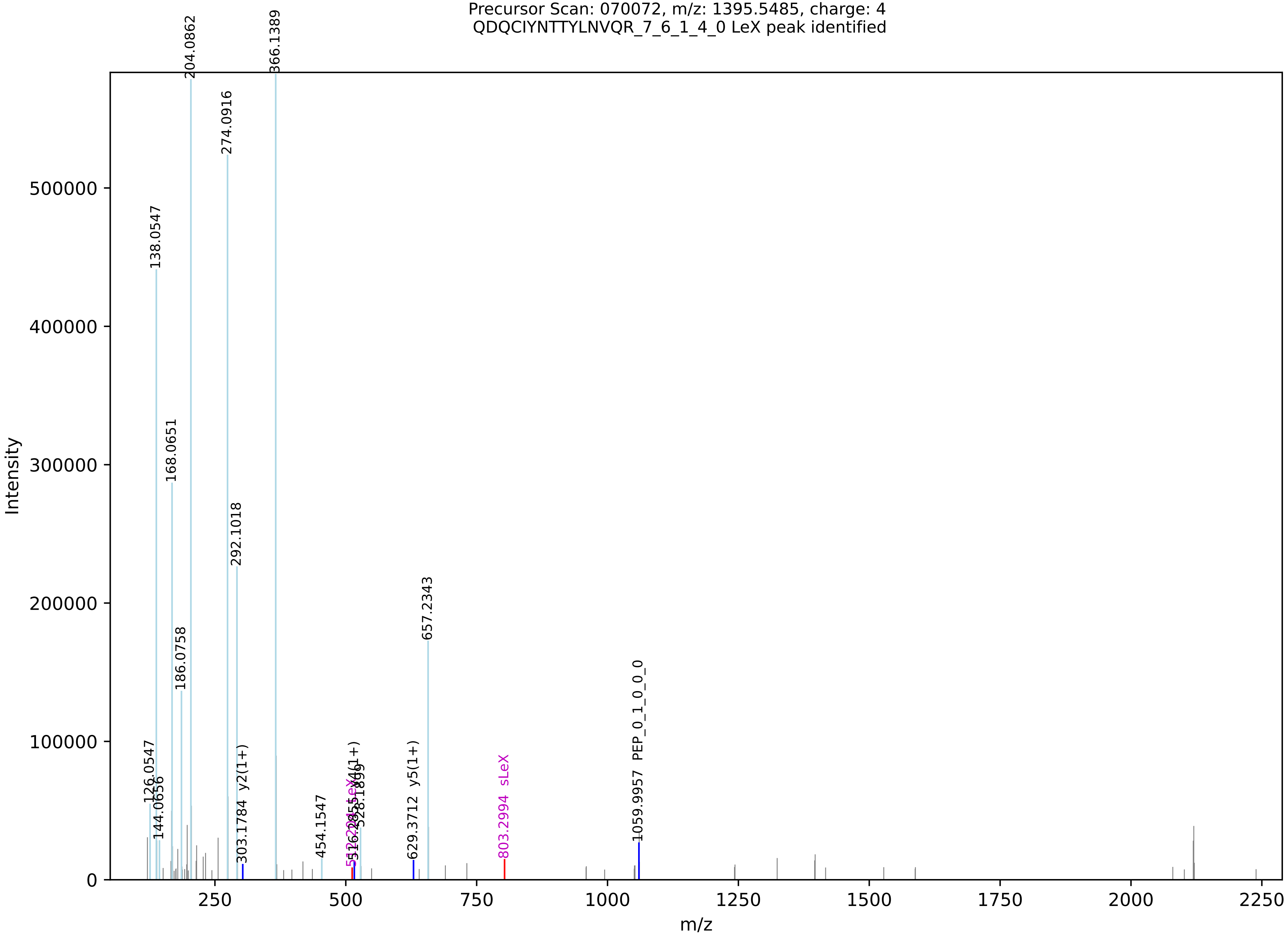

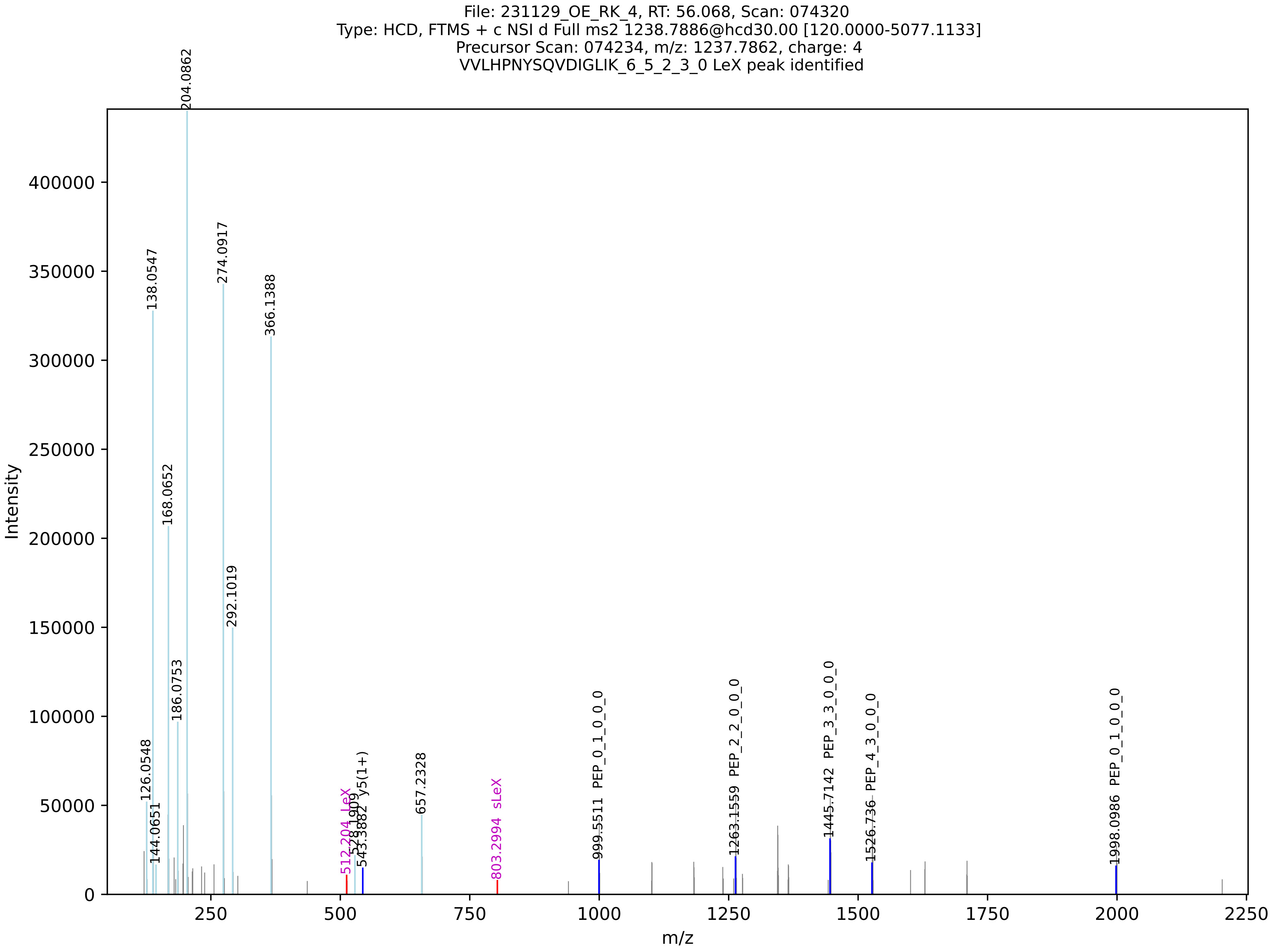
File: 231129_OE_RK_4, RT: 56.068, Scan: 074320
Type: HCD, FTMS + c NSI d Full ms2 1238.7886@hcd30.00 [120.0000-5077.1133]
Precursor Scan: 074234, m/z: 1237.7862, charge: 4
VVLHPNYSQVDIGLIK_6_5_2_3_0 LeX peak identified
Intensity
m/z
204.0862
138.0547
274.0917
366.1388
168.0652
292.1019
186.0753
126.0548
144.0651
512.204 LeX
528.1909
543.3882 y5(1+)
657.2328
803.2994 sLeX
999.5511 PEP_0_1_0_0_0_
1263.1559 PEP_2_2_0_0_0_
1445.7142 PEP_3_3_0_0_0_
1526.736 PEP_4_3_0_0_0_
1998.0986 PEP_0_1_0_0_0_
0
50000
100000
150000
200000
250000
300000
350000
400000
250
500
750
1000
1250
1500
1750
2000
2250

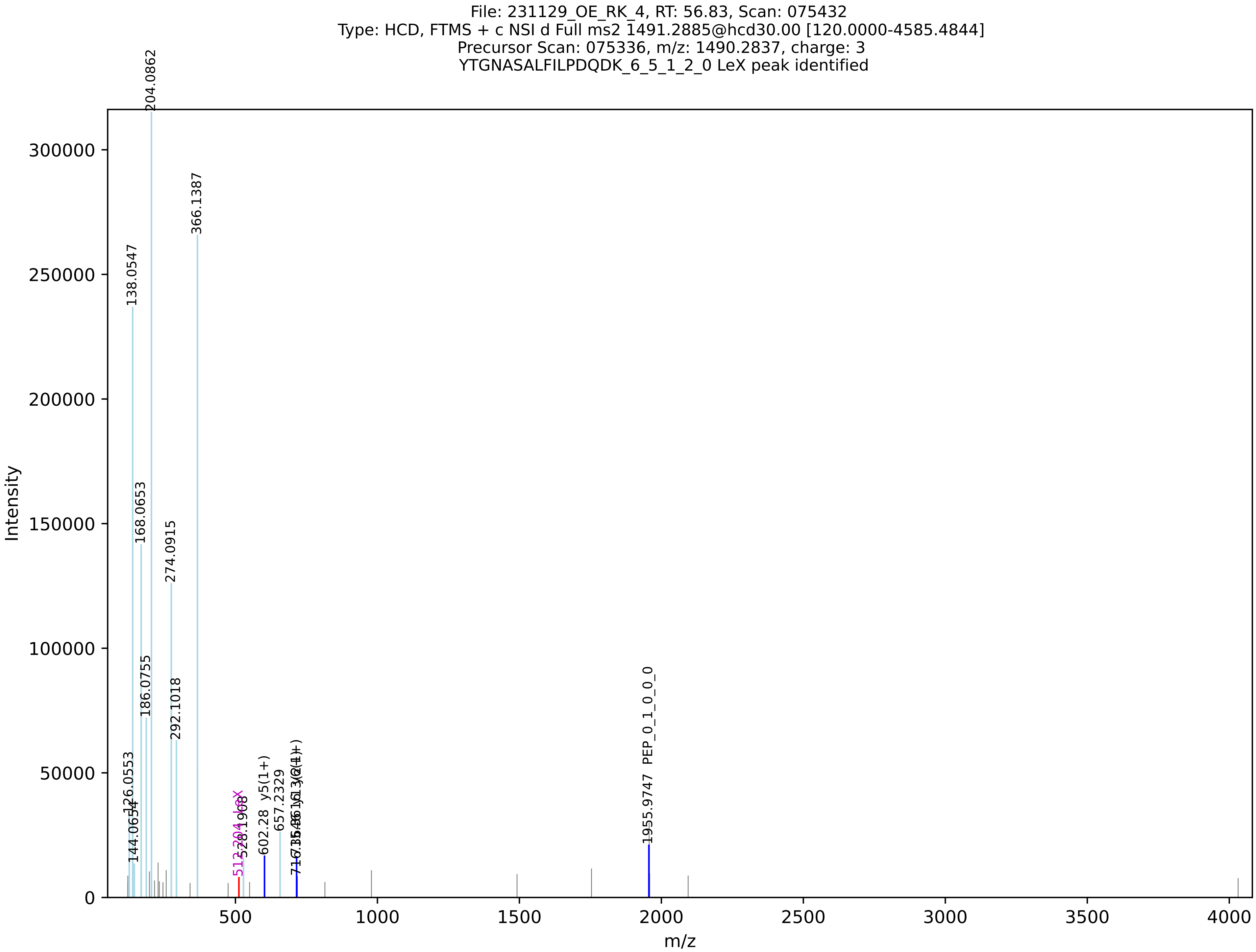
File: 231129_OE_RK_4, RT: 56.83, Scan: 075432
Type: HCD, FTMS + c NSI d Full ms2 1491.2885@hcd30.00 [120.0000-4585.4844]
Precursor Scan: 075336, m/z: 1490.2837, charge: 3
YTGNASALFILPDQDK_6_5_1_2_0 LeX peak identified
204.0862
366.1387
138.0547
168.0653
274.0915
186.0755
292.1018
126.0553
144.0654
512.204 LeX
528.1908
602.28 y5(1+)
657.2329
1955.9747 PEP_0_1_0_0_0
Intensity
m/z

File: 231129_OE_RK_4, RT: 61.511, Scan: 082244
Type: HCD, FTMS + c NSI d Full ms2 1588.3260@hcd30.00 [120.0000-4882.4199]
Precursor Scan: 082144, m/z: 1587.3237, charge: 3
YTGNASALFILPDQDK_6_5_1_3_0 LeX peak identified

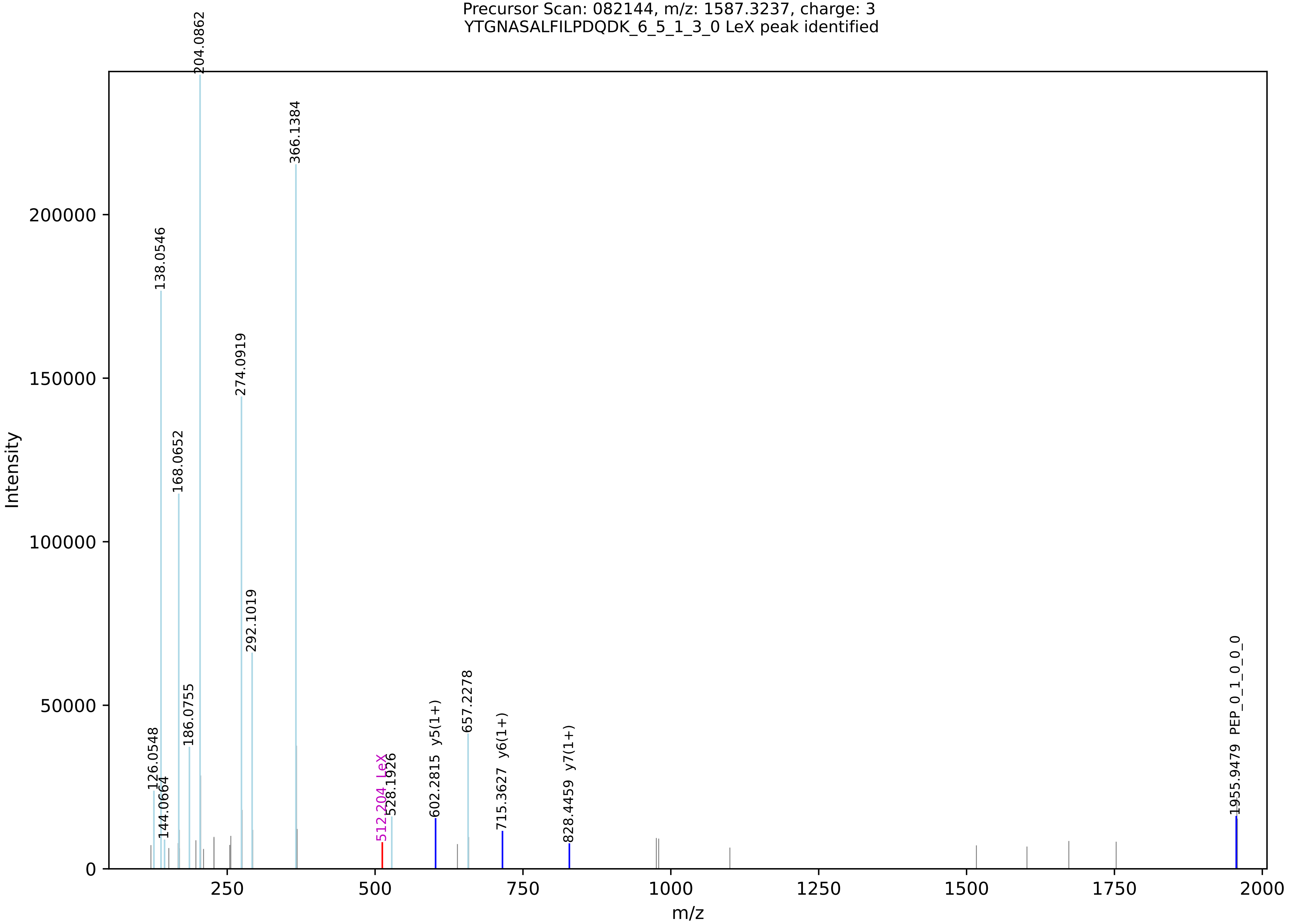

File: 231129_OE_RK_4, RT: 62.634, Scan: 083871
Type: HCD, FTMS + c NSI d Full ms2 1331.8119@hcd30.00 [120.0000-5456.6489]
Precursor Scan: 083784, m/z: 1331.8119, charge: 4
TEPMQVALHCTNGSVVYHEVLNAMECK_5_4_2_1_0 LeX peak identified

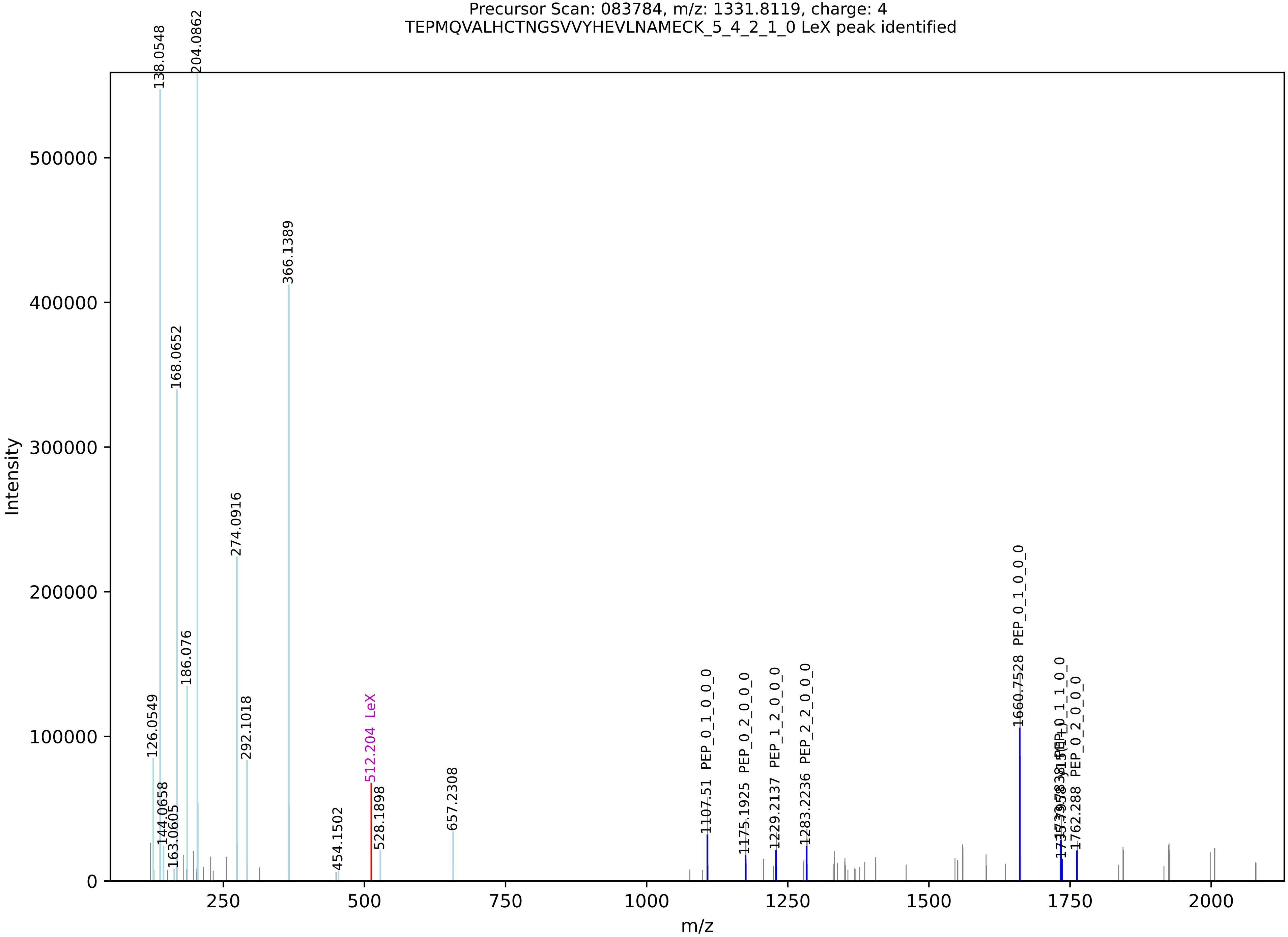

File: 231129_OE_RK_4, RT: 64.848, Scan: 087089
Type: HCD, FTMS + c NSI d Full ms2 1273.2148@hcd30.00 [120.0000-3918.1792]
Precursor Scan: 087004, m/z: 1272.5466, charge: 3
YLGNATAIFFLPDEGK_5_4_1_1_0 LeX peak identified

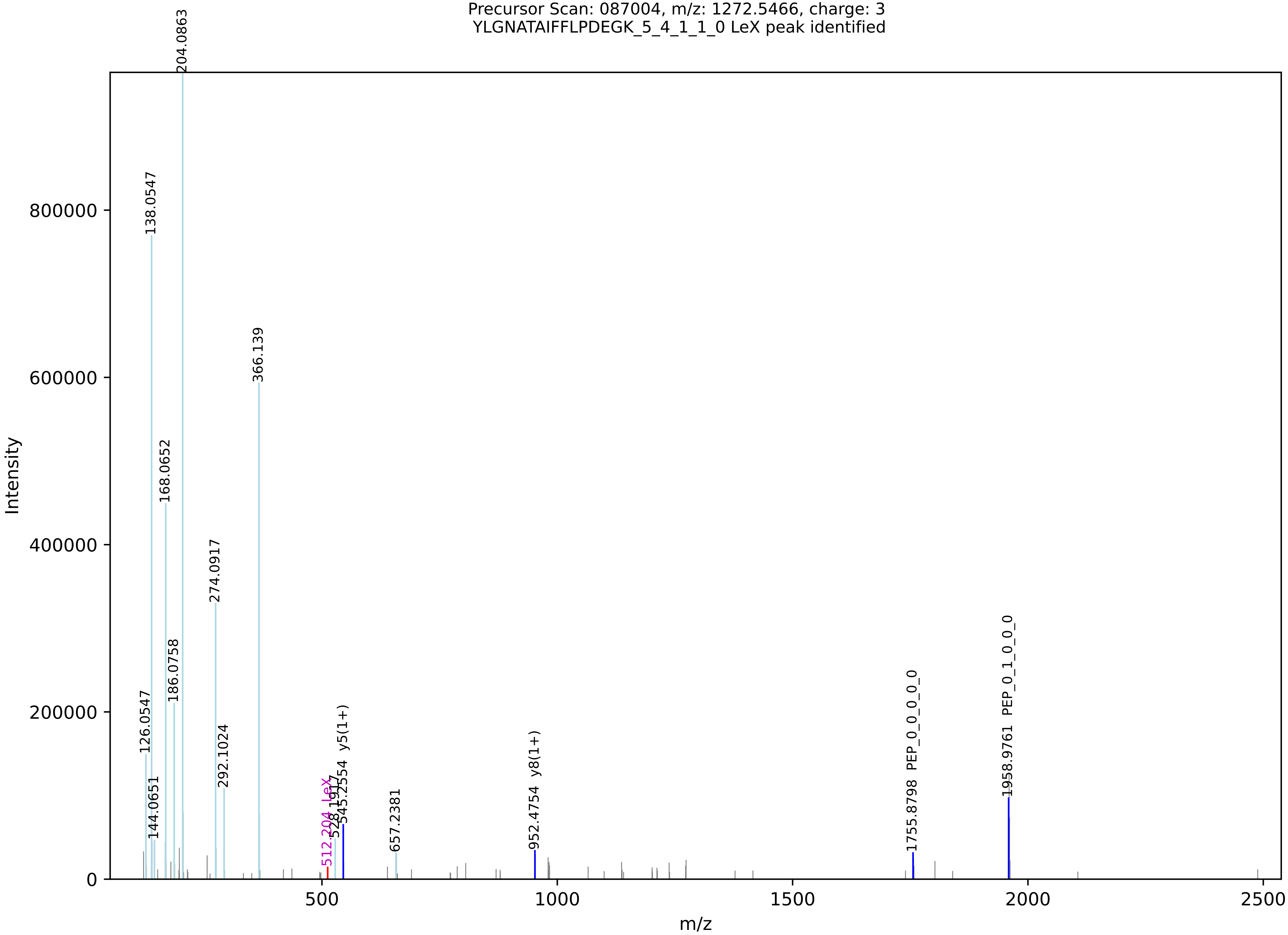

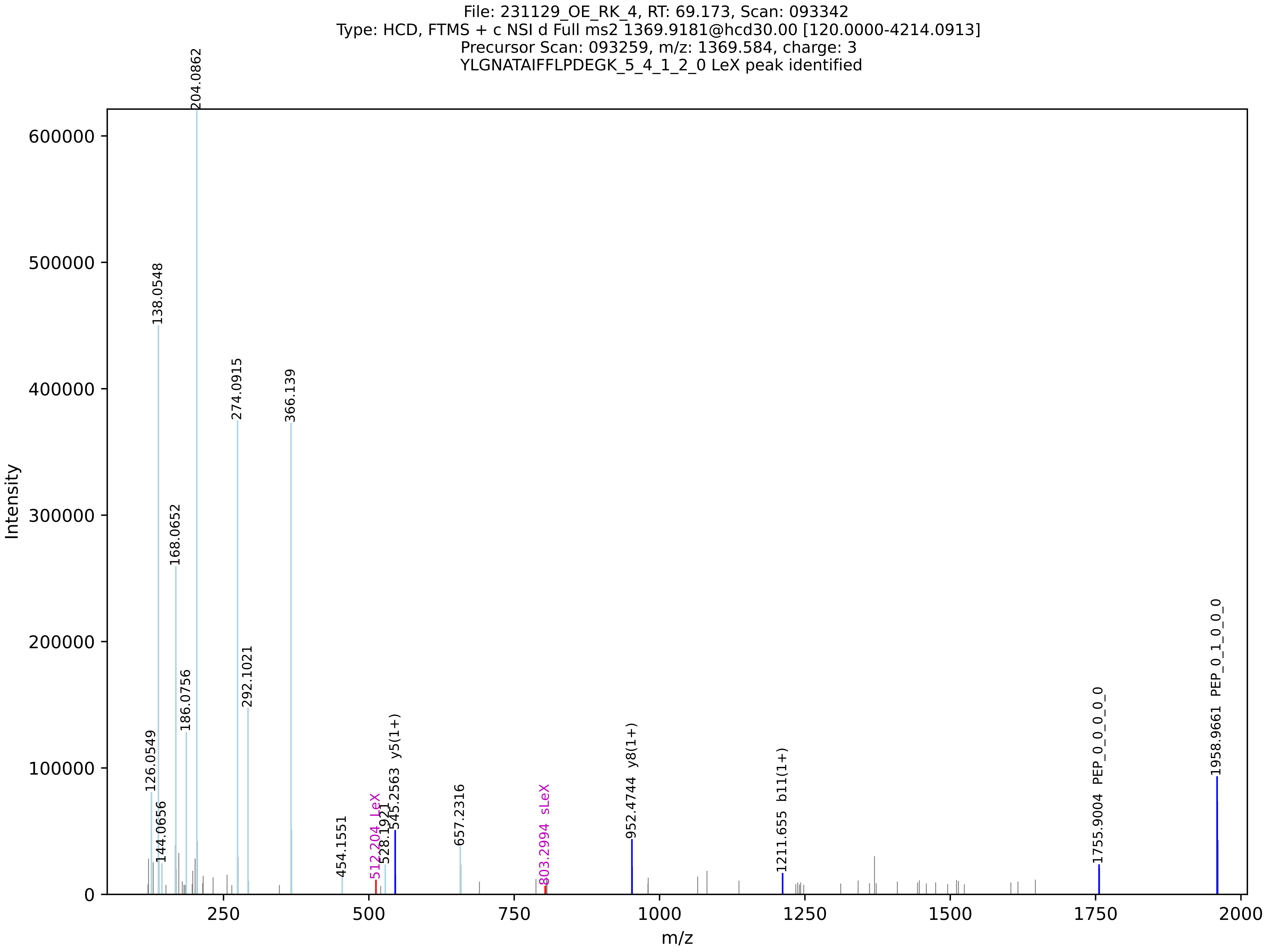
File: 231129_OE_RK_4, RT: 69.173, Scan: 093342
Type: HCD, FTMS + c NSI d Full ms2 1369.9181@hcd30.00 [120.0000-4214.0913]
Precursor Scan: 093259, m/z: 1369.584, charge: 3
YLGNATAIFFLPDEGK_5_4_1_2_0 LeX peak identified
Intensity
m/z
204.0862
138.0548
274.0915
366.139
168.0652
292.1021
186.0756
126.0549
144.0656
454.1551
512.204 LeX
528.1921
545.2563 y5(1+)
657.2316
803.2994 sLeX
952.4744 y8(1+)
1211.655 b11(1+)
1755.9004 PEP_0_0_0_0_0
1958.9661 PEP_0_1_0_0_0

File: 231129_OE_RK_4, RT: 81.874, Scan: 111813
Type: HCD, FTMS + c NSI d Full ms2 1759.7915@hcd30.00 [120.0000-5407.1040]
Precursor Scan: 111723, m/z: 1759.1182, charge: 3
VYIHPFHLVIHNESTCEQLAKANAGKPK_6_4_0_1_0 LeX peak identified

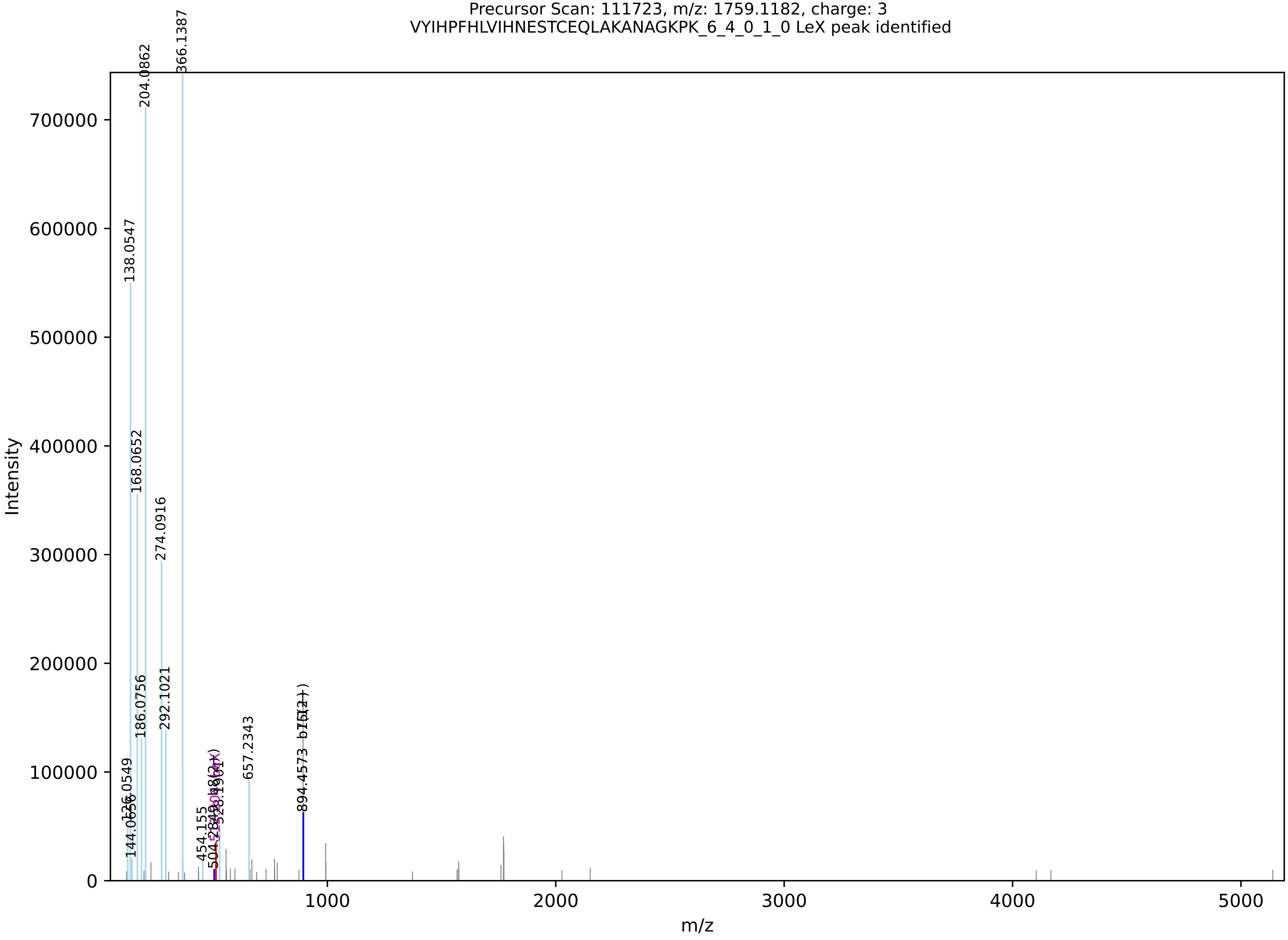

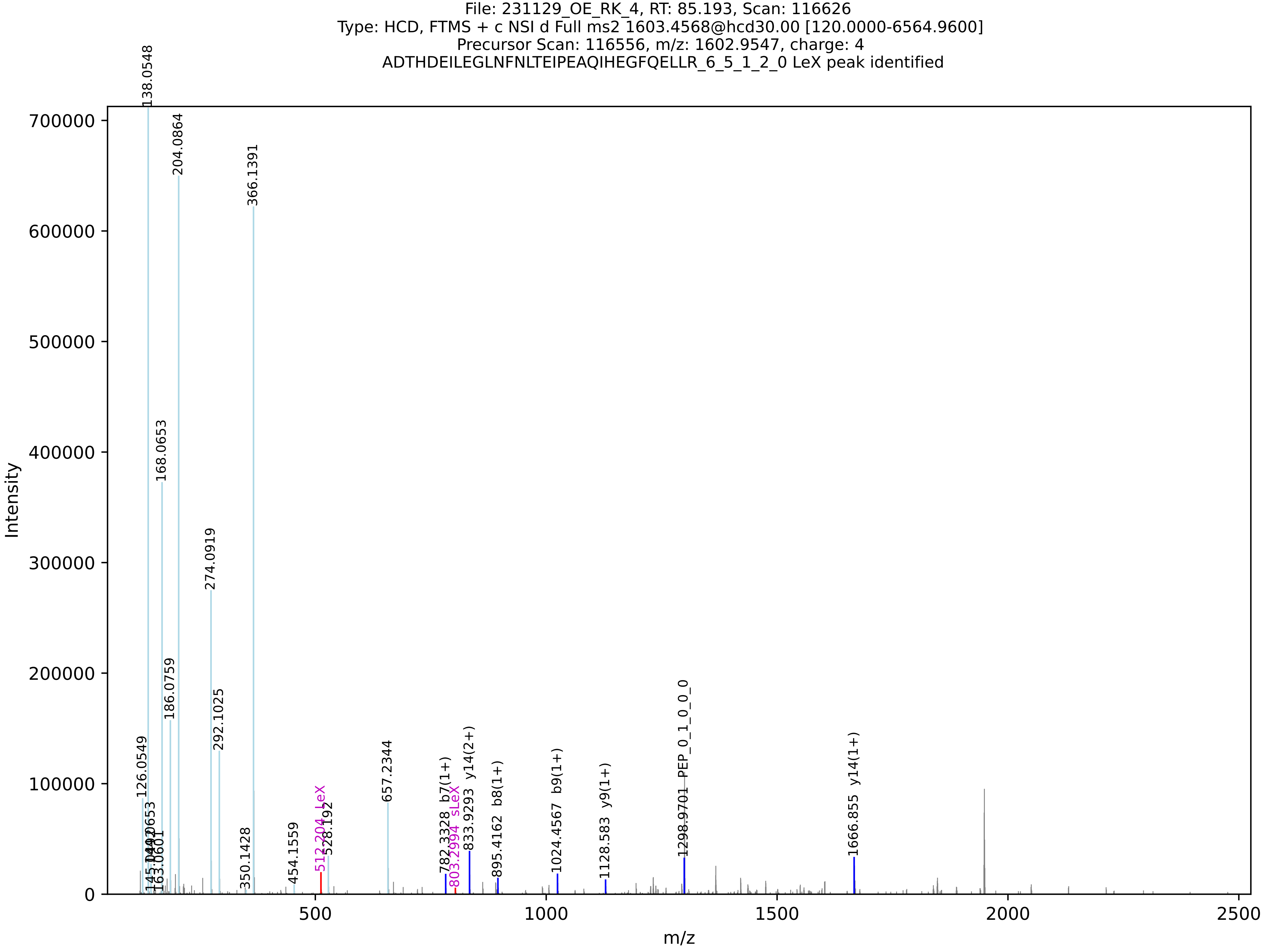
File: 231129_OE_RK_4, RT: 85.193, Scan: 116626
Type: HCD, FTMS + c NSI d Full ms2 1603.4568@hcd30.00 [120.0000-6564.9600]
Precursor Scan: 116556, m/z: 1602.9547, charge: 4
ADTHDEILEGLNFNLTEIPEAQIHEGFQELLR_6_5_1_2_0 LeX peak identified
Intensity
m/z
138.0548
204.0864
366.1391
168.0653
274.0919
186.0759
292.1025
126.0549
163.0601
350.1428
454.1559
512.204 LeX
528.192
657.2344
782.3328 b7(1+)
803.2994 sLeX
833.9293 y14(2+)
895.4162 b8(1+)
1024.4567 b9(1+)
1128.583 y9(1+)
1298.9701 PEP_0_1_0_0_0
1666.855 y14(1+)

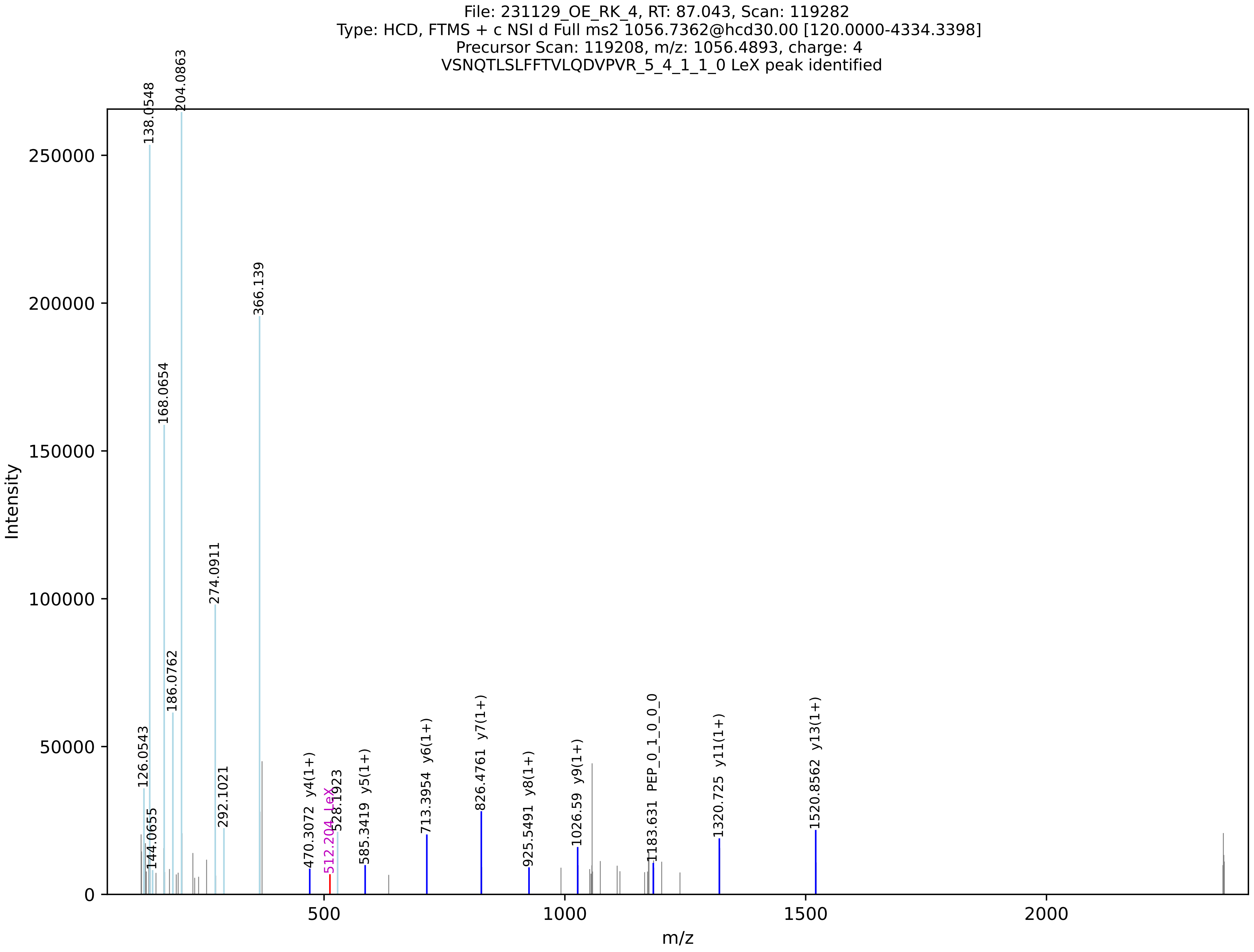
File: 231129_OE_RK_4, RT: 87.043, Scan: 119282
Type: HCD, FTMS + c NSI d Full ms2 1056.7362@hcd30.00 [120.0000-4334.3398]
Precursor Scan: 119208, m/z: 1056.4893, charge: 4
VSNQTLSLFFTVLQDVPVR_5_4_1_1_0 LeX peak identified
Intensity
m/z
0
50000
100000
150000
200000
250000
500
1000
1500
2000
138.0548
204.0863
366.139
168.0654
274.0911
186.0762
126.0543
144.0655
292.1021
470.3072 y4(1+)
512.204 LeX
528.1923
585.3419 y5(1+)
713.3954 y6(1+)
826.4761 y7(1+)
925.5491 y8(1+)
1026.59 y9(1+)
1183.631 PEP_0_1_0_0_0_
1320.725 y11(1+)
1520.8562 y13(1+)

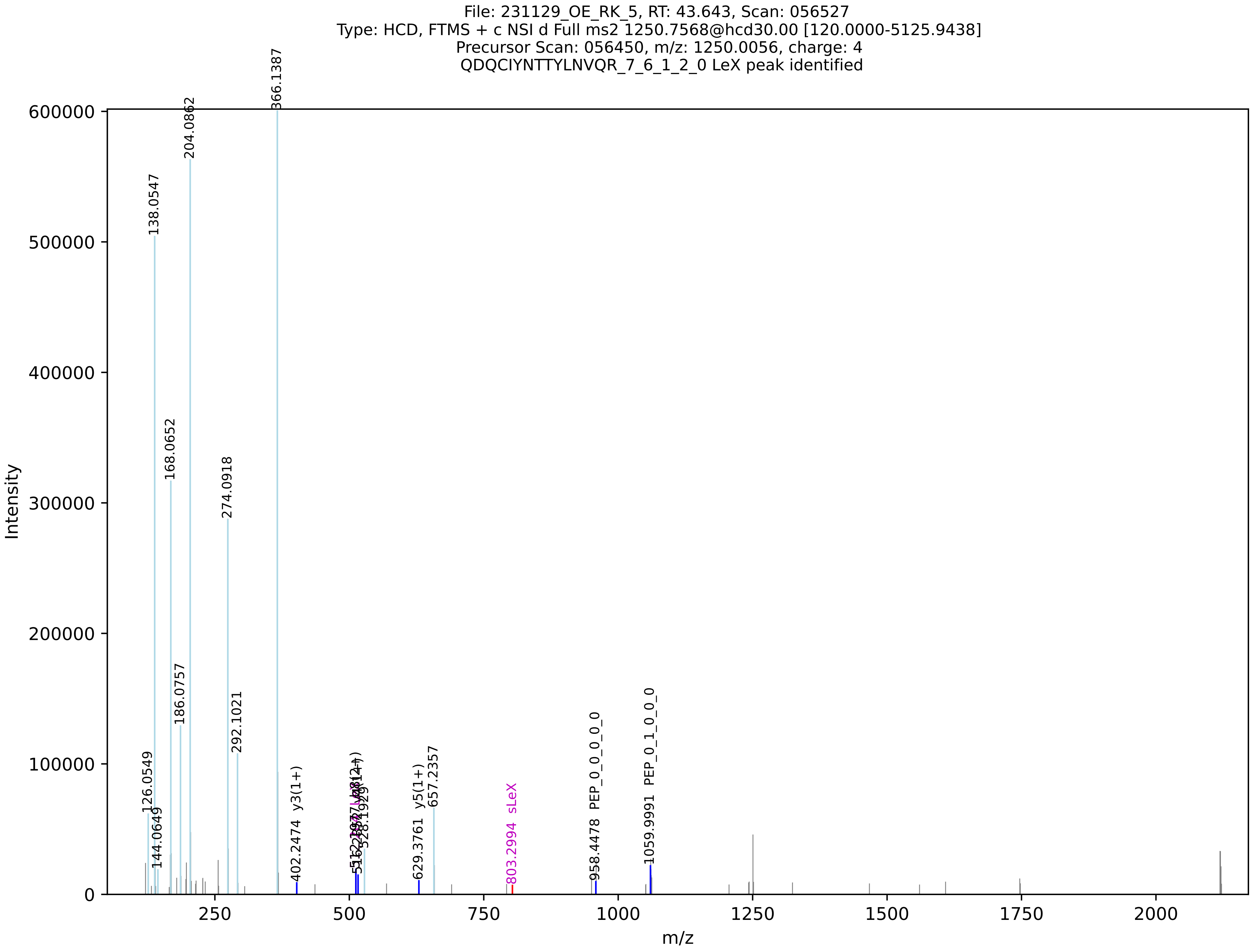
File: 231129_OE_RK_5, RT: 43.643, Scan: 056527
Type: HCD, FTMS + c NSI d Full ms2 1250.7568@hcd30.00 [120.0000-5125.9438]
Precursor Scan: 056450, m/z: 1250.0056, charge: 4
QDQCIYNTTYLNVQR_7_6_1_2_0 LeX peak identified
Intensity
m/z
366.1387
204.0862
138.0547
168.0652
274.0918
186.0757
292.1021
126.0549
144.0649
402.2474 y3(1+)
528.1929
629.3761 y5(1+)
657.2357
803.2994 sLeX
958.4478 PEP_0_0_0_0_0_
1059.9991 PEP_0_1_0_0_0_

File: 231129_OE_RK_5, RT: 44.058, Scan: 057126
Type: HCD, FTMS + c NSI d Full ms2 1250.5044@hcd30.00 [120.0000-5124.9141]
Precursor Scan: 057034, m/z: 1250.0018, charge: 4
QDQCIYNTTYLNVQR_7_6_1_2_0 LeX peak identified

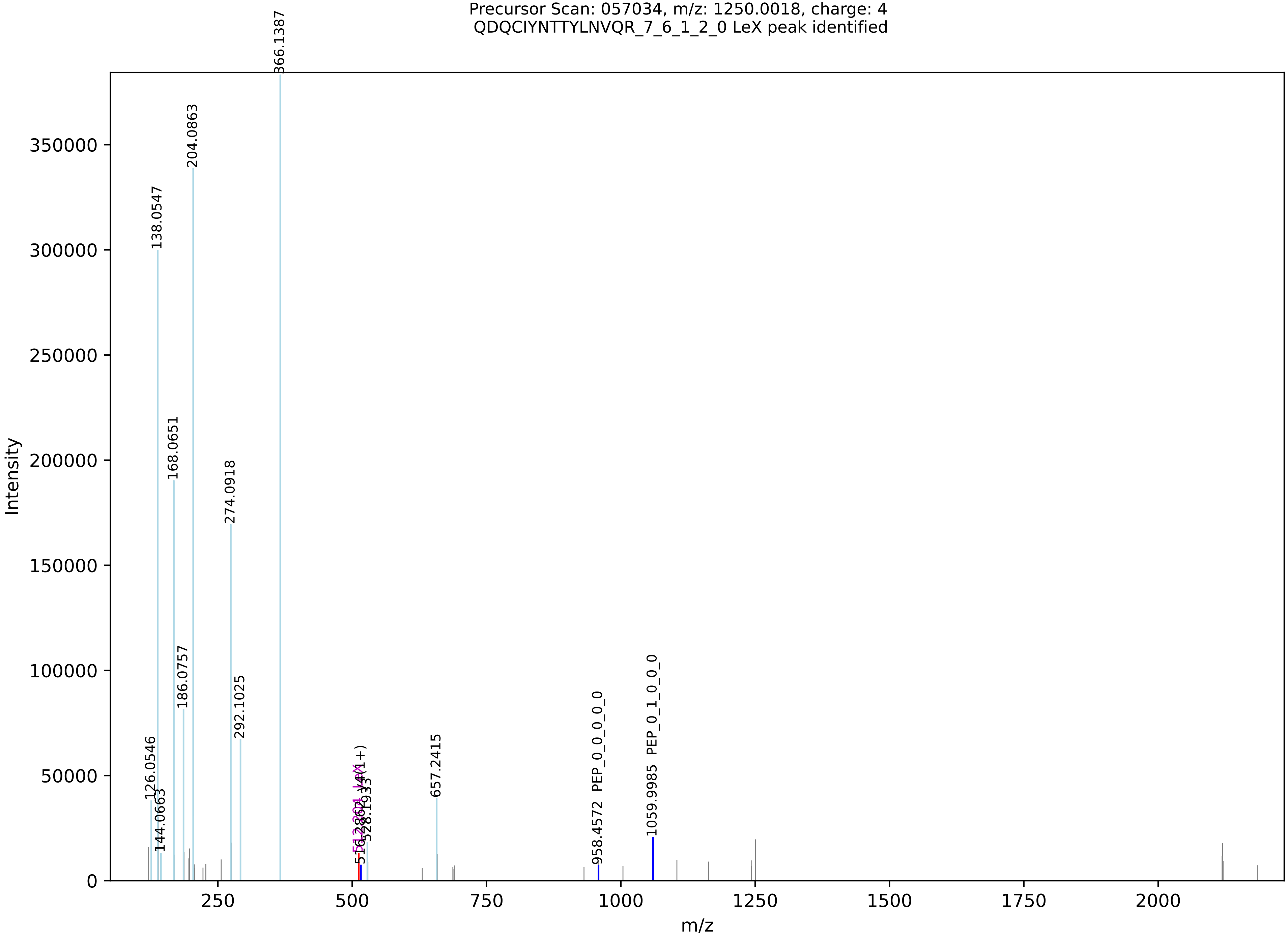

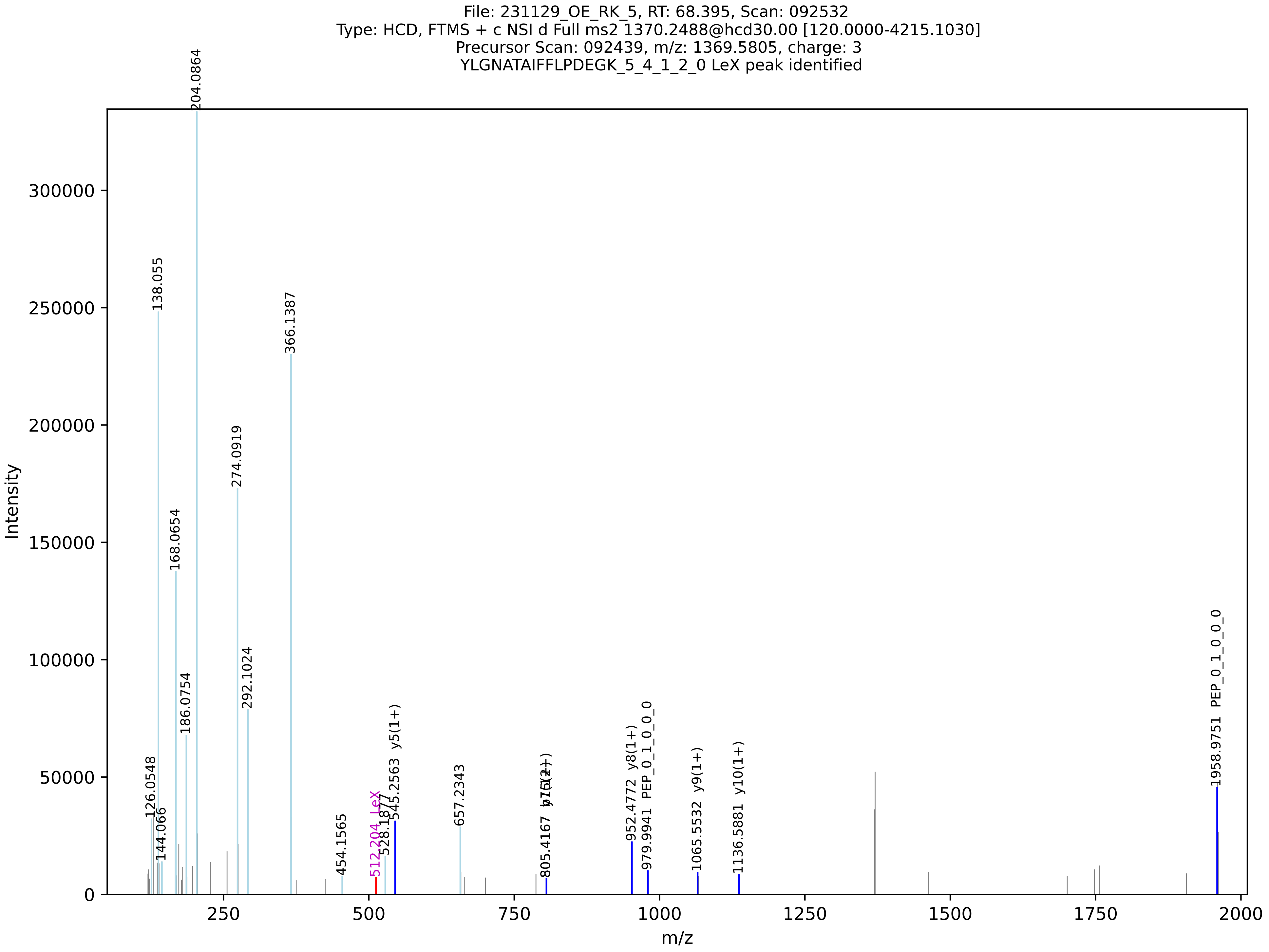
File: 231129_OE_RK_5, RT: 68.395, Scan: 092532
Type: HCD, FTMS + c NSI d Full ms2 1370.2488@hcd30.00 [120.0000-4215.1030]
Precursor Scan: 092439, m/z: 1369.5805, charge: 3
YLGNATAIFFLPDEGK_5_4_1_2_0 LeX peak identified
Intensity
m/z
0
50000
100000
150000
200000
250000
300000
250
500
750
1000
1250
1500
1750
2000
204.0864
138.055
366.1387
274.0919
168.0654
292.1024
186.0754
126.0548
144.066
454.1565
512.204 LeX
528.1877
545.2563 y5(1+)
657.2343
805.4167
952.4772 y8(1+)
979.9941 PEP_0_1_0_0_0
1065.5532 y9(1+)
1136.5881 y10(1+)
1958.9751 PEP_0_1_0_0_0

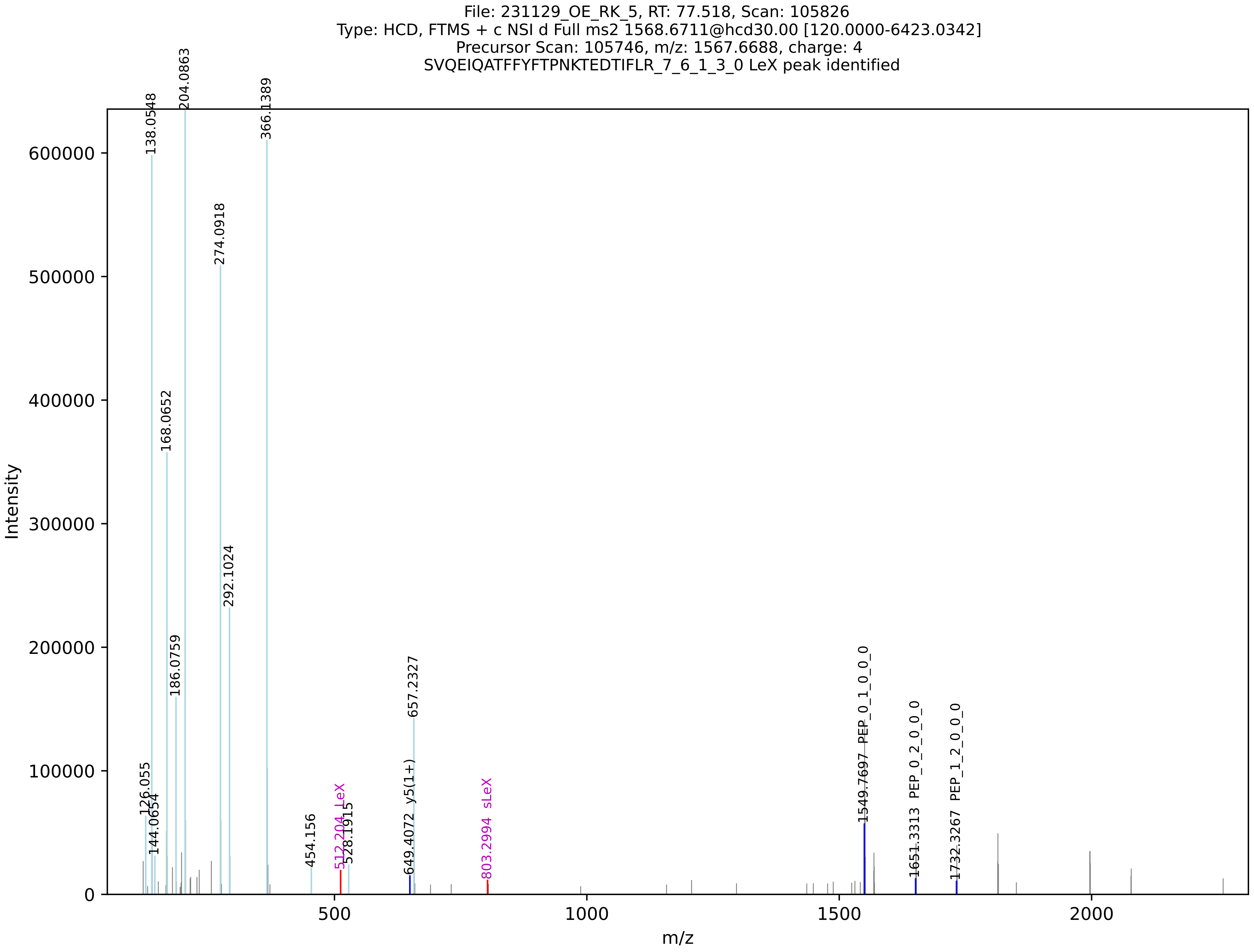
File: 231129_OE_RK_5, RT: 77.518, Scan: 105826
Type: HCD, FTMS + c NSI d Full ms2 1568.6711@hcd30.00 [120.0000-6423.0342]
Precursor Scan: 105746, m/z: 1567.6688, charge: 4
SVQEIQATFFYFTPNKTEDTIFLR_7_6_1_3_0 LeX peak identified
Intensity
m/z
0
100000
200000
300000
400000
500000
600000
500
1000
1500
2000
204.0863
138.0548
366.1389
274.0918
168.0652
292.1024
186.0759
126.055
144.0654
454.156
512.204 LeX
528.1915
649.4072 y5(1+)
657.2327
803.2994 sLeX
1549.7697 PEP_0_1_0_0_0_
1651.3313 PEP_0_2_0_0_0_
1732.3267 PEP_1_2_0_0_0_

File: 231129_OE_RK_6, RT: 45.247, Scan: 058486
Type: HCD, FTMS + c NSI d Full ms2 1483.6390@hcd30.00 [120.0000-4562.0776]
Precursor Scan: 058394, m/z: 1482.9708, charge: 3
QLRNSTLQCETINSDNEDLLAR_5_3_1_1_0 LeX peak identified

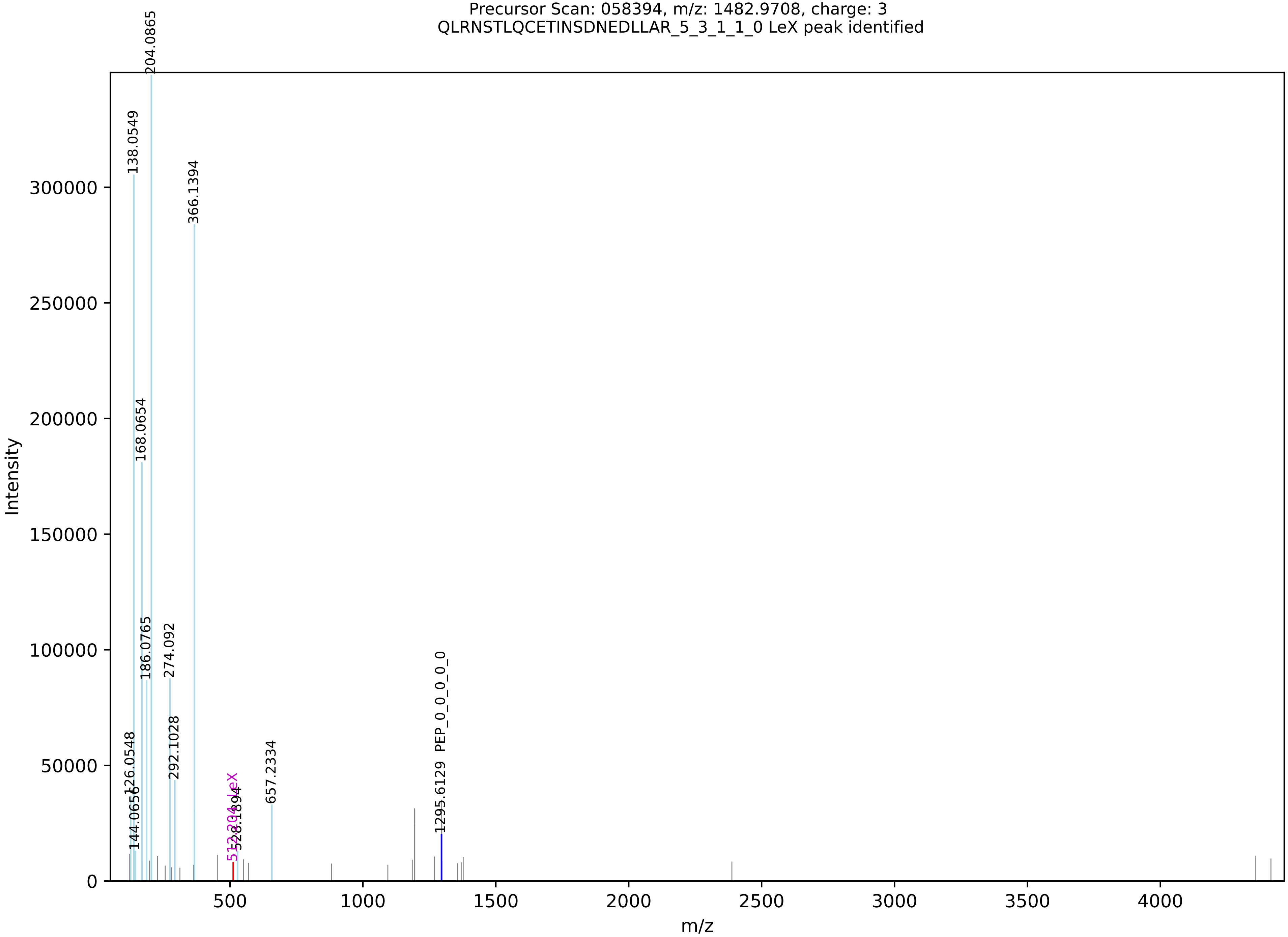

File: 231129_OE_RK_6, RT: 53.107, Scan: 069903
Type: HCD, FTMS + c NSI d Full ms2 1266.2111@hcd30.00 [120.0000-3896.7478]
Precursor Scan: 069810, m/z: 1265.877, charge: 3
ALPQPQNVTSLLGCTH_5_4_1_1_0 LeX peak identified

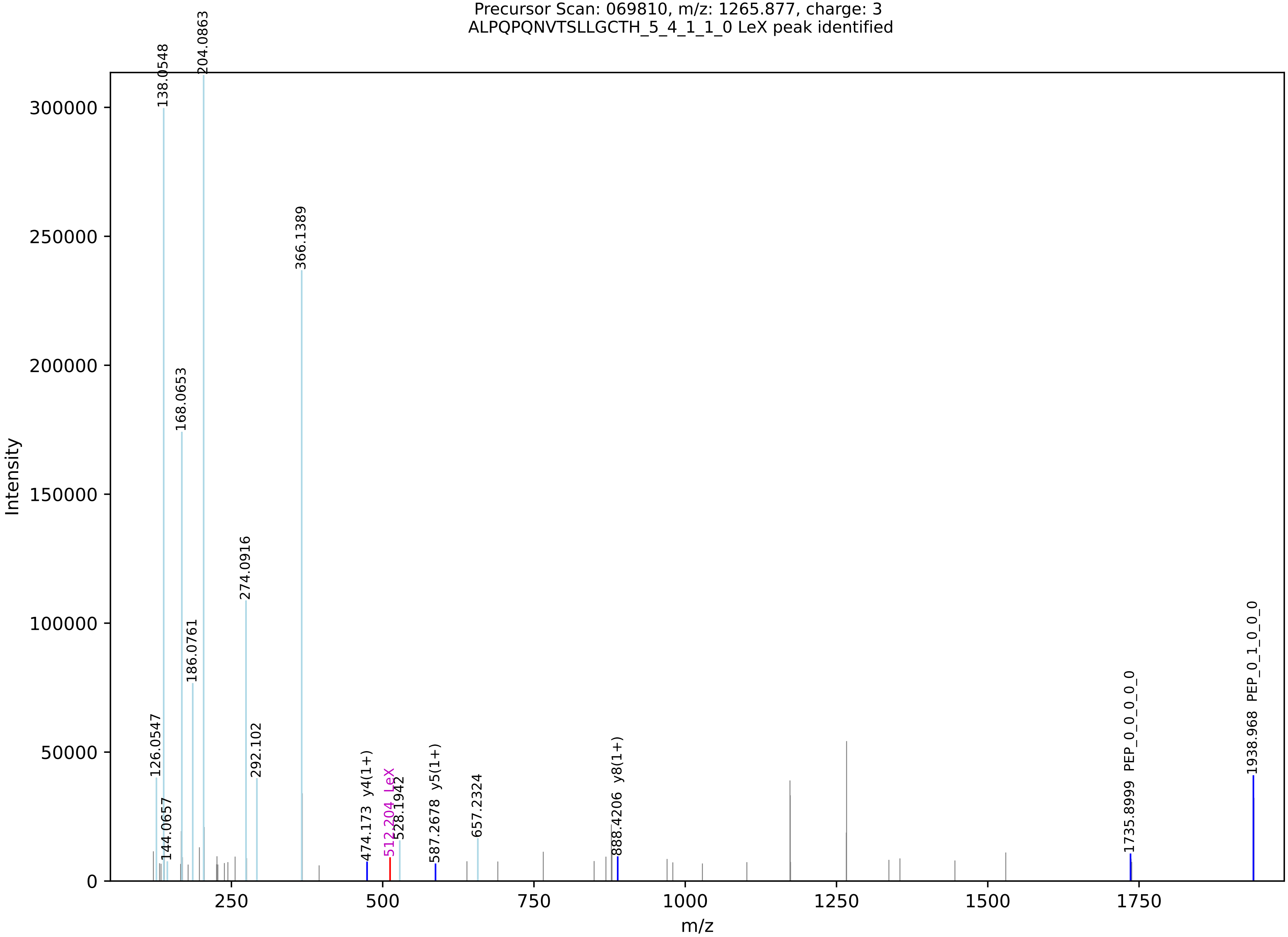

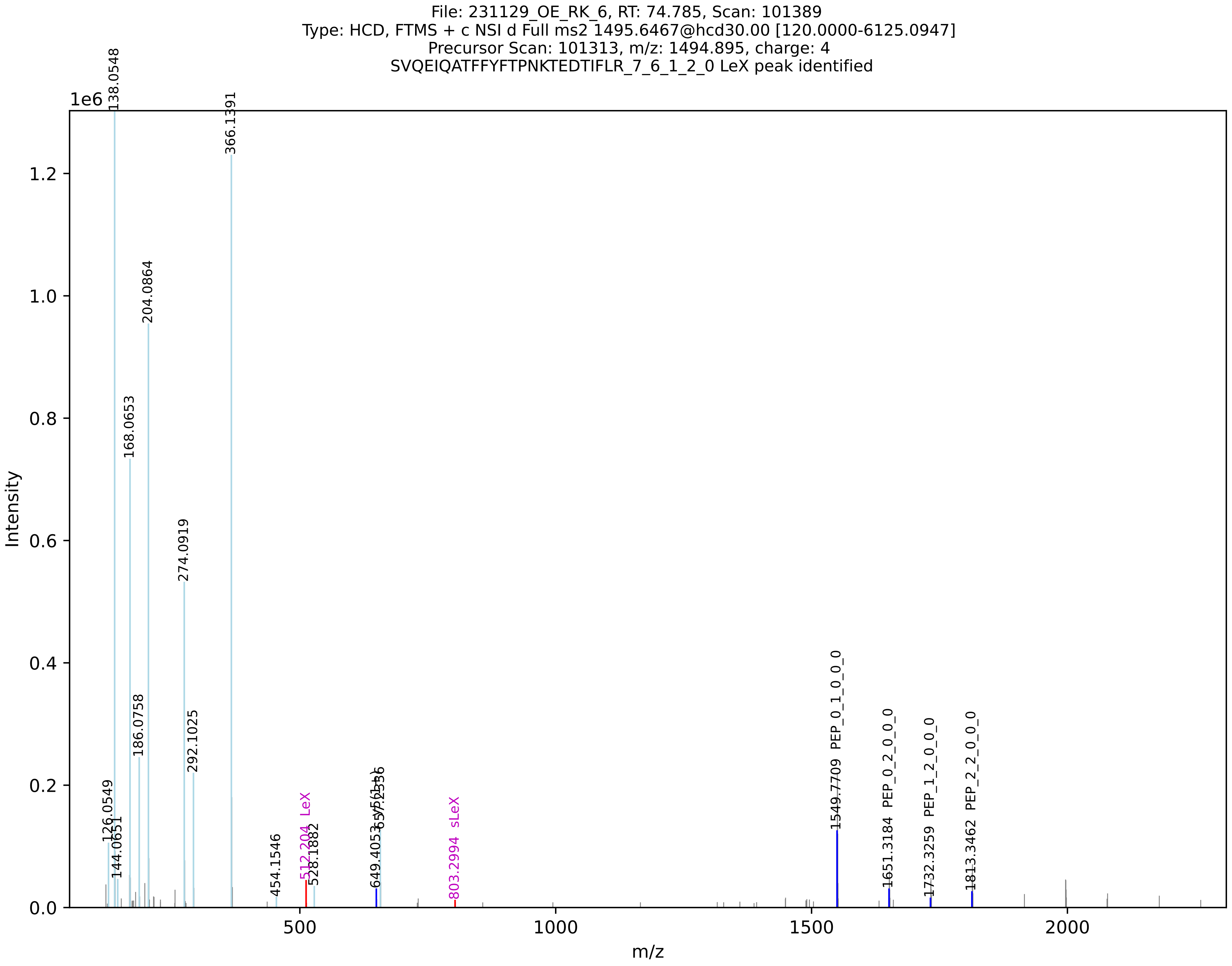
File: 231129_OE_RK_6, RT: 74.785, Scan: 101389
Type: HCD, FTMS + c NSI d Full ms2 1495.6467@hcd30.00 [120.0000-6125.0947]
Precursor Scan: 101313, m/z: 1494.895, charge: 4
SVQEIQATFFYFTPNKTEDTIFLR_7_6_1_2_0 LeX peak identified
1e6
Intensity
m/z
138.0548
366.1391
204.0864
168.0653
274.0919
186.0758
292.1025
126.0549
144.0651
454.1546
512.204 LeX
528.1882
649.4053
803.2994 sLeX
1549.7709 PEP_0_1_0_0_0
1651.3184 PEP_0_2_0_0_0
1732.3259 PEP_1_2_0_0_0
1813.3462 PEP_2_2_0_0_0

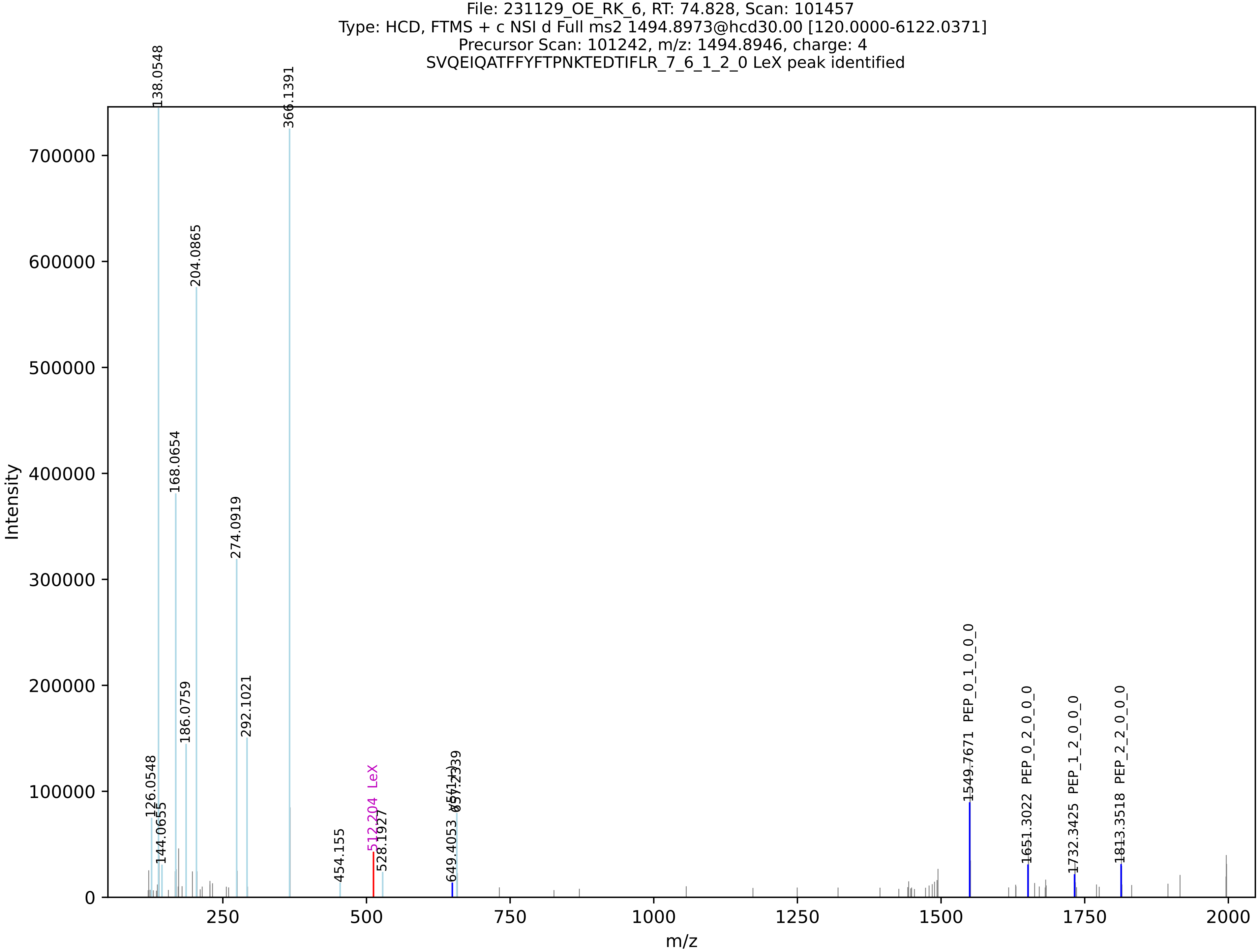

File: 231129_OE_RK_6, RT: 74.828, Scan: 101457
Type: HCD, FTMS + c NSI d Full ms2 1494.8973@hcd30.00 [120.0000-6122.0371]
Precursor Scan: 101242, m/z: 1494.8946, charge: 4
SVQEIQATFFYFTPNKTEDTIFLR_7_6_1_2_0 LeX peak identified
Intensity
m/z
138.0548
366.1391
204.0865
168.0654
274.0919
292.1021
186.0759
126.0548
144.0655
454.155
512.204 LeX
528.1927
649.4053 y5(1+)
657.2339
1549.7671 PEP_0_1_0_0_0
1651.3022 PEP_0_2_0_0_0
1732.3425 PEP_1_2_0_0_0
1813.3518 PEP_2_2_0_0_0

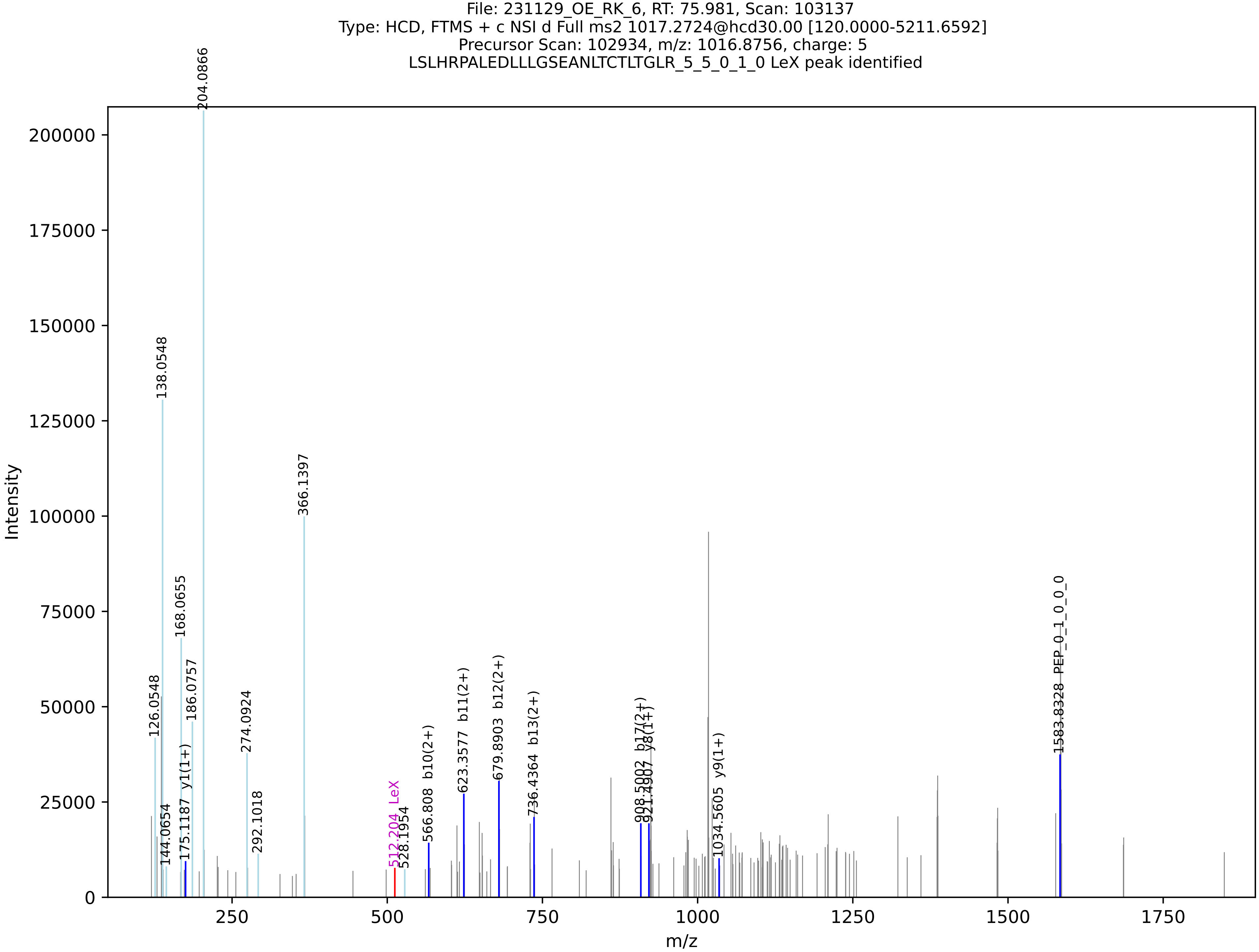
File: 231129_OE_RK_6, RT: 75.981, Scan: 103137
Type: HCD, FTMS + c NSI d Full ms2 1017.2724@hcd30.00 [120.0000-5211.6592]
Precursor Scan: 102934, m/z: 1016.8756, charge: 5
LSLHRPALEDLLLGSEANLTCTLTGLR_5_5_0_1_0 LeX peak identified
Intensity
m/z
204.0866
138.0548
366.1397
168.0655
186.0757
126.0548
274.0924
144.0654
175.1187 y1(1+)
292.1018
512.204 LeX
528.1954
566.808 b10(2+)
623.3577 b11(2+)
679.8903 b12(2+)
736.4364 b13(2+)
908.5002 b17(2+)
921.4907 y8(1+)
1034.5605 y9(1+)
1583.8328 PEP_0_1_0_0_0

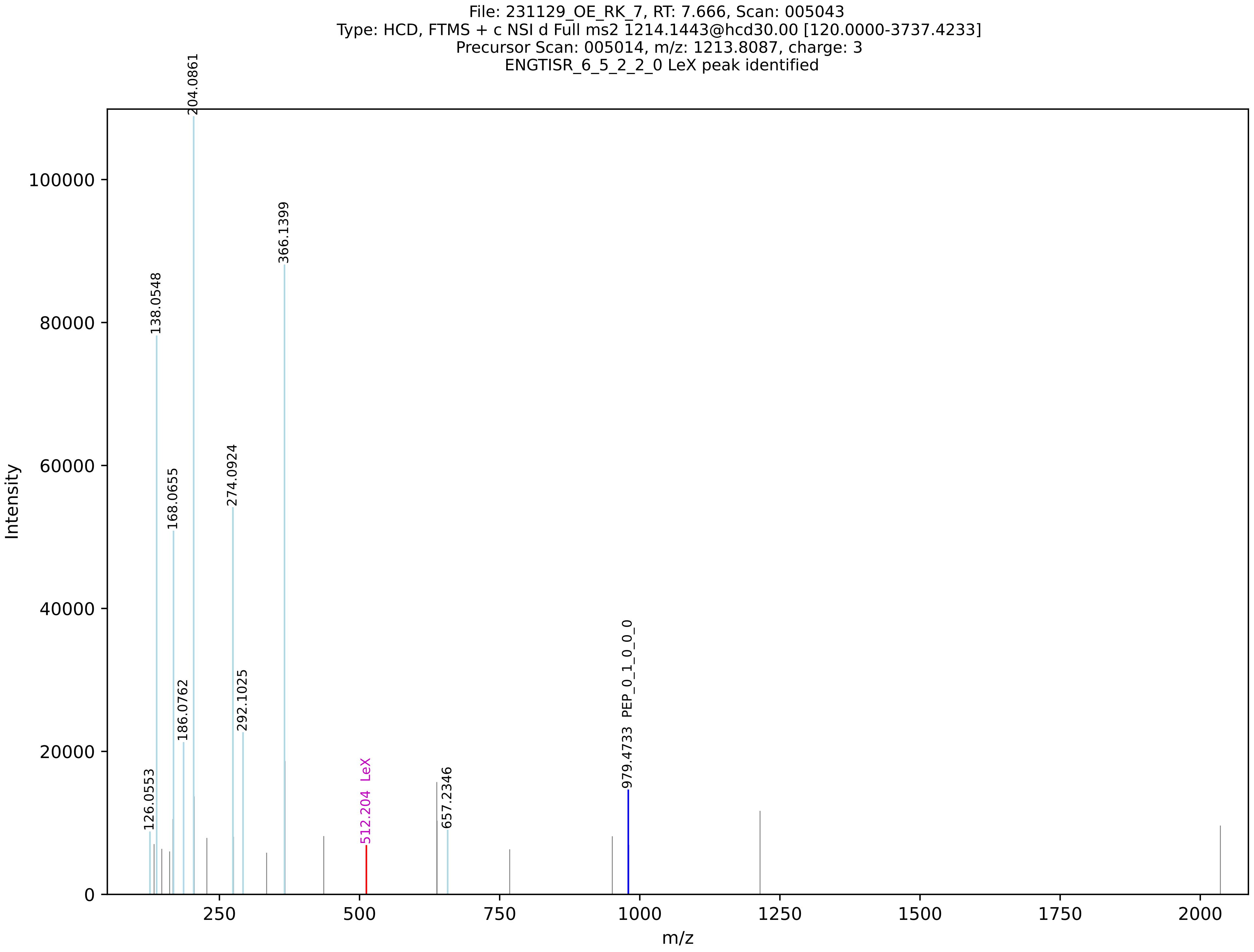

File: 231129_OE_RK_7, RT: 7.666, Scan: 005043
Type: HCD, FTMS + c NSI d Full ms2 1214.1443@hcd30.00 [120.0000-3737.4233]
Precursor Scan: 005014, m/z: 1213.8087, charge: 3
ENGTISR_6_5_2_2_0 LeX peak identified
Intensity
m/z
0
20000
40000
60000
80000
100000
250
500
750
1000
1250
1500
1750
2000
204.0861
366.1399
138.0548
168.0655
274.0924
186.0762
292.1025
126.0553
512.204 LeX
657.2346
979.4733 PEP_0_1_0_0_0

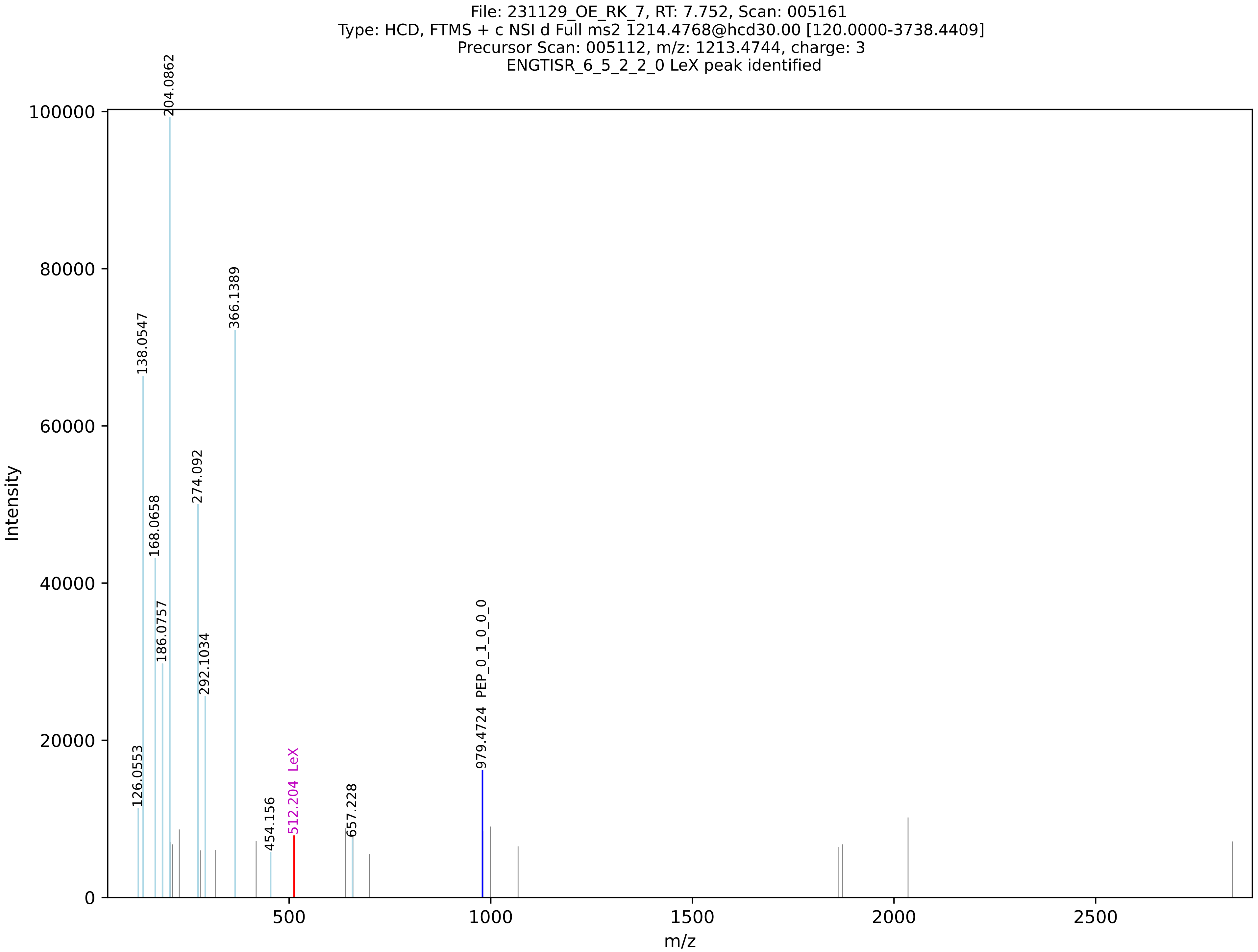
File: 231129_OE_RK_7, RT: 7.752, Scan: 005161
Type: HCD, FTMS + c NSI d Full ms2 1214.4768@hcd30.00 [120.0000-3738.4409]
Precursor Scan: 005112, m/z: 1213.4744, charge: 3
ENGTISR_6_5_2_2_0 LeX peak identified
Intensity
m/z
204.0862
366.1389
138.0547
274.092
168.0658
186.0757
292.1034
126.0553
454.156
512.204 LeX
657.228
979.4724 PEP_0_1_0_0_0
100000
80000
60000
40000
20000
0
500
1000
1500
2000
2500

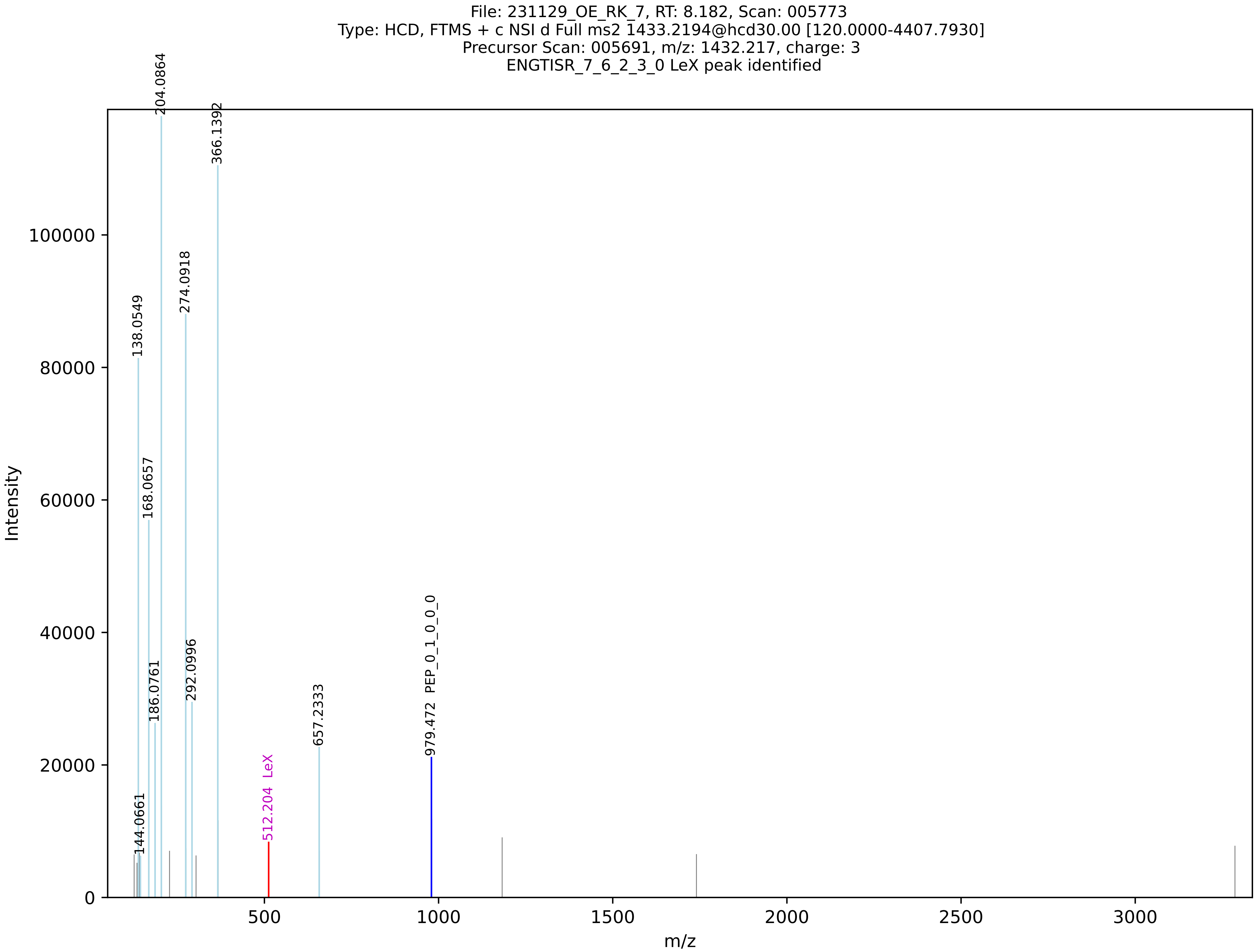
File: 231129_OE_RK_7, RT: 8.182, Scan: 005773
Type: HCD, FTMS + c NSI d Full ms2 1433.2194@hcd30.00 [120.0000-4407.7930]
Precursor Scan: 005691, m/z: 1432.217, charge: 3
ENGTISR_7_6_2_3_0 LeX peak identified
204.0864
366.1392
274.0918
138.0549
168.0657
186.0761
292.0996
144.0661
512.204 LeX
657.2333
979.472 PEP_0_1_0_0_0
Intensity
m/z
0
20000
40000
60000
80000
100000
500
1000
1500
2000
2500
3000

File: 231129_OE_RK_7, RT: 8.754, Scan: 006590
Type: HCD, FTMS + c NSI d Full ms2 1074.6638@hcd30.00 [120.0000-4407.4844]
Precursor Scan: 006514, m/z: 1074.1632, charge: 4
ENGTISR_7_6_0_4_0 LeX peak identified

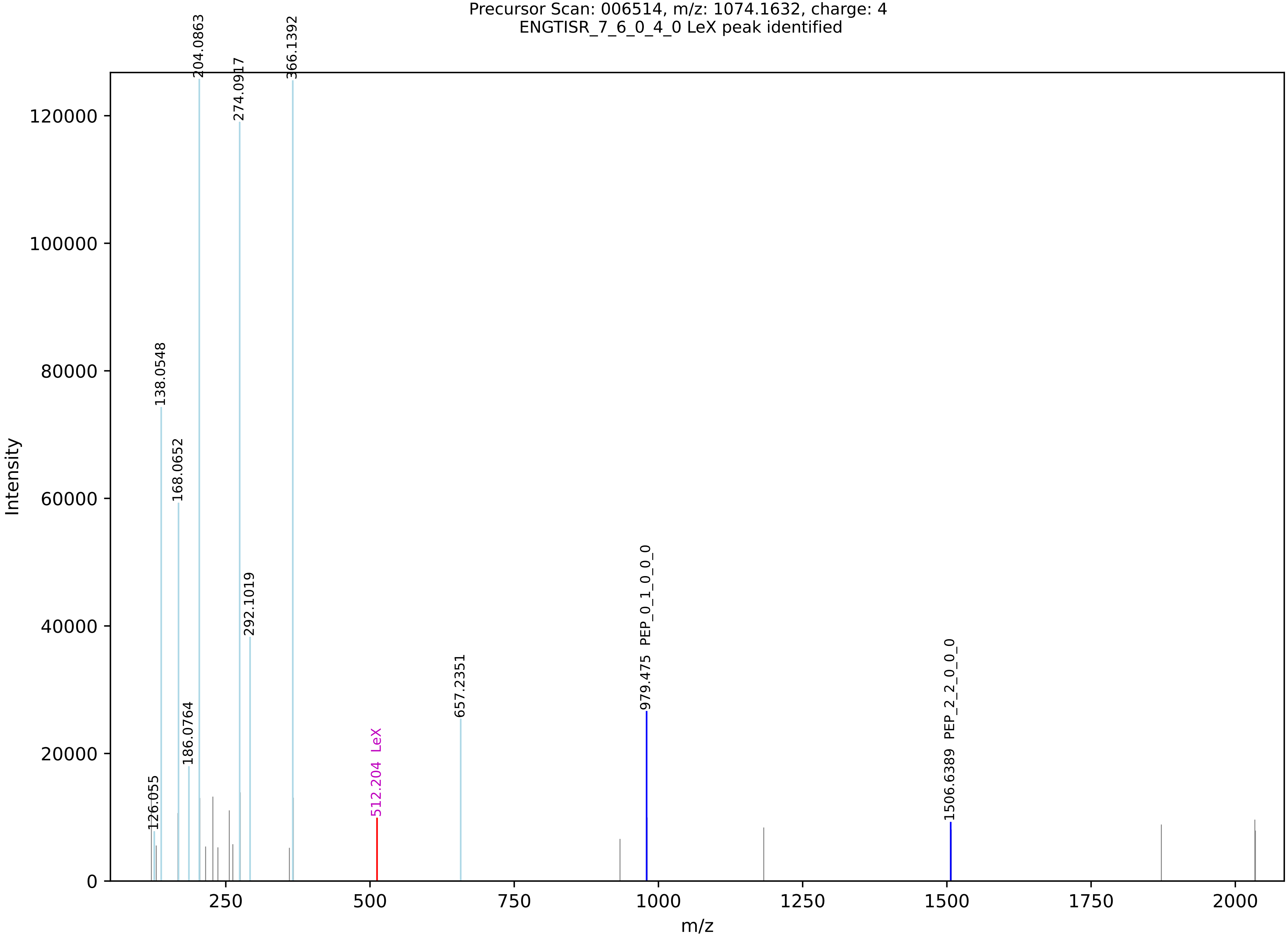

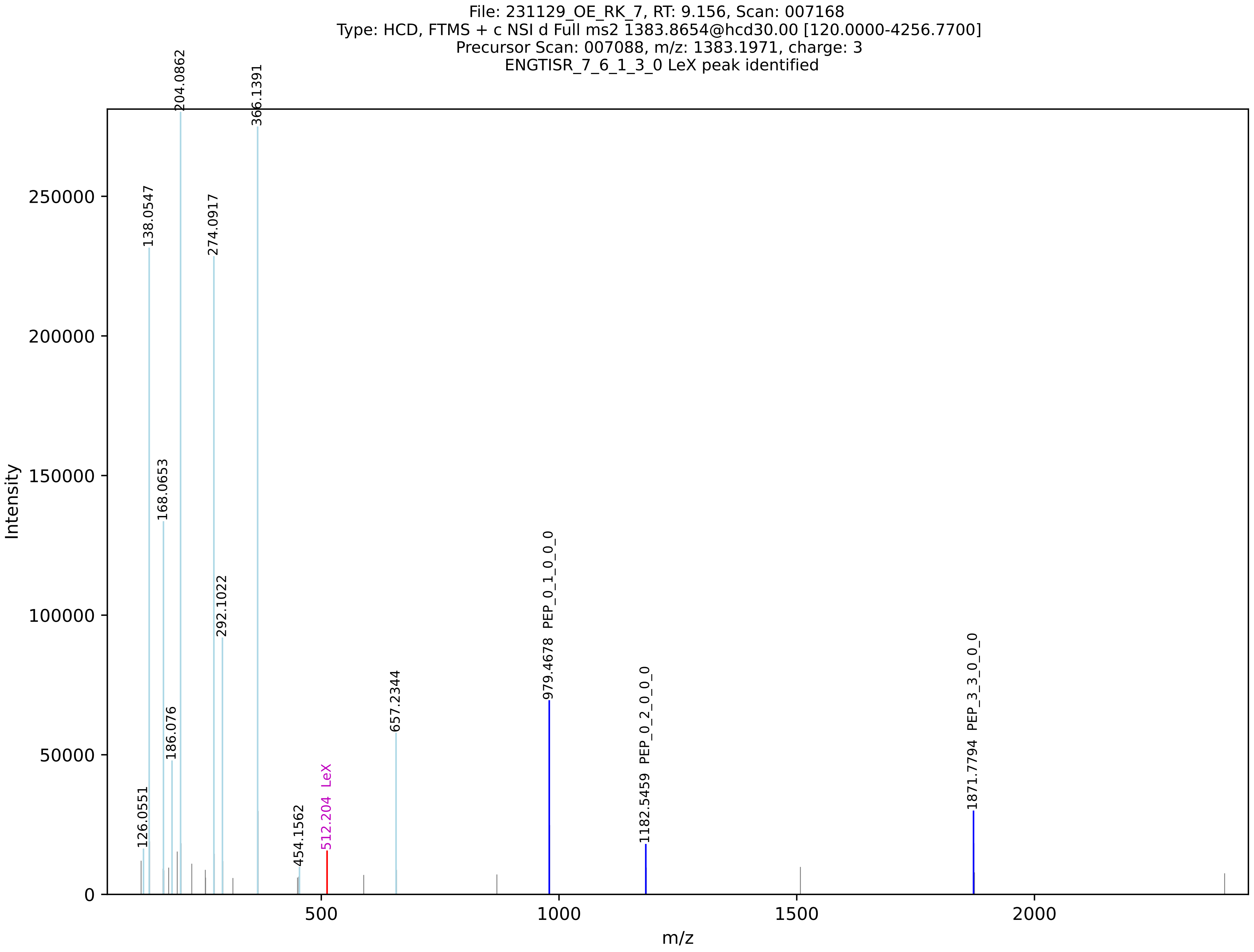

File: 231129_OE_RK_7, RT: 9.156, Scan: 007168
Type: HCD, FTMS + c NSI d Full ms2 1383.8654@hcd30.00 [120.0000-4256.7700]
Precursor Scan: 007088, m/z: 1383.1971, charge: 3
ENGTISR_7_6_1_3_0 LeX peak identified
204.0862
366.1391
138.0547
274.0917
168.0653
292.1022
186.076
126.0551
454.1562
512.204 LeX
657.2344
979.4678 PEP_0_1_0_0_0_
1182.5459 PEP_0_2_0_0_0_
1871.7794 PEP_3_3_0_0_0_
Intensity
250000
200000
150000
100000
50000
0
500
1000
1500
2000
m/z

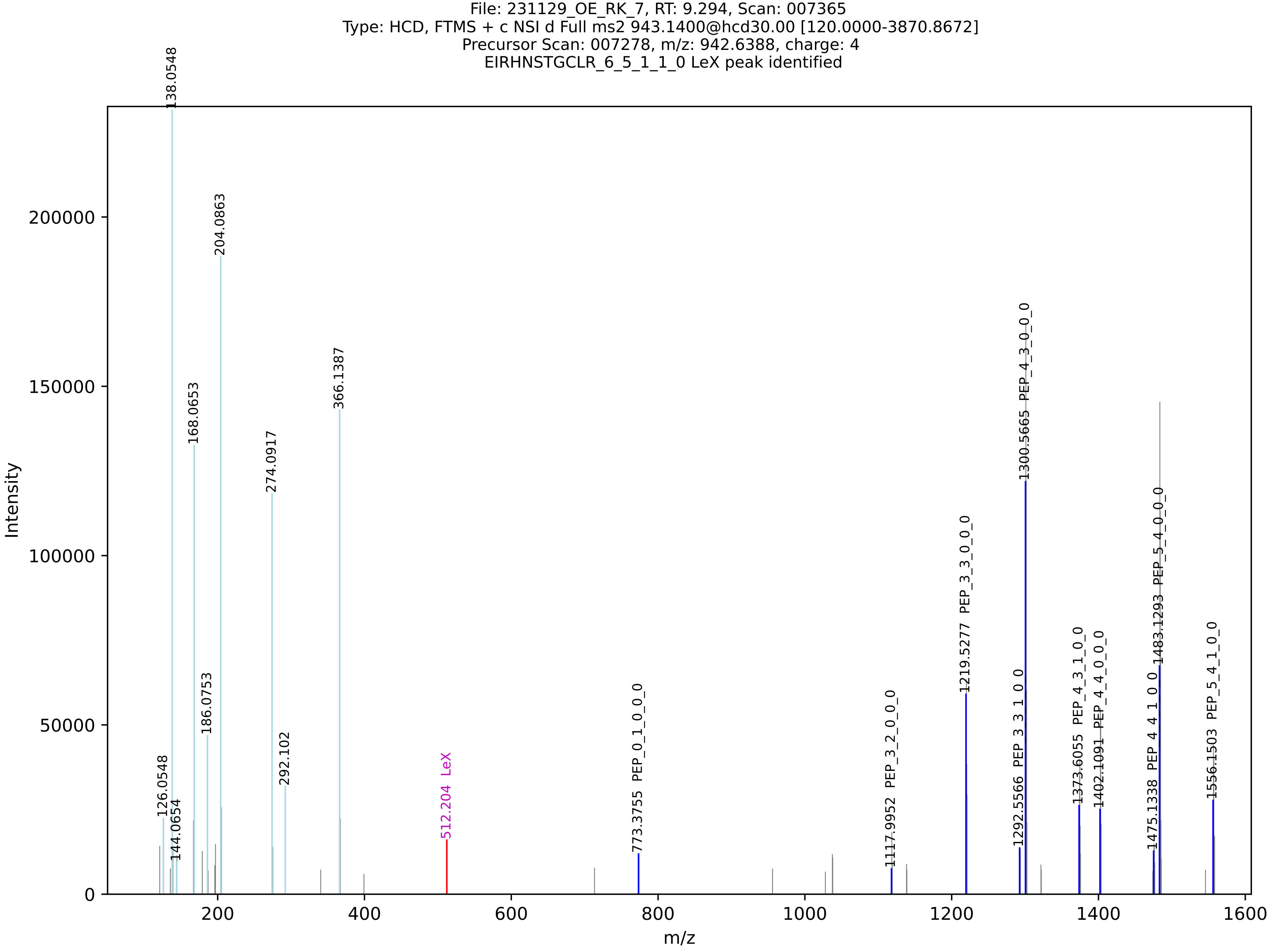
File: 231129_OE_RK_7, RT: 9.294, Scan: 007365
Type: HCD, FTMS + c NSI d Full ms2 943.1400@hcd30.00 [120.0000-3870.8672]
Precursor Scan: 007278, m/z: 942.6388, charge: 4
EIRHNSTGCLR_6_5_1_1_0 LeX peak identified
Intensity
m/z
138.0548
204.0863
366.1387
168.0653
274.0917
186.0753
292.102
126.0548
144.0654
512.204 LeX
773.3755 PEP_0_1_0_0_0
1117.9952 PEP_3_2_0_0_0
1219.5277 PEP_3_3_0_0_0
1292.5566 PEP_3_3_1_0_0
1300.5665 PEP_4_3_0_0_0
1373.6055 PEP_4_3_1_0_0
1402.1091 PEP_4_4_0_0_0
1475.1338 PEP_4_4_1_0_0
1483.1293 PEP_5_4_0_0_0
1556.1503 PEP_5_4_1_0_0

File: 231129_OE_RK_7, RT: 9.634, Scan: 007855
Type: HCD, FTMS + c NSI d Full ms2 1110.9250@hcd30.00 [120.0000-4555.4302]
Precursor Scan: 007777, m/z: 1110.6748, charge: 4
ENGTISR_7_6_1_4_0 LeX peak identified

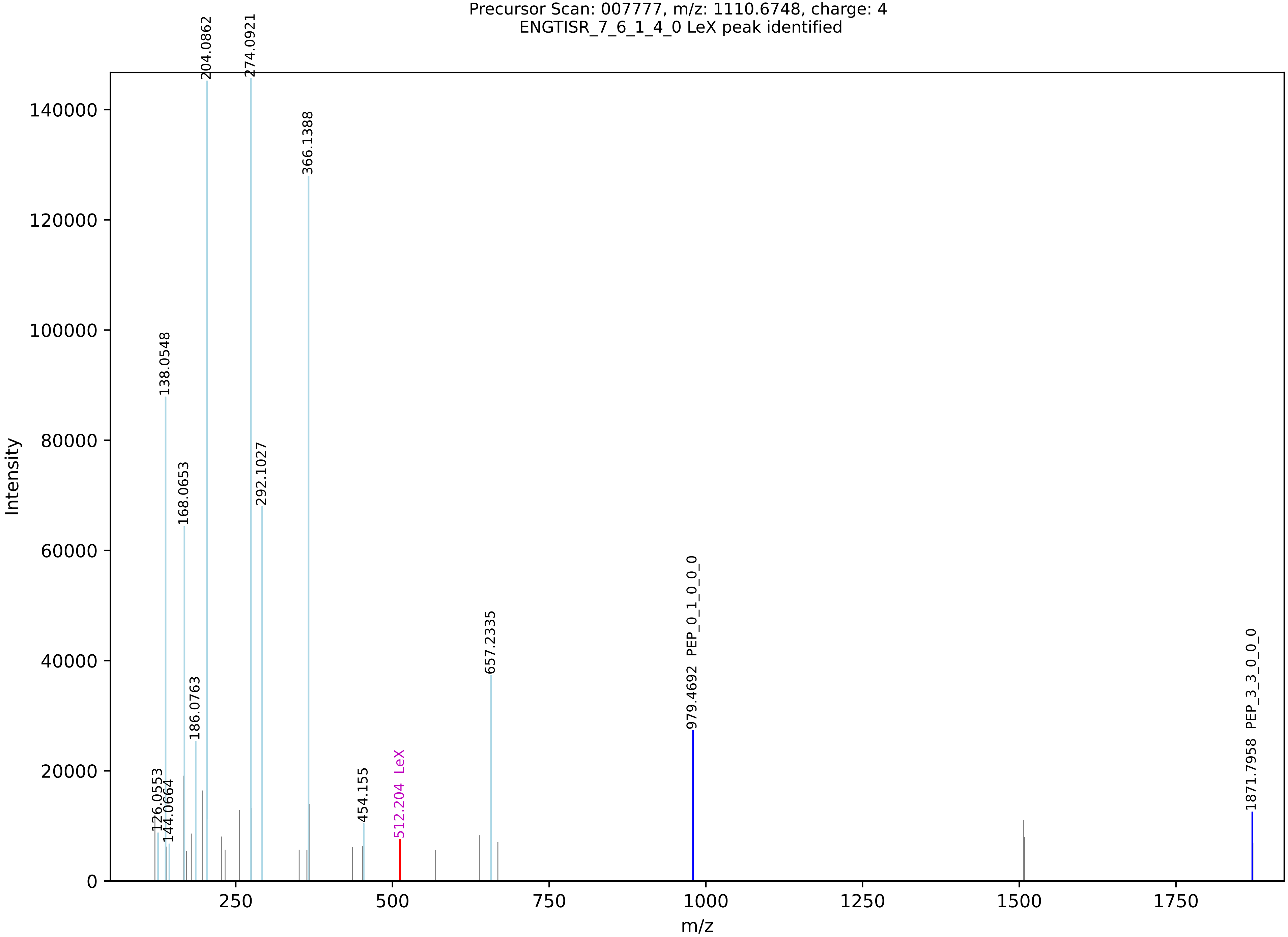

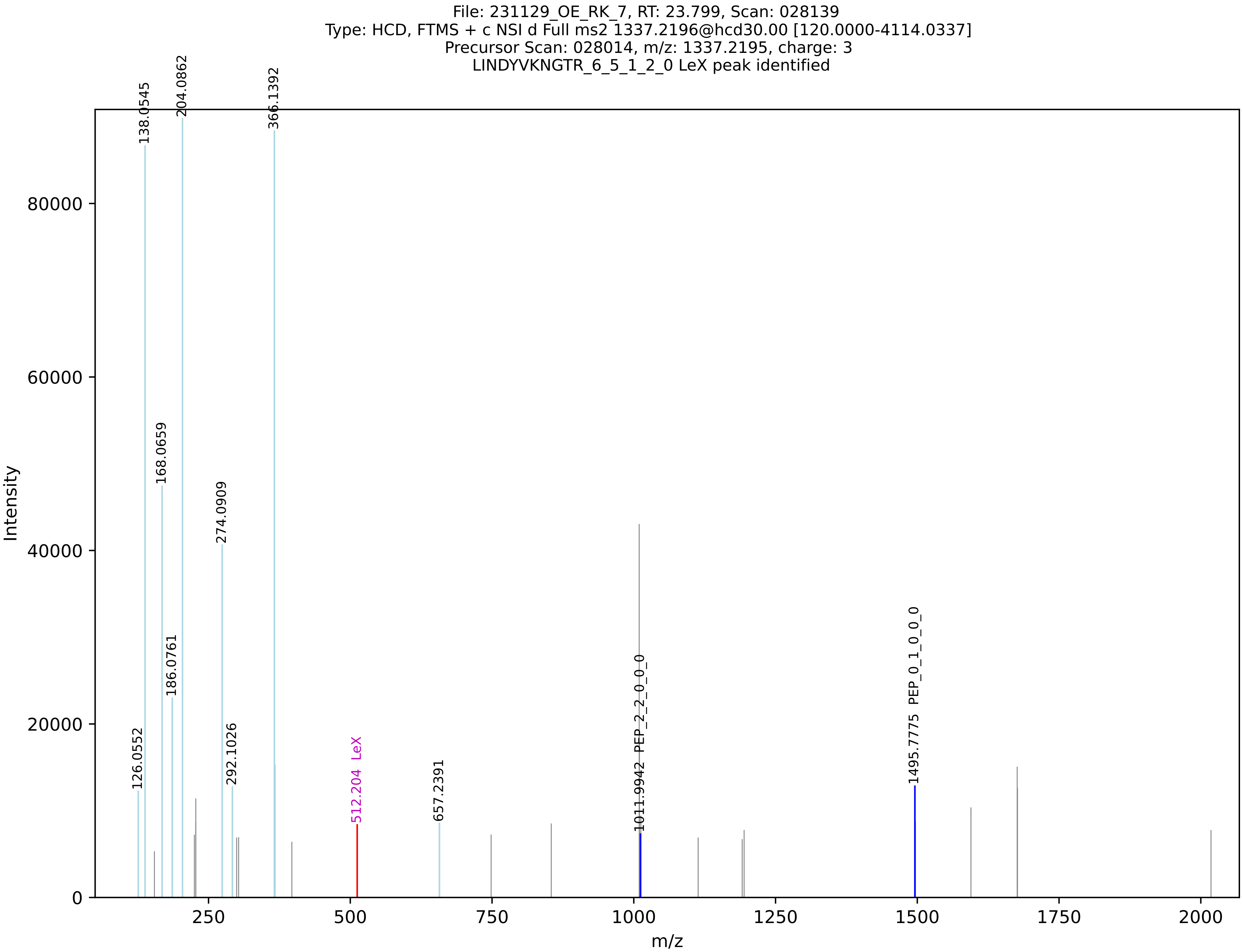
File: 231129_OE_RK_7, RT: 23.799, Scan: 028139
Type: HCD, FTMS + c NSI d Full ms2 1337.2196@hcd30.00 [120.0000-4114.0337]
Precursor Scan: 028014, m/z: 1337.2195, charge: 3
LINDYVKNGTR_6_5_1_2_0 LeX peak identified
Intensity
m/z
0
20000
40000
60000
80000
250
500
750
1000
1250
1500
1750
2000
126.0552
138.0545
168.0659
186.0761
204.0862
274.0909
292.1026
366.1392
512.204 LeX
657.2391
1011.9942 PEP_2_2_0_0_0
1495.7775 PEP_0_1_0_0_0

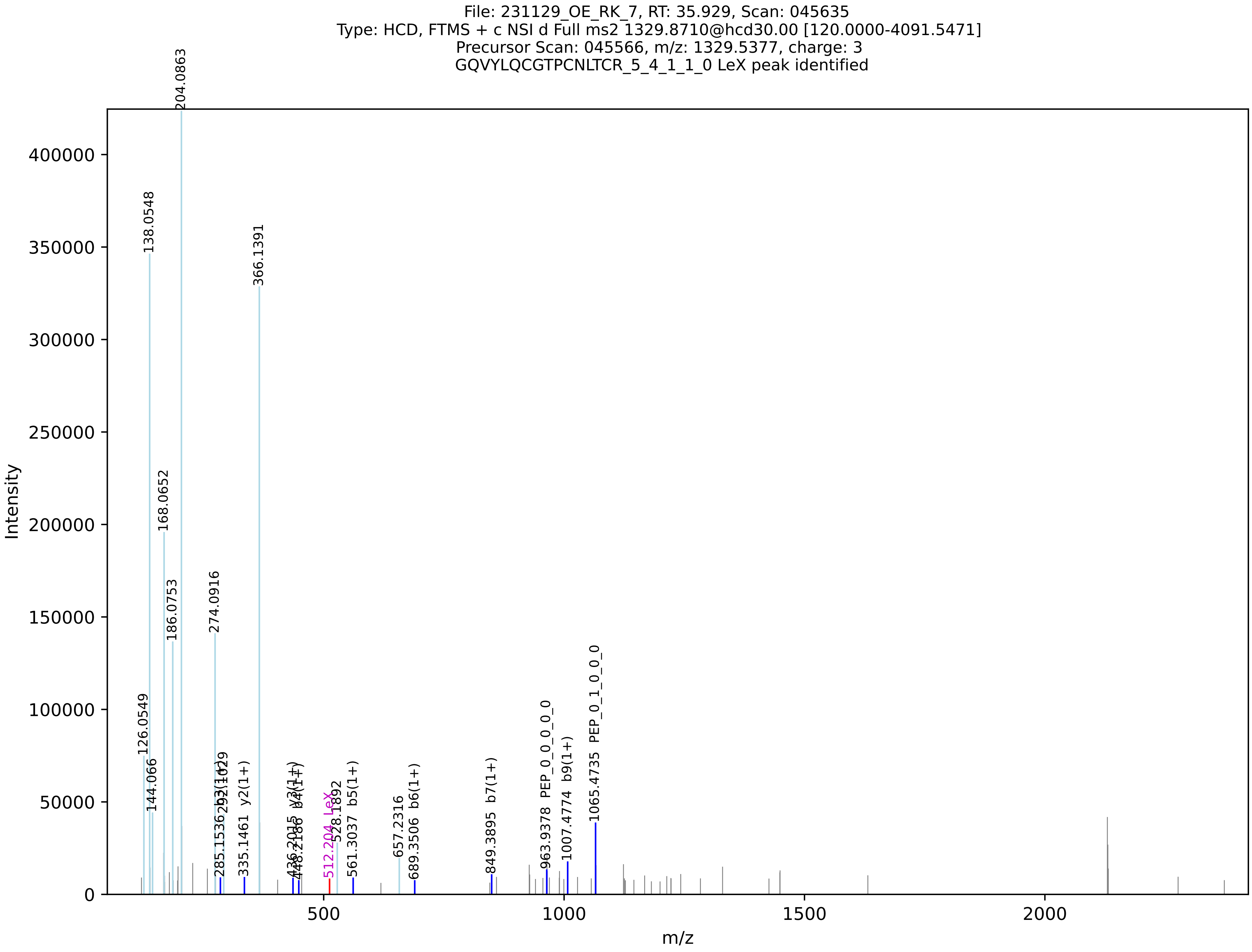

File: 231129_OE_RK_7, RT: 35.929, Scan: 045635
Type: HCD, FTMS + c NSI d Full ms2 1329.8710@hcd30.00 [120.0000-4091.5471]
Precursor Scan: 045566, m/z: 1329.5377, charge: 3
GQVYLQCGTPCNLTCR_5_4_1_1_0 LeX peak identified
Intensity
m/z
204.0863
138.0548
366.1391
168.0652
186.0753
274.0916
126.0549
144.066
335.1461 y2(1+)
512.204_LeX
528.1892
561.3037 b5(1+)
657.2316
689.3506 b6(1+)
849.3895 b7(1+)
963.9378 PEP_0_0_0_0_0
1007.4774 b9(1+)
1065.4735 PEP_0_1_0_0_0

File: 231129_OE_RK_7, RT: 43.061, Scan: 055996
Type: HCD, FTMS + c NSI d Full ms2 1287.0226@hcd30.00 [120.0000-5273.9077]
Precursor Scan: 055919, m/z: 1286.5222, charge: 4
QDQCIYNTTYLNVQR_7_6_2_2_0 LeX peak identified

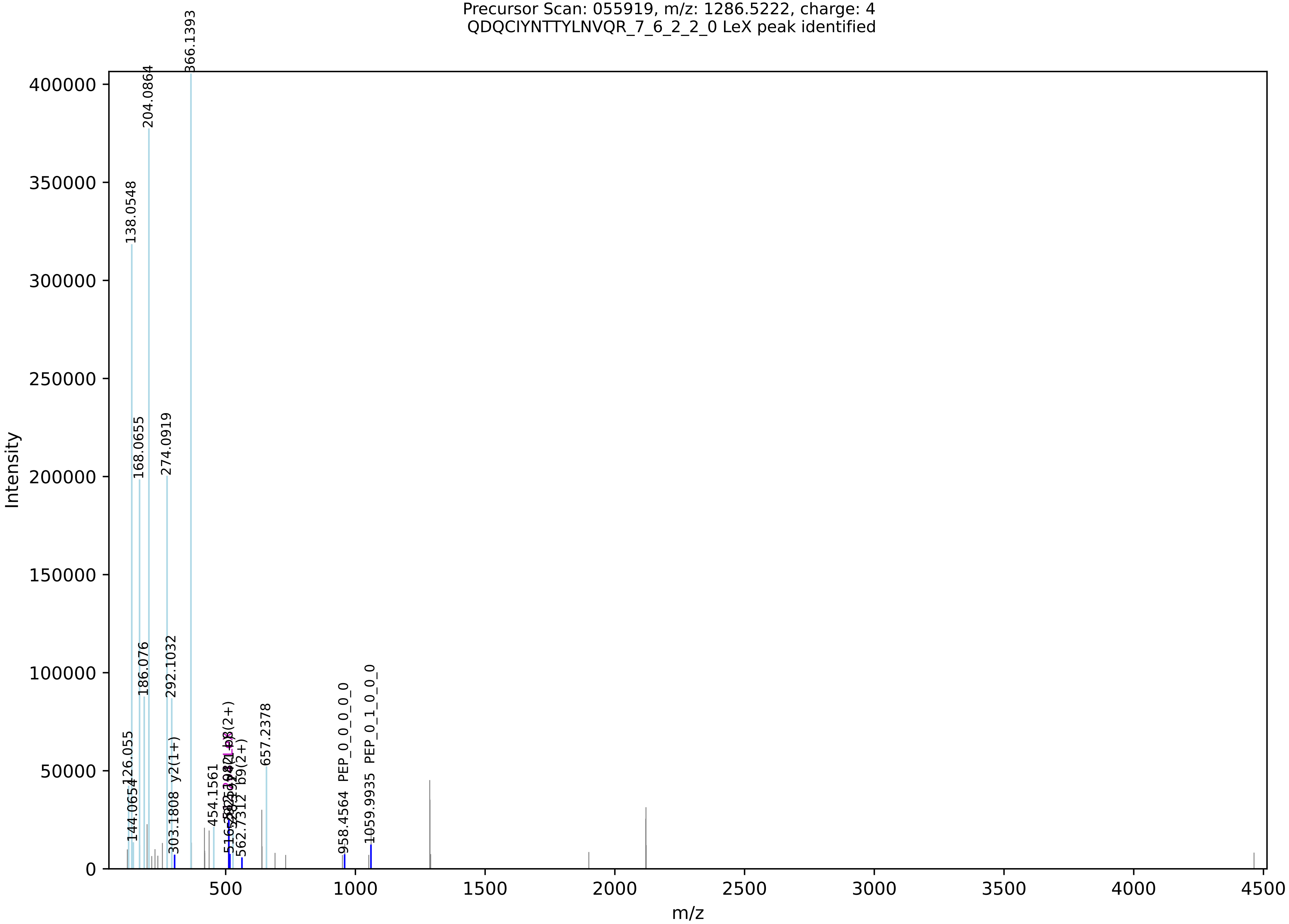

File: 231129_OE_RK_7, RT: 43.421, Scan: 056524
Type: HCD, FTMS + c NSI d Full ms2 1545.2932@hcd30.00 [120.0000-4750.7393]
Precursor Scan: 056436, m/z: 1544.6252, charge: 3
QDQCIYNTTYLNVQR_6_5_1_2_0 LeX peak identified

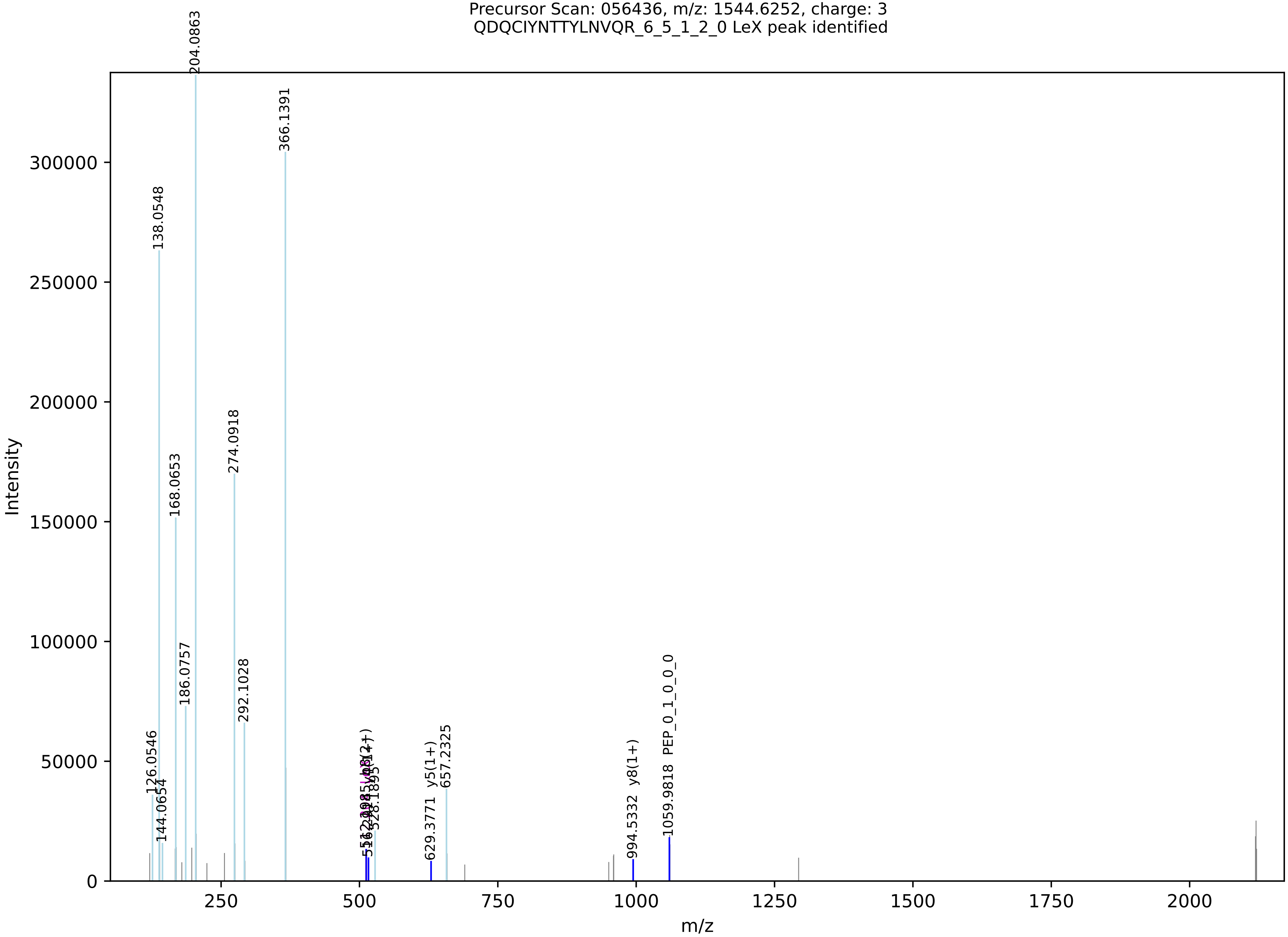

File: 231129_OE_RK_7, RT: 43.616, Scan: 056805
Type: HCD, FTMS + c NSI d Full ms2 1545.2936@hcd30.00 [120.0000-4750.7402]
Precursor Scan: 056727, m/z: 1544.6253, charge: 3
QDQCIYNTTYLNVQR_6_5_1_2_0 LeX peak identified

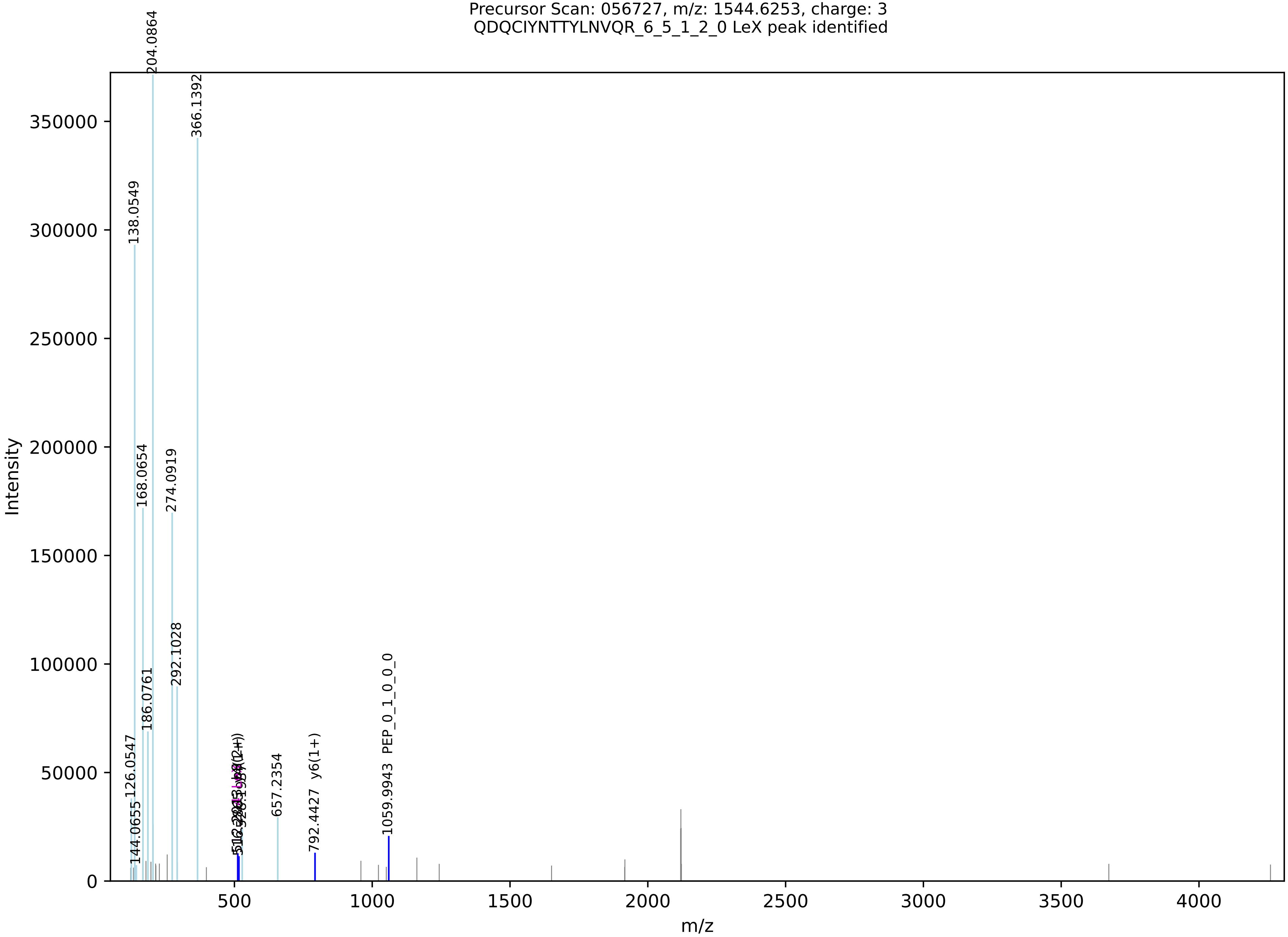

File: 231129_OE_RK_7, RT: 46.184, Scan: 060533
Type: HCD, FTMS + c NSI d Full ms2 1323.2802@hcd30.00 [120.0000-5421.8394]
Precursor Scan: 060454, m/z: 1322.7782, charge: 4
QDQCIYNTTYLNVQR_7_6_1_3_0 LeX peak identified

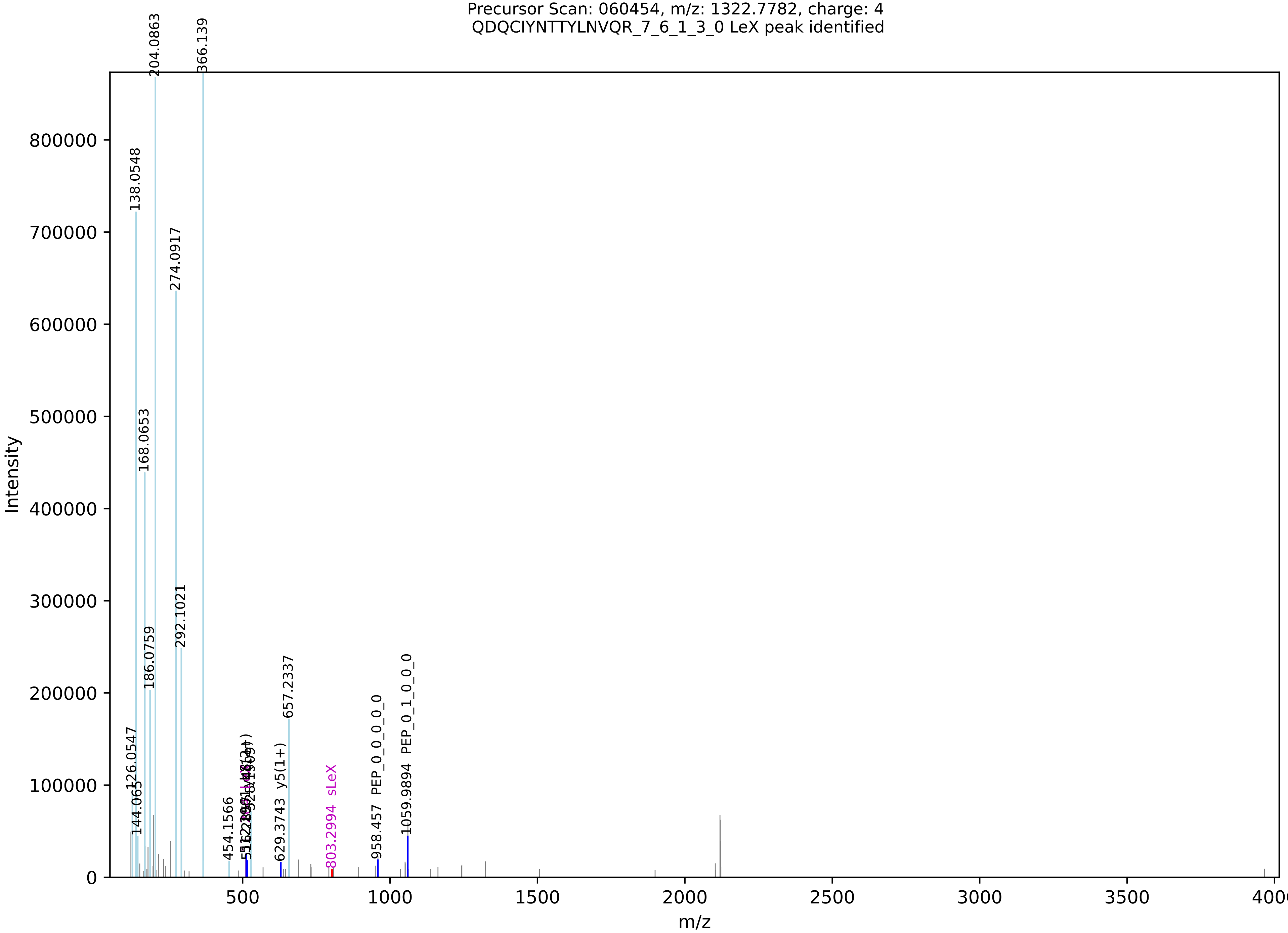

File: 231129_OE_RK_7, RT: 50.35, Scan: 066603
Type: HCD, FTMS + c NSI d Full ms2 1456.6563@hcd30.00 [120.0000-4479.5103]
Precursor Scan: 066516, m/z: 1455.988, charge: 3
VVLHPNYSQVDIGLIK_6_5_0_2_0 LeX peak identified

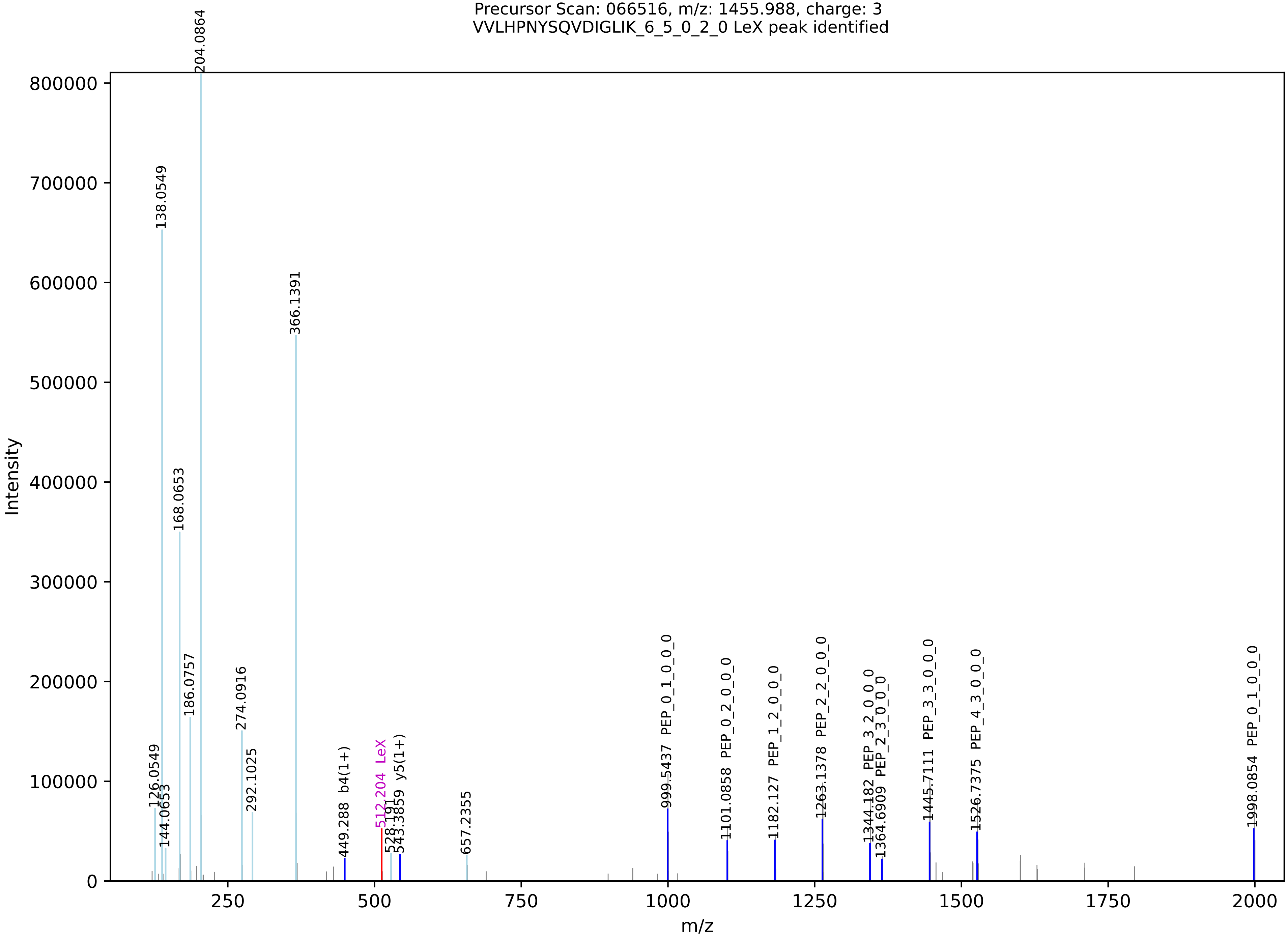

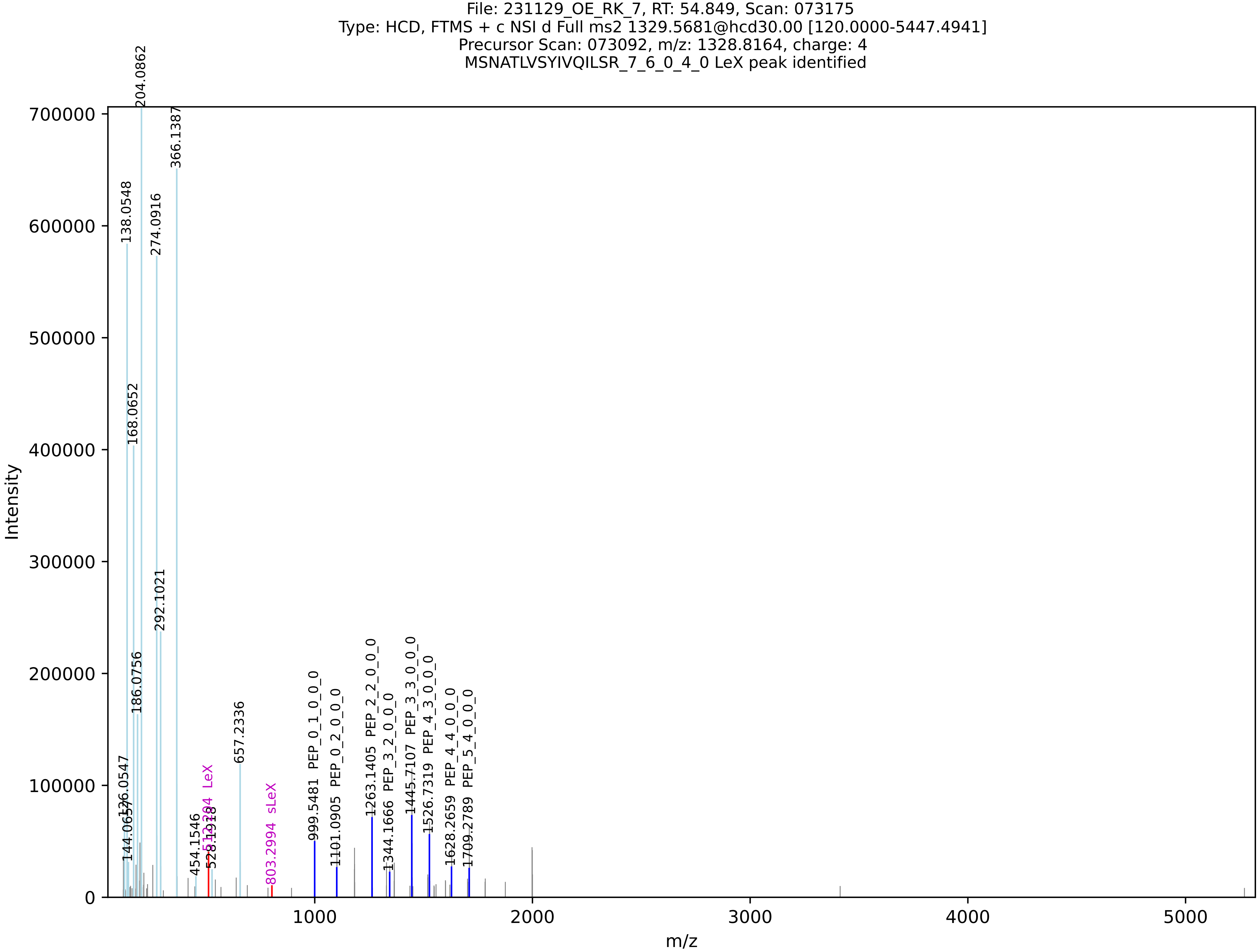

File: 231129_OE_RK_7, RT: 54.849, Scan: 073175
Type: HCD, FTMS + c NSI d Full ms2 1329.5681@hcd30.00 [120.0000-5447.4941]
Precursor Scan: 073092, m/z: 1328.8164, charge: 4
MSNATLVSYIVQILSR_7_6_0_4_0 LeX peak identified
Intensity
m/z
204.0862
366.1387
138.0548
274.0916
168.0652
292.1021
186.0756
126.0547
144.0657
454.1546
528.1918
LeX
803.2994 sLeX
657.2336
999.5481 PEP_0_1_0_0_0
1101.0905 PEP_0_2_0_0_0
1263.1405 PEP_2_2_0_0_0
1344.1666 PEP_3_2_0_0_0
1445.7107 PEP_3_3_0_0_0
1526.7319 PEP_4_3_0_0_0
1628.2659 PEP_4_4_0_0_0
1709.2789 PEP_5_4_0_0_0

File: 231129_OE_RK_7, RT: 55.4, Scan: 073982
Type: HCD, FTMS + c NSI d Full ms2 1238.5387@hcd30.00 [120.0000-5076.0938]
Precursor Scan: 073905, m/z: 1237.7869, charge: 4
VVLHPNYSQVDIGLIK_6_5_2_3_0 LeX peak identified

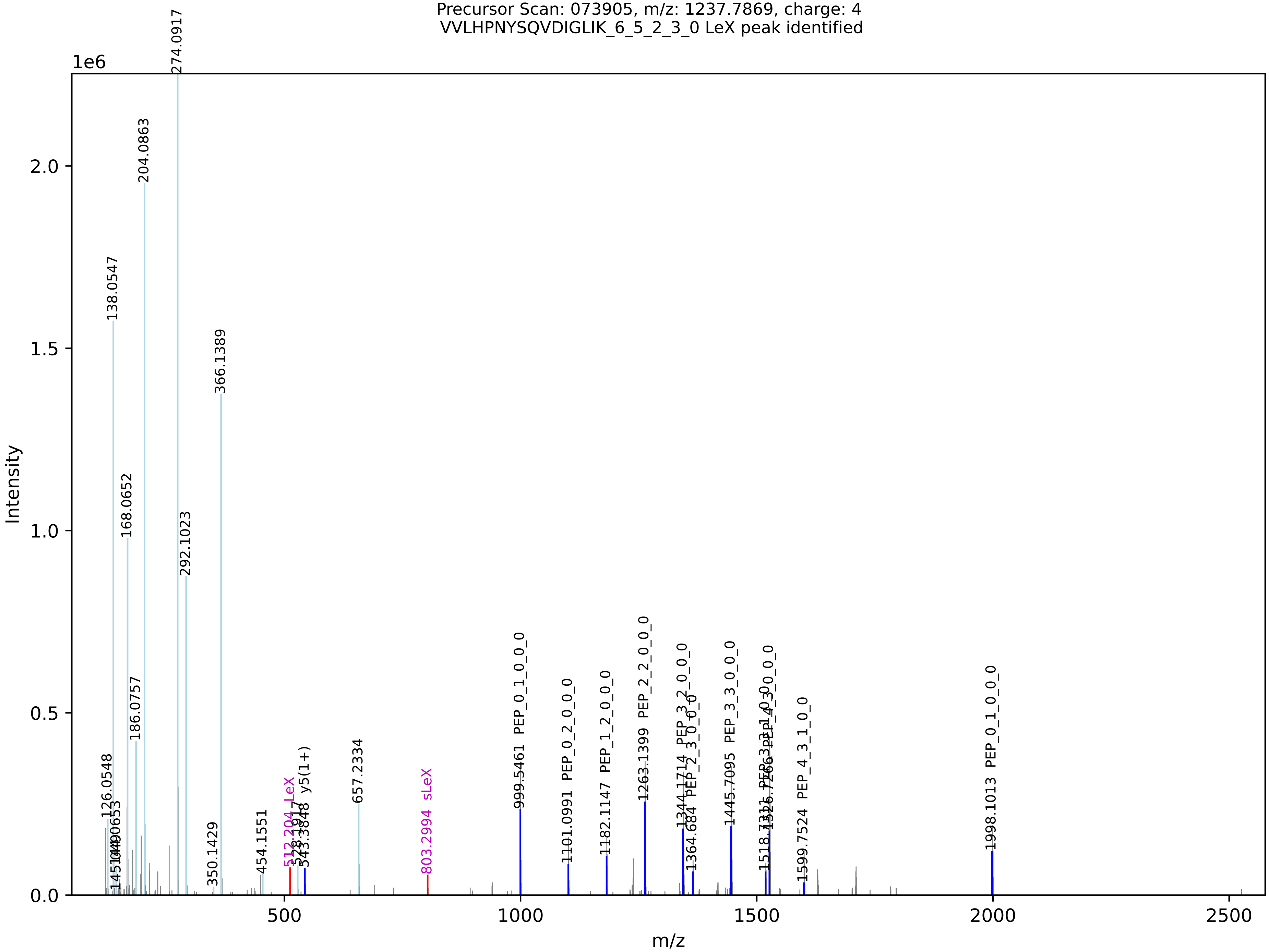

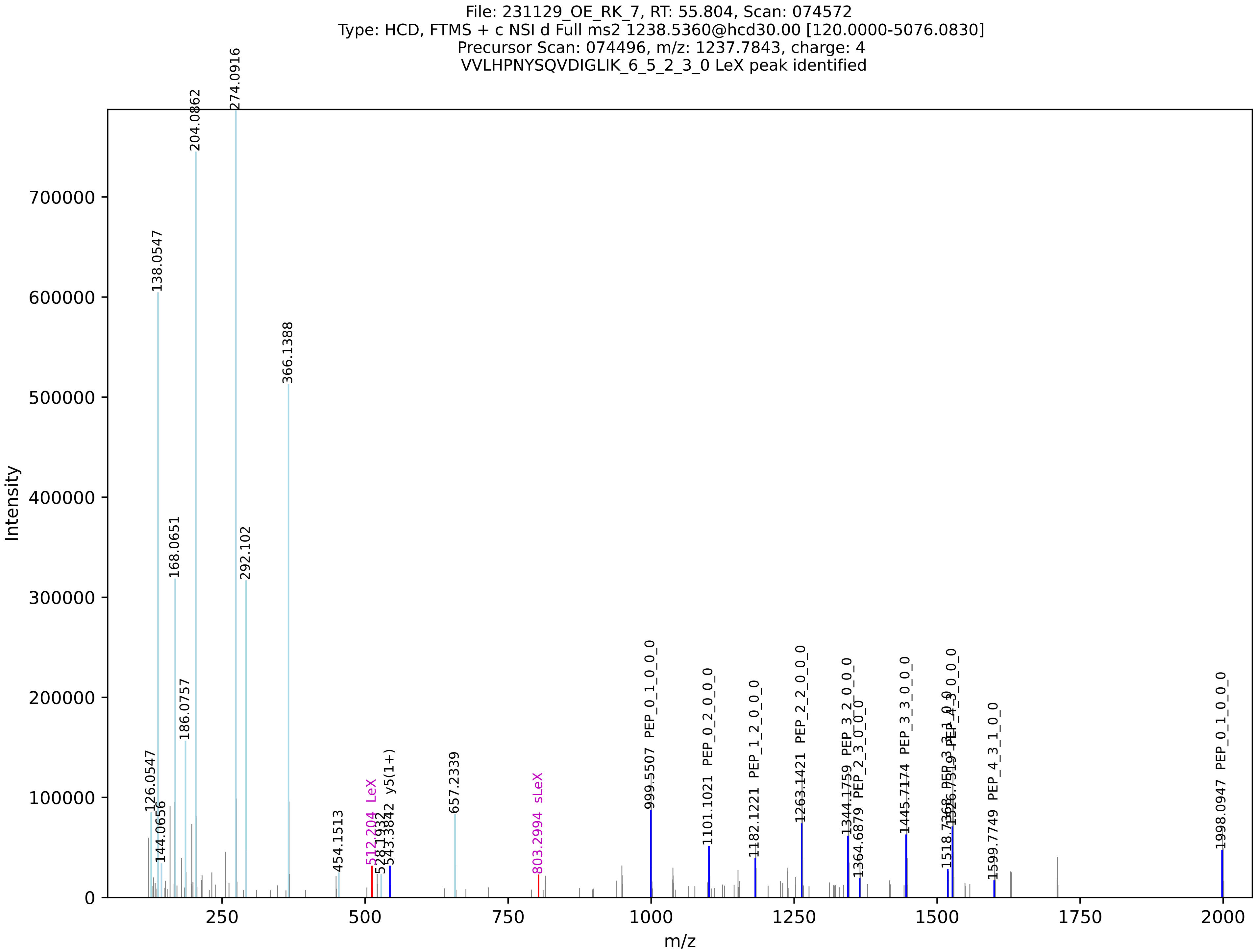
File: 231129_OE_RK_7, RT: 55.804, Scan: 074572
Type: HCD, FTMS + c NSI d Full ms2 1238.5360@hcd30.00 [120.0000-5076.0830]
Precursor Scan: 074496, m/z: 1237.7843, charge: 4
VVLHPNYSQVDIGLIK_6_5_2_3_0 LeX peak identified
Intensity
m/z
274.0916
204.0862
138.0547
366.1388
168.0651
292.102
186.0757
126.0547
144.0656
454.1513
512.204 LeX
543.3842 y5(1+)
657.2339
803.2994 sLeX
999.5507 PEP_0_1_0_0_0
1101.1021 PEP_0_2_0_0_0
1182.1221 PEP_1_2_0_0_0
1263.1421 PEP_2_2_0_0_0
1344.1759 PEP_3_2_0_0_0
1364.6879 PEP_2_3_0_0_0
1445.7174 PEP_3_3_0_0_0
1599.7749 PEP_4_3_1_0_0
1998.0947 PEP_0_1_0_0_0

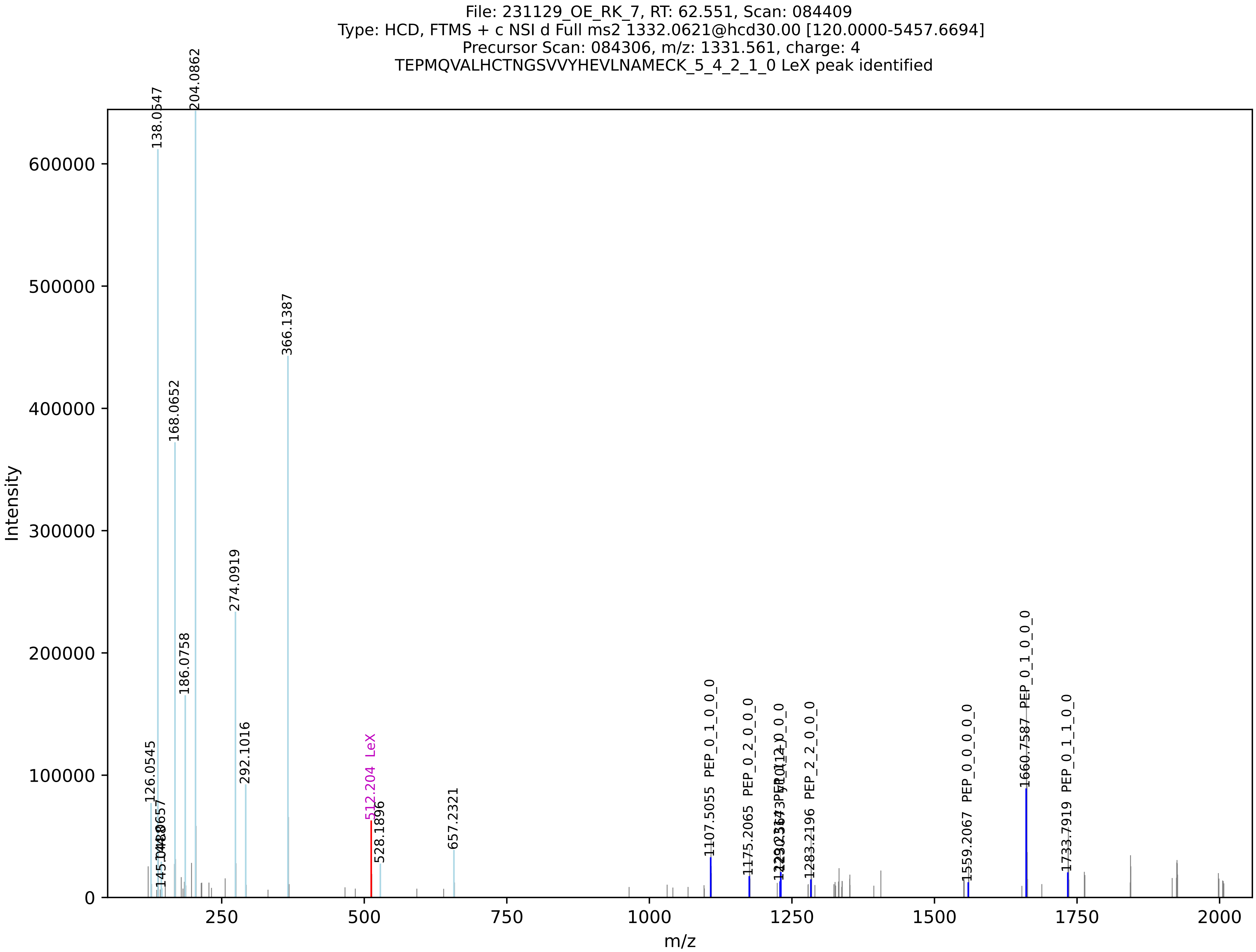
File: 231129_OE_RK_7, RT: 62.551, Scan: 084409
Type: HCD, FTMS + c NSI d Full ms2 1332.0621@hcd30.00 [120.0000-5457.6694]
Precursor Scan: 084306, m/z: 1331.561, charge: 4
TEPMQVALHCTNGSVVYHEVLNAMECK_5_4_2_1_0 LeX peak identified
Intensity
m/z
204.0862
138.0547
366.1387
168.0652
274.0919
186.0758
292.1016
126.0545
512.204 LeX
528.1896
657.2321
1107.5055 PEP_0_1_0_0_0
1175.2065 PEP_0_2_0_0_0
1283.2196 PEP_2_2_0_0_0
1559.2067 PEP_0_0_0_0_0
1660.7587 PEP_0_1_0_0_0
1733.7919 PEP_0_1_1_0_0

File: 231129_OE_RK_7, RT: 65.363, Scan: 088495
Type: HCD, FTMS + c NSI d Full ms2 1244.7333@hcd30.00 [120.0000-6371.7100]
Precursor Scan: 088412, m/z: 1244.3356, charge: 5
GLKFNLTETSEAEIHQSFQHLLR_7_6_2_3_0 LeX peak identified

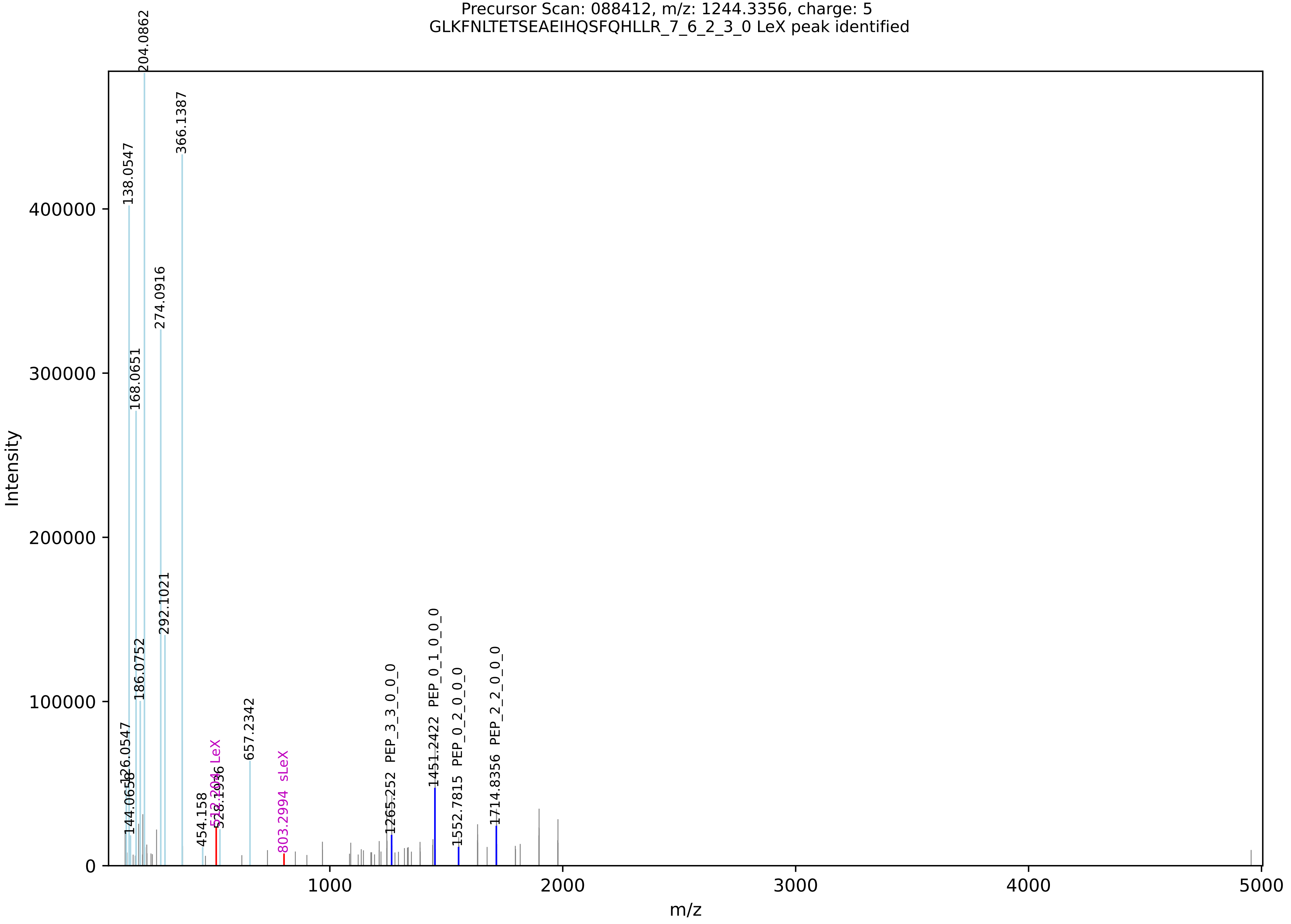

File: 231129_OE_RK_7, RT: 71.408, Scan: 097278
Type: HCD, FTMS + c NSI d Full ms2 1422.6216@hcd30.00 [120.0000-5827.1523]
Precursor Scan: 097197, m/z: 1422.1204, charge: 4
SVQEIQATFFYFTPNKTEDTIFLR_7_6_1_1_0 LeX peak identified

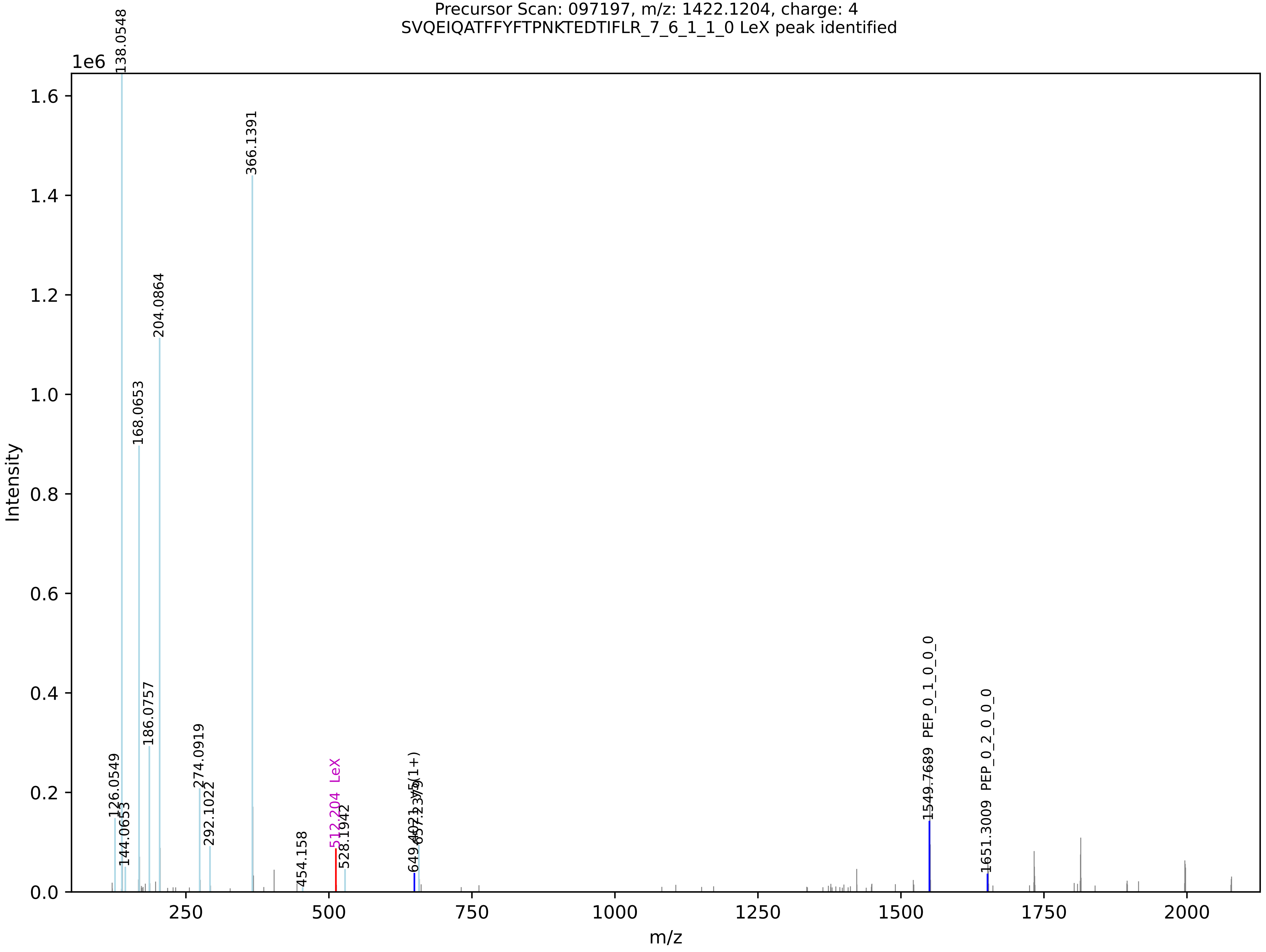

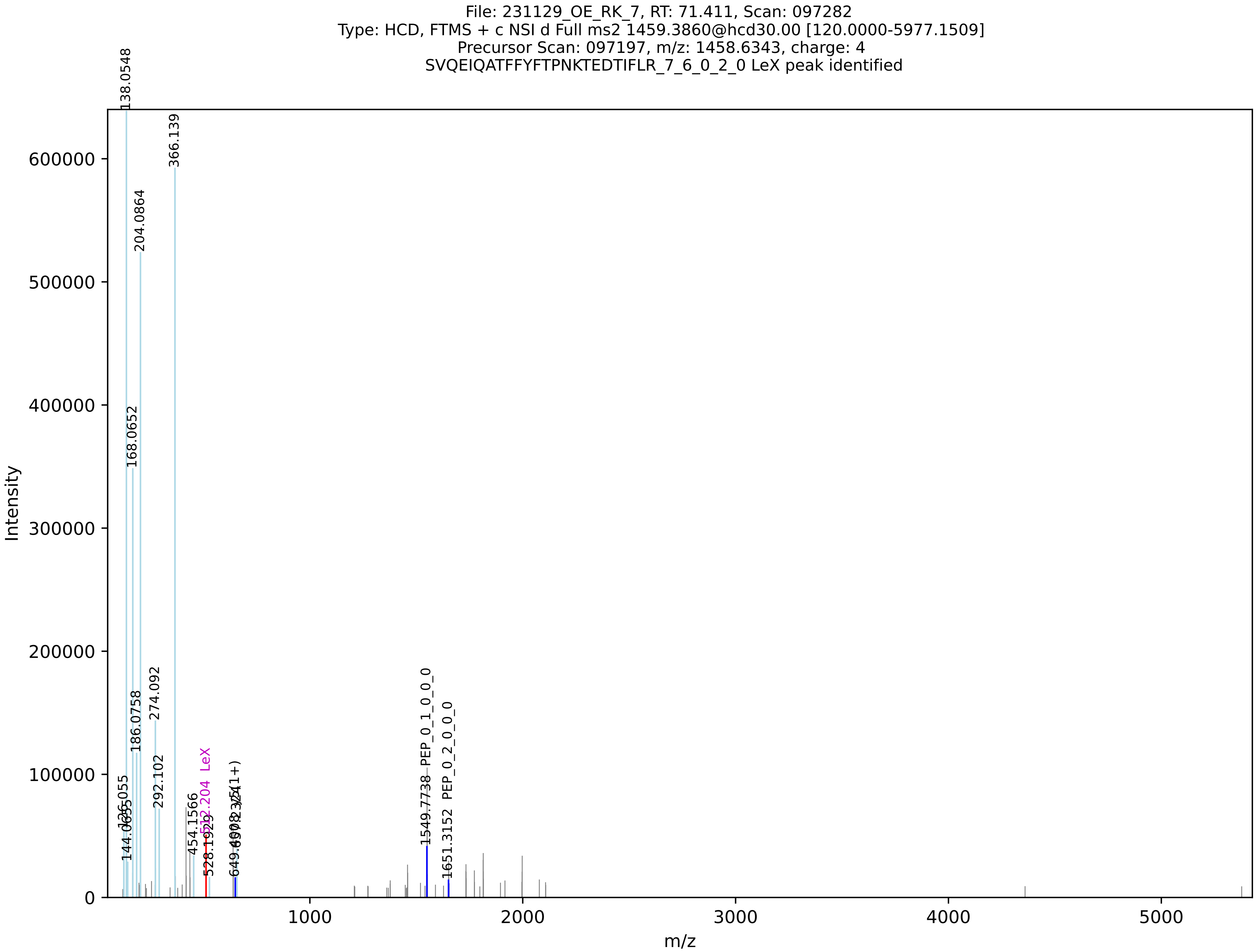
File: 231129_OE_RK_7, RT: 71.411, Scan: 097282
Type: HCD, FTMS + c NSI d Full ms2 1459.3860@hcd30.00 [120.0000-5977.1509]
Precursor Scan: 097197, m/z: 1458.6343, charge: 4
SVQEIQATFFYFTPNKTEDTIFLR_7_6_0_2_0 LeX peak identified
138.0548
366.139
204.0864
168.0652
186.0758
274.092
292.102
126.055
454.1566
LeX
1549.7738 PEP_0_1_0_0_0
1651.3152 PEP_0_2_0_0_0
Intensity
m/z
0
100000
200000
300000
400000
500000
600000
1000
2000
3000
4000
5000

File: 231129_OE_RK_7, RT: 74.179, Scan: 101296
Type: HCD, FTMS + c NSI d Full ms2 1495.3912@hcd30.00 [120.0000-6124.0522]
Precursor Scan: 101219, m/z: 1495.1428, charge: 4
SVQEIQATFFYFTPNKTEDTIFLR_7_6_1_2_0 LeX peak identified

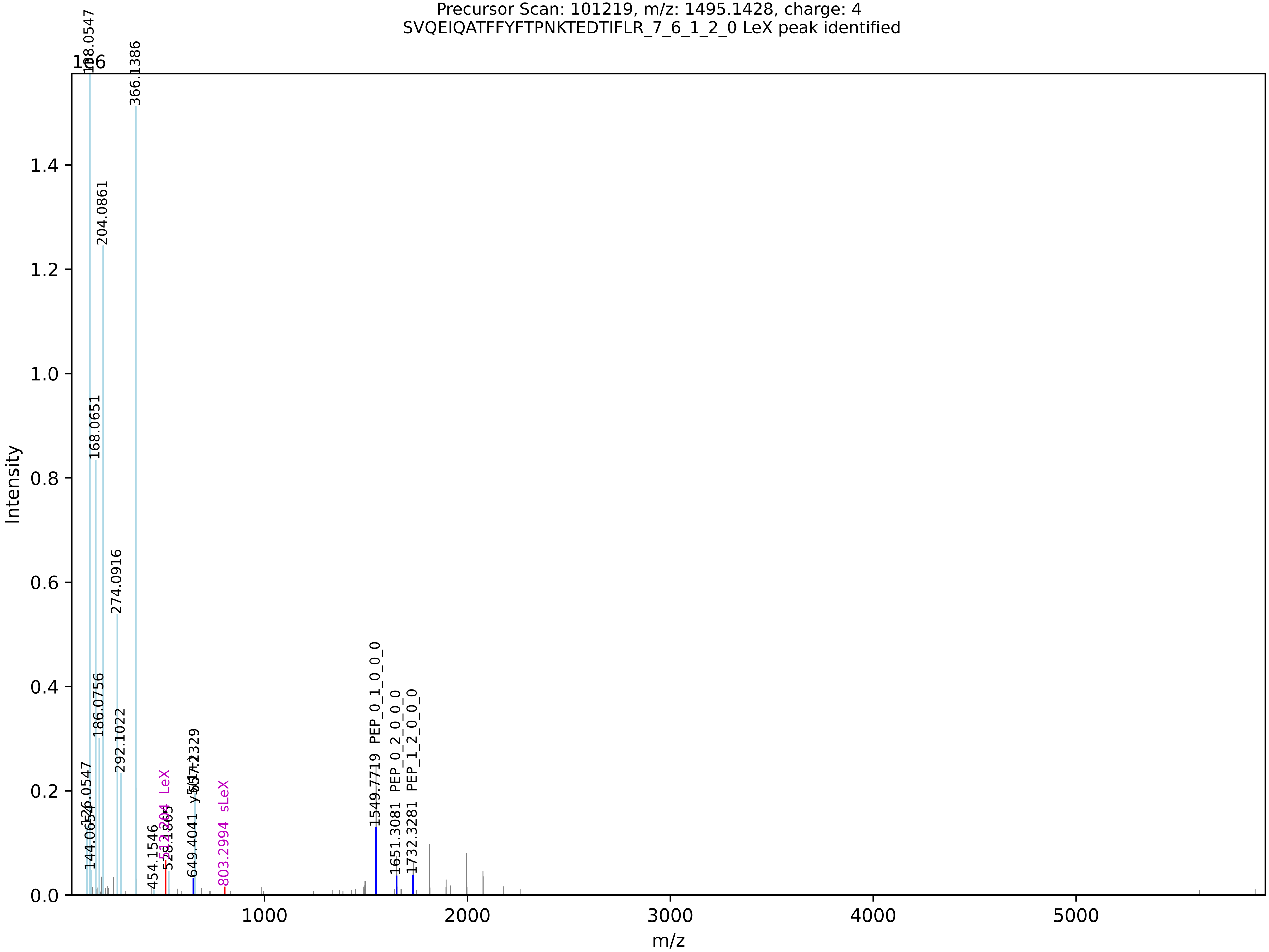

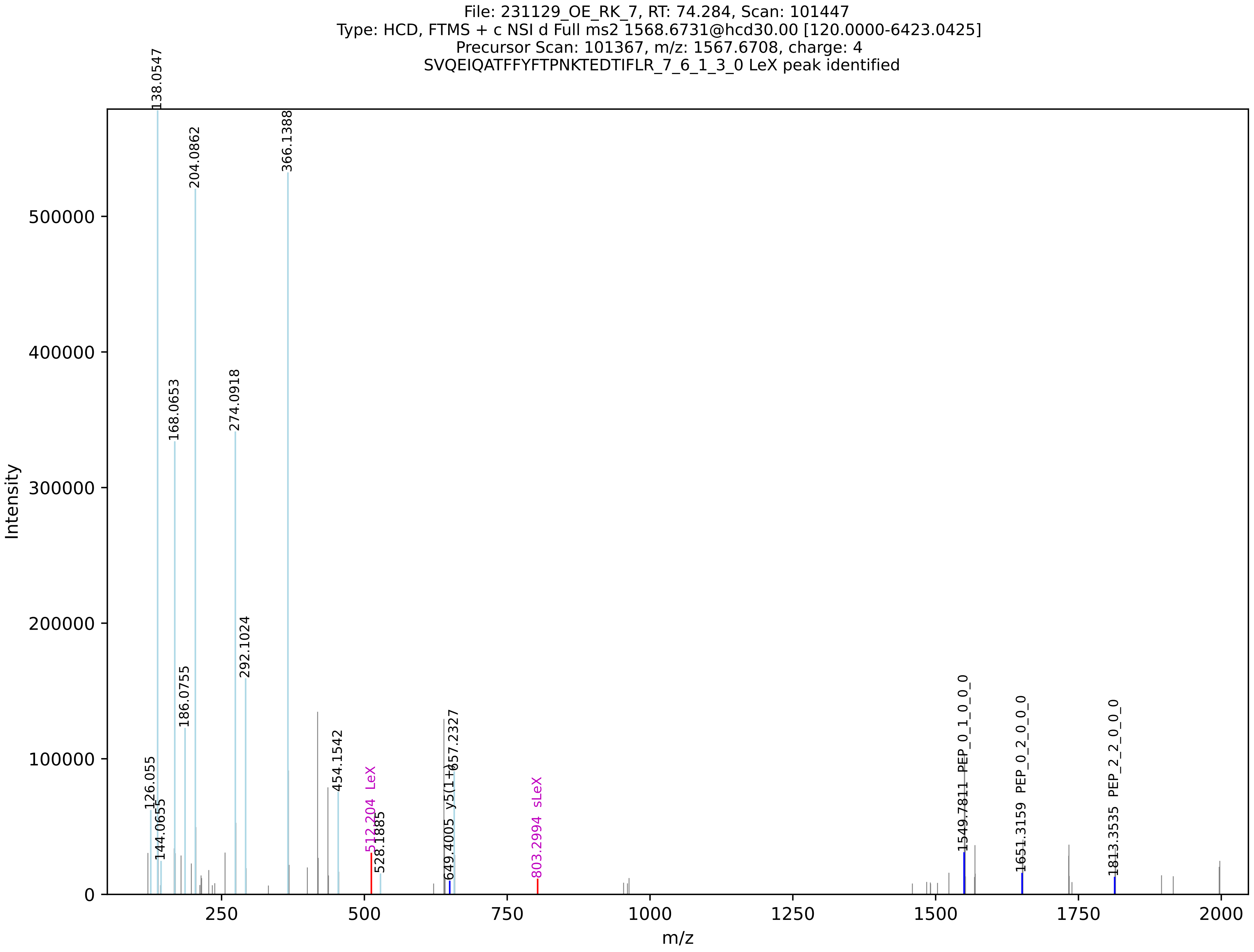
File: 231129_OE_RK_7, RT: 74.284, Scan: 101447
Type: HCD, FTMS + c NSI d Full ms2 1568.6731@hcd30.00 [120.0000-6423.0425]
Precursor Scan: 101367, m/z: 1567.6708, charge: 4
SVQEIQATFFYFTPNKTEDTIFLR_7_6_1_3_0 LeX peak identified
Intensity
m/z
138.0547
366.1388
204.0862
168.0653
274.0918
292.1024
186.0755
126.055
144.0655
454.1542
512.204 LeX
528.1885
649.4005 y5(1+)
657.2327
803.2994 sLeX
1549.7811 PEP_0_1_0_0_0
1651.3159 PEP_0_2_0_0_0
1813.3535 PEP_2_2_0_0_0
500000
400000
300000
200000
100000
0
250
500
750
1000
1250
1500
1750
2000

File: 231129_OE_RK_7, RT: 74.582, Scan: 101880
Type: HCD, FTMS + c NSI d Full ms2 1495.6458@hcd30.00 [120.0000-6125.0908]
Precursor Scan: 101805, m/z: 1494.894, charge: 4
SVQEIQATFFYFTPNKTEDTIFLR_7_6_1_2_0 LeX peak identified

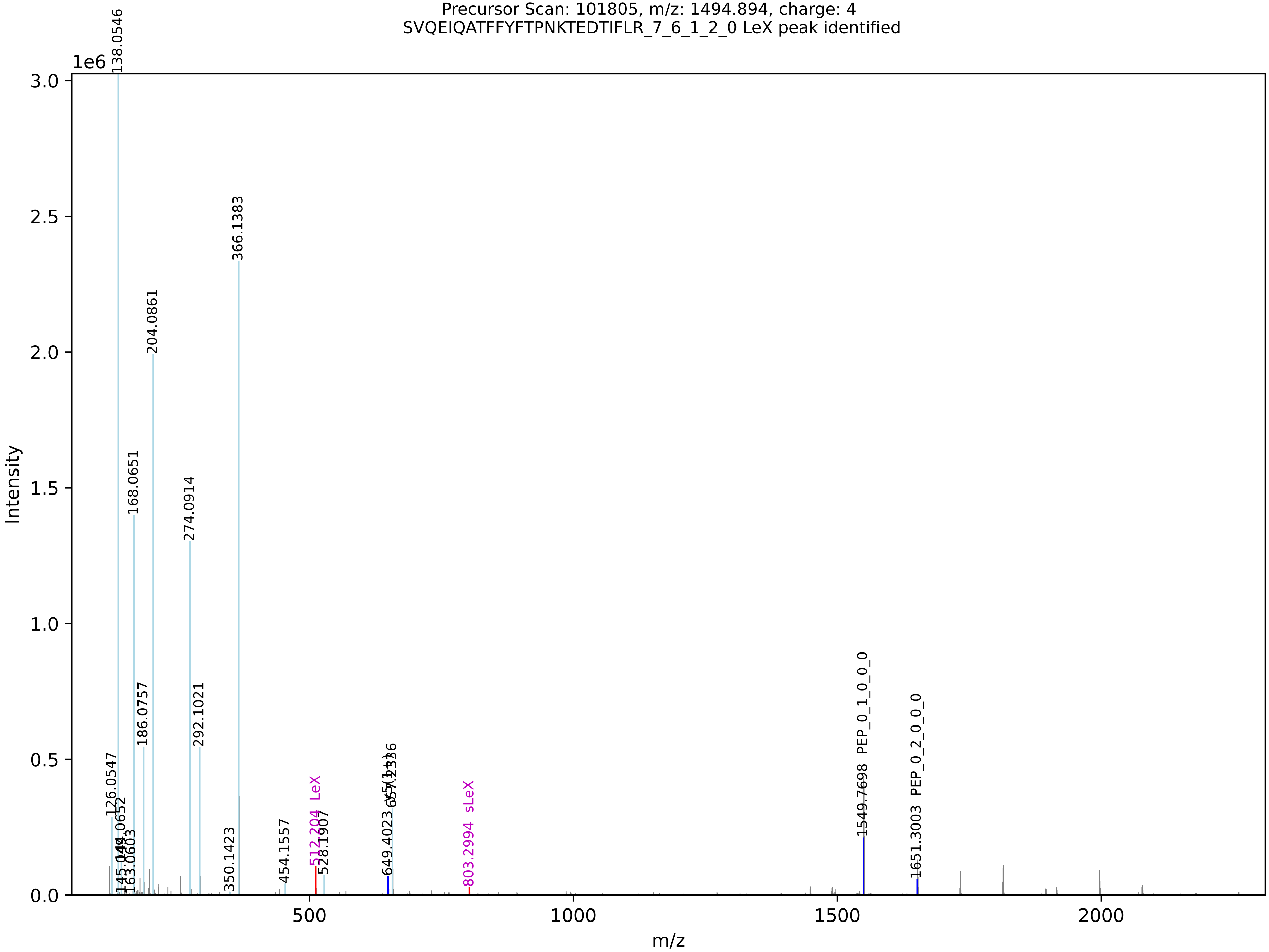

File: 231129_OE_RK_7, RT: 74.634, Scan: 101956
Type: HCD, FTMS + c NSI d Full ms2 1404.3654@hcd30.00 [120.0000-5752.6665]
Precursor Scan: 101879, m/z: 1403.6125, charge: 4
SVQEIQATFFYFTPNKTEDTIFLR_6_5_1_2_0 LeX peak identified

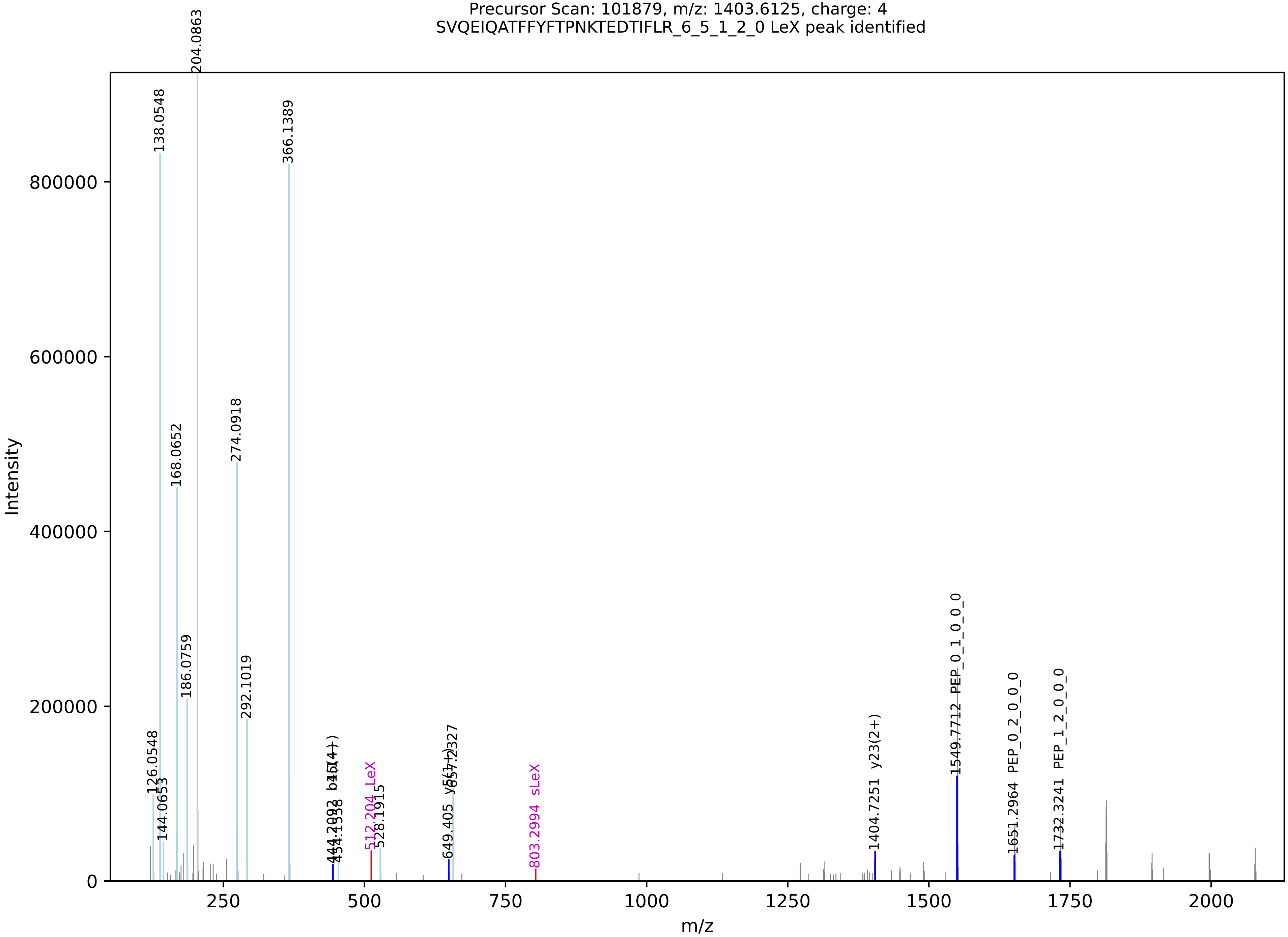

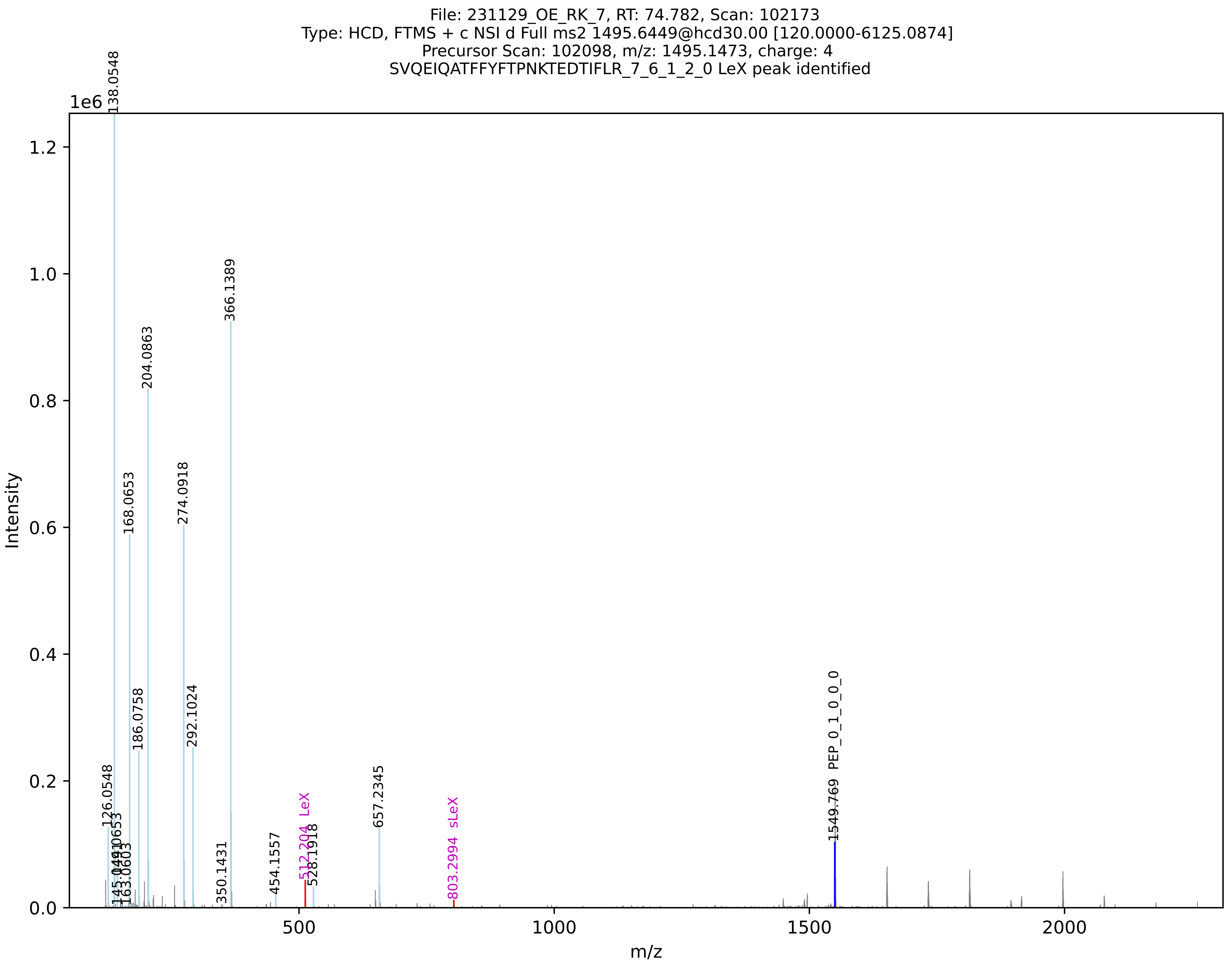
File: 231129_OE_RK_7, RT: 74.782, Scan: 102173
Type: HCD, FTMS + c NSI d Full ms2 1495.6449@hcd30.00 [120.0000-6125.0874]
Precursor Scan: 102098, m/z: 1495.1473, charge: 4
SVQEIQATFFYFTPNKTEDTIFLR_7_6_1_2_0 LeX peak identified
1e6
Intensity
m/z
138.0548
366.1389
204.0863
168.0653
274.0918
186.0758
292.1024
126.0548
163.0603
350.1431
454.1557
512.204 LeX
528.1918
657.2345
803.2994 sLeX
1549.769 PEP_0_1_0_0_0

File: 231129_OE_RK_7, RT: 75.239, Scan: 102842
Type: HCD, FTMS + c NSI d Full ms2 1404.3618@hcd30.00 [120.0000-5752.6519]
Precursor Scan: 102762, m/z: 1403.61, charge: 4
SVQEIQATFFYFTPNKTEDTIFLR_6_5_1_2_0 LeX peak identified

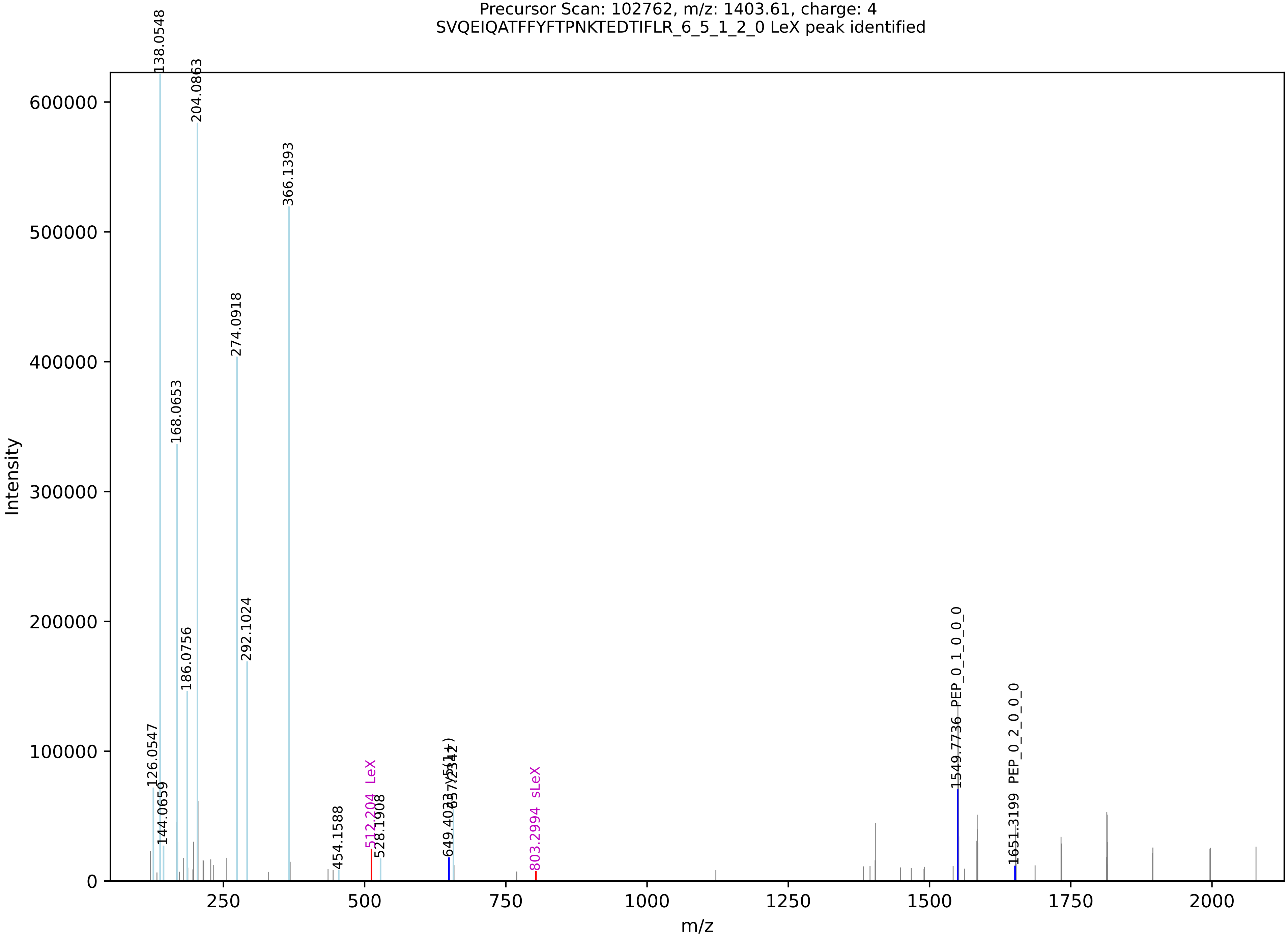

File: 231129_OE_RK_7, RT: 77.201, Scan: 105709
Type: HCD, FTMS + c NSI d Full ms2 1568.6693@hcd30.00 [120.0000-6423.0269]
Precursor Scan: 105634, m/z: 1567.667, charge: 4
SVQEIQATFFYFTPNKTEDTIFLR_7_6_1_3_0 LeX peak identified

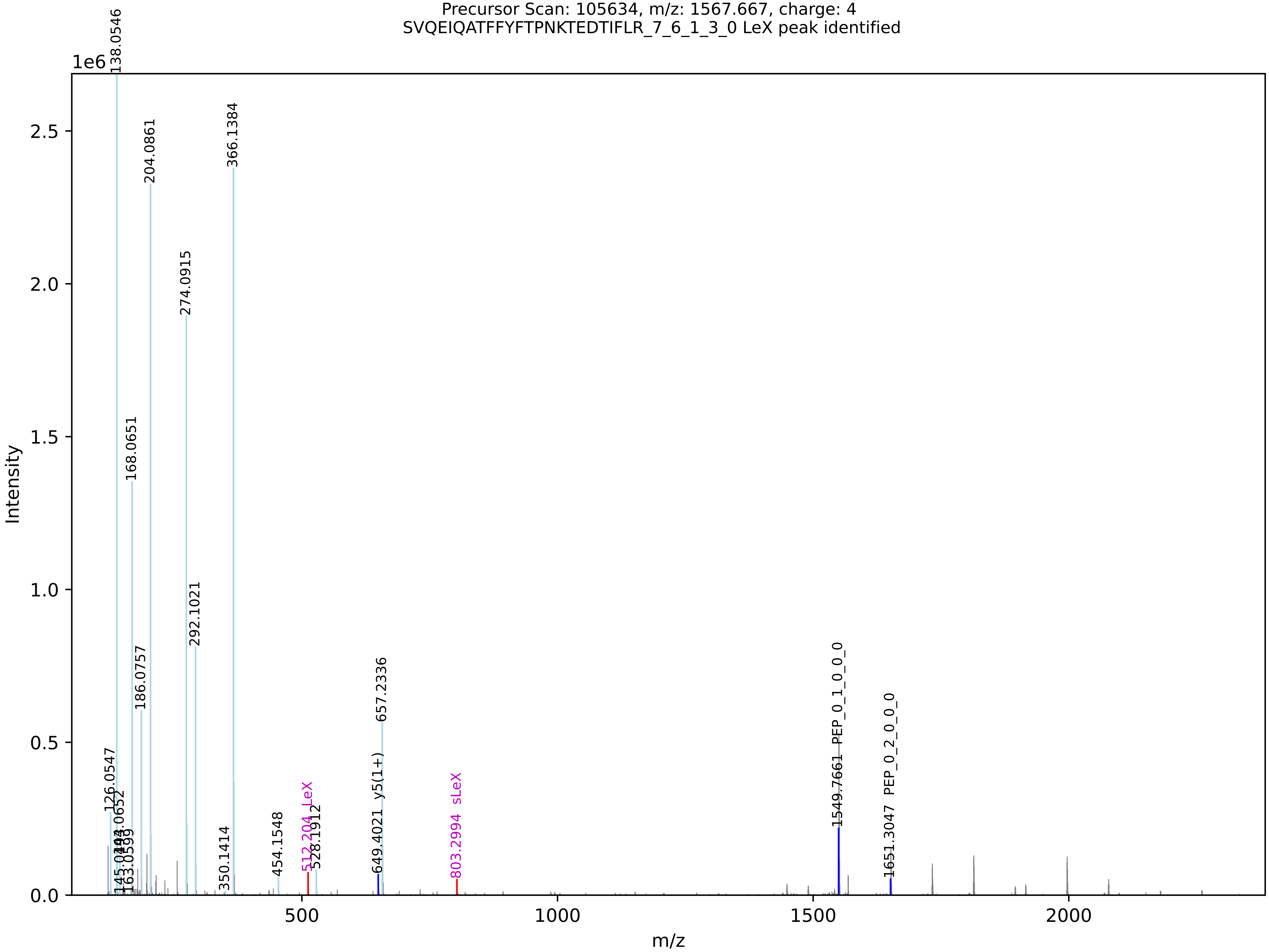

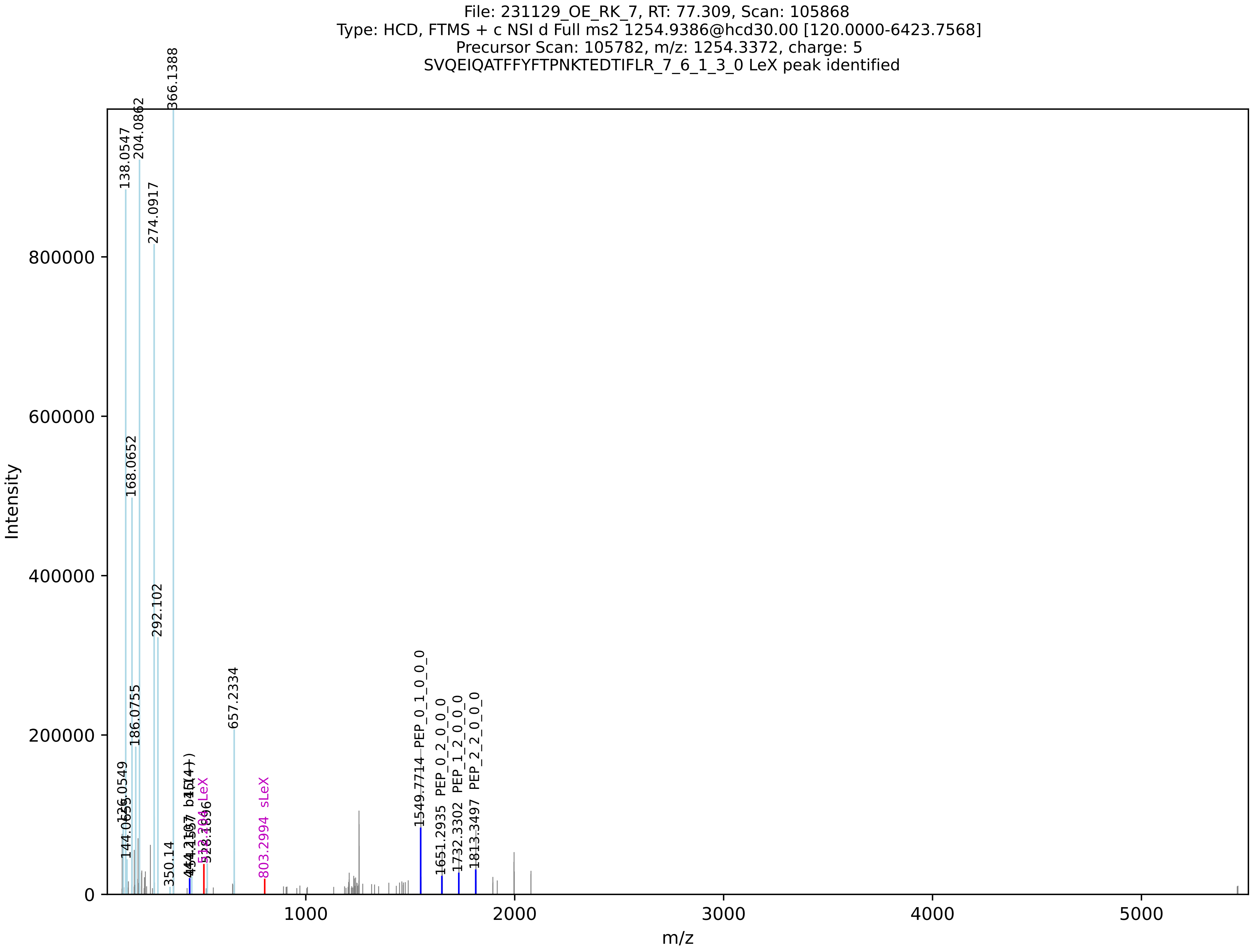

File: 231129_OE_RK_7, RT: 77.309, Scan: 105868
Type: HCD, FTMS + c NSI d Full ms2 1254.9386@hcd30.00 [120.0000-6423.7568]
Precursor Scan: 105782, m/z: 1254.3372, charge: 5
SVQEIQATFFYFTPNKTEDTIFLR_7_6_1_3_0 LeX peak identified
Intensity
m/z
366.1388
204.0862
138.0547
274.0917
168.0652
292.102
186.0755
657.2334
126.0549
144.0659
350.14
528.1896
803.2994 sLeX
1549.7714 PEP_0_1_0_0_0
1651.2935 PEP_0_2_0_0_0
1732.3302 PEP_1_2_0_0_0
1813.3497 PEP_2_2_0_0_0
0
200000
400000
600000
800000
1000
2000
3000
4000
5000

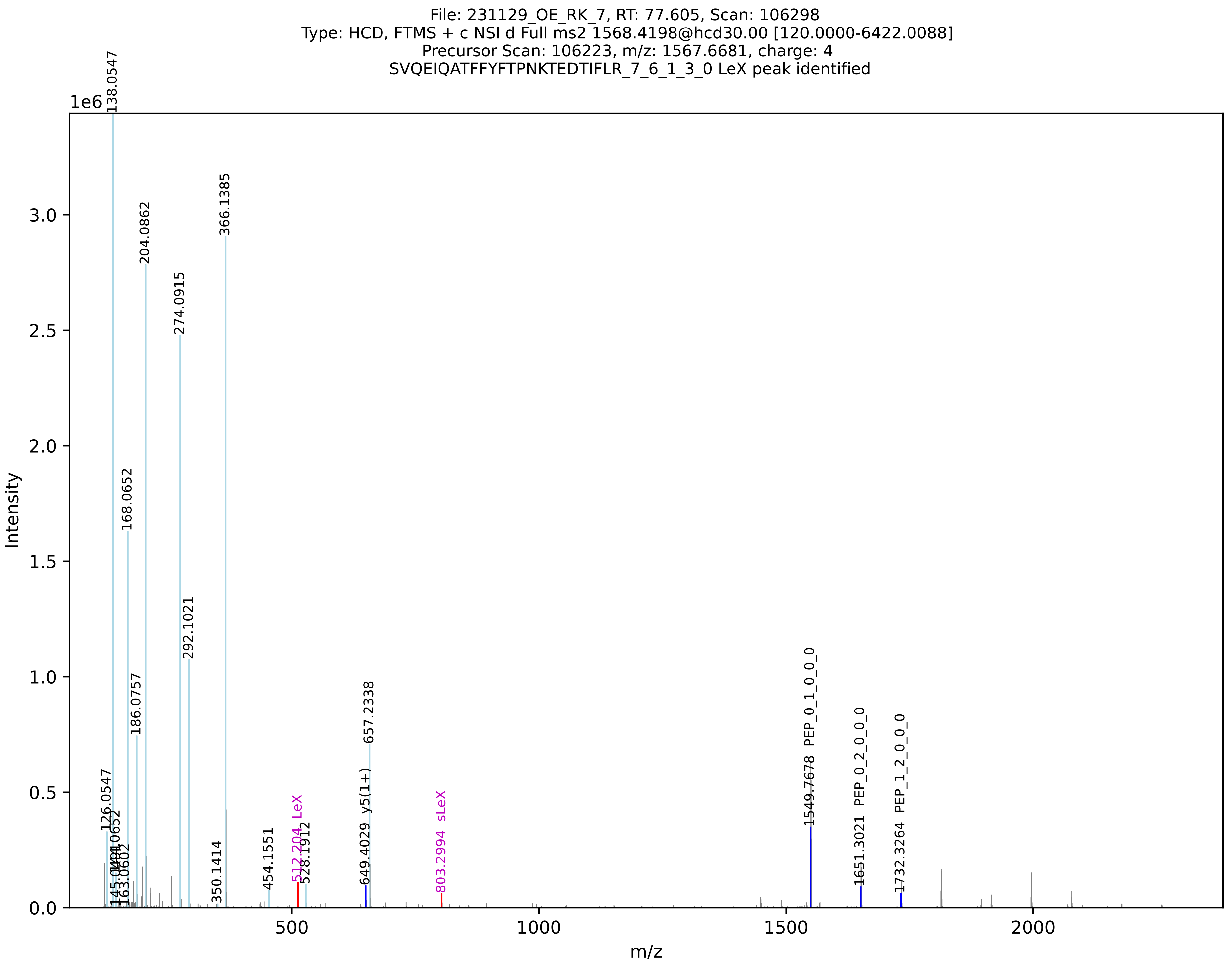
File: 231129_OE_RK_7, RT: 77.605, Scan: 106298
Type: HCD, FTMS + c NSI d Full ms2 1568.4198@hcd30.00 [120.0000-6422.0088]
Precursor Scan: 106223, m/z: 1567.6681, charge: 4
SVQEIQATFFYFTPNKTEDTIFLR_7_6_1_3_0 LeX peak identified
1e6
Intensity
m/z
138.0547
366.1385
204.0862
274.0915
168.0652
292.1021
186.0757
126.0547
350.1414
454.1551
512.204 LeX
528.1912
649.4029 y5(1+)
657.2338
803.2994 sLeX
1549.7678 PEP_0_1_0_0_0_
1651.3021 PEP_0_2_0_0_0_
1732.3264 PEP_1_2_0_0_0_

File: 231129_OE_RK_7, RT: 79.622, Scan: 109239
Type: HCD, FTMS + c NSI d Full ms2 1121.3208@hcd30.00 [120.0000-5742.3062]
Precursor Scan: 109164, m/z: 1120.9233, charge: 5
YLGNATAIFFLPDEGKLQHLENELTHDIITK_5_4_1_1_0 LeX peak identified

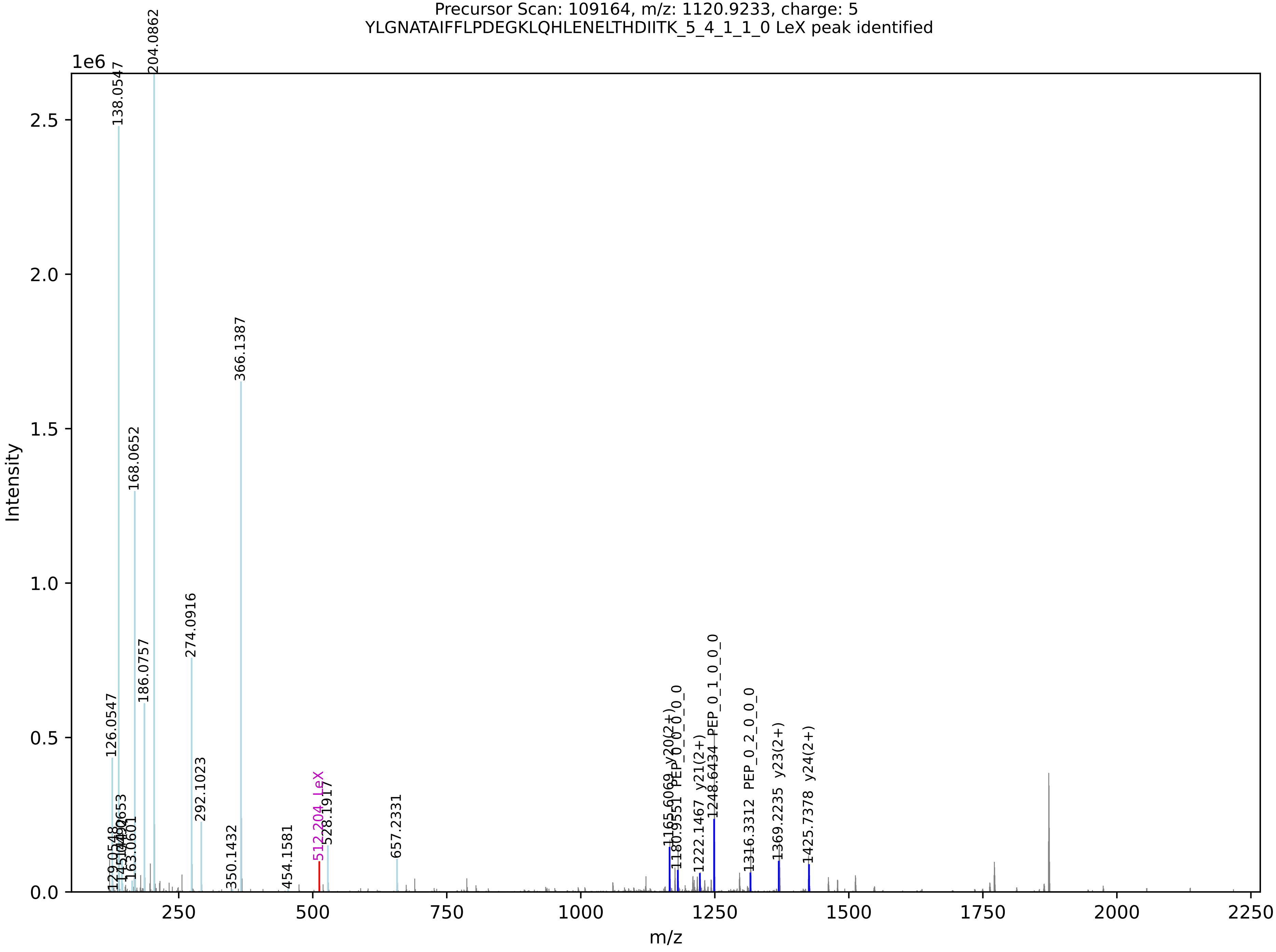

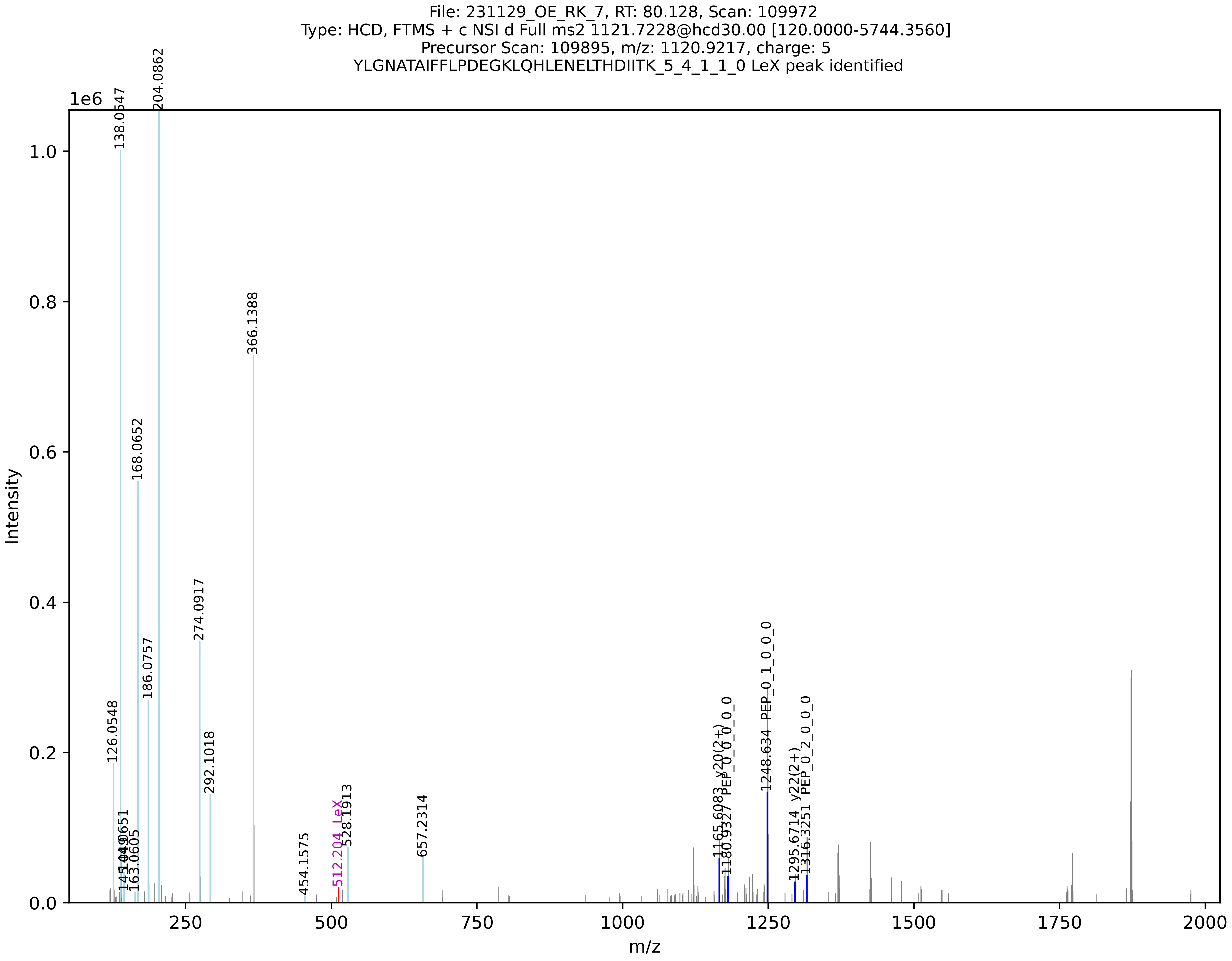
File: 231129_OE_RK_7, RT: 80.128, Scan: 109972
Type: HCD, FTMS + c NSI d Full ms2 1121.7228@hcd30.00 [120.0000-5744.3560]
Precursor Scan: 109895, m/z: 1120.9217, charge: 5
YLGNATAIFFLPDEGKLQHLENELTHDIITK_5_4_1_1_0 LeX peak identified
1e6
Intensity
m/z
204.0862
138.0547
366.1388
168.0652
274.0917
186.0757
126.0548
292.1018
163.0605
454.1575
512.204_LeX
528.1913
657.2314
1165.6083_y20(2+)
1180.9327_PEP_0_0_0_0
1248.634_PEP_0_1_0_0_0
1295.6714_y22(2+)
1316.3251_PEP_0_2_0_0_0

File: 231129_OE_RK_7, RT: 82.852, Scan: 113932
Type: HCD, FTMS + c NSI d Full ms2 1179.7449@hcd30.00 [120.0000-6040.2690]
Precursor Scan: 113859, m/z: 1179.1434, charge: 5
YLGNATAIFFLPDEGKLQHLENELTHDIITK_5_4_1_2_0 LeX peak identified

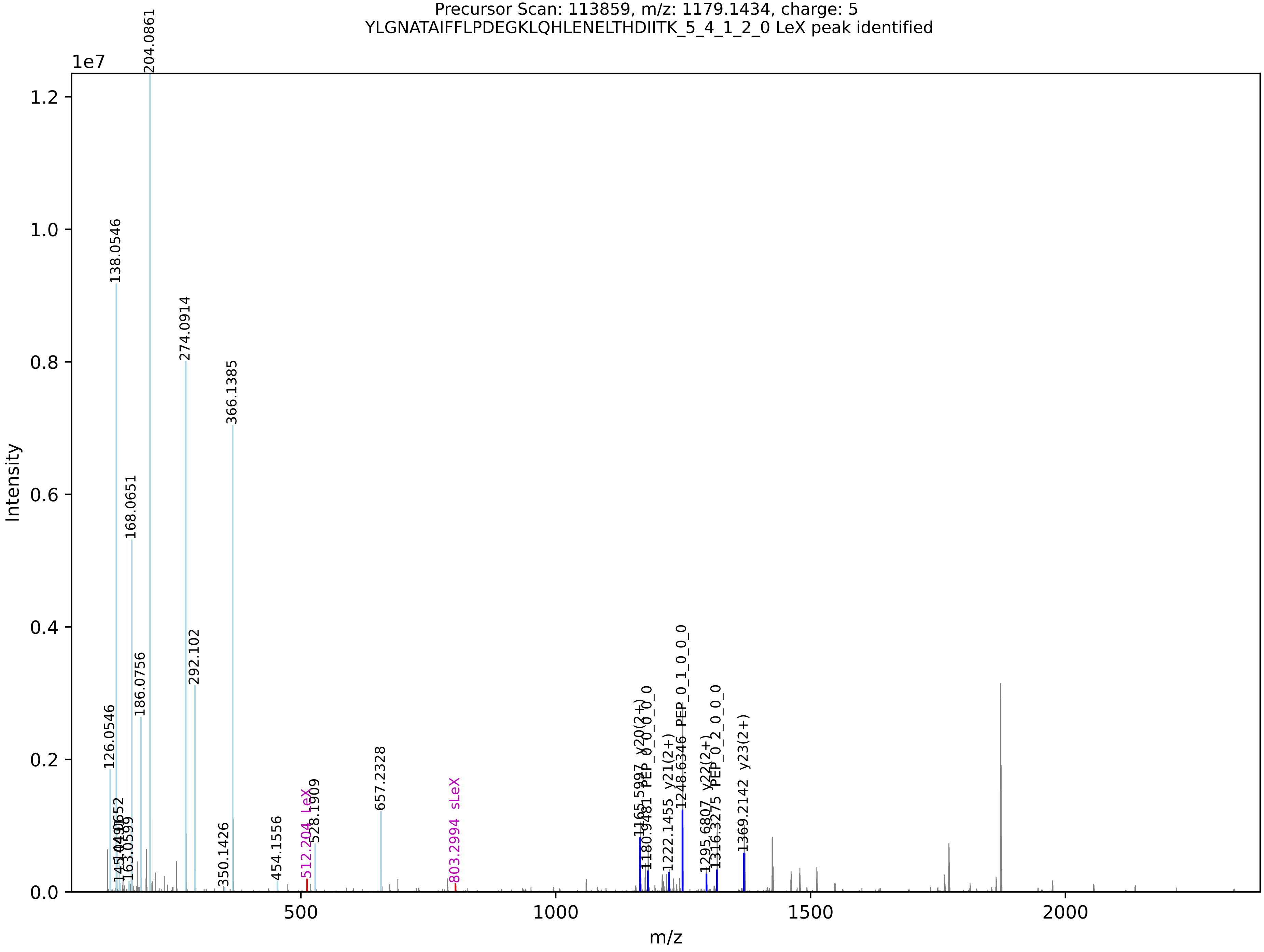

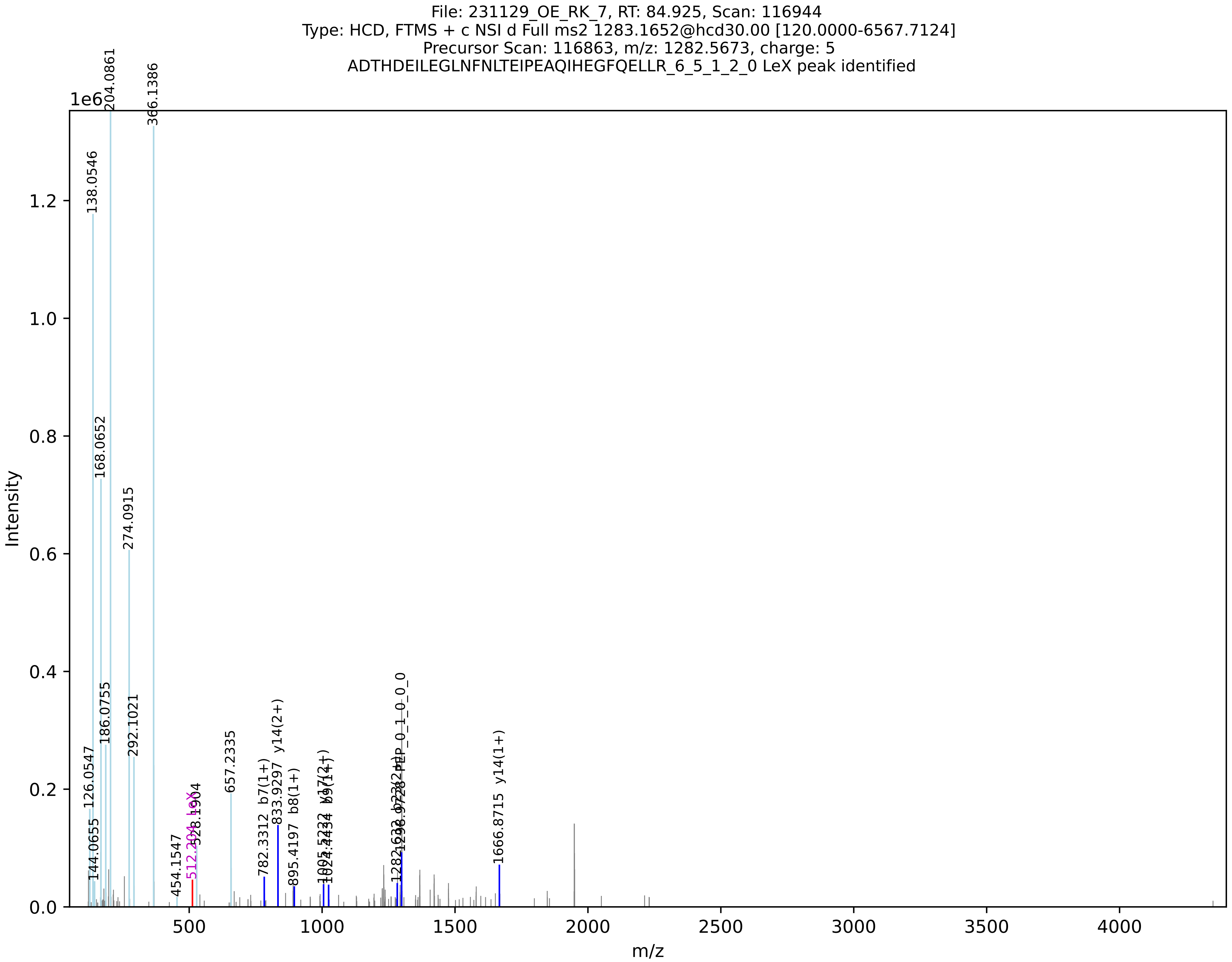
File: 231129_OE_RK_7, RT: 84.925, Scan: 116944
Type: HCD, FTMS + c NSI d Full ms2 1283.1652@hcd30.00 [120.0000-6567.7124]
Precursor Scan: 116863, m/z: 1282.5673, charge: 5
ADTHDEILEGLNFNLTEIPEAQIHEGFQELLR_6_5_1_2_0 LeX peak identified
1e6
204.0861
366.1386
138.0546
168.0652
274.0915
186.0755
292.1021
126.0547
144.0655
454.1547
512.204 LeX
528.1904
657.2335
782.3312 b7(1+)
833.9297 y14(2+)
895.4197 b8(1+)
1666.8715 y14(1+)
Intensity
m/z

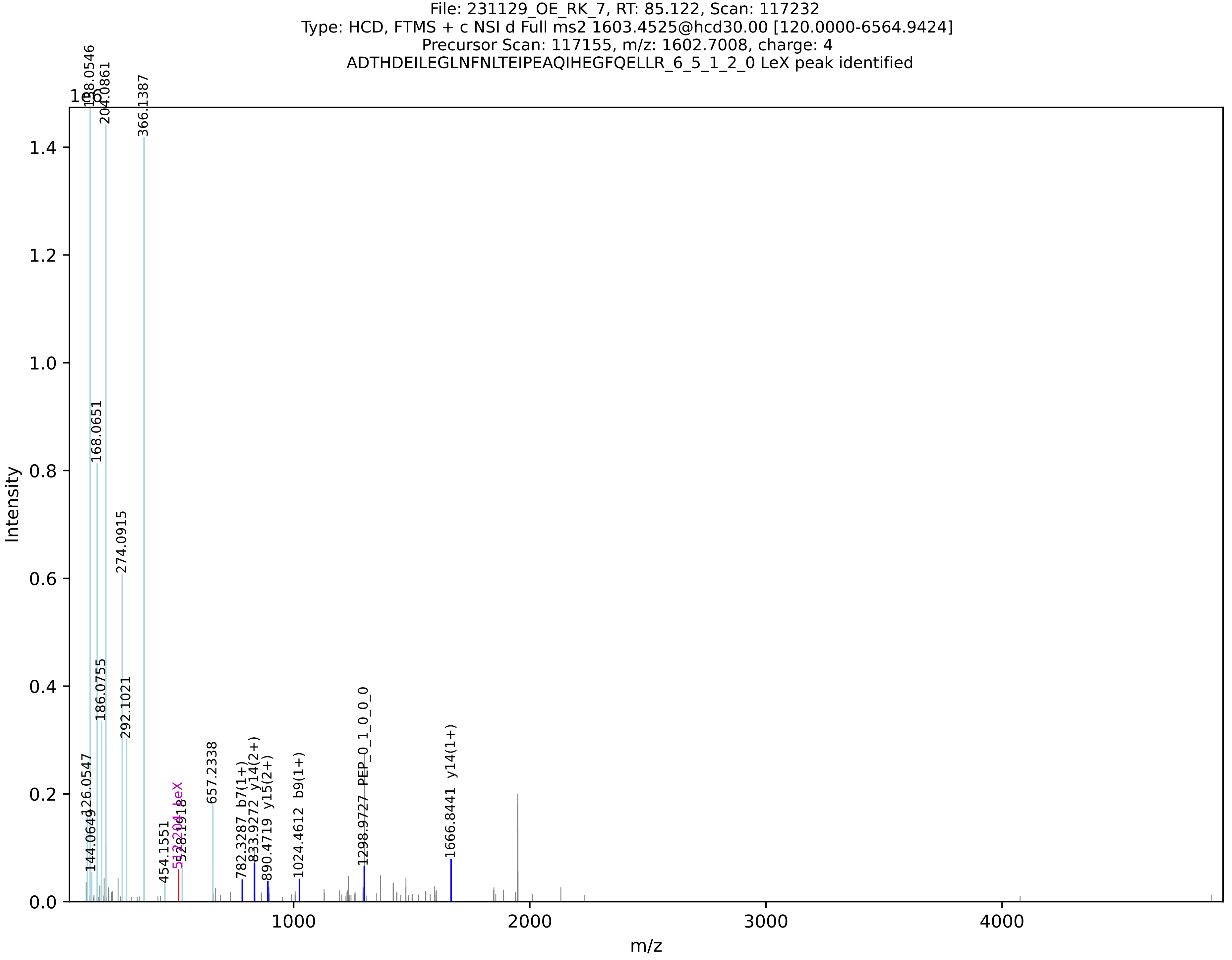
File: 231129_OE_RK_7, RT: 85.122, Scan: 117232
Type: HCD, FTMS + c NSI d Full ms2 1603.4525@hcd30.00 [120.0000-6564.9424]
Precursor Scan: 117155, m/z: 1602.7008, charge: 4
ADTHDEILEGLNFNLTEIPEAQIHEGFQELLR_6_5_1_2_0 LeX peak identified
1e6
138.0546
204.0861
366.1387
168.0651
274.0915
186.0755
292.1021
126.0547
144.0649
454.1551
512.204 LeX
528.1918
657.2338
782.3287 b7(1+)
833.9272 y14(2+)
890.4719 y15(2+)
1024.4612 b9(1+)
1298.9727 PEP_0_1_0_0_0
1666.8441 y14(1+)
Intensity
m/z

File: 231129_OE_RK_7, RT: 85.426, Scan: 117677
Type: HCD, FTMS + c NSI d Full ms2 1283.1633@hcd30.00 [120.0000-6567.7026]
Precursor Scan: 117598, m/z: 1282.3614, charge: 5
ADTHDEILEGLNFNLTEIPEAQIHEGFQELLR_6_5_1_2_0 LeX peak identified

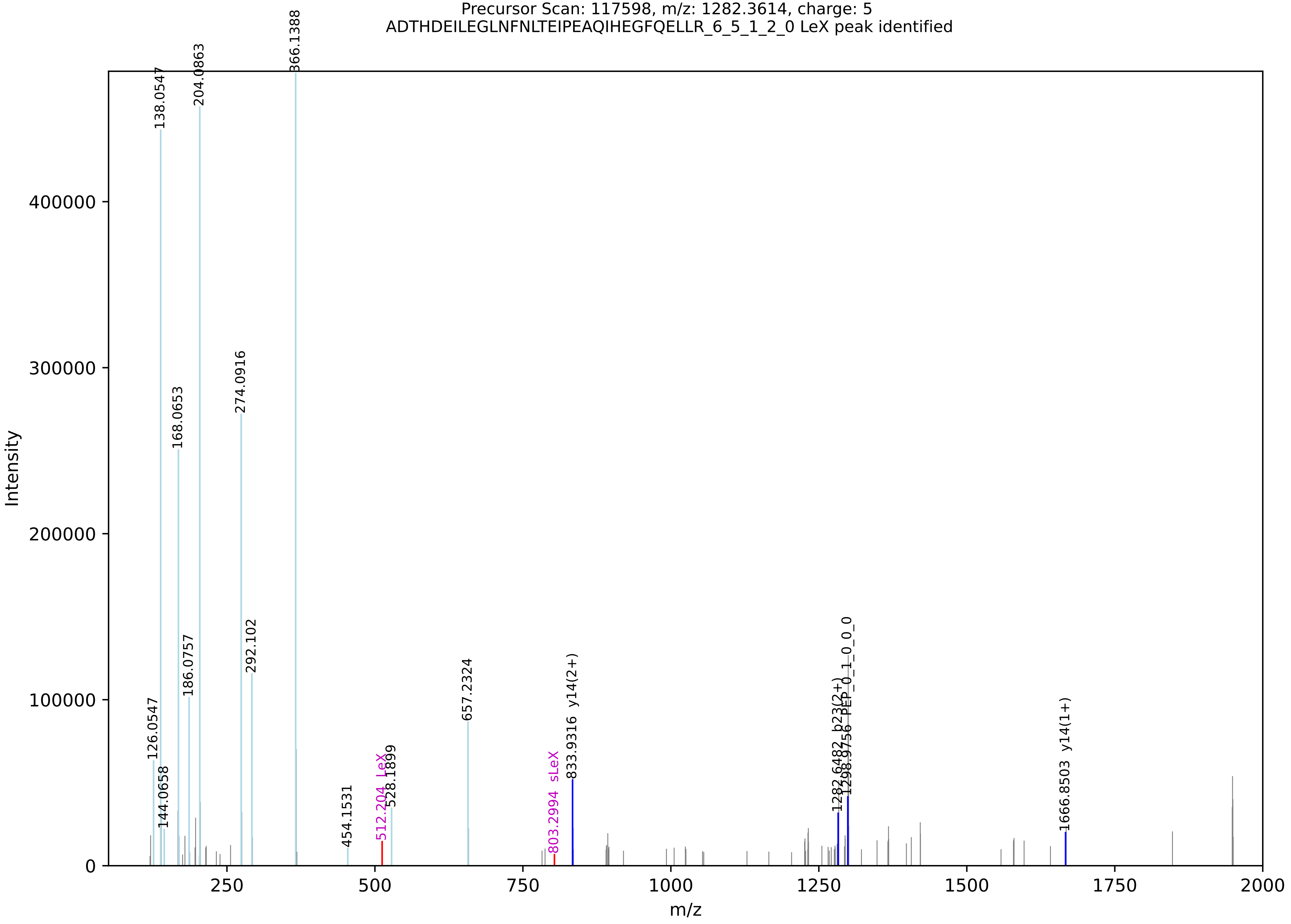

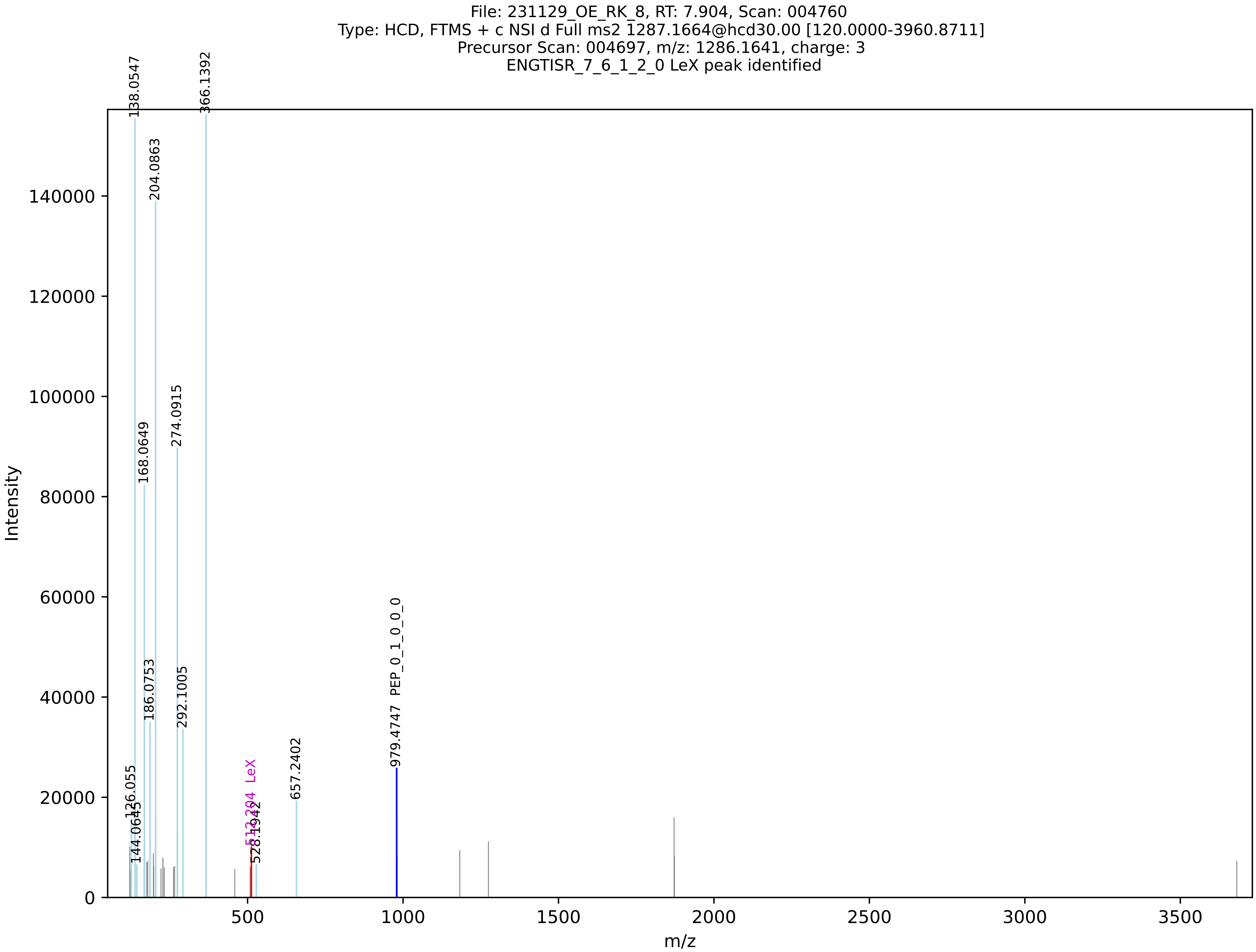

File: 231129_OE_RK_8, RT: 7.904, Scan: 004760
Type: HCD, FTMS + c NSI d Full ms2 1287.1664@hcd30.00 [120.0000-3960.8711]
Precursor Scan: 004697, m/z: 1286.1641, charge: 3
ENGTISR_7_6_1_2_0 LeX peak identified
138.0547
366.1392
204.0863
274.0915
168.0649
186.0753
292.1005
126.055
144.0645
512.204 LeX
528.1942
657.2402
979.4747 PEP_0_1_0_0_0
Intensity
m/z

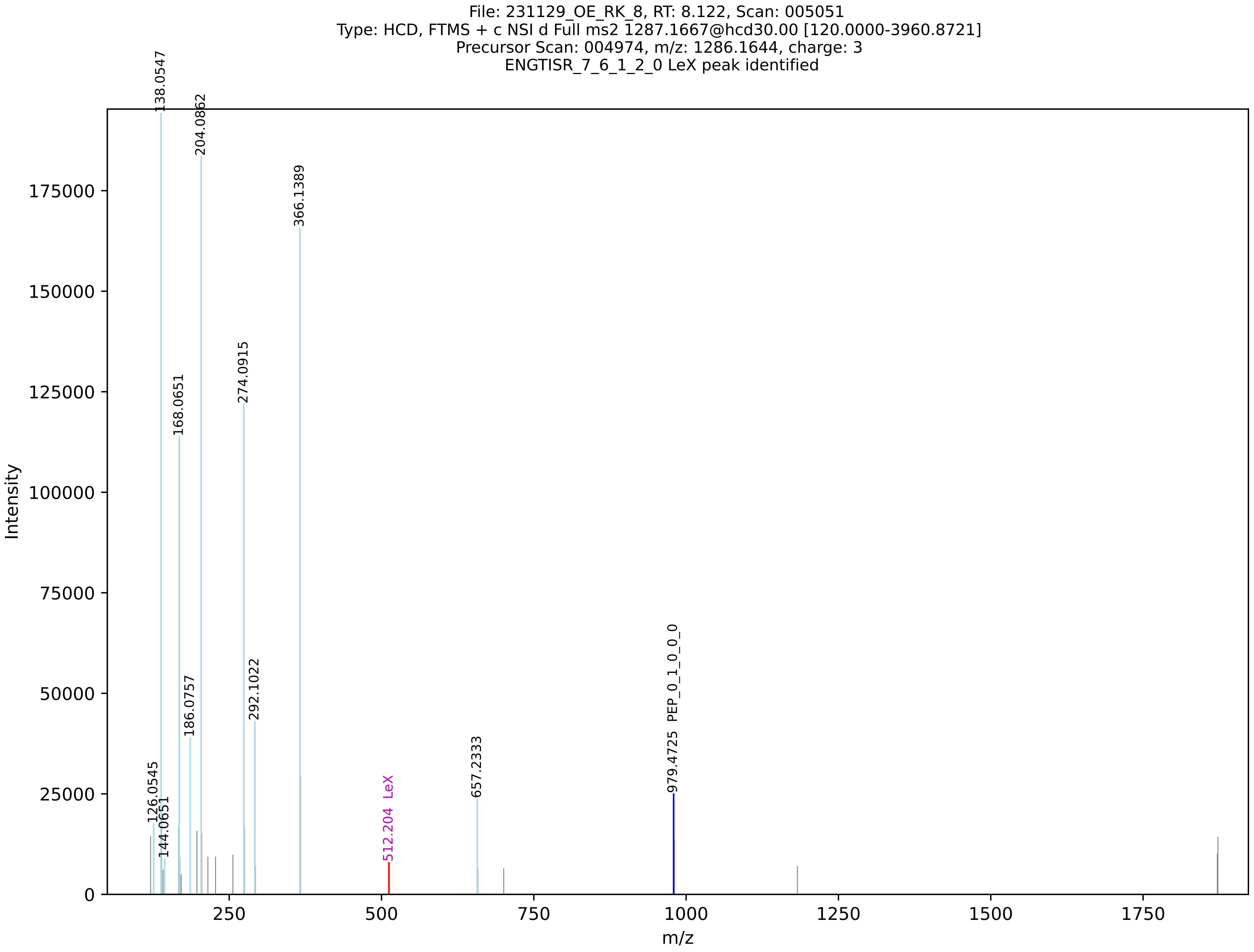
File: 231129_OE_RK_8, RT: 8.122, Scan: 005051
Type: HCD, FTMS + c NSI d Full ms2 1287.1667@hcd30.00 [120.0000-3960.8721]
Precursor Scan: 004974, m/z: 1286.1644, charge: 3
ENGTISR_7_6_1_2_0 LeX peak identified
138.0547
204.0862
366.1389
274.0915
168.0651
186.0757
292.1022
126.0545
144.0651
512.204 LeX
657.2333
979.4725 PEP_0_1_0_0_0
Intensity
m/z
0
25000
50000
75000
100000
125000
150000
175000
250
500
750
1000
1250
1500
1750

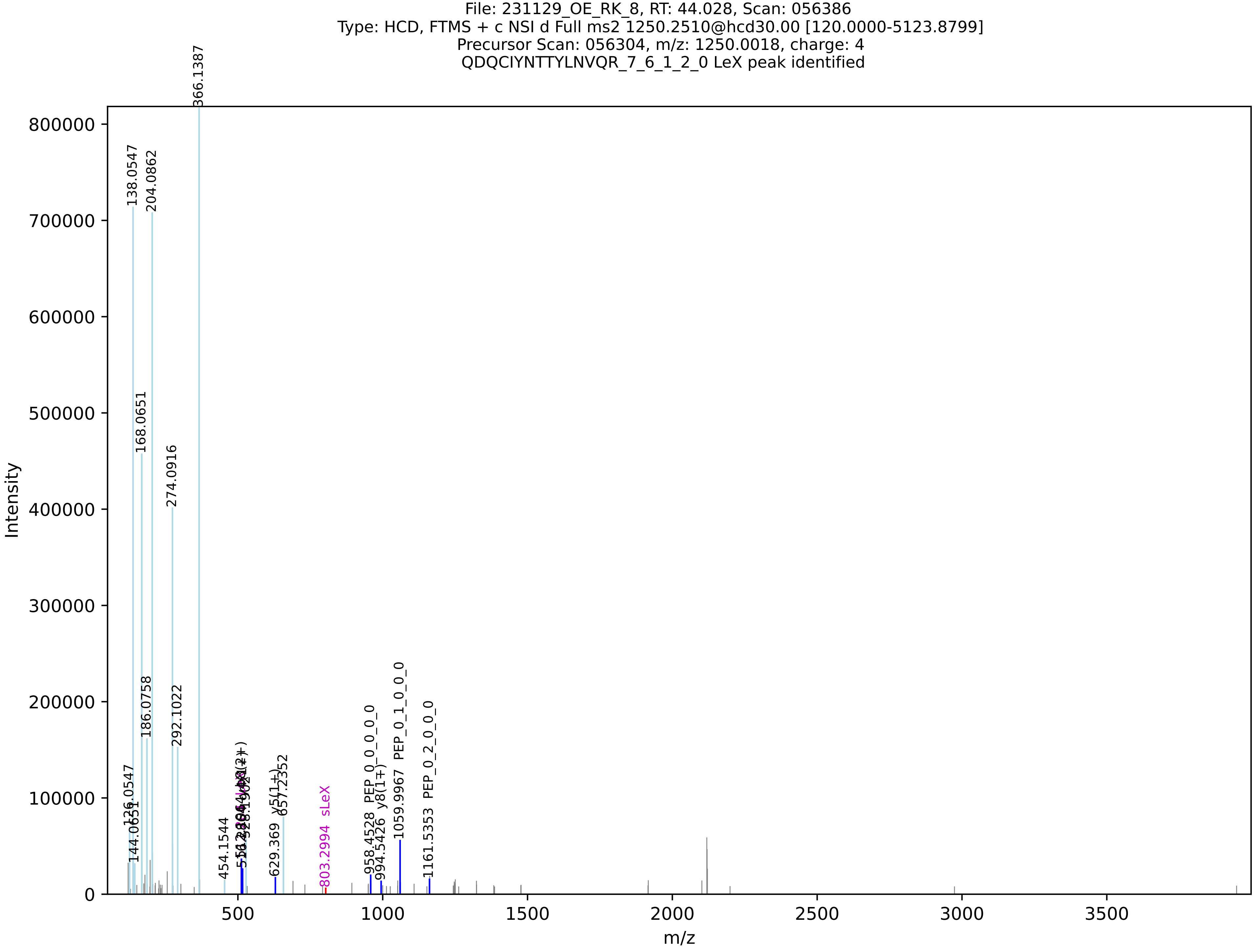

File: 231129_OE_RK_8, RT: 44.028, Scan: 056386
Type: HCD, FTMS + c NSI d Full ms2 1250.2510@hcd30.00 [120.0000-5123.8799]
Precursor Scan: 056304, m/z: 1250.0018, charge: 4
QDQCIYNTTYLNVQR_7_6_1_2_0 LeX peak identified
Intensity
m/z
366.1387
138.0547
204.0862
168.0651
274.0916
186.0758
292.1022
126.0547
144.0651
454.1544
528.1902
629.369 y5(1+)
657.2352
803.2994 sLeX
958.4528 PEP_0_0_0_0_
994.5426 y8(1+)
1059.9967 PEP_0_1_0_0_
1161.5353 PEP_0_2_0_0_

File: 231129_OE_RK_8, RT: 44.283, Scan: 056756
Type: HCD, FTMS + c NSI d Full ms2 1323.2788@hcd30.00 [120.0000-5421.8335]
Precursor Scan: 056672, m/z: 1322.7776, charge: 4
QDQCIYNTTYLNVQR_7_6_1_3_0 LeX peak identified

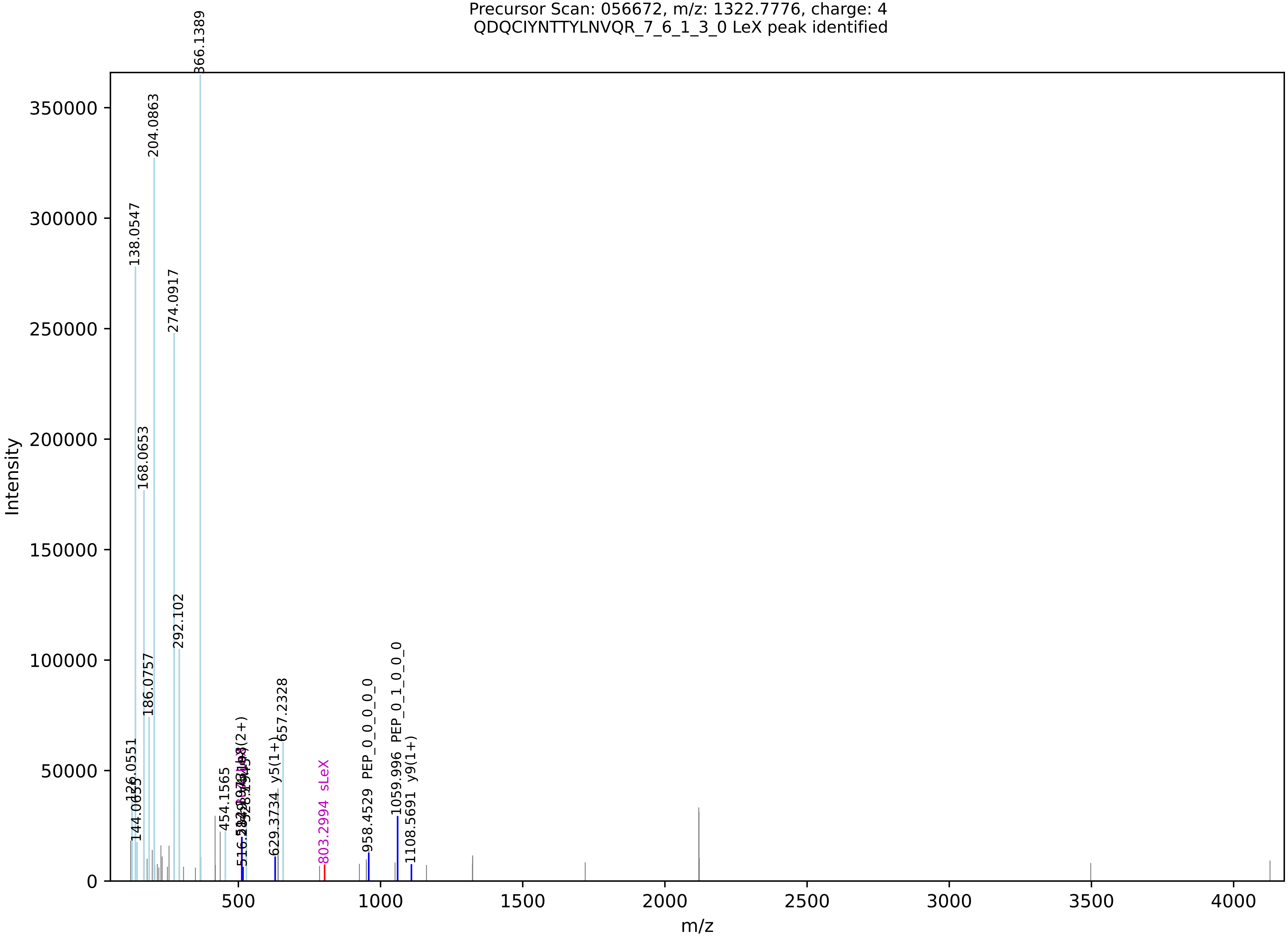

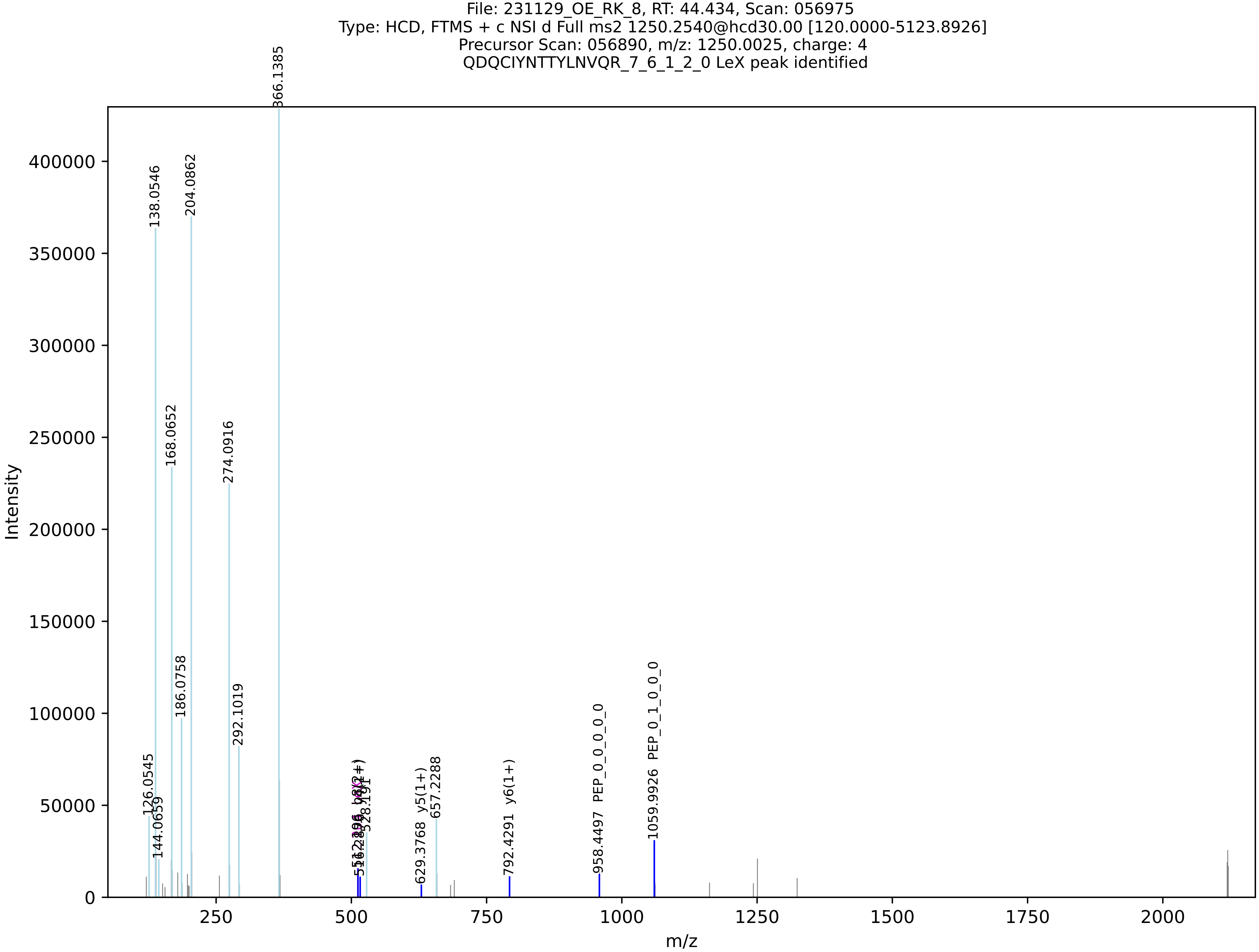

File: 231129_OE_RK_8, RT: 44.434, Scan: 056975
Type: HCD, FTMS + c NSI d Full ms2 1250.2540@hcd30.00 [120.0000-5123.8926]
Precursor Scan: 056890, m/z: 1250.0025, charge: 4
QDQCIYNTTYLNVQR_7_6_1_2_0 LeX peak identified
Intensity
m/z
366.1385
138.0546
204.0862
168.0652
274.0916
186.0758
292.1019
126.0545
144.0659
528.191
629.3768 y5(1+)
657.2288
792.4291 y6(1+)
958.4497 PEP_0_0_0_0_0
1059.9926 PEP_0_1_0_0_0

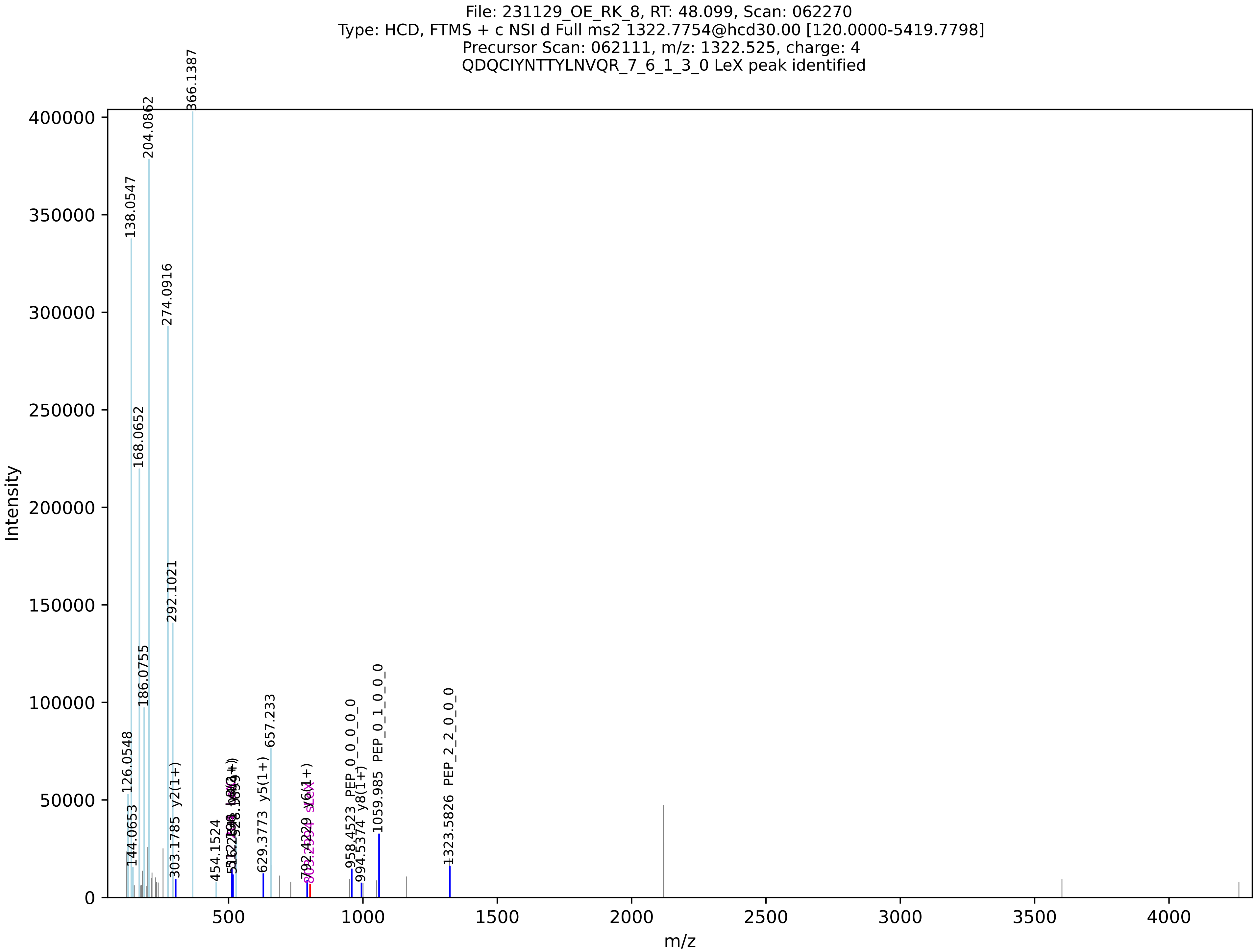
File: 231129_OE_RK_8, RT: 48.099, Scan: 062270
Type: HCD, FTMS + c NSI d Full ms2 1322.7754@hcd30.00 [120.0000-5419.7798]
Precursor Scan: 062111, m/z: 1322.525, charge: 4
QDQCIYNTTYLNVQR_7_6_1_3_0 LeX peak identified
Intensity
m/z
366.1387
204.0862
138.0547
274.0916
168.0652
292.1021
186.0755
126.0548
144.0653
303.1785 y2(1+)
454.1524
657.233
629.3773 y5(1+)
792.4229 y6(1+)
958.4523 PEP_0_0_0_0_0
994.5374 y8(1+)
1059.985 PEP_0_1_0_0_0
1323.5826 PEP_2_2_0_0_0
0
50000
100000
150000
200000
250000
300000
350000
400000
500
1000
1500
2000
2500
3000
3500
4000

File: 231129_OE_RK_8, RT: 49.582, Scan: 064427
Type: HCD, FTMS + c NSI d Full ms2 1105.4797@hcd30.00 [120.0000-4533.2134]
Precursor Scan: 064318, m/z: 1104.4722, charge: 4
RKPNYSSVDLSEVEWEDR_5_4_0_2_0 LeX peak identified

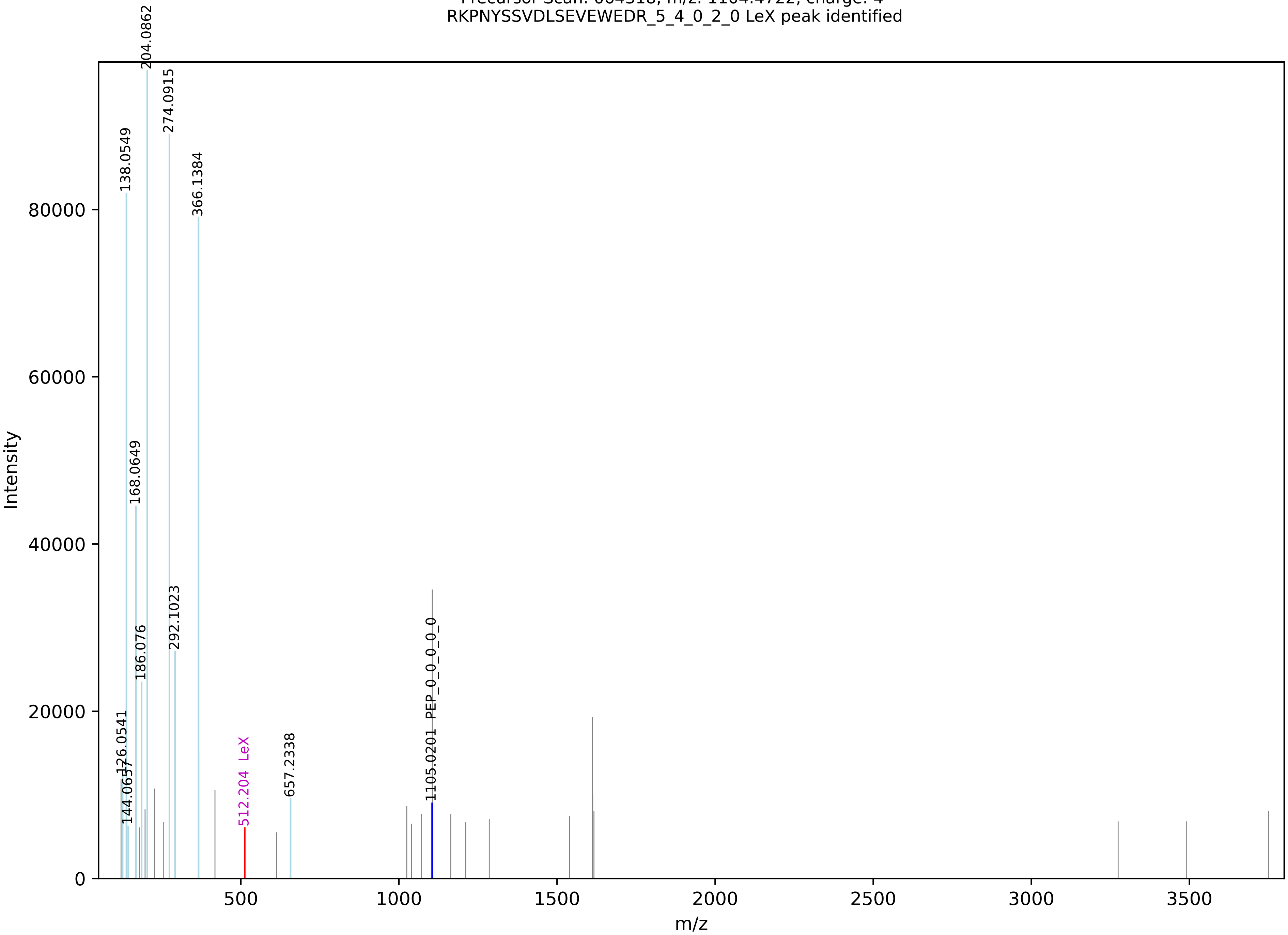

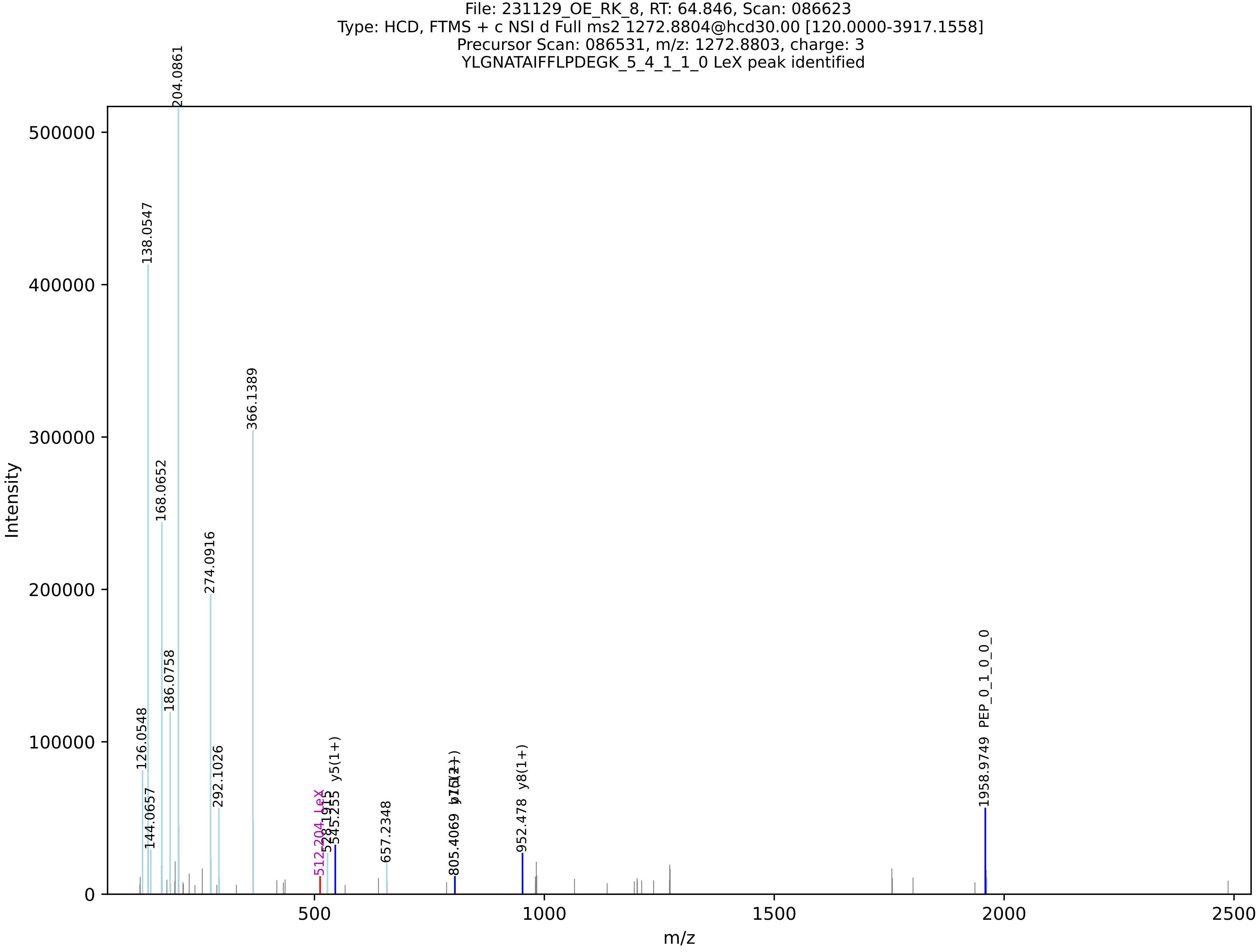

File: 231129_OE_RK_8, RT: 64.846, Scan: 086623
Type: HCD, FTMS + c NSI d Full ms2 1272.8804@hcd30.00 [120.0000-3917.1558]
Precursor Scan: 086531, m/z: 1272.8803, charge: 3
YLGNATAIFFLPDEGK_5_4_1_1_0 LeX peak identified
Intensity
m/z
204.0861
138.0547
366.1389
168.0652
274.0916
186.0758
126.0548
292.1026
144.0657
512.204 LeX
528.1915
545.255 y5(1+)
657.2348
805.4069
952.478 y8(1+)
1958.9749 PEP_0_1_0_0_0

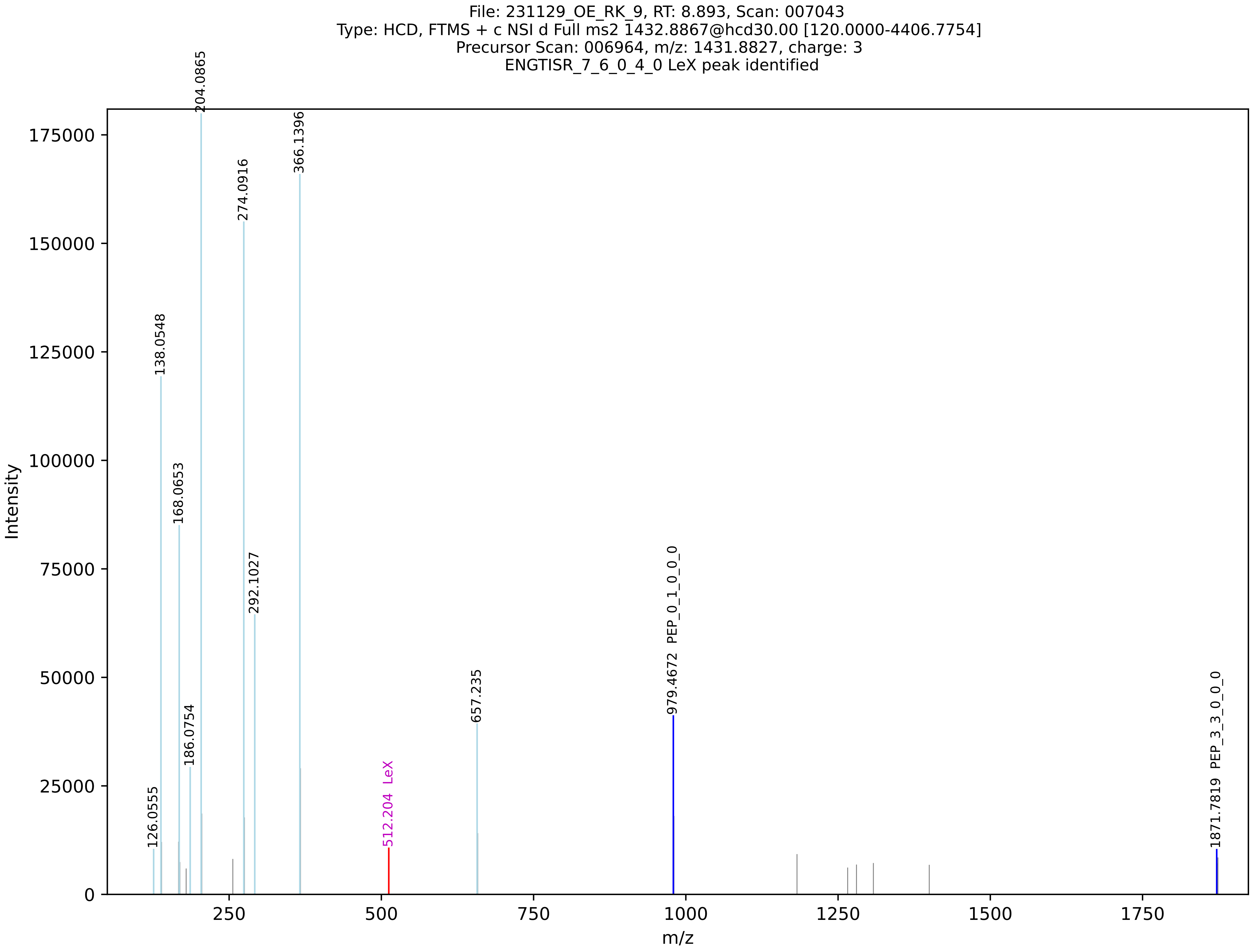
File: 231129_OE_RK_9, RT: 8.893, Scan: 007043
Type: HCD, FTMS + c NSI d Full ms2 1432.8867@hcd30.00 [120.0000-4406.7754]
Precursor Scan: 006964, m/z: 1431.8827, charge: 3
ENGTISR_7_6_0_4_0 LeX peak identified
Intensity
m/z
0
25000
50000
75000
100000
125000
150000
175000
250
500
750
1000
1250
1500
1750
204.0865
366.1396
274.0916
138.0548
168.0653
292.1027
979.4672 PEP_0_1_0_0_0
657.235
1871.7819 PEP_3_3_0_0_
186.0754
512.204 LeX
126.0555

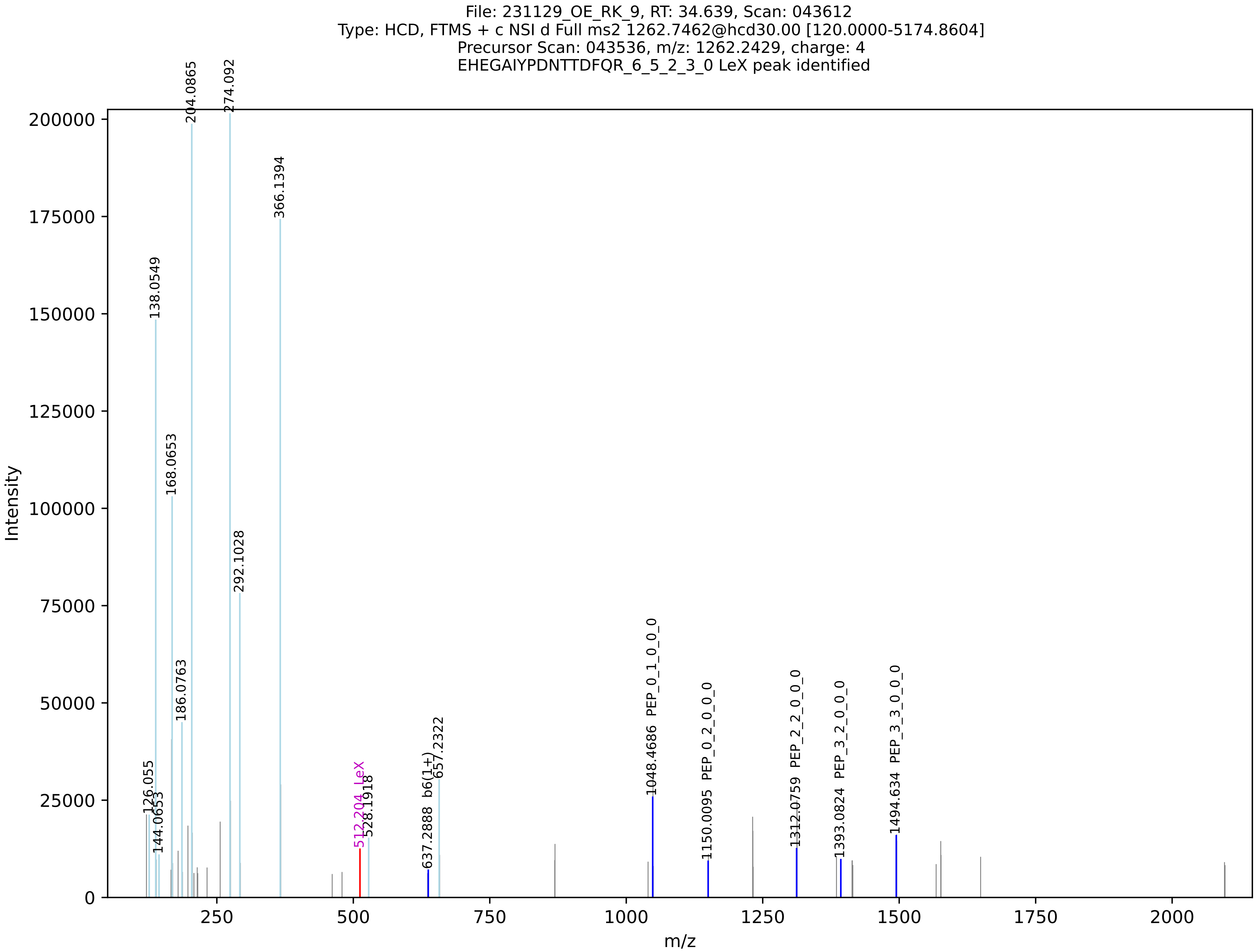

File: 231129_OE_RK_9, RT: 34.639, Scan: 043612
Type: HCD, FTMS + c NSI d Full ms2 1262.7462@hcd30.00 [120.0000-5174.8604]
Precursor Scan: 043536, m/z: 1262.2429, charge: 4
EHEGAIYPDNTTDFQR_6_5_2_3_0 LeX peak identified
Intensity
m/z
204.0865
274.092
366.1394
138.0549
168.0653
292.1028
186.0763
126.055
144.0653
512.204 LeX
528.1918
637.2888 b6(1+)
657.2322
1048.4686 PEP_0_1_0_0_0
1150.0095 PEP_0_2_0_0_0
1312.0759 PEP_2_2_0_0_0
1393.0824 PEP_3_2_0_0_0
1494.634 PEP_3_3_0_0_0

File: 231129_OE_RK_9, RT: 35.841, Scan: 045351
Type: HCD, FTMS + c NSI d Full ms2 1475.2606@hcd30.00 [120.0000-4536.4395]
Precursor Scan: 045261, m/z: 1474.2582, charge: 3
ITYSIVQTNCSK_6_5_1_3_0 LeX peak identified

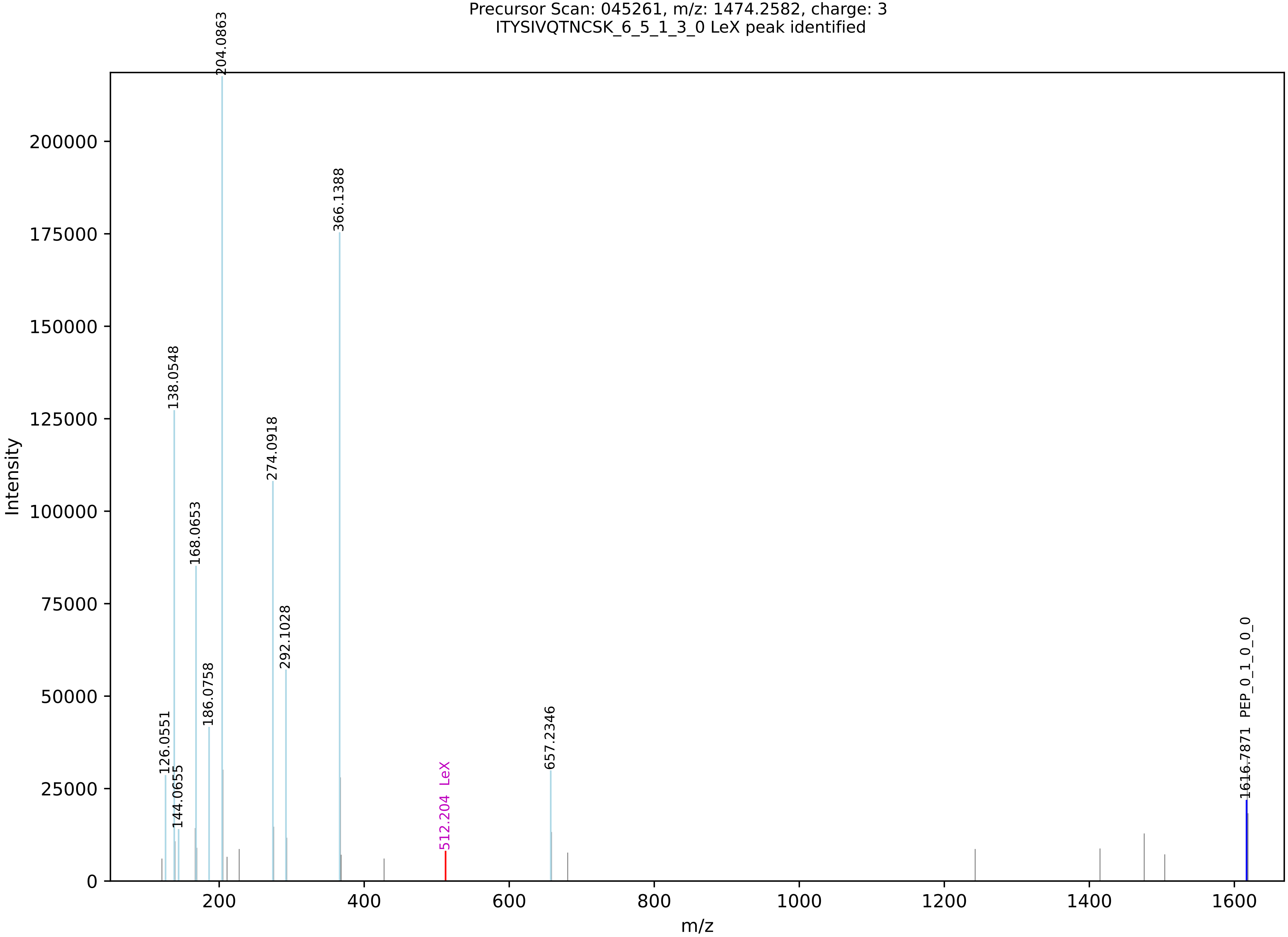

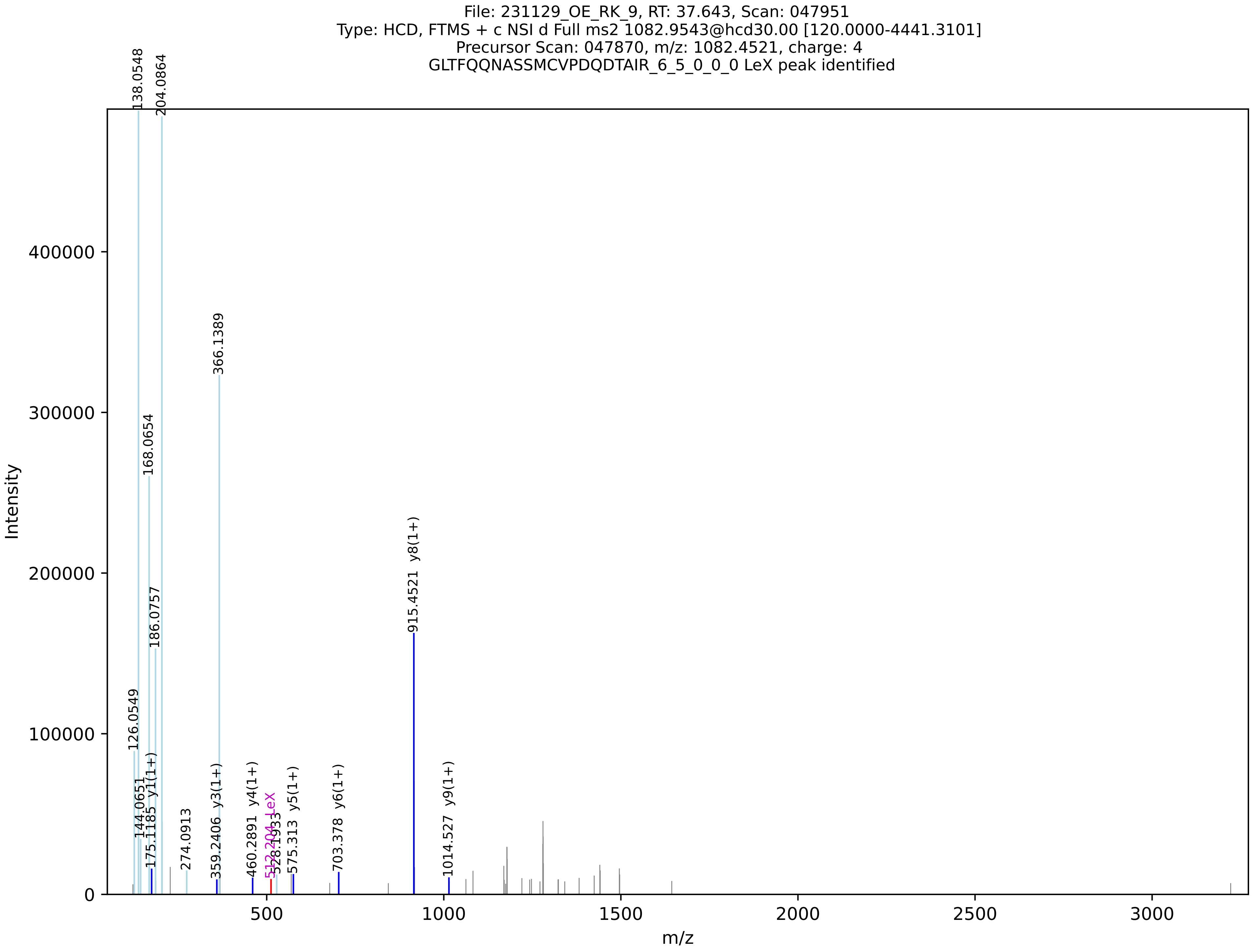
File: 231129_OE_RK_9, RT: 37.643, Scan: 047951
Type: HCD, FTMS + c NSI d Full ms2 1082.9543@hcd30.00 [120.0000-4441.3101]
Precursor Scan: 047870, m/z: 1082.4521, charge: 4
GLTFQQNASSMCVPDQDTAIR_6_5_0_0_0 LeX peak identified
138.0548
204.0864
366.1389
168.0654
186.0757
126.0549
144.0651
175.1185 y1(1+)
274.0913
359.2406 y3(1+)
460.2891 y4(1+)
512.204 LeX
528.1933
575.313 y5(1+)
703.378 y6(1+)
915.4521 y8(1+)
1014.527 y9(1+)
Intensity
m/z
0
100000
200000
300000
400000
500
1000
1500
2000
2500
3000

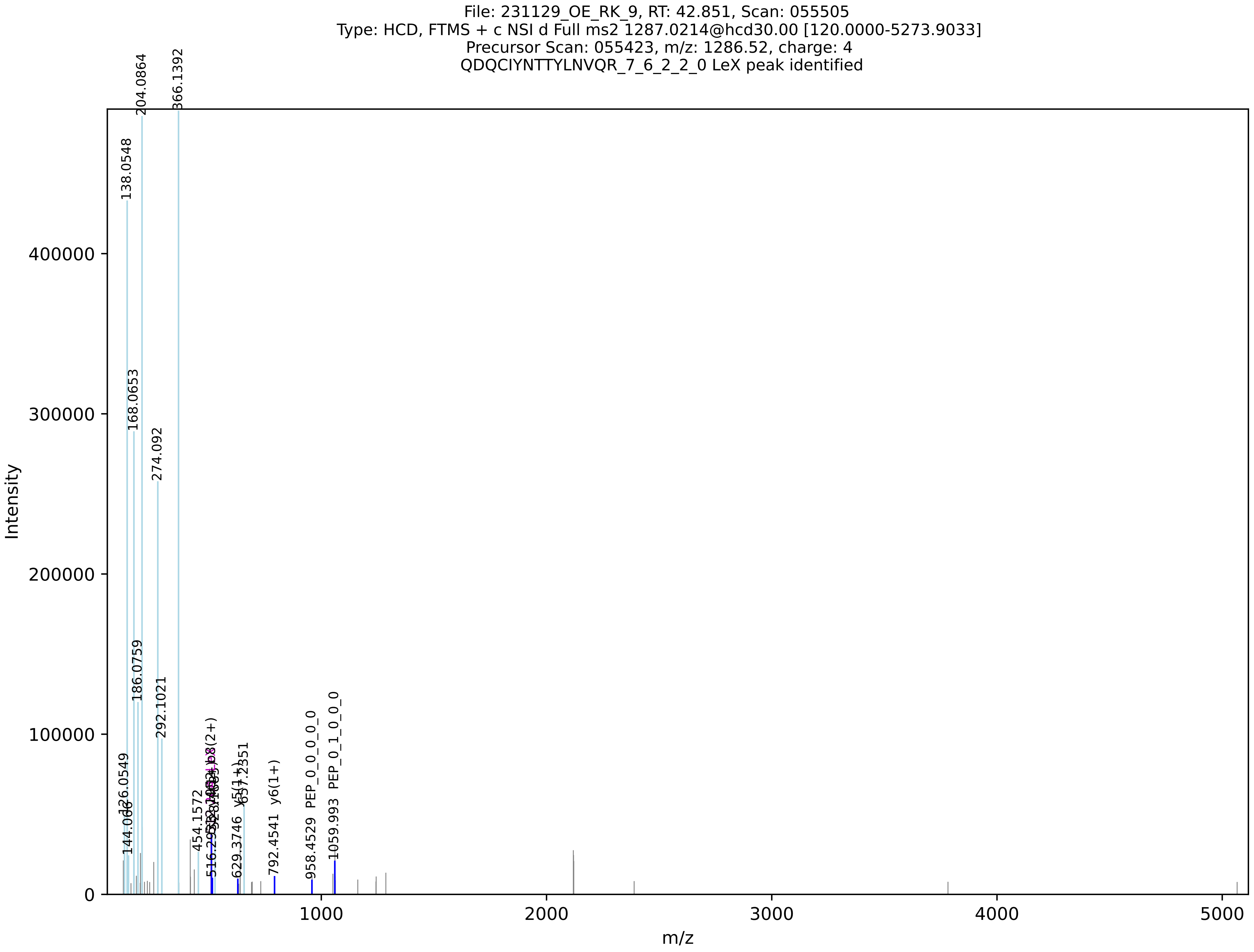

File: 231129_OE_RK_9, RT: 42.851, Scan: 055505
Type: HCD, FTMS + c NSI d Full ms2 1287.0214@hcd30.00 [120.0000-5273.9033]
Precursor Scan: 055423, m/z: 1286.52, charge: 4
QDQCIYNTTYLNVQR_7_6_2_2_0 LeX peak identified
Intensity
m/z
0
100000
200000
300000
400000
1000
2000
3000
4000
5000
204.0864
366.1392
138.0548
168.0653
274.092
186.0759
292.1021
126.0549
144.066
454.1572
629.3746 y5(1+)
657.2351
792.4541 y6(1+)
958.4529 PEP_0_0_0_0_0
1059.993 PEP_0_1_0_0_0

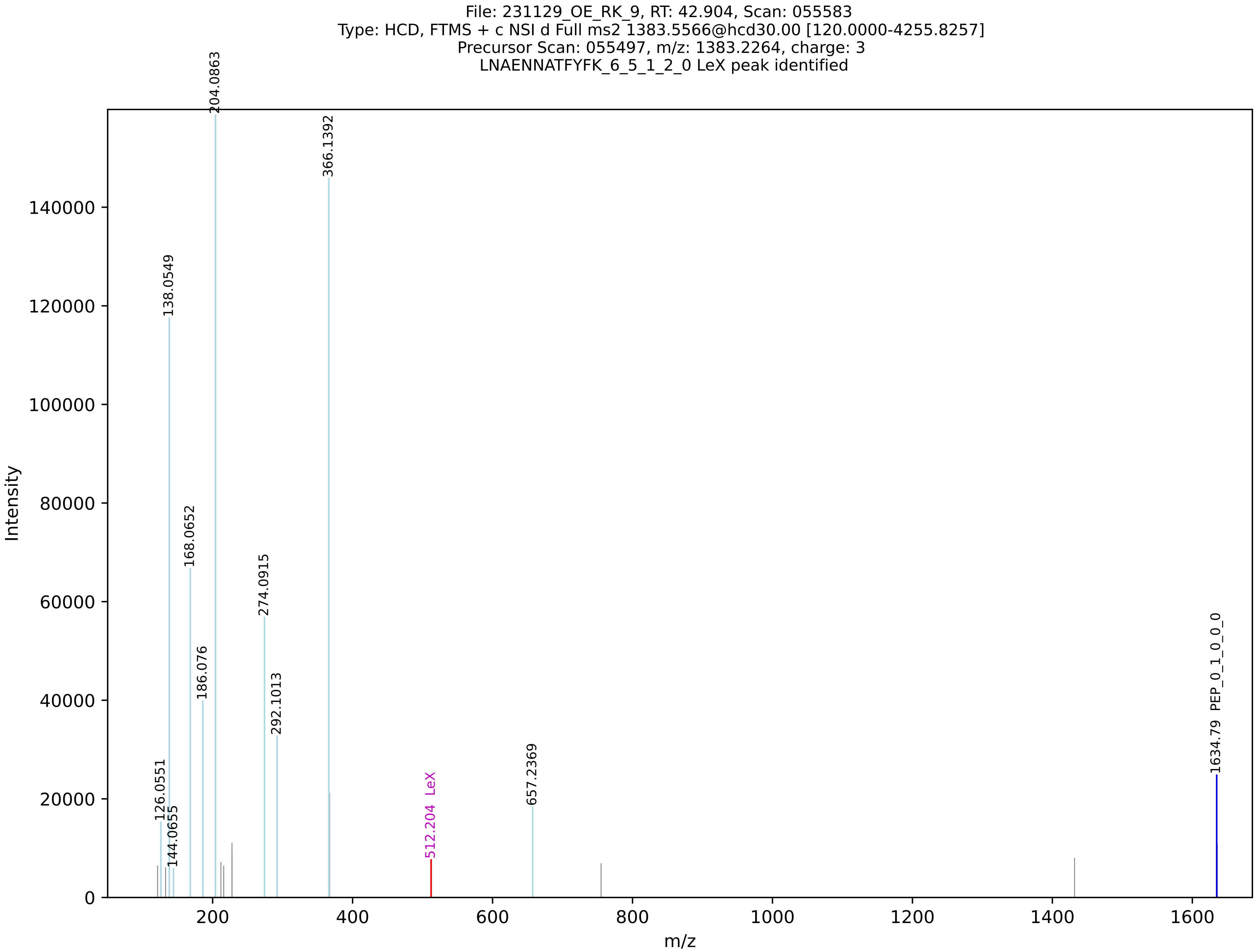
File: 231129_OE_RK_9, RT: 42.904, Scan: 055583
Type: HCD, FTMS + c NSI d Full ms2 1383.5566@hcd30.00 [120.0000-4255.8257]
Precursor Scan: 055497, m/z: 1383.2264, charge: 3
LNAENNATFYFK_6_5_1_2_0 LeX peak identified
Intensity
m/z
204.0863
366.1392
138.0549
168.0652
274.0915
186.076
292.1013
126.0551
144.0655
512.204 LeX
657.2369
1634.79 PEP_0_1_0_0_0
0
20000
40000
60000
80000
100000
120000
140000
200
400
600
800
1000
1200
1400
1600

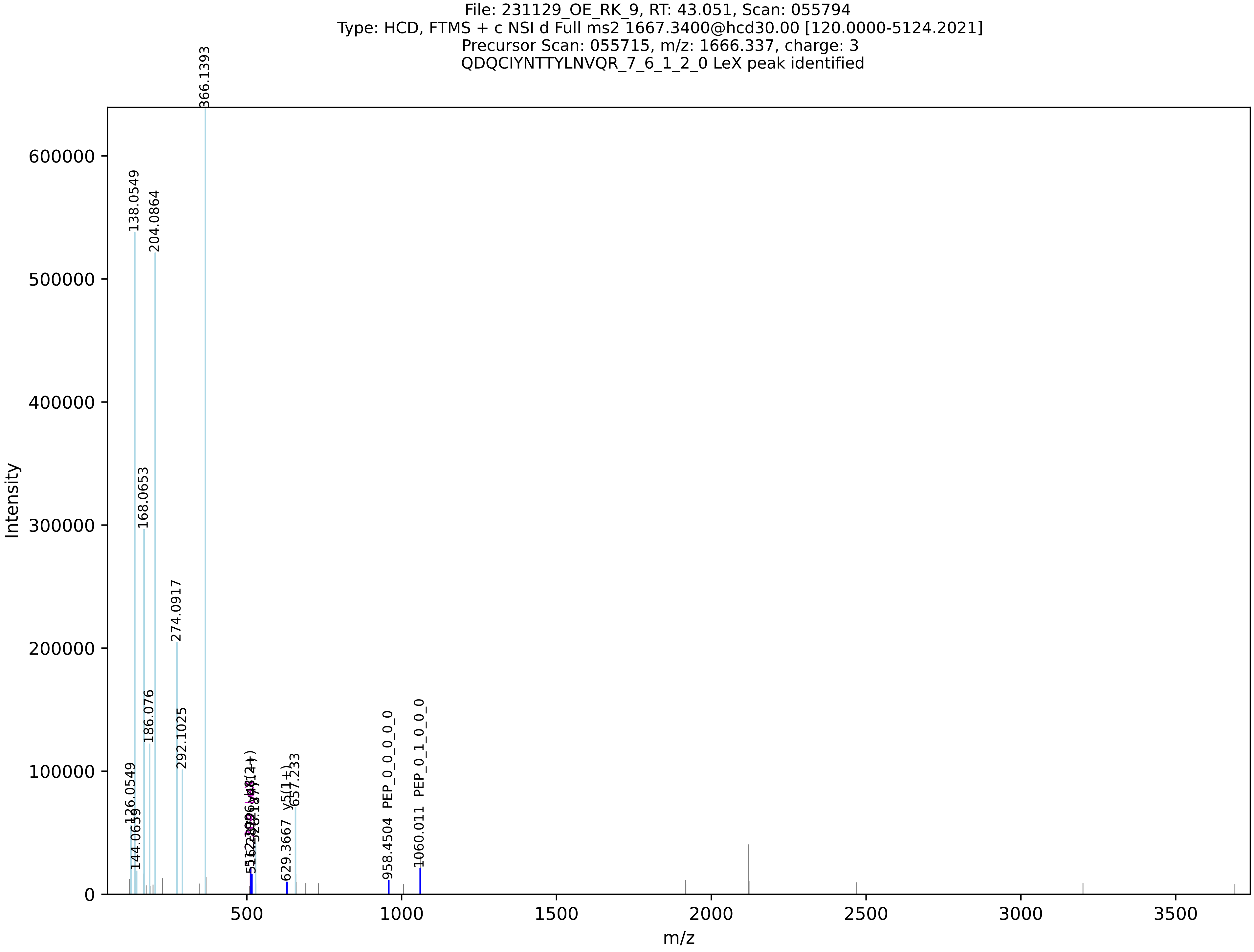
File: 231129_OE_RK_9, RT: 43.051, Scan: 055794
Type: HCD, FTMS + c NSI d Full ms2 1667.3400@hcd30.00 [120.0000-5124.2021]
Precursor Scan: 055715, m/z: 1666.337, charge: 3
QDQCIYNTTYLNVQR_7_6_1_2_0 LeX peak identified
366.1393
138.0549
204.0864
168.0653
274.0917
186.076
292.1025
126.0549
144.0659
657.233
629.3667 y5(1+)
958.4504 PEP_0_0_0_0_0_
1060.011 PEP_0_1_0_0_0_
Intensity
m/z
0
100000
200000
300000
400000
500000
600000
500
1000
1500
2000
2500
3000
3500

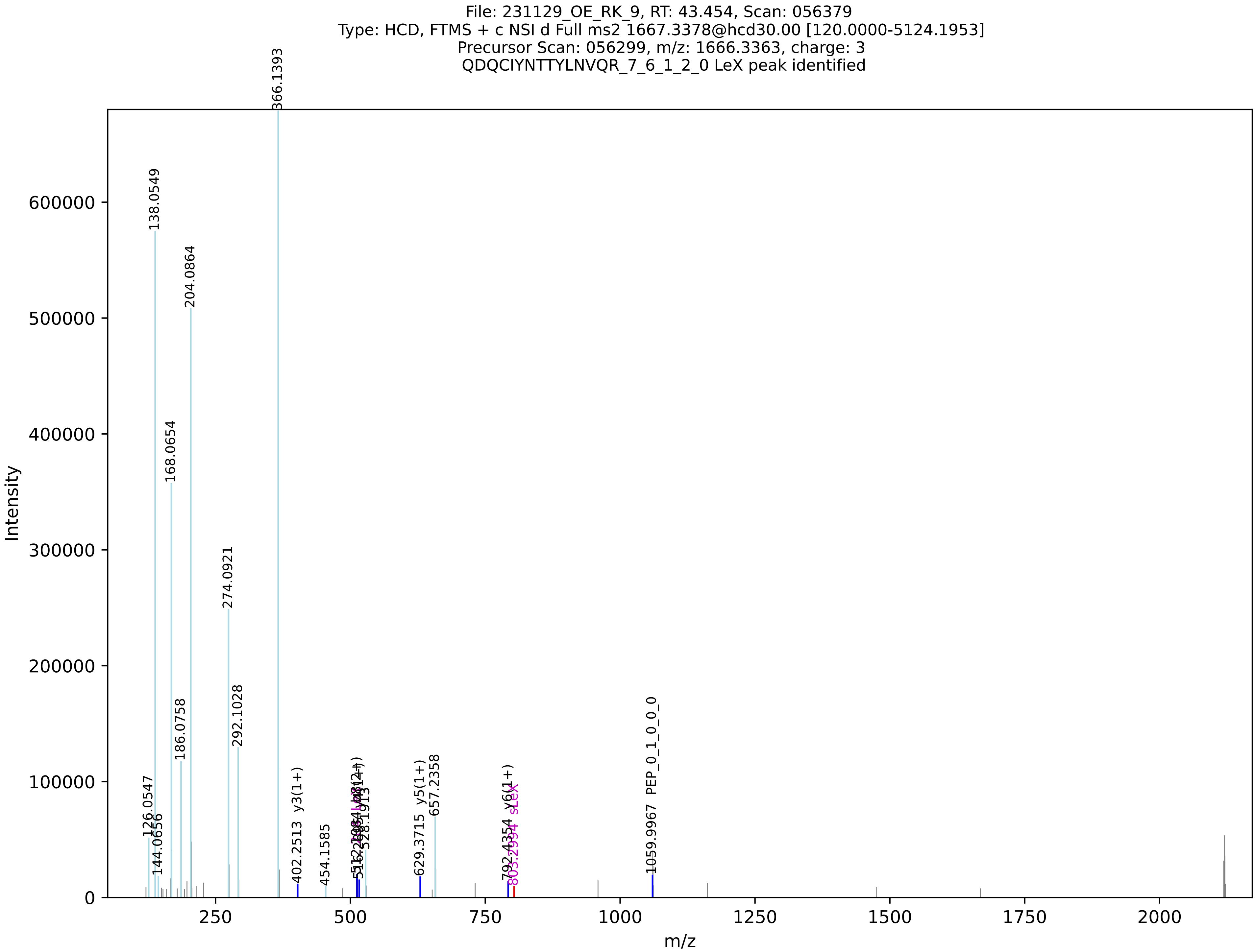
File: 231129_OE_RK_9, RT: 43.454, Scan: 056379
Type: HCD, FTMS + c NSI d Full ms2 1667.3378@hcd30.00 [120.0000-5124.1953]
Precursor Scan: 056299, m/z: 1666.3363, charge: 3
QDQCIYNTTYLNVQR_7_6_1_2_0 LeX peak identified
366.1393
138.0549
204.0864
168.0654
274.0921
292.1028
186.0758
126.0547
144.0656
402.2513 y3(1+)
454.1585
528.1913
629.3715 y5(1+)
657.2358
792.4354 y6(1+)
803.2994 sLeX
1059.9967 PEP_0_1_0_0_0_
Intensity
m/z
0
100000
200000
300000
400000
500000
600000
250
500
750
1000
1250
1500
1750
2000

File: 231129_OE_RK_9, RT: 43.654, Scan: 056669
Type: HCD, FTMS + c NSI d Full ms2 1667.0059@hcd30.00 [120.0000-5123.1797]
Precursor Scan: 056591, m/z: 1666.3374, charge: 3
QDQCIYNTTYLNVQR_7_6_1_2_0 LeX peak identified

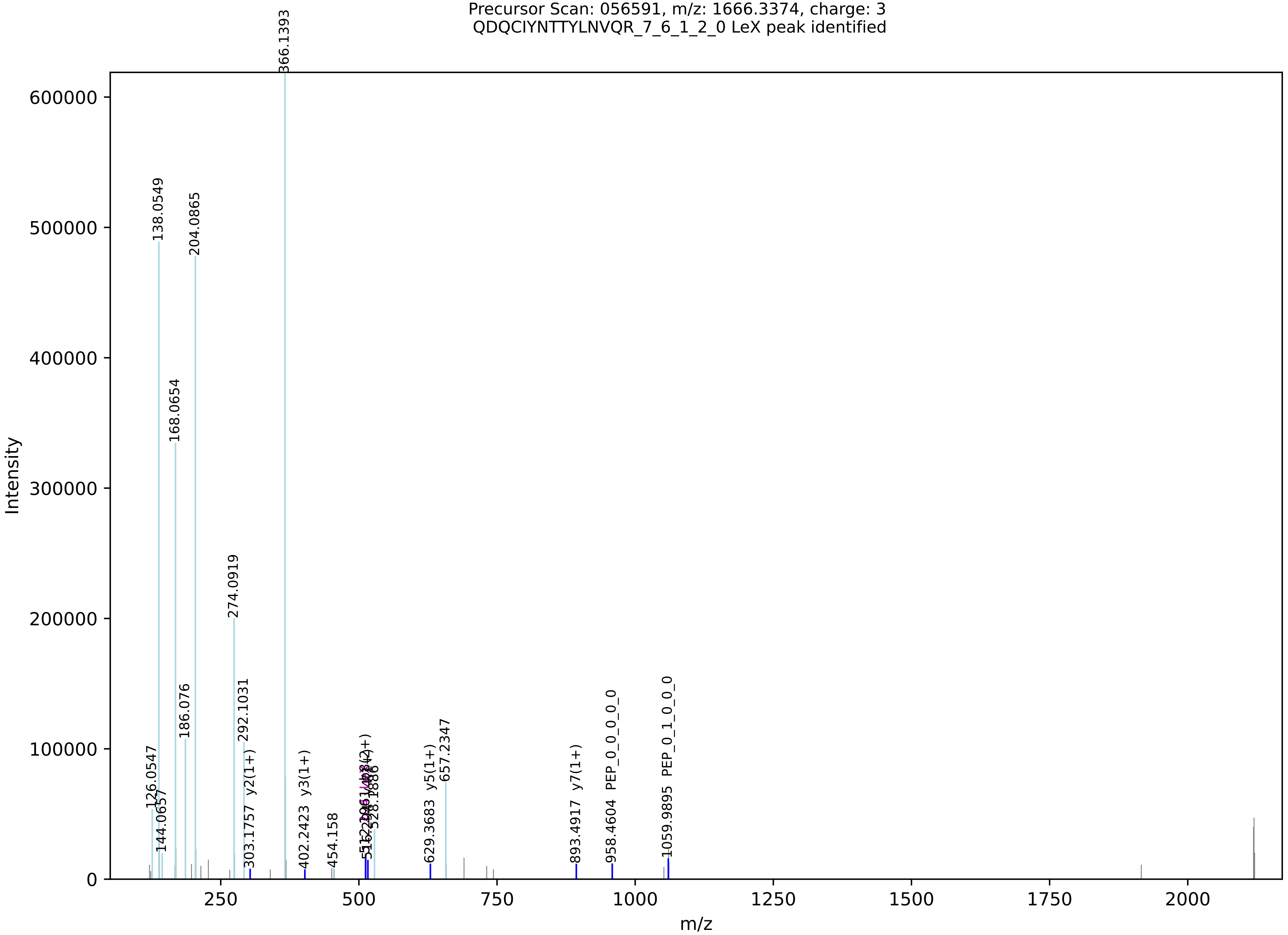

File: 231129_OE_RK_9, RT: 45.32, Scan: 059082
Type: HCD, FTMS + c NSI d Full ms2 1359.7950@hcd30.00 [120.0000-5570.8193]
Precursor Scan: 059004, m/z: 1359.0432, charge: 4
QDQCIYNTTYLNVQR_7_6_0_4_0 LeX peak identified

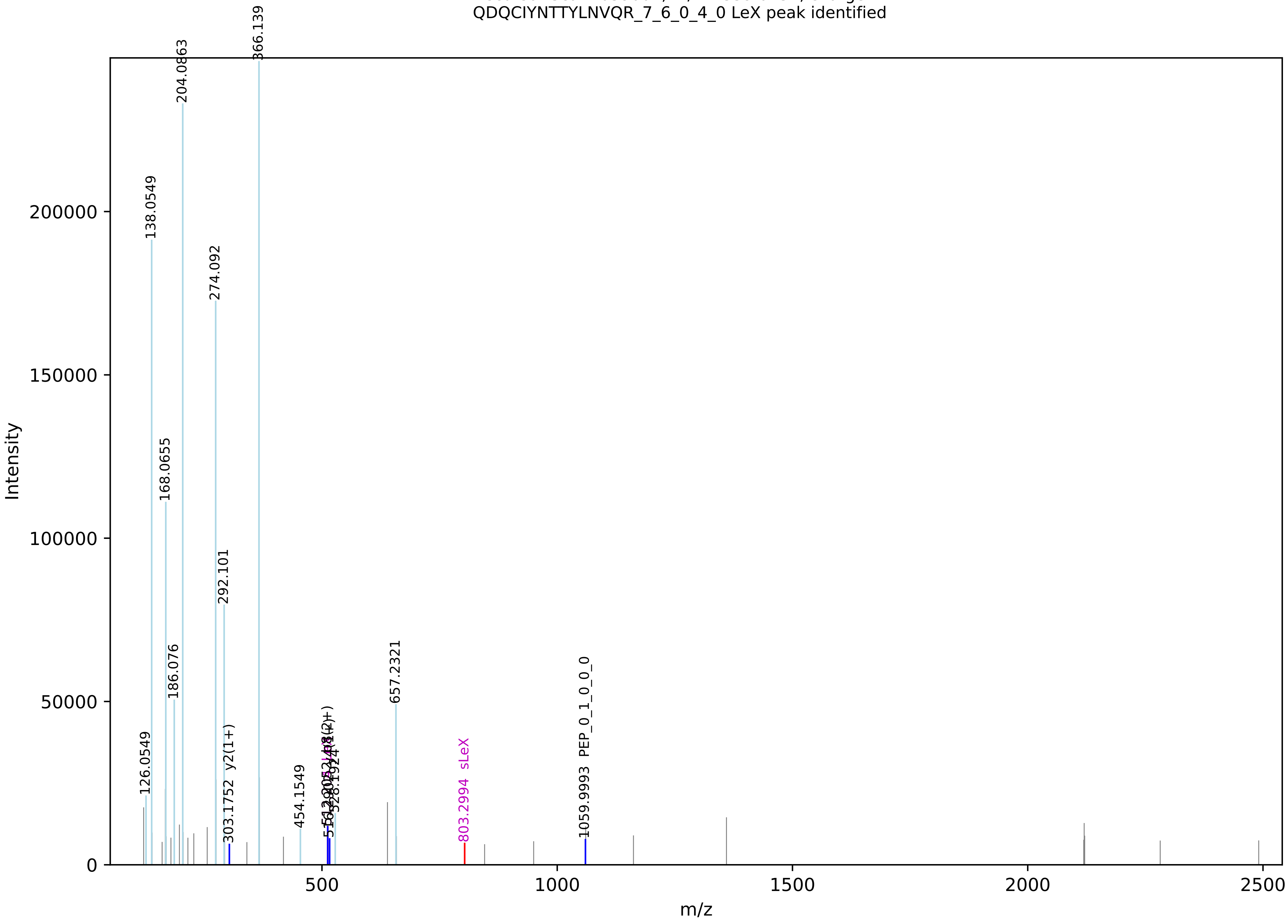

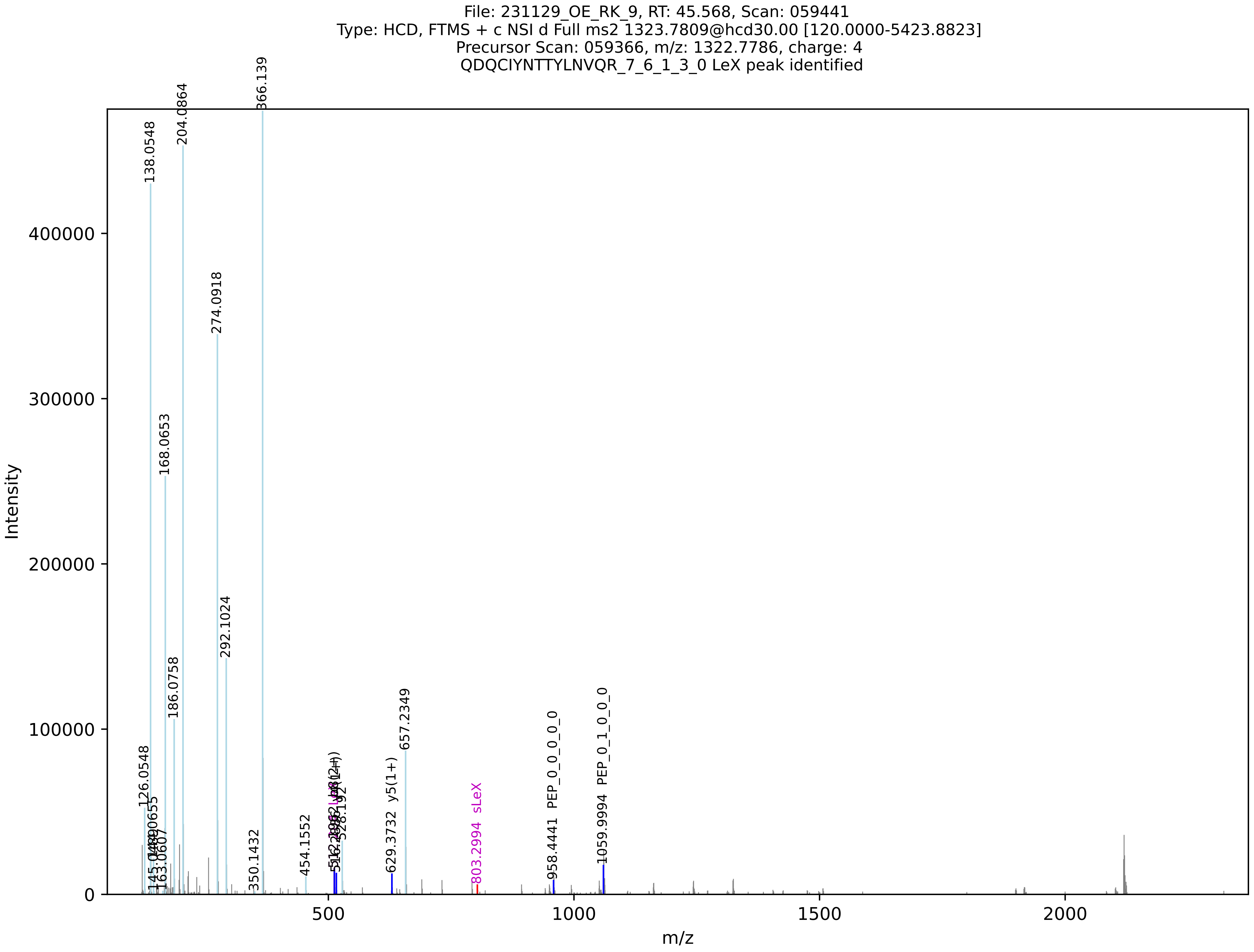
File: 231129_OE_RK_9, RT: 45.568, Scan: 059441
Type: HCD, FTMS + c NSI d Full ms2 1323.7809@hcd30.00 [120.0000-5423.8823]
Precursor Scan: 059366, m/z: 1322.7786, charge: 4
QDQCIYNTTYLNVQR_7_6_1_3_0 LeX peak identified
Intensity
m/z
366.139
204.0864
138.0548
274.0918
168.0653
292.1024
186.0758
126.0548
163.0607
350.1432
454.1552
528.192
629.3732 y5(1+)
657.2349
803.2994 sLeX
958.4441 PEP_0_0_0_0_0
1059.9994 PEP_0_1_0_0_0
0
100000
200000
300000
400000
500
1000
1500
2000

File: 231129_OE_RK_9, RT: 46.025, Scan: 060098
Type: HCD, FTMS + c NSI d Full ms2 1323.5300@hcd30.00 [120.0000-5422.8589]
Precursor Scan: 060021, m/z: 1322.7778, charge: 4
QDQCIYNTTYLNVQR_7_6_1_3_0 LeX peak identified

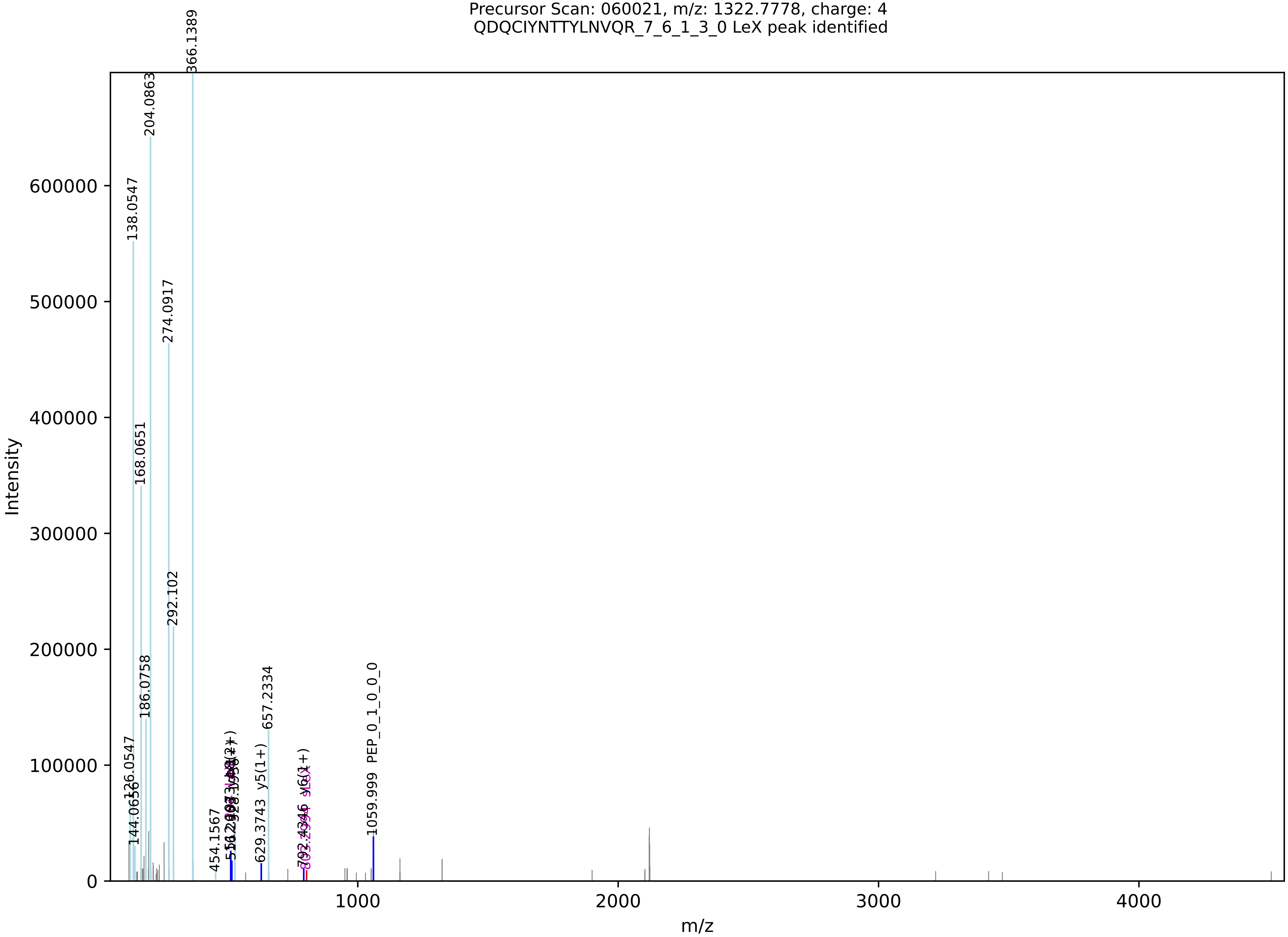

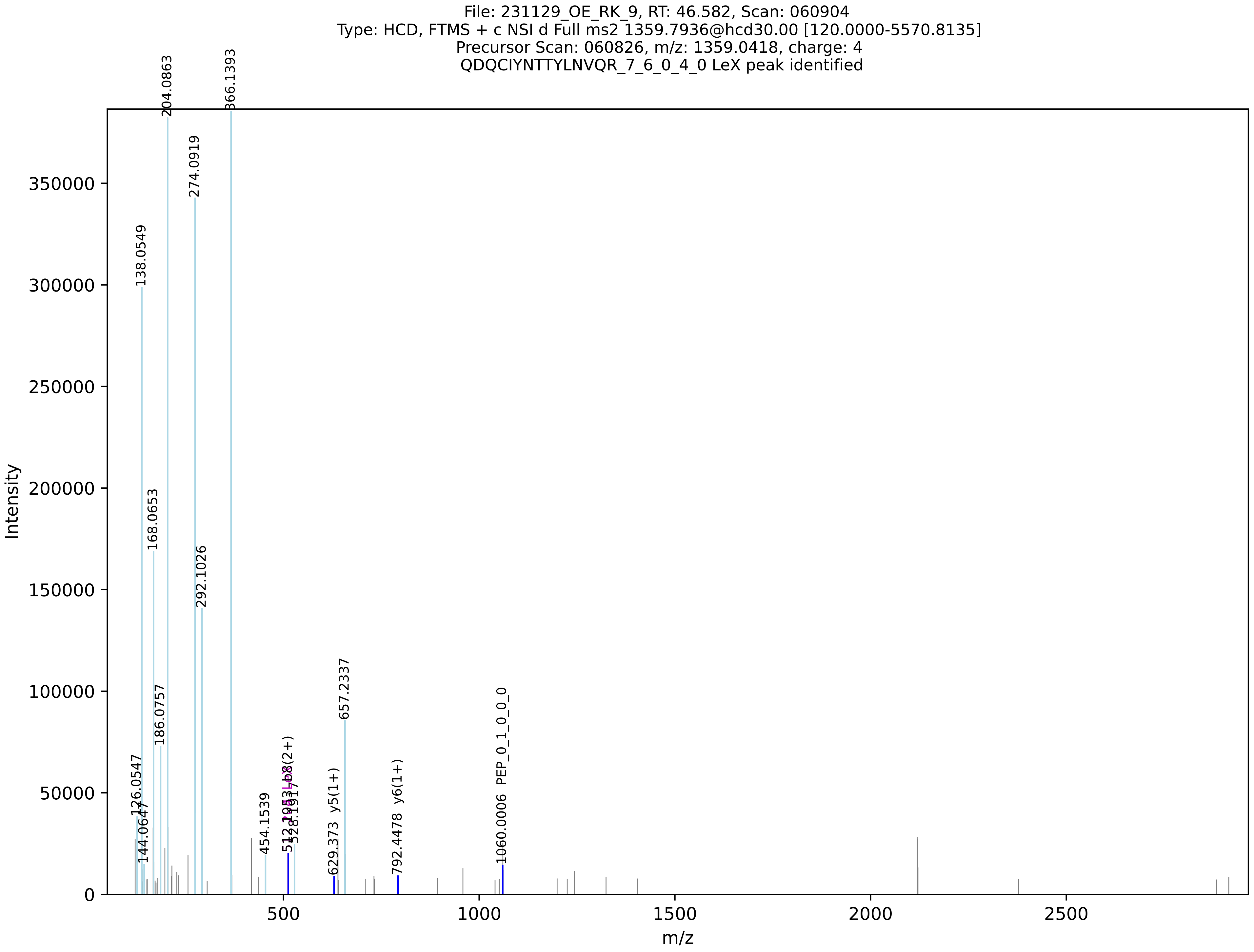
File: 231129_OE_RK_9, RT: 46.582, Scan: 060904
Type: HCD, FTMS + c NSI d Full ms2 1359.7936@hcd30.00 [120.0000-5570.8135]
Precursor Scan: 060826, m/z: 1359.0418, charge: 4
QDQCIYNTTYLNVQR_7_6_0_4_0 LeX peak identified
366.1393
204.0863
274.0919
138.0549
168.0653
292.1026
186.0757
657.2337
126.0547
144.0647
454.1539
512.2953 b8(2+)
LeX
528.1917
629.373 y5(1+)
792.4478 y6(1+)
1060.0006 PEP_0_1_0_0_0_
Intensity
350000
300000
250000
200000
150000
100000
50000
0
500
1000
1500
2000
2500
m/z

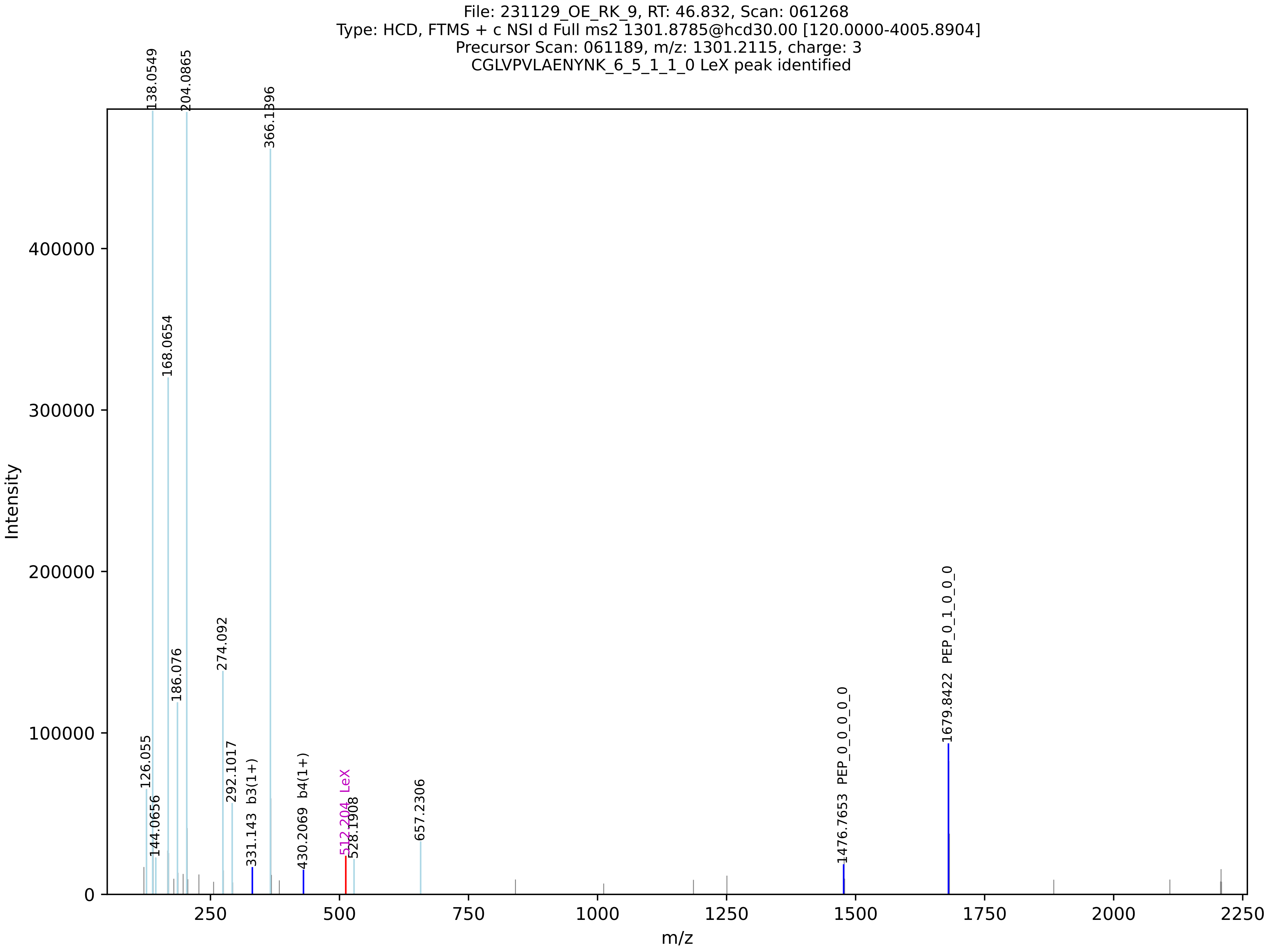
File: 231129_OE_RK_9, RT: 46.832, Scan: 061268
Type: HCD, FTMS + c NSI d Full ms2 1301.8785@hcd30.00 [120.0000-4005.8904]
Precursor Scan: 061189, m/z: 1301.2115, charge: 3
CGLVPVLAENYNK_6_5_1_1_0 LeX peak identified
138.0549
204.0865
366.1396
168.0654
186.076
274.092
126.055
144.0656
292.1017
331.143 b3(1+)
430.2069 b4(1+)
512.204 LeX
528.1908
657.2306
1476.7653 PEP_0_0_0_0_0
1679.8422 PEP_0_1_0_0_0
Intensity
m/z

File: 231129_OE_RK_9, RT: 47.328, Scan: 061991
Type: HCD, FTMS + c NSI d Full ms2 1179.8335@hcd30.00 [120.0000-3632.4324]
Precursor Scan: 061919, m/z: 1179.4993, charge: 3
CGLVPVLAENYNK_5_4_1_1_0 LeX peak identified

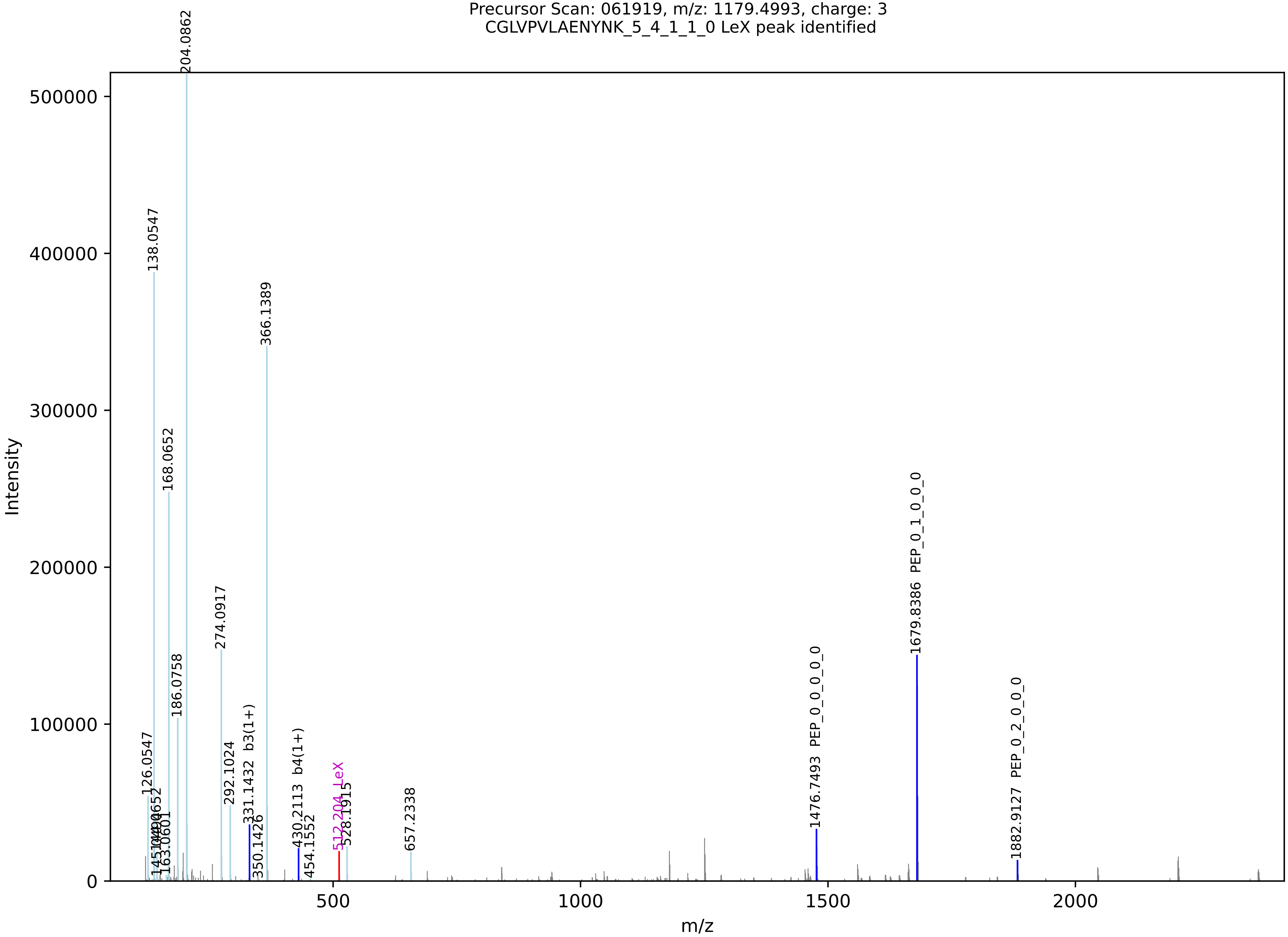

File: 231129_OE_RK_9, RT: 52.57, Scan: 069589
Type: HCD, FTMS + c NSI d Full ms2 1476.3044@hcd30.00 [120.0000-4539.6333]
Precursor Scan: 069485, m/z: 1475.6347, charge: 3
AALAAFNAQNNGSNFQLEEISR_5_4_1_1_0 LeX peak identified

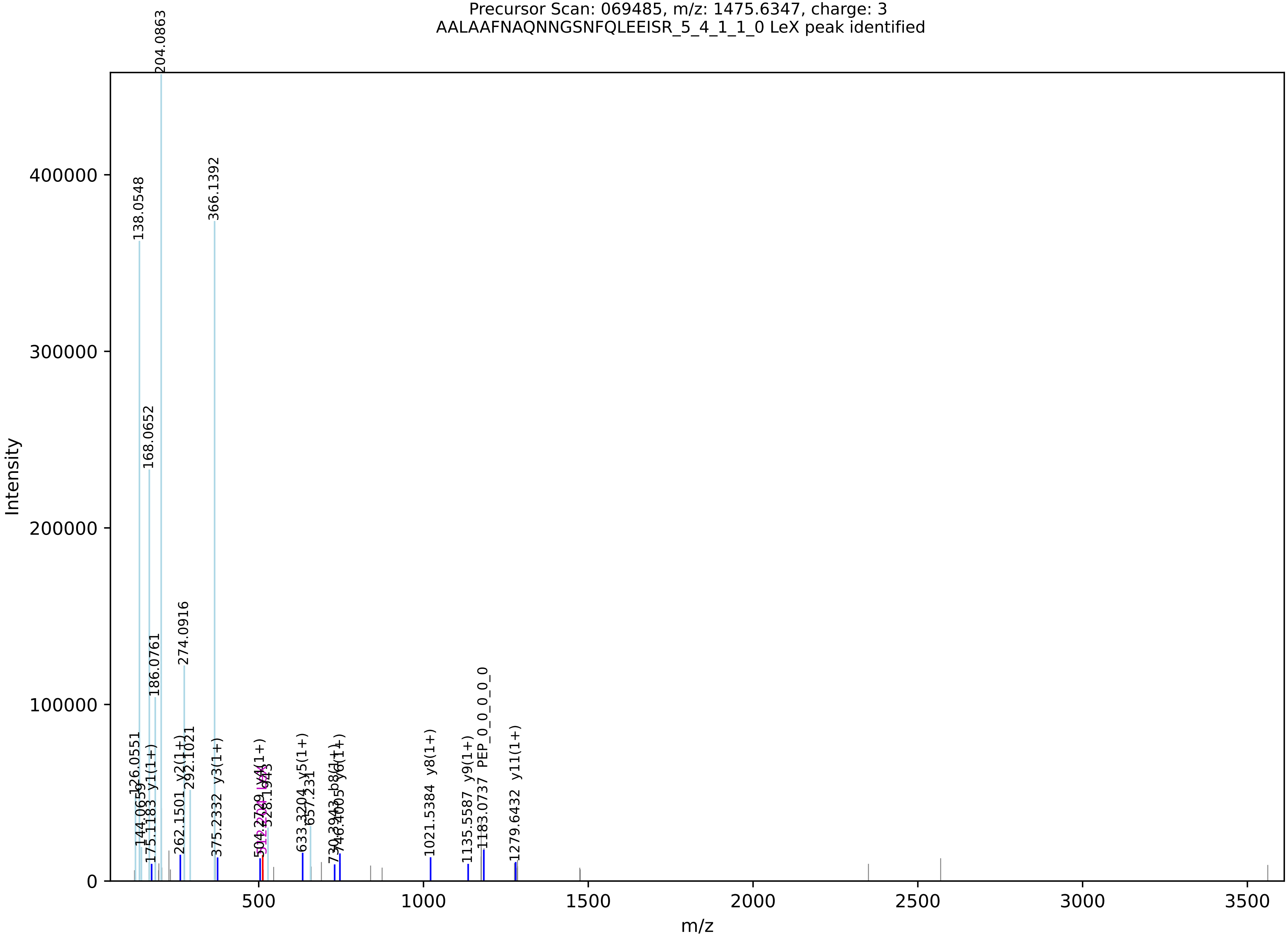

File: 231129_OE_RK_9, RT: 52.613, Scan: 069651
Type: HCD, FTMS + c NSI d Full ms2 1266.5438@hcd30.00 [120.0000-3897.7661]
Precursor Scan: 069559, m/z: 1265.8756, charge: 3
ALPQPQNVTSLLGCTH_5_4_1_1_0 LeX peak identified

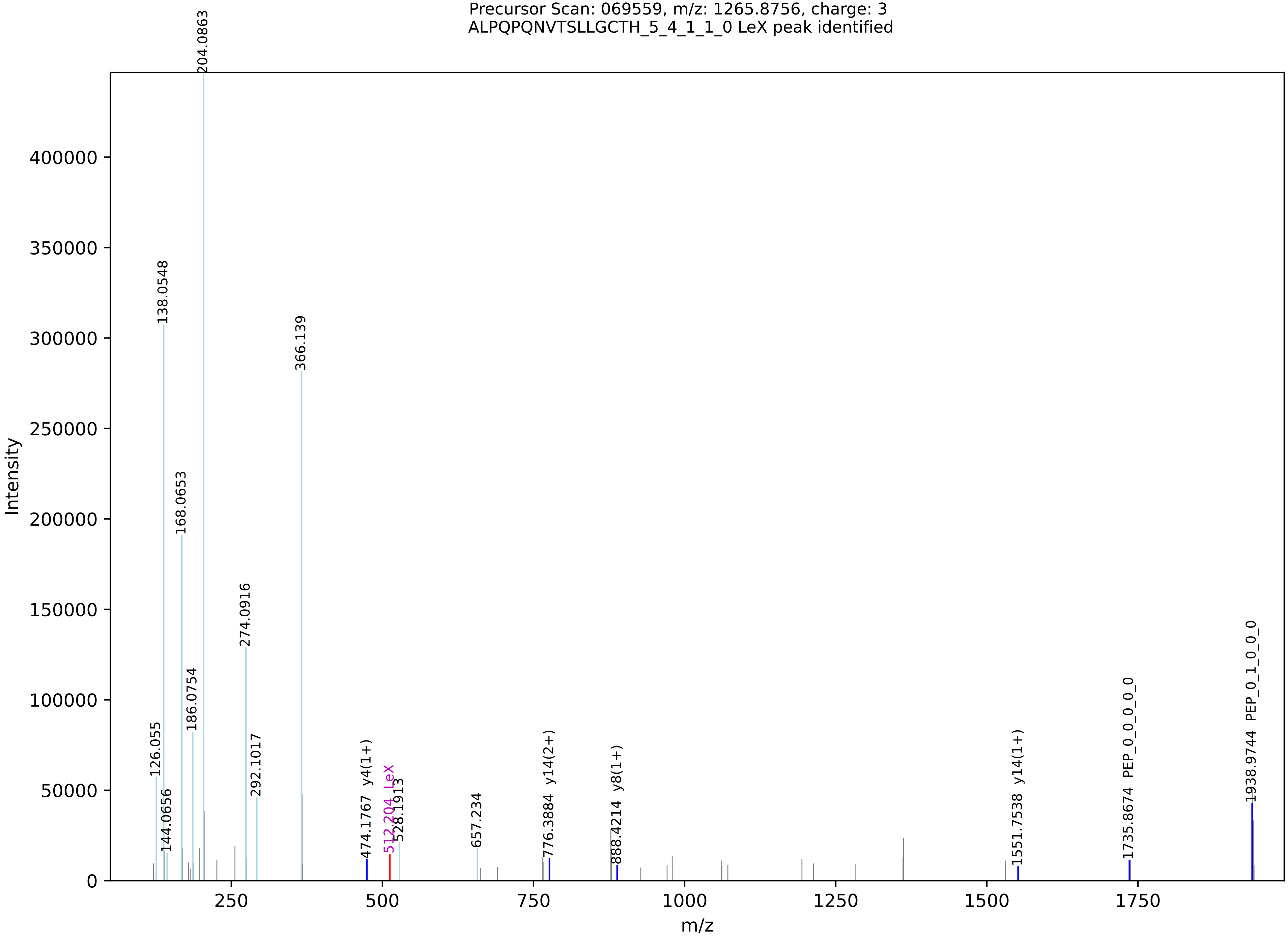

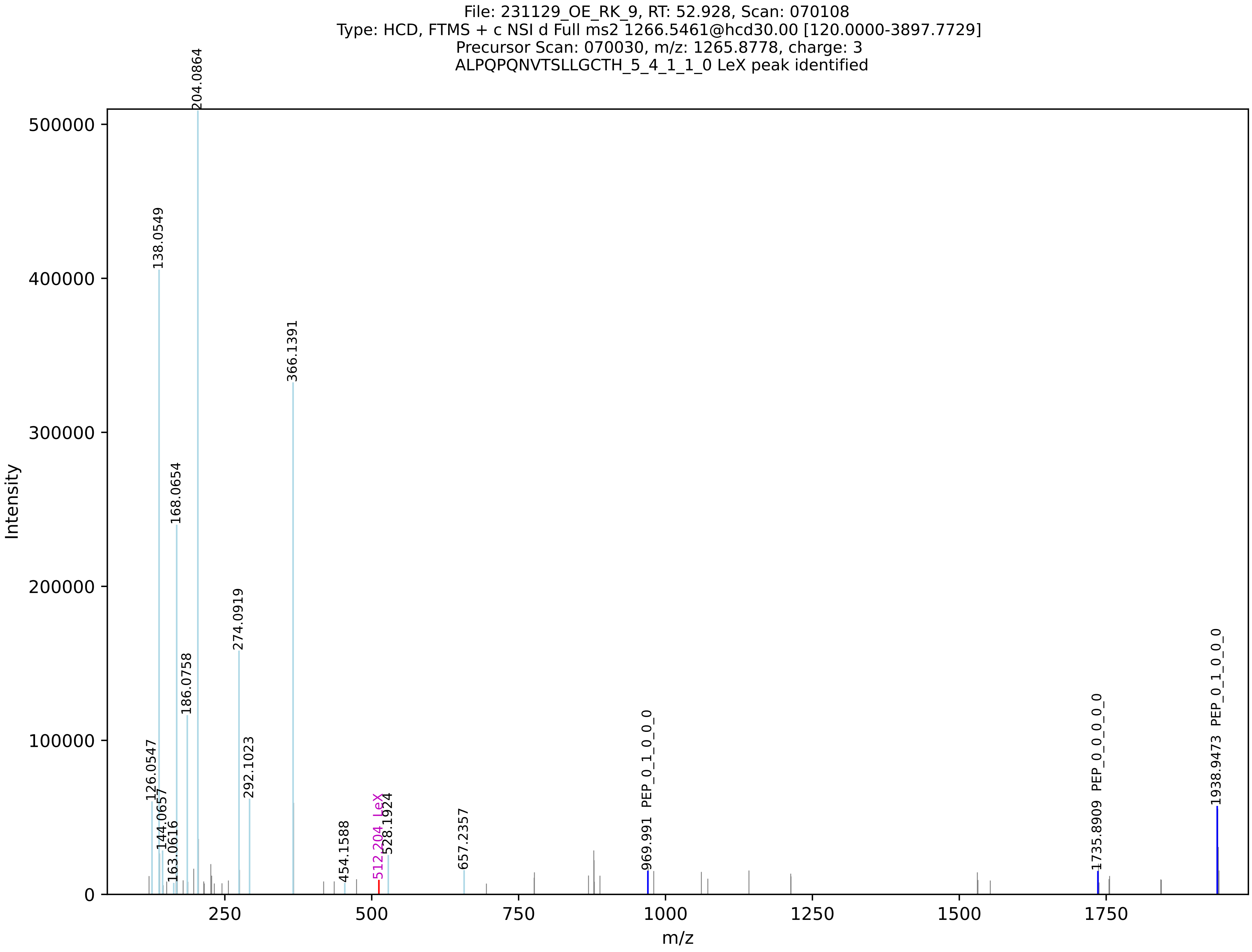
File: 231129_OE_RK_9, RT: 52.928, Scan: 070108
Type: HCD, FTMS + c NSI d Full ms2 1266.5461@hcd30.00 [120.0000-3897.7729]
Precursor Scan: 070030, m/z: 1265.8778, charge: 3
ALPQPQNVTSLLGCTH_5_4_1_1_0 LeX peak identified
Intensity
m/z
204.0864
138.0549
366.1391
168.0654
274.0919
186.0758
292.1023
126.0547
144.0657
163.0616
454.1588
512.204_LeX
528.1924
657.2357
969.991 PEP_0_1_0_0_0_
1735.8909 PEP_0_0_0_0_0_
1938.9473 PEP_0_1_0_0_0_

File: 231129_OE_RK_9, RT: 53.091, Scan: 070345
Type: HCD, FTMS + c NSI d Full ms2 1553.6854@hcd30.00 [120.0000-4776.4194]
Precursor Scan: 070249, m/z: 1553.3505, charge: 3
VVLHPNYSQVDIGLIK_6_5_0_3_0 LeX peak identified

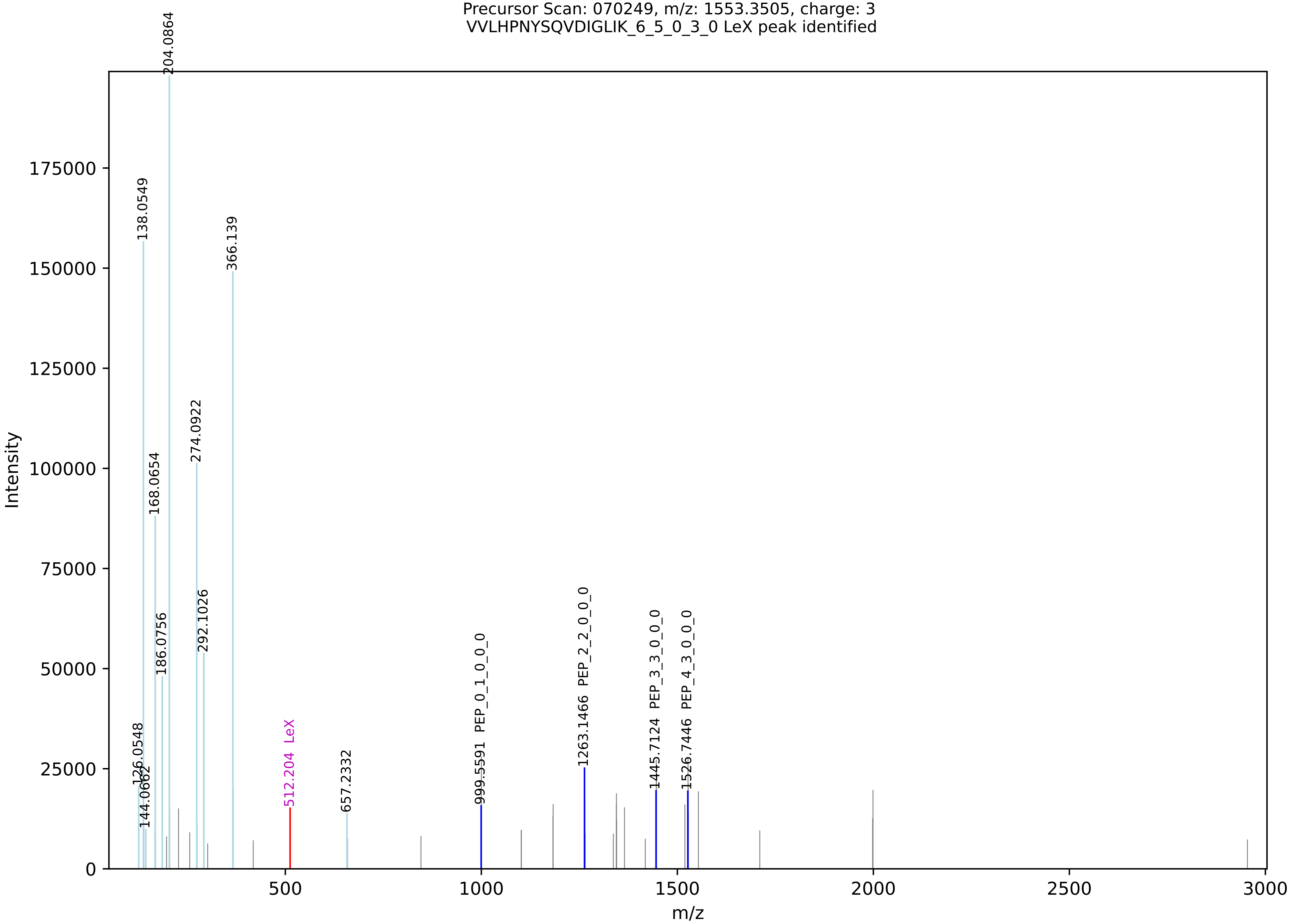

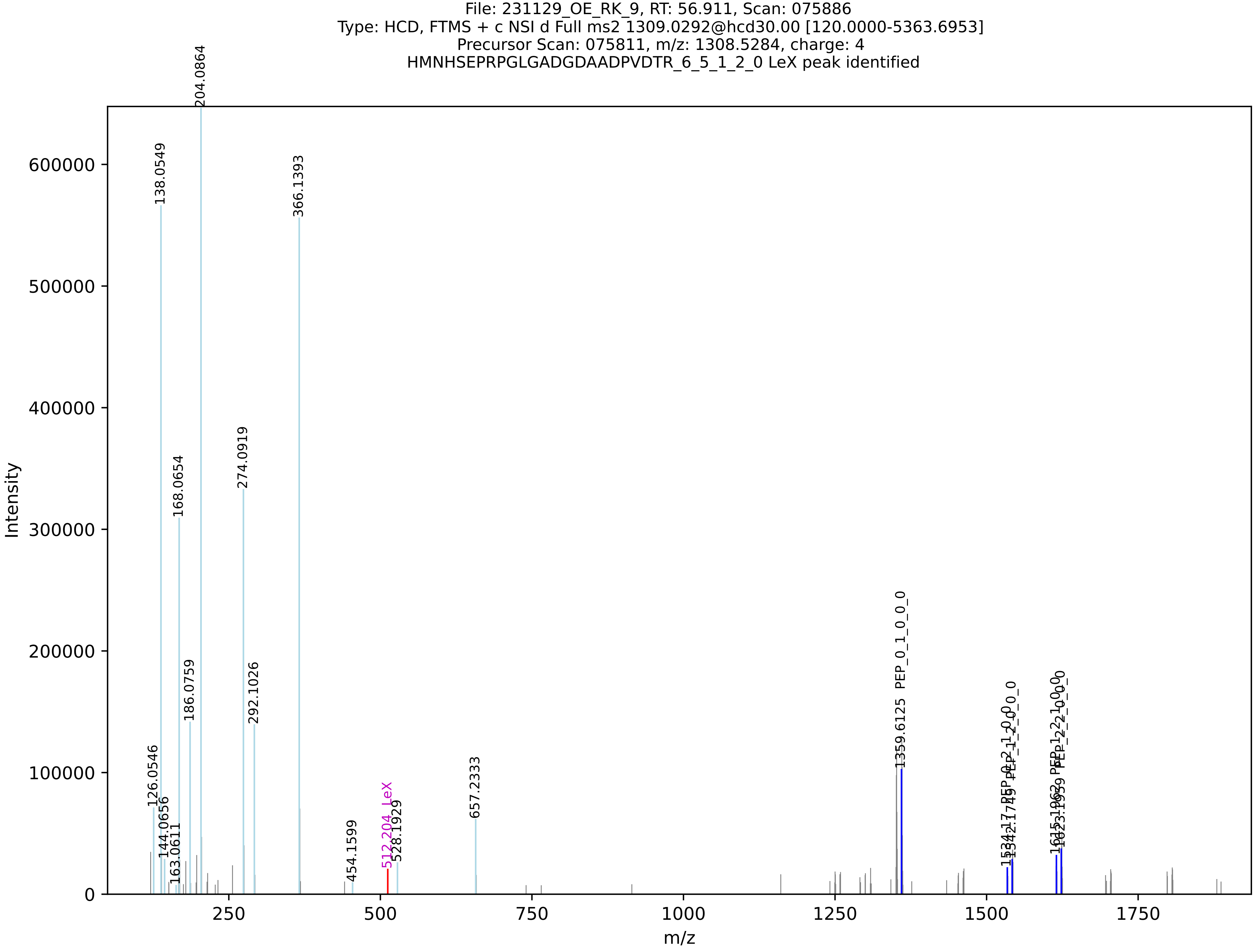
File: 231129_OE_RK_9, RT: 56.911, Scan: 075886
Type: HCD, FTMS + c NSI d Full ms2 1309.0292@hcd30.00 [120.0000-5363.6953]
Precursor Scan: 075811, m/z: 1308.5284, charge: 4
HMNHSEPRPGLGADGDAADPVDTR_6_5_1_2_0 LeX peak identified
Intensity
m/z
204.0864
138.0549
366.1393
274.0919
168.0654
186.0759
292.1026
126.0546
144.0656
163.0611
454.1599
512.204_LeX
528.1929
657.2333
1359.6125 PEP_0_1_0_0_0_
1534.1726 PEP_0_2_1_0_0
1542.1749 PEP_1_2_0_0_0_
1615.1962 PEP_1_2_1_0_0
1623.1959 PEP_2_2_0_0_0

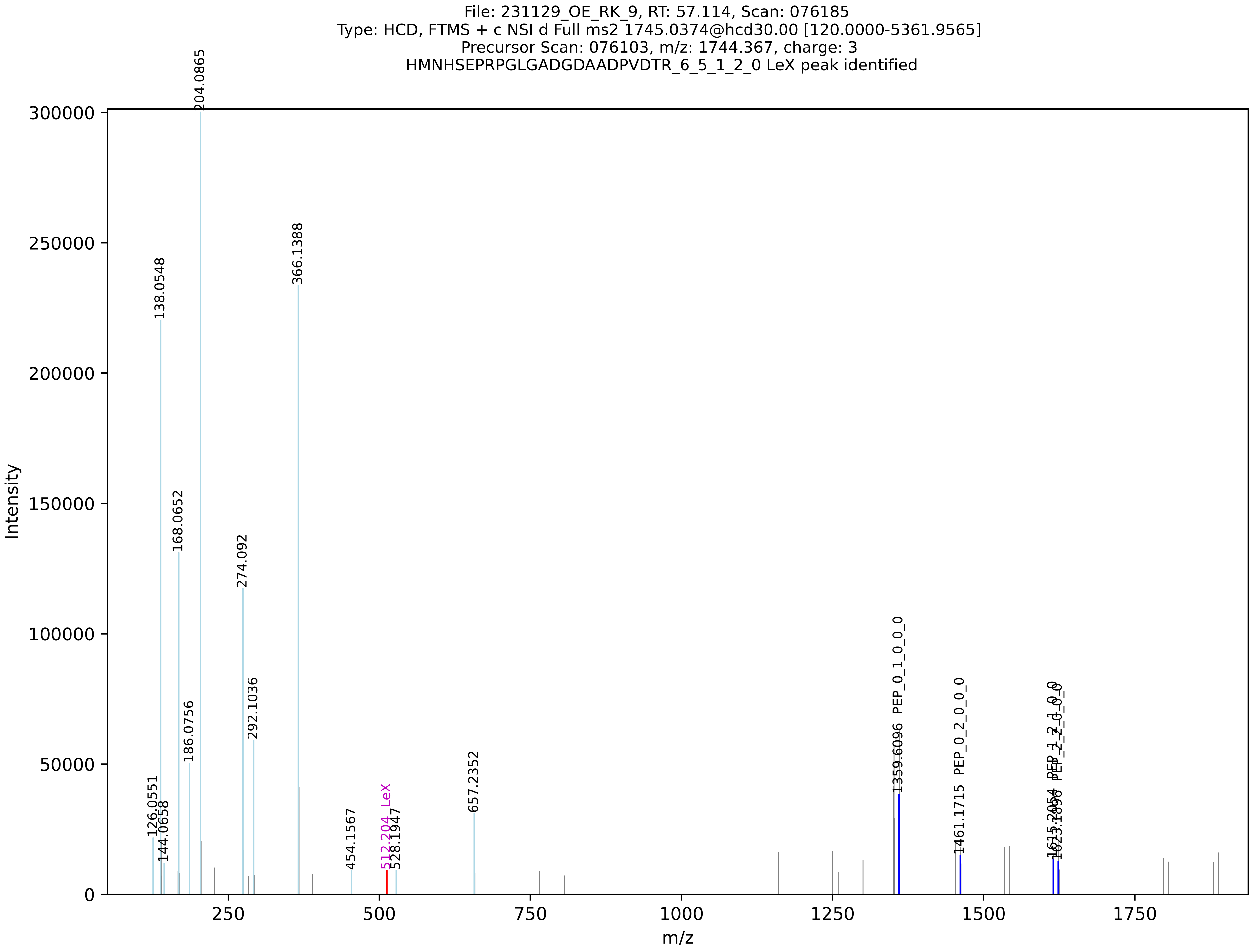
File: 231129_OE_RK_9, RT: 57.114, Scan: 076185
Type: HCD, FTMS + c NSI d Full ms2 1745.0374@hcd30.00 [120.0000-5361.9565]
Precursor Scan: 076103, m/z: 1744.367, charge: 3
HMNHSEPRPGLGADGDAADPVDTR_6_5_1_2_0 LeX peak identified
Intensity
m/z
204.0865
138.0548
366.1388
168.0652
274.092
292.1036
186.0756
126.0551
144.0658
454.1567
512.204_LeX
528.1947
657.2352
1359.6096 PEP_0_1_0_0_0
1461.1715 PEP_0_2_0_0_0
1615.2054
1623.1896
0
50000
100000
150000
200000
250000
300000
250
500
750
1000
1250
1500
1750

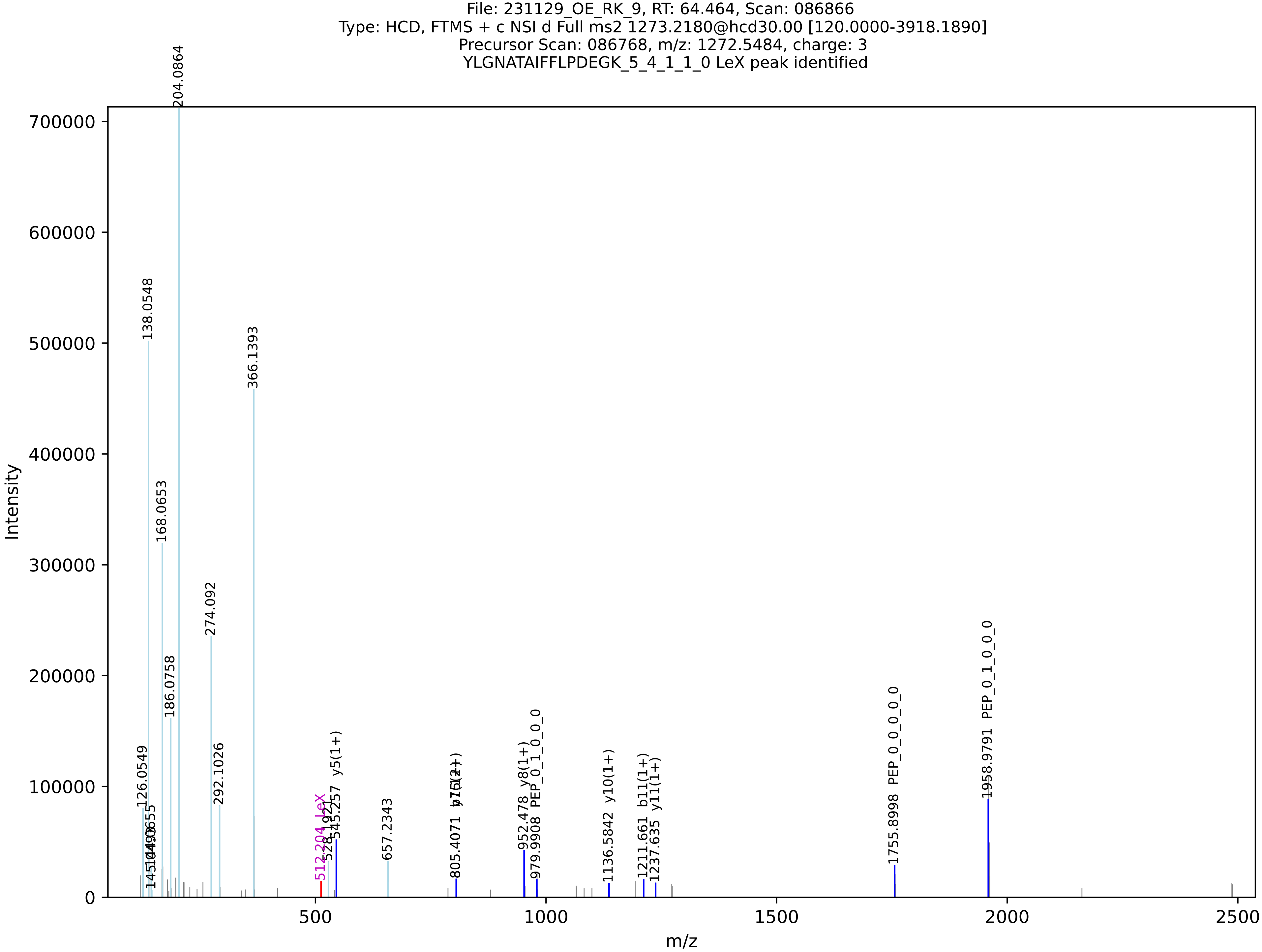
File: 231129_OE_RK_9, RT: 64.464, Scan: 086866
Type: HCD, FTMS + c NSI d Full ms2 1273.2180@hcd30.00 [120.0000-3918.1890]
Precursor Scan: 086768, m/z: 1272.5484, charge: 3
YLGNATAIFFLPDEGK_5_4_1_1_0 LeX peak identified
Intensity
m/z
204.0864
138.0548
366.1393
168.0653
274.092
186.0758
292.1026
126.0549
512.204 LeX
545.257 y5(1+)
657.2343
805.4071
952.478 y8(1+)
979.9908 PEP_0_1_0_0_0
1136.5842 y10(1+)
1211.661 b11(1+)
1237.635 y11(1+)
1755.8998 PEP_0_0_0_0_0
1958.9791 PEP_0_1_0_0_0

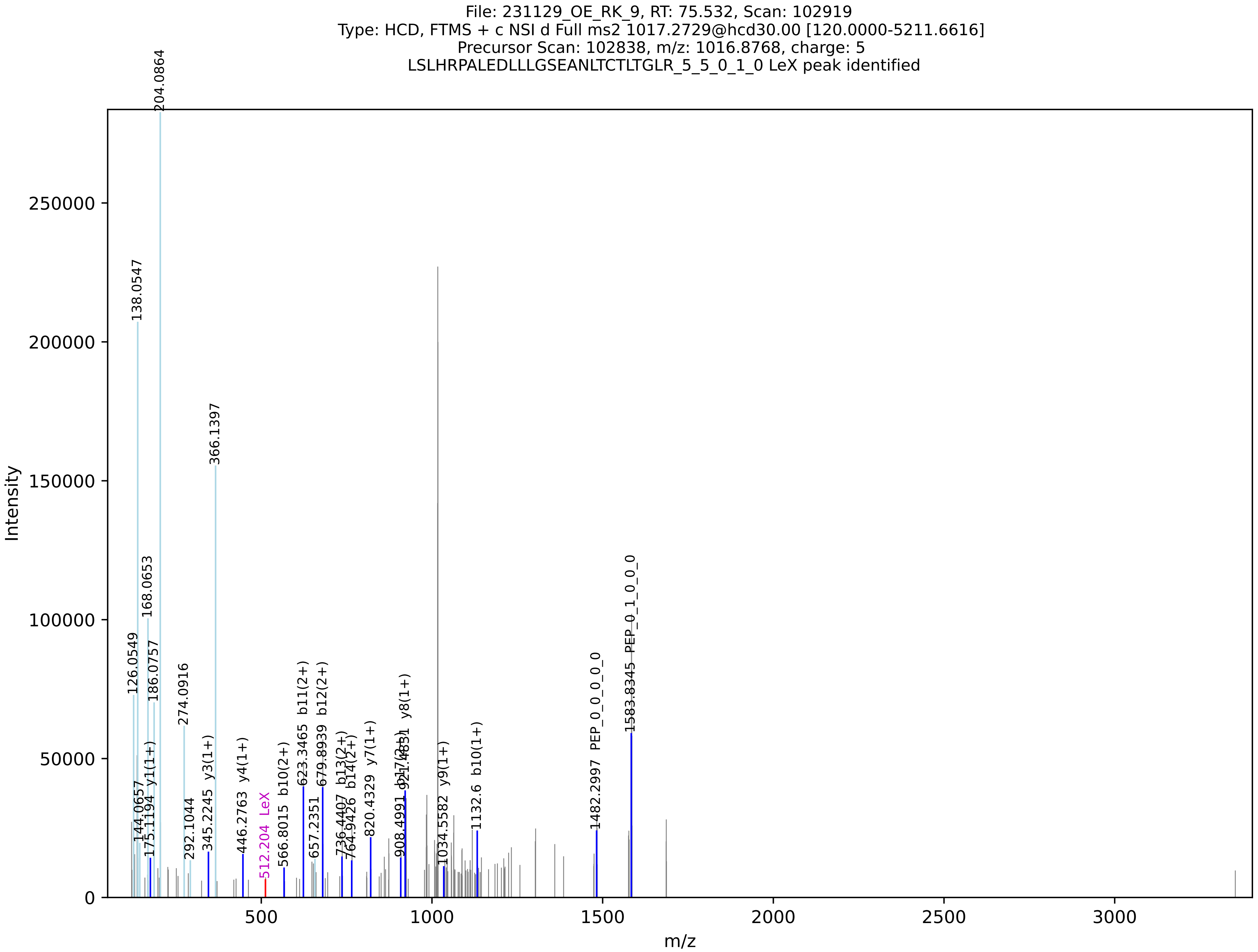
File: 231129_OE_RK_9, RT: 75.532, Scan: 102919
Type: HCD, FTMS + c NSI d Full ms2 1017.2729@hcd30.00 [120.0000-5211.6616]
Precursor Scan: 102838, m/z: 1016.8768, charge: 5
LSLHRPALEDLLLGSEANLTCTLTGLR_5_5_0_1_0 LeX peak identified
Intensity
m/z
204.0864
138.0547
366.1397
168.0653
126.0549
186.0757
274.0916
144.0657
175.1194 y1(1+)
292.1044
345.2245 y3(1+)
446.2763 y4(1+)
512.204 LeX
566.8015 b10(2+)
623.3465 b11(2+)
657.2351
679.8939 b12(2+)
736.4407 b13(2+)
764.9426 b14(2+)
820.4329 y7(1+)
908.4991 b17(2+)
921.4831 y8(1+)
1034.5582 y9(1+)
1132.6 b10(1+)
1482.2997 PEP_0_0_0_0_0
1583.8345 PEP_0_1_0_0_0

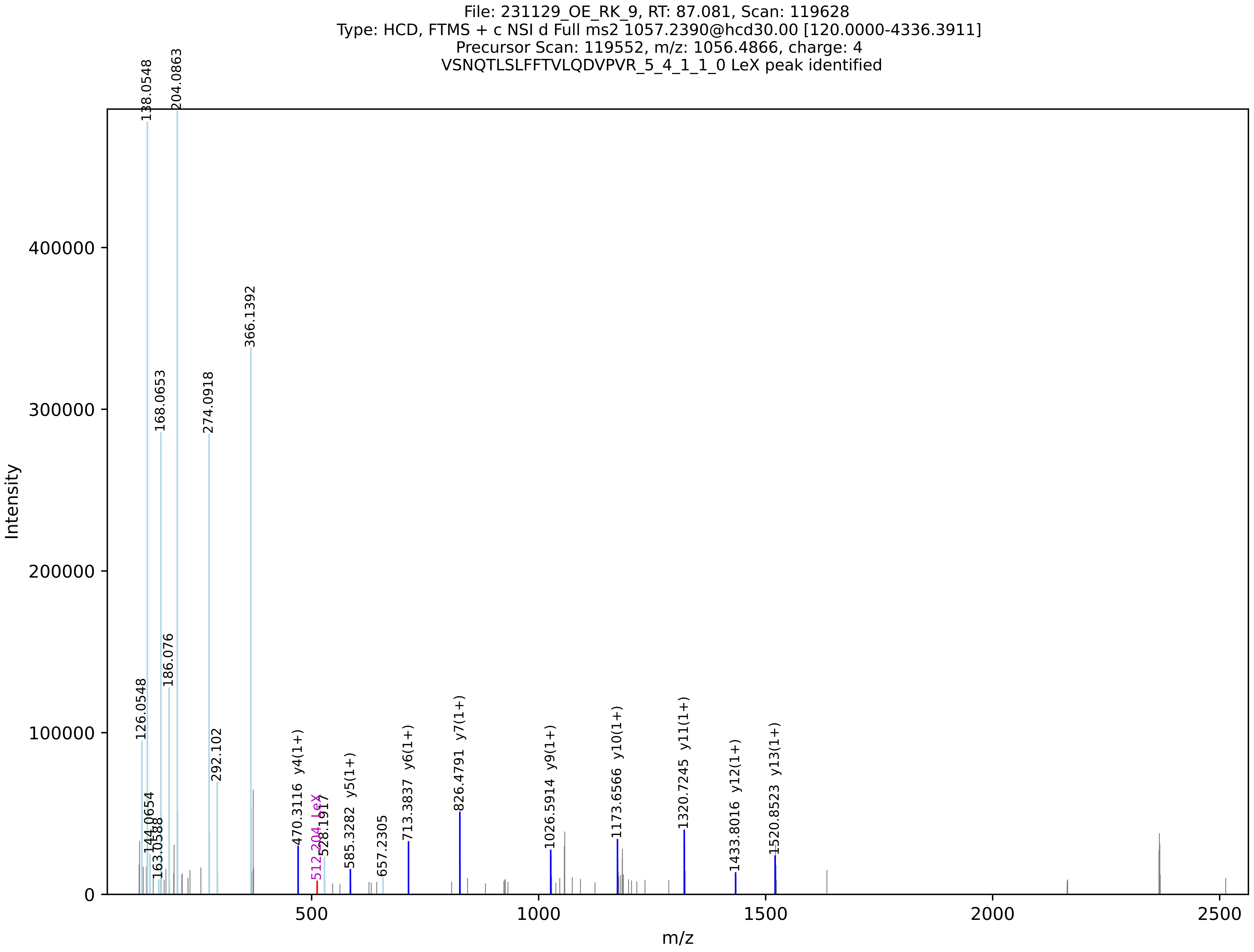

File: 231129_OE_RK_9, RT: 87.081, Scan: 119628
Type: HCD, FTMS + c NSI d Full ms2 1057.2390@hcd30.00 [120.0000-4336.3911]
Precursor Scan: 119552, m/z: 1056.4866, charge: 4
VSNQTLSLFFTVLQDVPVR_5_4_1_1_0 LeX peak identified
Intensity
m/z
138.0548
204.0863
366.1392
168.0653
274.0918
186.076
126.0548
292.102
144.0654
163.0588
470.3116 y4(1+)
512.204 LeX
528.1917
585.3282 y5(1+)
657.2305
713.3837 y6(1+)
826.4791 y7(1+)
1026.5914 y9(1+)
1173.6566 y10(1+)
1320.7245 y11(1+)
1433.8016 y12(1+)
1520.8523 y13(1+)

File: 231129_OE_RK_10, RT: 88.293, Scan: 121556
Type: HCD, FTMS + c NSI d Full ms2 1676.2310@hcd30.00 [120.0000-6861.8784]
Precursor Scan: 121485, m/z: 1675.7315, charge: 4
ADTHDEILEGLNFNLTEIPEAQIHEGFQELLR_6_5_1_3_0 LeX peak identified

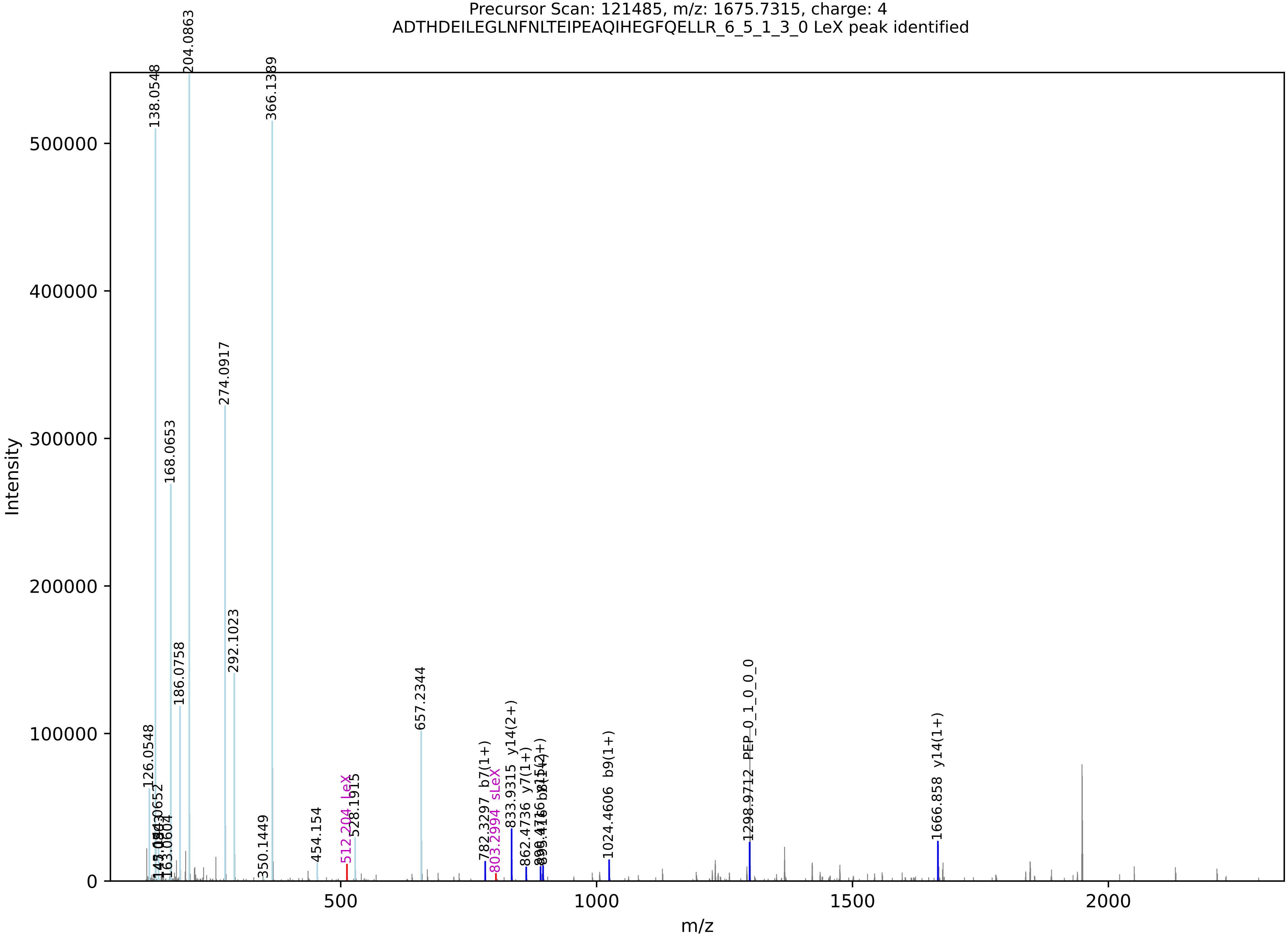

File: 231129_OE_RK_11, RT: 5.412, Scan: 001710
Type: HCD, FTMS + c NSI d Full ms2 1282.1543@hcd30.00 [120.0000-3945.5342]
Precursor Scan: 001691, m/z: 1281.8202, charge: 3
ENGTVSR_7_6_1_2_0 LeX peak identified

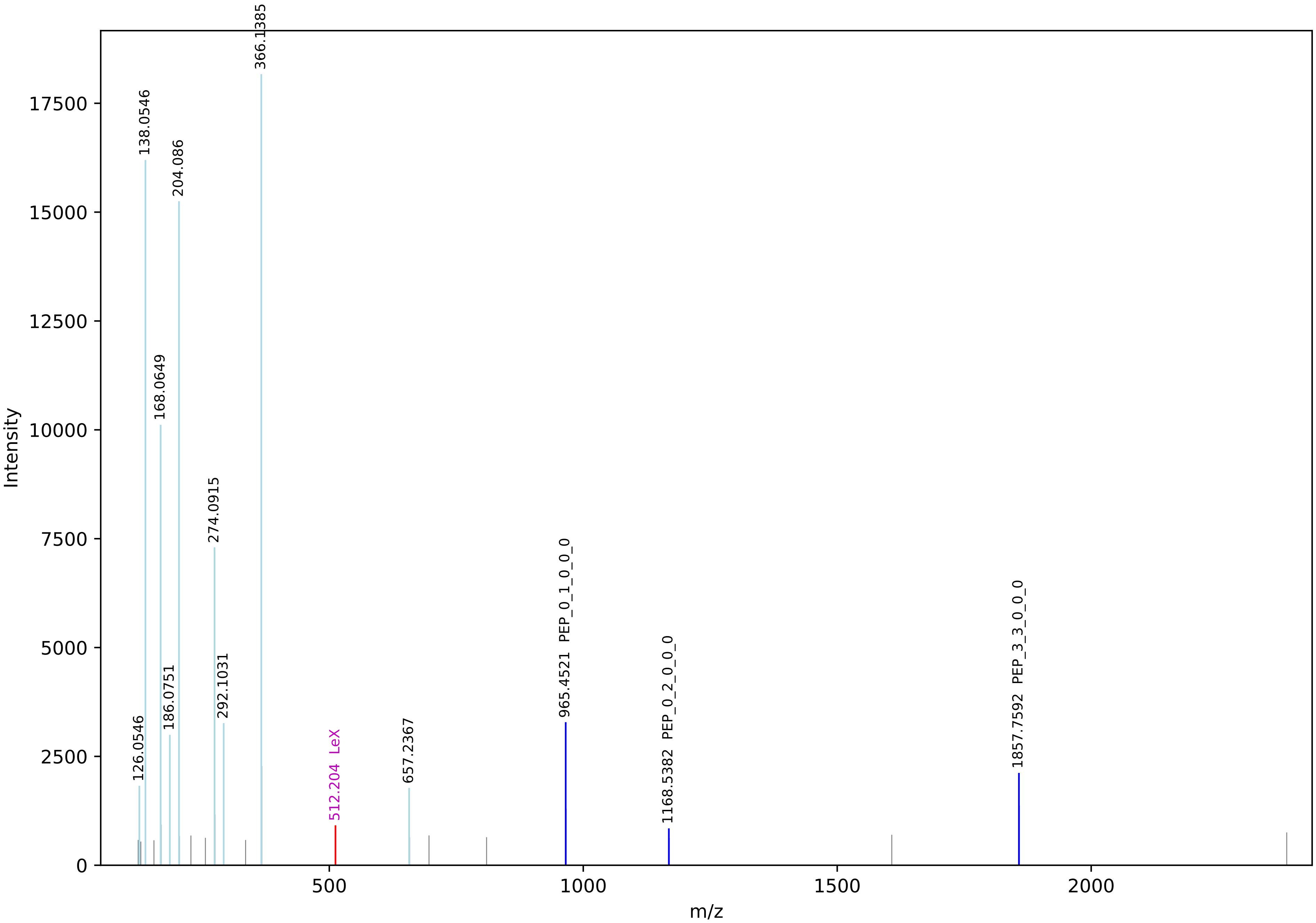

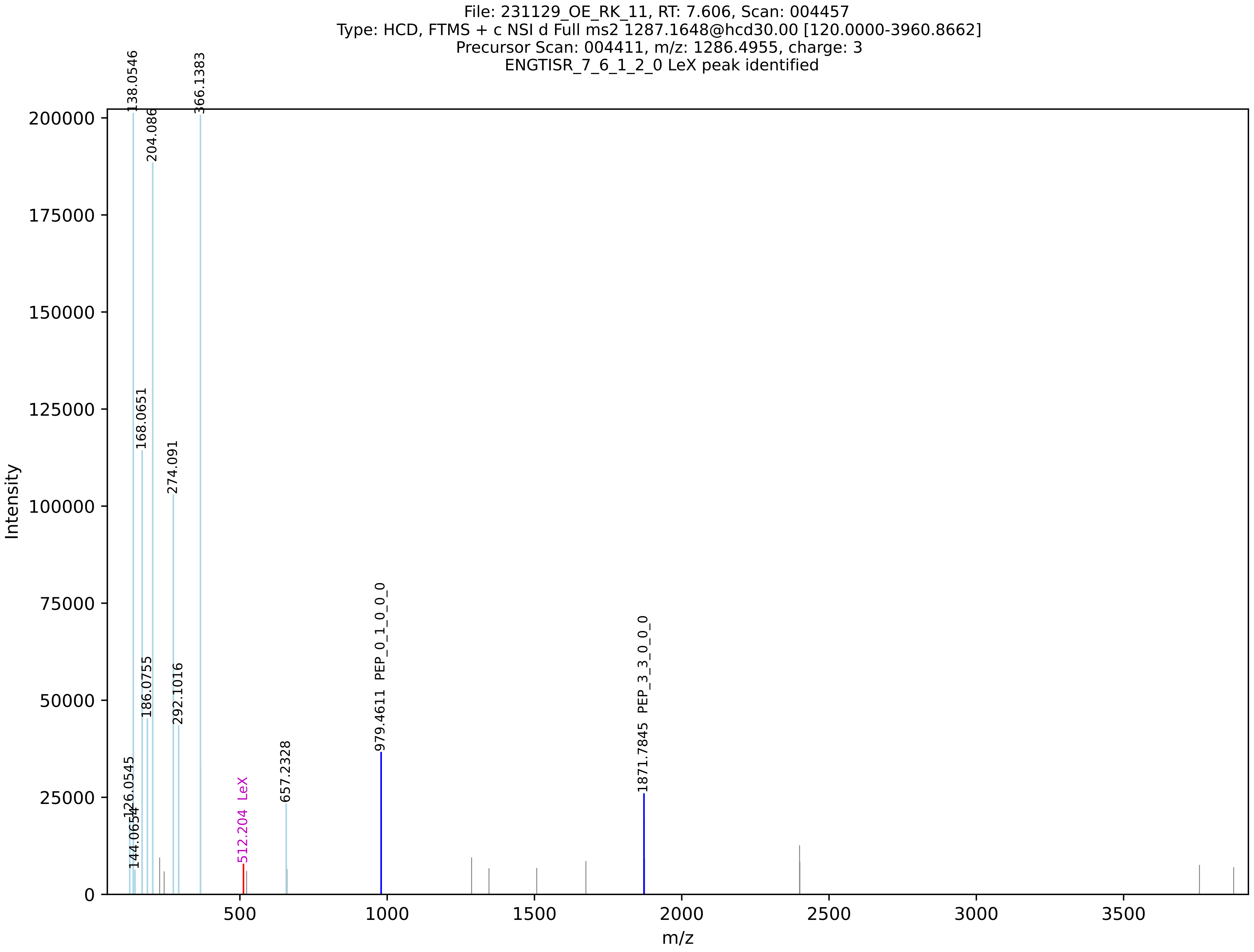

File: 231129_OE_RK_11, RT: 7.606, Scan: 004457
Type: HCD, FTMS + c NSI d Full ms2 1287.1648@hcd30.00 [120.0000-3960.8662]
Precursor Scan: 004411, m/z: 1286.4955, charge: 3
ENGTISR_7_6_1_2_0 LeX peak identified
Intensity
m/z
138.0546
366.1383
204.086
168.0651
274.091
186.0755
292.1016
126.0545
144.0654
512.204 LeX
657.2328
979.4611 PEP_0_1_0_0_0
1871.7845 PEP_3_3_0_0_0

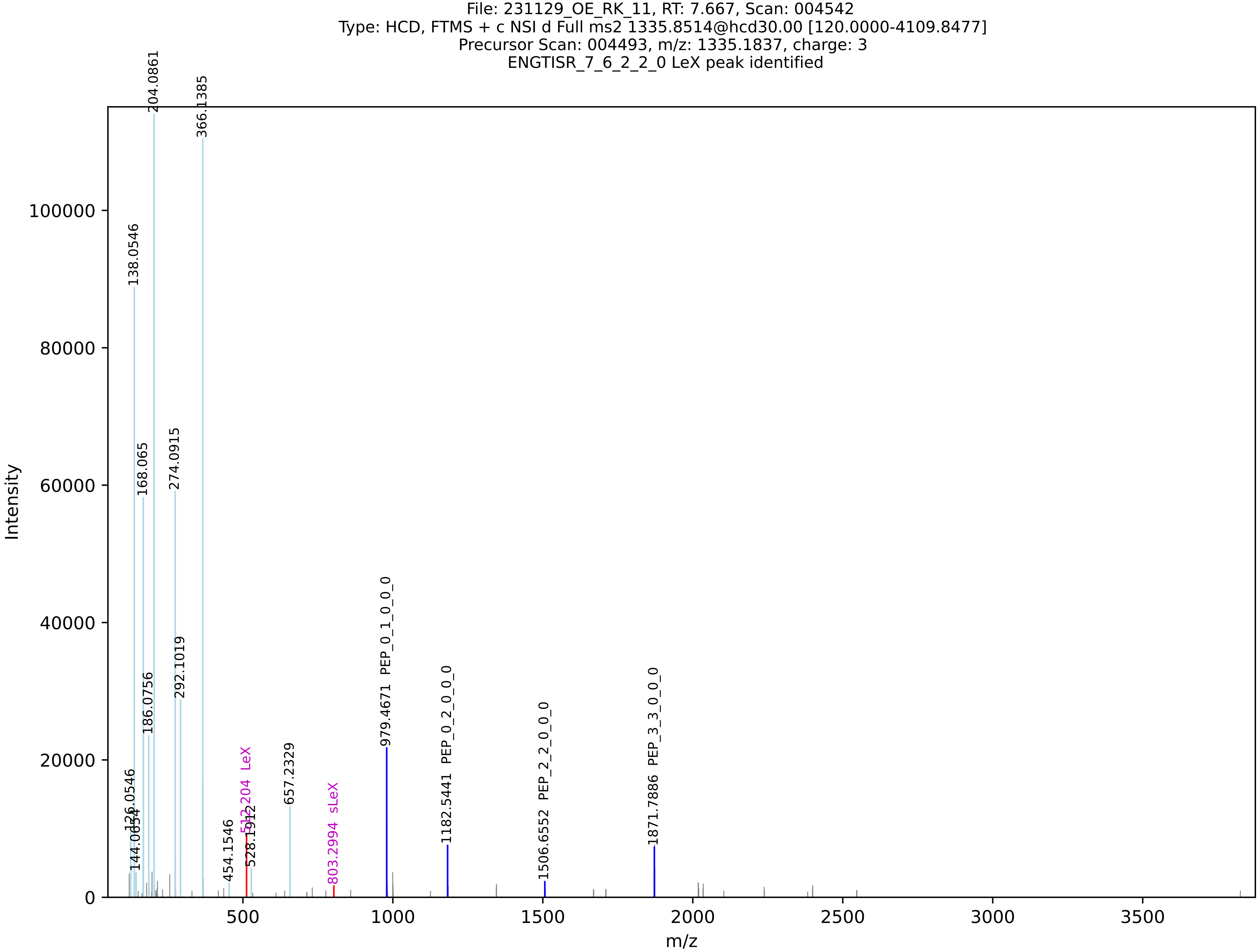
File: 231129_OE_RK_11, RT: 7.667, Scan: 004542
Type: HCD, FTMS + c NSI d Full ms2 1335.8514@hcd30.00 [120.0000-4109.8477]
Precursor Scan: 004493, m/z: 1335.1837, charge: 3
ENGTISR_7_6_2_2_0 LeX peak identified
Intensity
m/z
204.0861
366.1385
138.0546
168.065
274.0915
292.1019
186.0756
126.0546
144.0654
454.1546
512.204 LeX
528.1912
657.2329
803.2994 sLeX
979.4671 PEP_0_1_0_0_0
1182.5441 PEP_0_2_0_0_0
1506.6552 PEP_2_2_0_0_0
1871.7886 PEP_3_3_0_0_0

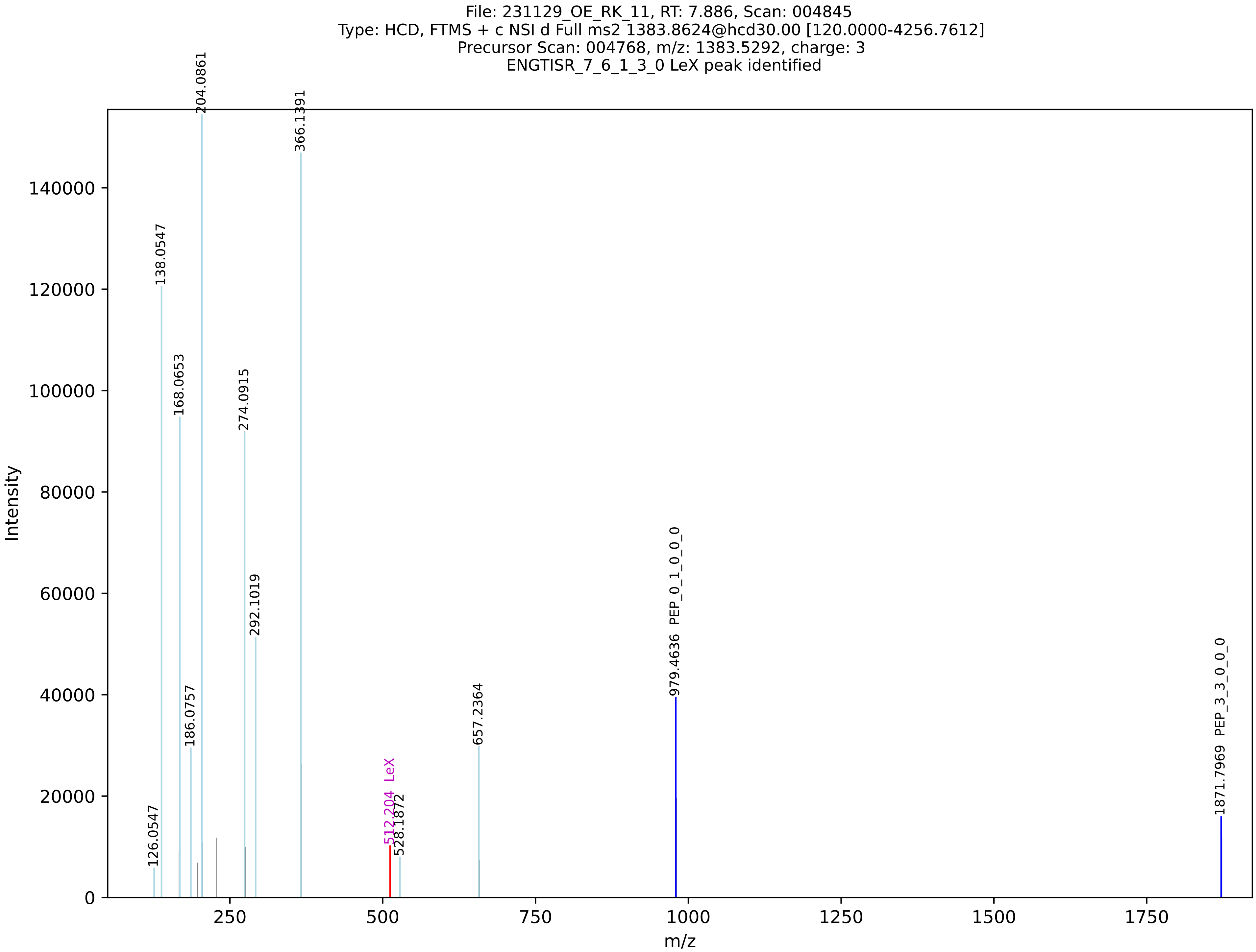
File: 231129_OE_RK_11, RT: 7.886, Scan: 004845
Type: HCD, FTMS + c NSI d Full ms2 1383.8624@hcd30.00 [120.0000-4256.7612]
Precursor Scan: 004768, m/z: 1383.5292, charge: 3
ENGTISR_7_6_1_3_0 LeX peak identified
Intensity
m/z
204.0861
366.1391
138.0547
168.0653
274.0915
292.1019
186.0757
126.0547
512.204 LeX
528.1872
657.2364
979.4636 PEP_0_1_0_0_0
1871.7969 PEP_3_3_0_0_0
0
20000
40000
60000
80000
100000
120000
140000
250
500
750
1000
1250
1500
1750

File: 231129_OE_RK_11, RT: 8.402, Scan: 005587
Type: HCD, FTMS + c NSI d Full ms2 1147.9379@hcd30.00 [120.0000-4706.4429]
Precursor Scan: 005506, m/z: 1147.1864, charge: 4
TPNASCMRK_9_8_1_1_0 LeX peak identified

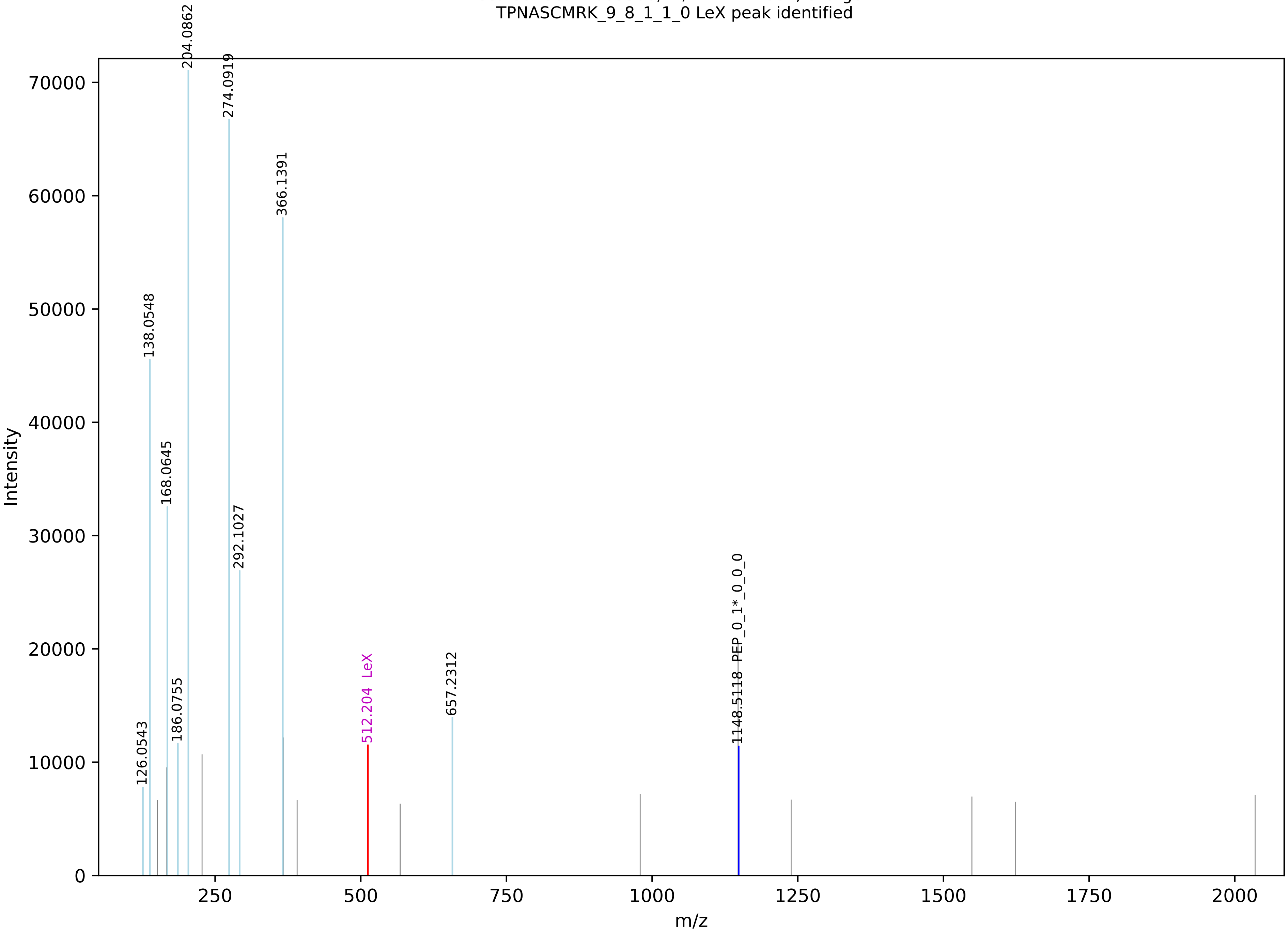

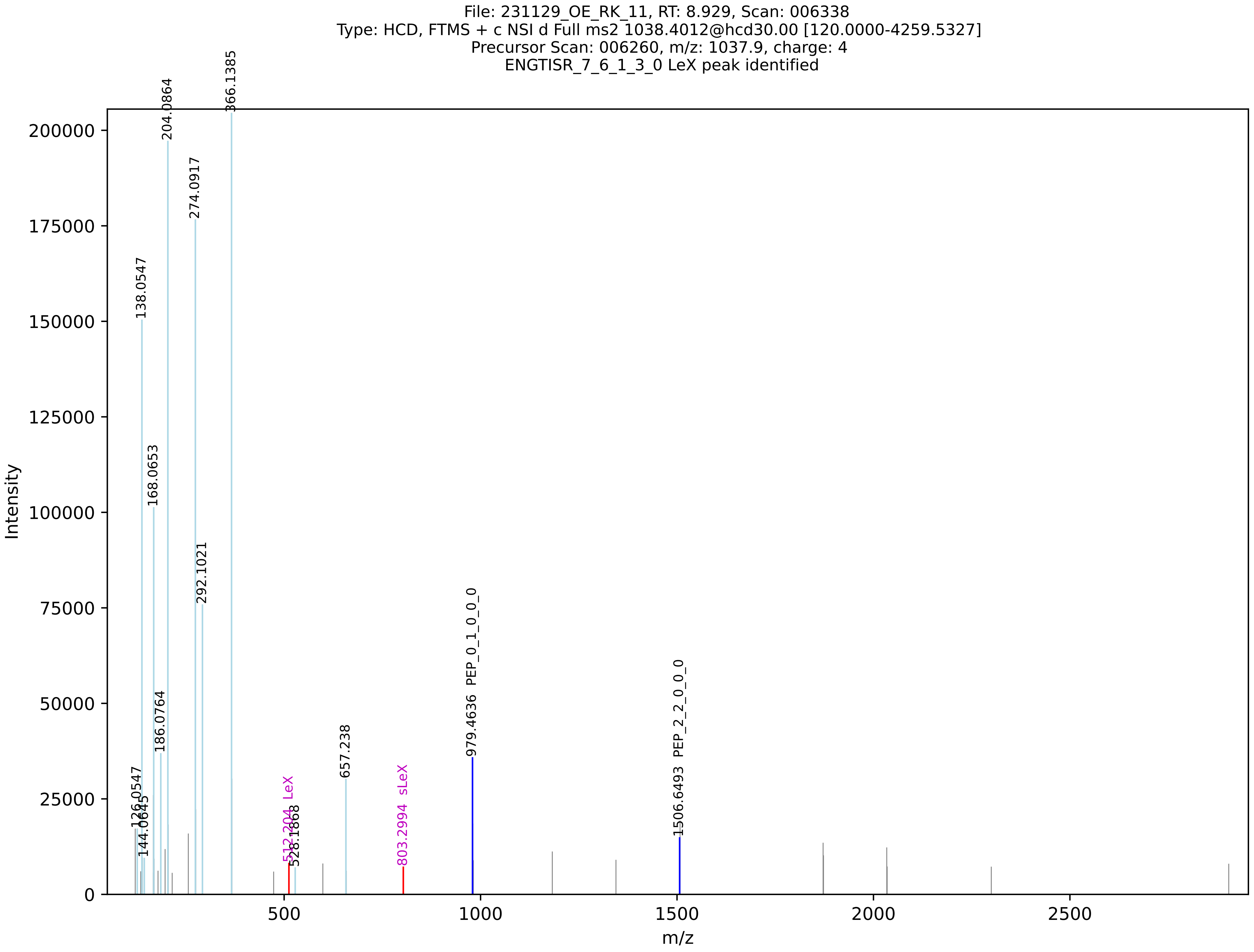

File: 231129_OE_RK_11, RT: 8.929, Scan: 006338
Type: HCD, FTMS + c NSI d Full ms2 1038.4012@hcd30.00 [120.0000-4259.5327]
Precursor Scan: 006260, m/z: 1037.9, charge: 4
ENGTISR_7_6_1_3_0 LeX peak identified
Intensity
m/z
366.1385
204.0864
274.0917
138.0547
168.0653
292.1021
186.0764
126.0547
144.0645
512.204 LeX
528.1868
657.238
803.2994 sLeX
979.4636 PEP_0_1_0_0_0_
1506.6493 PEP_2_2_0_0_0_

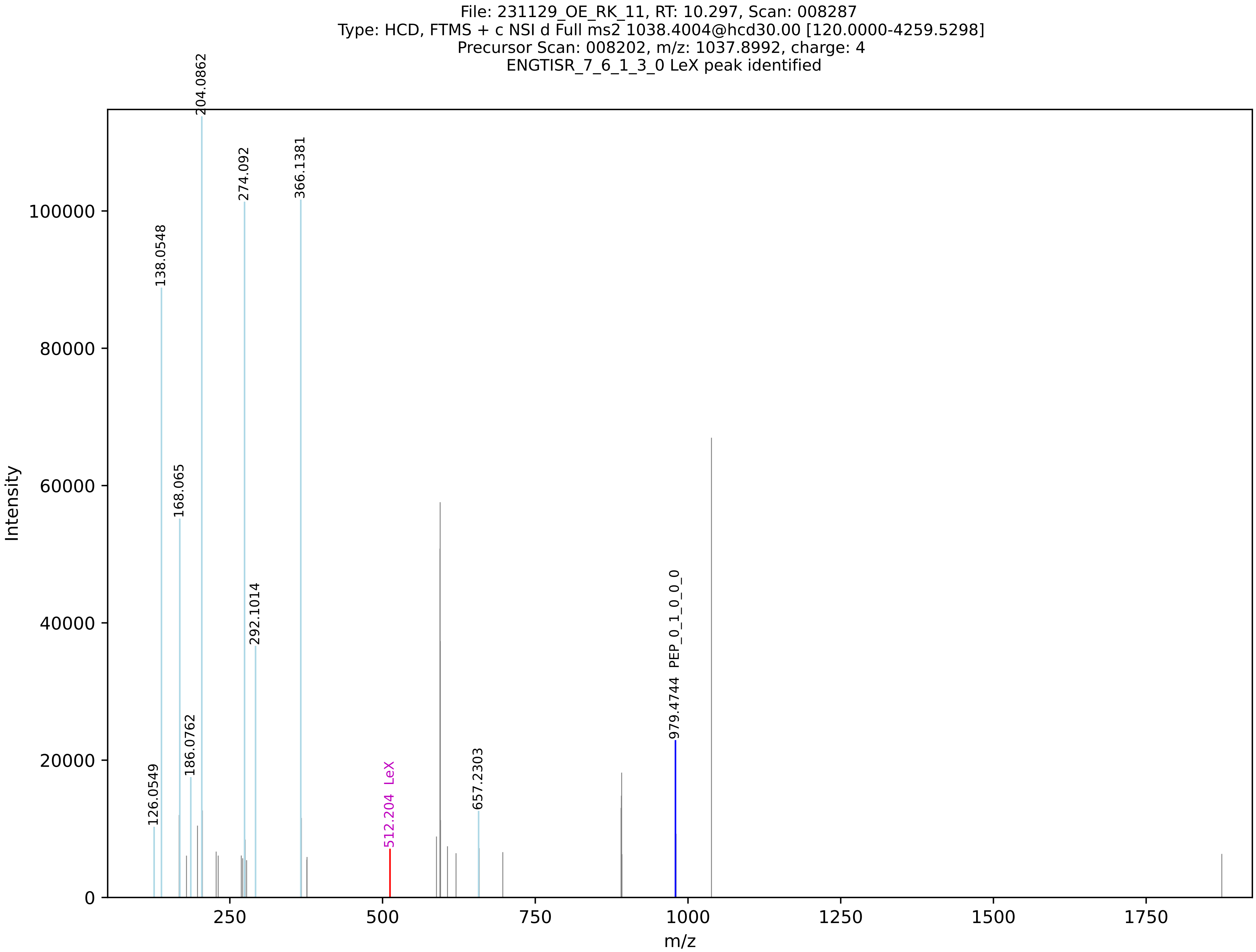
File: 231129_OE_RK_11, RT: 10.297, Scan: 008287
Type: HCD, FTMS + c NSI d Full ms2 1038.4004@hcd30.00 [120.0000-4259.5298]
Precursor Scan: 008202, m/z: 1037.8992, charge: 4
ENGTISR_7_6_1_3_0 LeX peak identified
204.0862
274.092
366.1381
138.0548
168.065
292.1014
186.0762
126.0549
512.204 LeX
657.2303
979.4744 PEP_0_1_0_0_0
Intensity
m/z
0
20000
40000
60000
80000
100000
250
500
750
1000
1250
1500
1750

File: 231129_OE_RK_11, RT: 10.931, Scan: 009197
Type: HCD, FTMS + c NSI d Full ms2 1383.8625@hcd30.00 [120.0000-4256.7612]
Precursor Scan: 009127, m/z: 1383.1943, charge: 3
ENGTISR_7_6_1_3_0 LeX peak identified

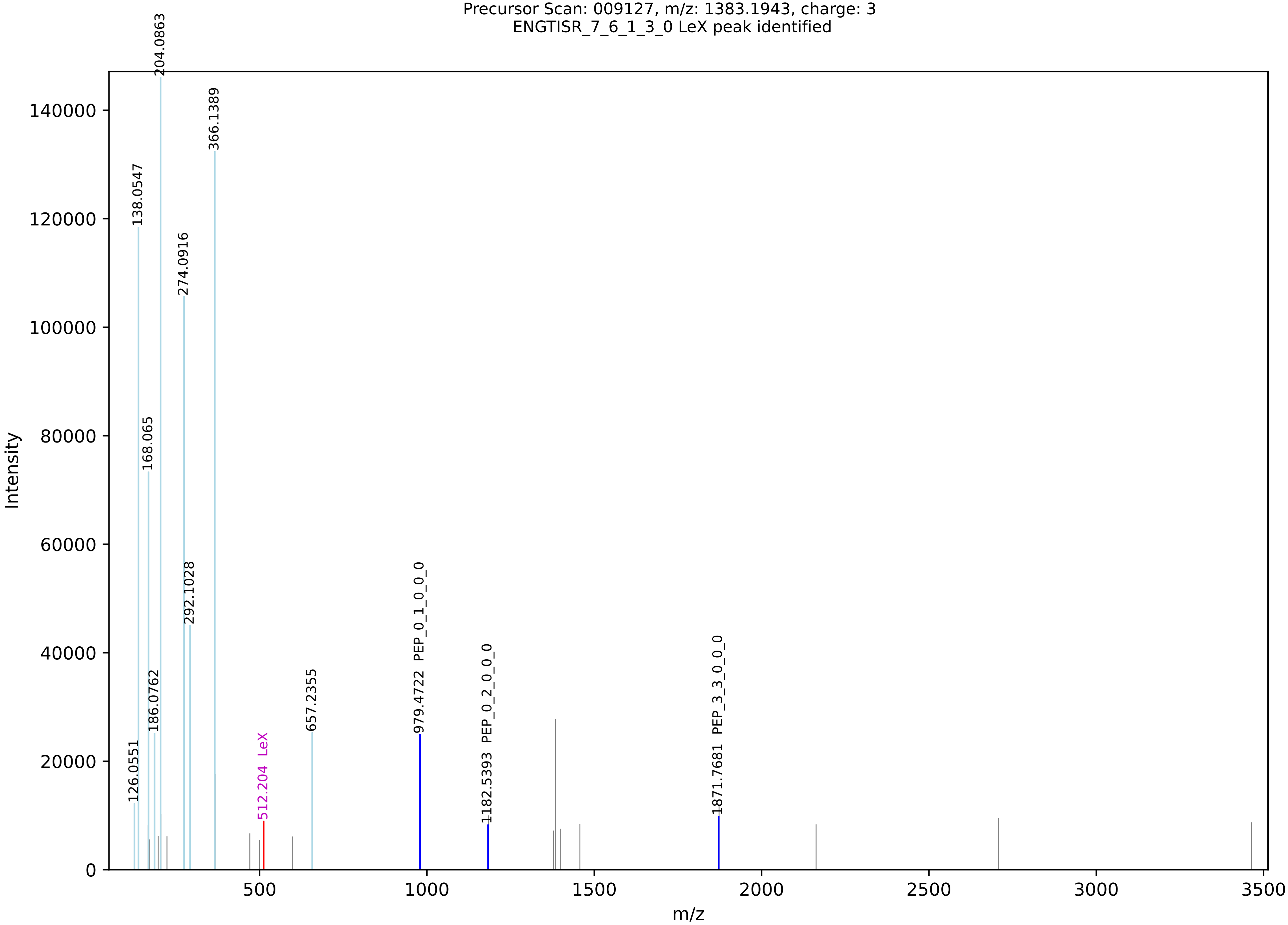

File: 231129_OE_RK_11, RT: 18.984, Scan: 020700
Type: HCD, FTMS + c NSI d Full ms2 1120.1029@hcd30.00 [120.0000-3449.6570]
Precursor Scan: 020630, m/z: 1119.4347, charge: 3
CINQSICEK_5_4_0_2_0 LeX peak identified

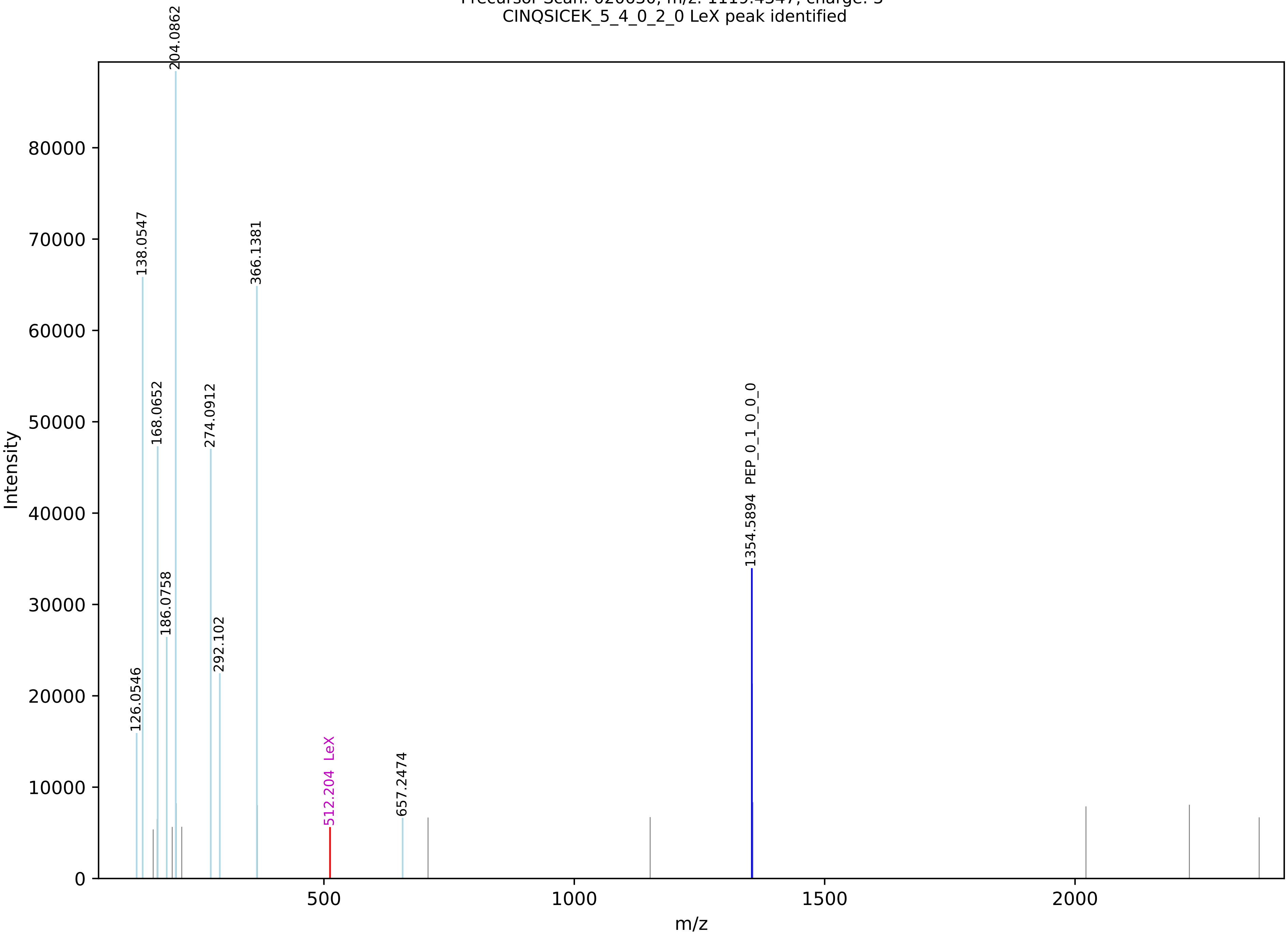

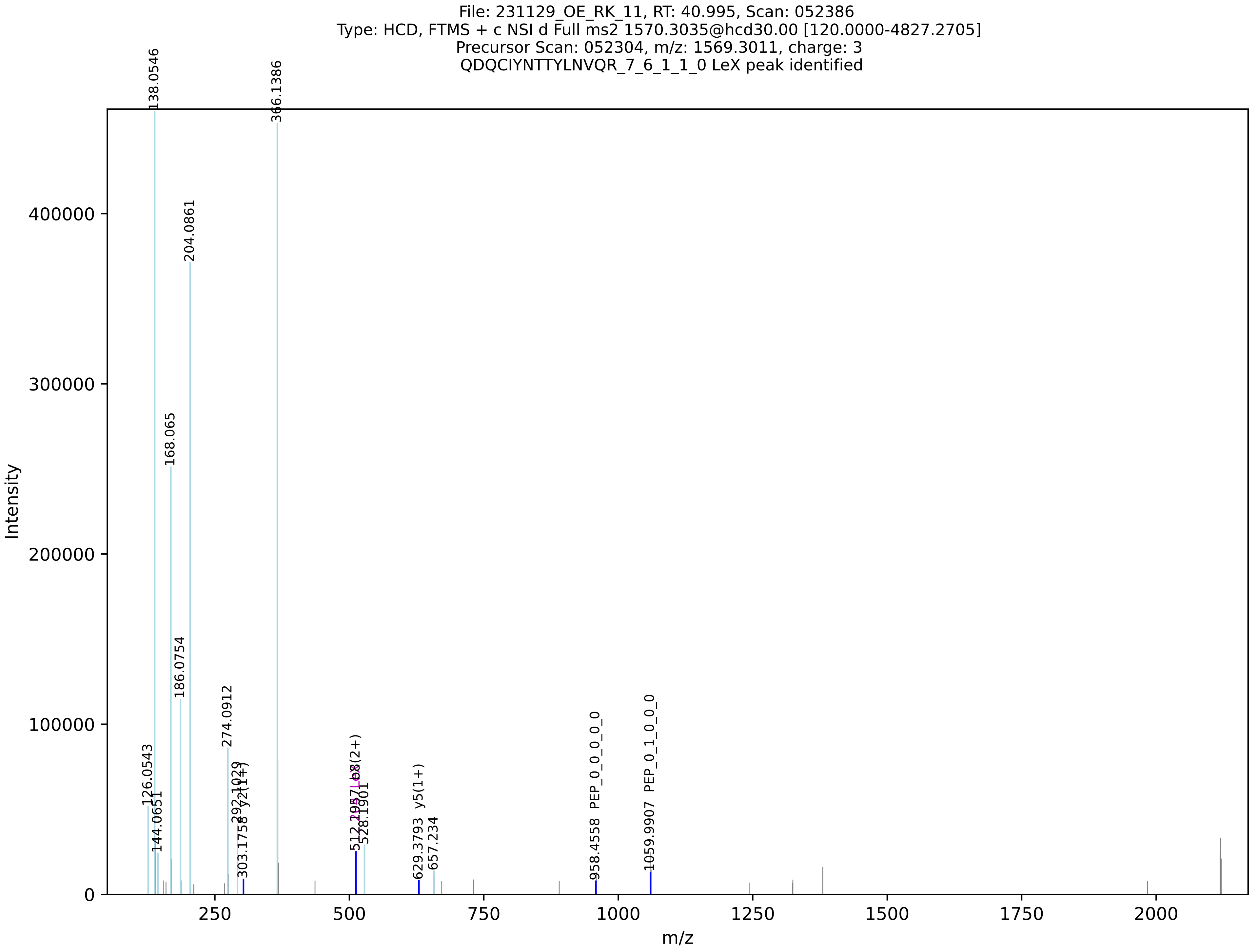
File: 231129_OE_RK_11, RT: 40.995, Scan: 052386
Type: HCD, FTMS + c NSI d Full ms2 1570.3035@hcd30.00 [120.0000-4827.2705]
Precursor Scan: 052304, m/z: 1569.3011, charge: 3
QDQCIYNTTYLNVQR_7_6_1_1_0 LeX peak identified
138.0546
366.1386
204.0861
168.065
186.0754
274.0912
126.0543
144.0651
292.1029
303.1758 y2(1+)
512.1957 b8(2+)
528.1901
629.3793 y5(1+)
657.234
958.4558 PEP_0_0_0_0_0_
1059.9907 PEP_0_1_0_0_0_
Intensity
m/z
0
100000
200000
300000
400000
250
500
750
1000
1250
1500
1750
2000

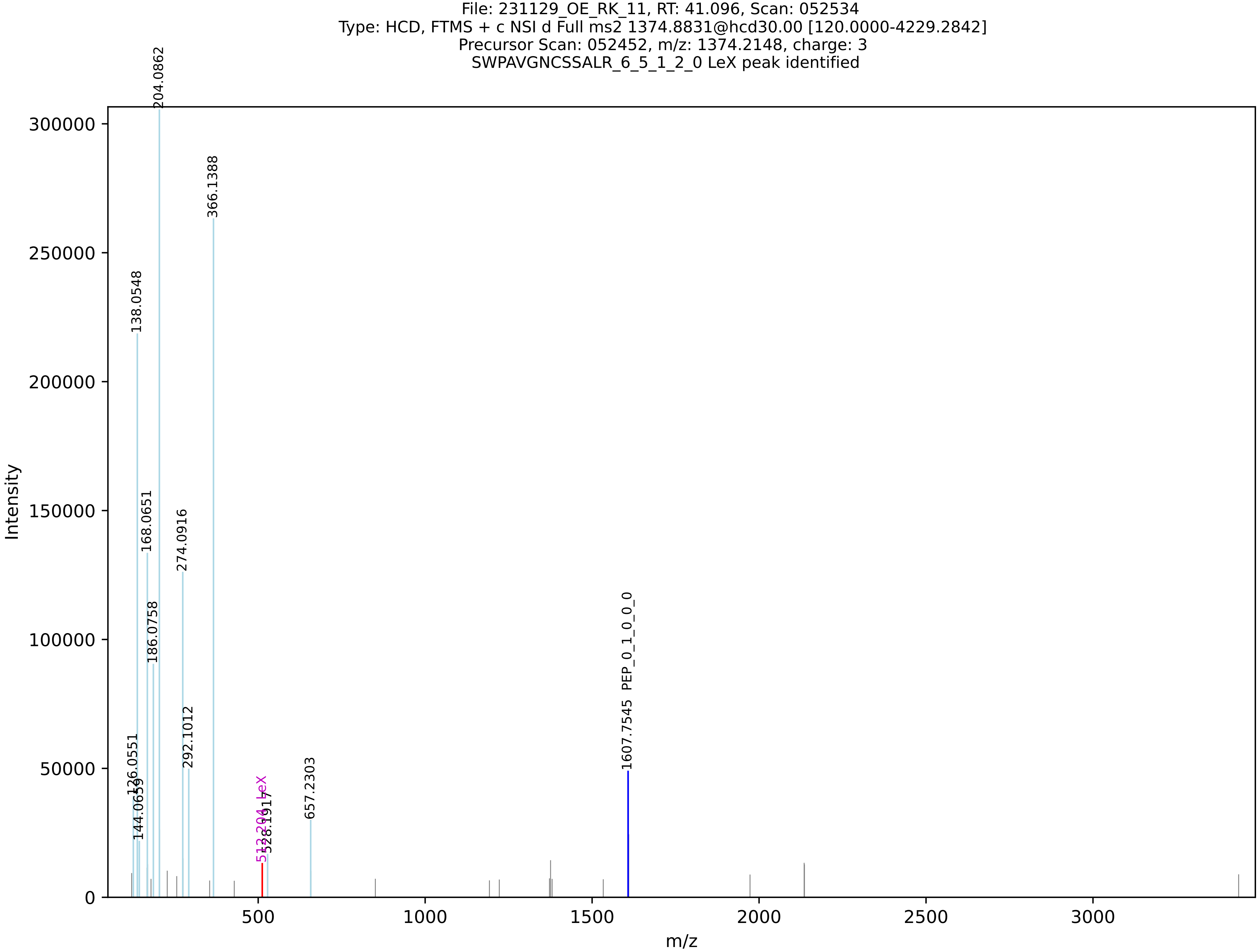
File: 231129_OE_RK_11, RT: 41.096, Scan: 052534
Type: HCD, FTMS + c NSI d Full ms2 1374.8831@hcd30.00 [120.0000-4229.2842]
Precursor Scan: 052452, m/z: 1374.2148, charge: 3
SWPAVGNCSSALR_6_5_1_2_0 LeX peak identified
Intensity
m/z
204.0862
366.1388
138.0548
168.0651
274.0916
186.0758
292.1012
126.0551
144.0659
512.204 LeX
528.1917
657.2303
1607.7545 PEP_0_1_0_0_0

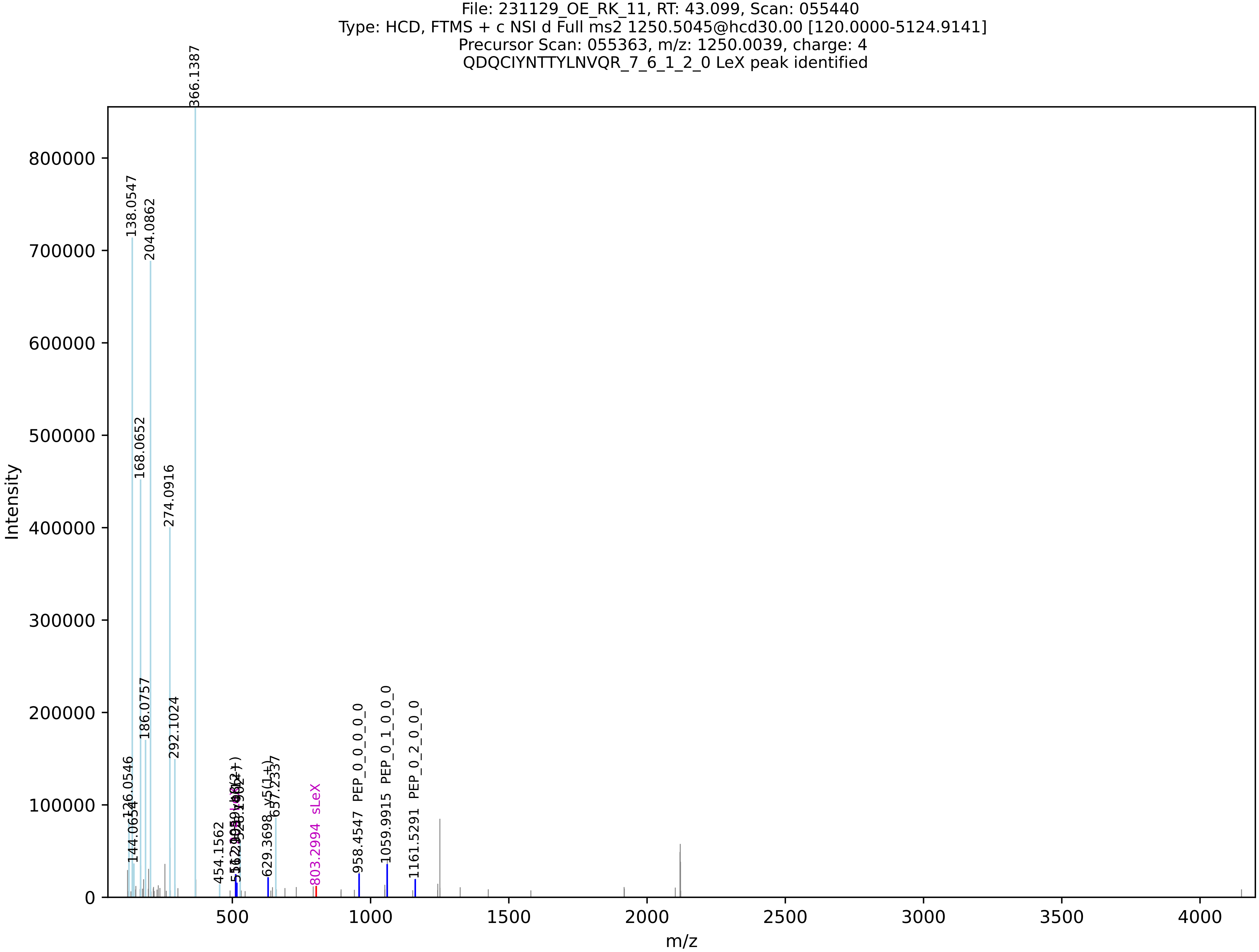

File: 231129_OE_RK_11, RT: 43.099, Scan: 055440
Type: HCD, FTMS + c NSI d Full ms2 1250.5045@hcd30.00 [120.0000-5124.9141]
Precursor Scan: 055363, m/z: 1250.0039, charge: 4
QDQCIYNTTYLNVQR_7_6_1_2_0 LeX peak identified
366.1387
138.0547
204.0862
168.0652
274.0916
186.0757
292.1024
126.0546
144.0654
454.1562
629.3698 y5(1+)
657.2337
803.2994 sLeX
958.4547 PEP_0_0_0_0_0
1059.9915 PEP_0_1_0_0_0
1161.5291 PEP_0_2_0_0_0
Intensity
m/z
0
100000
200000
300000
400000
500000
600000
700000
800000
500
1000
1500
2000
2500
3000
3500
4000

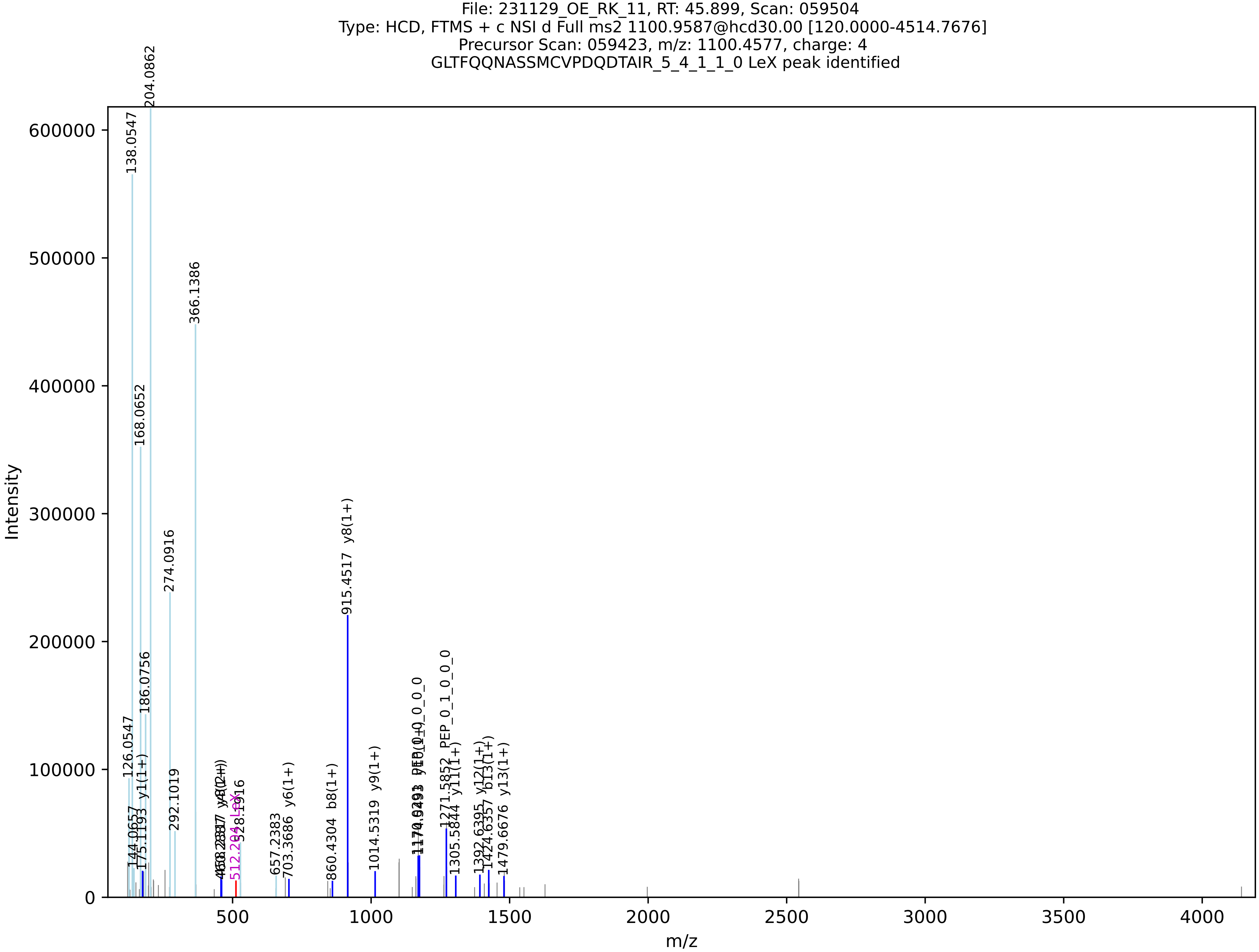
File: 231129_OE_RK_11, RT: 45.899, Scan: 059504
Type: HCD, FTMS + c NSI d Full ms2 1100.9587@hcd30.00 [120.0000-4514.7676]
Precursor Scan: 059423, m/z: 1100.4577, charge: 4
GLTFQQNASSMCVPDQDTAIR_5_4_1_1_0 LeX peak identified
Intensity
m/z
204.0862
138.0547
366.1386
168.0652
274.0916
186.0756
126.0547
144.0657
175.1193 y1(1+)
292.1019
512.264 LeX
528.1916
657.2383
703.3686 y6(1+)
860.4304 b8(1+)
915.4517 y8(1+)
1014.5319 y9(1+)
1271.5852 PEP_0_1_0_0_0_
1305.5844 y11(1+)
1392.6395 y12(1+)
1424.6357 b13(1+)
1479.6676 y13(1+)

File: 231129_OE_RK_11, RT: 47.648, Scan: 062045
Type: HCD, FTMS + c NSI d Full ms2 1152.0895@hcd30.00 [120.0000-5899.2266]
Precursor Scan: 061965, m/z: 1151.8894, charge: 5
CNIVTTEKTCILEILNSTK_7_6_2_3_0 LeX peak identified

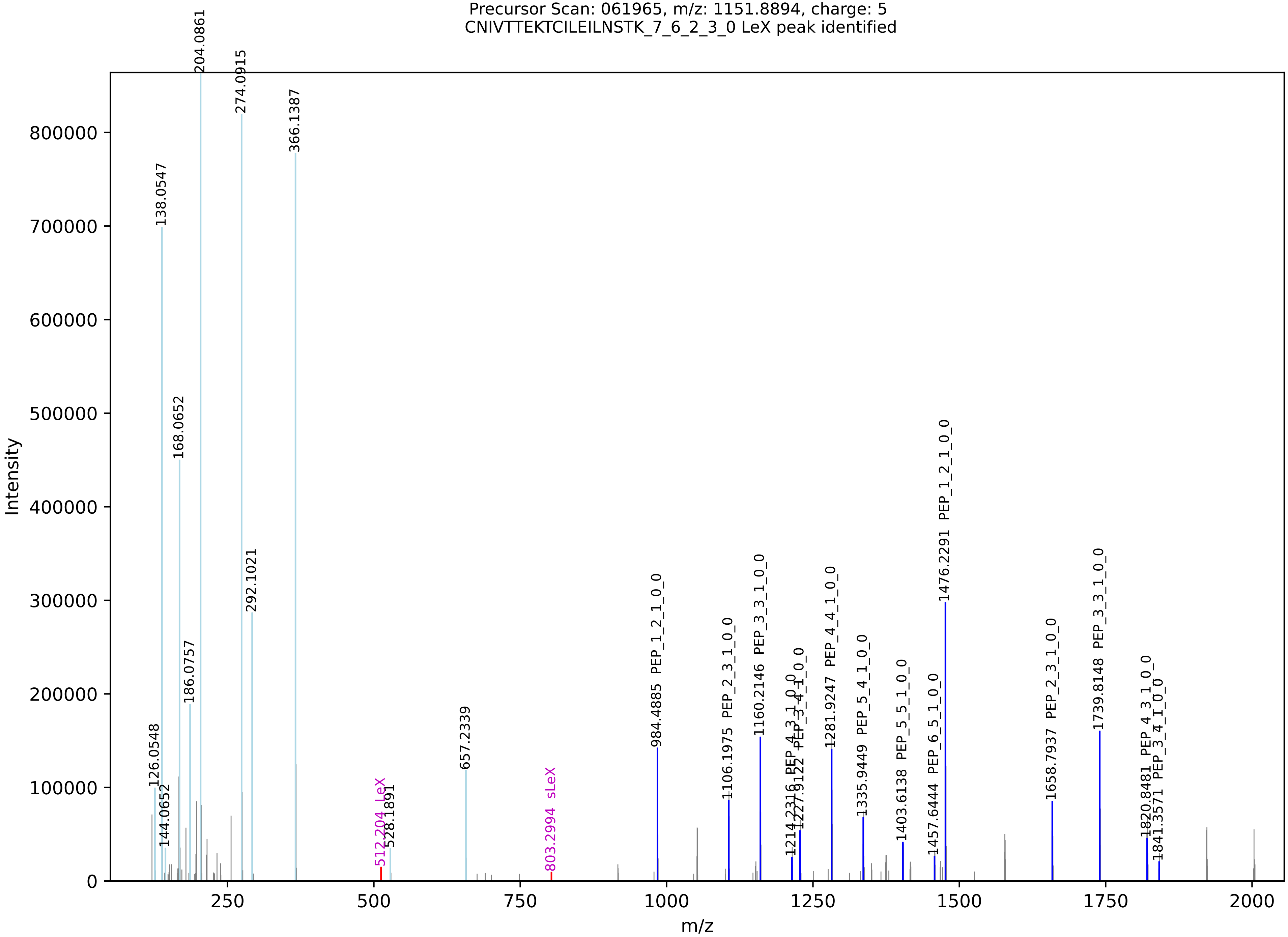

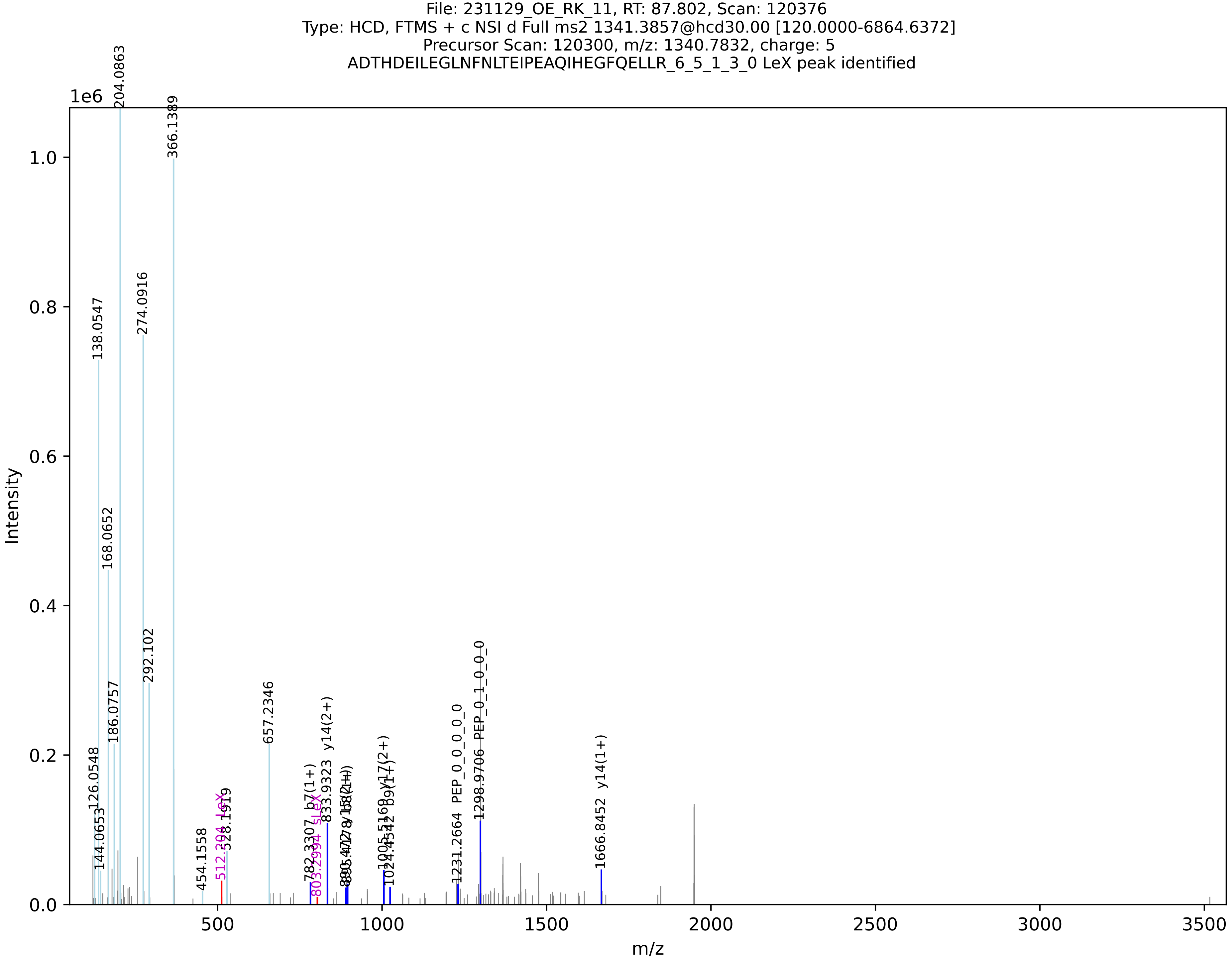
File: 231129_OE_RK_11, RT: 87.802, Scan: 120376
Type: HCD, FTMS + c NSI d Full ms2 1341.3857@hcd30.00 [120.0000-6864.6372]
Precursor Scan: 120300, m/z: 1340.7832, charge: 5
ADTHDEILEGLNFNLTEIPEAQIHEGFQELLR_6_5_1_3_0 LeX peak identified
1e6
Intensity
m/z
204.0863
366.1389
138.0547
274.0916
168.0652
292.102
186.0757
126.0548
144.0653
454.1558
512.204 LeX
528.1919
657.2346
782.3307 b7(1+)
803.2994 sLeX
833.9323 y14(2+)
1005.5169 y17(2+)
1024.4542 b9(1+)
1231.2664 PEP_0_0_0_0_0
1298.9706 PEP_0_1_0_0_0
1666.8452 y14(1+)

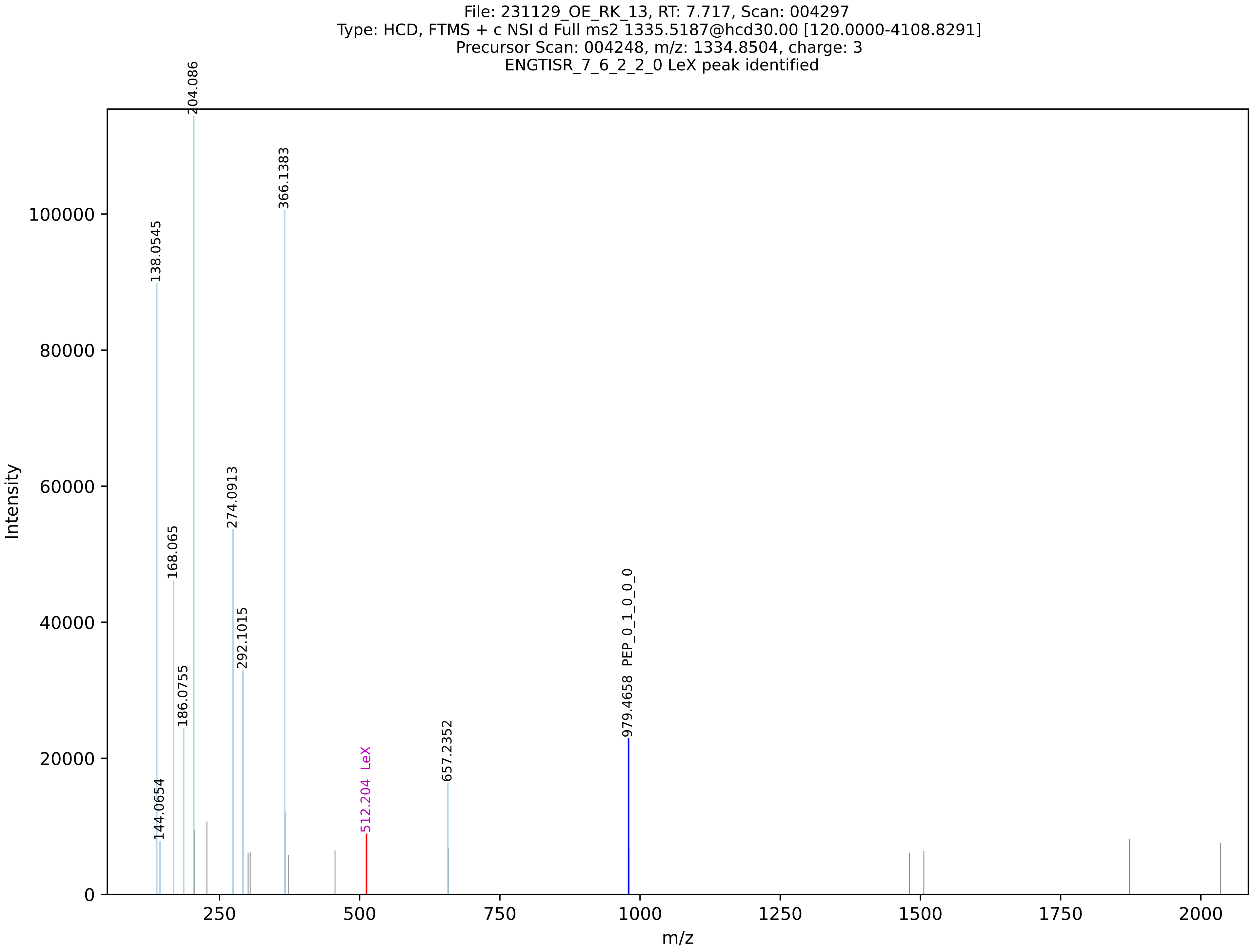
File: 231129_OE_RK_13, RT: 7.717, Scan: 004297
Type: HCD, FTMS + c NSI d Full ms2 1335.5187@hcd30.00 [120.0000-4108.8291]
Precursor Scan: 004248, m/z: 1334.8504, charge: 3
ENGTISR_7_6_2_2_0 LeX peak identified
Intensity
m/z
204.086
366.1383
138.0545
274.0913
168.065
292.1015
186.0755
144.0654
512.204 LeX
657.2352
979.4658 PEP_0_1_0_0_0
0
20000
40000
60000
80000
100000
250
500
750
1000
1250
1500
1750
2000

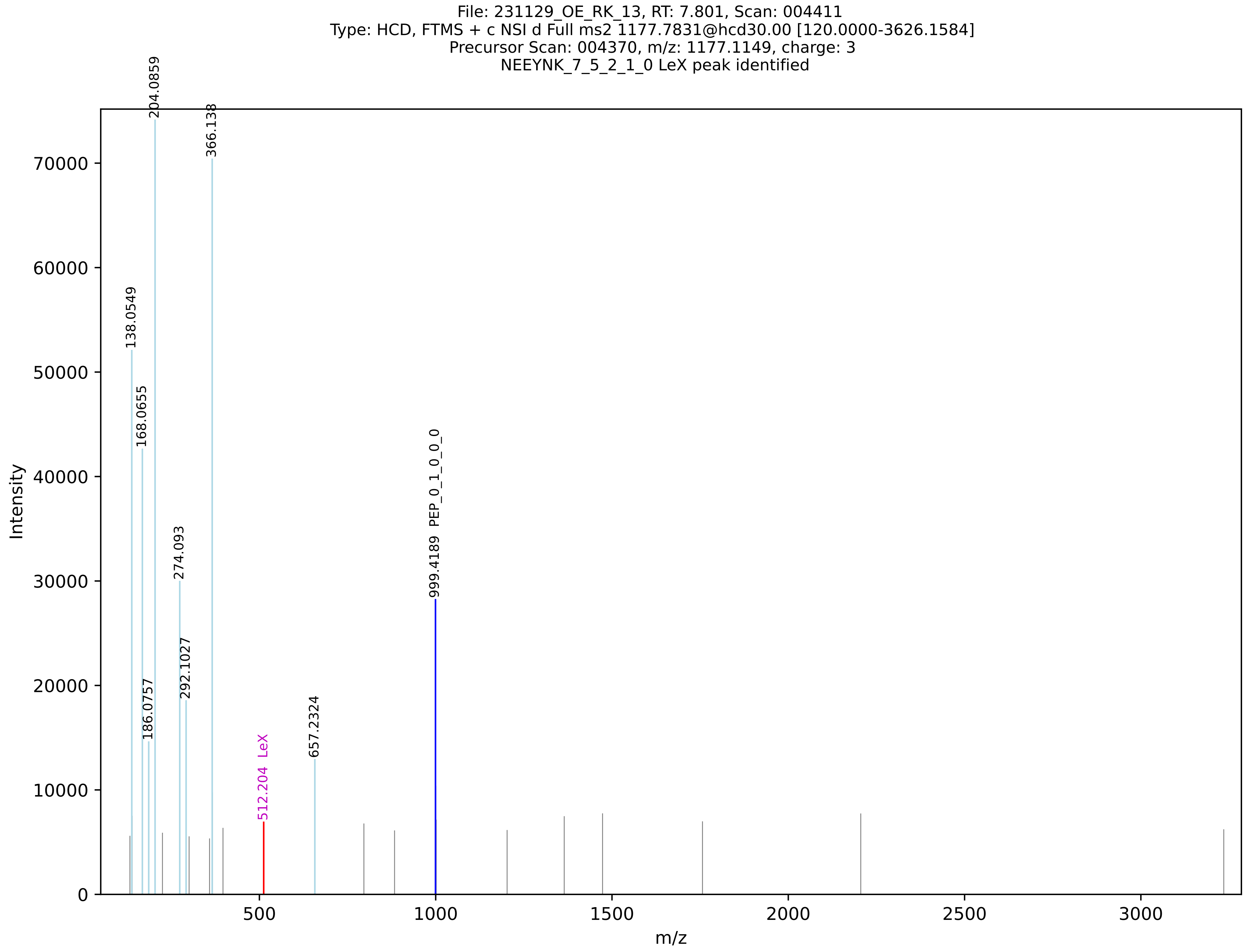

File: 231129_OE_RK_13, RT: 7.801, Scan: 004411
Type: HCD, FTMS + c NSI d Full ms2 1177.7831@hcd30.00 [120.0000-3626.1584]
Precursor Scan: 004370, m/z: 1177.1149, charge: 3
NEEYNK_7_5_2_1_0 LeX peak identified
204.0859
366.138
138.0549
168.0655
274.093
292.1027
186.0757
999.4189 PEP_0_1_0_0_0
657.2324
512.204 LeX
Intensity
m/z
0
10000
20000
30000
40000
50000
60000
70000
500
1000
1500
2000
2500
3000

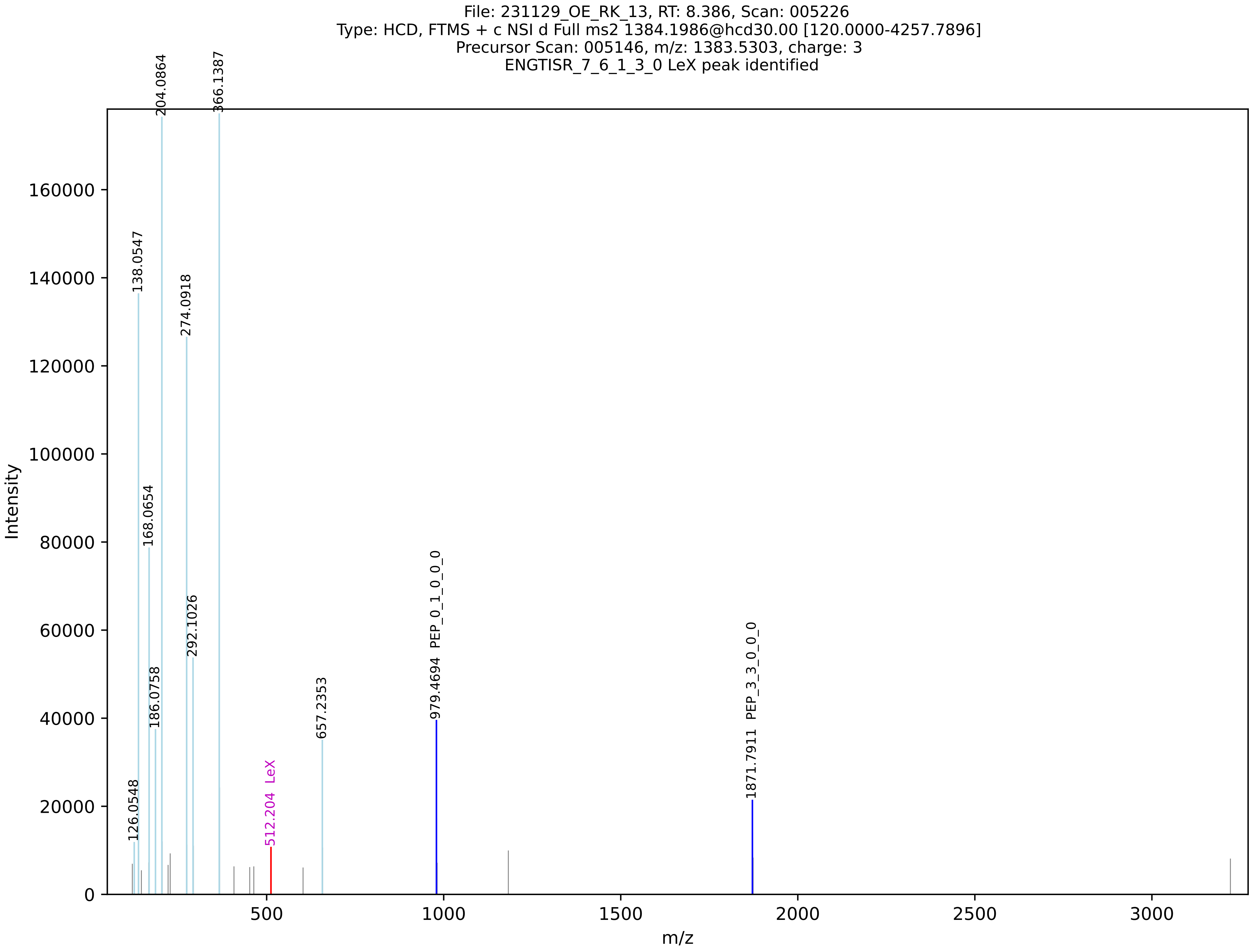
File: 231129_OE_RK_13, RT: 8.386, Scan: 005226
Type: HCD, FTMS + c NSI d Full ms2 1384.1986@hcd30.00 [120.0000-4257.7896]
Precursor Scan: 005146, m/z: 1383.5303, charge: 3
ENGTISR_7_6_1_3_0 LeX peak identified
Intensity
m/z
204.0864
366.1387
138.0547
274.0918
168.0654
292.1026
186.0758
126.0548
512.204 LeX
657.2353
979.4694 PEP_0_1_0_0_0
1871.7911 PEP_3_3_0_0_0

File: 231129_OE_RK_13, RT: 8.435, Scan: 005295
Type: HCD, FTMS + c NSI d Full ms2 1481.2313@hcd30.00 [120.0000-4554.7095]
Precursor Scan: 005219, m/z: 1480.5639, charge: 3
ENGTISR_7_6_1_4_0 LeX peak identified

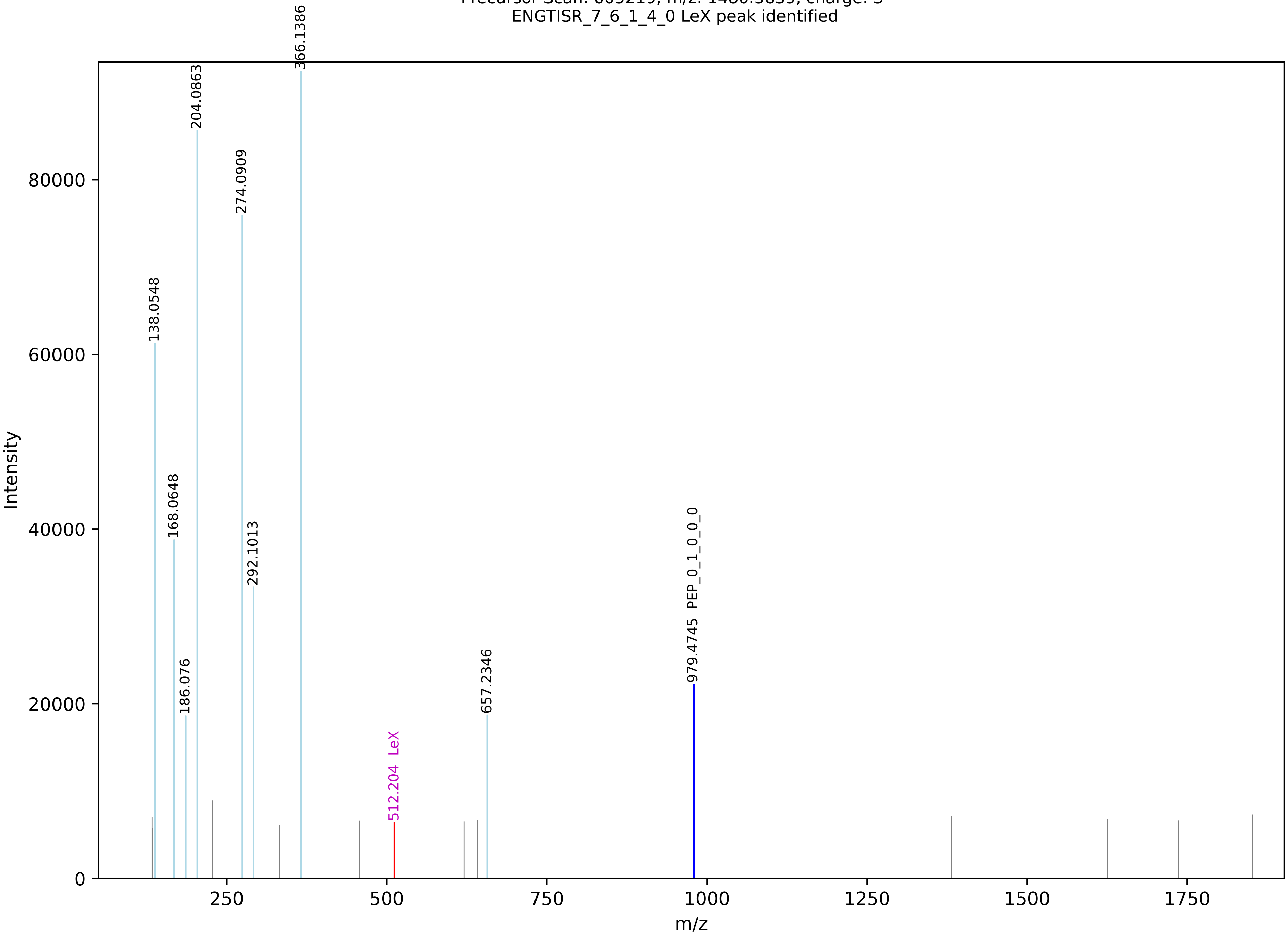

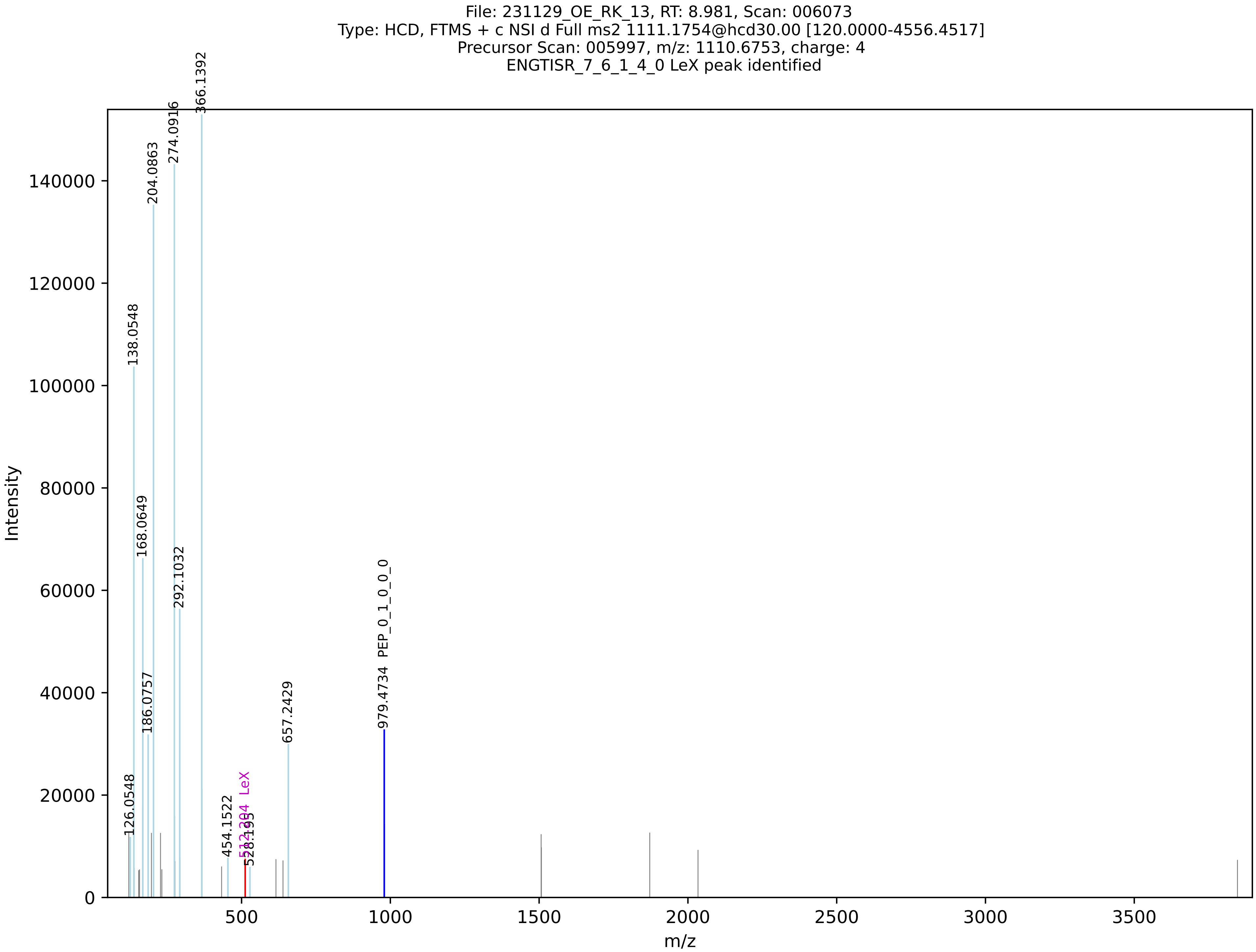
File: 231129_OE_RK_13, RT: 8.981, Scan: 006073
Type: HCD, FTMS + c NSI d Full ms2 1111.1754@hcd30.00 [120.0000-4556.4517]
Precursor Scan: 005997, m/z: 1110.6753, charge: 4
ENGTISR_7_6_1_4_0 LeX peak identified
366.1392
274.0916
204.0863
138.0548
168.0649
292.1032
186.0757
126.0548
454.1522
512.204 LeX
528.195
657.2429
979.4734 PEP_0_1_0_0_0
Intensity
m/z
0
20000
40000
60000
80000
100000
120000
140000
500
1000
1500
2000
2500
3000
3500

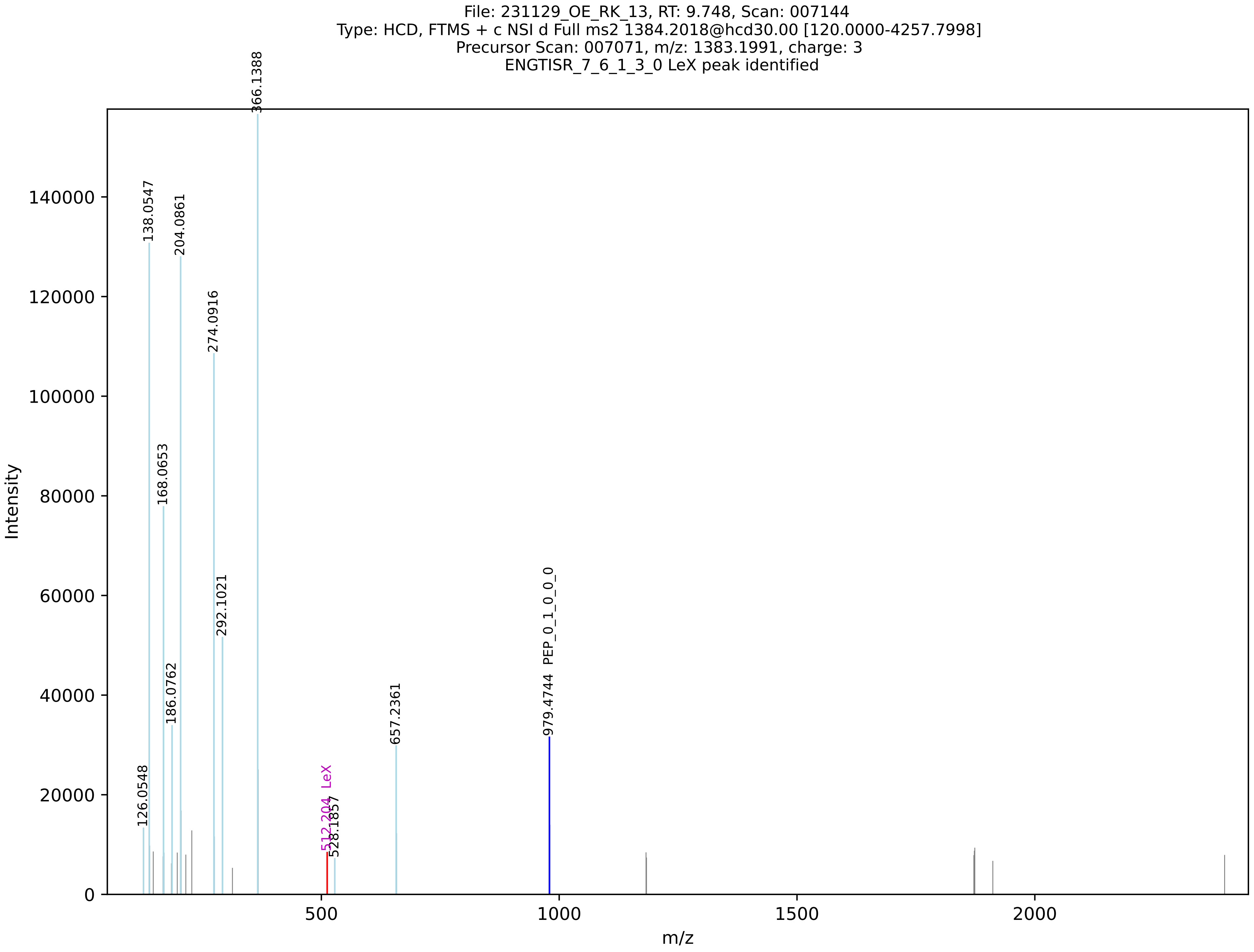
File: 231129_OE_RK_13, RT: 9.748, Scan: 007144
Type: HCD, FTMS + c NSI d Full ms2 1384.2018@hcd30.00 [120.0000-4257.7998]
Precursor Scan: 007071, m/z: 1383.1991, charge: 3
ENGTISR_7_6_1_3_0 LeX peak identified
Intensity
m/z
366.1388
138.0547
204.0861
274.0916
168.0653
292.1021
186.0762
126.0548
512.204 LeX
528.1857
657.2361
979.4744 PEP_0_1_0_0_0_
0
20000
40000
60000
80000
100000
120000
140000
500
1000
1500
2000

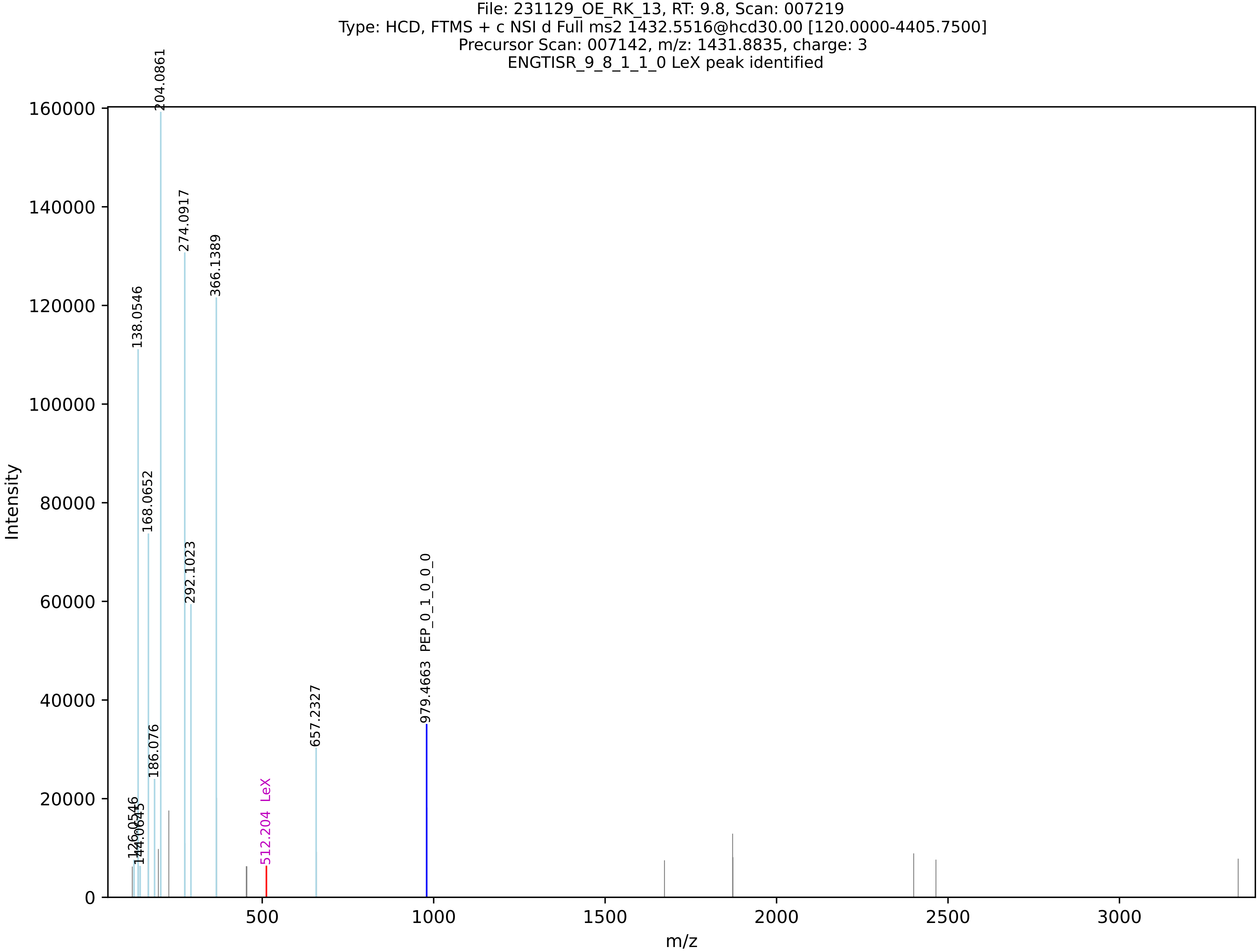

File: 231129_OE_RK_13, RT: 9.8, Scan: 007219
Type: HCD, FTMS + c NSI d Full ms2 1432.5516@hcd30.00 [120.0000-4405.7500]
Precursor Scan: 007142, m/z: 1431.8835, charge: 3
ENGTISR_9_8_1_1_0 LeX peak identified
Intensity
m/z
204.0861
274.0917
366.1389
138.0546
168.0652
292.1023
979.4663 PEP_0_1_0_0_0
657.2327
186.076
512.204 LeX
126.0546
144.0645

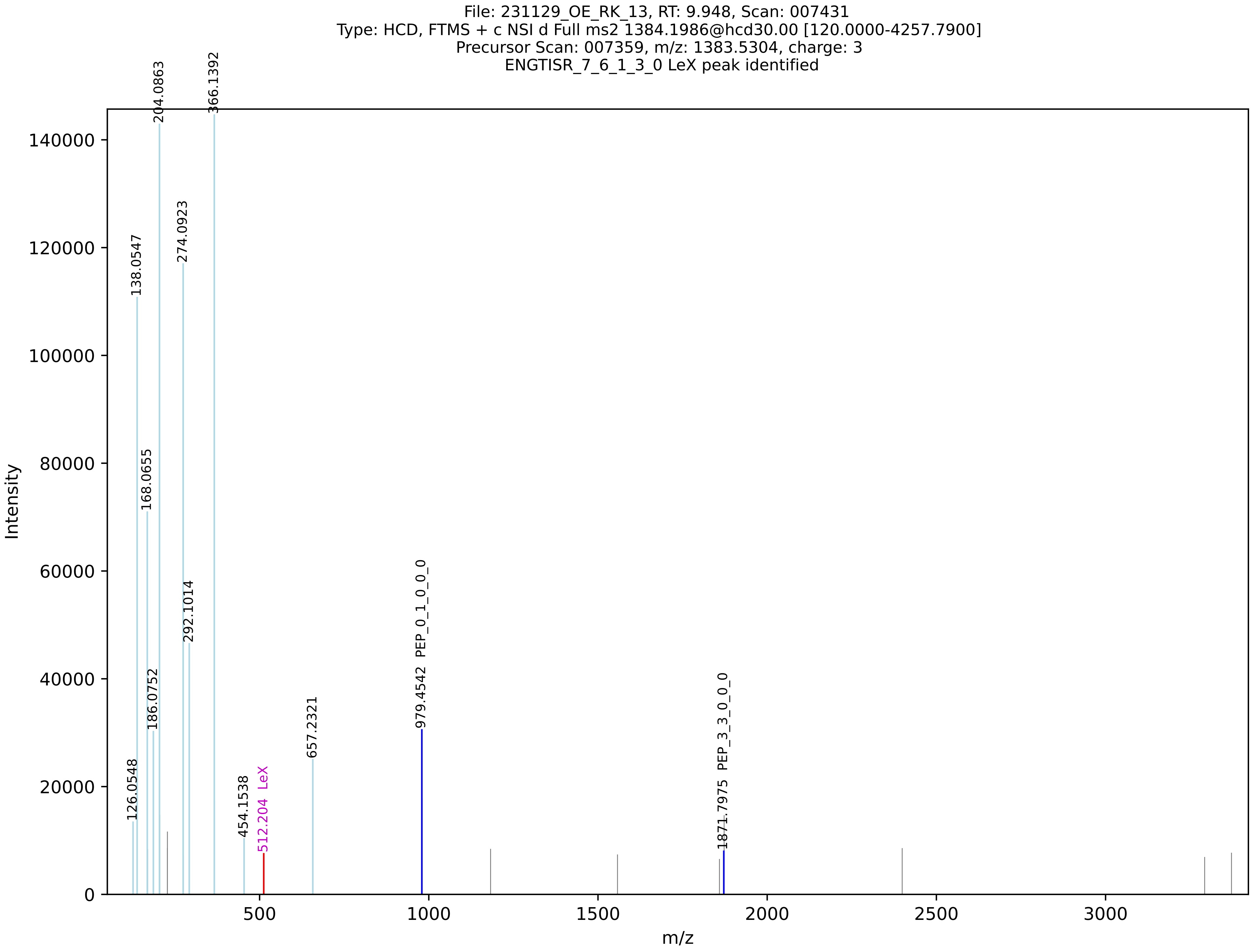

File: 231129_OE_RK_13, RT: 9.948, Scan: 007431
Type: HCD, FTMS + c NSI d Full ms2 1384.1986@hcd30.00 [120.0000-4257.7900]
Precursor Scan: 007359, m/z: 1383.5304, charge: 3
ENGTISR_7_6_1_3_0 LeX peak identified
Intensity
m/z
0
20000
40000
60000
80000
100000
120000
140000
500
1000
1500
2000
2500
3000
126.0548
138.0547
168.0655
186.0752
204.0863
274.0923
292.1014
366.1392
454.1538
512.204 LeX
657.2321
979.4542 PEP_0_1_0_0_0
1871.7975 PEP_3_3_0_0_0

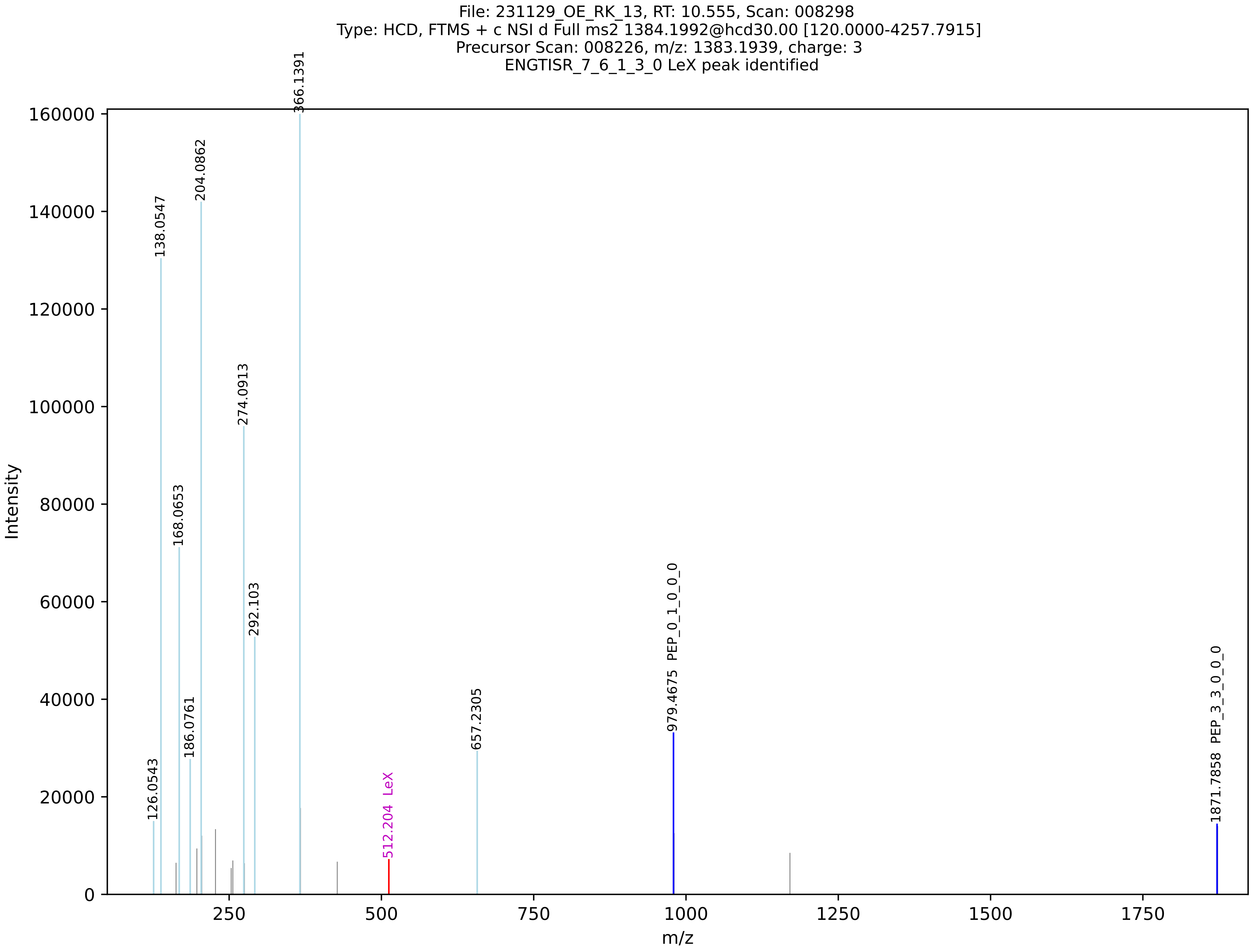

File: 231129_OE_RK_13, RT: 10.555, Scan: 008298
Type: HCD, FTMS + c NSI d Full ms2 1384.1992@hcd30.00 [120.0000-4257.7915]
Precursor Scan: 008226, m/z: 1383.1939, charge: 3
ENGTISR_7_6_1_3_0 LeX peak identified
Intensity
m/z
366.1391
204.0862
138.0547
274.0913
168.0653
292.103
979.4675 PEP_0_1_0_0_0
1871.7858 PEP_3_3_0_0_0
657.2305
186.0761
126.0543
512.204 LeX

File: 231129_OE_RK_13, RT: 11.823, Scan: 010106
Type: HCD, FTMS + c NSI d Full ms2 1111.1754@hcd30.00 [120.0000-4556.4517]
Precursor Scan: 010030, m/z: 1110.6749, charge: 4
ENGTISR_7_6_1_4_0 LeX peak identified

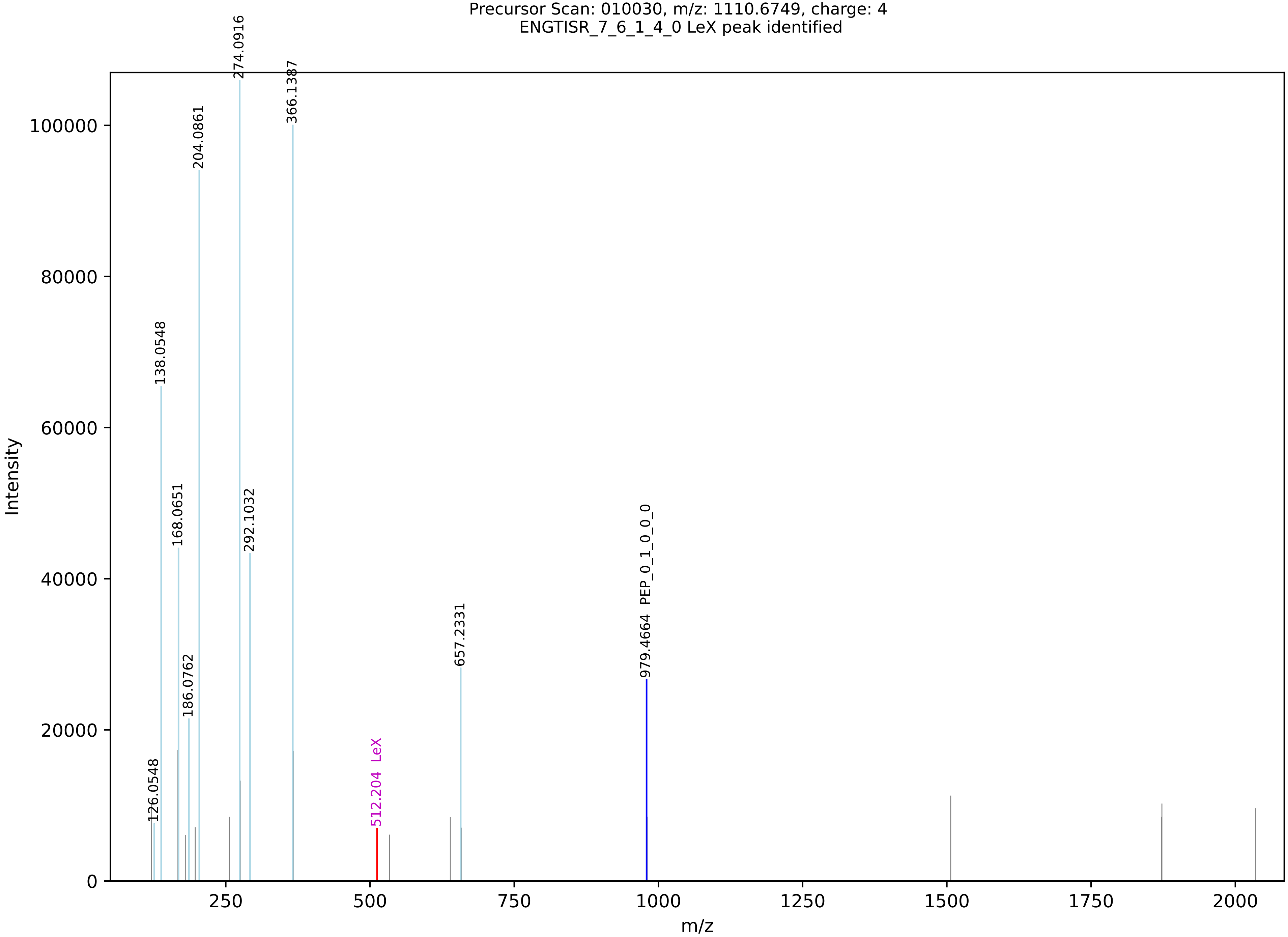

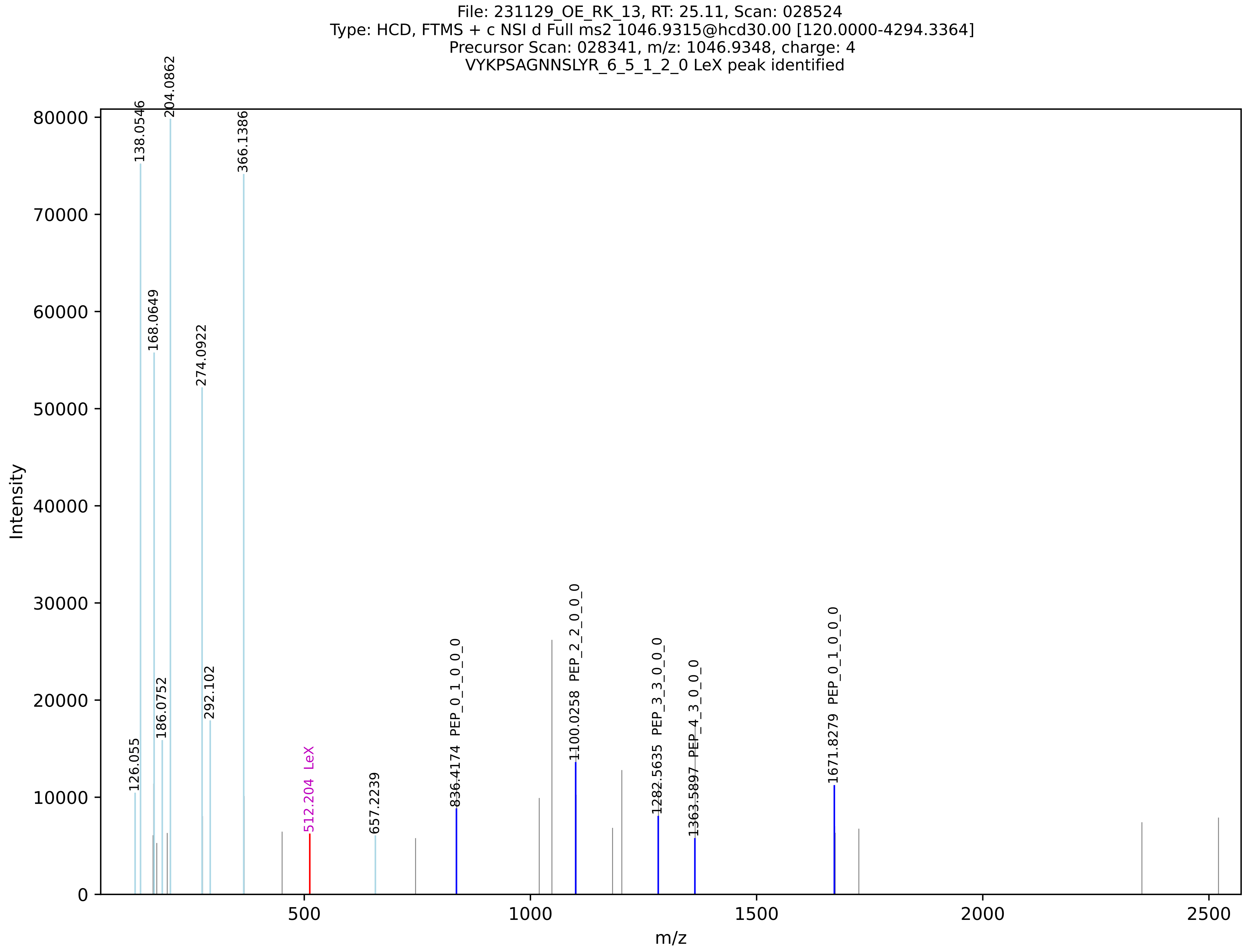

File: 231129_OE_RK_13, RT: 25.11, Scan: 028524
Type: HCD, FTMS + c NSI d Full ms2 1046.9315@hcd30.00 [120.0000-4294.3364]
Precursor Scan: 028341, m/z: 1046.9348, charge: 4
VYKPSAGNNSLYR_6_5_1_2_0 LeX peak identified
Intensity
m/z
0
10000
20000
30000
40000
50000
60000
70000
80000
500
1000
1500
2000
2500
126.055
138.0546
168.0649
186.0752
204.0862
274.0922
292.102
366.1386
512.204 LeX
657.2239
836.4174 PEP_0_1_0_0_0
1100.0258 PEP_2_2_0_0_0
1282.5635 PEP_3_3_0_0_0
1363.5897 PEP_4_3_0_0_0
1671.8279 PEP_0_1_0_0_0

File: 231129_OE_RK_13, RT: 41.288, Scan: 051857
Type: HCD, FTMS + c NSI d Full ms2 1177.7307@hcd30.00 [120.0000-4827.9976]
Precursor Scan: 051778, m/z: 1177.2288, charge: 4
QDQCIYNTTYLNVQR_7_6_1_1_0 LeX peak identified

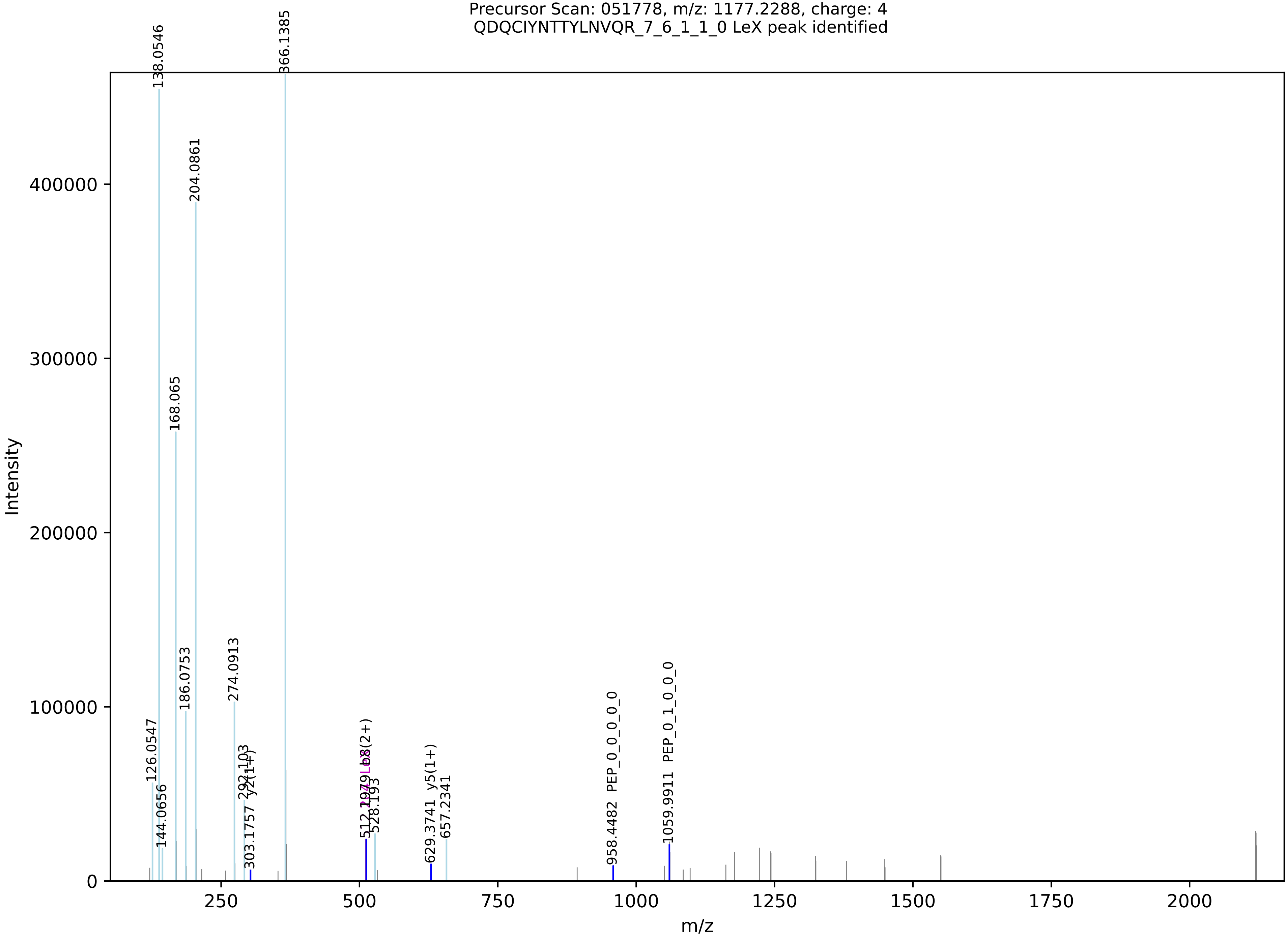

File: 231129_OE_RK_13, RT: 43.266, Scan: 054740
Type: HCD, FTMS + c NSI d Full ms2 1250.7554@hcd30.00 [120.0000-5125.9380]
Precursor Scan: 054660, m/z: 1250.0037, charge: 4
QDQCIYNTTYLNVQR_7_6_1_2_0 LeX peak identified

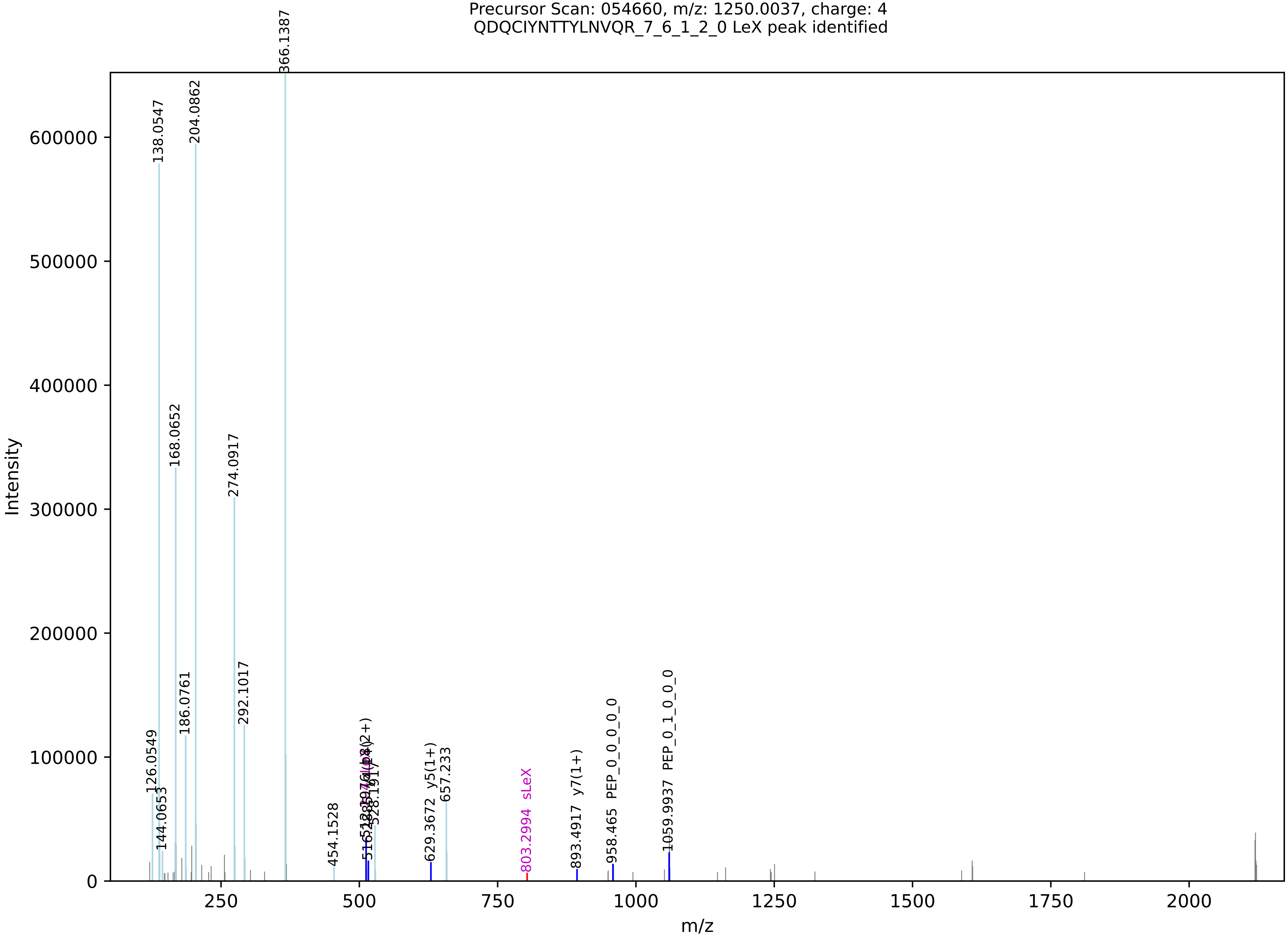

File: 231129_OE_RK_13, RT: 43.567, Scan: 055178
Type: HCD, FTMS + c NSI d Full ms2 1545.2937@hcd30.00 [120.0000-4750.7407]
Precursor Scan: 055103, m/z: 1544.6252, charge: 3
QDQCIYNTTYLNVQR_6_5_1_2_0 LeX peak identified

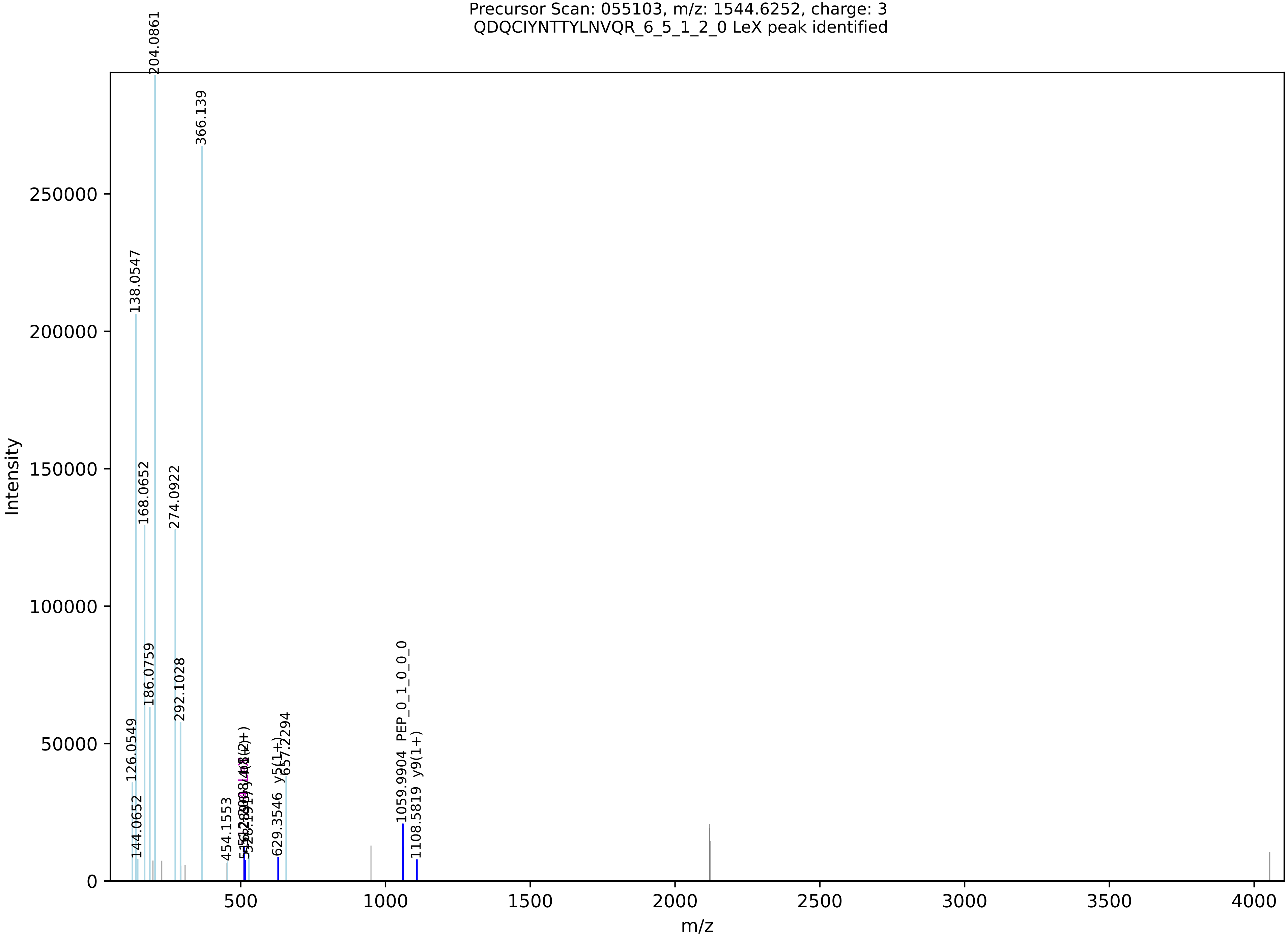

File: 231129_OE_RK_13, RT: 43.568, Scan: 055180
Type: HCD, FTMS + c NSI d Full ms2 1159.2218@hcd30.00 [120.0000-4752.4810]
Precursor Scan: 055103, m/z: 1158.7208, charge: 4
QDQCIYNTTYLNVQR_6_5_1_2_0 LeX peak identified

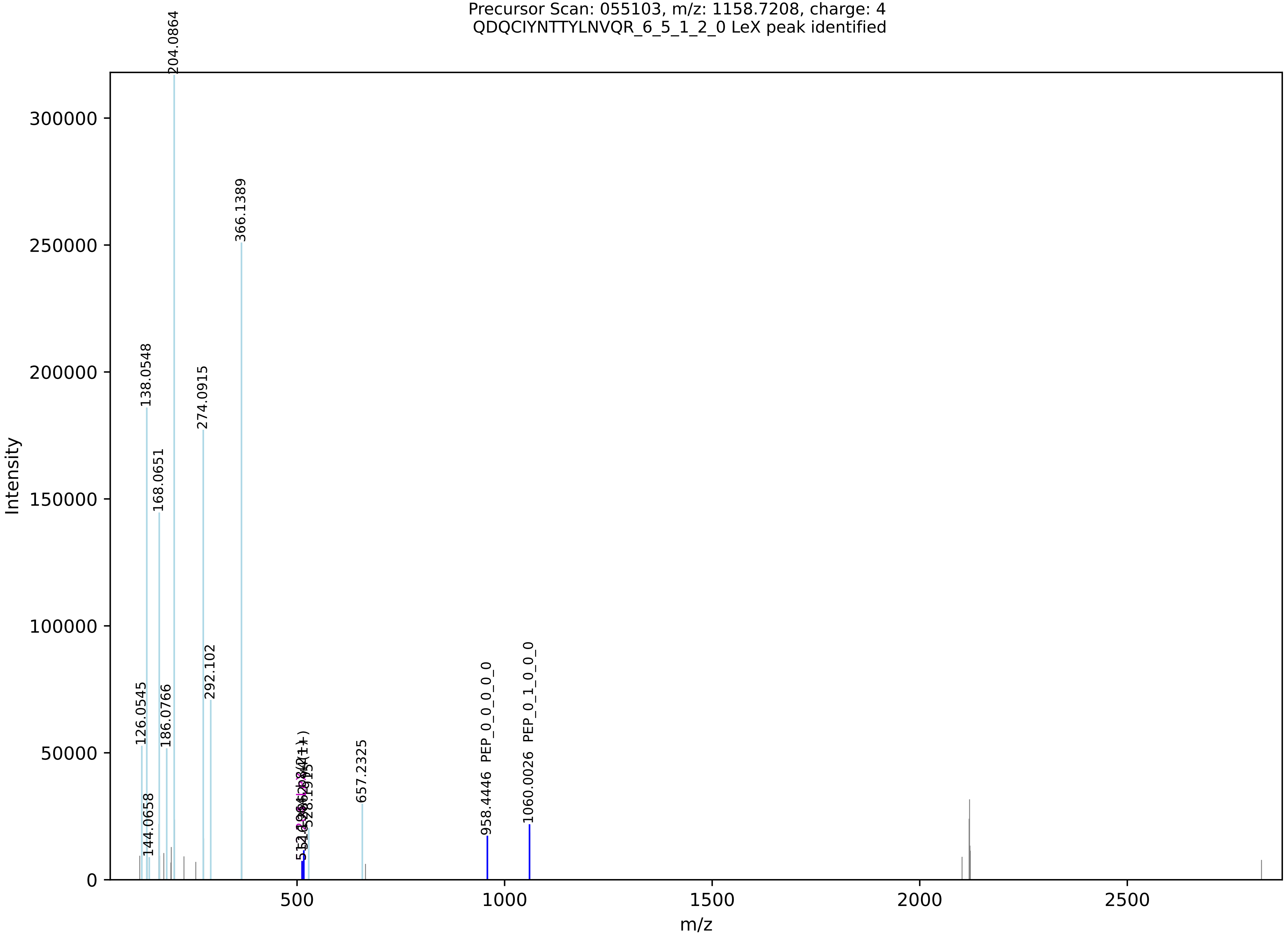

File: 231129_OE_RK_13, RT: 43.77, Scan: 055473
Type: HCD, FTMS + c NSI d Full ms2 1159.4730@hcd30.00 [120.0000-4753.5059]
Precursor Scan: 055395, m/z: 1158.7211, charge: 4
QDQCIYNTTYLNVQR_6_5_1_2_0 LeX peak identified

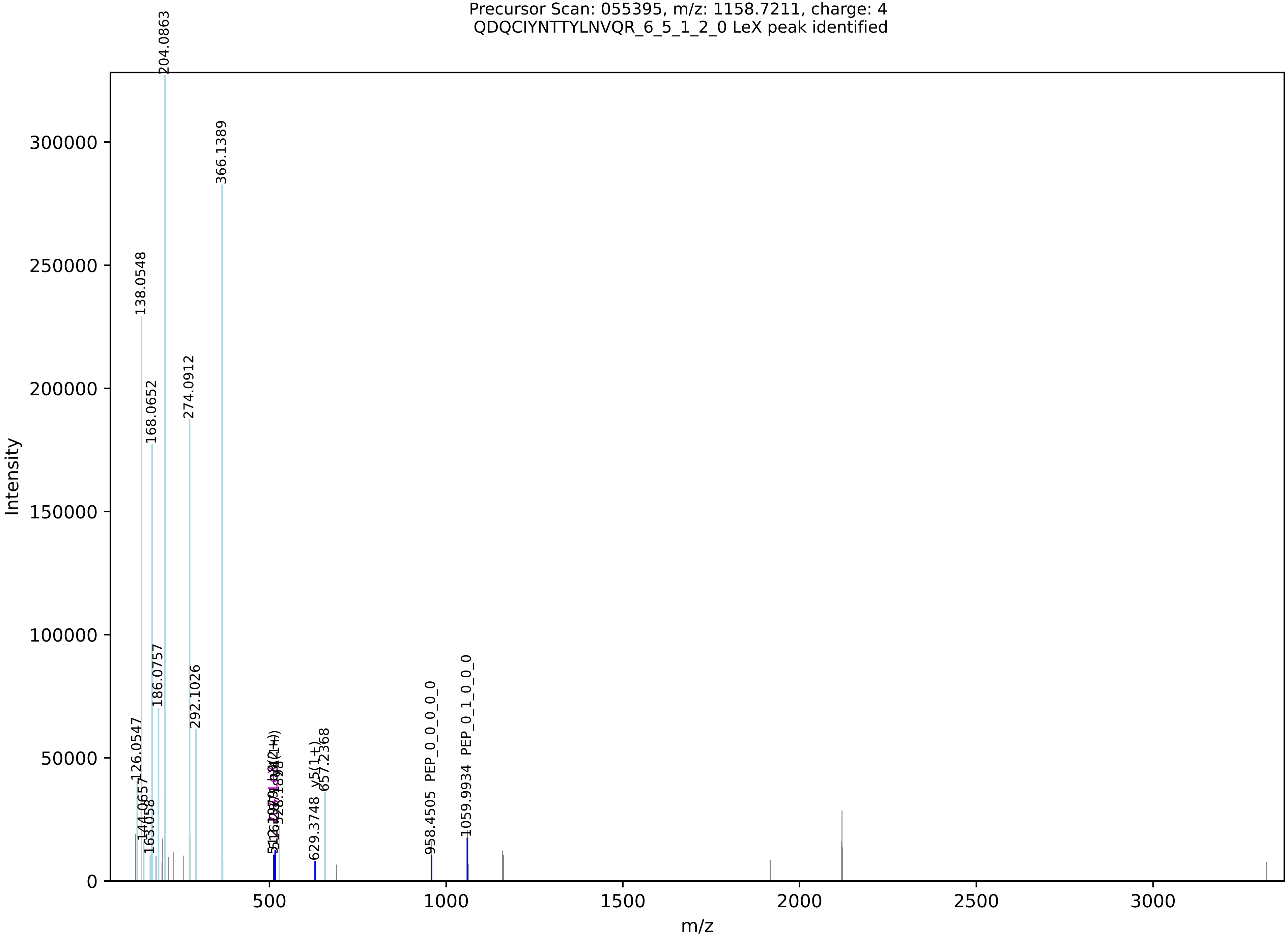

File: 231129_OE_RK_13, RT: 43.772, Scan: 055475
Type: HCD, FTMS + c NSI d Full ms2 1545.2935@hcd30.00 [120.0000-4750.7402]
Precursor Scan: 055395, m/z: 1544.6257, charge: 3
QDQCIYNTTYLNVQR_6_5_1_2_0 LeX peak identified

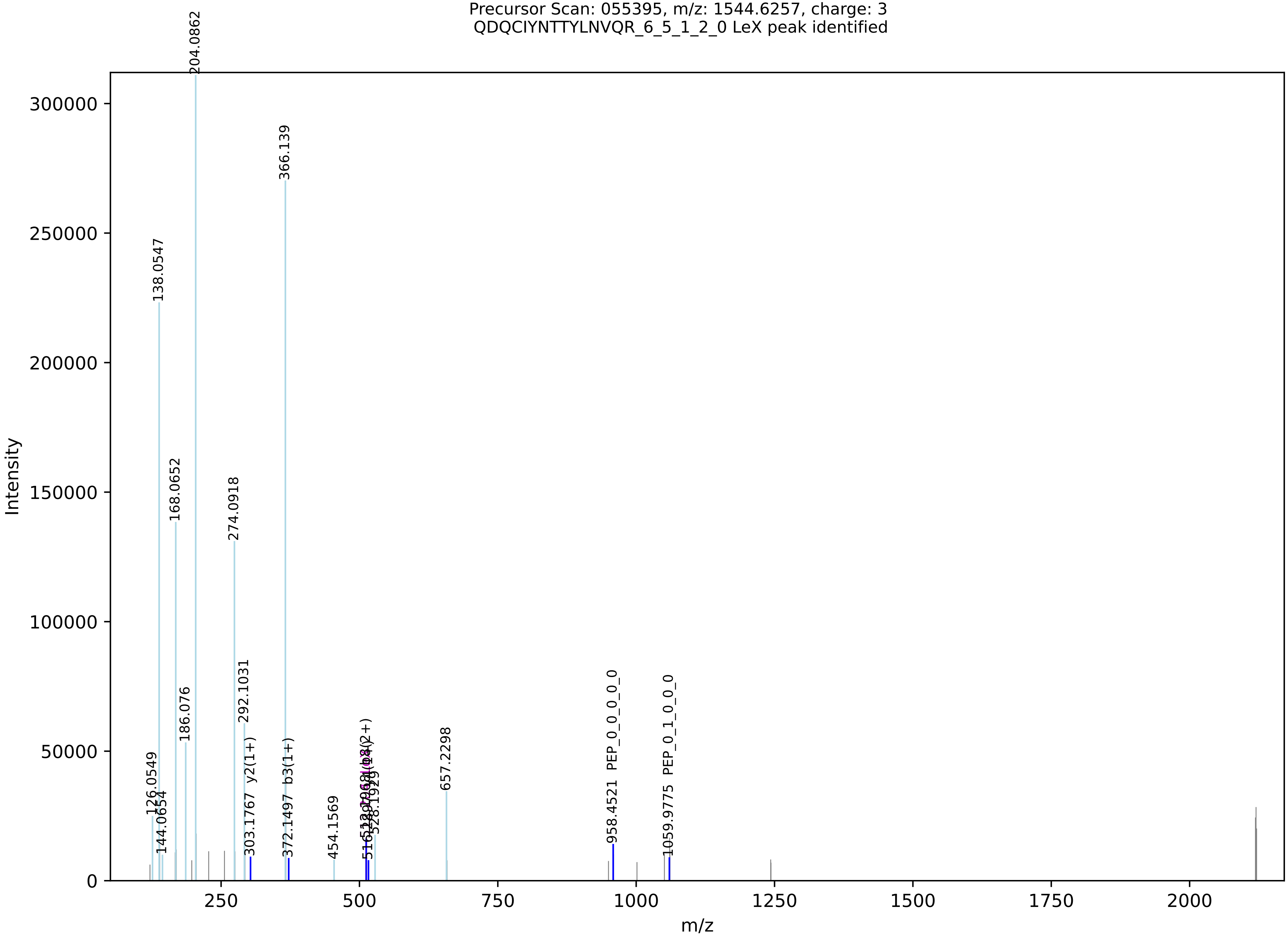

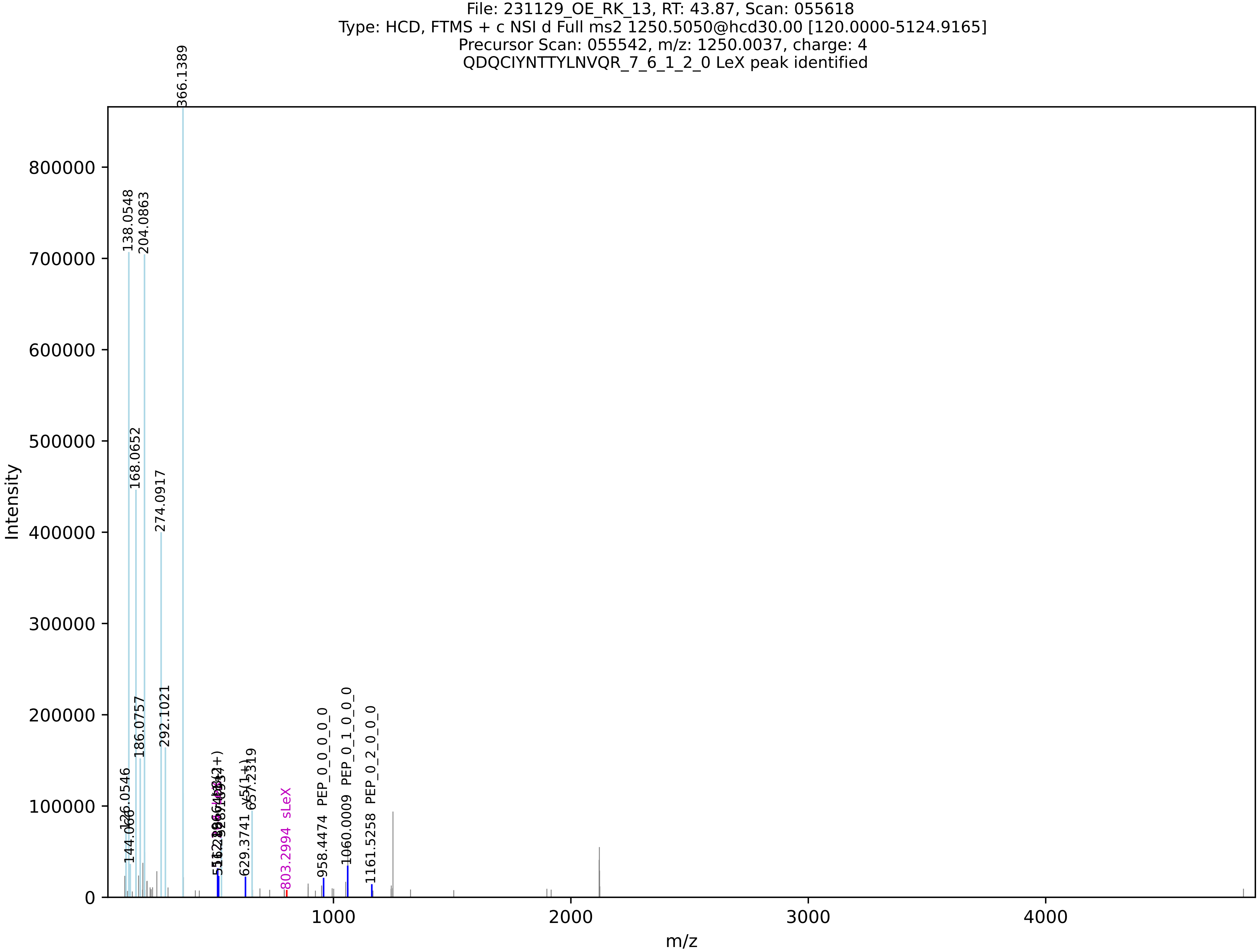

File: 231129_OE_RK_13, RT: 43.87, Scan: 055618
Type: HCD, FTMS + c NSI d Full ms2 1250.5050@hcd30.00 [120.0000-5124.9165]
Precursor Scan: 055542, m/z: 1250.0037, charge: 4
QDQCIYNTTYLNVQR_7_6_1_2_0 LeX peak identified
366.1389
138.0548
204.0863
168.0652
274.0917
186.0757
292.1021
126.0546
144.0660
629.3741 y5(1+)
657.2319
803.2994 sLeX
958.4474 PEP_0_0_0_0_0
1060.0009 PEP_0_1_0_0_0
1161.5258 PEP_0_2_0_0_0
Intensity
m/z
0
100000
200000
300000
400000
500000
600000
700000
800000
1000
2000
3000
4000

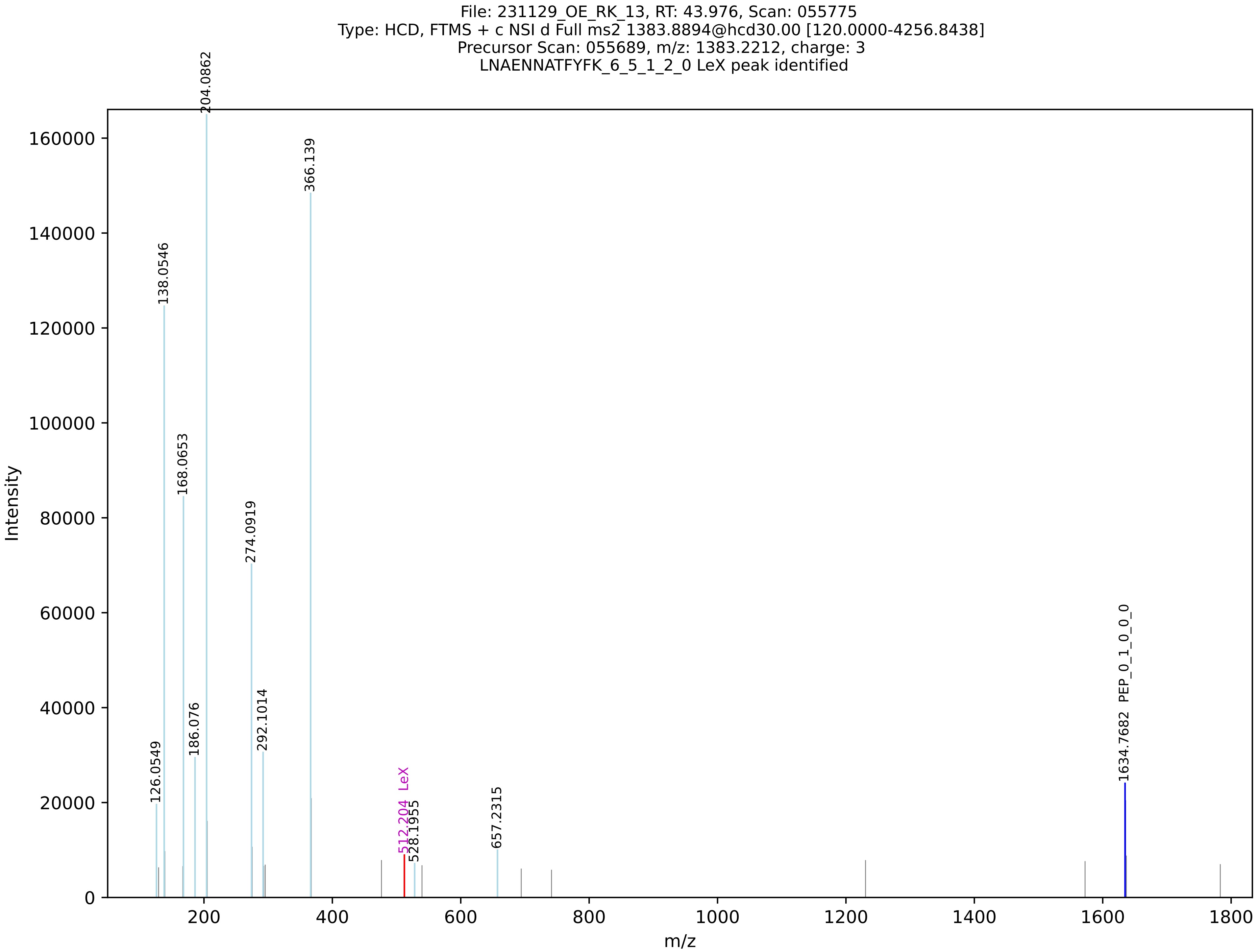

File: 231129_OE_RK_13, RT: 43.976, Scan: 055775
Type: HCD, FTMS + c NSI d Full ms2 1383.8894@hcd30.00 [120.0000-4256.8438]
Precursor Scan: 055689, m/z: 1383.2212, charge: 3
LNAENNATFYFK_6_5_1_2_0 LeX peak identified
Intensity
m/z
126.0549
138.0546
168.0653
186.076
204.0862
274.0919
292.1014
366.139
512.204 LeX
528.1955
657.2315
1634.7682 PEP_0_1_0_0_0

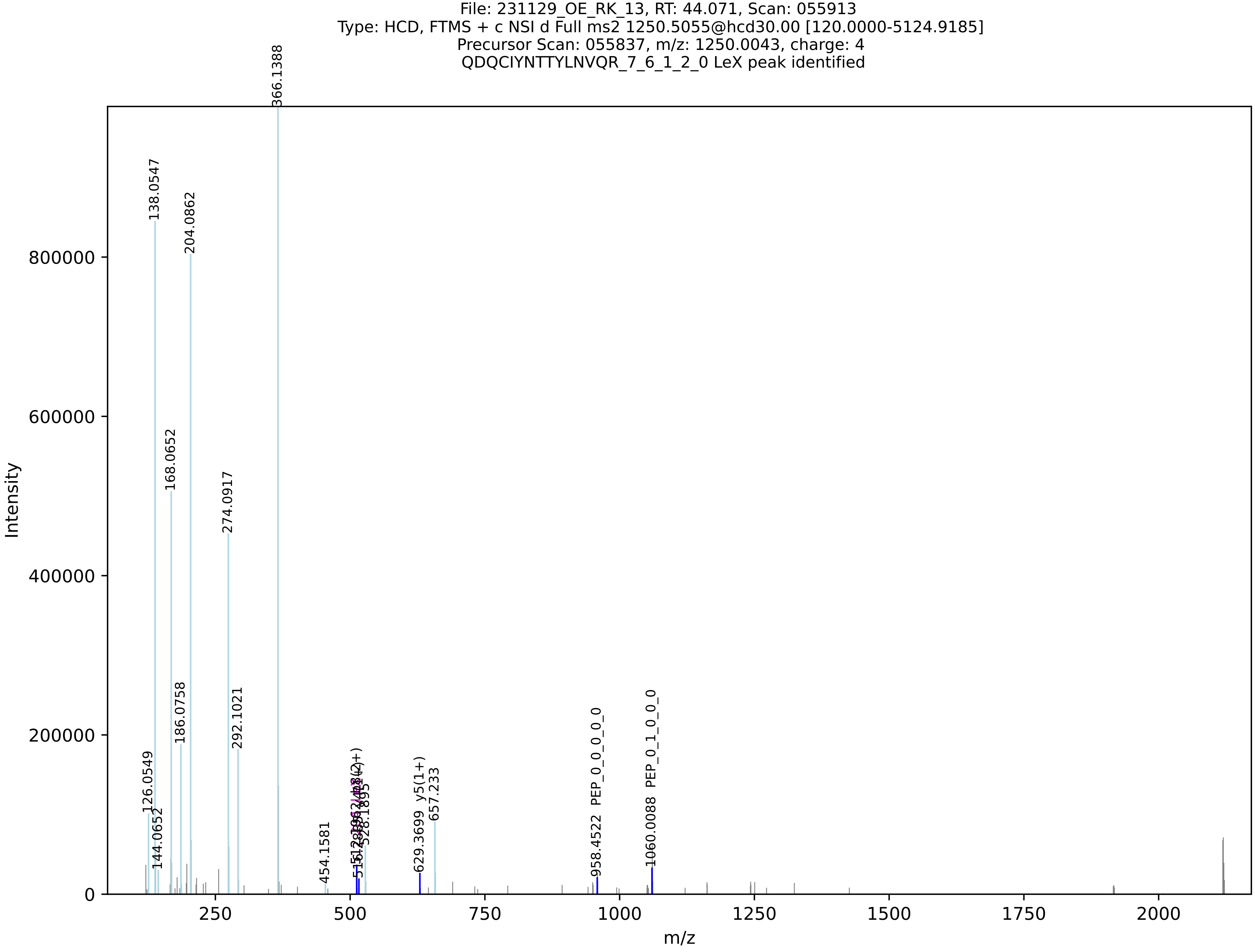
File: 231129_OE_RK_13, RT: 44.071, Scan: 055913
Type: HCD, FTMS + c NSI d Full ms2 1250.5055@hcd30.00 [120.0000-5124.9185]
Precursor Scan: 055837, m/z: 1250.0043, charge: 4
QDQCIYNTTYLNVQR_7_6_1_2_0 LeX peak identified
366.1388
138.0547
204.0862
168.0652
274.0917
186.0758
292.1021
126.0549
144.0652
454.1581
528.1895
629.3699 y5(1+)
657.233
958.4522 PEP_0_0_0_0_0_
1060.0088 PEP_0_1_0_0_0_
Intensity
800000
600000
400000
200000
0
250
500
750
1000
1250
1500
1750
2000
m/z

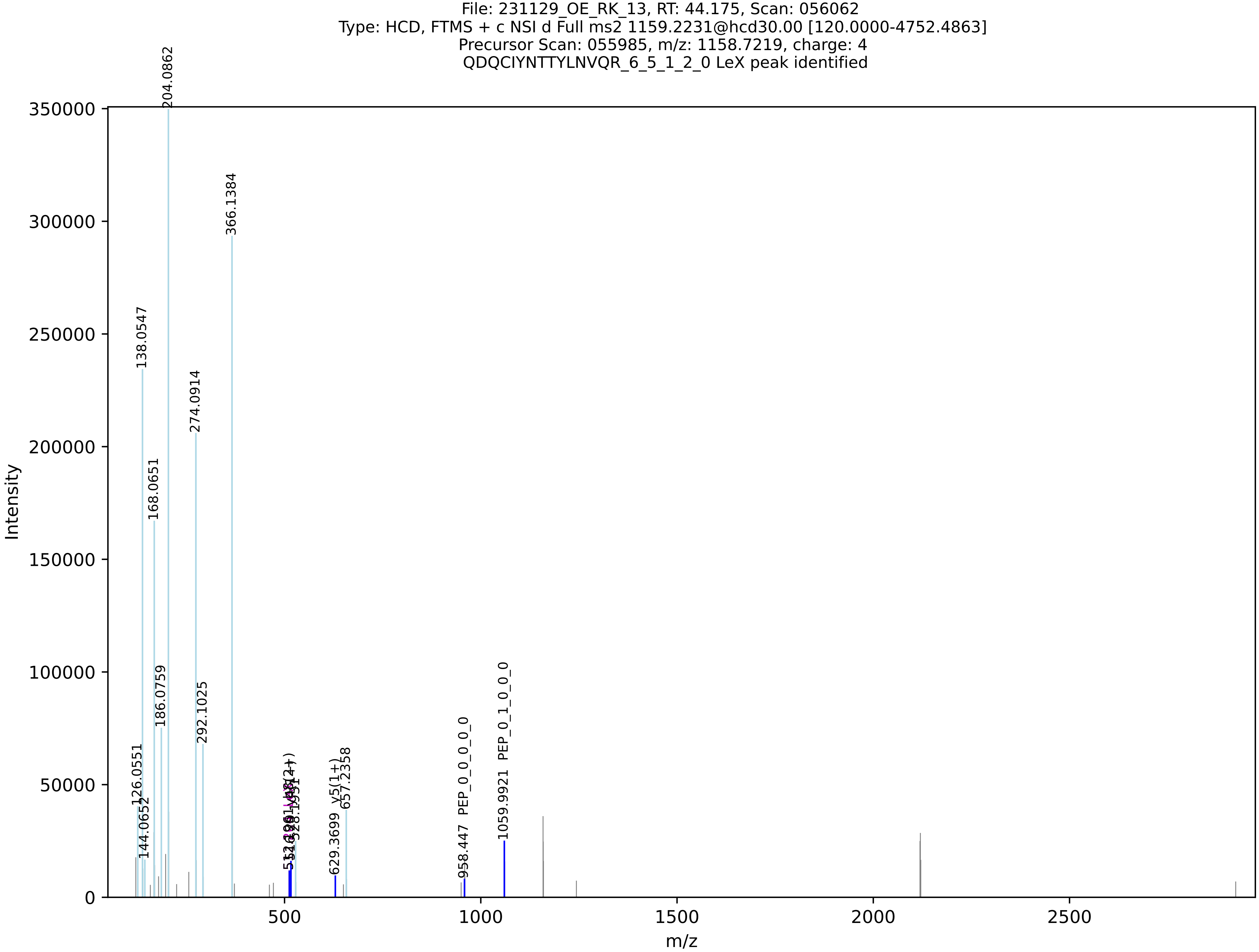
File: 231129_OE_RK_13, RT: 44.175, Scan: 056062
Type: HCD, FTMS + c NSI d Full ms2 1159.2231@hcd30.00 [120.0000-4752.4863]
Precursor Scan: 055985, m/z: 1158.7219, charge: 4
QDQCIYNTTYLNVQR_6_5_1_2_0 LeX peak identified
204.0862
366.1384
138.0547
274.0914
168.0651
186.0759
292.1025
126.0551
144.0652
629.3699
y5(1+)
657.2358
958.447
PEP_0_0_0_0_0
1059.9921
PEP_0_1_0_0_0
Intensity
m/z
0
50000
100000
150000
200000
250000
300000
350000
500
1000
1500
2000
2500

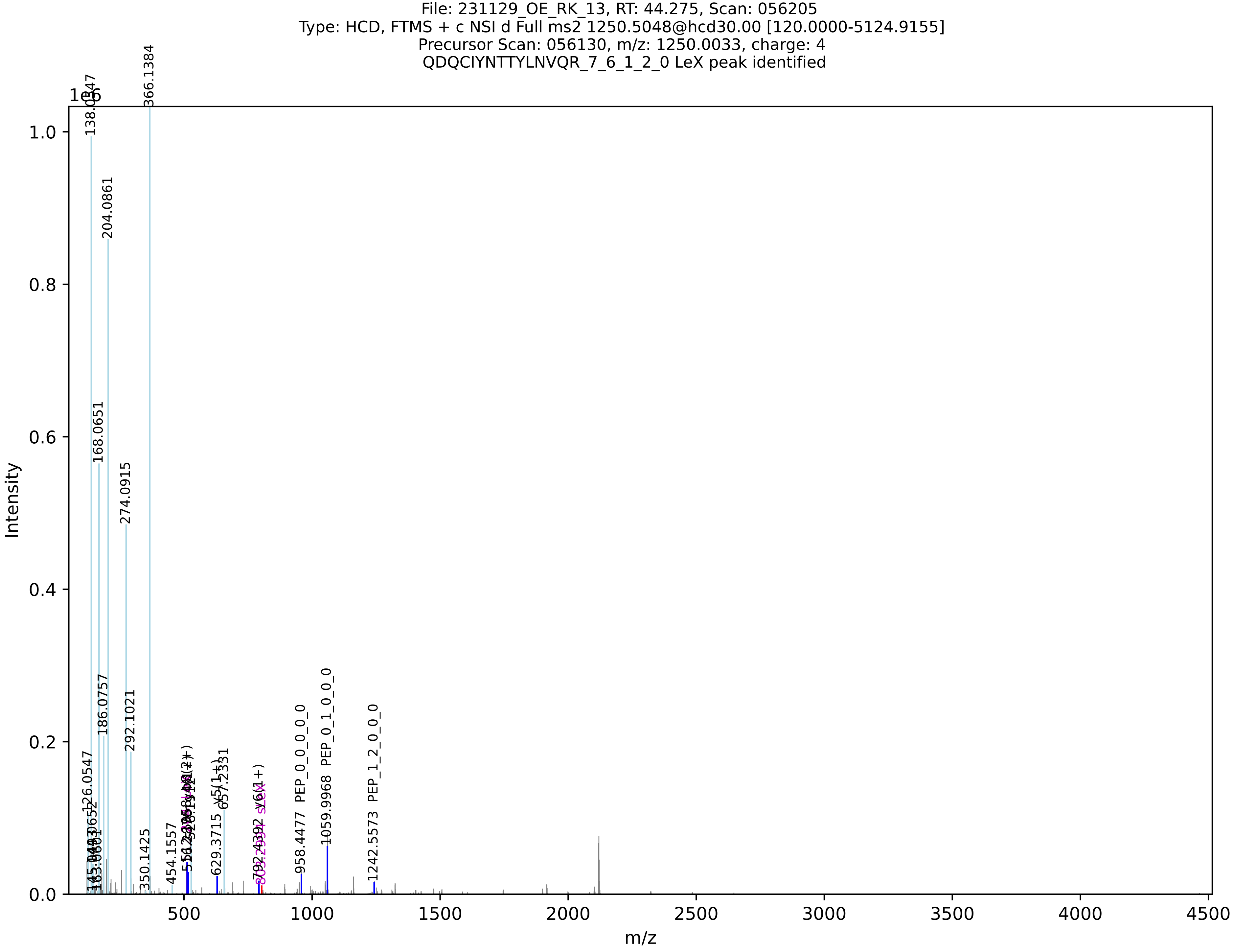
File: 231129_OE_RK_13, RT: 44.275, Scan: 056205
Type: HCD, FTMS + c NSI d Full ms2 1250.5048@hcd30.00 [120.0000-5124.9155]
Precursor Scan: 056130, m/z: 1250.0033, charge: 4
QDQCIYNTTYLNVQR_7_6_1_2_0 LeX peak identified
1e6
Intensity
m/z
366.1384
204.0861
168.0651
274.0915
186.0757
292.1021
126.0547
350.1425
454.1557
629.3715 y5(1+)
657.2331
792.4392 y6(1+)
958.4477 PEP_0_0_0_0_0
1059.9968 PEP_0_1_0_0_0
1242.5573 PEP_1_2_0_0_0

File: 231129_OE_RK_13, RT: 44.324, Scan: 056277
Type: HCD, FTMS + c NSI d Full ms2 1287.0172@hcd30.00 [120.0000-5273.8862]
Precursor Scan: 056204, m/z: 1286.5161, charge: 4
QDQCIYNTTYLNVQR_7_6_2_2_0 LeX peak identified

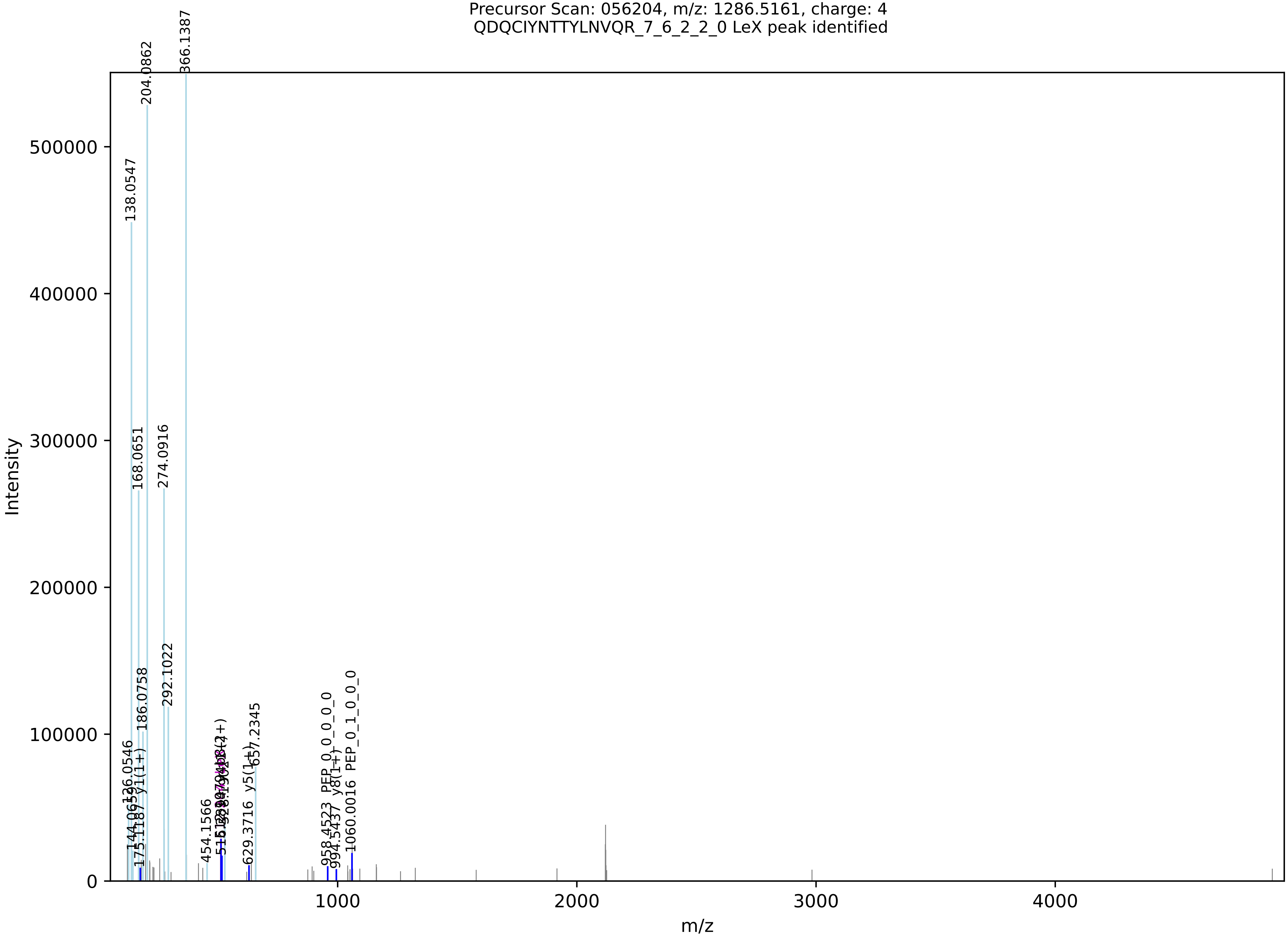

File: 231129_OE_RK_13, RT: 44.375, Scan: 056352
Type: HCD, FTMS + c NSI d Full ms2 1159.2219@hcd30.00 [120.0000-4752.4814]
Precursor Scan: 056275, m/z: 1158.7213, charge: 4
QDQCIYNTTYLNVQR_6_5_1_2_0 LeX peak identified

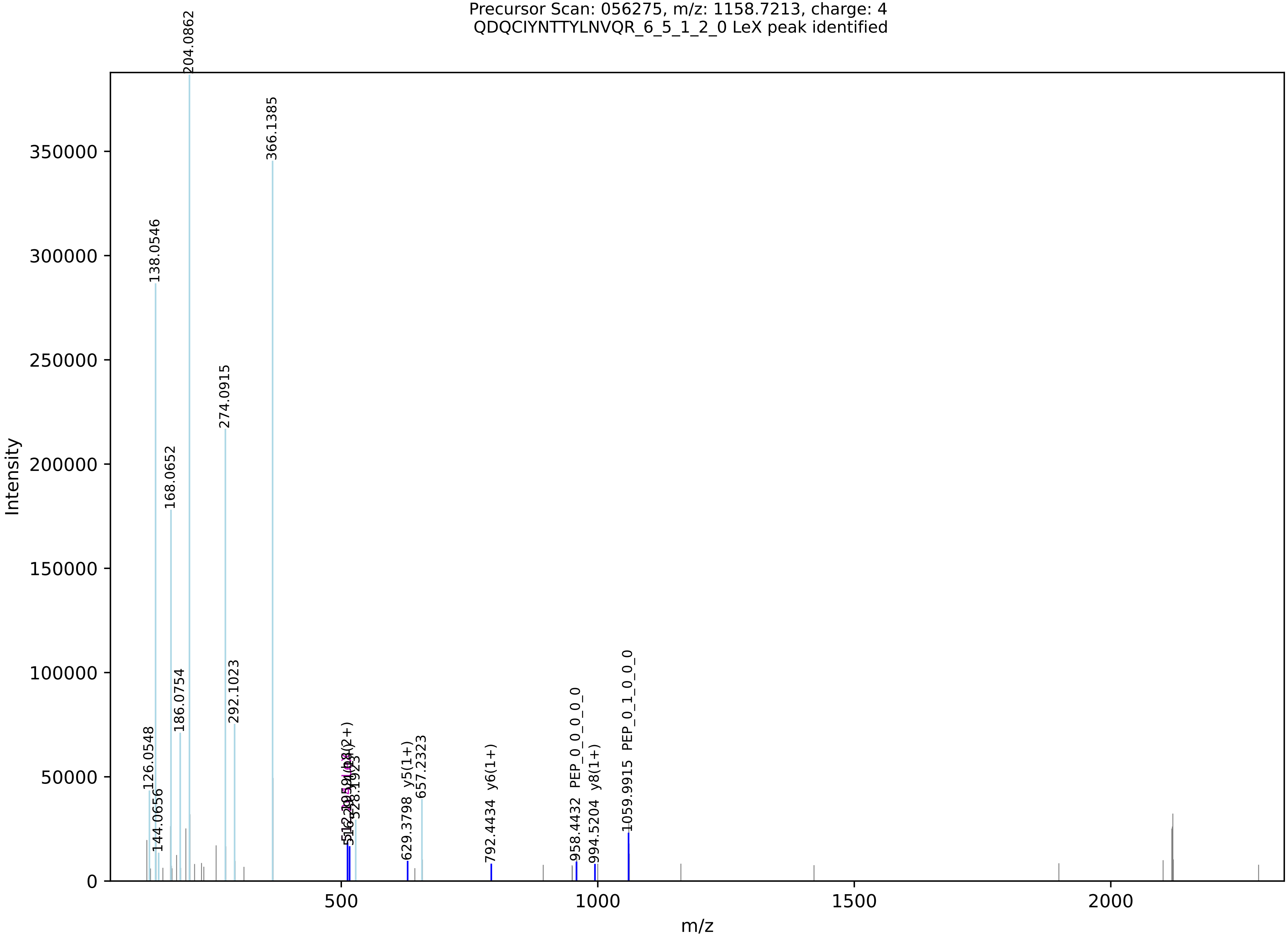

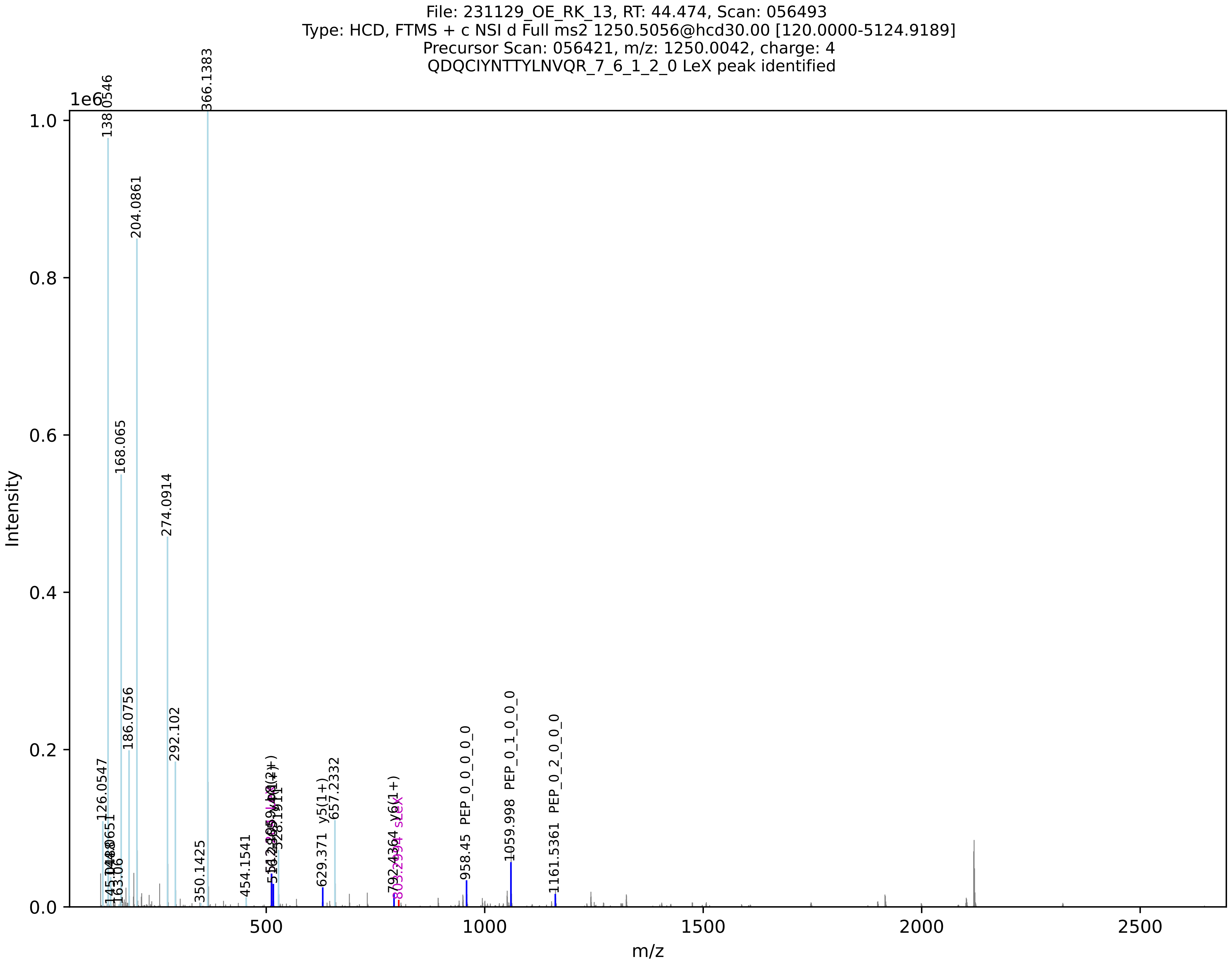
File: 231129_OE_RK_13, RT: 44.474, Scan: 056493
Type: HCD, FTMS + c NSI d Full ms2 1250.5056@hcd30.00 [120.0000-5124.9189]
Precursor Scan: 056421, m/z: 1250.0042, charge: 4
QDQCIYNTTYLNVQR_7_6_1_2_0 LeX peak identified
1e6
Intensity
m/z
0.0
0.2
0.4
0.6
0.8
1.0
500
1000
1500
2000
2500
366.1383
204.0861
168.065
274.0914
186.0756
292.102
126.0547
350.1425
454.1541
629.371
y5(1+)
657.2332
792.4364
y6(1+)
958.45
PEP_0_0_0_0_0
1059.998
PEP_0_1_0_0_0
1161.5361
PEP_0_2_0_0_0

File: 231129_OE_RK_13, RT: 44.527, Scan: 056567
Type: HCD, FTMS + c NSI d Full ms2 1287.0190@hcd30.00 [120.0000-5273.8936]
Precursor Scan: 056492, m/z: 1286.2673, charge: 4
QDQCIYNTTYLNVQR_7_6_0_3_0 LeX peak identified

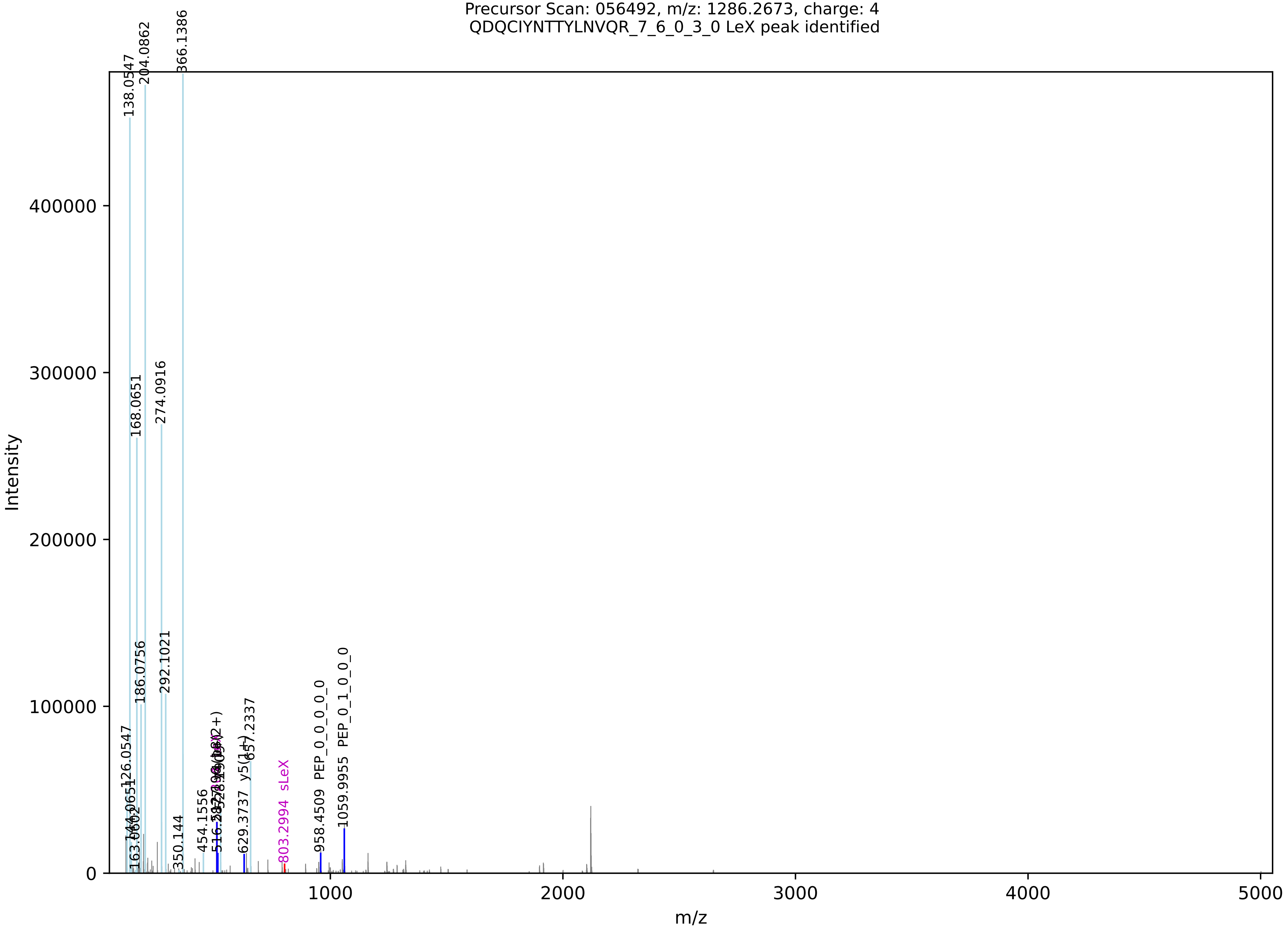

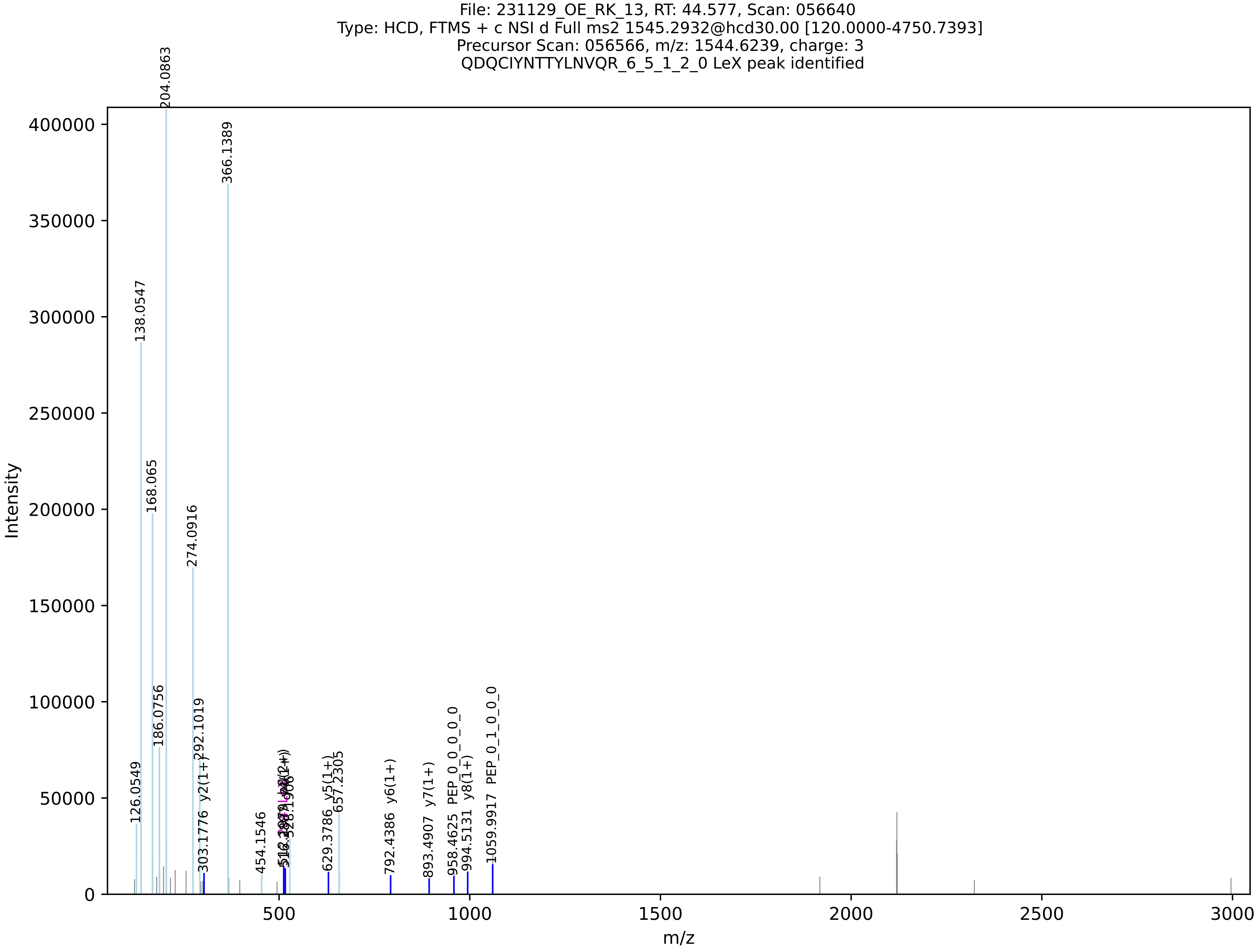
File: 231129_OE_RK_13, RT: 44.577, Scan: 056640
Type: HCD, FTMS + c NSI d Full ms2 1545.2932@hcd30.00 [120.0000-4750.7393]
Precursor Scan: 056566, m/z: 1544.6239, charge: 3
QDQCIYNTTYLNVQR_6_5_1_2_0 LeX peak identified
Intensity
m/z
204.0863
366.1389
138.0547
168.065
274.0916
186.0756
292.1019
126.0549
303.1776 y2(1+)
454.1546
629.3786 y5(1+)
657.2305
792.4386 y6(1+)
893.4907 y7(1+)
958.4625 PEP_0_0_0_0_0
994.5131 y8(1+)
1059.9917 PEP_0_1_0_0_0
0
50000
100000
150000
200000
250000
300000
350000
400000
500
1000
1500
2000
2500
3000

File: 231129_OE_RK_13, RT: 44.578, Scan: 056642
Type: HCD, FTMS + c NSI d Full ms2 1159.2220@hcd30.00 [120.0000-4752.4819]
Precursor Scan: 056566, m/z: 1158.7216, charge: 4
QDQCIYNTTYLNVQR_6_5_1_2_0 LeX peak identified

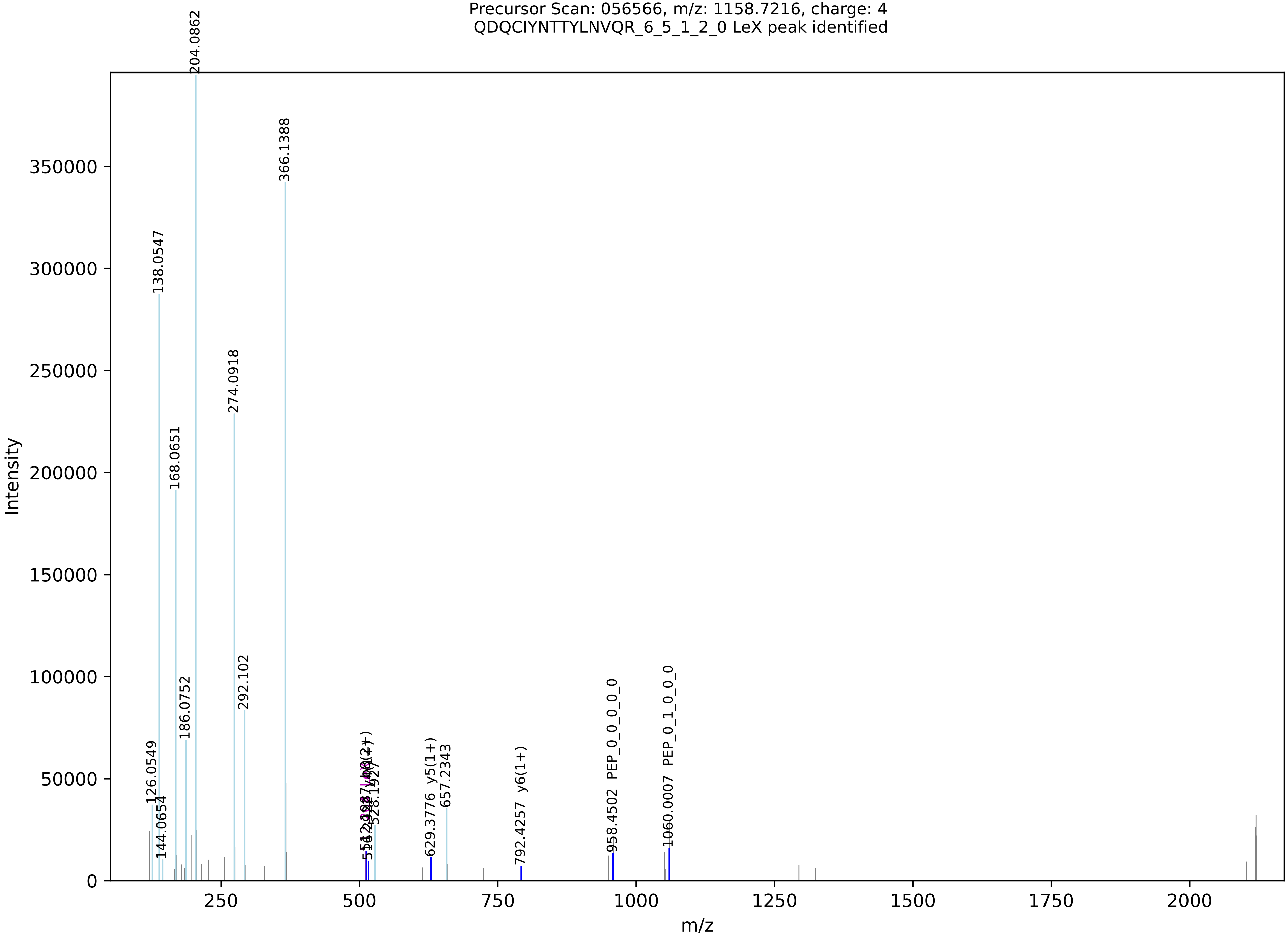

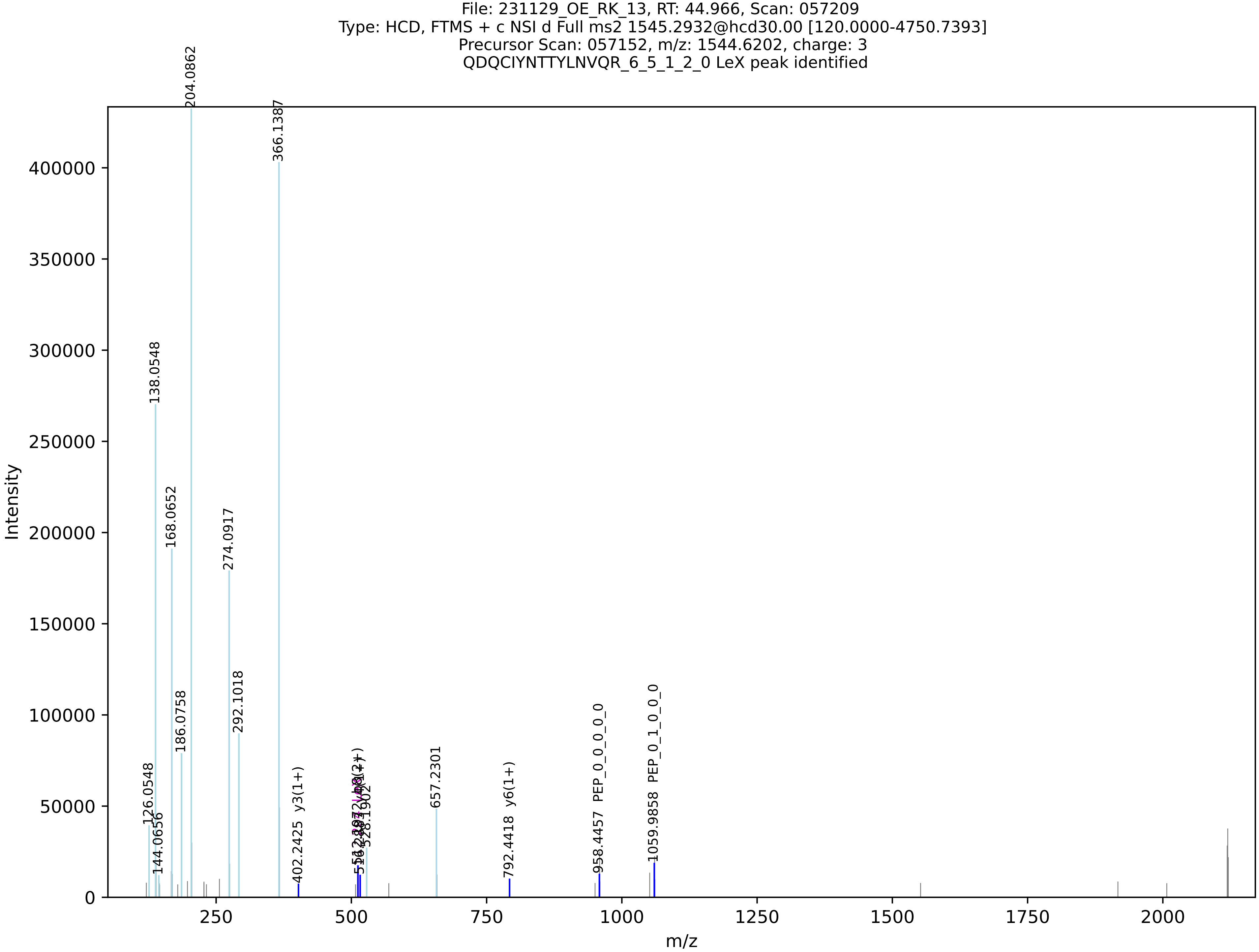
File: 231129_OE_RK_13, RT: 44.966, Scan: 057209
Type: HCD, FTMS + c NSI d Full ms2 1545.2932@hcd30.00 [120.0000-4750.7393]
Precursor Scan: 057152, m/z: 1544.6202, charge: 3
QDQCIYNTTYLNVQR_6_5_1_2_0 LeX peak identified
Intensity
m/z
0
50000
100000
150000
200000
250000
300000
350000
400000
250
500
750
1000
1250
1500
1750
2000
204.0862
366.1387
138.0548
168.0652
274.0917
292.1018
186.0758
126.0548
144.0656
402.2425 y3(1+)
528.1902
657.2301
792.4418 y6(1+)
958.4457 PEP_0_0_0_0_0_
1059.9858 PEP_0_1_0_0_0_

File: 231129_OE_RK_13, RT: 45.148, Scan: 057470
Type: HCD, FTMS + c NSI d Full ms2 1159.2203@hcd30.00 [120.0000-4752.4751]
Precursor Scan: 057402, m/z: 1158.718, charge: 4
QDQCIYNTTYLNVQR_6_5_1_2_0 LeX peak identified

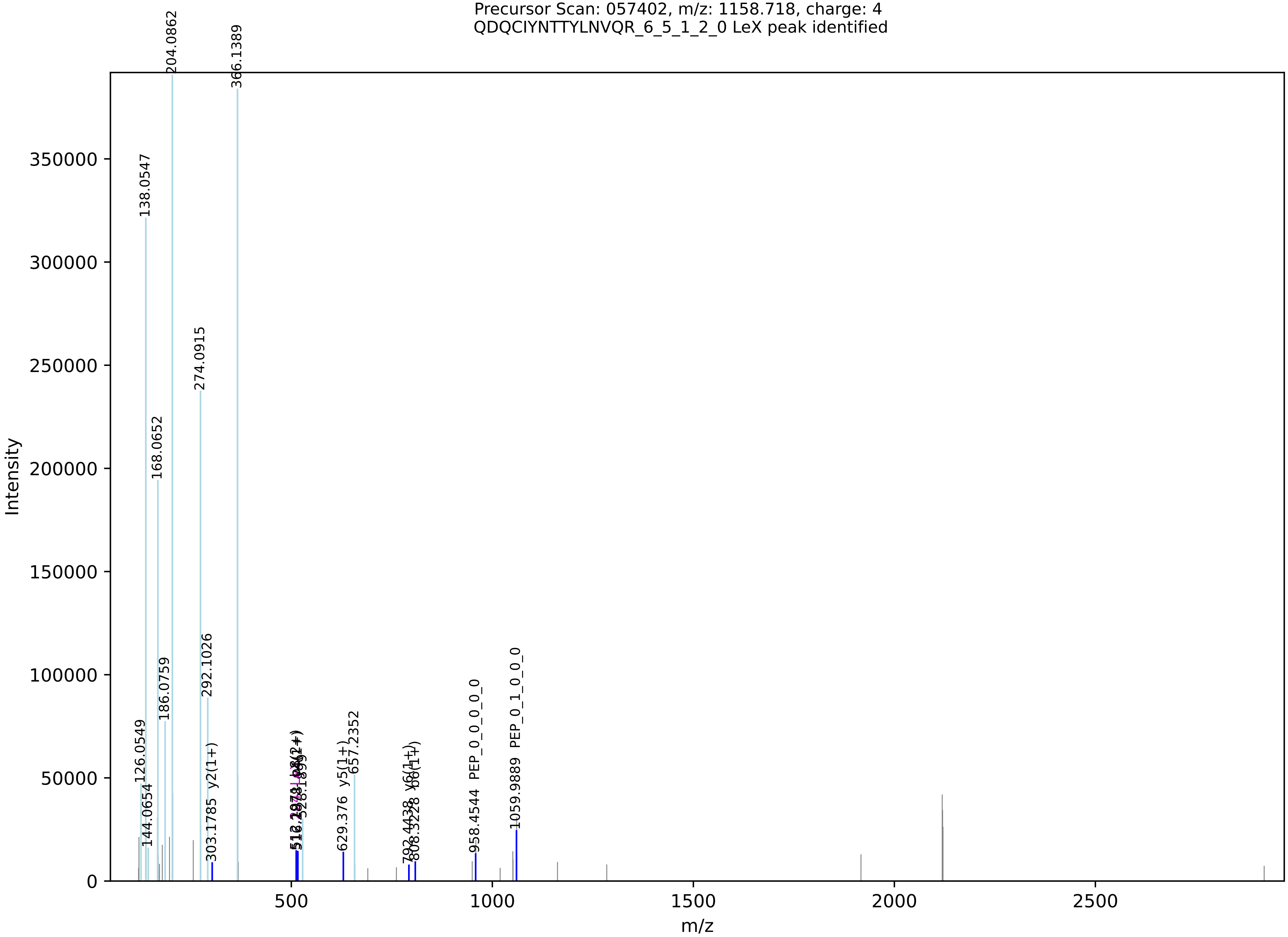

File: 231129_OE_RK_13, RT: 45.201, Scan: 057547
Type: HCD, FTMS + c NSI d Full ms2 1287.0160@hcd30.00 [120.0000-5273.8809]
Precursor Scan: 057467, m/z: 1286.2626, charge: 4
QDQCIYNTTYLNVQR_7_6_0_3_0 LeX peak identified

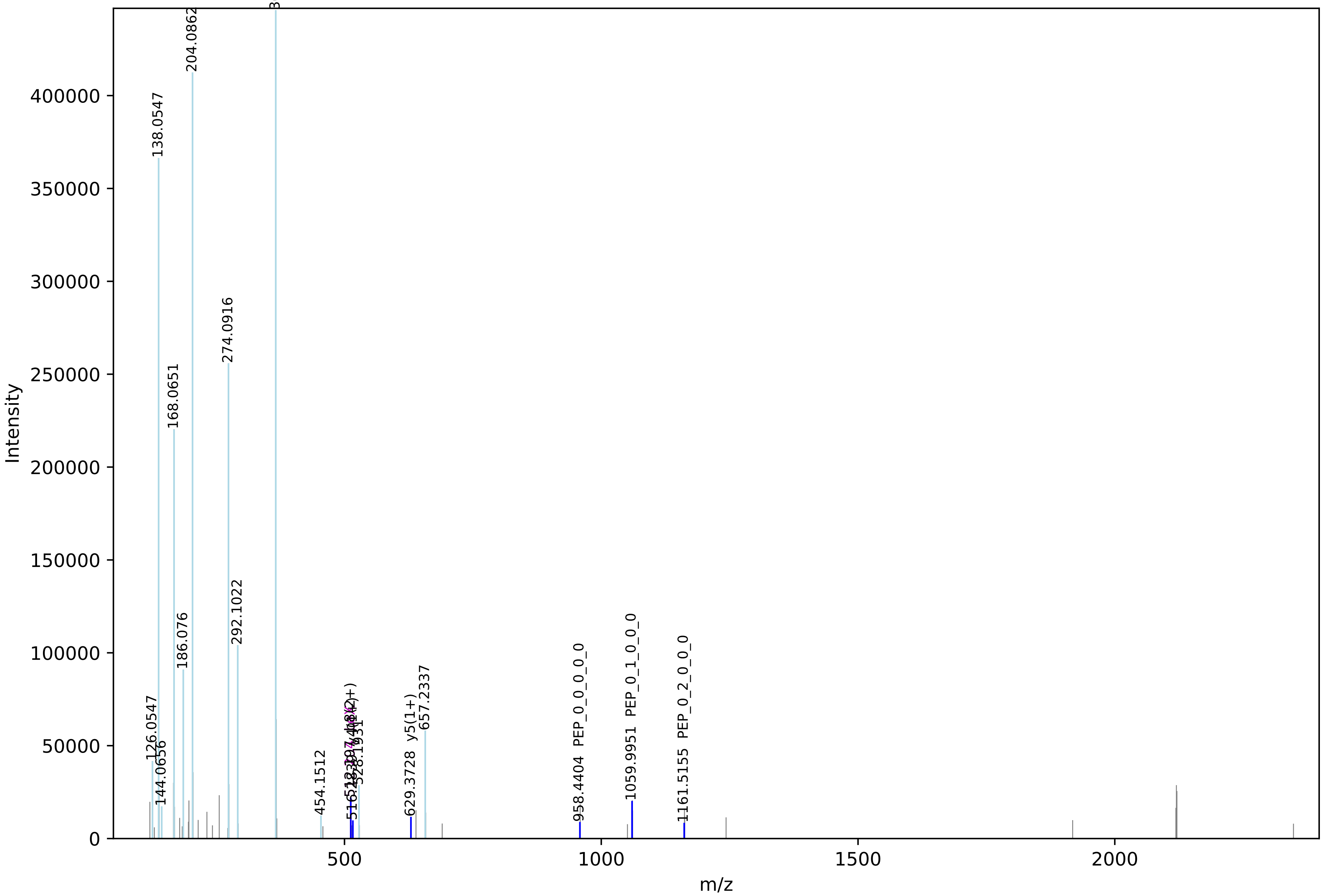

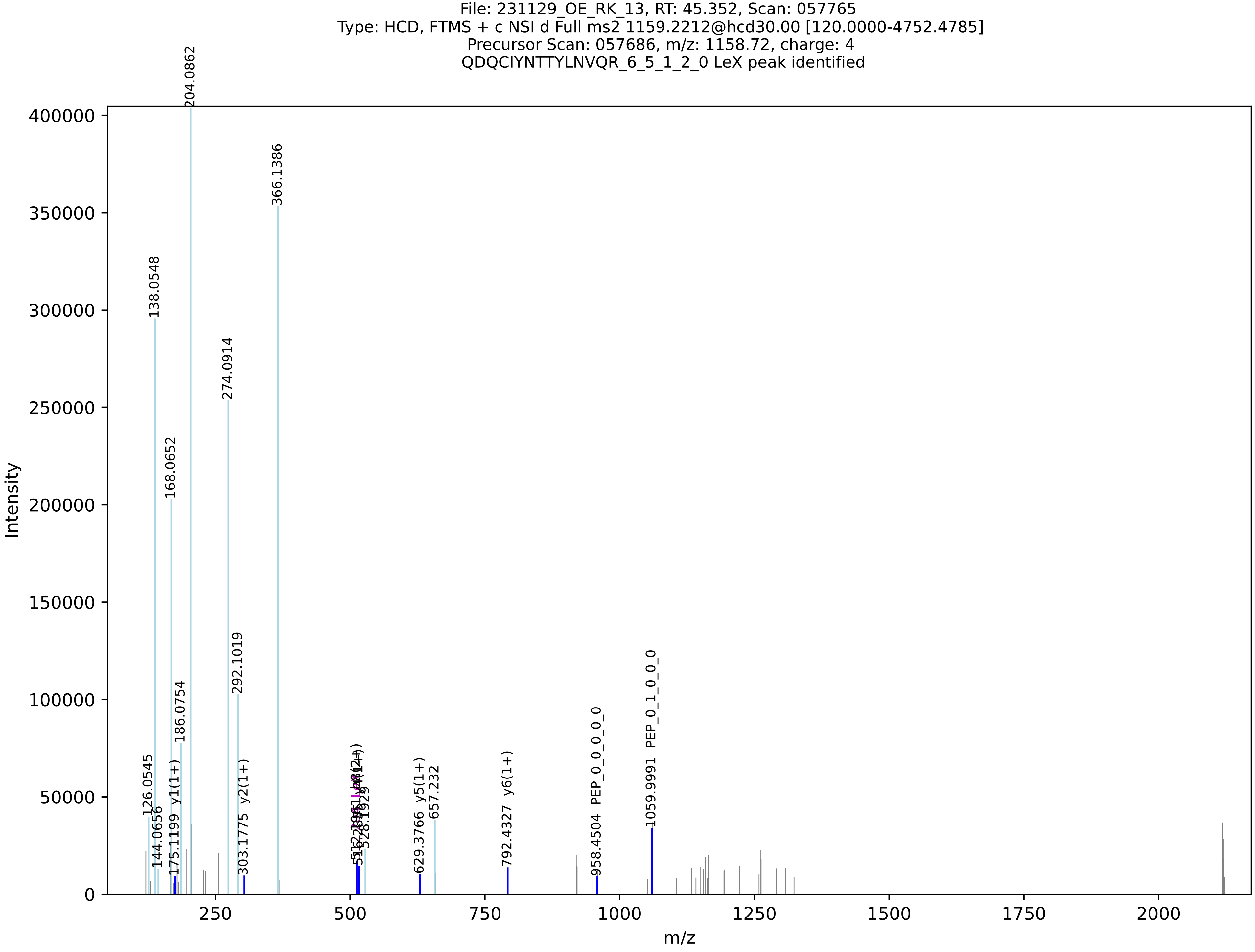
File: 231129_OE_RK_13, RT: 45.352, Scan: 057765
Type: HCD, FTMS + c NSI d Full ms2 1159.2212@hcd30.00 [120.0000-4752.4785]
Precursor Scan: 057686, m/z: 1158.72, charge: 4
QDQCIYNTTYLNVQR_6_5_1_2_0 LeX peak identified
Intensity
m/z
204.0862
366.1386
138.0548
274.0914
168.0652
292.1019
186.0754
126.0545
144.0656
175.1199 y1(1+)
303.1775 y2(1+)
528.1929
629.3766 y5(1+)
657.232
792.4327 y6(1+)
958.4504 PEP_0_0_0_0_0
1059.9991 PEP_0_1_0_0_0

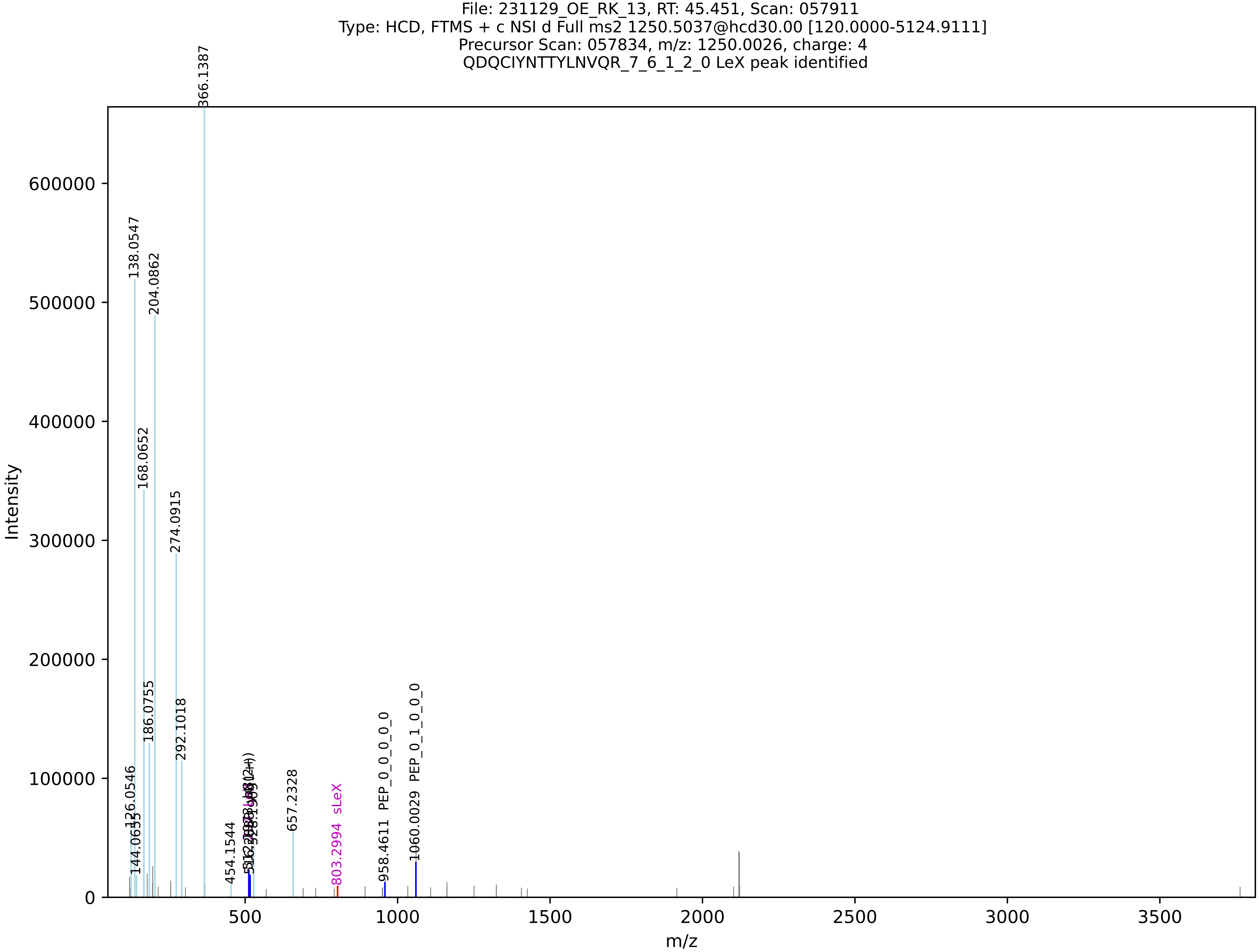

File: 231129_OE_RK_13, RT: 45.451, Scan: 057911
Type: HCD, FTMS + c NSI d Full ms2 1250.5037@hcd30.00 [120.0000-5124.9111]
Precursor Scan: 057834, m/z: 1250.0026, charge: 4
QDQCIYNTTYLNVQR_7_6_1_2_0 LeX peak identified
366.1387
138.0547
204.0862
168.0652
274.0915
186.0755
292.1018
126.0546
144.0655
454.1544
657.2328
803.2994 sLeX
958.4611 PEP_0_0_0_0_0
1060.0029 PEP_0_1_0_0_0
Intensity
m/z

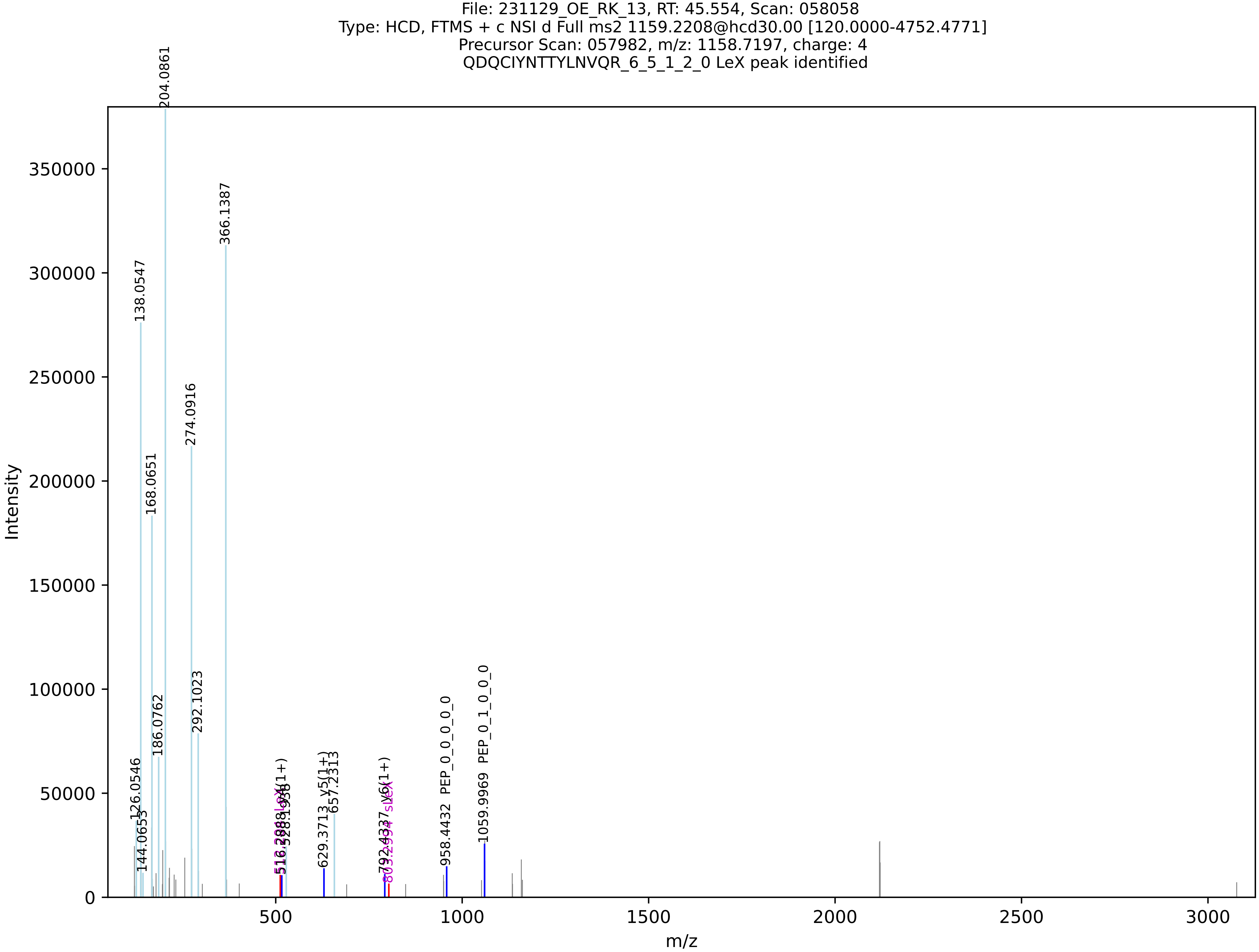
File: 231129_OE_RK_13, RT: 45.554, Scan: 058058
Type: HCD, FTMS + c NSI d Full ms2 1159.2208@hcd30.00 [120.0000-4752.4771]
Precursor Scan: 057982, m/z: 1158.7197, charge: 4
QDQCIYNTTYLNVQR_6_5_1_2_0 LeX peak identified
Intensity
m/z
204.0861
366.1387
138.0547
274.0916
168.0651
292.1023
186.0762
126.0546
144.0653
528.1938
629.3713 y5(1+)
657.2313
792.4337 y6(1+)
958.4432 PEP_0_0_0_0_0
1059.9969 PEP_0_1_0_0_0

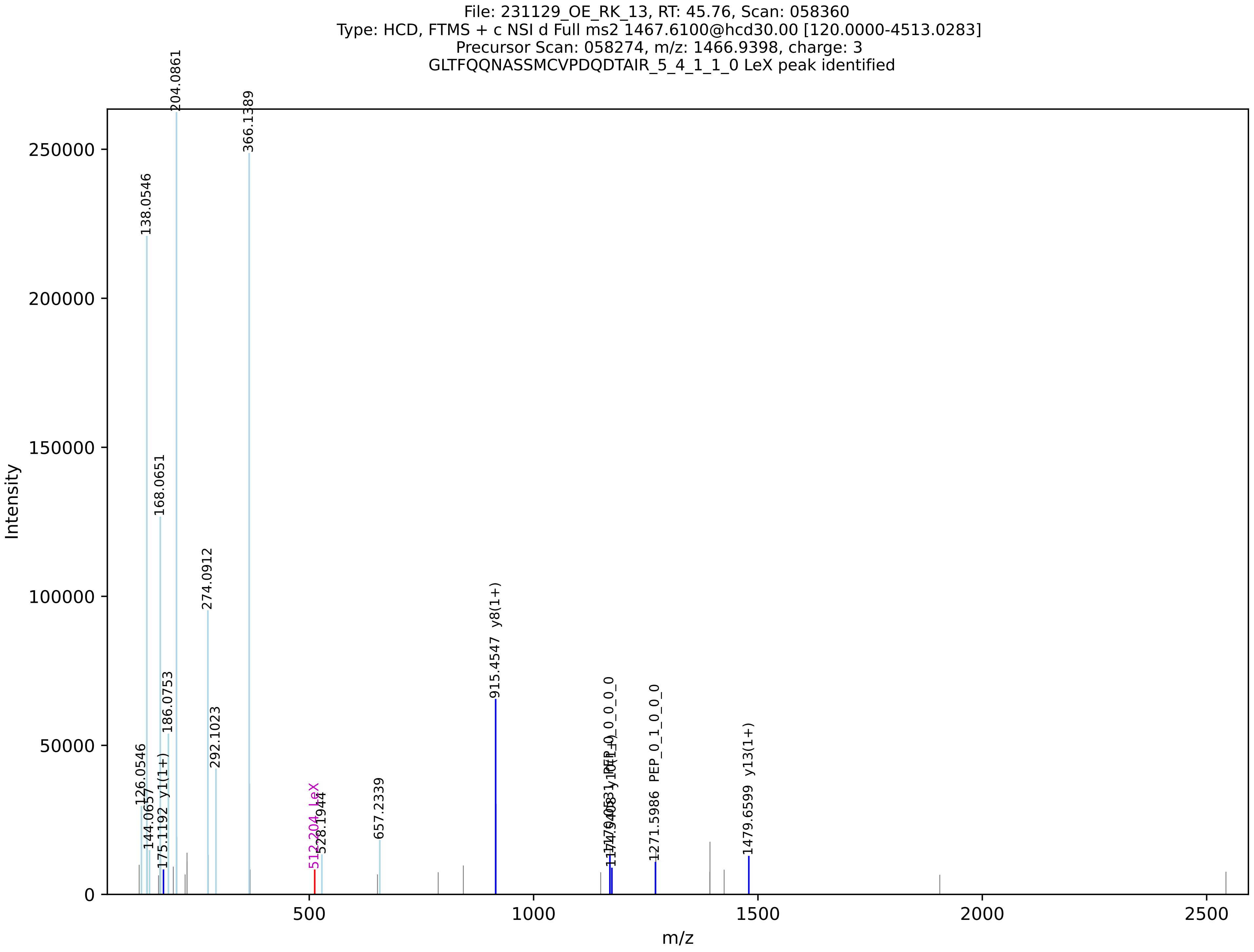
File: 231129_OE_RK_13, RT: 45.76, Scan: 058360
Type: HCD, FTMS + c NSI d Full ms2 1467.6100@hcd30.00 [120.0000-4513.0283]
Precursor Scan: 058274, m/z: 1466.9398, charge: 3
GLTFQQNASSMCVPDQDTAIR_5_4_1_1_0 LeX peak identified
Intensity
m/z
204.0861
366.1389
138.0546
168.0651
274.0912
186.0753
292.1023
126.0546
144.0657
175.1192 y1(1+)
512.204 LeX
528.1944
657.2339
915.4547 y8(1+)
1170.0531 PEP_0_0_0_0_0
1174.5405 y10(1+)
1271.5986 PEP_0_1_0_0_0
1479.6599 y13(1+)
0
50000
100000
150000
200000
250000
500
1000
1500
2000
2500

File: 231129_OE_RK_13, RT: 45.959, Scan: 058647
Type: HCD, FTMS + c NSI d Full ms2 1323.5294@hcd30.00 [120.0000-5422.8560]
Precursor Scan: 058570, m/z: 1322.7777, charge: 4
QDQCIYNTTYLNVQR_7_6_1_3_0 LeX peak identified

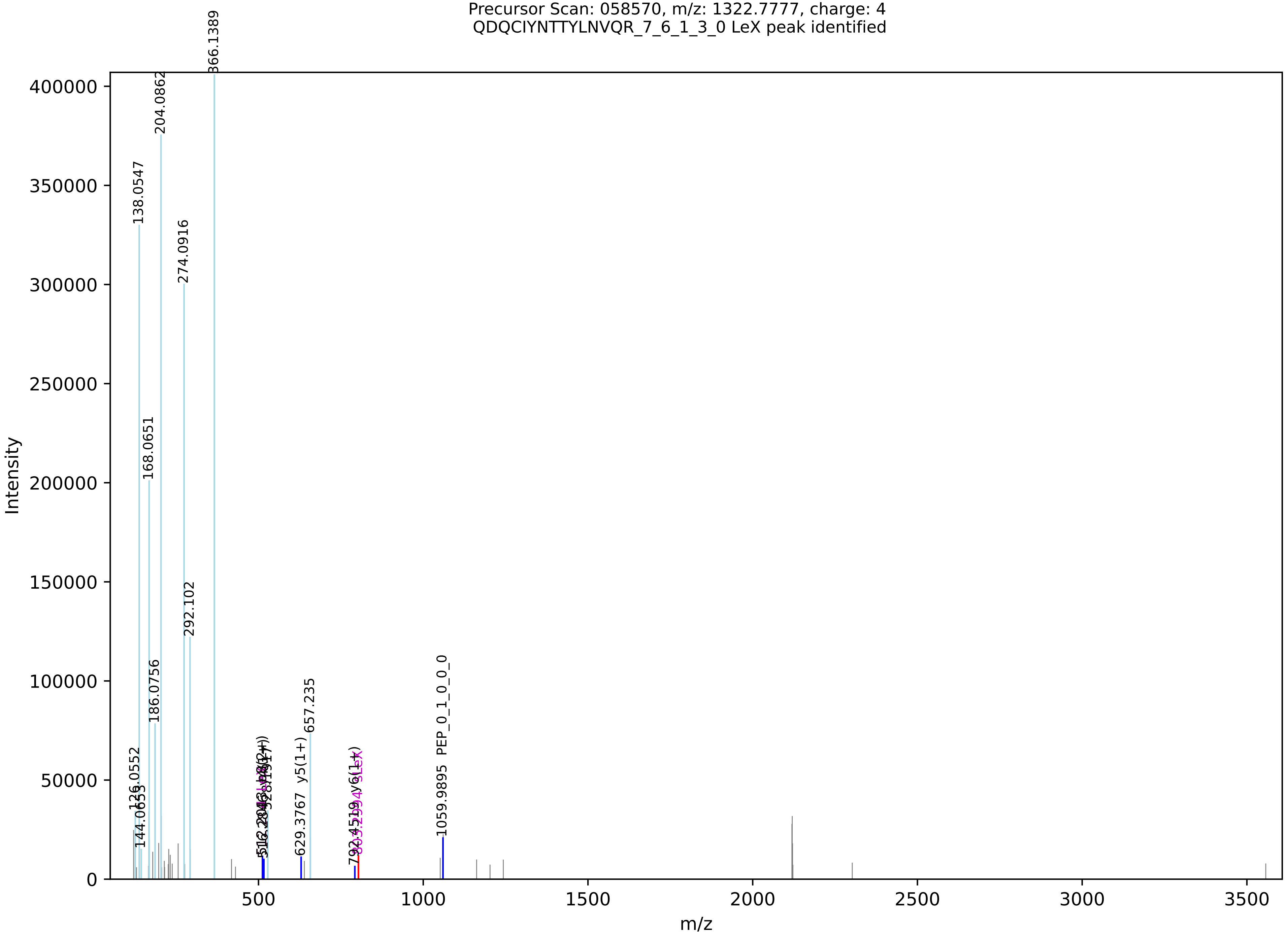

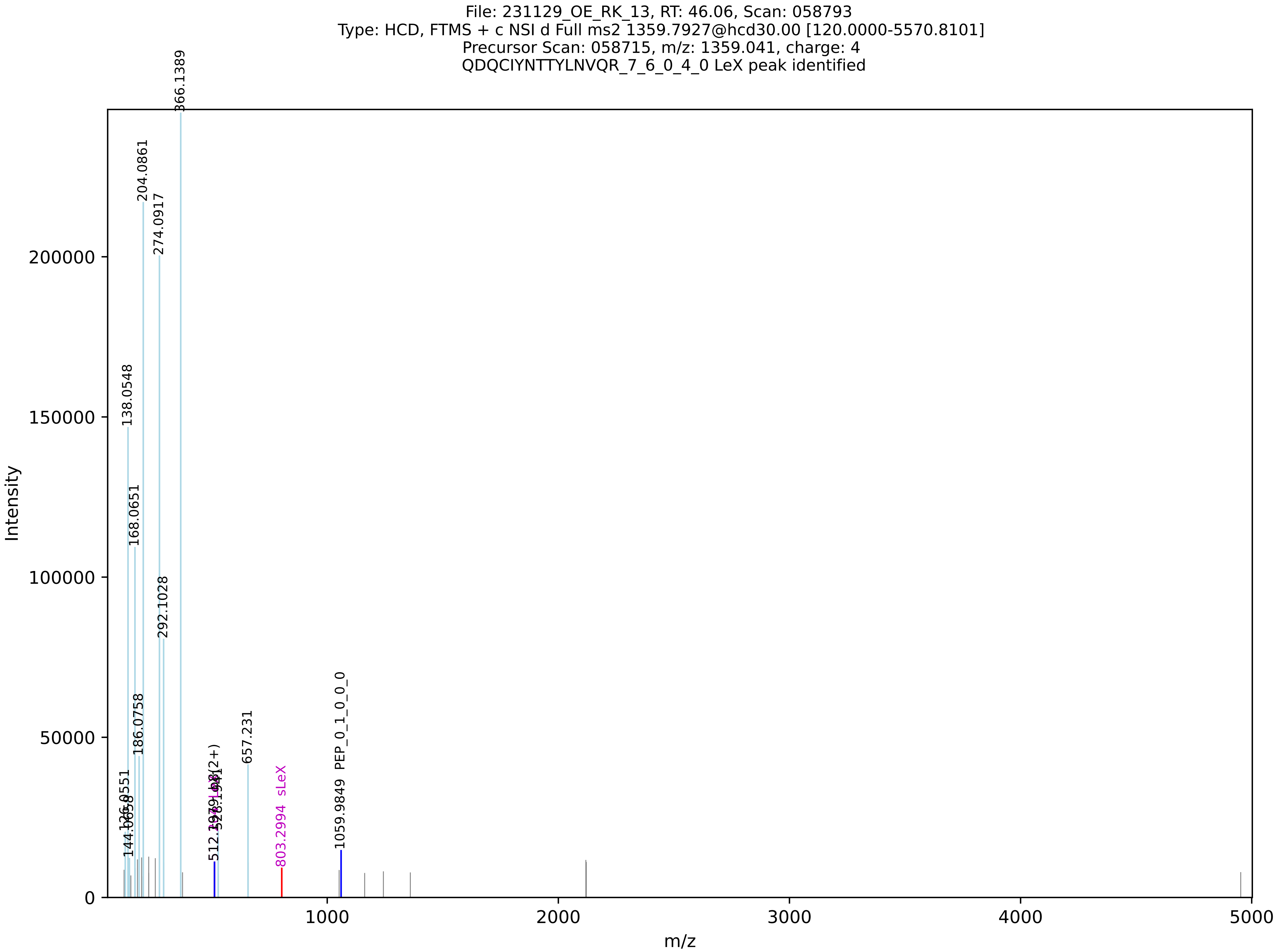
File: 231129_OE_RK_13, RT: 46.06, Scan: 058793
Type: HCD, FTMS + c NSI d Full ms2 1359.7927@hcd30.00 [120.0000-5570.8101]
Precursor Scan: 058715, m/z: 1359.041, charge: 4
QDQCIYNTTYLNVQR_7_6_0_4_0 LeX peak identified
366.1389
204.0861
274.0917
138.0548
168.0651
292.1028
186.0758
657.231
1059.9849 PEP 0_1_0_0_0
803.2994 sLeX
Intensity
m/z
0
50000
100000
150000
200000
1000
2000
3000
4000
5000

File: 231129_OE_RK_13, RT: 46.719, Scan: 059741
Type: HCD, FTMS + c NSI d Full ms2 1359.7933@hcd30.00 [120.0000-5570.8130]
Precursor Scan: 059658, m/z: 1359.2881, charge: 4
QDQCIYNTTYLNVQR_7_6_0_4_0 LeX peak identified

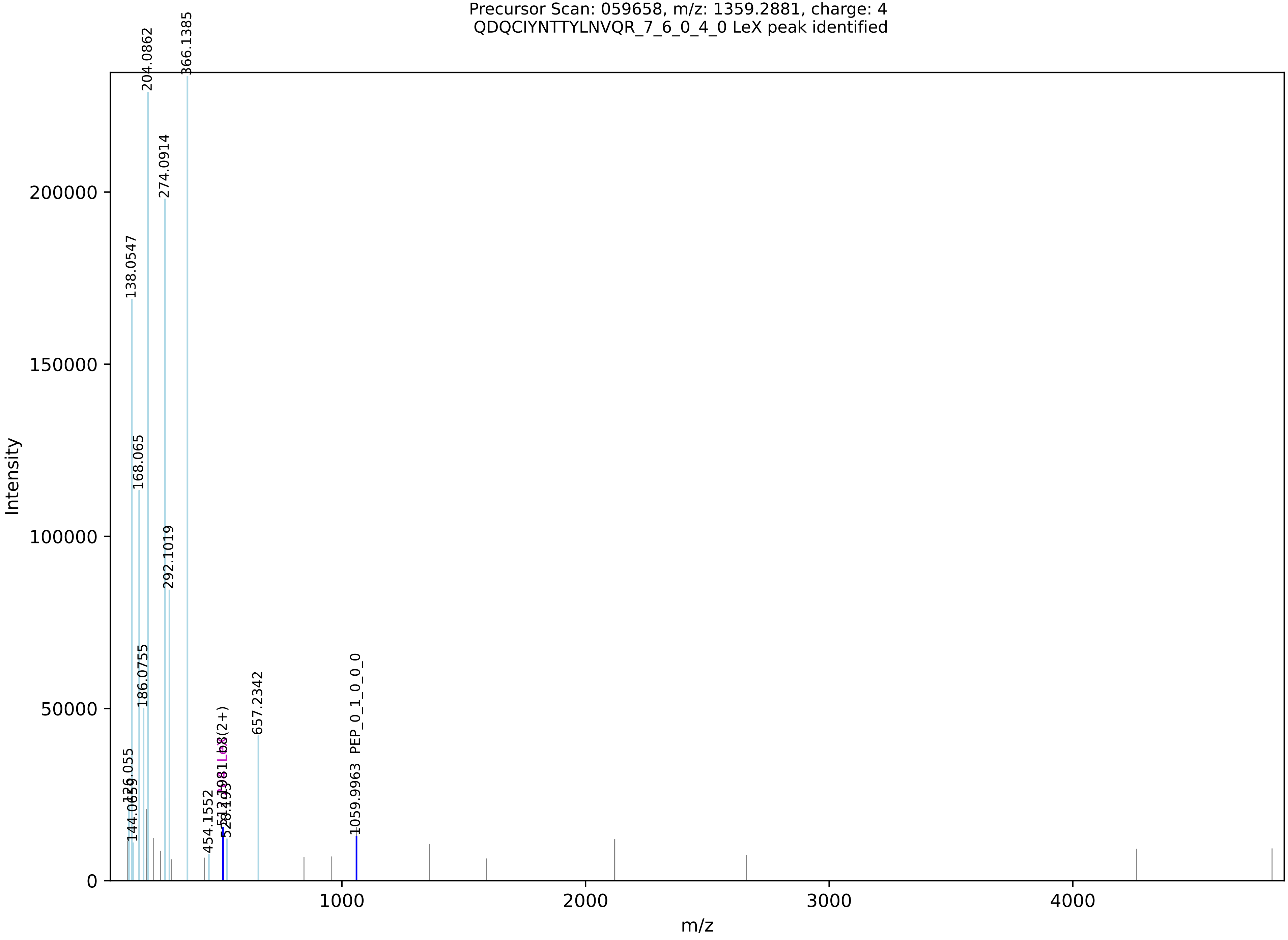

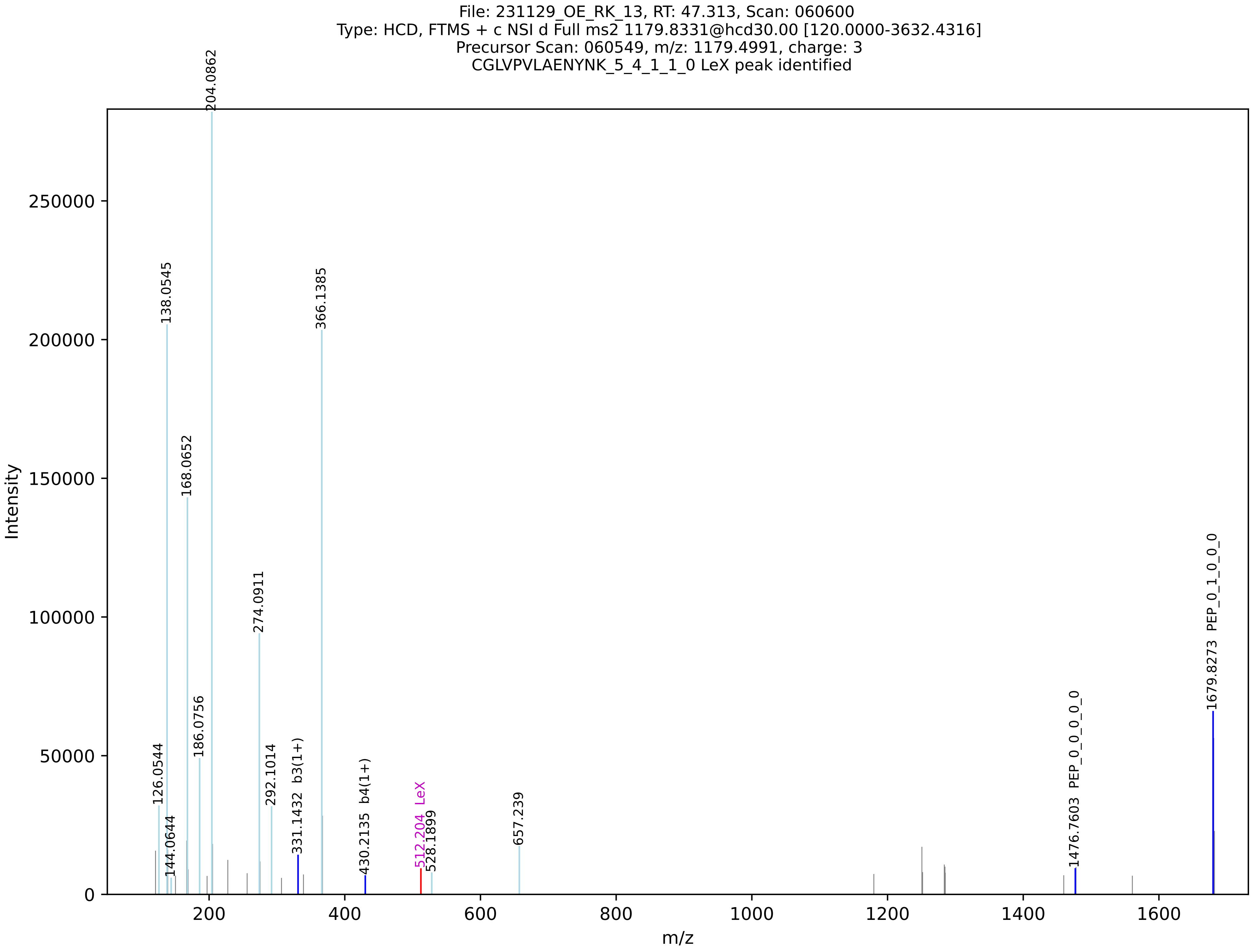

File: 231129_OE_RK_13, RT: 47.313, Scan: 060600
Type: HCD, FTMS + c NSI d Full ms2 1179.8331@hcd30.00 [120.0000-3632.4316]
Precursor Scan: 060549, m/z: 1179.4991, charge: 3
CGLVPVLAENYNK_5_4_1_1_0 LeX peak identified
Intensity
m/z
0
50000
100000
150000
200000
250000
200
400
600
800
1000
1200
1400
1600
204.0862
138.0545
366.1385
168.0652
274.0911
186.0756
126.0544
144.0644
292.1014
331.1432 b3(1+)
430.2135 b4(1+)
512.204 LeX
528.1899
657.239
1476.7603 PEP_0_0_0_0_0
1679.8273 PEP_0_1_0_0_0

File: 231129_OE_RK_13, RT: 48.604, Scan: 062461
Type: HCD, FTMS + c NSI d Full ms2 1323.5288@hcd30.00 [120.0000-5422.8535]
Precursor Scan: 062382, m/z: 1322.7746, charge: 4
QDQCIYNTTYLNVQR_7_6_1_3_0 LeX peak identified

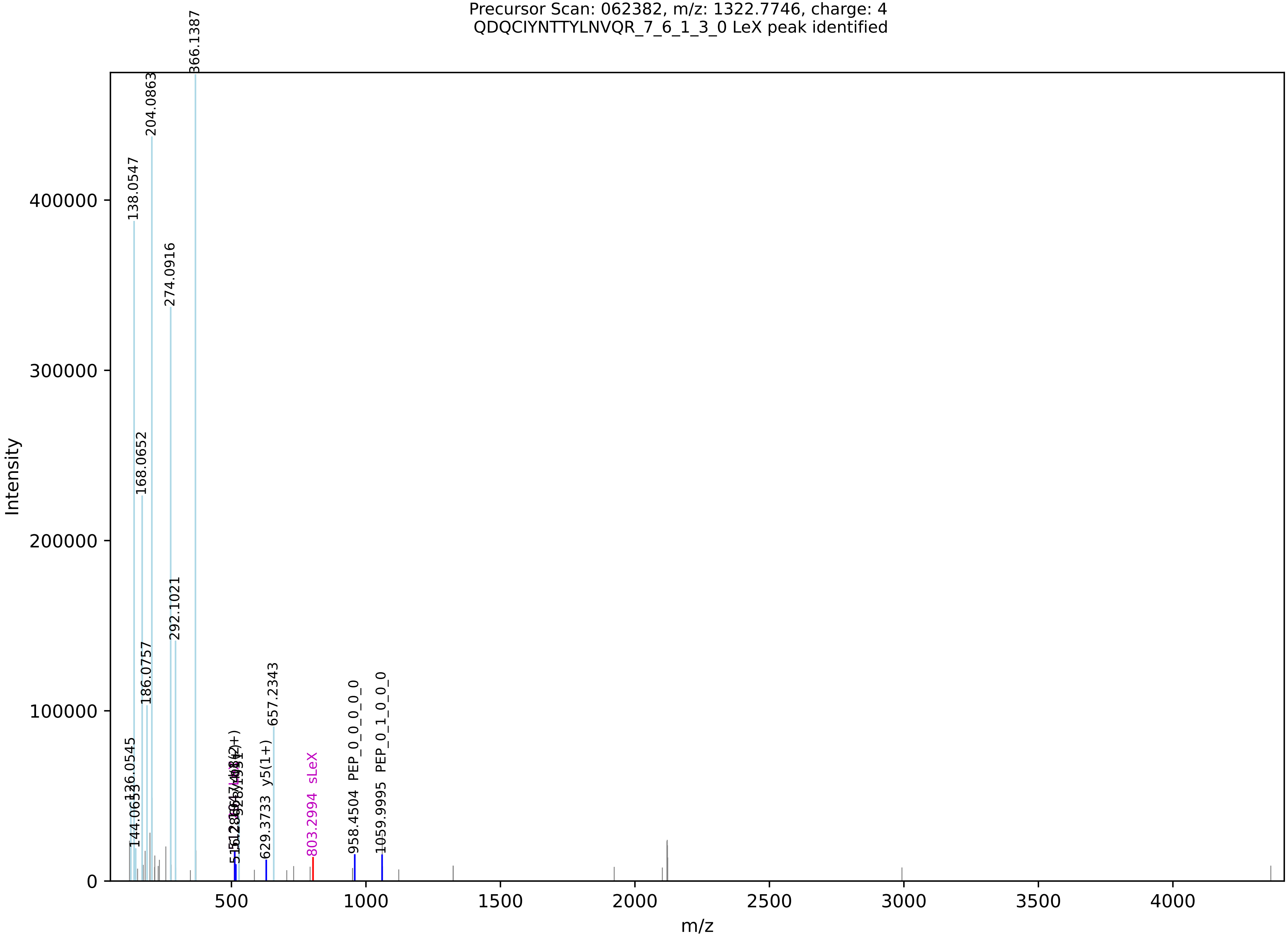

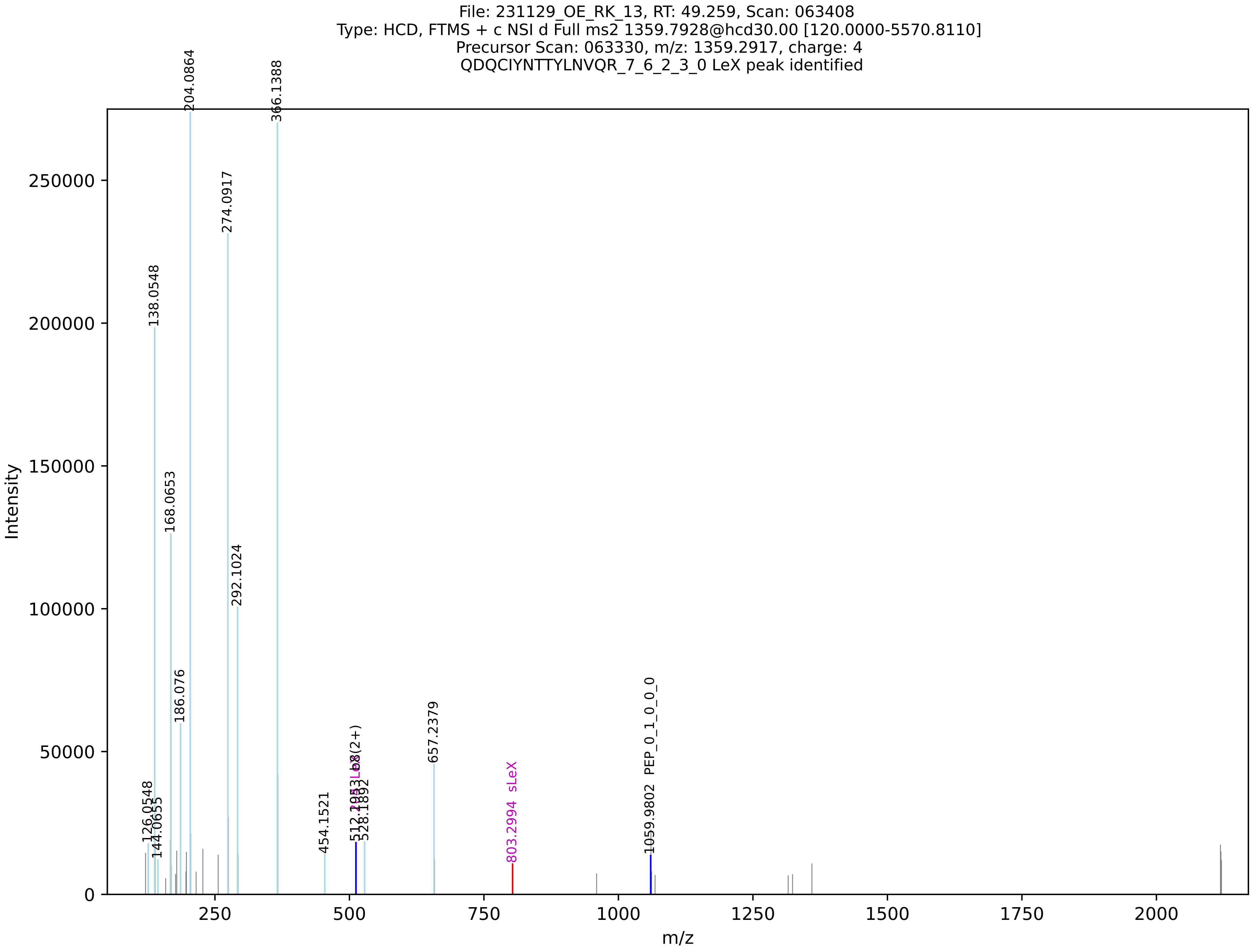

File: 231129_OE_RK_13, RT: 49.259, Scan: 063408
Type: HCD, FTMS + c NSI d Full ms2 1359.7928@hcd30.00 [120.0000-5570.8110]
Precursor Scan: 063330, m/z: 1359.2917, charge: 4
QDQCIYNTTYLNVQR_7_6_2_3_0 LeX peak identified
Intensity
m/z
204.0864
366.1388
274.0917
138.0548
168.0653
292.1024
186.076
126.0548
144.0655
454.1521
512.1953
LeX
b3(2+)
528.1892
657.2379
803.2994
sLeX
1059.9802
PEP_0_1_0_0_0_
0
50000
100000
150000
200000
250000
250
500
750
1000
1250
1500
1750
2000

File: 231129_OE_RK_13, RT: 51.384, Scan: 066485
Type: HCD, FTMS + c NSI d Full ms2 1232.2471@hcd30.00 [120.0000-5050.4243]
Precursor Scan: 066397, m/z: 1231.4954, charge: 4
QDQCIYNTTYLNVQR_6_5_1_3_0 LeX peak identified

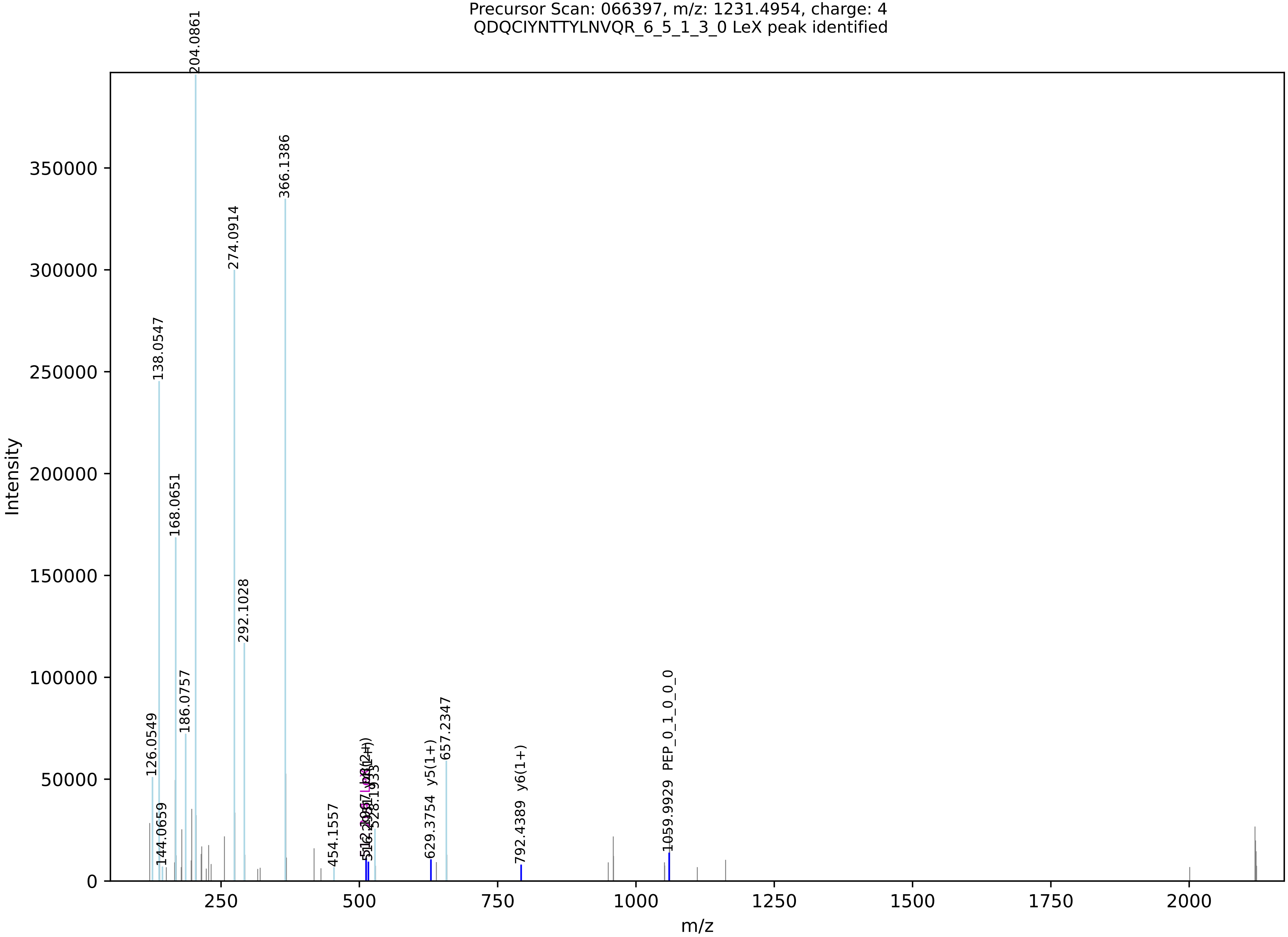

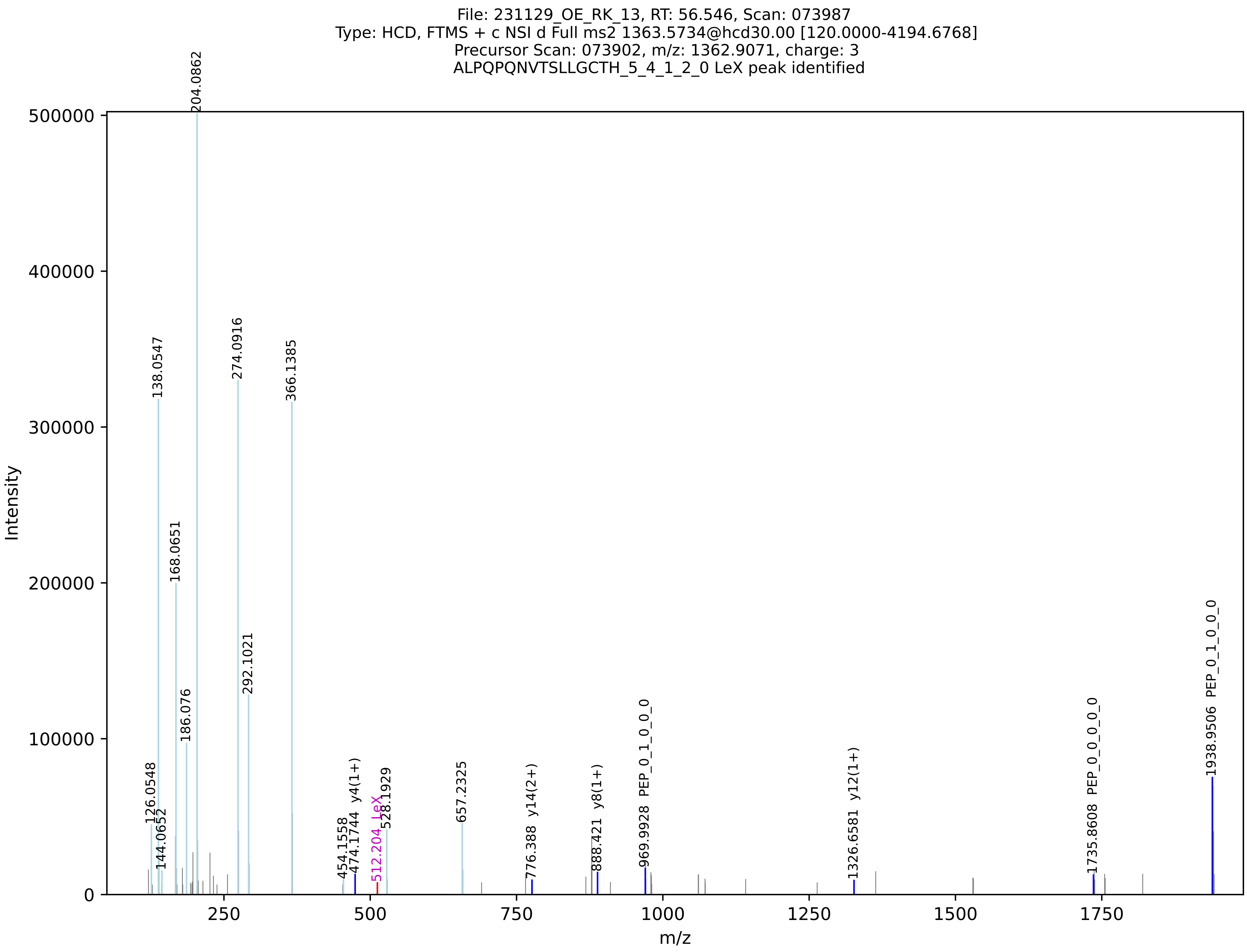
File: 231129_OE_RK_13, RT: 56.546, Scan: 073987
Type: HCD, FTMS + c NSI d Full ms2 1363.5734@hcd30.00 [120.0000-4194.6768]
Precursor Scan: 073902, m/z: 1362.9071, charge: 3
ALPQPQNVTSLLGCTH_5_4_1_2_0 LeX peak identified
Intensity
m/z
204.0862
138.0547
274.0916
366.1385
168.0651
292.1021
186.076
126.0548
144.0652
454.1558
474.1744 y4(1+)
512.204 LeX
528.1929
657.2325
776.388 y14(2+)
888.421 y8(1+)
969.9928 PEP_0_1_0_0_0
1326.6581 y12(1+)
1735.8608 PEP_0_0_0_0_0
1938.9506 PEP_0_1_0_0_0

File: 231129_OE_RK_13, RT: 64.804, Scan: 085969
Type: HCD, FTMS + c NSI d Full ms2 1273.2178@hcd30.00 [120.0000-3918.1885]
Precursor Scan: 085875, m/z: 1272.5496, charge: 3
YLGNATAIFFLPDEGK_5_4_1_1_0 LeX peak identified

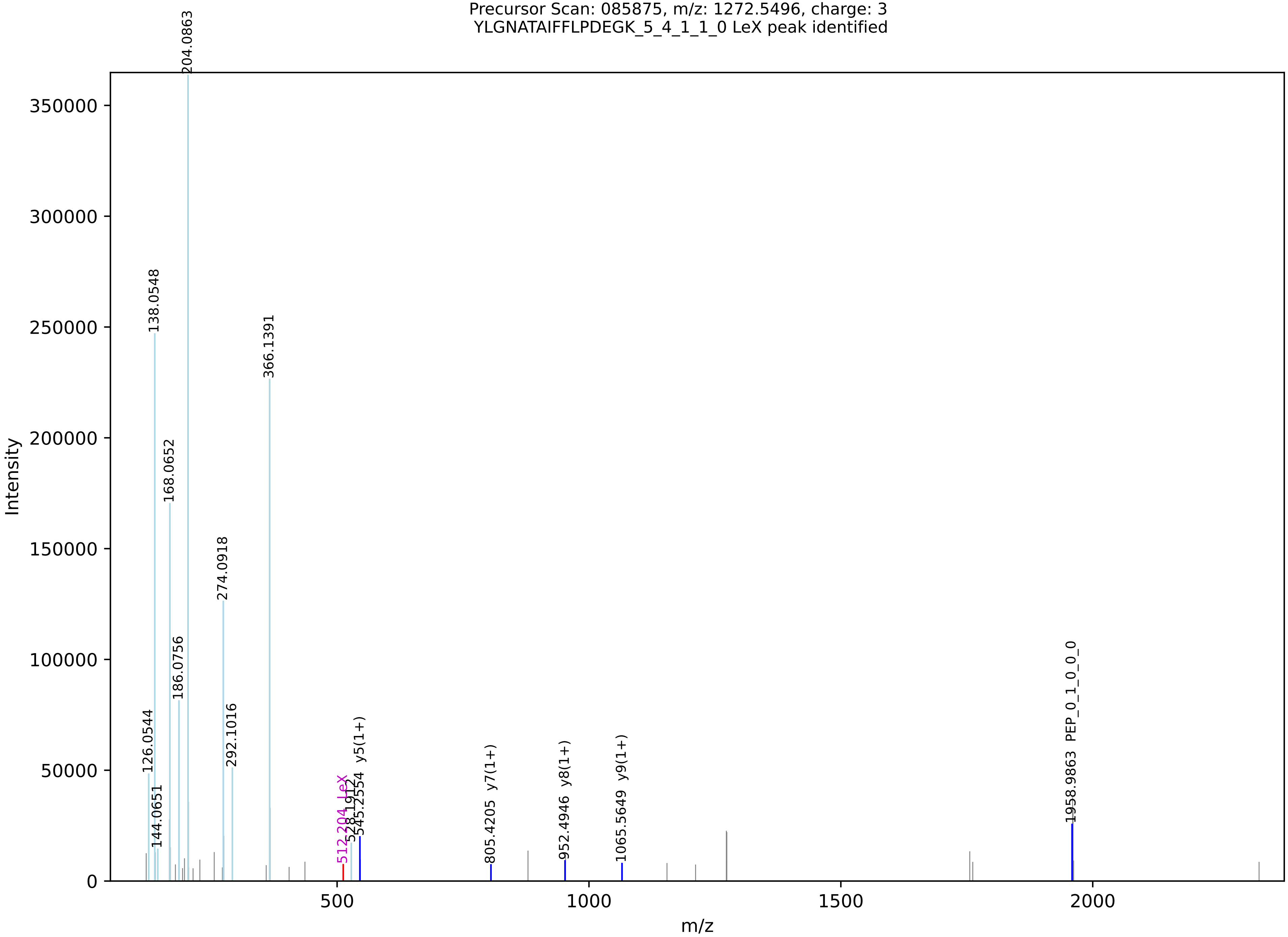

File: 231129_OE_RK_13, RT: 65.004, Scan: 086262
Type: HCD, FTMS + c NSI d Full ms2 1273.2147@hcd30.00 [120.0000-3918.1792]
Precursor Scan: 086171, m/z: 1272.5465, charge: 3
YLGNATAIFFLPDEGK_5_4_1_1_0 LeX peak identified

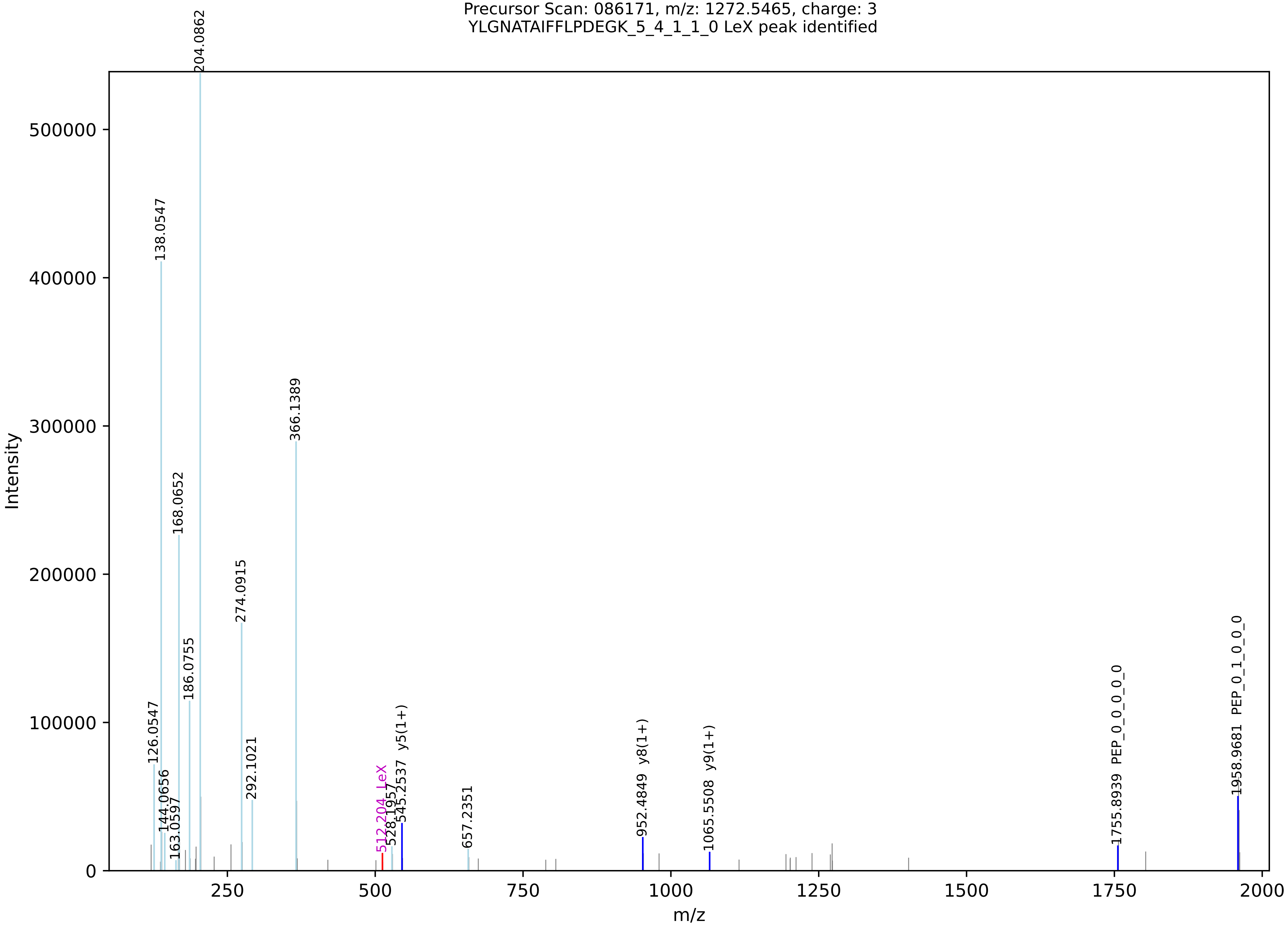

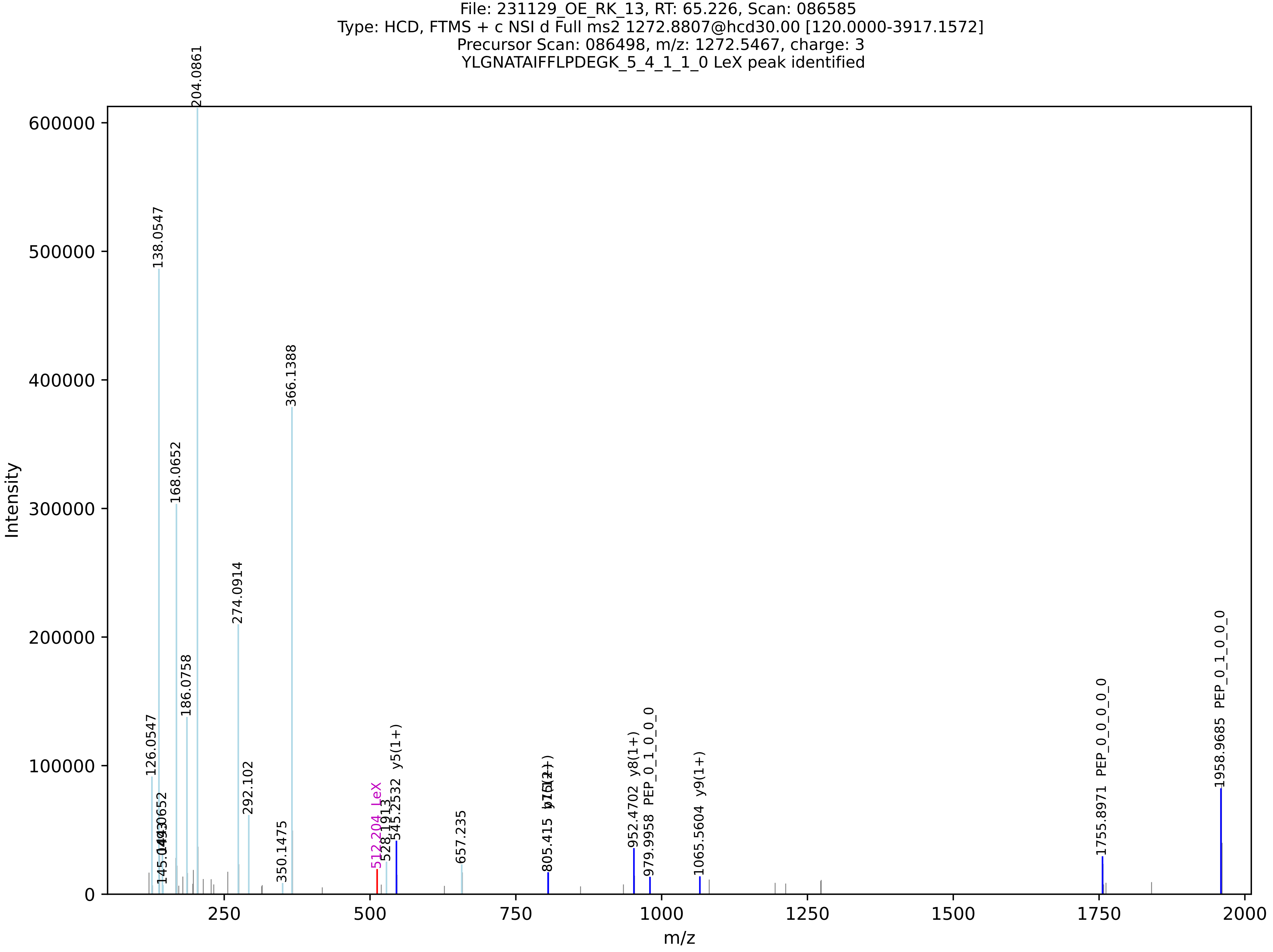
File: 231129_OE_RK_13, RT: 65.226, Scan: 086585
Type: HCD, FTMS + c NSI d Full ms2 1272.8807@hcd30.00 [120.0000-3917.1572]
Precursor Scan: 086498, m/z: 1272.5467, charge: 3
YLGNATAIFFLPDEGK_5_4_1_1_0 LeX peak identified
Intensity
m/z
204.0861
138.0547
366.1388
168.0652
274.0914
186.0758
126.0547
292.102
350.1475
512.204 LeX
528.1913
545.2532 y5(1+)
657.235
952.4702 y8(1+)
979.9958 PEP_0_1_0_0_0
1065.5604 y9(1+)
1755.8971 PEP_0_0_0_0_0
1958.9685 PEP_0_1_0_0_0

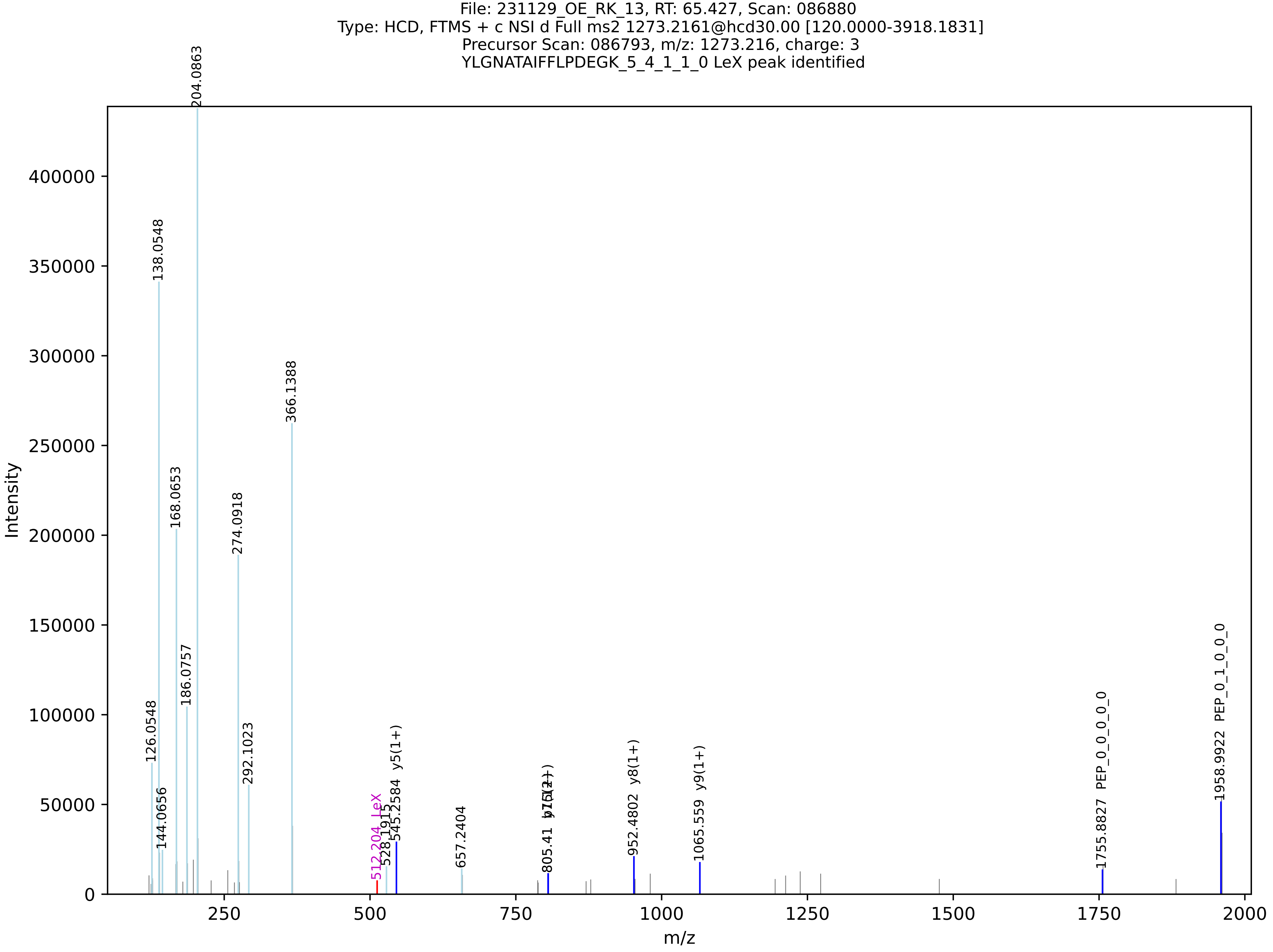
File: 231129_OE_RK_13, RT: 65.427, Scan: 086880
Type: HCD, FTMS + c NSI d Full ms2 1273.2161@hcd30.00 [120.0000-3918.1831]
Precursor Scan: 086793, m/z: 1273.216, charge: 3
YLGNATAIFFLPDEGK_5_4_1_1_0 LeX peak identified
Intensity
m/z
204.0863
138.0548
168.0653
274.0918
366.1388
186.0757
126.0548
144.0656
292.1023
512.204 LeX
528.1915
545.2584 y5(1+)
657.2404
805.41
952.4802 y8(1+)
1065.559 y9(1+)
1755.8827 PEP_0_0_0_0_0
1958.9922 PEP_0_1_0_0_0
0
50000
100000
150000
200000
250000
300000
350000
400000
250
500
750
1000
1250
1500
1750
2000

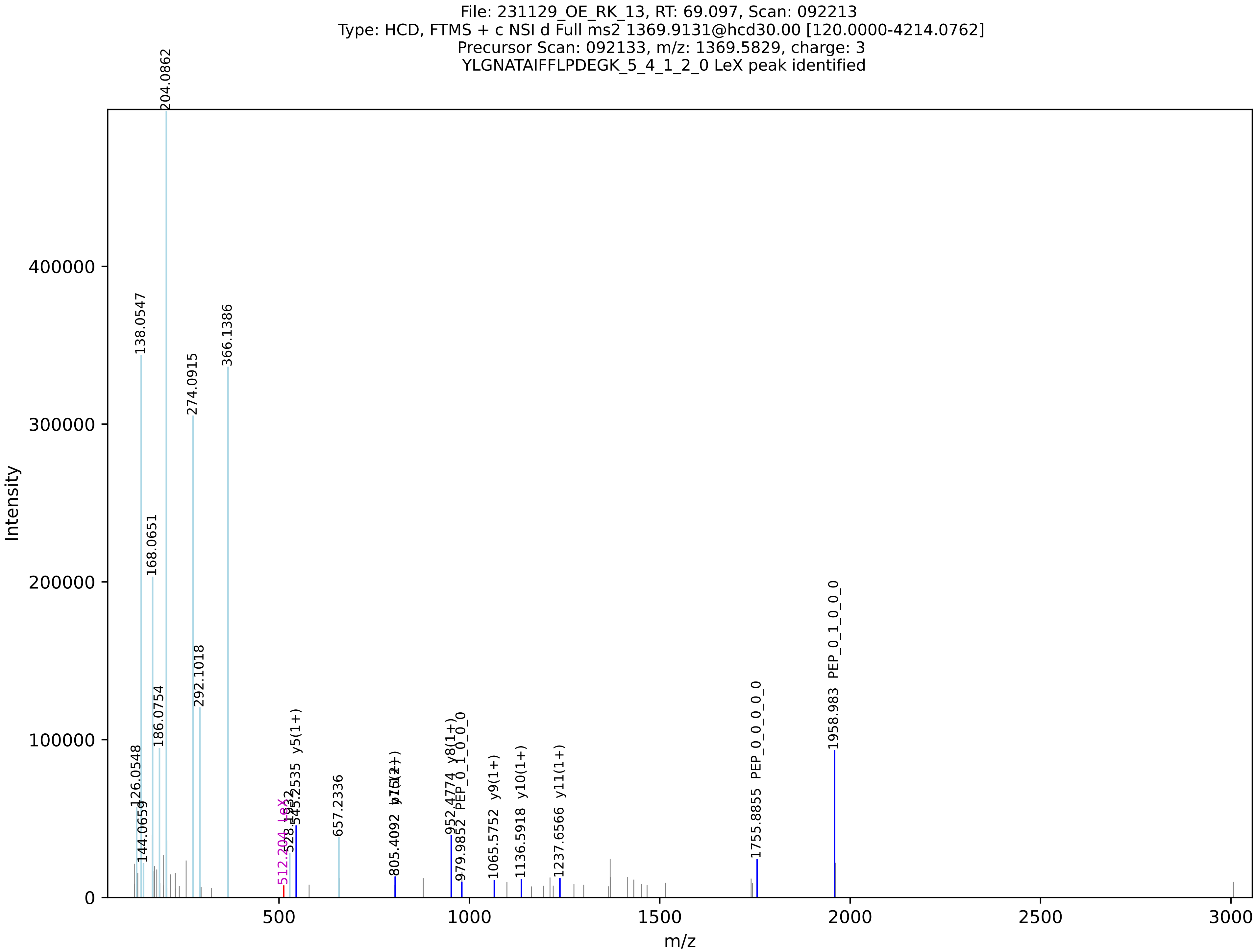

File: 231129_OE_RK_13, RT: 69.097, Scan: 092213
Type: HCD, FTMS + c NSI d Full ms2 1369.9131@hcd30.00 [120.0000-4214.0762]
Precursor Scan: 092133, m/z: 1369.5829, charge: 3
YLGNATAIFFLPDEGK_5_4_1_2_0 LeX peak identified
204.0862
138.0547
366.1386
274.0915
168.0651
292.1018
186.0754
126.0548
144.0659
512.204 LeX
545.2535 y5(1+)
657.2336
805.4092
952.4774 y8(1+)
979.9852 PEP_0_1_0_0_0
1065.5752 y9(1+)
1136.5918 y10(1+)
1237.6566 y11(1+)
1755.8855 PEP_0_0_0_0_0
1958.983 PEP_0_1_0_0_0
Intensity
0
100000
200000
300000
400000
m/z
500
1000
1500
2000
2500
3000

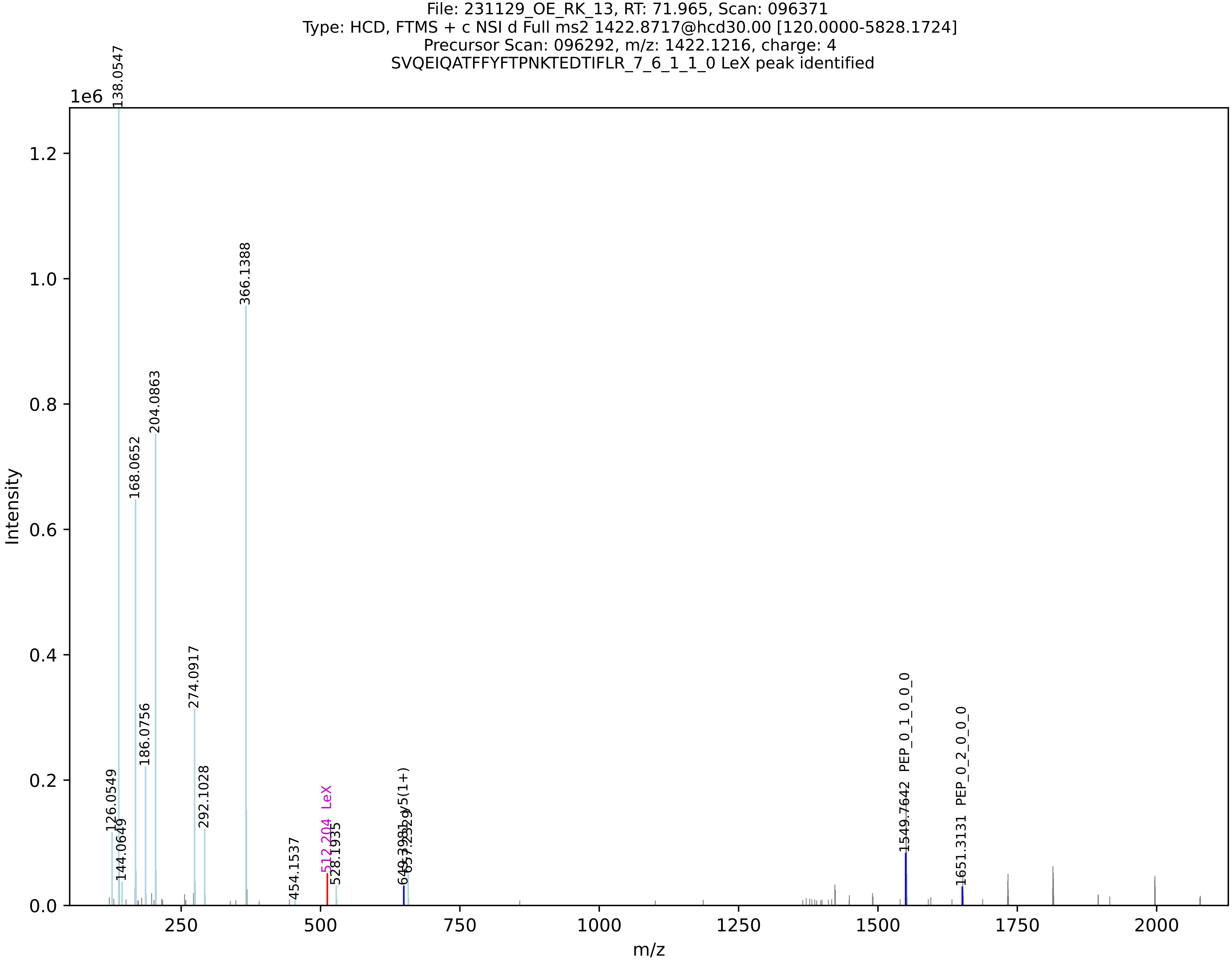
File: 231129_OE_RK_13, RT: 71.965, Scan: 096371
Type: HCD, FTMS + c NSI d Full ms2 1422.8717@hcd30.00 [120.0000-5828.1724]
Precursor Scan: 096292, m/z: 1422.1216, charge: 4
SVQEIQATFFYFTPNKTEDTIFLR_7_6_1_1_0 LeX peak identified
1e6
Intensity
m/z
138.0547
366.1388
204.0863
168.0652
274.0917
186.0756
292.1028
126.0549
144.0649
454.1537
512.204 LeX
528.1935
649.3981 y5(1+)
1549.7642 PEP_0_1_0_0_0
1651.3131 PEP_0_2_0_0_0

File: 231129_OE_RK_14, RT: 4.985, Scan: 001342
Type: HCD, FTMS + c NSI d Full ms2 1185.1313@hcd30.00 [120.0000-3648.6438]
Precursor Scan: 001335, m/z: 1184.7972, charge: 3
ENGTVSR_7_6_1_1_0 LeX peak identified

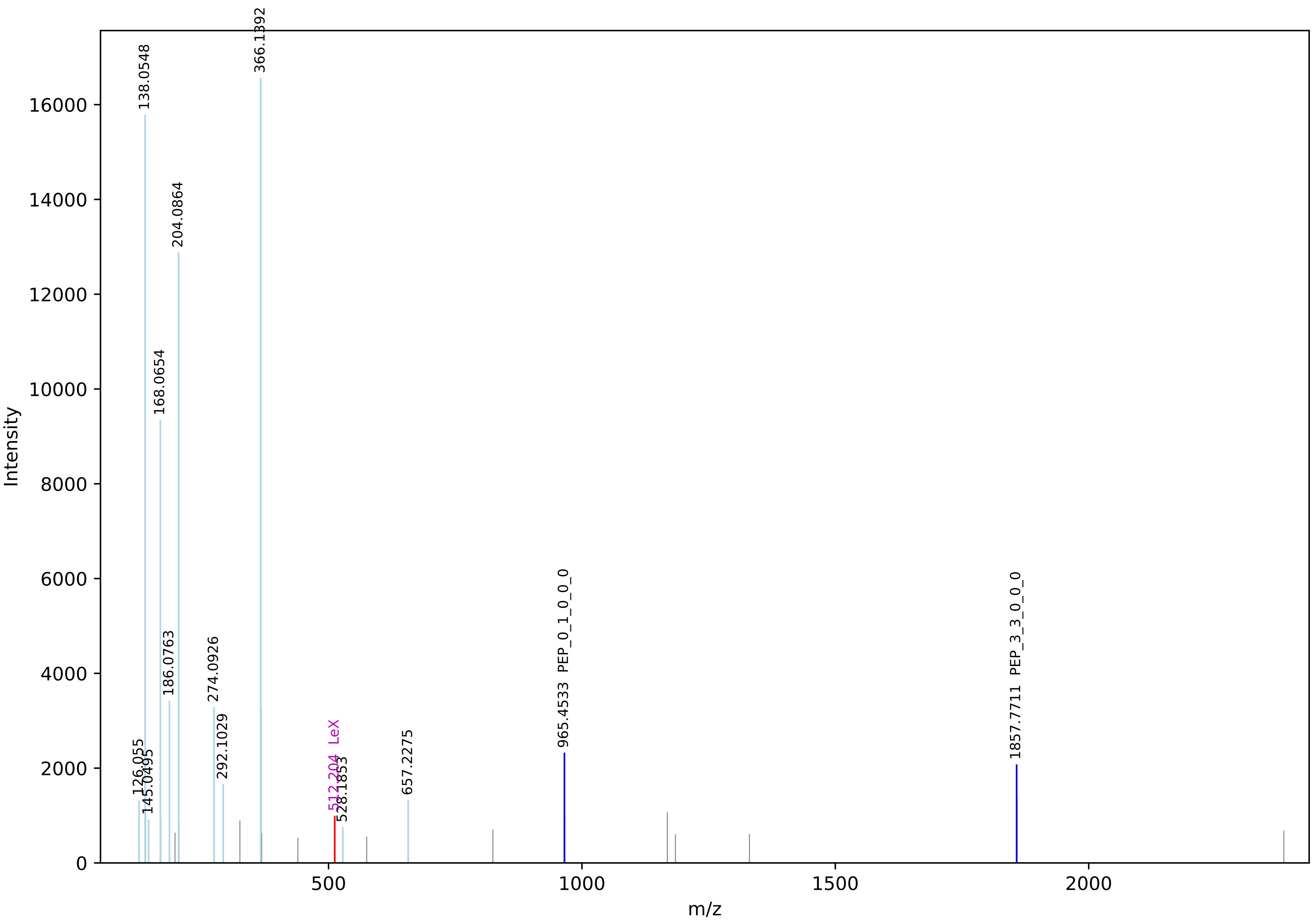

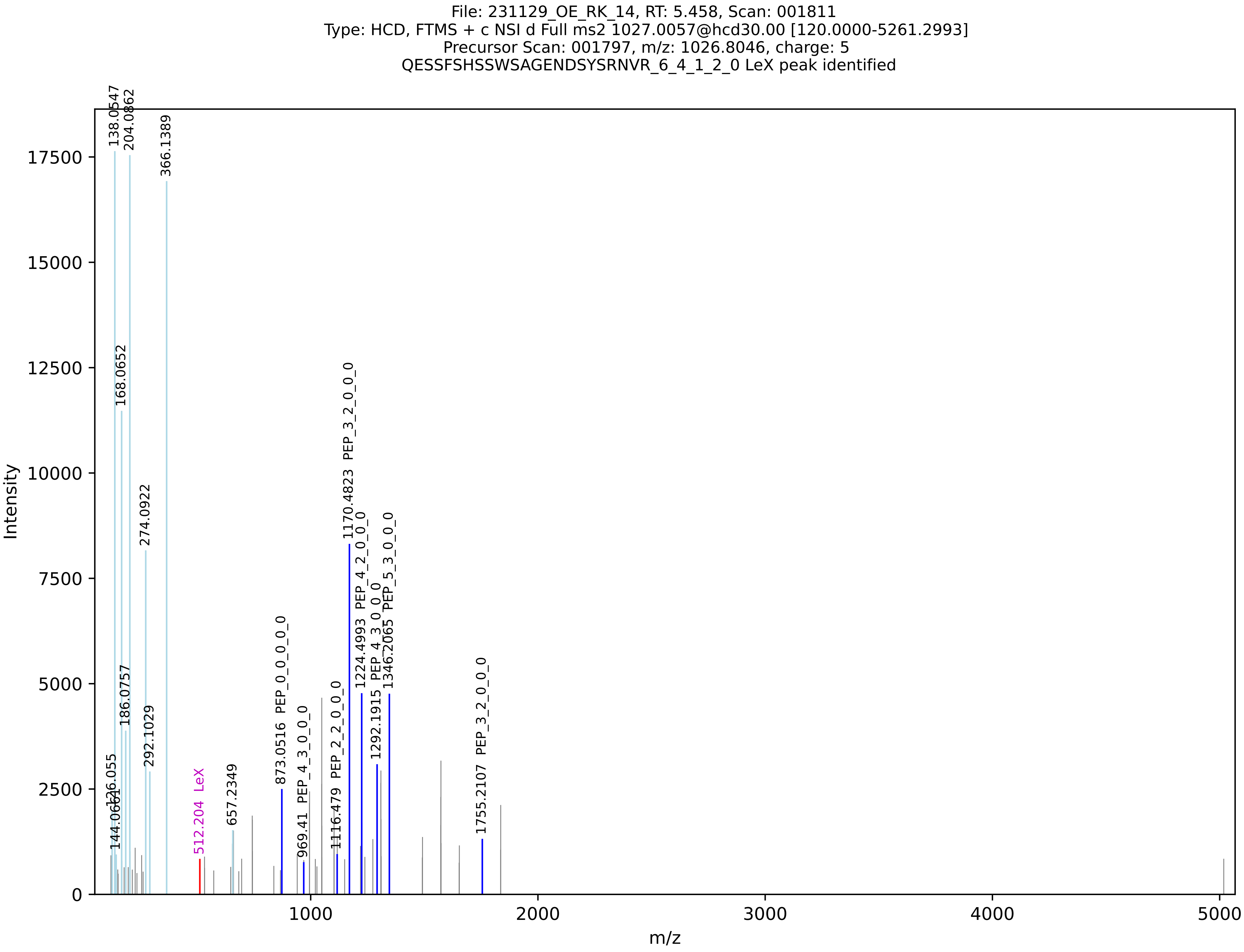

File: 231129_OE_RK_14, RT: 5.458, Scan: 001811
Type: HCD, FTMS + c NSI d Full ms2 1027.0057@hcd30.00 [120.0000-5261.2993]
Precursor Scan: 001797, m/z: 1026.8046, charge: 5
QESSFSHSSWSAGENDSYSRNVR_6_4_1_2_0 LeX peak identified
Intensity
m/z
138.0547
204.0862
366.1389
168.0652
274.0922
186.0757
292.1029
126.055
144.0661
512.204 LeX
657.2349
873.0516 PEP_0_0_0_0_0
969.41 PEP_4_3_0_0_0
1116.479 PEP_2_2_0_0_0
1170.4823 PEP_3_2_0_0_0
1224.4993 PEP_4_2_0_0_0
1292.1915 PEP_4_3_0_0_0
1346.2065 PEP_5_3_0_0_0
1755.2107 PEP_3_2_0_0_0

File: 231129_OE_RK_14, RT: 6.581, Scan: 003307
Type: HCD, FTMS + c NSI d Full ms2 1190.4218@hcd30.00 [120.0000-3664.8325]
Precursor Scan: 003221, m/z: 1190.4218, charge: 3
ENGTISR_7_6_1_1_0 LeX peak identified

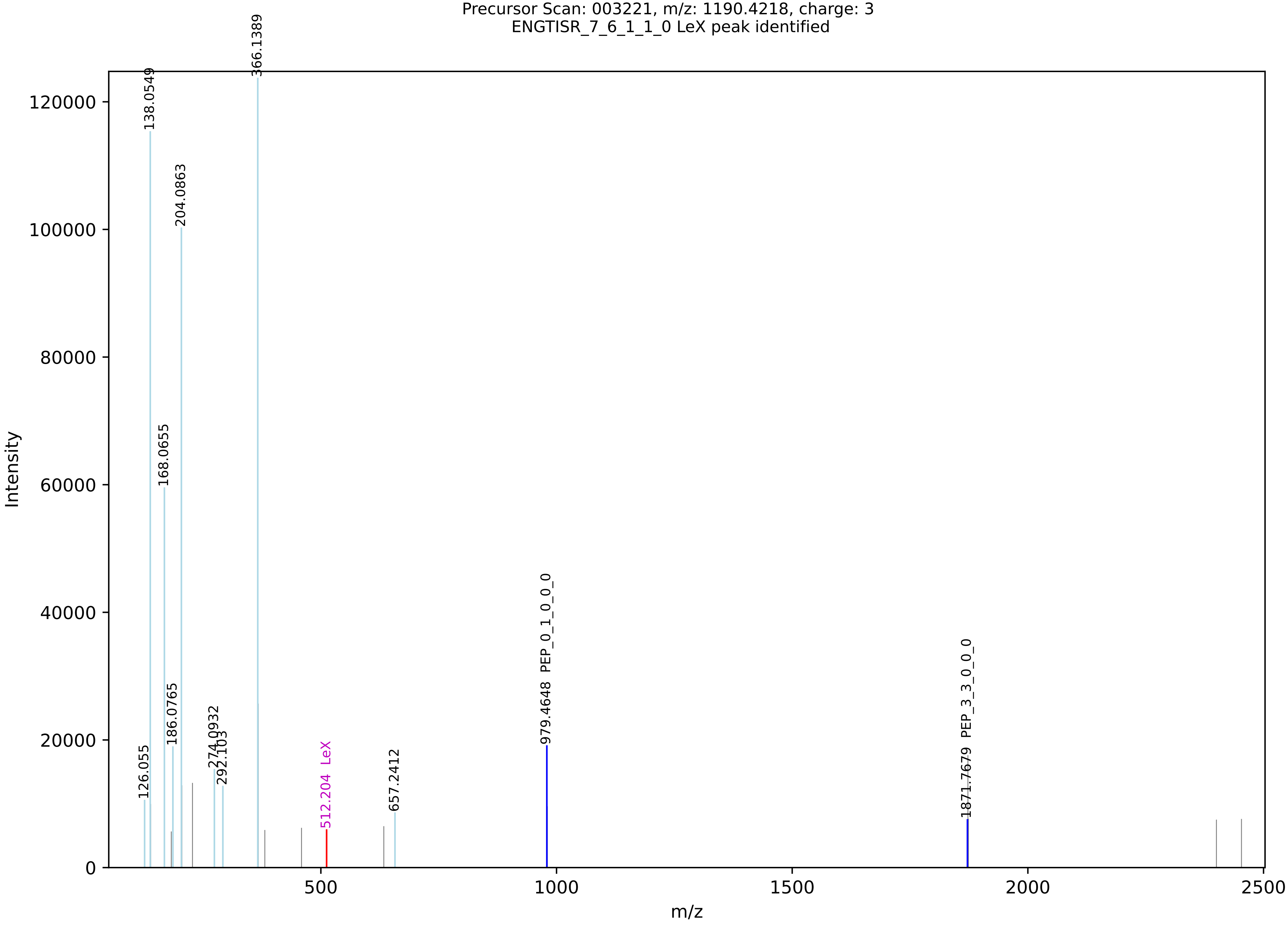

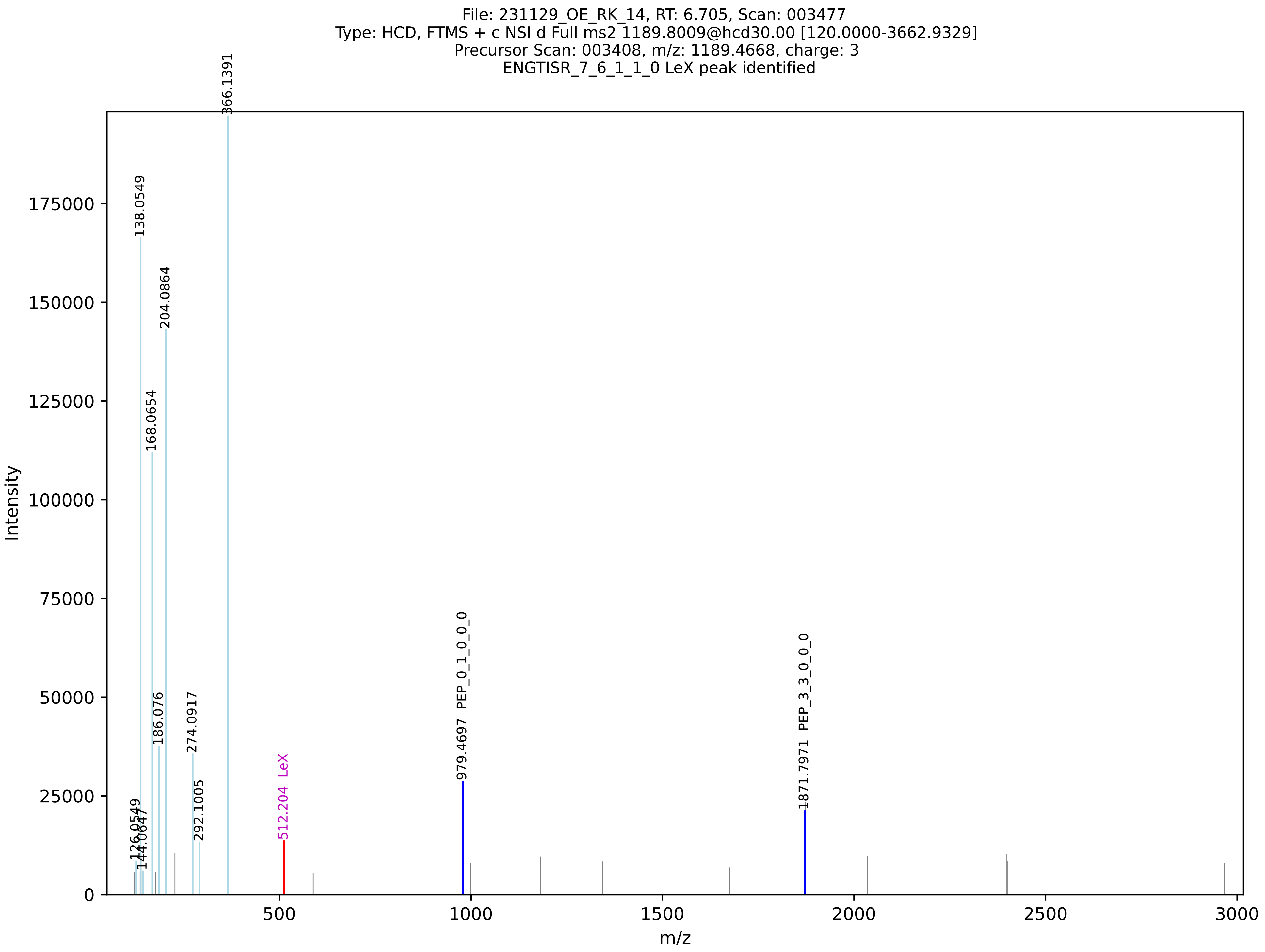

File: 231129_OE_RK_14, RT: 6.705, Scan: 003477
Type: HCD, FTMS + c NSI d Full ms2 1189.8009@hcd30.00 [120.0000-3662.9329]
Precursor Scan: 003408, m/z: 1189.4668, charge: 3
ENGTISR_7_6_1_1_0 LeX peak identified
366.1391
138.0549
204.0864
168.0654
186.076
274.0917
292.1005
126.0549
144.0647
512.204 LeX
979.4697 PEP_0_1_0_0_0
1871.7971 PEP_3_3_0_0_0
Intensity
m/z
0
25000
50000
75000
100000
125000
150000
175000
500
1000
1500
2000
2500
3000

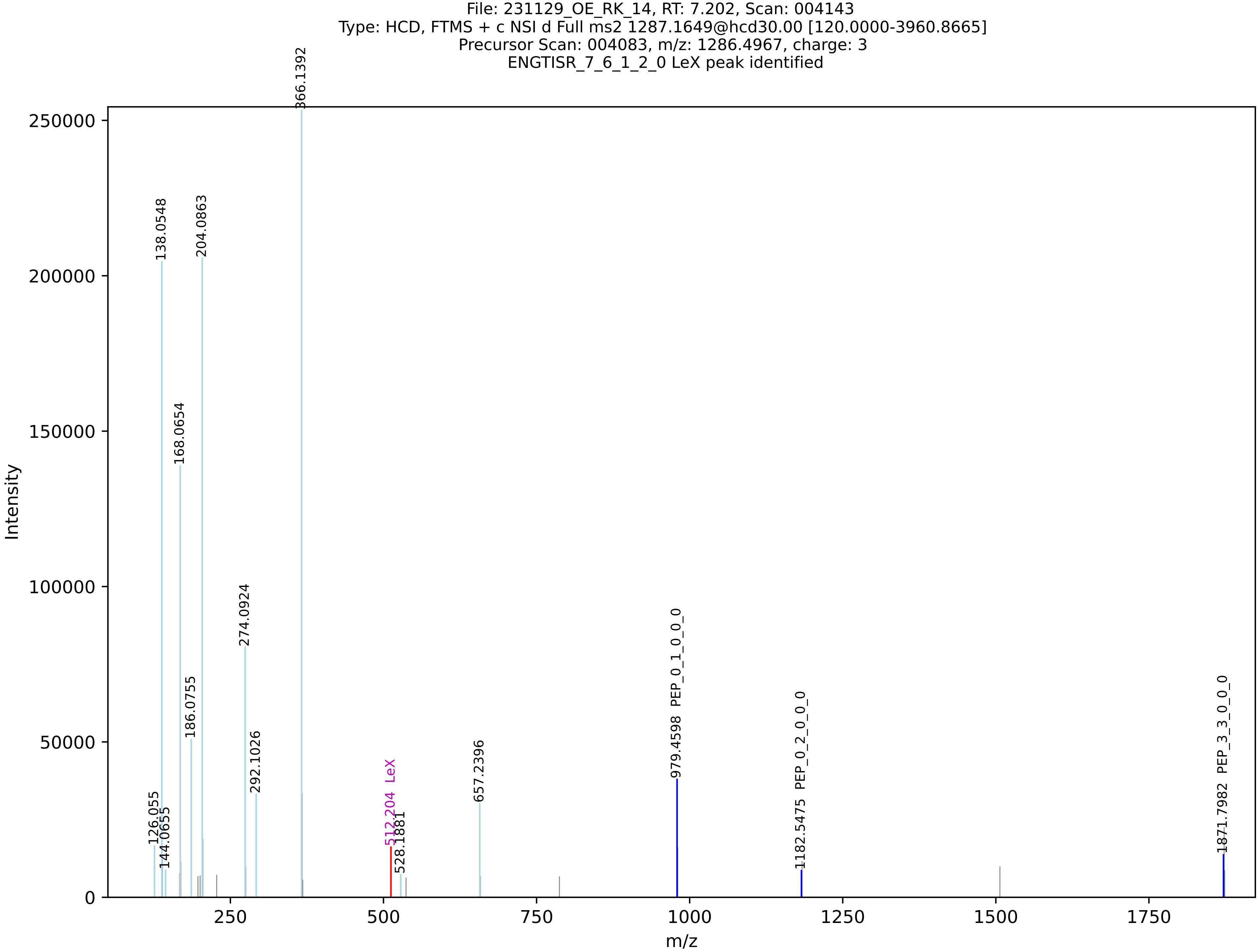
File: 231129_OE_RK_14, RT: 7.202, Scan: 004143
Type: HCD, FTMS + c NSI d Full ms2 1287.1649@hcd30.00 [120.0000-3960.8665]
Precursor Scan: 004083, m/z: 1286.4967, charge: 3
ENGTISR_7_6_1_2_0 LeX peak identified
Intensity
m/z
366.1392
138.0548
204.0863
168.0654
274.0924
186.0755
292.1026
126.055
144.0655
512.204 LeX
528.1881
657.2396
979.4598 PEP_0_1_0_0_0
1182.5475 PEP_0_2_0_0_0
1871.7982 PEP_3_3_0_0_0
0
50000
100000
150000
200000
250000
250
500
750
1000
1250
1500
1750

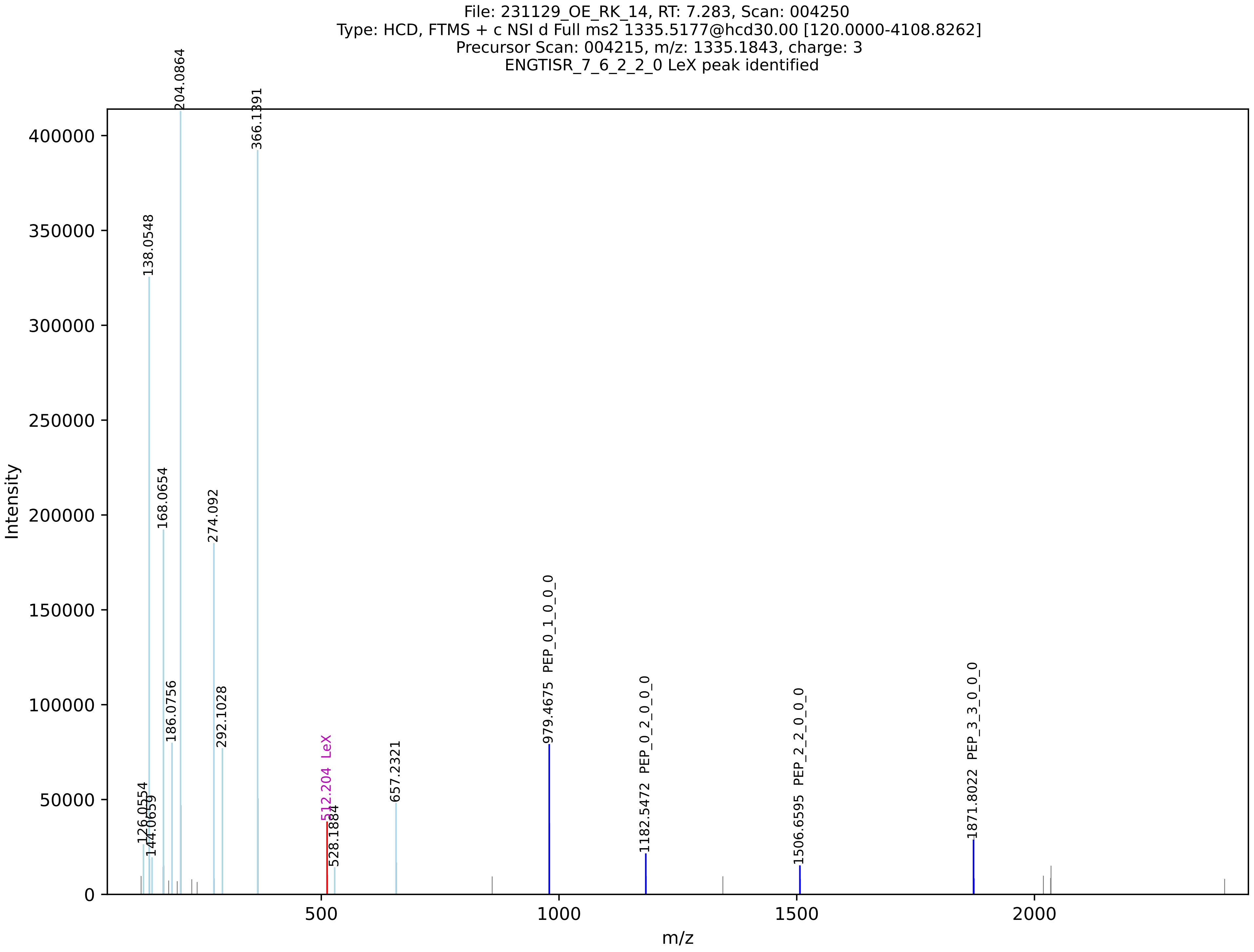
File: 231129_OE_RK_14, RT: 7.283, Scan: 004250
Type: HCD, FTMS + c NSI d Full ms2 1335.5177@hcd30.00 [120.0000-4108.8262]
Precursor Scan: 004215, m/z: 1335.1843, charge: 3
ENGTISR_7_6_2_2_0 LeX peak identified
Intensity
m/z
204.0864
366.1391
138.0548
168.0654
274.092
186.0756
292.1028
126.0554
144.0659
512.204 LeX
528.1884
657.2321
979.4675 PEP_0_1_0_0_0
1182.5472 PEP_0_2_0_0_0
1506.6595 PEP_2_2_0_0_0
1871.8022 PEP_3_3_0_0_0

File: 231129_OE_RK_14, RT: 7.43, Scan: 004450
Type: HCD, FTMS + c NSI d Full ms2 965.6273@hcd30.00 [120.0000-3962.6152]
Precursor Scan: 004411, m/z: 965.1257, charge: 4
ENGTISR_7_6_1_2_0 LeX peak identified

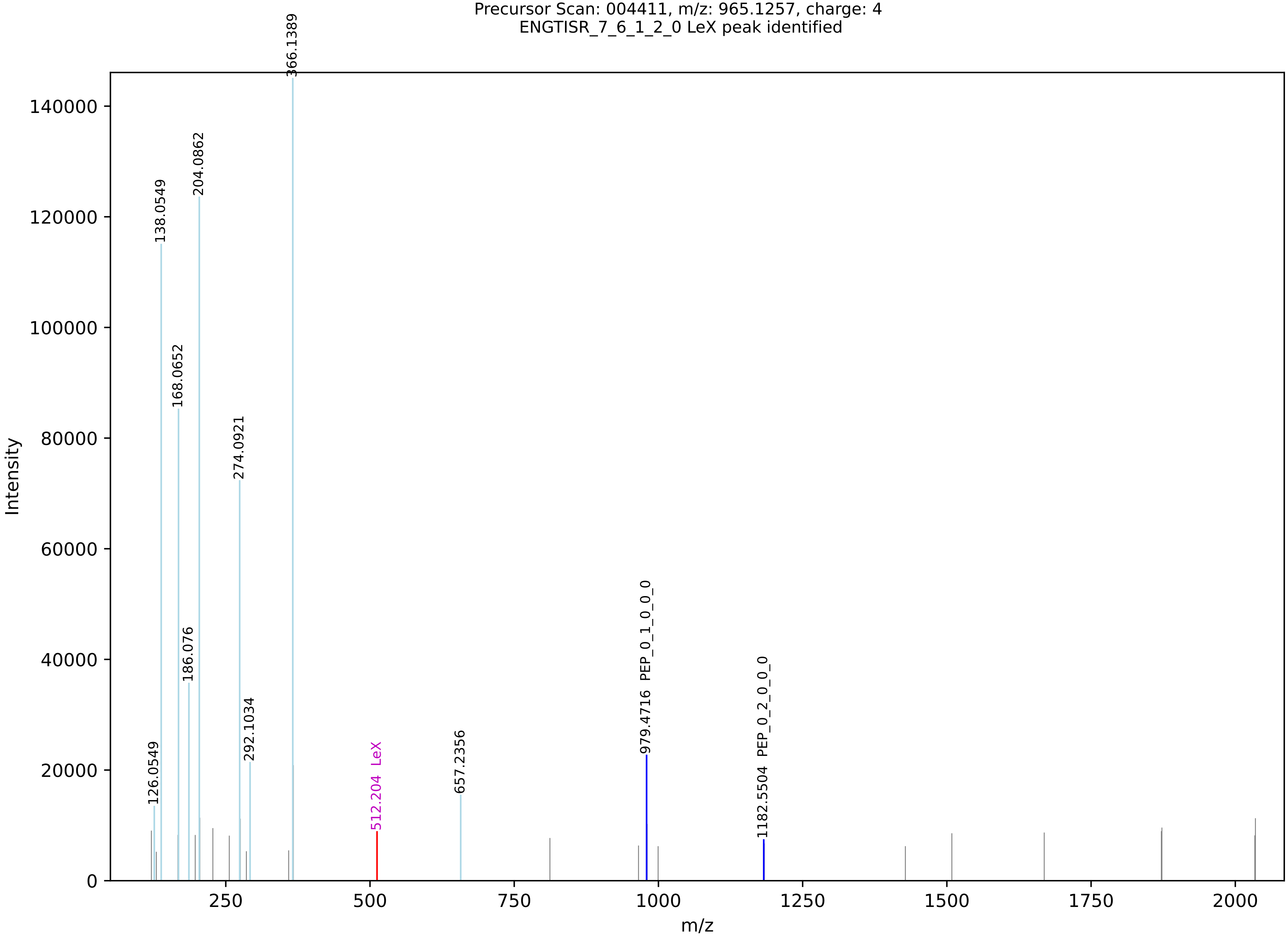

File: 231129_OE_RK_14, RT: 7.515, Scan: 004564
Type: HCD, FTMS + c NSI d Full ms2 1214.1432@hcd30.00 [120.0000-3737.4202]
Precursor Scan: 004523, m/z: 1213.4737, charge: 3
ENGTISR_6_5_2_2_0 LeX peak identified

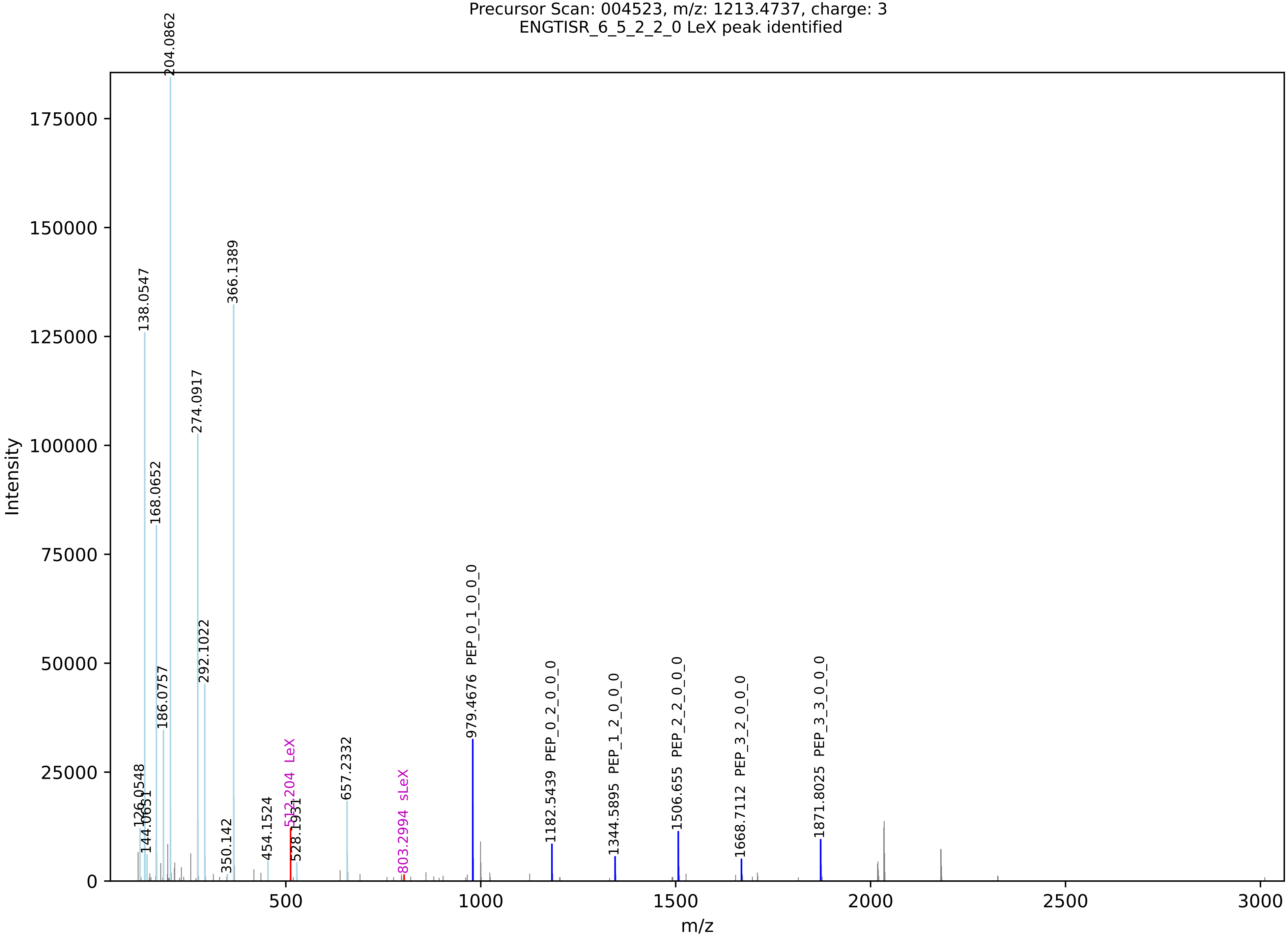

File: 231129_OE_RK_14, RT: 7.749, Scan: 004892
Type: HCD, FTMS + c NSI d Full ms2 1038.4021@hcd30.00 [120.0000-4259.5361]
Precursor Scan: 004842, m/z: 1037.9026, charge: 4
ENGTISR_7_6_1_3_0 LeX peak identified

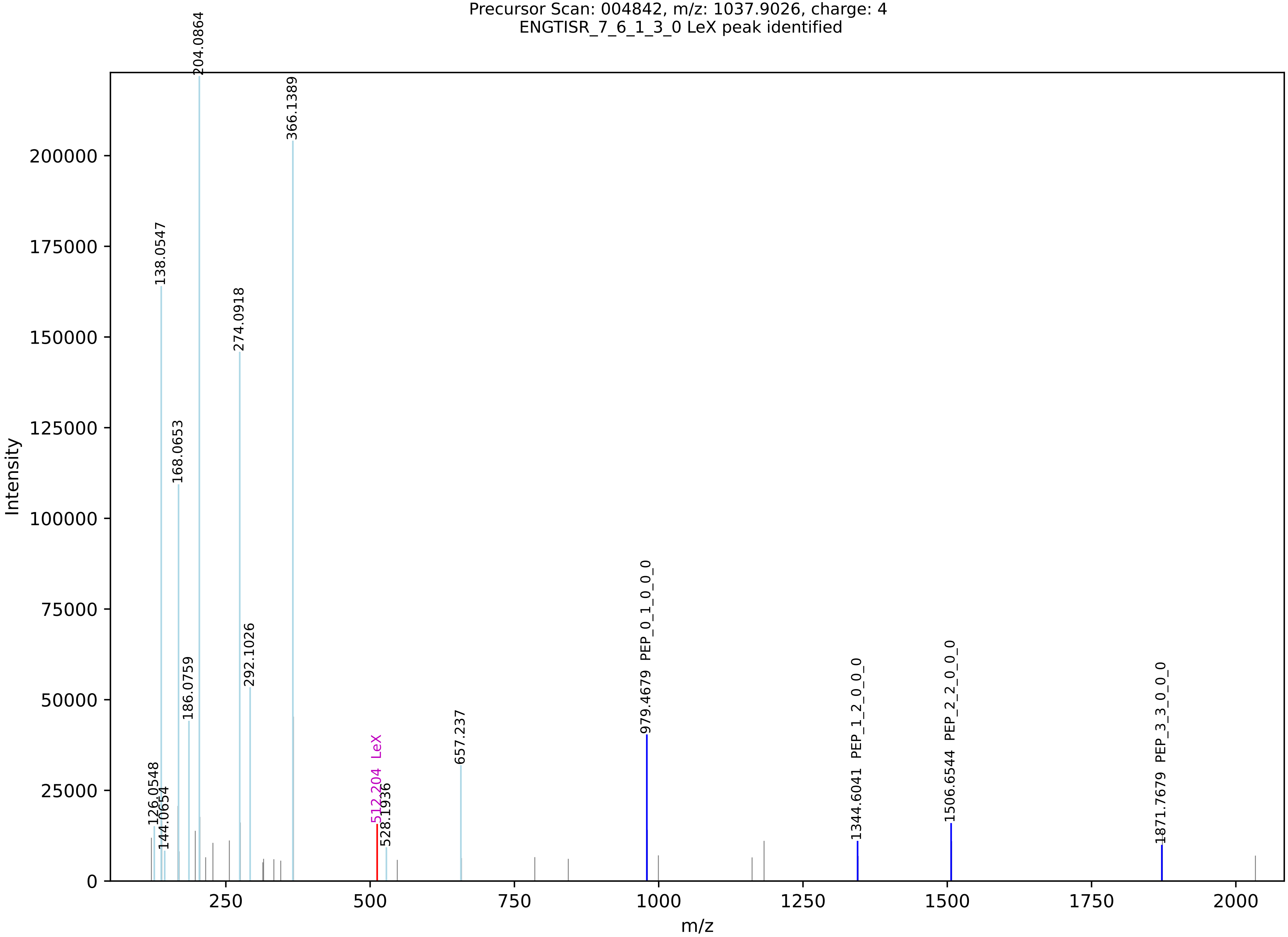

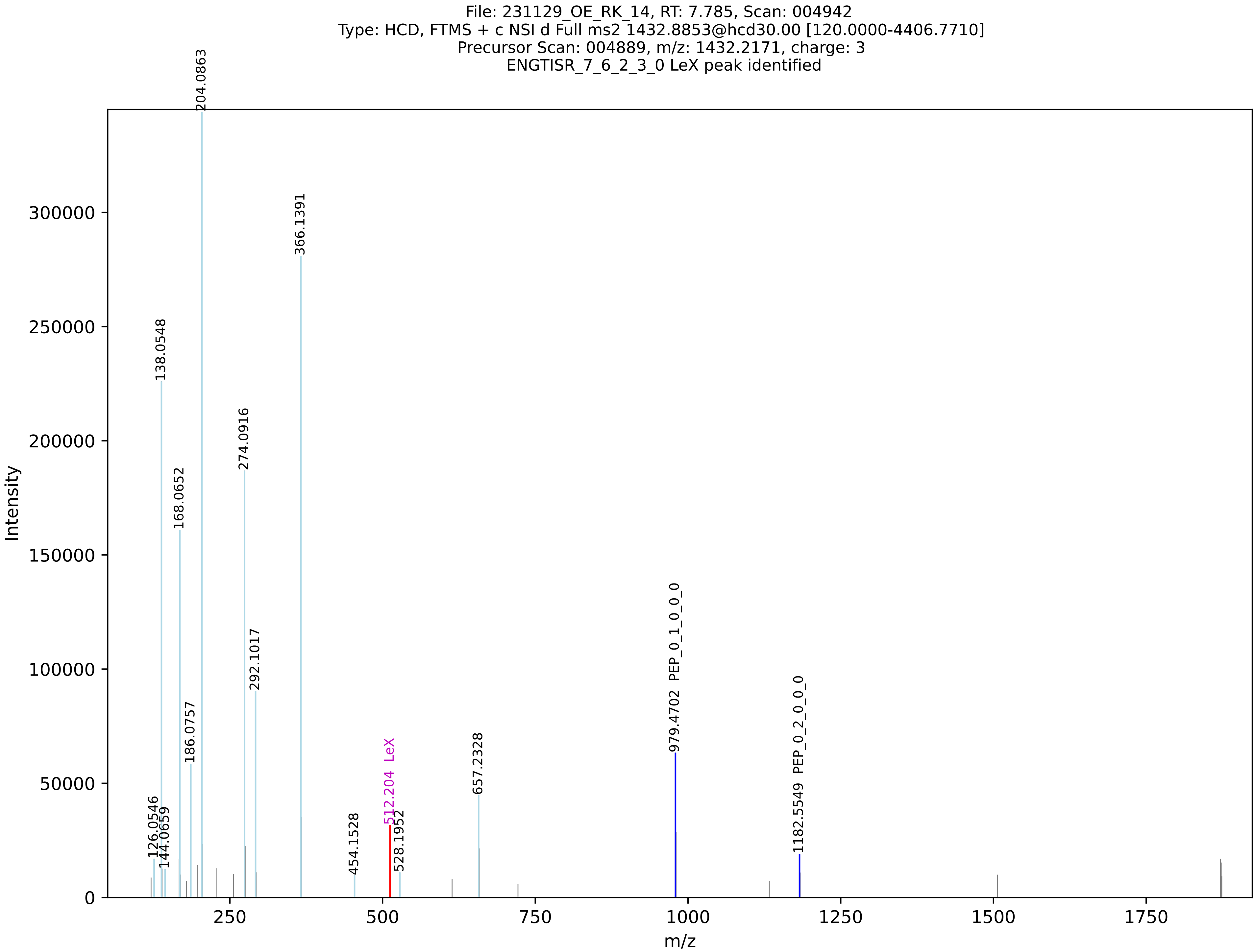
File: 231129_OE_RK_14, RT: 7.785, Scan: 004942
Type: HCD, FTMS + c NSI d Full ms2 1432.8853@hcd30.00 [120.0000-4406.7710]
Precursor Scan: 004889, m/z: 1432.2171, charge: 3
ENGTISR_7_6_2_3_0 LeX peak identified
Intensity
m/z
204.0863
366.1391
138.0548
274.0916
168.0652
292.1017
186.0757
126.0546
144.0659
454.1528
512.204 LeX
528.1952
657.2328
979.4702 PEP_0_1_0_0_0
1182.5549 PEP_0_2_0_0_0

File: 231129_OE_RK_14, RT: 7.788, Scan: 004947
Type: HCD, FTMS + c NSI d Full ms2 1481.5724@hcd30.00 [120.0000-4555.7534]
Precursor Scan: 004889, m/z: 1480.5698, charge: 3
ENGTISR_7_6_1_4_0 LeX peak identified

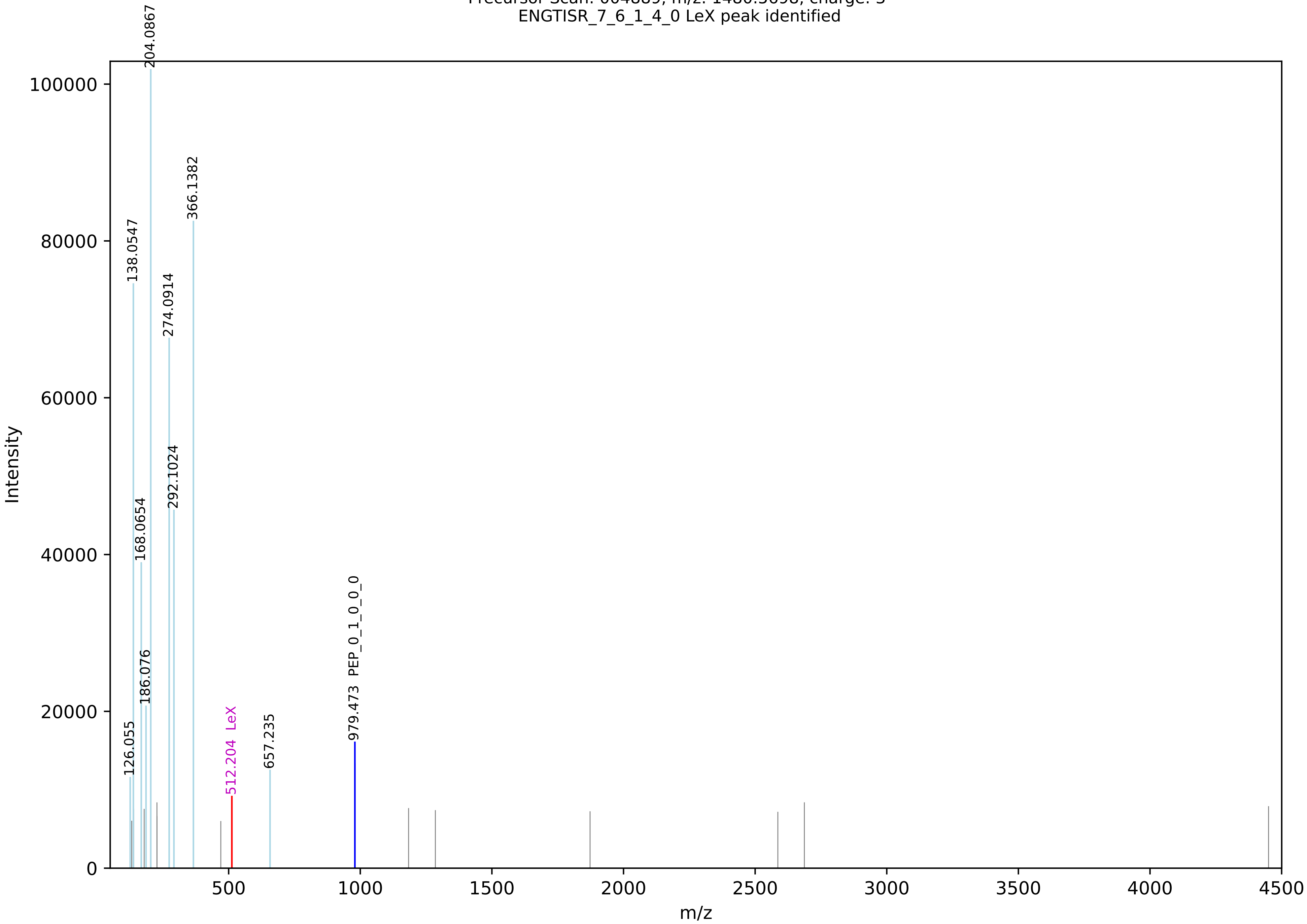

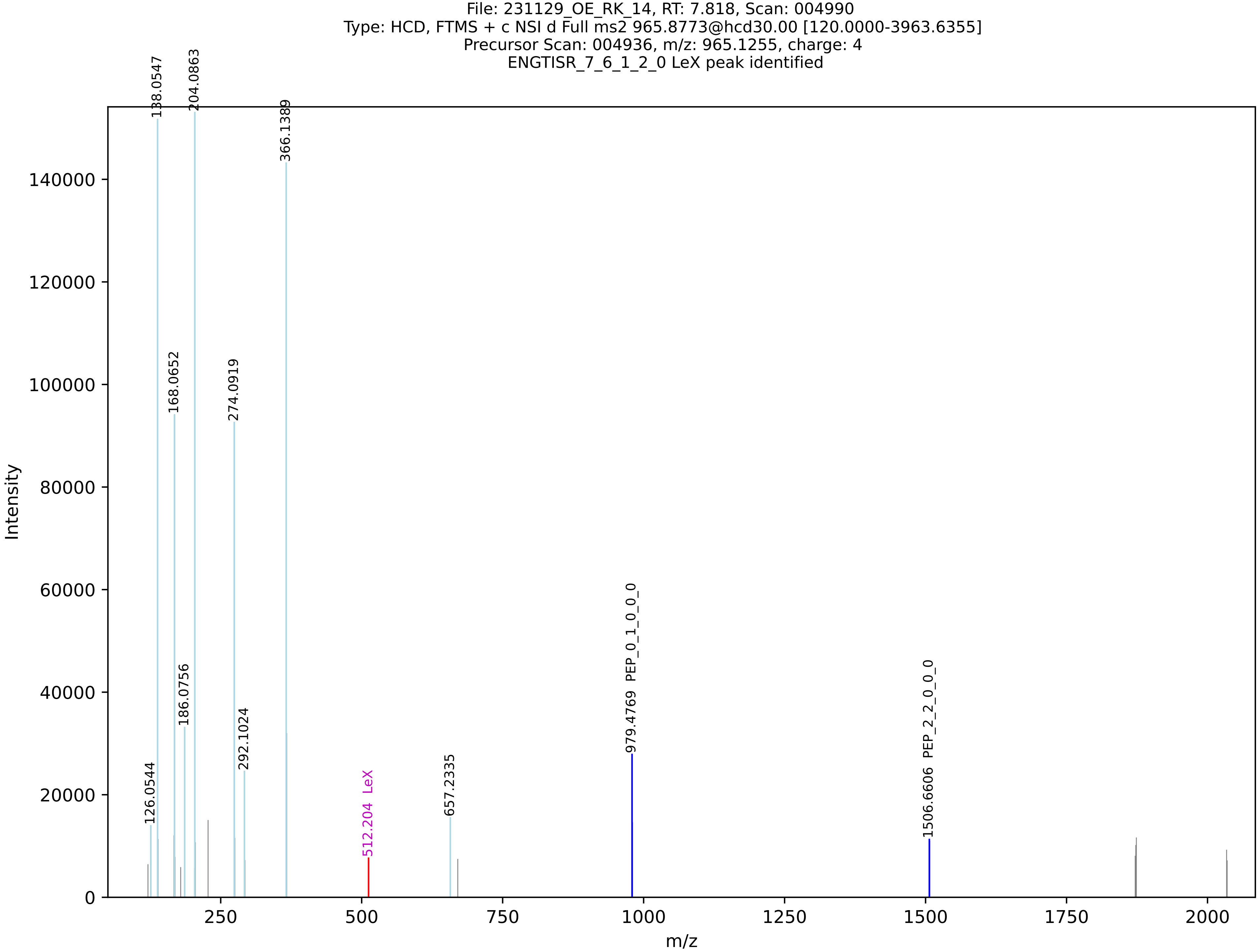

File: 231129_OE_RK_14, RT: 7.818, Scan: 004990
Type: HCD, FTMS + c NSI d Full ms2 965.8773@hcd30.00 [120.0000-3963.6355]
Precursor Scan: 004936, m/z: 965.1255, charge: 4
ENGTISR_7_6_1_2_0 LeX peak identified
138.0547
204.0863
366.1389
168.0652
274.0919
186.0756
292.1024
126.0544
512.204 LeX
657.2335
979.4769 PEP_0_1_0_0_0
1506.6606 PEP_2_2_0_0_0
Intensity
m/z
0
20000
40000
60000
80000
100000
120000
140000
250
500
750
1000
1250
1500
1750
2000

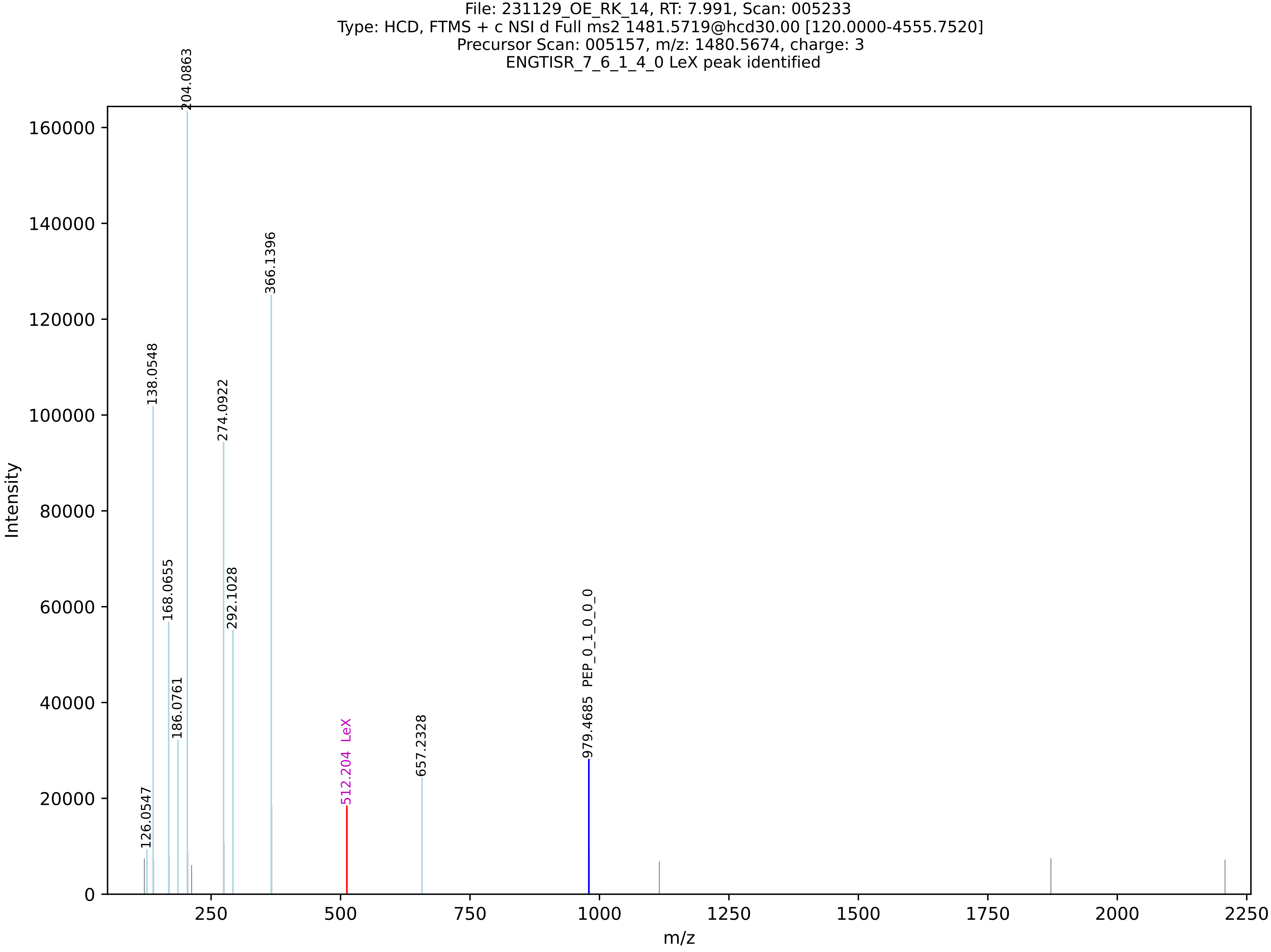
File: 231129_OE_RK_14, RT: 7.991, Scan: 005233
Type: HCD, FTMS + c NSI d Full ms2 1481.5719@hcd30.00 [120.0000-4555.7520]
Precursor Scan: 005157, m/z: 1480.5674, charge: 3
ENGTISR_7_6_1_4_0 LeX peak identified
Intensity
m/z
204.0863
366.1396
138.0548
274.0922
168.0655
292.1028
186.0761
126.0547
512.204 LeX
657.2328
979.4685 PEP_0_1_0_0_0
0
20000
40000
60000
80000
100000
120000
140000
160000
250
500
750
1000
1250
1500
1750
2000
2250

File: 231129_OE_RK_14, RT: 8.1, Scan: 005384
Type: HCD, FTMS + c NSI d Full ms2 1213.8073@hcd30.00 [120.0000-3736.3921]
Precursor Scan: 005333, m/z: 1213.139, charge: 3
ENGTISR_6_5_2_2_0 LeX peak identified

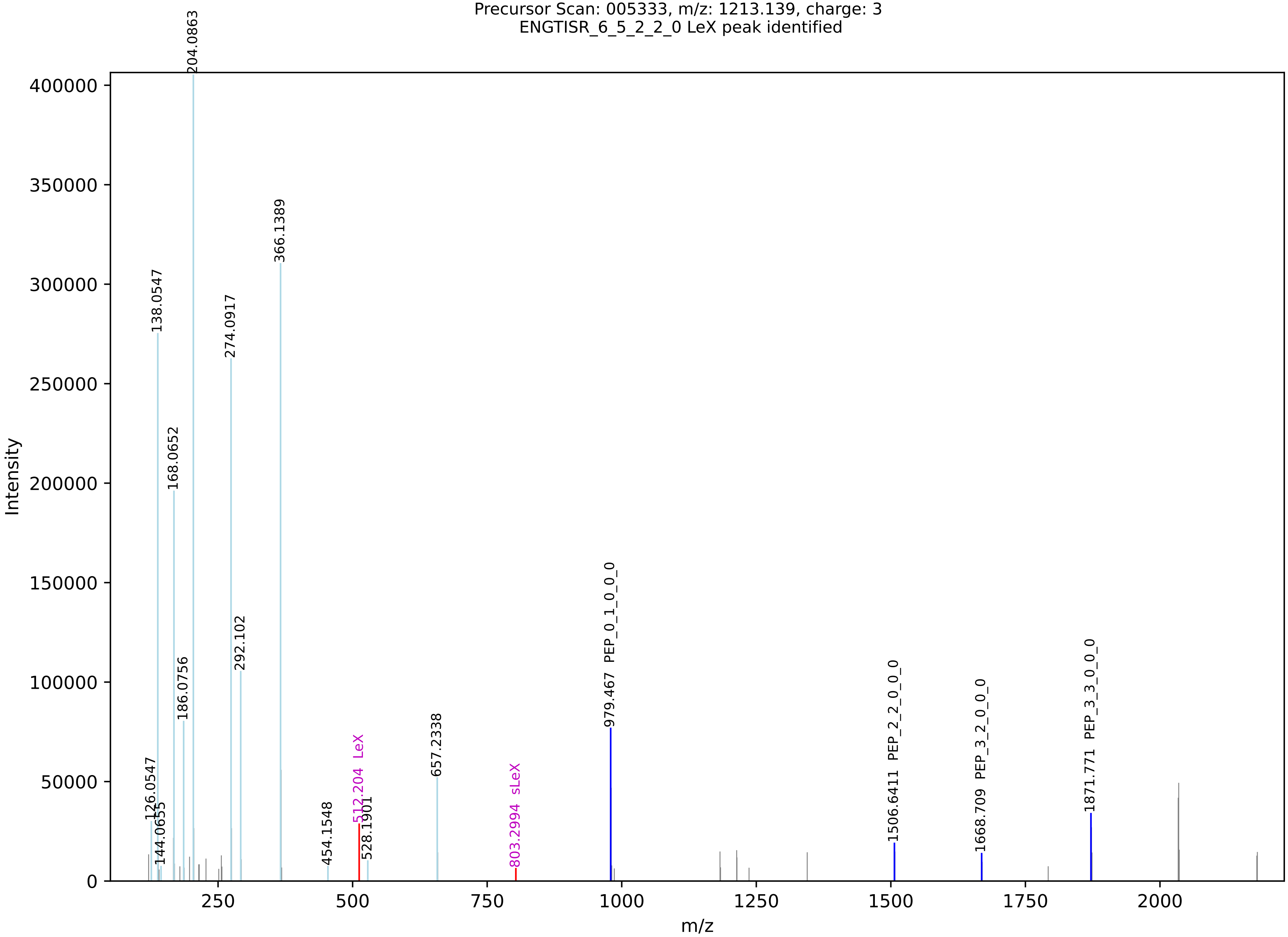

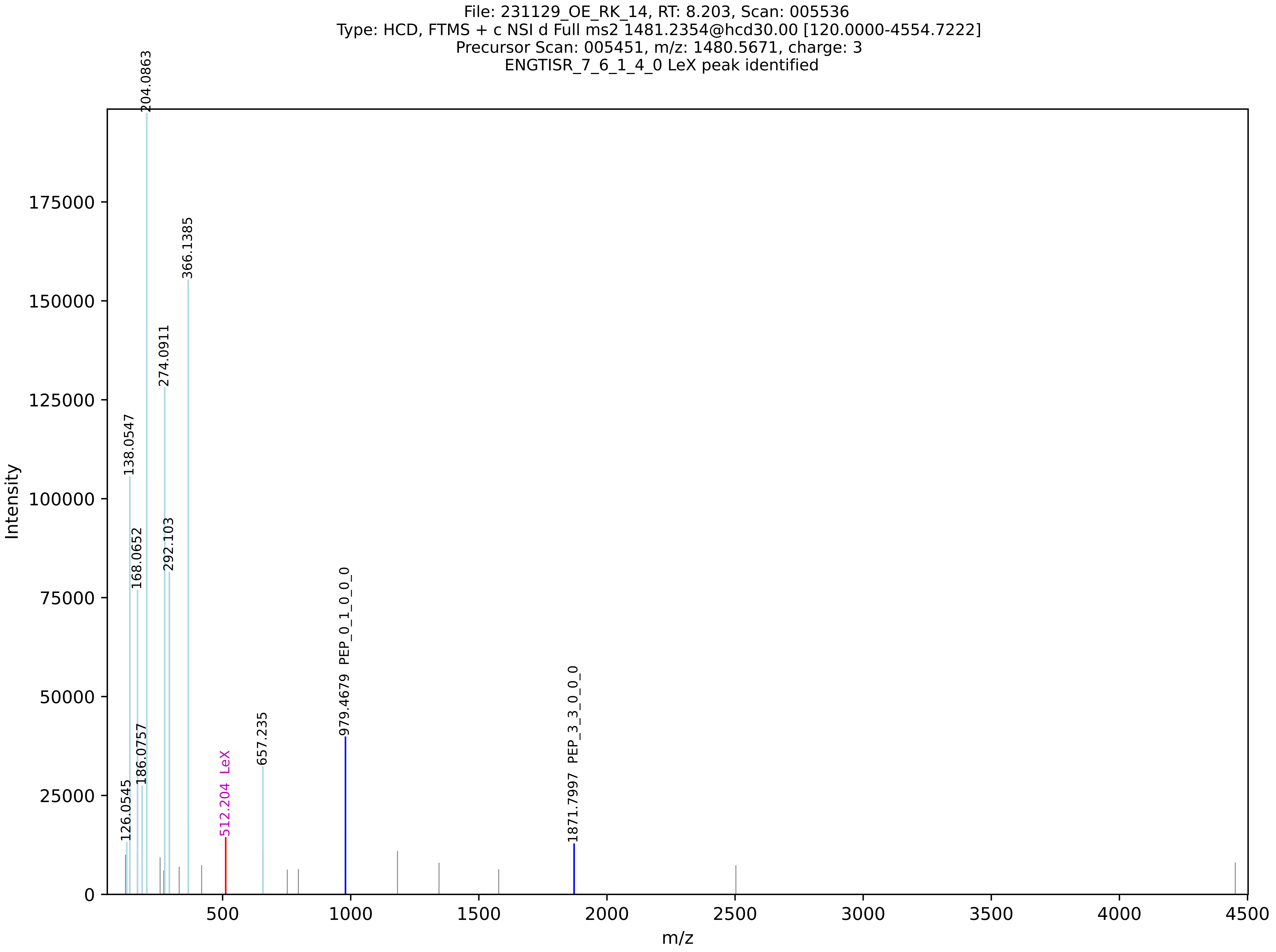
File: 231129_OE_RK_14, RT: 8.203, Scan: 005536
Type: HCD, FTMS + c NSI d Full ms2 1481.2354@hcd30.00 [120.0000-4554.7222]
Precursor Scan: 005451, m/z: 1480.5671, charge: 3
ENGTISR_7_6_1_4_0 LeX peak identified
Intensity
m/z
204.0863
366.1385
274.0911
138.0547
168.0652
292.103
186.0757
126.0545
512.204 LeX
657.235
979.4679 PEP_0_1_0_0_0_
1871.7997 PEP_3_3_0_0_0
0
25000
50000
75000
100000
125000
150000
175000
500
1000
1500
2000
2500
3000
3500
4000
4500

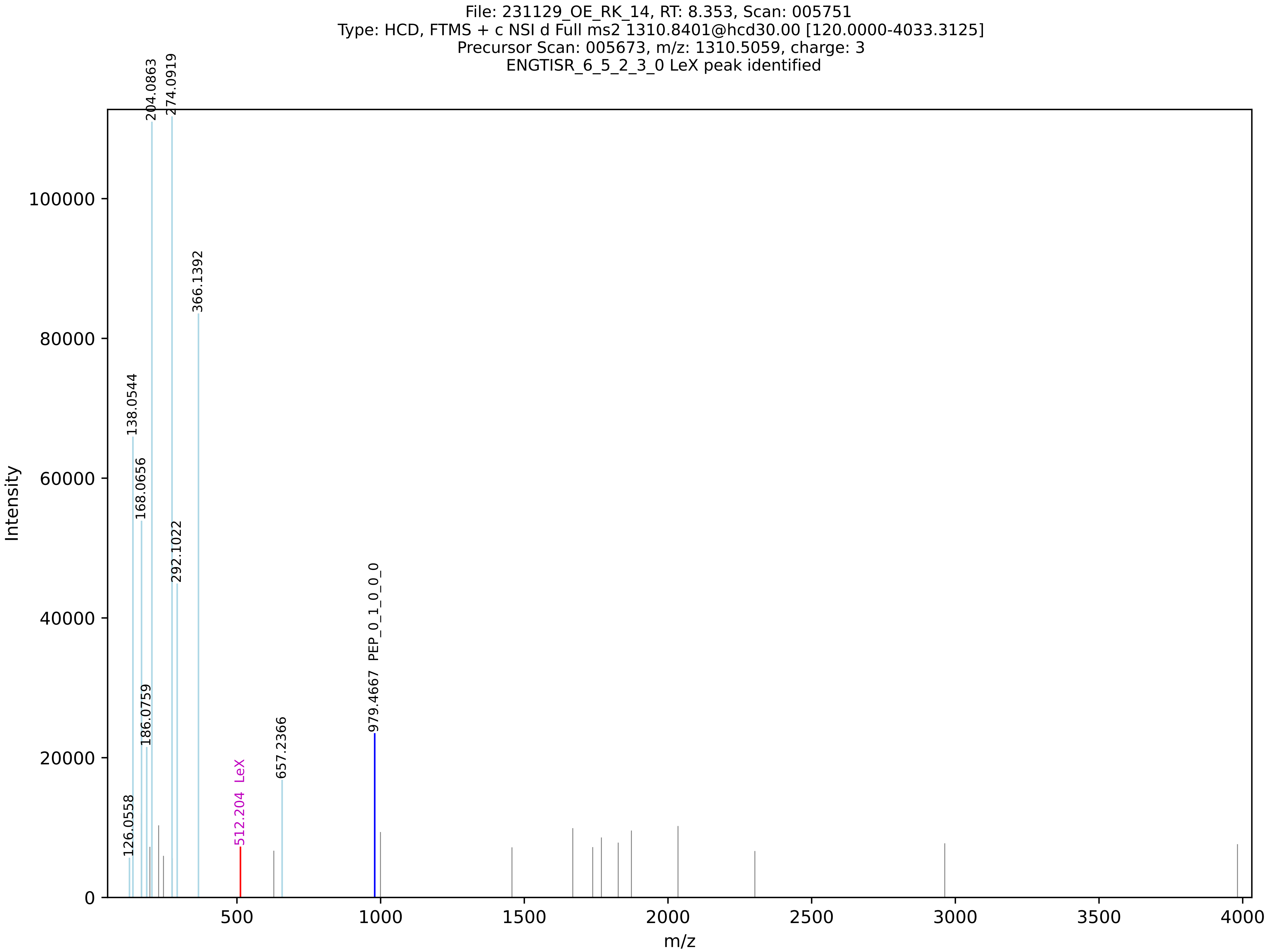
File: 231129_OE_RK_14, RT: 8.353, Scan: 005751
Type: HCD, FTMS + c NSI d Full ms2 1310.8401@hcd30.00 [120.0000-4033.3125]
Precursor Scan: 005673, m/z: 1310.5059, charge: 3
ENGTISR_6_5_2_3_0 LeX peak identified
204.0863
274.0919
366.1392
138.0544
168.0656
292.1022
PEP_0_1_0_0_0_
979.4667
186.0759
657.2366
512.204 LeX
126.0558
Intensity
m/z
0
20000
40000
60000
80000
100000
500
1000
1500
2000
2500
3000
3500
4000

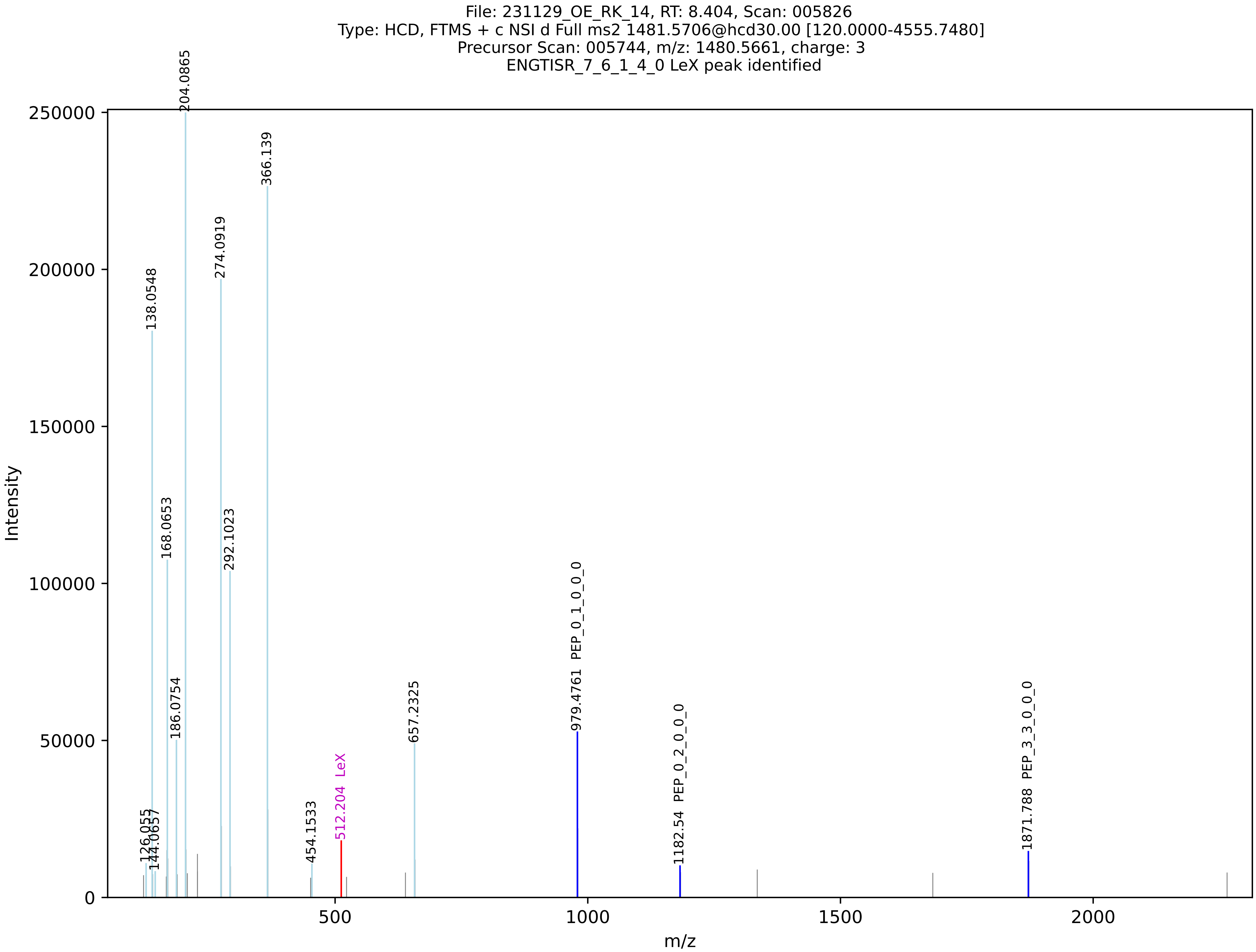

File: 231129_OE_RK_14, RT: 8.404, Scan: 005826
Type: HCD, FTMS + c NSI d Full ms2 1481.5706@hcd30.00 [120.0000-4555.7480]
Precursor Scan: 005744, m/z: 1480.5661, charge: 3
ENGTISR_7_6_1_4_0 LeX peak identified
Intensity
m/z
204.0865
366.139
274.0919
138.0548
168.0653
292.1023
186.0754
126.055
144.0657
454.1533
512.204 LeX
657.2325
979.4761 PEP_0_1_0_0_0
1182.54 PEP_0_2_0_0_0
1871.788 PEP_3_3_0_0_0

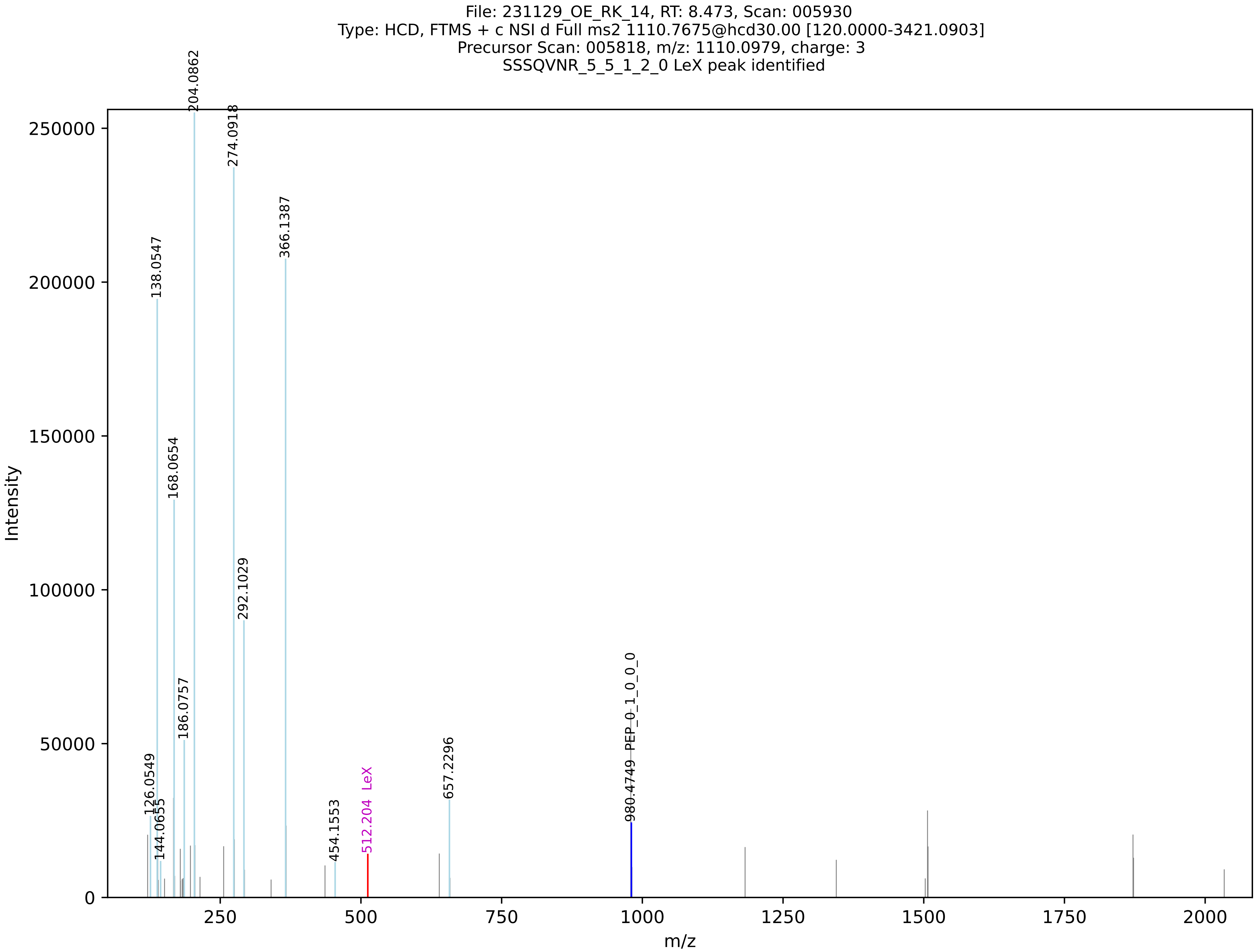
File: 231129_OE_RK_14, RT: 8.473, Scan: 005930
Type: HCD, FTMS + c NSI d Full ms2 1110.7675@hcd30.00 [120.0000-3421.0903]
Precursor Scan: 005818, m/z: 1110.0979, charge: 3
SSSQVNR_5_5_1_2_0 LeX peak identified
204.0862
274.0918
366.1387
138.0547
168.0654
292.1029
186.0757
126.0549
144.0655
454.1553
512.204 LeX
657.2296
980.4749 PEP_0_1_0_0_
Intensity
m/z
0
50000
100000
150000
200000
250000
250
500
750
1000
1250
1500
1750
2000

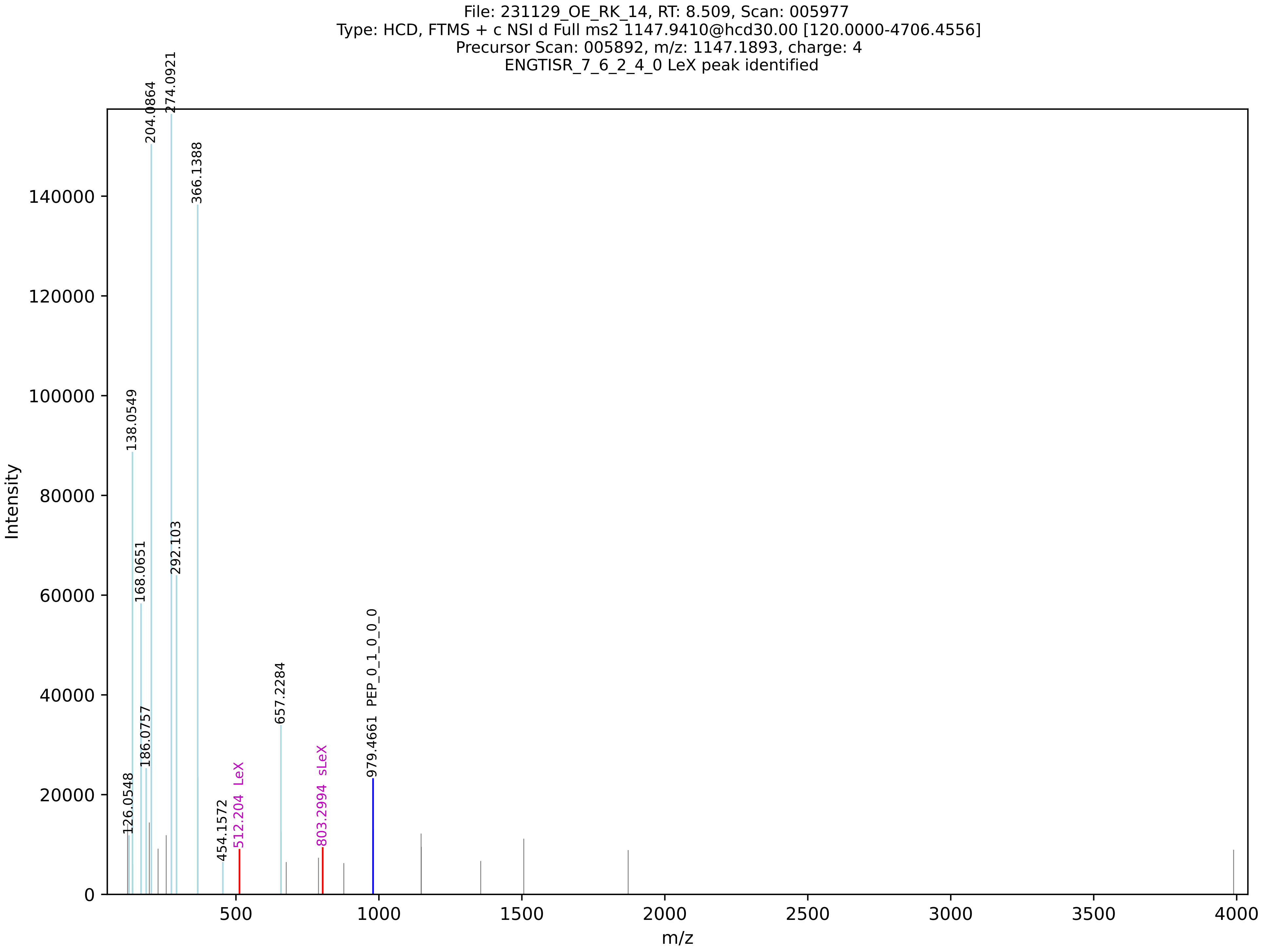

File: 231129_OE_RK_14, RT: 8.509, Scan: 005977
Type: HCD, FTMS + c NSI d Full ms2 1147.9410@hcd30.00 [120.0000-4706.4556]
Precursor Scan: 005892, m/z: 1147.1893, charge: 4
ENGTISR_7_6_2_4_0 LeX peak identified
Intensity
m/z
274.0921
204.0864
366.1388
138.0549
292.103
168.0651
186.0757
126.0548
454.1572
512.204 LeX
657.2284
803.2994 sLeX
979.4661 PEP_0_1_0_0_0

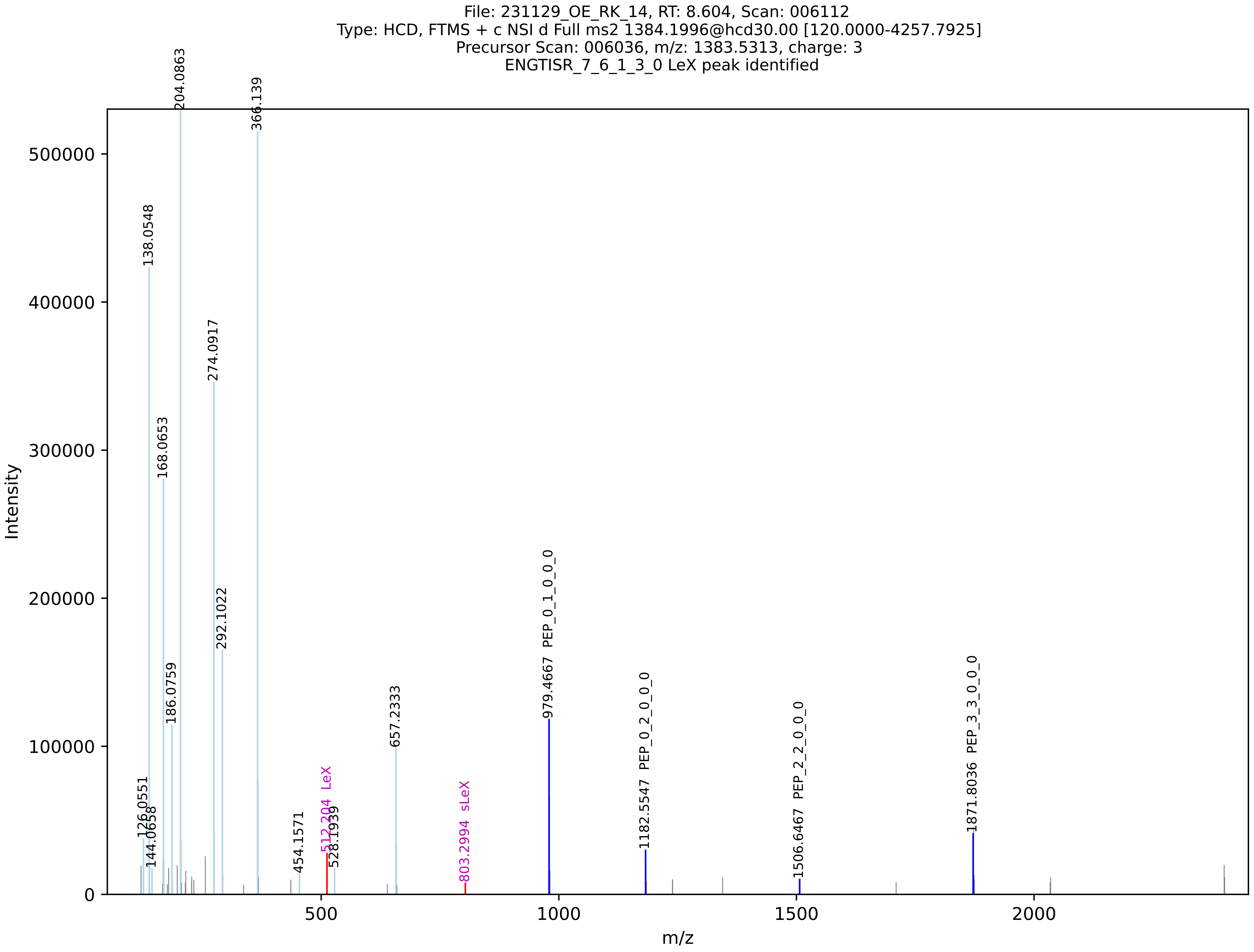
File: 231129_OE_RK_14, RT: 8.604, Scan: 006112
Type: HCD, FTMS + c NSI d Full ms2 1384.1996@hcd30.00 [120.0000-4257.7925]
Precursor Scan: 006036, m/z: 1383.5313, charge: 3
ENGTISR_7_6_1_3_0 LeX peak identified
Intensity
m/z
204.0863
366.139
138.0548
274.0917
168.0653
292.1022
186.0759
126.0551
144.0658
454.1571
512.204 LeX
528.1939
657.2333
803.2994 sLeX
979.4667 PEP_0_1_0_0_0
1182.5547 PEP_0_2_0_0_0
1506.6467 PEP_2_2_0_0_0
1871.8036 PEP_3_3_0_0_0
0
100000
200000
300000
400000
500000
500
1000
1500
2000

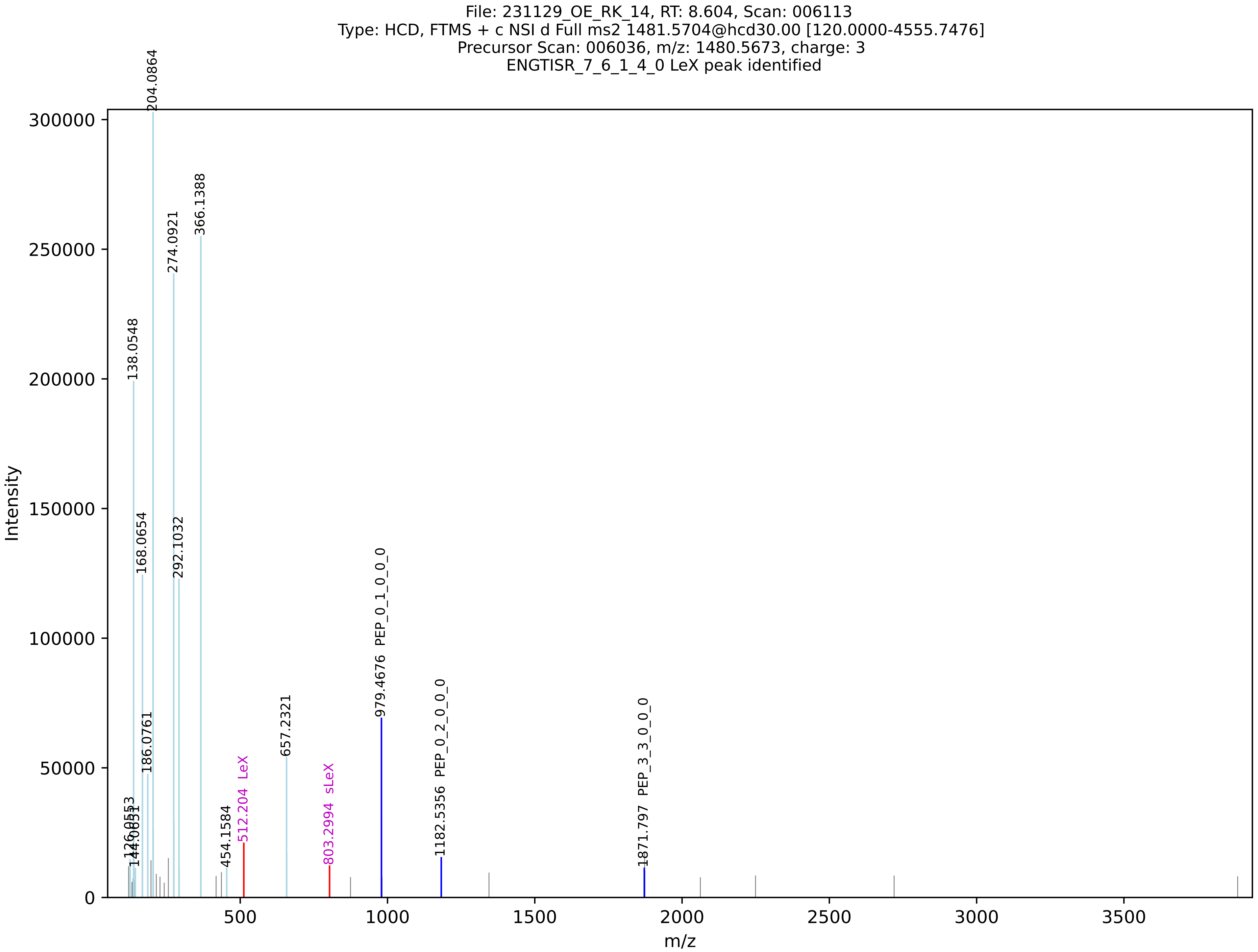

File: 231129_OE_RK_14, RT: 8.604, Scan: 006113
Type: HCD, FTMS + c NSI d Full ms2 1481.5704@hcd30.00 [120.0000-4555.7476]
Precursor Scan: 006036, m/z: 1480.5673, charge: 3
ENGTISR_7_6_1_4_0 LeX peak identified
Intensity
m/z
204.0864
366.1388
274.0921
138.0548
168.0654
292.1032
186.0761
126.0553
144.0651
454.1584
512.204 LeX
657.2321
803.2994 sLeX
979.4676 PEP_0_1_0_0_0
1182.5356 PEP_0_2_0_0_0
1871.797 PEP_3_3_0_0_0

File: 231129_OE_RK_14, RT: 8.605, Scan: 006114
Type: HCD, FTMS + c NSI d Full ms2 1111.1780@hcd30.00 [120.0000-4556.4624]
Precursor Scan: 006036, m/z: 1110.6773, charge: 4
ENGTISR_7_6_1_4_0 LeX peak identified

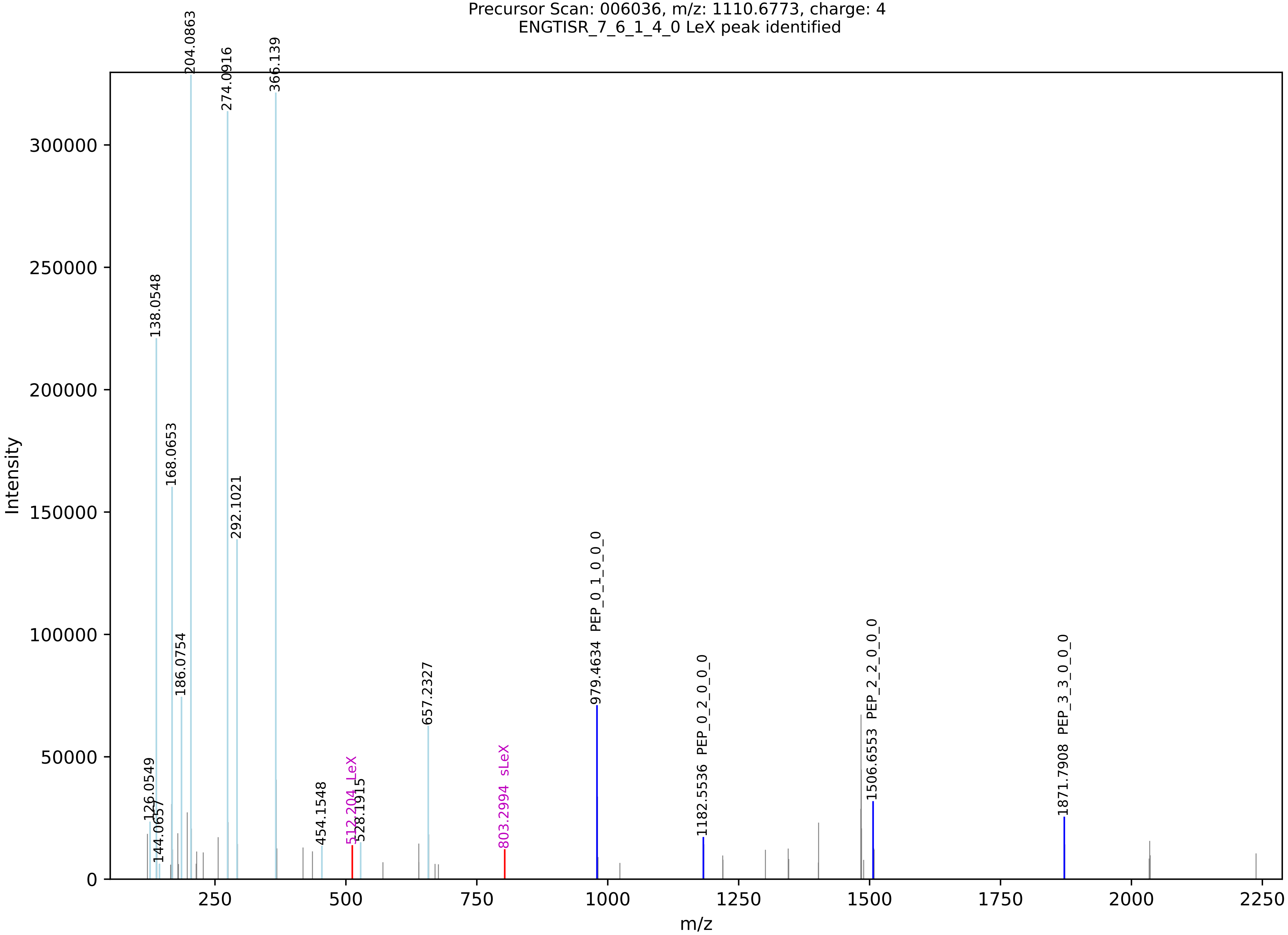

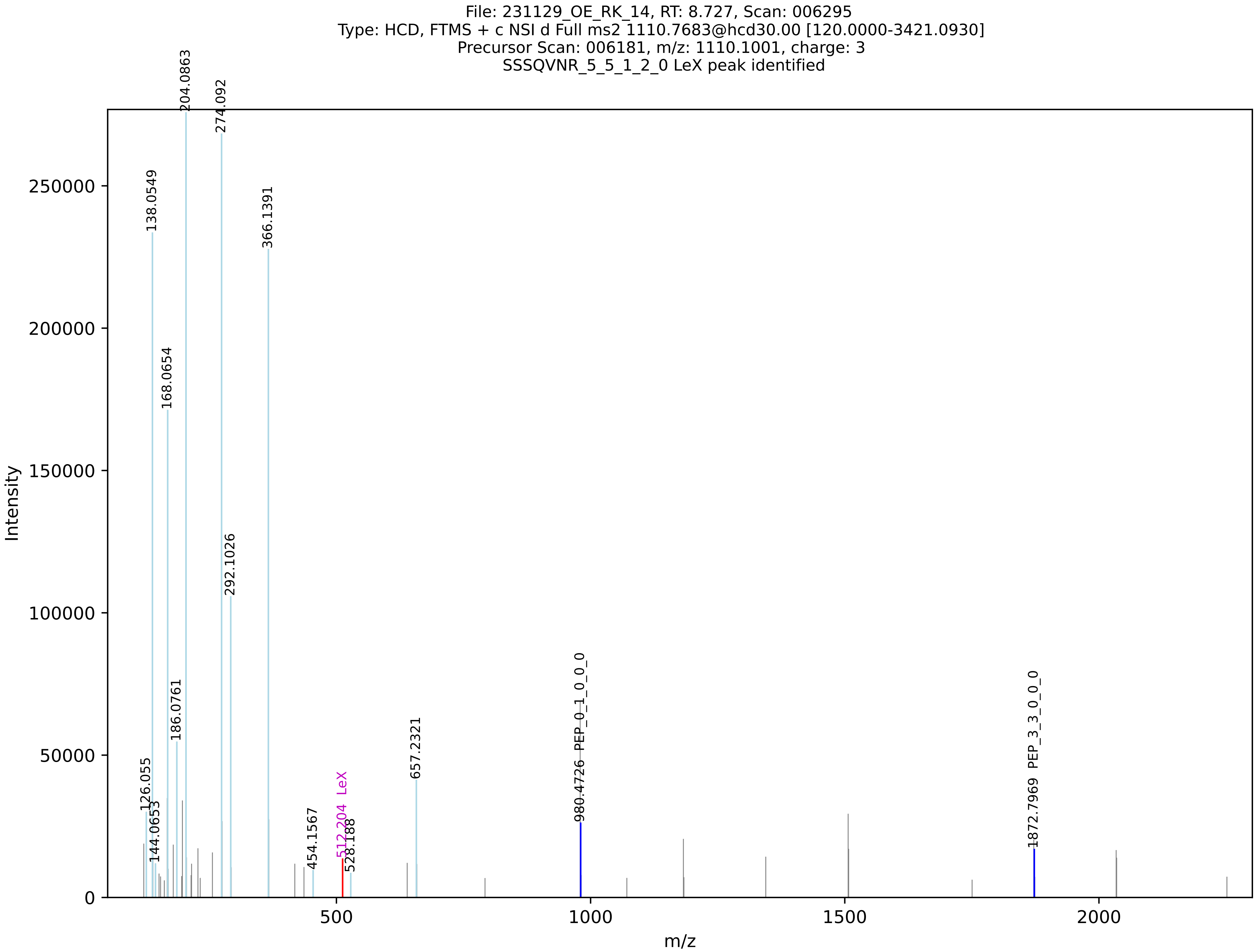
File: 231129_OE_RK_14, RT: 8.727, Scan: 006295
Type: HCD, FTMS + c NSI d Full ms2 1110.7683@hcd30.00 [120.0000-3421.0930]
Precursor Scan: 006181, m/z: 1110.1001, charge: 3
SSSQVNR_5_5_1_2_0 LeX peak identified
Intensity
m/z
204.0863
274.092
138.0549
366.1391
168.0654
292.1026
186.0761
126.055
144.0653
454.1567
512.204 LeX
528.188
657.2321
980.4726 PEP_0_1_0_0_0
1872.7969 PEP_3_3_0_0_0
0
50000
100000
150000
200000
250000
500
1000
1500
2000

File: 231129_OE_RK_14, RT: 8.907, Scan: 006550
Type: HCD, FTMS + c NSI d Full ms2 1432.8850@hcd30.00 [120.0000-4406.7700]
Precursor Scan: 006474, m/z: 1431.8816, charge: 3
ENGTISR_7_6_2_3_0 LeX peak identified

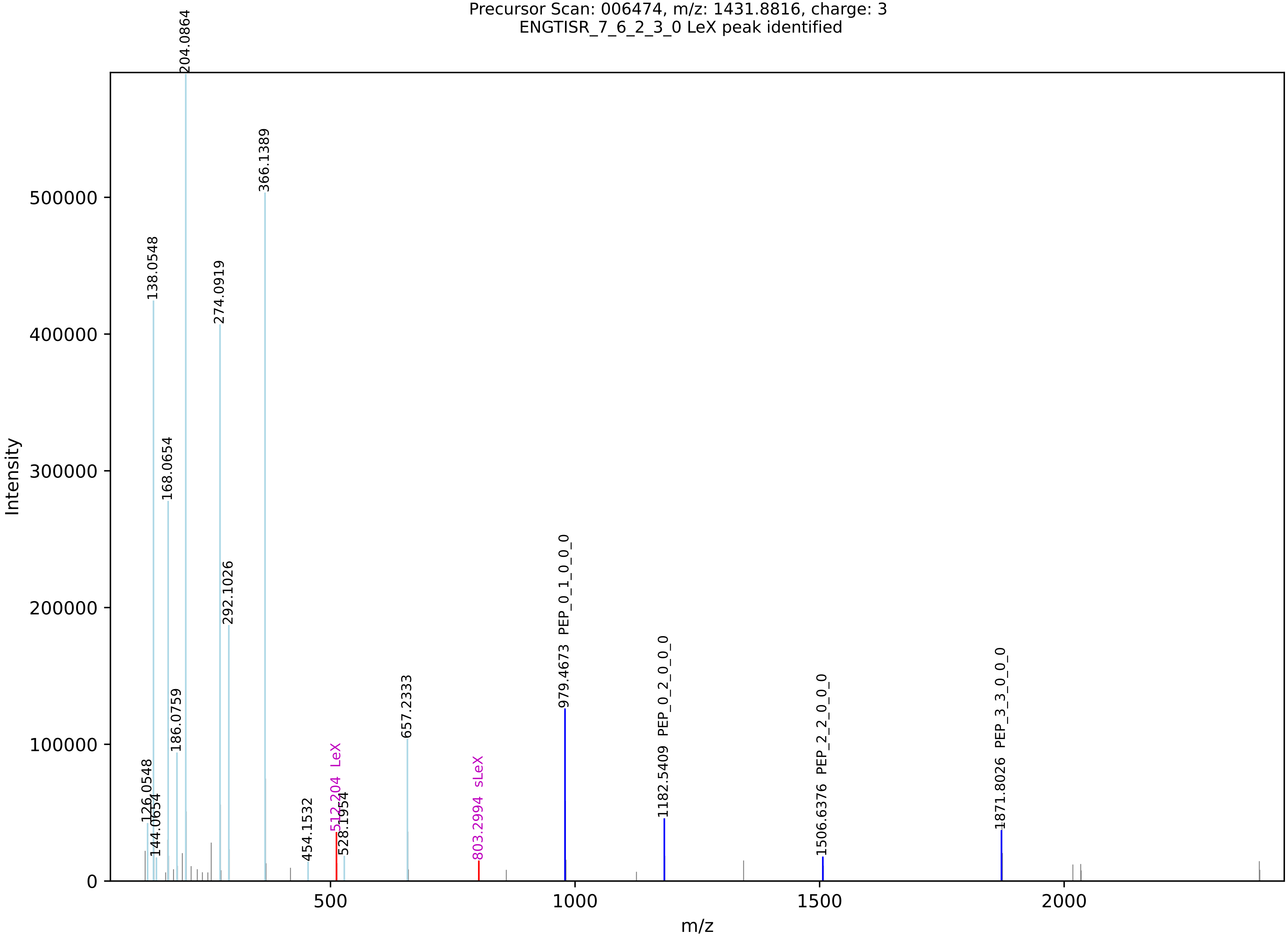

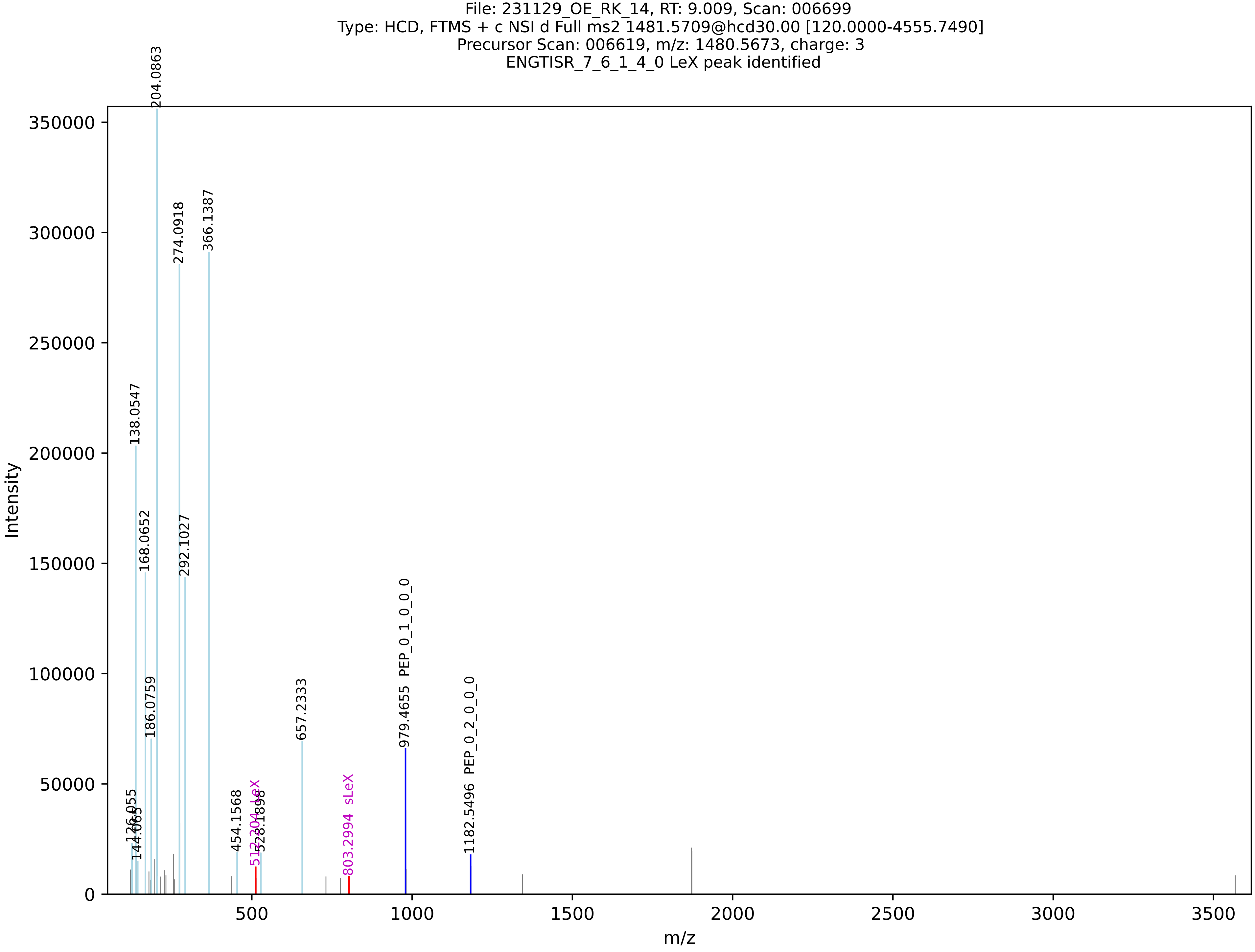
File: 231129_OE_RK_14, RT: 9.009, Scan: 006699
Type: HCD, FTMS + c NSI d Full ms2 1481.5709@hcd30.00 [120.0000-4555.7490]
Precursor Scan: 006619, m/z: 1480.5673, charge: 3
ENGTISR_7_6_1_4_0 LeX peak identified
204.0863
274.0918
366.1387
138.0547
168.0652
292.1027
186.0759
657.2333
979.4655 PEP_0_1_0_0_0
1182.5496 PEP_0_2_0_0_0
803.2994 sLeX
512.204 LeX
528.1898
454.1568
126.055
144.065
Intensity
m/z
350000
300000
250000
200000
150000
100000
50000
0
500
1000
1500
2000
2500
3000
3500

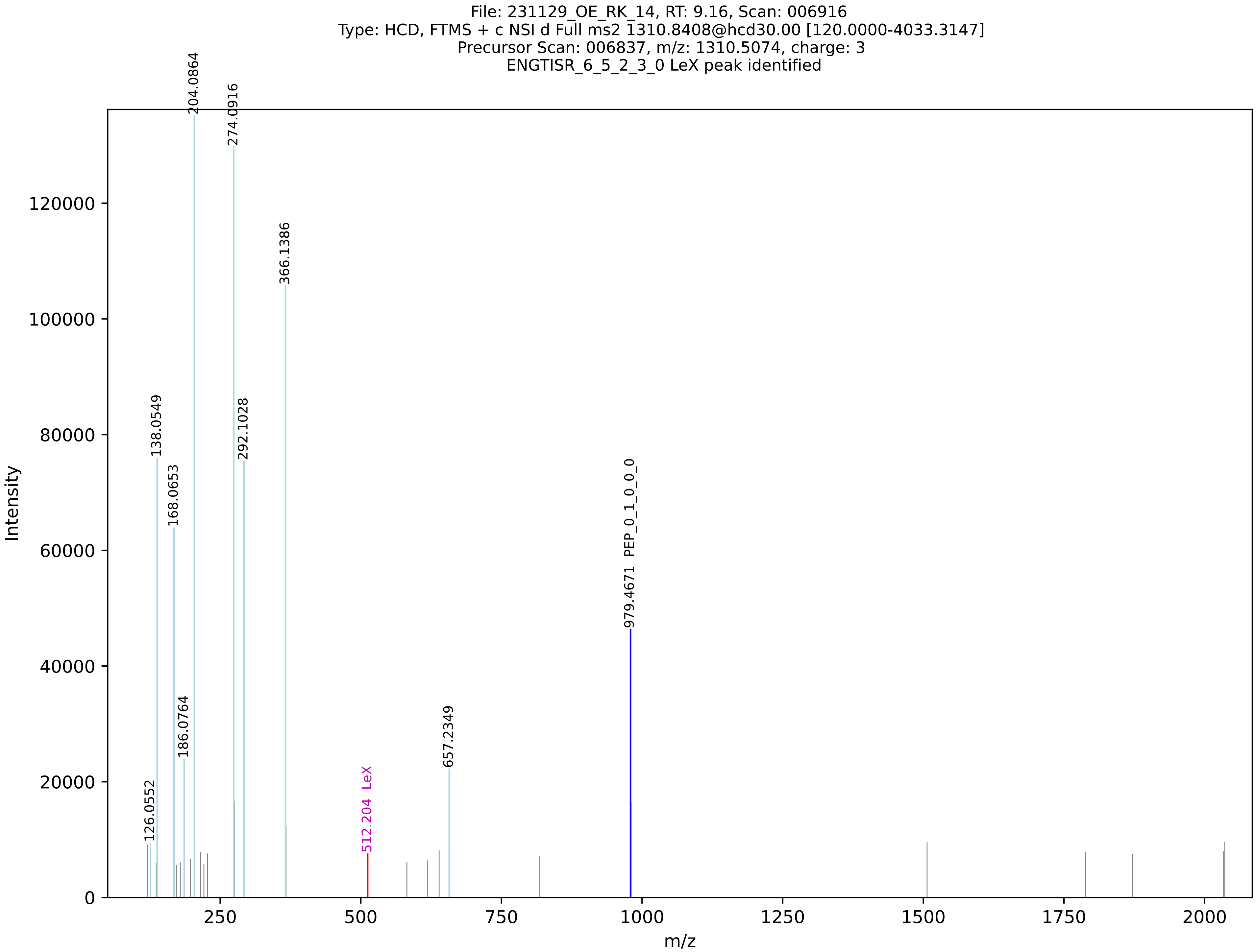

File: 231129_OE_RK_14, RT: 9.16, Scan: 006916
Type: HCD, FTMS + c NSI d Full ms2 1310.8408@hcd30.00 [120.0000-4033.3147]
Precursor Scan: 006837, m/z: 1310.5074, charge: 3
ENGTISR_6_5_2_3_0 LeX peak identified
204.0864
274.0916
366.1386
138.0549
292.1028
168.0653
979.4671 PEP_0_1_0_0_0
186.0764
657.2349
512.204 LeX
126.0552
Intensity
m/z

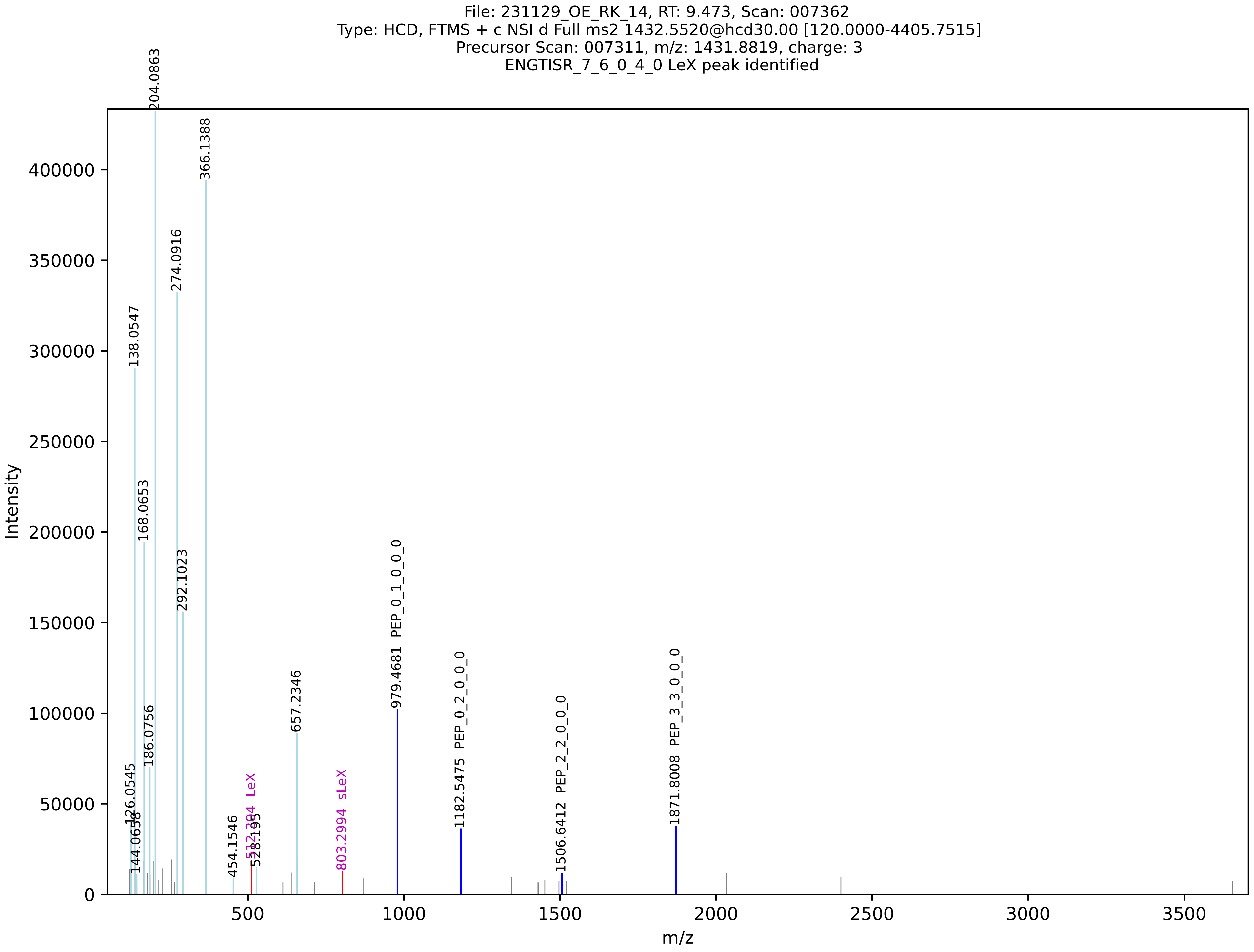

File: 231129_OE_RK_14, RT: 9.473, Scan: 007362
Type: HCD, FTMS + c NSI d Full ms2 1432.5520@hcd30.00 [120.0000-4405.7515]
Precursor Scan: 007311, m/z: 1431.8819, charge: 3
ENGTISR_7_6_0_4_0 LeX peak identified
Intensity
m/z
204.0863
366.1388
274.0916
138.0547
168.0653
292.1023
186.0756
126.0545
144.0658
454.1546
512.2004 LeX
528.195
657.2346
803.2994 sLeX
979.4681 PEP_0_1_0_0_0
1182.5475 PEP_0_2_0_0_0
1506.6412 PEP_2_2_0_0_0
1871.8008 PEP_3_3_0_0_0

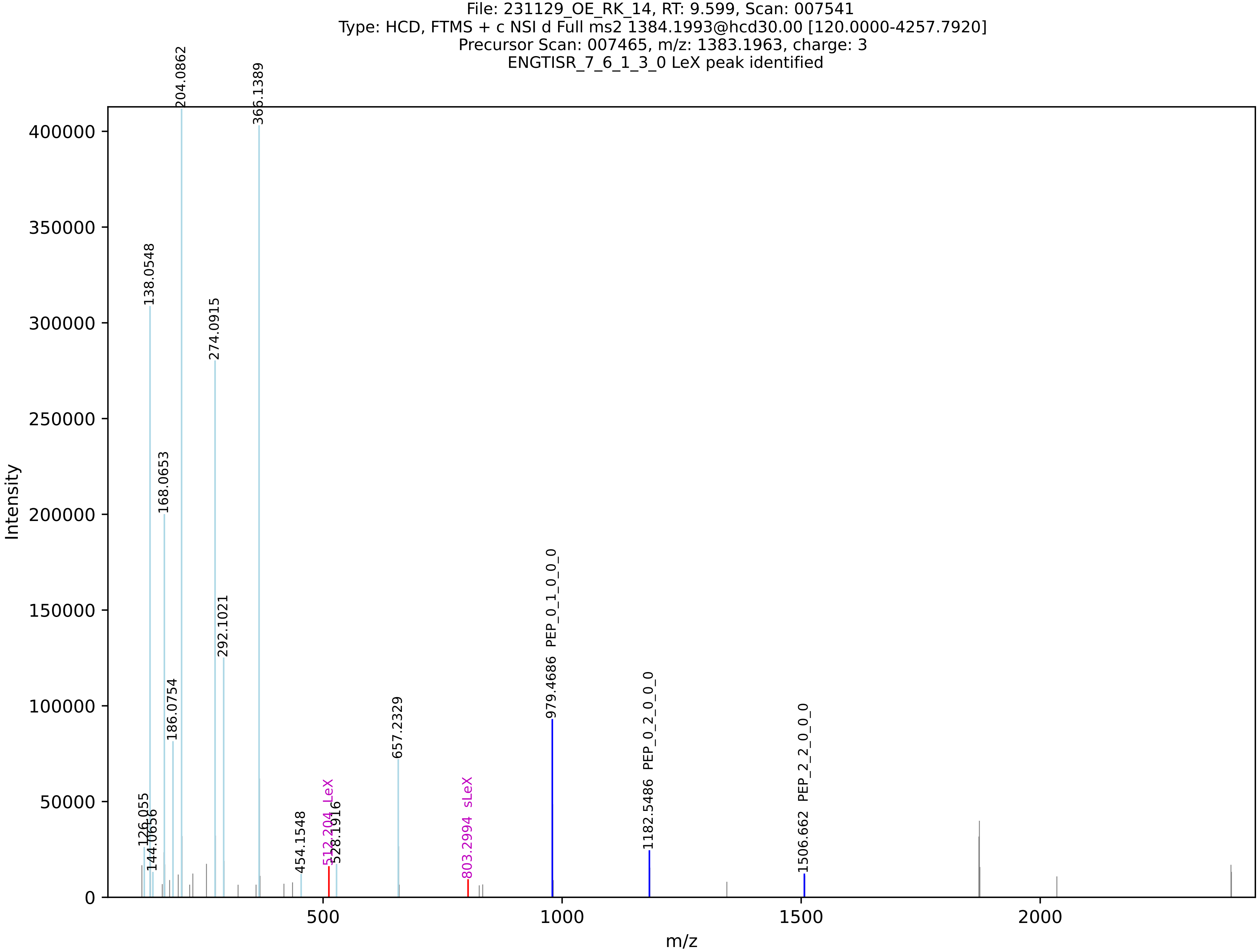
File: 231129_OE_RK_14, RT: 9.599, Scan: 007541
Type: HCD, FTMS + c NSI d Full ms2 1384.1993@hcd30.00 [120.0000-4257.7920]
Precursor Scan: 007465, m/z: 1383.1963, charge: 3
ENGTISR_7_6_1_3_0 LeX peak identified
Intensity
m/z
204.0862
366.1389
138.0548
274.0915
168.0653
292.1021
186.0754
126.055
144.0656
454.1548
512.204 LeX
528.1916
657.2329
803.2994 sLeX
979.4686 PEP_0_1_0_0_0
1182.5486 PEP_0_2_0_0_0
1506.662 PEP_2_2_0_0_0

File: 231129_OE_RK_14, RT: 9.649, Scan: 007614
Type: HCD, FTMS + c NSI d Full ms2 1432.5497@hcd30.00 [120.0000-4405.7441]
Precursor Scan: 007537, m/z: 1431.8811, charge: 3
ENGTISR_7_6_2_3_0 LeX peak identified

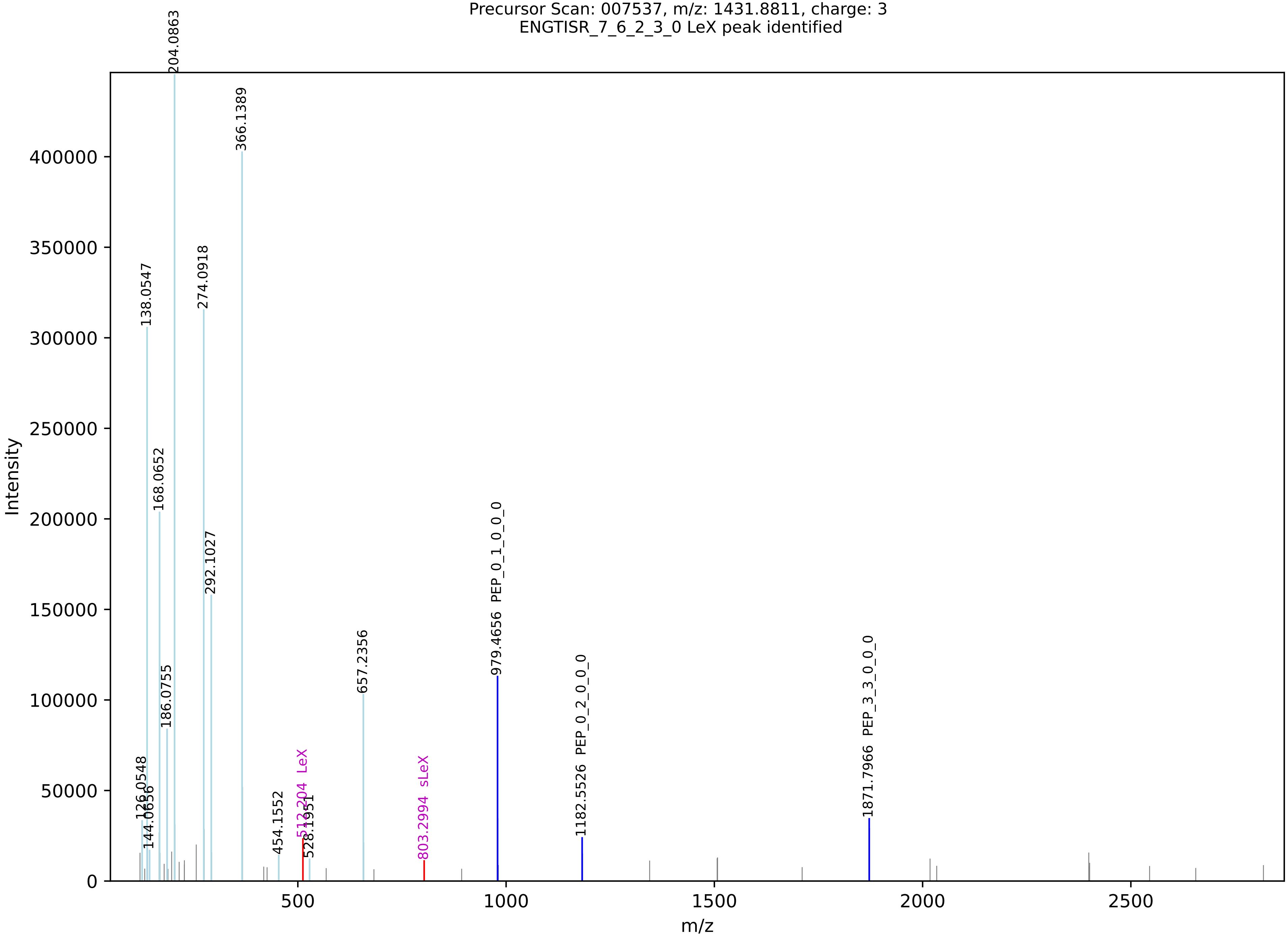

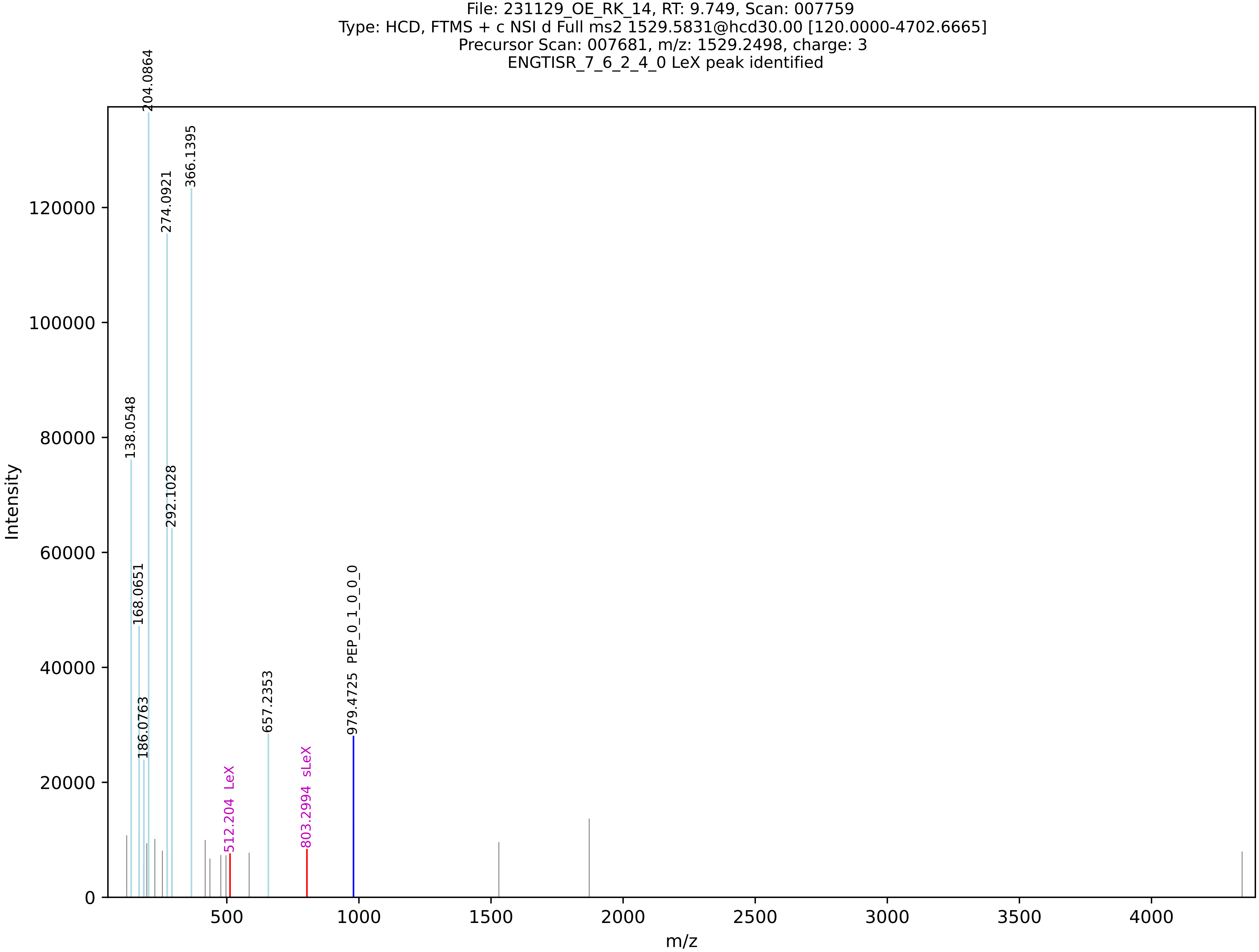
File: 231129_OE_RK_14, RT: 9.749, Scan: 007759
Type: HCD, FTMS + c NSI d Full ms2 1529.5831@hcd30.00 [120.0000-4702.6665]
Precursor Scan: 007681, m/z: 1529.2498, charge: 3
ENGTISR_7_6_2_4_0 LeX peak identified
204.0864
366.1395
274.0921
138.0548
292.1028
168.0651
186.0763
657.2353
979.4725 PEP_0_1_0_0_0_
512.204 LeX
803.2994 sLeX
Intensity
m/z
0
20000
40000
60000
80000
100000
120000
500
1000
1500
2000
2500
3000
3500
4000

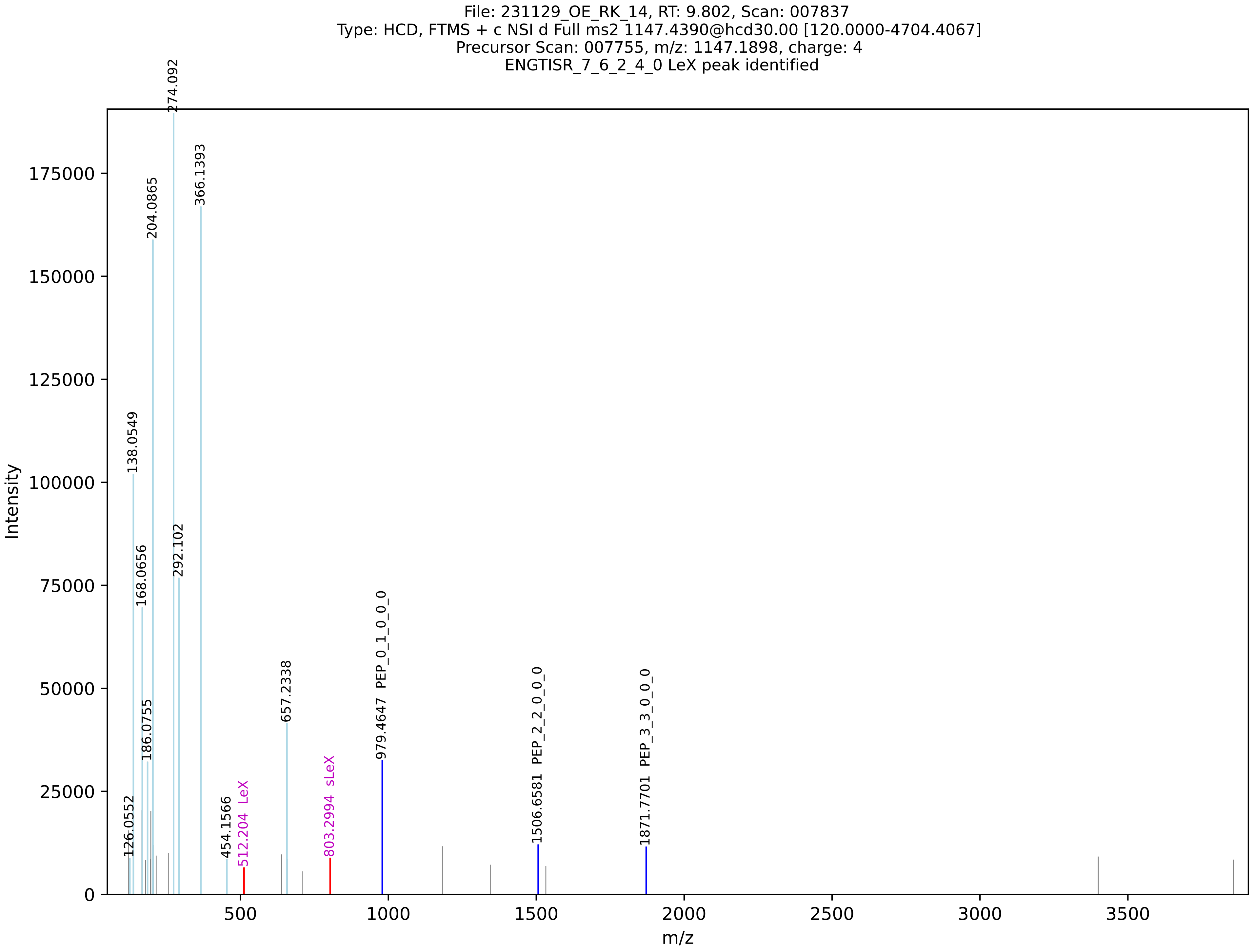

File: 231129_OE_RK_14, RT: 9.802, Scan: 007837
Type: HCD, FTMS + c NSI d Full ms2 1147.4390@hcd30.00 [120.0000-4704.4067]
Precursor Scan: 007755, m/z: 1147.1898, charge: 4
ENGTISR_7_6_2_4_0 LeX peak identified
Intensity
m/z
274.092
366.1393
204.0865
138.0549
168.0656
292.102
186.0755
126.0552
454.1566
512.204 LeX
657.2338
803.2994 sLeX
979.4647 PEP_0_1_0_0_0
1506.6581 PEP_2_2_0_0_0
1871.7701 PEP_3_3_0_0_0

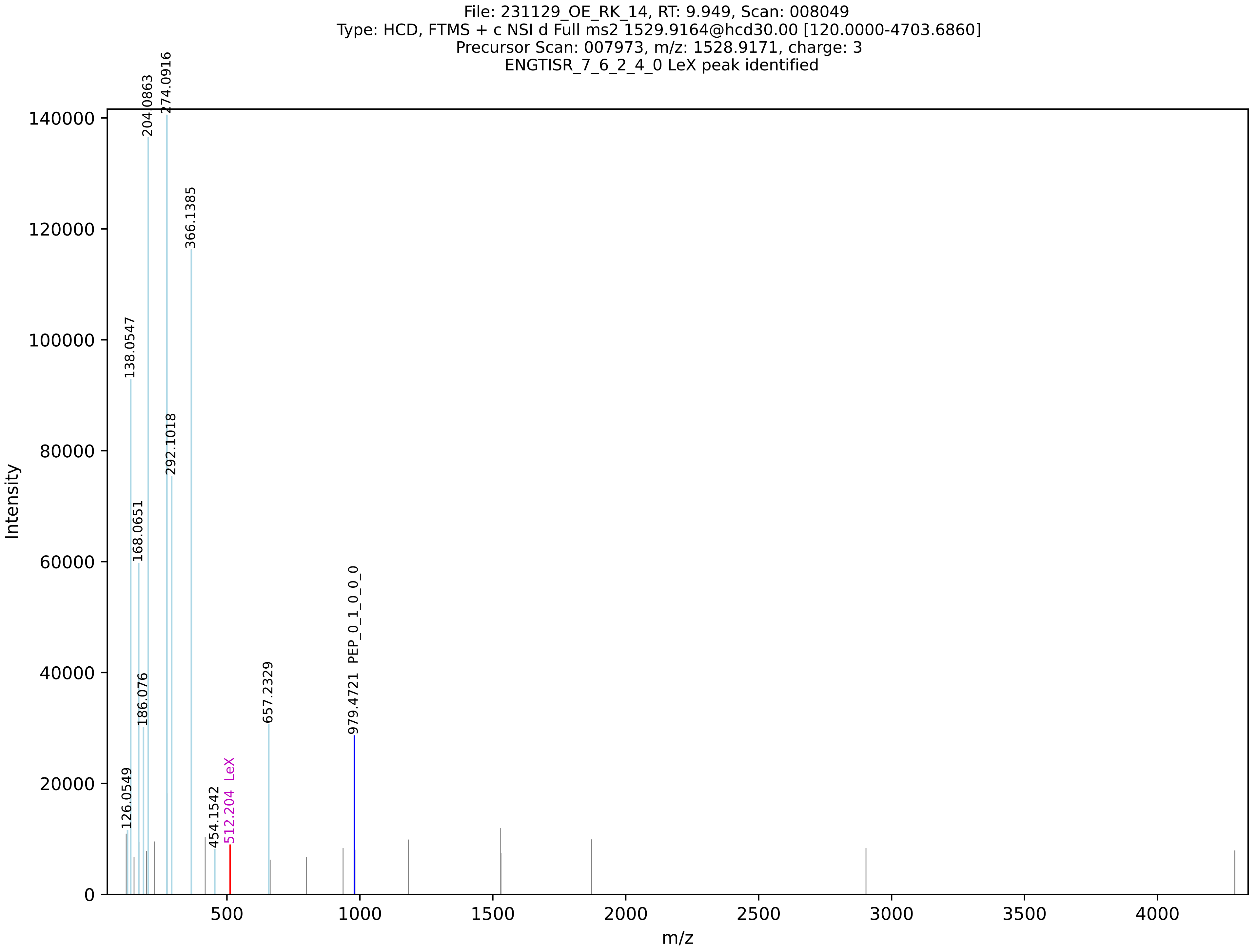
File: 231129_OE_RK_14, RT: 9.949, Scan: 008049
Type: HCD, FTMS + c NSI d Full ms2 1529.9164@hcd30.00 [120.0000-4703.6860]
Precursor Scan: 007973, m/z: 1528.9171, charge: 3
ENGTISR_7_6_2_4_0 LeX peak identified
Intensity
m/z
274.0916
204.0863
366.1385
138.0547
292.1018
168.0651
186.076
126.0549
454.1542
512.204 LeX
657.2329
979.4721 PEP_0_1_0_0_0_
0
20000
40000
60000
80000
100000
120000
140000
500
1000
1500
2000
2500
3000
3500
4000

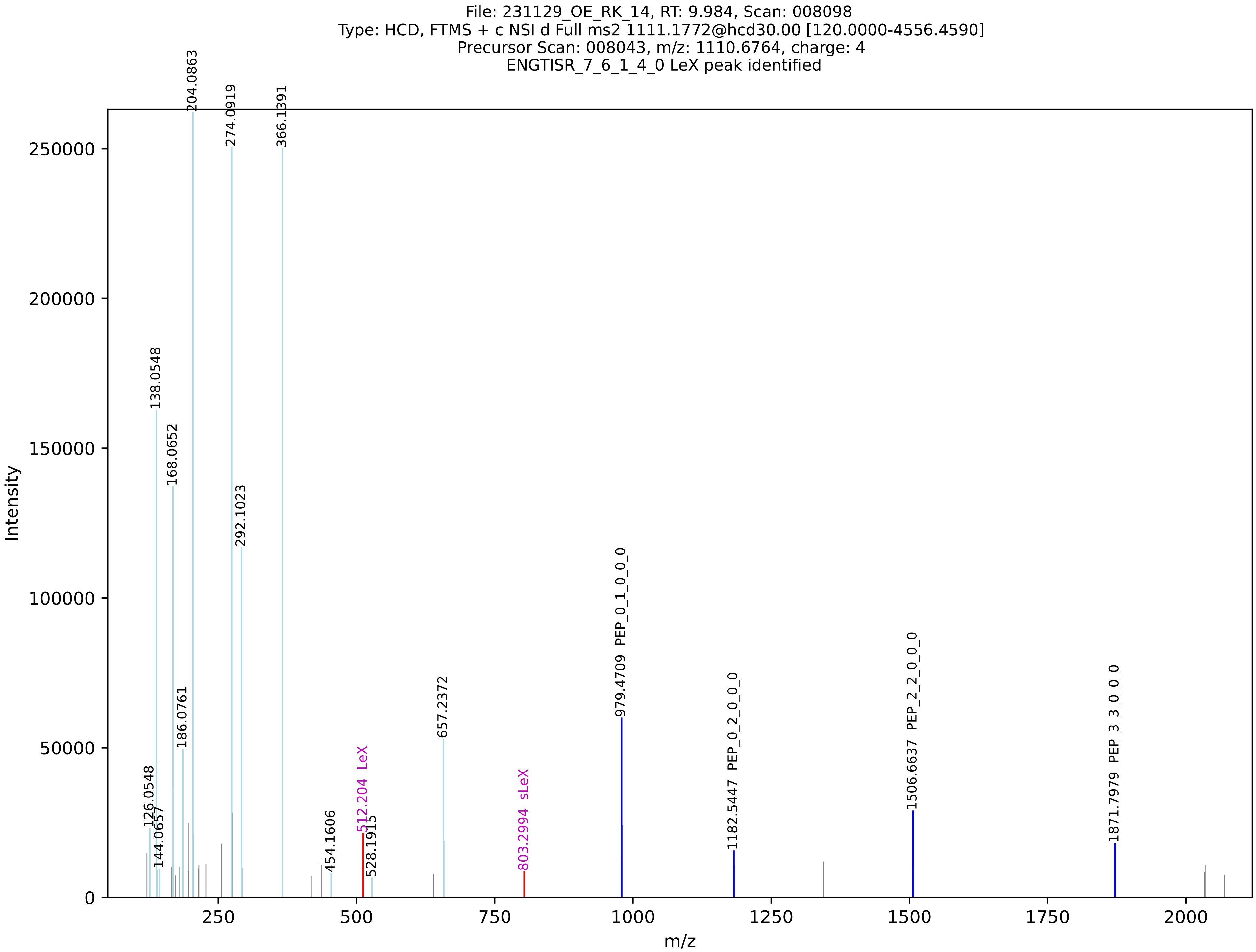
File: 231129_OE_RK_14, RT: 9.984, Scan: 008098
Type: HCD, FTMS + c NSI d Full ms2 1111.1772@hcd30.00 [120.0000-4556.4590]
Precursor Scan: 008043, m/z: 1110.6764, charge: 4
ENGTISR_7_6_1_4_0 LeX peak identified
Intensity
m/z
204.0863
274.0919
366.1391
138.0548
168.0652
292.1023
186.0761
126.0548
144.0657
454.1606
512.204 LeX
528.1915
657.2372
803.2994 sLeX
979.4709 PEP_0_1_0_0_0
1182.5447 PEP_0_2_0_0_0
1506.6637 PEP_2_2_0_0_0
1871.7979 PEP_3_3_0_0_0

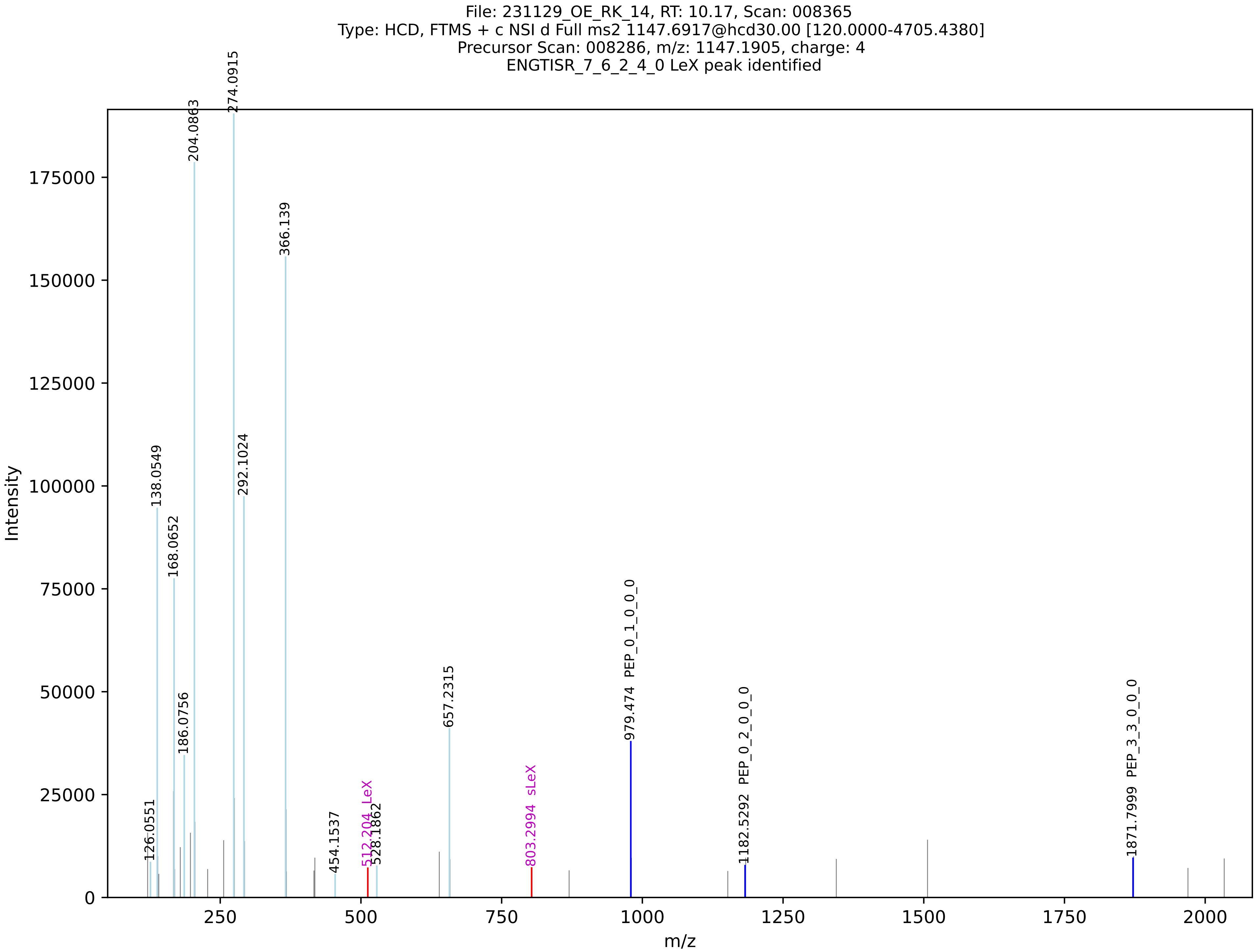
File: 231129_OE_RK_14, RT: 10.17, Scan: 008365
Type: HCD, FTMS + c NSI d Full ms2 1147.6917@hcd30.00 [120.0000-4705.4380]
Precursor Scan: 008286, m/z: 1147.1905, charge: 4
ENGTISR_7_6_2_4_0 LeX peak identified
Intensity
m/z
274.0915
204.0863
366.139
292.1024
138.0549
168.0652
186.0756
126.0551
454.1537
512.204 LeX
528.1862
657.2315
803.2994 sLeX
979.474 PEP_0_1_0_0_0
1182.5292 PEP_0_2_0_0_0
1871.7999 PEP_3_3_0_0_0

File: 231129_OE_RK_14, RT: 10.86, Scan: 009358
Type: HCD, FTMS + c NSI d Full ms2 1038.1511@hcd30.00 [120.0000-4258.5127]
Precursor Scan: 009276, m/z: 1037.9002, charge: 4
ENGTISR_7_6_1_3_0 LeX peak identified

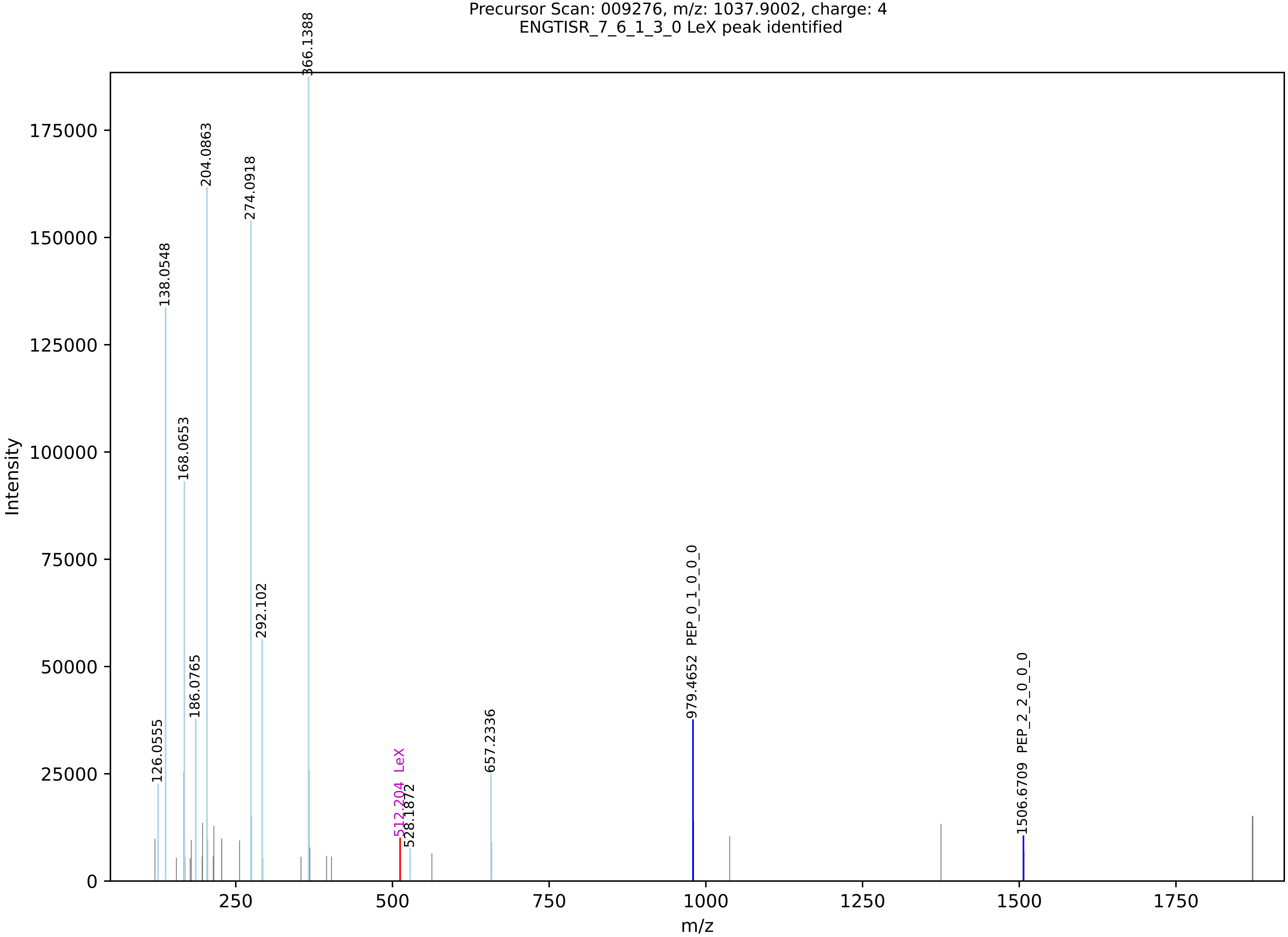

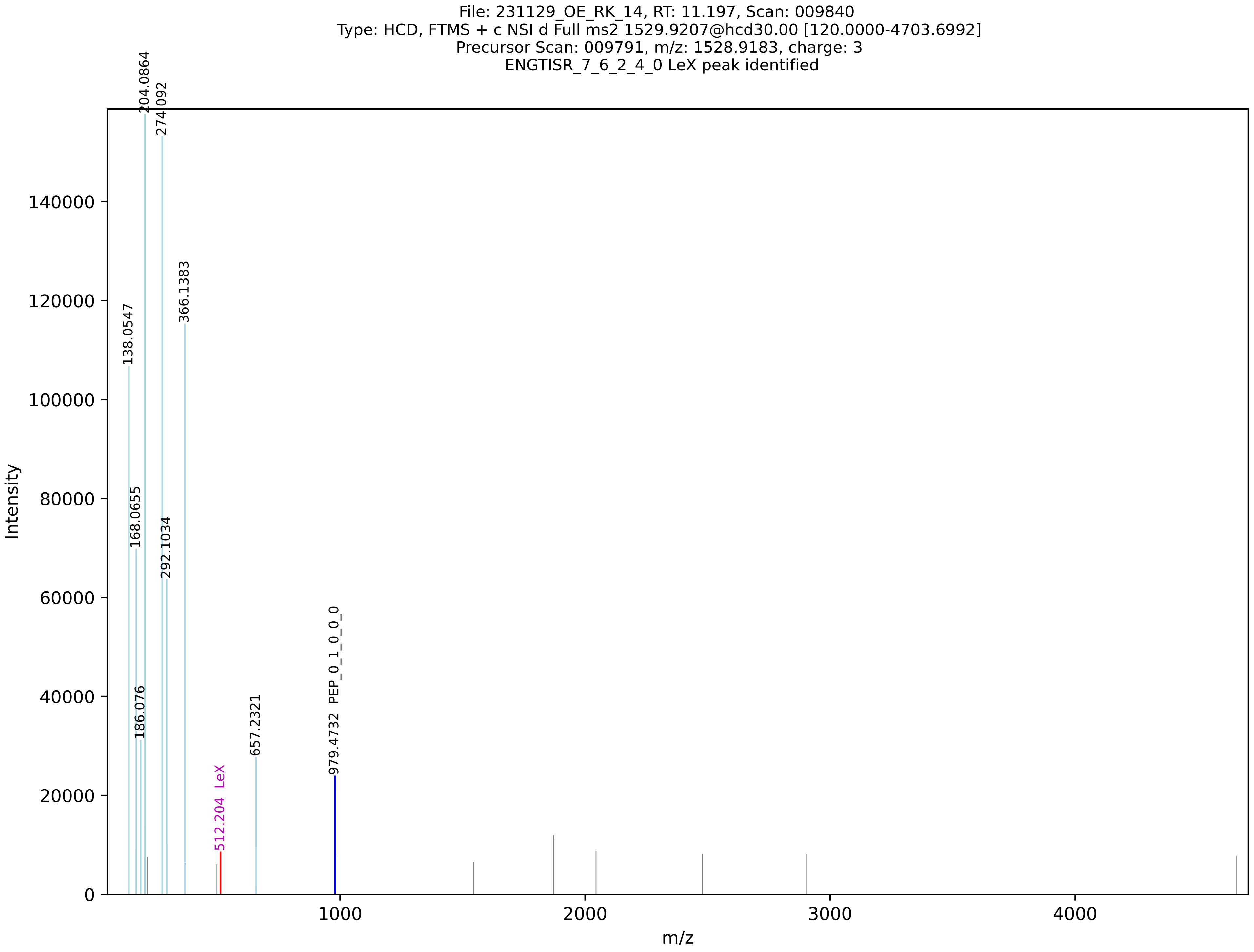

File: 231129_OE_RK_14, RT: 11.197, Scan: 009840
Type: HCD, FTMS + c NSI d Full ms2 1529.9207@hcd30.00 [120.0000-4703.6992]
Precursor Scan: 009791, m/z: 1528.9183, charge: 3
ENGTISR_7_6_2_4_0 LeX peak identified
Intensity
m/z
204.0864
274.092
366.1383
138.0547
168.0655
292.1034
186.076
657.2321
512.204 LeX
979.4732 PEP_0_1_0_0_0
0
20000
40000
60000
80000
100000
120000
140000
1000
2000
3000
4000

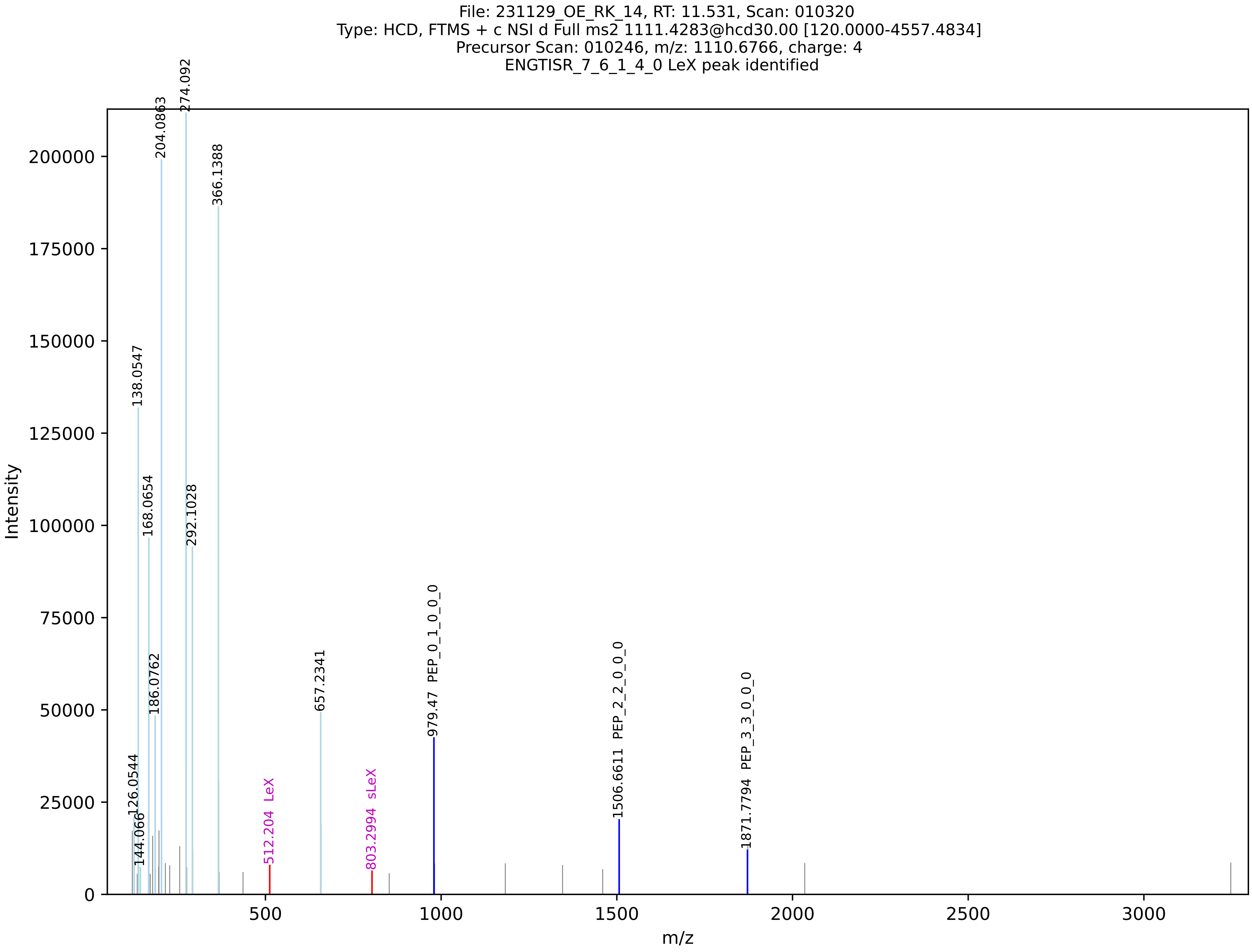

File: 231129_OE_RK_14, RT: 11.531, Scan: 010320
Type: HCD, FTMS + c NSI d Full ms2 1111.4283@hcd30.00 [120.0000-4557.4834]
Precursor Scan: 010246, m/z: 1110.6766, charge: 4
ENGTISR_7_6_1_4_0 LeX peak identified
274.092
204.0863
366.1388
138.0547
168.0654
292.1028
186.0762
657.2341
126.0544
144.066
512.204 LeX
803.2994 sLeX
979.47 PEP_0_1_0_0_0
1506.6611 PEP_2_2_0_0_0
1871.7794 PEP_3_3_0_0_0
Intensity
m/z

File: 231129_OE_RK_14, RT: 12.231, Scan: 011311
Type: HCD, FTMS + c NSI d Full ms2 1432.8842@hcd30.00 [120.0000-4406.7676]
Precursor Scan: 011274, m/z: 1432.2142, charge: 3
ENGTISR_7_6_0_4_0 LeX peak identified

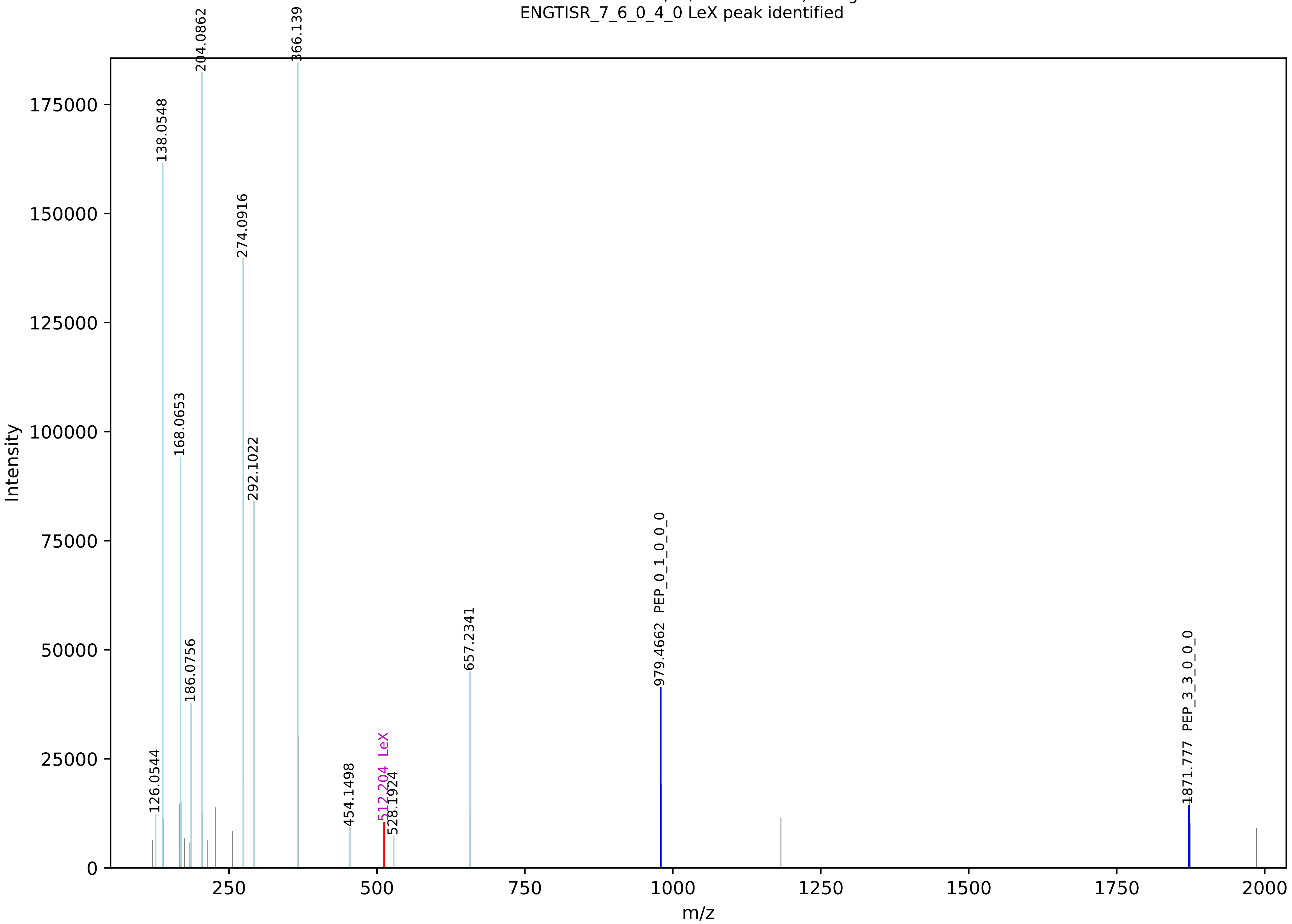

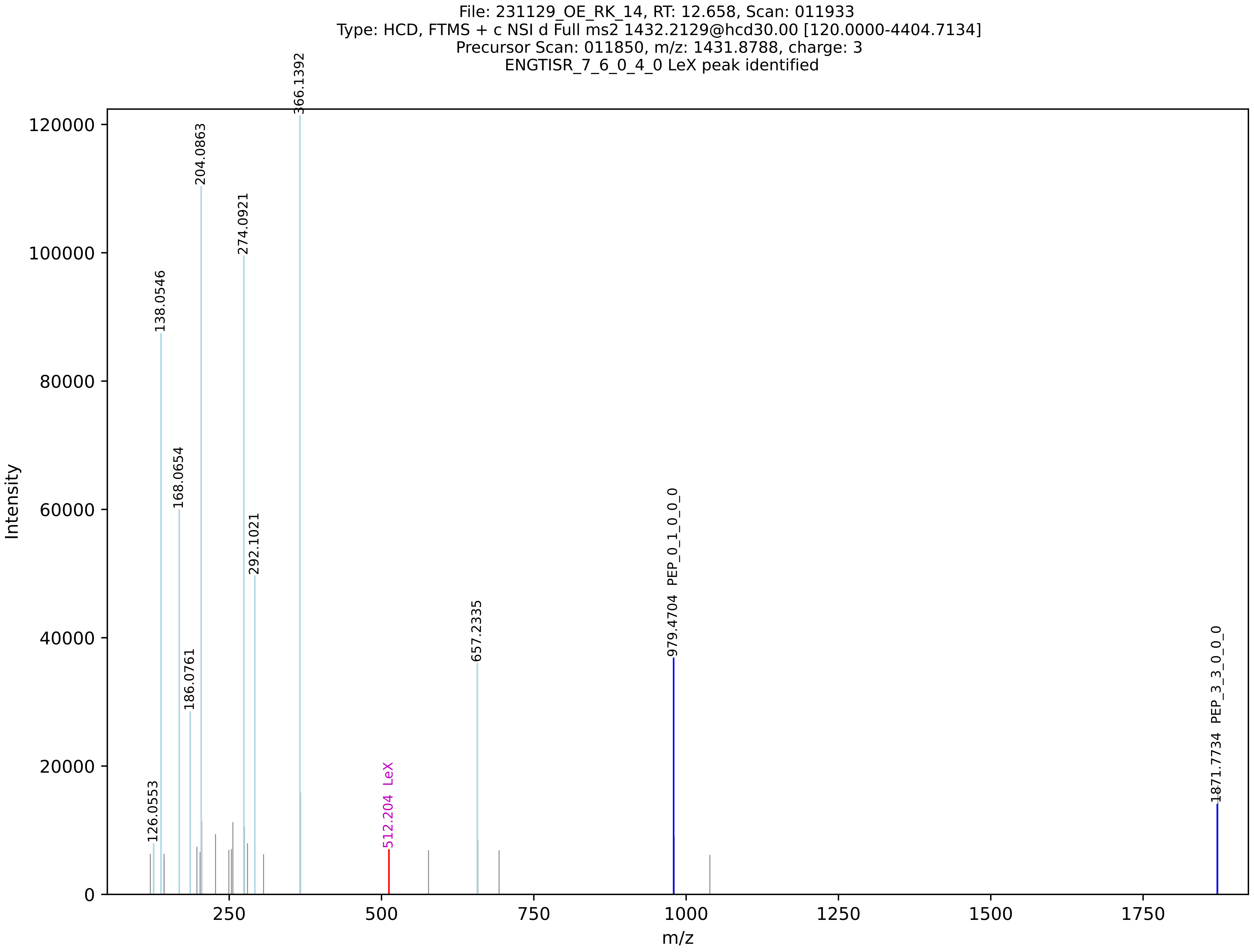
File: 231129_OE_RK_14, RT: 12.658, Scan: 011933
Type: HCD, FTMS + c NSI d Full ms2 1432.2129@hcd30.00 [120.0000-4404.7134]
Precursor Scan: 011850, m/z: 1431.8788, charge: 3
ENGTISR_7_6_0_4_0 LeX peak identified
Intensity
m/z
366.1392
204.0863
274.0921
138.0546
168.0654
292.1021
186.0761
126.0553
512.204 LeX
657.2335
979.4704 PEP_0_1_0_0_0
1871.7734 PEP_3_3_0_0_0
0
20000
40000
60000
80000
100000
120000
250
500
750
1000
1250
1500
1750

File: 231129_OE_RK_14, RT: 14.312, Scan: 014298
Type: HCD, FTMS + c NSI d Full ms2 1147.6909@hcd30.00 [120.0000-4705.4346]
Precursor Scan: 014216, m/z: 1147.1897, charge: 4
ENGTISR_7_6_2_4_0 LeX peak identified

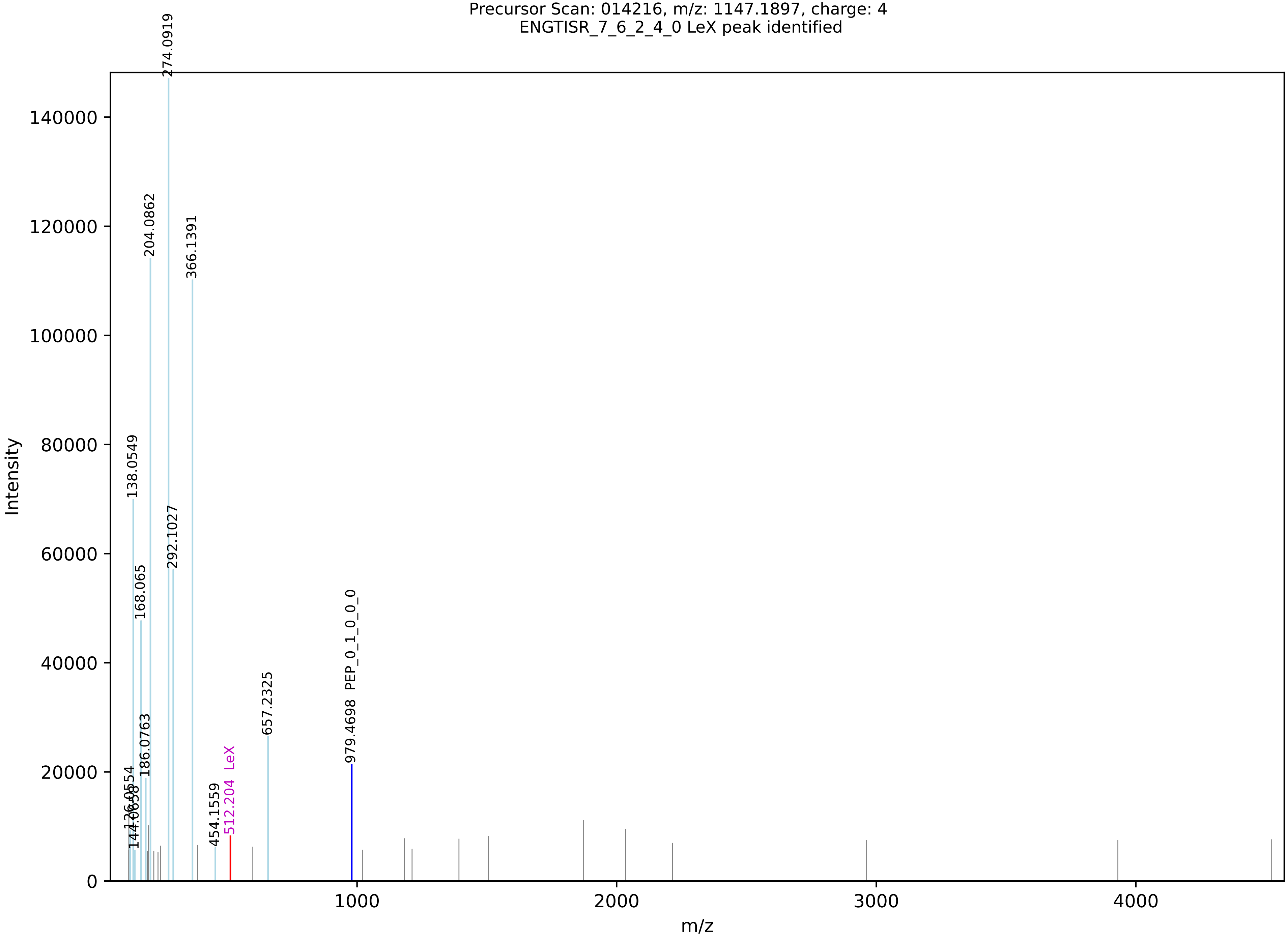

File: 231129_OE_RK_14, RT: 14.506, Scan: 014580
Type: HCD, FTMS + c NSI d Full ms2 1147.6899@hcd30.00 [120.0000-4705.4312]
Precursor Scan: 014502, m/z: 1147.1888, charge: 4
ENGTISR_7_6_2_4_0 LeX peak identified

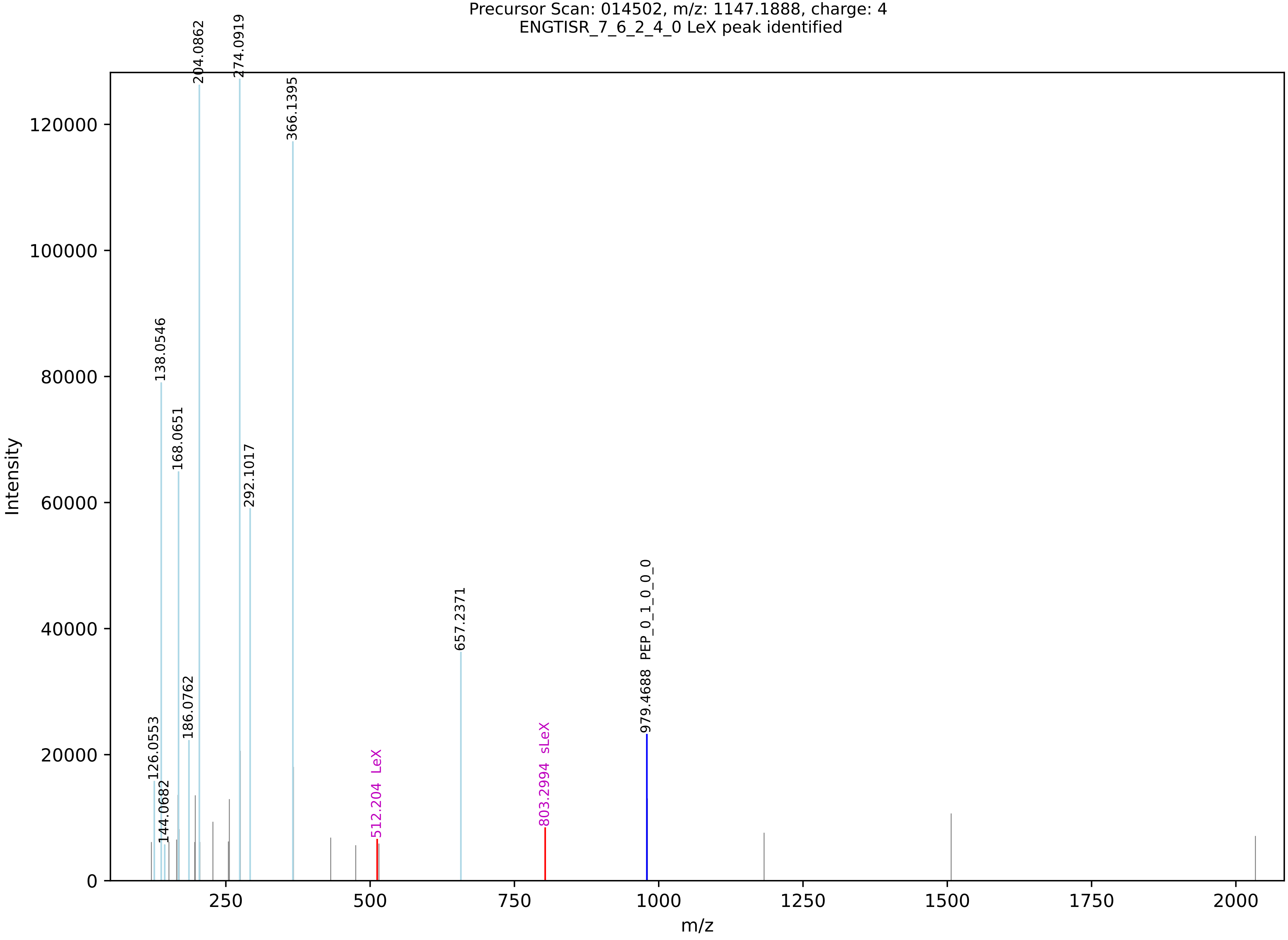

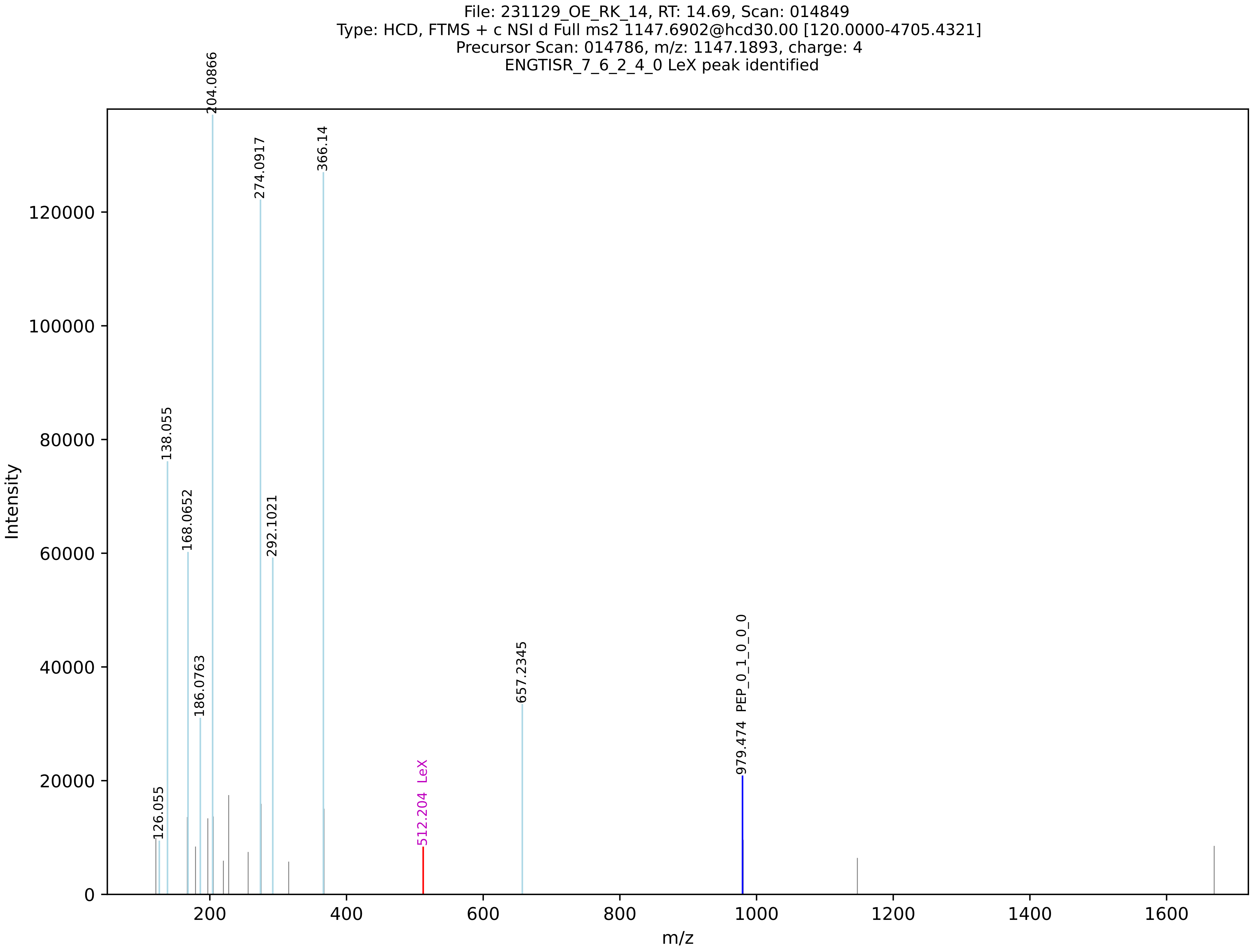
File: 231129_OE_RK_14, RT: 14.69, Scan: 014849
Type: HCD, FTMS + c NSI d Full ms2 1147.6902@hcd30.00 [120.0000-4705.4321]
Precursor Scan: 014786, m/z: 1147.1893, charge: 4
ENGTISR_7_6_2_4_0 LeX peak identified
204.0866
274.0917
366.14
138.055
168.0652
292.1021
186.0763
126.055
657.2345
979.474 PEP_0_1_0_0_0
512.204 LeX
Intensity
m/z
0
20000
40000
60000
80000
100000
120000
200
400
600
800
1000
1200
1400
1600

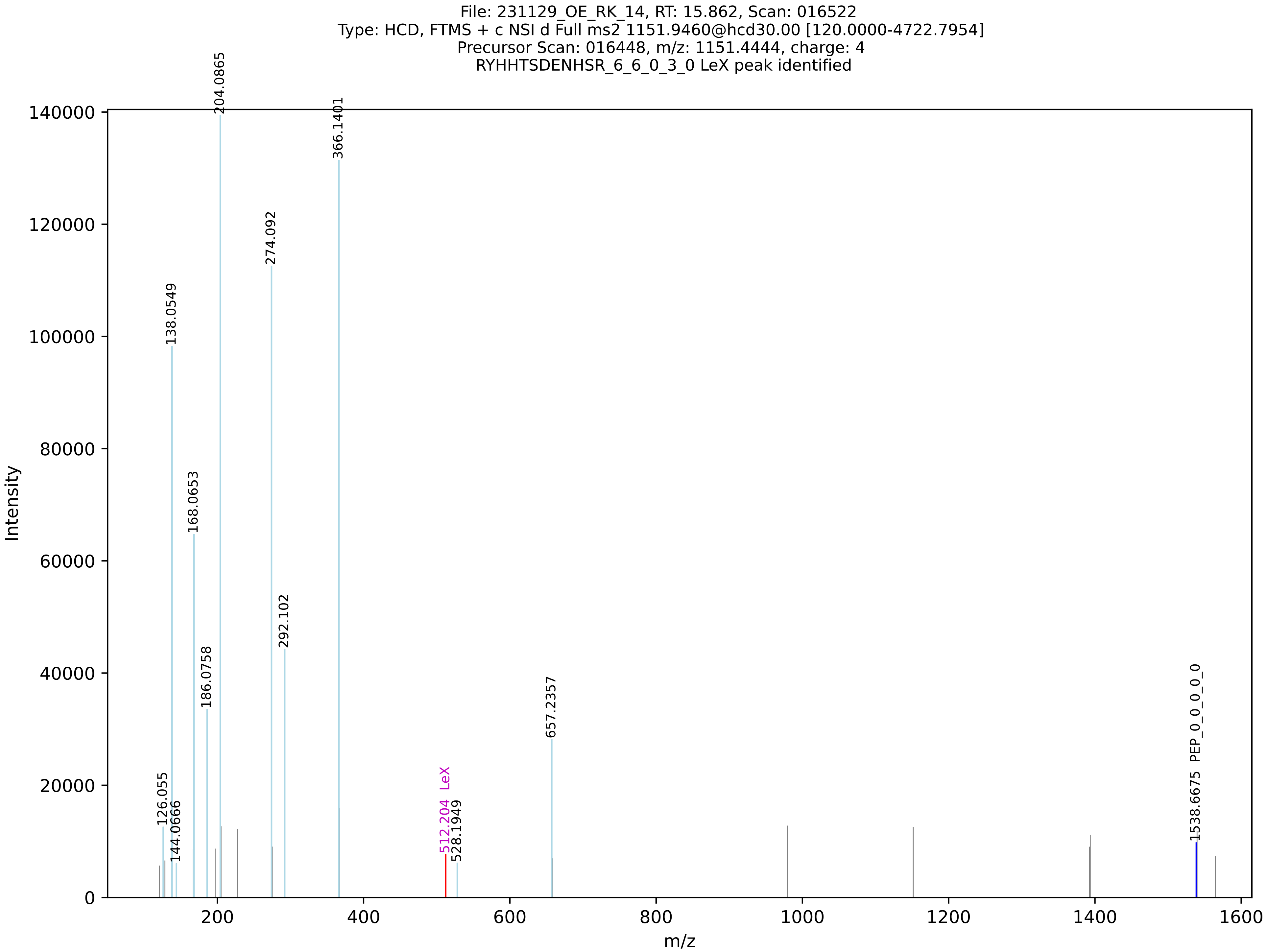
File: 231129_OE_RK_14, RT: 15.862, Scan: 016522
Type: HCD, FTMS + c NSI d Full ms2 1151.9460@hcd30.00 [120.0000-4722.7954]
Precursor Scan: 016448, m/z: 1151.4444, charge: 4
RYHHTSDENHSR_6_6_0_3_0 LeX peak identified
Intensity
m/z
0
20000
40000
60000
80000
100000
120000
140000
200
400
600
800
1000
1200
1400
1600
204.0865
366.1401
274.092
138.0549
168.0653
292.102
186.0758
657.2357
126.055
144.0666
512.204 LeX
528.1949
1538.6675 PEP_0_0_0_0_0

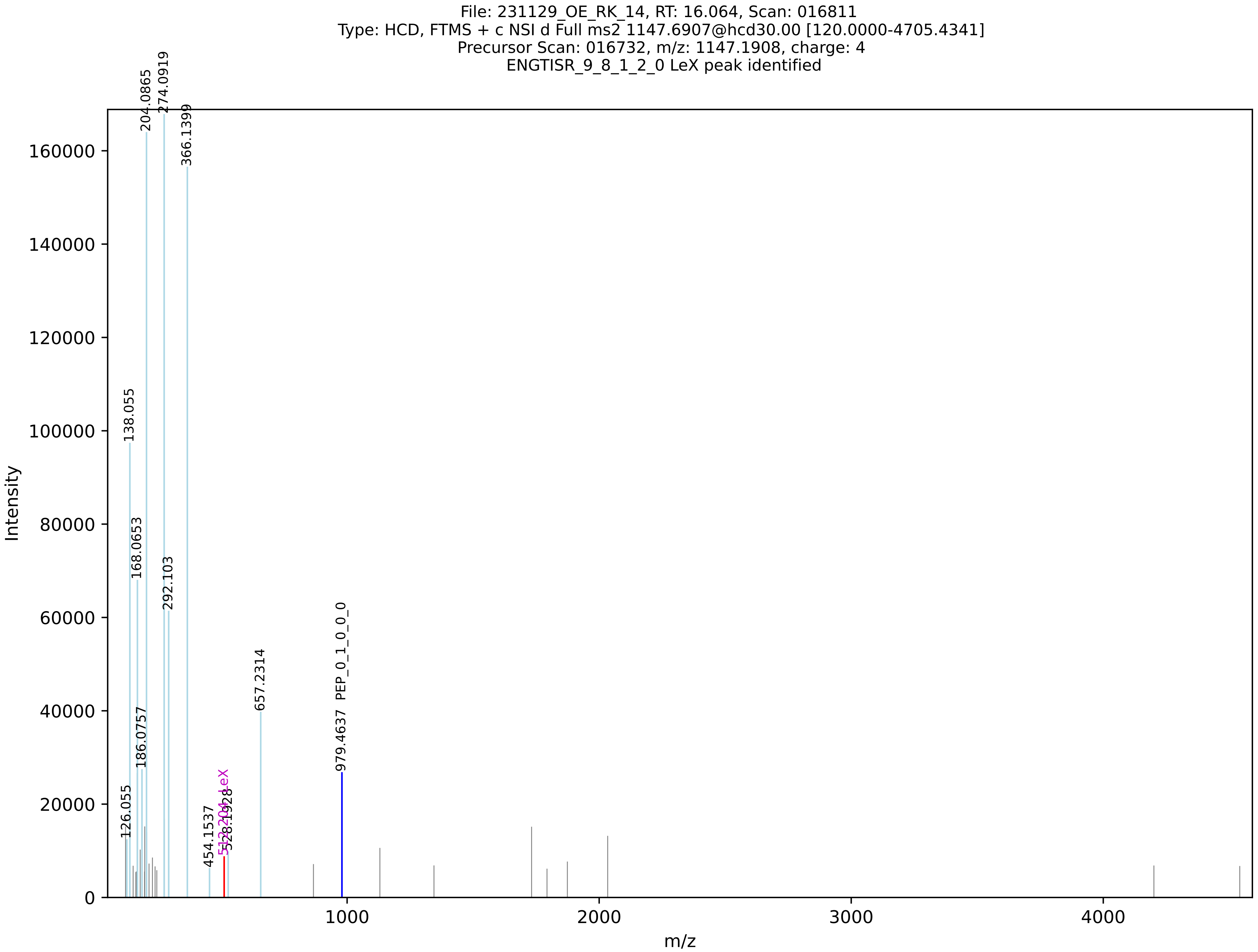

File: 231129_OE_RK_14, RT: 16.064, Scan: 016811
Type: HCD, FTMS + c NSI d Full ms2 1147.6907@hcd30.00 [120.0000-4705.4341]
Precursor Scan: 016732, m/z: 1147.1908, charge: 4
ENGTISR_9_8_1_2_0 LeX peak identified
Intensity
m/z
204.0865
274.0919
366.1399
138.055
168.0653
292.103
186.0757
126.055
454.1537
512.204 LeX
528.1928
657.2314
979.4637 PEP_0_1_0_0_0

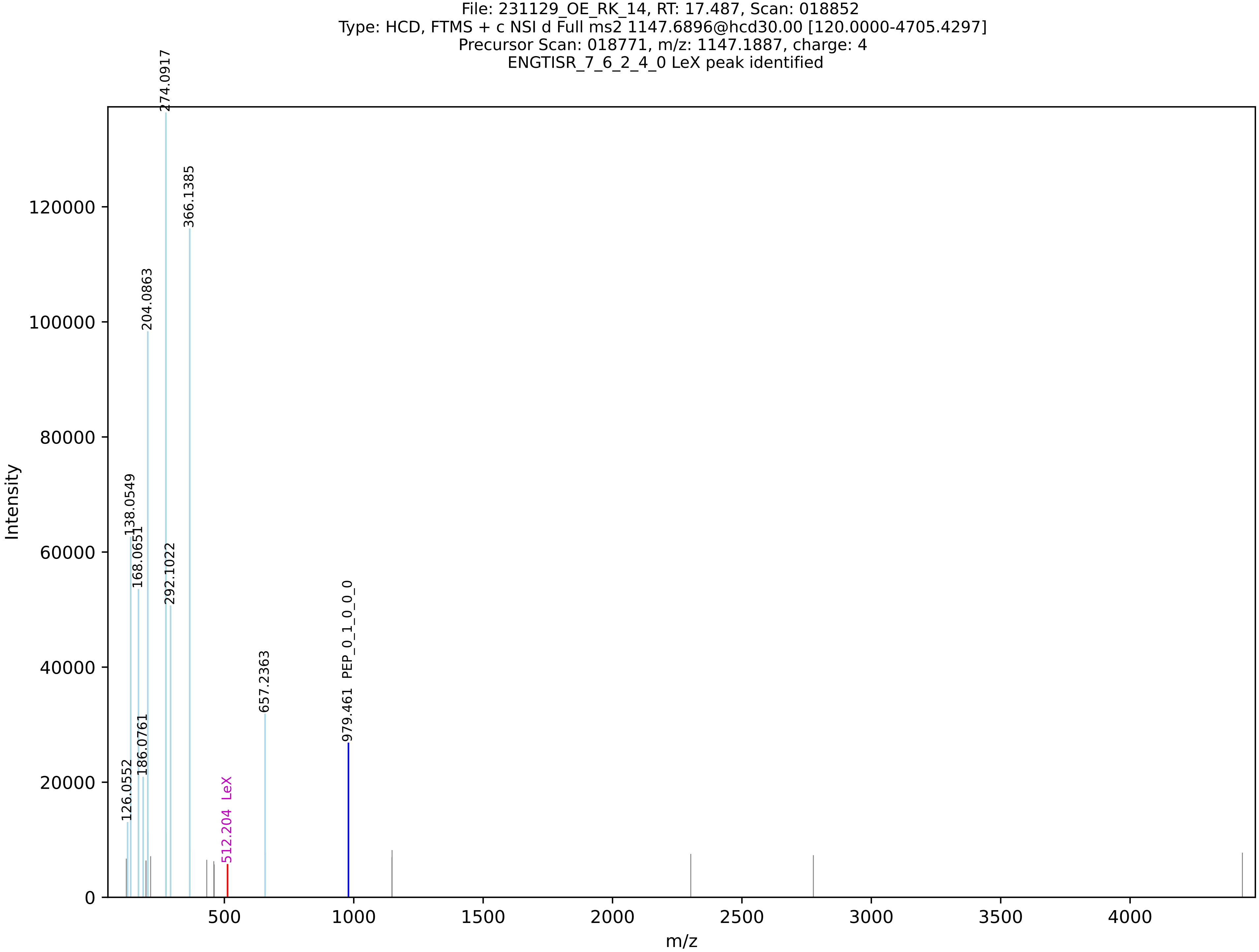
File: 231129_OE_RK_14, RT: 17.487, Scan: 018852
Type: HCD, FTMS + c NSI d Full ms2 1147.6896@hcd30.00 [120.0000-4705.4297]
Precursor Scan: 018771, m/z: 1147.1887, charge: 4
ENGTISR_7_6_2_4_0 LeX peak identified
274.0917
366.1385
204.0863
138.0549
168.0651
292.1022
PEP_0_1_0_0_0
657.2363
979.461
186.0761
126.0552
LeX
512.204
Intensity
m/z
0
20000
40000
60000
80000
100000
120000
500
1000
1500
2000
2500
3000
3500
4000

File: 231129_OE_RK_14, RT: 17.536, Scan: 018922
Type: HCD, FTMS + c NSI d Full ms2 1529.9174@hcd30.00 [120.0000-4703.6890]
Precursor Scan: 018845, m/z: 1529.2491, charge: 3
ENGTISR_7_6_2_4_0 LeX peak identified

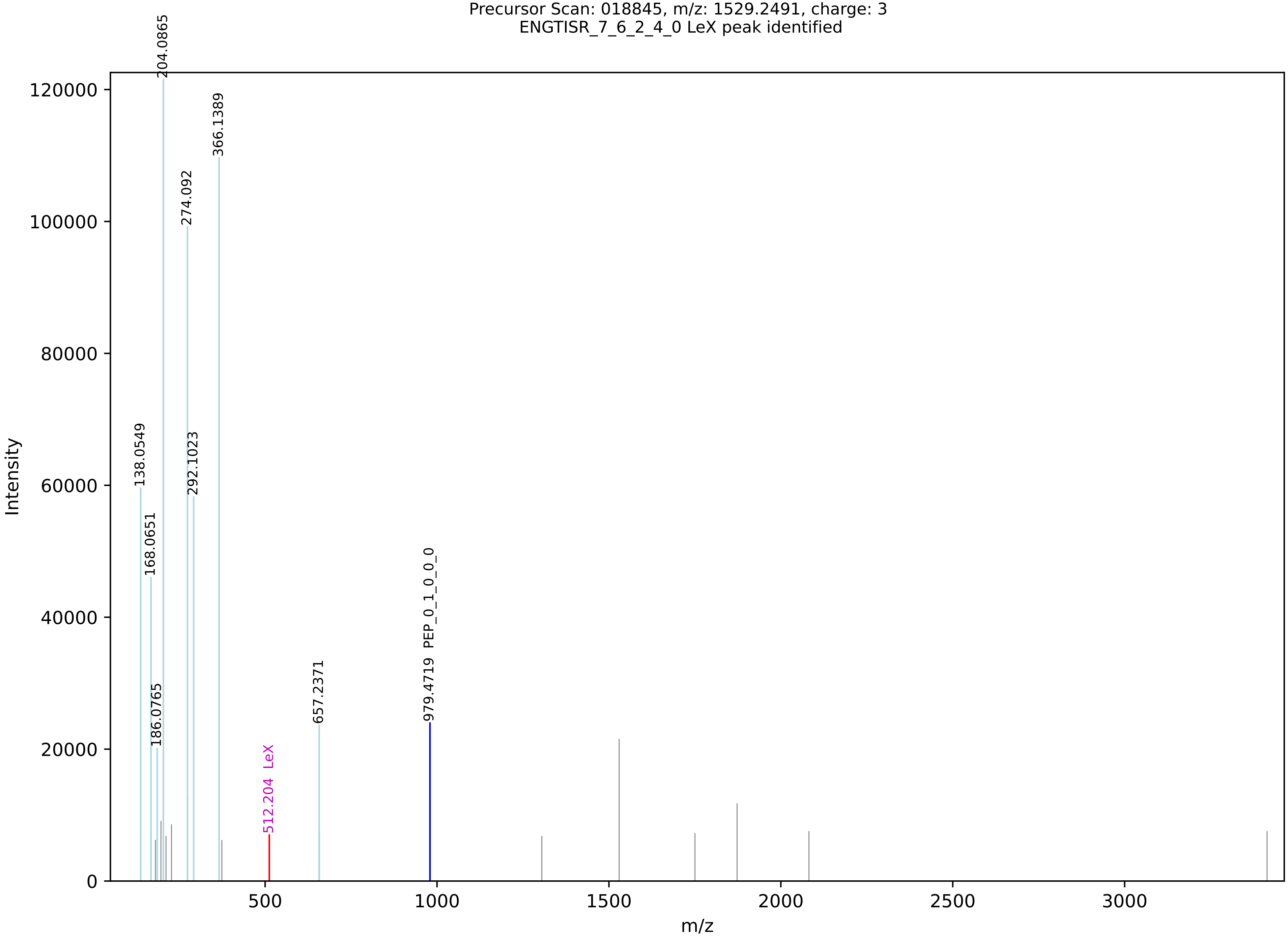

File: 231129_OE_RK_14, RT: 17.638, Scan: 019071
Type: HCD, FTMS + c NSI d Full ms2 1110.9250@hcd30.00 [120.0000-4555.4302]
Precursor Scan: 018992, m/z: 1110.6731, charge: 4
ENGTISR_7_6_1_4_0 LeX peak identified

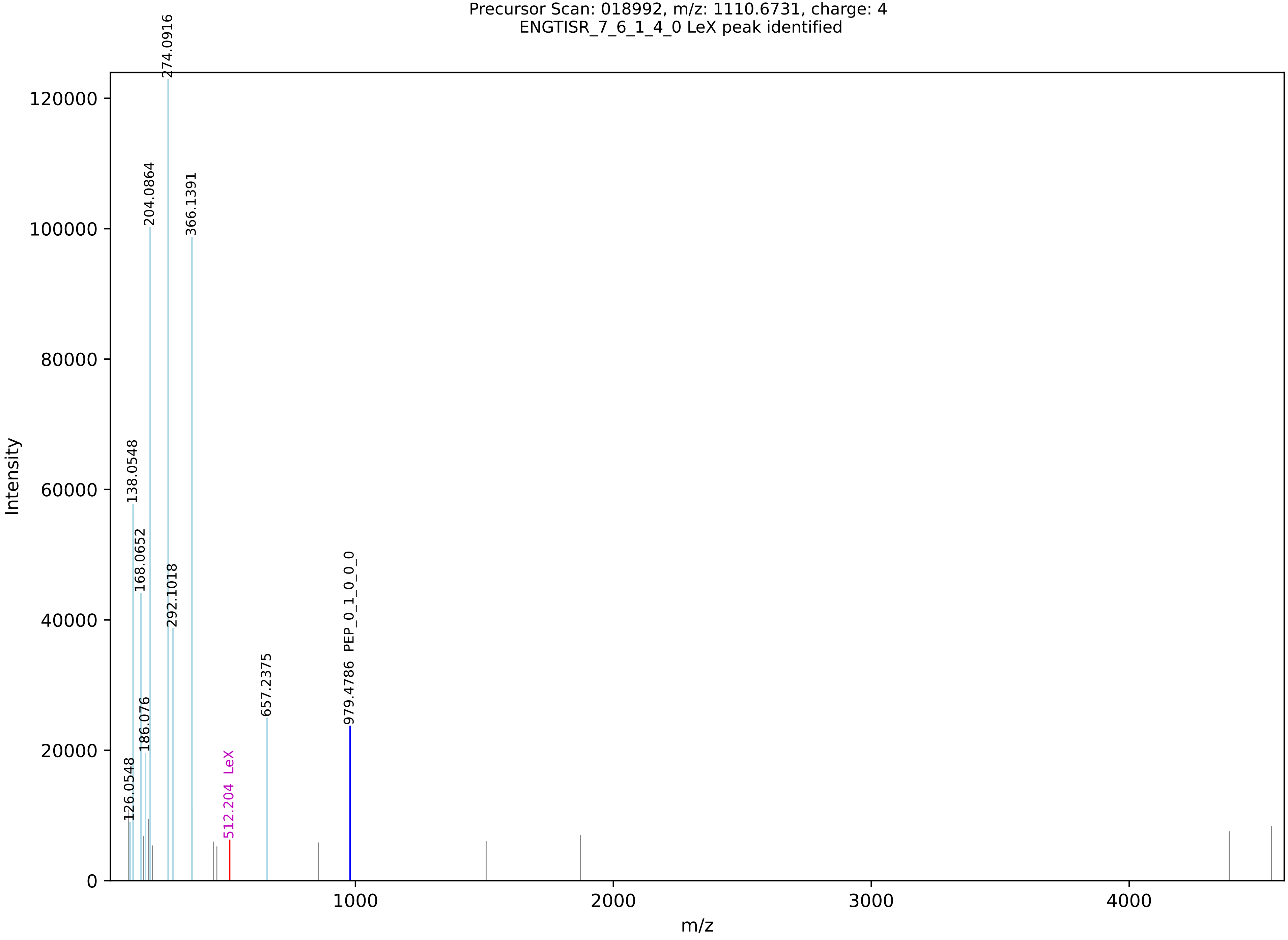

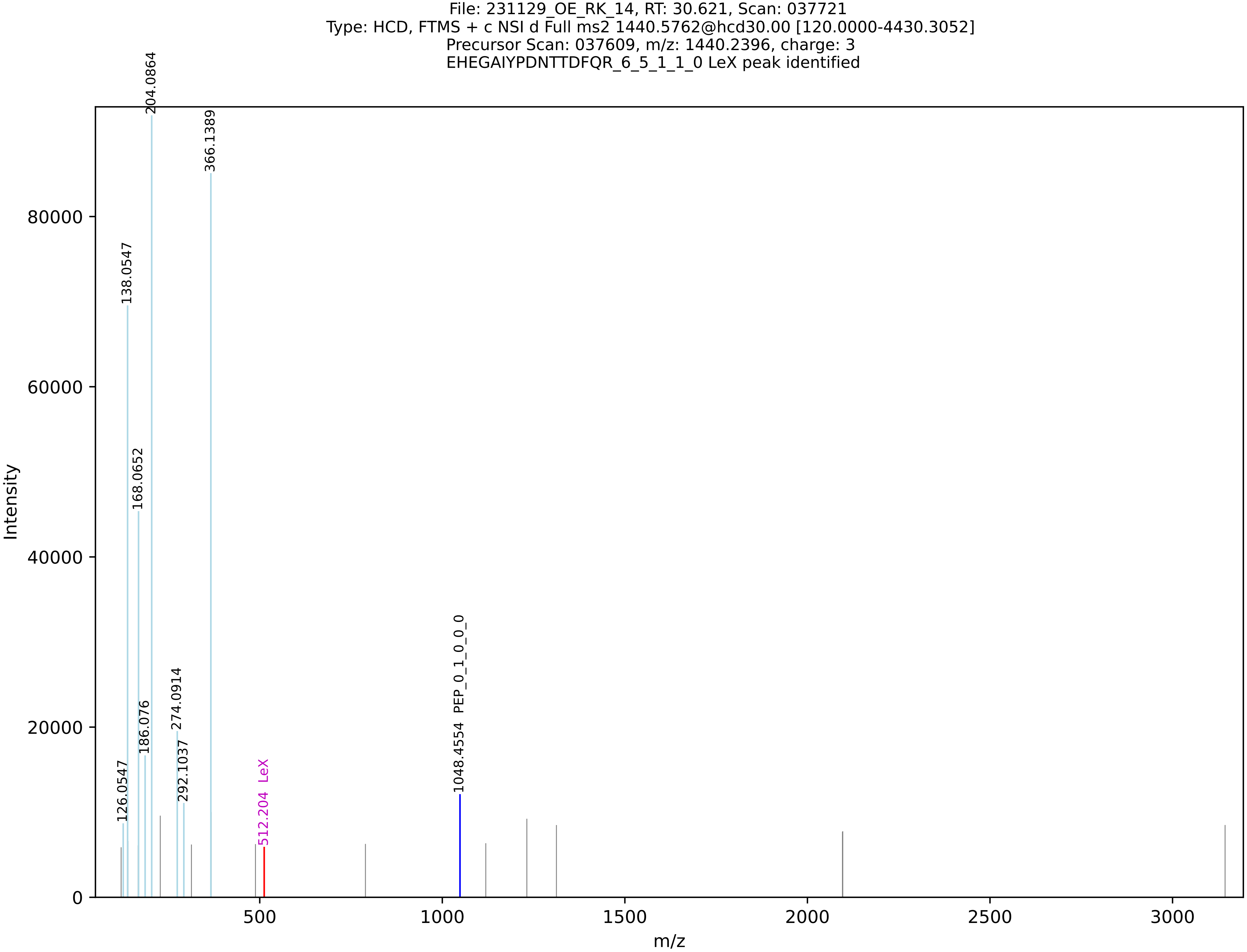

File: 231129_OE_RK_14, RT: 30.621, Scan: 037721
Type: HCD, FTMS + c NSI d Full ms2 1440.5762@hcd30.00 [120.0000-4430.3052]
Precursor Scan: 037609, m/z: 1440.2396, charge: 3
EHEGAIYPDNTTDFQR_6_5_1_1_0 LeX peak identified
Intensity
m/z
0
20000
40000
60000
80000
500
1000
1500
2000
2500
3000
204.0864
366.1389
138.0547
168.0652
186.076
274.0914
292.1037
126.0547
512.204 LeX
1048.4554 PEP_0_1_0_0_0

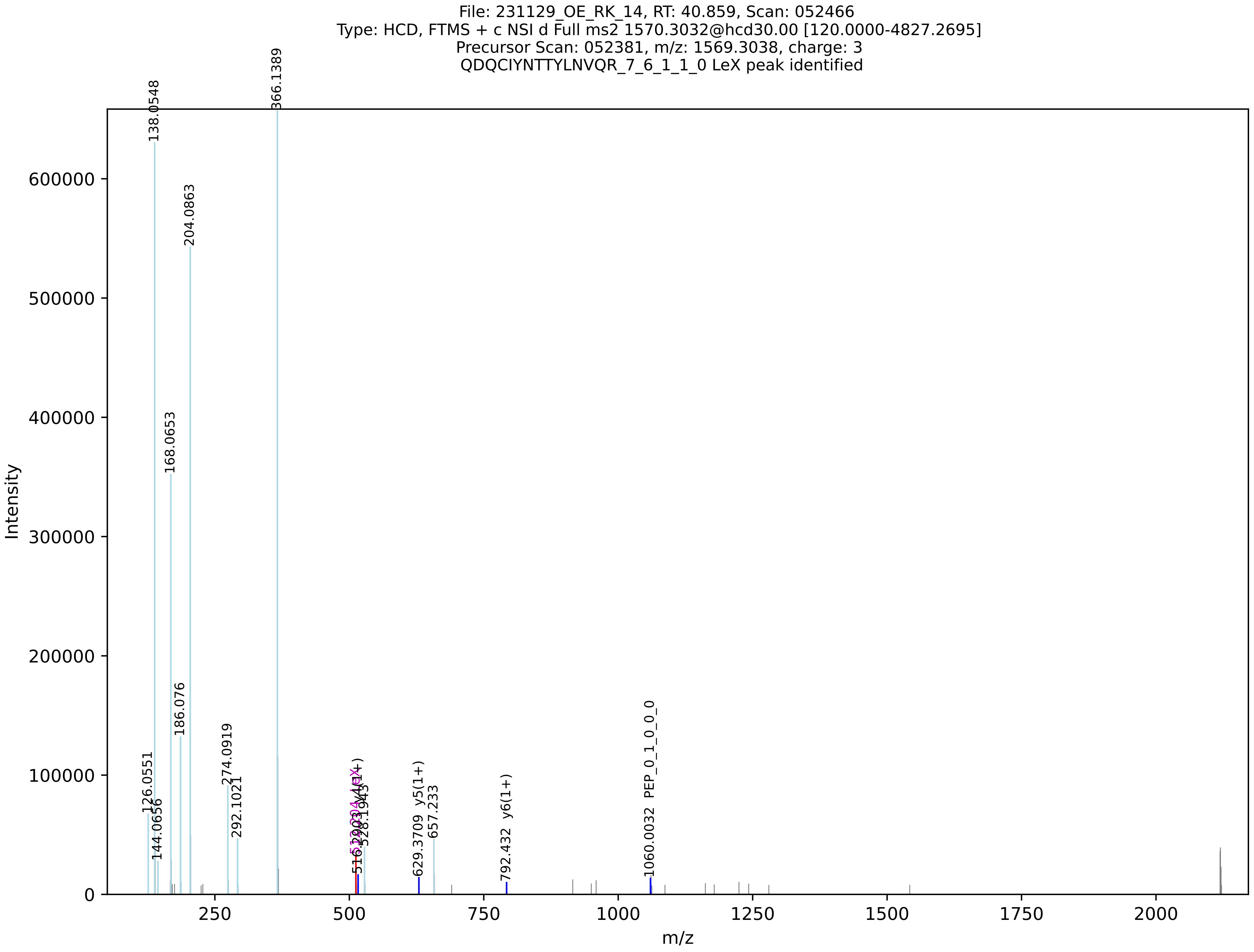
File: 231129_OE_RK_14, RT: 40.859, Scan: 052466
Type: HCD, FTMS + c NSI d Full ms2 1570.3032@hcd30.00 [120.0000-4827.2695]
Precursor Scan: 052381, m/z: 1569.3038, charge: 3
QDQCIYNTTYLNVQR_7_6_1_1_0 LeX peak identified
Intensity
m/z
138.0548
366.1389
204.0863
168.0653
186.076
126.0551
144.0656
274.0919
292.1021
528.1943
629.3709 y5(1+)
657.233
792.432 y6(1+)
1060.0032 PEP_0_1_0_0_0_
0
100000
200000
300000
400000
500000
600000
250
500
750
1000
1250
1500
1750
2000

File: 231129_OE_RK_14, RT: 40.993, Scan: 052663
Type: HCD, FTMS + c NSI d Full ms2 1618.3192@hcd30.00 [120.0000-4974.1987]
Precursor Scan: 052522, m/z: 1617.9912, charge: 3
QDQCIYNTTYLNVQR_7_6_2_1_0 LeX peak identified

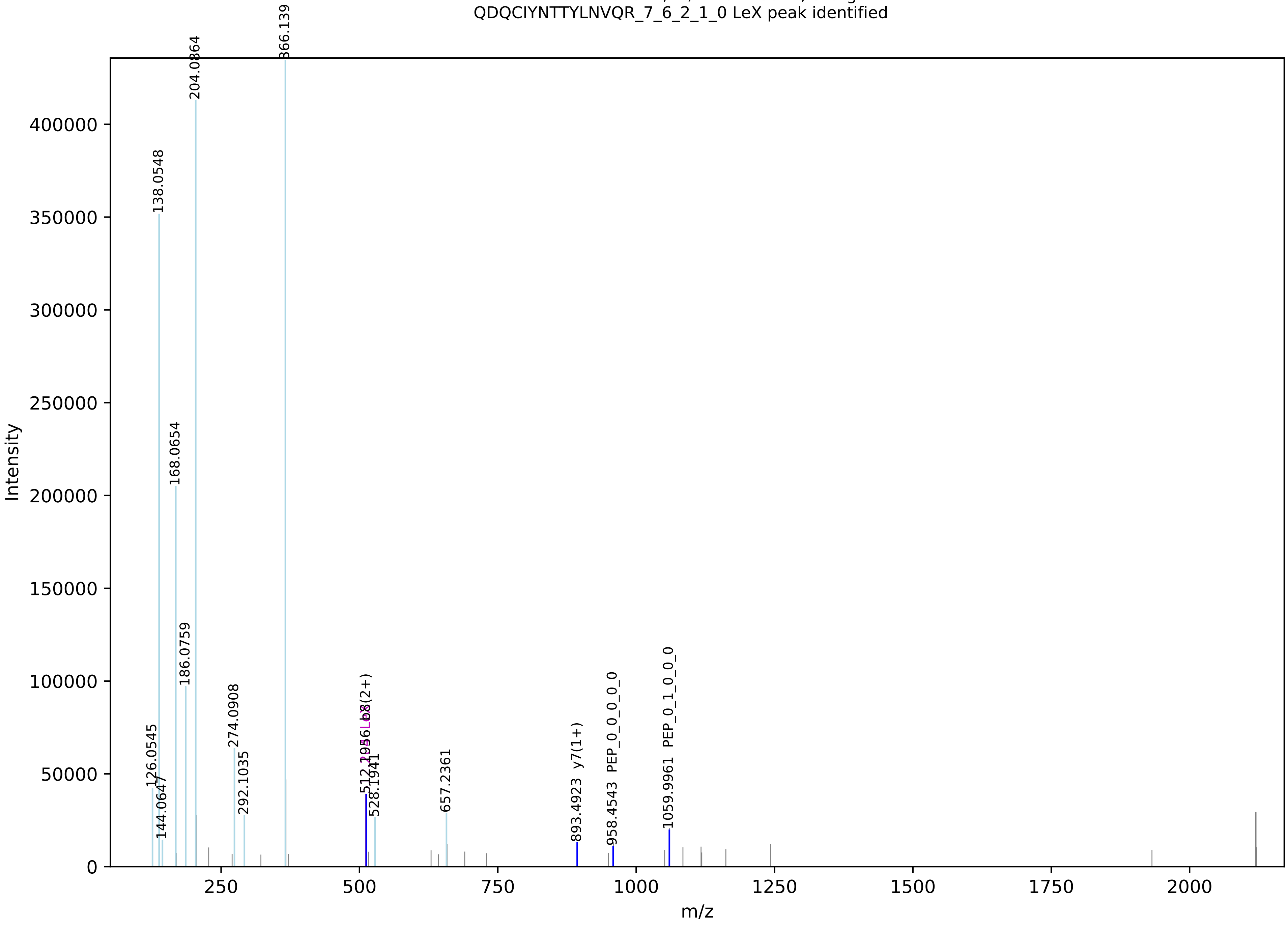

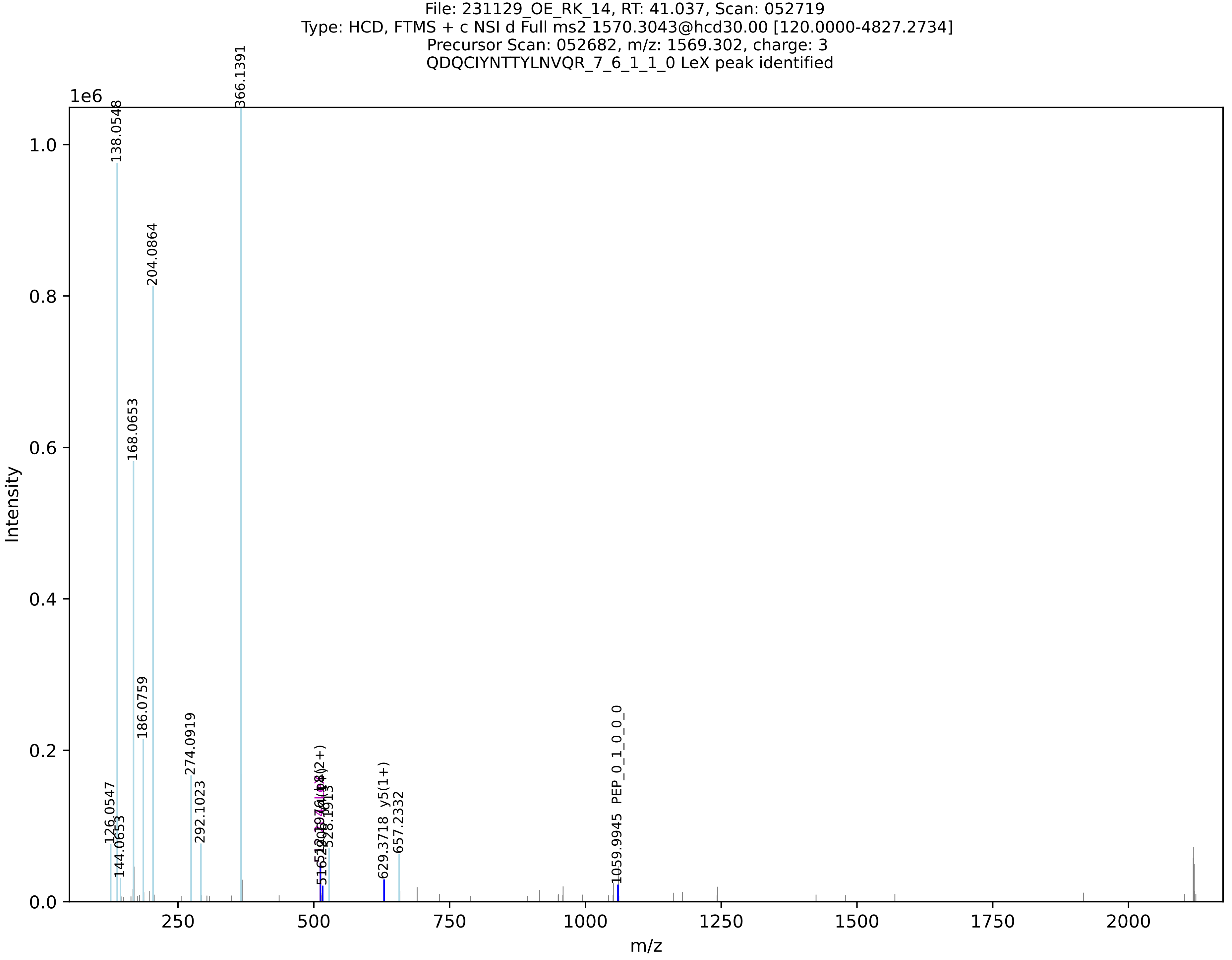
File: 231129_OE_RK_14, RT: 41.037, Scan: 052719
Type: HCD, FTMS + c NSI d Full ms2 1570.3043@hcd30.00 [120.0000-4827.2734]
Precursor Scan: 052682, m/z: 1569.302, charge: 3
QDQCIYNTTYLNVQR_7_6_1_1_0 LeX peak identified
1e6
Intensity
m/z
366.1391
138.0548
204.0864
168.0653
186.0759
274.0919
292.1023
126.0547
144.0653
528.1913
629.3718 y5(1+)
657.2332
1059.9945 PEP_0_1_0_0_0_

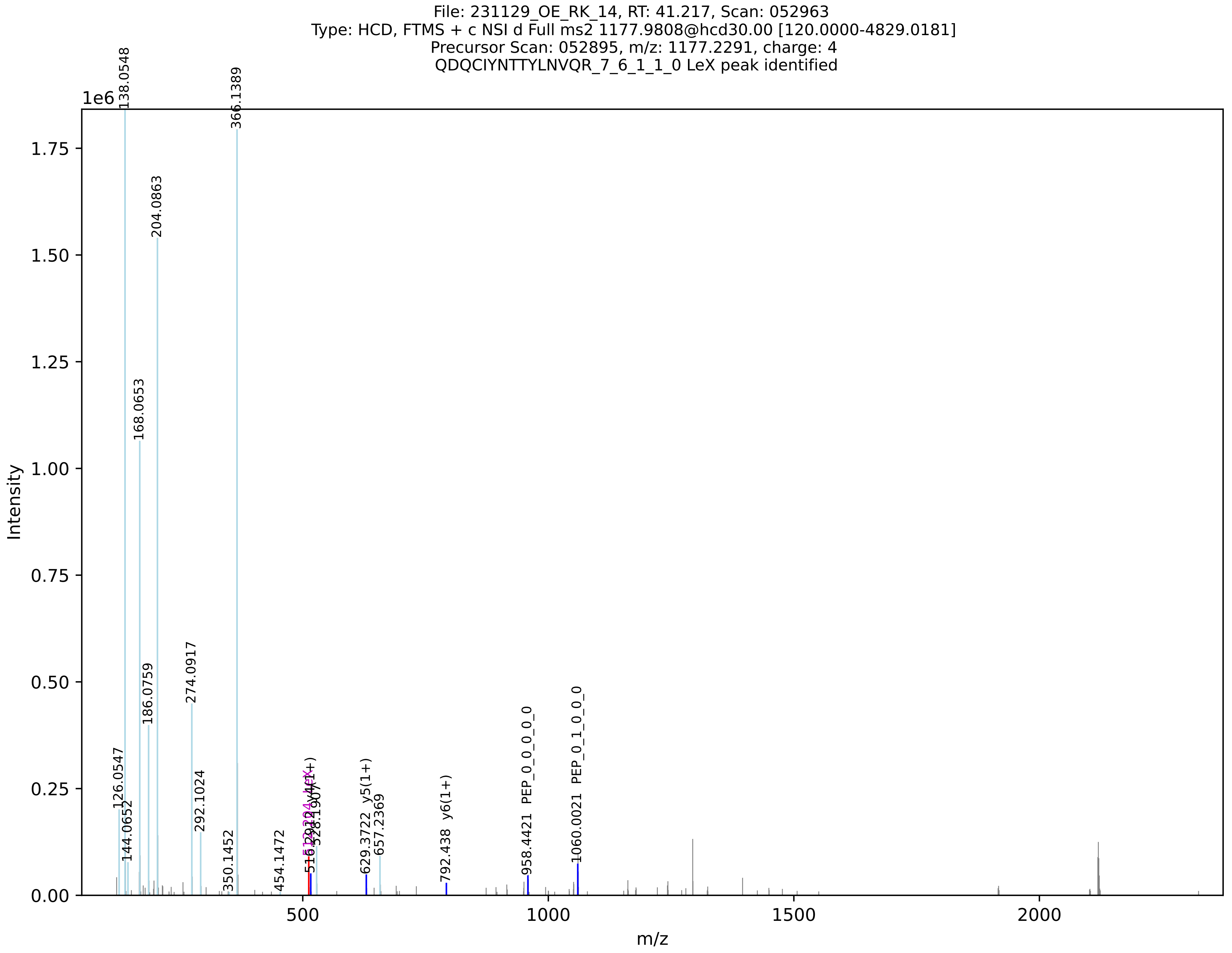

File: 231129_OE_RK_14, RT: 41.217, Scan: 052963
Type: HCD, FTMS + c NSI d Full ms2 1177.9808@hcd30.00 [120.0000-4829.0181]
Precursor Scan: 052895, m/z: 1177.2291, charge: 4
QDQCIYNTTYLNVQR_7_6_1_1_0 LeX peak identified
1e6
Intensity
m/z
138.0548
366.1389
204.0863
168.0653
186.0759
274.0917
126.0547
144.0652
292.1024
350.1452
454.1472
629.3722 y5(1+)
657.2369
792.438 y6(1+)
958.4421 PEP_0_0_0_0_0
1060.0021 PEP_0_1_0_0_0

File: 231129_OE_RK_14, RT: 43.981, Scan: 056969
Type: HCD, FTMS + c NSI d Full ms2 1250.5050@hcd30.00 [120.0000-5124.9165]
Precursor Scan: 056892, m/z: 1250.0039, charge: 4
QDQCIYNTTYLNVQR_7_6_1_2_0 LeX peak identified

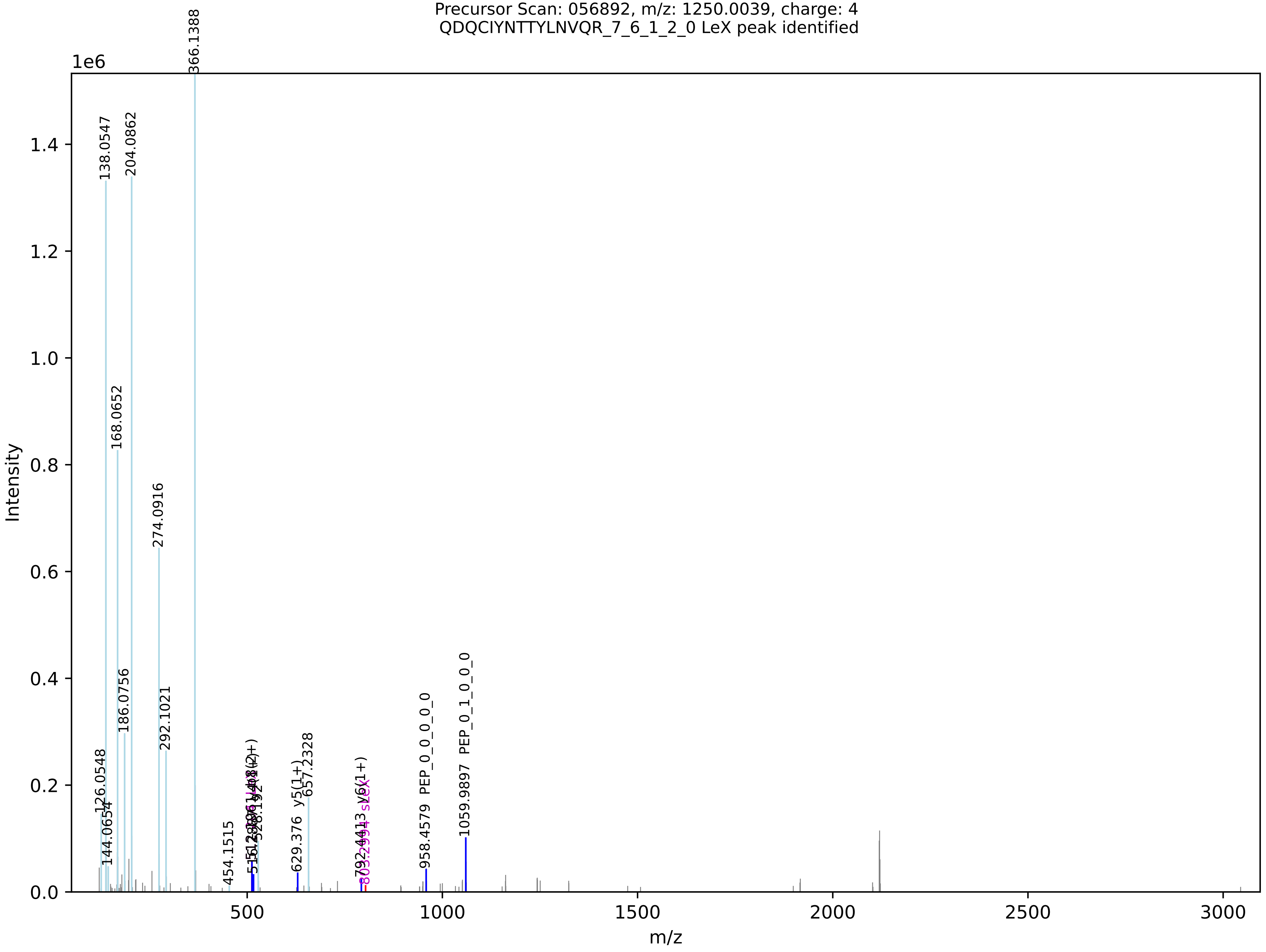

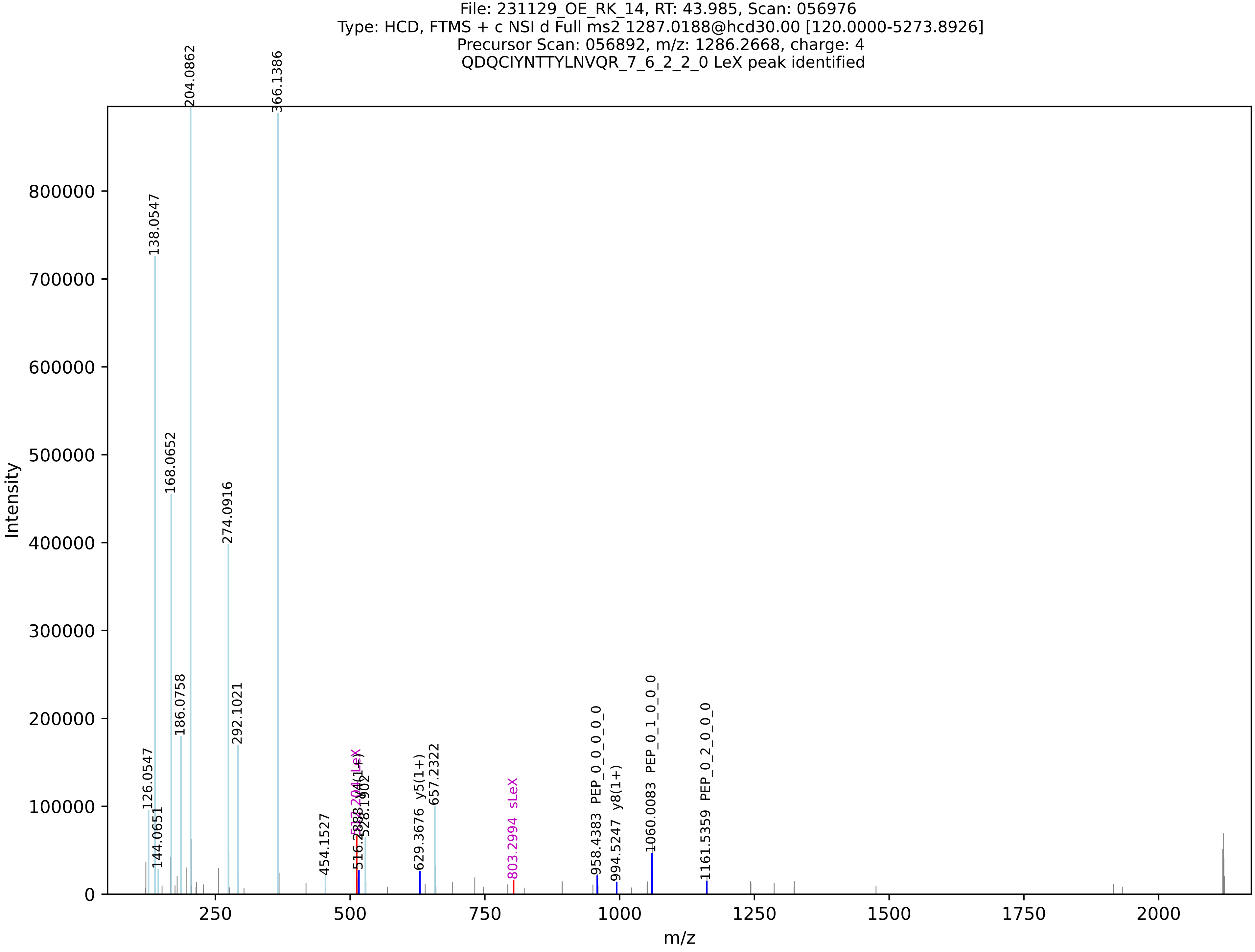
File: 231129_OE_RK_14, RT: 43.985, Scan: 056976
Type: HCD, FTMS + c NSI d Full ms2 1287.0188@hcd30.00 [120.0000-5273.8926]
Precursor Scan: 056892, m/z: 1286.2668, charge: 4
QDQCIYNTTYLNVQR_7_6_2_2_0 LeX peak identified
Intensity
m/z
204.0862
366.1386
138.0547
168.0652
274.0916
186.0758
292.1021
126.0547
144.0651
454.1527
LeX
516.2883 y4(1+)
528.1902
629.3676 y5(1+)
657.2322
803.2994 sLeX
958.4383 PEP_0_0_0_0_0
994.5247 y8(1+)
1060.0083 PEP_0_1_0_0_0
1161.5359 PEP_0_2_0_0_0

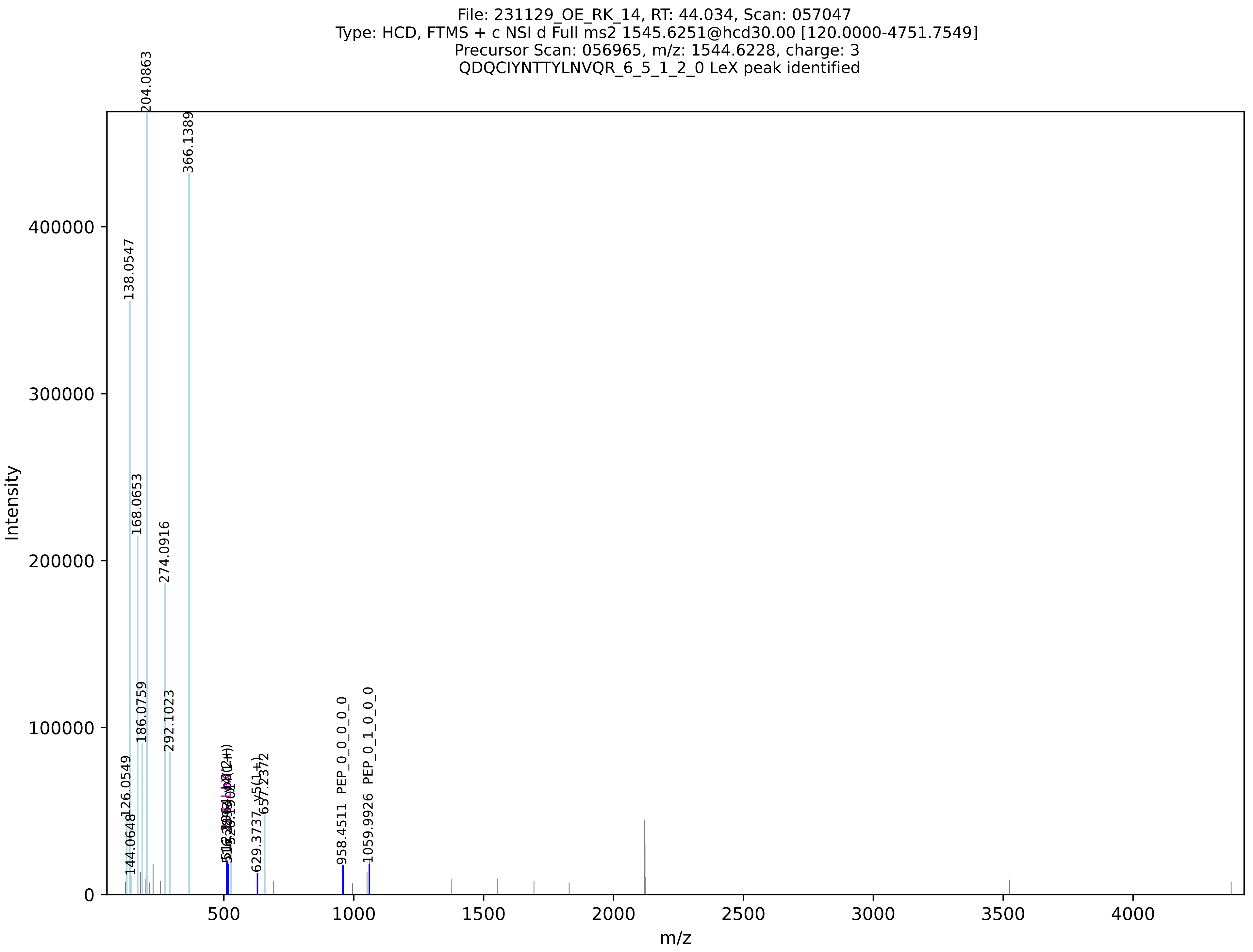
File: 231129_OE_RK_14, RT: 44.034, Scan: 057047
Type: HCD, FTMS + c NSI d Full ms2 1545.6251@hcd30.00 [120.0000-4751.7549]
Precursor Scan: 056965, m/z: 1544.6228, charge: 3
QDQCIYNTTYLNVQR_6_5_1_2_0 LeX peak identified
Intensity
m/z
204.0863
366.1389
138.0547
168.0653
274.0916
186.0759
292.1023
126.0549
144.0648
629.3737_y5(1+)
657.2372
958.4511 PEP_0_0_0_0_0
1059.9926 PEP_0_1_0_0_0
0
100000
200000
300000
400000
500
1000
1500
2000
2500
3000
3500
4000

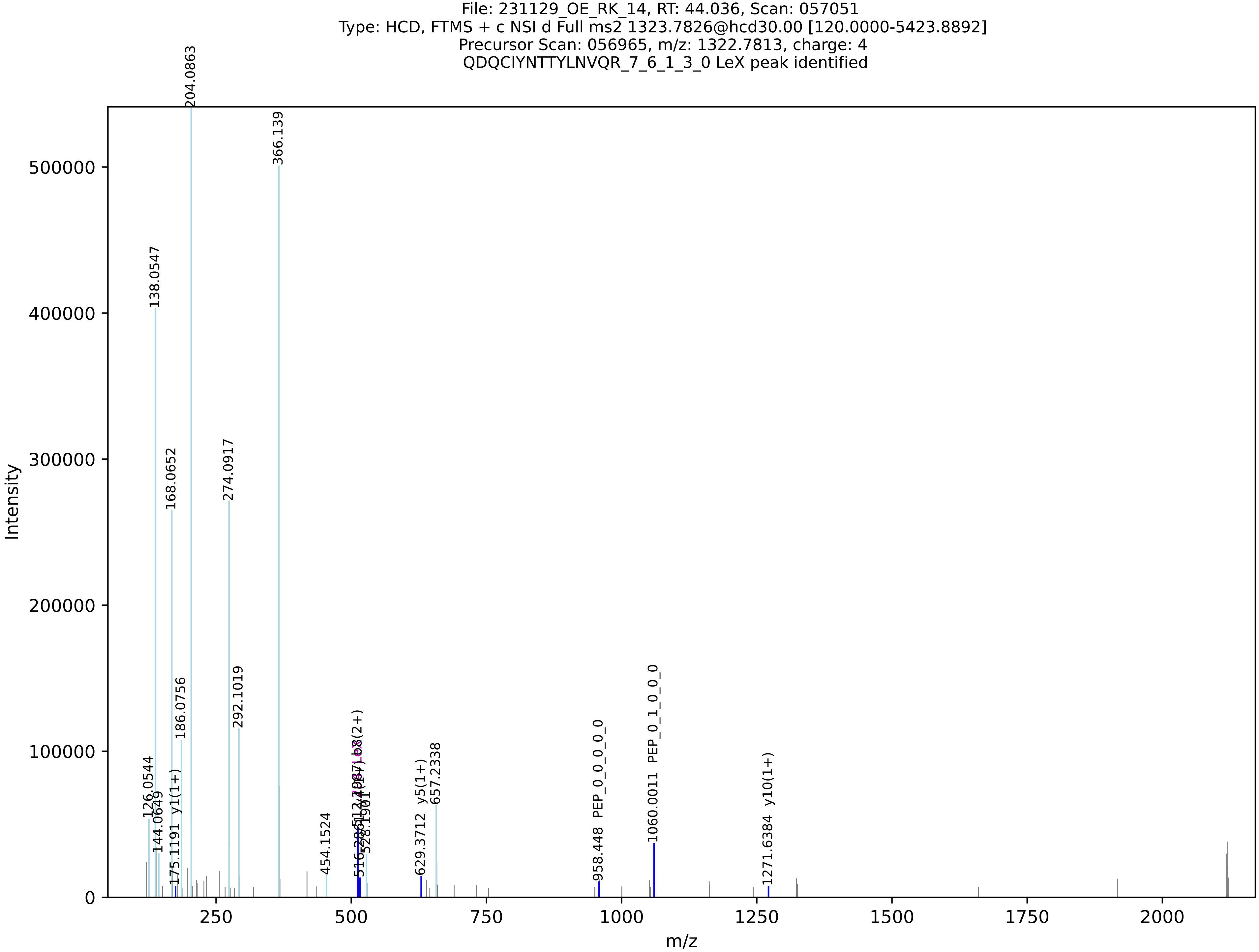
File: 231129_OE_RK_14, RT: 44.036, Scan: 057051
Type: HCD, FTMS + c NSI d Full ms2 1323.7826@hcd30.00 [120.0000-5423.8892]
Precursor Scan: 056965, m/z: 1322.7813, charge: 4
QDQCIYNTTYLNVQR_7_6_1_3_0 LeX peak identified
Intensity
m/z
204.0863
366.139
138.0547
168.0652
274.0917
186.0756
292.1019
126.0544
144.0649
175.1191 y1(1+)
454.1524
528.1901
629.3712 y5(1+)
657.2338
958.448 PEP_0_0_0_0_0
1060.0011 PEP_0_1_0_0_0
1271.6384 y10(1+)

File: 231129_OE_RK_14, RT: 44.181, Scan: 057262
Type: HCD, FTMS + c NSI d Full ms2 1250.5043@hcd30.00 [120.0000-5124.9136]
Precursor Scan: 057185, m/z: 1250.0031, charge: 4
QDQCIYNTTYLNVQR_7_6_1_2_0 LeX peak identified

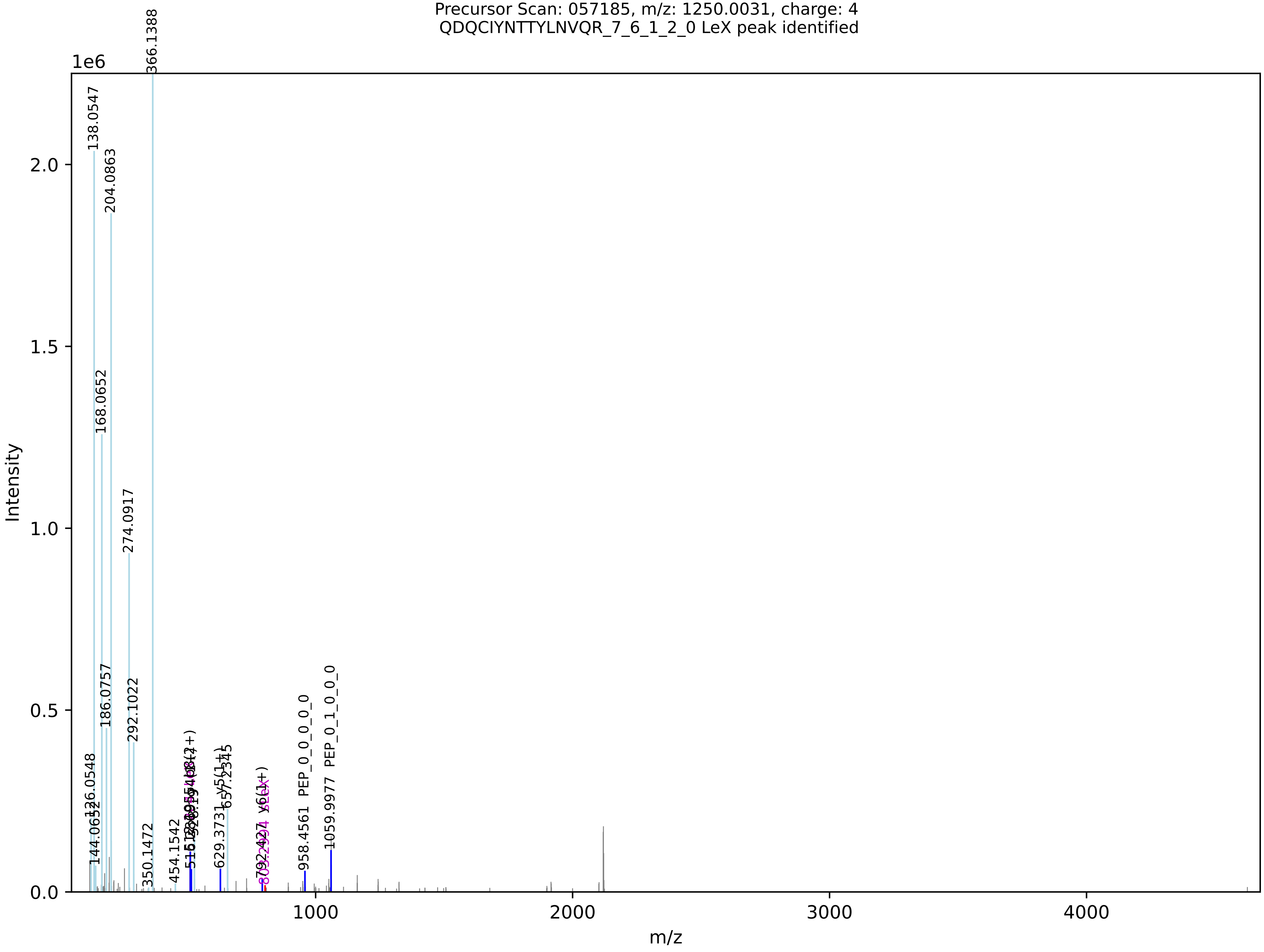

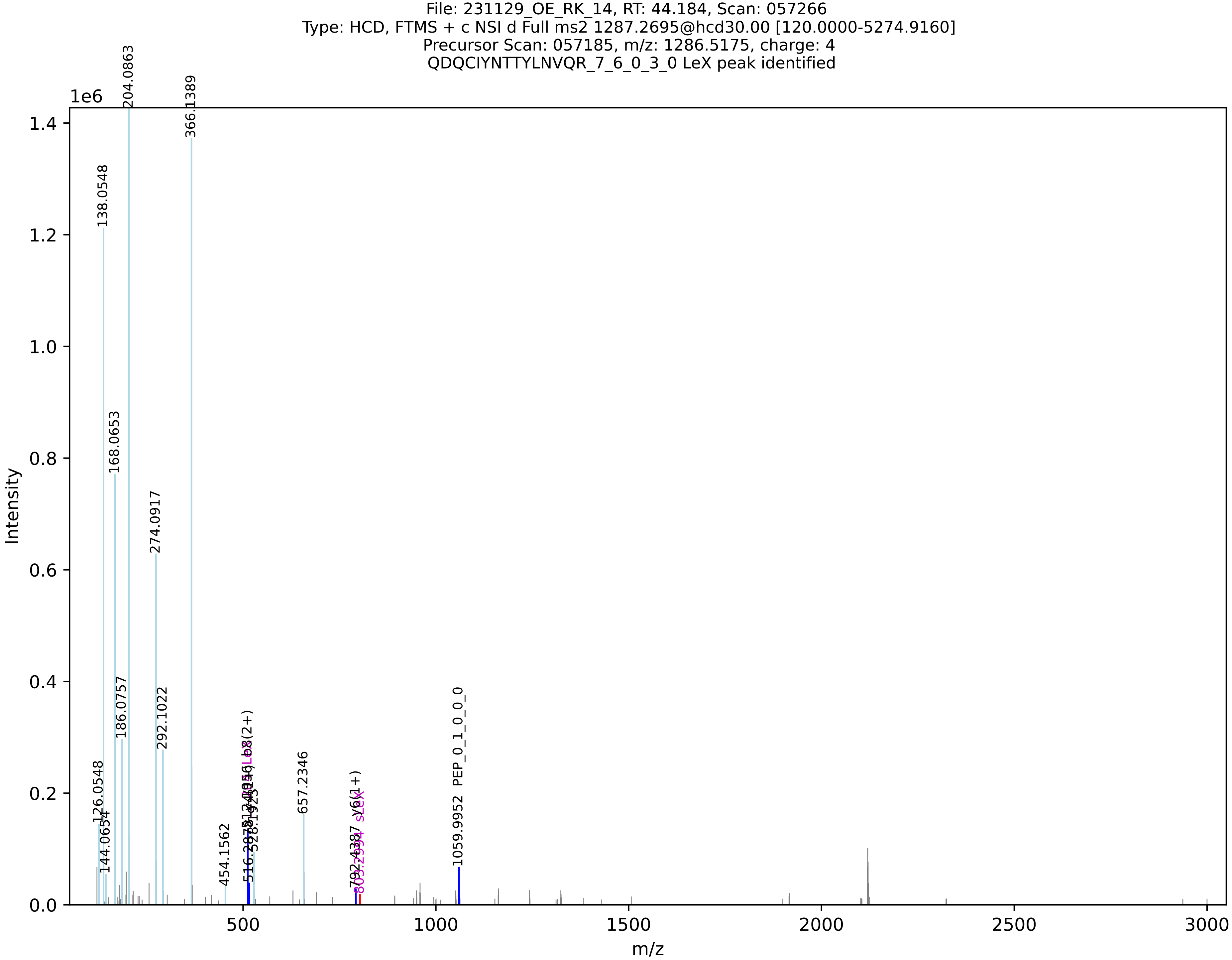
File: 231129_OE_RK_14, RT: 44.184, Scan: 057266
Type: HCD, FTMS + c NSI d Full ms2 1287.2695@hcd30.00 [120.0000-5274.9160]
Precursor Scan: 057185, m/z: 1286.5175, charge: 4
QDQCIYNTTYLNVQR_7_6_0_3_0 LeX peak identified
1e6
204.0863
366.1389
138.0548
168.0653
274.0917
186.0757
292.1022
126.0548
144.0654
454.1562
657.2346
528.1925
792.4387 y6(1+)
1059.9952 PEP_0_1_0_0_0
Intensity
m/z
0.0
0.2
0.4
0.6
0.8
1.0
1.2
1.4
500
1000
1500
2000
2500
3000

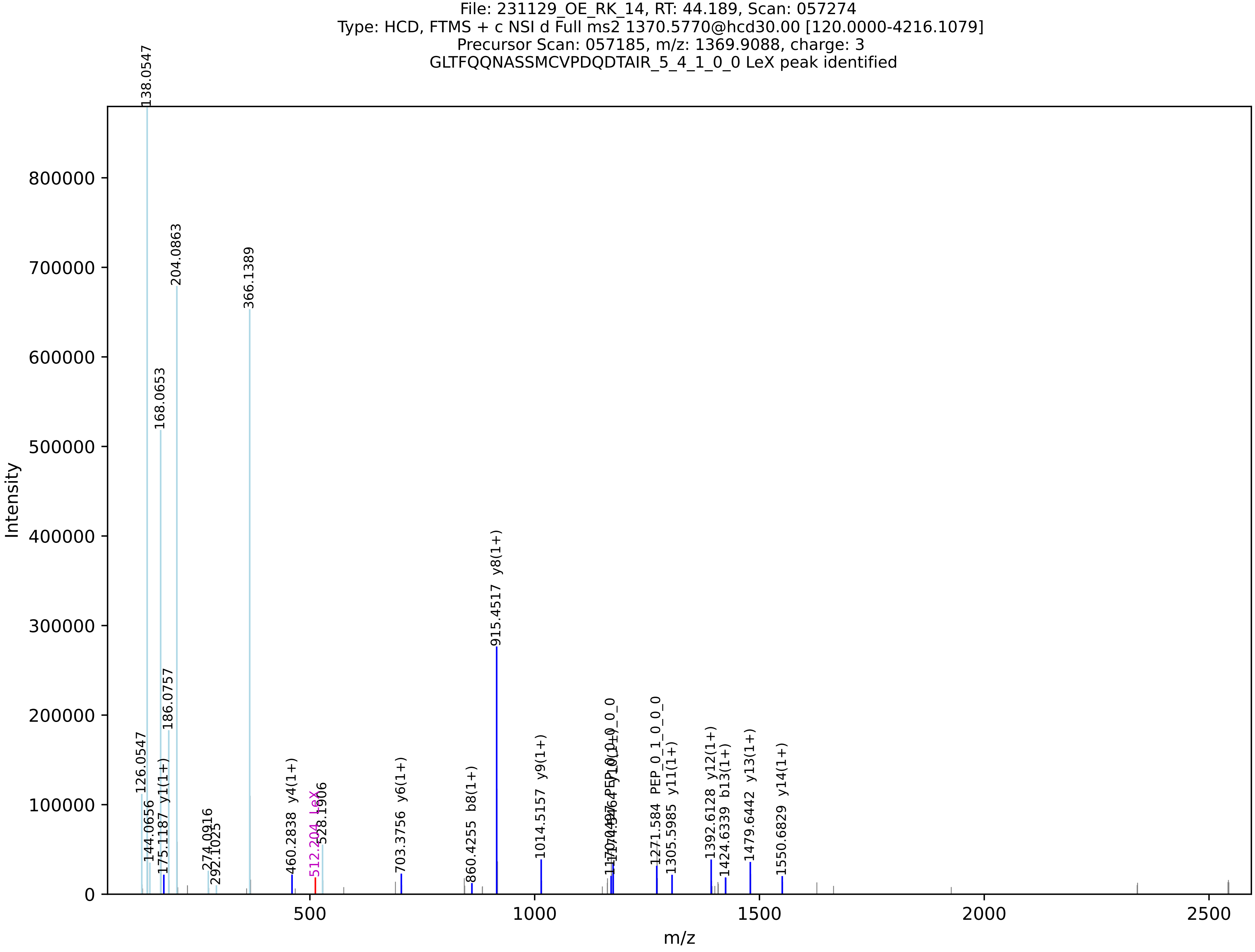

File: 231129_OE_RK_14, RT: 44.189, Scan: 057274
Type: HCD, FTMS + c NSI d Full ms2 1370.5770@hcd30.00 [120.0000-4216.1079]
Precursor Scan: 057185, m/z: 1369.9088, charge: 3
GLTFQQNASSMCVPDQDTAIR_5_4_1_0_0 LeX peak identified
Intensity
m/z
138.0547
204.0863
366.1389
168.0653
186.0757
126.0547
144.0656
175.1187 y1(1+)
274.0916
292.1025
460.2838 y4(1+)
512.204 LeX
528.1906
703.3756 y6(1+)
860.4255 b8(1+)
915.4517 y8(1+)
1014.5157 y9(1+)
1271.584 PEP_0_1_0_0_0_
1305.5985 y11(1+)
1392.6128 y12(1+)
1424.6339 b13(1+)
1479.6442 y13(1+)
1550.6829 y14(1+)

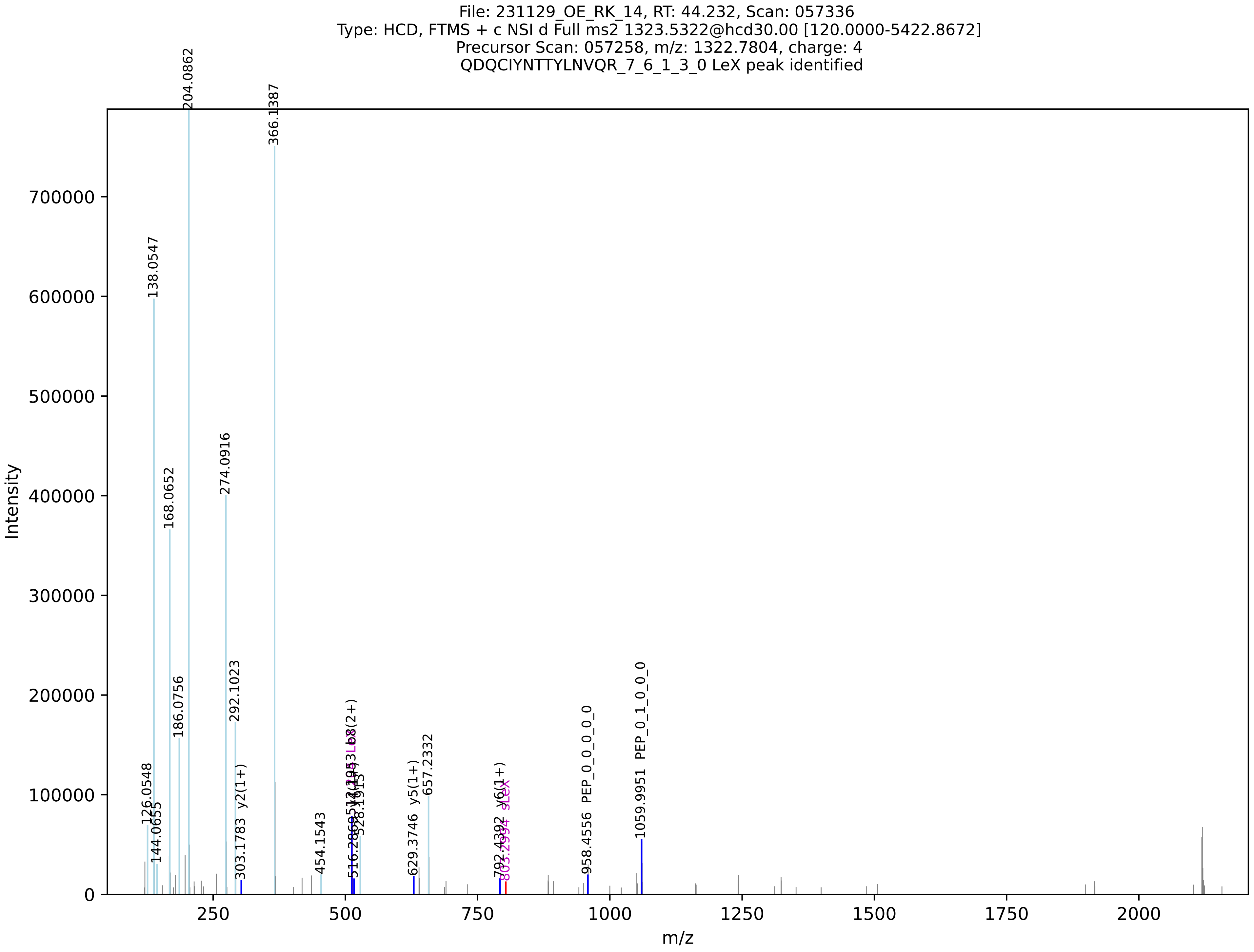
File: 231129_OE_RK_14, RT: 44.232, Scan: 057336
Type: HCD, FTMS + c NSI d Full ms2 1323.5322@hcd30.00 [120.0000-5422.8672]
Precursor Scan: 057258, m/z: 1322.7804, charge: 4
QDQCIYNTTYLNVQR_7_6_1_3_0 LeX peak identified
Intensity
m/z
0
100000
200000
300000
400000
500000
600000
700000
250
500
750
1000
1250
1500
1750
2000
126.0548
138.0547
144.0655
168.0652
186.0756
204.0862
274.0916
292.1023
303.1783 y2(1+)
366.1387
454.1543
528.1913
629.3746 y5(1+)
657.2332
792.4392 y6(1+)
803.2994 sLeX
958.4556 PEP_0_0_0_0_0
1059.9951 PEP_0_1_0_0_0

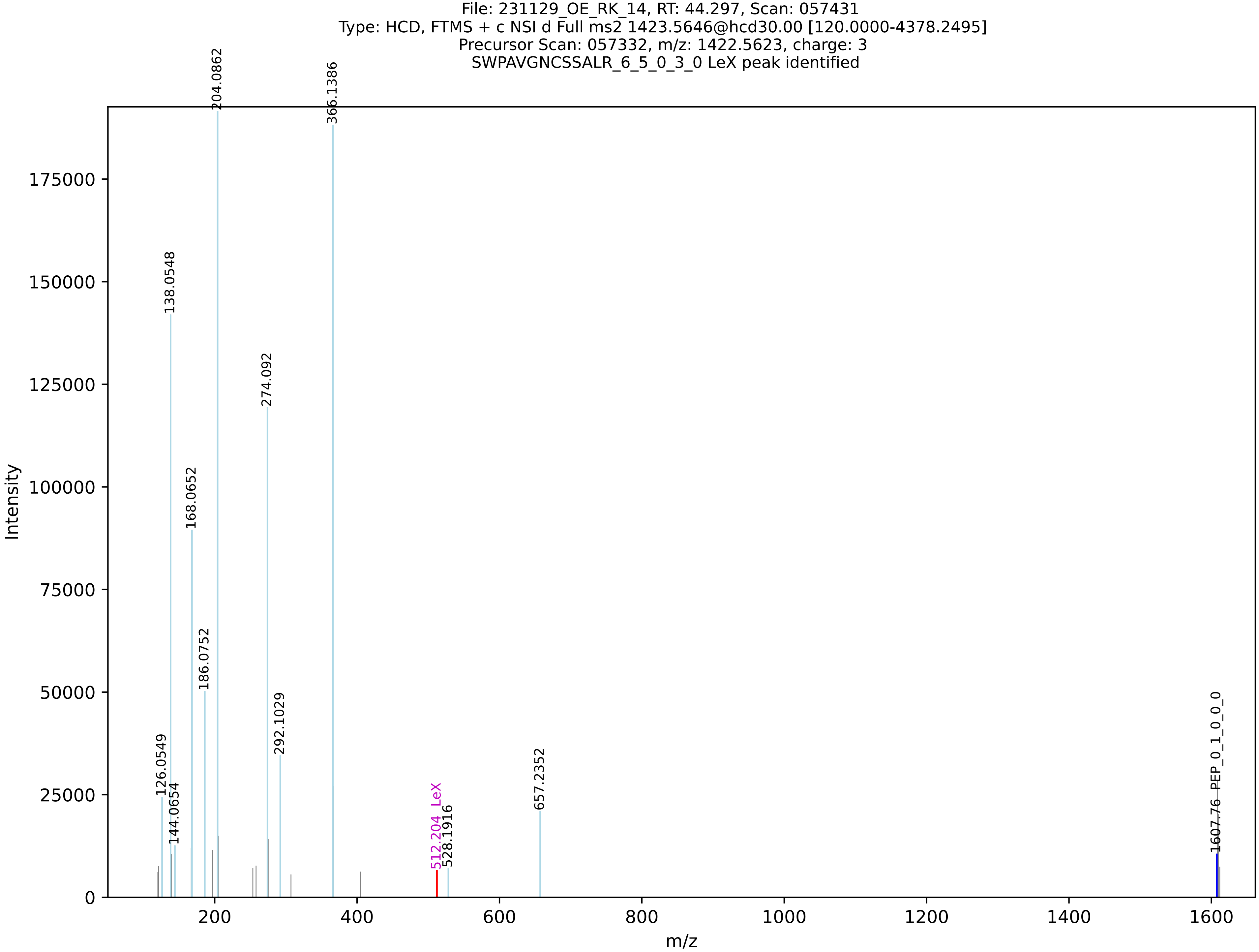

File: 231129_OE_RK_14, RT: 44.297, Scan: 057431
Type: HCD, FTMS + c NSI d Full ms2 1423.5646@hcd30.00 [120.0000-4378.2495]
Precursor Scan: 057332, m/z: 1422.5623, charge: 3
SWPAVGNCSSALR_6_5_0_3_0 LeX peak identified
204.0862
366.1386
138.0548
274.092
168.0652
186.0752
292.1029
126.0549
144.0654
512.204_LeX
528.1916
657.2352
1607.76_PEP_0_1_0_0_0
Intensity
m/z
0
25000
50000
75000
100000
125000
150000
175000
200
400
600
800
1000
1200
1400
1600

File: 231129_OE_RK_14, RT: 44.382, Scan: 057552
Type: HCD, FTMS + c NSI d Full ms2 1250.5054@hcd30.00 [120.0000-5124.9180]
Precursor Scan: 057477, m/z: 1250.0042, charge: 4
QDQCIYNTTYLNVQR_7_6_1_2_0 LeX peak identified

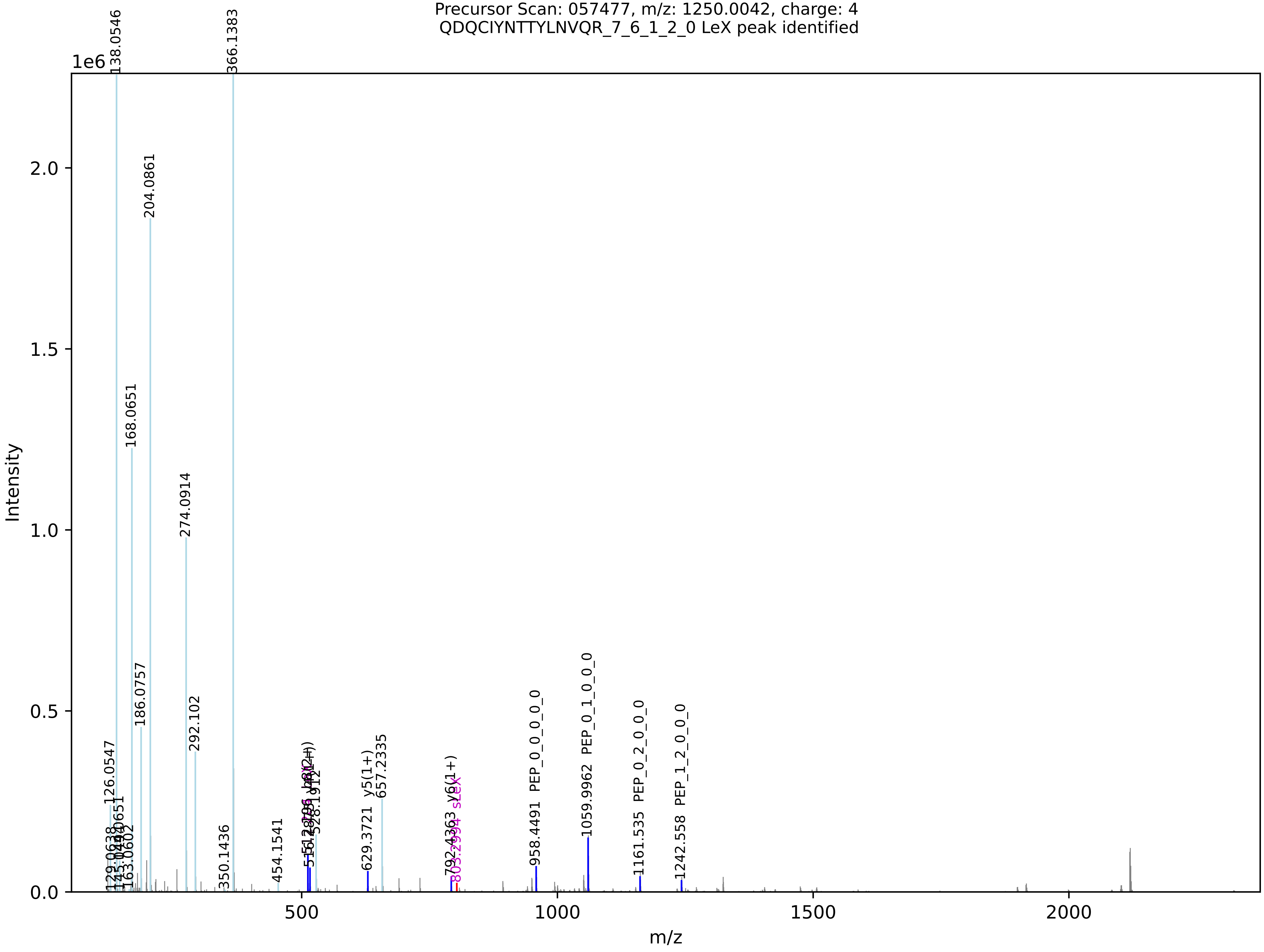

File: 231129_OE_RK_14, RT: 44.384, Scan: 057555
Type: HCD, FTMS + c NSI d Full ms2 1287.0197@hcd30.00 [120.0000-5273.8960]
Precursor Scan: 057477, m/z: 1286.518, charge: 4
QDQCIYNTTYLNVQR_7_6_0_3_0 LeX peak identified

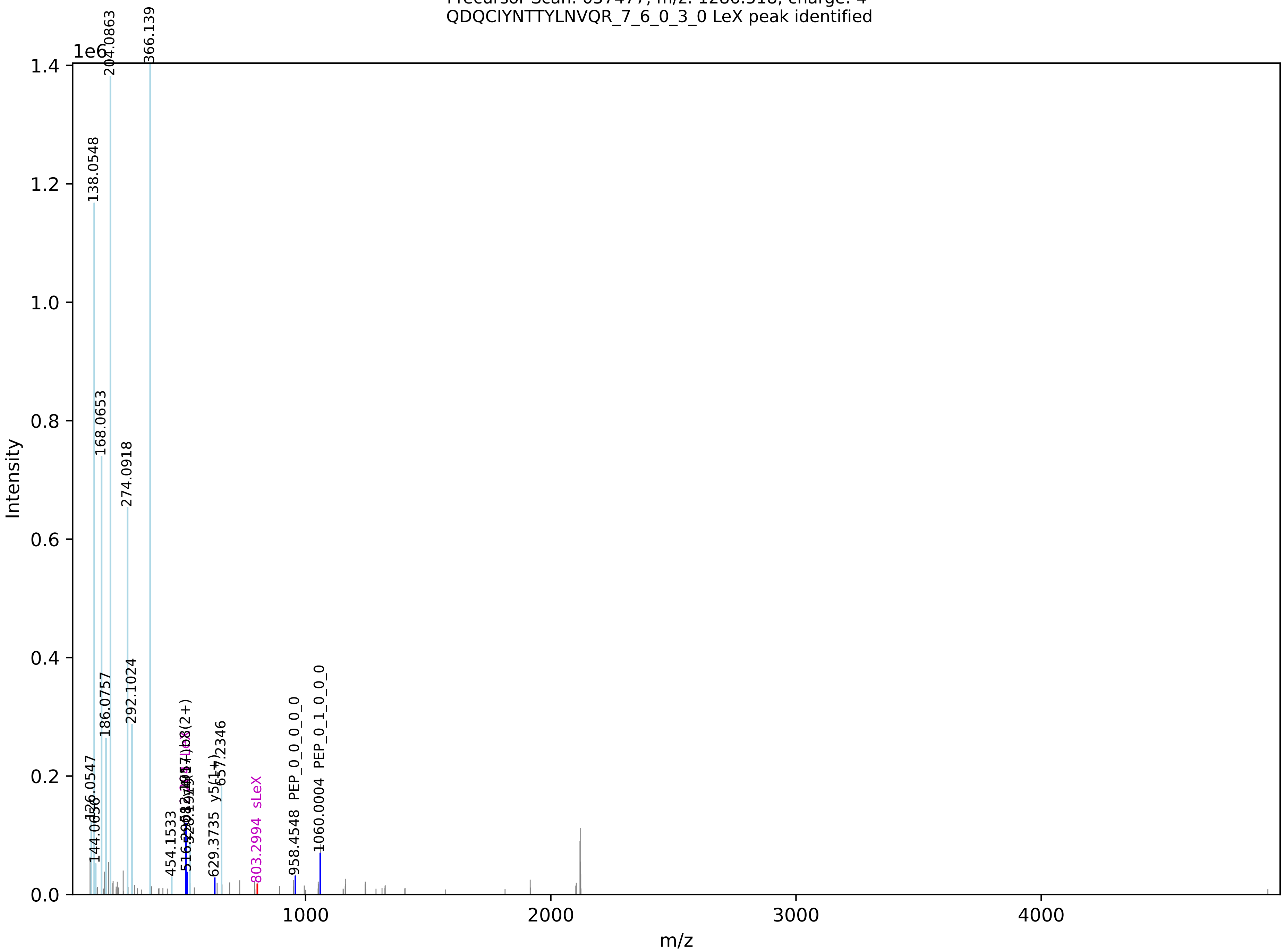

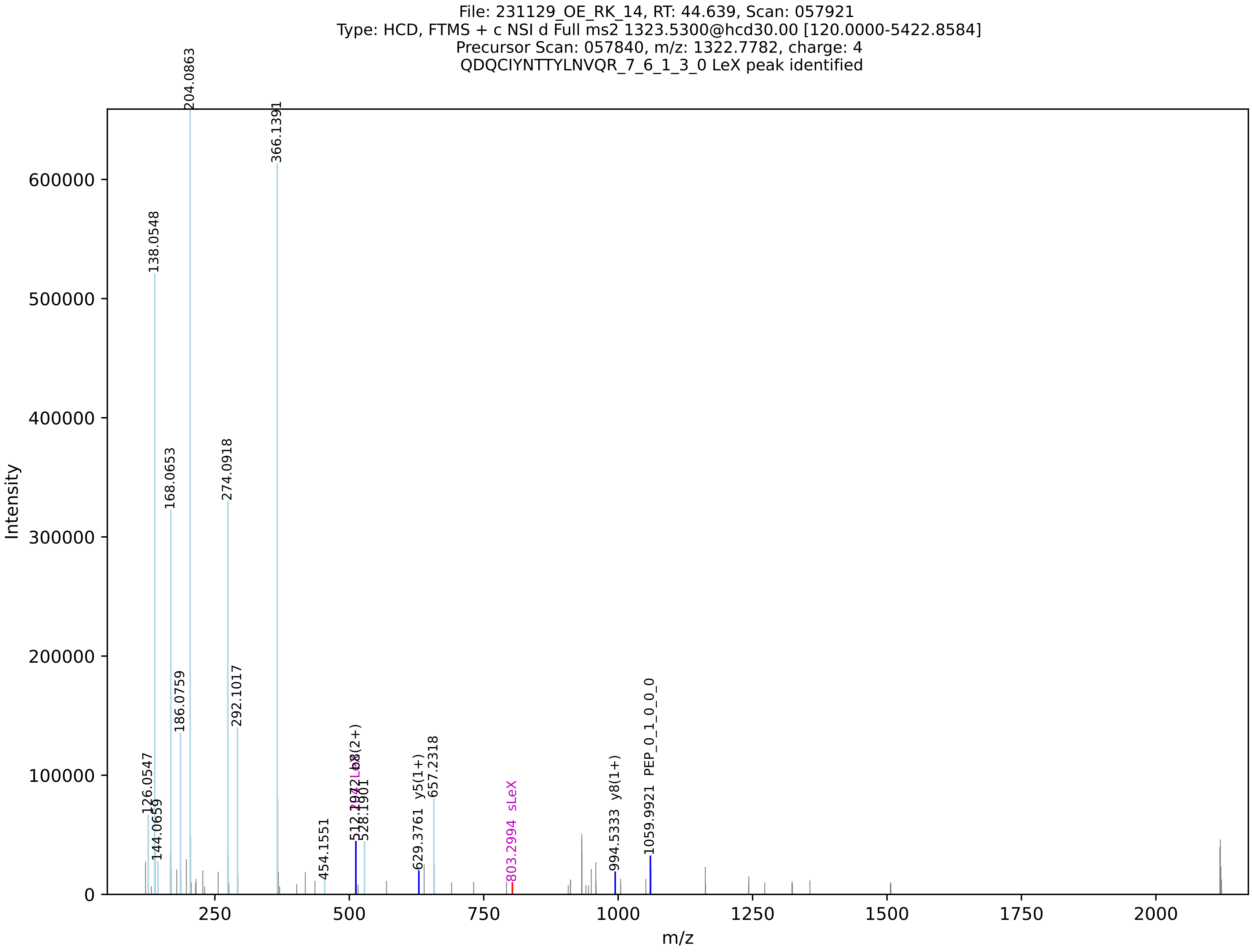
File: 231129_OE_RK_14, RT: 44.639, Scan: 057921
Type: HCD, FTMS + c NSI d Full ms2 1323.5300@hcd30.00 [120.0000-5422.8584]
Precursor Scan: 057840, m/z: 1322.7782, charge: 4
QDQCIYNTTYLNVQR_7_6_1_3_0 LeX peak identified
Intensity
m/z
0
100000
200000
300000
400000
500000
600000
250
500
750
1000
1250
1500
1750
2000
204.0863
366.1391
138.0548
168.0653
274.0918
186.0759
292.1017
126.0547
144.0659
454.1551
b3(2+)
LeX
528.1901
629.3761
y5(1+)
657.2318
803.2994
sLeX
994.5333
y8(1+)
1059.9921
PEP_0_1_0_0_0

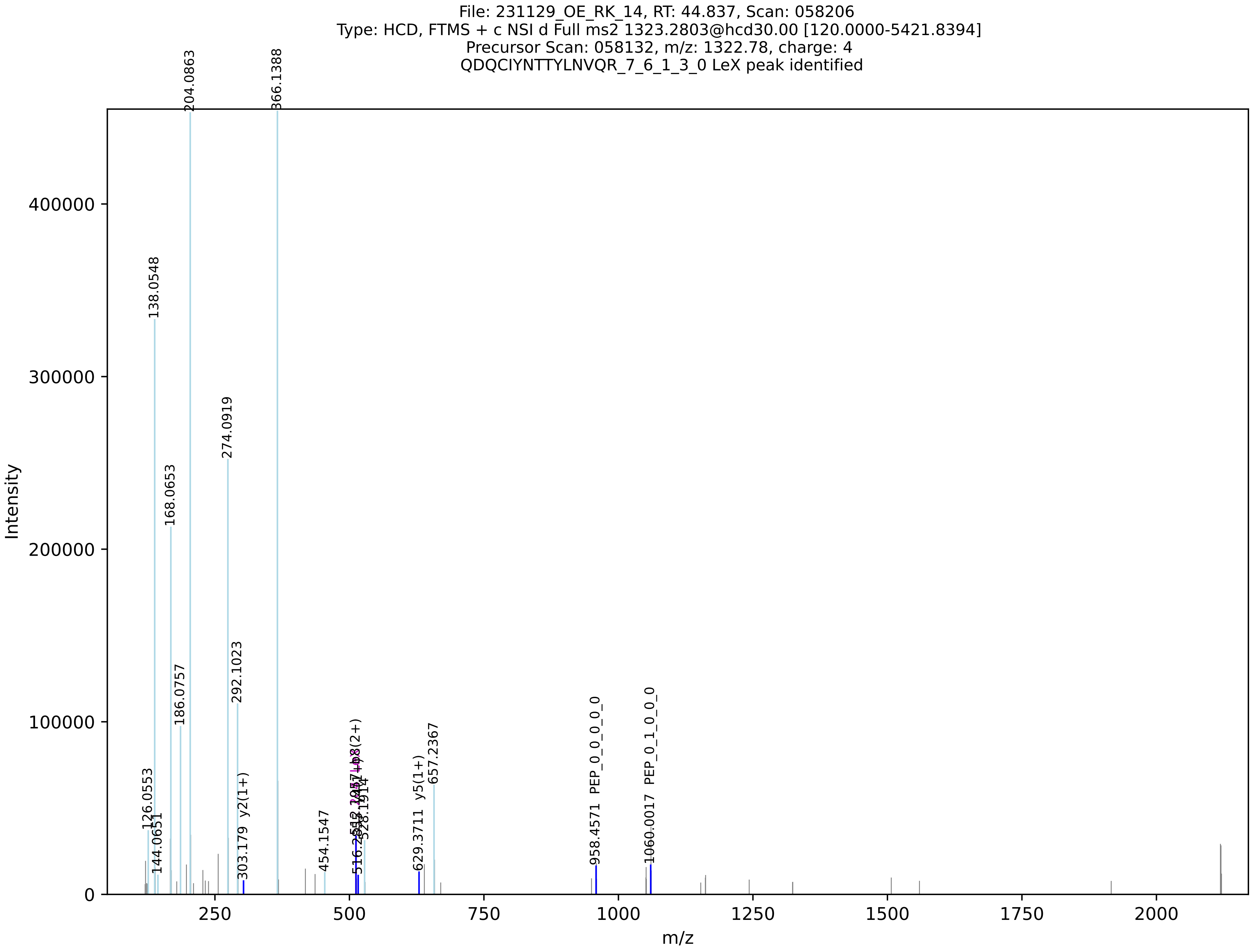

File: 231129_OE_RK_14, RT: 44.837, Scan: 058206
Type: HCD, FTMS + c NSI d Full ms2 1323.2803@hcd30.00 [120.0000-5421.8394]
Precursor Scan: 058132, m/z: 1322.78, charge: 4
QDQCIYNTTYLNVQR_7_6_1_3_0 LeX peak identified
Intensity
m/z
0
100000
200000
300000
400000
250
500
750
1000
1250
1500
1750
2000
204.0863
366.1388
138.0548
274.0919
168.0653
292.1023
186.0757
126.0553
144.0651
303.179 y2(1+)
454.1547
516.2
528.1914
629.3711 y5(1+)
657.2367
958.4571 PEP_0_0_0_0_0
1060.0017 PEP_0_1_0_0_0

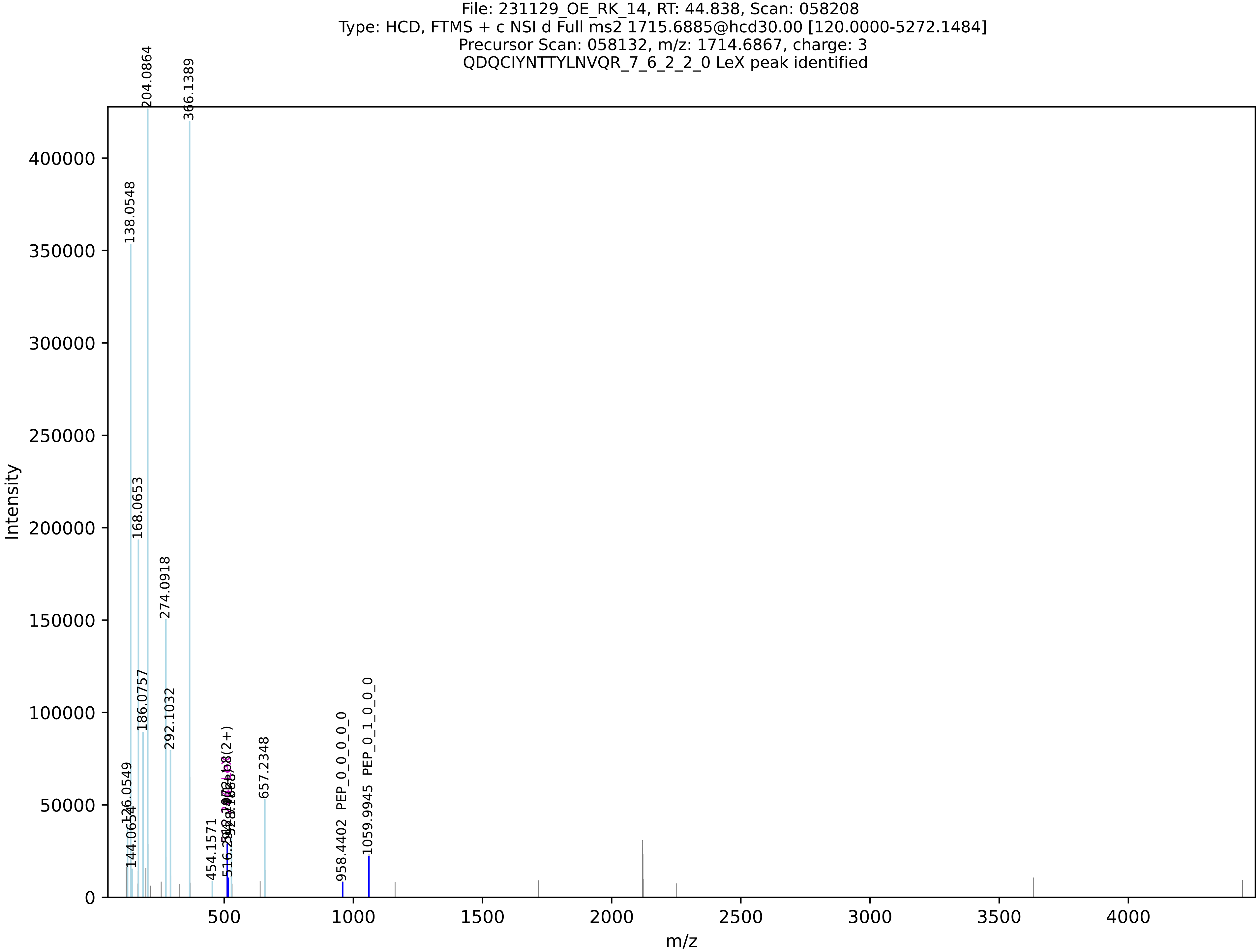
File: 231129_OE_RK_14, RT: 44.838, Scan: 058208
Type: HCD, FTMS + c NSI d Full ms2 1715.6885@hcd30.00 [120.0000-5272.1484]
Precursor Scan: 058132, m/z: 1714.6867, charge: 3
QDQCIYNTTYLNVQR_7_6_2_2_0 LeX peak identified
204.0864
366.1389
138.0548
168.0653
274.0918
186.0757
292.1032
126.0549
144.0654
454.1571
657.2348
958.4402 PEP_0_0_0_0_0
1059.9945 PEP_0_1_0_0_0
Intensity
m/z
0
50000
100000
150000
200000
250000
300000
350000
400000
500
1000
1500
2000
2500
3000
3500
4000

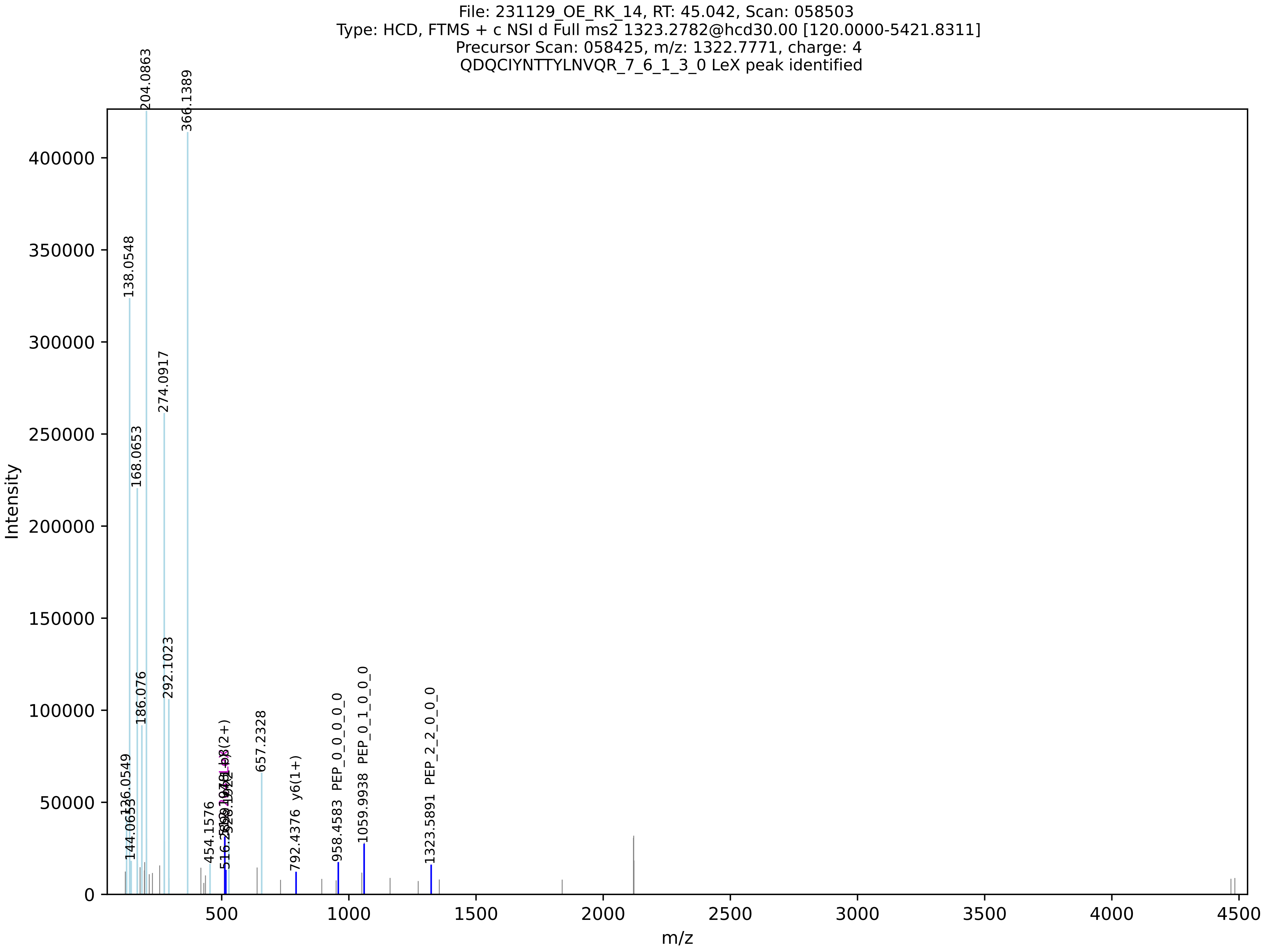
File: 231129_OE_RK_14, RT: 45.042, Scan: 058503
Type: HCD, FTMS + c NSI d Full ms2 1323.2782@hcd30.00 [120.0000-5421.8311]
Precursor Scan: 058425, m/z: 1322.7771, charge: 4
QDQCIYNTTYLNVQR_7_6_1_3_0 LeX peak identified
204.0863
366.1389
138.0548
274.0917
168.0653
292.1023
186.076
126.0549
144.0655
454.1576
657.2328
792.4376 y6(1+)
958.4583 PEP_0_0_0_0_0
1059.9938 PEP_0_1_0_0_0
1323.5891 PEP_2_2_0_0_0
Intensity
0
50000
100000
150000
200000
250000
300000
350000
400000
m/z
500
1000
1500
2000
2500
3000
3500
4000
4500

File: 231129_OE_RK_14, RT: 45.192, Scan: 058722
Type: HCD, FTMS + c NSI d Full ms2 1667.3362@hcd30.00 [120.0000-5124.1904]
Precursor Scan: 058642, m/z: 1666.3342, charge: 3
QDQCIYNTTYLNVQR_7_6_1_2_0 LeX peak identified

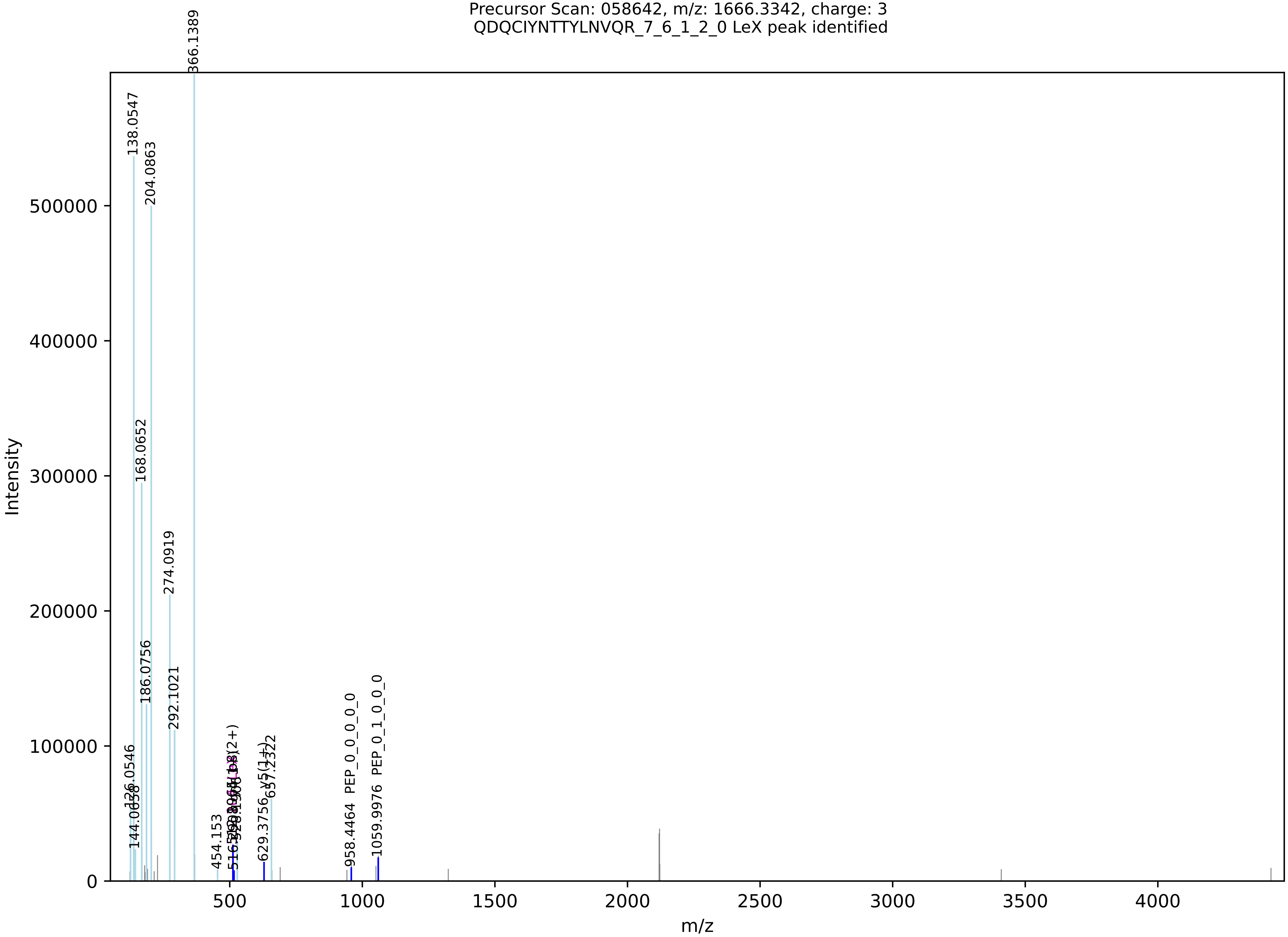

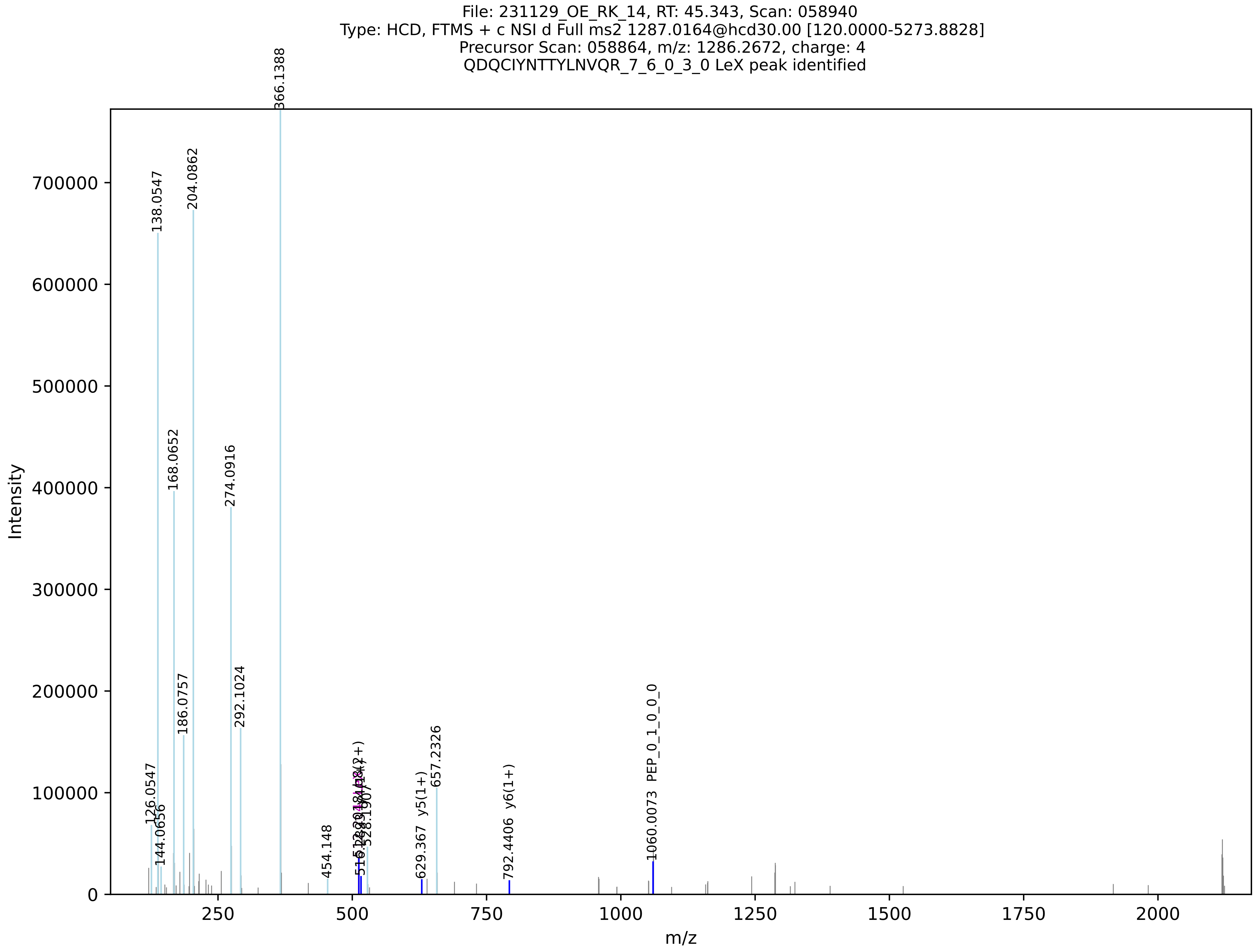
File: 231129_OE_RK_14, RT: 45.343, Scan: 058940
Type: HCD, FTMS + c NSI d Full ms2 1287.0164@hcd30.00 [120.0000-5273.8828]
Precursor Scan: 058864, m/z: 1286.2672, charge: 4
QDQCIYNTTYLNVQR_7_6_0_3_0 LeX peak identified
Intensity
m/z
366.1388
204.0862
138.0547
168.0652
274.0916
186.0757
292.1024
126.0547
144.0656
454.148
528.1907
629.367 y5(1+)
657.2326
792.4406 y6(1+)
1060.0073 PEP_0_1_0_0_0
0
100000
200000
300000
400000
500000
600000
700000
250
500
750
1000
1250
1500
1750
2000

File: 231129_OE_RK_14, RT: 46.214, Scan: 060203
Type: HCD, FTMS + c NSI d Full ms2 1642.6613@hcd30.00 [120.0000-5048.6851]
Precursor Scan: 060107, m/z: 1641.6559, charge: 3
QDQCIYNTTYLNVQR_8_7_0_1_0 LeX peak identified

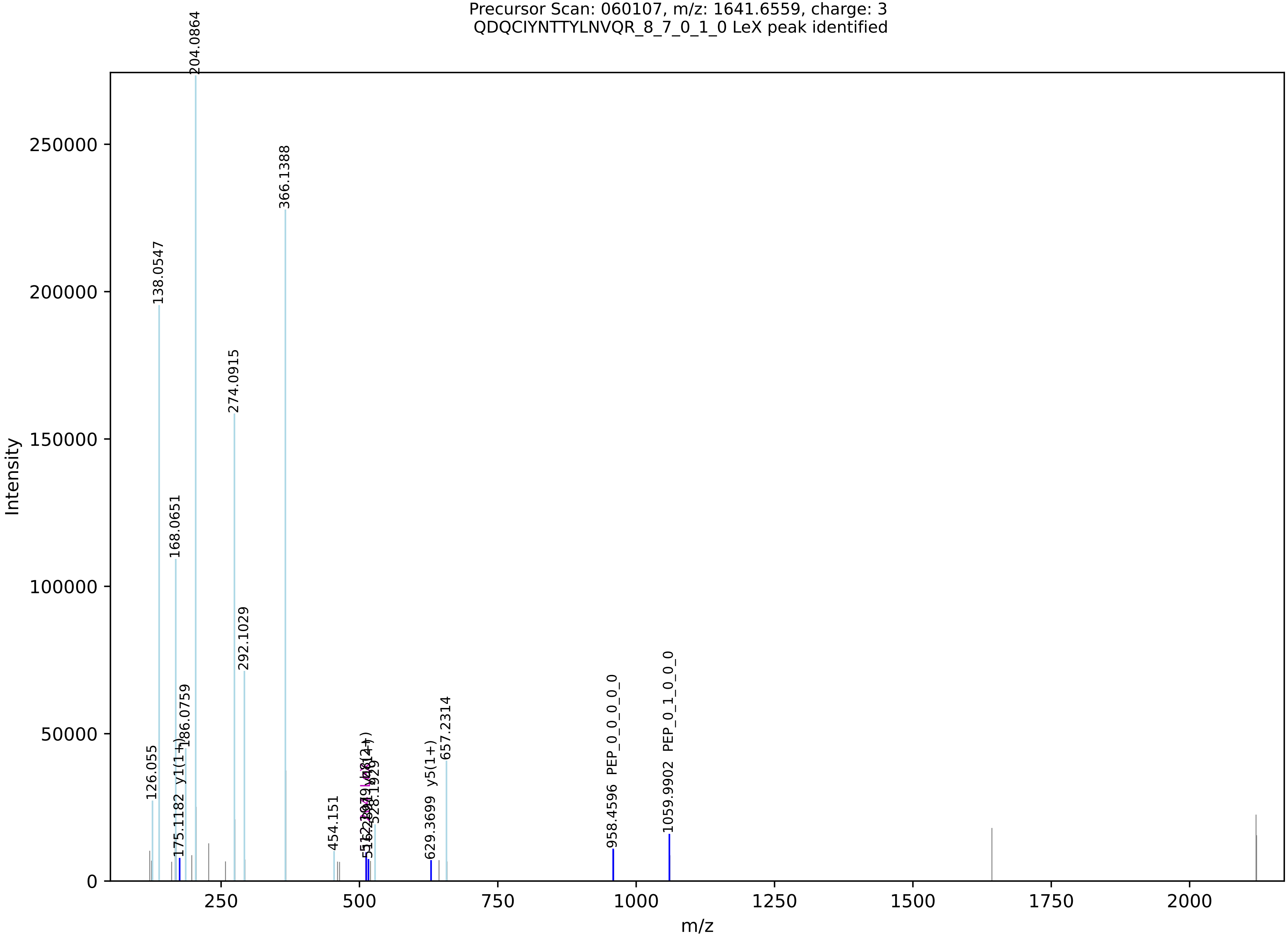

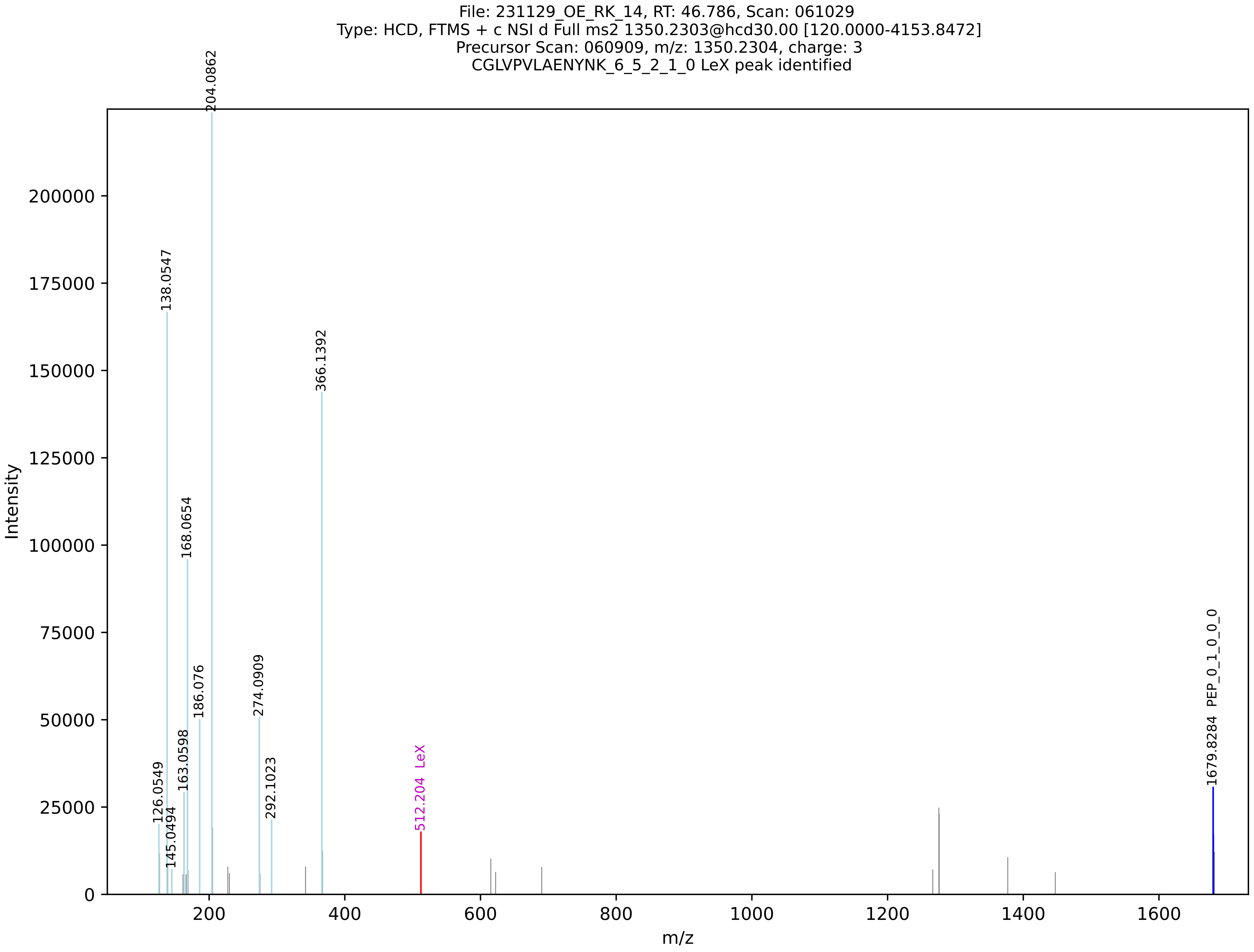
File: 231129_OE_RK_14, RT: 46.786, Scan: 061029
Type: HCD, FTMS + c NSI d Full ms2 1350.2303@hcd30.00 [120.0000-4153.8472]
Precursor Scan: 060909, m/z: 1350.2304, charge: 3
CGLVPVLAENYNK_6_5_2_1_0 LeX peak identified
Intensity
m/z
204.0862
138.0547
366.1392
168.0654
186.076
274.0909
163.0598
126.0549
292.1023
145.0494
512.204 LeX
1679.8284 PEP_0_1_0_0_0
0
25000
50000
75000
100000
125000
150000
175000
200000
200
400
600
800
1000
1200
1400
1600

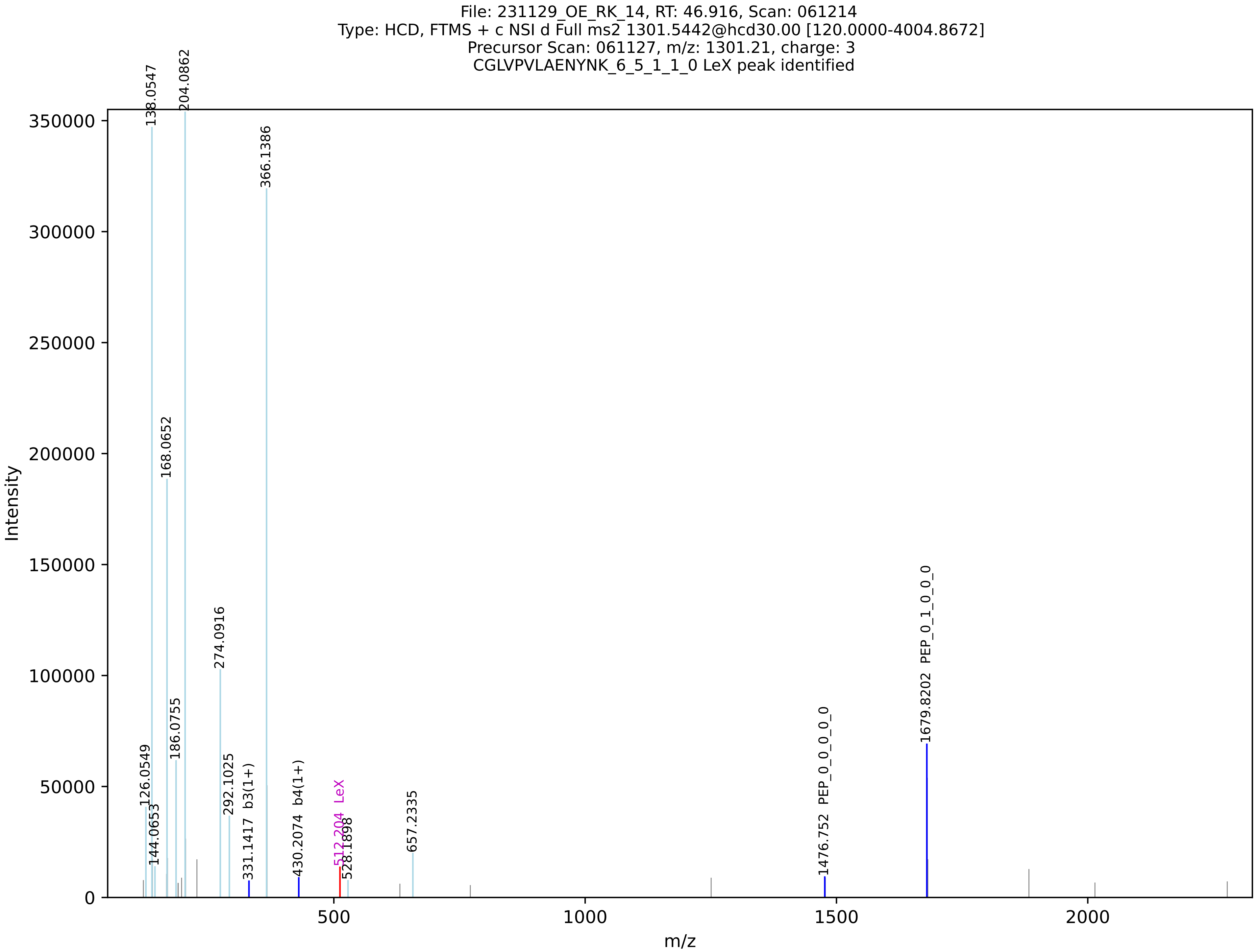
File: 231129_OE_RK_14, RT: 46.916, Scan: 061214
Type: HCD, FTMS + c NSI d Full ms2 1301.5442@hcd30.00 [120.0000-4004.8672]
Precursor Scan: 061127, m/z: 1301.21, charge: 3
CGLVPVLAENYNK_6_5_1_1_0 LeX peak identified
Intensity
m/z
138.0547
204.0862
366.1386
168.0652
274.0916
186.0755
126.0549
144.0653
292.1025
331.1417 b3(1+)
430.2074 b4(1+)
512.204 LeX
528.1898
657.2335
1476.752 PEP_0_0_0_0_0_
1679.8202 PEP_0_1_0_0_0_
0
50000
100000
150000
200000
250000
300000
350000
500
1000
1500
2000

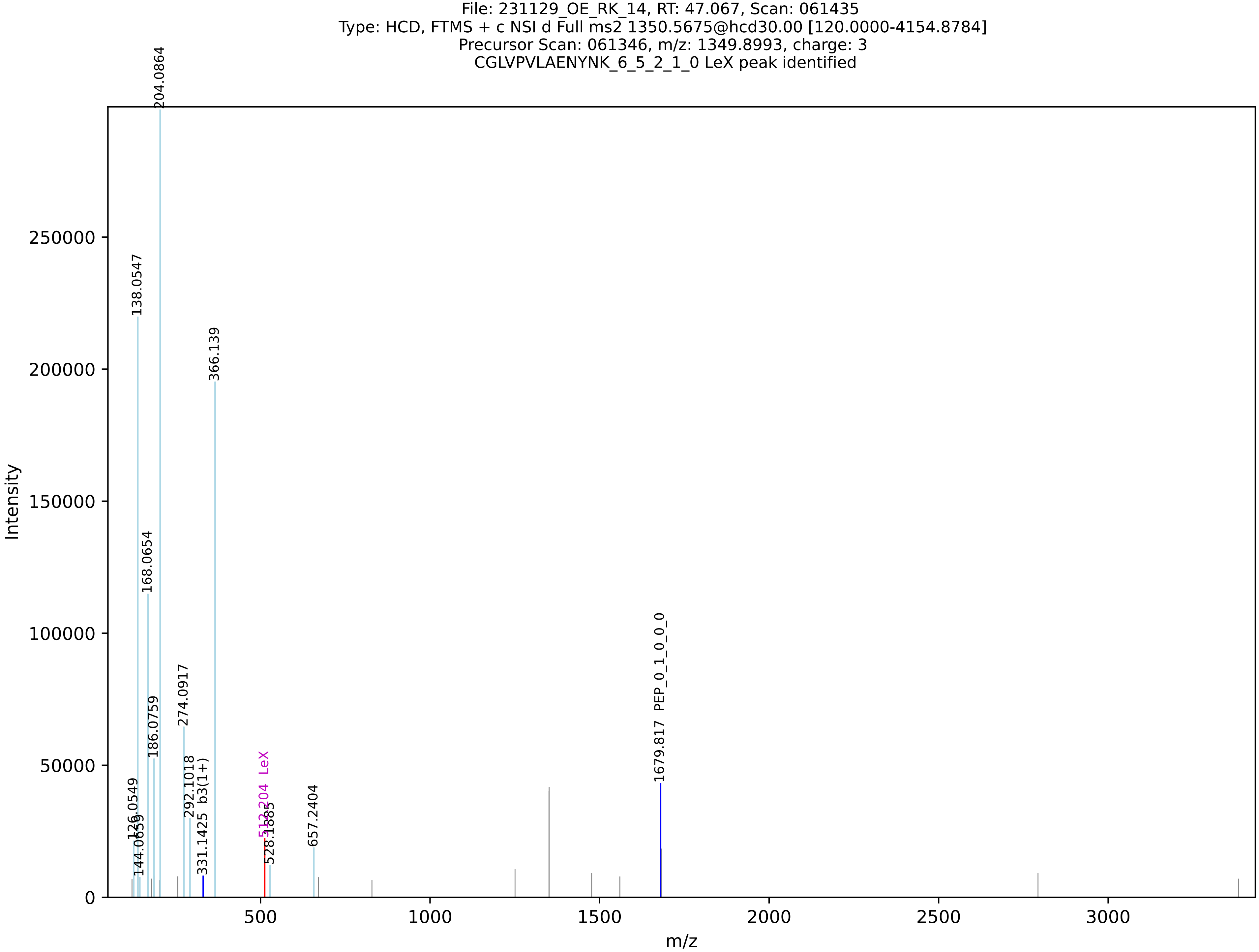

File: 231129_OE_RK_14, RT: 47.067, Scan: 061435
Type: HCD, FTMS + c NSI d Full ms2 1350.5675@hcd30.00 [120.0000-4154.8784]
Precursor Scan: 061346, m/z: 1349.8993, charge: 3
CGLVPVLAENYNK_6_5_2_1_0 LeX peak identified
Intensity
m/z
204.0864
138.0547
366.139
168.0654
186.0759
274.0917
292.1018
331.1425 b3(1+)
126.0549
144.0659
512.204 LeX
528.1885
657.2404
1679.817 PEP_0_1_0_0_0

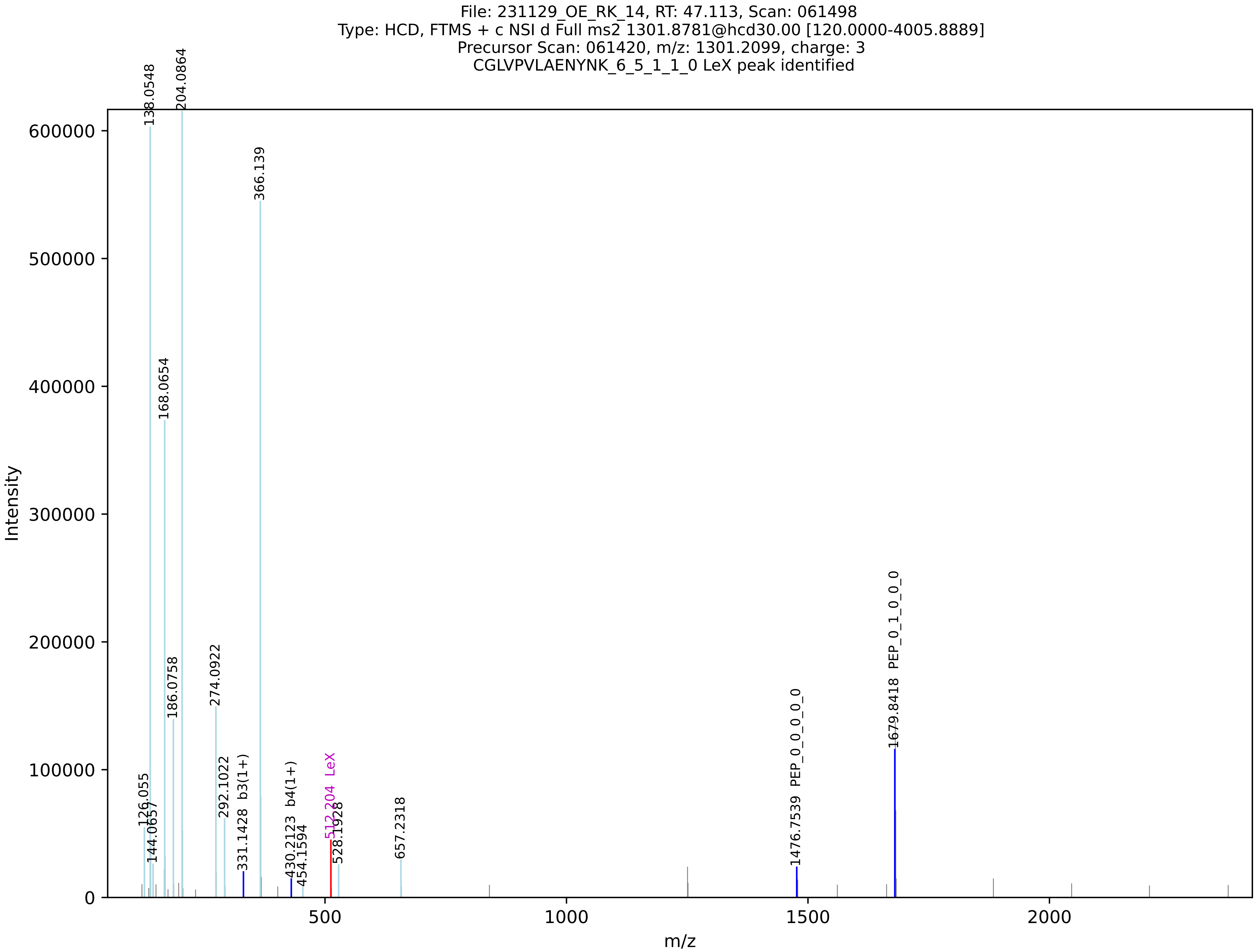
File: 231129_OE_RK_14, RT: 47.113, Scan: 061498
Type: HCD, FTMS + c NSI d Full ms2 1301.8781@hcd30.00 [120.0000-4005.8889]
Precursor Scan: 061420, m/z: 1301.2099, charge: 3
CGLVPVLAENYNK_6_5_1_1_0 LeX peak identified
Intensity
m/z
138.0548
204.0864
366.139
168.0654
186.0758
274.0922
126.055
144.0657
292.1022
331.1428 b3(1+)
430.2123 b4(1+)
454.1594
512.204 LeX
528.1928
657.2318
1476.7539 PEP_0_0_0_0_0
1679.8418 PEP_0_1_0_0_0

File: 231129_OE_RK_14, RT: 47.34, Scan: 061828
Type: HCD, FTMS + c NSI d Full ms2 1180.1687@hcd30.00 [120.0000-3633.4583]
Precursor Scan: 061764, m/z: 1179.5005, charge: 3
CGLVPVLAENYNK_5_4_1_1_0 LeX peak identified

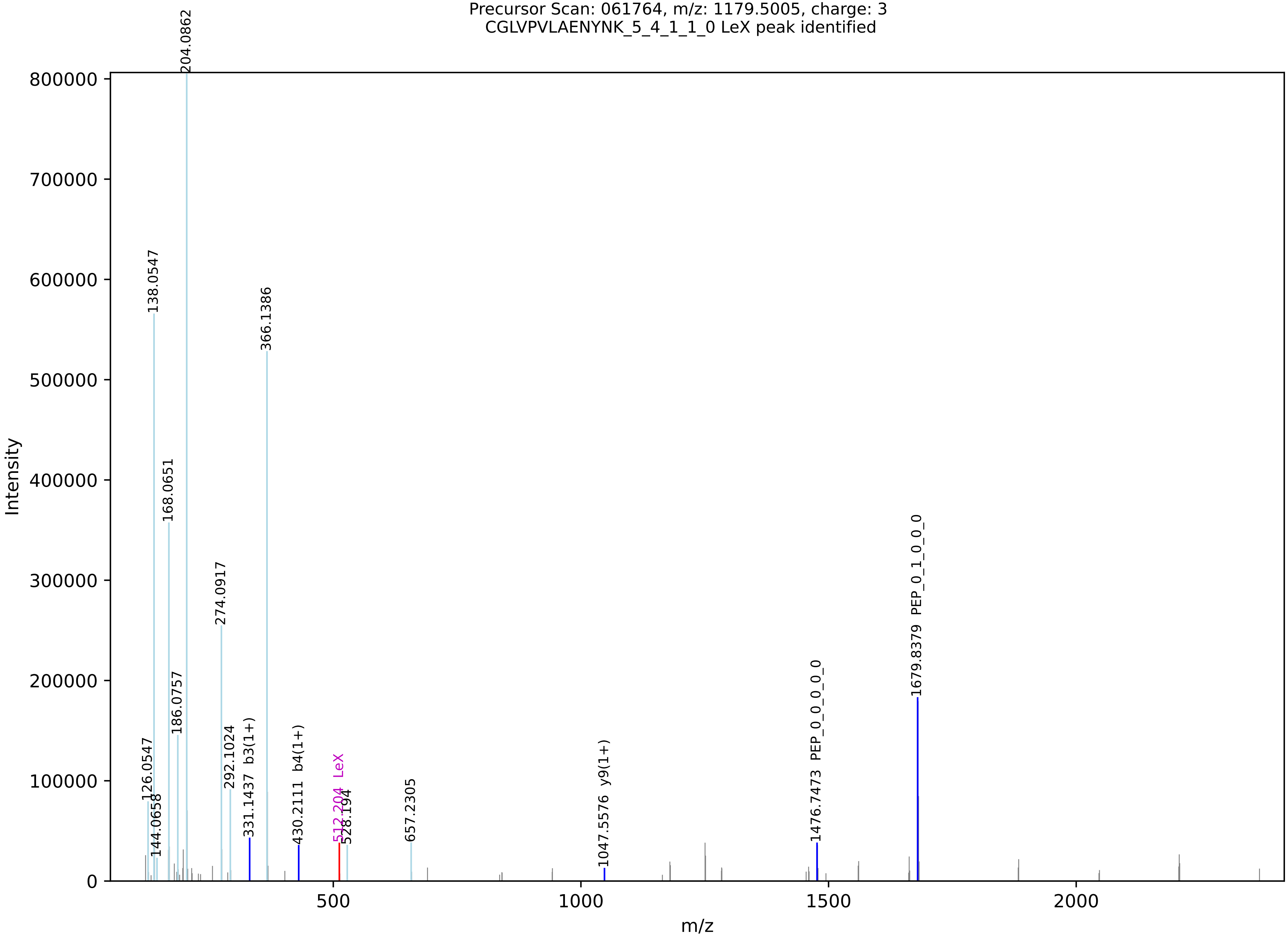

File: 231129_OE_RK_14, RT: 47.444, Scan: 061978
Type: HCD, FTMS + c NSI d Full ms2 1244.5201@hcd30.00 [120.0000-5100.4980]
Precursor Scan: 061892, m/z: 1243.7693, charge: 4
NSLTLQWKAPSDNGSK_7_6_0_3_0 LeX peak identified

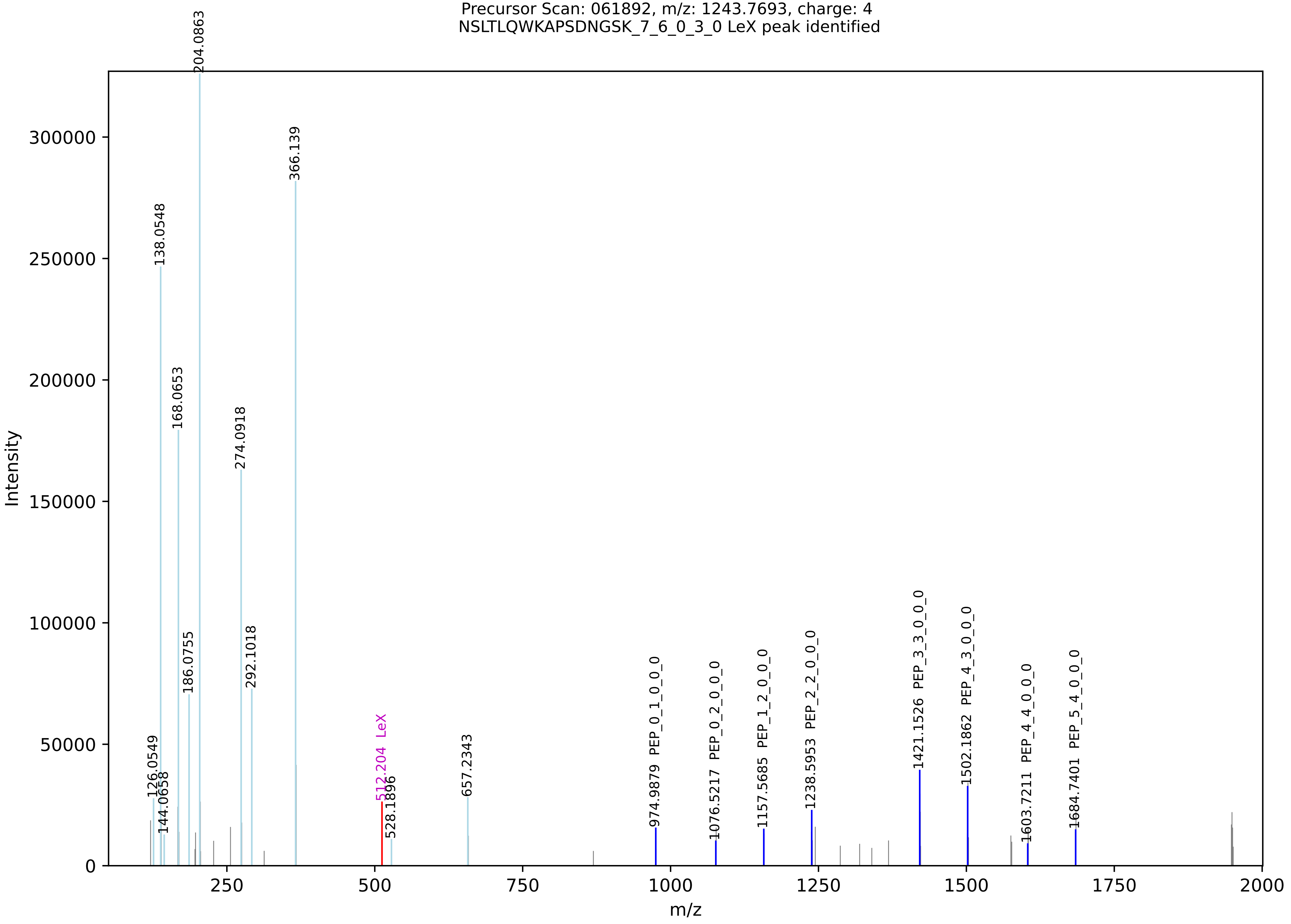

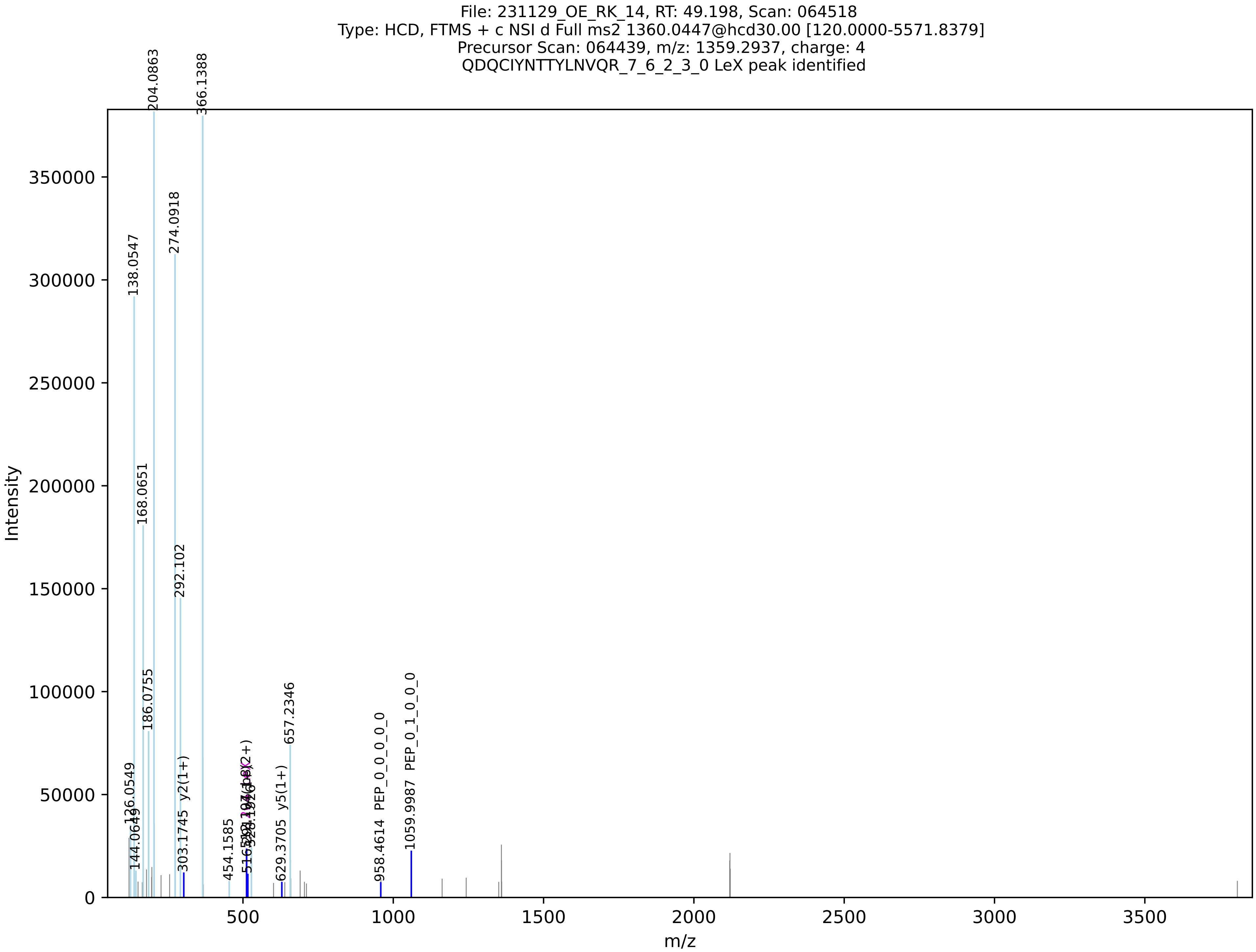
File: 231129_OE_RK_14, RT: 49.198, Scan: 064518
Type: HCD, FTMS + c NSI d Full ms2 1360.0447@hcd30.00 [120.0000-5571.8379]
Precursor Scan: 064439, m/z: 1359.2937, charge: 4
QDQCIYNTTYLNVQR_7_6_2_3_0 LeX peak identified
204.0863
366.1388
274.0918
138.0547
168.0651
292.102
186.0755
657.2346
136.0549
144.0649
303.1745 y2(1+)
454.1585
629.3705 y5(1+)
958.4614 PEP_0_0_0_0_0
1059.9987 PEP_0_1_0_0_0
Intensity
m/z
350000
300000
250000
200000
150000
100000
50000
0
500
1000
1500
2000
2500
3000
3500

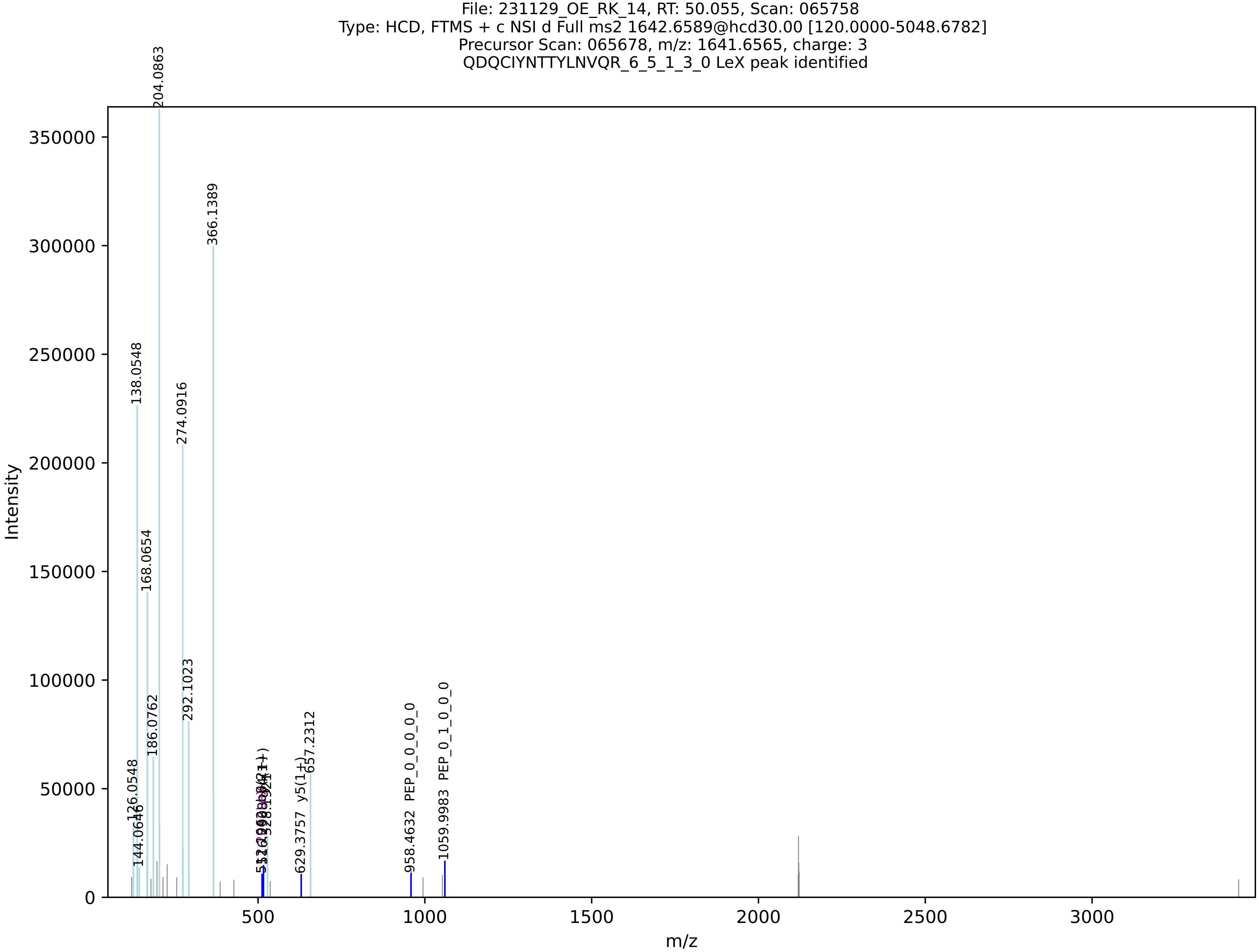
File: 231129_OE_RK_14, RT: 50.055, Scan: 065758
Type: HCD, FTMS + c NSI d Full ms2 1642.6589@hcd30.00 [120.0000-5048.6782]
Precursor Scan: 065678, m/z: 1641.6565, charge: 3
QDQCIYNTTYLNVQR_6_5_1_3_0 LeX peak identified
204.0863
366.1389
138.0548
274.0916
168.0654
292.1023
186.0762
126.0548
144.0646
629.3757 y5(1+)
657.2312
958.4632 PEP_0_0_0_0_0
1059.9983 PEP_0_1_0_0_0
Intensity
m/z
0
50000
100000
150000
200000
250000
300000
350000
500
1000
1500
2000
2500
3000

File: 231129_OE_RK_14, RT: 50.468, Scan: 066362
Type: HCD, FTMS + c NSI d Full ms2 1627.0468@hcd30.00 [120.0000-5000.9053]
Precursor Scan: 066267, m/z: 1626.3811, charge: 3
VVLHPNYSQVDIGLIK_7_6_1_2_0 LeX peak identified

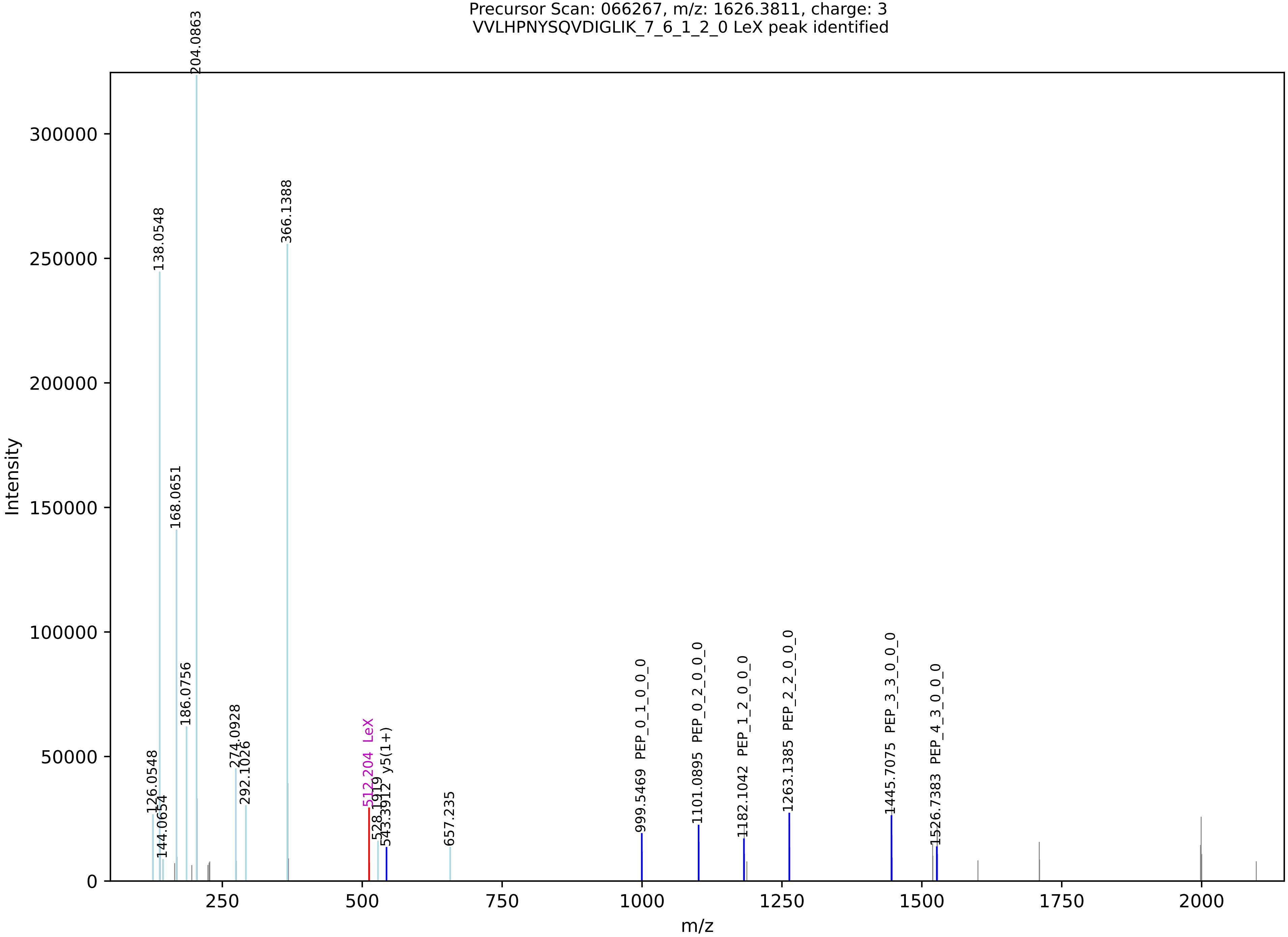

File: 231129_OE_RK_14, RT: 50.512, Scan: 066425
Type: HCD, FTMS + c NSI d Full ms2 1231.9941@hcd30.00 [120.0000-5049.3921]
Precursor Scan: 066341, m/z: 1231.4943, charge: 4
QDQCIYNTTYLNVQR_6_5_1_3_0 LeX peak identified

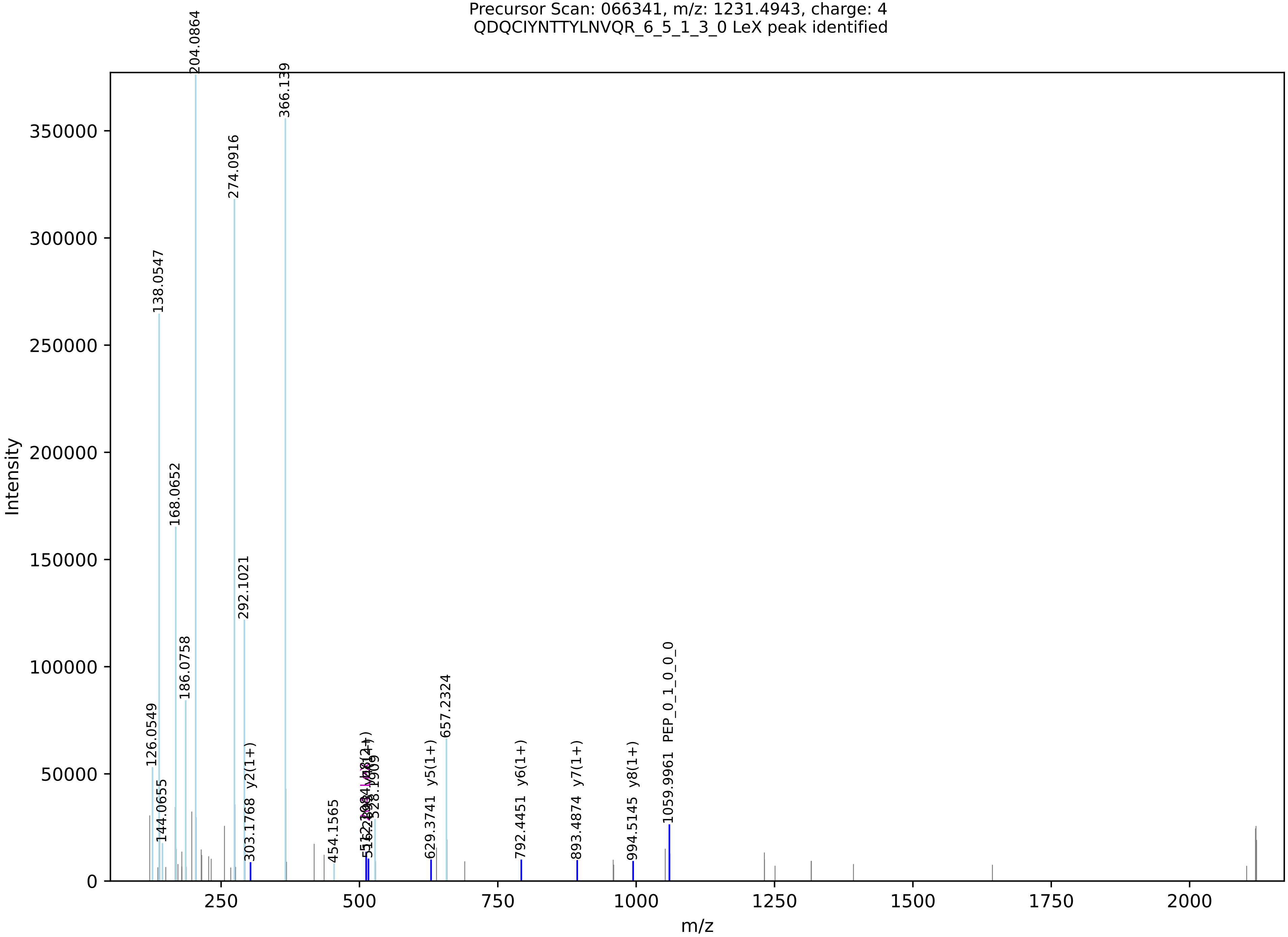

File: 231129_OE_RK_14, RT: 50.513, Scan: 066426
Type: HCD, FTMS + c NSI d Full ms2 1184.2754@hcd30.00 [120.0000-4854.6997]
Precursor Scan: 066341, m/z: 1183.5236, charge: 4
VVLHPNYSQVDIGLIK_7_6_2_1_0 LeX peak identified

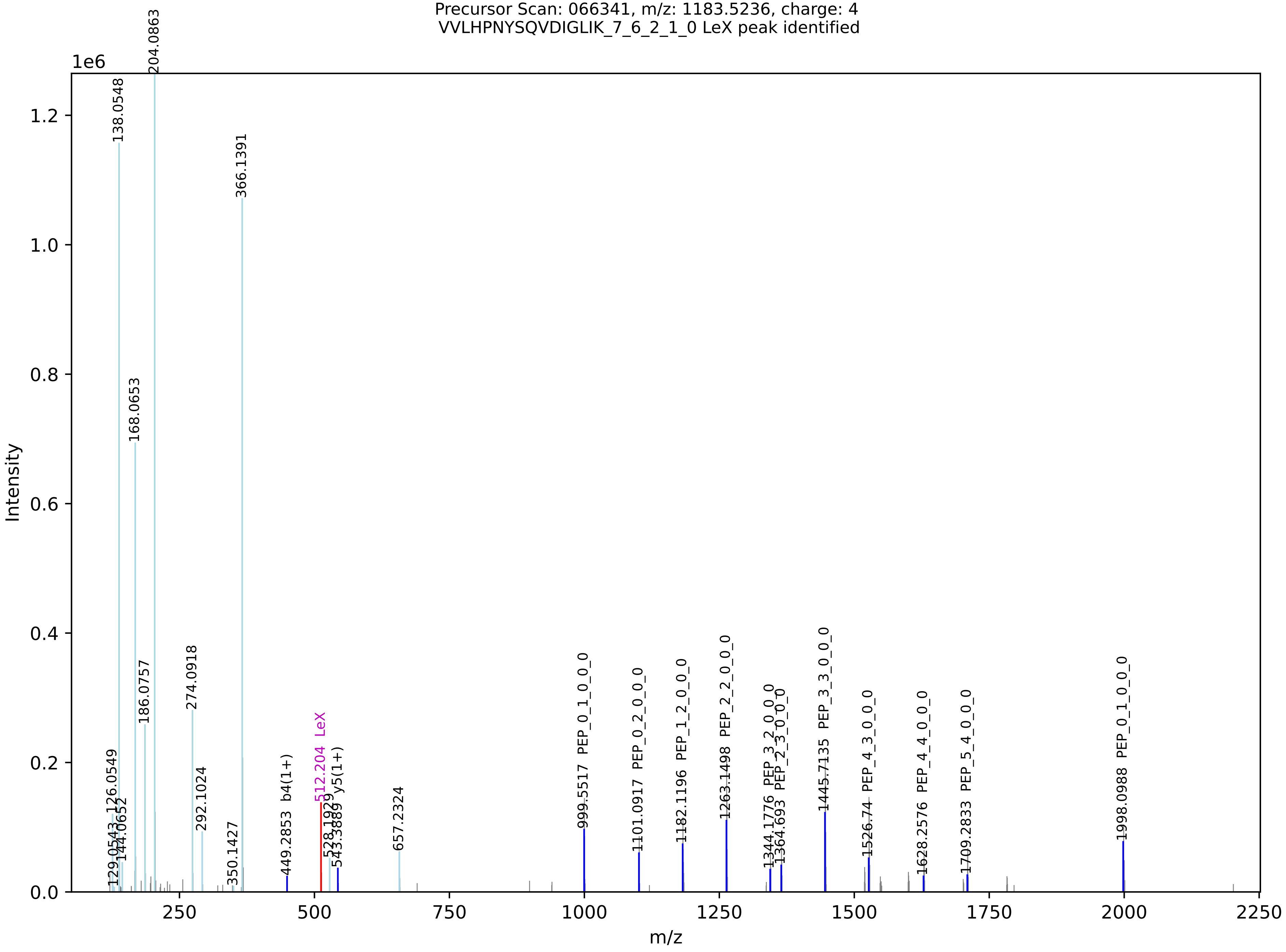

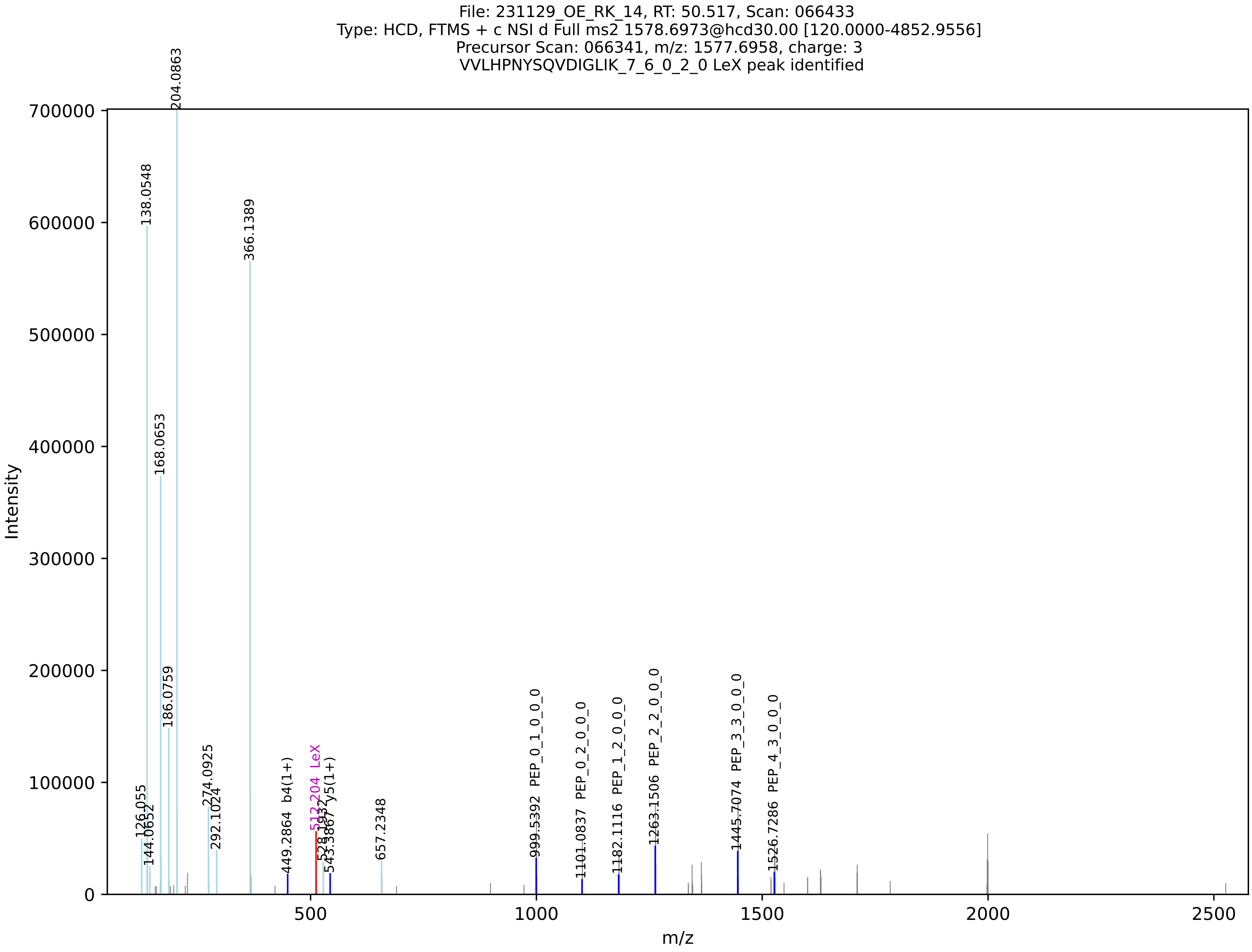
File: 231129_OE_RK_14, RT: 50.517, Scan: 066433
Type: HCD, FTMS + c NSI d Full ms2 1578.6973@hcd30.00 [120.0000-4852.9556]
Precursor Scan: 066341, m/z: 1577.6958, charge: 3
VVLHPNYSQVDIGLIK_7_6_0_2_0 LeX peak identified
Intensity
m/z
204.0863
138.0548
366.1389
168.0653
186.0759
274.0925
292.1024
126.055
144.0652
449.2864 b4(1+)
512.204 LeX
543.3867 y5(1+)
657.2348
999.5392 PEP_0_1_0_0_0
1101.0837 PEP_0_2_0_0_0
1182.1116 PEP_1_2_0_0_0
1263.1506 PEP_2_2_0_0_0
1445.7074 PEP_3_3_0_0_0
1526.7286 PEP_4_3_0_0_0

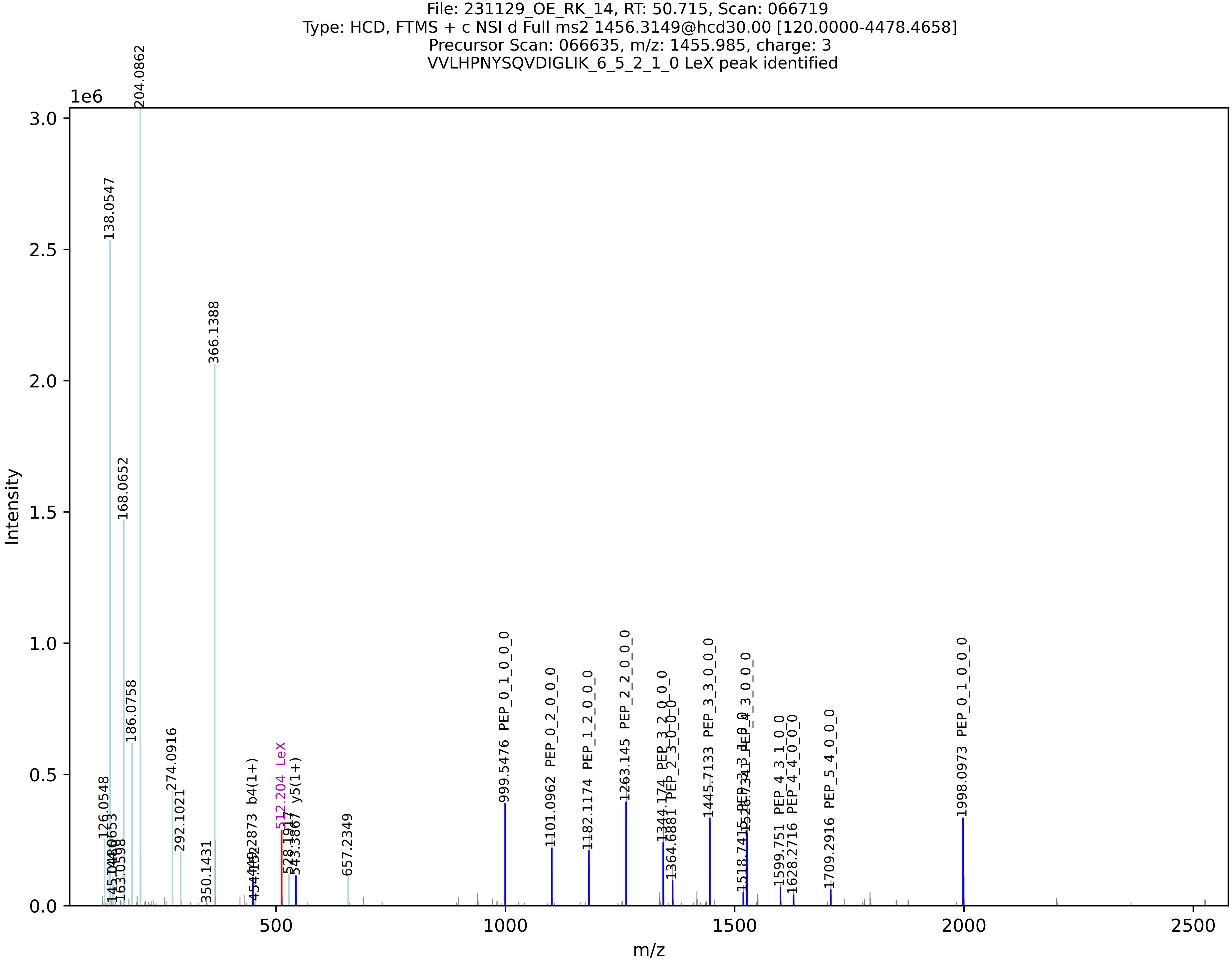
File: 231129_OE_RK_14, RT: 50.715, Scan: 066719
Type: HCD, FTMS + c NSI d Full ms2 1456.3149@hcd30.00 [120.0000-4478.4658]
Precursor Scan: 066635, m/z: 1455.985, charge: 3
VVLHPNYSQVDIGLIK_6_5_2_1_0 LeX peak identified
1e6
Intensity
m/z
204.0862
138.0547
366.1388
168.0652
186.0758
274.0916
292.1021
126.0548
350.1431
454.2873 b4(1+)
512.204 LeX
543.3867 y5(1+)
657.2349
999.5476 PEP_0_1_0_0_0
1101.0962 PEP_0_2_0_0_0
1182.1174 PEP_1_2_0_0_0
1263.145 PEP_2_2_0_0_0
1364.6881 PEP_2_3_0_0_0
1445.7133 PEP_3_3_0_0_0
1599.751 PEP_4_3_1_0_0
1628.2716 PEP_4_4_0_0_0
1709.2916 PEP_5_4_0_0_0
1998.0973 PEP_0_1_0_0_0

File: 231129_OE_RK_14, RT: 52.719, Scan: 069627
Type: HCD, FTMS + c NSI d Full ms2 1476.3004@hcd30.00 [120.0000-4539.6211]
Precursor Scan: 069554, m/z: 1476.3004, charge: 3
AALAAFNAQNNGSNFQLEEISR_5_4_1_1_0 LeX peak identified

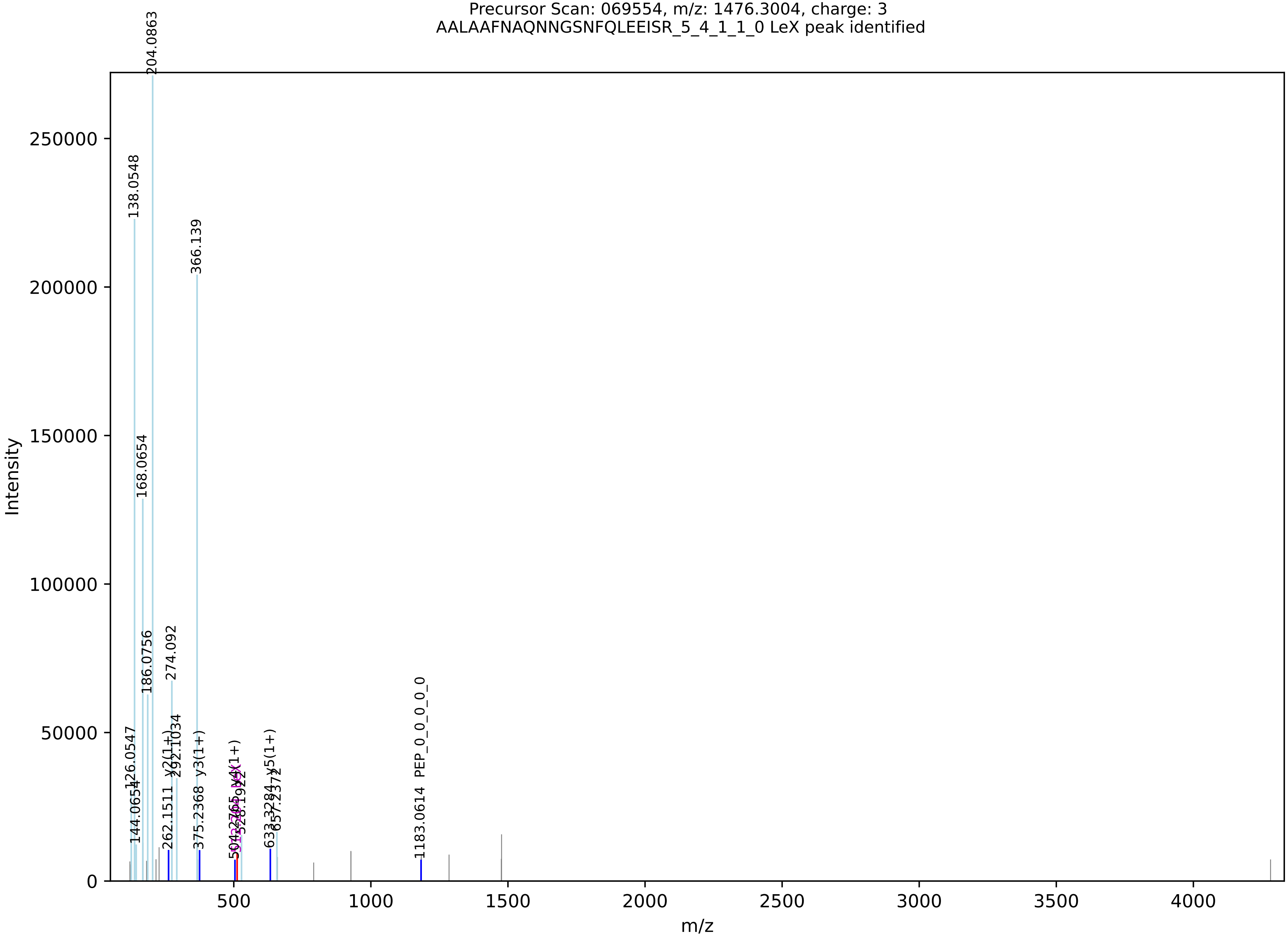

File: 231129_OE_RK_14, RT: 53.009, Scan: 070047
Type: HCD, FTMS + c NSI d Full ms2 1266.5454@hcd30.00 [120.0000-3897.7708]
Precursor Scan: 069971, m/z: 1265.877, charge: 3
ALPQPQNVTSLLGCTH_5_4_1_1_0 LeX peak identified

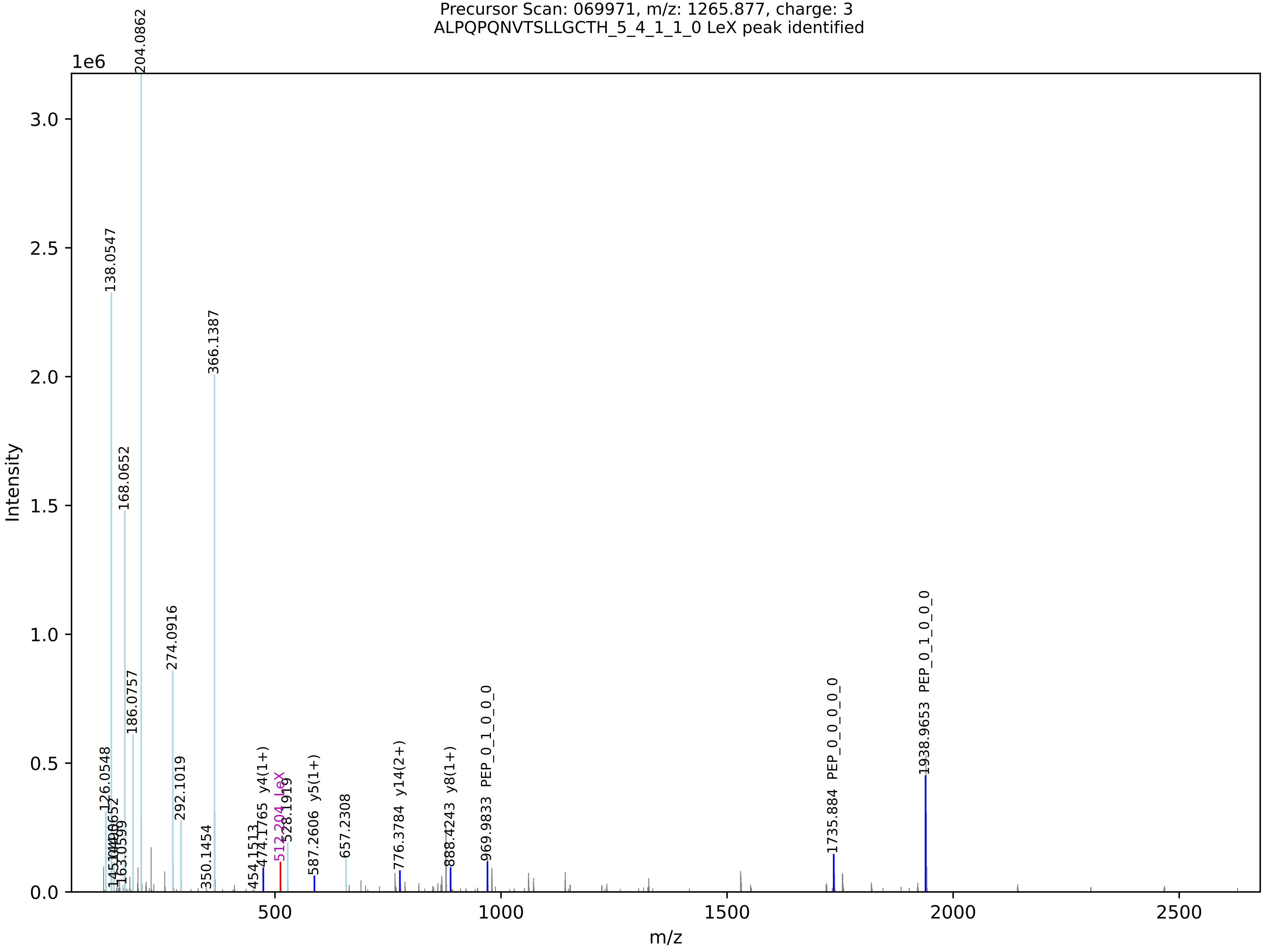

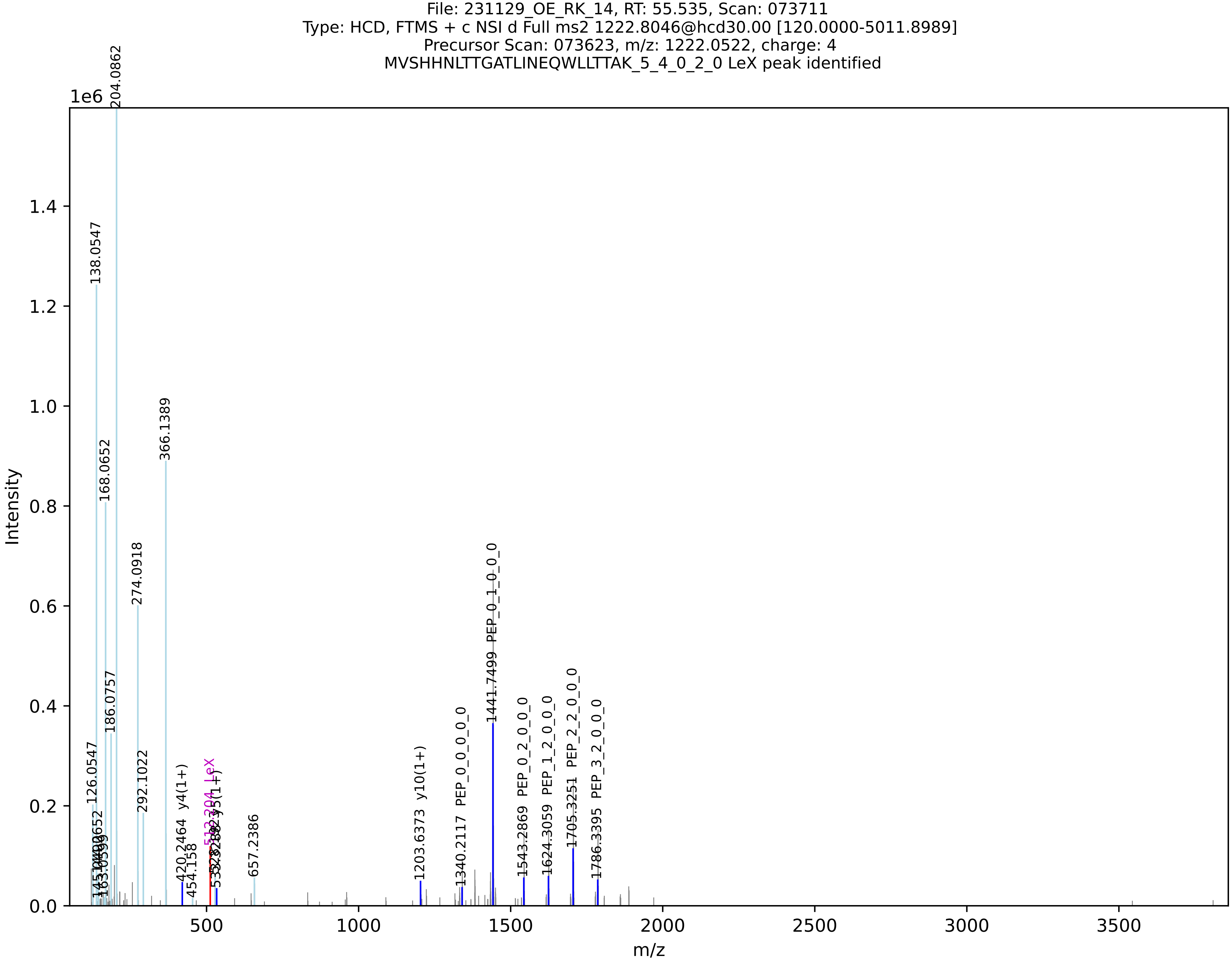

File: 231129_OE_RK_14, RT: 55.535, Scan: 073711
Type: HCD, FTMS + c NSI d Full ms2 1222.8046@hcd30.00 [120.0000-5011.8989]
Precursor Scan: 073623, m/z: 1222.0522, charge: 4
MVSHHNLTTGATLINEQWLLTTAK_5_4_0_2_0 LeX peak identified
1e6
Intensity
m/z
204.0862
138.0547
168.0652
366.1389
274.0918
186.0757
126.0547
292.1022
420.2464 y4(1+)
454.158
512.204 LeX
657.2386
1203.6373 y10(1+)
1340.2117 PEP_0_0_0_0_0
1441.7499 PEP_0_1_0_0_0
1543.2869 PEP_0_2_0_0_0
1624.3059 PEP_1_2_0_0_0
1705.3251 PEP_2_2_0_0_0
1786.3395 PEP_3_2_0_0_0

File: 231129_OE_RK_14, RT: 65.085, Scan: 087589
Type: HCD, FTMS + c NSI d Full ms2 1273.2162@hcd30.00 [120.0000-3918.1836]
Precursor Scan: 087513, m/z: 1272.548, charge: 3
YLGNATAIFFLPDEGK_5_4_1_1_0 LeX peak identified

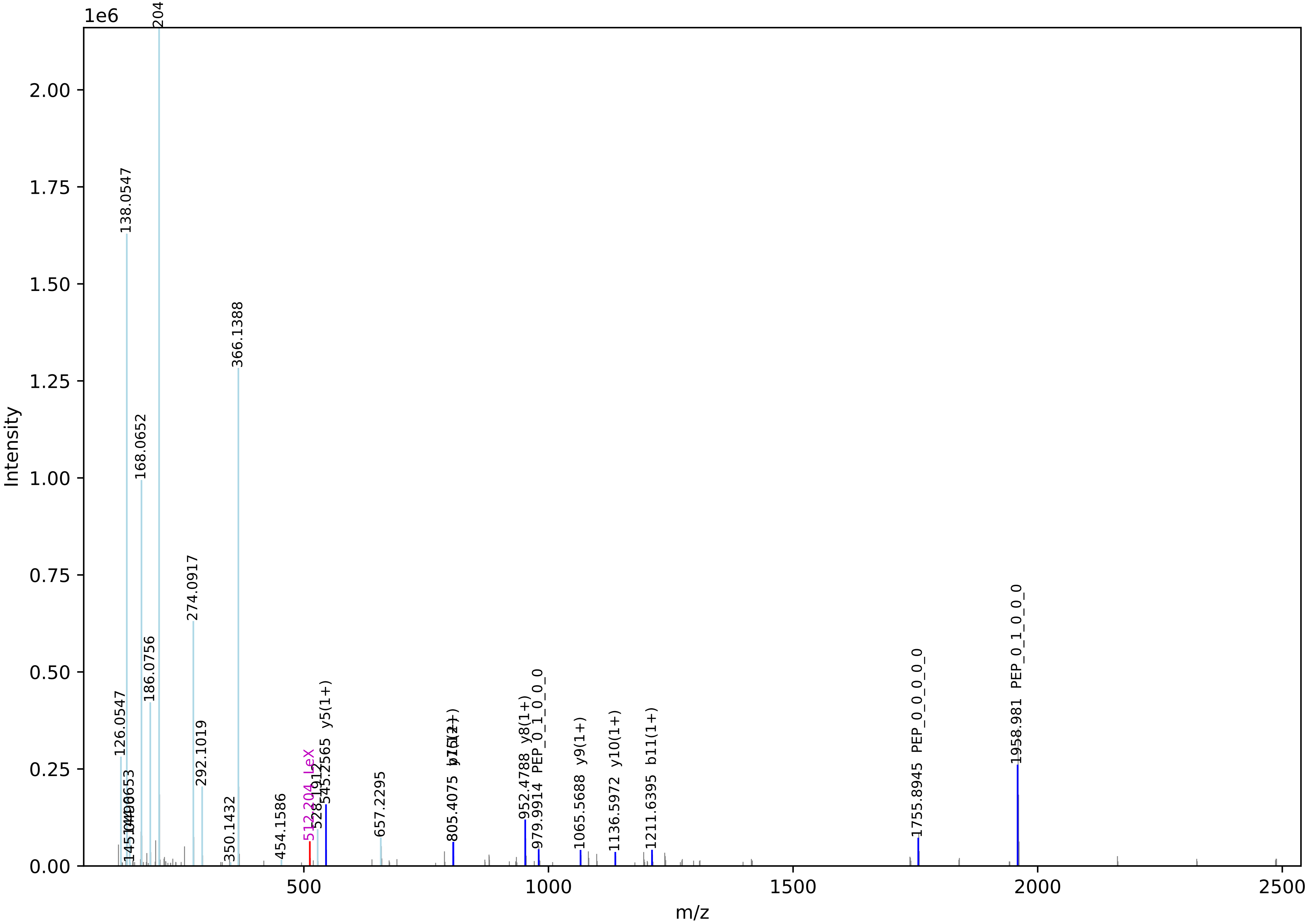

File: 231129_OE_RK_14, RT: 71.196, Scan: 096480
Type: HCD, FTMS + c NSI d Full ms2 1495.9006@hcd30.00 [120.0000-6126.1309]
Precursor Scan: 096402, m/z: 1495.1489, charge: 4
SVQEIQATFFYFTPNKTEDTIFLR_7_6_1_2_0 LeX peak identified

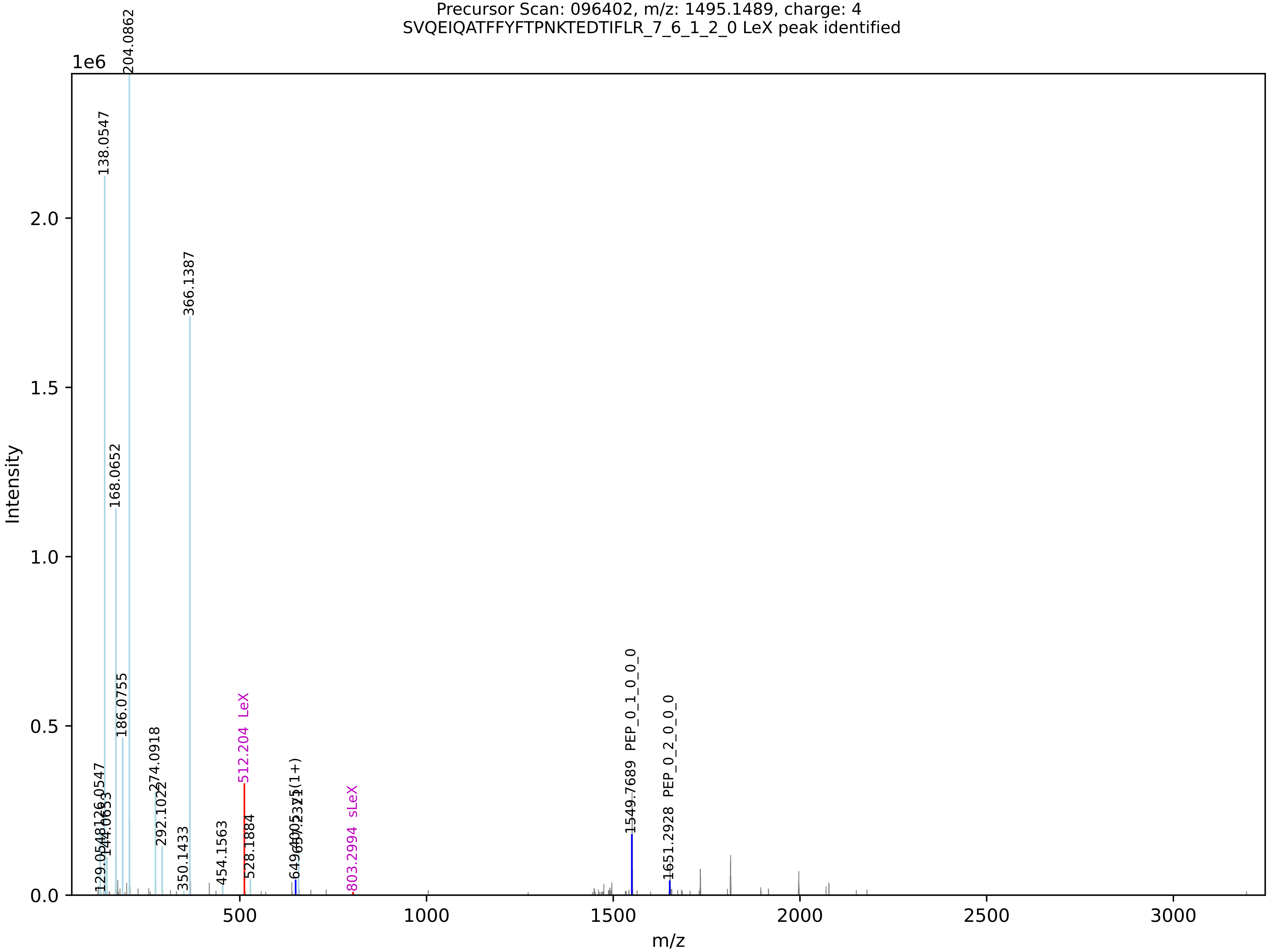

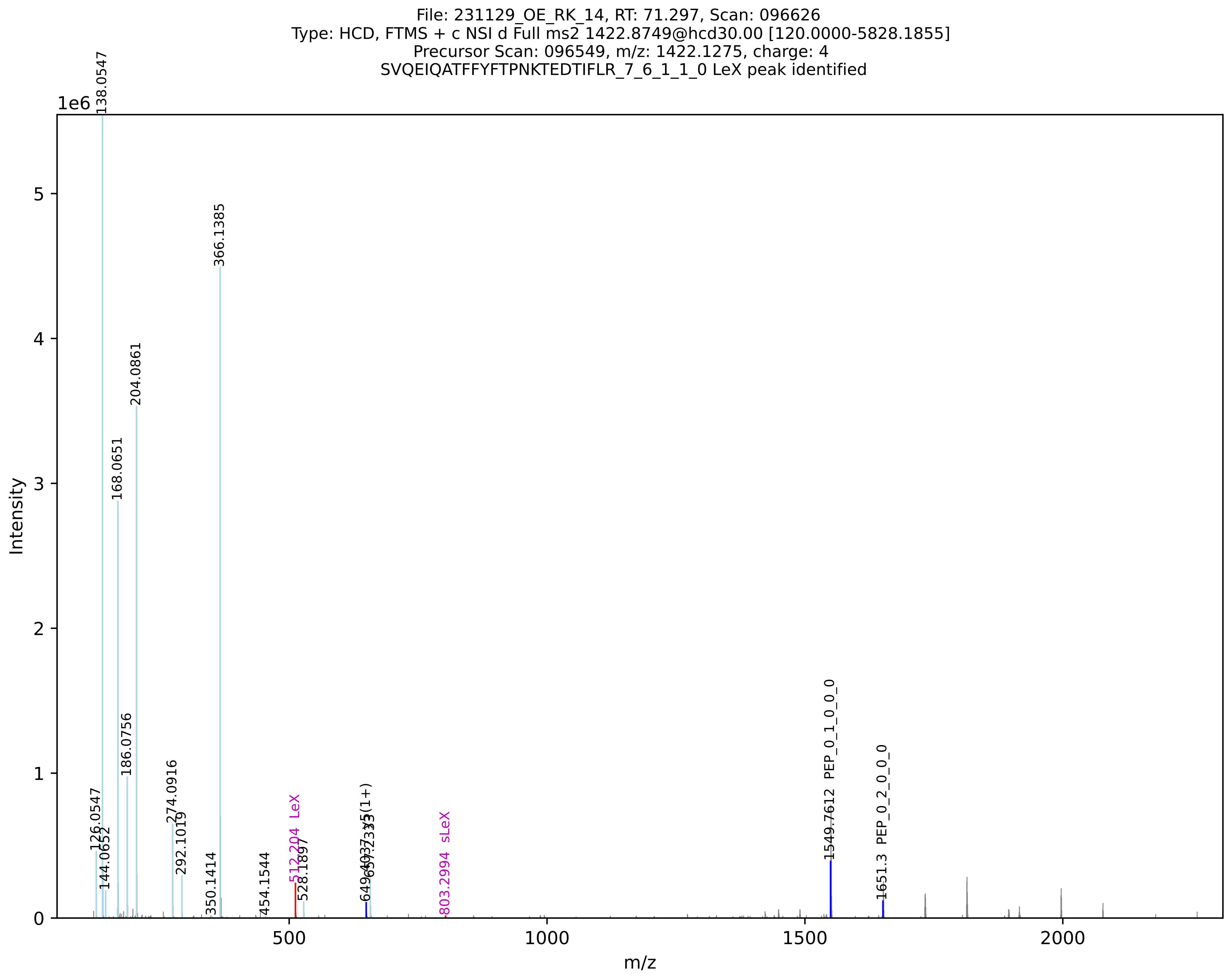

File: 231129_OE_RK_14, RT: 71.297, Scan: 096626
Type: HCD, FTMS + c NSI d Full ms2 1422.8749@hcd30.00 [120.0000-5828.1855]
Precursor Scan: 096549, m/z: 1422.1275, charge: 4
SVQEIQATFFYFTPNKTEDTIFLR_7_6_1_1_0 LeX peak identified
1e6
Intensity
m/z
138.0547
366.1385
204.0861
168.0651
186.0756
274.0916
126.0547
292.1019
144.0652
350.1414
454.1544
512.204 LeX
528.1897
649.4037 y5(1+)
657.2333
803.2994 sLeX
1549.7612 PEP_0_1_0_0_0
1651.3 PEP_0_2_0_0_0

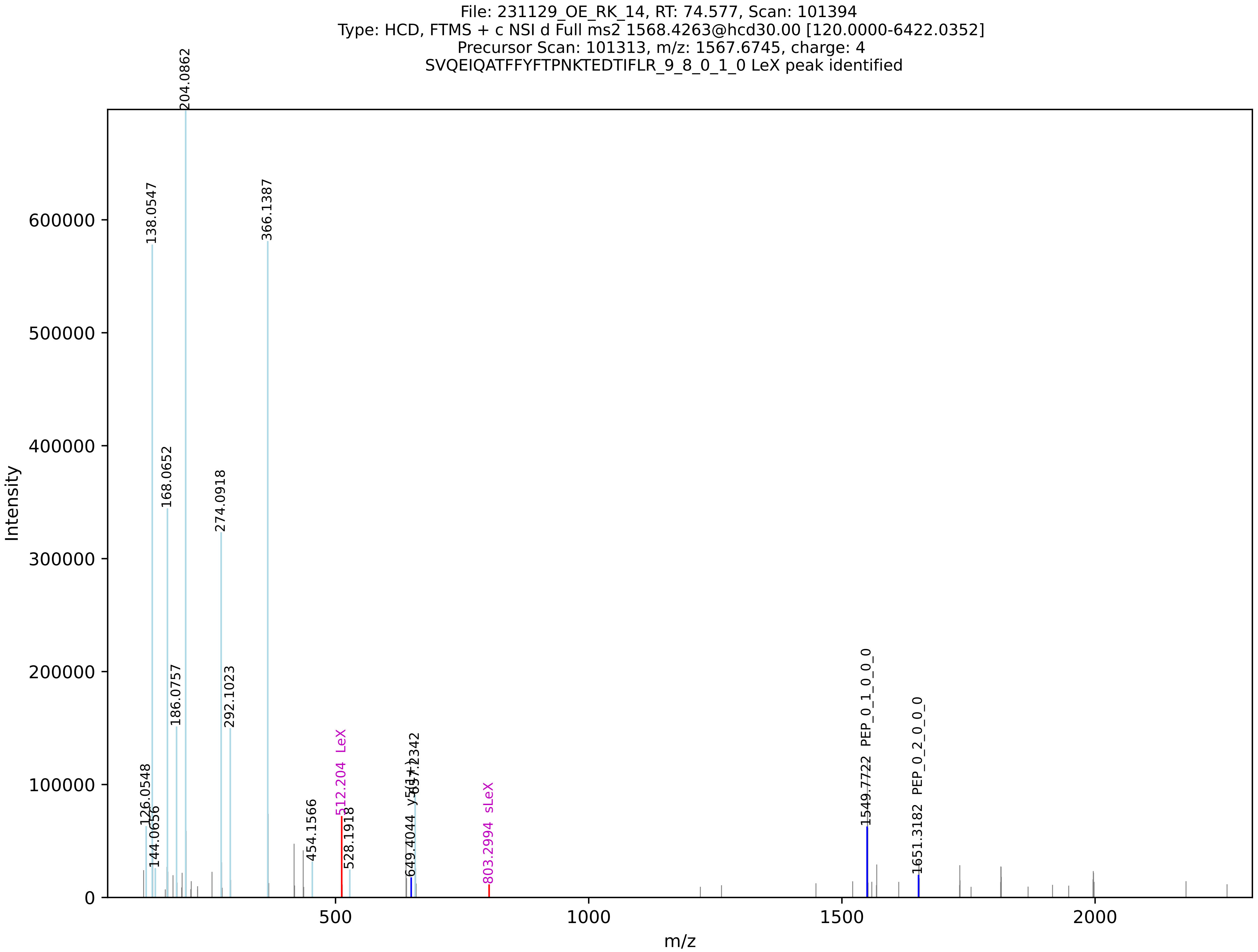
File: 231129_OE_RK_14, RT: 74.577, Scan: 101394
Type: HCD, FTMS + c NSI d Full ms2 1568.4263@hcd30.00 [120.0000-6422.0352]
Precursor Scan: 101313, m/z: 1567.6745, charge: 4
SVQEIQATFFYFTPNKTEDTIFLR_9_8_0_1_0 LeX peak identified
Intensity
m/z
204.0862
138.0547
366.1387
168.0652
274.0918
186.0757
292.1023
126.0548
144.0656
454.1566
512.204 LeX
528.1918
649.4044
657.2342
803.2994 sLeX
1549.7722 PEP_0_1_0_0_0
1651.3182 PEP_0_2_0_0_0
0
100000
200000
300000
400000
500000
600000
500
1000
1500
2000

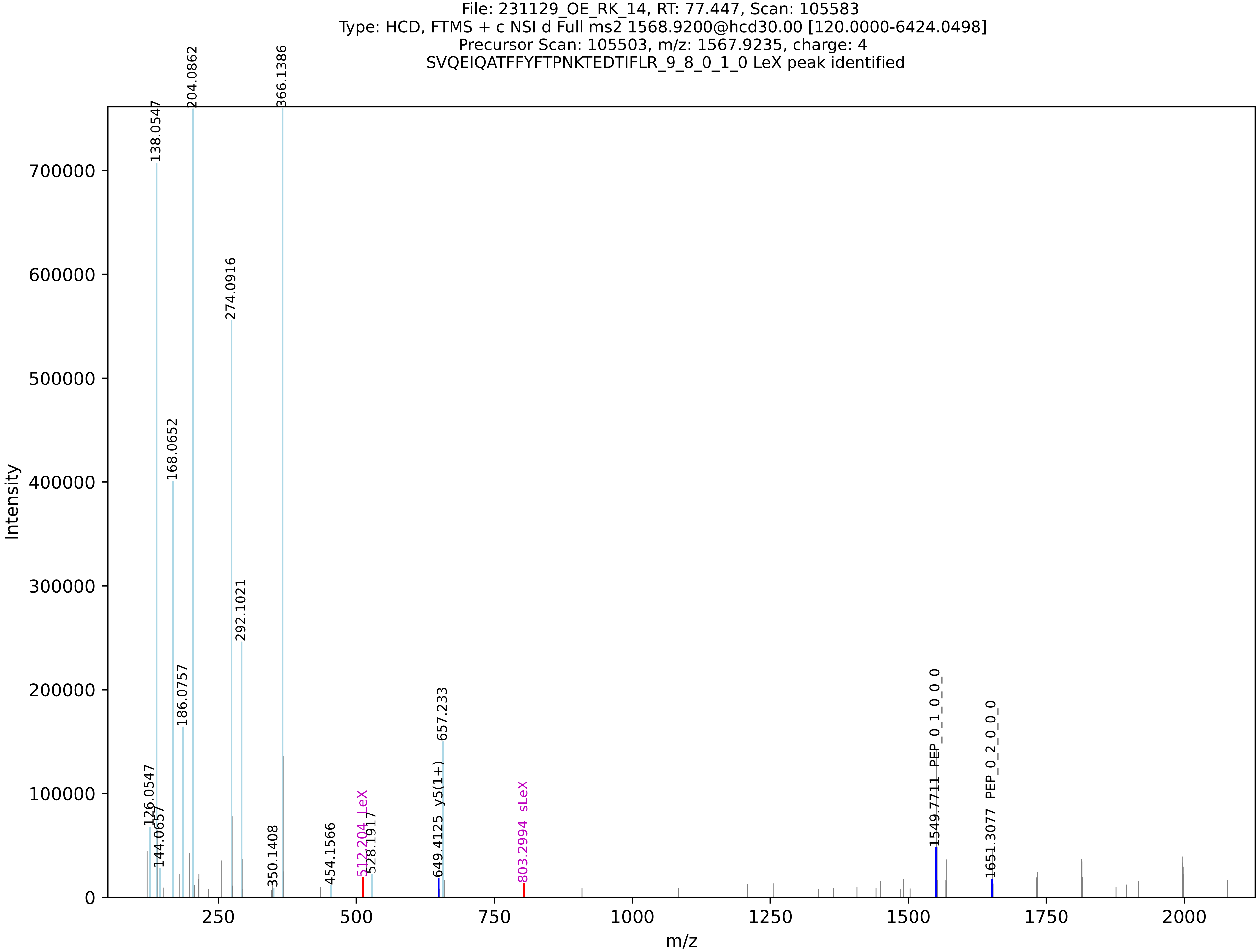
File: 231129_OE_RK_14, RT: 77.447, Scan: 105583
Type: HCD, FTMS + c NSI d Full ms2 1568.9200@hcd30.00 [120.0000-6424.0498]
Precursor Scan: 105503, m/z: 1567.9235, charge: 4
SVQEIQATFFYFTPNKTEDTIFLR_9_8_0_1_0 LeX peak identified
Intensity
m/z
204.0862
366.1386
138.0547
274.0916
168.0652
292.1021
186.0757
126.0547
144.0657
350.1408
454.1566
512.204 LeX
528.1917
649.4125 y5(1+)
657.233
803.2994 sLeX
1549.7711 PEP_0_1_0_0_0
1651.3077 PEP_0_2_0_0_0

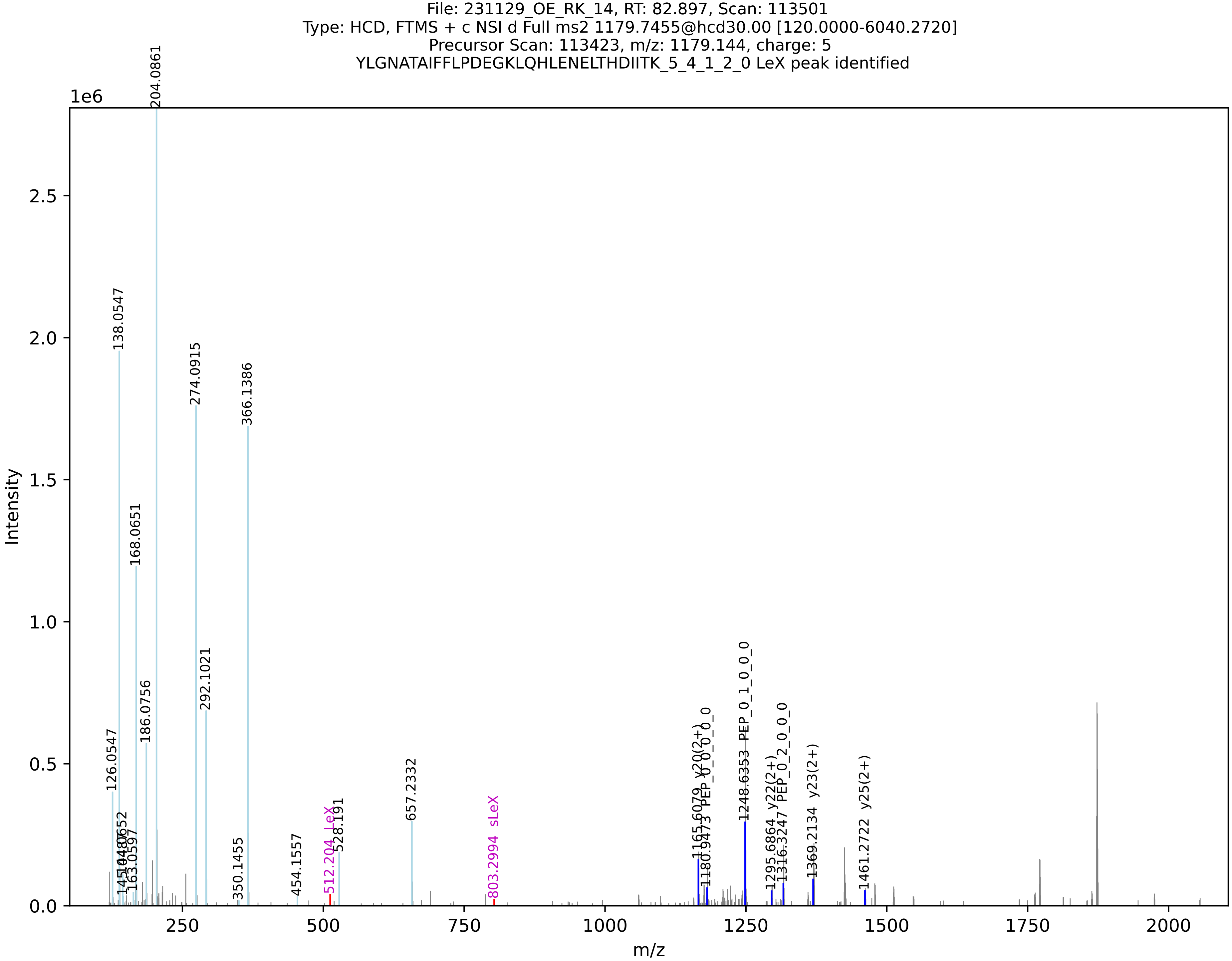

File: 231129_OE_RK_14, RT: 82.897, Scan: 113501
Type: HCD, FTMS + c NSI d Full ms2 1179.7455@hcd30.00 [120.0000-6040.2720]
Precursor Scan: 113423, m/z: 1179.144, charge: 5
YLGNATAIFFLPDEGKLQHLENELTHDIITK_5_4_1_2_0 LeX peak identified
1e6
Intensity
m/z
204.0861
138.0547
274.0915
366.1386
168.0651
292.1021
186.0756
126.0547
163.0597
350.1455
454.1557
512.204 LeX
528.191
657.2332
803.2994 sLeX
1165.6079 y20(2+)
1180.9473 PEP_0_0_0_0
1248.6353 PEP_0_1_0_0_0
1295.6864 y22(2+)
1316.3247 PEP_0_2_0_0_0
1369.2134 y23(2+)
1461.2722 y25(2+)

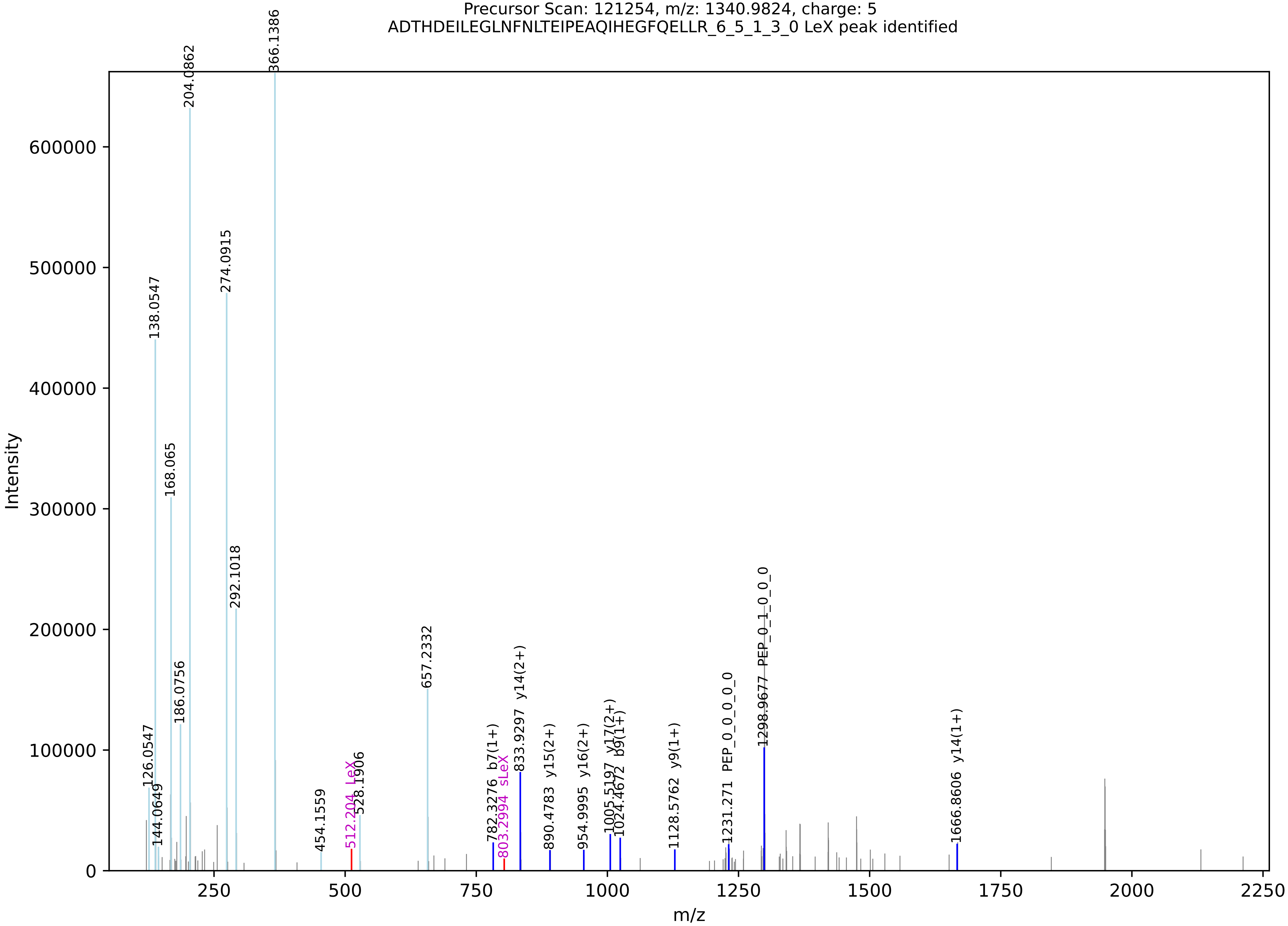

File: 231129_OE_RK_14, RT: 88.312, Scan: 121306
Type: HCD, FTMS + c NSI d Full ms2 1340.9824@hcd30.00 [120.0000-6862.5801]
Precursor Scan: 121254, m/z: 1340.9824, charge: 5
ADTHDEILEGLNFNLTEIPEAQIHEGFQELLR_6_5_1_3_0 LeX peak identified
Intensity
m/z
366.1386
204.0862
274.0915
138.0547
168.065
292.1018
186.0756
126.0547
144.0649
454.1559
512.204 LeX
528.1906
657.2332
782.3276 b7(1+)
803.2994 sLeX
833.9297 y14(2+)
890.4783 y15(2+)
954.9995 y16(2+)
1005.5197 y17(2+)
1024.4672 b9(1+)
1128.5762 y9(1+)
1231.271 PEP_0_0_0_0_0
1298.9677 PEP_0_1_0_0_0
1666.8606 y14(1+)

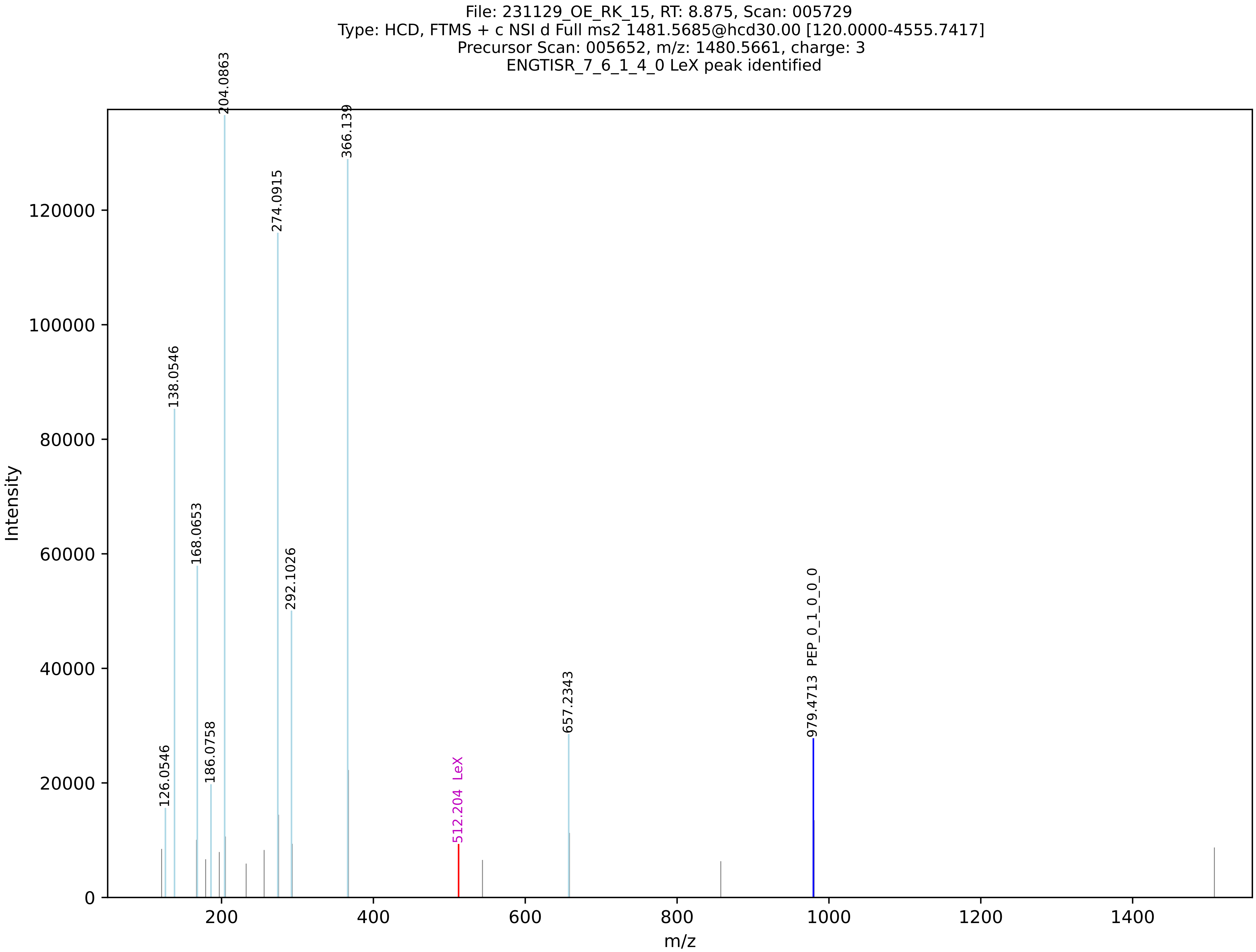

File: 231129_OE_RK_15, RT: 8.875, Scan: 005729
Type: HCD, FTMS + c NSI d Full ms2 1481.5685@hcd30.00 [120.0000-4555.7417]
Precursor Scan: 005652, m/z: 1480.5661, charge: 3
ENGTISR_7_6_1_4_0 LeX peak identified
204.0863
366.139
274.0915
138.0546
168.0653
292.1026
979.4713 PEP_0_1_0_0_0_
657.2343
512.204 LeX
186.0758
126.0546
Intensity
m/z
0
20000
40000
60000
80000
100000
120000
200
400
600
800
1000
1200
1400

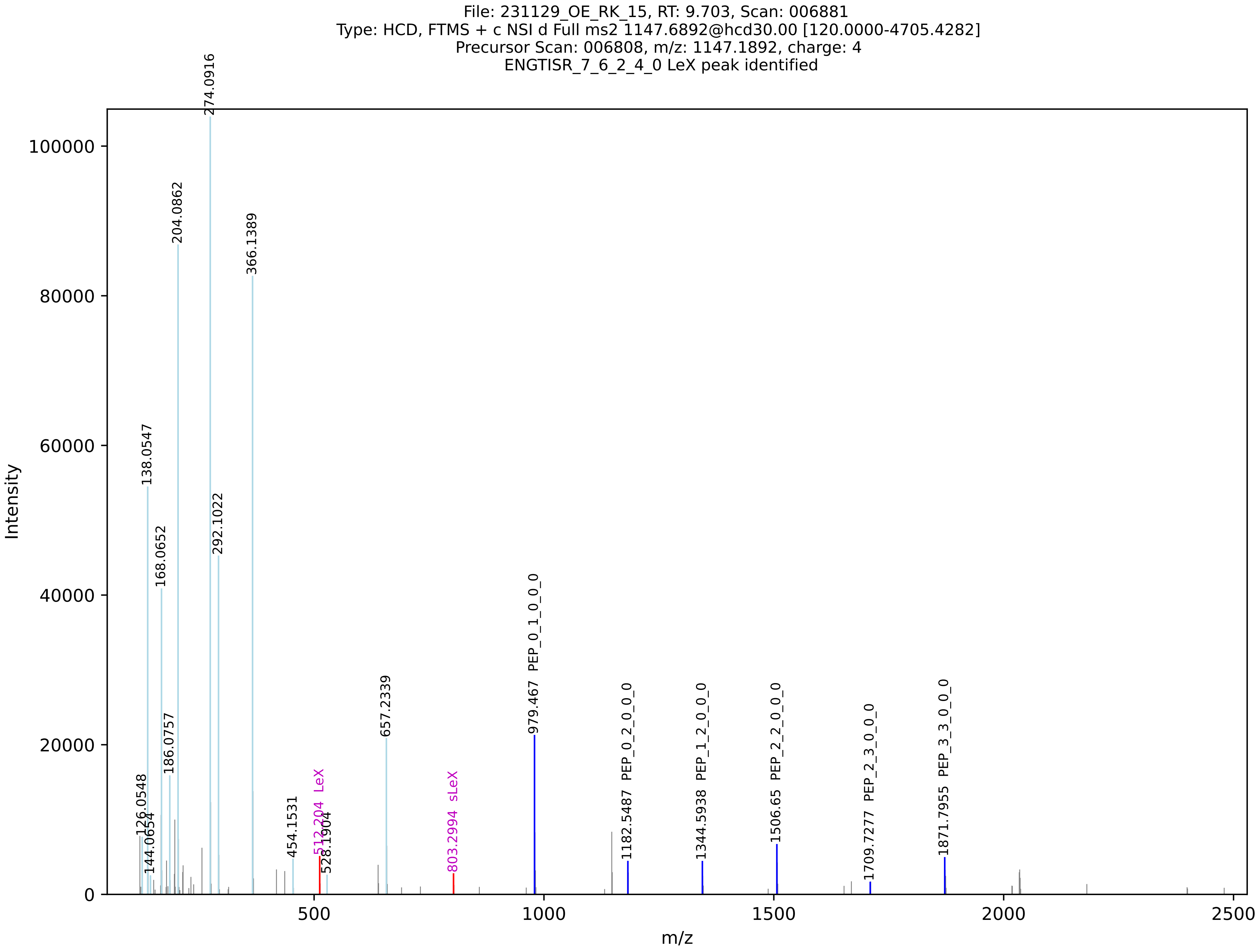
File: 231129_OE_RK_15, RT: 9.703, Scan: 006881
Type: HCD, FTMS + c NSI d Full ms2 1147.6892@hcd30.00 [120.0000-4705.4282]
Precursor Scan: 006808, m/z: 1147.1892, charge: 4
ENGTISR_7_6_2_4_0 LeX peak identified
Intensity
m/z
274.0916
204.0862
366.1389
138.0547
292.1022
168.0652
186.0757
126.0548
144.0654
454.1531
512.204 LeX
528.1904
657.2339
803.2994 sLeX
979.467 PEP_0_1_0_0_0
1182.5487 PEP_0_2_0_0_0
1344.5938 PEP_1_2_0_0_0
1506.65 PEP_2_2_0_0_0
1709.7277 PEP_2_3_0_0_0
1871.7955 PEP_3_3_0_0_0

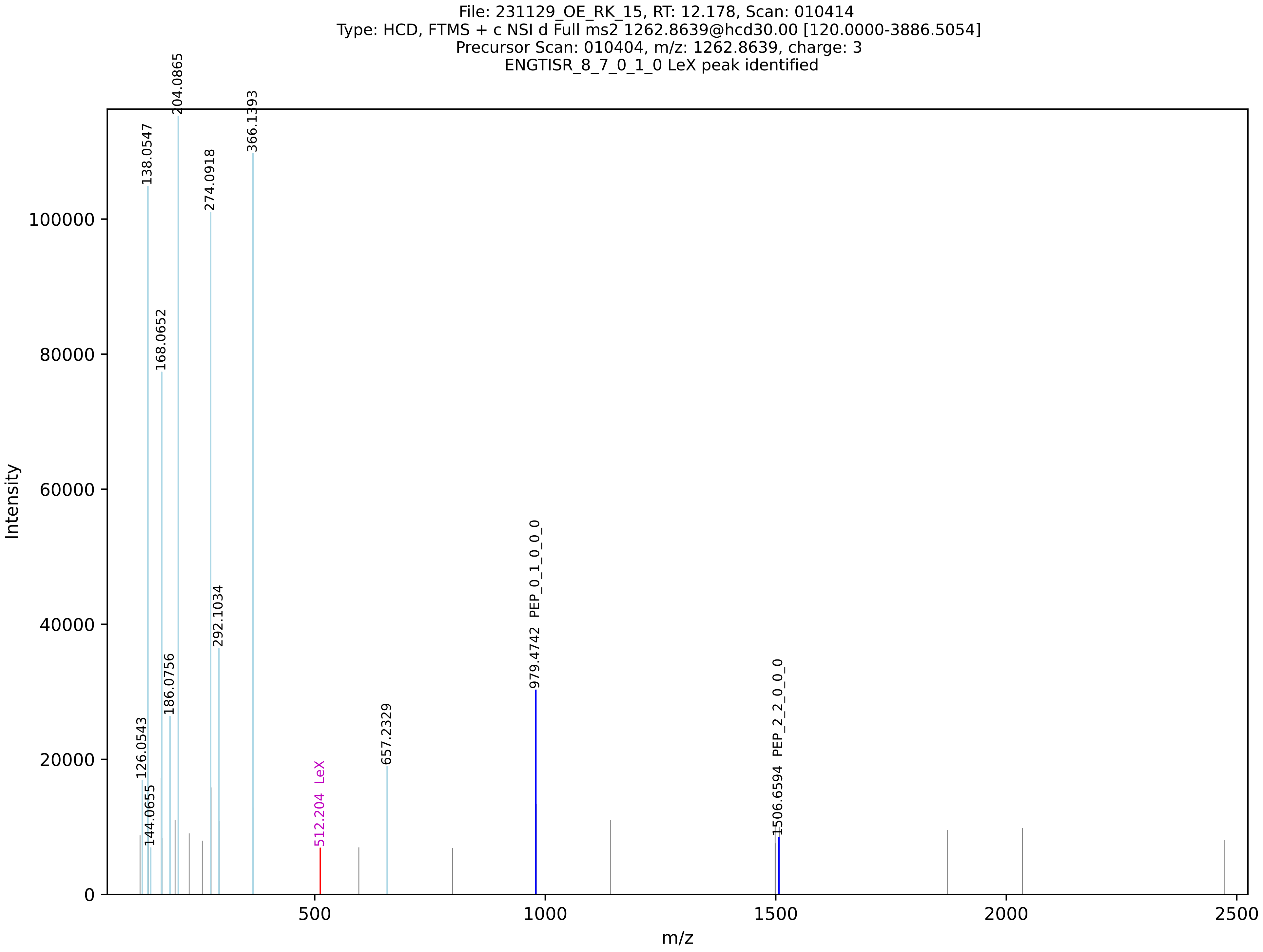
File: 231129_OE_RK_15, RT: 12.178, Scan: 010414
Type: HCD, FTMS + c NSI d Full ms2 1262.8639@hcd30.00 [120.0000-3886.5054]
Precursor Scan: 010404, m/z: 1262.8639, charge: 3
ENGTISR_8_7_0_1_0 LeX peak identified
204.0865
366.1393
138.0547
274.0918
168.0652
292.1034
186.0756
126.0543
144.0655
512.204 LeX
657.2329
979.4742 PEP_0_1_0_0_0
1506.6594 PEP_2_2_0_0_0
Intensity
m/z
0
20000
40000
60000
80000
100000
500
1000
1500
2000
2500

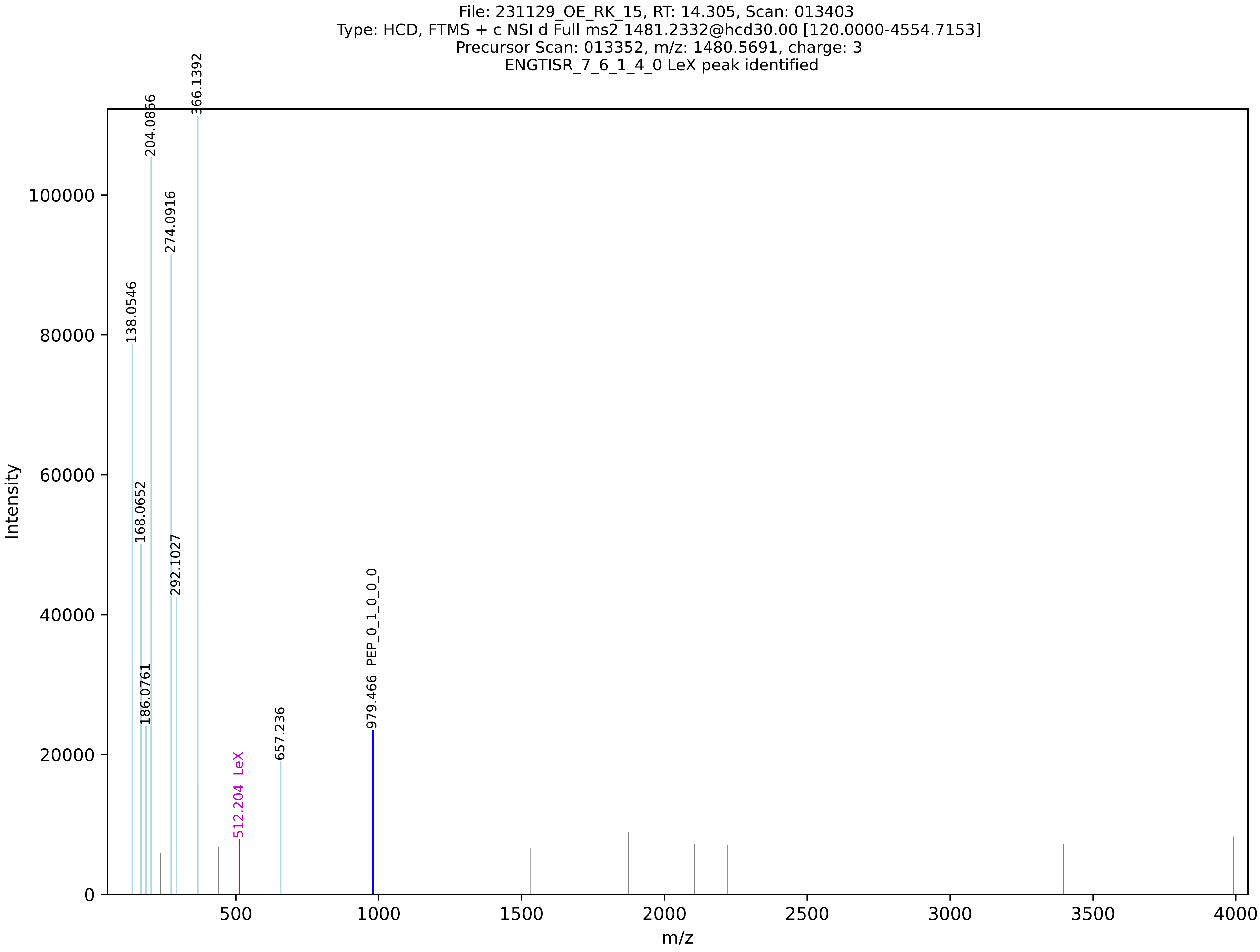

File: 231129_OE_RK_15, RT: 14.305, Scan: 013403
Type: HCD, FTMS + c NSI d Full ms2 1481.2332@hcd30.00 [120.0000-4554.7153]
Precursor Scan: 013352, m/z: 1480.5691, charge: 3
ENGTISR_7_6_1_4_0 LeX peak identified
Intensity
m/z
366.1392
204.0866
274.0916
138.0546
168.0652
292.1027
186.0761
512.204 LeX
657.236
979.466 PEP_0_1_0_0_0_

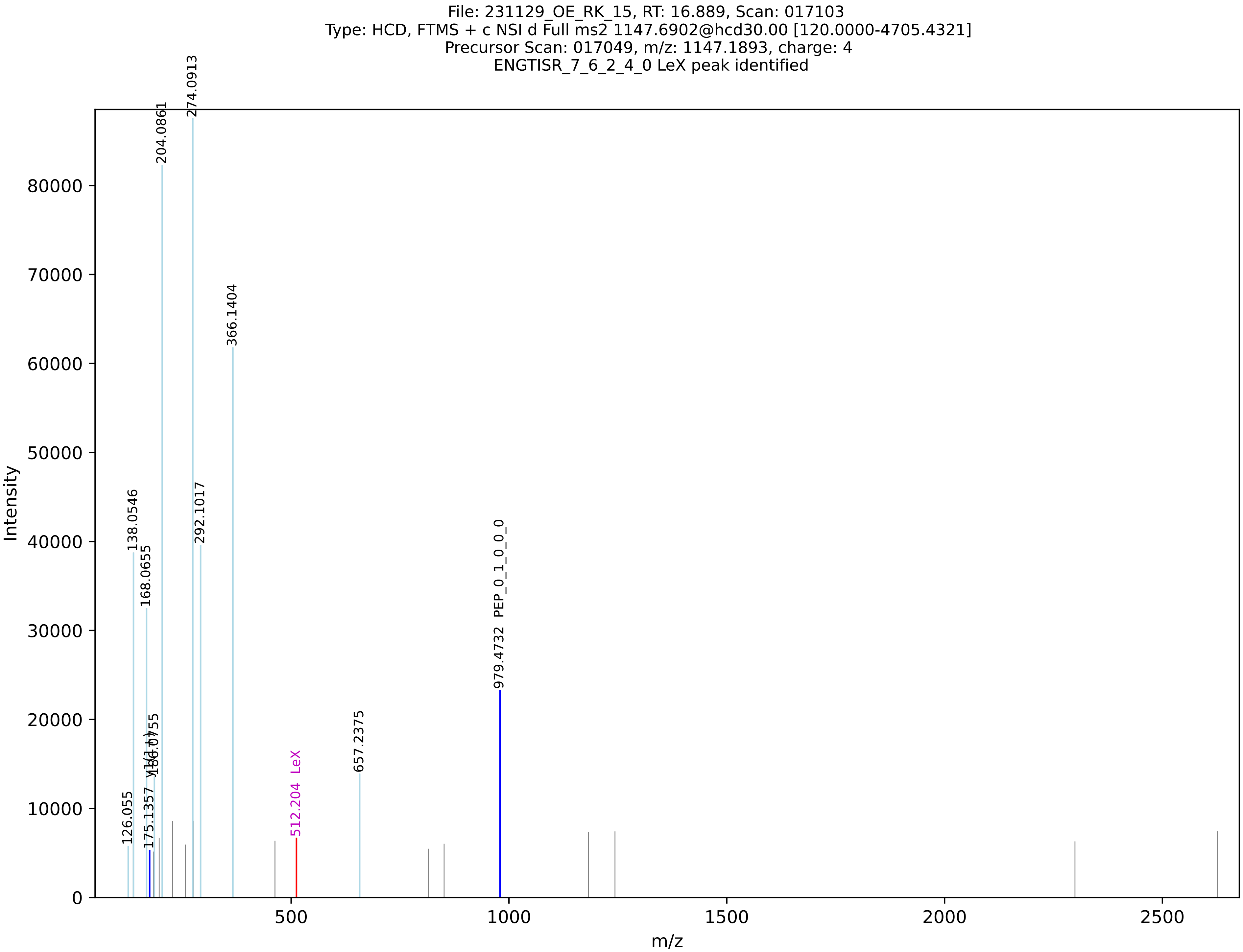

File: 231129_OE_RK_15, RT: 16.889, Scan: 017103
Type: HCD, FTMS + c NSI d Full ms2 1147.6902@hcd30.00 [120.0000-4705.4321]
Precursor Scan: 017049, m/z: 1147.1893, charge: 4
ENGTISR_7_6_2_4_0 LeX peak identified
Intensity
m/z
0
10000
20000
30000
40000
50000
60000
70000
80000
500
1000
1500
2000
2500
274.0913
204.0861
366.1404
138.0546
292.1017
168.0655
979.4732 PEP_0_1_0_0_0_
186.0755
y1(1+)
657.2375
512.204 LeX
126.055
175.1357

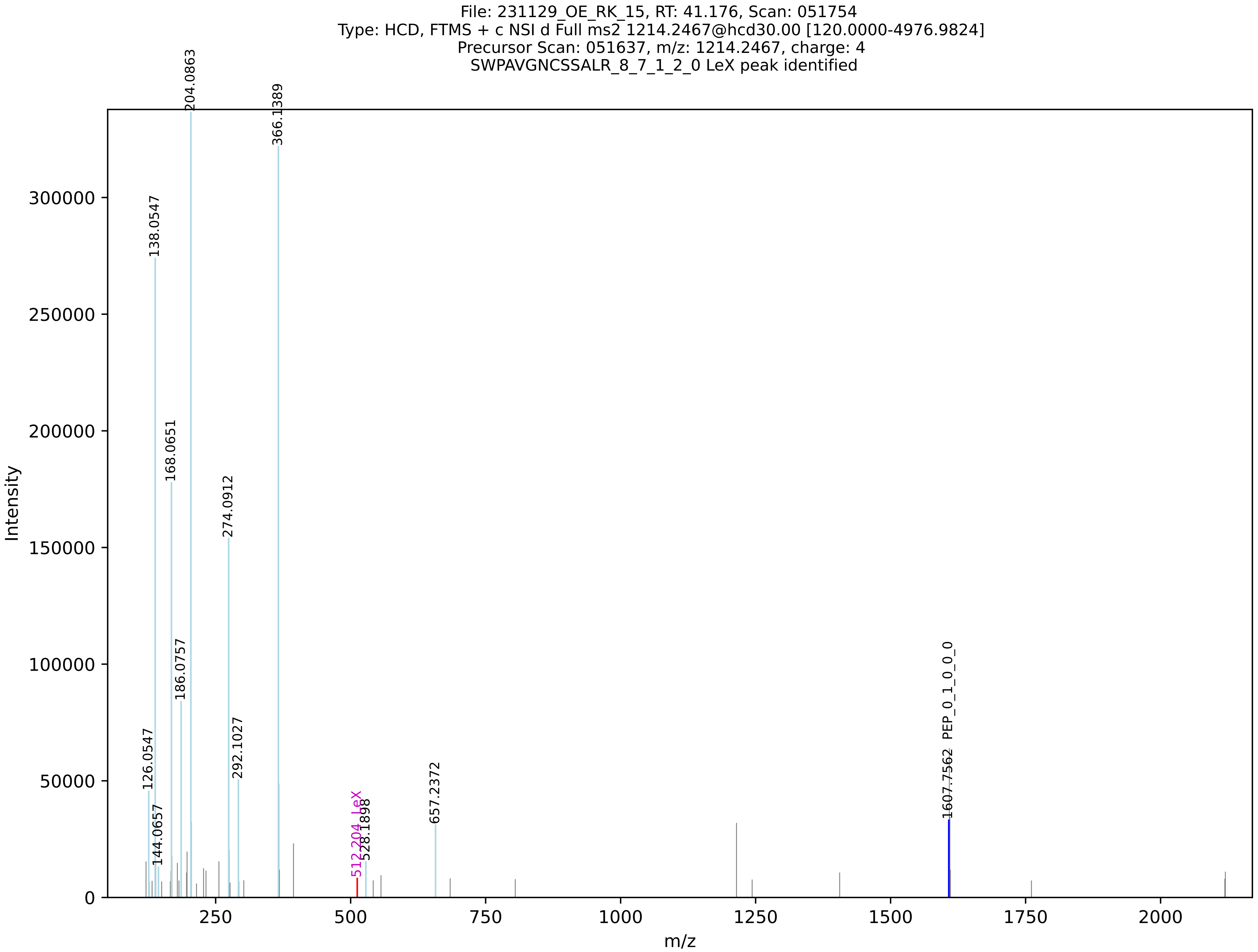

File: 231129_OE_RK_15, RT: 41.176, Scan: 051754
Type: HCD, FTMS + c NSI d Full ms2 1214.2467@hcd30.00 [120.0000-4976.9824]
Precursor Scan: 051637, m/z: 1214.2467, charge: 4
SWPAVGNCSSALR_8_7_1_2_0 LeX peak identified
Intensity
m/z
204.0863
366.1389
138.0547
168.0651
274.0912
186.0757
126.0547
292.1027
144.0657
512.204_LeX
528.1898
657.2372
1607.7562
PEP_0_1_0_0_0_

File: 231129_OE_RK_15, RT: 41.502, Scan: 052228
Type: HCD, FTMS + c NSI d Full ms2 1177.7322@hcd30.00 [120.0000-4828.0029]
Precursor Scan: 052149, m/z: 1177.2307, charge: 4
QDQCIYNTTYLNVQR_7_6_1_1_0 LeX peak identified

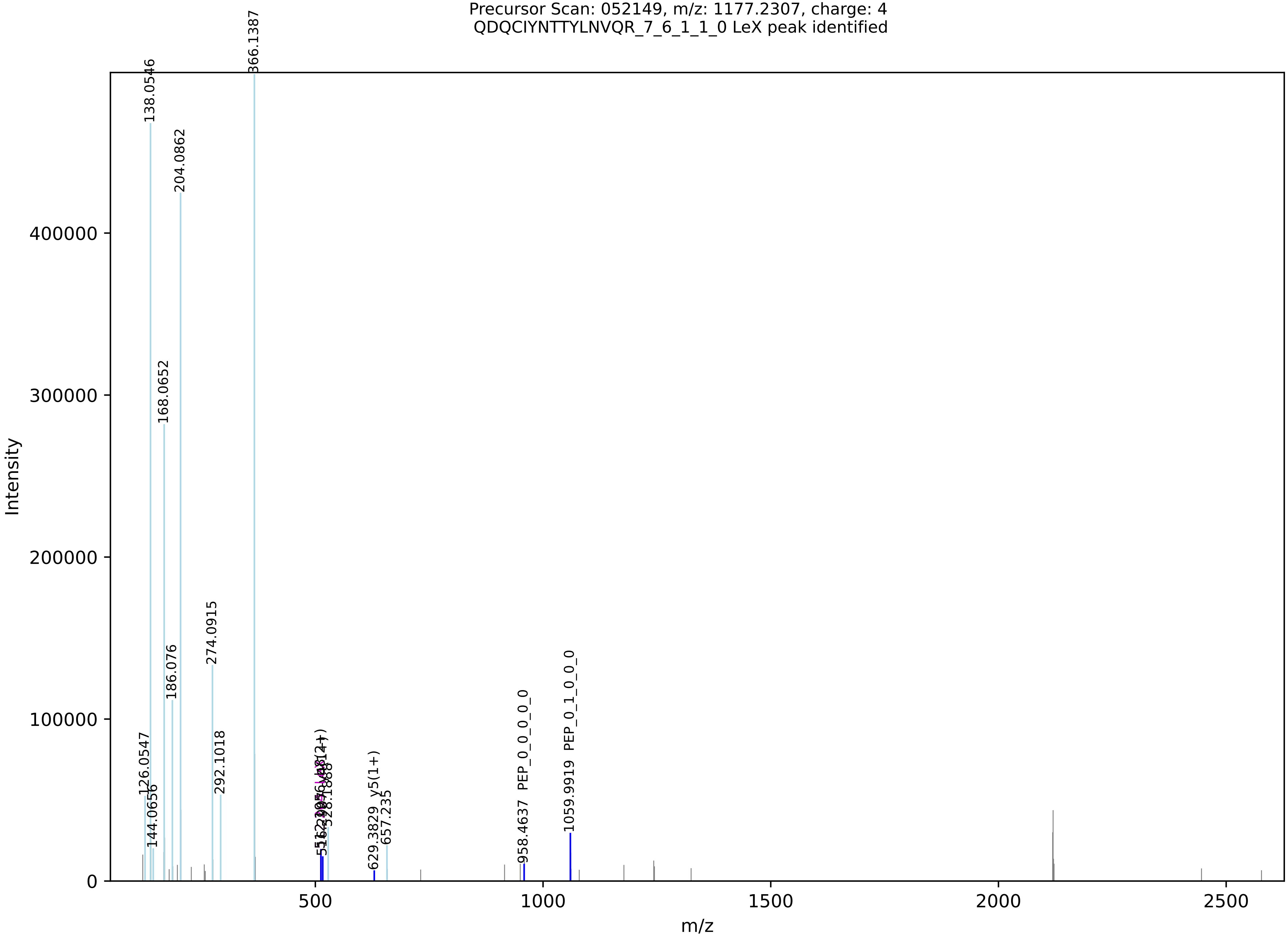

File: 231129_OE_RK_15, RT: 43.816, Scan: 055583
Type: HCD, FTMS + c NSI d Full ms2 1159.2223@hcd30.00 [120.0000-4752.4829]
Precursor Scan: 055504, m/z: 1158.7212, charge: 4
QDQCIYNTTYLNVQR_6_5_1_2_0 LeX peak identified

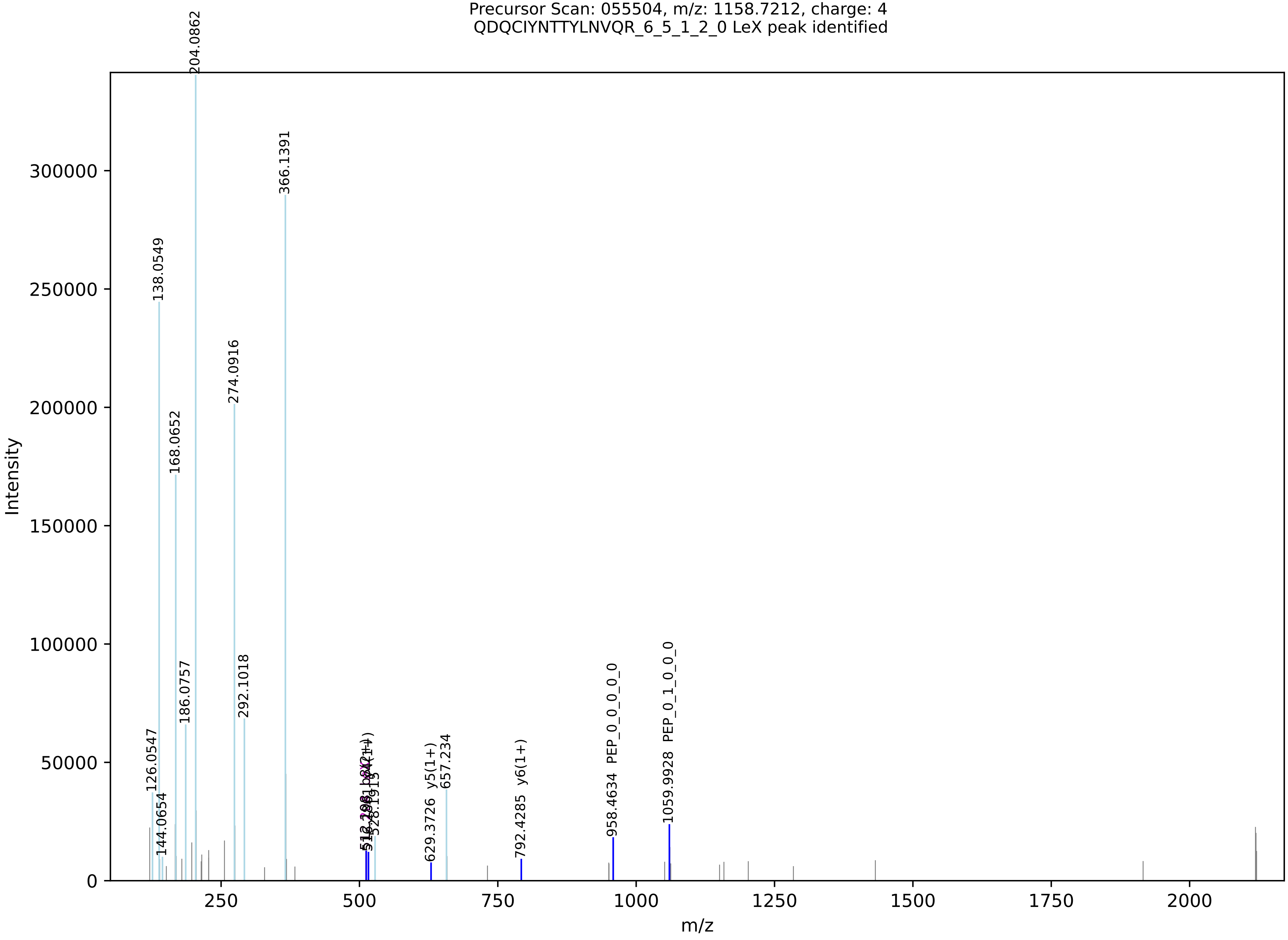

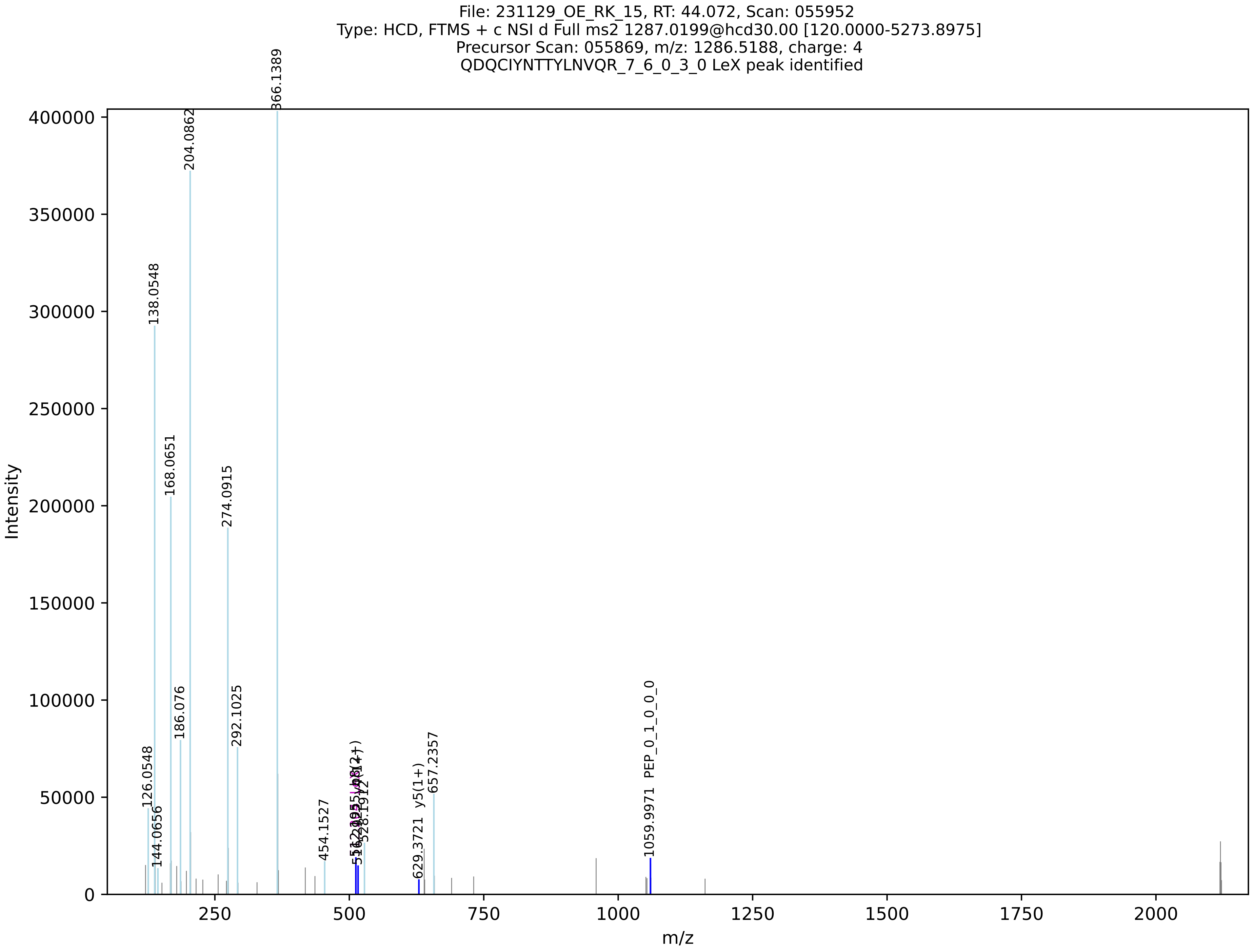

File: 231129_OE_RK_15, RT: 44.072, Scan: 055952
Type: HCD, FTMS + c NSI d Full ms2 1287.0199@hcd30.00 [120.0000-5273.8975]
Precursor Scan: 055869, m/z: 1286.5188, charge: 4
QDQCIYNTTYLNVQR_7_6_0_3_0 LeX peak identified
Intensity
m/z
366.1389
204.0862
138.0548
168.0651
274.0915
186.076
292.1025
126.0548
144.0656
454.1527
528.1912
629.3721
y5(1+)
657.2357
1059.9971
PEP_0_1_0_0_0

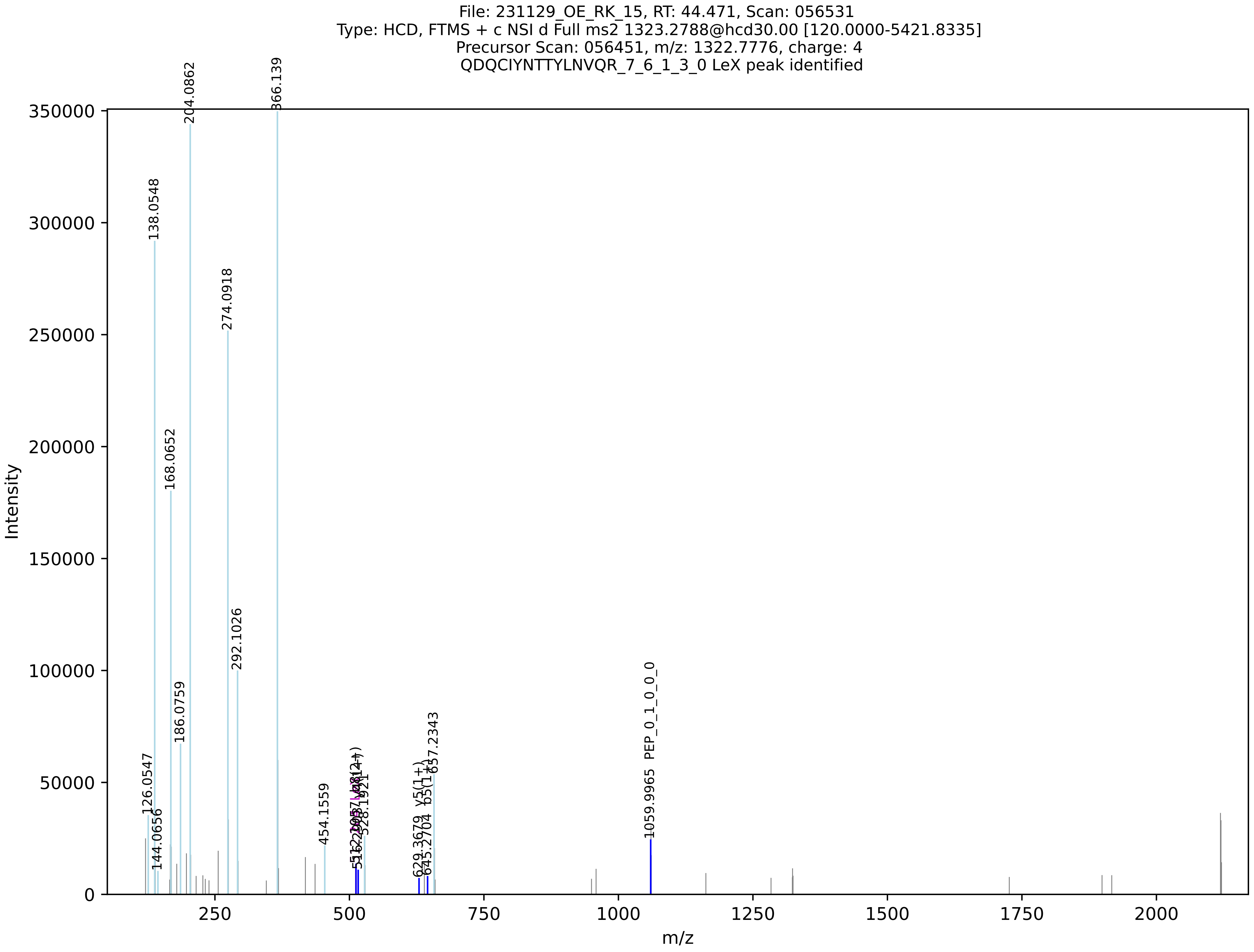

File: 231129_OE_RK_15, RT: 44.471, Scan: 056531
Type: HCD, FTMS + c NSI d Full ms2 1323.2788@hcd30.00 [120.0000-5421.8335]
Precursor Scan: 056451, m/z: 1322.7776, charge: 4
QDQCIYNTTYLNVQR_7_6_1_3_0 LeX peak identified
Intensity
m/z
0
50000
100000
150000
200000
250000
300000
350000
250
500
750
1000
1250
1500
1750
2000
366.139
204.0862
138.0548
274.0918
168.0652
292.1026
186.0759
126.0547
144.0656
454.1559
528.1921
629.3679
645.2704
y5(1+)
b5(1+)
657.2343
1059.9965
PEP_0_1_0_0_0

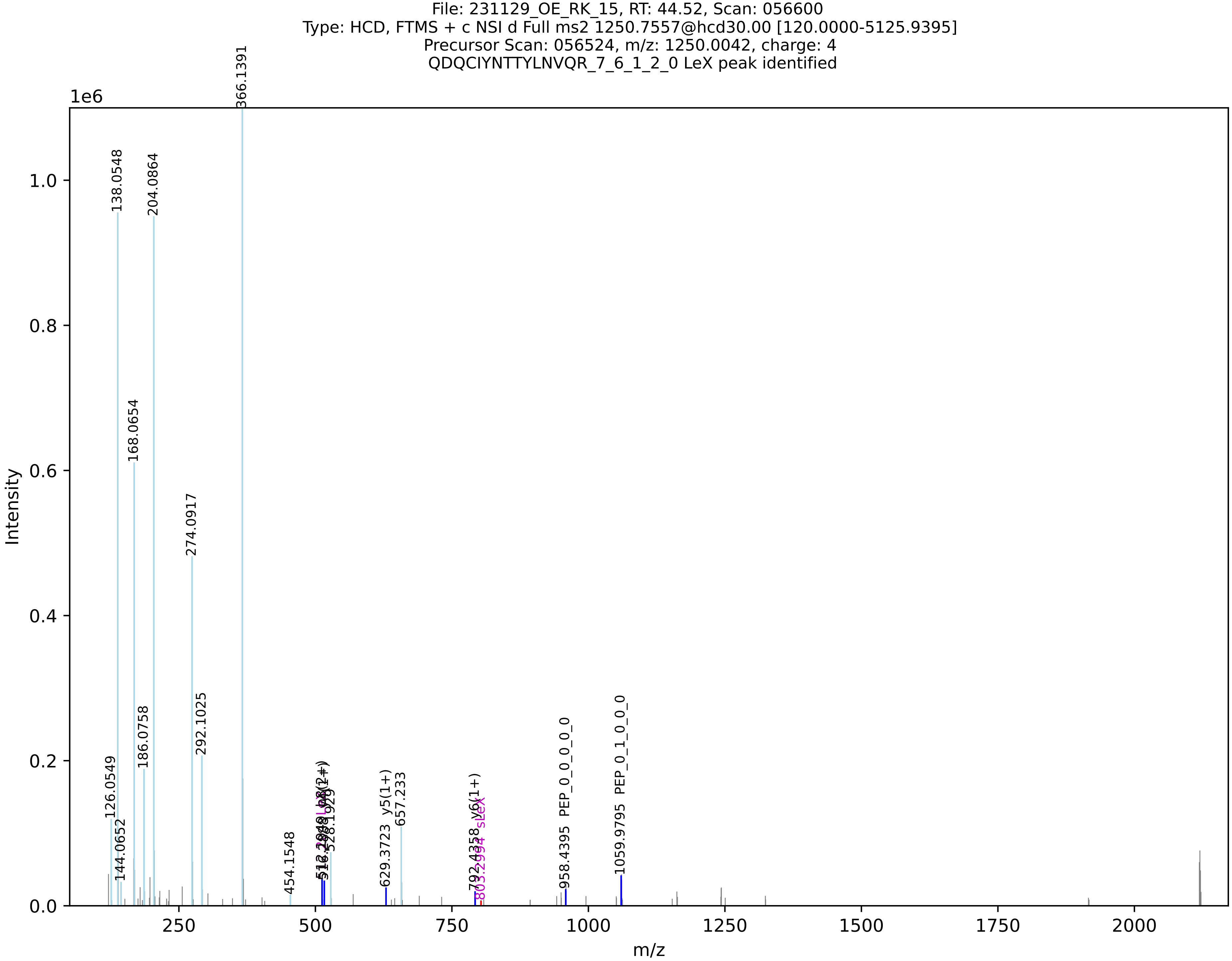
File: 231129_OE_RK_15, RT: 44.52, Scan: 056600
Type: HCD, FTMS + c NSI d Full ms2 1250.7557@hcd30.00 [120.0000-5125.9395]
Precursor Scan: 056524, m/z: 1250.0042, charge: 4
QDQCIYNTTYLNVQR_7_6_1_2_0 LeX peak identified
1e6
Intensity
m/z
366.1391
138.0548
204.0864
168.0654
274.0917
292.1025
186.0758
126.0549
144.0652
454.1548
528.1929
629.3723 y5(1+)
657.233
792.4358 y6(1+)
803.2994 sLeX
958.4395 PEP_0_0_0_0_0
1059.9795 PEP_0_1_0_0_0
0.0
0.2
0.4
0.6
0.8
1.0
250
500
750
1000
1250
1500
1750
2000

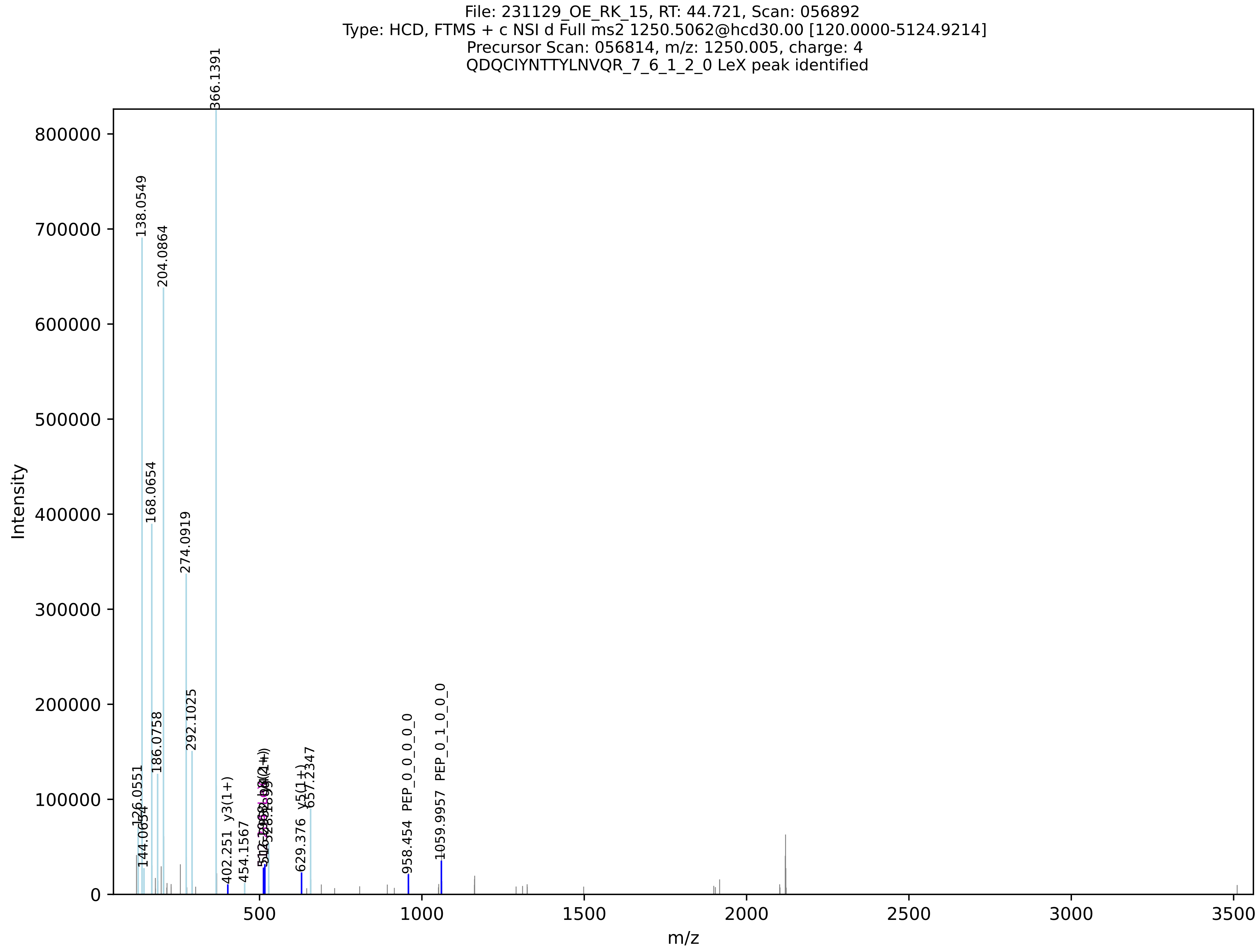
File: 231129_OE_RK_15, RT: 44.721, Scan: 056892
Type: HCD, FTMS + c NSI d Full ms2 1250.5062@hcd30.00 [120.0000-5124.9214]
Precursor Scan: 056814, m/z: 1250.005, charge: 4
QDQCIYNTTYLNVQR_7_6_1_2_0 LeX peak identified
Intensity
m/z
366.1391
138.0549
204.0864
168.0654
274.0919
292.1025
186.0758
126.0551
144.0654
402.251 y3(1+)
454.1567
629.376 y5(1+)
657.2347
958.454 PEP_0_0_0_0_0_
1059.9957 PEP_0_1_0_0_0_

File: 231129_OE_RK_15, RT: 46.127, Scan: 058930
Type: HCD, FTMS + c NSI d Full ms2 1323.2782@hcd30.00 [120.0000-5421.8311]
Precursor Scan: 058852, m/z: 1322.7755, charge: 4
QDQCIYNTTYLNVQR_7_6_1_3_0 LeX peak identified

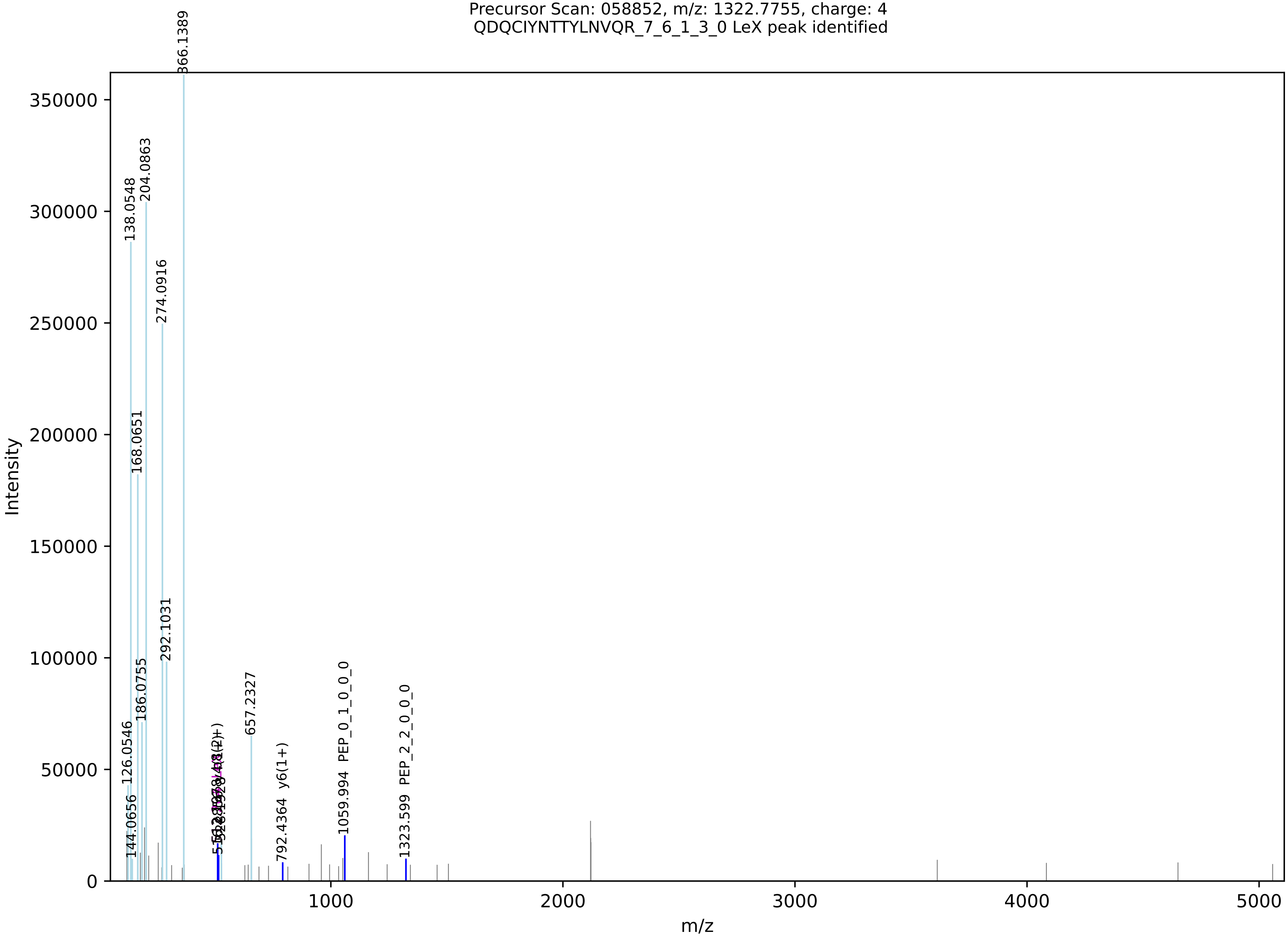

File: 231129_OE_RK_15, RT: 48.558, Scan: 062448
Type: HCD, FTMS + c NSI d Full ms2 1396.0537@hcd30.00 [120.0000-5718.7554]
Precursor Scan: 062376, m/z: 1395.5525, charge: 4
QDQCIYNTTYLNVQR_7_6_1_4_0 LeX peak identified

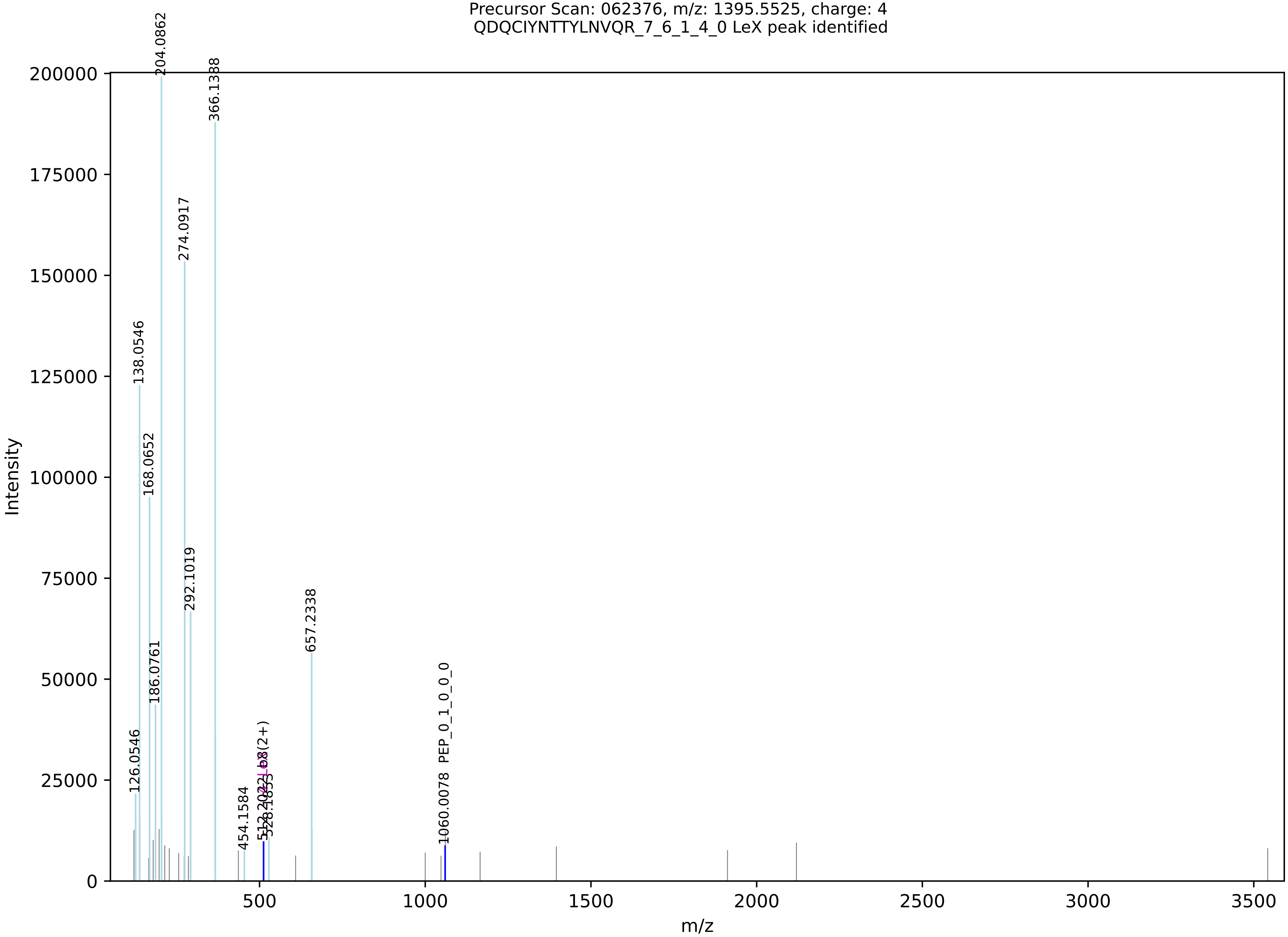

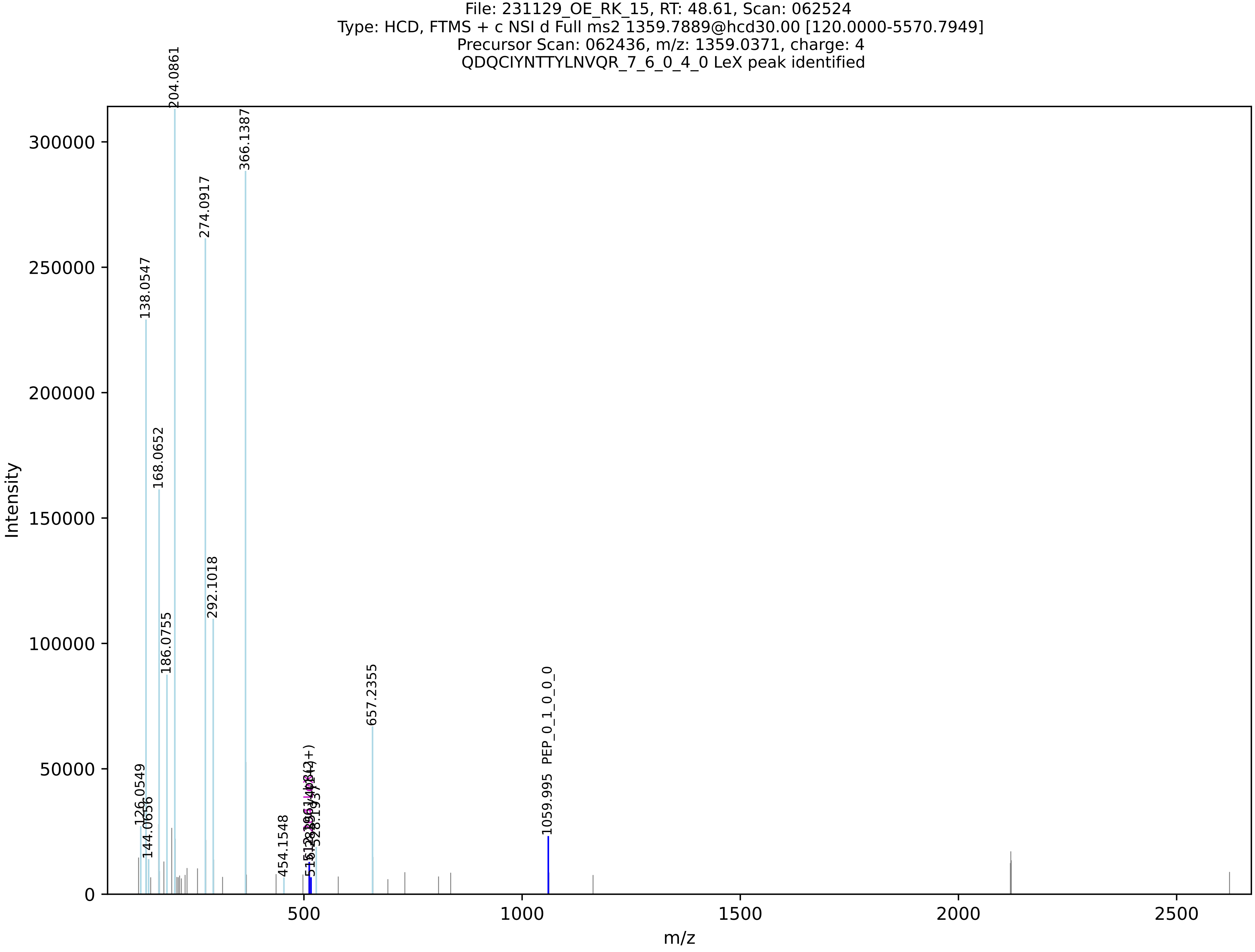

File: 231129_OE_RK_15, RT: 48.61, Scan: 062524
Type: HCD, FTMS + c NSI d Full ms2 1359.7889@hcd30.00 [120.0000-5570.7949]
Precursor Scan: 062436, m/z: 1359.0371, charge: 4
QDQCIYNTTYLNVQR_7_6_0_4_0 LeX peak identified
204.0861
366.1387
274.0917
138.0547
168.0652
292.1018
186.0755
657.2355
1059.995 PEP_0_1_0_0_0
126.0549
144.0656
454.1548
528.1937
Intensity
m/z
0
50000
100000
150000
200000
250000
300000
500
1000
1500
2000
2500

File: 231129_OE_RK_15, RT: 49.464, Scan: 063755
Type: HCD, FTMS + c NSI d Full ms2 1232.2460@hcd30.00 [120.0000-5050.4194]
Precursor Scan: 063676, m/z: 1231.4942, charge: 4
QDQCIYNTTYLNVQR_6_5_1_3_0 LeX peak identified

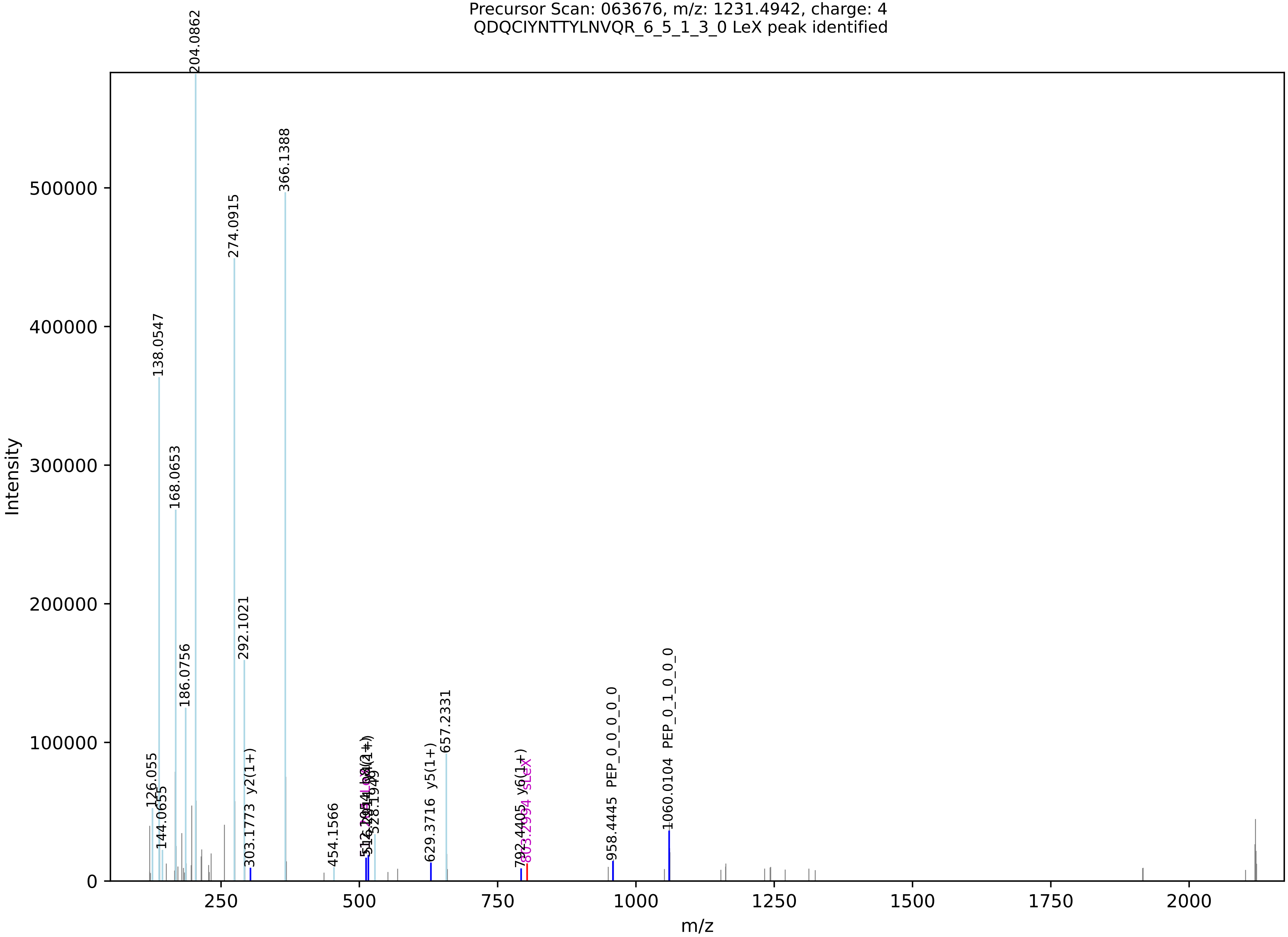

File: 231129_OE_RK_15, RT: 49.615, Scan: 063974
Type: HCD, FTMS + c NSI d Full ms2 1323.2786@hcd30.00 [120.0000-5421.8325]
Precursor Scan: 063894, m/z: 1322.7759, charge: 4
QDQCIYNTTYLNVQR_7_6_1_3_0 LeX peak identified

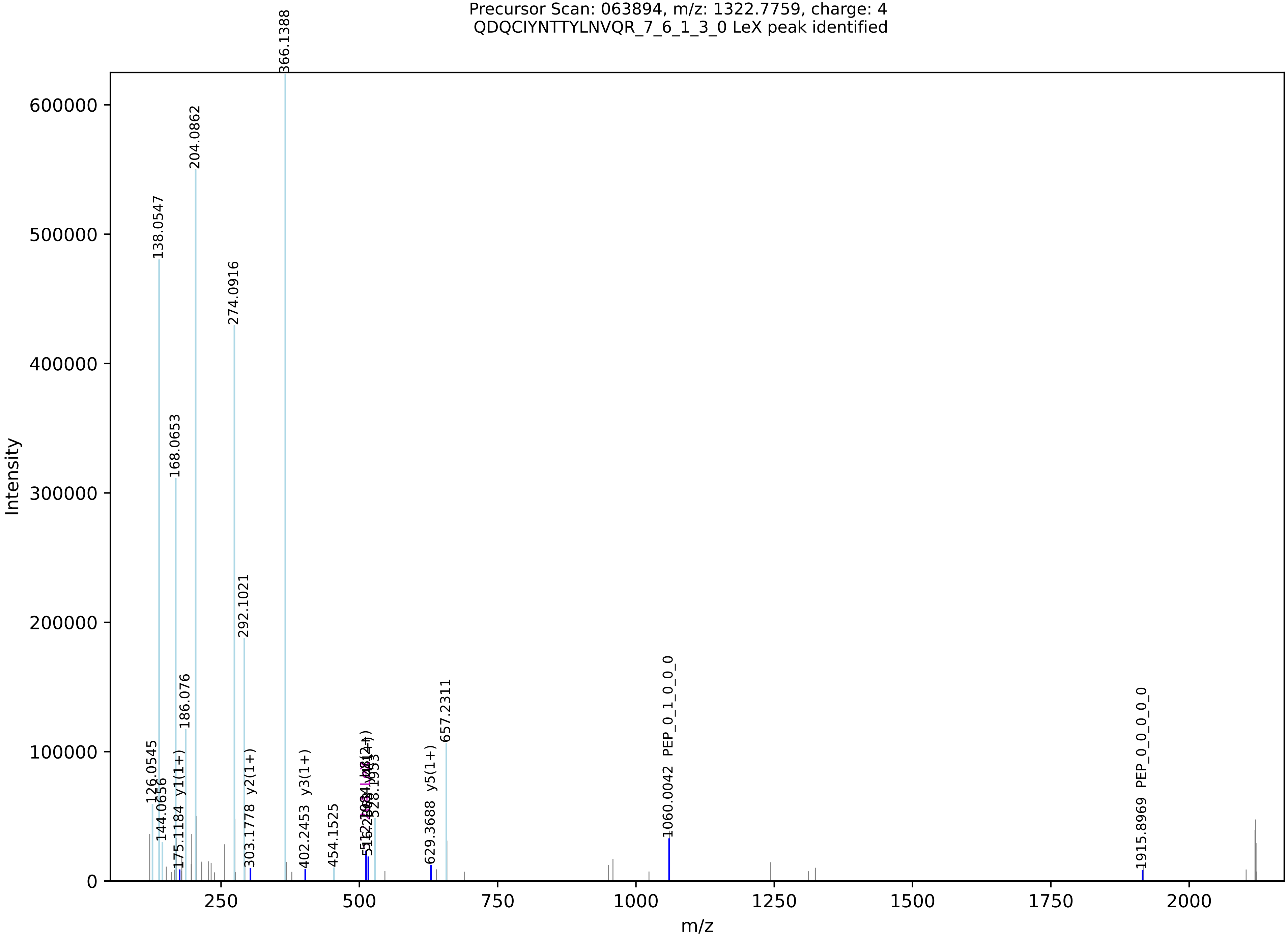

File: 231129_OE_RK_15, RT: 50.015, Scan: 064551
Type: HCD, FTMS + c NSI d Full ms2 1323.2782@hcd30.00 [120.0000-5421.8311]
Precursor Scan: 064476, m/z: 1322.7755, charge: 4
QDQCIYNTTYLNVQR_7_6_1_3_0 LeX peak identified

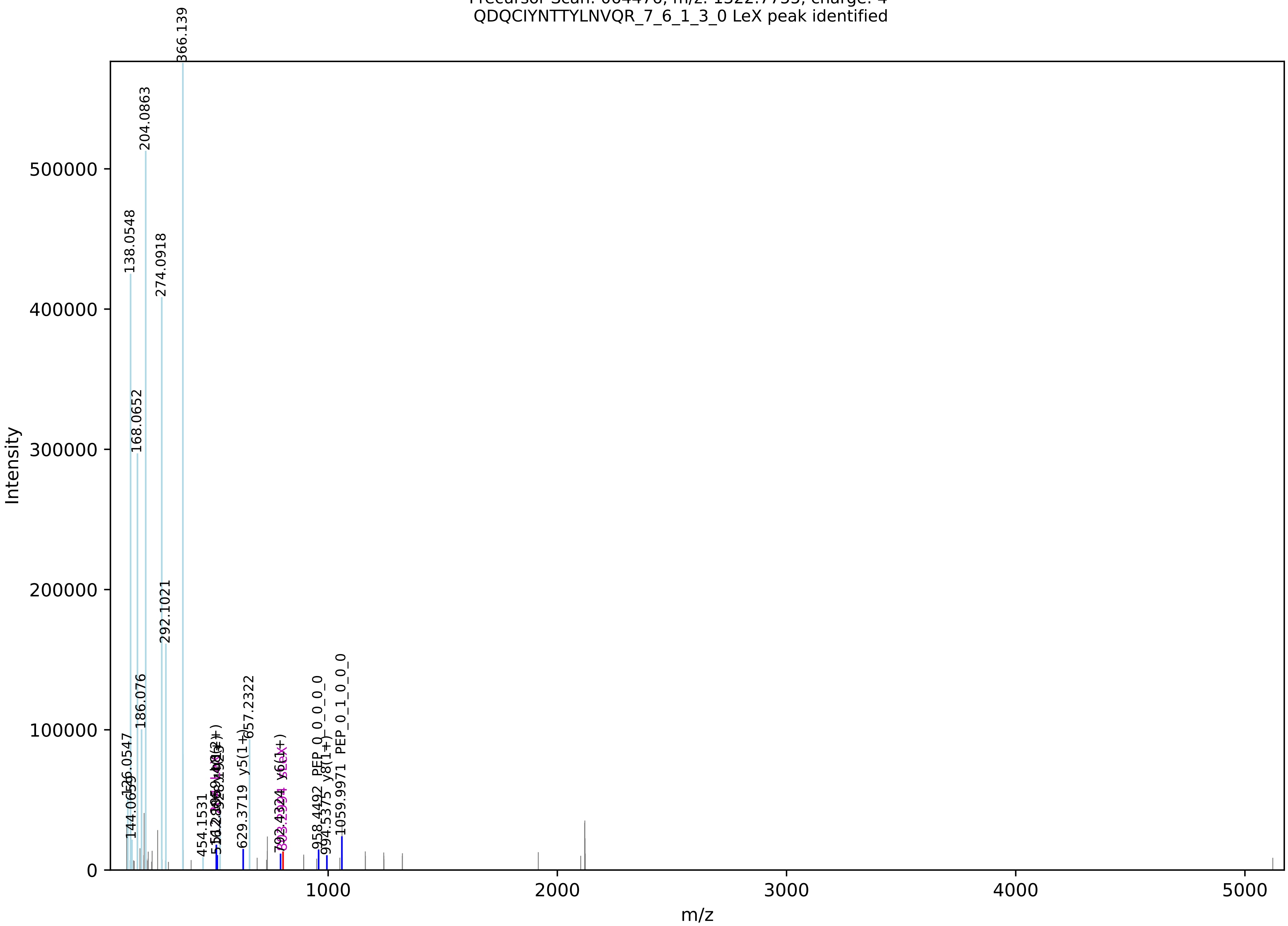

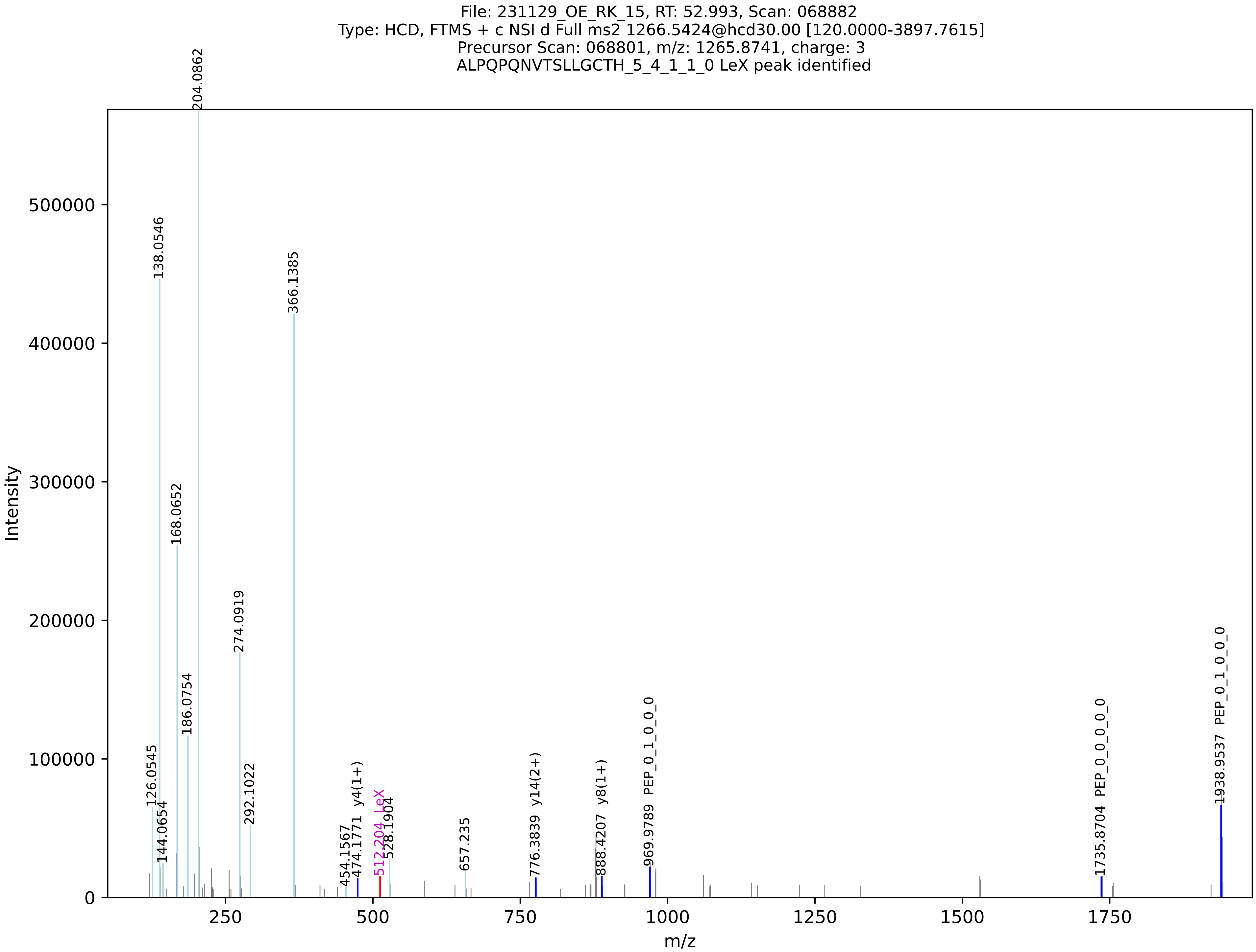

File: 231129_OE_RK_15, RT: 52.993, Scan: 068882
Type: HCD, FTMS + c NSI d Full ms2 1266.5424@hcd30.00 [120.0000-3897.7615]
Precursor Scan: 068801, m/z: 1265.8741, charge: 3
ALPQPQNVTSLLGCTH_5_4_1_1_0 LeX peak identified
Intensity
m/z
204.0862
138.0546
366.1385
168.0652
274.0919
186.0754
126.0545
144.0654
292.1022
454.1567
474.1771 y4(1+)
512.204_LeX
528.1904
657.235
776.3839 y14(2+)
888.4207 y8(1+)
969.9789 PEP_0_1_0_0_0
1735.8704 PEP_0_0_0_0_0
1938.9537 PEP_0_1_0_0_0

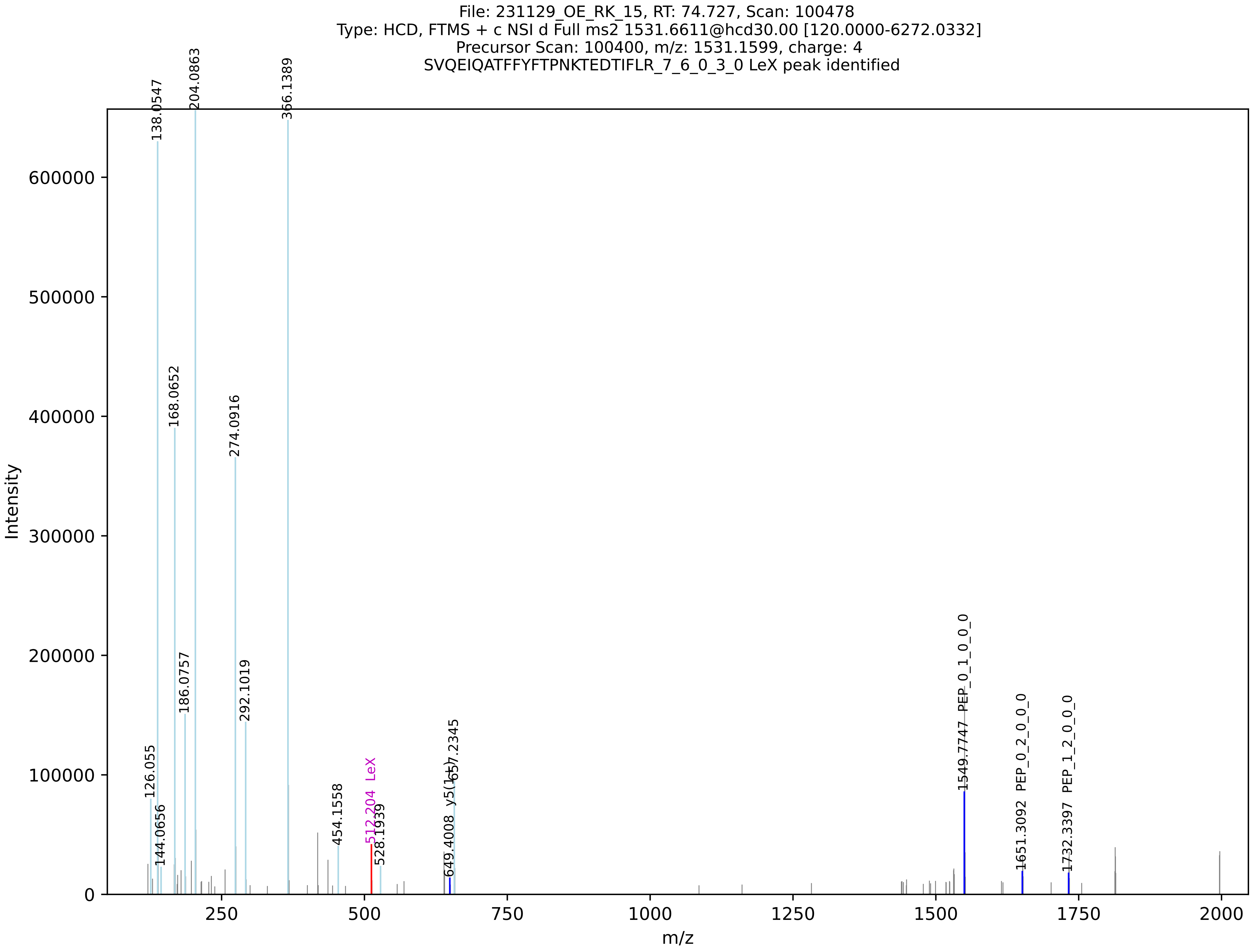

File: 231129_OE_RK_15, RT: 74.727, Scan: 100478
Type: HCD, FTMS + c NSI d Full ms2 1531.6611@hcd30.00 [120.0000-6272.0332]
Precursor Scan: 100400, m/z: 1531.1599, charge: 4
SVQEIQATFFYFTPNKTEDTIFLR_7_6_0_3_0 LeX peak identified
Intensity
m/z
138.0547
204.0863
366.1389
168.0652
274.0916
186.0757
292.1019
126.055
144.0656
454.1558
512.204 LeX
528.1939
649.4008 y5(1+)
657.2345
1549.7747 PEP_0_1_0_0_0
1651.3092 PEP_0_2_0_0_0
1732.3397 PEP_1_2_0_0_0

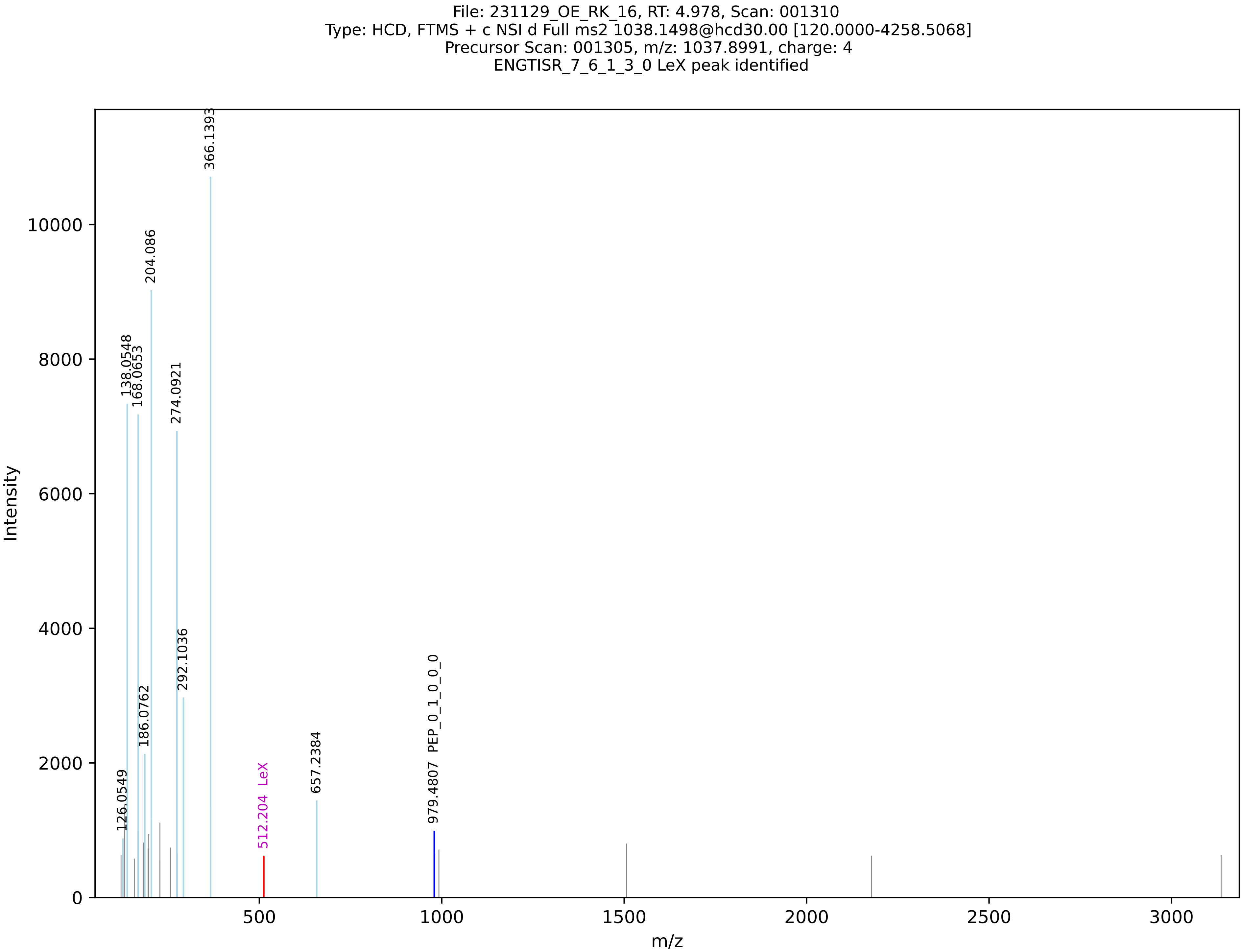

File: 231129_OE_RK_16, RT: 4.978, Scan: 001310
Type: HCD, FTMS + c NSI d Full ms2 1038.1498@hcd30.00 [120.0000-4258.5068]
Precursor Scan: 001305, m/z: 1037.8991, charge: 4
ENGTISR_7_6_1_3_0 LeX peak identified
Intensity
m/z
0
2000
4000
6000
8000
10000
500
1000
1500
2000
2500
3000
366.1393
204.086
138.0548
168.0653
274.0921
292.1036
186.0762
126.0549
512.204 LeX
657.2384
979.4807 PEP_0_1_0_0_0

File: 231129_OE_RK_16, RT: 5.076, Scan: 001401
Type: HCD, FTMS + c NSI d Full ms2 1433.2196@hcd30.00 [120.0000-4407.7939]
Precursor Scan: 001390, m/z: 1431.8791, charge: 3
ENGTISR_9_8_1_1_0 LeX peak identified

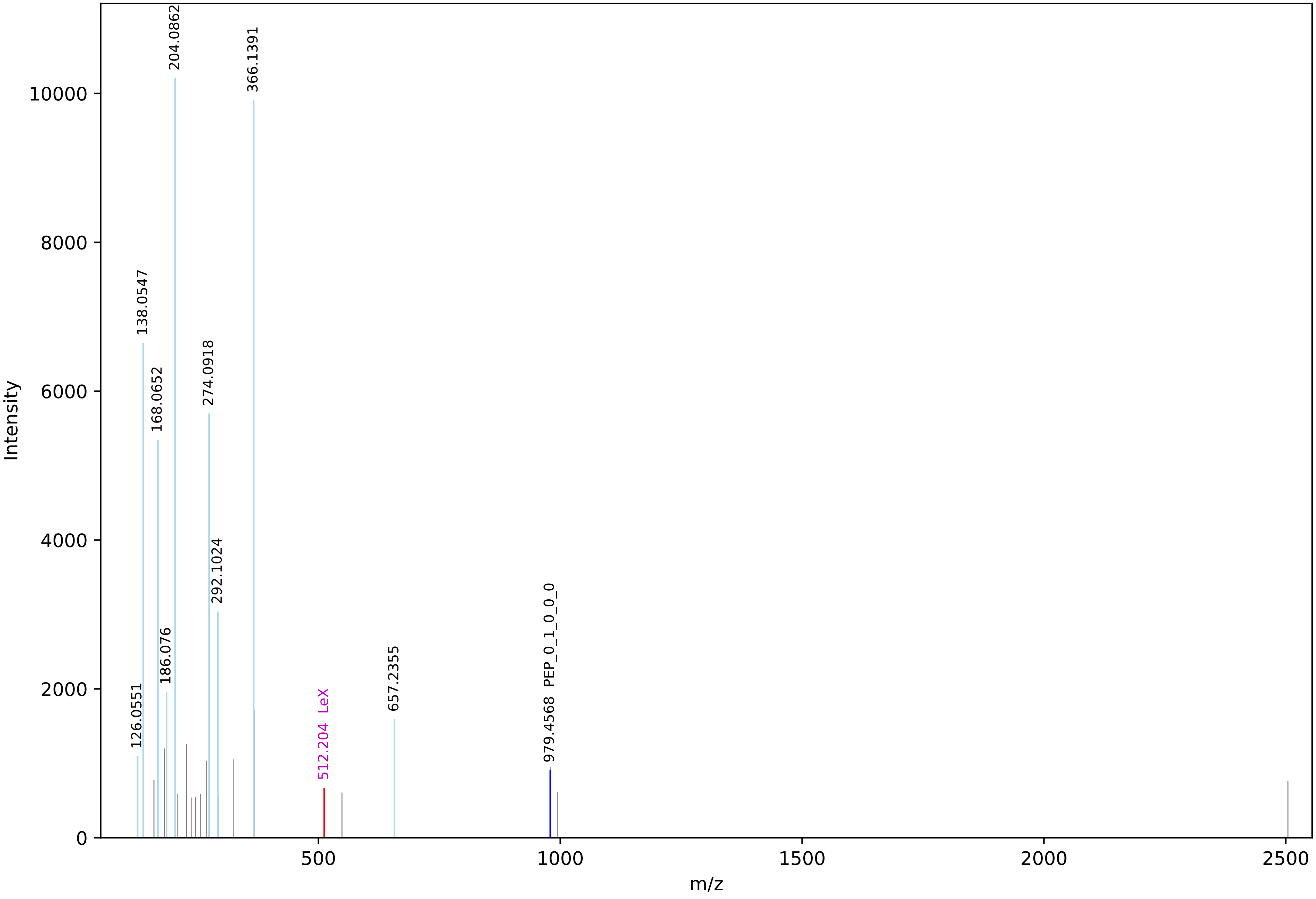

File: 231129_OE_RK_16, RT: 6.623, Scan: 003165
Type: HCD, FTMS + c NSI d Full ms2 1189.8018@hcd30.00 [120.0000-3662.9355]
Precursor Scan: 003117, m/z: 1189.4677, charge: 3
ENGTISR_7_6_1_1_0 LeX peak identified

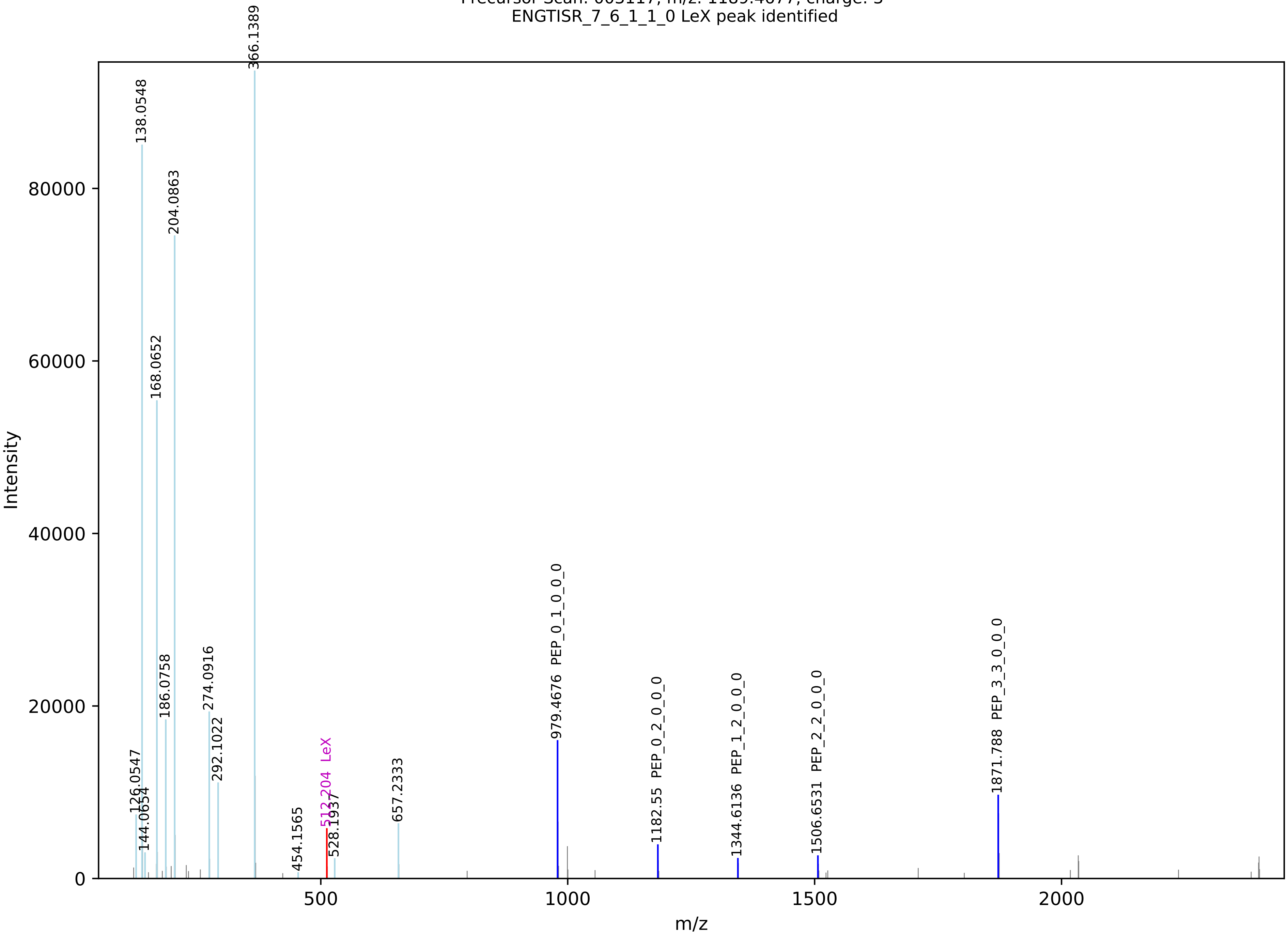

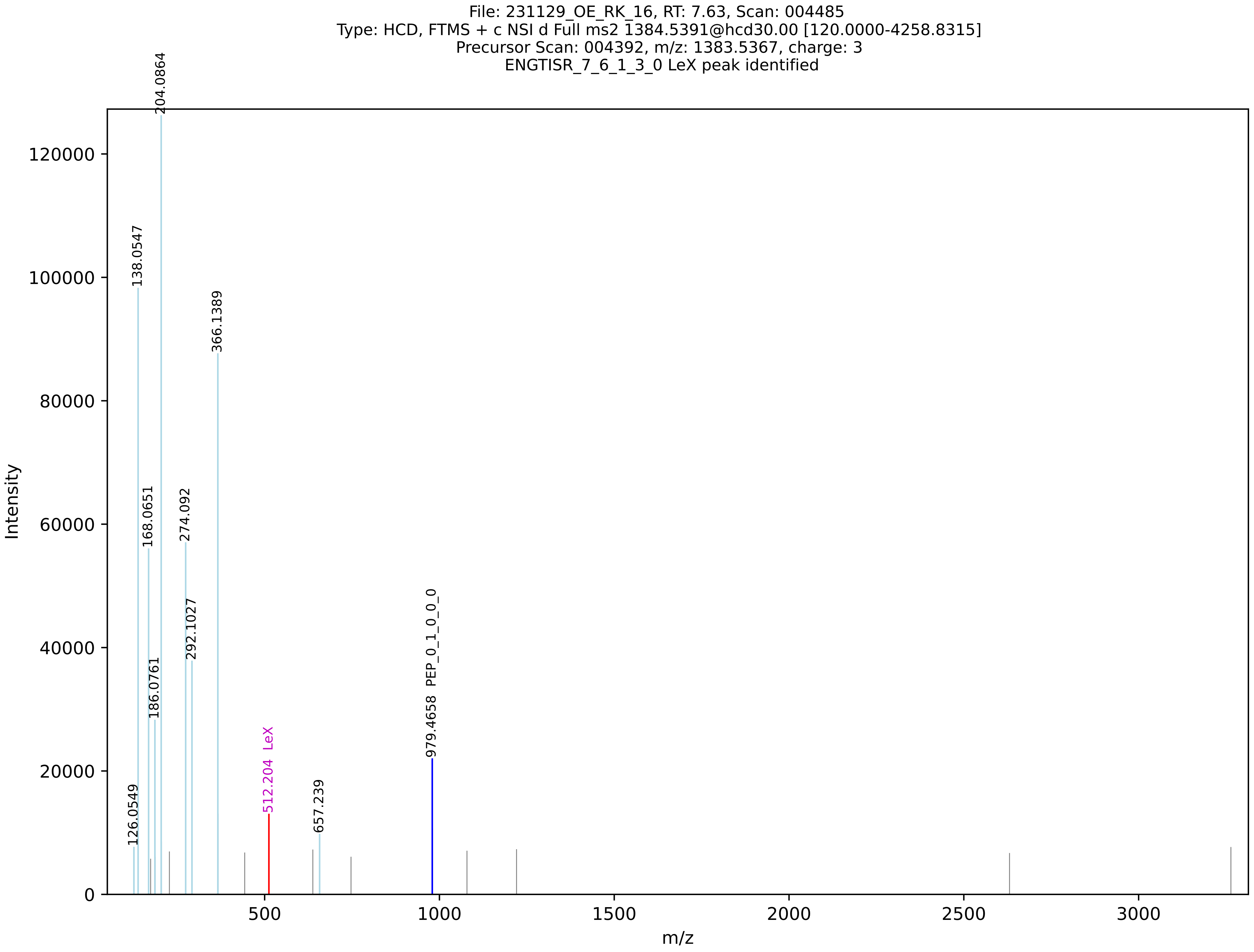
File: 231129_OE_RK_16, RT: 7.63, Scan: 004485
Type: HCD, FTMS + c NSI d Full ms2 1384.5391@hcd30.00 [120.0000-4258.8315]
Precursor Scan: 004392, m/z: 1383.5367, charge: 3
ENGTISR_7_6_1_3_0 LeX peak identified
204.0864
138.0547
366.1389
168.0651
274.092
292.1027
186.0761
979.4658 PEP_0_1_0_0_0_
512.204 LeX
657.239
126.0549
Intensity
m/z
0
20000
40000
60000
80000
100000
120000
500
1000
1500
2000
2500
3000

File: 231129_OE_RK_16, RT: 7.69, Scan: 004551
Type: HCD, FTMS + c NSI d Full ms2 1165.4563@hcd30.00 [120.0000-3588.4382]
Precursor Scan: 004529, m/z: 1164.788, charge: 3
ENGTISR_6_5_1_2_0 LeX peak identified

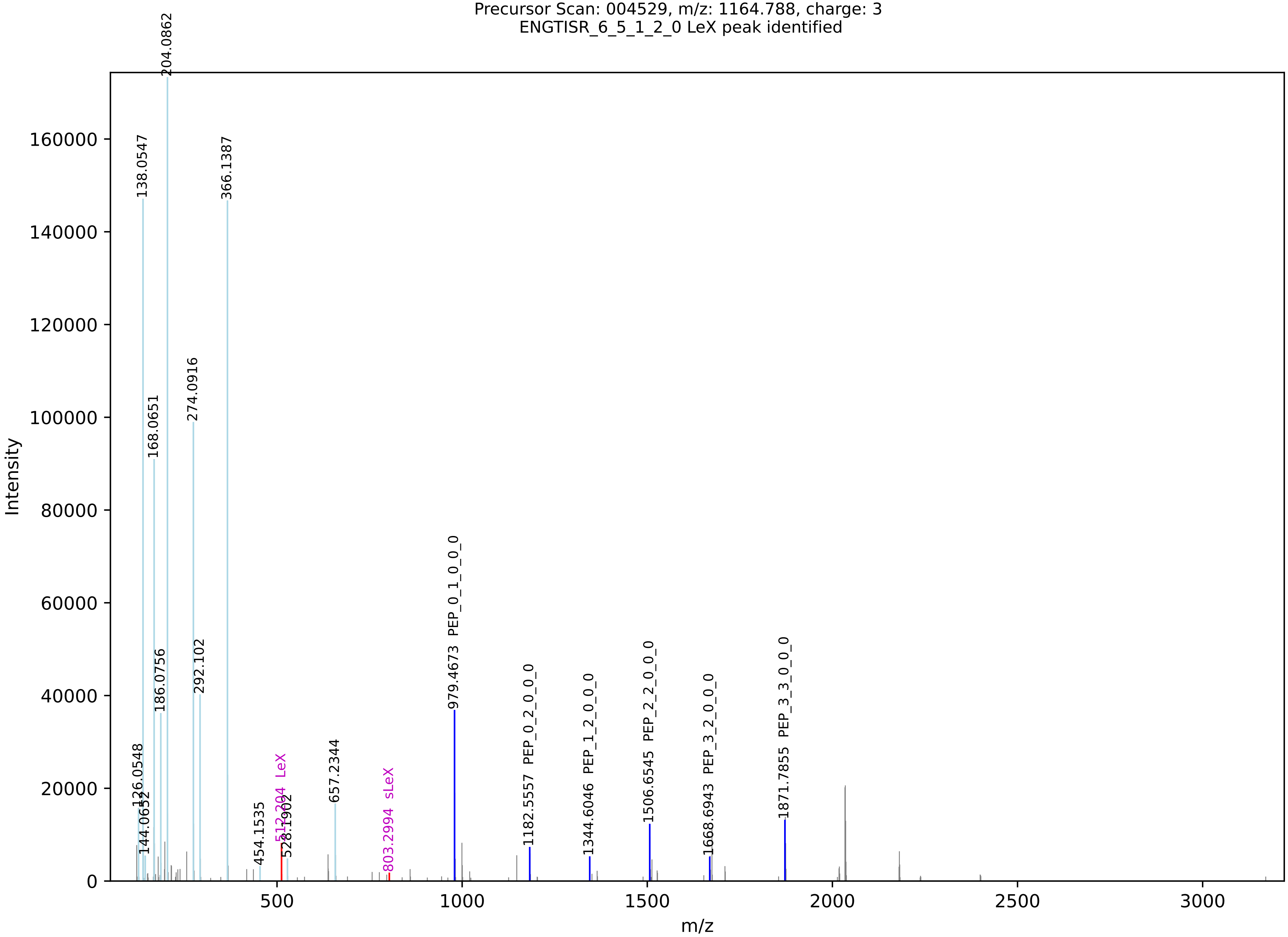

File: 231129_OE_RK_16, RT: 8.925, Scan: 006311
Type: HCD, FTMS + c NSI d Full ms2 1147.6880@hcd30.00 [120.0000-4705.4229]
Precursor Scan: 006231, m/z: 1147.1874, charge: 4
ENGTISR_7_6_2_4_0 LeX peak identified

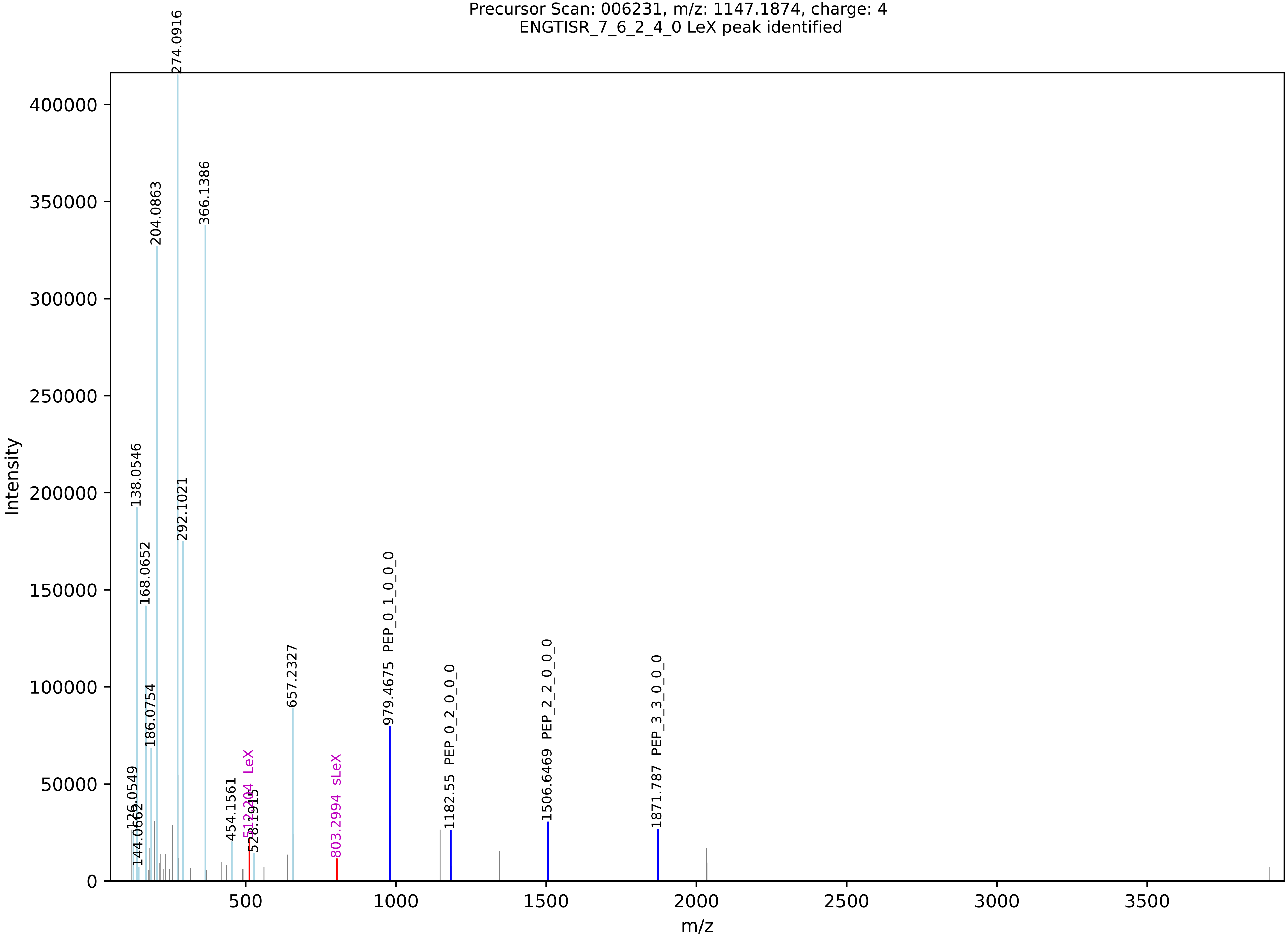

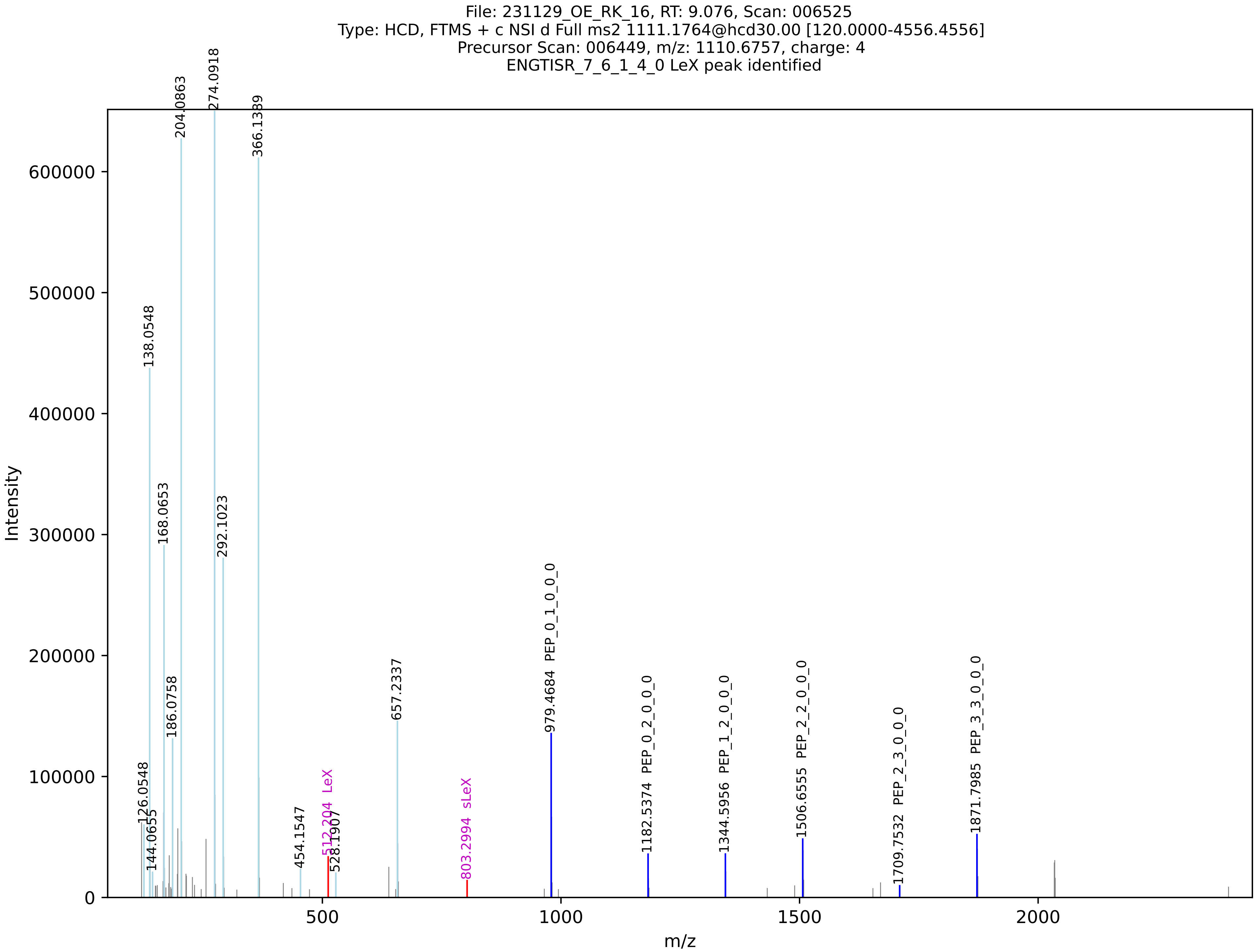
File: 231129_OE_RK_16, RT: 9.076, Scan: 006525
Type: HCD, FTMS + c NSI d Full ms2 1111.1764@hcd30.00 [120.0000-4556.4556]
Precursor Scan: 006449, m/z: 1110.6757, charge: 4
ENGTISR_7_6_1_4_0 LeX peak identified
Intensity
m/z
274.0918
204.0863
366.1389
138.0548
168.0653
292.1023
186.0758
126.0548
144.0655
454.1547
512.204 LeX
528.1907
657.2337
803.2994 sLeX
979.4684 PEP_0_1_0_0_0
1182.5374 PEP_0_2_0_0_0
1344.5956 PEP_1_2_0_0_0
1506.6555 PEP_2_2_0_0_0
1709.7532 PEP_2_3_0_0_0
1871.7985 PEP_3_3_0_0_0

File: 231129_OE_RK_16, RT: 9.273, Scan: 006811
Type: HCD, FTMS + c NSI d Full ms2 1111.4268@hcd30.00 [120.0000-4557.4775]
Precursor Scan: 006738, m/z: 1110.6753, charge: 4
ENGTISR_7_6_1_4_0 LeX peak identified

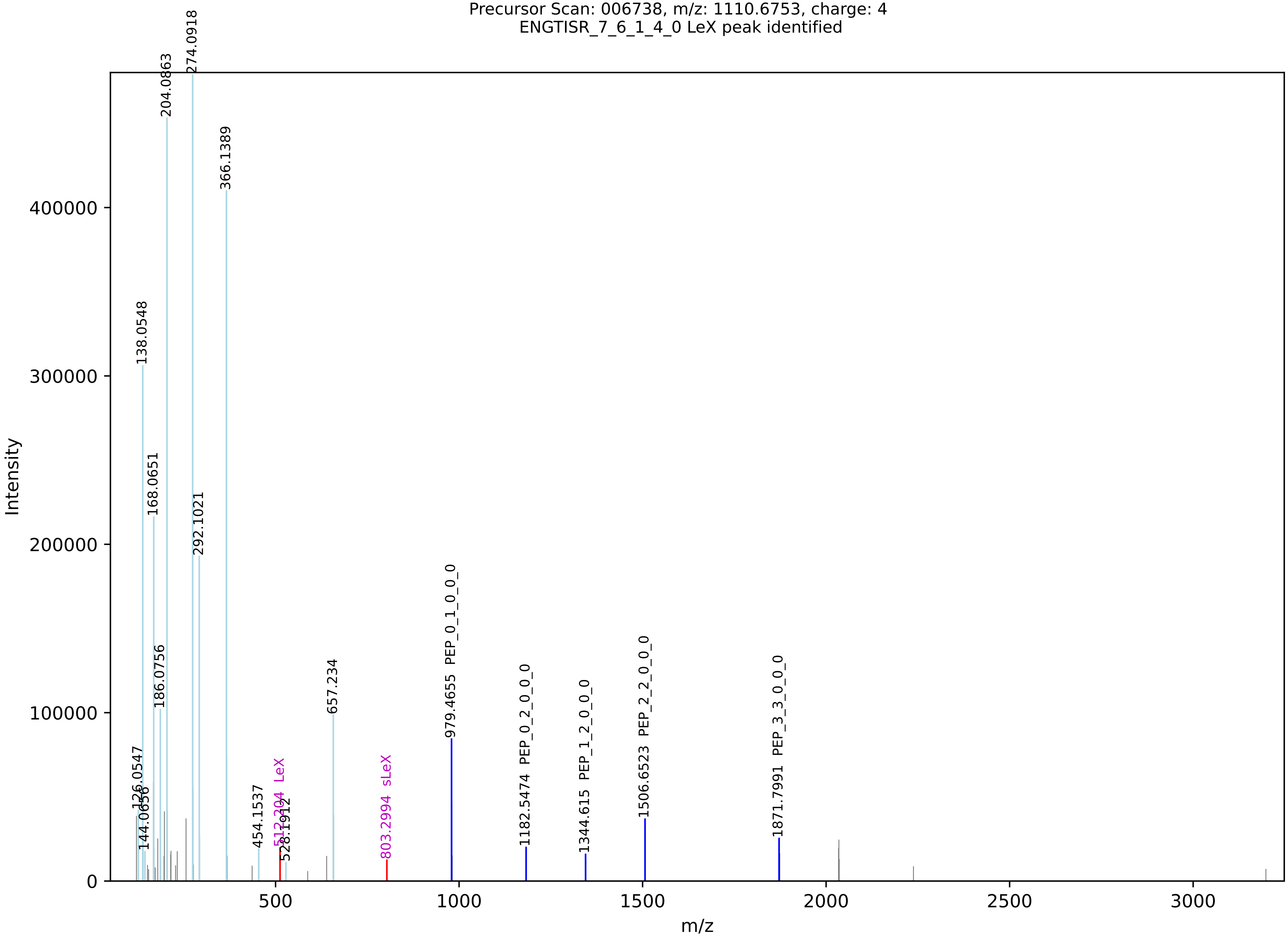

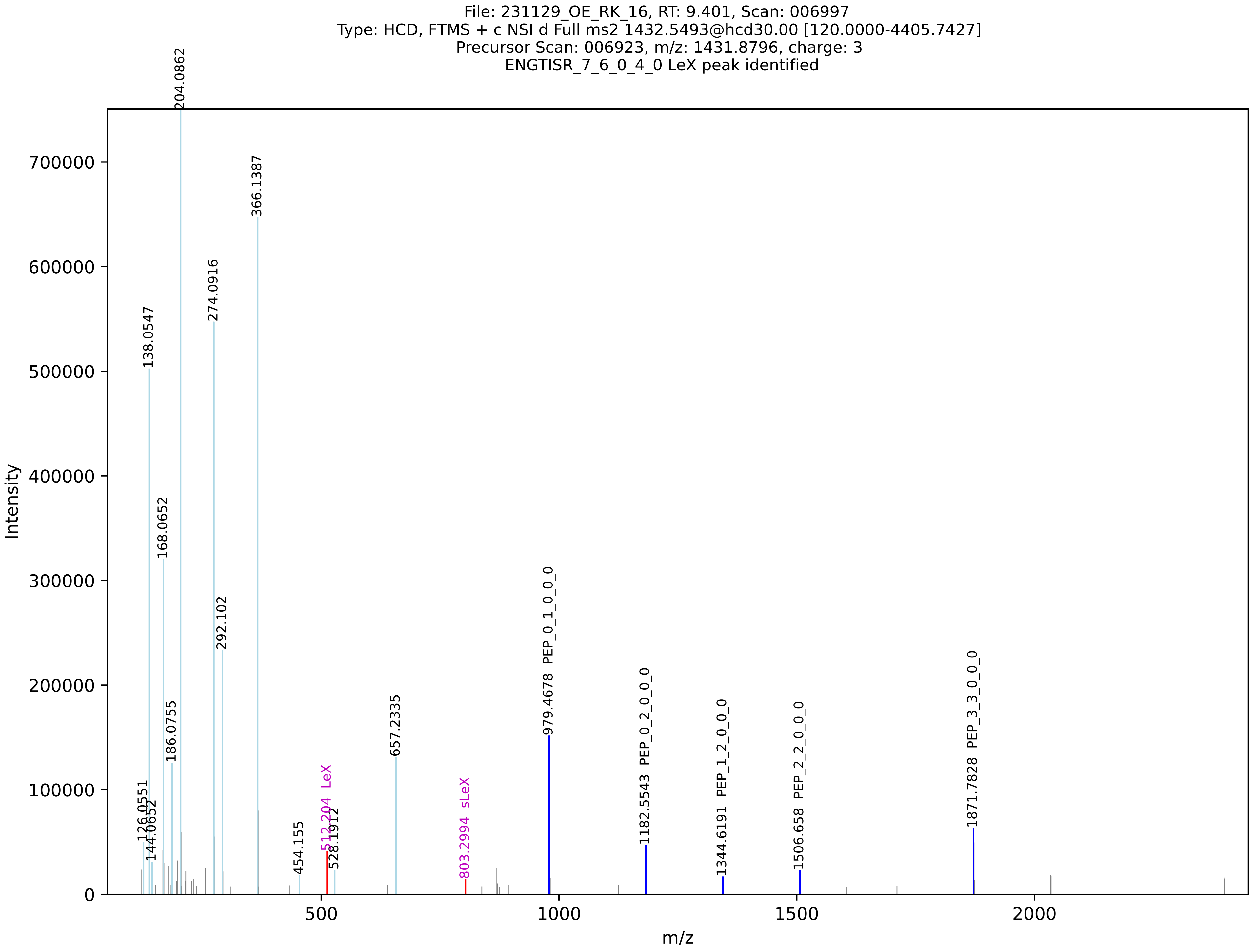

File: 231129_OE_RK_16, RT: 9.401, Scan: 006997
Type: HCD, FTMS + c NSI d Full ms2 1432.5493@hcd30.00 [120.0000-4405.7427]
Precursor Scan: 006923, m/z: 1431.8796, charge: 3
ENGTISR_7_6_0_4_0 LeX peak identified
Intensity
m/z
204.0862
366.1387
274.0916
138.0547
168.0652
292.102
186.0755
126.0551
144.0652
454.155
512.204 LeX
528.1912
657.2335
803.2994 sLeX
979.4678 PEP_0_1_0_0_0
1182.5543 PEP_0_2_0_0_0
1344.6191 PEP_1_2_0_0_0
1506.658 PEP_2_2_0_0_0
1871.7828 PEP_3_3_0_0_0

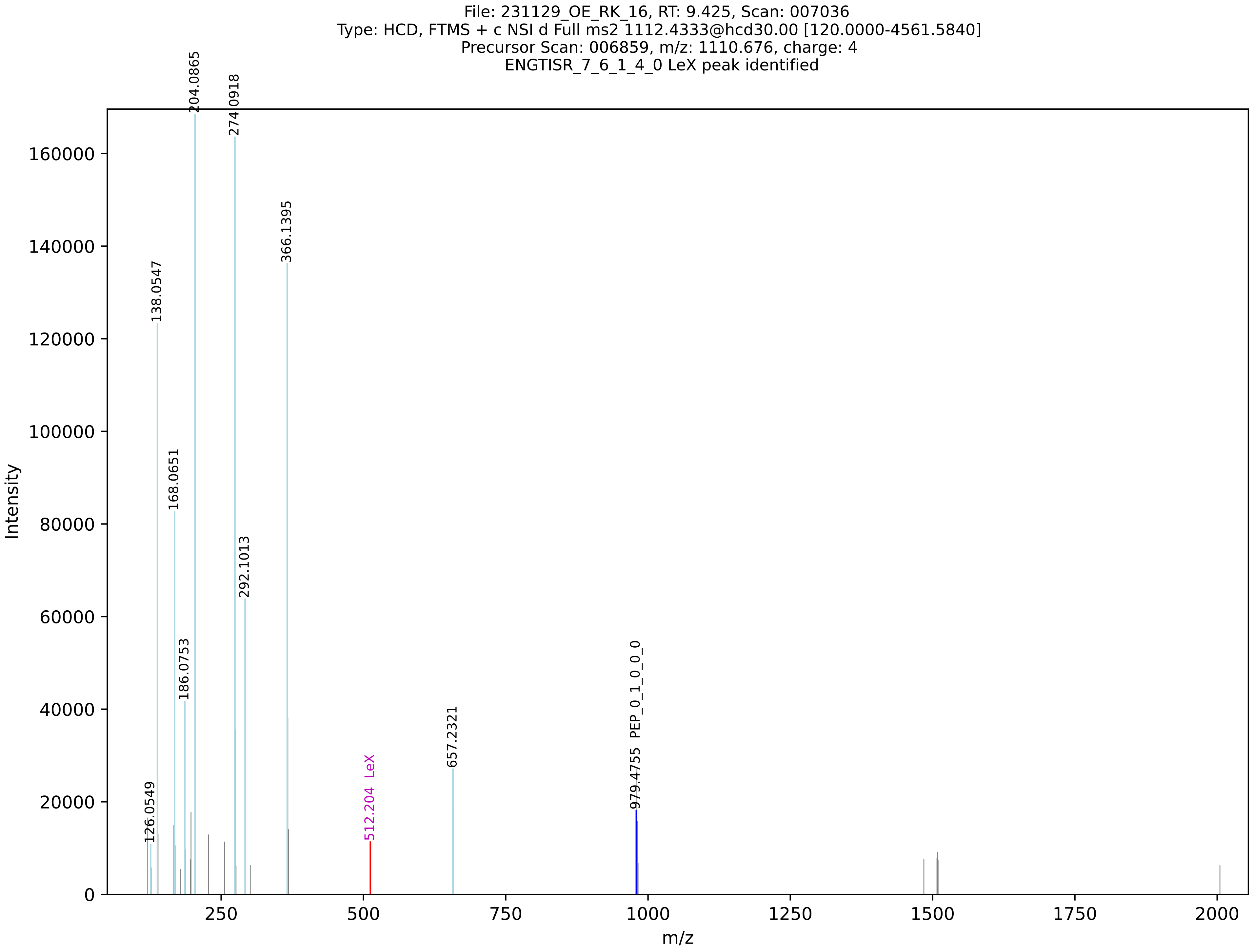
File: 231129_OE_RK_16, RT: 9.425, Scan: 007036
Type: HCD, FTMS + c NSI d Full ms2 1112.4333@hcd30.00 [120.0000-4561.5840]
Precursor Scan: 006859, m/z: 1110.676, charge: 4
ENGTISR_7_6_1_4_0 LeX peak identified
Intensity
m/z
204.0865
274.0918
366.1395
138.0547
168.0651
292.1013
186.0753
126.0549
512.204 LeX
657.2321
979.4755 PEP_0_1_0_0_0
0
20000
40000
60000
80000
100000
120000
140000
160000
250
500
750
1000
1250
1500
1750
2000

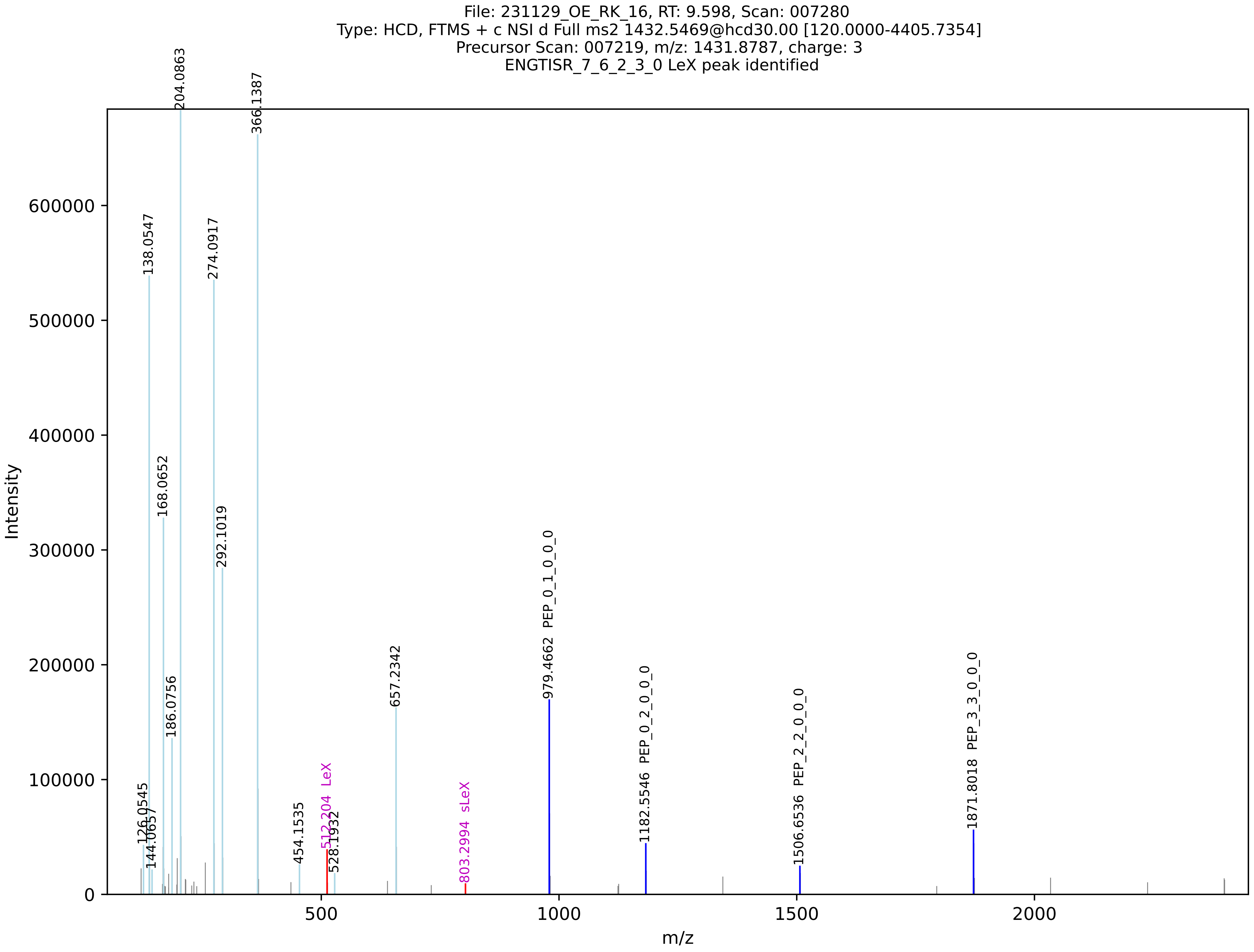

File: 231129_OE_RK_16, RT: 9.598, Scan: 007280
Type: HCD, FTMS + c NSI d Full ms2 1432.5469@hcd30.00 [120.0000-4405.7354]
Precursor Scan: 007219, m/z: 1431.8787, charge: 3
ENGTISR_7_6_2_3_0 LeX peak identified
Intensity
m/z
204.0863
366.1387
138.0547
274.0917
168.0652
292.1019
186.0756
126.0545
144.0657
454.1535
512.204 LeX
528.1932
657.2342
803.2994 sLeX
979.4662 PEP_0_1_0_0_0
1182.5546 PEP_0_2_0_0_0
1506.6536 PEP_2_2_0_0_0
1871.8018 PEP_3_3_0_0_0

File: 231129_OE_RK_16, RT: 9.672, Scan: 007392
Type: HCD, FTMS + c NSI d Full ms2 1112.6814@hcd30.00 [120.0000-4562.5962]
Precursor Scan: 007219, m/z: 1110.6741, charge: 4
ENGTISR_7_6_1_4_0 LeX peak identified

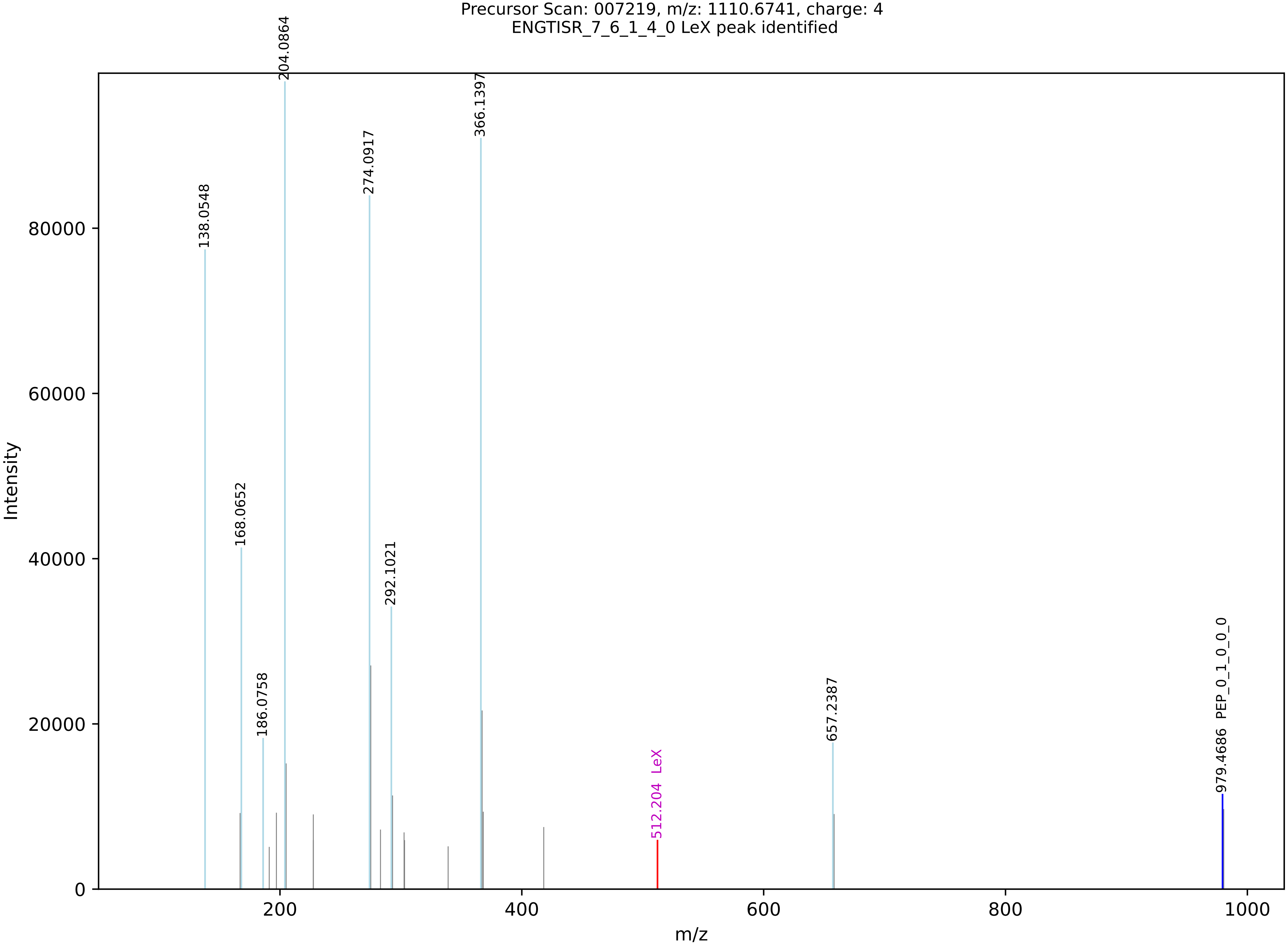

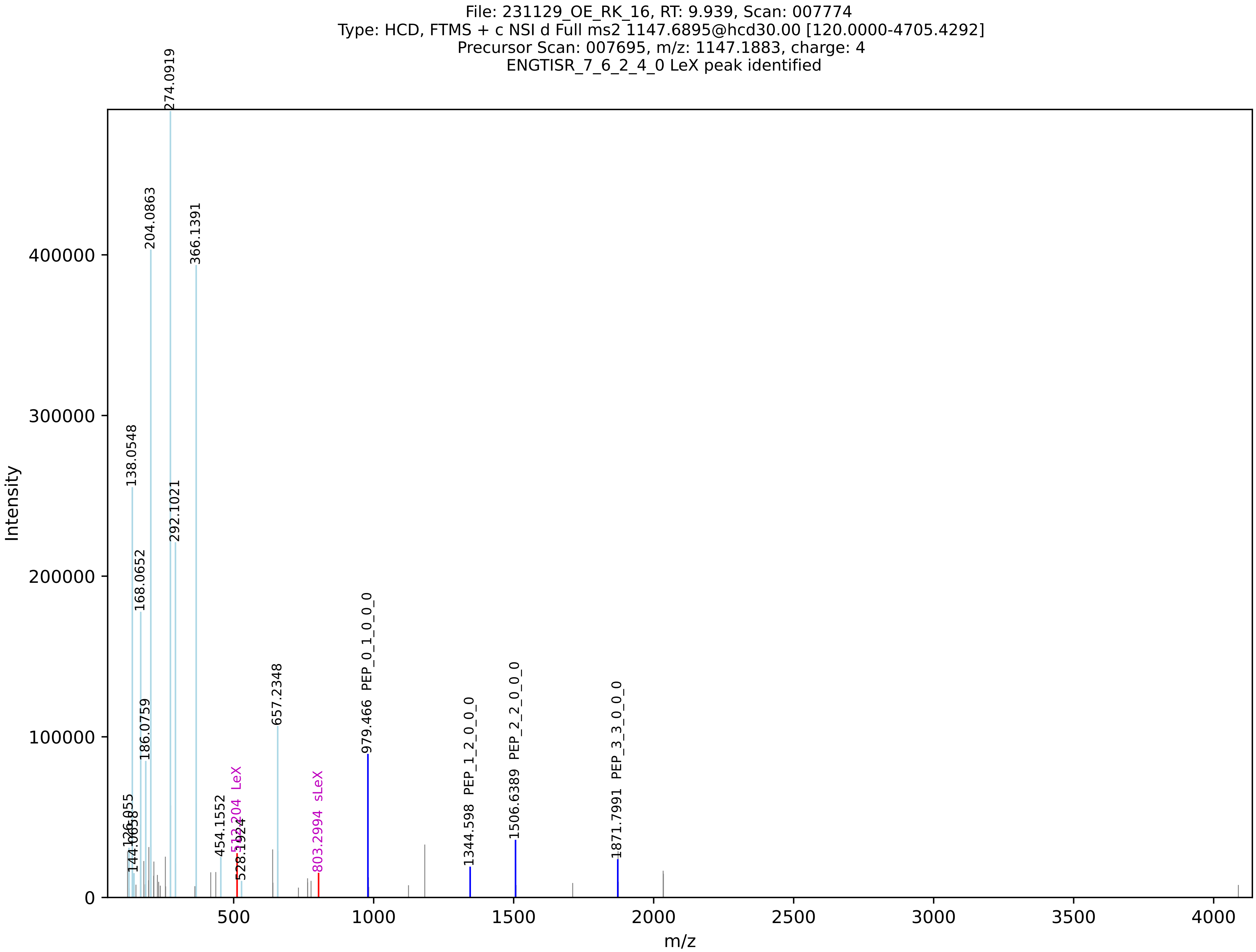

File: 231129_OE_RK_16, RT: 9.939, Scan: 007774
Type: HCD, FTMS + c NSI d Full ms2 1147.6895@hcd30.00 [120.0000-4705.4292]
Precursor Scan: 007695, m/z: 1147.1883, charge: 4
ENGTISR_7_6_2_4_0 LeX peak identified
Intensity
m/z
274.0919
204.0863
366.1391
138.0548
292.1021
168.0652
186.0759
657.2348
979.466 PEP_0_1_0_0_
1344.598 PEP_1_2_0_0_0
1506.6389 PEP_2_2_0_0_0
1871.7991 PEP_3_3_0_0_
454.1552
512.204 LeX
528.1924
803.2994 sLeX
126.055
144.0658
0
100000
200000
300000
400000
500
1000
1500
2000
2500
3000
3500
4000

File: 231129_OE_RK_16, RT: 10.084, Scan: 007984
Type: HCD, FTMS + c NSI d Full ms2 1310.8413@hcd30.00 [120.0000-4033.3167]
Precursor Scan: 007907, m/z: 1310.5072, charge: 3
ENGTISR_6_5_2_3_0 LeX peak identified

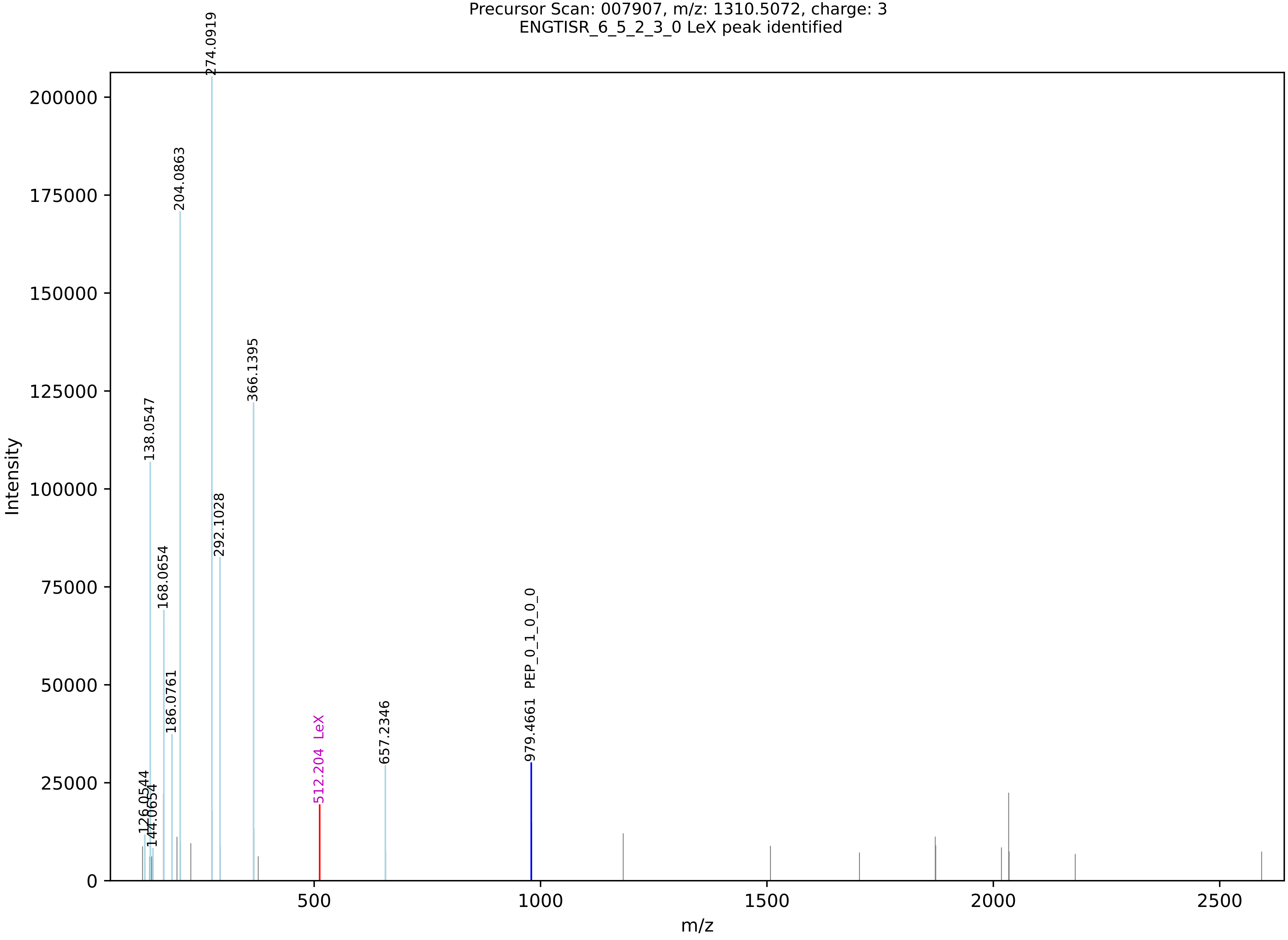

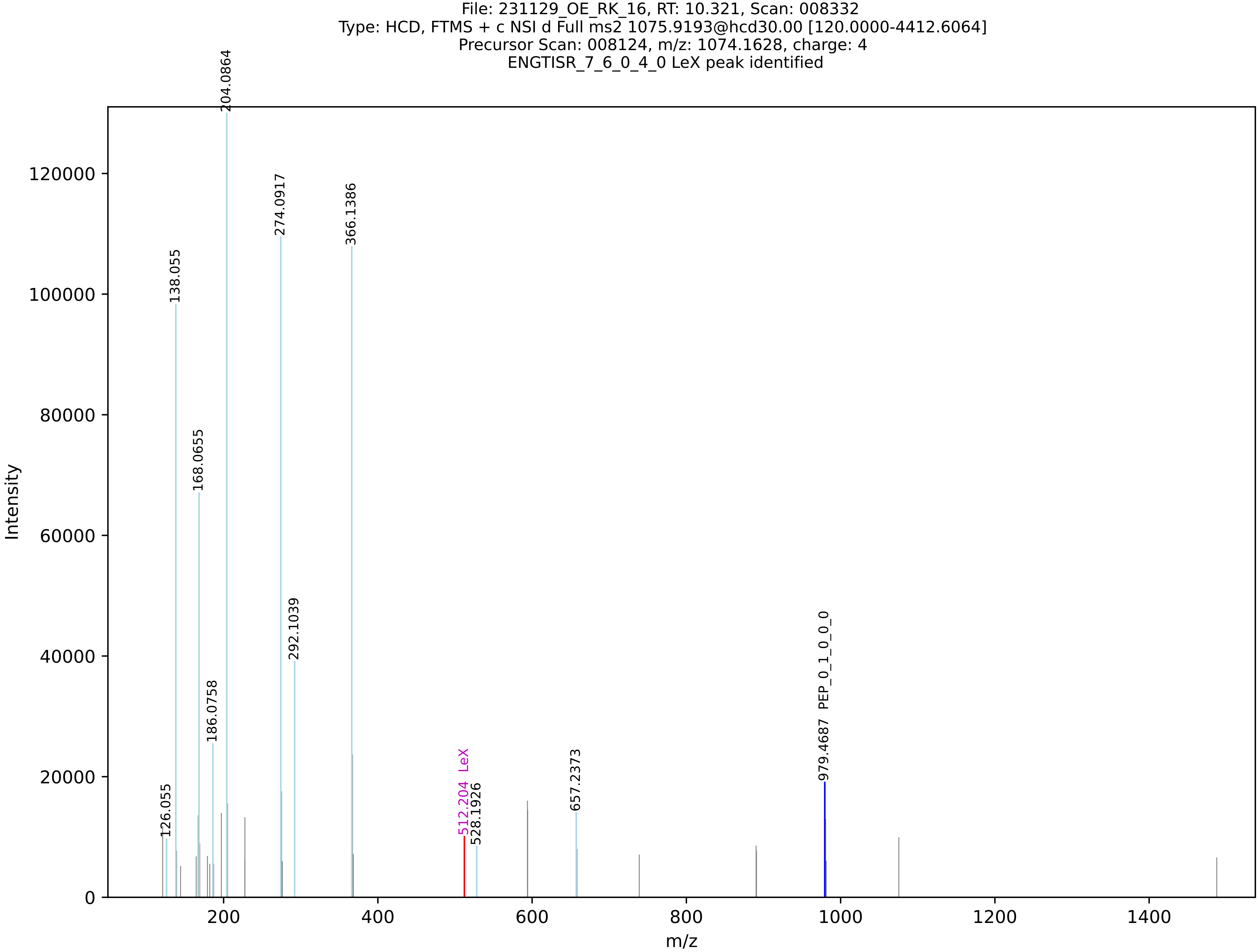
File: 231129_OE_RK_16, RT: 10.321, Scan: 008332
Type: HCD, FTMS + c NSI d Full ms2 1075.9193@hcd30.00 [120.0000-4412.6064]
Precursor Scan: 008124, m/z: 1074.1628, charge: 4
ENGTISR_7_6_0_4_0 LeX peak identified
Intensity
m/z
204.0864
274.0917
366.1386
138.055
168.0655
292.1039
186.0758
126.055
512.204 LeX
528.1926
657.2373
979.4687 PEP_0_1_0_0_0_
0
20000
40000
60000
80000
100000
120000
200
400
600
800
1000
1200
1400

File: 231129_OE_RK_16, RT: 10.588, Scan: 008712
Type: HCD, FTMS + c NSI d Full ms2 1147.6909@hcd30.00 [120.0000-4705.4346]
Precursor Scan: 008634, m/z: 1146.4703, charge: 4
ENGTISR_7_6_2_4_0 LeX peak identified

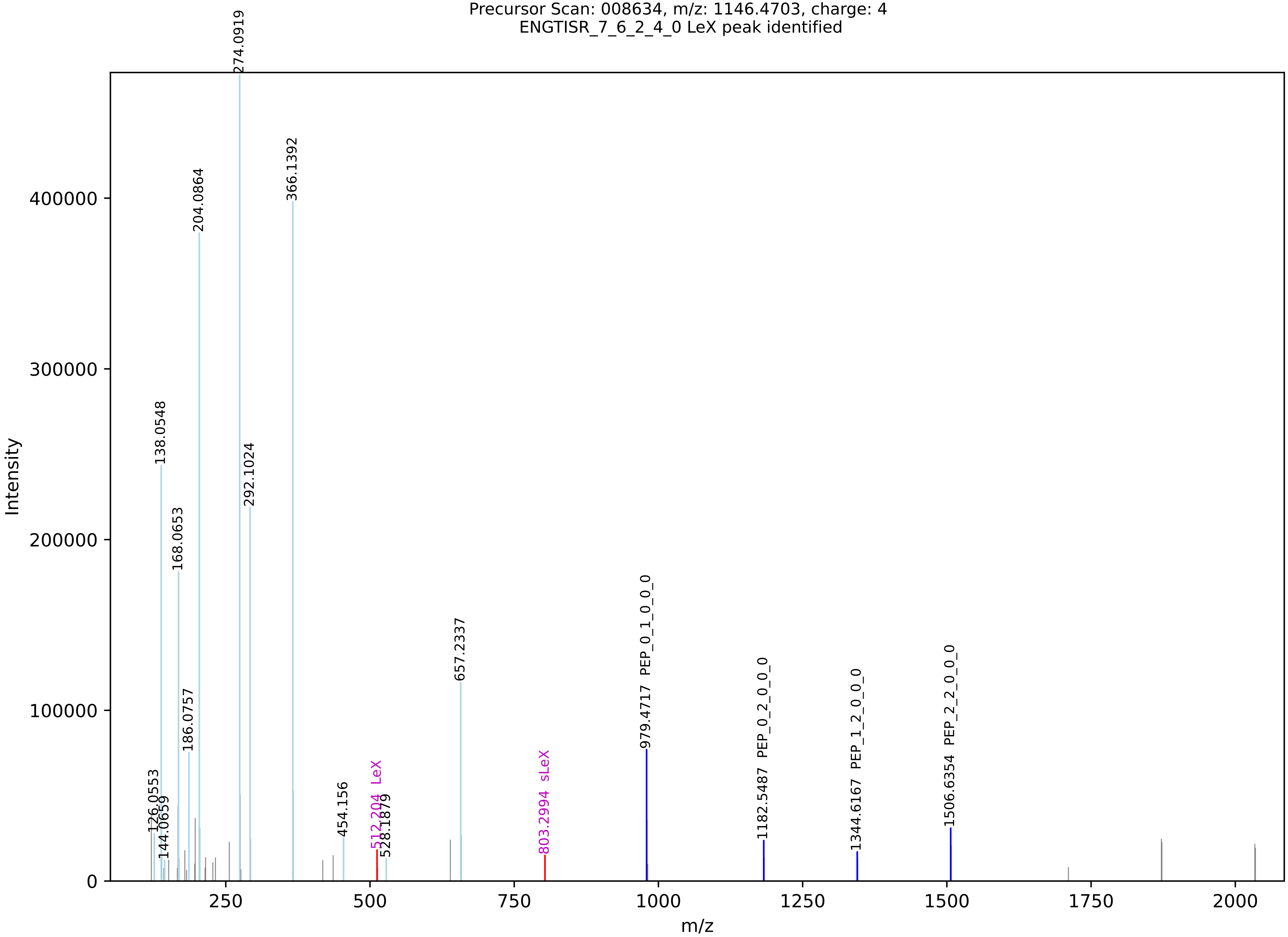

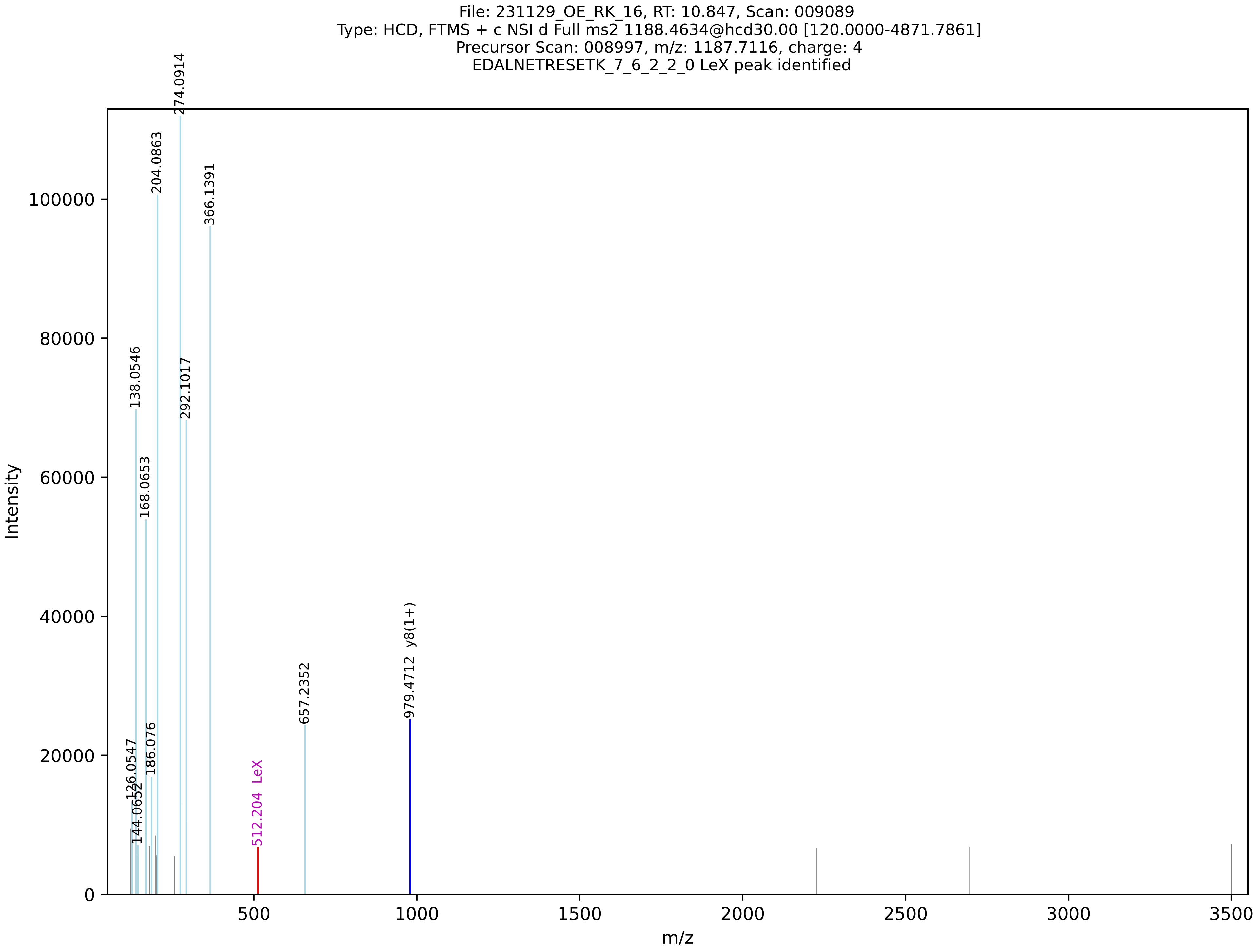
File: 231129_OE_RK_16, RT: 10.847, Scan: 009089
Type: HCD, FTMS + c NSI d Full ms2 1188.4634@hcd30.00 [120.0000-4871.7861]
Precursor Scan: 008997, m/z: 1187.7116, charge: 4
EDALNETRESETK_7_6_2_2_0 LeX peak identified
Intensity
m/z
274.0914
204.0863
366.1391
138.0546
292.1017
168.0653
186.076
126.0547
144.0652
512.204 LeX
657.2352
979.4712 y8(1+)
0
20000
40000
60000
80000
100000
500
1000
1500
2000
2500
3000
3500

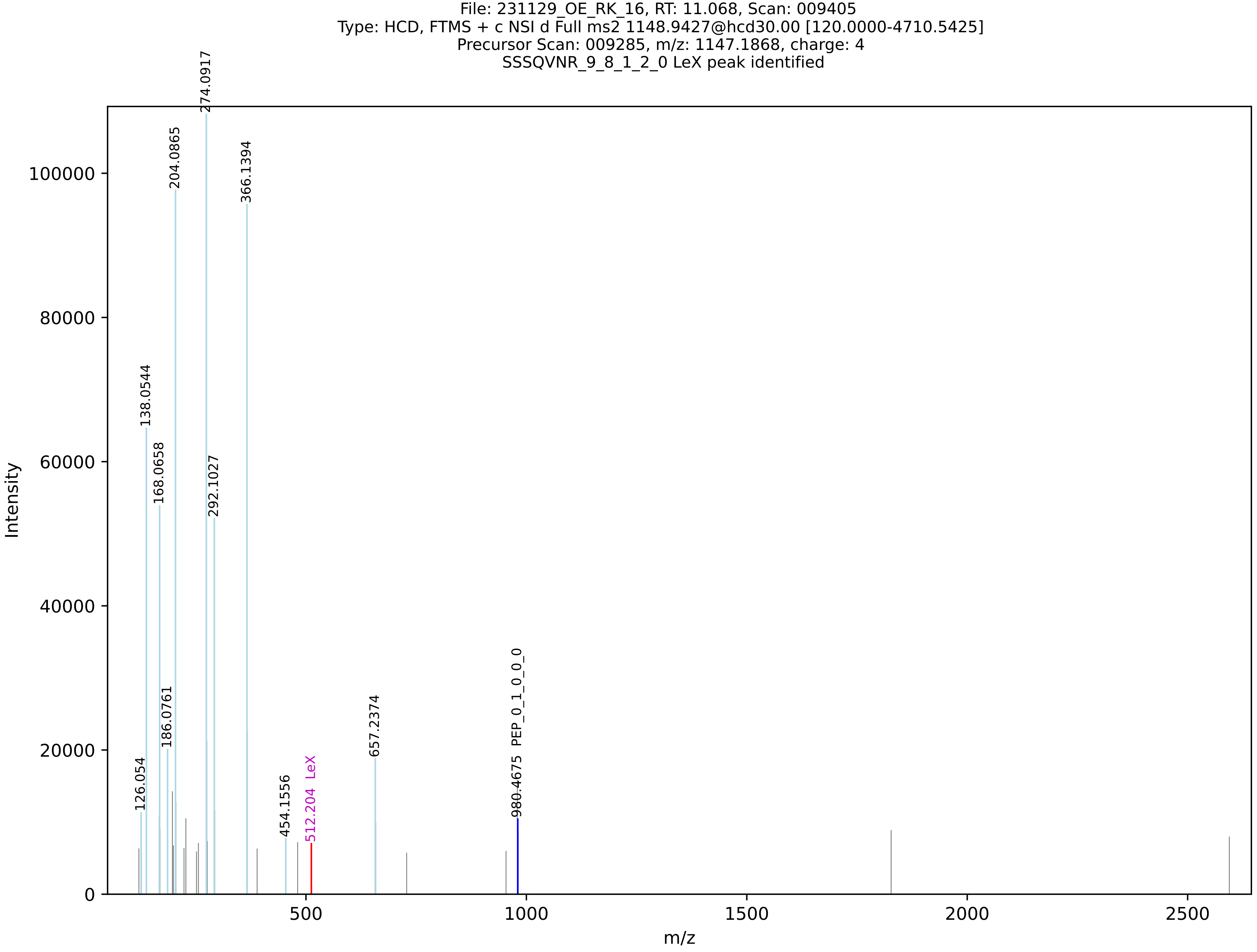

File: 231129_OE_RK_16, RT: 11.068, Scan: 009405
Type: HCD, FTMS + c NSI d Full ms2 1148.9427@hcd30.00 [120.0000-4710.5425]
Precursor Scan: 009285, m/z: 1147.1868, charge: 4
SSSQVNR_9_8_1_2_0 LeX peak identified
Intensity
m/z
274.0917
204.0865
366.1394
138.0544
168.0658
292.1027
186.0761
126.054
454.1556
512.204 LeX
657.2374
980.4675 PEP_0_1_0_0_0

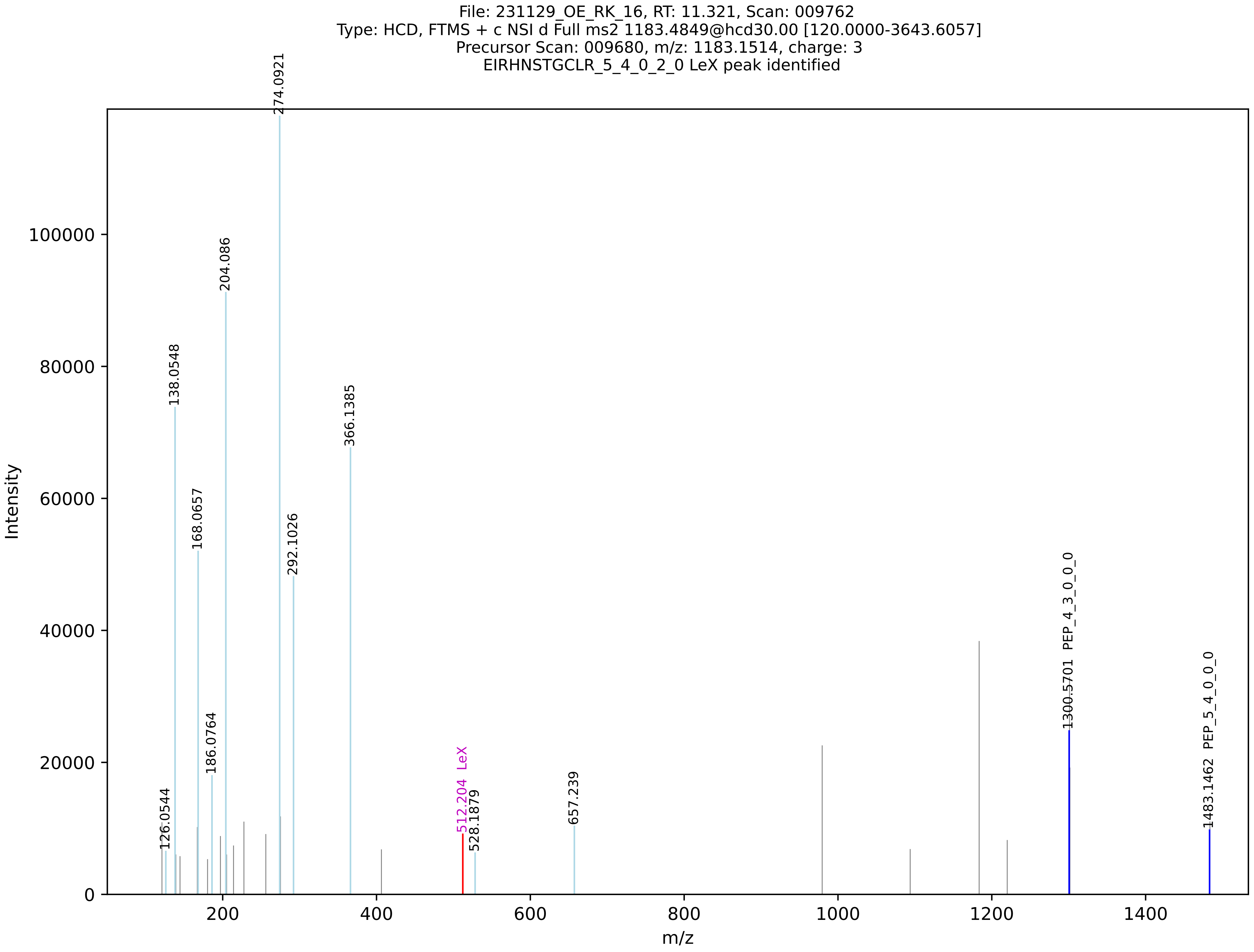
File: 231129_OE_RK_16, RT: 11.321, Scan: 009762
Type: HCD, FTMS + c NSI d Full ms2 1183.4849@hcd30.00 [120.0000-3643.6057]
Precursor Scan: 009680, m/z: 1183.1514, charge: 3
EIRHNSTGCLR_5_4_0_2_0 LeX peak identified
Intensity
m/z
274.0921
204.086
138.0548
168.0657
366.1385
292.1026
186.0764
126.0544
512.204 LeX
528.1879
657.239
1300.5701 PEP_4_3_0_0_0
1483.1462 PEP_5_4_0_0_0
0
20000
40000
60000
80000
100000
200
400
600
800
1000
1200
1400

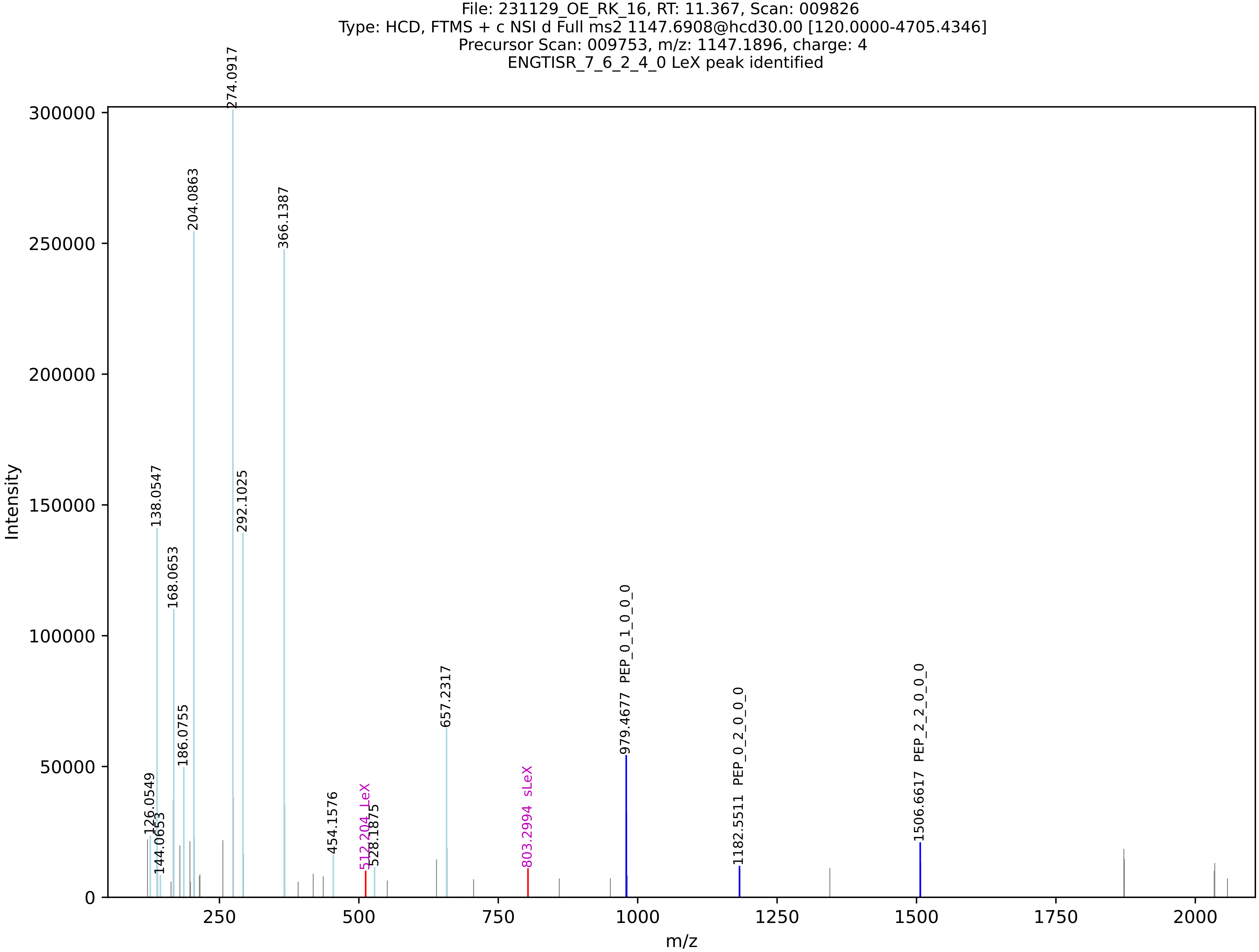
File: 231129_OE_RK_16, RT: 11.367, Scan: 009826
Type: HCD, FTMS + c NSI d Full ms2 1147.6908@hcd30.00 [120.0000-4705.4346]
Precursor Scan: 009753, m/z: 1147.1896, charge: 4
ENGTISR_7_6_2_4_0 LeX peak identified
Intensity
m/z
274.0917
204.0863
366.1387
138.0547
292.1025
168.0653
186.0755
126.0549
144.0653
454.1576
512.204_LeX
528.1875
657.2317
803.2994 sLeX
979.4677 PEP_0_1_0_0_0_
1182.5511 PEP_0_2_0_0_0
1506.6617 PEP_2_2_0_0_0

File: 231129_OE_RK_16, RT: 12.273, Scan: 011103
Type: HCD, FTMS + c NSI d Full ms2 1116.1798@hcd30.00 [120.0000-4576.8696]
Precursor Scan: 010982, m/z: 1114.6785, charge: 4
EDALNETRESETK_7_6_0_2_0 LeX peak identified

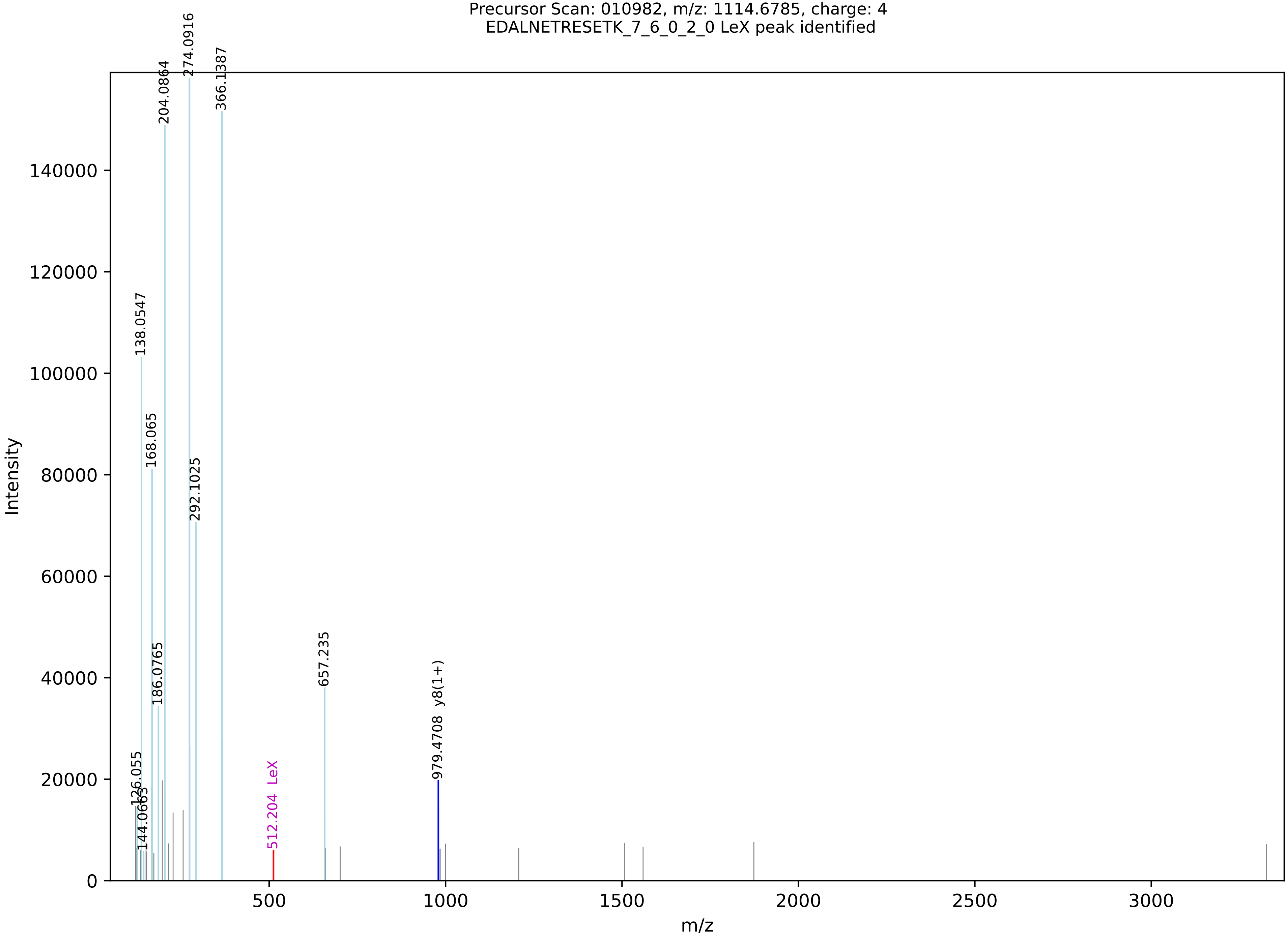

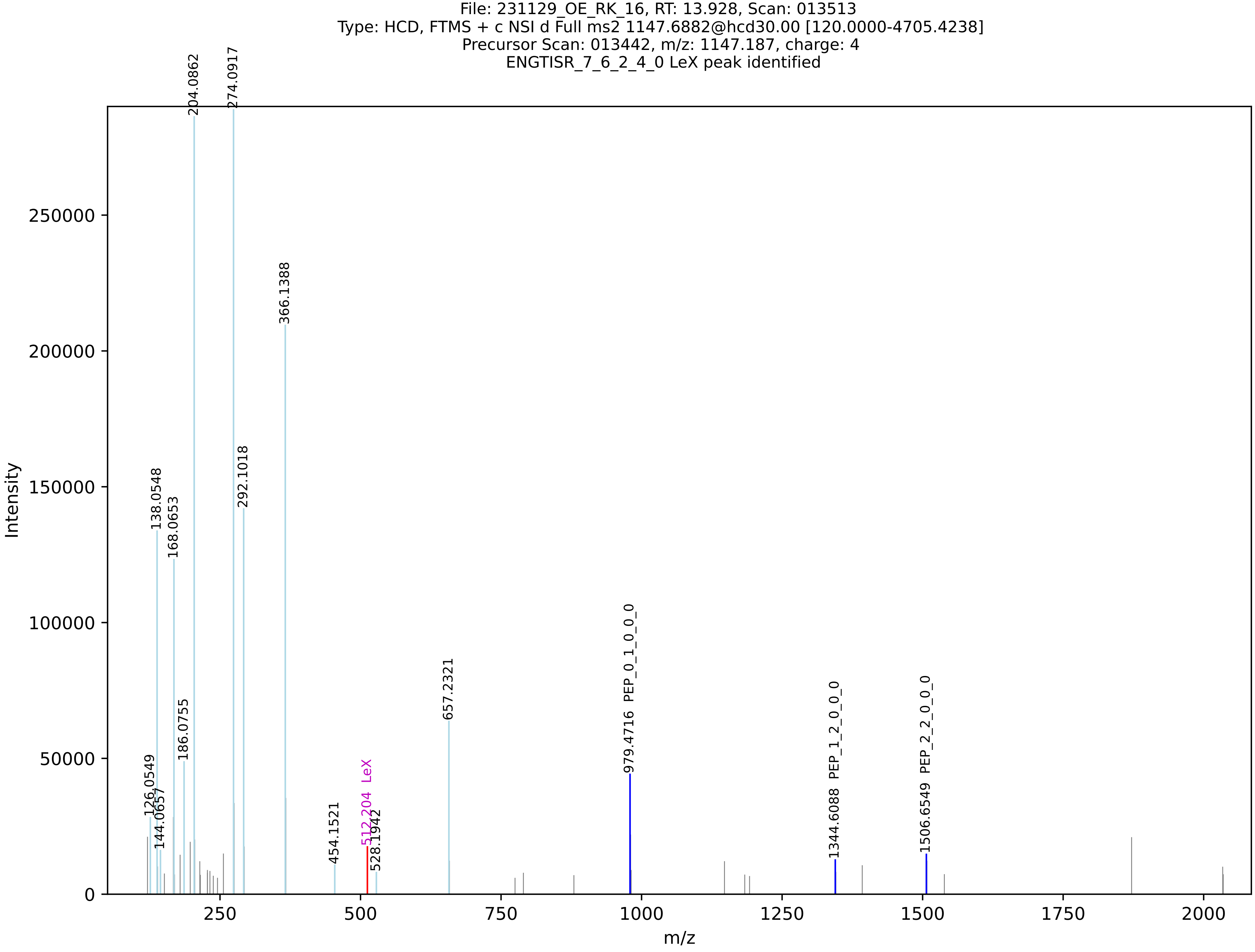

File: 231129_OE_RK_16, RT: 13.928, Scan: 013513
Type: HCD, FTMS + c NSI d Full ms2 1147.6882@hcd30.00 [120.0000-4705.4238]
Precursor Scan: 013442, m/z: 1147.187, charge: 4
ENGTISR_7_6_2_4_0 LeX peak identified
Intensity
m/z
204.0862
274.0917
366.1388
292.1018
138.0548
168.0653
186.0755
126.0549
144.0657
454.1521
512.204 LeX
528.1942
657.2321
979.4716 PEP_0_1_0_0_0
1344.6088 PEP_1_2_0_0_0
1506.6549 PEP_2_2_0_0_0

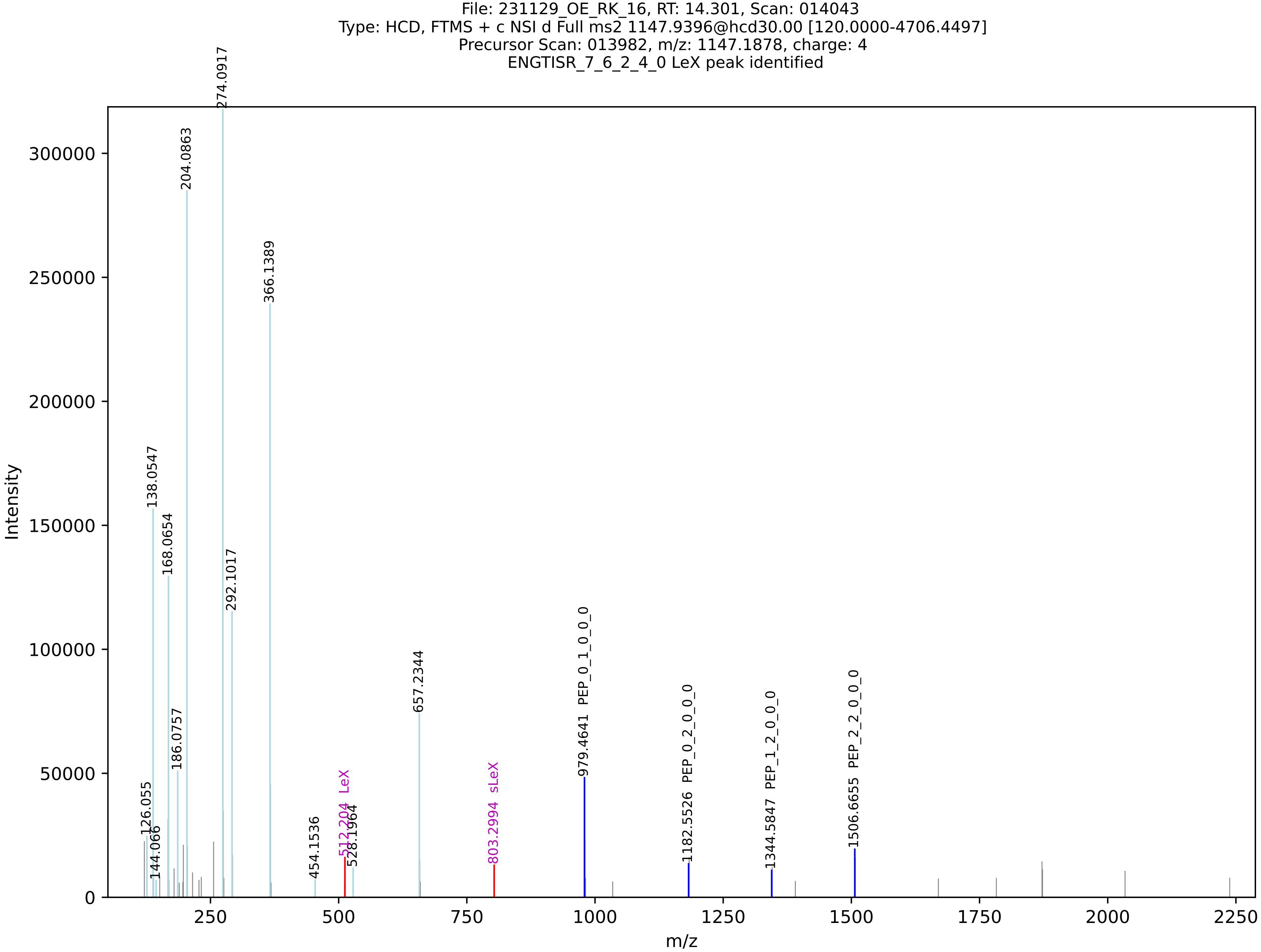
File: 231129_OE_RK_16, RT: 14.301, Scan: 014043
Type: HCD, FTMS + c NSI d Full ms2 1147.9396@hcd30.00 [120.0000-4706.4497]
Precursor Scan: 013982, m/z: 1147.1878, charge: 4
ENGTISR_7_6_2_4_0 LeX peak identified
Intensity
m/z
274.0917
204.0863
366.1389
138.0547
168.0654
292.1017
186.0757
126.055
144.066
454.1536
512.204 LeX
528.1964
657.2344
803.2994 sLeX
979.4641 PEP_0_1_0_0_0_
1182.5526 PEP_0_2_0_0_0_
1344.5847 PEP_1_2_0_0_0_
1506.6655 PEP_2_2_0_0_0_

File: 231129_OE_RK_16, RT: 14.577, Scan: 014442
Type: HCD, FTMS + c NSI d Full ms2 1530.2511@hcd30.00 [120.0000-4704.7104]
Precursor Scan: 014375, m/z: 1529.2487, charge: 3
ENGTISR_7_6_2_4_0 LeX peak identified

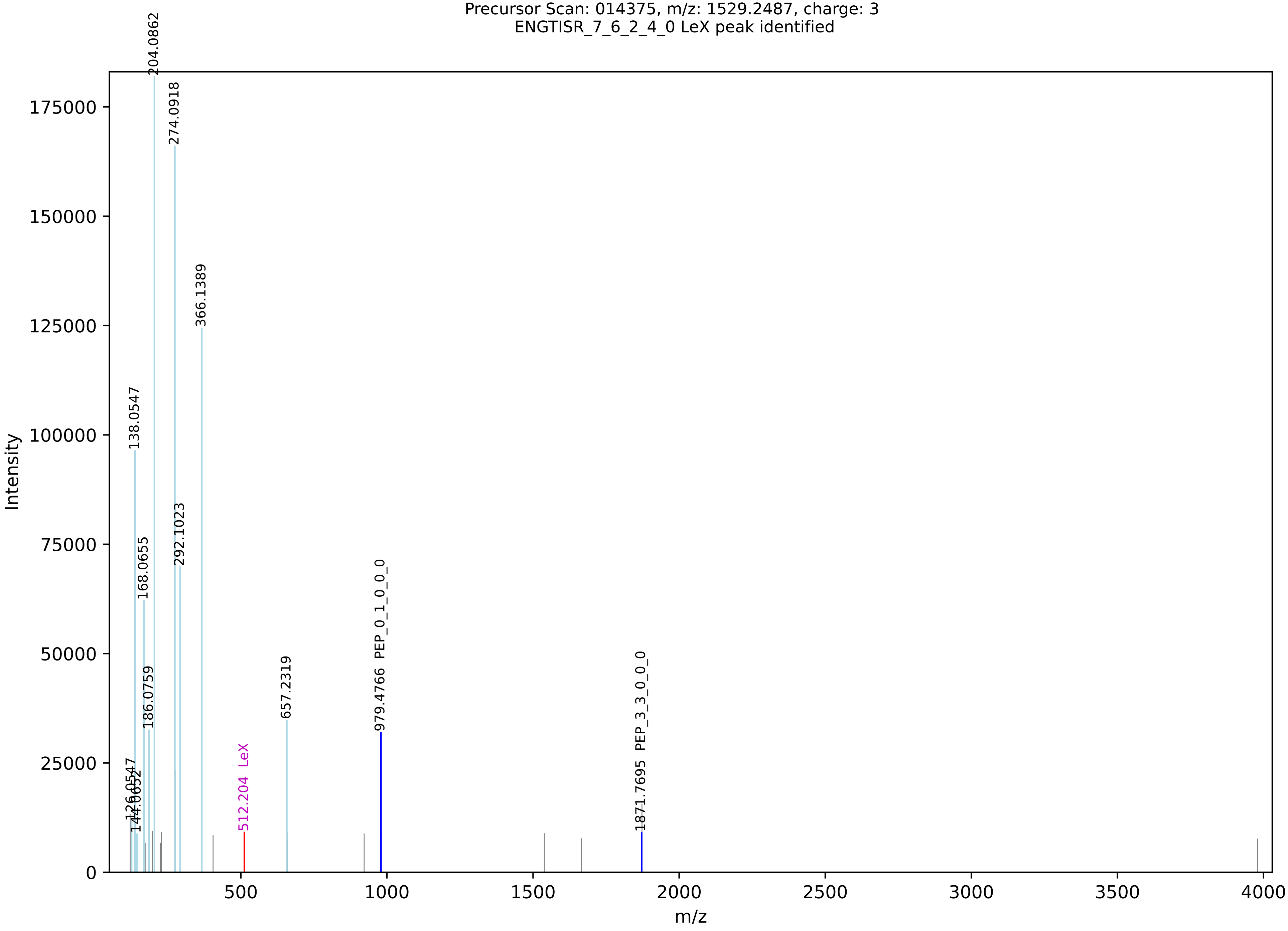

File: 231129_OE_RK_16, RT: 14.659, Scan: 014562
Type: HCD, FTMS + c NSI d Full ms2 1481.5654@hcd30.00 [120.0000-4555.7324]
Precursor Scan: 014492, m/z: 1480.2293, charge: 3
ENGTISR_7_6_1_4_0 LeX peak identified

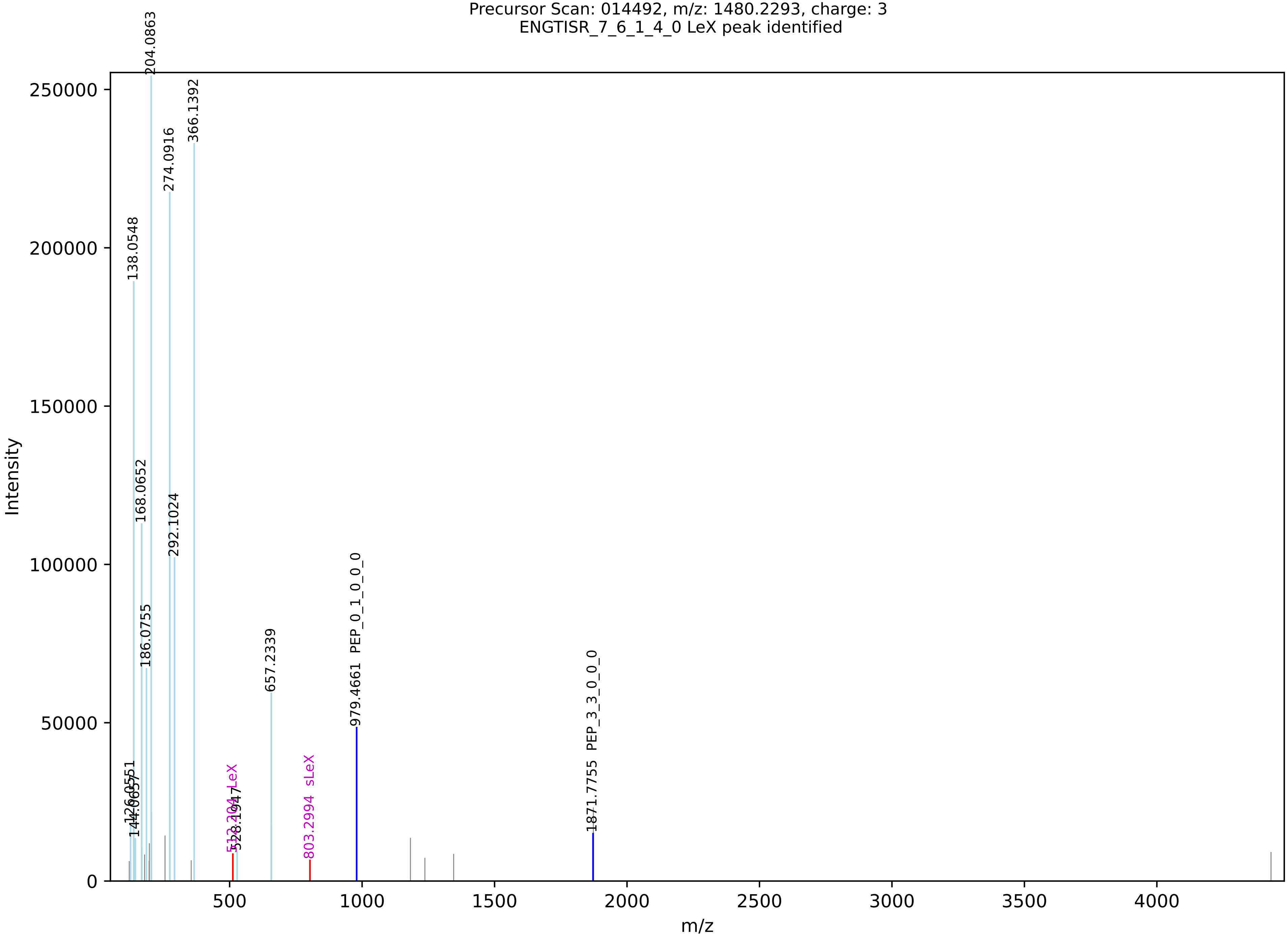

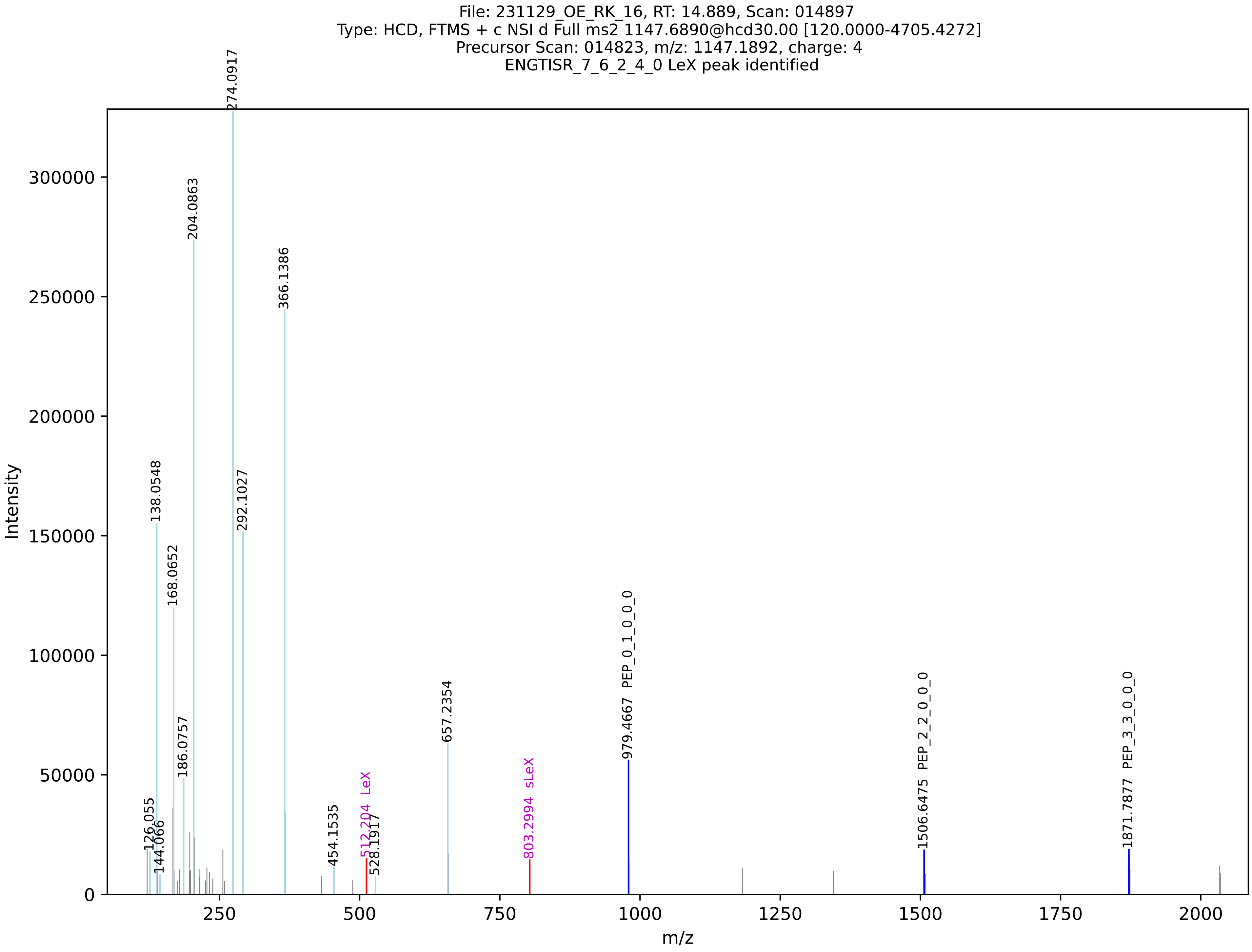

File: 231129_OE_RK_16, RT: 14.889, Scan: 014897
Type: HCD, FTMS + c NSI d Full ms2 1147.6890@hcd30.00 [120.0000-4705.4272]
Precursor Scan: 014823, m/z: 1147.1892, charge: 4
ENGTISR_7_6_2_4_0 LeX peak identified
Intensity
m/z
274.0917
204.0863
366.1386
138.0548
292.1027
168.0652
186.0757
126.055
144.066
454.1535
512.204 LeX
528.1917
657.2354
803.2994 sLeX
979.4667 PEP_0_1_0_0_0
1506.6475 PEP_2_2_0_0_0
1871.7877 PEP_3_3_0_0_0

File: 231129_OE_RK_16, RT: 15.727, Scan: 016104
Type: HCD, FTMS + c NSI d Full ms2 1147.6907@hcd30.00 [120.0000-4705.4336]
Precursor Scan: 016031, m/z: 1147.1895, charge: 4
ENGTISR_7_6_2_4_0 LeX peak identified

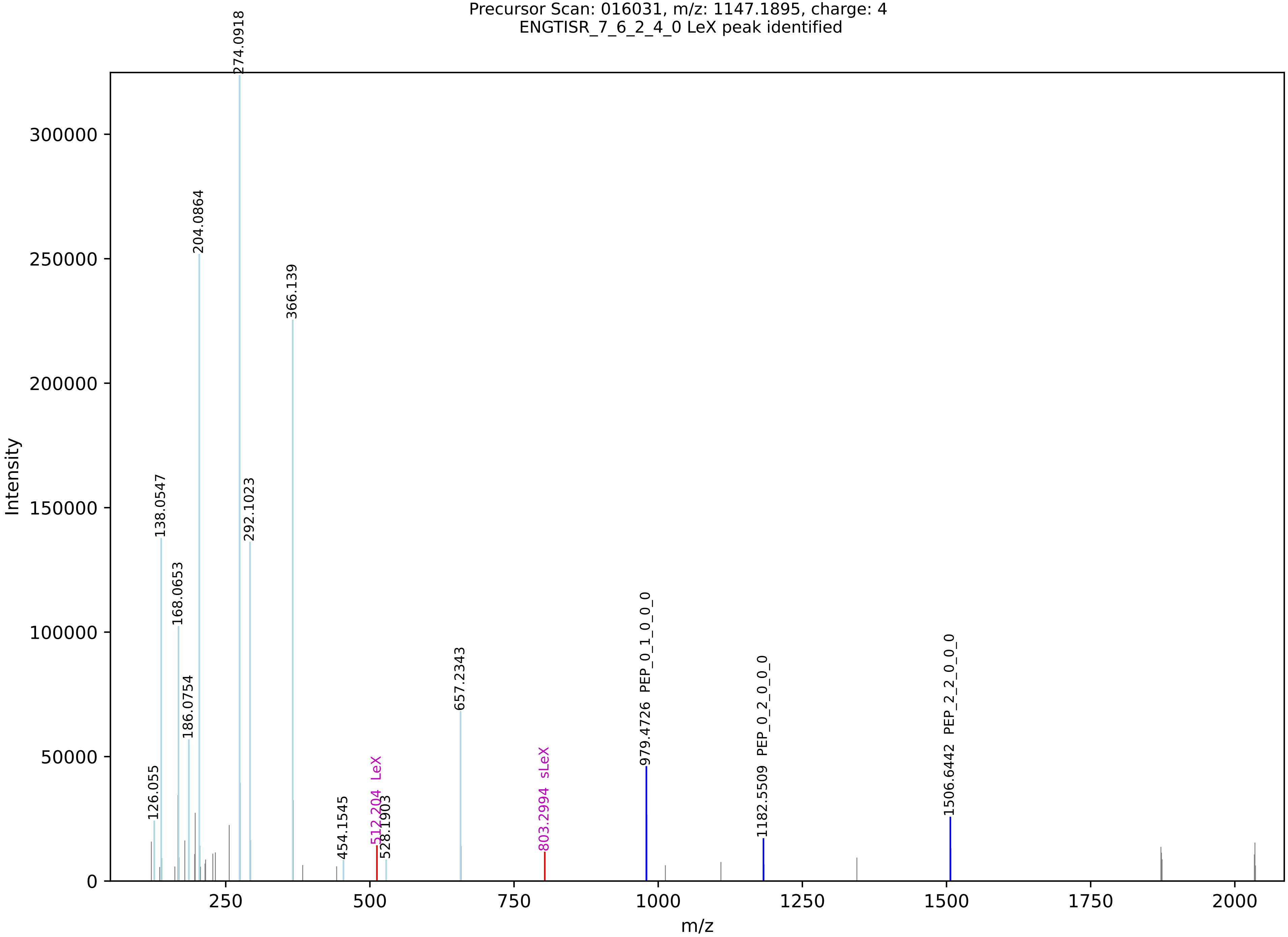

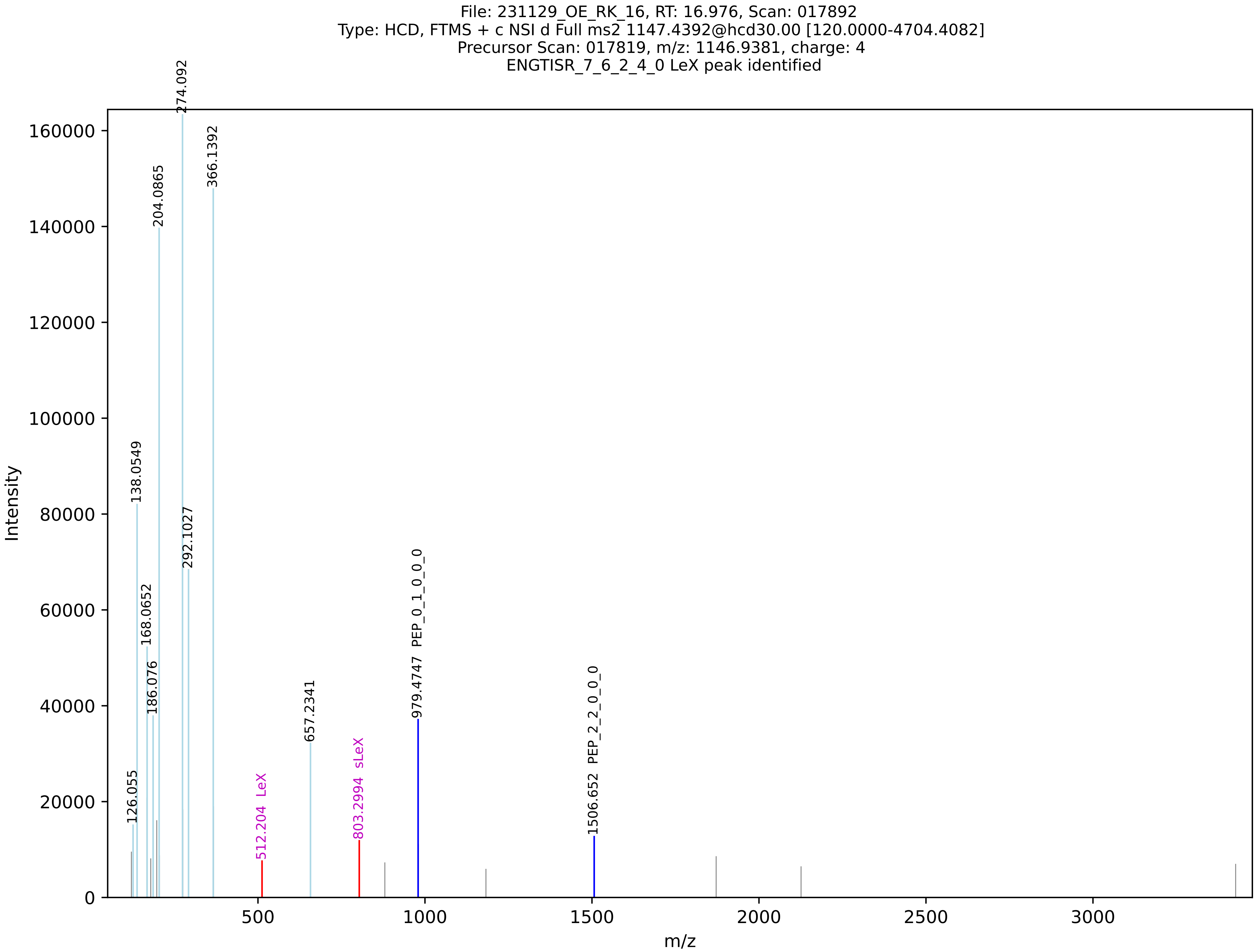

File: 231129_OE_RK_16, RT: 16.976, Scan: 017892
Type: HCD, FTMS + c NSI d Full ms2 1147.4392@hcd30.00 [120.0000-4704.4082]
Precursor Scan: 017819, m/z: 1146.9381, charge: 4
ENGTISR_7_6_2_4_0 LeX peak identified
Intensity
m/z
274.092
366.1392
204.0865
138.0549
292.1027
168.0652
186.076
126.055
657.2341
512.204 LeX
803.2994 sLeX
979.4747 PEP_0_1_0_0_0
1506.652 PEP_2_2_0_0_0
0
20000
40000
60000
80000
100000
120000
140000
160000
500
1000
1500
2000
2500
3000

File: 231129_OE_RK_16, RT: 17.16, Scan: 018153
Type: HCD, FTMS + c NSI d Full ms2 1147.6906@hcd30.00 [120.0000-4705.4331]
Precursor Scan: 018102, m/z: 1147.1893, charge: 4
ENGTISR_7_6_2_4_0 LeX peak identified

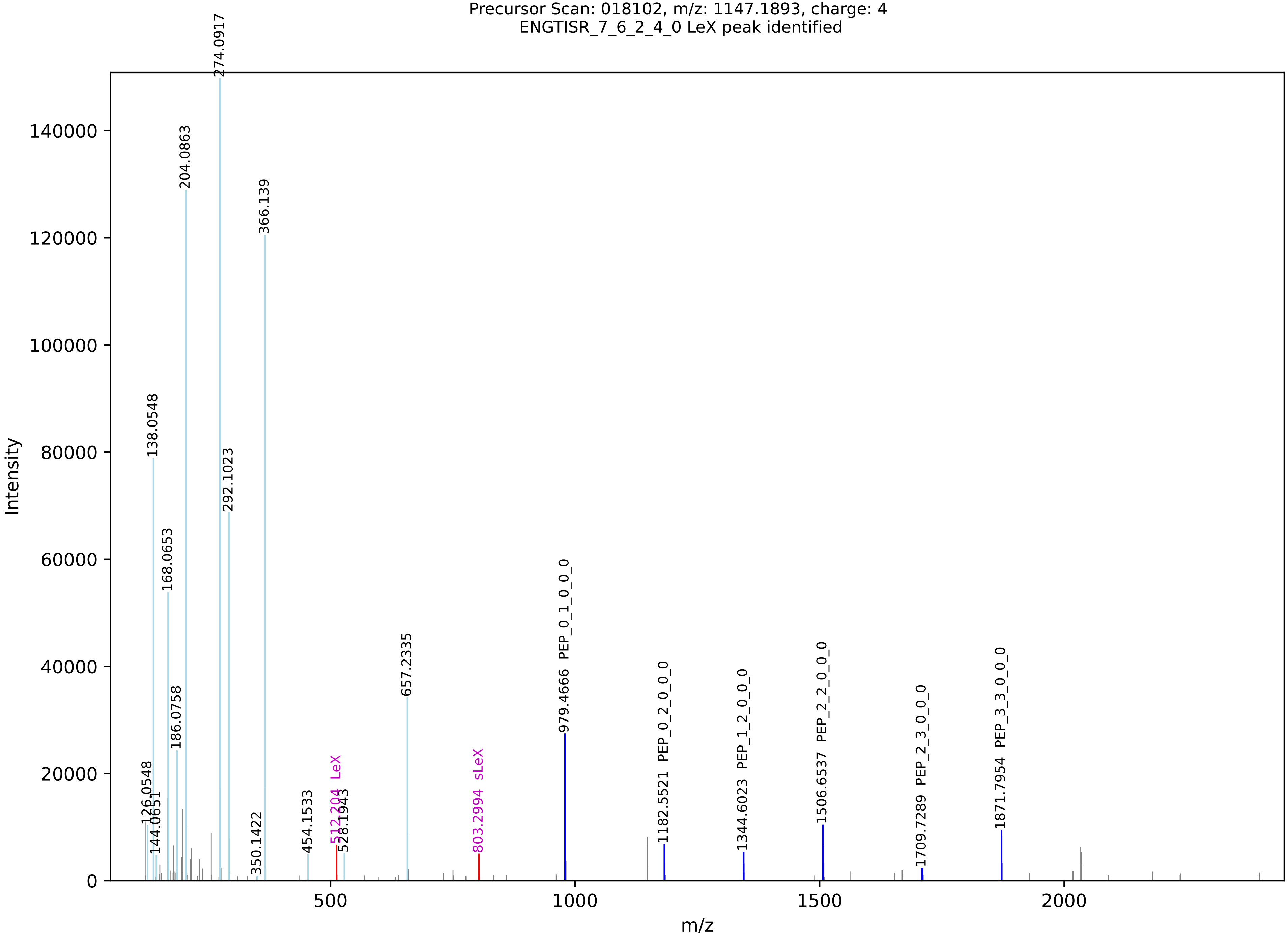

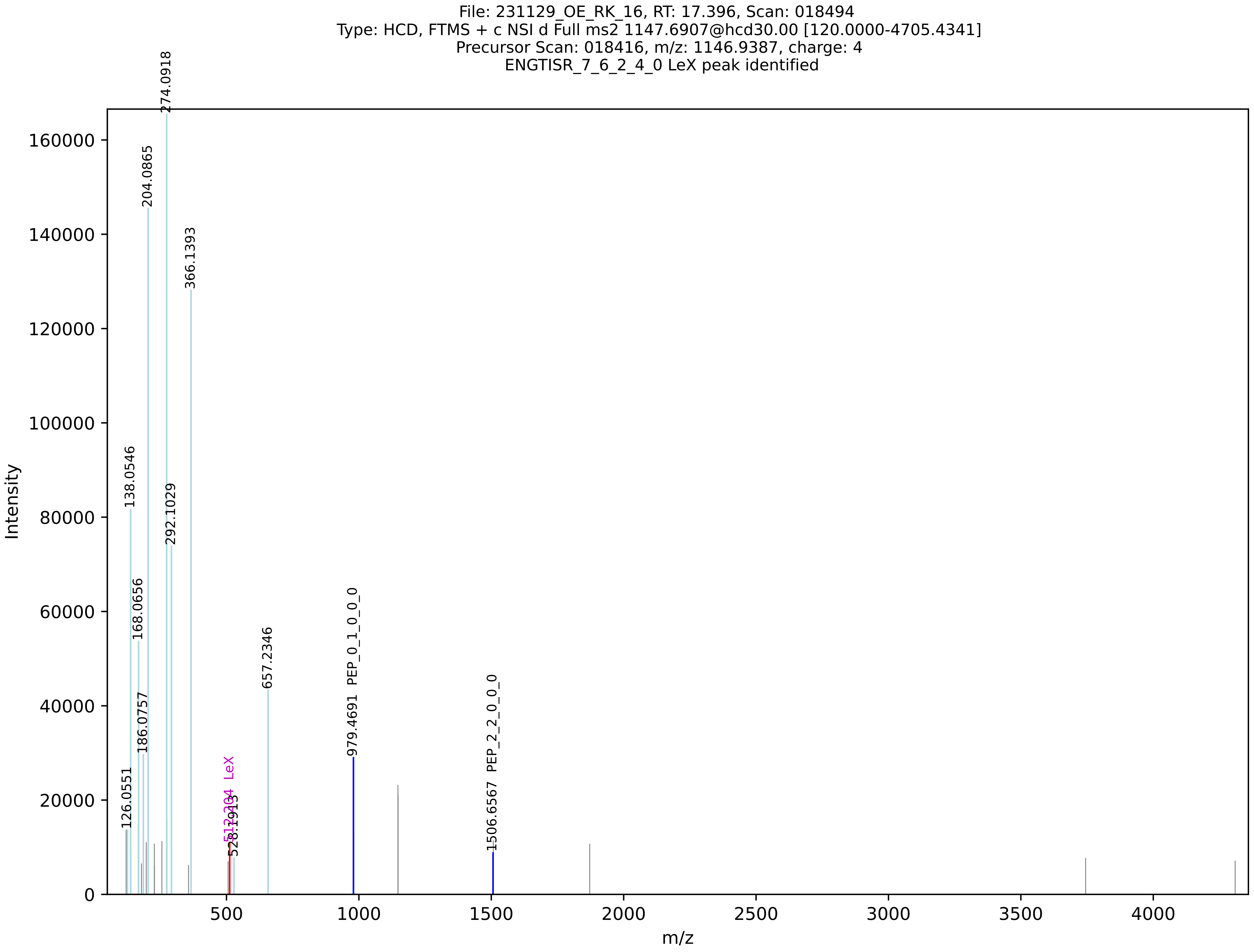
File: 231129_OE_RK_16, RT: 17.396, Scan: 018494
Type: HCD, FTMS + c NSI d Full ms2 1147.6907@hcd30.00 [120.0000-4705.4341]
Precursor Scan: 018416, m/z: 1146.9387, charge: 4
ENGTISR_7_6_2_4_0 LeX peak identified
Intensity
m/z
274.0918
204.0865
366.1393
138.0546
292.1029
168.0656
657.2346
979.4691 PEP_0_1_0_0_0
1506.6567 PEP_2_2_0_0_0
186.0757
126.0551
512.2204 LeX
528.1913
0
20000
40000
60000
80000
100000
120000
140000
160000
500
1000
1500
2000
2500
3000
3500
4000

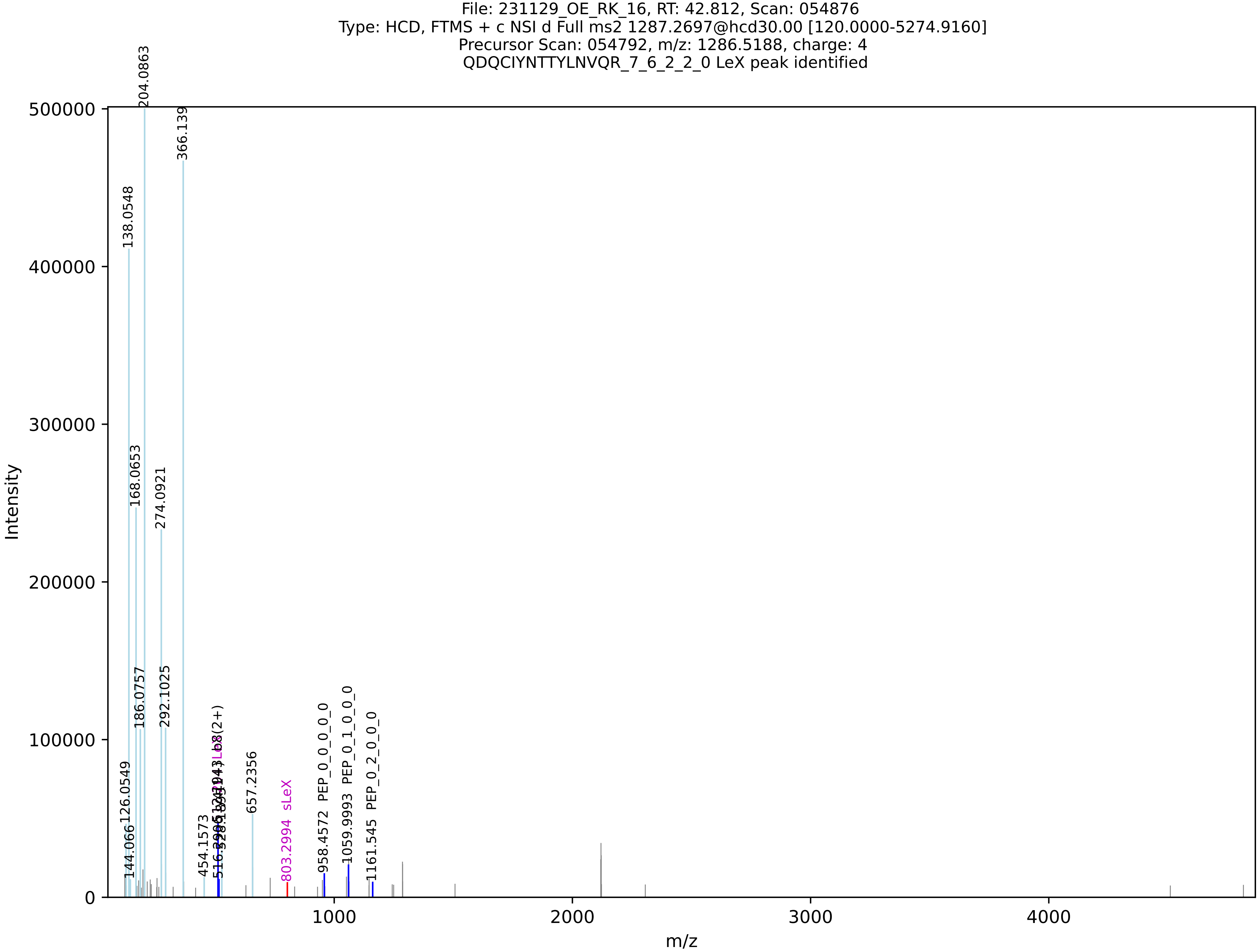

File: 231129_OE_RK_16, RT: 42.812, Scan: 054876
Type: HCD, FTMS + c NSI d Full ms2 1287.2697@hcd30.00 [120.0000-5274.9160]
Precursor Scan: 054792, m/z: 1286.5188, charge: 4
QDQCIYNTTYLNVQR_7_6_2_2_0 LeX peak identified
Intensity
m/z
204.0863
366.139
138.0548
168.0653
274.0921
186.0757
292.1025
126.0549
144.066
454.1573
657.2356
803.2994 sLeX
958.4572 PEP_0_0_0_0_0
1059.9993 PEP_0_1_0_0_0
1161.545 PEP_0_2_0_0_0

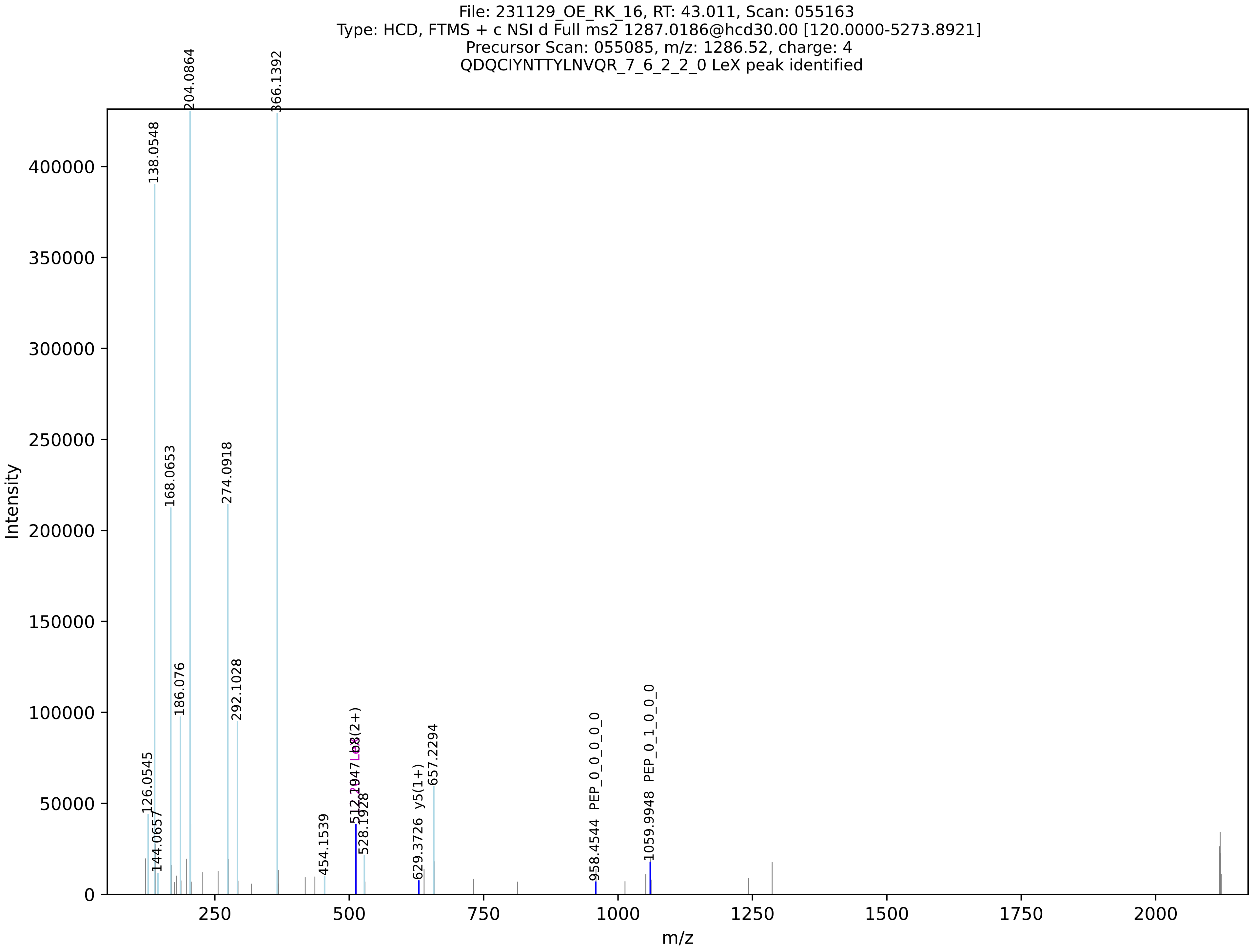
File: 231129_OE_RK_16, RT: 43.011, Scan: 055163
Type: HCD, FTMS + c NSI d Full ms2 1287.0186@hcd30.00 [120.0000-5273.8921]
Precursor Scan: 055085, m/z: 1286.52, charge: 4
QDQCIYNTTYLNVQR_7_6_2_2_0 LeX peak identified
Intensity
m/z
204.0864
366.1392
138.0548
168.0653
274.0918
186.076
292.1028
126.0545
144.0657
454.1539
512.1947
LeX
b3(2+)
528.1928
629.3726
y5(1+)
657.2294
958.4544
PEP_0_0_0_0_0
1059.9948
PEP_0_1_0_0_0

File: 231129_OE_RK_16, RT: 43.064, Scan: 055241
Type: HCD, FTMS + c NSI d Full ms2 1715.6880@hcd30.00 [120.0000-5272.1475]
Precursor Scan: 055156, m/z: 1714.6864, charge: 3
QDQCIYNTTYLNVQR_7_6_0_3_0 LeX peak identified

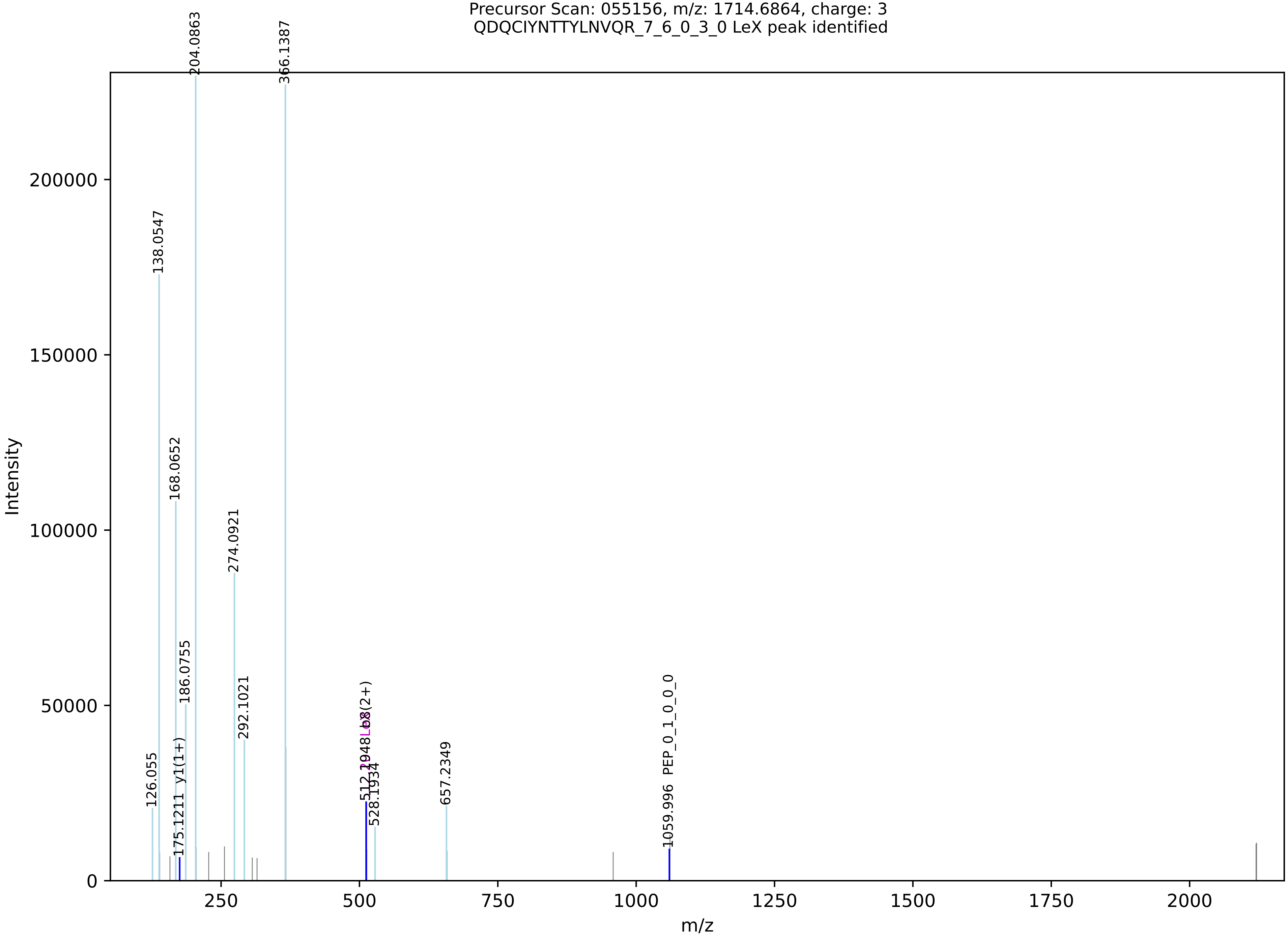

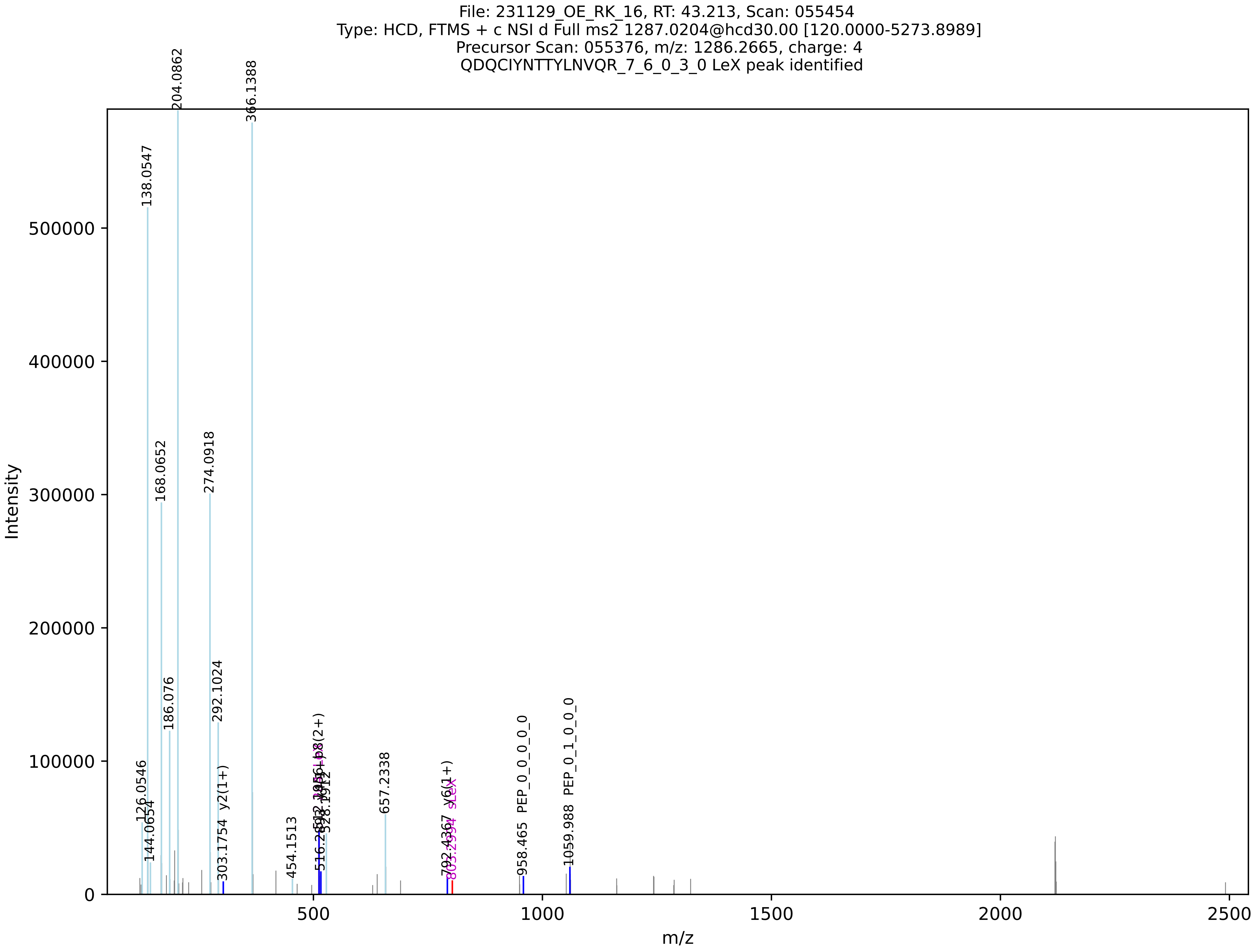

File: 231129_OE_RK_16, RT: 43.213, Scan: 055454
Type: HCD, FTMS + c NSI d Full ms2 1287.0204@hcd30.00 [120.0000-5273.8989]
Precursor Scan: 055376, m/z: 1286.2665, charge: 4
QDQCIYNTTYLNVQR_7_6_0_3_0 LeX peak identified
204.0862
366.1388
138.0547
168.0652
274.0918
186.076
292.1024
126.0546
144.0654
y2(1+)
303.1754
454.1513
528.1912
657.2338
y6(1+)
sLeX
958.465
PEP_0_0_0_0_0
1059.988
PEP_0_1_0_0_0
Intensity
m/z

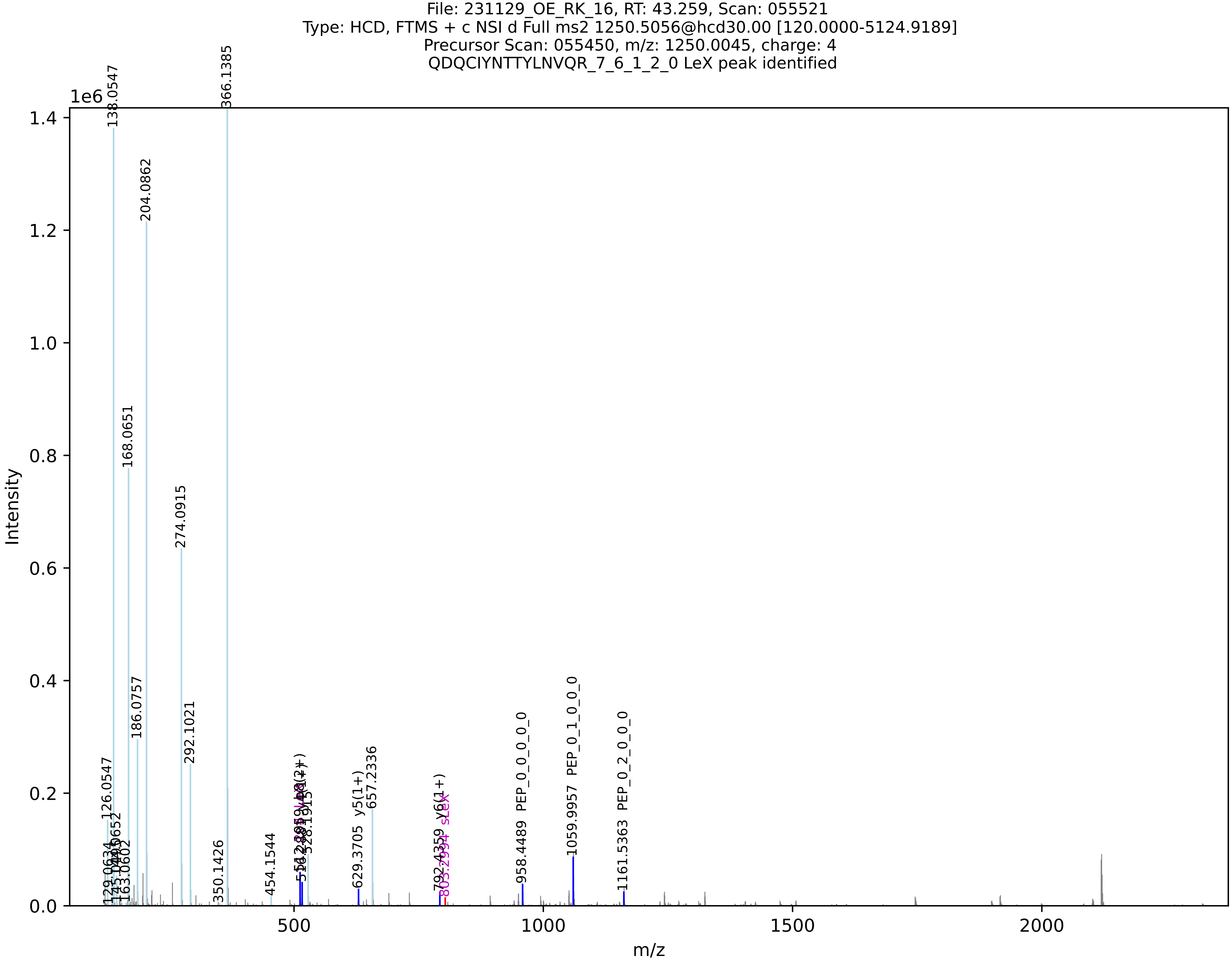
File: 231129_OE_RK_16, RT: 43.259, Scan: 055521
Type: HCD, FTMS + c NSI d Full ms2 1250.5056@hcd30.00 [120.0000-5124.9189]
Precursor Scan: 055450, m/z: 1250.0045, charge: 4
QDQCIYNTTYLNVQR_7_6_1_2_0 LeX peak identified
1e6
Intensity
m/z
366.1385
138.0547
204.0862
168.0651
274.0915
186.0757
292.1021
126.0547
350.1426
454.1544
629.3705 y5(1+)
657.2336
792.4359 y6(1+)
958.4489 PEP_0_0_0_0_0_
1059.9957 PEP_0_1_0_0_0_
1161.5363 PEP_0_2_0_0_0_

File: 231129_OE_RK_16, RT: 43.411, Scan: 055737
Type: HCD, FTMS + c NSI d Full ms2 1287.2690@hcd30.00 [120.0000-5274.9136]
Precursor Scan: 055663, m/z: 1286.5194, charge: 4
QDQCIYNTTYLNVQR_7_6_2_2_0 LeX peak identified

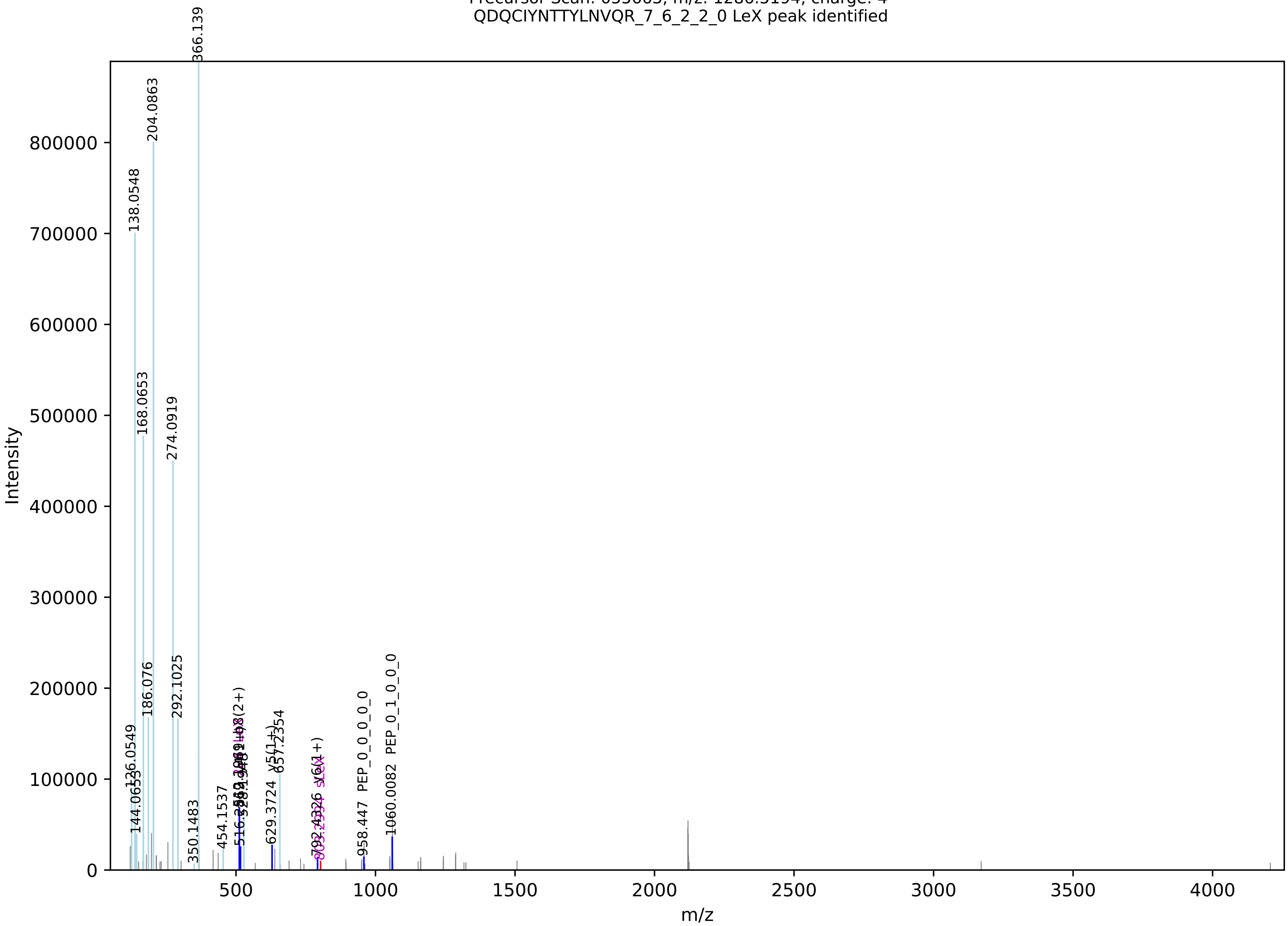

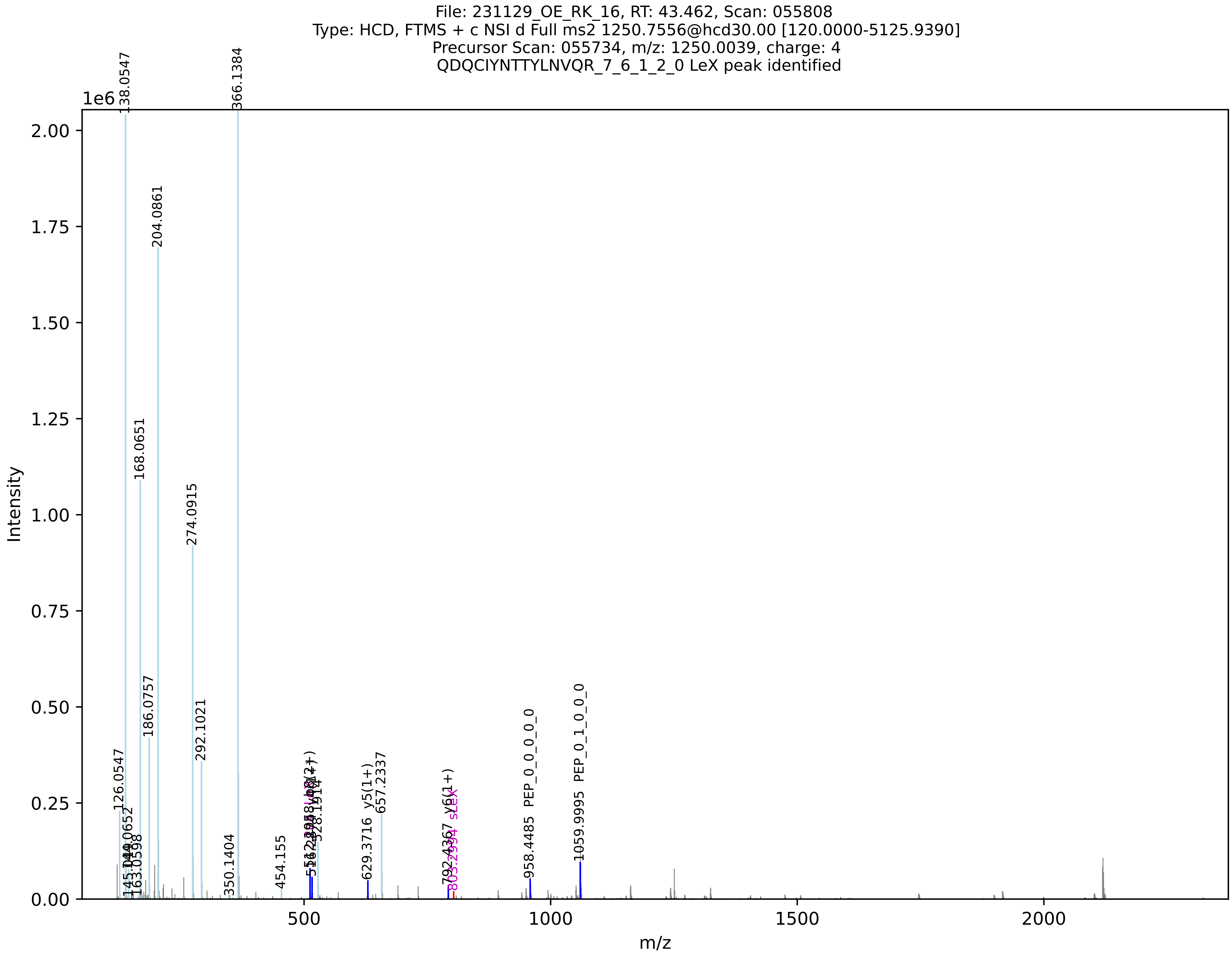
File: 231129_OE_RK_16, RT: 43.462, Scan: 055808
Type: HCD, FTMS + c NSI d Full ms2 1250.7556@hcd30.00 [120.0000-5125.9390]
Precursor Scan: 055734, m/z: 1250.0039, charge: 4
QDQCIYNTTYLNVQR_7_6_1_2_0 LeX peak identified
1e6
Intensity
m/z
138.0547
366.1384
204.0861
168.0651
274.0915
186.0757
292.1021
126.0547
163.0598
350.1404
454.155
629.3716 y5(1+)
657.2337
792.4367 y6(1+)
sLeX
958.4485 PEP_0_0_0_0_0
1059.9995 PEP_0_1_0_0_0

File: 231129_OE_RK_16, RT: 43.662, Scan: 056101
Type: HCD, FTMS + c NSI d Full ms2 1250.5056@hcd30.00 [120.0000-5124.9189]
Precursor Scan: 056026, m/z: 1250.0044, charge: 4
QDQCIYNTTYLNVQR_7_6_1_2_0 LeX peak identified

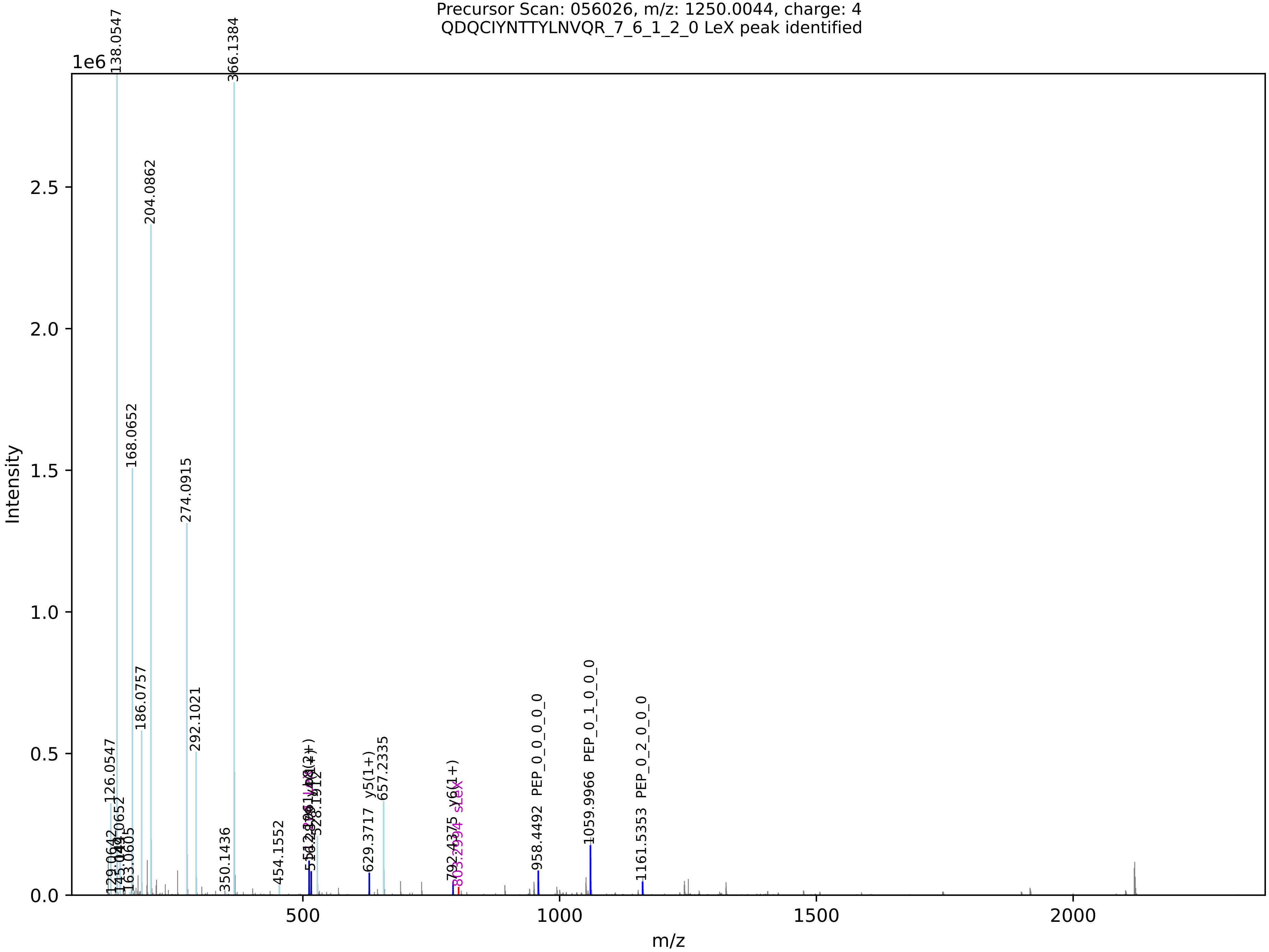

File: 231129_OE_RK_16, RT: 43.865, Scan: 056395
Type: HCD, FTMS + c NSI d Full ms2 1250.7552@hcd30.00 [120.0000-5125.9375]
Precursor Scan: 056319, m/z: 1250.0034, charge: 4
QDQCIYNTTYLNVQR_7_6_1_2_0 LeX peak identified

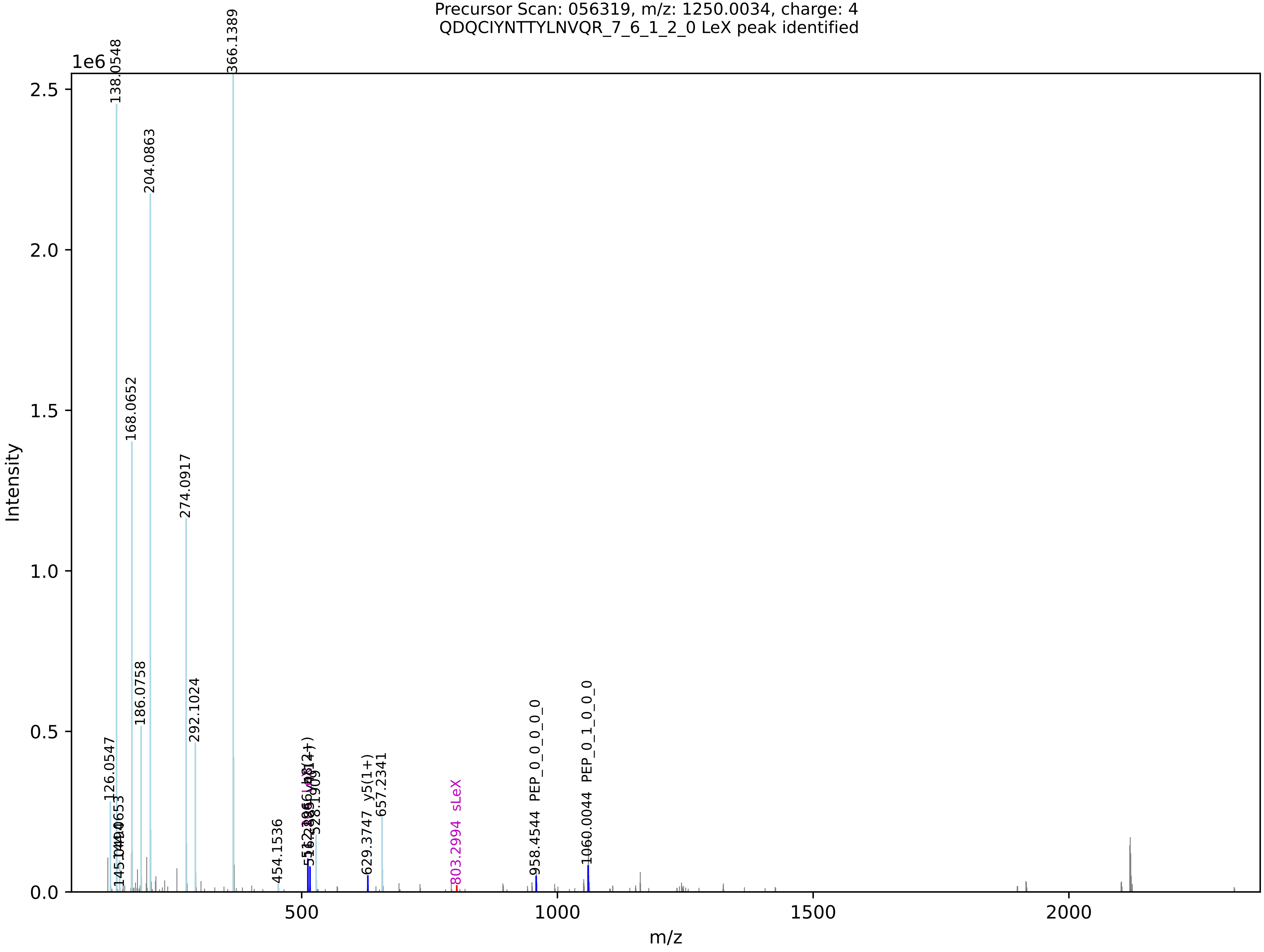

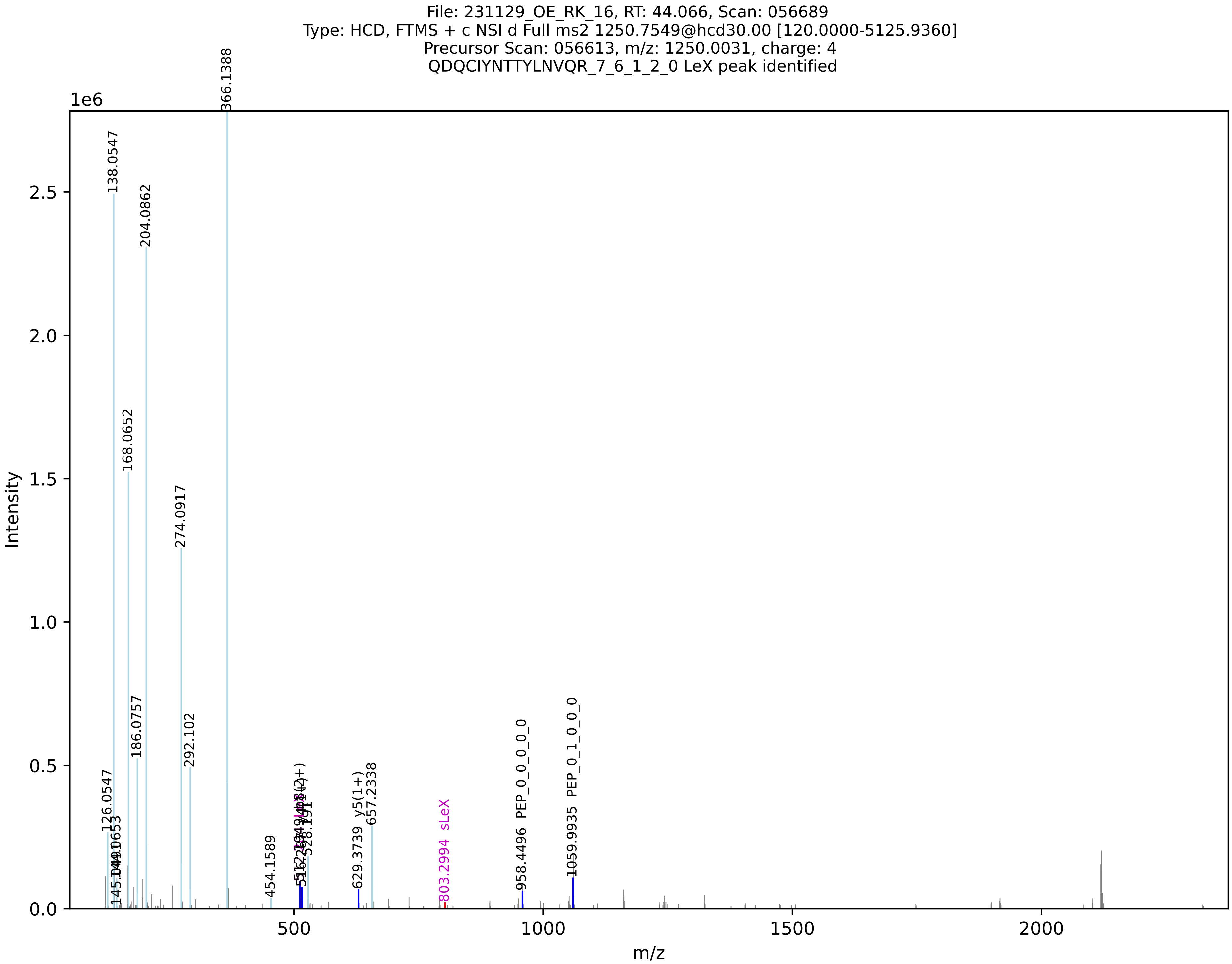
File: 231129_OE_RK_16, RT: 44.066, Scan: 056689
Type: HCD, FTMS + c NSI d Full ms2 1250.7549@hcd30.00 [120.0000-5125.9360]
Precursor Scan: 056613, m/z: 1250.0031, charge: 4
QDQCIYNTTYLNVQR_7_6_1_2_0 LeX peak identified
1e6
Intensity
m/z
366.1388
138.0547
204.0862
168.0652
274.0917
186.0757
292.102
126.0547
454.1589
629.3739 y5(1+)
657.2338
803.2994 sLeX
958.4496 PEP_0_0_0_0_0_
1059.9935 PEP_0_1_0_0_0_

File: 231129_OE_RK_16, RT: 44.116, Scan: 056763
Type: HCD, FTMS + c NSI d Full ms2 1667.0033@hcd30.00 [120.0000-5123.1724]
Precursor Scan: 056687, m/z: 1666.3351, charge: 3
QDQCIYNTTYLNVQR_7_6_1_2_0 LeX peak identified

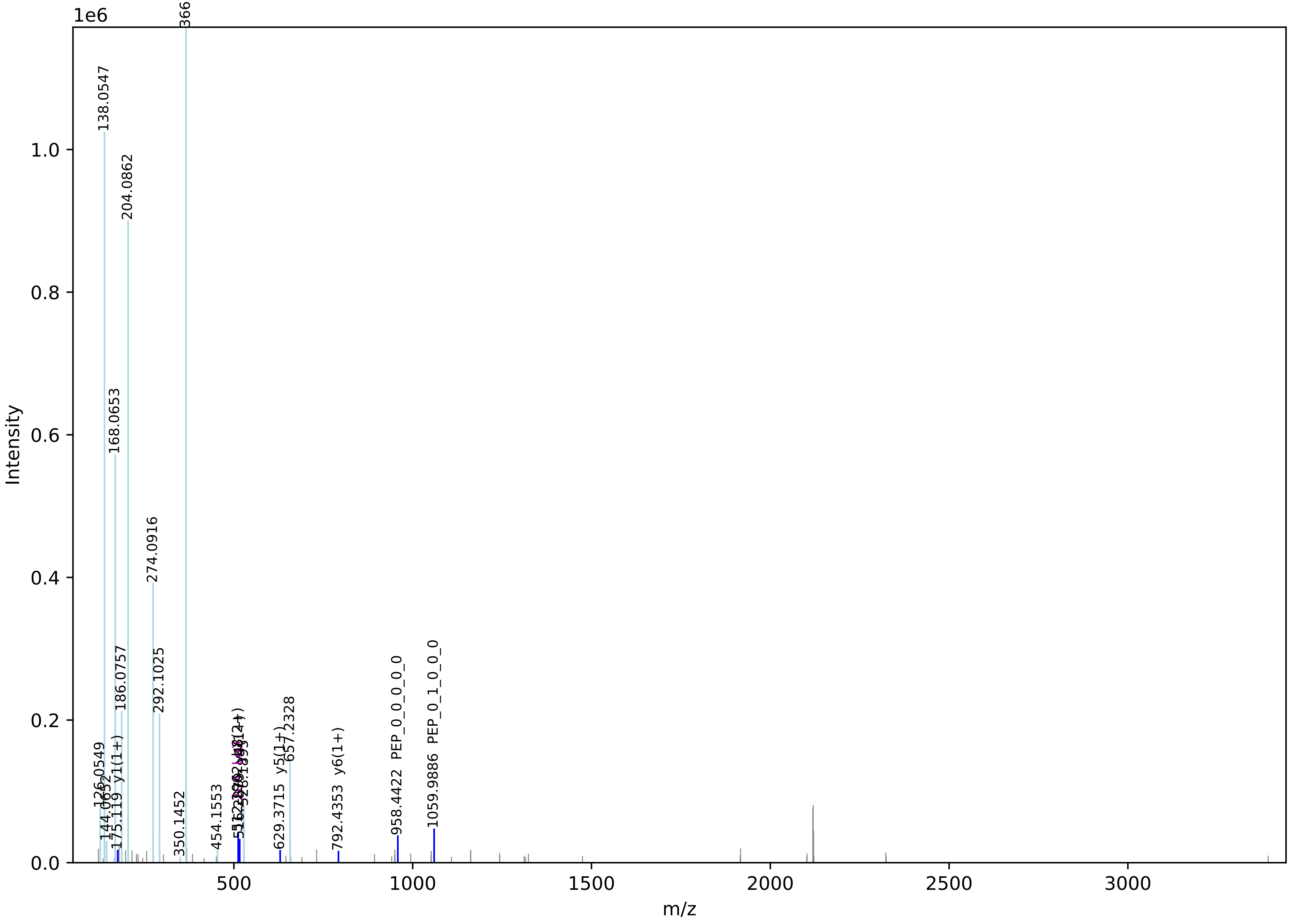

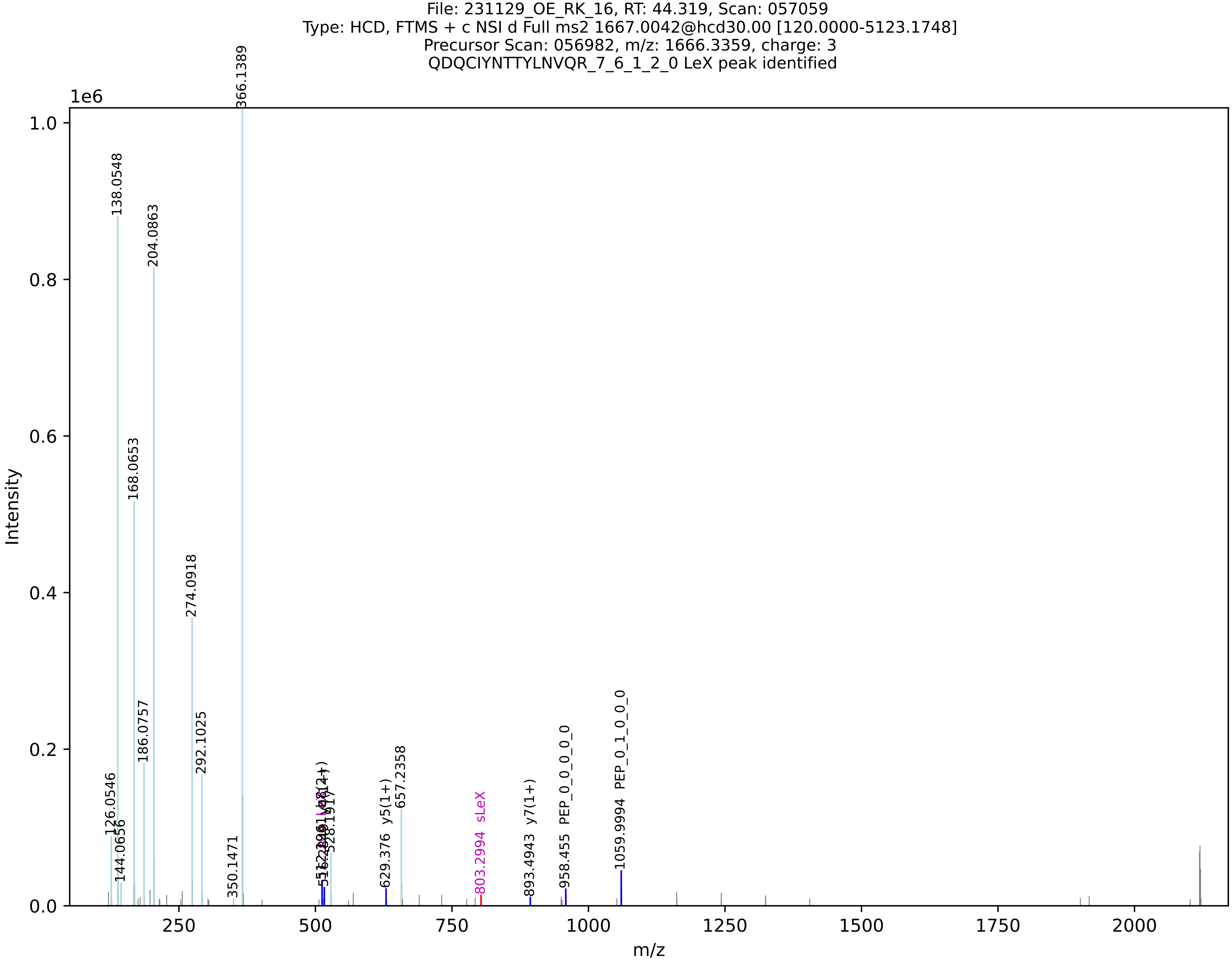

File: 231129_OE_RK_16, RT: 44.319, Scan: 057059
Type: HCD, FTMS + c NSI d Full ms2 1667.0042@hcd30.00 [120.0000-5123.1748]
Precursor Scan: 056982, m/z: 1666.3359, charge: 3
QDQCIYNTTYLNVQR_7_6_1_2_0 LeX peak identified
1e6
Intensity
m/z
366.1389
138.0548
204.0863
168.0653
274.0918
186.0757
292.1025
126.0546
144.0656
350.1471
528.1917
629.376 y5(1+)
657.2358
803.2994 sLeX
893.4943 y7(1+)
958.455 PEP_0_0_0_0_0
1059.9994 PEP_0_1_0_0_0

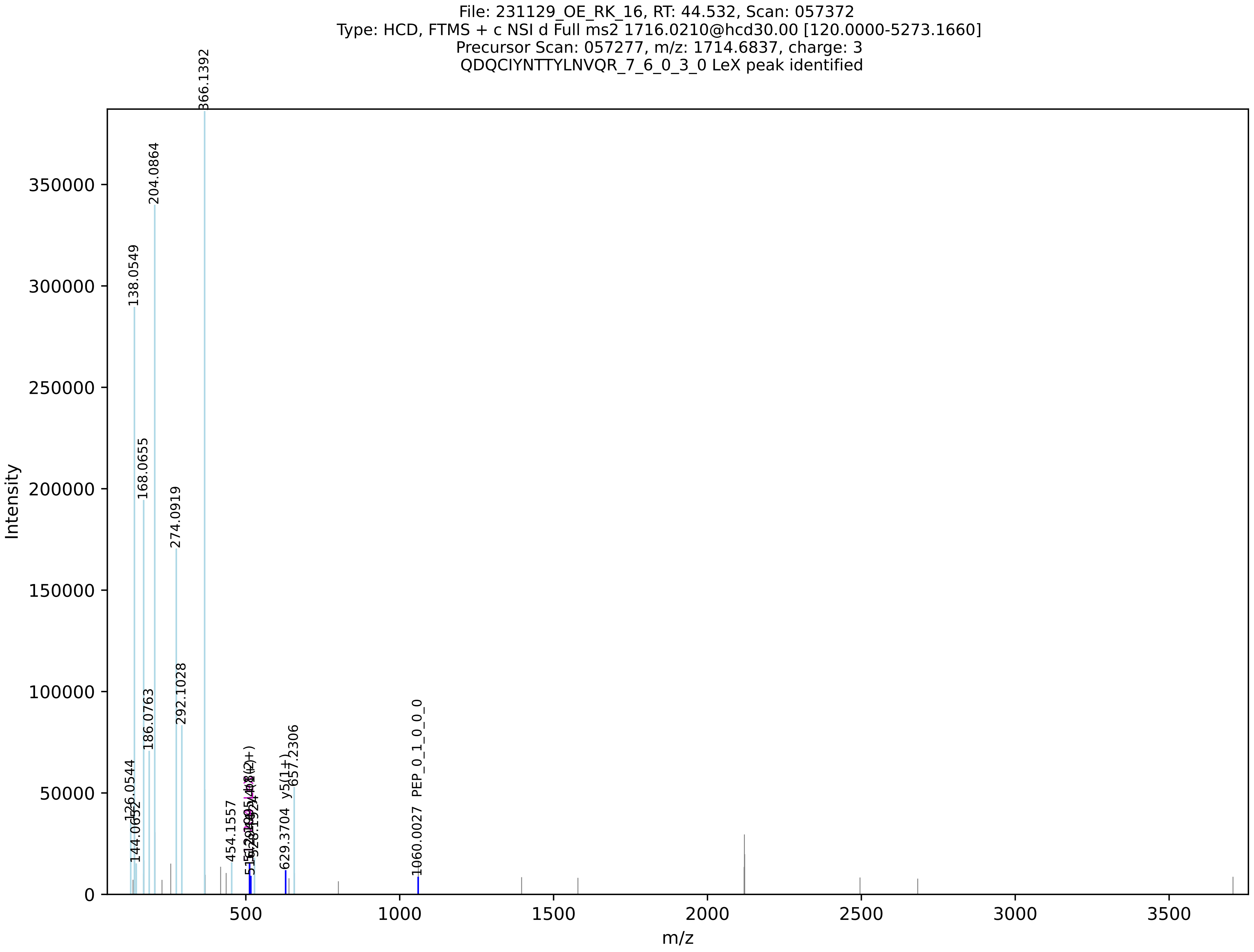
File: 231129_OE_RK_16, RT: 44.532, Scan: 057372
Type: HCD, FTMS + c NSI d Full ms2 1716.0210@hcd30.00 [120.0000-5273.1660]
Precursor Scan: 057277, m/z: 1714.6837, charge: 3
QDQCIYNTTYLNVQR_7_6_0_3_0 LeX peak identified
366.1392
204.0864
138.0549
168.0655
274.0919
292.1028
186.0763
126.0544
144.0652
454.1557
528.1924
629.3704
y5(1+)
657.2306
1060.0027
PEP_0_1_0_0_0_
Intensity
m/z
0
50000
100000
150000
200000
250000
300000
350000
500
1000
1500
2000
2500
3000
3500

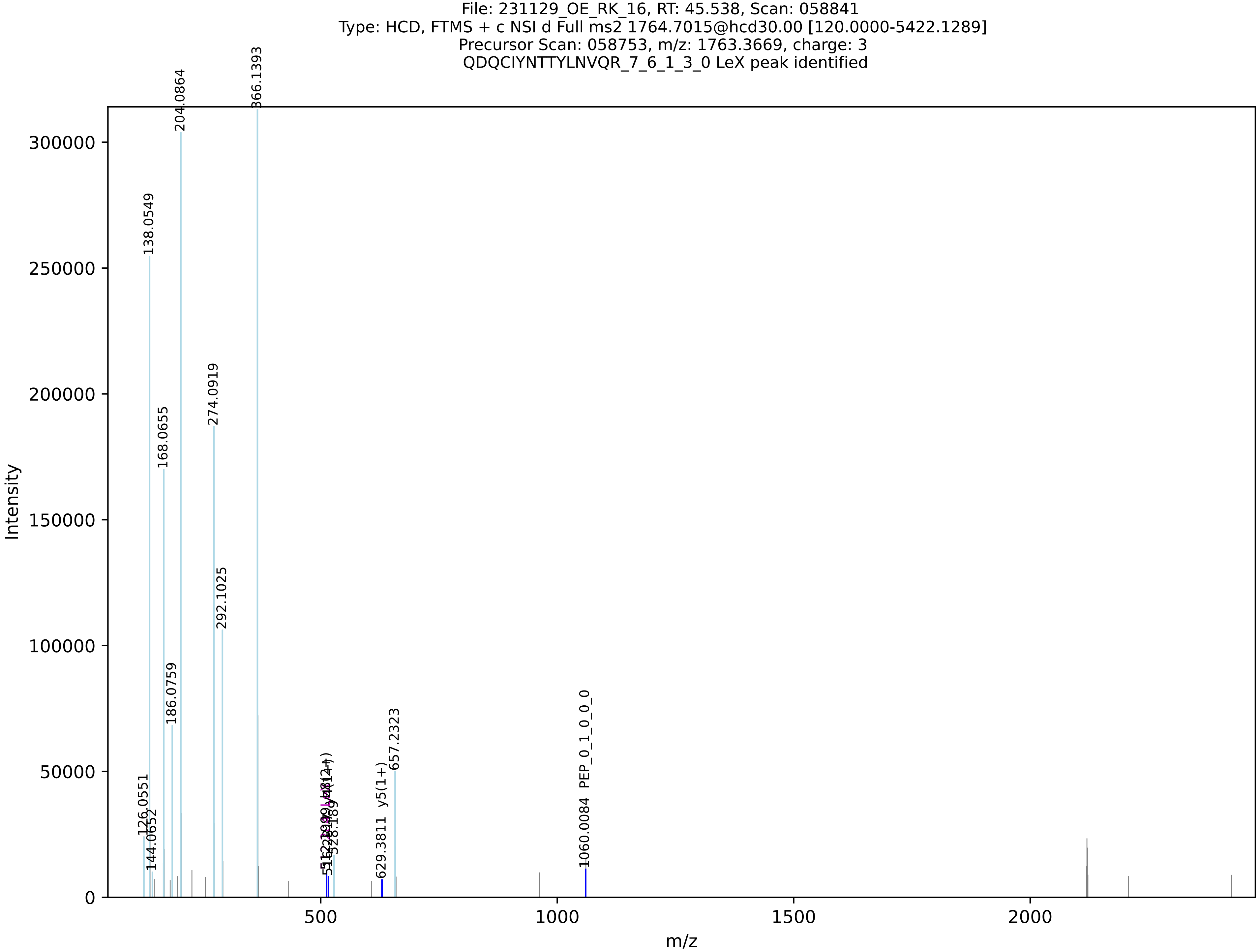
File: 231129_OE_RK_16, RT: 45.538, Scan: 058841
Type: HCD, FTMS + c NSI d Full ms2 1764.7015@hcd30.00 [120.0000-5422.1289]
Precursor Scan: 058753, m/z: 1763.3669, charge: 3
QDQCIYNTTYLNVQR_7_6_1_3_0 LeX peak identified
366.1393
204.0864
138.0549
274.0919
168.0655
292.1025
186.0759
126.0551
144.0652
657.2323
y5(1+)
629.3811
PEP_0_1_0_0_0
1060.0084
Intensity
m/z
0
50000
100000
150000
200000
250000
300000
500
1000
1500
2000

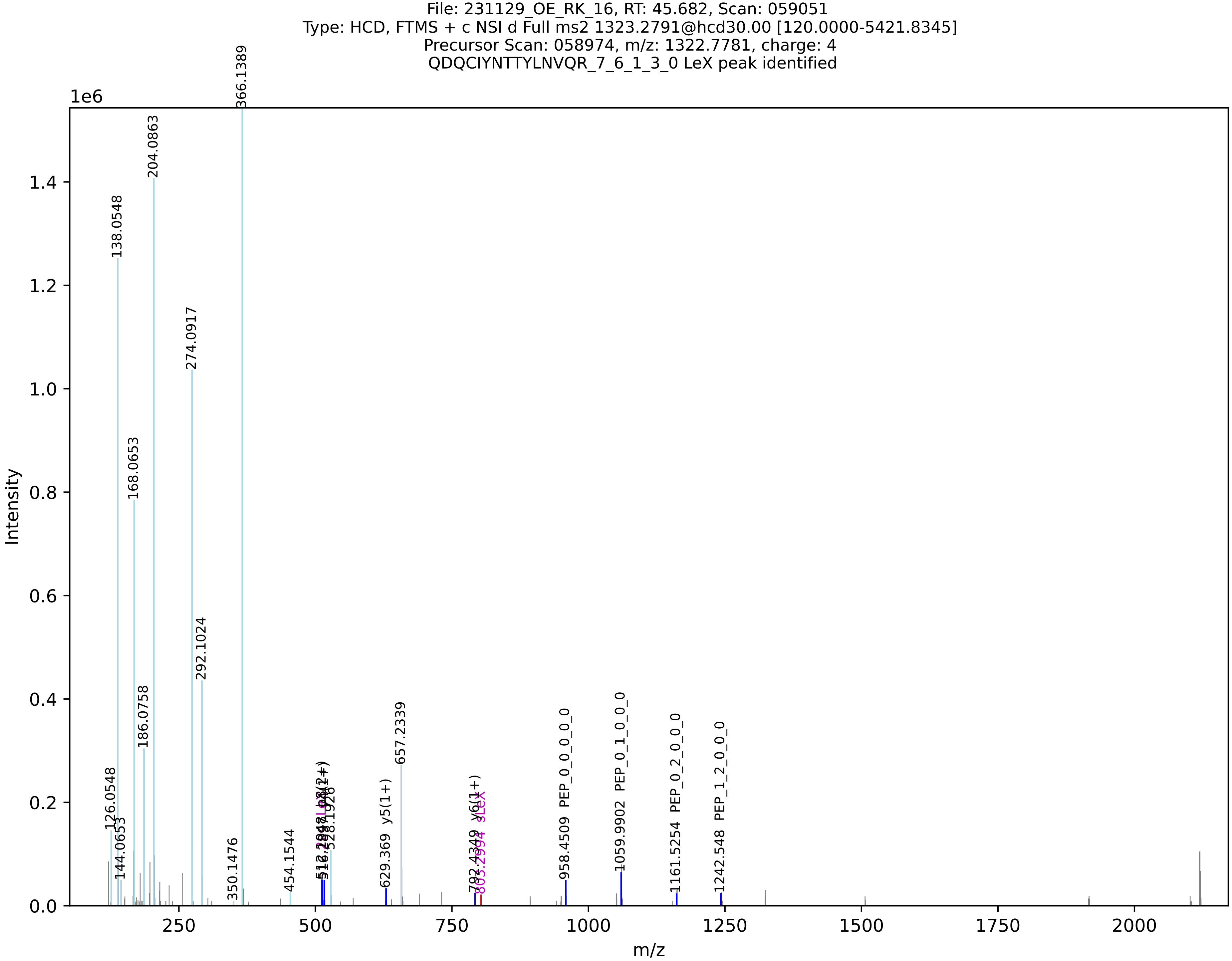
File: 231129_OE_RK_16, RT: 45.682, Scan: 059051
Type: HCD, FTMS + c NSI d Full ms2 1323.2791@hcd30.00 [120.0000-5421.8345]
Precursor Scan: 058974, m/z: 1322.7781, charge: 4
QDQCIYNTTYLNVQR_7_6_1_3_0 LeX peak identified
1e6
Intensity
m/z
366.1389
204.0863
138.0548
274.0917
168.0653
292.1024
186.0758
126.0548
144.0653
350.1476
454.1544
528.1926
629.369 y5(1+)
657.2339
792.4349 y6(1+)
803.2994 sLeX
958.4509 PEP_0_0_0_0_0_
1059.9902 PEP_0_1_0_0_0_
1161.5254 PEP_0_2_0_0_0
1242.548 PEP_1_2_0_0_0_

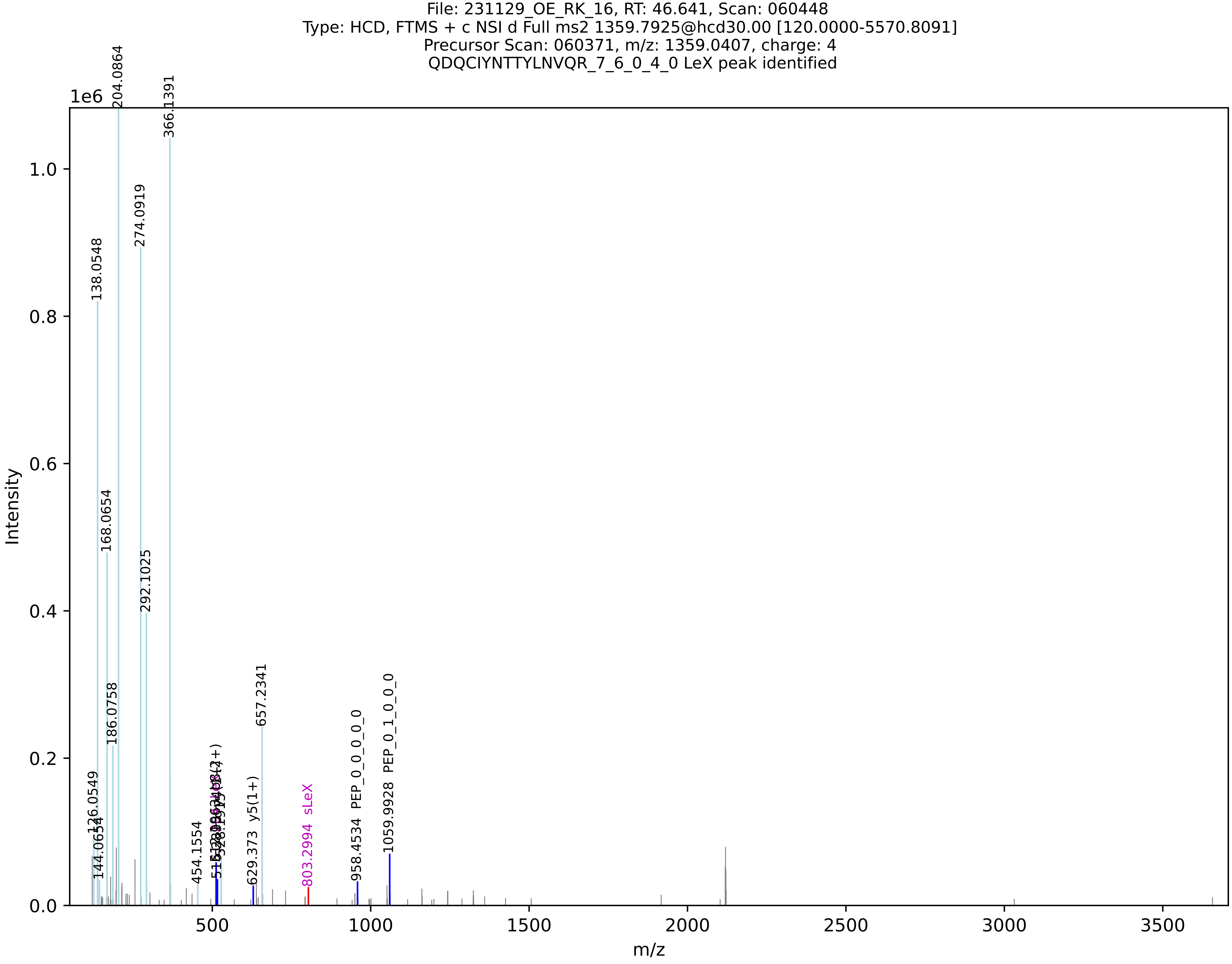

File: 231129_OE_RK_16, RT: 46.641, Scan: 060448
Type: HCD, FTMS + c NSI d Full ms2 1359.7925@hcd30.00 [120.0000-5570.8091]
Precursor Scan: 060371, m/z: 1359.0407, charge: 4
QDQCIYNTTYLNVQR_7_6_0_4_0 LeX peak identified
1e6
Intensity
m/z
204.0864
366.1391
274.0919
138.0548
168.0654
292.1025
186.0758
657.2341
126.0549
144.0654
454.1554
629.373 y5(1+)
803.2994 sLeX
958.4534 PEP_0_0_0_0_0_
1059.9928 PEP_0_1_0_0_0_

File: 231129_OE_RK_16, RT: 47.756, Scan: 062054
Type: HCD, FTMS + c NSI d Full ms2 1396.3019@hcd30.00 [120.0000-5719.7676]
Precursor Scan: 061962, m/z: 1395.5501, charge: 4
QDQCIYNTTYLNVQR_7_6_1_4_0 LeX peak identified

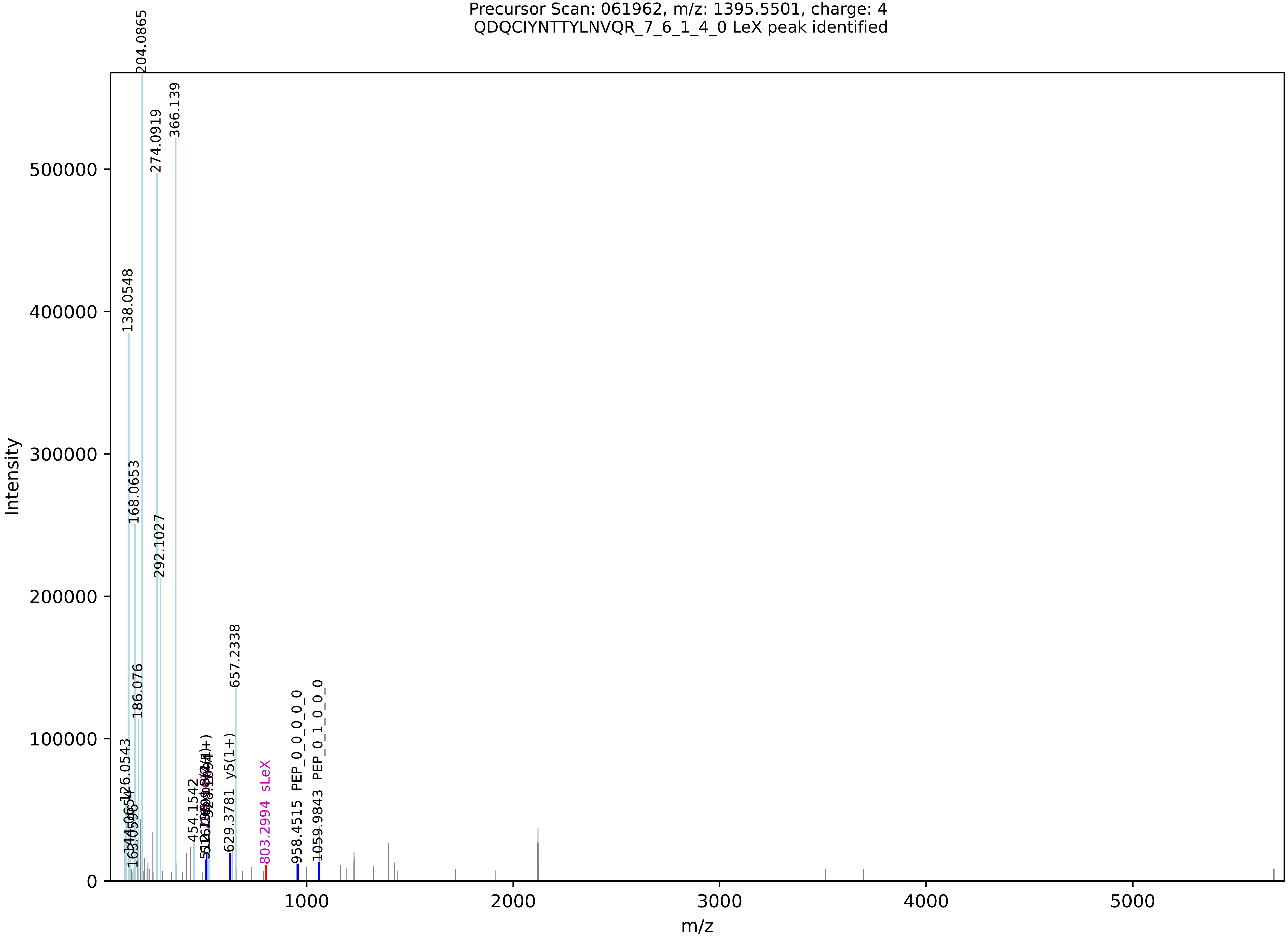

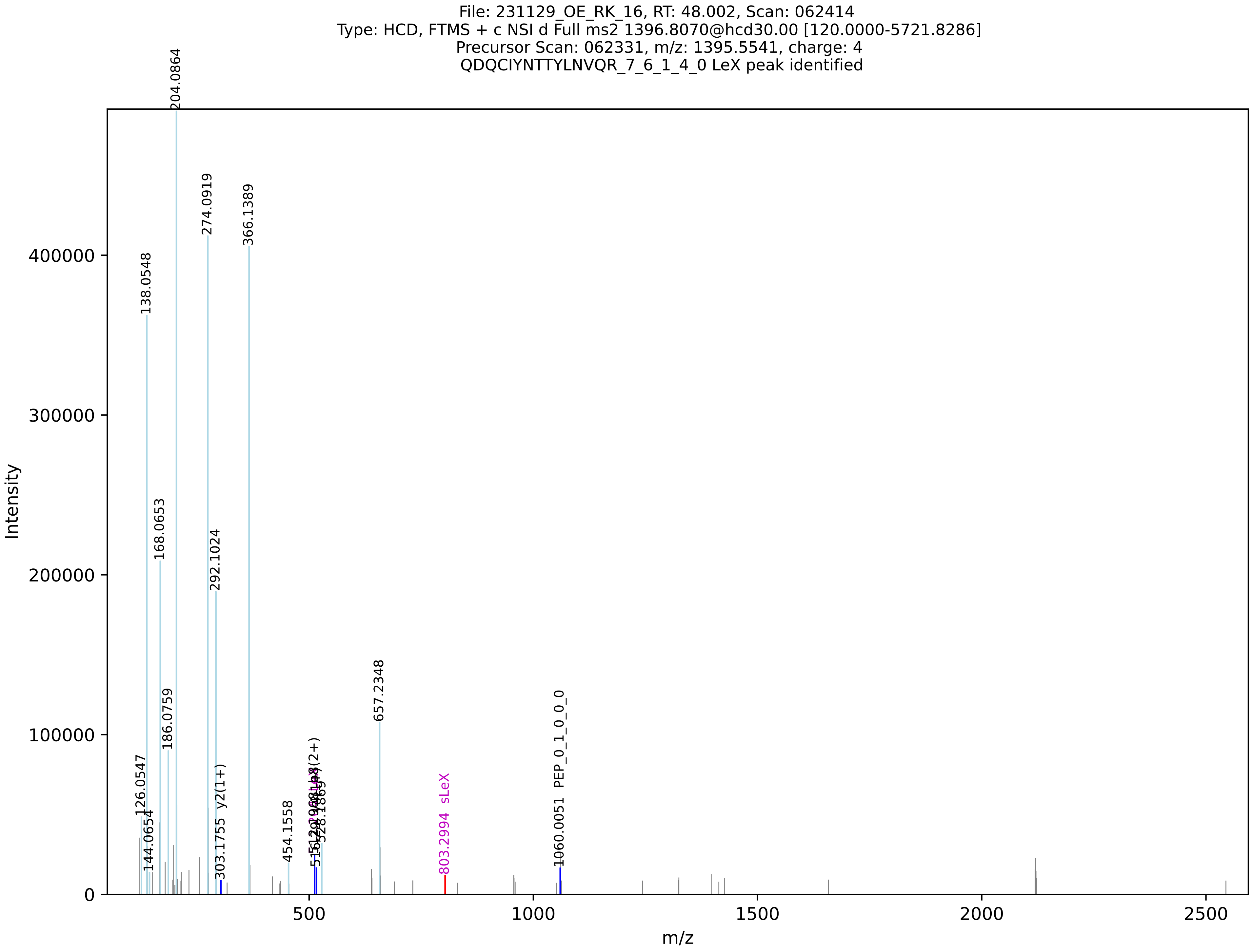
File: 231129_OE_RK_16, RT: 48.002, Scan: 062414
Type: HCD, FTMS + c NSI d Full ms2 1396.8070@hcd30.00 [120.0000-5721.8286]
Precursor Scan: 062331, m/z: 1395.5541, charge: 4
QDQCIYNTTYLNVQR_7_6_1_4_0 LeX peak identified
204.0864
274.0919
366.1389
138.0548
168.0653
292.1024
186.0759
126.0547
144.0654
303.1755 y2(1+)
454.1558
528.1869
657.2348
803.2994 sLeX
1060.0051 PEP_0_1_0_0_0_
Intensity
m/z
0
100000
200000
300000
400000
500
1000
1500
2000
2500

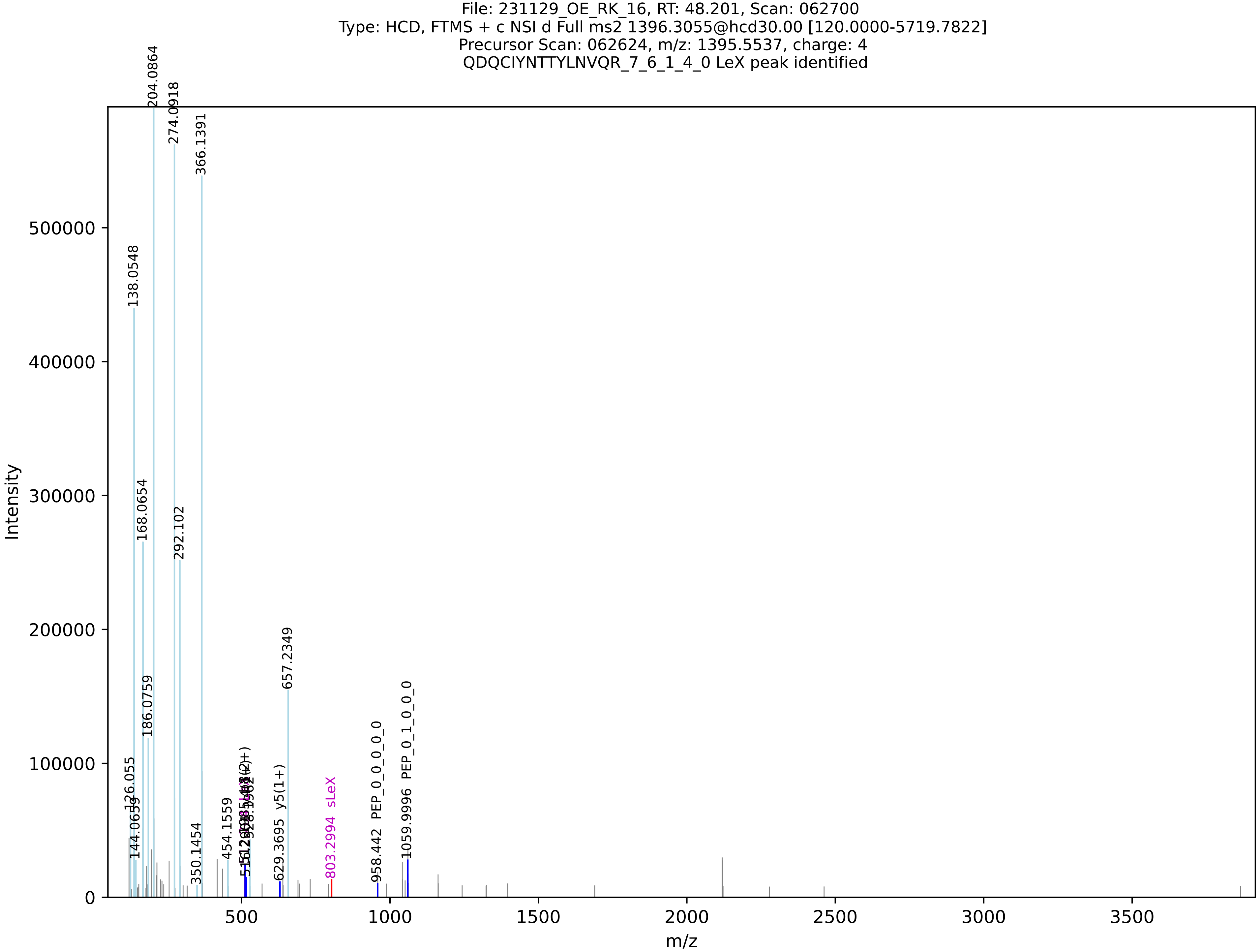
File: 231129_OE_RK_16, RT: 48.201, Scan: 062700
Type: HCD, FTMS + c NSI d Full ms2 1396.3055@hcd30.00 [120.0000-5719.7822]
Precursor Scan: 062624, m/z: 1395.5537, charge: 4
QDQCIYNTTYLNVQR_7_6_1_4_0 LeX peak identified
Intensity
m/z
204.0864
274.0918
366.1391
138.0548
168.0654
292.102
186.0759
126.055
144.0659
350.1454
454.1559
657.2349
629.3695 y5(1+)
803.2994 sLeX
958.442 PEP_0_0_0_0_0
1059.9996 PEP_0_1_0_0_0
0
100000
200000
300000
400000
500000
500
1000
1500
2000
2500
3000
3500

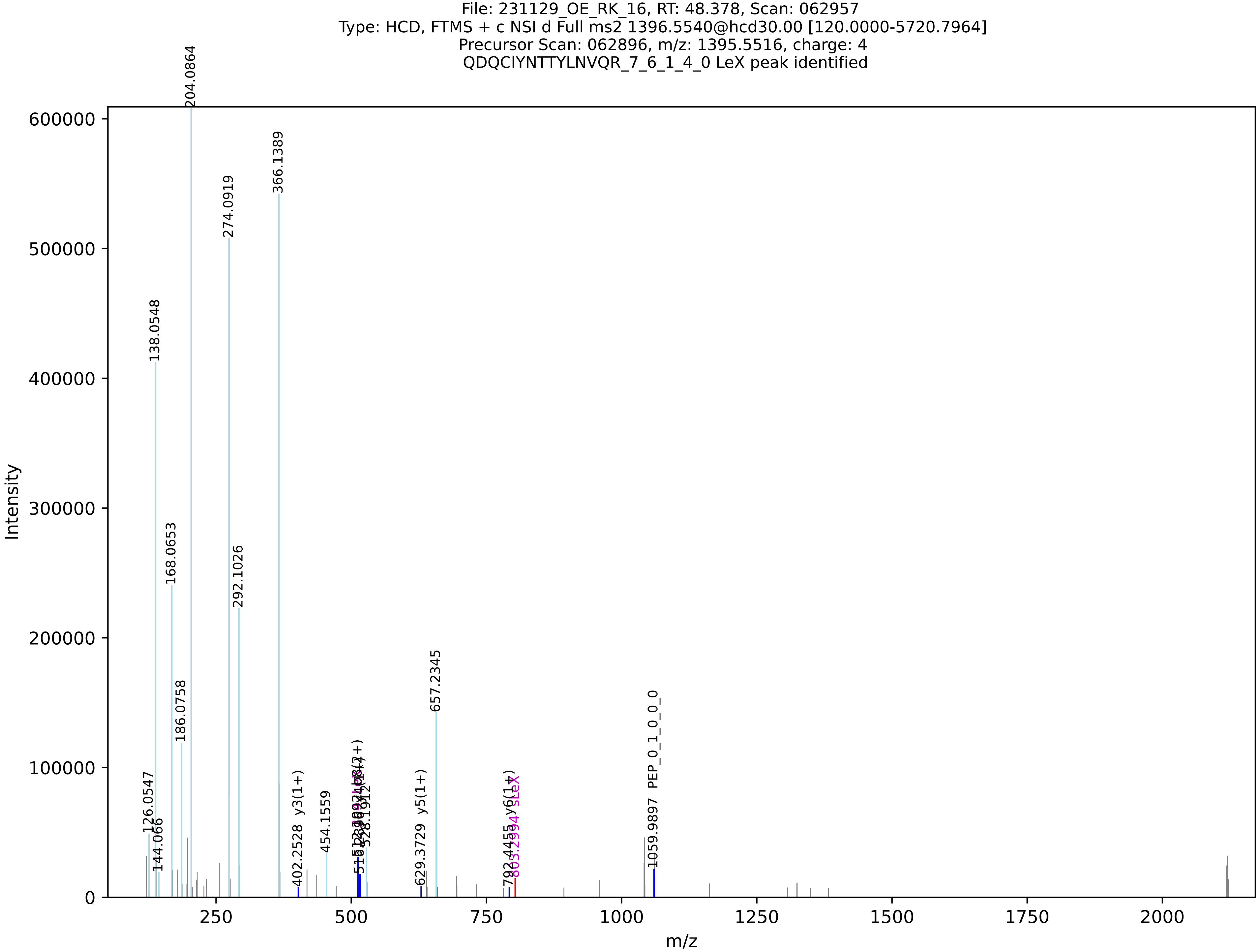
File: 231129_OE_RK_16, RT: 48.378, Scan: 062957
Type: HCD, FTMS + c NSI d Full ms2 1396.5540@hcd30.00 [120.0000-5720.7964]
Precursor Scan: 062896, m/z: 1395.5516, charge: 4
QDQCIYNTTYLNVQR_7_6_1_4_0 LeX peak identified
Intensity
m/z
204.0864
366.1389
274.0919
138.0548
168.0653
292.1026
186.0758
657.2345
126.0547
144.066
402.2528 y3(1+)
454.1559
528.1912
629.3729 y5(1+)
792.4455 y6(1+)
803.2994 SLeX
1059.9897 PEP_0_1_0_0_0

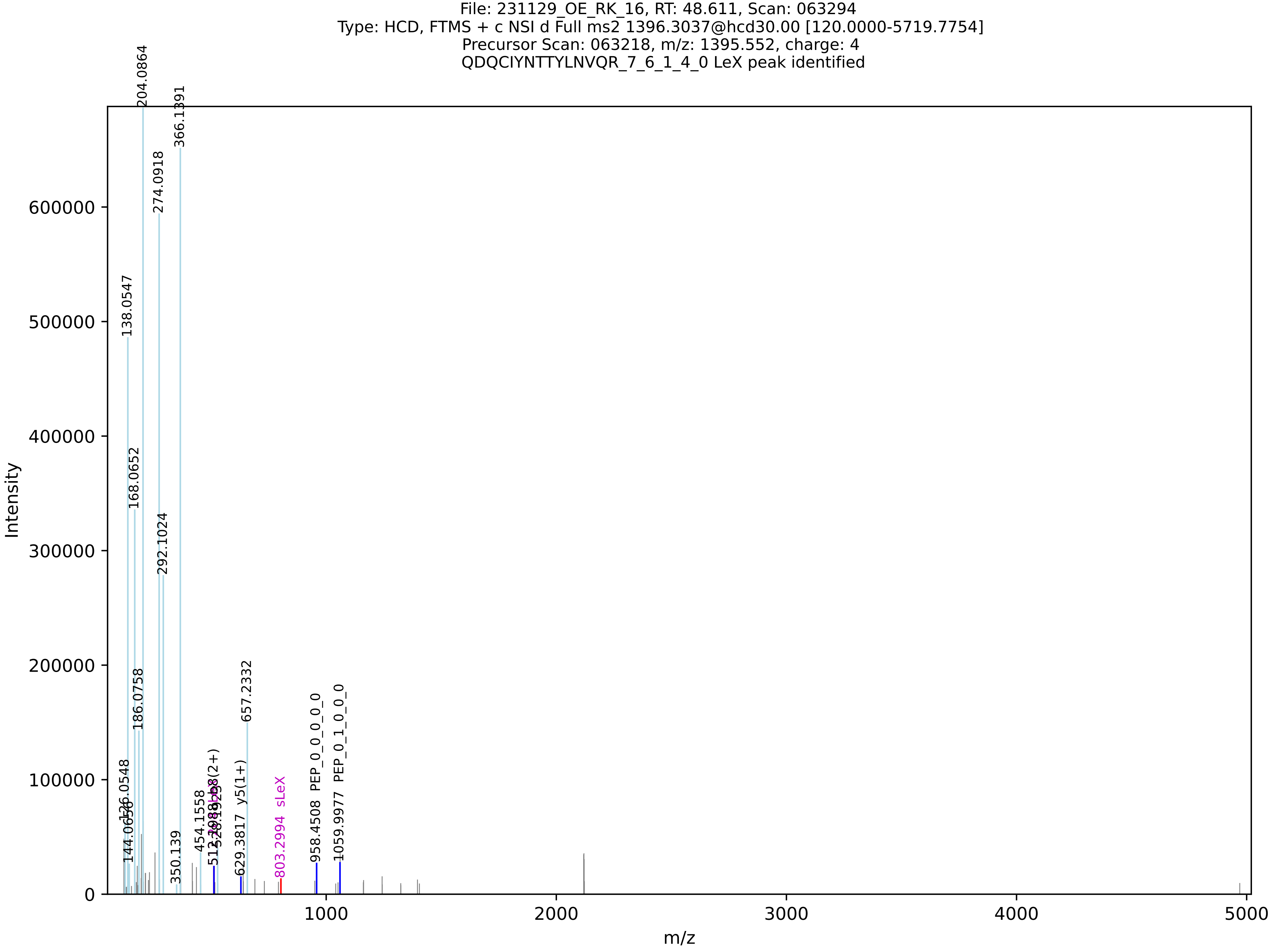
File: 231129_OE_RK_16, RT: 48.611, Scan: 063294
Type: HCD, FTMS + c NSI d Full ms2 1396.3037@hcd30.00 [120.0000-5719.7754]
Precursor Scan: 063218, m/z: 1395.552, charge: 4
QDQCIYNTTYLNVQR_7_6_1_4_0 LeX peak identified
204.0864
366.1391
274.0918
138.0547
168.0652
292.1024
186.0758
657.2332
126.0548
144.0656
350.139
454.1558
629.3817 y5(1+)
803.2994 sLeX
958.4508 PEP_0_0_0_0_0
1059.9977 PEP_0_1_0_0_0
Intensity
m/z
0
100000
200000
300000
400000
500000
600000
1000
2000
3000
4000
5000

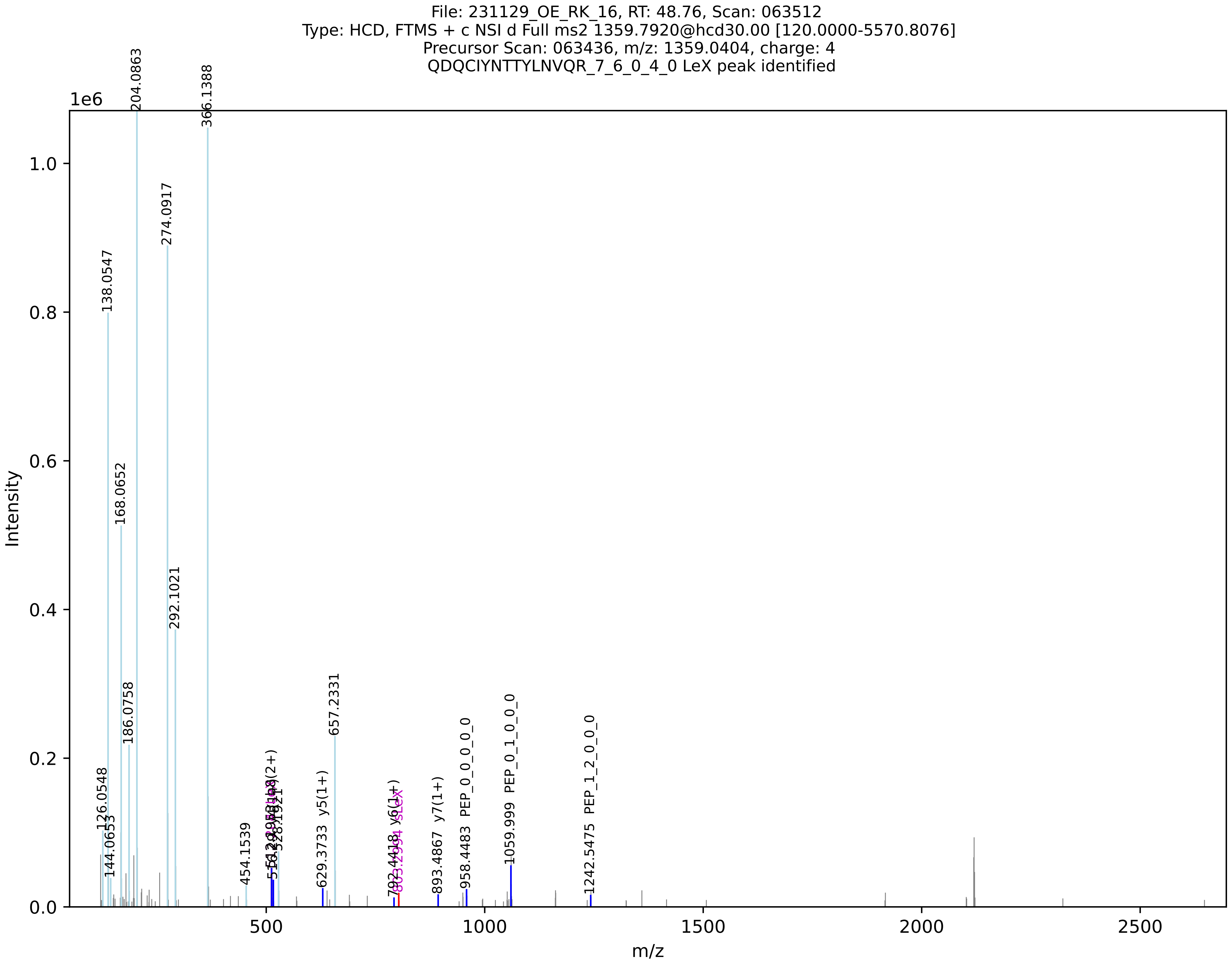

File: 231129_OE_RK_16, RT: 48.76, Scan: 063512
Type: HCD, FTMS + c NSI d Full ms2 1359.7920@hcd30.00 [120.0000-5570.8076]
Precursor Scan: 063436, m/z: 1359.0404, charge: 4
QDQCIYNTTYLNVQR_7_6_0_4_0 LeX peak identified
1e6
Intensity
m/z
204.0863
366.1388
274.0917
138.0547
168.0652
292.1021
186.0758
657.2331
126.0548
144.0653
454.1539
528.1921
629.3733 y5(1+)
792.4418 y6(1+)
803.2994 sLeX
893.4867 y7(1+)
958.4483 PEP_0_0_0_0_0
1059.999 PEP_0_1_0_0_0
1242.5475 PEP_1_2_0_0_0

File: 231129_OE_RK_16, RT: 48.813, Scan: 063590
Type: HCD, FTMS + c NSI d Full ms2 1764.3684@hcd30.00 [120.0000-5421.1094]
Precursor Scan: 063510, m/z: 1763.3661, charge: 3
QDQCIYNTTYLNVQR_7_6_1_3_0 LeX peak identified

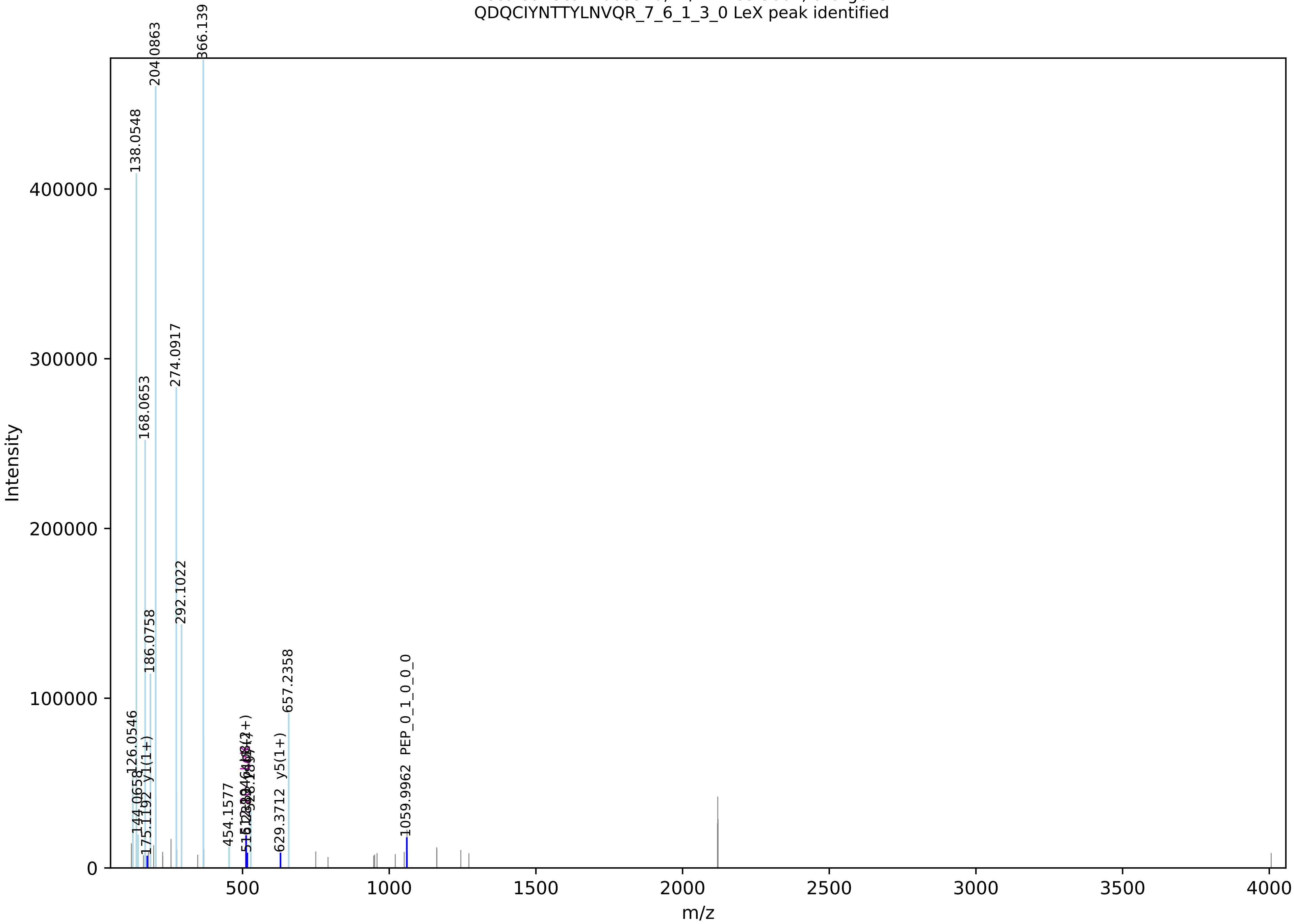

File: 231129_OE_RK_16, RT: 49.066, Scan: 063961
Type: HCD, FTMS + c NSI d Full ms2 1432.5663@hcd30.00 [120.0000-5867.7261]
Precursor Scan: 063880, m/z: 1432.0651, charge: 4
QDQCIYNTTYLNVQR_7_6_2_4_0 LeX peak identified

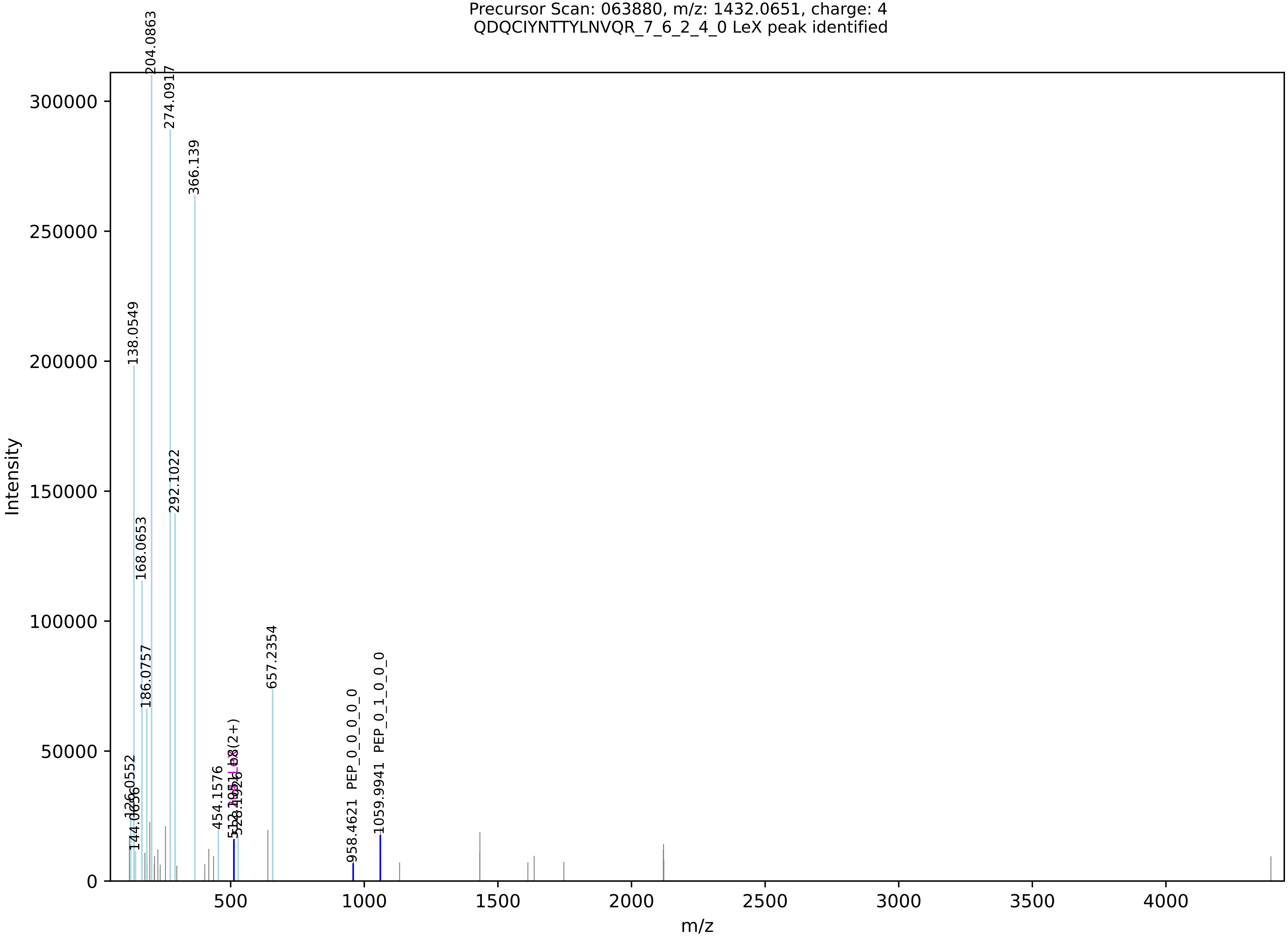

File: 231129_OE_RK_16, RT: 49.166, Scan: 064105
Type: HCD, FTMS + c NSI d Full ms2 1359.7911@hcd30.00 [120.0000-5570.8037]
Precursor Scan: 064028, m/z: 1359.0393, charge: 4
QDQCIYNTTYLNVQR_7_6_0_4_0 LeX peak identified

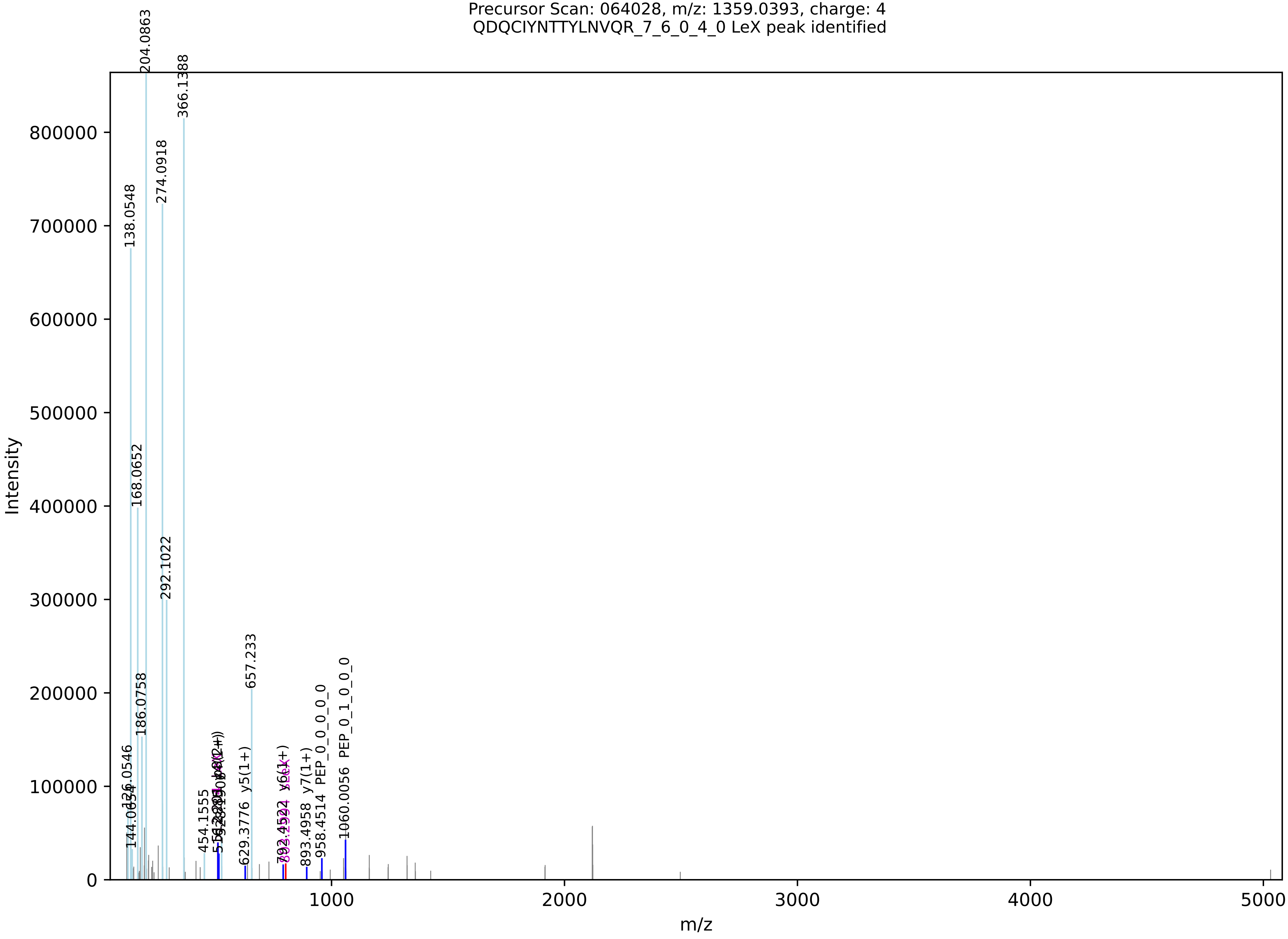

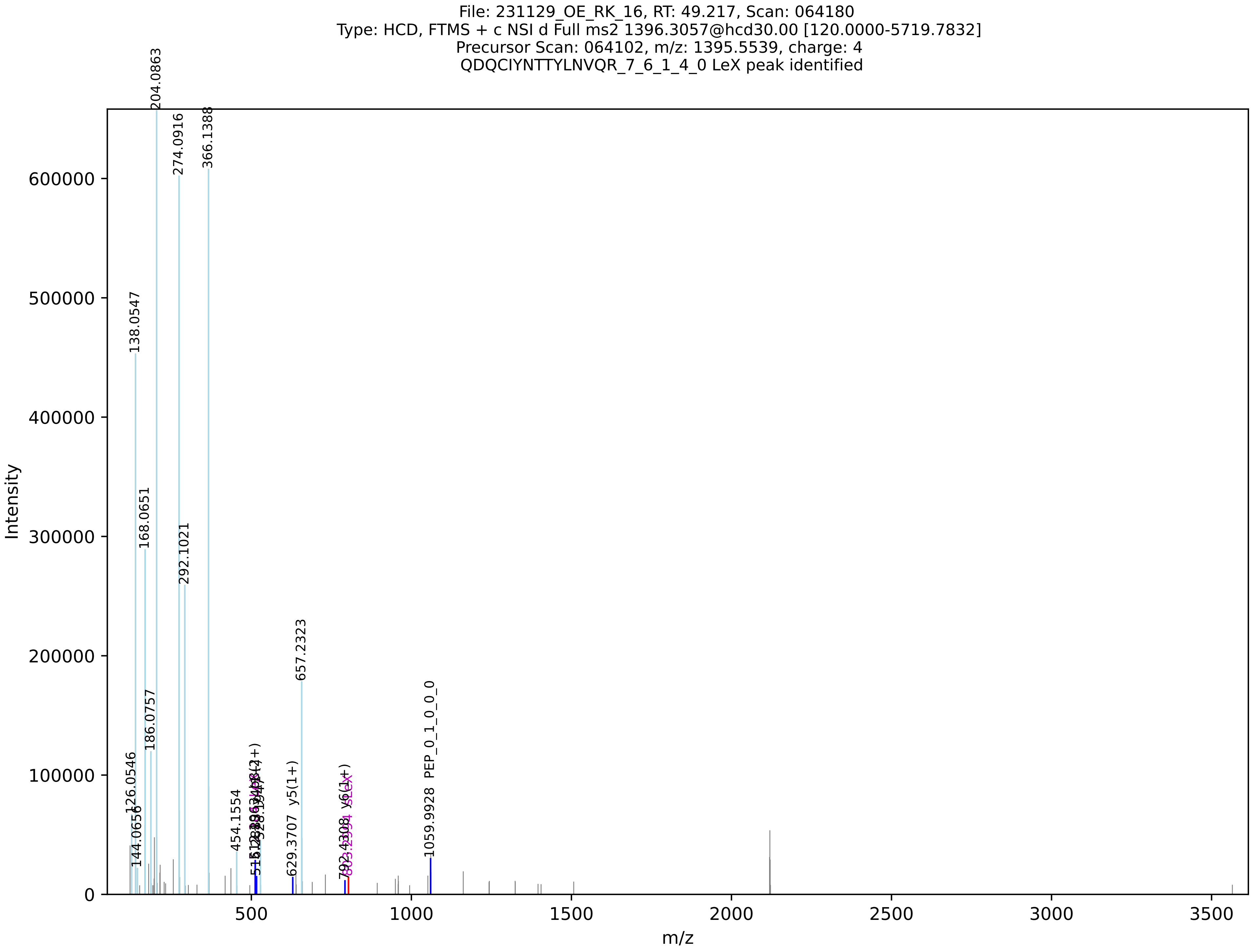
File: 231129_OE_RK_16, RT: 49.217, Scan: 064180
Type: HCD, FTMS + c NSI d Full ms2 1396.3057@hcd30.00 [120.0000-5719.7832]
Precursor Scan: 064102, m/z: 1395.5539, charge: 4
QDQCIYNTTYLNVQR_7_6_1_4_0 LeX peak identified
204.0863
274.0916
366.1388
138.0547
168.0651
292.1021
657.2323
186.0757
126.0546
144.0656
454.1554
629.3707 y5(1+)
792.4308 y6(1+)
1059.9928 PEP_0_1_0_0_0
Intensity
m/z
0
100000
200000
300000
400000
500000
600000
500
1000
1500
2000
2500
3000
3500

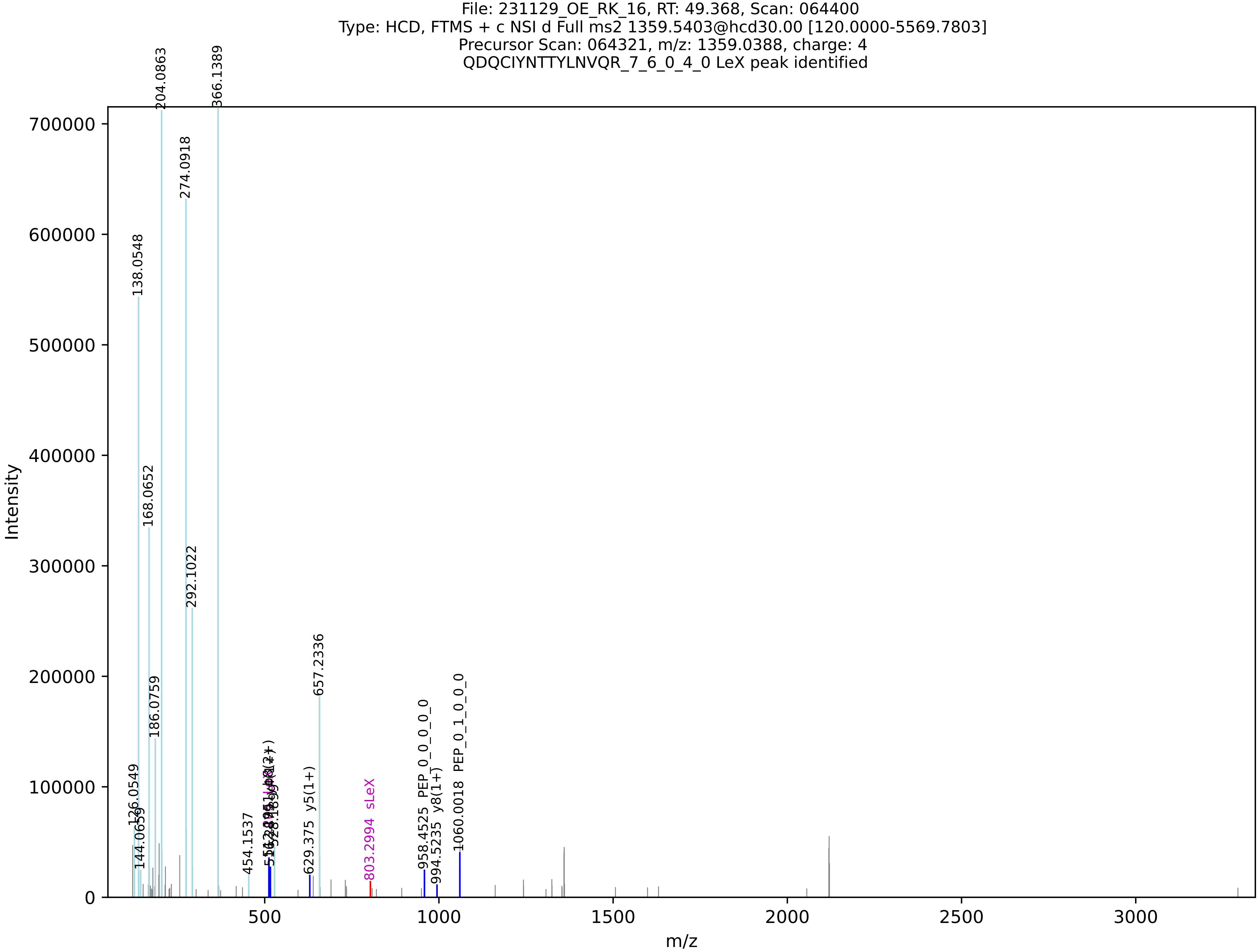

File: 231129_OE_RK_16, RT: 49.368, Scan: 064400
Type: HCD, FTMS + c NSI d Full ms2 1359.5403@hcd30.00 [120.0000-5569.7803]
Precursor Scan: 064321, m/z: 1359.0388, charge: 4
QDQCIYNTTYLNVQR_7_6_0_4_0 LeX peak identified
204.0863
366.1389
274.0918
138.0548
168.0652
292.1022
186.0759
126.0549
144.0659
454.1537
629.375 y5(1+)
657.2336
803.2994 sLeX
958.4525 PEP_0_0_0_0_0
994.5235 y8(1+)
1060.0018 PEP_0_1_0_0_0
Intensity
m/z

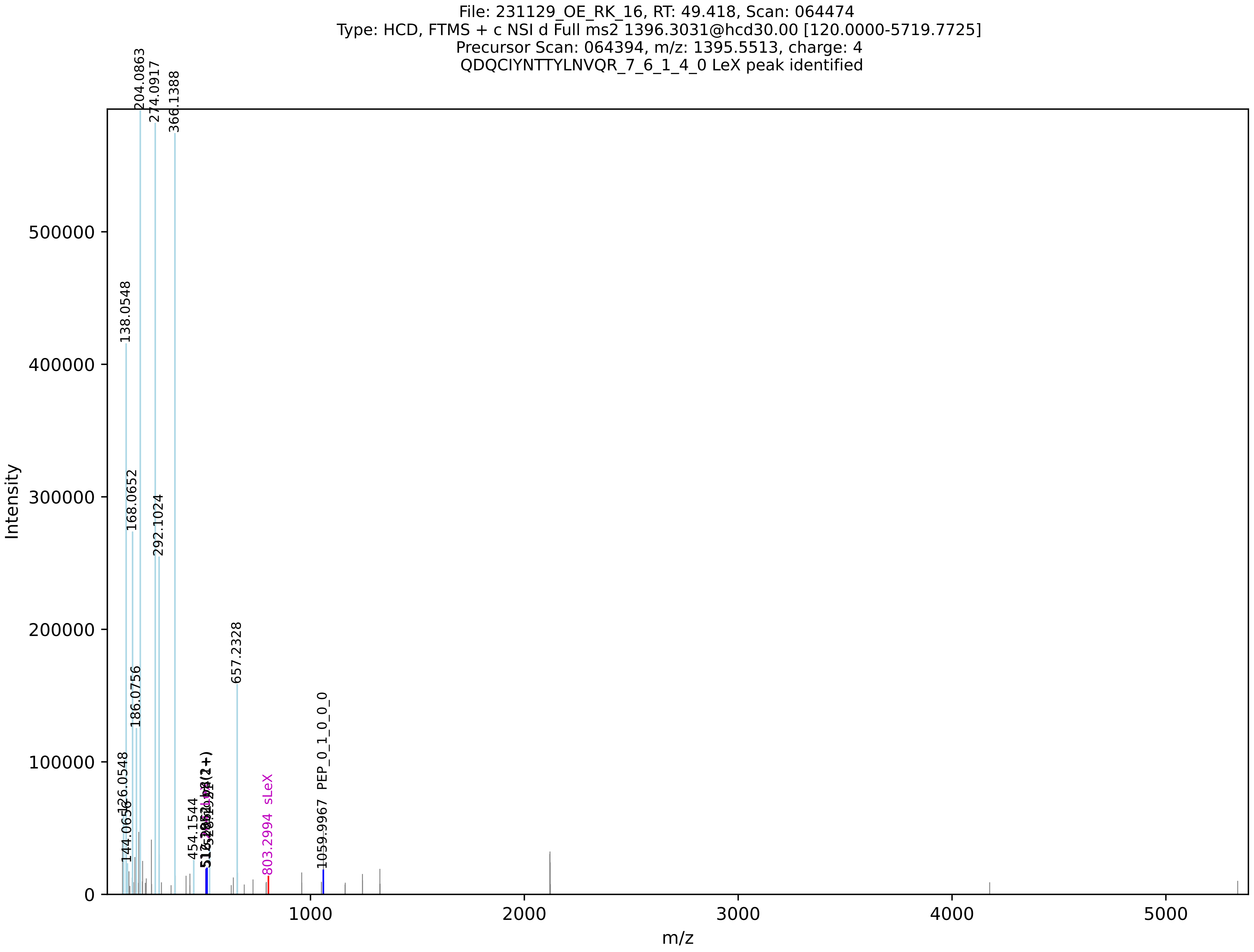

File: 231129_OE_RK_16, RT: 49.418, Scan: 064474
Type: HCD, FTMS + c NSI d Full ms2 1396.3031@hcd30.00 [120.0000-5719.7725]
Precursor Scan: 064394, m/z: 1395.5513, charge: 4
QDQCIYNTTYLNVQR_7_6_1_4_0 LeX peak identified
Intensity
m/z
0
100000
200000
300000
400000
500000
1000
2000
3000
4000
5000
204.0863
274.0917
366.1388
138.0548
168.0652
292.1024
186.0756
657.2328
126.0548
144.0656
454.1544
803.2994 sLeX
1059.9967 PEP_0_1_0_0_0_

File: 231129_OE_RK_16, RT: 49.82, Scan: 065062
Type: HCD, FTMS + c NSI d Full ms2 1396.3042@hcd30.00 [120.0000-5719.7773]
Precursor Scan: 064984, m/z: 1395.5519, charge: 4
LDVEEPDSANSSFYSTR_7_6_1_4_0 LeX peak identified

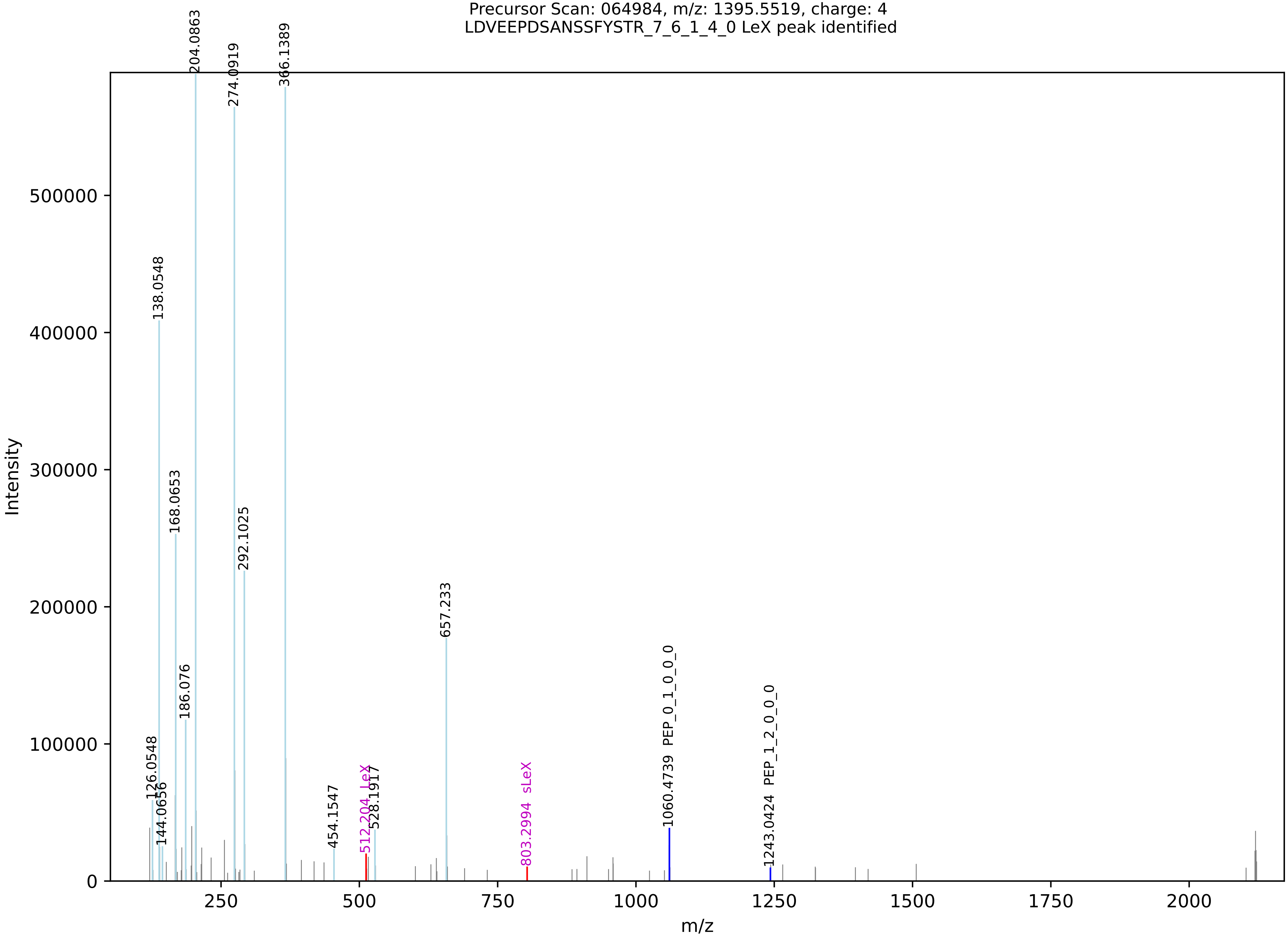

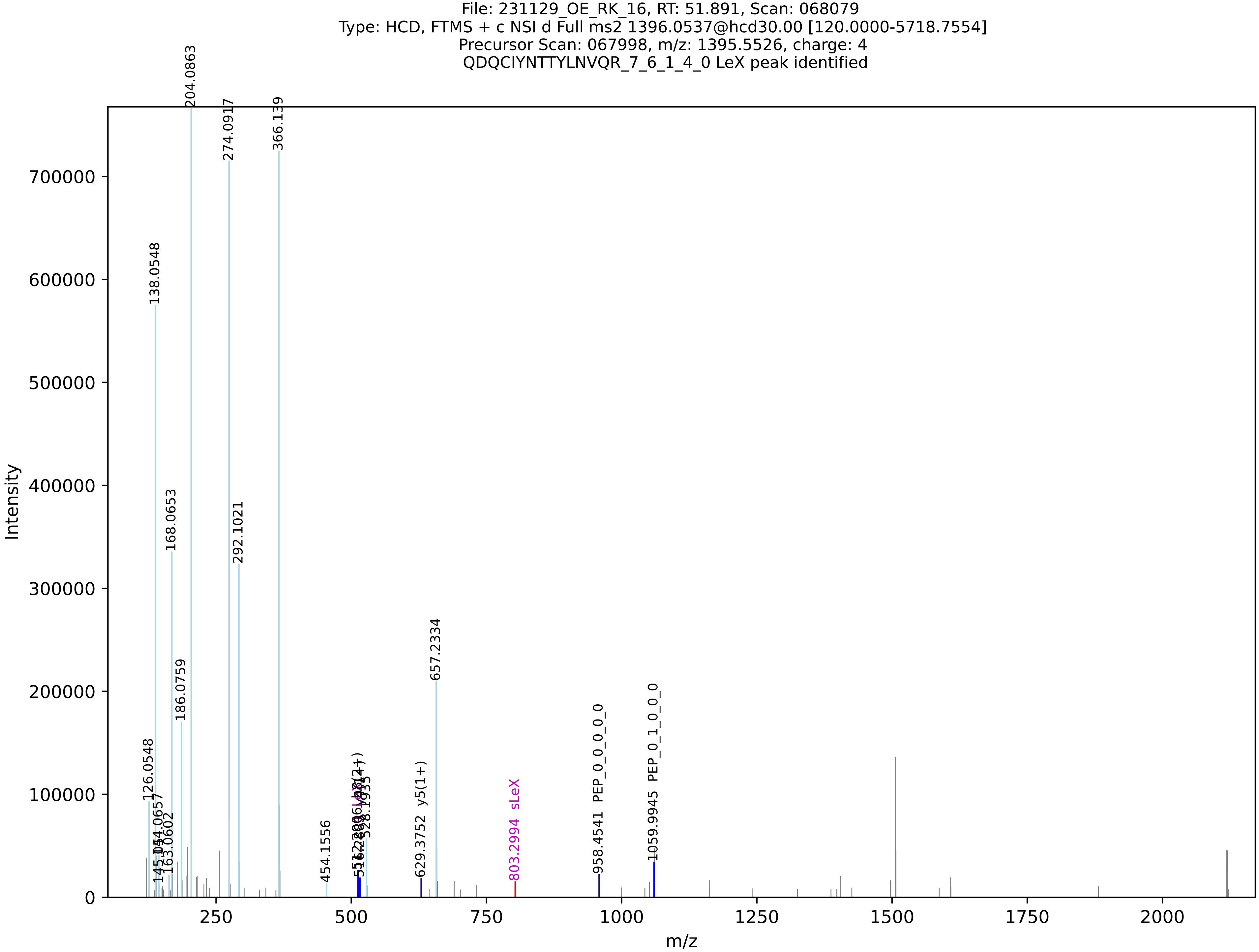
File: 231129_OE_RK_16, RT: 51.891, Scan: 068079
Type: HCD, FTMS + c NSI d Full ms2 1396.0537@hcd30.00 [120.0000-5718.7554]
Precursor Scan: 067998, m/z: 1395.5526, charge: 4
QDQCIYNTTYLNVQR_7_6_1_4_0 LeX peak identified
Intensity
m/z
204.0863
274.0917
366.139
138.0548
168.0653
292.1021
186.0759
126.0548
657.2334
454.1556
629.3752 y5(1+)
803.2994 sLeX
958.4541 PEP_0_0_0_0_0_
1059.9945 PEP_0_1_0_0_0_
528.1935
163.0602

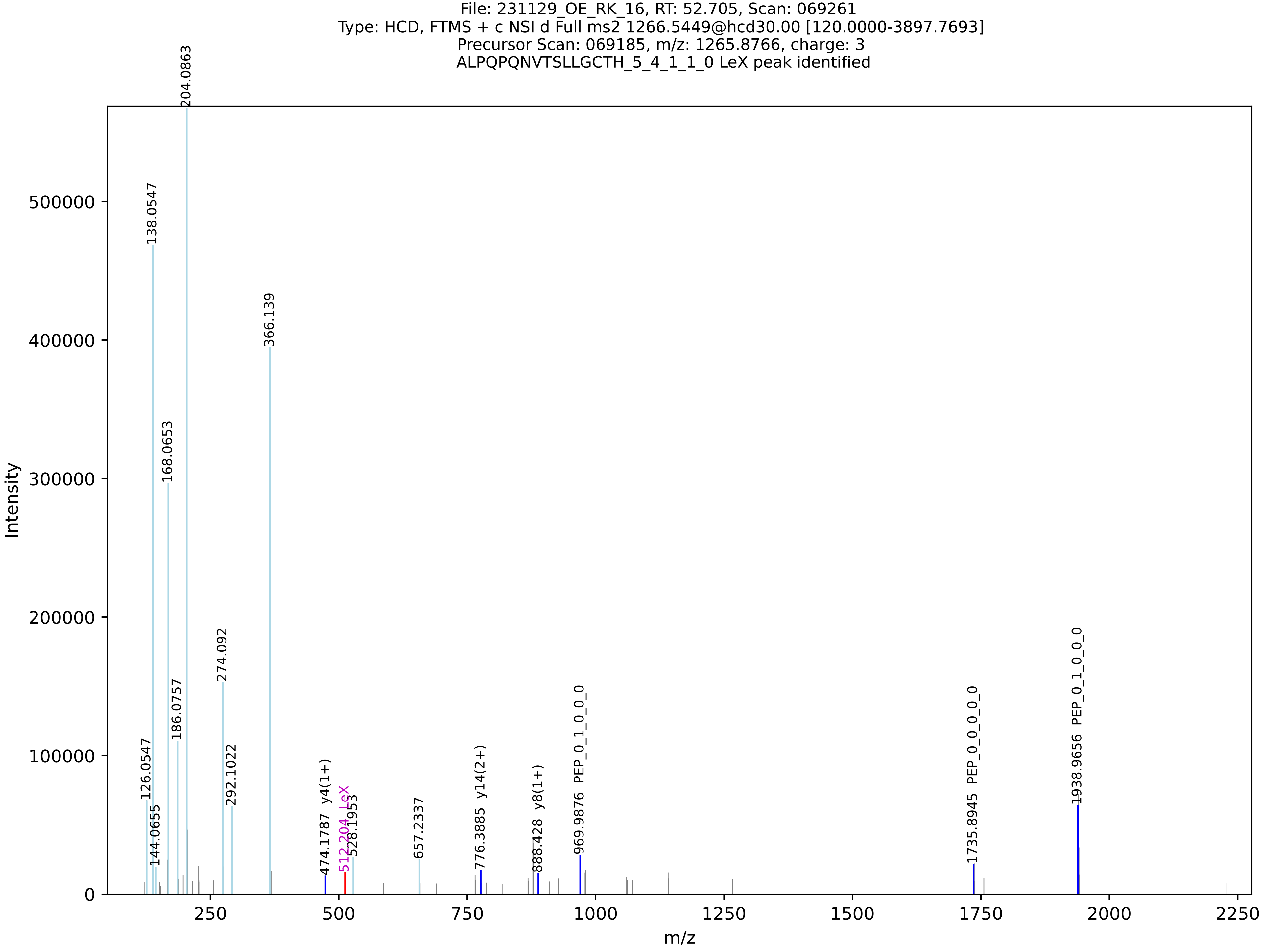
File: 231129_OE_RK_16, RT: 52.705, Scan: 069261
Type: HCD, FTMS + c NSI d Full ms2 1266.5449@hcd30.00 [120.0000-3897.7693]
Precursor Scan: 069185, m/z: 1265.8766, charge: 3
ALPQPQNVTSLLGCTH_5_4_1_1_0 LeX peak identified
Intensity
m/z
204.0863
138.0547
366.139
168.0653
274.092
186.0757
292.1022
126.0547
144.0655
474.1787 y4(1+)
512.204 LeX
528.1953
657.2337
776.3885 y14(2+)
888.428 y8(1+)
969.9876 PEP_0_1_0_0_0
1735.8945 PEP_0_0_0_0_0
1938.9656 PEP_0_1_0_0_0

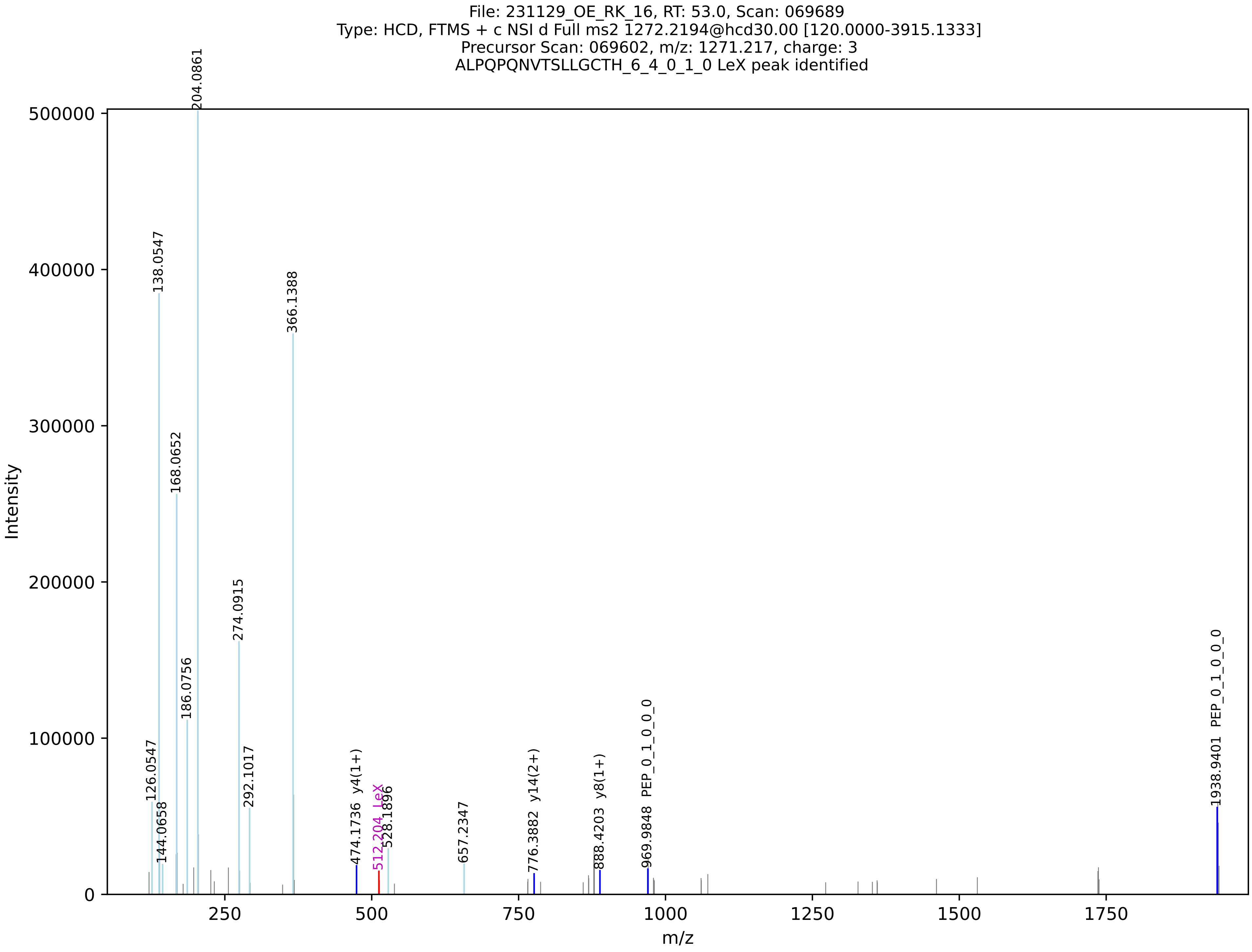
File: 231129_OE_RK_16, RT: 53.0, Scan: 069689
Type: HCD, FTMS + c NSI d Full ms2 1272.2194@hcd30.00 [120.0000-3915.1333]
Precursor Scan: 069602, m/z: 1271.217, charge: 3
ALPQPQNVTSLLGCTH_6_4_0_1_0 LeX peak identified
Intensity
m/z
204.0861
138.0547
366.1388
168.0652
274.0915
186.0756
126.0547
144.0658
292.1017
474.1736 y4(1+)
512.204_LeX
528.1896
657.2347
776.3882 y14(2+)
888.4203 y8(1+)
969.9848 PEP_0_1_0_0_0
1938.9401 PEP_0_1_0_0_0
0
100000
200000
300000
400000
500000
250
500
750
1000
1250
1500
1750

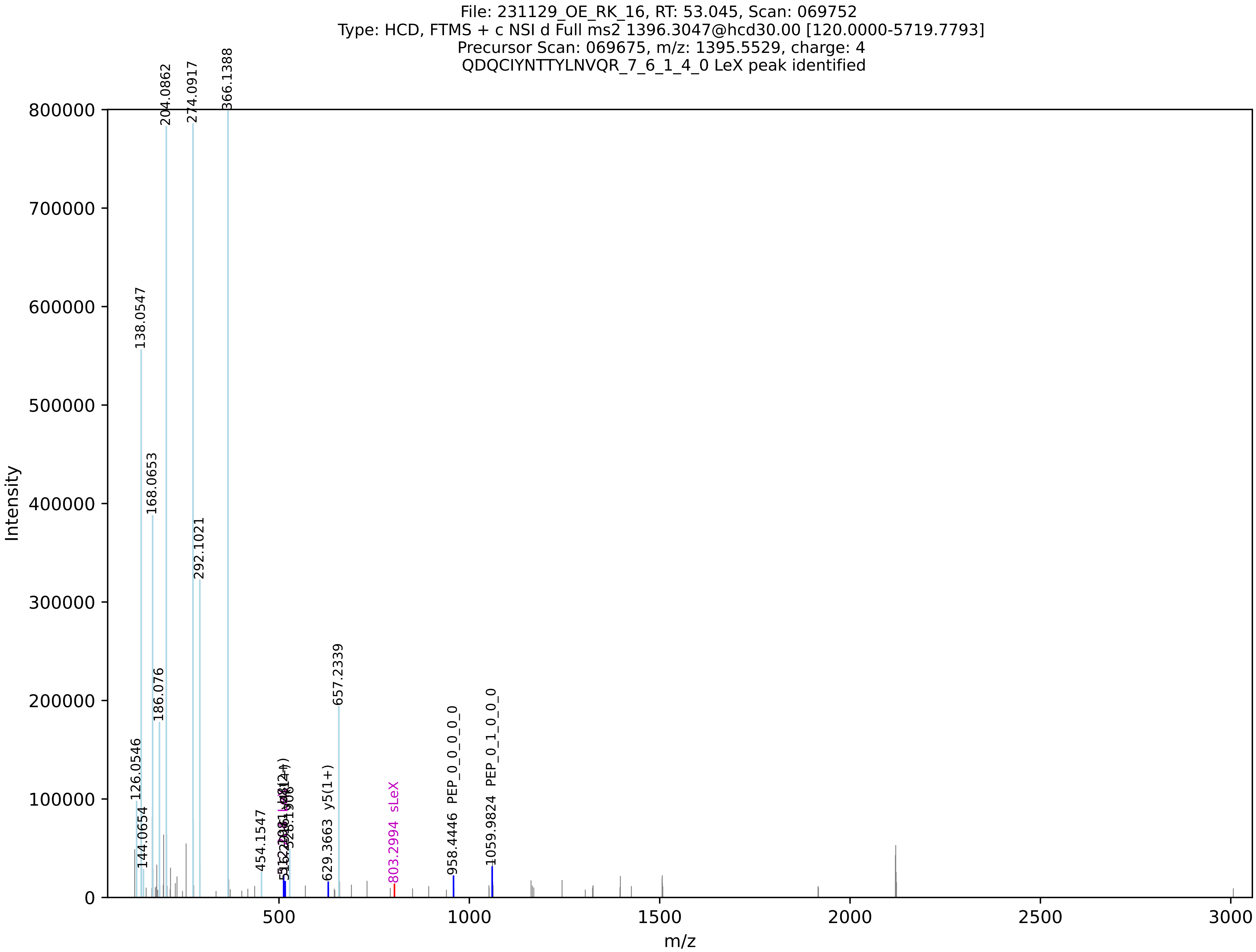
File: 231129_OE_RK_16, RT: 53.045, Scan: 069752
Type: HCD, FTMS + c NSI d Full ms2 1396.3047@hcd30.00 [120.0000-5719.7793]
Precursor Scan: 069675, m/z: 1395.5529, charge: 4
QDQCIYNTTYLNVQR_7_6_1_4_0 LeX peak identified
Intensity
m/z
204.0862
274.0917
366.1388
138.0547
168.0653
292.1021
186.076
126.0546
144.0654
454.1547
657.2339
629.3663 y5(1+)
803.2994 sLeX
958.4446 PEP_0_0_0_0_0_
1059.9824 PEP_0_1_0_0_0_

File: 231129_OE_RK_16, RT: 53.095, Scan: 069825
Type: HCD, FTMS + c NSI d Full ms2 1266.5447@hcd30.00 [120.0000-3897.7688]
Precursor Scan: 069749, m/z: 1265.8761, charge: 3
ALPQPQNVTSLLGCTH_5_4_1_1_0 LeX peak identified

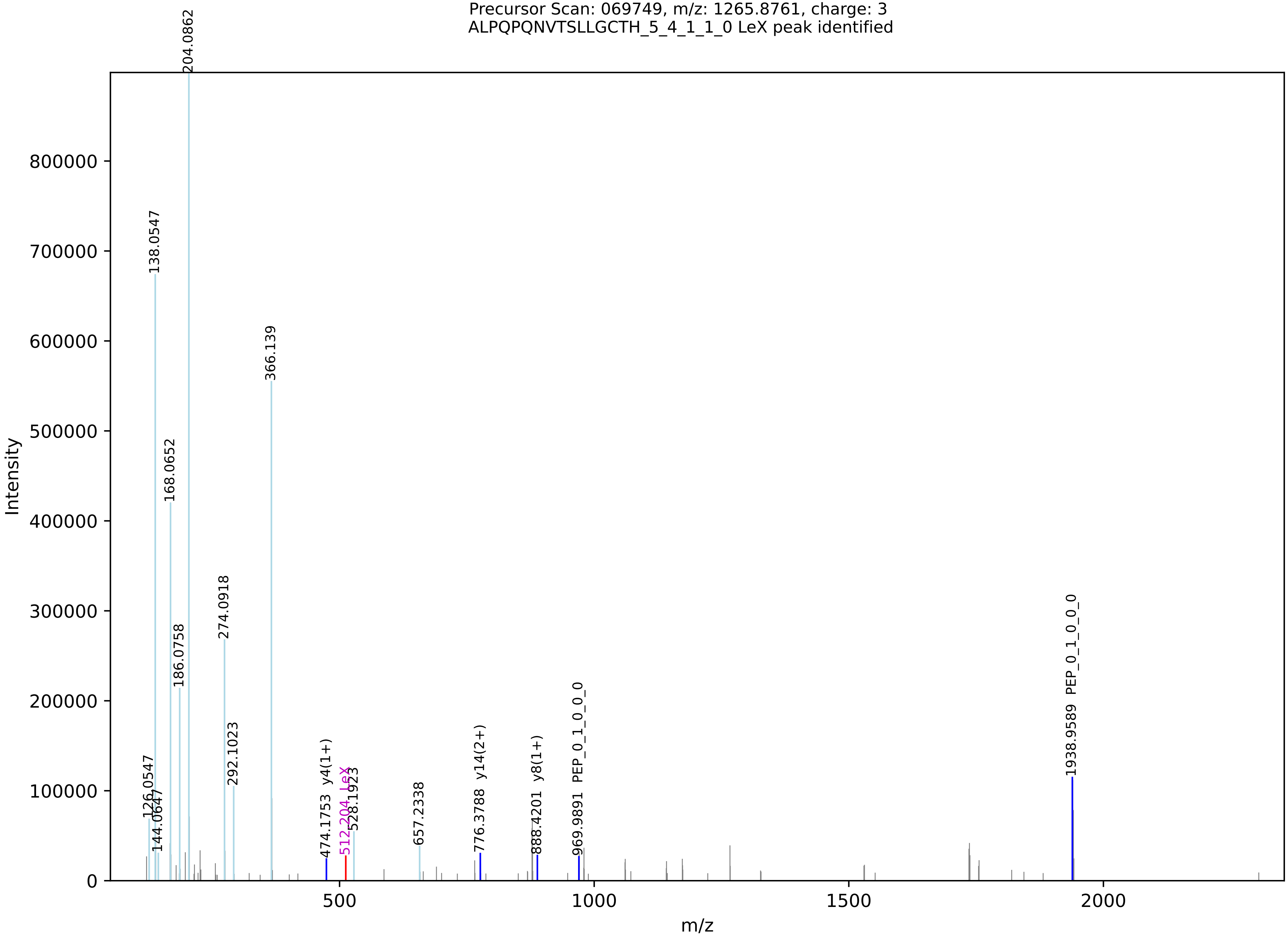

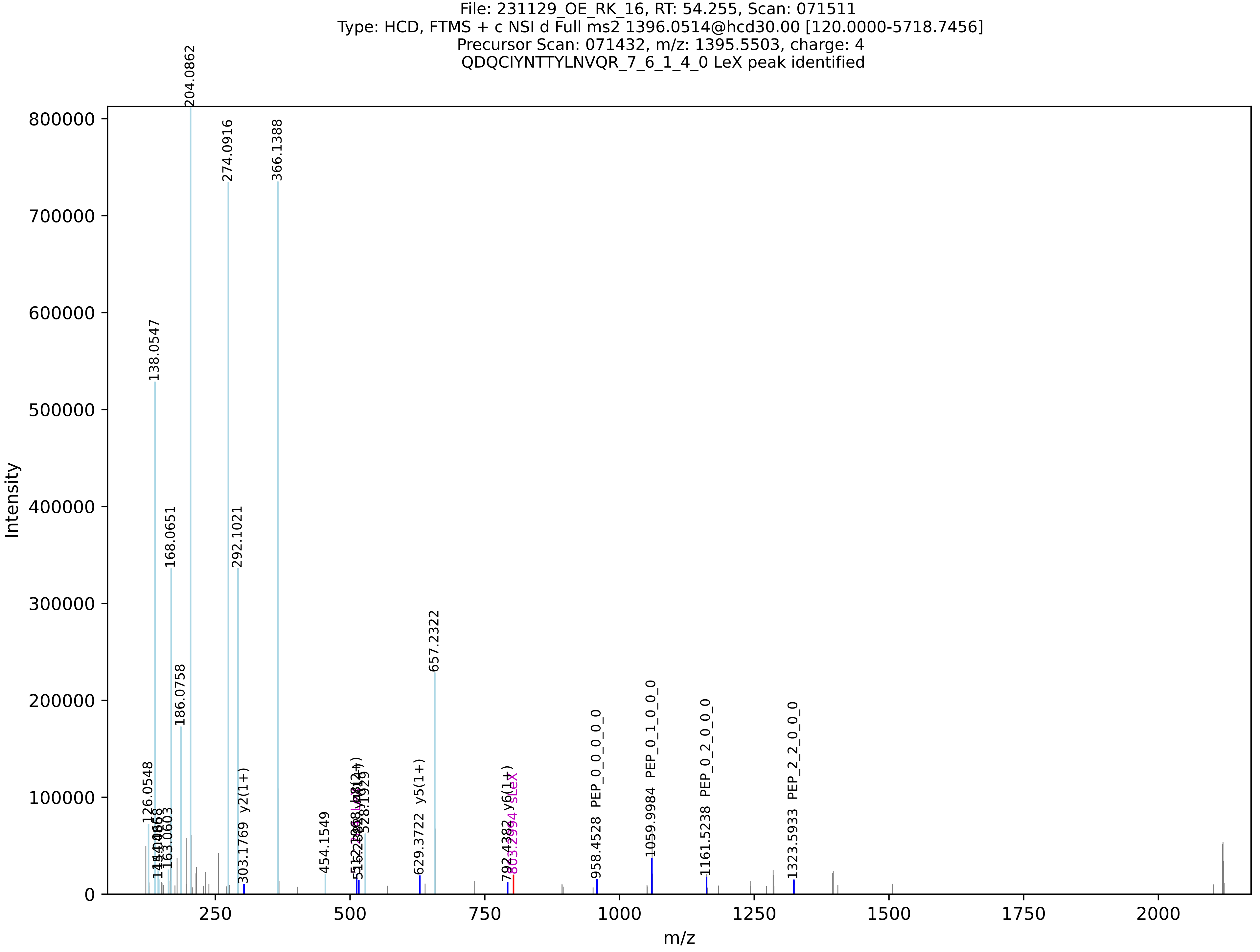

File: 231129_OE_RK_16, RT: 54.255, Scan: 071511
Type: HCD, FTMS + c NSI d Full ms2 1396.0514@hcd30.00 [120.0000-5718.7456]
Precursor Scan: 071432, m/z: 1395.5503, charge: 4
QDQCIYNTTYLNVQR_7_6_1_4_0 LeX peak identified
Intensity
m/z
204.0862
274.0916
366.1388
138.0547
168.0651
292.1021
186.0758
657.2322
126.0548
163.0603
303.1769 y2(1+)
454.1549
528.1929
629.3722 y5(1+)
792.4382 y6(1+)
803.2994 SLeX
958.4528 PEP_0_0_0_0_0
1059.9984 PEP_0_1_0_0_0
1161.5238 PEP_0_2_0_0_0
1323.5933 PEP_2_2_0_0_0

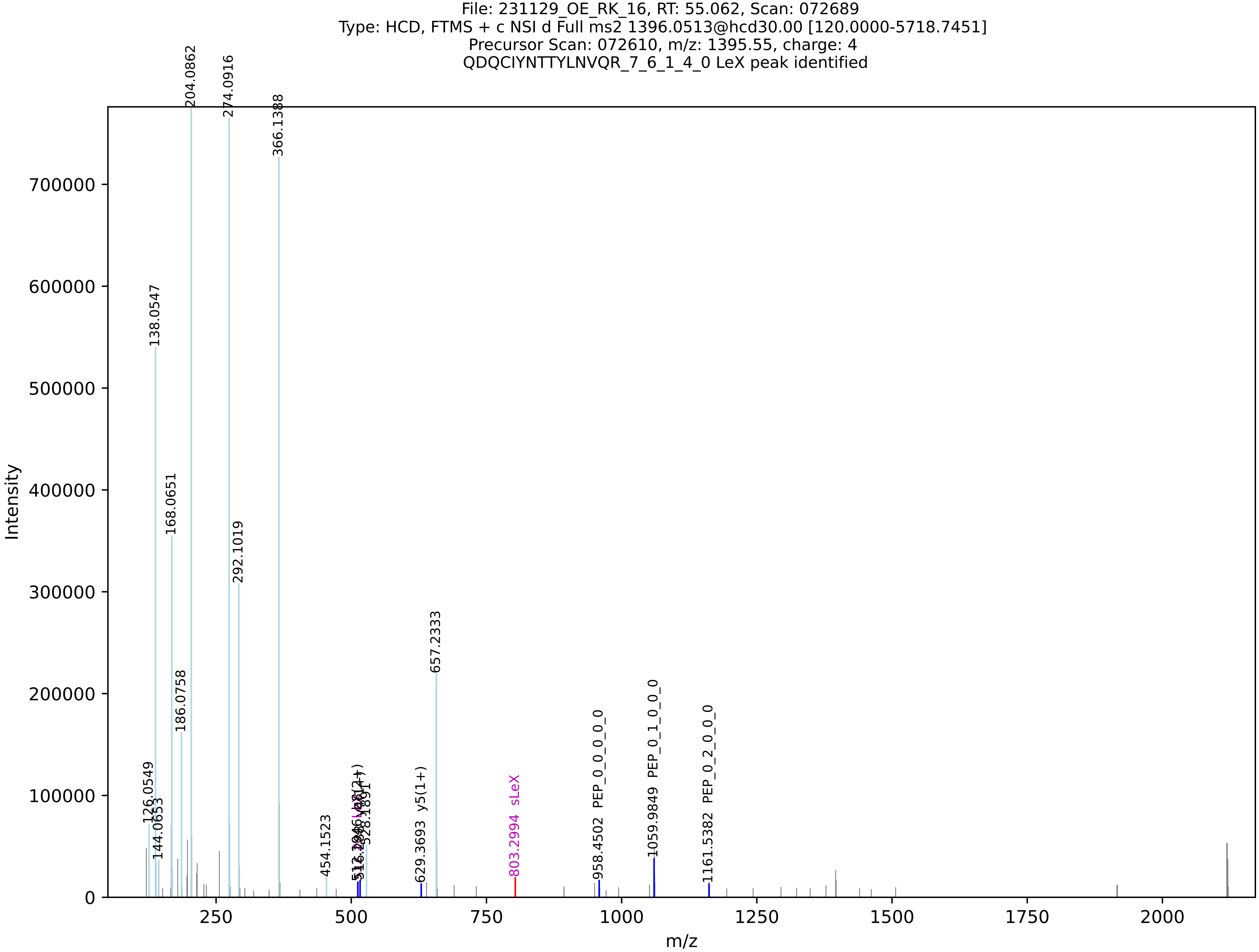
File: 231129_OE_RK_16, RT: 55.062, Scan: 072689
Type: HCD, FTMS + c NSI d Full ms2 1396.0513@hcd30.00 [120.0000-5718.7451]
Precursor Scan: 072610, m/z: 1395.55, charge: 4
QDQCIYNTTYLNVQR_7_6_1_4_0 LeX peak identified
Intensity
m/z
204.0862
274.0916
366.1388
138.0547
168.0651
292.1019
186.0758
657.2333
126.0549
144.0653
454.1523
629.3693 y5(1+)
803.2994 sLeX
958.4502 PEP_0_0_0_0_0
1059.9849 PEP_0_1_0_0_0
1161.5382 PEP_0_2_0_0_0
528.1891

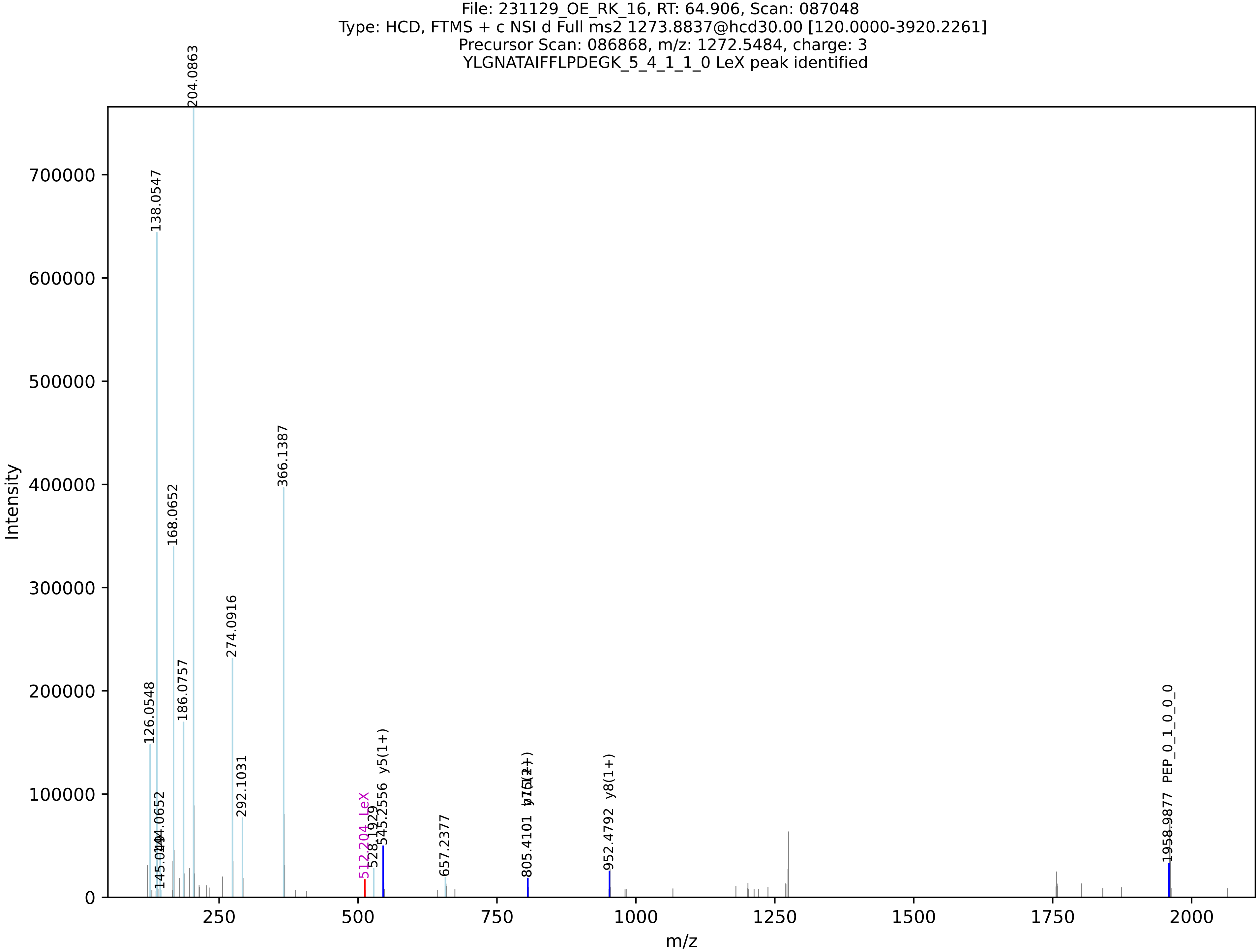
File: 231129_OE_RK_16, RT: 64.906, Scan: 087048
Type: HCD, FTMS + c NSI d Full ms2 1273.8837@hcd30.00 [120.0000-3920.2261]
Precursor Scan: 086868, m/z: 1272.5484, charge: 3
YLGNATAIFFLPDEGK_5_4_1_1_0 LeX peak identified
Intensity
m/z
204.0863
138.0547
366.1387
168.0652
274.0916
186.0757
126.0548
292.1031
144.0652
512.204_LeX
545.2556 y5(1+)
657.2377
805.4101
952.4792 y8(1+)
1958.9877 PEP_0_1_0_0_0

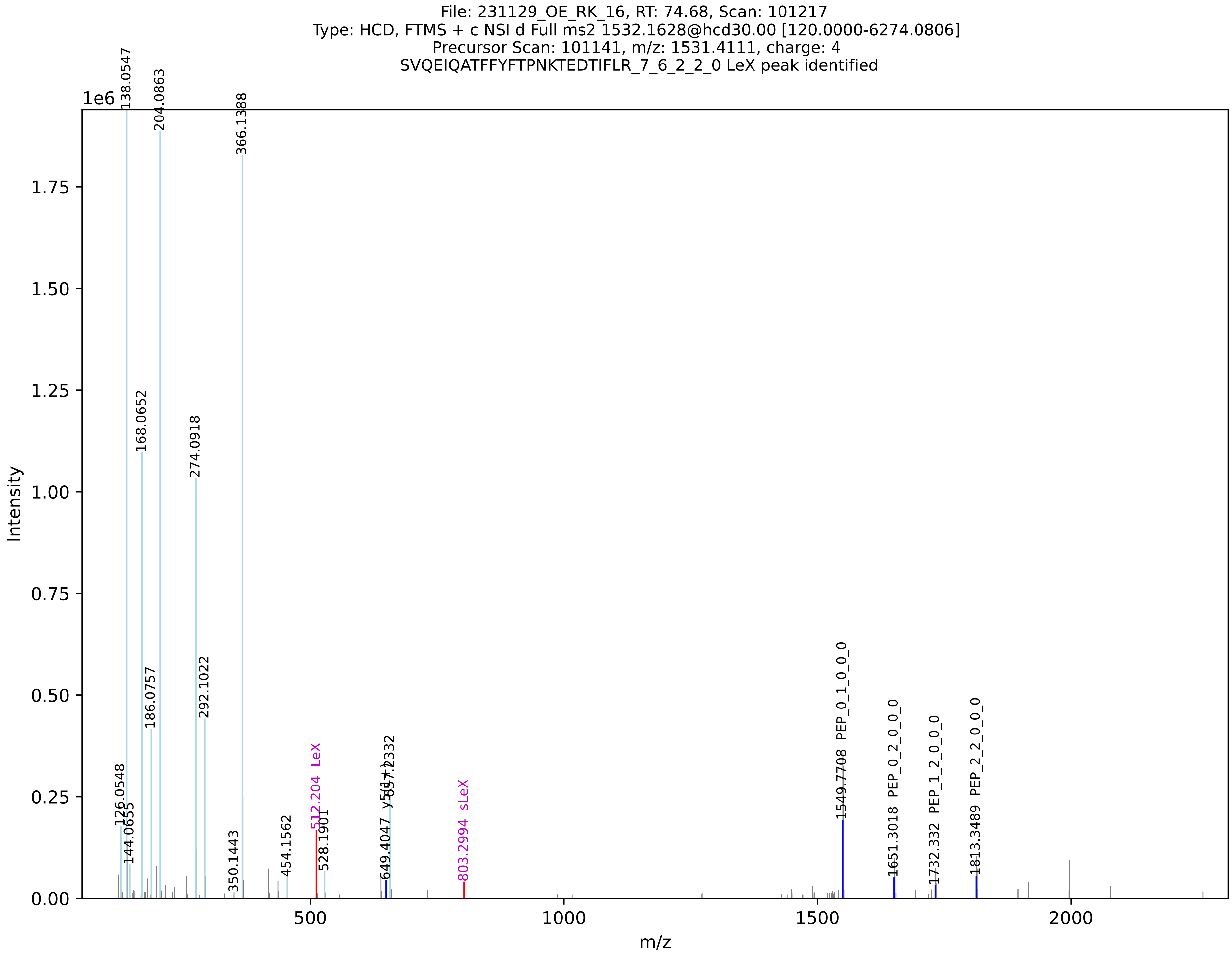

File: 231129_OE_RK_16, RT: 74.68, Scan: 101217
Type: HCD, FTMS + c NSI d Full ms2 1532.1628@hcd30.00 [120.0000-6274.0806]
Precursor Scan: 101141, m/z: 1531.4111, charge: 4
SVQEIQATFFYFTPNKTEDTIFLR_7_6_2_2_0 LeX peak identified
1e6
Intensity
m/z
0.00
0.25
0.50
0.75
1.00
1.25
1.50
1.75
500
1000
1500
2000
138.0547
204.0863
366.1388
168.0652
274.0918
186.0757
292.1022
126.0548
144.0655
350.1443
454.1562
512.204 LeX
528.1901
649.4047
657.2332
803.2994 sLeX
1549.7708 PEP_0_1_0_0_0
1651.3018 PEP_0_2_0_0_0
1732.332 PEP_1_2_0_0_0
1813.3489 PEP_2_2_0_0_0

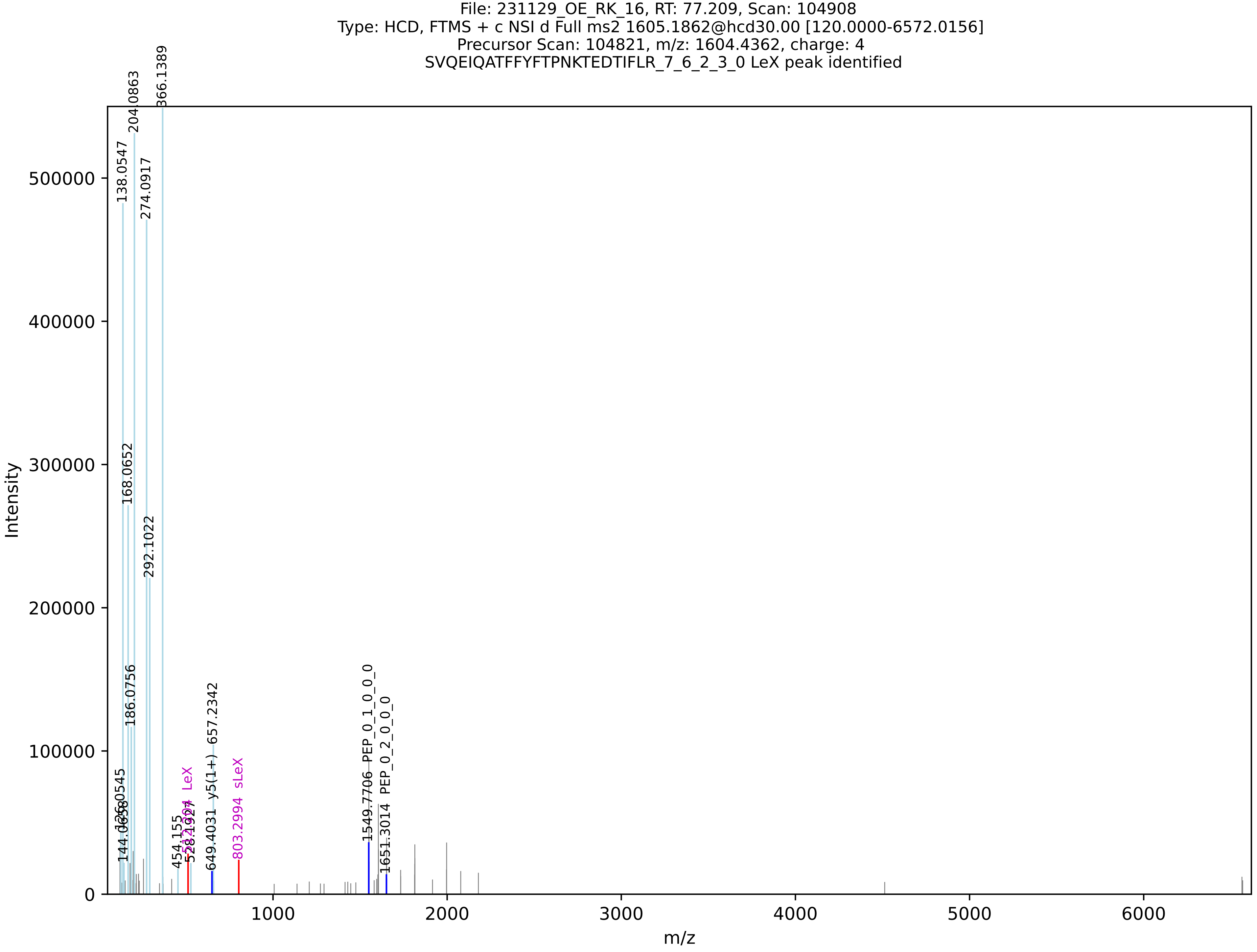
File: 231129_OE_RK_16, RT: 77.209, Scan: 104908
Type: HCD, FTMS + c NSI d Full ms2 1605.1862@hcd30.00 [120.0000-6572.0156]
Precursor Scan: 104821, m/z: 1604.4362, charge: 4
SVQEIQATFFYFTPNKTEDTIFLR_7_6_2_3_0 LeX peak identified
Intensity
m/z
366.1389
204.0863
138.0547
274.0917
168.0652
292.1022
186.0756
144.0658
657.2342
y5(1+)
649.4031
LeX
528.1927
454.155
sLeX
803.2994
1549.7706_PEP_0_1_0_0_0
1651.3014_PEP_0_2_0_0_0

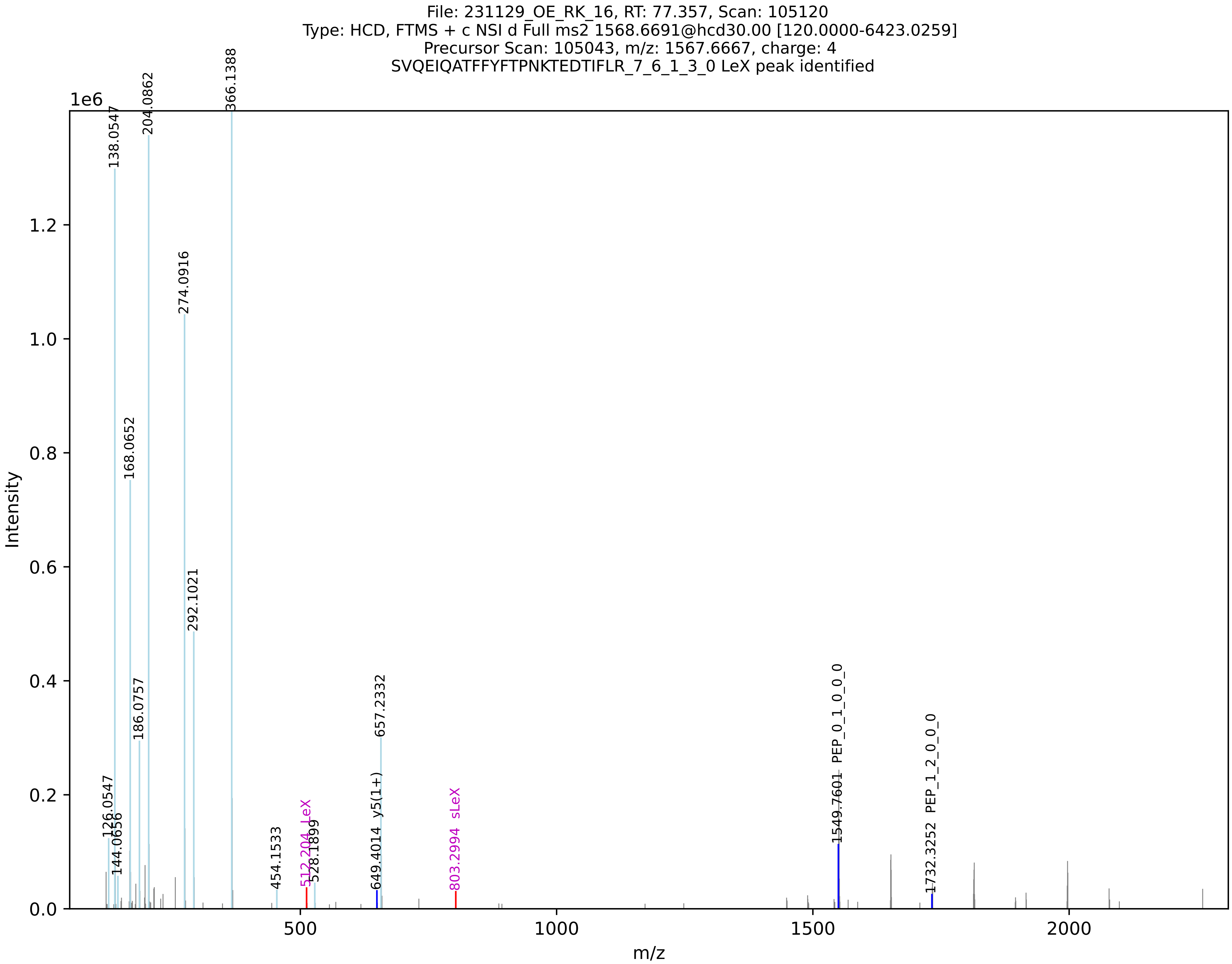

File: 231129_OE_RK_16, RT: 77.357, Scan: 105120
Type: HCD, FTMS + c NSI d Full ms2 1568.6691@hcd30.00 [120.0000-6423.0259]
Precursor Scan: 105043, m/z: 1567.6667, charge: 4
SVQEIQATFFYFTPNKTEDTIFLR_7_6_1_3_0 LeX peak identified
1e6
Intensity
m/z
366.1388
204.0862
138.0547
274.0916
168.0652
292.1021
186.0757
126.0547
144.0656
454.1533
512.204 LeX
528.1899
657.2332
649.4014 y5(1+)
803.2994 sLeX
1549.7601 PEP_0_1_0_0_0
1732.3252 PEP_1_2_0_0_0

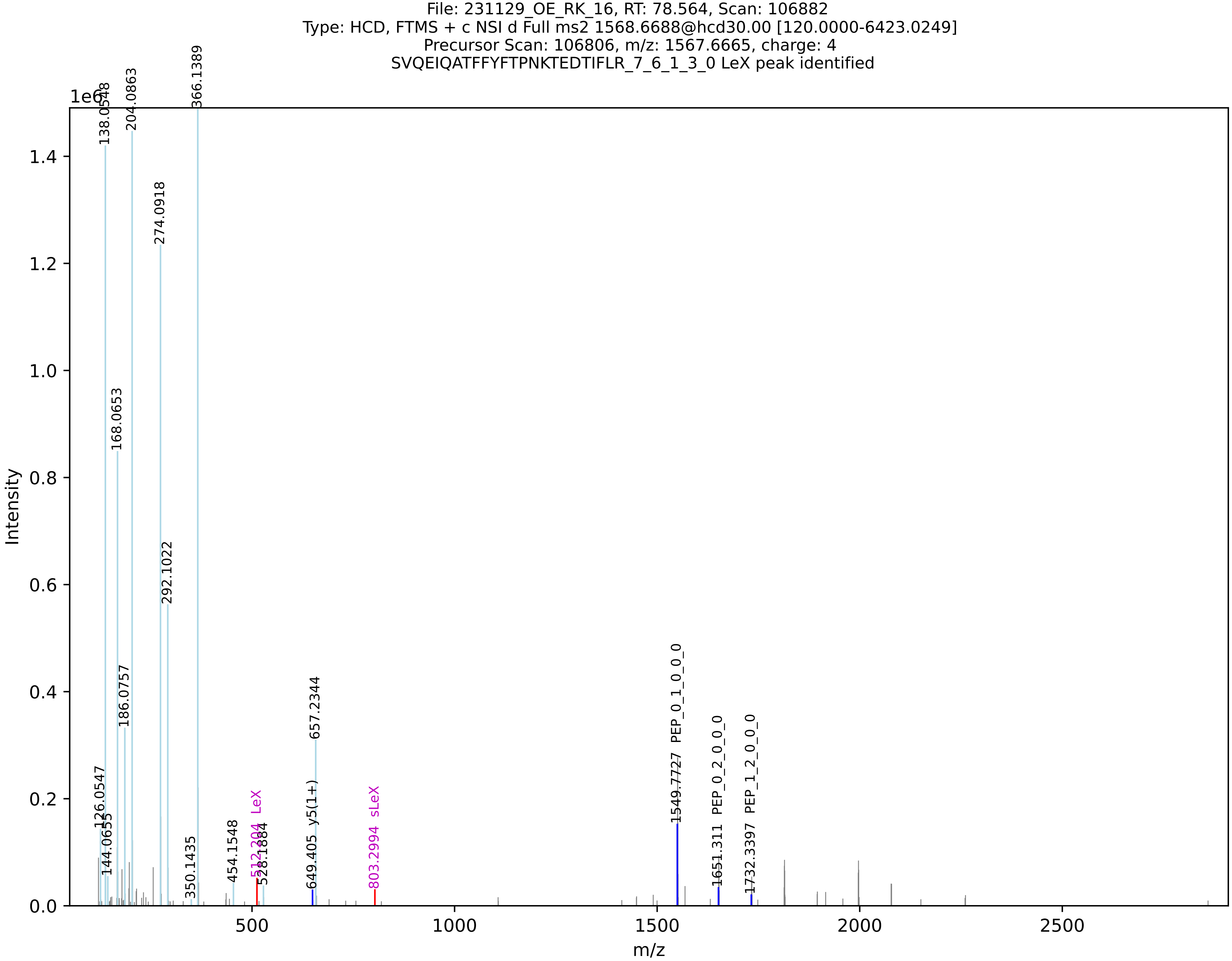
File: 231129_OE_RK_16, RT: 78.564, Scan: 106882
Type: HCD, FTMS + c NSI d Full ms2 1568.6688@hcd30.00 [120.0000-6423.0249]
Precursor Scan: 106806, m/z: 1567.6665, charge: 4
SVQEIQATFFYFTPNKTEDTIFLR_7_6_1_3_0 LeX peak identified
1e6
Intensity
m/z
138.0548
204.0863
366.1389
168.0653
274.0918
292.1022
186.0757
126.0547
144.0655
350.1435
454.1548
512.204 LeX
528.1884
649.405 y5(1+)
657.2344
803.2994 sLeX
1549.7727 PEP_0_1_0_0_0
1651.311 PEP_0_2_0_0_0
1732.3397 PEP_1_2_0_0_0

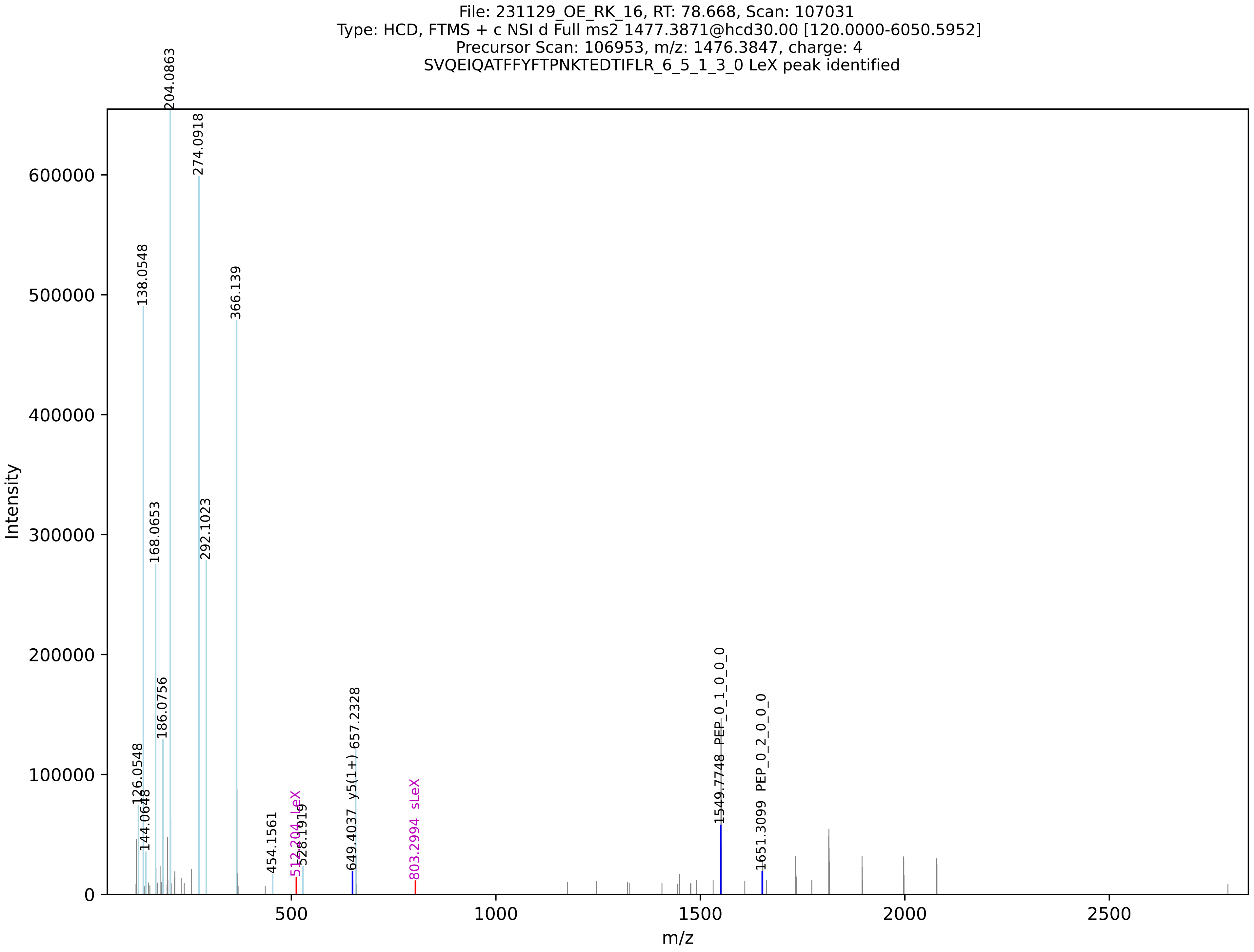

File: 231129_OE_RK_16, RT: 78.668, Scan: 107031
Type: HCD, FTMS + c NSI d Full ms2 1477.3871@hcd30.00 [120.0000-6050.5952]
Precursor Scan: 106953, m/z: 1476.3847, charge: 4
SVQEIQATFFYFTPNKTEDTIFLR_6_5_1_3_0 LeX peak identified
Intensity
m/z
0
100000
200000
300000
400000
500000
600000
500
1000
1500
2000
2500
204.0863
274.0918
138.0548
366.139
168.0653
292.1023
186.0756
126.0548
144.0648
454.1561
512.204 LeX
528.1919
649.4037 y5(1+) 657.2328
803.2994 sLeX
1549.7748 PEP_0_1_0_0_0
1651.3099 PEP_0_2_0_0_0

File: 231129_OE_RK_16, RT: 85.691, Scan: 117208
Type: HCD, FTMS + c NSI d Full ms2 1603.7047@hcd30.00 [120.0000-6565.9712]
Precursor Scan: 117127, m/z: 1602.7023, charge: 4
ADTHDEILEGLNFNLTEIPEAQIHEGFQELLR_6_5_1_2_0 LeX peak identified

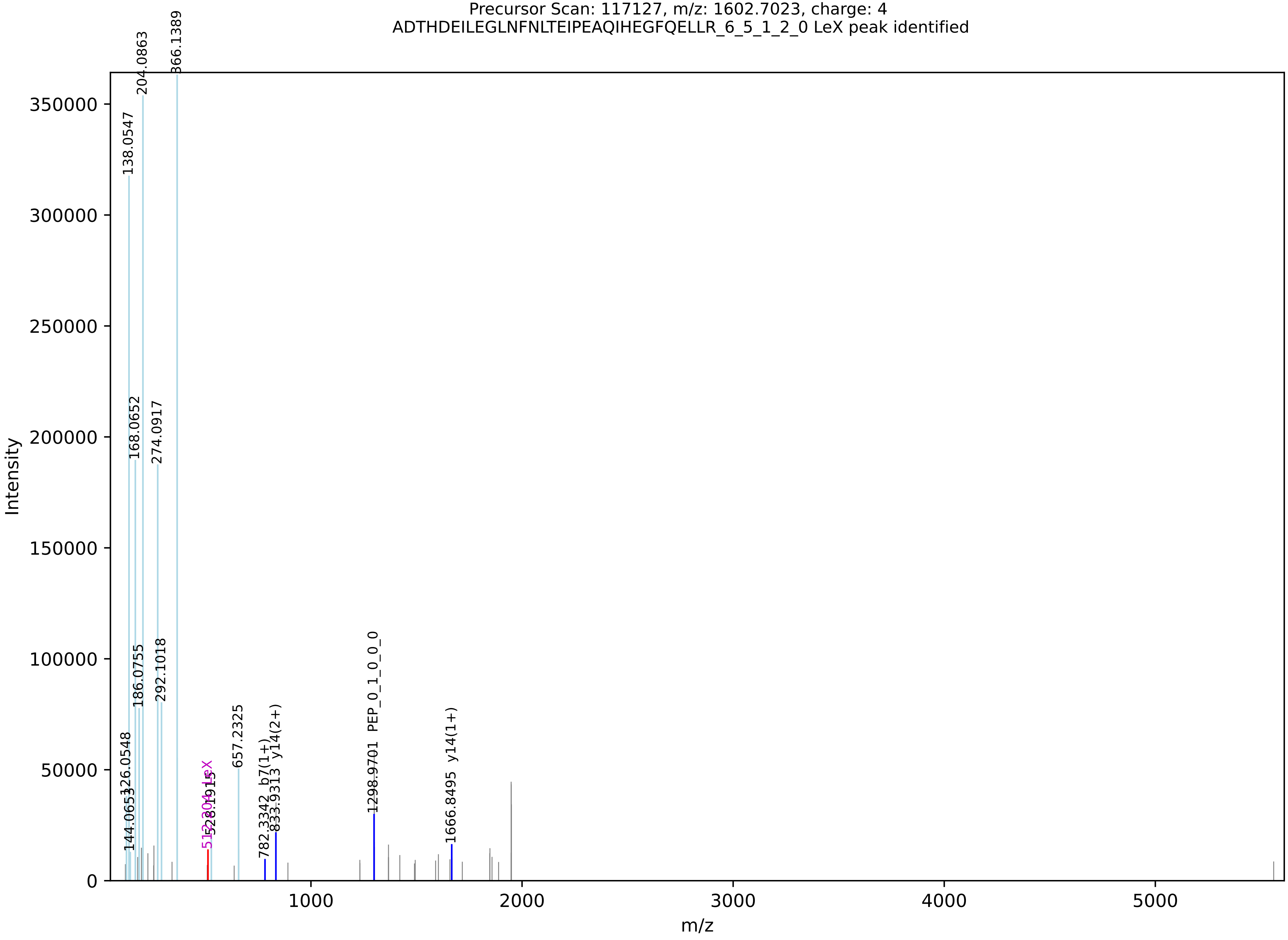

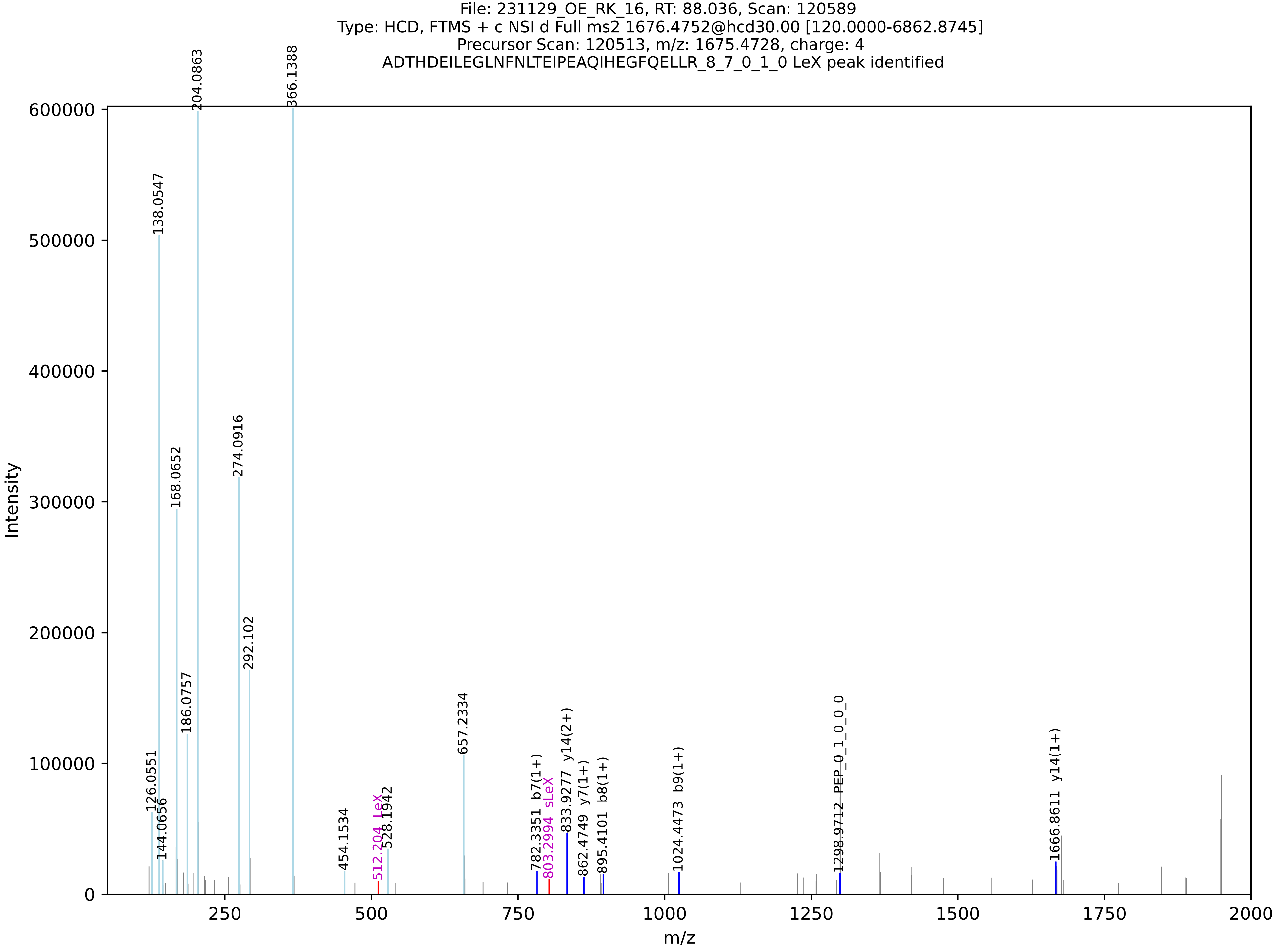
File: 231129_OE_RK_16, RT: 88.036, Scan: 120589
Type: HCD, FTMS + c NSI d Full ms2 1676.4752@hcd30.00 [120.0000-6862.8745]
Precursor Scan: 120513, m/z: 1675.4728, charge: 4
ADTHDEILEGLNFNLTEIPEAQIHEGFQELLR_8_7_0_1_0 LeX peak identified
Intensity
m/z
204.0863
366.1388
138.0547
168.0652
274.0916
292.102
186.0757
126.0551
144.0656
454.1534
512.204 LeX
528.1942
657.2334
782.3351 b7(1+)
803.2994 sLeX
833.9277 y14(2+)
862.4749 y7(1+)
895.4101 b8(1+)
1024.4473 b9(1+)
1298.9712 PEP_0_1_0_0_0
1666.8611 y14(1+)

File: 231129_OE_RK_16, RT: 88.241, Scan: 120884
Type: HCD, FTMS + c NSI d Full ms2 1341.3828@hcd30.00 [120.0000-6864.6226]
Precursor Scan: 120826, m/z: 1340.581, charge: 5
ADTHDEILEGLNFNLTEIPEAQIHEGFQELLR_6_5_1_3_0 LeX peak identified

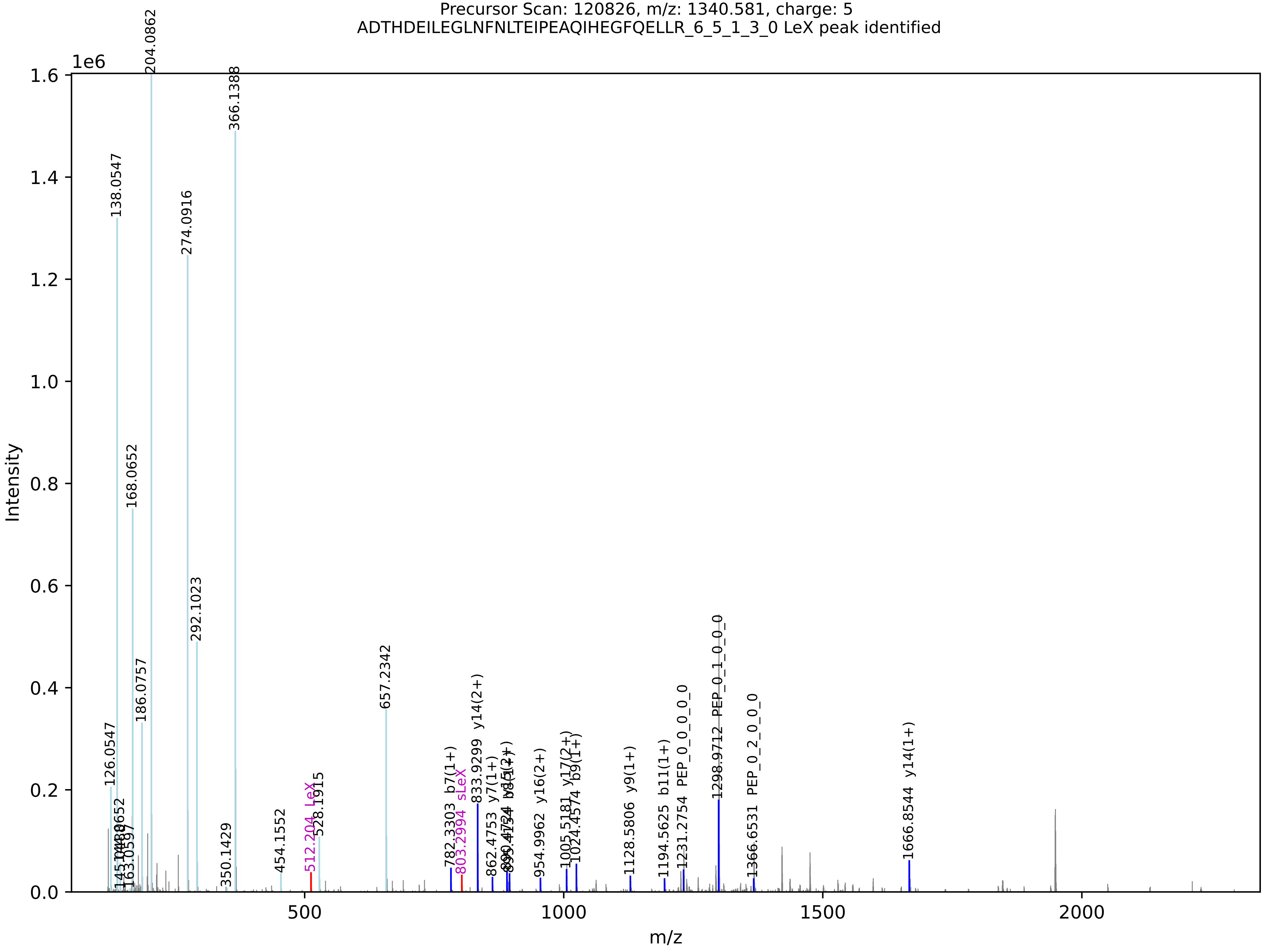

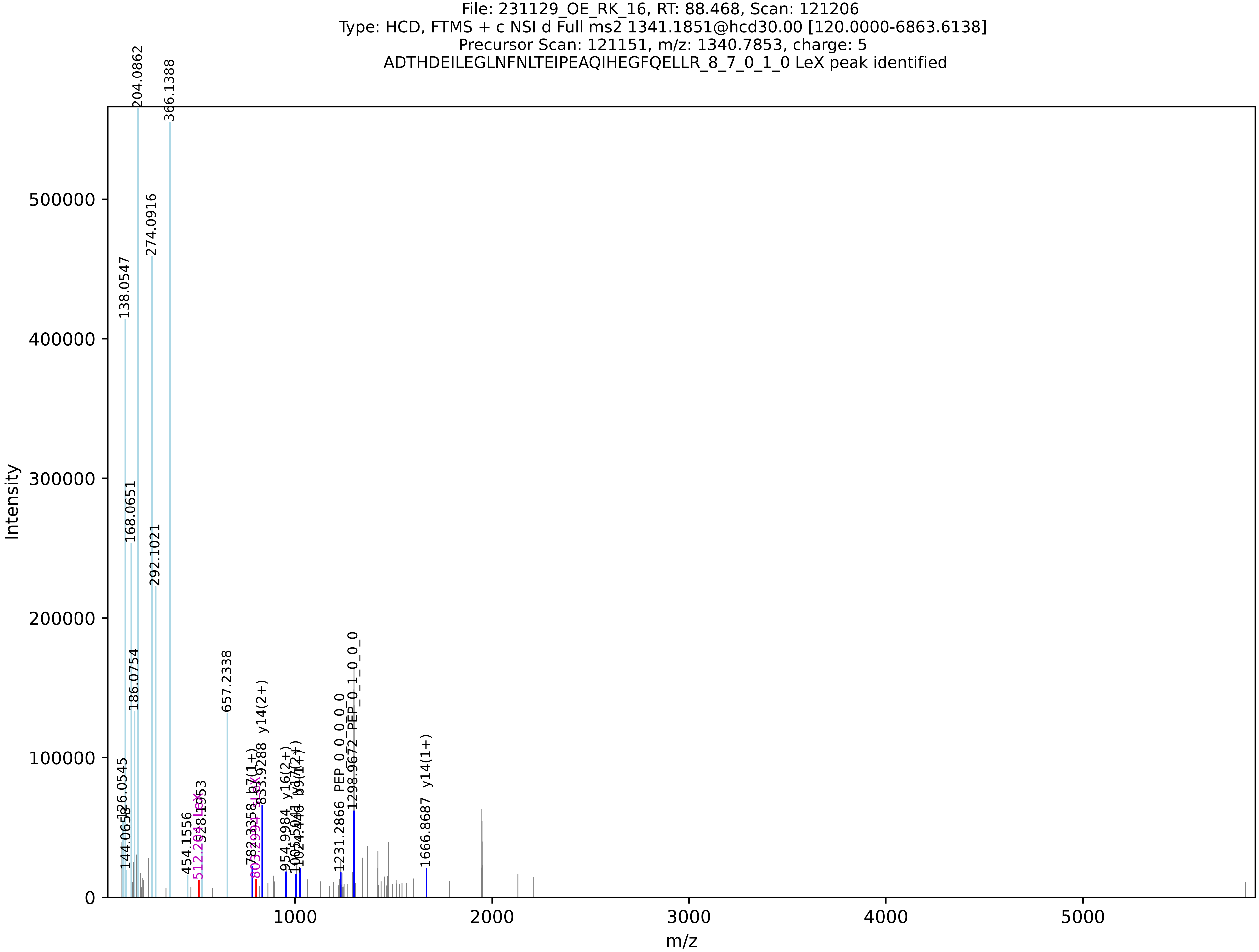
File: 231129_OE_RK_16, RT: 88.468, Scan: 121206
Type: HCD, FTMS + c NSI d Full ms2 1341.1851@hcd30.00 [120.0000-6863.6138]
Precursor Scan: 121151, m/z: 1340.7853, charge: 5
ADTHDEILEGLNFNLTEIPEAQIHEGFQELLR_8_7_0_1_0 LeX peak identified
204.0862
366.1388
274.0916
138.0547
168.0651
292.1021
186.0754
657.2338
126.0545
144.0658
454.1556
528.1953
512.2[illegible] LeX
782.3358 b7(1+)
803.2934 LeX
833.9288 y14(2+)
954.9984 y16(2+)
1231.2866 PEP_0_0_0_0_0
1298.9672 PEP_0_1_0_0_0
1666.8687 y14(1+)
Intensity
m/z
0
100000
200000
300000
400000
500000
1000
2000
3000
4000
5000

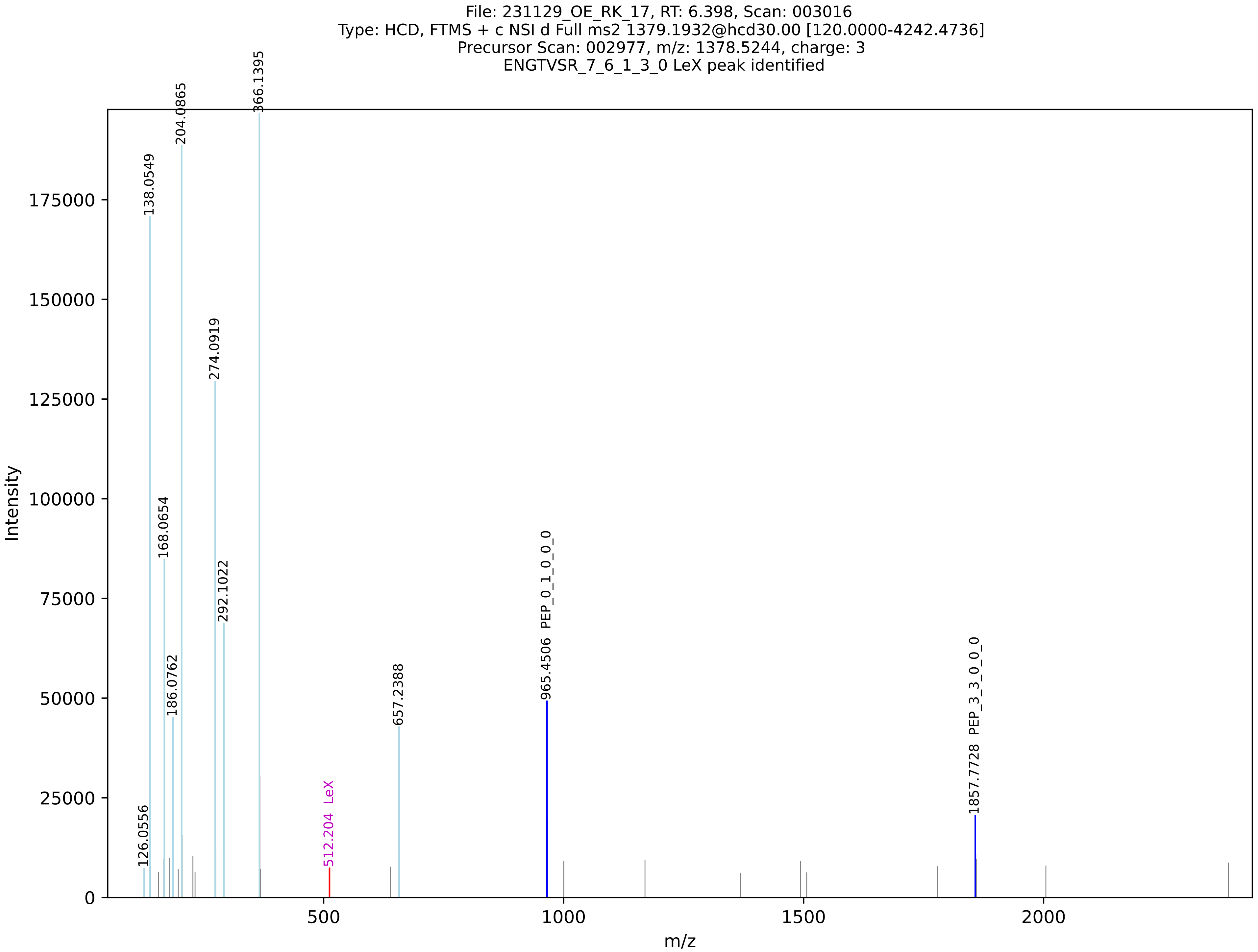
File: 231129_OE_RK_17, RT: 6.398, Scan: 003016
Type: HCD, FTMS + c NSI d Full ms2 1379.1932@hcd30.00 [120.0000-4242.4736]
Precursor Scan: 002977, m/z: 1378.5244, charge: 3
ENGTVSR_7_6_1_3_0 LeX peak identified
Intensity
m/z
126.0556
138.0549
168.0654
186.0762
204.0865
274.0919
292.1022
366.1395
512.204 LeX
657.2388
965.4506 PEP_0_1_0_0_0
1857.7728 PEP_3_3_0_0_0
0
25000
50000
75000
100000
125000
150000
175000
500
1000
1500
2000

File: 231129_OE_RK_17, RT: 6.574, Scan: 003252
Type: HCD, FTMS + c NSI d Full ms2 1379.1920@hcd30.00 [120.0000-4242.4692]
Precursor Scan: 003219, m/z: 1378.5244, charge: 3
ENGTVSR_7_6_1_3_0 LeX peak identified

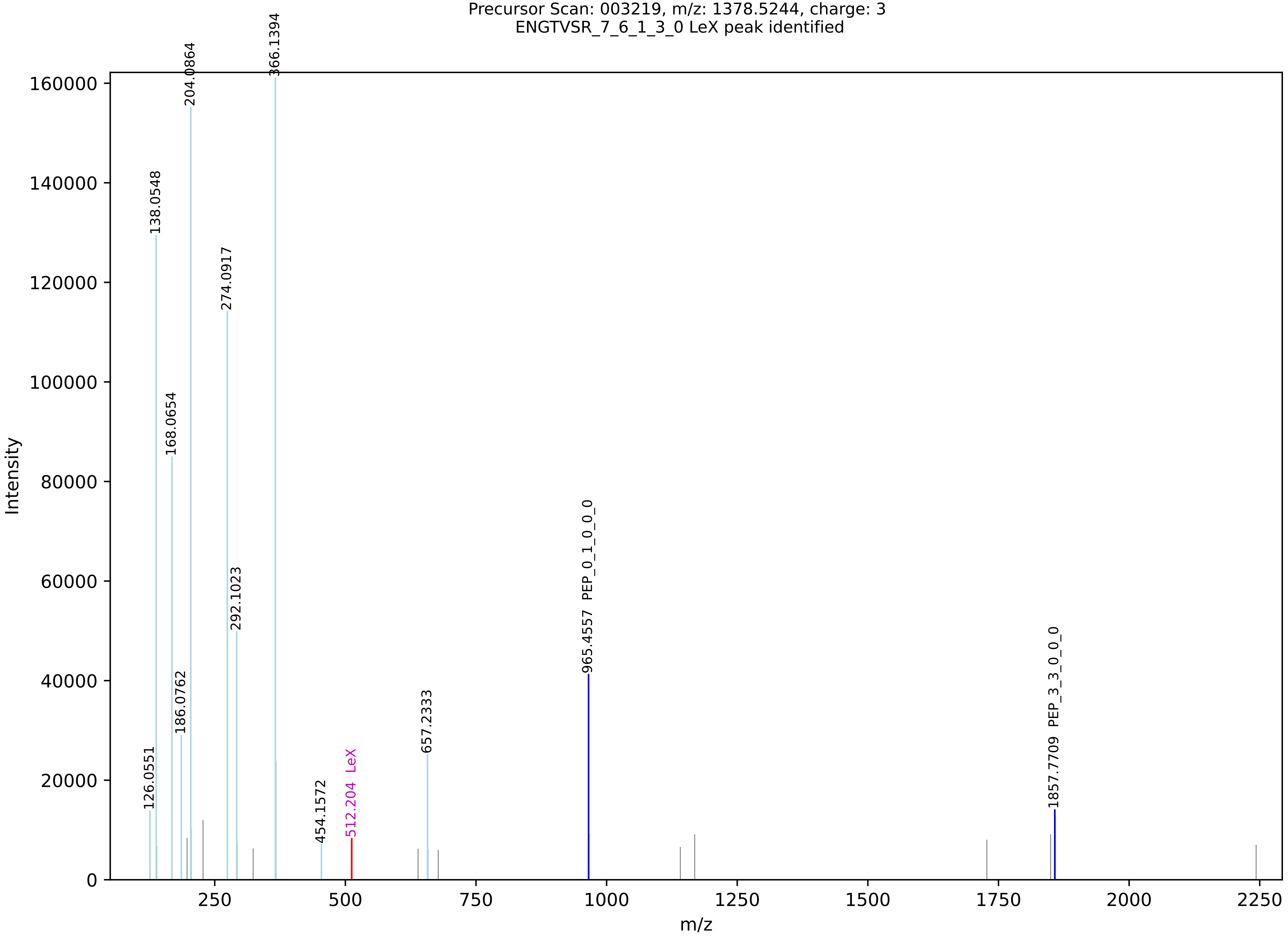

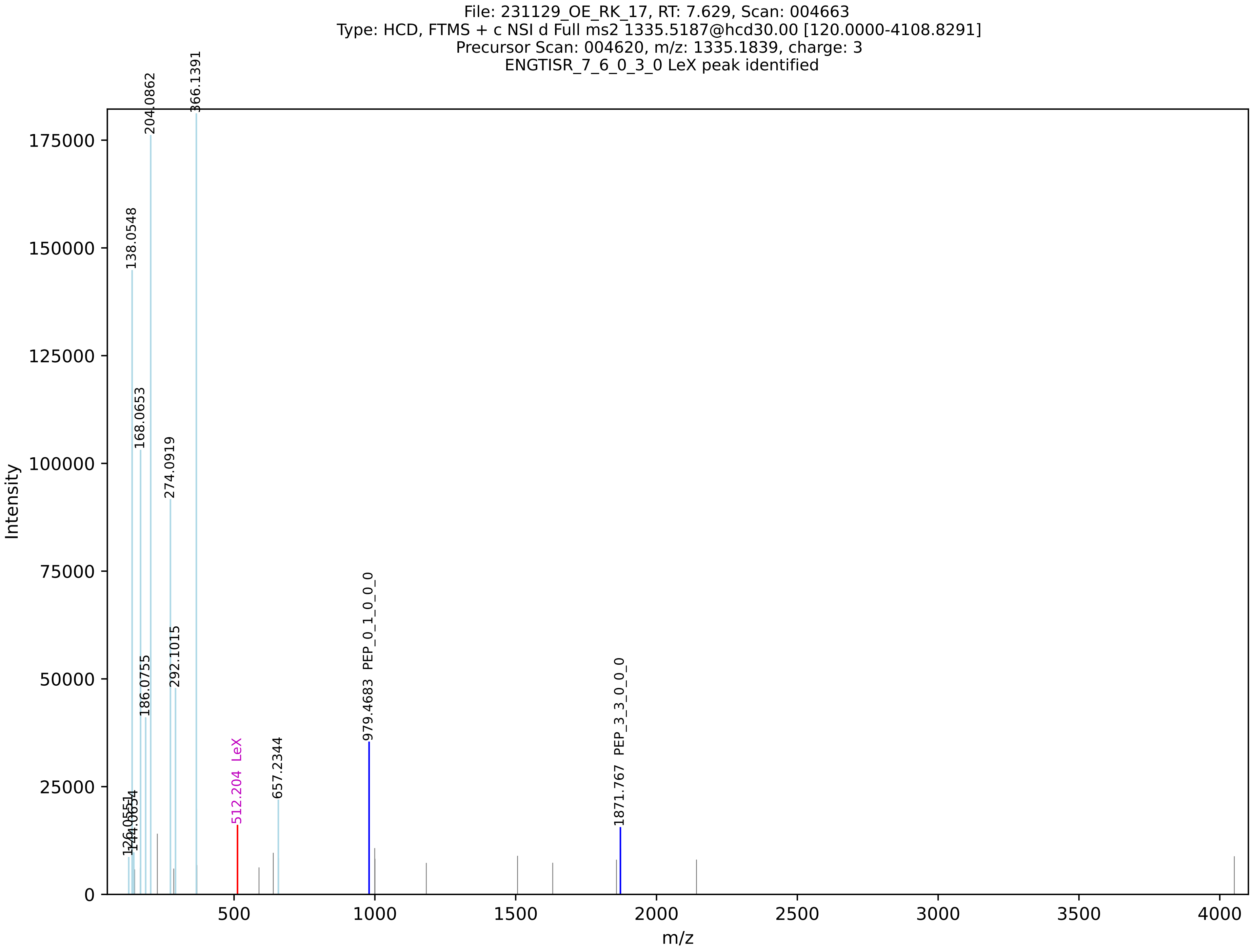
File: 231129_OE_RK_17, RT: 7.629, Scan: 004663
Type: HCD, FTMS + c NSI d Full ms2 1335.5187@hcd30.00 [120.0000-4108.8291]
Precursor Scan: 004620, m/z: 1335.1839, charge: 3
ENGTISR_7_6_0_3_0 LeX peak identified
Intensity
m/z
366.1391
204.0862
138.0548
168.0653
274.0919
292.1015
186.0755
126.0551
144.0654
512.204 LeX
657.2344
979.4683 PEP_0_1_0_0_0
1871.767 PEP_3_3_0_0_0
0
25000
50000
75000
100000
125000
150000
175000
500
1000
1500
2000
2500
3000
3500
4000

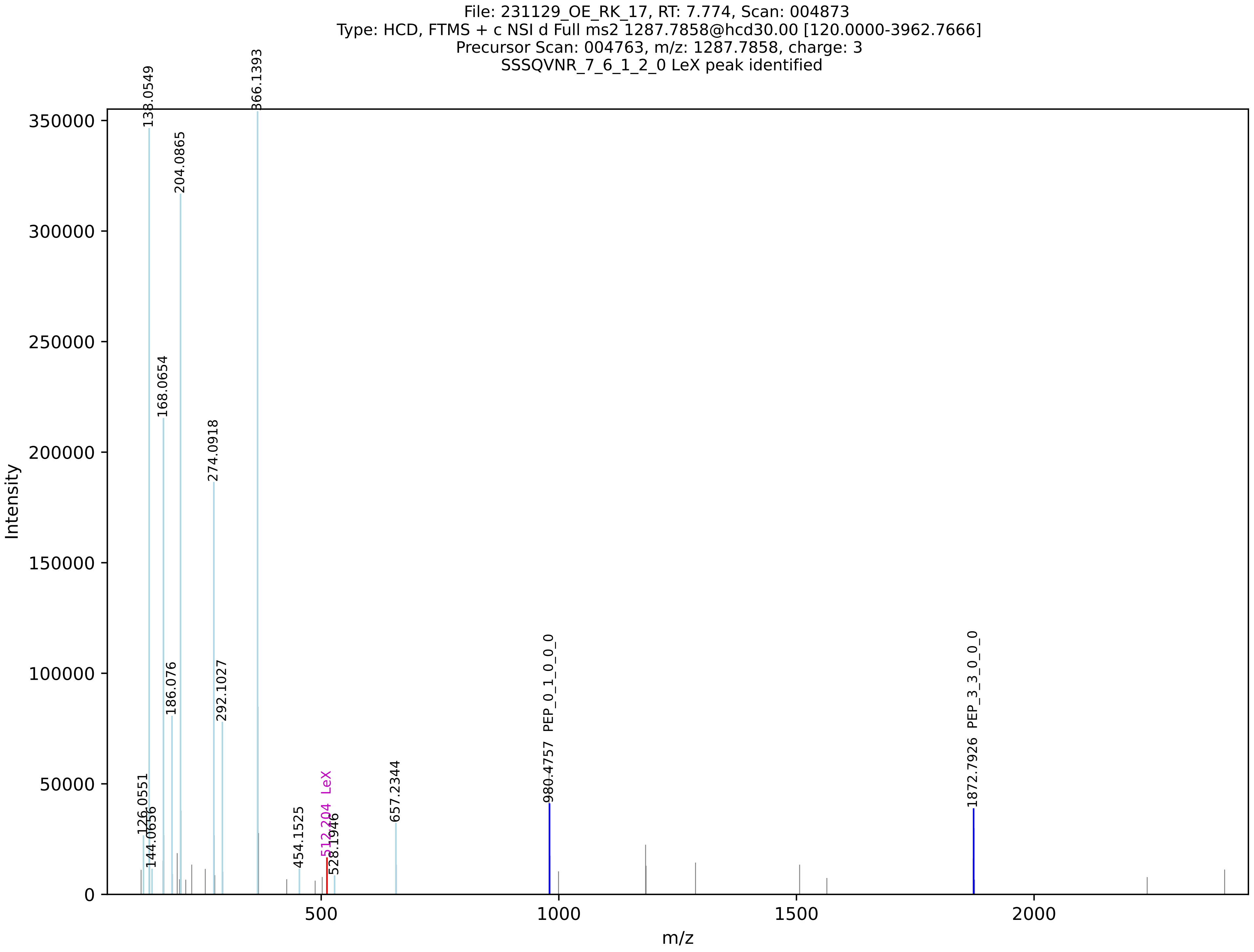
File: 231129_OE_RK_17, RT: 7.774, Scan: 004873
Type: HCD, FTMS + c NSI d Full ms2 1287.7858@hcd30.00 [120.0000-3962.7666]
Precursor Scan: 004763, m/z: 1287.7858, charge: 3
SSSQVNR_7_6_1_2_0 LeX peak identified
Intensity
m/z
138.0549
366.1393
204.0865
168.0654
274.0918
186.076
292.1027
126.0551
144.0656
454.1525
512.204 LeX
528.1946
657.2344
980.4757 PEP_0_1_0_0_0
1872.7926 PEP_3_3_0_0_0
0
50000
100000
150000
200000
250000
300000
350000
500
1000
1500
2000

File: 231129_OE_RK_17, RT: 7.983, Scan: 005167
Type: HCD, FTMS + c NSI d Full ms2 1432.8861@hcd30.00 [120.0000-4406.7734]
Precursor Scan: 005085, m/z: 1432.2179, charge: 3
ENGTISR_7_6_2_3_0 LeX peak identified

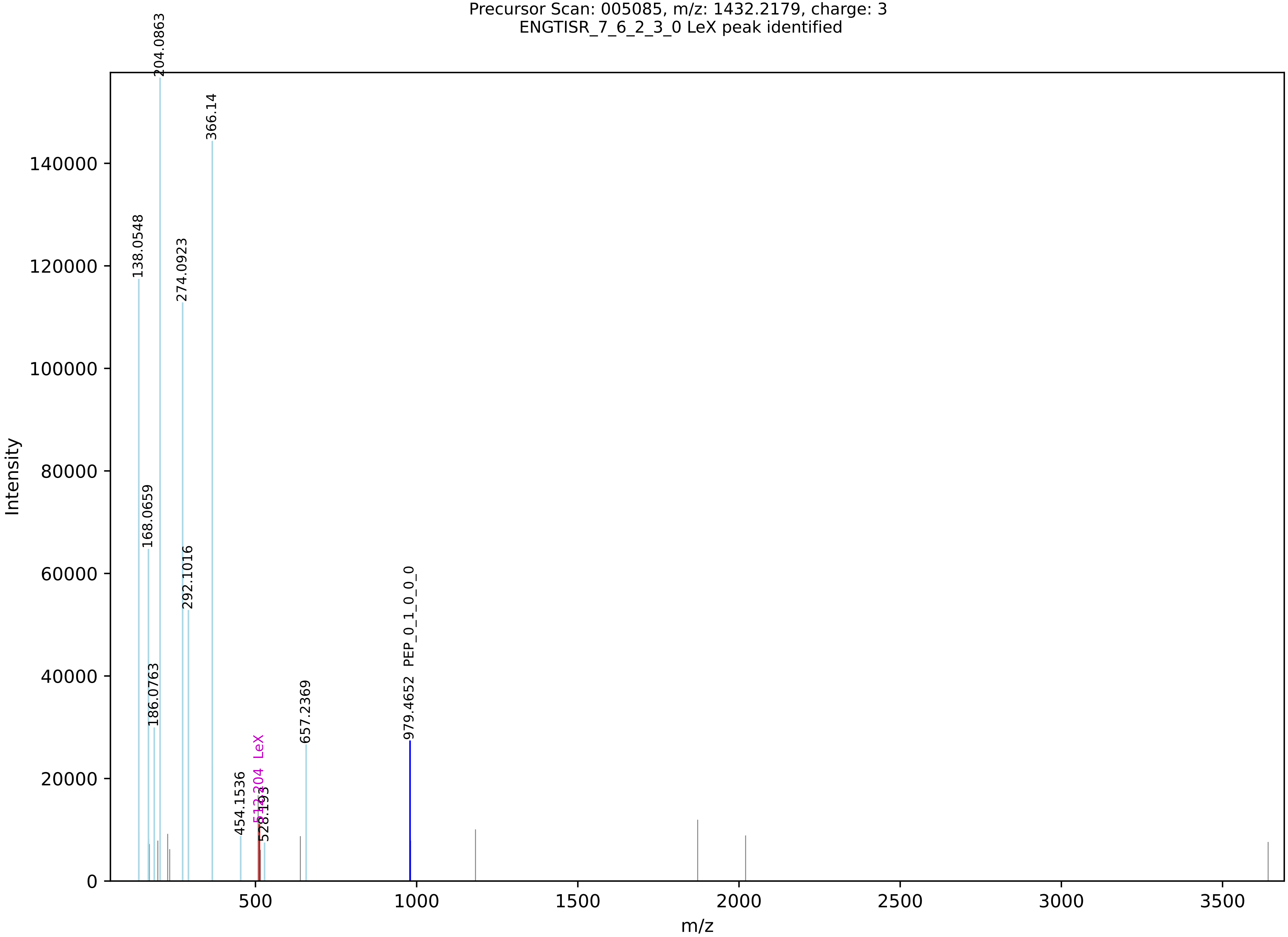

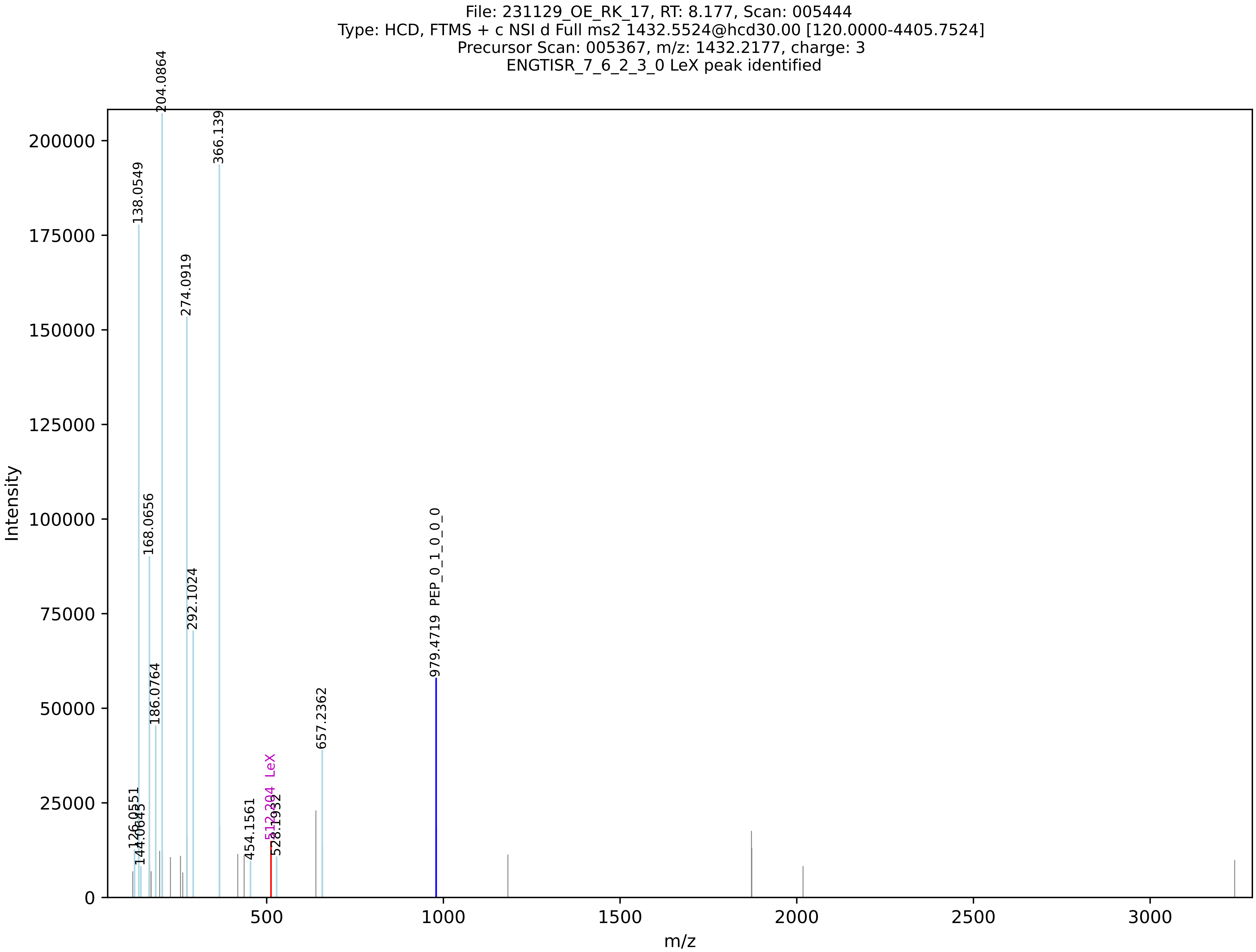
File: 231129_OE_RK_17, RT: 8.177, Scan: 005444
Type: HCD, FTMS + c NSI d Full ms2 1432.5524@hcd30.00 [120.0000-4405.7524]
Precursor Scan: 005367, m/z: 1432.2177, charge: 3
ENGTISR_7_6_2_3_0 LeX peak identified
Intensity
m/z
204.0864
366.139
138.0549
274.0919
168.0656
292.1024
186.0764
PEP_0_1_0_0_0
979.4719
657.2362
LeX
512.204
528.1932
454.1561
126.0551
144.0645

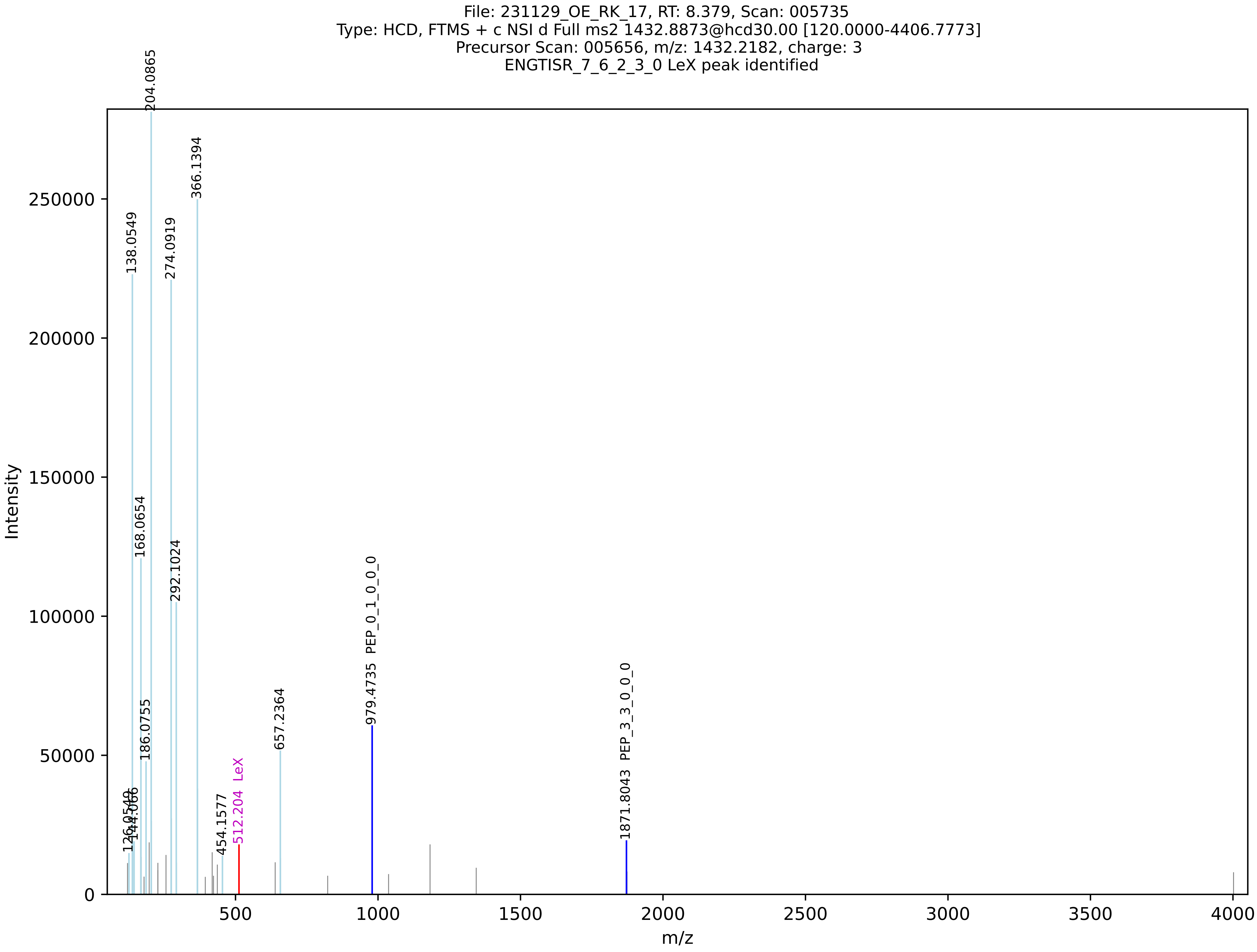
File: 231129_OE_RK_17, RT: 8.379, Scan: 005735
Type: HCD, FTMS + c NSI d Full ms2 1432.8873@hcd30.00 [120.0000-4406.7773]
Precursor Scan: 005656, m/z: 1432.2182, charge: 3
ENGTISR_7_6_2_3_0 LeX peak identified
Intensity
m/z
204.0865
366.1394
138.0549
274.0919
168.0654
292.1024
186.0755
126.0549
144.066
454.1577
512.204 LeX
657.2364
979.4735 PEP_0_1_0_0_0_
1871.8043 PEP_3_3_0_0_0
0
50000
100000
150000
200000
250000
500
1000
1500
2000
2500
3000
3500
4000

File: 231129_OE_RK_17, RT: 8.738, Scan: 006249
Type: HCD, FTMS + c NSI d Full ms2 1166.4468@hcd30.00 [120.0000-4781.9590]
Precursor Scan: 006161, m/z: 1165.6934, charge: 4
ENGTISR_8_7_0_4_0 LeX peak identified

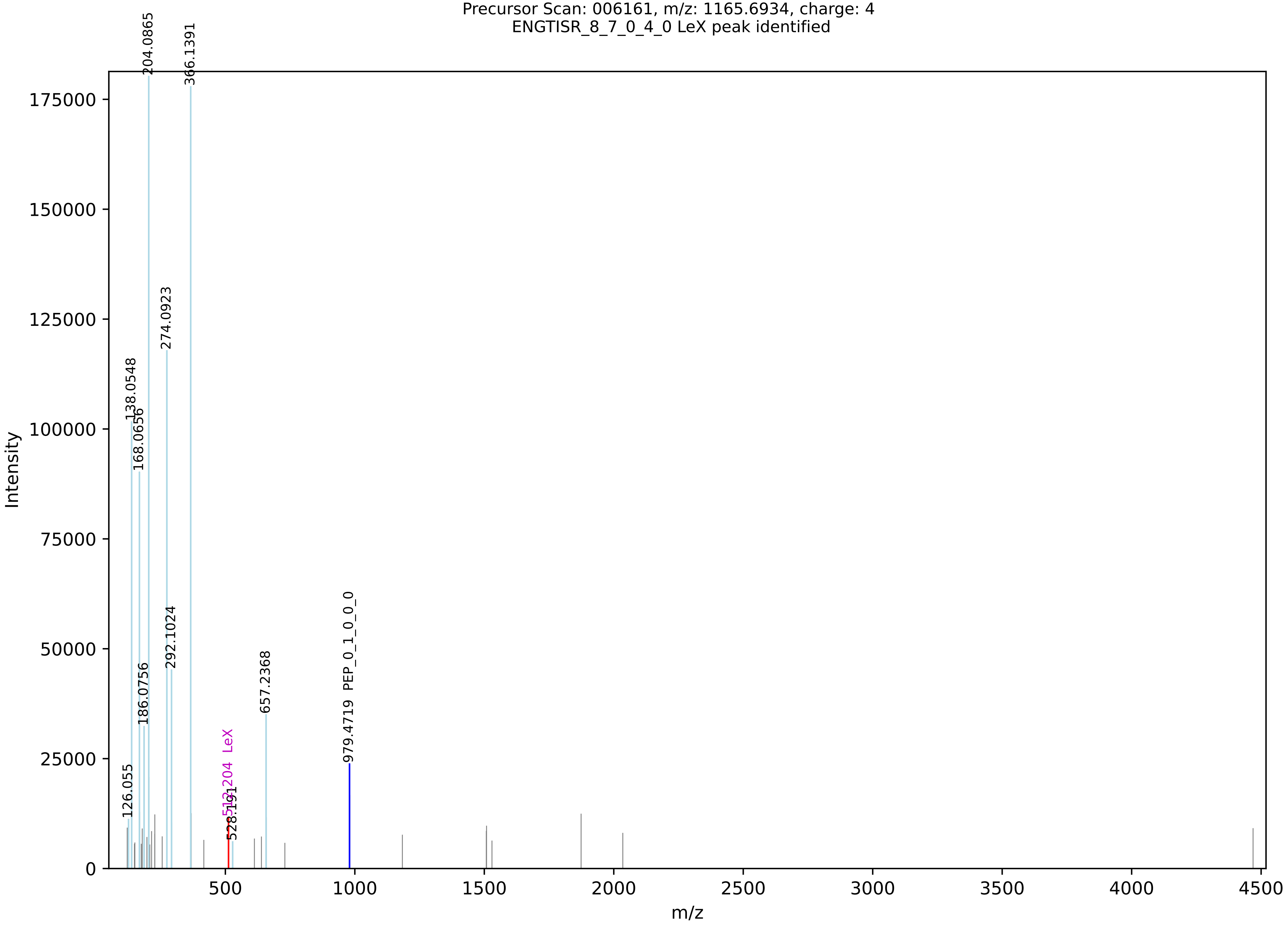

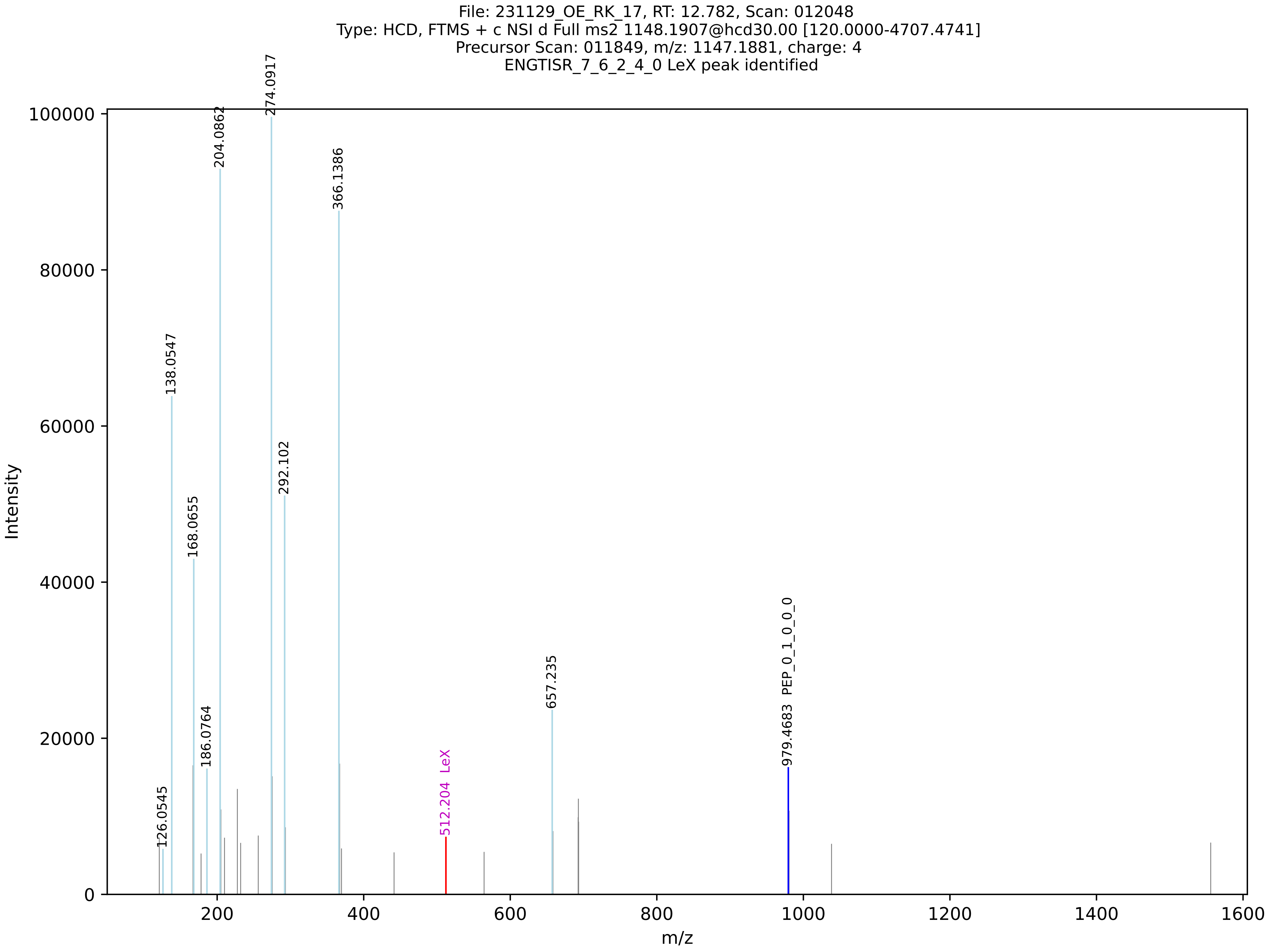

File: 231129_OE_RK_17, RT: 12.782, Scan: 012048
Type: HCD, FTMS + c NSI d Full ms2 1148.1907@hcd30.00 [120.0000-4707.4741]
Precursor Scan: 011849, m/z: 1147.1881, charge: 4
ENGTISR_7_6_2_4_0 LeX peak identified
Intensity
m/z
0
20000
40000
60000
80000
100000
200
400
600
800
1000
1200
1400
1600
126.0545
138.0547
168.0655
186.0764
204.0862
274.0917
292.102
366.1386
512.204 LeX
657.235
979.4683 PEP_0_1_0_0_

File: 231129_OE_RK_17, RT: 40.982, Scan: 052482
Type: HCD, FTMS + c NSI d Full ms2 1177.9806@hcd30.00 [120.0000-4829.0171]
Precursor Scan: 052405, m/z: 1177.2287, charge: 4
QDQCIYNTTYLNVQR_7_6_1_1_0 LeX peak identified

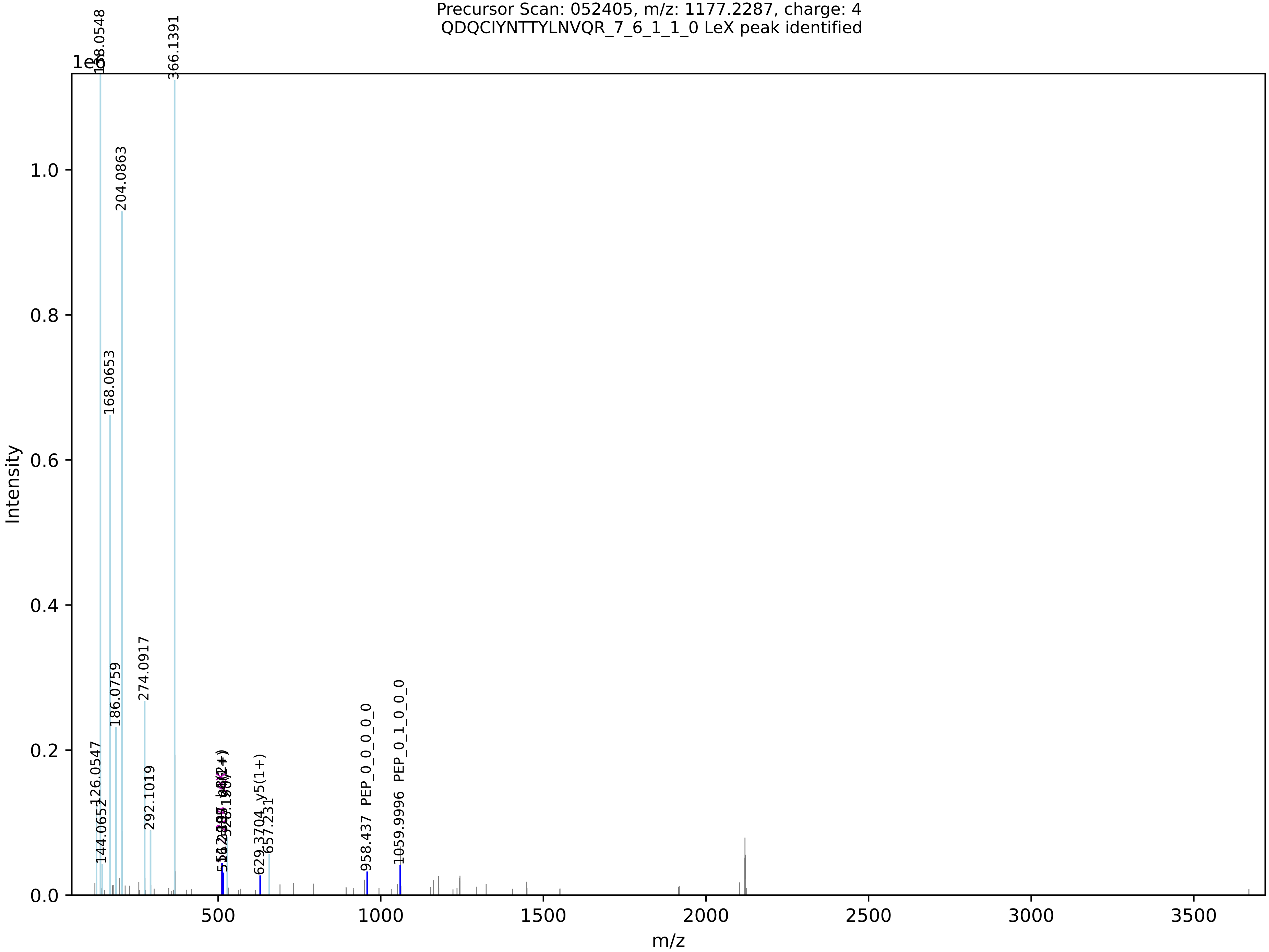

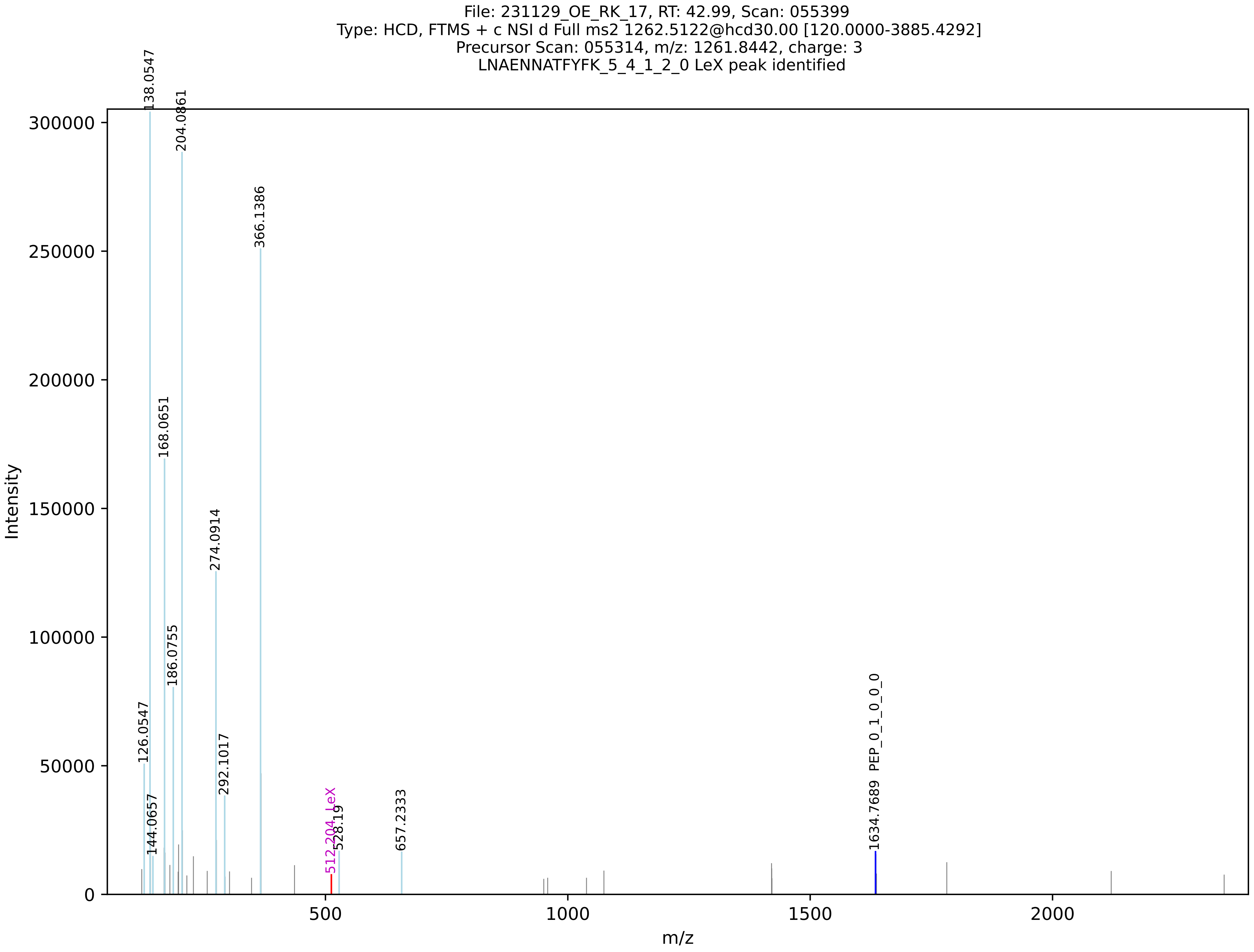
File: 231129_OE_RK_17, RT: 42.99, Scan: 055399
Type: HCD, FTMS + c NSI d Full ms2 1262.5122@hcd30.00 [120.0000-3885.4292]
Precursor Scan: 055314, m/z: 1261.8442, charge: 3
LNAENNATFYFK_5_4_1_2_0 LeX peak identified
Intensity
m/z
138.0547
204.0861
366.1386
168.0651
274.0914
186.0755
126.0547
292.1017
144.0657
512.204_LeX
528.19
657.2333
1634.7689 PEP_0_1_0_0_0_
0
50000
100000
150000
200000
250000
300000
500
1000
1500
2000

File: 231129_OE_RK_17, RT: 43.082, Scan: 055532
Type: HCD, FTMS + c NSI d Full ms2 1250.5051@hcd30.00 [120.0000-5124.9170]
Precursor Scan: 055457, m/z: 1250.0039, charge: 4
QDQCIYNTTYLNVQR_7_6_1_2_0 LeX peak identified

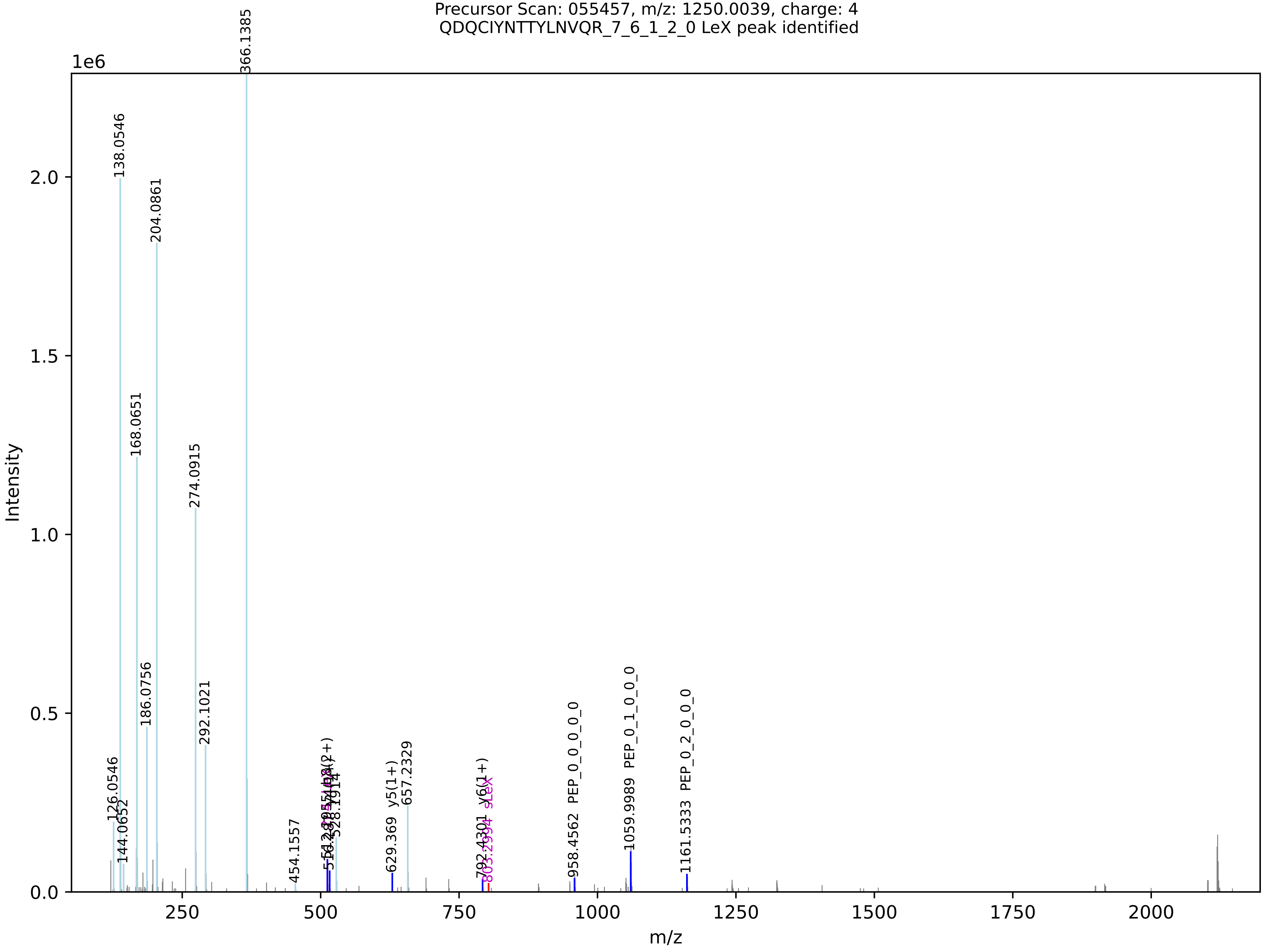

File: 231129_OE_RK_17, RT: 43.436, Scan: 056049
Type: HCD, FTMS + c NSI d Full ms2 1544.9576@hcd30.00 [120.0000-4749.7124]
Precursor Scan: 055971, m/z: 1544.6237, charge: 3
QDQCIYNTTYLNVQR_6_5_1_2_0 LeX peak identified

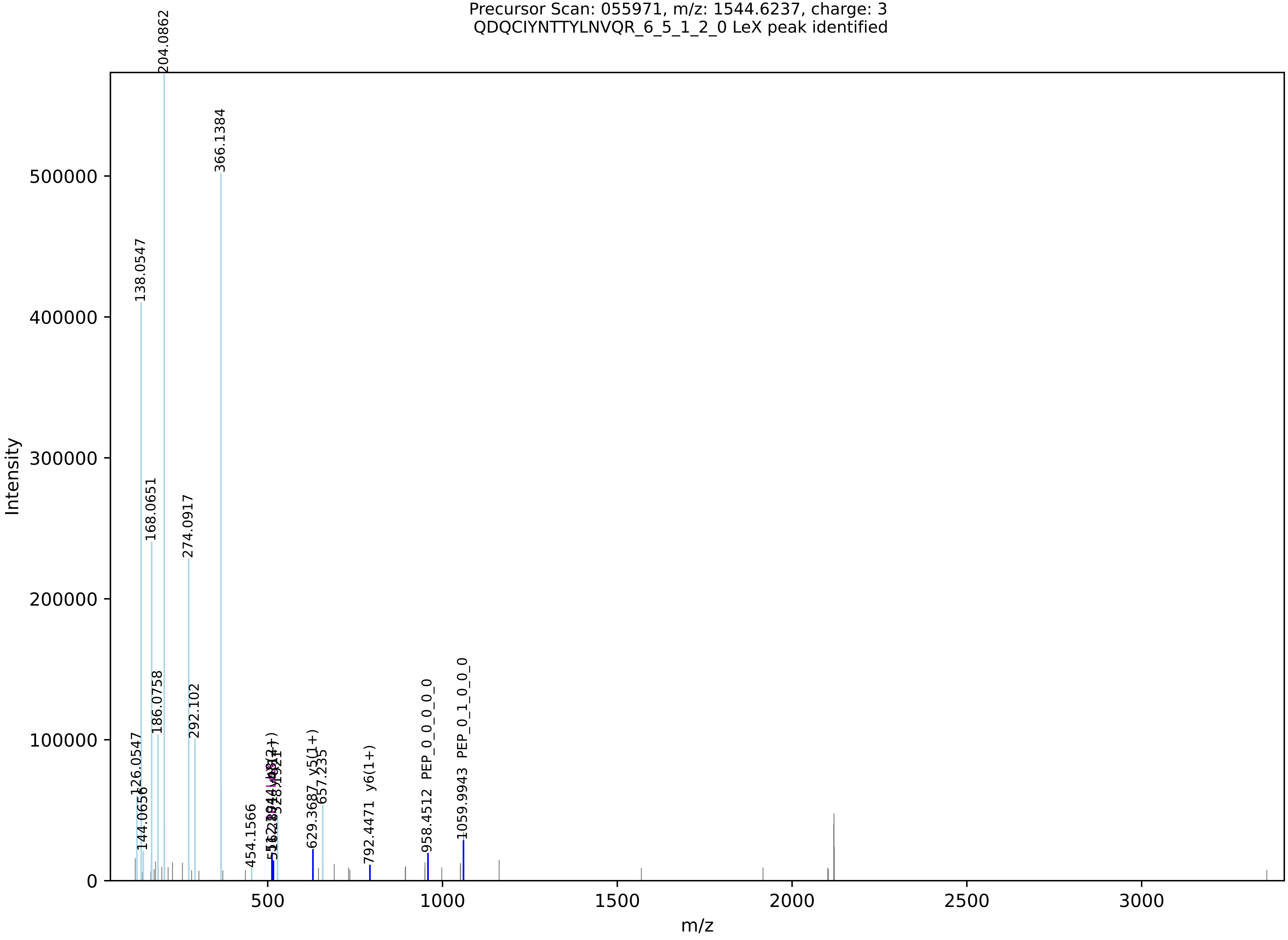

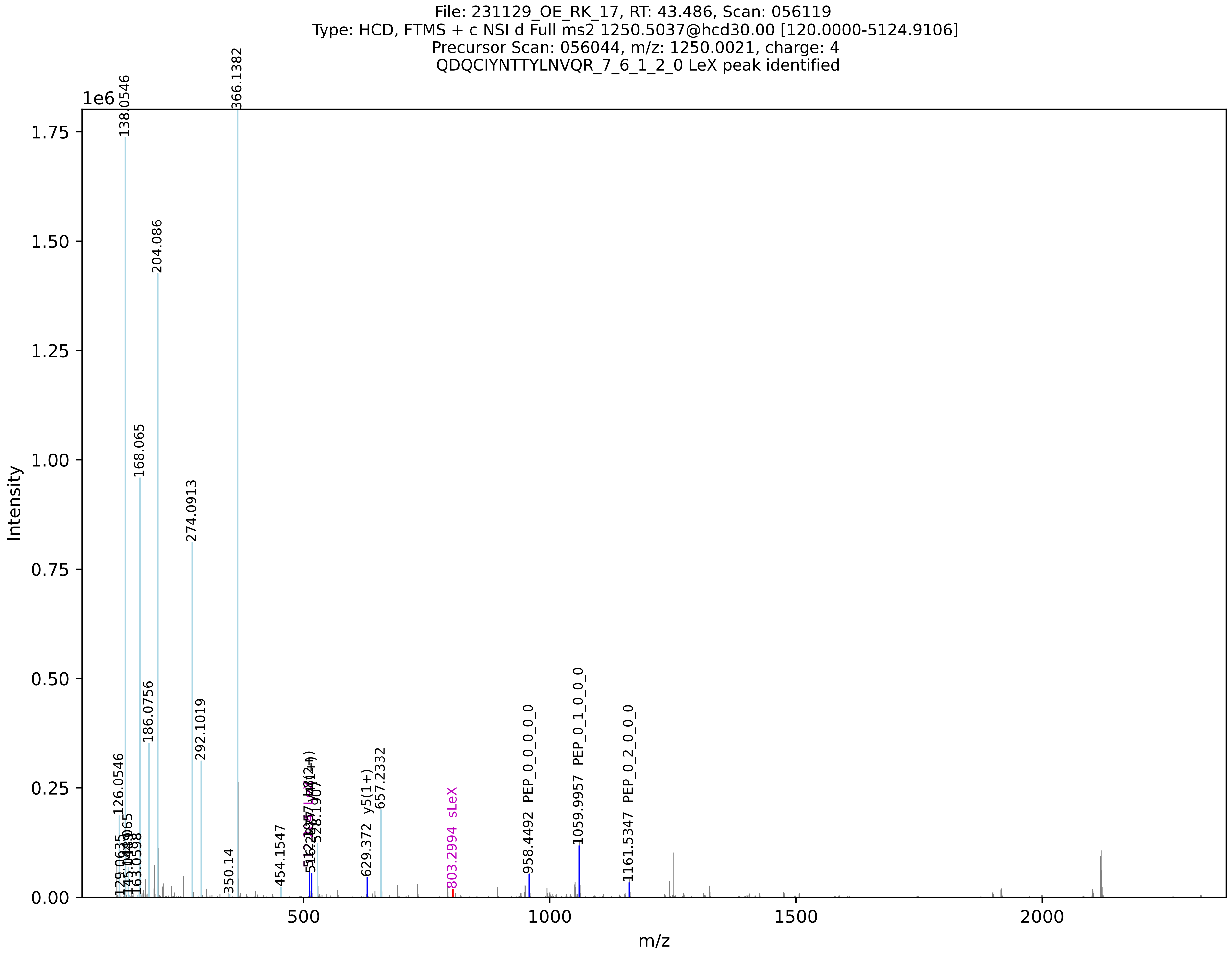
File: 231129_OE_RK_17, RT: 43.486, Scan: 056119
Type: HCD, FTMS + c NSI d Full ms2 1250.5037@hcd30.00 [120.0000-5124.9106]
Precursor Scan: 056044, m/z: 1250.0021, charge: 4
QDQCIYNTTYLNVQR_7_6_1_2_0 LeX peak identified
1e6
Intensity
m/z
0.00
0.25
0.50
0.75
1.00
1.25
1.50
1.75
500
1000
1500
2000
366.1382
204.086
168.065
274.0913
186.0756
292.1019
126.0546
350.14
454.1547
629.372 y5(1+)
657.2332
803.2994 sLeX
958.4492 PEP_0_0_0_0_0
1059.9957 PEP_0_1_0_0_0
1161.5347 PEP_0_2_0_0_0

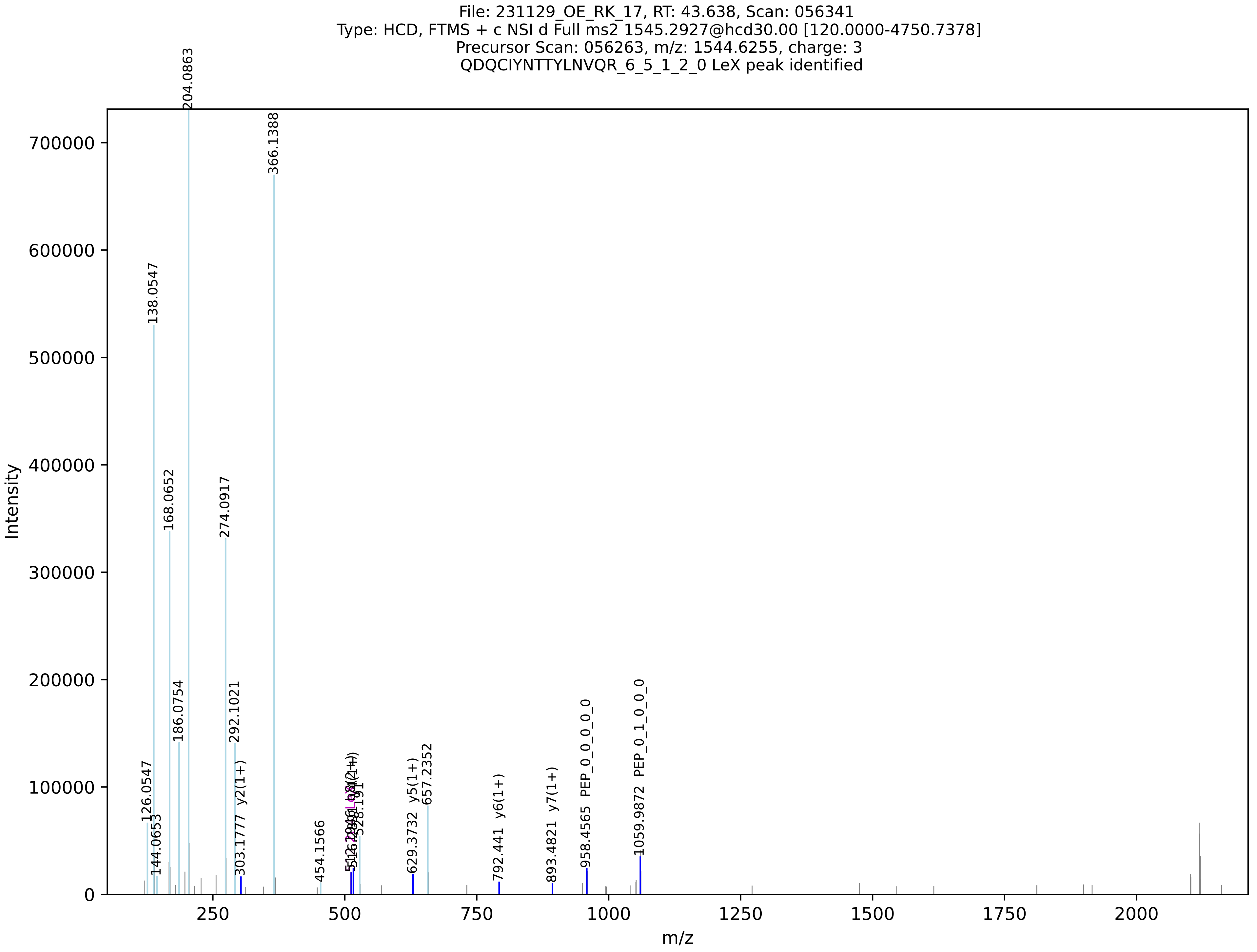
File: 231129_OE_RK_17, RT: 43.638, Scan: 056341
Type: HCD, FTMS + c NSI d Full ms2 1545.2927@hcd30.00 [120.0000-4750.7378]
Precursor Scan: 056263, m/z: 1544.6255, charge: 3
QDQCIYNTTYLNVQR_6_5_1_2_0 LeX peak identified
Intensity
m/z
204.0863
366.1388
138.0547
168.0652
274.0917
186.0754
292.1021
126.0547
144.0653
303.1777 y2(1+)
454.1566
528.191
629.3732 y5(1+)
657.2352
792.441 y6(1+)
893.4821 y7(1+)
958.4565 PEP_0_0_0_0_0
1059.9872 PEP_0_1_0_0_0

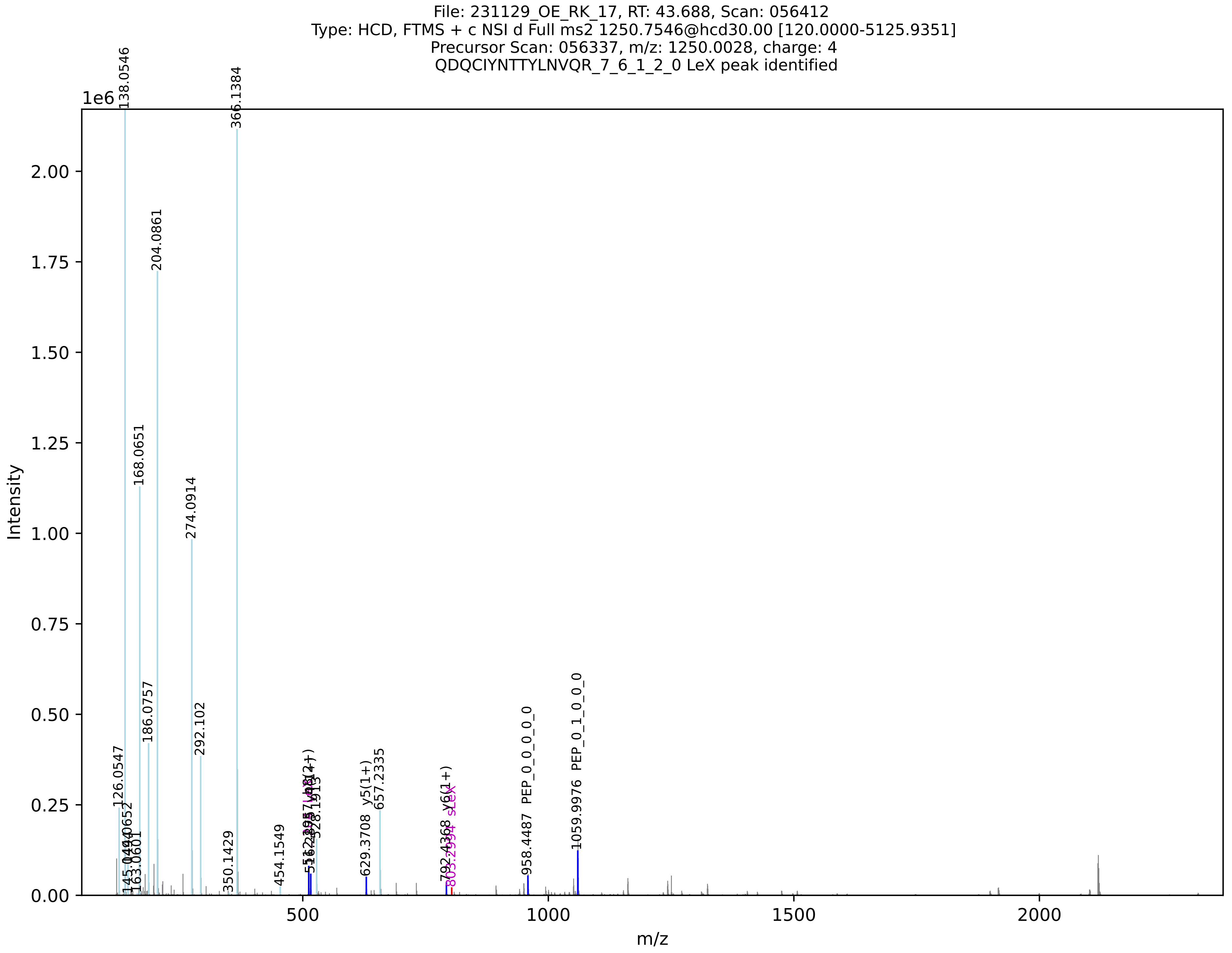
File: 231129_OE_RK_17, RT: 43.688, Scan: 056412
Type: HCD, FTMS + c NSI d Full ms2 1250.7546@hcd30.00 [120.0000-5125.9351]
Precursor Scan: 056337, m/z: 1250.0028, charge: 4
QDQCIYNTTYLNVQR_7_6_1_2_0 LeX peak identified
1e6
Intensity
m/z
138.0546
366.1384
204.0861
168.0651
274.0914
186.0757
292.102
126.0547
163.0601
350.1429
454.1549
657.2335
y5(1+)
629.3708
y6(1+)
792.4368
sLeX
PEP_0_0_0_0_0
958.4487
PEP_0_1_0_0_0
1059.9976

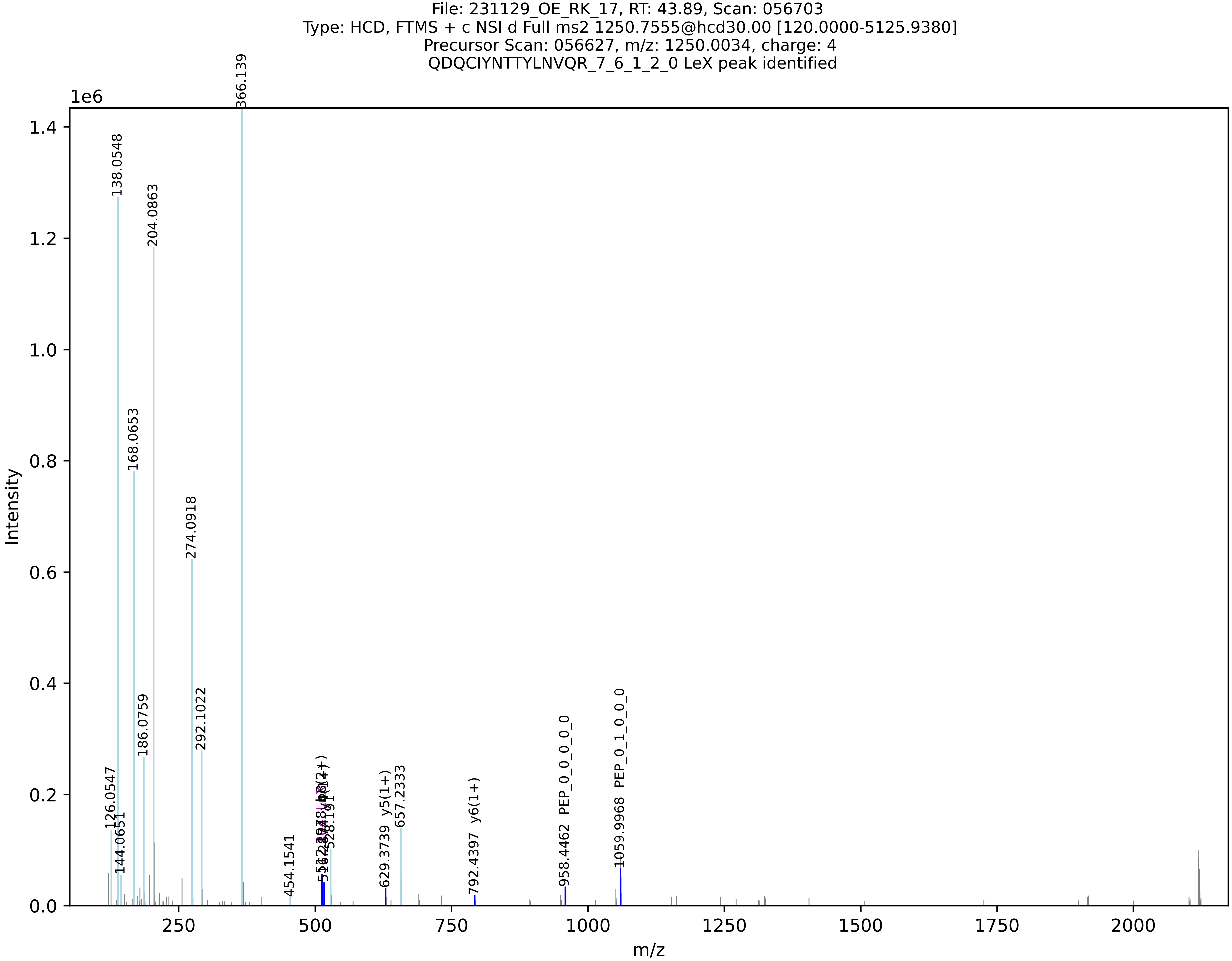

File: 231129_OE_RK_17, RT: 43.89, Scan: 056703
Type: HCD, FTMS + c NSI d Full ms2 1250.7555@hcd30.00 [120.0000-5125.9380]
Precursor Scan: 056627, m/z: 1250.0034, charge: 4
QDQCIYNTTYLNVQR_7_6_1_2_0 LeX peak identified
1e6
Intensity
m/z
366.139
138.0548
204.0863
168.0653
274.0918
186.0759
292.1022
126.0547
144.0651
454.1541
528.191
629.3739 y5(1+)
657.2333
792.4397 y6(1+)
958.4462 PEP_0_0_0_0_0
1059.9968 PEP_0_1_0_0_0
0.0
0.2
0.4
0.6
0.8
1.0
1.2
1.4
250
500
750
1000
1250
1500
1750
2000

File: 231129_OE_RK_17, RT: 43.945, Scan: 056781
Type: HCD, FTMS + c NSI d Full ms2 1159.4733@hcd30.00 [120.0000-4753.5068]
Precursor Scan: 056701, m/z: 1158.7201, charge: 4
QDQCIYNTTYLNVQR_6_5_1_2_0 LeX peak identified

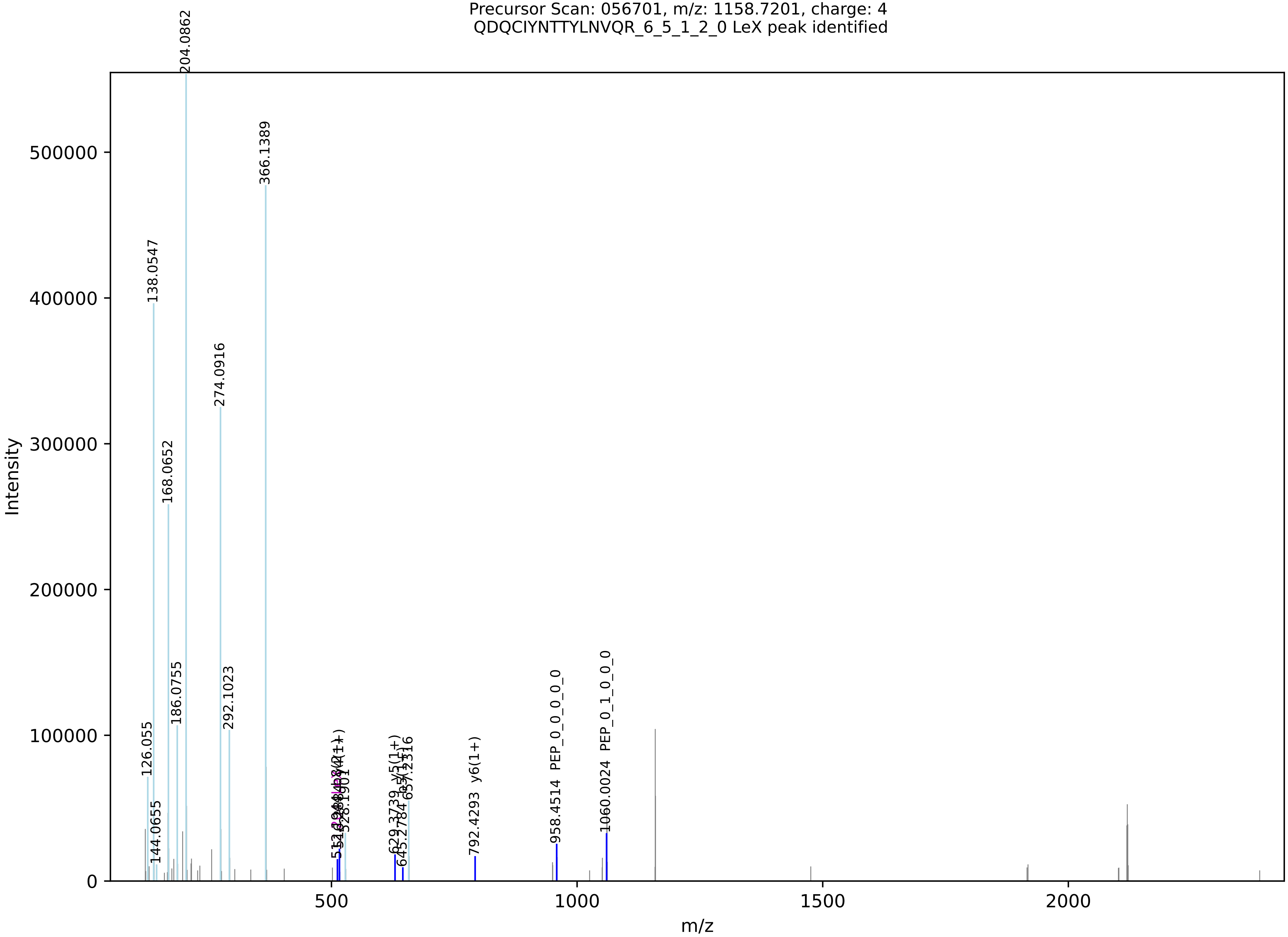

File: 231129_OE_RK_17, RT: 44.091, Scan: 056991
Type: HCD, FTMS + c NSI d Full ms2 1250.5048@hcd30.00 [120.0000-5124.9150]
Precursor Scan: 056915, m/z: 1250.005, charge: 4
QDQCIYNTTYLNVQR_7_6_1_2_0 LeX peak identified

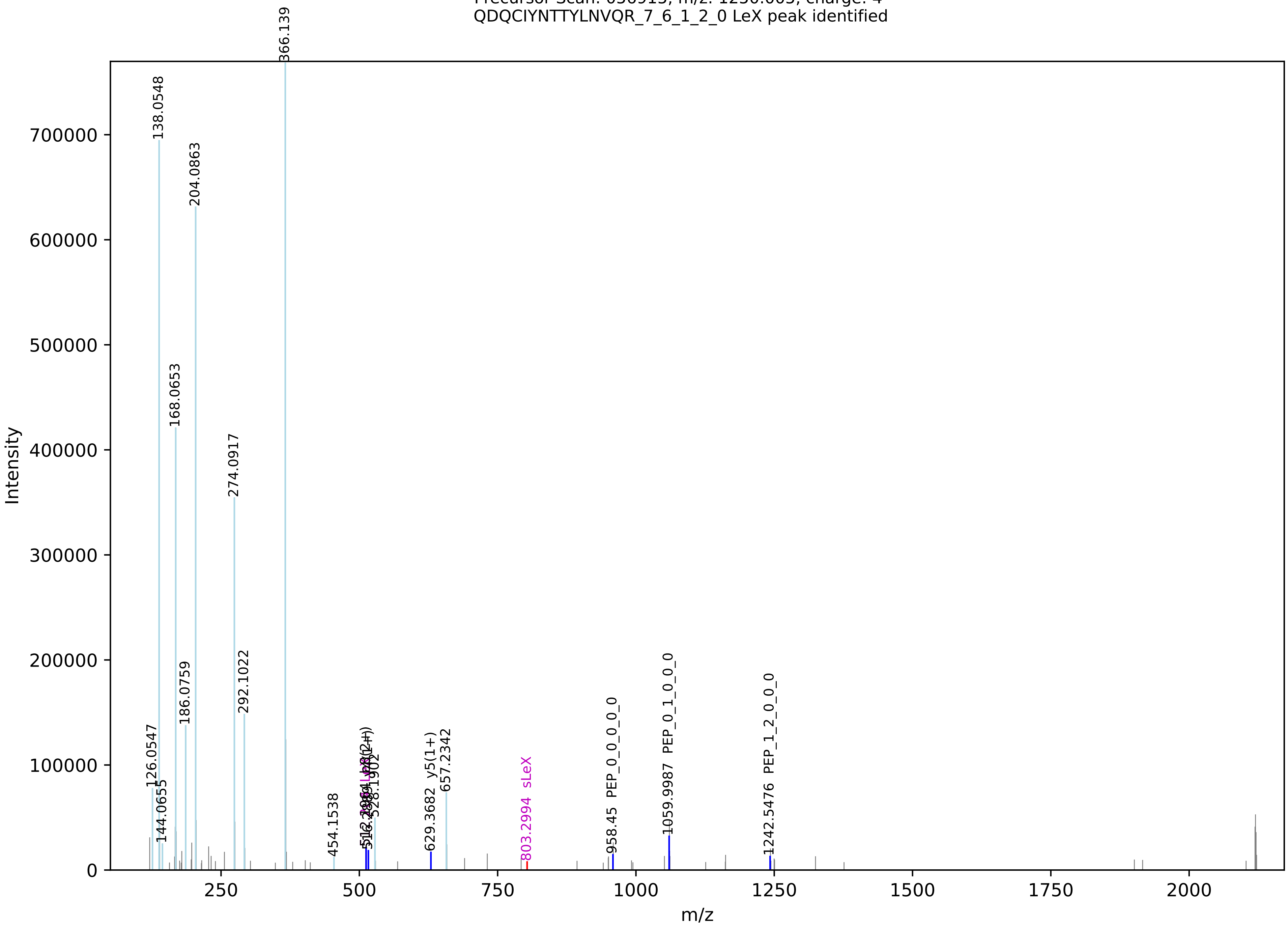

File: 231129_OE_RK_17, RT: 44.244, Scan: 057214
Type: HCD, FTMS + c NSI d Full ms2 1159.2224@hcd30.00 [120.0000-4752.4834]
Precursor Scan: 057134, m/z: 1158.7213, charge: 4
QDQCIYNTTYLNVQR_6_5_1_2_0 LeX peak identified

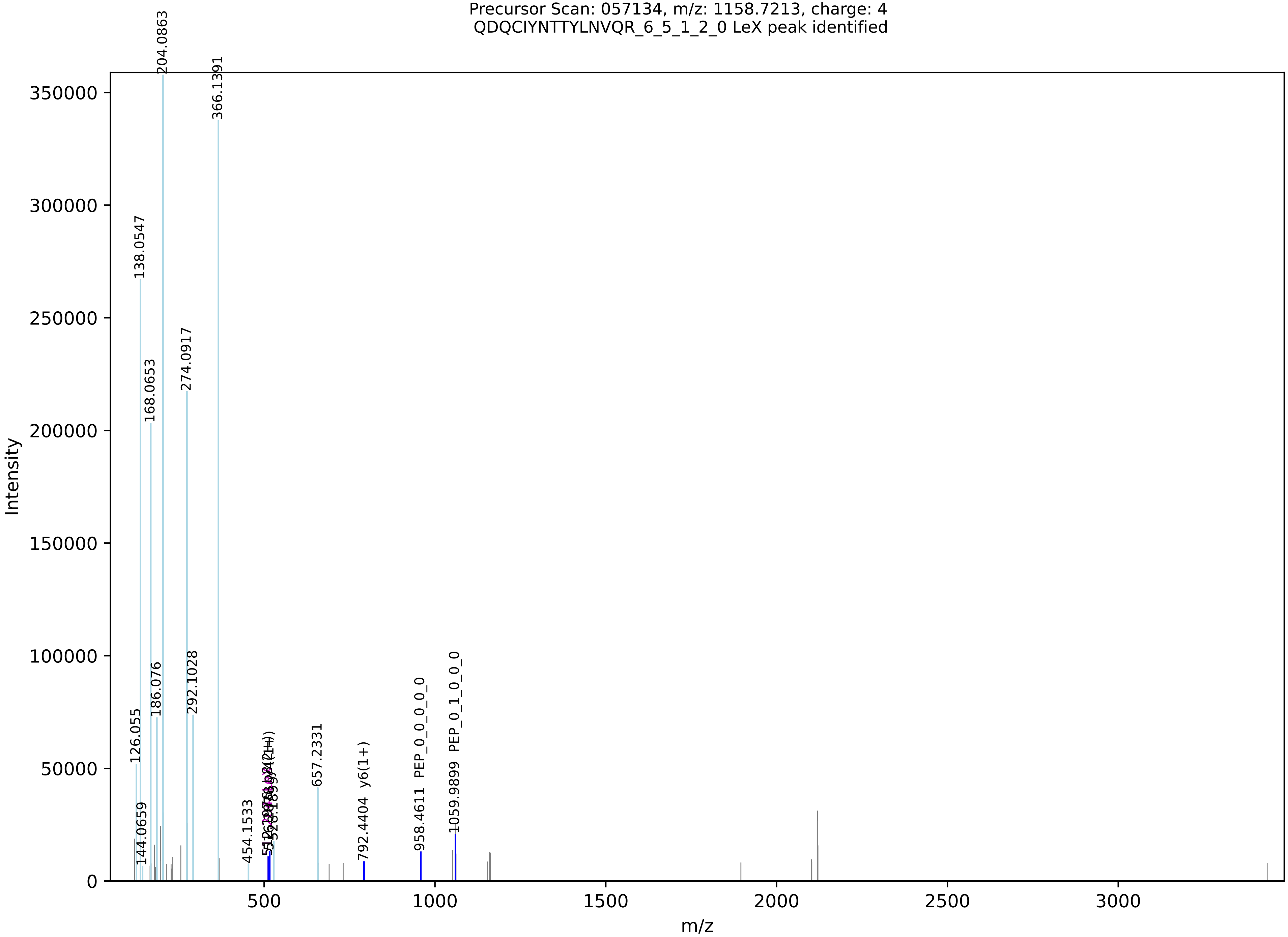

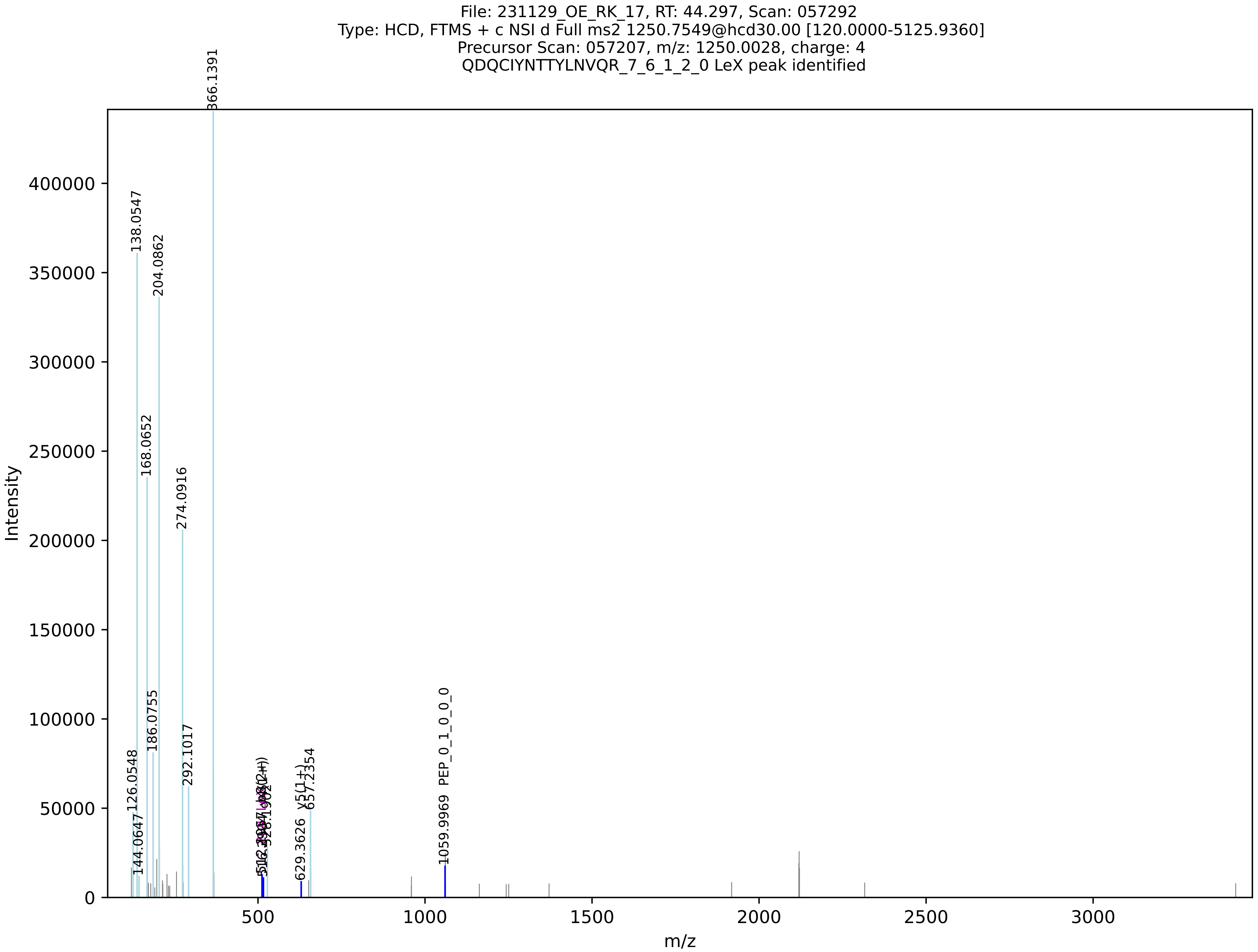

File: 231129_OE_RK_17, RT: 44.297, Scan: 057292
Type: HCD, FTMS + c NSI d Full ms2 1250.7549@hcd30.00 [120.0000-5125.9360]
Precursor Scan: 057207, m/z: 1250.0028, charge: 4
QDQCIYNTTYLNVQR_7_6_1_2_0 LeX peak identified
Intensity
m/z
366.1391
138.0547
204.0862
168.0652
274.0916
186.0755
292.1017
126.0548
144.0647
y5(1+)
657.2354
629.3626
PEP_0_1_0_0_0
1059.9969

File: 231129_OE_RK_17, RT: 44.698, Scan: 057873
Type: HCD, FTMS + c NSI d Full ms2 1323.2760@hcd30.00 [120.0000-5421.8223]
Precursor Scan: 057796, m/z: 1323.0259, charge: 4
QDQCIYNTTYLNVQR_7_6_1_3_0 LeX peak identified

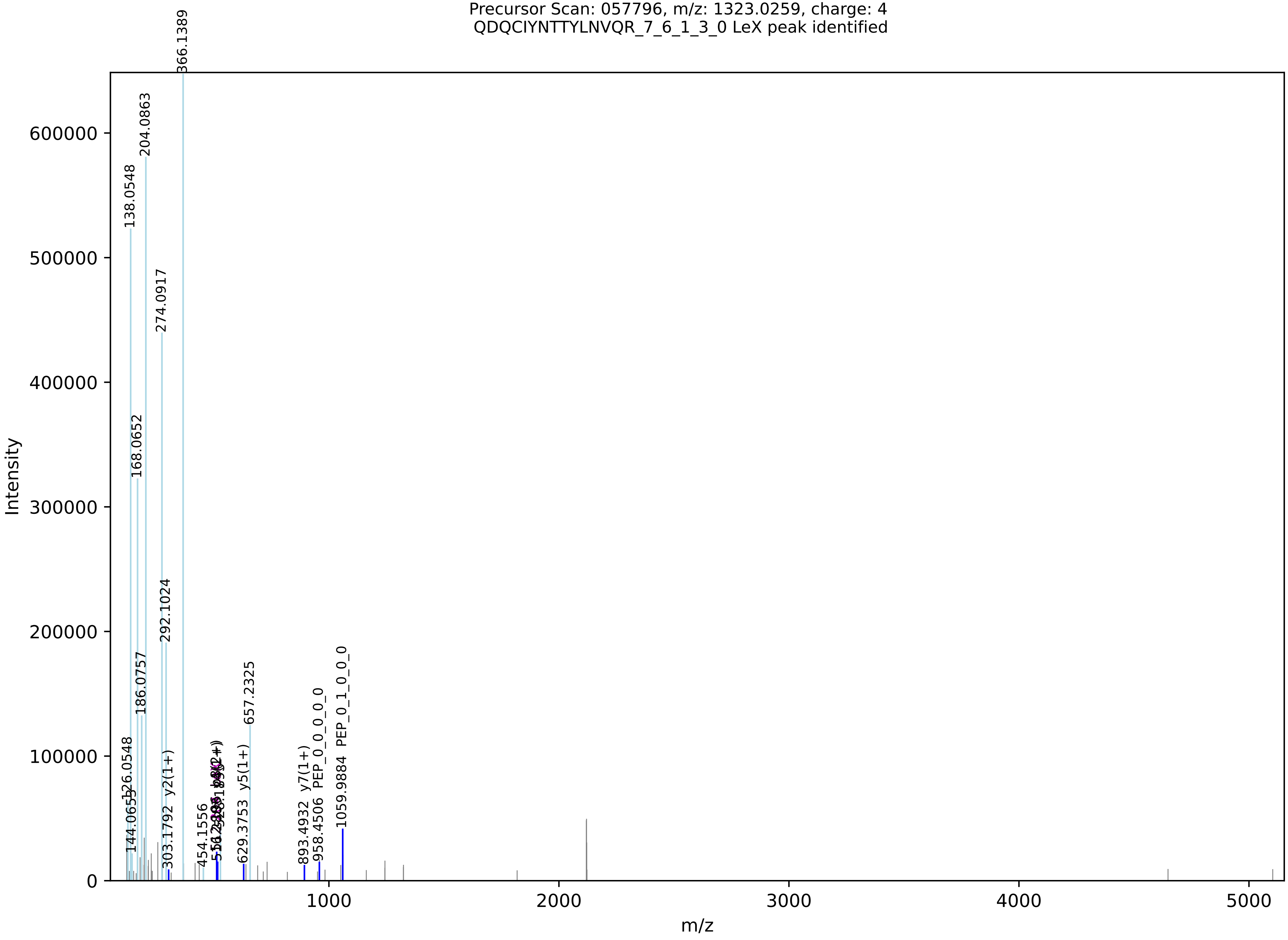

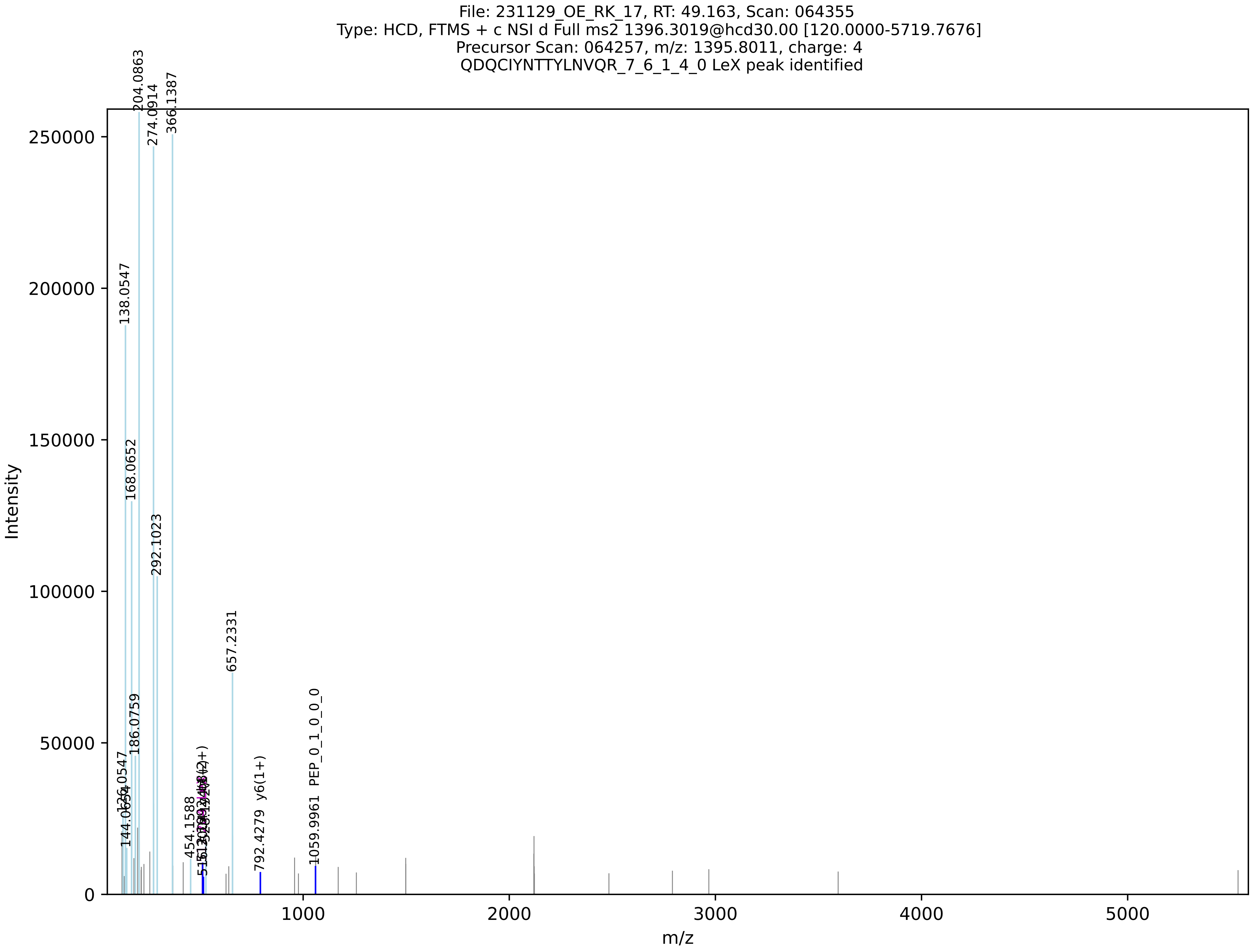
File: 231129_OE_RK_17, RT: 49.163, Scan: 064355
Type: HCD, FTMS + c NSI d Full ms2 1396.3019@hcd30.00 [120.0000-5719.7676]
Precursor Scan: 064257, m/z: 1395.8011, charge: 4
QDQCIYNTTYLNVQR_7_6_1_4_0 LeX peak identified
204.0863
274.0914
366.1387
138.0547
168.0652
292.1023
657.2331
186.0759
126.0547
144.0651
454.1588
792.4279 y6(1+)
1059.9961 PEP_0_1_0_0_0
Intensity
m/z
0
50000
100000
150000
200000
250000
1000
2000
3000
4000
5000

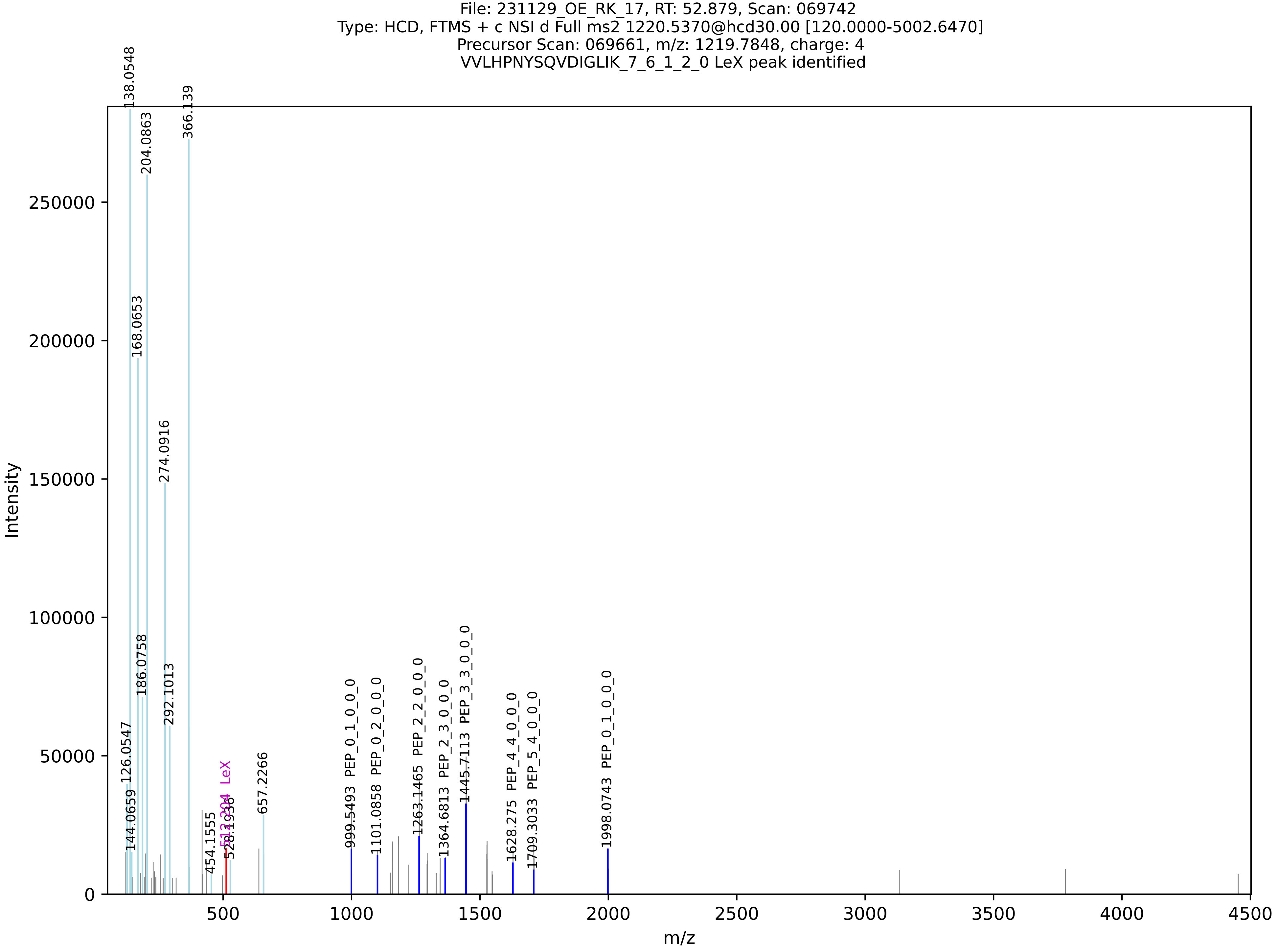
File: 231129_OE_RK_17, RT: 52.879, Scan: 069742
Type: HCD, FTMS + c NSI d Full ms2 1220.5370@hcd30.00 [120.0000-5002.6470]
Precursor Scan: 069661, m/z: 1219.7848, charge: 4
VVLHPNYSQVDIGLIK_7_6_1_2_0 LeX peak identified
Intensity
m/z
138.0548
366.139
204.0863
168.0653
274.0916
186.0758
292.1013
126.0547
144.0659
454.1555
512.204 LeX
528.1936
657.2266
999.5493 PEP_0_1_0_0_0
1101.0858 PEP_0_2_0_0_0
1263.1465 PEP_2_2_0_0_0
1364.6813 PEP_2_3_0_0_0
1445.7113 PEP_3_3_0_0_0
1628.275 PEP_4_4_0_0_0
1709.3033 PEP_5_4_0_0_0
1998.0743 PEP_0_1_0_0_0

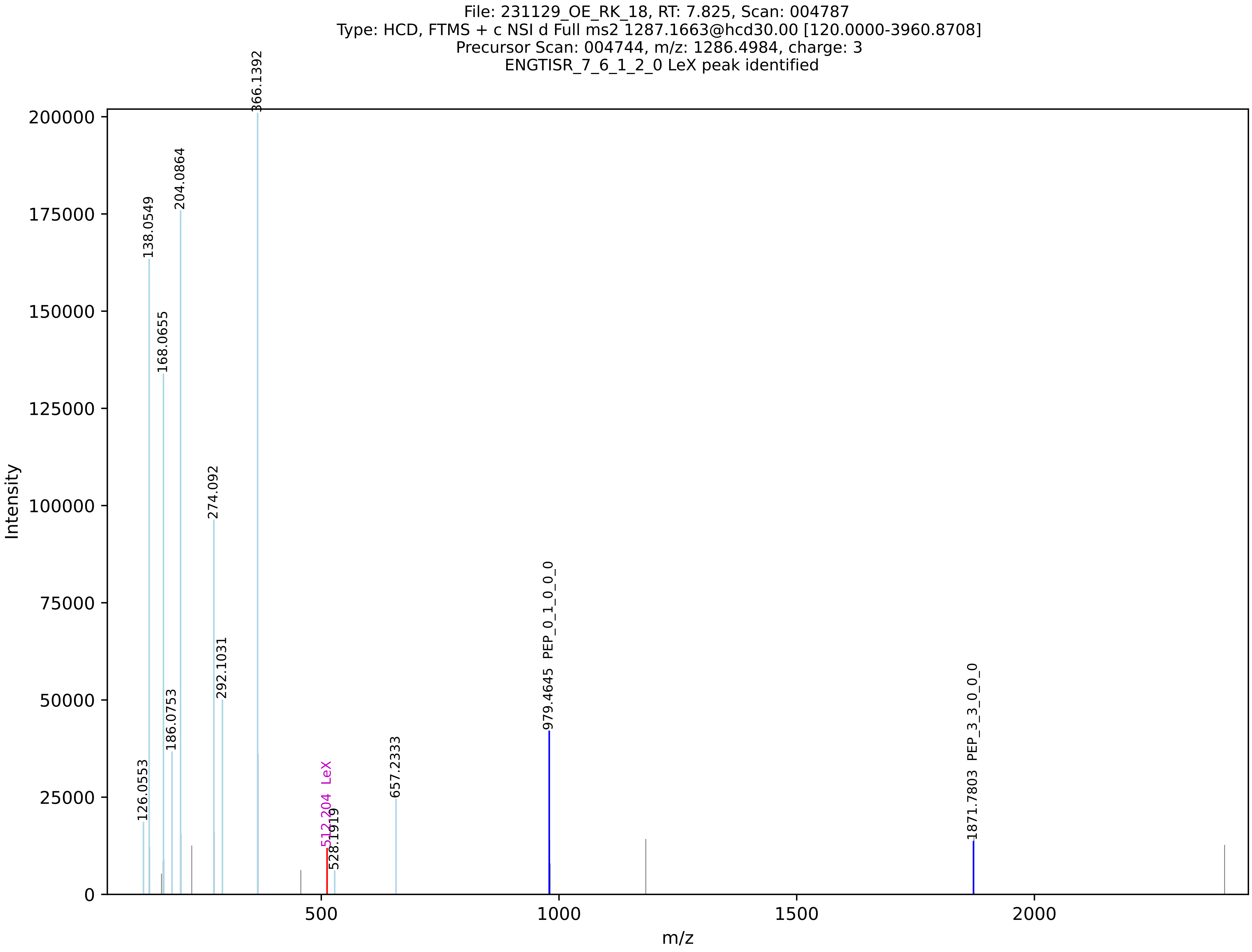
File: 231129_OE_RK_18, RT: 7.825, Scan: 004787
Type: HCD, FTMS + c NSI d Full ms2 1287.1663@hcd30.00 [120.0000-3960.8708]
Precursor Scan: 004744, m/z: 1286.4984, charge: 3
ENGTISR_7_6_1_2_0 LeX peak identified
Intensity
m/z
0
25000
50000
75000
100000
125000
150000
175000
200000
500
1000
1500
2000
366.1392
204.0864
138.0549
168.0655
274.092
292.1031
186.0753
126.0553
512.204 LeX
528.1919
657.2333
979.4645 PEP_0_1_0_0_0
1871.7803 PEP_3_3_0_0_0

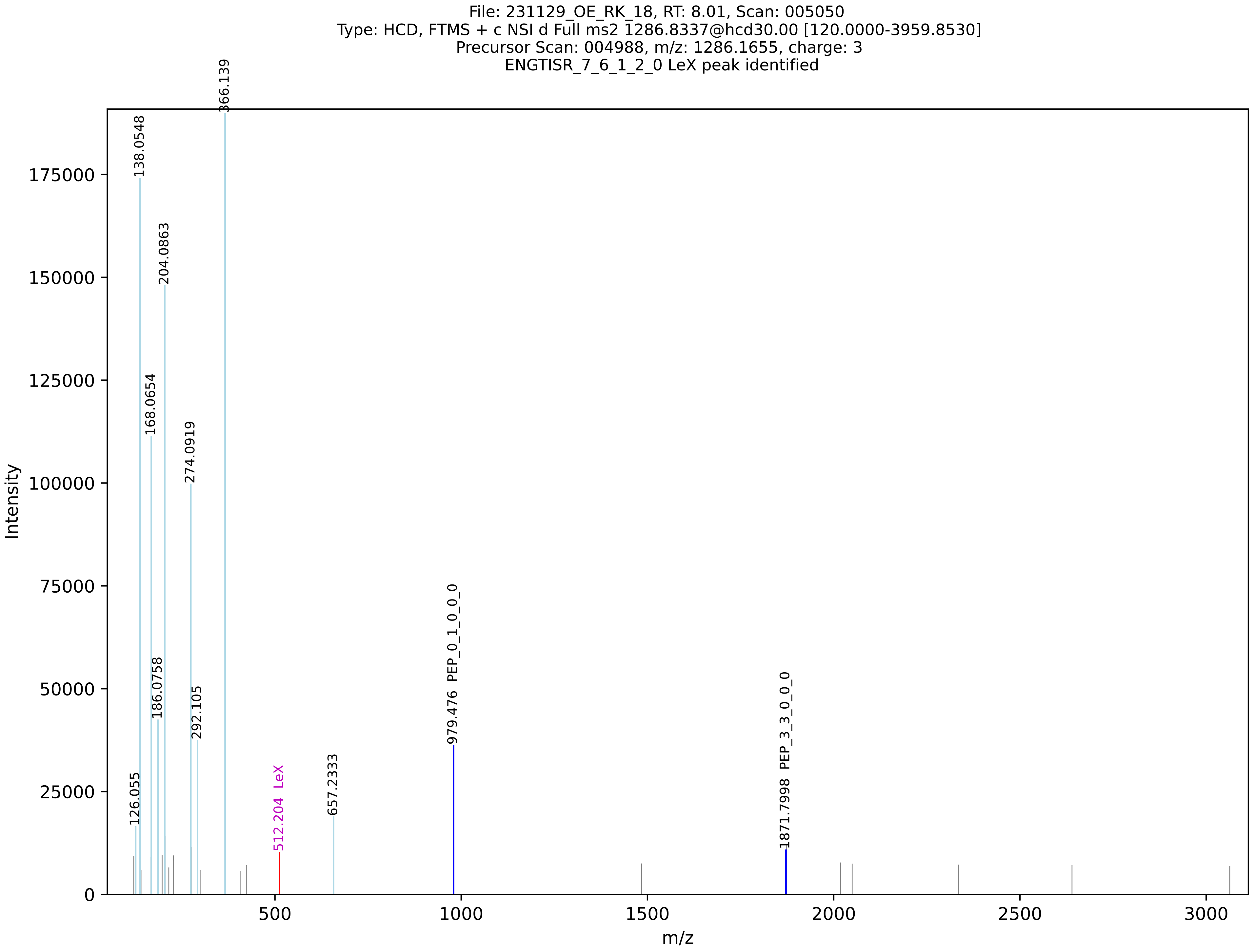

File: 231129_OE_RK_18, RT: 8.01, Scan: 005050
Type: HCD, FTMS + c NSI d Full ms2 1286.8337@hcd30.00 [120.0000-3959.8530]
Precursor Scan: 004988, m/z: 1286.1655, charge: 3
ENGTISR_7_6_1_2_0 LeX peak identified
366.139
138.0548
204.0863
168.0654
274.0919
186.0758
292.105
126.055
512.204 LeX
657.2333
979.476 PEP_0_1_0_0_0
1871.7998 PEP_3_3_0_0_0
Intensity
m/z
0
25000
50000
75000
100000
125000
150000
175000
500
1000
1500
2000
2500
3000

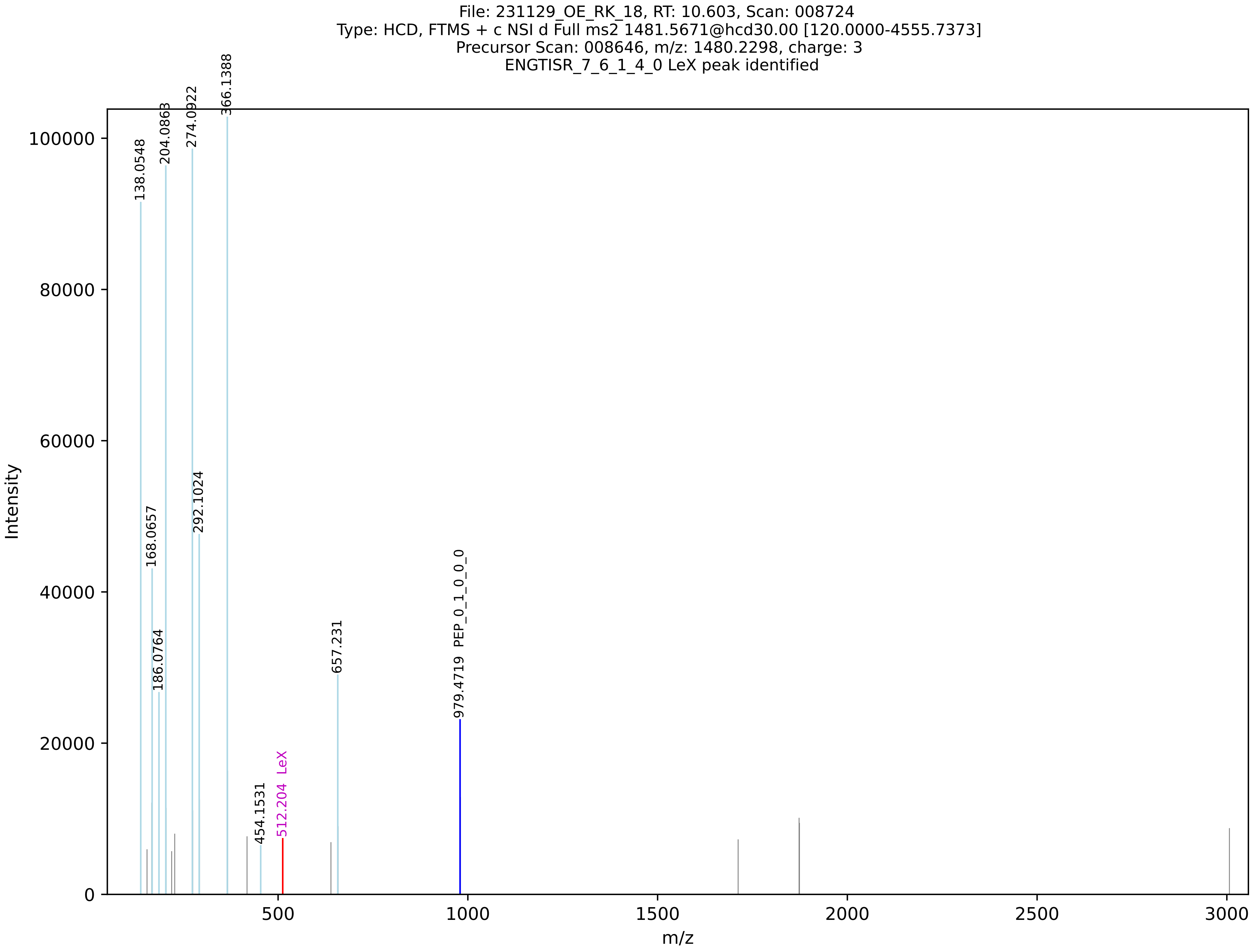
File: 231129_OE_RK_18, RT: 10.603, Scan: 008724
Type: HCD, FTMS + c NSI d Full ms2 1481.5671@hcd30.00 [120.0000-4555.7373]
Precursor Scan: 008646, m/z: 1480.2298, charge: 3
ENGTISR_7_6_1_4_0 LeX peak identified
Intensity
m/z
0
20000
40000
60000
80000
100000
500
1000
1500
2000
2500
3000
138.0548
168.0657
186.0764
204.0863
274.0922
292.1024
366.1388
454.1531
512.204 LeX
657.231
979.4719 PEP_0_1_0_0_0

File: 231129_OE_RK_18, RT: 48.67, Scan: 062655
Type: HCD, FTMS + c NSI d Full ms2 1323.5272@hcd30.00 [120.0000-5422.8472]
Precursor Scan: 062567, m/z: 1322.781, charge: 4
QDQCIYNTTYLNVQR_7_6_1_3_0 LeX peak identified

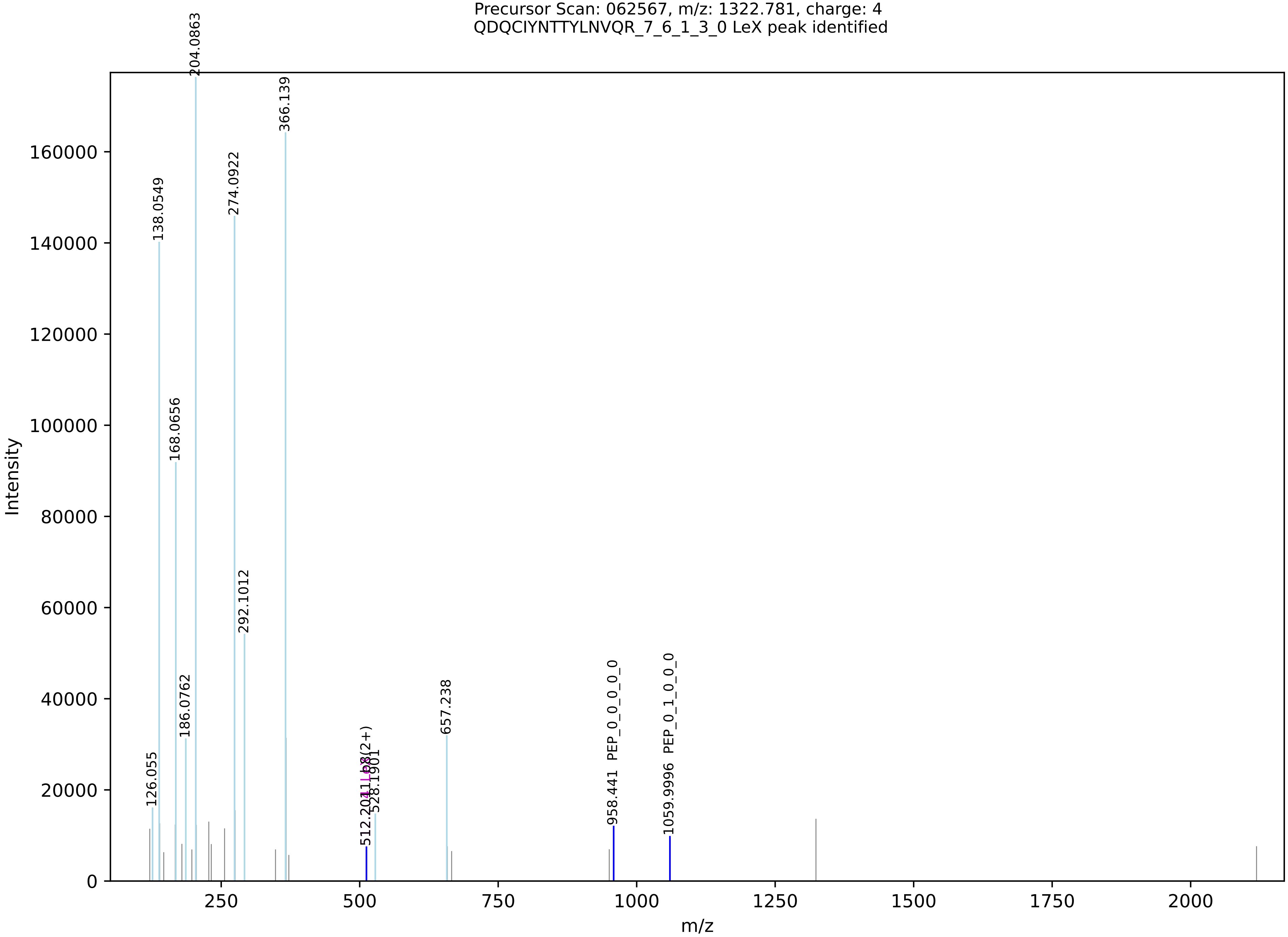

File: 231129_OE_RK_18, RT: 50.923, Scan: 065918
Type: HCD, FTMS + c NSI d Full ms2 1056.2274@hcd30.00 [120.0000-4332.2637]
Precursor Scan: 065834, m/z: 1055.7253, charge: 4
VVLHPNYSQVDIGLIK_6_5_1_1_0 LeX peak identified

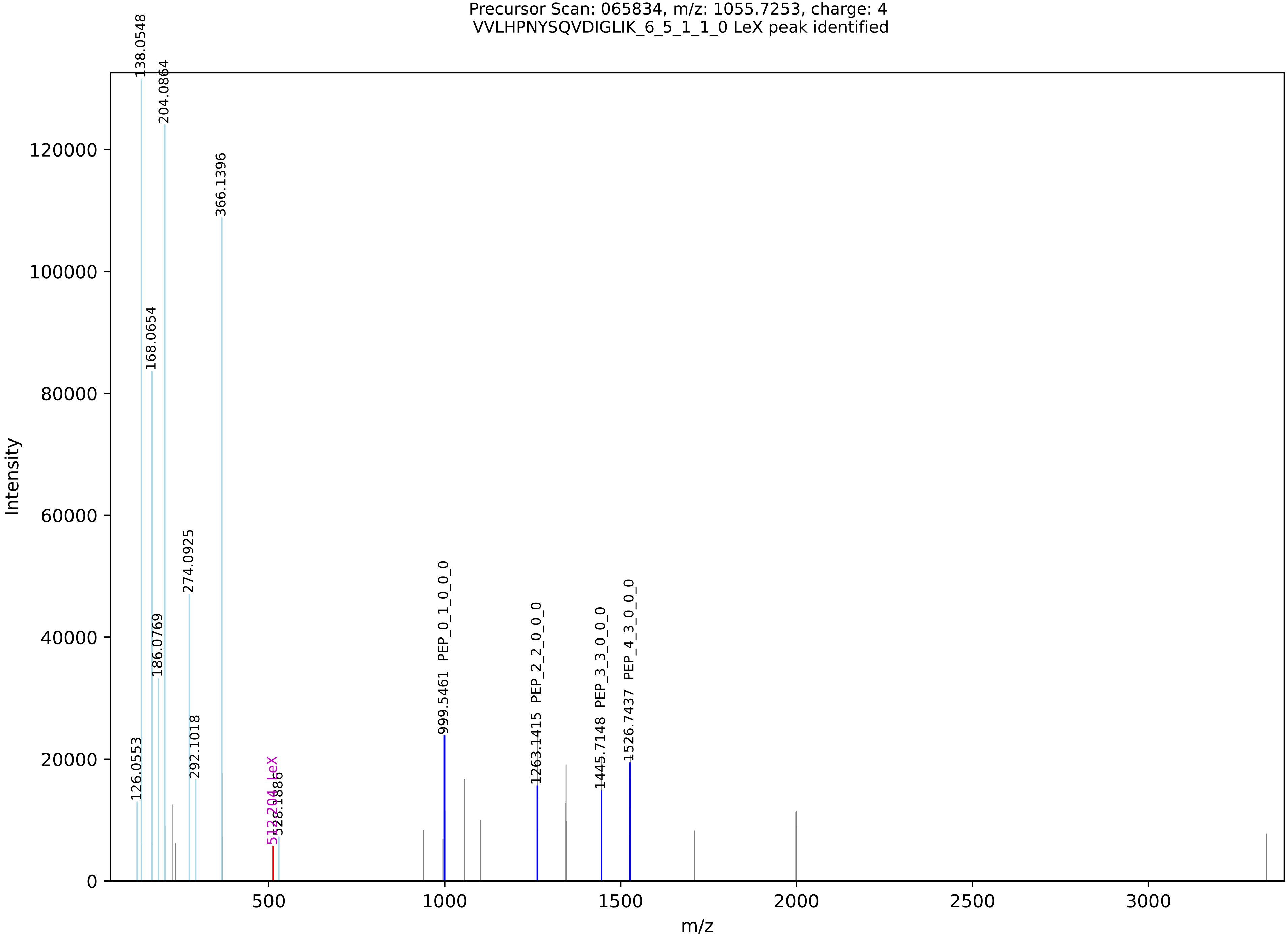

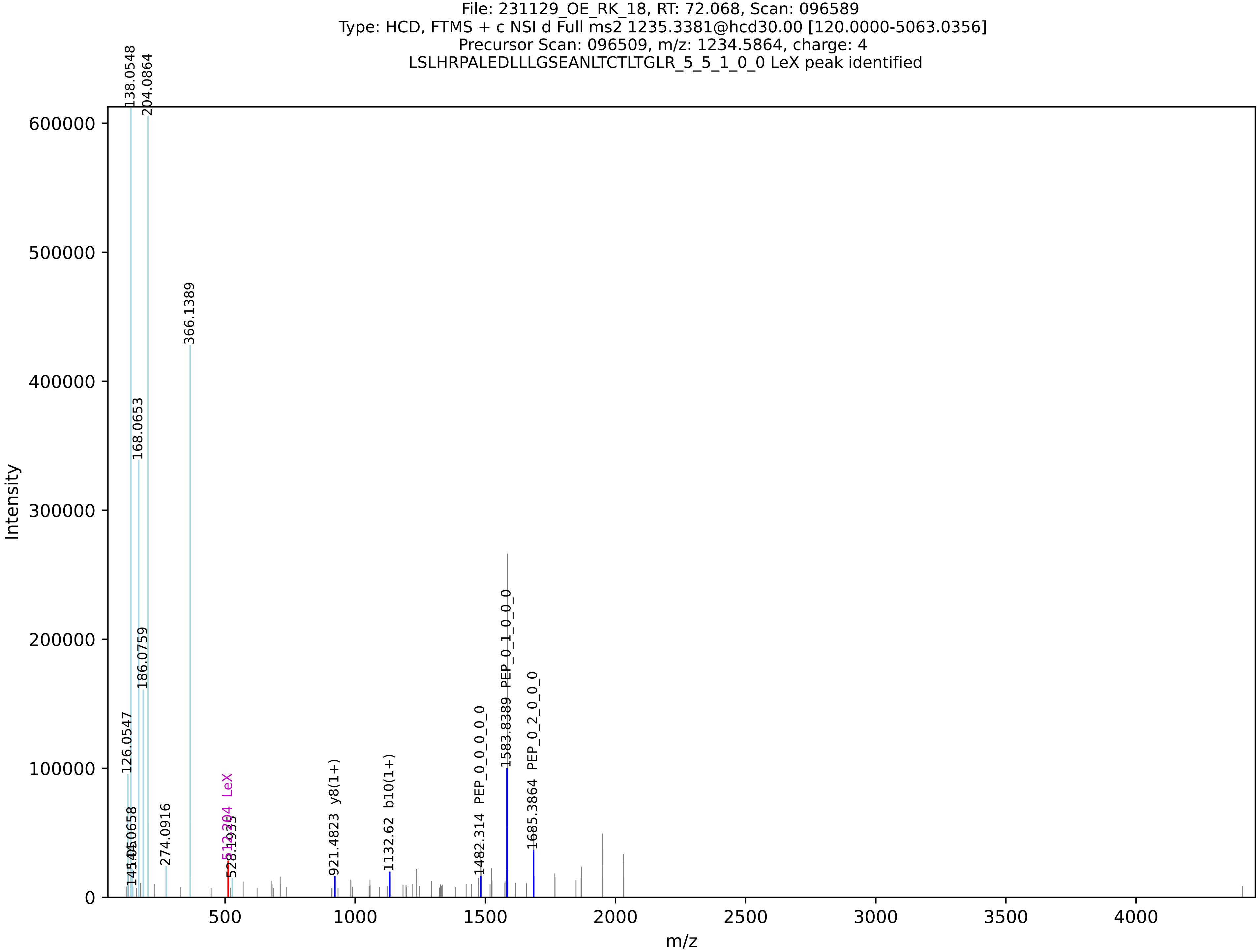
File: 231129_OE_RK_18, RT: 72.068, Scan: 096589
Type: HCD, FTMS + c NSI d Full ms2 1235.3381@hcd30.00 [120.0000-5063.0356]
Precursor Scan: 096509, m/z: 1234.5864, charge: 4
LSLHRPALEDLLLGSEANLTCTLTGLR_5_5_1_0_0 LeX peak identified
138.0548
204.0864
366.1389
168.0653
186.0759
126.0547
274.0916
512.204 LeX
528.1935
921.4823 y8(1+)
1132.62 b10(1+)
1482.314 PEP_0_0_0_0_0
1583.8389 PEP_0_1_0_0_0
1685.3864 PEP_0_2_0_0_0
Intensity
m/z

File: 231129_OE_RK_18, RT: 75.443, Scan: 101500
Type: HCD, FTMS + c NSI d Full ms2 1017.2778@hcd30.00 [120.0000-5211.6865]
Precursor Scan: 101421, m/z: 1016.8788, charge: 5
LSLHRPALEDLLLGSEANLTCTLTGLR_5_5_0_1_0 LeX peak identified

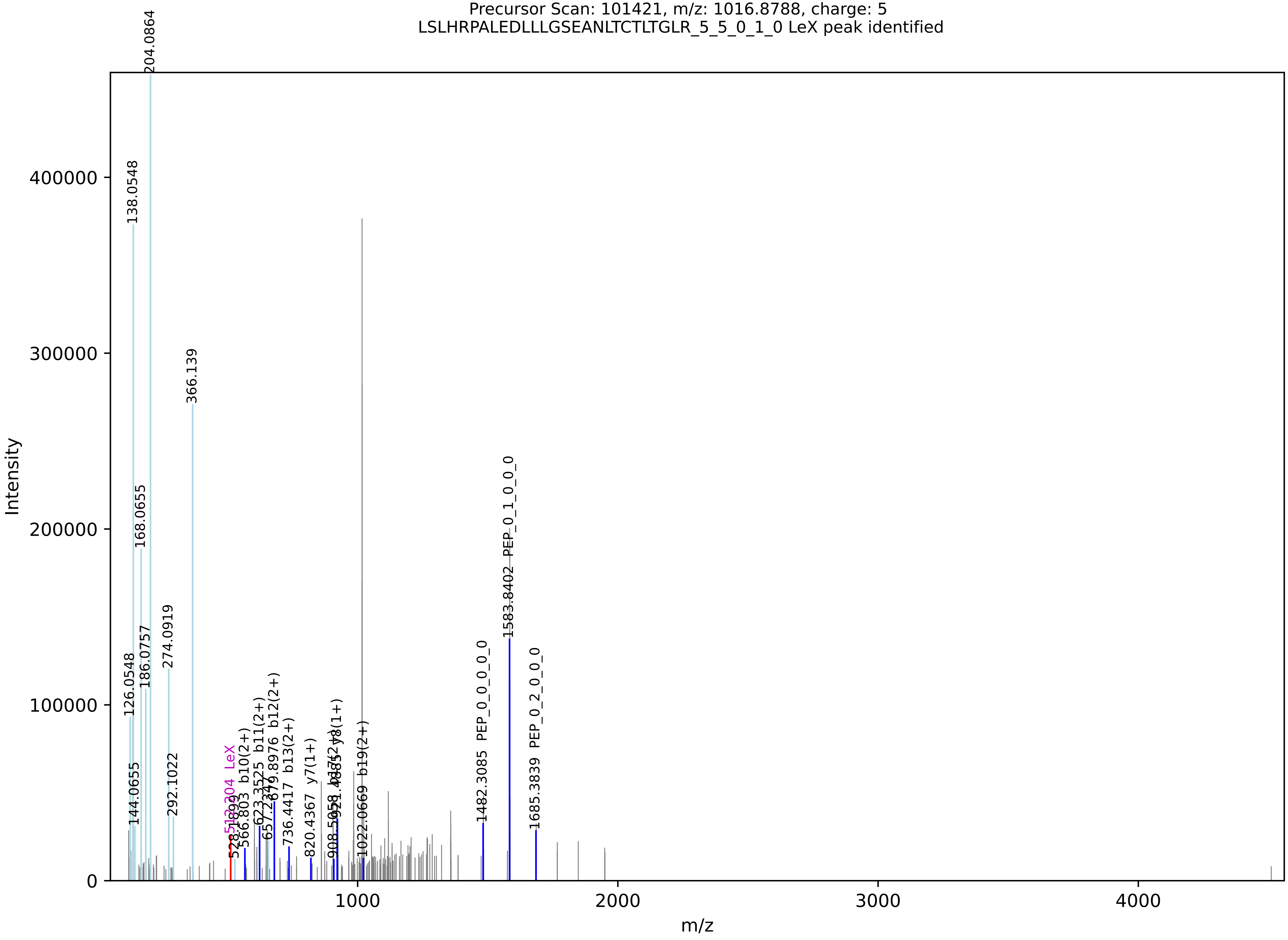

File: 231129_OE_RK_19, RT: 7.222, Scan: 002845
Type: HCD, FTMS + c NSI d Full ms2 1335.1857@hcd30.00 [120.0000-4107.8101]
Precursor Scan: 002836, m/z: 1334.8516, charge: 3
ENGTISR_7_6_0_3_0 LeX peak identified

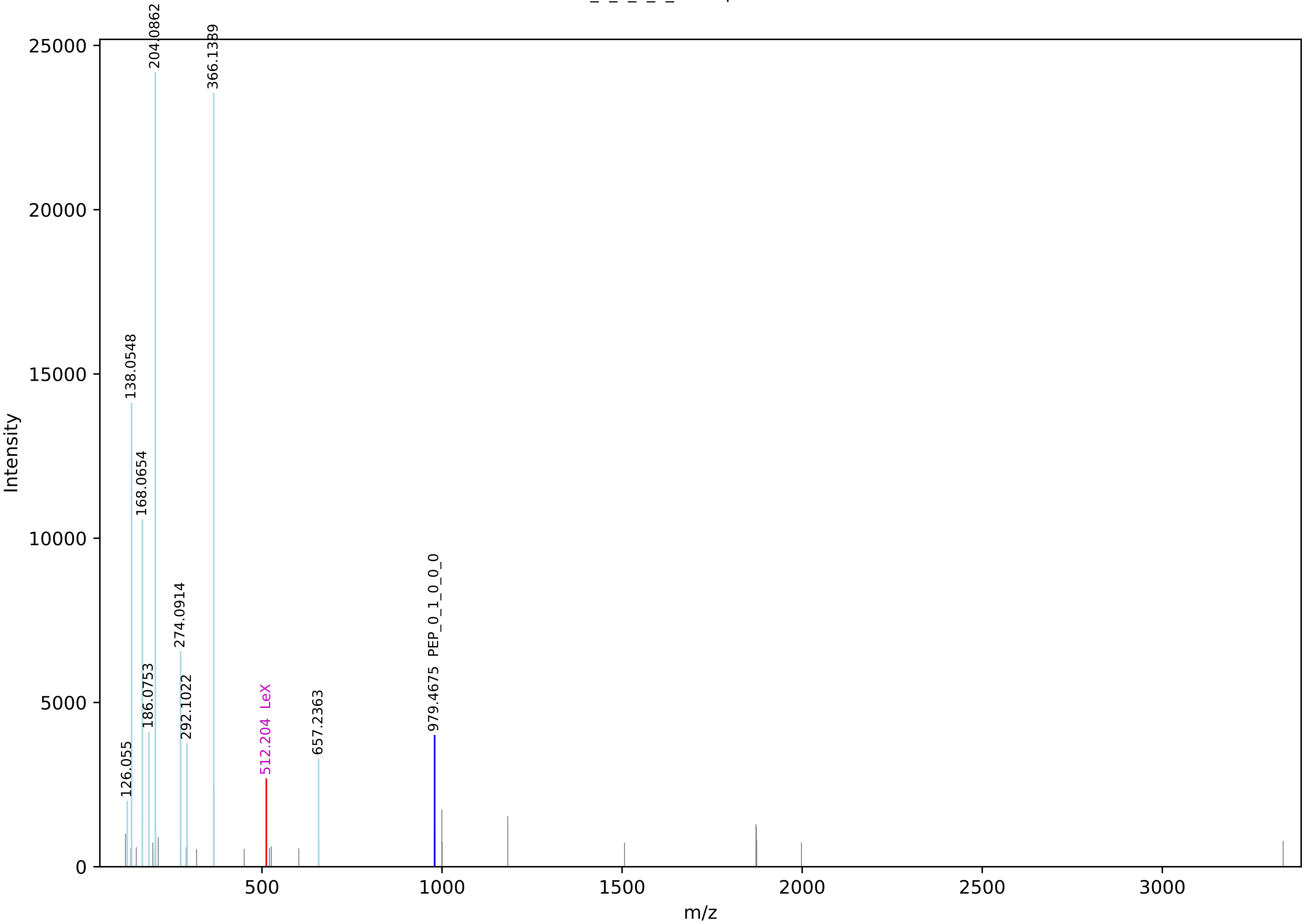

File: 231129_OE_RK_19, RT: 7.878, Scan: 003653
Type: HCD, FTMS + c NSI d Full ms2 1384.2030@hcd30.00 [120.0000-4257.8032]
Precursor Scan: 003617, m/z: 1383.534, charge: 3
ENGTISR_7_6_1_3_0 LeX peak identified

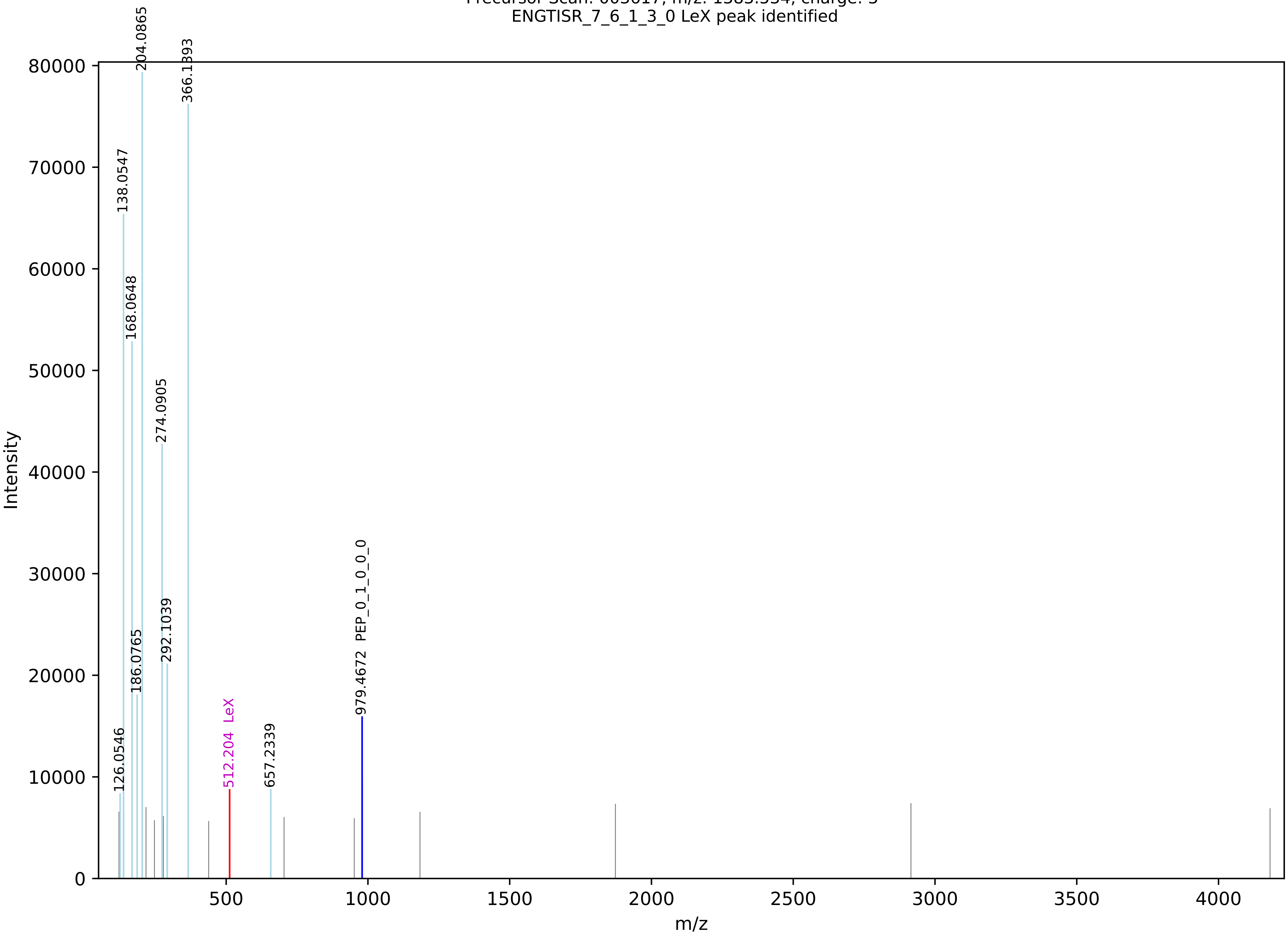

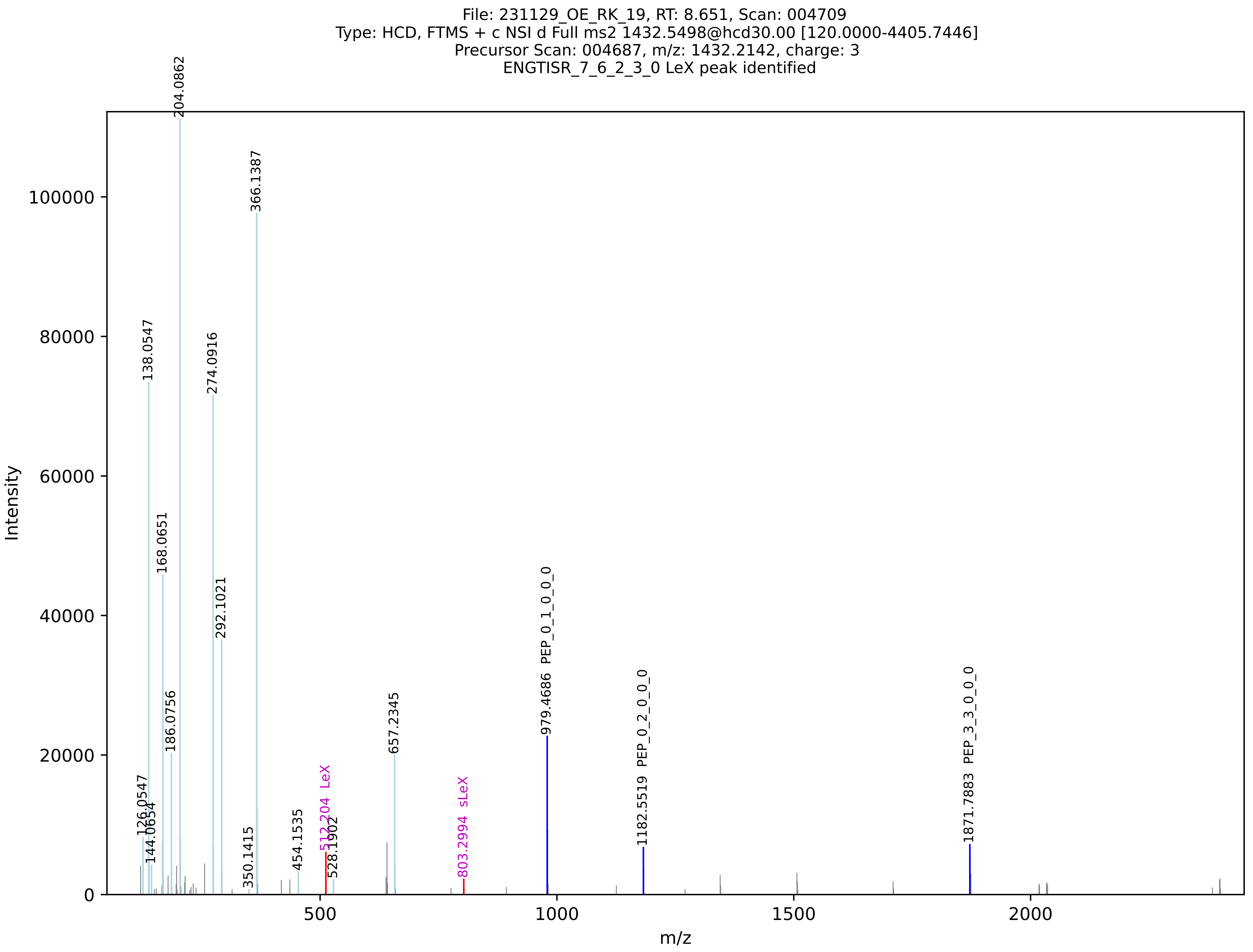

File: 231129_OE_RK_19, RT: 8.651, Scan: 004709
Type: HCD, FTMS + c NSI d Full ms2 1432.5498@hcd30.00 [120.0000-4405.7446]
Precursor Scan: 004687, m/z: 1432.2142, charge: 3
ENGTISR_7_6_2_3_0 LeX peak identified
Intensity
m/z
204.0862
366.1387
138.0547
274.0916
168.0651
292.1021
186.0756
126.0547
144.0654
350.1415
454.1535
512.204 LeX
528.1902
657.2345
803.2994 sLeX
979.4686 PEP_0_1_0_0_0_
1182.5519 PEP_0_2_0_0_0
1871.7883 PEP_3_3_0_0_0_

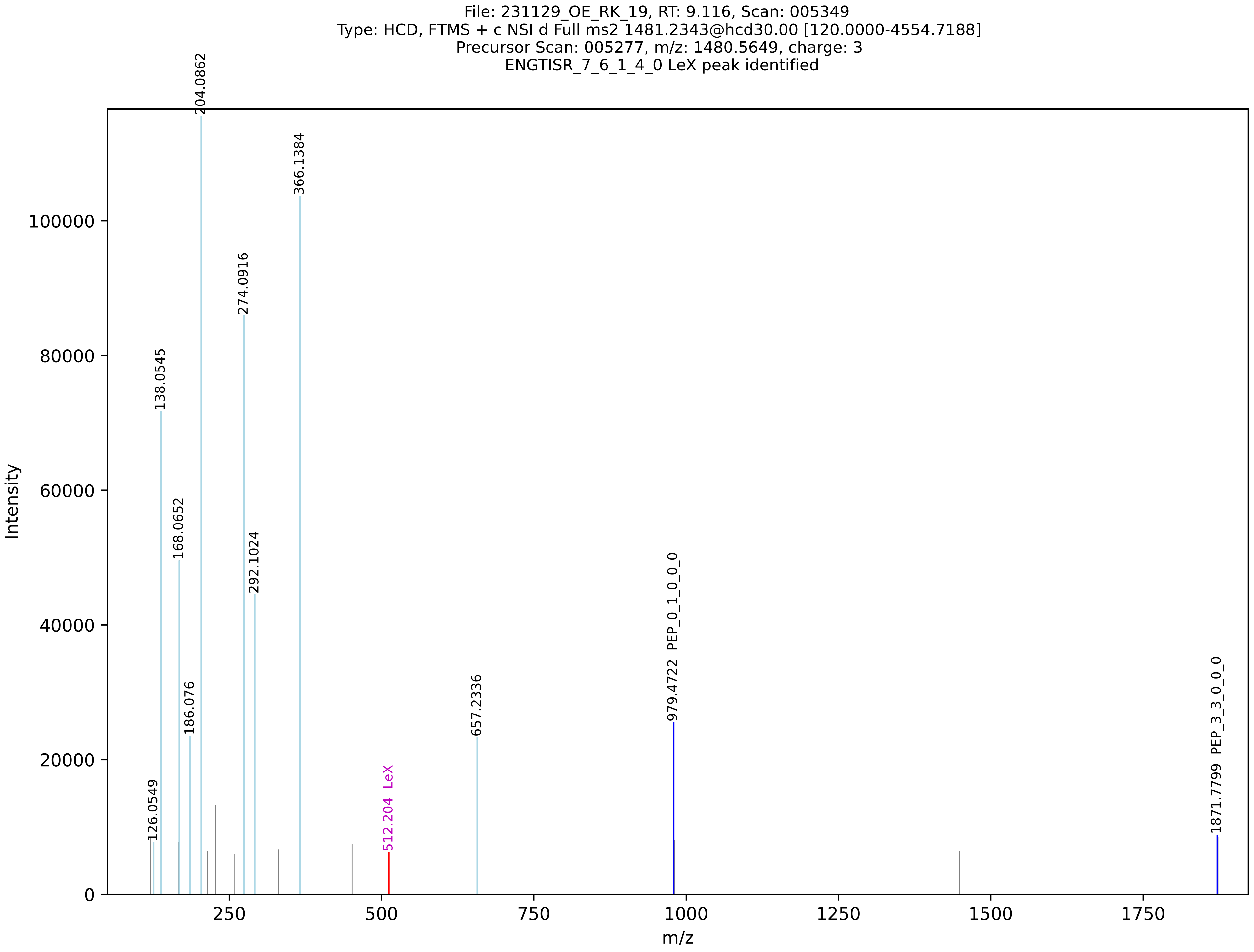

File: 231129_OE_RK_19, RT: 9.116, Scan: 005349
Type: HCD, FTMS + c NSI d Full ms2 1481.2343@hcd30.00 [120.0000-4554.7188]
Precursor Scan: 005277, m/z: 1480.5649, charge: 3
ENGTISR_7_6_1_4_0 LeX peak identified
Intensity
m/z
204.0862
366.1384
274.0916
138.0545
168.0652
292.1024
186.076
126.0549
512.204 LeX
657.2336
979.4722 PEP_0_1_0_0_0
1871.7799 PEP_3_3_0_0_0

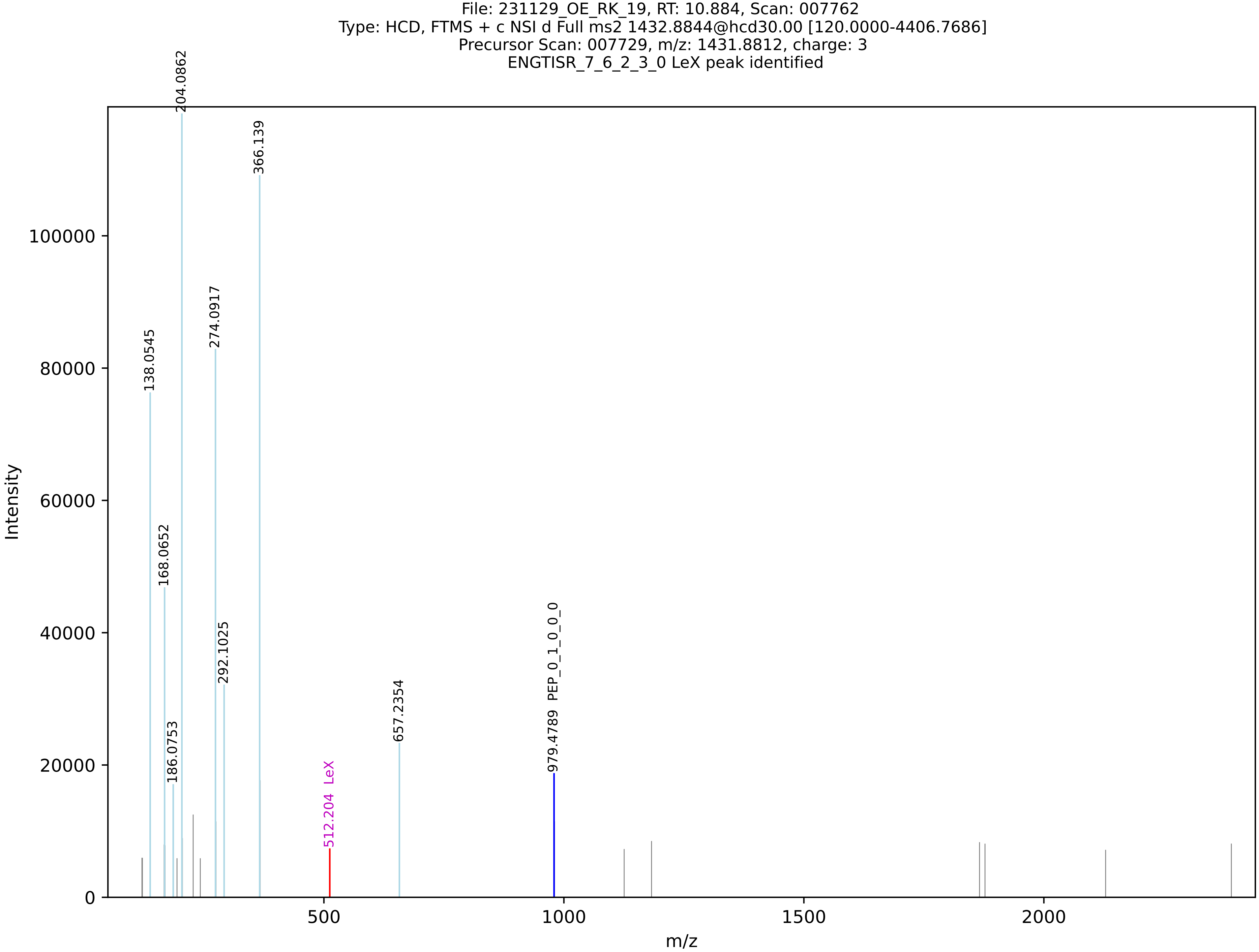
File: 231129_OE_RK_19, RT: 10.884, Scan: 007762
Type: HCD, FTMS + c NSI d Full ms2 1432.8844@hcd30.00 [120.0000-4406.7686]
Precursor Scan: 007729, m/z: 1431.8812, charge: 3
ENGTISR_7_6_2_3_0 LeX peak identified
204.0862
366.139
274.0917
138.0545
168.0652
292.1025
186.0753
657.2354
512.204 LeX
979.4789 PEP_0_1_0_0_0_
Intensity
m/z
0
20000
40000
60000
80000
100000
500
1000
1500
2000

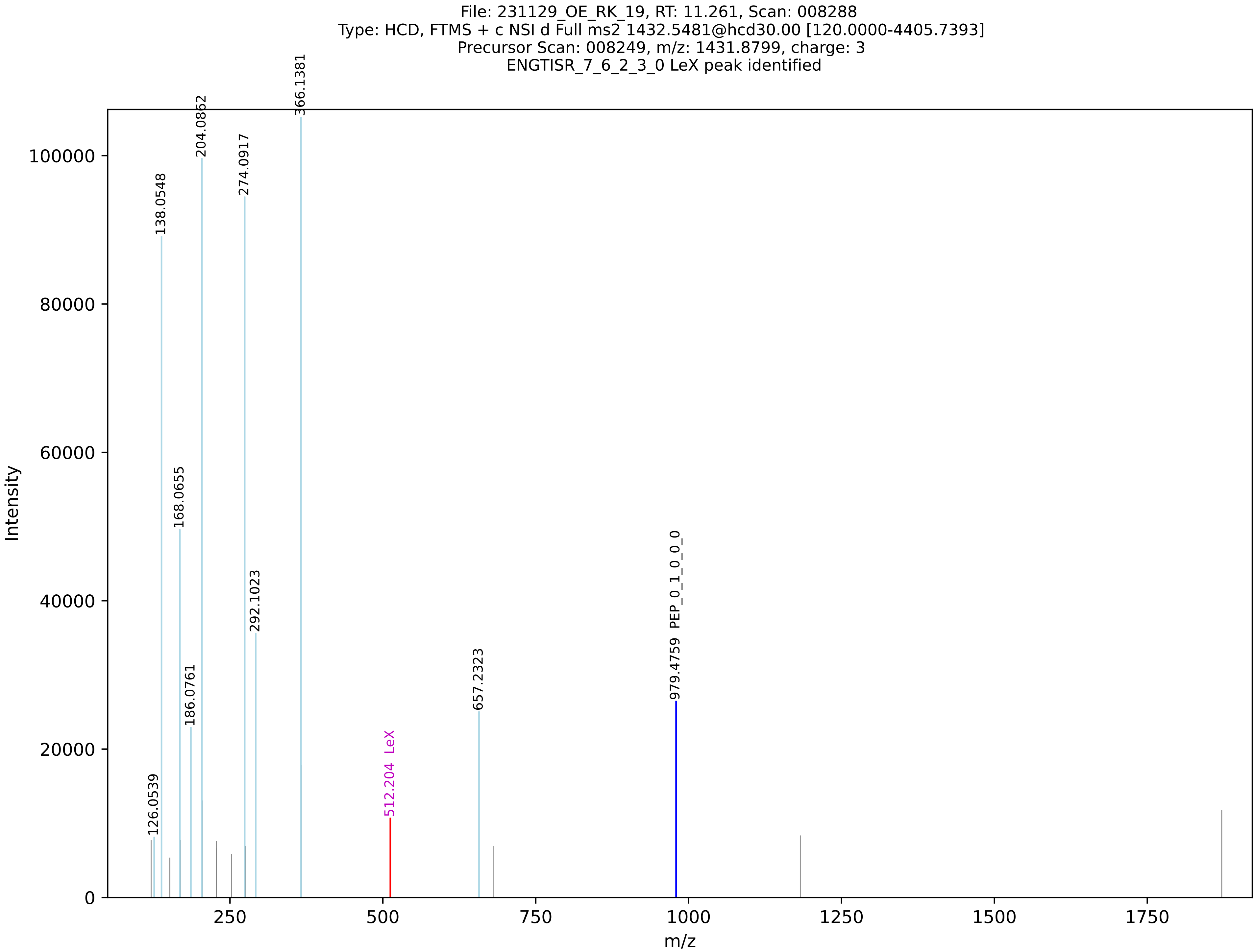
File: 231129_OE_RK_19, RT: 11.261, Scan: 008288
Type: HCD, FTMS + c NSI d Full ms2 1432.5481@hcd30.00 [120.0000-4405.7393]
Precursor Scan: 008249, m/z: 1431.8799, charge: 3
ENGTISR_7_6_2_3_0 LeX peak identified
Intensity
m/z
366.1381
204.0862
274.0917
138.0548
168.0655
292.1023
186.0761
126.0539
512.204 LeX
657.2323
979.4759 PEP_0_1_0_0_0
0
20000
40000
60000
80000
100000
250
500
750
1000
1250
1500
1750

File: 231129_OE_RK_19, RT: 11.636, Scan: 008825
Type: HCD, FTMS + c NSI d Full ms2 1432.8831@hcd30.00 [120.0000-4406.7642]
Precursor Scan: 008745, m/z: 1431.8802, charge: 3
ENGTISR_7_6_2_3_0 LeX peak identified

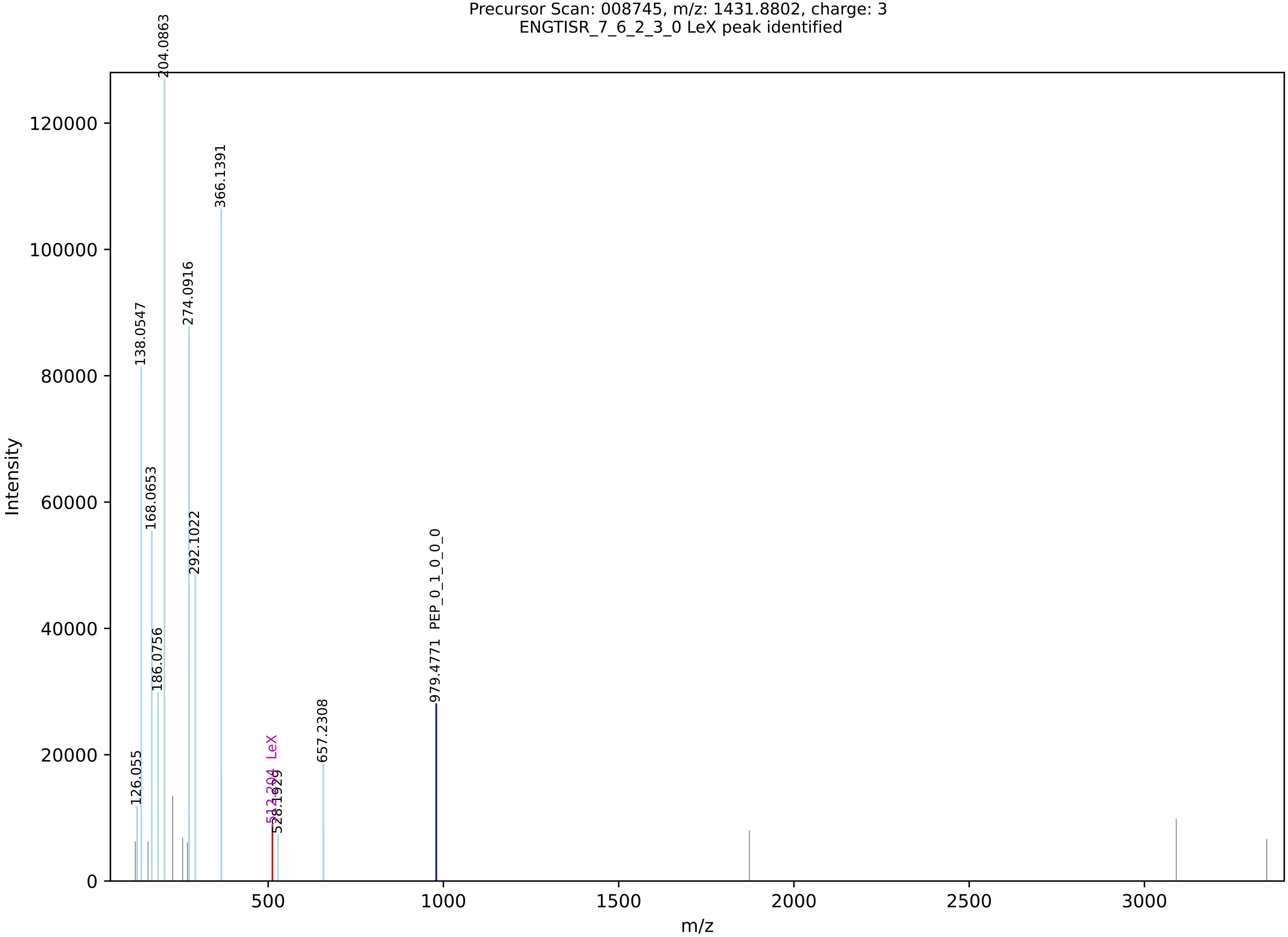

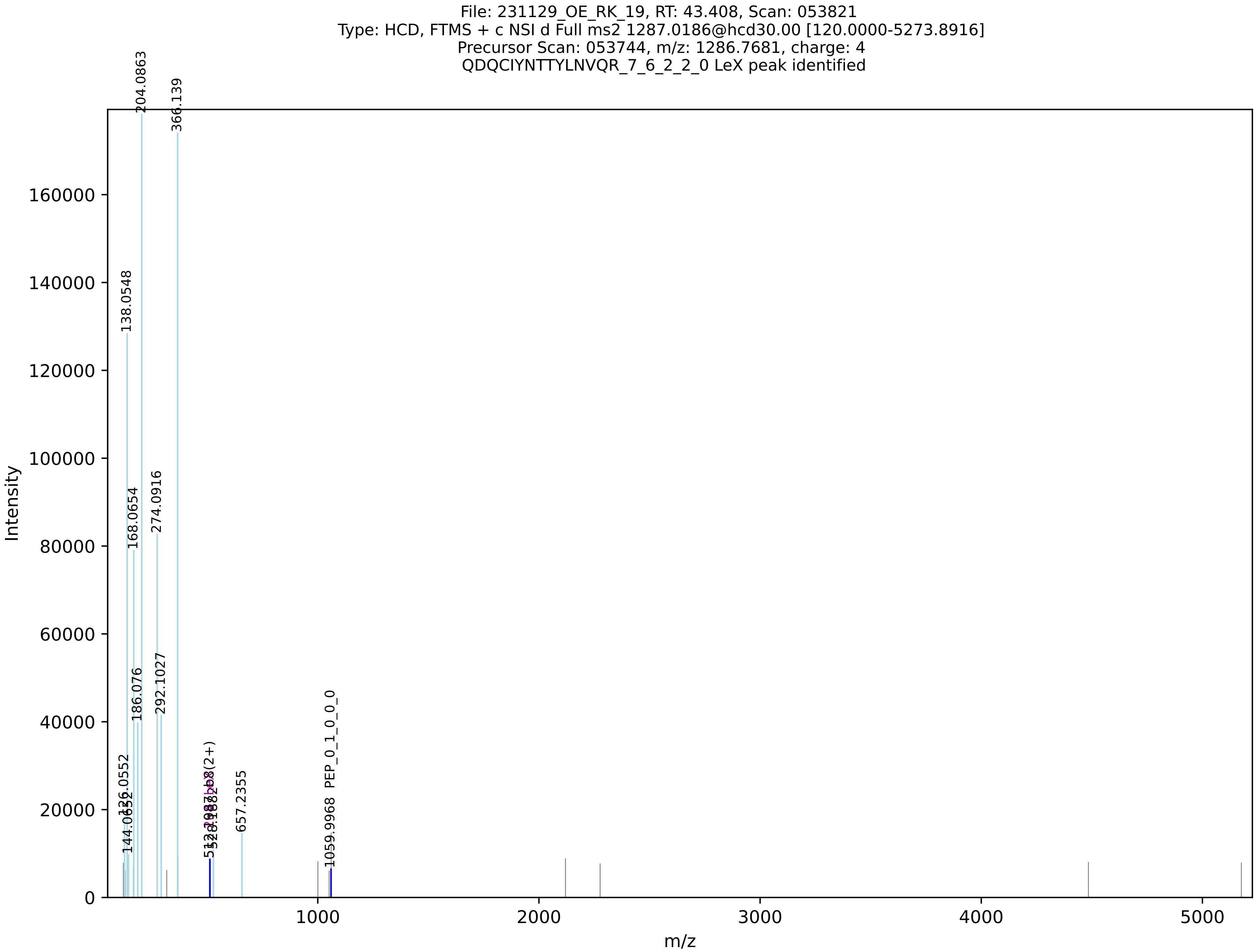
File: 231129_OE_RK_19, RT: 43.408, Scan: 053821
Type: HCD, FTMS + c NSI d Full ms2 1287.0186@hcd30.00 [120.0000-5273.8916]
Precursor Scan: 053744, m/z: 1286.7681, charge: 4
QDQCIYNTTYLNVQR_7_6_2_2_0 LeX peak identified
Intensity
m/z
0
20000
40000
60000
80000
100000
120000
140000
160000
1000
2000
3000
4000
5000
204.0863
366.139
138.0548
168.0654
274.0916
186.076
292.1027
126.0552
144.0652
512.2087 b8(2+)
528.1882
657.2355
1059.9968 PEP_0_1_0_0_0

File: 231129_OE_RK_19, RT: 44.164, Scan: 054928
Type: HCD, FTMS + c NSI d Full ms2 1287.0209@hcd30.00 [120.0000-5273.9014]
Precursor Scan: 054850, m/z: 1286.2688, charge: 4
QDQCIYNTTYLNVQR_7_6_0_3_0 LeX peak identified

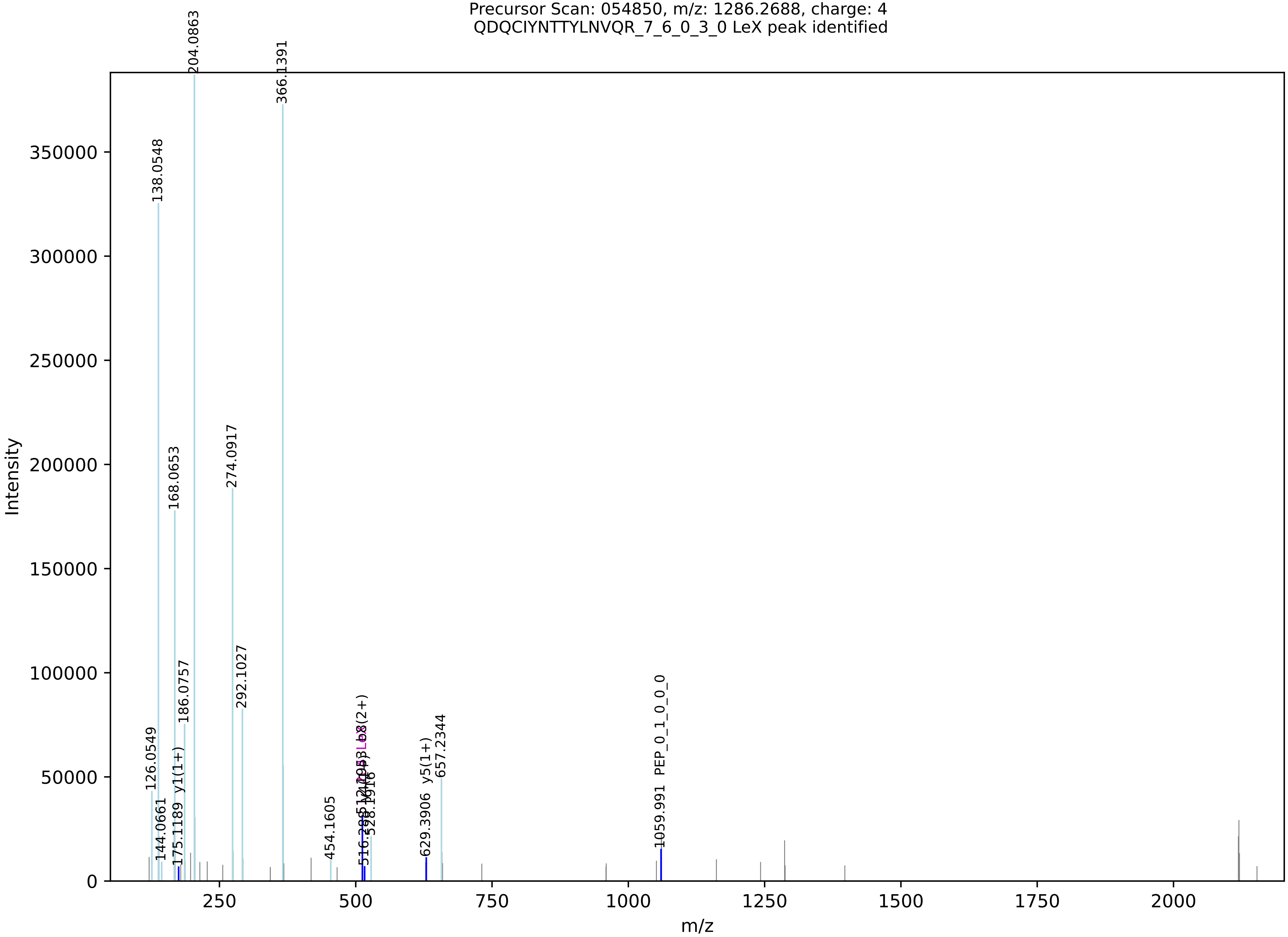

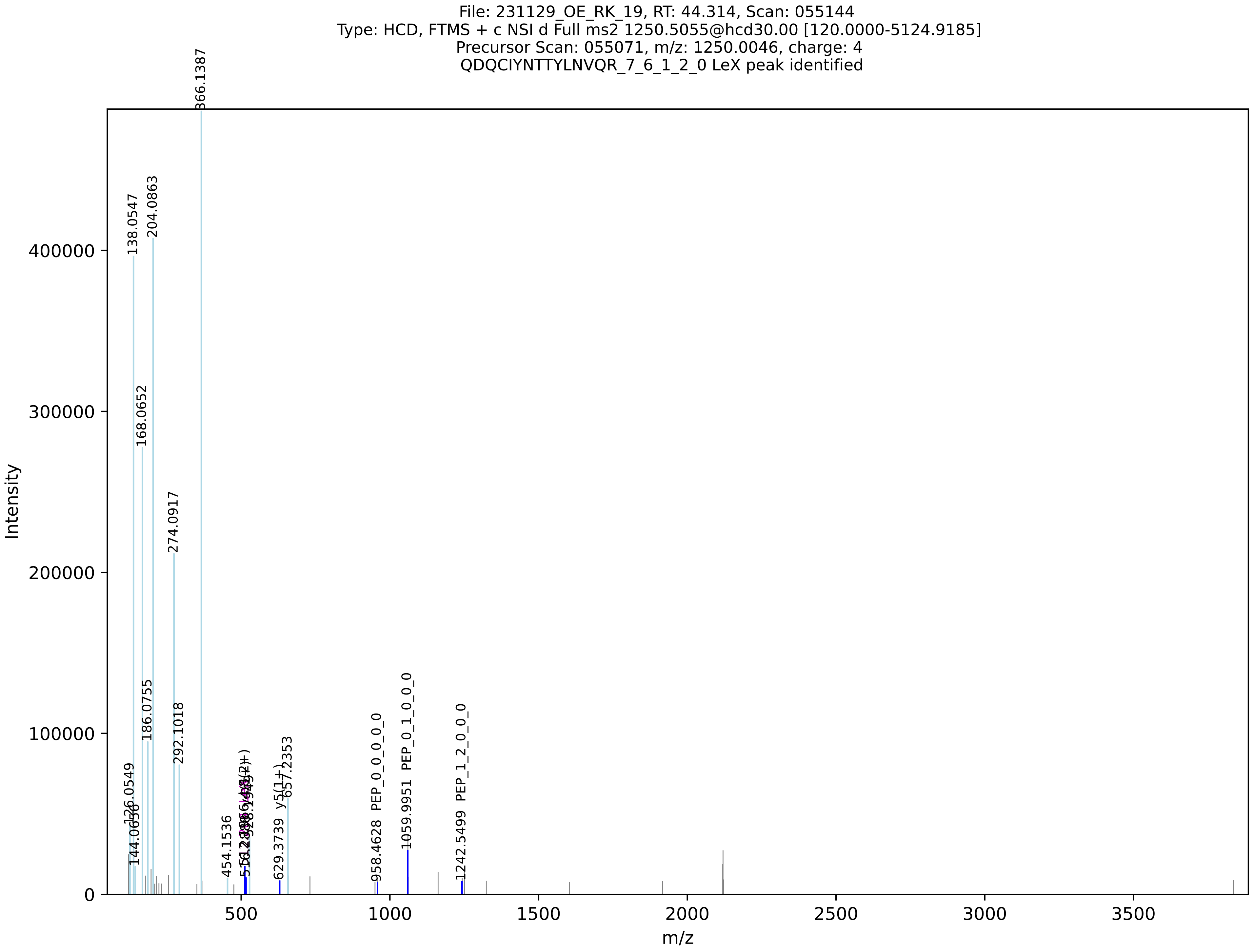
File: 231129_OE_RK_19, RT: 44.314, Scan: 055144
Type: HCD, FTMS + c NSI d Full ms2 1250.5055@hcd30.00 [120.0000-5124.9185]
Precursor Scan: 055071, m/z: 1250.0046, charge: 4
QDQCIYNTTYLNVQR_7_6_1_2_0 LeX peak identified
366.1387
138.0547
204.0863
168.0652
274.0917
186.0755
292.1018
126.0549
144.0656
454.1536
629.3739 y5(1+)
657.2353
958.4628 PEP_0_0_0_0_0_
1059.9951 PEP_0_1_0_0_0_
1242.5499 PEP_1_2_0_0_0_
Intensity
0
100000
200000
300000
400000
500
1000
1500
2000
2500
3000
3500
m/z

File: 231129_OE_RK_19, RT: 44.567, Scan: 055512
Type: HCD, FTMS + c NSI d Full ms2 1287.2694@hcd30.00 [120.0000-5274.9155]
Precursor Scan: 055436, m/z: 1286.2668, charge: 4
QDQCIYNTTYLNVQR_7_6_0_3_0 LeX peak identified

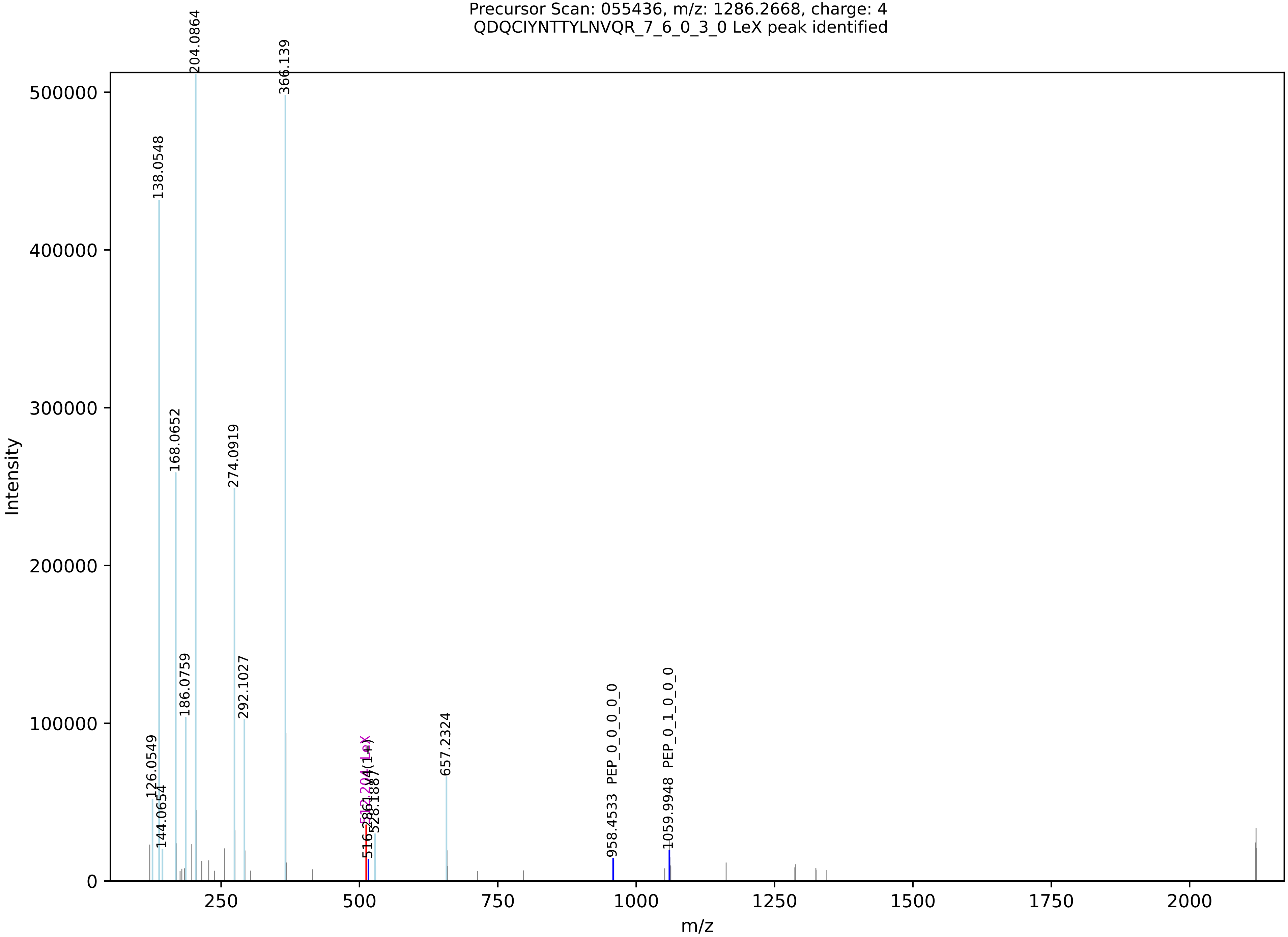

File: 231129_OE_RK_19, RT: 44.883, Scan: 055969
Type: HCD, FTMS + c NSI d Full ms2 1360.0457@hcd30.00 [120.0000-5571.8423]
Precursor Scan: 055873, m/z: 1359.2939, charge: 4
QDQCIYNTTYLNVQR_7_6_2_3_0 LeX peak identified

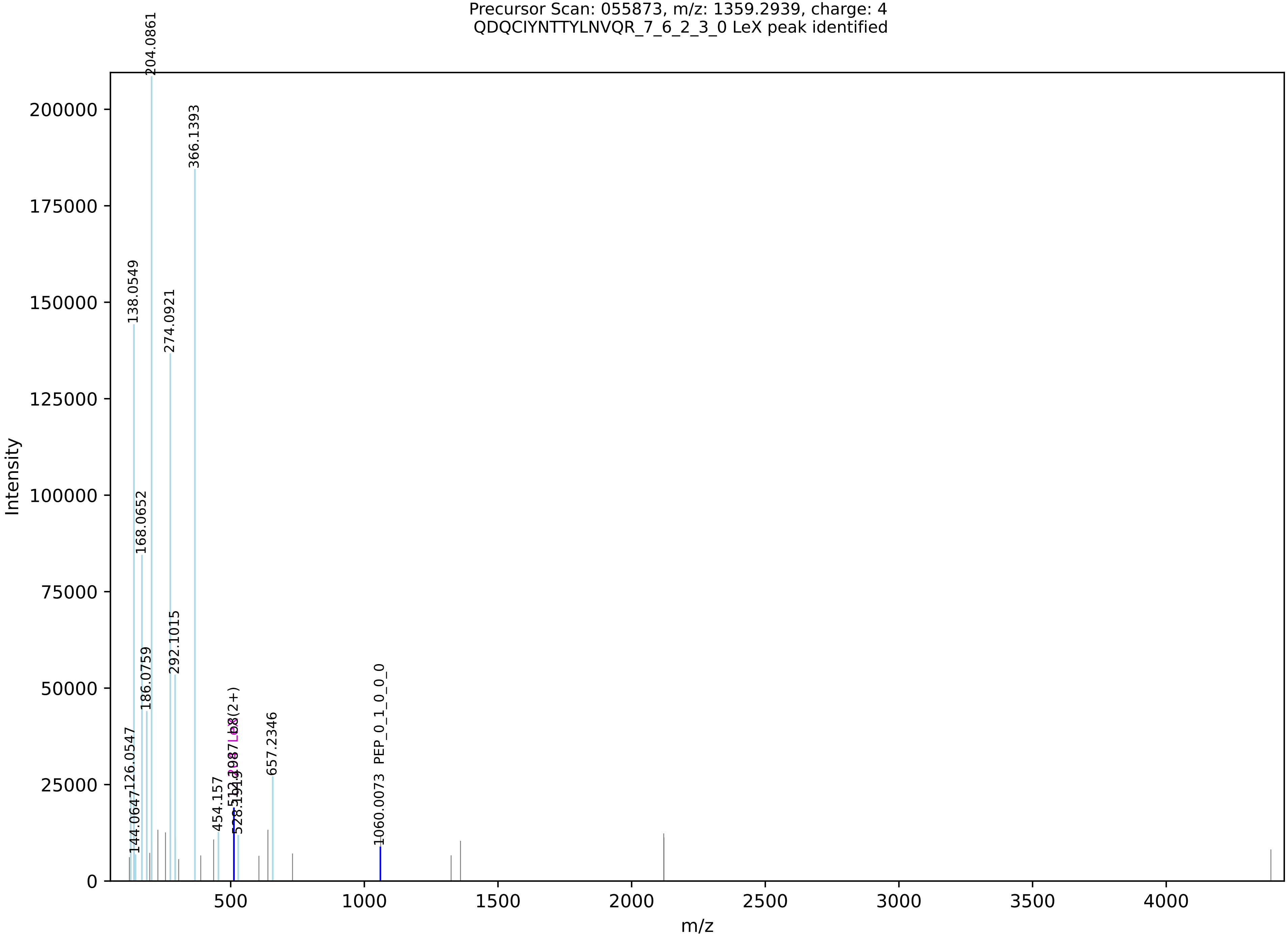

File: 231129_OE_RK_19, RT: 44.972, Scan: 056097
Type: HCD, FTMS + c NSI d Full ms2 1287.0183@hcd30.00 [120.0000-5273.8906]
Precursor Scan: 056018, m/z: 1286.2674, charge: 4
QDQCIYNTTYLNVQR_7_6_0_3_0 LeX peak identified

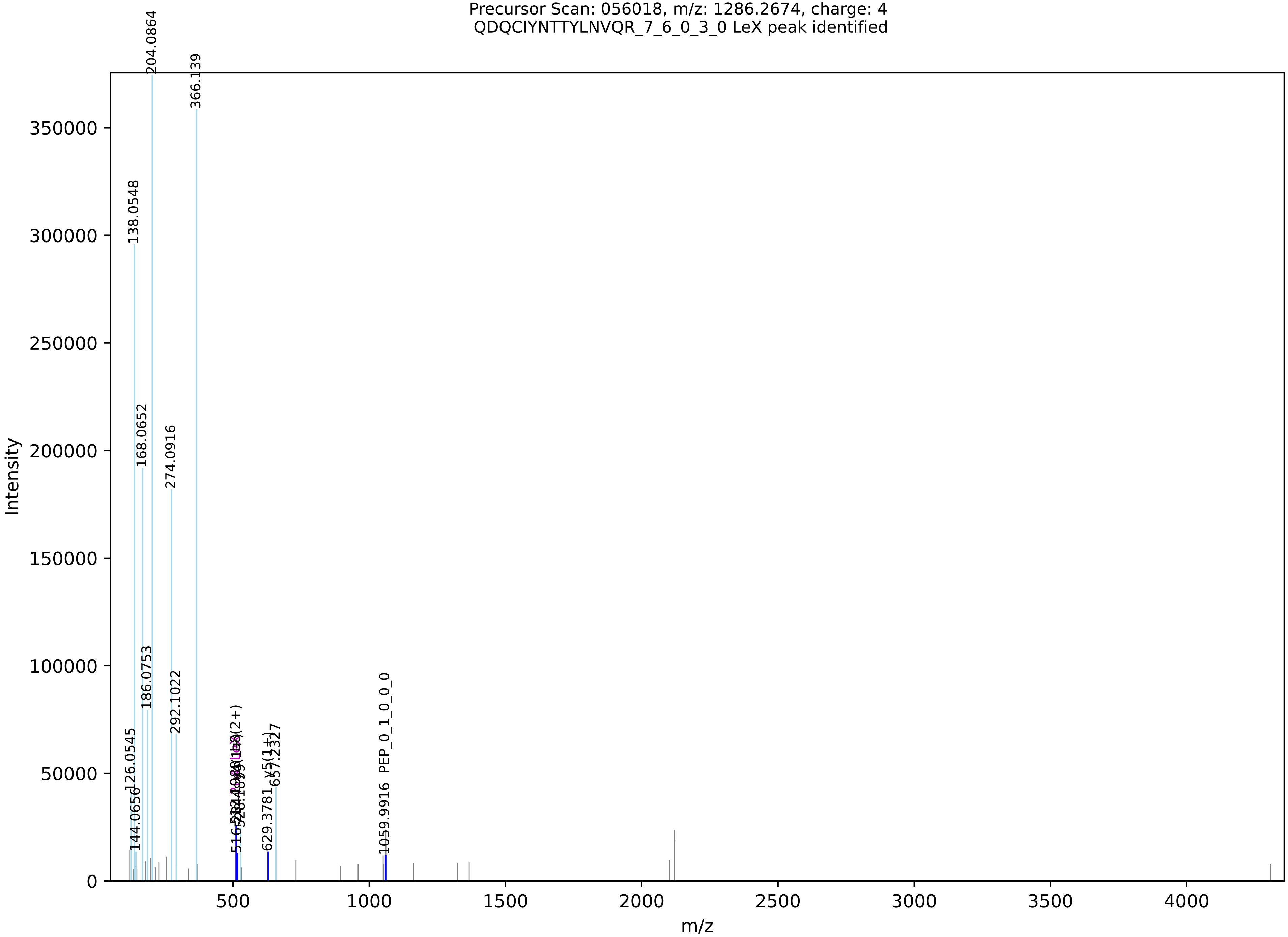

File: 231129_OE_RK_19, RT: 45.352, Scan: 056653
Type: HCD, FTMS + c NSI d Full ms2 1287.5209@hcd30.00 [120.0000-5275.9414]
Precursor Scan: 056571, m/z: 1286.5185, charge: 4
QDQCIYNTTYLNVQR_7_6_2_2_0 LeX peak identified

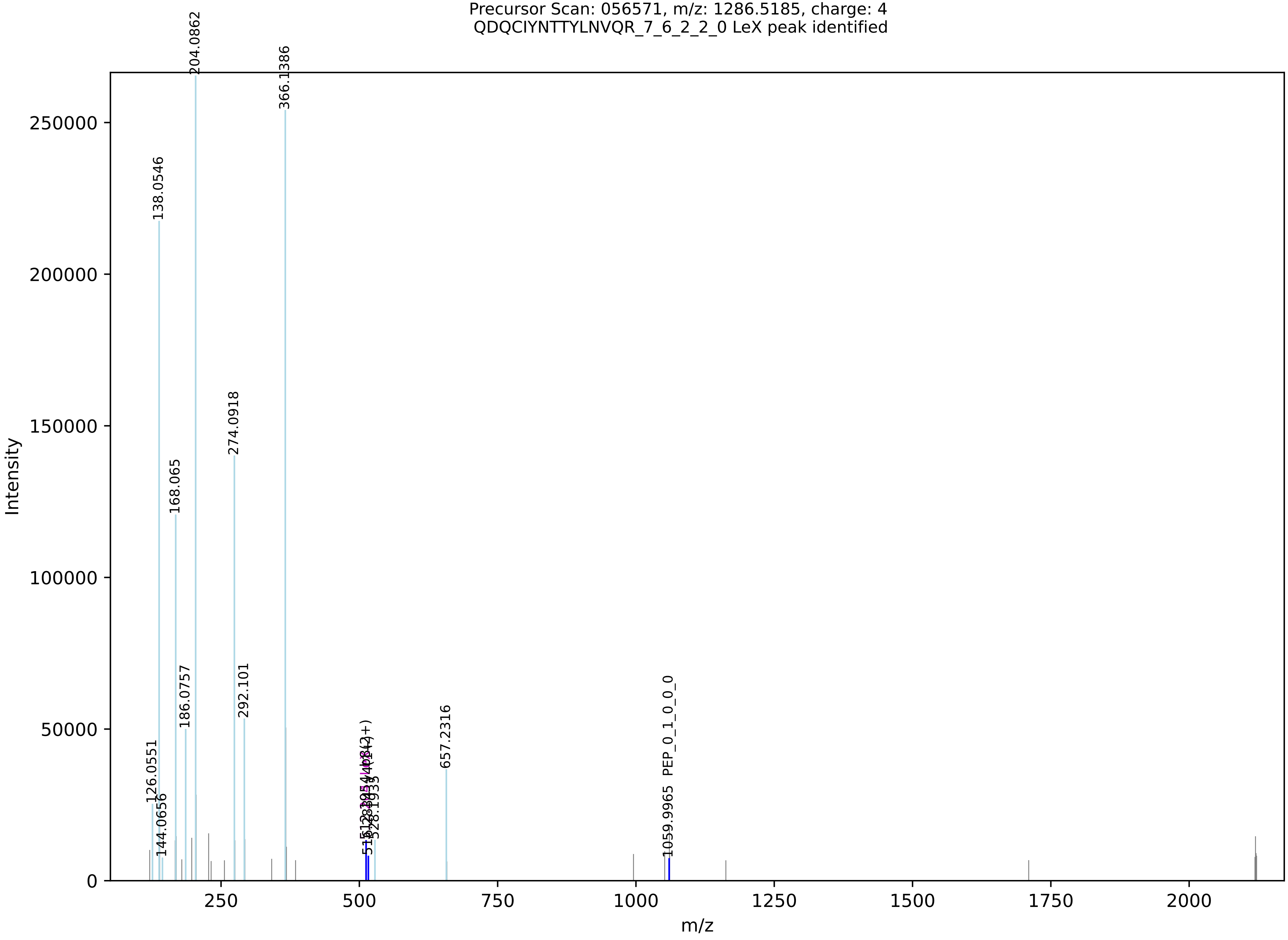

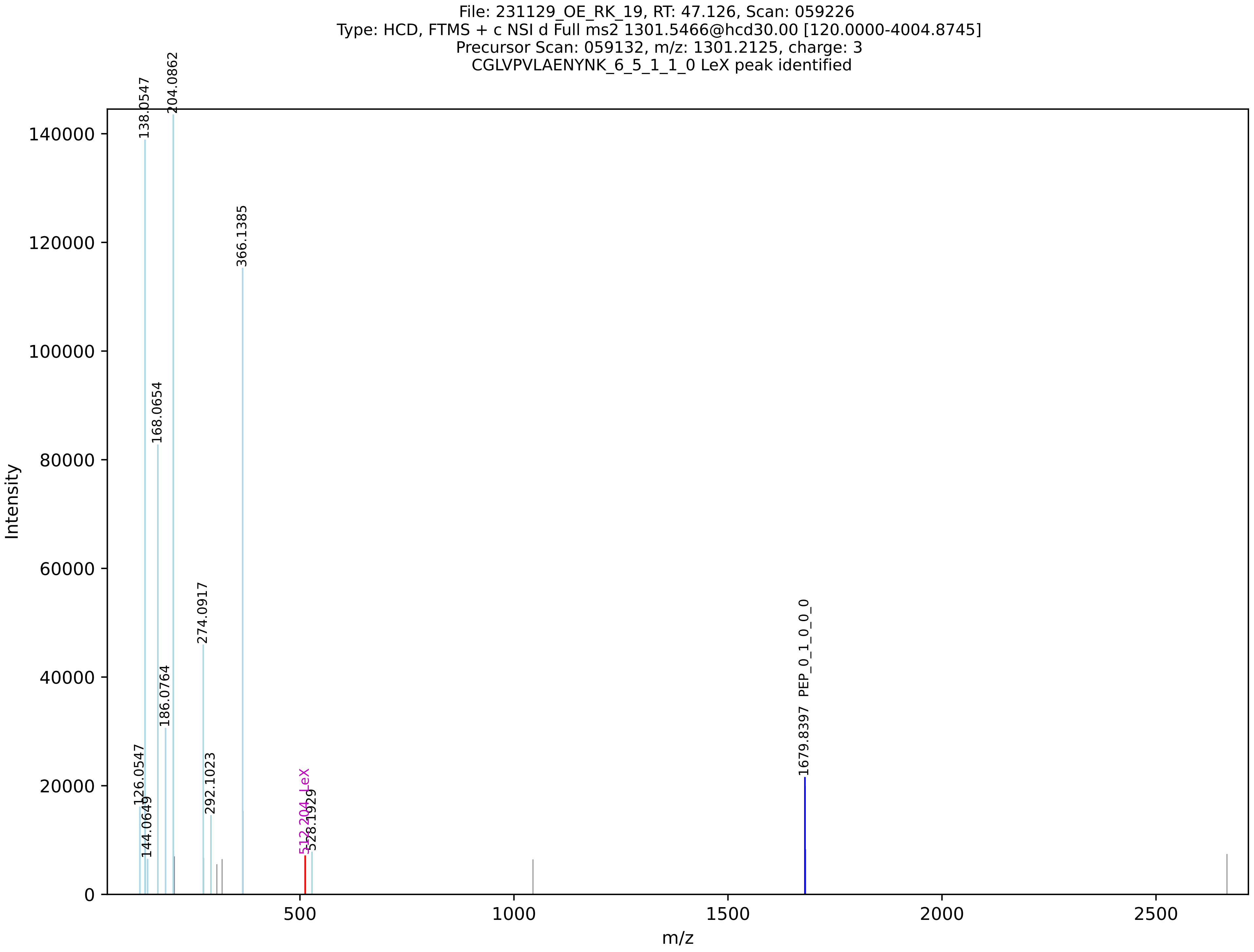
File: 231129_OE_RK_19, RT: 47.126, Scan: 059226
Type: HCD, FTMS + c NSI d Full ms2 1301.5466@hcd30.00 [120.0000-4004.8745]
Precursor Scan: 059132, m/z: 1301.2125, charge: 3
CGLVPVLAENYNK_6_5_1_1_0 LeX peak identified
138.0547
204.0862
366.1385
168.0654
186.0764
274.0917
126.0547
144.0649
292.1023
512.204_LeX
528.1929
1679.8397 PEP_0_1_0_0_0
Intensity
m/z
0
20000
40000
60000
80000
100000
120000
140000
500
1000
1500
2000
2500

File: 231129_OE_RK_19, RT: 48.377, Scan: 061043
Type: HCD, FTMS + c NSI d Full ms2 1396.3073@hcd30.00 [120.0000-5719.7896]
Precursor Scan: 060957, m/z: 1395.5555, charge: 4
QDQCIYNTTYLNVQR_7_6_1_4_0 LeX peak identified

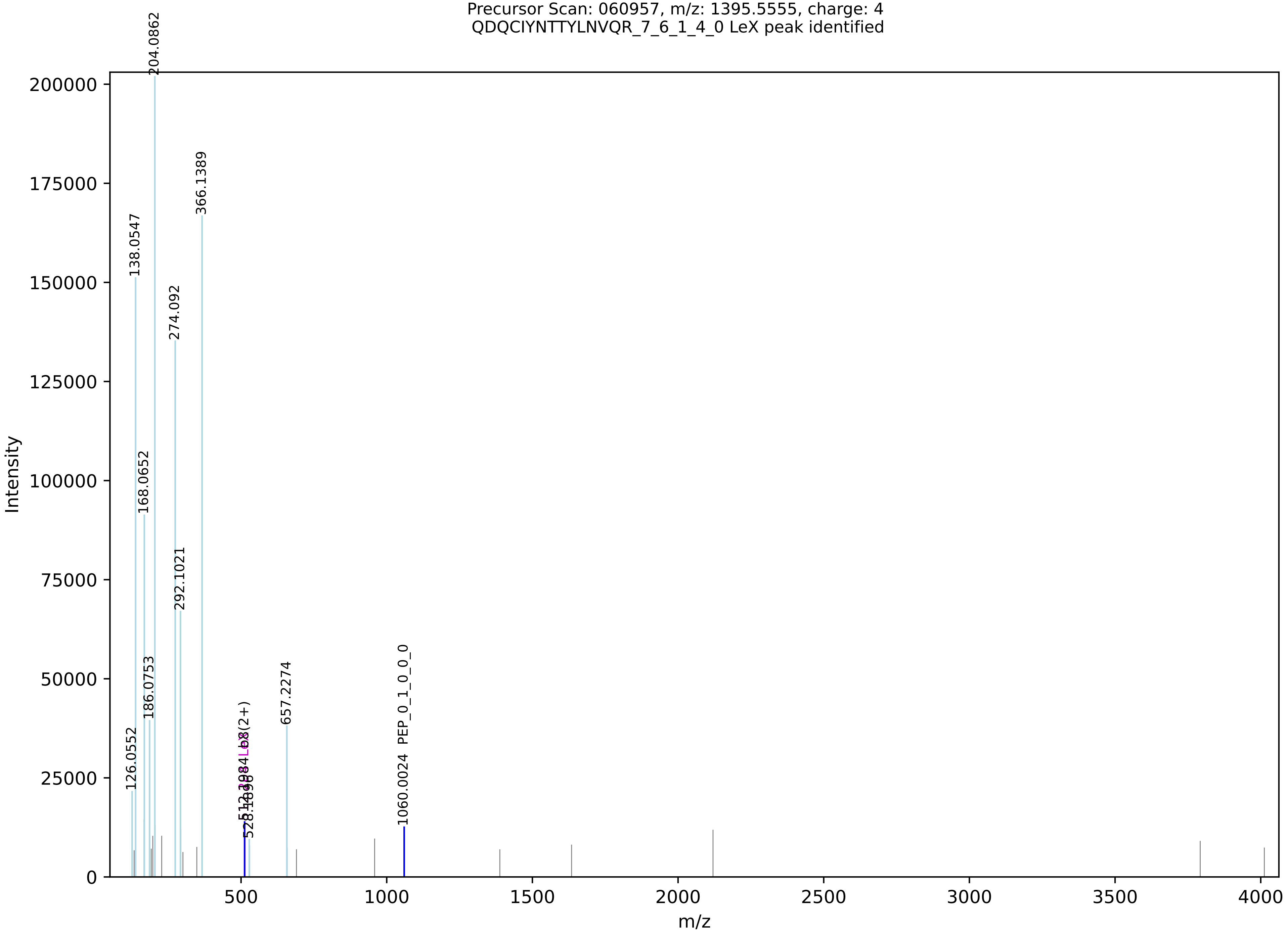

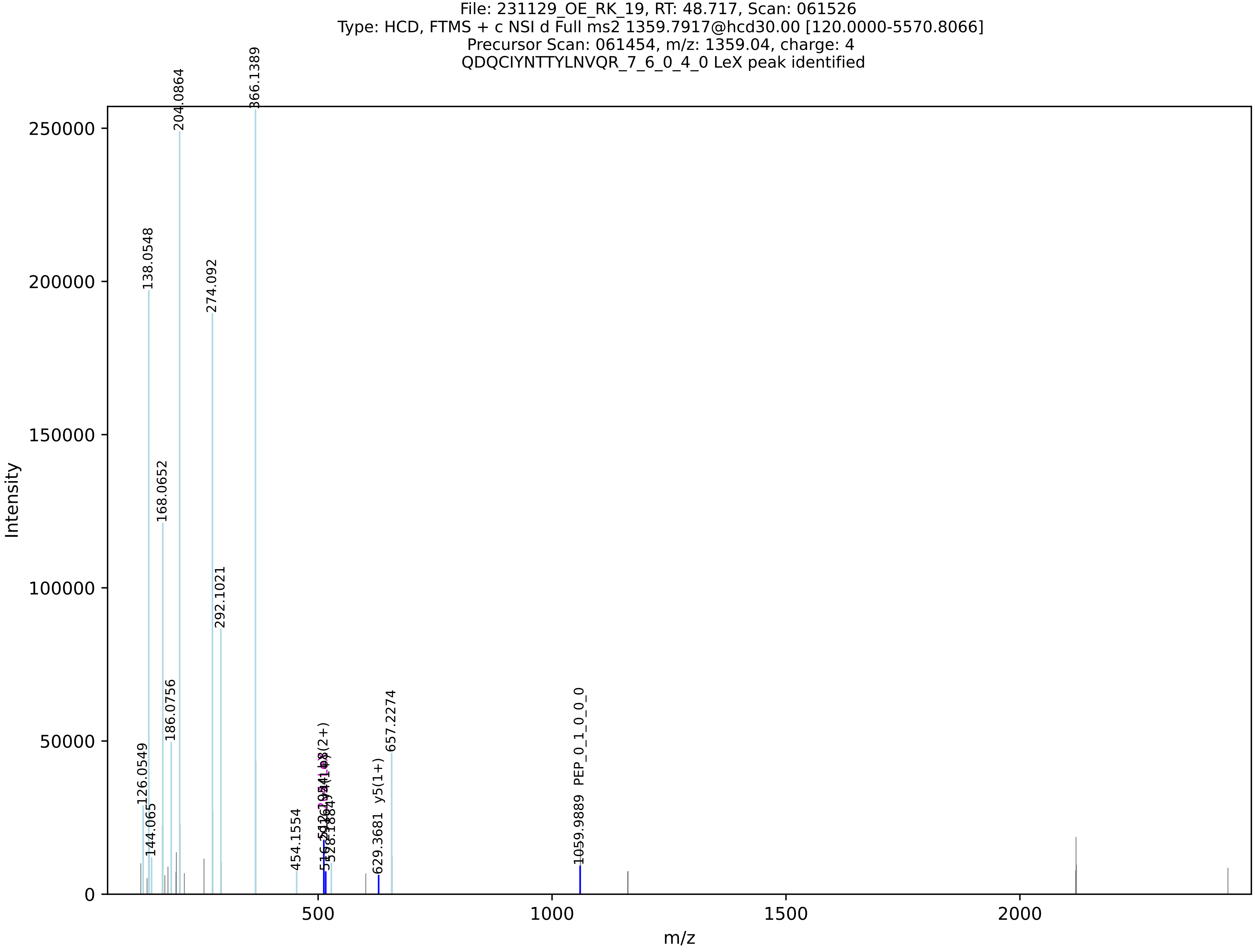
File: 231129_OE_RK_19, RT: 48.717, Scan: 061526
Type: HCD, FTMS + c NSI d Full ms2 1359.7917@hcd30.00 [120.0000-5570.8066]
Precursor Scan: 061454, m/z: 1359.04, charge: 4
QDQCIYNTTYLNVQR_7_6_0_4_0 LeX peak identified
Intensity
m/z
0
50000
100000
150000
200000
250000
500
1000
1500
2000
366.1389
204.0864
138.0548
274.092
168.0652
292.1021
186.0756
126.0549
144.065
454.1554
528.1884
629.3681 y5(1+)
657.2274
1059.9889 PEP_0_1_0_0_0

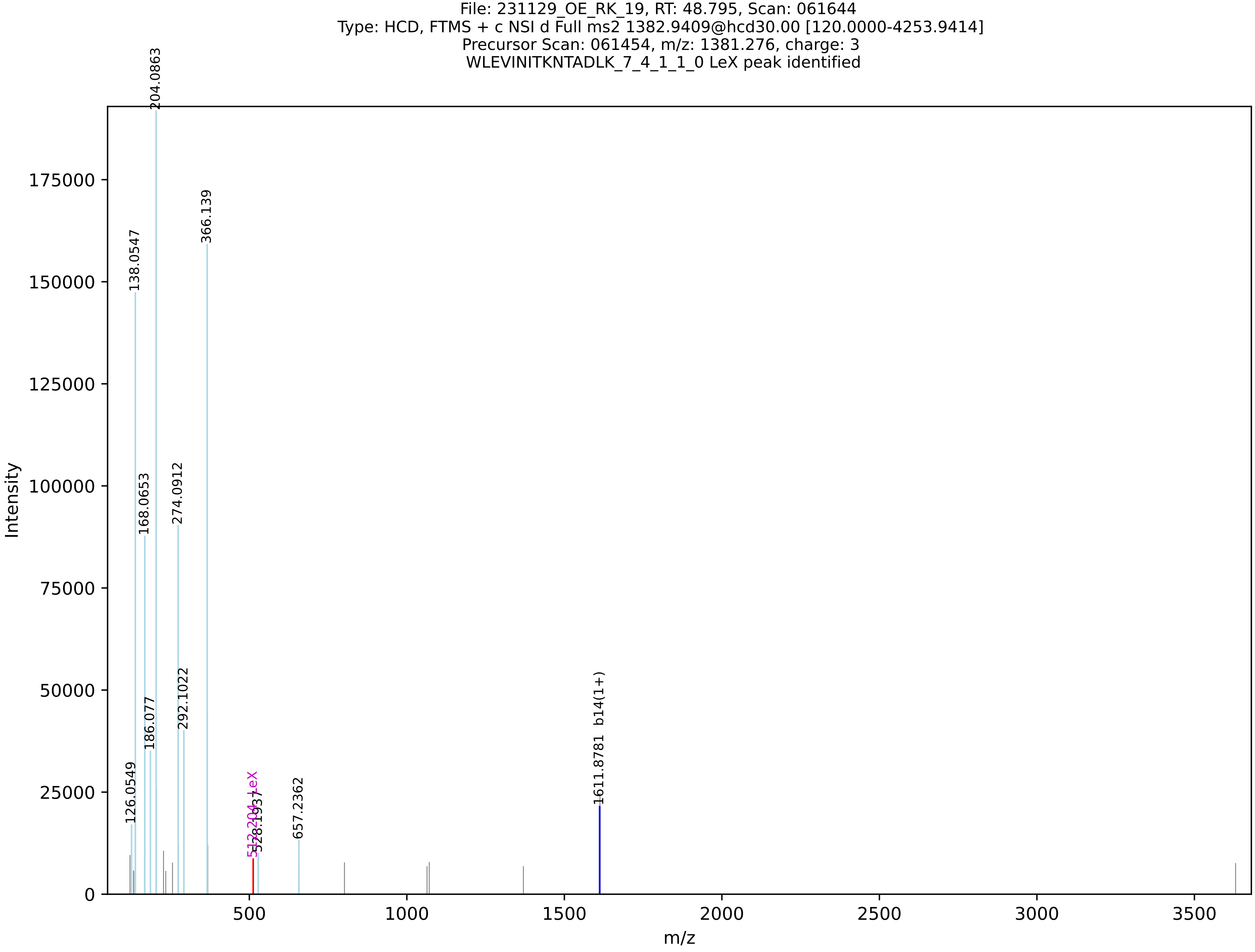

File: 231129_OE_RK_19, RT: 48.795, Scan: 061644
Type: HCD, FTMS + c NSI d Full ms2 1382.9409@hcd30.00 [120.0000-4253.9414]
Precursor Scan: 061454, m/z: 1381.276, charge: 3
WLEVINITKNTADLK_7_4_1_1_0 LeX peak identified
204.0863
366.139
138.0547
168.0653
274.0912
292.1022
186.077
126.0549
512.204 LeX
528.1937
657.2362
1611.8781 b14(1+)
Intensity
m/z

File: 231129_OE_RK_19, RT: 48.919, Scan: 061822
Type: HCD, FTMS + c NSI d Full ms2 1359.7927@hcd30.00 [120.0000-5570.8105]
Precursor Scan: 061732, m/z: 1359.041, charge: 4
QDQCIYNTTYLNVQR_7_6_0_4_0 LeX peak identified

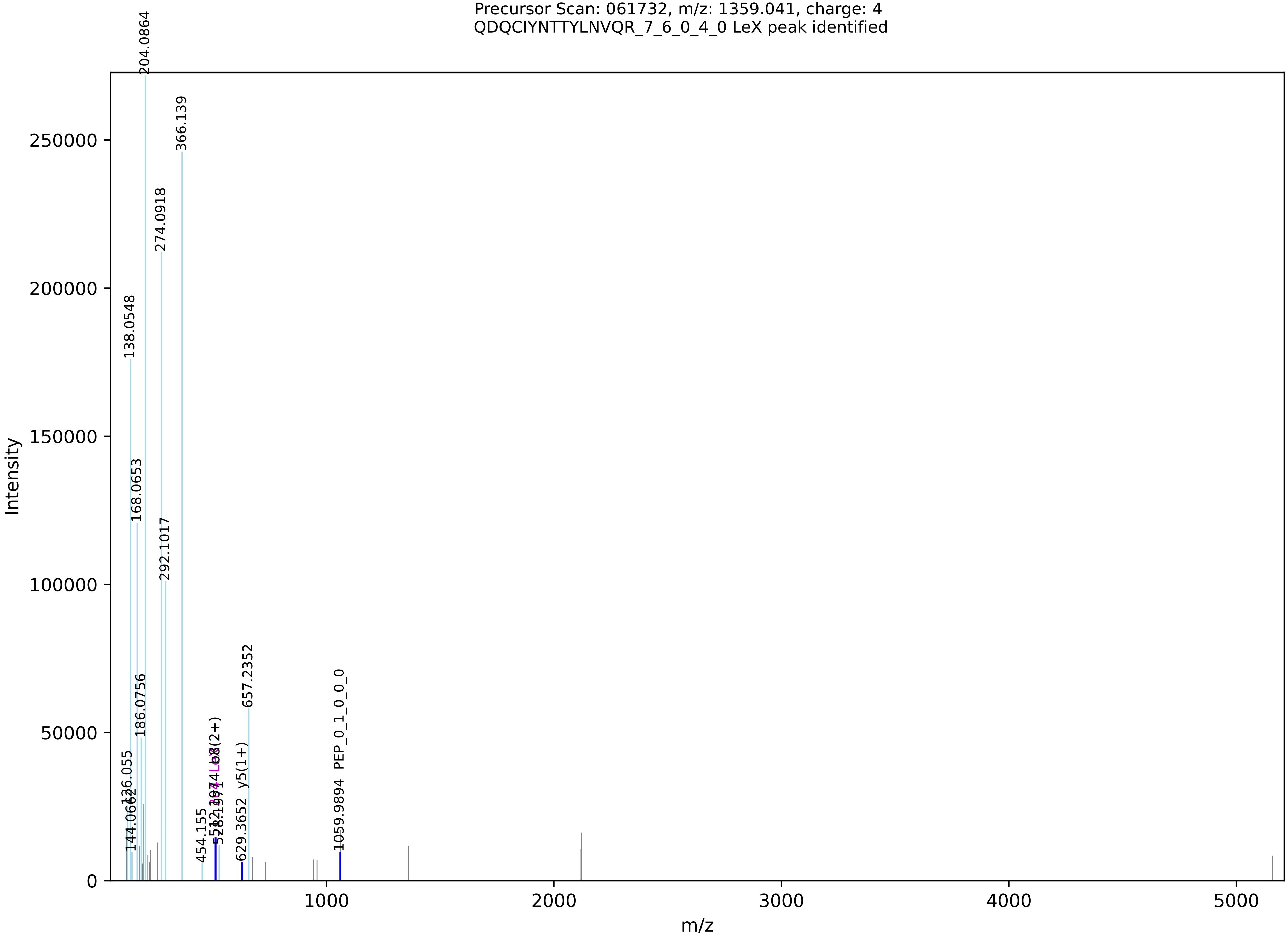

File: 231129_OE_RK_19, RT: 49.368, Scan: 062476
Type: HCD, FTMS + c NSI d Full ms2 1359.7937@hcd30.00 [120.0000-5570.8145]
Precursor Scan: 062397, m/z: 1359.0419, charge: 4
QDQCIYNTTYLNVQR_7_6_0_4_0 LeX peak identified

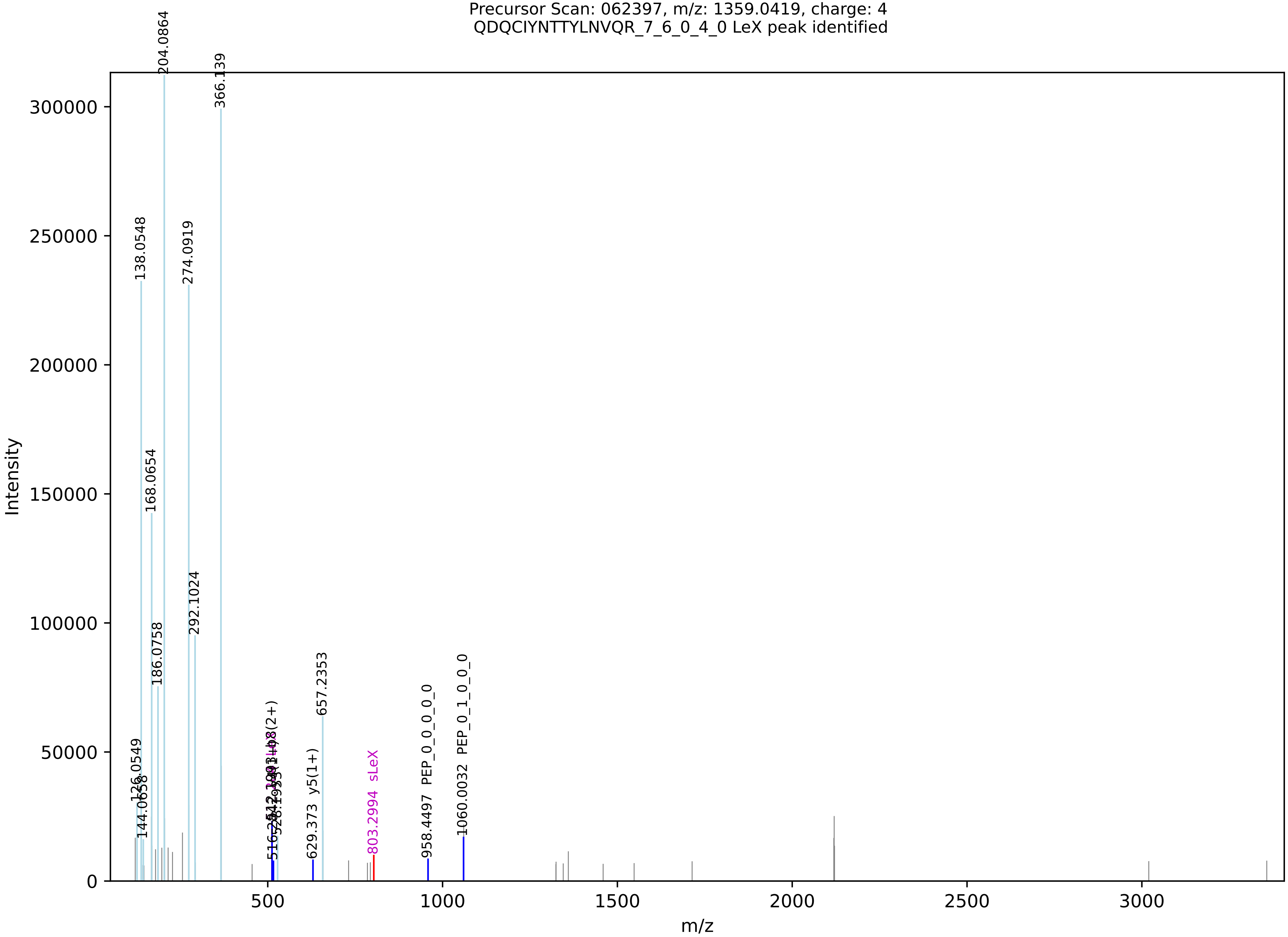

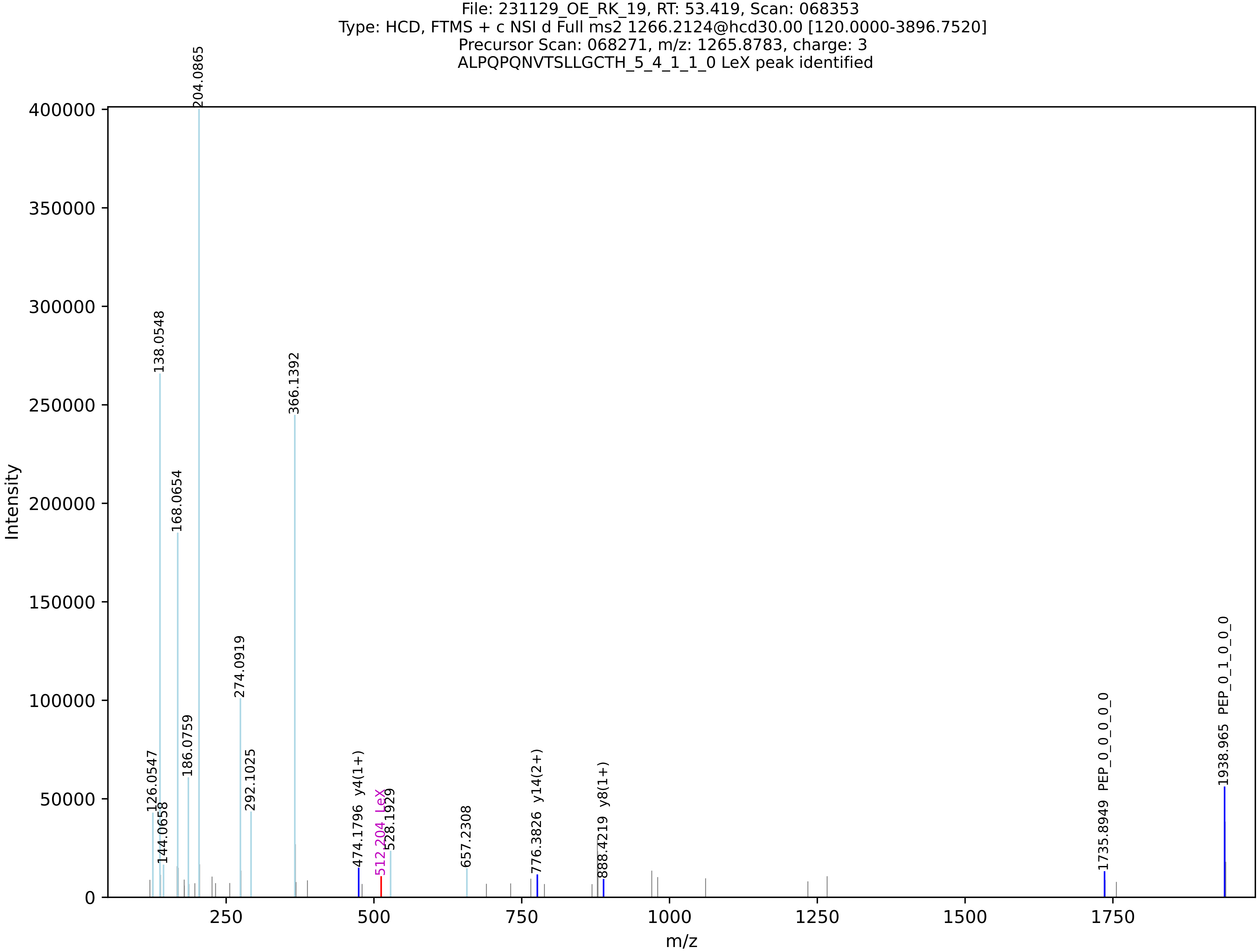
File: 231129_OE_RK_19, RT: 53.419, Scan: 068353
Type: HCD, FTMS + c NSI d Full ms2 1266.2124@hcd30.00 [120.0000-3896.7520]
Precursor Scan: 068271, m/z: 1265.8783, charge: 3
ALPQPQNVTSLLGCTH_5_4_1_1_0 LeX peak identified
Intensity
m/z
204.0865
138.0548
366.1392
168.0654
274.0919
186.0759
126.0547
144.0658
292.1025
474.1796 y4(1+)
512.204 LeX
528.1929
657.2308
776.3826 y14(2+)
888.4219 y8(1+)
1735.8949 PEP_0_0_0_0_0
1938.965 PEP_0_1_0_0_0

File: 231129_OE_RK_19, RT: 55.696, Scan: 071651
Type: HCD, FTMS + c NSI d Full ms2 1468.9264@hcd30.00 [120.0000-4517.0571]
Precursor Scan: 071559, m/z: 1467.9249, charge: 3
NGSLFAFRGQYCYELDEK_5_4_0_2_0 LeX peak identified

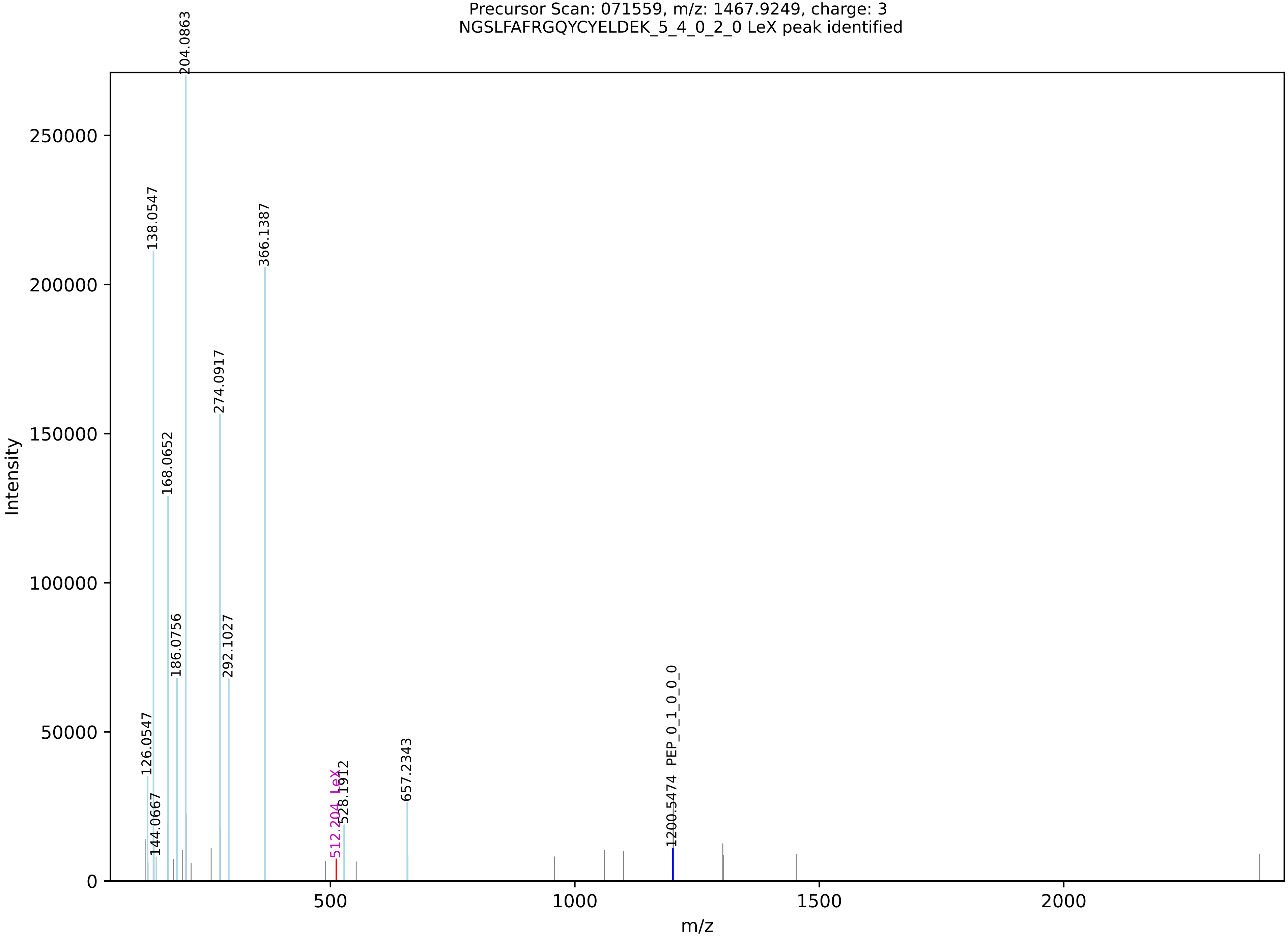

File: 231129_OE_RK_20, RT: 5.872, Scan: 001966
Type: HCD, FTMS + c NSI d Full ms2 953.0322@hcd30.00 [120.0000-2938.4204]
Precursor Scan: 001938, m/z: 952.6981, charge: 3
NEEYNK_5_4_1_1_0 LeX peak identified

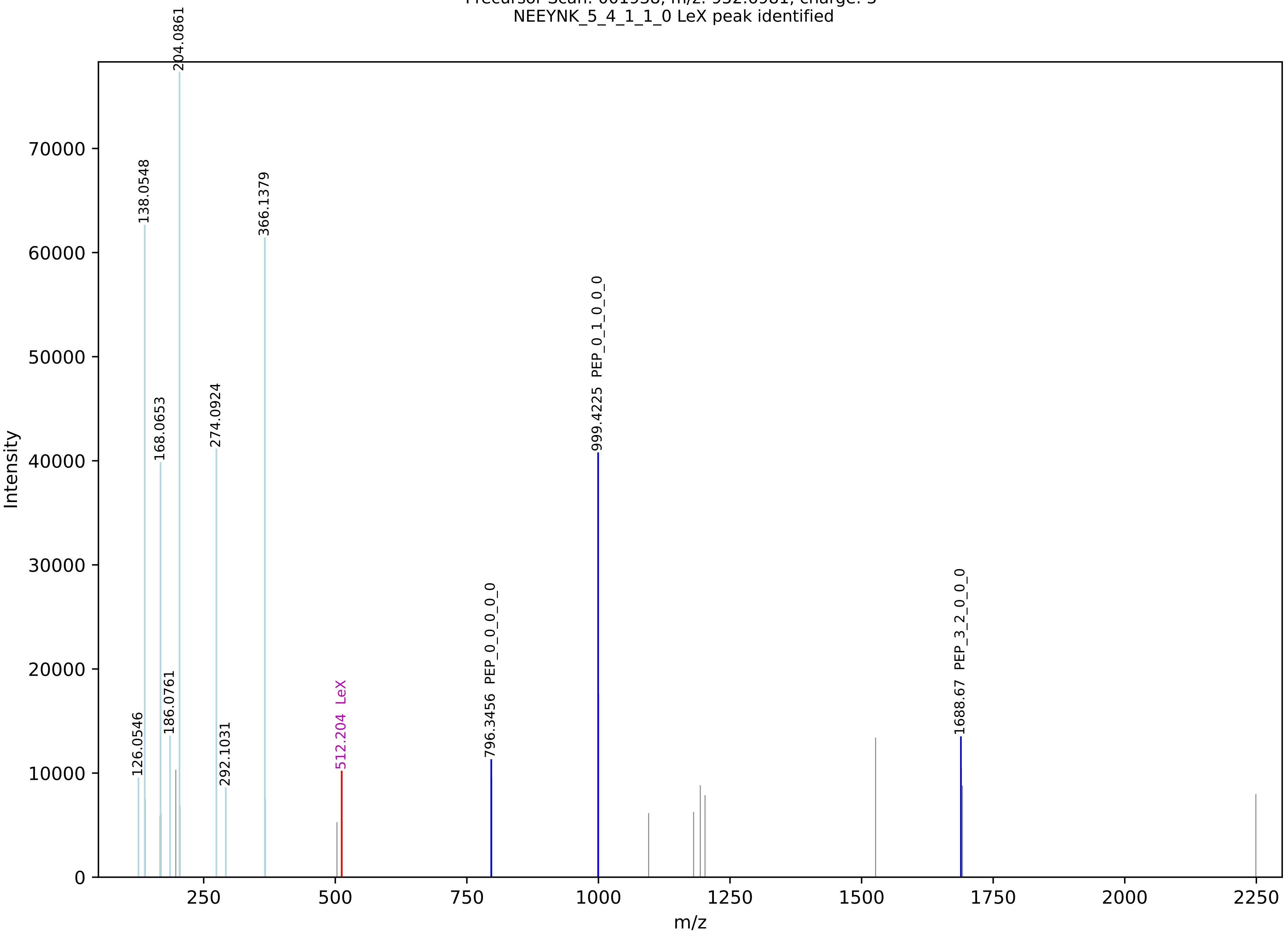

File: 231129_OE_RK_20, RT: 7.57, Scan: 004046
Type: HCD, FTMS + c NSI d Full ms2 1336.1866@hcd30.00 [120.0000-4110.8730]
Precursor Scan: 004014, m/z: 1335.1843, charge: 3
ENGTISR_7_6_2_2_0 LeX peak identified

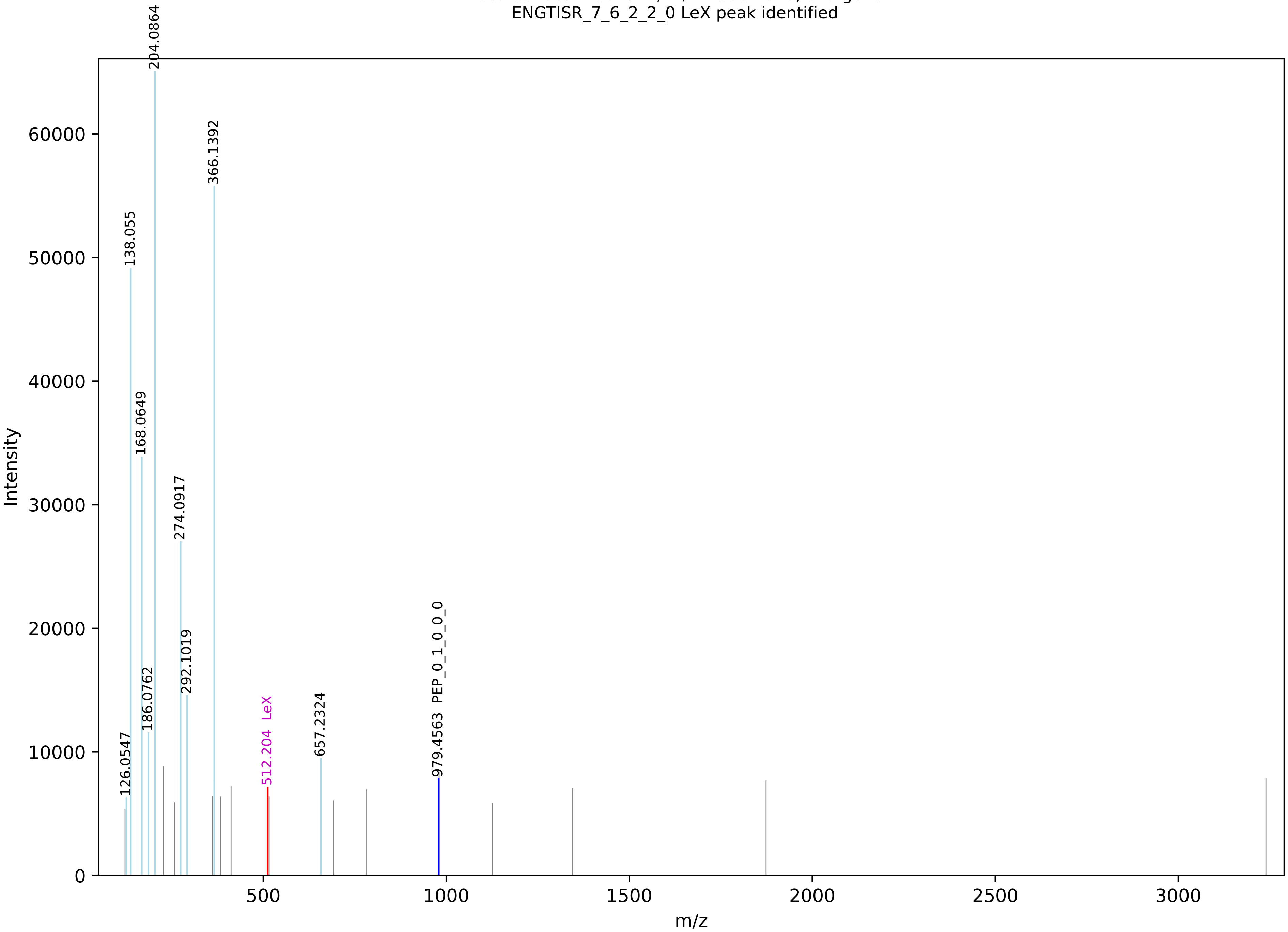

File: 231129_OE_RK_20, RT: 7.751, Scan: 004295
Type: HCD, FTMS + c NSI d Full ms2 1335.8503@hcd30.00 [120.0000-4109.8442]
Precursor Scan: 004250, m/z: 1335.1822, charge: 3
ENGTISR_7_6_2_2_0 LeX peak identified

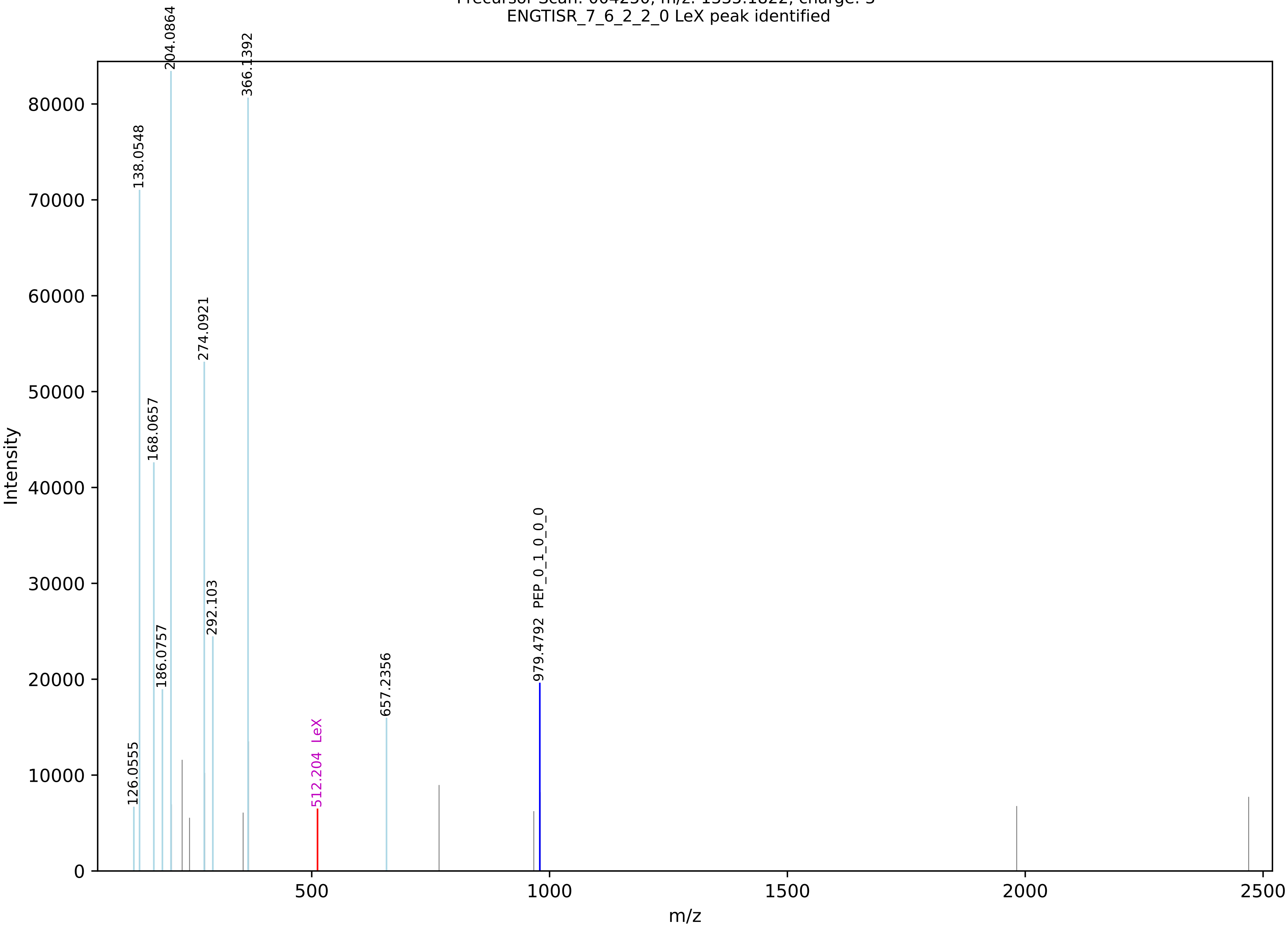

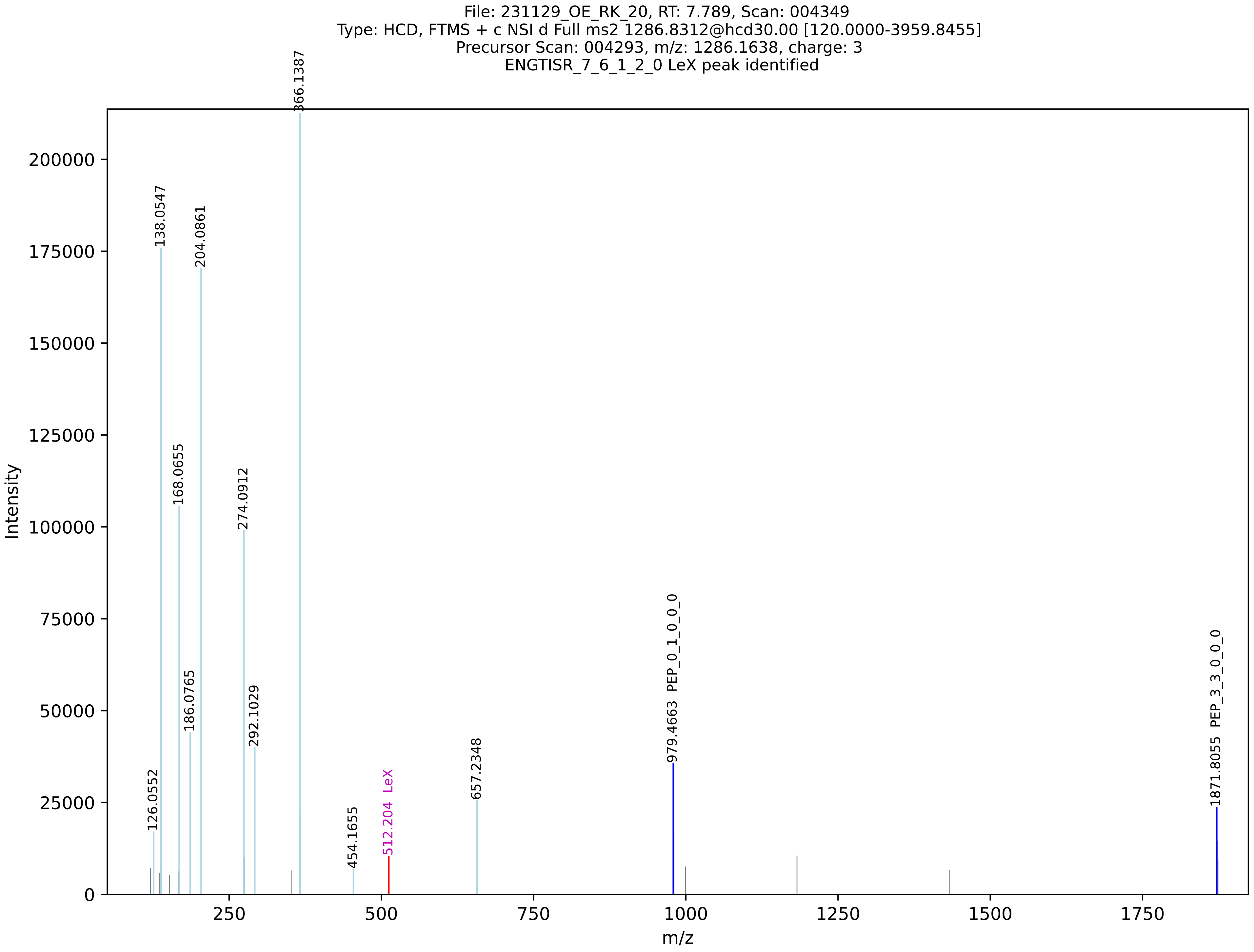

File: 231129_OE_RK_20, RT: 7.789, Scan: 004349
Type: HCD, FTMS + c NSI d Full ms2 1286.8312@hcd30.00 [120.0000-3959.8455]
Precursor Scan: 004293, m/z: 1286.1638, charge: 3
ENGTISR_7_6_1_2_0 LeX peak identified
Intensity
m/z
366.1387
138.0547
204.0861
168.0655
274.0912
186.0765
292.1029
126.0552
454.1655
512.204 LeX
657.2348
979.4663 PEP_0_1_0_0_0
1871.8055 PEP_3_3_0_0_0

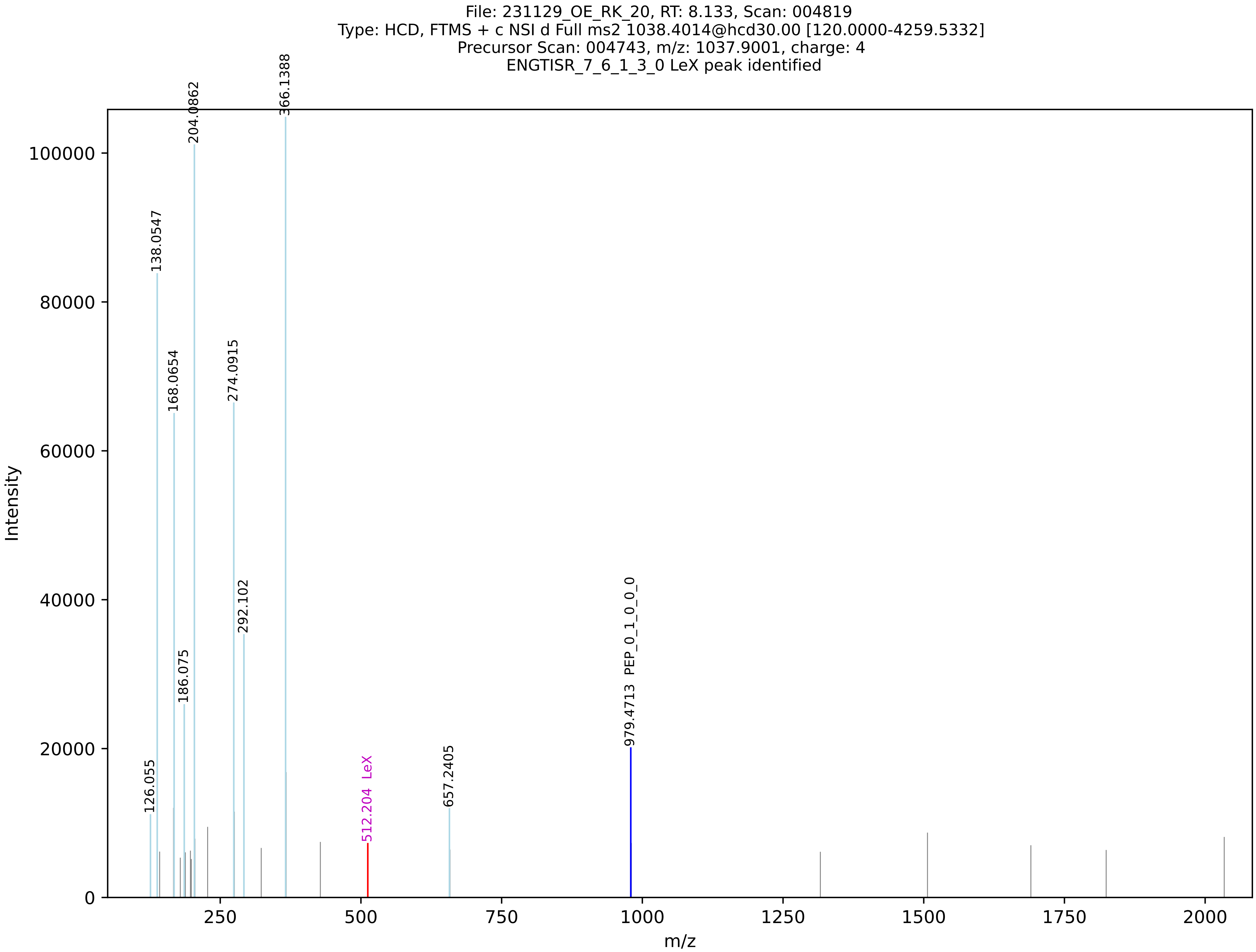

File: 231129_OE_RK_20, RT: 8.133, Scan: 004819
Type: HCD, FTMS + c NSI d Full ms2 1038.4014@hcd30.00 [120.0000-4259.5332]
Precursor Scan: 004743, m/z: 1037.9001, charge: 4
ENGTISR_7_6_1_3_0 LeX peak identified
Intensity
m/z
366.1388
204.0862
138.0547
168.0654
274.0915
292.102
186.075
126.055
512.204 LeX
657.2405
979.4713 PEP_0_1_0_0_0
0
20000
40000
60000
80000
100000
250
500
750
1000
1250
1500
1750
2000

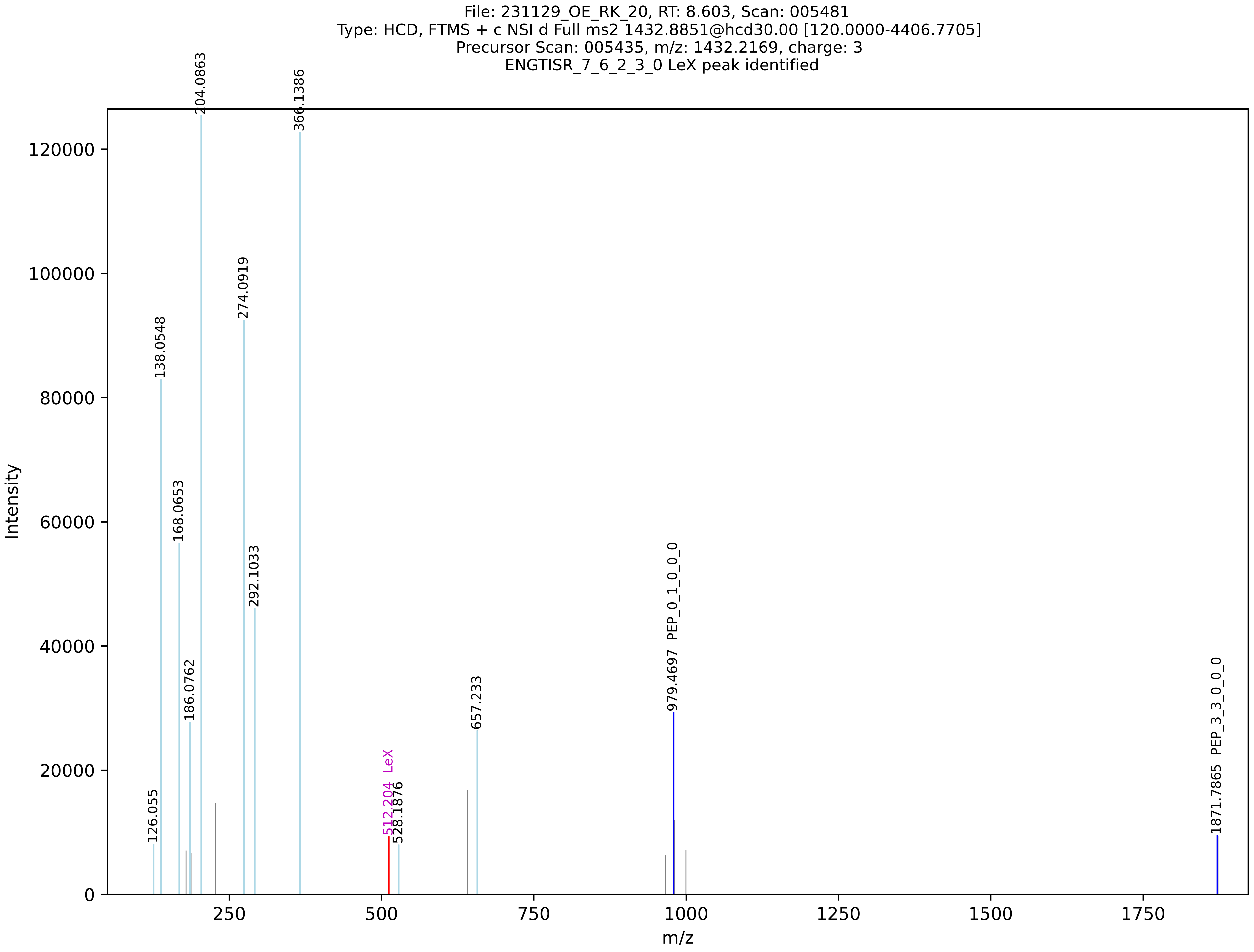
File: 231129_OE_RK_20, RT: 8.603, Scan: 005481
Type: HCD, FTMS + c NSI d Full ms2 1432.8851@hcd30.00 [120.0000-4406.7705]
Precursor Scan: 005435, m/z: 1432.2169, charge: 3
ENGTISR_7_6_2_3_0 LeX peak identified
Intensity
m/z
0
20000
40000
60000
80000
100000
120000
250
500
750
1000
1250
1500
1750
126.055
138.0548
168.0653
186.0762
204.0863
274.0919
292.1033
366.1386
512.204 LeX
528.1876
657.233
979.4697 PEP_0_1_0_0_0
1871.7865 PEP_3_3_0_0_0

File: 231129_OE_RK_20, RT: 9.021, Scan: 006066
Type: HCD, FTMS + c NSI d Full ms2 1311.1750@hcd30.00 [120.0000-4034.3376]
Precursor Scan: 005984, m/z: 1310.5068, charge: 3
ENGTISR_6_5_2_3_0 LeX peak identified

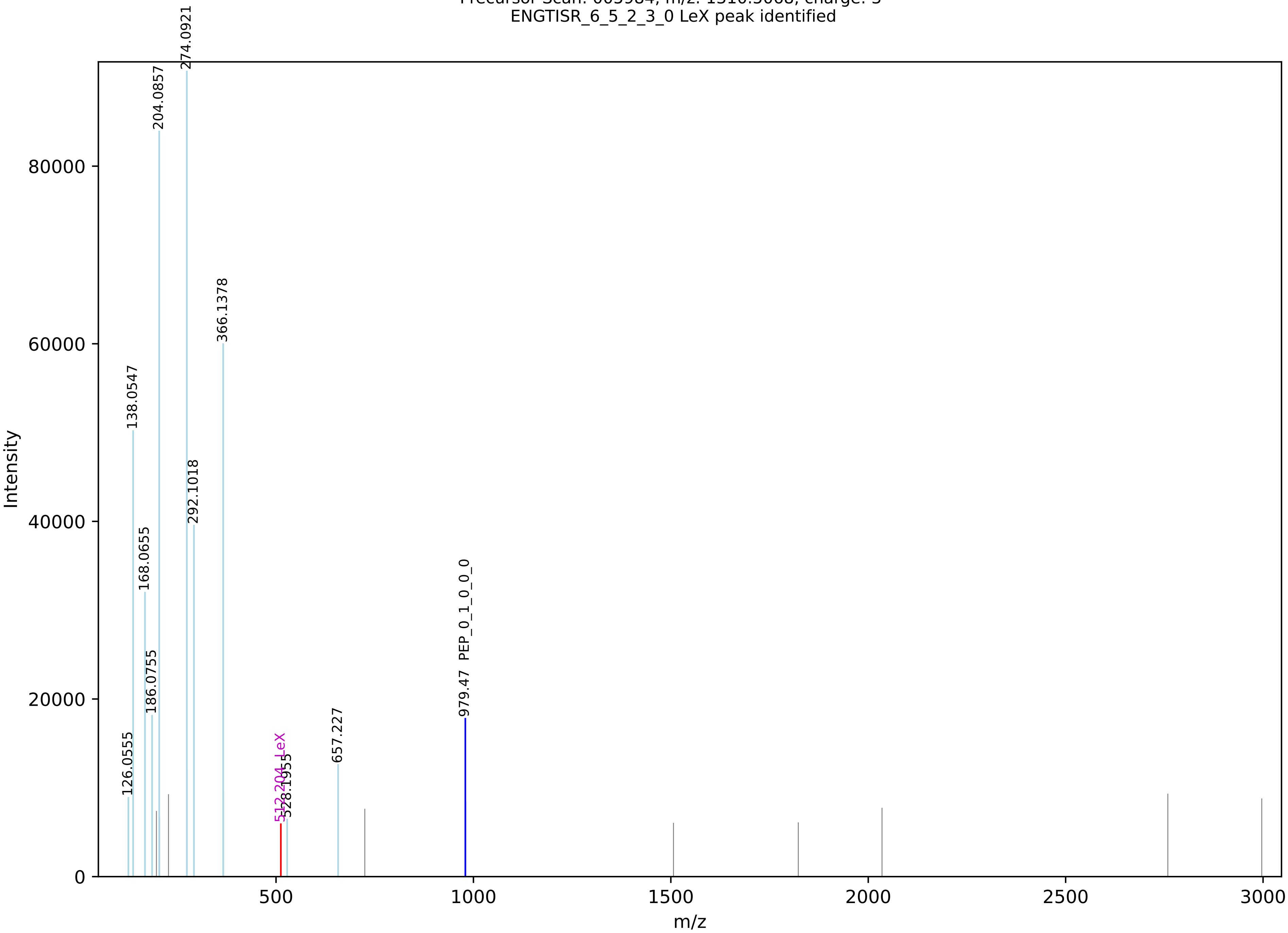

File: 231129_OE_RK_20, RT: 12.411, Scan: 010835
Type: HCD, FTMS + c NSI d Full ms2 1147.6891@hcd30.00 [120.0000-4705.4272]
Precursor Scan: 010777, m/z: 1147.1879, charge: 4
ENGTISR_7_6_2_4_0 LeX peak identified

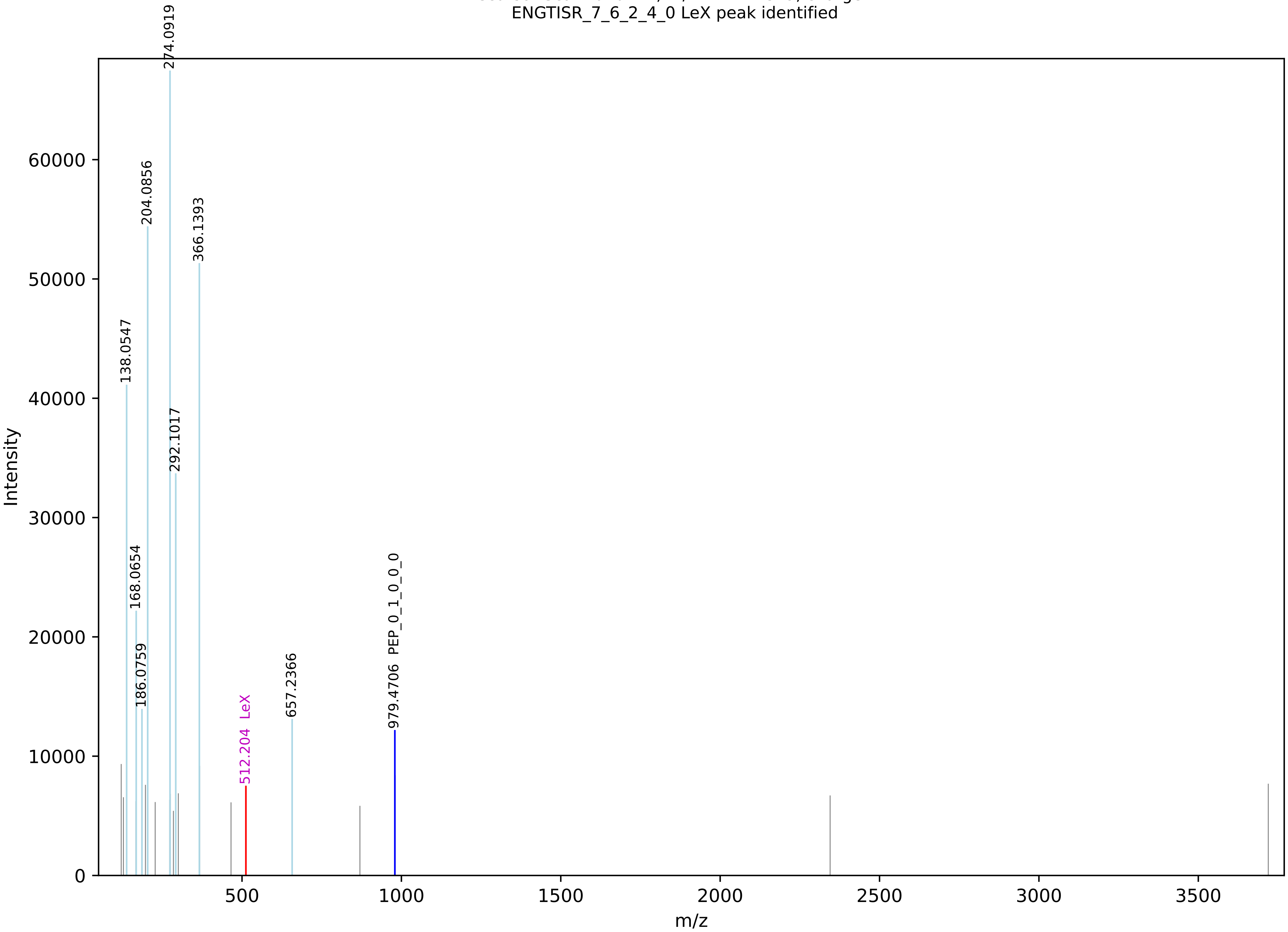

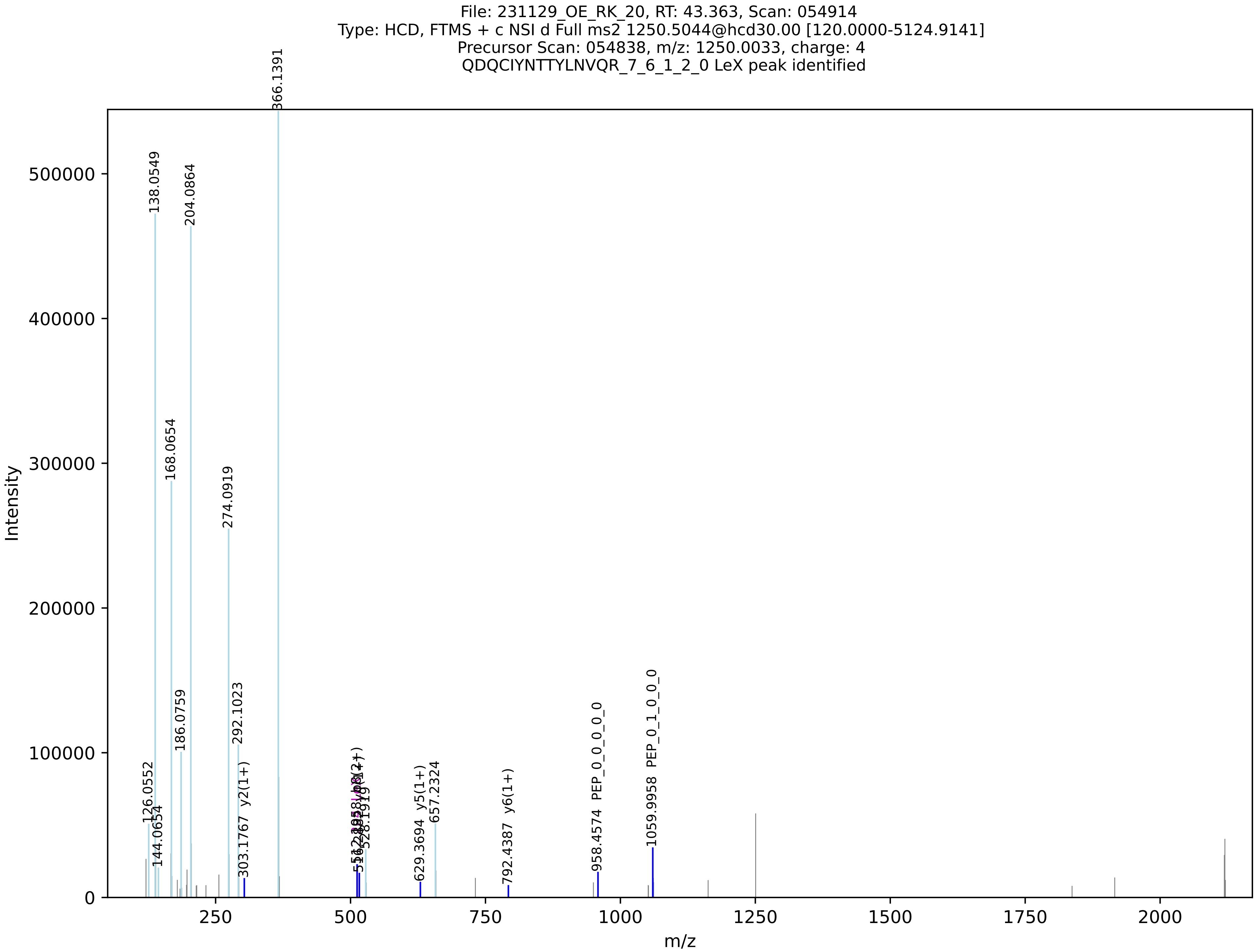
File: 231129_OE_RK_20, RT: 43.363, Scan: 054914
Type: HCD, FTMS + c NSI d Full ms2 1250.5044@hcd30.00 [120.0000-5124.9141]
Precursor Scan: 054838, m/z: 1250.0033, charge: 4
QDQCIYNTTYLNVQR_7_6_1_2_0 LeX peak identified
Intensity
m/z
366.1391
138.0549
204.0864
168.0654
274.0919
186.0759
292.1023
126.0552
144.0654
303.1767 y2(1+)
528.1919
629.3694 y5(1+)
657.2324
792.4387 y6(1+)
958.4574 PEP_0_0_0_0_0_
1059.9958 PEP_0_1_0_0_0_
0
100000
200000
300000
400000
500000
250
500
750
1000
1250
1500
1750
2000

File: 231129_OE_RK_20, RT: 43.667, Scan: 055354
Type: HCD, FTMS + c NSI d Full ms2 1667.0034@hcd30.00 [120.0000-5123.1724]
Precursor Scan: 055275, m/z: 1666.3372, charge: 3
QDQCIYNTTYLNVQR_7_6_1_2_0 LeX peak identified

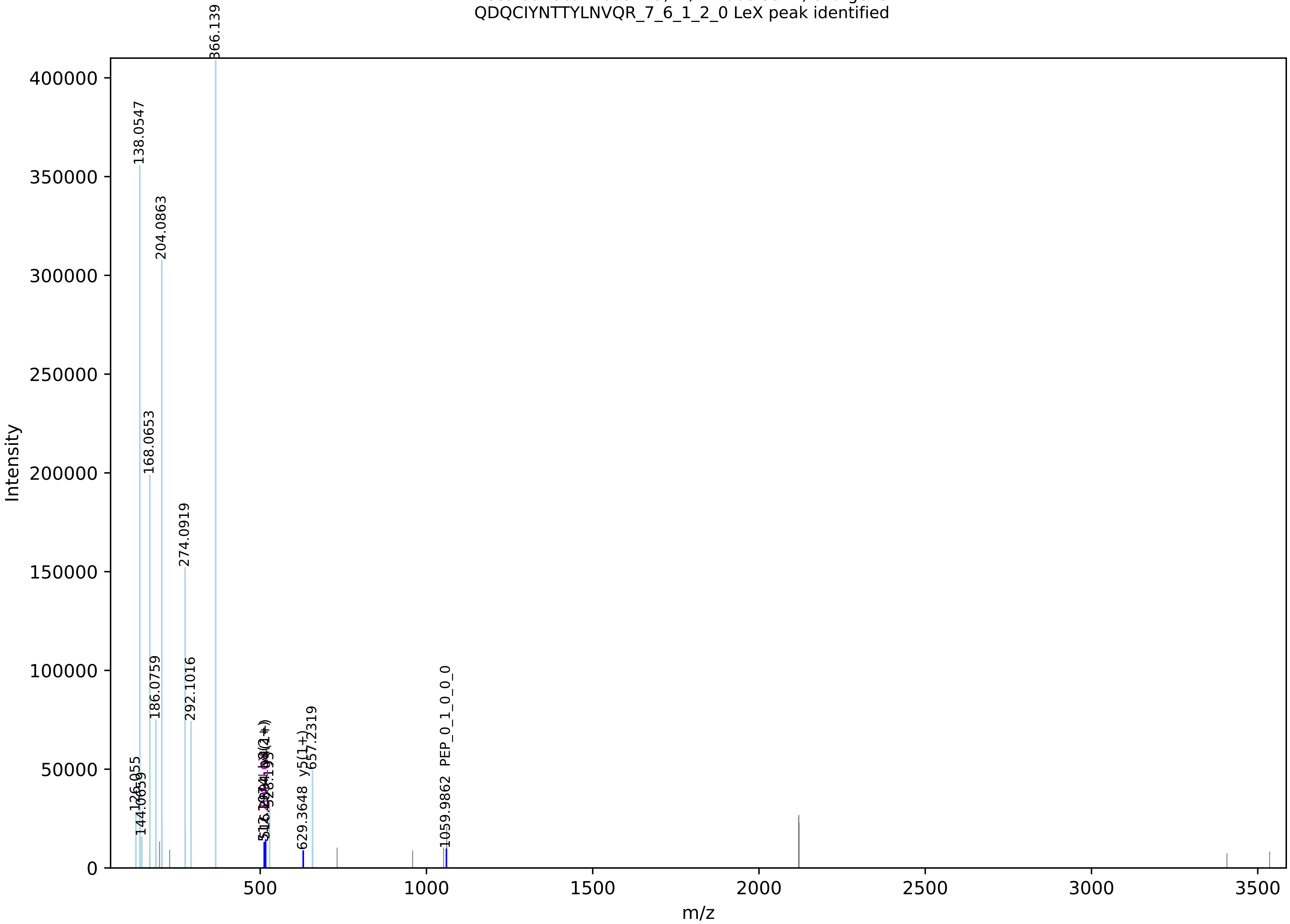

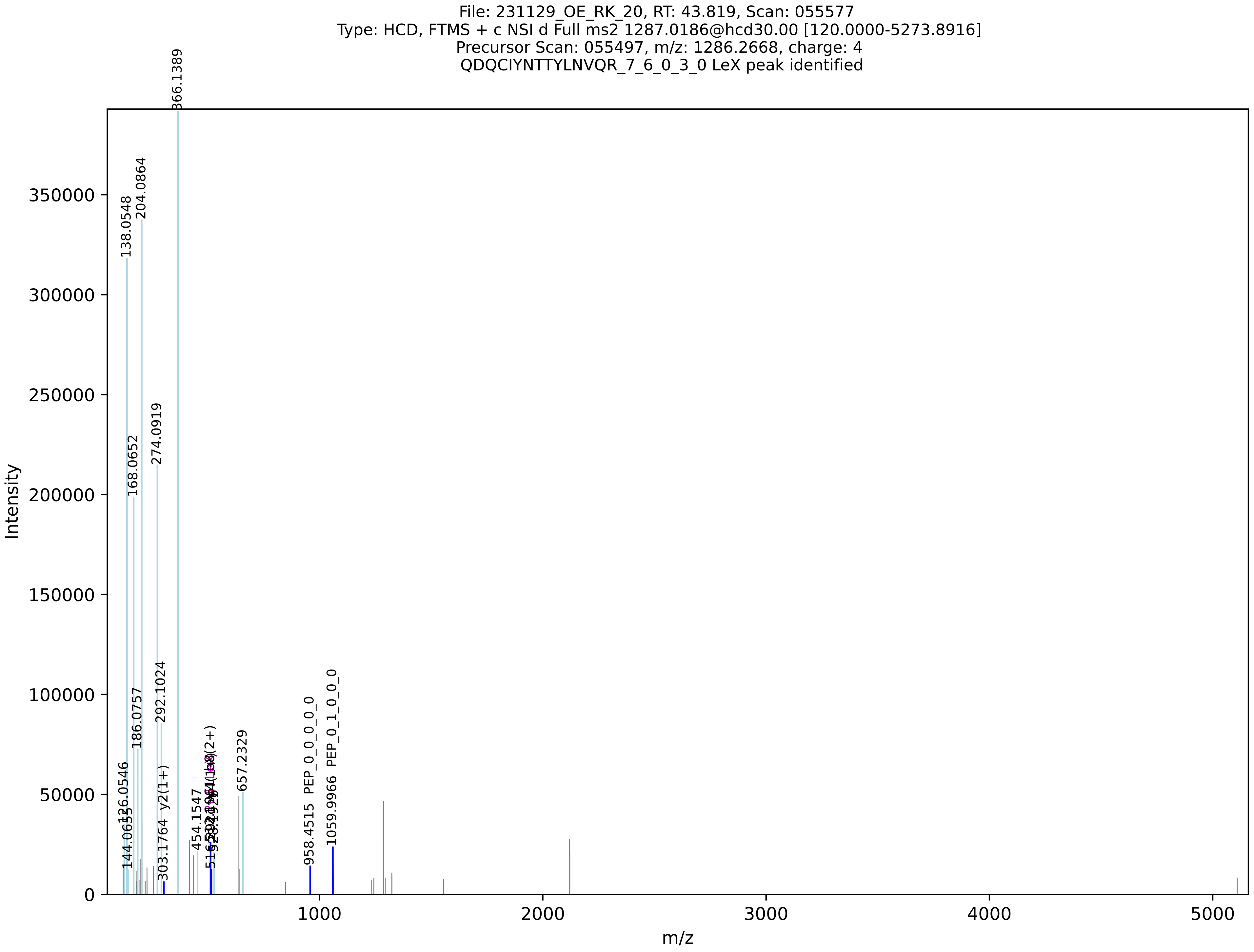
File: 231129_OE_RK_20, RT: 43.819, Scan: 055577
Type: HCD, FTMS + c NSI d Full ms2 1287.0186@hcd30.00 [120.0000-5273.8916]
Precursor Scan: 055497, m/z: 1286.2668, charge: 4
QDQCIYNTTYLNVQR_7_6_0_3_0 LeX peak identified
366.1389
204.0864
138.0548
274.0919
168.0652
292.1024
186.0757
126.0546
144.0655
303.1764 y2(1+)
454.1547
657.2329
958.4515 PEP_0_0_0_0_0
1059.9966 PEP_0_1_0_0_0
Intensity
m/z
0
50000
100000
150000
200000
250000
300000
350000
1000
2000
3000
4000
5000

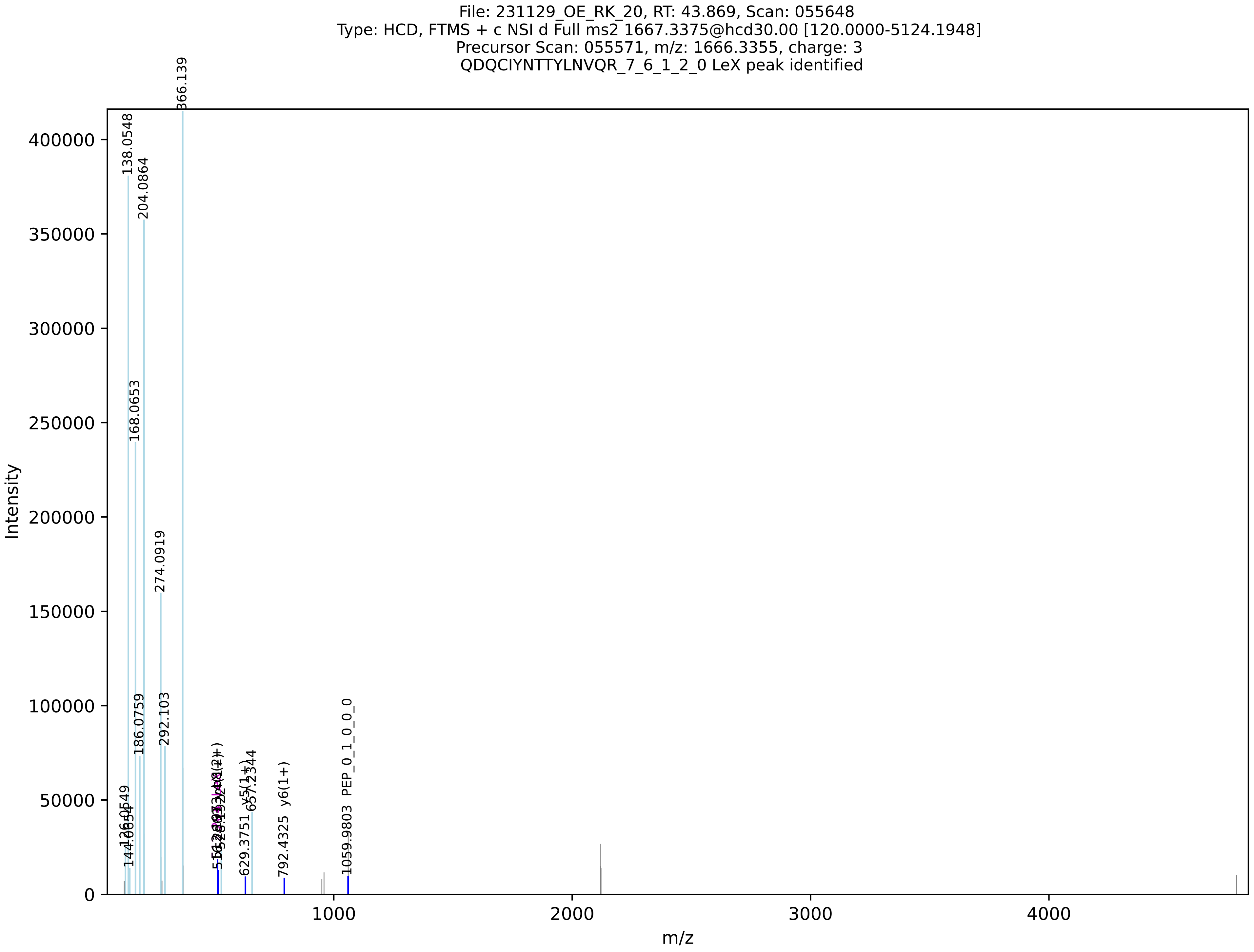
File: 231129_OE_RK_20, RT: 43.869, Scan: 055648
Type: HCD, FTMS + c NSI d Full ms2 1667.3375@hcd30.00 [120.0000-5124.1948]
Precursor Scan: 055571, m/z: 1666.3355, charge: 3
QDQCIYNTTYLNVQR_7_6_1_2_0 LeX peak identified
Intensity
m/z
366.139
138.0548
204.0864
168.0653
274.0919
186.0759
292.103
126.0549
144.0654
629.3751 y5(1+)
657.2344
792.4325 y6(1+)
1059.9803 PEP_0_1_0_0_0

File: 231129_OE_RK_20, RT: 43.969, Scan: 055794
Type: HCD, FTMS + c NSI d Full ms2 1250.5054@hcd30.00 [120.0000-5124.9180]
Precursor Scan: 055716, m/z: 1250.0041, charge: 4
QDQCIYNTTYLNVQR_7_6_1_2_0 LeX peak identified

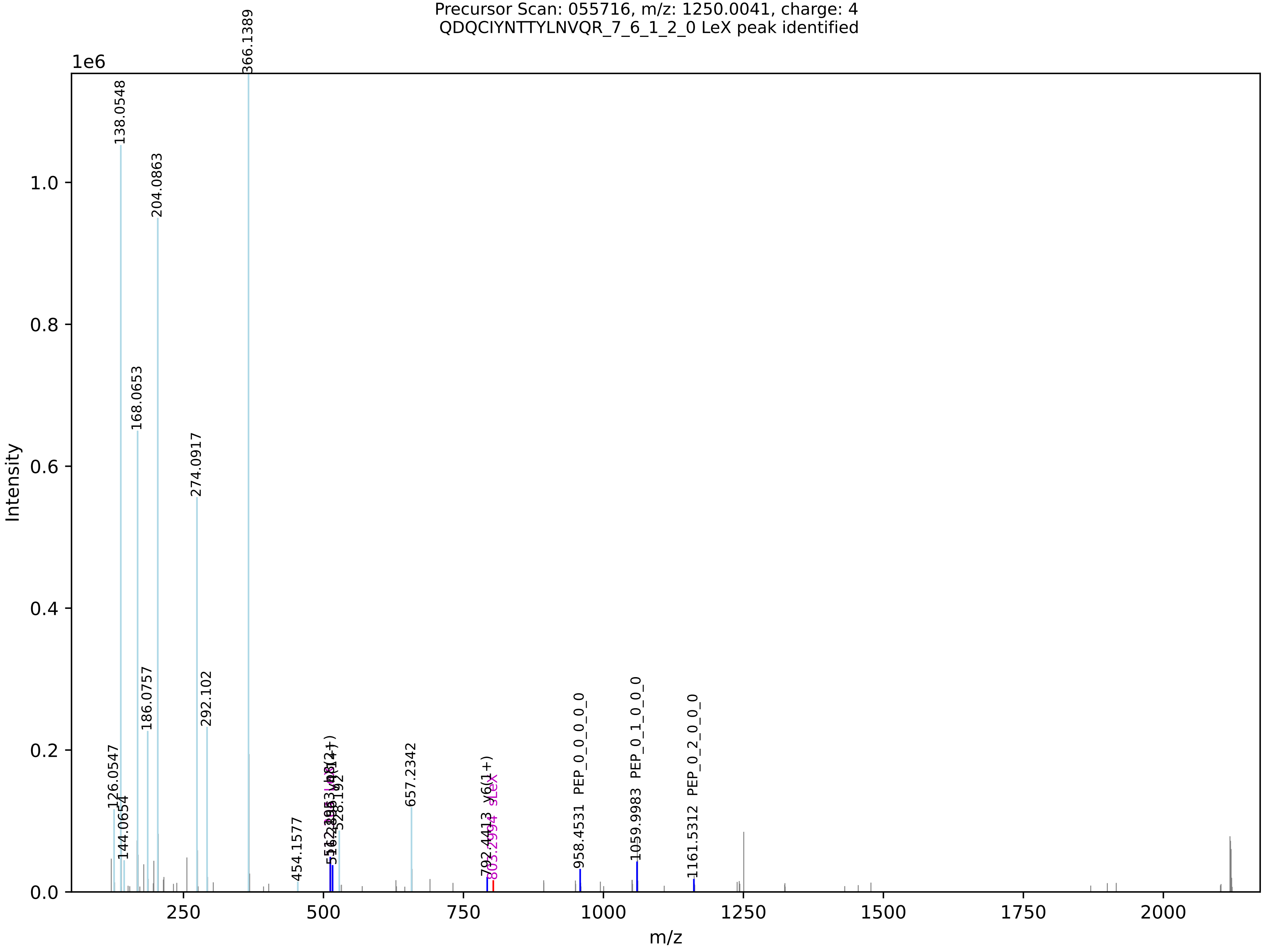

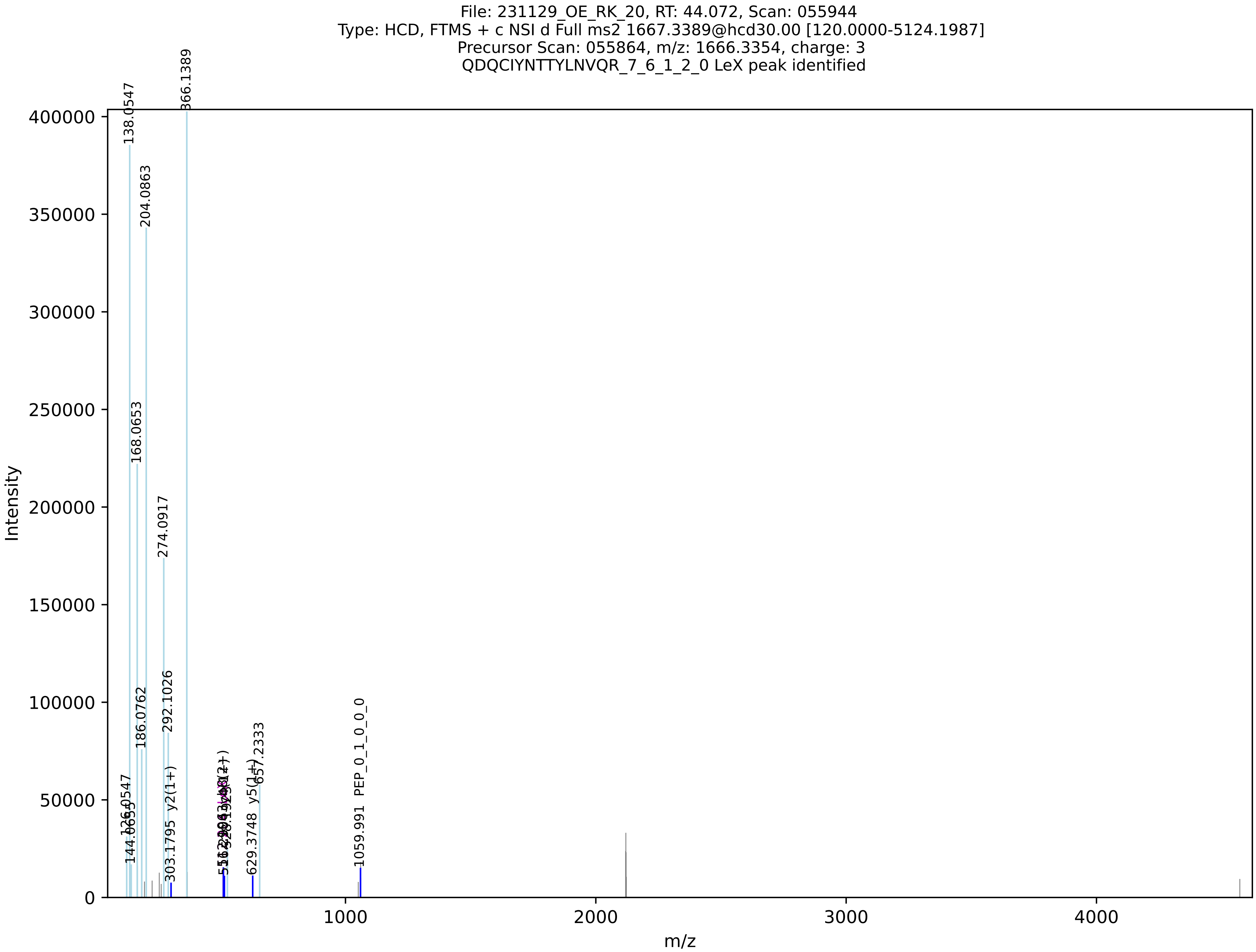
File: 231129_OE_RK_20, RT: 44.072, Scan: 055944
Type: HCD, FTMS + c NSI d Full ms2 1667.3389@hcd30.00 [120.0000-5124.1987]
Precursor Scan: 055864, m/z: 1666.3354, charge: 3
QDQCIYNTTYLNVQR_7_6_1_2_0 LeX peak identified
Intensity
m/z
366.1389
138.0547
204.0863
168.0653
274.0917
292.1026
186.0762
144.0655
303.1795 y2(1+)
629.3748 y5(1+)
657.2333
1059.991 PEP 0_1_0_0_0_

File: 231129_OE_RK_20, RT: 44.073, Scan: 055946
Type: HCD, FTMS + c NSI d Full ms2 1286.7662@hcd30.00 [120.0000-5272.8623]
Precursor Scan: 055864, m/z: 1286.2651, charge: 4
QDQCIYNTTYLNVQR_7_6_0_3_0 LeX peak identified

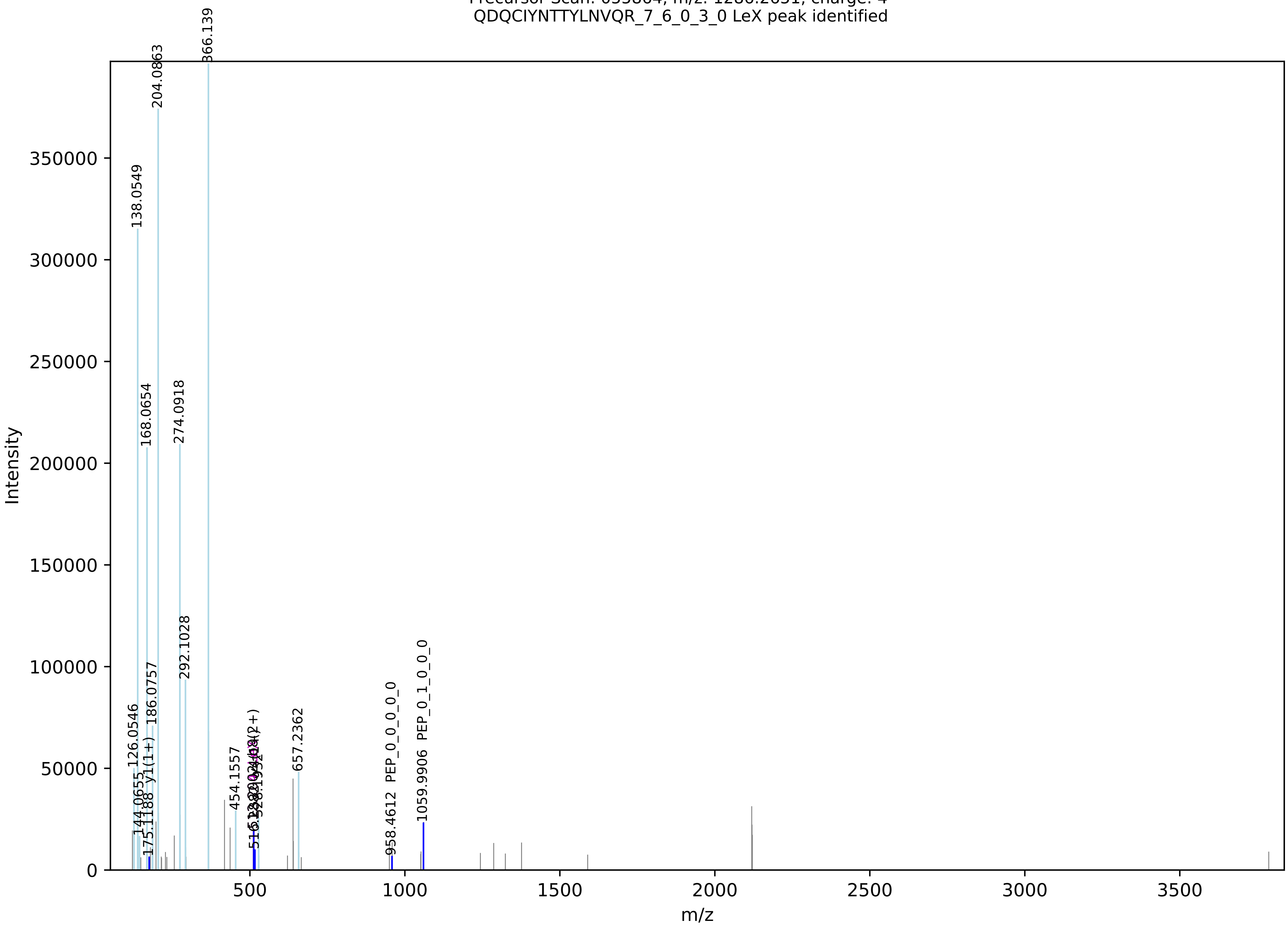

File: 231129_OE_RK_20, RT: 44.575, Scan: 056671
Type: HCD, FTMS + c NSI d Full ms2 1250.5048@hcd30.00 [120.0000-5124.9155]
Precursor Scan: 056594, m/z: 1250.0036, charge: 4
QDQCIYNTTYLNVQR_7_6_1_2_0 LeX peak identified

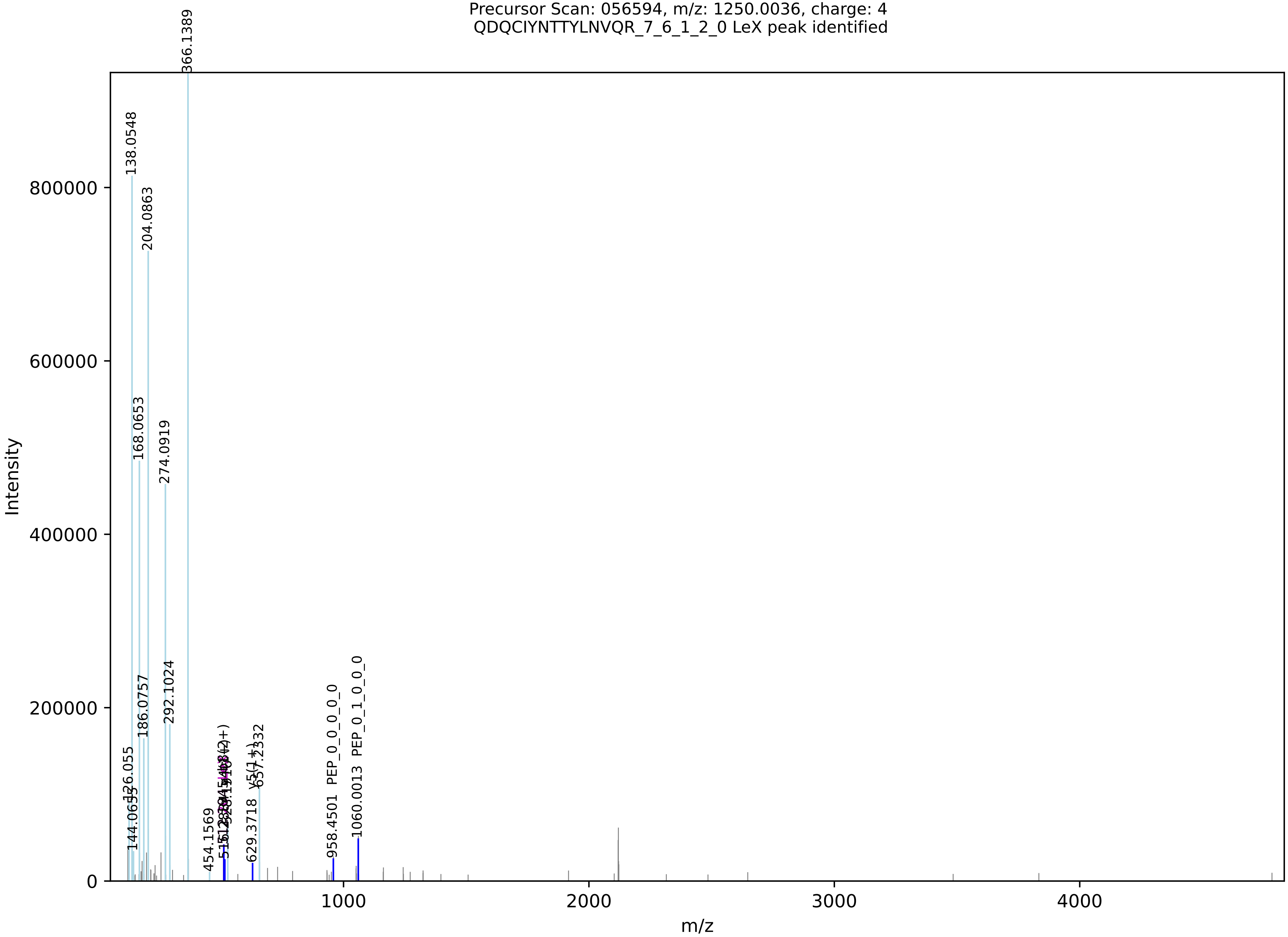

File: 231129_OE_RK_20, RT: 44.828, Scan: 057038
Type: HCD, FTMS + c NSI d Full ms2 1250.7554@hcd30.00 [120.0000-5125.9380]
Precursor Scan: 056961, m/z: 1250.0061, charge: 4
QDQCIYNTTYLNVQR_7_6_1_2_0 LeX peak identified

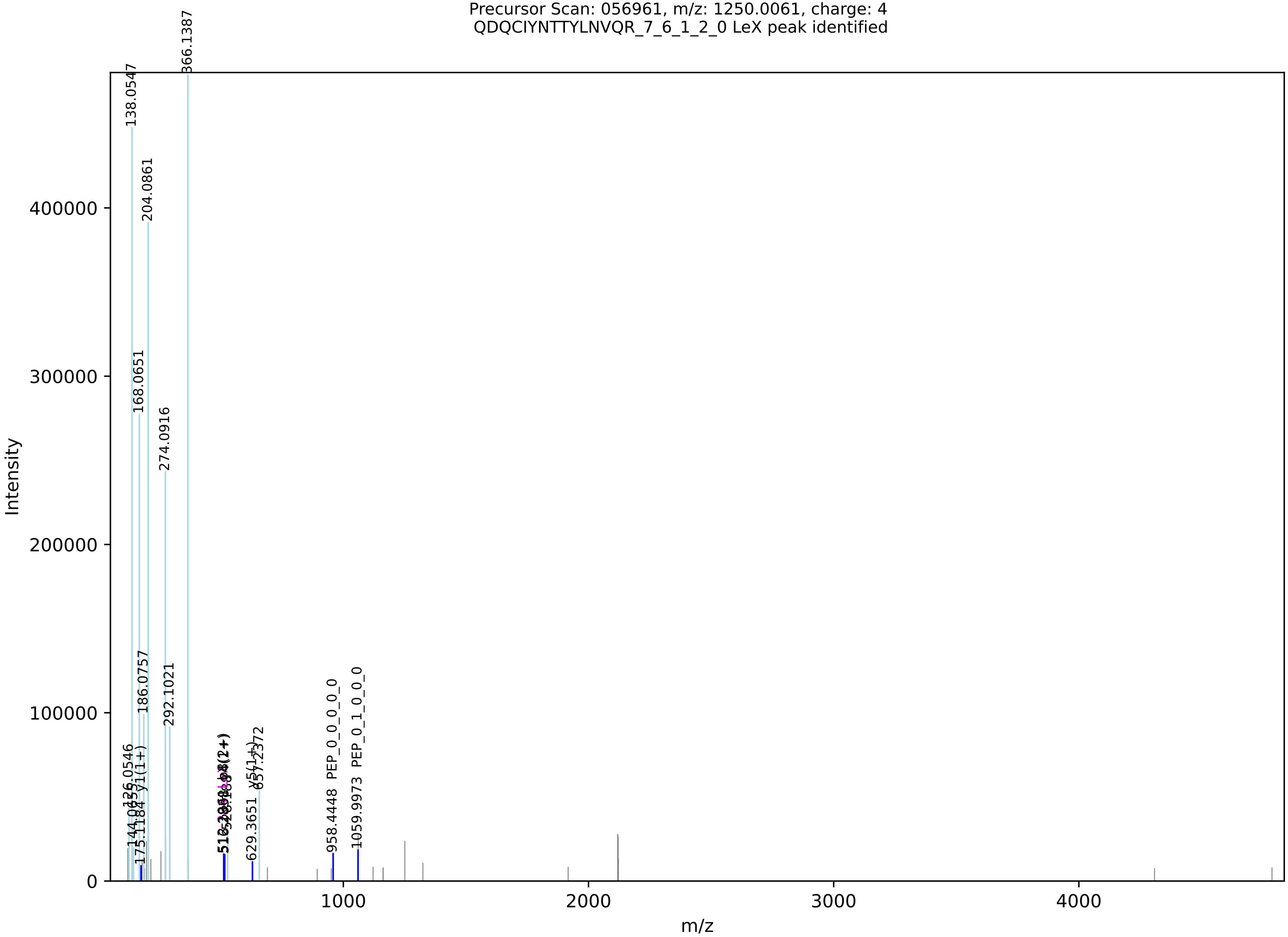

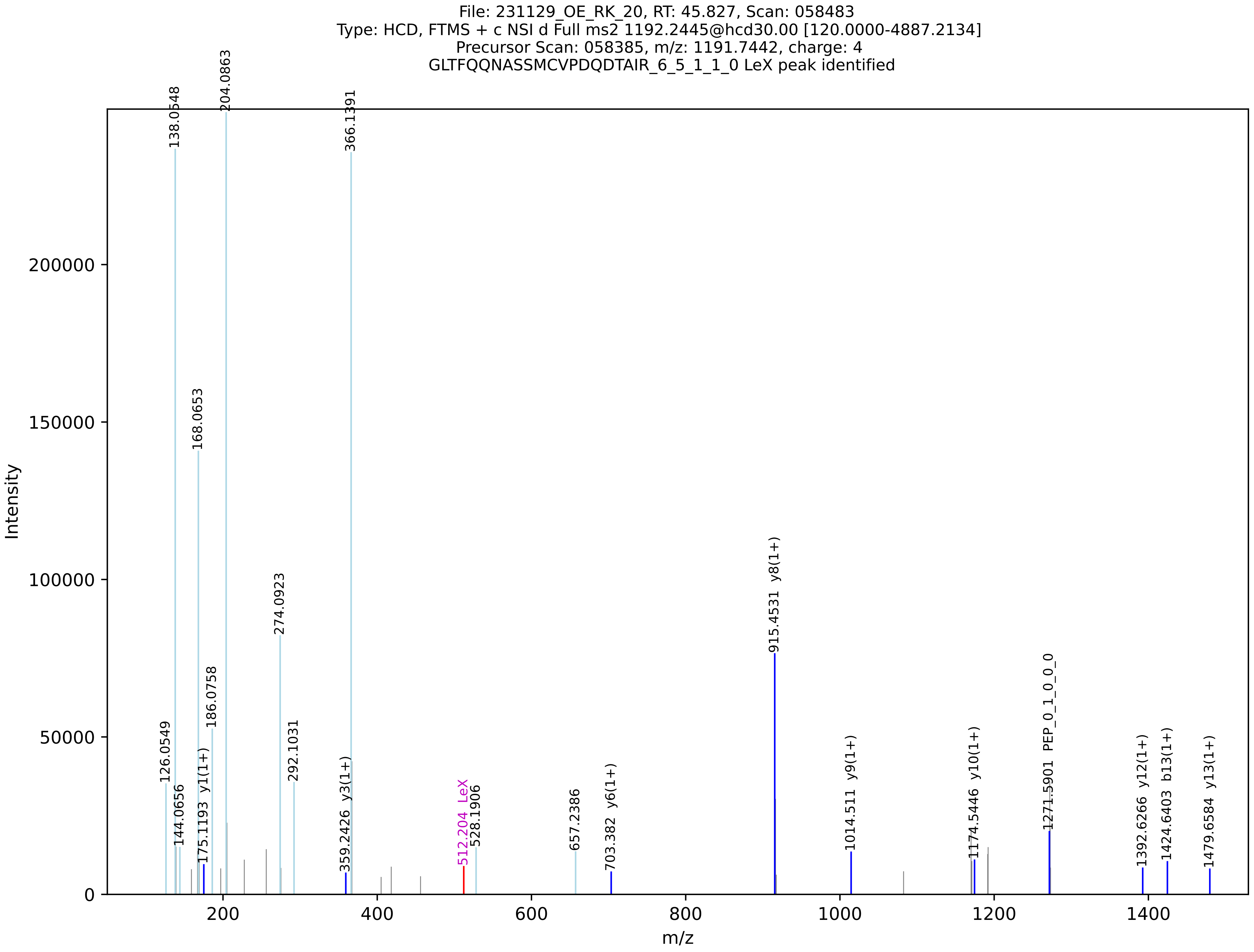
File: 231129_OE_RK_20, RT: 45.827, Scan: 058483
Type: HCD, FTMS + c NSI d Full ms2 1192.2445@hcd30.00 [120.0000-4887.2134]
Precursor Scan: 058385, m/z: 1191.7442, charge: 4
GLTFQQNASSMCVPDQDTAIR_6_5_1_1_0 LeX peak identified
Intensity
m/z
0
50000
100000
150000
200000
200
400
600
800
1000
1200
1400
126.0549
138.0548
144.0656
168.0653
175.1193 y1(1+)
186.0758
204.0863
274.0923
292.1031
359.2426 y3(1+)
366.1391
512.204 LeX
528.1906
657.2386
703.382 y6(1+)
915.4531 y8(1+)
1014.511 y9(1+)
1174.5446 y10(1+)
1271.5901 PEP_0_1_0_0_0
1392.6266 y12(1+)
1424.6403 b13(1+)
1479.6584 y13(1+)

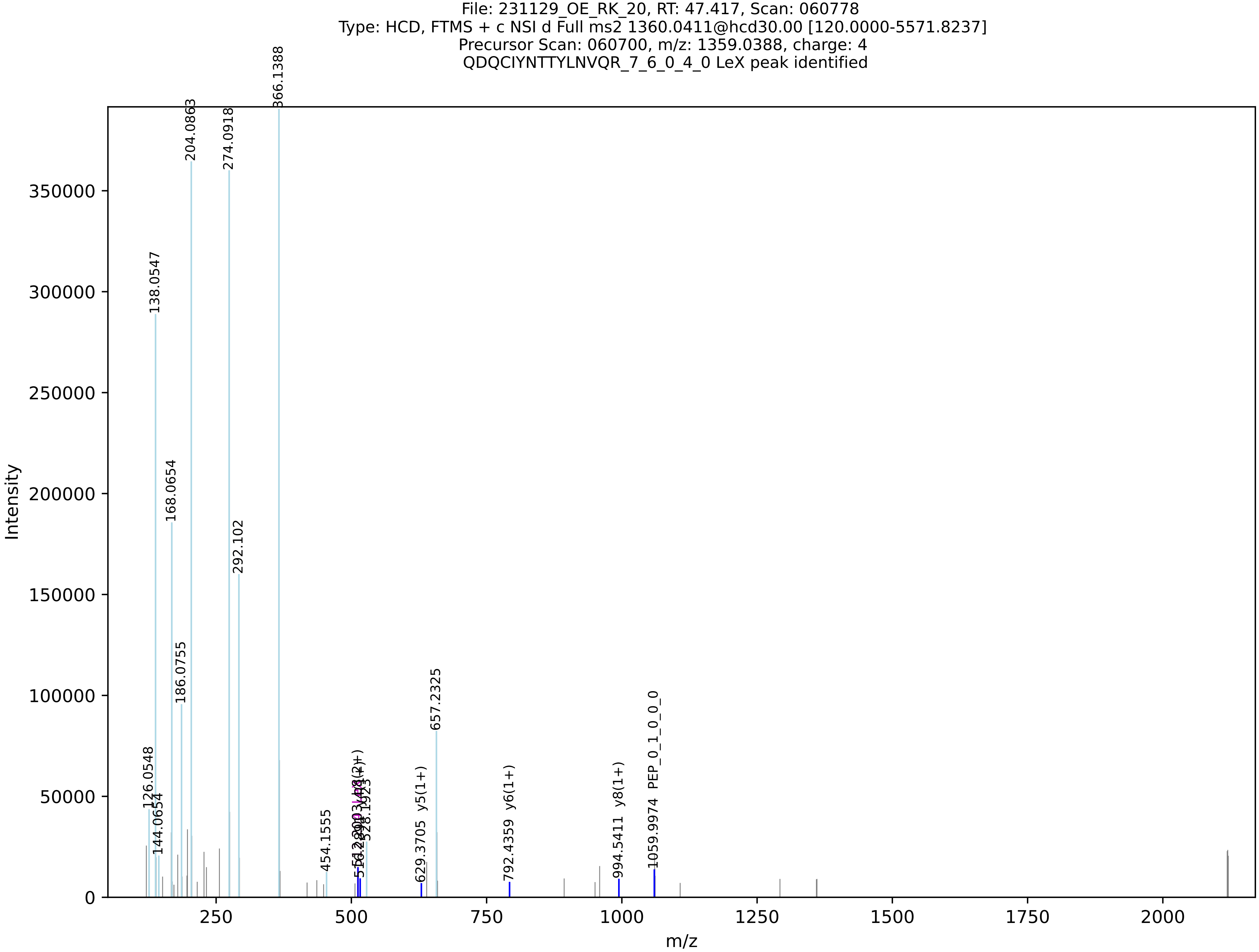

File: 231129_OE_RK_20, RT: 47.417, Scan: 060778
Type: HCD, FTMS + c NSI d Full ms2 1360.0411@hcd30.00 [120.0000-5571.8237]
Precursor Scan: 060700, m/z: 1359.0388, charge: 4
QDQCIYNTTYLNVQR_7_6_0_4_0 LeX peak identified
Intensity
m/z
366.1388
204.0863
274.0918
138.0547
168.0654
292.102
186.0755
126.0548
144.0654
454.1555
528.1923
657.2325
629.3705 y5(1+)
792.4359 y6(1+)
994.5411 y8(1+)
1059.9974 PEP_0_1_0_0_0

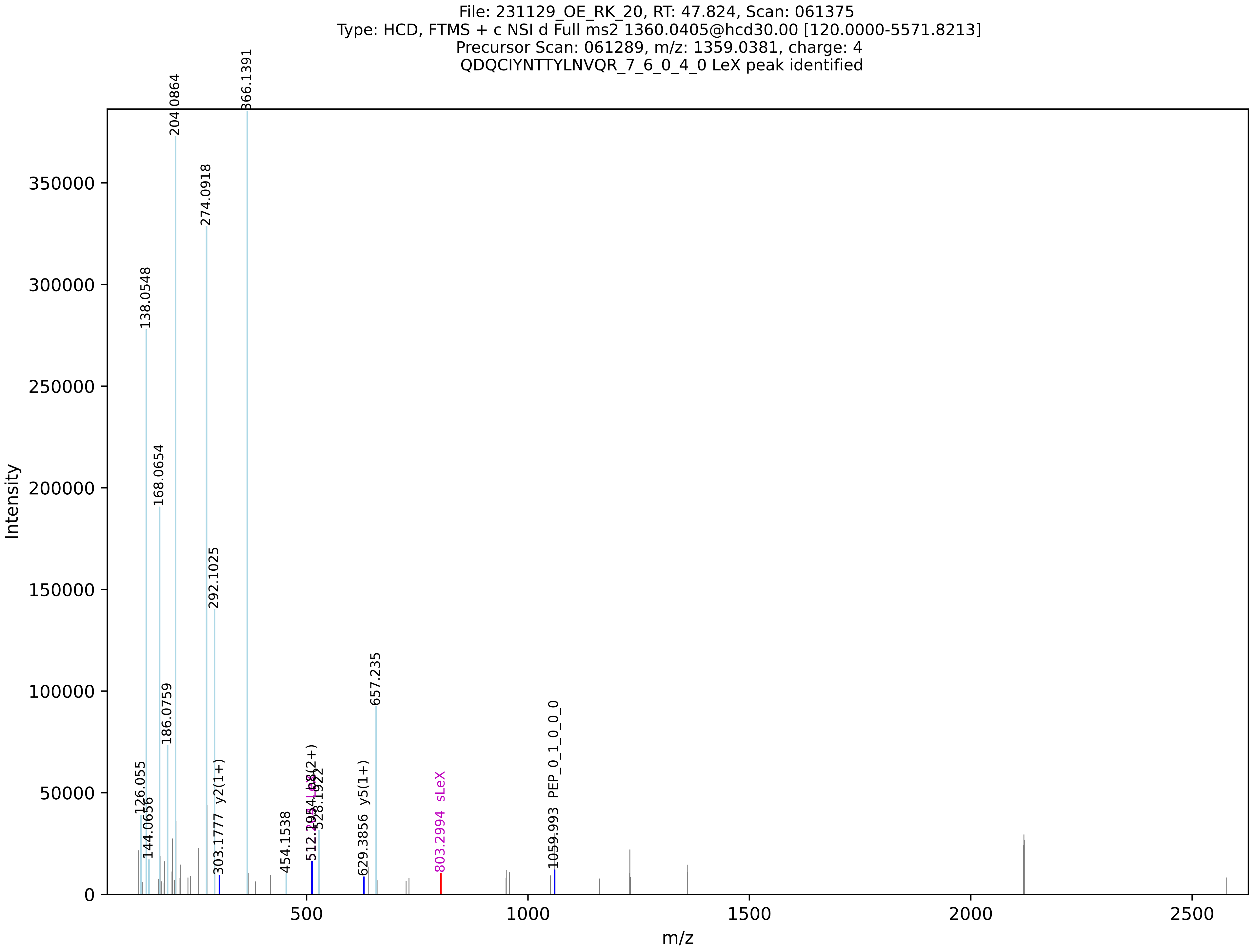
File: 231129_OE_RK_20, RT: 47.824, Scan: 061375
Type: HCD, FTMS + c NSI d Full ms2 1360.0405@hcd30.00 [120.0000-5571.8213]
Precursor Scan: 061289, m/z: 1359.0381, charge: 4
QDQCIYNTTYLNVQR_7_6_0_4_0 LeX peak identified
Intensity
m/z
0
50000
100000
150000
200000
250000
300000
350000
500
1000
1500
2000
2500
366.1391
204.0864
274.0918
138.0548
168.0654
292.1025
186.0759
126.055
144.0656
303.1777 y2(1+)
454.1538
512.1954 b8(2+)
528.1922
629.3856 y5(1+)
657.235
803.2994 sLeX
1059.993 PEP_0_1_0_0_0

File: 231129_OE_RK_20, RT: 48.023, Scan: 061662
Type: HCD, FTMS + c NSI d Full ms2 1359.7885@hcd30.00 [120.0000-5570.7930]
Precursor Scan: 061580, m/z: 1359.0367, charge: 4
QDQCIYNTTYLNVQR_7_6_0_4_0 LeX peak identified

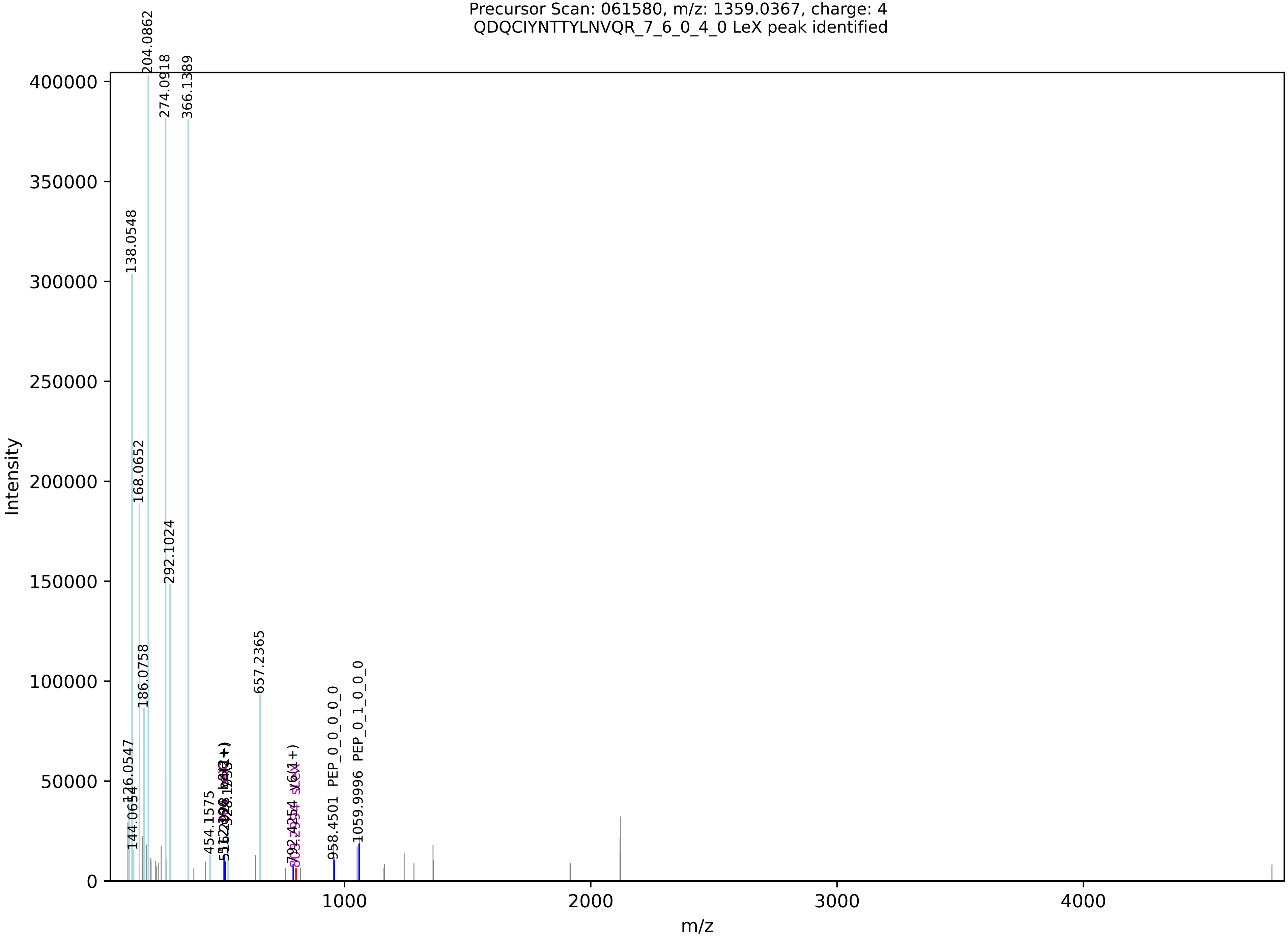

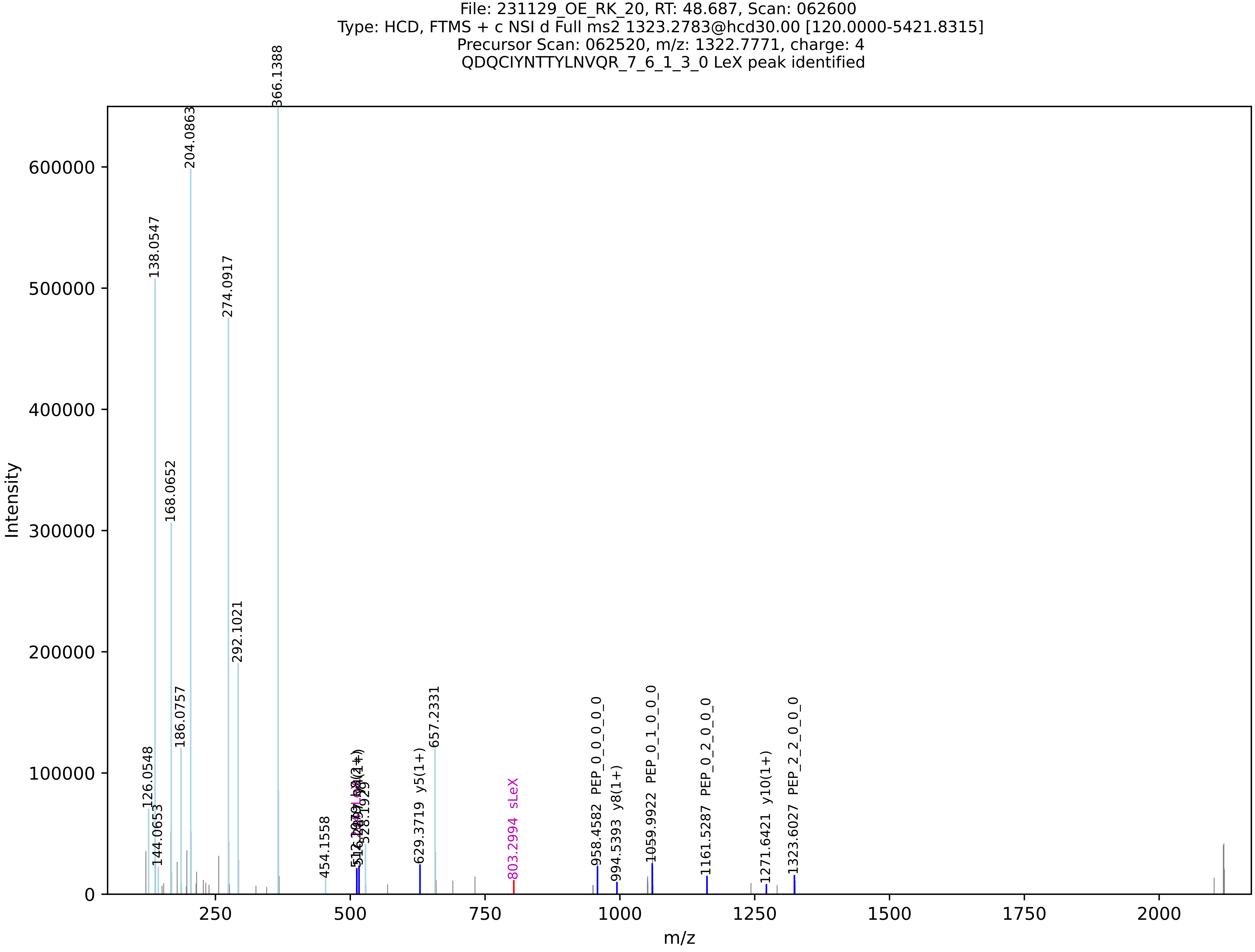
File: 231129_OE_RK_20, RT: 48.687, Scan: 062600
Type: HCD, FTMS + c NSI d Full ms2 1323.2783@hcd30.00 [120.0000-5421.8315]
Precursor Scan: 062520, m/z: 1322.7771, charge: 4
QDQCIYNTTYLNVQR_7_6_1_3_0 LeX peak identified
Intensity
m/z
126.0548
138.0547
144.0653
168.0652
186.0757
204.0863
274.0917
292.1021
366.1388
454.1558
528.1929
629.3719 y5(1+)
657.2331
803.2994 sLeX
958.4582 PEP_0_0_0_0_0
994.5393 y8(1+)
1059.9922 PEP_0_1_0_0_0
1161.5287 PEP_0_2_0_0_0
1271.6421 y10(1+)
1323.6027 PEP_2_2_0_0_0

File: 231129_OE_RK_20, RT: 48.939, Scan: 062969
Type: HCD, FTMS + c NSI d Full ms2 1359.5408@hcd30.00 [120.0000-5569.7822]
Precursor Scan: 062890, m/z: 1359.0396, charge: 4
QDQCIYNTTYLNVQR_7_6_0_4_0 LeX peak identified

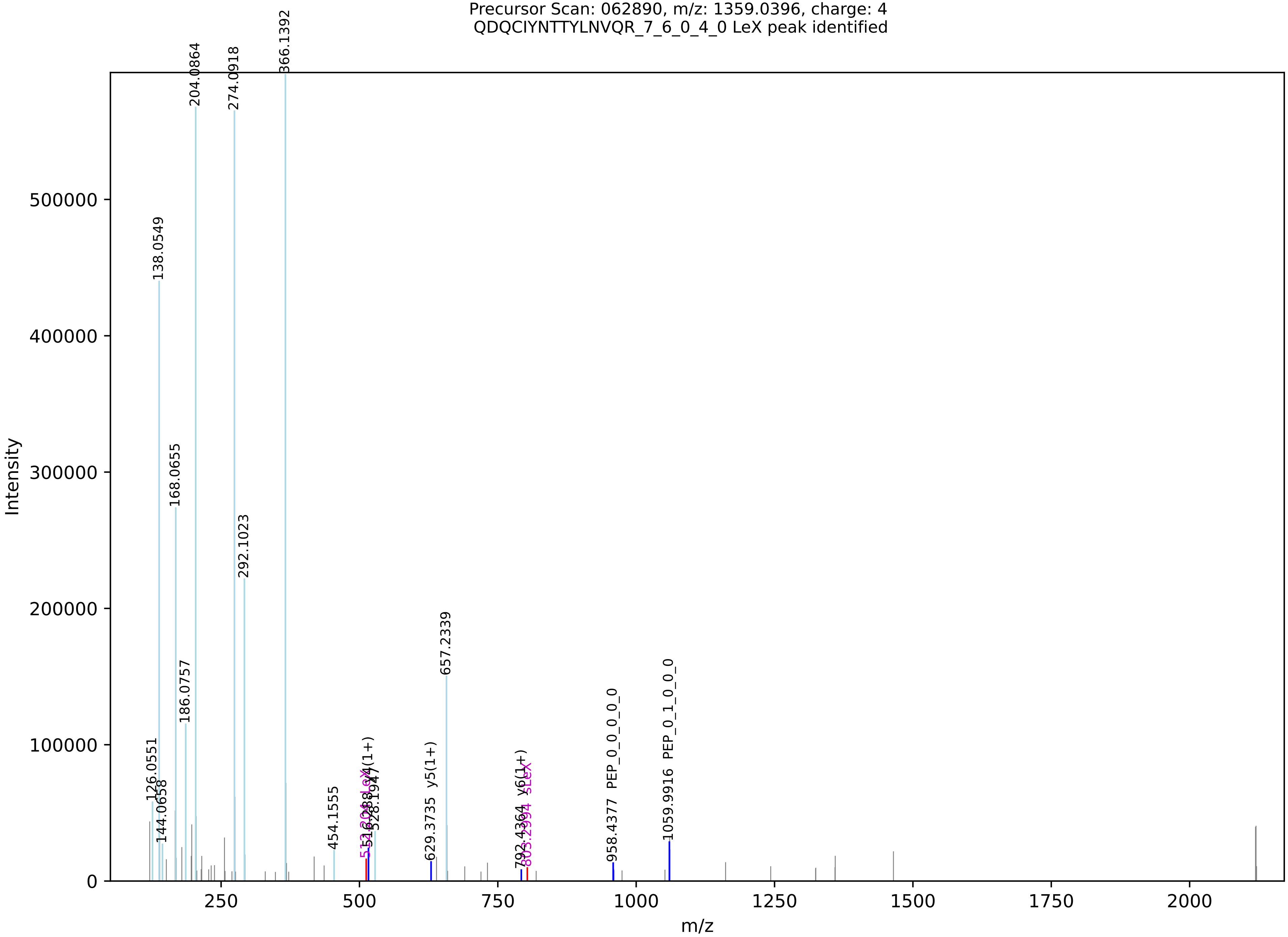

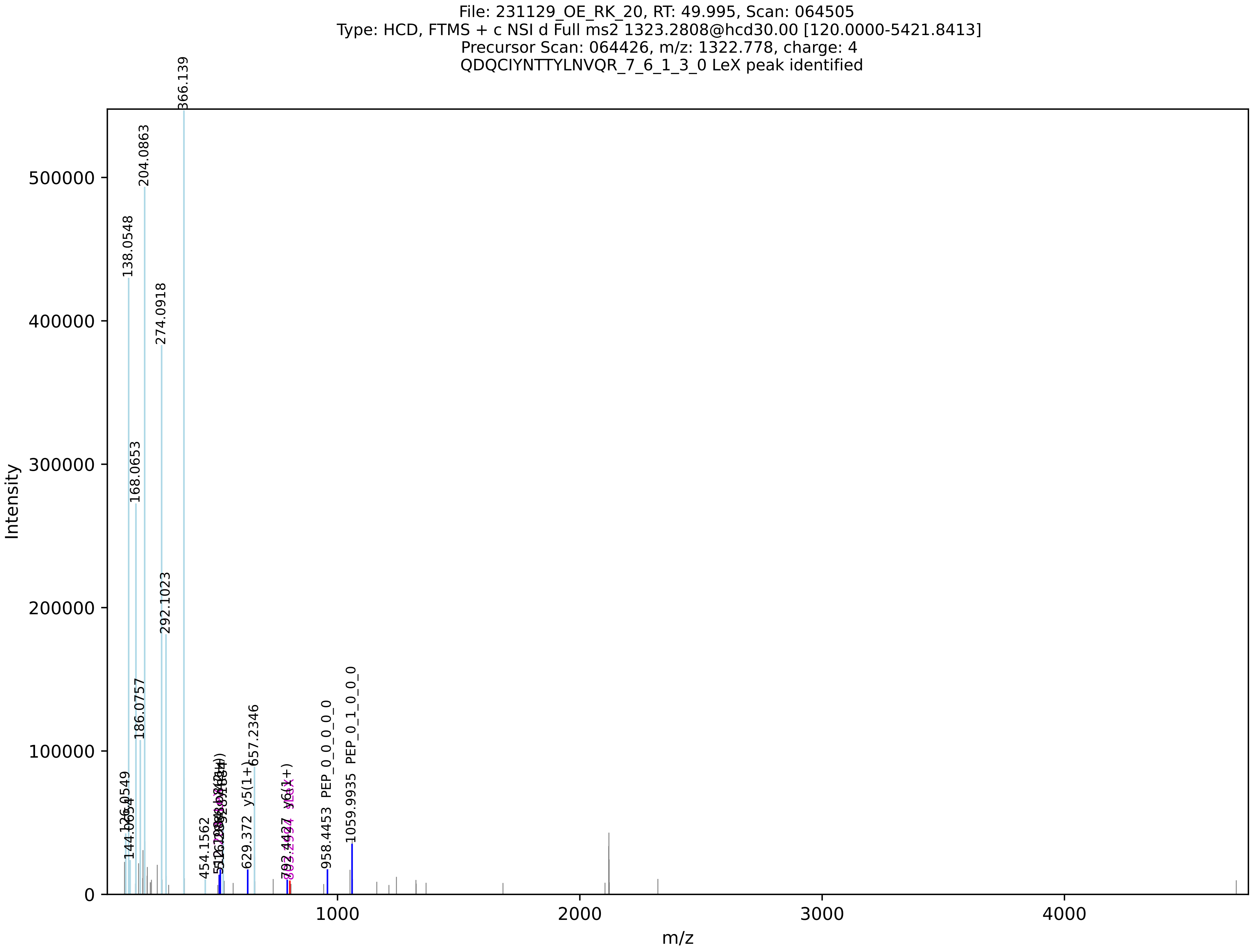

File: 231129_OE_RK_20, RT: 49.995, Scan: 064505
Type: HCD, FTMS + c NSI d Full ms2 1323.2808@hcd30.00 [120.0000-5421.8413]
Precursor Scan: 064426, m/z: 1322.778, charge: 4
QDQCIYNTTYLNVQR_7_6_1_3_0 LeX peak identified
Intensity
m/z
0
100000
200000
300000
400000
500000
1000
2000
3000
4000
366.139
204.0863
138.0548
274.0918
168.0653
292.1023
186.0757
126.0549
144.0654
454.1562
629.372 y5(1+)
657.2346
792.4427 y6(1+)
958.4453 PEP_0_0_0_0_0_
1059.9935 PEP_0_1_0_0_0_

File: 231129_OE_RK_20, RT: 64.885, Scan: 086128
Type: HCD, FTMS + c NSI d Full ms2 1273.2151@hcd30.00 [120.0000-3918.1802]
Precursor Scan: 086029, m/z: 1272.8815, charge: 3
YLGNATAIFFLPDEGK_5_4_1_1_0 LeX peak identified

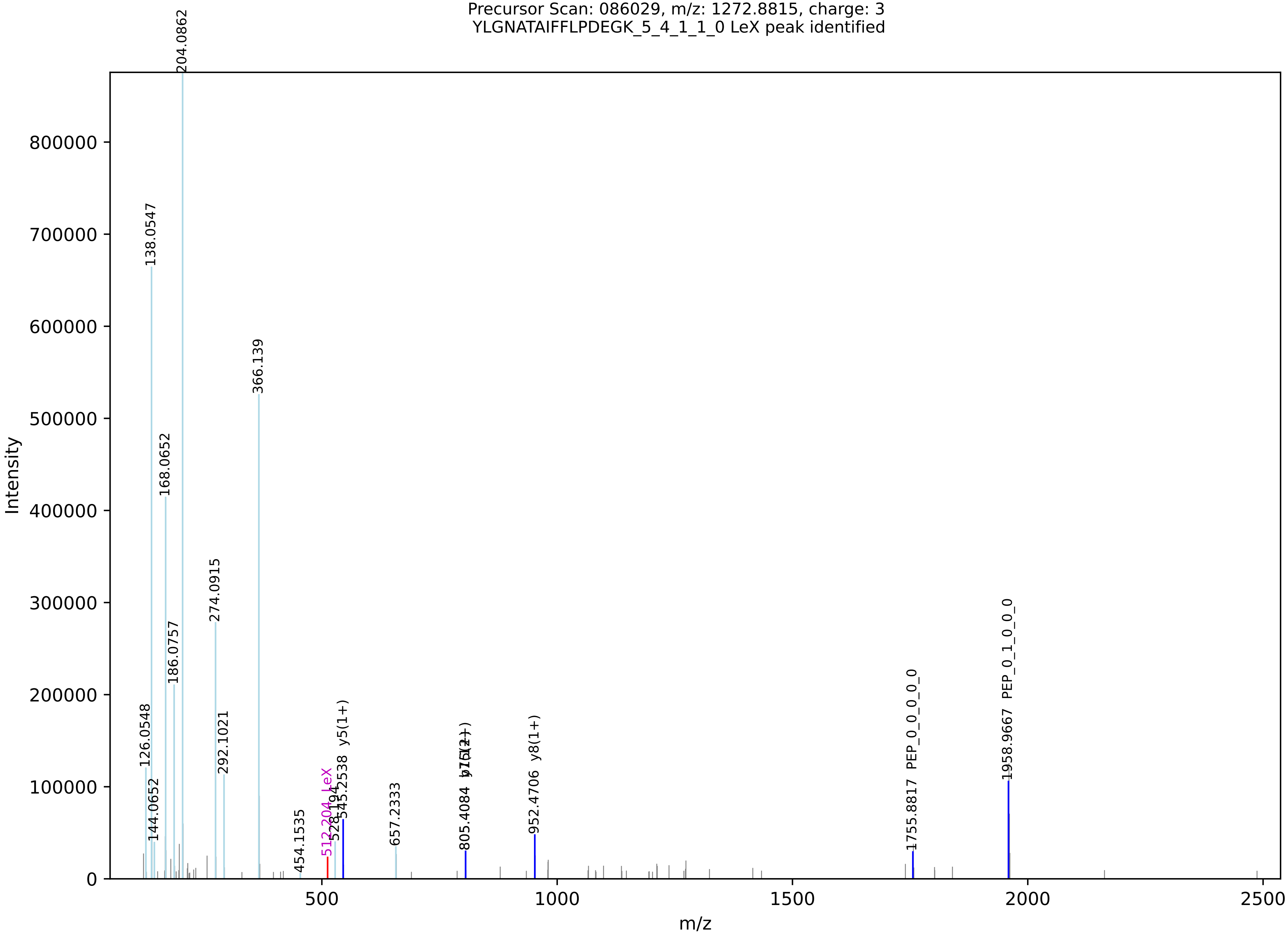

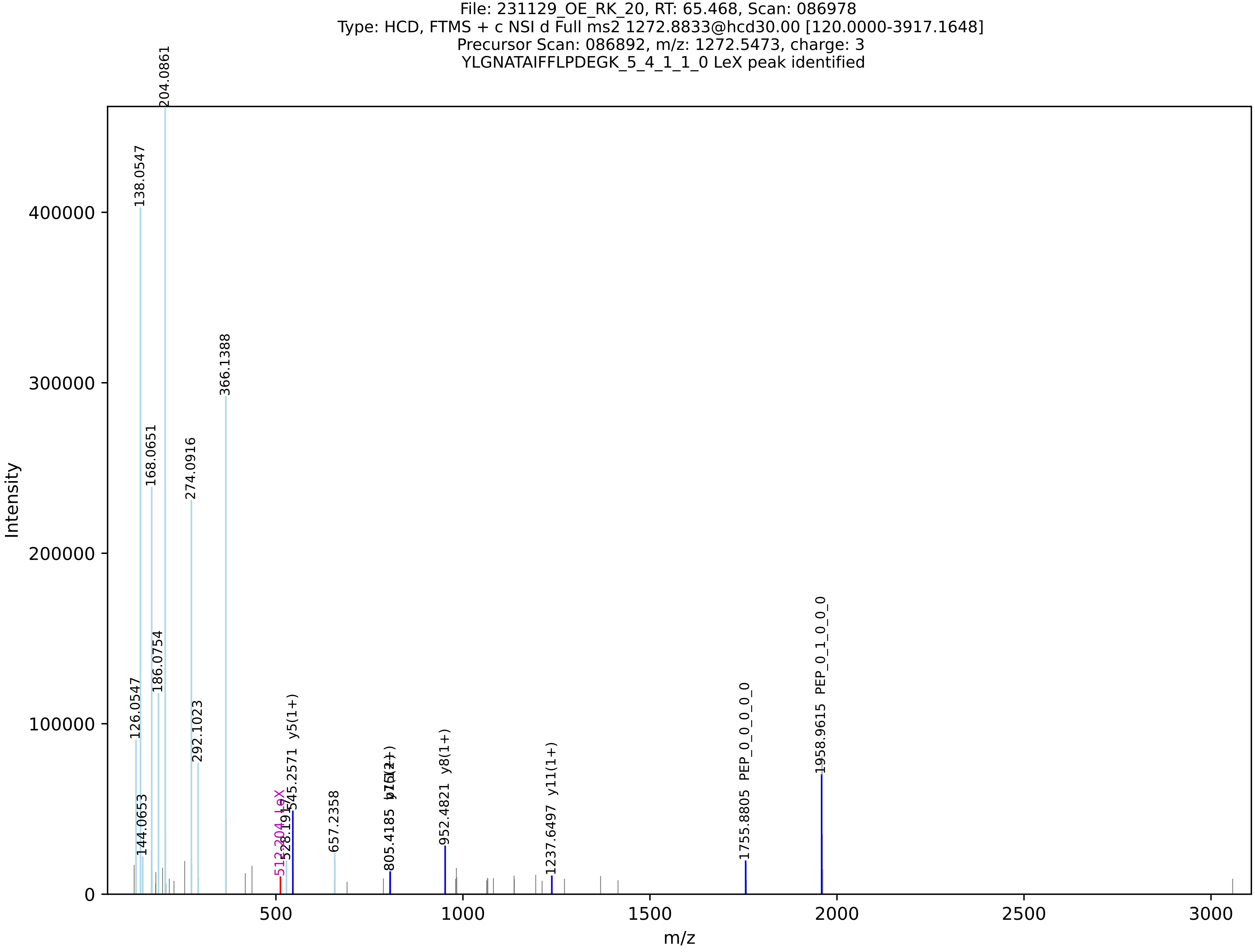
File: 231129_OE_RK_20, RT: 65.468, Scan: 086978
Type: HCD, FTMS + c NSI d Full ms2 1272.8833@hcd30.00 [120.0000-3917.1648]
Precursor Scan: 086892, m/z: 1272.5473, charge: 3
YLGNATAIFFLPDEGK_5_4_1_1_0 LeX peak identified
Intensity
m/z
204.0861
138.0547
168.0651
274.0916
366.1388
186.0754
126.0547
292.1023
144.0653
512.204 LeX
545.2571 y5(1+)
657.2358
805.4185
952.4821 y8(1+)
1237.6497 y11(1+)
1755.8805 PEP_0_0_0_0_0
1958.9615 PEP_0_1_0_0_0
0
100000
200000
300000
400000
500
1000
1500
2000
2500
3000

File: 231129_OE_RK_20, RT: 71.62, Scan: 095956
Type: HCD, FTMS + c NSI d Full ms2 1422.8724@hcd30.00 [120.0000-5828.1753]
Precursor Scan: 095880, m/z: 1422.1206, charge: 4
SVQEIQATFFYFTPNKTEDTIFLR_7_6_1_1_0 LeX peak identified

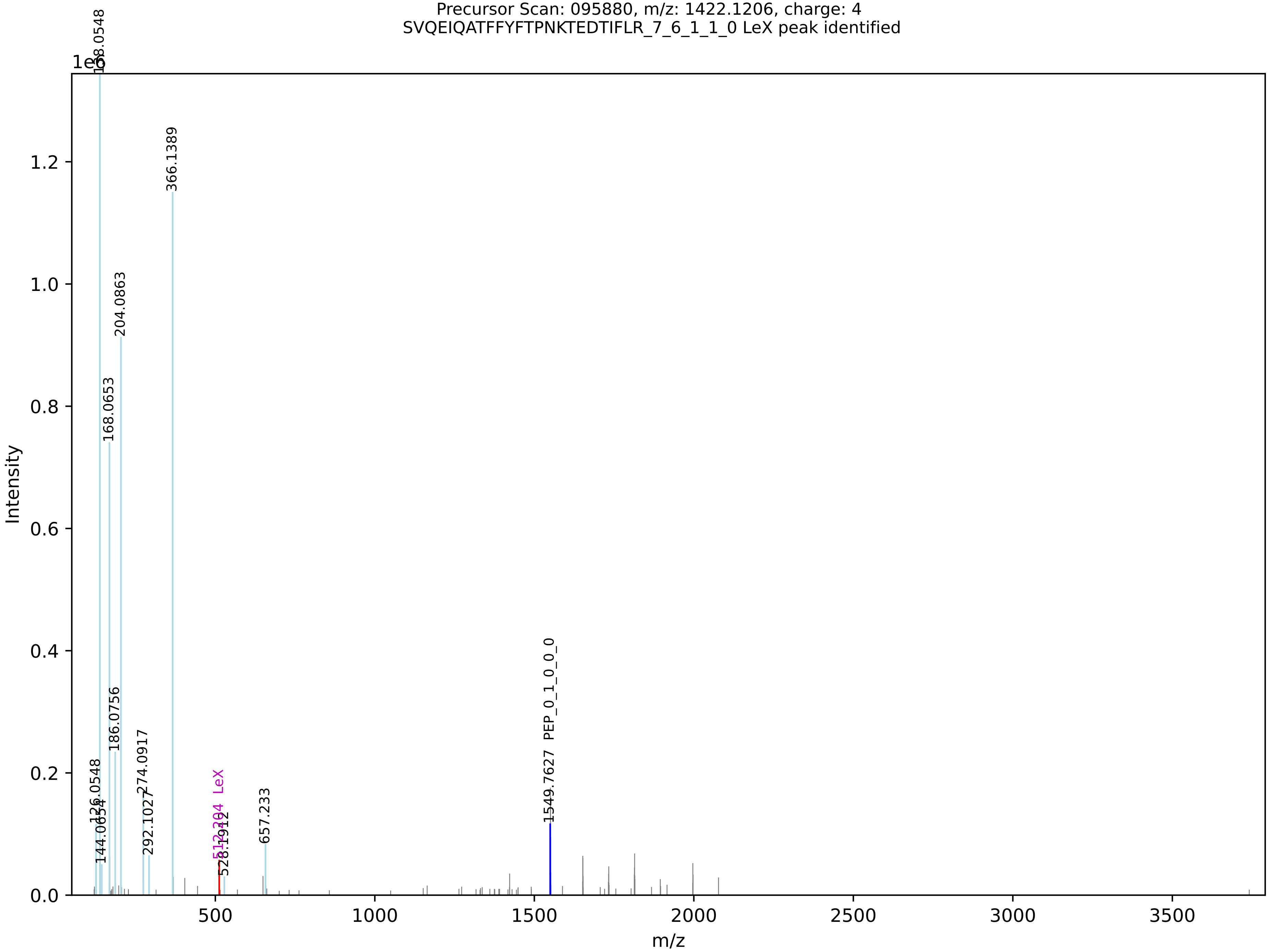

File: 231129_OE_RK_21, RT: 7.184, Scan: 004504
Type: HCD, FTMS + c NSI d Full ms2 1220.8009@hcd30.00 [120.0000-3757.7927]
Precursor Scan: 004460, m/z: 1220.4668, charge: 3
NEEYNK_6_5_0_3_0 LeX peak identified

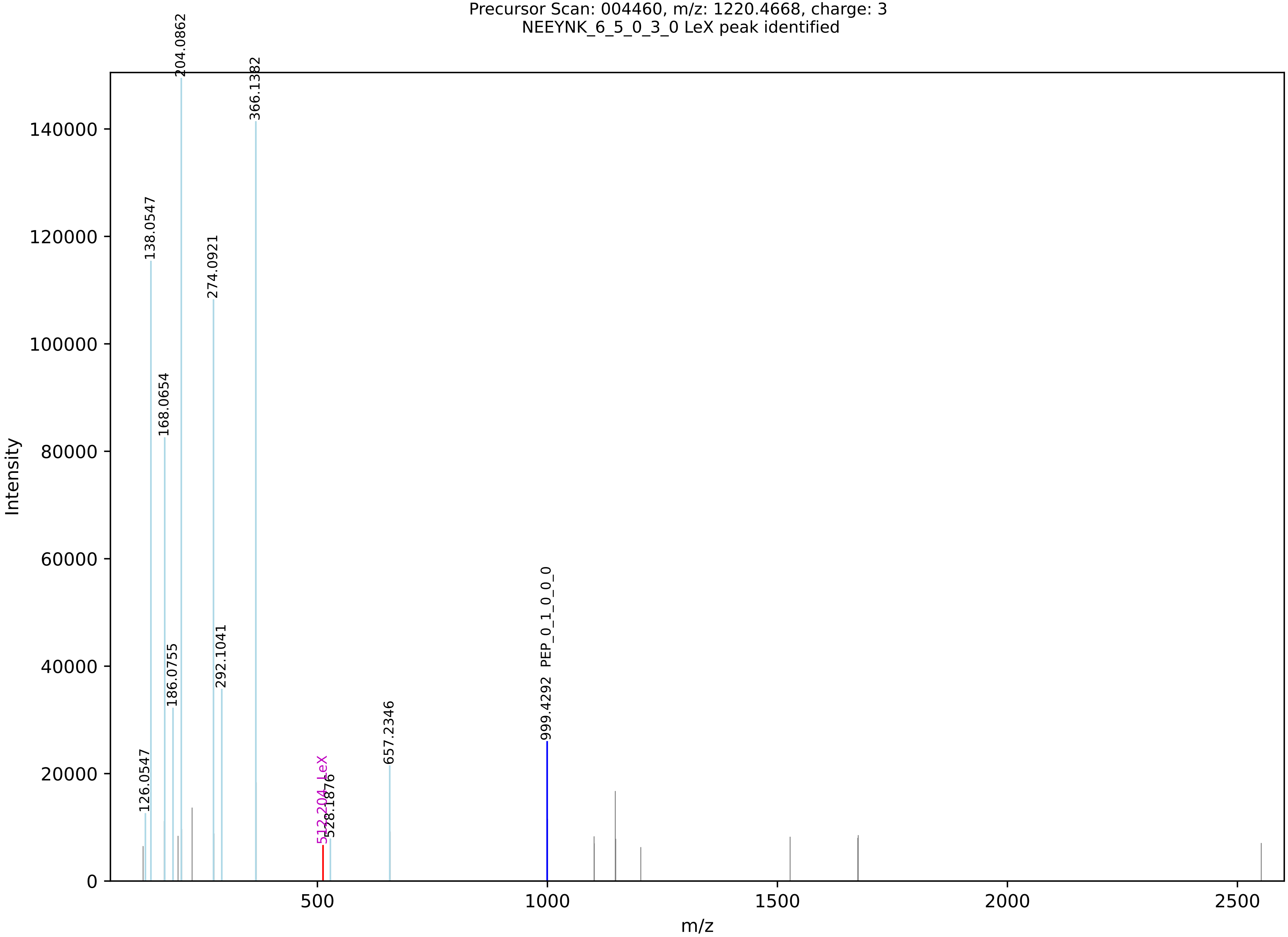

File: 231129_OE_RK_21, RT: 7.274, Scan: 004625
Type: HCD, FTMS + c NSI d Full ms2 1335.8521@hcd30.00 [120.0000-4109.8491]
Precursor Scan: 004583, m/z: 1334.8497, charge: 3
ENGTISR_7_6_0_3_0 LeX peak identified

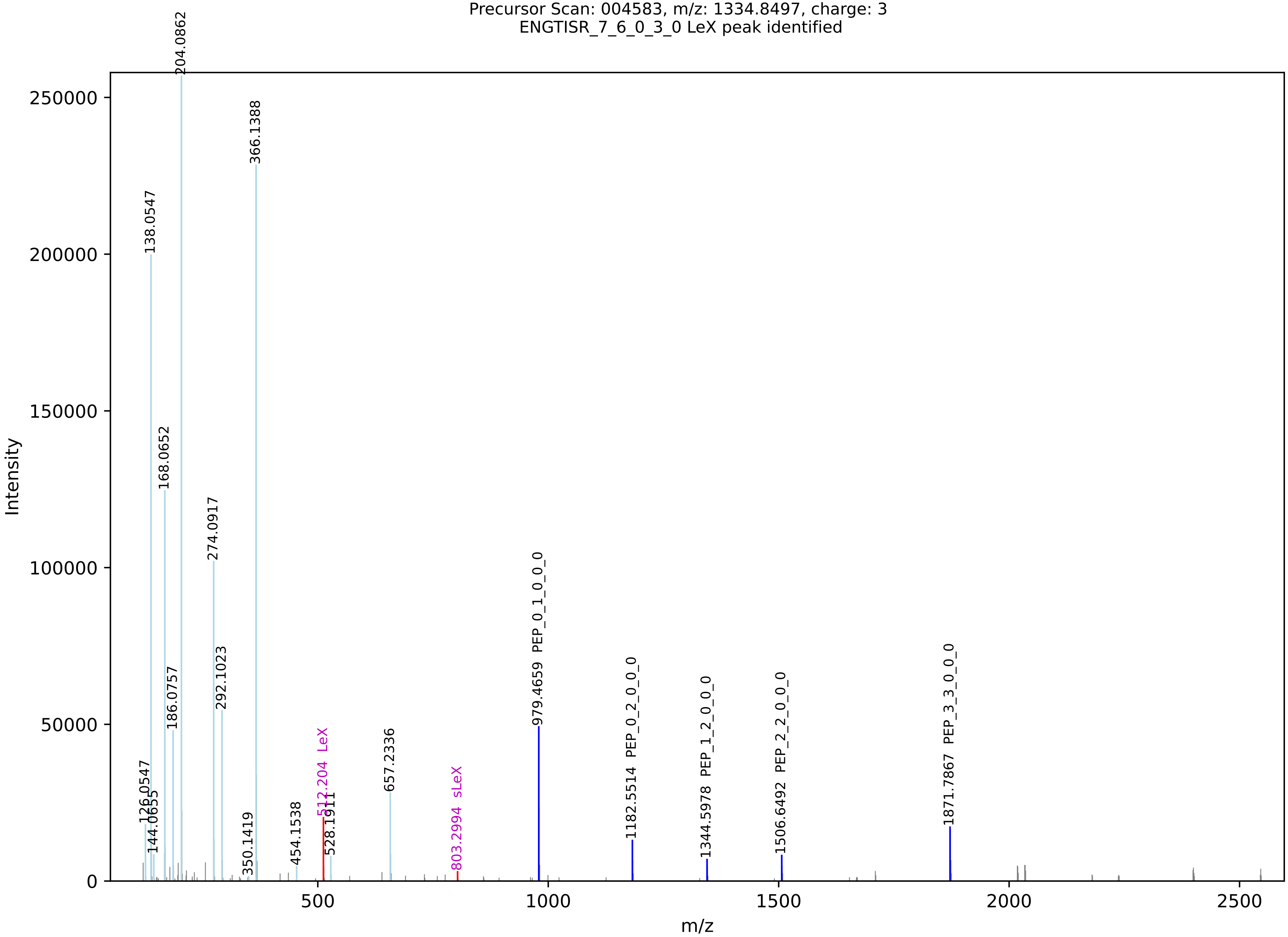

File: 231129_OE_RK_21, RT: 7.8, Scan: 005366
Type: HCD, FTMS + c NSI d Full ms2 1038.4038@hcd30.00 [120.0000-4259.5435]
Precursor Scan: 005323, m/z: 1037.9029, charge: 4
ENGTISR_7_6_1_3_0 LeX peak identified

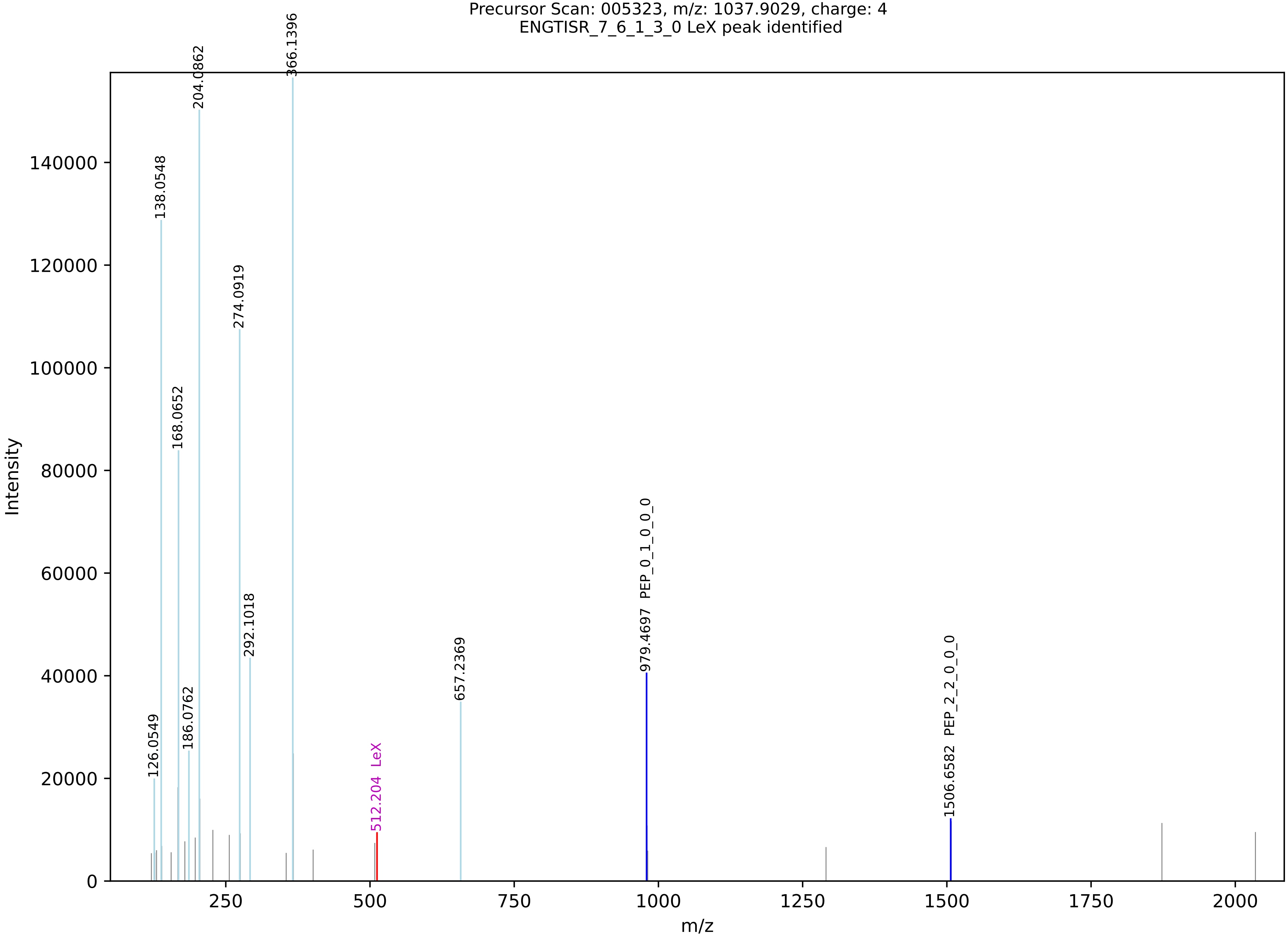

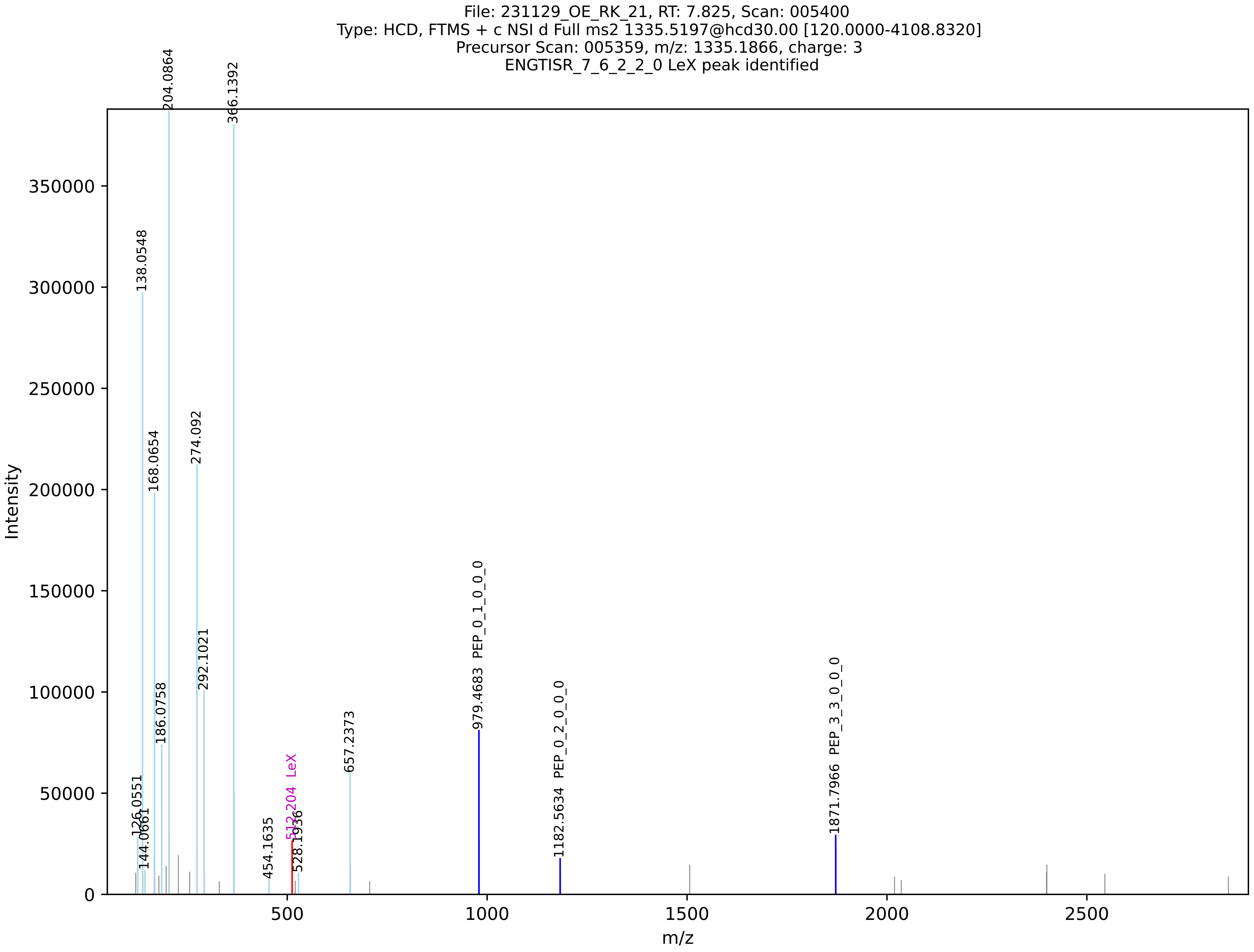

File: 231129_OE_RK_21, RT: 7.825, Scan: 005400
Type: HCD, FTMS + c NSI d Full ms2 1335.5197@hcd30.00 [120.0000-4108.8320]
Precursor Scan: 005359, m/z: 1335.1866, charge: 3
ENGTISR_7_6_2_2_0 LeX peak identified
Intensity
m/z
204.0864
366.1392
138.0548
168.0654
274.092
292.1021
186.0758
126.0551
144.0661
454.1635
512.204 LeX
528.1936
657.2373
979.4683 PEP_0_1_0_0_0
1182.5634 PEP_0_2_0_0_0
1871.7966 PEP_3_3_0_0_0

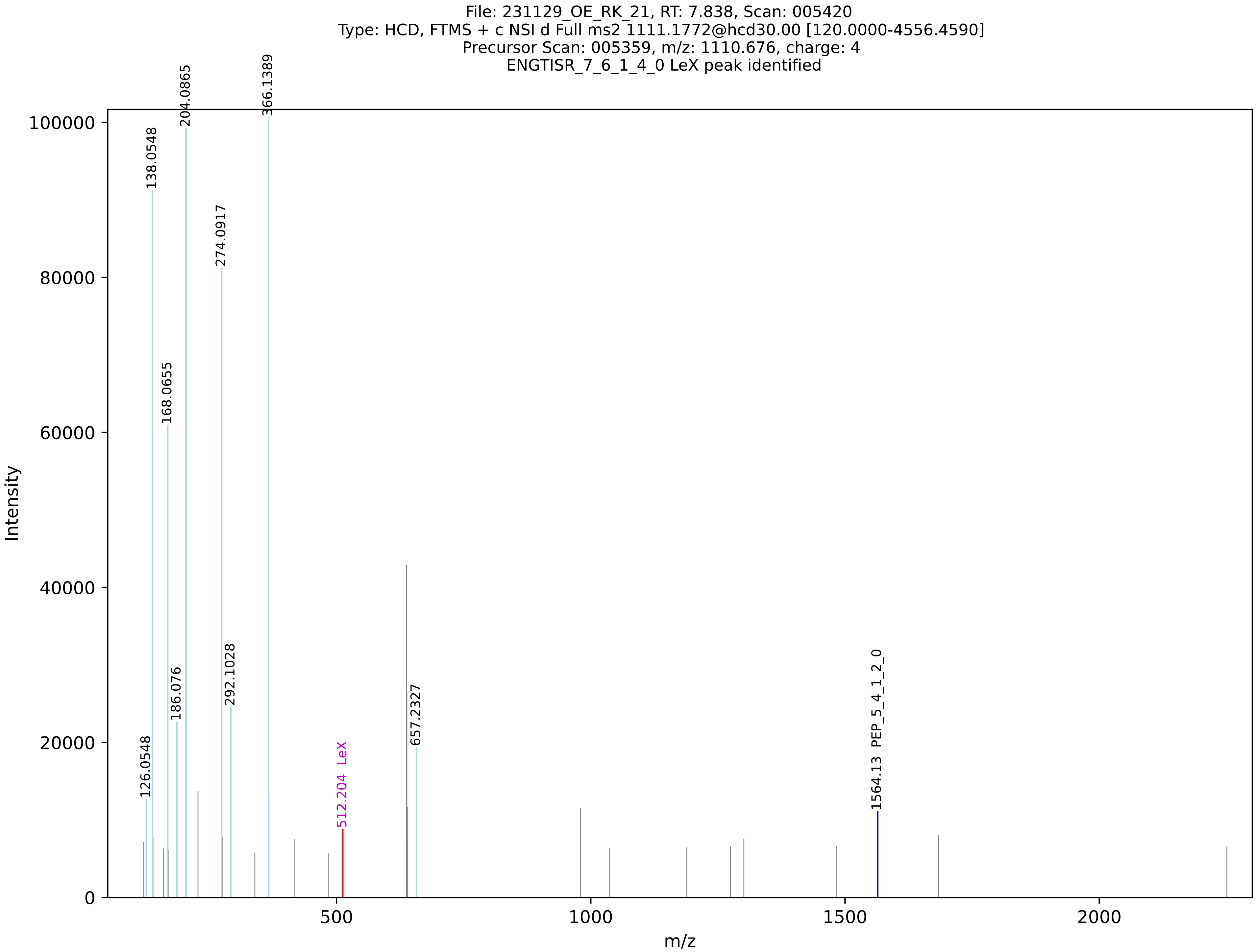

File: 231129_OE_RK_21, RT: 7.838, Scan: 005420
Type: HCD, FTMS + c NSI d Full ms2 1111.1772@hcd30.00 [120.0000-4556.4590]
Precursor Scan: 005359, m/z: 1110.676, charge: 4
ENGTISR_7_6_1_4_0 LeX peak identified
Intensity
m/z
126.0548
138.0548
168.0655
186.076
204.0865
274.0917
292.1028
366.1389
512.204 LeX
657.2327
1564.13 PEP_5_4_1_2_0

File: 231129_OE_RK_21, RT: 7.847, Scan: 005435
Type: HCD, FTMS + c NSI d Full ms2 1214.4756@hcd30.00 [120.0000-3738.4373]
Precursor Scan: 005323, m/z: 1213.1355, charge: 3
ENGTISR_6_5_0_3_0 LeX peak identified

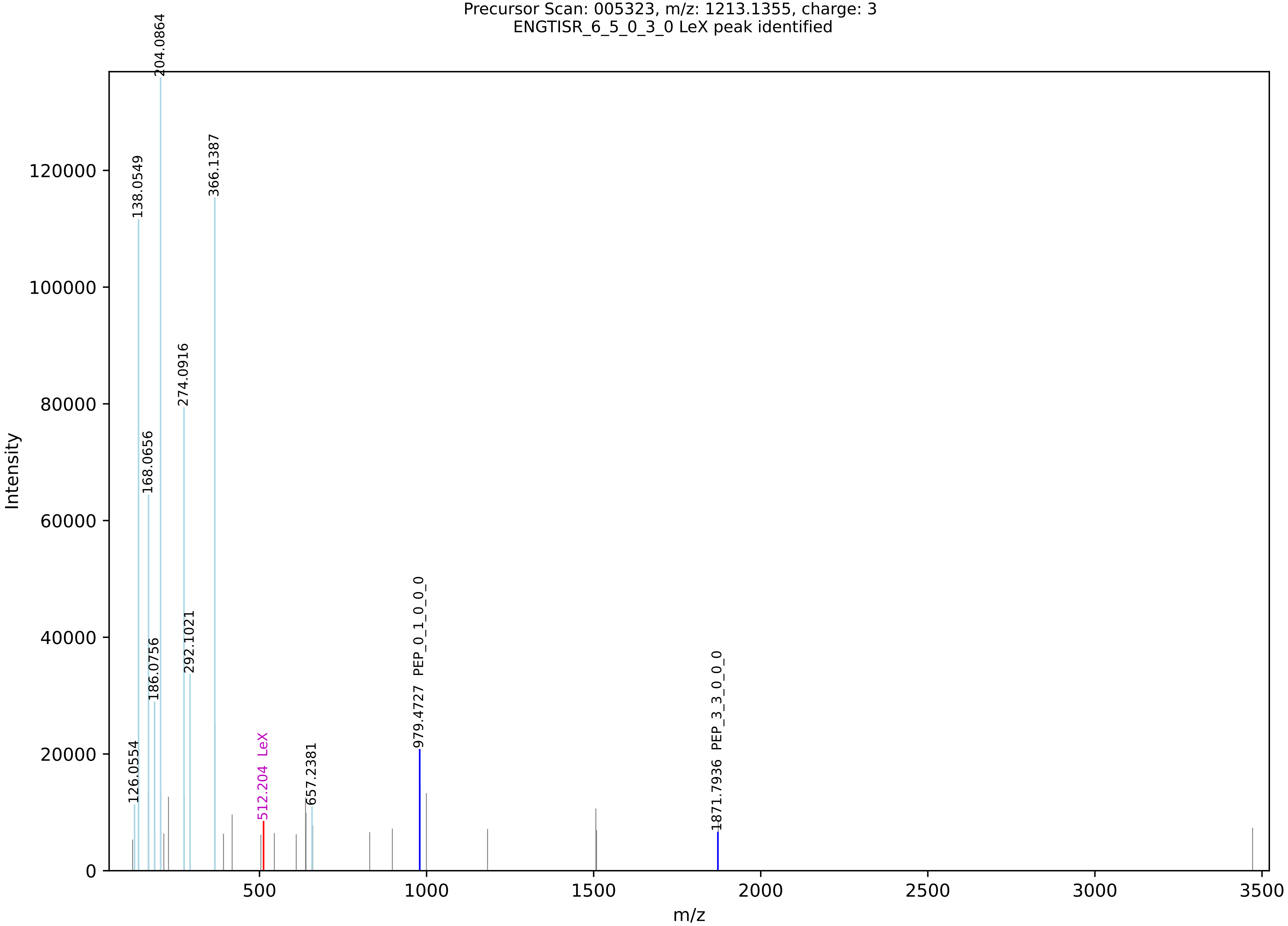

File: 231129_OE_RK_21, RT: 7.892, Scan: 005500
Type: HCD, FTMS + c NSI d Full ms2 1383.2030@hcd30.00 [120.0000-4254.7432]
Precursor Scan: 005359, m/z: 1383.5347, charge: 3
ENGTISR_7_6_1_3_0 LeX peak identified

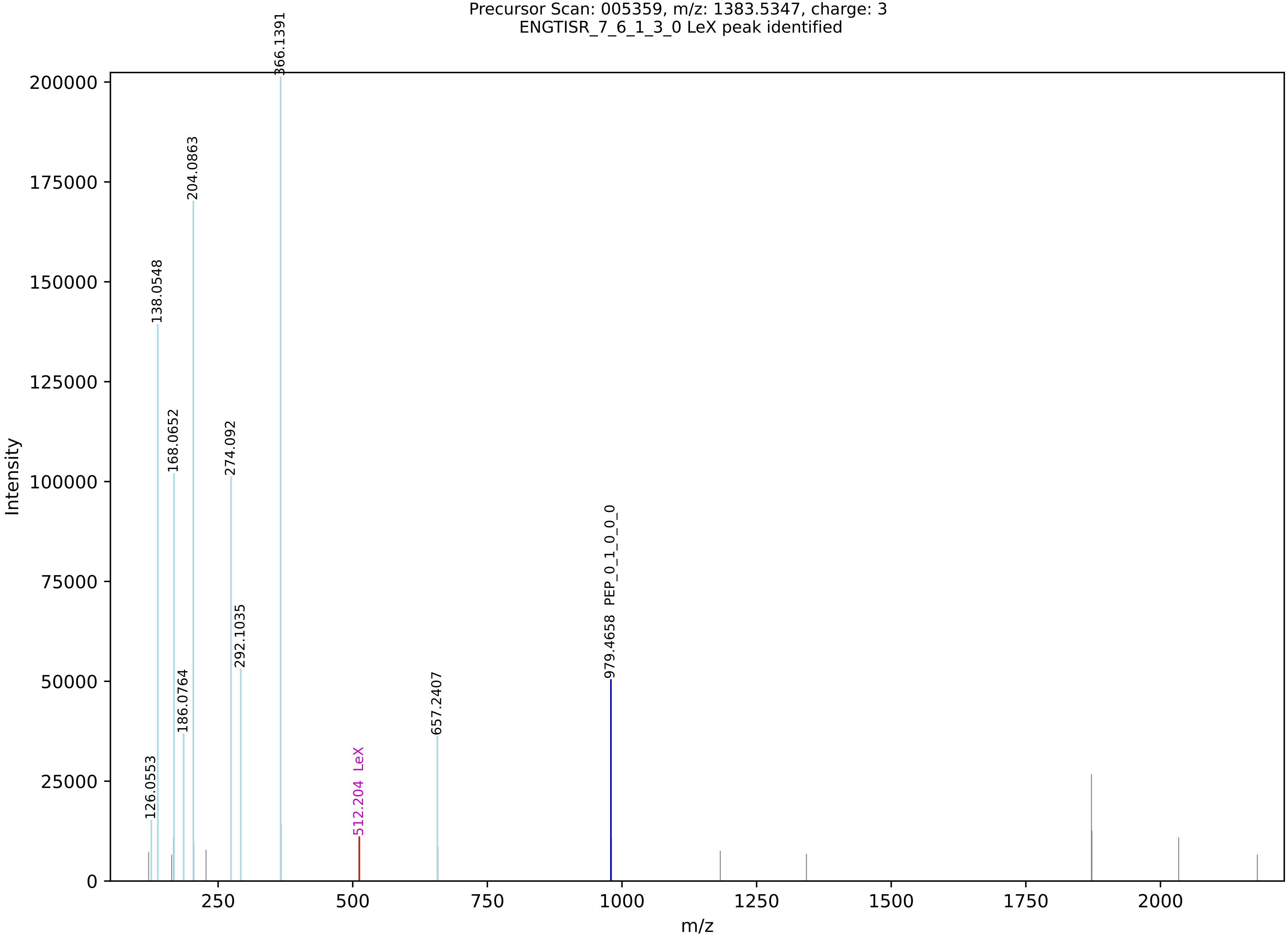

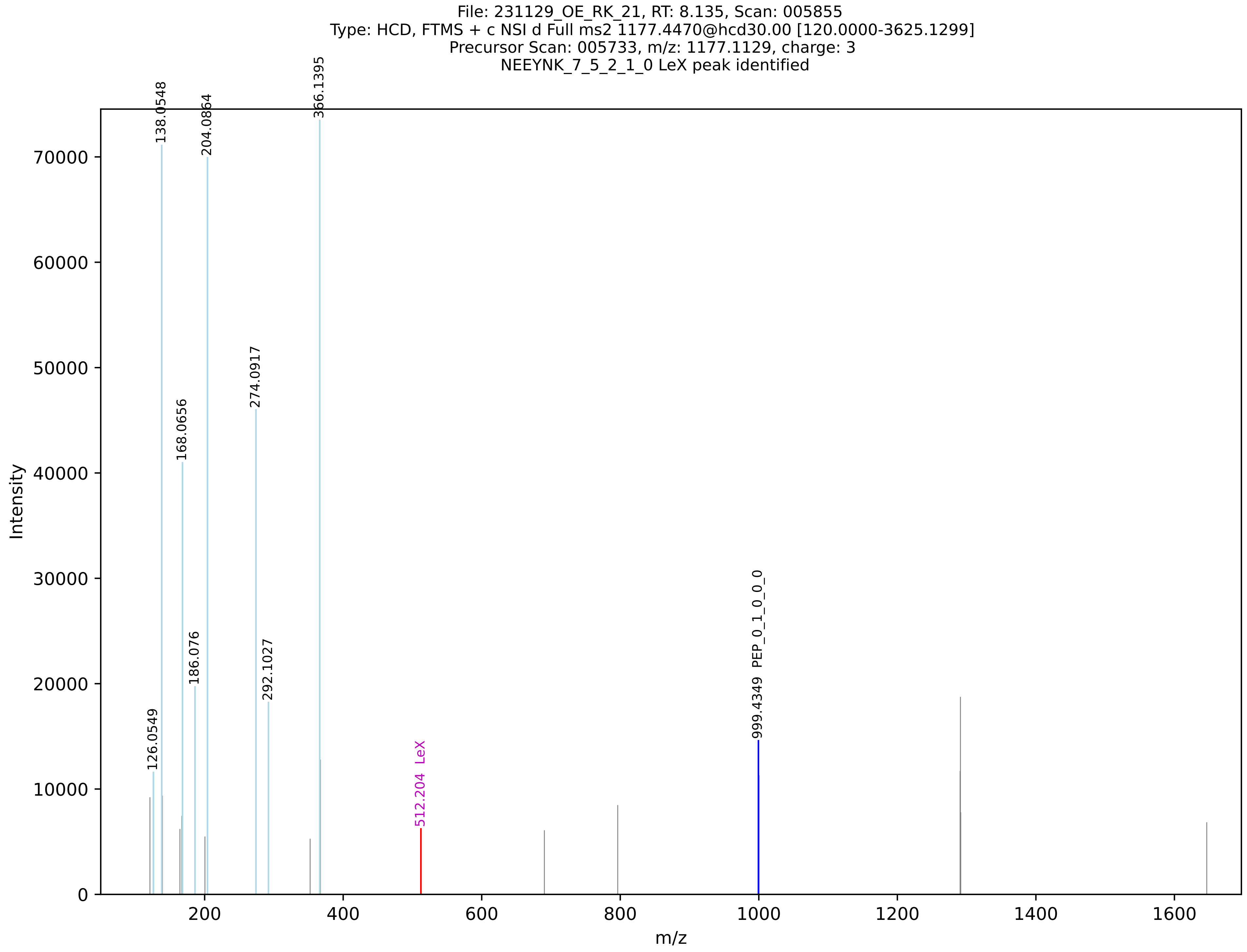
File: 231129_OE_RK_21, RT: 8.135, Scan: 005855
Type: HCD, FTMS + c NSI d Full ms2 1177.4470@hcd30.00 [120.0000-3625.1299]
Precursor Scan: 005733, m/z: 1177.1129, charge: 3
NEEYNK_7_5_2_1_0 LeX peak identified
Intensity
m/z
138.0548
204.0864
366.1395
274.0917
168.0656
186.076
292.1027
126.0549
512.204 LeX
999.4349 PEP_0_1_0_0_0

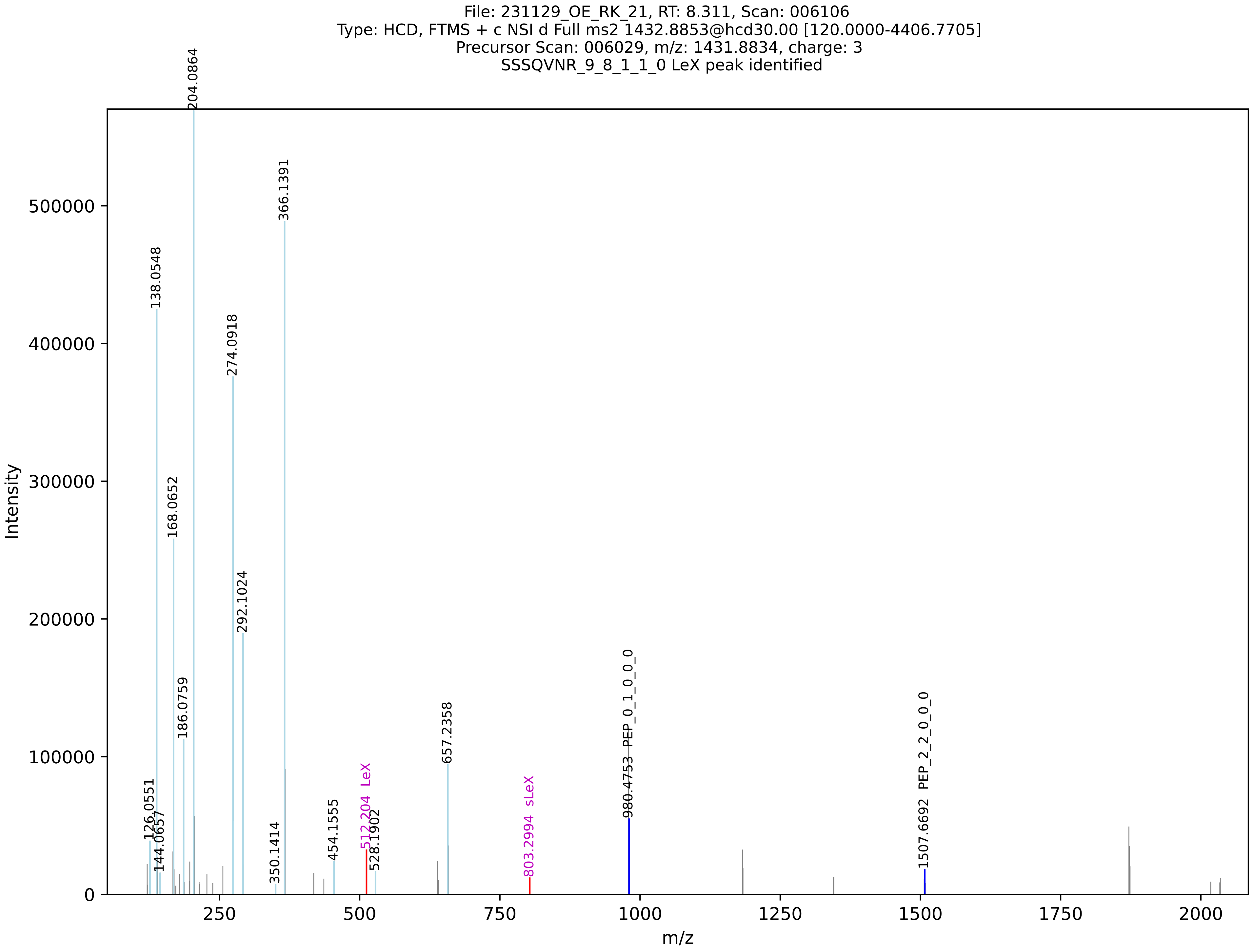

File: 231129_OE_RK_21, RT: 8.311, Scan: 006106
Type: HCD, FTMS + c NSI d Full ms2 1432.8853@hcd30.00 [120.0000-4406.7705]
Precursor Scan: 006029, m/z: 1431.8834, charge: 3
SSSQVNR_9_8_1_1_0 LeX peak identified
Intensity
m/z
204.0864
366.1391
138.0548
274.0918
168.0652
292.1024
186.0759
126.0551
144.0657
350.1414
454.1555
512.204 LeX
528.1902
657.2358
803.2994 sLeX
980.4753 PEP_0_1_0_0_0
1507.6692 PEP_2_2_0_0_0

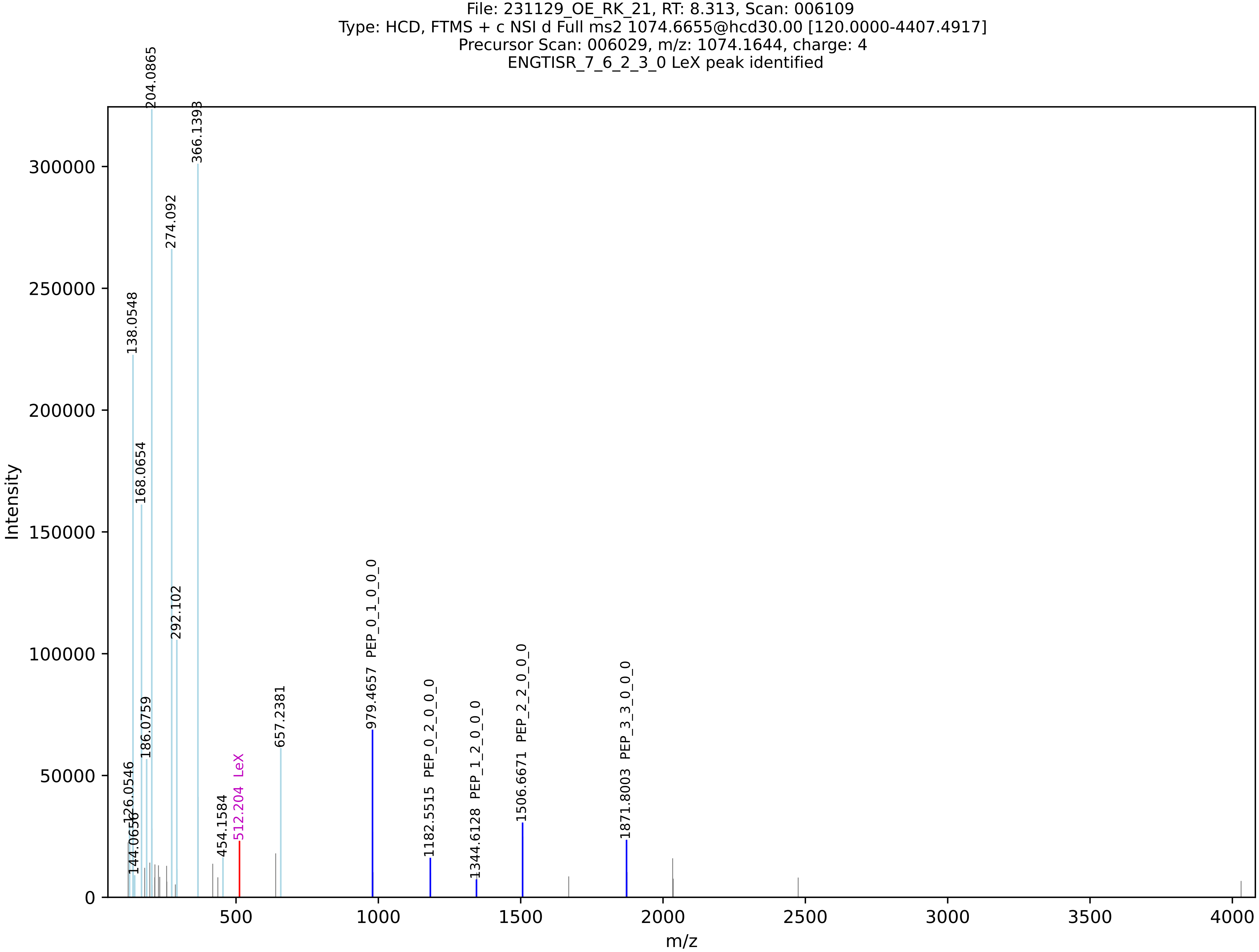

File: 231129_OE_RK_21, RT: 8.313, Scan: 006109
Type: HCD, FTMS + c NSI d Full ms2 1074.6655@hcd30.00 [120.0000-4407.4917]
Precursor Scan: 006029, m/z: 1074.1644, charge: 4
ENGTISR_7_6_2_3_0 LeX peak identified
Intensity
m/z
204.0865
366.1393
274.092
138.0548
168.0654
292.102
186.0759
126.0546
144.0656
454.1584
512.204 LeX
657.2381
979.4657 PEP_0_1_0_0_0
1182.5515 PEP_0_2_0_0_0
1344.6128 PEP_1_2_0_0_0
1506.6671 PEP_2_2_0_0_0
1871.8003 PEP_3_3_0_0_0

File: 231129_OE_RK_21, RT: 8.665, Scan: 006611
Type: HCD, FTMS + c NSI d Full ms2 1481.2345@hcd30.00 [120.0000-4554.7197]
Precursor Scan: 006534, m/z: 1480.5673, charge: 3
ENGTISR_7_6_1_4_0 LeX peak identified

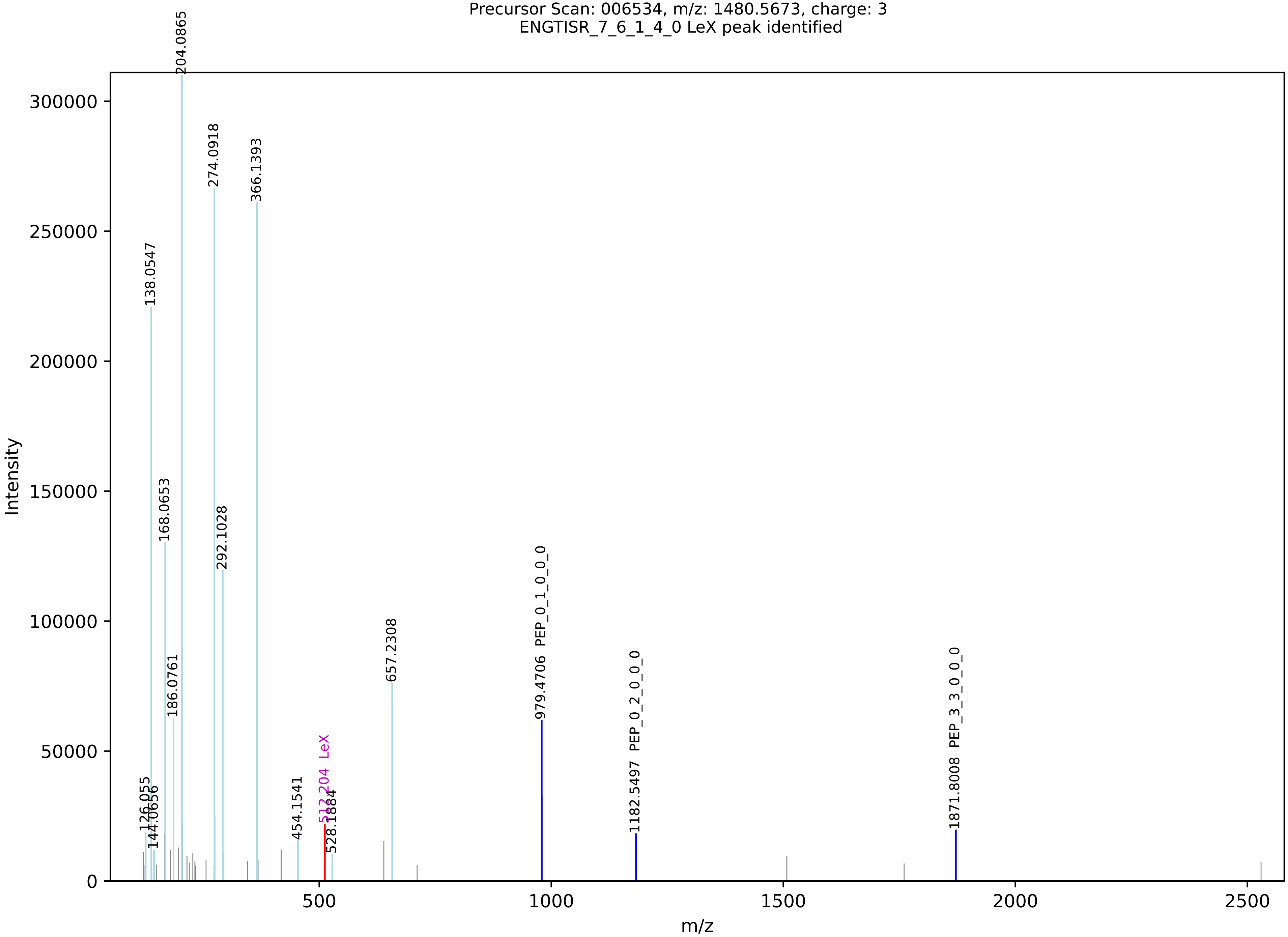

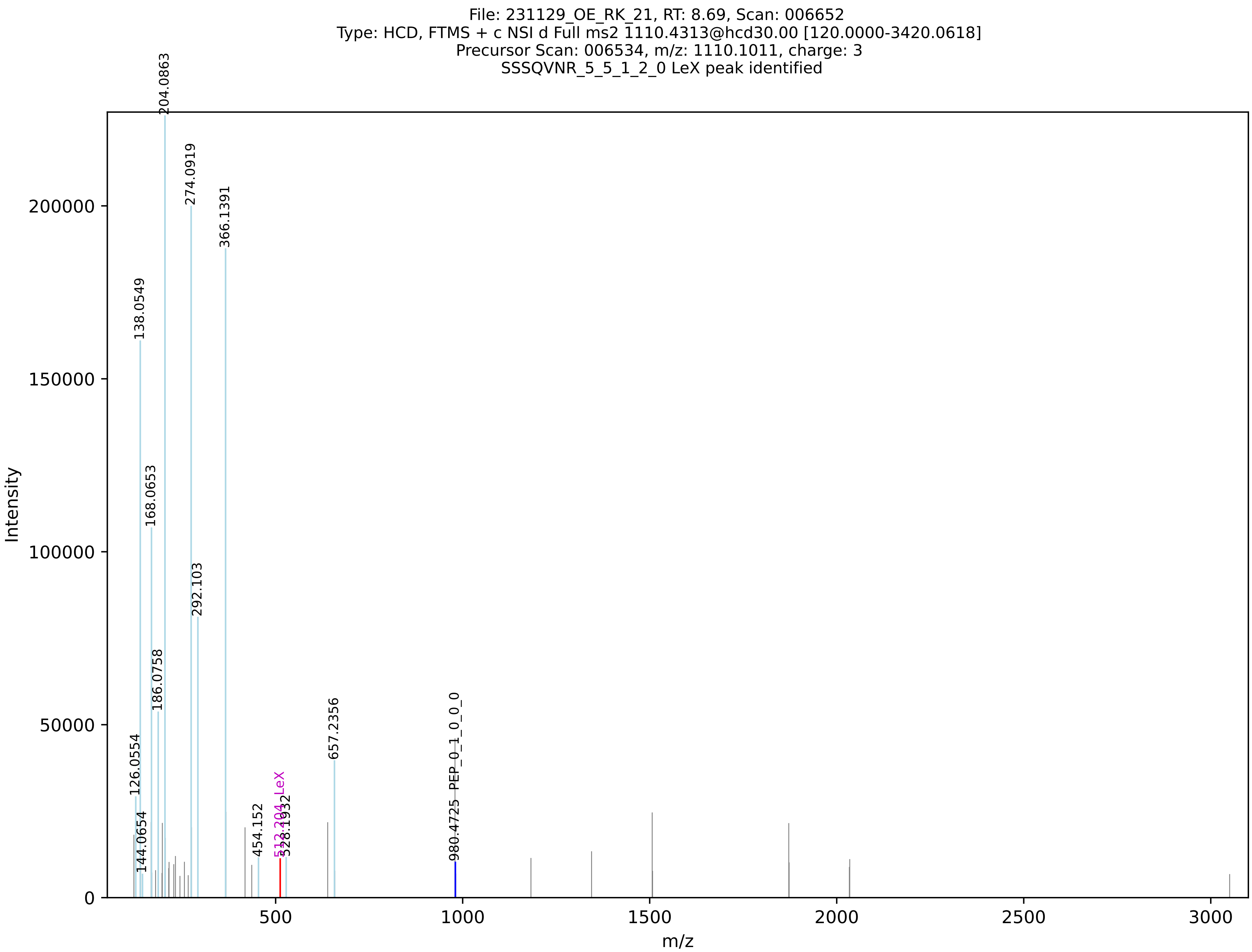
File: 231129_OE_RK_21, RT: 8.69, Scan: 006652
Type: HCD, FTMS + c NSI d Full ms2 1110.4313@hcd30.00 [120.0000-3420.0618]
Precursor Scan: 006534, m/z: 1110.1011, charge: 3
SSSQVNR_5_5_1_2_0 LeX peak identified
Intensity
m/z
204.0863
274.0919
366.1391
138.0549
168.0653
292.103
186.0758
126.0554
144.0654
454.152
512.204_LeX
528.1932
657.2356
980.4725_PEP_0_1_0_0_0
0
50000
100000
150000
200000
500
1000
1500
2000
2500
3000

File: 231129_OE_RK_21, RT: 8.714, Scan: 006684
Type: HCD, FTMS + c NSI d Full ms2 1074.6649@hcd30.00 [120.0000-4407.4893]
Precursor Scan: 006608, m/z: 1074.1642, charge: 4
ENGTISR_7_6_2_3_0 LeX peak identified

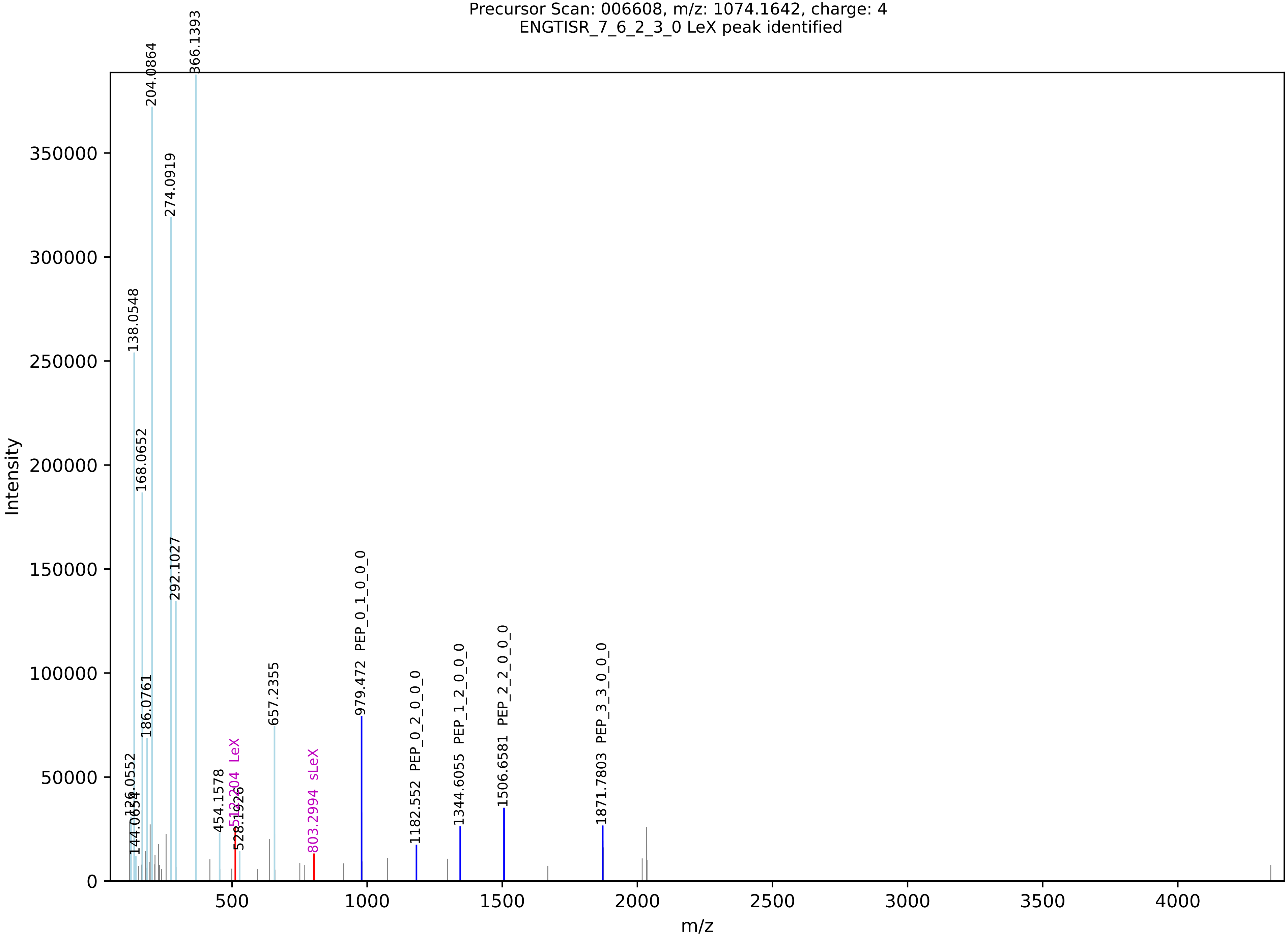

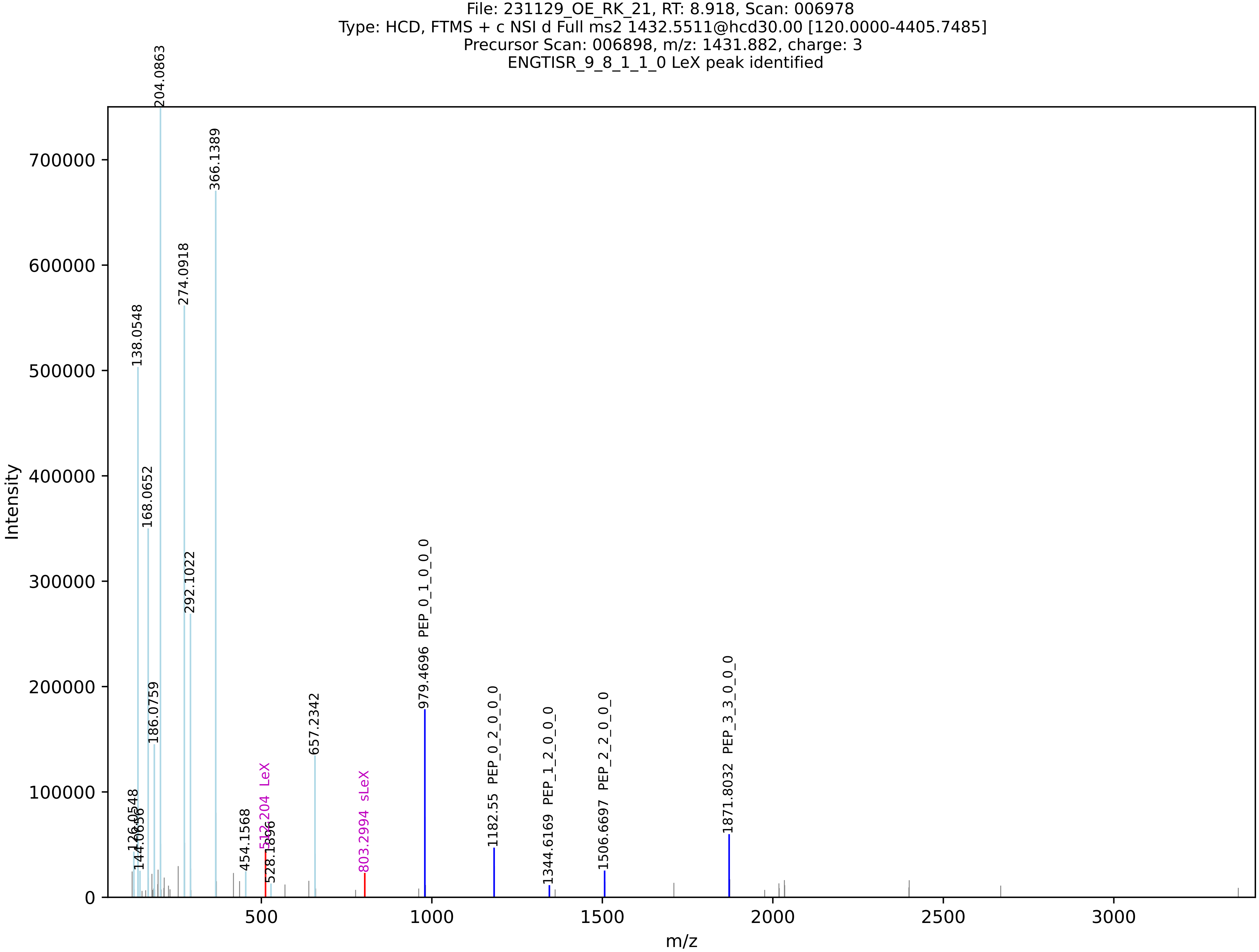
File: 231129_OE_RK_21, RT: 8.918, Scan: 006978
Type: HCD, FTMS + c NSI d Full ms2 1432.5511@hcd30.00 [120.0000-4405.7485]
Precursor Scan: 006898, m/z: 1431.882, charge: 3
ENGTISR_9_8_1_1_0 LeX peak identified
Intensity
m/z
204.0863
366.1389
274.0918
138.0548
168.0652
292.1022
186.0759
126.0548
144.0656
454.1568
512.204 LeX
528.1896
657.2342
803.2994 sLeX
979.4696 PEP_0_1_0_0_0
1182.55 PEP_0_2_0_0_0
1344.6169 PEP_1_2_0_0_0
1506.6697 PEP_2_2_0_0_0
1871.8032 PEP_3_3_0_0_0

File: 231129_OE_RK_21, RT: 9.066, Scan: 007194
Type: HCD, FTMS + c NSI d Full ms2 1111.1760@hcd30.00 [120.0000-4556.4541]
Precursor Scan: 007118, m/z: 1110.6753, charge: 4
ENGTISR_7_6_1_4_0 LeX peak identified

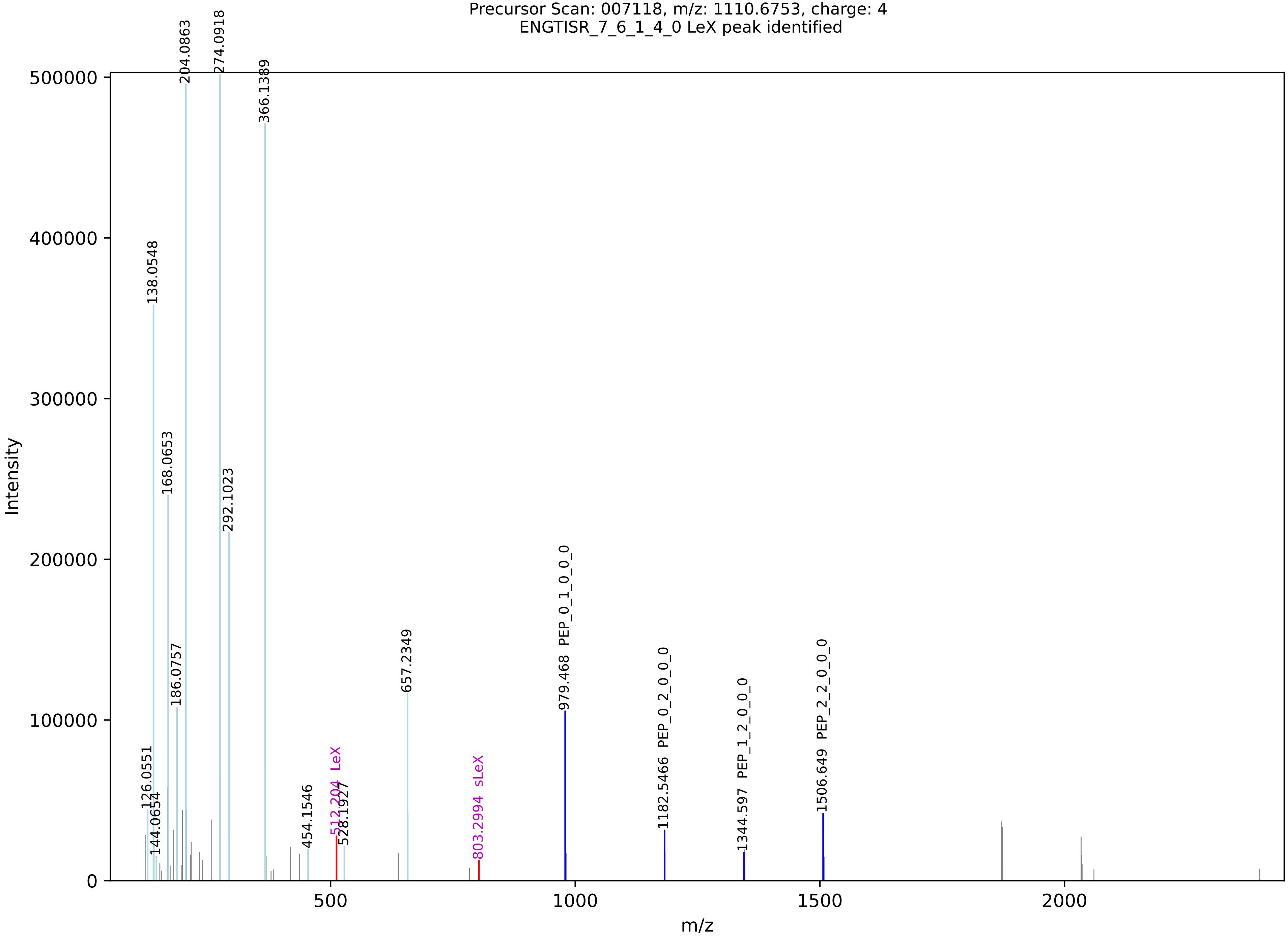

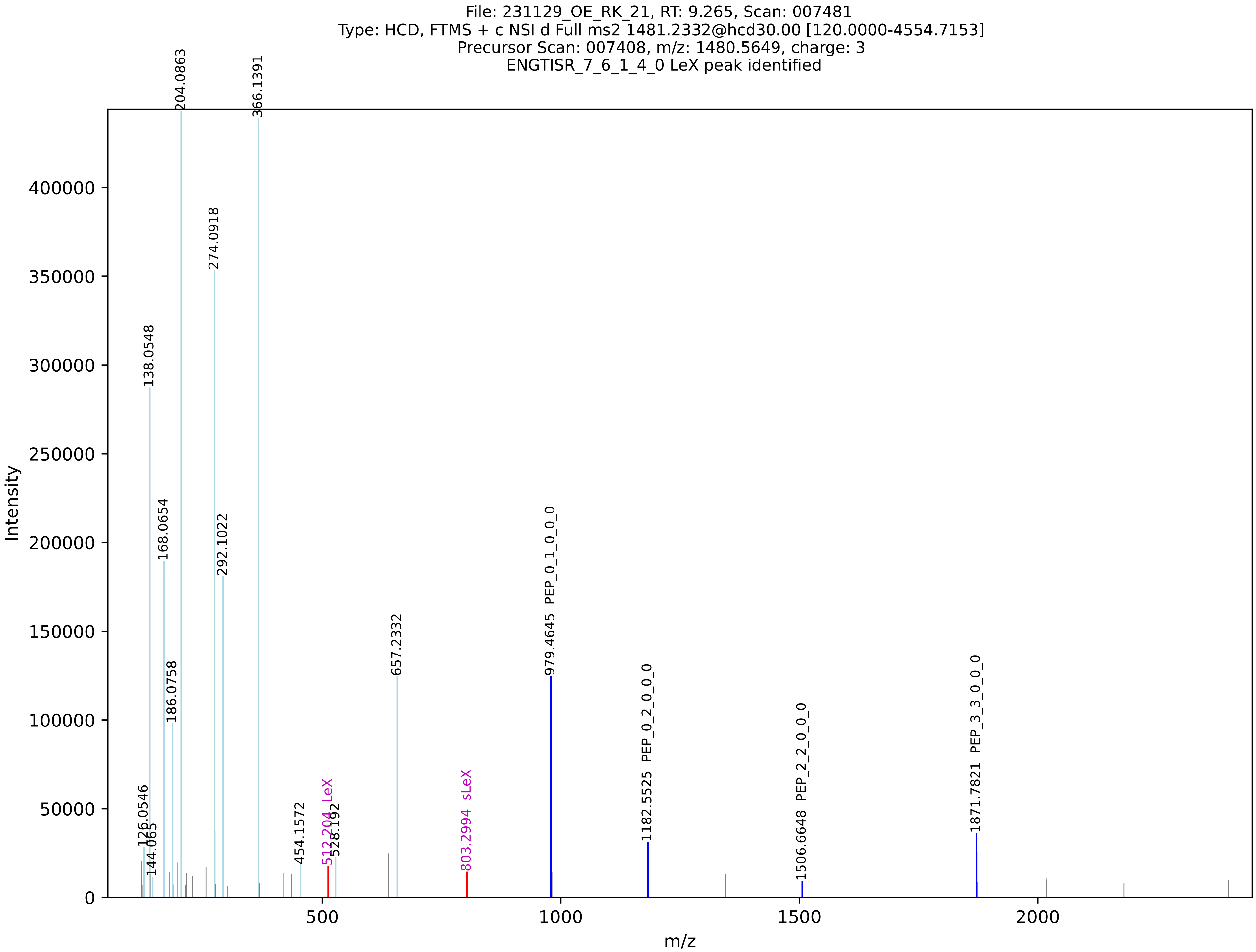
File: 231129_OE_RK_21, RT: 9.265, Scan: 007481
Type: HCD, FTMS + c NSI d Full ms2 1481.2332@hcd30.00 [120.0000-4554.7153]
Precursor Scan: 007408, m/z: 1480.5649, charge: 3
ENGTISR_7_6_1_4_0 LeX peak identified
204.0863
366.1391
274.0918
138.0548
168.0654
292.1022
186.0758
657.2332
979.4645 PEP_0_1_0_0_0
1182.5525 PEP_0_2_0_0_0
1506.6648 PEP_2_2_0_0_0
1871.7821 PEP_3_3_0_0_0
126.0546
144.065
454.1572
512.204 LeX
528.192
803.2994 sLeX
Intensity
m/z
0
50000
100000
150000
200000
250000
300000
350000
400000
500
1000
1500
2000

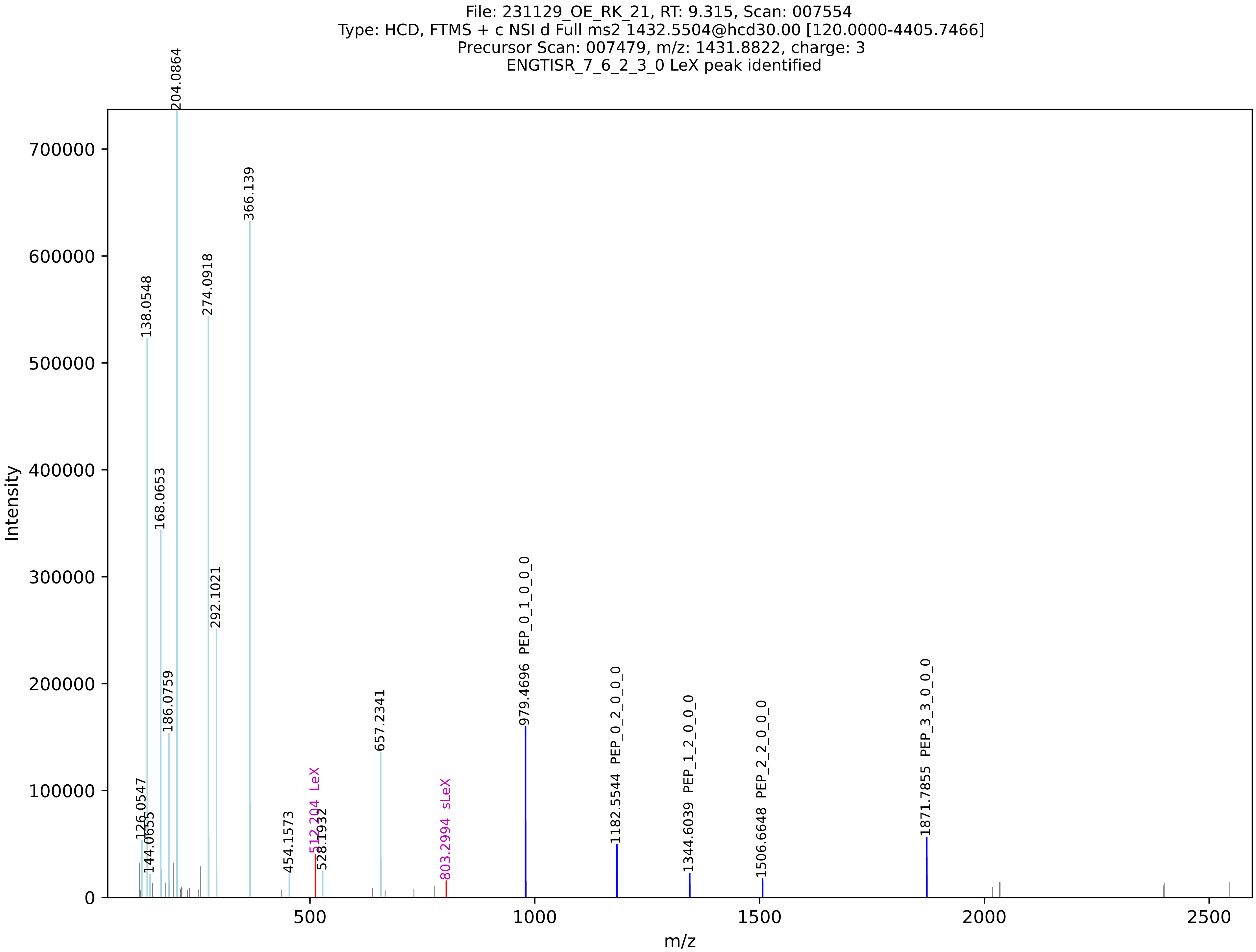

File: 231129_OE_RK_21, RT: 9.315, Scan: 007554
Type: HCD, FTMS + c NSI d Full ms2 1432.5504@hcd30.00 [120.0000-4405.7466]
Precursor Scan: 007479, m/z: 1431.8822, charge: 3
ENGTISR_7_6_2_3_0 LeX peak identified
Intensity
m/z
204.0864
366.139
274.0918
138.0548
168.0653
292.1021
186.0759
126.0547
144.0655
454.1573
512.204 LeX
528.1932
657.2341
803.2994 sLeX
979.4696 PEP_0_1_0_0_0
1182.5544 PEP_0_2_0_0_0
1344.6039 PEP_1_2_0_0_0
1506.6648 PEP_2_2_0_0_0
1871.7855 PEP_3_3_0_0_0

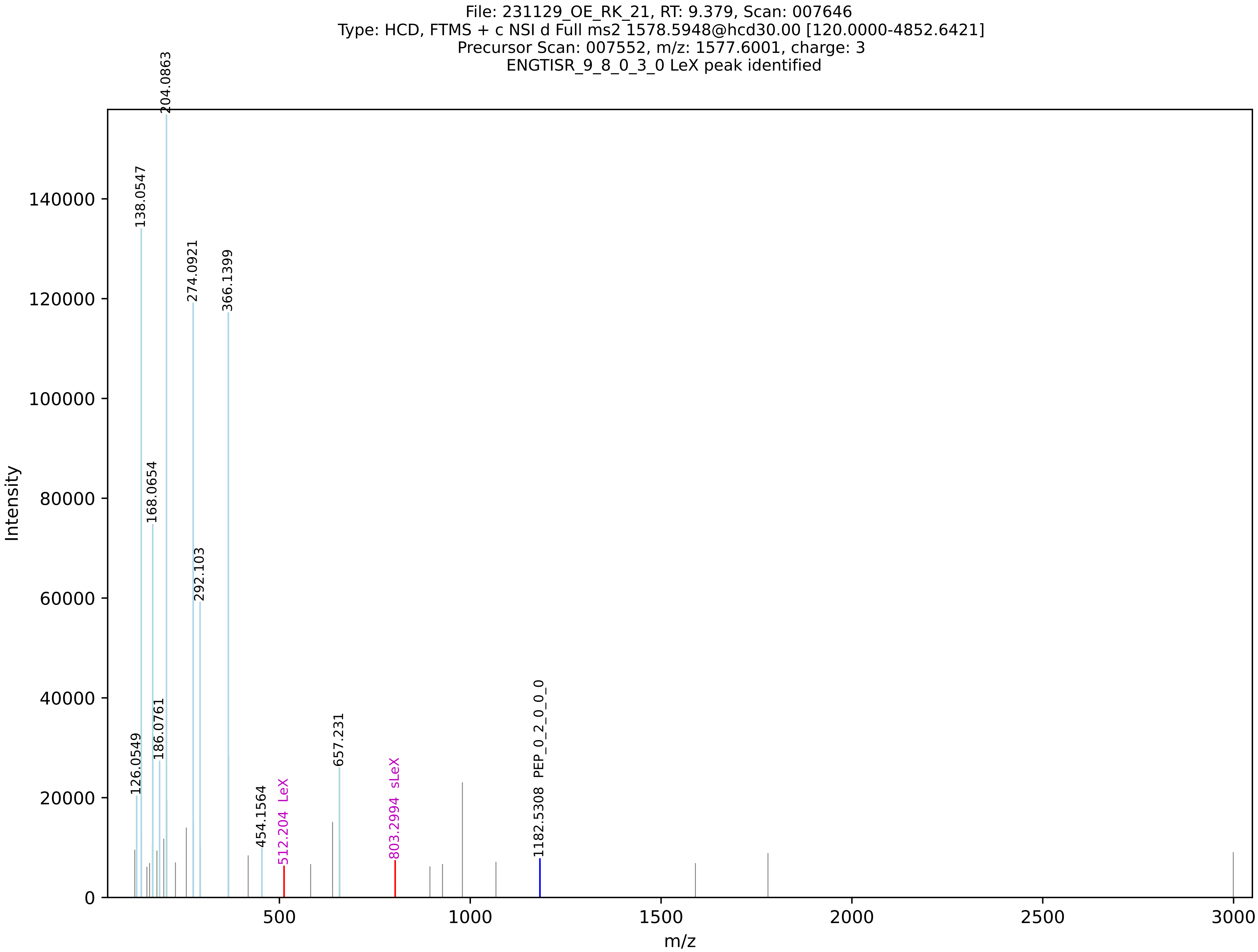
File: 231129_OE_RK_21, RT: 9.379, Scan: 007646
Type: HCD, FTMS + c NSI d Full ms2 1578.5948@hcd30.00 [120.0000-4852.6421]
Precursor Scan: 007552, m/z: 1577.6001, charge: 3
ENGTISR_9_8_0_3_0 LeX peak identified
Intensity
m/z
204.0863
138.0547
274.0921
366.1399
168.0654
292.103
186.0761
126.0549
454.1564
512.204 LeX
657.231
803.2994 sLeX
1182.5308 PEP_0_2_0_0_0
0
20000
40000
60000
80000
100000
120000
140000
500
1000
1500
2000
2500
3000

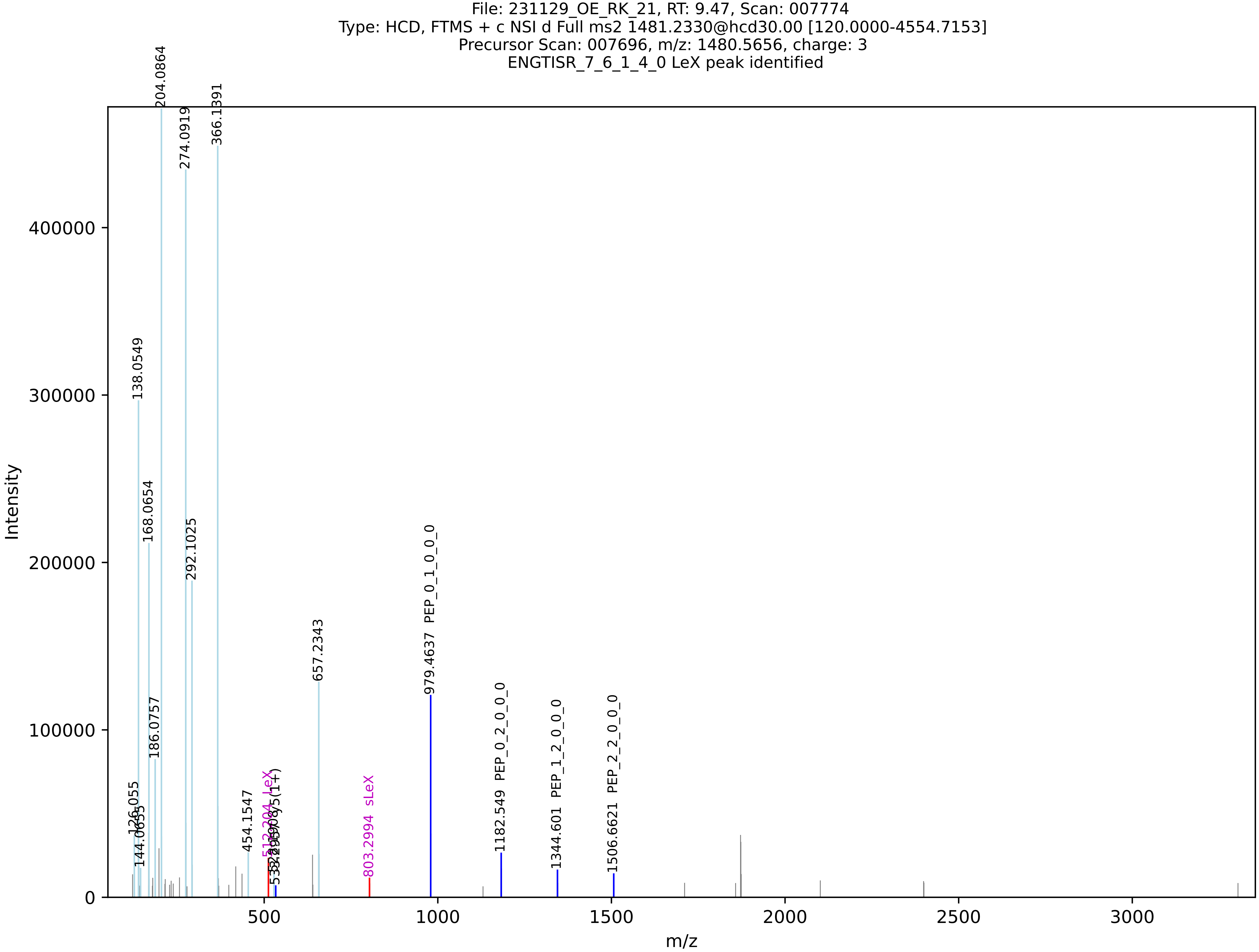
File: 231129_OE_RK_21, RT: 9.47, Scan: 007774
Type: HCD, FTMS + c NSI d Full ms2 1481.2330@hcd30.00 [120.0000-4554.7153]
Precursor Scan: 007696, m/z: 1480.5656, charge: 3
ENGTISR_7_6_1_4_0 LeX peak identified
204.0864
366.1391
274.0919
138.0549
168.0654
292.1025
186.0757
144.0655
454.1547
657.2343
979.4637 PEP_0_1_0_0_0
803.2994 sLeX
1182.549 PEP_0_2_0_0_0
1344.601 PEP_1_2_0_0_0
1506.6621 PEP_2_2_0_0_0
Intensity
m/z
0
100000
200000
300000
400000
500
1000
1500
2000
2500
3000

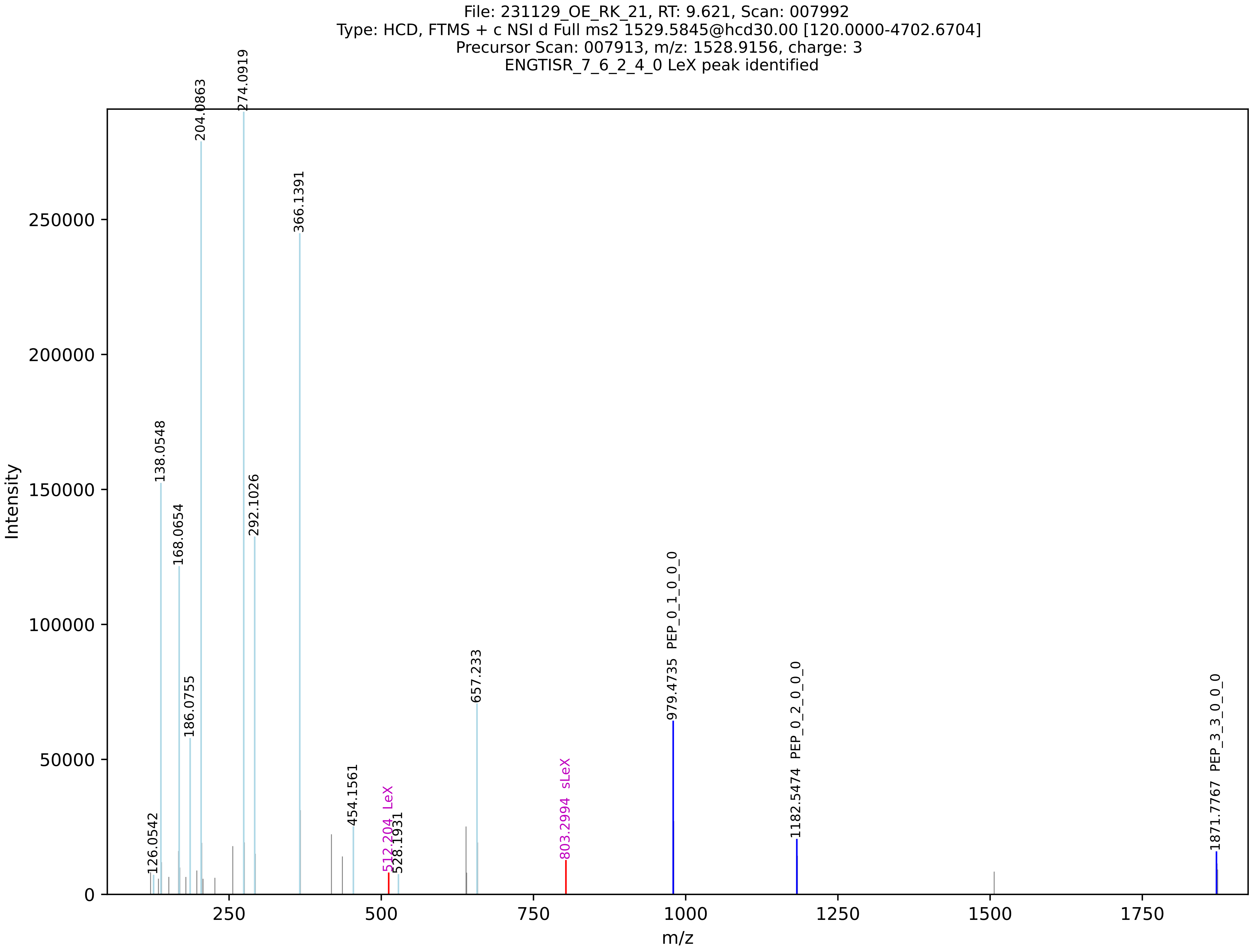

File: 231129_OE_RK_21, RT: 9.621, Scan: 007992
Type: HCD, FTMS + c NSI d Full ms2 1529.5845@hcd30.00 [120.0000-4702.6704]
Precursor Scan: 007913, m/z: 1528.9156, charge: 3
ENGTISR_7_6_2_4_0 LeX peak identified
Intensity
m/z
126.0542
138.0548
168.0654
186.0755
204.0863
274.0919
292.1026
366.1391
454.1561
512.204 LeX
528.1931
657.233
803.2994 sLeX
979.4735 PEP_0_1_0_0_0
1182.5474 PEP_0_2_0_0_0
1871.7767 PEP_3_3_0_0_0

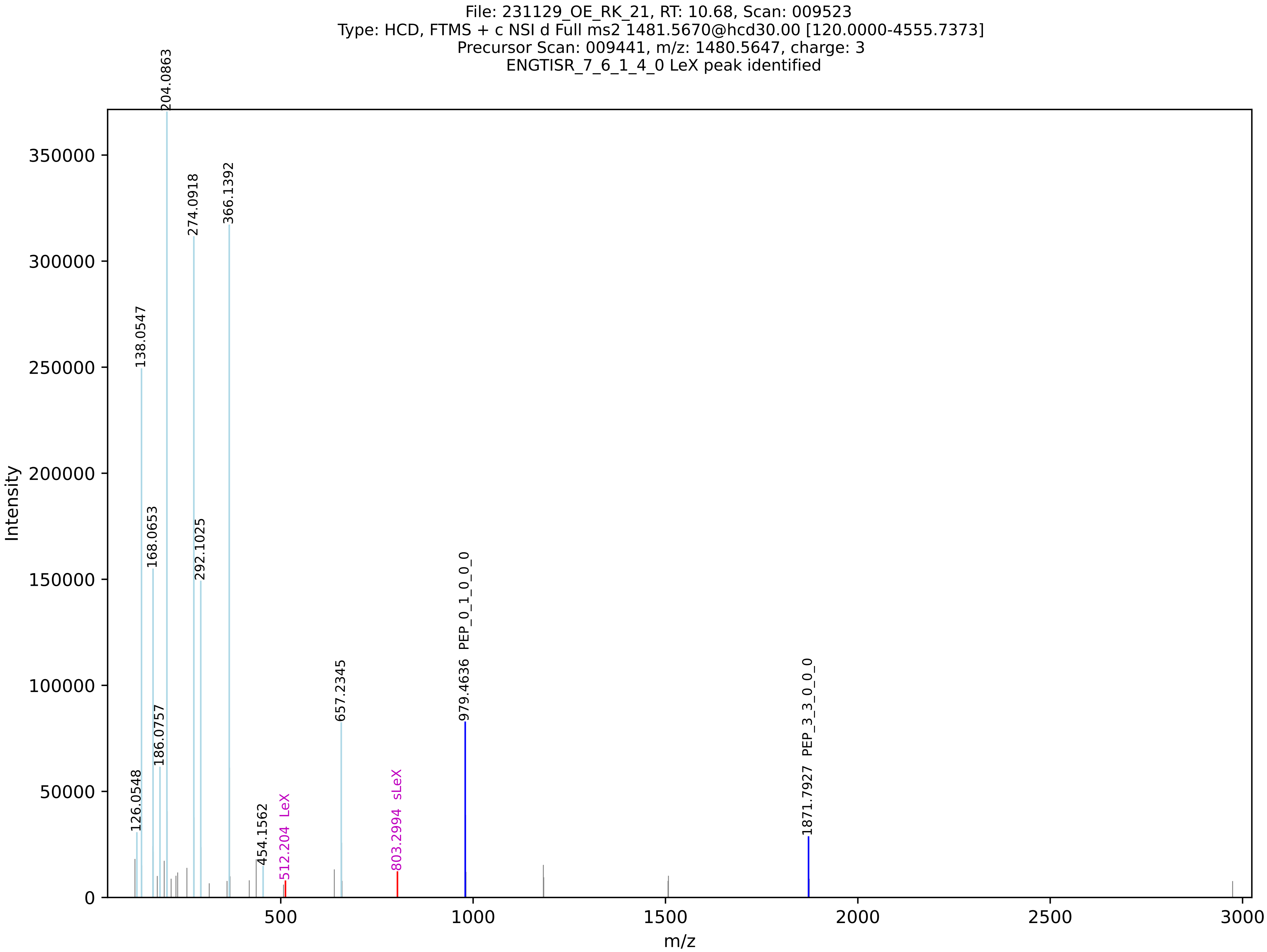
File: 231129_OE_RK_21, RT: 10.68, Scan: 009523
Type: HCD, FTMS + c NSI d Full ms2 1481.5670@hcd30.00 [120.0000-4555.7373]
Precursor Scan: 009441, m/z: 1480.5647, charge: 3
ENGTISR_7_6_1_4_0 LeX peak identified
Intensity
m/z
204.0863
274.0918
366.1392
138.0547
168.0653
292.1025
186.0757
126.0548
454.1562
512.204 LeX
657.2345
803.2994 sLeX
979.4636 PEP_0_1_0_0_0_
1871.7927 PEP_3_3_0_0_0_
350000
300000
250000
200000
150000
100000
50000
0
500
1000
1500
2000
2500
3000

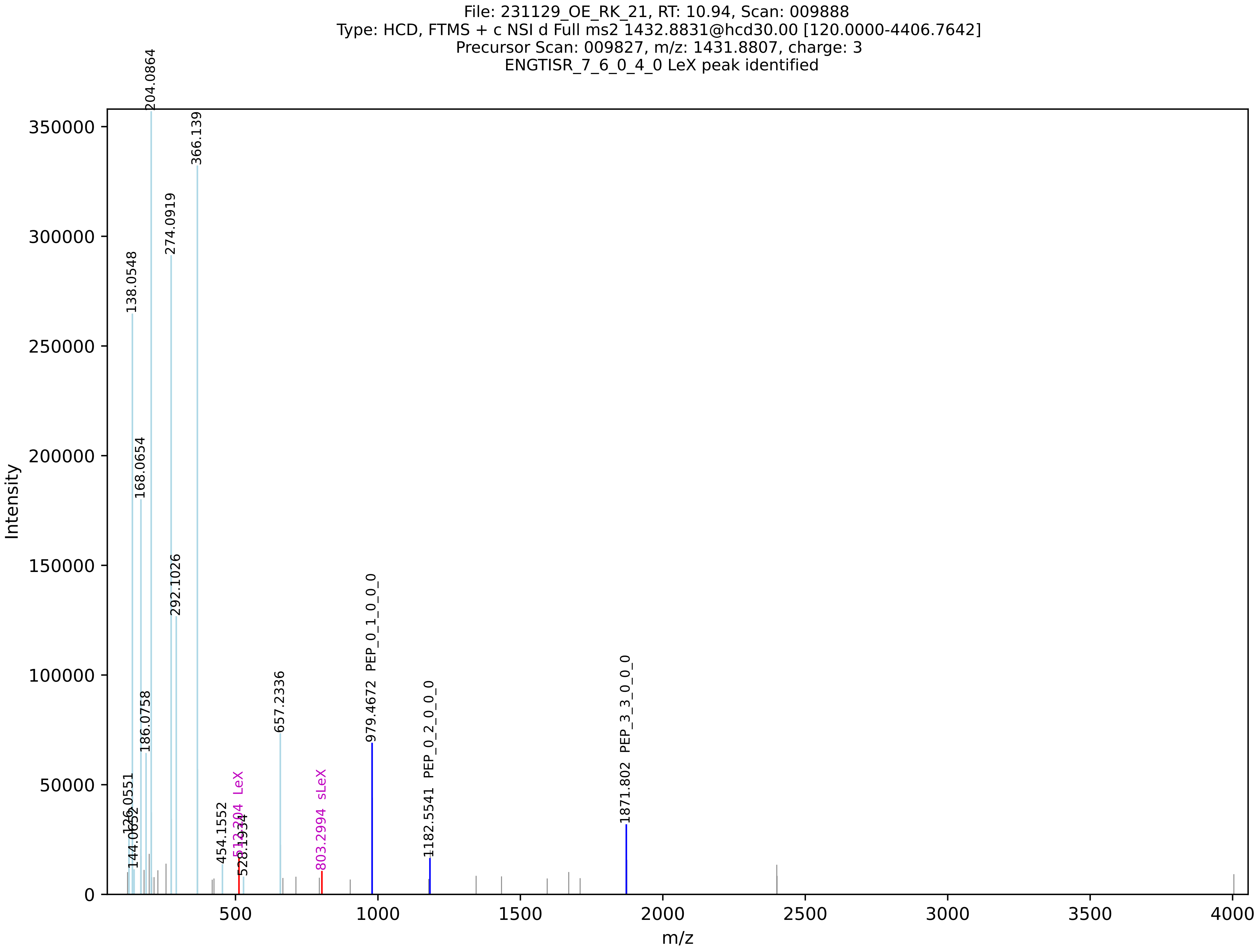
File: 231129_OE_RK_21, RT: 10.94, Scan: 009888
Type: HCD, FTMS + c NSI d Full ms2 1432.8831@hcd30.00 [120.0000-4406.7642]
Precursor Scan: 009827, m/z: 1431.8807, charge: 3
ENGTISR_7_6_0_4_0 LeX peak identified
Intensity
m/z
204.0864
366.139
274.0919
138.0548
168.0654
292.1026
186.0758
657.2336
979.4672 PEP_0_1_0_0_0_
1182.5541 PEP_0_2_0_0_0
1871.802 PEP_3_3_0_0_0
126.0551
144.0652
454.1552
512.204 LeX
528.1934
803.2994 sLeX
0
50000
100000
150000
200000
250000
300000
350000
500
1000
1500
2000
2500
3000
3500
4000

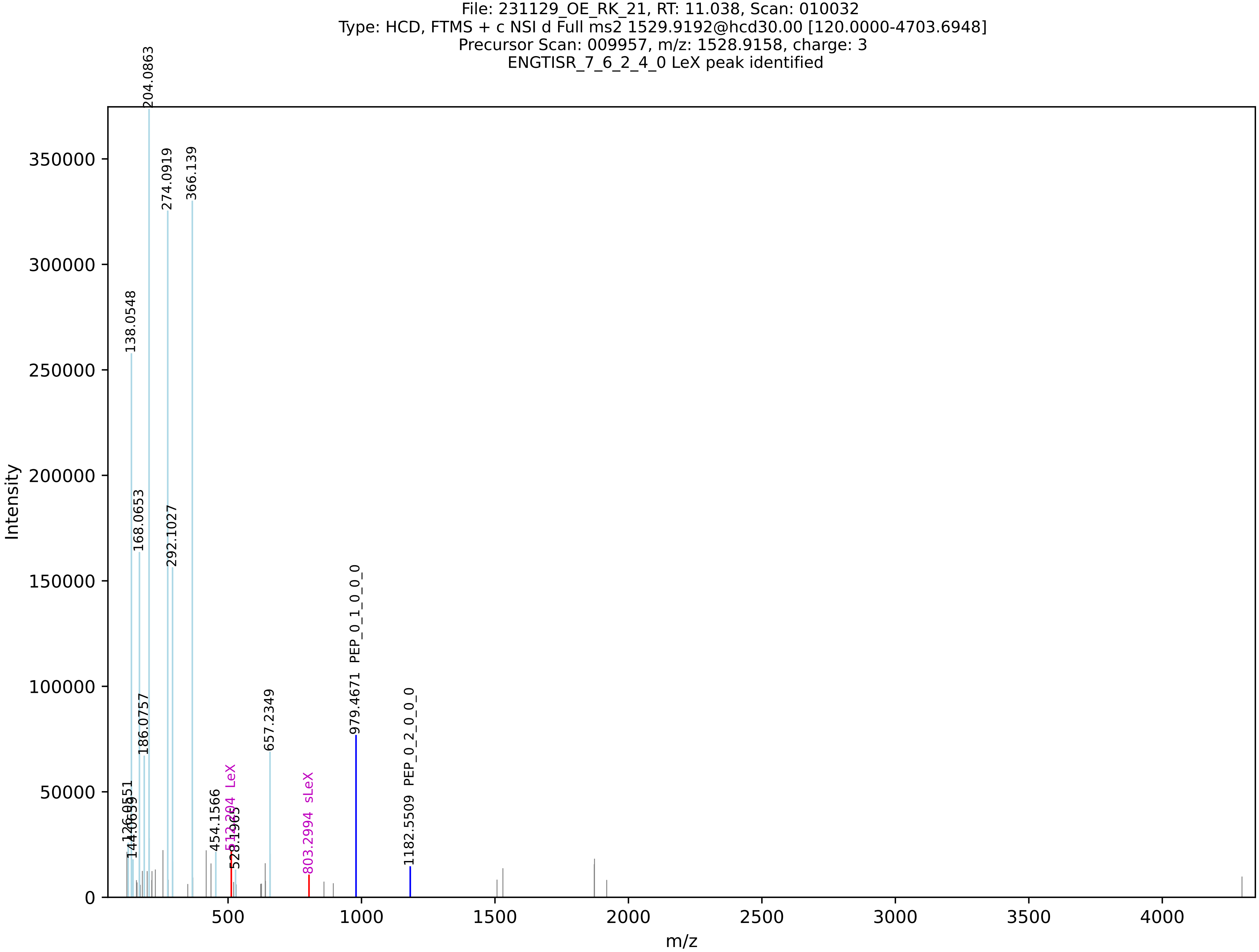

File: 231129_OE_RK_21, RT: 11.038, Scan: 010032
Type: HCD, FTMS + c NSI d Full ms2 1529.9192@hcd30.00 [120.0000-4703.6948]
Precursor Scan: 009957, m/z: 1528.9158, charge: 3
ENGTISR_7_6_2_4_0 LeX peak identified
Intensity
m/z
204.0863
274.0919
366.139
138.0548
168.0653
292.1027
186.0757
657.2349
979.4671 PEP_0_1_0_0_0
1182.5509 PEP_0_2_0_0_0
454.1566
528.1965
803.2994 sLeX
LeX

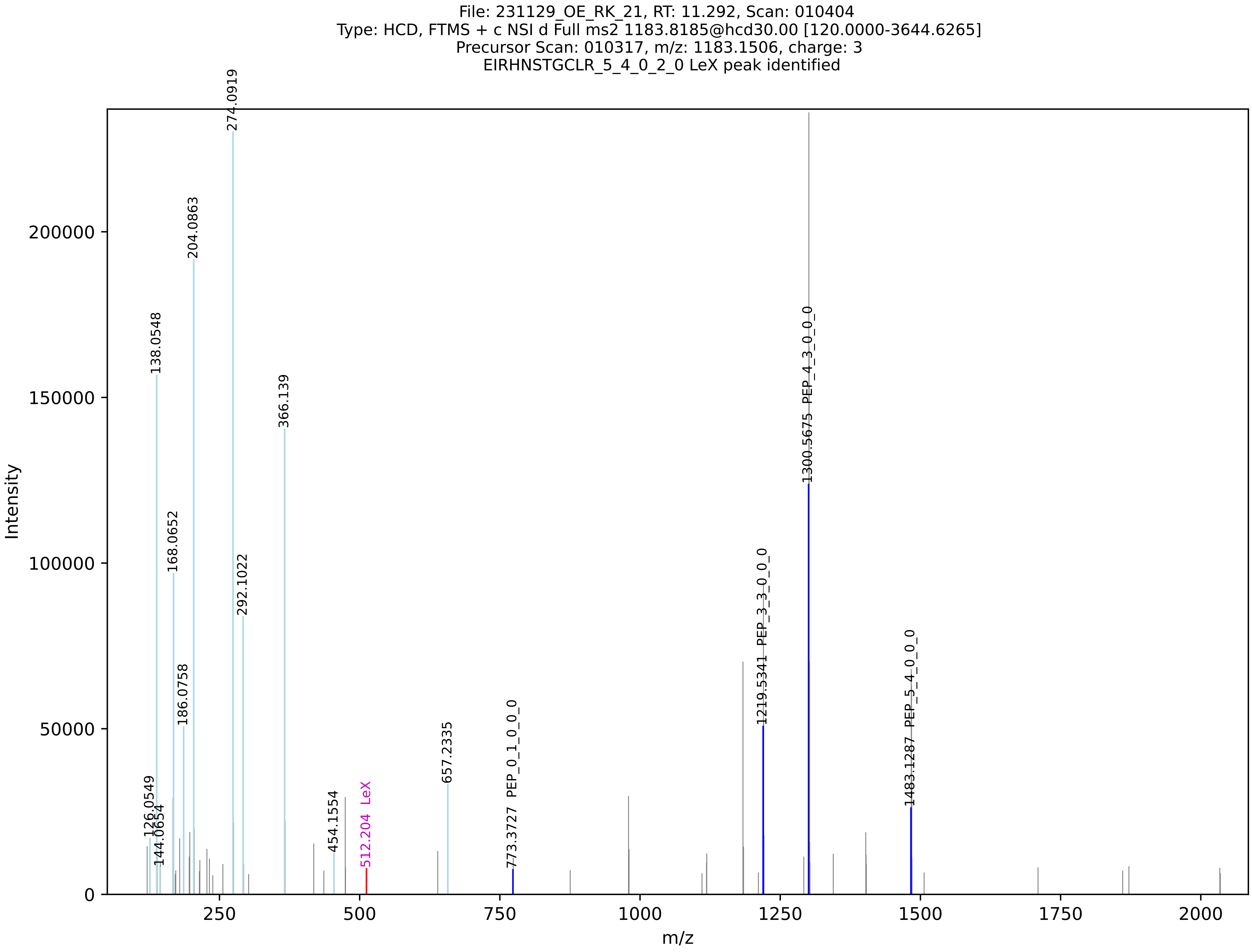
File: 231129_OE_RK_21, RT: 11.292, Scan: 010404
Type: HCD, FTMS + c NSI d Full ms2 1183.8185@hcd30.00 [120.0000-3644.6265]
Precursor Scan: 010317, m/z: 1183.1506, charge: 3
EIRHNSTGCLR_5_4_0_2_0 LeX peak identified
Intensity
m/z
274.0919
204.0863
138.0548
366.139
168.0652
292.1022
186.0758
126.0549
144.0654
454.1554
512.204 LeX
657.2335
773.3727 PEP_0_1_0_0_0_
1219.5341 PEP_3_3_0_0_0
1300.5675 PEP_4_3_0_0_0
1483.1287 PEP_5_4_0_0_0
0
50000
100000
150000
200000
250
500
750
1000
1250
1500
1750
2000

File: 231129_OE_RK_21, RT: 11.493, Scan: 010698
Type: HCD, FTMS + c NSI d Full ms2 1529.5854@hcd30.00 [120.0000-4702.6733]
Precursor Scan: 010613, m/z: 1528.9172, charge: 3
ENGTISR_7_6_2_4_0 LeX peak identified

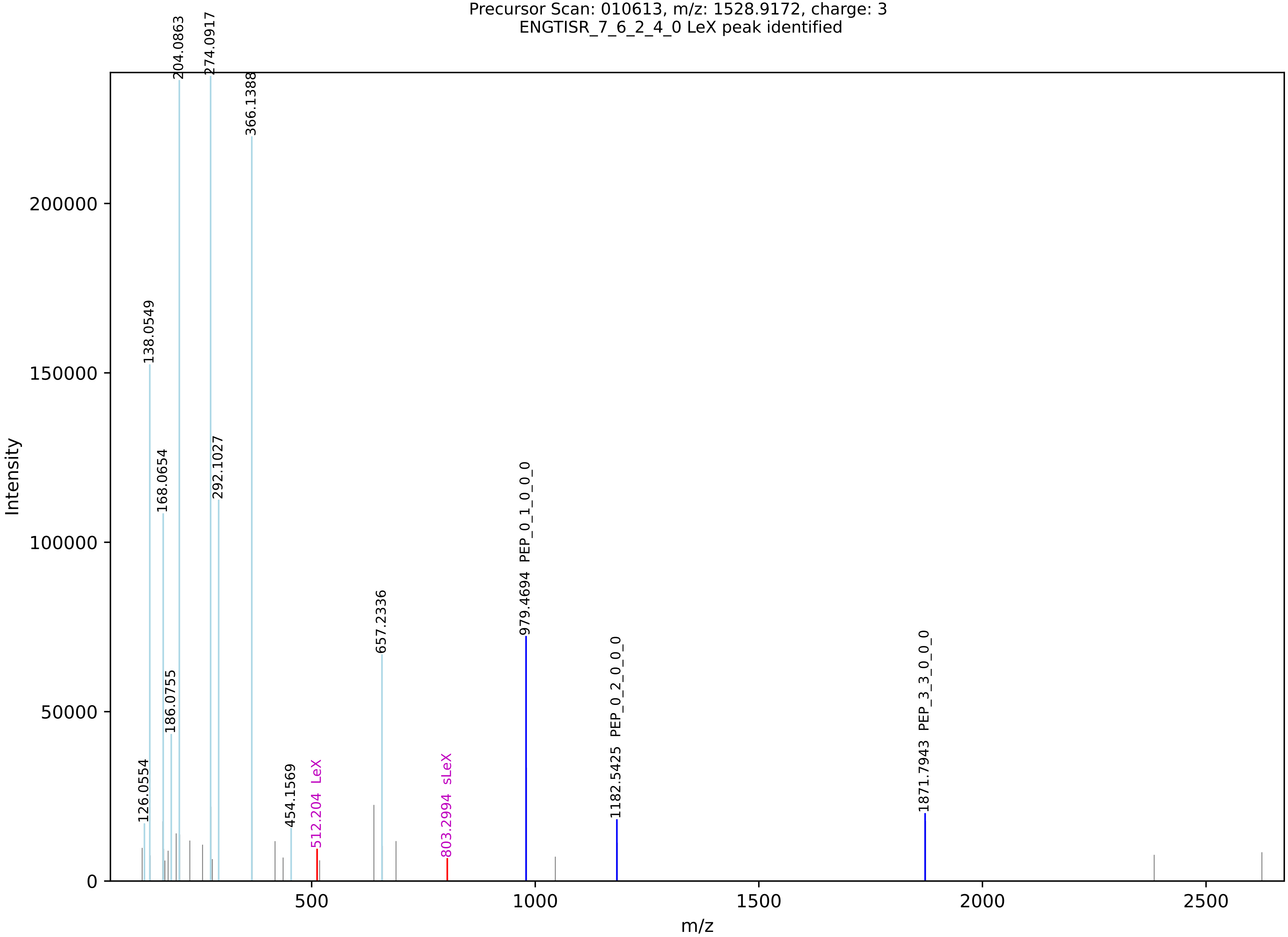

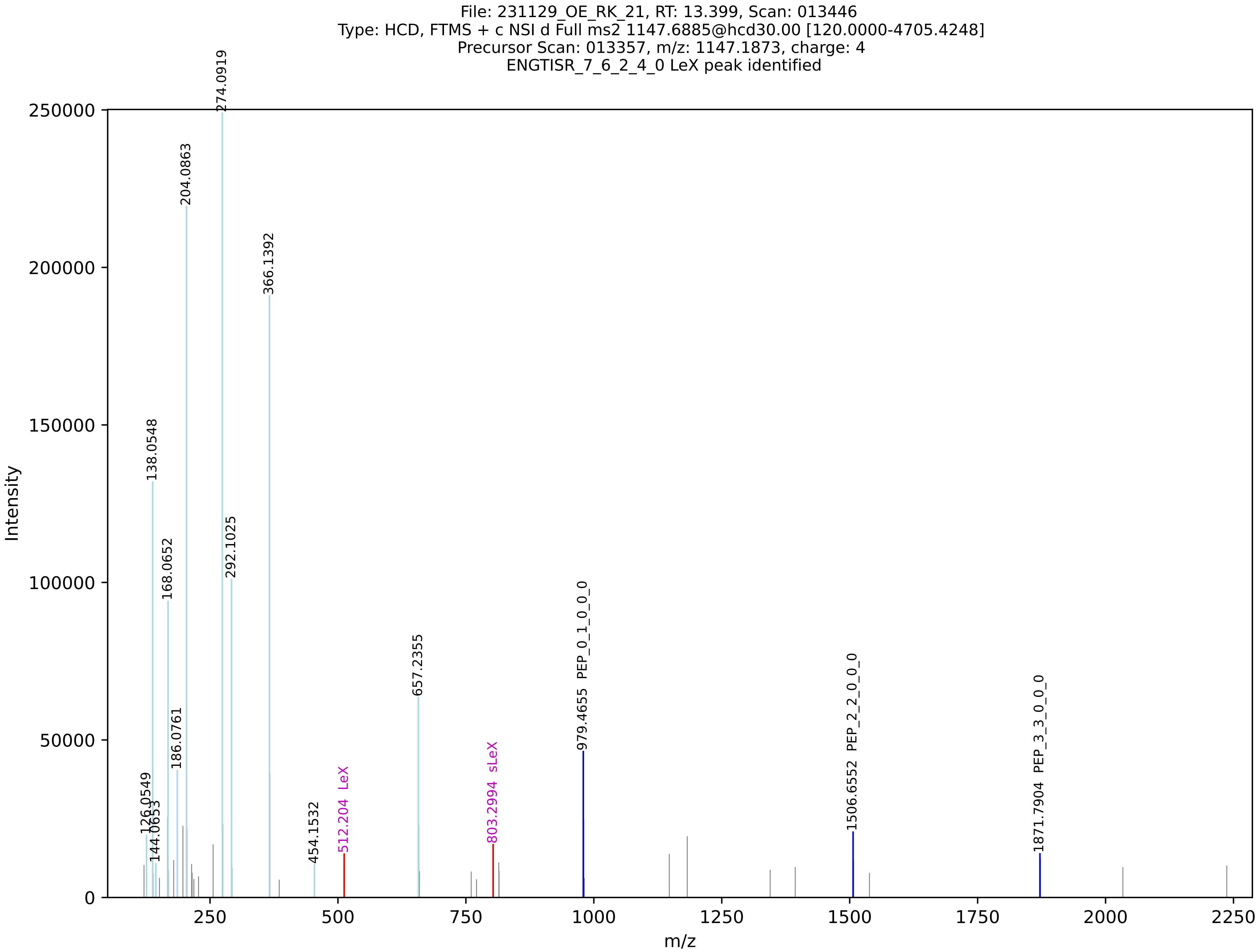
File: 231129_OE_RK_21, RT: 13.399, Scan: 013446
Type: HCD, FTMS + c NSI d Full ms2 1147.6885@hcd30.00 [120.0000-4705.4248]
Precursor Scan: 013357, m/z: 1147.1873, charge: 4
ENGTISR_7_6_2_4_0 LeX peak identified
274.0919
204.0863
366.1392
138.0548
292.1025
168.0652
186.0761
126.0549
144.0653
454.1532
512.204 LeX
657.2355
803.2994 sLeX
979.4655 PEP_0_1_0_0_
1506.6552 PEP_2_2_0_0_0
1871.7904 PEP_3_3_0_0_0
Intensity
m/z
0
50000
100000
150000
200000
250000
250
500
750
1000
1250
1500
1750
2000
2250

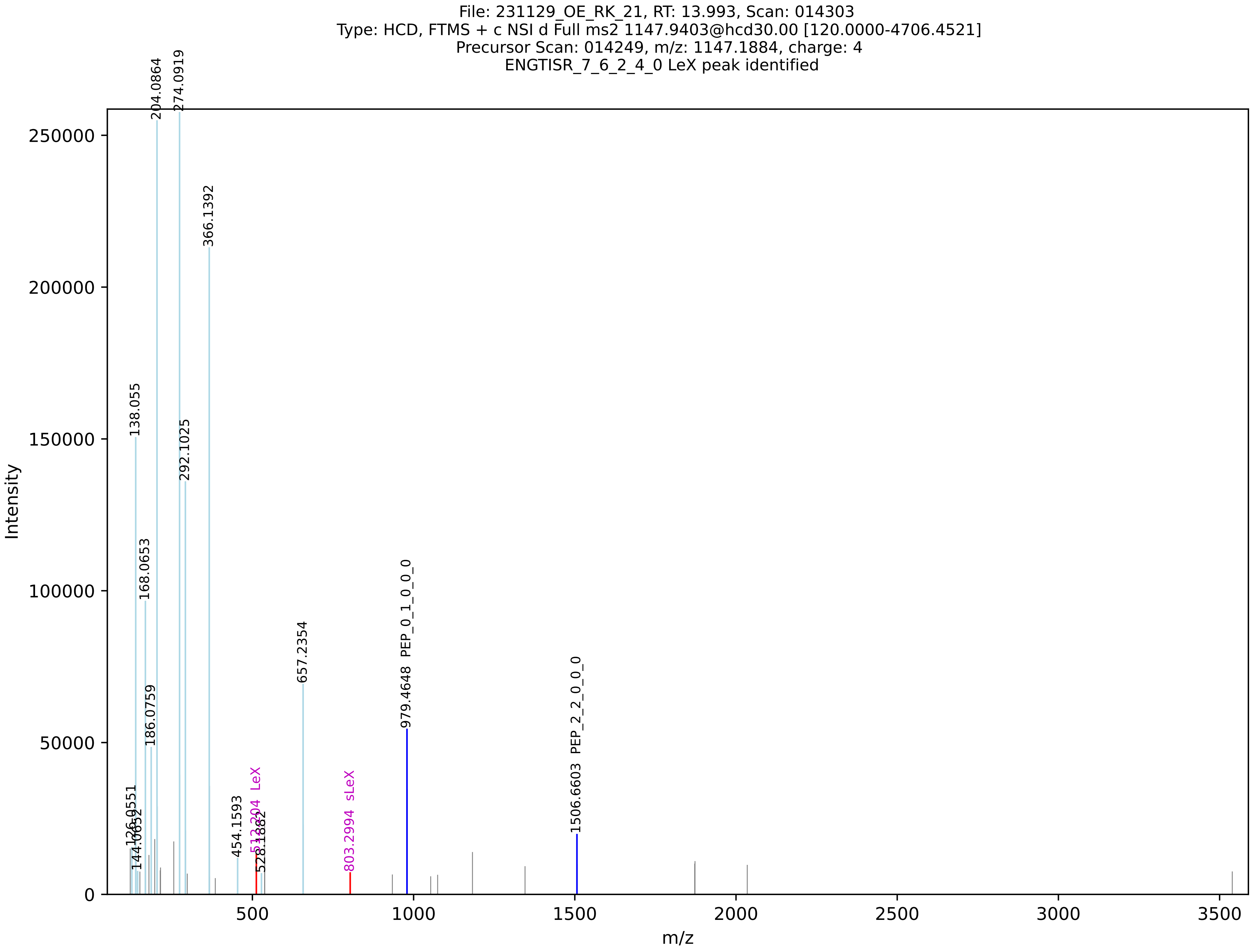

File: 231129_OE_RK_21, RT: 13.993, Scan: 014303
Type: HCD, FTMS + c NSI d Full ms2 1147.9403@hcd30.00 [120.0000-4706.4521]
Precursor Scan: 014249, m/z: 1147.1884, charge: 4
ENGTISR_7_6_2_4_0 LeX peak identified
Intensity
m/z
204.0864
274.0919
366.1392
138.055
292.1025
168.0653
186.0759
126.0551
144.0652
454.1593
512.204 LeX
528.1882
657.2354
803.2994 sLeX
979.4648 PEP_0_1_0_0_0
1506.6603 PEP_2_2_0_0_0

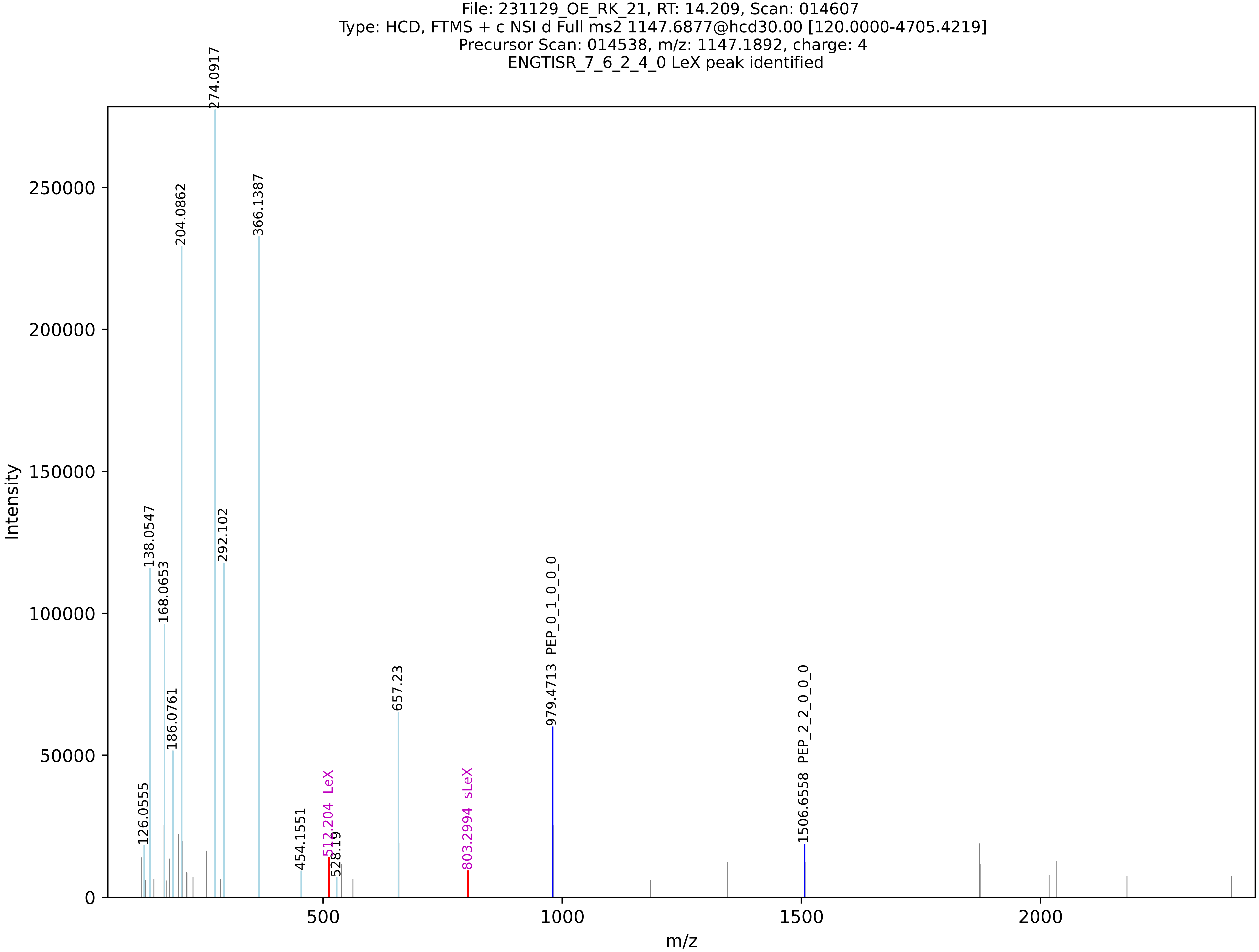

File: 231129_OE_RK_21, RT: 14.209, Scan: 014607
Type: HCD, FTMS + c NSI d Full ms2 1147.6877@hcd30.00 [120.0000-4705.4219]
Precursor Scan: 014538, m/z: 1147.1892, charge: 4
ENGTISR_7_6_2_4_0 LeX peak identified
Intensity
m/z
0
50000
100000
150000
200000
250000
500
1000
1500
2000
274.0917
366.1387
204.0862
138.0547
292.102
168.0653
186.0761
126.0555
454.1551
512.204 LeX
528.19
657.23
803.2994 sLeX
979.4713 PEP_0_1_0_0_0
1506.6558 PEP_2_2_0_0_0

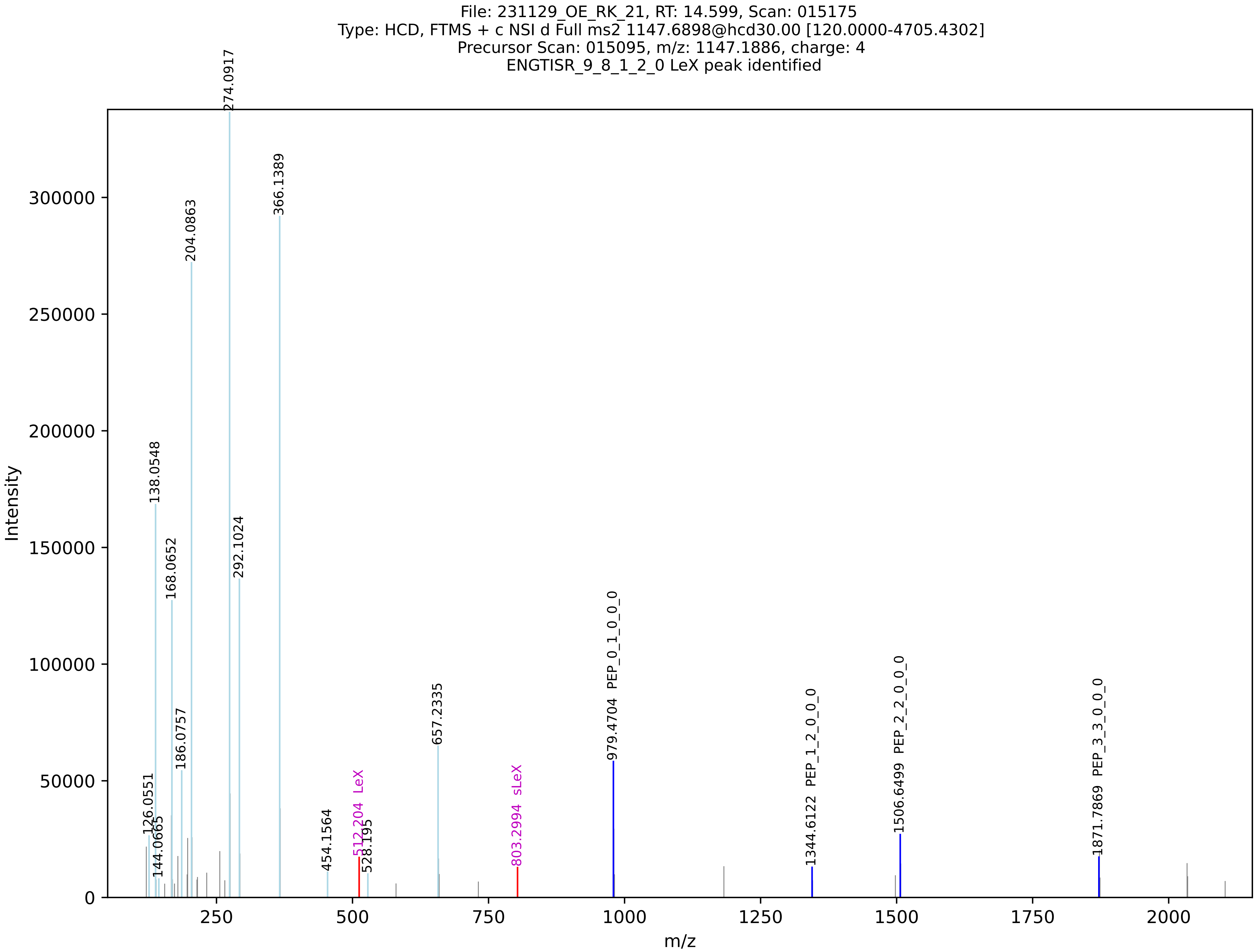
File: 231129_OE_RK_21, RT: 14.599, Scan: 015175
Type: HCD, FTMS + c NSI d Full ms2 1147.6898@hcd30.00 [120.0000-4705.4302]
Precursor Scan: 015095, m/z: 1147.1886, charge: 4
ENGTISR_9_8_1_2_0 LeX peak identified
Intensity
m/z
274.0917
366.1389
204.0863
138.0548
292.1024
168.0652
186.0757
126.0551
144.0665
454.1564
512.204 LeX
528.195
657.2335
803.2994 sLeX
979.4704 PEP_0_1_0_0_0
1344.6122 PEP_1_2_0_0_0
1506.6499 PEP_2_2_0_0_0
1871.7869 PEP_3_3_0_0_0

File: 231129_OE_RK_21, RT: 15.504, Scan: 016487
Type: HCD, FTMS + c NSI d Full ms2 1529.9192@hcd30.00 [120.0000-4703.6948]
Precursor Scan: 016414, m/z: 1529.2516, charge: 3
SSSQVNR_9_8_1_2_0 LeX peak identified

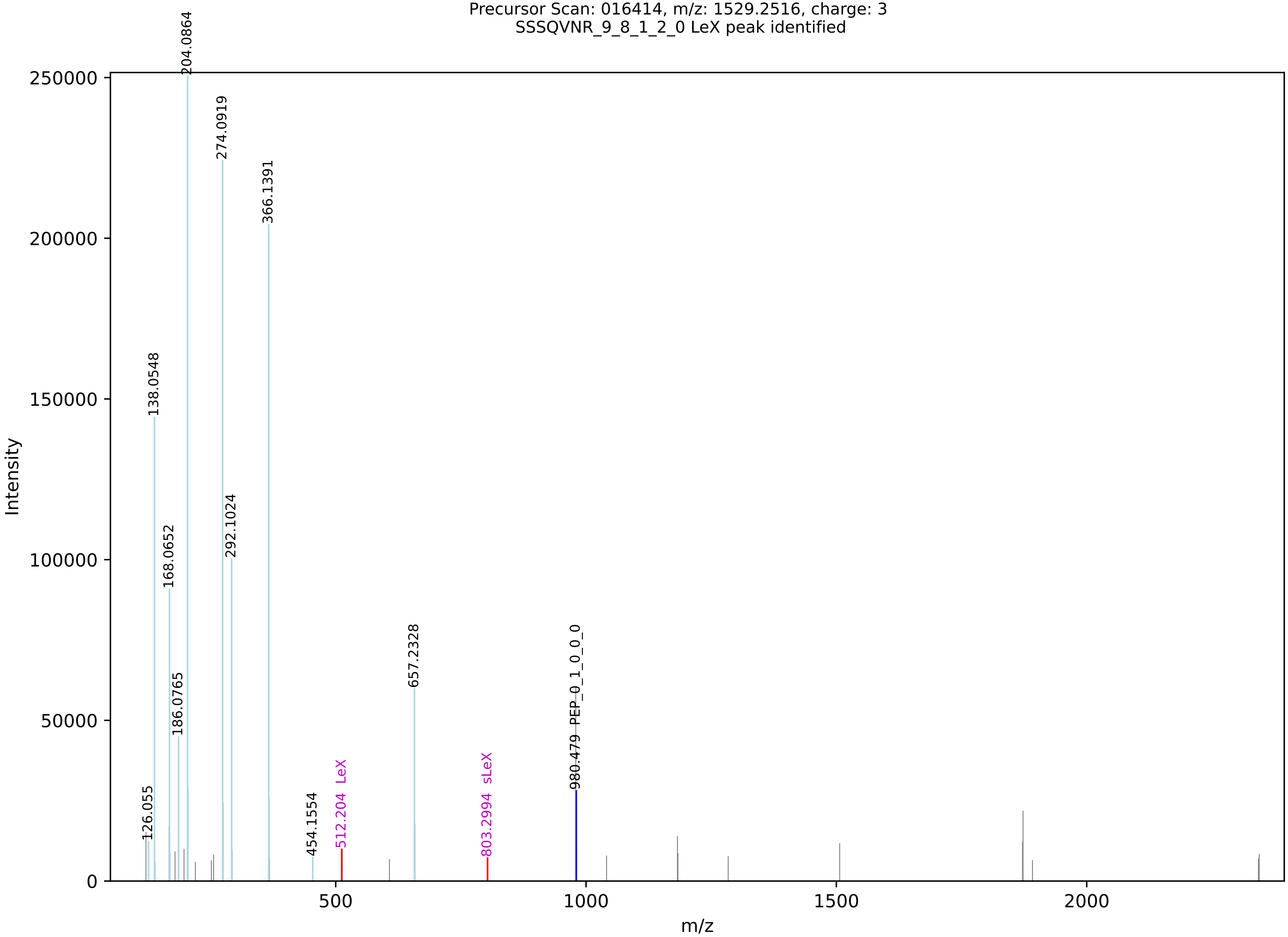

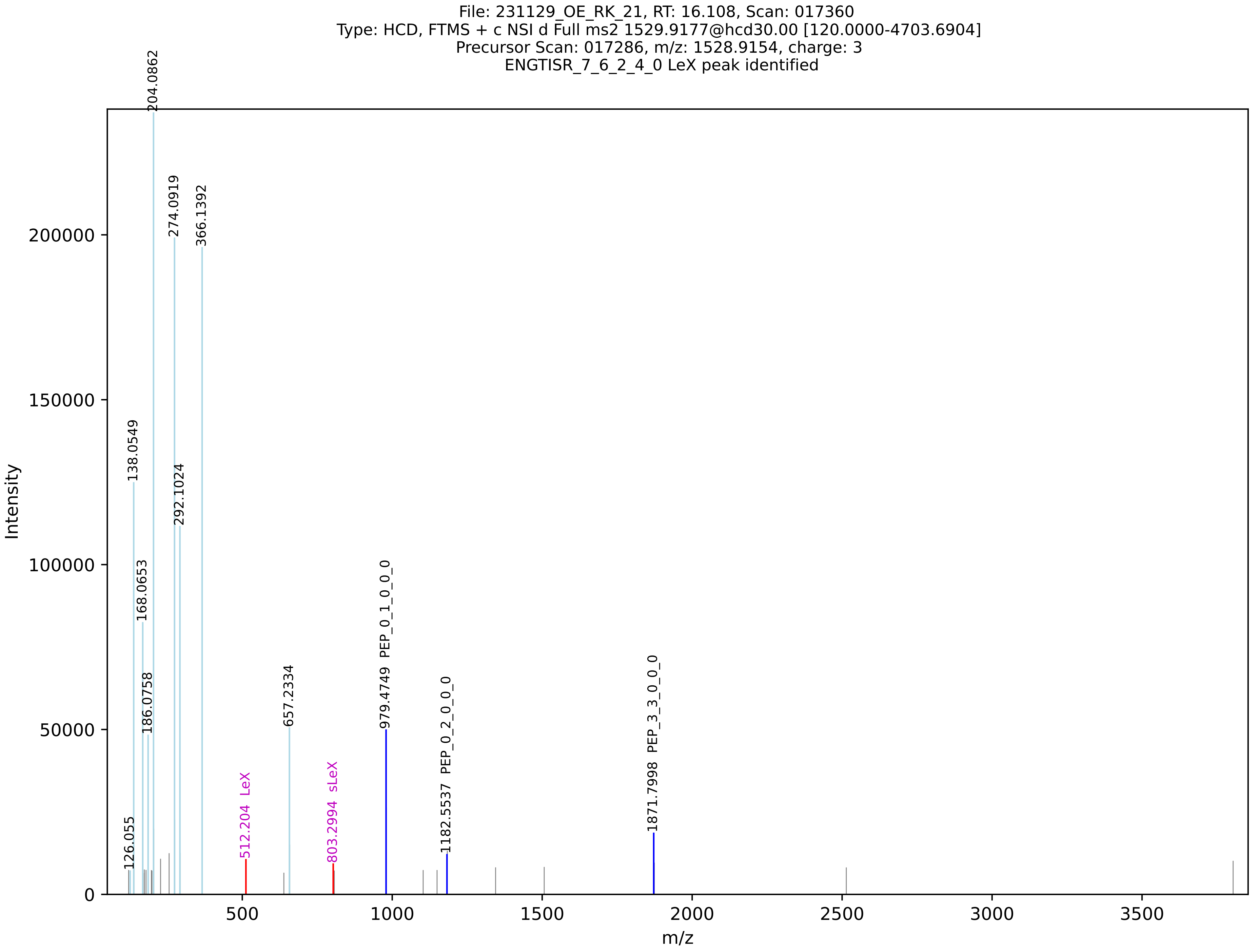

File: 231129_OE_RK_21, RT: 16.108, Scan: 017360
Type: HCD, FTMS + c NSI d Full ms2 1529.9177@hcd30.00 [120.0000-4703.6904]
Precursor Scan: 017286, m/z: 1528.9154, charge: 3
ENGTISR_7_6_2_4_0 LeX peak identified
Intensity
m/z
204.0862
274.0919
366.1392
138.0549
292.1024
168.0653
186.0758
126.055
512.204 LeX
657.2334
803.2994 sLeX
979.4749 PEP_0_1_0_0_0
1182.5537 PEP_0_2_0_0_0
1871.7998 PEP_3_3_0_0_0

File: 231129_OE_RK_21, RT: 16.359, Scan: 017722
Type: HCD, FTMS + c NSI d Full ms2 1147.6902@hcd30.00 [120.0000-4705.4321]
Precursor Scan: 017647, m/z: 1147.1889, charge: 4
ENGTISR_7_6_2_4_0 LeX peak identified

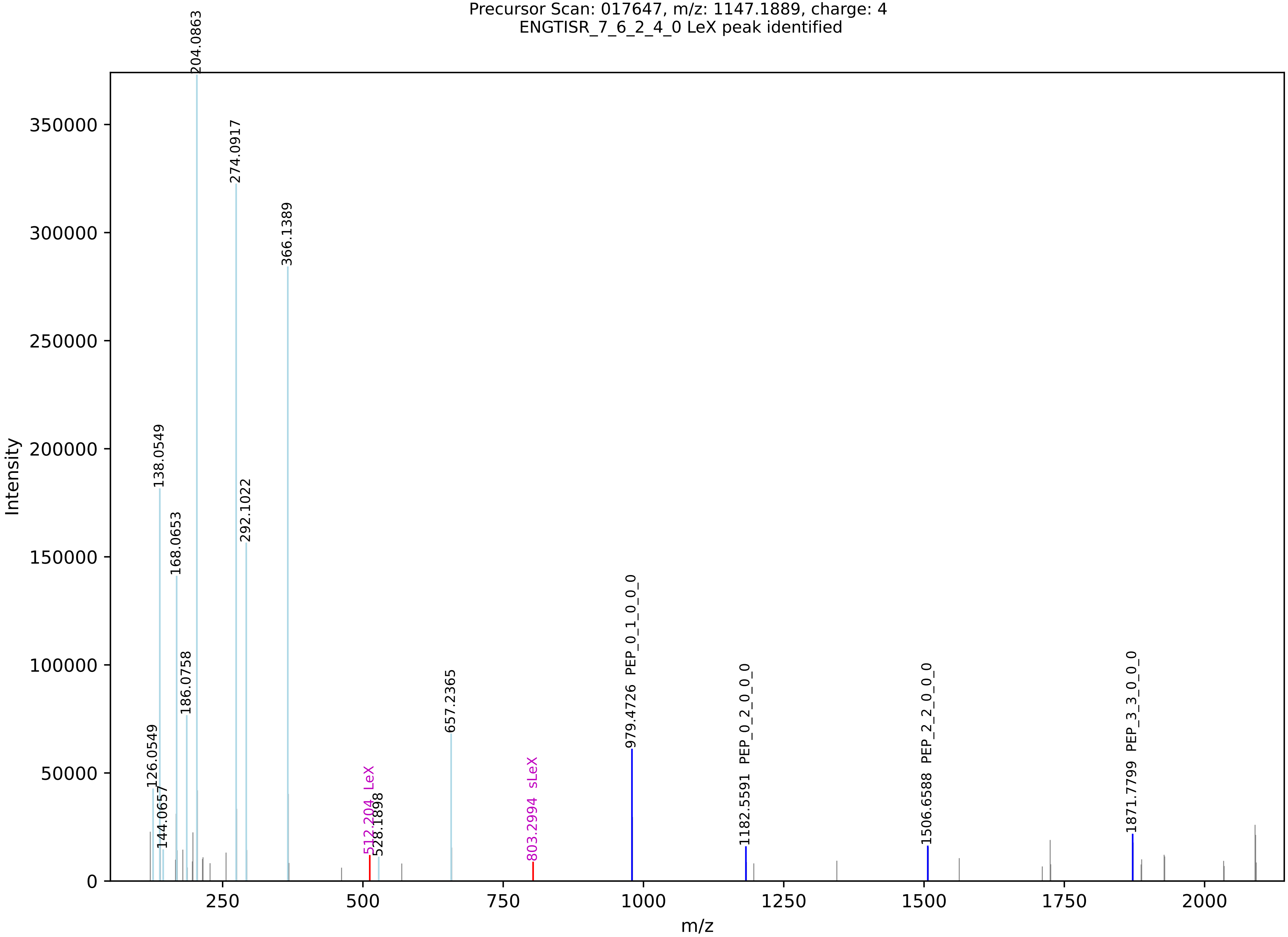

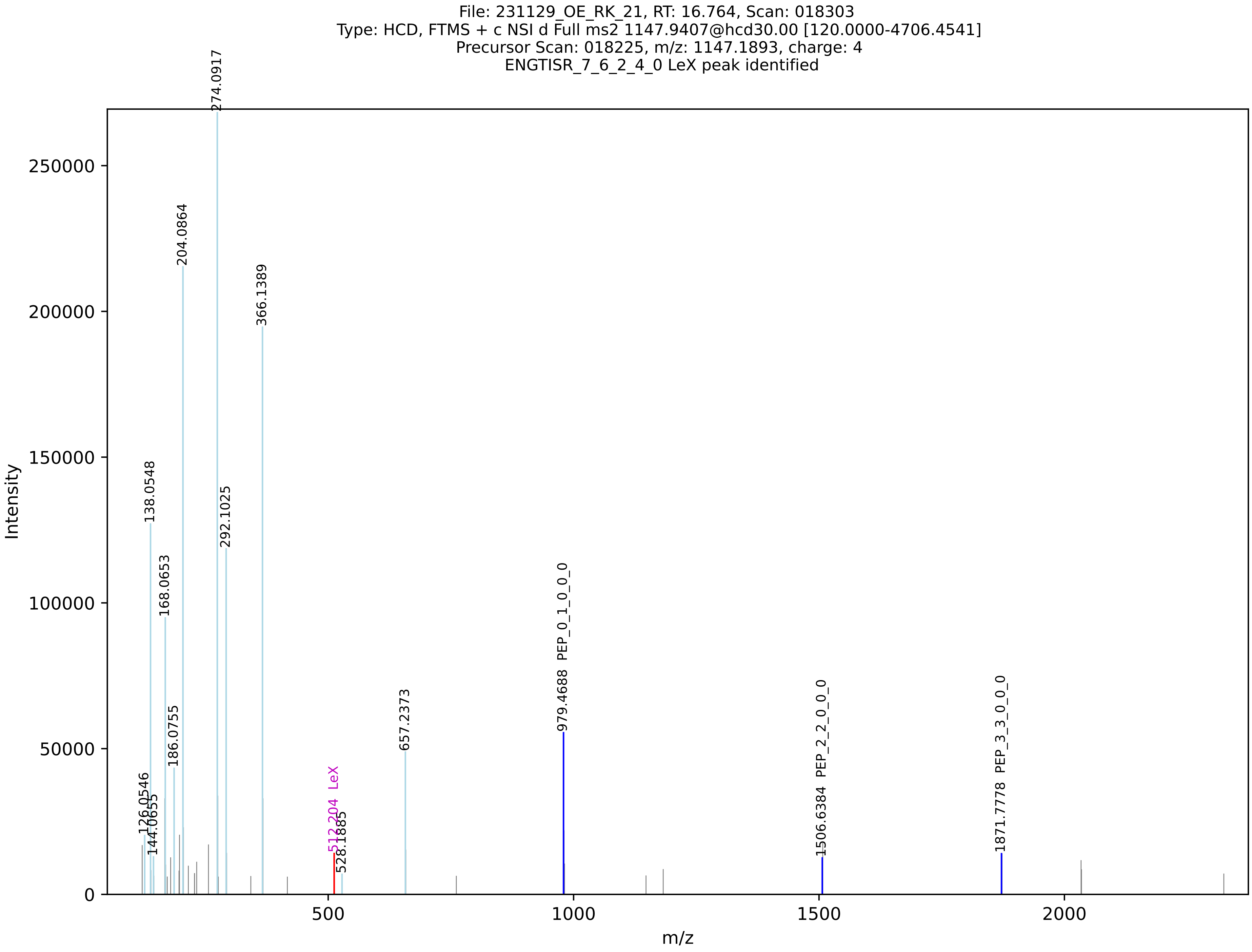
File: 231129_OE_RK_21, RT: 16.764, Scan: 018303
Type: HCD, FTMS + c NSI d Full ms2 1147.9407@hcd30.00 [120.0000-4706.4541]
Precursor Scan: 018225, m/z: 1147.1893, charge: 4
ENGTISR_7_6_2_4_0 LeX peak identified
Intensity
m/z
274.0917
204.0864
366.1389
138.0548
292.1025
168.0653
186.0755
126.0546
144.0655
512.204 LeX
528.1885
657.2373
979.4688 PEP_0_1_0_0_
1506.6384 PEP_2_2_0_0_0_
1871.7778 PEP_3_3_0_0_0_
0
50000
100000
150000
200000
250000
500
1000
1500
2000

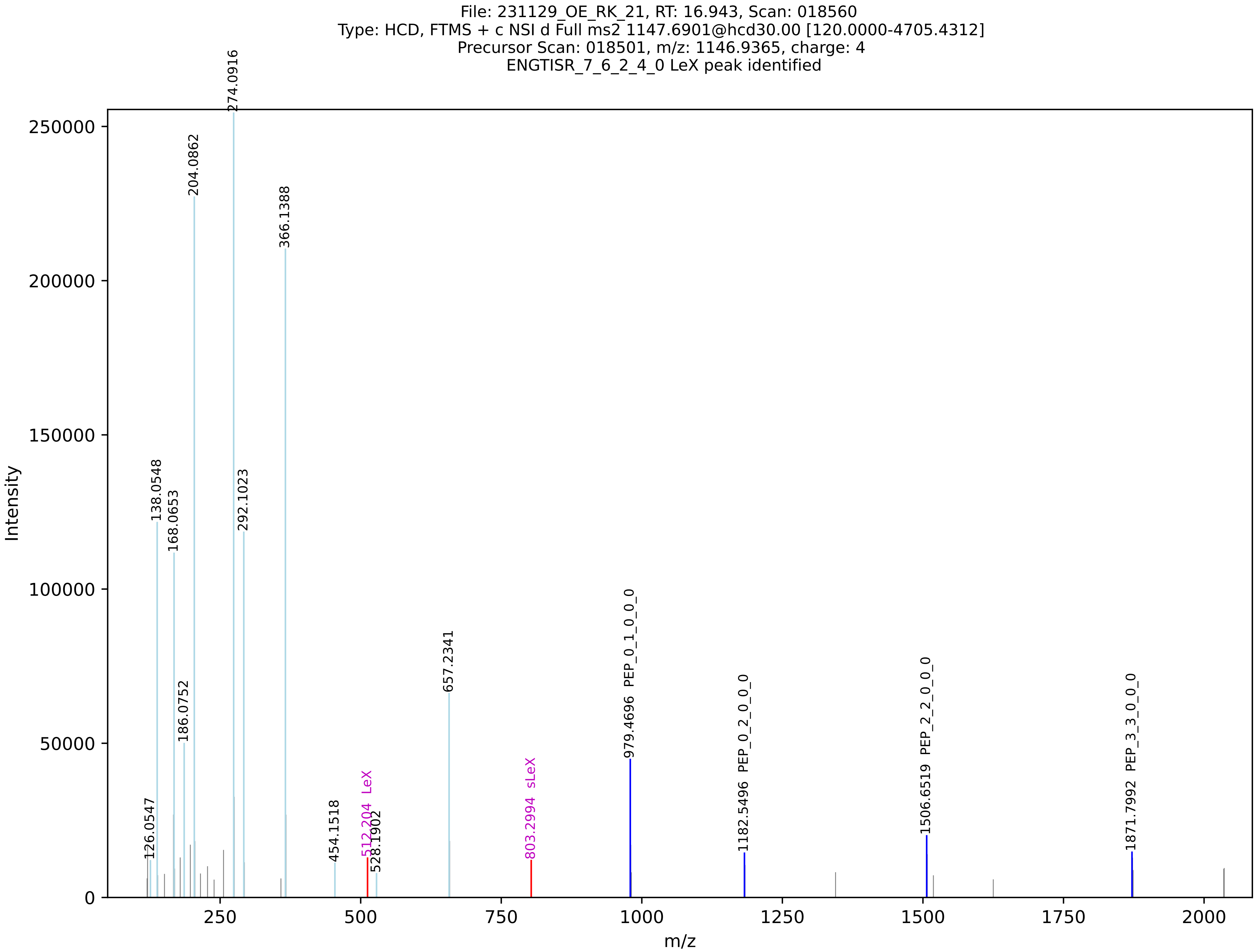
File: 231129_OE_RK_21, RT: 16.943, Scan: 018560
Type: HCD, FTMS + c NSI d Full ms2 1147.6901@hcd30.00 [120.0000-4705.4312]
Precursor Scan: 018501, m/z: 1146.9365, charge: 4
ENGTISR_7_6_2_4_0 LeX peak identified
Intensity
m/z
274.0916
204.0862
366.1388
138.0548
168.0653
292.1023
186.0752
126.0547
454.1518
512.204 LeX
528.1902
657.2341
803.2994 sLeX
979.4696 PEP_0_1_0_0_0
1182.5496 PEP_0_2_0_0_0
1506.6519 PEP_2_2_0_0_0
1871.7992 PEP_3_3_0_0_0

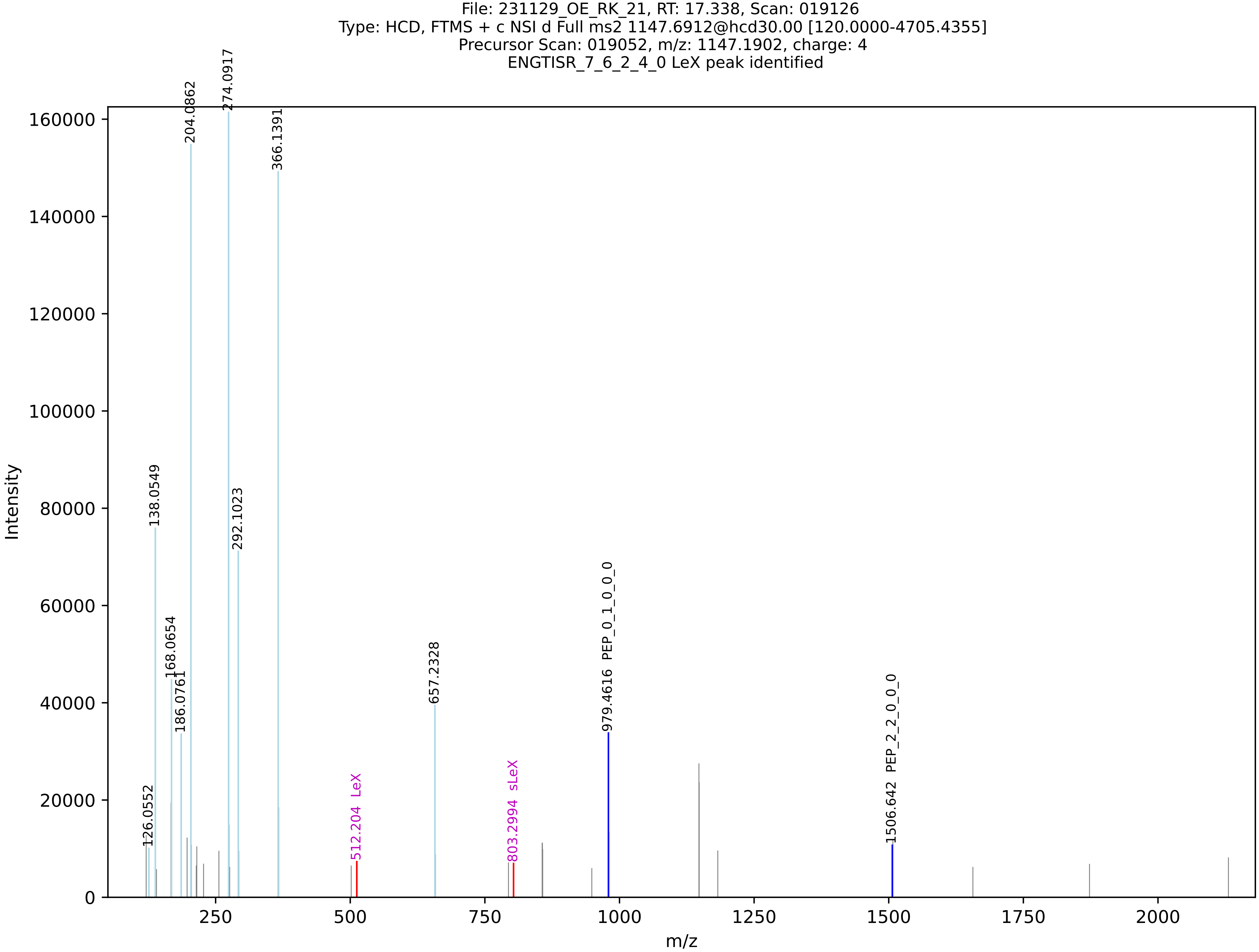
File: 231129_OE_RK_21, RT: 17.338, Scan: 019126
Type: HCD, FTMS + c NSI d Full ms2 1147.6912@hcd30.00 [120.0000-4705.4355]
Precursor Scan: 019052, m/z: 1147.1902, charge: 4
ENGTISR_7_6_2_4_0 LeX peak identified
Intensity
m/z
204.0862
274.0917
366.1391
138.0549
292.1023
168.0654
186.0761
126.0552
512.204 LeX
657.2328
803.2994 sLeX
979.4616 PEP_0_1_0_0_0
1506.642 PEP_2_2_0_0_0

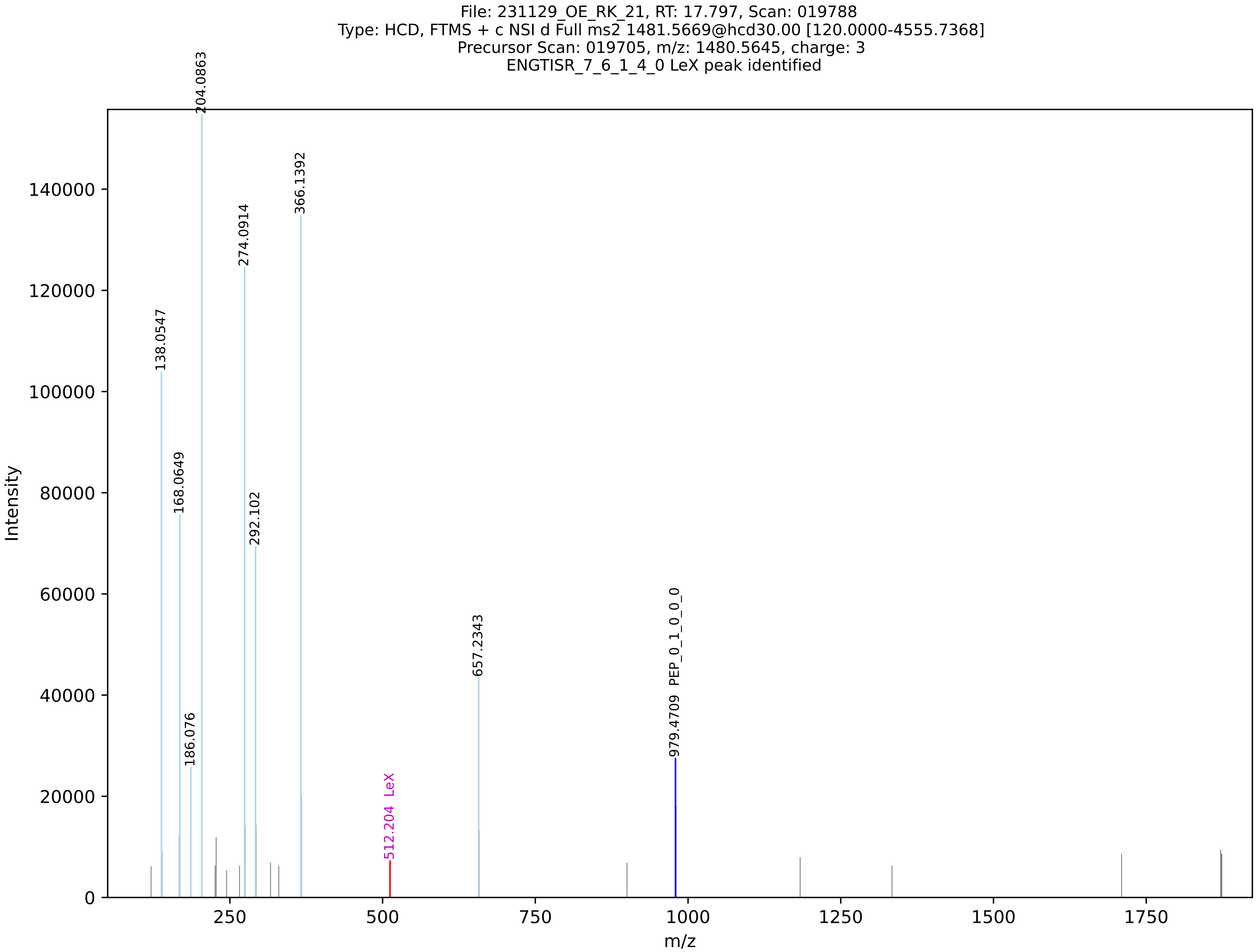

File: 231129_OE_RK_21, RT: 17.797, Scan: 019788
Type: HCD, FTMS + c NSI d Full ms2 1481.5669@hcd30.00 [120.0000-4555.7368]
Precursor Scan: 019705, m/z: 1480.5645, charge: 3
ENGTISR_7_6_1_4_0 LeX peak identified
Intensity
m/z
204.0863
366.1392
274.0914
138.0547
168.0649
292.102
186.076
512.204 LeX
657.2343
979.4709 PEP_0_1_0_0_0_

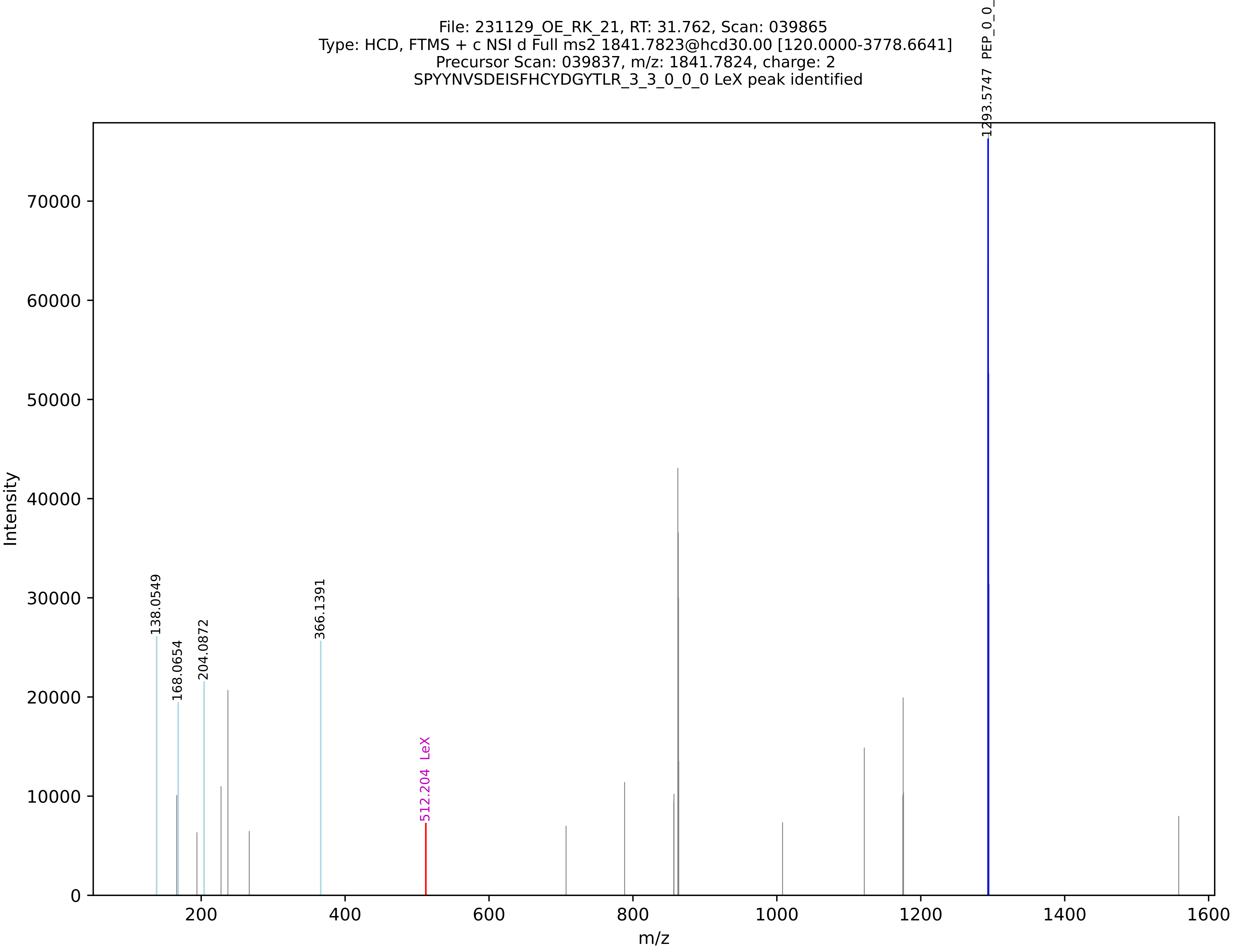
File: 231129_OE_RK_21, RT: 31.762, Scan: 039865
Type: HCD, FTMS + c NSI d Full ms2 1841.7823@hcd30.00 [120.0000-3778.6641]
Precursor Scan: 039837, m/z: 1841.7824, charge: 2
SPYYNVSDEISFHCYDGYTLR_3_3_0_0_0 LeX peak identified
1293.5747 PEP_0_0_
138.0549
168.0654
204.0872
366.1391
512.204 LeX
Intensity
m/z
0
10000
20000
30000
40000
50000
60000
70000
200
400
600
800
1000
1200
1400
1600

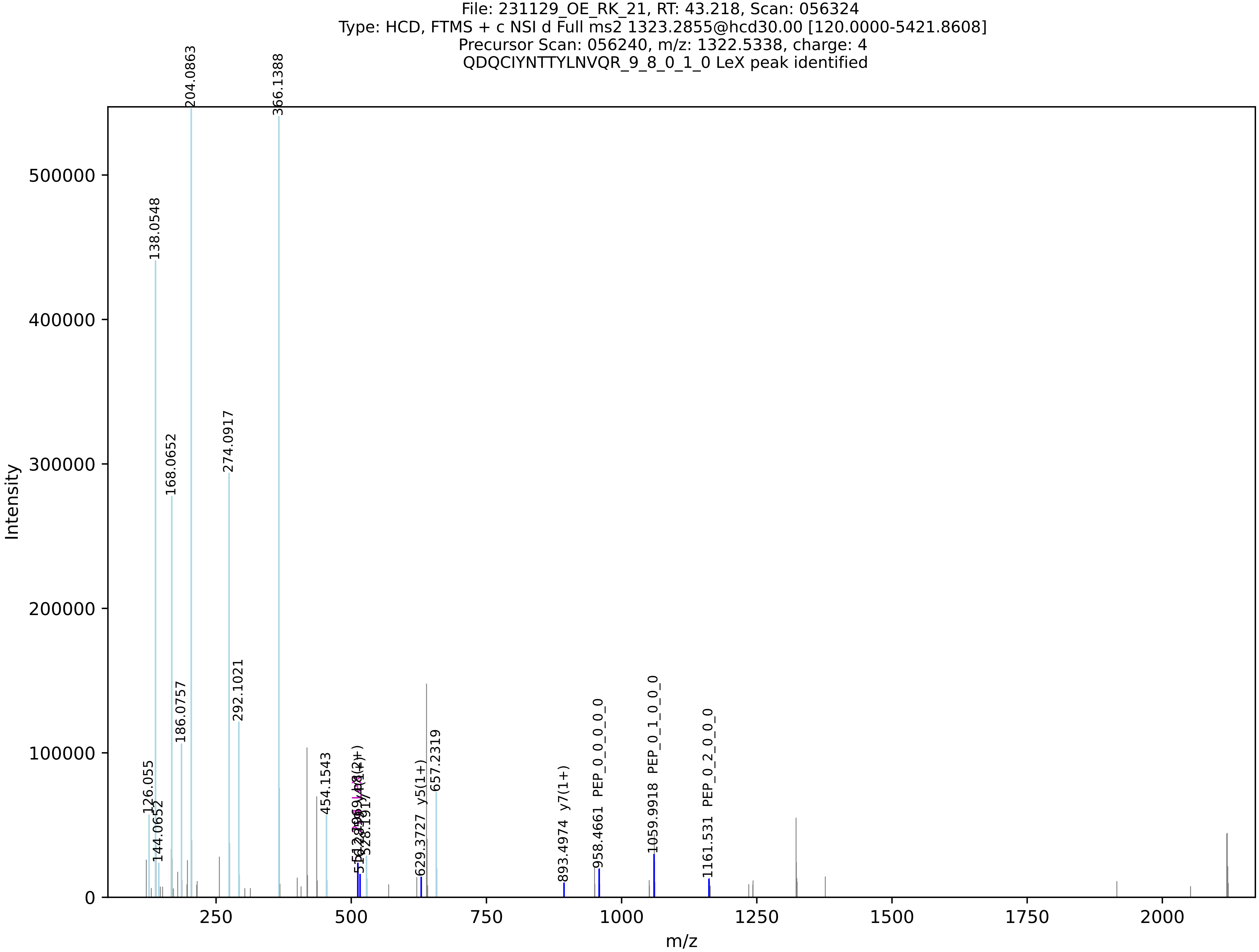
File: 231129_OE_RK_21, RT: 43.218, Scan: 056324
Type: HCD, FTMS + c NSI d Full ms2 1323.2855@hcd30.00 [120.0000-5421.8608]
Precursor Scan: 056240, m/z: 1322.5338, charge: 4
QDQCIYNTTYLNVQR_9_8_0_1_0 LeX peak identified
204.0863
366.1388
138.0548
168.0652
274.0917
292.1021
186.0757
126.055
144.0652
454.1543
629.3727 y5(1+)
657.2319
893.4974 y7(1+)
958.4661 PEP_0_0_0_0_0
1059.9918 PEP_0_1_0_0_0
1161.531 PEP_0_2_0_0_0
Intensity
m/z
0
100000
200000
300000
400000
500000
250
500
750
1000
1250
1500
1750
2000

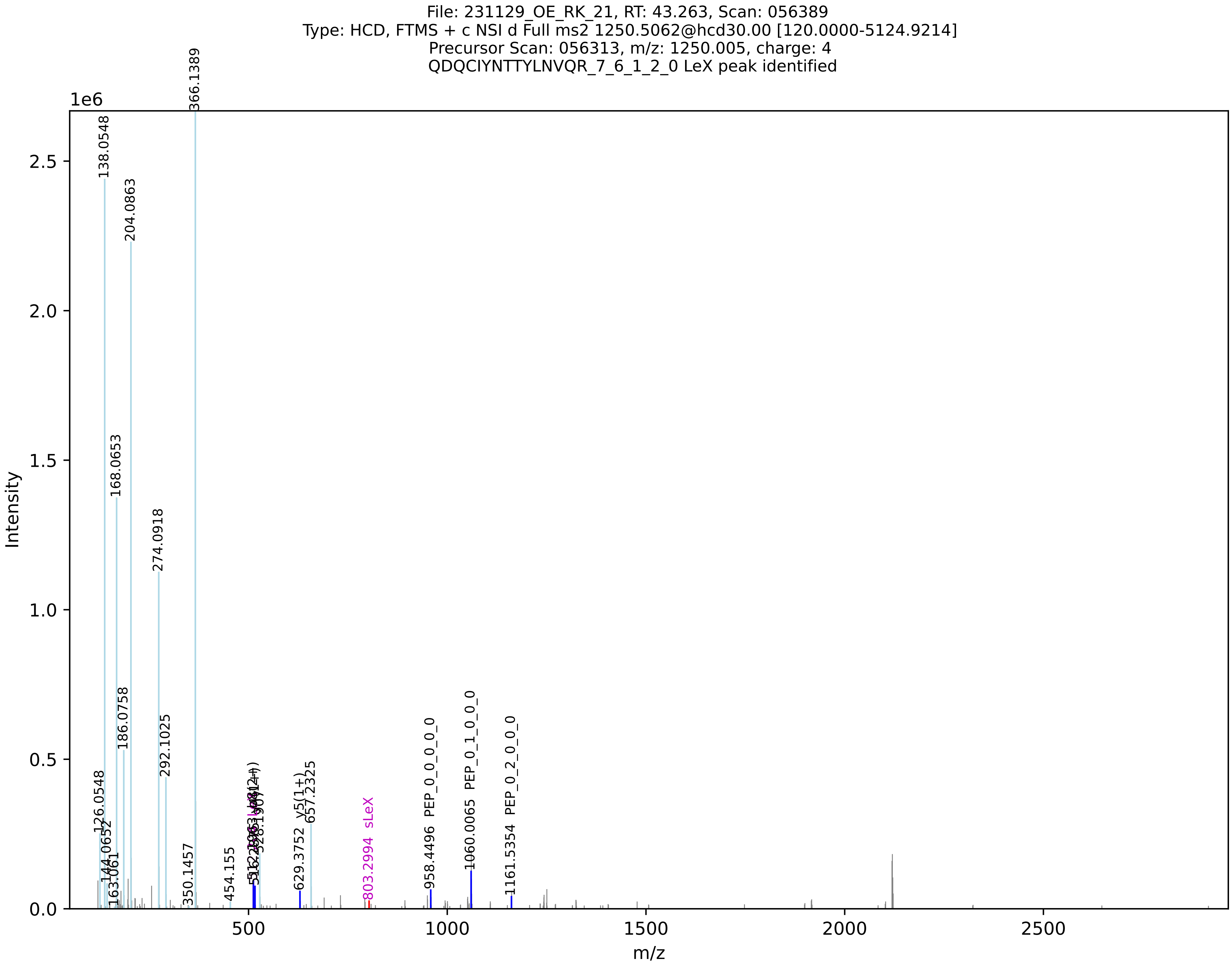

File: 231129_OE_RK_21, RT: 43.263, Scan: 056389
Type: HCD, FTMS + c NSI d Full ms2 1250.5062@hcd30.00 [120.0000-5124.9214]
Precursor Scan: 056313, m/z: 1250.005, charge: 4
QDQCIYNTTYLNVQR_7_6_1_2_0 LeX peak identified
1e6
Intensity
m/z
366.1389
138.0548
204.0863
168.0653
274.0918
186.0758
292.1025
126.0548
144.0652
163.061
350.1457
454.155
528.1907
629.3752 y5(1+)
657.2325
803.2994 sLeX
958.4496 PEP_0_0_0_0_0
1060.0065 PEP_0_1_0_0_0
1161.5354 PEP_0_2_0_0_0

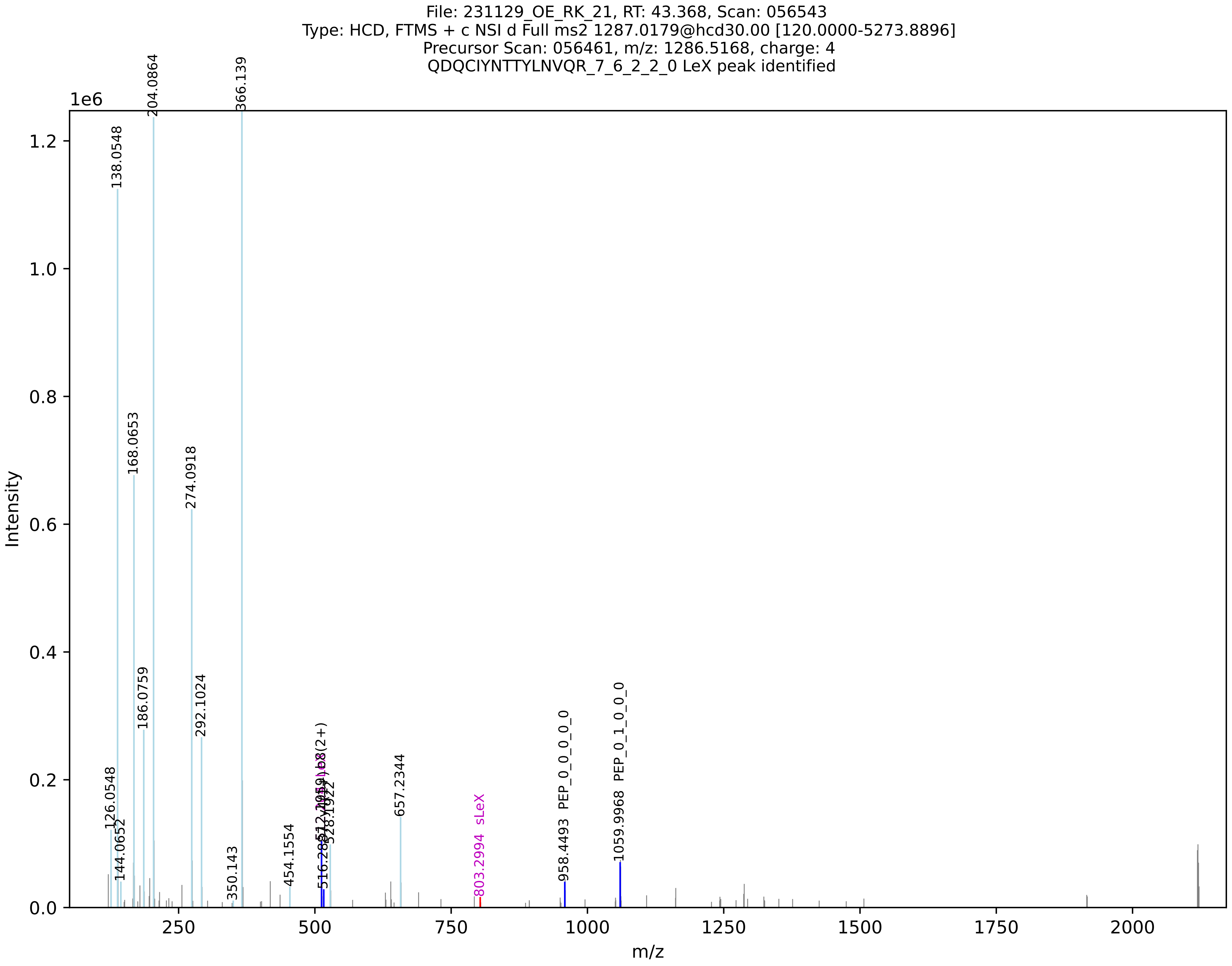
File: 231129_OE_RK_21, RT: 43.368, Scan: 056543
Type: HCD, FTMS + c NSI d Full ms2 1287.0179@hcd30.00 [120.0000-5273.8896]
Precursor Scan: 056461, m/z: 1286.5168, charge: 4
QDQCIYNTTYLNVQR_7_6_2_2_0 LeX peak identified
1e6
Intensity
m/z
204.0864
366.139
138.0548
168.0653
274.0918
186.0759
292.1024
126.0548
144.0652
350.143
454.1554
528.1922
657.2344
803.2994 sLeX
958.4493 PEP_0_0_0_0_0_
1059.9968 PEP_0_1_0_0_0_

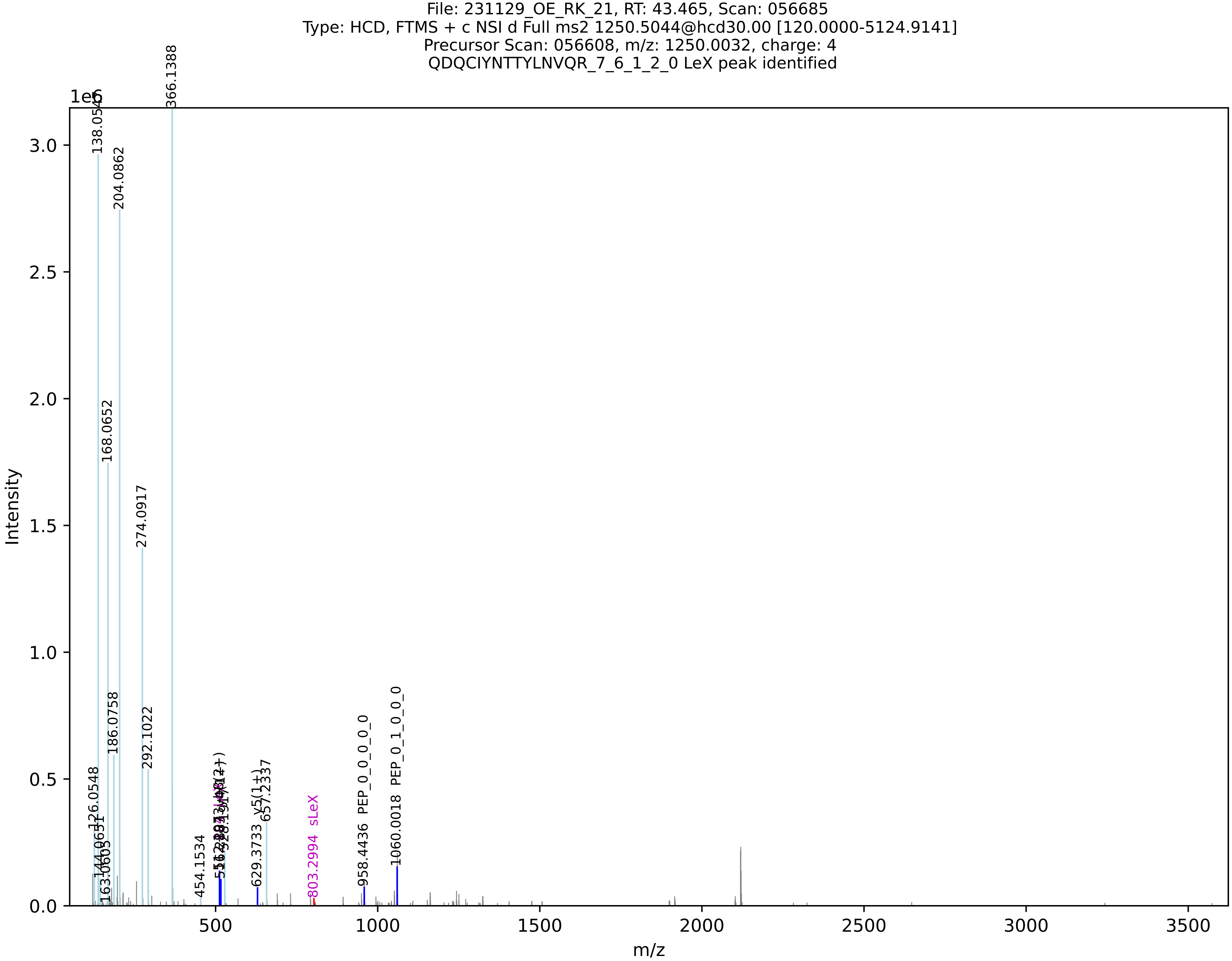
File: 231129_OE_RK_21, RT: 43.465, Scan: 056685
Type: HCD, FTMS + c NSI d Full ms2 1250.5044@hcd30.00 [120.0000-5124.9141]
Precursor Scan: 056608, m/z: 1250.0032, charge: 4
QDQCIYNTTYLNVQR_7_6_1_2_0 LeX peak identified
1e6
Intensity
m/z
366.1388
138.0545
204.0862
168.0652
274.0917
186.0758
292.1022
126.0548
144.0651
163.0605
454.1534
629.3733 y5(1+)
657.2337
803.2994 sLeX
958.4436 PEP_0_0_0_0_0
1060.0018 PEP_0_1_0_0_0

File: 231129_OE_RK_21, RT: 43.475, Scan: 056701
Type: HCD, FTMS + c NSI d Full ms2 1545.2939@hcd30.00 [120.0000-4750.7412]
Precursor Scan: 056608, m/z: 1544.6257, charge: 3
QDQCIYNTTYLNVQR_6_5_1_2_0 LeX peak identified

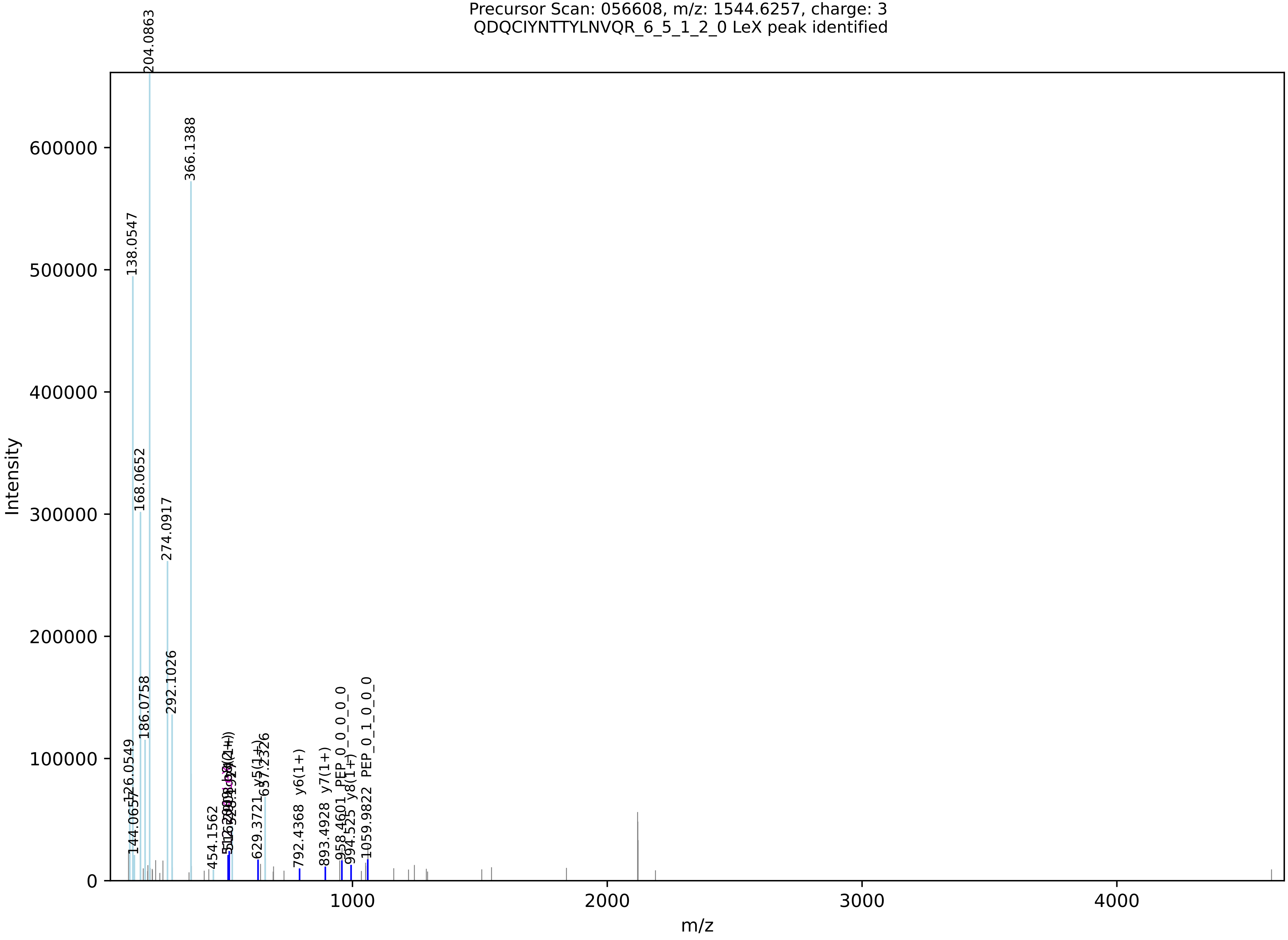

File: 231129_OE_RK_21, RT: 43.667, Scan: 056977
Type: HCD, FTMS + c NSI d Full ms2 1250.5051@hcd30.00 [120.0000-5124.9170]
Precursor Scan: 056899, m/z: 1250.0041, charge: 4
QDQCIYNTTYLNVQR_7_6_1_2_0 LeX peak identified

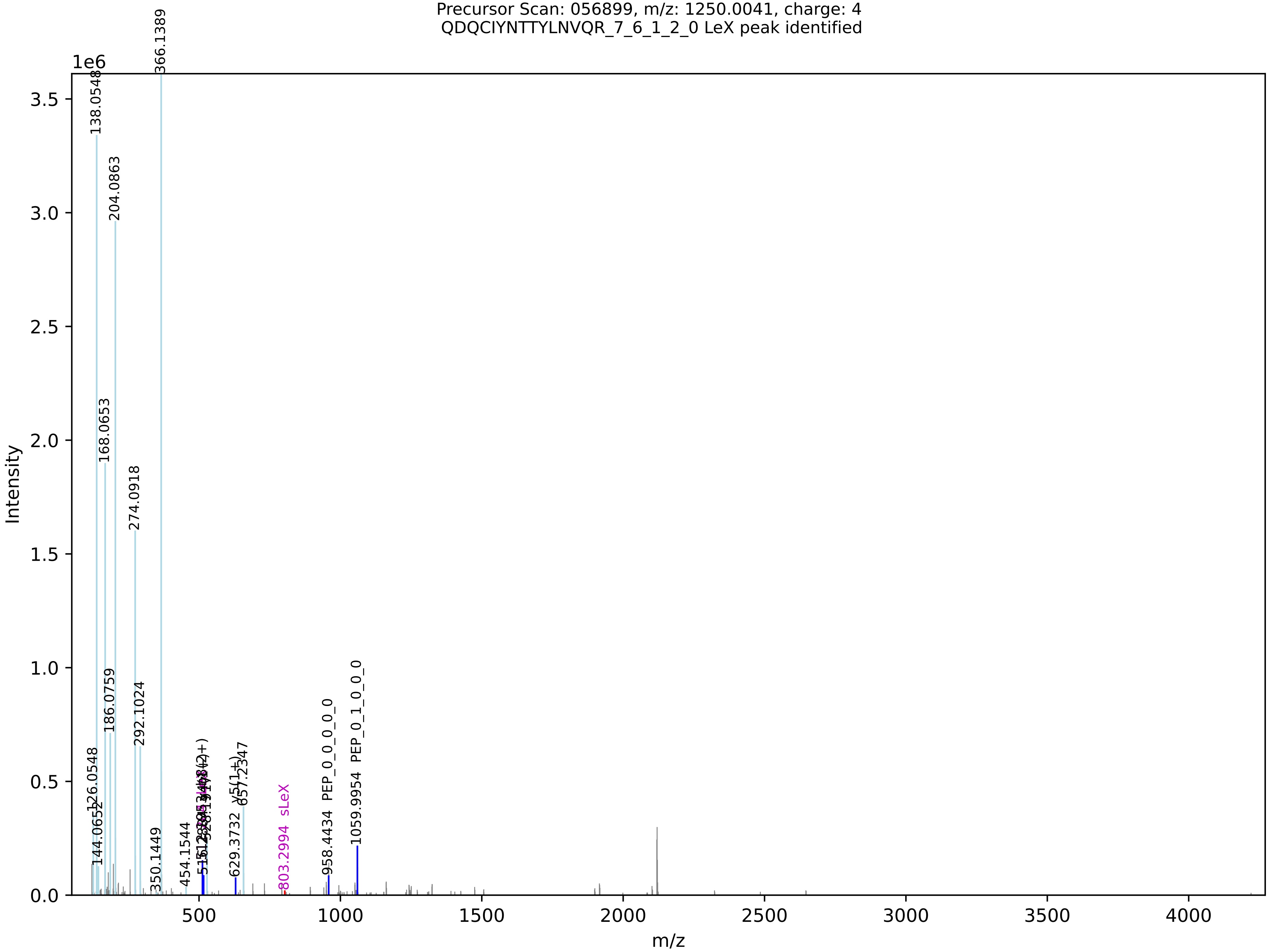

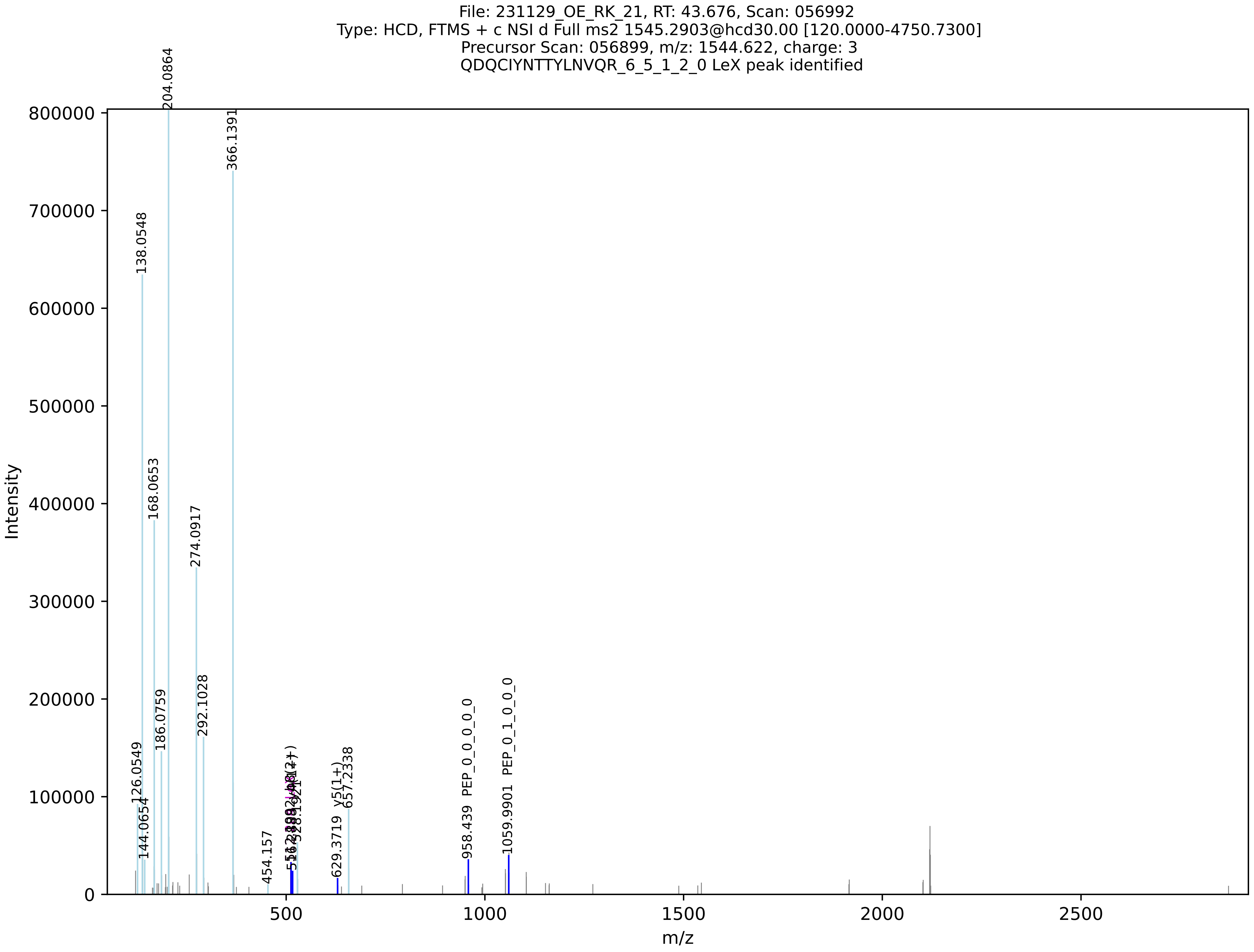
File: 231129_OE_RK_21, RT: 43.676, Scan: 056992
Type: HCD, FTMS + c NSI d Full ms2 1545.2903@hcd30.00 [120.0000-4750.7300]
Precursor Scan: 056899, m/z: 1544.622, charge: 3
QDQCIYNTTYLNVQR_6_5_1_2_0 LeX peak identified
Intensity
m/z
204.0864
366.1391
138.0548
168.0653
274.0917
292.1028
186.0759
126.0549
144.0654
454.157
629.3719
y5(1+)
657.2338
958.439 PEP_0_0_0_0_0
1059.9901 PEP_0_1_0_0_0
0
100000
200000
300000
400000
500000
600000
700000
800000
500
1000
1500
2000
2500

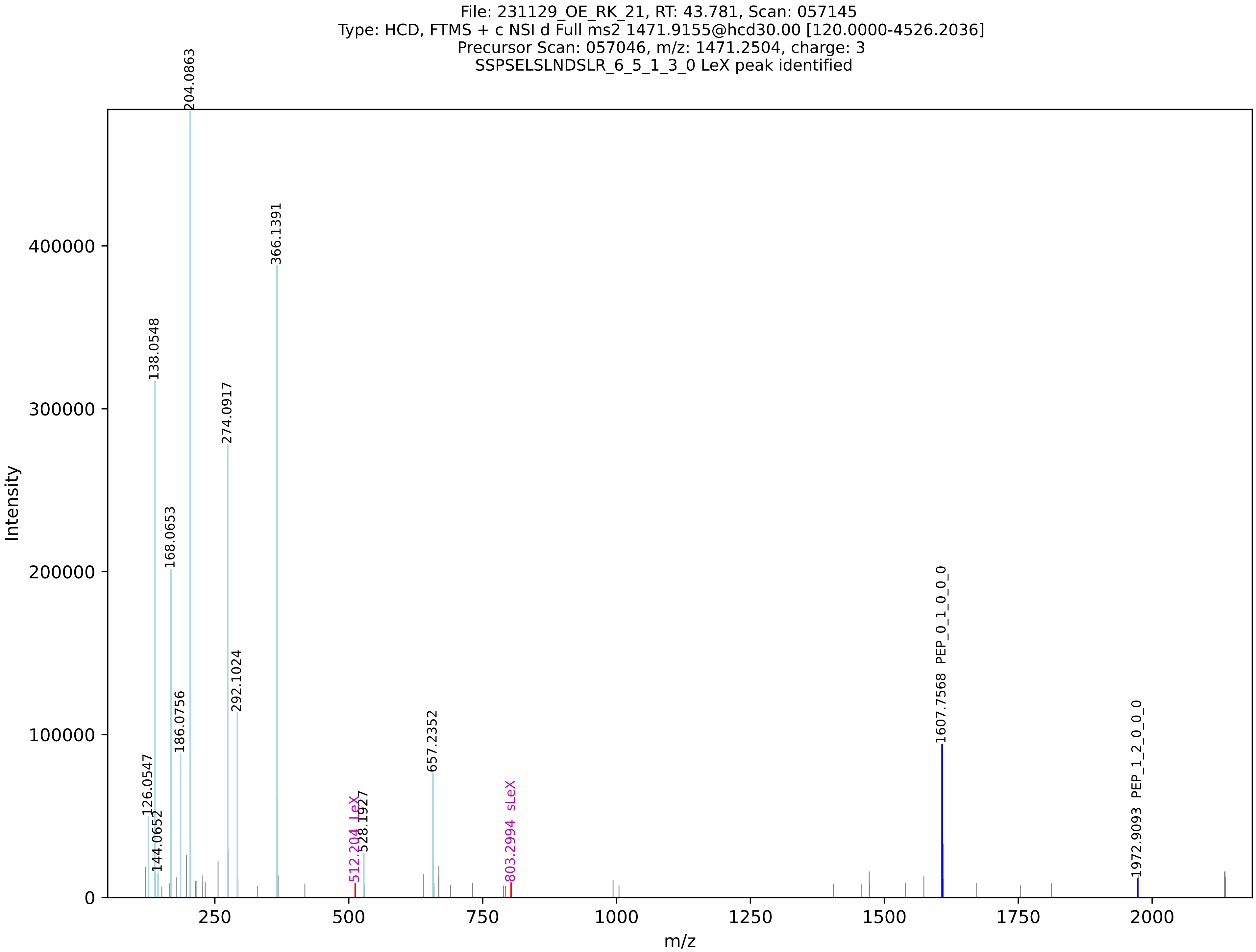
File: 231129_OE_RK_21, RT: 43.781, Scan: 057145
Type: HCD, FTMS + c NSI d Full ms2 1471.9155@hcd30.00 [120.0000-4526.2036]
Precursor Scan: 057046, m/z: 1471.2504, charge: 3
SSPSELSLNDSLR_6_5_1_3_0 LeX peak identified
204.0863
366.1391
138.0548
274.0917
168.0653
292.1024
186.0756
126.0547
144.0652
512.204 LeX
528.1927
657.2352
803.2994 sLeX
1607.7568 PEP_0_1_0_0_0
1972.9093 PEP_1_2_0_0_0
Intensity
m/z
0
100000
200000
300000
400000
250
500
750
1000
1250
1500
1750
2000

File: 231129_OE_RK_21, RT: 43.866, Scan: 057268
Type: HCD, FTMS + c NSI d Full ms2 1250.5059@hcd30.00 [120.0000-5124.9199]
Precursor Scan: 057192, m/z: 1250.0042, charge: 4
QDQCIYNTTYLNVQR_7_6_1_2_0 LeX peak identified

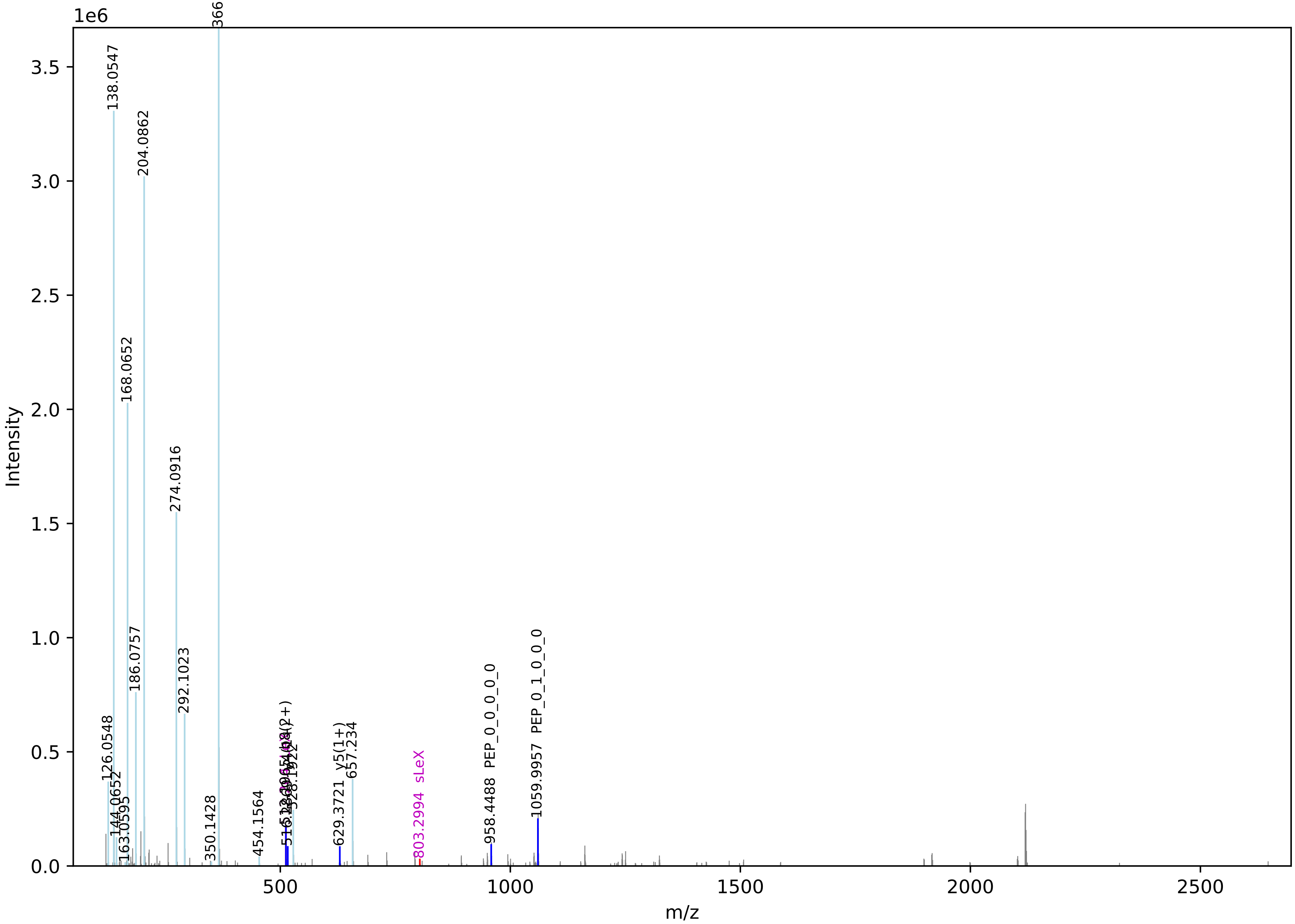

File: 231129_OE_RK_21, RT: 43.875, Scan: 057282
Type: HCD, FTMS + c NSI d Full ms2 1159.2223@hcd30.00 [120.0000-4752.4829]
Precursor Scan: 057192, m/z: 1158.7221, charge: 4
QDQCIYNTTYLNVQR_6_5_1_2_0 LeX peak identified

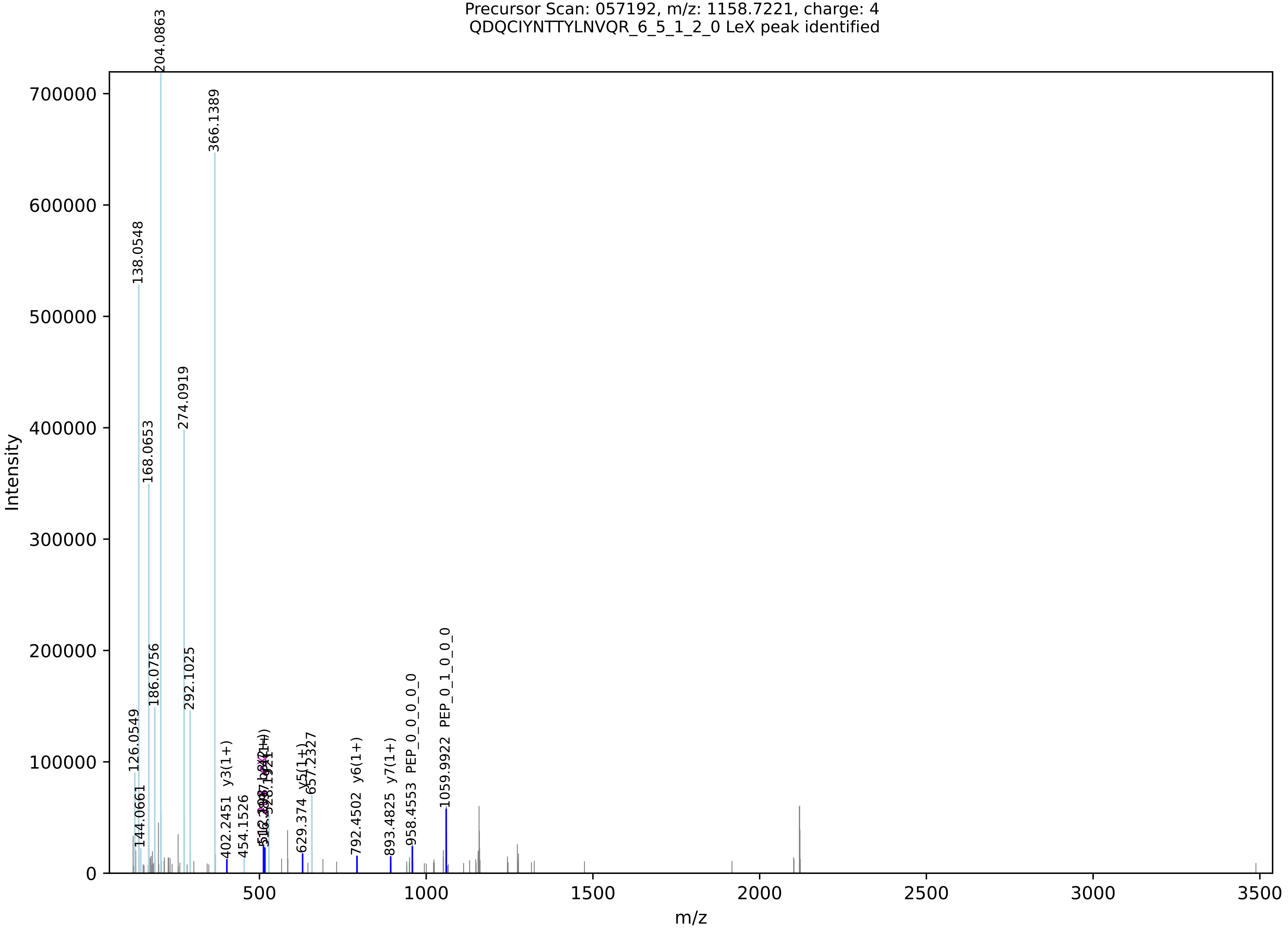

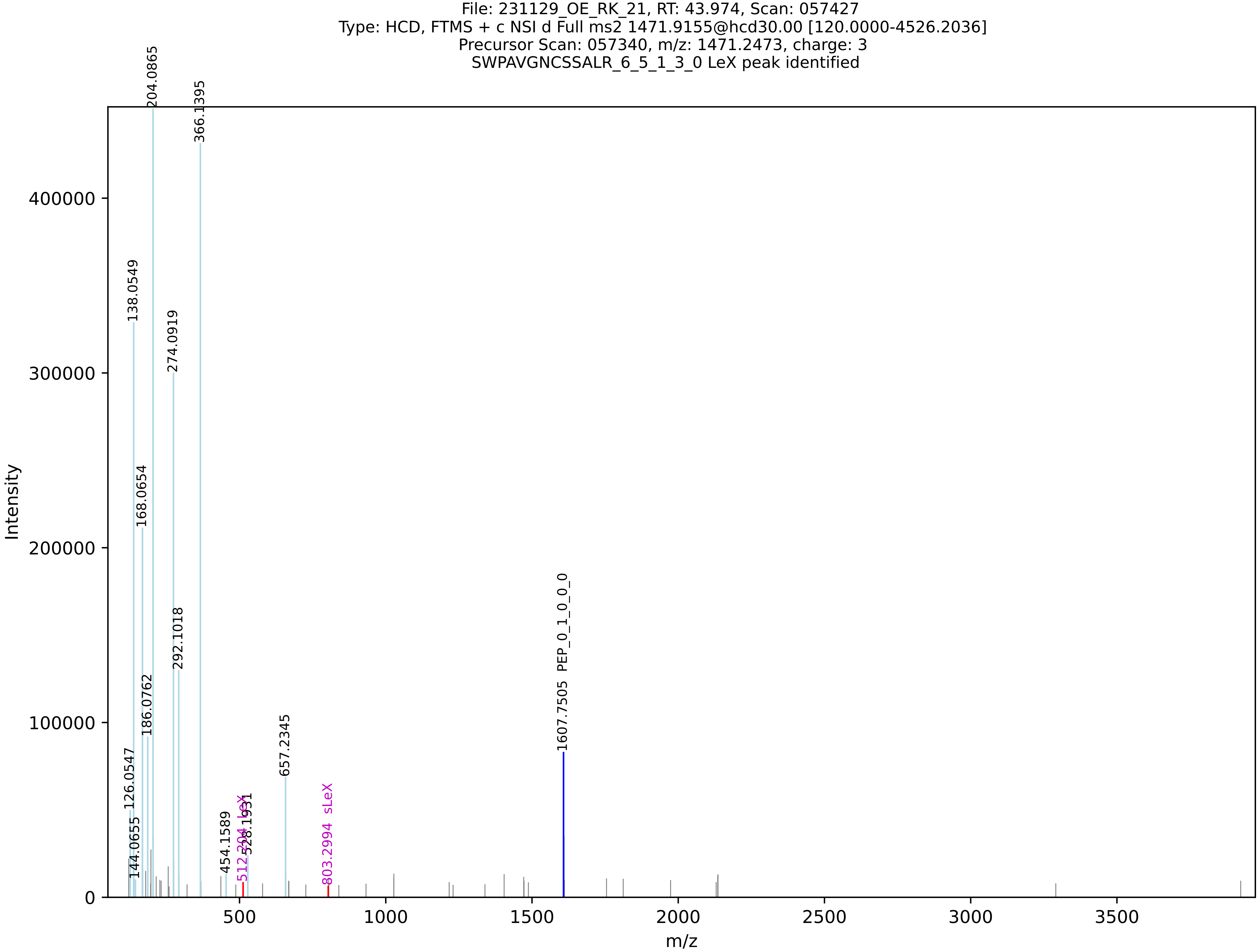

File: 231129_OE_RK_21, RT: 43.974, Scan: 057427
Type: HCD, FTMS + c NSI d Full ms2 1471.9155@hcd30.00 [120.0000-4526.2036]
Precursor Scan: 057340, m/z: 1471.2473, charge: 3
SWPAVGNCSSALR_6_5_1_3_0 LeX peak identified
Intensity
m/z
0
100000
200000
300000
400000
500
1000
1500
2000
2500
3000
3500
204.0865
366.1395
138.0549
274.0919
168.0654
292.1018
186.0762
126.0547
144.0655
454.1589
528.1931
LeX
657.2345
803.2994 sLeX
1607.7505 PEP_0_1_0_0_0

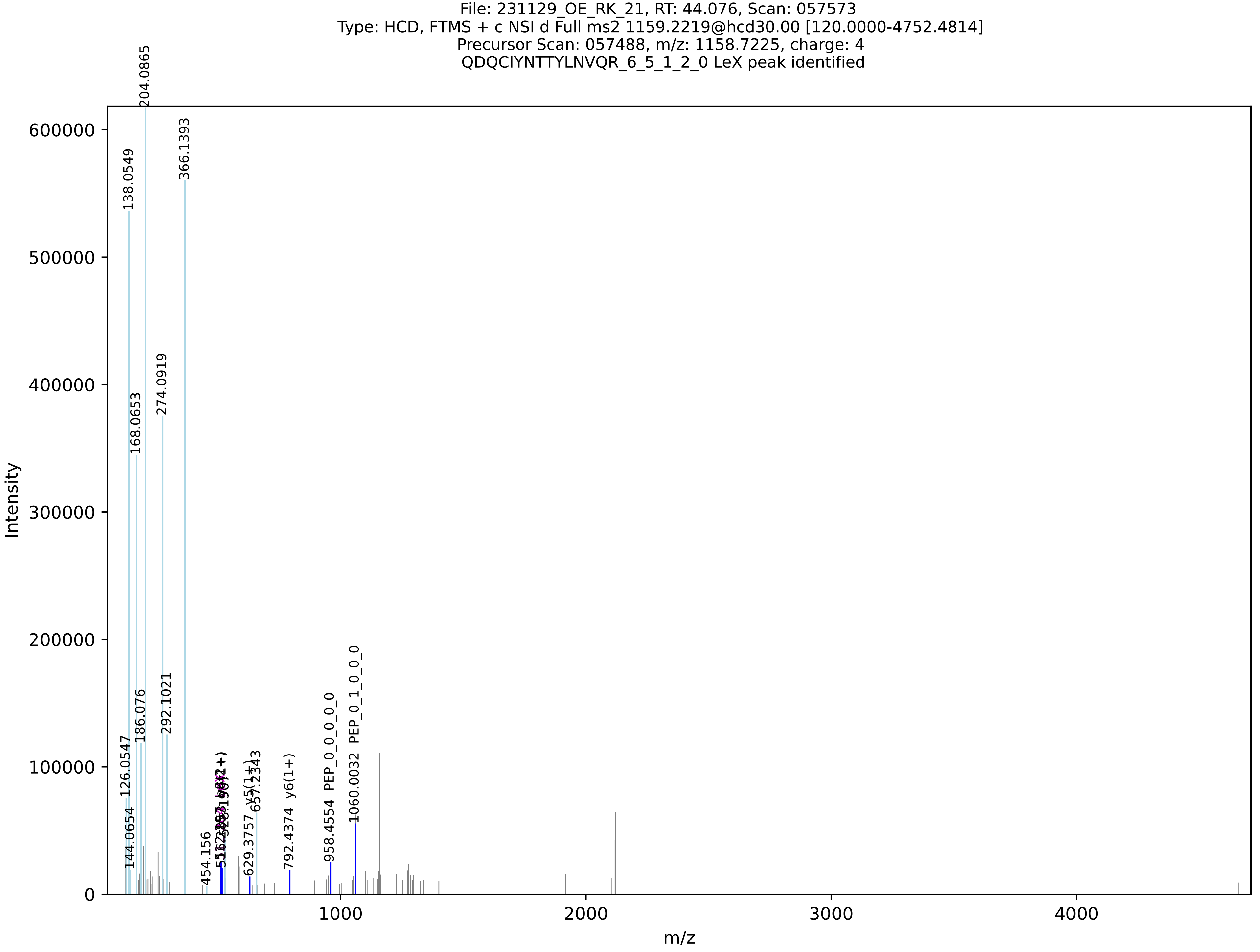
File: 231129_OE_RK_21, RT: 44.076, Scan: 057573
Type: HCD, FTMS + c NSI d Full ms2 1159.2219@hcd30.00 [120.0000-4752.4814]
Precursor Scan: 057488, m/z: 1158.7225, charge: 4
QDQCIYNTTYLNVQR_6_5_1_2_0 LeX peak identified
Intensity
m/z
204.0865
366.1393
138.0549
274.0919
168.0653
292.1021
186.076
126.0547
144.0654
454.156
629.3757 y5(1+)
657.2343
792.4374 y6(1+)
958.4554 PEP_0_0_0_0_0
1060.0032 PEP_0_1_0_0_0

File: 231129_OE_RK_21, RT: 44.277, Scan: 057867
Type: HCD, FTMS + c NSI d Full ms2 1250.2522@hcd30.00 [120.0000-5123.8848]
Precursor Scan: 057780, m/z: 1250.003, charge: 4
QDQCIYNTTYLNVQR_7_6_1_2_0 LeX peak identified

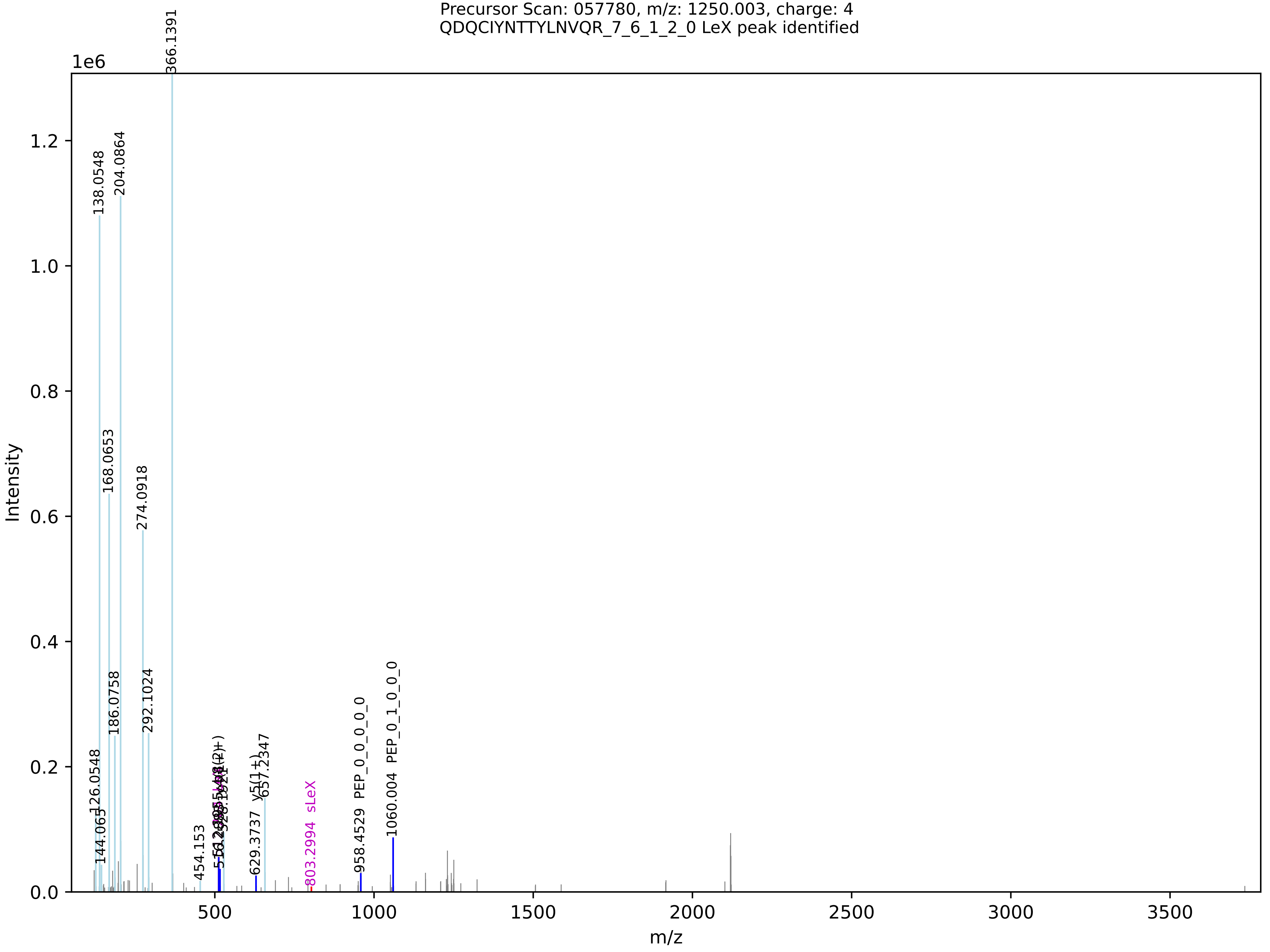

File: 231129_OE_RK_21, RT: 45.582, Scan: 059776
Type: HCD, FTMS + c NSI d Full ms2 1764.3678@hcd30.00 [120.0000-5421.1074]
Precursor Scan: 059684, m/z: 1763.3682, charge: 3
QDQCIYNTTYLNVQR_7_6_1_3_0 LeX peak identified

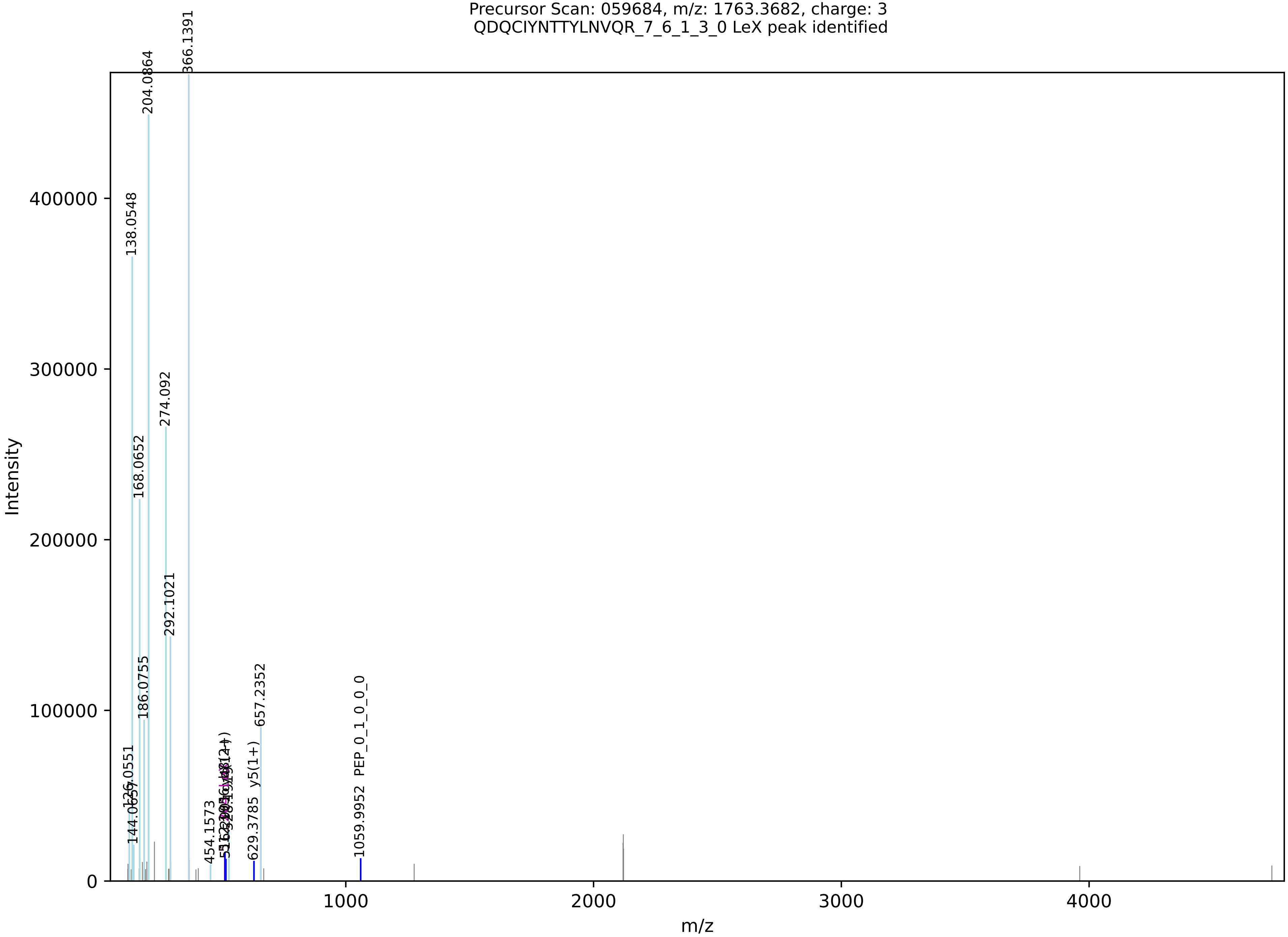

File: 231129_OE_RK_21, RT: 47.14, Scan: 062058
Type: HCD, FTMS + c NSI d Full ms2 1323.2761@hcd30.00 [120.0000-5421.8228]
Precursor Scan: 061976, m/z: 1322.7771, charge: 4
QDQCIYNTTYLNVQR_7_6_1_3_0 LeX peak identified

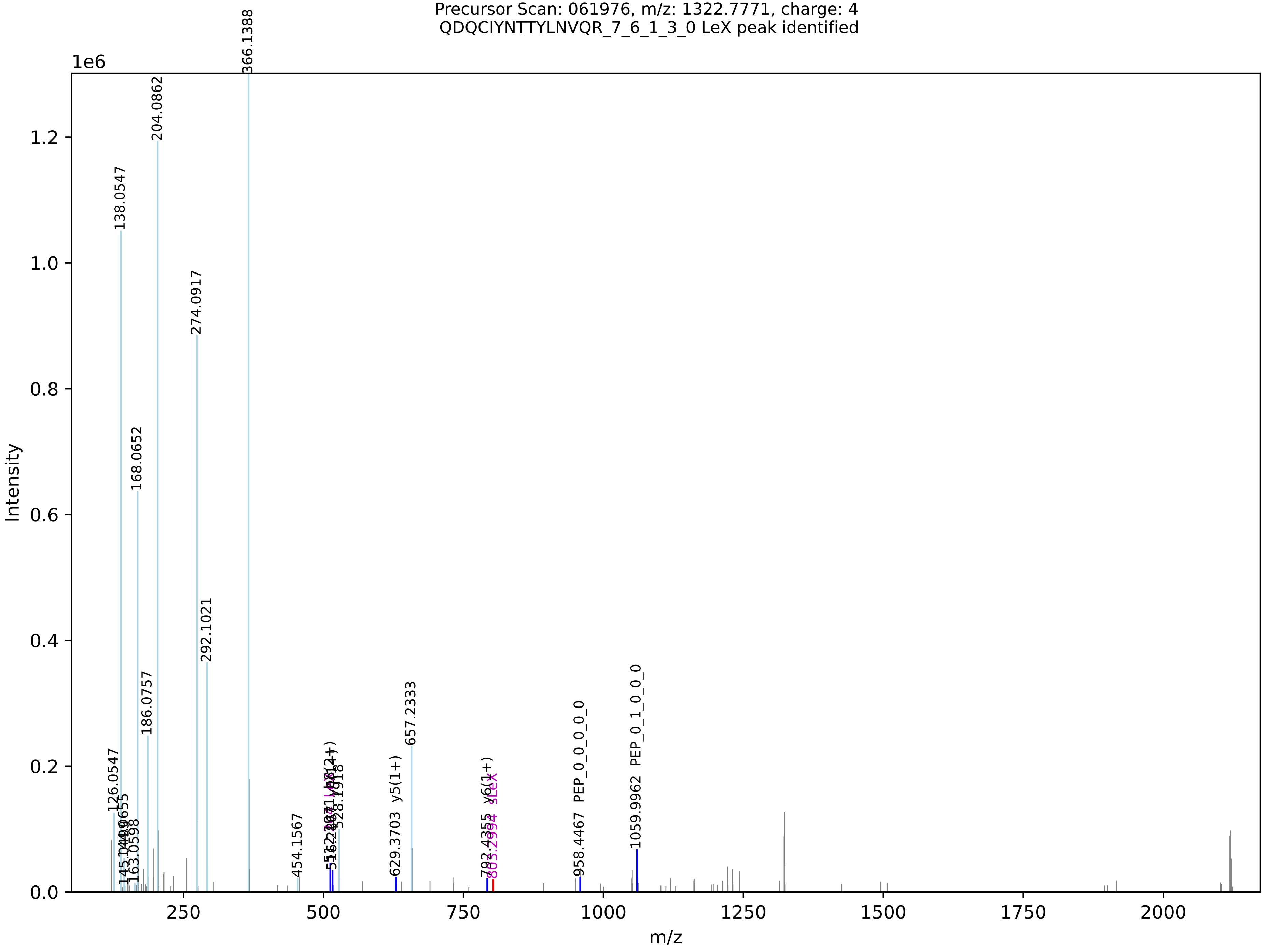

File: 231129_OE_RK_21, RT: 47.473, Scan: 062544
Type: HCD, FTMS + c NSI d Full ms2 1360.0435@hcd30.00 [120.0000-5571.8330]
Precursor Scan: 062483, m/z: 1359.7927, charge: 4
QDQCIYNTTYLNVQR_7_6_2_3_0 LeX peak identified

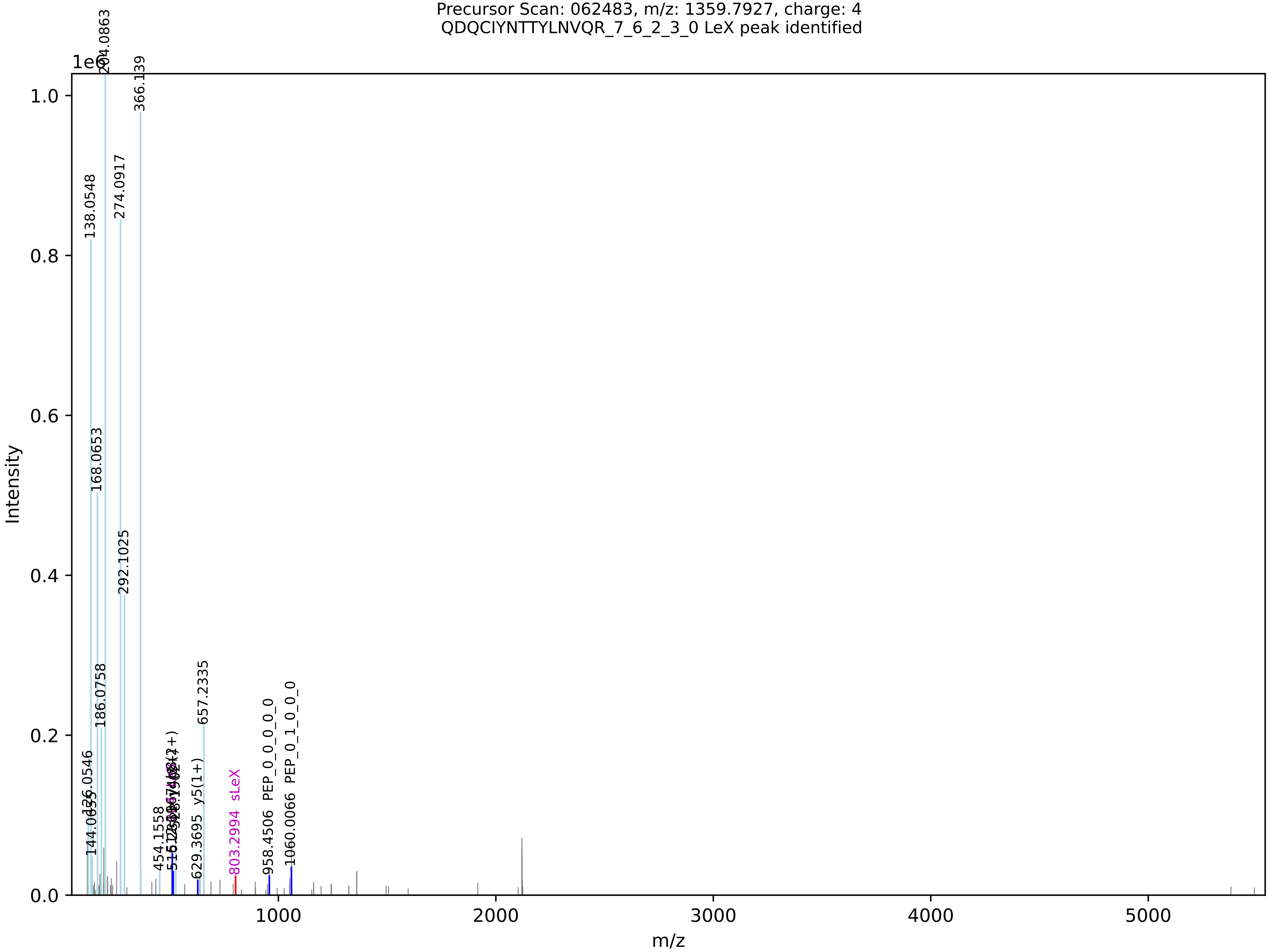

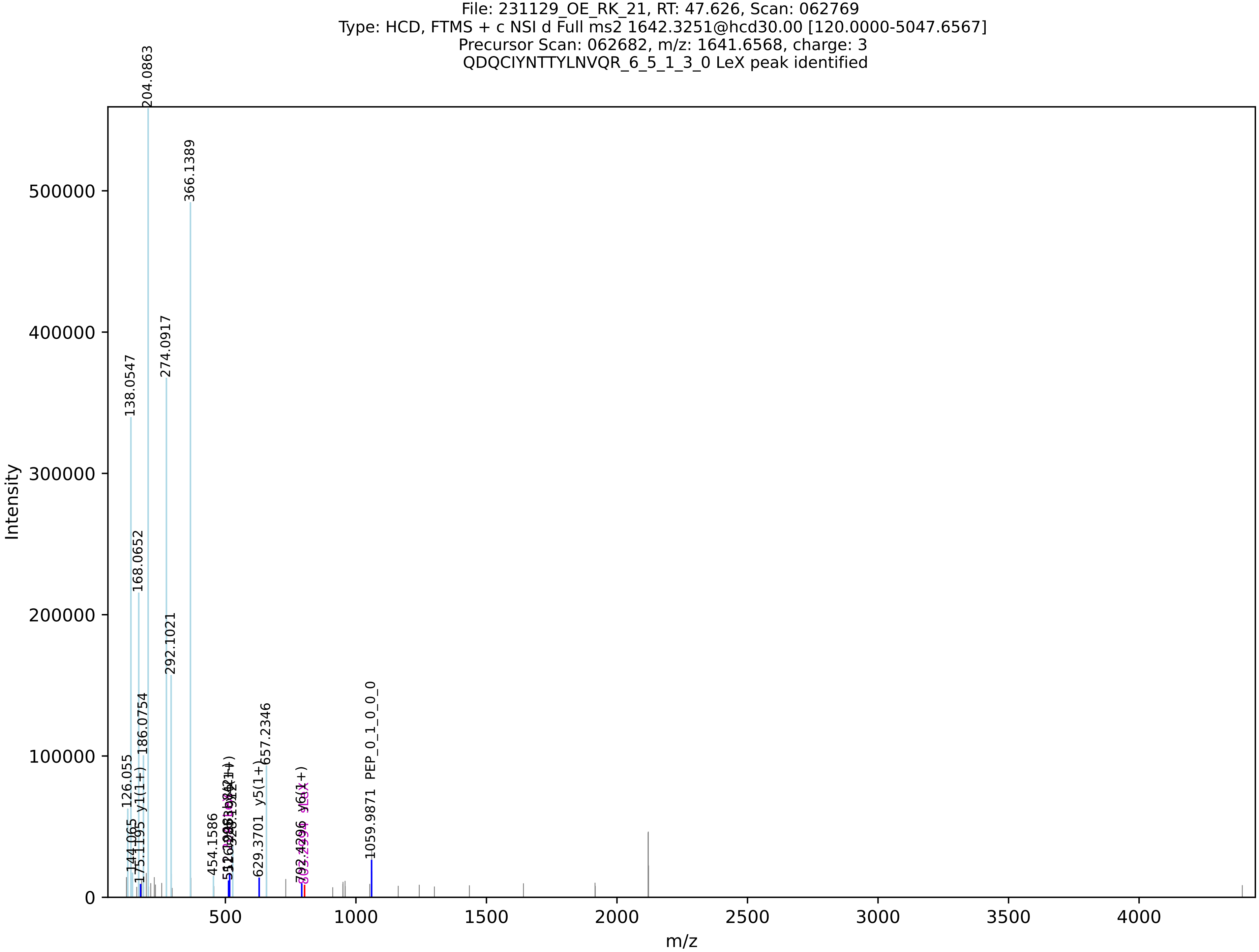

File: 231129_OE_RK_21, RT: 47.626, Scan: 062769
Type: HCD, FTMS + c NSI d Full ms2 1642.3251@hcd30.00 [120.0000-5047.6567]
Precursor Scan: 062682, m/z: 1641.6568, charge: 3
QDQCIYNTTYLNVQR_6_5_1_3_0 LeX peak identified
204.0863
366.1389
274.0917
138.0547
168.0652
292.1021
186.0754
126.055
y1(1+)
144.065
175.1195
454.1586
657.2346
629.3701
y5(1+)
792.4296
y6(1+)
1059.9871
PEP_0_1_0_0_0_
Intensity
m/z
0
100000
200000
300000
400000
500000
500
1000
1500
2000
2500
3000
3500
4000

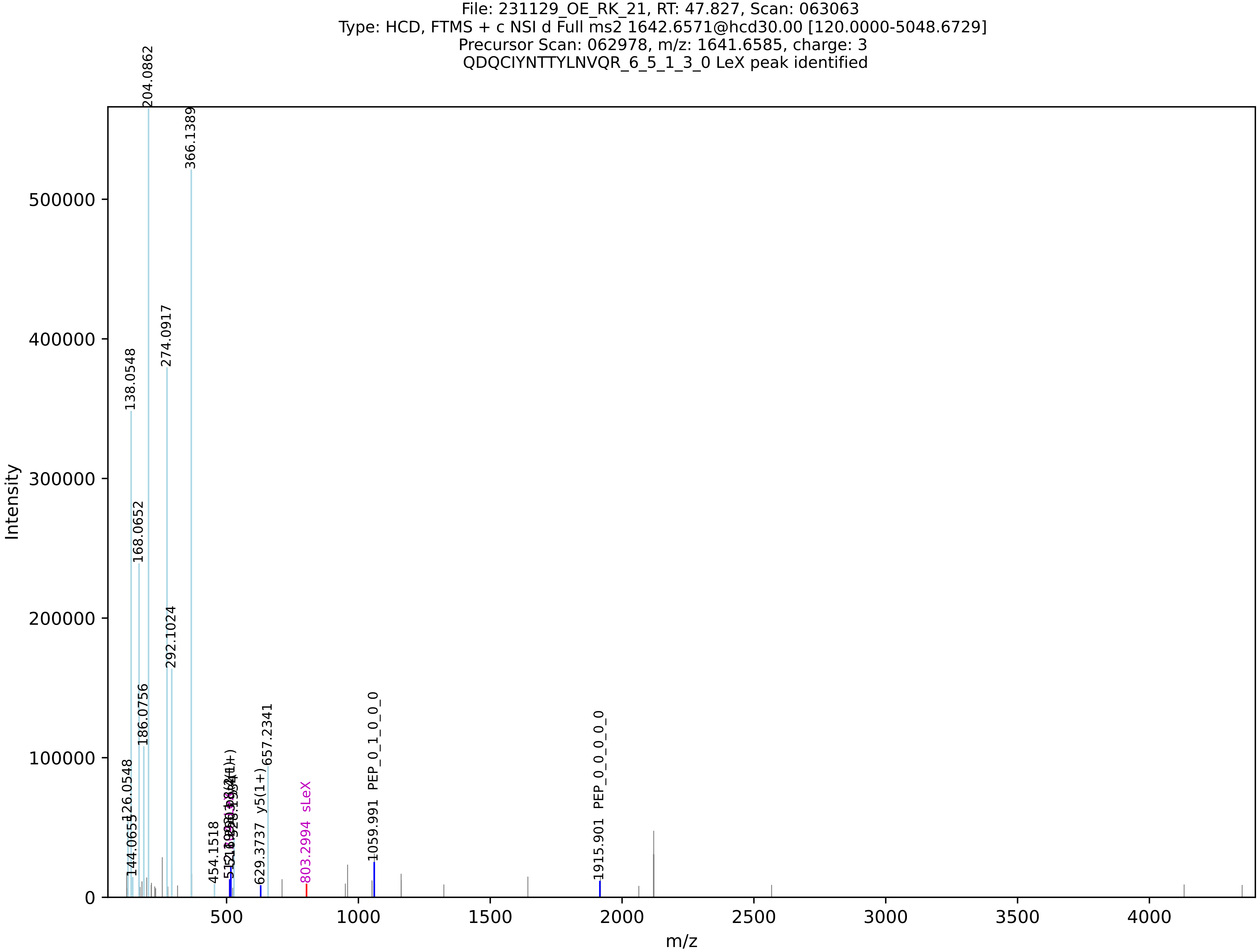
File: 231129_OE_RK_21, RT: 47.827, Scan: 063063
Type: HCD, FTMS + c NSI d Full ms2 1642.6571@hcd30.00 [120.0000-5048.6729]
Precursor Scan: 062978, m/z: 1641.6585, charge: 3
QDQCIYNTTYLNVQR_6_5_1_3_0 LeX peak identified
Intensity
m/z
0
100000
200000
300000
400000
500000
500
1000
1500
2000
2500
3000
3500
4000
204.0862
366.1389
274.0917
138.0548
168.0652
292.1024
186.0756
126.0548
144.0655
454.1518
657.2341
629.3737 y5(1+)
803.2994 sLeX
1059.991 PEP_0_1_0_0_0
1915.901 PEP_0_0_0_0_0

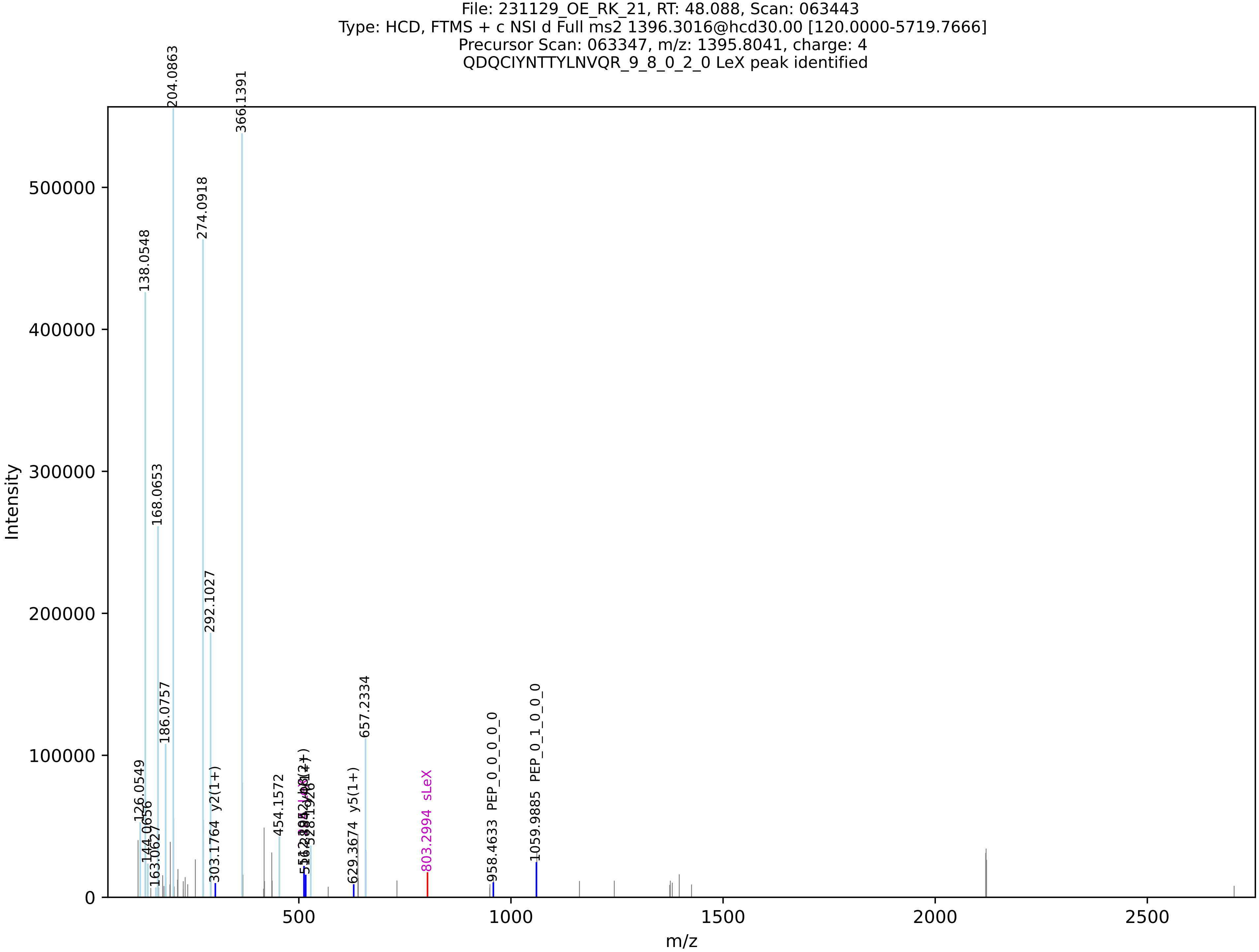
File: 231129_OE_RK_21, RT: 48.088, Scan: 063443
Type: HCD, FTMS + c NSI d Full ms2 1396.3016@hcd30.00 [120.0000-5719.7666]
Precursor Scan: 063347, m/z: 1395.8041, charge: 4
QDQCIYNTTYLNVQR_9_8_0_2_0 LeX peak identified
204.0863
366.1391
274.0918
138.0548
168.0653
292.1027
186.0757
657.2334
126.0549
144.0656
163.0627
303.1764 y2(1+)
454.1572
629.3674 y5(1+)
803.2994 sLeX
958.4633 PEP_0_0_0_0_0
1059.9885 PEP_0_1_0_0_0
Intensity
m/z
0
100000
200000
300000
400000
500000
500
1000
1500
2000
2500

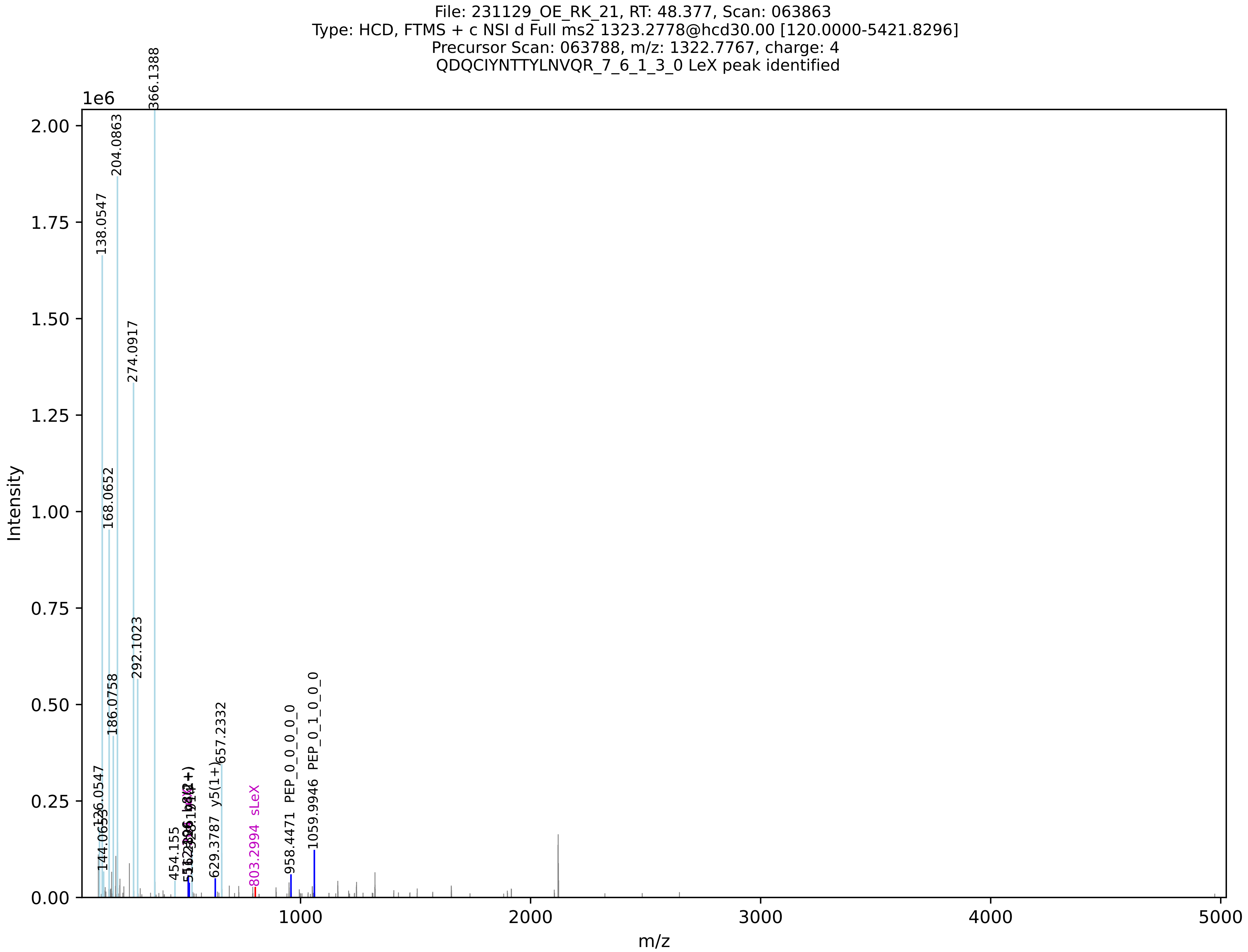

File: 231129_OE_RK_21, RT: 48.377, Scan: 063863
Type: HCD, FTMS + c NSI d Full ms2 1323.2778@hcd30.00 [120.0000-5421.8296]
Precursor Scan: 063788, m/z: 1322.7767, charge: 4
QDQCIYNTTYLNVQR_7_6_1_3_0 LeX peak identified
1e6
Intensity
m/z
366.1388
204.0863
138.0547
274.0917
168.0652
292.1023
186.0758
657.2332
126.0547
144.0655
454.155
629.3787 y5(1+)
803.2994 sLeX
958.4471 PEP_0_0_0_0_0
1059.9946 PEP_0_1_0_0_0

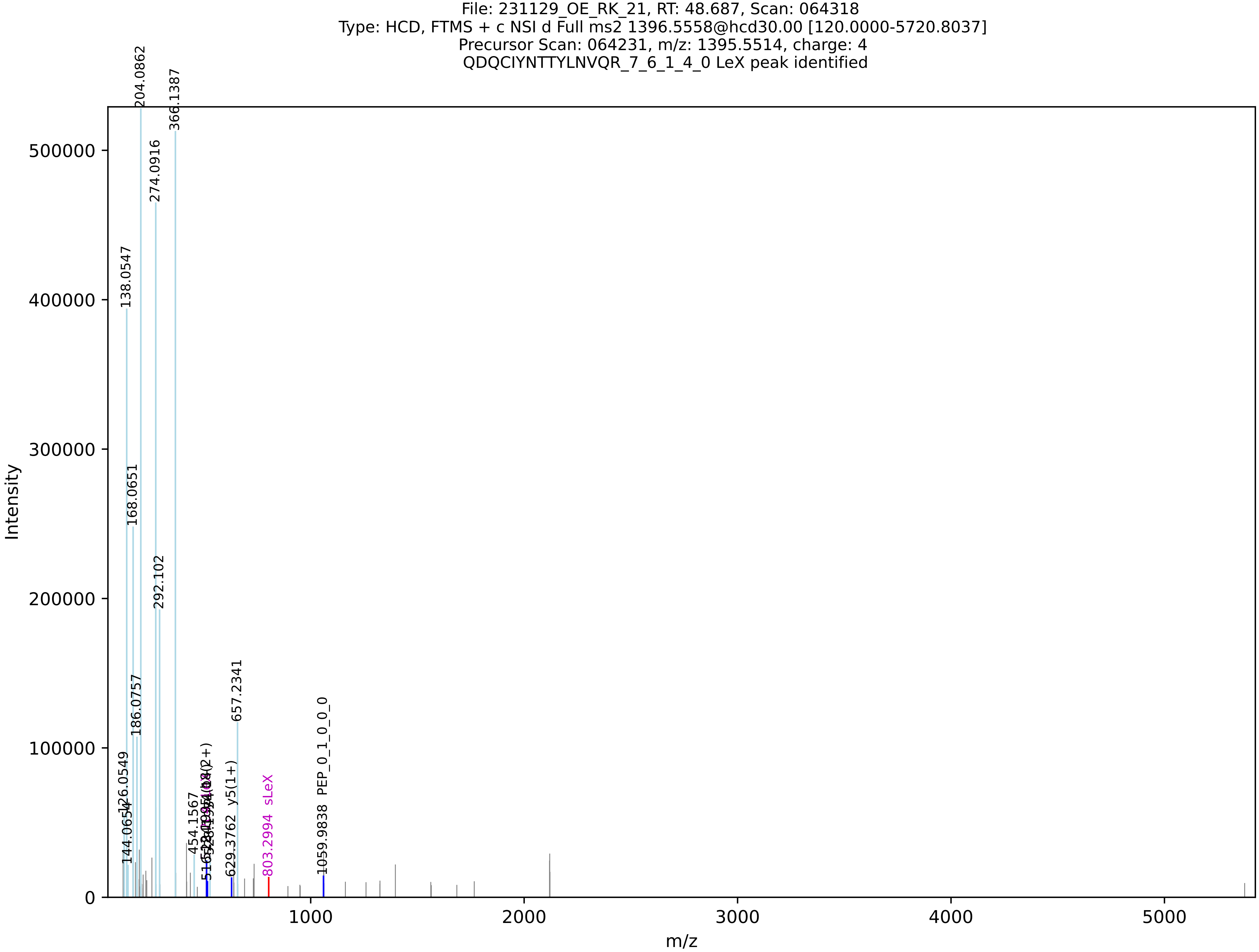

File: 231129_OE_RK_21, RT: 48.687, Scan: 064318
Type: HCD, FTMS + c NSI d Full ms2 1396.5558@hcd30.00 [120.0000-5720.8037]
Precursor Scan: 064231, m/z: 1395.5514, charge: 4
QDQCIYNTTYLNVQR_7_6_1_4_0 LeX peak identified
Intensity
m/z
204.0862
366.1387
274.0916
138.0547
168.0651
292.102
186.0757
657.2341
126.0549
144.0654
454.1567
629.3762 y5(1+)
803.2994 sLeX
1059.9838 PEP_0_1_0_0_0_

File: 231129_OE_RK_21, RT: 48.736, Scan: 064388
Type: HCD, FTMS + c NSI d Full ms2 1764.3688@hcd30.00 [120.0000-5421.1104]
Precursor Scan: 064304, m/z: 1763.3657, charge: 3
QDQCIYNTTYLNVQR_7_6_1_3_0 LeX peak identified

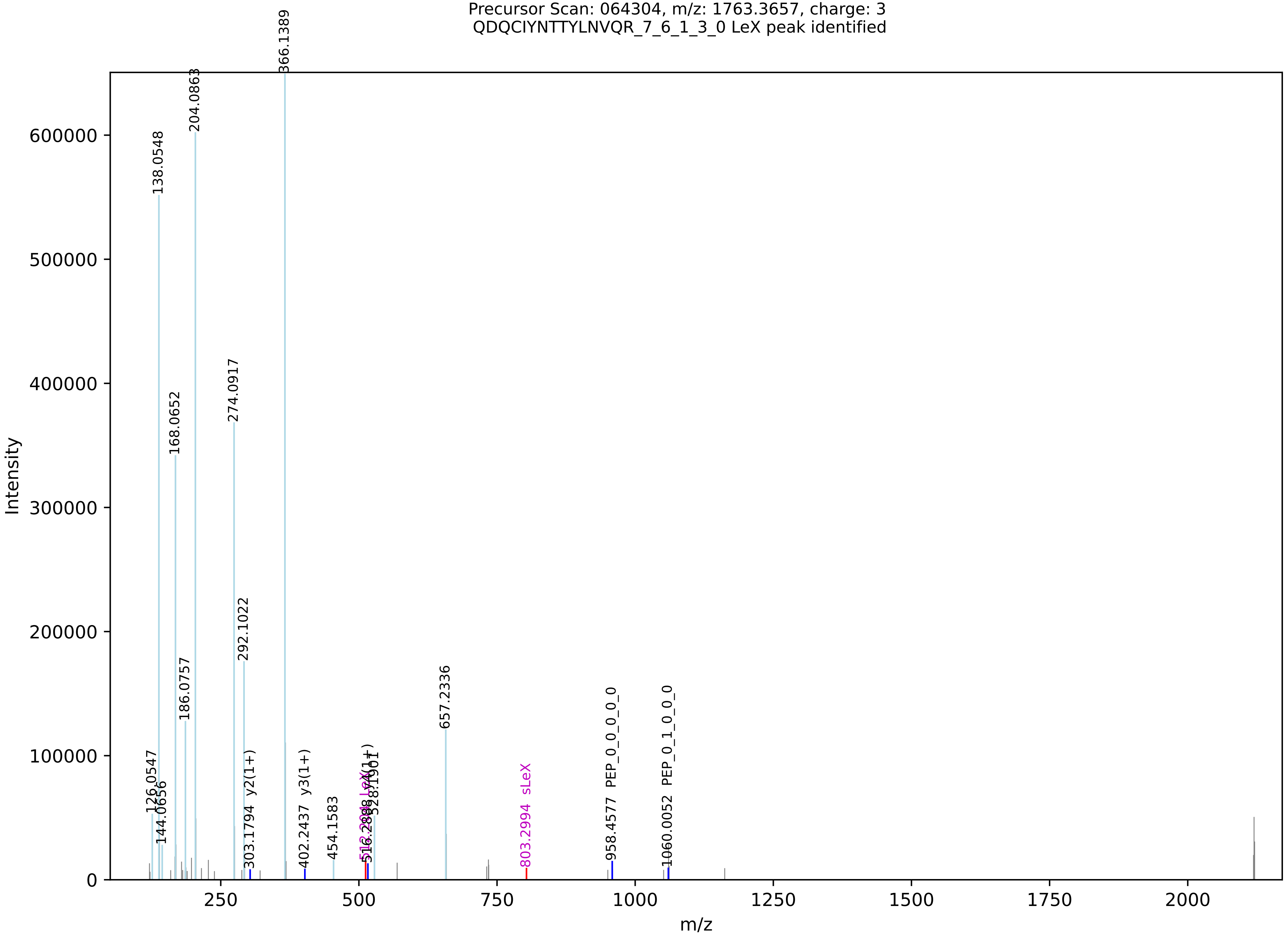

File: 231129_OE_RK_21, RT: 48.884, Scan: 064597
Type: HCD, FTMS + c NSI d Full ms2 1360.0435@hcd30.00 [120.0000-5571.8330]
Precursor Scan: 064522, m/z: 1359.2917, charge: 4
QDQCIYNTTYLNVQR_7_6_2_3_0 LeX peak identified

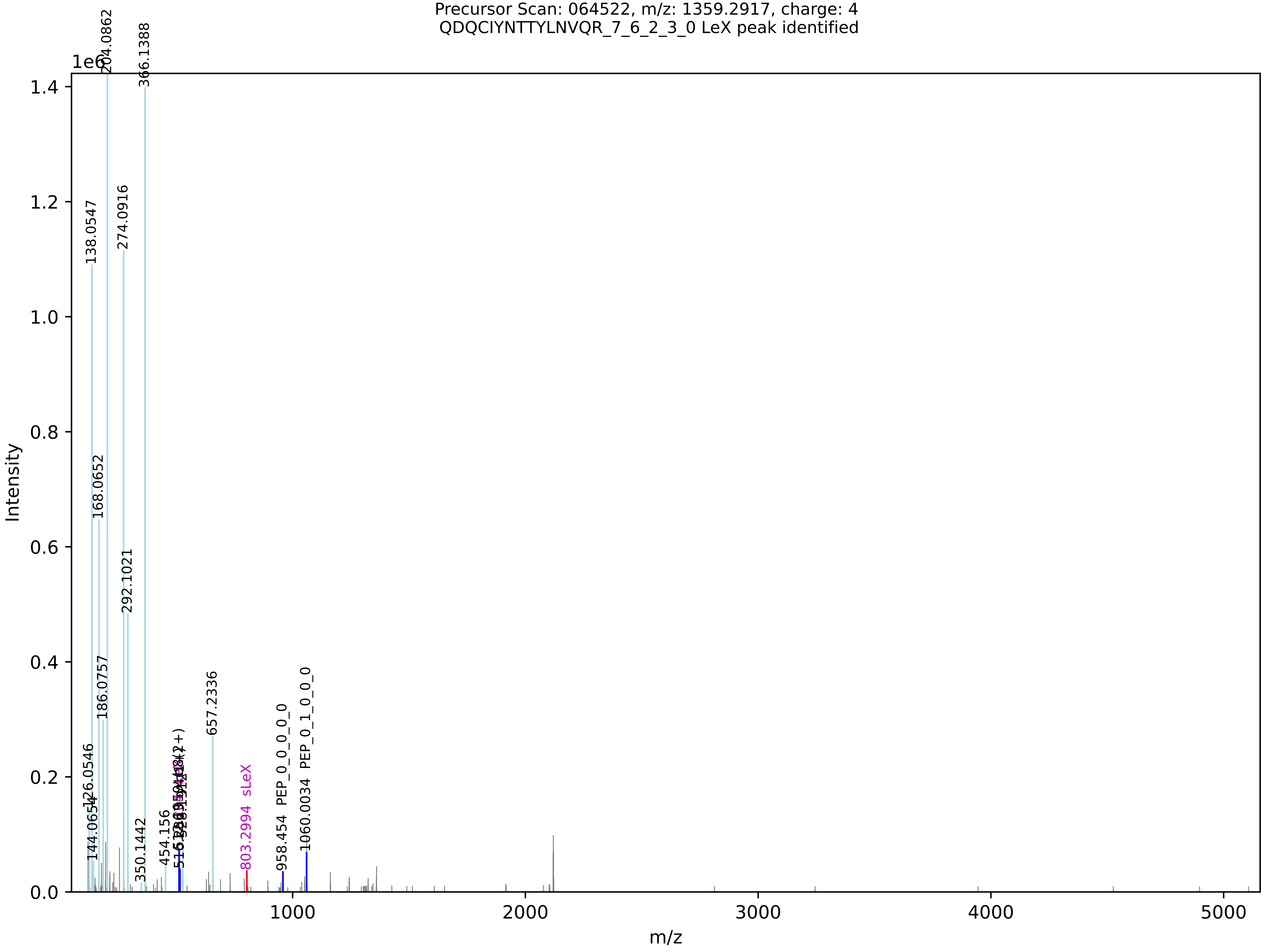

File: 231129_OE_RK_21, RT: 49.291, Scan: 065187
Type: HCD, FTMS + c NSI d Full ms2 1396.0535@hcd30.00 [120.0000-5718.7539]
Precursor Scan: 065100, m/z: 1395.5523, charge: 4
QDQCIYNTTYLNVQR_7_6_1_4_0 LeX peak identified

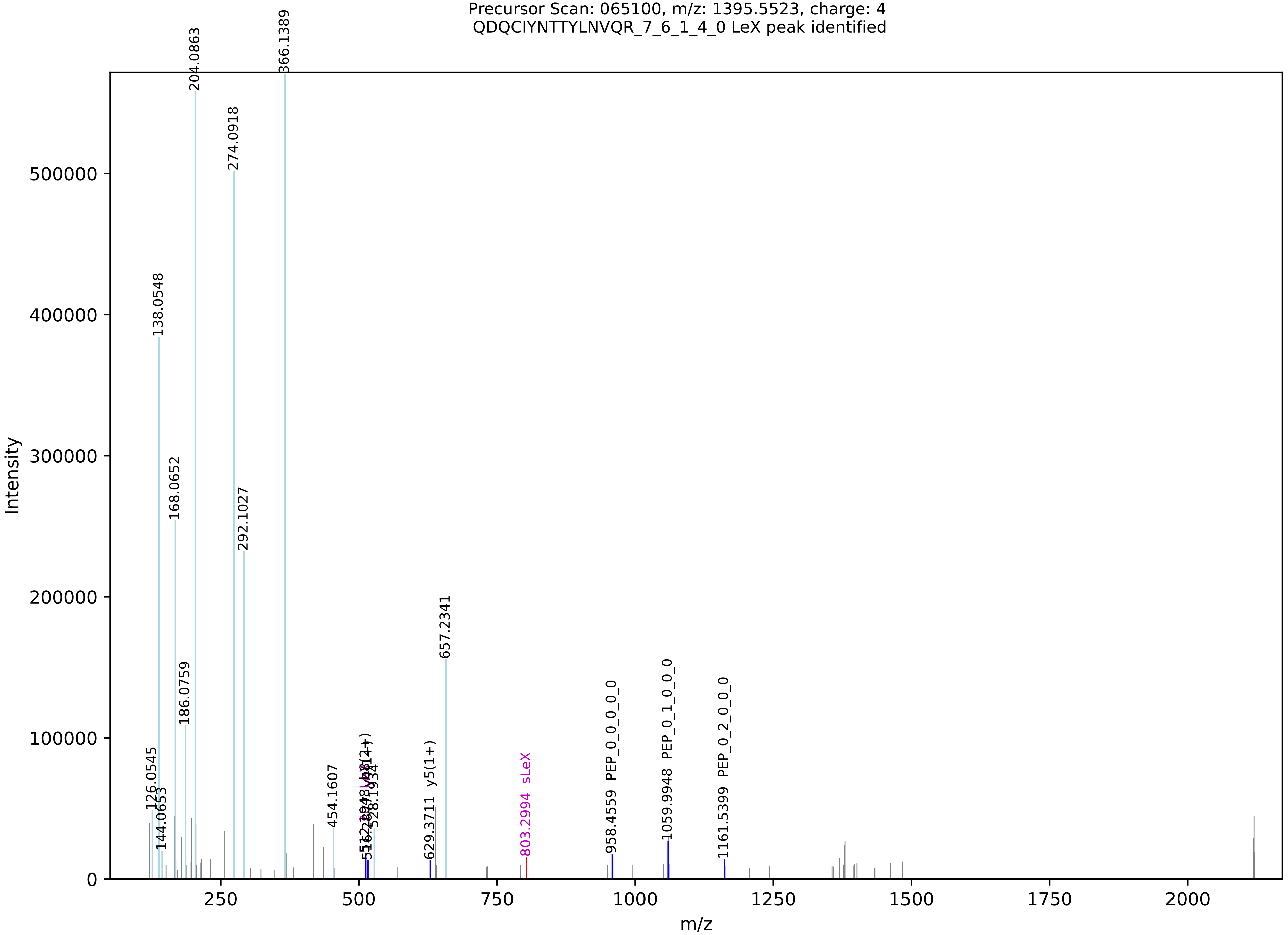

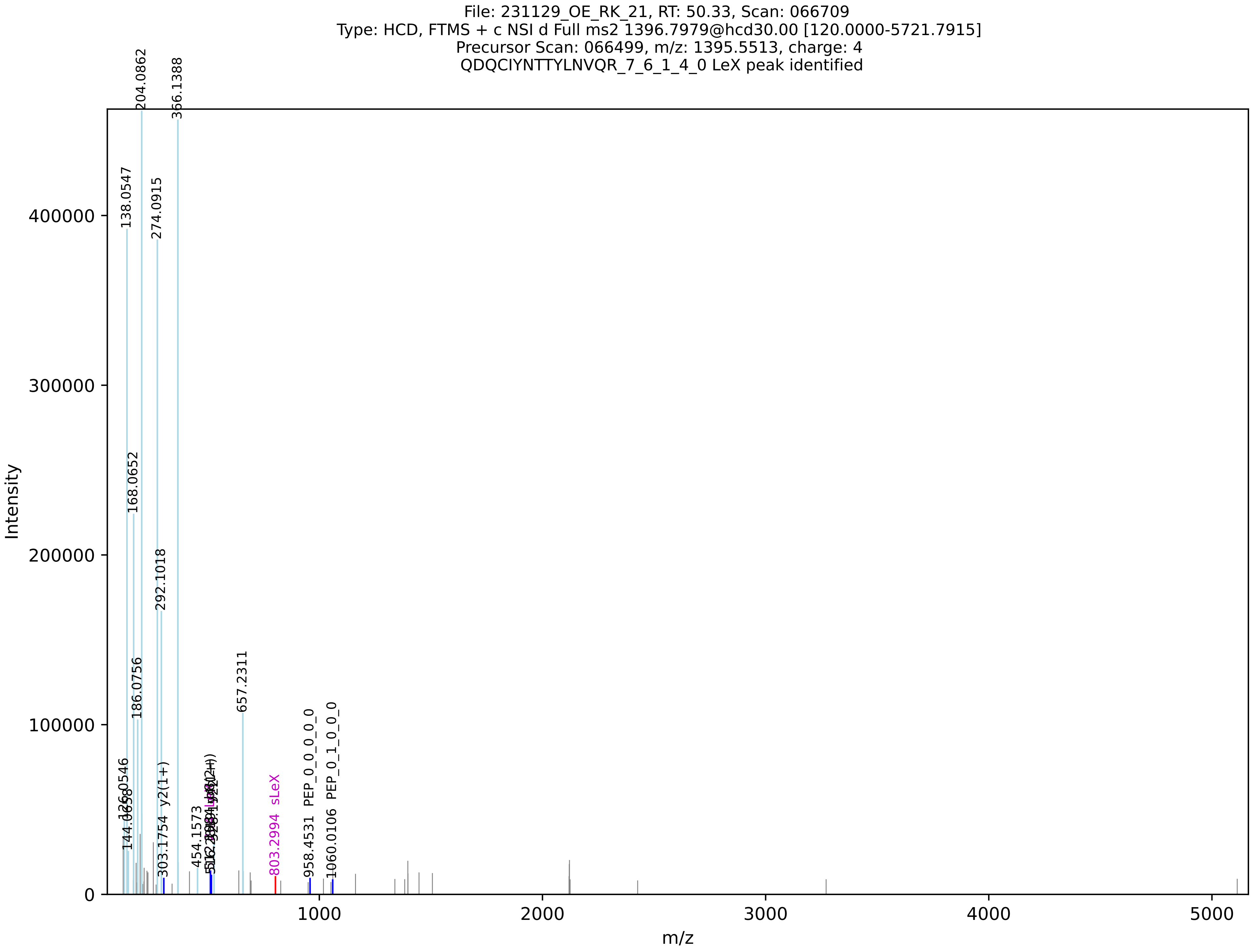

File: 231129_OE_RK_21, RT: 50.33, Scan: 066709
Type: HCD, FTMS + c NSI d Full ms2 1396.7979@hcd30.00 [120.0000-5721.7915]
Precursor Scan: 066499, m/z: 1395.5513, charge: 4
QDQCIYNTTYLNVQR_7_6_1_4_0 LeX peak identified
204.0862
366.1388
138.0547
274.0915
168.0652
292.1018
186.0756
657.2311
126.0546
144.0658
303.1754 y2(1+)
454.1573
803.2994 sLeX
958.4531 PEP_0_0_0_0_0
1060.0106 PEP_0_1_0_0_0
Intensity
m/z
0
100000
200000
300000
400000
1000
2000
3000
4000
5000

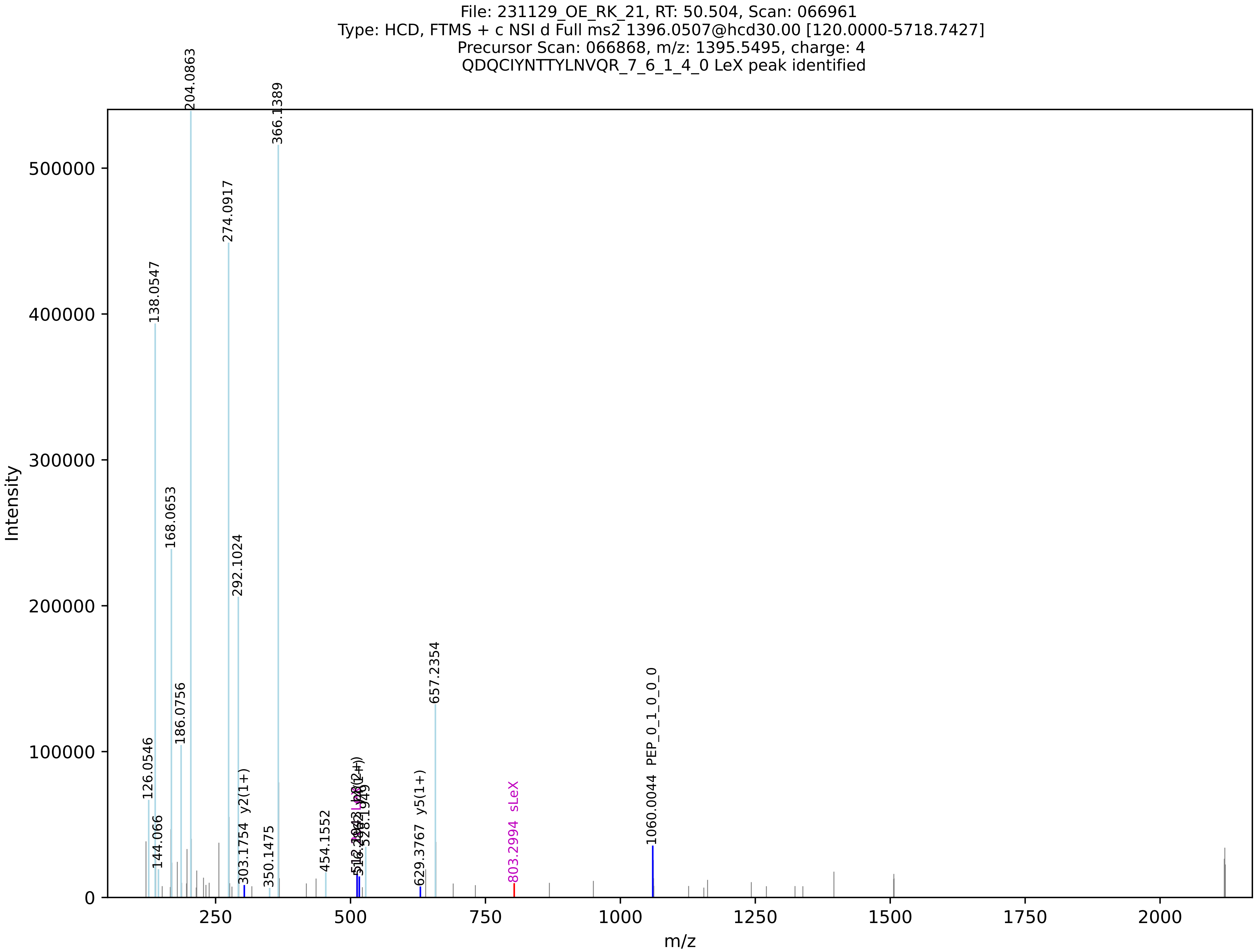

File: 231129_OE_RK_21, RT: 50.504, Scan: 066961
Type: HCD, FTMS + c NSI d Full ms2 1396.0507@hcd30.00 [120.0000-5718.7427]
Precursor Scan: 066868, m/z: 1395.5495, charge: 4
QDQCIYNTTYLNVQR_7_6_1_4_0 LeX peak identified
Intensity
m/z
204.0863
366.1389
274.0917
138.0547
168.0653
292.1024
657.2354
186.0756
126.0546
144.066
303.1754 y2(1+)
350.1475
454.1552
528.1949
629.3767 y5(1+)
803.2994 sLeX
1060.0044 PEP_0_1_0_0_0_

File: 231129_OE_RK_21, RT: 52.426, Scan: 069763
Type: HCD, FTMS + c NSI d Full ms2 1433.0679@hcd30.00 [120.0000-5869.7729]
Precursor Scan: 069672, m/z: 1432.0656, charge: 4
QDQCIYNTTYLNVQR_7_6_2_4_0 LeX peak identified

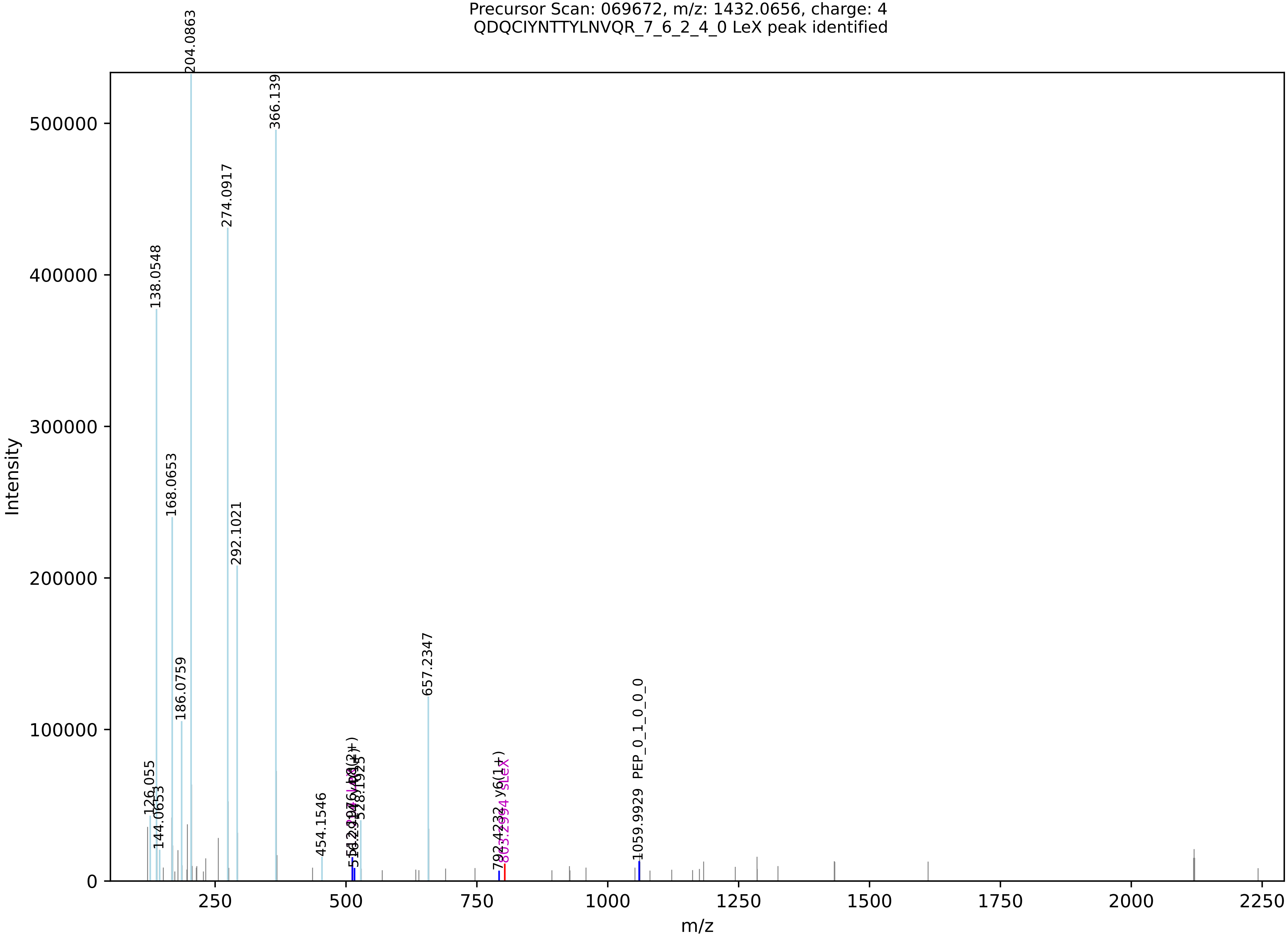

File: 231129_OE_RK_21, RT: 52.518, Scan: 069896
Type: HCD, FTMS + c NSI d Full ms2 1266.5460@hcd30.00 [120.0000-3897.7727]
Precursor Scan: 069820, m/z: 1265.8777, charge: 3
ALPQPQNVTSLLGCTH_5_4_1_1_0 LeX peak identified

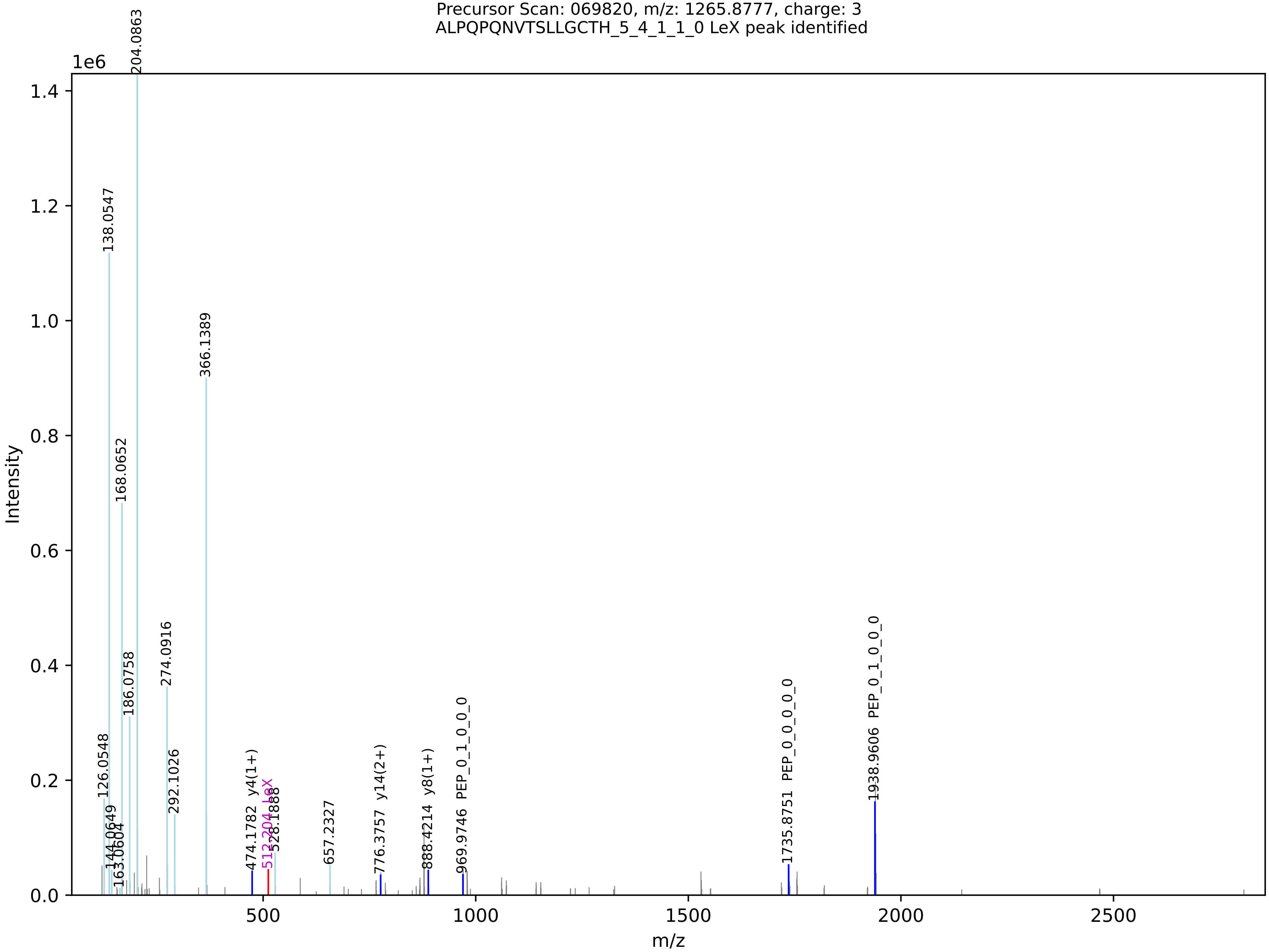

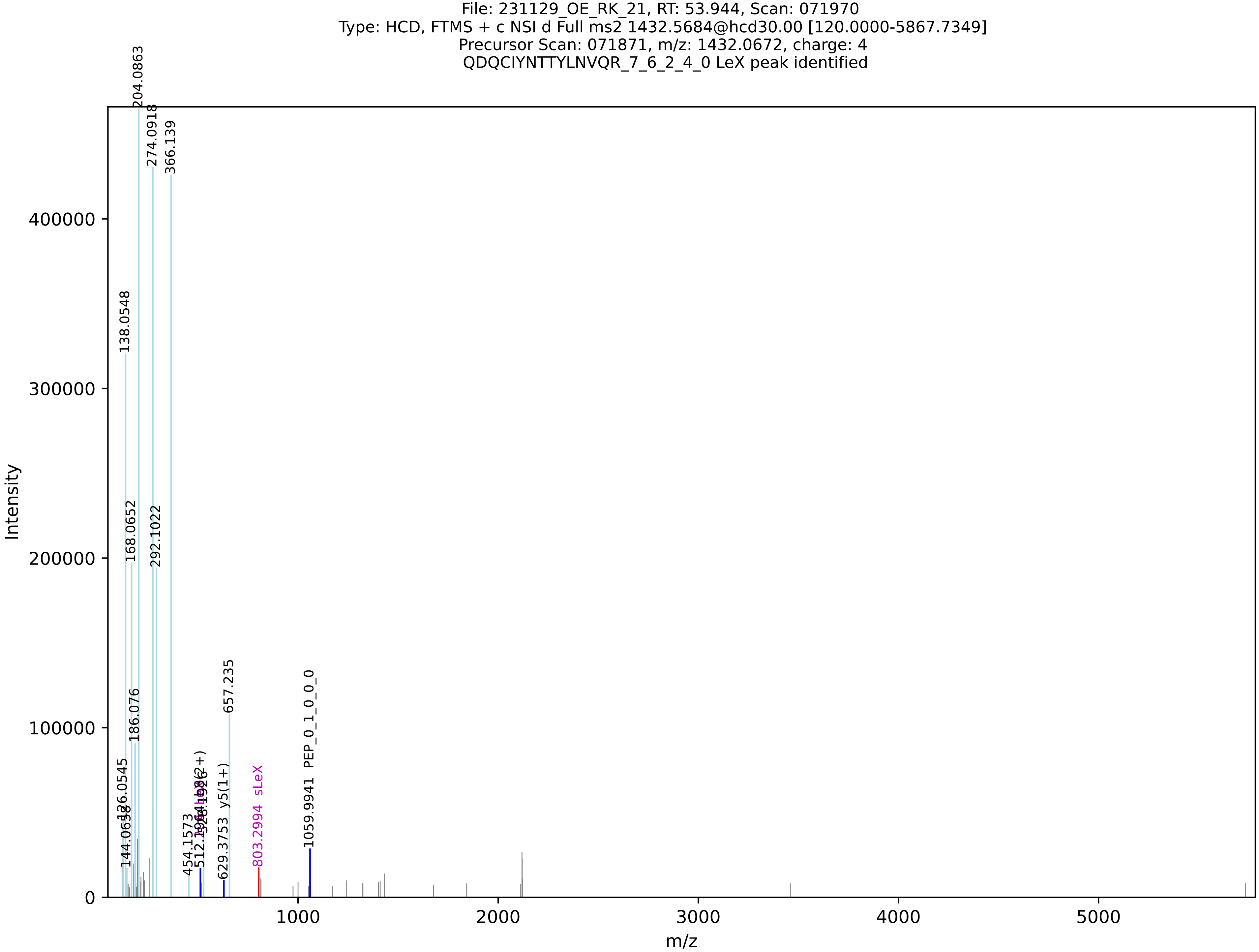

File: 231129_OE_RK_21, RT: 53.944, Scan: 071970
Type: HCD, FTMS + c NSI d Full ms2 1432.5684@hcd30.00 [120.0000-5867.7349]
Precursor Scan: 071871, m/z: 1432.0672, charge: 4
QDQCIYNTTYLNVQR_7_6_2_4_0 LeX peak identified
204.0863
274.0918
366.139
138.0548
168.0652
292.1022
186.076
657.235
PEP_0_1_0_0_0
1059.9941
803.2994 sLeX
629.3753 y5(1+)
454.1573
126.0545
144.0658
Intensity
m/z
0
100000
200000
300000
400000
1000
2000
3000
4000
5000

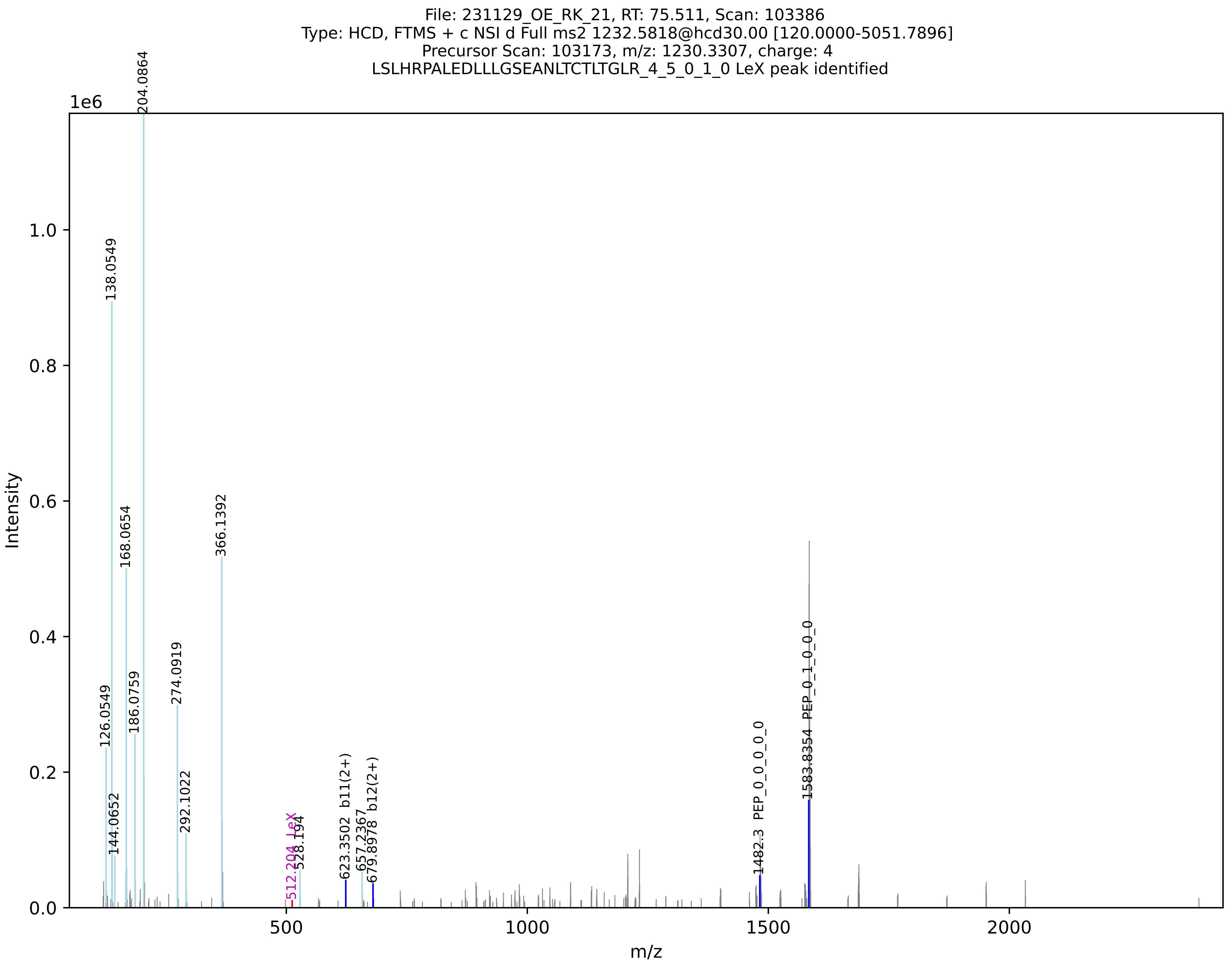

File: 231129_OE_RK_21, RT: 75.511, Scan: 103386
Type: HCD, FTMS + c NSI d Full ms2 1232.5818@hcd30.00 [120.0000-5051.7896]
Precursor Scan: 103173, m/z: 1230.3307, charge: 4
LSLHRPALEDLLLGSEANLTCTLTGLR_4_5_0_1_0 LeX peak identified
1e6
Intensity
m/z
204.0864
138.0549
168.0654
366.1392
274.0919
126.0549
186.0759
292.1022
144.0652
512.204 LeX
528.194
623.3502 b11(2+)
657.2367
679.8978 b12(2+)
1482.3 PEP_0_0_0_0_0
1583.8354 PEP_0_1_0_0_0

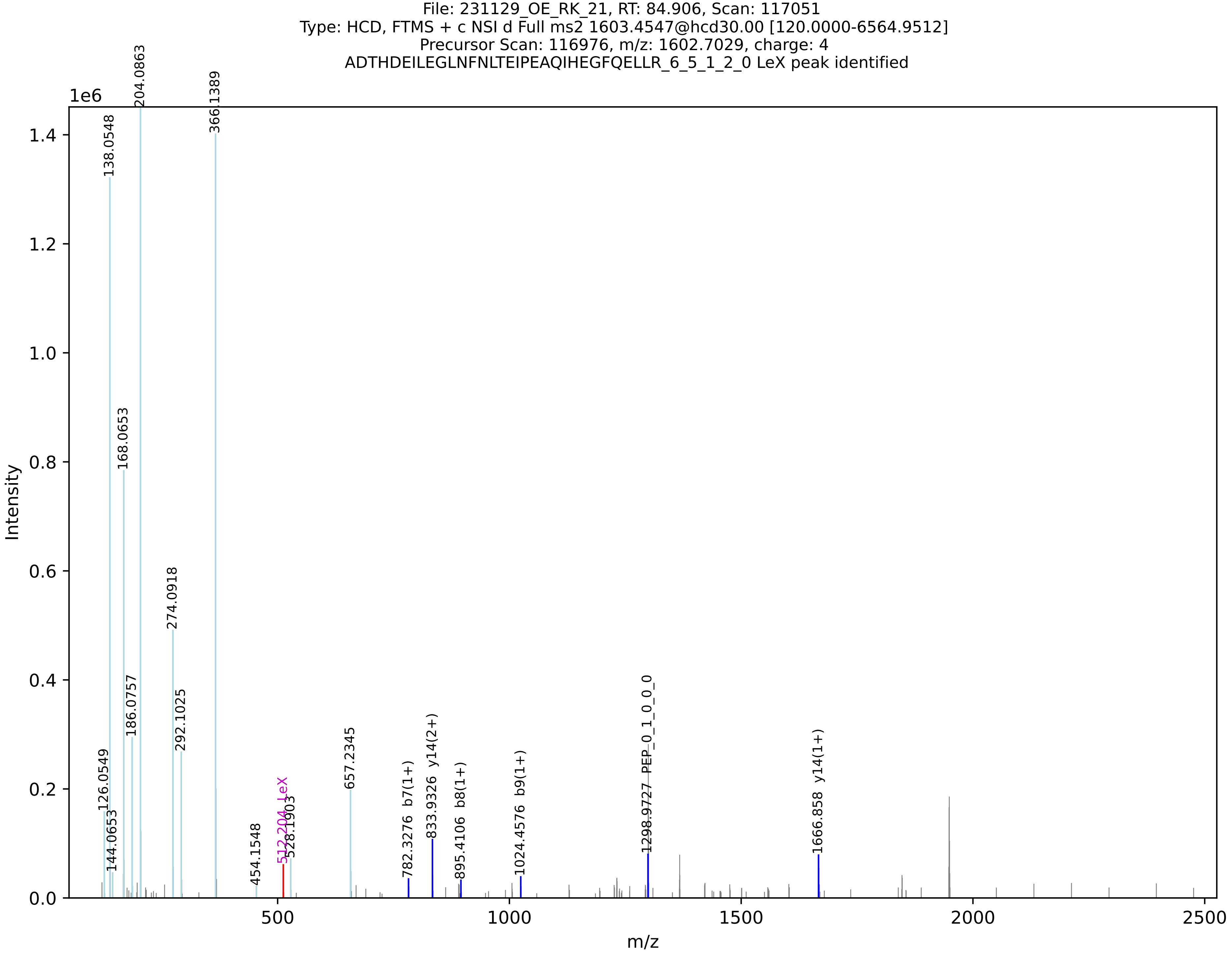

File: 231129_OE_RK_21, RT: 84.906, Scan: 117051
Type: HCD, FTMS + c NSI d Full ms2 1603.4547@hcd30.00 [120.0000-6564.9512]
Precursor Scan: 116976, m/z: 1602.7029, charge: 4
ADTHDEILEGLNFNLTEIPEAQIHEGFQELLR_6_5_1_2_0 LeX peak identified
1e6
Intensity
m/z
204.0863
366.1389
138.0548
168.0653
274.0918
186.0757
292.1025
126.0549
144.0653
454.1548
512.204 LeX
528.1903
657.2345
782.3276 b7(1+)
833.9326 y14(2+)
895.4106 b8(1+)
1024.4576 b9(1+)
1298.9727 PEP_0_1_0_0_0
1666.858 y14(1+)

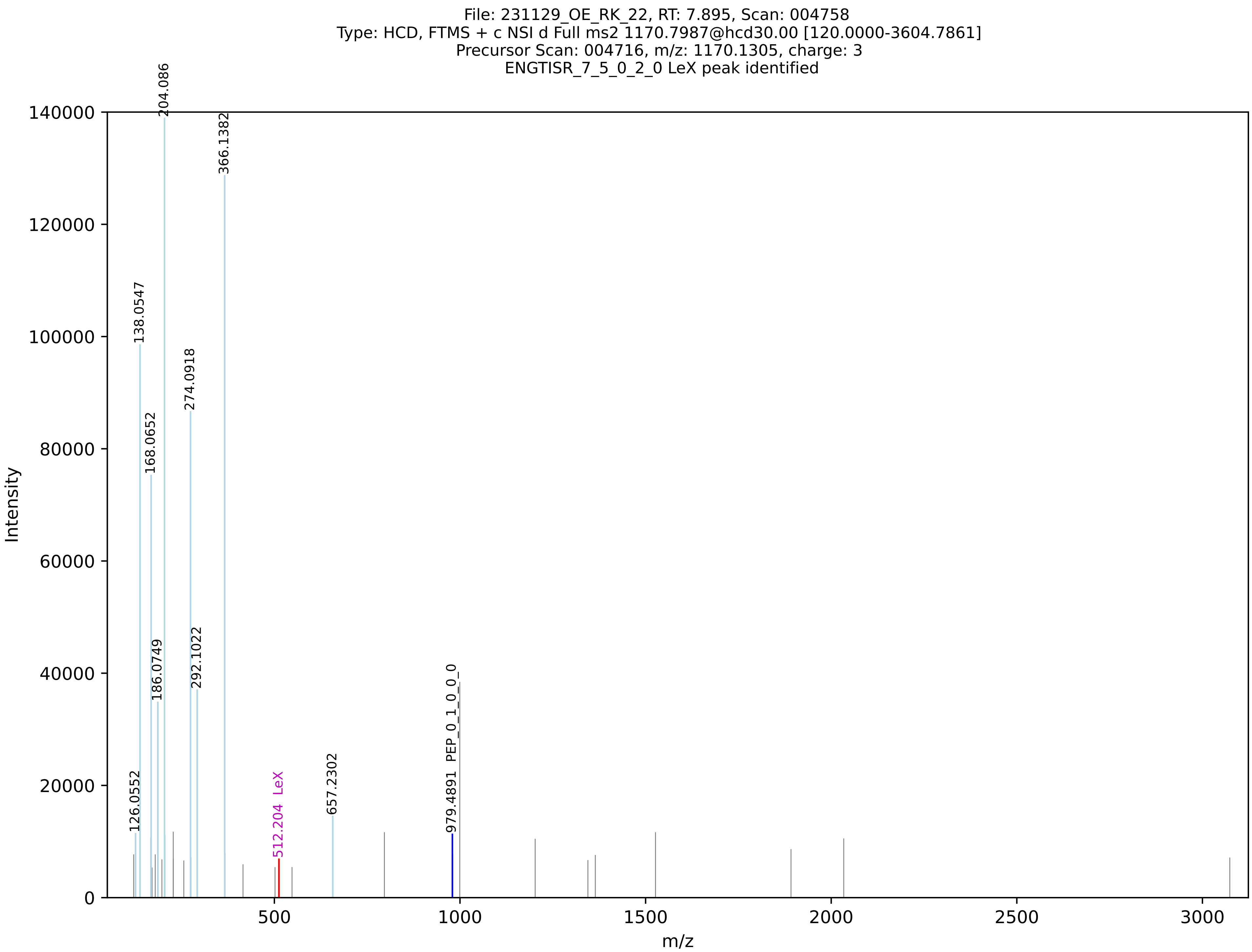
File: 231129_OE_RK_22, RT: 7.895, Scan: 004758
Type: HCD, FTMS + c NSI d Full ms2 1170.7987@hcd30.00 [120.0000-3604.7861]
Precursor Scan: 004716, m/z: 1170.1305, charge: 3
ENGTISR_7_5_0_2_0 LeX peak identified
Intensity
m/z
204.086
366.1382
138.0547
274.0918
168.0652
186.0749
292.1022
126.0552
512.204 LeX
657.2302
979.4891 PEP_0_1_0_0_0
0
20000
40000
60000
80000
100000
120000
140000
500
1000
1500
2000
2500
3000

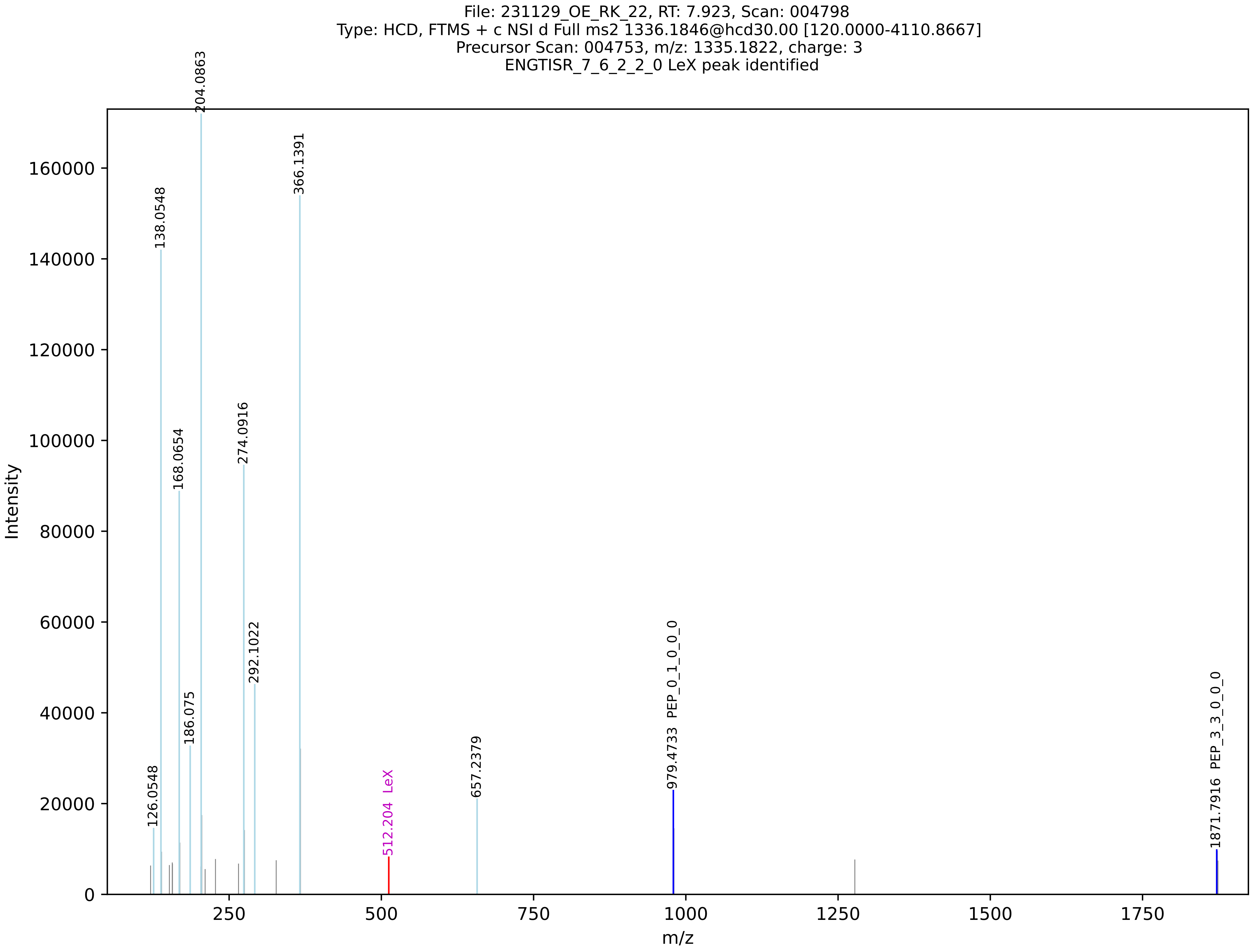

File: 231129_OE_RK_22, RT: 7.923, Scan: 004798
Type: HCD, FTMS + c NSI d Full ms2 1336.1846@hcd30.00 [120.0000-4110.8667]
Precursor Scan: 004753, m/z: 1335.1822, charge: 3
ENGTISR_7_6_2_2_0 LeX peak identified
204.0863
366.1391
138.0548
168.0654
274.0916
292.1022
186.075
126.0548
512.204 LeX
657.2379
979.4733 PEP_0_1_0_0_0
1871.7916 PEP_3_3_0_0_0
Intensity
m/z
0
20000
40000
60000
80000
100000
120000
140000
160000
250
500
750
1000
1250
1500
1750

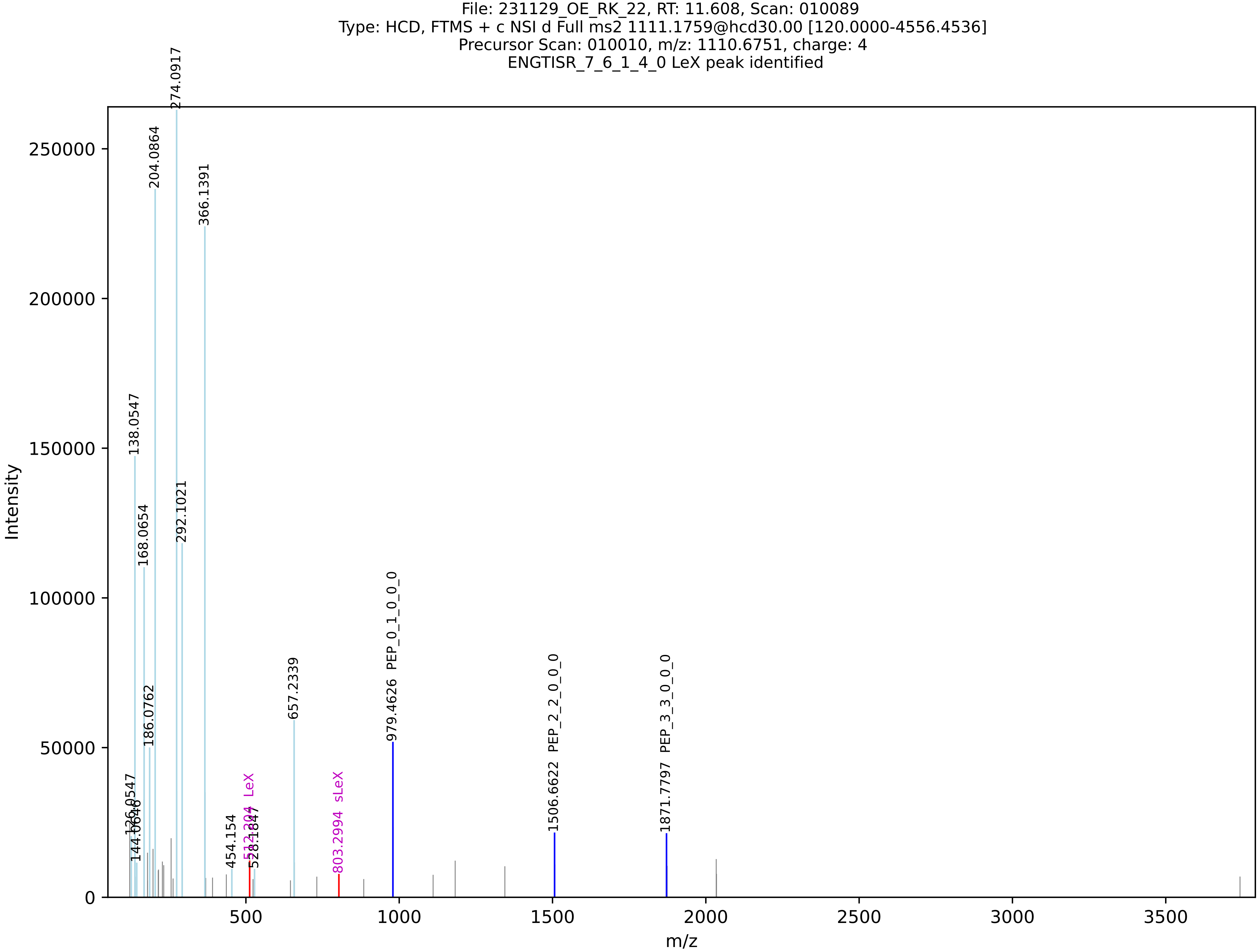

File: 231129_OE_RK_22, RT: 11.608, Scan: 010089
Type: HCD, FTMS + c NSI d Full ms2 1111.1759@hcd30.00 [120.0000-4556.4536]
Precursor Scan: 010010, m/z: 1110.6751, charge: 4
ENGTISR_7_6_1_4_0 LeX peak identified
Intensity
m/z
274.0917
204.0864
366.1391
138.0547
292.1021
168.0654
186.0762
126.0547
144.0648
454.154
512.204 LeX
528.1847
657.2339
803.2994 sLeX
979.4626 PEP_0_1_0_0_0
1506.6622 PEP_2_2_0_0_0
1871.7797 PEP_3_3_0_0_0

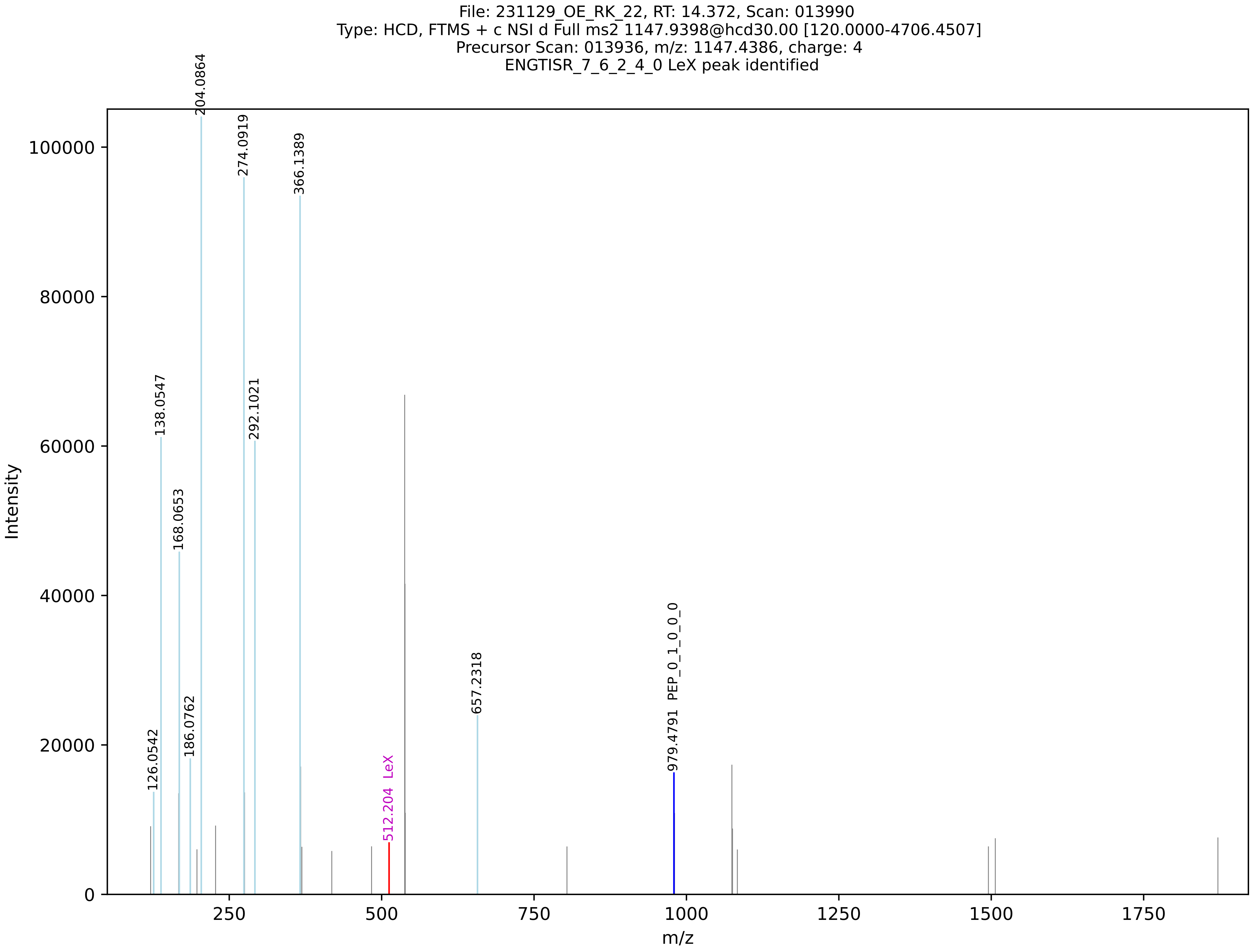

File: 231129_OE_RK_22, RT: 14.372, Scan: 013990
Type: HCD, FTMS + c NSI d Full ms2 1147.9398@hcd30.00 [120.0000-4706.4507]
Precursor Scan: 013936, m/z: 1147.4386, charge: 4
ENGTISR_7_6_2_4_0 LeX peak identified
204.0864
274.0919
366.1389
138.0547
292.1021
168.0653
186.0762
126.0542
512.204 LeX
657.2318
979.4791 PEP_0_1_0_0_0_
Intensity
m/z
0
20000
40000
60000
80000
100000
250
500
750
1000
1250
1500
1750

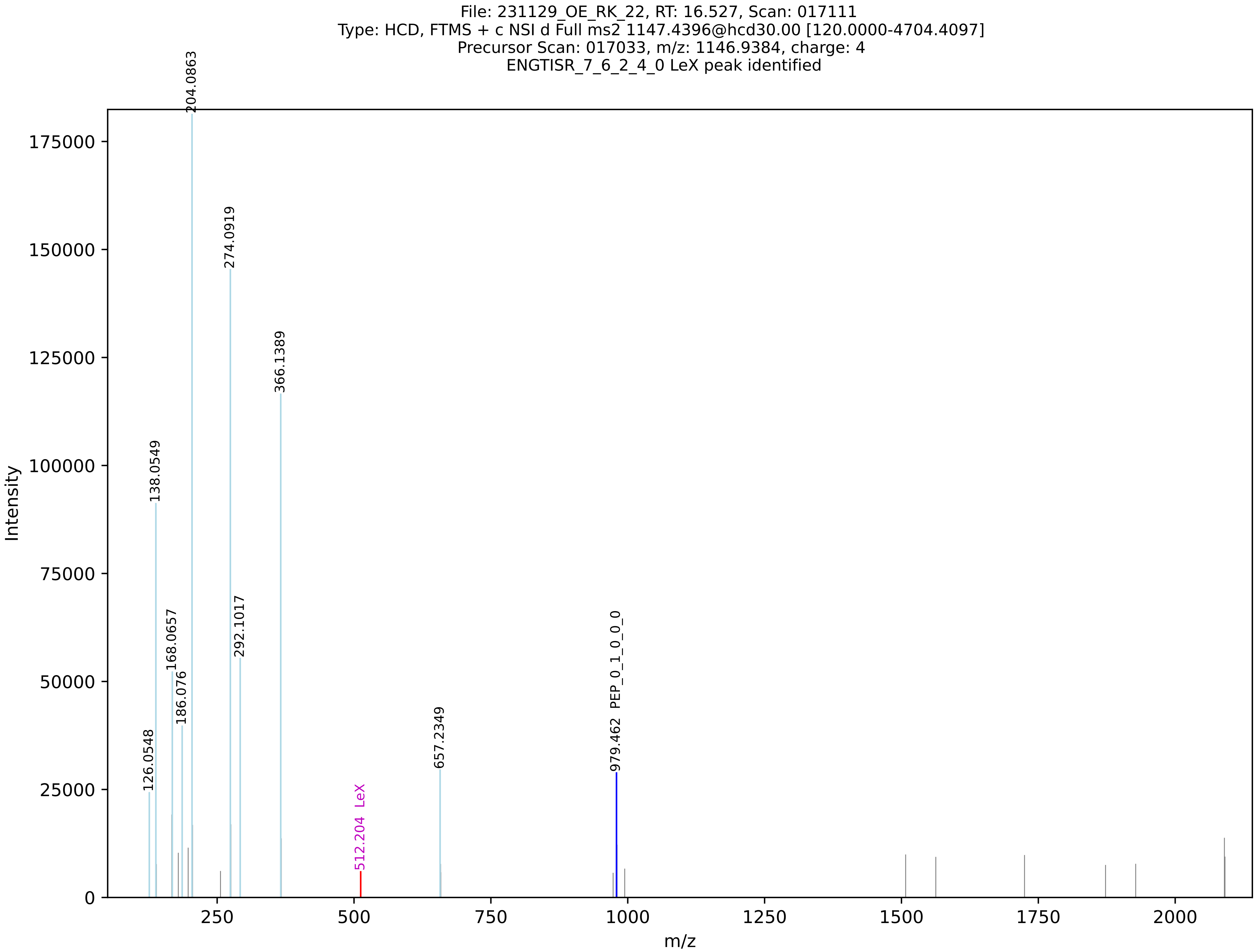
File: 231129_OE_RK_22, RT: 16.527, Scan: 017111
Type: HCD, FTMS + c NSI d Full ms2 1147.4396@hcd30.00 [120.0000-4704.4097]
Precursor Scan: 017033, m/z: 1146.9384, charge: 4
ENGTISR_7_6_2_4_0 LeX peak identified
Intensity
m/z
0
25000
50000
75000
100000
125000
150000
175000
250
500
750
1000
1250
1500
1750
2000
204.0863
274.0919
366.1389
138.0549
168.0657
292.1017
186.076
126.0548
512.204 LeX
657.2349
979.462 PEP_0_1_0_0_0

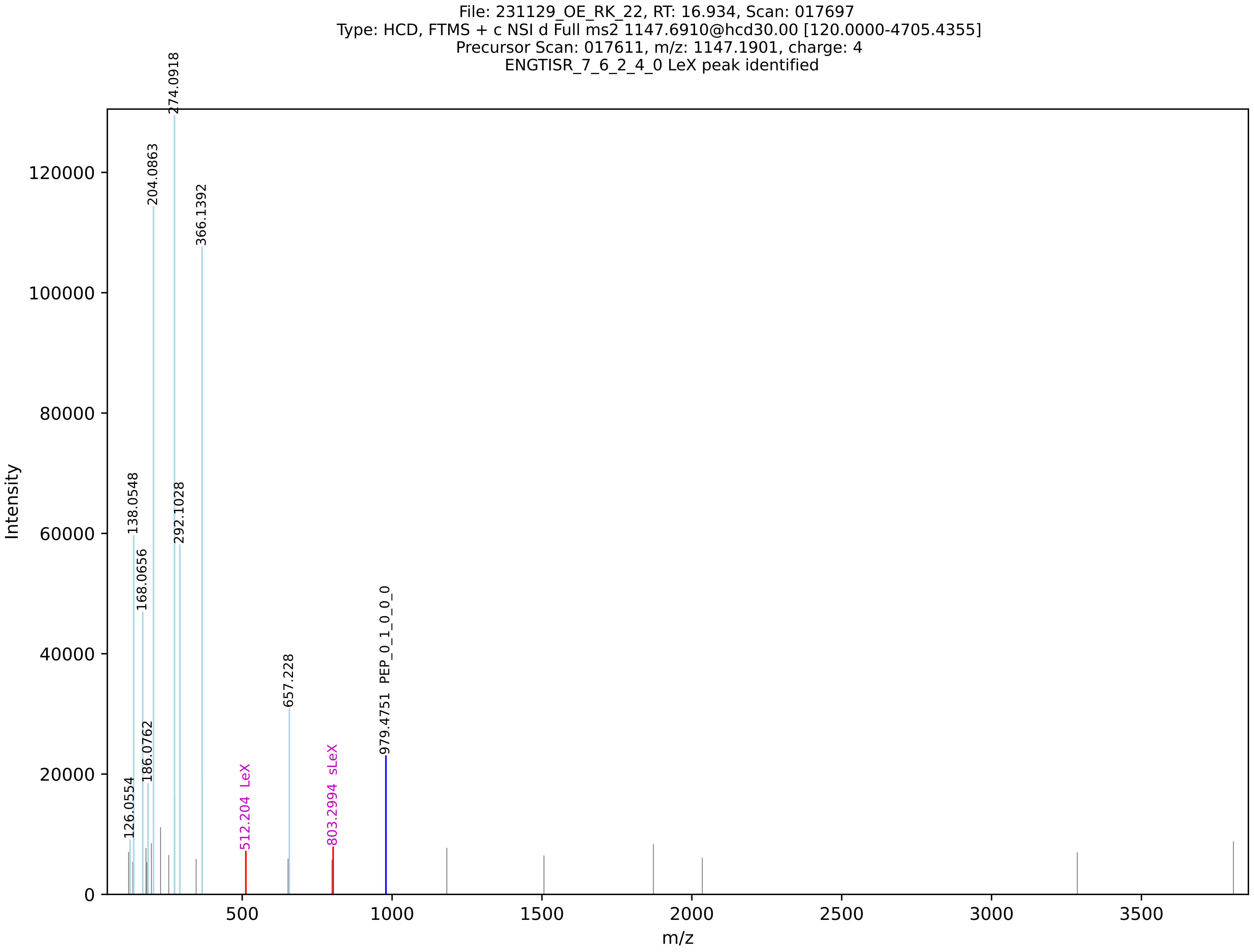
File: 231129_OE_RK_22, RT: 16.934, Scan: 017697
Type: HCD, FTMS + c NSI d Full ms2 1147.6910@hcd30.00 [120.0000-4705.4355]
Precursor Scan: 017611, m/z: 1147.1901, charge: 4
ENGTISR_7_6_2_4_0 LeX peak identified
274.0918
204.0863
366.1392
138.0548
292.1028
168.0656
186.0762
126.0554
657.228
979.4751 PEP_0_1_0_0_0_
512.204 LeX
803.2994 sLeX
Intensity
m/z
0
20000
40000
60000
80000
100000
120000
500
1000
1500
2000
2500
3000
3500

File: 231129_OE_RK_22, RT: 44.131, Scan: 056952
Type: HCD, FTMS + c NSI d Full ms2 1667.0027@hcd30.00 [120.0000-5123.1704]
Precursor Scan: 056870, m/z: 1666.3338, charge: 3
QDQCIYNTTYLNVQR_7_6_1_2_0 LeX peak identified

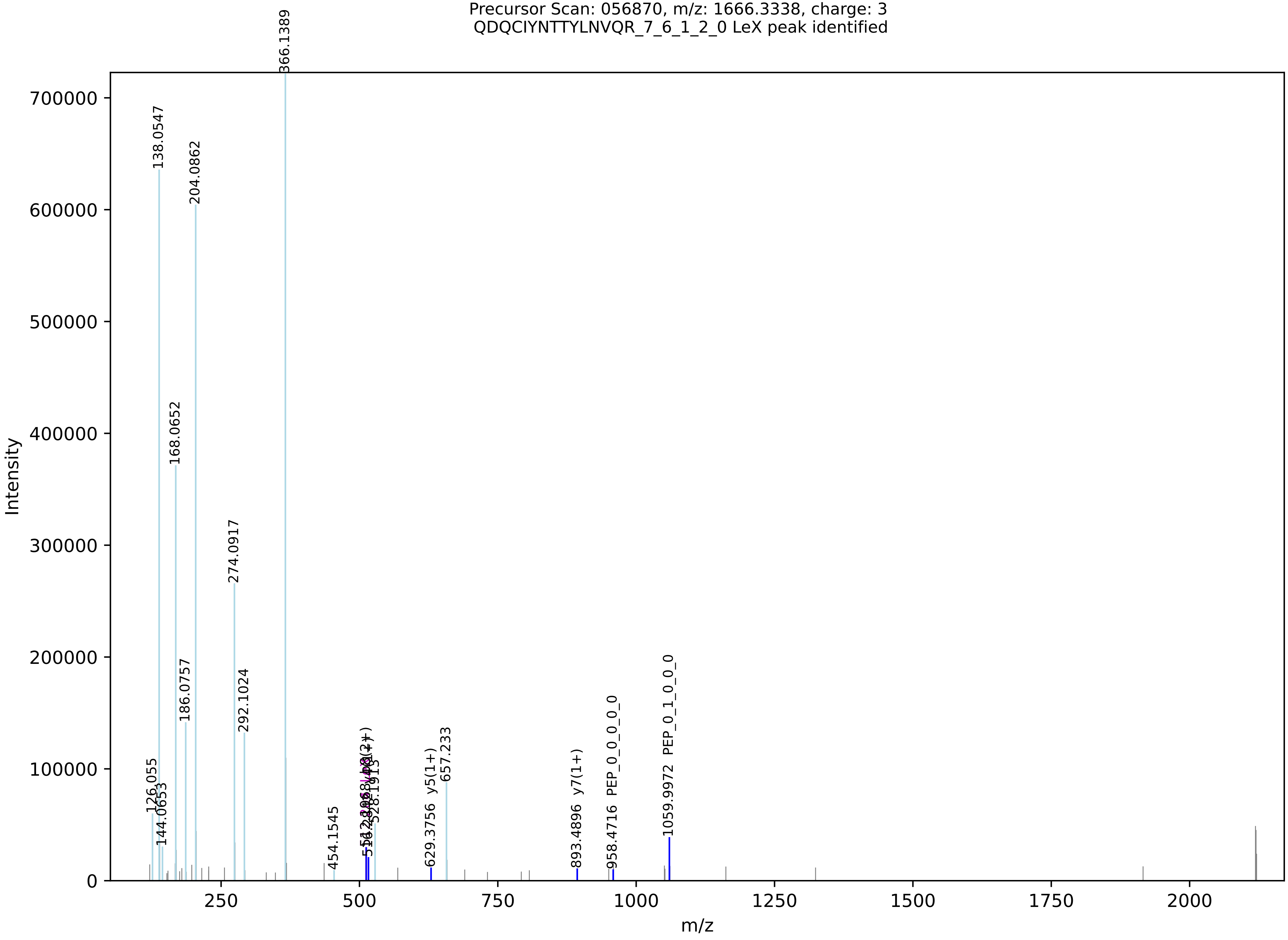

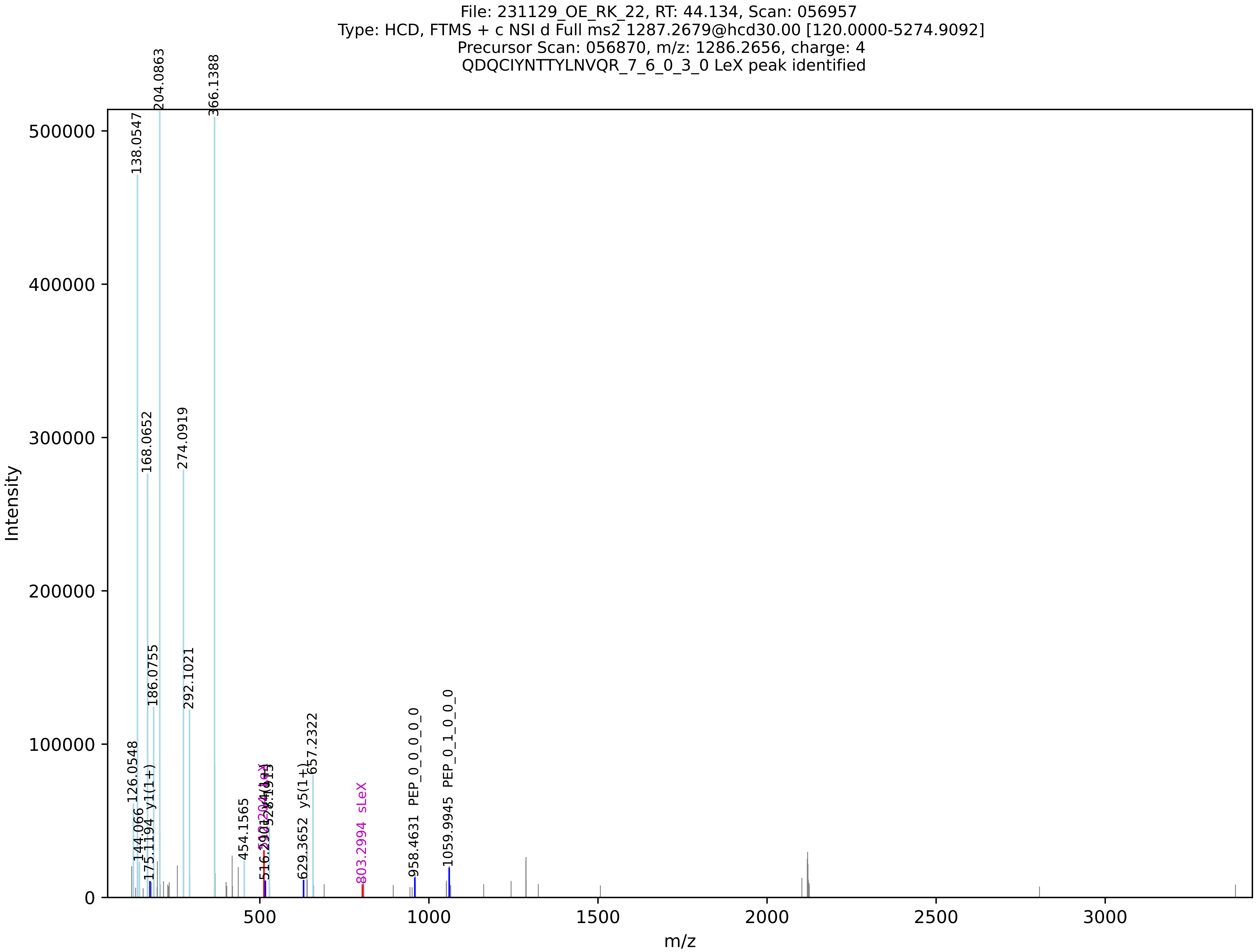

File: 231129_OE_RK_22, RT: 44.134, Scan: 056957
Type: HCD, FTMS + c NSI d Full ms2 1287.2679@hcd30.00 [120.0000-5274.9092]
Precursor Scan: 056870, m/z: 1286.2656, charge: 4
QDQCIYNTTYLNVQR_7_6_0_3_0 LeX peak identified
Intensity
m/z
0
100000
200000
300000
400000
500000
500
1000
1500
2000
2500
3000
204.0863
366.1388
138.0547
168.0652
274.0919
186.0755
292.1021
126.0548
144.066
175.1194 y1(1+)
454.1565
516.2903
528.1913
629.3652 y5(1+)
657.2322
803.2994 sLeX
958.4631 PEP_0_0_0_0_0
1059.9945 PEP_0_1_0_0_0

File: 231129_OE_RK_22, RT: 44.142, Scan: 056970
Type: HCD, FTMS + c NSI d Full ms2 1715.3582@hcd30.00 [120.0000-5271.1377]
Precursor Scan: 056870, m/z: 1714.685, charge: 3
QDQCIYNTTYLNVQR_7_6_0_3_0 LeX peak identified

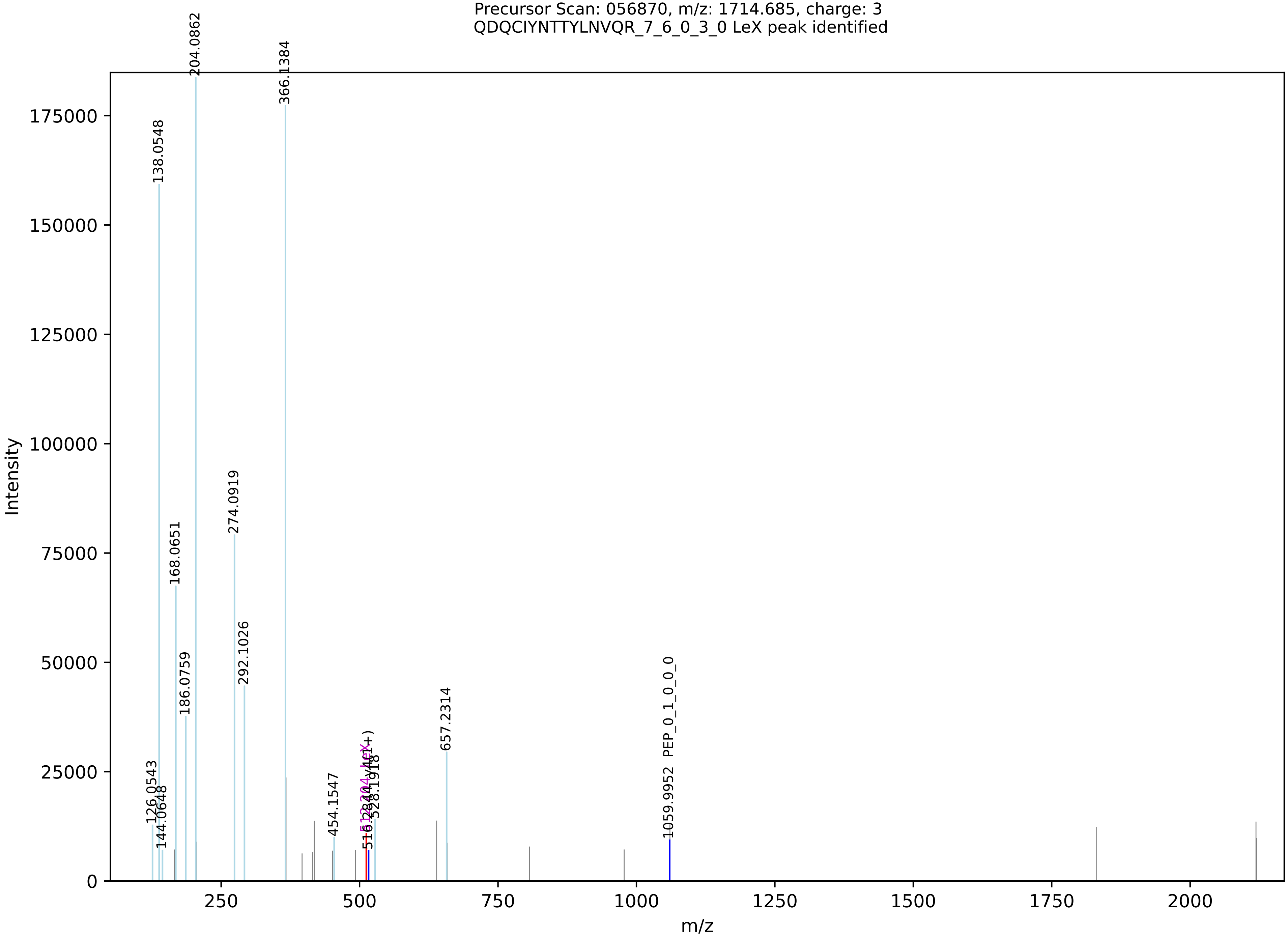

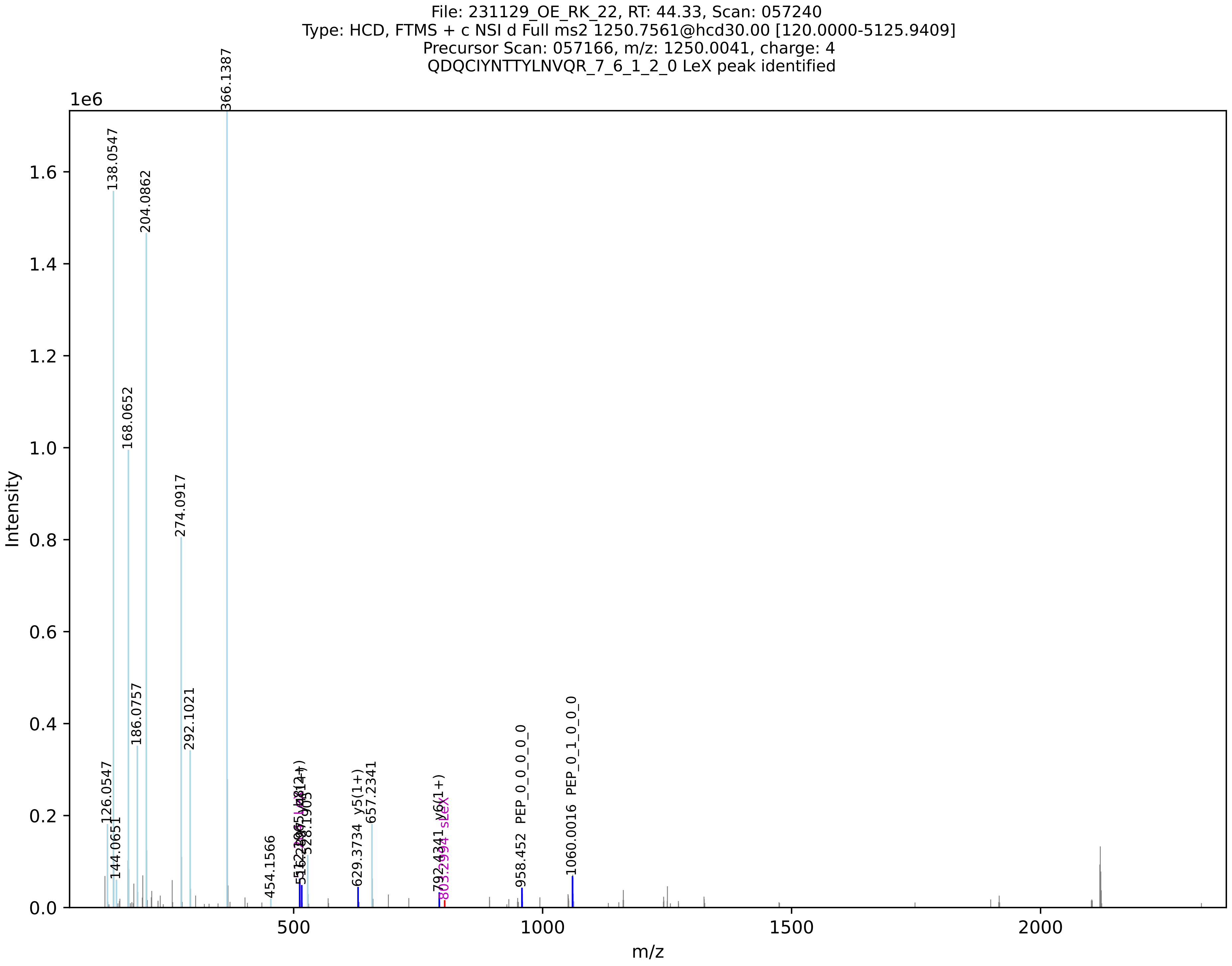
File: 231129_OE_RK_22, RT: 44.33, Scan: 057240
Type: HCD, FTMS + c NSI d Full ms2 1250.7561@hcd30.00 [120.0000-5125.9409]
Precursor Scan: 057166, m/z: 1250.0041, charge: 4
QDQCIYNTTYLNVQR_7_6_1_2_0 LeX peak identified
1e6
Intensity
m/z
366.1387
138.0547
204.0862
168.0652
274.0917
186.0757
292.1021
126.0547
144.0651
454.1566
629.3734 y5(1+)
657.2341
792.4341 y6(1+)
803.2994 sLeX
958.452 PEP_0_0_0_0_0
1060.0016 PEP_0_1_0_0_0

File: 231129_OE_RK_22, RT: 44.339, Scan: 057255
Type: HCD, FTMS + c NSI d Full ms2 1159.2239@hcd30.00 [120.0000-4752.4893]
Precursor Scan: 057166, m/z: 1158.7222, charge: 4
QDQCIYNTTYLNVQR_6_5_1_2_0 LeX peak identified

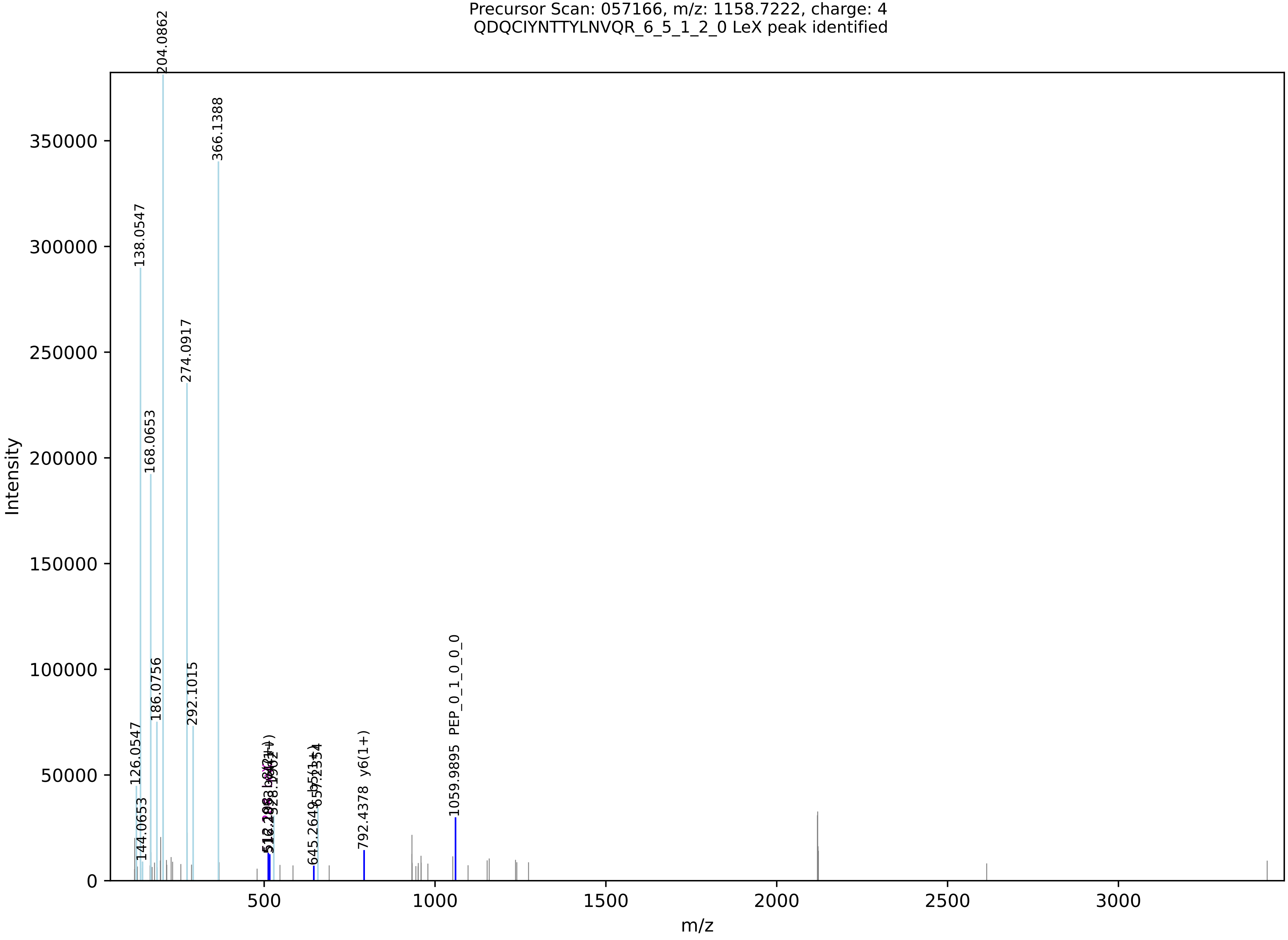

File: 231129_OE_RK_22, RT: 44.531, Scan: 057531
Type: HCD, FTMS + c NSI d Full ms2 1250.5037@hcd30.00 [120.0000-5124.9106]
Precursor Scan: 057454, m/z: 1250.0014, charge: 4
QDQCIYNTTYLNVQR_7_6_1_2_0 LeX peak identified

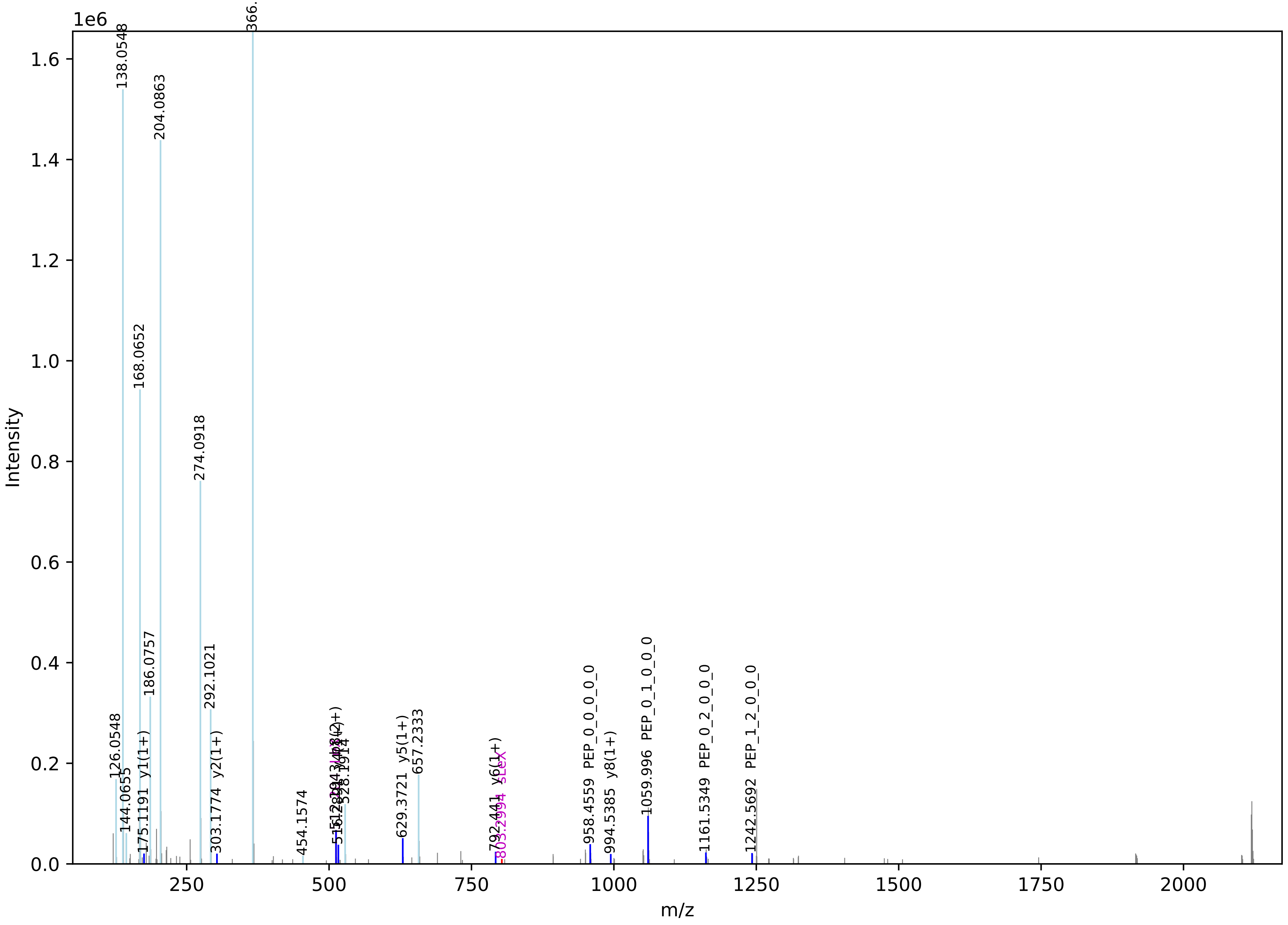

File: 231129_OE_RK_22, RT: 44.631, Scan: 057675
Type: HCD, FTMS + c NSI d Full ms2 1667.0035@hcd30.00 [120.0000-5123.1729]
Precursor Scan: 057602, m/z: 1666.3355, charge: 3
QDQCIYNTTYLNVQR_7_6_1_2_0 LeX peak identified

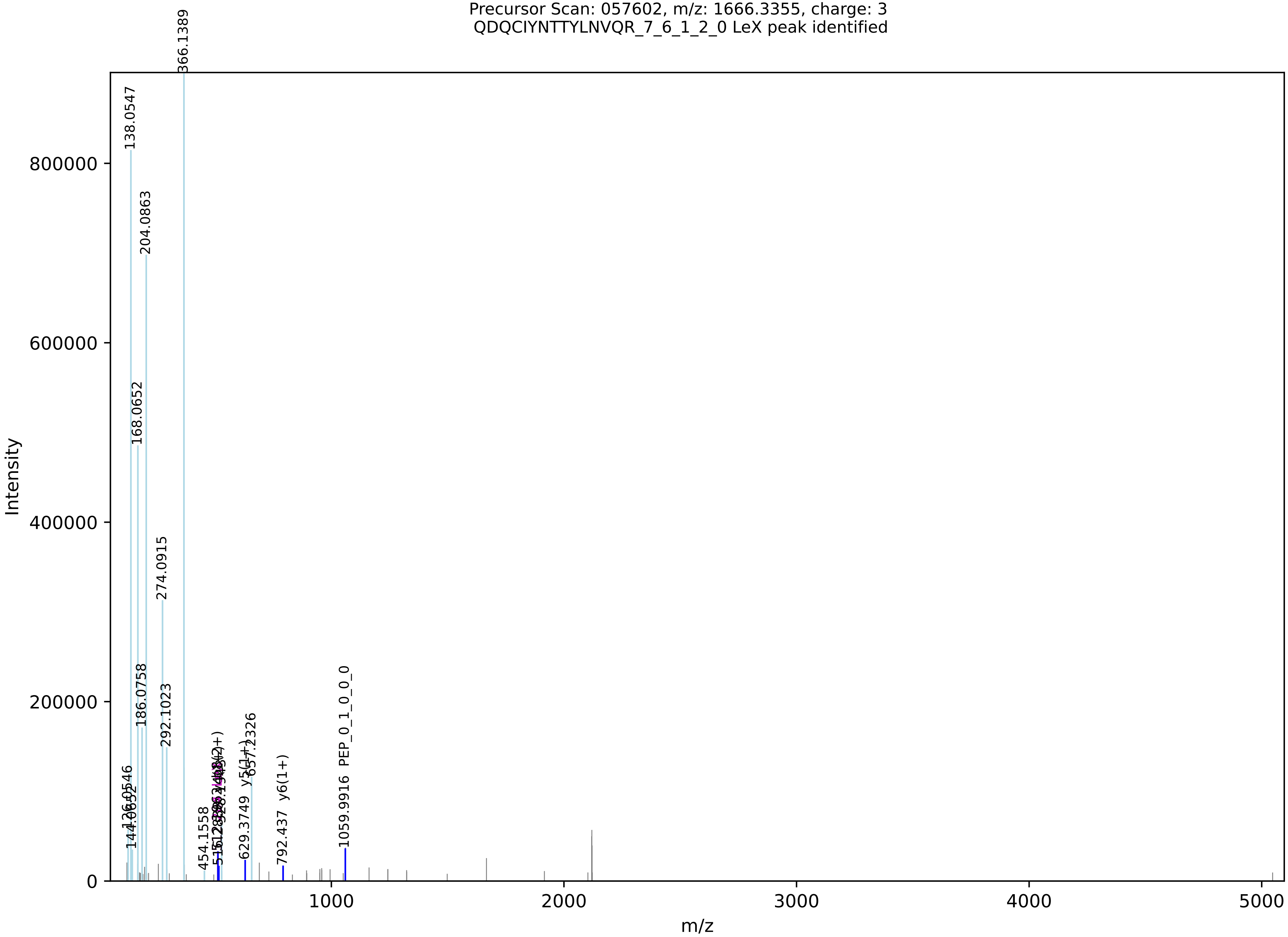

File: 231129_OE_RK_22, RT: 44.736, Scan: 057828
Type: HCD, FTMS + c NSI d Full ms2 1287.0143@hcd30.00 [120.0000-5273.8745]
Precursor Scan: 057747, m/z: 1286.2625, charge: 4
QDQCIYNTTYLNVQR_7_6_0_3_0 LeX peak identified

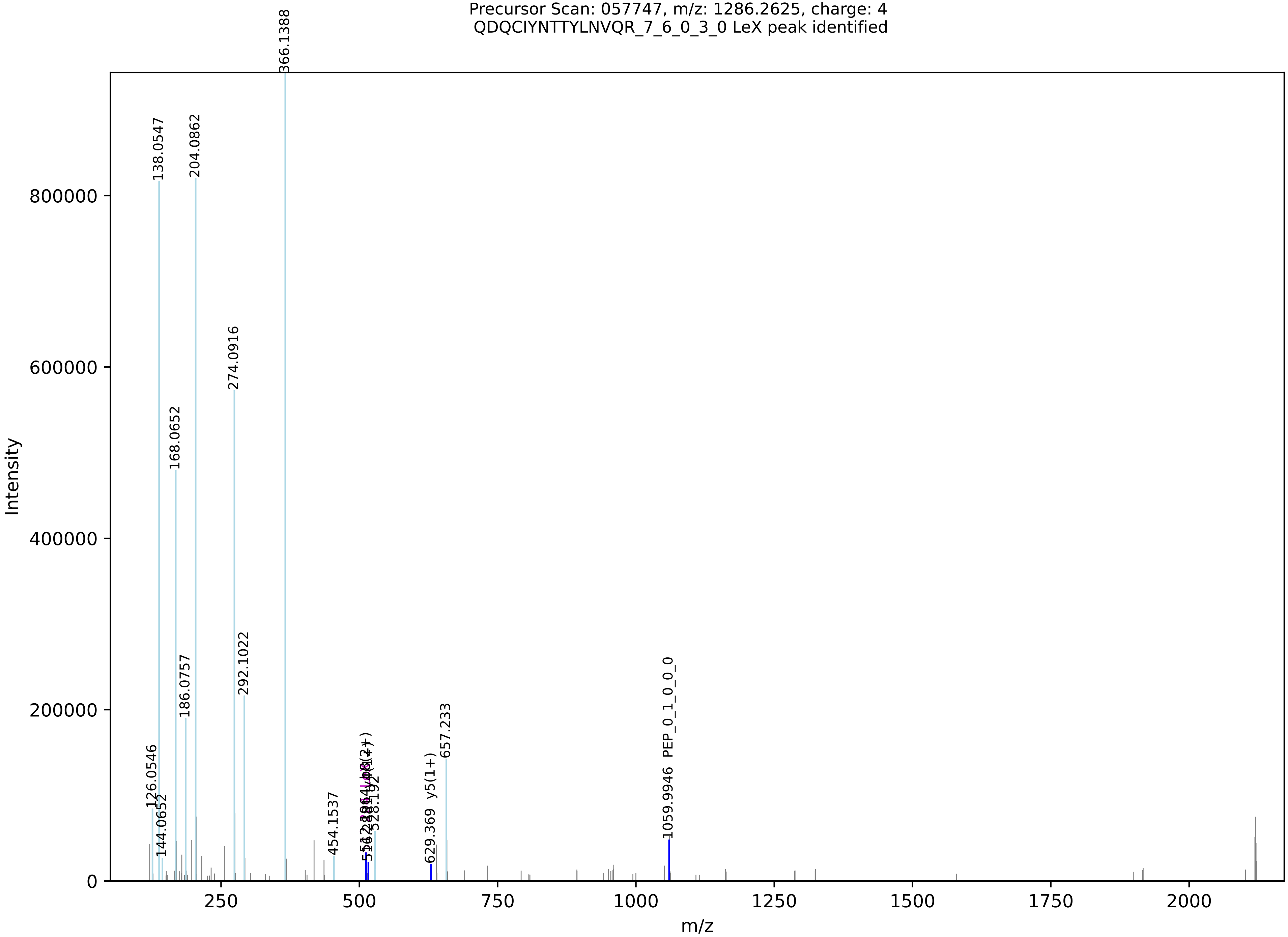

File: 231129_OE_RK_22, RT: 44.771, Scan: 057884
Type: HCD, FTMS + c NSI d Full ms2 1544.6223@hcd30.00 [120.0000-4748.6865]
Precursor Scan: 057673, m/z: 1544.6238, charge: 3
QDQCIYNTTYLNVQR_6_5_1_2_0 LeX peak identified

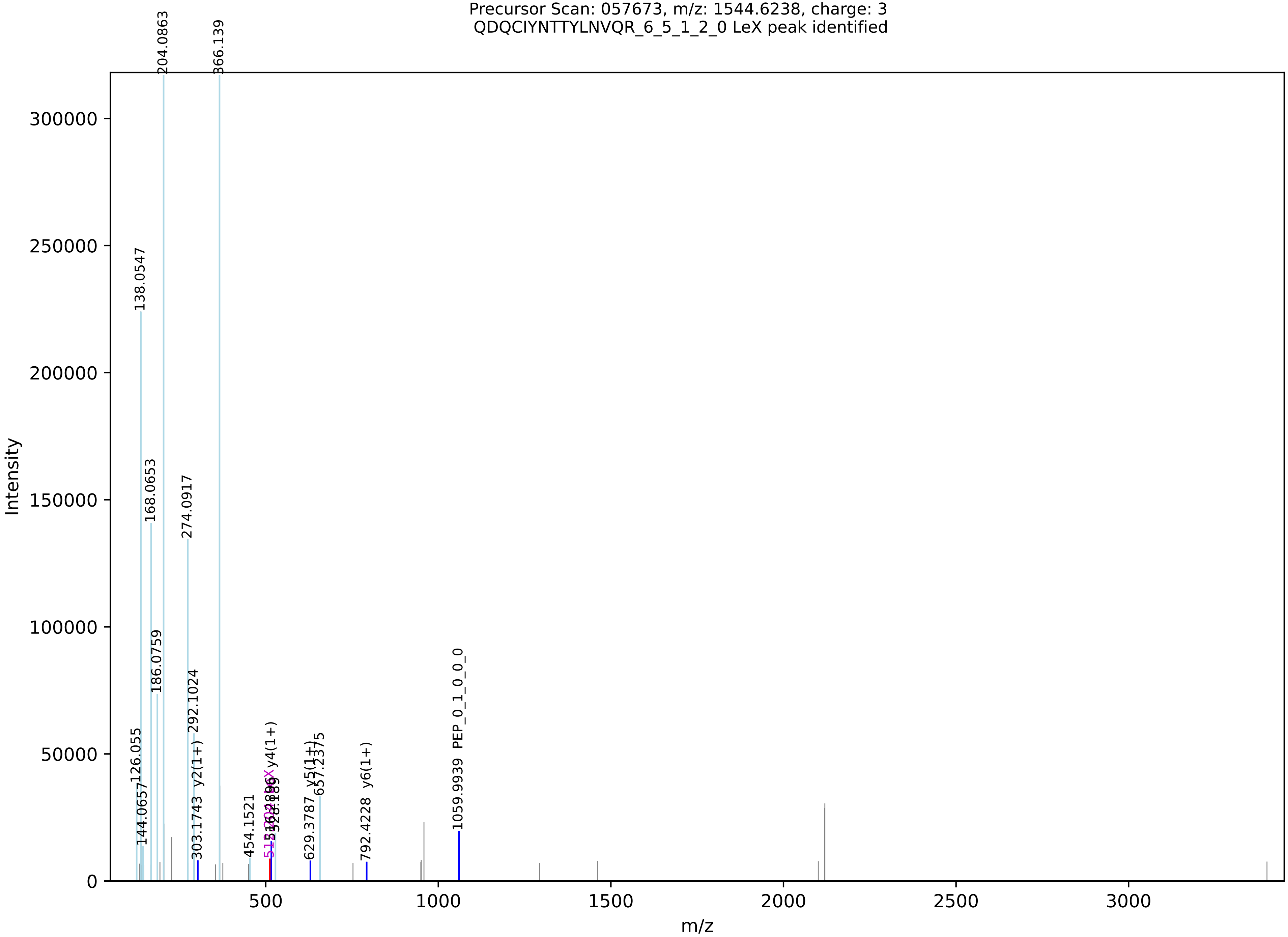

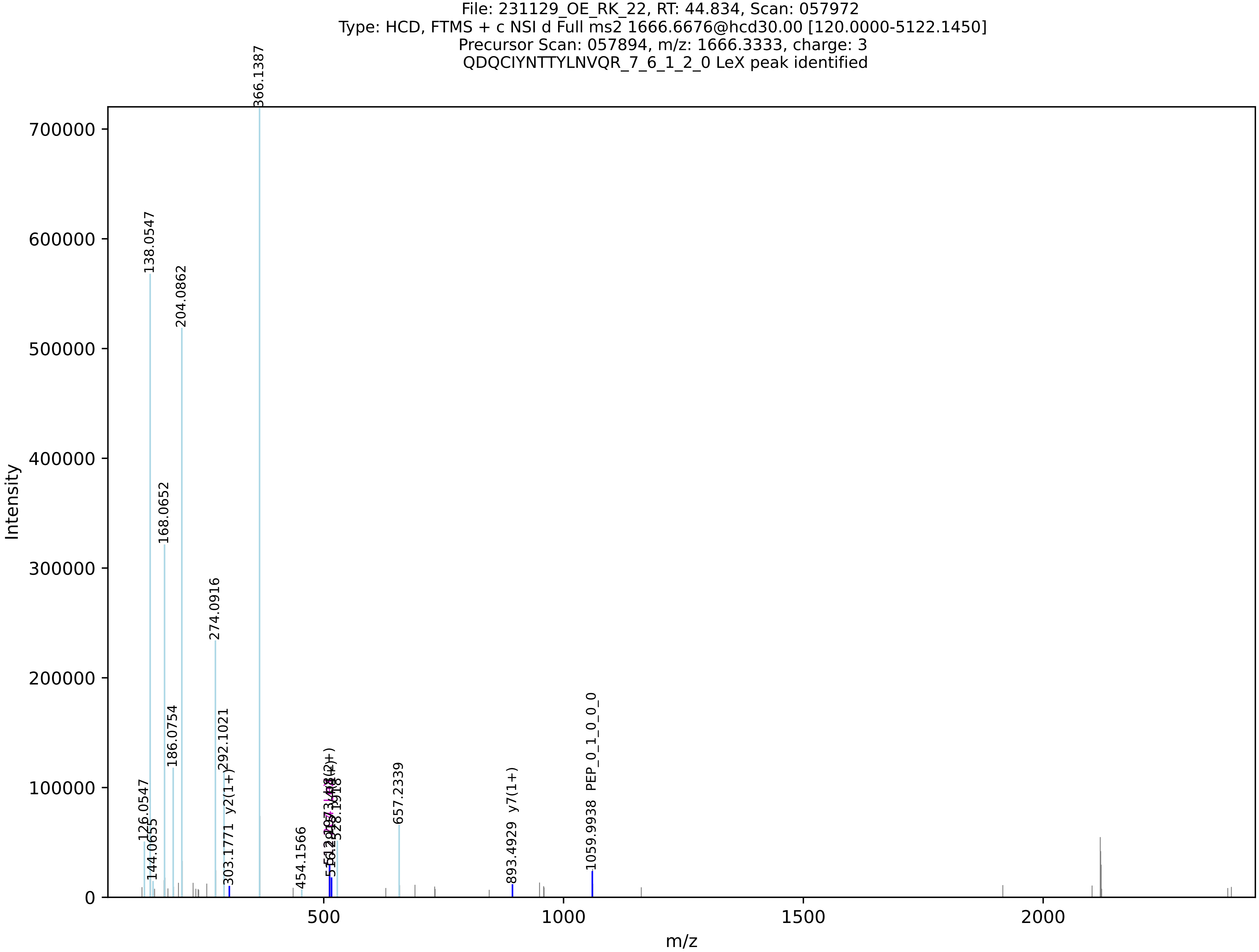

File: 231129_OE_RK_22, RT: 44.834, Scan: 057972
Type: HCD, FTMS + c NSI d Full ms2 1666.6676@hcd30.00 [120.0000-5122.1450]
Precursor Scan: 057894, m/z: 1666.3333, charge: 3
QDQCIYNTTYLNVQR_7_6_1_2_0 LeX peak identified
366.1387
138.0547
204.0862
168.0652
274.0916
186.0754
292.1021
126.0547
144.0655
303.1771 y2(1+)
454.1566
528.1918
657.2339
893.4929 y7(1+)
1059.9938 PEP_0_1_0_0_0_
Intensity
m/z
0
100000
200000
300000
400000
500000
600000
700000
500
1000
1500
2000

File: 231129_OE_RK_22, RT: 45.124, Scan: 058397
Type: HCD, FTMS + c NSI d Full ms2 1250.5033@hcd30.00 [120.0000-5124.9092]
Precursor Scan: 058324, m/z: 1250.0023, charge: 4
QDQCIYNTTYLNVQR_7_6_1_2_0 LeX peak identified

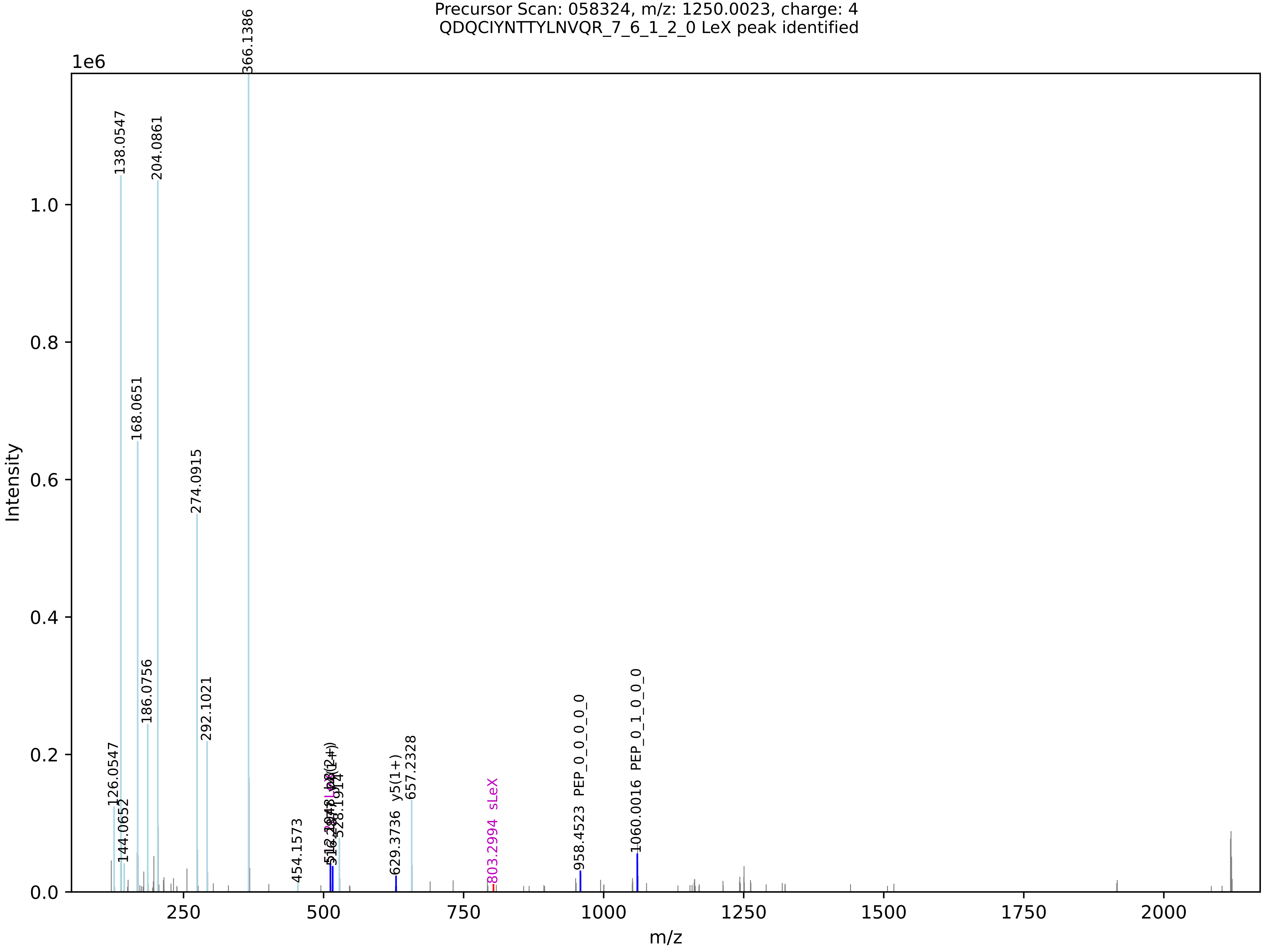

File: 231129_OE_RK_22, RT: 45.128, Scan: 058404
Type: HCD, FTMS + c NSI d Full ms2 1545.2909@hcd30.00 [120.0000-4750.7319]
Precursor Scan: 058324, m/z: 1545.2909, charge: 3
QDQCIYNTTYLNVQR_6_5_1_2_0 LeX peak identified

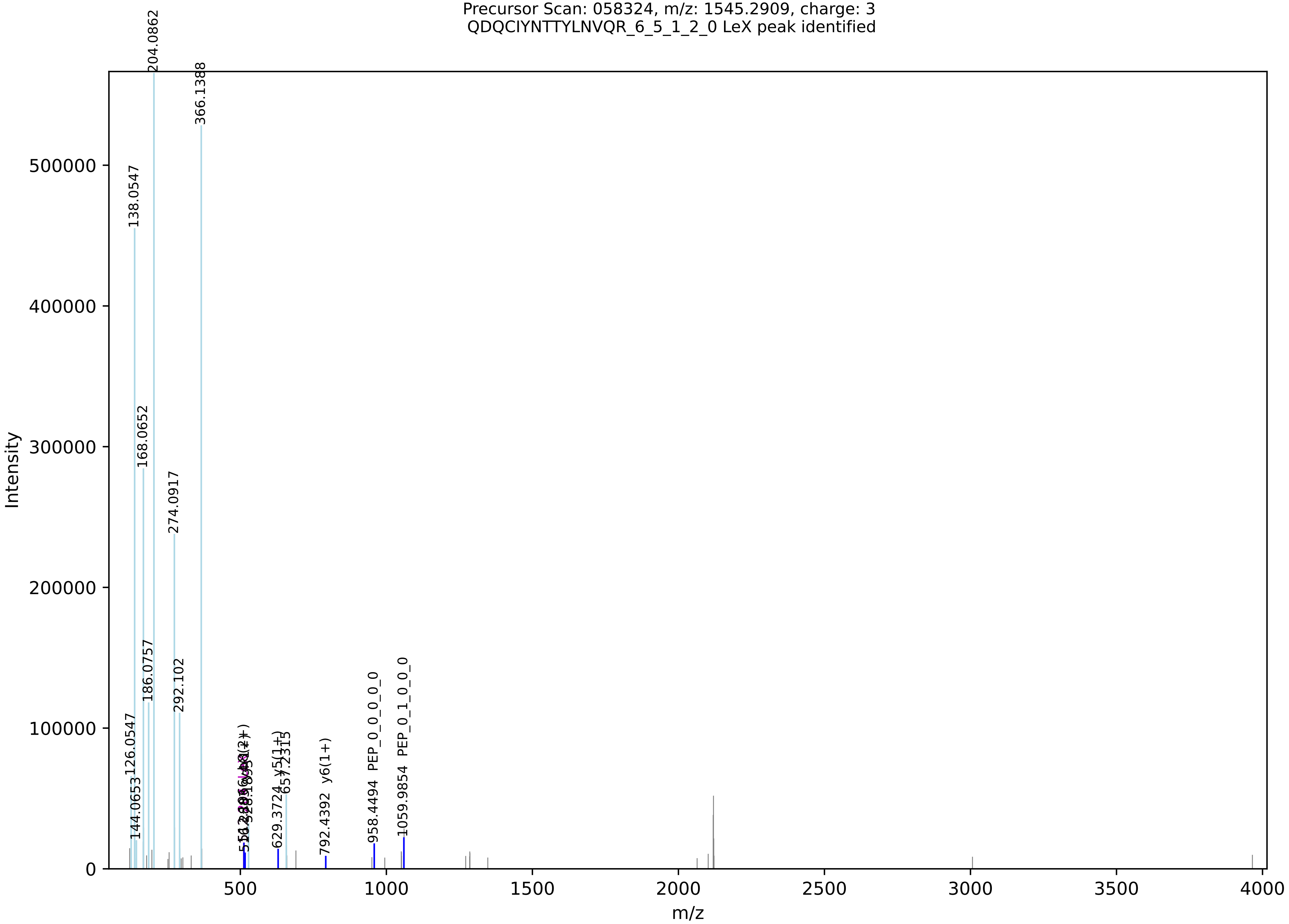

File: 231129_OE_RK_22, RT: 45.185, Scan: 058488
Type: HCD, FTMS + c NSI d Full ms2 1323.5244@hcd30.00 [120.0000-5422.8359]
Precursor Scan: 058390, m/z: 1322.7727, charge: 4
QDQCIYNTTYLNVQR_7_6_1_3_0 LeX peak identified

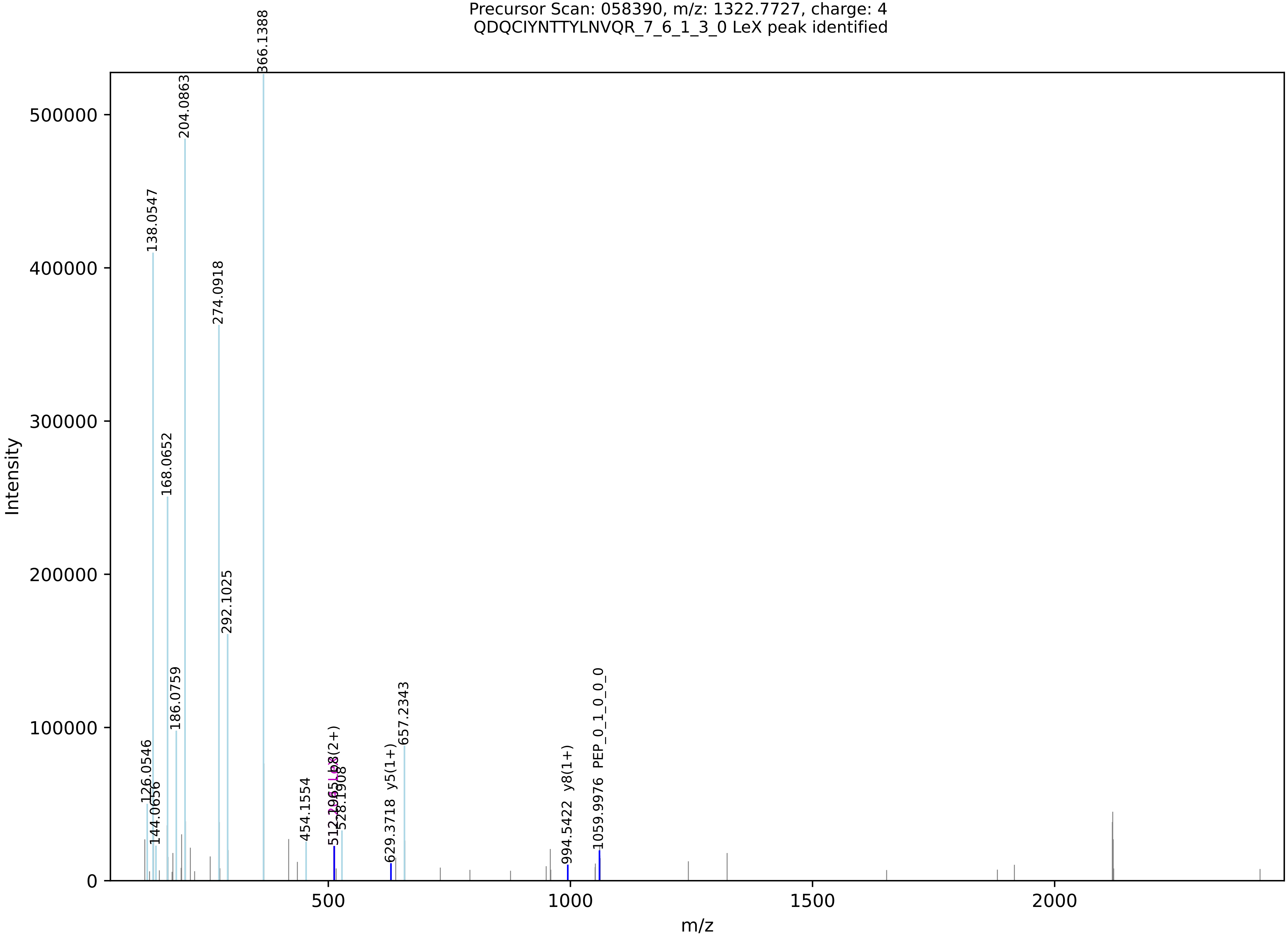

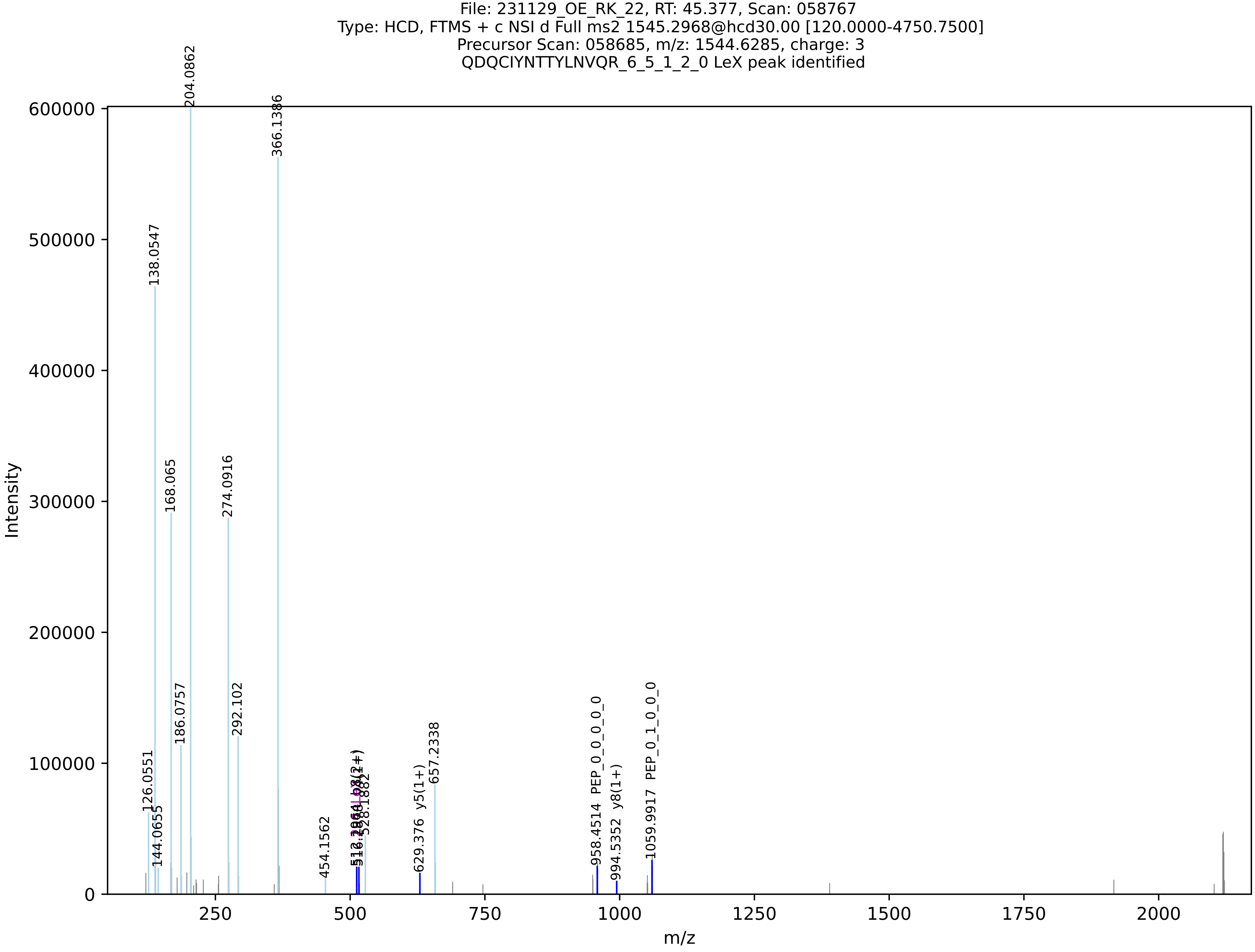

File: 231129_OE_RK_22, RT: 45.377, Scan: 058767
Type: HCD, FTMS + c NSI d Full ms2 1545.2968@hcd30.00 [120.0000-4750.7500]
Precursor Scan: 058685, m/z: 1544.6285, charge: 3
QDQCIYNTTYLNVQR_6_5_1_2_0 LeX peak identified
Intensity
m/z
0
100000
200000
300000
400000
500000
600000
250
500
750
1000
1250
1500
1750
2000
204.0862
366.1386
138.0547
168.065
274.0916
186.0757
292.102
126.0551
144.0655
454.1562
528.1882
629.376 y5(1+)
657.2338
958.4514 PEP_0_0_0_0_0
994.5352 y8(1+)
1059.9917 PEP_0_1_0_0_0

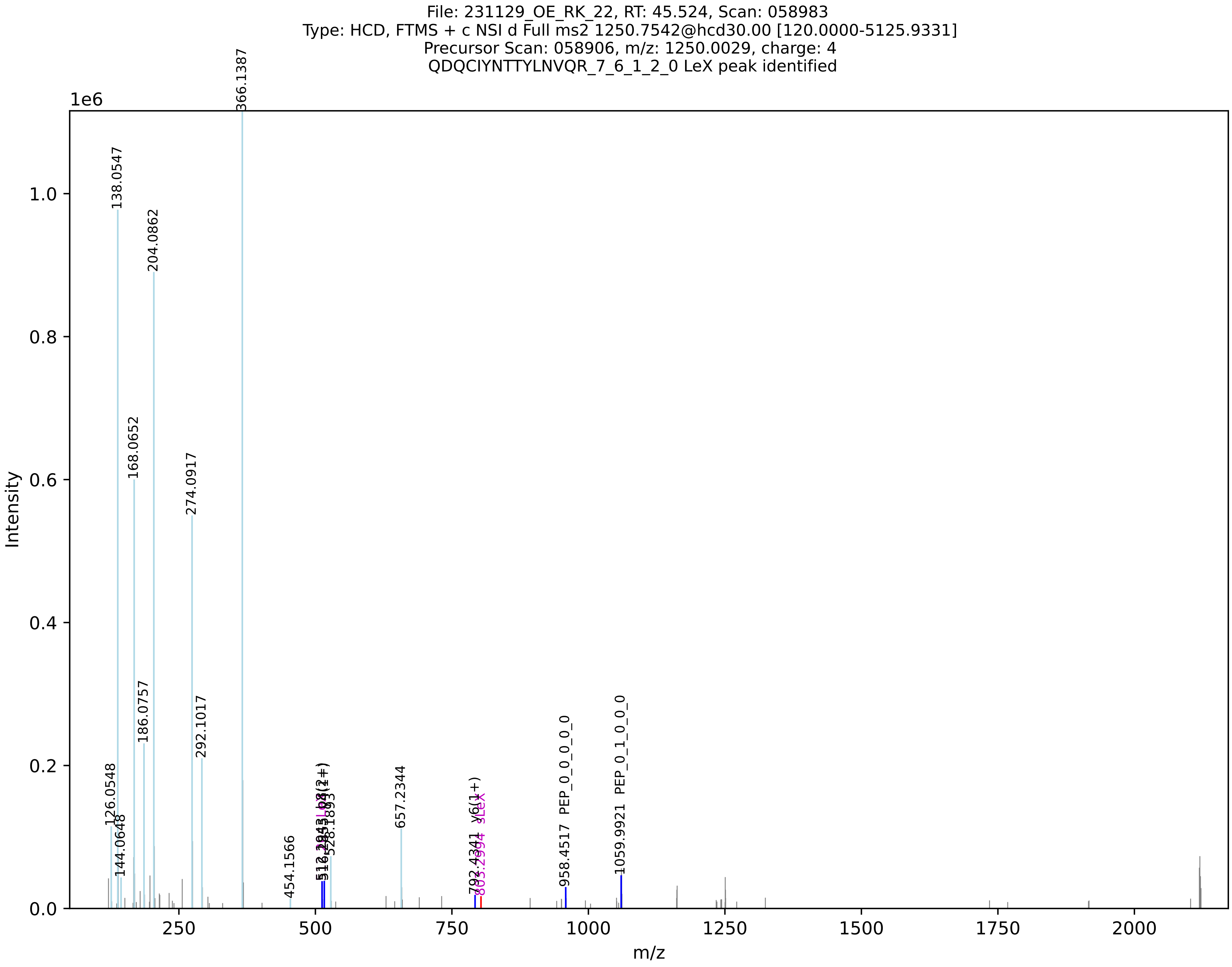
File: 231129_OE_RK_22, RT: 45.524, Scan: 058983
Type: HCD, FTMS + c NSI d Full ms2 1250.7542@hcd30.00 [120.0000-5125.9331]
Precursor Scan: 058906, m/z: 1250.0029, charge: 4
QDQCIYNTTYLNVQR_7_6_1_2_0 LeX peak identified
1e6
Intensity
m/z
366.1387
138.0547
204.0862
168.0652
274.0917
186.0757
292.1017
126.0548
144.0648
454.1566
528.1893
657.2344
792.4341 y6(1+)
803.2994 sLeX
958.4517 PEP_0_0_0_0_0
1059.9921 PEP_0_1_0_0_0

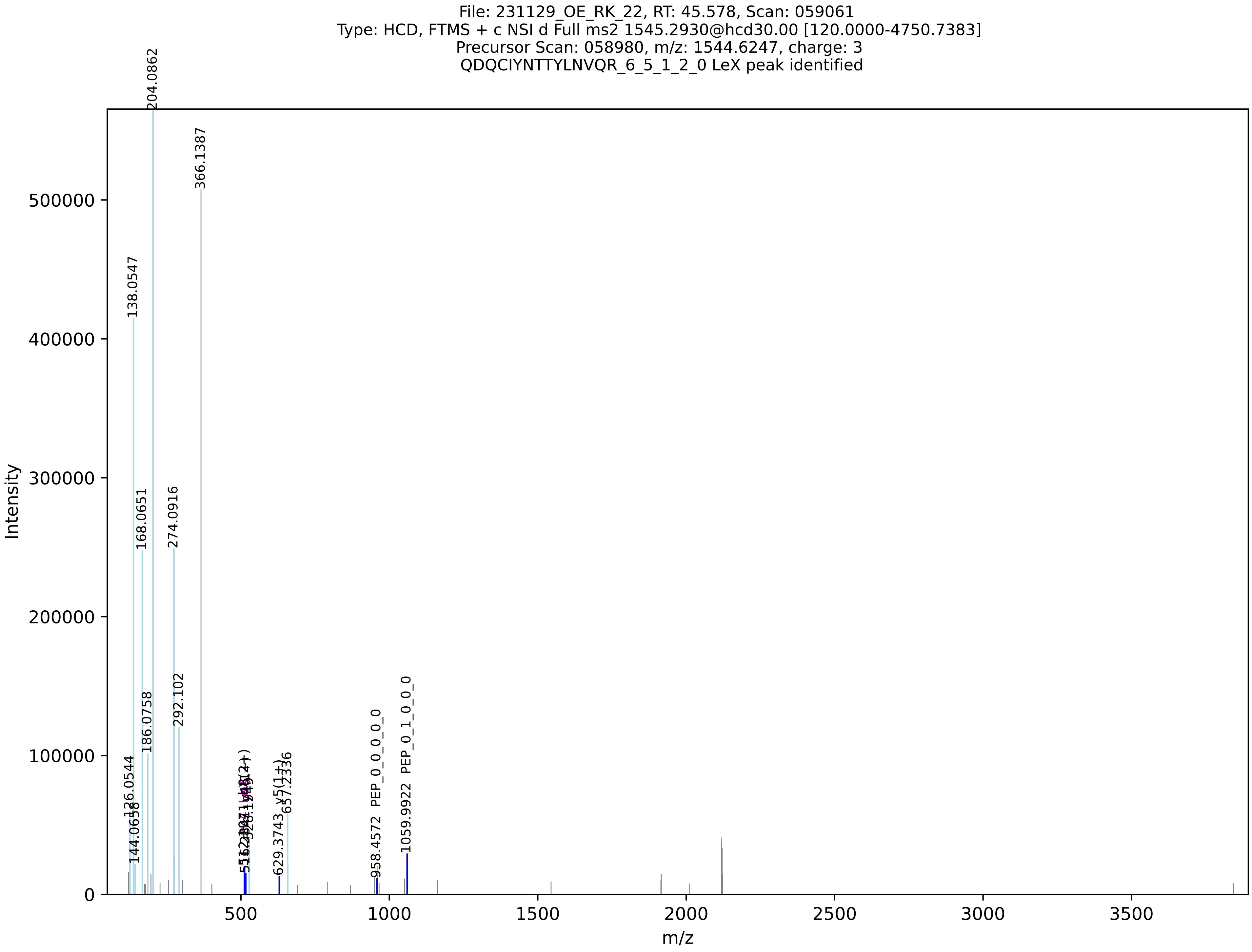
File: 231129_OE_RK_22, RT: 45.578, Scan: 059061
Type: HCD, FTMS + c NSI d Full ms2 1545.2930@hcd30.00 [120.0000-4750.7383]
Precursor Scan: 058980, m/z: 1544.6247, charge: 3
QDQCIYNTTYLNVQR_6_5_1_2_0 LeX peak identified
204.0862
366.1387
138.0547
168.0651
274.0916
186.0758
292.102
126.0544
144.0658
629.3743 y5(1+)
657.2336
958.4572 PEP_0_0_0_0_0
1059.9922 PEP_0_1_0_0_0
Intensity
m/z
0
100000
200000
300000
400000
500000
500
1000
1500
2000
2500
3000
3500

File: 231129_OE_RK_22, RT: 45.679, Scan: 059209
Type: HCD, FTMS + c NSI d Full ms2 1323.2782@hcd30.00 [120.0000-5421.8311]
Precursor Scan: 059128, m/z: 1323.2782, charge: 4
QDQCIYNTTYLNVQR_7_6_1_3_0 LeX peak identified

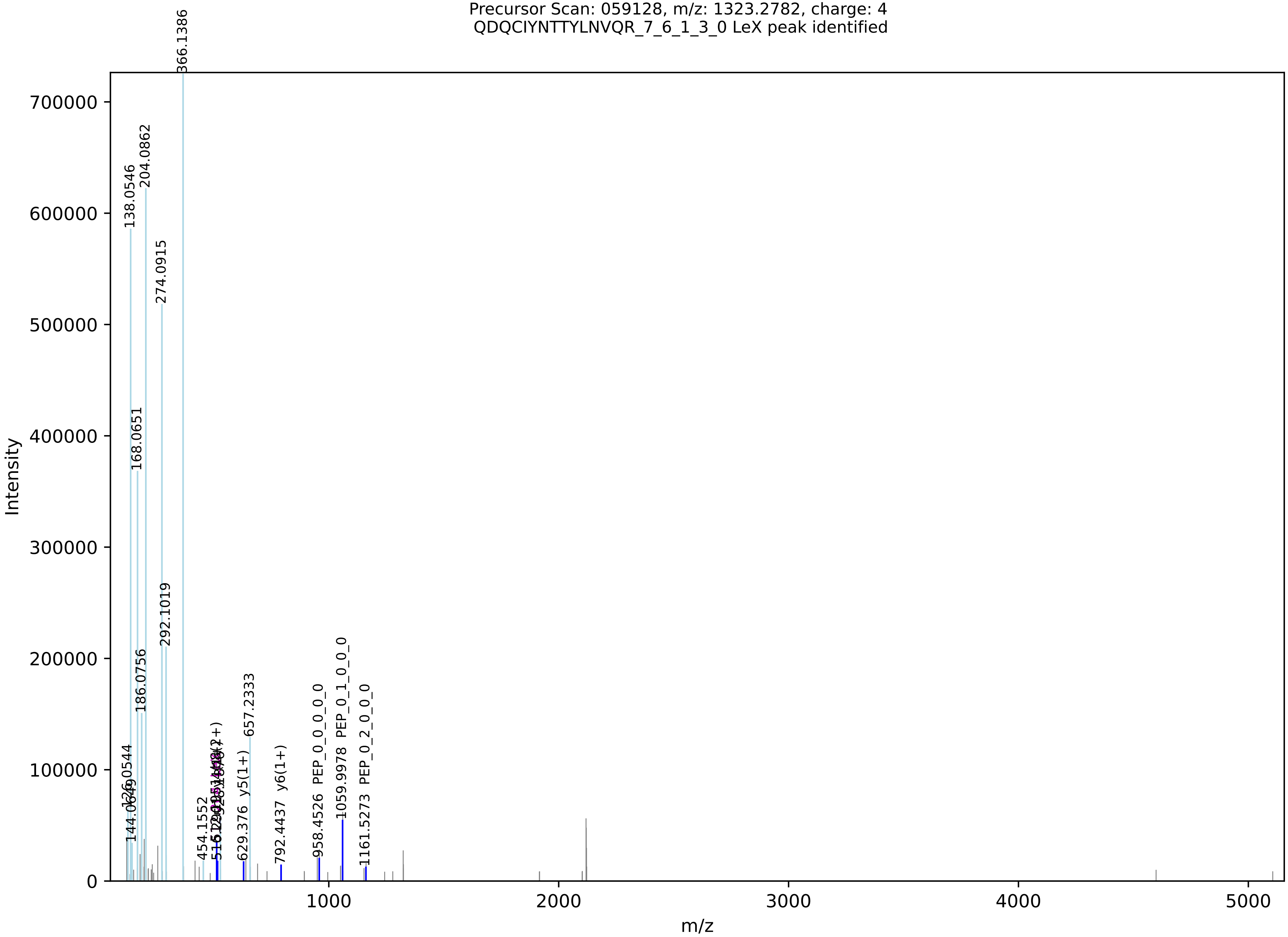

File: 231129_OE_RK_22, RT: 45.88, Scan: 059501
Type: HCD, FTMS + c NSI d Full ms2 1323.5286@hcd30.00 [120.0000-5422.8525]
Precursor Scan: 059423, m/z: 1322.7768, charge: 4
QDQCIYNTTYLNVQR_7_6_1_3_0 LeX peak identified

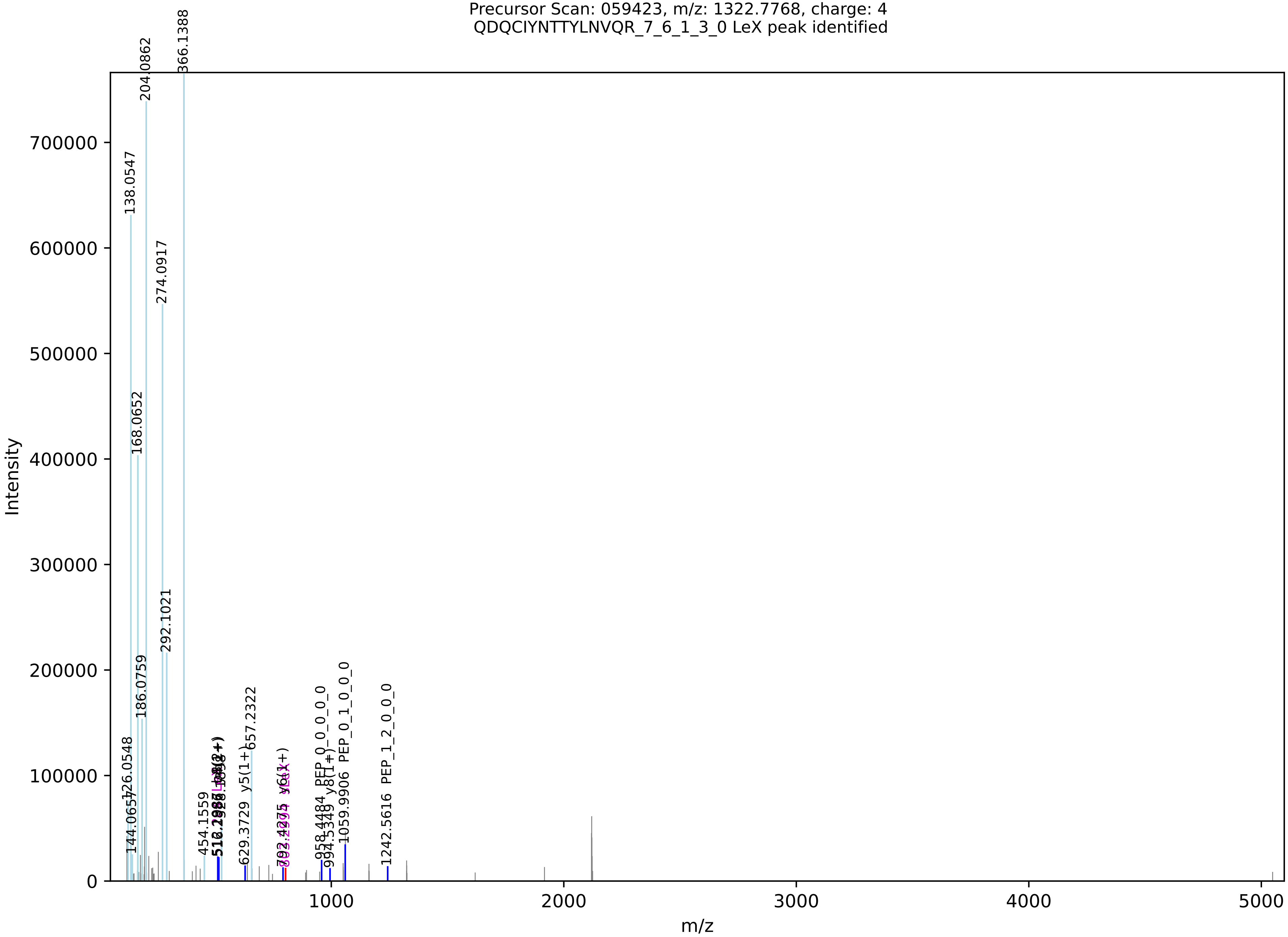

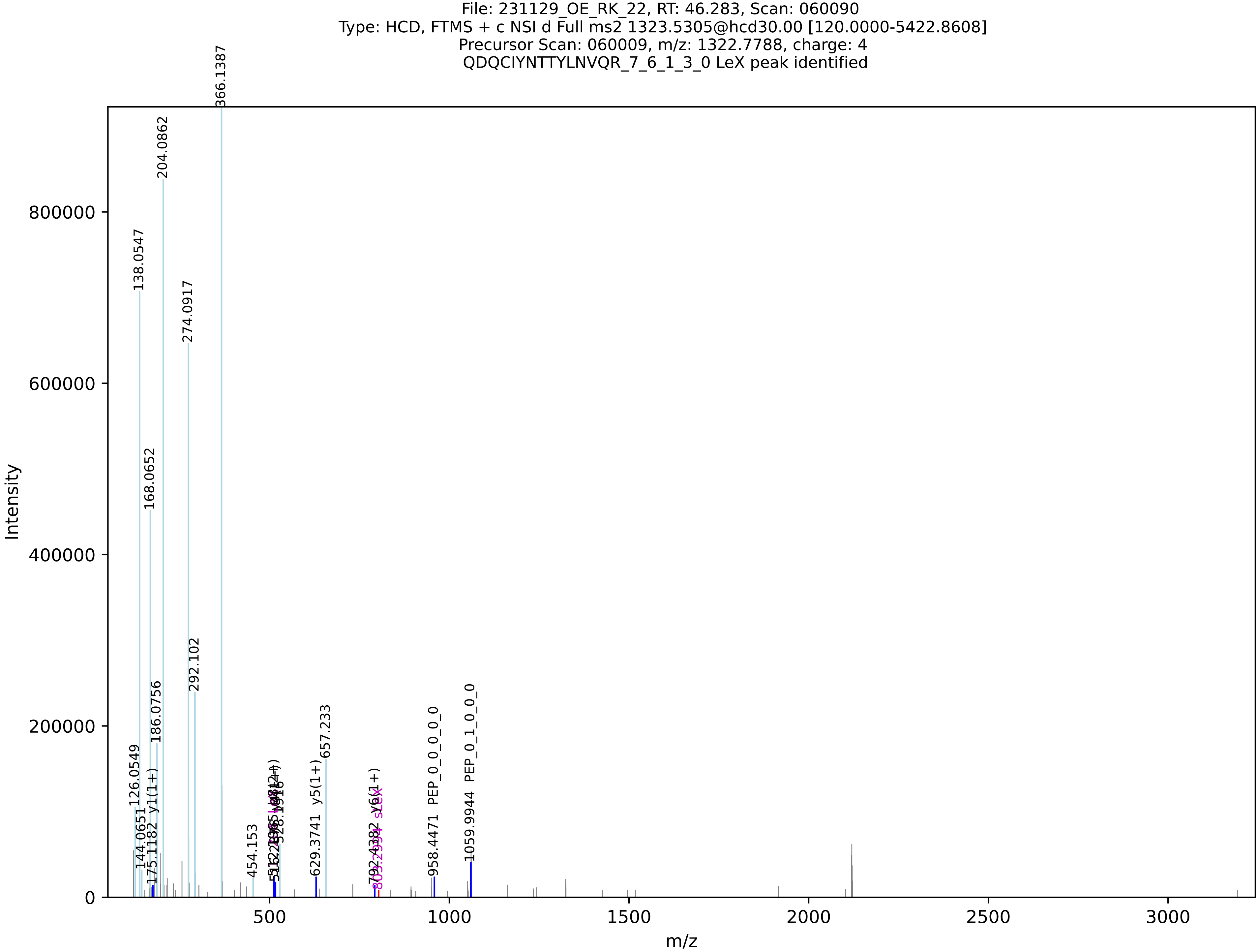

File: 231129_OE_RK_22, RT: 46.283, Scan: 060090
Type: HCD, FTMS + c NSI d Full ms2 1323.5305@hcd30.00 [120.0000-5422.8608]
Precursor Scan: 060009, m/z: 1322.7788, charge: 4
QDQCIYNTTYLNVQR_7_6_1_3_0 LeX peak identified
366.1387
204.0862
138.0547
274.0917
168.0652
292.102
186.0756
126.0549
144.0651
175.1182 y1(1+)
454.153
629.3741 y5(1+)
657.233
792.4382 y6(1+)
958.4471 PEP_0_0_0_0_0_
1059.9944 PEP_0_1_0_0_0_
Intensity
m/z
800000
600000
400000
200000
0
500
1000
1500
2000
2500
3000

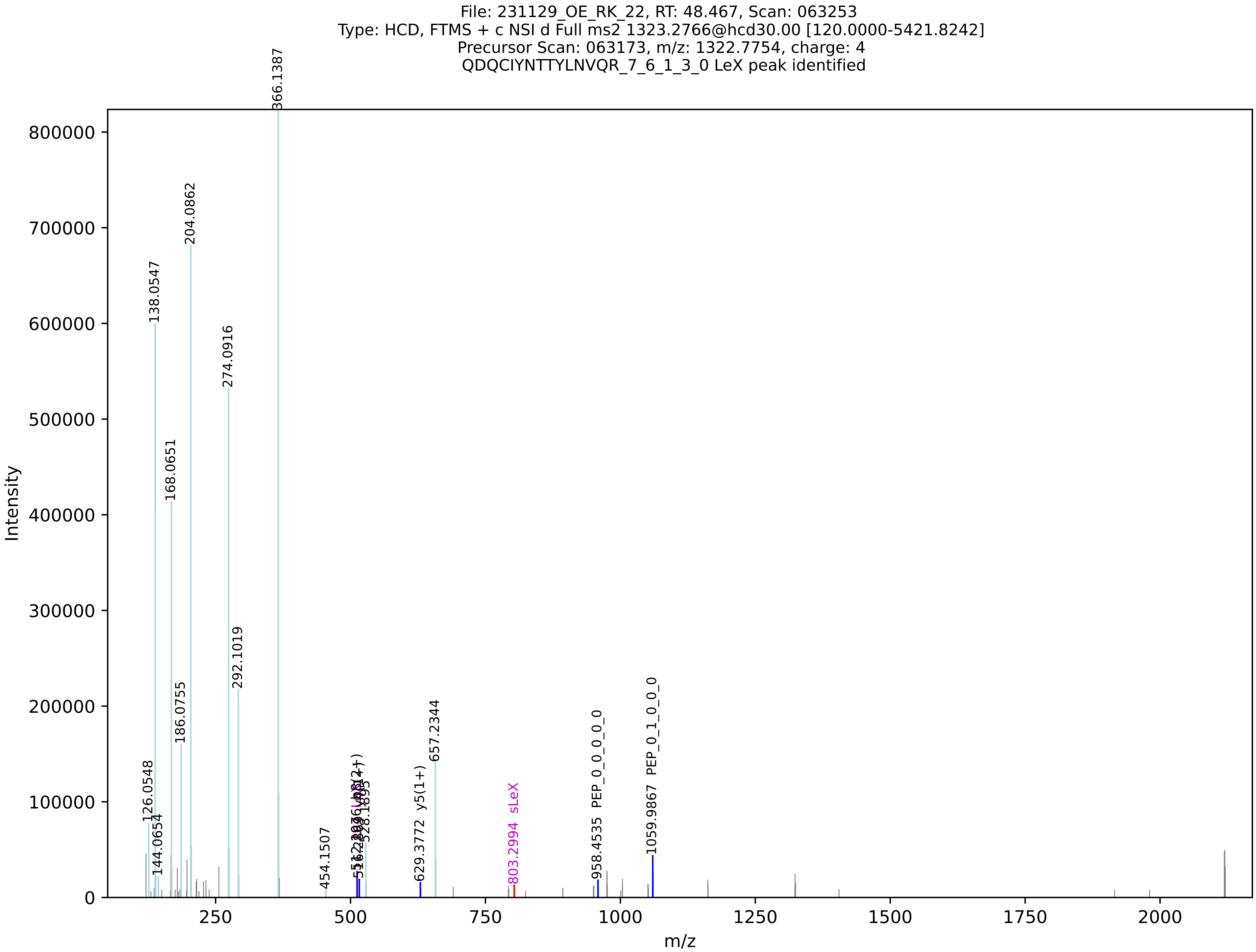
File: 231129_OE_RK_22, RT: 48.467, Scan: 063253
Type: HCD, FTMS + c NSI d Full ms2 1323.2766@hcd30.00 [120.0000-5421.8242]
Precursor Scan: 063173, m/z: 1322.7754, charge: 4
QDQCIYNTTYLNVQR_7_6_1_3_0 LeX peak identified
Intensity
m/z
0
100000
200000
300000
400000
500000
600000
700000
800000
250
500
750
1000
1250
1500
1750
2000
366.1387
204.0862
138.0547
274.0916
168.0651
292.1019
186.0755
126.0548
144.0654
454.1507
528.1895
629.3772 y5(1+)
657.2344
803.2994 sLeX
958.4535 PEP_0_0_0_0_0
1059.9867 PEP_0_1_0_0_0

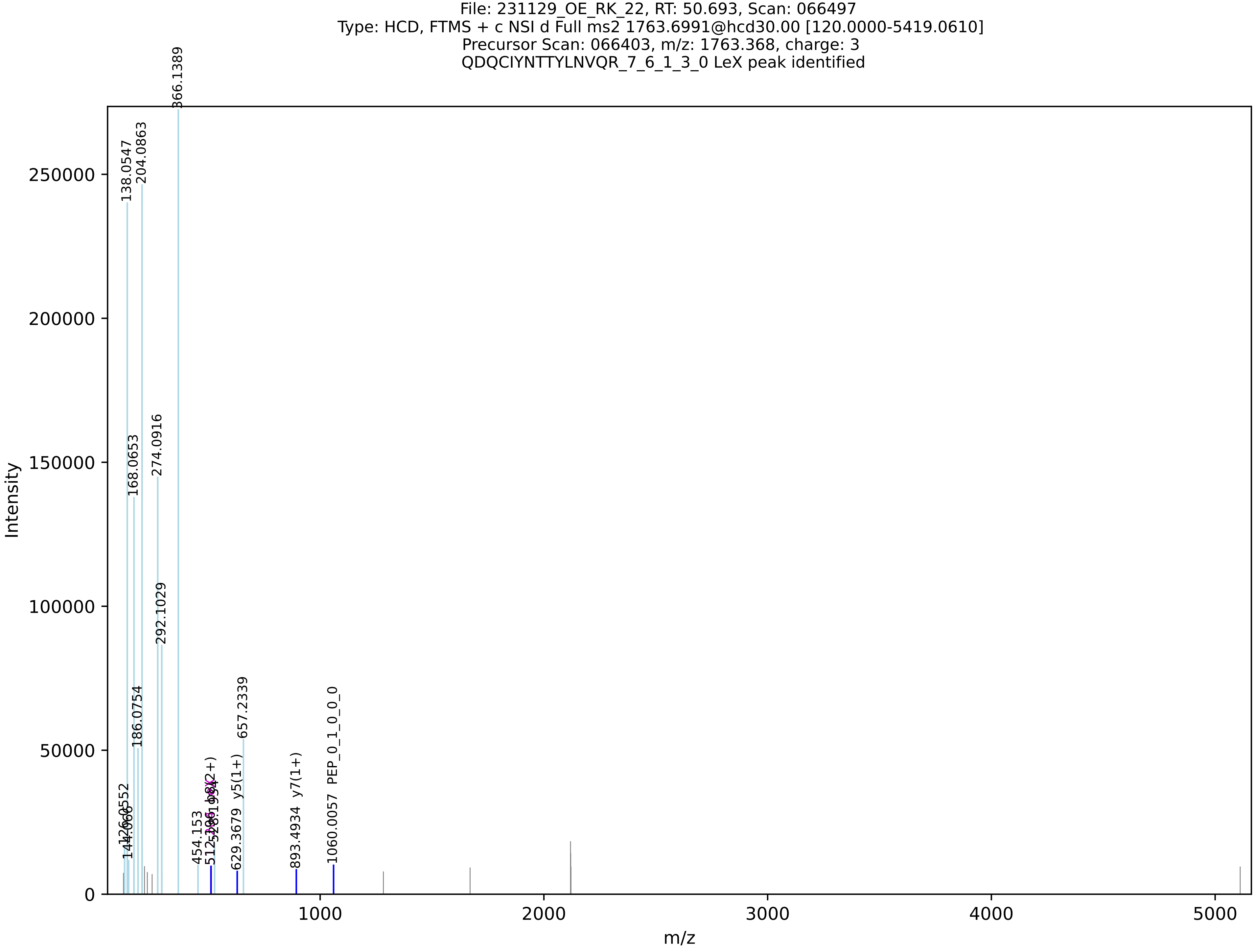
File: 231129_OE_RK_22, RT: 50.693, Scan: 066497
Type: HCD, FTMS + c NSI d Full ms2 1763.6991@hcd30.00 [120.0000-5419.0610]
Precursor Scan: 066403, m/z: 1763.368, charge: 3
QDQCIYNTTYLNVQR_7_6_1_3_0 LeX peak identified
Intensity
m/z
0
50000
100000
150000
200000
250000
1000
2000
3000
4000
5000
366.1389
138.0547
204.0863
168.0653
274.0916
292.1029
186.0754
657.2339
126.0552
144.066
454.153
629.3679 y5(1+)
893.4934 y7(1+)
1060.0057 PEP_0_1_0_0_0

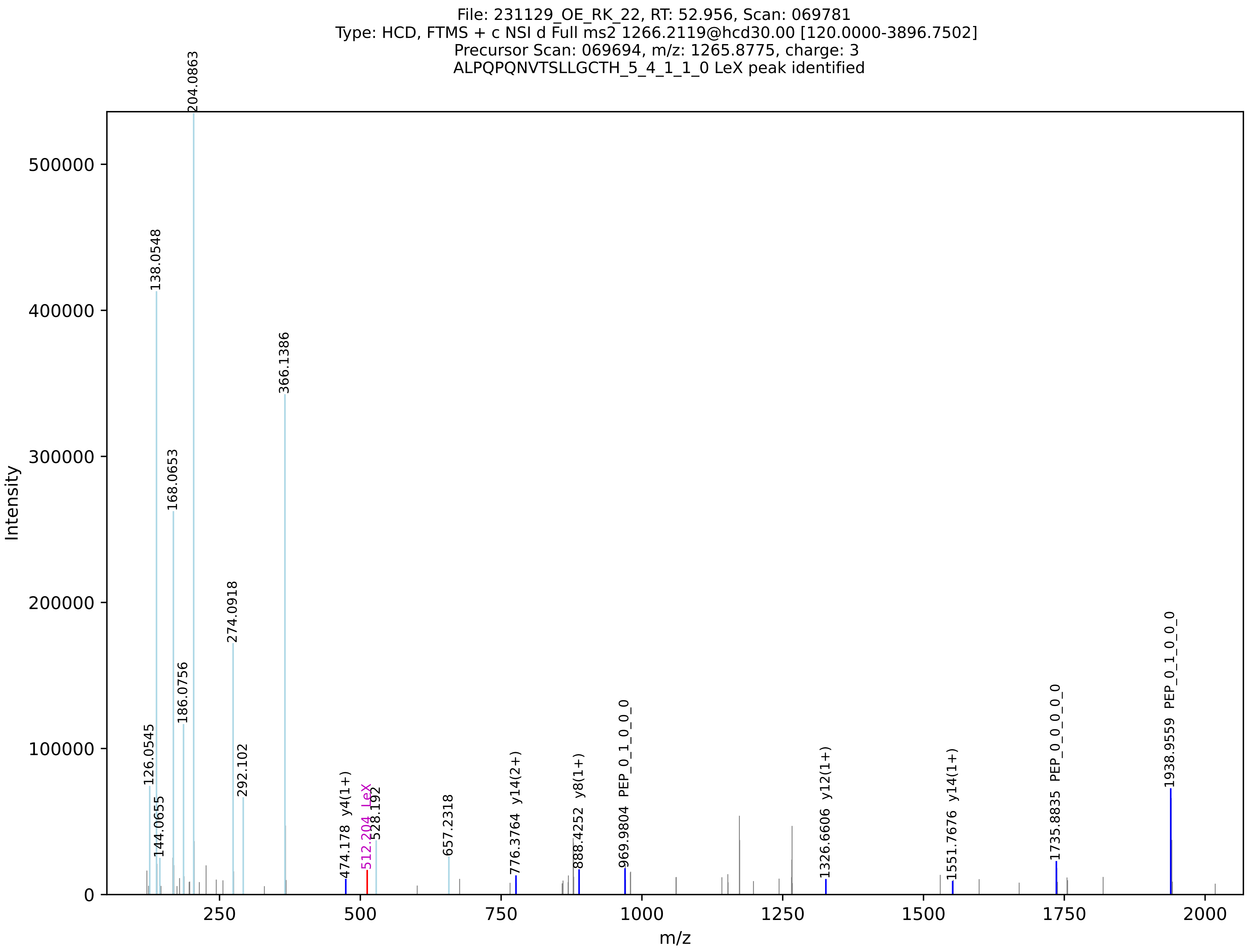
File: 231129_OE_RK_22, RT: 52.956, Scan: 069781
Type: HCD, FTMS + c NSI d Full ms2 1266.2119@hcd30.00 [120.0000-3896.7502]
Precursor Scan: 069694, m/z: 1265.8775, charge: 3
ALPQPQNVTSLLGCTH_5_4_1_1_0 LeX peak identified
Intensity
m/z
204.0863
138.0548
366.1386
168.0653
274.0918
186.0756
126.0545
292.102
144.0655
474.178 y4(1+)
512.204 LeX
528.192
657.2318
776.3764 y14(2+)
888.4252 y8(1+)
969.9804 PEP_0_1_0_0_0
1326.6606 y12(1+)
1551.7676 y14(1+)
1735.8835 PEP_0_0_0_0_0
1938.9559 PEP_0_1_0_0_0
0
100000
200000
300000
400000
500000
250
500
750
1000
1250
1500
1750
2000

File: 231129_OE_RK_22, RT: 53.157, Scan: 070074
Type: HCD, FTMS + c NSI d Full ms2 1266.2120@hcd30.00 [120.0000-3896.7510]
Precursor Scan: 069988, m/z: 1265.878, charge: 3
ALPQPQNVTSLLGCTH_5_4_1_1_0 LeX peak identified

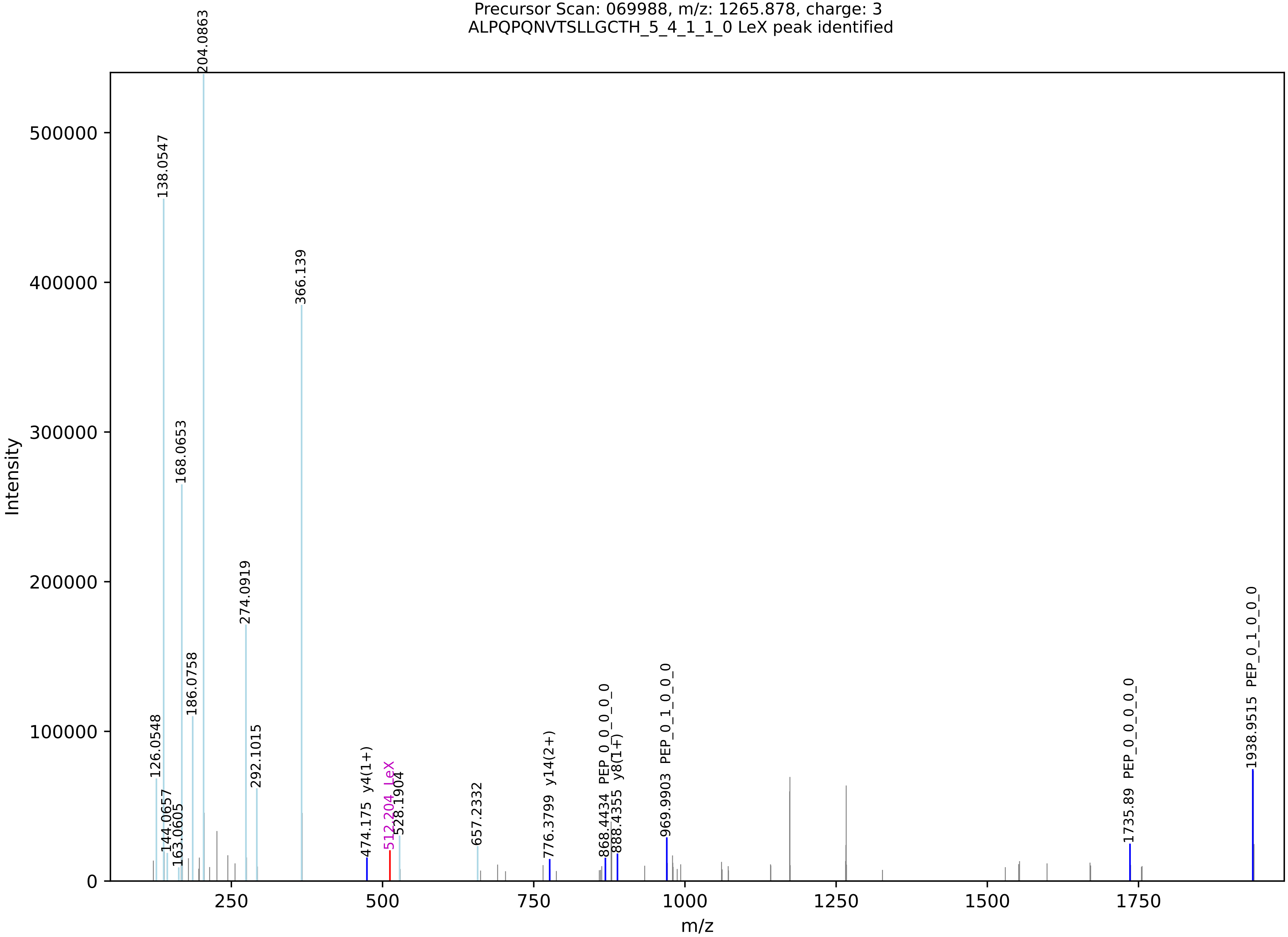

File: 231129_OE_RK_22, RT: 59.156, Scan: 078829
Type: HCD, FTMS + c NSI d Full ms2 1396.5553@hcd30.00 [120.0000-5720.8018]
Precursor Scan: 078738, m/z: 1395.5529, charge: 4
QDQCIYNTTYLNVQR_7_6_1_4_0 LeX peak identified

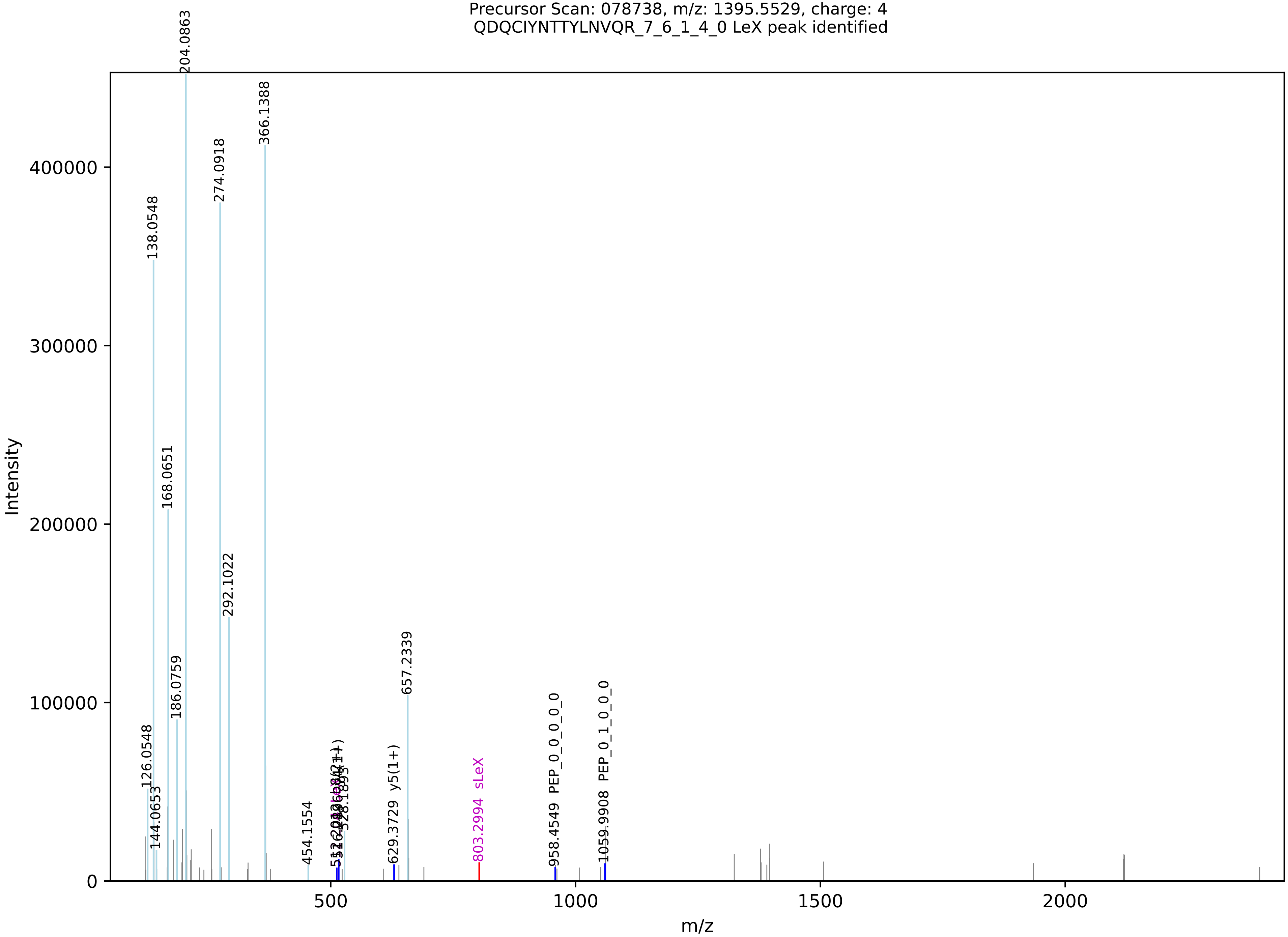

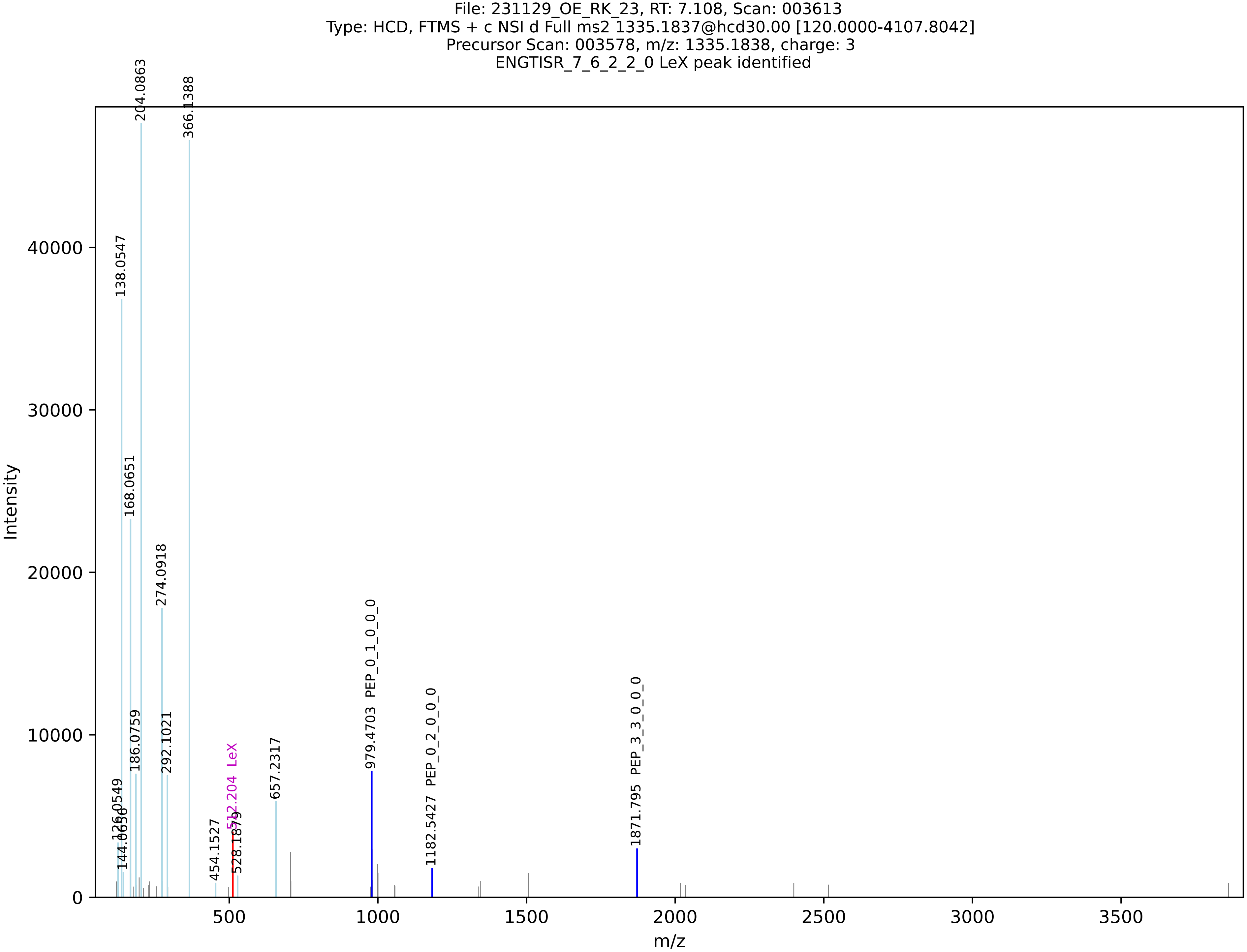
File: 231129_OE_RK_23, RT: 7.108, Scan: 003613
Type: HCD, FTMS + c NSI d Full ms2 1335.1837@hcd30.00 [120.0000-4107.8042]
Precursor Scan: 003578, m/z: 1335.1838, charge: 3
ENGTISR_7_6_2_2_0 LeX peak identified
Intensity
m/z
204.0863
366.1388
138.0547
168.0651
274.0918
186.0759
292.1021
126.0549
454.1527
512.204 LeX
528.1879
657.2317
979.4703 PEP_0_1_0_0_0
1182.5427 PEP_0_2_0_0_0
1871.795 PEP_3_3_0_0_0
0
10000
20000
30000
40000
500
1000
1500
2000
2500
3000
3500

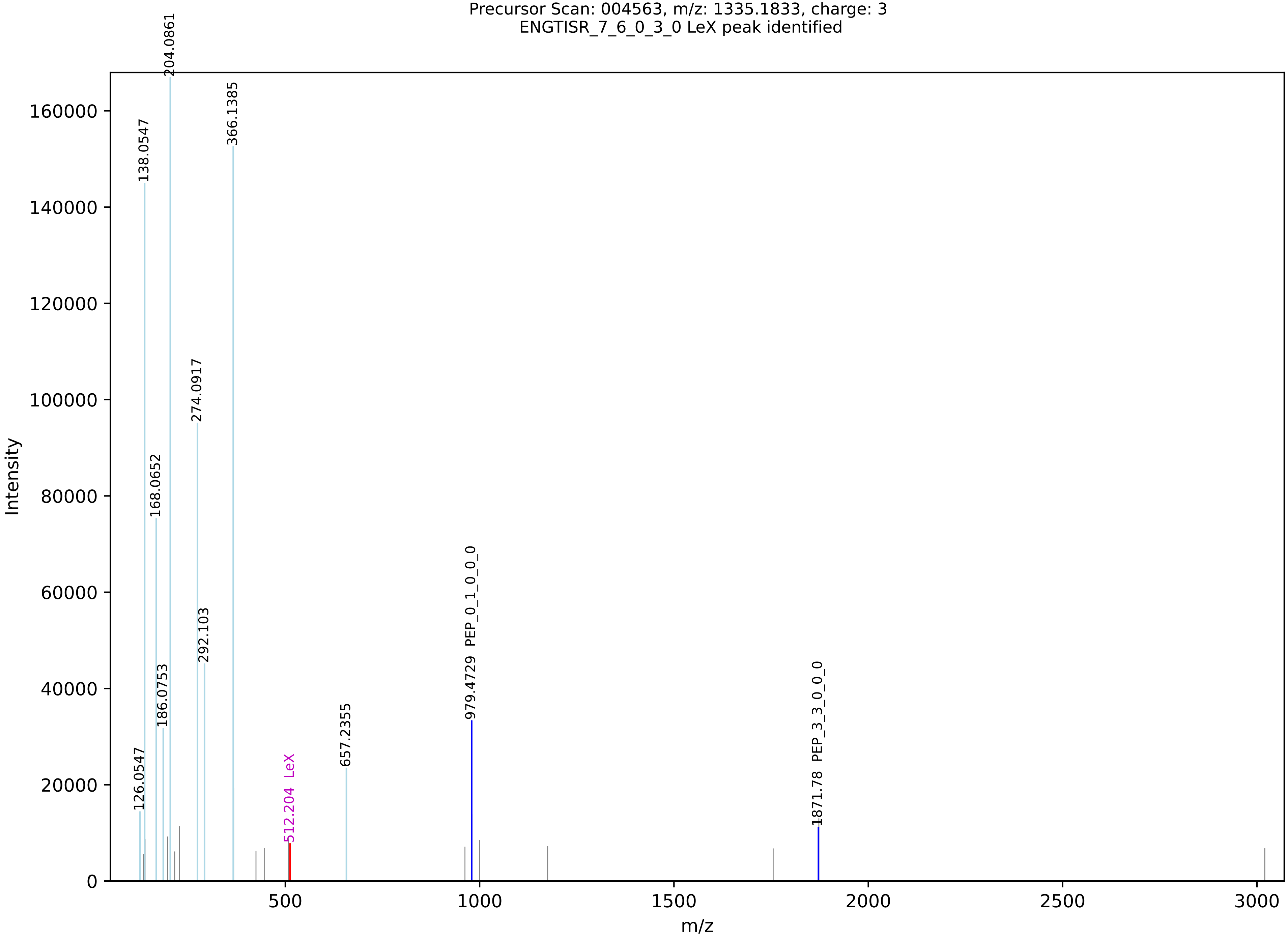

File: 231129_OE_RK_23, RT: 7.855, Scan: 004608
Type: HCD, FTMS + c NSI d Full ms2 1335.8516@hcd30.00 [120.0000-4109.8477]
Precursor Scan: 004563, m/z: 1335.1833, charge: 3
ENGTISR_7_6_0_3_0 LeX peak identified
Intensity
m/z
0
20000
40000
60000
80000
100000
120000
140000
160000
500
1000
1500
2000
2500
3000
204.0861
366.1385
138.0547
274.0917
168.0652
292.103
186.0753
126.0547
512.204 LeX
657.2355
979.4729 PEP_0_1_0_0_0
1871.78 PEP_3_3_0_0_0

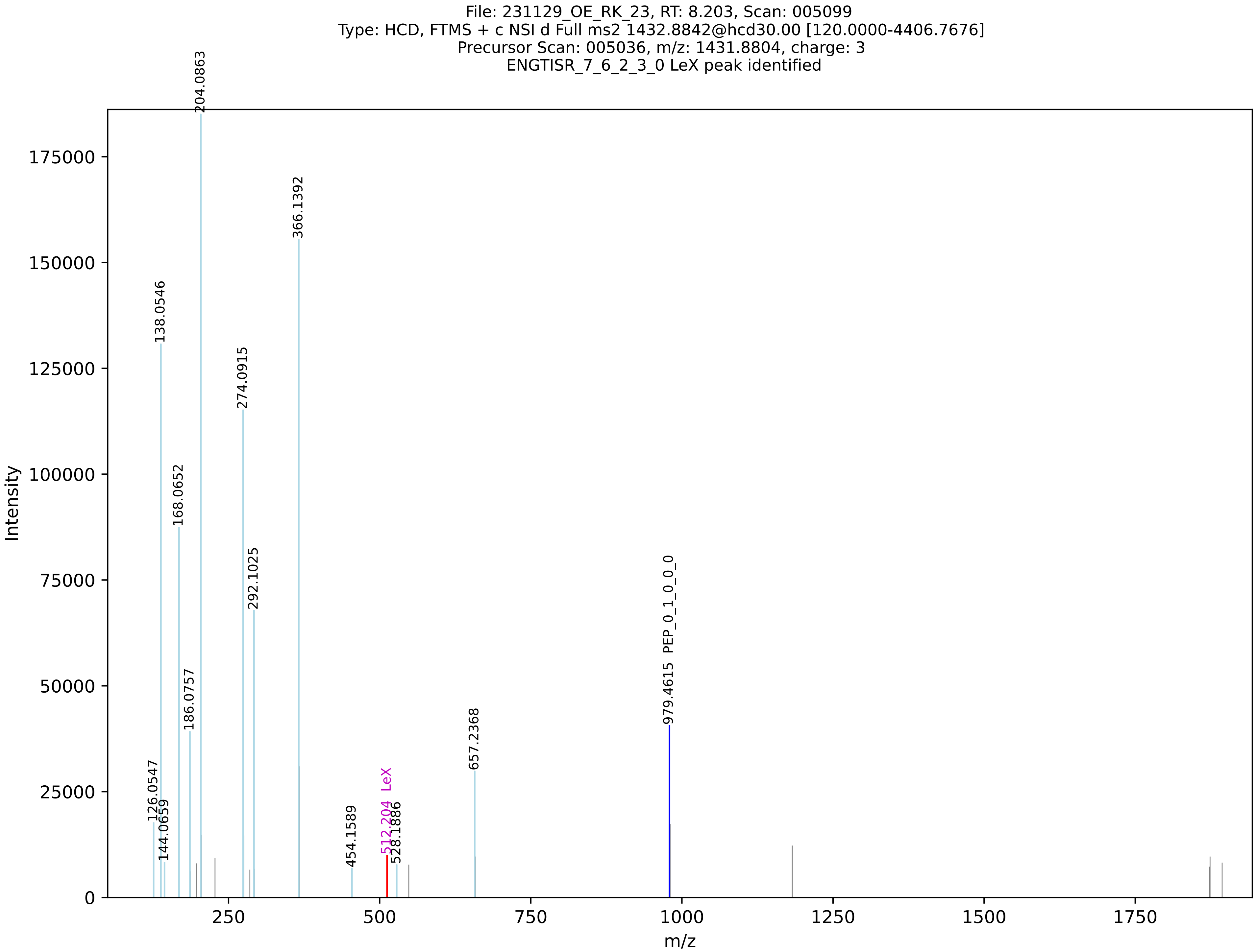
File: 231129_OE_RK_23, RT: 8.203, Scan: 005099
Type: HCD, FTMS + c NSI d Full ms2 1432.8842@hcd30.00 [120.0000-4406.7676]
Precursor Scan: 005036, m/z: 1431.8804, charge: 3
ENGTISR_7_6_2_3_0 LeX peak identified
Intensity
m/z
204.0863
366.1392
138.0546
274.0915
168.0652
292.1025
186.0757
126.0547
144.0659
454.1589
512.204 LeX
528.1886
657.2368
979.4615 PEP_0_1_0_0_0
0
25000
50000
75000
100000
125000
150000
175000
250
500
750
1000
1250
1500
1750

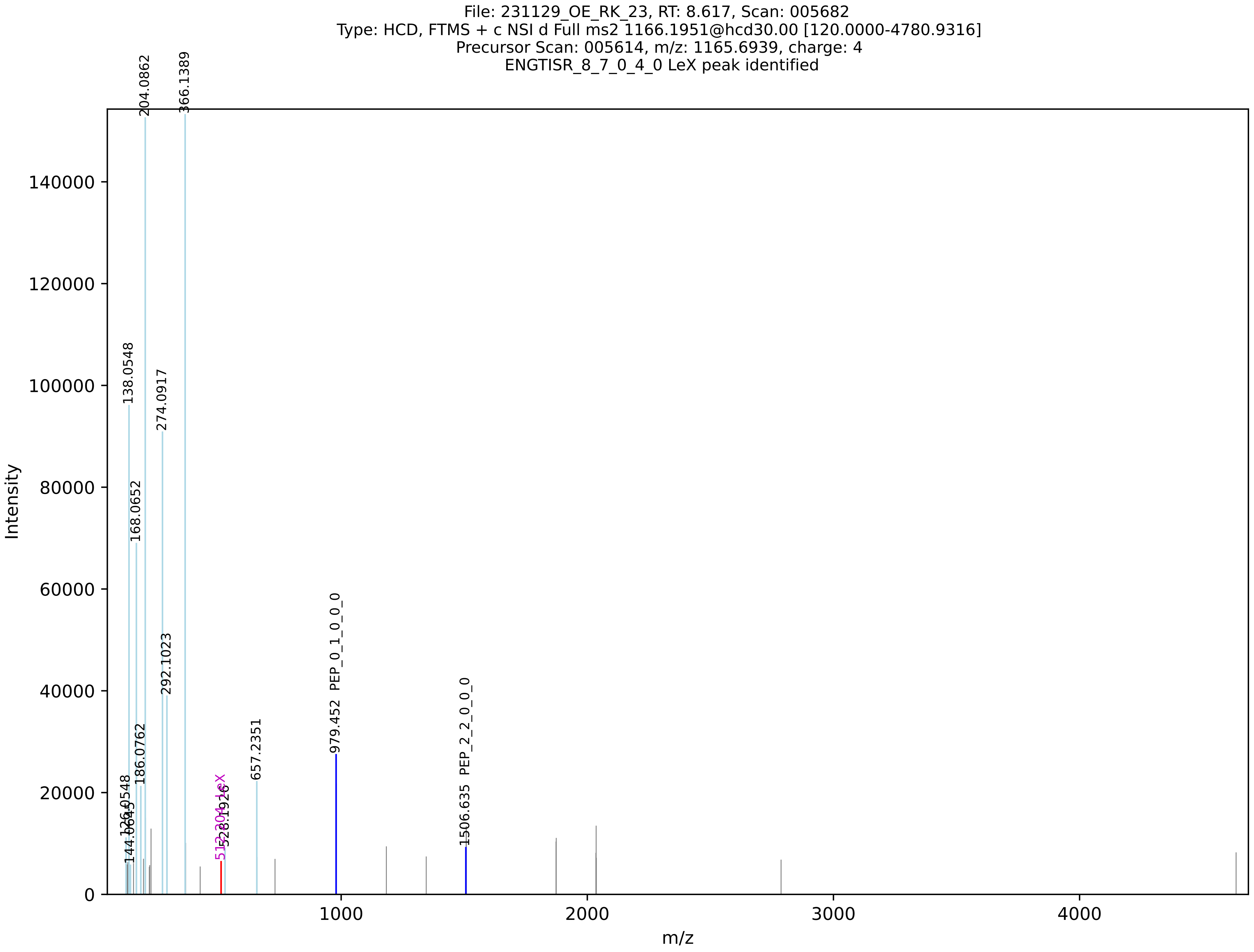
File: 231129_OE_RK_23, RT: 8.617, Scan: 005682
Type: HCD, FTMS + c NSI d Full ms2 1166.1951@hcd30.00 [120.0000-4780.9316]
Precursor Scan: 005614, m/z: 1165.6939, charge: 4
ENGTISR_8_7_0_4_0 LeX peak identified
Intensity
m/z
204.0862
366.1389
138.0548
274.0917
168.0652
292.1023
186.0762
126.0548
144.0645
512.204 LeX
528.1926
657.2351
979.452 PEP_0_1_0_0_0_
1506.635 PEP_2_2_0_0_0_
140000
120000
100000
80000
60000
40000
20000
0
1000
2000
3000
4000

File: 231129_OE_RK_23, RT: 9.842, Scan: 007403
Type: HCD, FTMS + c NSI d Full ms2 1147.6901@hcd30.00 [120.0000-4705.4316]
Precursor Scan: 007317, m/z: 1147.1894, charge: 4
ENGTISR_7_6_2_4_0 LeX peak identified

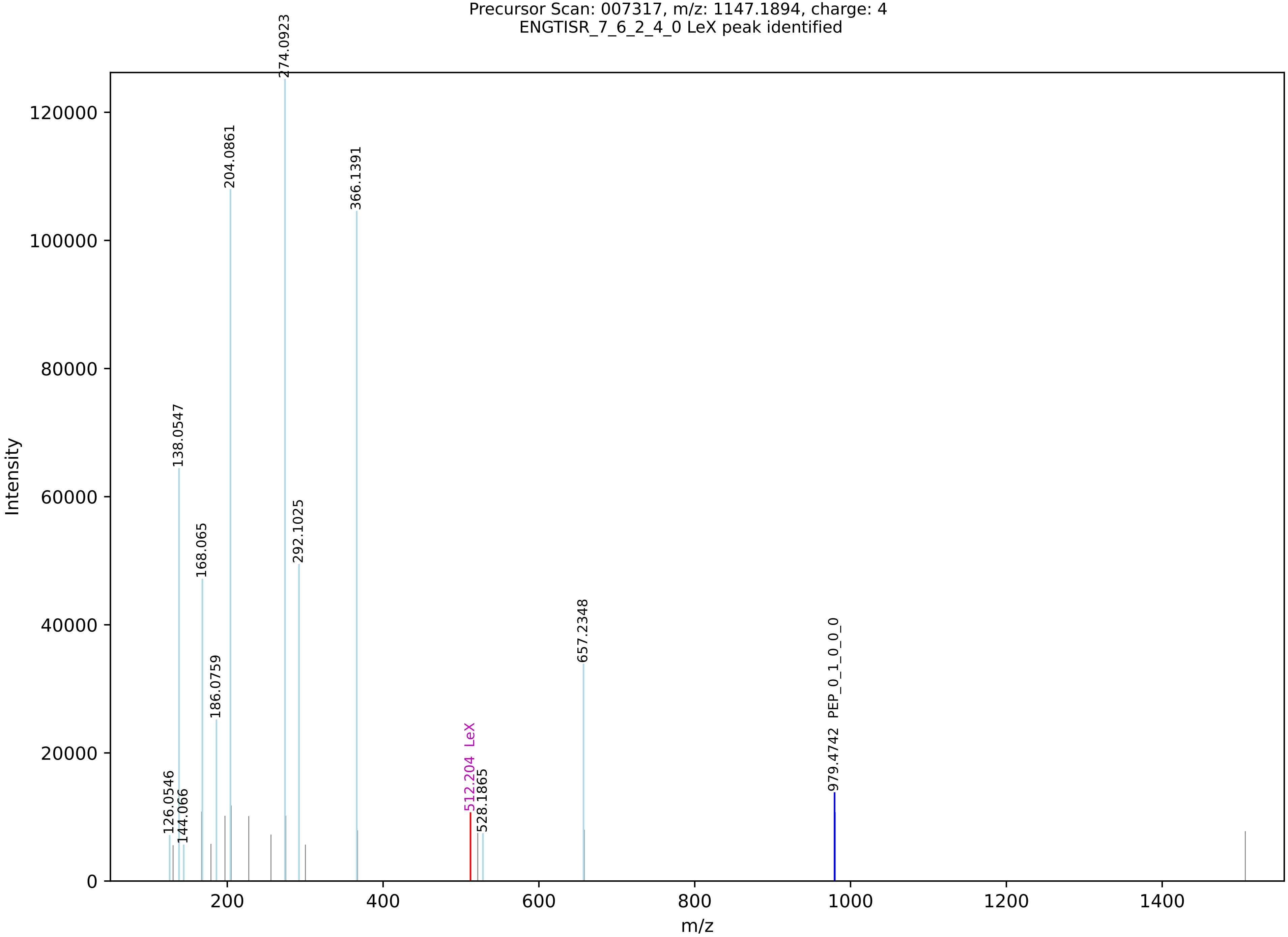

File: 231129_OE_RK_23, RT: 12.477, Scan: 011166
Type: HCD, FTMS + c NSI d Full ms2 1147.9397@hcd30.00 [120.0000-4706.4502]
Precursor Scan: 011078, m/z: 1147.188, charge: 4
ENGTISR_7_6_2_4_0 LeX peak identified

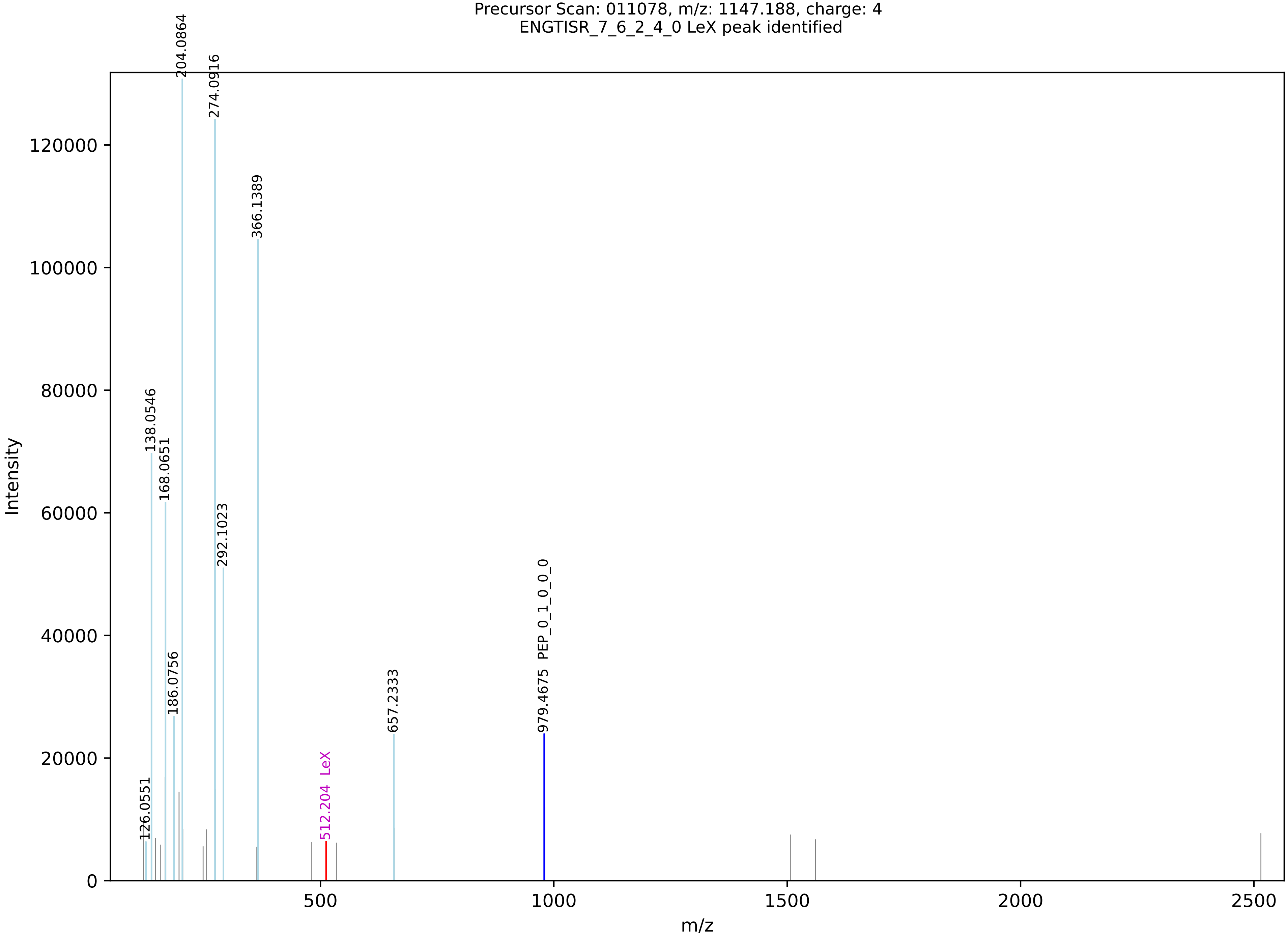

File: 231129_OE_RK_23, RT: 14.912, Scan: 014682
Type: HCD, FTMS + c NSI d Full ms2 1147.6897@hcd30.00 [120.0000-4705.4302]
Precursor Scan: 014598, m/z: 1146.9373, charge: 4
ENGTISR_7_6_2_4_0 LeX peak identified

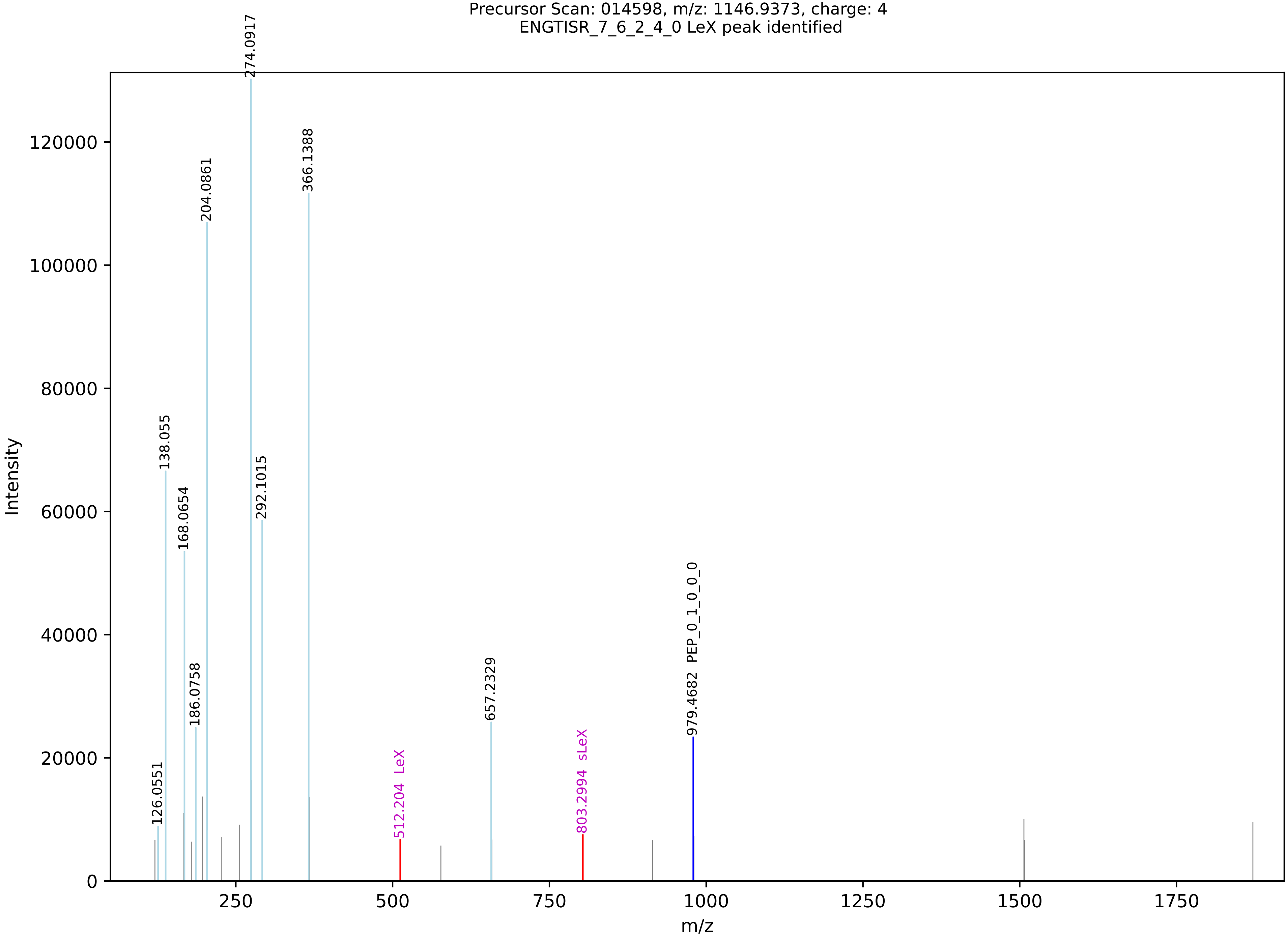

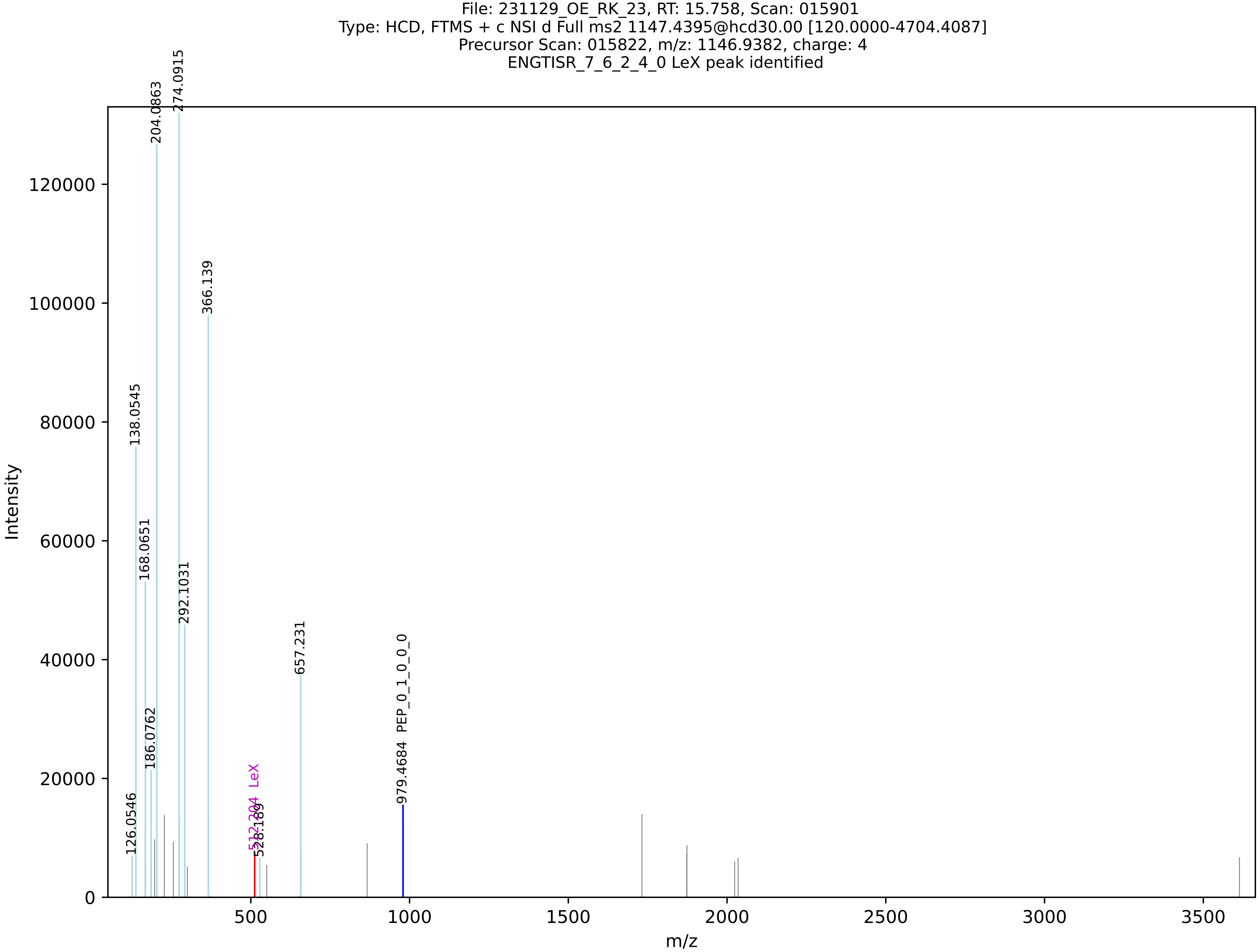
File: 231129_OE_RK_23, RT: 15.758, Scan: 015901
Type: HCD, FTMS + c NSI d Full ms2 1147.4395@hcd30.00 [120.0000-4704.4087]
Precursor Scan: 015822, m/z: 1146.9382, charge: 4
ENGTISR_7_6_2_4_0 LeX peak identified
274.0915
204.0863
366.139
138.0545
168.0651
292.1031
657.231
PEP_0_1_0_0_0
979.4684
186.0762
LeX
512.204
528.189
126.0546
Intensity
m/z
0
20000
40000
60000
80000
100000
120000
500
1000
1500
2000
2500
3000
3500

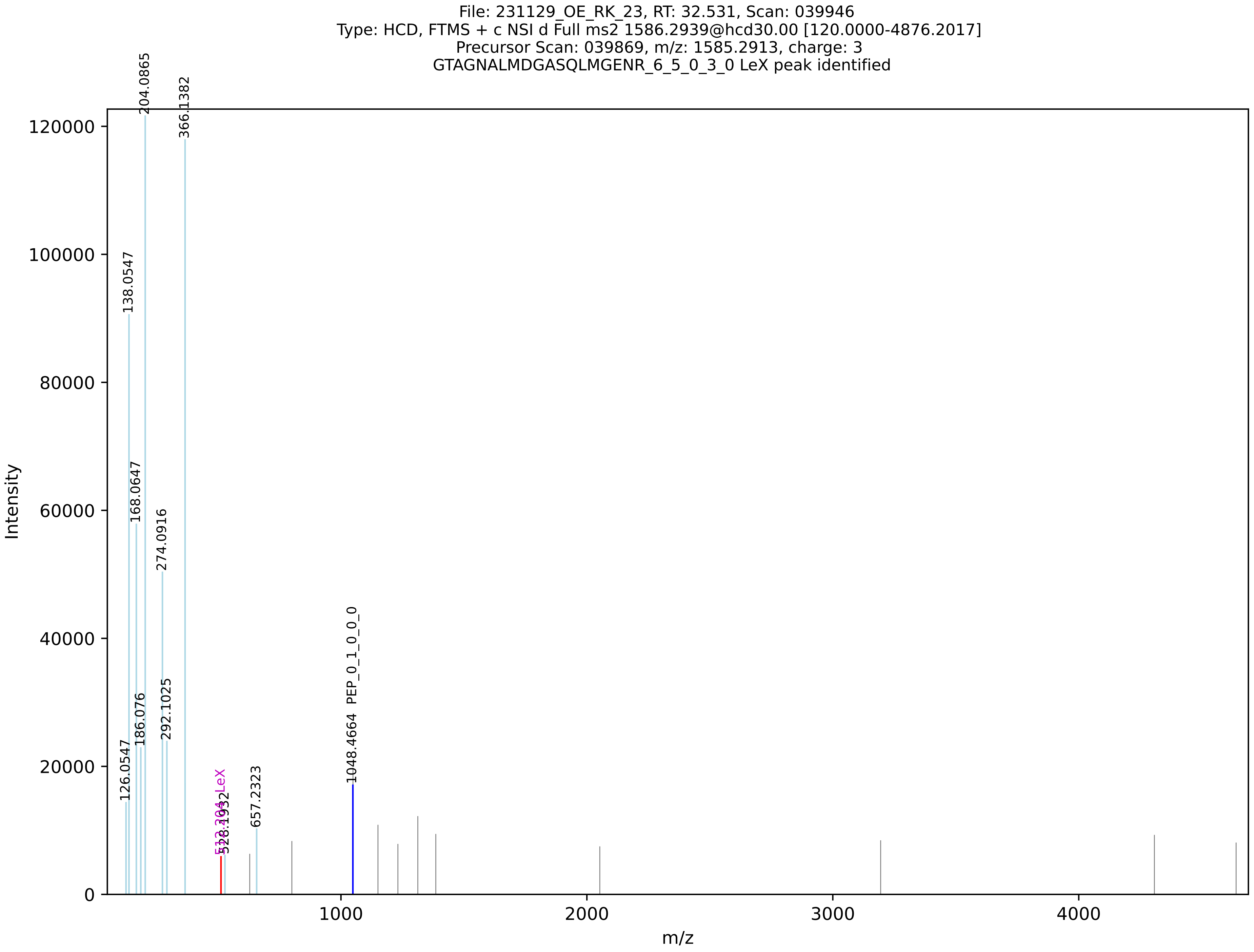

File: 231129_OE_RK_23, RT: 32.531, Scan: 039946
Type: HCD, FTMS + c NSI d Full ms2 1586.2939@hcd30.00 [120.0000-4876.2017]
Precursor Scan: 039869, m/z: 1585.2913, charge: 3
GTAGNALMDGASQLMGENR_6_5_0_3_0 LeX peak identified
204.0865
366.1382
138.0547
168.0647
274.0916
292.1025
186.076
126.0547
513.204_LeX
528.1932
657.2323
1048.4664 PEP_0_1_0_0_0
Intensity
m/z

File: 231129_OE_RK_23, RT: 41.044, Scan: 052290
Type: HCD, FTMS + c NSI d Full ms2 1570.3057@hcd30.00 [120.0000-4827.2773]
Precursor Scan: 052211, m/z: 1569.3046, charge: 3
QDQCIYNTTYLNVQR_7_6_1_1_0 LeX peak identified

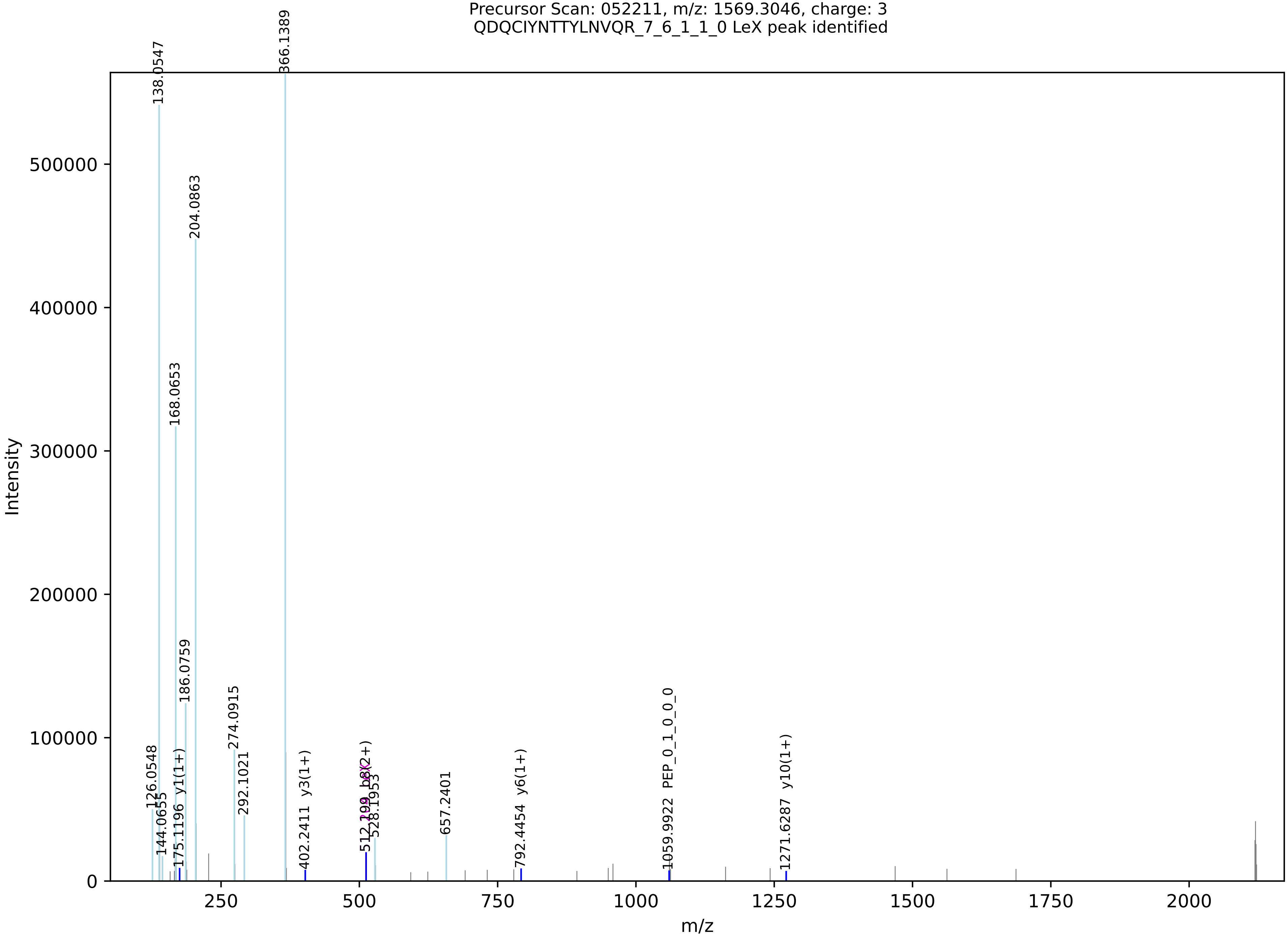

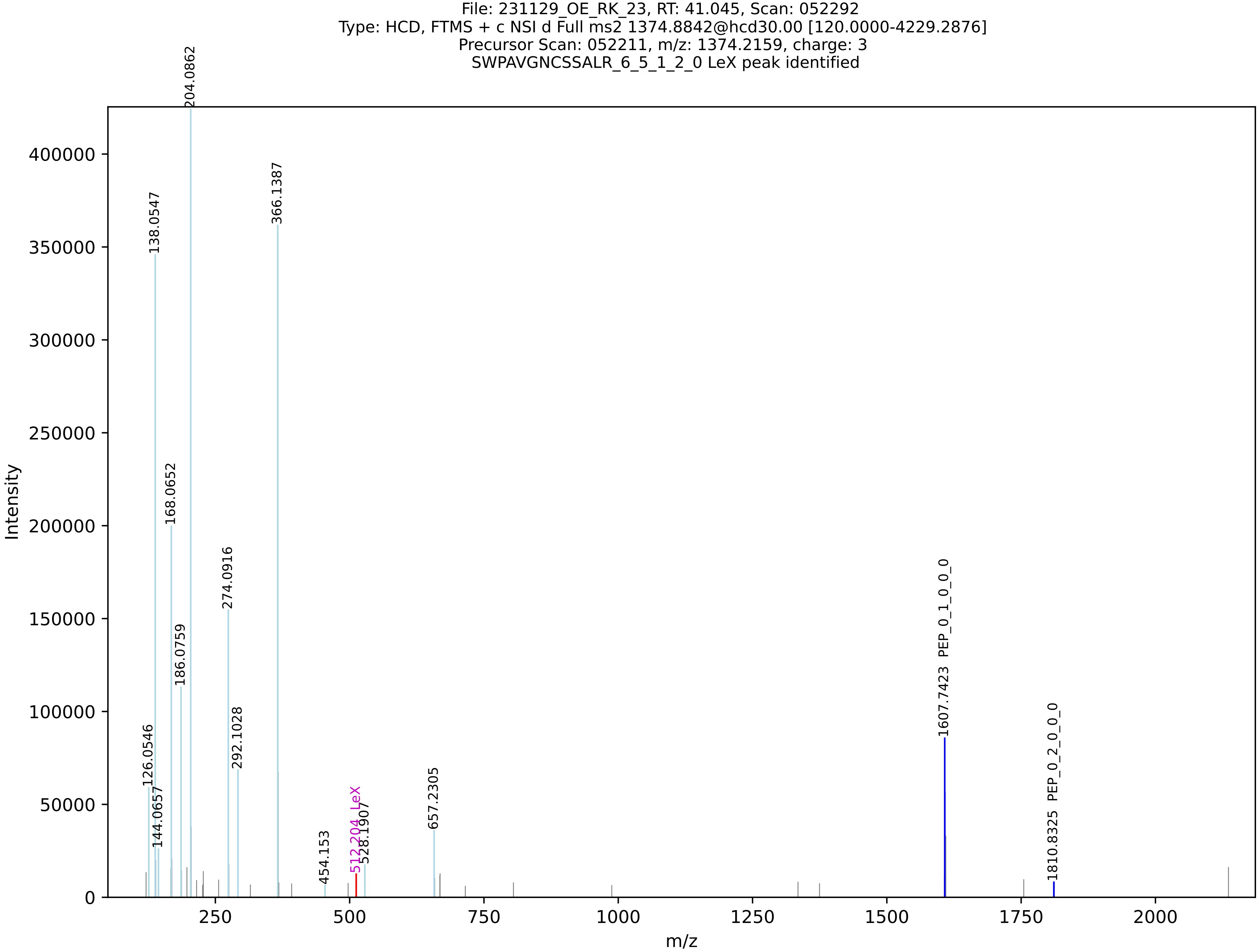

File: 231129_OE_RK_23, RT: 41.045, Scan: 052292
Type: HCD, FTMS + c NSI d Full ms2 1374.8842@hcd30.00 [120.0000-4229.2876]
Precursor Scan: 052211, m/z: 1374.2159, charge: 3
SWPAVGNCSSALR_6_5_1_2_0 LeX peak identified
Intensity
m/z
204.0862
366.1387
138.0547
168.0652
274.0916
186.0759
292.1028
126.0546
144.0657
454.153
512.204_LeX
528.1907
657.2305
1607.7423 PEP_0_1_0_0_0
1810.8325 PEP_0_2_0_0_0

File: 231129_OE_RK_23, RT: 41.245, Scan: 052582
Type: HCD, FTMS + c NSI d Full ms2 1569.9717@hcd30.00 [120.0000-4826.2554]
Precursor Scan: 052504, m/z: 1569.3035, charge: 3
QDQCIYNTTYLNVQR_7_6_1_1_0 LeX peak identified

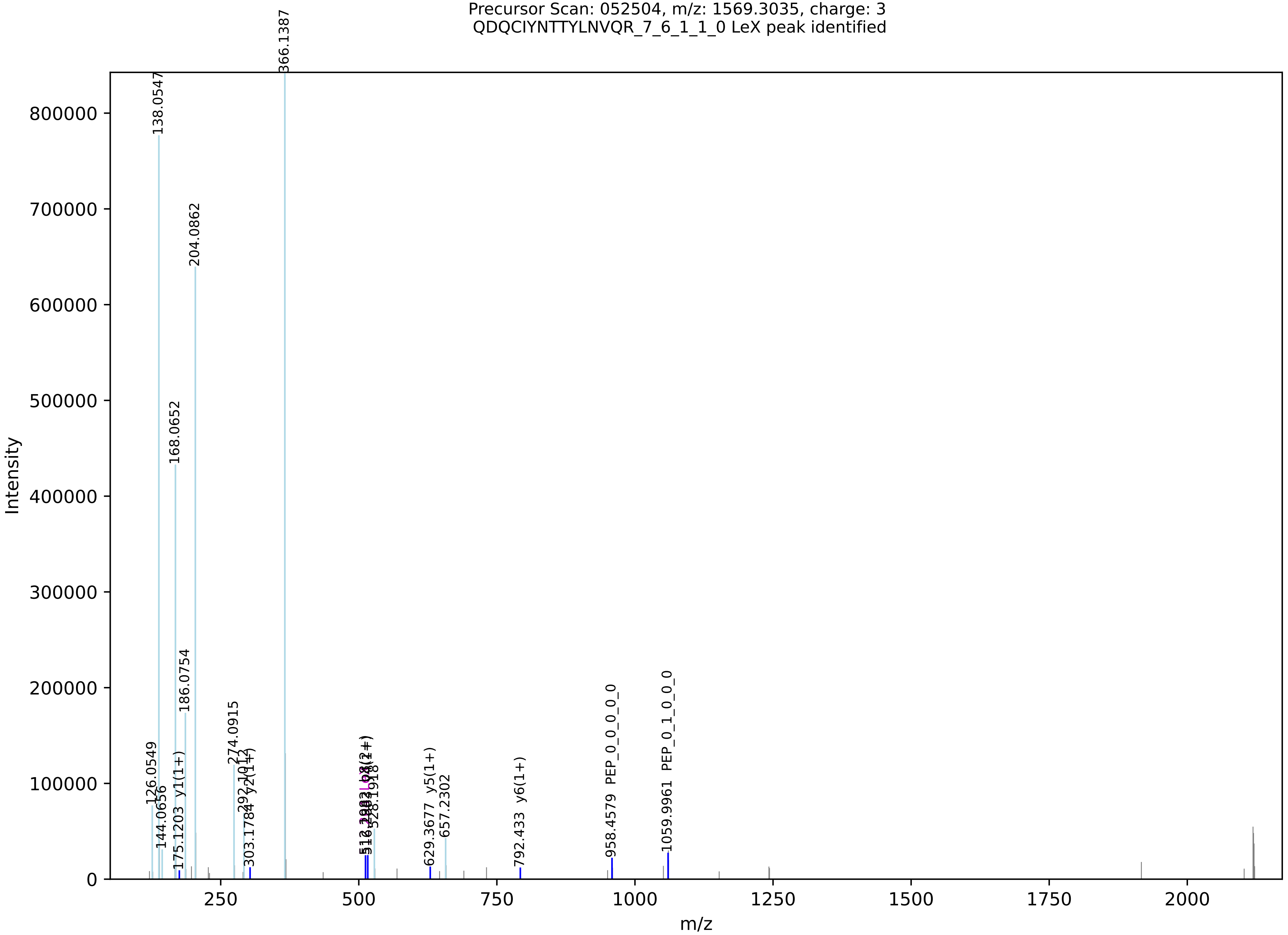

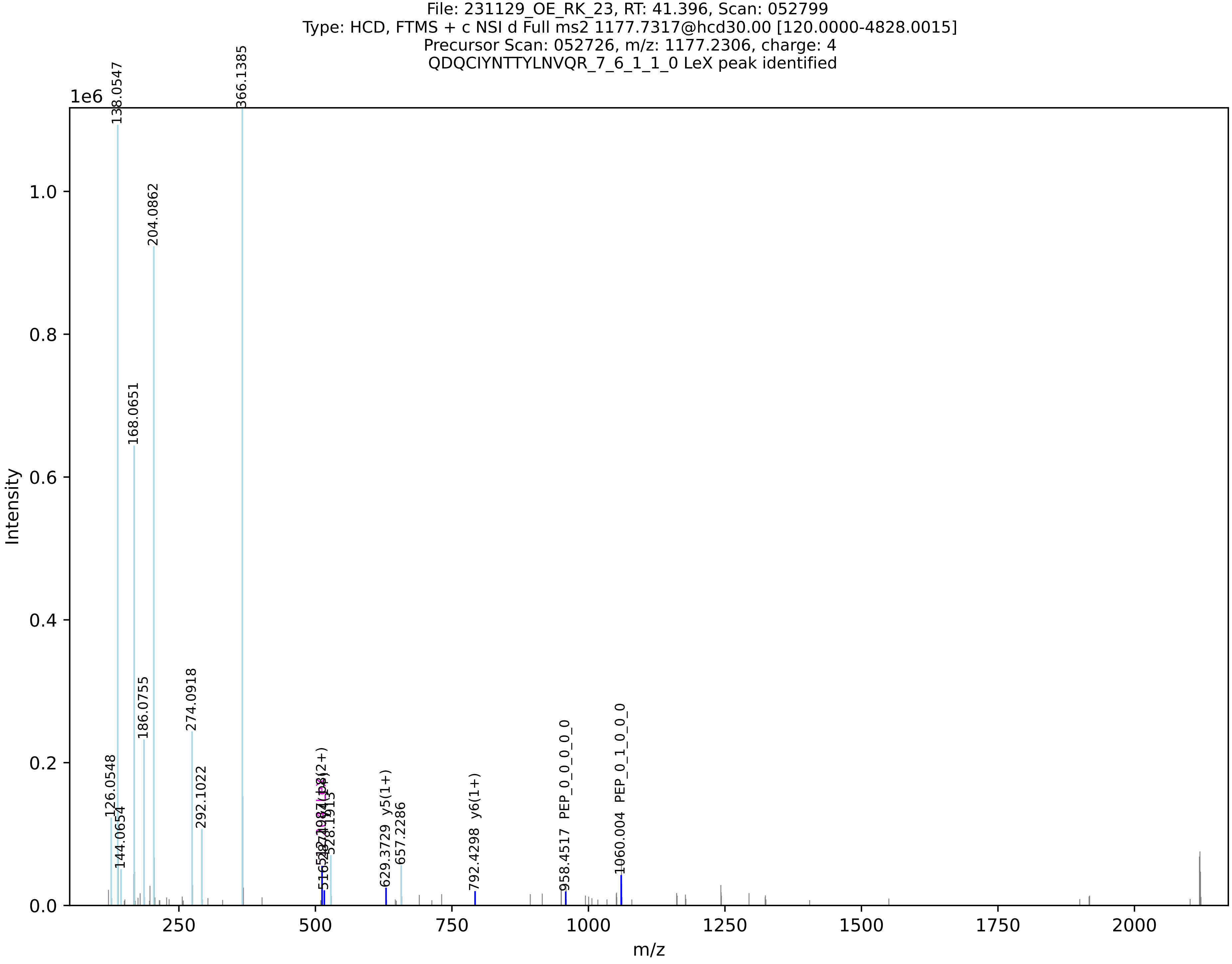
File: 231129_OE_RK_23, RT: 41.396, Scan: 052799
Type: HCD, FTMS + c NSI d Full ms2 1177.7317@hcd30.00 [120.0000-4828.0015]
Precursor Scan: 052726, m/z: 1177.2306, charge: 4
QDQCIYNTTYLNVQR_7_6_1_1_0 LeX peak identified
1e6
138.0547
366.1385
204.0862
168.0651
186.0755
274.0918
126.0548
144.0654
292.1022
528.1915
629.3729 y5(1+)
657.2286
792.4298 y6(1+)
958.4517 PEP_0_0_0_0_0_
1060.004 PEP_0_1_0_0_0_
Intensity
m/z
0.0
0.2
0.4
0.6
0.8
1.0
250
500
750
1000
1250
1500
1750
2000

File: 231129_OE_RK_23, RT: 43.867, Scan: 056379
Type: HCD, FTMS + c NSI d Full ms2 1287.2719@hcd30.00 [120.0000-5274.9253]
Precursor Scan: 056299, m/z: 1286.2695, charge: 4
QDQCIYNTTYLNVQR_7_6_2_2_0 LeX peak identified

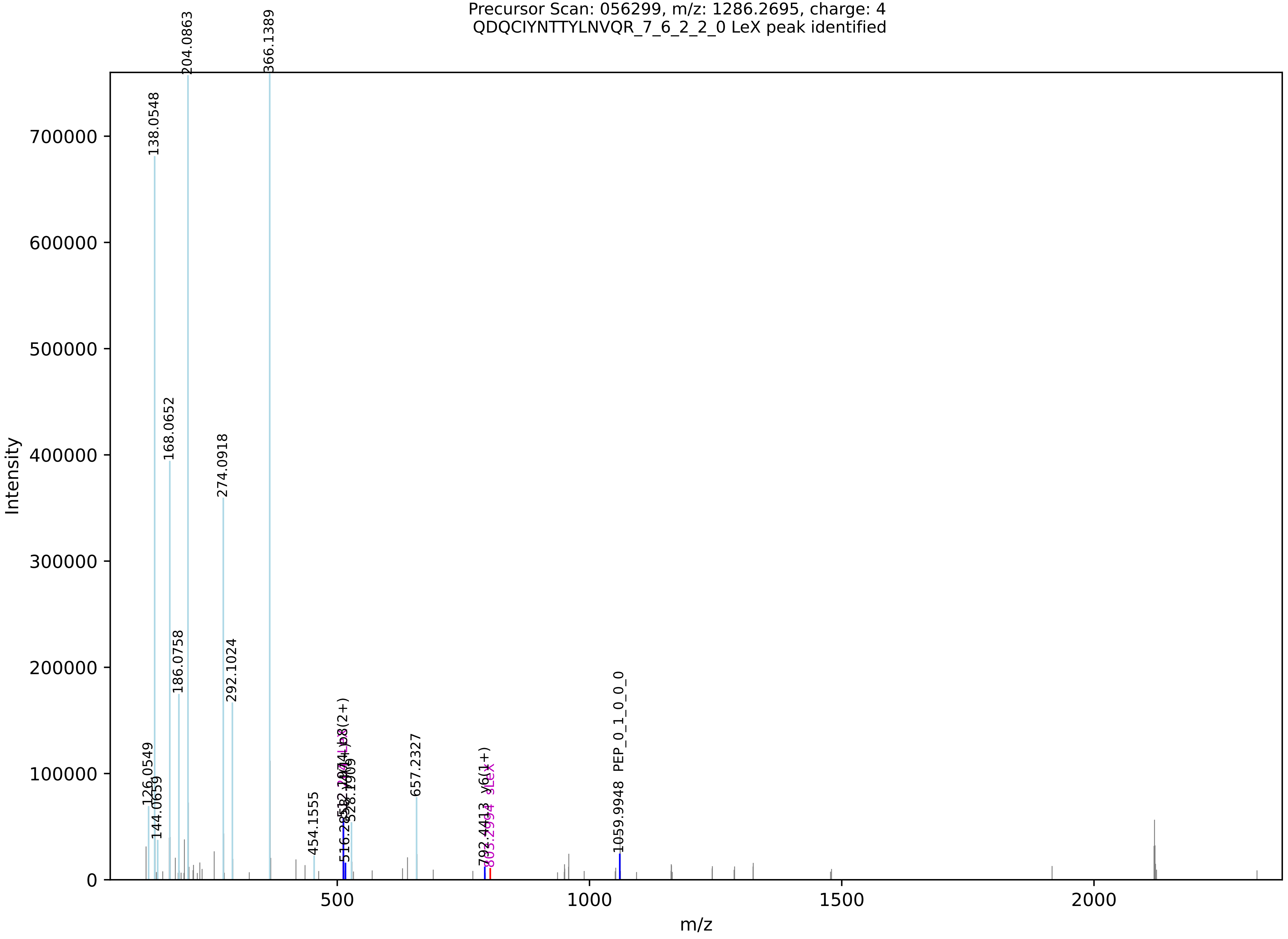

File: 231129_OE_RK_23, RT: 43.969, Scan: 056529
Type: HCD, FTMS + c NSI d Full ms2 1323.2859@hcd30.00 [120.0000-5421.8623]
Precursor Scan: 056445, m/z: 1323.0345, charge: 4
QDQCIYNTTYLNVQR_7_6_1_3_0 LeX peak identified

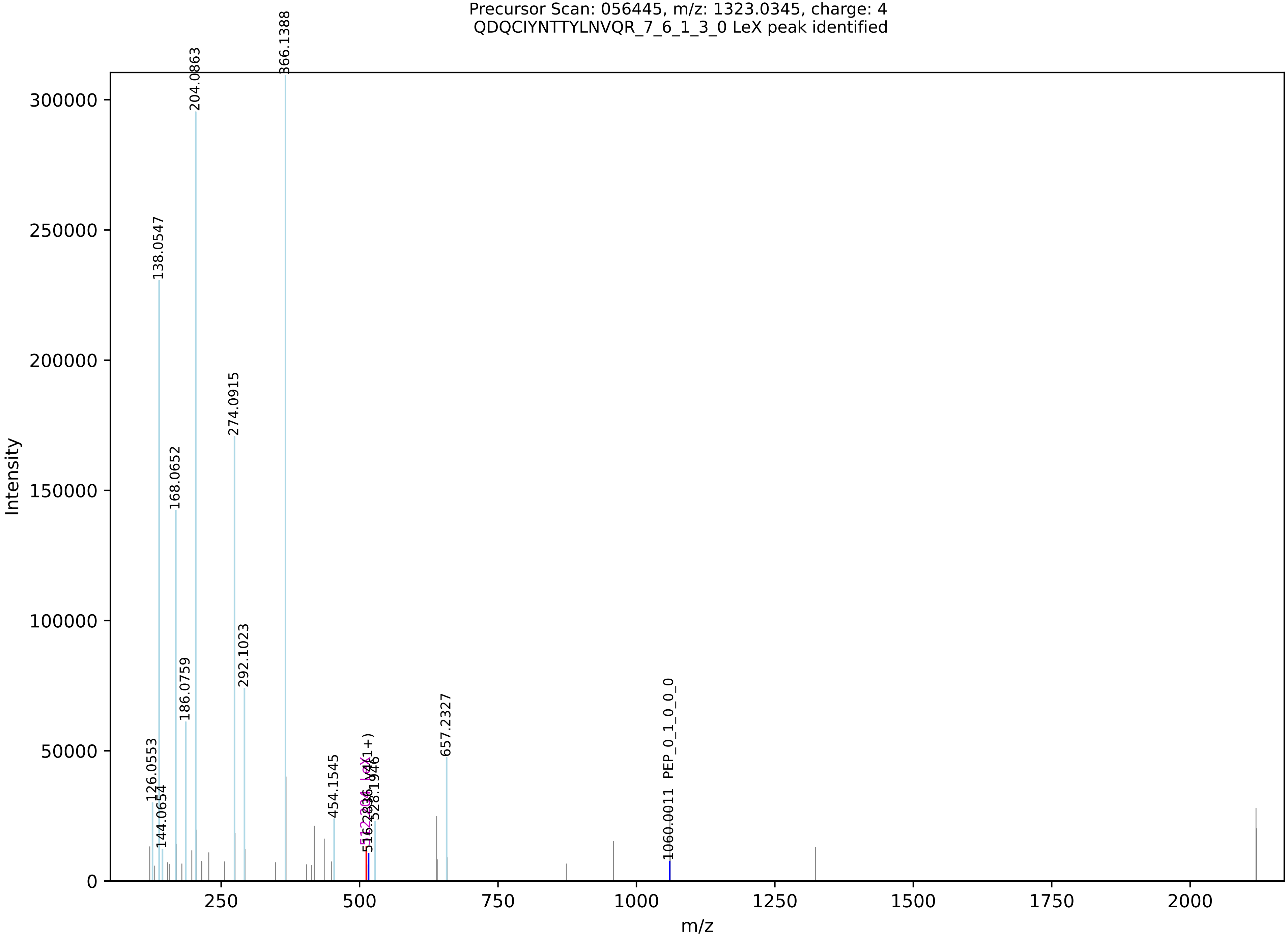

File: 231129_OE_RK_23, RT: 44.169, Scan: 056818
Type: HCD, FTMS + c NSI d Full ms2 1323.7832@hcd30.00 [120.0000-5423.8916]
Precursor Scan: 056741, m/z: 1322.7803, charge: 4
QDQCIYNTTYLNVQR_7_6_1_3_0 LeX peak identified

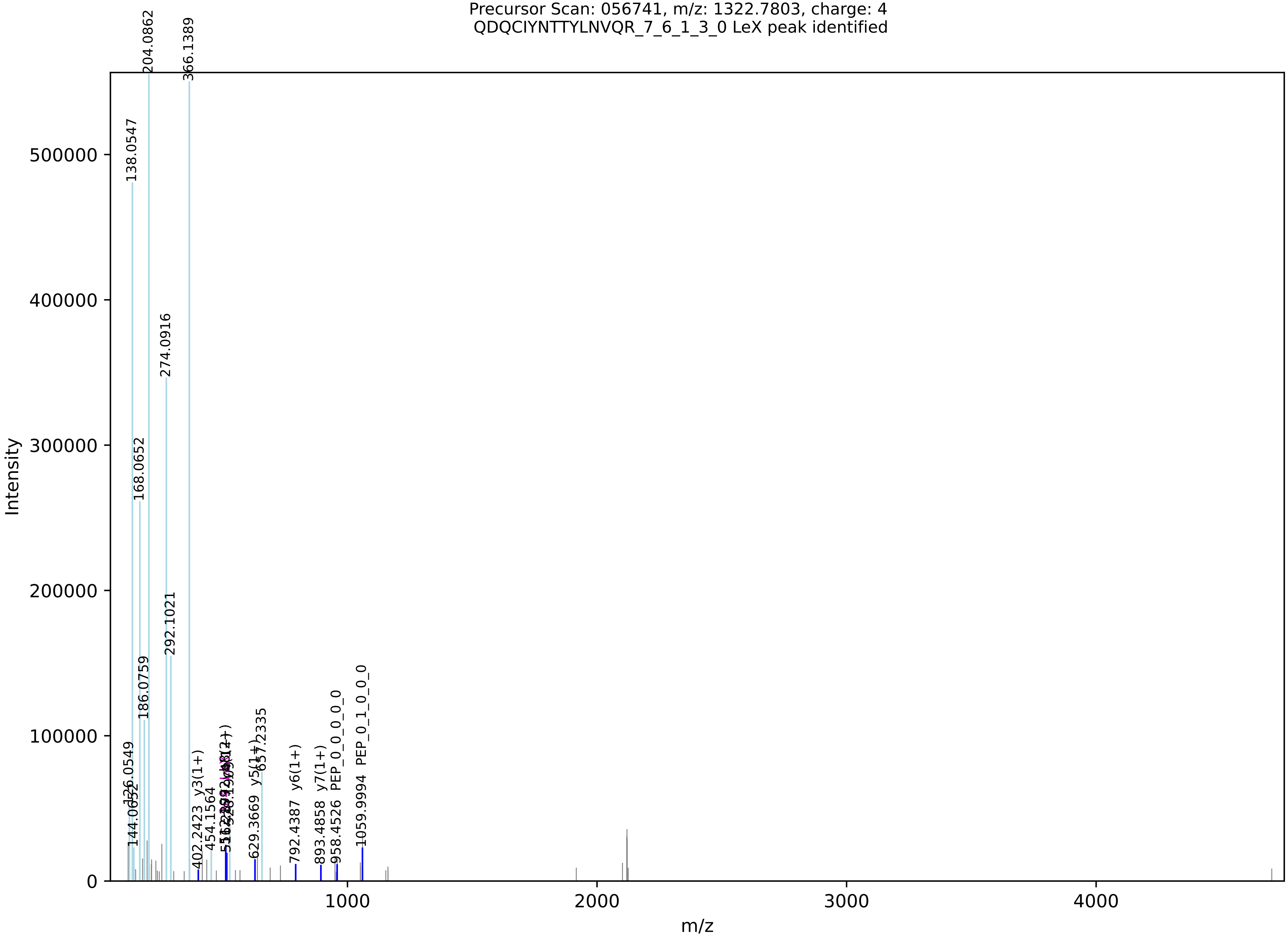

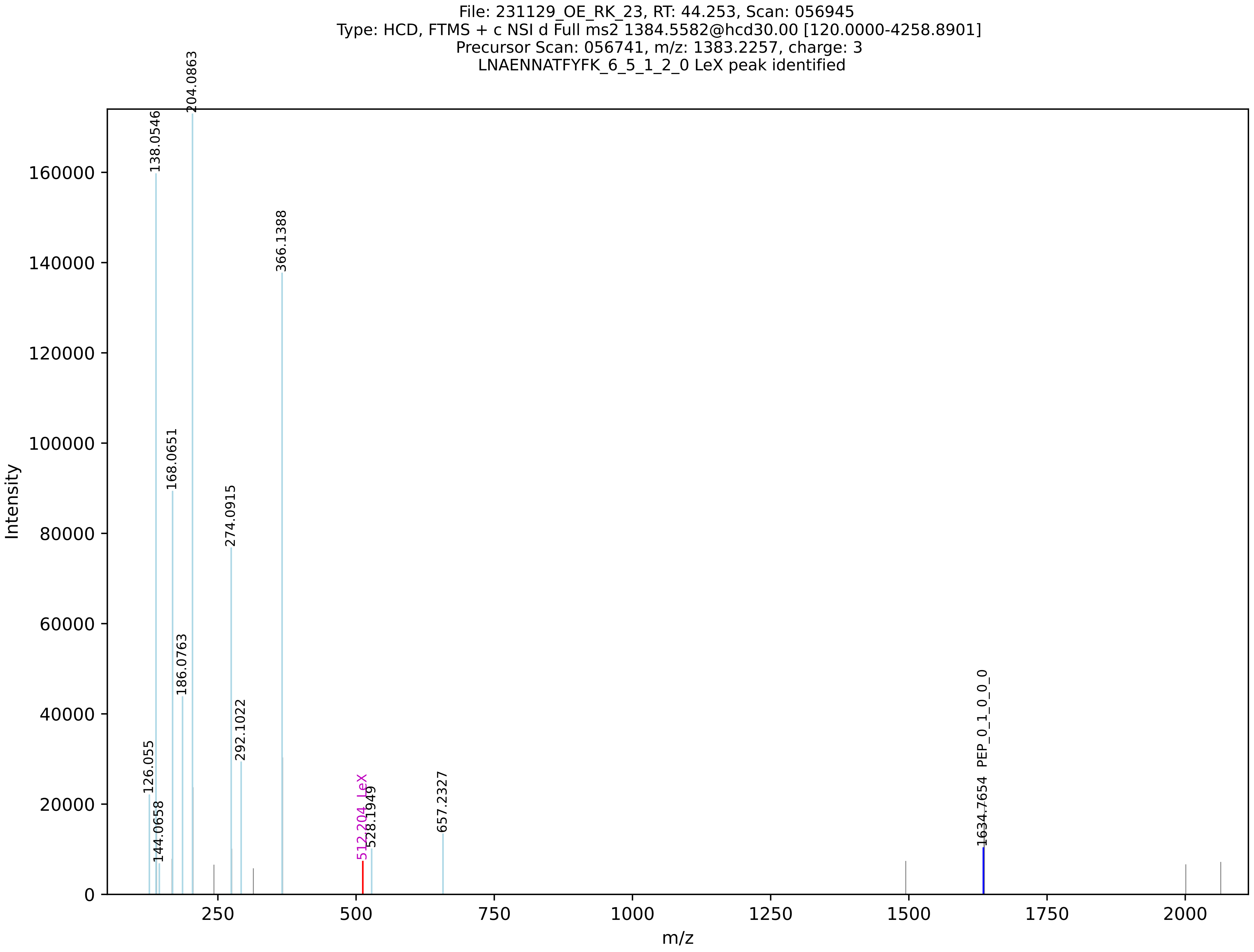
File: 231129_OE_RK_23, RT: 44.253, Scan: 056945
Type: HCD, FTMS + c NSI d Full ms2 1384.5582@hcd30.00 [120.0000-4258.8901]
Precursor Scan: 056741, m/z: 1383.2257, charge: 3
LNAENNATFYFK_6_5_1_2_0 LeX peak identified
Intensity
m/z
204.0863
138.0546
366.1388
168.0651
274.0915
186.0763
292.1022
126.055
144.0658
512.204_LeX
528.1949
657.2327
1634.7654 PEP_0_1_0_0_0
0
20000
40000
60000
80000
100000
120000
140000
160000
250
500
750
1000
1250
1500
1750
2000

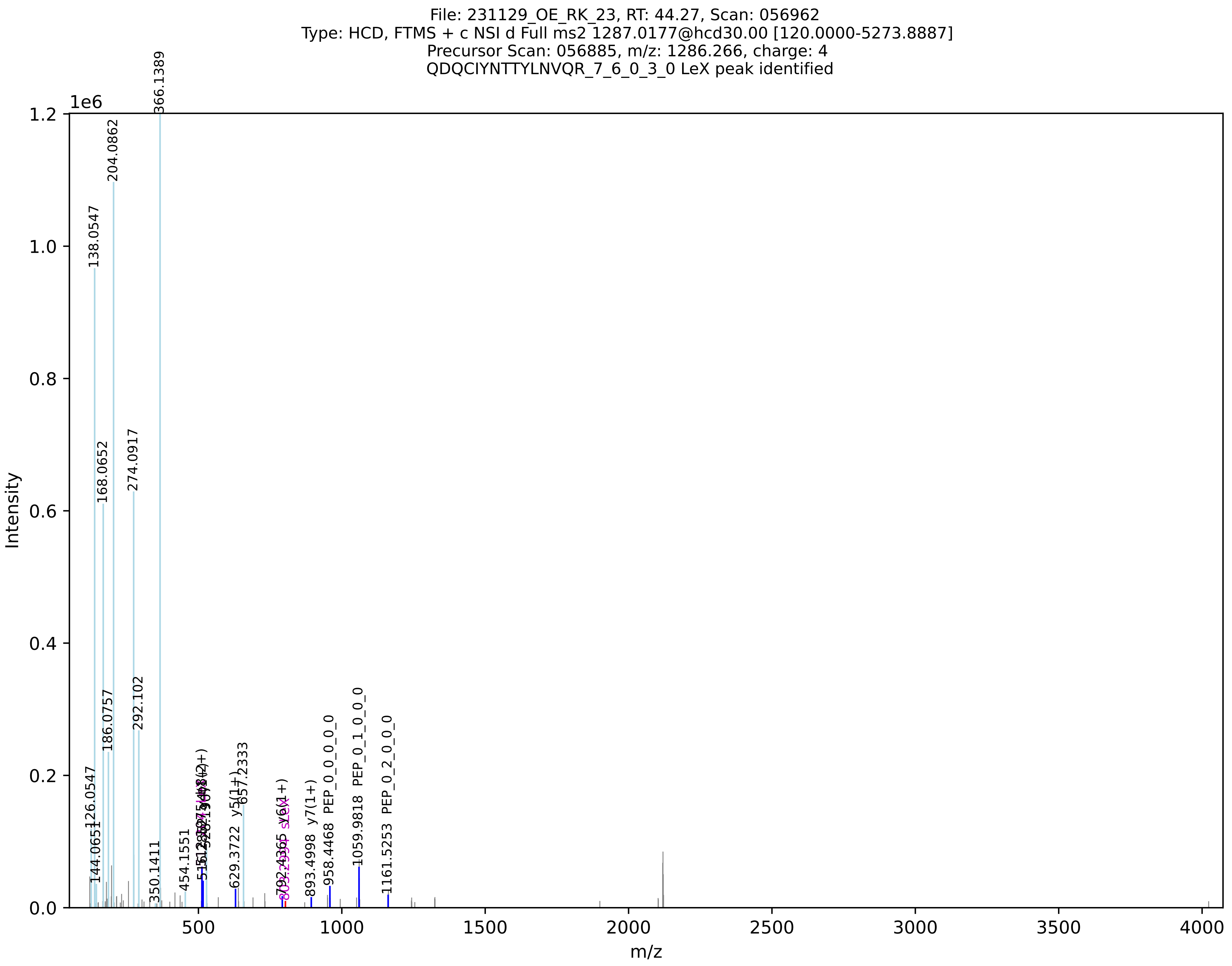
File: 231129_OE_RK_23, RT: 44.27, Scan: 056962
Type: HCD, FTMS + c NSI d Full ms2 1287.0177@hcd30.00 [120.0000-5273.8887]
Precursor Scan: 056885, m/z: 1286.266, charge: 4
QDQCIYNTTYLNVQR_7_6_0_3_0 LeX peak identified
1e6
Intensity
m/z
366.1389
204.0862
138.0547
168.0652
274.0917
292.102
186.0757
126.0547
144.0651
350.1411
454.1551
657.2333
y5(1+)
629.3722
y6(1+)
792.4365
sLeX
803.2934
y7(1+)
893.4998
PEP_0_0_0_0_0
958.4468
PEP_0_1_0_0_0
1059.9818
PEP_0_2_0_0_0
1161.5253

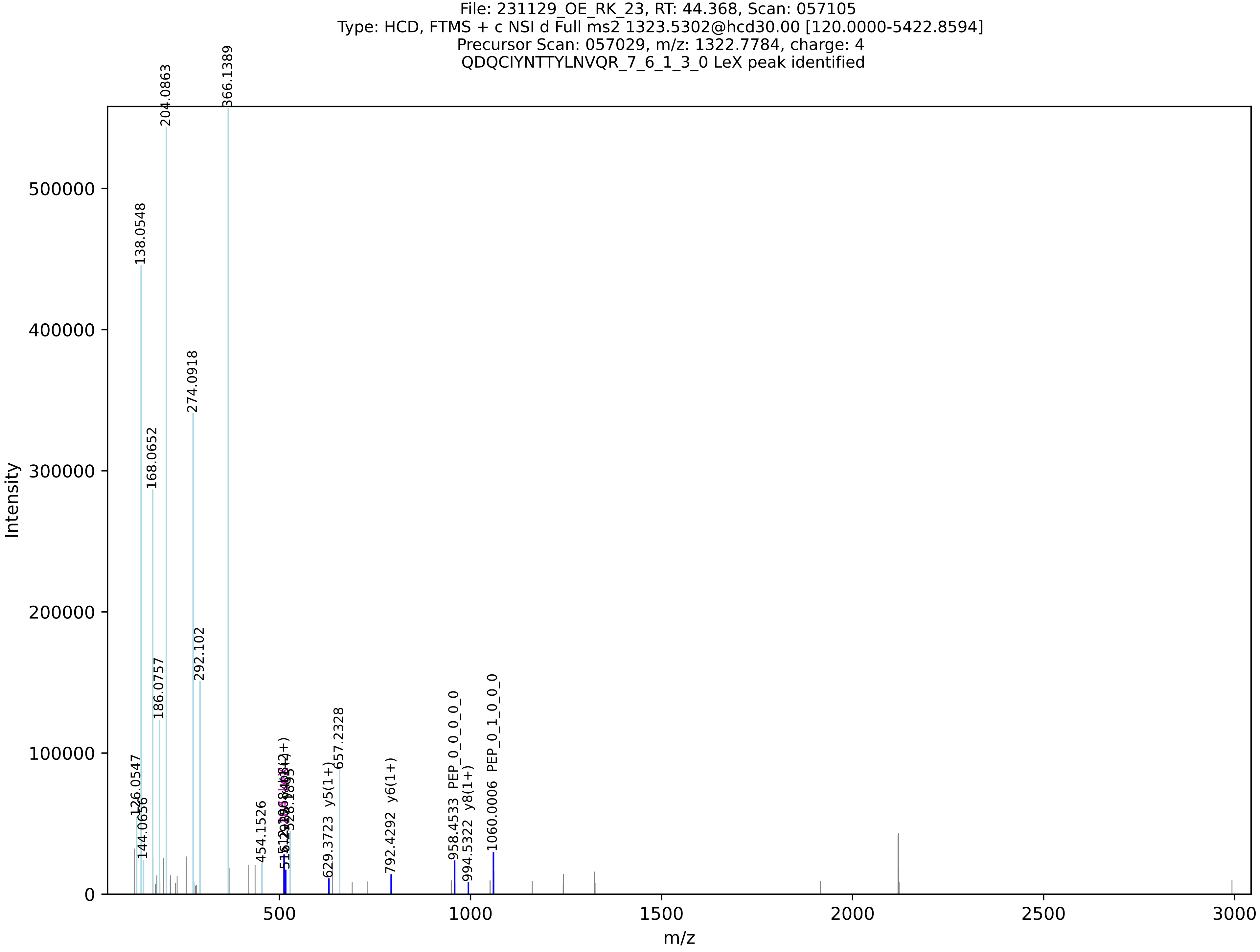

File: 231129_OE_RK_23, RT: 44.368, Scan: 057105
Type: HCD, FTMS + c NSI d Full ms2 1323.5302@hcd30.00 [120.0000-5422.8594]
Precursor Scan: 057029, m/z: 1322.7784, charge: 4
QDQCIYNTTYLNVQR_7_6_1_3_0 LeX peak identified
Intensity
m/z
366.1389
204.0863
138.0548
274.0918
168.0652
292.102
186.0757
126.0547
144.0656
454.1526
528.1895
629.3723 y5(1+)
657.2328
792.4292 y6(1+)
958.4533 PEP_0_0_0_0_0
994.5322 y8(1+)
1060.0006 PEP_0_1_0_0_0

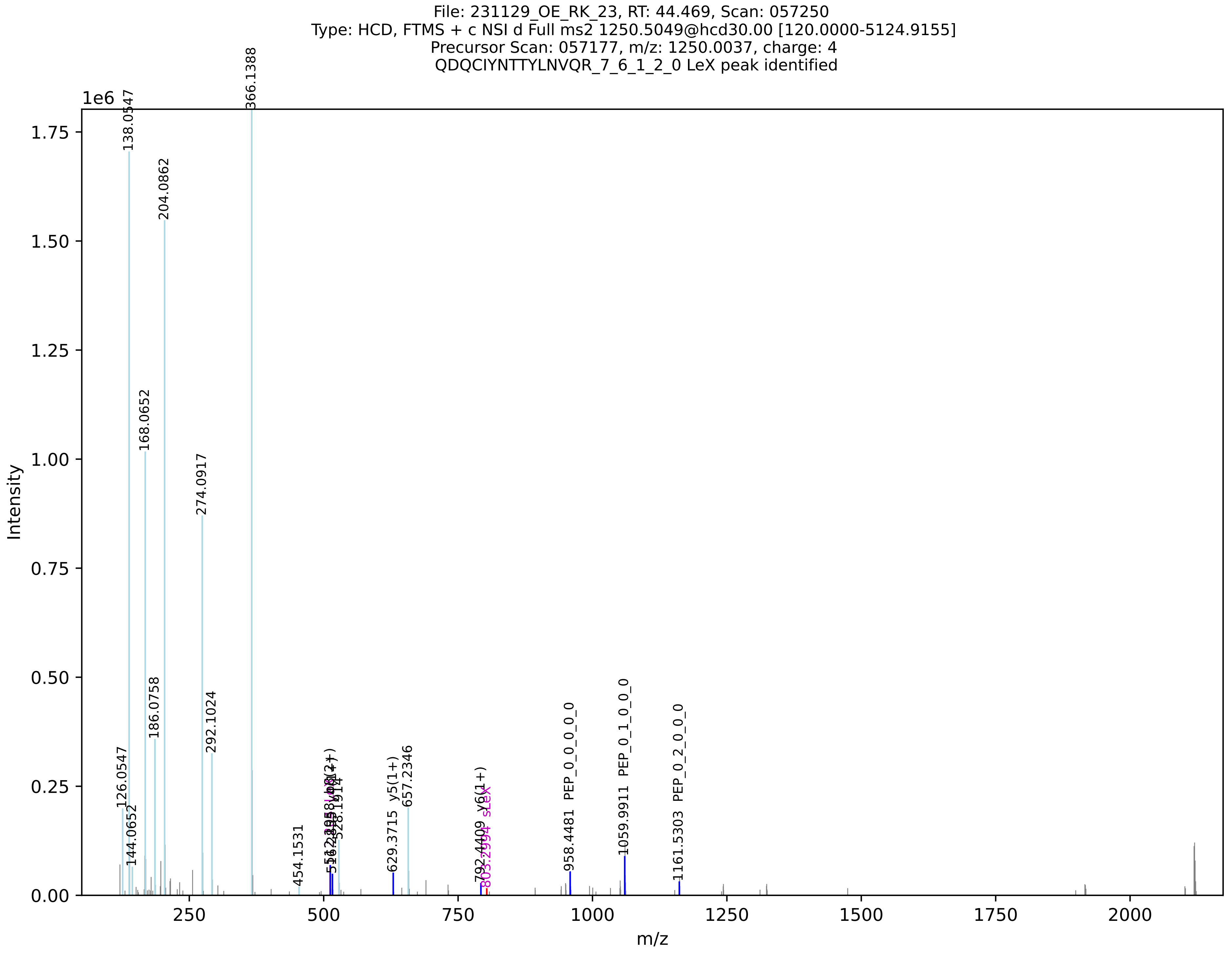
File: 231129_OE_RK_23, RT: 44.469, Scan: 057250
Type: HCD, FTMS + c NSI d Full ms2 1250.5049@hcd30.00 [120.0000-5124.9155]
Precursor Scan: 057177, m/z: 1250.0037, charge: 4
QDQCIYNTTYLNVQR_7_6_1_2_0 LeX peak identified
1e6
Intensity
m/z
366.1388
138.0547
204.0862
168.0652
274.0917
186.0758
292.1024
126.0547
144.0652
454.1531
528.1914
629.3715 y5(1+)
657.2346
792.4409 y6(1+)
803.2994 sLeX
958.4481 PEP_0_0_0_0_0
1059.9911 PEP_0_1_0_0_0
1161.5303 PEP_0_2_0_0_0

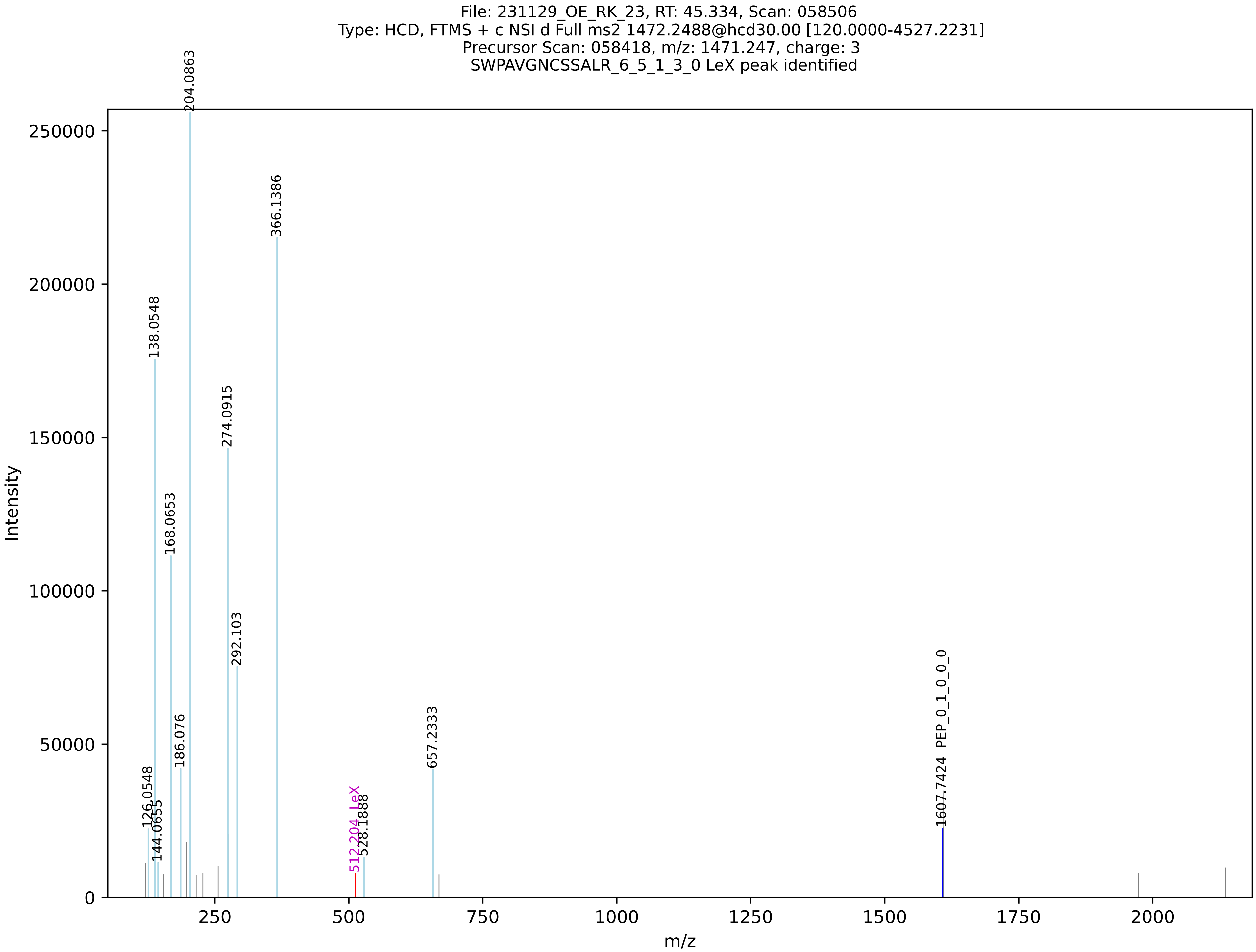
File: 231129_OE_RK_23, RT: 45.334, Scan: 058506
Type: HCD, FTMS + c NSI d Full ms2 1472.2488@hcd30.00 [120.0000-4527.2231]
Precursor Scan: 058418, m/z: 1471.247, charge: 3
SWPAVGNCSSALR_6_5_1_3_0 LeX peak identified
Intensity
m/z
204.0863
366.1386
138.0548
274.0915
168.0653
292.103
186.076
126.0548
144.0655
512.204_LeX
528.1888
657.2333
1607.7424 PEP_0_1_0_0_0
0
50000
100000
150000
200000
250000
250
500
750
1000
1250
1500
1750
2000

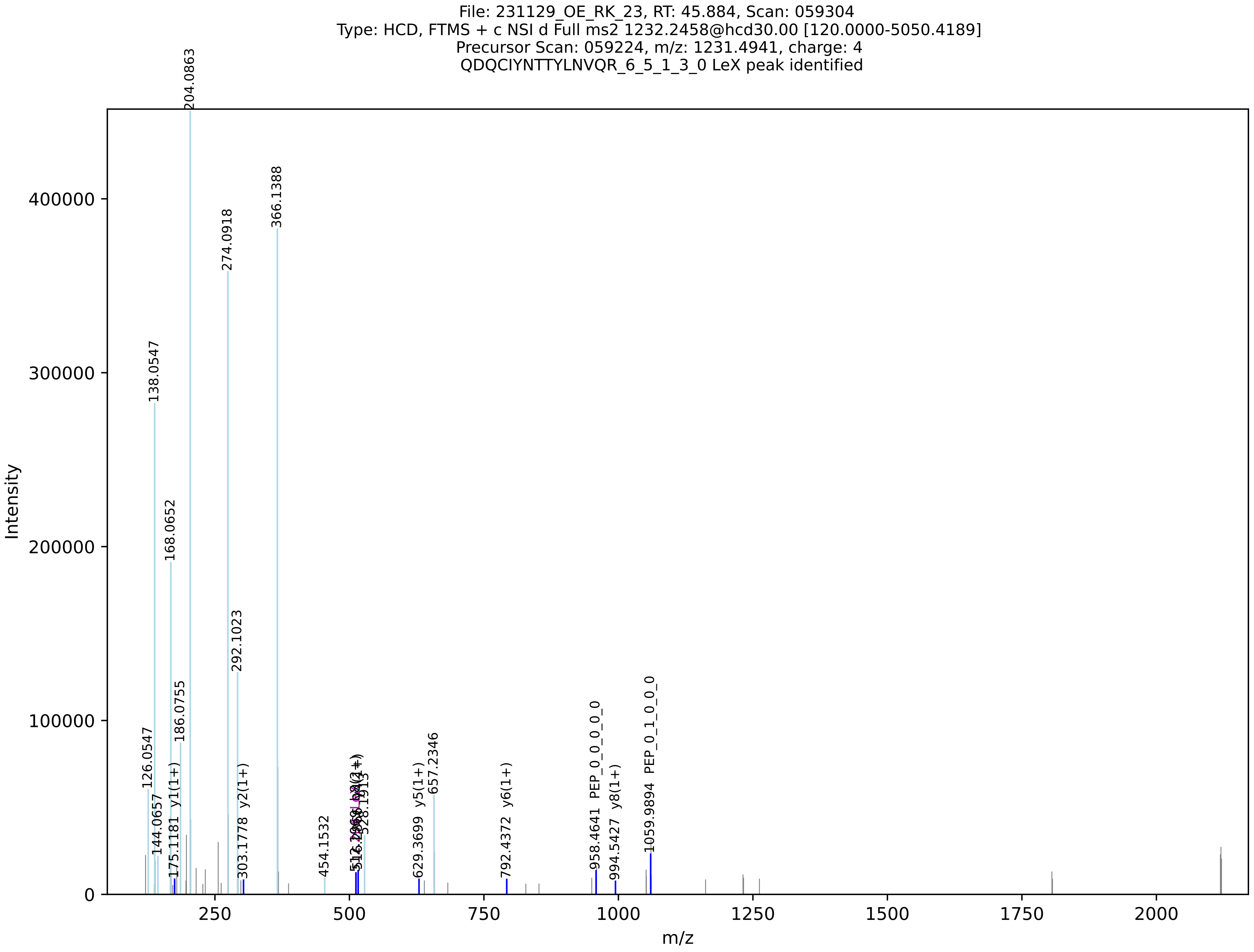
File: 231129_OE_RK_23, RT: 45.884, Scan: 059304
Type: HCD, FTMS + c NSI d Full ms2 1232.2458@hcd30.00 [120.0000-5050.4189]
Precursor Scan: 059224, m/z: 1231.4941, charge: 4
QDQCIYNTTYLNVQR_6_5_1_3_0 LeX peak identified
Intensity
m/z
0
100000
200000
300000
400000
250
500
750
1000
1250
1500
1750
2000
204.0863
366.1388
274.0918
138.0547
168.0652
292.1023
186.0755
126.0547
144.0657
175.1181 y1(1+)
303.1778 y2(1+)
454.1532
528.1915
629.3699 y5(1+)
657.2346
792.4372 y6(1+)
958.4641 PEP_0_0_0_0_0_
994.5427 y8(1+)
1059.9894 PEP_0_1_0_0_0_

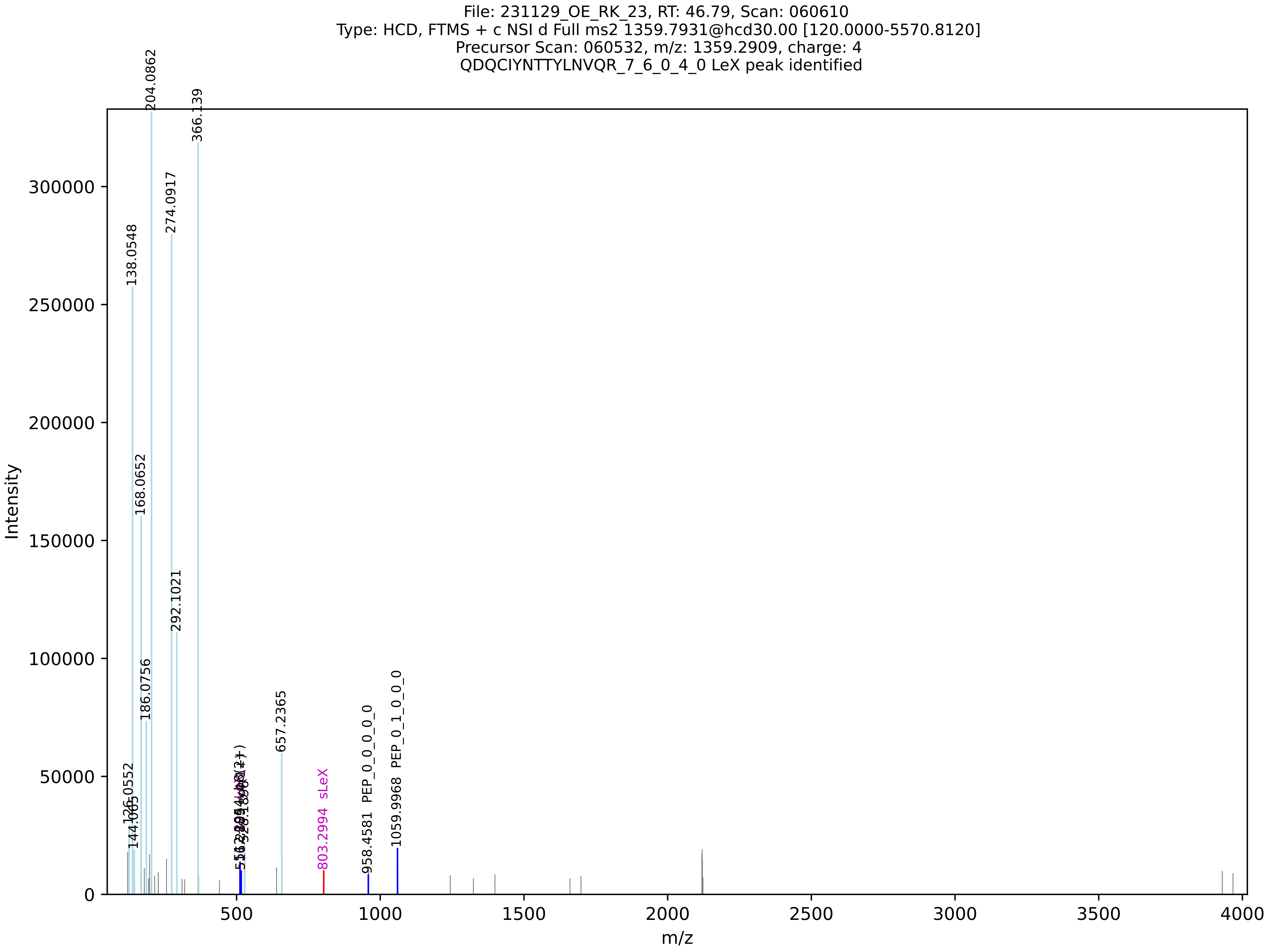
File: 231129_OE_RK_23, RT: 46.79, Scan: 060610
Type: HCD, FTMS + c NSI d Full ms2 1359.7931@hcd30.00 [120.0000-5570.8120]
Precursor Scan: 060532, m/z: 1359.2909, charge: 4
QDQCIYNTTYLNVQR_7_6_0_4_0 LeX peak identified
204.0862
366.139
274.0917
138.0548
168.0652
292.1021
186.0756
126.0552
144.065
657.2365
803.2994 sLeX
958.4581 PEP_0_0_0_0_0
1059.9968 PEP_0_1_0_0_0
Intensity
m/z
0
50000
100000
150000
200000
250000
300000
500
1000
1500
2000
2500
3000
3500
4000

File: 231129_OE_RK_23, RT: 46.993, Scan: 060906
Type: HCD, FTMS + c NSI d Full ms2 1360.0414@hcd30.00 [120.0000-5571.8247]
Precursor Scan: 060825, m/z: 1359.2896, charge: 4
QDQCIYNTTYLNVQR_7_6_0_4_0 LeX peak identified

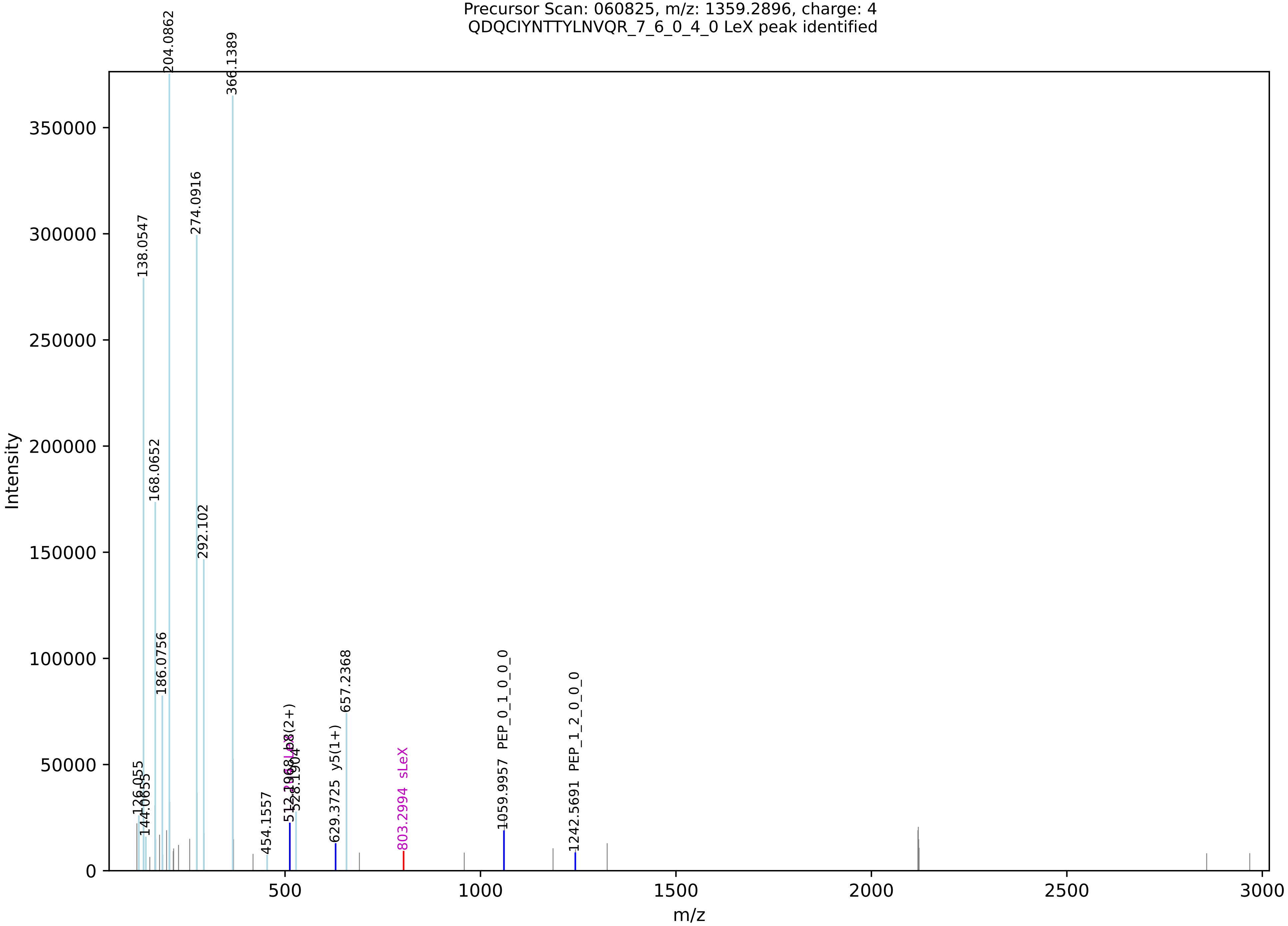

File: 231129_OE_RK_23, RT: 48.414, Scan: 062977
Type: HCD, FTMS + c NSI d Full ms2 1360.0406@hcd30.00 [120.0000-5571.8218]
Precursor Scan: 062893, m/z: 1359.0382, charge: 4
QDQCIYNTTYLNVQR_7_6_0_4_0 LeX peak identified

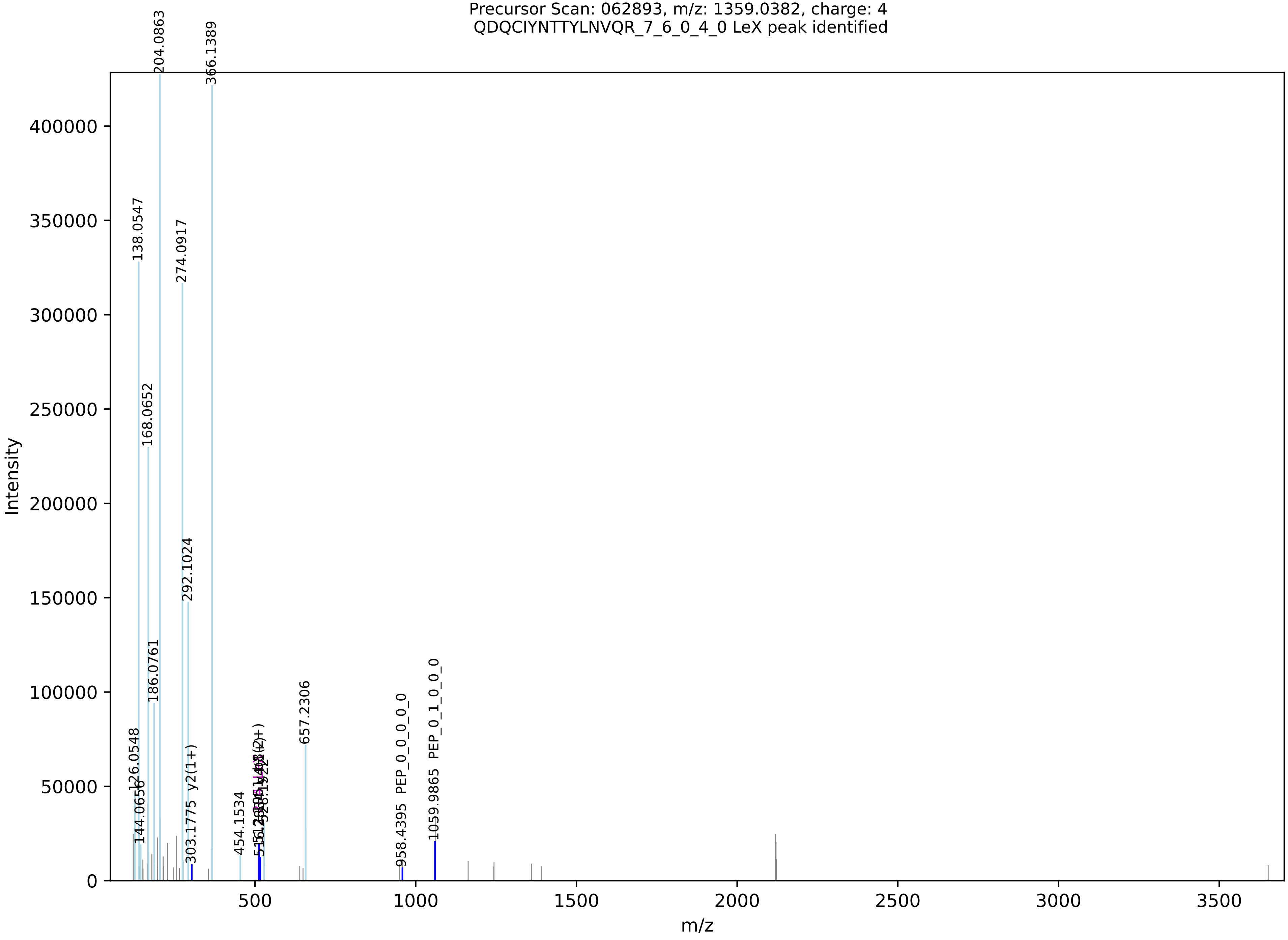

File: 231129_OE_RK_23, RT: 48.46, Scan: 063040
Type: HCD, FTMS + c NSI d Full ms2 1278.5328@hcd30.00 [120.0000-3934.4524]
Precursor Scan: 062958, m/z: 1277.8646, charge: 3
VCQDCPLLAPLNDTR_5_4_1_1_0 LeX peak identified

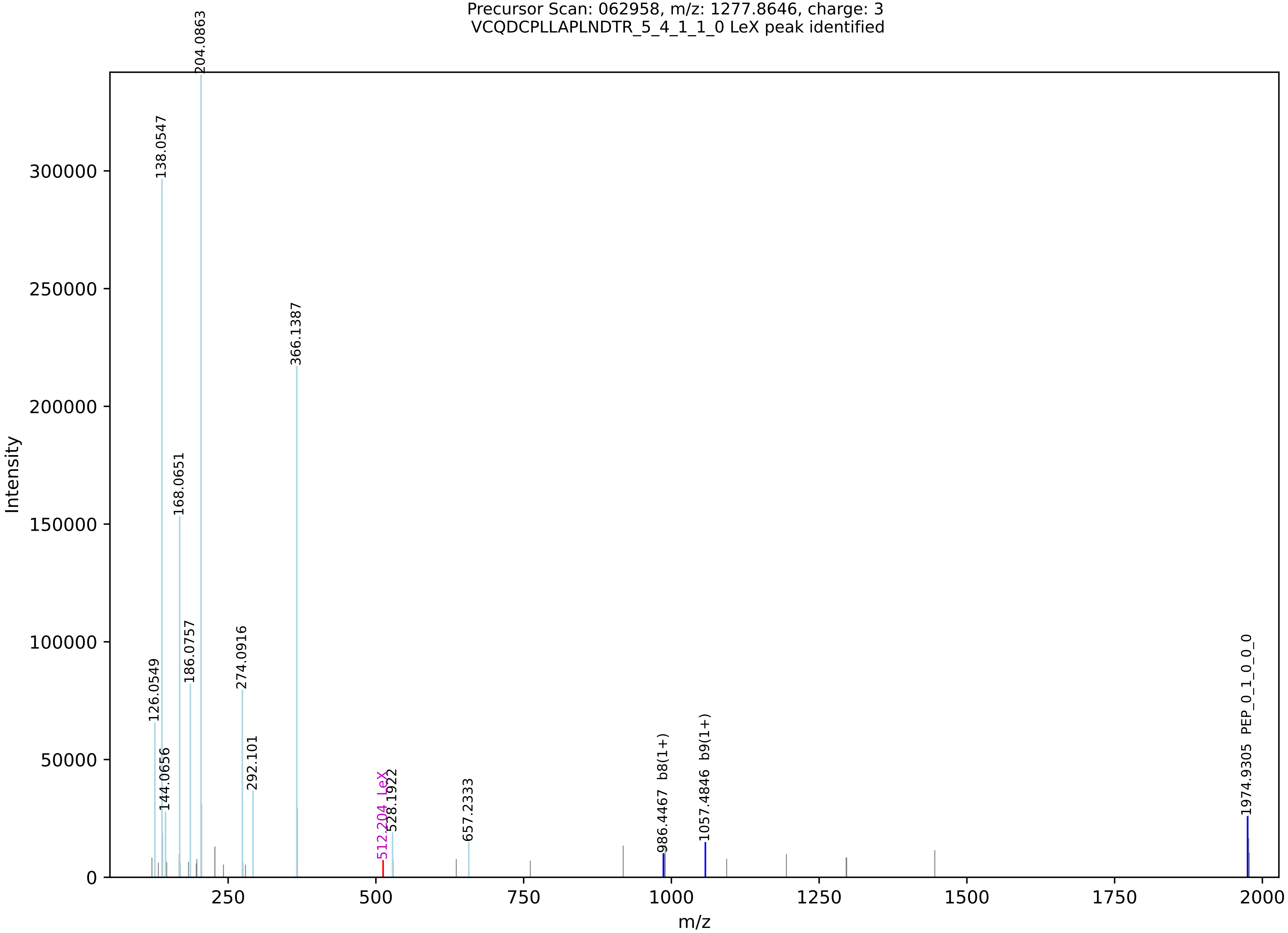

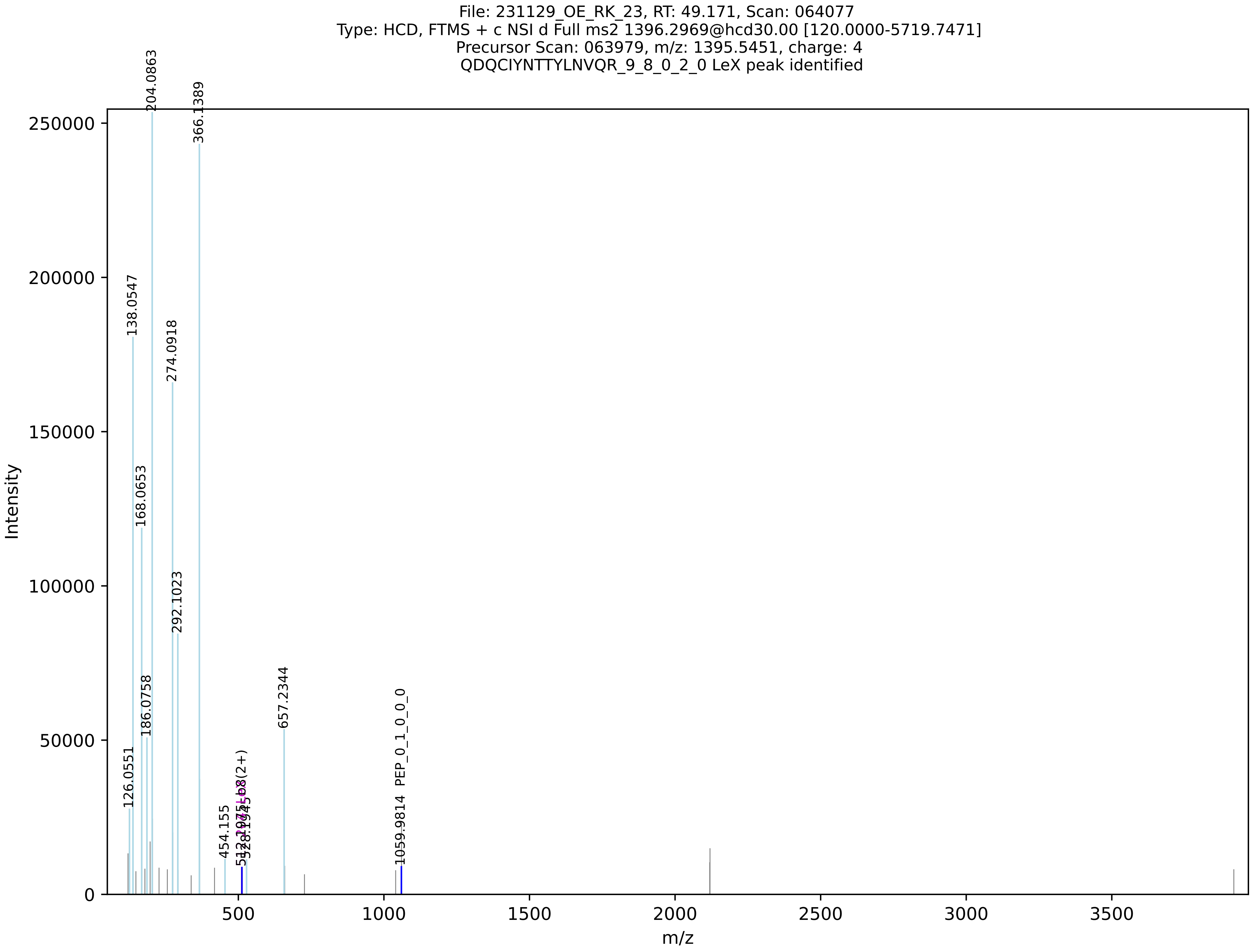

File: 231129_OE_RK_23, RT: 49.171, Scan: 064077
Type: HCD, FTMS + c NSI d Full ms2 1396.2969@hcd30.00 [120.0000-5719.7471]
Precursor Scan: 063979, m/z: 1395.5451, charge: 4
QDQCIYNTTYLNVQR_9_8_0_2_0 LeX peak identified
204.0863
366.1389
138.0547
274.0918
168.0653
292.1023
186.0758
657.2344
PEP_0_1_0_0_0
126.0551
454.155
1059.9814
Intensity
m/z

File: 231129_OE_RK_23, RT: 49.511, Scan: 064560
Type: HCD, FTMS + c NSI d Full ms2 1323.5288@hcd30.00 [120.0000-5422.8535]
Precursor Scan: 064486, m/z: 1322.777, charge: 4
QDQCIYNTTYLNVQR_7_6_1_3_0 LeX peak identified

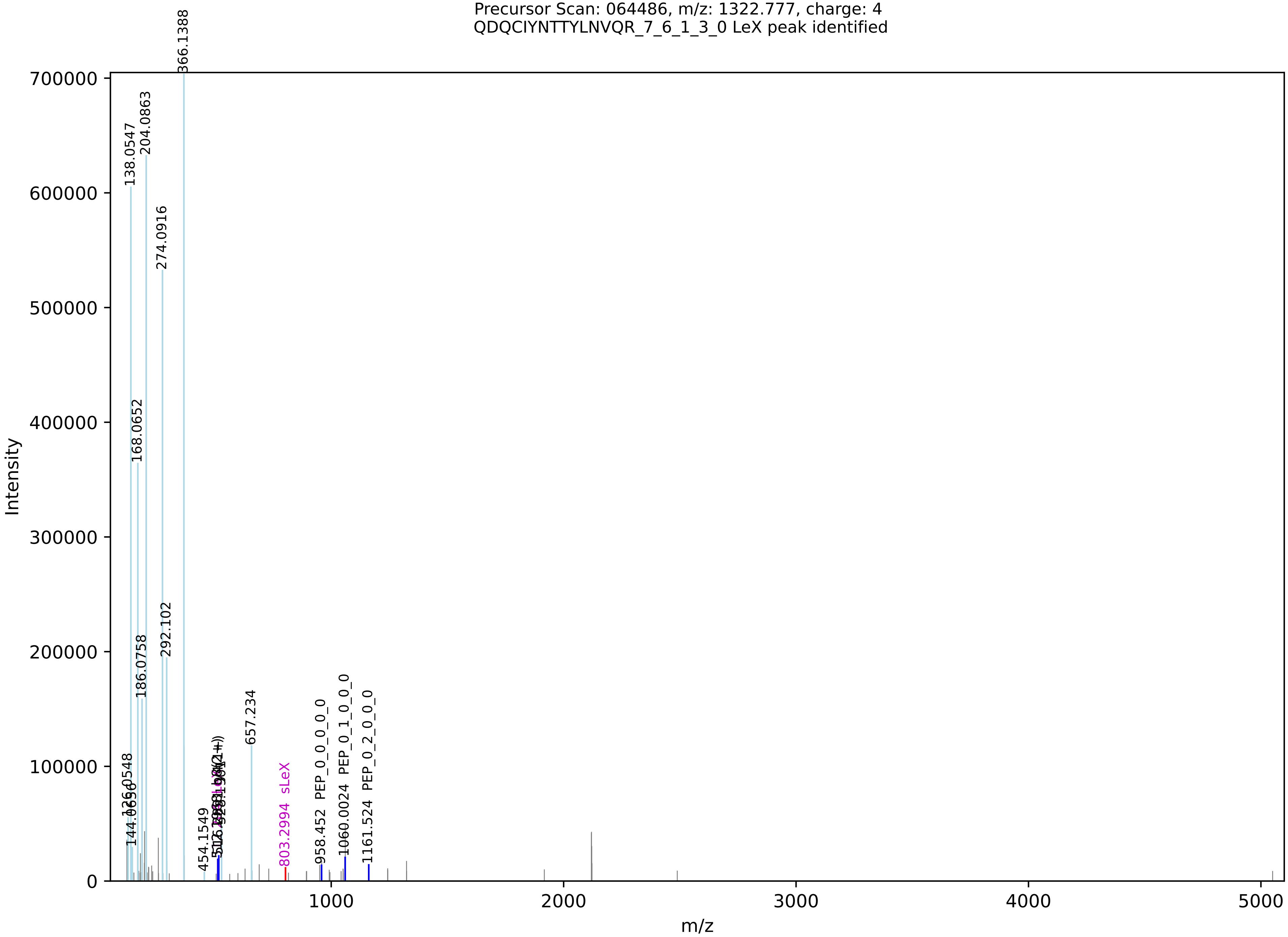

File: 231129_OE_RK_23, RT: 53.093, Scan: 069758
Type: HCD, FTMS + c NSI d Full ms2 1266.5446@hcd30.00 [120.0000-3897.7681]
Precursor Scan: 069681, m/z: 1265.8763, charge: 3
ALPQPQNVTSLLGCTH_5_4_1_1_0 LeX peak identified

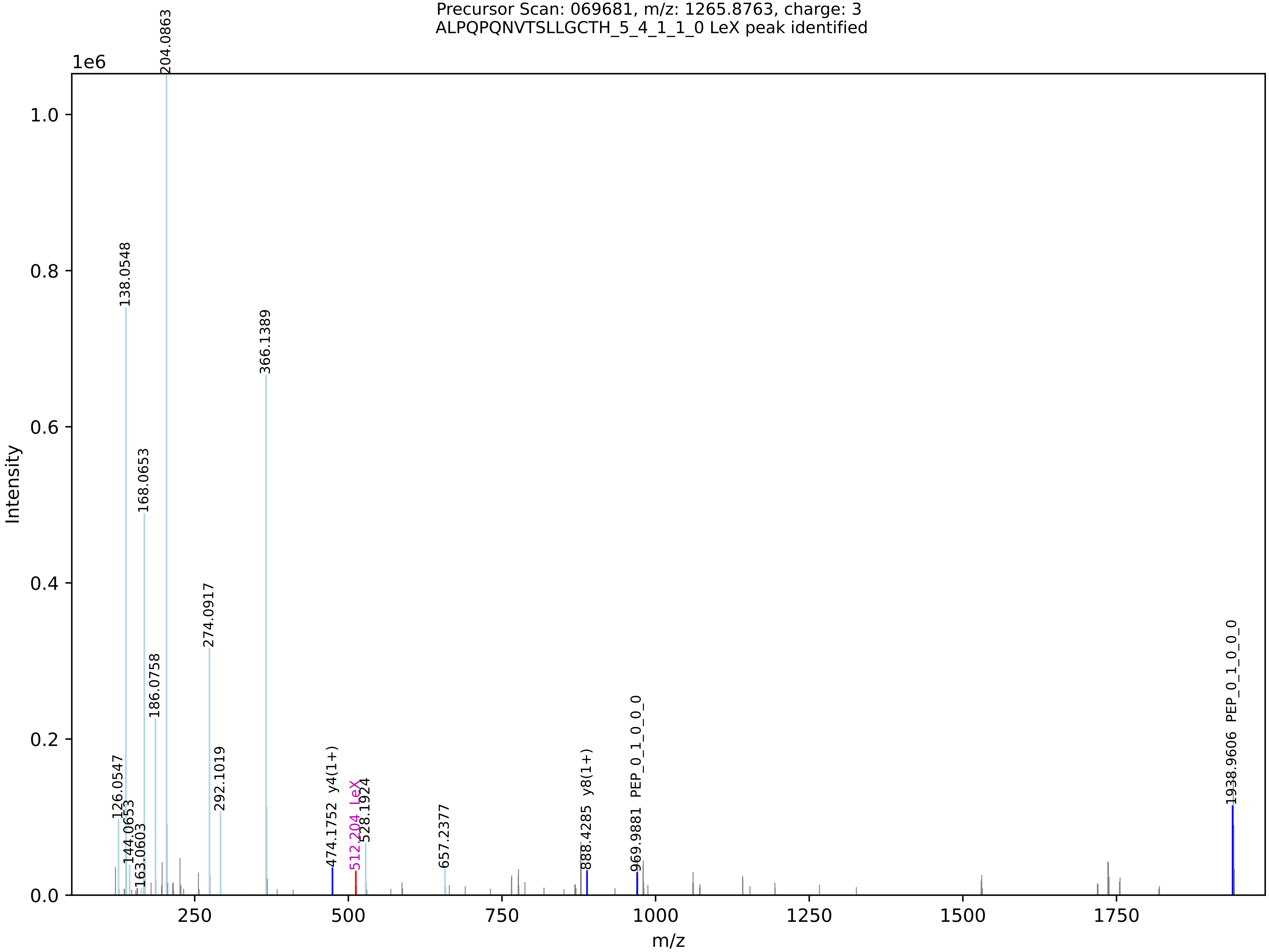

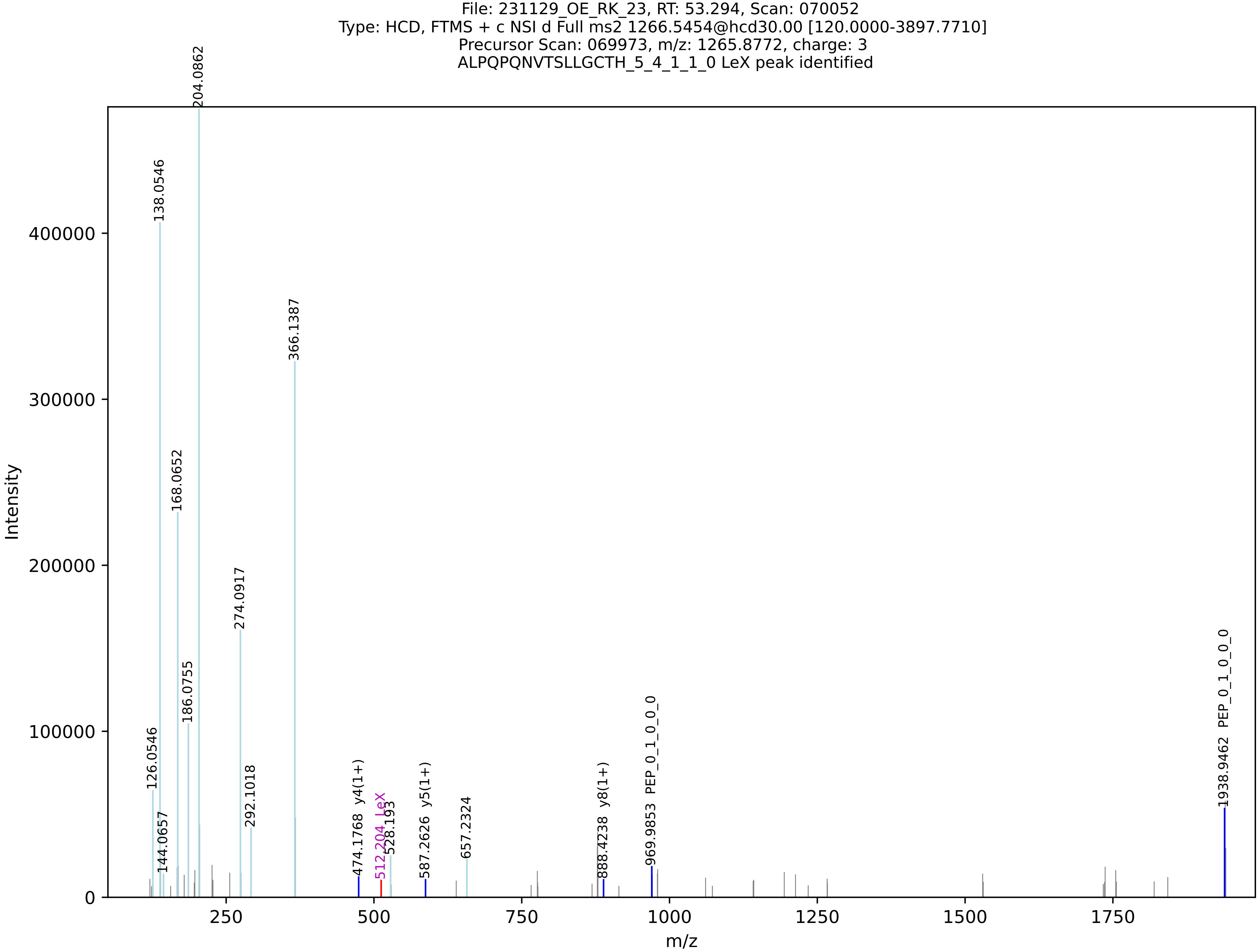

File: 231129_OE_RK_23, RT: 53.294, Scan: 070052
Type: HCD, FTMS + c NSI d Full ms2 1266.5454@hcd30.00 [120.0000-3897.7710]
Precursor Scan: 069973, m/z: 1265.8772, charge: 3
ALPQPQNVTSLLGCTH_5_4_1_1_0 LeX peak identified
Intensity
m/z
204.0862
138.0546
366.1387
168.0652
274.0917
186.0755
126.0546
292.1018
144.0657
474.1768 y4(1+)
512.204_LeX
528.193
587.2626 y5(1+)
657.2324
888.4238 y8(1+)
969.9853 PEP_0_1_0_0_0
1938.9462 PEP_0_1_0_0_0

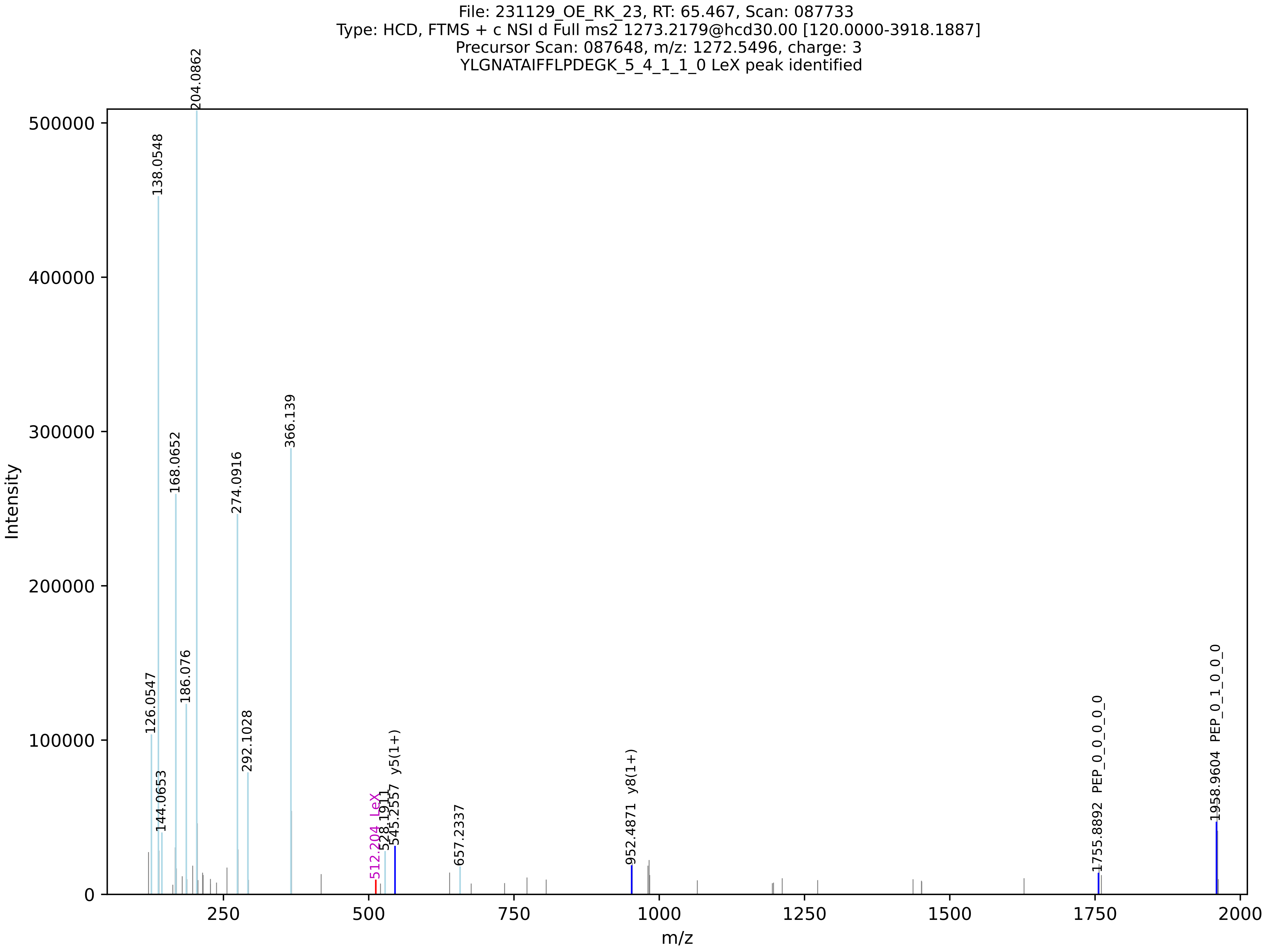
File: 231129_OE_RK_23, RT: 65.467, Scan: 087733
Type: HCD, FTMS + c NSI d Full ms2 1273.2179@hcd30.00 [120.0000-3918.1887]
Precursor Scan: 087648, m/z: 1272.5496, charge: 3
YLGNATAIFFLPDEGK_5_4_1_1_0 LeX peak identified
Intensity
m/z
204.0862
138.0548
168.0652
274.0916
366.139
186.076
126.0547
292.1028
144.0653
512.204 LeX
528.1911
545.2557 y5(1+)
657.2337
952.4871 y8(1+)
1755.8892 PEP_0_0_0_0_0
1958.9604 PEP_0_1_0_0_0
0
100000
200000
300000
400000
500000
250
500
750
1000
1250
1500
1750
2000

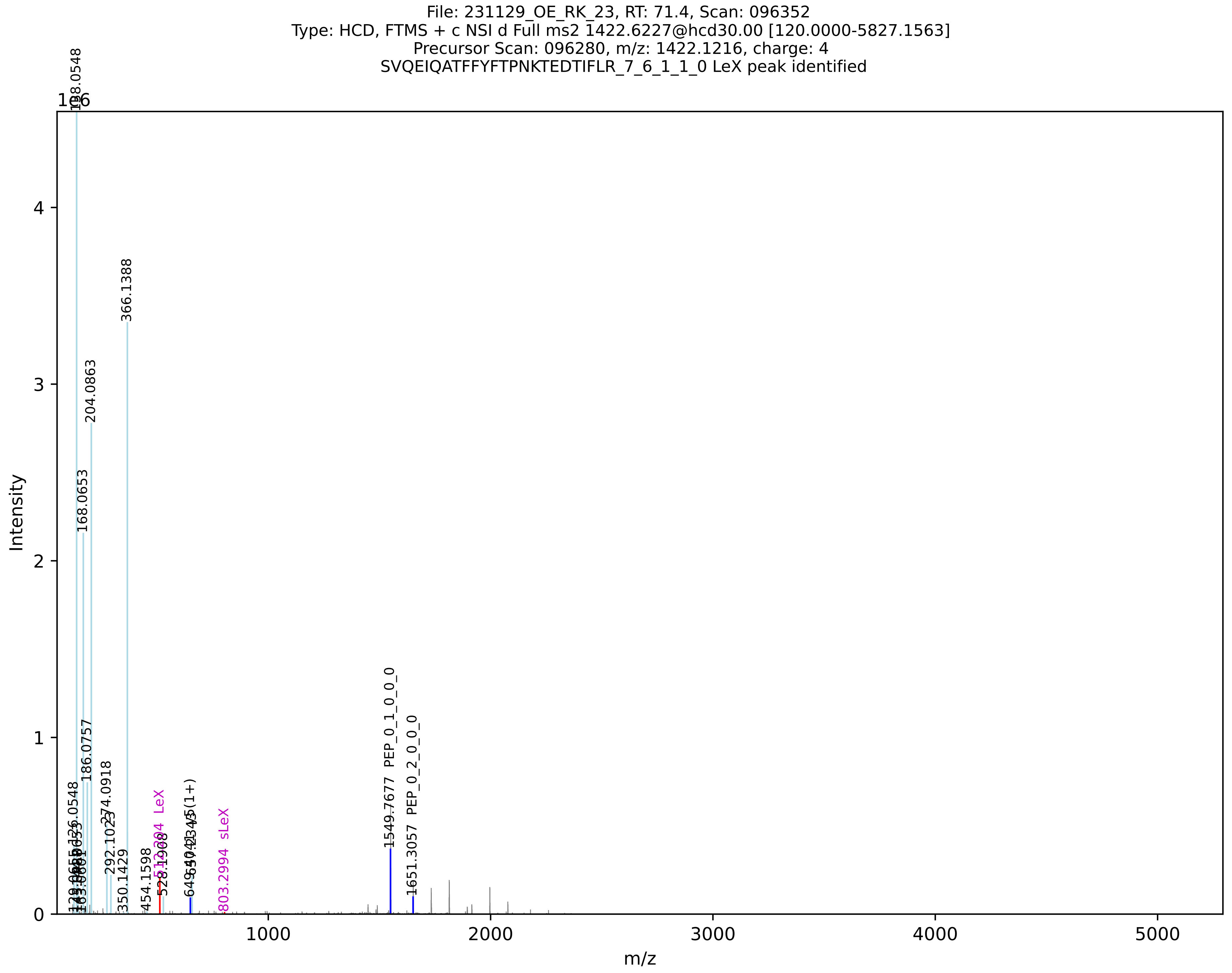
File: 231129_OE_RK_23, RT: 71.4, Scan: 096352
Type: HCD, FTMS + c NSI d Full ms2 1422.6227@hcd30.00 [120.0000-5827.1563]
Precursor Scan: 096280, m/z: 1422.1216, charge: 4
SVQEIQATFFYFTPNKTEDTIFLR_7_6_1_1_0 LeX peak identified
1e6
138.0548
366.1388
204.0863
168.0653
186.0757
126.0548
274.0918
292.1023
350.1429
454.1598
528.1908
LeX
sLeX
803.2994
1549.7677 PEP_0_1_0_0_0
1651.3057 PEP_0_2_0_0_0
Intensity
m/z
0
1
2
3
4
1000
2000
3000
4000
5000

File: 231129_OE_RK_23, RT: 75.637, Scan: 102521
Type: HCD, FTMS + c NSI d Full ms2 1016.8760@hcd30.00 [120.0000-5209.6377]
Precursor Scan: 102438, m/z: 1016.876, charge: 5
LSLHRPALEDLLLGSEANLTCTLTGLR_5_5_0_1_0 LeX peak identified

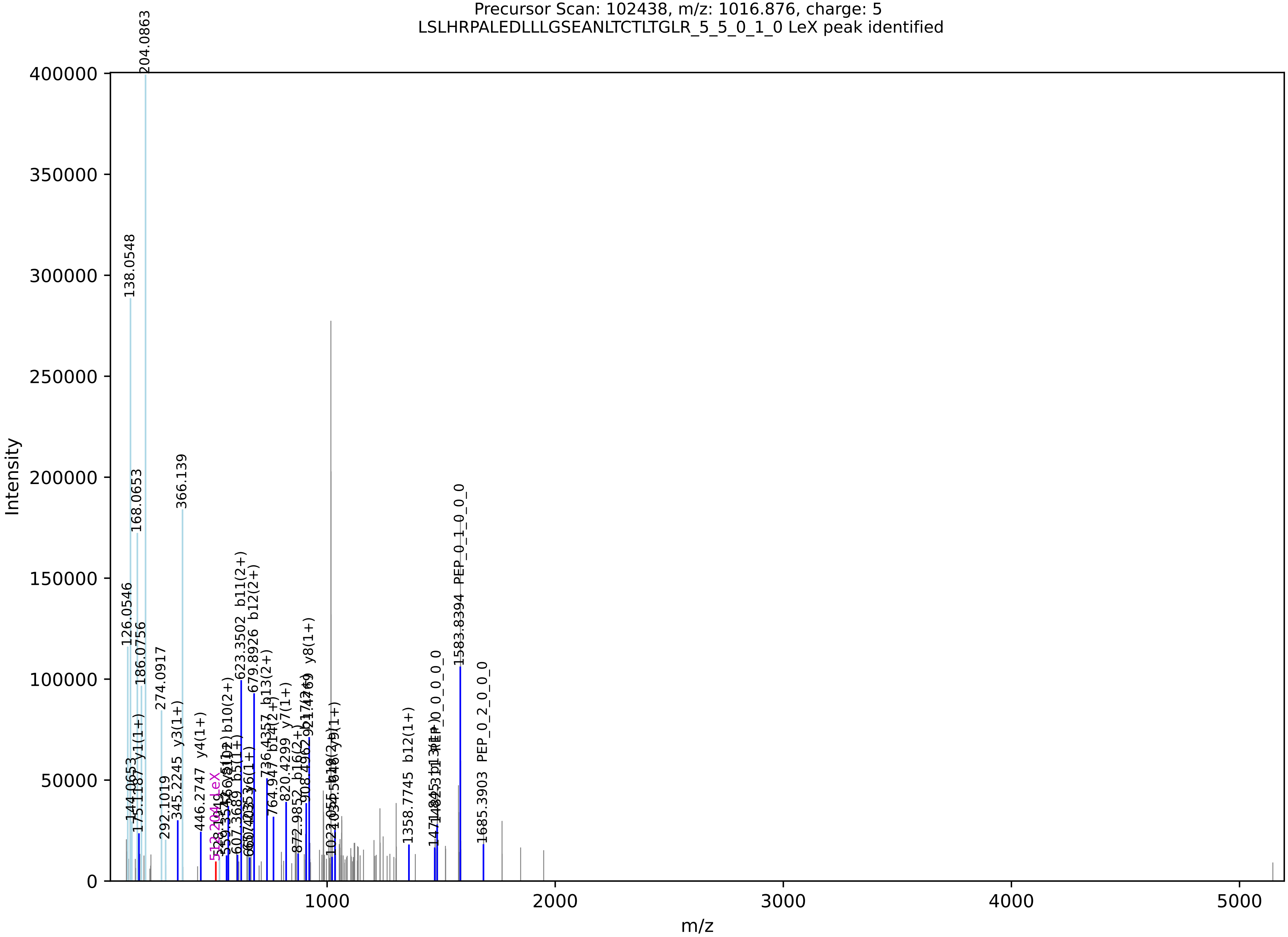

File: 231129_OE_RK_23, RT: 75.889, Scan: 102889
Type: HCD, FTMS + c NSI d Full ms2 1017.4786@hcd30.00 [120.0000-5212.7109]
Precursor Scan: 102807, m/z: 1016.877, charge: 5
LSLHRPALEDLLLGSEANLTCTLTGLR_5_5_0_1_0 LeX peak identified

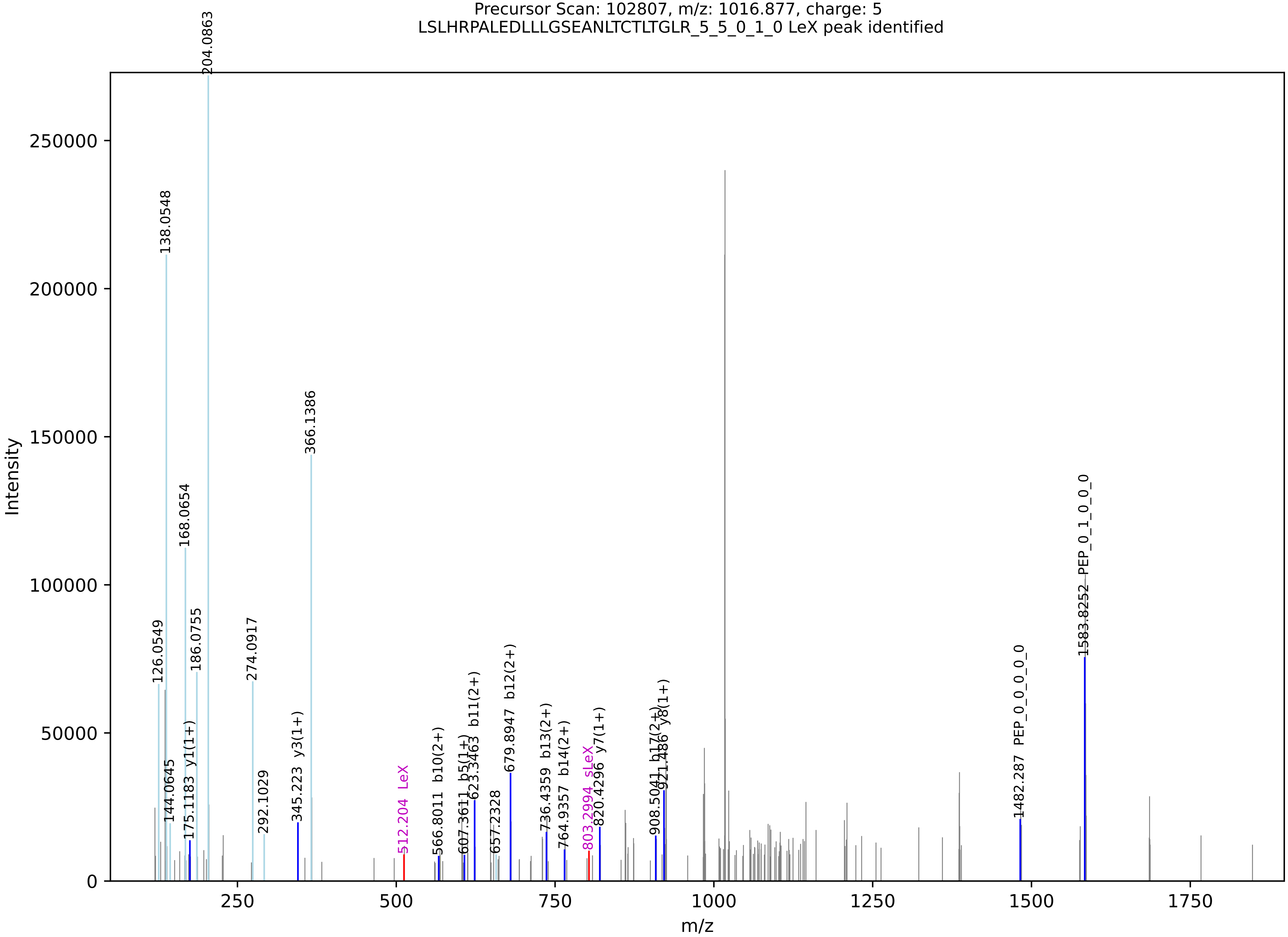

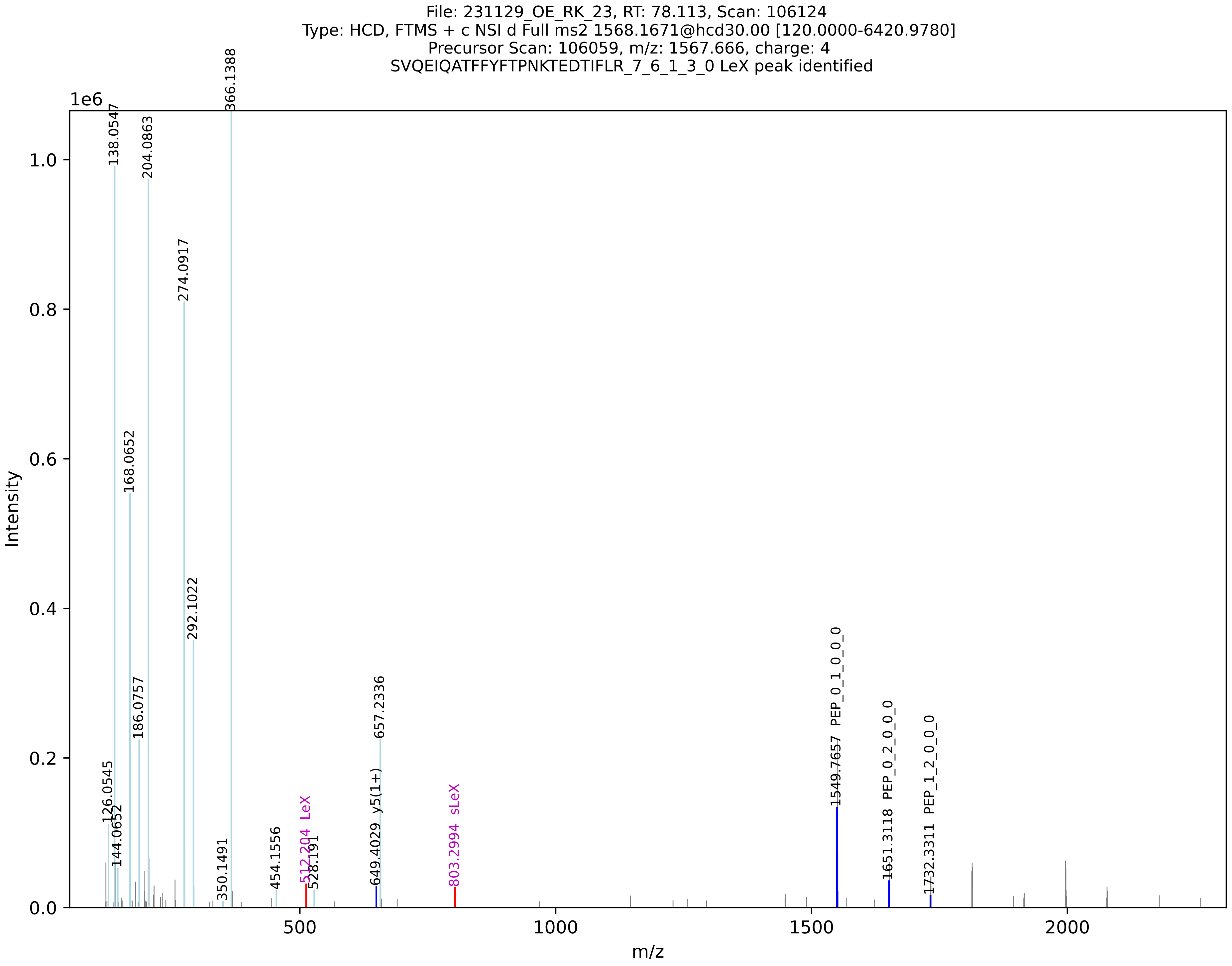
File: 231129_OE_RK_23, RT: 78.113, Scan: 106124
Type: HCD, FTMS + c NSI d Full ms2 1568.1671@hcd30.00 [120.0000-6420.9780]
Precursor Scan: 106059, m/z: 1567.666, charge: 4
SVQEIQATFFYFTPNKTEDTIFLR_7_6_1_3_0 LeX peak identified
1e6
Intensity
m/z
138.0547
204.0863
366.1388
274.0917
168.0652
292.1022
186.0757
126.0545
144.0652
350.1491
454.1556
512.204 LeX
528.191
649.4029 y5(1+)
657.2336
803.2994 sLeX
1549.7657 PEP_0_1_0_0_0
1651.3118 PEP_0_2_0_0_0
1732.3311 PEP_1_2_0_0_0

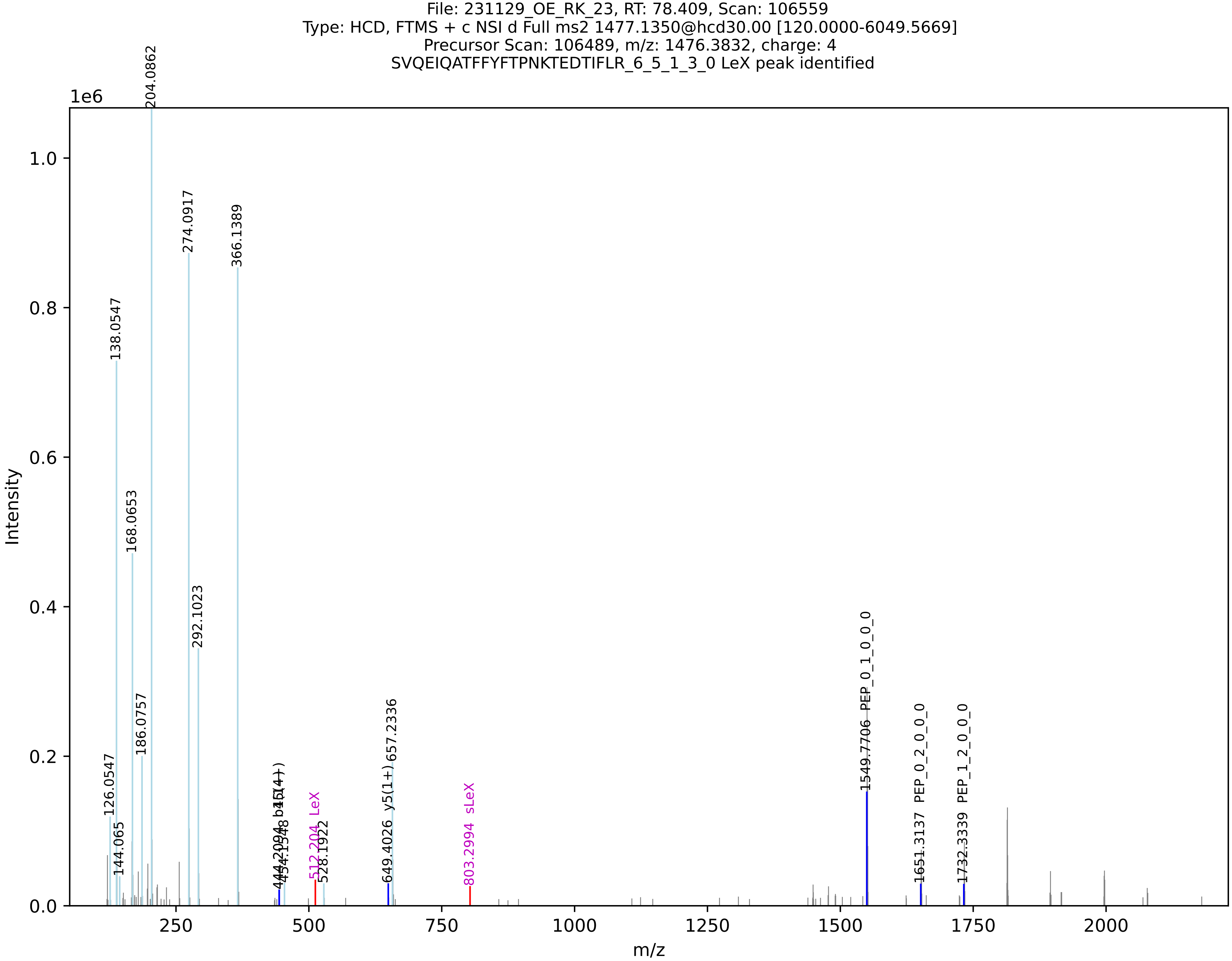
File: 231129_OE_RK_23, RT: 78.409, Scan: 106559
Type: HCD, FTMS + c NSI d Full ms2 1477.1350@hcd30.00 [120.0000-6049.5669]
Precursor Scan: 106489, m/z: 1476.3832, charge: 4
SVQEIQATFFYFTPNKTEDTIFLR_6_5_1_3_0 LeX peak identified
1e6
Intensity
m/z
204.0862
274.0917
366.1389
138.0547
168.0653
292.1023
186.0757
126.0547
144.065
528.1922
512.204 LeX
649.4026 y5(1+)
657.2336
803.2994 sLeX
1549.7706 PEP_0_1_0_0_0
1651.3137 PEP_0_2_0_0_0
1732.3339 PEP_1_2_0_0_0

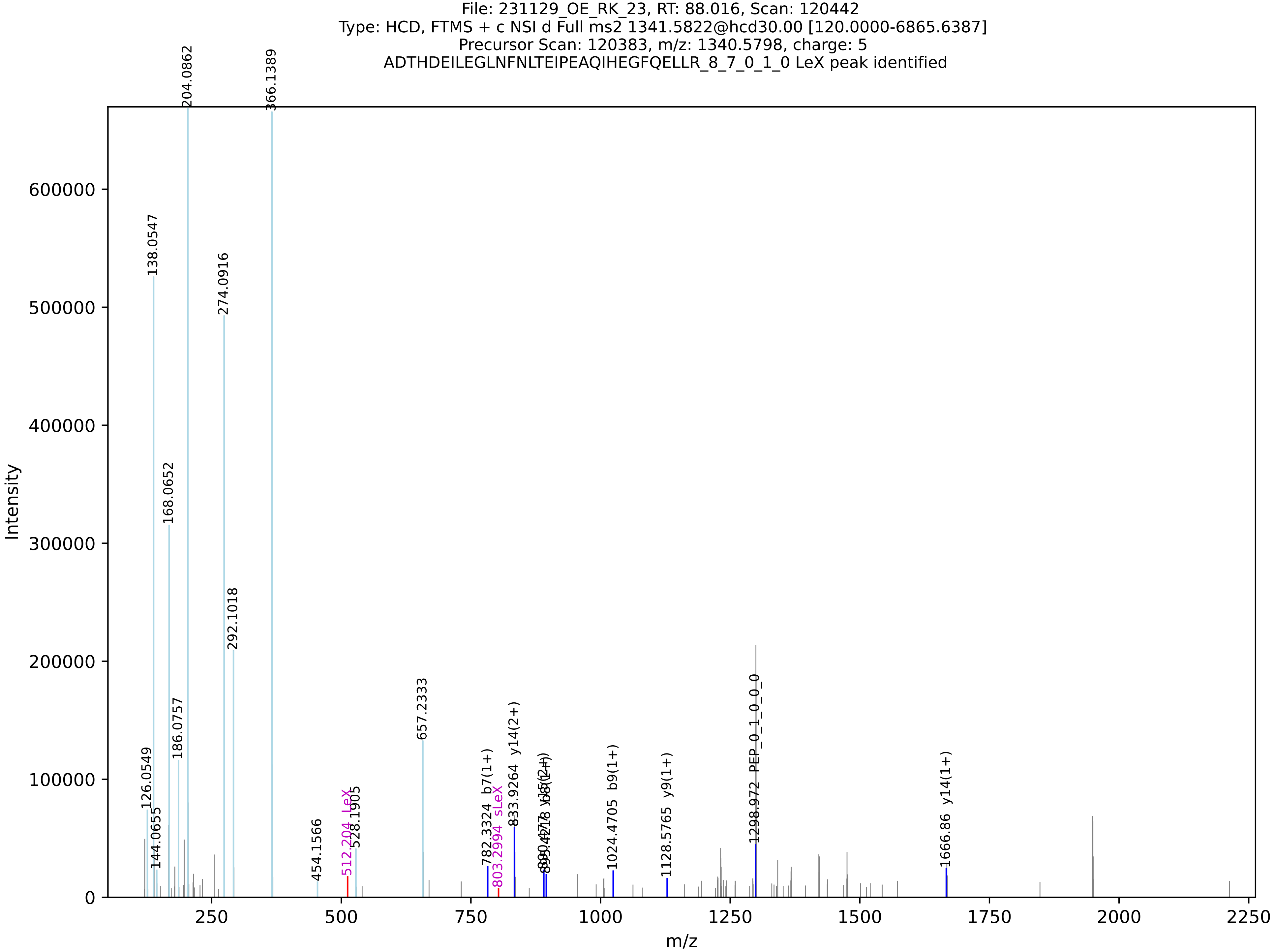

File: 231129_OE_RK_23, RT: 88.016, Scan: 120442
Type: HCD, FTMS + c NSI d Full ms2 1341.5822@hcd30.00 [120.0000-6865.6387]
Precursor Scan: 120383, m/z: 1340.5798, charge: 5
ADTHDEILEGLNFNLTEIPEAQIHEGFQELLR_8_7_0_1_0 LeX peak identified
Intensity
m/z
0
100000
200000
300000
400000
500000
600000
250
500
750
1000
1250
1500
1750
2000
2250
204.0862
366.1389
138.0547
274.0916
168.0652
292.1018
186.0757
126.0549
144.0655
454.1566
512.204 LeX
528.1905
657.2333
782.3324 b7(1+)
803.2994 sLeX
833.9264 y14(2+)
1024.4705 b9(1+)
1128.5765 y9(1+)
1298.972 PEP_0_1_0_0_0
1666.86 y14(1+)

File: 231129_OE_RK_24, RT: 8.446, Scan: 006199
Type: HCD, FTMS + c NSI d Full ms2 1165.9427@hcd30.00 [120.0000-4779.9023]
Precursor Scan: 006103, m/z: 1165.4564, charge: 4
ENGTISR_8_7_0_4_0 LeX peak identified

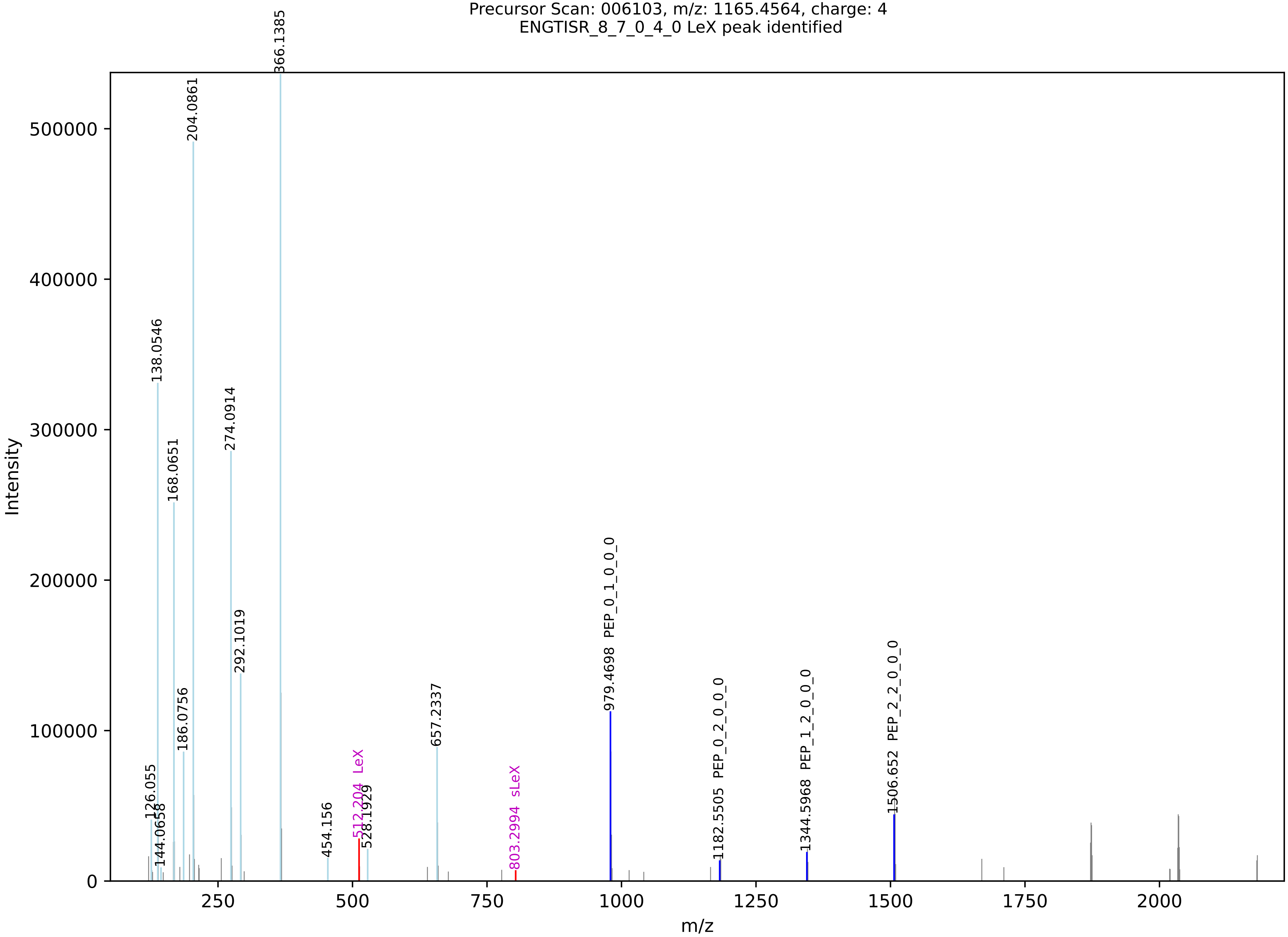

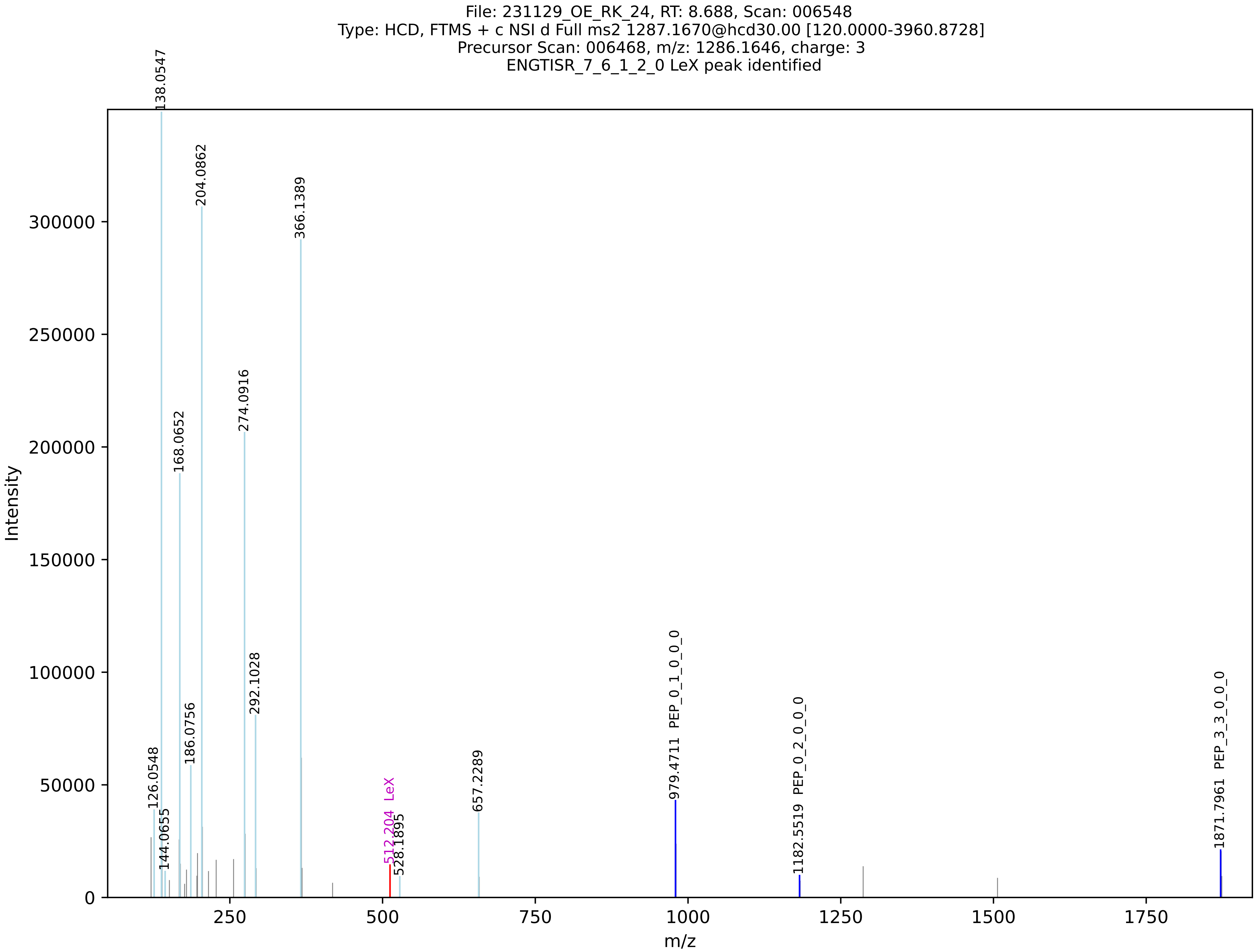
File: 231129_OE_RK_24, RT: 8.688, Scan: 006548
Type: HCD, FTMS + c NSI d Full ms2 1287.1670@hcd30.00 [120.0000-3960.8728]
Precursor Scan: 006468, m/z: 1286.1646, charge: 3
ENGTISR_7_6_1_2_0 LeX peak identified
138.0547
204.0862
366.1389
274.0916
168.0652
292.1028
186.0756
126.0548
144.0655
512.204 LeX
528.1895
657.2289
979.4711 PEP_0_1_0_0_0
1182.5519 PEP_0_2_0_0_0
1871.7961 PEP_3_3_0_0_0
Intensity
m/z

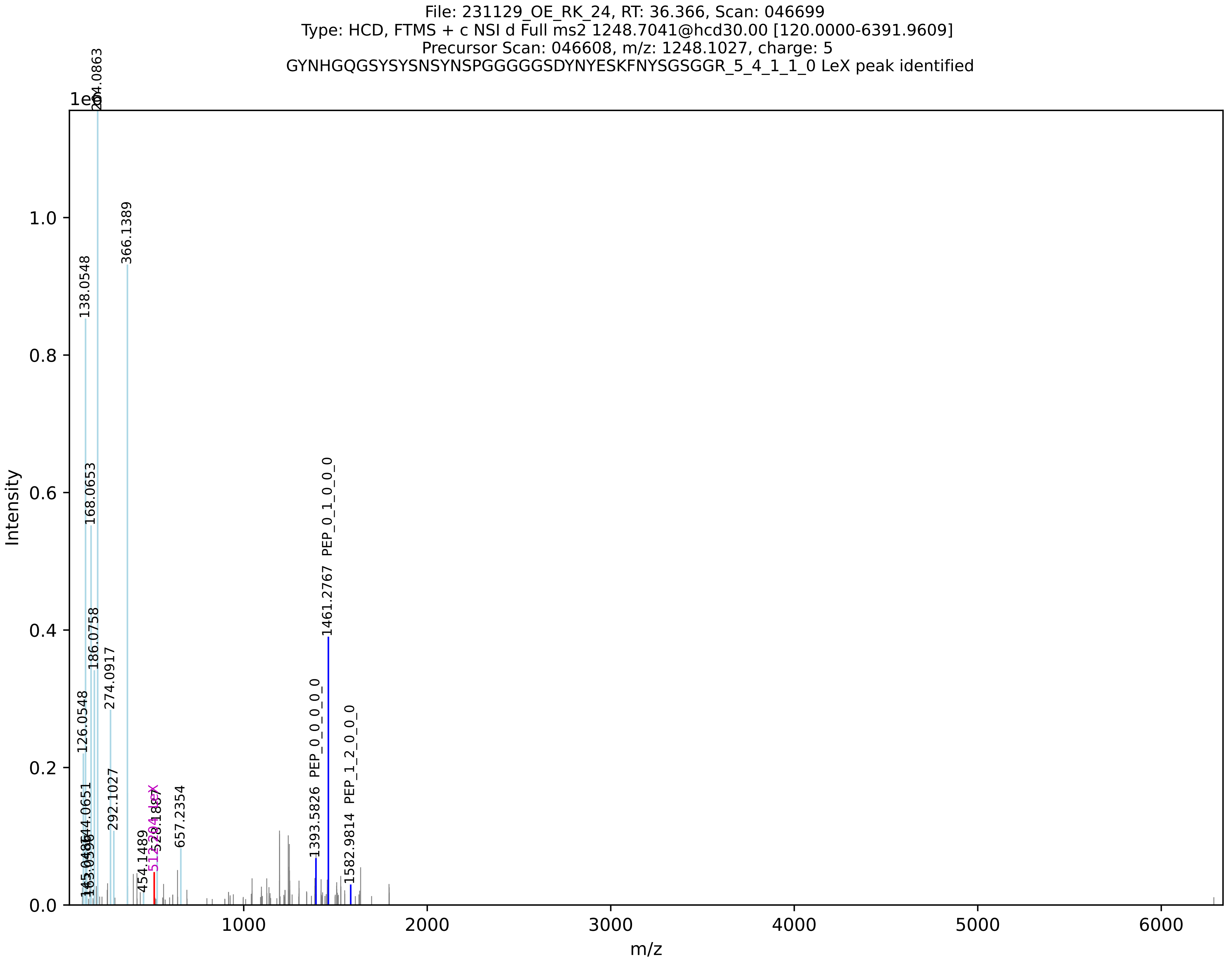

File: 231129_OE_RK_24, RT: 36.366, Scan: 046699
Type: HCD, FTMS + c NSI d Full ms2 1248.7041@hcd30.00 [120.0000-6391.9609]
Precursor Scan: 046608, m/z: 1248.1027, charge: 5
GYNHGQGSYSYSNSYNSPGGGGGSDYNYESKFNYSGSGGR_5_4_1_1_0 LeX peak identified
1e6
Intensity
m/z
138.0548
366.1389
168.0653
186.0758
274.0917
126.0548
292.1027
144.0651
454.1489
528.1887
657.2354
1393.5826 PEP_0_0_0_0_0
1461.2767 PEP_0_1_0_0_0
1582.9814 PEP_1_2_0_0_0

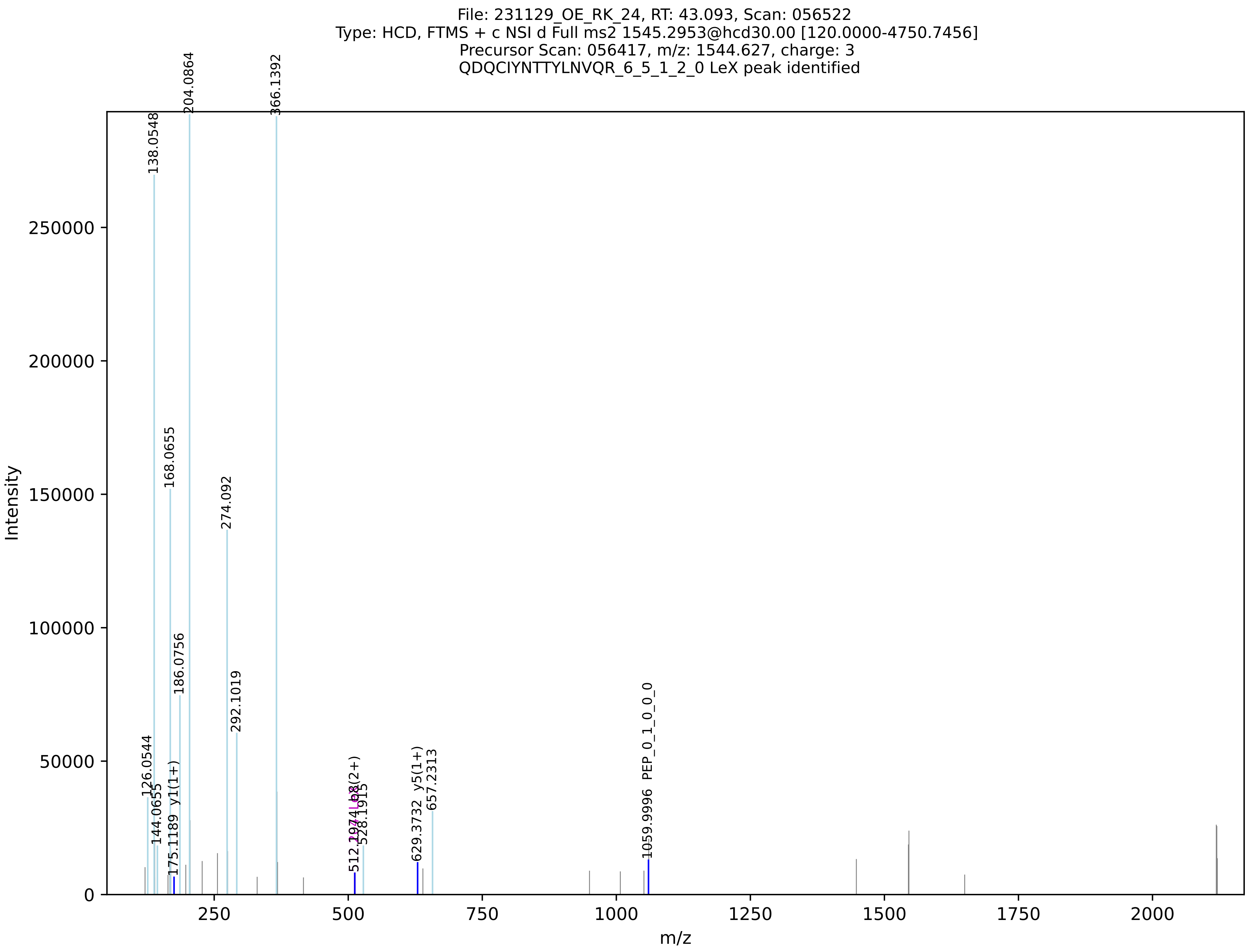

File: 231129_OE_RK_24, RT: 43.093, Scan: 056522
Type: HCD, FTMS + c NSI d Full ms2 1545.2953@hcd30.00 [120.0000-4750.7456]
Precursor Scan: 056417, m/z: 1544.627, charge: 3
QDQCIYNTTYLNVQR_6_5_1_2_0 LeX peak identified
Intensity
m/z
204.0864
366.1392
138.0548
168.0655
274.092
186.0756
292.1019
126.0544
144.0655
175.1189 y1(1+)
512.2974 b8(2+)
528.1915
629.3732 y5(1+)
657.2313
1059.9996 PEP_0_1_0_0_0
0
50000
100000
150000
200000
250000
250
500
750
1000
1250
1500
1750
2000

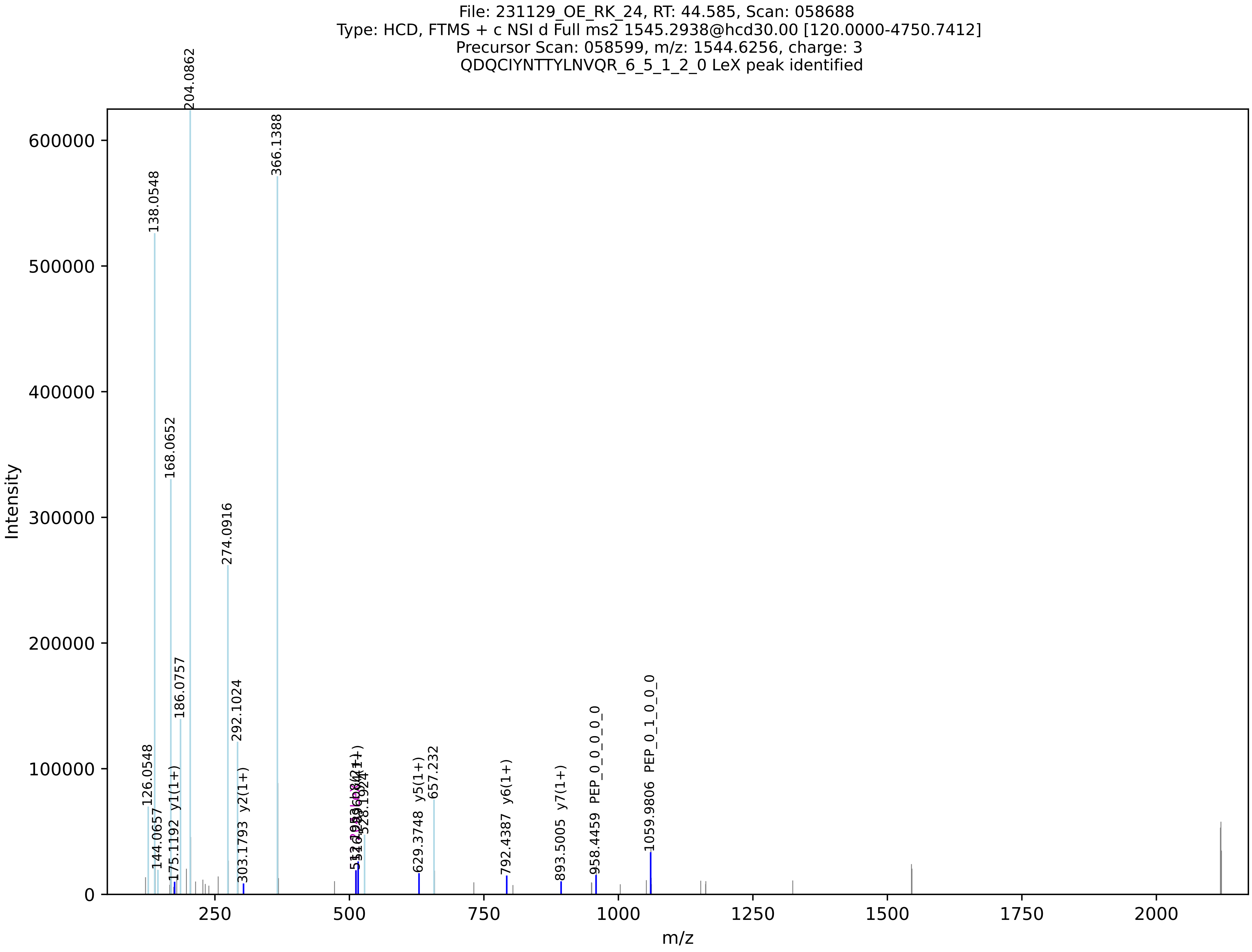
File: 231129_OE_RK_24, RT: 44.585, Scan: 058688
Type: HCD, FTMS + c NSI d Full ms2 1545.2938@hcd30.00 [120.0000-4750.7412]
Precursor Scan: 058599, m/z: 1544.6256, charge: 3
QDQCIYNTTYLNVQR_6_5_1_2_0 LeX peak identified
Intensity
m/z
204.0862
366.1388
138.0548
168.0652
274.0916
186.0757
292.1024
126.0548
144.0657
175.1192 y1(1+)
303.1793 y2(1+)
528.1924
629.3748 y5(1+)
657.232
792.4387 y6(1+)
893.5005 y7(1+)
958.4459 PEP_0_0_0_0_0
1059.9806 PEP_0_1_0_0_0

File: 231129_OE_RK_24, RT: 45.335, Scan: 059780
Type: HCD, FTMS + c NSI d Full ms2 1159.2208@hcd30.00 [120.0000-4752.4771]
Precursor Scan: 059696, m/z: 1158.7221, charge: 4
QDQCIYNTTYLNVQR_6_5_1_2_0 LeX peak identified

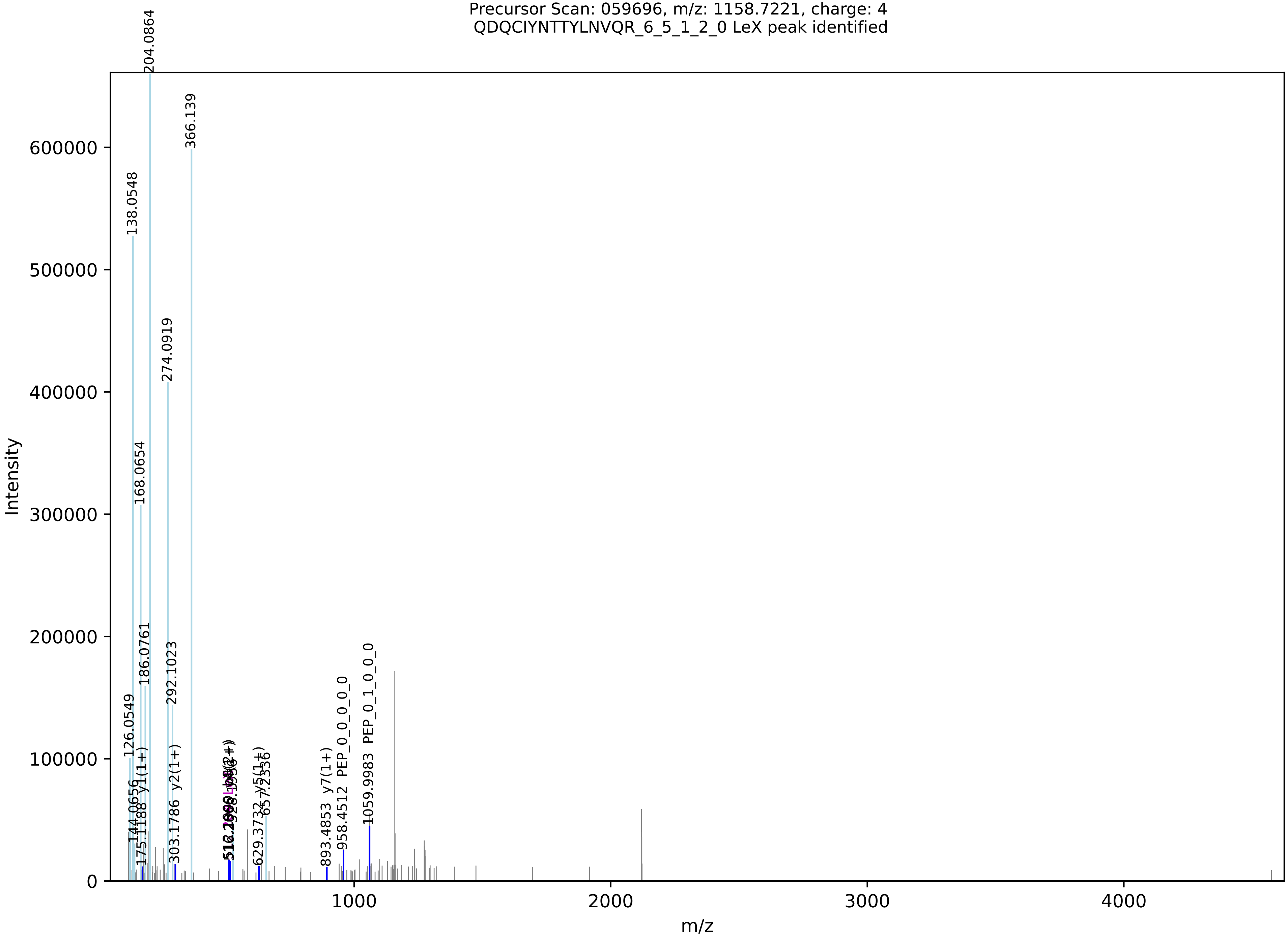

File: 231129_OE_RK_24, RT: 45.641, Scan: 060227
Type: HCD, FTMS + c NSI d Full ms2 1667.0031@hcd30.00 [120.0000-5123.1714]
Precursor Scan: 060140, m/z: 1666.3355, charge: 3
QDQCIYNTTYLNVQR_7_6_1_2_0 LeX peak identified

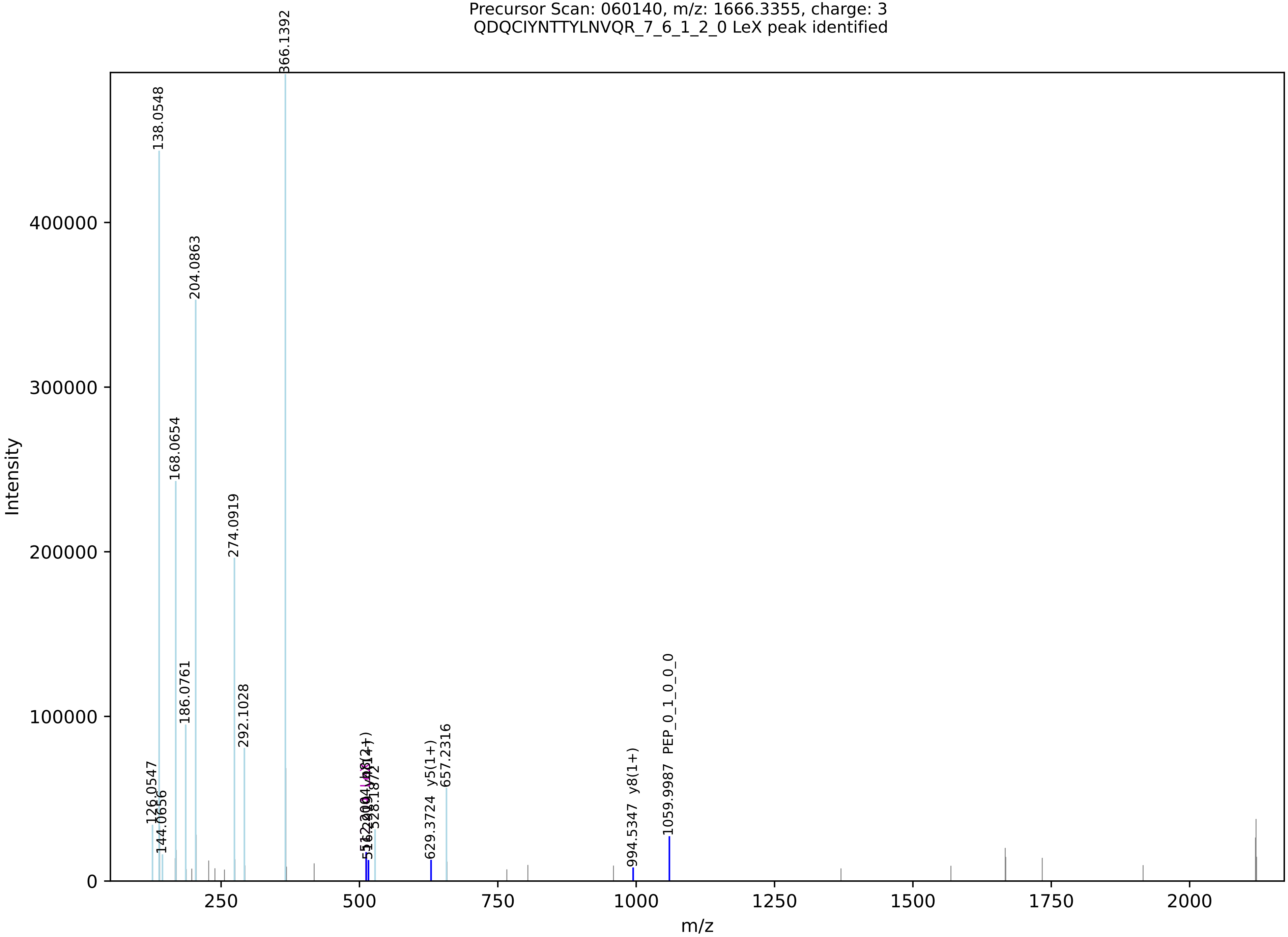

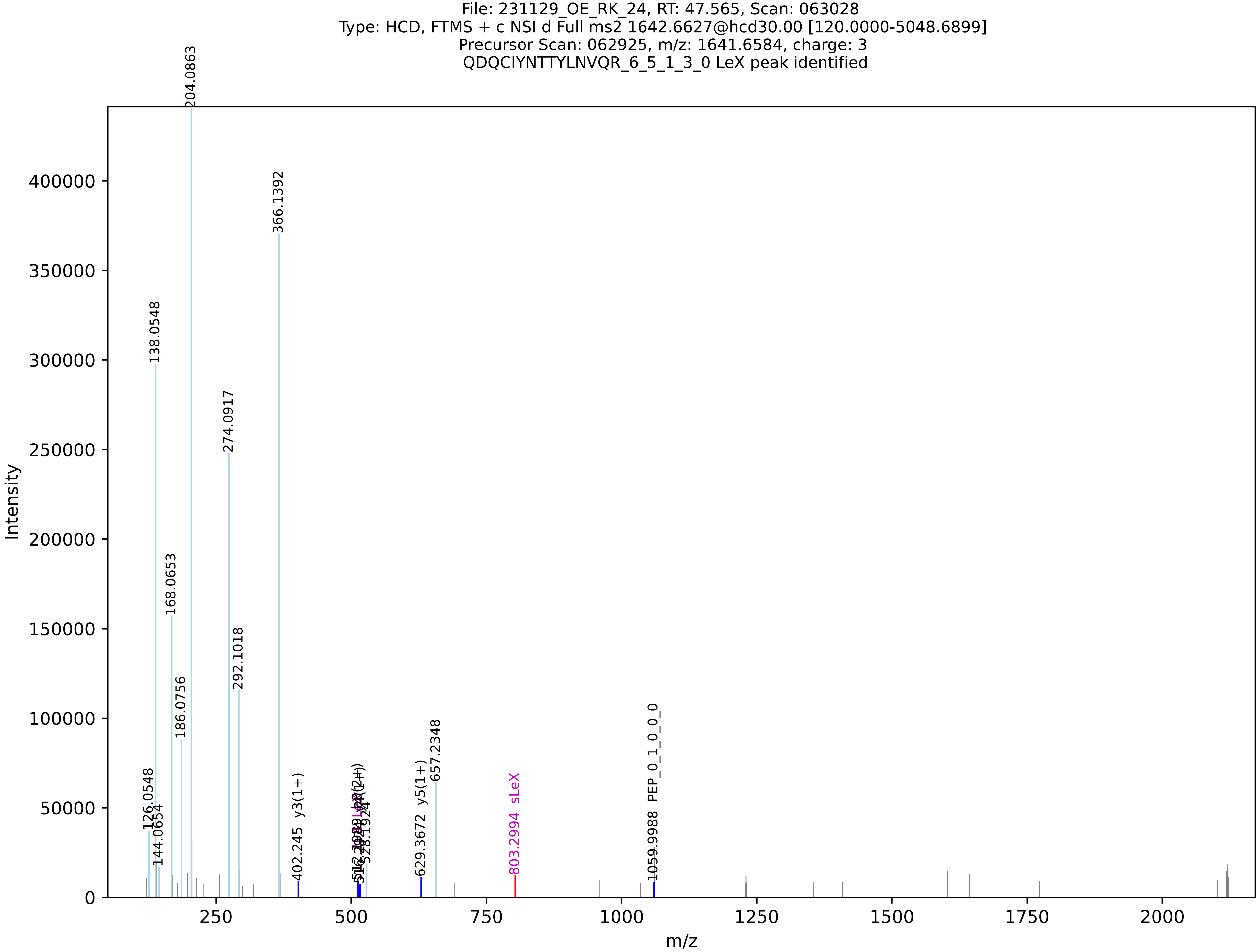
File: 231129_OE_RK_24, RT: 47.565, Scan: 063028
Type: HCD, FTMS + c NSI d Full ms2 1642.6627@hcd30.00 [120.0000-5048.6899]
Precursor Scan: 062925, m/z: 1641.6584, charge: 3
QDQCIYNTTYLNVQR_6_5_1_3_0 LeX peak identified
Intensity
m/z
204.0863
366.1392
138.0548
274.0917
168.0653
292.1018
186.0756
126.0548
144.0654
402.245 y3(1+)
528.1924
629.3672 y5(1+)
657.2348
803.2994 sLeX
1059.9988 PEP_0_1_0_0_0_
400000
350000
300000
250000
200000
150000
100000
50000
0
250
500
750
1000
1250
1500
1750
2000

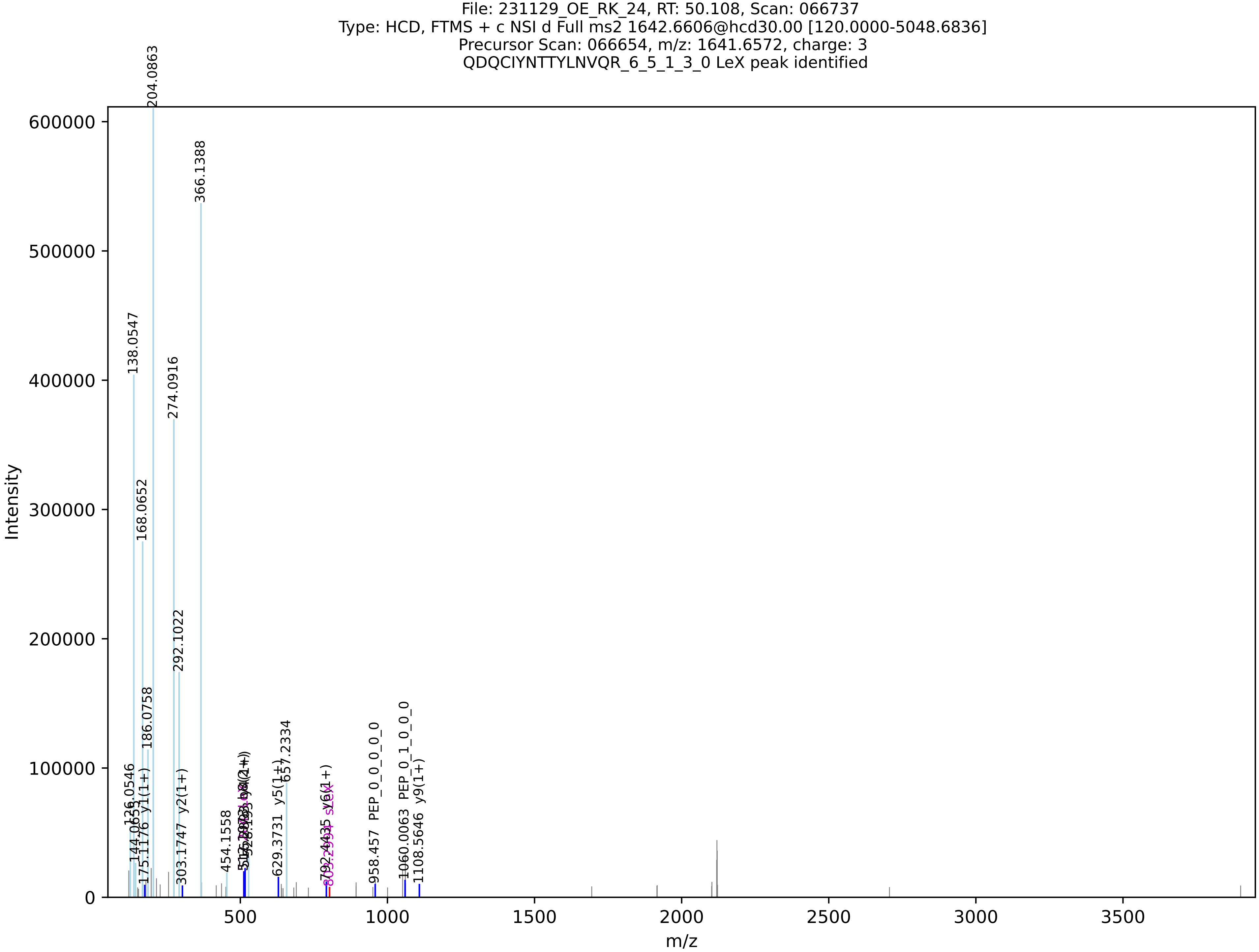

File: 231129_OE_RK_24, RT: 50.108, Scan: 066737
Type: HCD, FTMS + c NSI d Full ms2 1642.6606@hcd30.00 [120.0000-5048.6836]
Precursor Scan: 066654, m/z: 1641.6572, charge: 3
QDQCIYNTTYLNVQR_6_5_1_3_0 LeX peak identified
Intensity
m/z
204.0863
366.1388
138.0547
274.0916
168.0652
292.1022
186.0758
126.0546
144.0655
175.1176 y1(1+)
303.1747 y2(1+)
454.1558
629.3731 y5(1+)
657.2334
792.4435 y6(1+)
958.457 PEP_0_0_0_0_0
1060.0063 PEP_0_1_0_0_0
1108.5646 y9(1+)

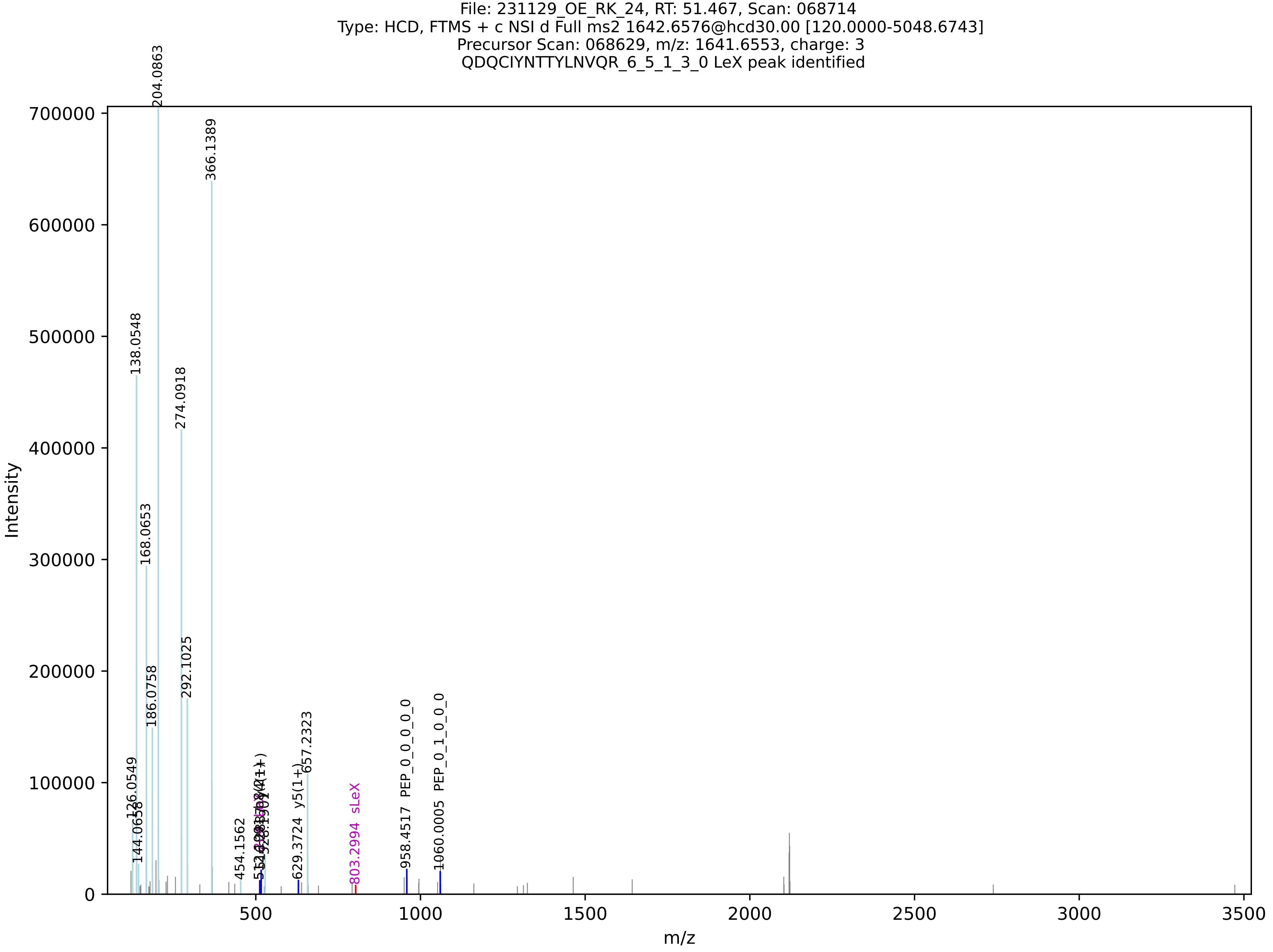
File: 231129_OE_RK_24, RT: 51.467, Scan: 068714
Type: HCD, FTMS + c NSI d Full ms2 1642.6576@hcd30.00 [120.0000-5048.6743]
Precursor Scan: 068629, m/z: 1641.6553, charge: 3
QDQCIYNTTYLNVQR_6_5_1_3_0 LeX peak identified
204.0863
366.1389
138.0548
274.0918
168.0653
292.1025
186.0758
126.0549
144.0658
454.1562
629.3724 y5(1+)
657.2323
803.2994 sLeX
958.4517 PEP_0_0_0_0_0_
1060.0005 PEP_0_1_0_0_0_
Intensity
m/z

File: 231129_OE_RK_24, RT: 51.719, Scan: 069080
Type: HCD, FTMS + c NSI d Full ms2 1323.5314@hcd30.00 [120.0000-5422.8638]
Precursor Scan: 068994, m/z: 1322.7796, charge: 4
QDQCIYNTTYLNVQR_7_6_1_3_0 LeX peak identified

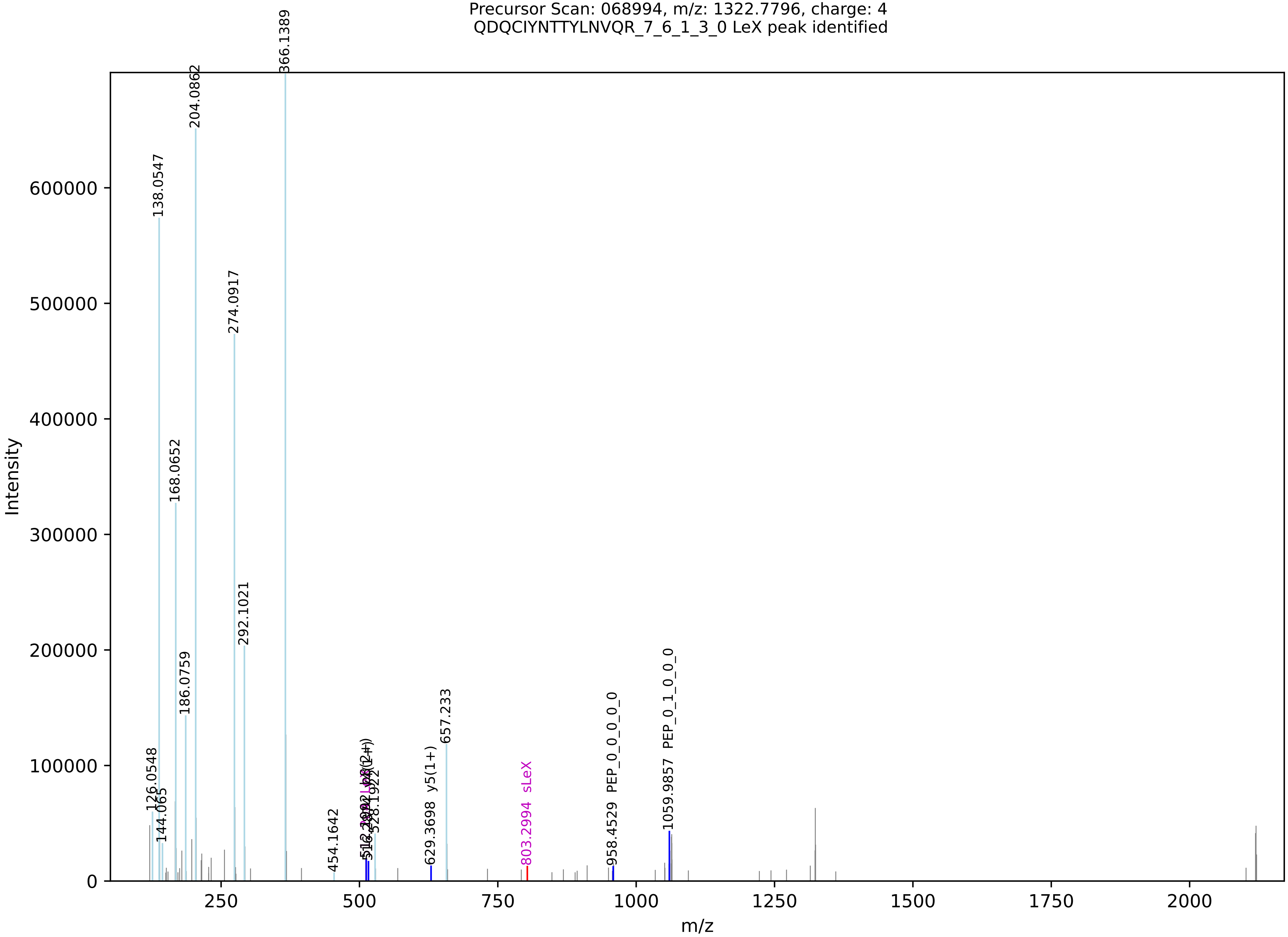

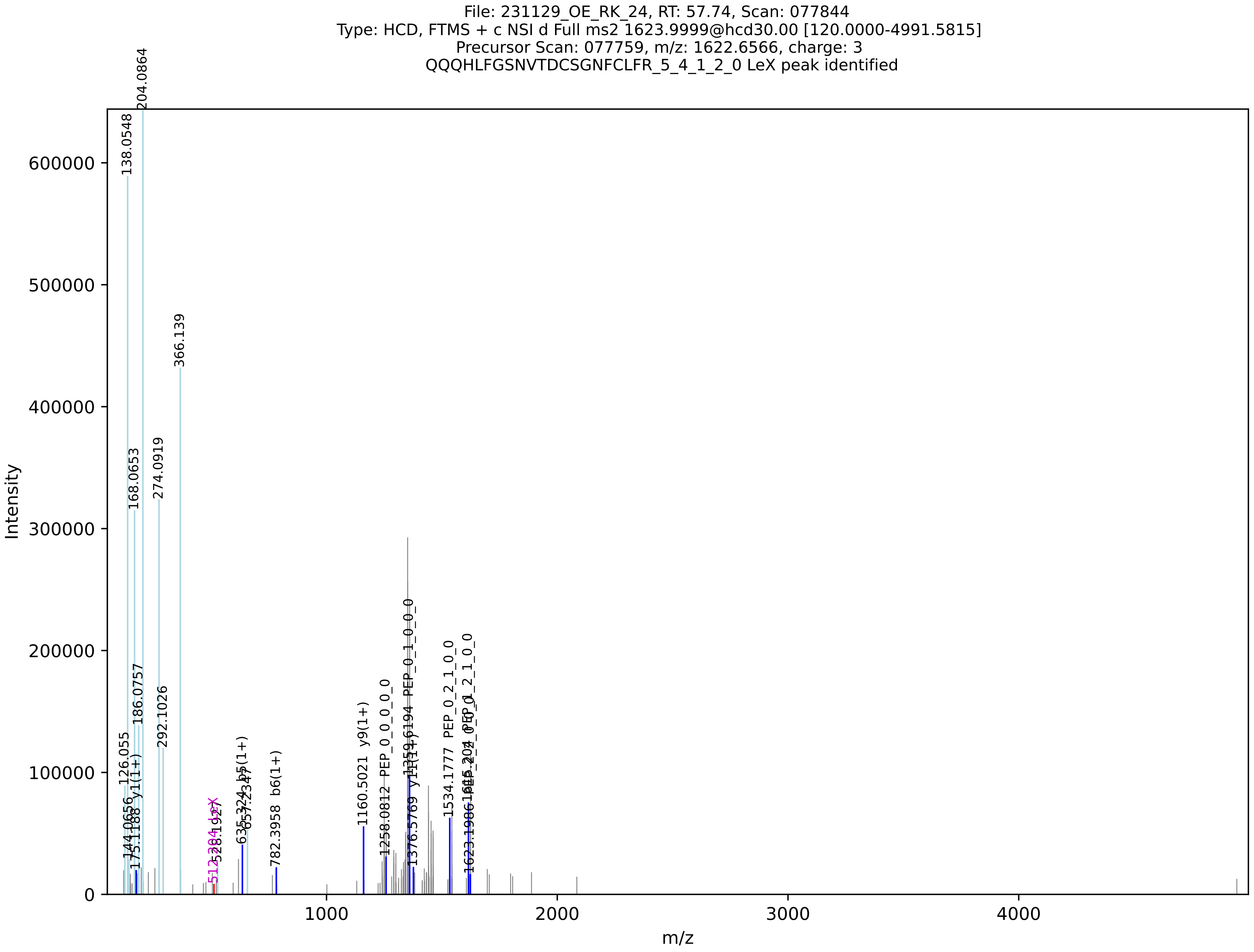

File: 231129_OE_RK_24, RT: 57.74, Scan: 077844
Type: HCD, FTMS + c NSI d Full ms2 1623.9999@hcd30.00 [120.0000-4991.5815]
Precursor Scan: 077759, m/z: 1622.6566, charge: 3
QQQHLFGSNVTDCSGNFCLFR_5_4_1_2_0 LeX peak identified
Intensity
m/z
204.0864
138.0548
366.139
168.0653
274.0919
186.0757
292.1026
126.055
144.0656
175.1188 y1(1+)
528.1927
657.2347
635.324 b5(1+)
782.3958 b6(1+)
1160.5021 y9(1+)
1258.0812 PEP_0_0_0_0_0
1359.6194 PEP_0_1_0_0_0
1376.5769 y11(1+)
1534.1777 PEP_0_2_1_0_0
1623.1986 PEP_1_2_1_0_0

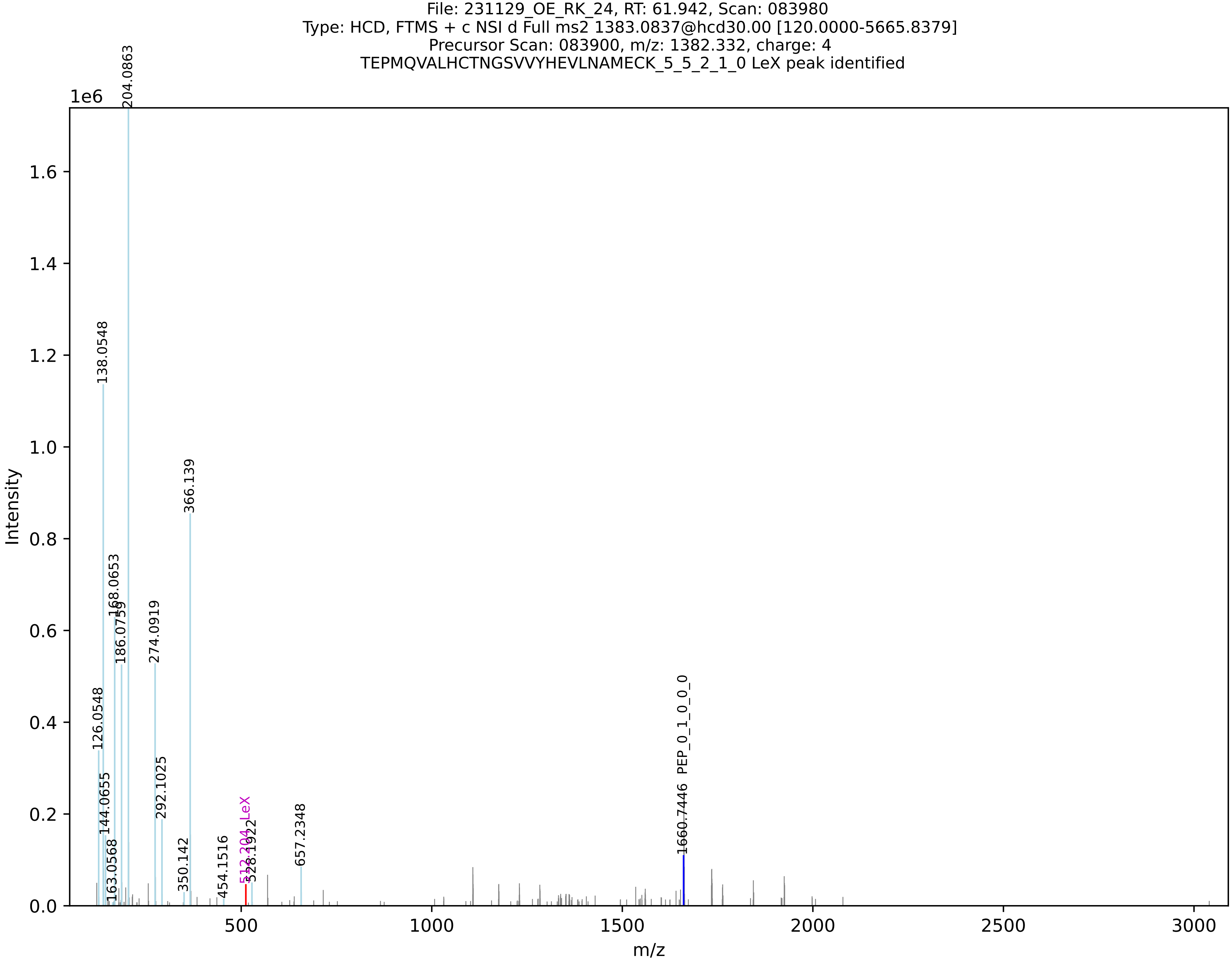
File: 231129_OE_RK_24, RT: 61.942, Scan: 083980
Type: HCD, FTMS + c NSI d Full ms2 1383.0837@hcd30.00 [120.0000-5665.8379]
Precursor Scan: 083900, m/z: 1382.332, charge: 4
TEPMQVALHCTNGSVVYHEVLNAMECK_5_5_2_1_0 LeX peak identified
1e6
Intensity
m/z
204.0863
138.0548
366.139
168.0653
186.0759
274.0919
126.0548
144.0655
292.1025
163.0568
350.142
454.1516
512.204_LeX
528.1922
657.2348
1660.7446 PEP_0_1_0_0_0

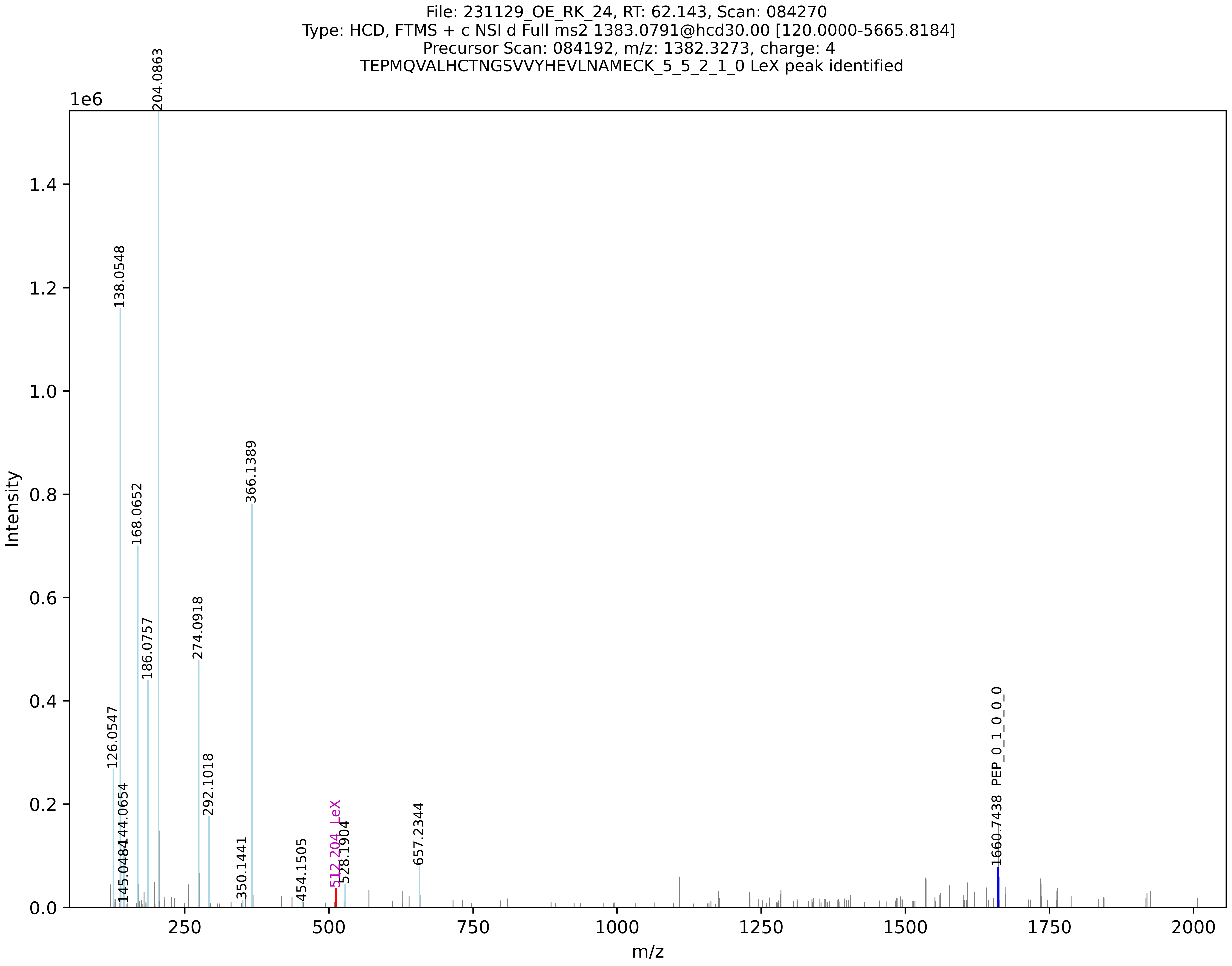

File: 231129_OE_RK_24, RT: 62.143, Scan: 084270
Type: HCD, FTMS + c NSI d Full ms2 1383.0791@hcd30.00 [120.0000-5665.8184]
Precursor Scan: 084192, m/z: 1382.3273, charge: 4
TEPMQVALHCTNGSVVYHEVLNAMECK_5_5_2_1_0 LeX peak identified
1e6
Intensity
m/z
204.0863
138.0548
168.0652
186.0757
126.0547
144.0654
145.0484
274.0918
292.1018
366.1389
350.1441
454.1505
512.204_LeX
528.1904
657.2344
1660.7438 PEP_0_1_0_0_0

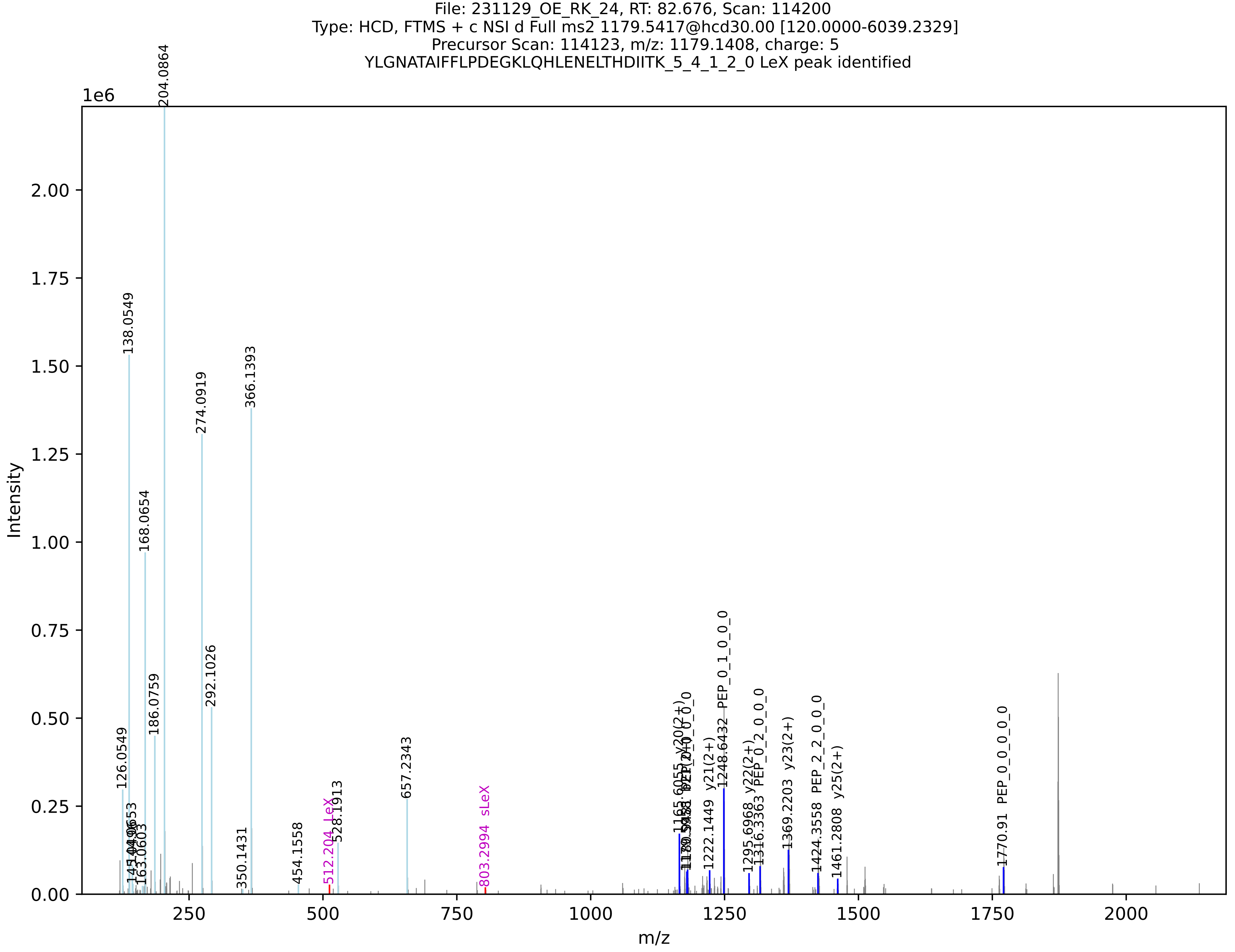
File: 231129_OE_RK_24, RT: 82.676, Scan: 114200
Type: HCD, FTMS + c NSI d Full ms2 1179.5417@hcd30.00 [120.0000-6039.2329]
Precursor Scan: 114123, m/z: 1179.1408, charge: 5
YLGNATAIFFLPDEGKLQHLENELTHDIITK_5_4_1_2_0 LeX peak identified
1e6
Intensity
m/z
204.0864
138.0549
366.1393
274.0919
168.0654
292.1026
186.0759
126.0549
163.0603
350.1431
454.1558
512.204 LeX
528.1913
657.2343
803.2994 sLeX
1165.6055 y20(2+)
PEP_1_0_0_0
1222.1449 y21(2+)
1248.6432 PEP_0_1_0_0_0
1295.6968 y22(2+)
1316.3363 PEP_0_2_0_0_0
1369.2203 y23(2+)
1424.3558 PEP_2_2_0_0_0
1461.2808 y25(2+)
1770.91 PEP_0_0_0_0_0

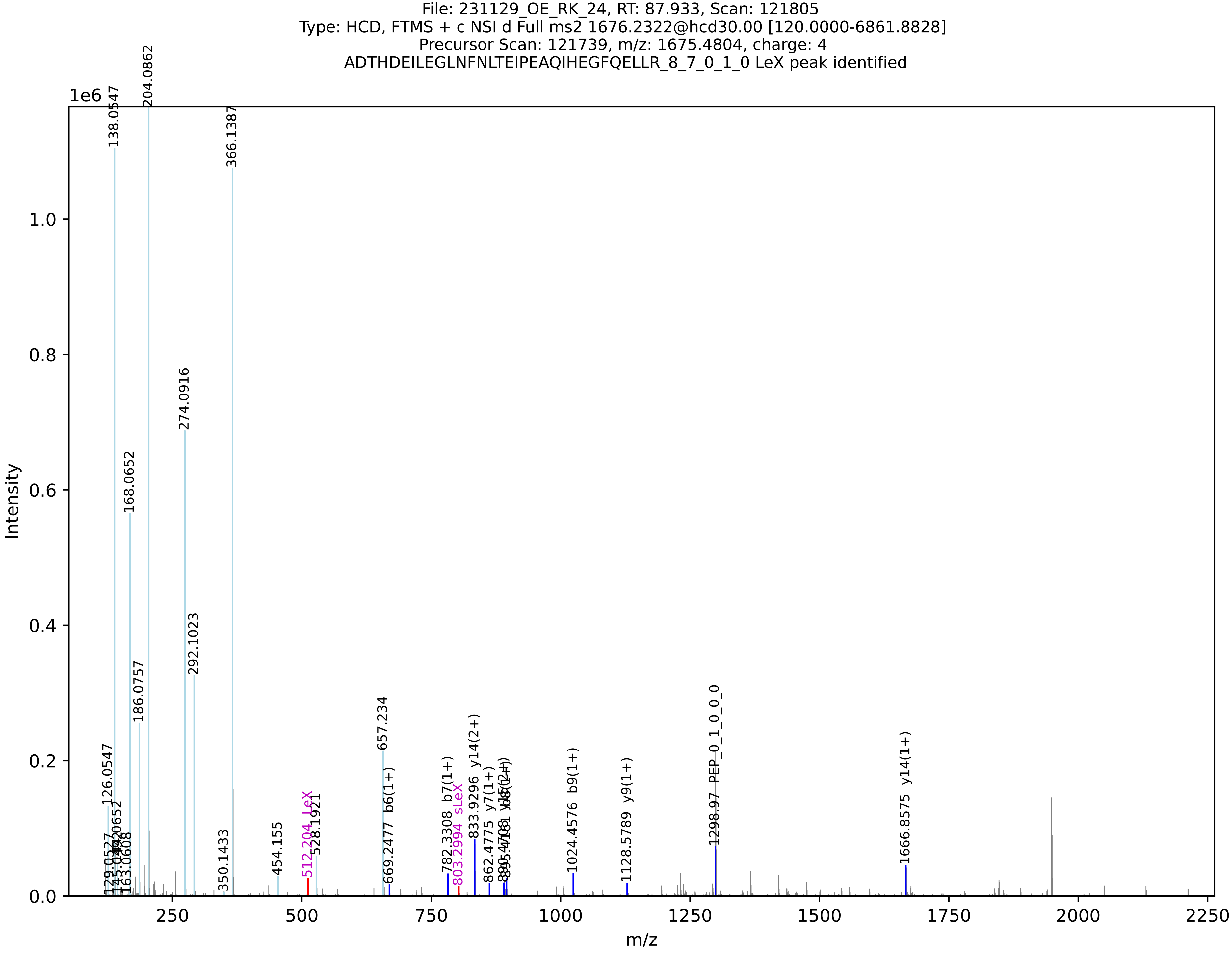
File: 231129_OE_RK_24, RT: 87.933, Scan: 121805
Type: HCD, FTMS + c NSI d Full ms2 1676.2322@hcd30.00 [120.0000-6861.8828]
Precursor Scan: 121739, m/z: 1675.4804, charge: 4
ADTHDEILEGLNFNLTEIPEAQIHEGFQELLR_8_7_0_1_0 LeX peak identified
1e6
Intensity
m/z
204.0862
138.0547
366.1387
274.0916
168.0652
292.1023
186.0757
126.0547
657.234
454.155
512.204 LeX
528.1921
350.1433
669.2477 b6(1+)
782.3308 b7(1+)
803.2994 sLeX
833.9296 y14(2+)
862.4775 y7(1+)
1024.4576 b9(1+)
1128.5789 y9(1+)
1298.97 PEP_0_1_0_0_0
1666.8575 y14(1+)

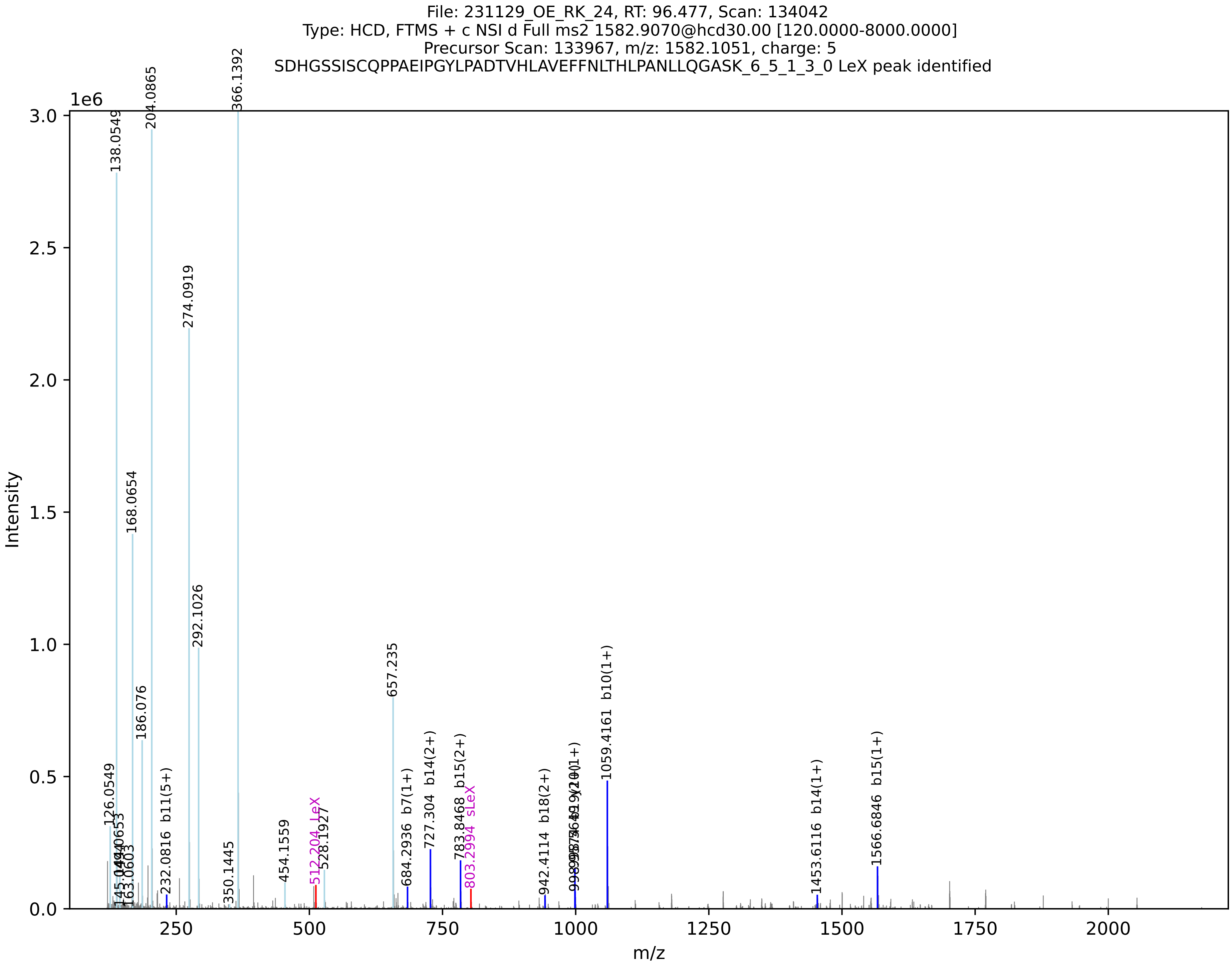
File: 231129_OE_RK_24, RT: 96.477, Scan: 134042
Type: HCD, FTMS + c NSI d Full ms2 1582.9070@hcd30.00 [120.0000-8000.0000]
Precursor Scan: 133967, m/z: 1582.1051, charge: 5
SDHGSSISCQPPAEIPGYLPADTVHLAVEFFNLTHLPANLLQGASK_6_5_1_3_0 LeX peak identified
1e6
Intensity
m/z
366.1392
204.0865
138.0549
274.0919
168.0654
292.1026
186.076
126.0549
163.0603
232.0816 b11(5+)
350.1445
454.1559
512.204 LeX
528.1927
657.235
684.2936 b7(1+)
727.304 b14(2+)
783.8468 b15(2+)
803.2994 sLeX
942.4114 b18(2+)
1059.4161 b10(1+)
1453.6116 b14(1+)
1566.6846 b15(1+)

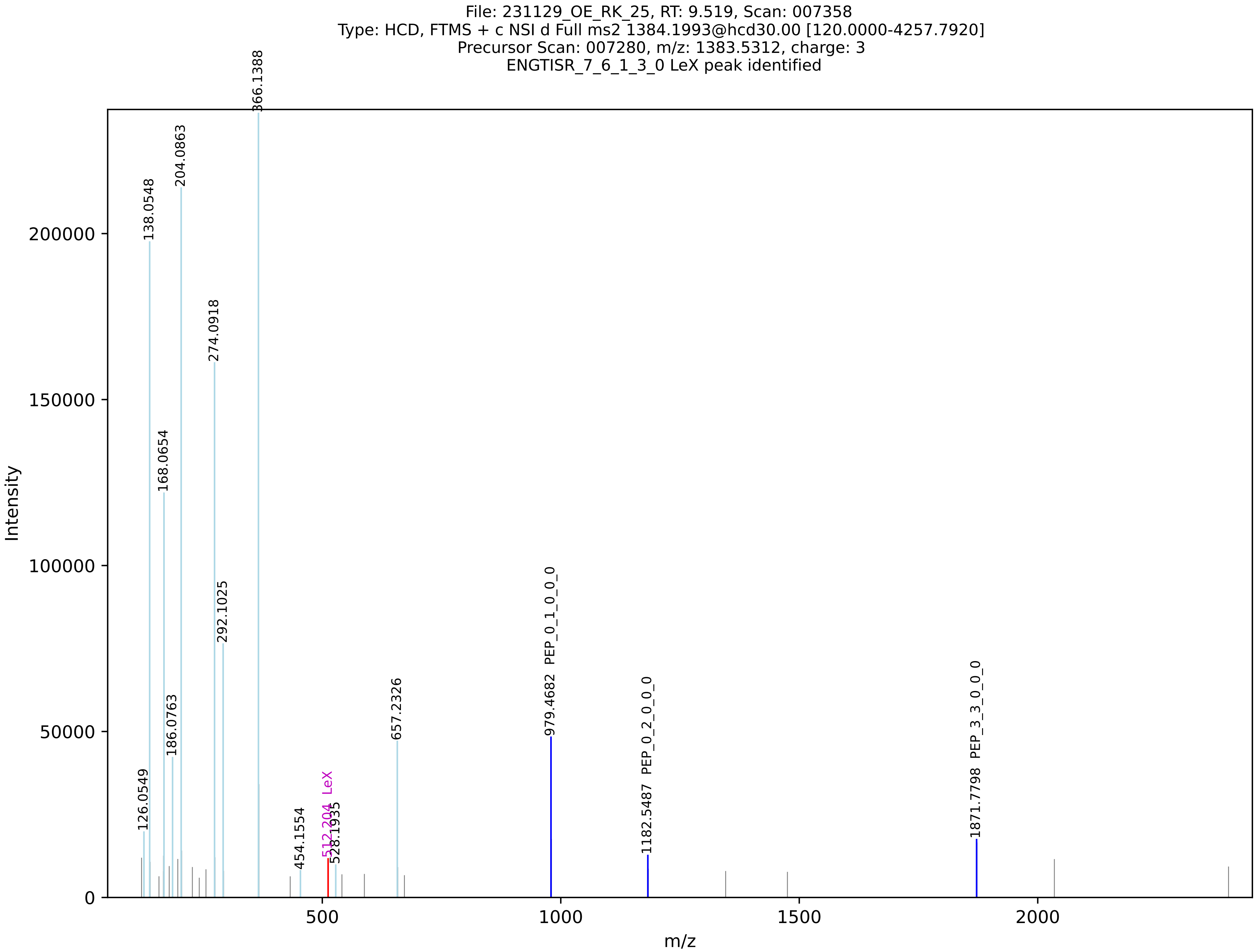
File: 231129_OE_RK_25, RT: 9.519, Scan: 007358
Type: HCD, FTMS + c NSI d Full ms2 1384.1993@hcd30.00 [120.0000-4257.7920]
Precursor Scan: 007280, m/z: 1383.5312, charge: 3
ENGTISR_7_6_1_3_0 LeX peak identified
Intensity
m/z
0
50000
100000
150000
200000
500
1000
1500
2000
366.1388
204.0863
138.0548
274.0918
168.0654
292.1025
186.0763
126.0549
454.1554
512.204 LeX
528.1935
657.2326
979.4682 PEP_0_1_0_0_0
1182.5487 PEP_0_2_0_0_0
1871.7798 PEP_3_3_0_0_0

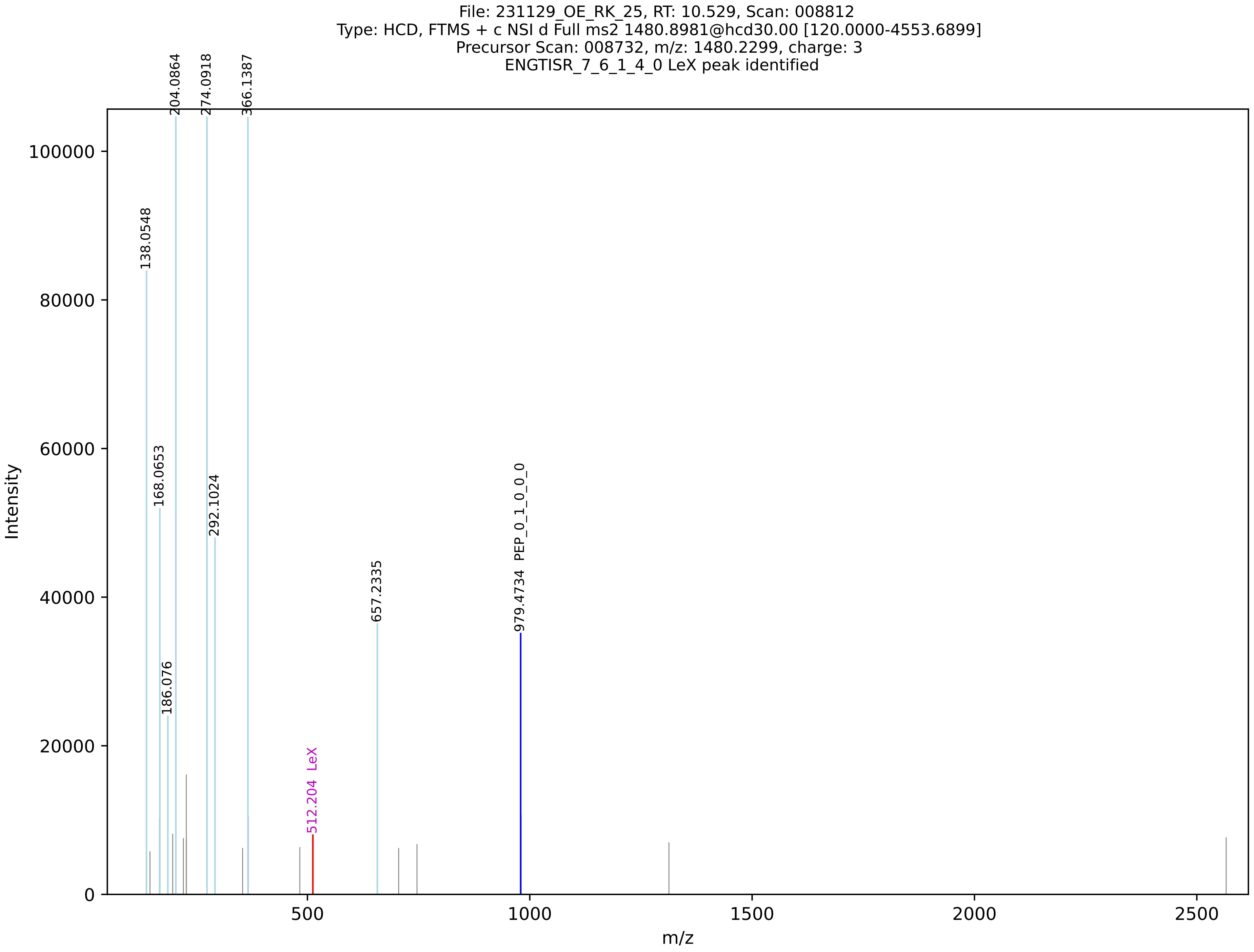

File: 231129_OE_RK_25, RT: 10.529, Scan: 008812
Type: HCD, FTMS + c NSI d Full ms2 1480.8981@hcd30.00 [120.0000-4553.6899]
Precursor Scan: 008732, m/z: 1480.2299, charge: 3
ENGTISR_7_6_1_4_0 LeX peak identified
Intensity
m/z
0
20000
40000
60000
80000
100000
500
1000
1500
2000
2500
138.0548
168.0653
186.076
204.0864
274.0918
292.1024
366.1387
512.204 LeX
657.2335
979.4734 PEP_0_1_0_0_0_

File: 231129_OE_RK_25, RT: 43.178, Scan: 055950
Type: HCD, FTMS + c NSI d Full ms2 1250.5066@hcd30.00 [120.0000-5124.9229]
Precursor Scan: 055874, m/z: 1250.0055, charge: 4
QDQCIYNTTYLNVQR_7_6_1_2_0 LeX peak identified

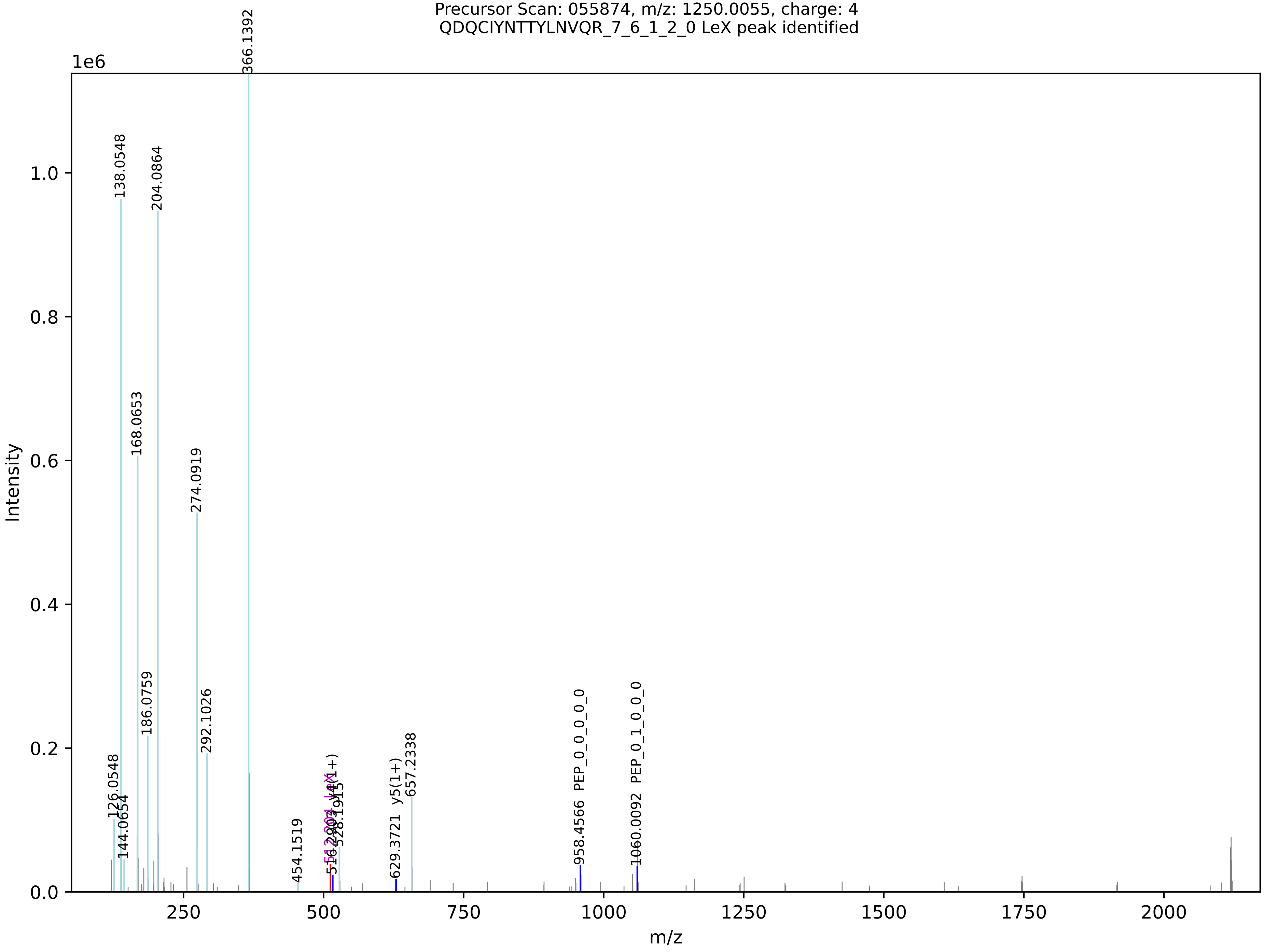

File: 231129_OE_RK_25, RT: 43.18, Scan: 055953
Type: HCD, FTMS + c NSI d Full ms2 1079.7224@hcd30.00 [120.0000-4428.1235]
Precursor Scan: 055874, m/z: 1079.221, charge: 4
ADGTVNQIEGEATPVNLTEPAK_5_4_1_1_0 LeX peak identified

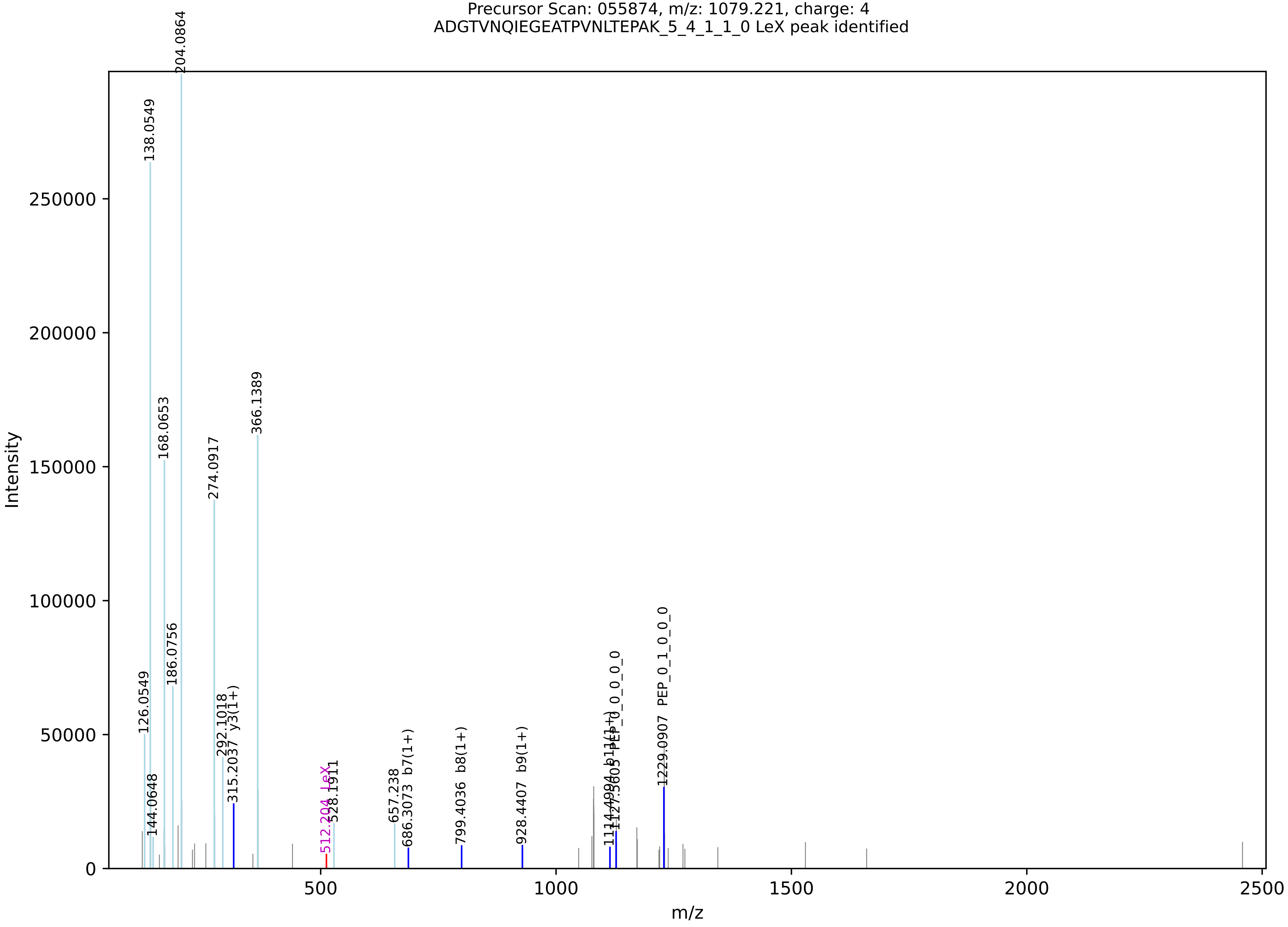

File: 231129_OE_RK_25, RT: 43.578, Scan: 056527
Type: HCD, FTMS + c NSI d Full ms2 1250.5056@hcd30.00 [120.0000-5124.9189]
Precursor Scan: 056451, m/z: 1250.0044, charge: 4
QDQCIYNTTYLNVQR_7_6_1_2_0 LeX peak identified

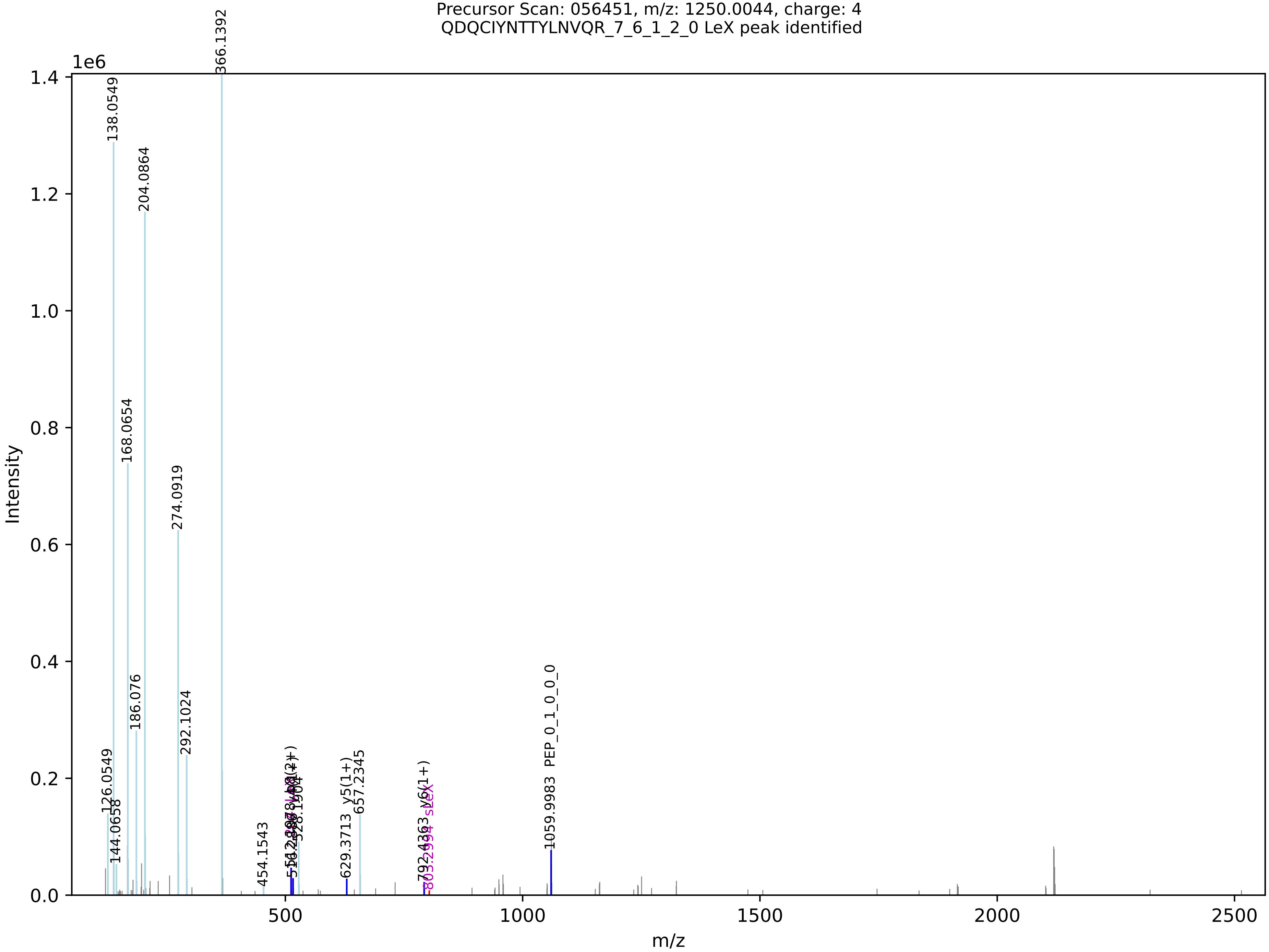

File: 231129_OE_RK_25, RT: 43.885, Scan: 056973
Type: HCD, FTMS + c NSI d Full ms2 1545.6295@hcd30.00 [120.0000-4751.7686]
Precursor Scan: 056892, m/z: 1544.2929, charge: 3
QDQCIYNTTYLNVQR_6_5_1_2_0 LeX peak identified

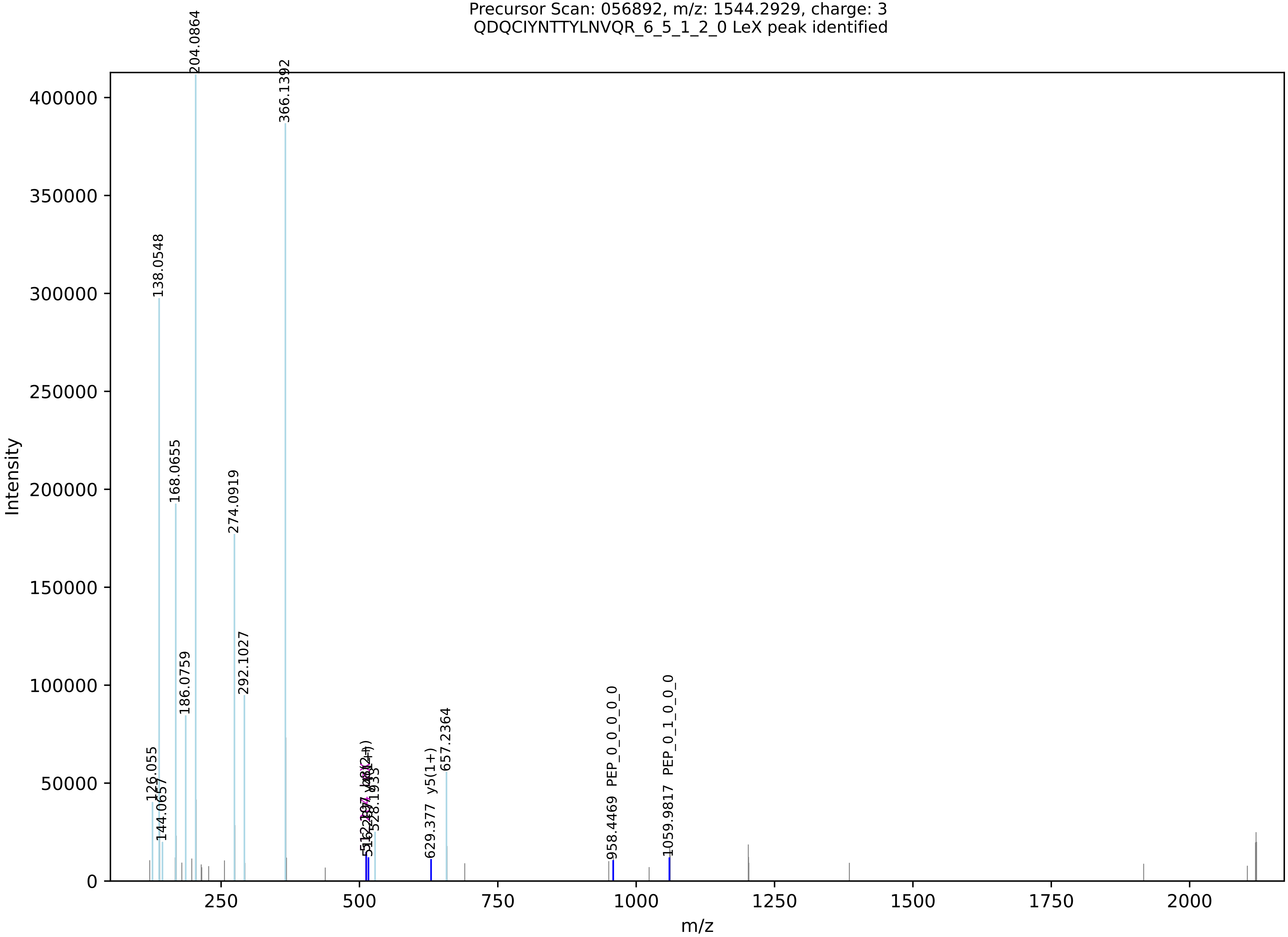

File: 231129_OE_RK_25, RT: 44.033, Scan: 057185
Type: HCD, FTMS + c NSI d Full ms2 1250.5059@hcd30.00 [120.0000-5124.9199]
Precursor Scan: 057111, m/z: 1249.7541, charge: 4
QDQCIYNTTYLNVQR_7_6_1_2_0 LeX peak identified

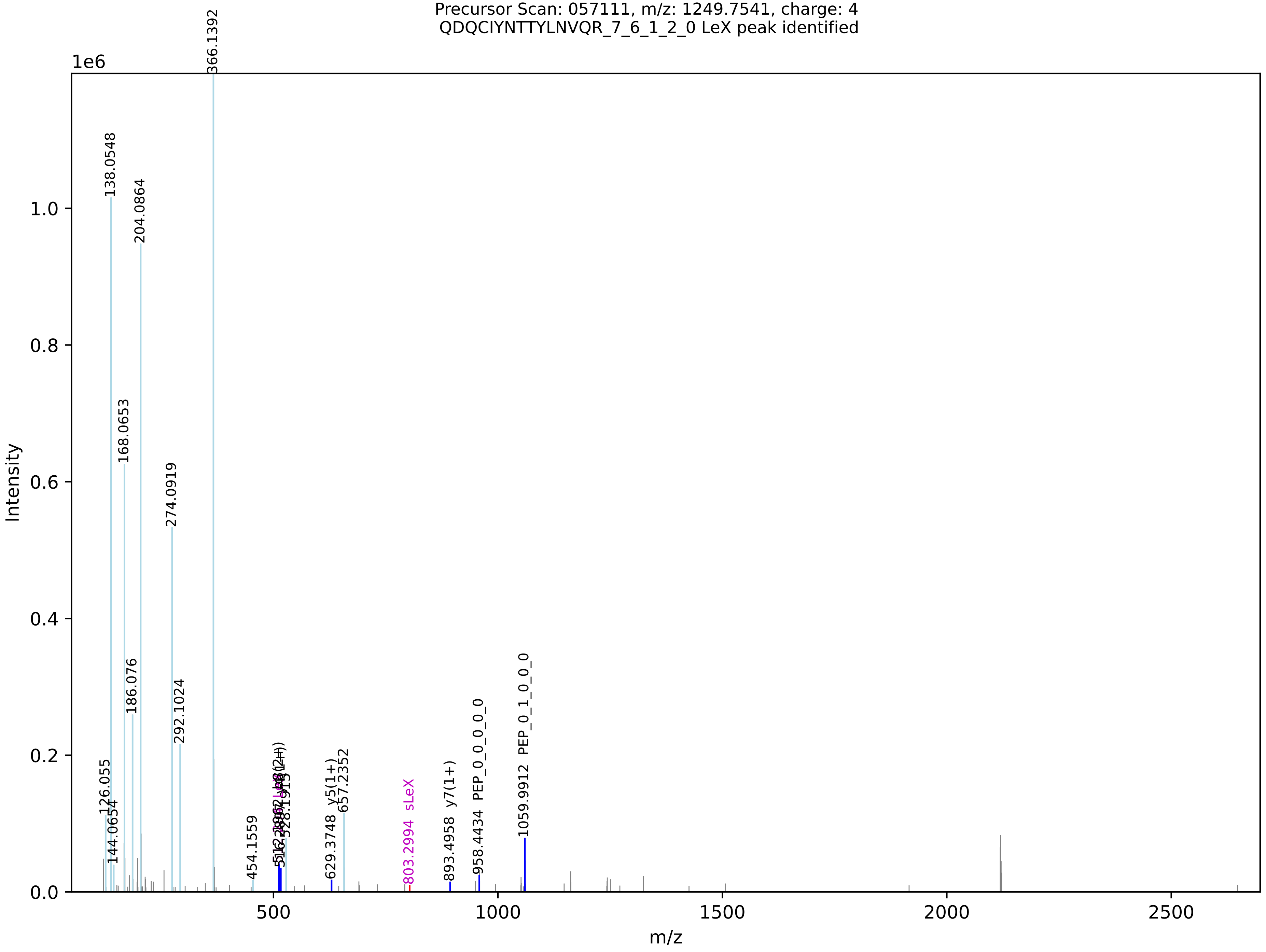

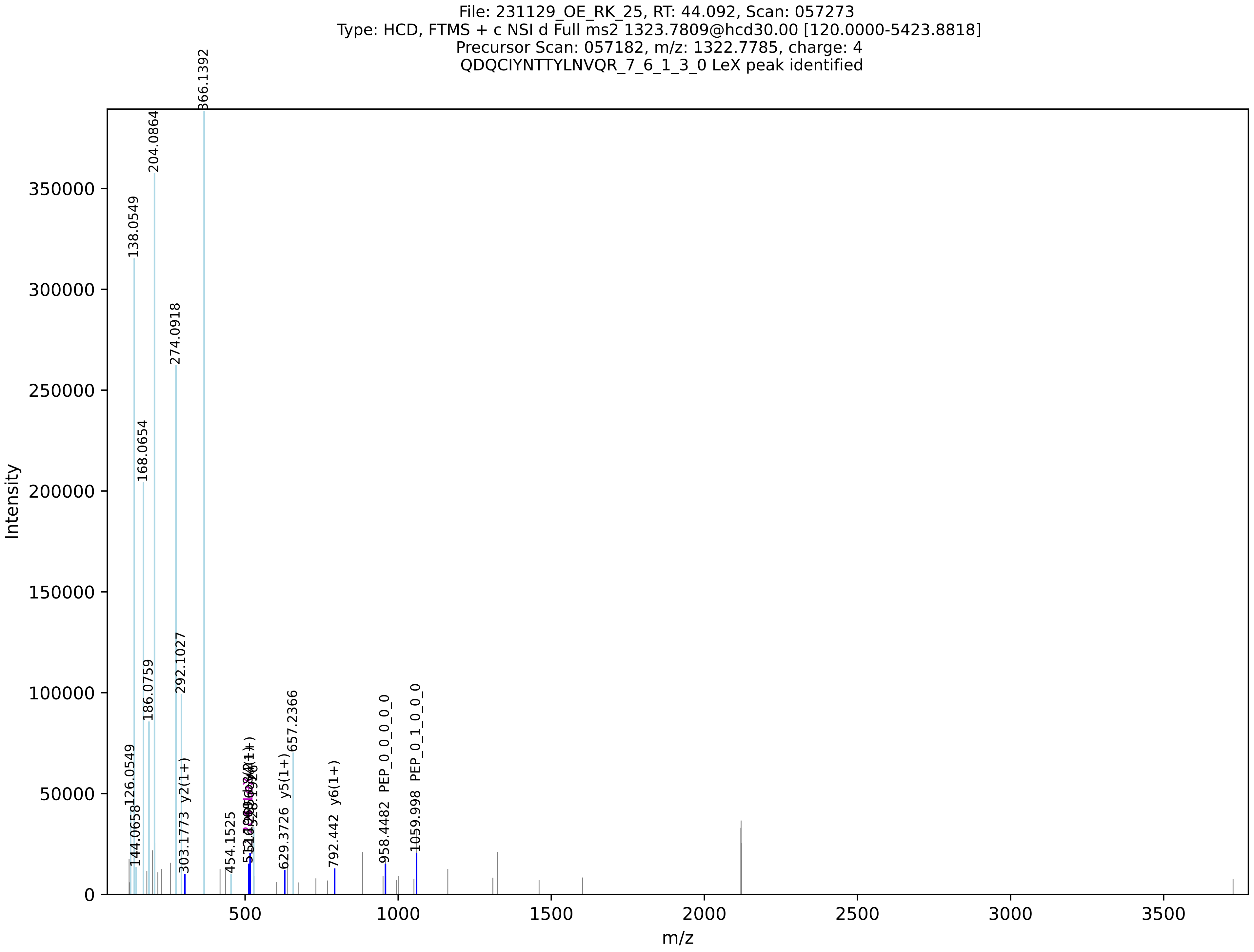

File: 231129_OE_RK_25, RT: 44.092, Scan: 057273
Type: HCD, FTMS + c NSI d Full ms2 1323.7809@hcd30.00 [120.0000-5423.8818]
Precursor Scan: 057182, m/z: 1322.7785, charge: 4
QDQCIYNTTYLNVQR_7_6_1_3_0 LeX peak identified
Intensity
m/z
366.1392
204.0864
138.0549
274.0918
168.0654
292.1027
186.0759
126.0549
144.0658
303.1773 y2(1+)
454.1525
528.1920
629.3726 y5(1+)
657.2366
792.442 y6(1+)
958.4482 PEP_0_0_0_0_0
1059.998 PEP_0_1_0_0_0

File: 231129_OE_RK_25, RT: 45.545, Scan: 059380
Type: HCD, FTMS + c NSI d Full ms2 1323.5309@hcd30.00 [120.0000-5422.8618]
Precursor Scan: 059299, m/z: 1322.7791, charge: 4
QDQCIYNTTYLNVQR_7_6_1_3_0 LeX peak identified

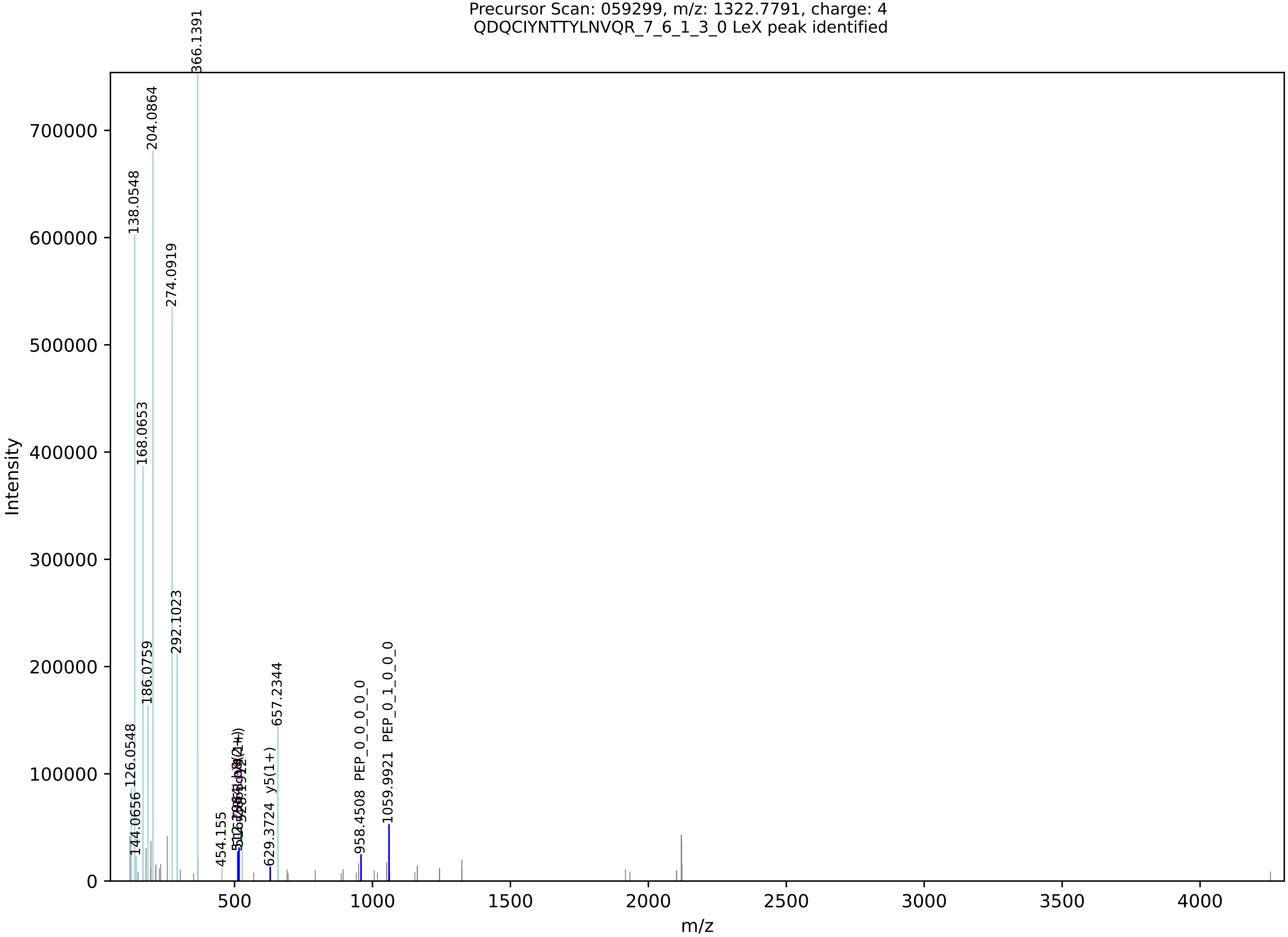

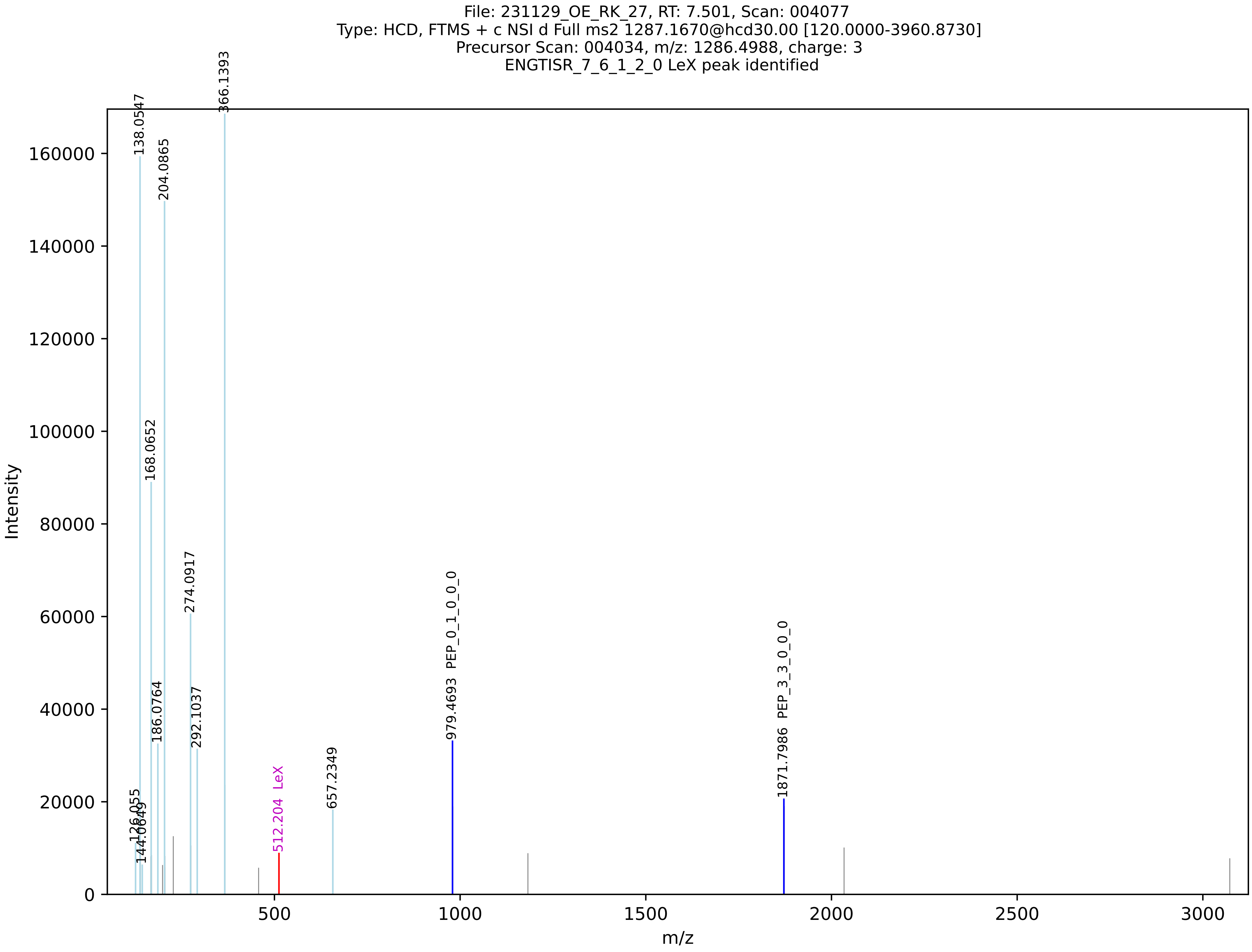

File: 231129_OE_RK_27, RT: 7.501, Scan: 004077
Type: HCD, FTMS + c NSI d Full ms2 1287.1670@hcd30.00 [120.0000-3960.8730]
Precursor Scan: 004034, m/z: 1286.4988, charge: 3
ENGTISR_7_6_1_2_0 LeX peak identified
Intensity
m/z
366.1393
138.0547
204.0865
168.0652
274.0917
186.0764
292.1037
126.055
144.0649
512.204 LeX
657.2349
979.4693 PEP_0_1_0_0_0
1871.7986 PEP_3_3_0_0_0

File: 231129_OE_RK_27, RT: 7.804, Scan: 004483
Type: HCD, FTMS + c NSI d Full ms2 1384.5360@hcd30.00 [120.0000-4258.8223]
Precursor Scan: 004431, m/z: 1383.5337, charge: 3
ENGTISR_7_6_1_3_0 LeX peak identified

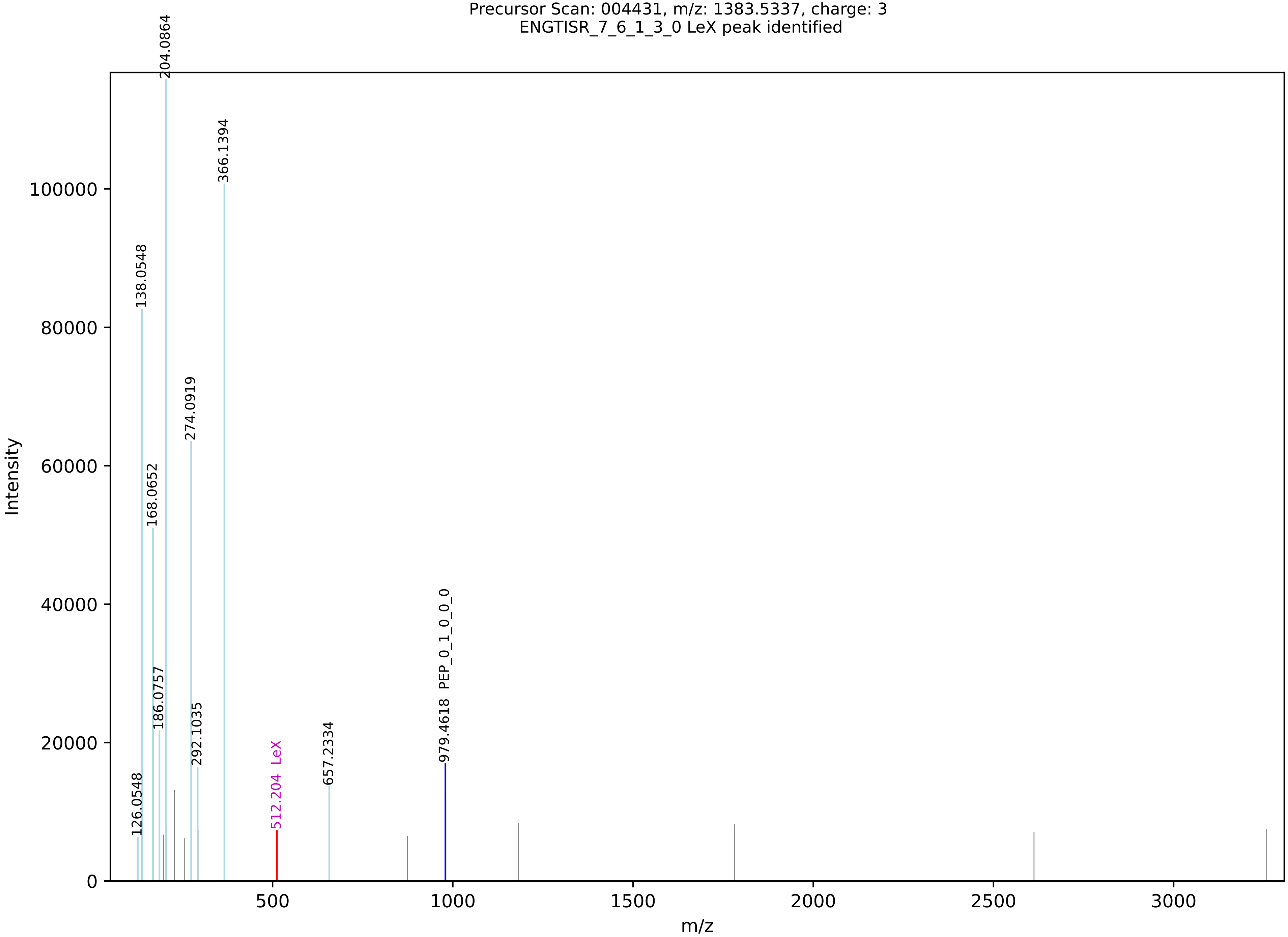

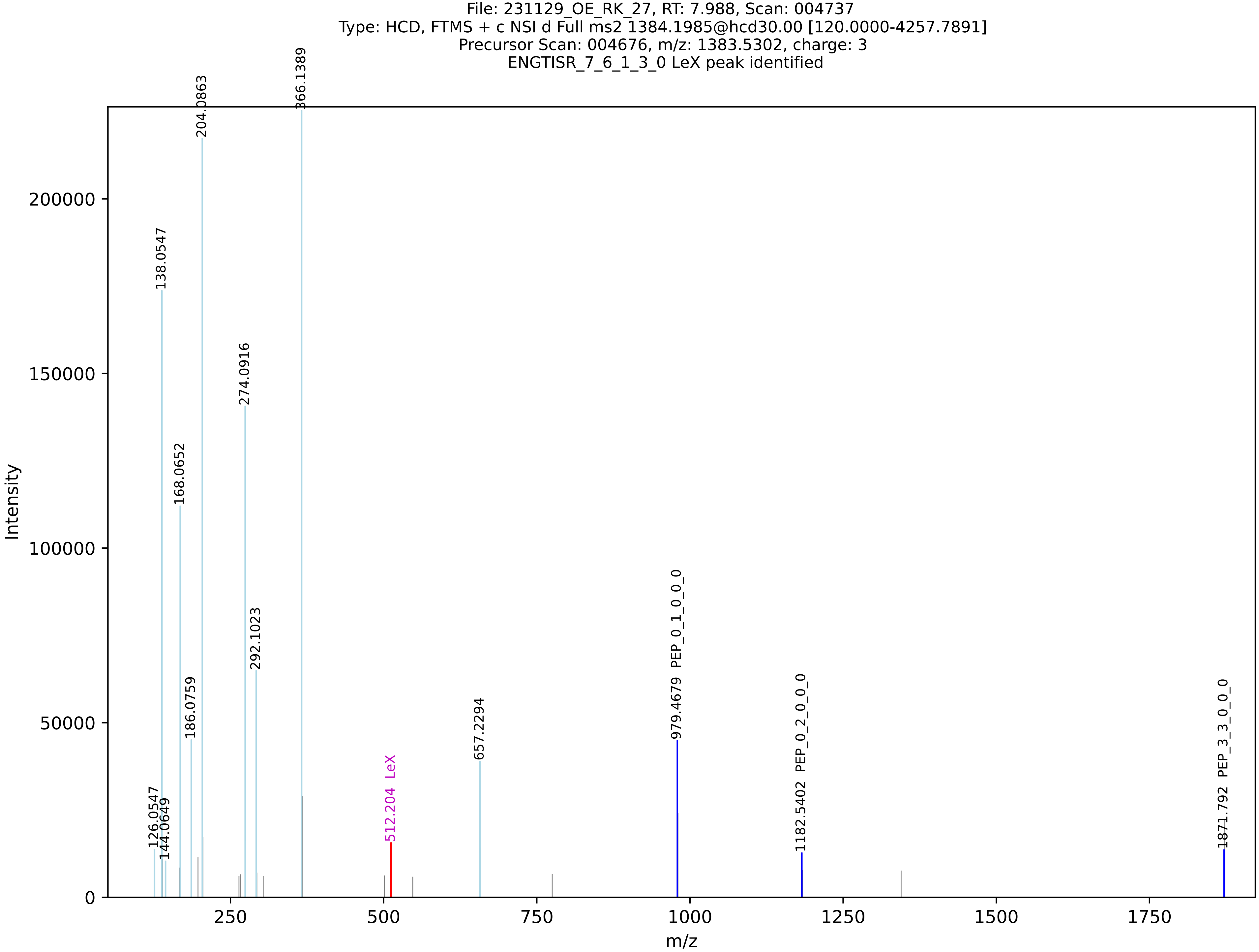
File: 231129_OE_RK_27, RT: 7.988, Scan: 004737
Type: HCD, FTMS + c NSI d Full ms2 1384.1985@hcd30.00 [120.0000-4257.7891]
Precursor Scan: 004676, m/z: 1383.5302, charge: 3
ENGTISR_7_6_1_3_0 LeX peak identified
Intensity
m/z
366.1389
204.0863
138.0547
274.0916
168.0652
292.1023
186.0759
126.0547
144.0649
512.204 LeX
657.2294
979.4679 PEP_0_1_0_0_0
1182.5402 PEP_0_2_0_0_0
1871.792 PEP_3_3_0_0_0
0
50000
100000
150000
200000
250
500
750
1000
1250
1500
1750

File: 231129_OE_RK_27, RT: 8.467, Scan: 005407
Type: HCD, FTMS + c NSI d Full ms2 1074.9148@hcd30.00 [120.0000-4408.5083]
Precursor Scan: 005346, m/z: 1074.413, charge: 4
ENGTISR_7_6_2_3_0 LeX peak identified

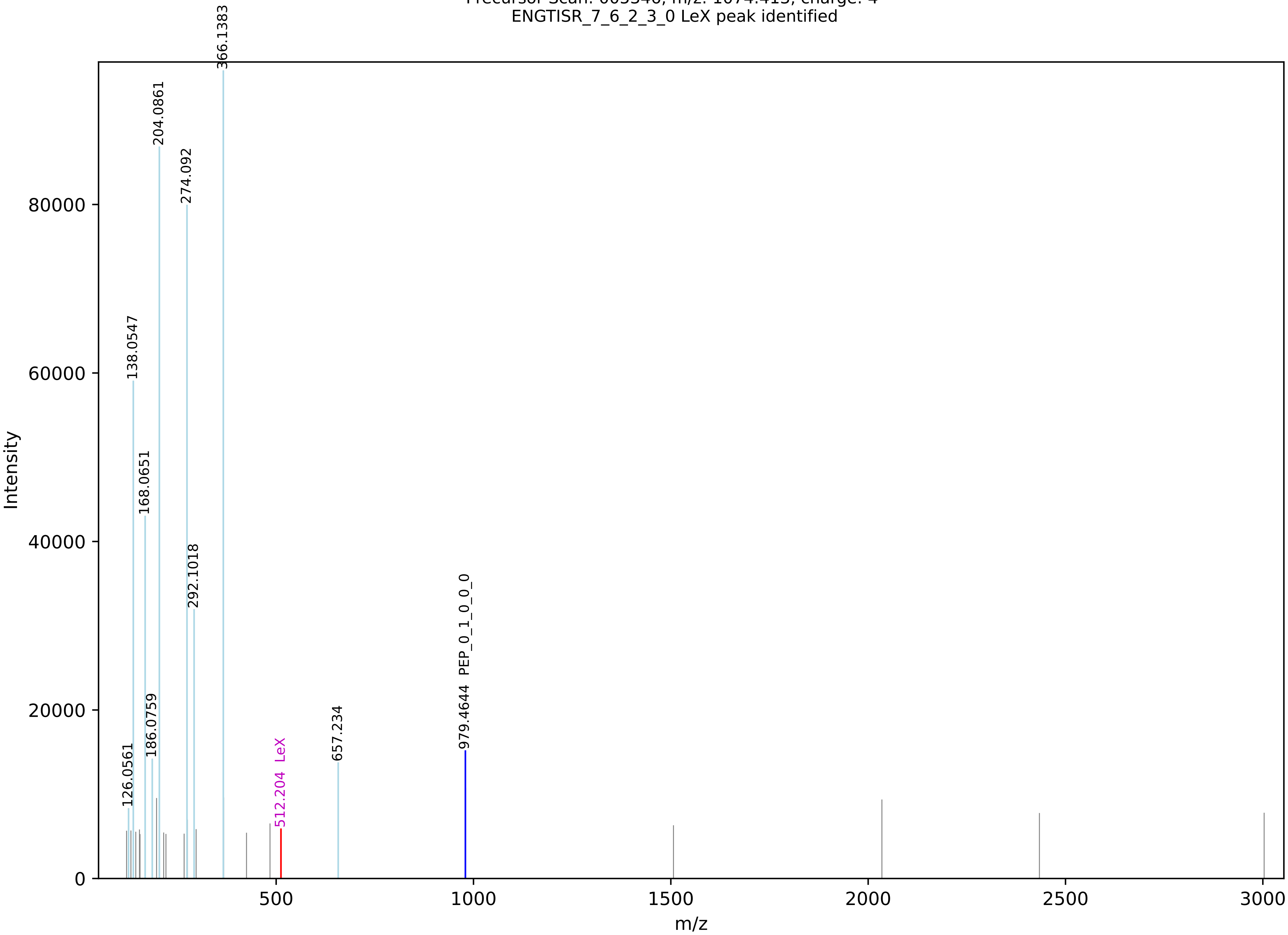

File: 231129_OE_RK_27, RT: 9.912, Scan: 007450
Type: HCD, FTMS + c NSI d Full ms2 1147.9395@hcd30.00 [120.0000-4706.4487]
Precursor Scan: 007371, m/z: 1147.1877, charge: 4
ENGTISR_7_6_2_4_0 LeX peak identified

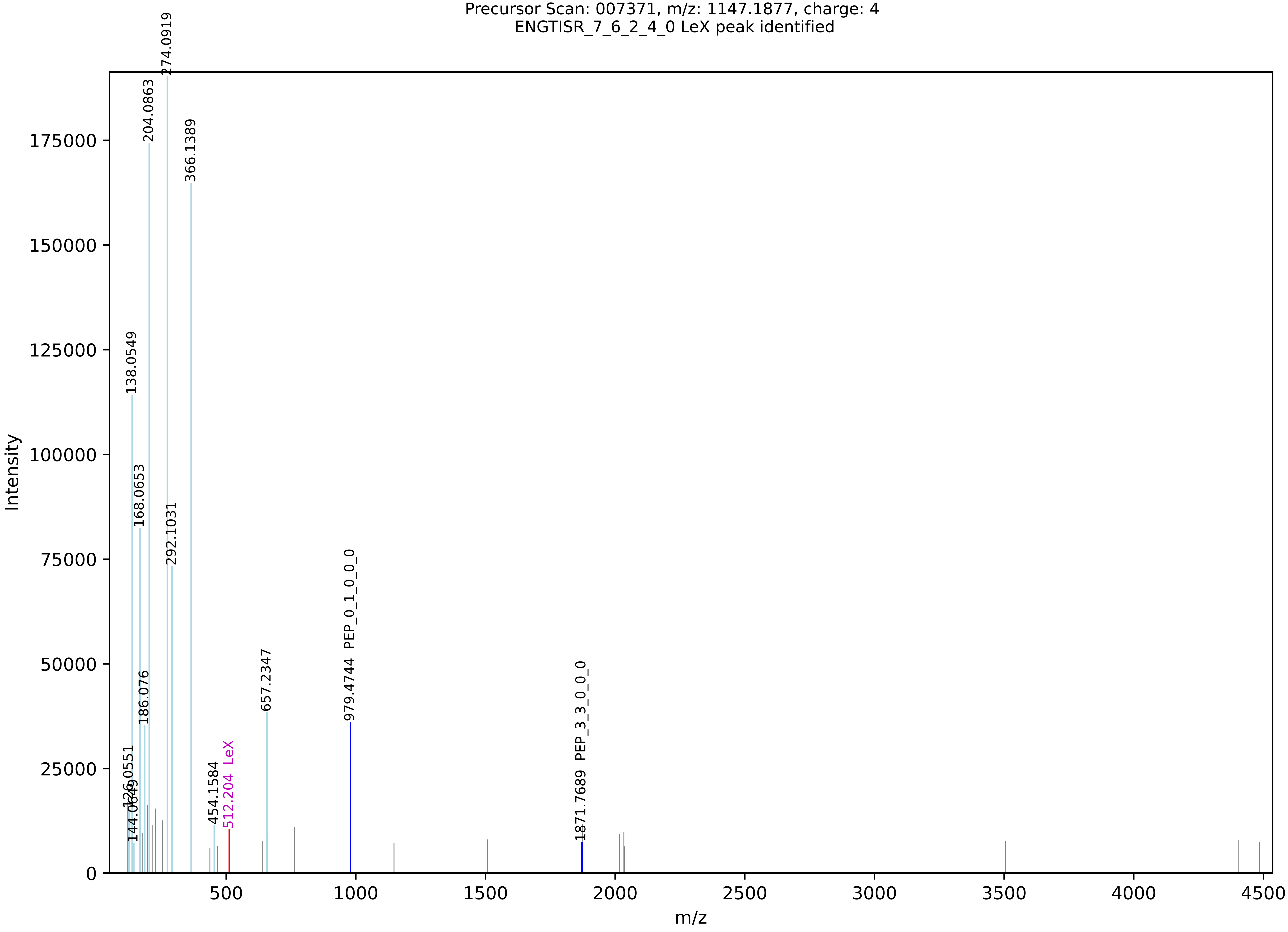

File: 231129_OE_RK_27, RT: 13.156, Scan: 012081
Type: HCD, FTMS + c NSI d Full ms2 1111.6782@hcd30.00 [120.0000-4558.5034]
Precursor Scan: 011904, m/z: 1110.6732, charge: 4
ENGTISR_7_6_1_4_0 LeX peak identified

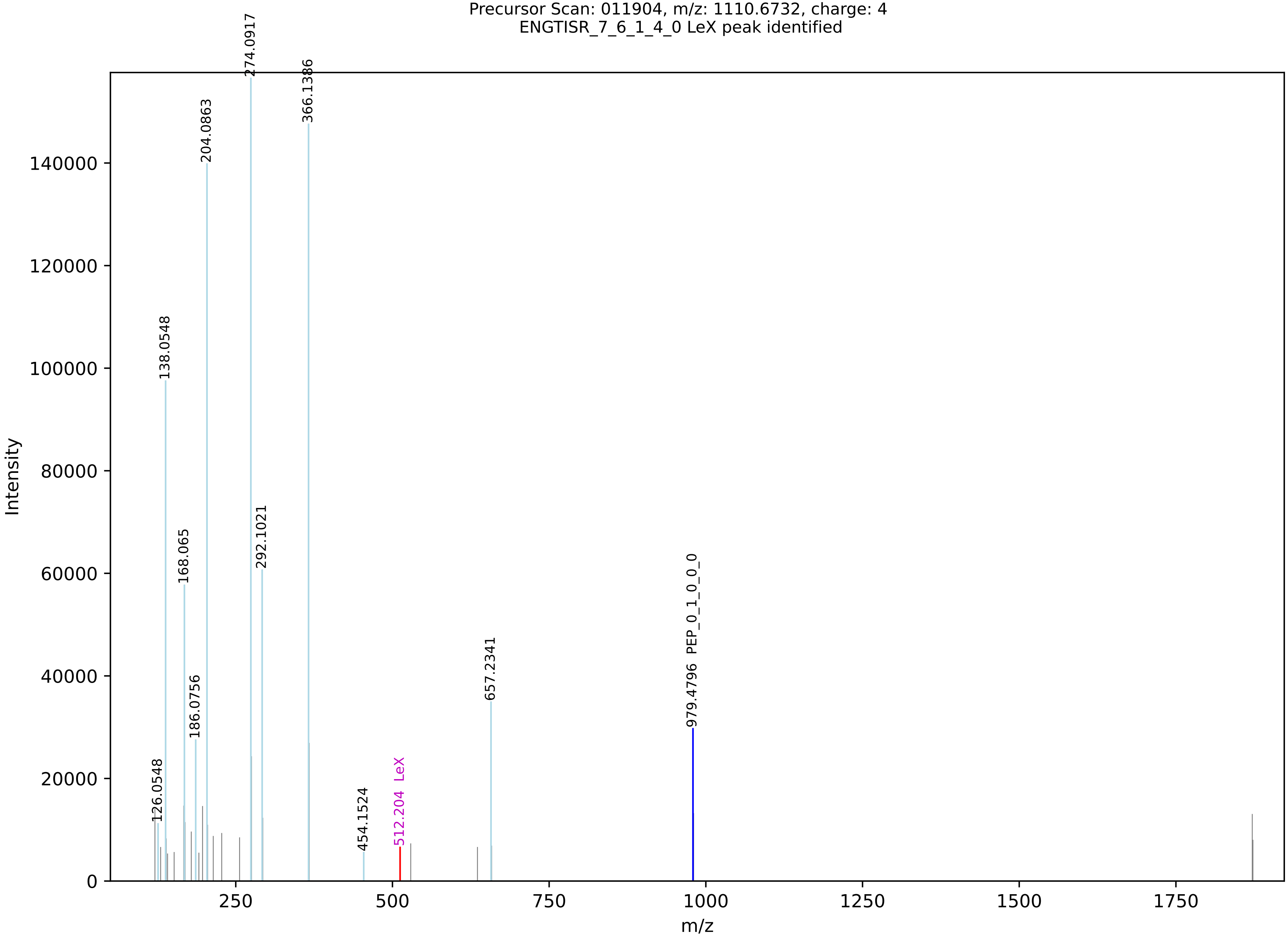

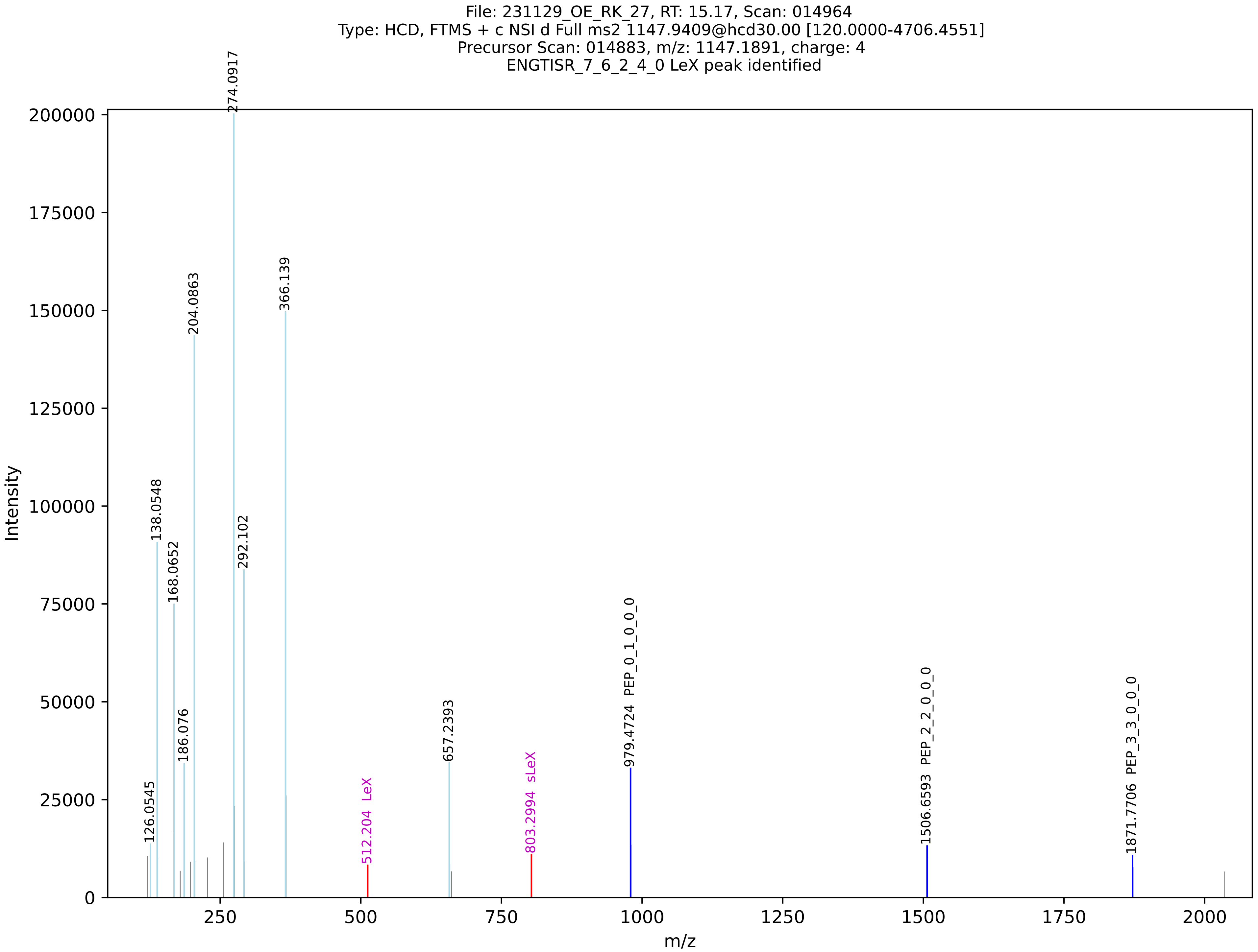

File: 231129_OE_RK_27, RT: 15.17, Scan: 014964
Type: HCD, FTMS + c NSI d Full ms2 1147.9409@hcd30.00 [120.0000-4706.4551]
Precursor Scan: 014883, m/z: 1147.1891, charge: 4
ENGTISR_7_6_2_4_0 LeX peak identified
Intensity
m/z
274.0917
366.139
204.0863
138.0548
292.102
168.0652
186.076
126.0545
512.204 LeX
657.2393
803.2994 sLeX
979.4724 PEP_0_1_0_0_0
1506.6593 PEP_2_2_0_0_0
1871.7706 PEP_3_3_0_0_0

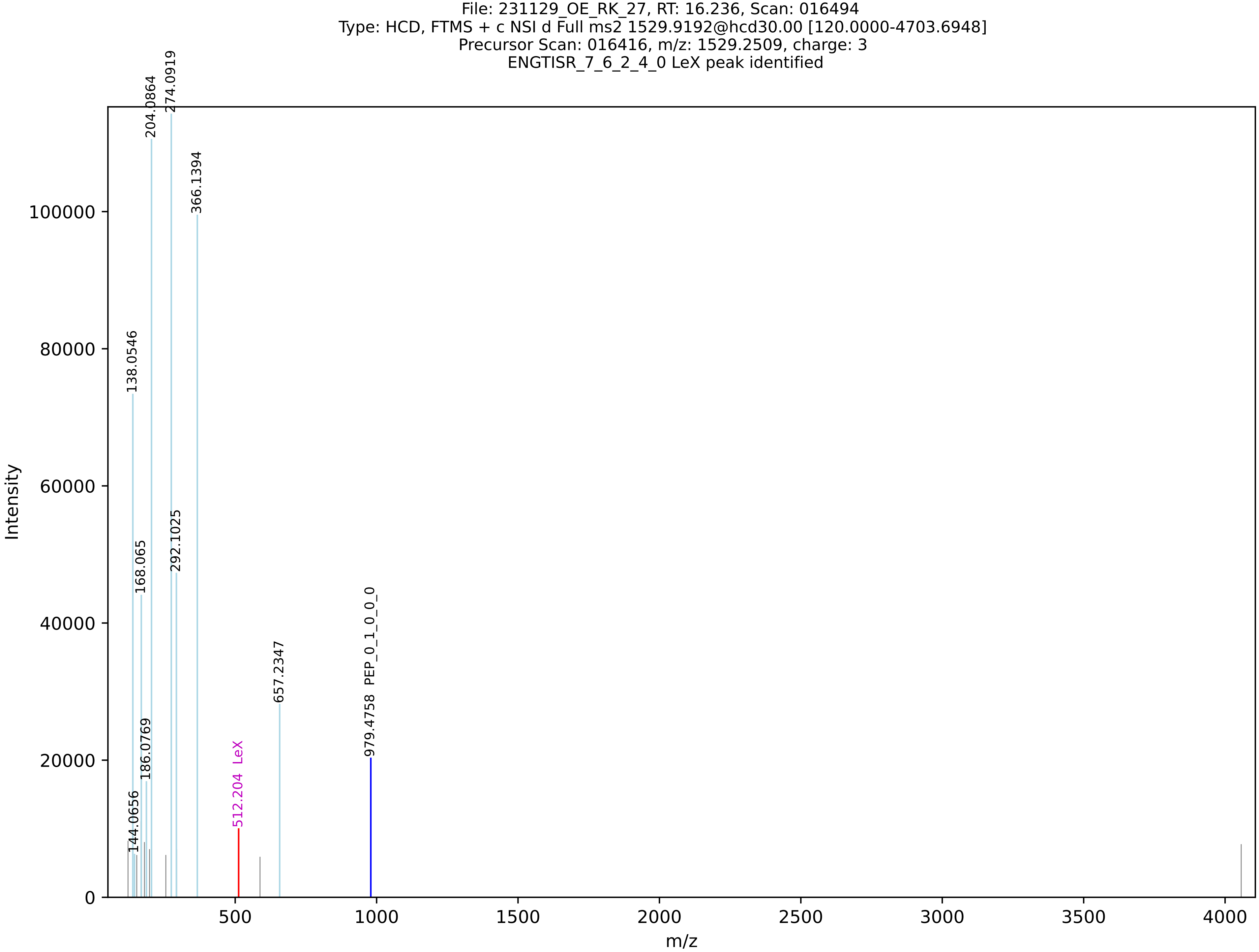
File: 231129_OE_RK_27, RT: 16.236, Scan: 016494
Type: HCD, FTMS + c NSI d Full ms2 1529.9192@hcd30.00 [120.0000-4703.6948]
Precursor Scan: 016416, m/z: 1529.2509, charge: 3
ENGTISR_7_6_2_4_0 LeX peak identified
274.0919
204.0864
366.1394
138.0546
292.1025
168.065
PEP_0_1_0_0_0
979.4758
657.2347
LeX
512.204
186.0769
144.0656
Intensity
m/z
0
20000
40000
60000
80000
100000
500
1000
1500
2000
2500
3000
3500
4000

File: 231129_OE_RK_27, RT: 17.341, Scan: 018072
Type: HCD, FTMS + c NSI d Full ms2 1147.6896@hcd30.00 [120.0000-4705.4292]
Precursor Scan: 018023, m/z: 1147.1877, charge: 4
ENGTISR_7_6_2_4_0 LeX peak identified

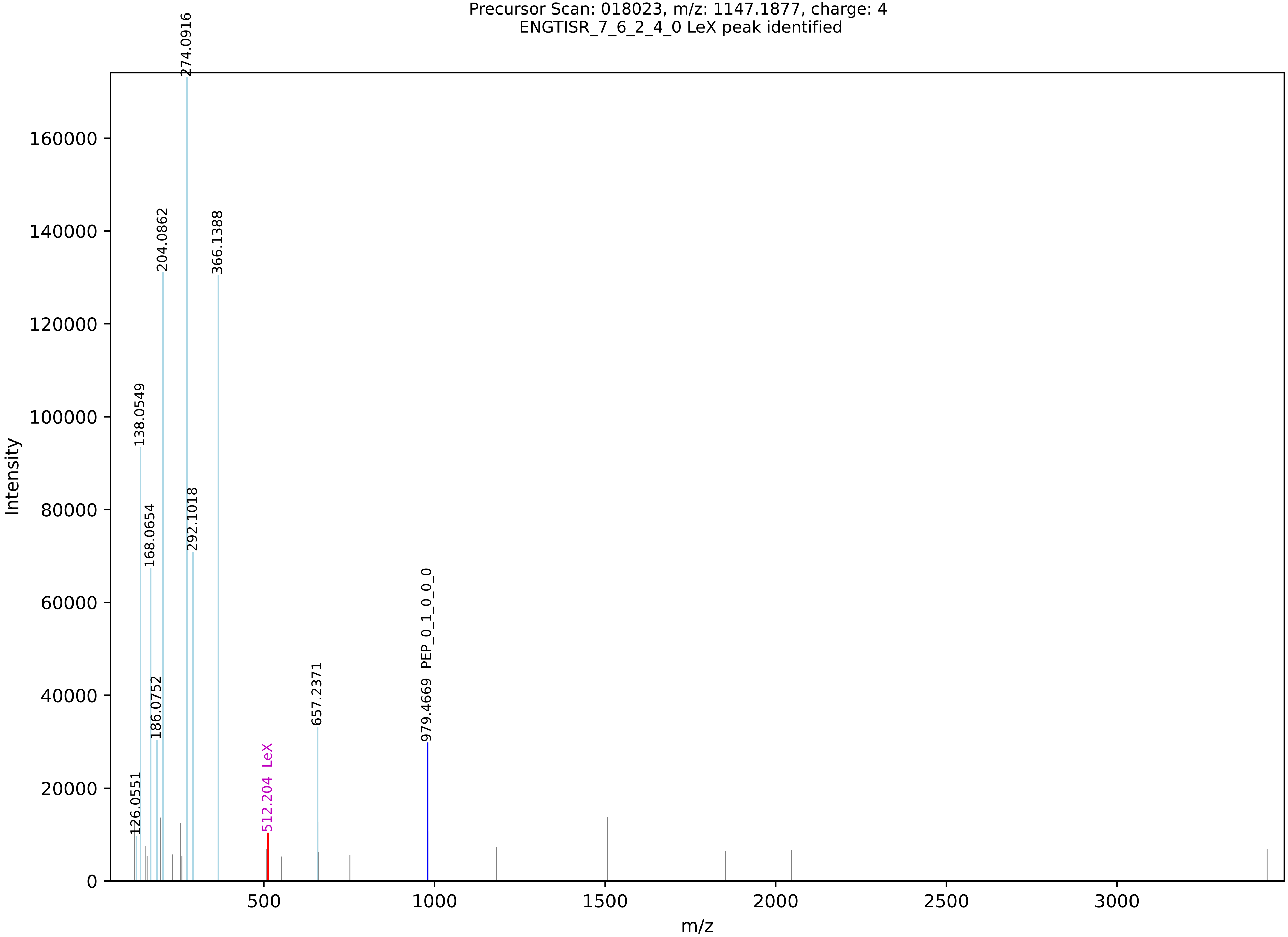

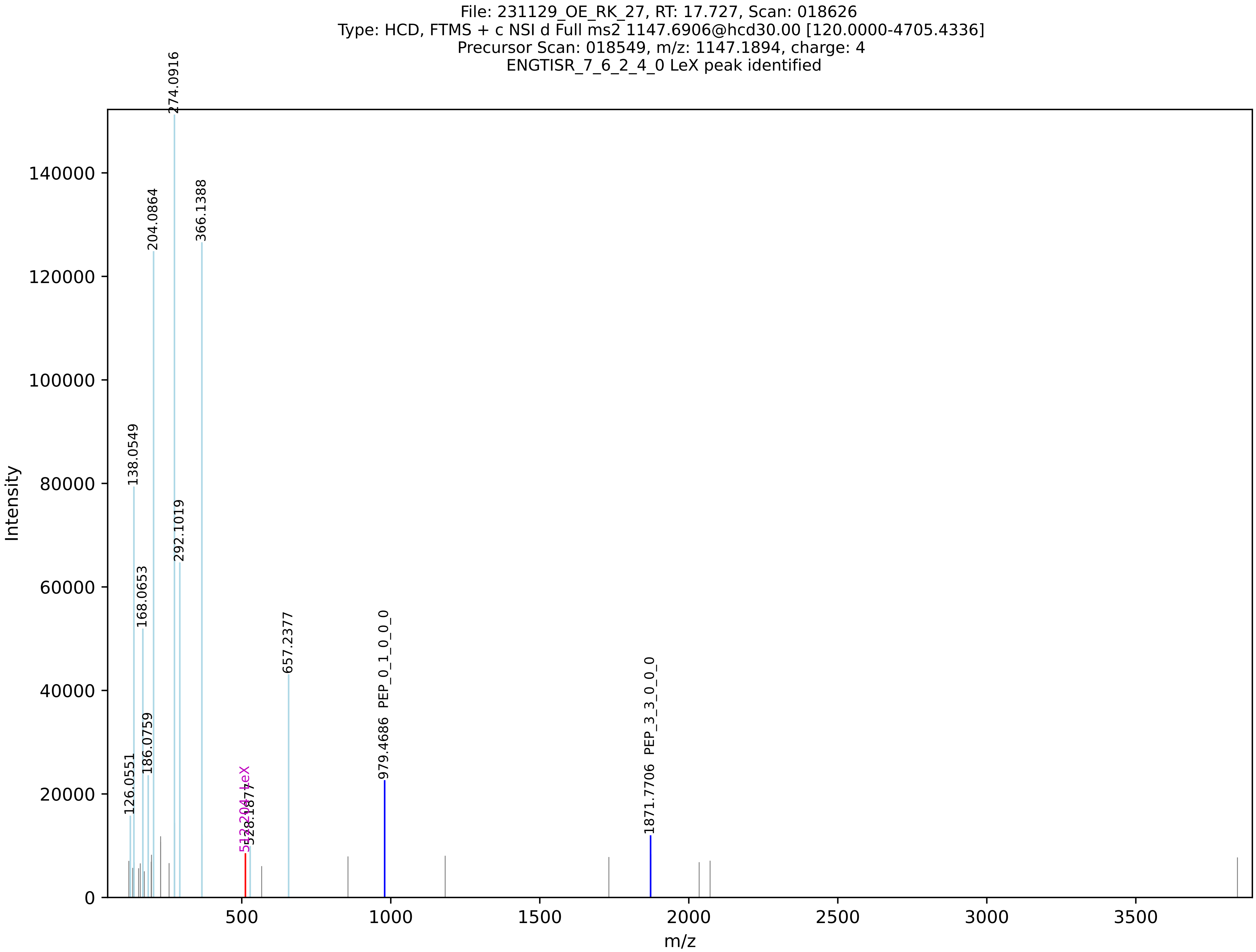
File: 231129_OE_RK_27, RT: 17.727, Scan: 018626
Type: HCD, FTMS + c NSI d Full ms2 1147.6906@hcd30.00 [120.0000-4705.4336]
Precursor Scan: 018549, m/z: 1147.1894, charge: 4
ENGTISR_7_6_2_4_0 LeX peak identified
274.0916
204.0864
366.1388
138.0549
292.1019
168.0653
657.2377
979.4686 PEP_0_1_0_0_0
1871.7706 PEP_3_3_0_0_0
186.0759
126.0551
512.204 LeX
528.1877
Intensity
m/z
0
20000
40000
60000
80000
100000
120000
140000
500
1000
1500
2000
2500
3000
3500

File: 231129_OE_RK_27, RT: 18.379, Scan: 019542
Type: HCD, FTMS + c NSI d Full ms2 1147.6897@hcd30.00 [120.0000-4705.4302]
Precursor Scan: 019461, m/z: 1146.938, charge: 4
ENGTISR_7_6_2_4_0 LeX peak identified

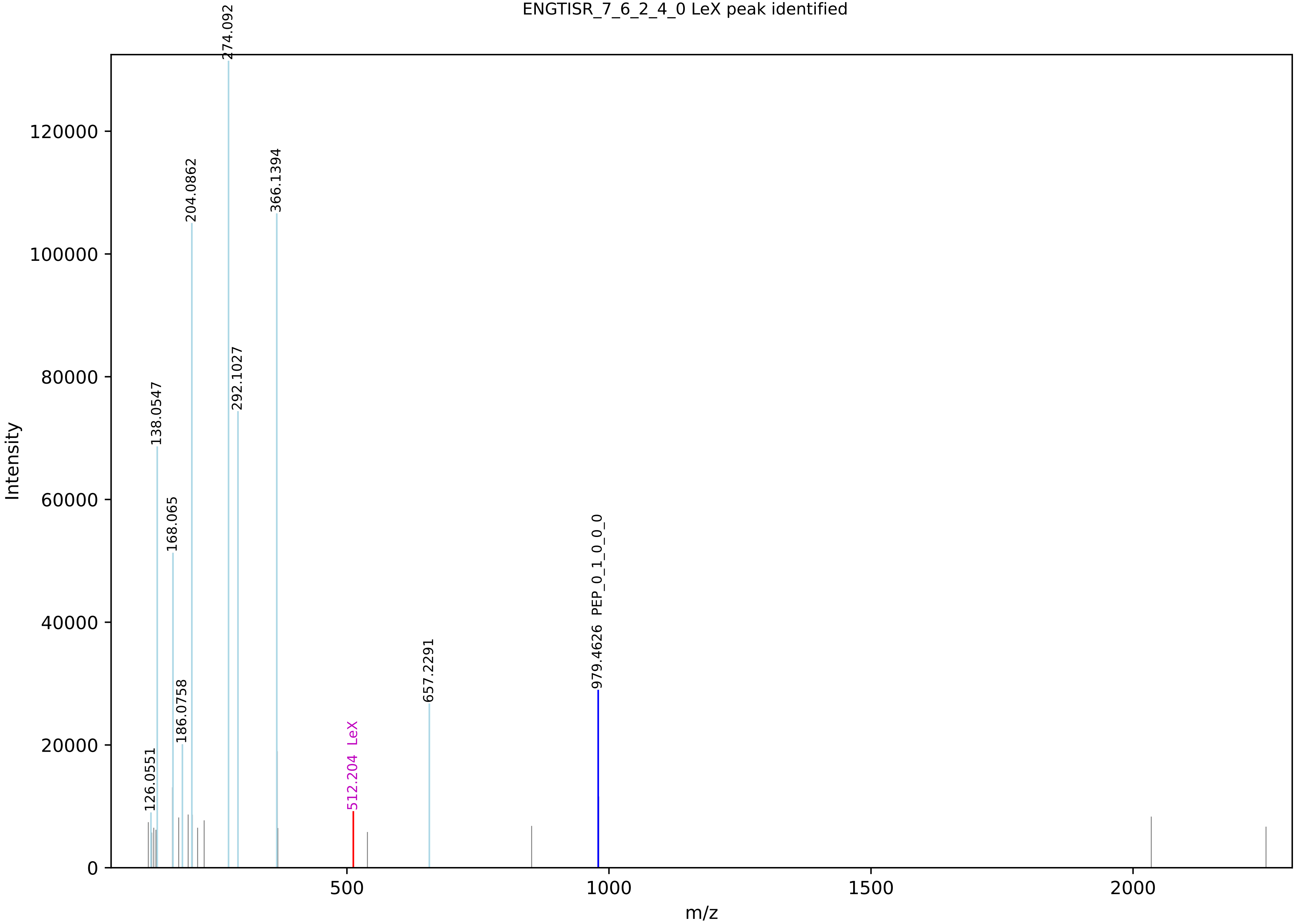

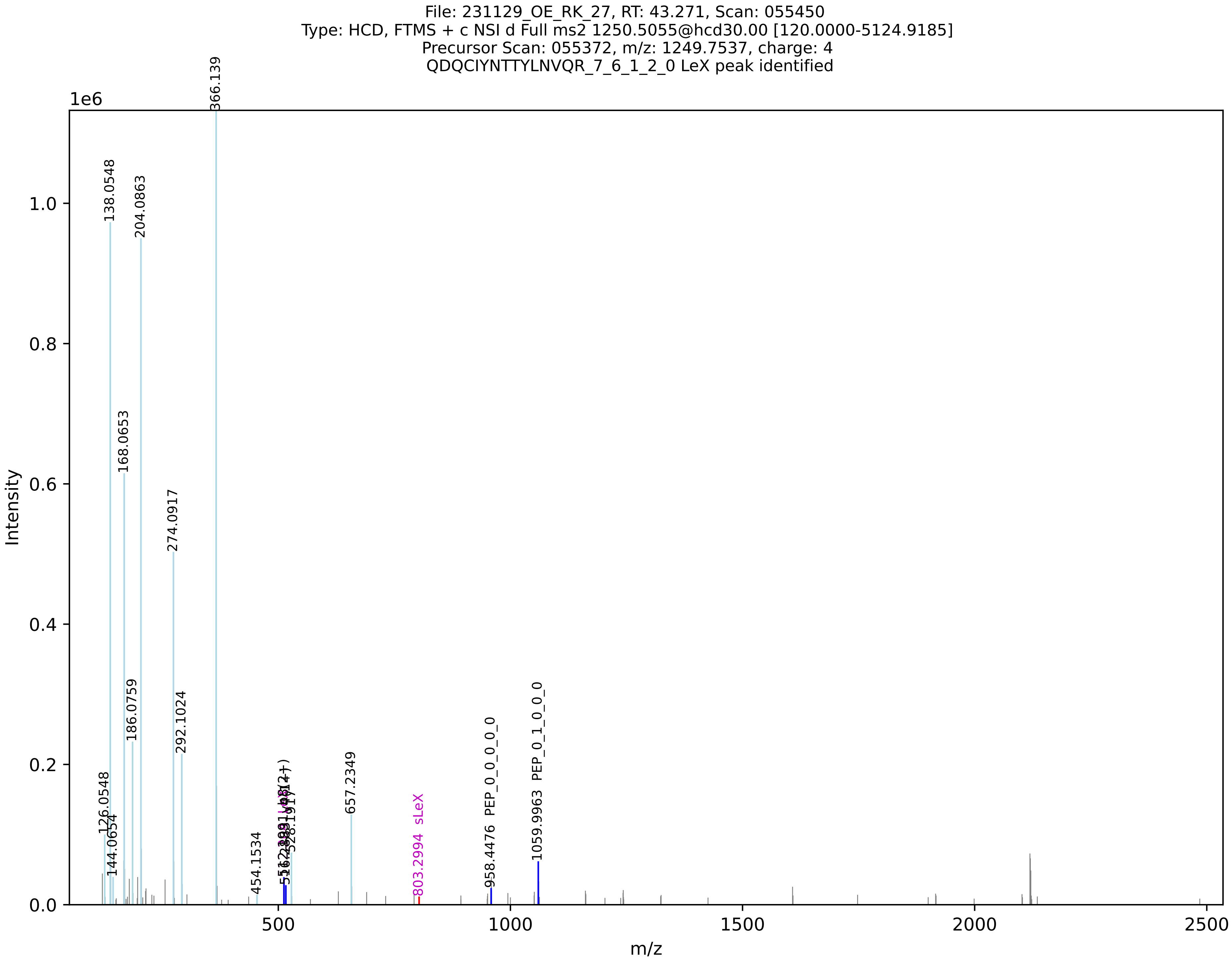
File: 231129_OE_RK_27, RT: 43.271, Scan: 055450
Type: HCD, FTMS + c NSI d Full ms2 1250.5055@hcd30.00 [120.0000-5124.9185]
Precursor Scan: 055372, m/z: 1249.7537, charge: 4
QDQCIYNTTYLNVQR_7_6_1_2_0 LeX peak identified
1e6
Intensity
m/z
366.139
138.0548
204.0863
168.0653
274.0917
186.0759
292.1024
126.0548
144.0654
454.1534
528.1917
657.2349
803.2994 sLeX
958.4476 PEP_0_0_0_0_0
1059.9963 PEP_0_1_0_0_0
0.0
0.2
0.4
0.6
0.8
1.0
500
1000
1500
2000
2500

File: 231129_OE_RK_27, RT: 44.076, Scan: 056613
Type: HCD, FTMS + c NSI d Full ms2 1250.5055@hcd30.00 [120.0000-5124.9180]
Precursor Scan: 056536, m/z: 1250.0043, charge: 4
QDQCIYNTTYLNVQR_7_6_1_2_0 LeX peak identified

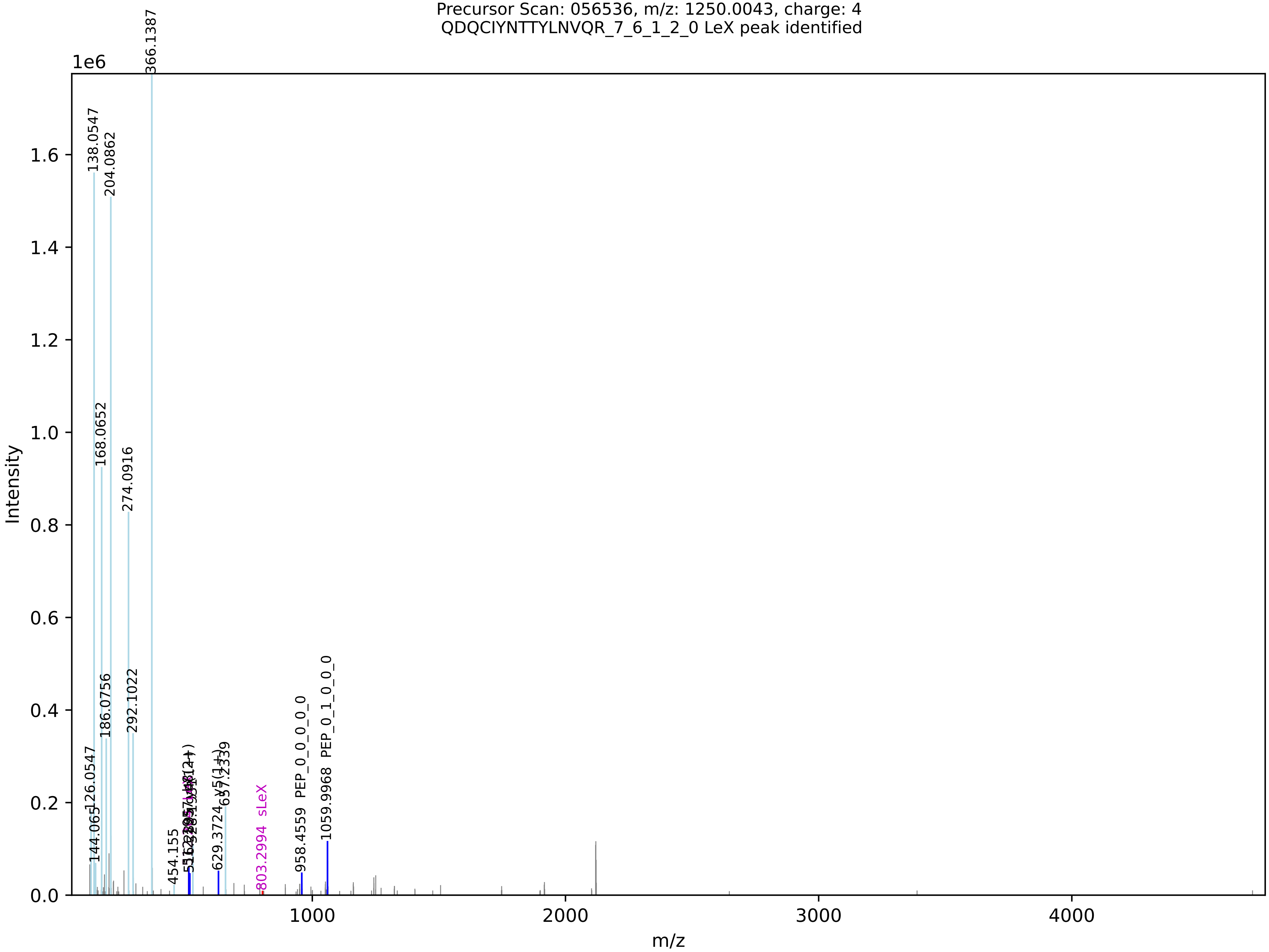

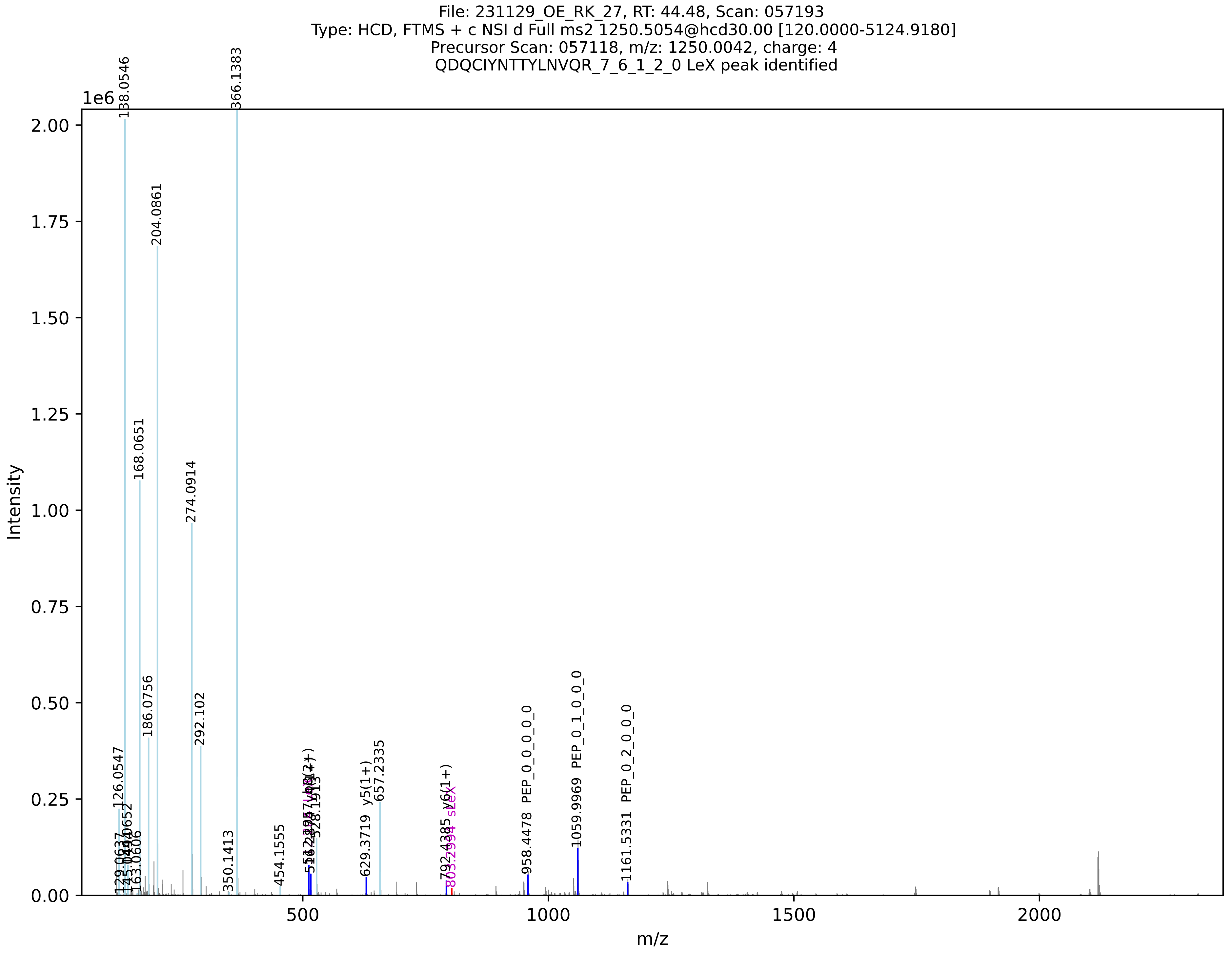

File: 231129_OE_RK_27, RT: 44.48, Scan: 057193
Type: HCD, FTMS + c NSI d Full ms2 1250.5054@hcd30.00 [120.0000-5124.9180]
Precursor Scan: 057118, m/z: 1250.0042, charge: 4
QDQCIYNTTYLNVQR_7_6_1_2_0 LeX peak identified
1e6
Intensity
m/z
138.0546
366.1383
204.0861
168.0651
274.0914
186.0756
292.102
126.0547
350.1413
454.1555
629.3719 y5(1+)
657.2335
792.4385 y6(1+)
803.2994 sLeX
958.4478 PEP_0_0_0_0_0
1059.9969 PEP_0_1_0_0_0
1161.5331 PEP_0_2_0_0_0

File: 231129_OE_RK_27, RT: 44.482, Scan: 057197
Type: HCD, FTMS + c NSI d Full ms2 1323.5297@hcd30.00 [120.0000-5422.8569]
Precursor Scan: 057118, m/z: 1322.5284, charge: 4
QDQCIYNTTYLNVQR_9_8_0_1_0 LeX peak identified

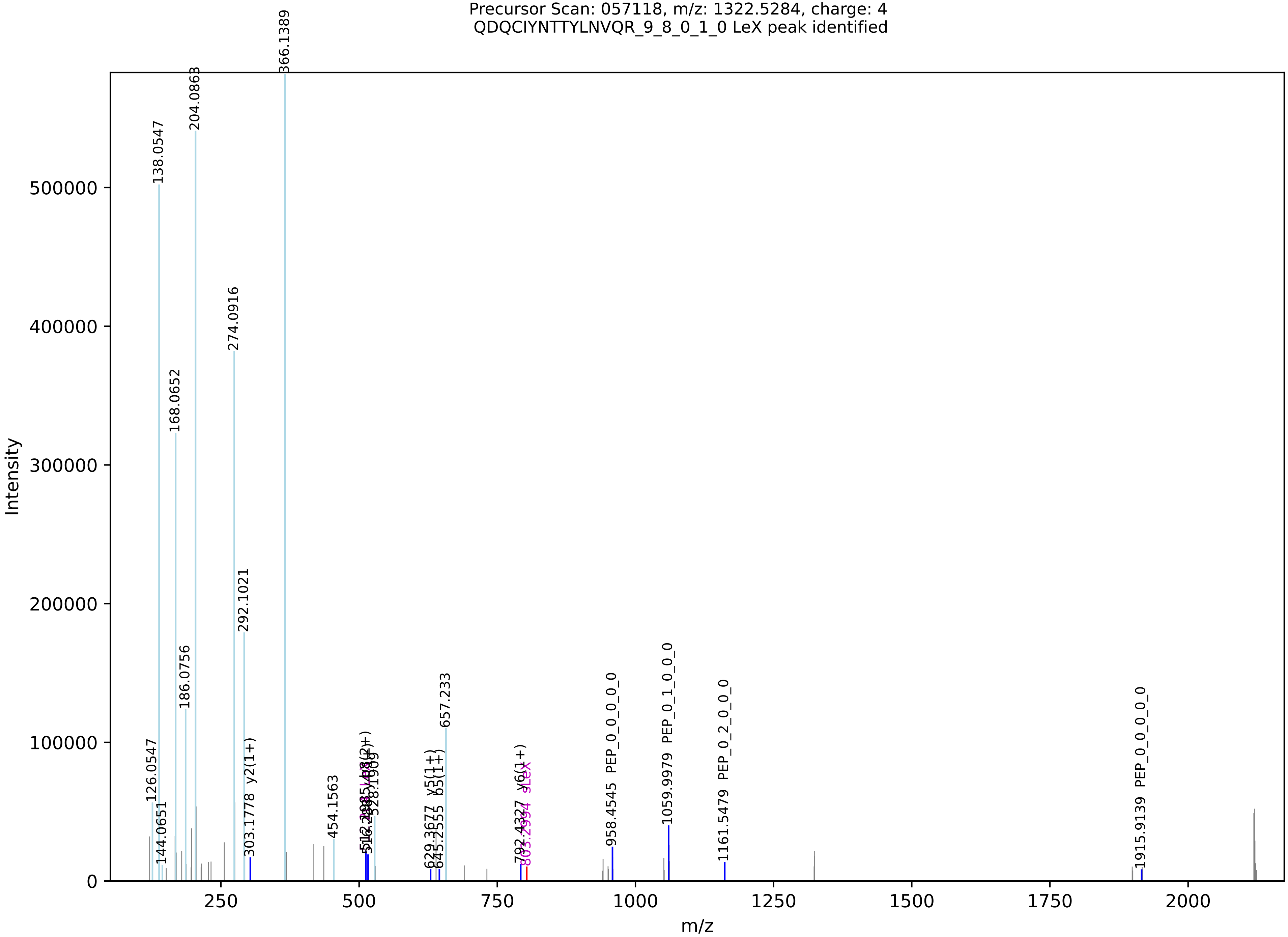

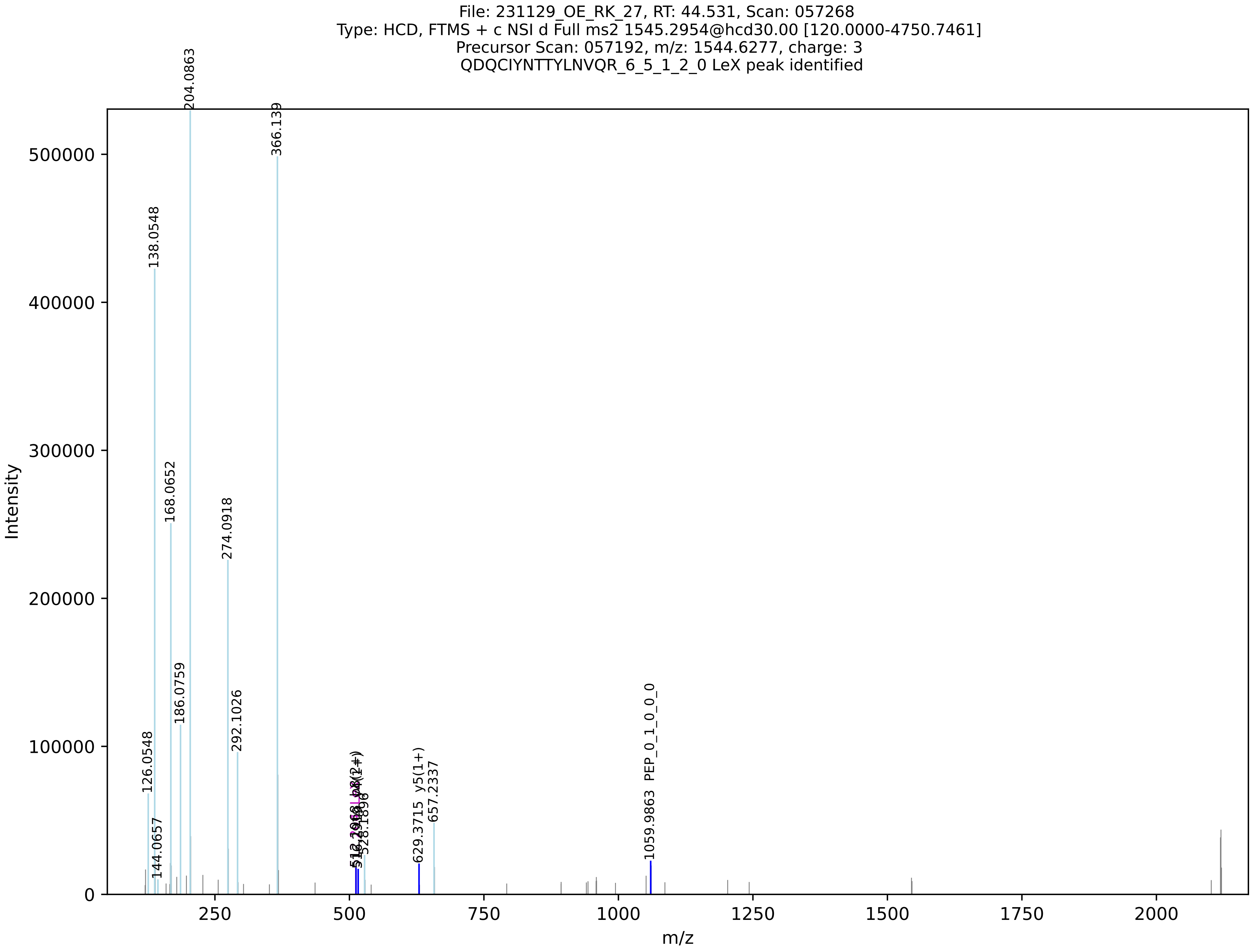
File: 231129_OE_RK_27, RT: 44.531, Scan: 057268
Type: HCD, FTMS + c NSI d Full ms2 1545.2954@hcd30.00 [120.0000-4750.7461]
Precursor Scan: 057192, m/z: 1544.6277, charge: 3
QDQCIYNTTYLNVQR_6_5_1_2_0 LeX peak identified
Intensity
m/z
204.0863
366.139
138.0548
168.0652
274.0918
186.0759
292.1026
126.0548
144.0657
528.1896
629.3715 y5(1+)
657.2337
1059.9863 PEP_0_1_0_0_0
0
100000
200000
300000
400000
500000
250
500
750
1000
1250
1500
1750
2000

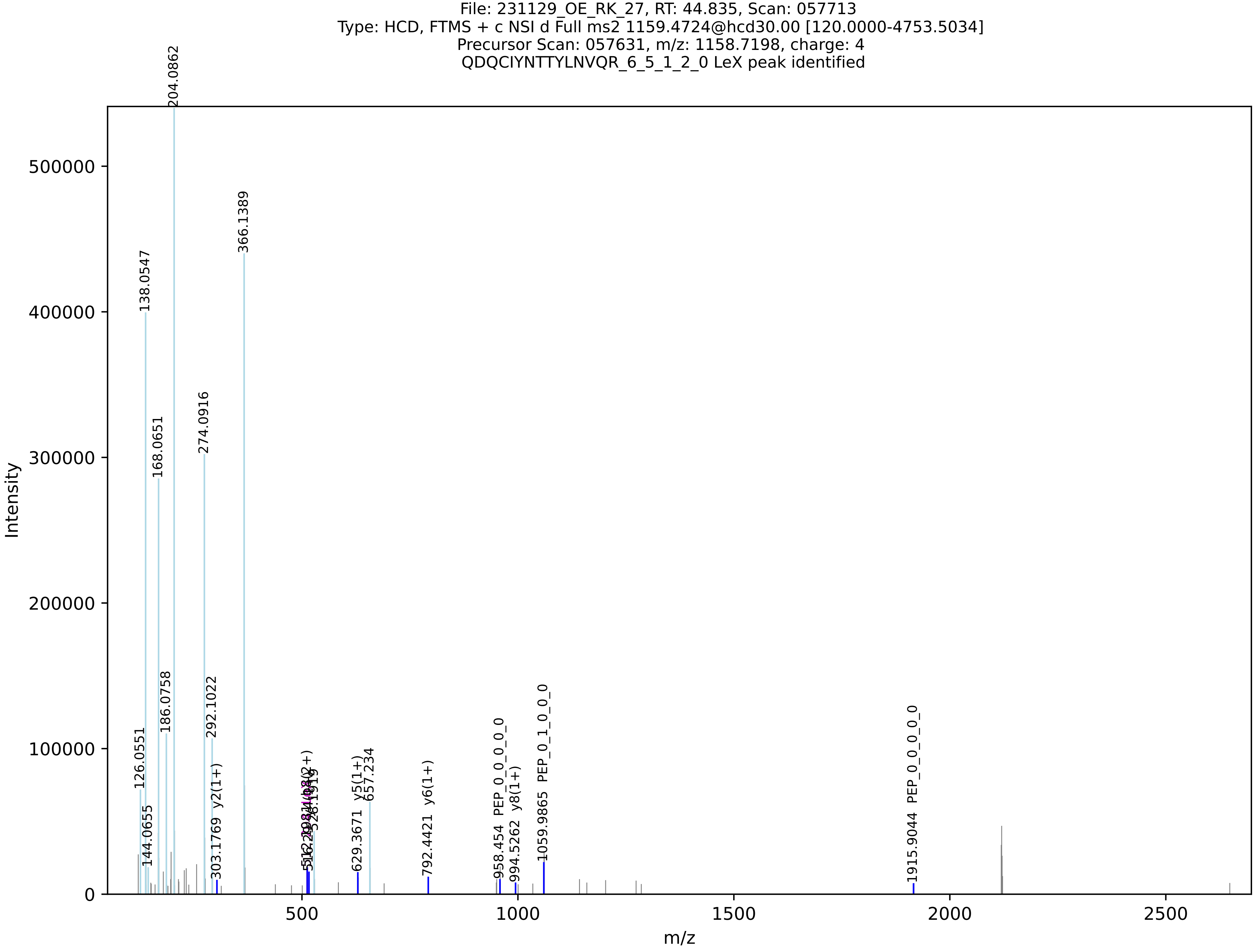
File: 231129_OE_RK_27, RT: 44.835, Scan: 057713
Type: HCD, FTMS + c NSI d Full ms2 1159.4724@hcd30.00 [120.0000-4753.5034]
Precursor Scan: 057631, m/z: 1158.7198, charge: 4
QDQCIYNTTYLNVQR_6_5_1_2_0 LeX peak identified
Intensity
m/z
204.0862
366.1389
138.0547
168.0651
274.0916
186.0758
292.1022
126.0551
144.0655
303.1769 y2(1+)
629.3671 y5(1+)
657.234
792.4421 y6(1+)
958.454 PEP_0_0_0_0_0
994.5262 y8(1+)
1059.9865 PEP_0_1_0_0_0
1915.9044 PEP_0_0_0_0_0

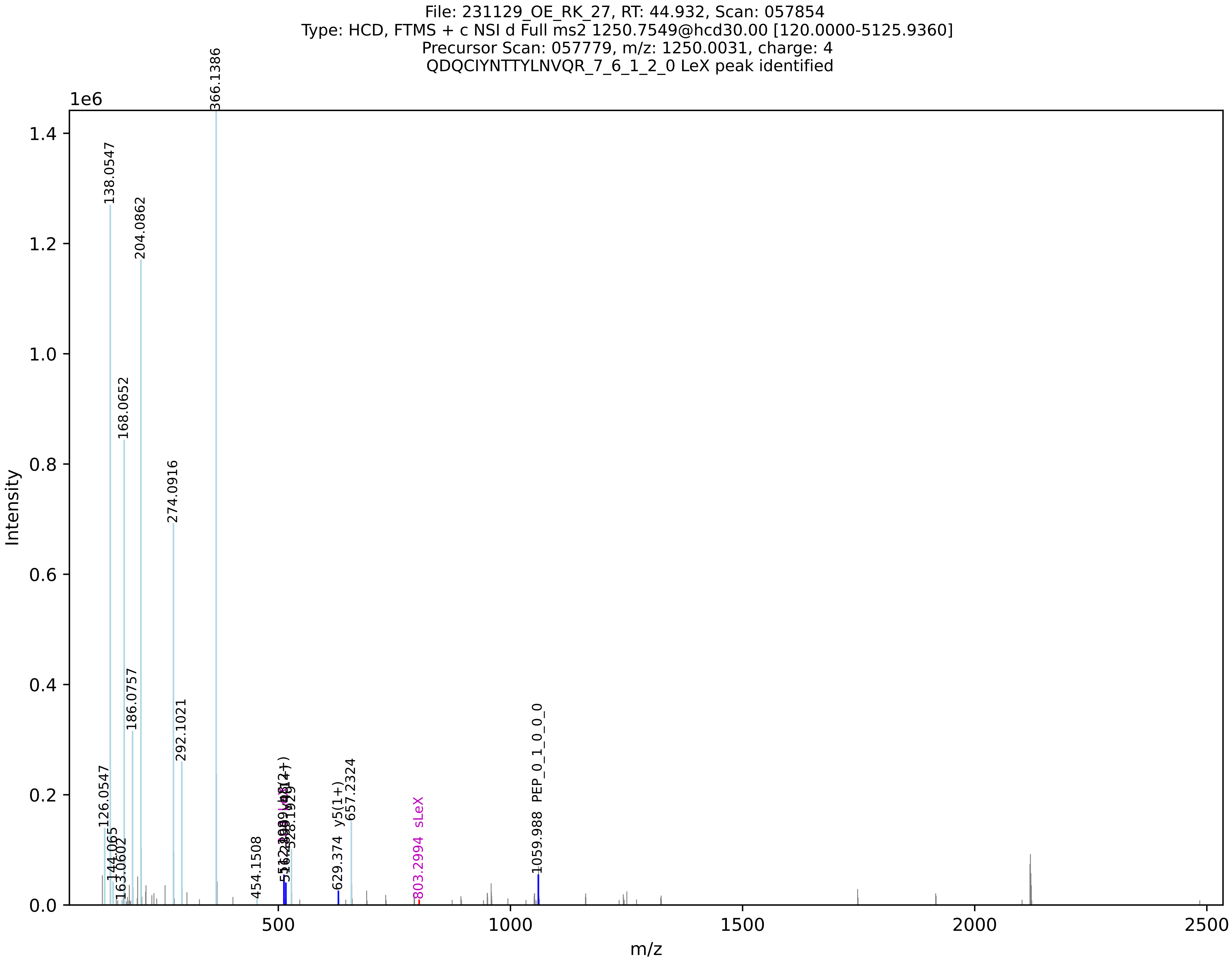

File: 231129_OE_RK_27, RT: 44.932, Scan: 057854
Type: HCD, FTMS + c NSI d Full ms2 1250.7549@hcd30.00 [120.0000-5125.9360]
Precursor Scan: 057779, m/z: 1250.0031, charge: 4
QDQCIYNTTYLNVQR_7_6_1_2_0 LeX peak identified
1e6
Intensity
m/z
366.1386
138.0547
204.0862
168.0652
274.0916
186.0757
292.1021
126.0547
144.065
163.0602
454.1508
528.1929
629.374 y5(1+)
657.2324
803.2994 sLeX
1059.988 PEP_0_1_0_0_0

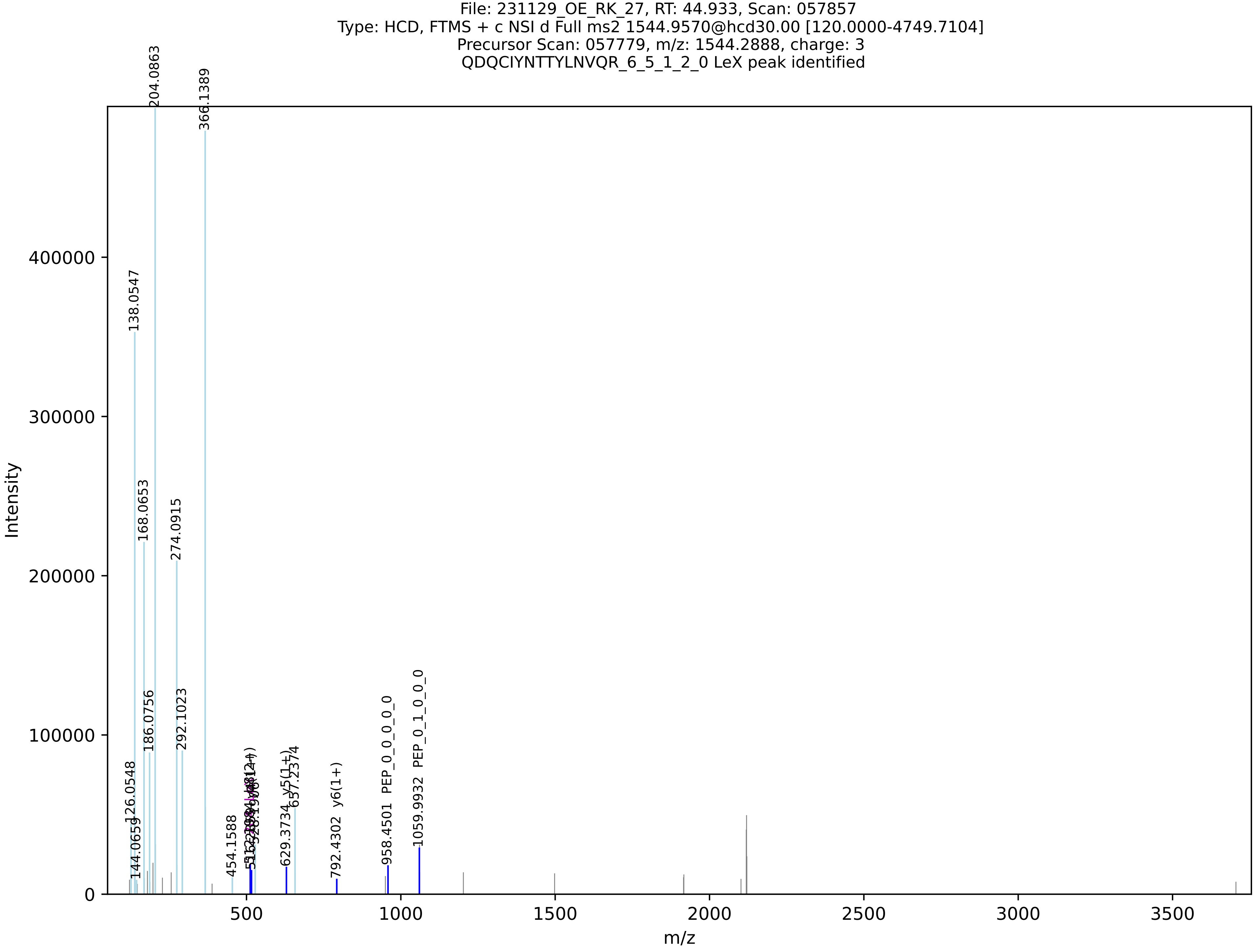

File: 231129_OE_RK_27, RT: 44.933, Scan: 057857
Type: HCD, FTMS + c NSI d Full ms2 1544.9570@hcd30.00 [120.0000-4749.7104]
Precursor Scan: 057779, m/z: 1544.2888, charge: 3
QDQCIYNTTYLNVQR_6_5_1_2_0 LeX peak identified
Intensity
m/z
204.0863
366.1389
138.0547
168.0653
274.0915
186.0756
292.1023
126.0548
144.0659
454.1588
629.3734 y5(1+)
657.2374
792.4302 y6(1+)
958.4501 PEP_0_0_0_0_0
1059.9932 PEP_0_1_0_0_0

File: 231129_OE_RK_27, RT: 45.131, Scan: 058145
Type: HCD, FTMS + c NSI d Full ms2 1250.5042@hcd30.00 [120.0000-5124.9131]
Precursor Scan: 058074, m/z: 1250.0031, charge: 4
QDQCIYNTTYLNVQR_7_6_1_2_0 LeX peak identified

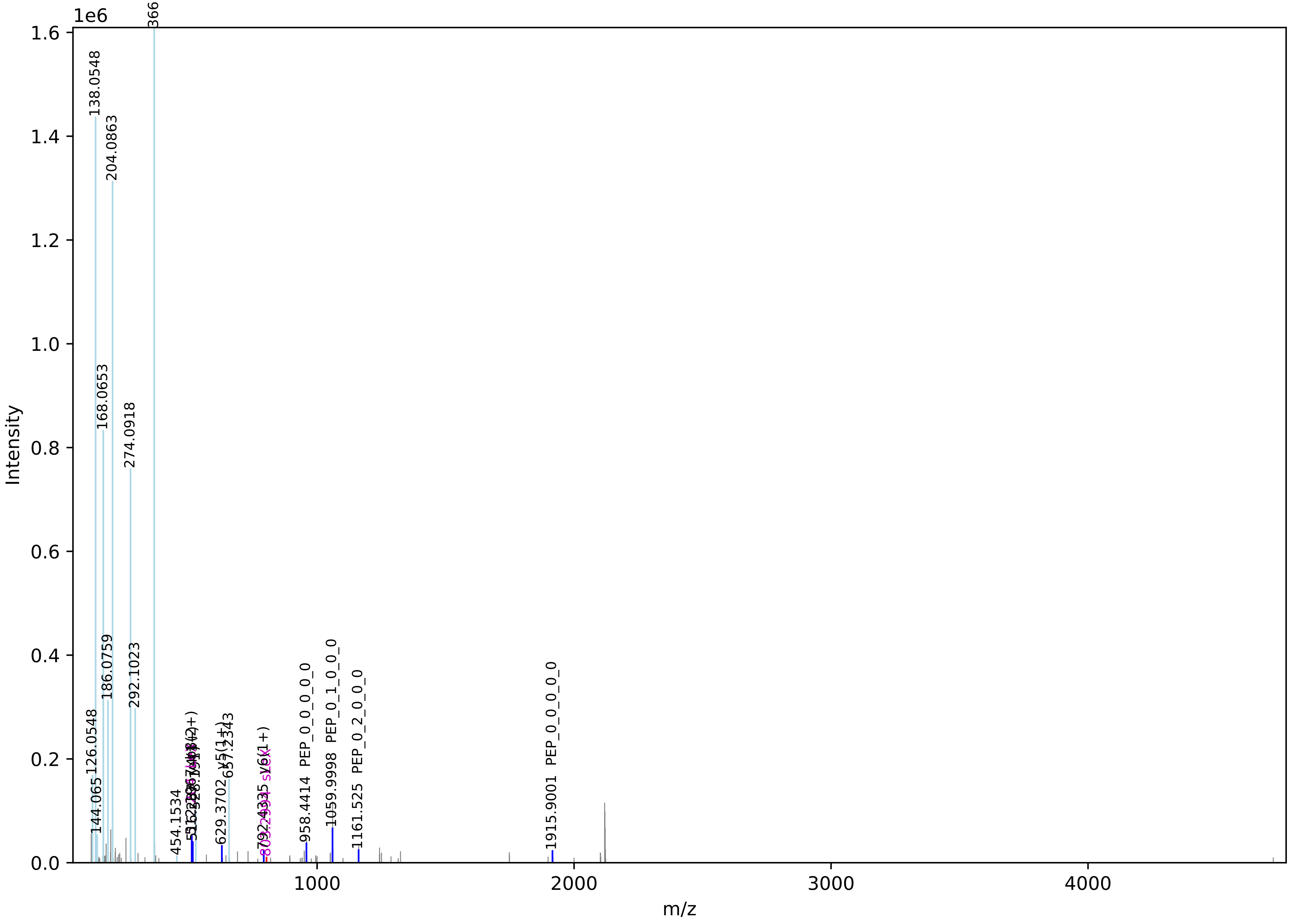

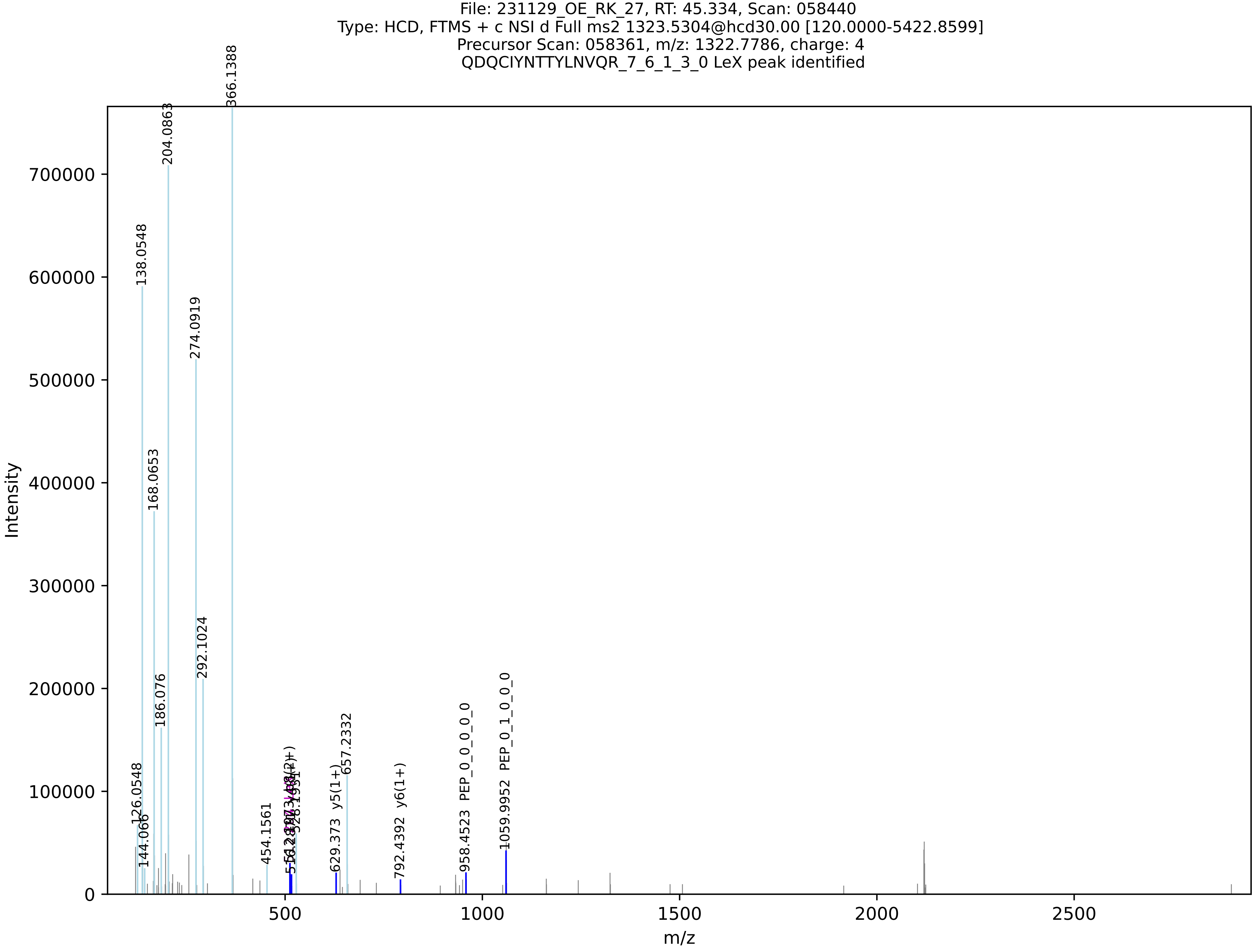

File: 231129_OE_RK_27, RT: 45.334, Scan: 058440
Type: HCD, FTMS + c NSI d Full ms2 1323.5304@hcd30.00 [120.0000-5422.8599]
Precursor Scan: 058361, m/z: 1322.7786, charge: 4
QDQCIYNTTYLNVQR_7_6_1_3_0 LeX peak identified
Intensity
m/z
366.1388
204.0863
138.0548
274.0919
168.0653
292.1024
186.076
126.0548
144.066
454.1561
657.2332
629.373 y5(1+)
792.4392 y6(1+)
958.4523 PEP_0_0_0_0_0_
1059.9952 PEP_0_1_0_0_0_

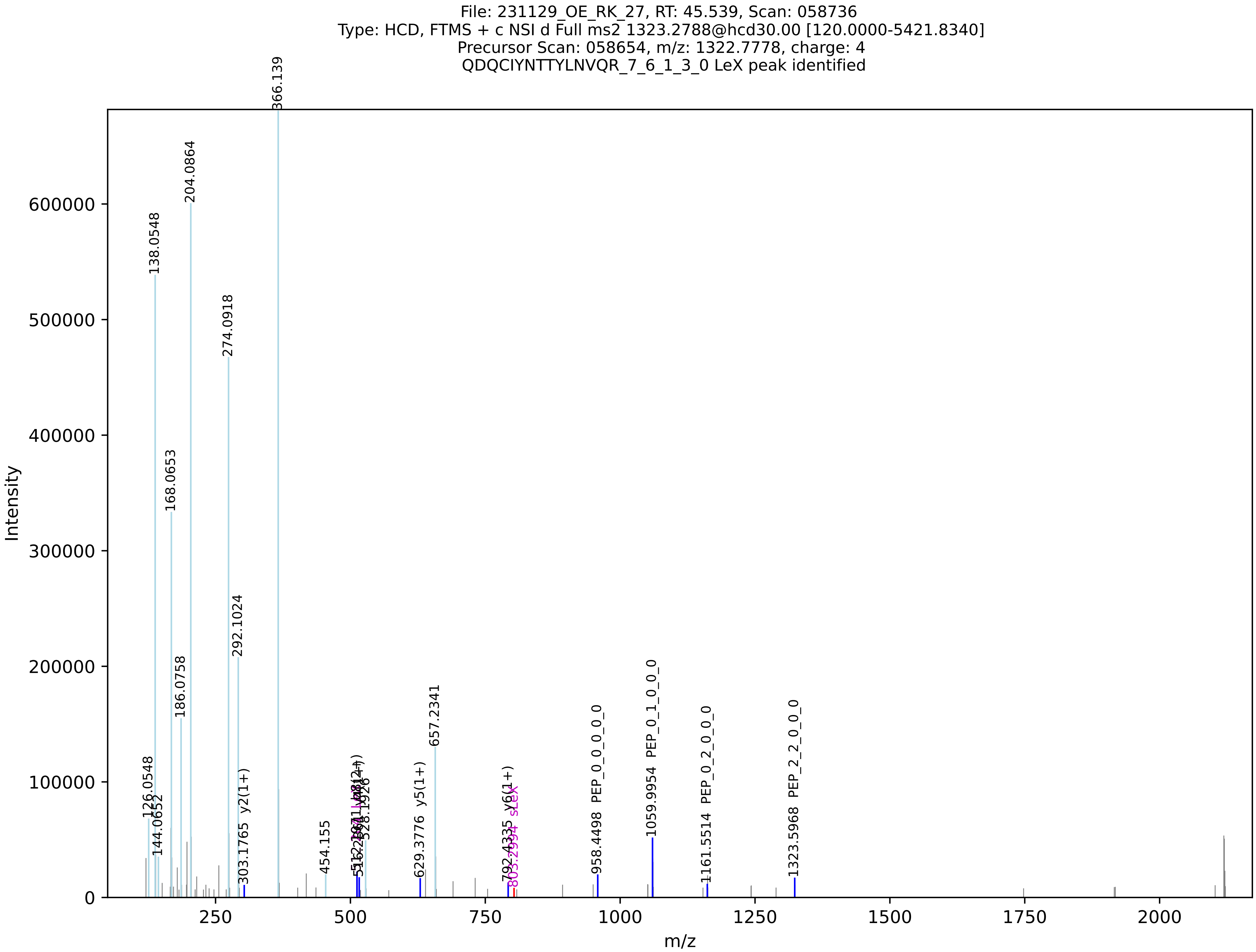
File: 231129_OE_RK_27, RT: 45.539, Scan: 058736
Type: HCD, FTMS + c NSI d Full ms2 1323.2788@hcd30.00 [120.0000-5421.8340]
Precursor Scan: 058654, m/z: 1322.7778, charge: 4
QDQCIYNTTYLNVQR_7_6_1_3_0 LeX peak identified
Intensity
m/z
366.139
204.0864
138.0548
274.0918
168.0653
292.1024
186.0758
126.0548
144.0652
303.1765 y2(1+)
454.155
528.1926
657.2341
629.3776 y5(1+)
792.4335 y6(1+)
803.2954 sLeX
958.4498 PEP_0_0_0_0_0
1059.9954 PEP_0_1_0_0_0
1161.5514 PEP_0_2_0_0_0
1323.5968 PEP_2_2_0_0_0

File: 231129_OE_RK_27, RT: 45.891, Scan: 059248
Type: HCD, FTMS + c NSI d Full ms2 1101.2096@hcd30.00 [120.0000-4515.7910]
Precursor Scan: 059166, m/z: 1100.4592, charge: 4
GLTFQQNASSMCVPDQDTAIR_5_4_1_1_0 LeX peak identified

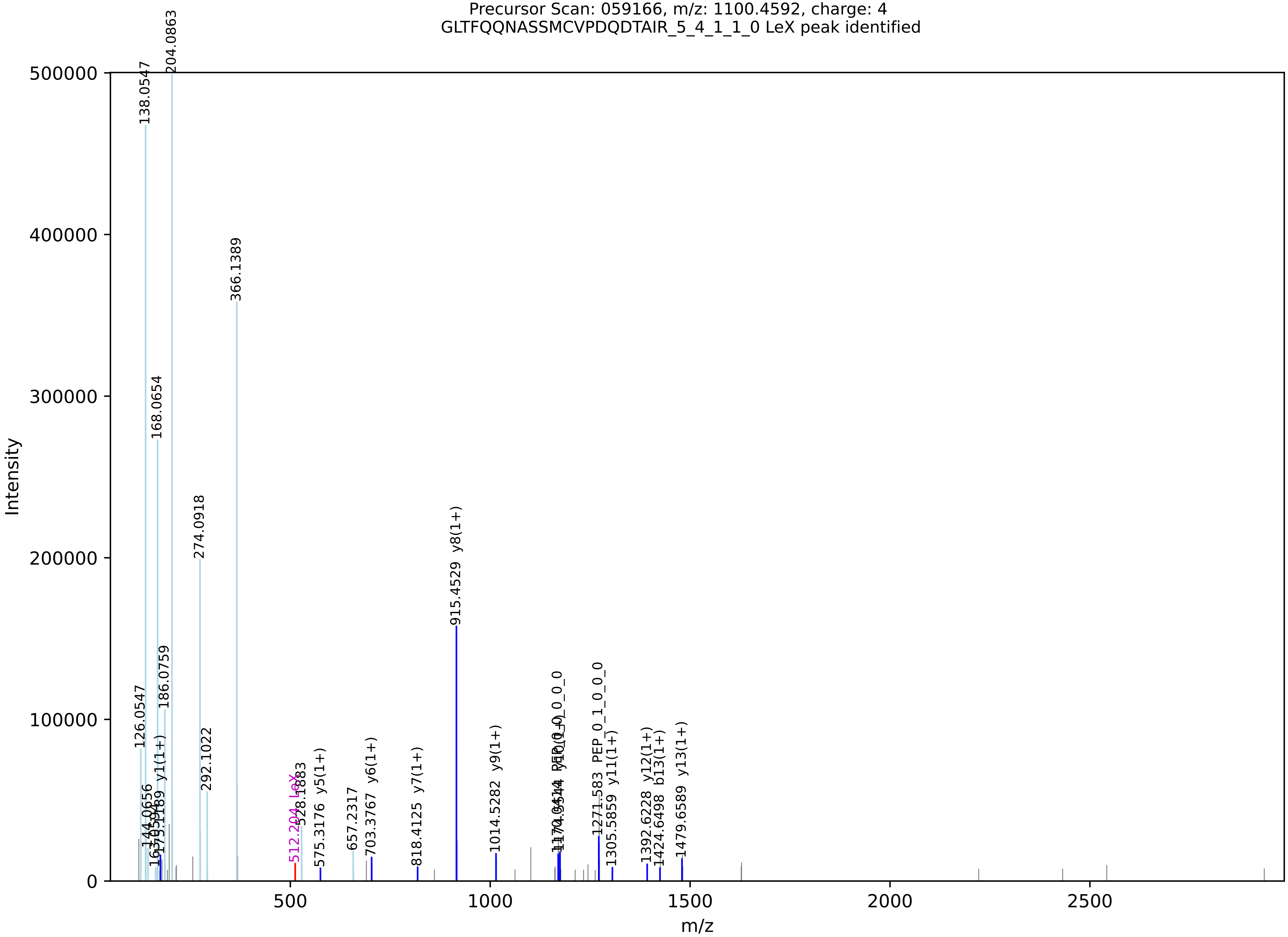

File: 231129_OE_RK_27, RT: 46.801, Scan: 060563
Type: HCD, FTMS + c NSI d Full ms2 1359.5411@hcd30.00 [120.0000-5569.7842]
Precursor Scan: 060478, m/z: 1359.04, charge: 4
QDQCIYNTTYLNVQR_7_6_0_4_0 LeX peak identified

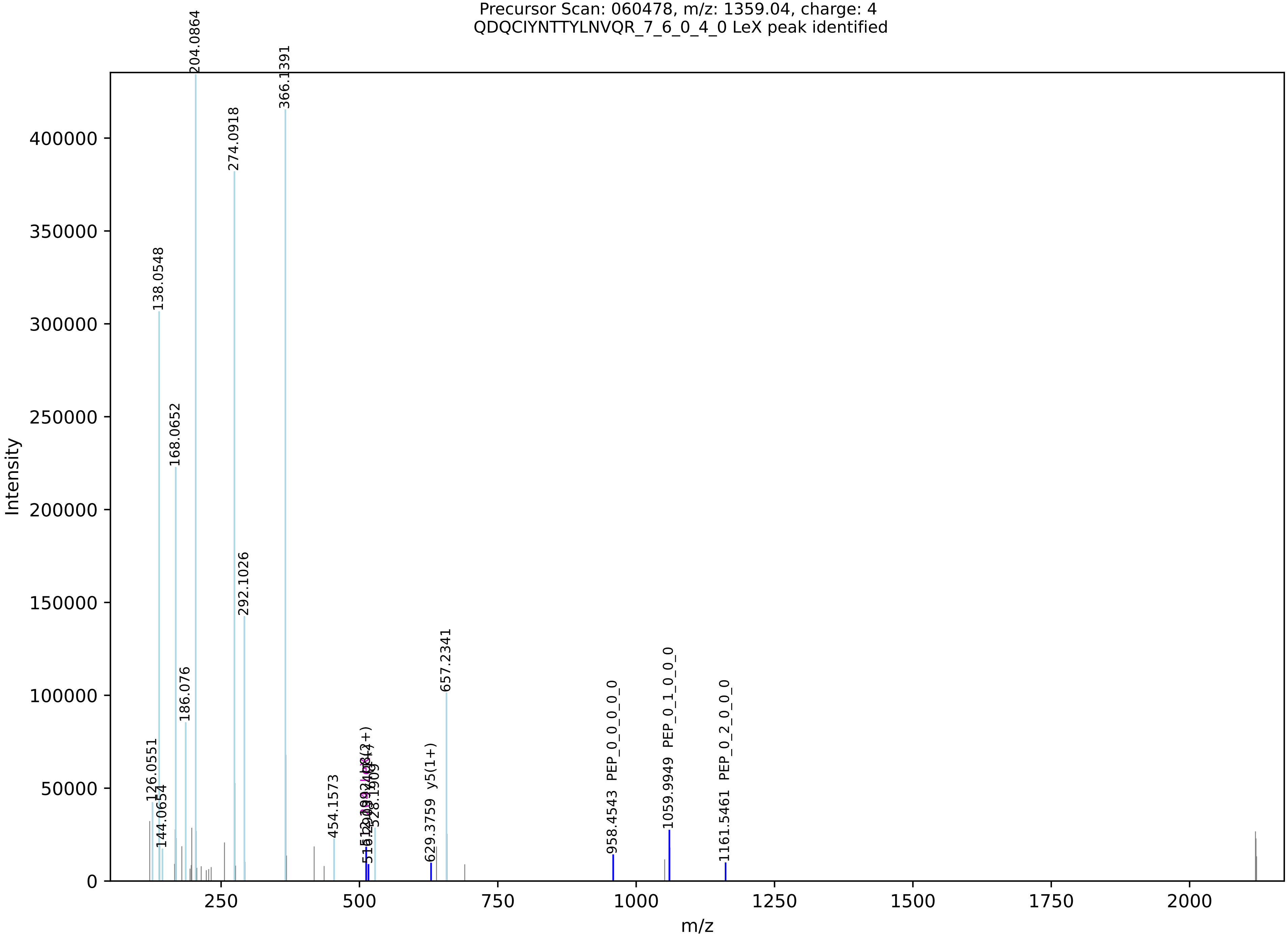

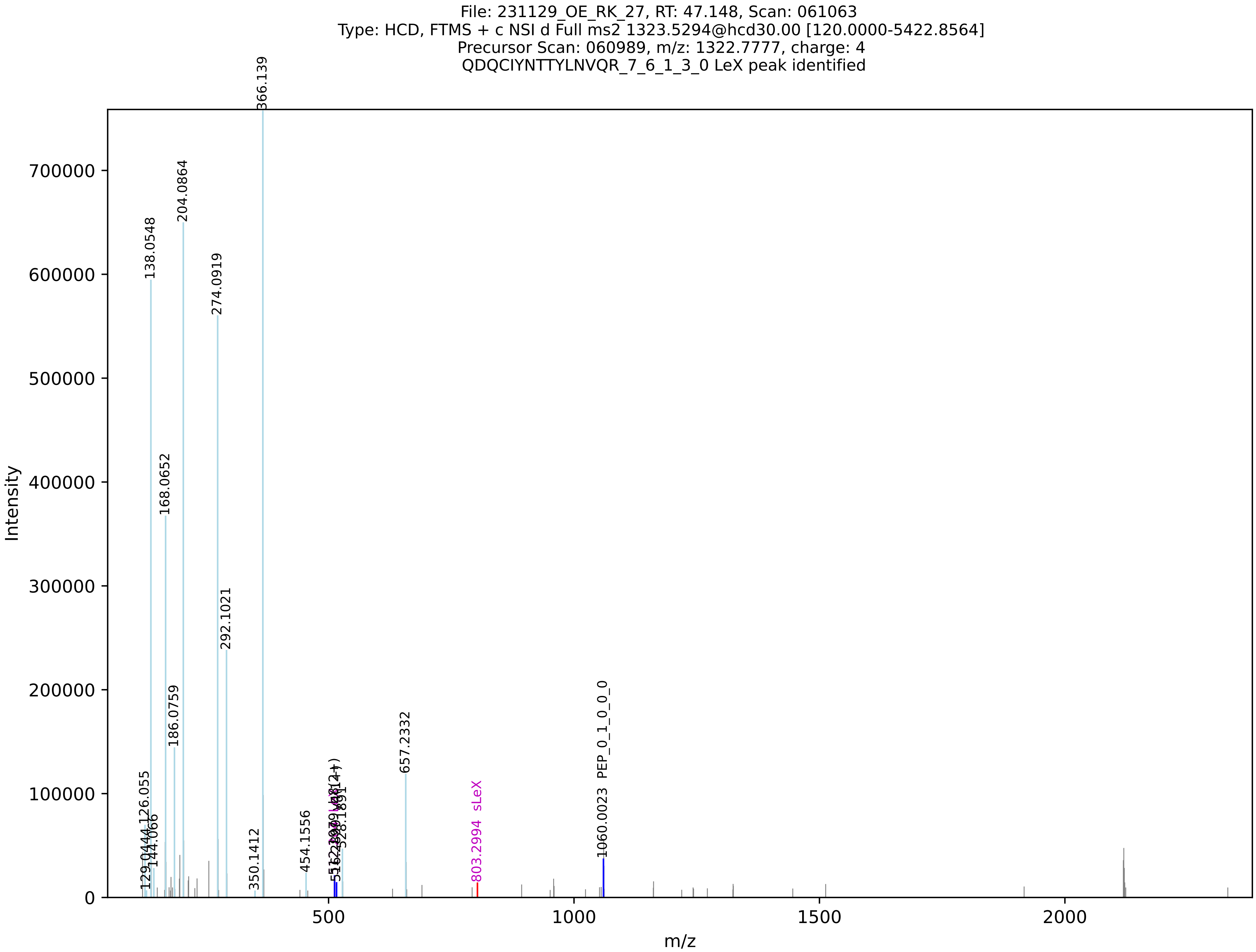
File: 231129_OE_RK_27, RT: 47.148, Scan: 061063
Type: HCD, FTMS + c NSI d Full ms2 1323.5294@hcd30.00 [120.0000-5422.8564]
Precursor Scan: 060989, m/z: 1322.7777, charge: 4
QDQCIYNTTYLNVQR_7_6_1_3_0 LeX peak identified
Intensity
m/z
366.139
204.0864
138.0548
274.0919
168.0652
292.1021
186.0759
126.055
144.066
129.0444
350.1412
454.1556
528.1891
657.2332
803.2994 sLeX
1060.0023 PEP_0_1_0_0_0
0
100000
200000
300000
400000
500000
600000
700000
500
1000
1500
2000

File: 231129_OE_RK_27, RT: 47.203, Scan: 061143
Type: HCD, FTMS + c NSI d Full ms2 1359.7921@hcd30.00 [120.0000-5570.8076]
Precursor Scan: 061060, m/z: 1359.0403, charge: 4
QDQCIYNTTYLNVQR_7_6_0_4_0 LeX peak identified

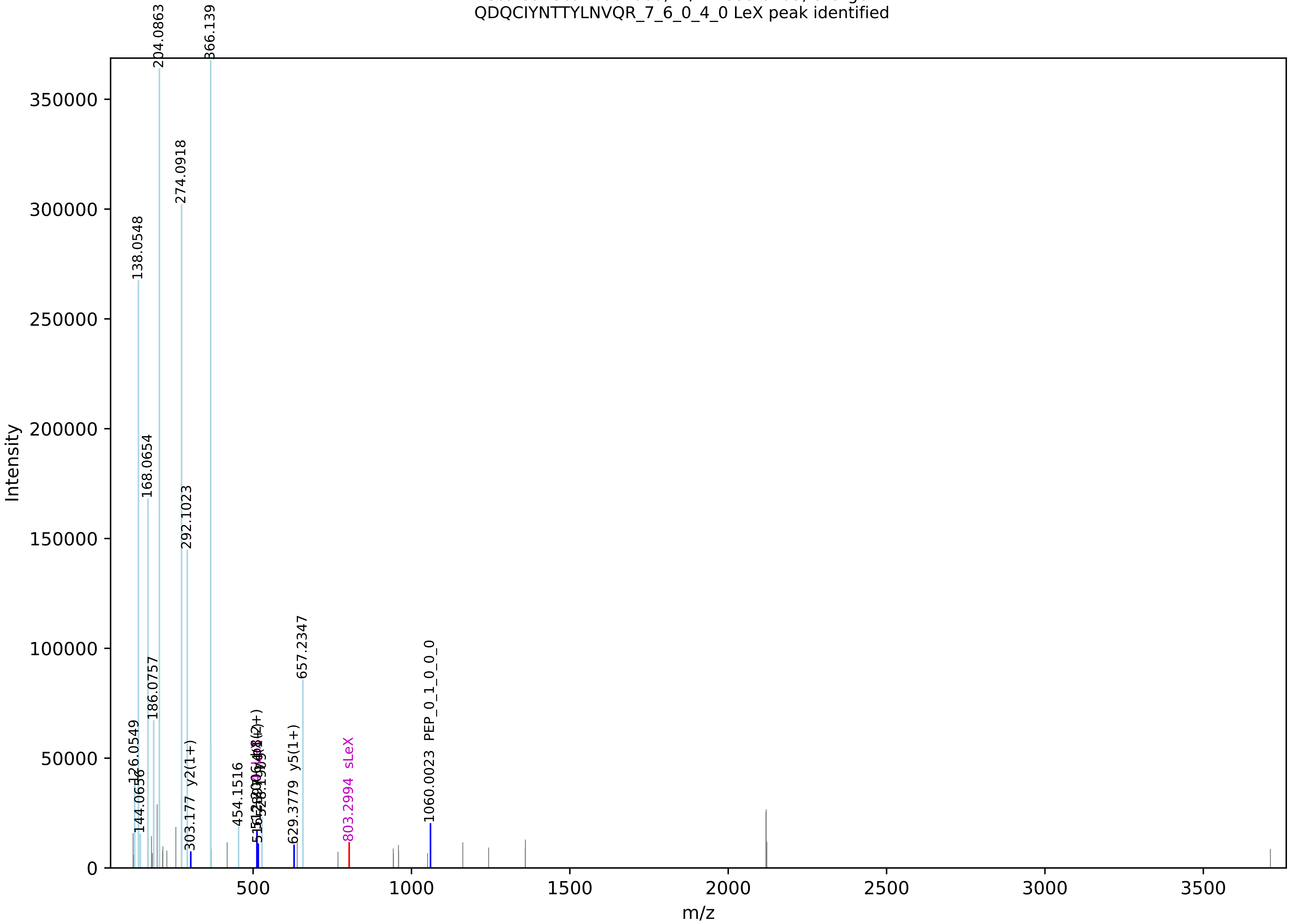

File: 231129_OE_RK_27, RT: 47.782, Scan: 061982
Type: HCD, FTMS + c NSI d Full ms2 1360.0426@hcd30.00 [120.0000-5571.8296]
Precursor Scan: 061894, m/z: 1359.0402, charge: 4
QDQCIYNTTYLNVQR_7_6_0_4_0 LeX peak identified

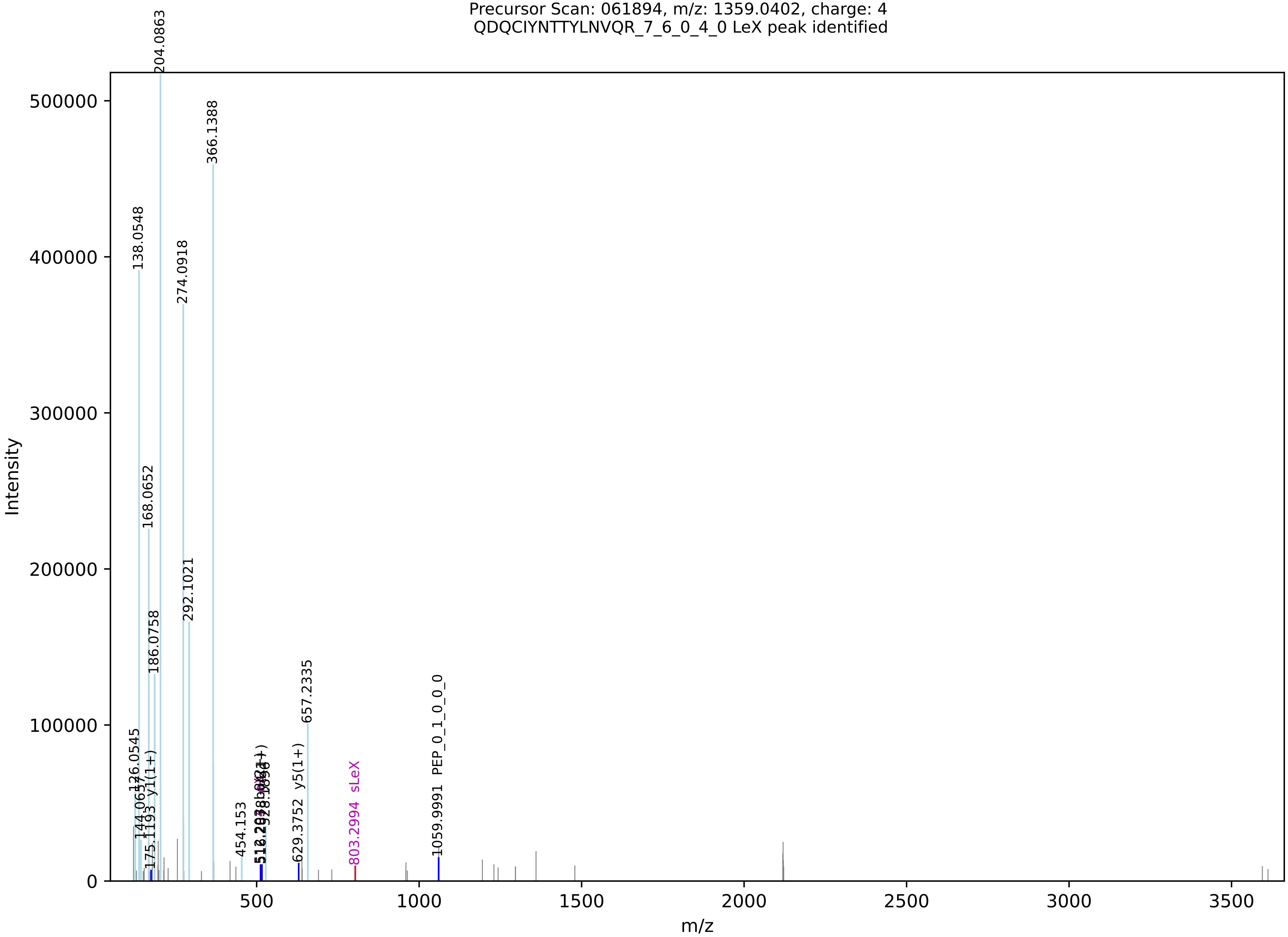

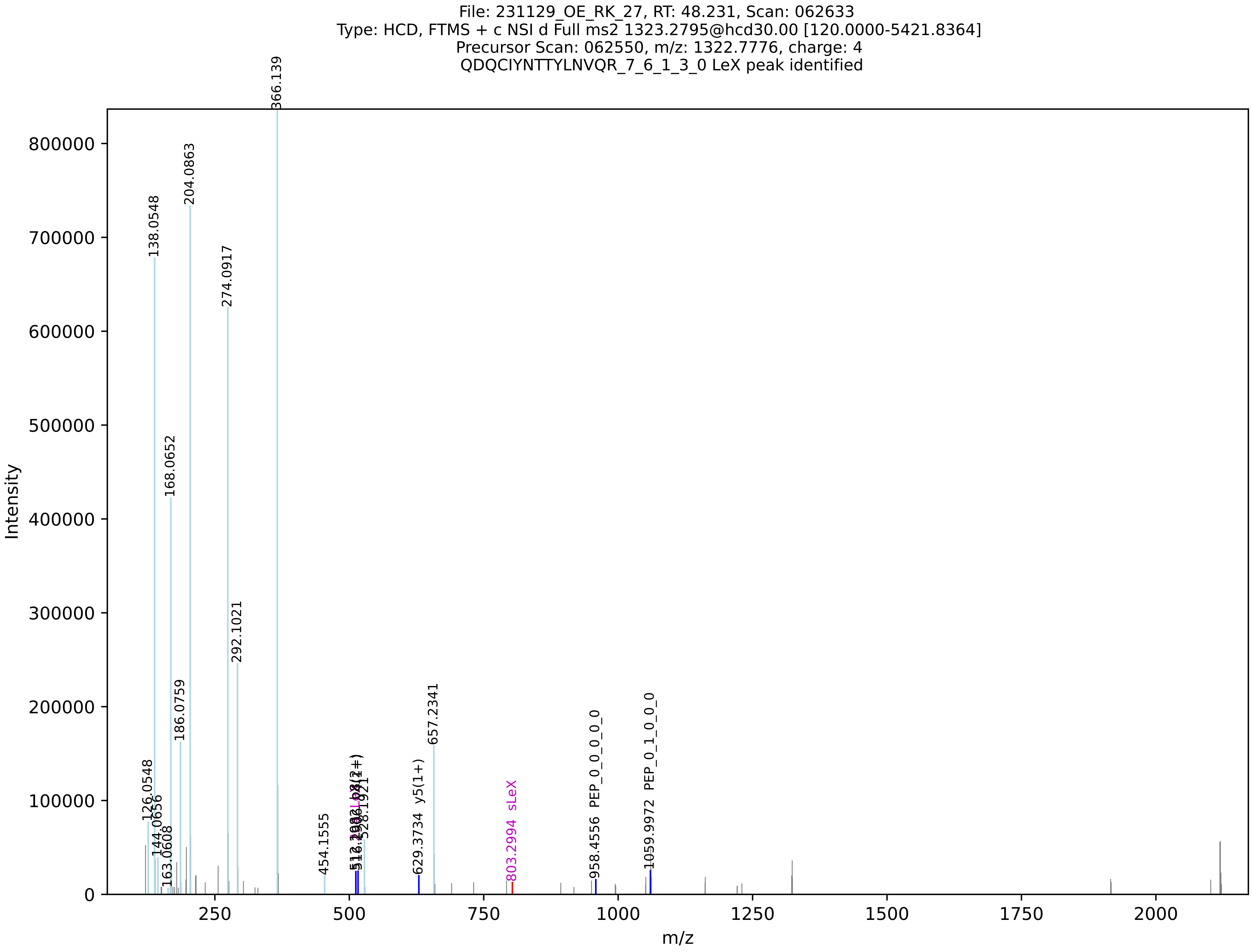

File: 231129_OE_RK_27, RT: 48.231, Scan: 062633
Type: HCD, FTMS + c NSI d Full ms2 1323.2795@hcd30.00 [120.0000-5421.8364]
Precursor Scan: 062550, m/z: 1322.7776, charge: 4
QDQCIYNTTYLNVQR_7_6_1_3_0 LeX peak identified
Intensity
m/z
366.139
204.0863
138.0548
274.0917
168.0652
292.1021
186.0759
126.0548
144.0656
163.0608
454.1555
528.1921
629.3734 y5(1+)
657.2341
803.2994 sLeX
958.4556 PEP_0_0_0_0_0_
1059.9972 PEP_0_1_0_0_0_

File: 231129_OE_RK_27, RT: 48.785, Scan: 063440
Type: HCD, FTMS + c NSI d Full ms2 1359.7928@hcd30.00 [120.0000-5570.8105]
Precursor Scan: 063354, m/z: 1359.0397, charge: 4
QDQCIYNTTYLNVQR_7_6_0_4_0 LeX peak identified

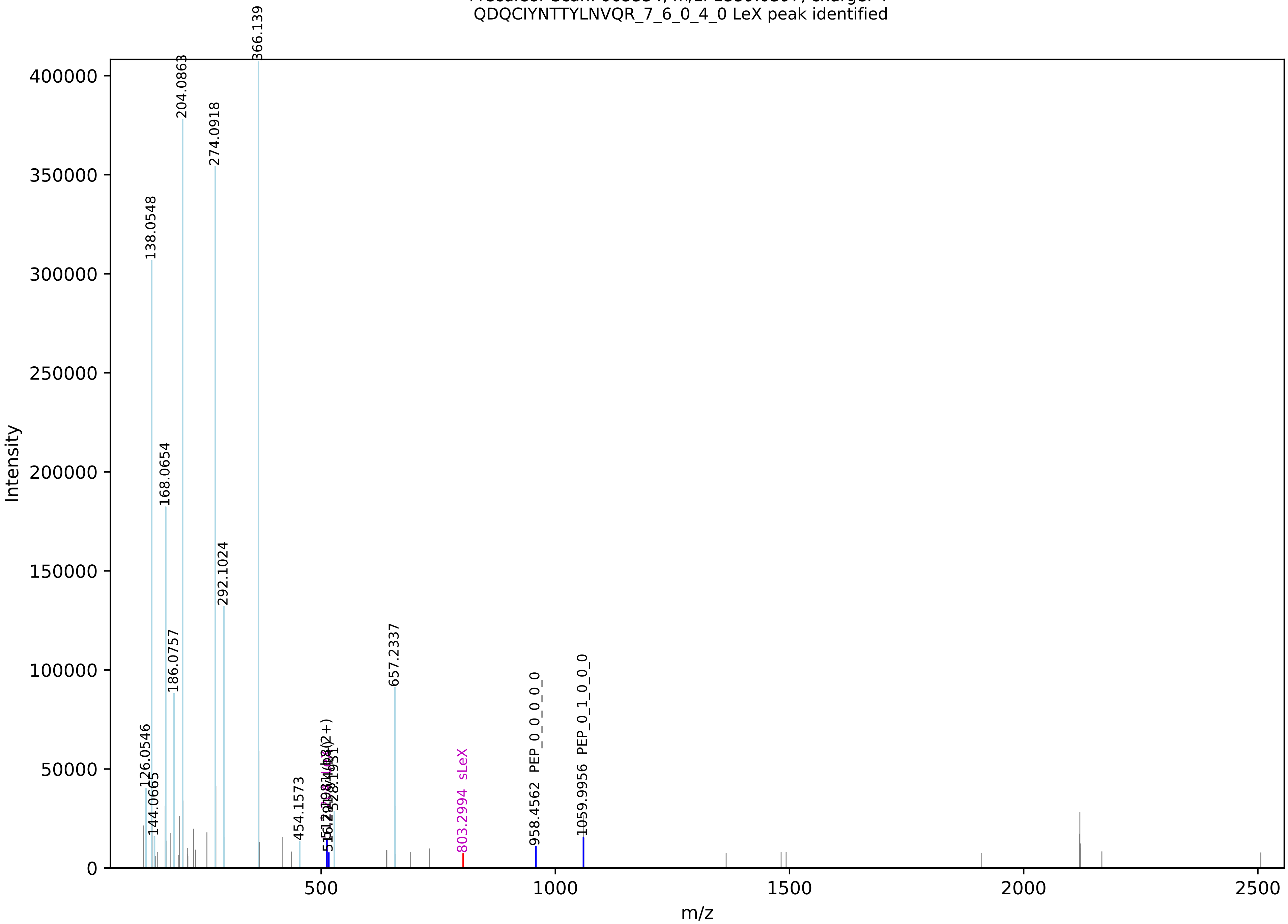

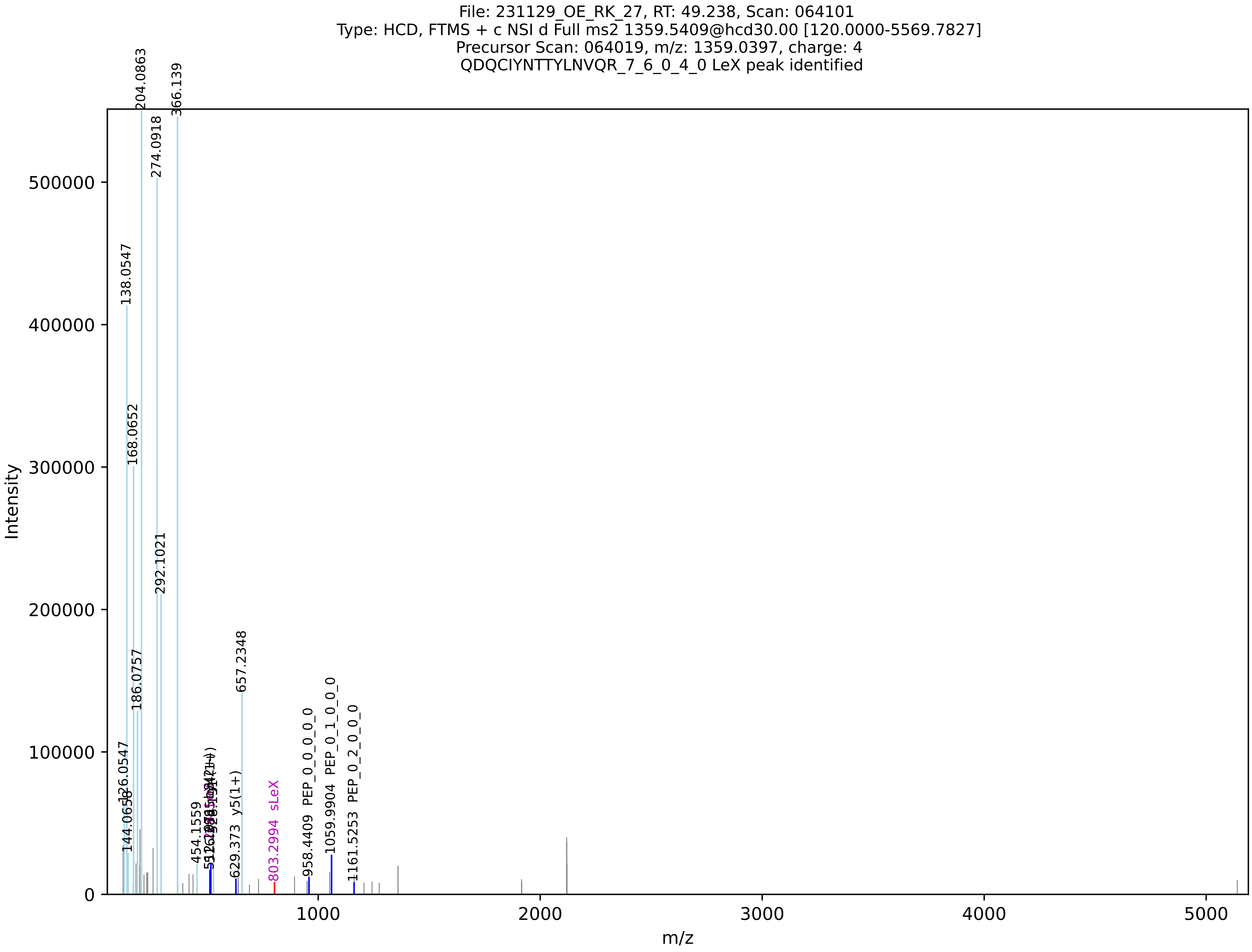
File: 231129_OE_RK_27, RT: 49.238, Scan: 064101
Type: HCD, FTMS + c NSI d Full ms2 1359.5409@hcd30.00 [120.0000-5569.7827]
Precursor Scan: 064019, m/z: 1359.0397, charge: 4
QDQCIYNTTYLNVQR_7_6_0_4_0 LeX peak identified
204.0863
366.139
274.0918
138.0547
168.0652
292.1021
186.0757
657.2348
126.0547
144.0658
454.1559
629.373 y5(1+)
803.2994 sLeX
958.4409 PEP_0_0_0_0_0_
1059.9904 PEP_0_1_0_0_0_
1161.5253 PEP_0_2_0_0_0_
Intensity
m/z
0
100000
200000
300000
400000
500000
1000
2000
3000
4000
5000

File: 231129_OE_RK_27, RT: 49.59, Scan: 064605
Type: HCD, FTMS + c NSI d Full ms2 1360.0425@hcd30.00 [120.0000-5571.8296]
Precursor Scan: 064525, m/z: 1359.2911, charge: 4
QDQCIYNTTYLNVQR_7_6_0_4_0 LeX peak identified

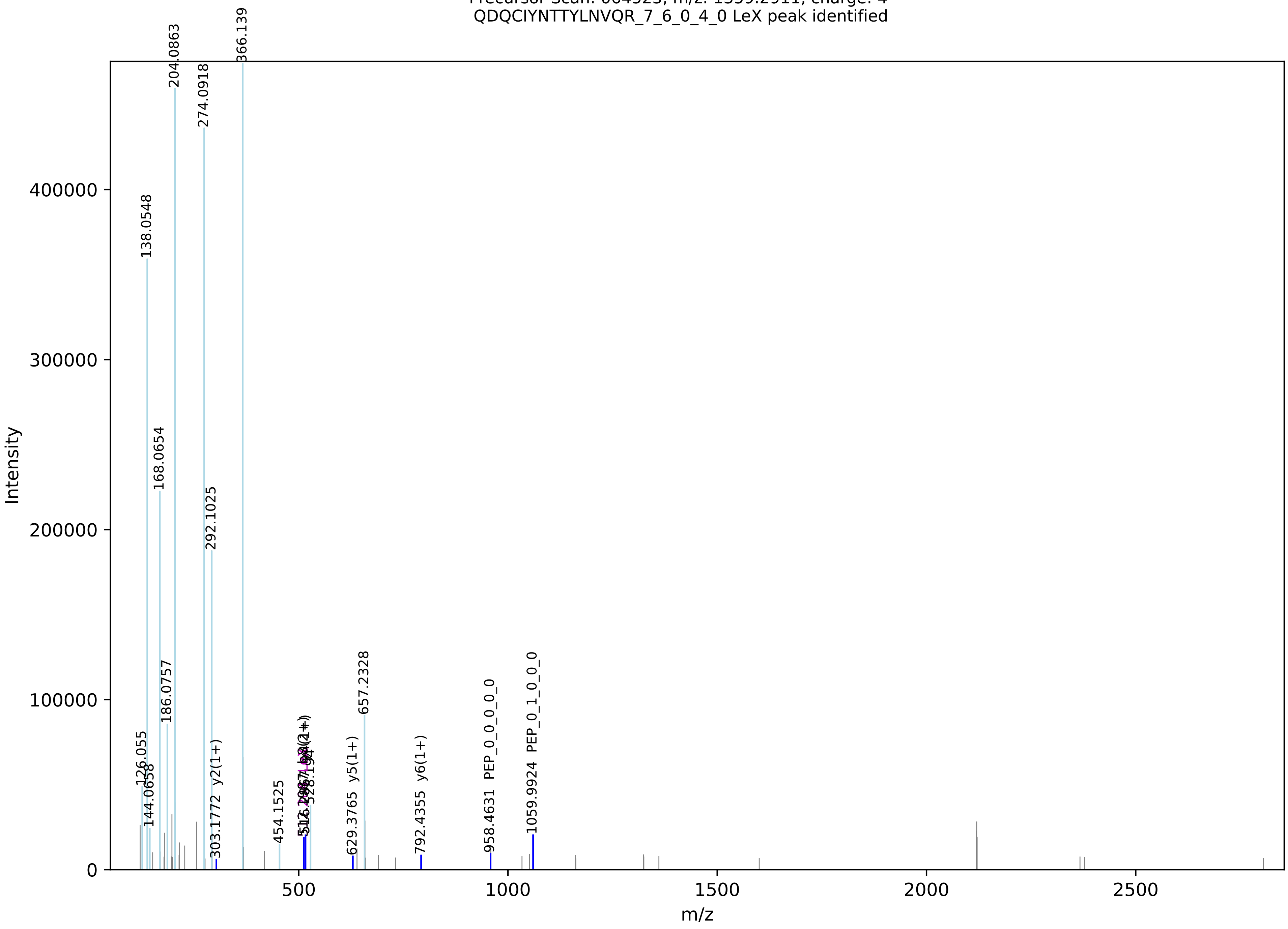

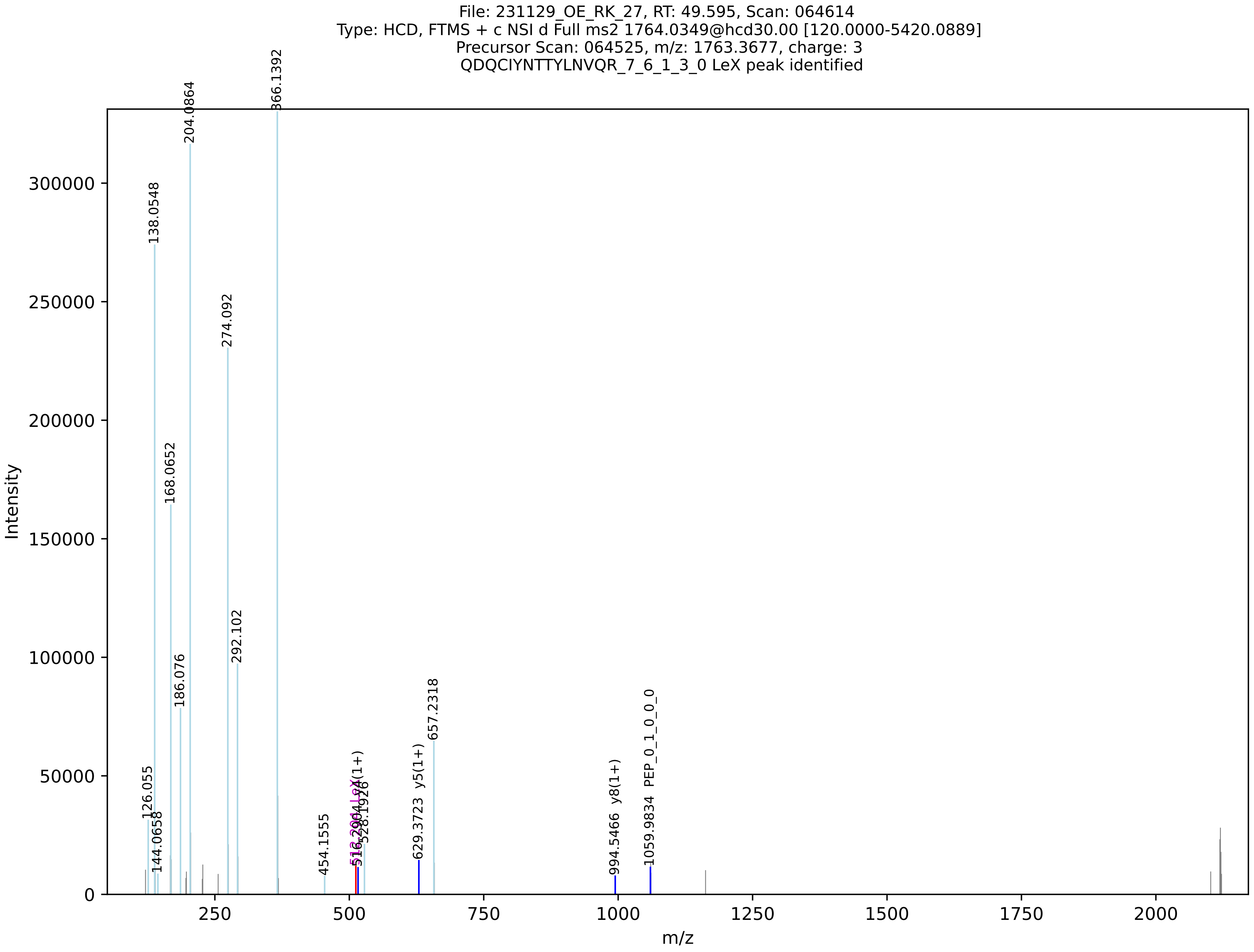

File: 231129_OE_RK_27, RT: 49.595, Scan: 064614
Type: HCD, FTMS + c NSI d Full ms2 1764.0349@hcd30.00 [120.0000-5420.0889]
Precursor Scan: 064525, m/z: 1763.3677, charge: 3
QDQCIYNTTYLNVQR_7_6_1_3_0 LeX peak identified
Intensity
m/z
366.1392
204.0864
138.0548
274.092
168.0652
292.102
186.076
126.055
144.0658
454.1555
512.204 LeX
516.2904 y4(1+)
528.1926
629.3723 y5(1+)
657.2318
994.5466 y8(1+)
1059.9834 PEP_0_1_0_0_0

File: 231129_OE_RK_27, RT: 49.642, Scan: 064682
Type: HCD, FTMS + c NSI d Full ms2 1396.3042@hcd30.00 [120.0000-5719.7769]
Precursor Scan: 064599, m/z: 1395.5524, charge: 4
QDQCIYNTTYLNVQR_7_6_1_4_0 LeX peak identified

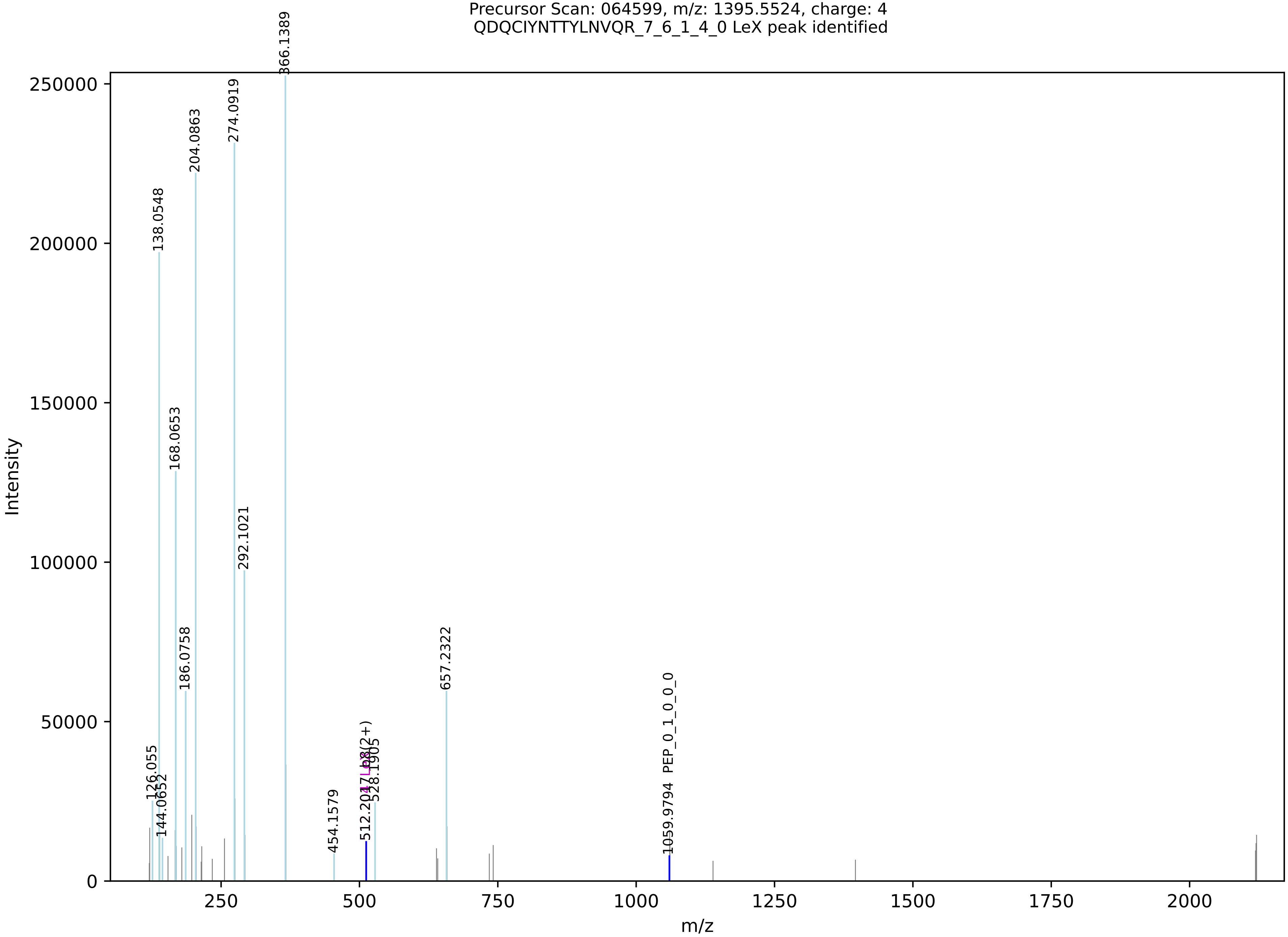

File: 231129_OE_RK_27, RT: 50.04, Scan: 065254
Type: HCD, FTMS + c NSI d Full ms2 1323.2782@hcd30.00 [120.0000-5421.8311]
Precursor Scan: 065180, m/z: 1322.5264, charge: 4
QDQCIYNTTYLNVQR_7_6_1_3_0 LeX peak identified

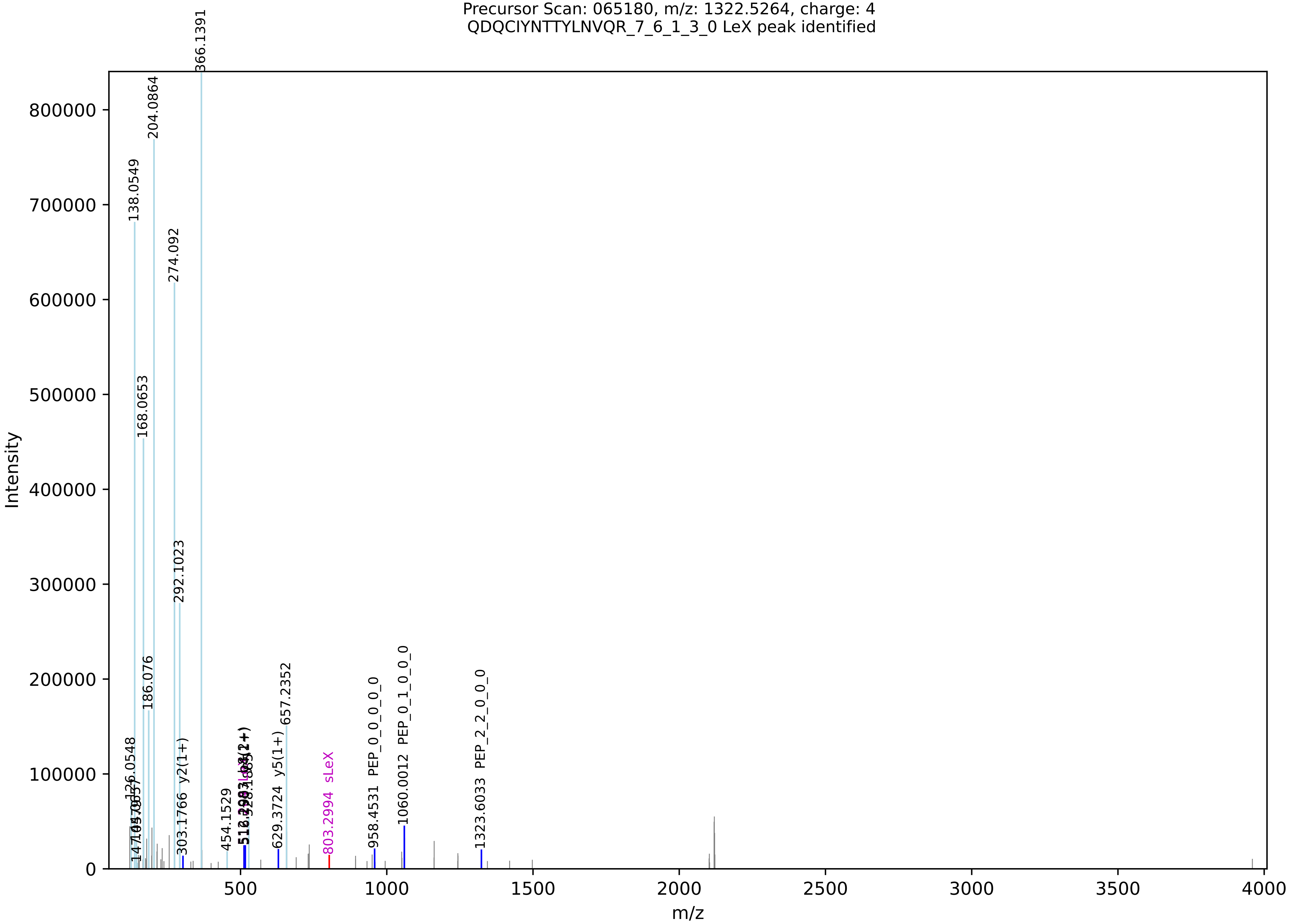

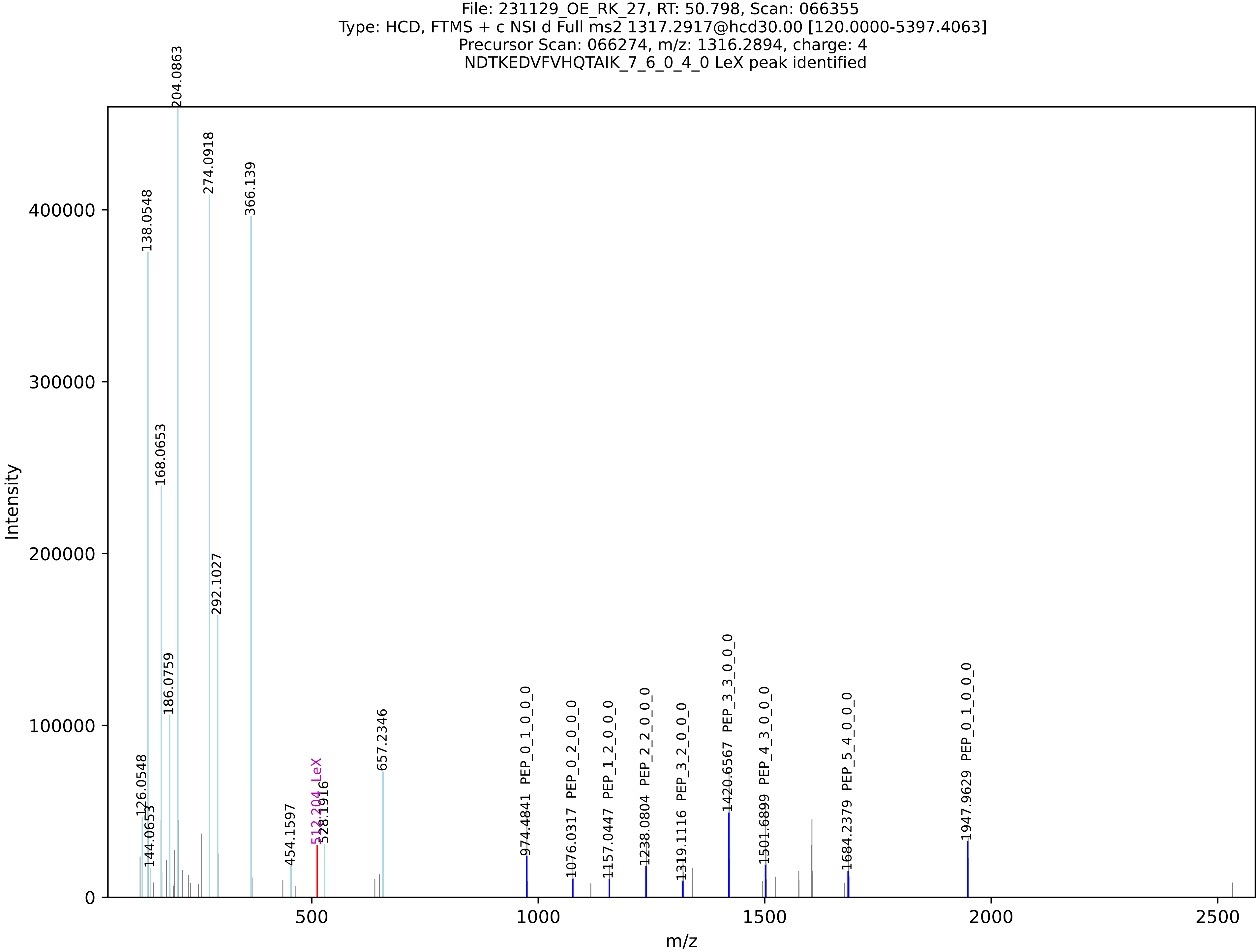
File: 231129_OE_RK_27, RT: 50.798, Scan: 066355
Type: HCD, FTMS + c NSI d Full ms2 1317.2917@hcd30.00 [120.0000-5397.4063]
Precursor Scan: 066274, m/z: 1316.2894, charge: 4
NDTKEDVFVHQTAIK_7_6_0_4_0 LeX peak identified
Intensity
m/z
204.0863
274.0918
366.139
138.0548
168.0653
292.1027
186.0759
126.0548
144.0653
454.1597
512.204 LeX
528.1916
657.2346
974.4841 PEP_0_1_0_0_0
1076.0317 PEP_0_2_0_0_0
1157.0447 PEP_1_2_0_0_0
1238.0804 PEP_2_2_0_0_0
1319.1116 PEP_3_2_0_0_0
1420.6567 PEP_3_3_0_0_0
1501.6899 PEP_4_3_0_0_0
1684.2379 PEP_5_4_0_0_0
1947.9629 PEP_0_1_0_0_0

File: 231129_OE_RK_27, RT: 50.805, Scan: 066366
Type: HCD, FTMS + c NSI d Full ms2 1641.9902@hcd30.00 [120.0000-5046.6318]
Precursor Scan: 066274, m/z: 1641.6551, charge: 3
QDQCIYNTTYLNVQR_6_5_1_3_0 LeX peak identified

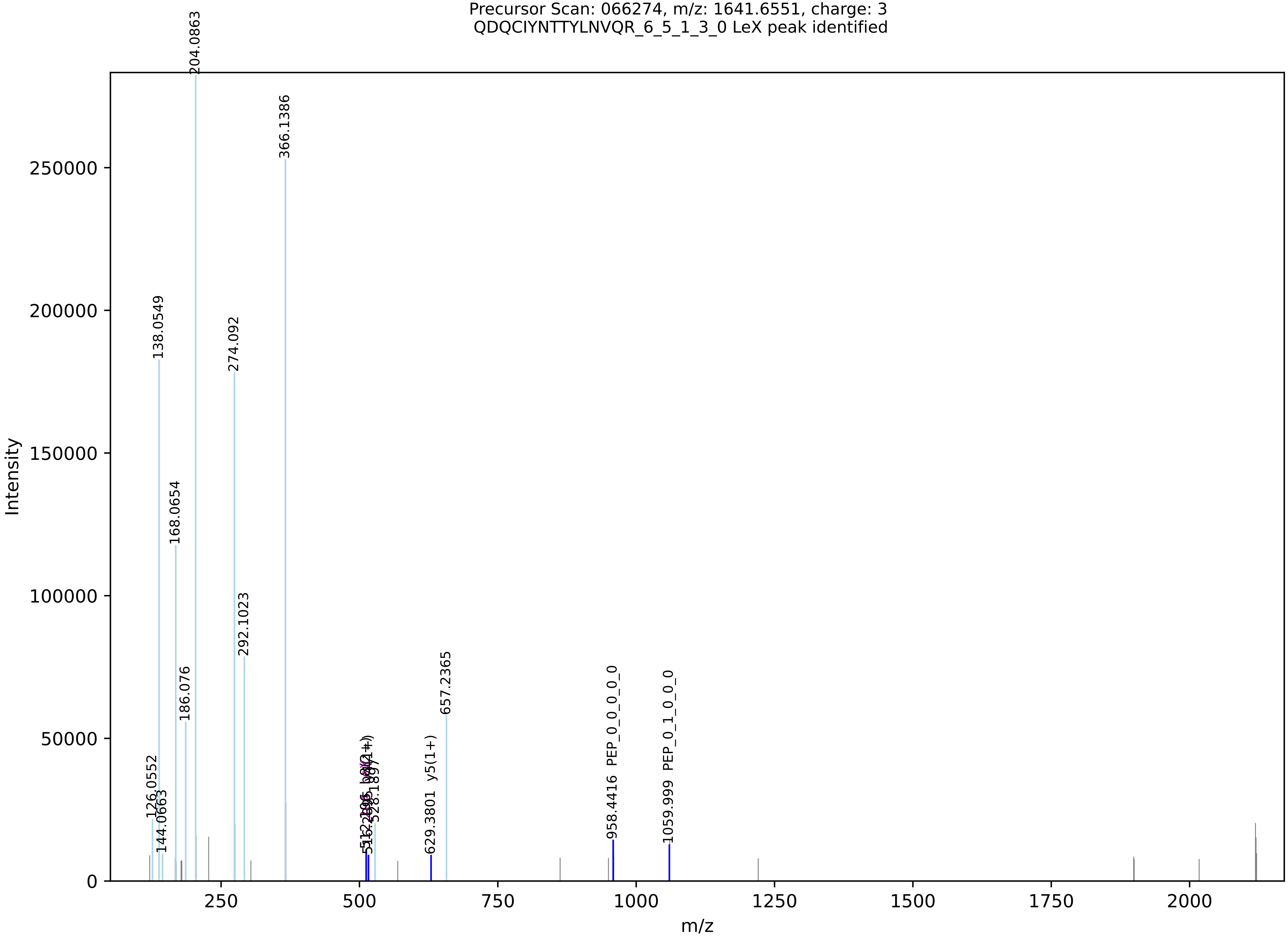

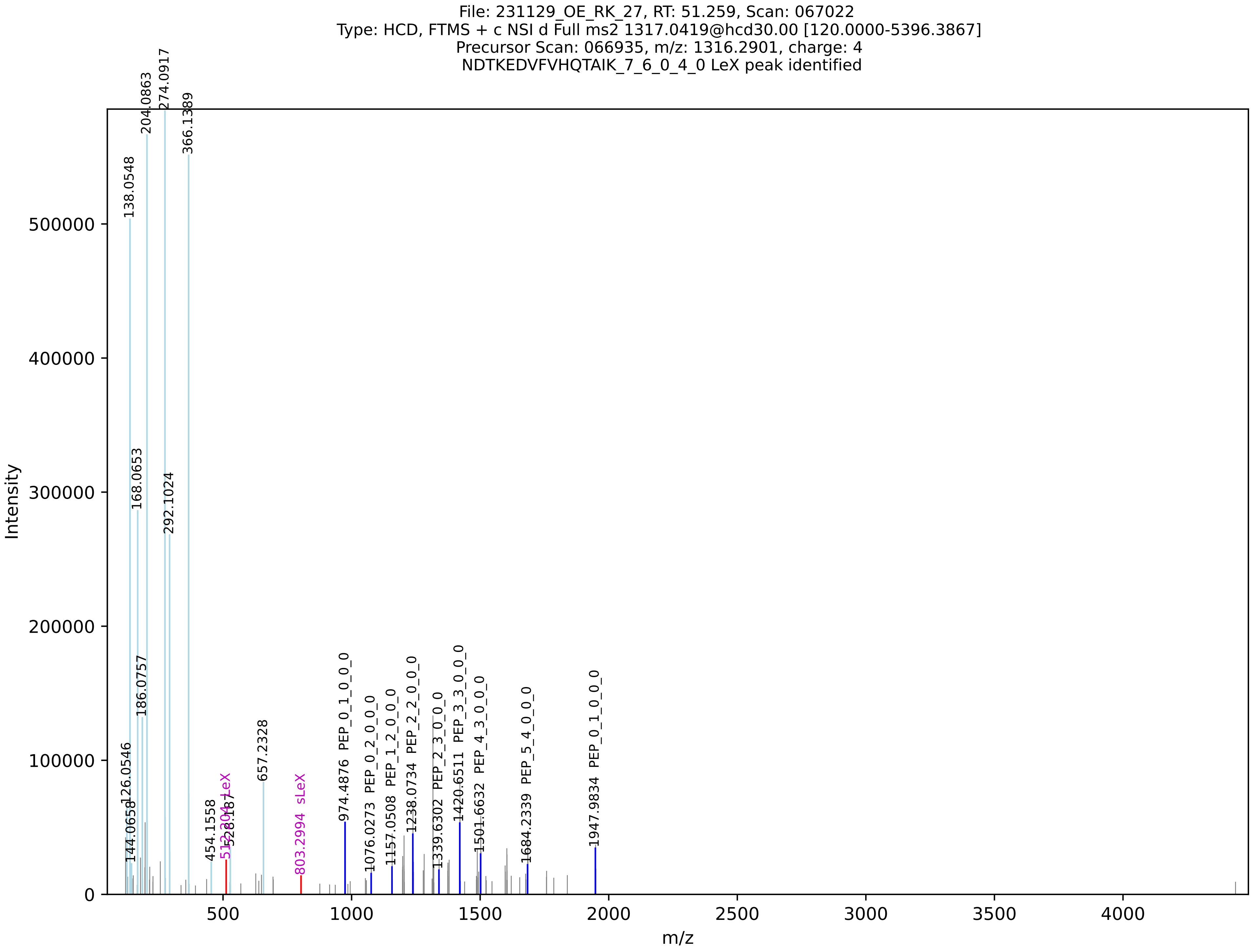

File: 231129_OE_RK_27, RT: 51.259, Scan: 067022
Type: HCD, FTMS + c NSI d Full ms2 1317.0419@hcd30.00 [120.0000-5396.3867]
Precursor Scan: 066935, m/z: 1316.2901, charge: 4
NDTKEDVFVHQTAIK_7_6_0_4_0 LeX peak identified
Intensity
m/z
274.0917
204.0863
366.1389
138.0548
168.0653
292.1024
186.0757
126.0546
144.0658
454.1558
528.187
657.2328
803.2994 sLeX
974.4876 PEP_0_1_0_0_0
1076.0273 PEP_0_2_0_0_0
1157.0508 PEP_1_2_0_0_0
1238.0734 PEP_2_2_0_0_0
1339.6302 PEP_2_3_0_0_0
1420.6511 PEP_3_3_0_0_0
1501.6632 PEP_4_3_0_0_0
1684.2339 PEP_5_4_0_0_0
1947.9834 PEP_0_1_0_0_0
0
100000
200000
300000
400000
500000
500
1000
1500
2000
2500
3000
3500
4000

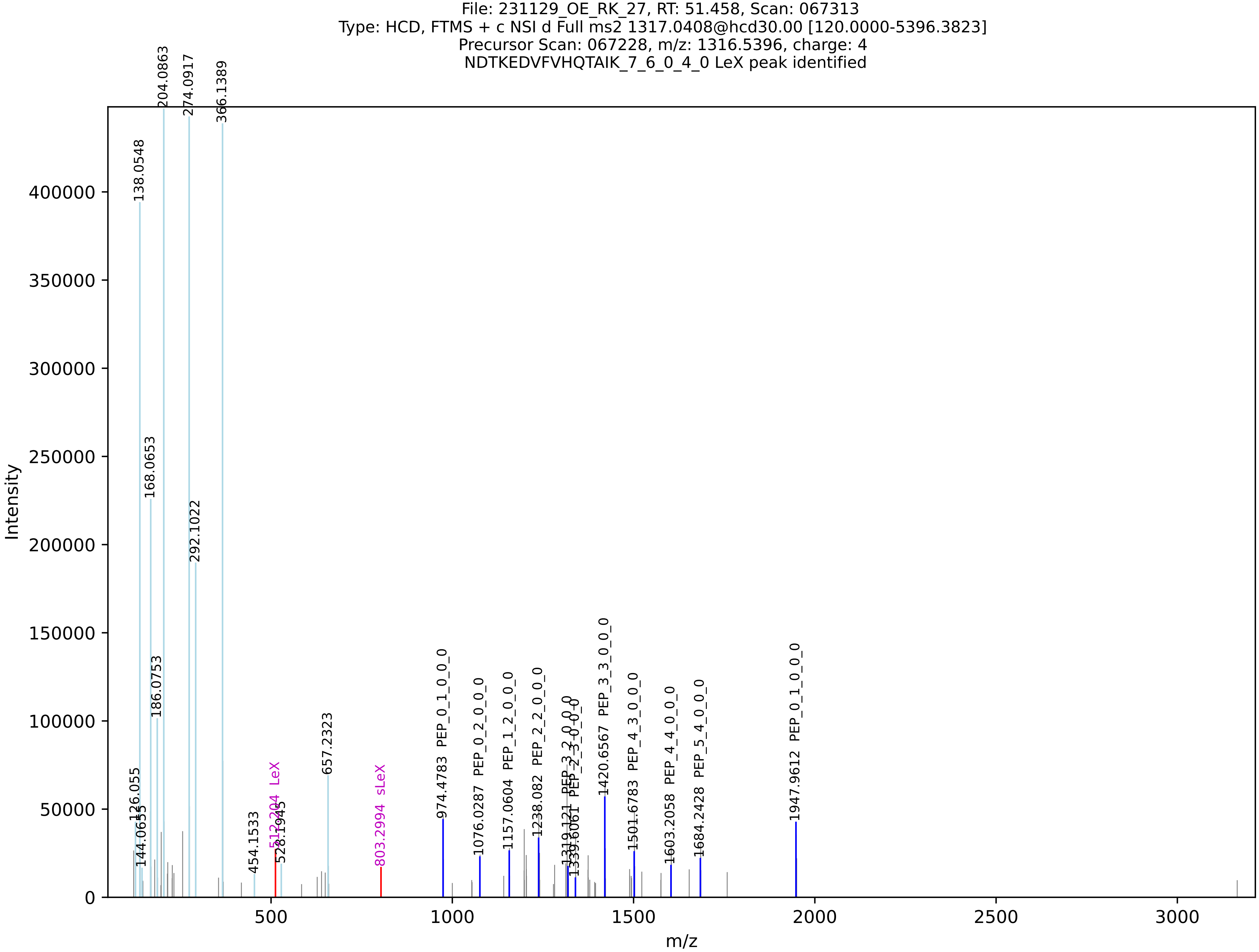

File: 231129_OE_RK_27, RT: 51.458, Scan: 067313
Type: HCD, FTMS + c NSI d Full ms2 1317.0408@hcd30.00 [120.0000-5396.3823]
Precursor Scan: 067228, m/z: 1316.5396, charge: 4
NDTKEDVFVHQTAIK_7_6_0_4_0 LeX peak identified
Intensity
m/z
204.0863
274.0917
366.1389
138.0548
168.0653
292.1022
186.0753
126.055
144.0655
454.1533
512.204 LeX
528.1945
657.2323
803.2994 sLeX
974.4783 PEP_0_1_0_0_0
1076.0287 PEP_0_2_0_0_0
1157.0604 PEP_1_2_0_0_0
1238.082 PEP_2_2_0_0_0
1319.121 PEP_3_2_0_0_0
1339.6061 PEP_2_3_0_0_0
1420.6567 PEP_3_3_0_0_0
1501.6783 PEP_4_3_0_0_0
1603.2058 PEP_4_4_0_0_0
1684.2428 PEP_5_4_0_0_0
1947.9612 PEP_0_1_0_0_0

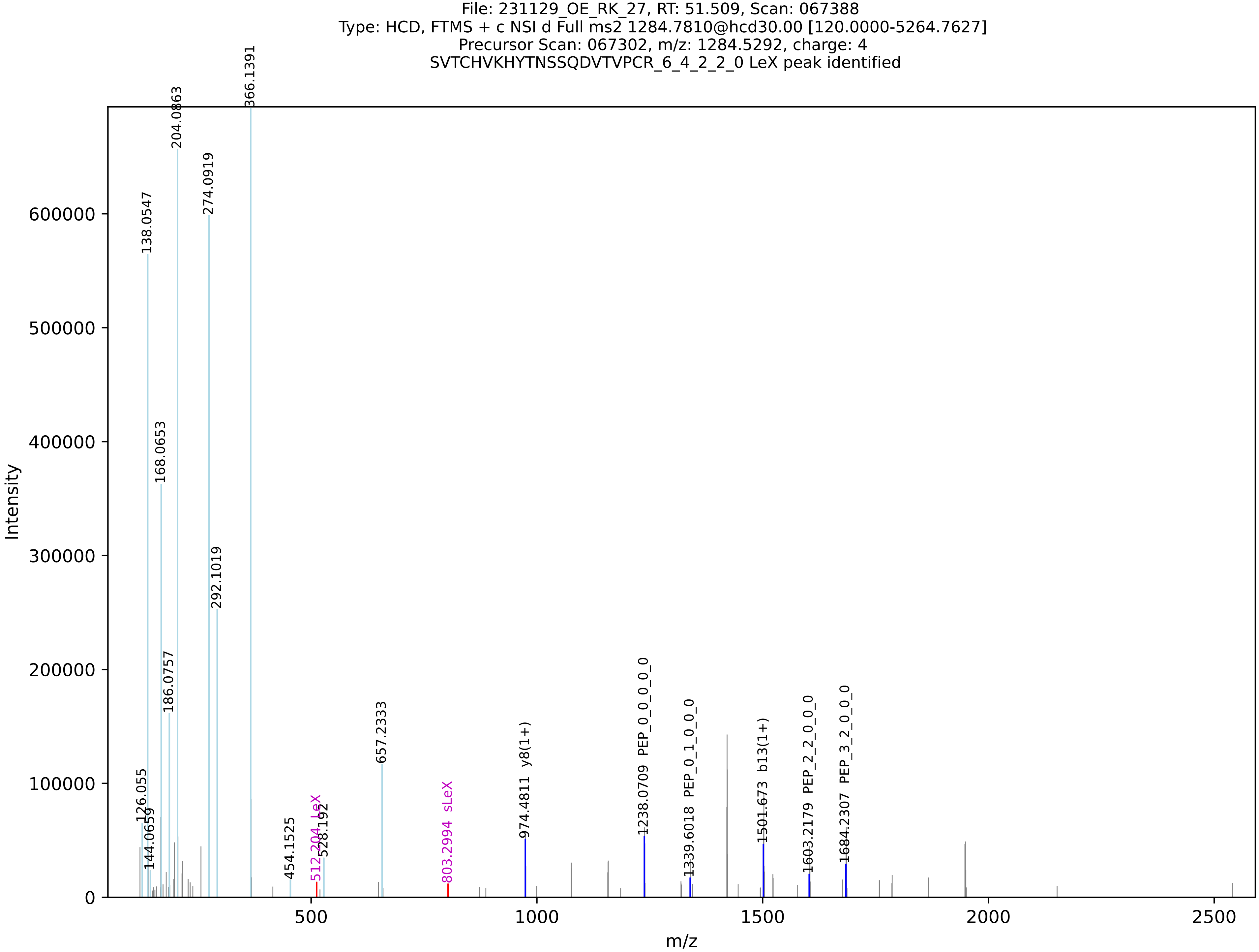
File: 231129_OE_RK_27, RT: 51.509, Scan: 067388
Type: HCD, FTMS + c NSI d Full ms2 1284.7810@hcd30.00 [120.0000-5264.7627]
Precursor Scan: 067302, m/z: 1284.5292, charge: 4
SVTCHVKHYTNSSQDVTVPCR_6_4_2_2_0 LeX peak identified
Intensity
m/z
366.1391
204.0863
274.0919
138.0547
168.0653
292.1019
186.0757
126.055
144.0659
454.1525
512.204 LeX
528.192
657.2333
803.2994 sLeX
974.4811 y8(1+)
1238.0709 PEP_0_0_0_0_0
1339.6018 PEP_0_1_0_0_0
1501.673 b13(1+)
1603.2179 PEP_2_2_0_0_0
1684.2307 PEP_3_2_0_0_0
0
100000
200000
300000
400000
500000
600000
500
1000
1500
2000
2500

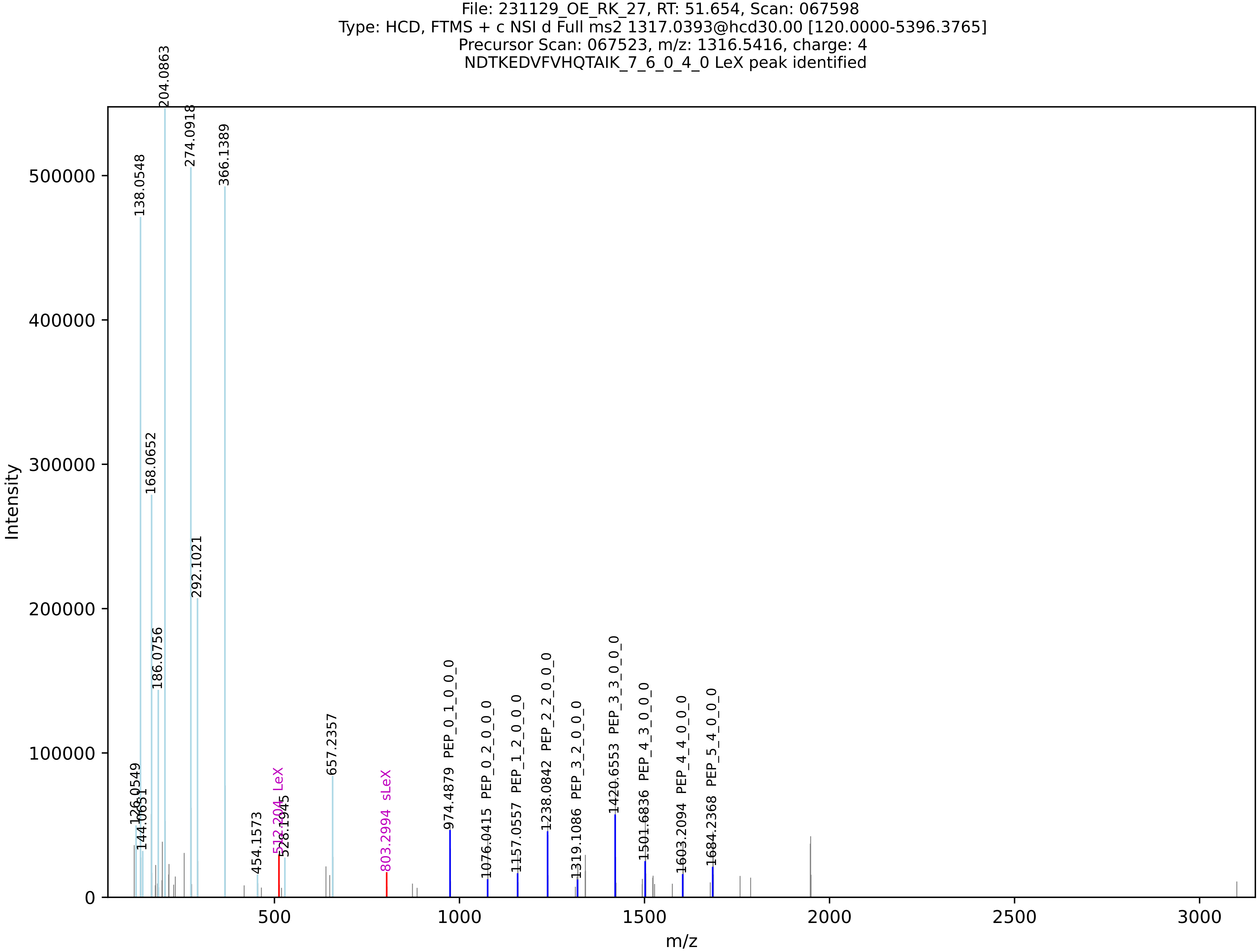

File: 231129_OE_RK_27, RT: 51.654, Scan: 067598
Type: HCD, FTMS + c NSI d Full ms2 1317.0393@hcd30.00 [120.0000-5396.3765]
Precursor Scan: 067523, m/z: 1316.5416, charge: 4
NDTKEDVFVHQTAIK_7_6_0_4_0 LeX peak identified
Intensity
m/z
204.0863
274.0918
366.1389
138.0548
168.0652
292.1021
186.0756
126.0549
144.0651
454.1573
512.204 LeX
528.1945
657.2357
803.2994 sLeX
974.4879 PEP_0_1_0_0_0
1076.0415 PEP_0_2_0_0_0
1157.0557 PEP_1_2_0_0_0
1238.0842 PEP_2_2_0_0_0
1319.1086 PEP_3_2_0_0_0
1420.6553 PEP_3_3_0_0_0
1501.6836 PEP_4_3_0_0_0
1603.2094 PEP_4_4_0_0_0
1684.2368 PEP_5_4_0_0_0

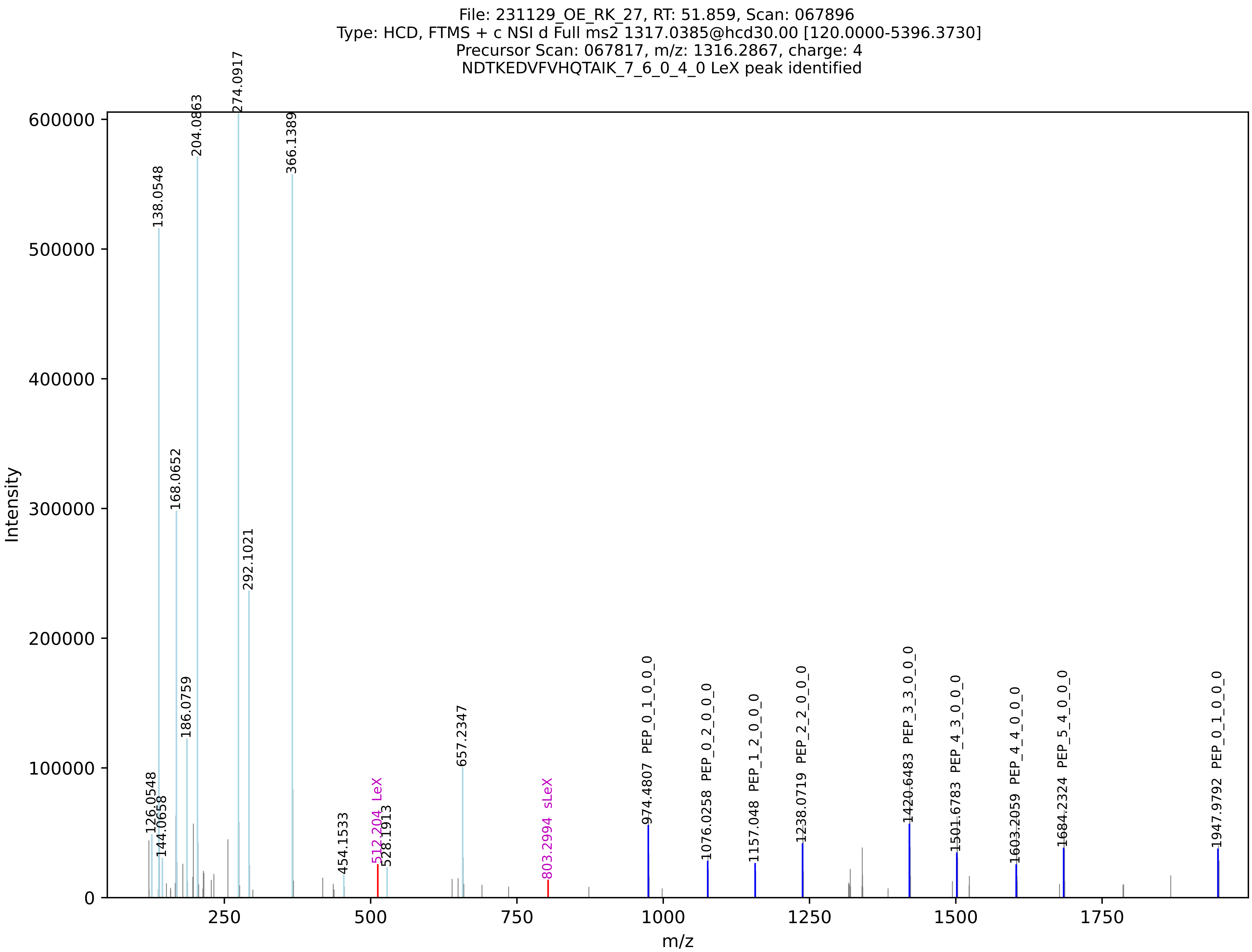

File: 231129_OE_RK_27, RT: 51.859, Scan: 067896
Type: HCD, FTMS + c NSI d Full ms2 1317.0385@hcd30.00 [120.0000-5396.3730]
Precursor Scan: 067817, m/z: 1316.2867, charge: 4
NDTKEDVFVHQTAIK_7_6_0_4_0 LeX peak identified
Intensity
m/z
274.0917
204.0863
366.1389
138.0548
168.0652
292.1021
186.0759
126.0548
144.0658
454.1533
512.204 LeX
528.1913
657.2347
803.2994 sLeX
974.4807 PEP_0_1_0_0_0
1076.0258 PEP_0_2_0_0_0
1157.048 PEP_1_2_0_0_0
1238.0719 PEP_2_2_0_0_0
1420.6483 PEP_3_3_0_0_0
1501.6783 PEP_4_3_0_0_0
1603.2059 PEP_4_4_0_0_0
1684.2324 PEP_5_4_0_0_0
1947.9792 PEP_0_1_0_0_0

File: 231129_OE_RK_27, RT: 53.39, Scan: 070126
Type: HCD, FTMS + c NSI d Full ms2 1266.5459@hcd30.00 [120.0000-3897.7725]
Precursor Scan: 070051, m/z: 1265.8777, charge: 3
ALPQPQNVTSLLGCTH_5_4_1_1_0 LeX peak identified

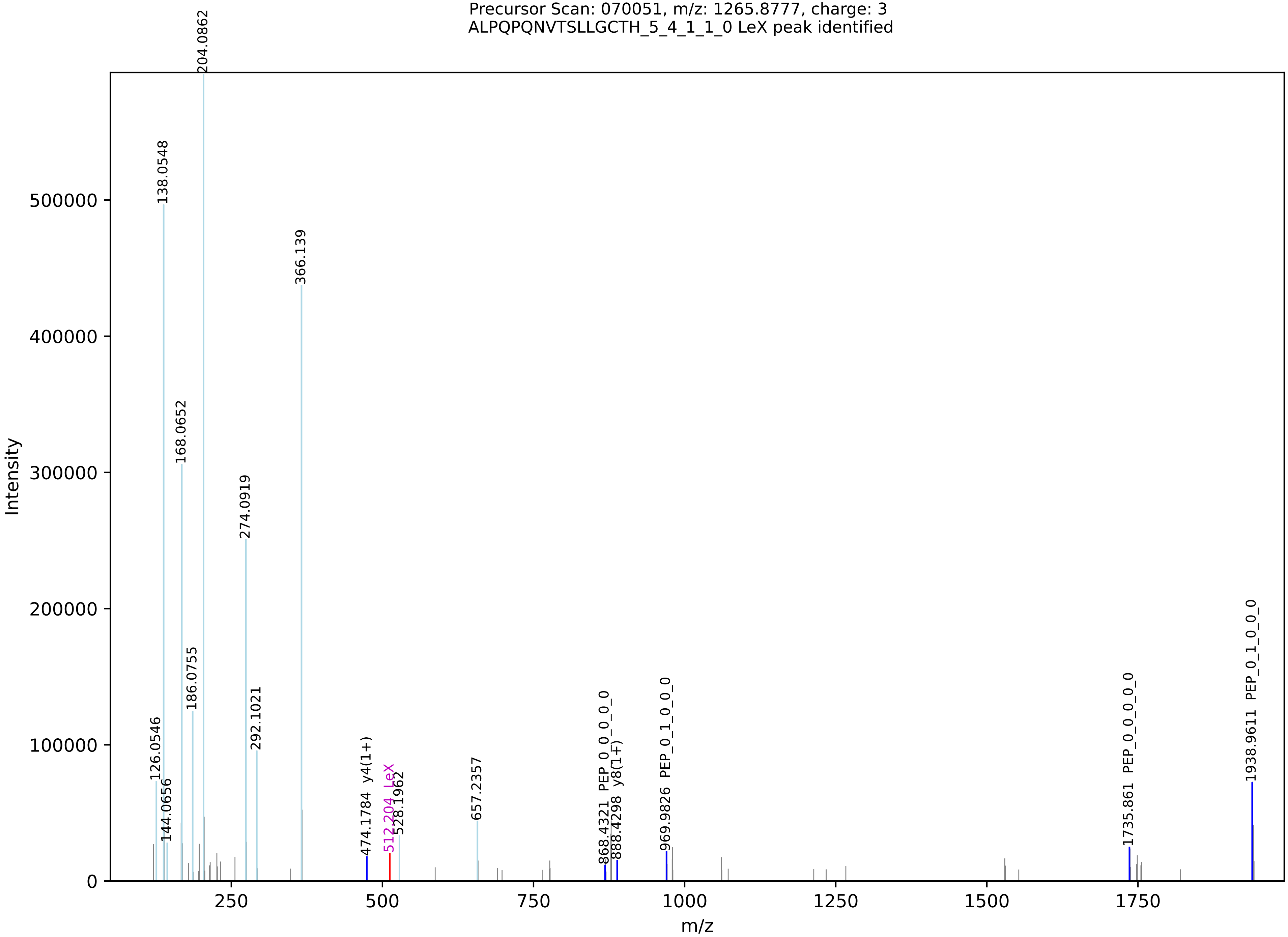

File: 231129_OE_RK_27, RT: 59.611, Scan: 079150
Type: HCD, FTMS + c NSI d Full ms2 1353.5543@hcd30.00 [120.0000-5545.3574]
Precursor Scan: 079072, m/z: 1352.8026, charge: 4
NDTKEDVFVHQTAIK_7_6_1_4_0 LeX peak identified

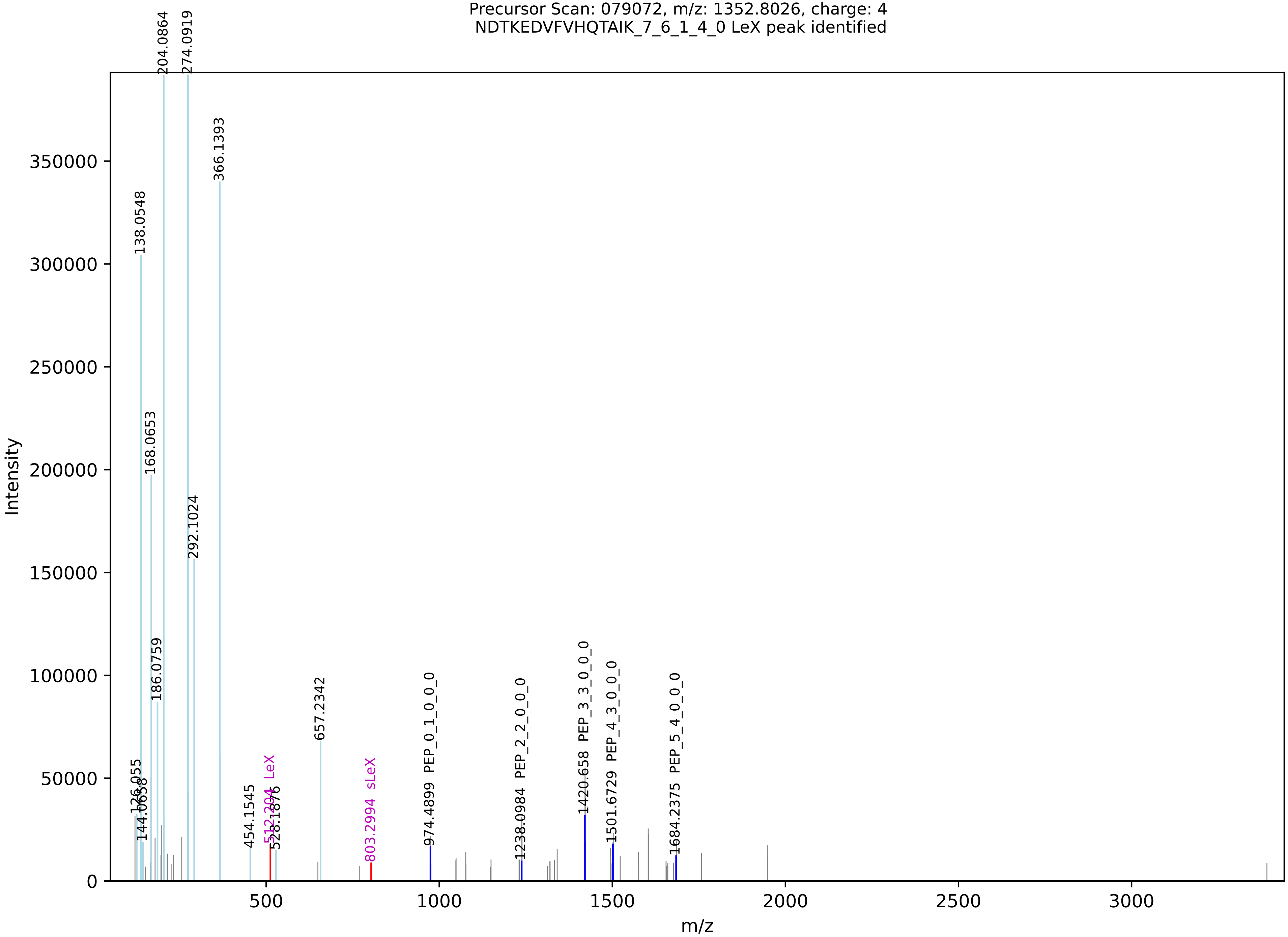

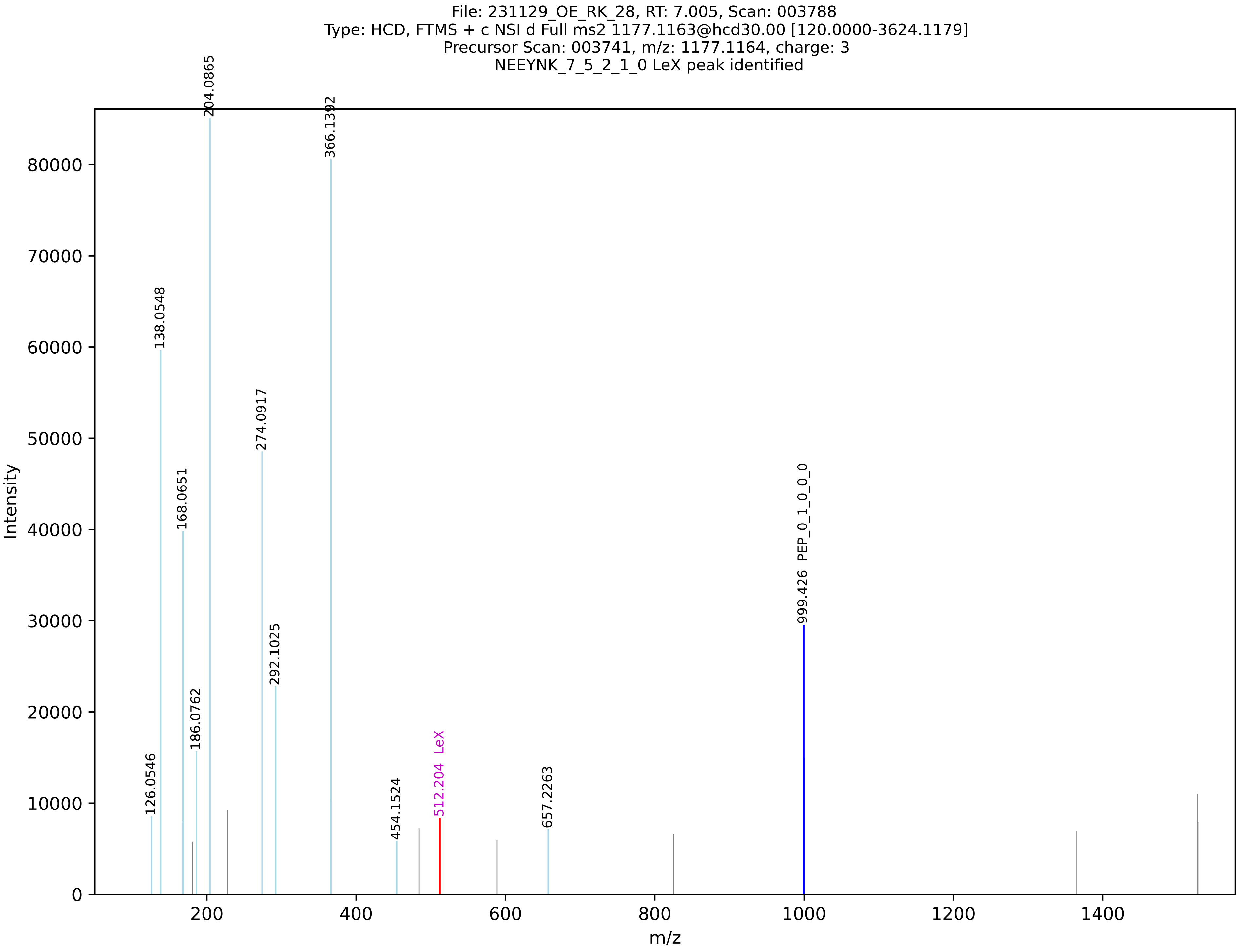
File: 231129_OE_RK_28, RT: 7.005, Scan: 003788
Type: HCD, FTMS + c NSI d Full ms2 1177.1163@hcd30.00 [120.0000-3624.1179]
Precursor Scan: 003741, m/z: 1177.1164, charge: 3
NEEYNK_7_5_2_1_0 LeX peak identified
Intensity
m/z
204.0865
366.1392
138.0548
274.0917
168.0651
999.426 PEP_0_1_0_0_0
292.1025
186.0762
512.204 LeX
126.0546
454.1524
657.2263
0
10000
20000
30000
40000
50000
60000
70000
80000
200
400
600
800
1000
1200
1400

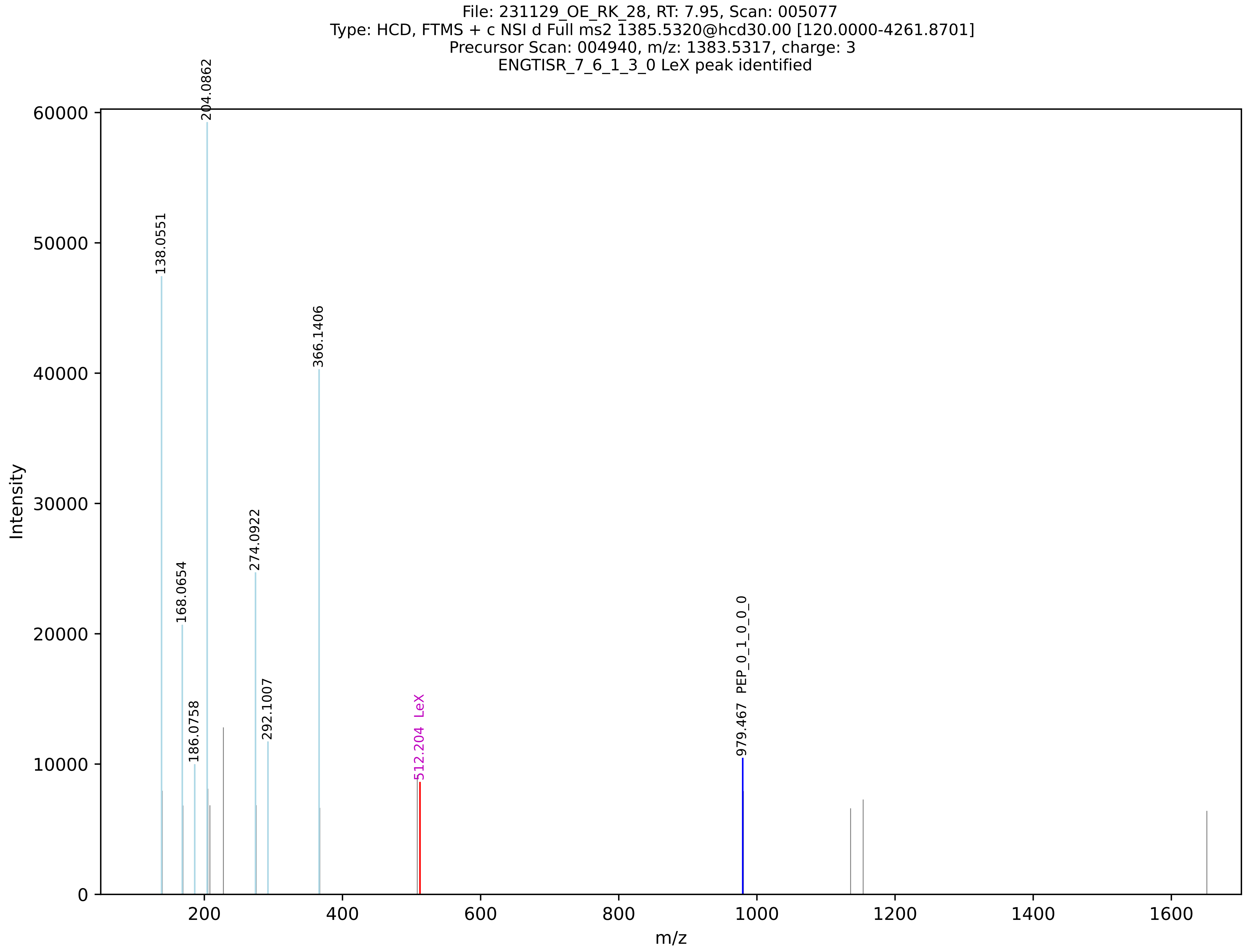
File: 231129_OE_RK_28, RT: 7.95, Scan: 005077
Type: HCD, FTMS + c NSI d Full ms2 1385.5320@hcd30.00 [120.0000-4261.8701]
Precursor Scan: 004940, m/z: 1383.5317, charge: 3
ENGTISR_7_6_1_3_0 LeX peak identified
Intensity
m/z
204.0862
138.0551
366.1406
274.0922
168.0654
186.0758
292.1007
512.204 LeX
979.467 PEP_0_1_0_0_0
0
10000
20000
30000
40000
50000
60000
200
400
600
800
1000
1200
1400
1600

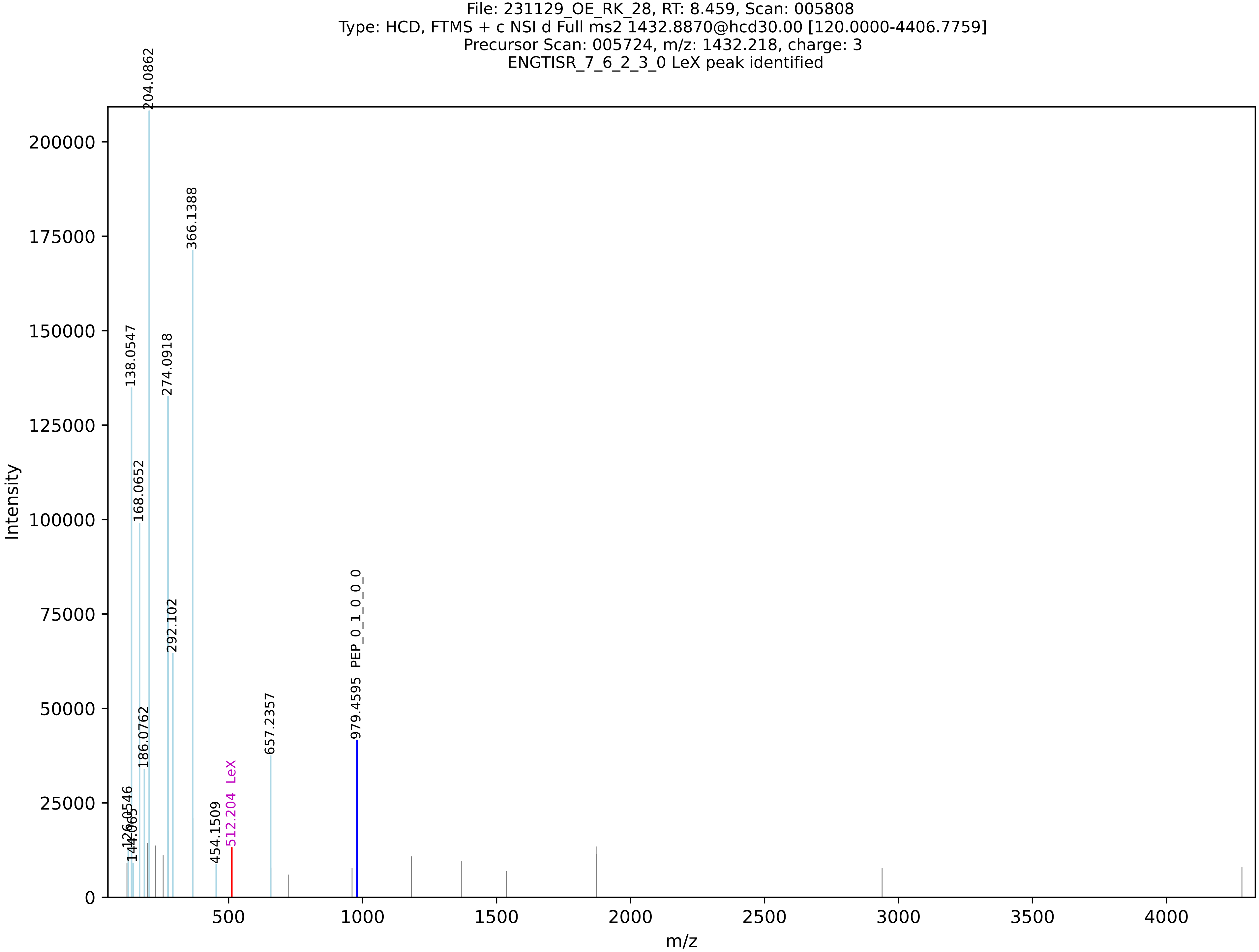
File: 231129_OE_RK_28, RT: 8.459, Scan: 005808
Type: HCD, FTMS + c NSI d Full ms2 1432.8870@hcd30.00 [120.0000-4406.7759]
Precursor Scan: 005724, m/z: 1432.218, charge: 3
ENGTISR_7_6_2_3_0 LeX peak identified
204.0862
366.1388
138.0547
274.0918
168.0652
292.102
PEP_0_1_0_0_0
979.4595
657.2357
186.0762
LeX
512.204
454.1509
126.0546
144.065
Intensity
m/z
0
25000
50000
75000
100000
125000
150000
175000
200000
500
1000
1500
2000
2500
3000
3500
4000

File: 231129_OE_RK_28, RT: 11.883, Scan: 010723
Type: HCD, FTMS + c NSI d Full ms2 1481.2341@hcd30.00 [120.0000-4554.7183]
Precursor Scan: 010643, m/z: 1480.2303, charge: 3
ENGTISR_7_6_1_4_0 LeX peak identified

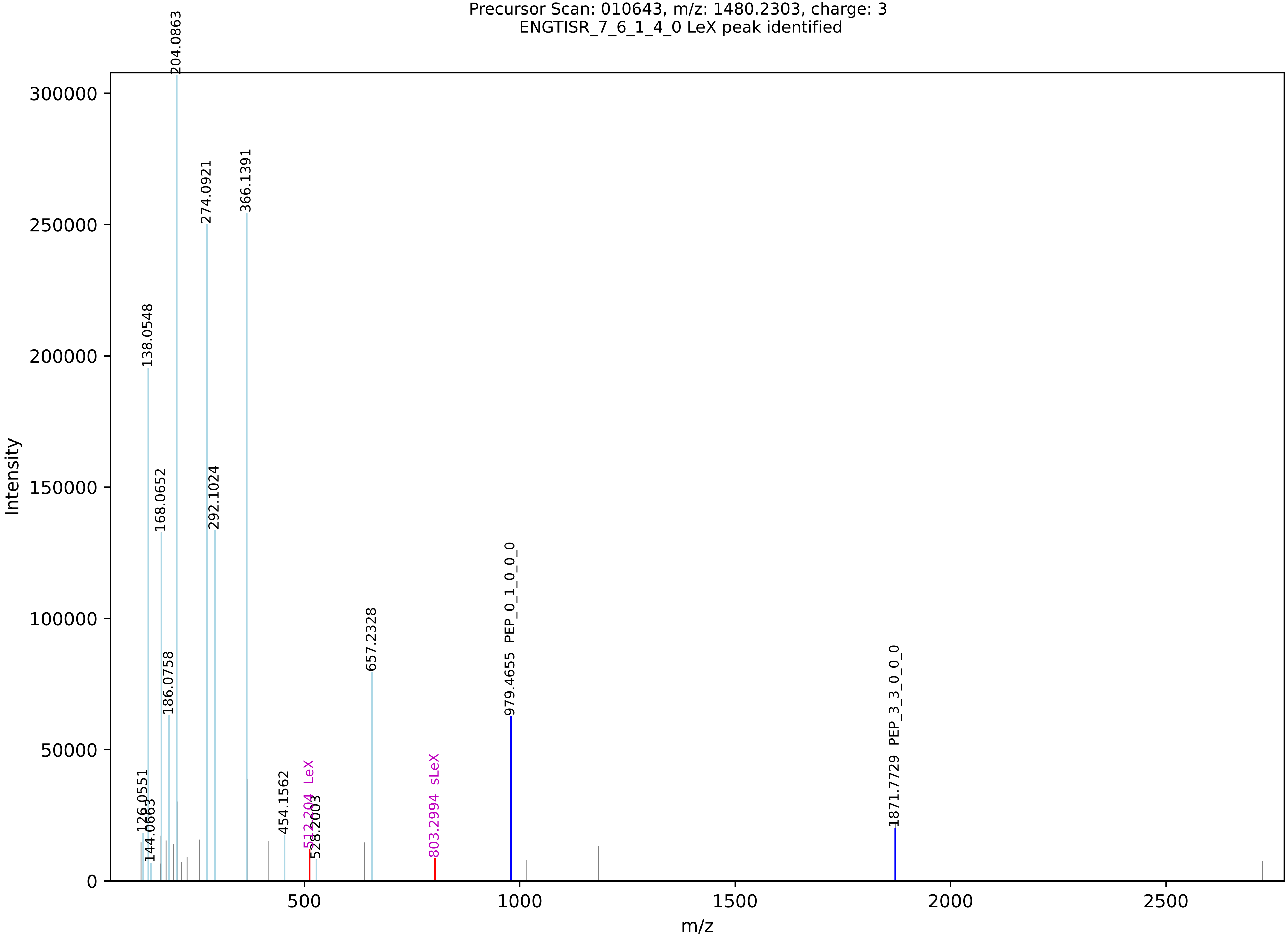

File: 231129_OE_RK_28, RT: 14.287, Scan: 014155
Type: HCD, FTMS + c NSI d Full ms2 1529.5836@hcd30.00 [120.0000-4702.6680]
Precursor Scan: 014084, m/z: 1528.916, charge: 3
ENGTISR_7_6_2_4_0 LeX peak identified

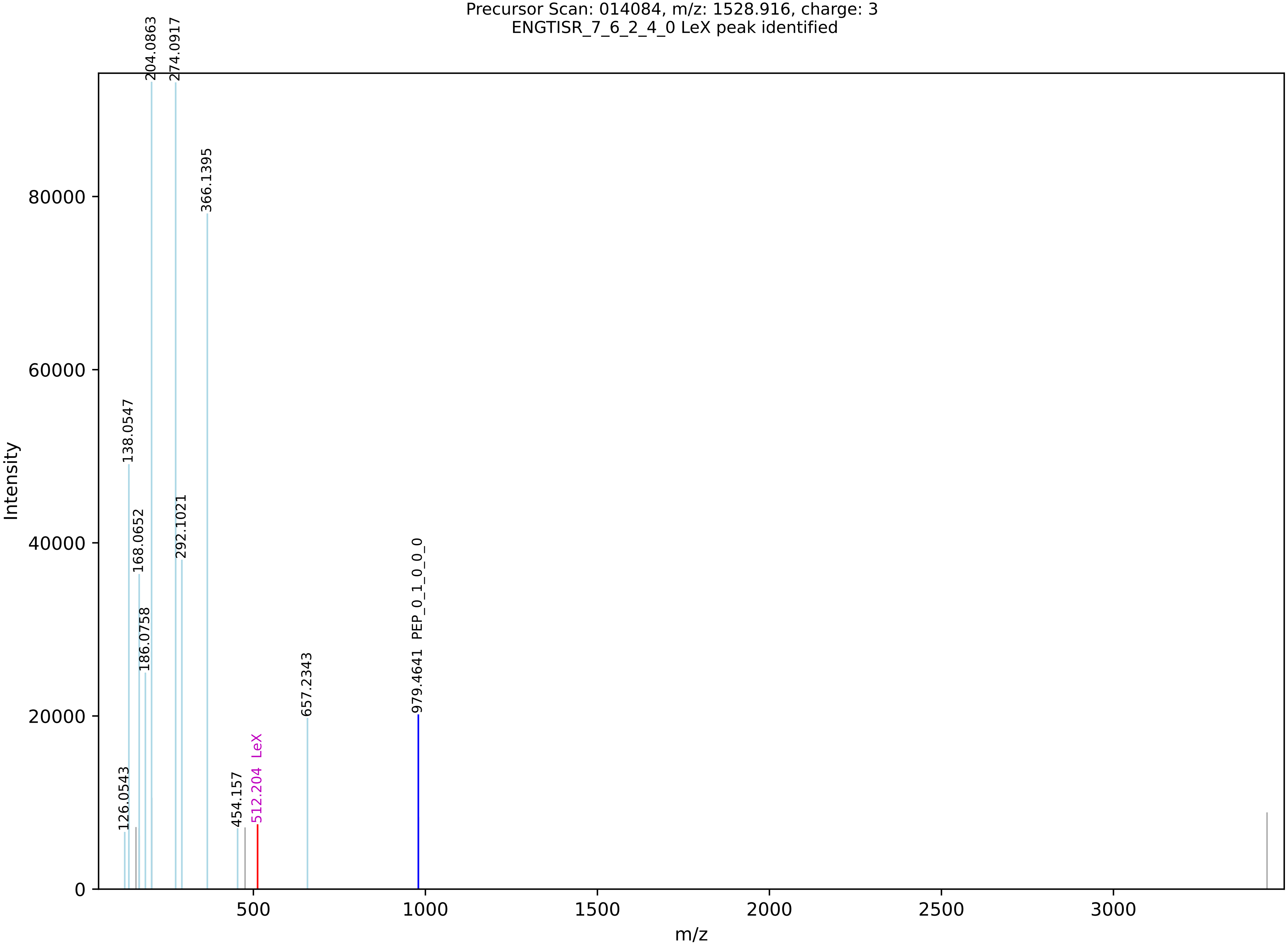

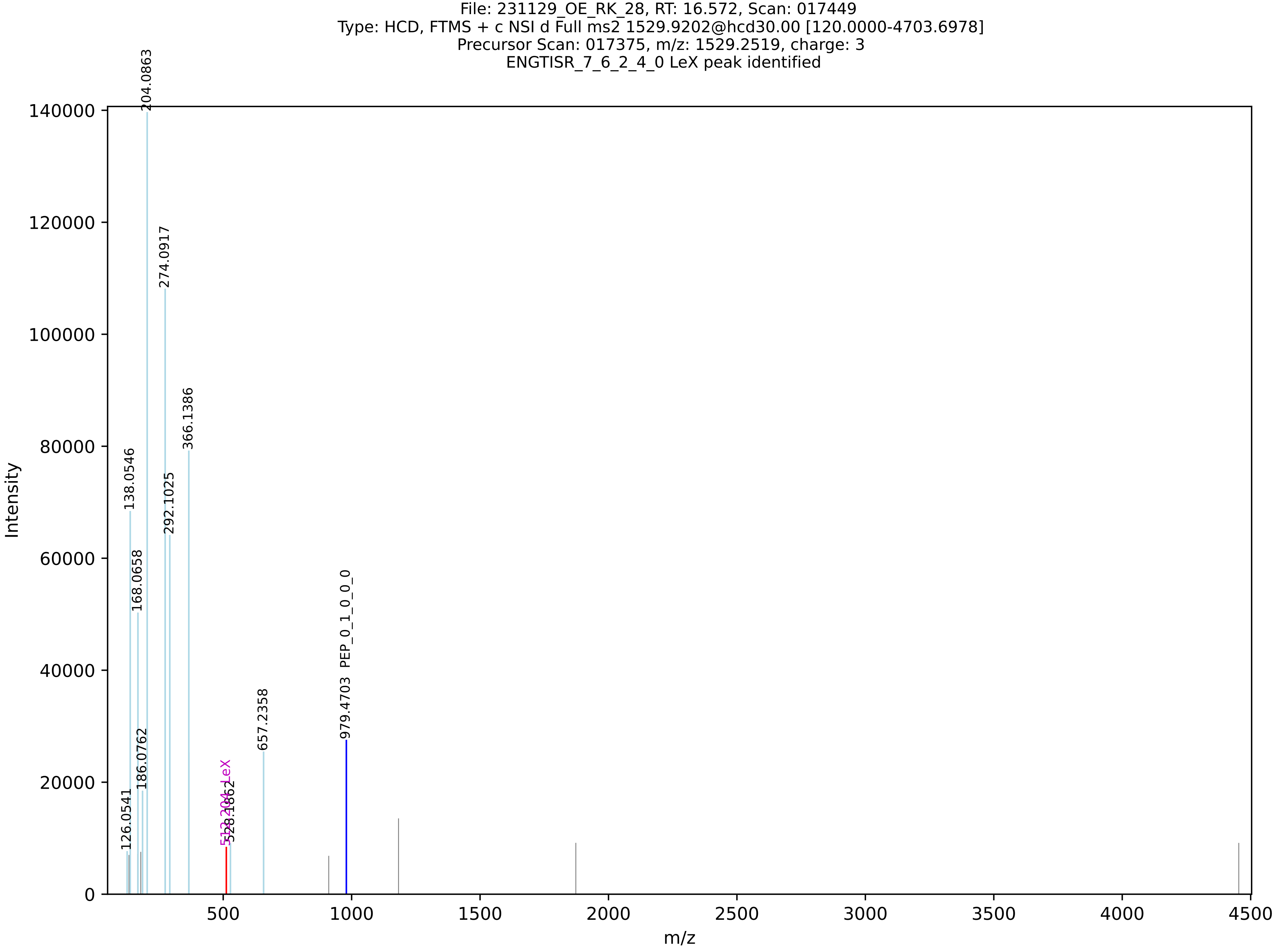

File: 231129_OE_RK_28, RT: 16.572, Scan: 017449
Type: HCD, FTMS + c NSI d Full ms2 1529.9202@hcd30.00 [120.0000-4703.6978]
Precursor Scan: 017375, m/z: 1529.2519, charge: 3
ENGTISR_7_6_2_4_0 LeX peak identified
Intensity
m/z
204.0863
274.0917
366.1386
138.0546
292.1025
168.0658
186.0762
126.0541
512.204_LeX
528.1862
657.2358
979.4703 PEP_0_1_0_0_0

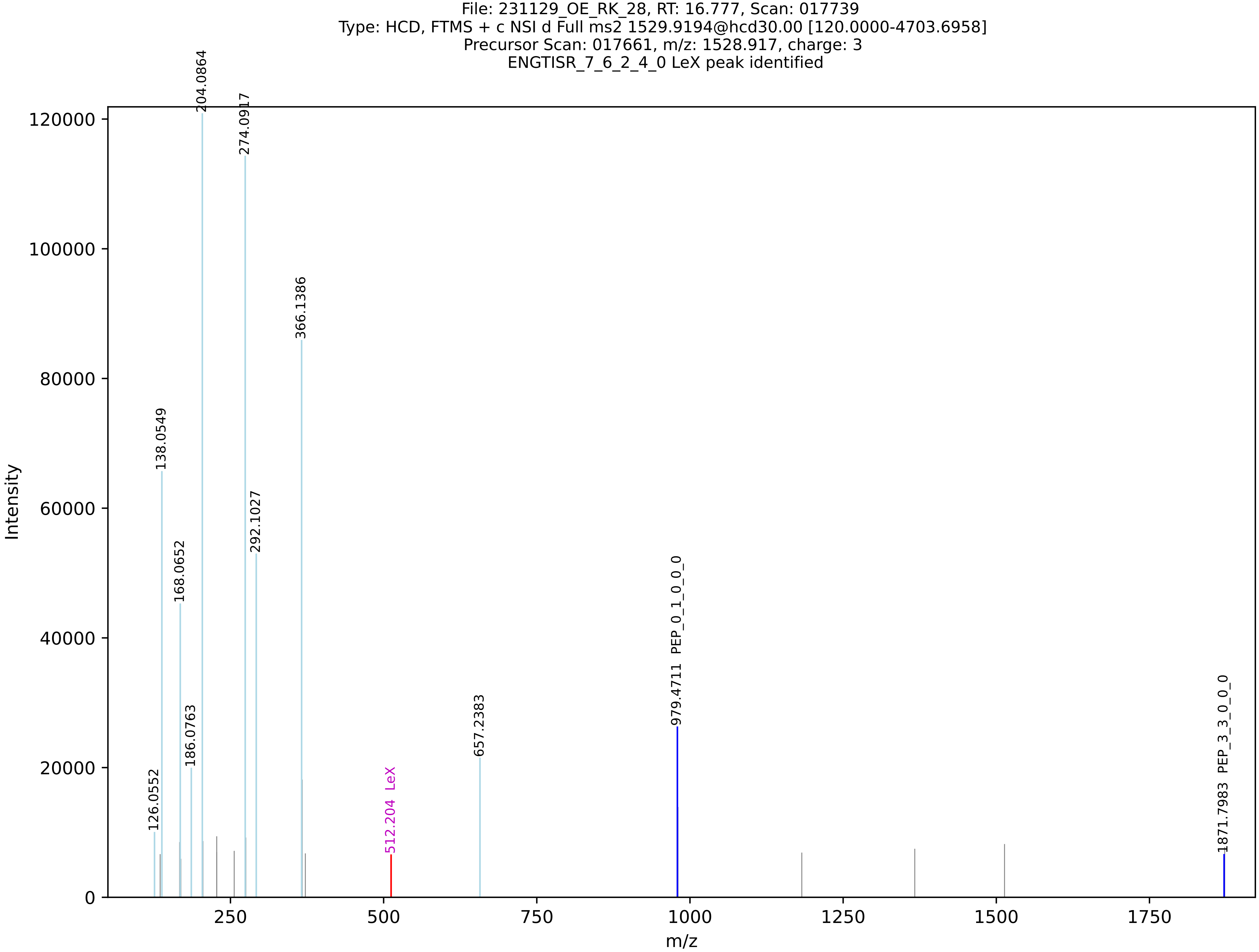

File: 231129_OE_RK_28, RT: 16.777, Scan: 017739
Type: HCD, FTMS + c NSI d Full ms2 1529.9194@hcd30.00 [120.0000-4703.6958]
Precursor Scan: 017661, m/z: 1528.917, charge: 3
ENGTISR_7_6_2_4_0 LeX peak identified
Intensity
m/z
0
20000
40000
60000
80000
100000
120000
250
500
750
1000
1250
1500
1750
126.0552
138.0549
168.0652
186.0763
204.0864
274.0917
292.1027
366.1386
512.204 LeX
657.2383
979.4711 PEP_0_1_0_0_0
1871.7983 PEP_3_3_0_0_0_

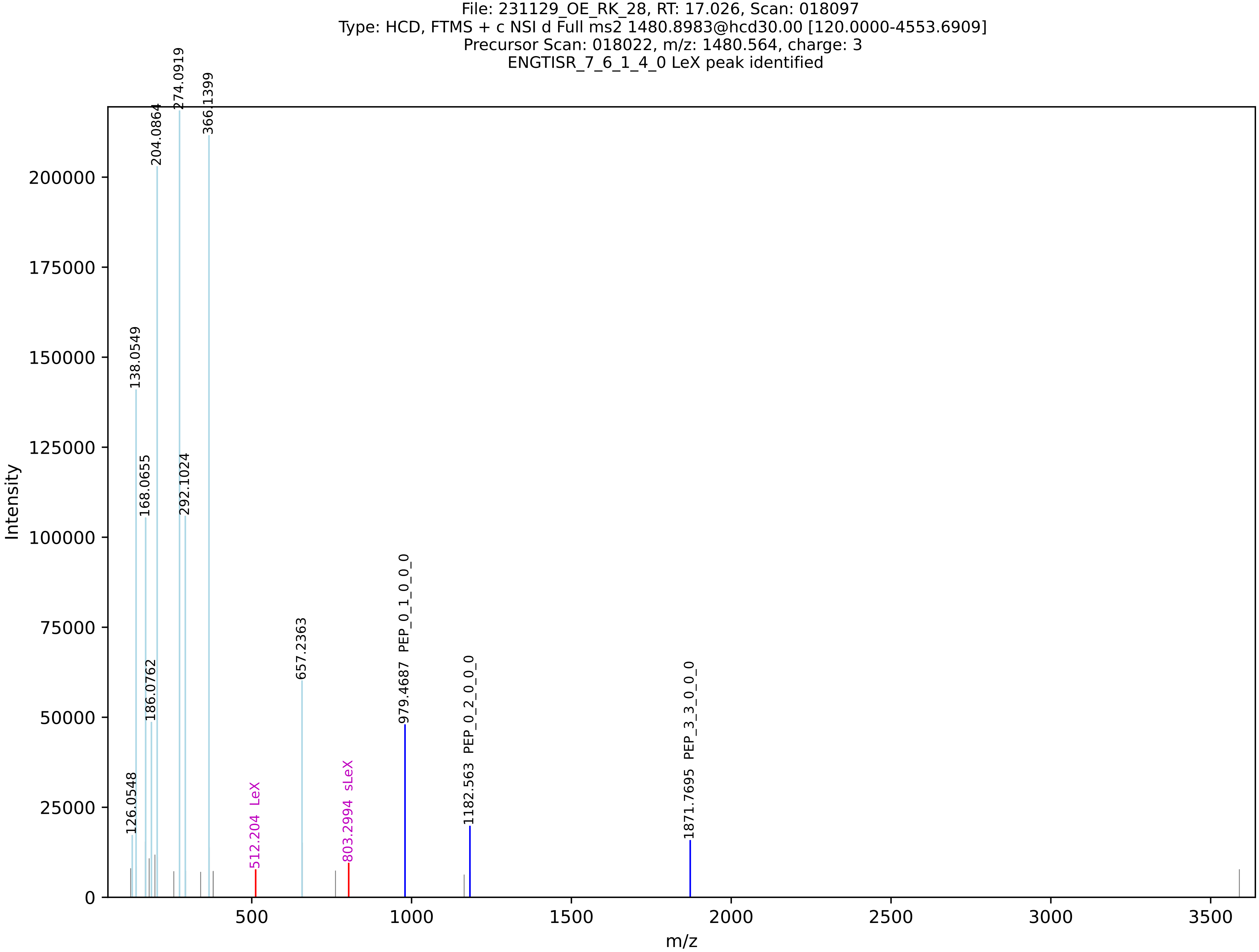

File: 231129_OE_RK_28, RT: 17.026, Scan: 018097
Type: HCD, FTMS + c NSI d Full ms2 1480.8983@hcd30.00 [120.0000-4553.6909]
Precursor Scan: 018022, m/z: 1480.564, charge: 3
ENGTISR_7_6_1_4_0 LeX peak identified
Intensity
m/z
274.0919
366.1399
204.0864
138.0549
168.0655
292.1024
657.2363
979.4687 PEP_0_1_0_0_
186.0762
1182.563 PEP_0_2_0_0_0
1871.7695 PEP_3_3_0_0_0
126.0548
512.204 LeX
803.2994 sLeX

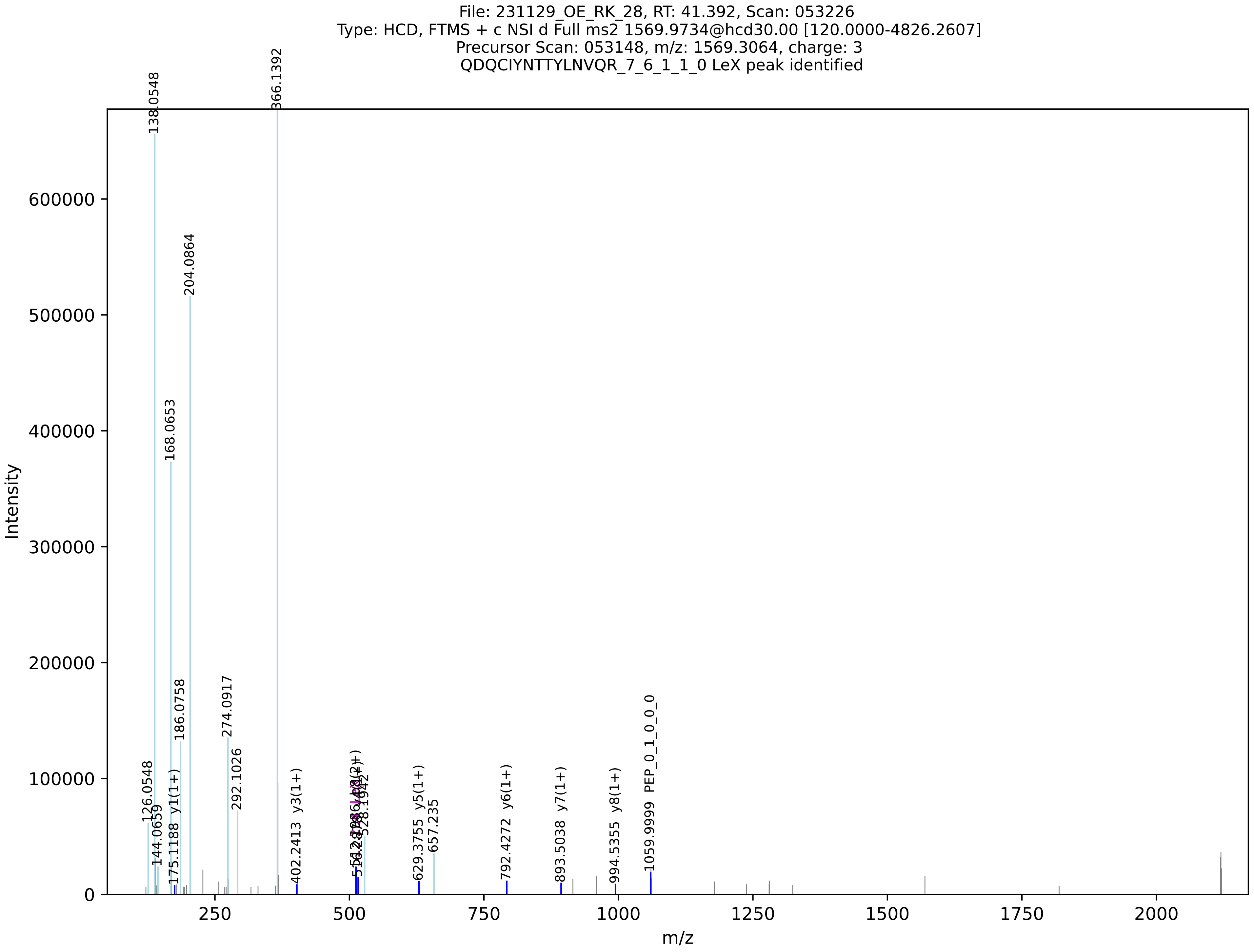
File: 231129_OE_RK_28, RT: 41.392, Scan: 053226
Type: HCD, FTMS + c NSI d Full ms2 1569.9734@hcd30.00 [120.0000-4826.2607]
Precursor Scan: 053148, m/z: 1569.3064, charge: 3
QDQCIYNTTYLNVQR_7_6_1_1_0 LeX peak identified
Intensity
m/z
366.1392
138.0548
204.0864
168.0653
186.0758
274.0917
292.1026
126.0548
144.0659
175.1188 y1(1+)
402.2413 y3(1+)
528.1942
629.3755 y5(1+)
657.235
792.4272 y6(1+)
893.5038 y7(1+)
994.5355 y8(1+)
1059.9999 PEP_0_1_0_0_0
600000
500000
400000
300000
200000
100000
0
250
500
750
1000
1250
1500
1750
2000

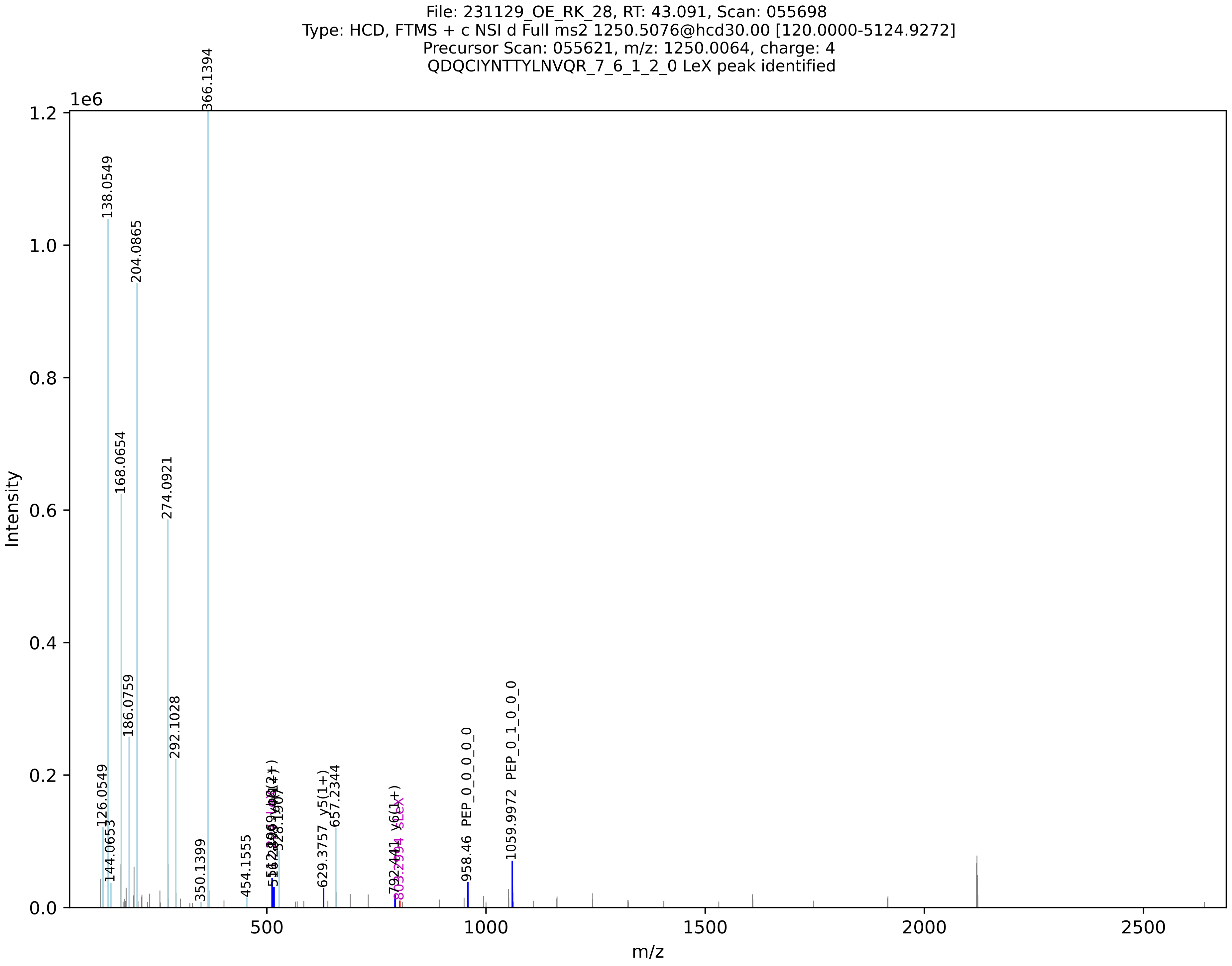
File: 231129_OE_RK_28, RT: 43.091, Scan: 055698
Type: HCD, FTMS + c NSI d Full ms2 1250.5076@hcd30.00 [120.0000-5124.9272]
Precursor Scan: 055621, m/z: 1250.0064, charge: 4
QDQCIYNTTYLNVQR_7_6_1_2_0 LeX peak identified
1e6
Intensity
m/z
366.1394
138.0549
204.0865
168.0654
274.0921
186.0759
292.1028
126.0549
144.0653
350.1399
454.1555
629.3757 y5(1+)
657.2344
792.441 y6(1+)
958.46 PEP_0_0_0_0_0
1059.9972 PEP_0_1_0_0_0

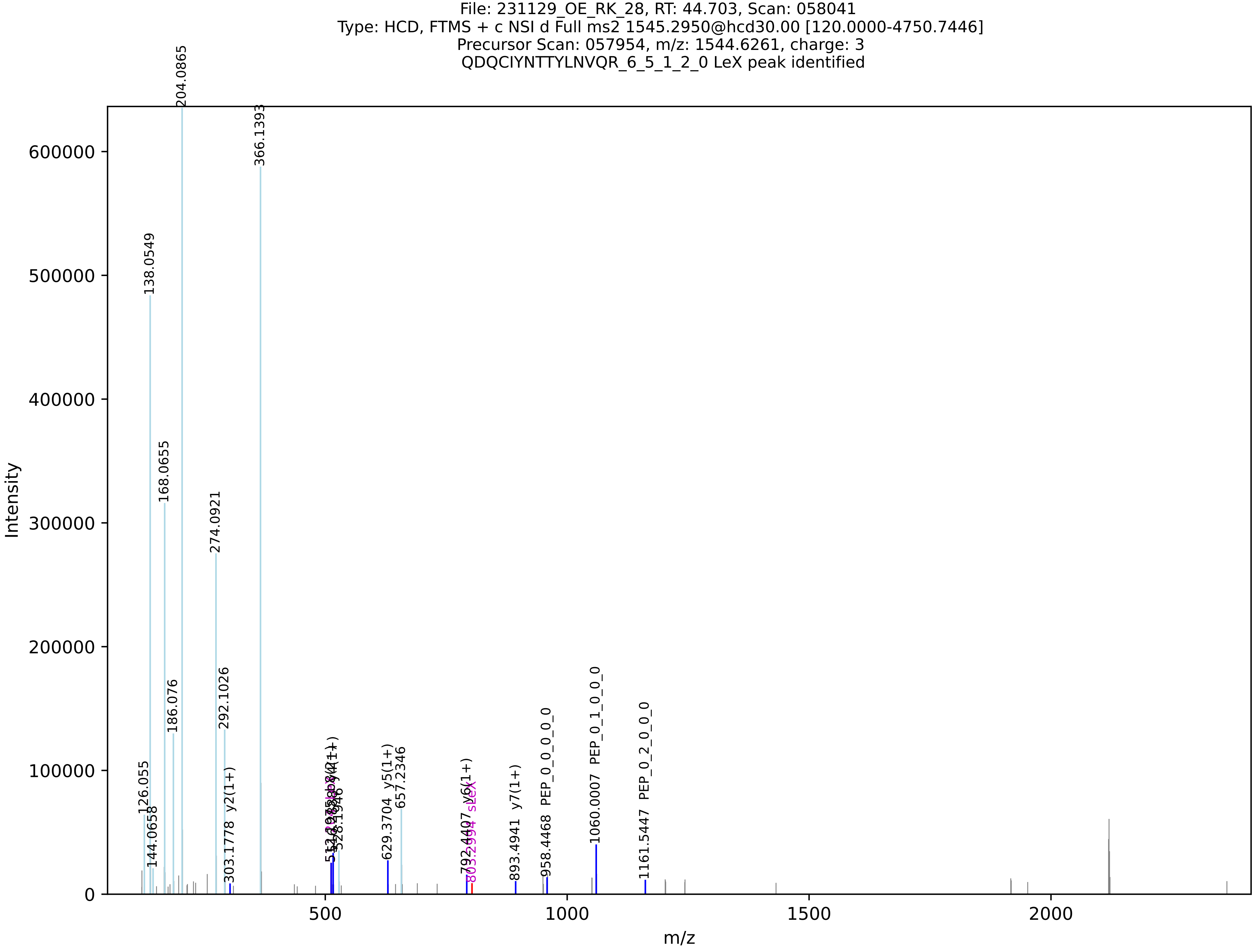

File: 231129_OE_RK_28, RT: 44.703, Scan: 058041
Type: HCD, FTMS + c NSI d Full ms2 1545.2950@hcd30.00 [120.0000-4750.7446]
Precursor Scan: 057954, m/z: 1544.6261, charge: 3
QDQCIYNTTYLNVQR_6_5_1_2_0 LeX peak identified
Intensity
m/z
204.0865
366.1393
138.0549
168.0655
274.0921
186.076
292.1026
126.055
144.0658
303.1778 y2(1+)
528.1946
629.3704 y5(1+)
657.2346
792.4407 y6(1+)
803.2994 sLeX
893.4941 y7(1+)
958.4468 PEP_0_0_0_0_0
1060.0007 PEP_0_1_0_0_0
1161.5447 PEP_0_2_0_0_0

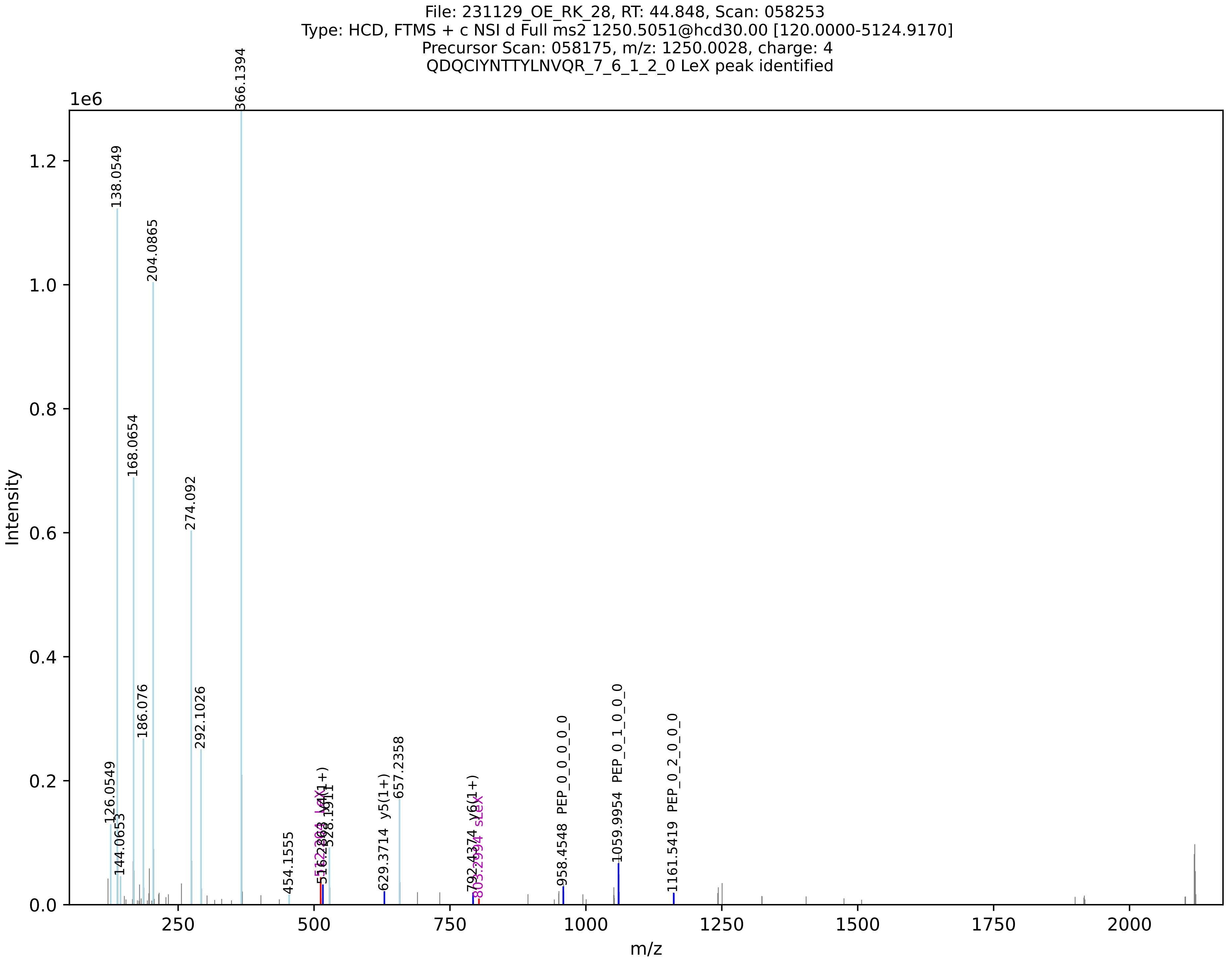

File: 231129_OE_RK_28, RT: 44.848, Scan: 058253
Type: HCD, FTMS + c NSI d Full ms2 1250.5051@hcd30.00 [120.0000-5124.9170]
Precursor Scan: 058175, m/z: 1250.0028, charge: 4
QDQCIYNTTYLNVQR_7_6_1_2_0 LeX peak identified
1e6
Intensity
m/z
366.1394
138.0549
204.0865
168.0654
274.092
186.076
292.1026
126.0549
144.0653
454.1555
512.2004 LeX
516.2863
528.1911
y4(1+)
629.3714 y5(1+)
657.2358
792.4374 y6(1+)
803.2994 sLeX
958.4548 PEP_0_0_0_0_0
1059.9954 PEP_0_1_0_0_0
1161.5419 PEP_0_2_0_0_0

File: 231129_OE_RK_28, RT: 44.901, Scan: 058330
Type: HCD, FTMS + c NSI d Full ms2 1545.2943@hcd30.00 [120.0000-4750.7427]
Precursor Scan: 058249, m/z: 1544.6257, charge: 3
QDQCIYNTTYLNVQR_6_5_1_2_0 LeX peak identified

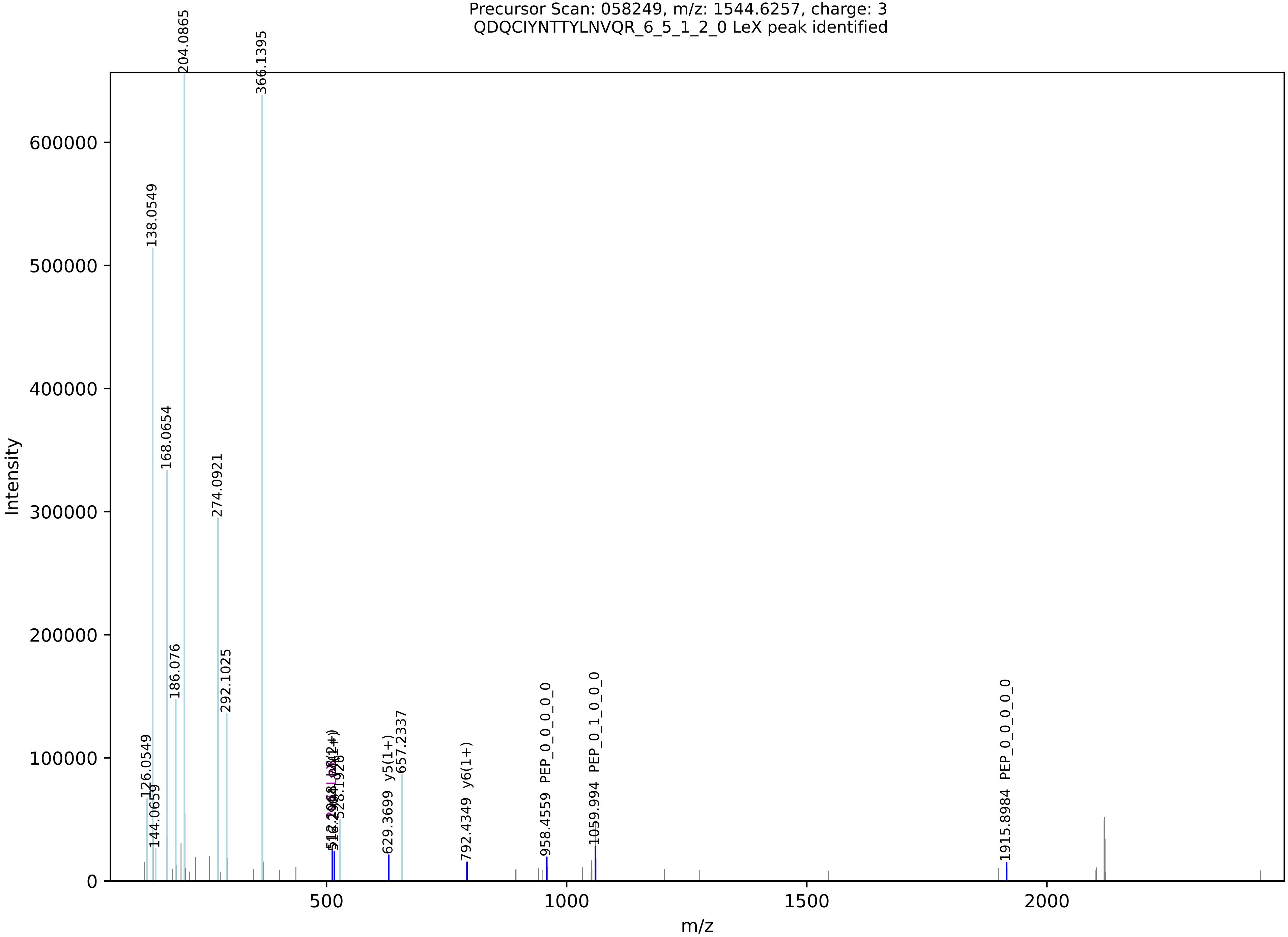

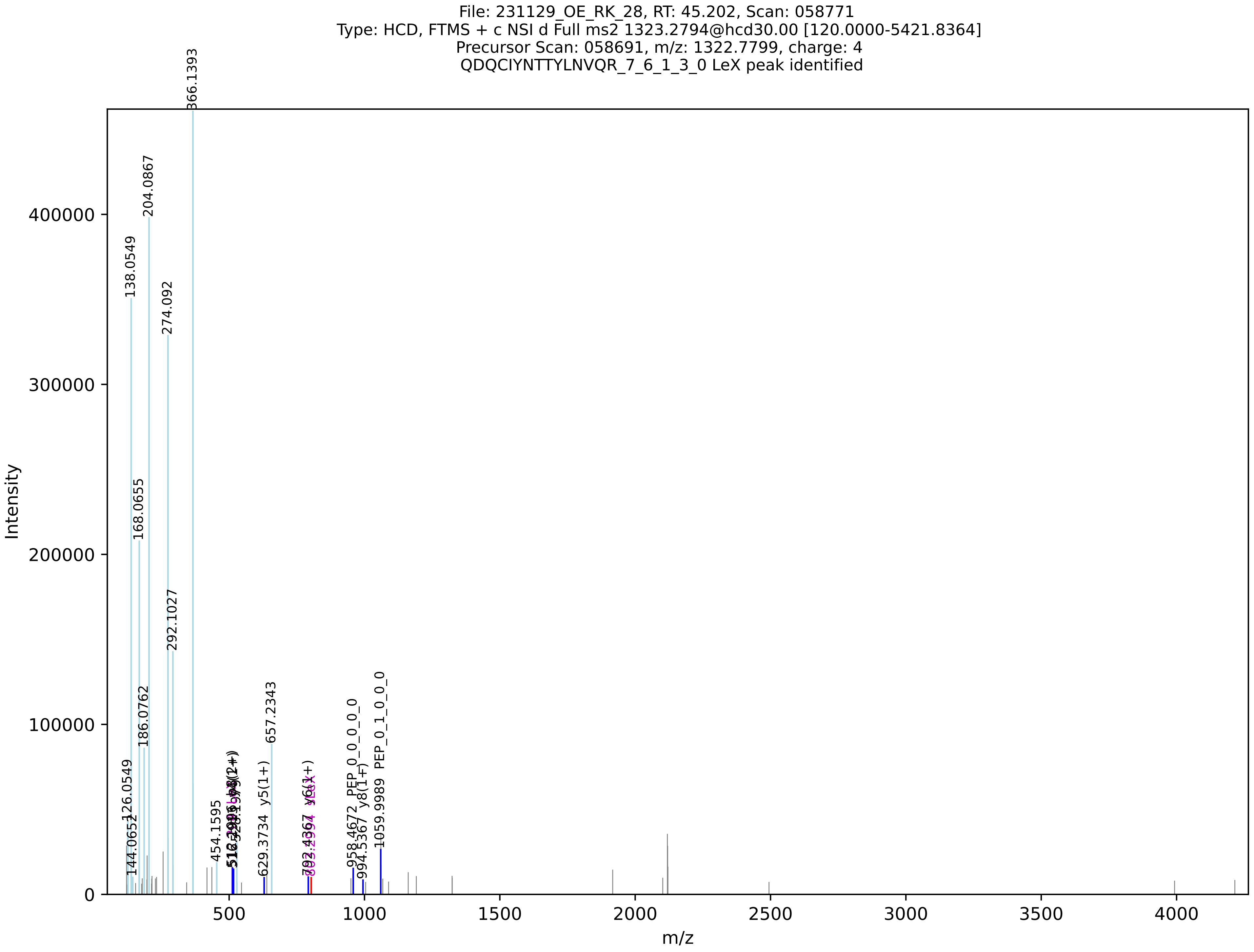
File: 231129_OE_RK_28, RT: 45.202, Scan: 058771
Type: HCD, FTMS + c NSI d Full ms2 1323.2794@hcd30.00 [120.0000-5421.8364]
Precursor Scan: 058691, m/z: 1322.7799, charge: 4
QDQCIYNTTYLNVQR_7_6_1_3_0 LeX peak identified
Intensity
m/z
366.1393
204.0867
138.0549
274.092
168.0655
292.1027
186.0762
657.2343
126.0549
144.0652
454.1595
629.3734 y5(1+)
792.4367 y6(1+)
958.4672 PEP_0_0_0_0_0
994.5367 y8(1+)
1059.9989 PEP_0_1_0_0_0
0
100000
200000
300000
400000
500
1000
1500
2000
2500
3000
3500
4000

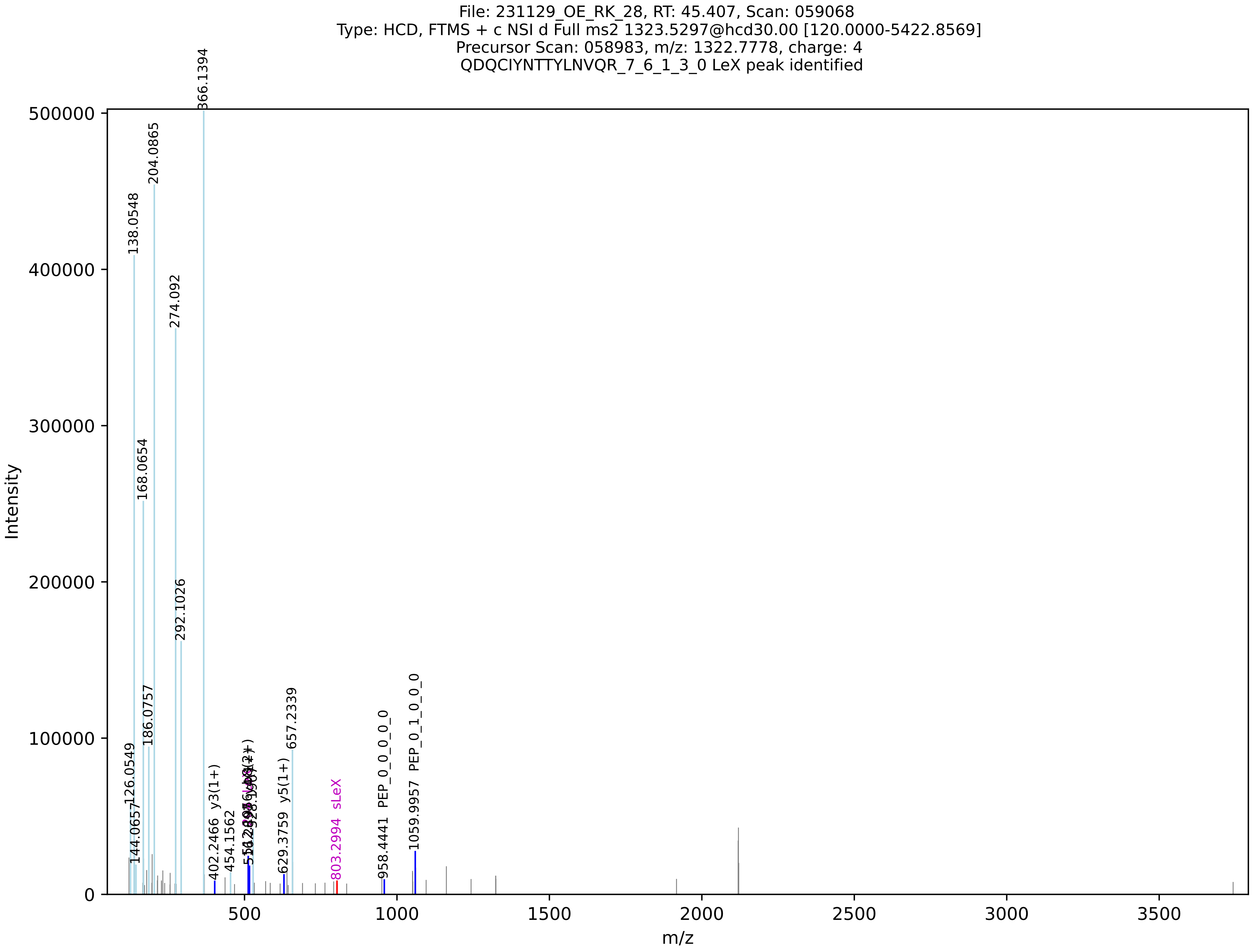
File: 231129_OE_RK_28, RT: 45.407, Scan: 059068
Type: HCD, FTMS + c NSI d Full ms2 1323.5297@hcd30.00 [120.0000-5422.8569]
Precursor Scan: 058983, m/z: 1322.7778, charge: 4
QDQCIYNTTYLNVQR_7_6_1_3_0 LeX peak identified
Intensity
m/z
366.1394
204.0865
138.0548
274.092
168.0654
292.1026
186.0757
126.0549
144.0657
402.2466 y3(1+)
454.1562
629.3759 y5(1+)
657.2339
803.2994 sLeX
958.4441 PEP_0_0_0_0_0
1059.9957 PEP_0_1_0_0_0
0
100000
200000
300000
400000
500000
500
1000
1500
2000
2500
3000
3500

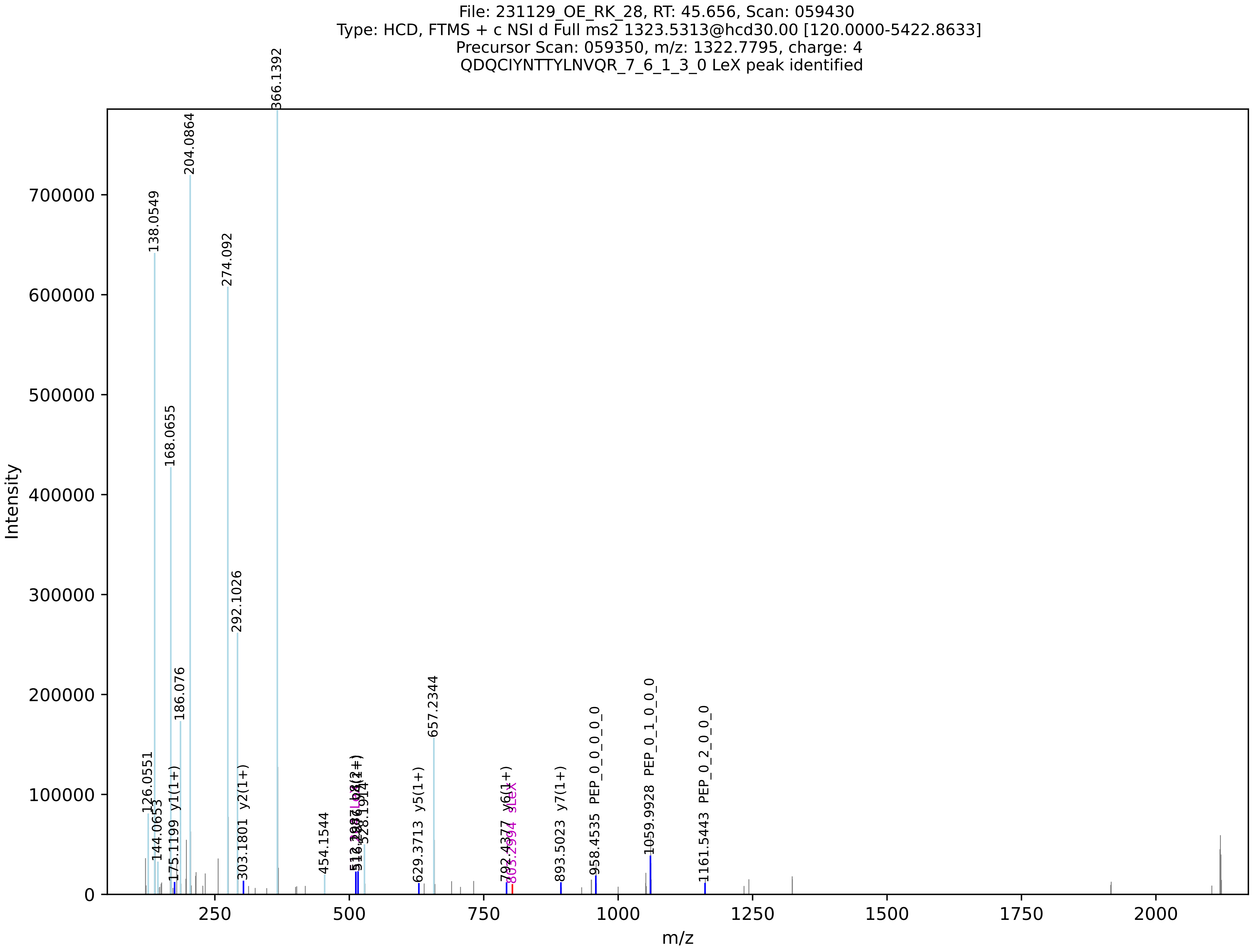
File: 231129_OE_RK_28, RT: 45.656, Scan: 059430
Type: HCD, FTMS + c NSI d Full ms2 1323.5313@hcd30.00 [120.0000-5422.8633]
Precursor Scan: 059350, m/z: 1322.7795, charge: 4
QDQCIYNTTYLNVQR_7_6_1_3_0 LeX peak identified
Intensity
m/z
366.1392
204.0864
138.0549
274.092
168.0655
292.1026
186.076
657.2344
126.0551
144.0653
175.1199 y1(1+)
303.1801 y2(1+)
454.1544
528.1914
629.3713 y5(1+)
792.4377 y6(1+)
803.2994 sLeX
893.5023 y7(1+)
958.4535 PEP_0_0_0_0_0
1059.9928 PEP_0_1_0_0_0
1161.5443 PEP_0_2_0_0_0
0
100000
200000
300000
400000
500000
600000
700000
250
500
750
1000
1250
1500
1750
2000

File: 231129_OE_RK_28, RT: 45.715, Scan: 059517
Type: HCD, FTMS + c NSI d Full ms2 1360.0425@hcd30.00 [120.0000-5571.8291]
Precursor Scan: 059424, m/z: 1359.0401, charge: 4
QDQCIYNTTYLNVQR_7_6_0_4_0 LeX peak identified

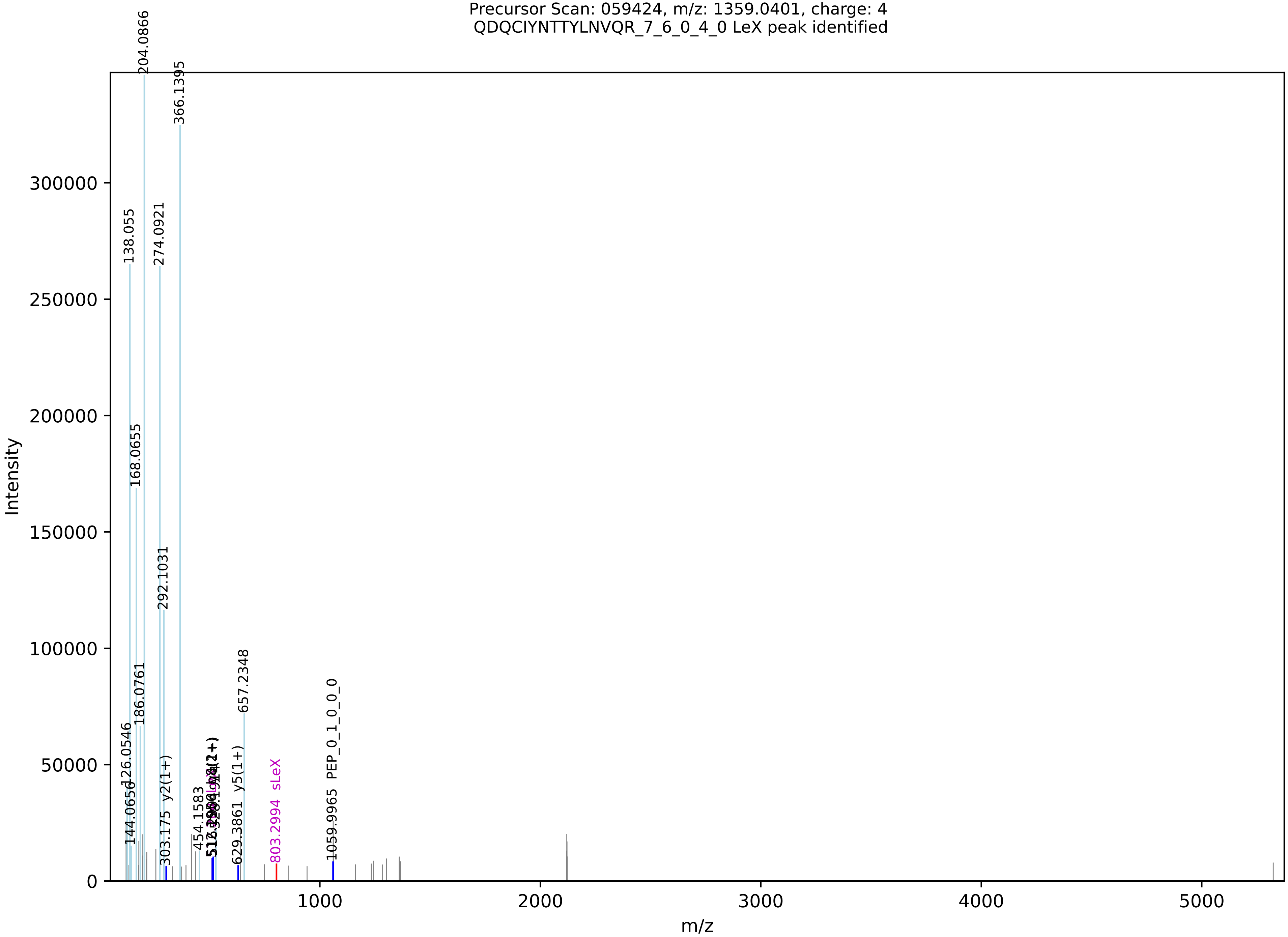

File: 231129_OE_RK_28, RT: 45.958, Scan: 059868
Type: HCD, FTMS + c NSI d Full ms2 1323.5295@hcd30.00 [120.0000-5422.8569]
Precursor Scan: 059789, m/z: 1322.7778, charge: 4
QDQCIYNTTYLNVQR_7_6_1_3_0 LeX peak identified

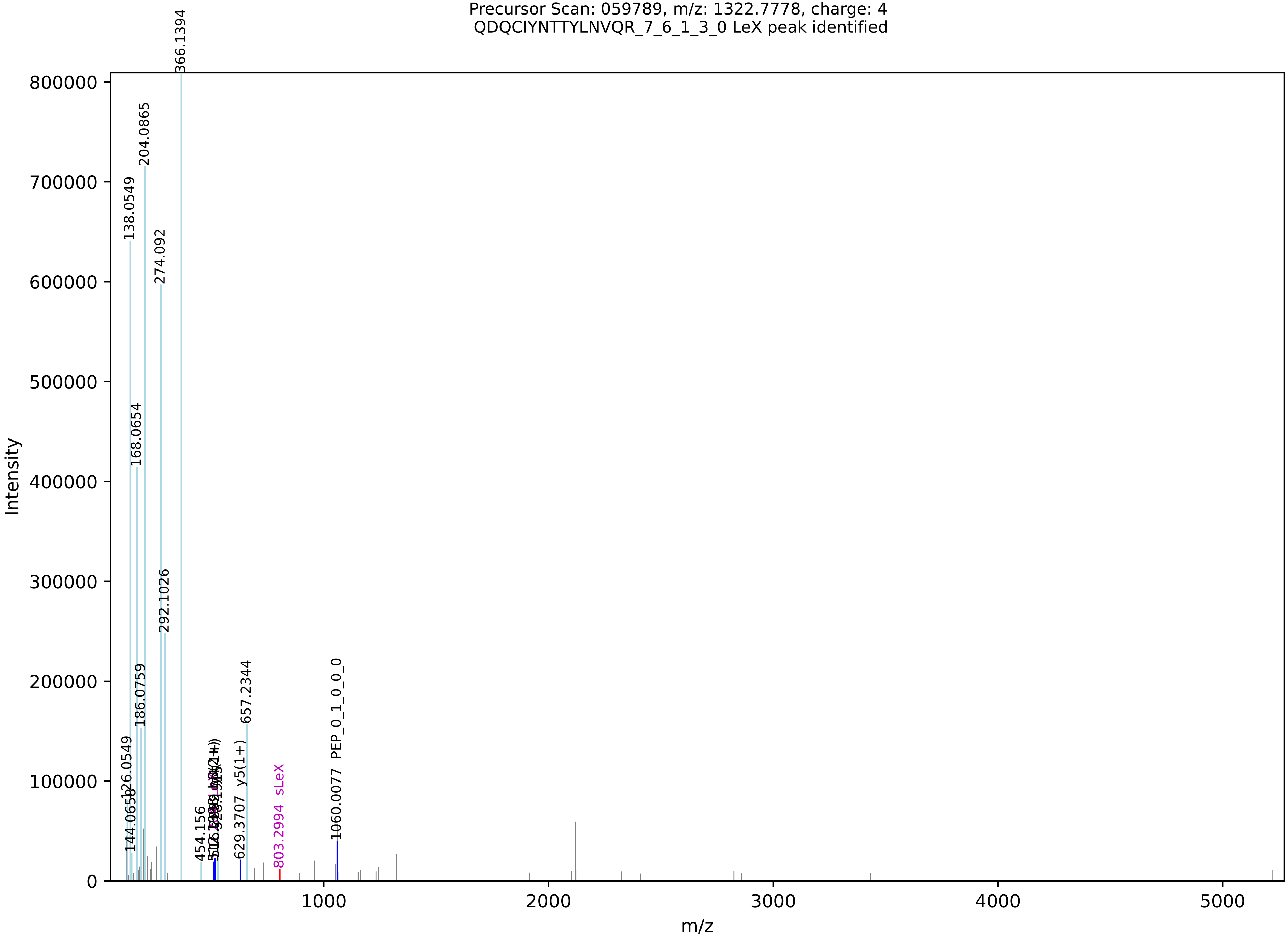

File: 231129_OE_RK_28, RT: 46.77, Scan: 061045
Type: HCD, FTMS + c NSI d Full ms2 1359.7913@hcd30.00 [120.0000-5570.8042]
Precursor Scan: 060962, m/z: 1359.0395, charge: 4
QDQCIYNTTYLNVQR_7_6_0_4_0 LeX peak identified

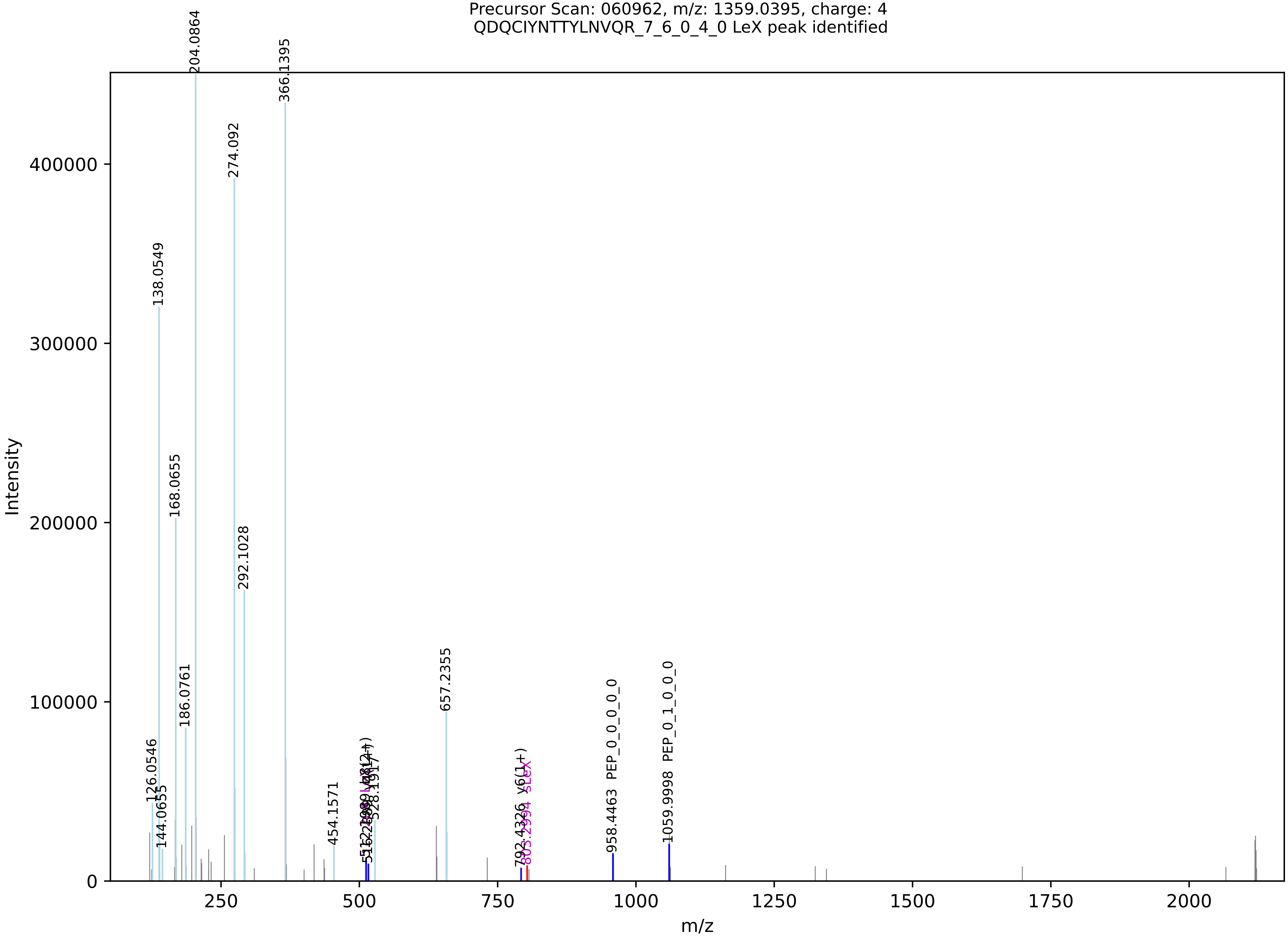

File: 231129_OE_RK_28, RT: 47.023, Scan: 061409
Type: HCD, FTMS + c NSI d Full ms2 1642.3259@hcd30.00 [120.0000-5047.6592]
Precursor Scan: 061325, m/z: 1641.6577, charge: 3
QDQCIYNTTYLNVQR_6_5_1_3_0 LeX peak identified

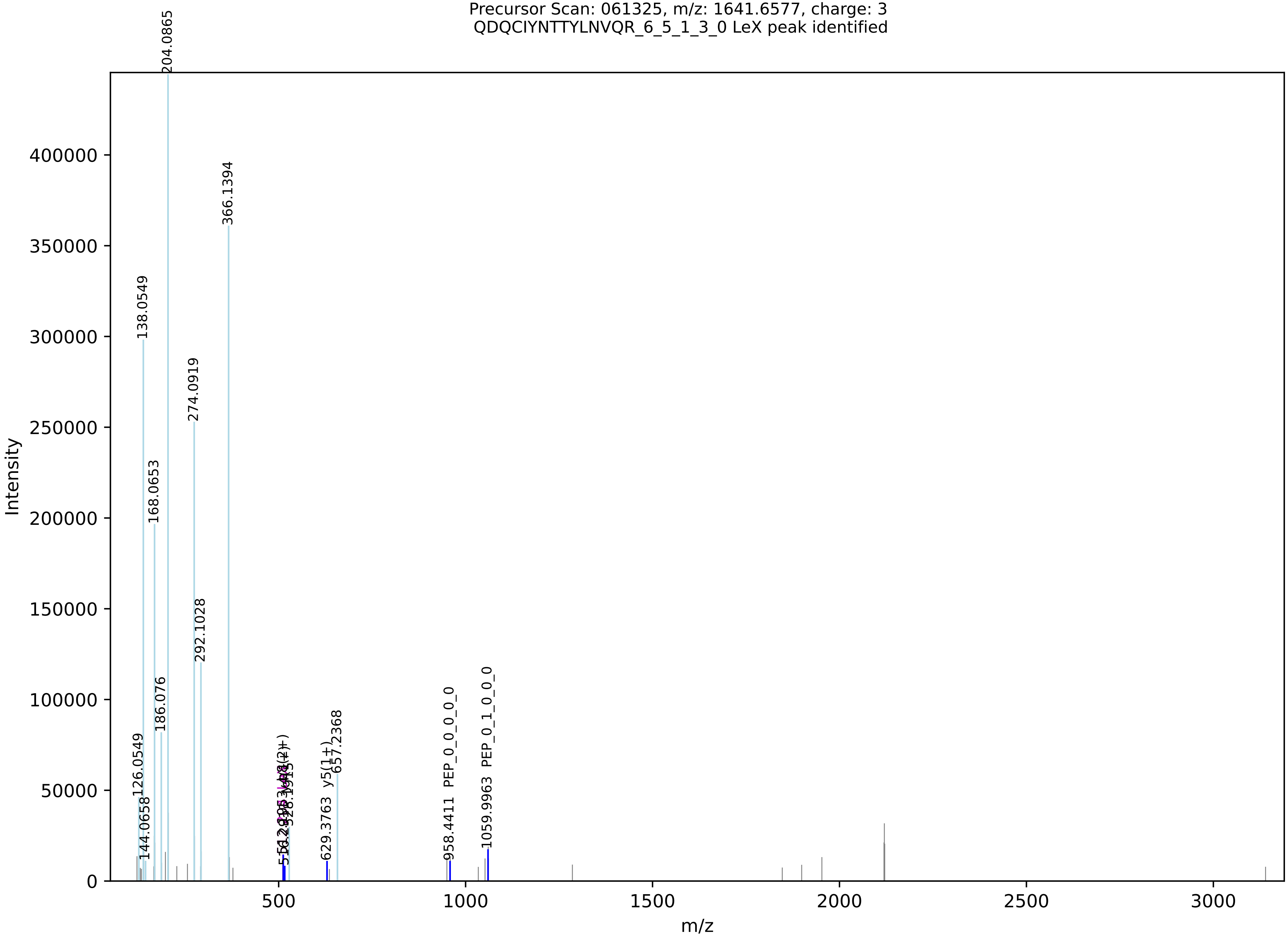

File: 231129_OE_RK_28, RT: 47.431, Scan: 061996
Type: HCD, FTMS + c NSI d Full ms2 1642.6603@hcd30.00 [120.0000-5048.6821]
Precursor Scan: 061913, m/z: 1641.6579, charge: 3
QDQCIYNTTYLNVQR_6_5_1_3_0 LeX peak identified

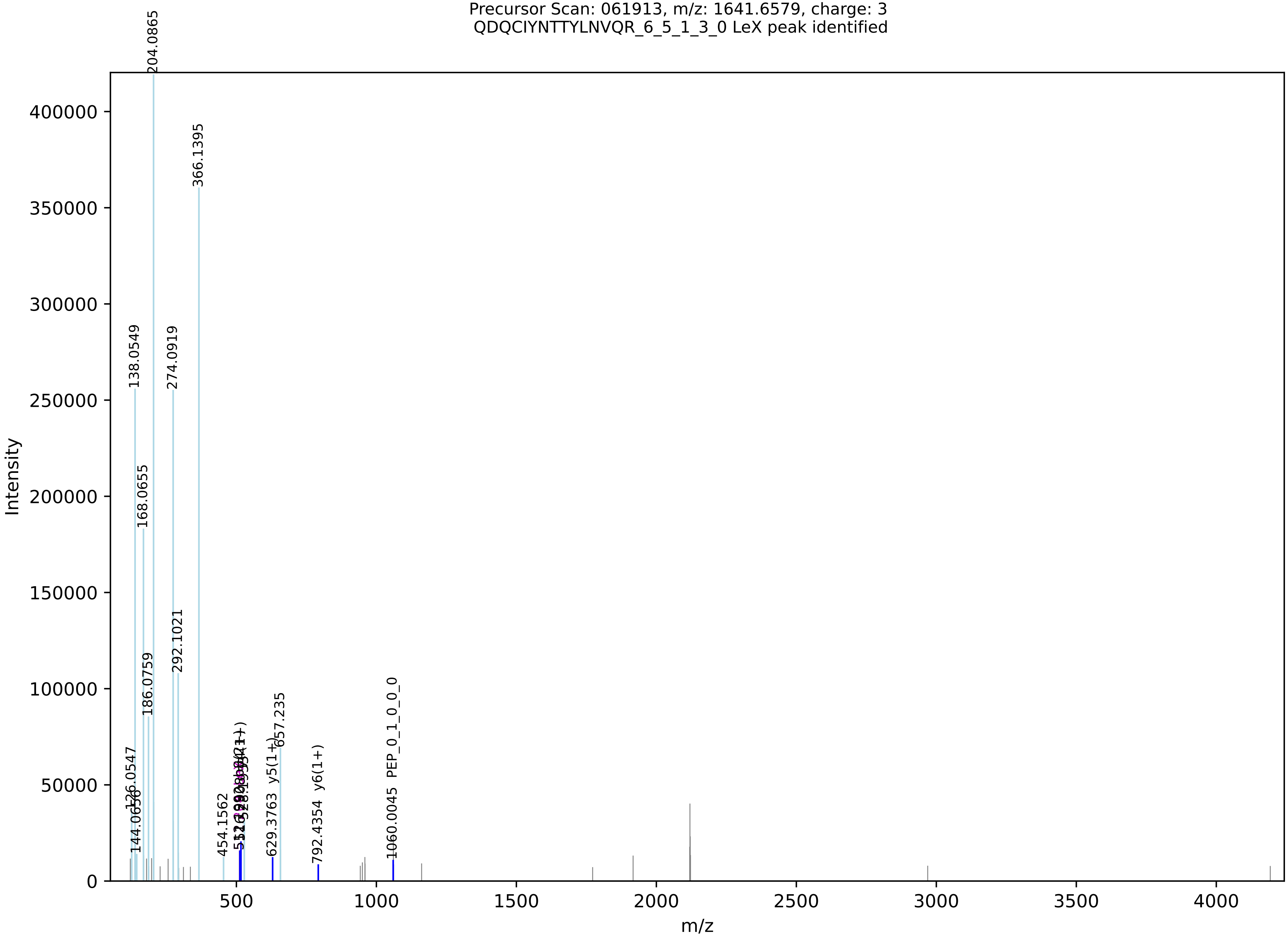

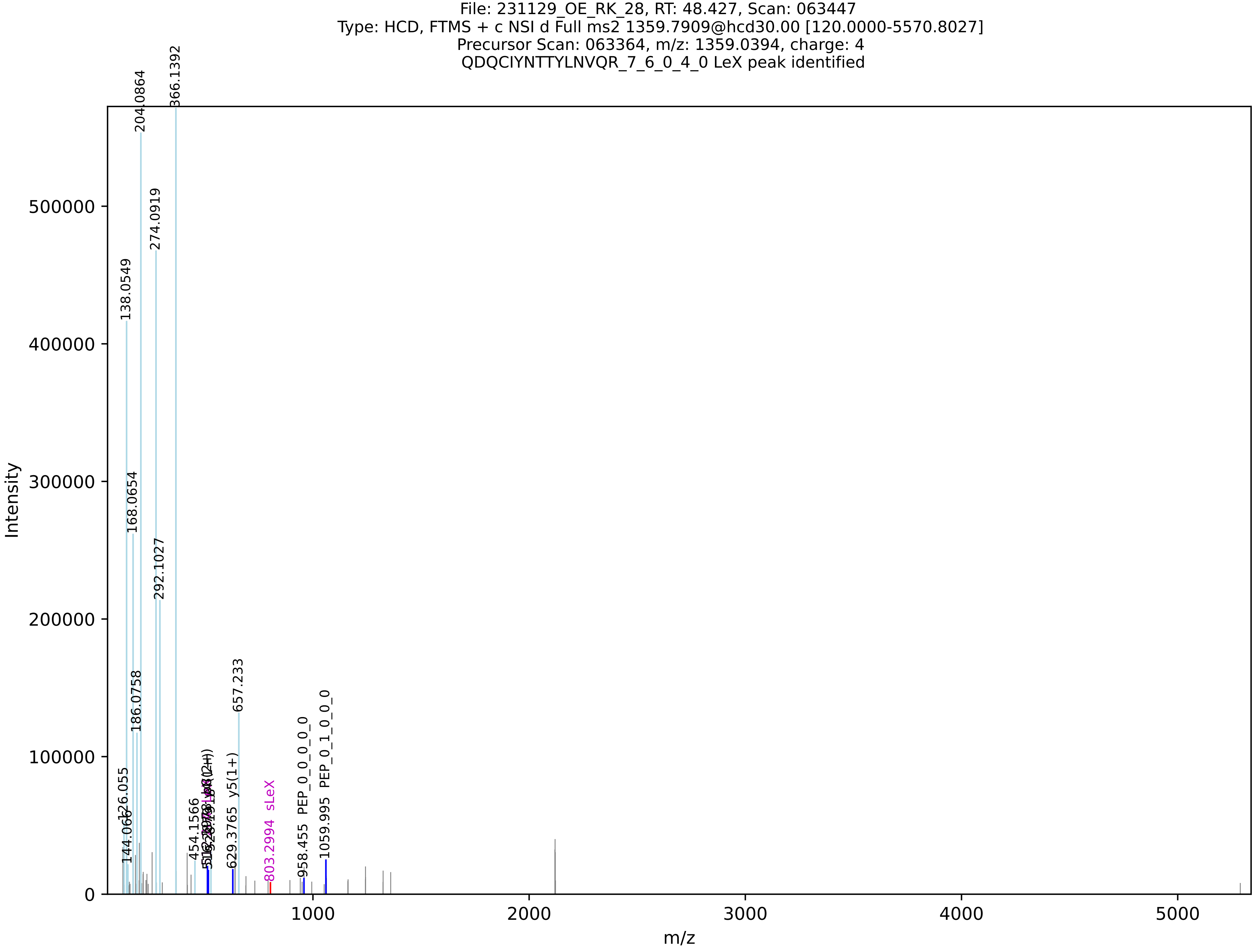
File: 231129_OE_RK_28, RT: 48.427, Scan: 063447
Type: HCD, FTMS + c NSI d Full ms2 1359.7909@hcd30.00 [120.0000-5570.8027]
Precursor Scan: 063364, m/z: 1359.0394, charge: 4
QDQCIYNTTYLNVQR_7_6_0_4_0 LeX peak identified
Intensity
m/z
366.1392
204.0864
274.0919
138.0549
168.0654
292.1027
186.0758
657.233
126.055
144.066
454.1566
629.3765 y5(1+)
803.2994 sLeX
958.455 PEP_0_0_0_0_0
1059.995 PEP_0_1_0_0_0

File: 231129_OE_RK_28, RT: 48.576, Scan: 063665
Type: HCD, FTMS + c NSI d Full ms2 1323.5294@hcd30.00 [120.0000-5422.8560]
Precursor Scan: 063586, m/z: 1322.7776, charge: 4
QDQCIYNTTYLNVQR_7_6_1_3_0 LeX peak identified

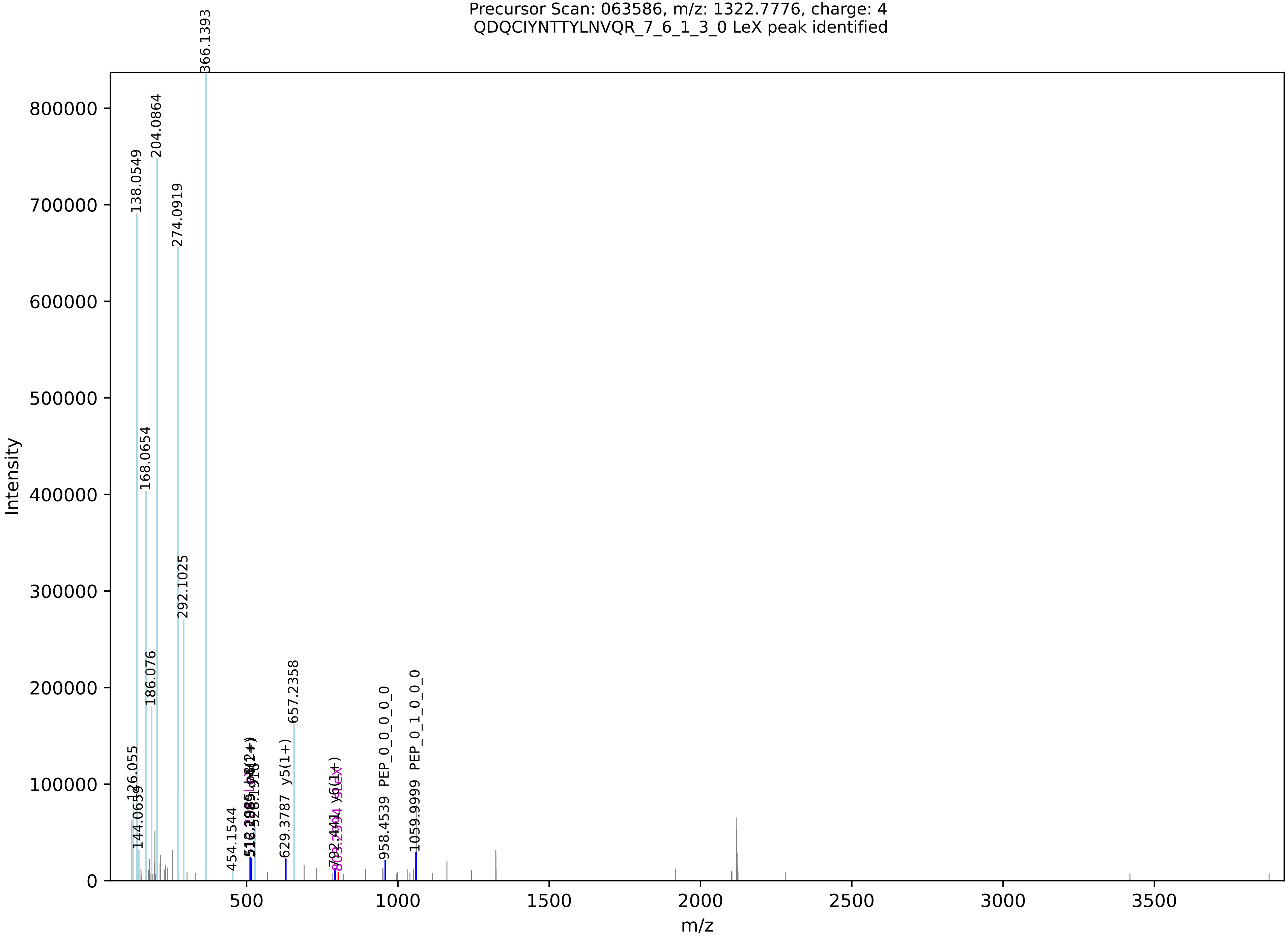

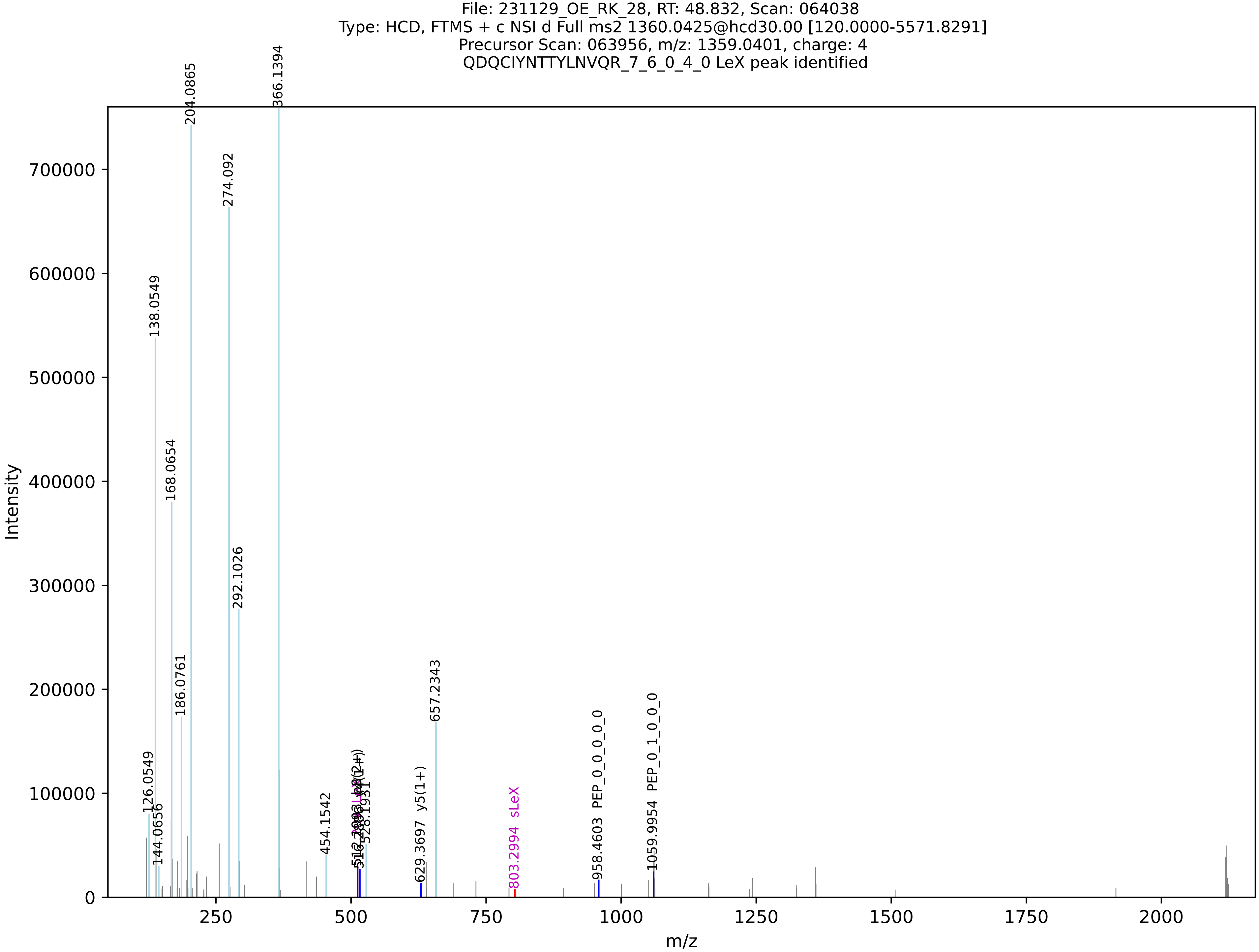

File: 231129_OE_RK_28, RT: 48.832, Scan: 064038
Type: HCD, FTMS + c NSI d Full ms2 1360.0425@hcd30.00 [120.0000-5571.8291]
Precursor Scan: 063956, m/z: 1359.0401, charge: 4
QDQCIYNTTYLNVQR_7_6_0_4_0 LeX peak identified
Intensity
m/z
366.1394
204.0865
274.092
138.0549
168.0654
292.1026
186.0761
657.2343
126.0549
144.0656
454.1542
528.1931
629.3697 y5(1+)
803.2994 sLeX
958.4603 PEP_0_0_0_0_0_
1059.9954 PEP_0_1_0_0_0_

File: 231129_OE_RK_28, RT: 49.888, Scan: 065566
Type: HCD, FTMS + c NSI d Full ms2 1323.5292@hcd30.00 [120.0000-5422.8550]
Precursor Scan: 065487, m/z: 1322.7776, charge: 4
QDQCIYNTTYLNVQR_7_6_1_3_0 LeX peak identified

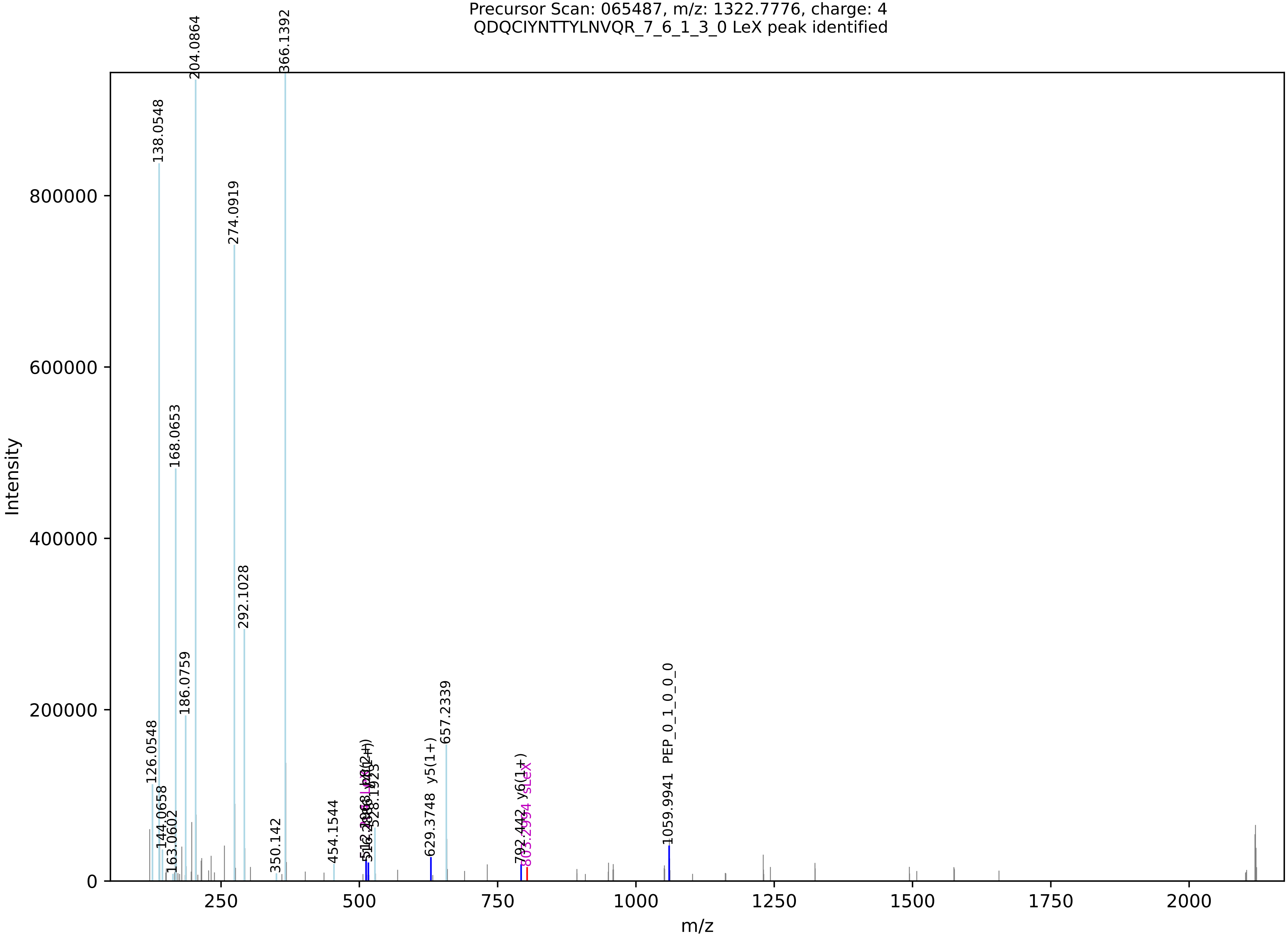

File: 231129_OE_RK_28, RT: 51.503, Scan: 067915
Type: HCD, FTMS + c NSI d Full ms2 1323.2769@hcd30.00 [120.0000-5421.8257]
Precursor Scan: 067836, m/z: 1323.0281, charge: 4
QDQCIYNTTYLNVQR_7_6_1_3_0 LeX peak identified

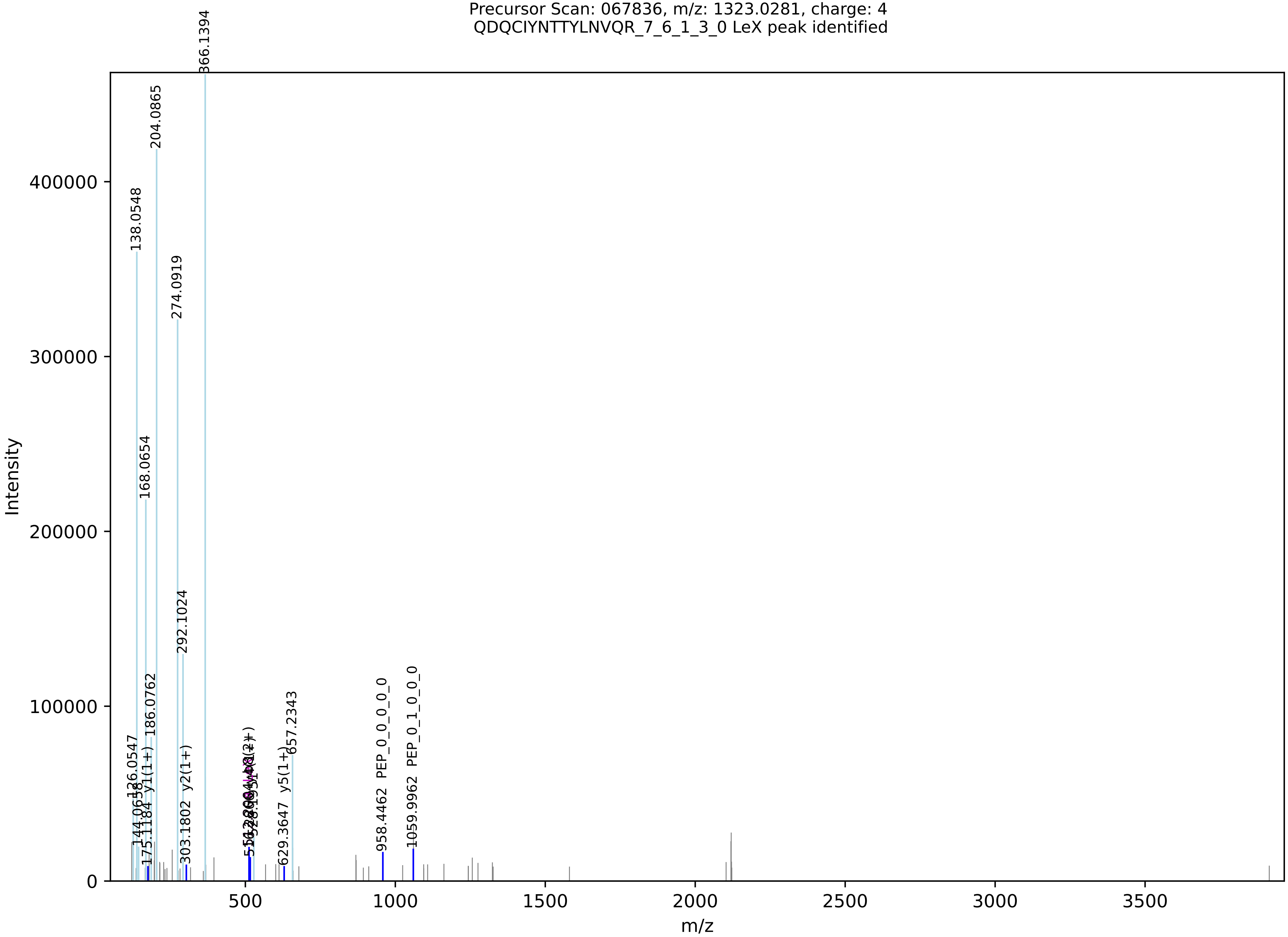

File: 231129_OE_RK_28, RT: 52.698, Scan: 069628
Type: HCD, FTMS + c NSI d Full ms2 1271.8770@hcd30.00 [120.0000-3914.0857]
Precursor Scan: 069530, m/z: 1271.2088, charge: 3
ALPQPQNVTSLLGCTH_6_4_0_1_0 LeX peak identified

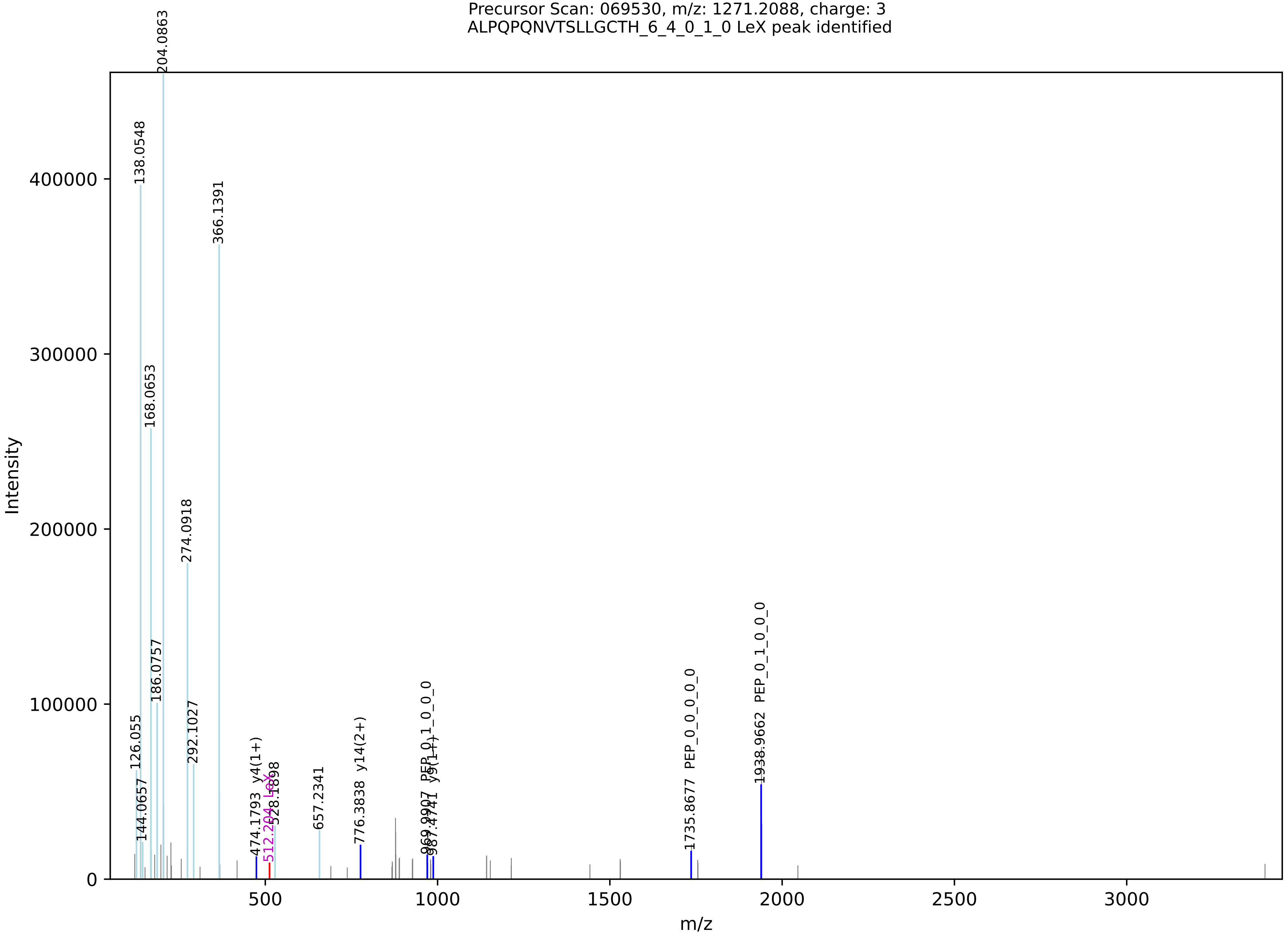

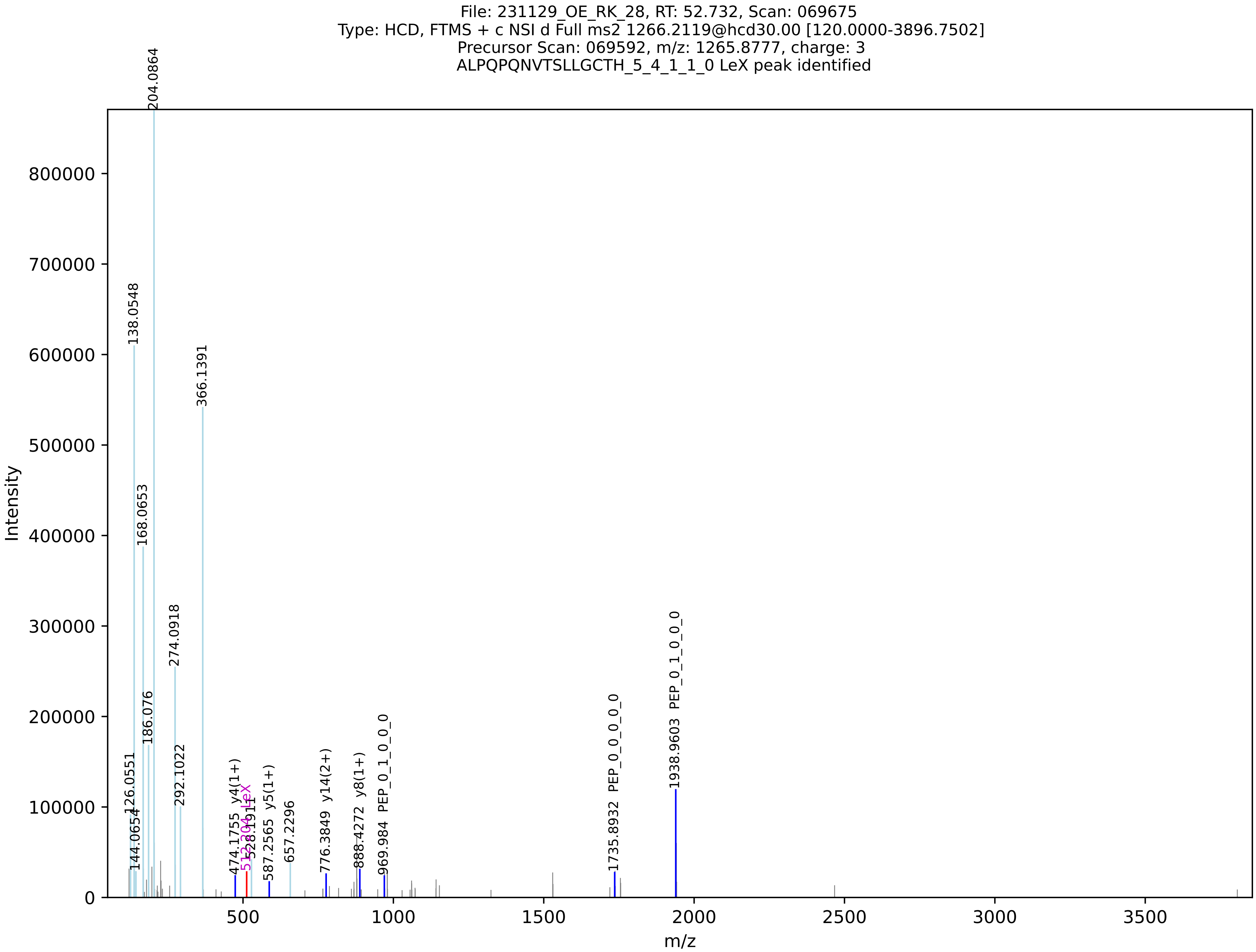
File: 231129_OE_RK_28, RT: 52.732, Scan: 069675
Type: HCD, FTMS + c NSI d Full ms2 1266.2119@hcd30.00 [120.0000-3896.7502]
Precursor Scan: 069592, m/z: 1265.8777, charge: 3
ALPQPQNVTSLLGCTH_5_4_1_1_0 LeX peak identified
Intensity
m/z
204.0864
138.0548
366.1391
168.0653
274.0918
186.076
292.1022
126.0551
144.0654
474.1755 y4(1+)
512.204 LeX
528.1911
587.2565 y5(1+)
657.2296
776.3849 y14(2+)
888.4272 y8(1+)
969.984 PEP_0_1_0_0_0
1735.8932 PEP_0_0_0_0_0
1938.9603 PEP_0_1_0_0_0
0
100000
200000
300000
400000
500000
600000
700000
800000
500
1000
1500
2000
2500
3000
3500

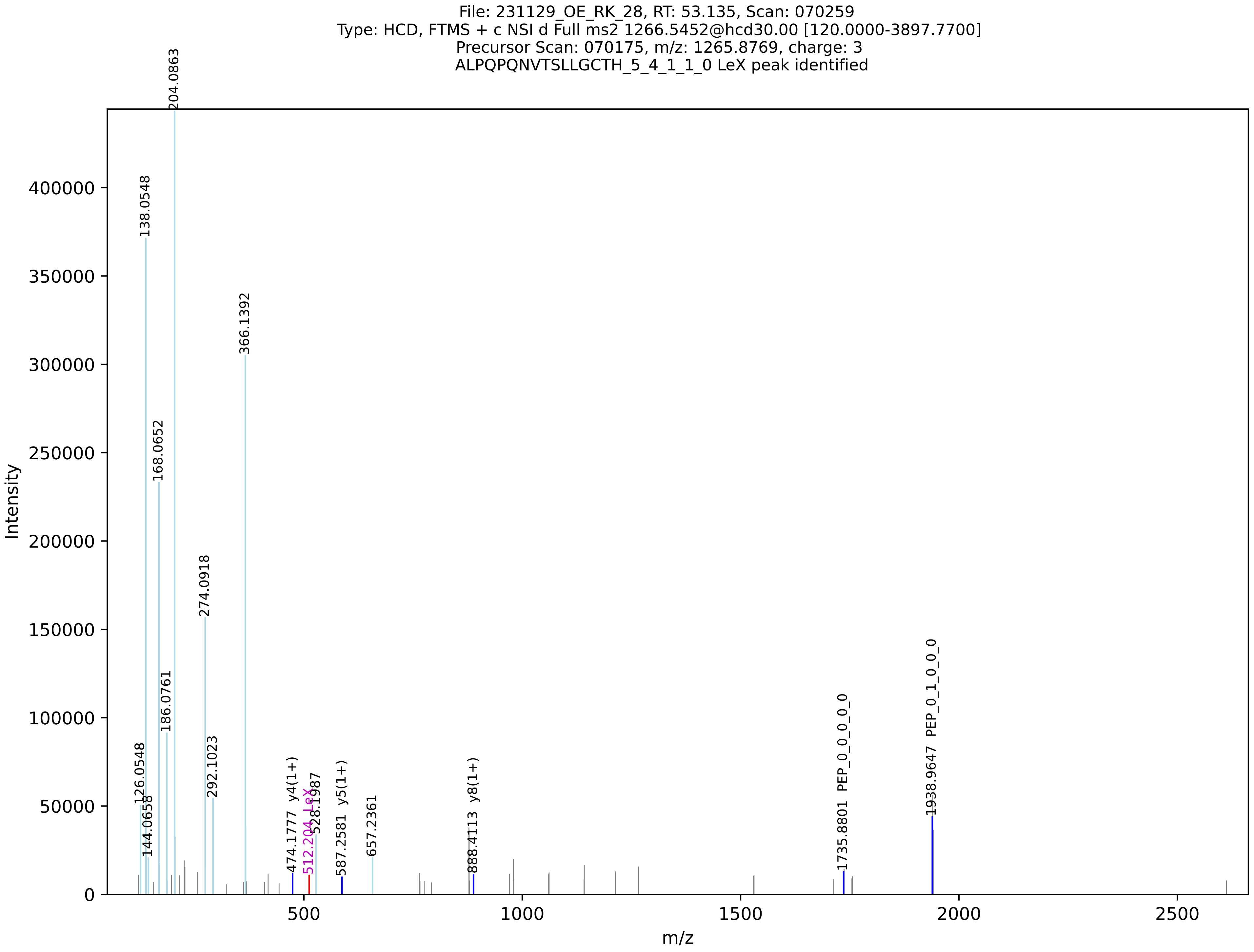

File: 231129_OE_RK_28, RT: 53.135, Scan: 070259
Type: HCD, FTMS + c NSI d Full ms2 1266.5452@hcd30.00 [120.0000-3897.7700]
Precursor Scan: 070175, m/z: 1265.8769, charge: 3
ALPQPQNVTSLLGCTH_5_4_1_1_0 LeX peak identified
Intensity
m/z
204.0863
138.0548
366.1392
168.0652
274.0918
186.0761
292.1023
126.0548
144.0658
474.1777 y4(1+)
512.204_LeX
528.1987
587.2581 y5(1+)
657.2361
888.4113 y8(1+)
1735.8801 PEP_0_0_0_0_0
1938.9647 PEP_0_1_0_0_0

File: 231129_OE_RK_28, RT: 62.661, Scan: 084104
Type: HCD, FTMS + c NSI d Full ms2 1332.3135@hcd30.00 [120.0000-5458.6953]
Precursor Scan: 084013, m/z: 1331.5617, charge: 4
TEPMQVALHCTNGSVVYHEVLNAMECK_5_4_2_1_0 LeX peak identified

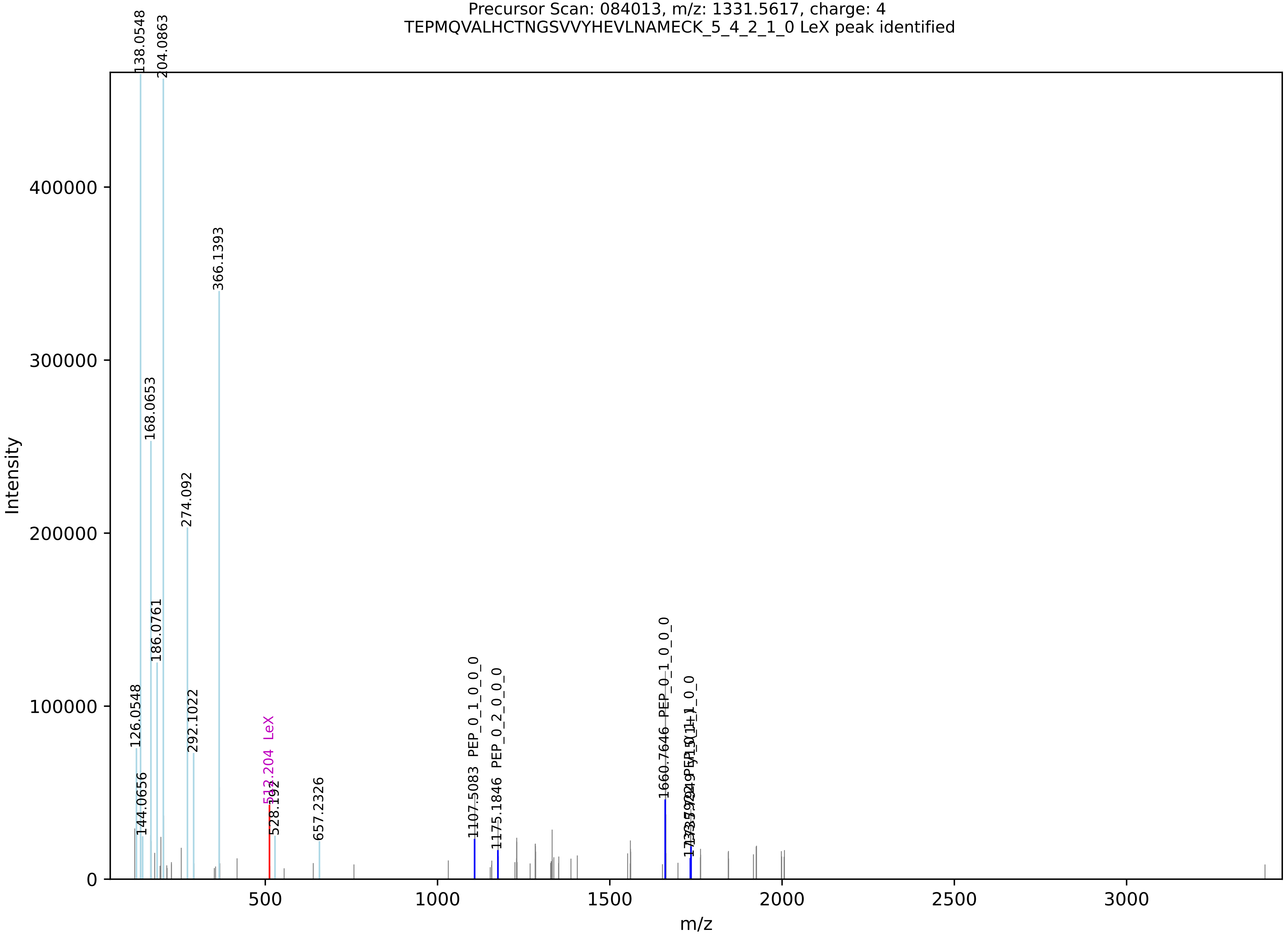

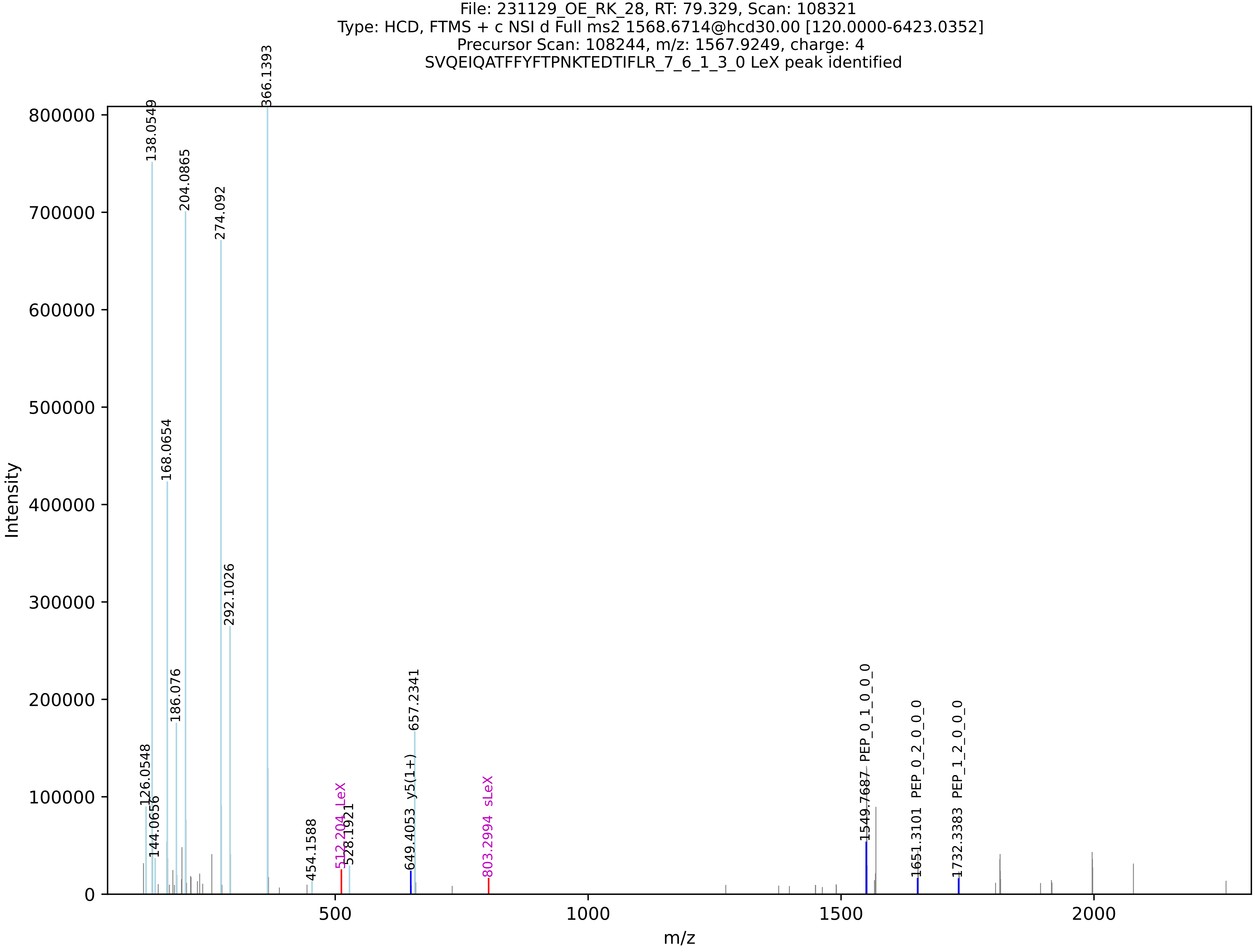
File: 231129_OE_RK_28, RT: 79.329, Scan: 108321
Type: HCD, FTMS + c NSI d Full ms2 1568.6714@hcd30.00 [120.0000-6423.0352]
Precursor Scan: 108244, m/z: 1567.9249, charge: 4
SVQEIQATFFYFTPNKTEDTIFLR_7_6_1_3_0 LeX peak identified
Intensity
m/z
366.1393
138.0549
204.0865
274.092
168.0654
292.1026
186.076
126.0548
144.0656
454.1588
512.204 LeX
528.1921
649.4053 y5(1+)
657.2341
803.2994 sLeX
1549.7687 PEP_0_1_0_0_0
1651.3101 PEP_0_2_0_0_0
1732.3383 PEP_1_2_0_0_0

File: 231129_OE_RK_29, RT: 42.847, Scan: 055244
Type: HCD, FTMS + c NSI d Full ms2 1250.7560@hcd30.00 [120.0000-5125.9404]
Precursor Scan: 055164, m/z: 1250.0042, charge: 4
QDQCIYNTTYLNVQR_7_6_1_2_0 LeX peak identified

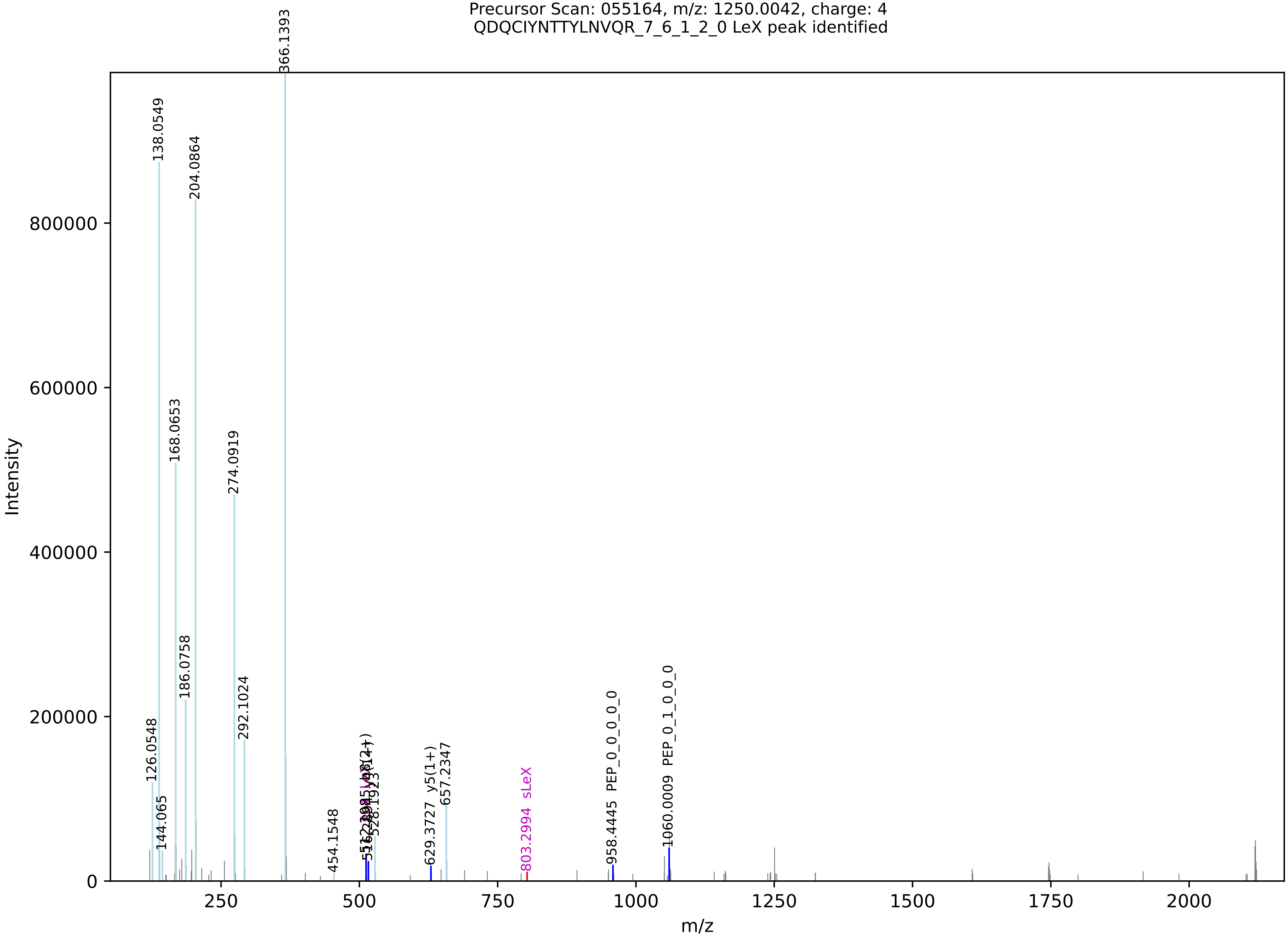

File: 231129_OE_RK_29, RT: 43.101, Scan: 055617
Type: HCD, FTMS + c NSI d Full ms2 1286.7712@hcd30.00 [120.0000-5272.8828]
Precursor Scan: 055531, m/z: 1286.2701, charge: 4
QDQCIYNTTYLNVQR_7_6_0_3_0 LeX peak identified

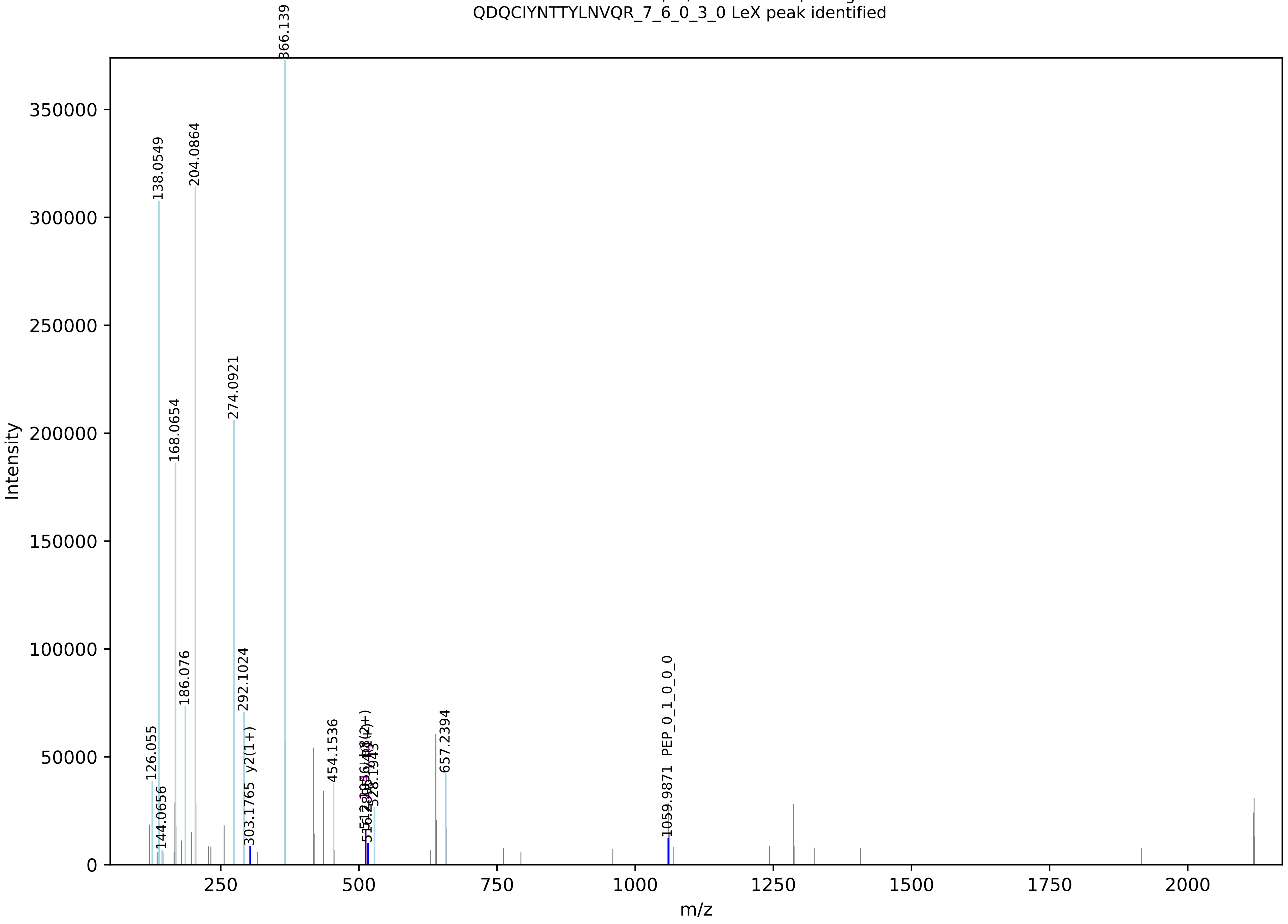

File: 231129_OE_RK_29, RT: 43.351, Scan: 055979
Type: HCD, FTMS + c NSI d Full ms2 1545.6283@hcd30.00 [120.0000-4751.7646]
Precursor Scan: 055901, m/z: 1544.6261, charge: 3
QDQCIYNTTYLNVQR_6_5_1_2_0 LeX peak identified

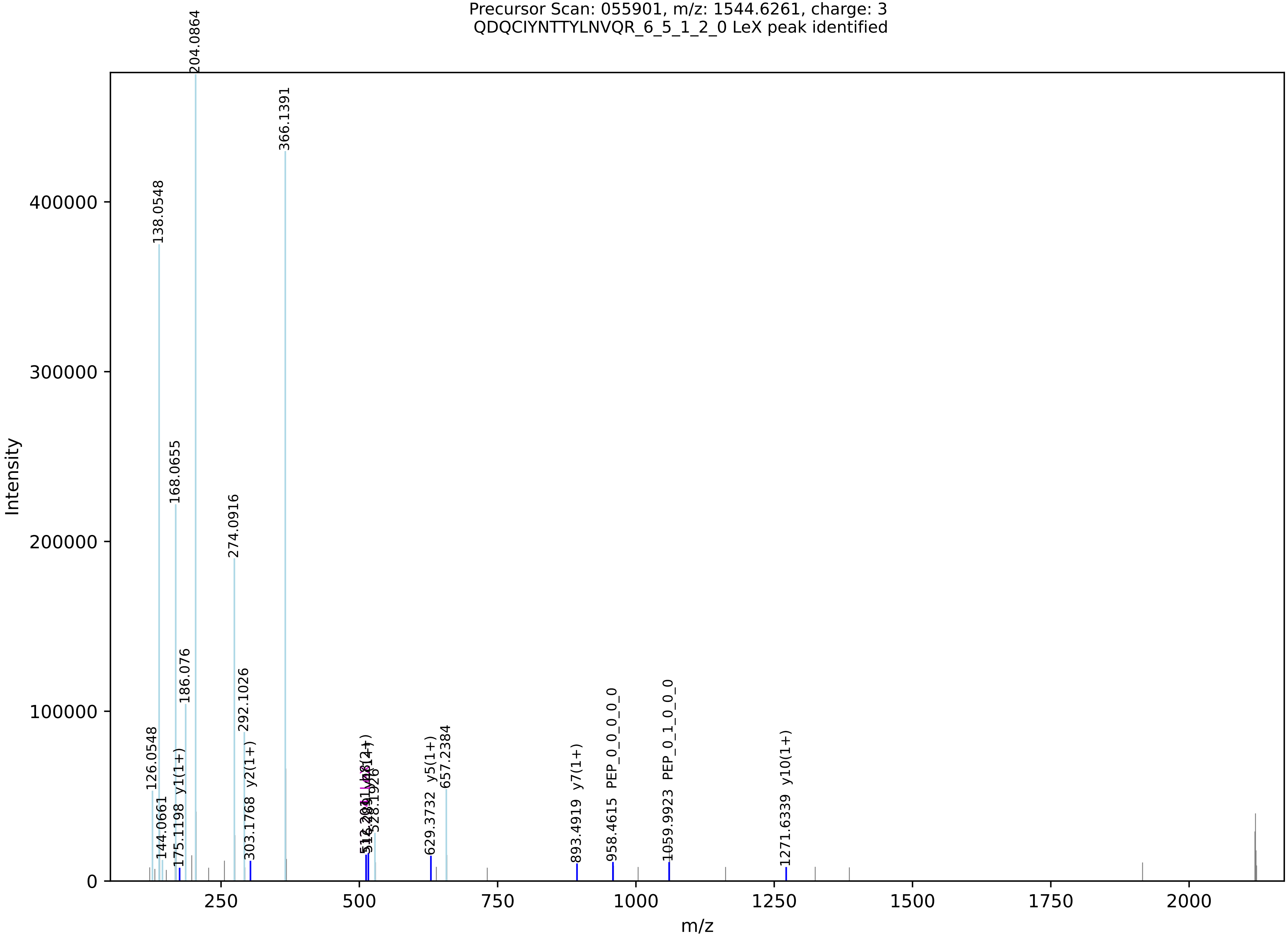

File: 231129_OE_RK_29, RT: 43.955, Scan: 056854
Type: HCD, FTMS + c NSI d Full ms2 1545.2944@hcd30.00 [120.0000-4750.7427]
Precursor Scan: 056777, m/z: 1544.6273, charge: 3
QDQCIYNTTYLNVQR_6_5_1_2_0 LeX peak identified

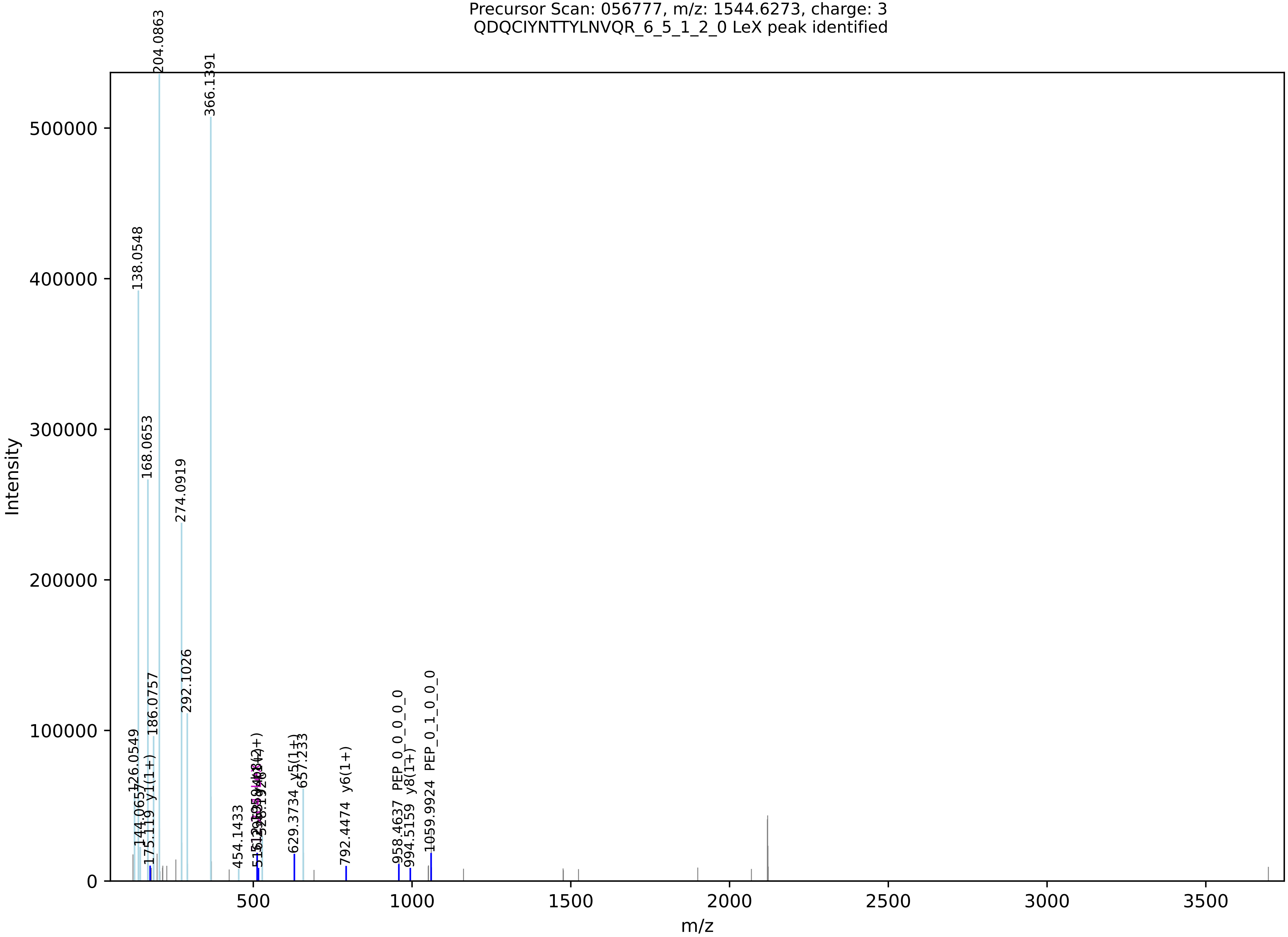

File: 231129_OE_RK_29, RT: 44.269, Scan: 057309
Type: HCD, FTMS + c NSI d Full ms2 1028.4363@hcd30.00 [120.0000-4218.8760]
Precursor Scan: 057217, m/z: 1027.6839, charge: 4
GLTFQQNASSMCVPDQDTAIR_5_4_1_0_0 LeX peak identified

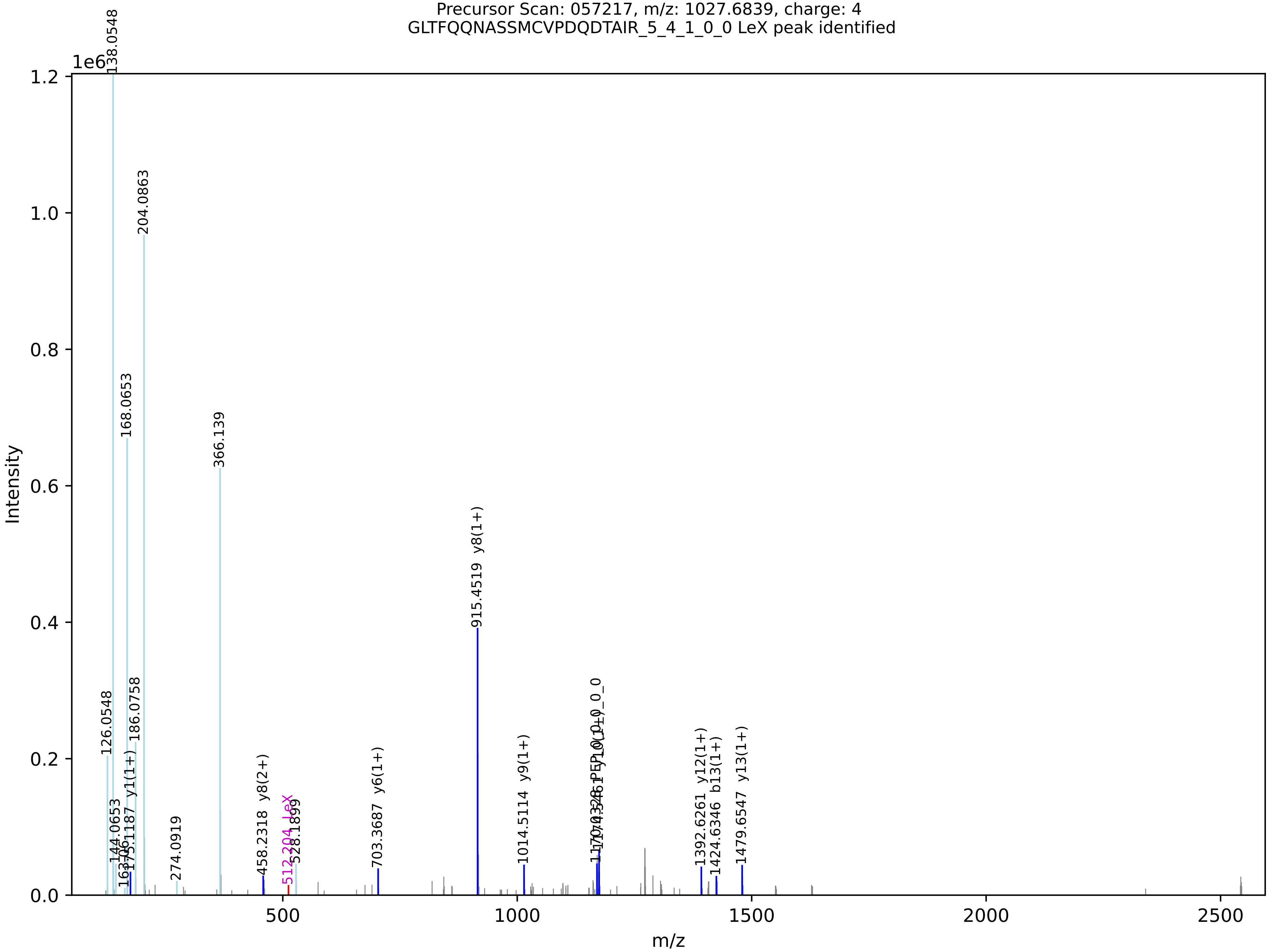

File: 231129_OE_RK_29, RT: 45.739, Scan: 059439
Type: HCD, FTMS + c NSI d Full ms2 1188.4944@hcd30.00 [120.0000-4871.9131]
Precursor Scan: 059335, m/z: 1187.9963, charge: 4
GLTFQQNASSMCVPDQDTAIR_5_5_2_1_0 LeX peak identified

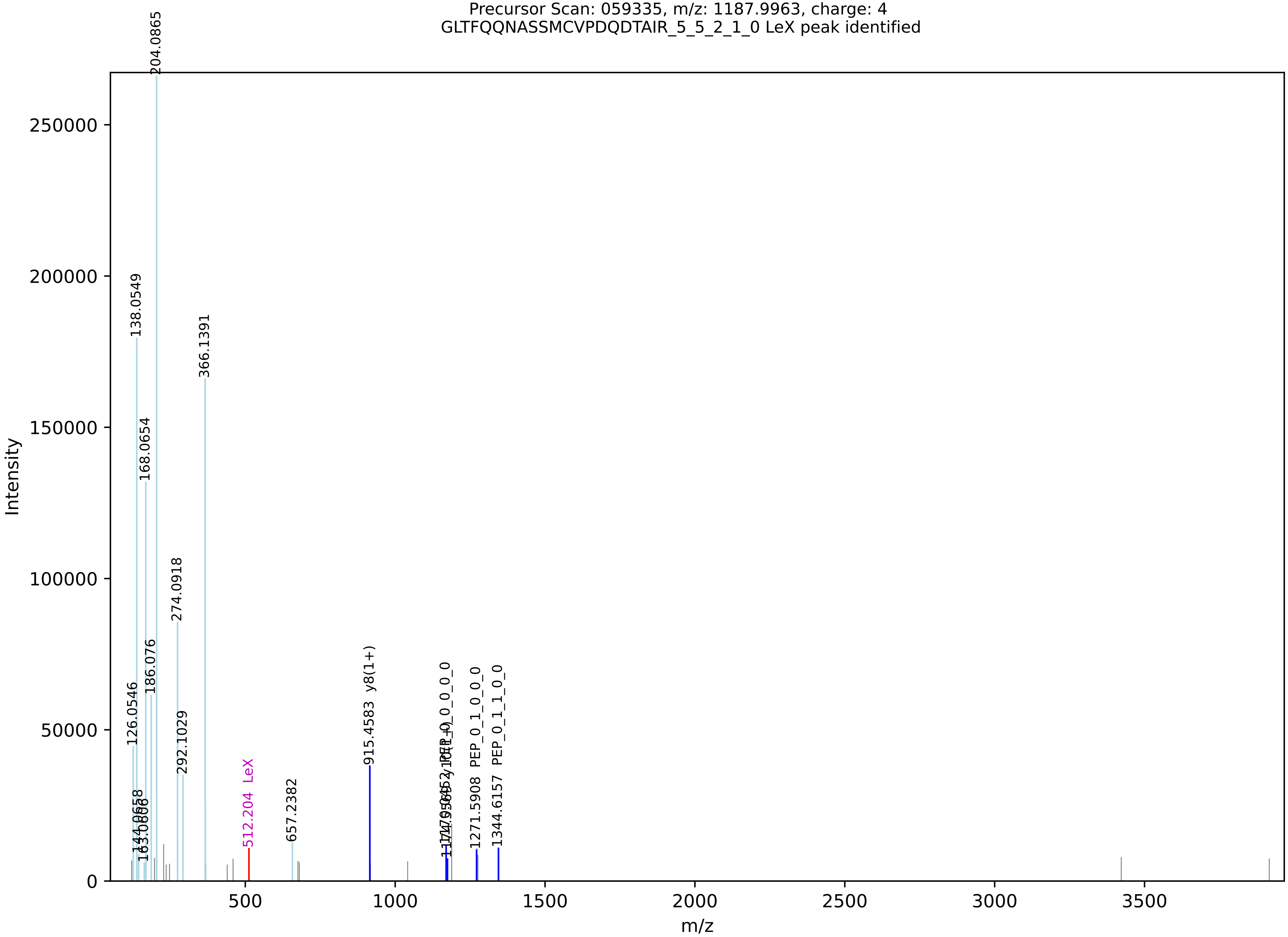

File: 231129_OE_RK_29, RT: 52.991, Scan: 069945
Type: HCD, FTMS + c NSI d Full ms2 1266.2106@hcd30.00 [120.0000-3896.7461]
Precursor Scan: 069865, m/z: 1265.8764, charge: 3
ALPQPQNVTSLLGCTH_5_4_1_1_0 LeX peak identified

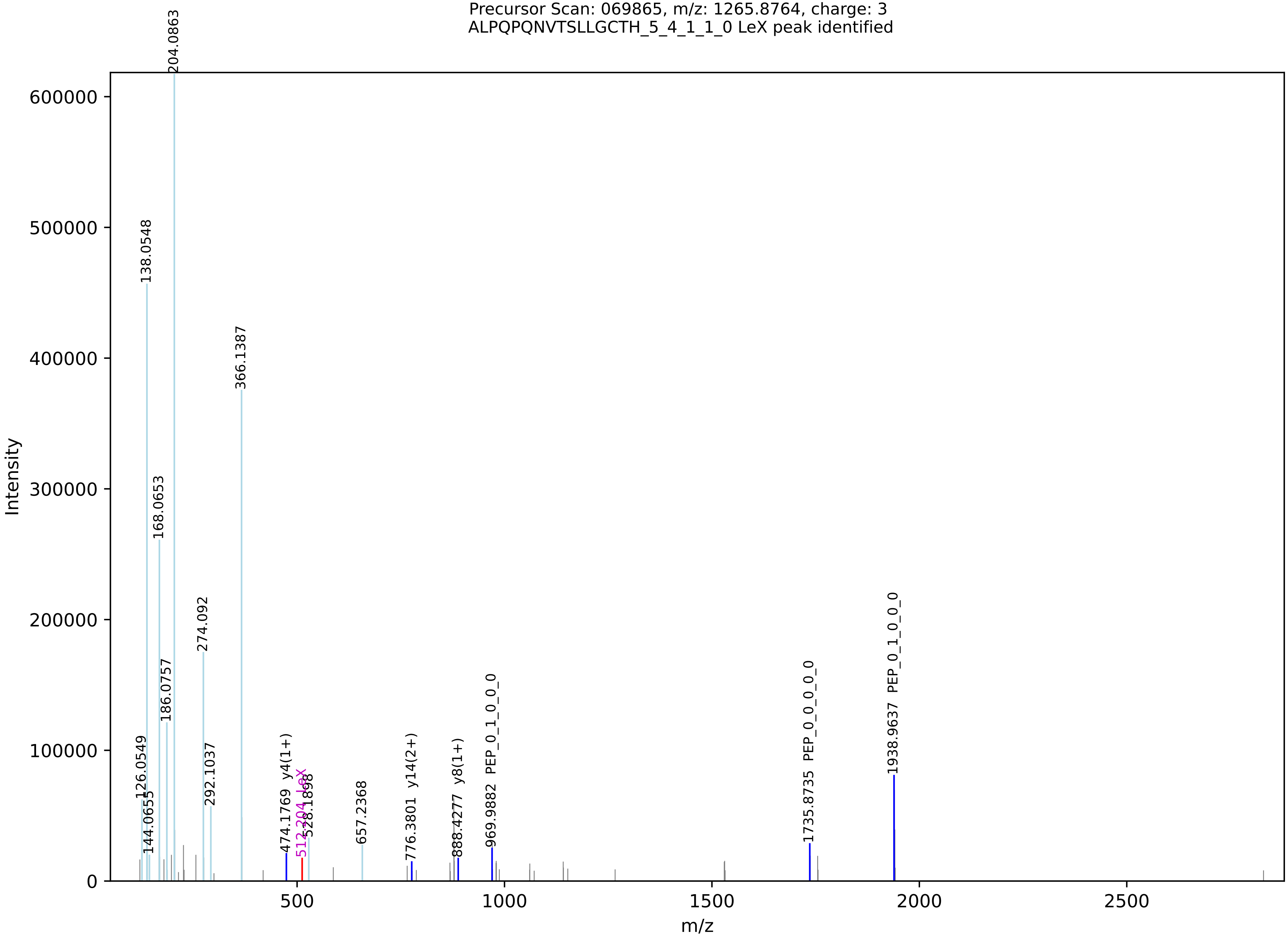

File: 231129_OE_RK_29, RT: 53.188, Scan: 070232
Type: HCD, FTMS + c NSI d Full ms2 1266.2109@hcd30.00 [120.0000-3896.7476]
Precursor Scan: 070155, m/z: 1265.8768, charge: 3
ALPQPQNVTSLLGCTH_5_4_1_1_0 LeX peak identified

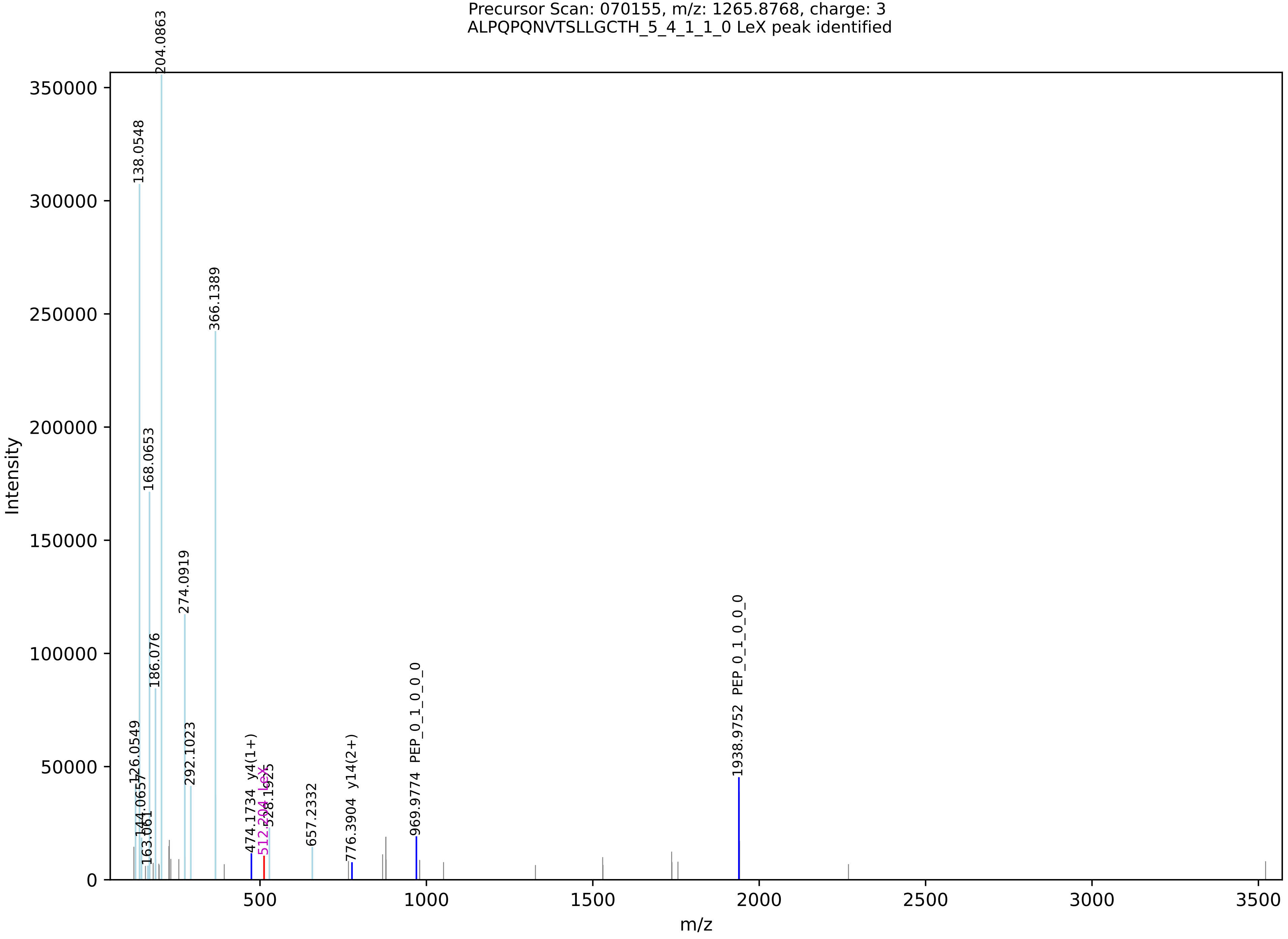

File: 231129_OE_RK_29, RT: 59.712, Scan: 079731
Type: HCD, FTMS + c NSI d Full ms2 1381.8026@hcd30.00 [120.0000-5660.6108]
Precursor Scan: 079658, m/z: 1381.3015, charge: 4
HMNHSEPRPGLGADGDAADPVDTR_6_5_1_3_0 LeX peak identified

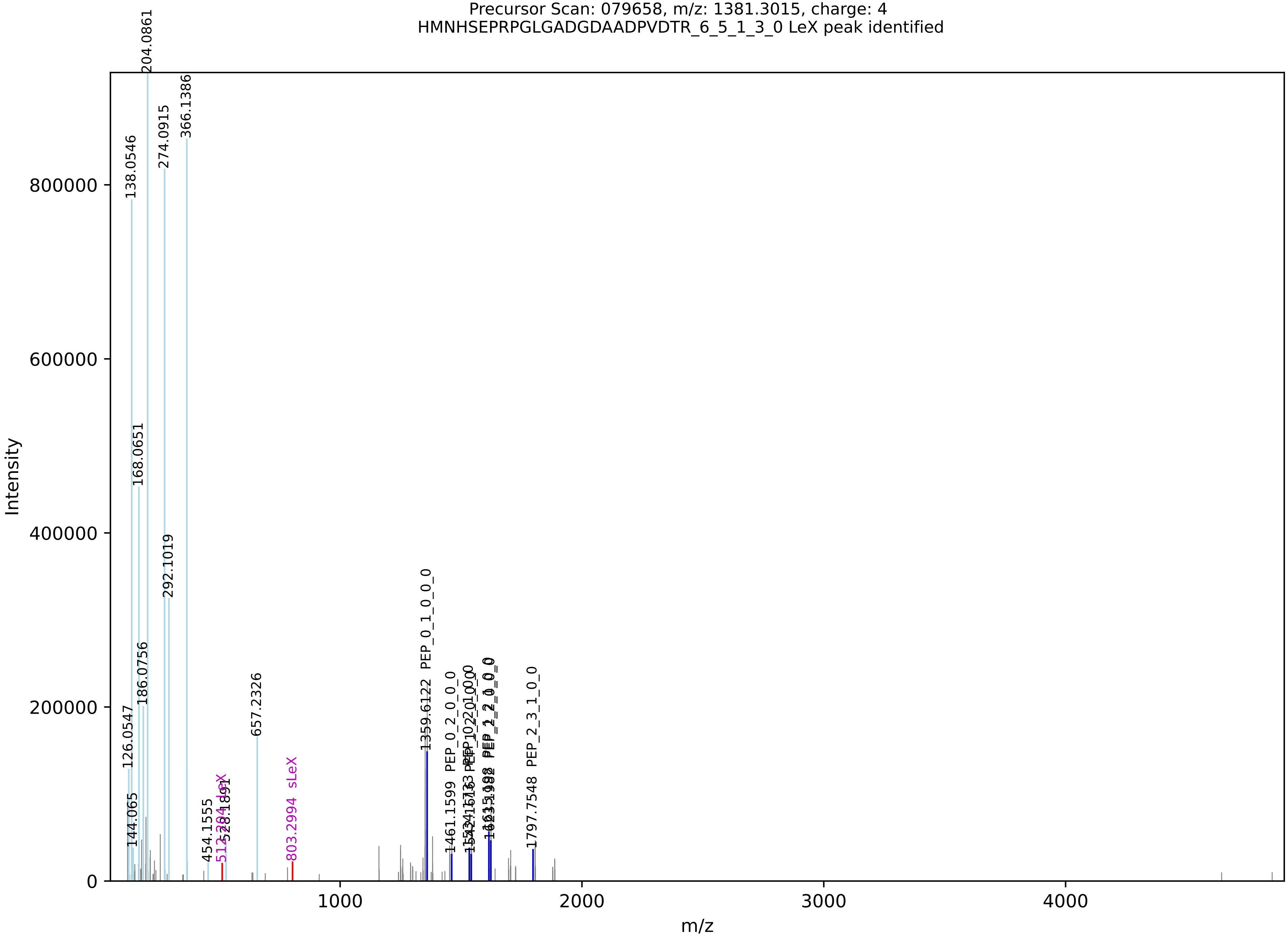

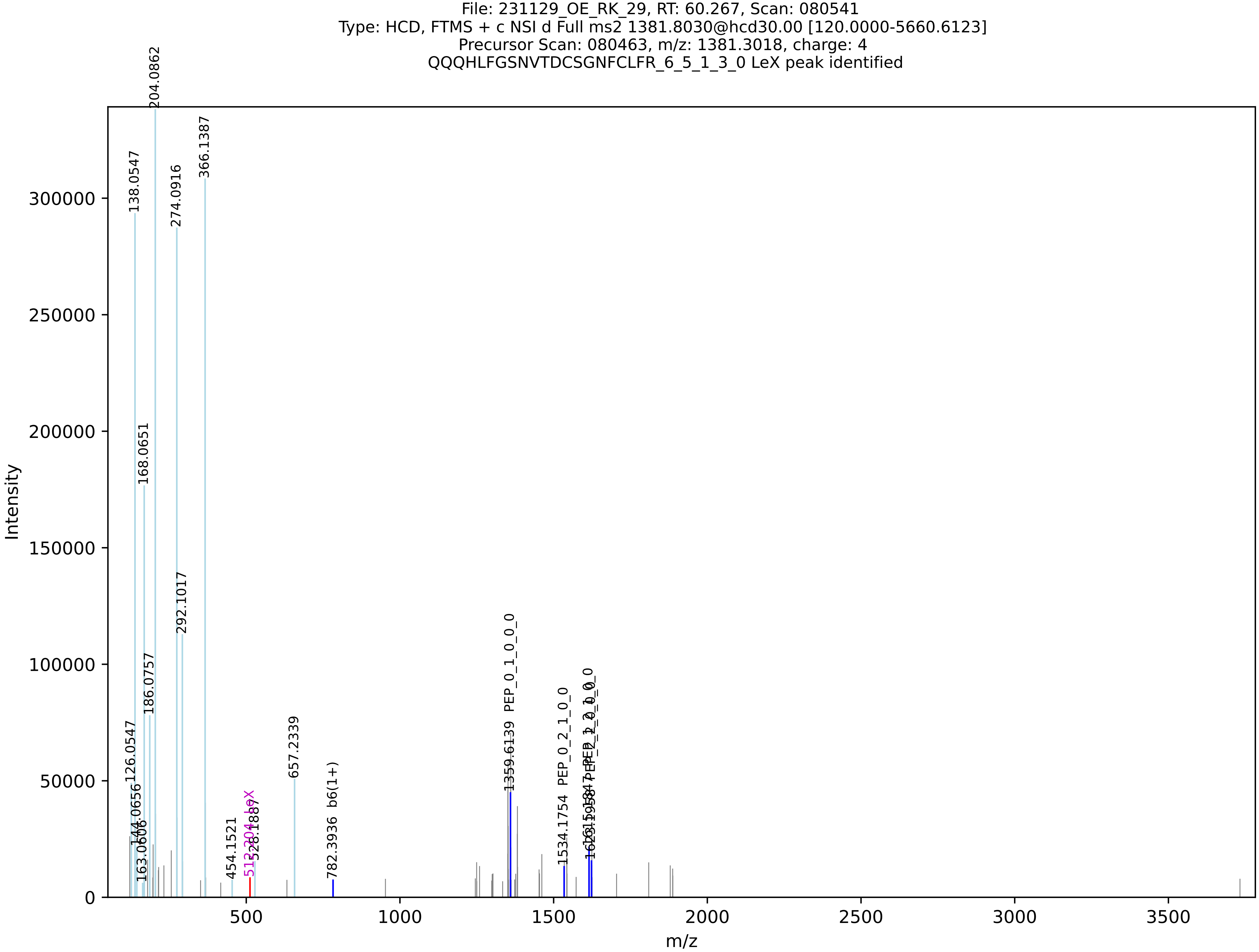

File: 231129_OE_RK_29, RT: 60.267, Scan: 080541
Type: HCD, FTMS + c NSI d Full ms2 1381.8030@hcd30.00 [120.0000-5660.6123]
Precursor Scan: 080463, m/z: 1381.3018, charge: 4
QQQHLFGSNVTDCSGNFCLFR_6_5_1_3_0 LeX peak identified
Intensity
m/z
204.0862
366.1387
138.0547
274.0916
168.0651
292.1017
186.0757
126.0547
144.0656
163.0606
454.1521
512.204 LeX
528.1887
657.2339
782.3936 b6(1+)
1359.6139 PEP_0_1_0_0_0
1534.1754 PEP_0_2_1_0_0
0
50000
100000
150000
200000
250000
300000
500
1000
1500
2000
2500
3000
3500

File: 231129_OE_RK_29, RT: 62.893, Scan: 084361
Type: HCD, FTMS + c NSI d Full ms2 1332.3129@hcd30.00 [120.0000-5458.6924]
Precursor Scan: 084276, m/z: 1331.561, charge: 4
TEPMQVALHCTNGSVVYHEVLNAMECK_5_4_2_1_0 LeX peak identified

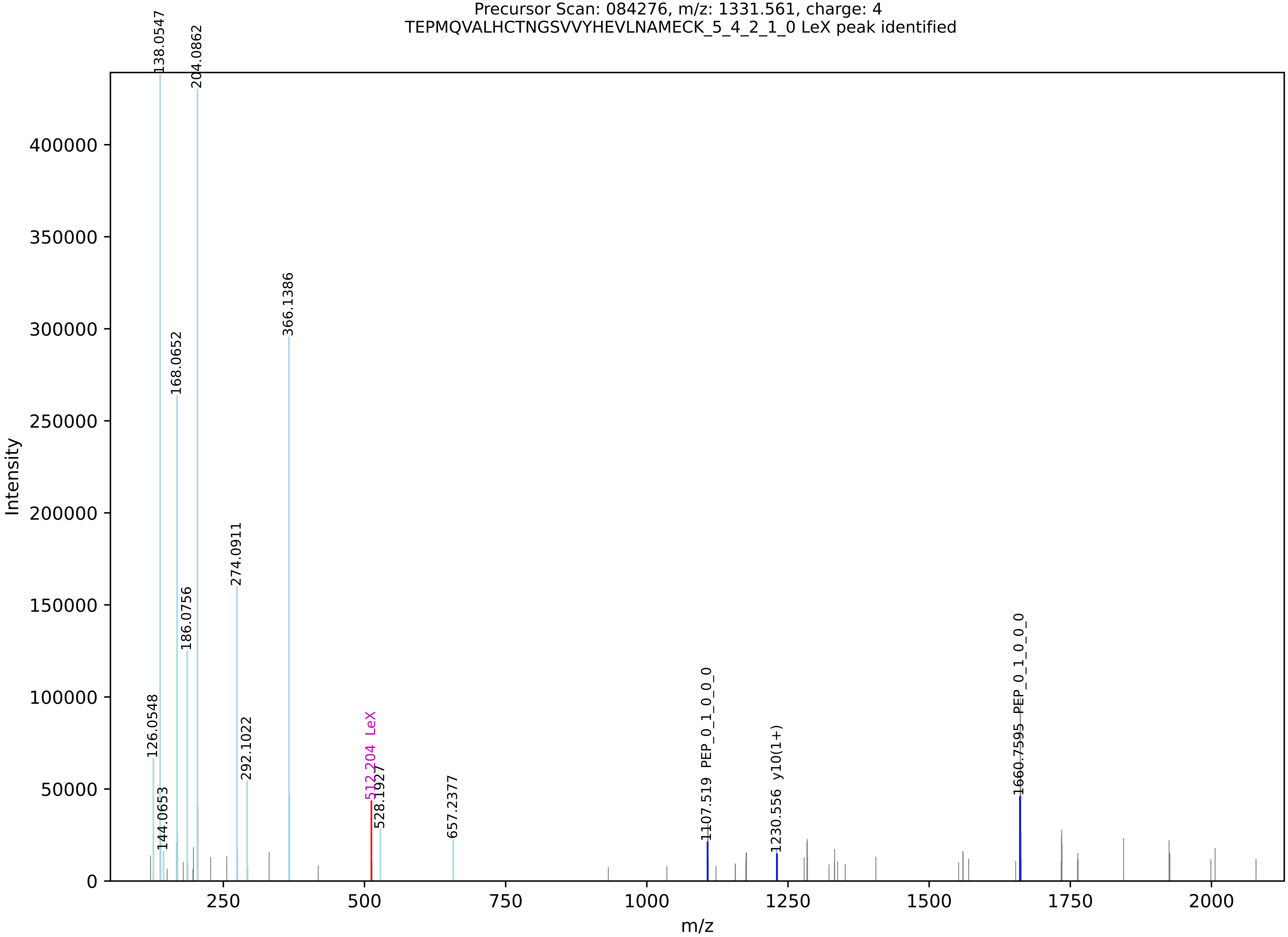

File: 231129_OE_RK_29, RT: 78.183, Scan: 106588
Type: HCD, FTMS + c NSI d Full ms2 1299.5637@hcd30.00 [120.0000-5325.0762]
Precursor Scan: 106375, m/z: 1299.3146, charge: 4
VYIHPFHLVIHNESTCEQLAK_6_4_2_2_0 LeX peak identified

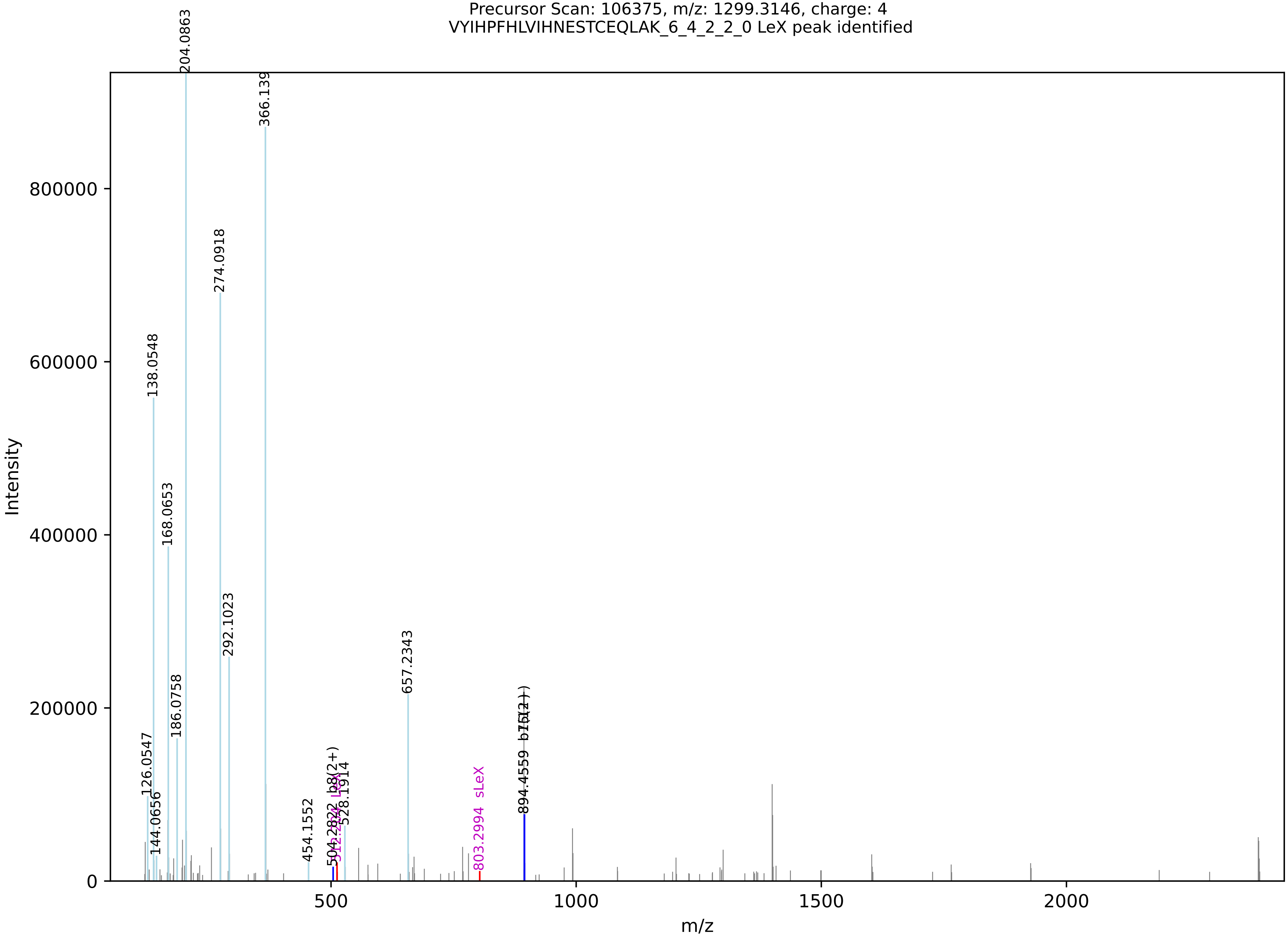

File: 231129_OE_RK_30, RT: 5.868, Scan: 002196
Type: HCD, FTMS + c NSI d Full ms2 1379.5277@hcd30.00 [120.0000-4243.4966]
Precursor Scan: 002169, m/z: 1378.8595, charge: 3
ENGTVSR_7_6_1_3_0 LeX peak identified

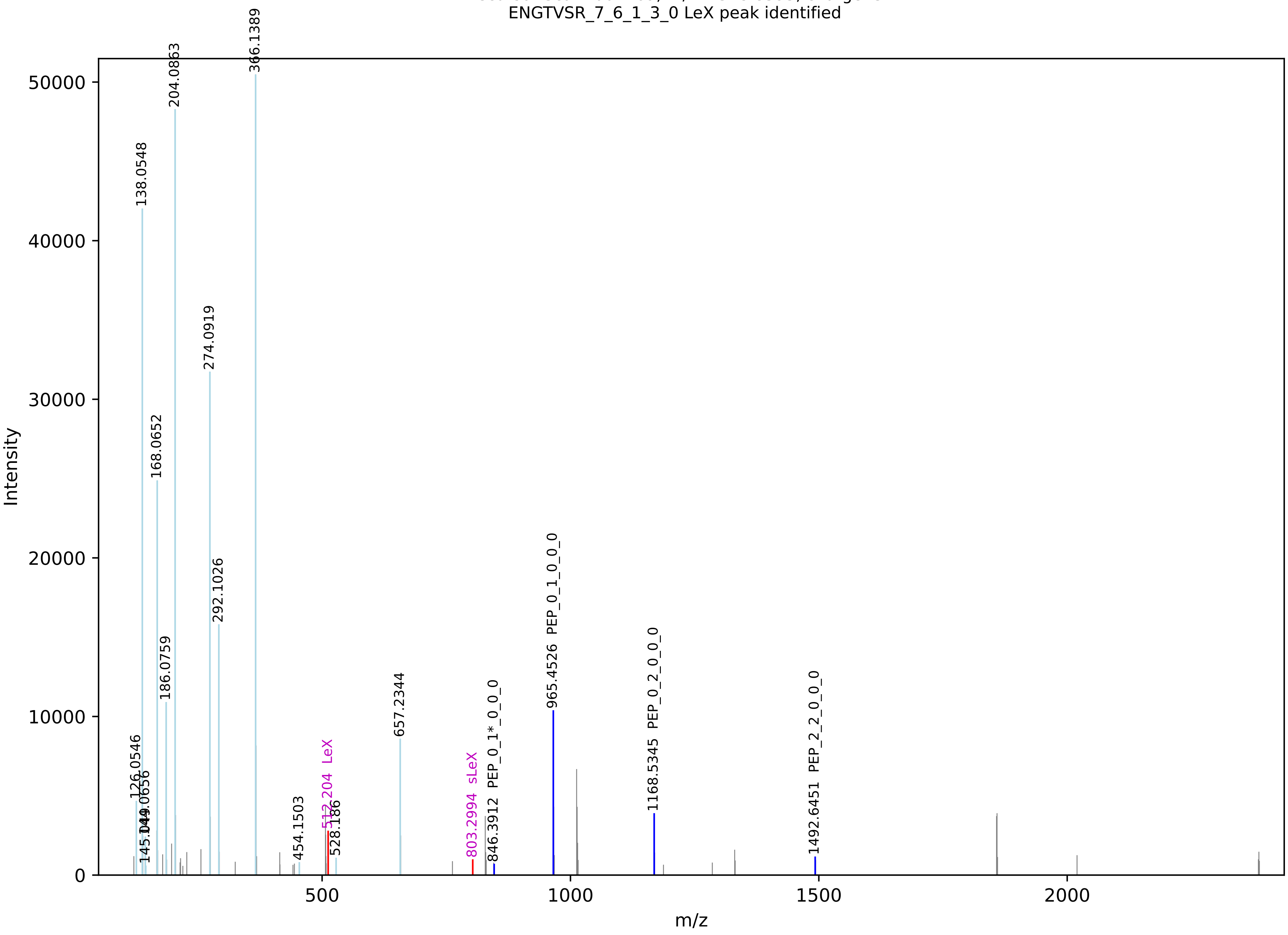

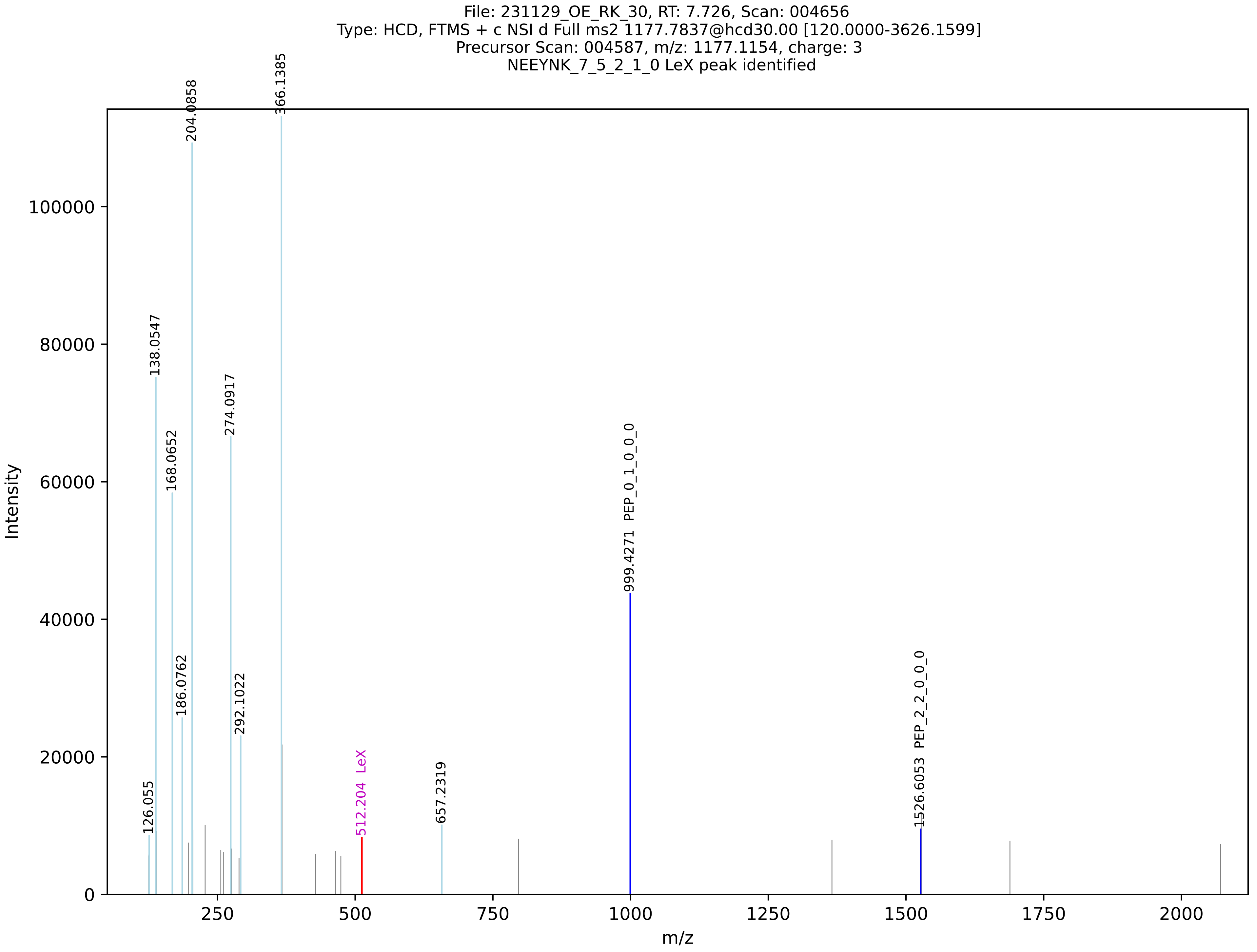
File: 231129_OE_RK_30, RT: 7.726, Scan: 004656
Type: HCD, FTMS + c NSI d Full ms2 1177.7837@hcd30.00 [120.0000-3626.1599]
Precursor Scan: 004587, m/z: 1177.1154, charge: 3
NEEYNK_7_5_2_1_0 LeX peak identified
Intensity
m/z
126.055
138.0547
168.0652
186.0762
204.0858
274.0917
292.1022
366.1385
512.204 LeX
657.2319
999.4271 PEP_0_1_0_0_0
1526.6053 PEP_2_2_0_0_0
0
20000
40000
60000
80000
100000
250
500
750
1000
1250
1500
1750
2000

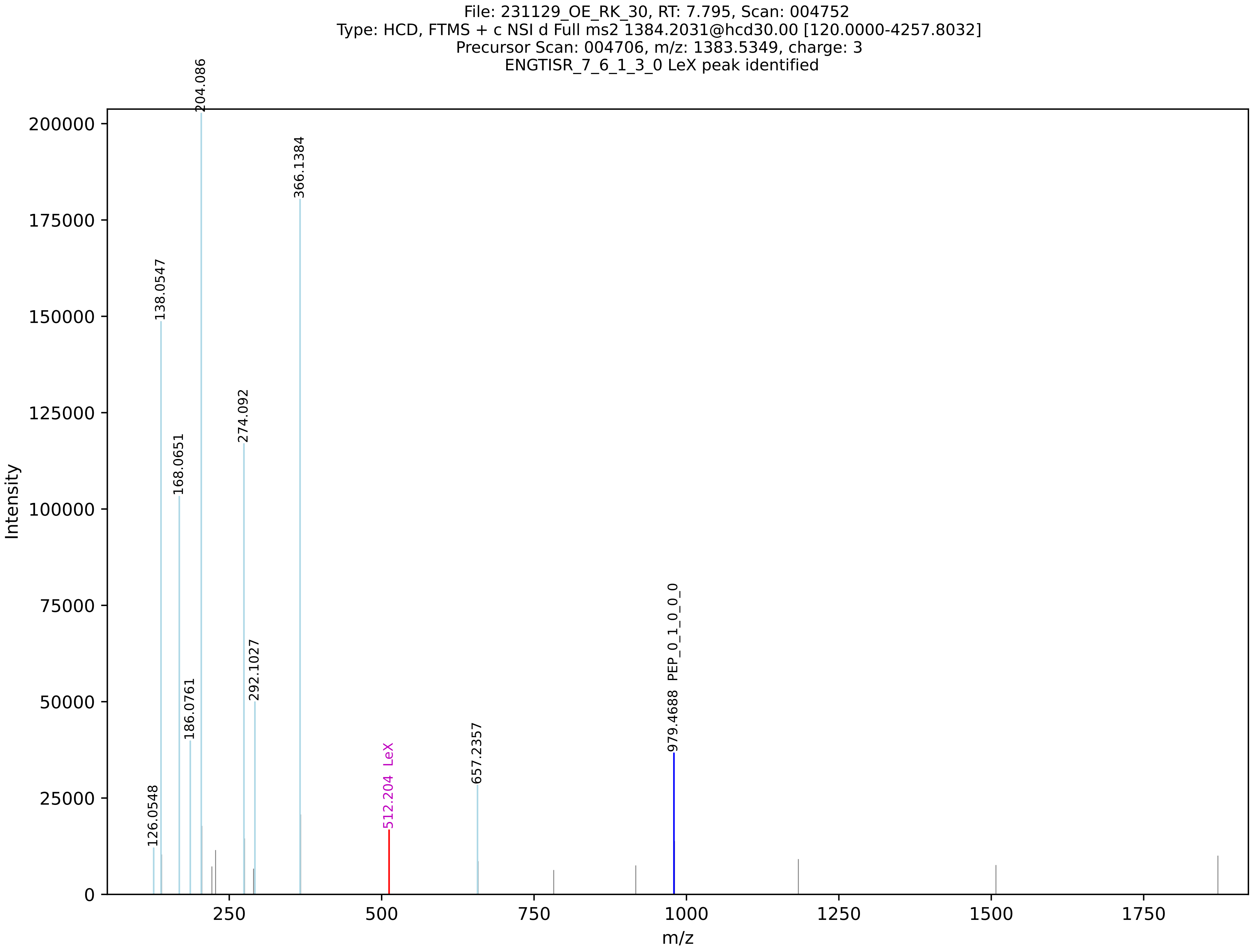
File: 231129_OE_RK_30, RT: 7.795, Scan: 004752
Type: HCD, FTMS + c NSI d Full ms2 1384.2031@hcd30.00 [120.0000-4257.8032]
Precursor Scan: 004706, m/z: 1383.5349, charge: 3
ENGTISR_7_6_1_3_0 LeX peak identified
Intensity
m/z
204.086
366.1384
138.0547
274.092
168.0651
292.1027
186.0761
126.0548
512.204 LeX
657.2357
979.4688 PEP_0_1_0_0_0
0
25000
50000
75000
100000
125000
150000
175000
200000
250
500
750
1000
1250
1500
1750

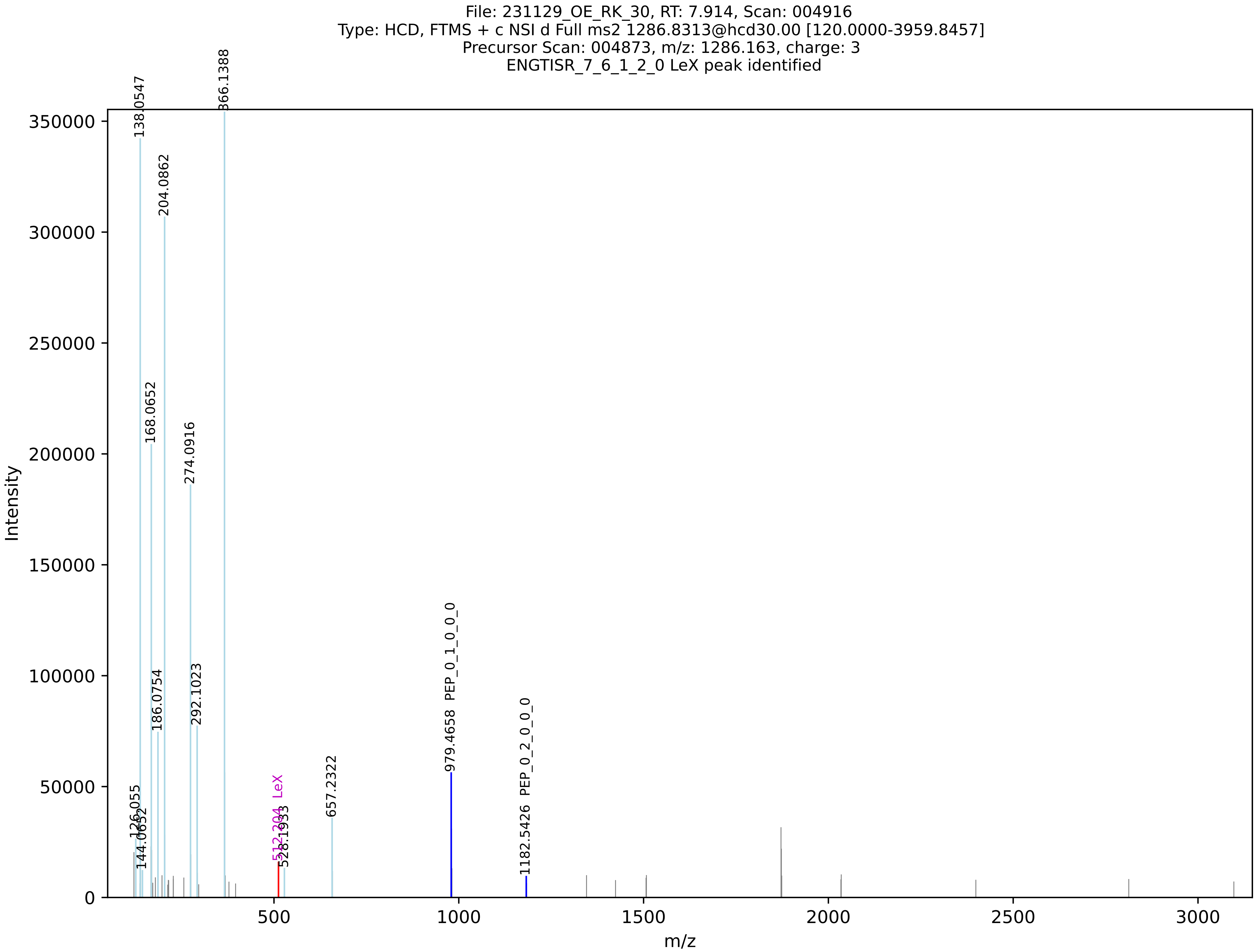
File: 231129_OE_RK_30, RT: 7.914, Scan: 004916
Type: HCD, FTMS + c NSI d Full ms2 1286.8313@hcd30.00 [120.0000-3959.8457]
Precursor Scan: 004873, m/z: 1286.163, charge: 3
ENGTISR_7_6_1_2_0 LeX peak identified
Intensity
m/z
138.0547
366.1388
204.0862
168.0652
274.0916
186.0754
292.1023
126.055
144.0652
512.204 LeX
528.1933
657.2322
979.4658 PEP_0_1_0_0_0_
1182.5426 PEP_0_2_0_0_0_
350000
300000
250000
200000
150000
100000
50000
0
500
1000
1500
2000
2500
3000

File: 231129_OE_RK_30, RT: 7.978, Scan: 005003
Type: HCD, FTMS + c NSI d Full ms2 1384.2002@hcd30.00 [120.0000-4257.7949]
Precursor Scan: 004954, m/z: 1383.532, charge: 3
ENGTISR_7_6_1_3_0 LeX peak identified

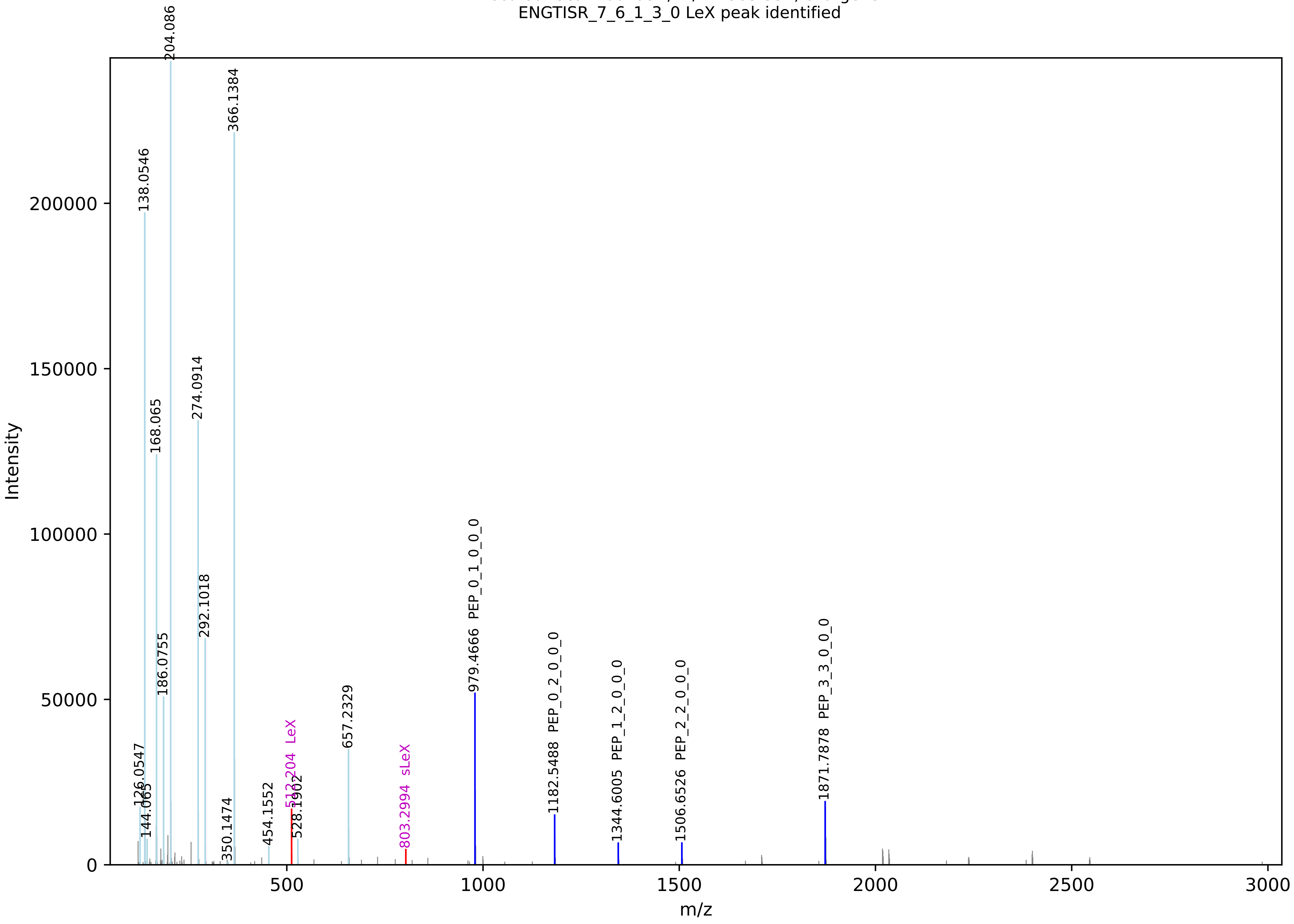

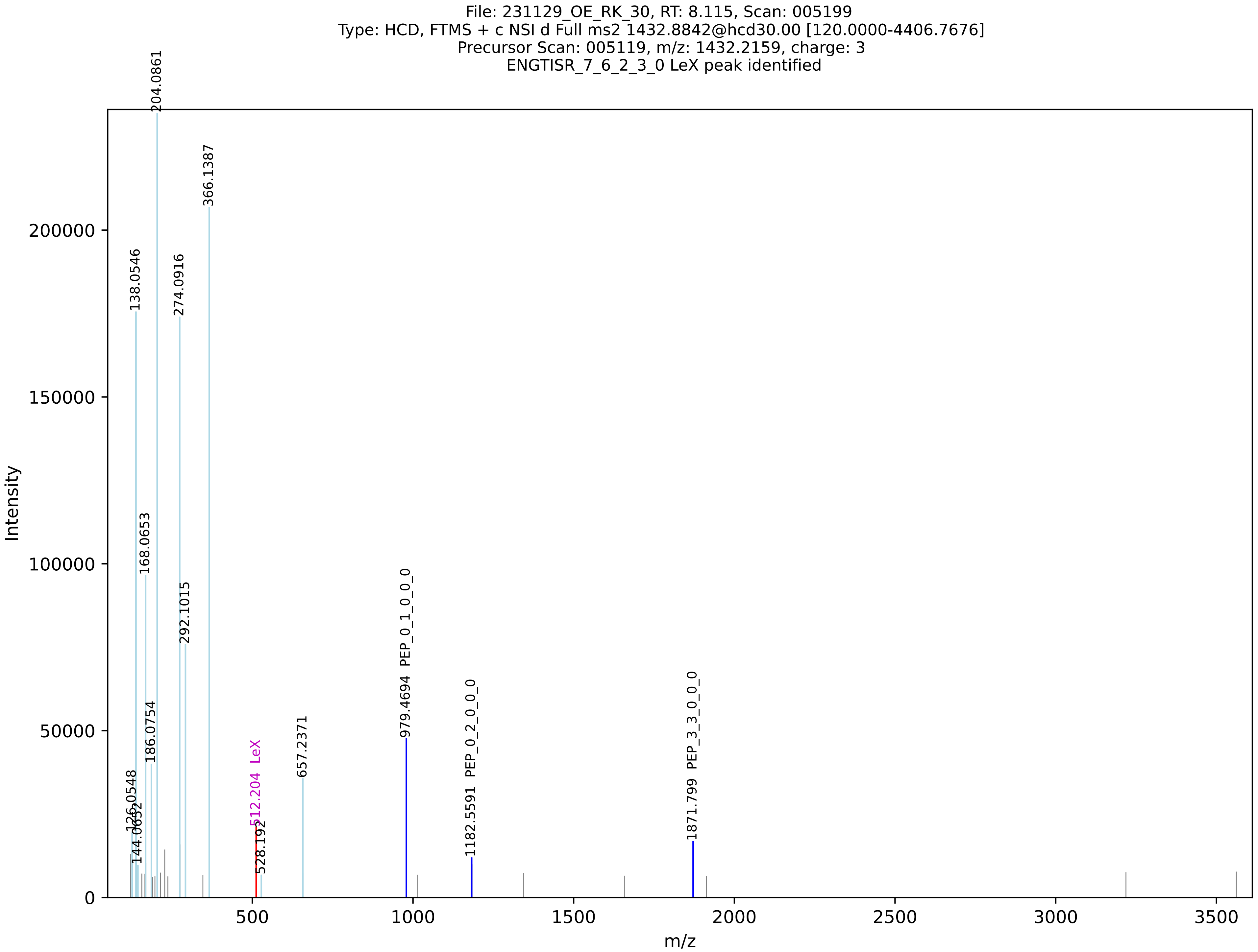

File: 231129_OE_RK_30, RT: 8.115, Scan: 005199
Type: HCD, FTMS + c NSI d Full ms2 1432.8842@hcd30.00 [120.0000-4406.7676]
Precursor Scan: 005119, m/z: 1432.2159, charge: 3
ENGTISR_7_6_2_3_0 LeX peak identified
Intensity
m/z
204.0861
366.1387
138.0546
274.0916
168.0653
292.1015
186.0754
126.0548
144.0652
512.204 LeX
528.192
657.2371
979.4694 PEP_0_1_0_0_0
1182.5591 PEP_0_2_0_0_0
1871.799 PEP_3_3_0_0_0
0
50000
100000
150000
200000
500
1000
1500
2000
2500
3000
3500

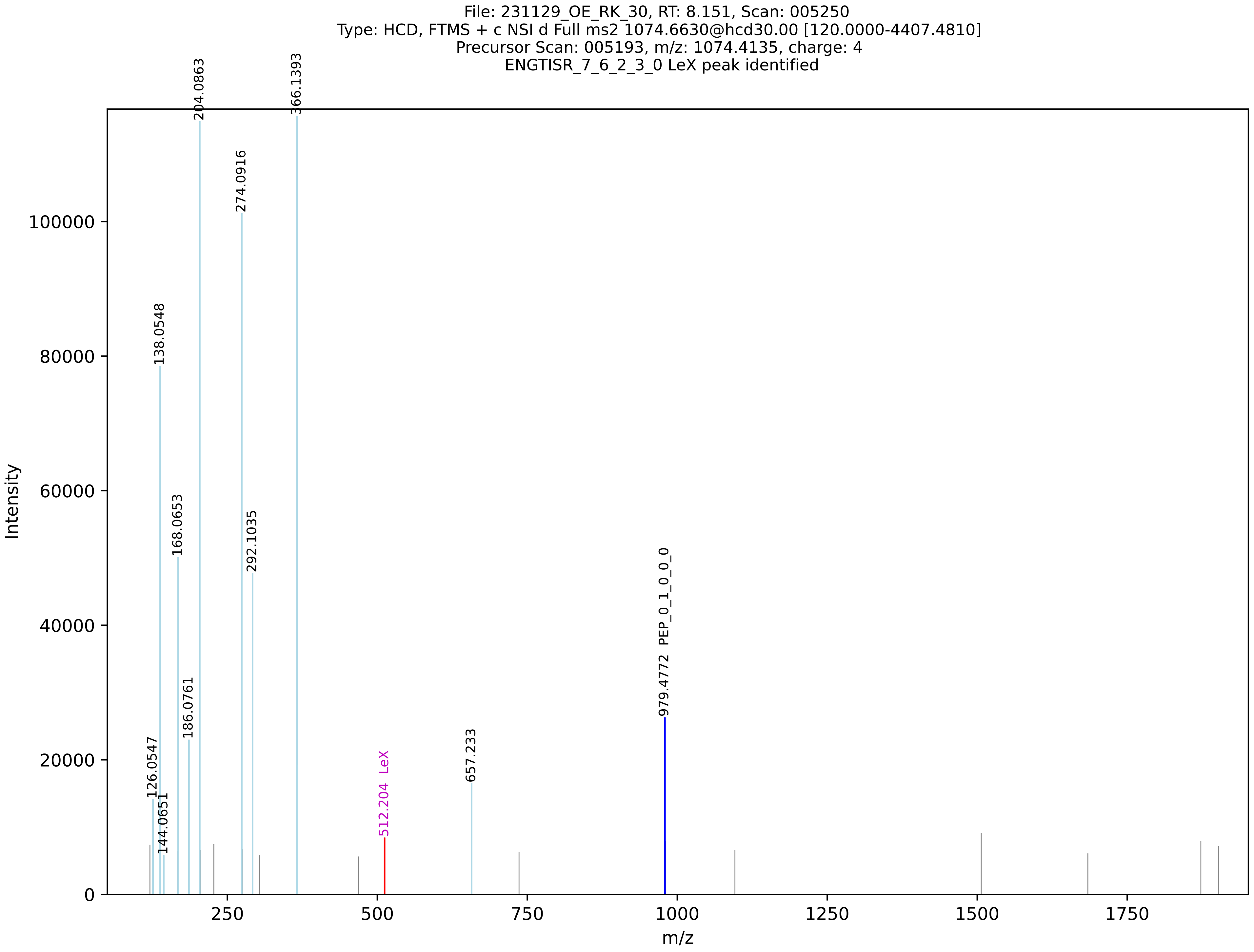
File: 231129_OE_RK_30, RT: 8.151, Scan: 005250
Type: HCD, FTMS + c NSI d Full ms2 1074.6630@hcd30.00 [120.0000-4407.4810]
Precursor Scan: 005193, m/z: 1074.4135, charge: 4
ENGTISR_7_6_2_3_0 LeX peak identified
Intensity
m/z
204.0863
366.1393
274.0916
138.0548
168.0653
292.1035
186.0761
126.0547
144.0651
512.204 LeX
657.233
979.4772 PEP_0_1_0_0_0
0
20000
40000
60000
80000
100000
250
500
750
1000
1250
1500
1750

File: 231129_OE_RK_30, RT: 8.301, Scan: 005467
Type: HCD, FTMS + c NSI d Full ms2 1432.5490@hcd30.00 [120.0000-4405.7417]
Precursor Scan: 005388, m/z: 1431.8811, charge: 3
ENGTISR_7_6_2_3_0 LeX peak identified

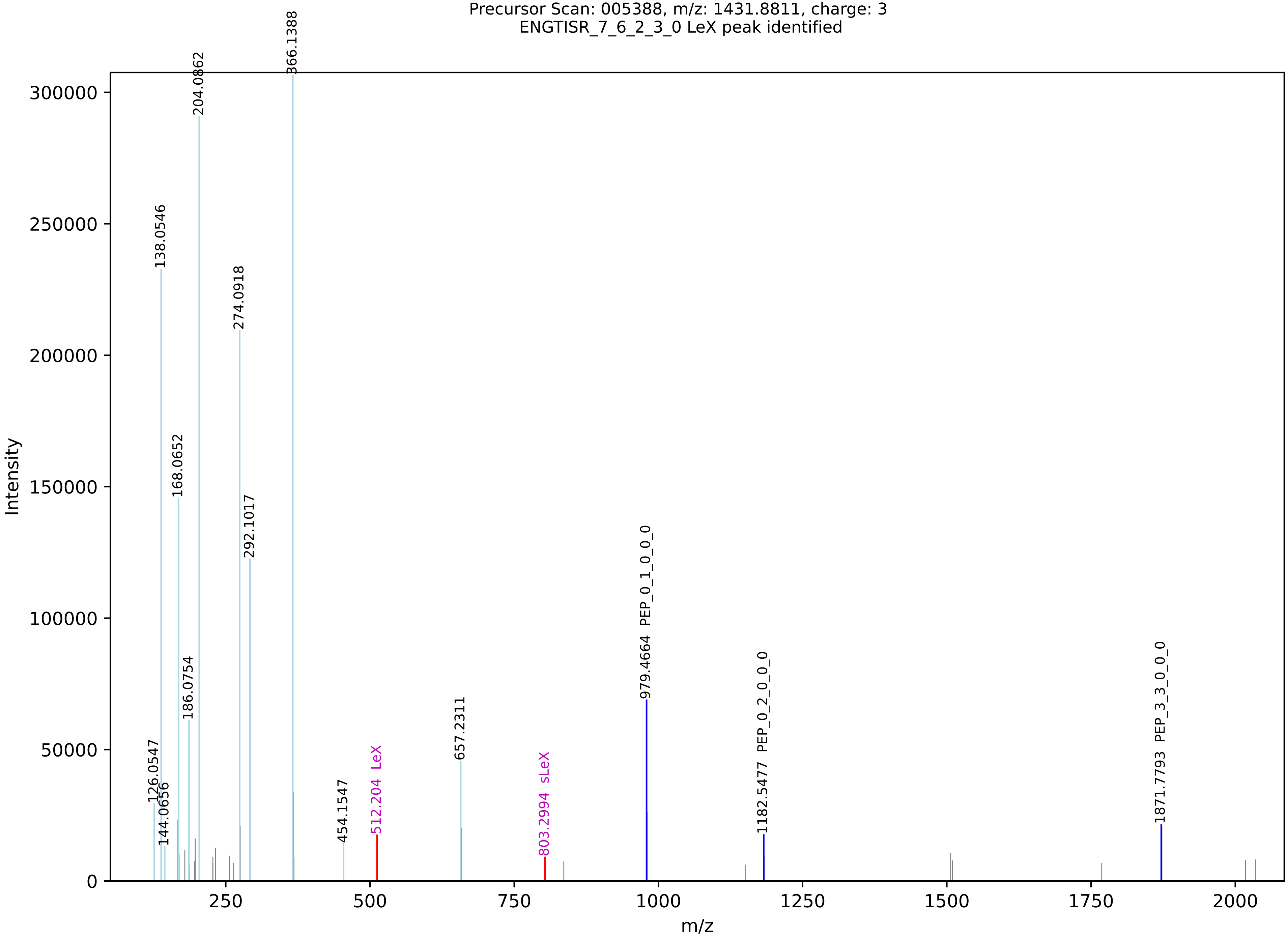

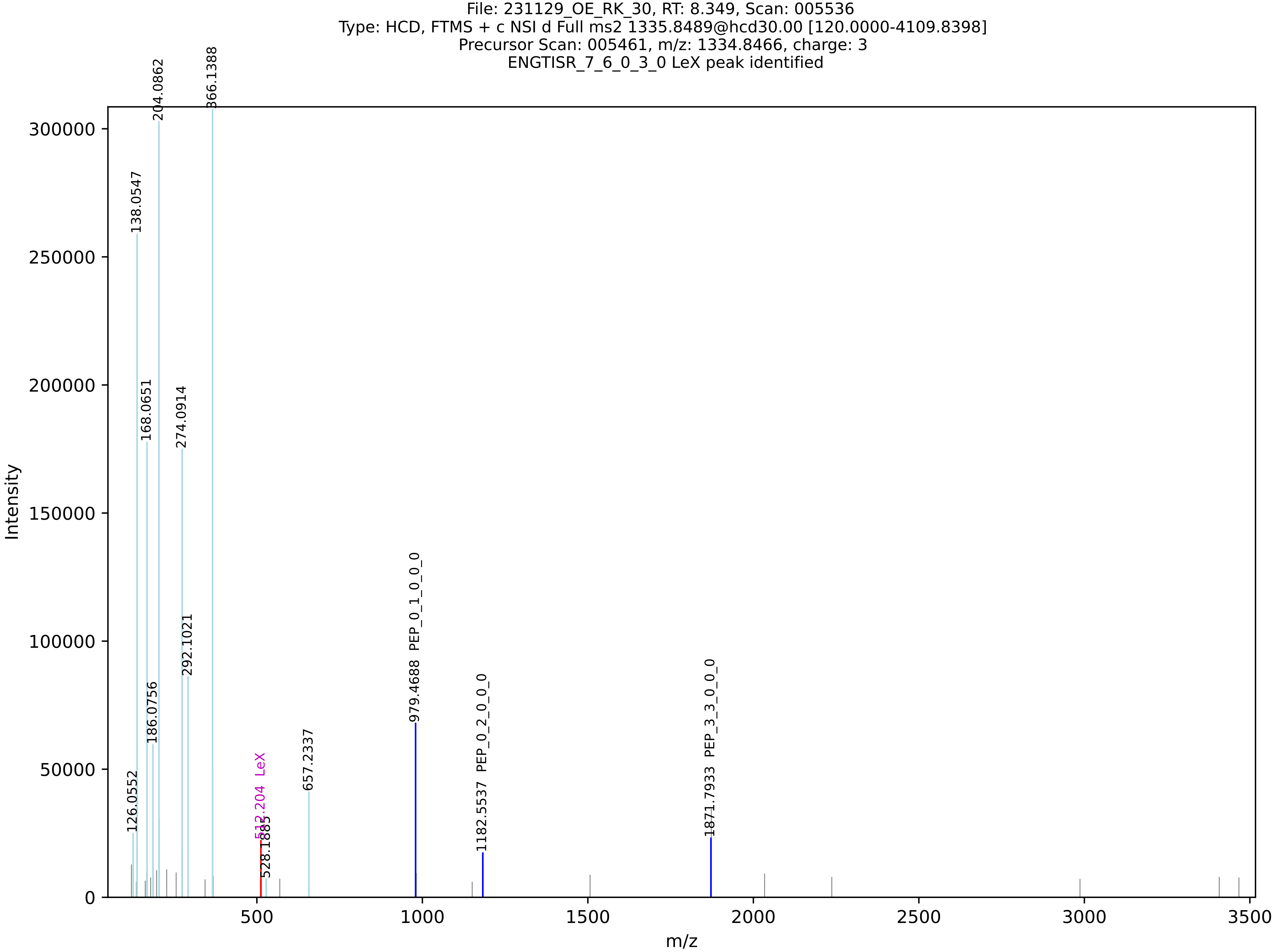

File: 231129_OE_RK_30, RT: 8.349, Scan: 005536
Type: HCD, FTMS + c NSI d Full ms2 1335.8489@hcd30.00 [120.0000-4109.8398]
Precursor Scan: 005461, m/z: 1334.8466, charge: 3
ENGTISR_7_6_0_3_0 LeX peak identified
Intensity
m/z
366.1388
204.0862
138.0547
168.0651
274.0914
292.1021
186.0756
126.0552
512.204 LeX
528.1885
657.2337
979.4688 PEP_0_1_0_0_0_
1182.5537 PEP_0_2_0_0_0
1871.7933 PEP_3_3_0_0_0
0
50000
100000
150000
200000
250000
300000
500
1000
1500
2000
2500
3000
3500

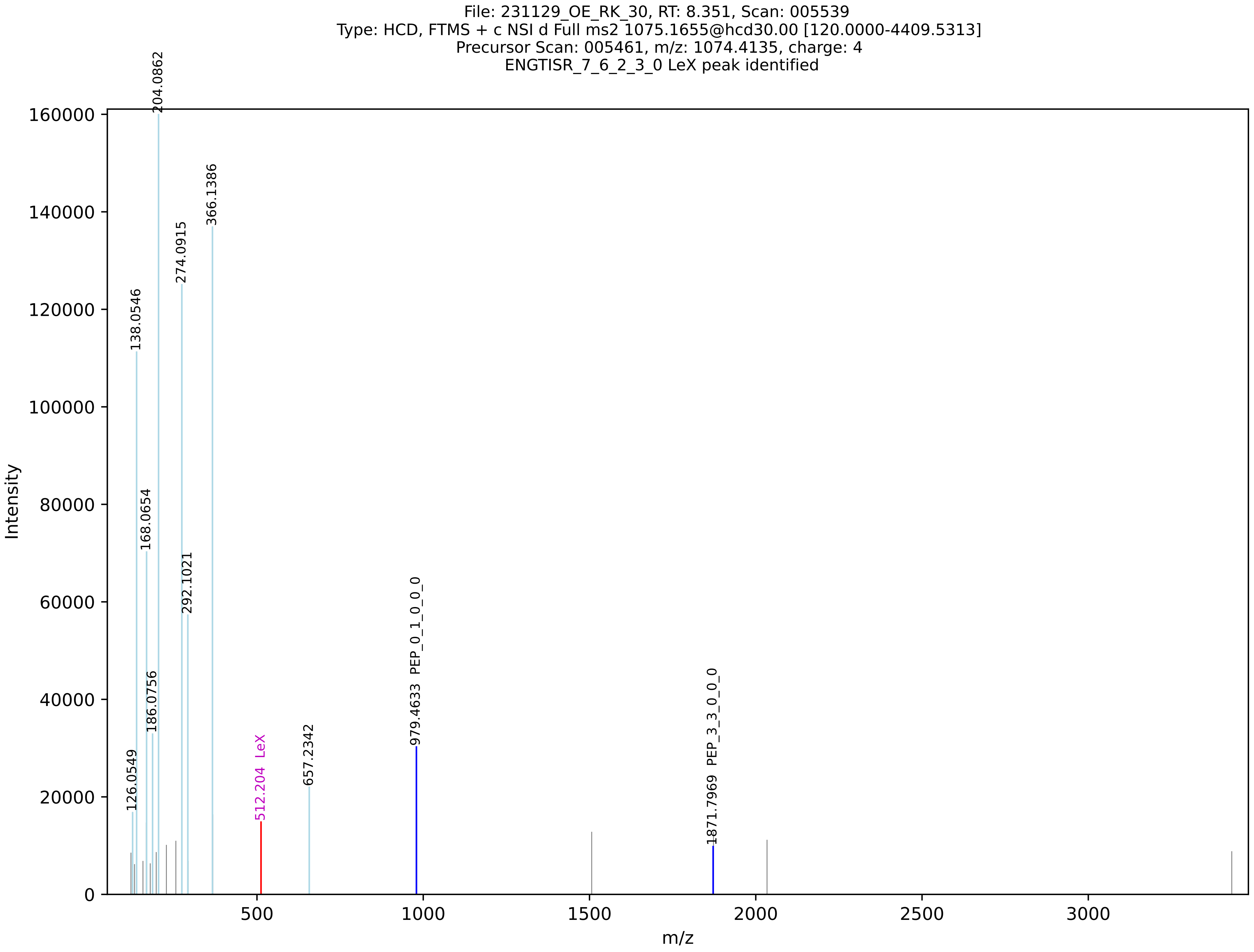
File: 231129_OE_RK_30, RT: 8.351, Scan: 005539
Type: HCD, FTMS + c NSI d Full ms2 1075.1655@hcd30.00 [120.0000-4409.5313]
Precursor Scan: 005461, m/z: 1074.4135, charge: 4
ENGTISR_7_6_2_3_0 LeX peak identified
Intensity
m/z
204.0862
366.1386
274.0915
138.0546
168.0654
292.1021
186.0756
126.0549
512.204 LeX
657.2342
979.4633 PEP_0_1_0_0_0_
1871.7969 PEP_3_3_0_0_0_
0
20000
40000
60000
80000
100000
120000
140000
160000
500
1000
1500
2000
2500
3000

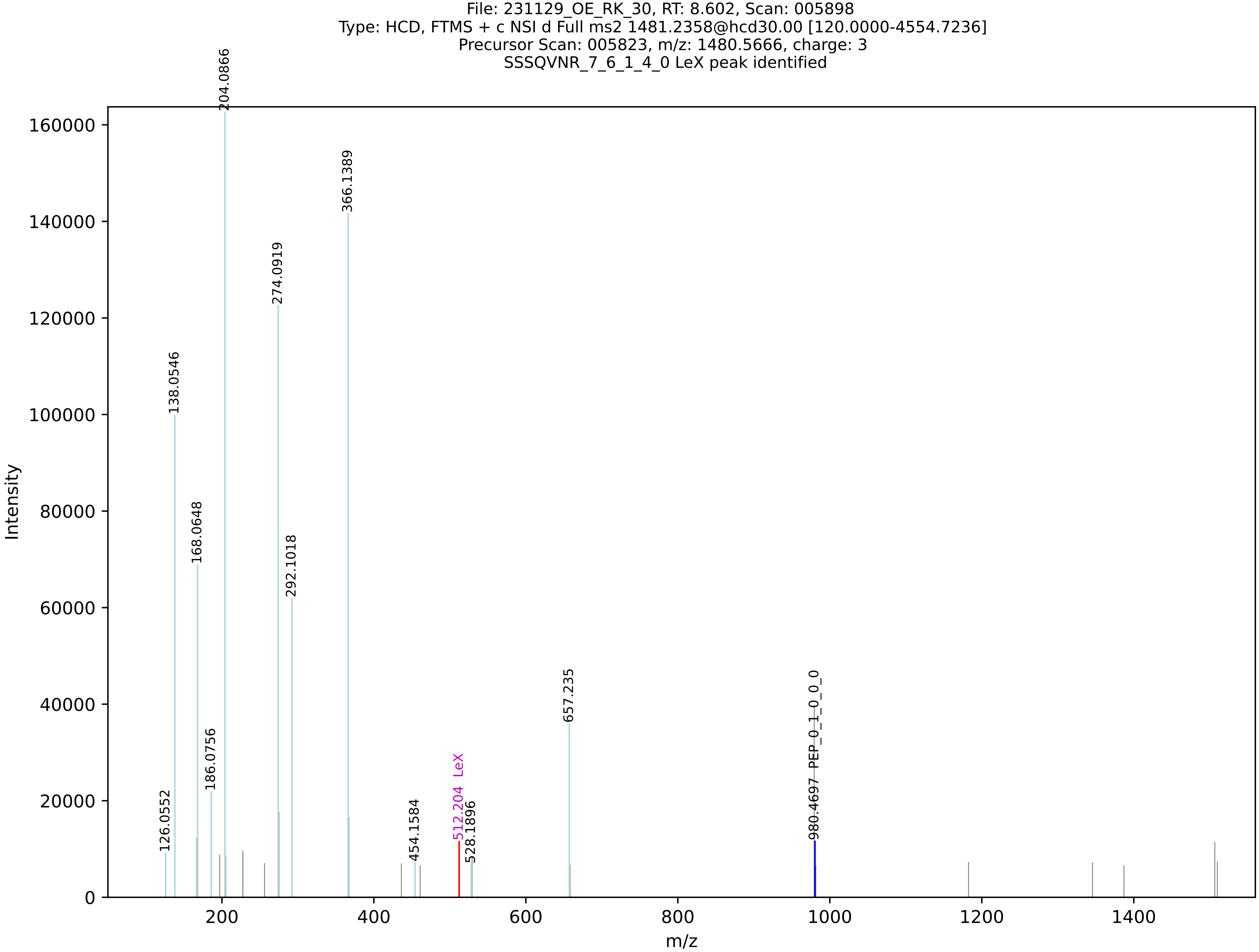
File: 231129_OE_RK_30, RT: 8.602, Scan: 005898
Type: HCD, FTMS + c NSI d Full ms2 1481.2358@hcd30.00 [120.0000-4554.7236]
Precursor Scan: 005823, m/z: 1480.5666, charge: 3
SSSQVNR_7_6_1_4_0 LeX peak identified
Intensity
m/z
126.0552
138.0546
168.0648
186.0756
204.0866
274.0919
292.1018
366.1389
454.1584
512.204 LeX
528.1896
657.235
980.4697 PEP_0_1_0_0_0

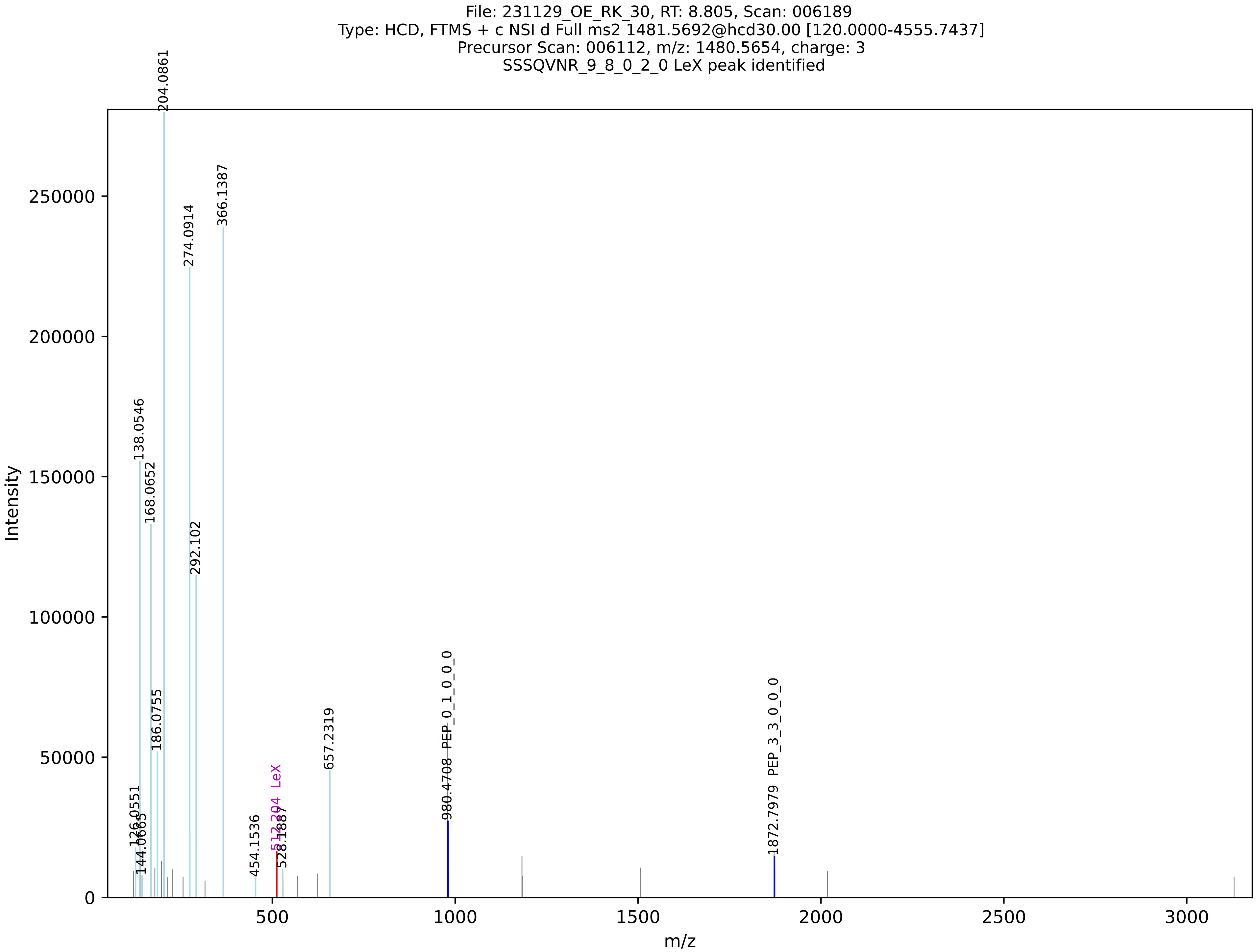
File: 231129_OE_RK_30, RT: 8.805, Scan: 006189
Type: HCD, FTMS + c NSI d Full ms2 1481.5692@hcd30.00 [120.0000-4555.7437]
Precursor Scan: 006112, m/z: 1480.5654, charge: 3
SSSQVNR_9_8_0_2_0 LeX peak identified
Intensity
m/z
0
50000
100000
150000
200000
250000
500
1000
1500
2000
2500
3000
204.0861
366.1387
274.0914
138.0546
168.0652
292.102
186.0755
126.0551
144.0665
454.1536
LeX
528.1887
657.2319
980.4708 PEP_0_1_0_0_0_
1872.7979 PEP_3_3_0_0_0_

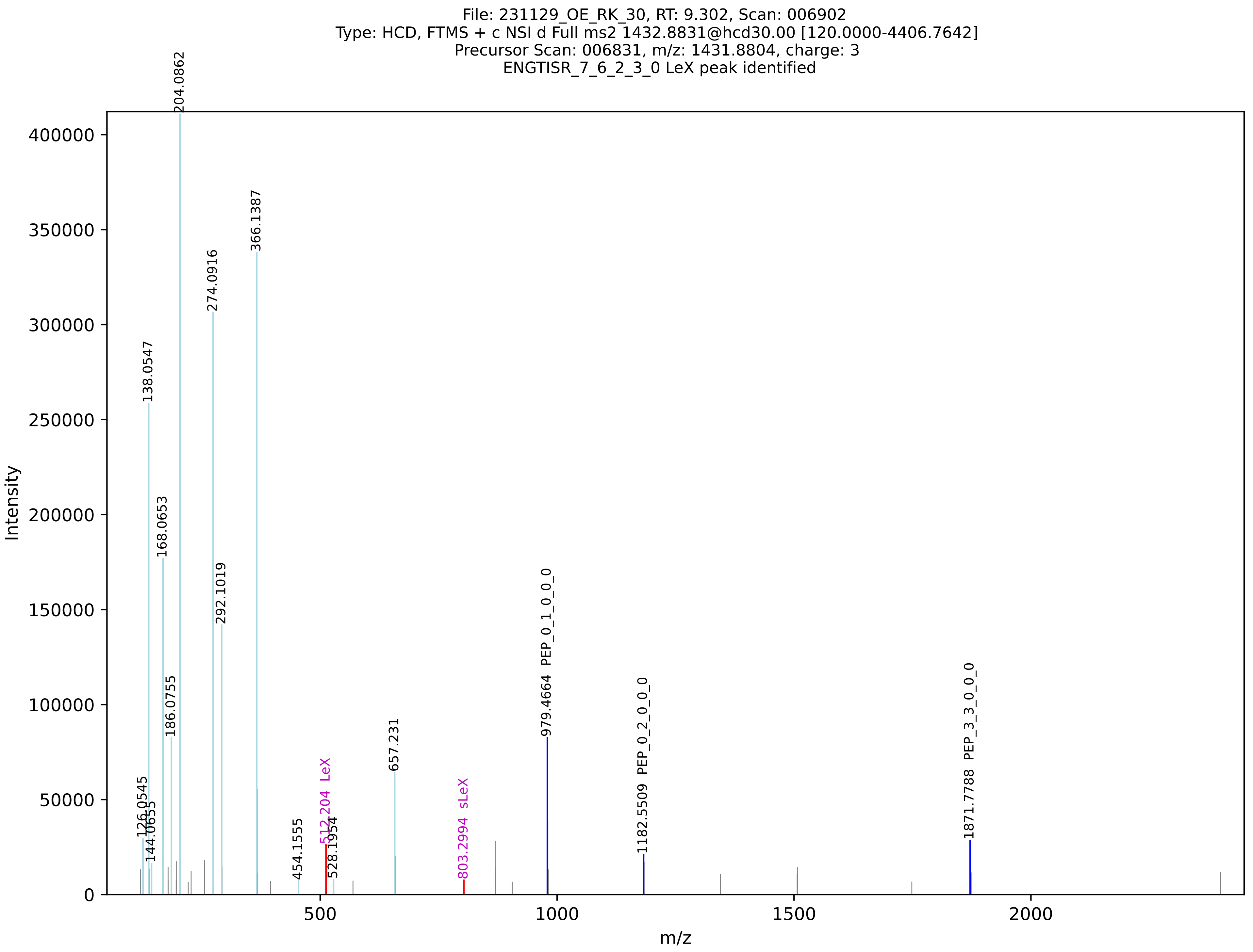
File: 231129_OE_RK_30, RT: 9.302, Scan: 006902
Type: HCD, FTMS + c NSI d Full ms2 1432.8831@hcd30.00 [120.0000-4406.7642]
Precursor Scan: 006831, m/z: 1431.8804, charge: 3
ENGTISR_7_6_2_3_0 LeX peak identified
Intensity
m/z
204.0862
366.1387
274.0916
138.0547
168.0653
292.1019
186.0755
126.0545
144.0655
454.1555
512.204 LeX
528.1954
657.231
803.2994 sLeX
979.4664 PEP_0_1_0_0_0
1182.5509 PEP_0_2_0_0_0
1871.7788 PEP_3_3_0_0_0

File: 231129_OE_RK_30, RT: 9.442, Scan: 007096
Type: HCD, FTMS + c NSI d Full ms2 942.8884@hcd30.00 [120.0000-3869.8406]
Precursor Scan: 007030, m/z: 942.6387, charge: 4
EIRHNSTGCLR_6_5_1_1_0 LeX peak identified

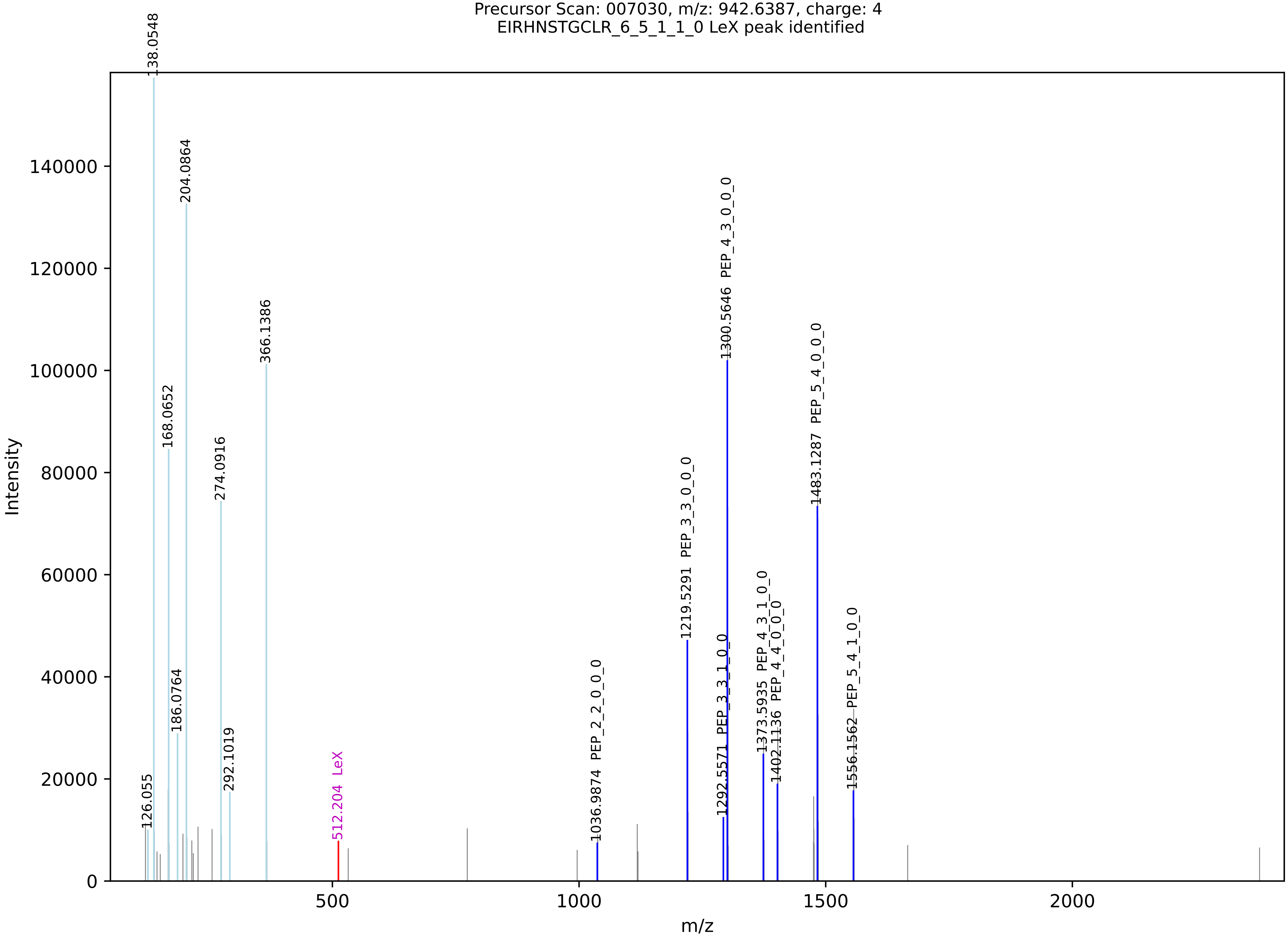

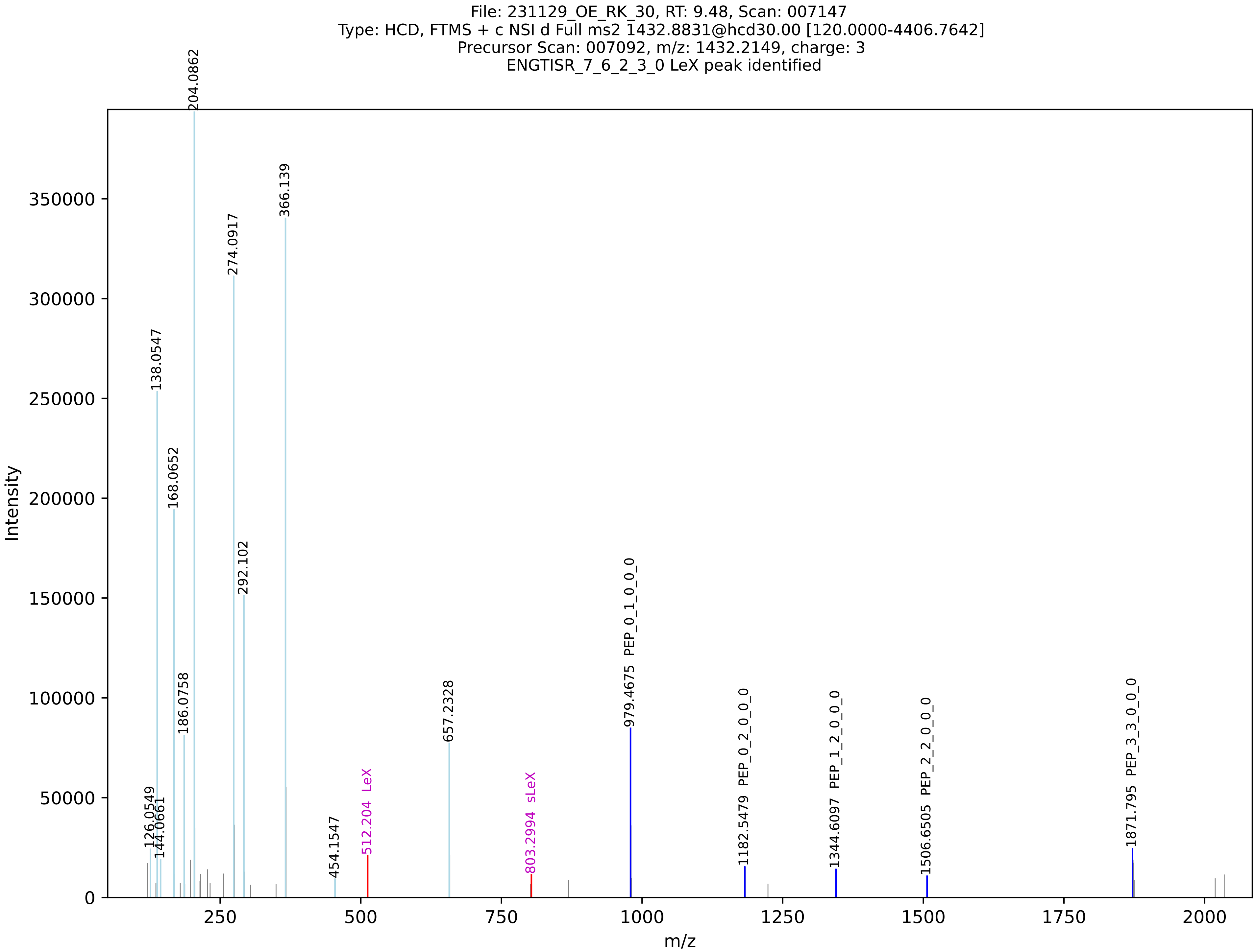

File: 231129_OE_RK_30, RT: 9.48, Scan: 007147
Type: HCD, FTMS + c NSI d Full ms2 1432.8831@hcd30.00 [120.0000-4406.7642]
Precursor Scan: 007092, m/z: 1432.2149, charge: 3
ENGTISR_7_6_2_3_0 LeX peak identified
Intensity
m/z
204.0862
366.139
274.0917
138.0547
168.0652
292.102
186.0758
126.0549
144.0661
454.1547
512.204 LeX
657.2328
803.2994 sLeX
979.4675 PEP_0_1_0_0_0
1182.5479 PEP_0_2_0_0_0
1344.6097 PEP_1_2_0_0_0
1506.6505 PEP_2_2_0_0_0
1871.795 PEP_3_3_0_0_0

File: 231129_OE_RK_30, RT: 9.481, Scan: 007149
Type: HCD, FTMS + c NSI d Full ms2 1111.4269@hcd30.00 [120.0000-4557.4775]
Precursor Scan: 007092, m/z: 1110.6758, charge: 4
ENGTISR_7_6_1_4_0 LeX peak identified

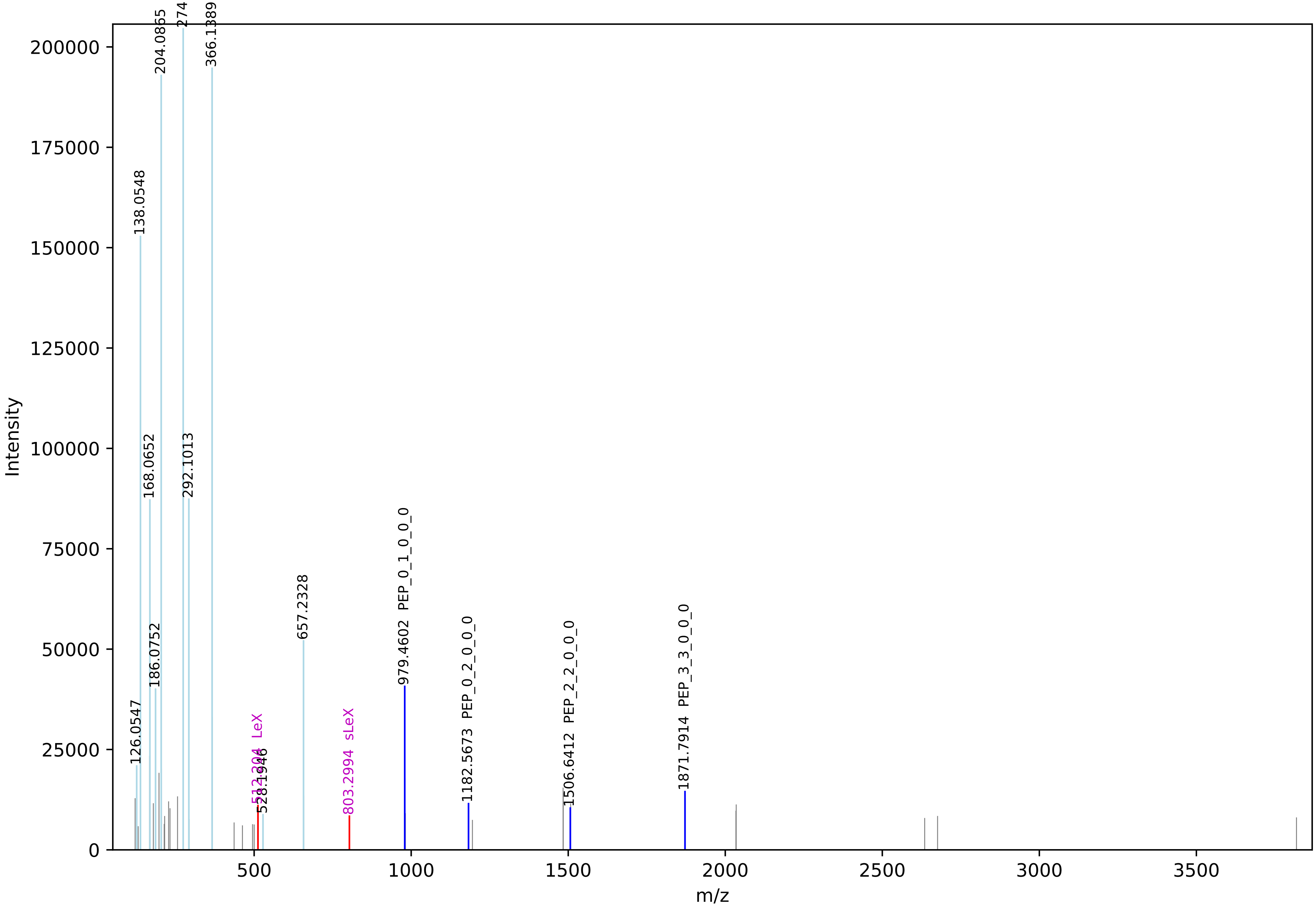

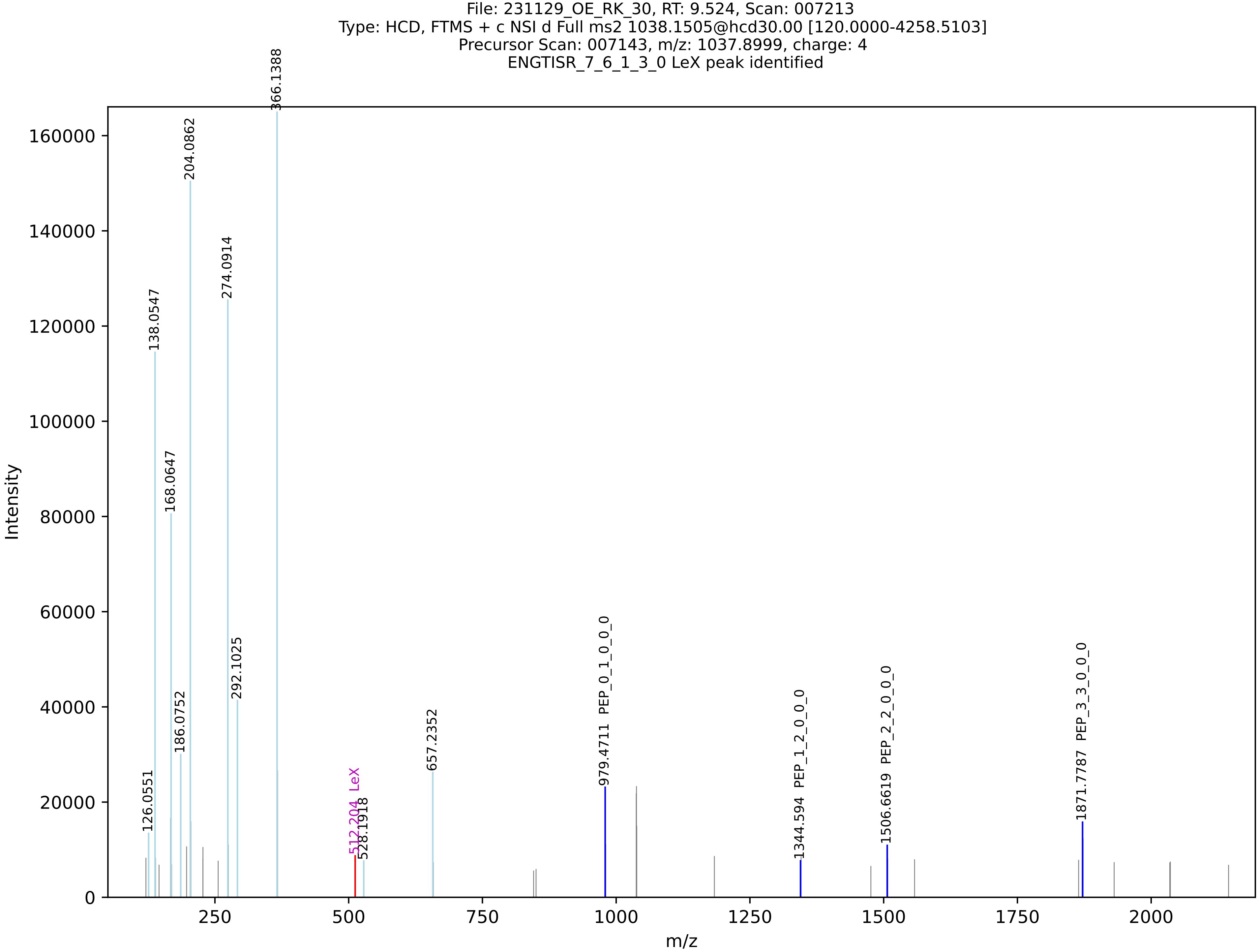
File: 231129_OE_RK_30, RT: 9.524, Scan: 007213
Type: HCD, FTMS + c NSI d Full ms2 1038.1505@hcd30.00 [120.0000-4258.5103]
Precursor Scan: 007143, m/z: 1037.8999, charge: 4
ENGTISR_7_6_1_3_0 LeX peak identified
Intensity
m/z
366.1388
204.0862
274.0914
138.0547
168.0647
292.1025
186.0752
126.0551
512.204 LeX
528.1918
657.2352
979.4711 PEP_0_1_0_0_0
1344.594 PEP_1_2_0_0_0
1506.6619 PEP_2_2_0_0_0
1871.7787 PEP_3_3_0_0_0

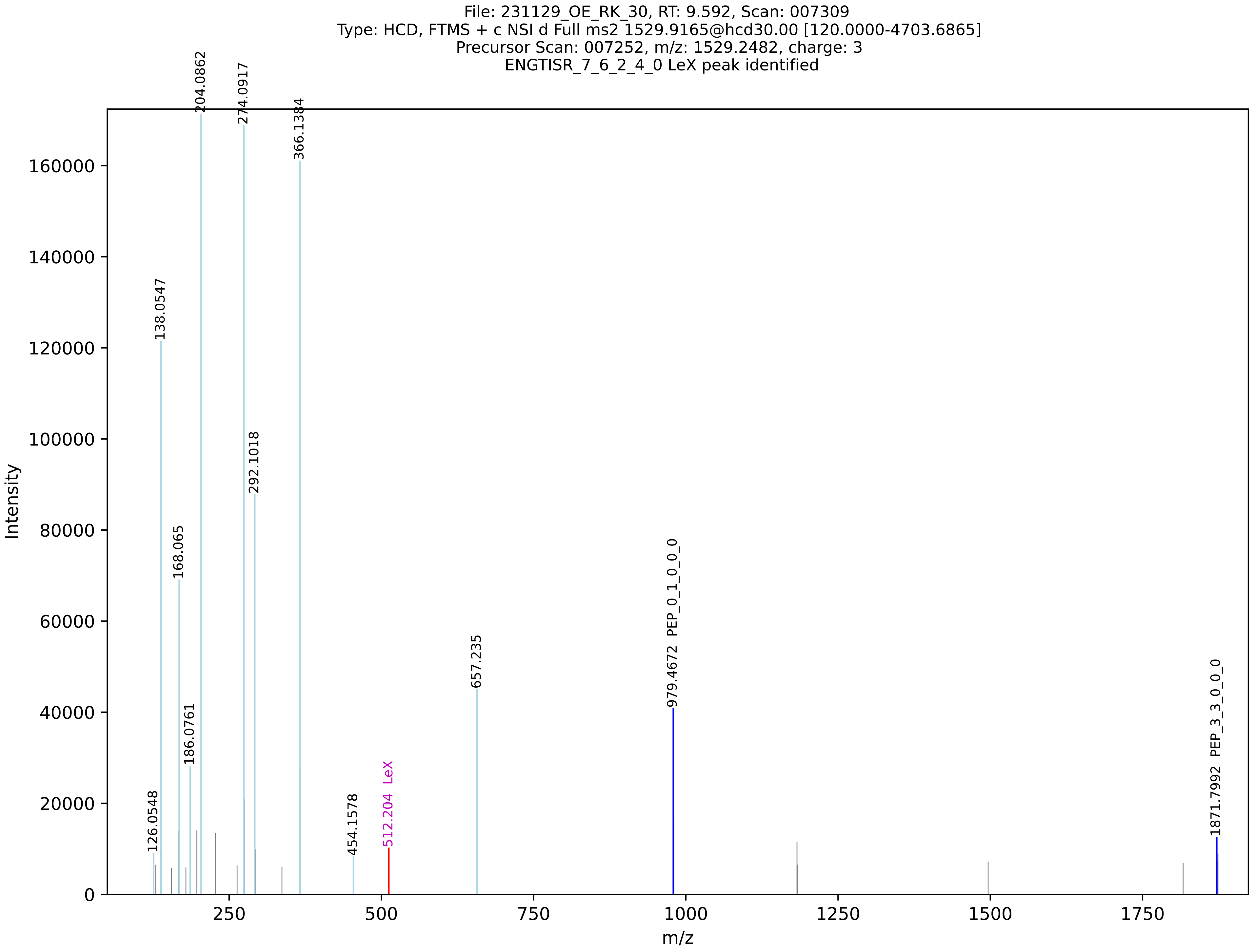
File: 231129_OE_RK_30, RT: 9.592, Scan: 007309
Type: HCD, FTMS + c NSI d Full ms2 1529.9165@hcd30.00 [120.0000-4703.6865]
Precursor Scan: 007252, m/z: 1529.2482, charge: 3
ENGTISR_7_6_2_4_0 LeX peak identified
Intensity
m/z
204.0862
274.0917
366.1384
138.0547
292.1018
168.065
186.0761
126.0548
454.1578
512.204 LeX
657.235
979.4672 PEP_0_1_0_0_0
1871.7992 PEP_3_3_0_0_0
0
20000
40000
60000
80000
100000
120000
140000
160000
250
500
750
1000
1250
1500
1750

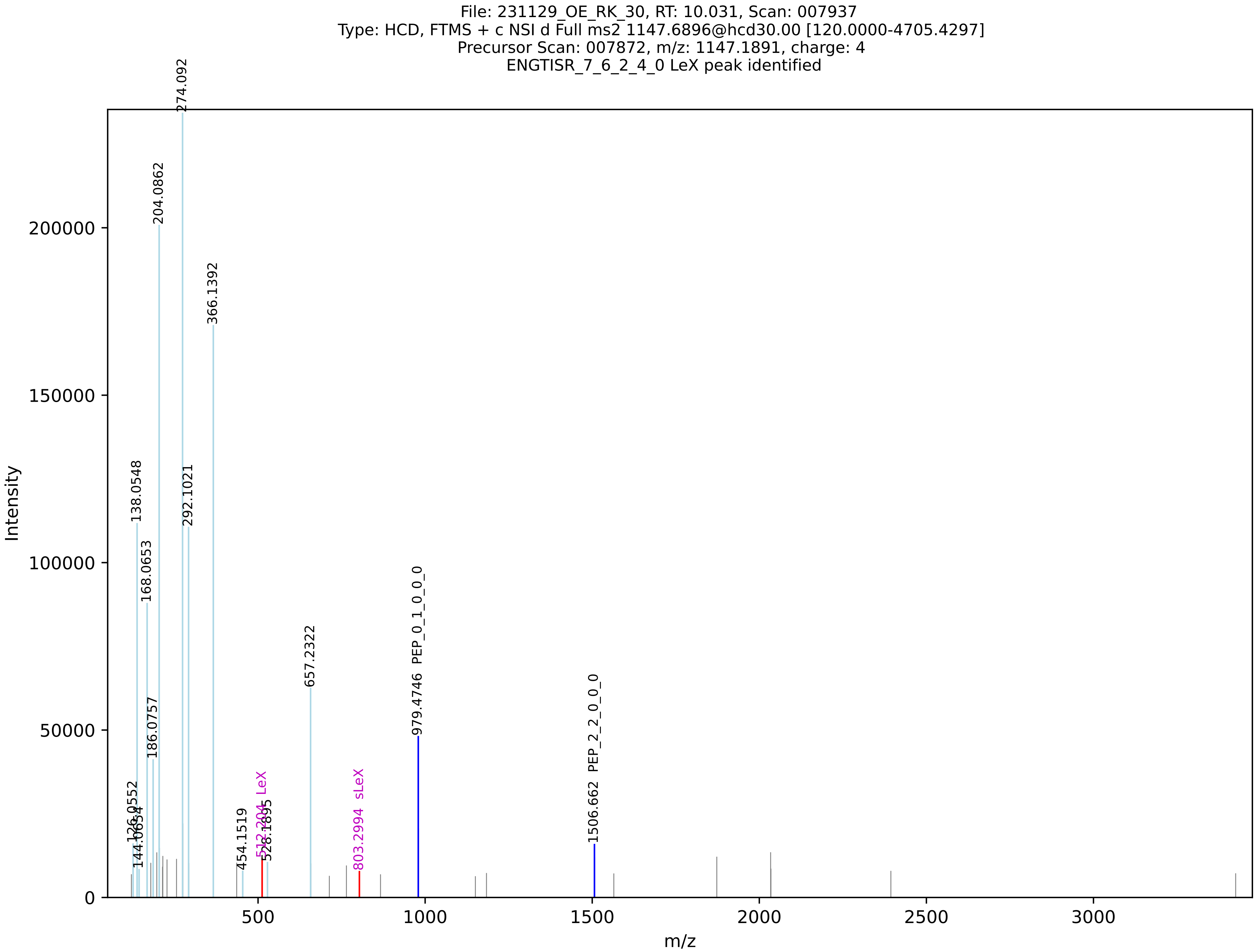
File: 231129_OE_RK_30, RT: 10.031, Scan: 007937
Type: HCD, FTMS + c NSI d Full ms2 1147.6896@hcd30.00 [120.0000-4705.4297]
Precursor Scan: 007872, m/z: 1147.1891, charge: 4
ENGTISR_7_6_2_4_0 LeX peak identified
274.092
204.0862
366.1392
138.0548
292.1021
168.0653
186.0757
126.0552
144.0654
454.1519
512.204 LeX
528.1895
657.2322
803.2994 sLeX
979.4746 PEP_0_1_0_0_0
1506.662 PEP_2_2_0_0_0
Intensity
m/z

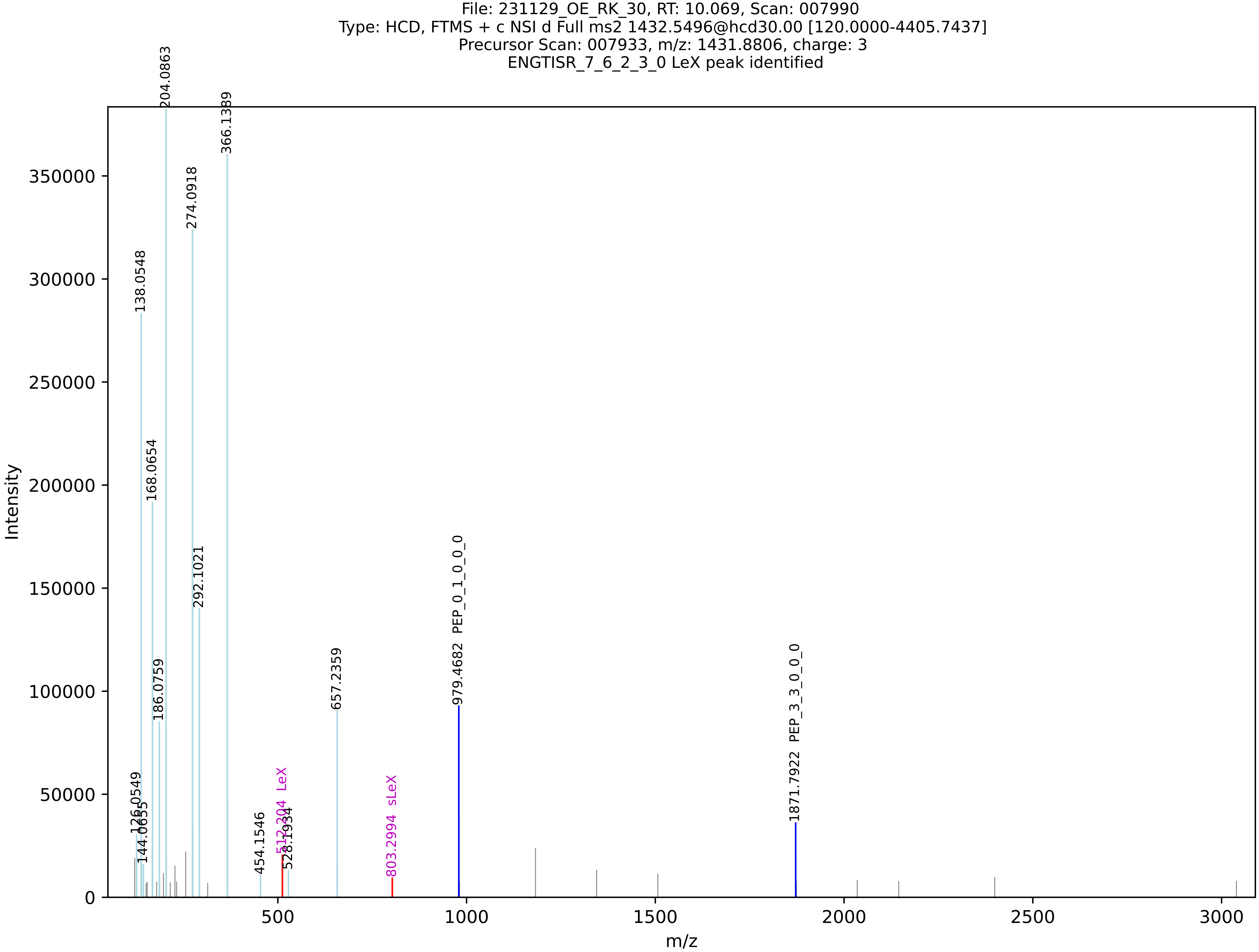
File: 231129_OE_RK_30, RT: 10.069, Scan: 007990
Type: HCD, FTMS + c NSI d Full ms2 1432.5496@hcd30.00 [120.0000-4405.7437]
Precursor Scan: 007933, m/z: 1431.8806, charge: 3
ENGTISR_7_6_2_3_0 LeX peak identified
Intensity
m/z
0
50000
100000
150000
200000
250000
300000
350000
500
1000
1500
2000
2500
3000
204.0863
366.1389
274.0918
138.0548
168.0654
292.1021
186.0759
126.0549
144.0655
454.1546
512.204 LeX
528.1934
657.2359
803.2994 sLeX
979.4682 PEP_0_1_0_0_0_
1871.7922 PEP_3_3_0_0_0_

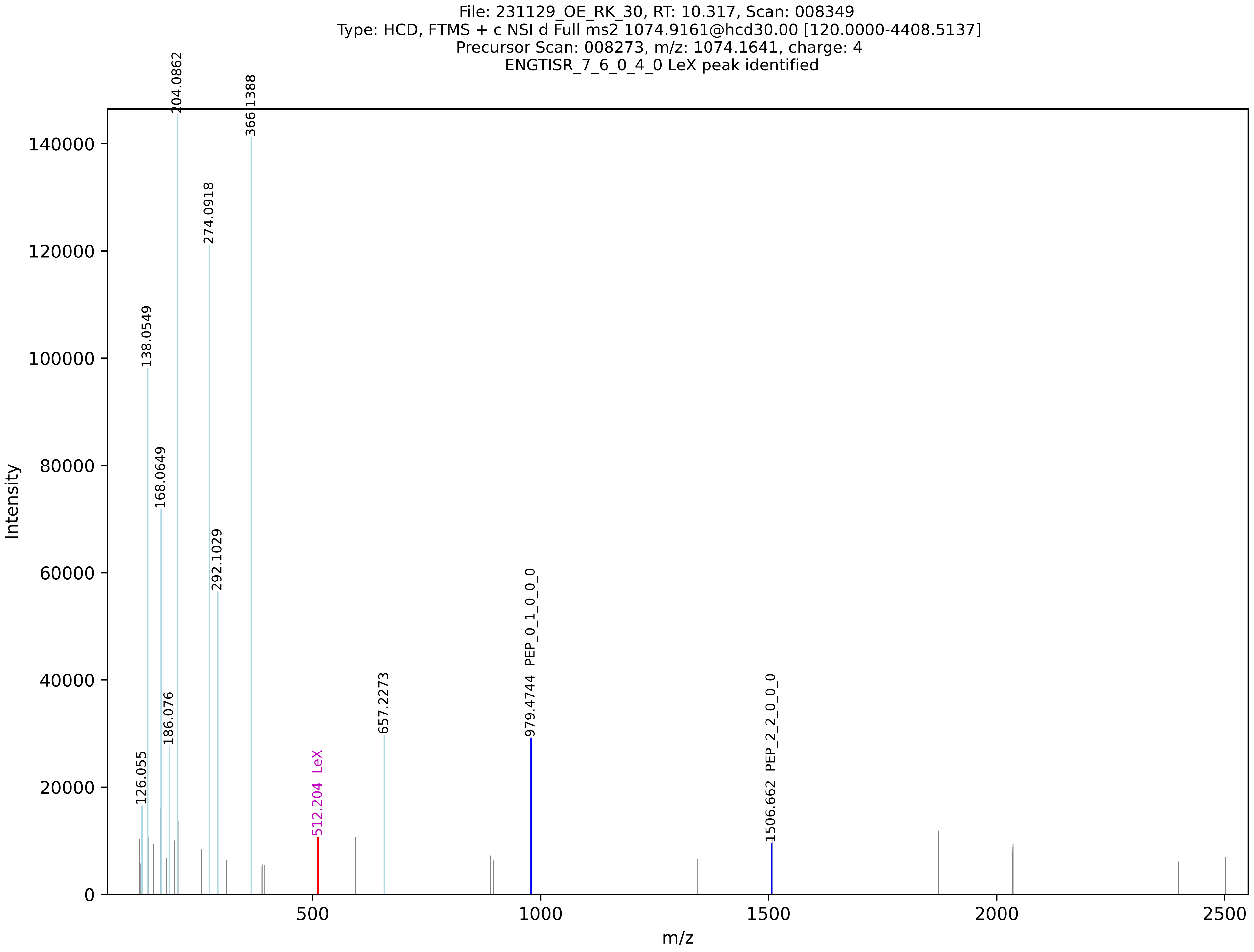

File: 231129_OE_RK_30, RT: 10.317, Scan: 008349
Type: HCD, FTMS + c NSI d Full ms2 1074.9161@hcd30.00 [120.0000-4408.5137]
Precursor Scan: 008273, m/z: 1074.1641, charge: 4
ENGTISR_7_6_0_4_0 LeX peak identified
204.0862
366.1388
274.0918
138.0549
168.0649
292.1029
186.076
126.055
512.204 LeX
657.2273
979.4744 PEP_0_1_0_0_0_
1506.662 PEP_2_2_0_0_0_
Intensity
m/z
0
20000
40000
60000
80000
100000
120000
140000
500
1000
1500
2000
2500

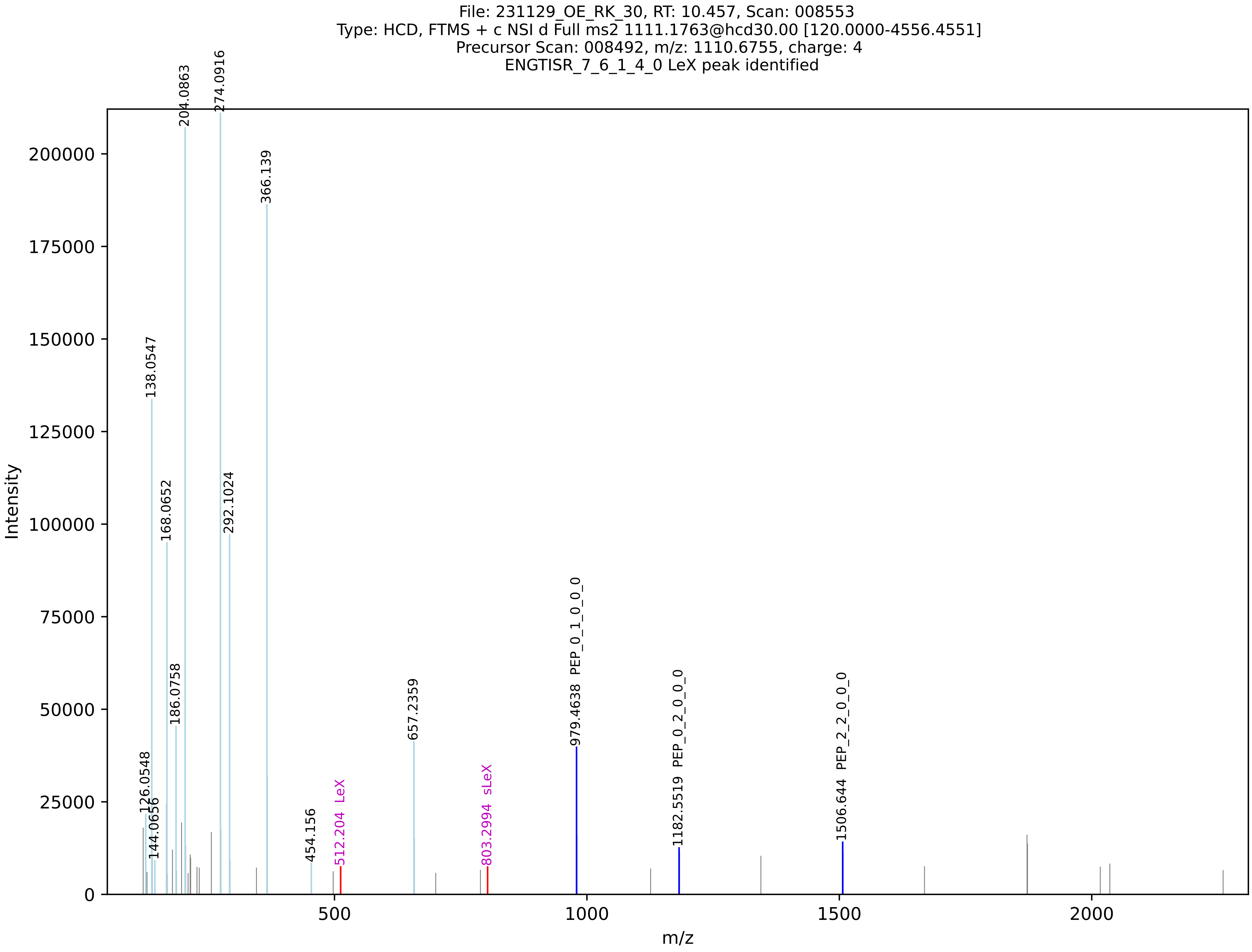

File: 231129_OE_RK_30, RT: 10.457, Scan: 008553
Type: HCD, FTMS + c NSI d Full ms2 1111.1763@hcd30.00 [120.0000-4556.4551]
Precursor Scan: 008492, m/z: 1110.6755, charge: 4
ENGTISR_7_6_1_4_0 LeX peak identified
Intensity
m/z
204.0863
274.0916
366.139
138.0547
168.0652
292.1024
186.0758
126.0548
144.0656
454.156
512.204 LeX
657.2359
803.2994 sLeX
979.4638 PEP_0_1_0_0_0
1182.5519 PEP_0_2_0_0_0
1506.644 PEP_2_2_0_0_0

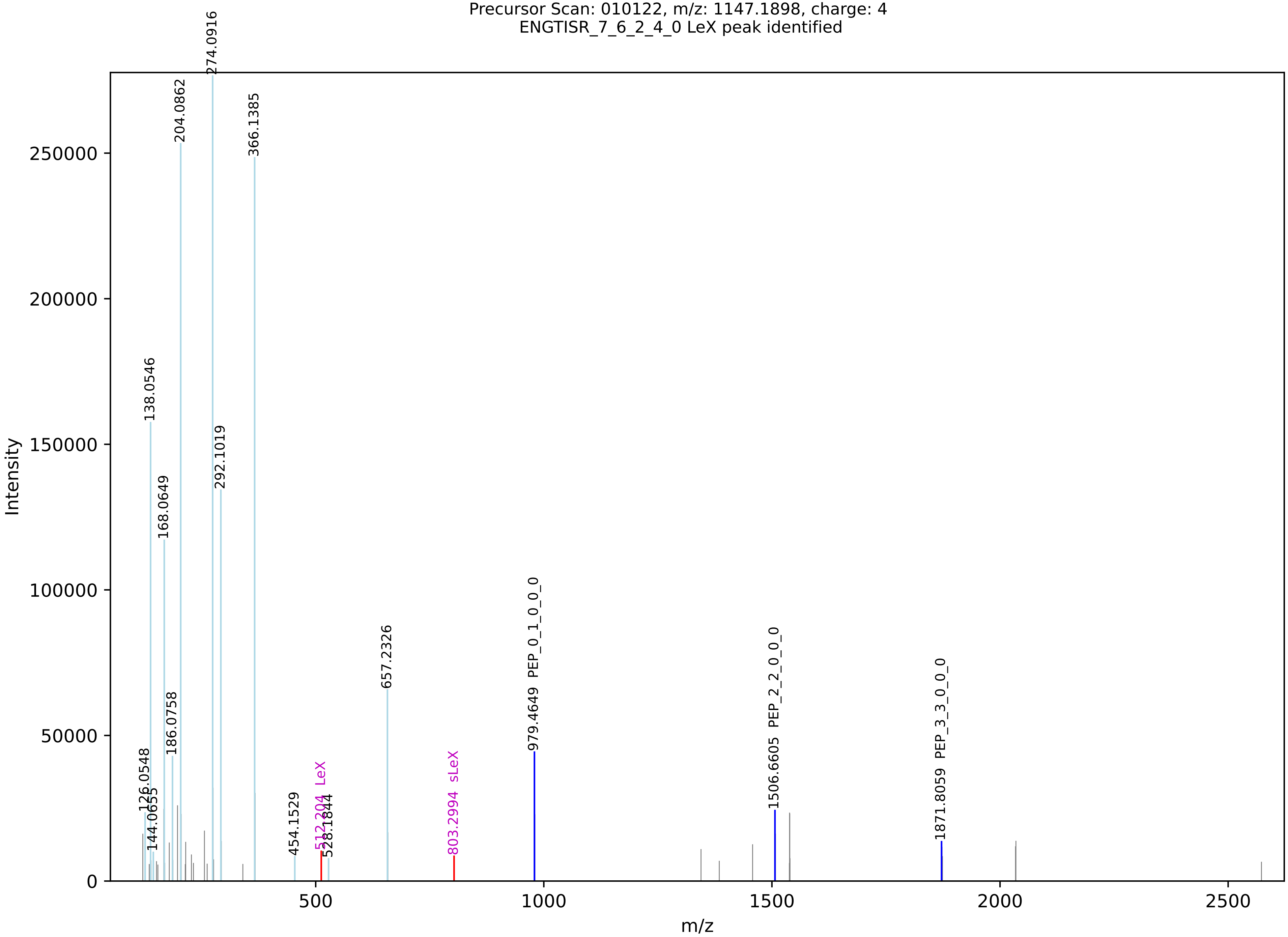
File: 231129_OE_RK_30, RT: 11.604, Scan: 010199
Type: HCD, FTMS + c NSI d Full ms2 1147.6888@hcd30.00 [120.0000-4705.4268]
Precursor Scan: 010122, m/z: 1147.1898, charge: 4
ENGTISR_7_6_2_4_0 LeX peak identified
Intensity
m/z
274.0916
204.0862
366.1385
138.0546
292.1019
168.0649
186.0758
126.0548
144.0655
454.1529
512.204 LeX
528.1844
657.2326
803.2994 sLeX
979.4649 PEP_0_1_0_0_0
1506.6605 PEP_2_2_0_0_0
1871.8059 PEP_3_3_0_0_0
0
50000
100000
150000
200000
250000
500
1000
1500
2000
2500

File: 231129_OE_RK_30, RT: 11.655, Scan: 010274
Type: HCD, FTMS + c NSI d Full ms2 1432.5494@hcd30.00 [120.0000-4405.7432]
Precursor Scan: 010194, m/z: 1431.881, charge: 3
ENGTISR_7_6_0_4_0 LeX peak identified

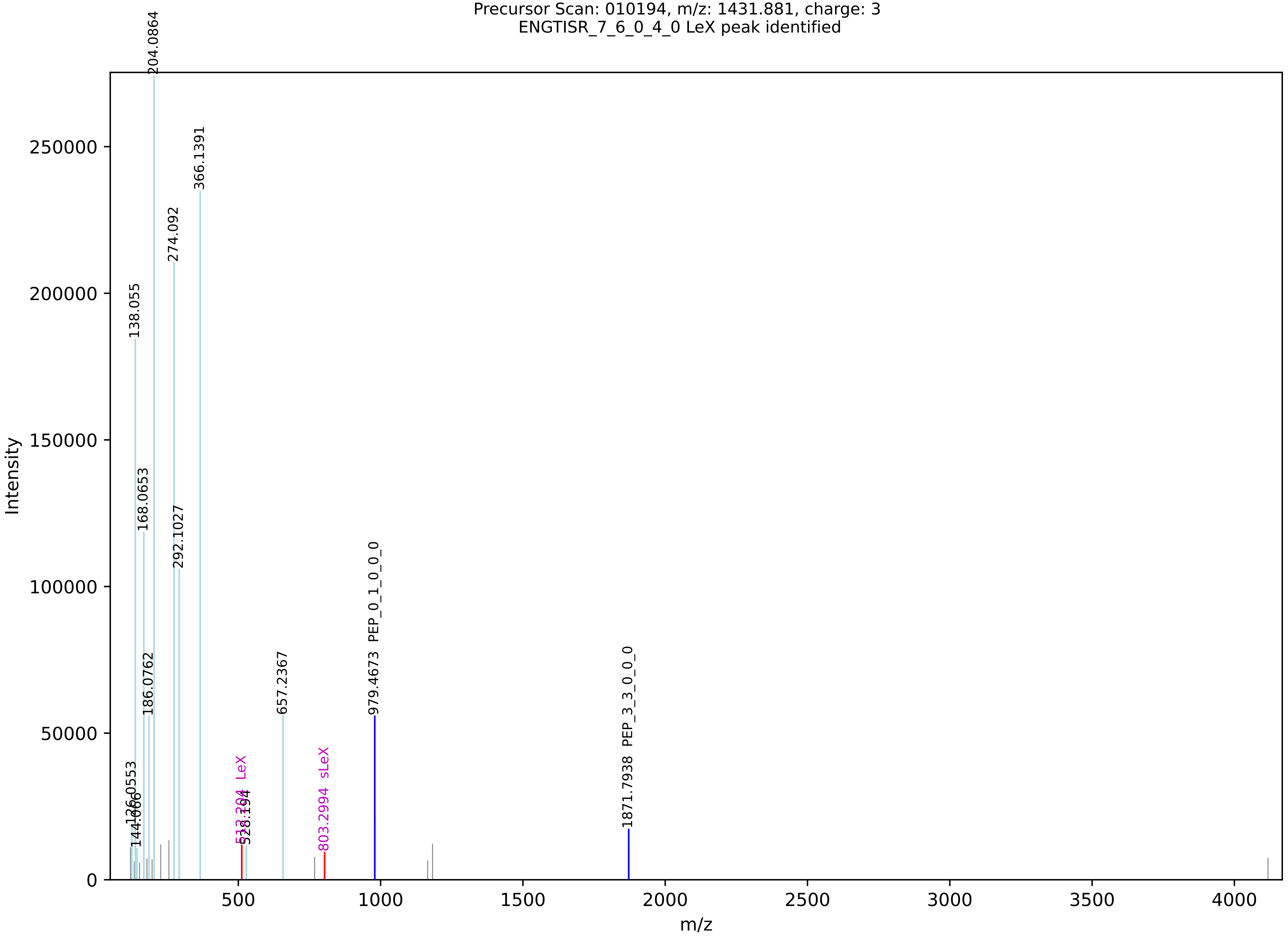

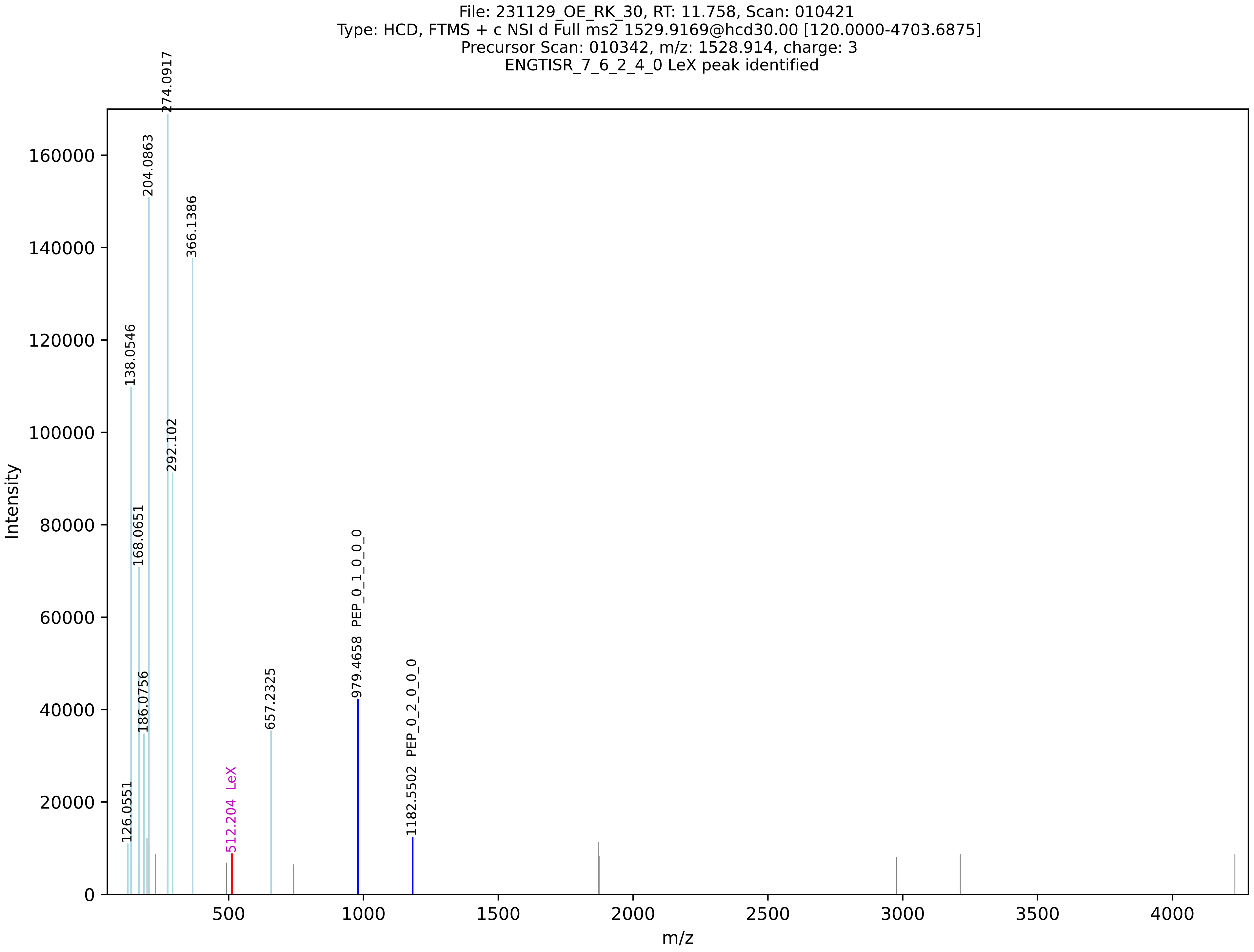
File: 231129_OE_RK_30, RT: 11.758, Scan: 010421
Type: HCD, FTMS + c NSI d Full ms2 1529.9169@hcd30.00 [120.0000-4703.6875]
Precursor Scan: 010342, m/z: 1528.914, charge: 3
ENGTISR_7_6_2_4_0 LeX peak identified
274.0917
204.0863
366.1386
138.0546
292.102
168.0651
PEP_0_1_0_0_0
979.4658
PEP_0_2_0_0_0
186.0756
657.2325
LeX
512.204
1182.5502
126.0551
Intensity
m/z

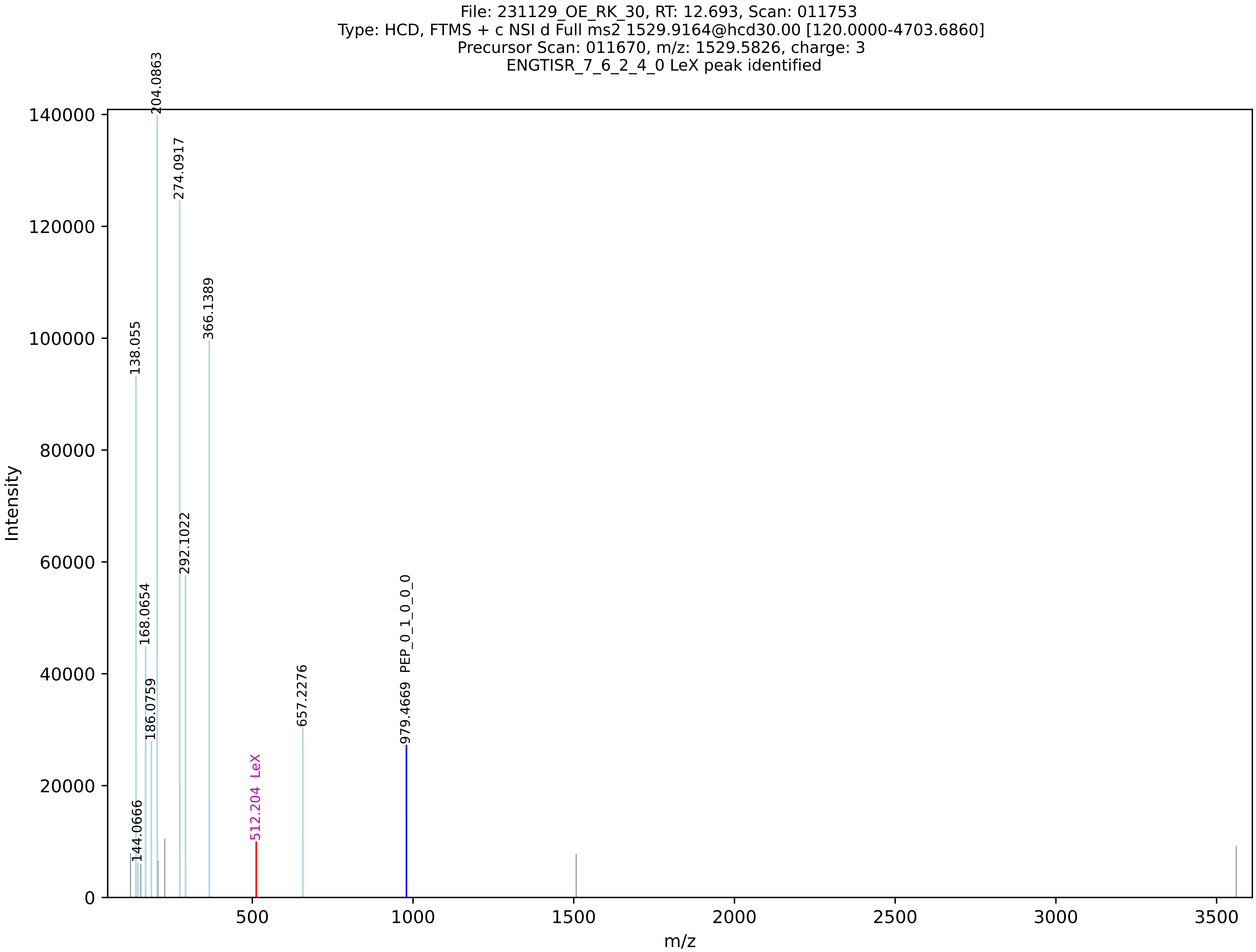
File: 231129_OE_RK_30, RT: 12.693, Scan: 011753
Type: HCD, FTMS + c NSI d Full ms2 1529.9164@hcd30.00 [120.0000-4703.6860]
Precursor Scan: 011670, m/z: 1529.5826, charge: 3
ENGTISR_7_6_2_4_0 LeX peak identified
Intensity
m/z
0
20000
40000
60000
80000
100000
120000
140000
500
1000
1500
2000
2500
3000
3500
204.0863
274.0917
366.1389
138.055
292.1022
168.0654
186.0759
144.0666
512.204 LeX
657.2276
979.4669 PEP_0_1_0_0_0_

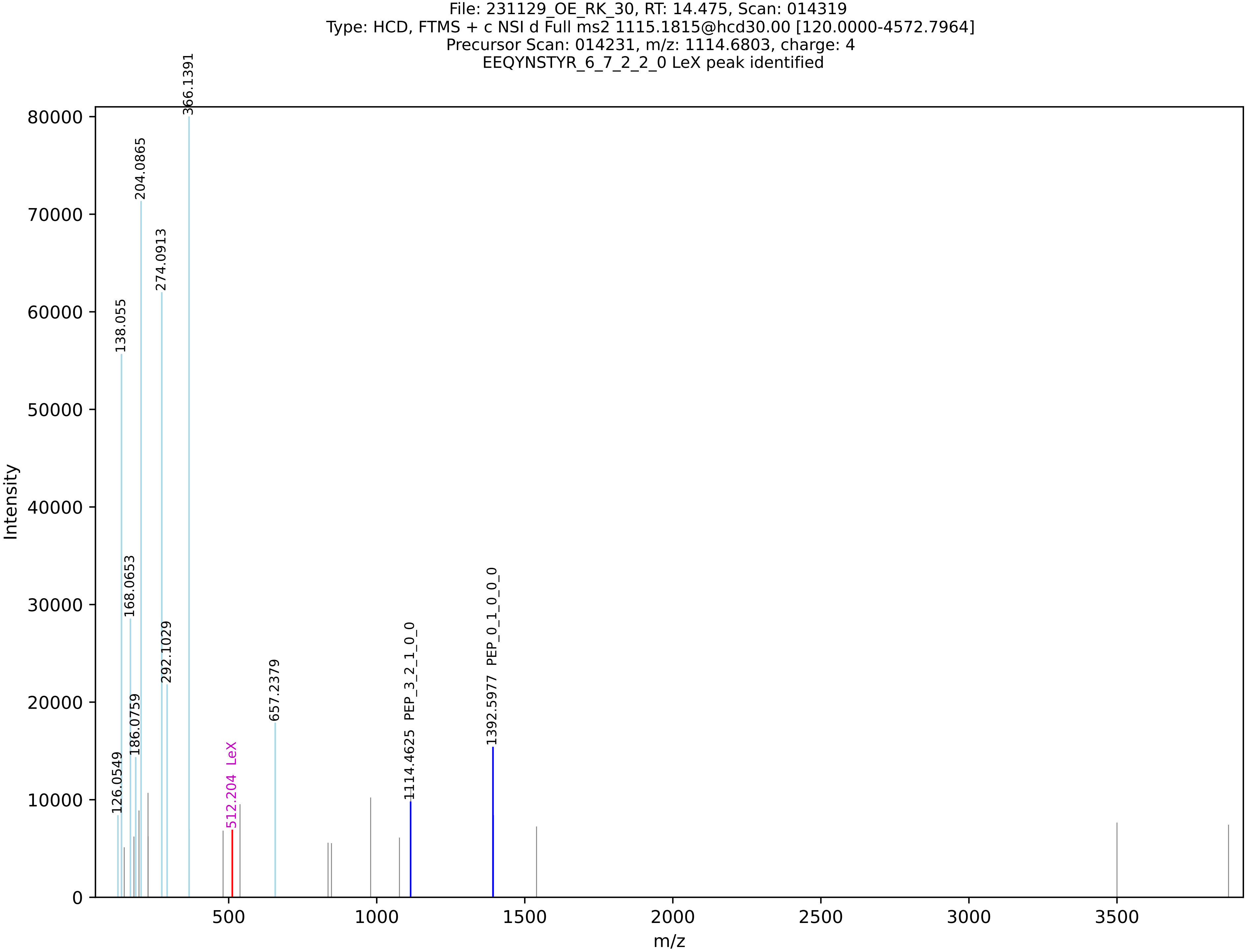

File: 231129_OE_RK_30, RT: 14.475, Scan: 014319
Type: HCD, FTMS + c NSI d Full ms2 1115.1815@hcd30.00 [120.0000-4572.7964]
Precursor Scan: 014231, m/z: 1114.6803, charge: 4
EEQYNSTYR_6_7_2_2_0 LeX peak identified
Intensity
m/z
366.1391
204.0865
274.0913
138.055
168.0653
292.1029
186.0759
126.0549
512.204 LeX
657.2379
1114.4625 PEP_3_2_1_0_0
1392.5977 PEP_0_1_0_0_0
0
10000
20000
30000
40000
50000
60000
70000
80000
500
1000
1500
2000
2500
3000
3500

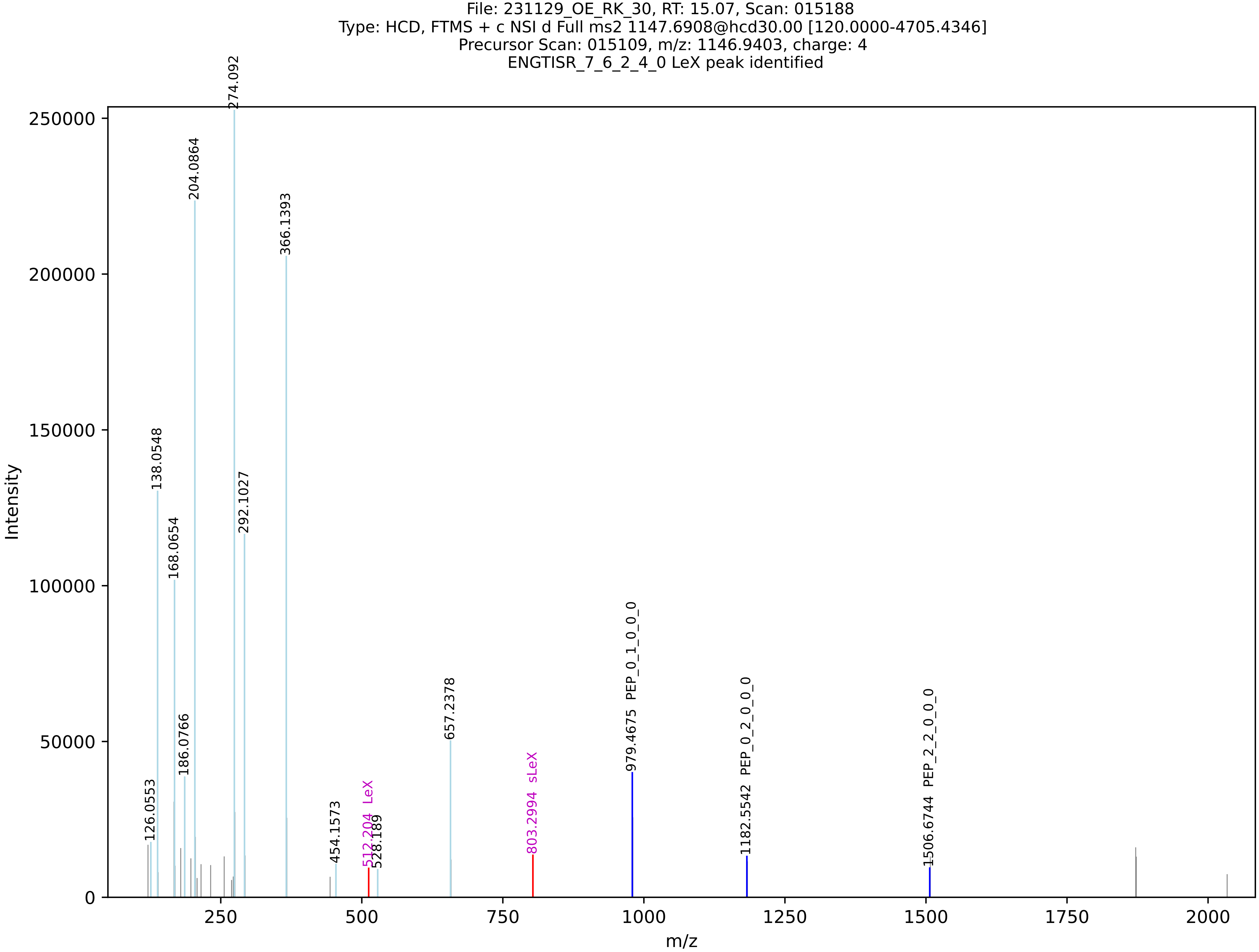

File: 231129_OE_RK_30, RT: 15.07, Scan: 015188
Type: HCD, FTMS + c NSI d Full ms2 1147.6908@hcd30.00 [120.0000-4705.4346]
Precursor Scan: 015109, m/z: 1146.9403, charge: 4
ENGTISR_7_6_2_4_0 LeX peak identified
Intensity
m/z
274.092
204.0864
366.1393
138.0548
292.1027
168.0654
186.0766
126.0553
454.1573
512.204 LeX
528.189
657.2378
803.2994 sLeX
979.4675 PEP_0_1_0_0_0
1182.5542 PEP_0_2_0_0_0
1506.6744 PEP_2_2_0_0_0

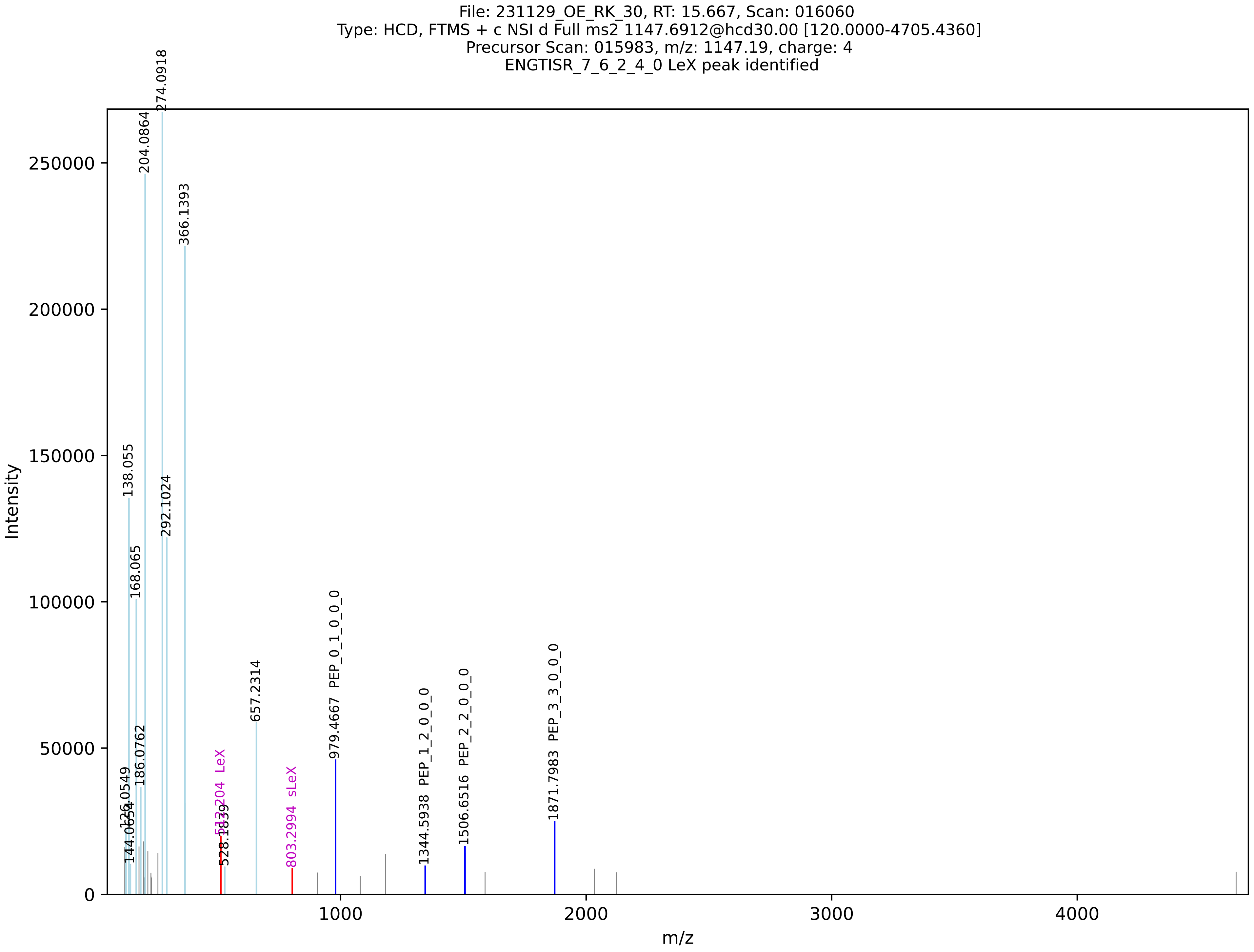

File: 231129_OE_RK_30, RT: 15.667, Scan: 016060
Type: HCD, FTMS + c NSI d Full ms2 1147.6912@hcd30.00 [120.0000-4705.4360]
Precursor Scan: 015983, m/z: 1147.19, charge: 4
ENGTISR_7_6_2_4_0 LeX peak identified
Intensity
m/z
274.0918
204.0864
366.1393
138.055
292.1024
168.065
186.0762
126.0549
144.0654
528.1839
512.204 LeX
803.2994 sLeX
657.2314
979.4667 PEP_0_1_0_0_0
1344.5938 PEP_1_2_0_0_0
1506.6516 PEP_2_2_0_0_0
1871.7983 PEP_3_3_0_0_0

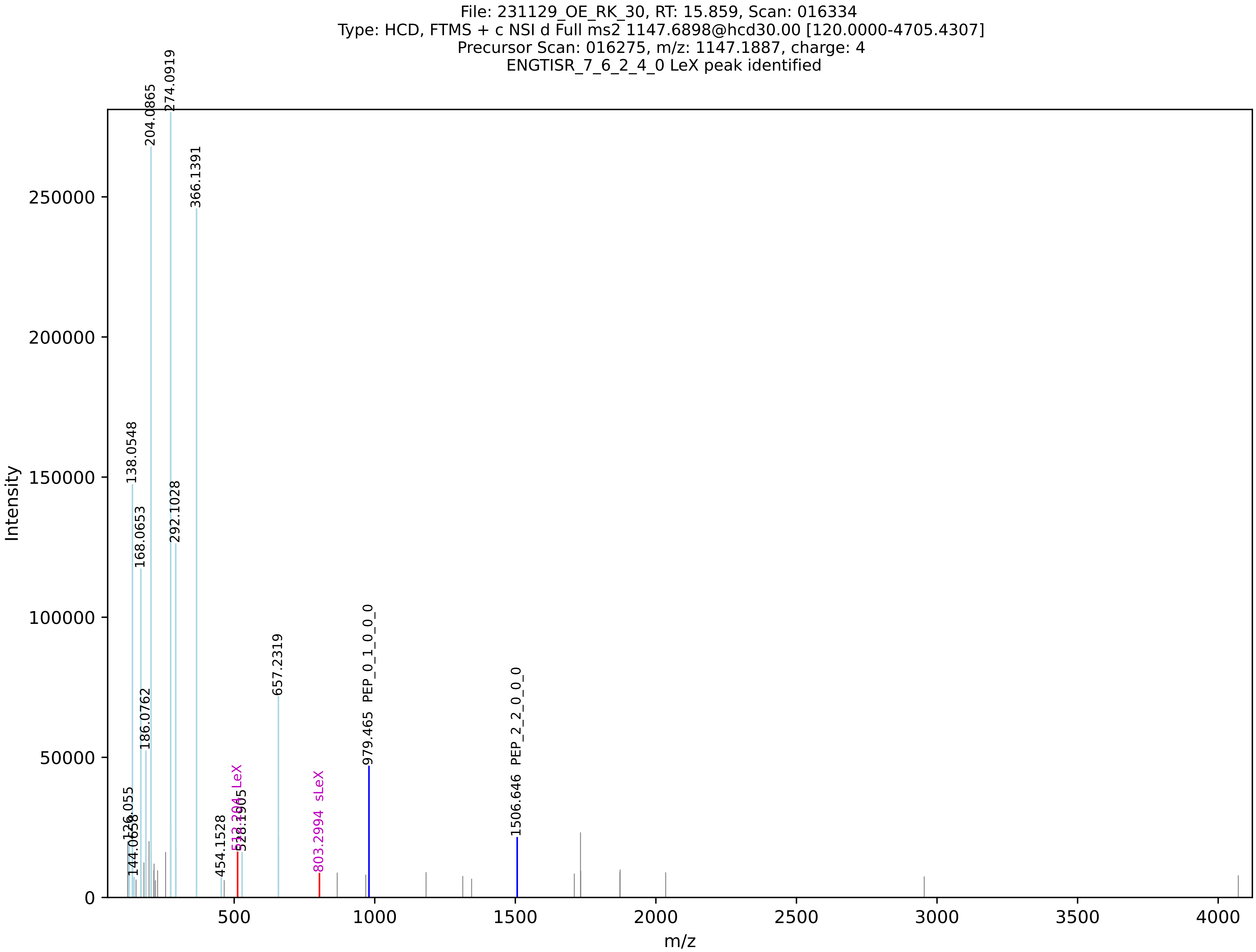

File: 231129_OE_RK_30, RT: 15.859, Scan: 016334
Type: HCD, FTMS + c NSI d Full ms2 1147.6898@hcd30.00 [120.0000-4705.4307]
Precursor Scan: 016275, m/z: 1147.1887, charge: 4
ENGTISR_7_6_2_4_0 LeX peak identified
Intensity
m/z
274.0919
204.0865
366.1391
138.0548
292.1028
168.0653
186.0762
126.055
144.0658
454.1528
512.204 LeX
528.1905
657.2319
803.2994 sLeX
979.465 PEP_0_1_0_0_0_
1506.646 PEP_2_2_0_0_0_

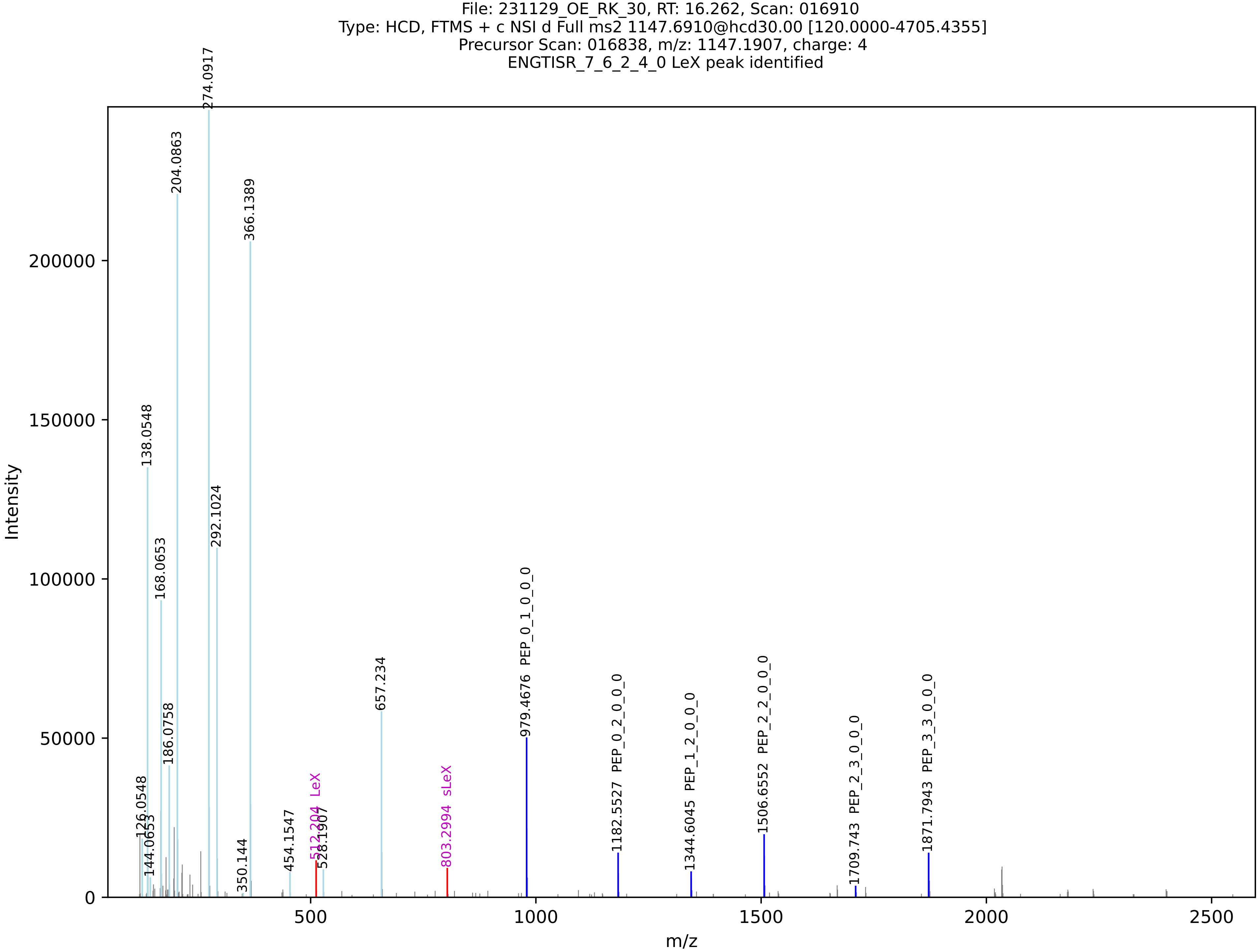
File: 231129_OE_RK_30, RT: 16.262, Scan: 016910
Type: HCD, FTMS + c NSI d Full ms2 1147.6910@hcd30.00 [120.0000-4705.4355]
Precursor Scan: 016838, m/z: 1147.1907, charge: 4
ENGTISR_7_6_2_4_0 LeX peak identified
Intensity
m/z
274.0917
204.0863
366.1389
138.0548
292.1024
168.0653
186.0758
126.0548
144.0653
350.144
454.1547
512.204 LeX
528.1907
657.234
803.2994 sLeX
979.4676 PEP_0_1_0_0_0
1182.5527 PEP_0_2_0_0_0
1344.6045 PEP_1_2_0_0_0
1506.6552 PEP_2_2_0_0_0
1709.743 PEP_2_3_0_0_0
1871.7943 PEP_3_3_0_0_0
0
50000
100000
150000
200000
500
1000
1500
2000
2500

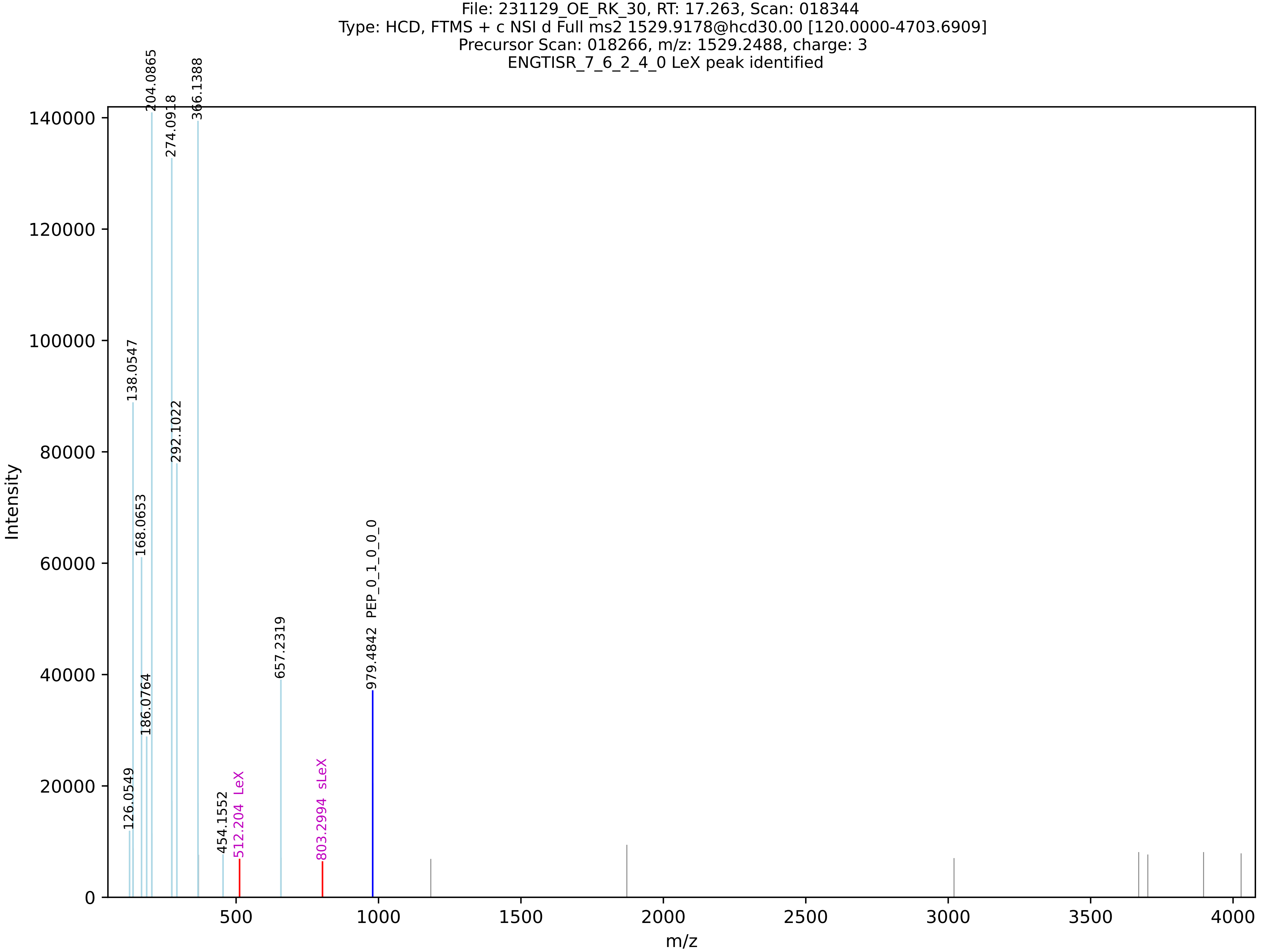
File: 231129_OE_RK_30, RT: 17.263, Scan: 018344
Type: HCD, FTMS + c NSI d Full ms2 1529.9178@hcd30.00 [120.0000-4703.6909]
Precursor Scan: 018266, m/z: 1529.2488, charge: 3
ENGTISR_7_6_2_4_0 LeX peak identified
Intensity
m/z
204.0865
366.1388
274.0918
138.0547
292.1022
168.0653
186.0764
126.0549
454.1552
512.204 LeX
657.2319
803.2994 sLeX
979.4842 PEP_0_1_0_0_0_

File: 231129_OE_RK_30, RT: 17.461, Scan: 018629
Type: HCD, FTMS + c NSI d Full ms2 1529.9186@hcd30.00 [120.0000-4703.6929]
Precursor Scan: 018553, m/z: 1529.2502, charge: 3
ENGTISR_7_6_2_4_0 LeX peak identified

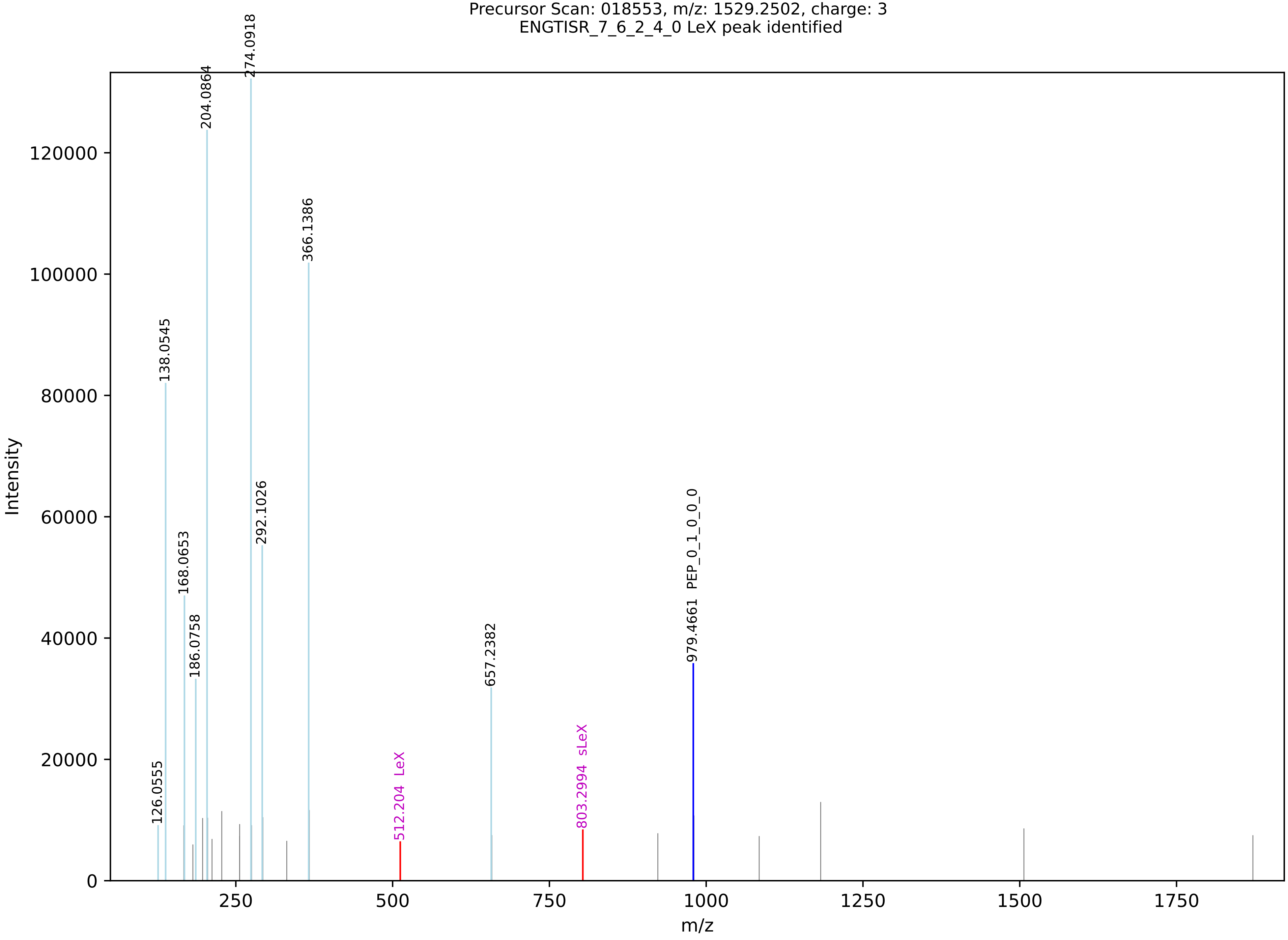

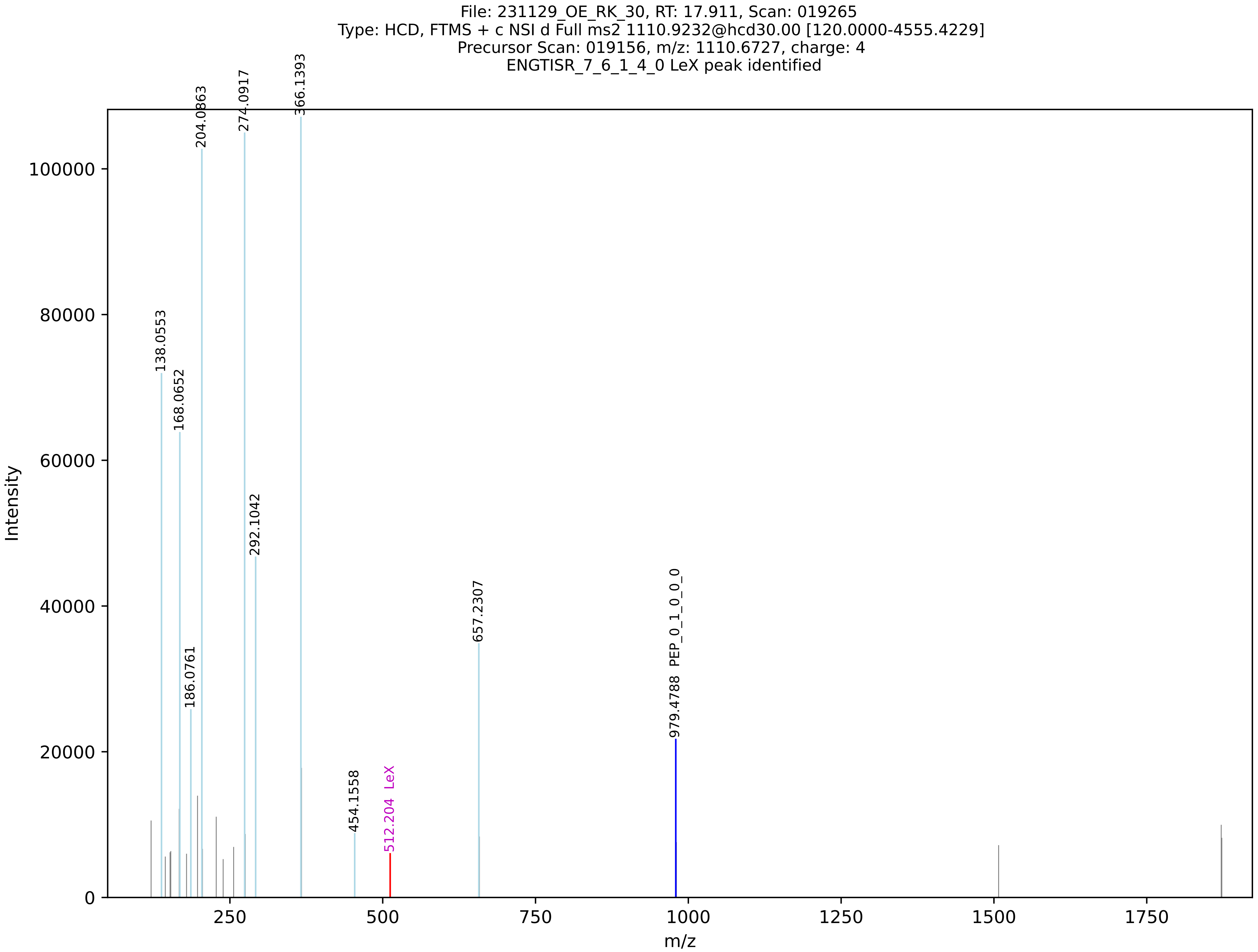
File: 231129_OE_RK_30, RT: 17.911, Scan: 019265
Type: HCD, FTMS + c NSI d Full ms2 1110.9232@hcd30.00 [120.0000-4555.4229]
Precursor Scan: 019156, m/z: 1110.6727, charge: 4
ENGTISR_7_6_1_4_0 LeX peak identified
Intensity
m/z
138.0553
168.0652
186.0761
204.0863
274.0917
292.1042
366.1393
454.1558
512.204 LeX
657.2307
979.4788 PEP_0_1_0_0_0
0
20000
40000
60000
80000
100000
250
500
750
1000
1250
1500
1750

File: 231129_OE_RK_30, RT: 18.05, Scan: 019458
Type: HCD, FTMS + c NSI d Full ms2 1529.9158@hcd30.00 [120.0000-4703.6846]
Precursor Scan: 019400, m/z: 1529.2476, charge: 3
ENGTISR_7_6_2_4_0 LeX peak identified

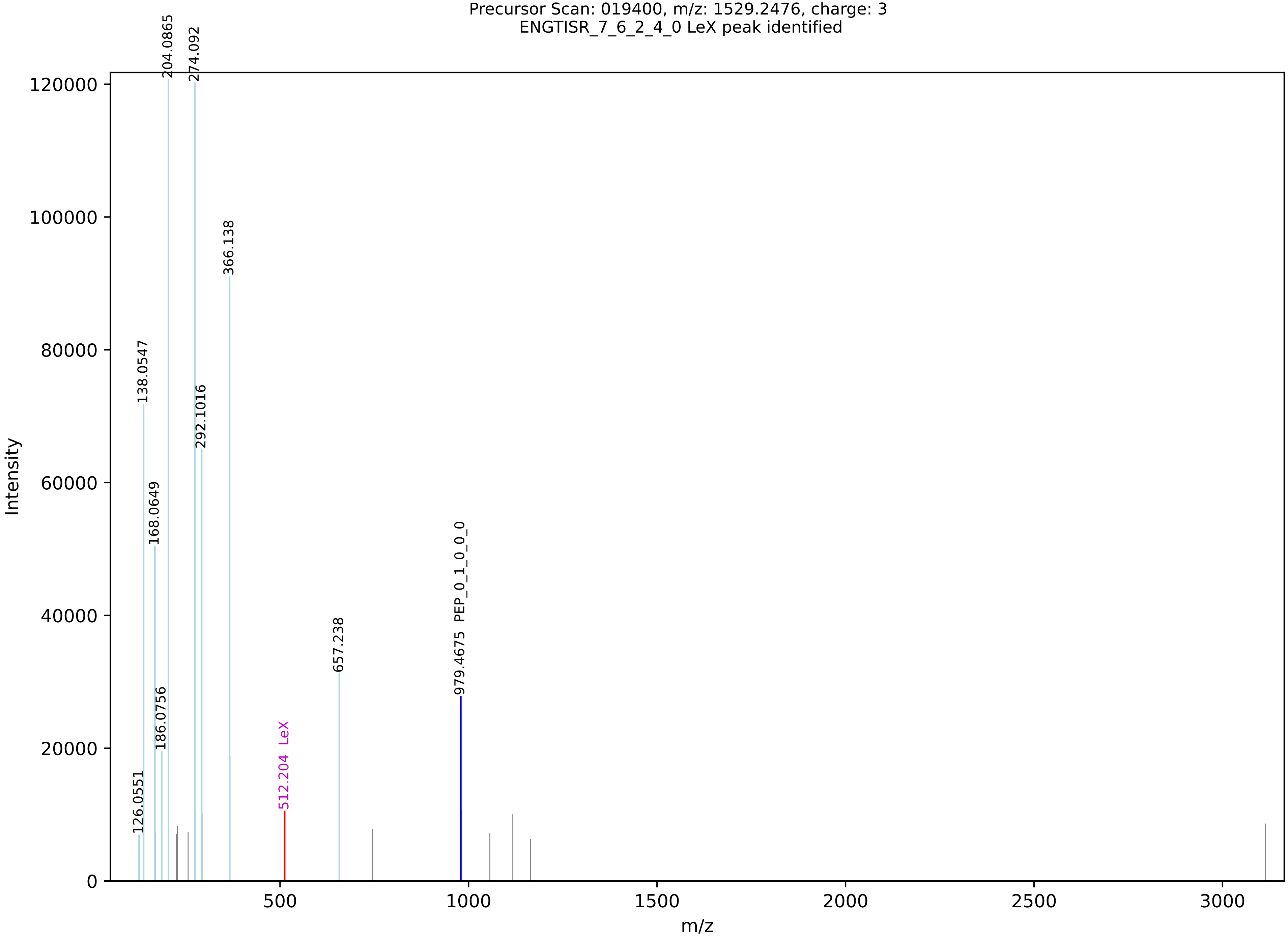

File: 231129_OE_RK_30, RT: 18.558, Scan: 020188
Type: HCD, FTMS + c NSI d Full ms2 1147.6914@hcd30.00 [120.0000-4705.4365]
Precursor Scan: 020109, m/z: 1147.1902, charge: 4
ENGTISR_7_6_2_4_0 LeX peak identified

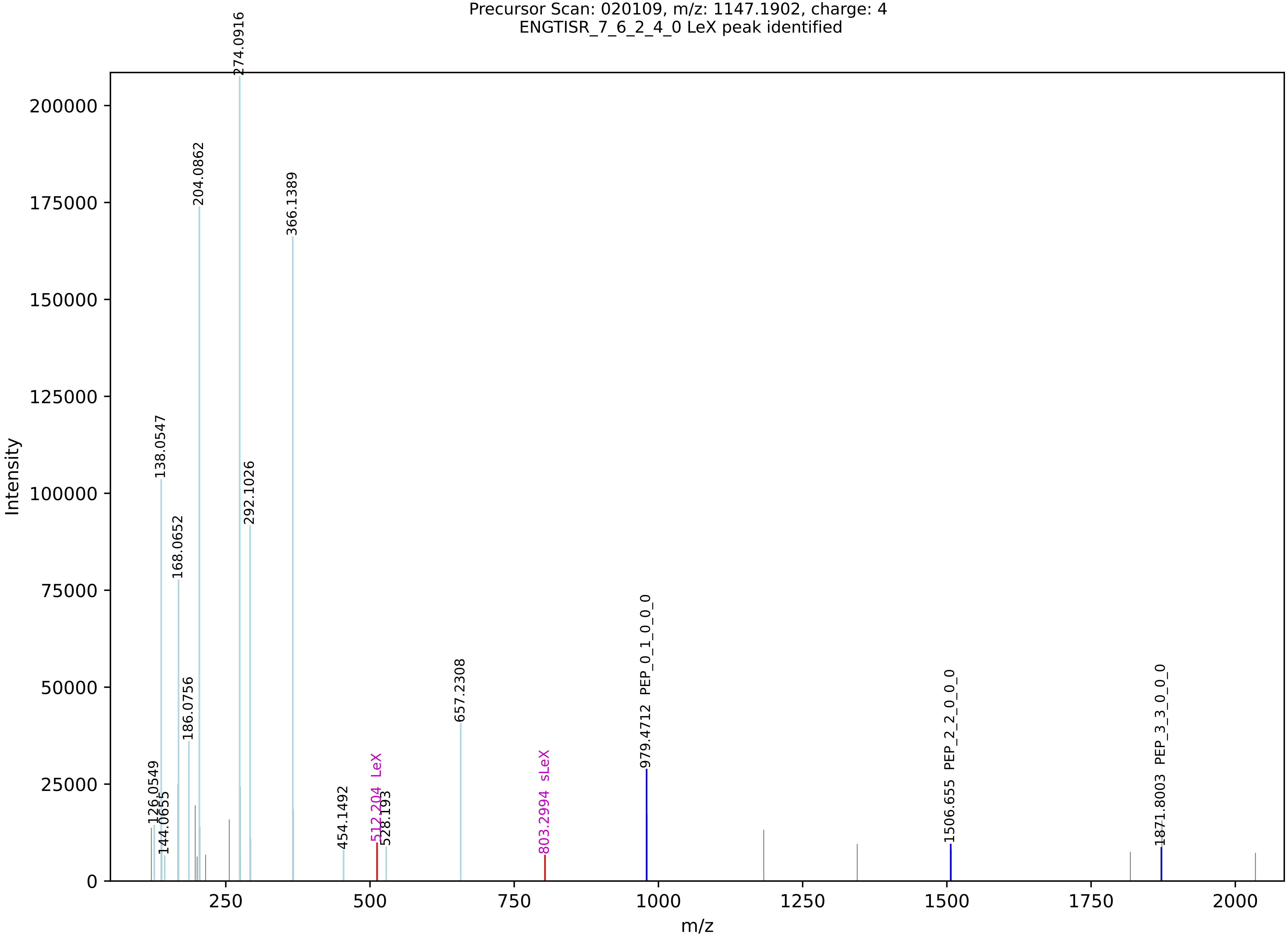

File: 231129_OE_RK_30, RT: 18.766, Scan: 020485
Type: HCD, FTMS + c NSI d Full ms2 1147.4390@hcd30.00 [120.0000-4704.4072]
Precursor Scan: 020399, m/z: 1147.1902, charge: 4
ENGTISR_7_6_2_4_0 LeX peak identified

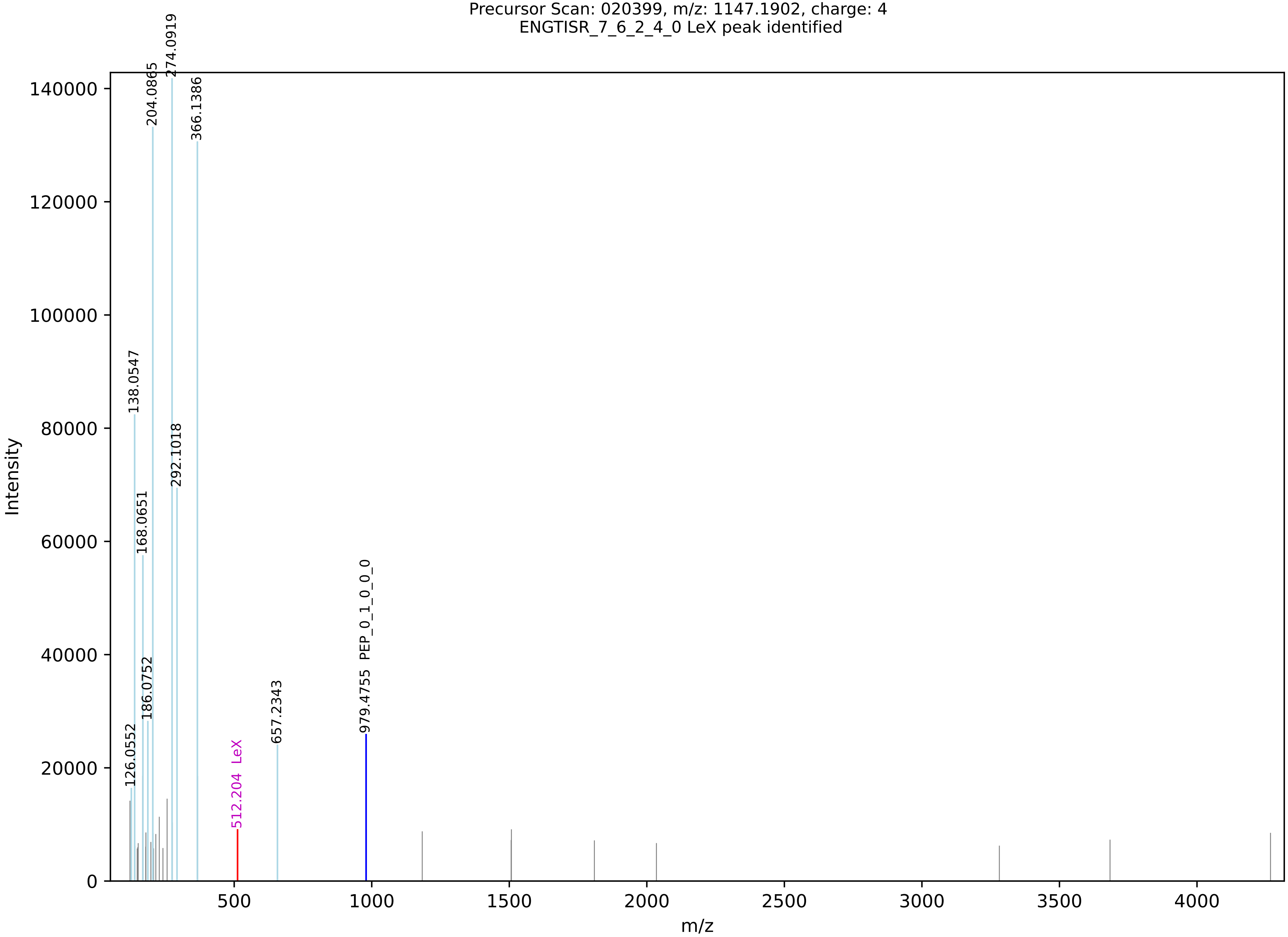

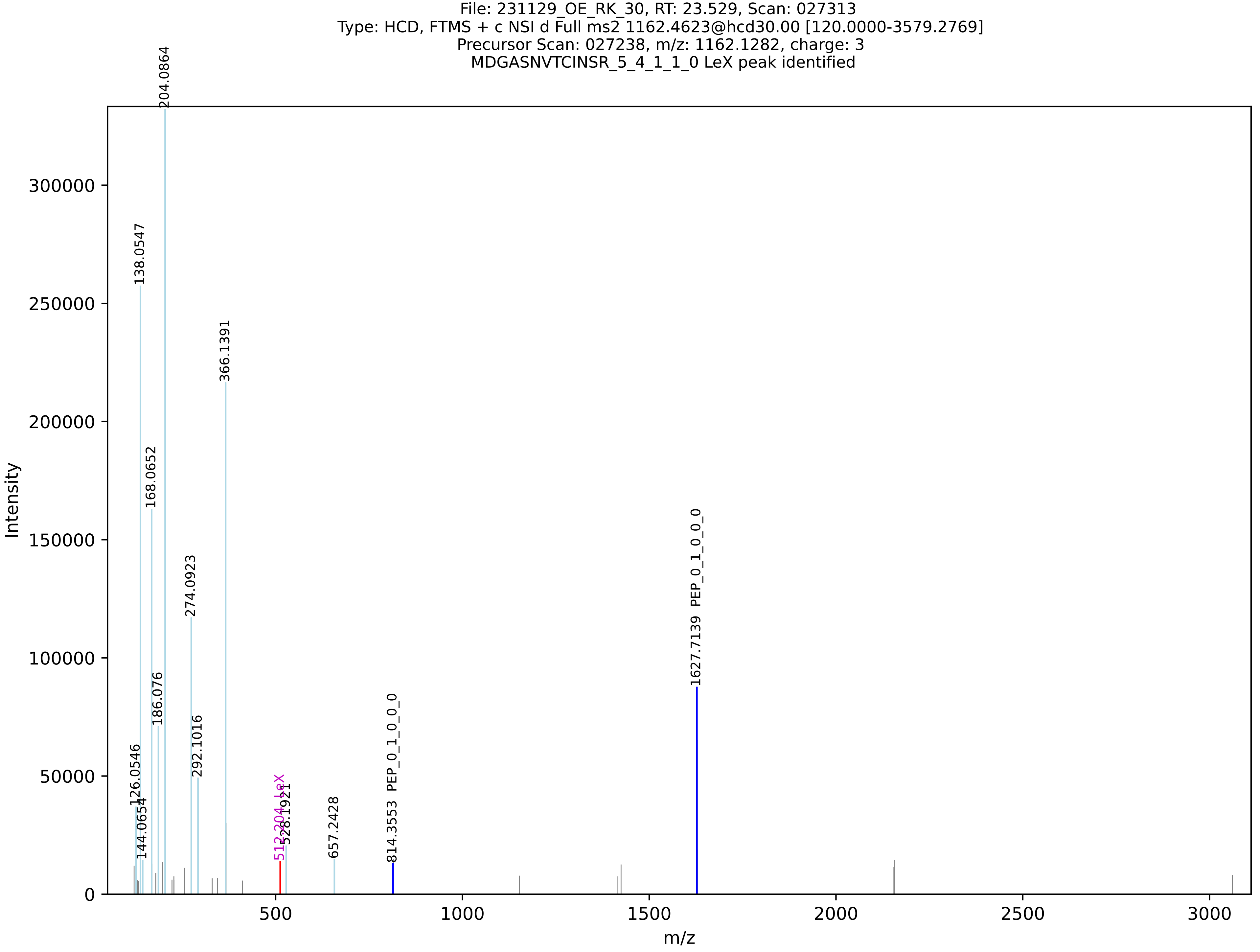
File: 231129_OE_RK_30, RT: 23.529, Scan: 027313
Type: HCD, FTMS + c NSI d Full ms2 1162.4623@hcd30.00 [120.0000-3579.2769]
Precursor Scan: 027238, m/z: 1162.1282, charge: 3
MDGASNVTCINSR_5_4_1_1_0 LeX peak identified
204.0864
138.0547
366.1391
168.0652
274.0923
186.076
292.1016
126.0546
144.0654
512.204 LeX
528.1921
657.2428
814.3553 PEP_0_1_0_0_0
1627.7139 PEP_0_1_0_0_0
Intensity
m/z

File: 231129_OE_RK_30, RT: 24.198, Scan: 028278
Type: HCD, FTMS + c NSI d Full ms2 1306.5105@hcd30.00 [120.0000-4020.0640]
Precursor Scan: 028182, m/z: 1306.1764, charge: 3
DTFVNASR_6_5_1_3_0 LeX peak identified

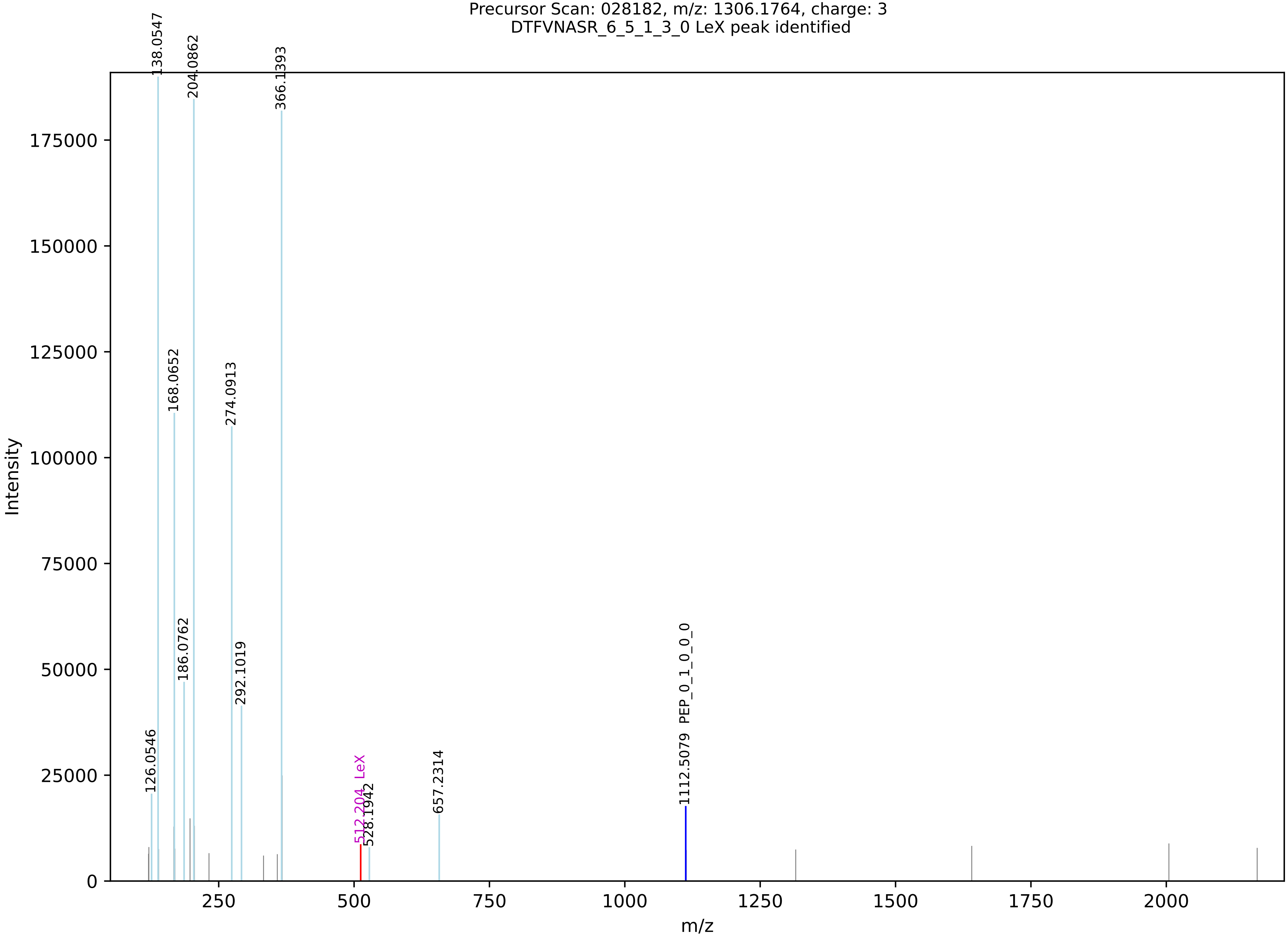

File: 231129_OE_RK_30, RT: 24.389, Scan: 028550
Type: HCD, FTMS + c NSI d Full ms2 1314.8621@hcd30.00 [120.0000-4045.6199]
Precursor Scan: 028472, m/z: 1314.1944, charge: 3
LQNNENNISCVER_5_4_1_2_0 LeX peak identified

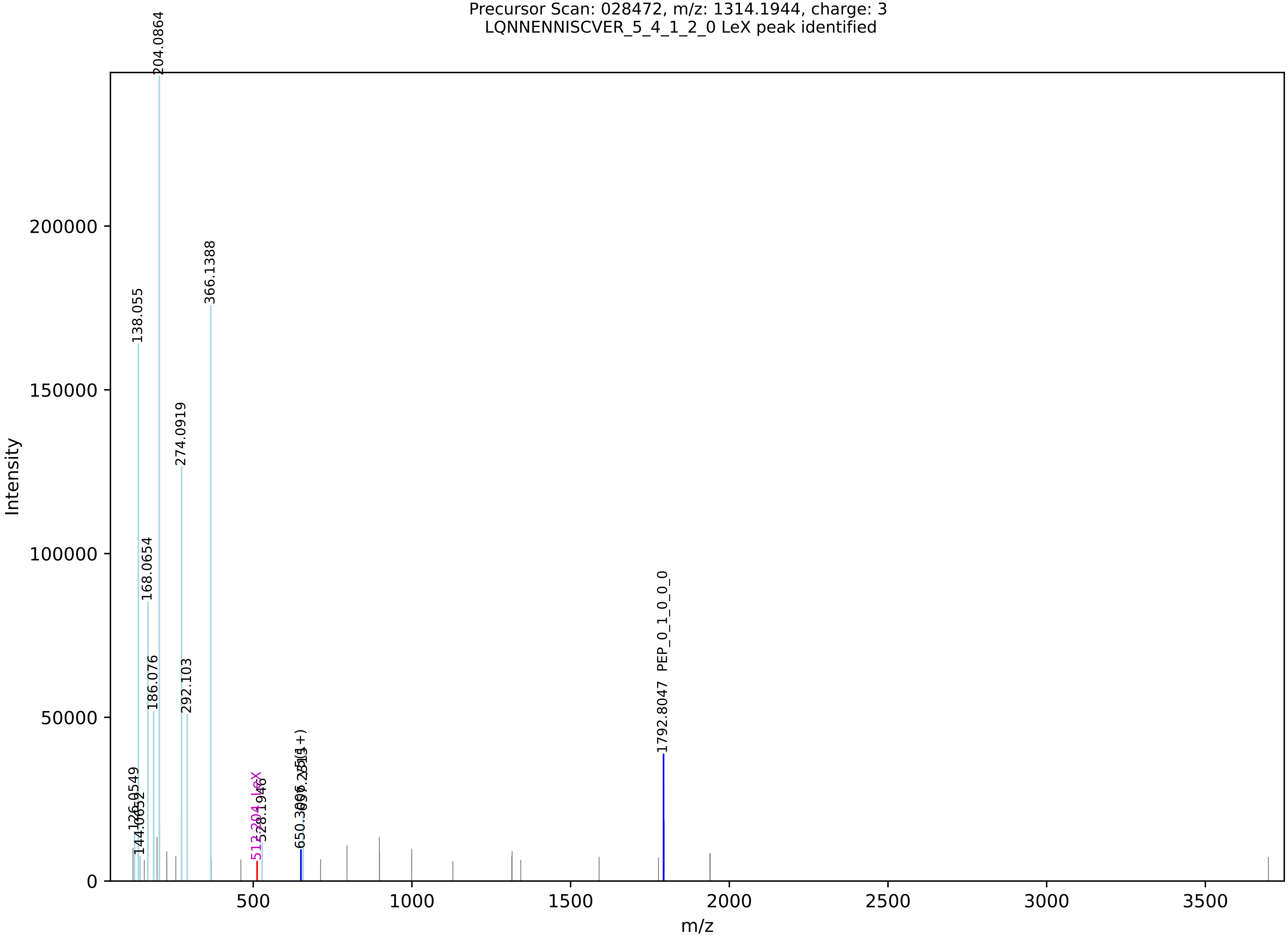

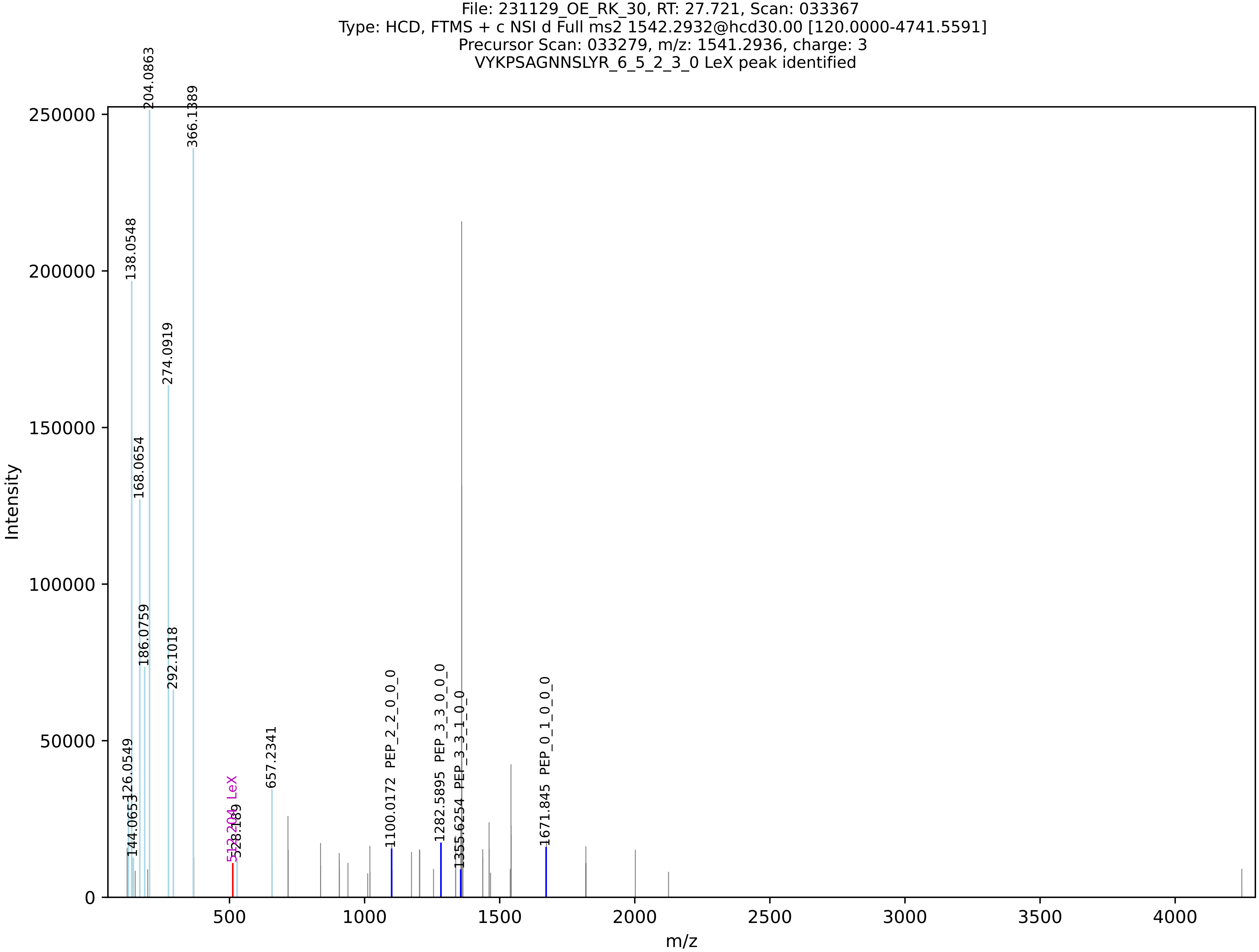
File: 231129_OE_RK_30, RT: 27.721, Scan: 033367
Type: HCD, FTMS + c NSI d Full ms2 1542.2932@hcd30.00 [120.0000-4741.5591]
Precursor Scan: 033279, m/z: 1541.2936, charge: 3
VYKPSAGNNSLYR_6_5_2_3_0 LeX peak identified
Intensity
m/z
204.0863
366.1389
138.0548
274.0919
168.0654
186.0759
292.1018
126.0549
144.0653
512.204 LeX
528.189
657.2341
1100.0172 PEP_2_2_0_0_0_
1282.5895 PEP_3_3_0_0_0_
1355.6254 PEP_3_3_1_0_0_
1671.845 PEP_0_1_0_0_0_

File: 231129_OE_RK_30, RT: 33.047, Scan: 041053
Type: HCD, FTMS + c NSI d Full ms2 1597.3749@hcd30.00 [120.0000-6540.1455]
Precursor Scan: 040912, m/z: 1595.6211, charge: 4
QQQHLFGSNVTDCSGNFCLFR_7_7_1_4_0 LeX peak identified

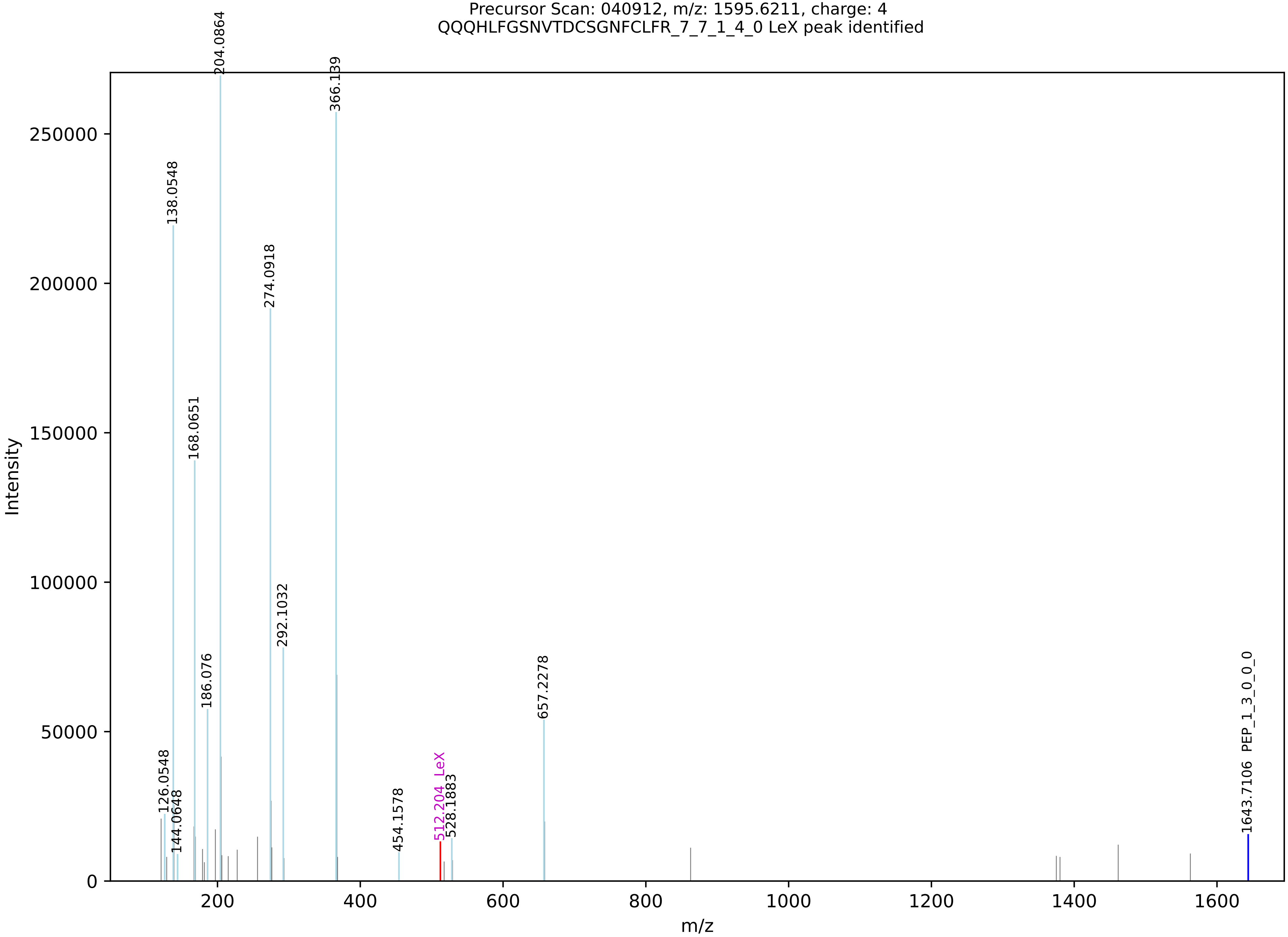

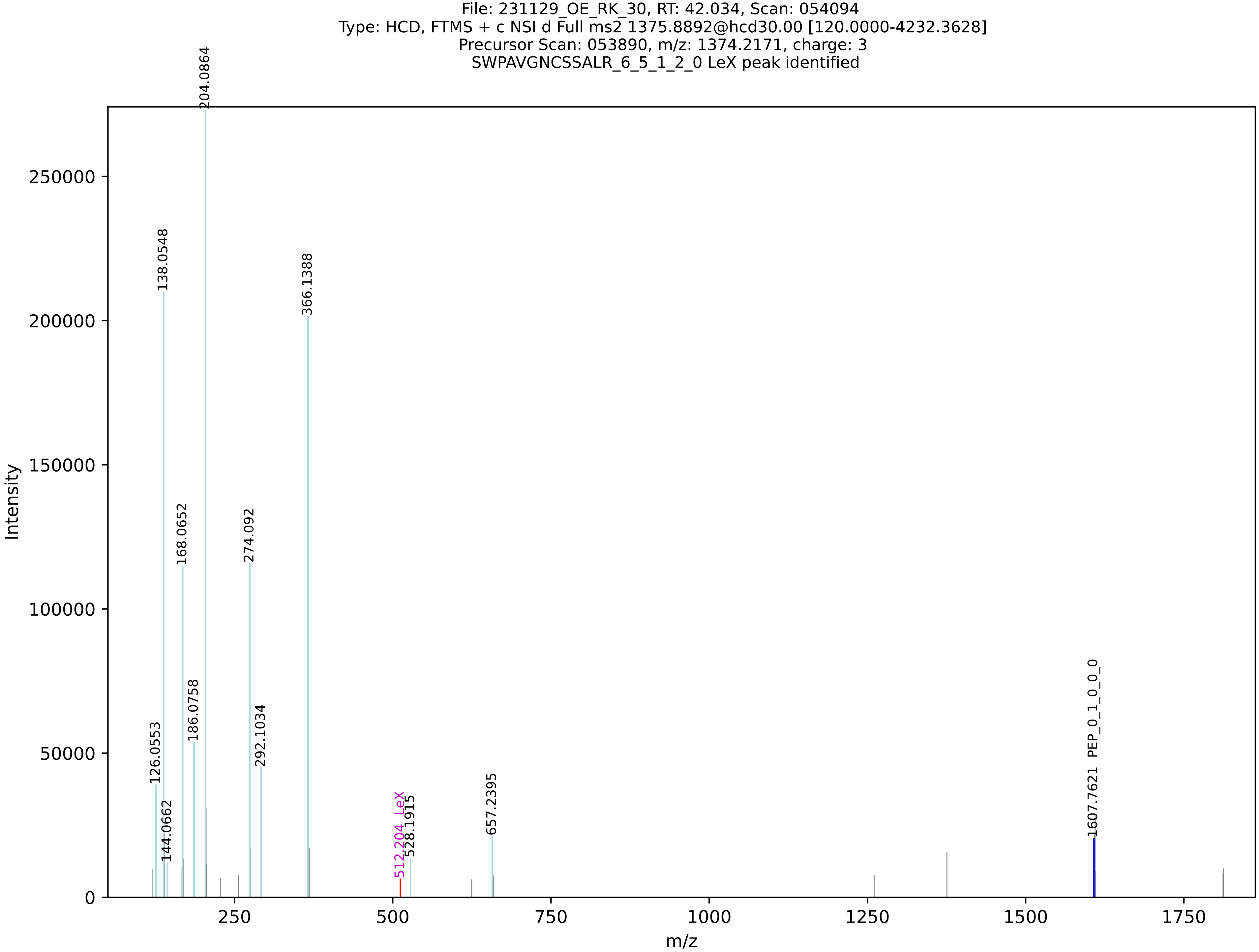
File: 231129_OE_RK_30, RT: 42.034, Scan: 054094
Type: HCD, FTMS + c NSI d Full ms2 1375.8892@hcd30.00 [120.0000-4232.3628]
Precursor Scan: 053890, m/z: 1374.2171, charge: 3
SWPAVGNCSSALR_6_5_1_2_0 LeX peak identified
Intensity
m/z
204.0864
138.0548
366.1388
168.0652
274.092
186.0758
292.1034
126.0553
144.0662
512.204_LeX
528.1915
657.2395
1607.7621
PEP_0_1_0_0_0
0
50000
100000
150000
200000
250000
250
500
750
1000
1250
1500
1750

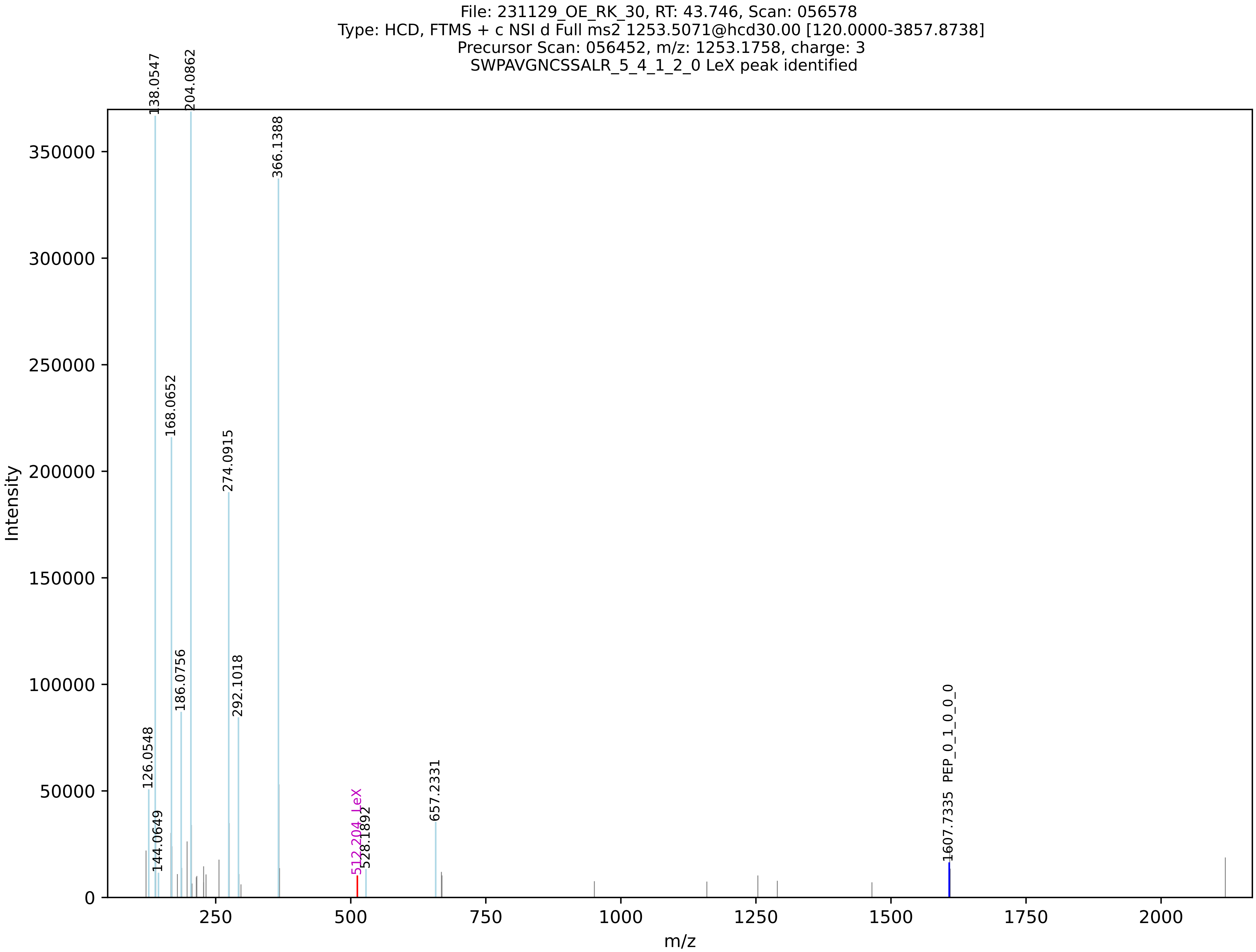
File: 231129_OE_RK_30, RT: 43.746, Scan: 056578
Type: HCD, FTMS + c NSI d Full ms2 1253.5071@hcd30.00 [120.0000-3857.8738]
Precursor Scan: 056452, m/z: 1253.1758, charge: 3
SWPAVGNCSSALR_5_4_1_2_0 LeX peak identified
138.0547
204.0862
366.1388
168.0652
274.0915
186.0756
292.1018
126.0548
144.0649
512.204_LeX
528.1892
657.2331
1607.7335 PEP_0_1_0_0_0
Intensity
m/z
0
50000
100000
150000
200000
250000
300000
350000
250
500
750
1000
1250
1500
1750
2000

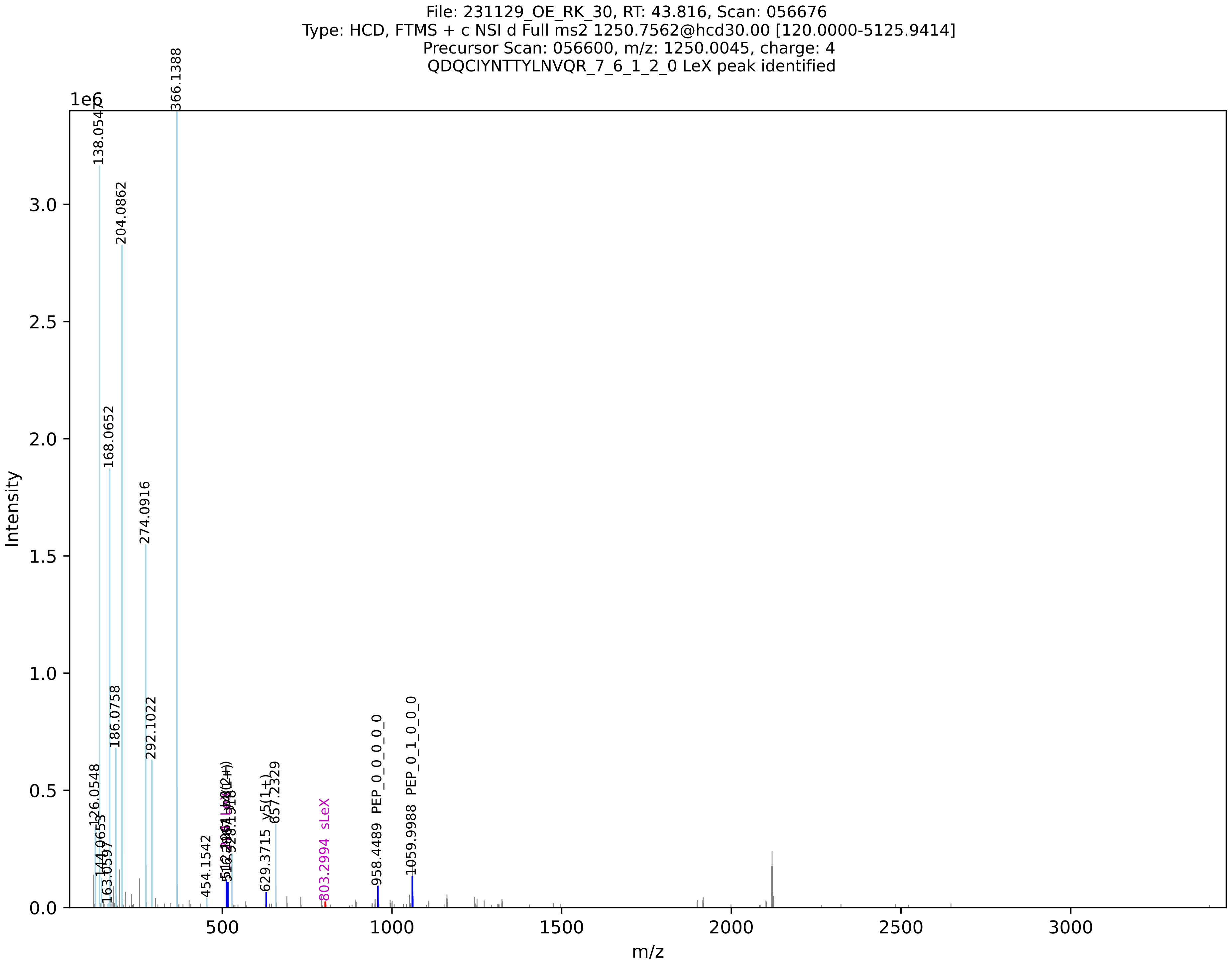
File: 231129_OE_RK_30, RT: 43.816, Scan: 056676
Type: HCD, FTMS + c NSI d Full ms2 1250.7562@hcd30.00 [120.0000-5125.9414]
Precursor Scan: 056600, m/z: 1250.0045, charge: 4
QDQCIYNTTYLNVQR_7_6_1_2_0 LeX peak identified
1e6
Intensity
m/z
366.1388
138.0547
204.0862
168.0652
274.0916
186.0758
292.1022
126.0548
144.0655
163.0597
454.1542
528.1918
629.3715 y5(1+)
657.2329
803.2994 sLeX
958.4489 PEP_0_0_0_0_0
1059.9988 PEP_0_1_0_0_0
0.0
0.5
1.0
1.5
2.0
2.5
3.0
500
1000
1500
2000
2500
3000

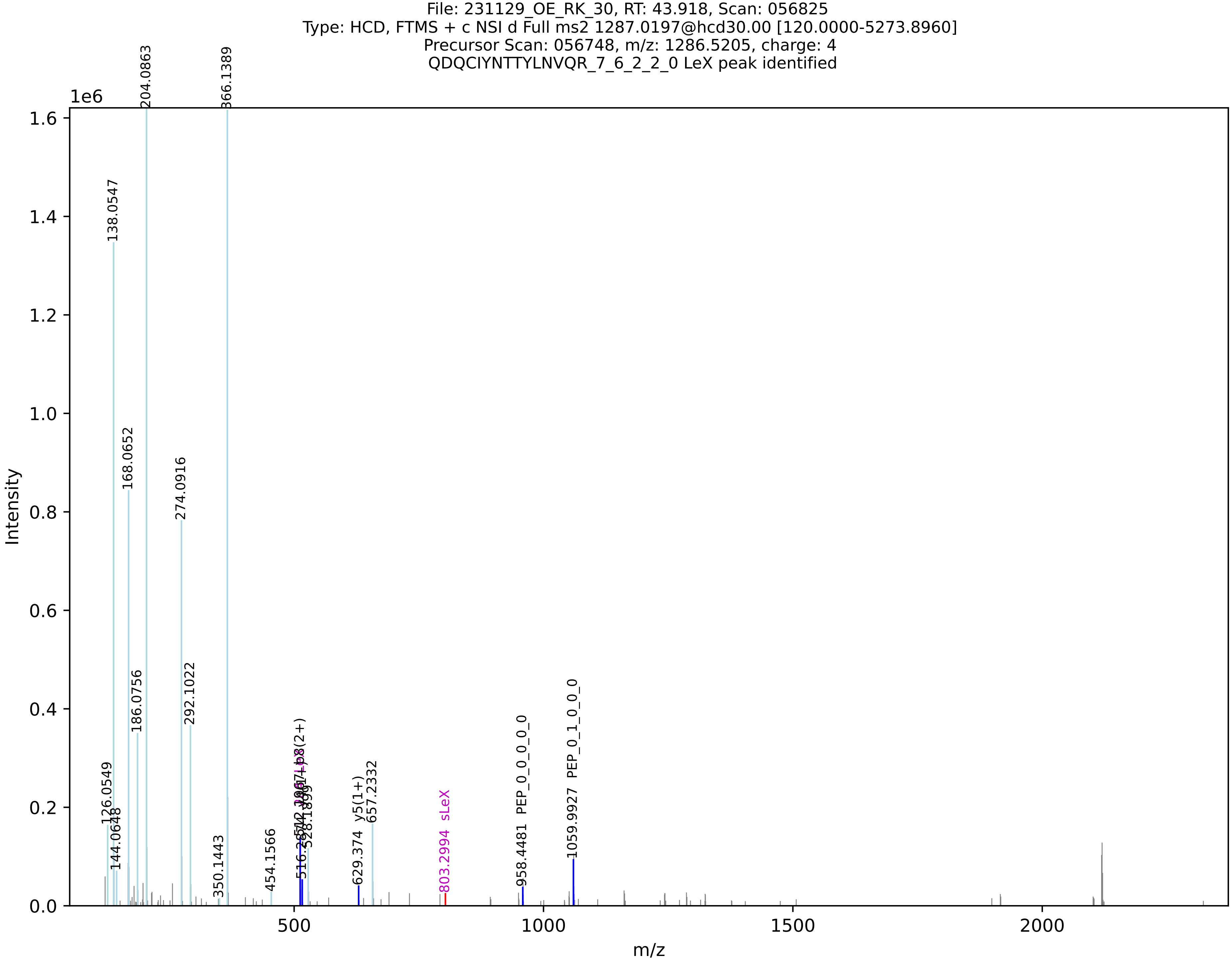
File: 231129_OE_RK_30, RT: 43.918, Scan: 056825
Type: HCD, FTMS + c NSI d Full ms2 1287.0197@hcd30.00 [120.0000-5273.8960]
Precursor Scan: 056748, m/z: 1286.5205, charge: 4
QDQCIYNTTYLNVQR_7_6_2_2_0 LeX peak identified
1e6
Intensity
m/z
204.0863
366.1389
138.0547
168.0652
274.0916
186.0756
292.1022
126.0549
144.0648
350.1443
454.1566
528.1899
b8(2+)
629.374 y5(1+)
657.2332
803.2994 sLeX
958.4481 PEP_0_0_0_0_0
1059.9927 PEP_0_1_0_0_0
0.0
0.2
0.4
0.6
0.8
1.0
1.2
1.4
1.6
500
1000
1500
2000

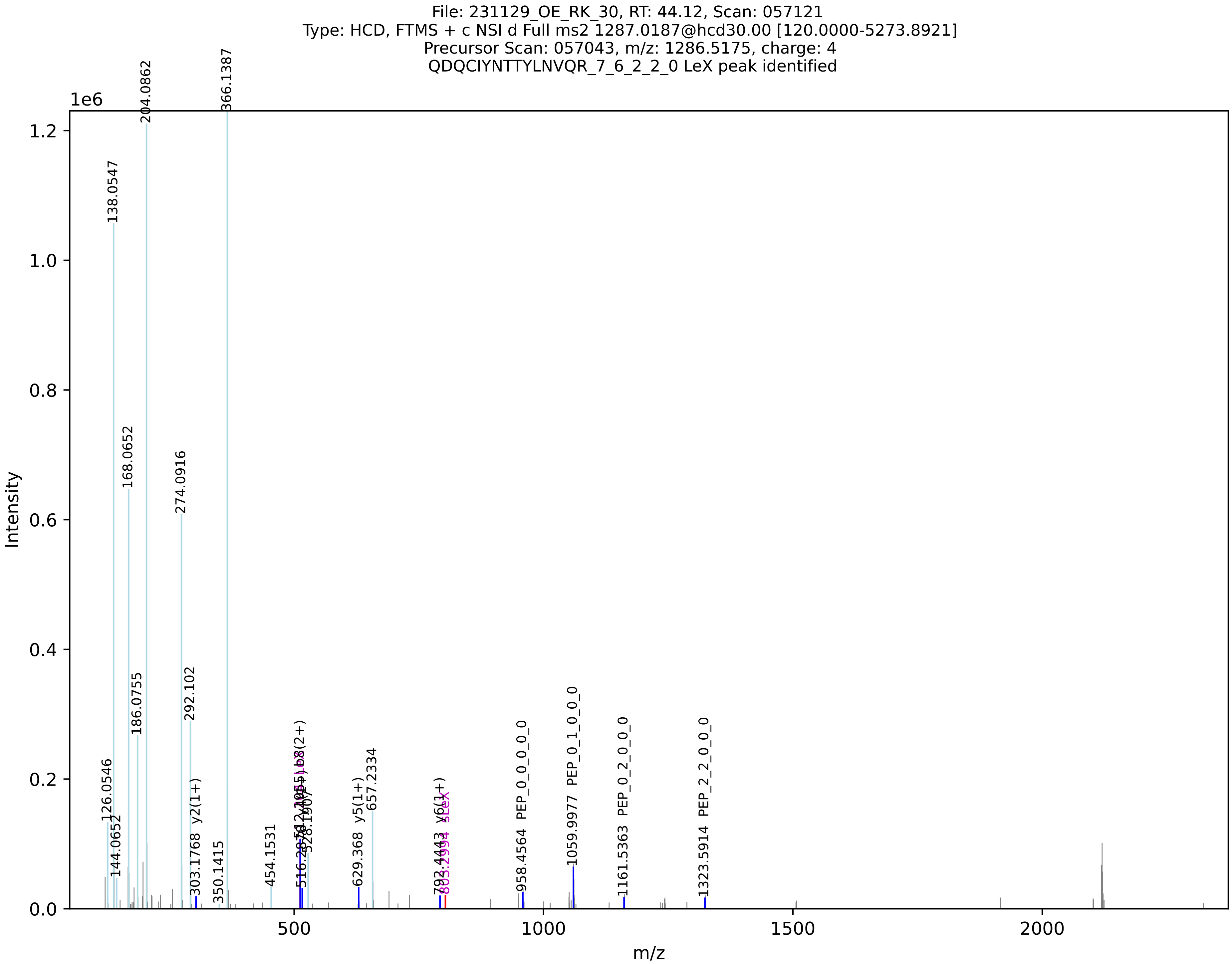

File: 231129_OE_RK_30, RT: 44.12, Scan: 057121
Type: HCD, FTMS + c NSI d Full ms2 1287.0187@hcd30.00 [120.0000-5273.8921]
Precursor Scan: 057043, m/z: 1286.5175, charge: 4
QDQCIYNTTYLNVQR_7_6_2_2_0 LeX peak identified
1e6
Intensity
m/z
366.1387
204.0862
138.0547
168.0652
274.0916
186.0755
292.102
126.0546
144.0652
303.1768 y2(1+)
350.1415
454.1531
528.1907
629.368 y5(1+)
657.2334
792.4443 y6(1+)
803.2994 sLeX
958.4564 PEP_0_0_0_0_0
1059.9977 PEP_0_1_0_0_0
1161.5363 PEP_0_2_0_0_0
1323.5914 PEP_2_2_0_0_0

File: 231129_OE_RK_30, RT: 44.163, Scan: 057184
Type: HCD, FTMS + c NSI d Full ms2 1715.6887@hcd30.00 [120.0000-5272.1494]
Precursor Scan: 057117, m/z: 1715.0205, charge: 3
QDQCIYNTTYLNVQR_7_6_2_2_0 LeX peak identified

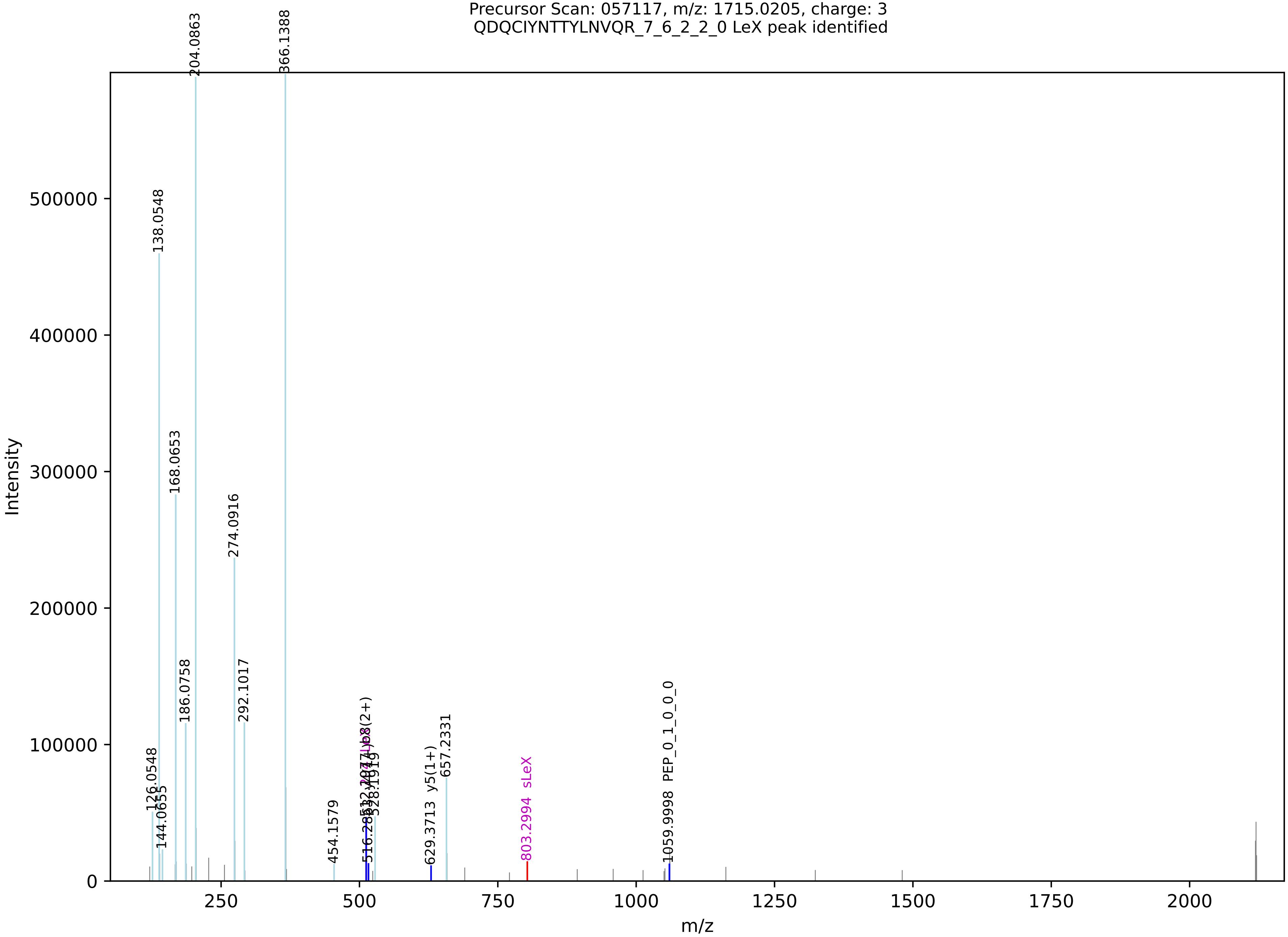

File: 231129_OE_RK_30, RT: 44.173, Scan: 057200
Type: HCD, FTMS + c NSI d Full ms2 1262.1794@hcd30.00 [120.0000-3884.4109]
Precursor Scan: 057117, m/z: 1261.5112, charge: 3
LNAENNATFYFK_5_4_1_2_0 LeX peak identified

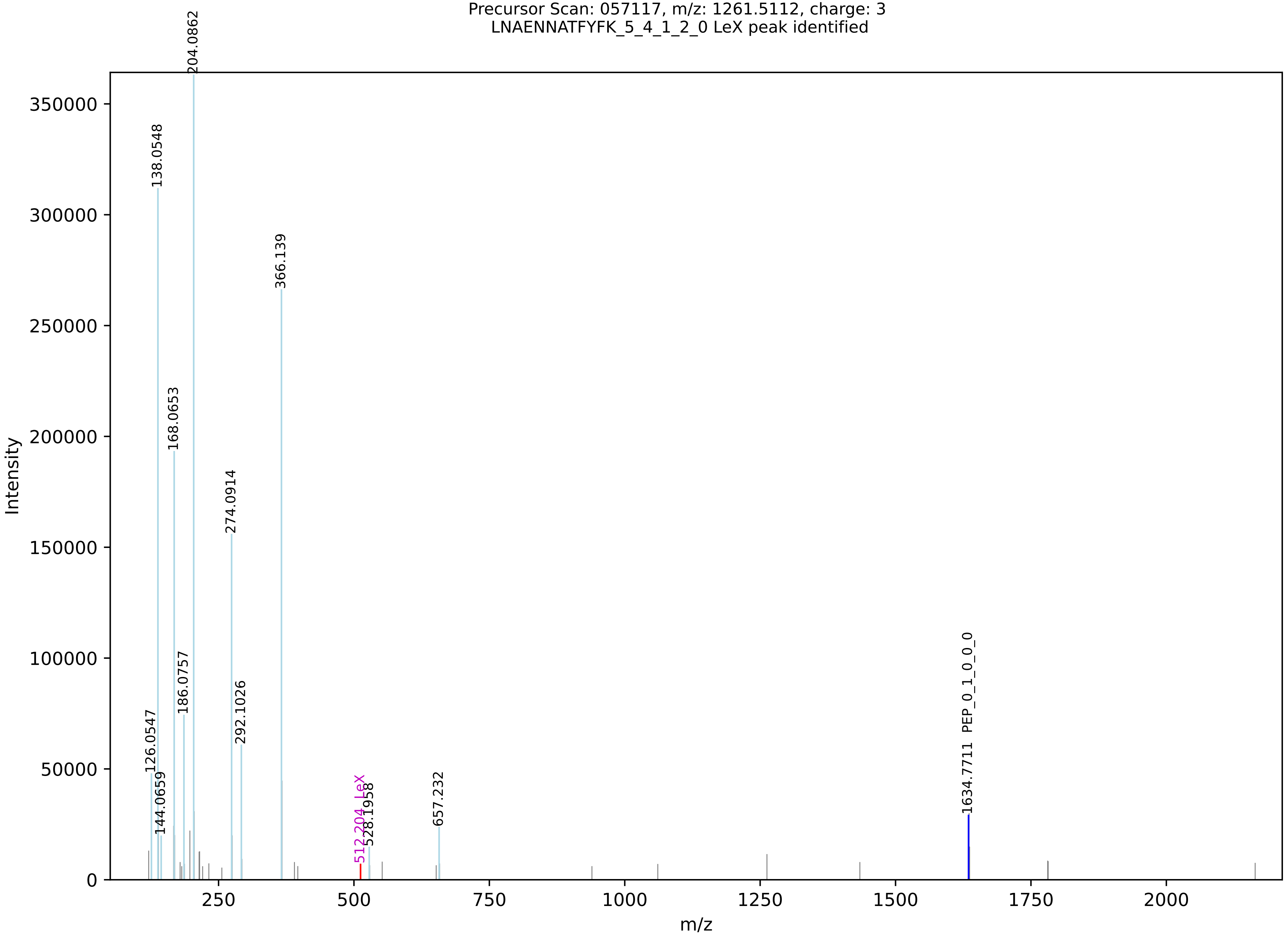

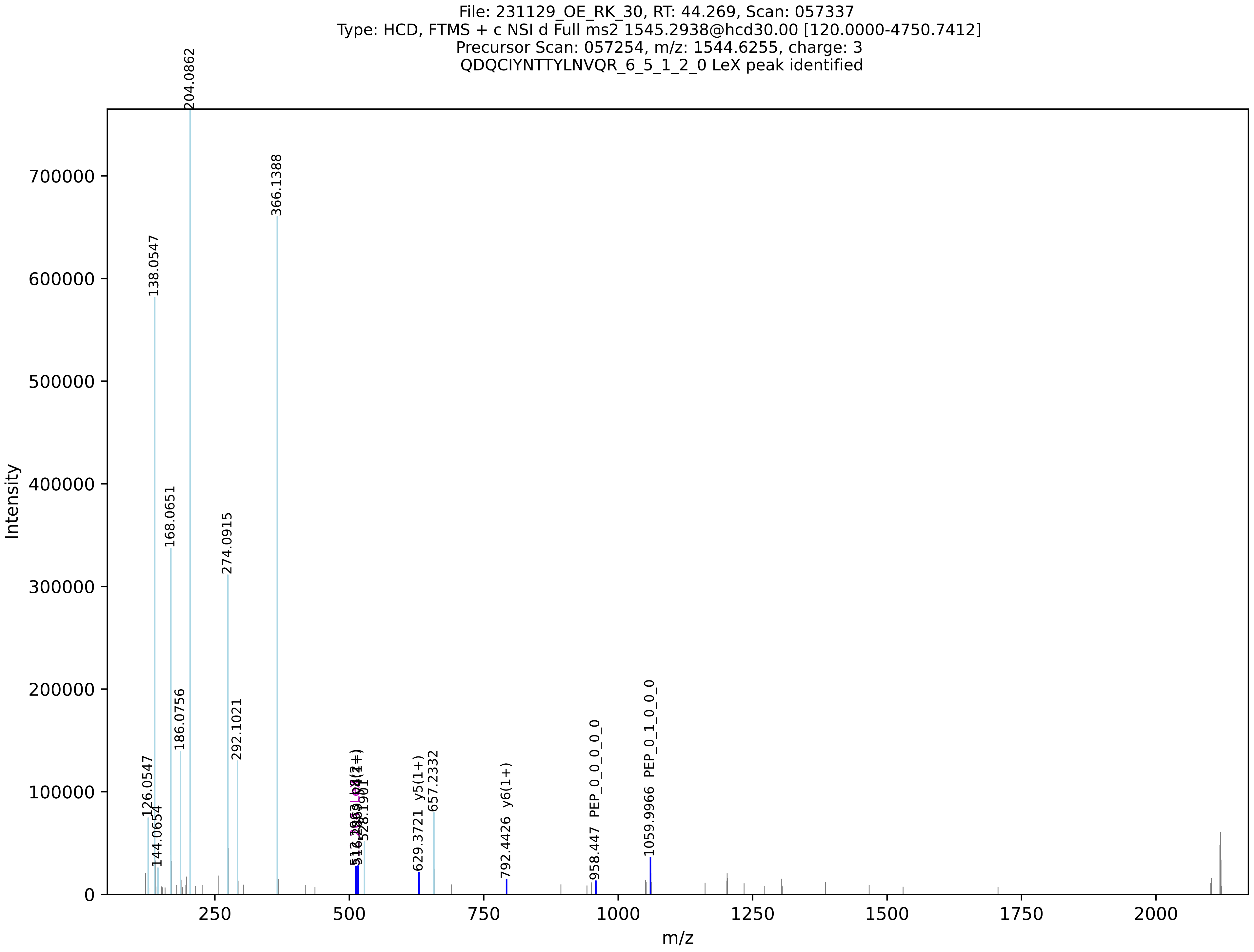
File: 231129_OE_RK_30, RT: 44.269, Scan: 057337
Type: HCD, FTMS + c NSI d Full ms2 1545.2938@hcd30.00 [120.0000-4750.7412]
Precursor Scan: 057254, m/z: 1544.6255, charge: 3
QDQCIYNTTYLNVQR_6_5_1_2_0 LeX peak identified
204.0862
366.1388
138.0547
168.0651
274.0915
186.0756
292.1021
126.0547
144.0654
528.1901
629.3721 y5(1+)
657.2332
792.4426 y6(1+)
958.447 PEP_0_0_0_0_0
1059.9966 PEP_0_1_0_0_0
Intensity
m/z
0
100000
200000
300000
400000
500000
600000
700000
250
500
750
1000
1250
1500
1750
2000

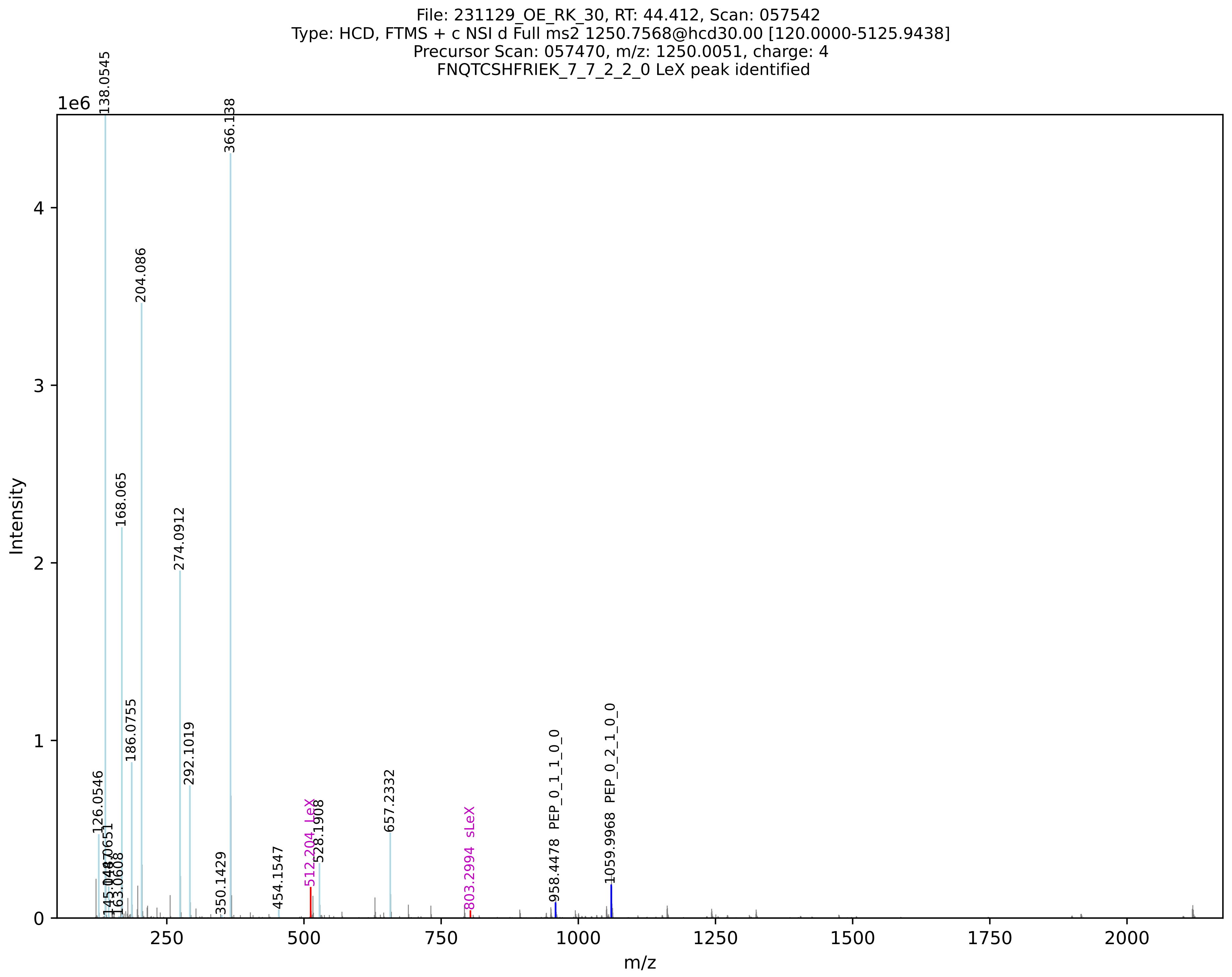
File: 231129_OE_RK_30, RT: 44.412, Scan: 057542
Type: HCD, FTMS + c NSI d Full ms2 1250.7568@hcd30.00 [120.0000-5125.9438]
Precursor Scan: 057470, m/z: 1250.0051, charge: 4
FNQTCSHFRIEK_7_7_2_2_0 LeX peak identified
1e6
Intensity
m/z
138.0545
366.138
204.086
168.065
274.0912
186.0755
292.1019
126.0546
163.0608
350.1429
454.1547
512.204 LeX
528.1908
657.2332
803.2994 sLeX
958.4478 PEP_0_1_1_0_0
1059.9968 PEP_0_2_1_0_0

File: 231129_OE_RK_30, RT: 44.414, Scan: 057545
Type: HCD, FTMS + c NSI d Full ms2 1323.5320@hcd30.00 [120.0000-5422.8662]
Precursor Scan: 057470, m/z: 1322.7808, charge: 4
QDQCIYNTTYLNVQR_7_6_1_3_0 LeX peak identified

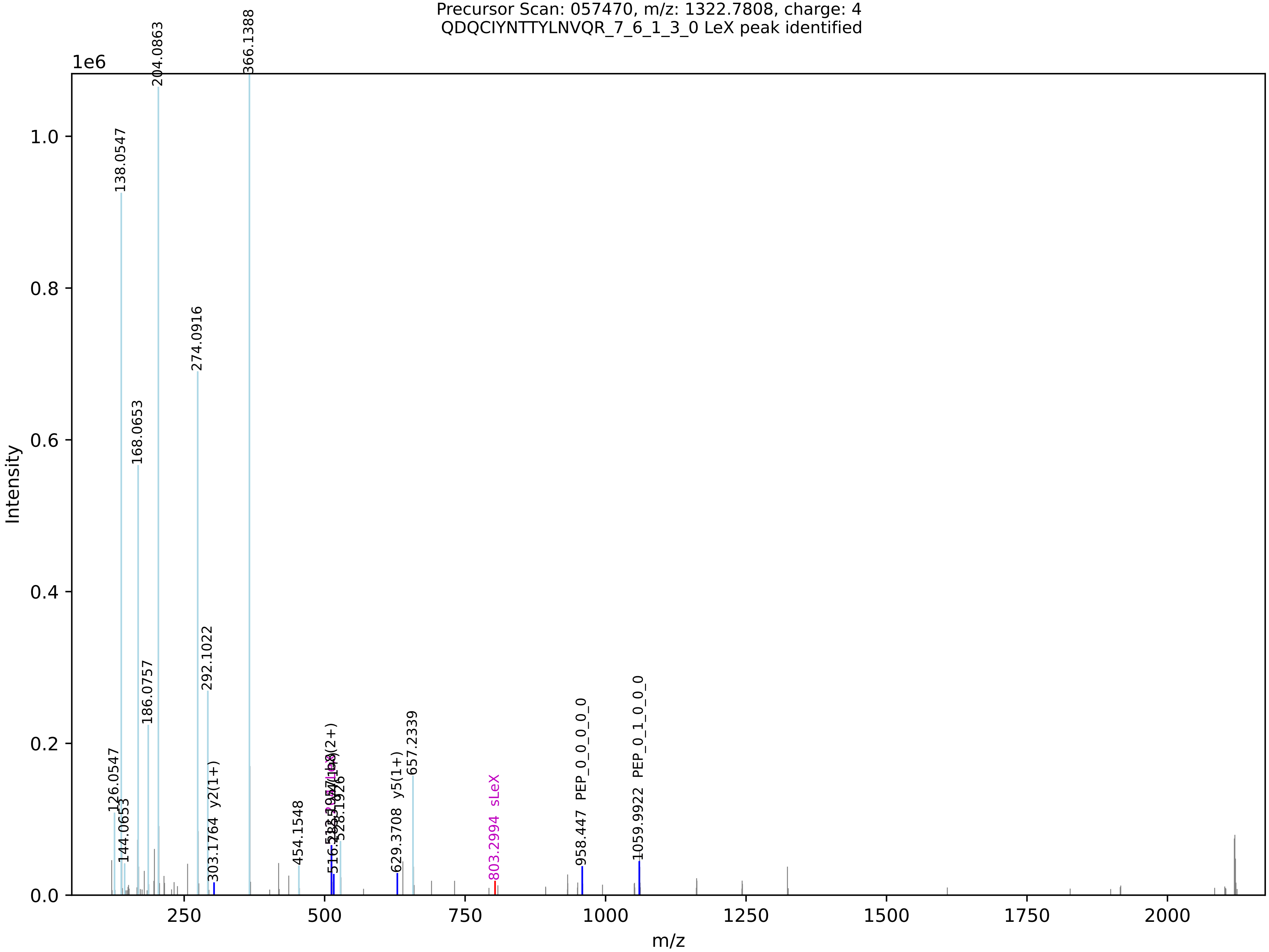

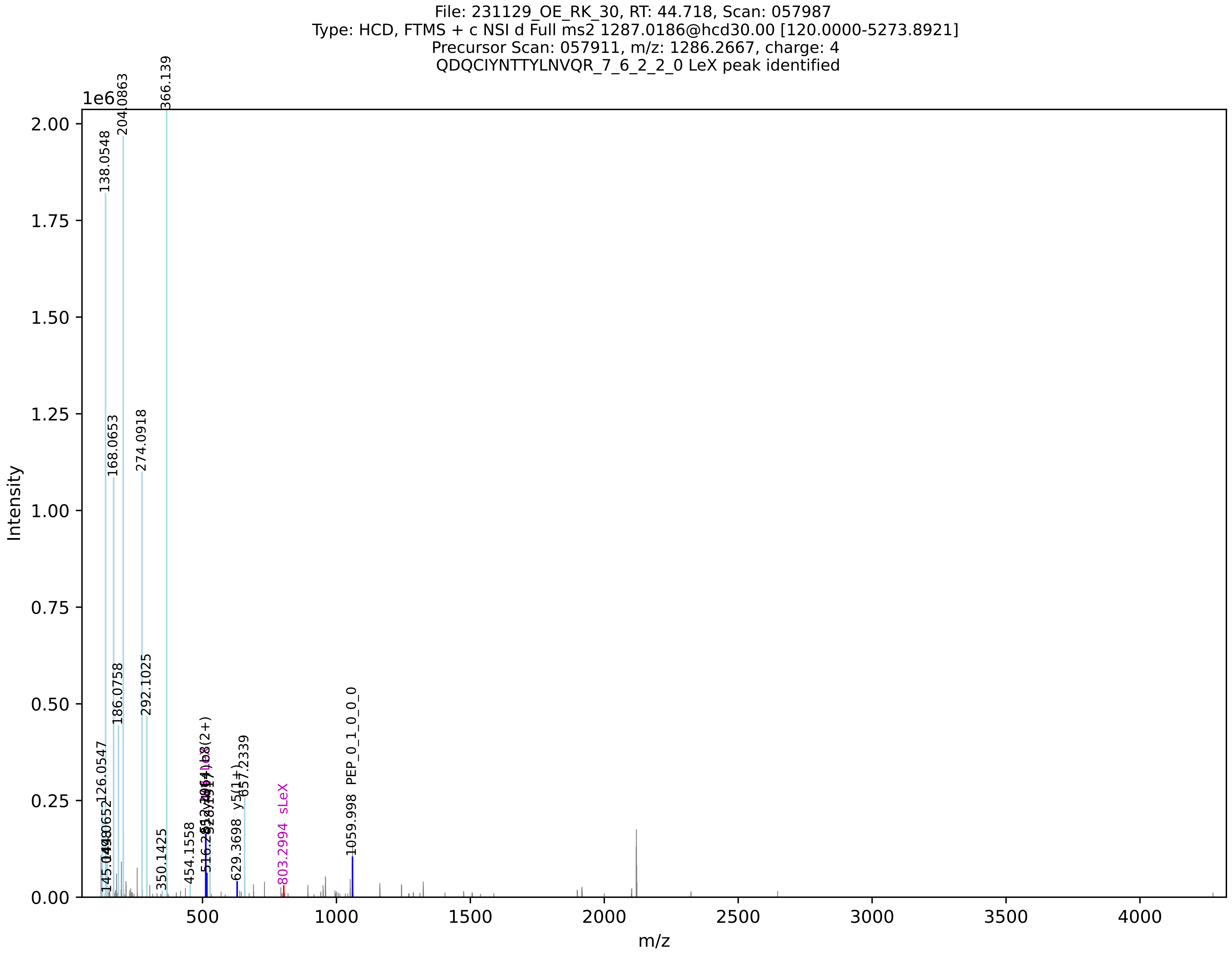
File: 231129_OE_RK_30, RT: 44.718, Scan: 057987
Type: HCD, FTMS + c NSI d Full ms2 1287.0186@hcd30.00 [120.0000-5273.8921]
Precursor Scan: 057911, m/z: 1286.2667, charge: 4
QDQCIYNTTYLNVQR_7_6_2_2_0 LeX peak identified
1e6
Intensity
m/z
366.139
204.0863
138.0548
168.0653
274.0918
186.0758
292.1025
126.0547
350.1425
454.1558
629.3698 y5(1+)
657.2339
803.2994 sLeX
1059.998 PEP_0_1_0_0_0

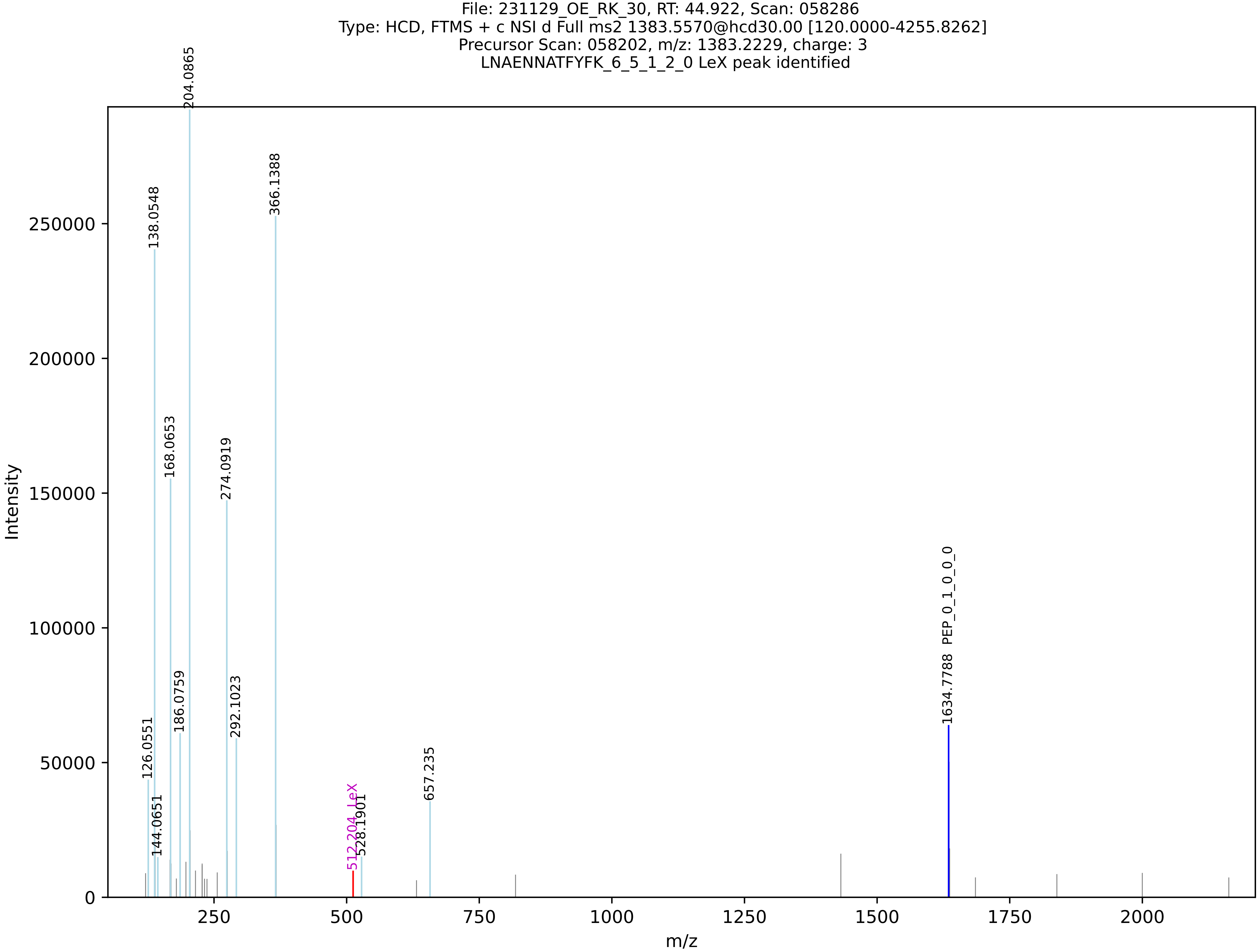
File: 231129_OE_RK_30, RT: 44.922, Scan: 058286
Type: HCD, FTMS + c NSI d Full ms2 1383.5570@hcd30.00 [120.0000-4255.8262]
Precursor Scan: 058202, m/z: 1383.2229, charge: 3
LNAENNATFYFK_6_5_1_2_0 LeX peak identified
204.0865
366.1388
138.0548
168.0653
274.0919
186.0759
292.1023
126.0551
144.0651
512.204_LeX
528.1901
657.235
1634.7788 PEP_0_1_0_0_0
Intensity
m/z
0
50000
100000
150000
200000
250000
250
500
750
1000
1250
1500
1750
2000

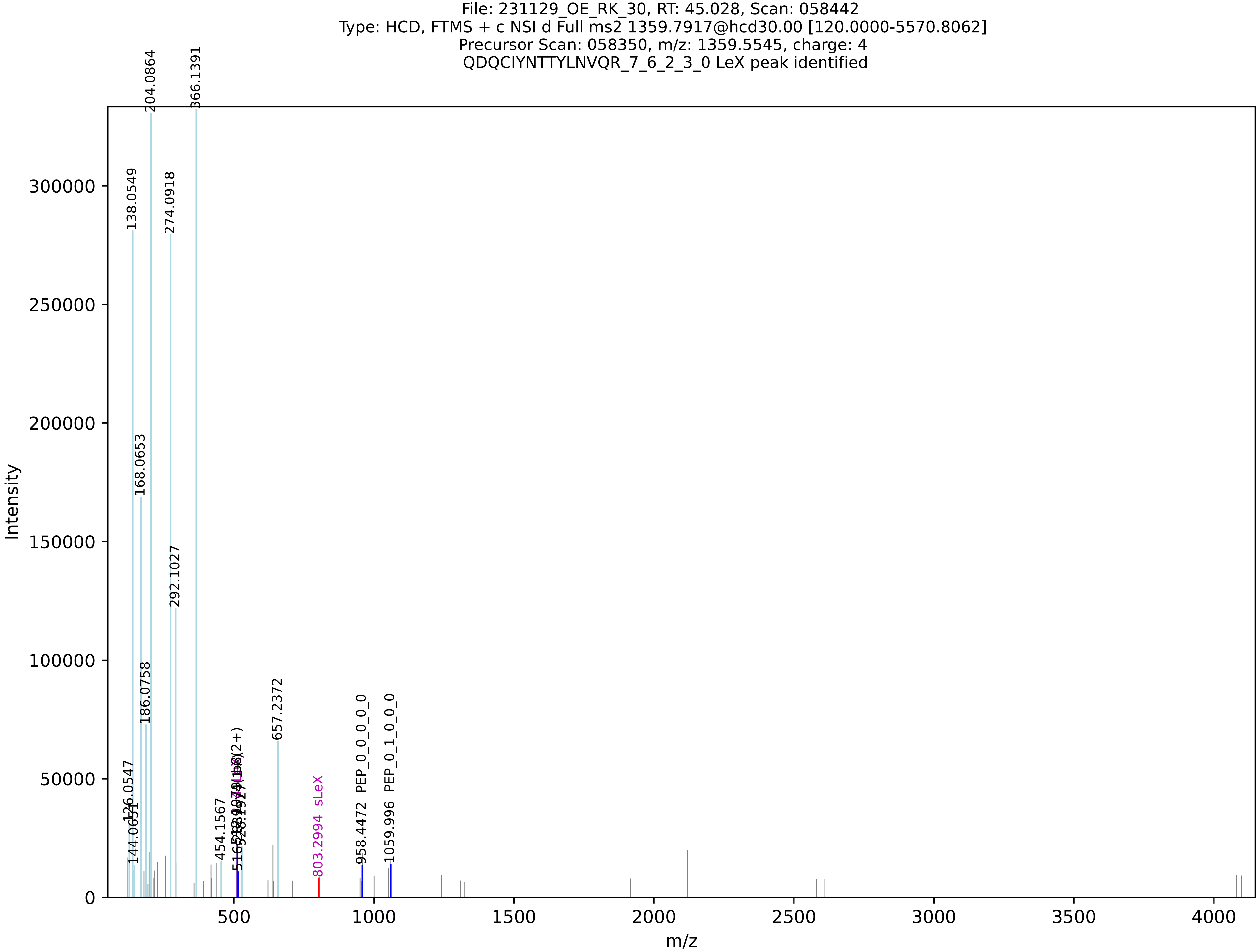
File: 231129_OE_RK_30, RT: 45.028, Scan: 058442
Type: HCD, FTMS + c NSI d Full ms2 1359.7917@hcd30.00 [120.0000-5570.8062]
Precursor Scan: 058350, m/z: 1359.5545, charge: 4
QDQCIYNTTYLNVQR_7_6_2_3_0 LeX peak identified
Intensity
m/z
204.0864
366.1391
138.0549
274.0918
168.0653
292.1027
186.0758
657.2372
126.0547
144.0651
454.1567
803.2994 sLeX
958.4472 PEP_0_0_0_0_0_
1059.996 PEP_0_1_0_0_0_

File: 231129_OE_RK_30, RT: 45.125, Scan: 058581
Type: HCD, FTMS + c NSI d Full ms2 1383.8894@hcd30.00 [120.0000-4256.8438]
Precursor Scan: 058497, m/z: 1383.2242, charge: 3
LNAENNATFYFK_6_5_1_2_0 LeX peak identified

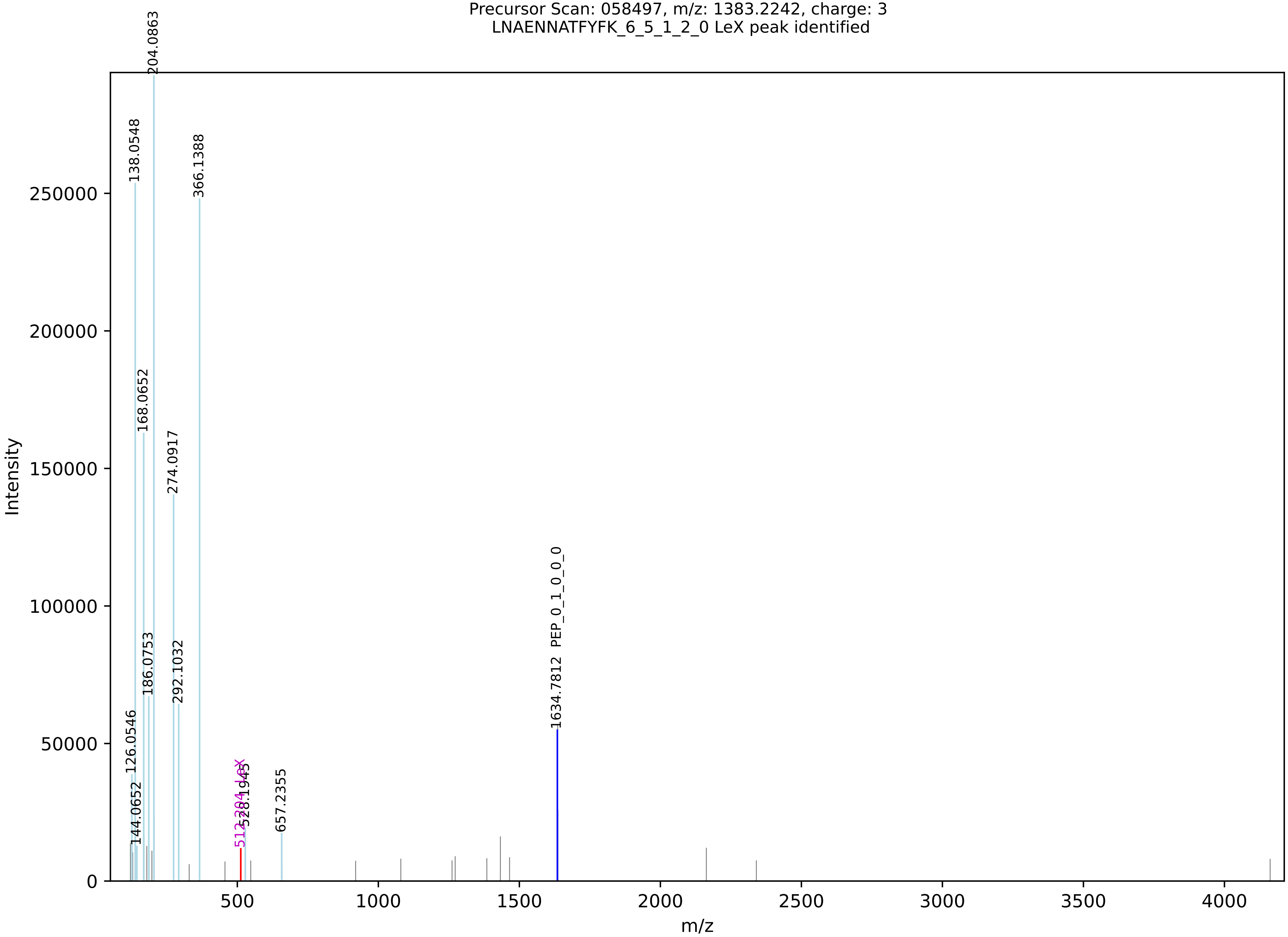

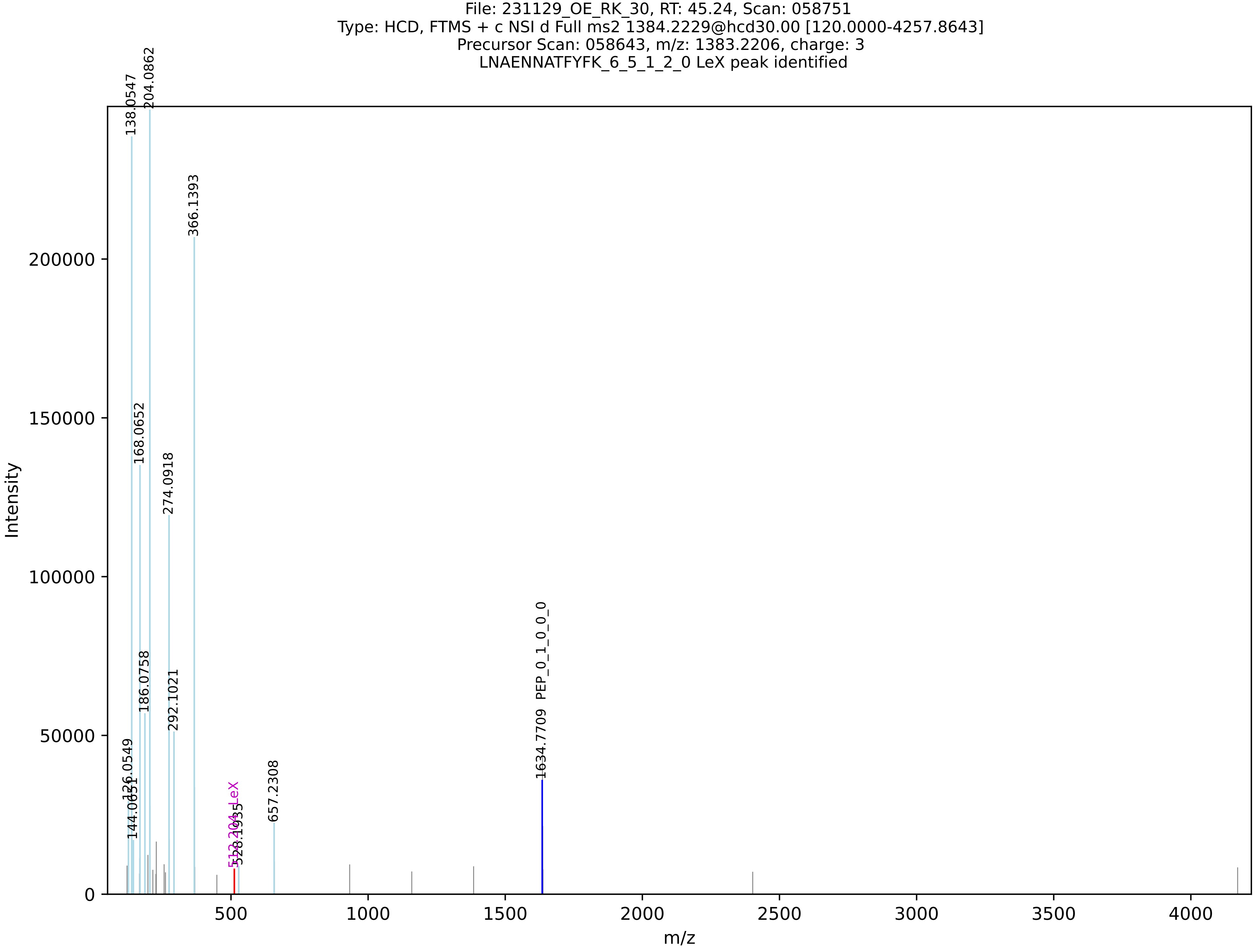
File: 231129_OE_RK_30, RT: 45.24, Scan: 058751
Type: HCD, FTMS + c NSI d Full ms2 1384.2229@hcd30.00 [120.0000-4257.8643]
Precursor Scan: 058643, m/z: 1383.2206, charge: 3
LNAENNATFYFK_6_5_1_2_0 LeX peak identified
Intensity
m/z
138.0547
204.0862
366.1393
168.0652
274.0918
186.0758
292.1021
126.0549
144.0651
512.204 LeX
528.1935
657.2308
1634.7709 PEP_0_1_0_0_0
0
50000
100000
150000
200000
500
1000
1500
2000
2500
3000
3500
4000

File: 231129_OE_RK_30, RT: 45.275, Scan: 058800
Type: HCD, FTMS + c NSI d Full ms2 1545.2949@hcd30.00 [120.0000-4750.7441]
Precursor Scan: 058717, m/z: 1544.6266, charge: 3
QDQCIYNTTYLNVQR_6_5_1_2_0 LeX peak identified

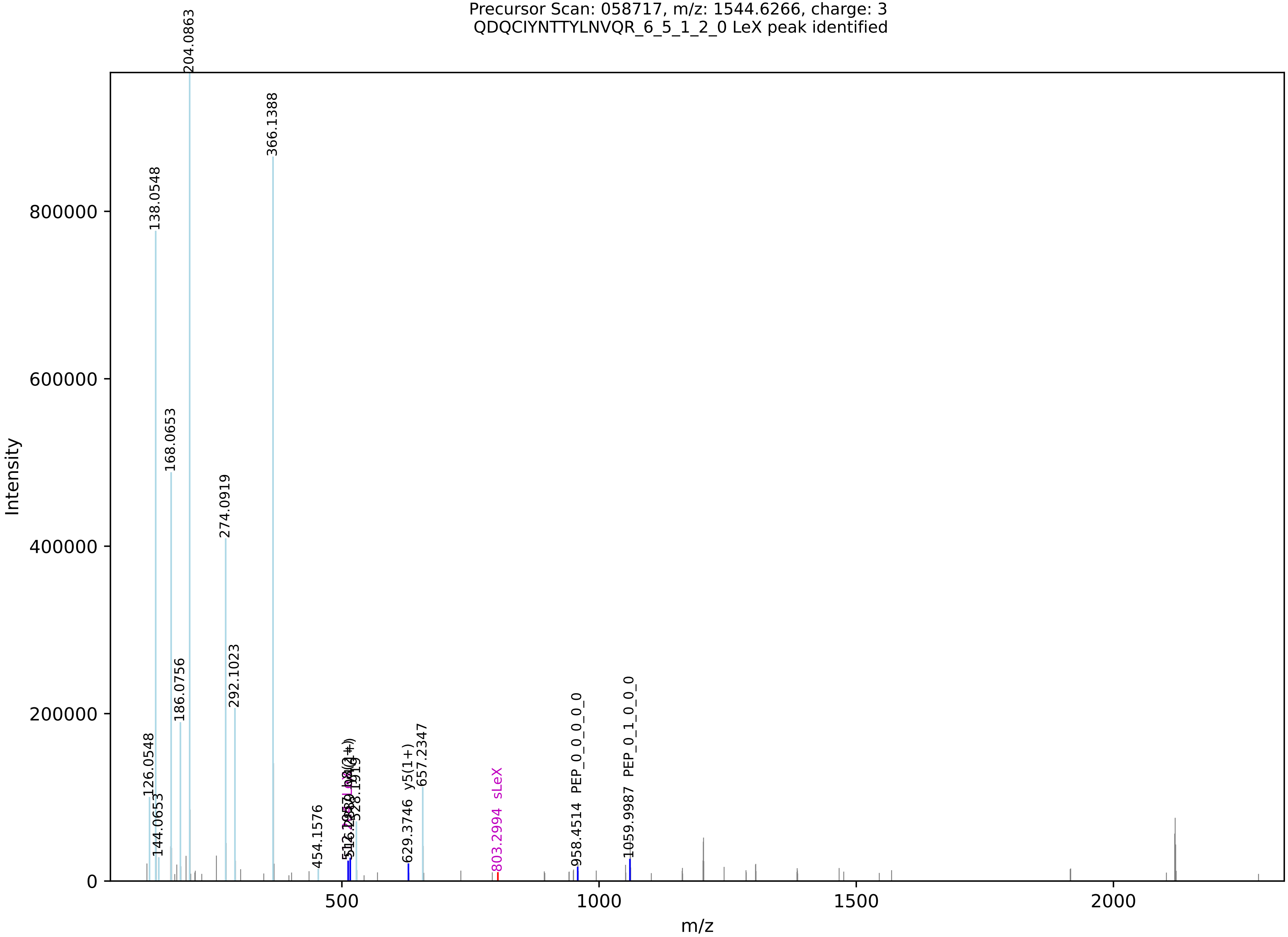

File: 231129_OE_RK_30, RT: 45.825, Scan: 059603
Type: HCD, FTMS + c NSI d Full ms2 1250.5049@hcd30.00 [120.0000-5124.9160]
Precursor Scan: 059526, m/z: 1250.0033, charge: 4
QDQCIYNTTYLNVQR_7_6_1_2_0 LeX peak identified

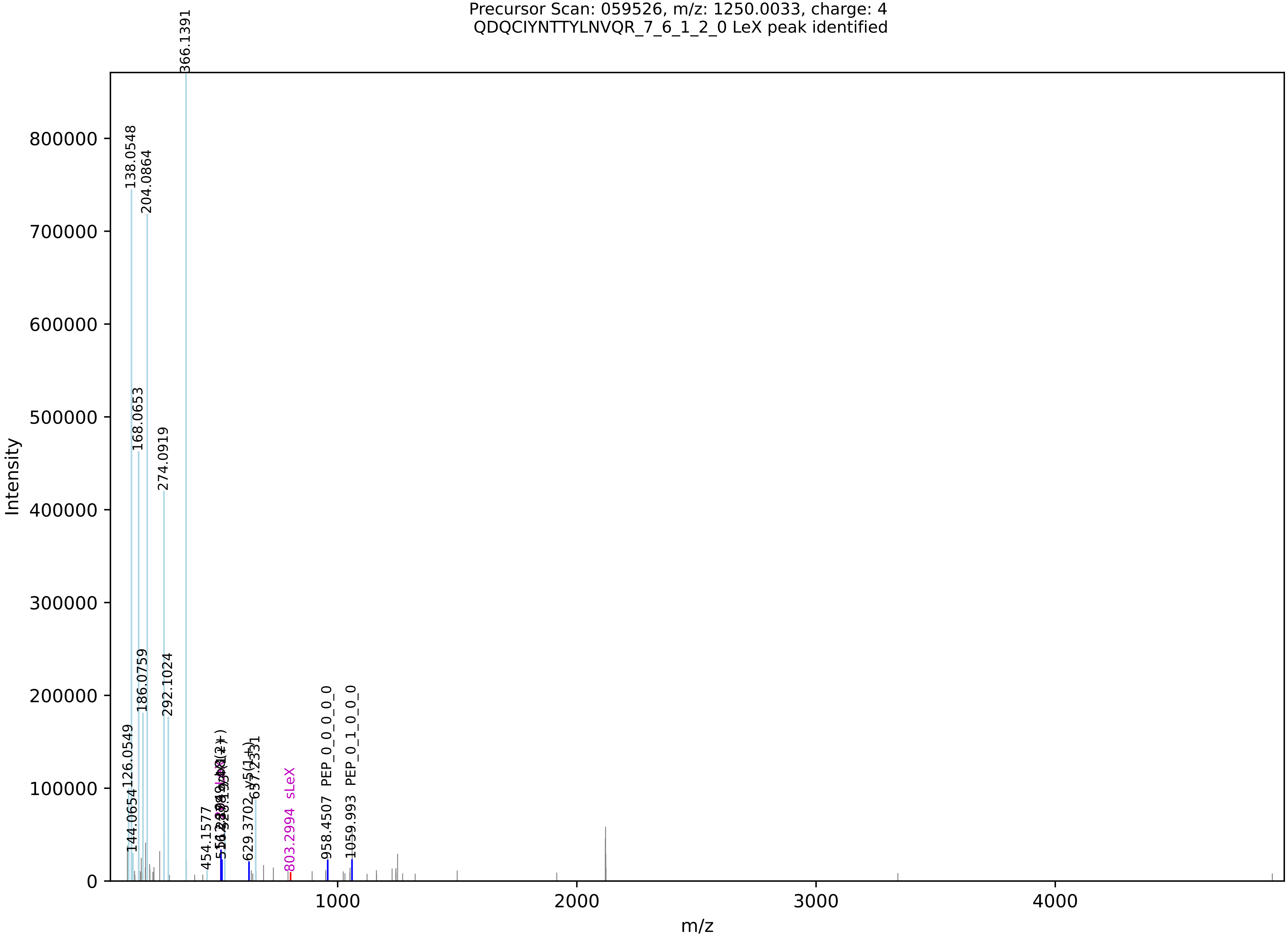

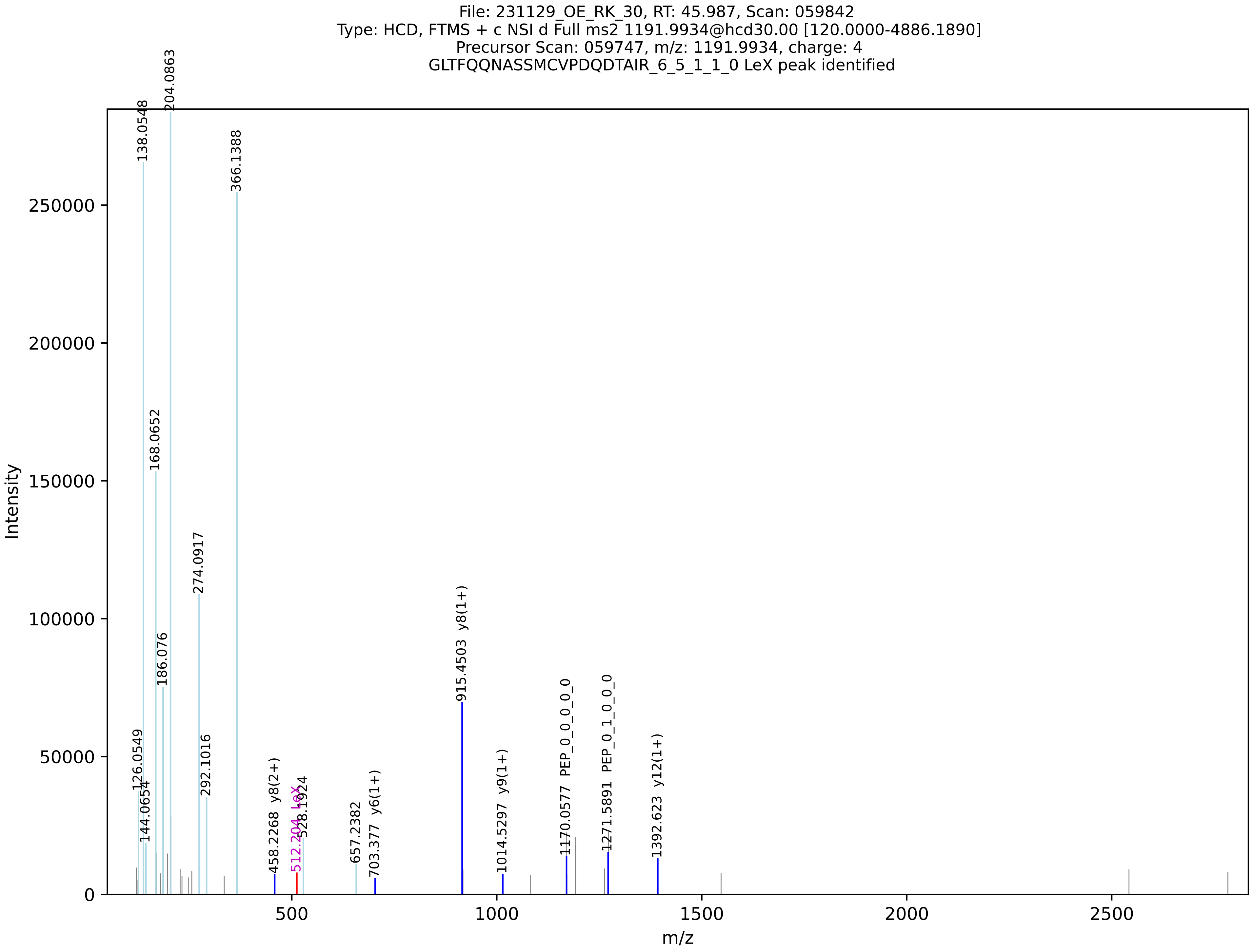
File: 231129_OE_RK_30, RT: 45.987, Scan: 059842
Type: HCD, FTMS + c NSI d Full ms2 1191.9934@hcd30.00 [120.0000-4886.1890]
Precursor Scan: 059747, m/z: 1191.9934, charge: 4
GLTFQQNASSMCVPDQDTAIR_6_5_1_1_0 LeX peak identified
Intensity
m/z
204.0863
138.0548
366.1388
168.0652
274.0917
186.076
126.0549
144.0654
292.1016
458.2268 y8(2+)
512.204 LeX
528.1924
657.2382
703.377 y6(1+)
915.4503 y8(1+)
1014.5297 y9(1+)
1170.0577 PEP_0_0_0_0_0
1271.5891 PEP_0_1_0_0_0
1392.623 y12(1+)

File: 231129_OE_RK_30, RT: 46.993, Scan: 061296
Type: HCD, FTMS + c NSI d Full ms2 1642.3264@hcd30.00 [120.0000-5047.6611]
Precursor Scan: 061205, m/z: 1641.6571, charge: 3
QDQCIYNTTYLNVQR_6_5_1_3_0 LeX peak identified

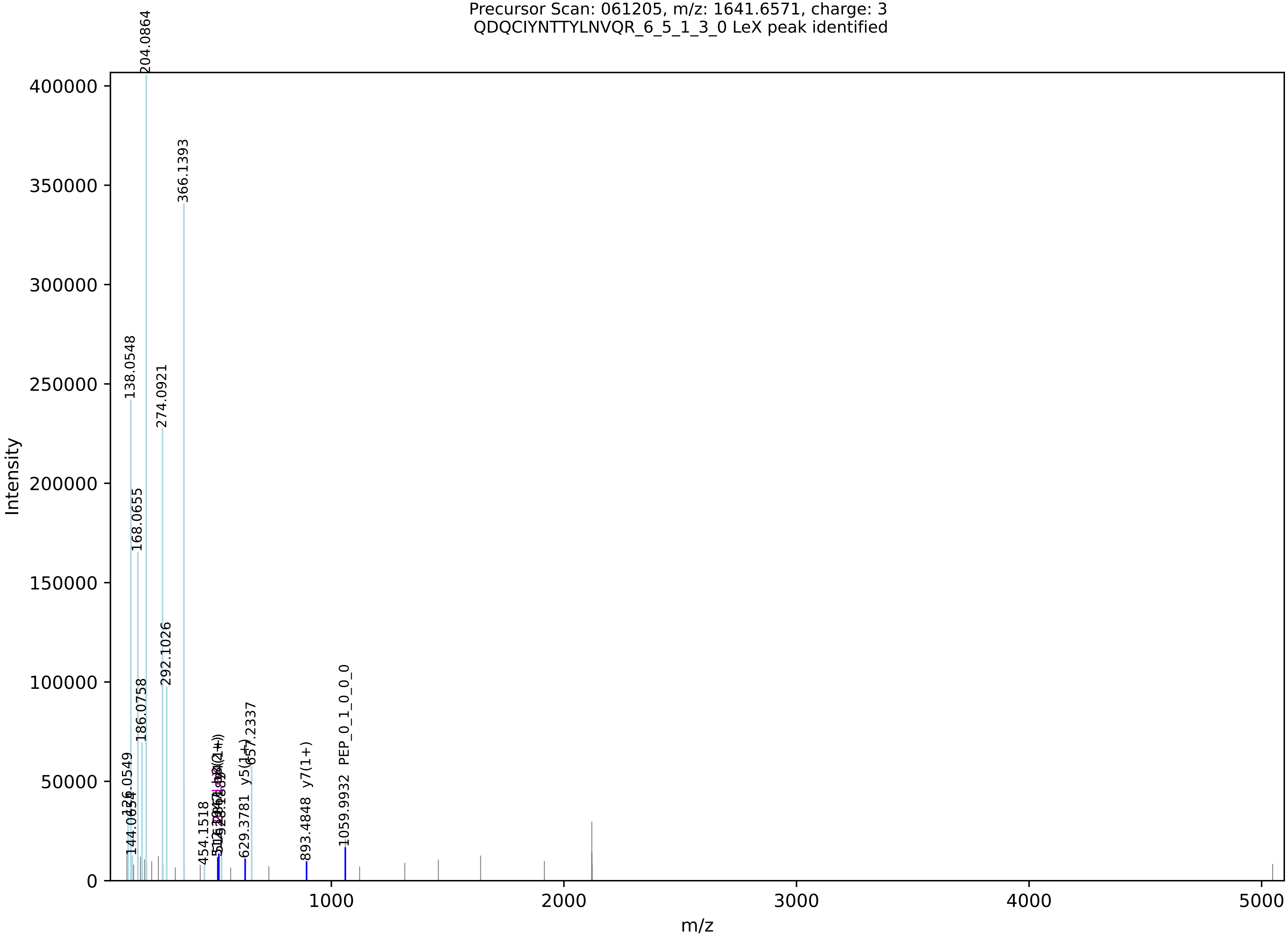

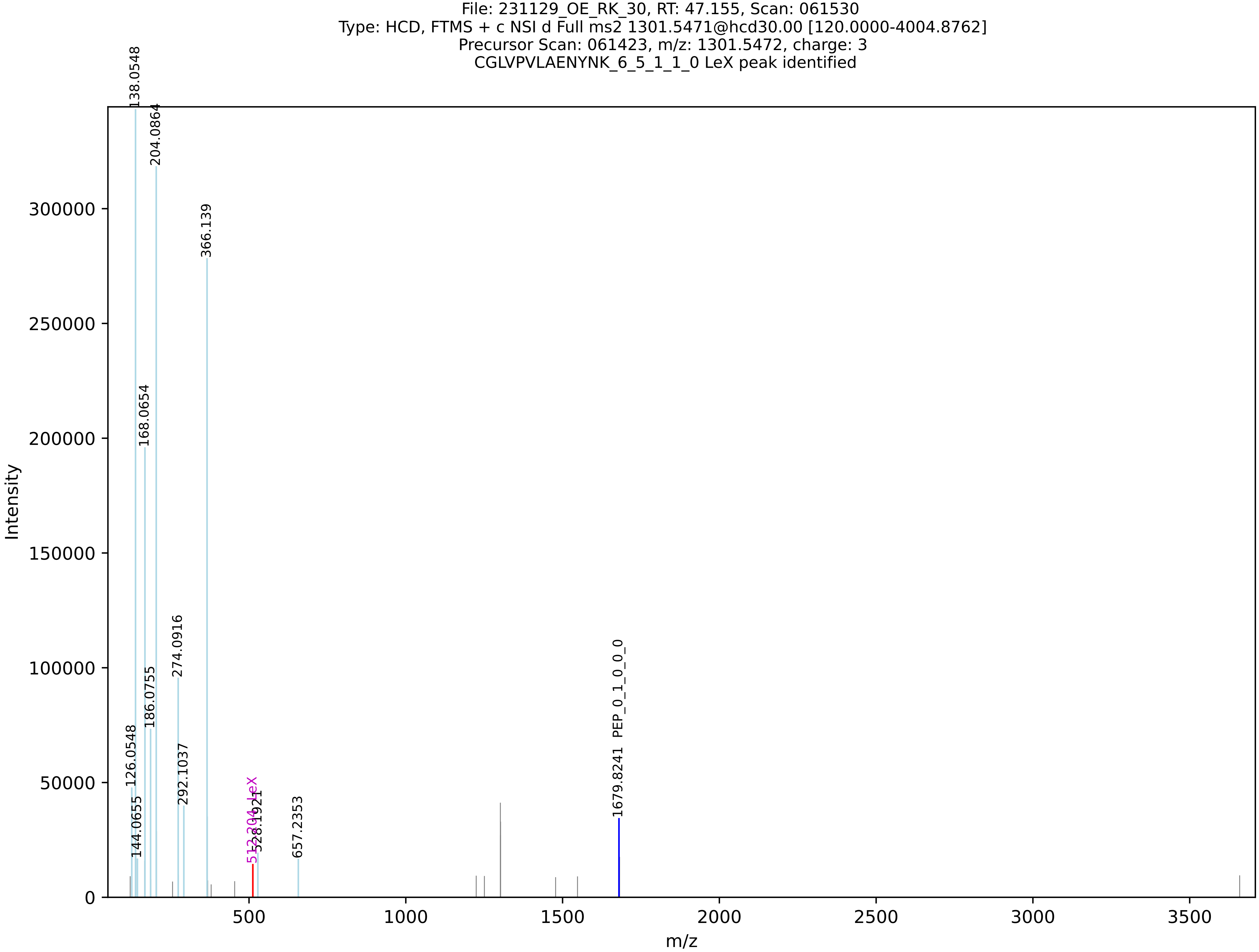

File: 231129_OE_RK_30, RT: 47.155, Scan: 061530
Type: HCD, FTMS + c NSI d Full ms2 1301.5471@hcd30.00 [120.0000-4004.8762]
Precursor Scan: 061423, m/z: 1301.5472, charge: 3
CGLVPVLAENYNK_6_5_1_1_0 LeX peak identified
138.0548
204.0864
366.139
168.0654
274.0916
186.0755
126.0548
292.1037
144.0655
512.204_LeX
528.1921
657.2353
1679.8241 PEP_0_1_0_0_0
Intensity
m/z
0
50000
100000
150000
200000
250000
300000
500
1000
1500
2000
2500
3000
3500

File: 231129_OE_RK_30, RT: 47.286, Scan: 061718
Type: HCD, FTMS + c NSI d Full ms2 1323.5286@hcd30.00 [120.0000-5422.8525]
Precursor Scan: 061642, m/z: 1322.7768, charge: 4
QDQCIYNTTYLNVQR_7_6_1_3_0 LeX peak identified

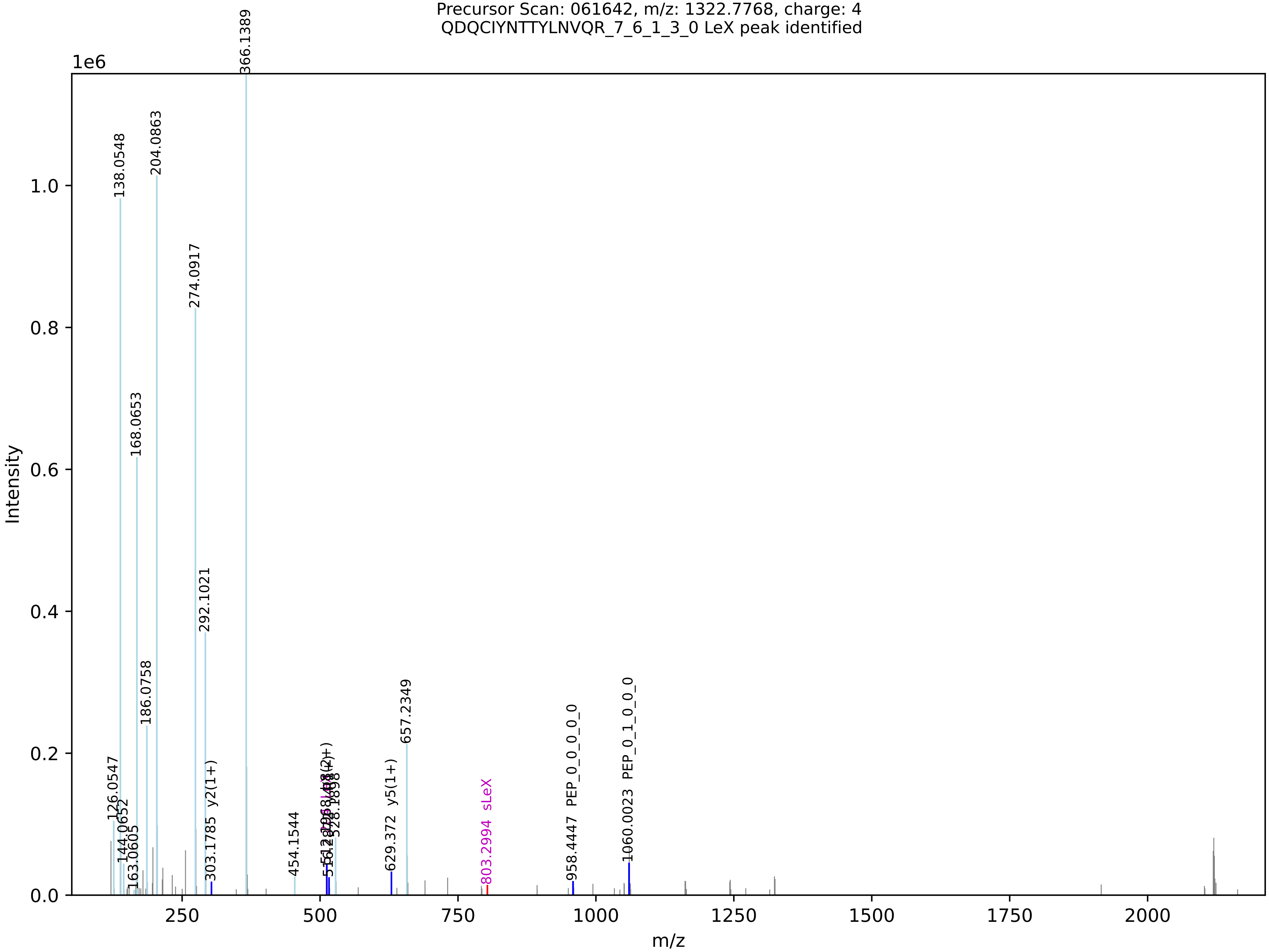

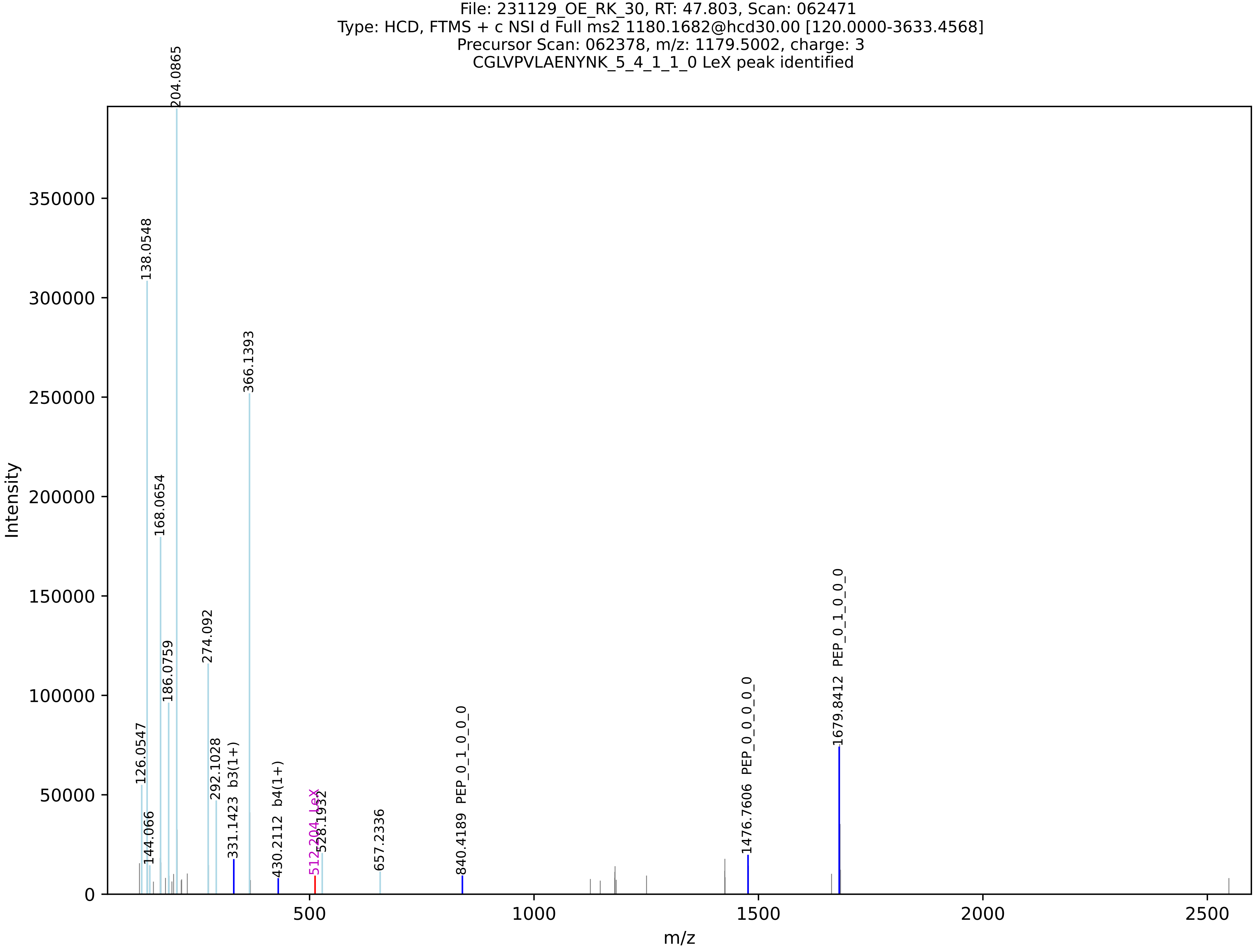
File: 231129_OE_RK_30, RT: 47.803, Scan: 062471
Type: HCD, FTMS + c NSI d Full ms2 1180.1682@hcd30.00 [120.0000-3633.4568]
Precursor Scan: 062378, m/z: 1179.5002, charge: 3
CGLVPVLAENYNK_5_4_1_1_0 LeX peak identified
204.0865
138.0548
366.1393
168.0654
274.092
186.0759
126.0547
144.066
292.1028
331.1423 b3(1+)
430.2112 b4(1+)
512.204 LeX
528.1932
657.2336
840.4189 PEP_0_1_0_0_0
1476.7606 PEP_0_0_0_0_0
1679.8412 PEP_0_1_0_0_0
Intensity
m/z
0
50000
100000
150000
200000
250000
300000
350000
500
1000
1500
2000
2500

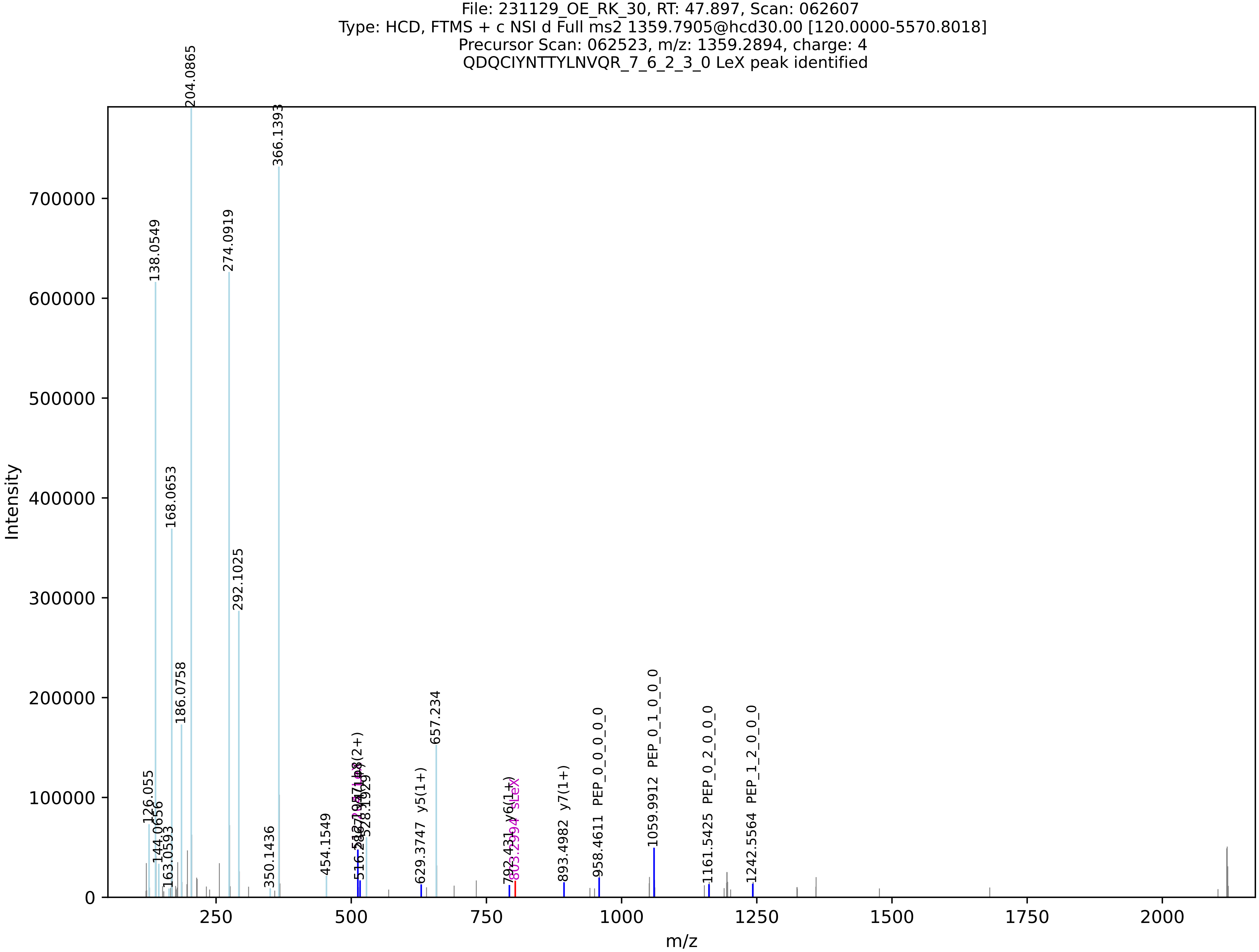

File: 231129_OE_RK_30, RT: 47.897, Scan: 062607
Type: HCD, FTMS + c NSI d Full ms2 1359.7905@hcd30.00 [120.0000-5570.8018]
Precursor Scan: 062523, m/z: 1359.2894, charge: 4
QDQCIYNTTYLNVQR_7_6_2_3_0 LeX peak identified
Intensity
m/z
0
100000
200000
300000
400000
500000
600000
700000
250
500
750
1000
1250
1500
1750
2000
204.0865
366.1393
138.0549
274.0919
168.0653
292.1025
186.0758
126.055
144.0656
163.0593
350.1436
454.1549
528.1929
629.3747 y5(1+)
657.234
893.4982 y7(1+)
958.4611 PEP_0_0_0_0_0
1059.9912 PEP_0_1_0_0_0
1161.5425 PEP_0_2_0_0_0
1242.5564 PEP_1_2_0_0_0

File: 231129_OE_RK_30, RT: 47.998, Scan: 062756
Type: HCD, FTMS + c NSI d Full ms2 1564.6344@hcd30.00 [120.0000-4809.9233]
Precursor Scan: 062669, m/z: 1563.6321, charge: 3
LANLTQGEDQYYLR_6_5_1_3_0 LeX peak identified

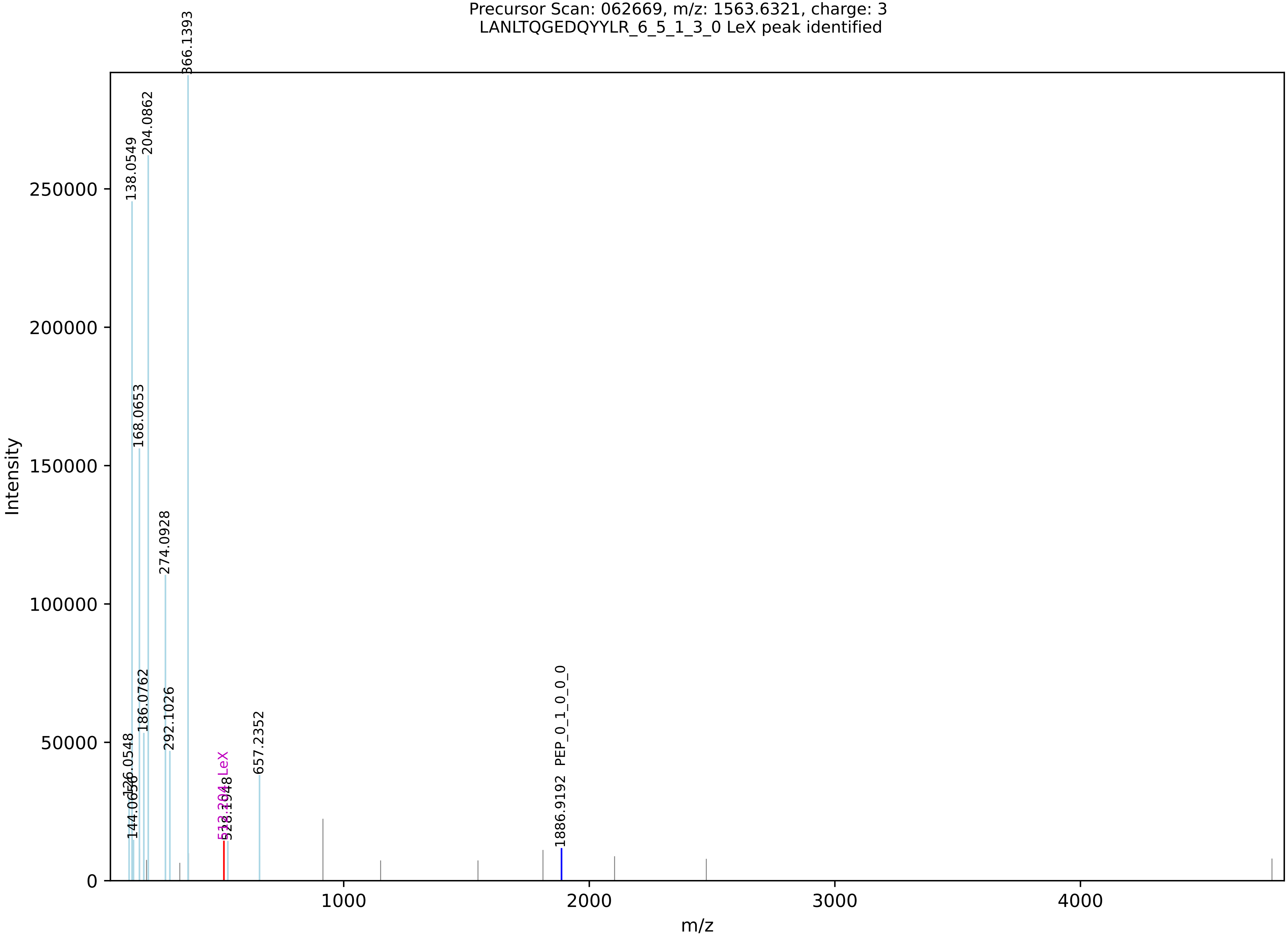

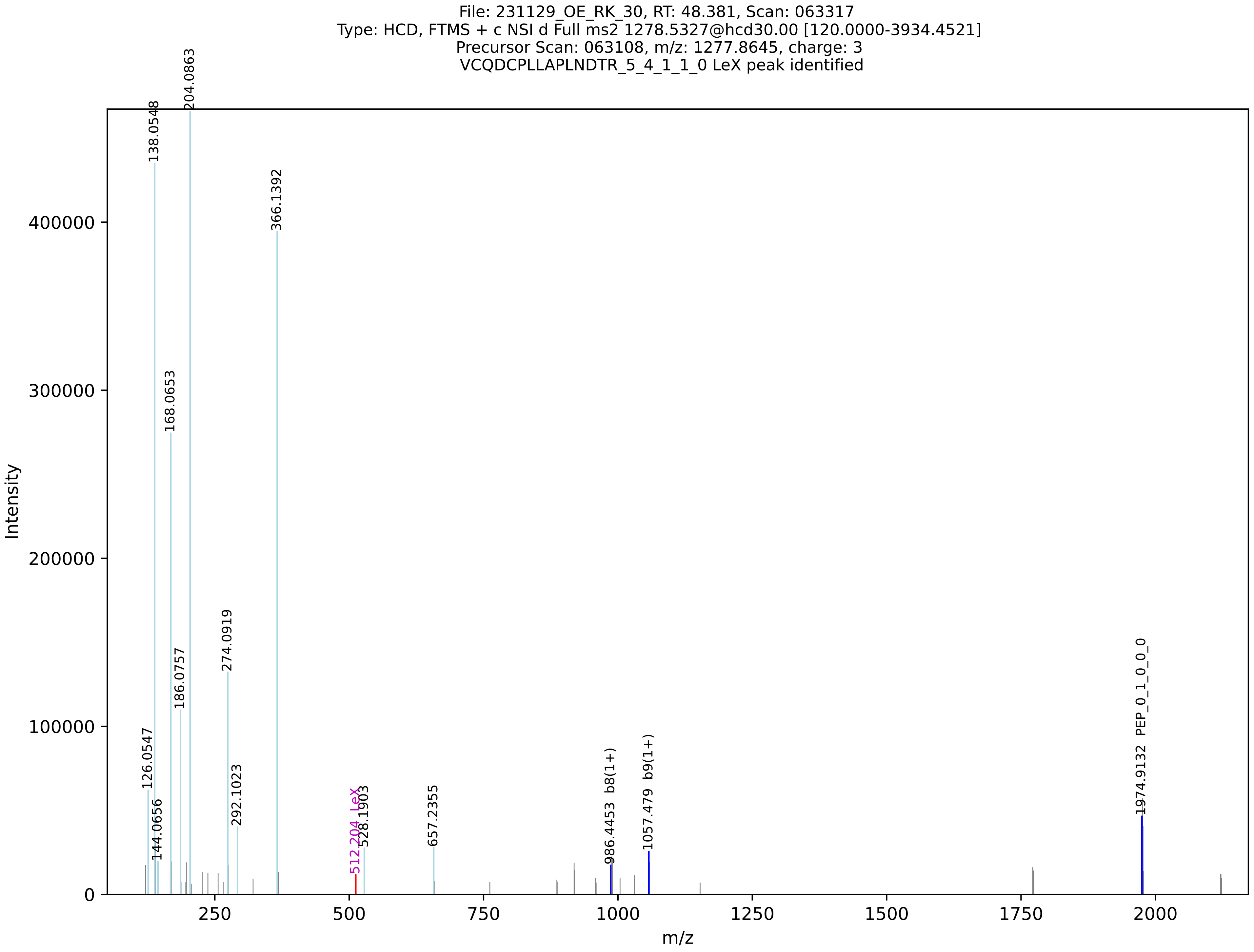

File: 231129_OE_RK_30, RT: 48.381, Scan: 063317
Type: HCD, FTMS + c NSI d Full ms2 1278.5327@hcd30.00 [120.0000-3934.4521]
Precursor Scan: 063108, m/z: 1277.8645, charge: 3
VCQDCPLLAPLNDTR_5_4_1_1_0 LeX peak identified
Intensity
m/z
0
100000
200000
300000
400000
250
500
750
1000
1250
1500
1750
2000
204.0863
138.0548
366.1392
168.0653
274.0919
186.0757
126.0547
144.0656
292.1023
512.204 LeX
528.1903
657.2355
986.4453 b8(1+)
1057.479 b9(1+)
1974.9132 PEP_0_1_0_0_0

File: 231129_OE_RK_30, RT: 48.888, Scan: 064050
Type: HCD, FTMS + c NSI d Full ms2 1323.5295@hcd30.00 [120.0000-5422.8569]
Precursor Scan: 063971, m/z: 1322.7777, charge: 4
QDQCIYNTTYLNVQR_7_6_1_3_0 LeX peak identified

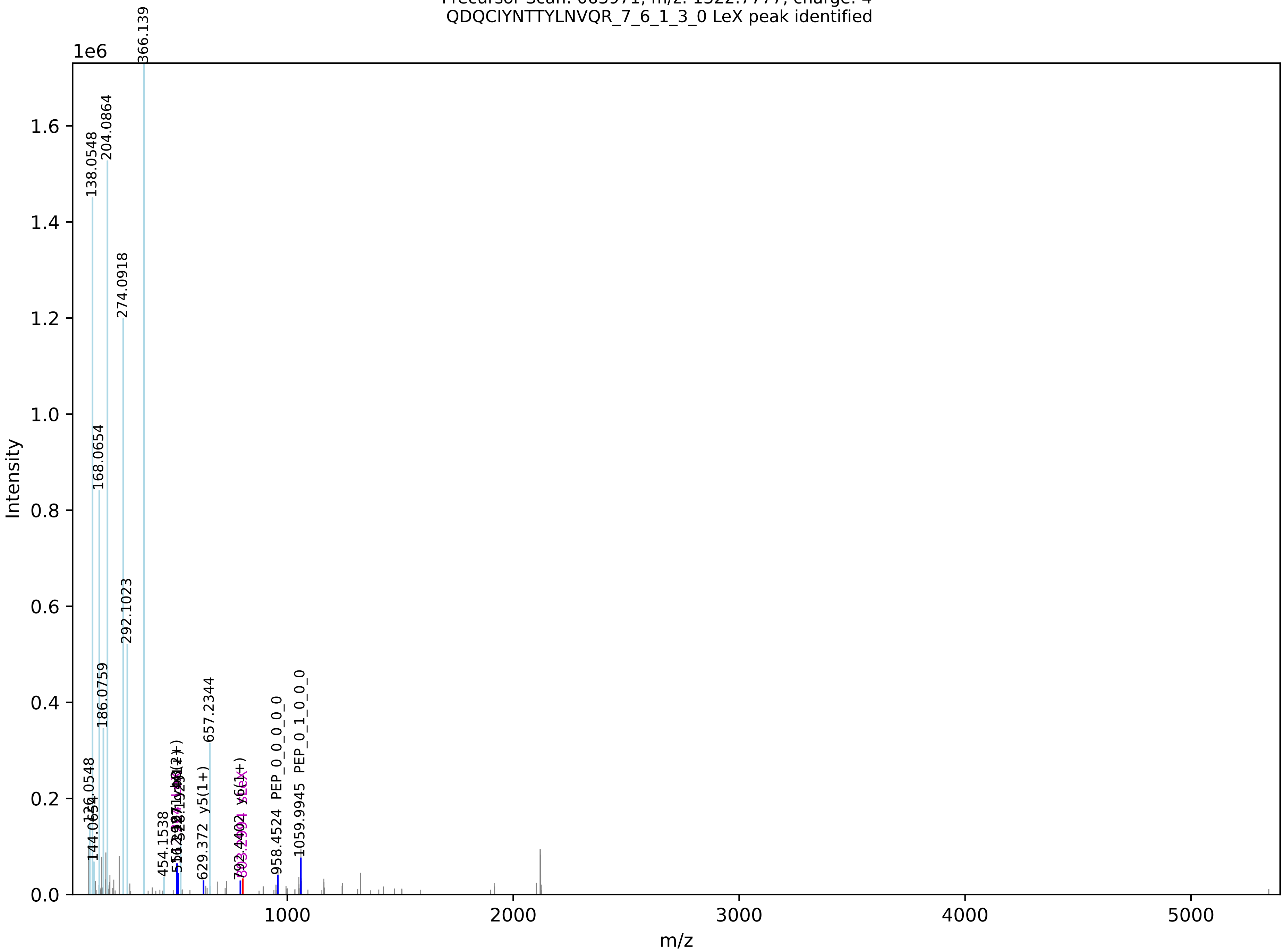

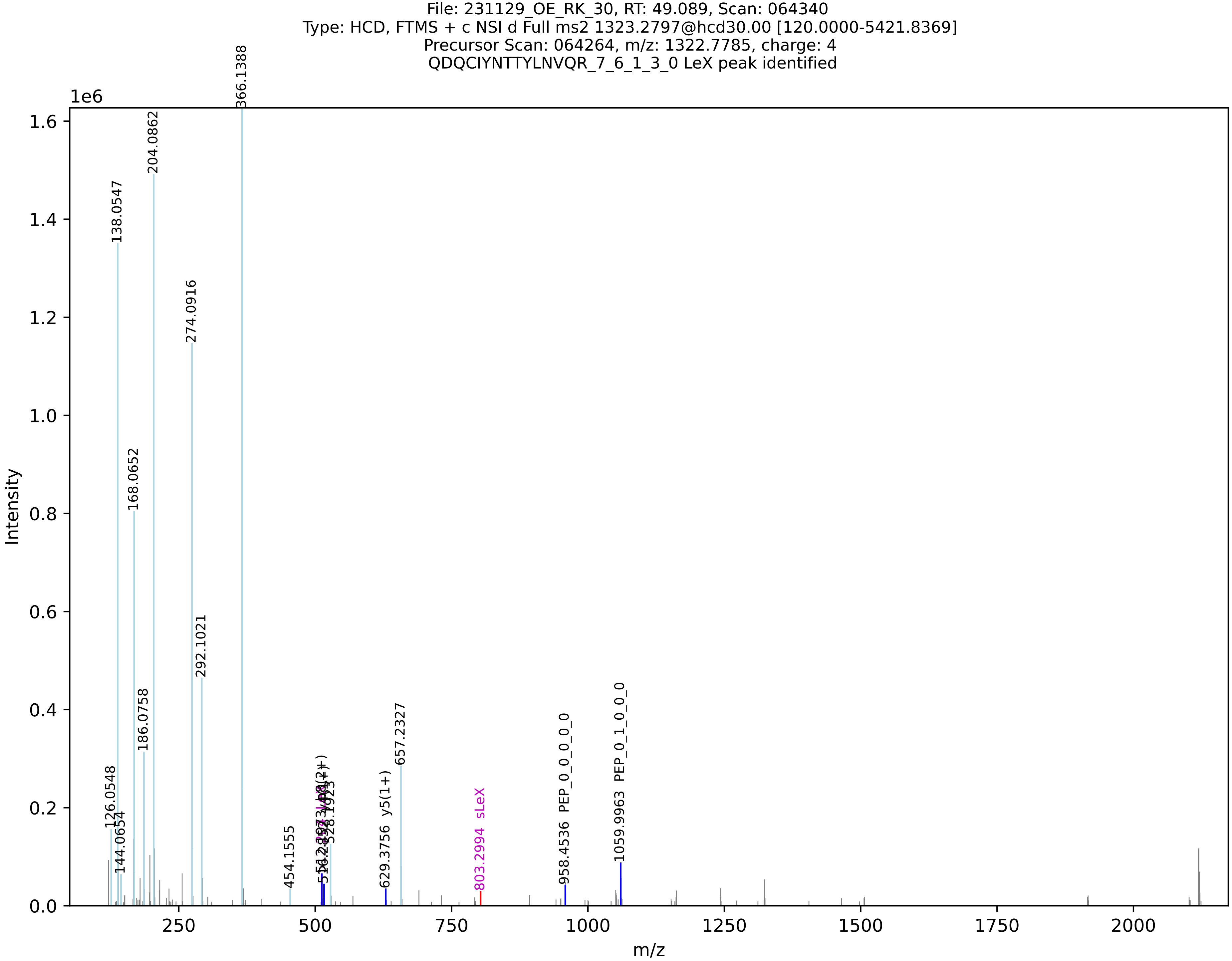
File: 231129_OE_RK_30, RT: 49.089, Scan: 064340
Type: HCD, FTMS + c NSI d Full ms2 1323.2797@hcd30.00 [120.0000-5421.8369]
Precursor Scan: 064264, m/z: 1322.7785, charge: 4
QDQCIYNTTYLNVQR_7_6_1_3_0 LeX peak identified
1e6
Intensity
m/z
366.1388
204.0862
138.0547
274.0916
168.0652
292.1021
186.0758
126.0548
144.0654
454.1555
657.2327
629.3756 y5(1+)
803.2994 sLeX
958.4536 PEP_0_0_0_0_0
1059.9963 PEP_0_1_0_0_0

File: 231129_OE_RK_30, RT: 49.145, Scan: 064423
Type: HCD, FTMS + c NSI d Full ms2 1764.3698@hcd30.00 [120.0000-5421.1138]
Precursor Scan: 064337, m/z: 1763.3677, charge: 3
QDQCIYNTTYLNVQR_7_6_1_3_0 LeX peak identified

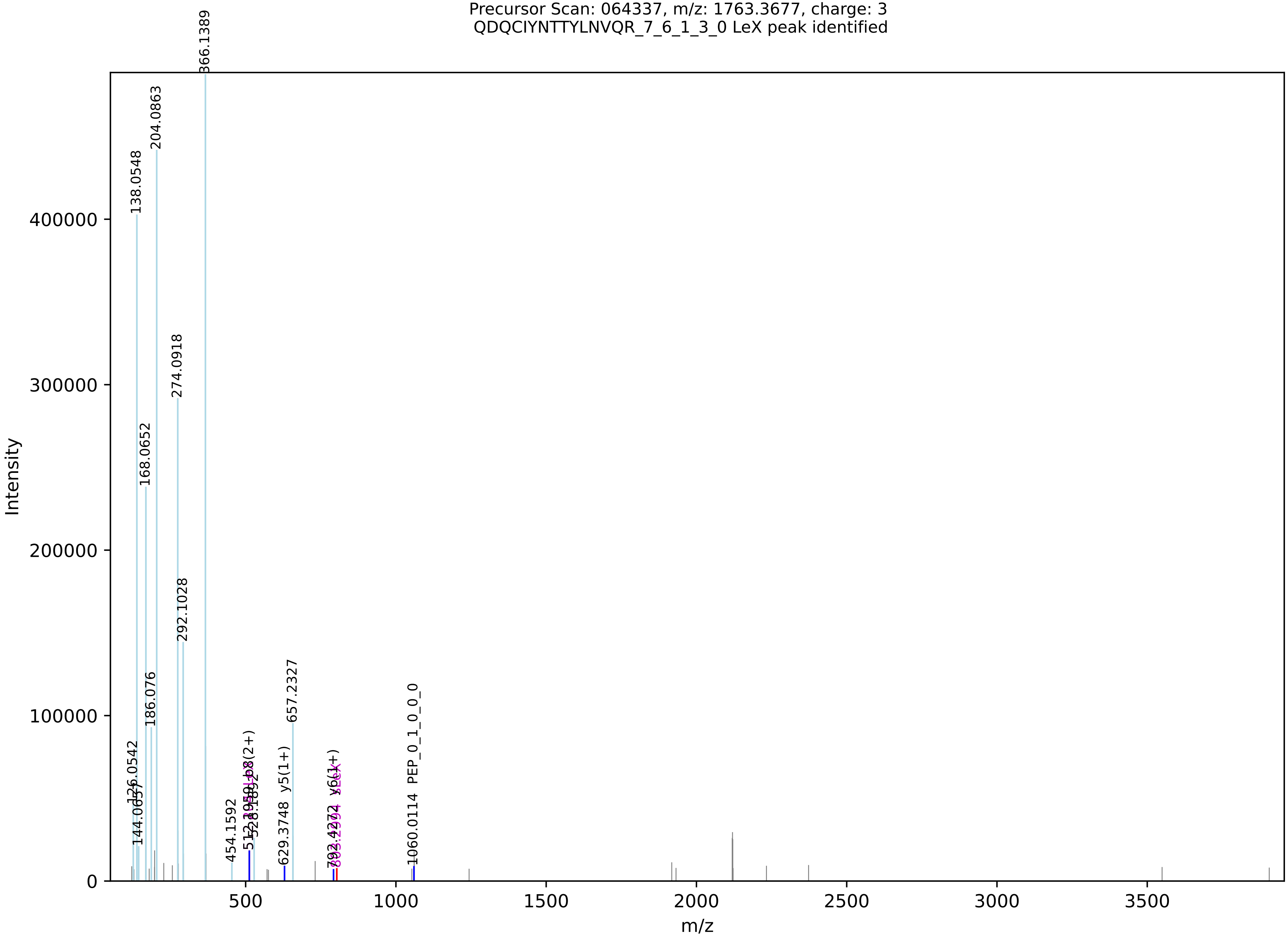

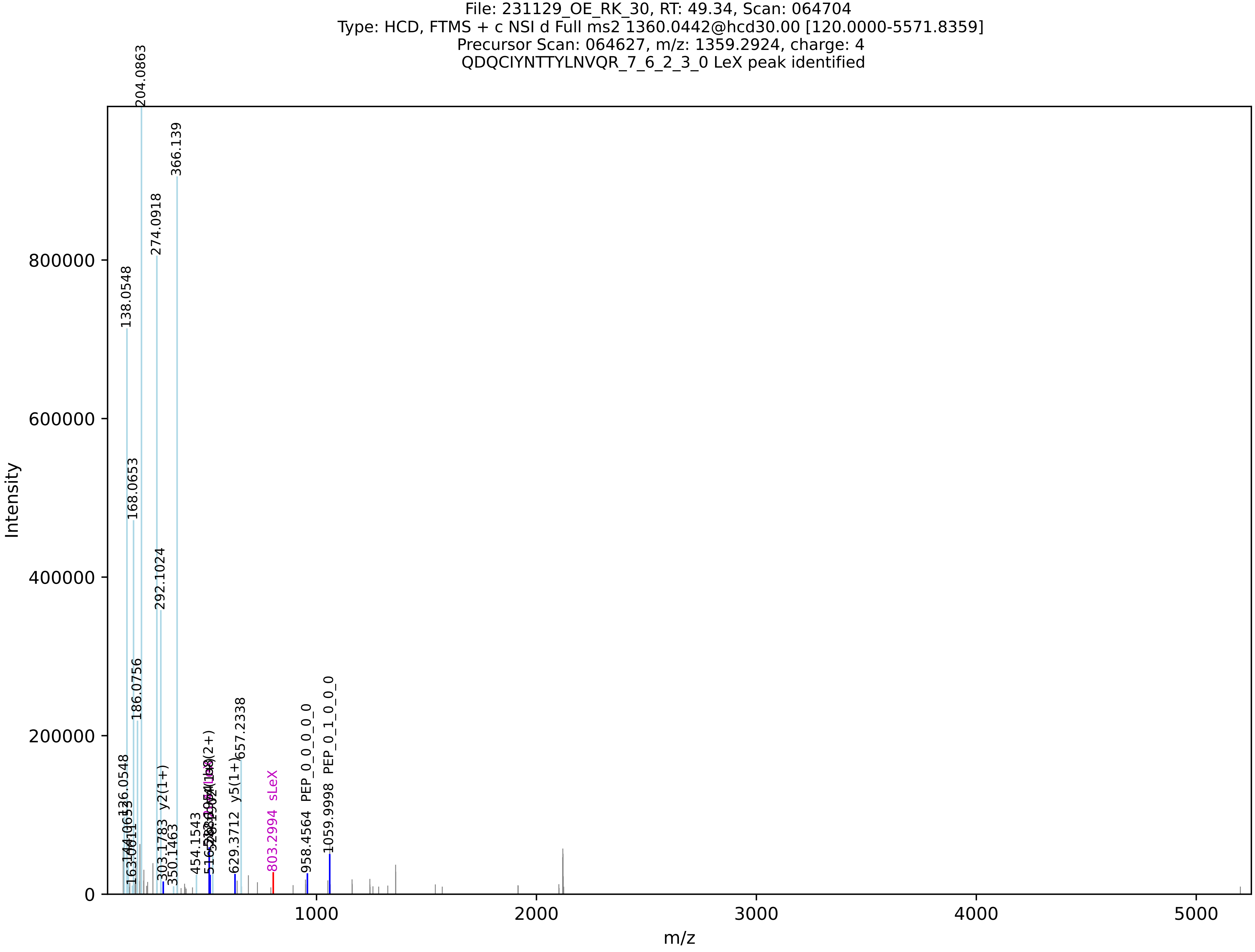
File: 231129_OE_RK_30, RT: 49.34, Scan: 064704
Type: HCD, FTMS + c NSI d Full ms2 1360.0442@hcd30.00 [120.0000-5571.8359]
Precursor Scan: 064627, m/z: 1359.2924, charge: 4
QDQCIYNTTYLNVQR_7_6_2_3_0 LeX peak identified
Intensity
m/z
0
200000
400000
600000
800000
1000
2000
3000
4000
5000
204.0863
366.139
274.0918
138.0548
168.0653
292.1024
186.0756
126.0548
144.0653
163.0611
303.1783 y2(1+)
350.1463
454.1543
657.2338
629.3712 y5(1+)
803.2994 sLeX
958.4564 PEP_0_0_0_0_0_
1059.9998 PEP_0_1_0_0_0_

File: 231129_OE_RK_30, RT: 50.458, Scan: 066331
Type: HCD, FTMS + c NSI d Full ms2 1578.3662@hcd30.00 [120.0000-4851.9424]
Precursor Scan: 066239, m/z: 1577.3619, charge: 3
VVLHPNYSQVDIGLIK_7_6_0_2_0 LeX peak identified

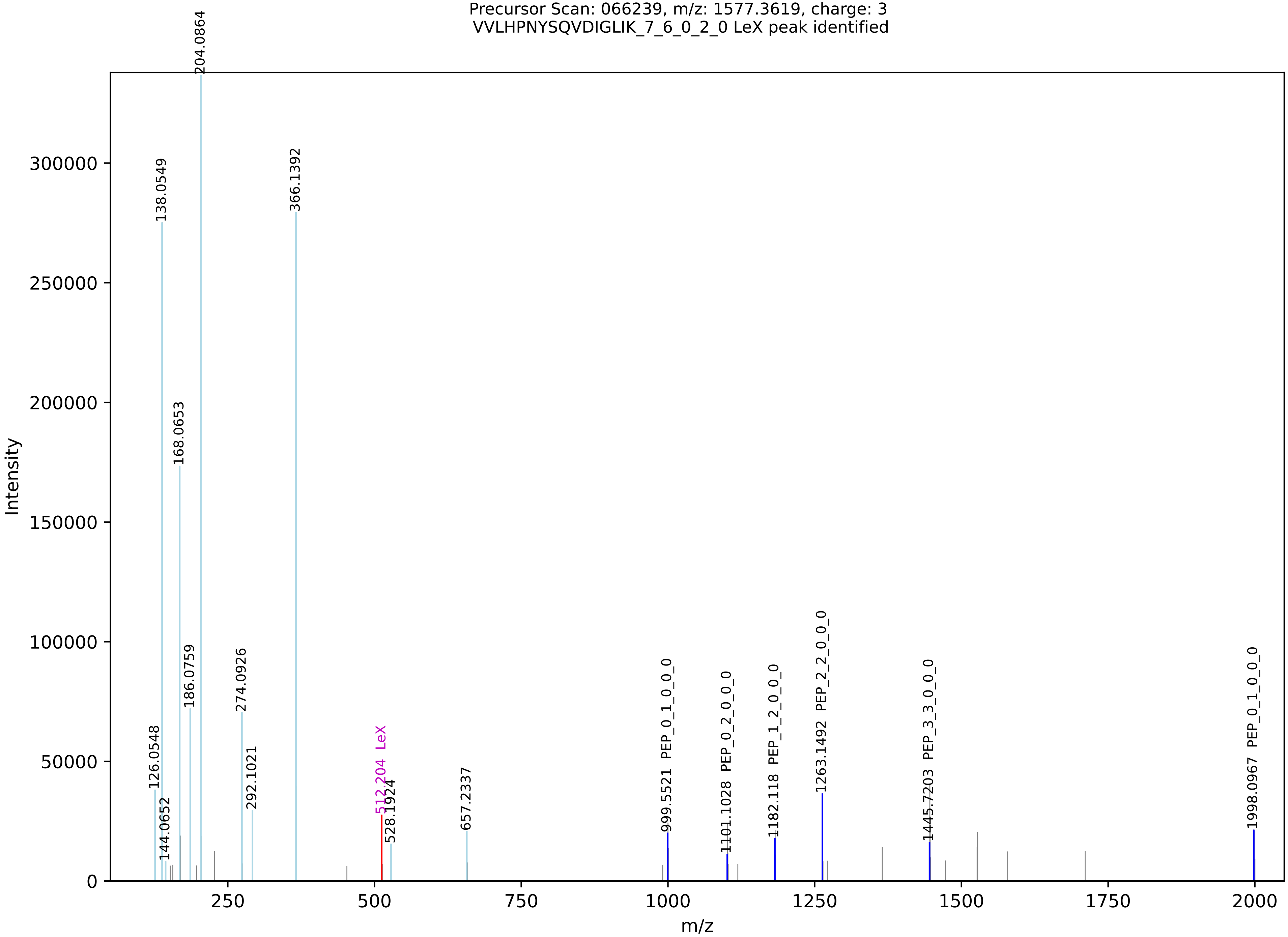

File: 231129_OE_RK_30, RT: 50.556, Scan: 066475
Type: HCD, FTMS + c NSI d Full ms2 1764.0371@hcd30.00 [120.0000-5420.0957]
Precursor Scan: 066384, m/z: 1763.368, charge: 3
QDQCIYNTTYLNVQR_7_6_1_3_0 LeX peak identified

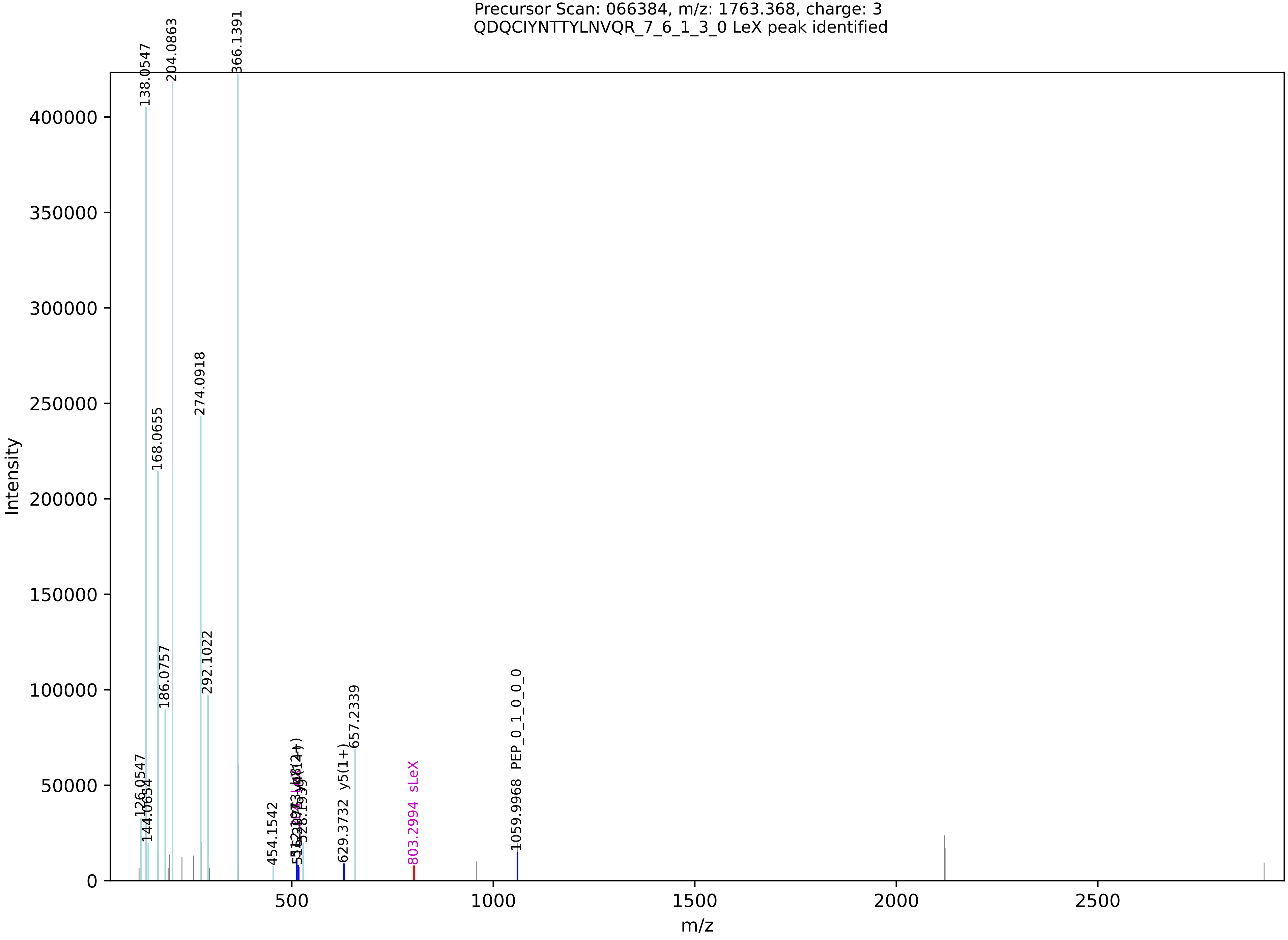

File: 231129_OE_RK_30, RT: 50.702, Scan: 066688
Type: HCD, FTMS + c NSI d Full ms2 1232.2427@hcd30.00 [120.0000-5050.4063]
Precursor Scan: 066605, m/z: 1231.4968, charge: 4
QDQCIYNTTYLNVQR_6_5_1_3_0 LeX peak identified

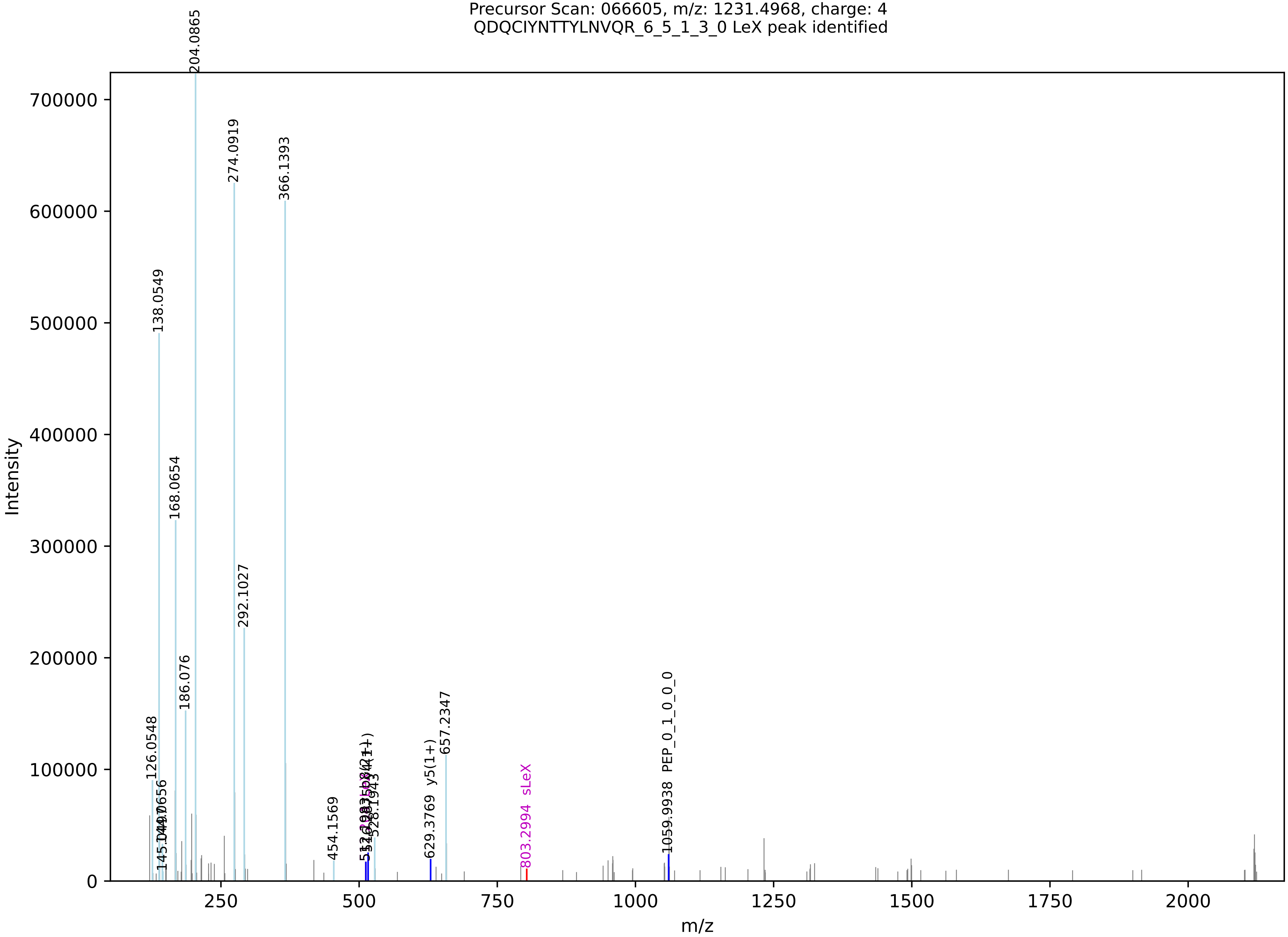

File: 231129_OE_RK_30, RT: 52.558, Scan: 069383
Type: HCD, FTMS + c NSI d Full ms2 1504.6770@hcd30.00 [120.0000-7697.4229]
Precursor Scan: 069287, m/z: 1503.8718, charge: 5
VFAKPKITYVENQTAMELEEQVTLTCEASGDPIPSITWR_6_6_2_2_0 LeX peak identified

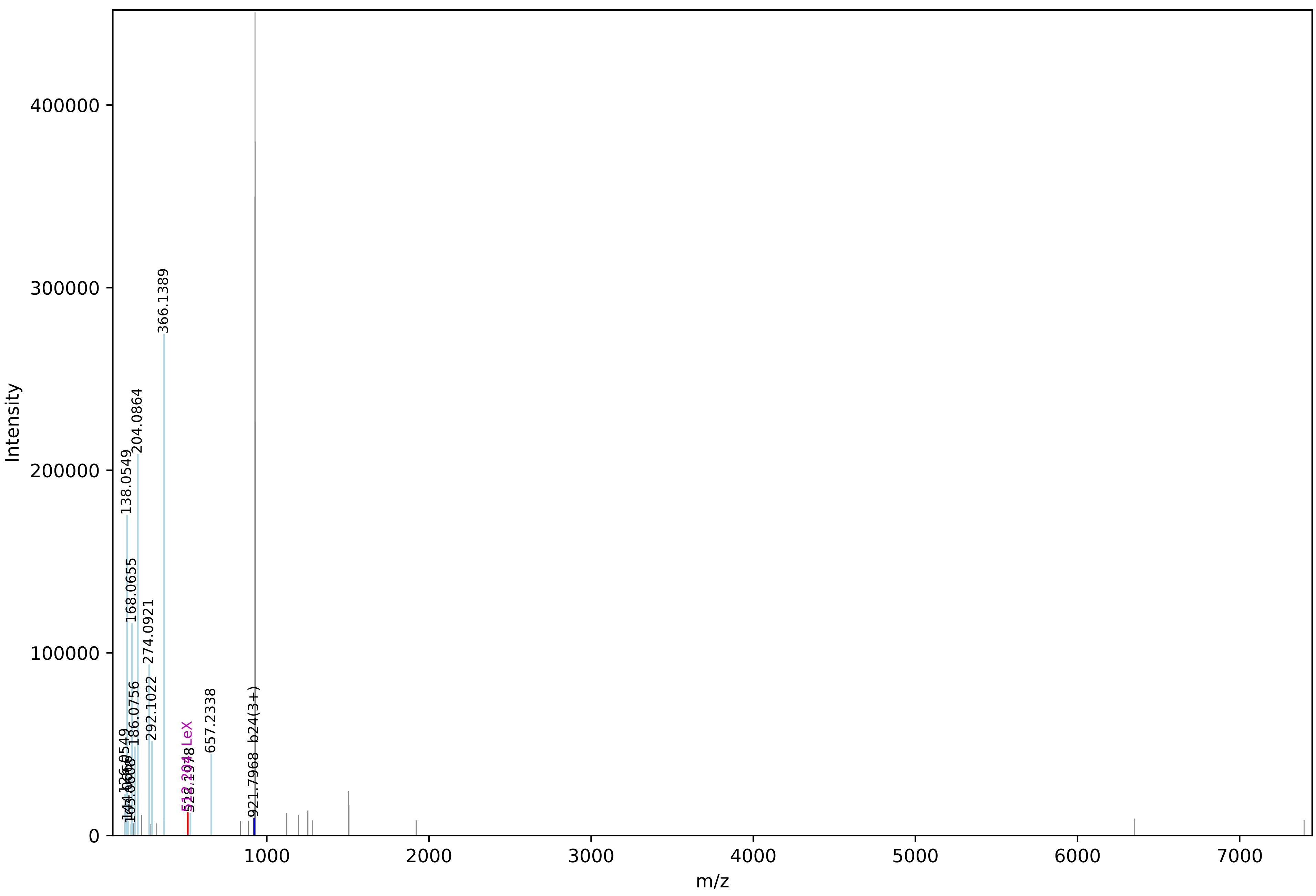

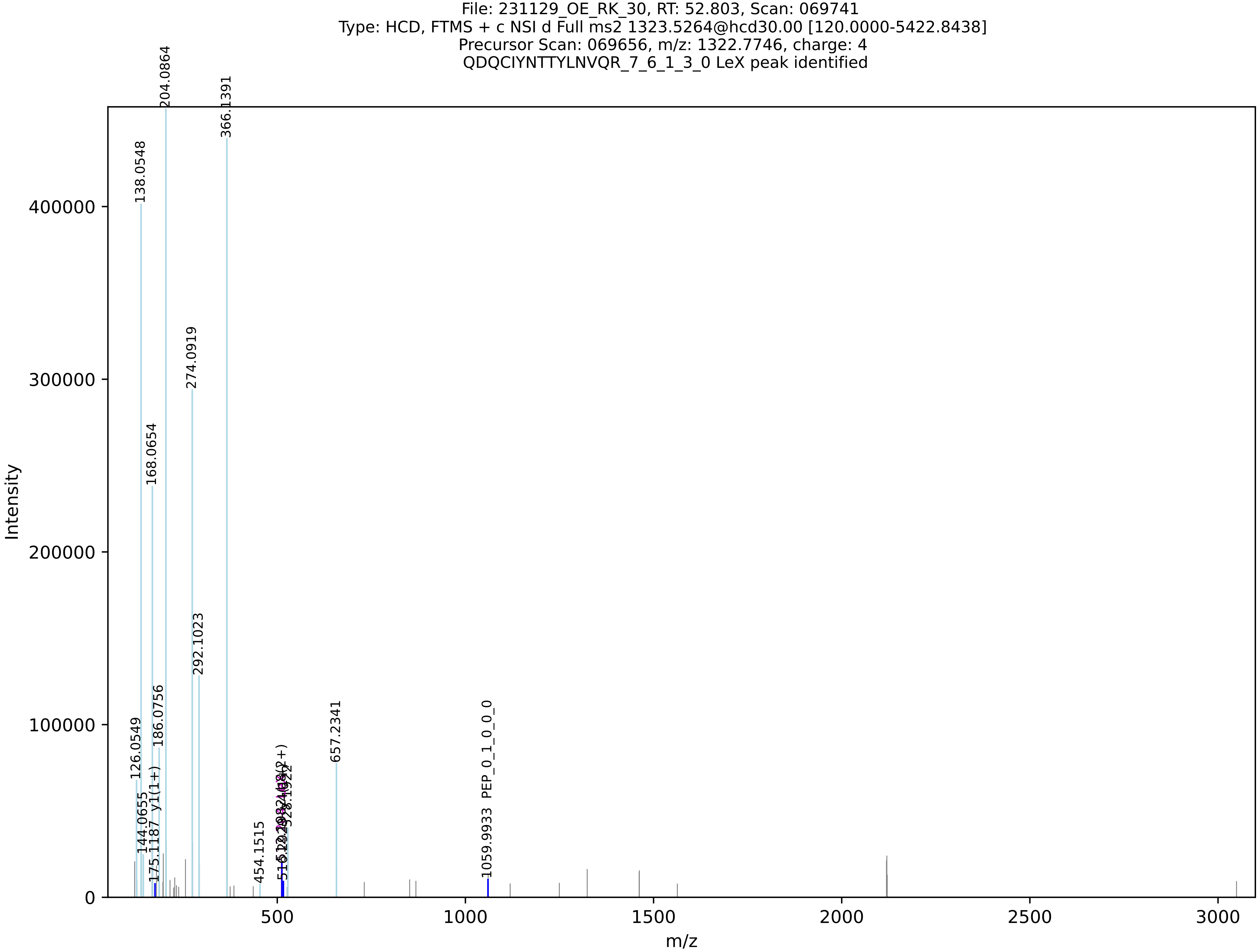

File: 231129_OE_RK_30, RT: 52.803, Scan: 069741
Type: HCD, FTMS + c NSI d Full ms2 1323.5264@hcd30.00 [120.0000-5422.8438]
Precursor Scan: 069656, m/z: 1322.7746, charge: 4
QDQCIYNTTYLNVQR_7_6_1_3_0 LeX peak identified
Intensity
m/z
204.0864
366.1391
138.0548
274.0919
168.0654
292.1023
186.0756
126.0549
144.0655
175.1187 y1(1+)
454.1515
657.2341
1059.9933 PEP_0_1_0_0_0_
0
100000
200000
300000
400000
500
1000
1500
2000
2500
3000

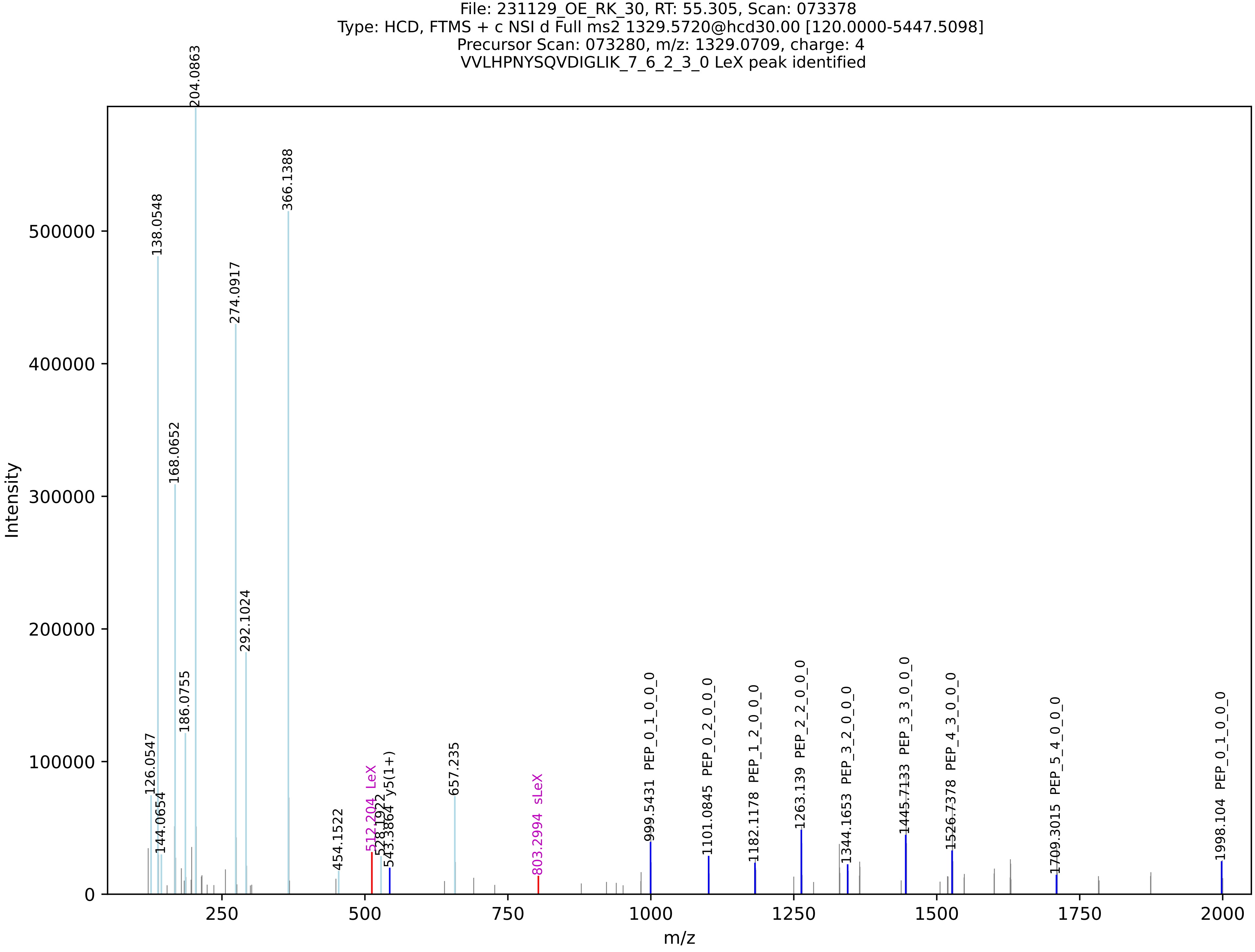
File: 231129_OE_RK_30, RT: 55.305, Scan: 073378
Type: HCD, FTMS + c NSI d Full ms2 1329.5720@hcd30.00 [120.0000-5447.5098]
Precursor Scan: 073280, m/z: 1329.0709, charge: 4
VVLHPNYSQVDIGLIK_7_6_2_3_0 LeX peak identified
Intensity
m/z
204.0863
366.1388
138.0548
274.0917
168.0652
292.1024
186.0755
126.0547
144.0654
454.1522
512.204 LeX
528.1922
543.3864 y5(1+)
657.235
803.2994 sLeX
999.5431 PEP_0_1_0_0_0
1101.0845 PEP_0_2_0_0_0
1182.1178 PEP_1_2_0_0_0
1263.139 PEP_2_2_0_0_0
1344.1653 PEP_3_2_0_0_0
1445.7133 PEP_3_3_0_0_0
1526.7378 PEP_4_3_0_0_0
1709.3015 PEP_5_4_0_0_0
1998.104 PEP_0_1_0_0_0

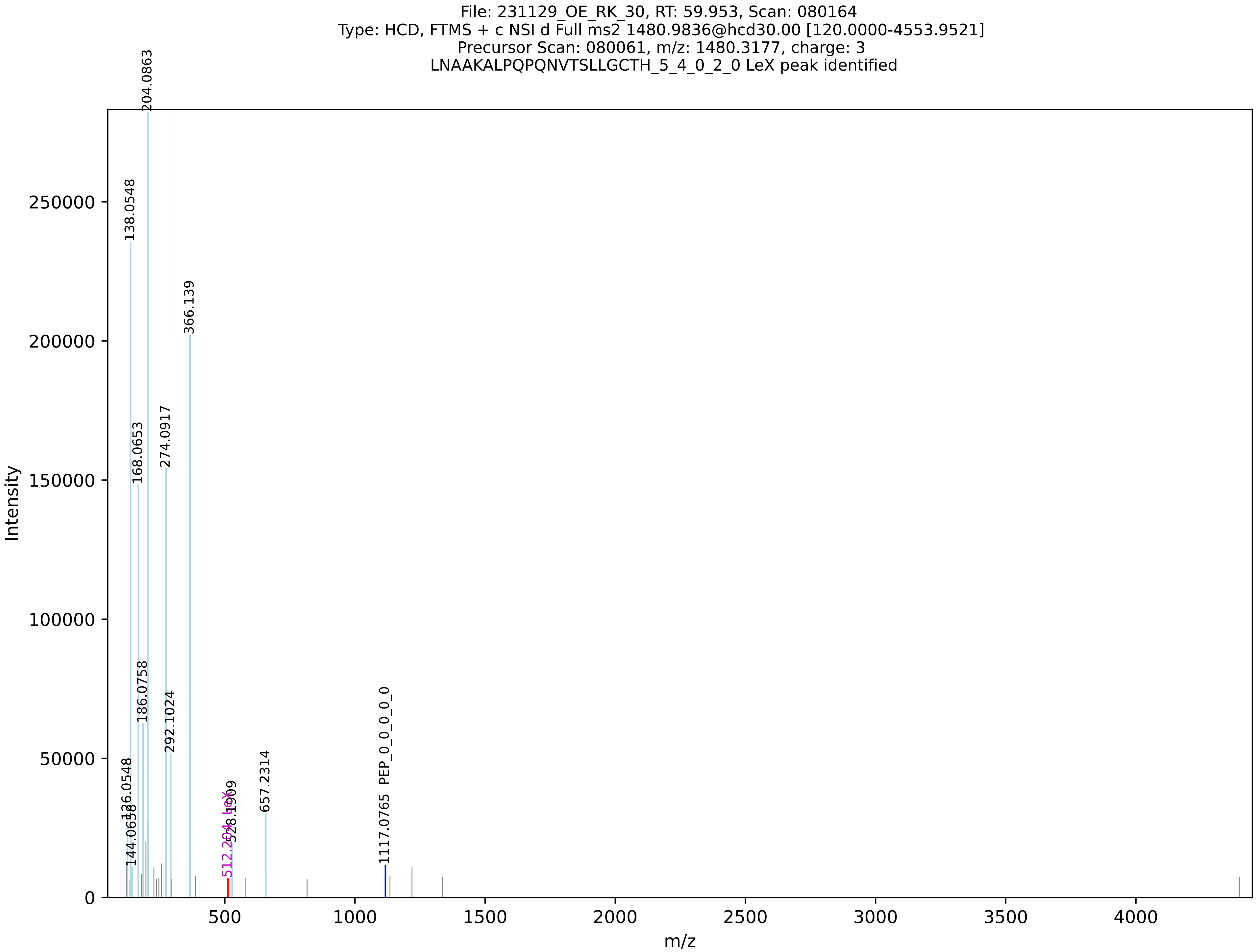

File: 231129_OE_RK_30, RT: 59.953, Scan: 080164
Type: HCD, FTMS + c NSI d Full ms2 1480.9836@hcd30.00 [120.0000-4553.9521]
Precursor Scan: 080061, m/z: 1480.3177, charge: 3
LNAAKALPQPQNVTSLLGCTH_5_4_0_2_0 LeX peak identified
Intensity
m/z
204.0863
138.0548
366.139
168.0653
274.0917
186.0758
292.1024
126.0548
144.0658
512.204_LeX
528.1909
657.2314
1117.0765
PEP_0_0_0_0_0

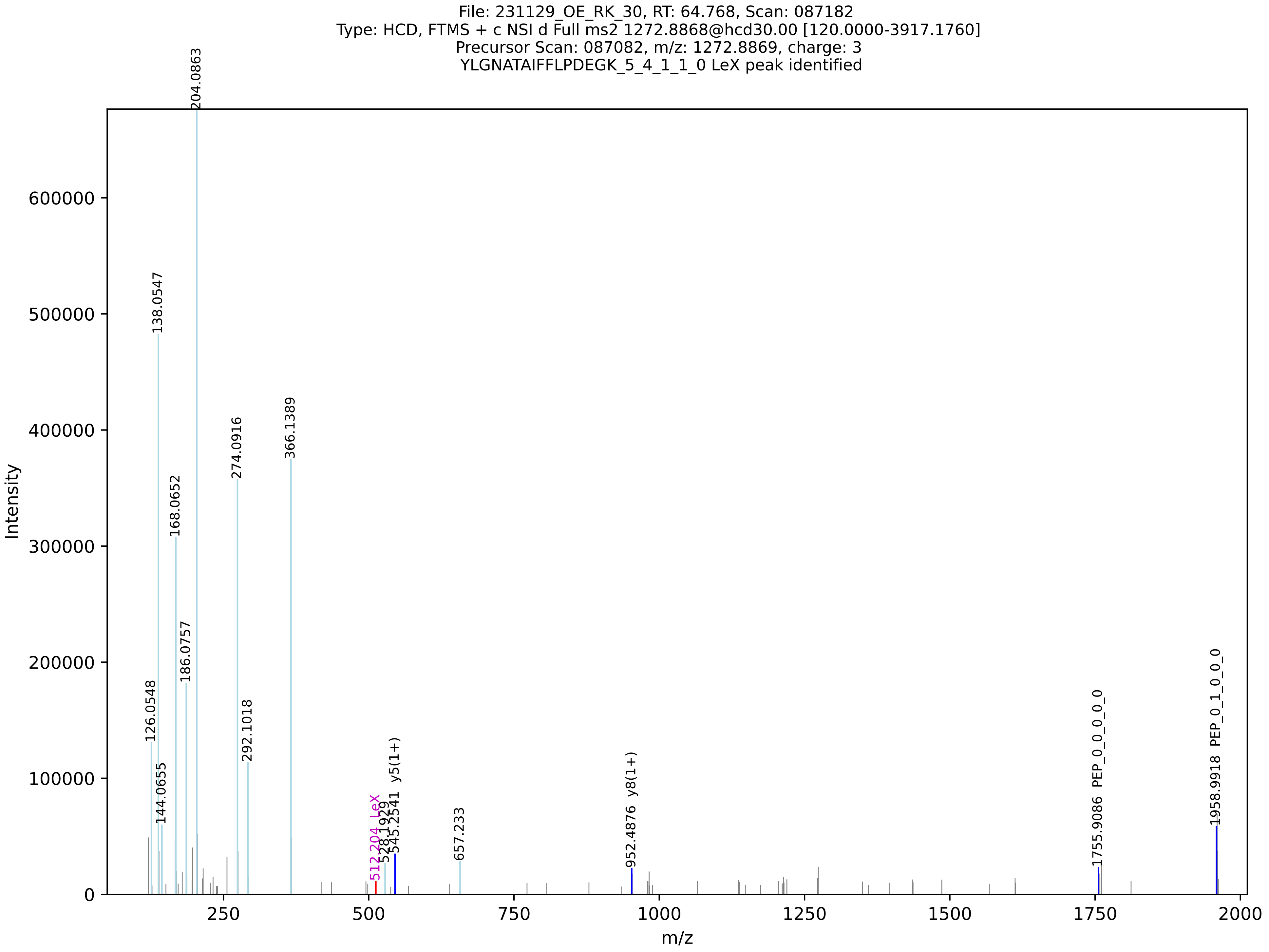
File: 231129_OE_RK_30, RT: 64.768, Scan: 087182
Type: HCD, FTMS + c NSI d Full ms2 1272.8868@hcd30.00 [120.0000-3917.1760]
Precursor Scan: 087082, m/z: 1272.8869, charge: 3
YLGNATAIFFLPDEGK_5_4_1_1_0 LeX peak identified
Intensity
m/z
204.0863
138.0547
168.0652
186.0757
126.0548
144.0655
274.0916
292.1018
366.1389
512.204 LeX
528.1929
545.2541 y5(1+)
657.233
952.4876 y8(1+)
1755.9086 PEP_0_0_0_0_0_
1958.9918 PEP_0_1_0_0_0

File: 231129_OE_RK_30, RT: 82.903, Scan: 113639
Type: HCD, FTMS + c NSI d Full ms2 1179.9449@hcd30.00 [120.0000-6041.2891]
Precursor Scan: 113560, m/z: 1179.143, charge: 5
YLGNATAIFFLPDEGKLQHLENELTHDIITK_5_4_1_2_0 LeX peak identified

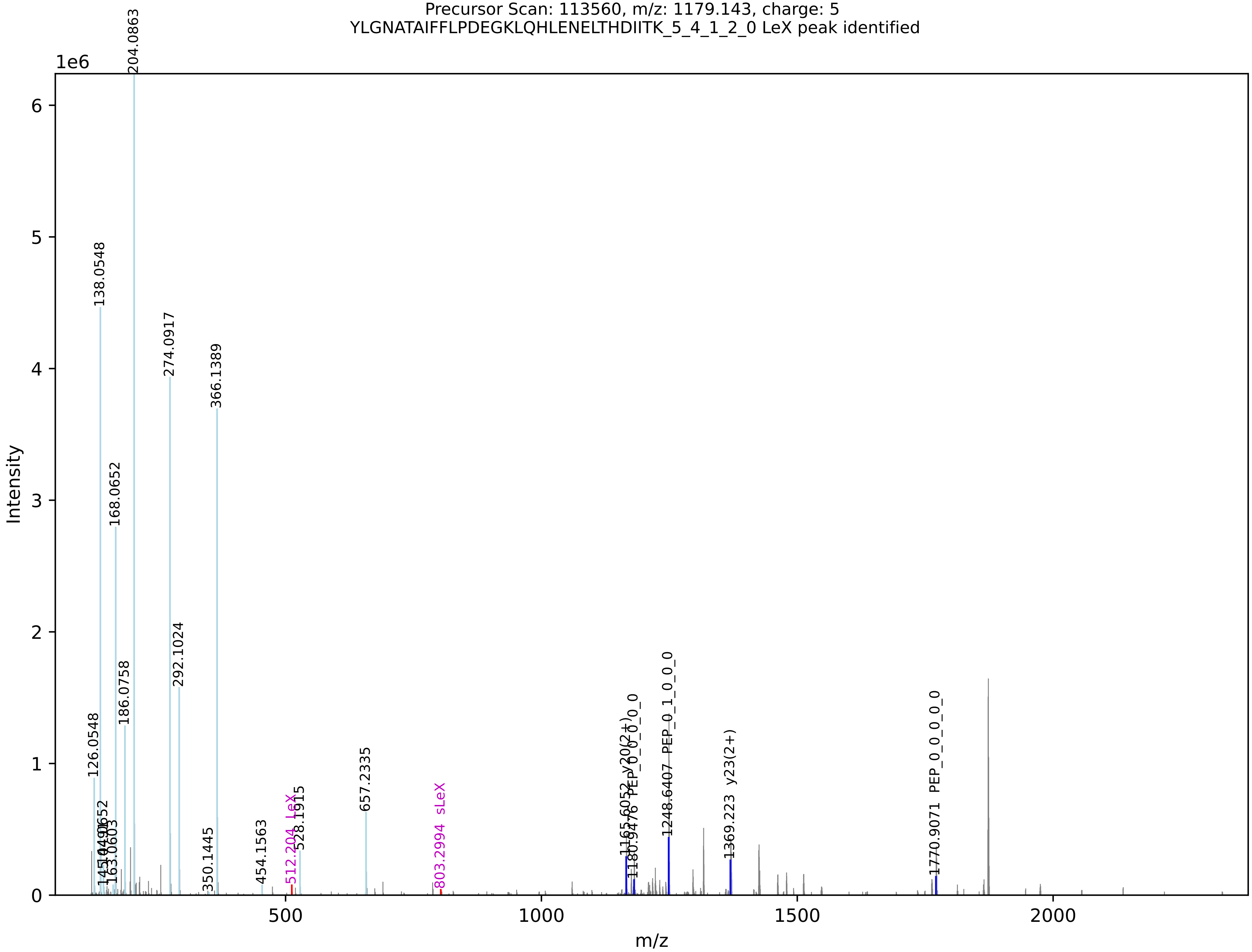

File: 231129_OE_RK_30, RT: 83.094, Scan: 113925
Type: HCD, FTMS + c NSI d Full ms2 1178.9572@hcd30.00 [120.0000-6036.2515]
Precursor Scan: 113708, m/z: 1179.1395, charge: 5
YLGNATAIFFLPDEGKLQHLENELTHDIITK_5_4_1_2_0 LeX peak identified

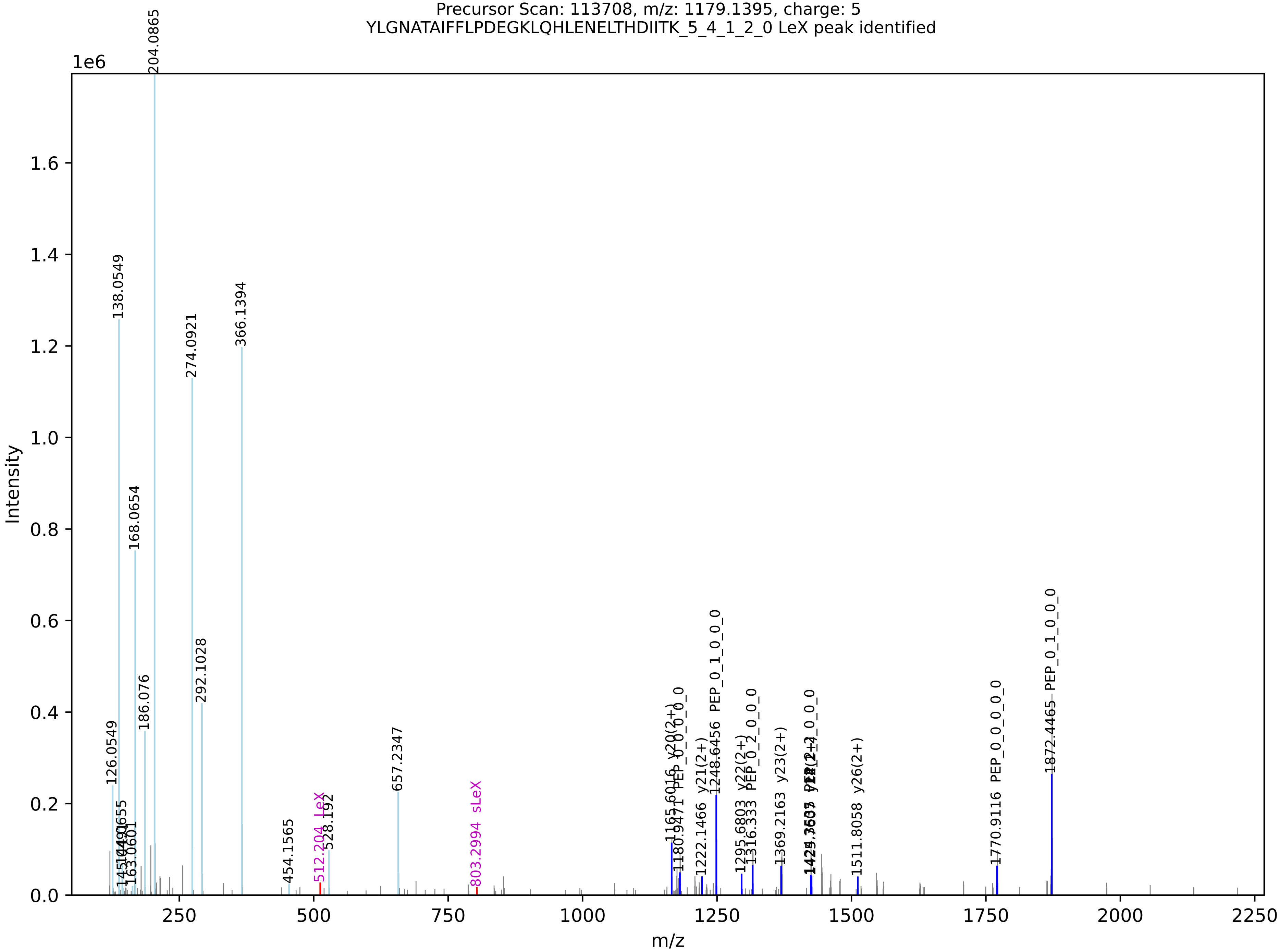

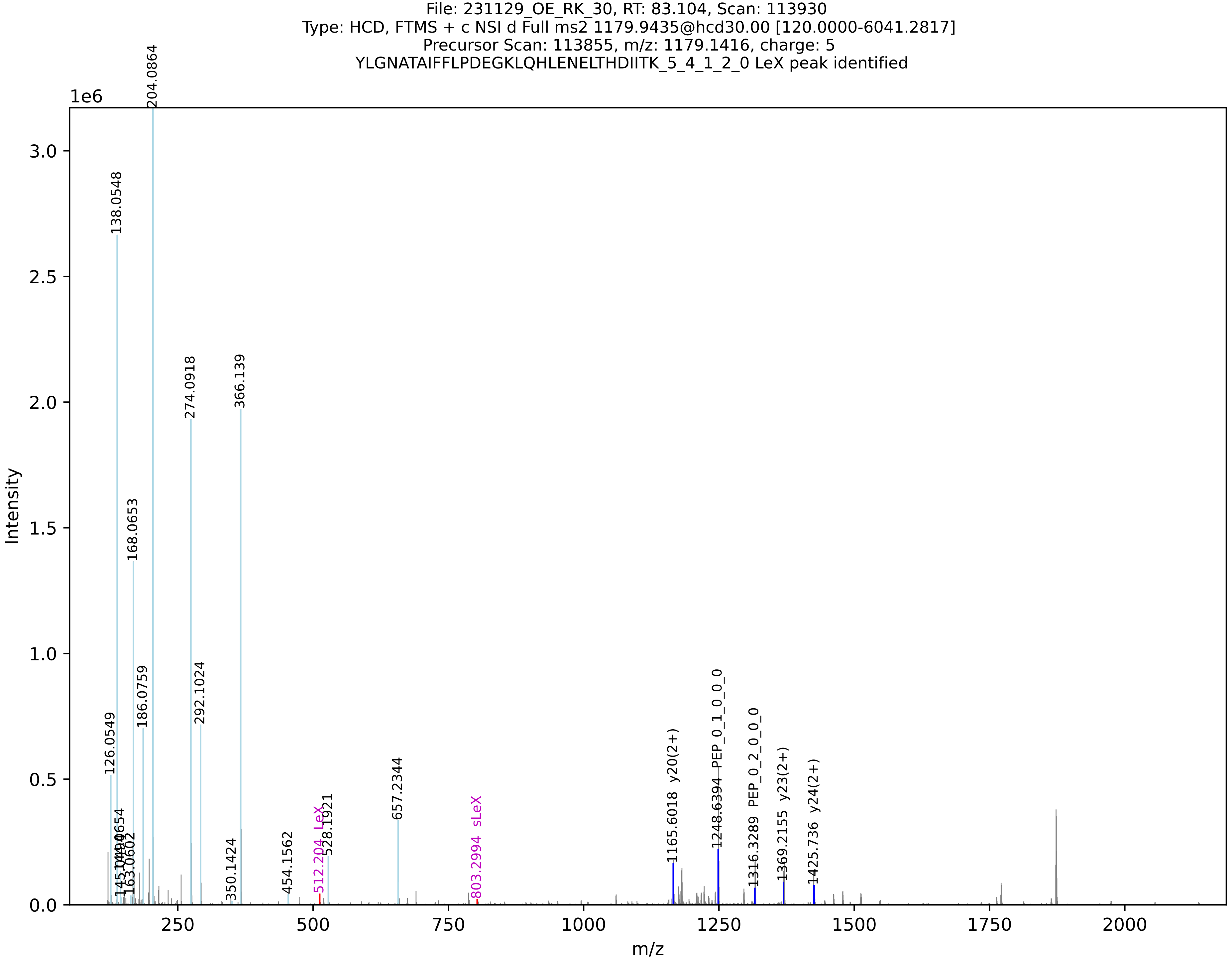
File: 231129_OE_RK_30, RT: 83.104, Scan: 113930
Type: HCD, FTMS + c NSI d Full ms2 1179.9435@hcd30.00 [120.0000-6041.2817]
Precursor Scan: 113855, m/z: 1179.1416, charge: 5
YLGNATAIFFLPDEGKLQHLENELTHDIITK_5_4_1_2_0 LeX peak identified
1e6
Intensity
m/z
204.0864
138.0548
274.0918
366.139
168.0653
186.0759
292.1024
126.0549
657.2344
350.1424
454.1562
512.204 LeX
528.1921
803.2994 sLeX
163.0602
1165.6018 y20(2+)
1248.6394 PEP_0_1_0_0_0
1316.3289 PEP_0_2_0_0_0
1369.2155 y23(2+)
1425.736 y24(2+)

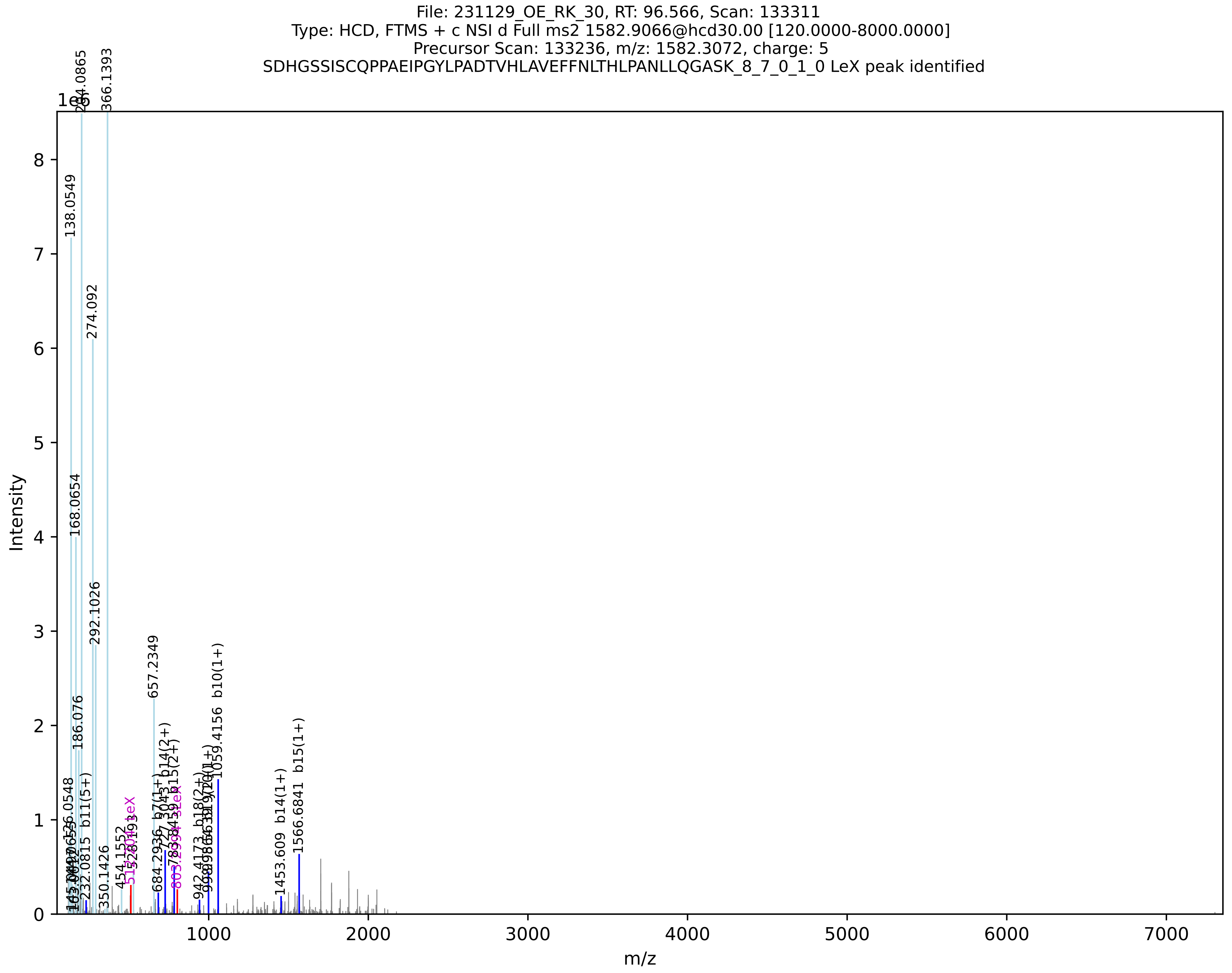
File: 231129_OE_RK_30, RT: 96.566, Scan: 133311
Type: HCD, FTMS + c NSI d Full ms2 1582.9066@hcd30.00 [120.0000-8000.0000]
Precursor Scan: 133236, m/z: 1582.3072, charge: 5
SDHGSSISCQPPAEIPGYLPADTVHLAVEFFNLTHLPANLLQGASK_8_7_0_1_0 LeX peak identified
1e6
366.1393
138.0549
274.092
168.0654
292.1026
186.076
657.2349
1059.4156 b10(1+)
1566.6841 b15(1+)
1453.609 b14(1+)
942.4173 b18(2+)
684.2936 b7(1+)
232.0815
350.1426
454.1552
b11(5+)
Intensity
m/z
0
1
2
3
4
5
6
7
8
1000
2000
3000
4000
5000
6000
7000

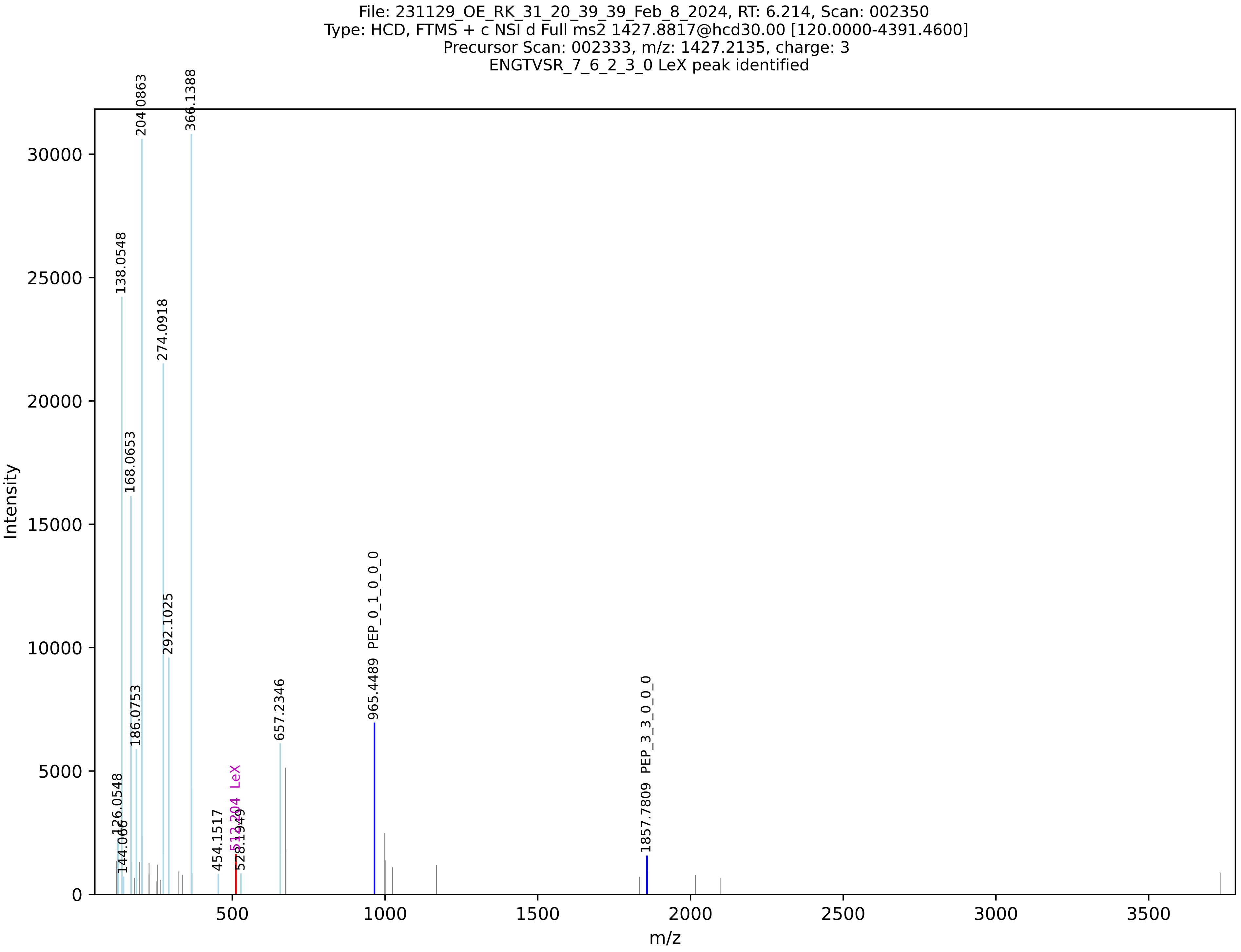
File: 231129_OE_RK_31_20_39_39_Feb_8_2024, RT: 6.214, Scan: 002350
Type: HCD, FTMS + c NSI d Full ms2 1427.8817@hcd30.00 [120.0000-4391.4600]
Precursor Scan: 002333, m/z: 1427.2135, charge: 3
ENGTVSR_7_6_2_3_0 LeX peak identified
204.0863
366.1388
138.0548
274.0918
168.0653
292.1025
186.0753
126.0548
144.066
454.1517
512.204 LeX
528.1949
657.2346
965.4489 PEP_0_1_0_0_0
1857.7809 PEP_3_3_0_0_0
Intensity
m/z

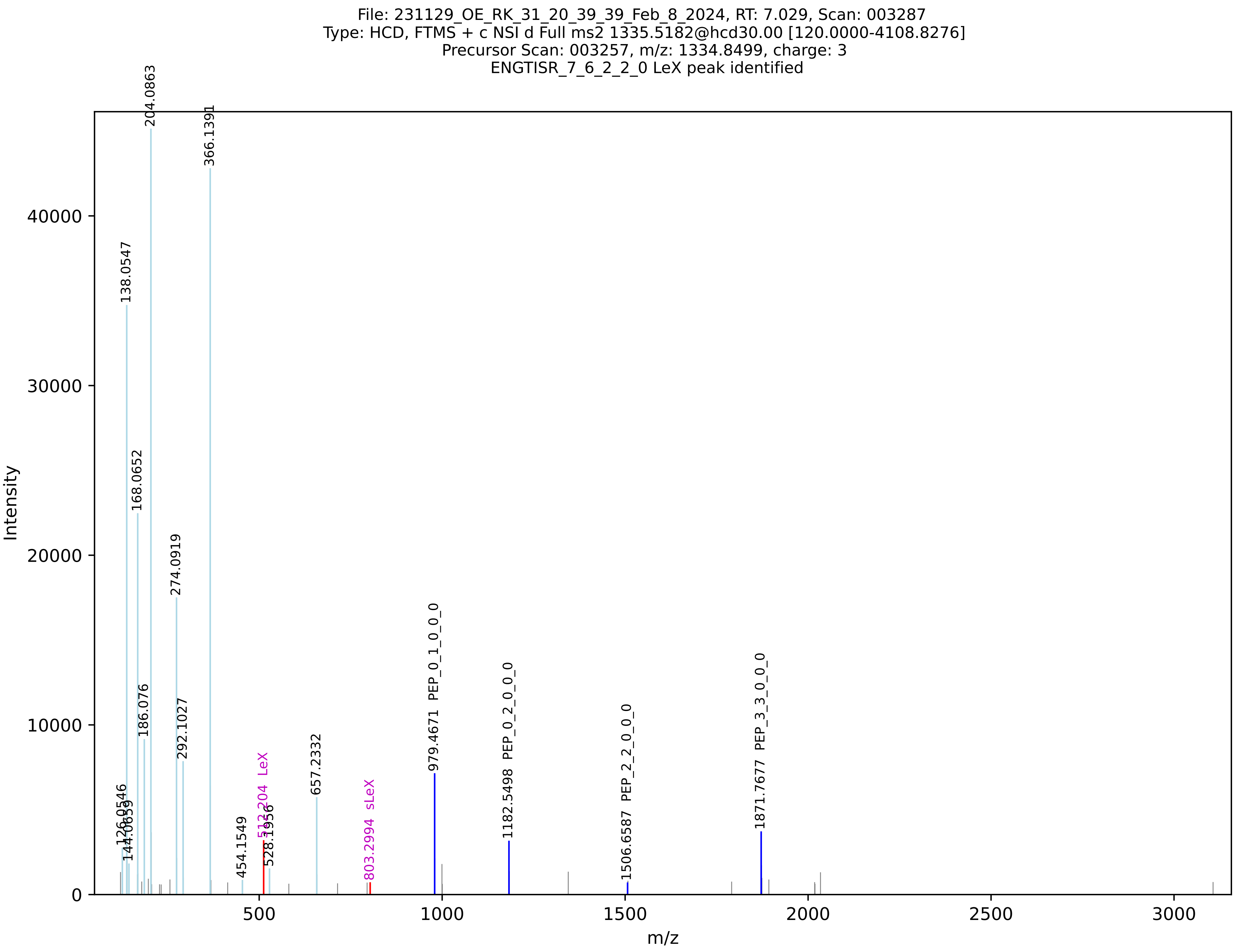
File: 231129_OE_RK_31_20_39_39_Feb_8_2024, RT: 7.029, Scan: 003287
Type: HCD, FTMS + c NSI d Full ms2 1335.5182@hcd30.00 [120.0000-4108.8276]
Precursor Scan: 003257, m/z: 1334.8499, charge: 3
ENGTISR_7_6_2_2_0 LeX peak identified
Intensity
m/z
204.0863
366.1391
138.0547
168.0652
274.0919
186.076
292.1027
126.0546
144.0659
454.1549
512.204 LeX
528.1956
657.2332
803.2994 sLeX
979.4671 PEP_0_1_0_0_0
1182.5498 PEP_0_2_0_0_0
1506.6587 PEP_2_2_0_0_0
1871.7677 PEP_3_3_0_0_0

File: 231129_OE_RK_31_20_39_39_Feb_8_2024, RT: 7.206, Scan: 003514
Type: HCD, FTMS + c NSI d Full ms2 1335.5182@hcd30.00 [120.0000-4108.8276]
Precursor Scan: 003486, m/z: 1334.85, charge: 3
ENGTISR_7_6_2_2_0 LeX peak identified

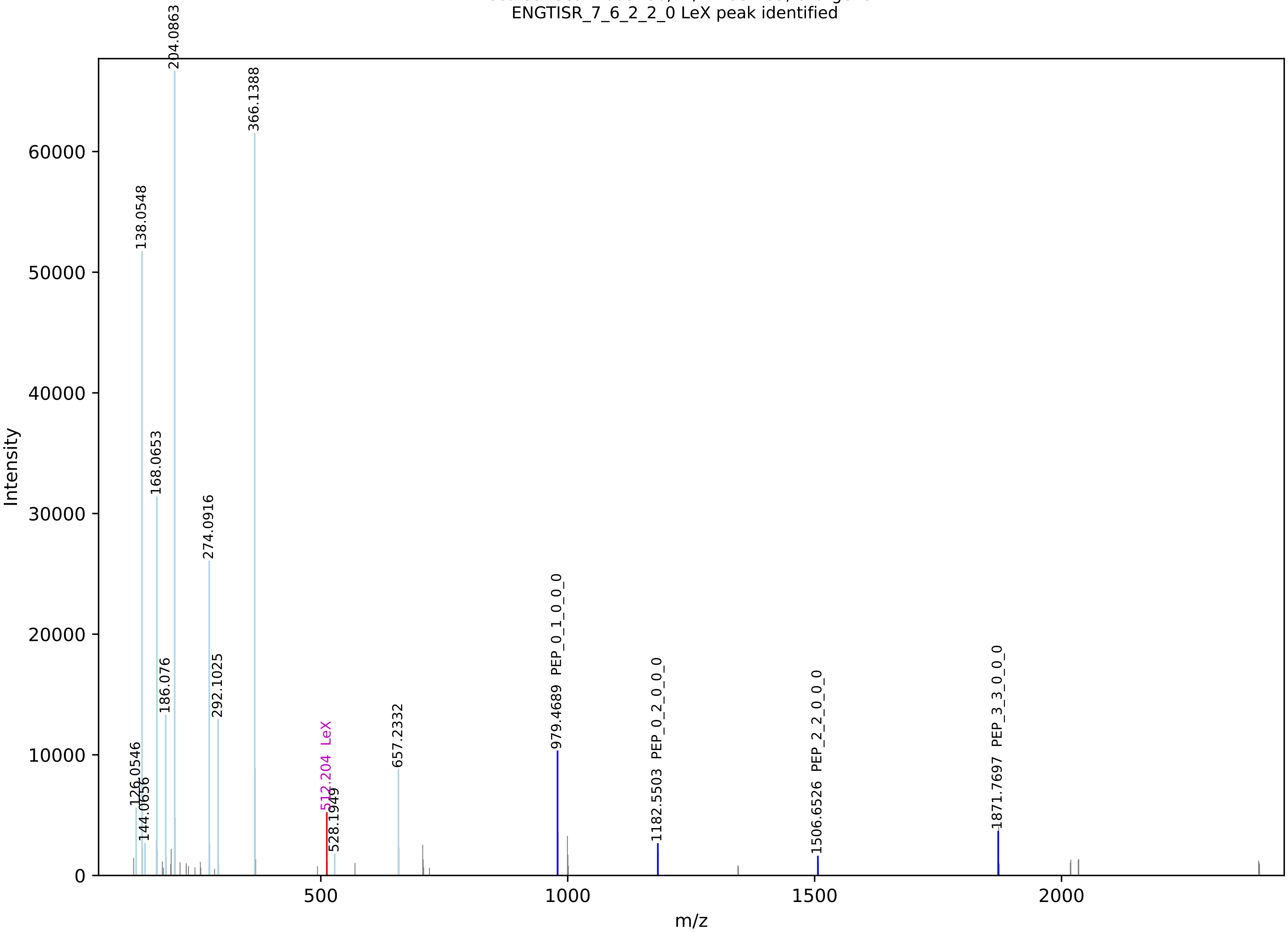

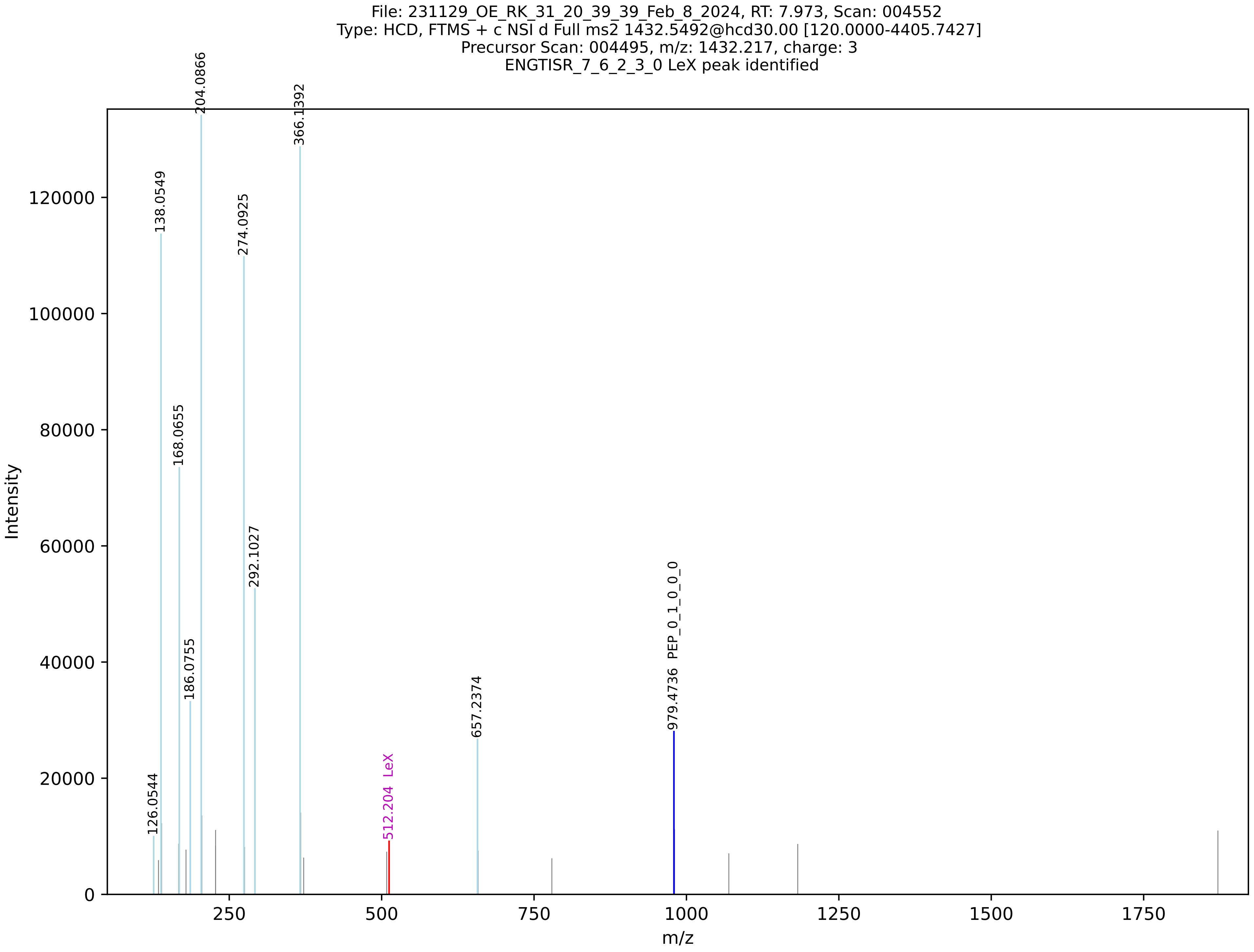
File: 231129_OE_RK_31_20_39_39_Feb_8_2024, RT: 7.973, Scan: 004552
Type: HCD, FTMS + c NSI d Full ms2 1432.5492@hcd30.00 [120.0000-4405.7427]
Precursor Scan: 004495, m/z: 1432.217, charge: 3
ENGTISR_7_6_2_3_0 LeX peak identified
204.0866
366.1392
138.0549
274.0925
168.0655
292.1027
186.0755
657.2374
979.4736 PEP_0_1_0_0_0
512.204 LeX
126.0544
Intensity
m/z
0
20000
40000
60000
80000
100000
120000
250
500
750
1000
1250
1500
1750

File: 231129_OE_RK_31_20_39_39_Feb_8_2024, RT: 8.077, Scan: 004699
Type: HCD, FTMS + c NSI d Full ms2 1038.4020@hcd30.00 [120.0000-4259.5361]
Precursor Scan: 004645, m/z: 1037.9003, charge: 4
ENGTISR_7_6_1_3_0 LeX peak identified

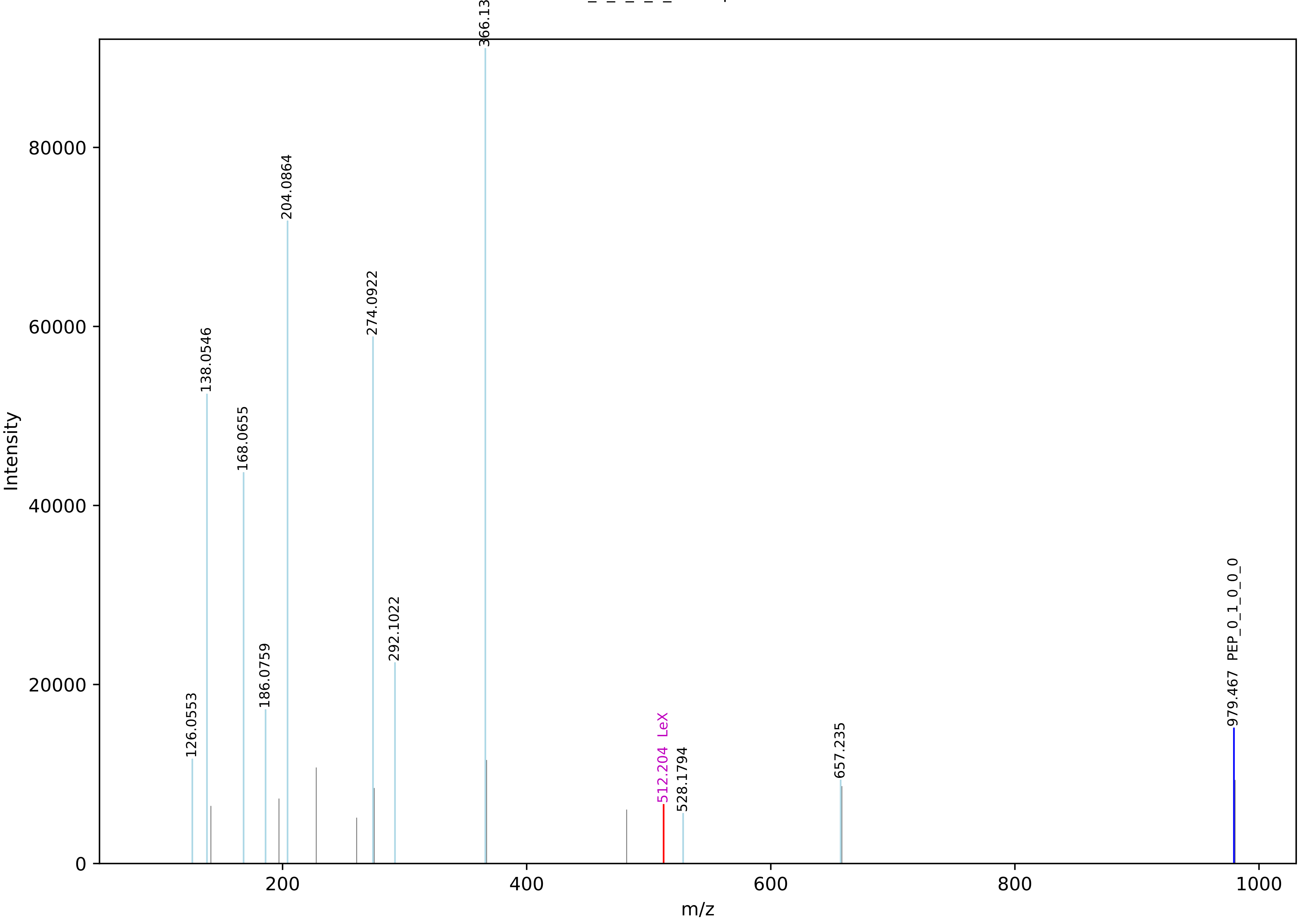

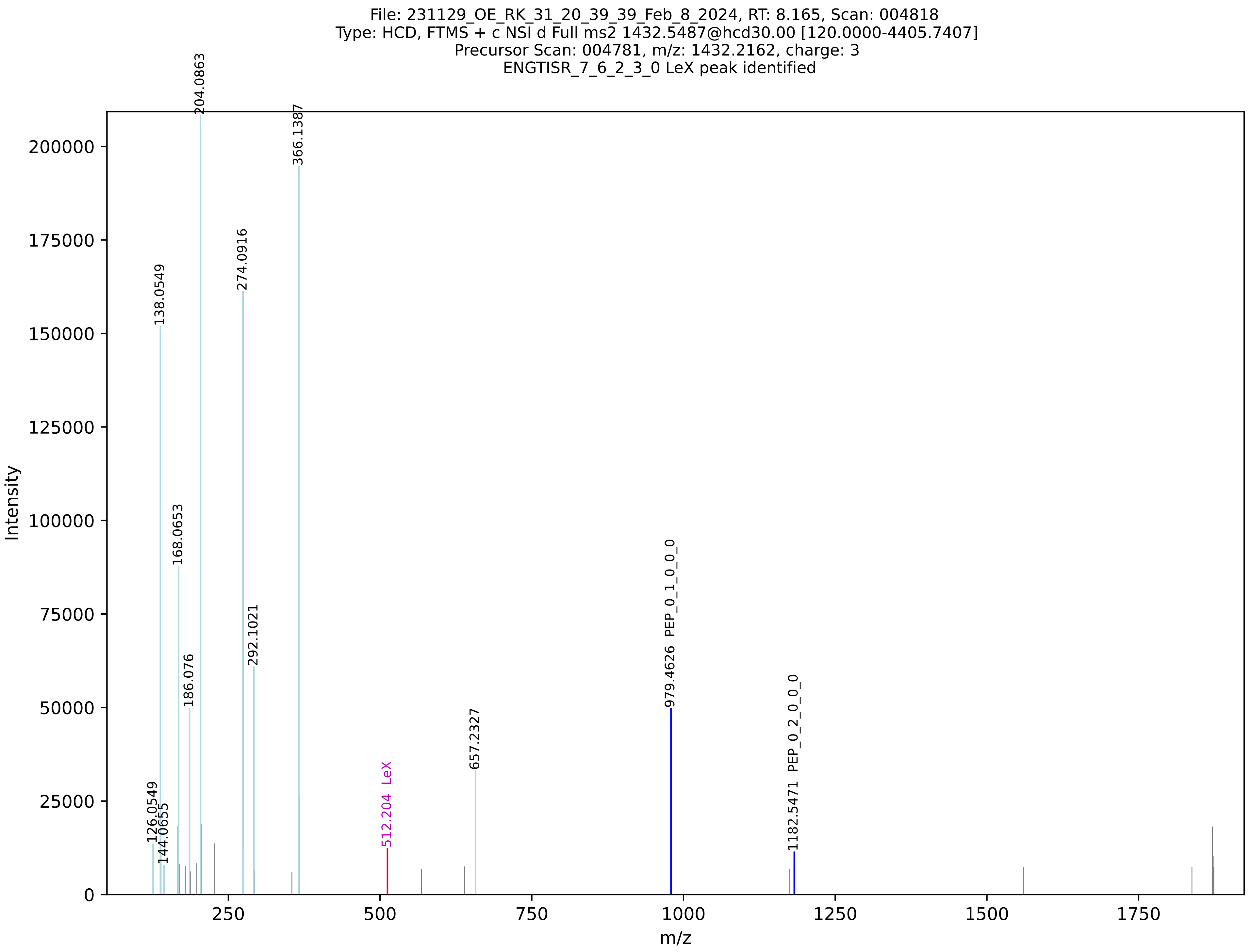
File: 231129_OE_RK_31_20_39_39_Feb_8_2024, RT: 8.165, Scan: 004818
Type: HCD, FTMS + c NSI d Full ms2 1432.5487@hcd30.00 [120.0000-4405.7407]
Precursor Scan: 004781, m/z: 1432.2162, charge: 3
ENGTISR_7_6_2_3_0 LeX peak identified
Intensity
m/z
204.0863
366.1387
274.0916
138.0549
168.0653
292.1021
186.076
657.2327
126.0549
144.0655
512.204 LeX
979.4626 PEP_0_1_0_0_0
1182.5471 PEP_0_2_0_0_0
0
25000
50000
75000
100000
125000
150000
175000
200000
250
500
750
1000
1250
1500
1750

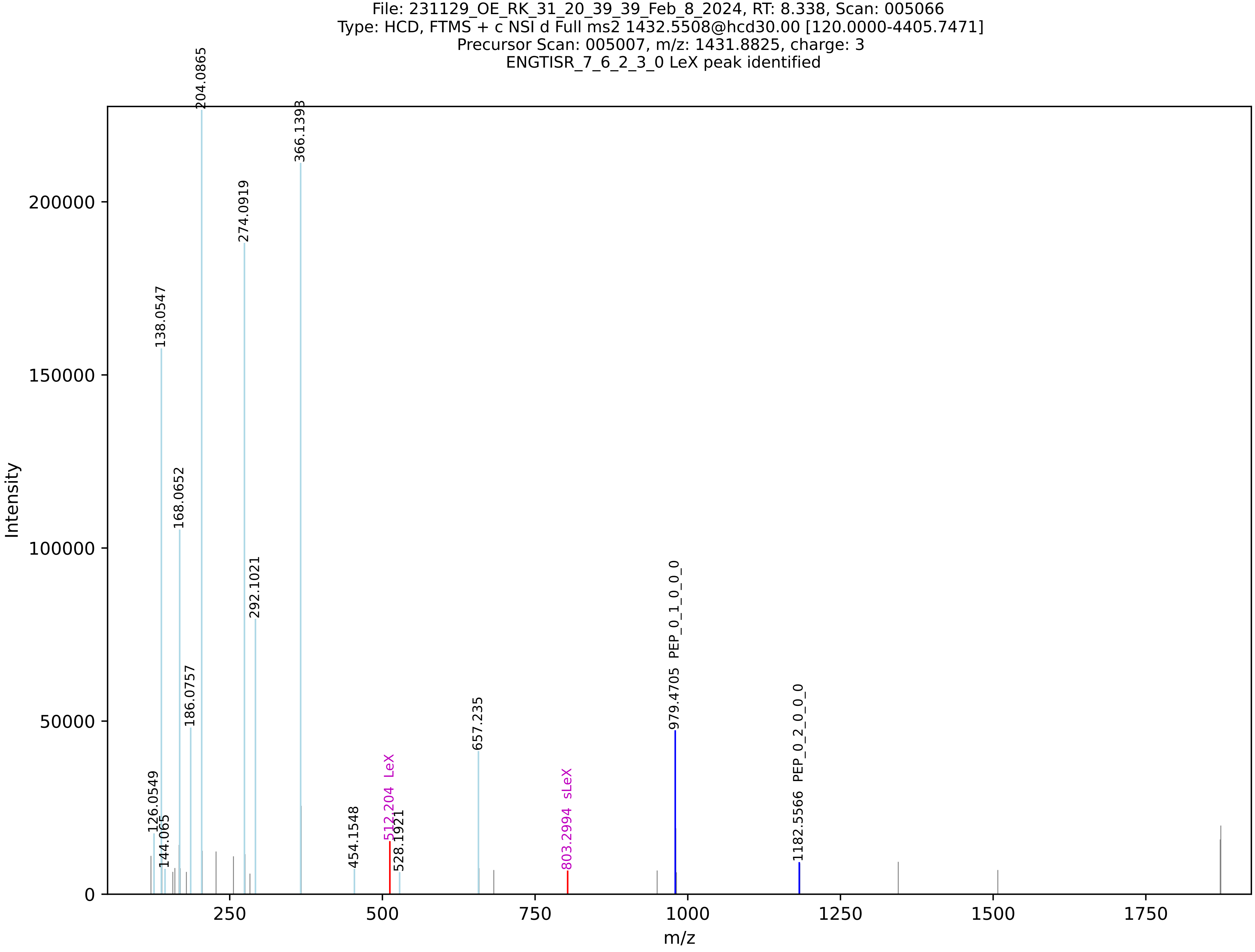

File: 231129_OE_RK_31_20_39_39_Feb_8_2024, RT: 8.338, Scan: 005066
Type: HCD, FTMS + c NSI d Full ms2 1432.5508@hcd30.00 [120.0000-4405.7471]
Precursor Scan: 005007, m/z: 1431.8825, charge: 3
ENGTISR_7_6_2_3_0 LeX peak identified
Intensity
m/z
204.0865
366.1393
274.0919
138.0547
168.0652
292.1021
186.0757
126.0549
144.065
454.1548
512.204 LeX
528.1921
657.235
803.2994 sLeX
979.4705 PEP_0_1_0_0_0
1182.5566 PEP_0_2_0_0_0

File: 231129_OE_RK_31_20_39_39_Feb_8_2024, RT: 8.341, Scan: 005070
Type: HCD, FTMS + c NSI d Full ms2 1481.5704@hcd30.00 [120.0000-4555.7476]
Precursor Scan: 005007, m/z: 1480.5669, charge: 3
ENGTISR_7_6_1_4_0 LeX peak identified

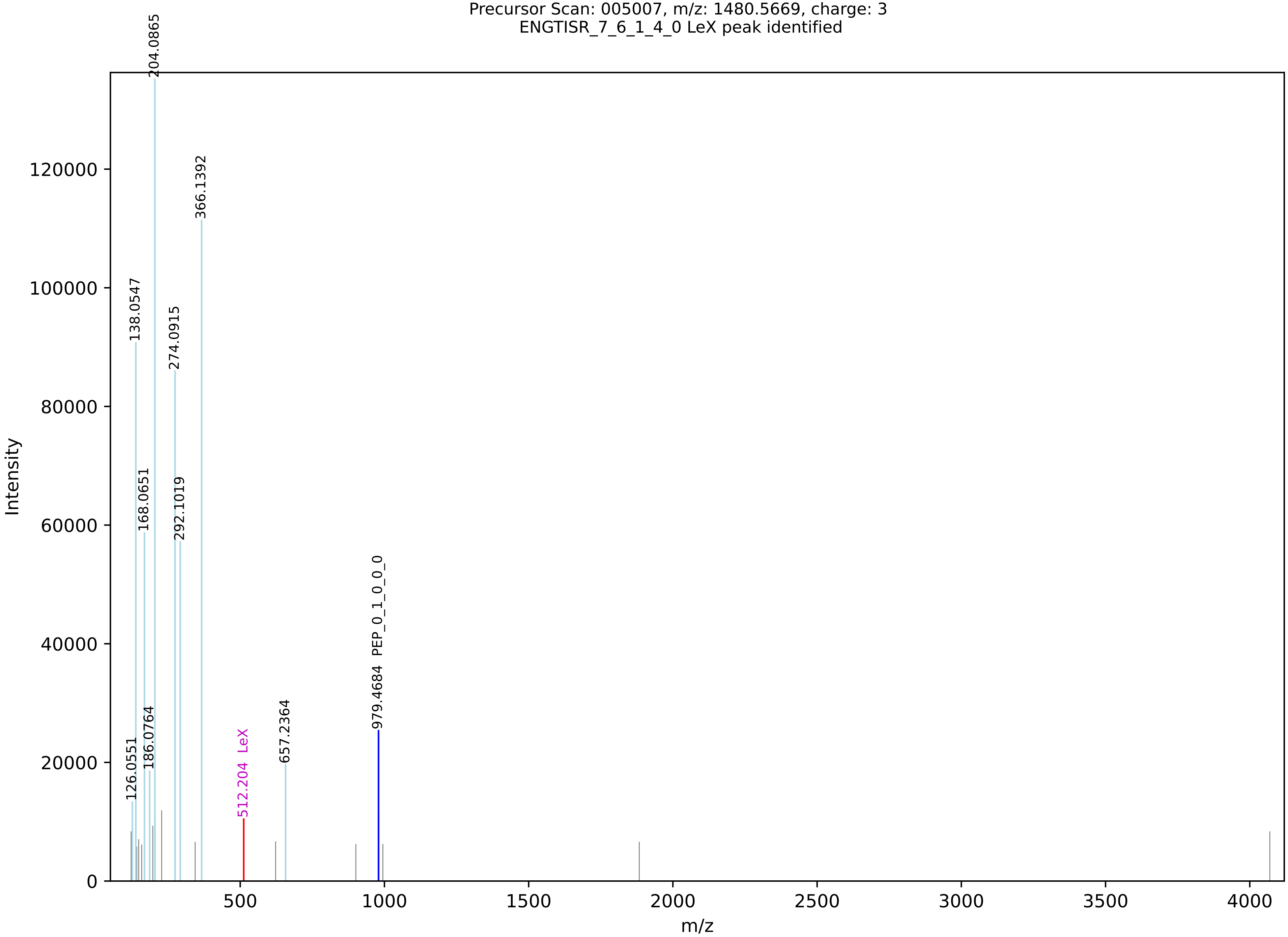

File: 231129_OE_RK_31_20_39_39_Feb_8_2024, RT: 8.341, Scan: 005071
Type: HCD, FTMS + c NSI d Full ms2 1214.1418@hcd30.00 [120.0000-3737.4160]
Precursor Scan: 005007, m/z: 1213.1395, charge: 3
ENGTISR_6_5_0_3_0 LeX peak identified

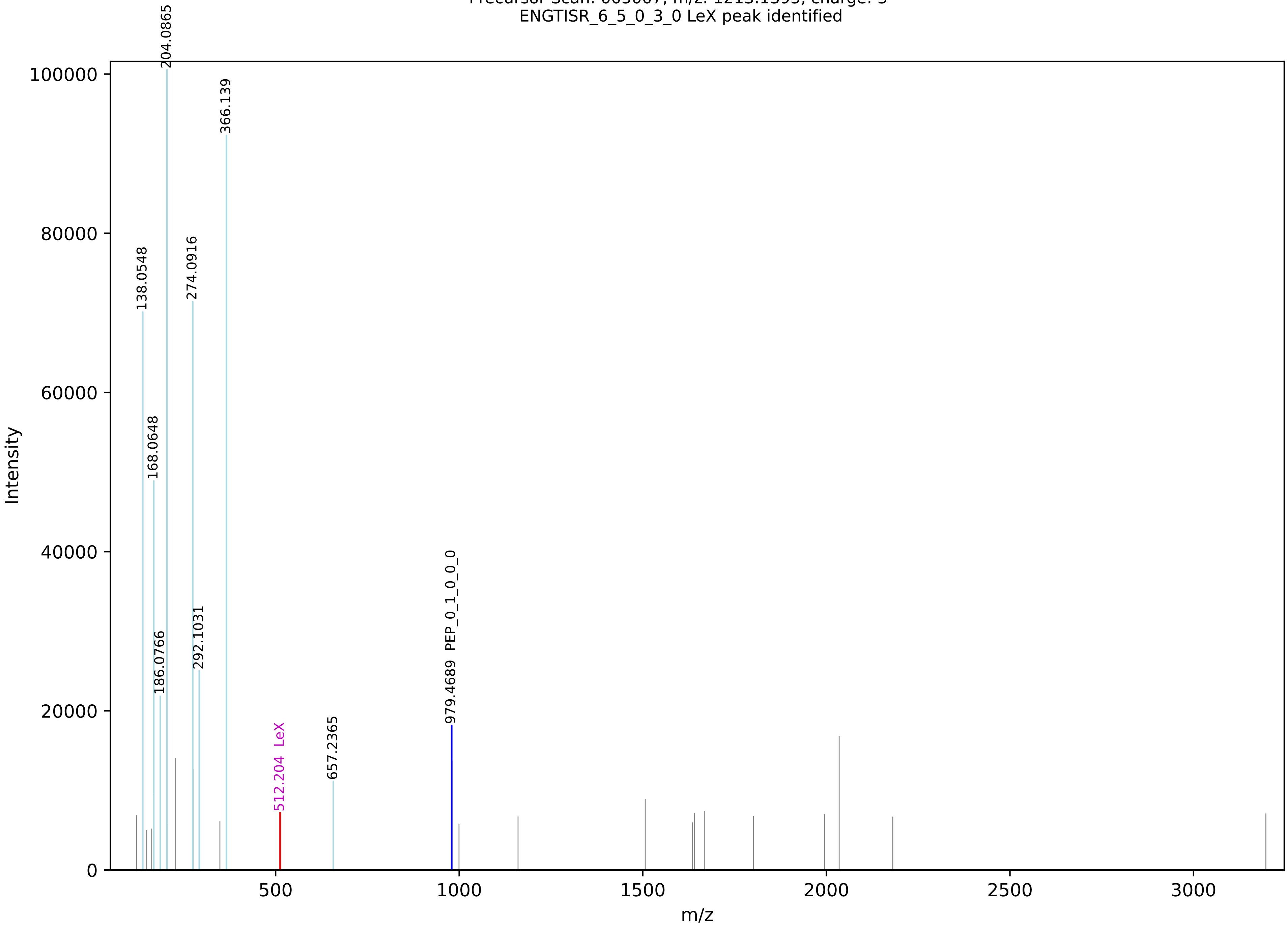

File: 231129_OE_RK_31_20_39_39_Feb_8_2024, RT: 8.482, Scan: 005271
Type: HCD, FTMS + c NSI d Full ms2 1074.9153@hcd30.00 [120.0000-4408.5103]
Precursor Scan: 005196, m/z: 1074.4141, charge: 4
ENGTISR_7_6_2_3_0 LeX peak identified

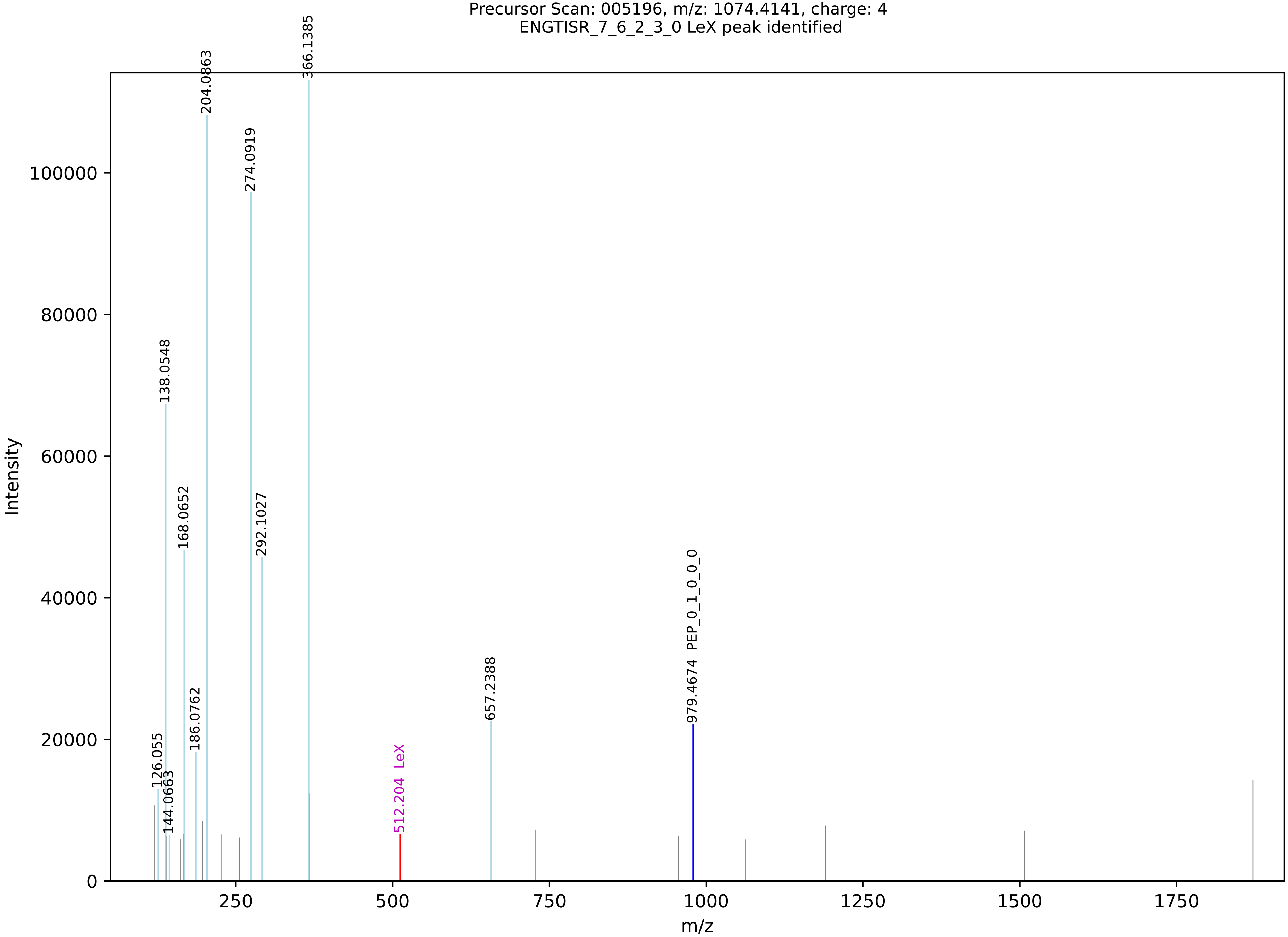

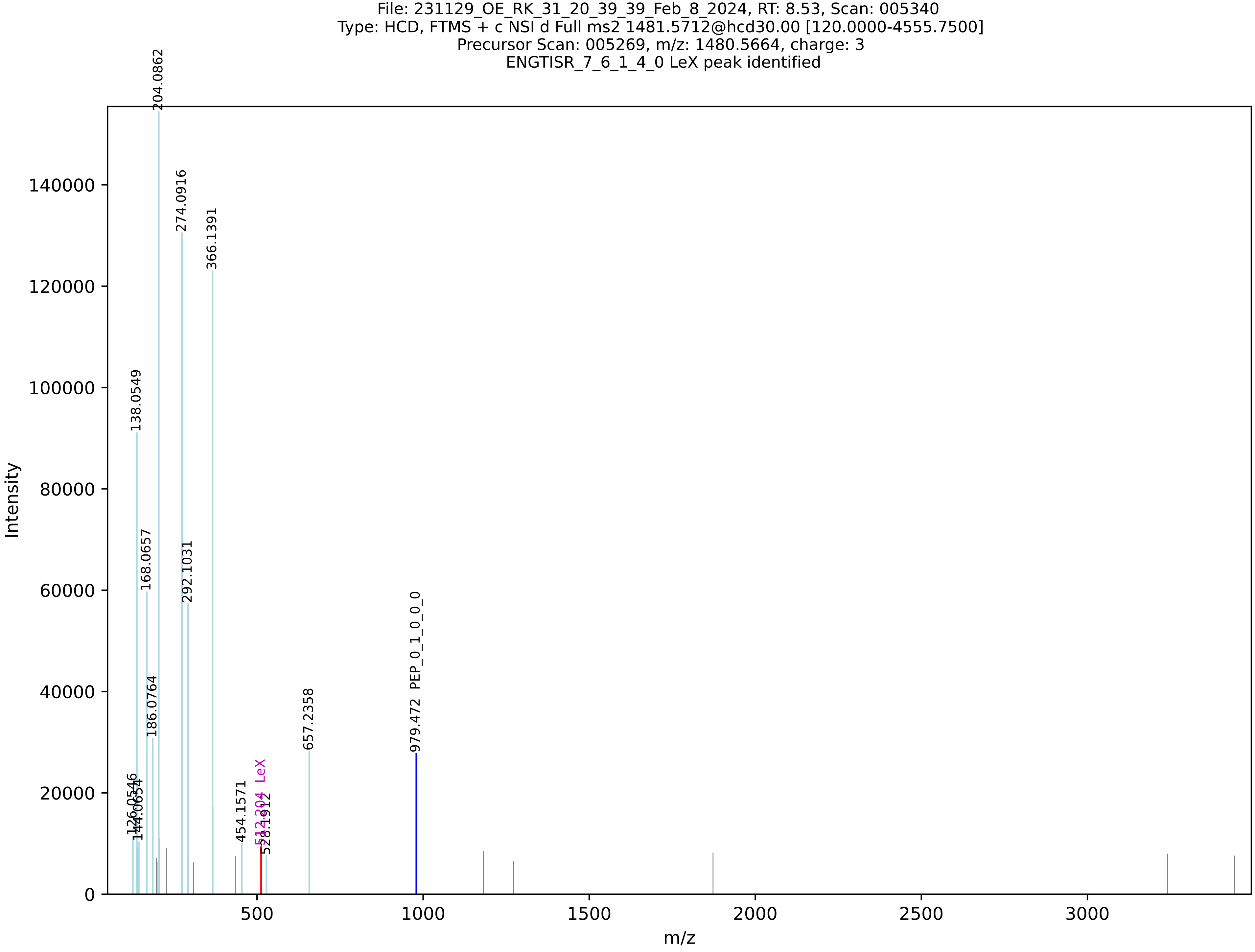

File: 231129_OE_RK_31_20_39_39_Feb_8_2024, RT: 8.53, Scan: 005340
Type: HCD, FTMS + c NSI d Full ms2 1481.5712@hcd30.00 [120.0000-4555.7500]
Precursor Scan: 005269, m/z: 1480.5664, charge: 3
ENGTISR_7_6_1_4_0 LeX peak identified
204.0862
274.0916
366.1391
138.0549
168.0657
292.1031
979.472 PEP_0_1_0_0_0_
186.0764
657.2358
LeX
126.0546
144.0654
454.1571
512.204
528.1912
Intensity
m/z
0
20000
40000
60000
80000
100000
120000
140000
500
1000
1500
2000
2500
3000

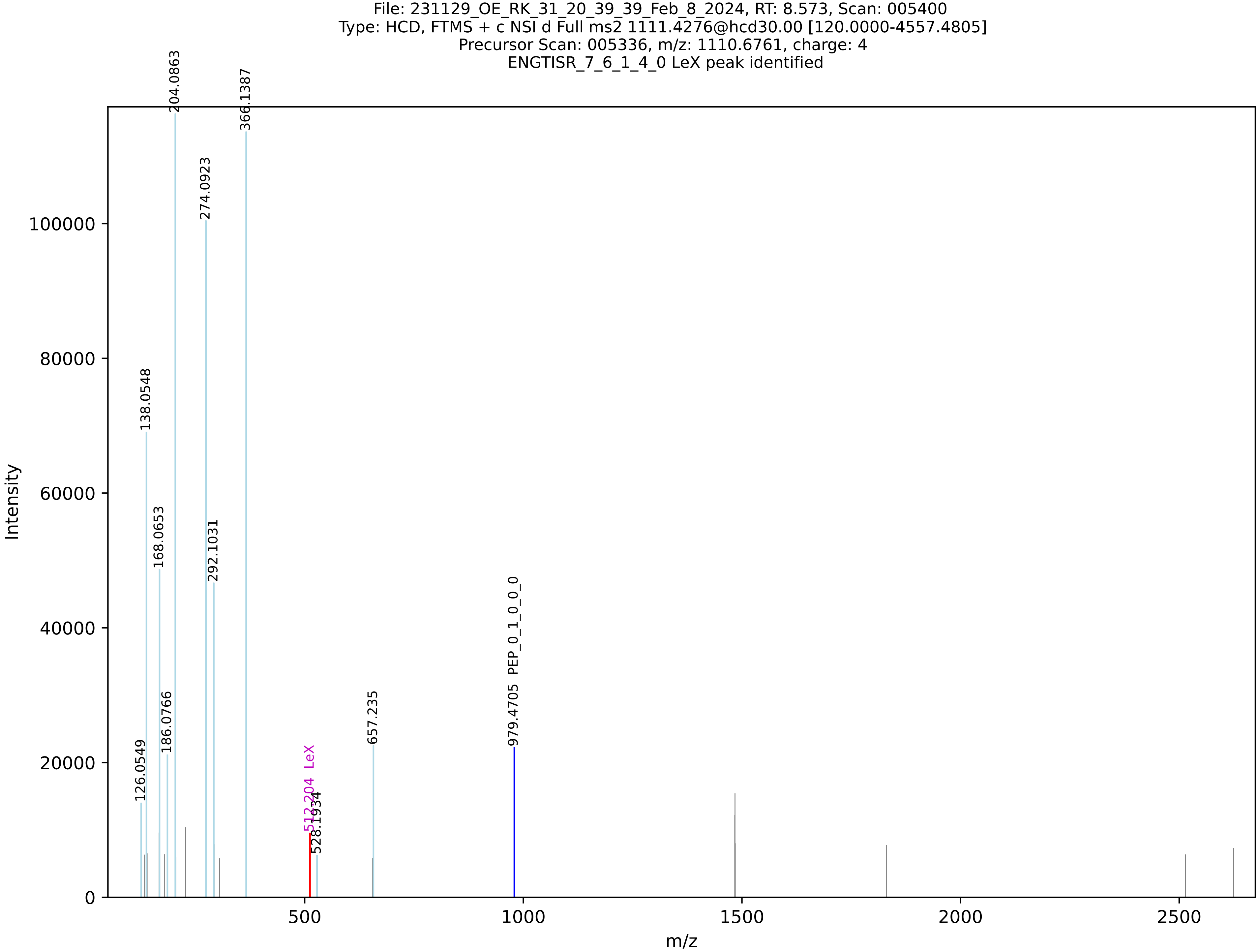
File: 231129_OE_RK_31_20_39_39_Feb_8_2024, RT: 8.573, Scan: 005400
Type: HCD, FTMS + c NSI d Full ms2 1111.4276@hcd30.00 [120.0000-4557.4805]
Precursor Scan: 005336, m/z: 1110.6761, charge: 4
ENGTISR_7_6_1_4_0 LeX peak identified
204.0863
366.1387
274.0923
138.0548
168.0653
292.1031
186.0766
126.0549
512.204 LeX
528.1934
657.235
979.4705 PEP_0_1_0_0_0
Intensity
m/z
0
20000
40000
60000
80000
100000
500
1000
1500
2000
2500

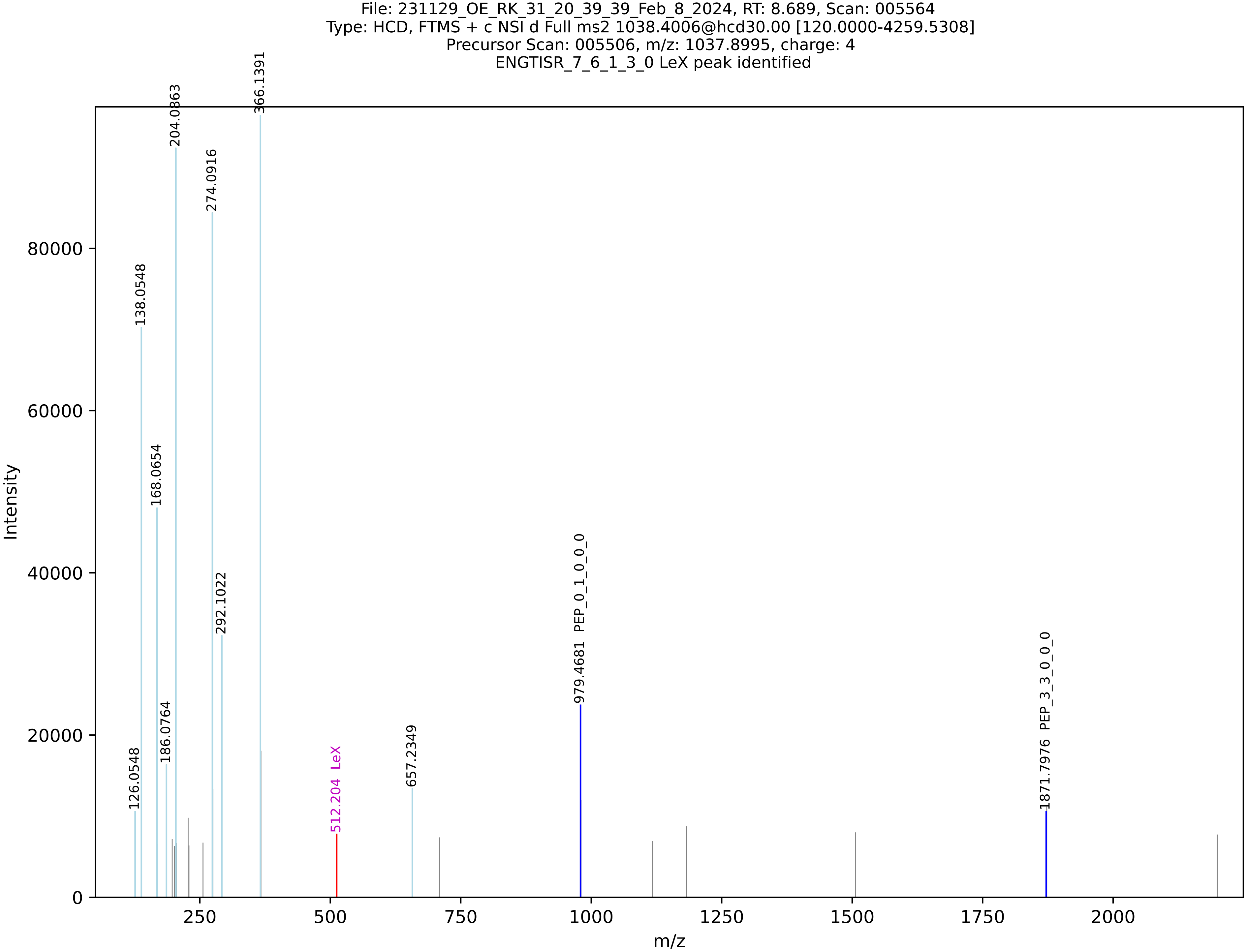
File: 231129_OE_RK_31_20_39_39_Feb_8_2024, RT: 8.689, Scan: 005564
Type: HCD, FTMS + c NSI d Full ms2 1038.4006@hcd30.00 [120.0000-4259.5308]
Precursor Scan: 005506, m/z: 1037.8995, charge: 4
ENGTISR_7_6_1_3_0 LeX peak identified
Intensity
m/z
0
20000
40000
60000
80000
250
500
750
1000
1250
1500
1750
2000
366.1391
204.0863
274.0916
138.0548
168.0654
292.1022
186.0764
126.0548
512.204 LeX
657.2349
979.4681 PEP_0_1_0_0_0_
1871.7976 PEP_3_3_0_0_0_

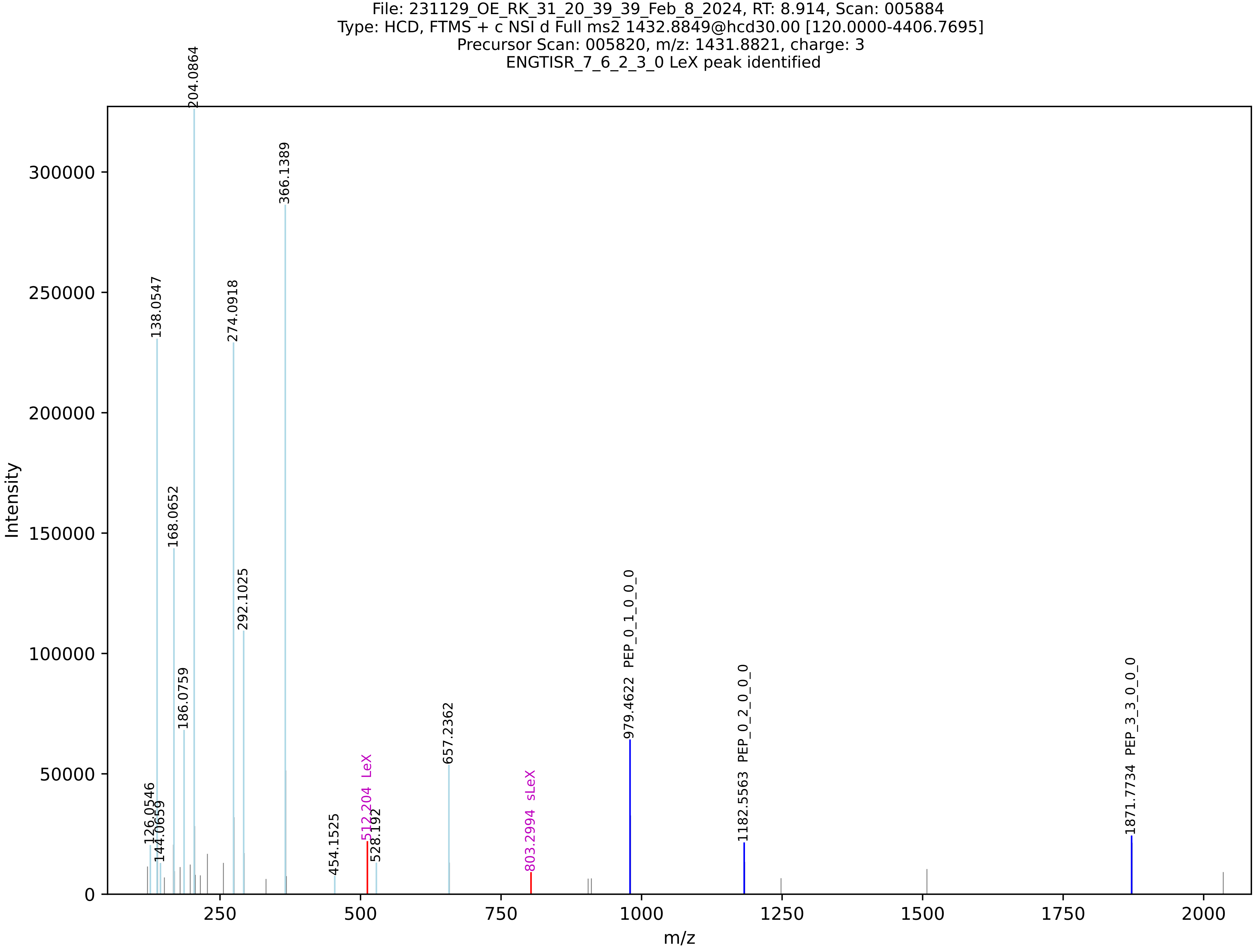

File: 231129_OE_RK_31_20_39_39_Feb_8_2024, RT: 8.914, Scan: 005884
Type: HCD, FTMS + c NSI d Full ms2 1432.8849@hcd30.00 [120.0000-4406.7695]
Precursor Scan: 005820, m/z: 1431.8821, charge: 3
ENGTISR_7_6_2_3_0 LeX peak identified
Intensity
m/z
204.0864
366.1389
138.0547
274.0918
168.0652
292.1025
186.0759
126.0546
144.0659
454.1525
512.204 LeX
528.192
657.2362
803.2994 sLeX
979.4622 PEP_0_1_0_0_0
1182.5563 PEP_0_2_0_0_0
1871.7734 PEP_3_3_0_0_0

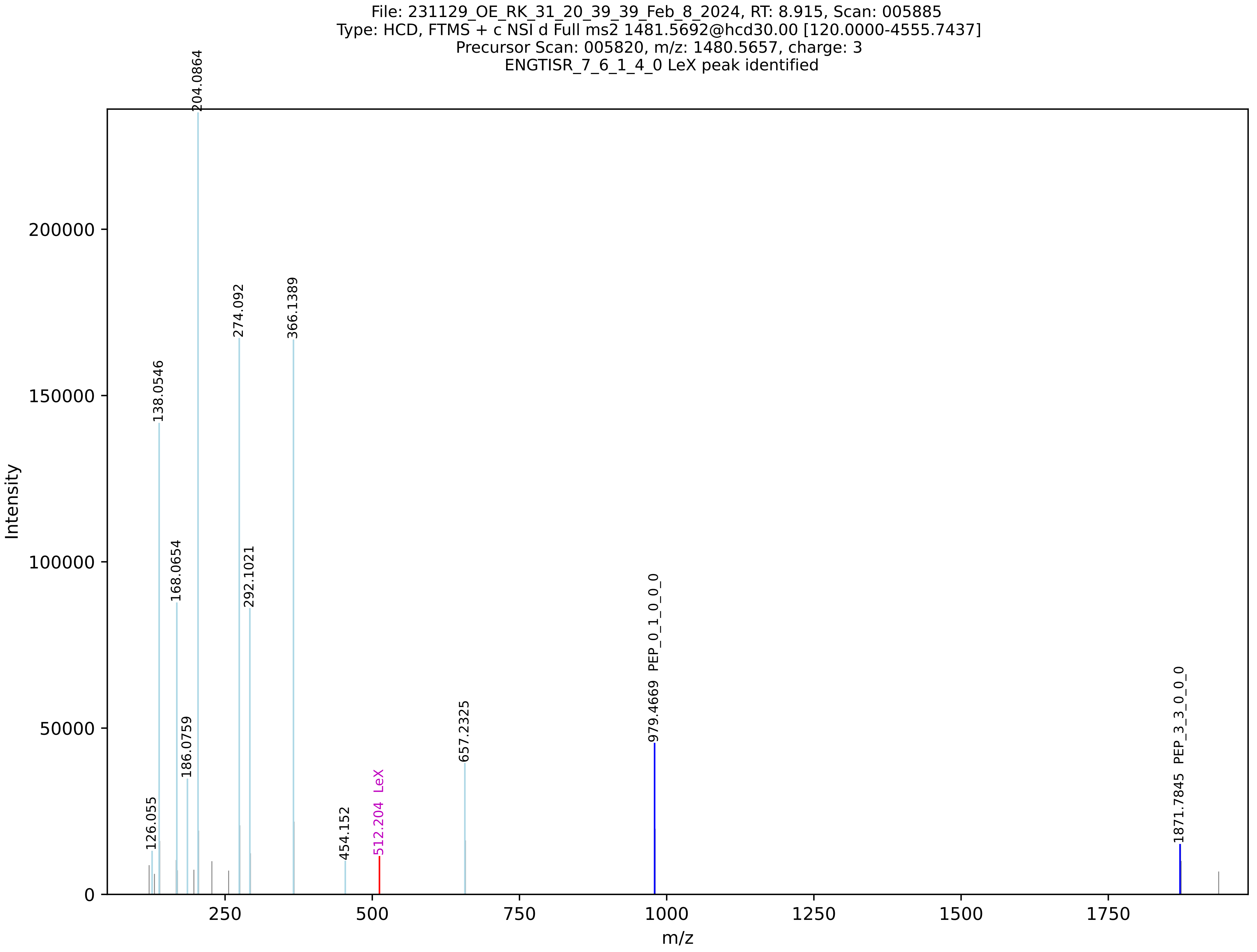
File: 231129_OE_RK_31_20_39_39_Feb_8_2024, RT: 8.915, Scan: 005885
Type: HCD, FTMS + c NSI d Full ms2 1481.5692@hcd30.00 [120.0000-4555.7437]
Precursor Scan: 005820, m/z: 1480.5657, charge: 3
ENGTISR_7_6_1_4_0 LeX peak identified
Intensity
m/z
204.0864
274.092
366.1389
138.0546
168.0654
292.1021
979.4669 PEP_0_1_0_0_0
1871.7845 PEP_3_3_0_0_0
657.2325
186.0759
512.204 LeX
126.055
454.152
0
50000
100000
150000
200000
250
500
750
1000
1250
1500
1750

File: 231129_OE_RK_31_20_39_39_Feb_8_2024, RT: 9.101, Scan: 006149
Type: HCD, FTMS + c NSI d Full ms2 1432.8838@hcd30.00 [120.0000-4406.7666]
Precursor Scan: 006076, m/z: 1431.8815, charge: 3
ENGTISR_7_6_2_3_0 LeX peak identified

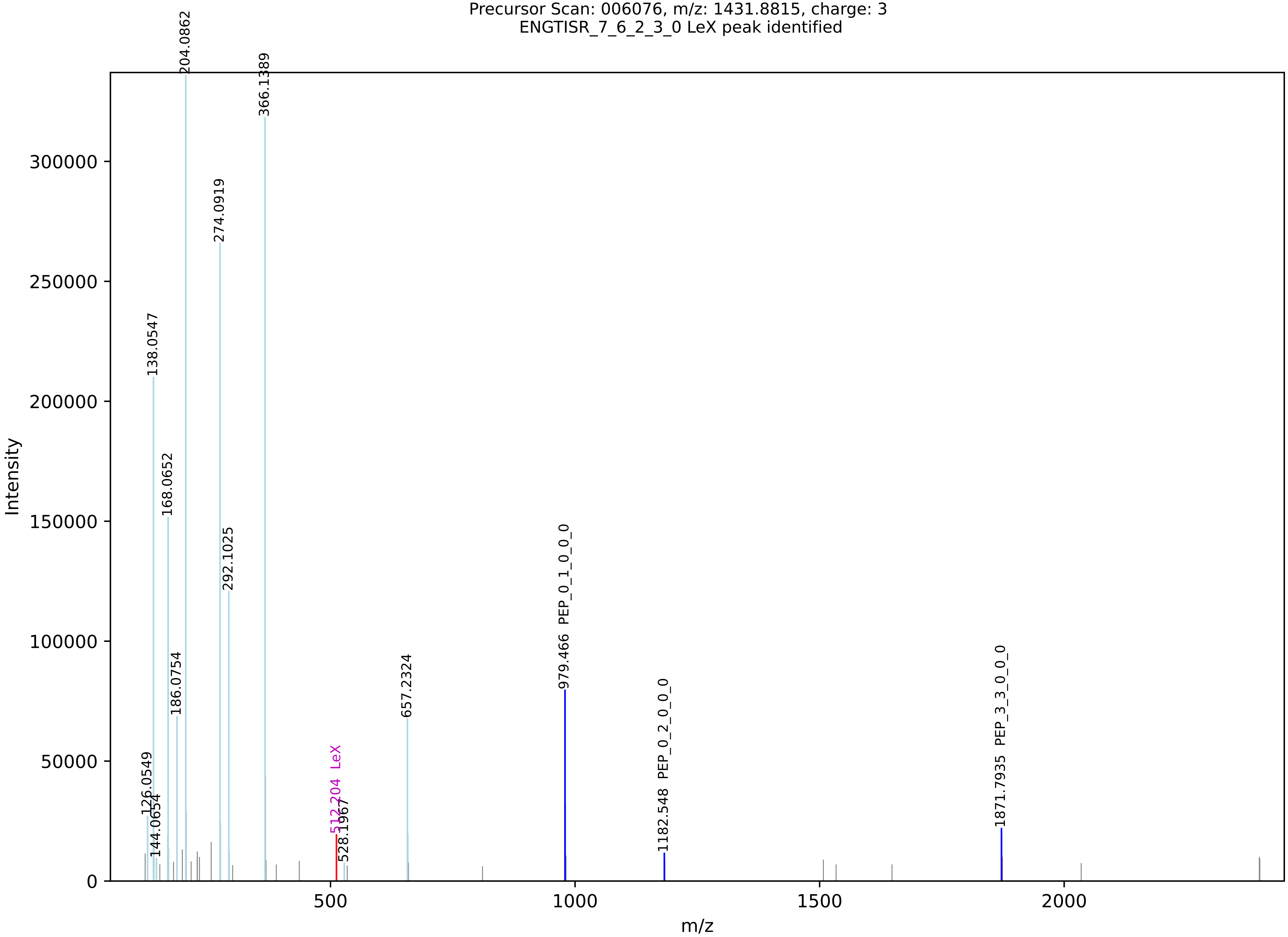

File: 231129_OE_RK_31_20_39_39_Feb_8_2024, RT: 9.102, Scan: 006150
Type: HCD, FTMS + c NSI d Full ms2 1481.2355@hcd30.00 [120.0000-4554.7227]
Precursor Scan: 006076, m/z: 1480.5654, charge: 3
ENGTISR_7_6_1_4_0 LeX peak identified

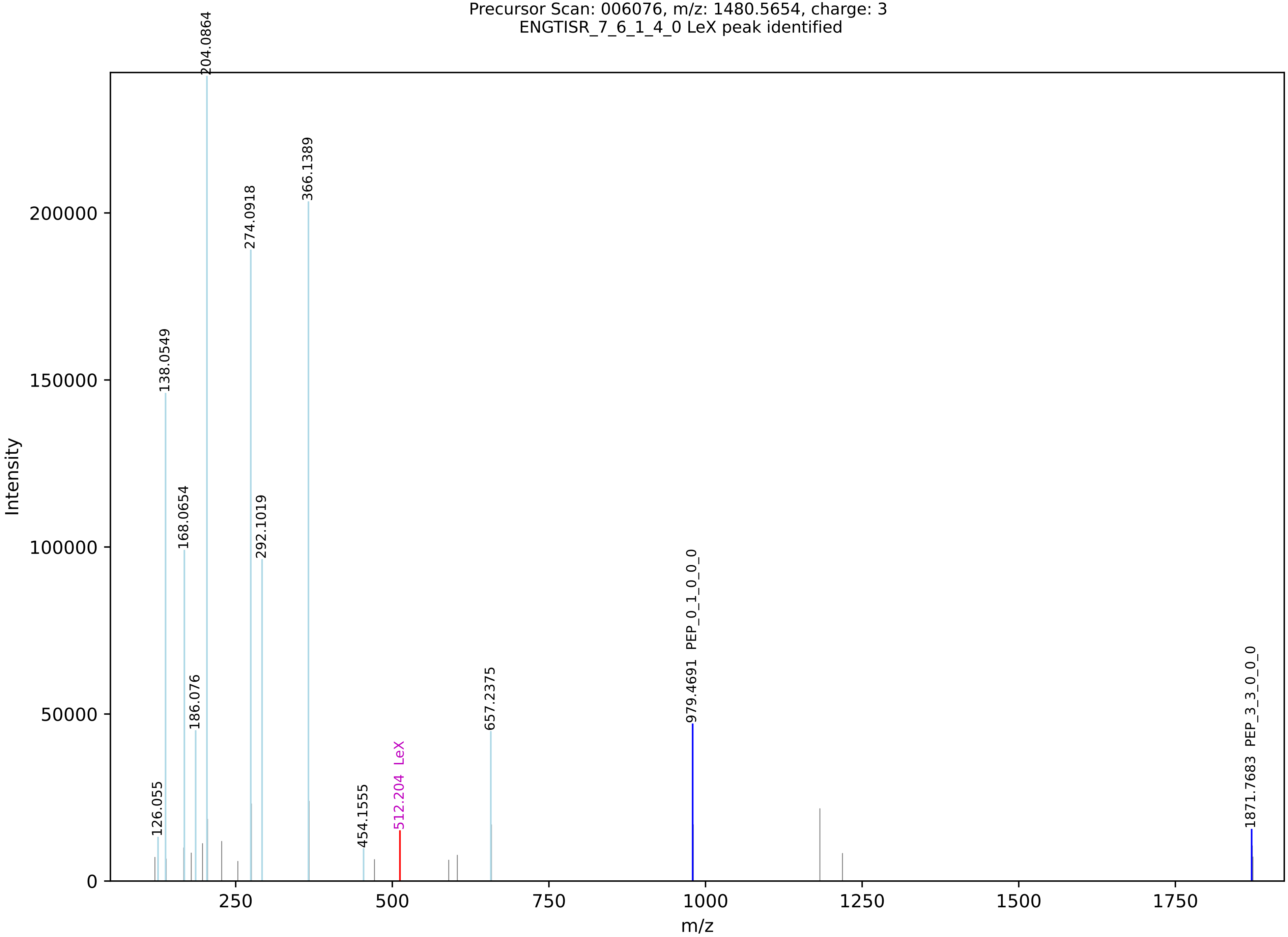

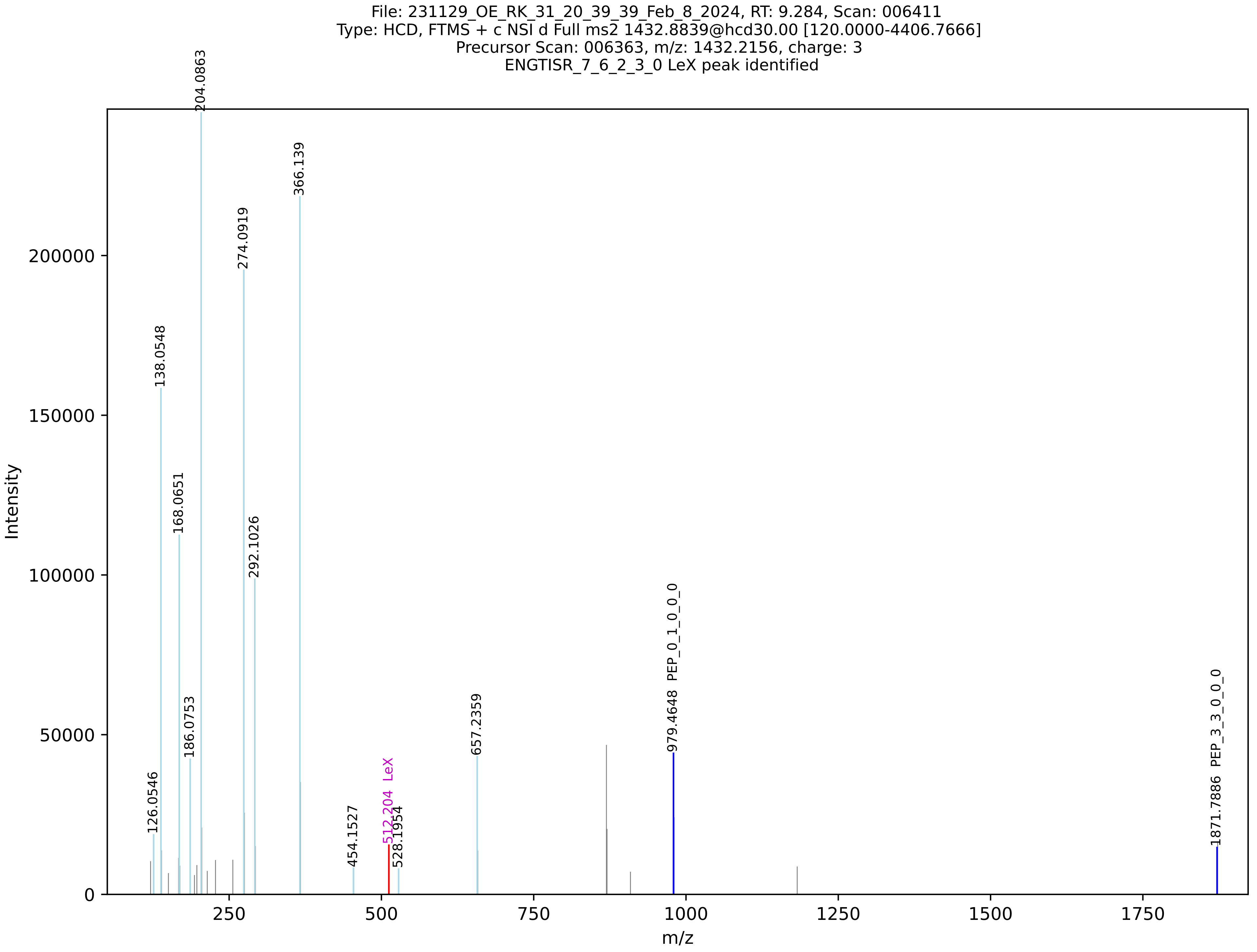
File: 231129_OE_RK_31_20_39_39_Feb_8_2024, RT: 9.284, Scan: 006411
Type: HCD, FTMS + c NSI d Full ms2 1432.8839@hcd30.00 [120.0000-4406.7666]
Precursor Scan: 006363, m/z: 1432.2156, charge: 3
ENGTISR_7_6_2_3_0 LeX peak identified
204.0863
366.139
274.0919
138.0548
168.0651
292.1026
186.0753
126.0546
454.1527
512.204 LeX
528.1954
657.2359
979.4648 PEP_0_1_0_0_0
1871.7886 PEP_3_3_0_0_0
Intensity
m/z
0
50000
100000
150000
200000
250
500
750
1000
1250
1500
1750

File: 231129_OE_RK_31_20_39_39_Feb_8_2024, RT: 9.383, Scan: 006533
Type: HCD, FTMS + c NSI d Full ms2 1111.1755@hcd30.00 [120.0000-4556.4521]
Precursor Scan: 006501, m/z: 1110.675, charge: 4
ENGTISR_7_6_1_4_0 LeX peak identified

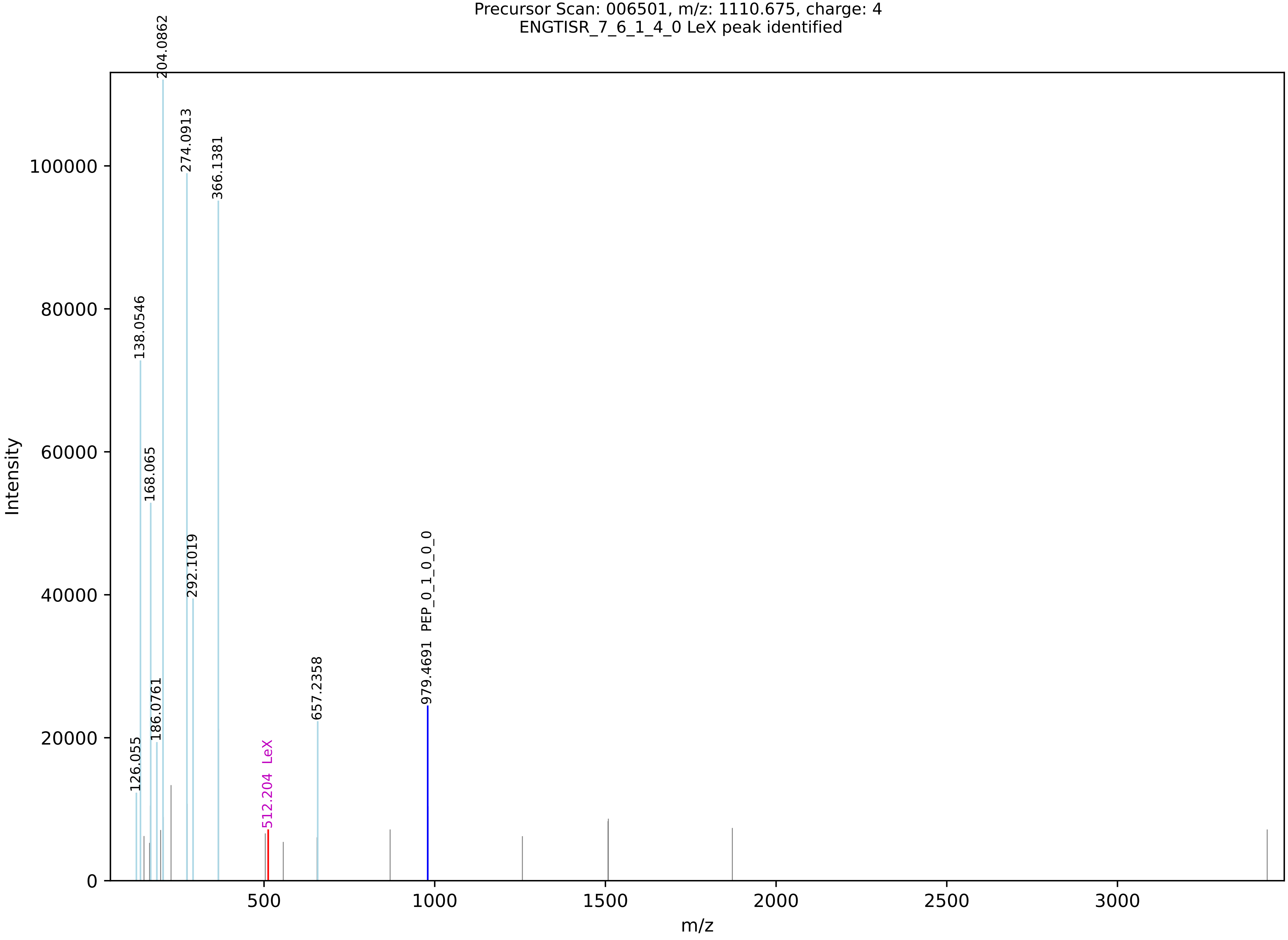

File: 231129_OE_RK_31_20_39_39_Feb_8_2024, RT: 9.499, Scan: 006689
Type: HCD, FTMS + c NSI d Full ms2 1481.2332@hcd30.00 [120.0000-4554.7153]
Precursor Scan: 006648, m/z: 1480.5649, charge: 3
ENGTISR_7_6_1_4_0 LeX peak identified

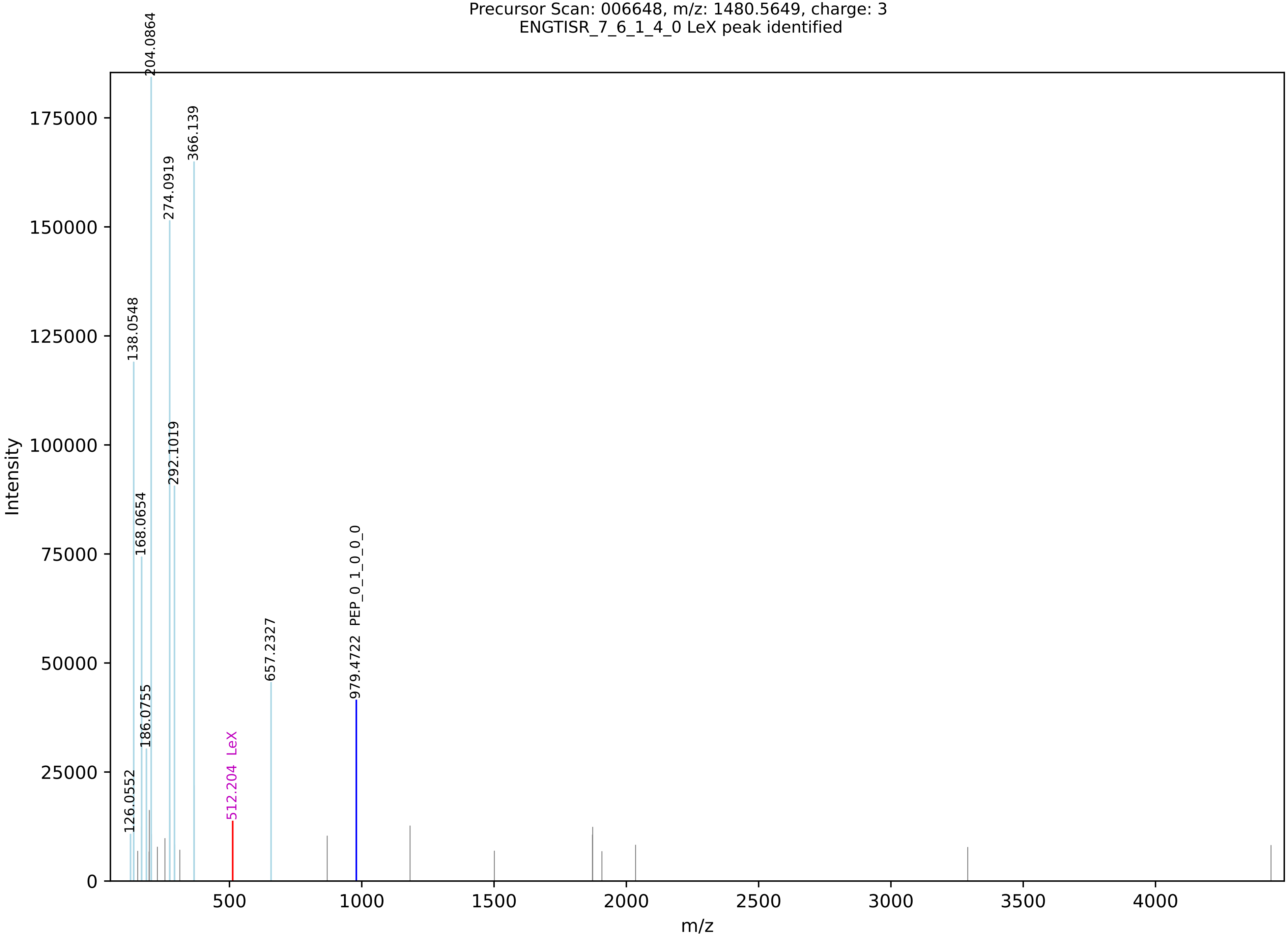

File: 231129_OE_RK_31_20_39_39_Feb_8_2024, RT: 9.691, Scan: 006953
Type: HCD, FTMS + c NSI d Full ms2 1432.8829@hcd30.00 [120.0000-4406.7637]
Precursor Scan: 006897, m/z: 1431.8796, charge: 3
ENGTISR_7_6_2_3_0 LeX peak identified

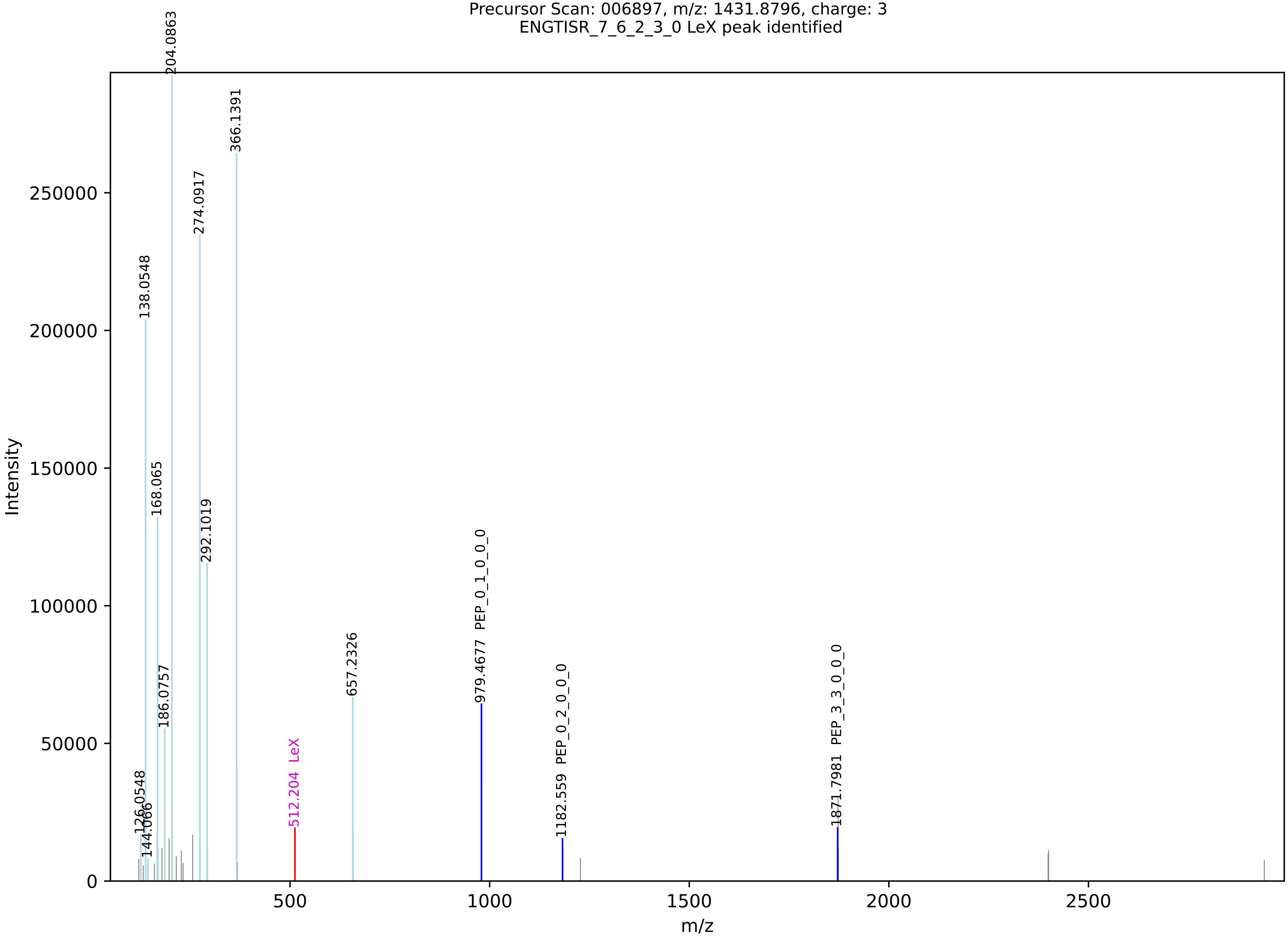

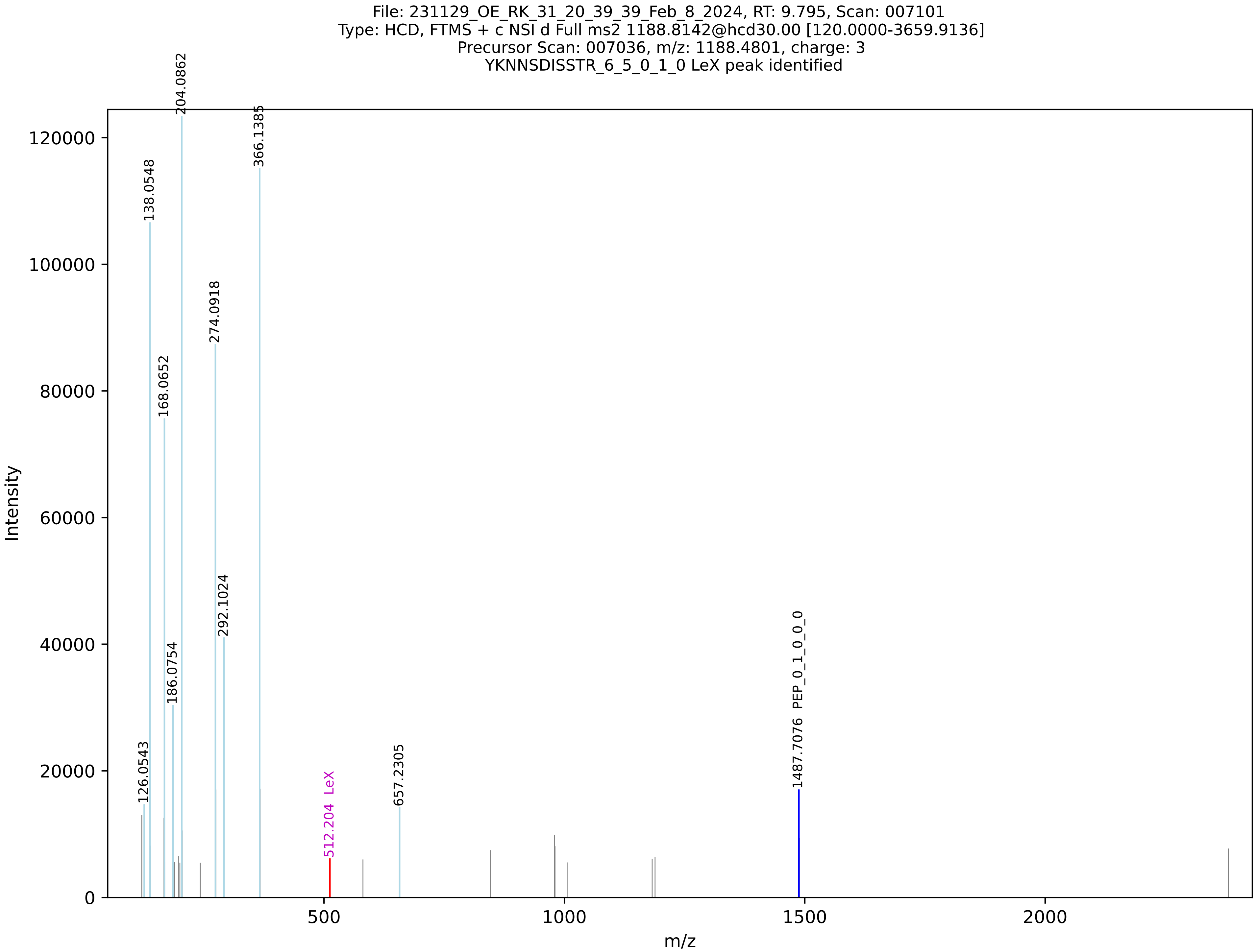
File: 231129_OE_RK_31_20_39_39_Feb_8_2024, RT: 9.795, Scan: 007101
Type: HCD, FTMS + c NSI d Full ms2 1188.8142@hcd30.00 [120.0000-3659.9136]
Precursor Scan: 007036, m/z: 1188.4801, charge: 3
YKNNSDISSTR_6_5_0_1_0 LeX peak identified
Intensity
m/z
204.0862
366.1385
138.0548
274.0918
168.0652
292.1024
186.0754
126.0543
512.204 LeX
657.2305
1487.7076 PEP_0_1_0_0_0

File: 231129_OE_RK_31_20_39_39_Feb_8_2024, RT: 10.027, Scan: 007433
Type: HCD, FTMS + c NSI d Full ms2 1038.1494@hcd30.00 [120.0000-4258.5059]
Precursor Scan: 007381, m/z: 1037.8998, charge: 4
ENGTISR_7_6_1_3_0 LeX peak identified

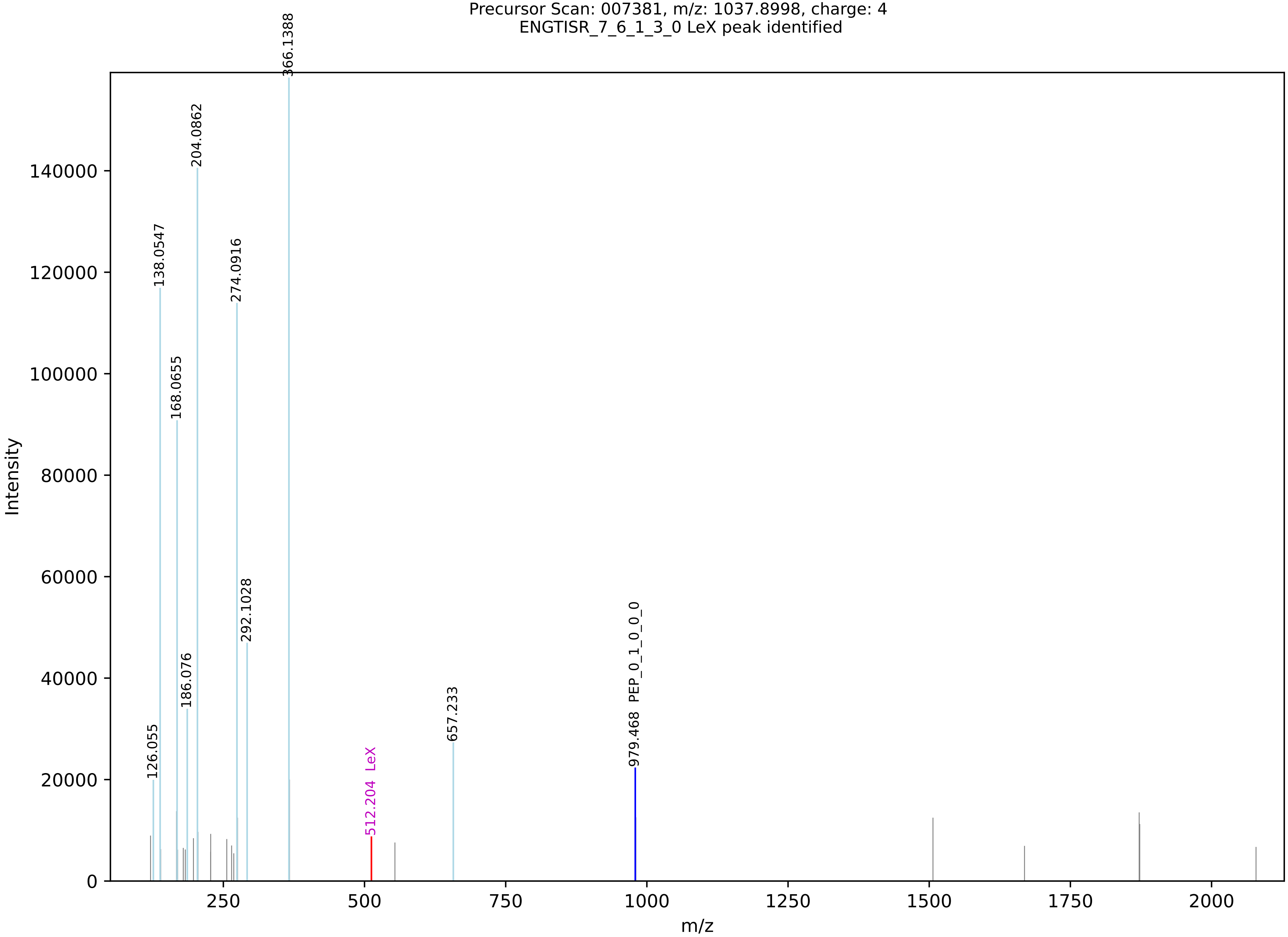

File: 231129_OE_RK_31_20_39_39_Feb_8_2024, RT: 10.142, Scan: 007598
Type: HCD, FTMS + c NSI d Full ms2 924.3780@hcd30.00 [120.0000-3794.3181]
Precursor Scan: 007508, m/z: 923.8768, charge: 4
YKNNSDISSTR_5_5_0_2_0 LeX peak identified

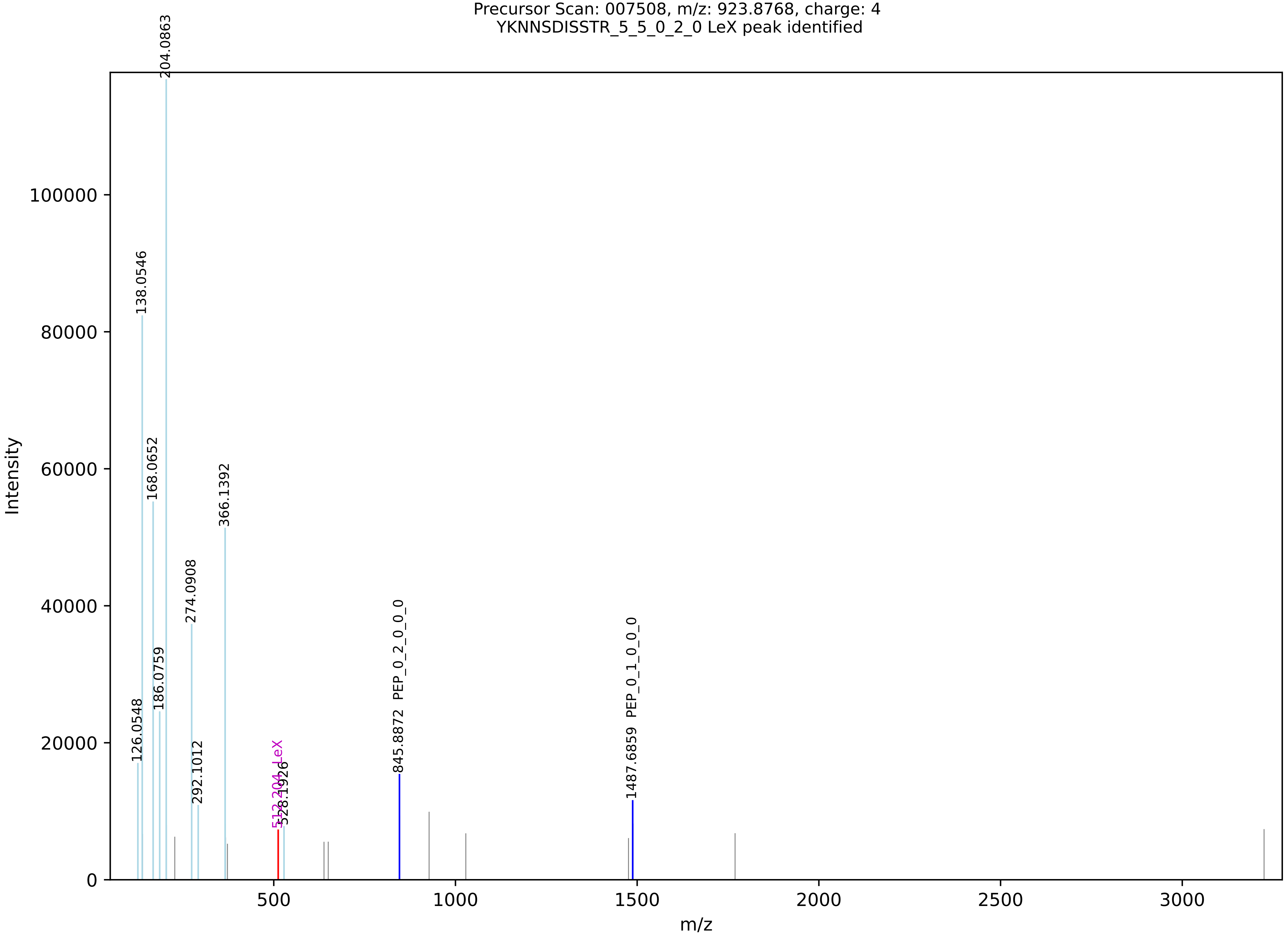

File: 231129_OE_RK_31_20_39_39_Feb_8_2024, RT: 10.335, Scan: 007876
Type: HCD, FTMS + c NSI d Full ms2 1115.7891@hcd30.00 [120.0000-3436.4568]
Precursor Scan: 007788, m/z: 1115.455, charge: 3
YKNNSDISSTR_5_4_1_1_0 LeX peak identified

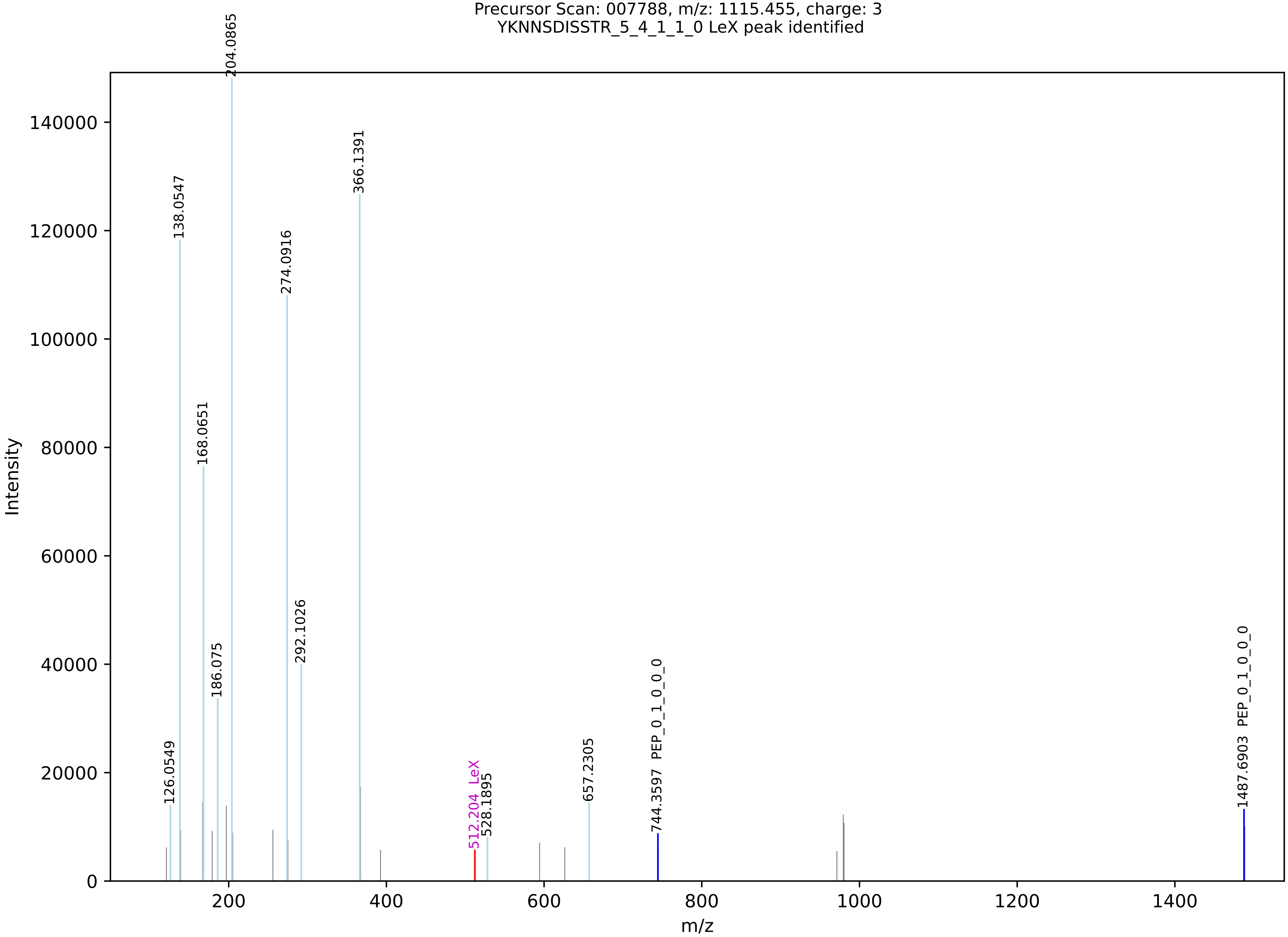

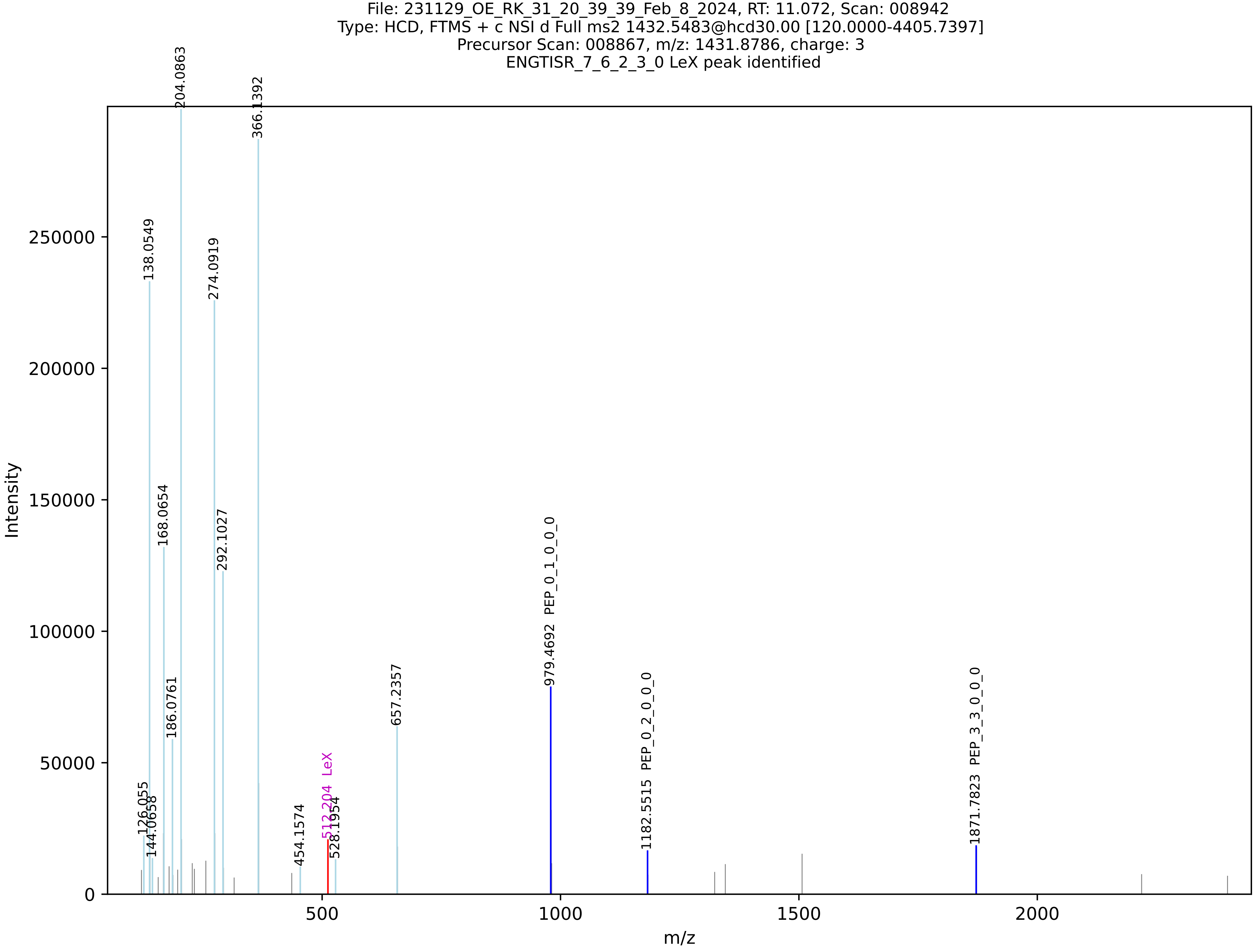

File: 231129_OE_RK_31_20_39_39_Feb_8_2024, RT: 11.072, Scan: 008942
Type: HCD, FTMS + c NSI d Full ms2 1432.5483@hcd30.00 [120.0000-4405.7397]
Precursor Scan: 008867, m/z: 1431.8786, charge: 3
ENGTISR_7_6_2_3_0 LeX peak identified
204.0863
366.1392
138.0549
274.0919
168.0654
292.1027
186.0761
126.055
144.0658
454.1574
512.204 LeX
528.1954
657.2357
979.4692 PEP_0_1_0_0_0
1182.5515 PEP_0_2_0_0_0
1871.7823 PEP_3_3_0_0_0
Intensity
m/z

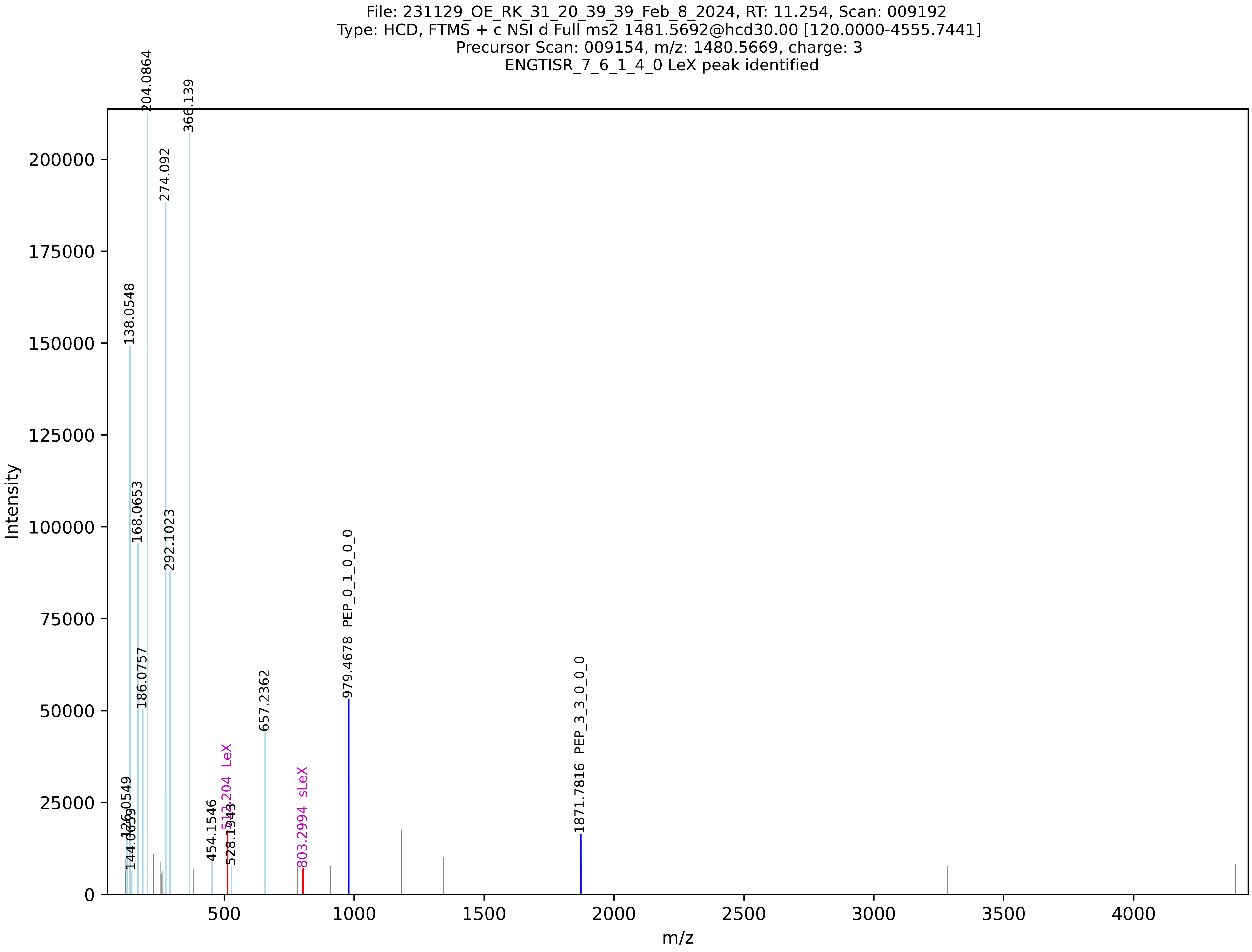
File: 231129_OE_RK_31_20_39_39_Feb_8_2024, RT: 11.254, Scan: 009192
Type: HCD, FTMS + c NSI d Full ms2 1481.5692@hcd30.00 [120.0000-4555.7441]
Precursor Scan: 009154, m/z: 1480.5669, charge: 3
ENGTISR_7_6_1_4_0 LeX peak identified
Intensity
m/z
204.0864
366.139
274.092
138.0548
168.0653
292.1023
186.0757
657.2362
979.4678 PEP_0_1_0_0_0
1871.7816 PEP_3_3_0_0_0
512.204 LeX
803.2994 sLeX
454.1546
528.1943
126.0549
144.0659

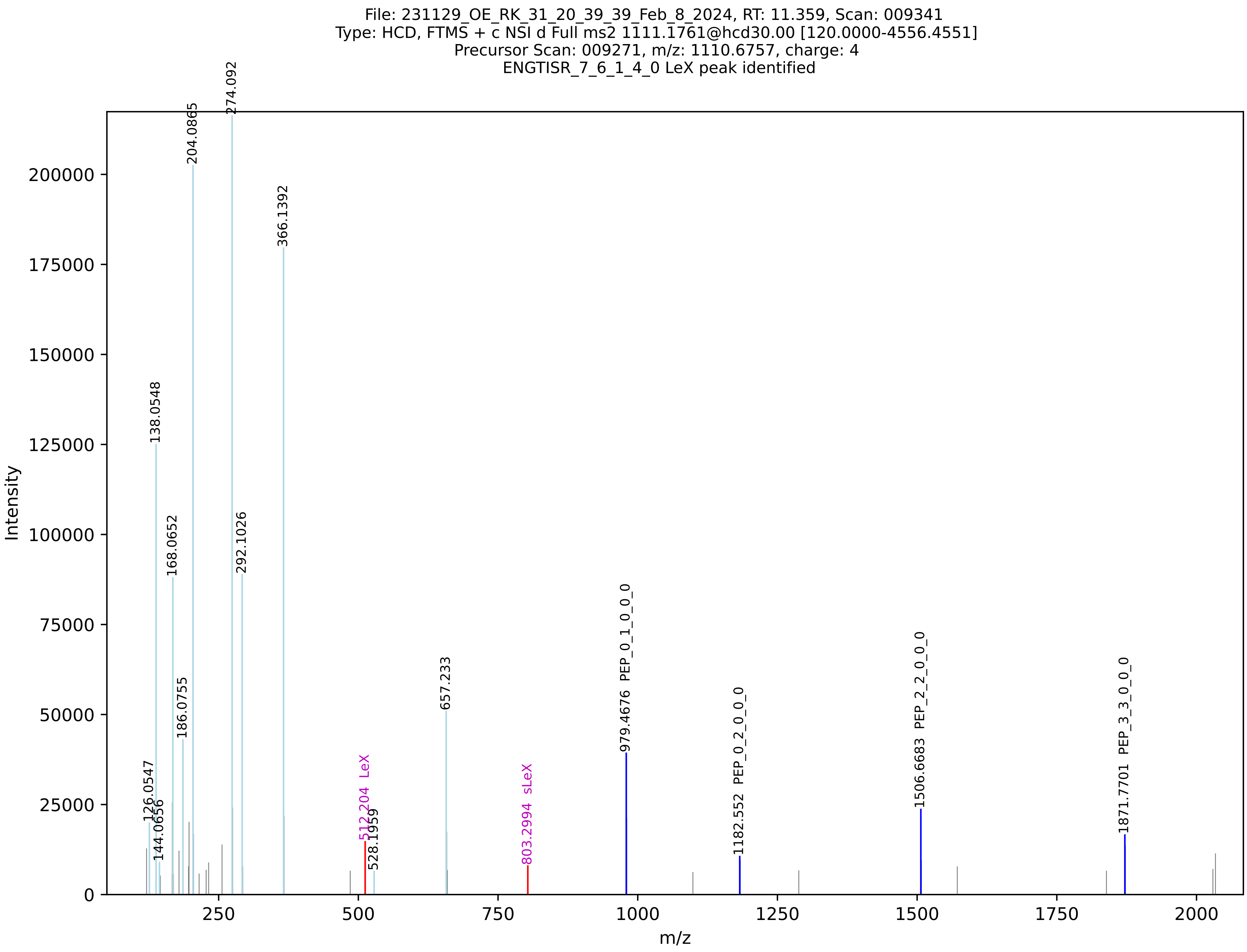

File: 231129_OE_RK_31_20_39_39_Feb_8_2024, RT: 11.359, Scan: 009341
Type: HCD, FTMS + c NSI d Full ms2 1111.1761@hcd30.00 [120.0000-4556.4551]
Precursor Scan: 009271, m/z: 1110.6757, charge: 4
ENGTISR_7_6_1_4_0 LeX peak identified
Intensity
m/z
274.092
204.0865
366.1392
138.0548
168.0652
292.1026
186.0755
126.0547
144.0656
512.204 LeX
528.1959
657.233
803.2994 sLeX
979.4676 PEP_0_1_0_0_0
1182.552 PEP_0_2_0_0_0
1506.6683 PEP_2_2_0_0_0
1871.7701 PEP_3_3_0_0_0

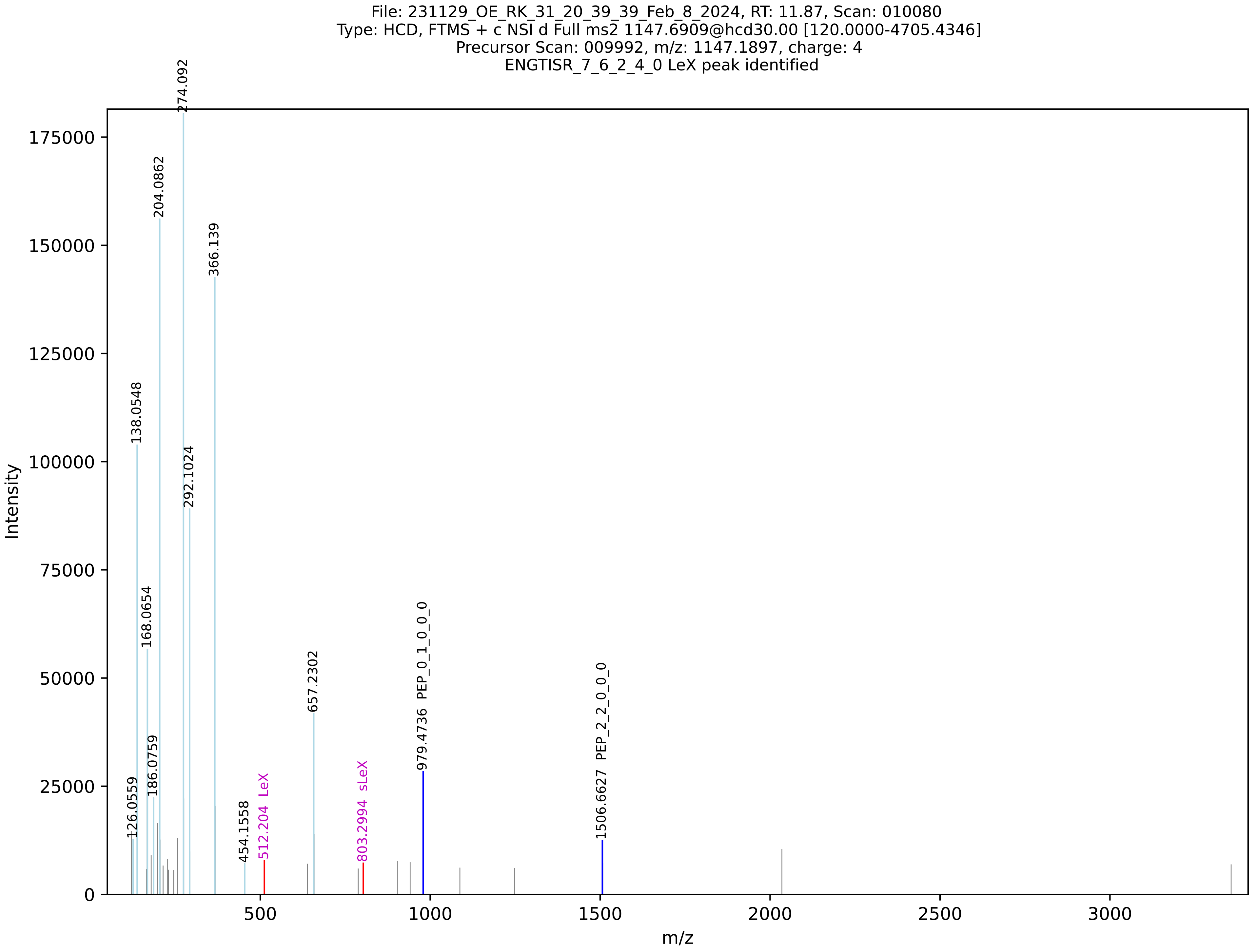
File: 231129_OE_RK_31_20_39_39_Feb_8_2024, RT: 11.87, Scan: 010080
Type: HCD, FTMS + c NSI d Full ms2 1147.6909@hcd30.00 [120.0000-4705.4346]
Precursor Scan: 009992, m/z: 1147.1897, charge: 4
ENGTISR_7_6_2_4_0 LeX peak identified
Intensity
m/z
274.092
204.0862
366.139
138.0548
292.1024
168.0654
186.0759
126.0559
454.1558
512.204 LeX
657.2302
803.2994 sLeX
979.4736 PEP_0_1_0_0_0
1506.6627 PEP_2_2_0_0_0
175000
150000
125000
100000
75000
50000
25000
0
500
1000
1500
2000
2500
3000

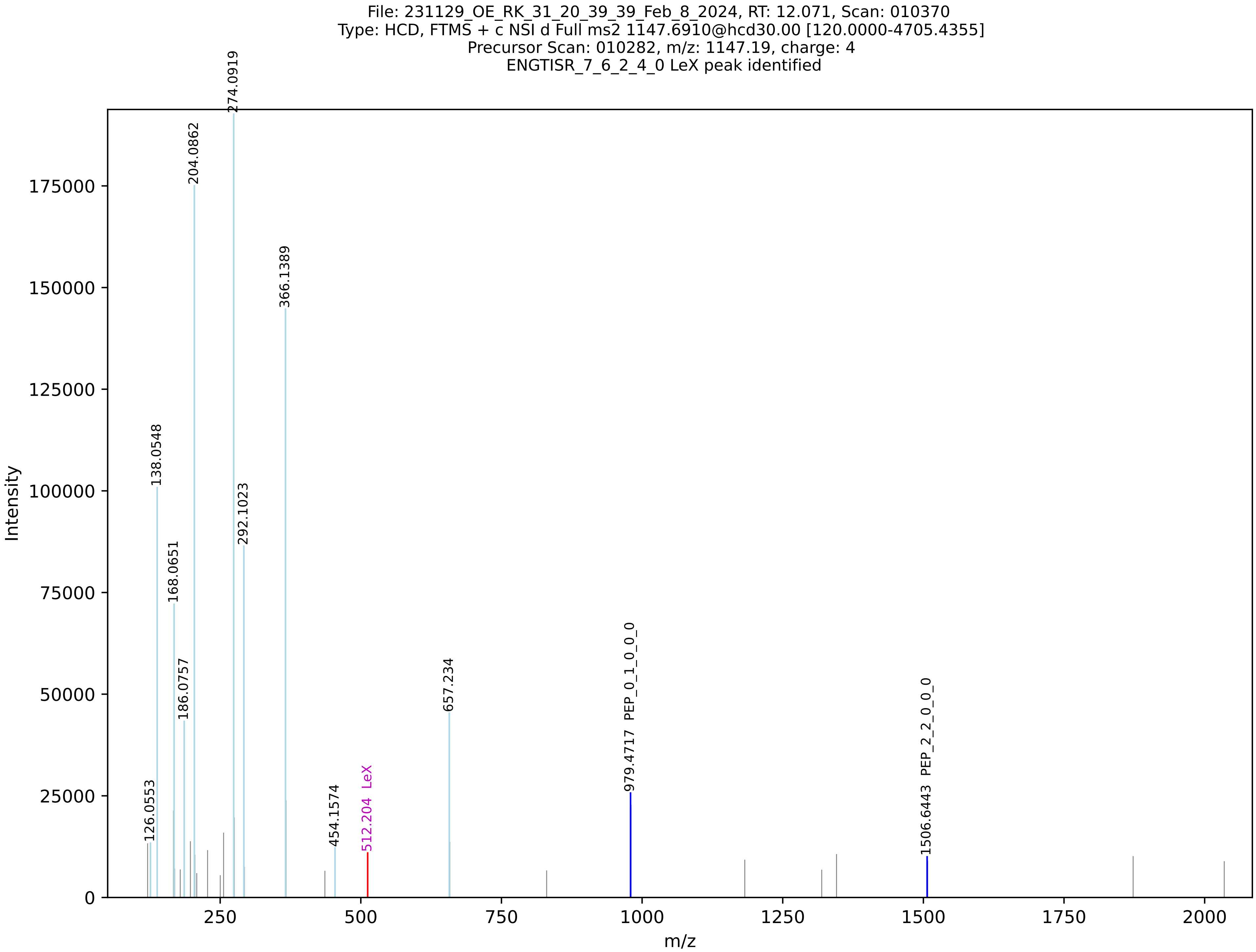

File: 231129_OE_RK_31_20_39_39_Feb_8_2024, RT: 12.071, Scan: 010370
Type: HCD, FTMS + c NSI d Full ms2 1147.6910@hcd30.00 [120.0000-4705.4355]
Precursor Scan: 010282, m/z: 1147.19, charge: 4
ENGTISR_7_6_2_4_0 LeX peak identified
Intensity
m/z
274.0919
204.0862
366.1389
138.0548
292.1023
168.0651
186.0757
657.234
979.4717 PEP_0_1_0_0_0
1506.6443 PEP_2_2_0_0_0
512.204 LeX
454.1574
126.0553

File: 231129_OE_RK_31_20_39_39_Feb_8_2024, RT: 12.243, Scan: 010604
Type: HCD, FTMS + c NSI d Full ms2 1432.8818@hcd30.00 [120.0000-4406.7603]
Precursor Scan: 010591, m/z: 1431.8795, charge: 3
SSSQVNR_7_6_0_4_0 LeX peak identified

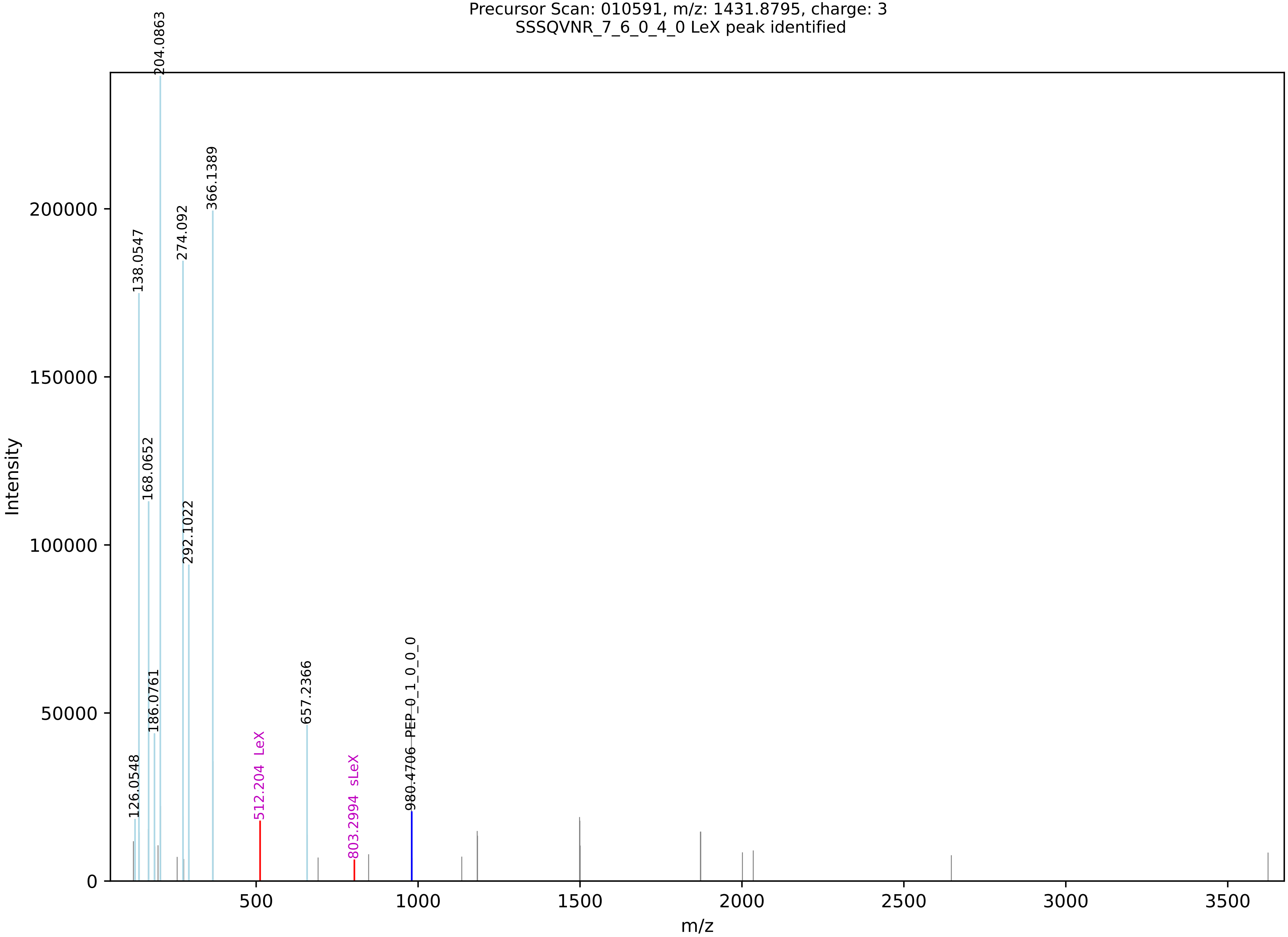

File: 231129_OE_RK_31_20_39_39_Feb_8_2024, RT: 12.292, Scan: 010661
Type: HCD, FTMS + c NSI d Full ms2 1481.8989@hcd30.00 [120.0000-4556.7529]
Precursor Scan: 010620, m/z: 1480.5642, charge: 3
ENGTISR_7_6_1_4_0 LeX peak identified

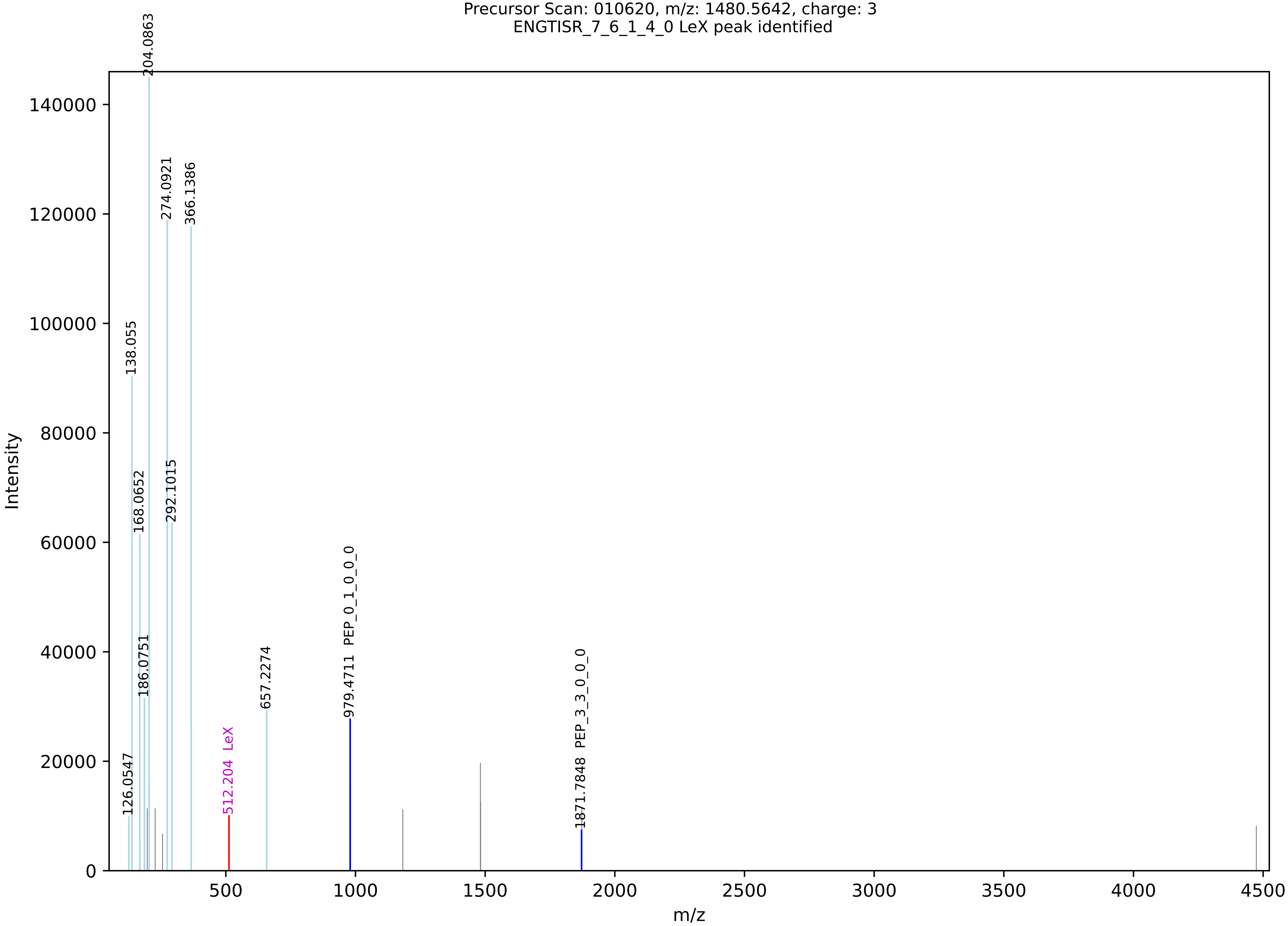

File: 231129_OE_RK_31_20_39_39_Feb_8_2024, RT: 14.852, Scan: 014336
Type: HCD, FTMS + c NSI d Full ms2 1147.9399@hcd30.00 [120.0000-4706.4512]
Precursor Scan: 014253, m/z: 1147.1882, charge: 4
ENGTISR_7_6_2_4_0 LeX peak identified

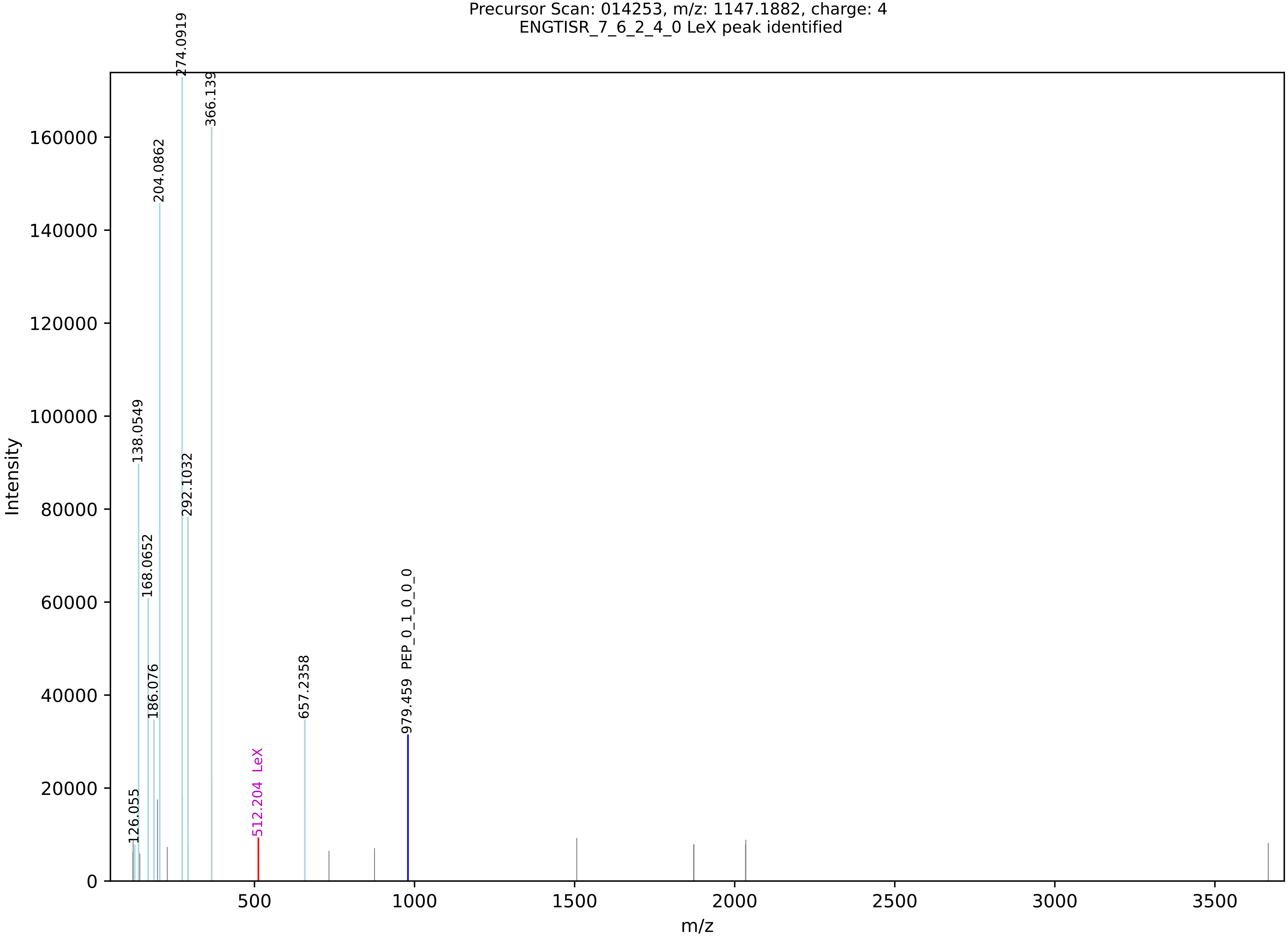

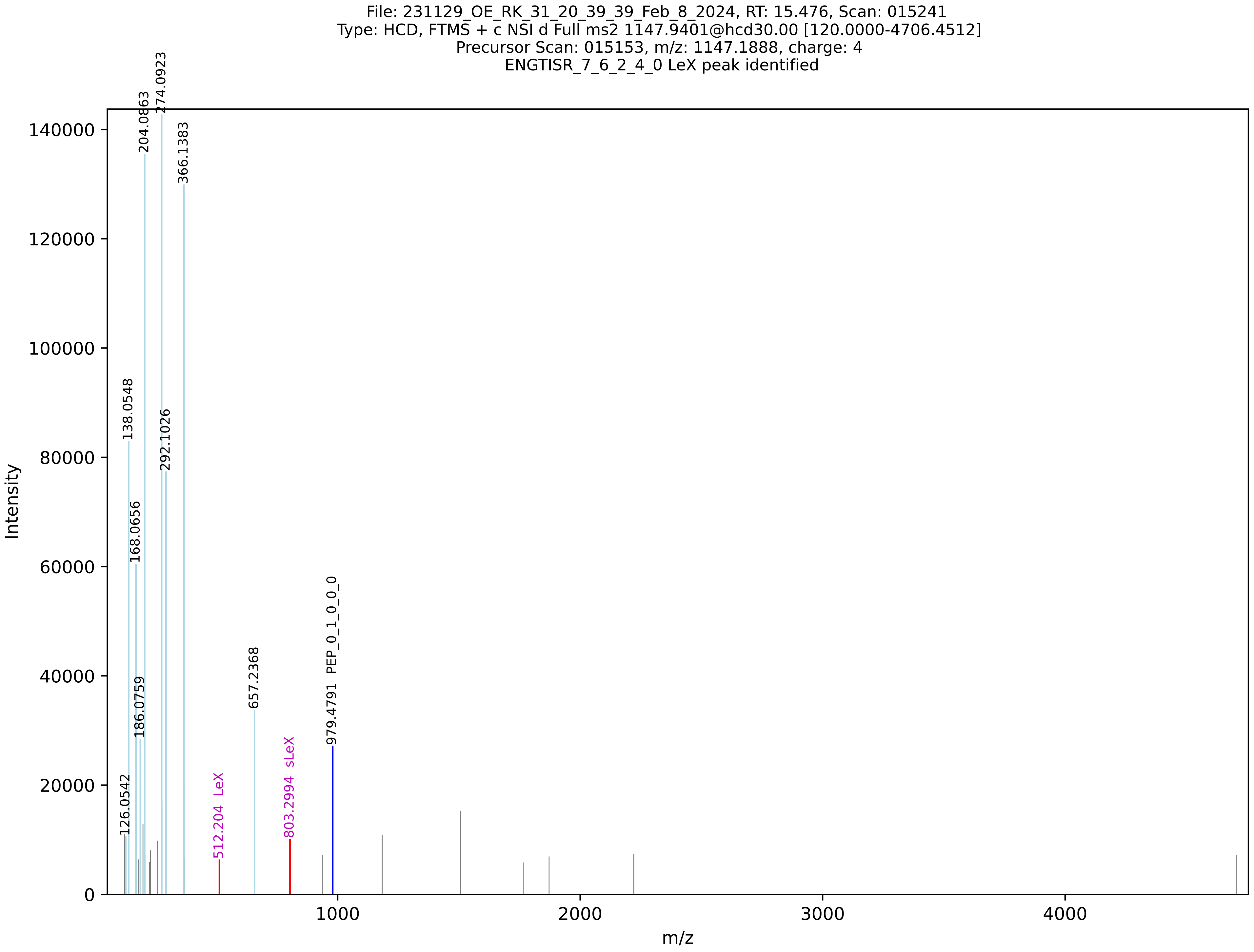
File: 231129_OE_RK_31_20_39_39_Feb_8_2024, RT: 15.476, Scan: 015241
Type: HCD, FTMS + c NSI d Full ms2 1147.9401@hcd30.00 [120.0000-4706.4512]
Precursor Scan: 015153, m/z: 1147.1888, charge: 4
ENGTISR_7_6_2_4_0 LeX peak identified
Intensity
m/z
274.0923
204.0863
366.1383
138.0548
292.1026
168.0656
186.0759
126.0542
657.2368
979.4791 PEP_0_1_0_0_0_
512.204 LeX
803.2994 sLeX
0
20000
40000
60000
80000
100000
120000
140000
1000
2000
3000
4000

File: 231129_OE_RK_31_20_39_39_Feb_8_2024, RT: 19.12, Scan: 020413
Type: HCD, FTMS + c NSI d Full ms2 1147.6884@hcd30.00 [120.0000-4705.4248]
Precursor Scan: 020342, m/z: 1147.1876, charge: 4
ENGTISR_7_6_2_4_0 LeX peak identified

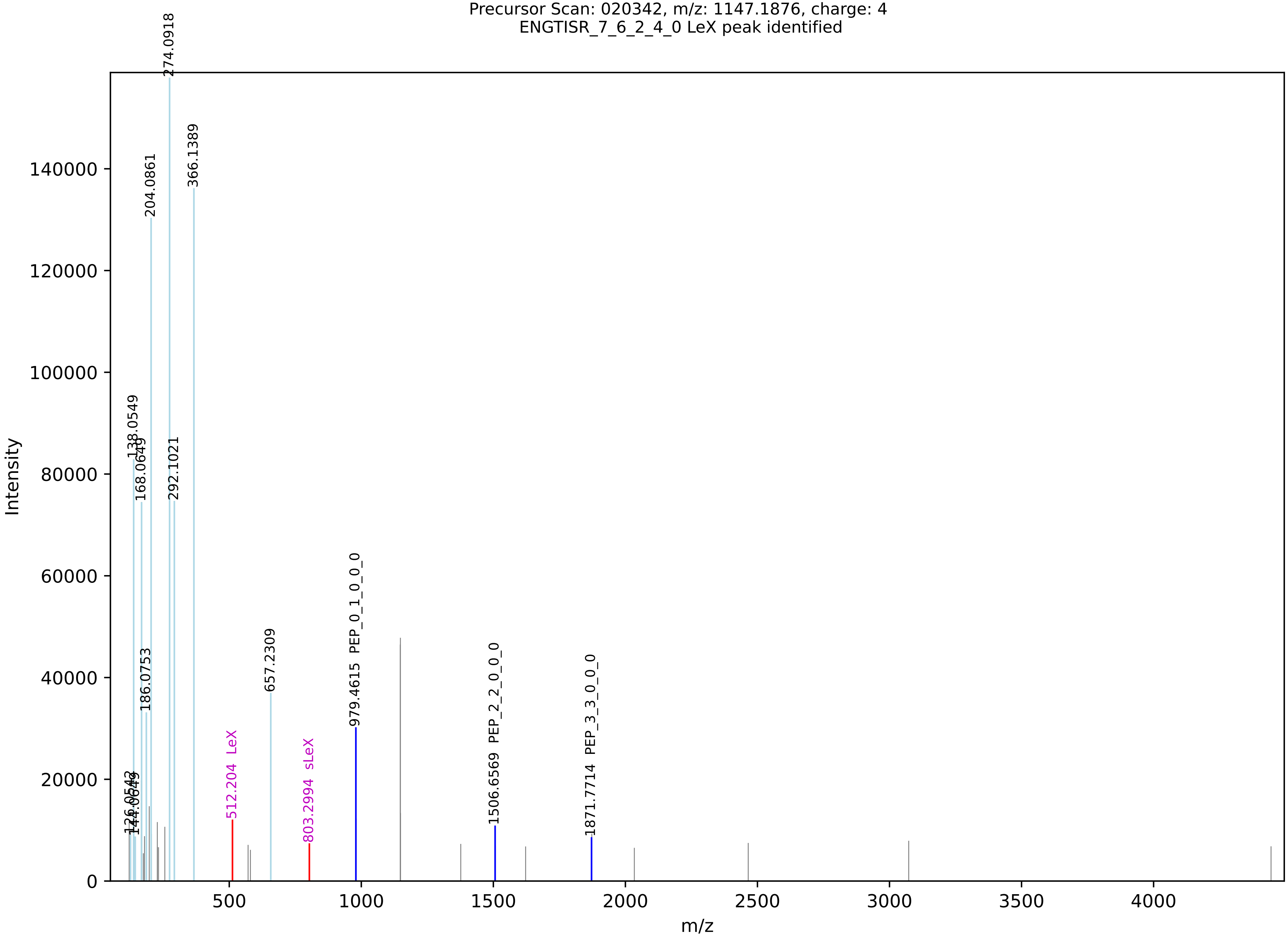

File: 231129_OE_RK_31_20_39_39_Feb_8_2024, RT: 20.154, Scan: 021869
Type: HCD, FTMS + c NSI d Full ms2 1147.6891@hcd30.00 [120.0000-4705.4272]
Precursor Scan: 021791, m/z: 1146.937, charge: 4
ENGTISR_7_6_2_4_0 LeX peak identified

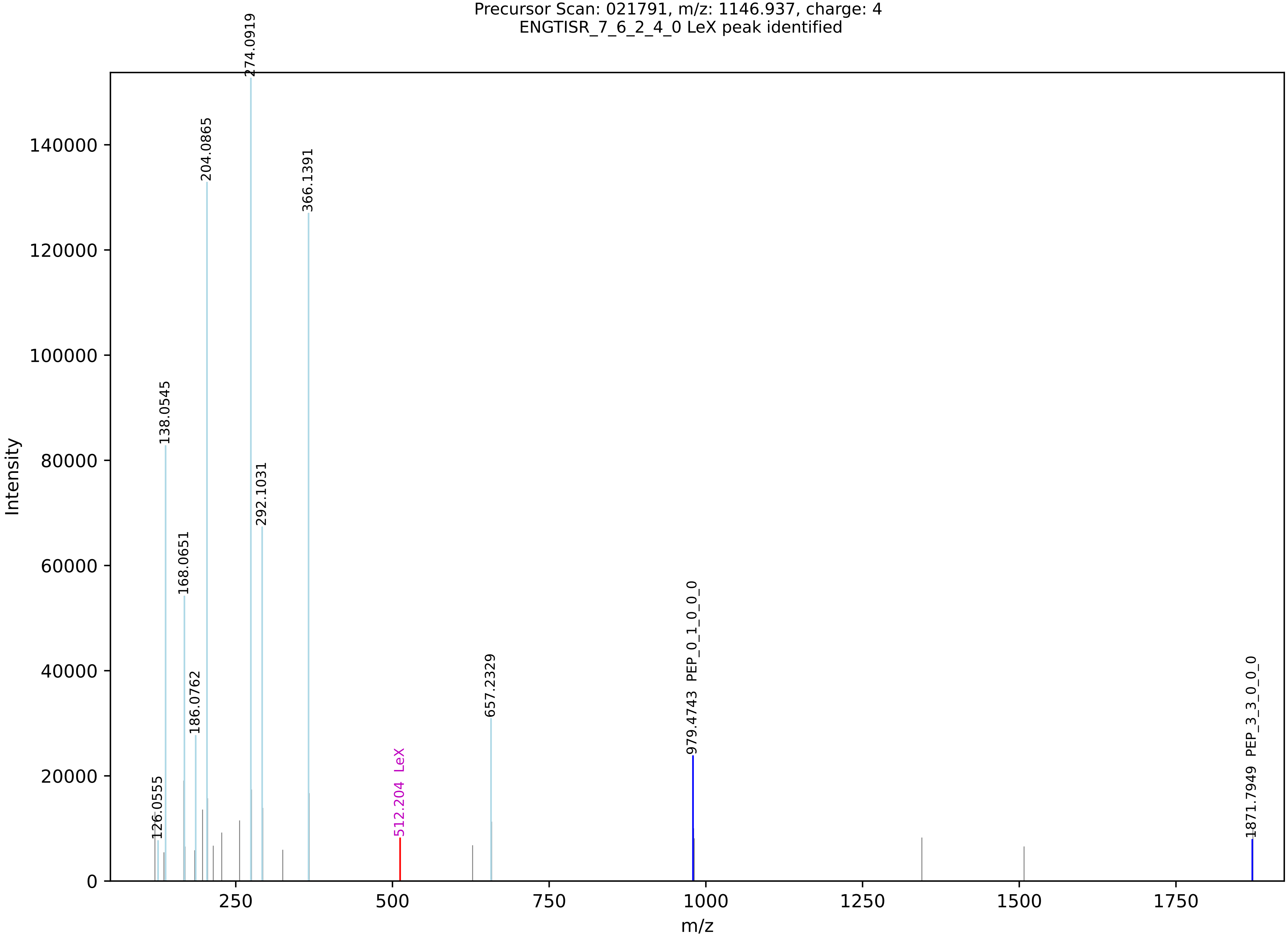

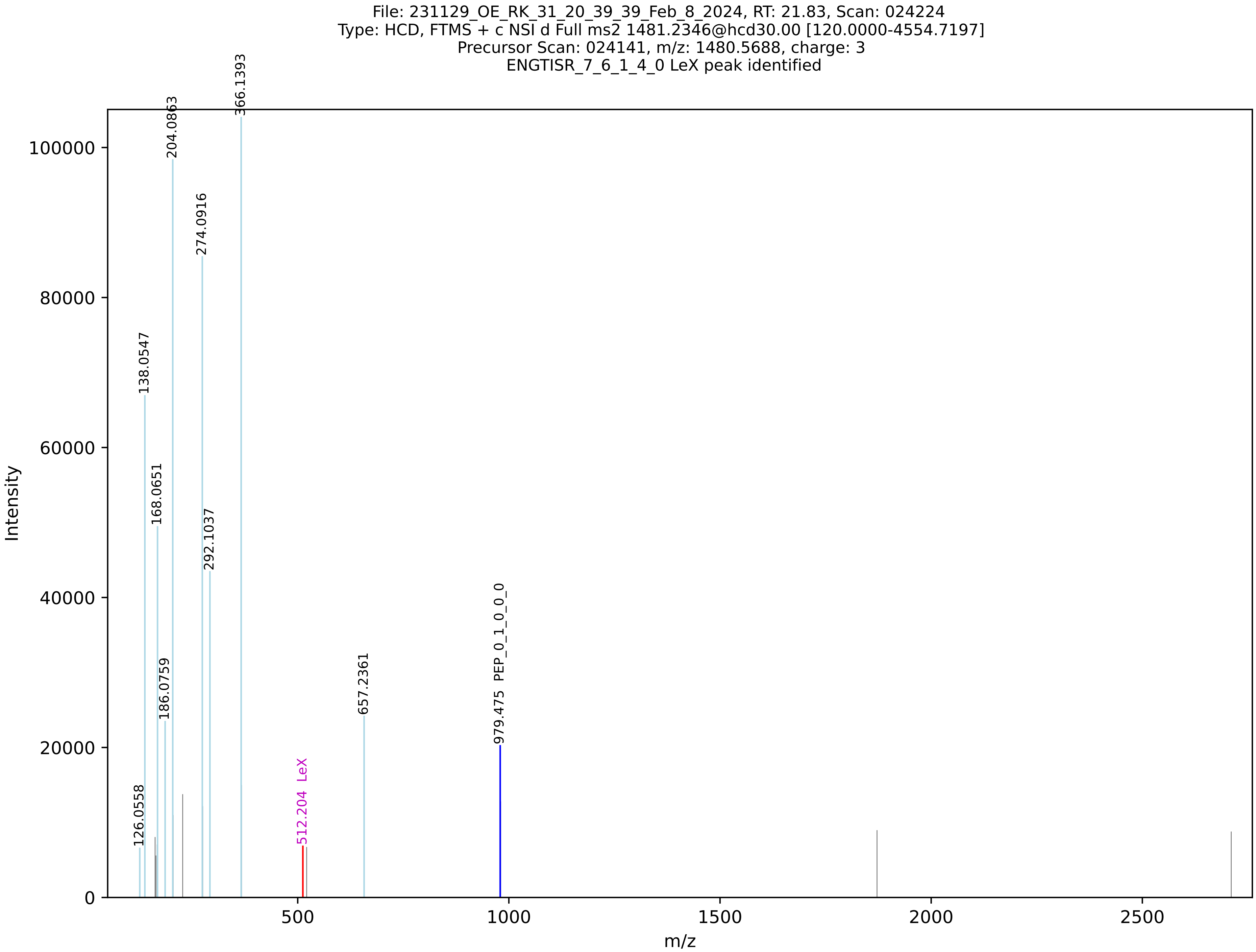
File: 231129_OE_RK_31_20_39_39_Feb_8_2024, RT: 21.83, Scan: 024224
Type: HCD, FTMS + c NSI d Full ms2 1481.2346@hcd30.00 [120.0000-4554.7197]
Precursor Scan: 024141, m/z: 1480.5688, charge: 3
ENGTISR_7_6_1_4_0 LeX peak identified
366.1393
204.0863
274.0916
138.0547
168.0651
292.1037
186.0759
126.0558
512.204 LeX
657.2361
979.475 PEP_0_1_0_0_0
Intensity
m/z
0
20000
40000
60000
80000
100000
500
1000
1500
2000
2500

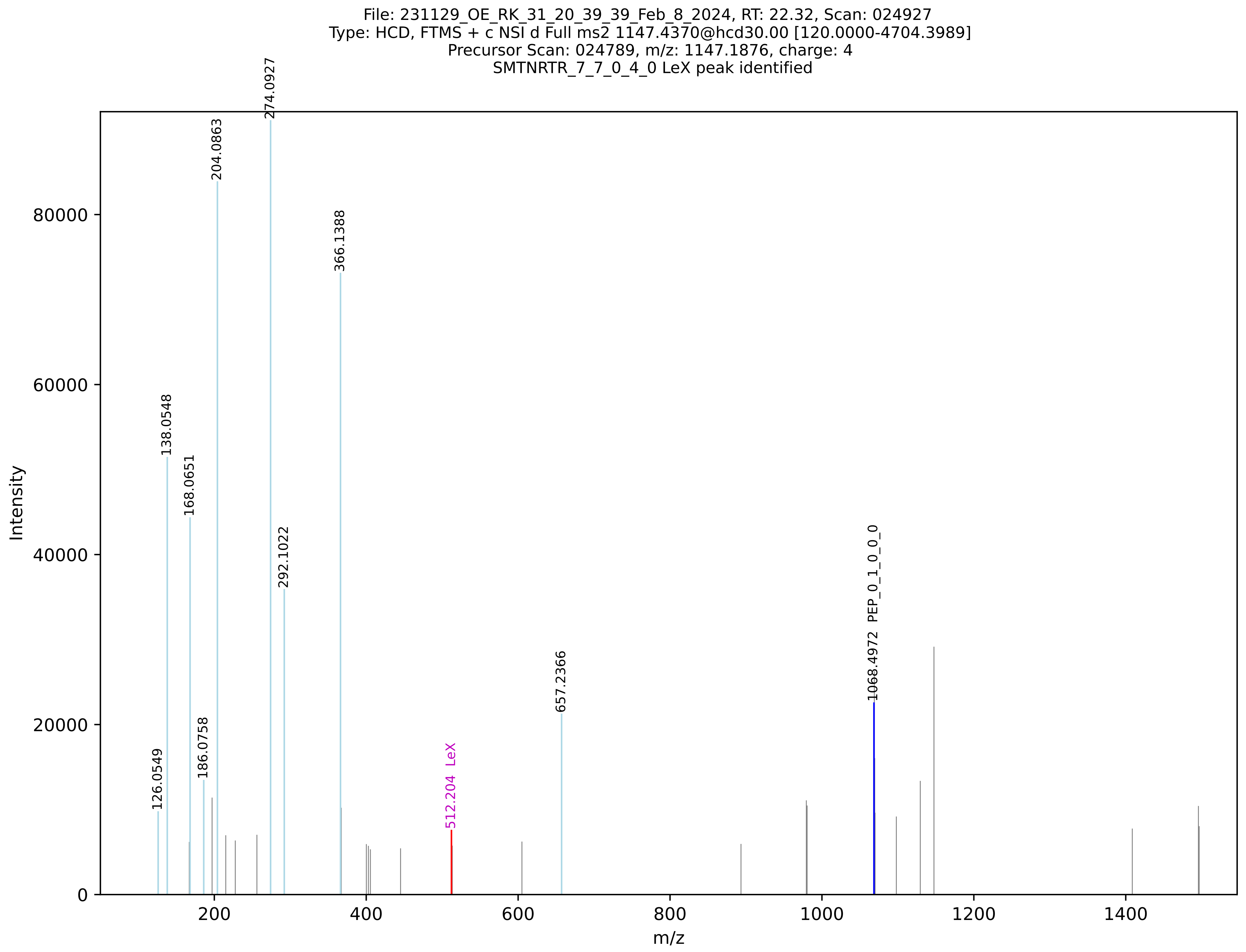
File: 231129_OE_RK_31_20_39_39_Feb_8_2024, RT: 22.32, Scan: 024927
Type: HCD, FTMS + c NSI d Full ms2 1147.4370@hcd30.00 [120.0000-4704.3989]
Precursor Scan: 024789, m/z: 1147.1876, charge: 4
SMTNRTR_7_7_0_4_0 LeX peak identified
Intensity
m/z
0
20000
40000
60000
80000
200
400
600
800
1000
1200
1400
126.0549
138.0548
168.0651
186.0758
204.0863
274.0927
292.1022
366.1388
512.204 LeX
657.2366
1068.4972 PEP_0_1_0_0_0

File: 231129_OE_RK_31_20_39_39_Feb_8_2024, RT: 39.62, Scan: 049891
Type: HCD, FTMS + c NSI d Full ms2 1104.7076@hcd30.00 [120.0000-4530.0635]
Precursor Scan: 049794, m/z: 1104.4571, charge: 4
GLTFQQNASSMCVPDQDTAIR_6_4_0_1_0 LeX peak identified

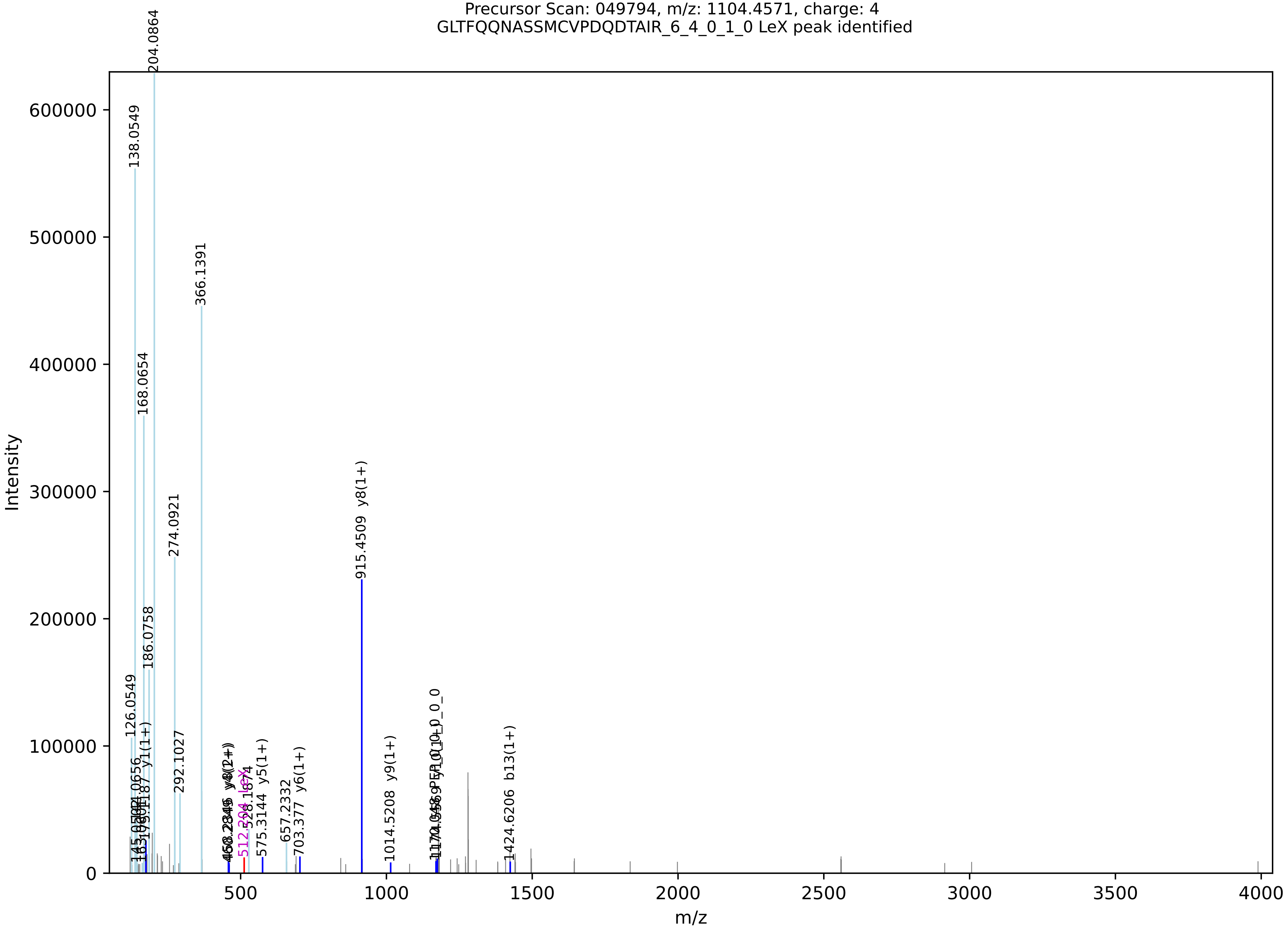

File: 231129_OE_RK_31_20_39_39_Feb_8_2024, RT: 43.274, Scan: 055207
Type: HCD, FTMS + c NSI d Full ms2 1287.0193@hcd30.00 [120.0000-5273.8945]
Precursor Scan: 055127, m/z: 1286.5192, charge: 4
QDQCIYNTTYLNVQR_7_6_2_2_0 LeX peak identified

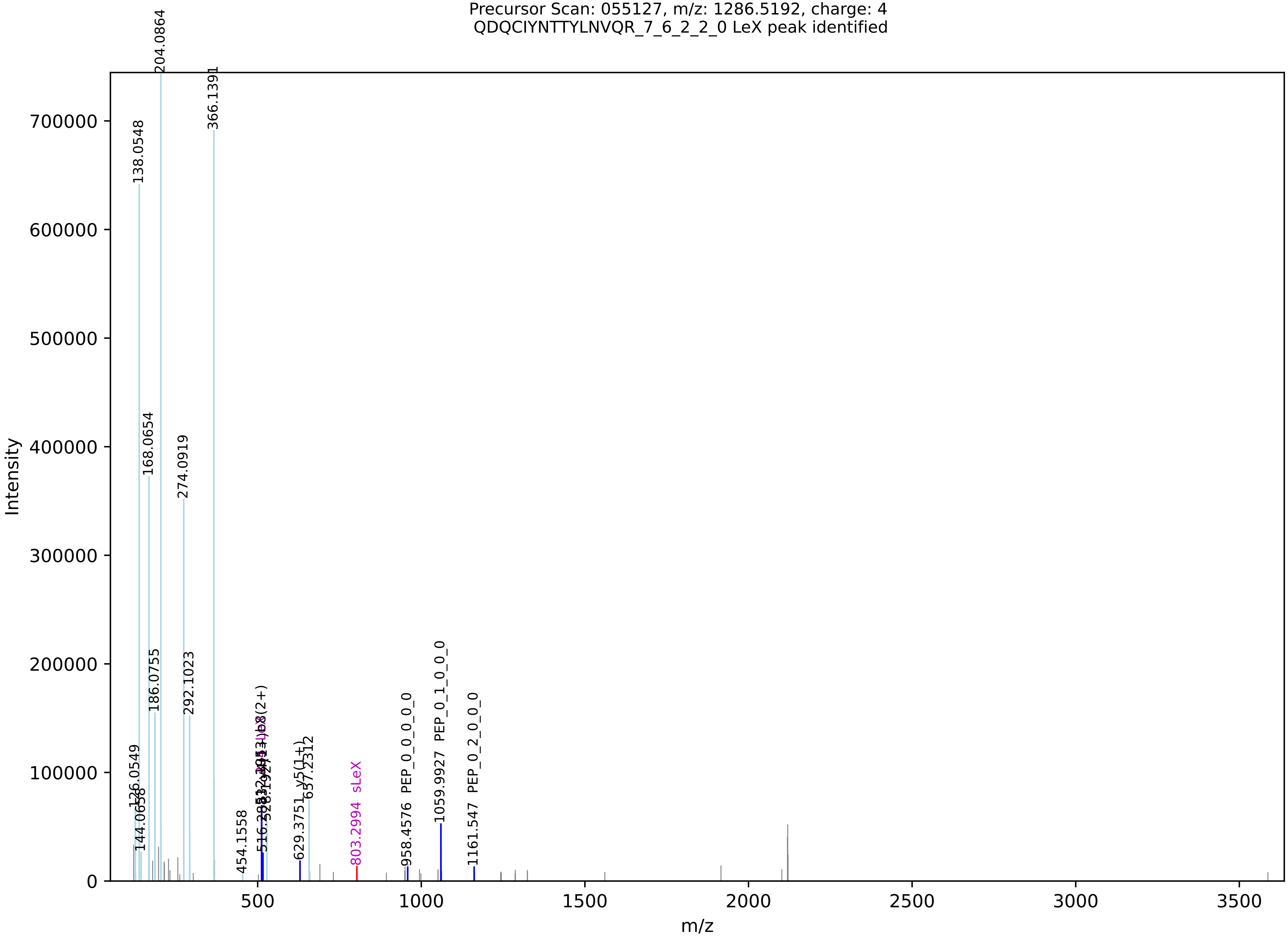

File: 231129_OE_RK_31_20_39_39_Feb_8_2024, RT: 44.783, Scan: 057391
Type: HCD, FTMS + c NSI d Full ms2 1250.7551@hcd30.00 [120.0000-5125.9365]
Precursor Scan: 057317, m/z: 1250.004, charge: 4
QDQCIYNTTYLNVQR_7_6_1_2_0 LeX peak identified

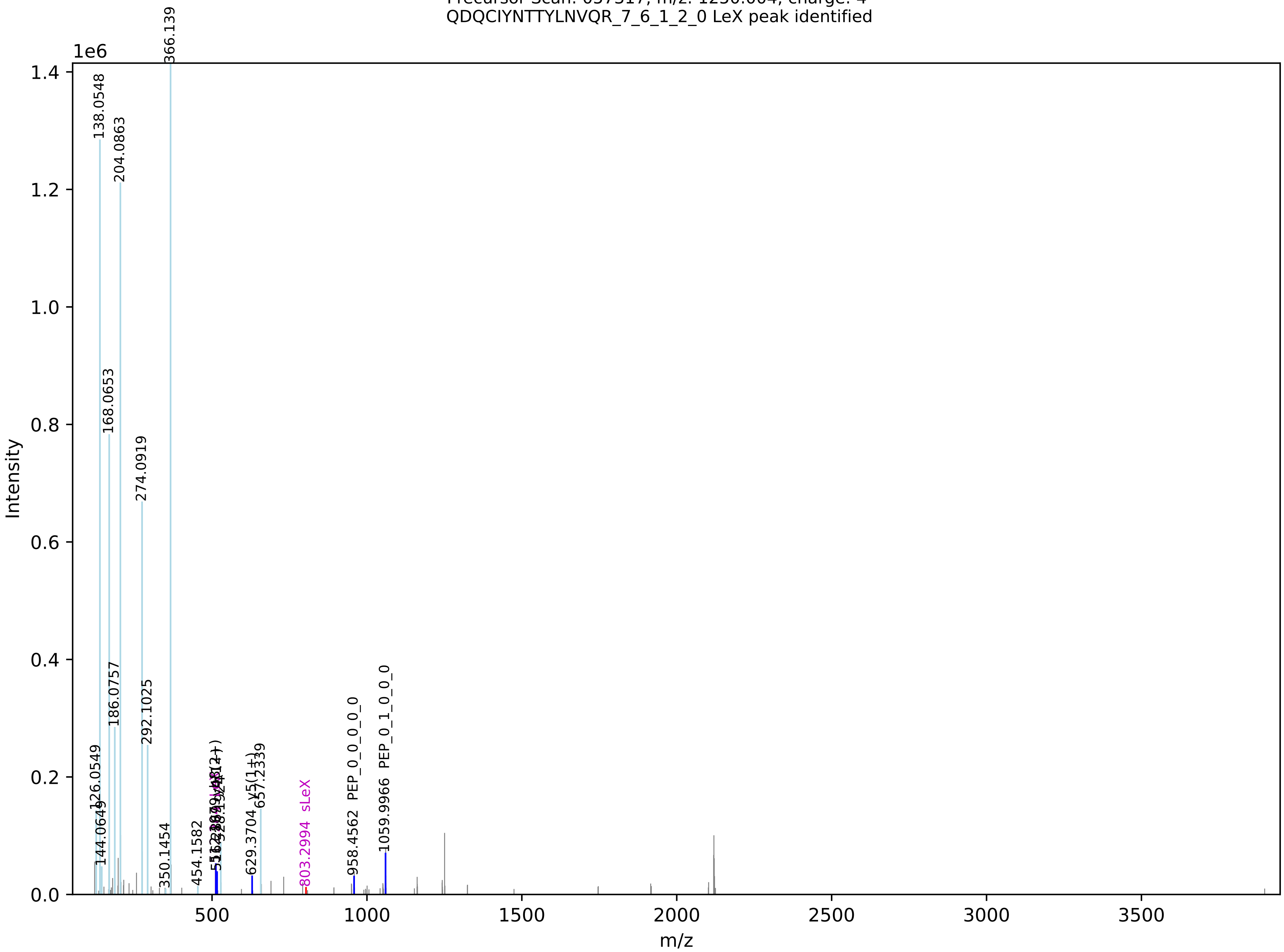

File: 231129_OE_RK_31_20_39_39_Feb_8_2024, RT: 44.888, Scan: 057543
Type: HCD, FTMS + c NSI d Full ms2 1545.2926@hcd30.00 [120.0000-4750.7373]
Precursor Scan: 057462, m/z: 1544.6244, charge: 3
QDQCIYNTTYLNVQR_6_5_1_2_0 LeX peak identified

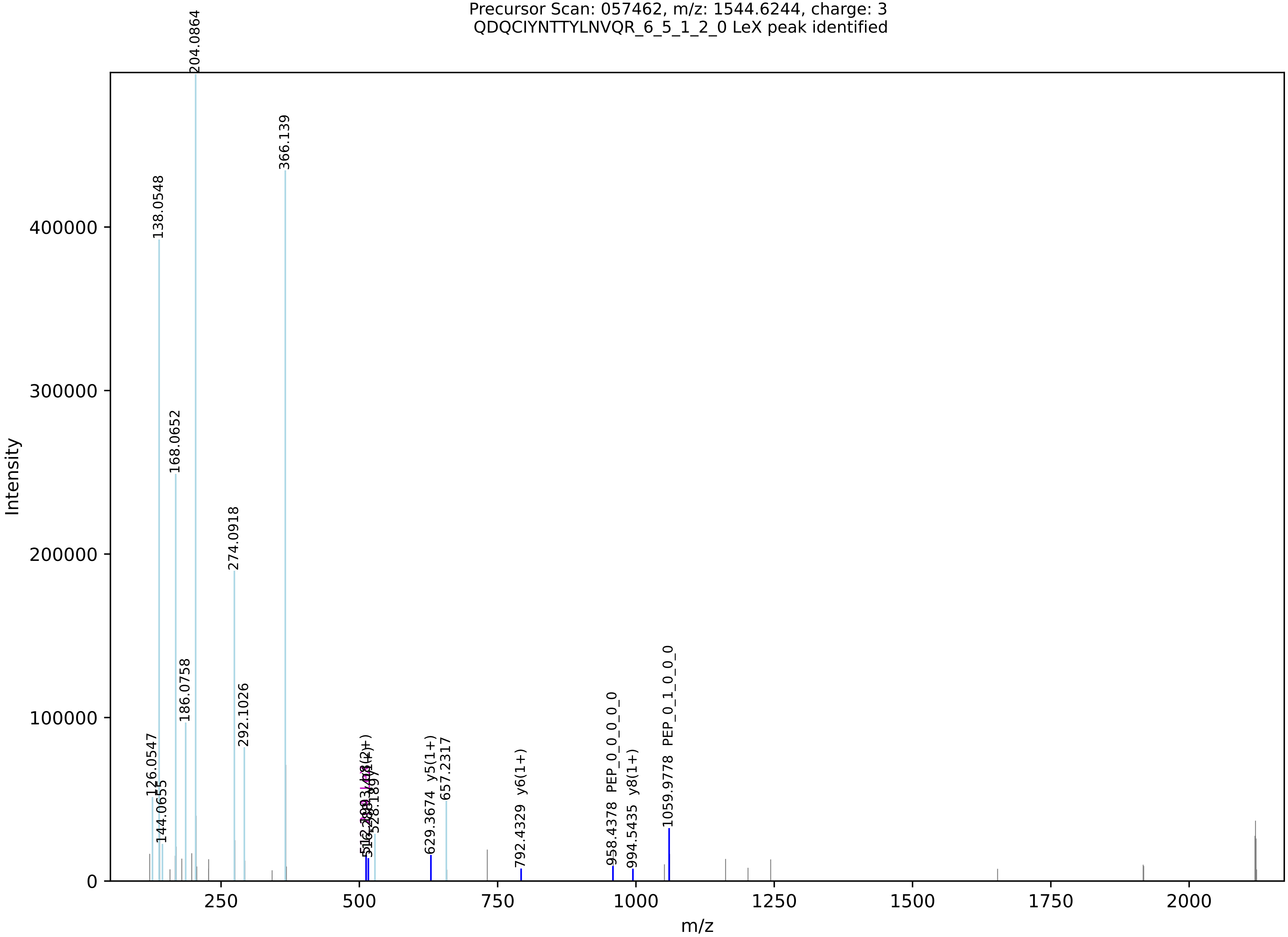

File: 231129_OE_RK_31_20_39_39_Feb_8_2024, RT: 45.034, Scan: 057756
Type: HCD, FTMS + c NSI d Full ms2 1287.2688@hcd30.00 [120.0000-5274.9126]
Precursor Scan: 057681, m/z: 1286.5183, charge: 4
QDQCIYNTTYLNVQR_7_6_2_2_0 LeX peak identified

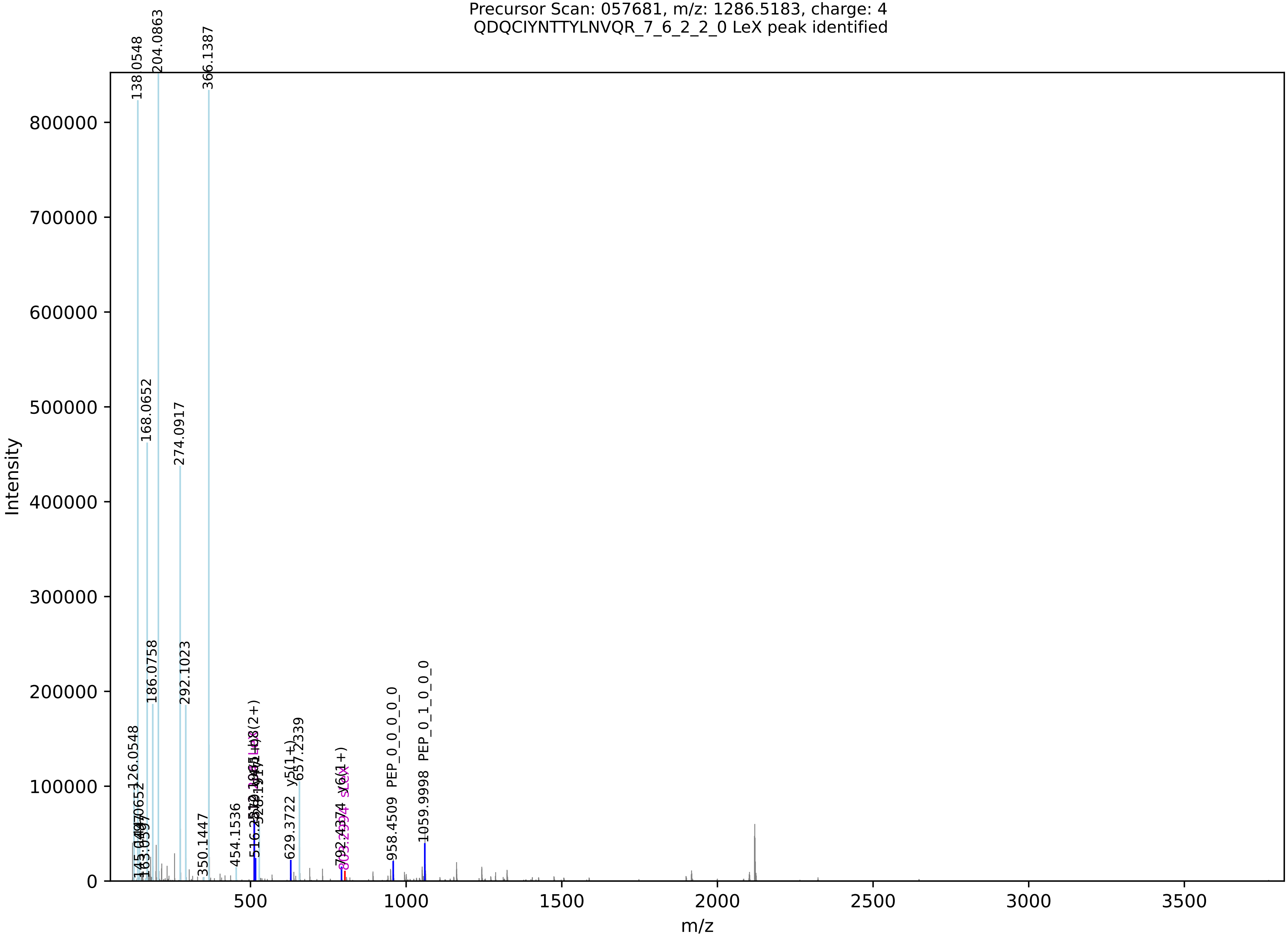

File: 231129_OE_RK_31_20_39_39_Feb_8_2024, RT: 45.14, Scan: 057912
Type: HCD, FTMS + c NSI d Full ms2 1360.0441@hcd30.00 [120.0000-5571.8354]
Precursor Scan: 057829, m/z: 1359.289, charge: 4
QDQCIYNTTYLNVQR_7_6_2_3_0 LeX peak identified

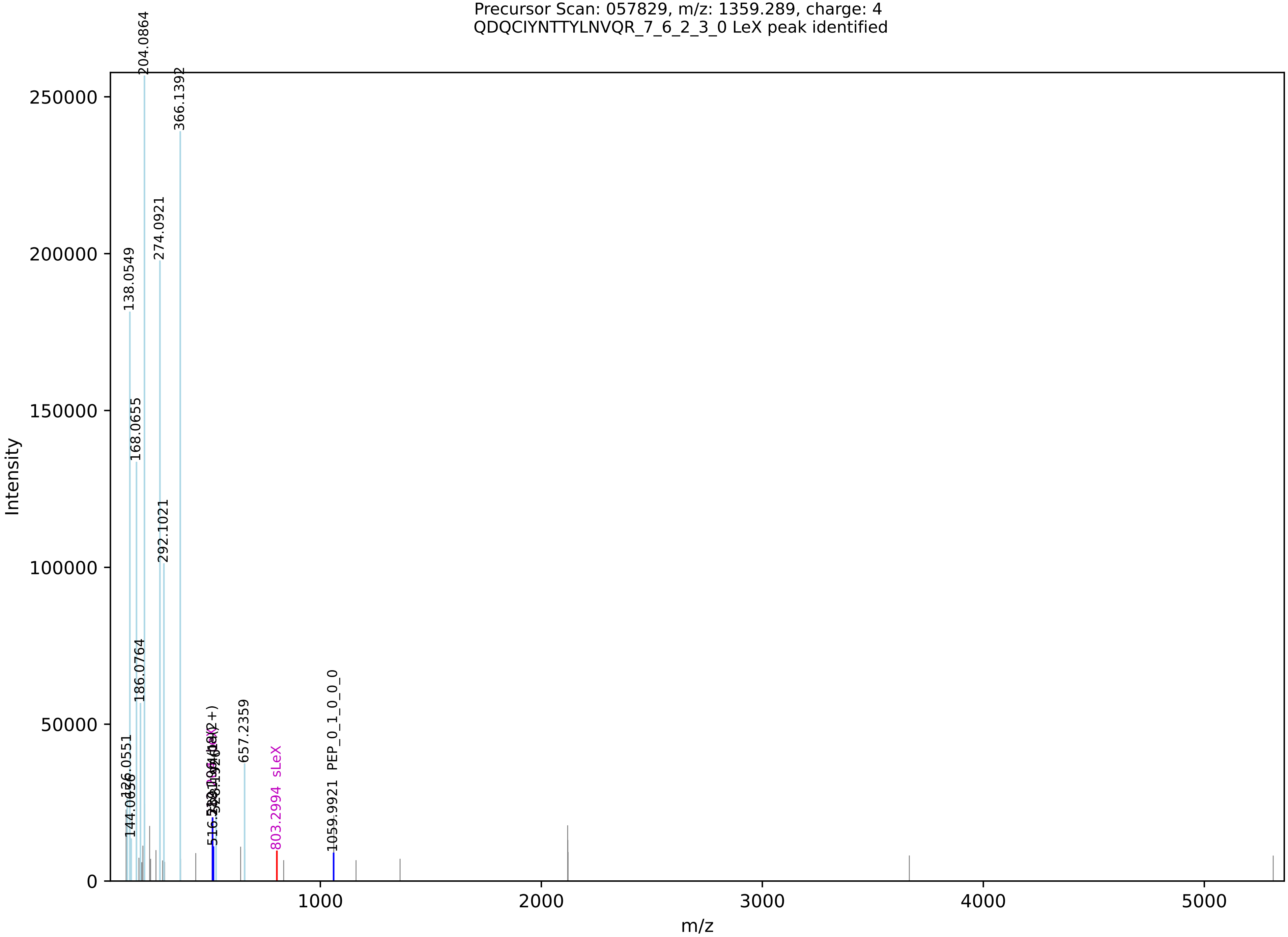

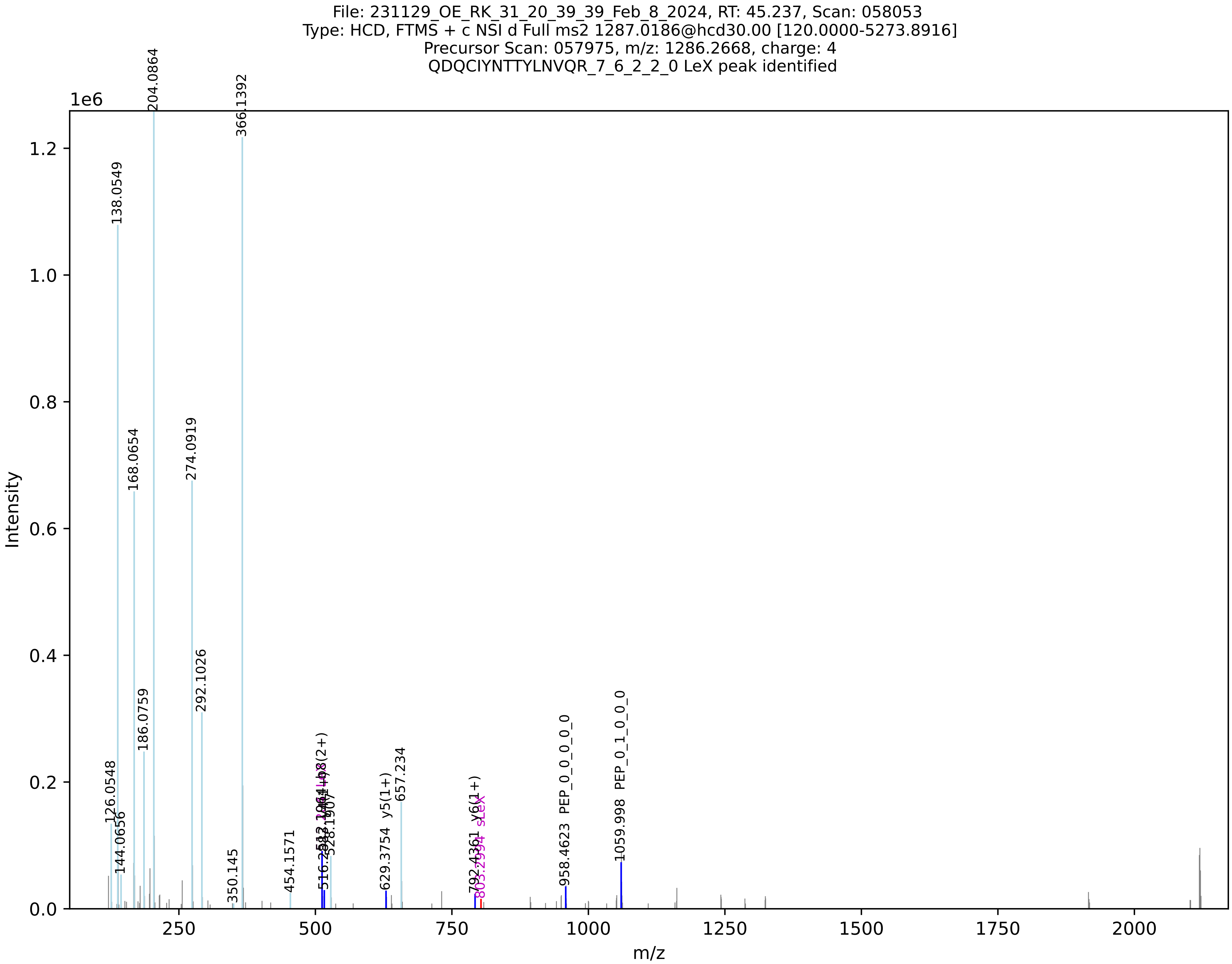
File: 231129_OE_RK_31_20_39_39_Feb_8_2024, RT: 45.237, Scan: 058053
Type: HCD, FTMS + c NSI d Full ms2 1287.0186@hcd30.00 [120.0000-5273.8916]
Precursor Scan: 057975, m/z: 1286.2668, charge: 4
QDQCIYNTTYLNVQR_7_6_2_2_0 LeX peak identified
1e6
Intensity
m/z
0.0
0.2
0.4
0.6
0.8
1.0
1.2
250
500
750
1000
1250
1500
1750
2000
204.0864
366.1392
138.0549
168.0654
274.0919
292.1026
186.0759
126.0548
144.0656
350.145
454.1571
528.1907
629.3754
y5(1+)
657.234
792.4361
y6(1+)
sLeX
958.4623
PEP_0_0_0_0_0
1059.998
PEP_0_1_0_0_0

File: 231129_OE_RK_31_20_39_39_Feb_8_2024, RT: 45.294, Scan: 058135
Type: HCD, FTMS + c NSI d Full ms2 1471.9161@hcd30.00 [120.0000-4526.2056]
Precursor Scan: 058049, m/z: 1471.2479, charge: 3
SWPAVGNCSSALR_6_5_1_3_0 LeX peak identified

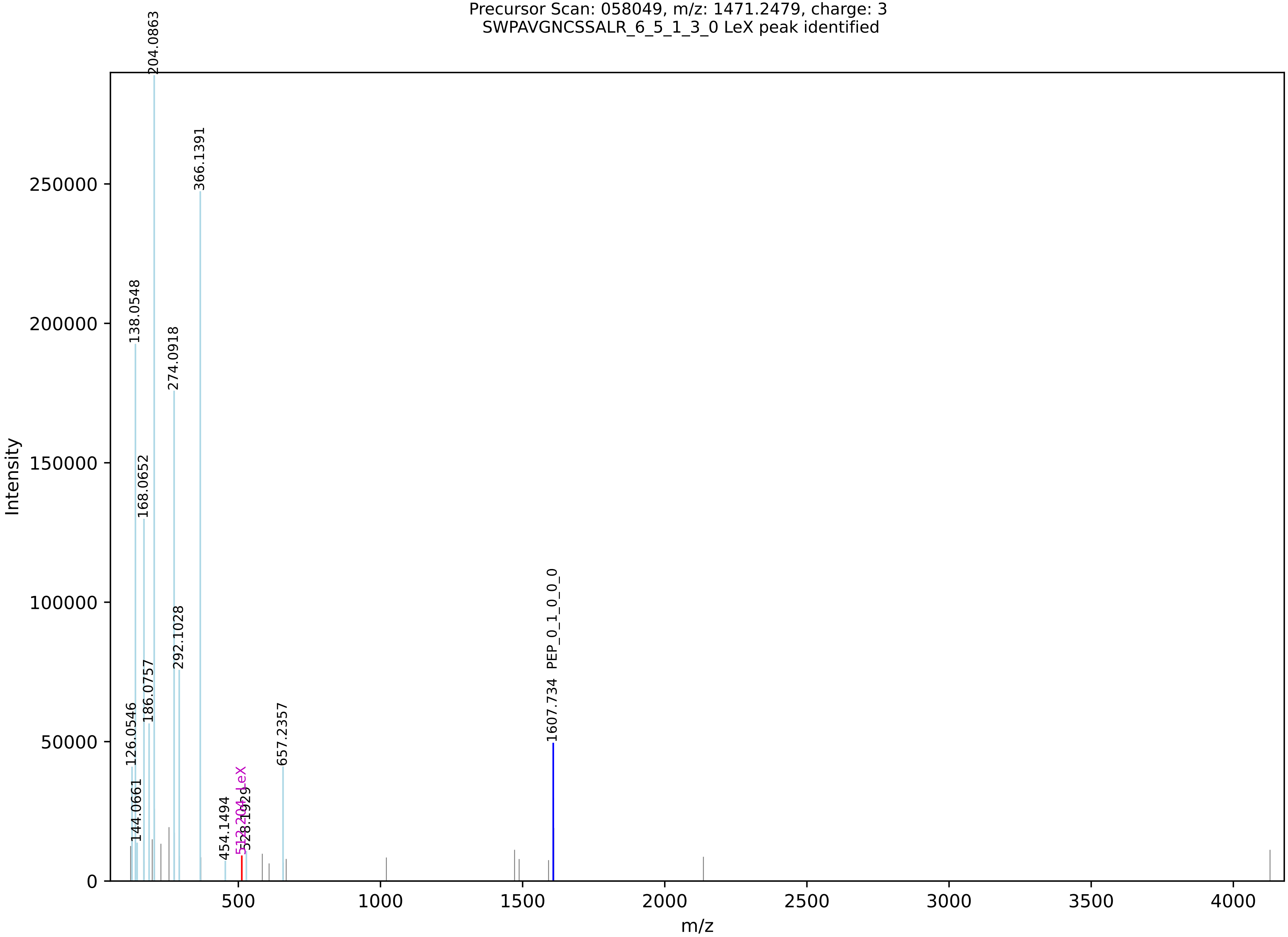

File: 231129_OE_RK_31_20_39_39_Feb_8_2024, RT: 45.491, Scan: 058421
Type: HCD, FTMS + c NSI d Full ms2 1545.2919@hcd30.00 [120.0000-4750.7354]
Precursor Scan: 058341, m/z: 1544.6237, charge: 3
QDQCIYNTTYLNVQR_6_5_1_2_0 LeX peak identified

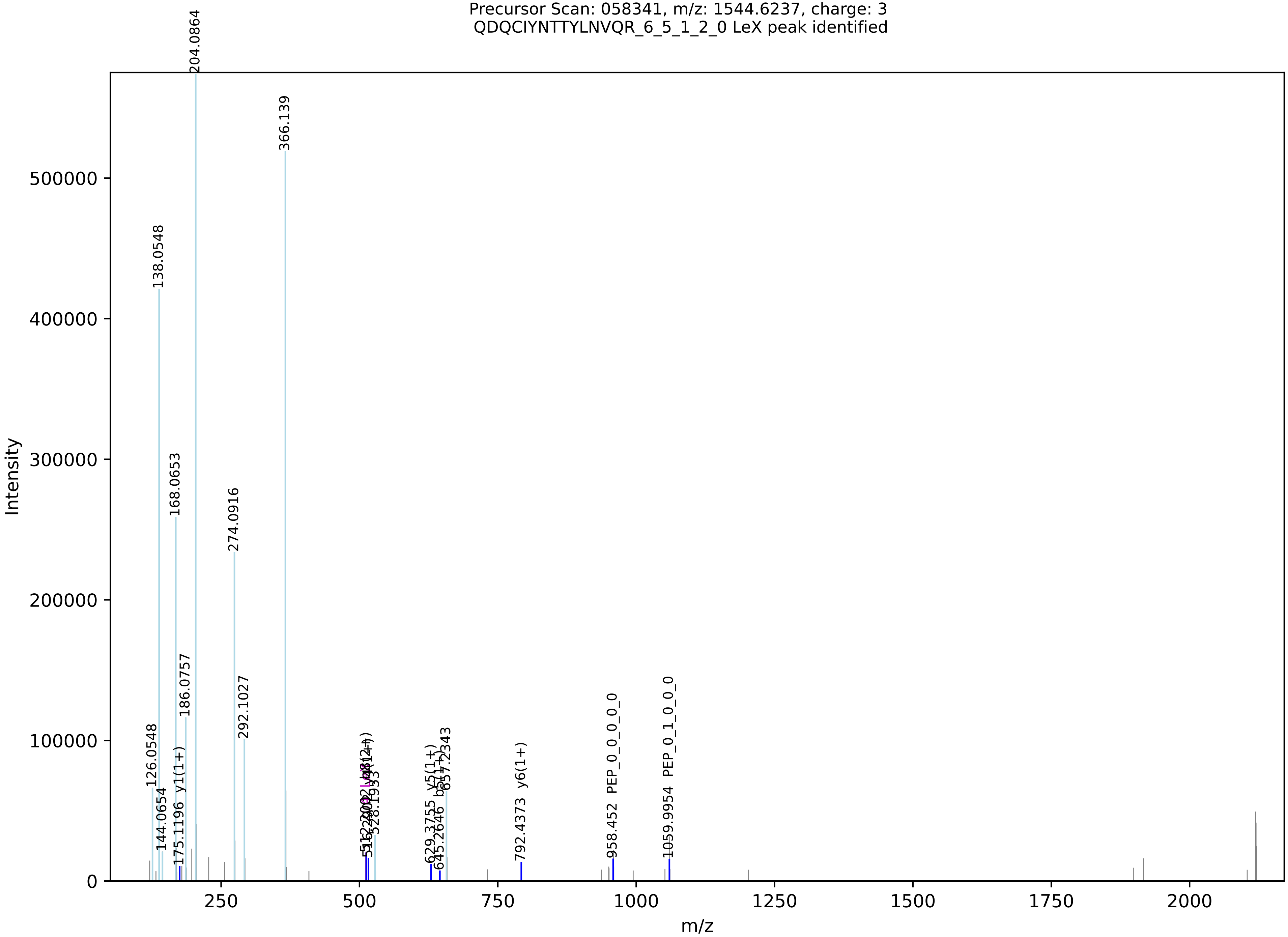

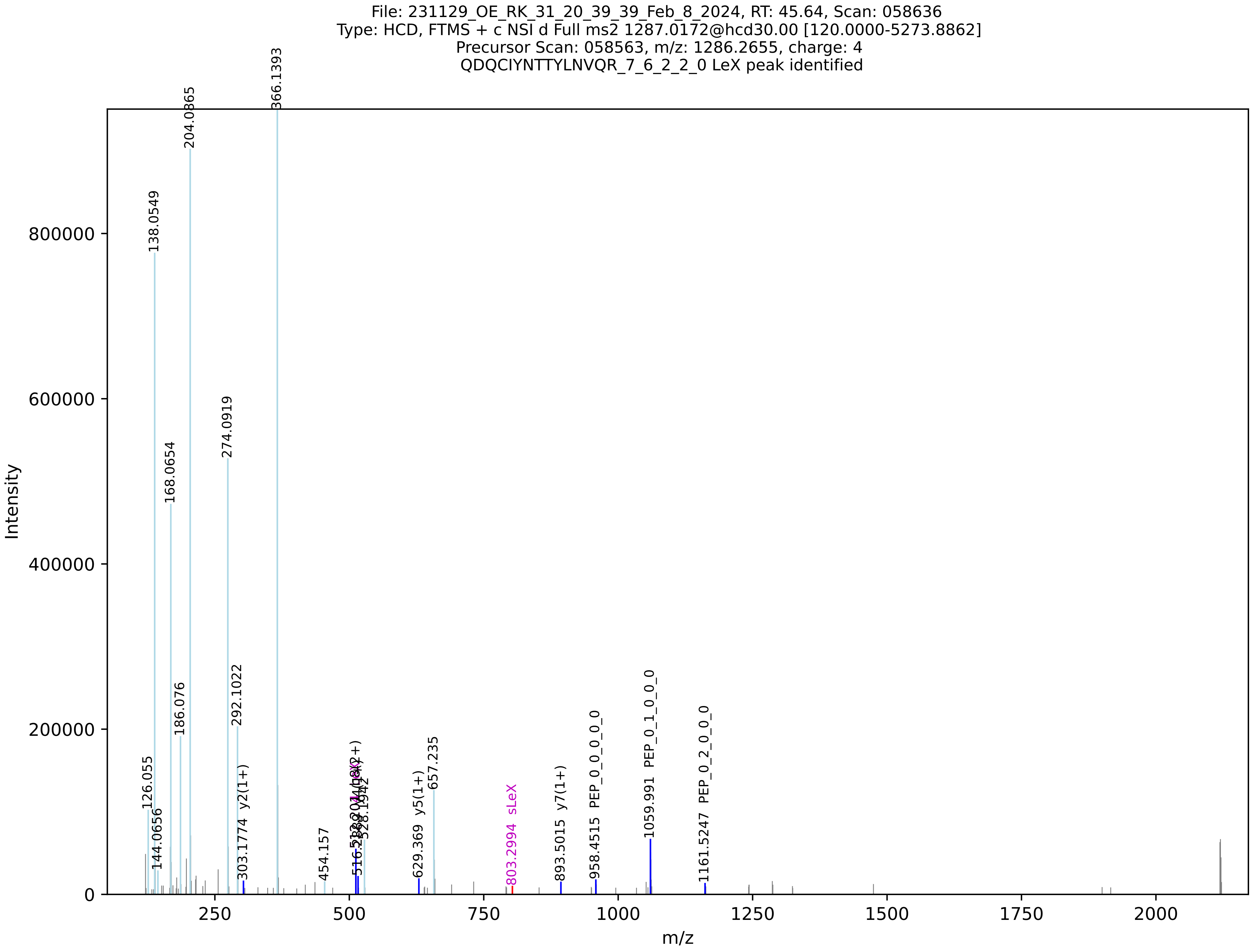
File: 231129_OE_RK_31_20_39_39_Feb_8_2024, RT: 45.64, Scan: 058636
Type: HCD, FTMS + c NSI d Full ms2 1287.0172@hcd30.00 [120.0000-5273.8862]
Precursor Scan: 058563, m/z: 1286.2655, charge: 4
QDQCIYNTTYLNVQR_7_6_2_2_0 LeX peak identified
Intensity
m/z
0
200000
400000
600000
800000
250
500
750
1000
1250
1500
1750
2000
366.1393
204.0865
138.0549
168.0654
274.0919
292.1022
186.076
126.055
144.0656
303.1774 y2(1+)
454.157
528.1942
629.369 y5(1+)
657.235
803.2994 sLeX
893.5015 y7(1+)
958.4515 PEP_0_0_0_0_0
1059.991 PEP_0_1_0_0_0
1161.5247 PEP_0_2_0_0_0

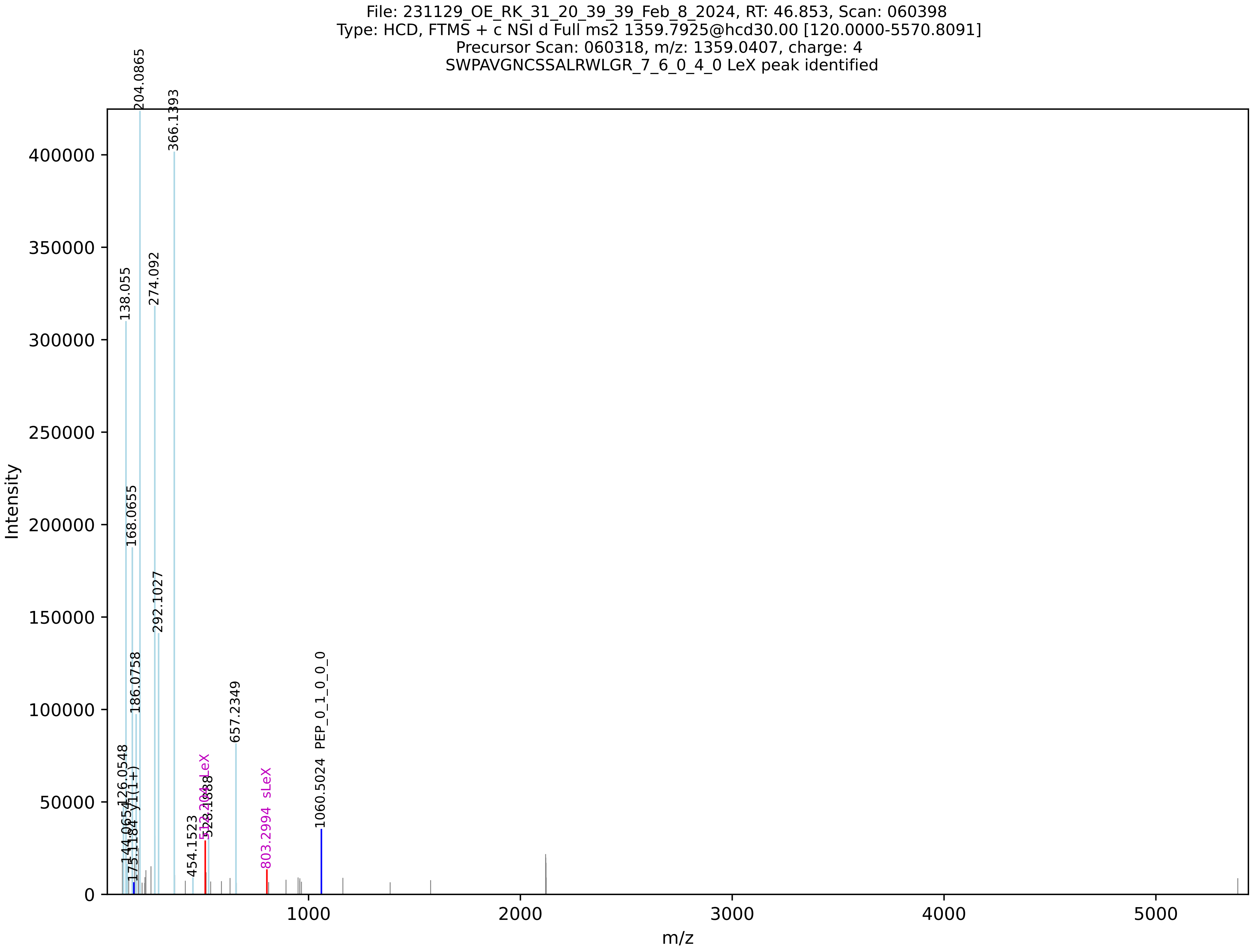
File: 231129_OE_RK_31_20_39_39_Feb_8_2024, RT: 46.853, Scan: 060398
Type: HCD, FTMS + c NSI d Full ms2 1359.7925@hcd30.00 [120.0000-5570.8091]
Precursor Scan: 060318, m/z: 1359.0407, charge: 4
SWPAVGNCSSALRWLGR_7_6_0_4_0 LeX peak identified
204.0865
366.1393
274.092
138.055
168.0655
292.1027
186.0758
657.2349
PEP_0_1_0_0_
1060.5024
126.0548
y1(1+)
144.0654
175.1184
454.1523
528.1888
LeX
803.2994 sLeX
Intensity
m/z
0
50000
100000
150000
200000
250000
300000
350000
400000
1000
2000
3000
4000
5000

File: 231129_OE_RK_31_20_39_39_Feb_8_2024, RT: 47.626, Scan: 061515
Type: HCD, FTMS + c NSI d Full ms2 1359.7886@hcd30.00 [120.0000-5570.7930]
Precursor Scan: 061434, m/z: 1359.2874, charge: 4
QDQCIYNTTYLNVQR_7_6_2_3_0 LeX peak identified

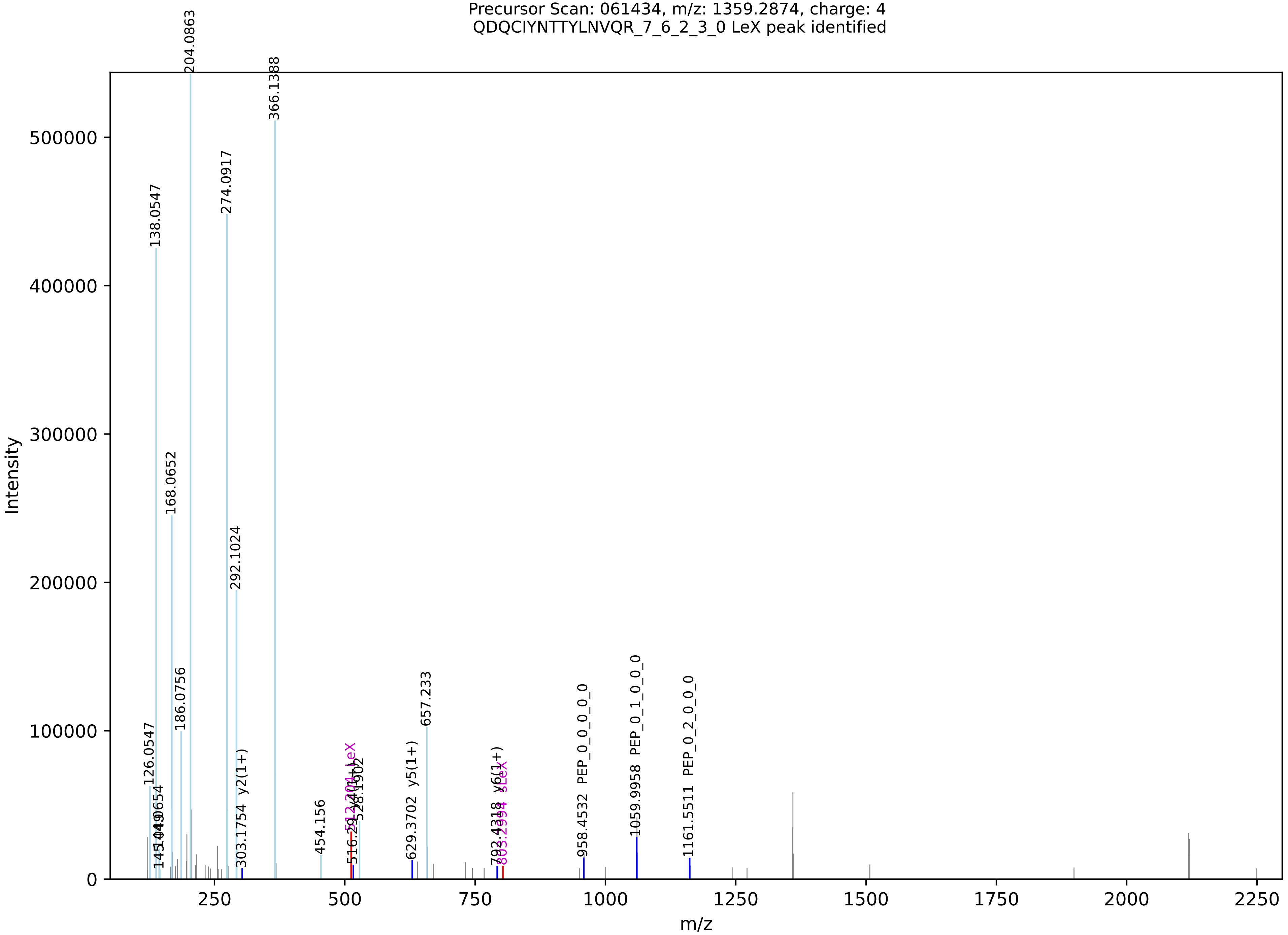

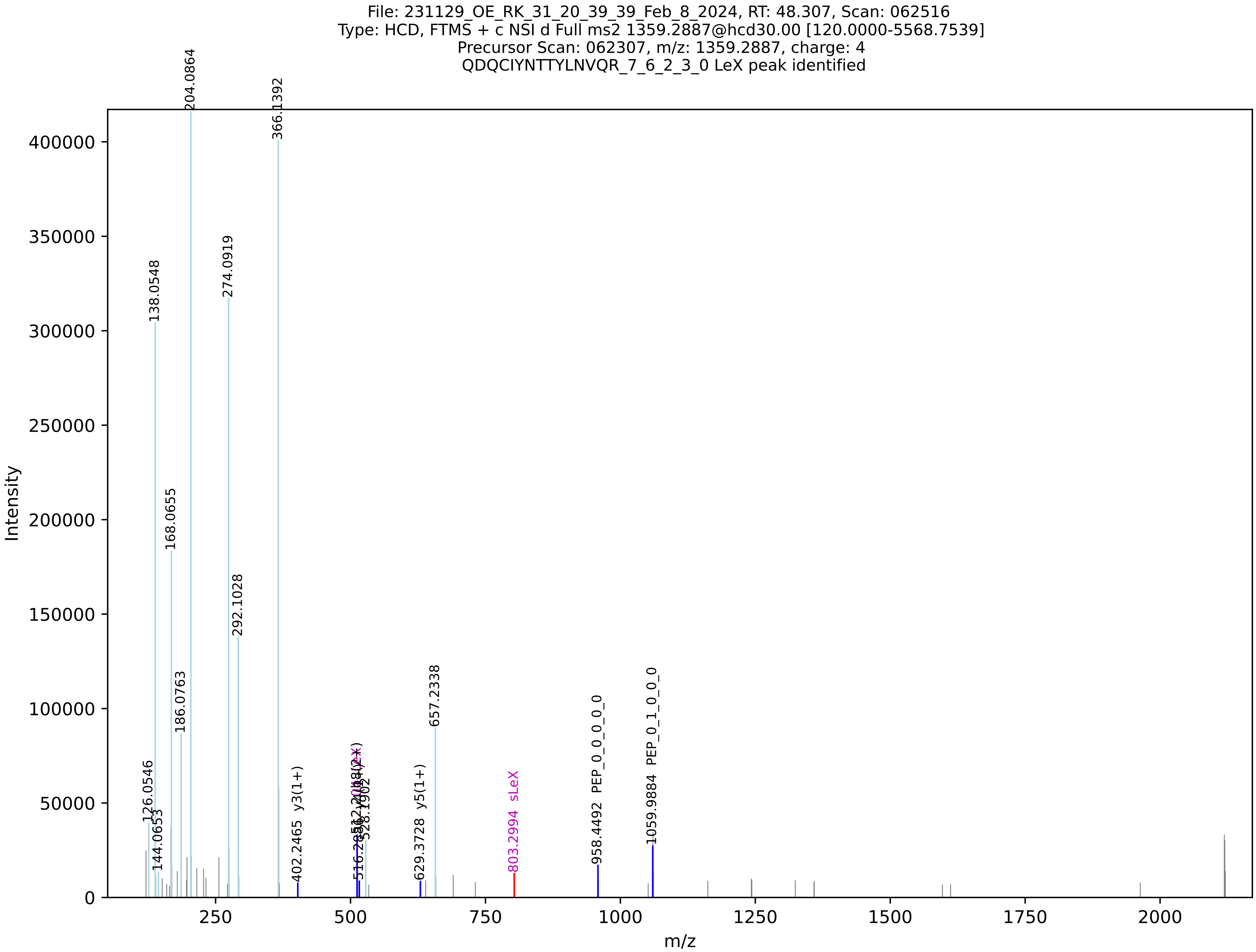
File: 231129_OE_RK_31_20_39_39_Feb_8_2024, RT: 48.307, Scan: 062516
Type: HCD, FTMS + c NSI d Full ms2 1359.2887@hcd30.00 [120.0000-5568.7539]
Precursor Scan: 062307, m/z: 1359.2887, charge: 4
QDQCIYNTTYLNVQR_7_6_2_3_0 LeX peak identified
Intensity
m/z
204.0864
366.1392
138.0548
274.0919
168.0655
292.1028
186.0763
657.2338
126.0546
144.0653
402.2465 y3(1+)
528.1902
629.3728 y5(1+)
803.2994 sLeX
958.4492 PEP_0_0_0_0_0_
1059.9884 PEP_0_1_0_0_0_
0
50000
100000
150000
200000
250000
300000
350000
400000
250
500
750
1000
1250
1500
1750
2000

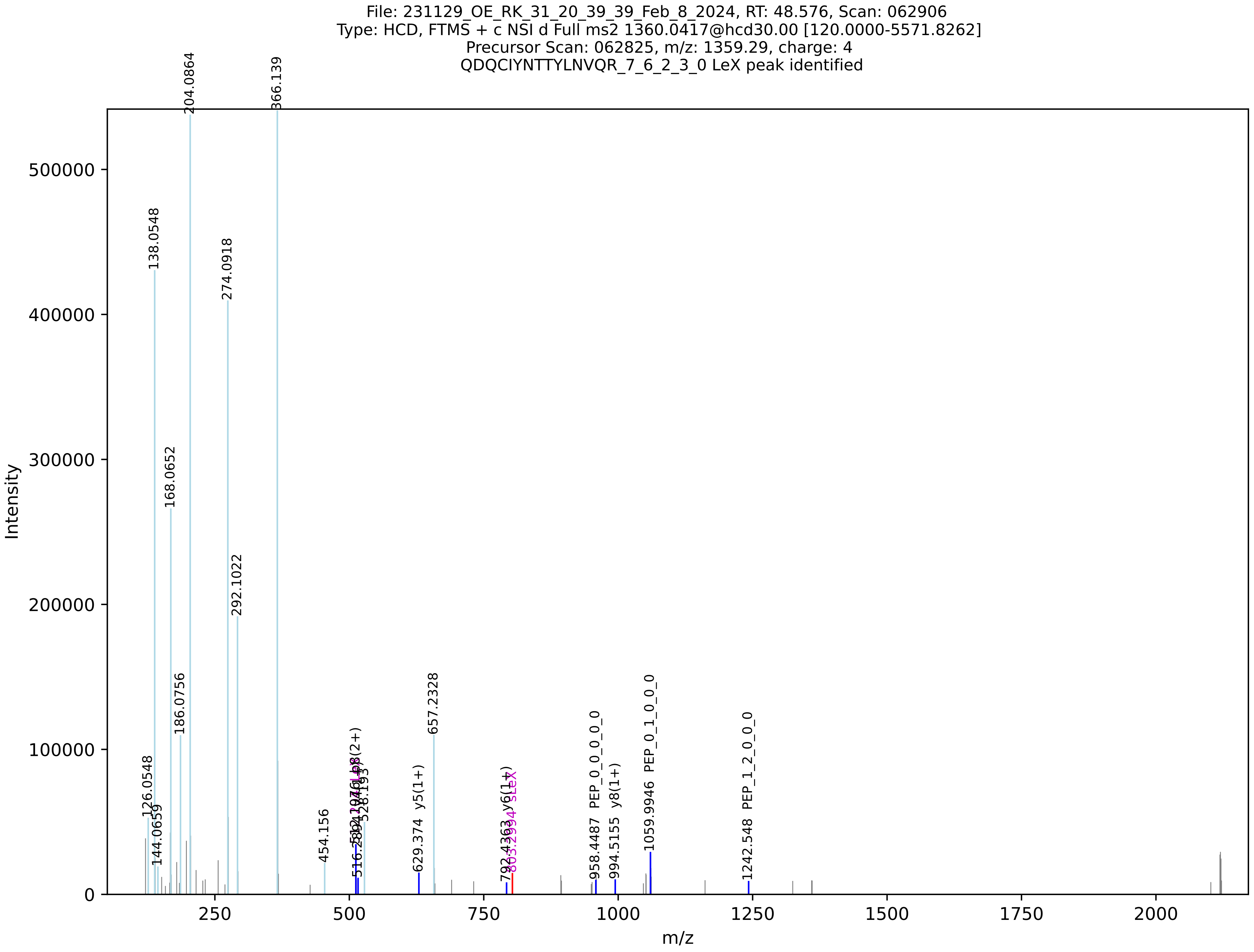
File: 231129_OE_RK_31_20_39_39_Feb_8_2024, RT: 48.576, Scan: 062906
Type: HCD, FTMS + c NSI d Full ms2 1360.0417@hcd30.00 [120.0000-5571.8262]
Precursor Scan: 062825, m/z: 1359.29, charge: 4
QDQCIYNTTYLNVQR_7_6_2_3_0 LeX peak identified
Intensity
m/z
0
100000
200000
300000
400000
500000
250
500
750
1000
1250
1500
1750
2000
204.0864
366.139
138.0548
274.0918
168.0652
292.1022
186.0756
126.0548
144.0659
454.156
528.193
629.374 y5(1+)
657.2328
792.4363 y6(1+)
803.2994 sLeX
958.4487 PEP_0_0_0_0_0
994.5155 y8(1+)
1059.9946 PEP_0_1_0_0_0
1242.548 PEP_1_2_0_0_0

File: 231129_OE_RK_31_20_39_39_Feb_8_2024, RT: 49.035, Scan: 063575
Type: HCD, FTMS + c NSI d Full ms2 1396.5519@hcd30.00 [120.0000-5720.7881]
Precursor Scan: 063488, m/z: 1395.5496, charge: 4
QDQCIYNTTYLNVQR_7_6_1_4_0 LeX peak identified

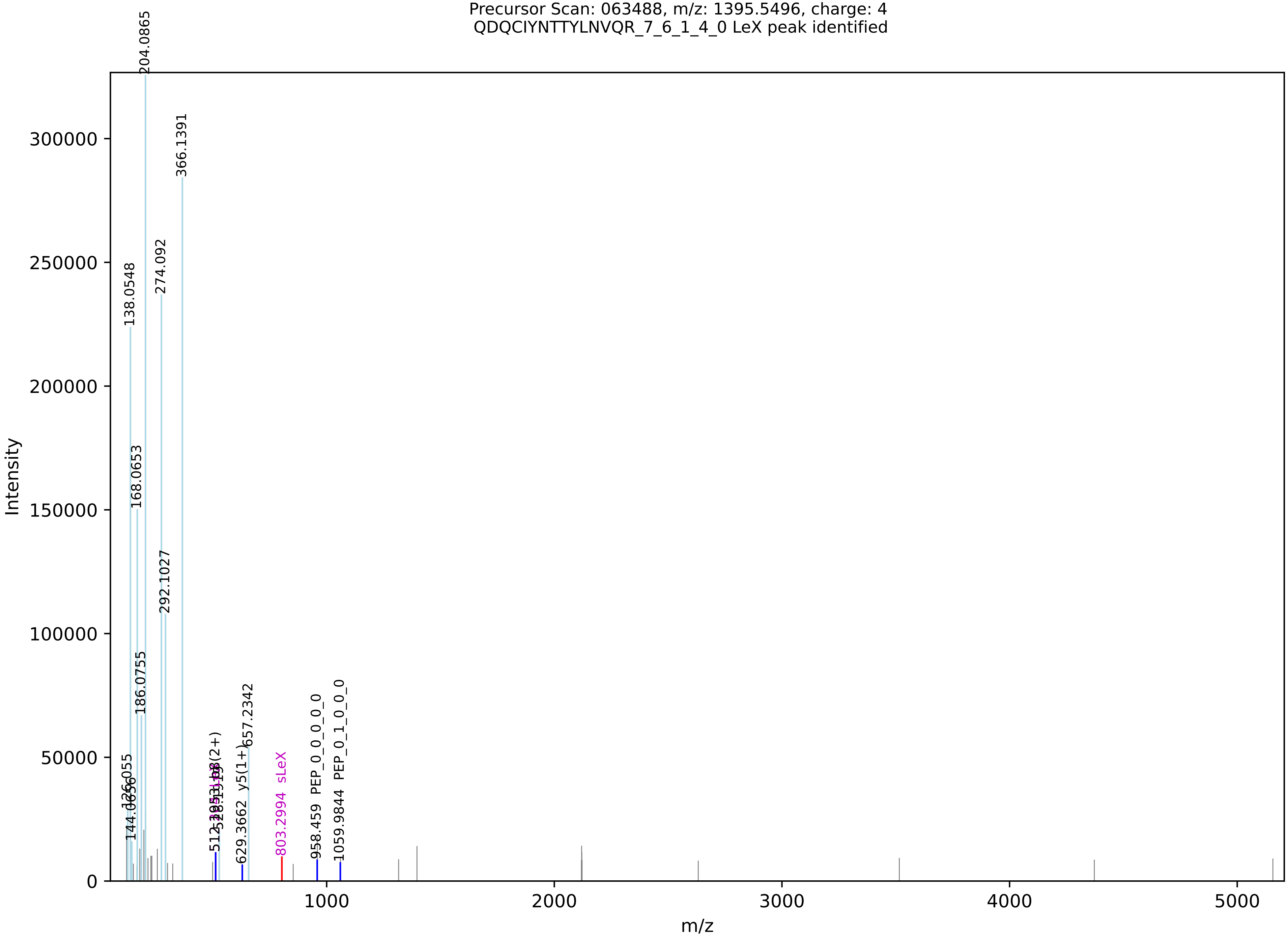

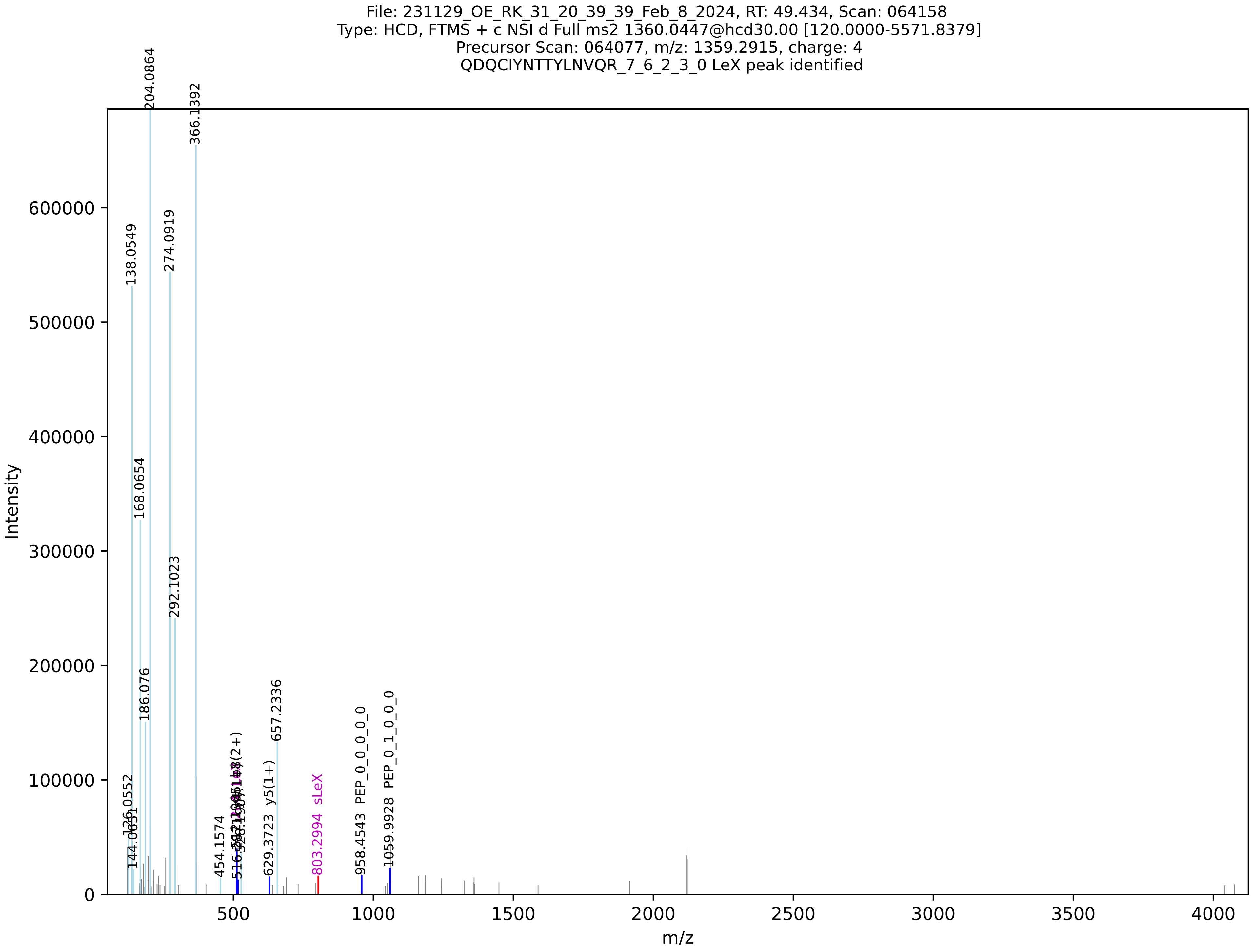
File: 231129_OE_RK_31_20_39_39_Feb_8_2024, RT: 49.434, Scan: 064158
Type: HCD, FTMS + c NSI d Full ms2 1360.0447@hcd30.00 [120.0000-5571.8379]
Precursor Scan: 064077, m/z: 1359.2915, charge: 4
QDQCIYNTTYLNVQR_7_6_2_3_0 LeX peak identified
204.0864
366.1392
274.0919
138.0549
168.0654
292.1023
186.076
657.2336
126.0552
454.1574
629.3723 y5(1+)
803.2994 sLeX
958.4543 PEP_0_0_0_0_0
1059.9928 PEP_0_1_0_0_0_
Intensity
m/z
0
100000
200000
300000
400000
500000
600000
500
1000
1500
2000
2500
3000
3500
4000

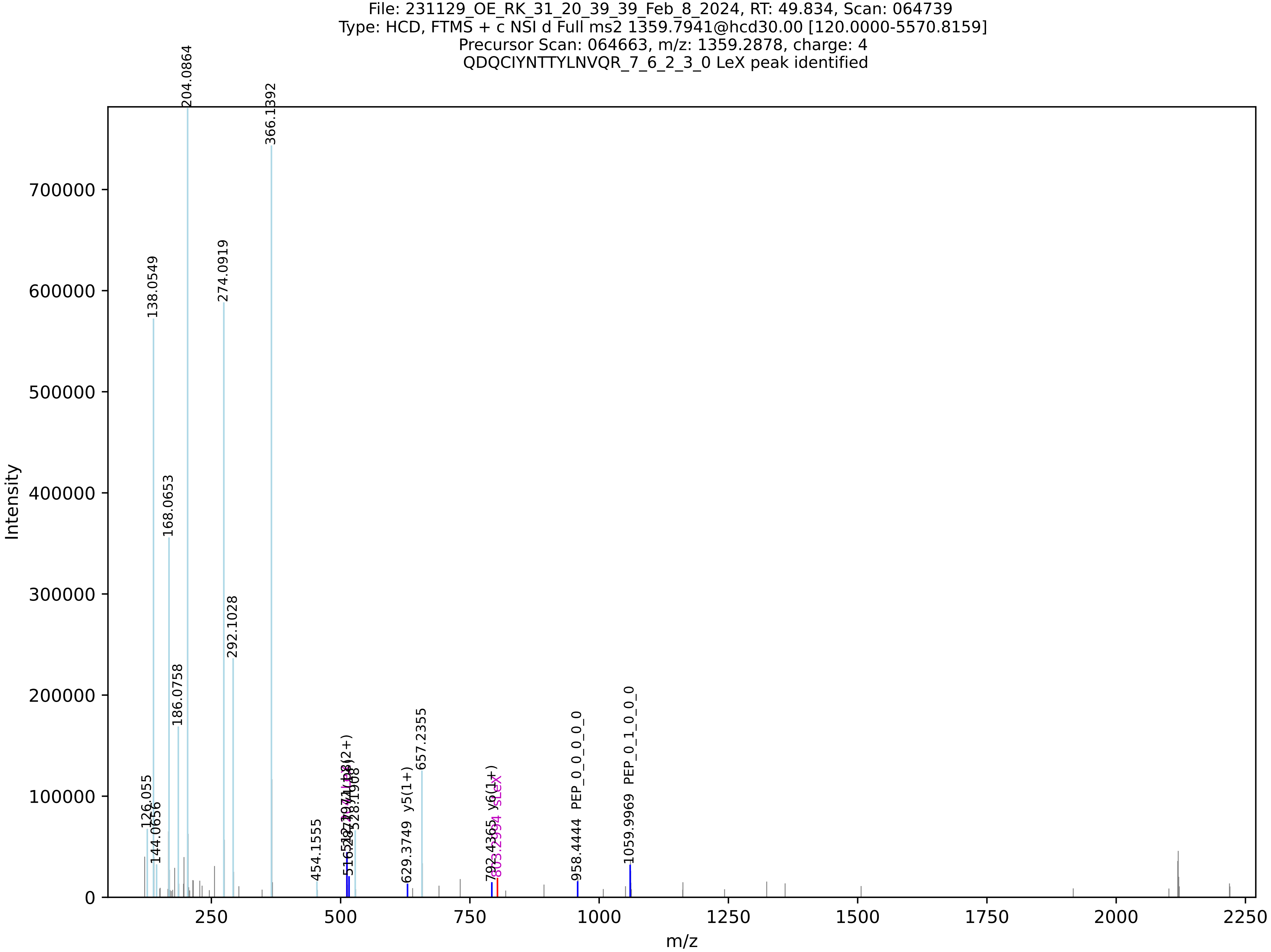
File: 231129_OE_RK_31_20_39_39_Feb_8_2024, RT: 49.834, Scan: 064739
Type: HCD, FTMS + c NSI d Full ms2 1359.7941@hcd30.00 [120.0000-5570.8159]
Precursor Scan: 064663, m/z: 1359.2878, charge: 4
QDQCIYNTTYLNVQR_7_6_2_3_0 LeX peak identified
Intensity
m/z
0
100000
200000
300000
400000
500000
600000
700000
250
500
750
1000
1250
1500
1750
2000
2250
204.0864
366.1392
138.0549
274.0919
168.0653
292.1028
186.0758
126.055
144.0656
454.1555
528.1908
629.3749 y5(1+)
657.2355
792.4365 y6(1+)
803.2994 sLeX
958.4444 PEP_0_0_0_0_0
1059.9969 PEP_0_1_0_0_0

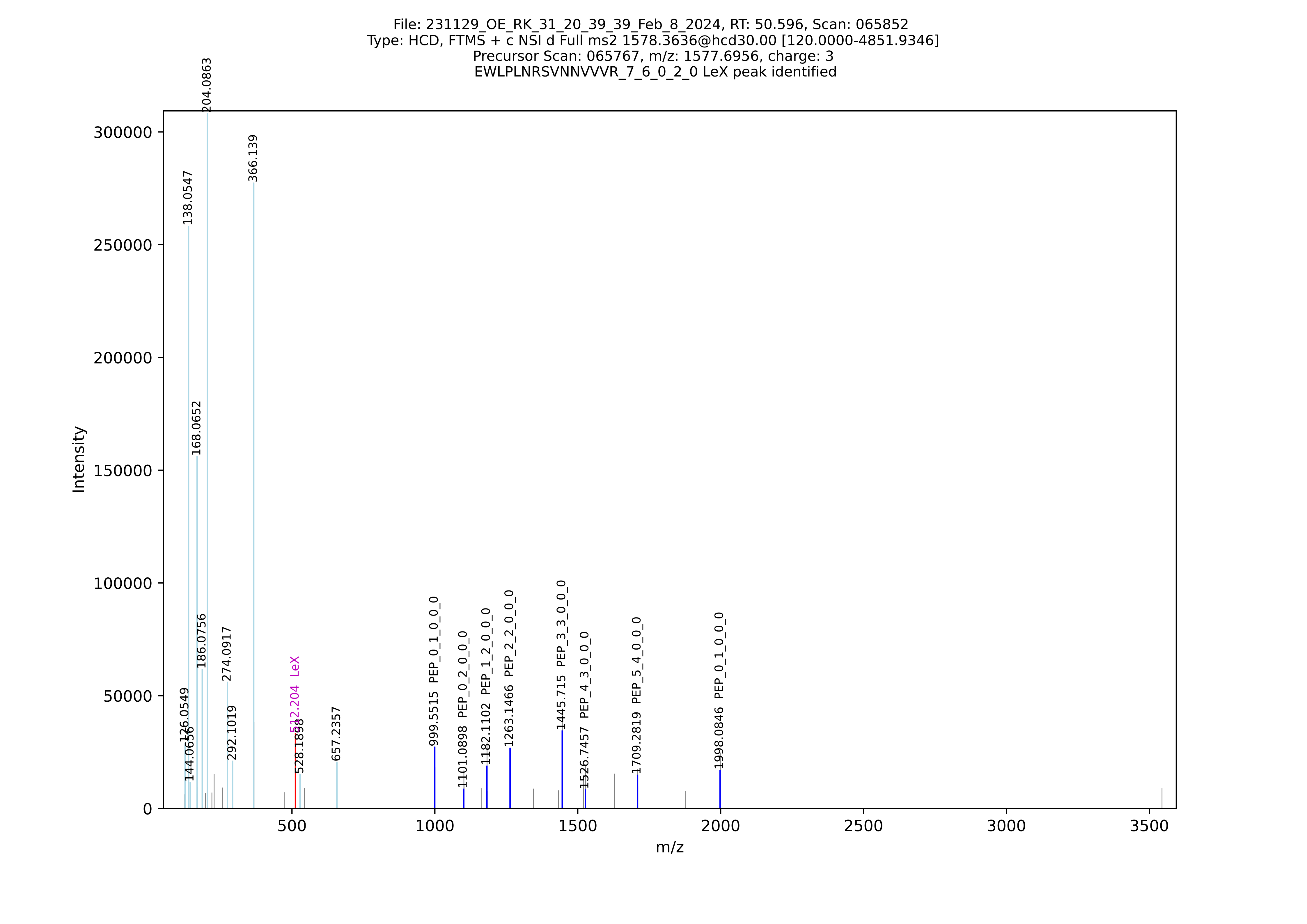
File: 231129_OE_RK_31_20_39_39_Feb_8_2024, RT: 50.596, Scan: 065852
Type: HCD, FTMS + c NSI d Full ms2 1578.3636@hcd30.00 [120.0000-4851.9346]
Precursor Scan: 065767, m/z: 1577.6956, charge: 3
EWLPLNRSVNNVVVR_7_6_0_2_0 LeX peak identified
Intensity
m/z
204.0863
366.139
138.0547
168.0652
186.0756
274.0917
126.0549
144.0656
292.1019
512.204 LeX
528.1898
657.2357
999.5515 PEP_0_1_0_0_0
1101.0898 PEP_0_2_0_0_0
1182.1102 PEP_1_2_0_0_0
1263.1466 PEP_2_2_0_0_0
1445.715 PEP_3_3_0_0_0
1526.7457 PEP_4_3_0_0_0
1709.2819 PEP_5_4_0_0_0
1998.0846 PEP_0_1_0_0_0

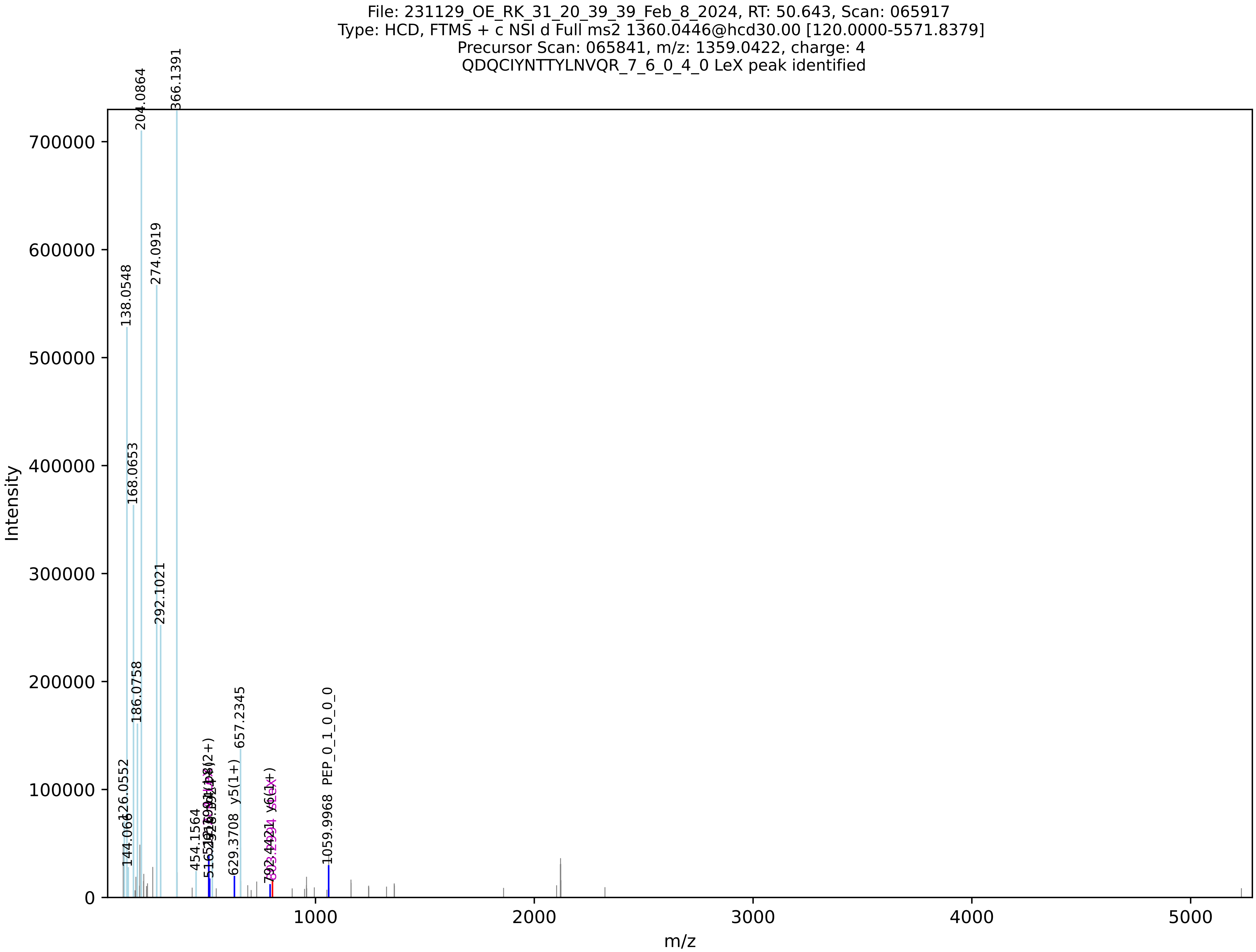
File: 231129_OE_RK_31_20_39_39_Feb_8_2024, RT: 50.643, Scan: 065917
Type: HCD, FTMS + c NSI d Full ms2 1360.0446@hcd30.00 [120.0000-5571.8379]
Precursor Scan: 065841, m/z: 1359.0422, charge: 4
QDQCIYNTTYLNVQR_7_6_0_4_0 LeX peak identified
366.1391
204.0864
274.0919
138.0548
168.0653
292.1021
186.0758
657.2345
126.0552
144.066
454.1564
629.3708 y5(1+)
1059.9968 PEP_0_1_0_0_0
Intensity
m/z

File: 231129_OE_RK_31_20_39_39_Feb_8_2024, RT: 50.85, Scan: 066222
Type: HCD, FTMS + c NSI d Full ms2 1092.7407@hcd30.00 [120.0000-4481.2383]
Precursor Scan: 066134, m/z: 1092.2396, charge: 4
EWLPLNRSVNNVVVR_6_5_0_2_0 LeX peak identified

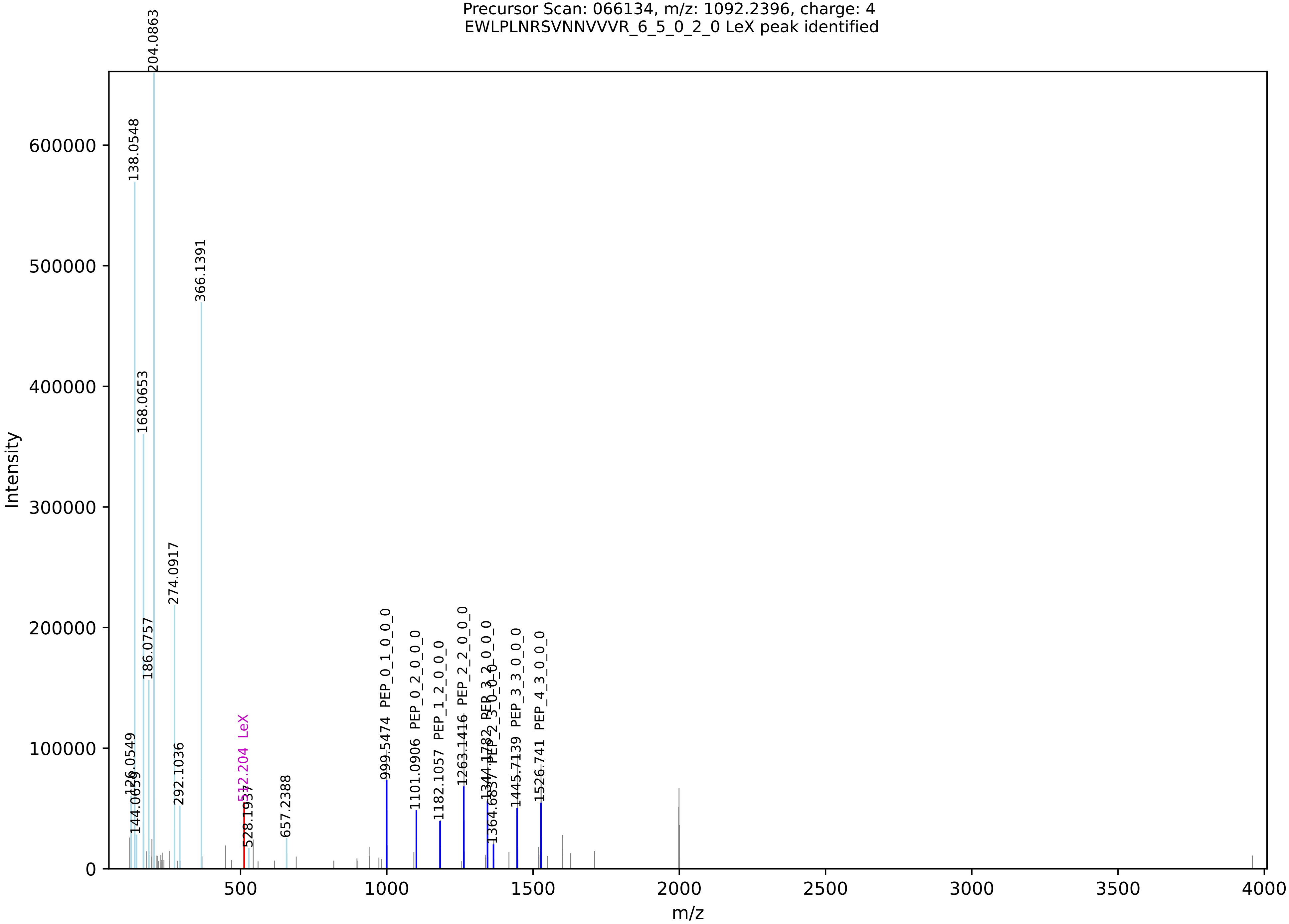

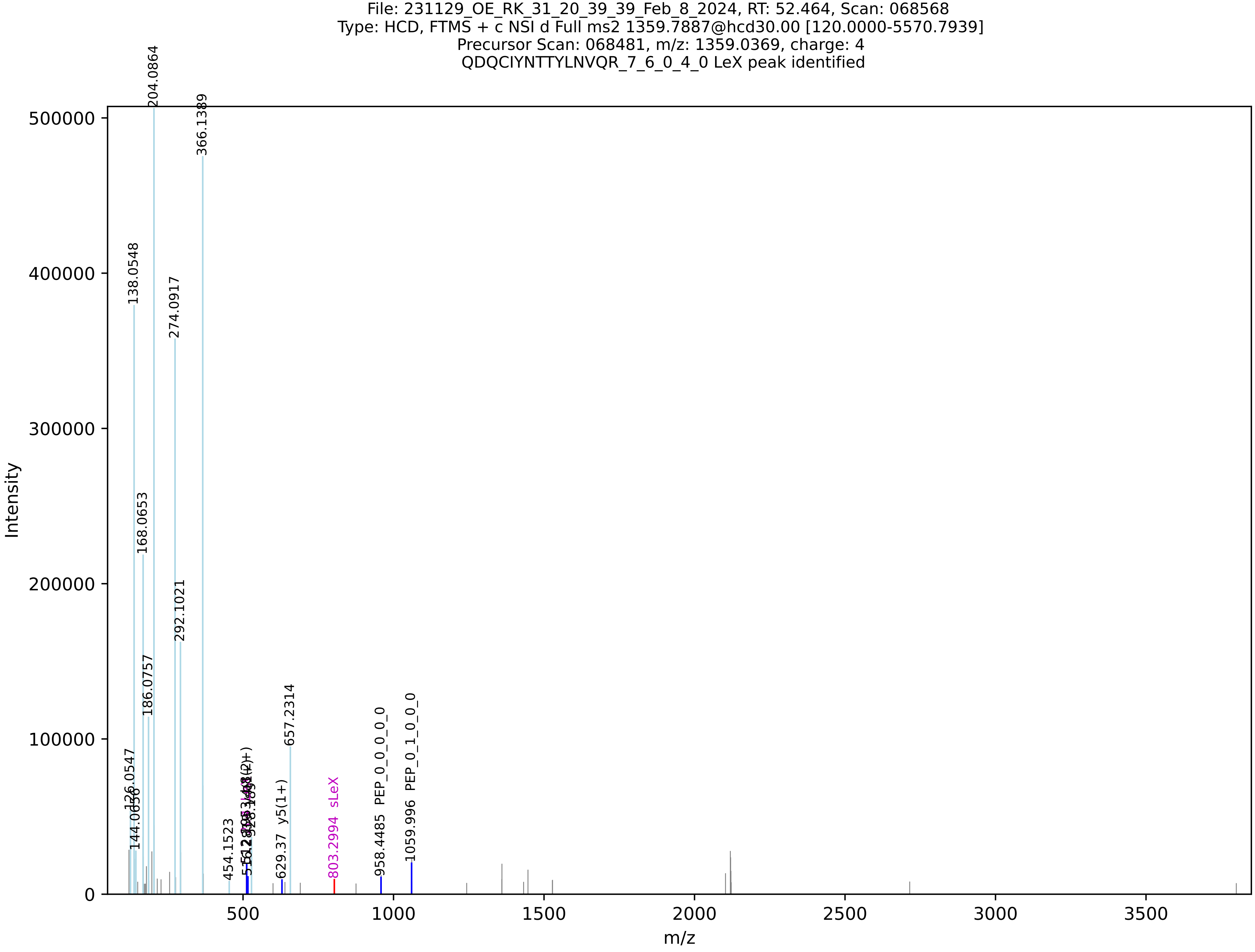
File: 231129_OE_RK_31_20_39_39_Feb_8_2024, RT: 52.464, Scan: 068568
Type: HCD, FTMS + c NSI d Full ms2 1359.7887@hcd30.00 [120.0000-5570.7939]
Precursor Scan: 068481, m/z: 1359.0369, charge: 4
QDQCIYNTTYLNVQR_7_6_0_4_0 LeX peak identified
204.0864
366.1389
138.0548
274.0917
168.0653
292.1021
186.0757
126.0547
144.0658
454.1523
657.2314
629.37 y5(1+)
803.2994 sLeX
958.4485 PEP_0_0_0_0_0_
1059.996 PEP_0_1_0_0_0_
Intensity
m/z
0
100000
200000
300000
400000
500000
500
1000
1500
2000
2500
3000
3500

File: 231129_OE_RK_31_20_39_39_Feb_8_2024, RT: 52.83, Scan: 069092
Type: HCD, FTMS + c NSI d Full ms2 1266.5425@hcd30.00 [120.0000-3897.7622]
Precursor Scan: 069021, m/z: 1265.8743, charge: 3
ALPQPQNVTSLLGCTH_5_4_1_1_0 LeX peak identified

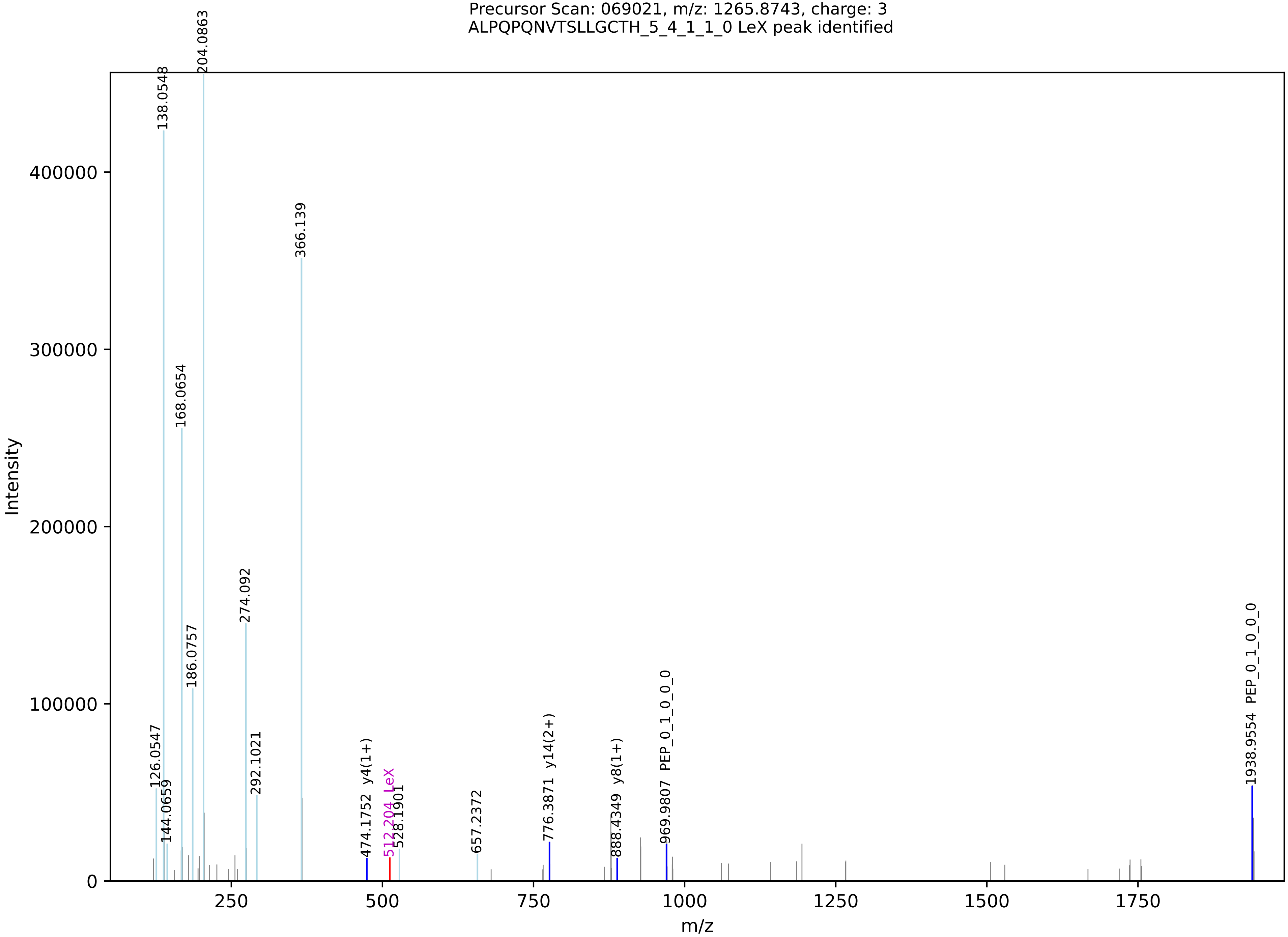

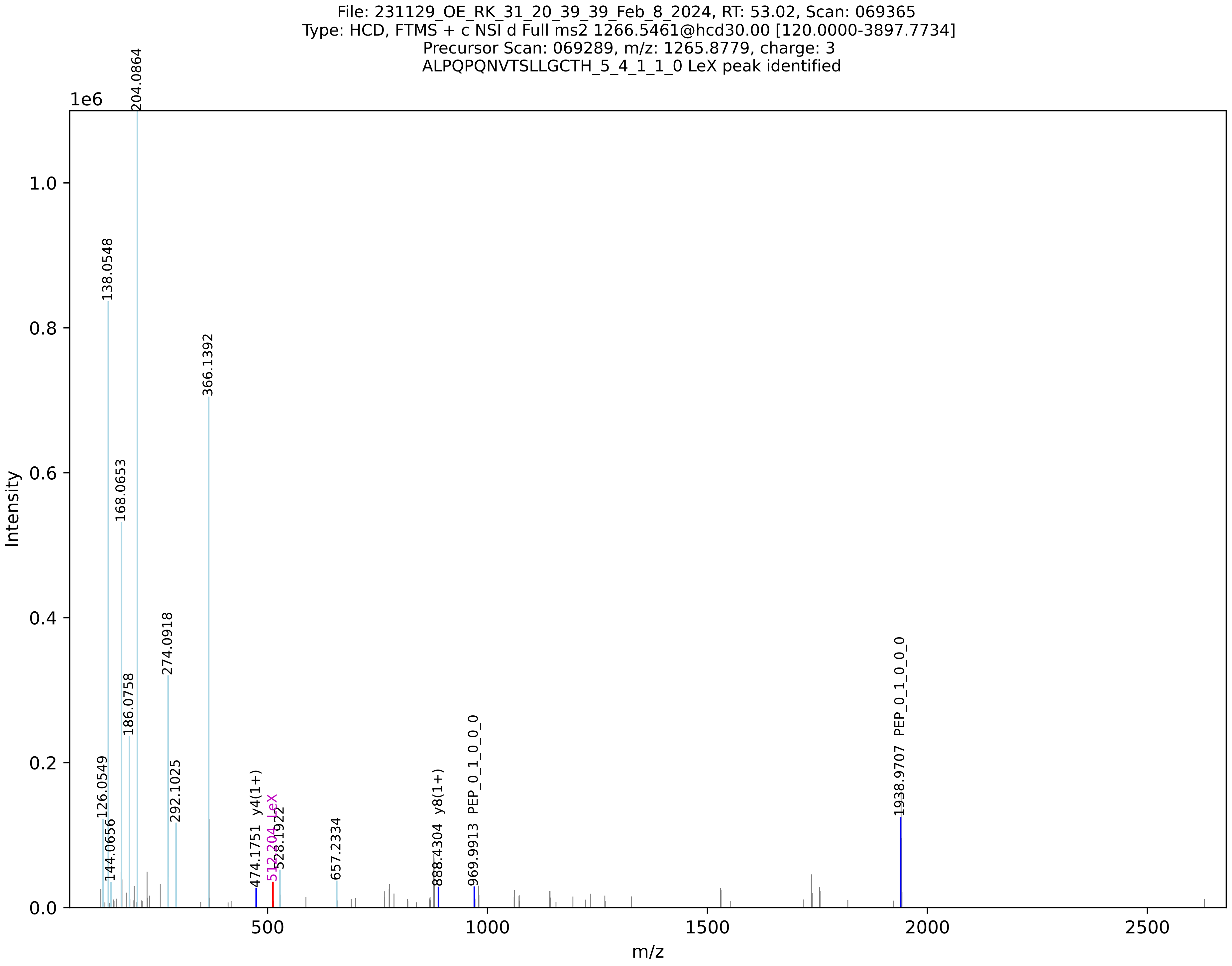

File: 231129_OE_RK_31_20_39_39_Feb_8_2024, RT: 53.02, Scan: 069365
Type: HCD, FTMS + c NSI d Full ms2 1266.5461@hcd30.00 [120.0000-3897.7734]
Precursor Scan: 069289, m/z: 1265.8779, charge: 3
ALPQPQNVTSLLGCTH_5_4_1_1_0 LeX peak identified
1e6
Intensity
m/z
204.0864
138.0548
168.0653
366.1392
274.0918
186.0758
126.0549
144.0656
292.1025
474.1751 y4(1+)
512.204_LeX
528.1922
657.2334
888.4304 y8(1+)
969.9913 PEP_0_1_0_0_0
1938.9707 PEP_0_1_0_0_0

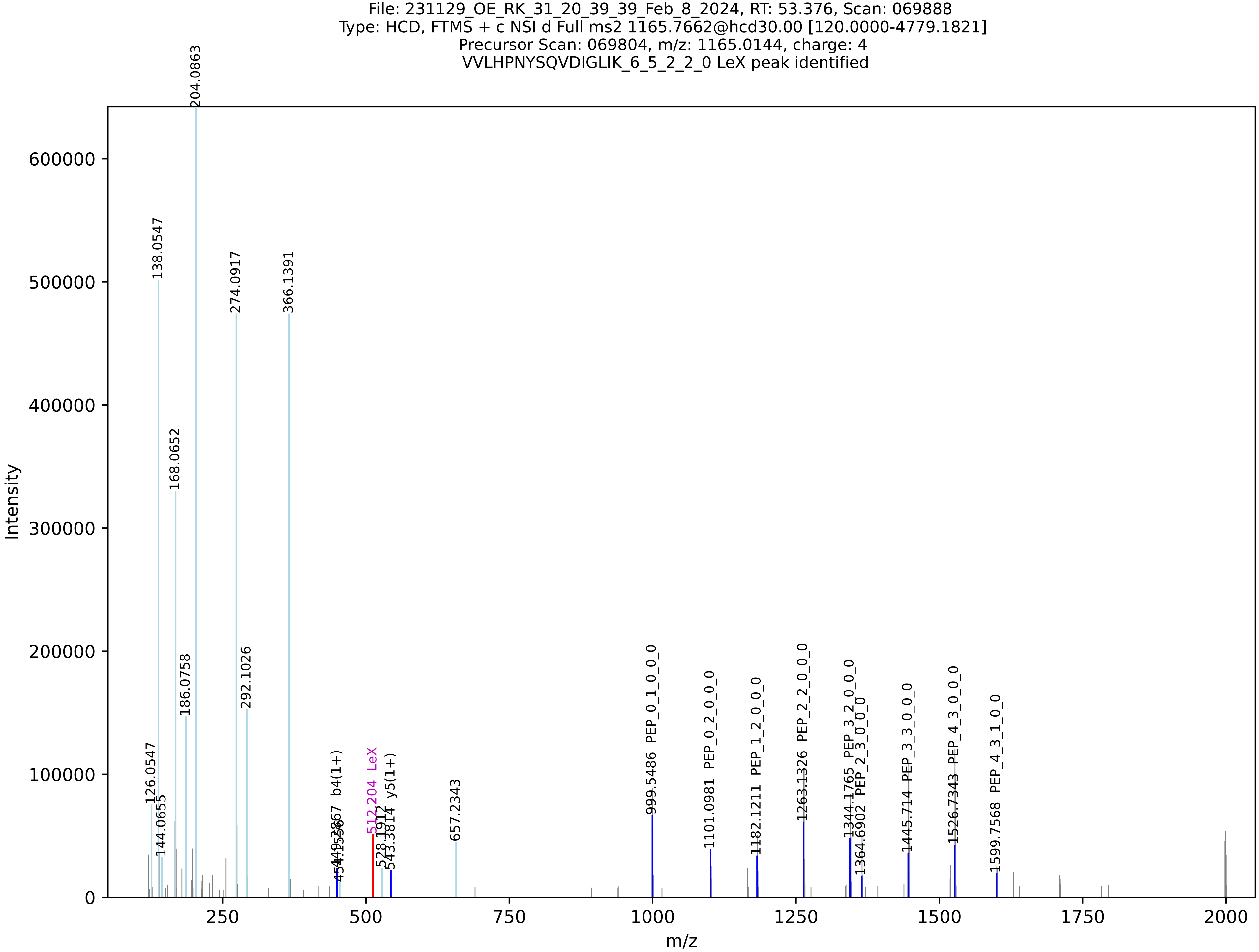
File: 231129_OE_RK_31_20_39_39_Feb_8_2024, RT: 53.376, Scan: 069888
Type: HCD, FTMS + c NSI d Full ms2 1165.7662@hcd30.00 [120.0000-4779.1821]
Precursor Scan: 069804, m/z: 1165.0144, charge: 4
VVLHPNYSQVDIGLIK_6_5_2_2_0 LeX peak identified
Intensity
m/z
204.0863
138.0547
168.0652
274.0917
366.1391
186.0758
292.1026
126.0547
144.0655
b4(1+)
512.204 LeX
528.1912
543.3814 y5(1+)
657.2343
999.5486 PEP_0_1_0_0_0
1101.0981 PEP_0_2_0_0_0
1182.1211 PEP_1_2_0_0_0
1263.1326 PEP_2_2_0_0_0
1344.1765 PEP_3_2_0_0_0
1364.6902 PEP_2_3_0_0_0
1445.714 PEP_3_3_0_0_0
1526.7343 PEP_4_3_0_0_0
1599.7568 PEP_4_3_1_0_0

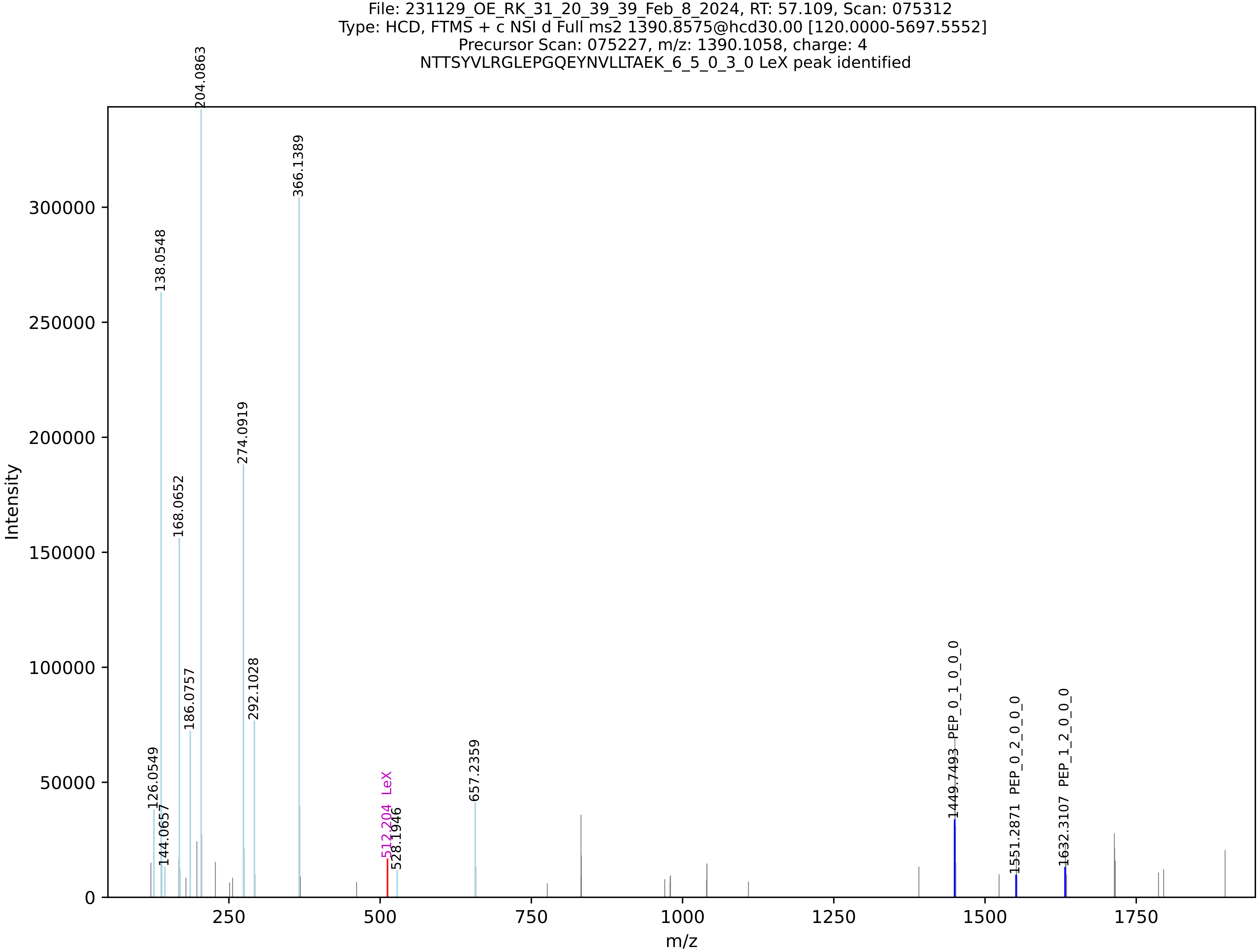
File: 231129_OE_RK_31_20_39_39_Feb_8_2024, RT: 57.109, Scan: 075312
Type: HCD, FTMS + c NSI d Full ms2 1390.8575@hcd30.00 [120.0000-5697.5552]
Precursor Scan: 075227, m/z: 1390.1058, charge: 4
NTTSYVLRGLEPGQEYNVLLTAEK_6_5_0_3_0 LeX peak identified
Intensity
m/z
204.0863
366.1389
138.0548
274.0919
168.0652
186.0757
292.1028
126.0549
144.0657
512.204 LeX
528.1946
657.2359
1449.7493_PEP_0_1_0_0_0
1551.2871_PEP_0_2_0_0_0
1632.3107_PEP_1_2_0_0_0

File: 231129_OE_RK_31_20_39_39_Feb_8_2024, RT: 63.535, Scan: 084653
Type: HCD, FTMS + c NSI d Full ms2 1114.2358@hcd30.00 [120.0000-4568.9380]
Precursor Scan: 084557, m/z: 1113.7346, charge: 4
VLSNNSDANLELINTWVAK_5_4_1_2_0 LeX peak identified

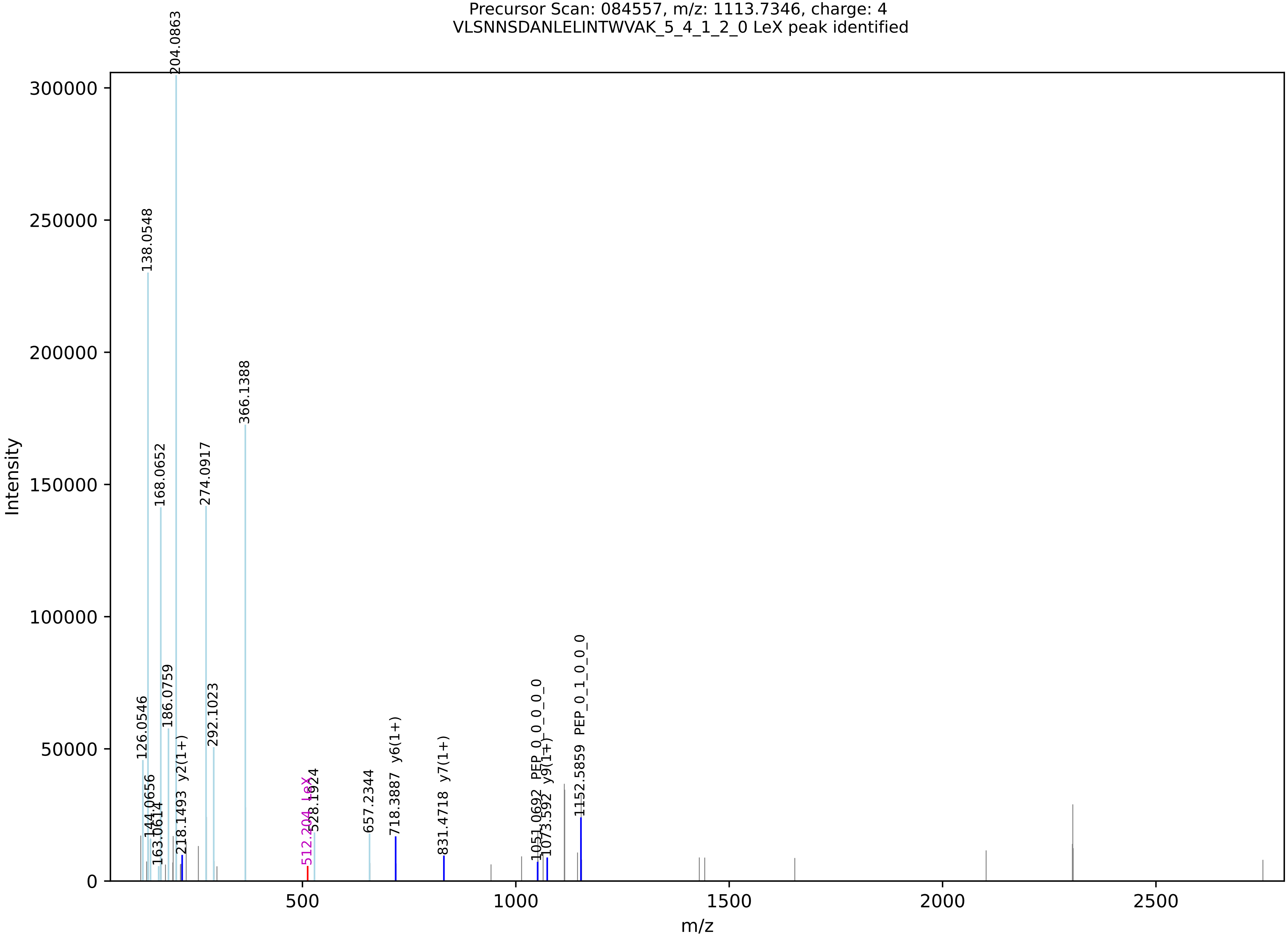

File: 231129_OE_RK_31_20_39_39_Feb_8_2024, RT: 65.017, Scan: 086803
Type: HCD, FTMS + c NSI d Full ms2 1273.2139@hcd30.00 [120.0000-3918.1763]
Precursor Scan: 086735, m/z: 1273.2138, charge: 3
NSSPTNFSKLISNGYK_5_4_1_1_0 LeX peak identified

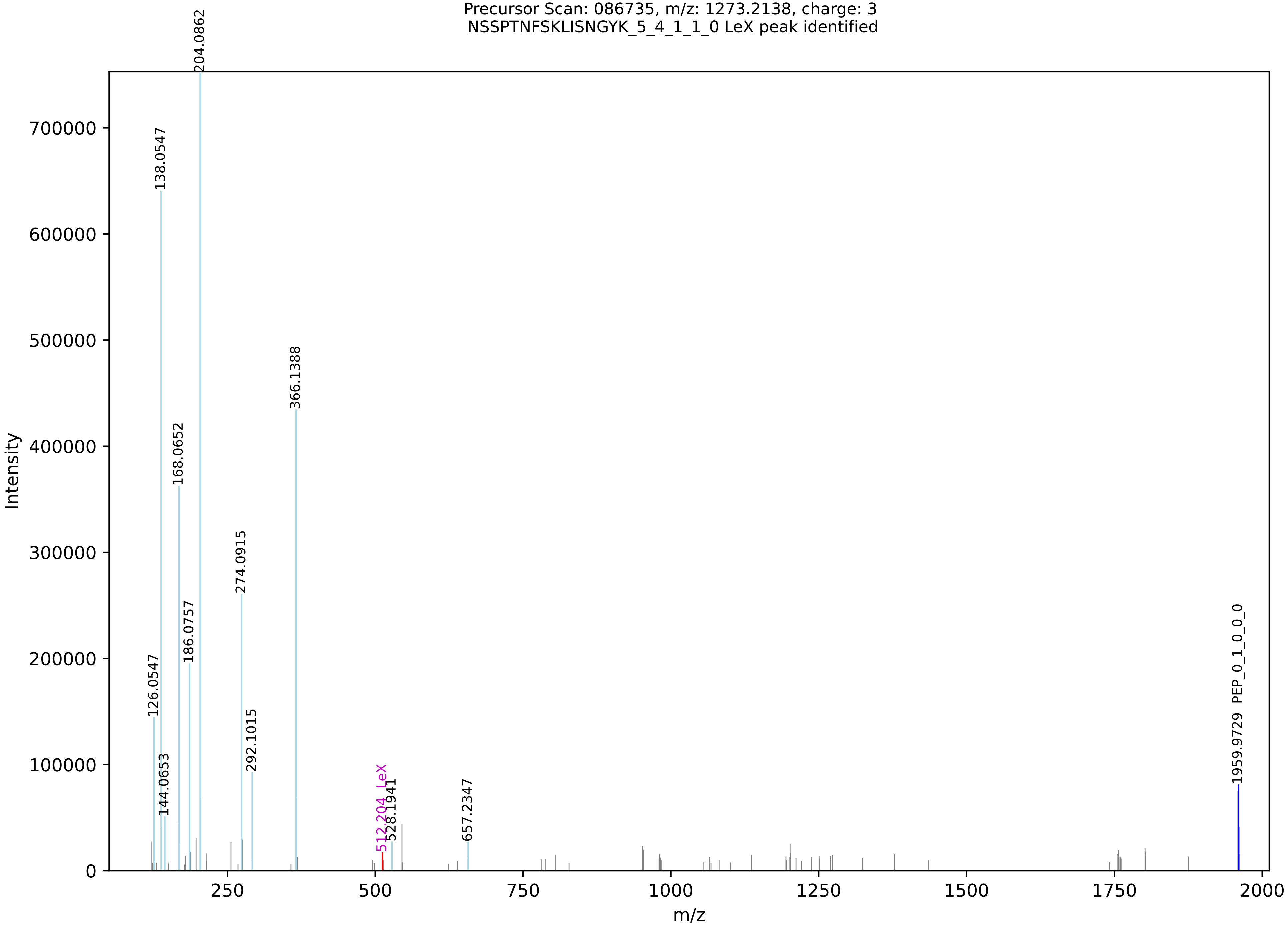

File: 231129_OE_RK_31_20_39_39_Feb_8_2024, RT: 65.201, Scan: 087071
Type: HCD, FTMS + c NSI d Full ms2 1273.2172@hcd30.00 [120.0000-3918.1868]
Precursor Scan: 086987, m/z: 1272.549, charge: 3
YLGNATAIFFLPDEGK_5_4_1_1_0 LeX peak identified

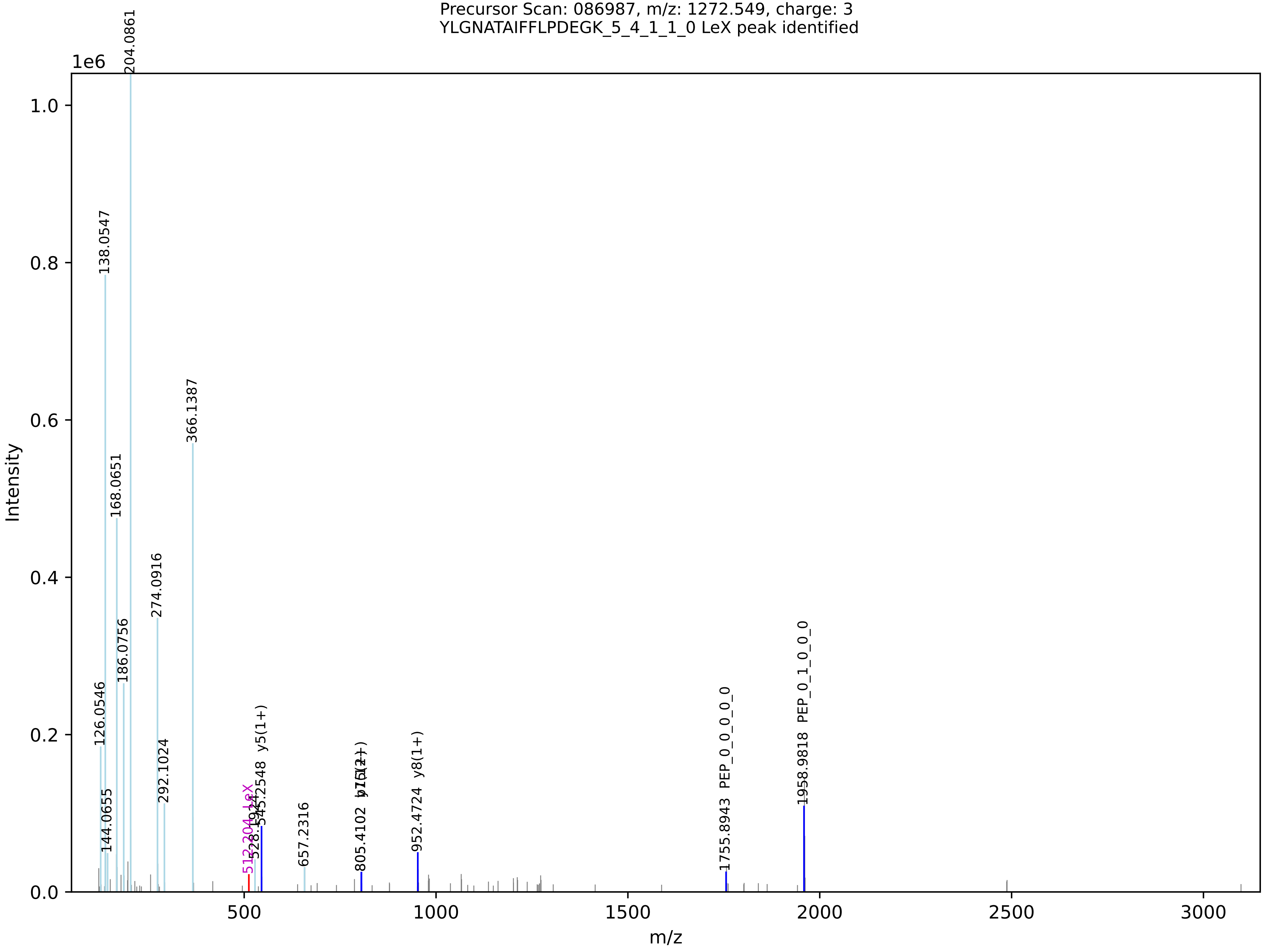

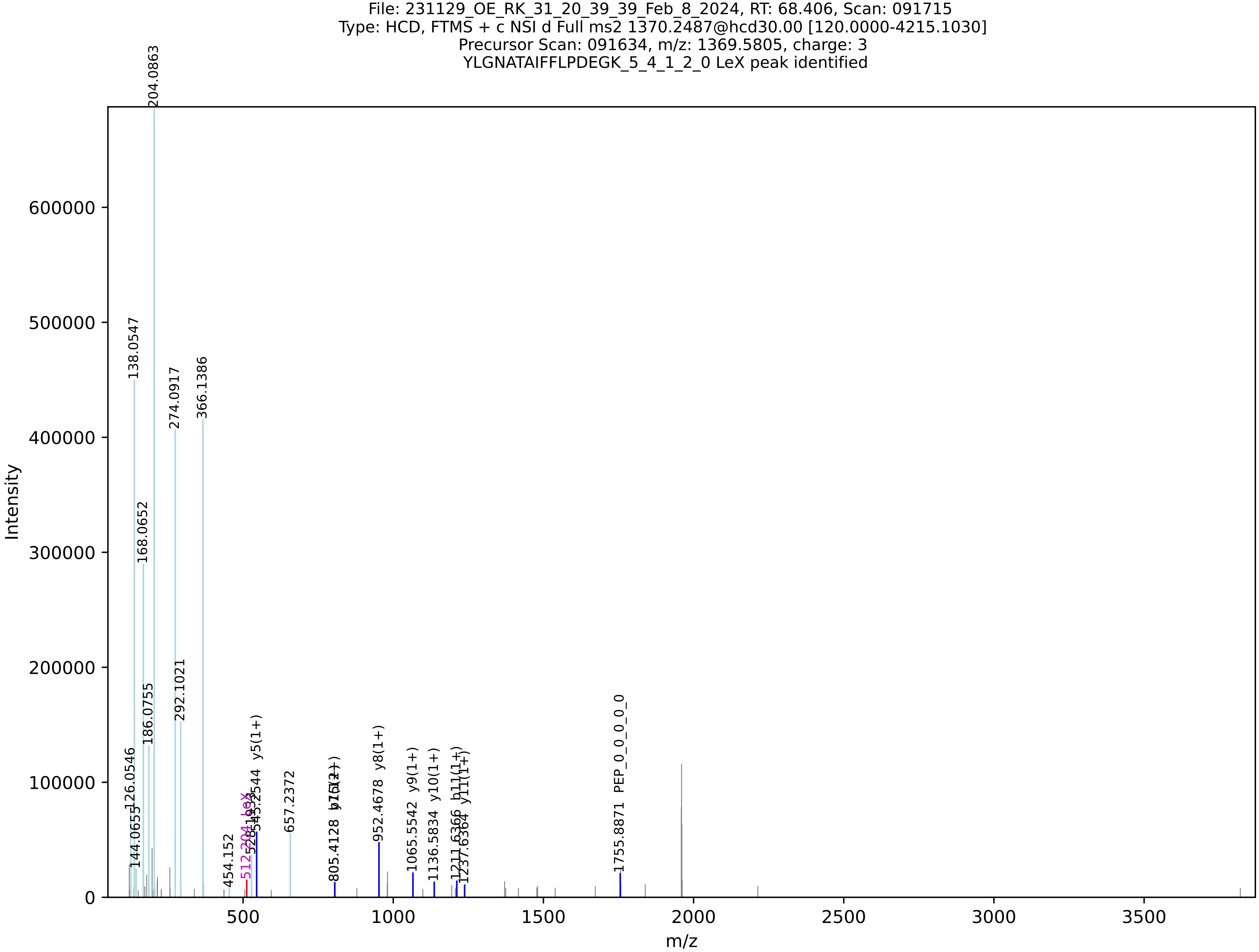

File: 231129_OE_RK_31_20_39_39_Feb_8_2024, RT: 68.406, Scan: 091715
Type: HCD, FTMS + c NSI d Full ms2 1370.2487@hcd30.00 [120.0000-4215.1030]
Precursor Scan: 091634, m/z: 1369.5805, charge: 3
YLGNATAIFFLPDEGK_5_4_1_2_0 LeX peak identified
Intensity
m/z
204.0863
138.0547
274.0917
366.1386
168.0652
292.1021
186.0755
126.0546
144.0655
454.152
512.204 LeX
543.2544 y5(1+)
657.2372
805.4128
952.4678 y8(1+)
1065.5542 y9(1+)
1136.5834 y10(1+)
1211.6366 b11(1+)
1237.6364 y11(1+)
1755.8871 PEP_0_0_0_0_0

File: 231129_OE_RK_31_20_39_39_Feb_8_2024, RT: 71.477, Scan: 096175
Type: HCD, FTMS + c NSI d Full ms2 1459.1410@hcd30.00 [120.0000-5976.1514]
Precursor Scan: 096101, m/z: 1458.6399, charge: 4
SVQEIQATFFYFTPNKTEDTIFLR_7_6_2_1_0 LeX peak identified

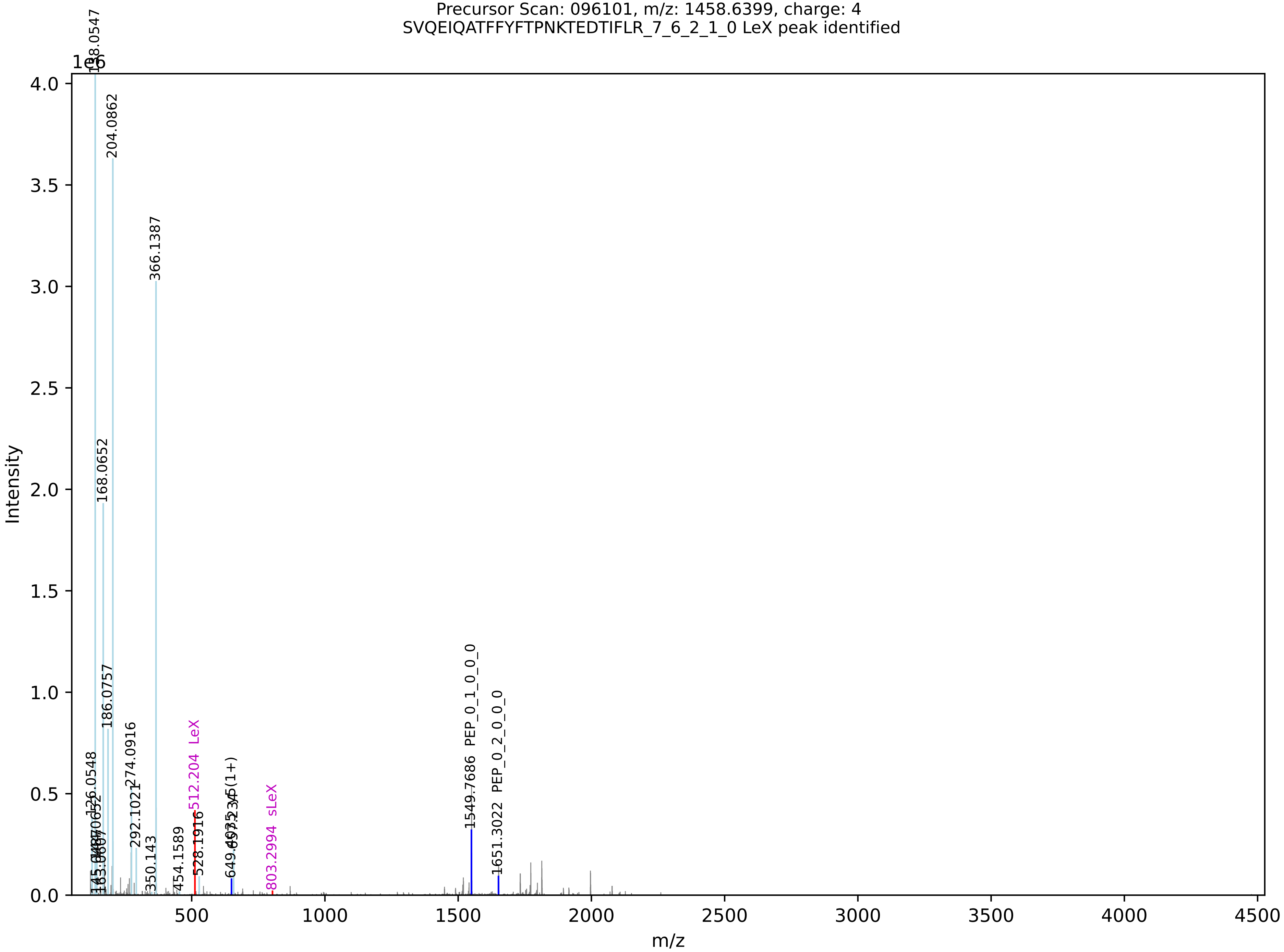

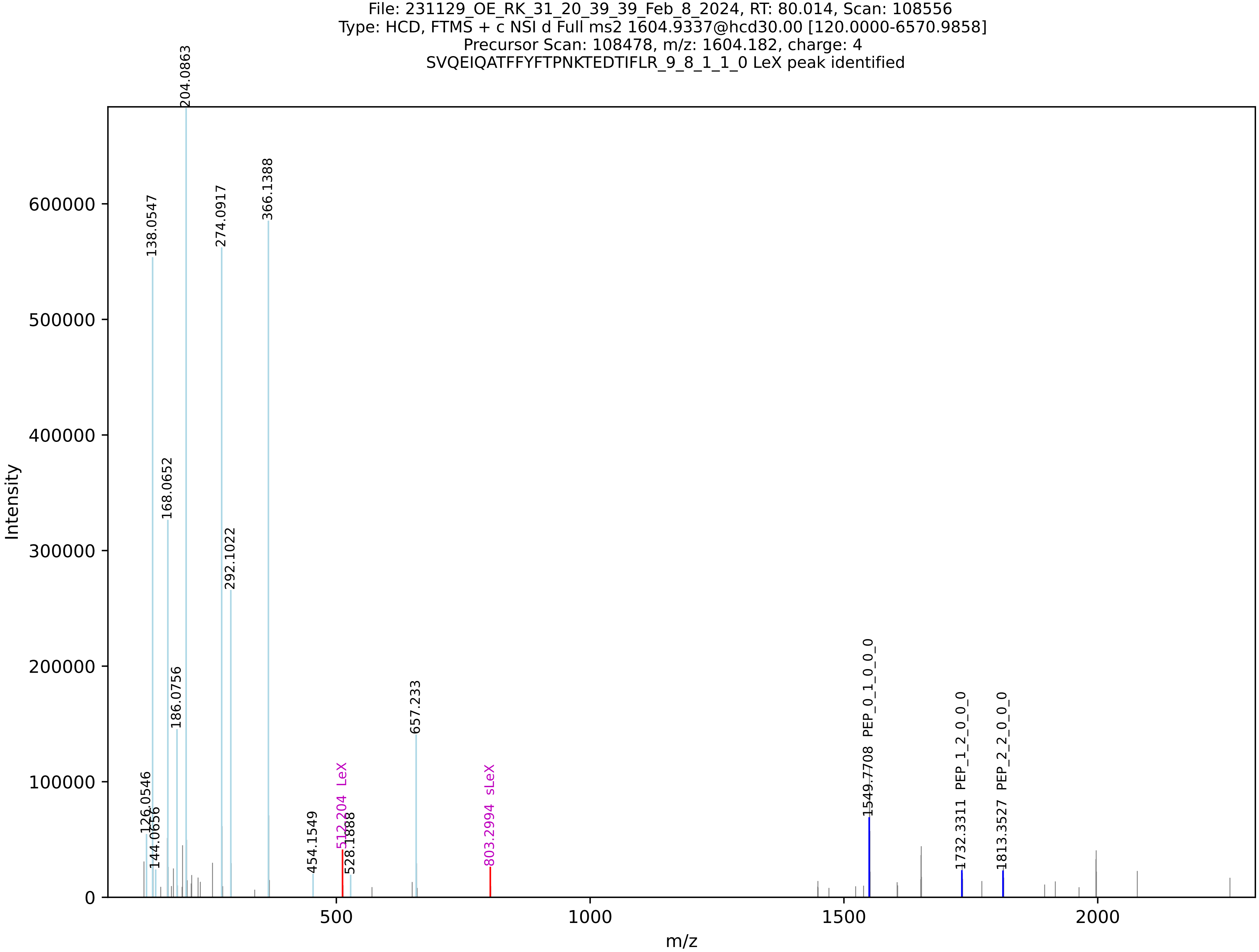
File: 231129_OE_RK_31_20_39_39_Feb_8_2024, RT: 80.014, Scan: 108556
Type: HCD, FTMS + c NSI d Full ms2 1604.9337@hcd30.00 [120.0000-6570.9858]
Precursor Scan: 108478, m/z: 1604.182, charge: 4
SVQEIQATFFYFTPNKTEDTIFLR_9_8_1_1_0 LeX peak identified
Intensity
m/z
204.0863
138.0547
274.0917
366.1388
168.0652
292.1022
186.0756
657.233
126.0546
144.0656
454.1549
512.204 LeX
528.1888
803.2994 sLeX
1549.7708 PEP_0_1_0_0_0
1732.3311 PEP_1_2_0_0_0
1813.3527 PEP_2_2_0_0_0
0
100000
200000
300000
400000
500000
600000
500
1000
1500
2000

File: 231129_OE_RK_32_6ul, RT: 43.242, Scan: 052695
Type: HCD, FTMS + c NSI d Full ms2 1545.2922@hcd30.00 [120.0000-4750.7363]
Precursor Scan: 052611, m/z: 1544.6237, charge: 3
QDQCIYNTTYLNVQR_6_5_1_2_0 LeX peak identified

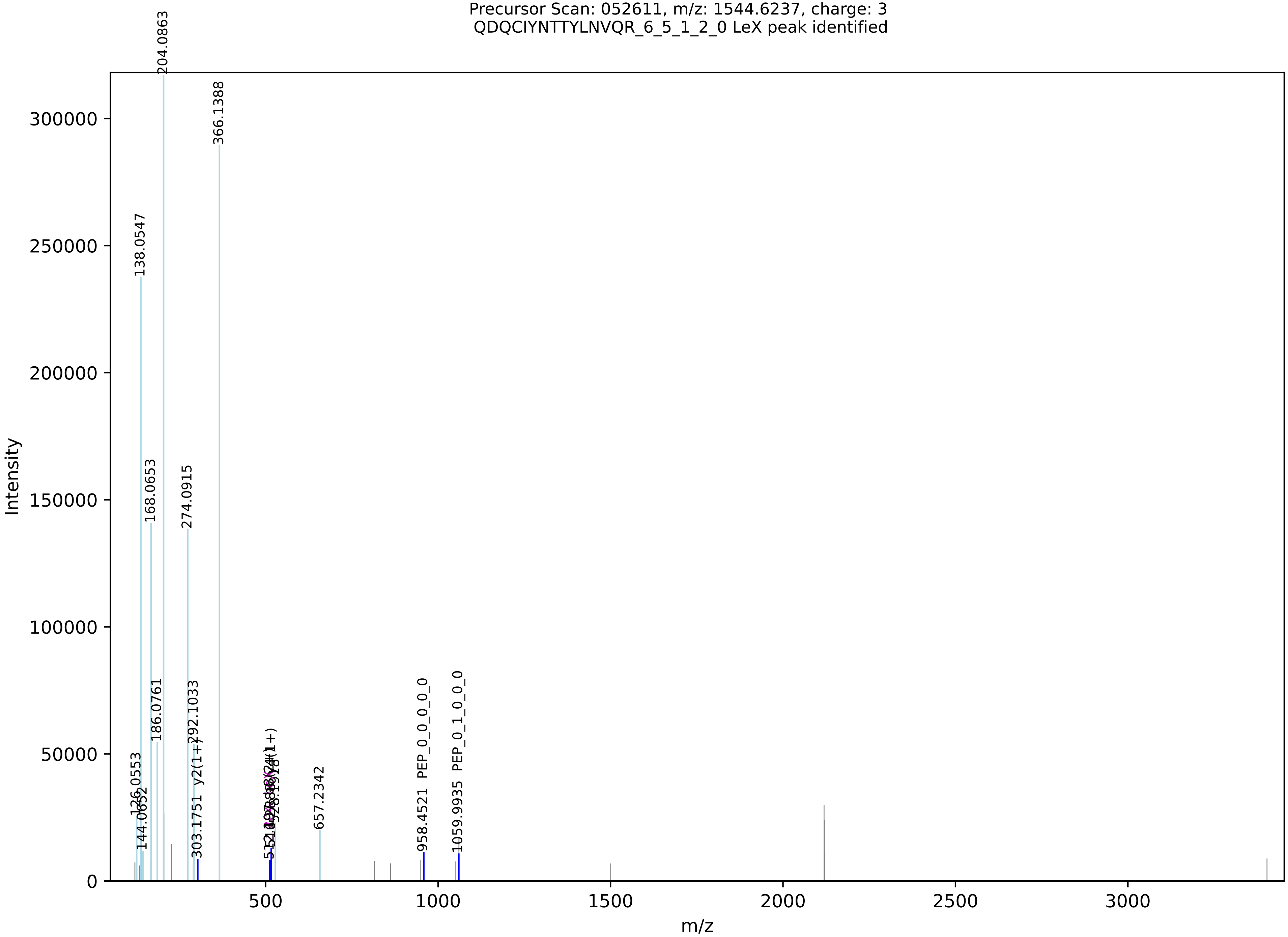

File: 231129_OE_RK_32_6ul, RT: 43.539, Scan: 053123
Type: HCD, FTMS + c NSI d Full ms2 1159.2212@hcd30.00 [120.0000-4752.4785]
Precursor Scan: 053042, m/z: 1158.7204, charge: 4
QDQCIYNTTYLNVQR_6_5_1_2_0 LeX peak identified

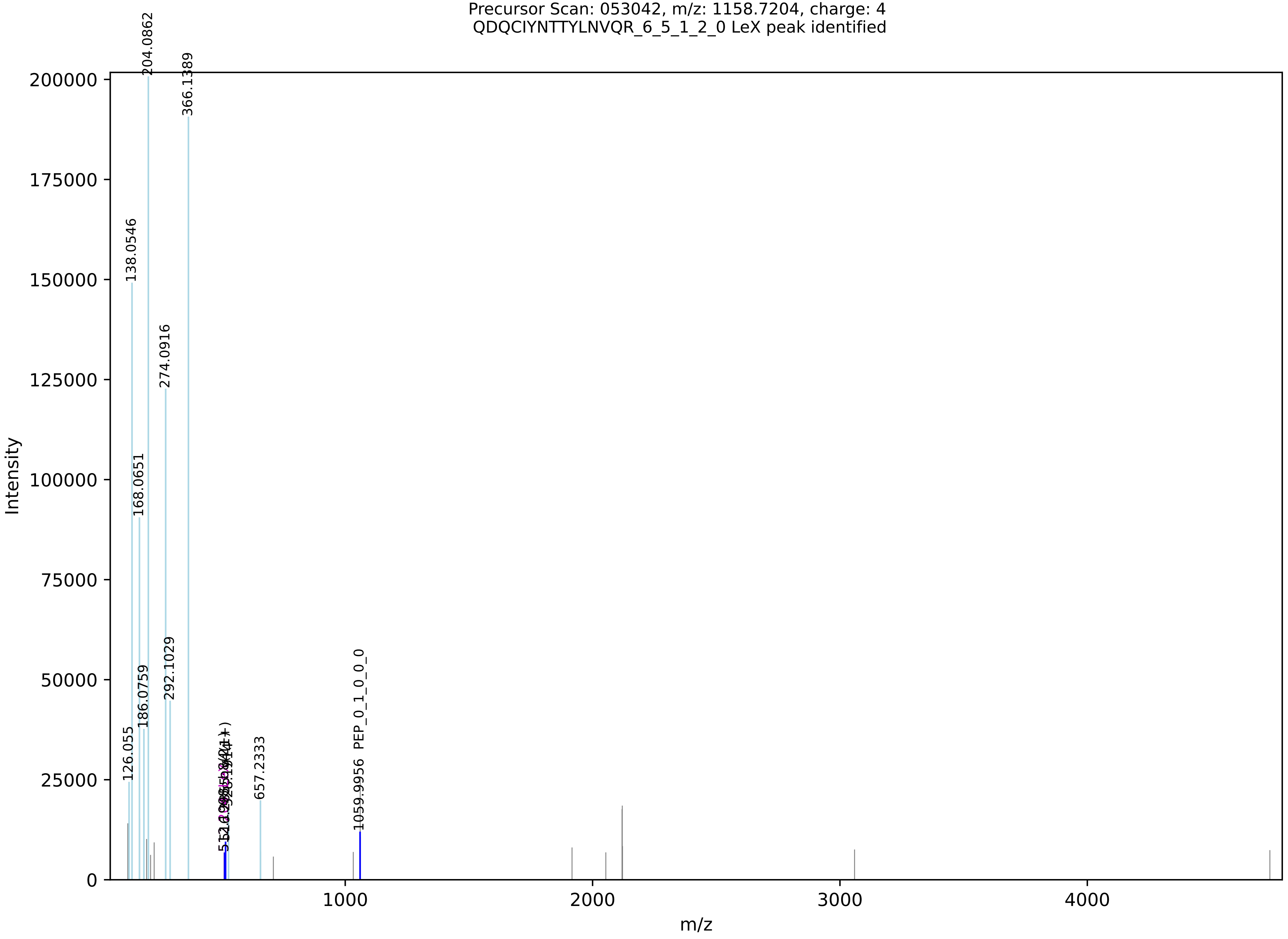

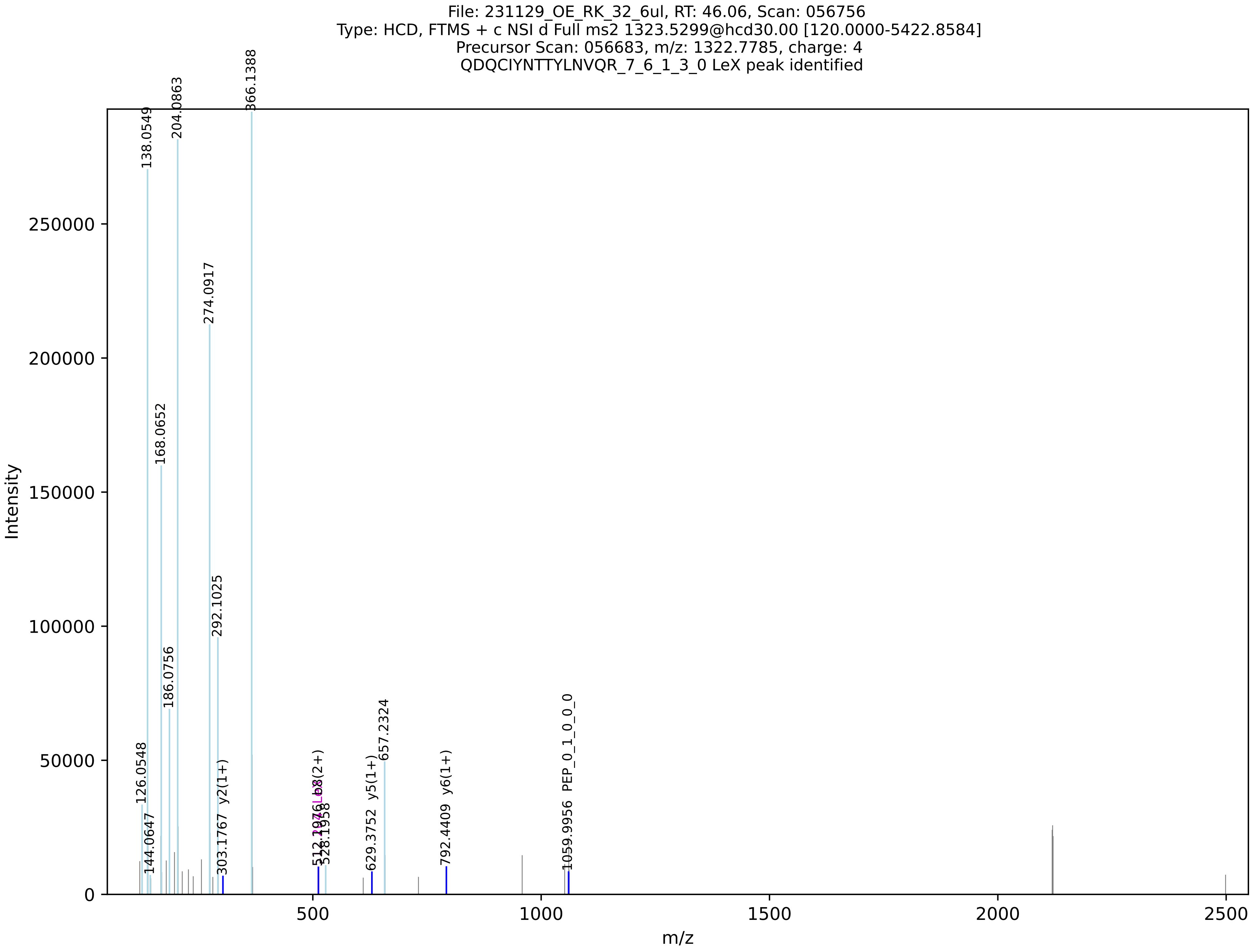
File: 231129_OE_RK_32_6ul, RT: 46.06, Scan: 056756
Type: HCD, FTMS + c NSI d Full ms2 1323.5299@hcd30.00 [120.0000-5422.8584]
Precursor Scan: 056683, m/z: 1322.7785, charge: 4
QDQCIYNTTYLNVQR_7_6_1_3_0 LeX peak identified
Intensity
m/z
366.1388
204.0863
138.0549
274.0917
168.0652
292.1025
186.0756
126.0548
144.0647
303.1767 y2(1+)
512.2976 b8(2+)
528.1958
629.3752 y5(1+)
657.2324
792.4409 y6(1+)
1059.9956 PEP_0_1_0_0_0

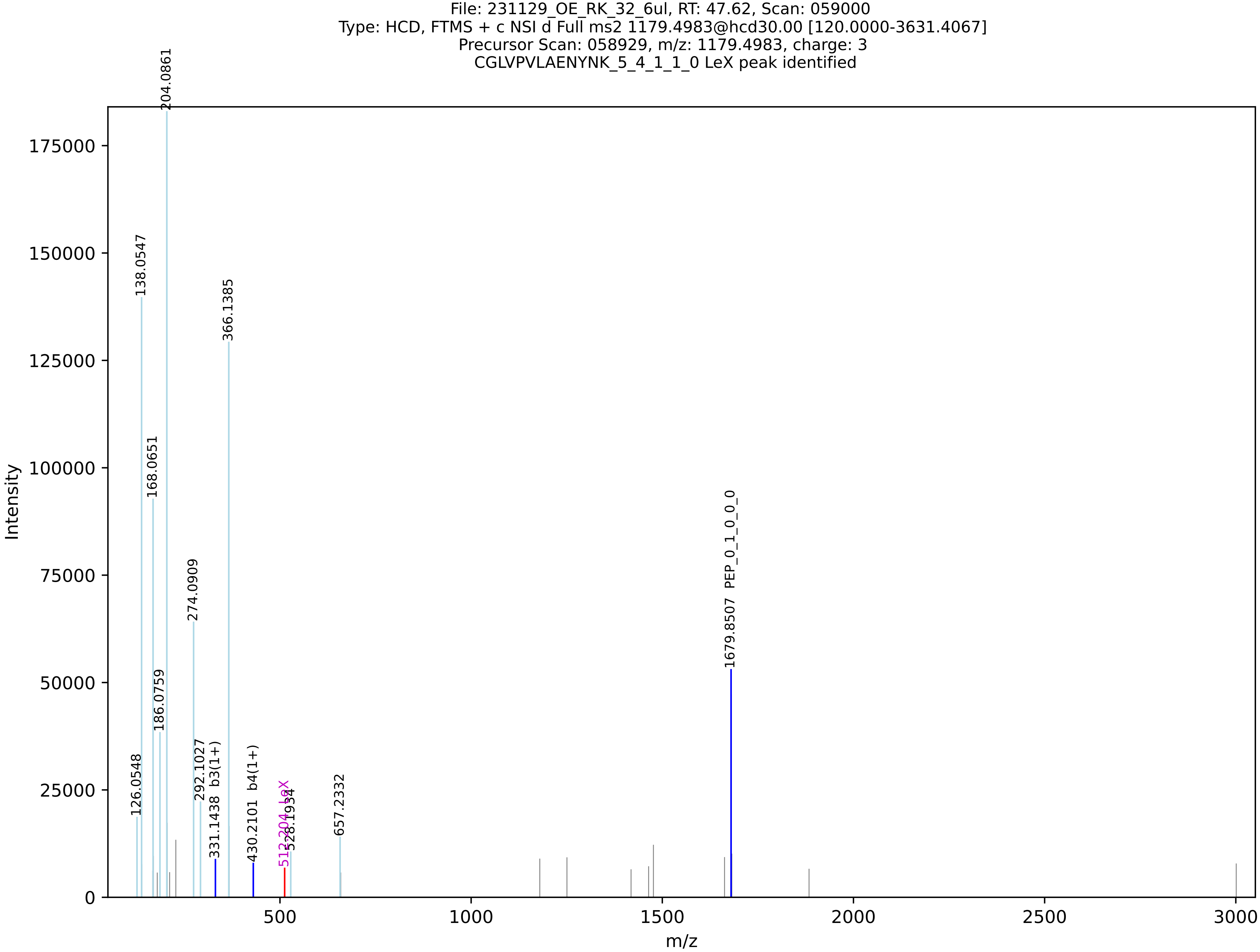
File: 231129_OE_RK_32_6ul, RT: 47.62, Scan: 059000
Type: HCD, FTMS + c NSI d Full ms2 1179.4983@hcd30.00 [120.0000-3631.4067]
Precursor Scan: 058929, m/z: 1179.4983, charge: 3
CGLVPVLAENYNK_5_4_1_1_0 LeX peak identified
Intensity
m/z
204.0861
138.0547
366.1385
168.0651
274.0909
186.0759
126.0548
292.1027
331.1438 b3(1+)
430.2101 b4(1+)
512.204 LeX
528.1934
657.2332
1679.8507 PEP_0_1_0_0_0
0
25000
50000
75000
100000
125000
150000
175000
500
1000
1500
2000
2500
3000

File: 231129_OE_RK_32_6ul, RT: 64.949, Scan: 083978
Type: HCD, FTMS + c NSI d Full ms2 1273.2169@hcd30.00 [120.0000-3918.1855]
Precursor Scan: 083889, m/z: 1272.8827, charge: 3
YLGNATAIFFLPDEGK_5_4_1_1_0 LeX peak identified

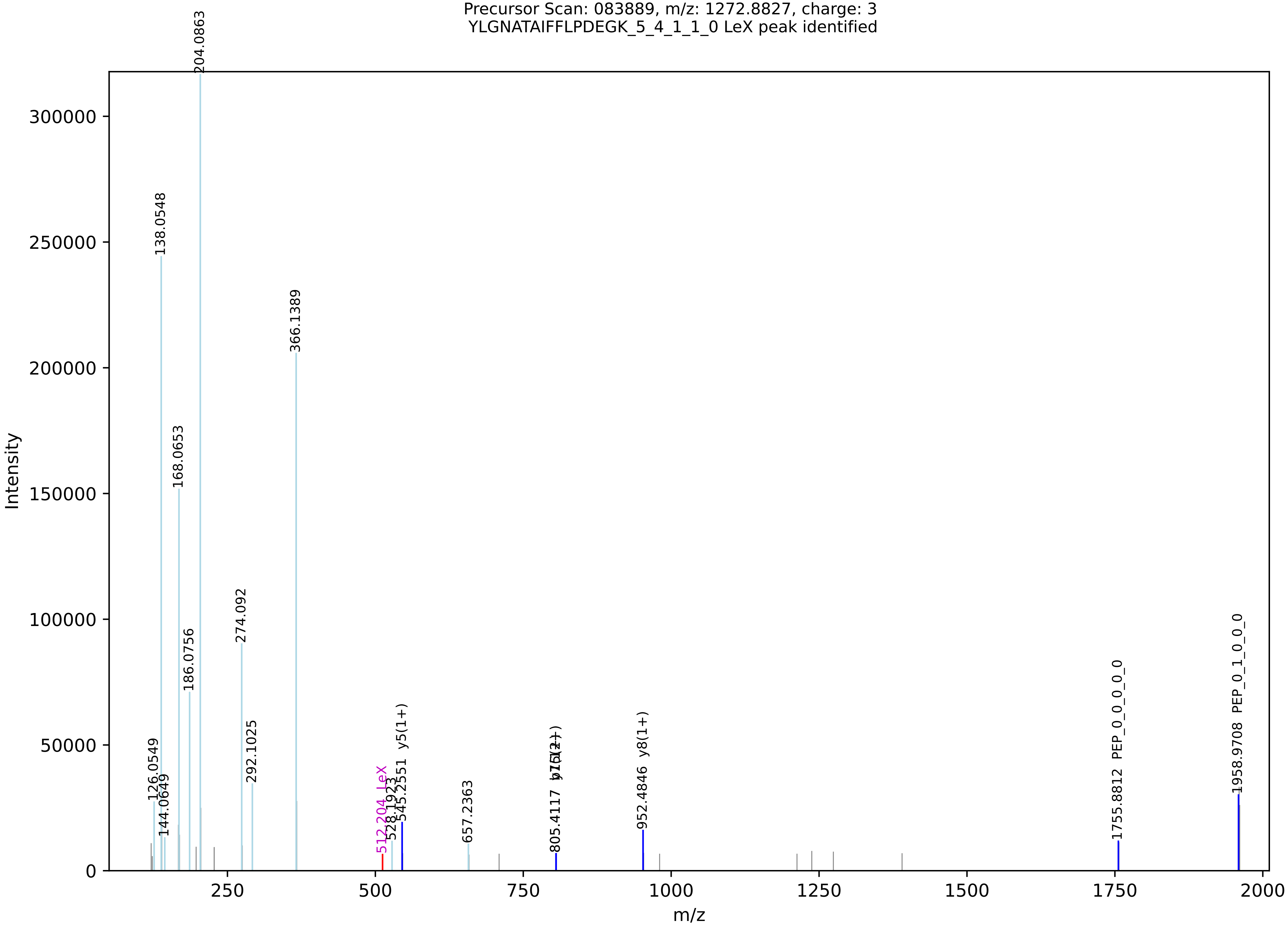

File: 231129_OE_RK_33, RT: 5.357, Scan: 001528
Type: HCD, FTMS + c NSI d Full ms2 1282.1575@hcd30.00 [120.0000-3945.5439]
Precursor Scan: 001516, m/z: 1282.1575, charge: 3
ENGTVSR_7_6_1_2_0 LeX peak identified

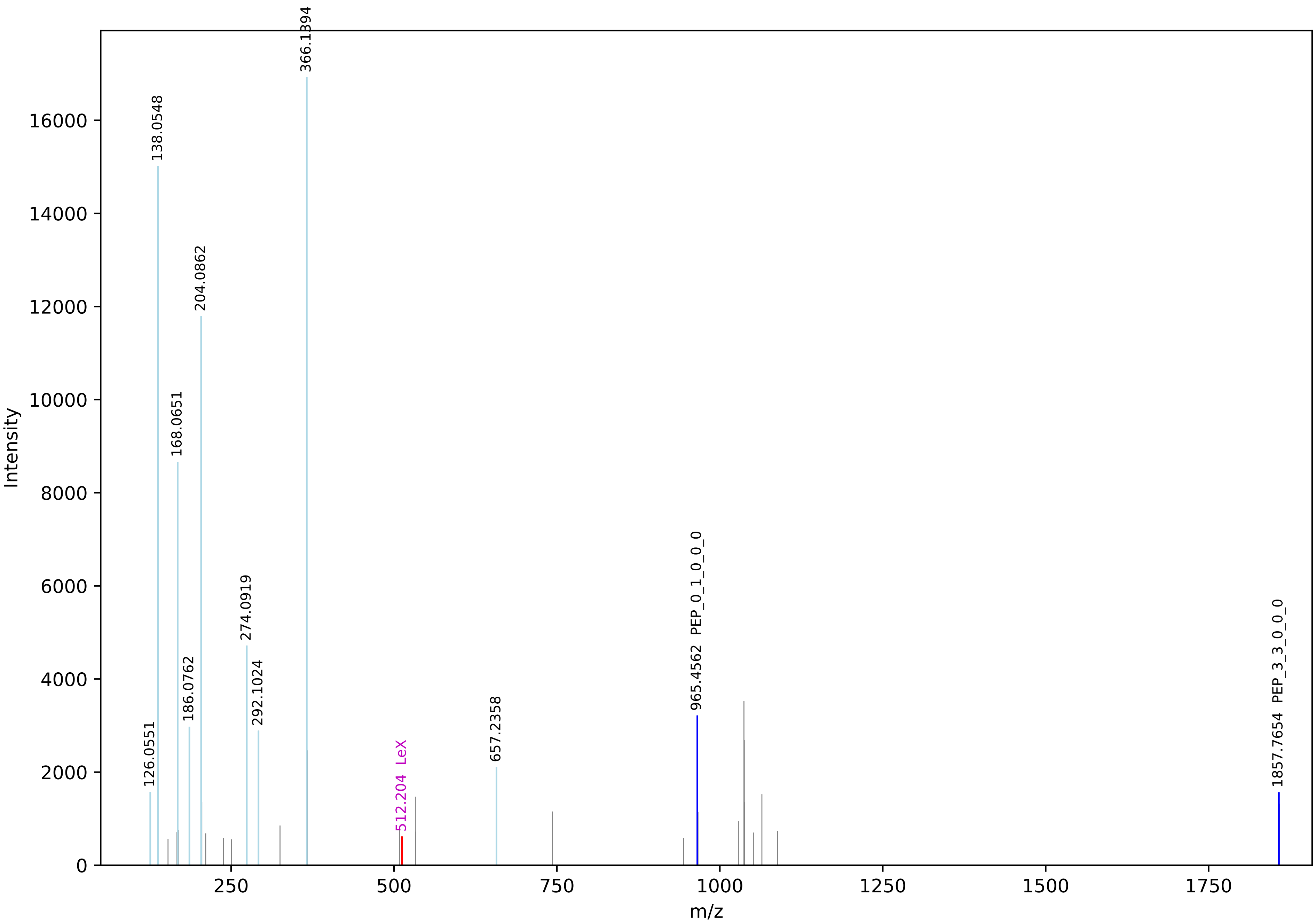

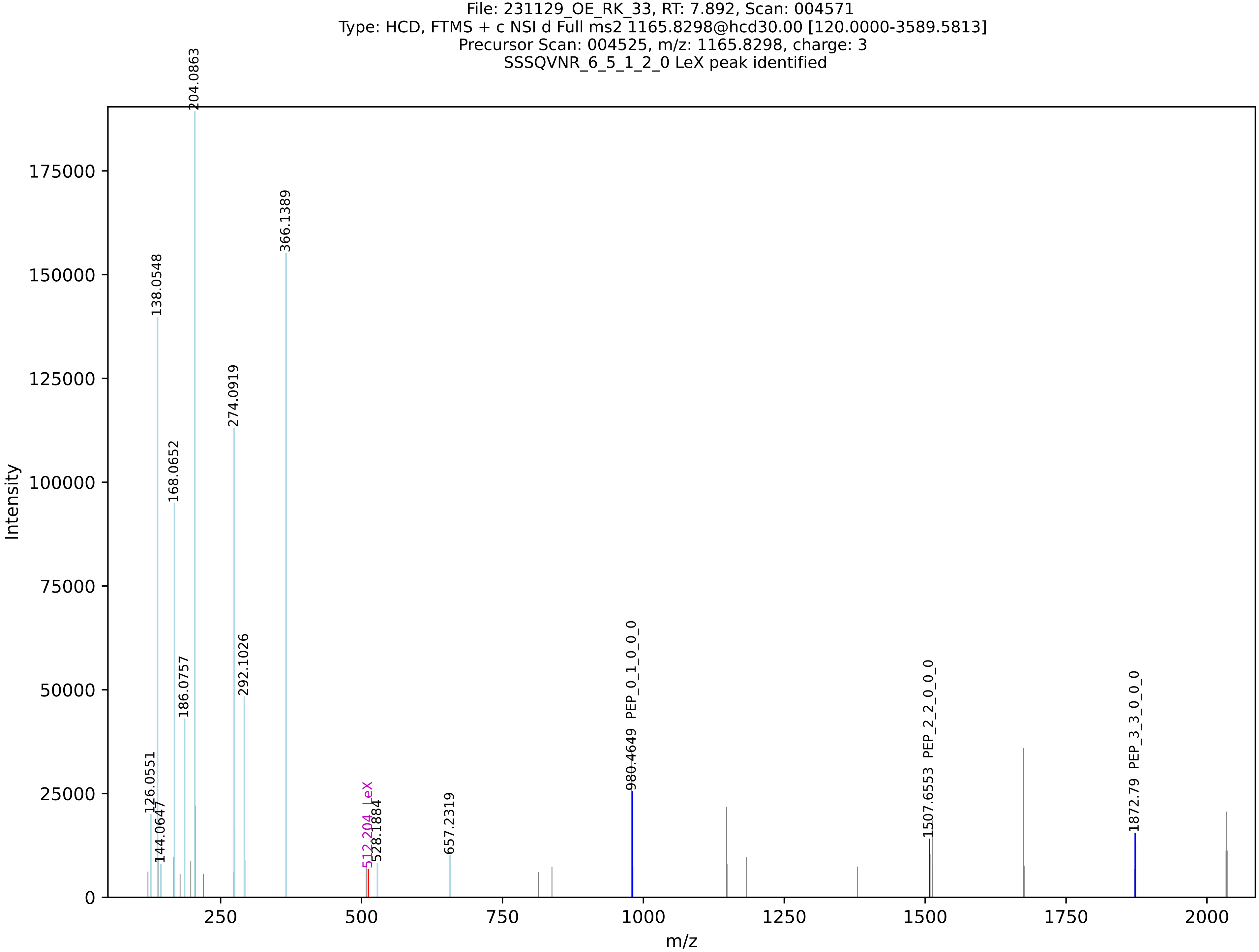

File: 231129_OE_RK_33, RT: 7.892, Scan: 004571
Type: HCD, FTMS + c NSI d Full ms2 1165.8298@hcd30.00 [120.0000-3589.5813]
Precursor Scan: 004525, m/z: 1165.8298, charge: 3
SSSQVNR_6_5_1_2_0 LeX peak identified
Intensity
m/z
204.0863
366.1389
138.0548
274.0919
168.0652
292.1026
186.0757
126.0551
144.0647
512.204_LeX
528.1884
657.2319
980.4649 PEP_0_1_0_0_0
1507.6553 PEP_2_2_0_0_0
1872.79 PEP_3_3_0_0_0

File: 231129_OE_RK_33, RT: 10.052, Scan: 007618
Type: HCD, FTMS + c NSI d Full ms2 1384.1993@hcd30.00 [120.0000-4257.7920]
Precursor Scan: 007540, m/z: 1383.5311, charge: 3
ENGTISR_7_6_1_3_0 LeX peak identified

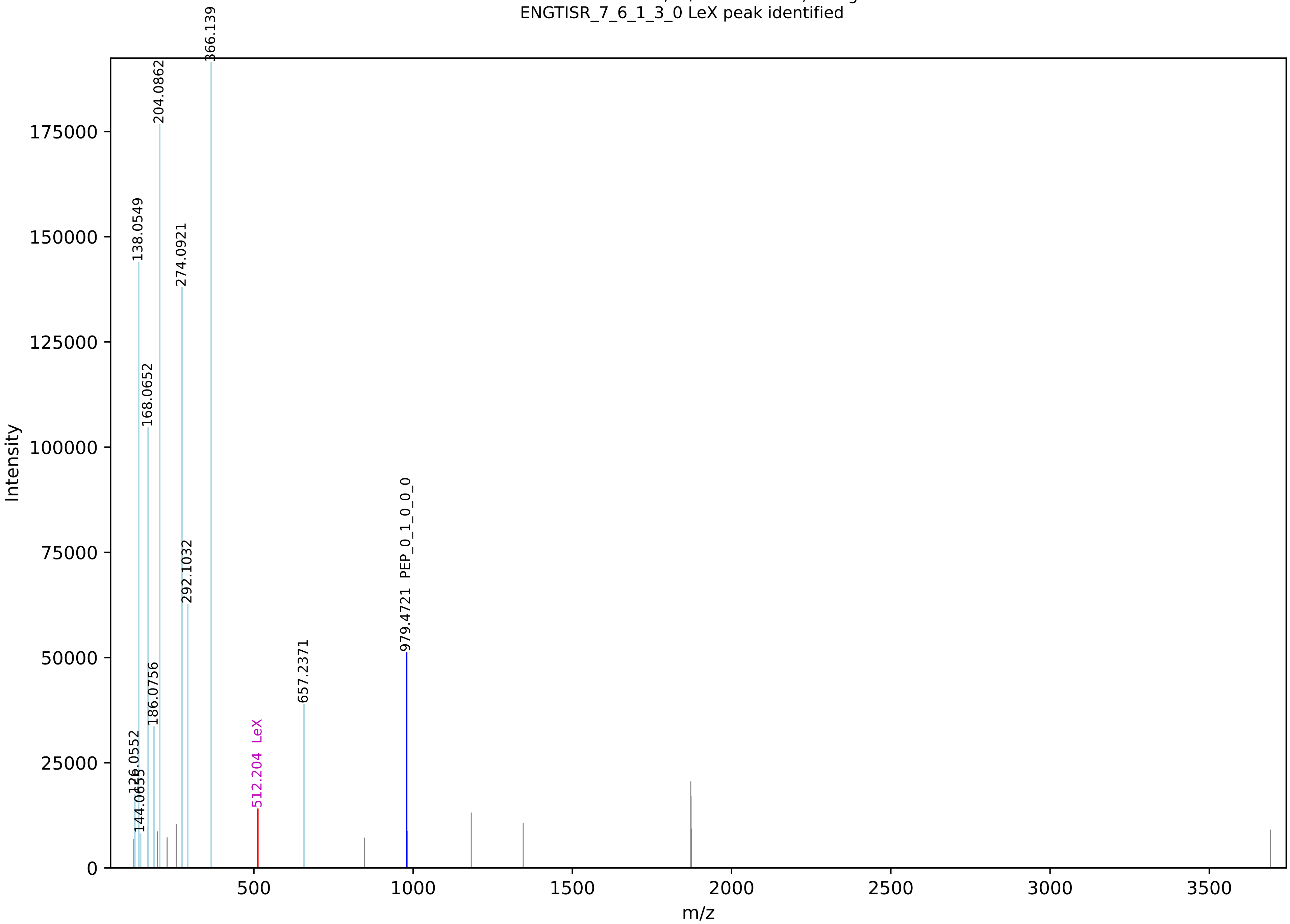

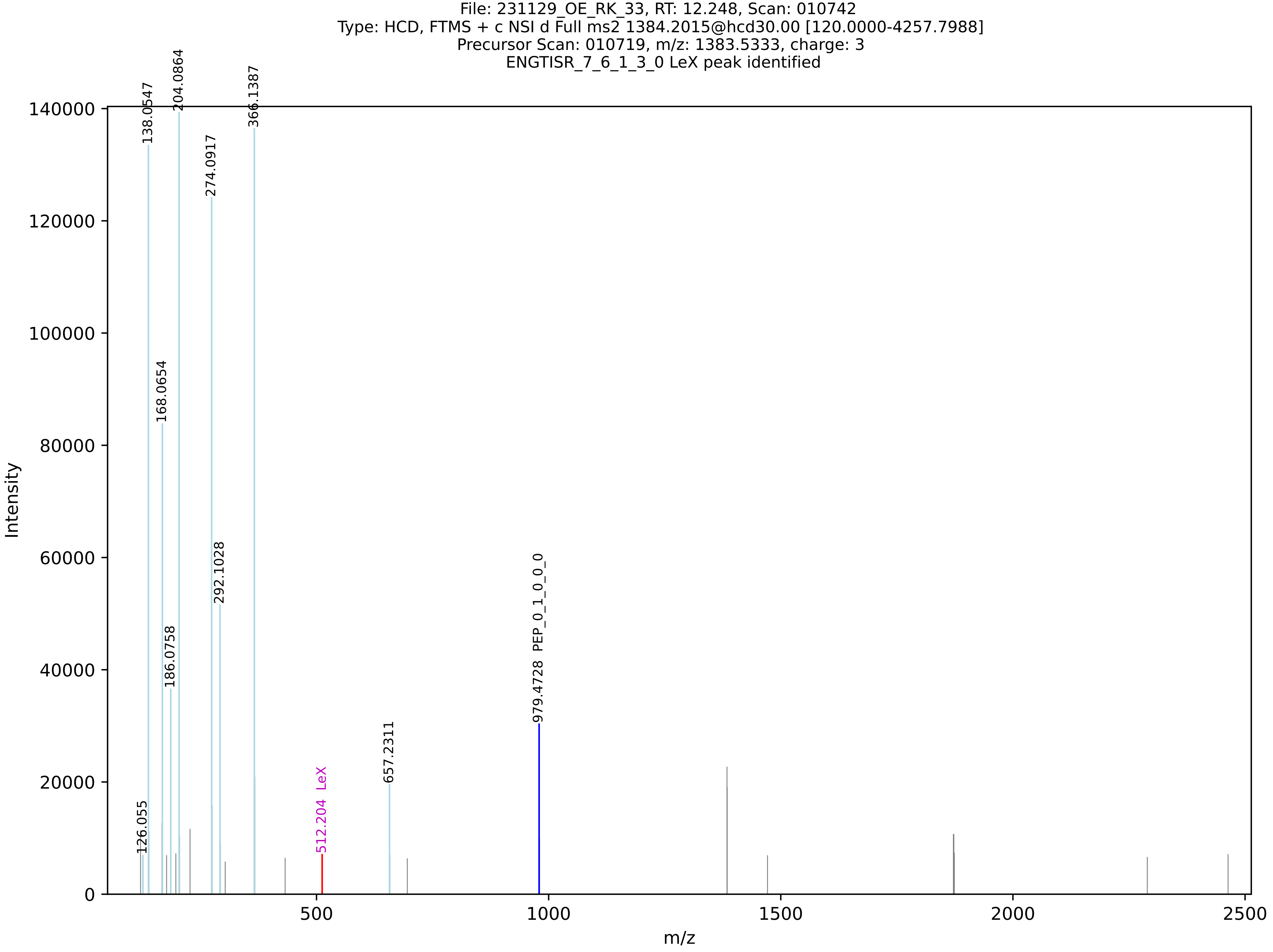
File: 231129_OE_RK_33, RT: 12.248, Scan: 010742
Type: HCD, FTMS + c NSI d Full ms2 1384.2015@hcd30.00 [120.0000-4257.7988]
Precursor Scan: 010719, m/z: 1383.5333, charge: 3
ENGTISR_7_6_1_3_0 LeX peak identified
Intensity
m/z
138.0547
204.0864
366.1387
274.0917
168.0654
292.1028
186.0758
979.4728 PEP_0_1_0_0_0_
657.2311
512.204 LeX
126.055
140000
120000
100000
80000
60000
40000
20000
0
500
1000
1500
2000
2500

File: 231129_OE_RK_33, RT: 43.694, Scan: 056296
Type: HCD, FTMS + c NSI d Full ms2 1250.5079@hcd30.00 [120.0000-5124.9282]
Precursor Scan: 056217, m/z: 1249.7562, charge: 4
QDQCIYNTTYLNVQR_7_6_1_2_0 LeX peak identified

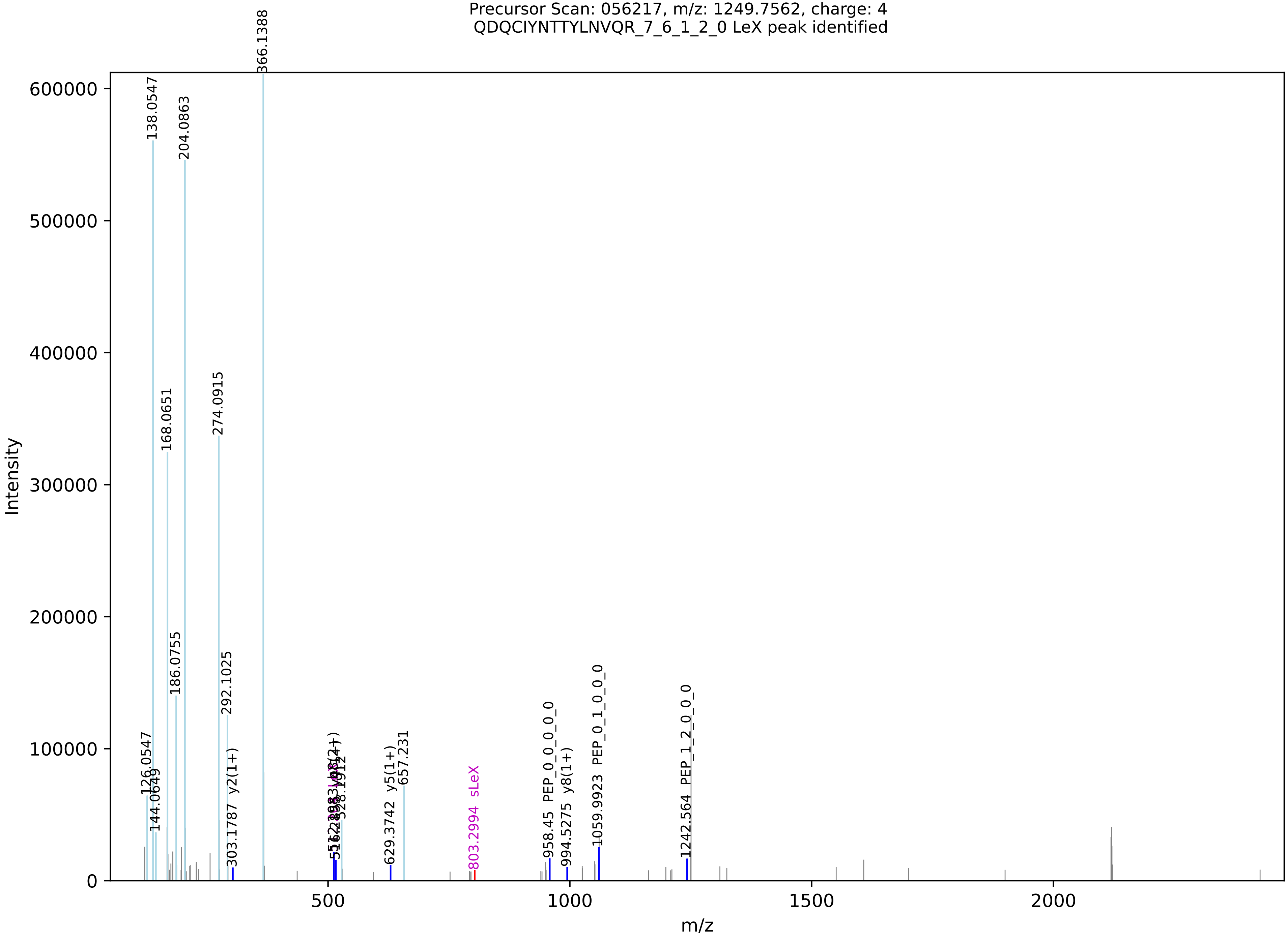

File: 231129_OE_RK_33, RT: 43.891, Scan: 056581
Type: HCD, FTMS + c NSI d Full ms2 1250.5077@hcd30.00 [120.0000-5124.9272]
Precursor Scan: 056503, m/z: 1249.7552, charge: 4
QDQCIYNTTYLNVQR_7_6_1_2_0 LeX peak identified

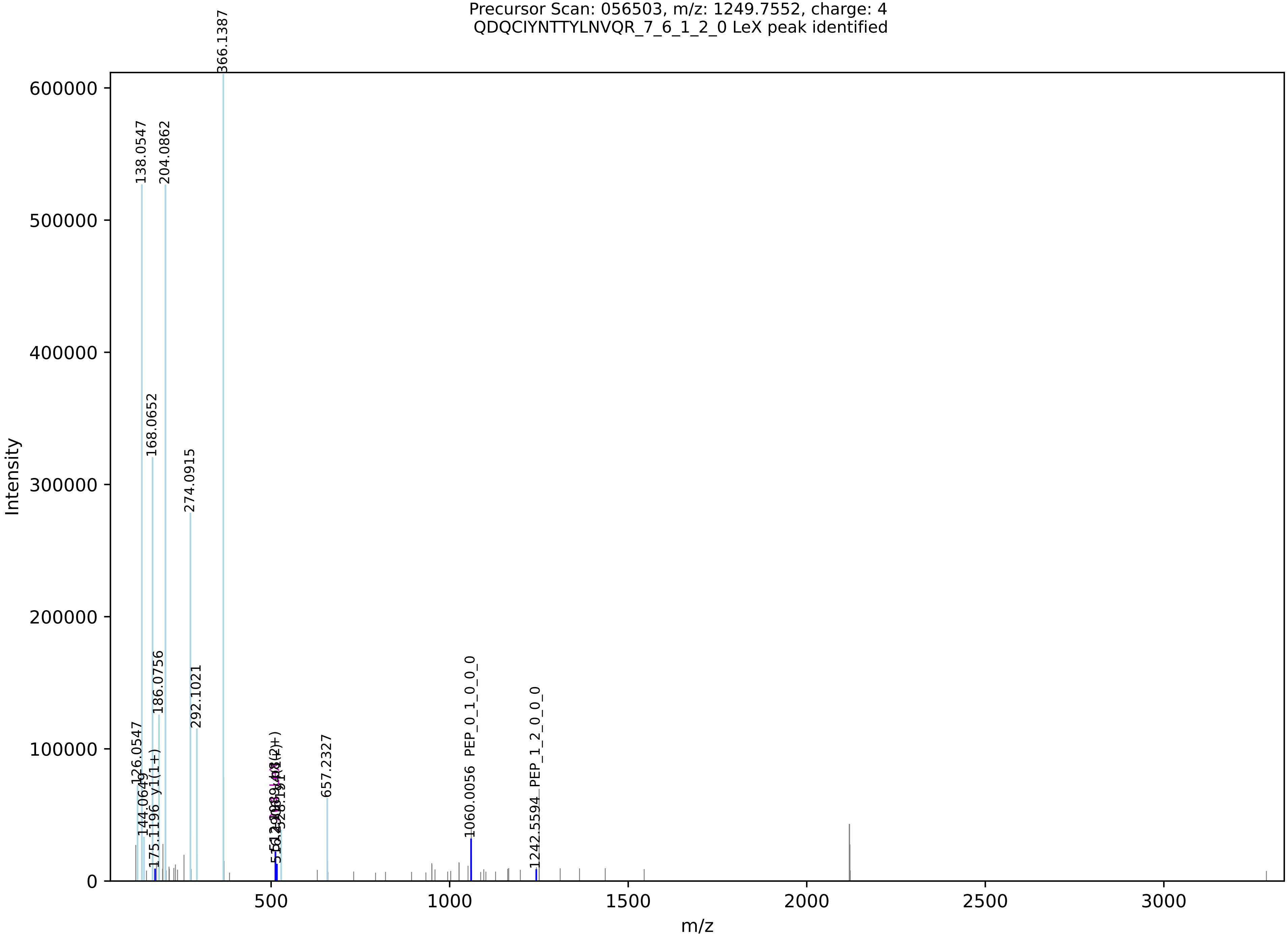

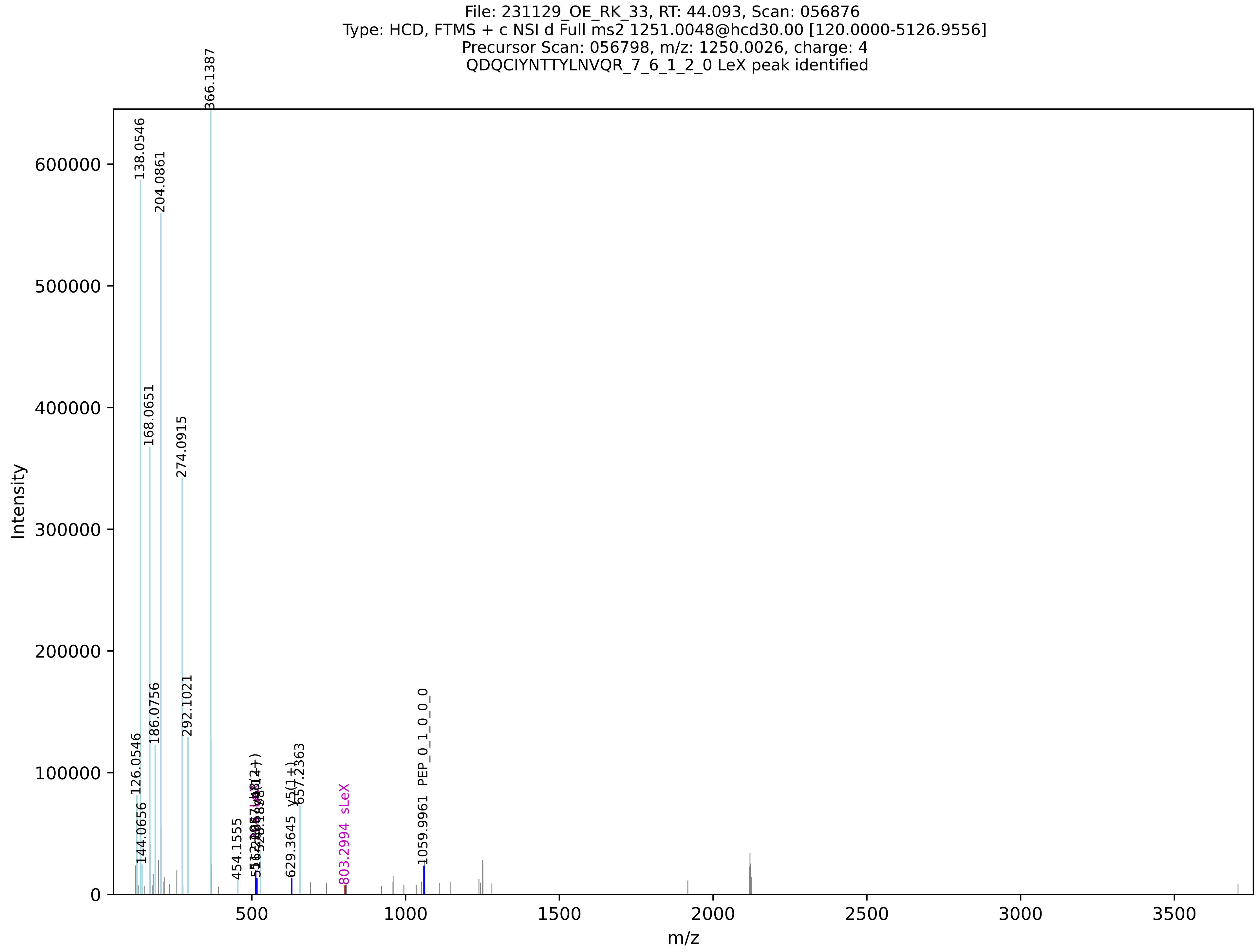
File: 231129_OE_RK_33, RT: 44.093, Scan: 056876
Type: HCD, FTMS + c NSI d Full ms2 1251.0048@hcd30.00 [120.0000-5126.9556]
Precursor Scan: 056798, m/z: 1250.0026, charge: 4
QDQCIYNTTYLNVQR_7_6_1_2_0 LeX peak identified
366.1387
138.0546
204.0861
168.0651
274.0915
292.1021
186.0756
126.0546
144.0656
454.1555
629.3645 y5(1+)
657.2363
803.2994 sLeX
1059.9961 PEP_0_1_0_0_0
Intensity
m/z
0
100000
200000
300000
400000
500000
600000
500
1000
1500
2000
2500
3000
3500

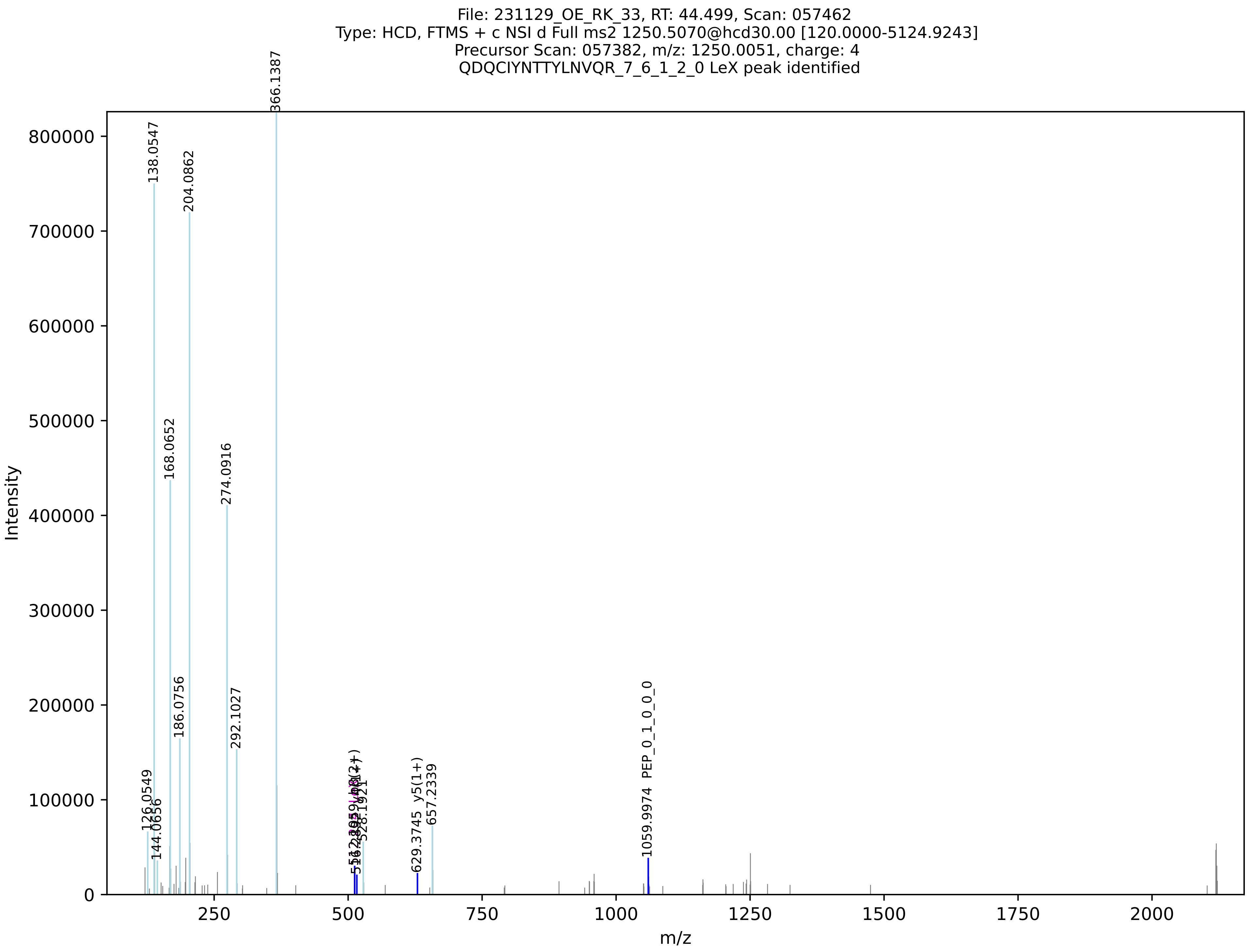
File: 231129_OE_RK_33, RT: 44.499, Scan: 057462
Type: HCD, FTMS + c NSI d Full ms2 1250.5070@hcd30.00 [120.0000-5124.9243]
Precursor Scan: 057382, m/z: 1250.0051, charge: 4
QDQCIYNTTYLNVQR_7_6_1_2_0 LeX peak identified
Intensity
m/z
366.1387
138.0547
204.0862
168.0652
274.0916
186.0756
292.1027
126.0549
144.0656
528.1921
629.3745 y5(1+)
657.2339
1059.9974 PEP_0_1_0_0_0

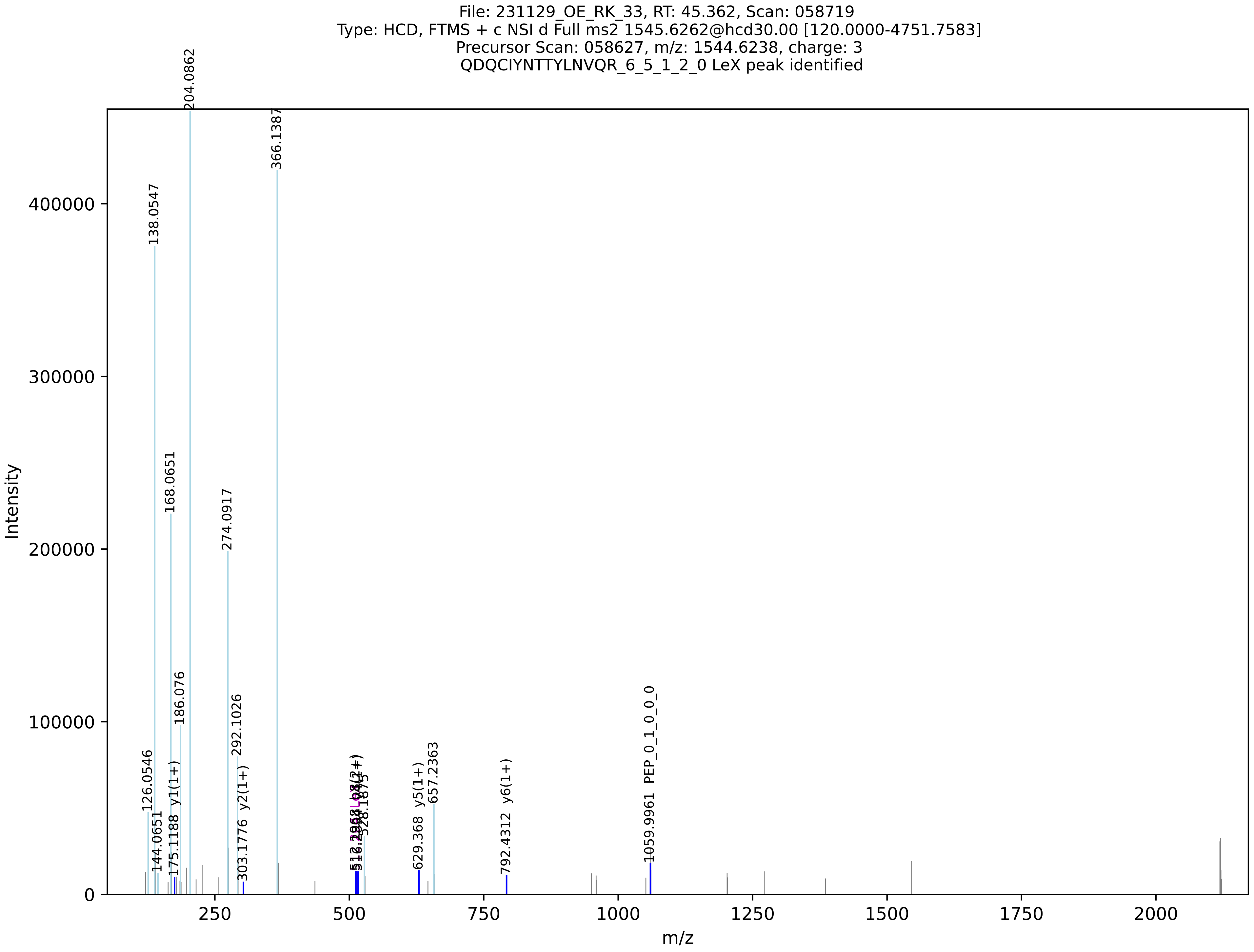

File: 231129_OE_RK_33, RT: 45.362, Scan: 058719
Type: HCD, FTMS + c NSI d Full ms2 1545.6262@hcd30.00 [120.0000-4751.7583]
Precursor Scan: 058627, m/z: 1544.6238, charge: 3
QDQCIYNTTYLNVQR_6_5_1_2_0 LeX peak identified
Intensity
m/z
204.0862
366.1387
138.0547
168.0651
274.0917
186.076
292.1026
126.0546
144.0651
175.1188 y1(1+)
303.1776 y2(1+)
528.1875
629.368 y5(1+)
657.2363
792.4312 y6(1+)
1059.9961 PEP_0_1_0_0_0

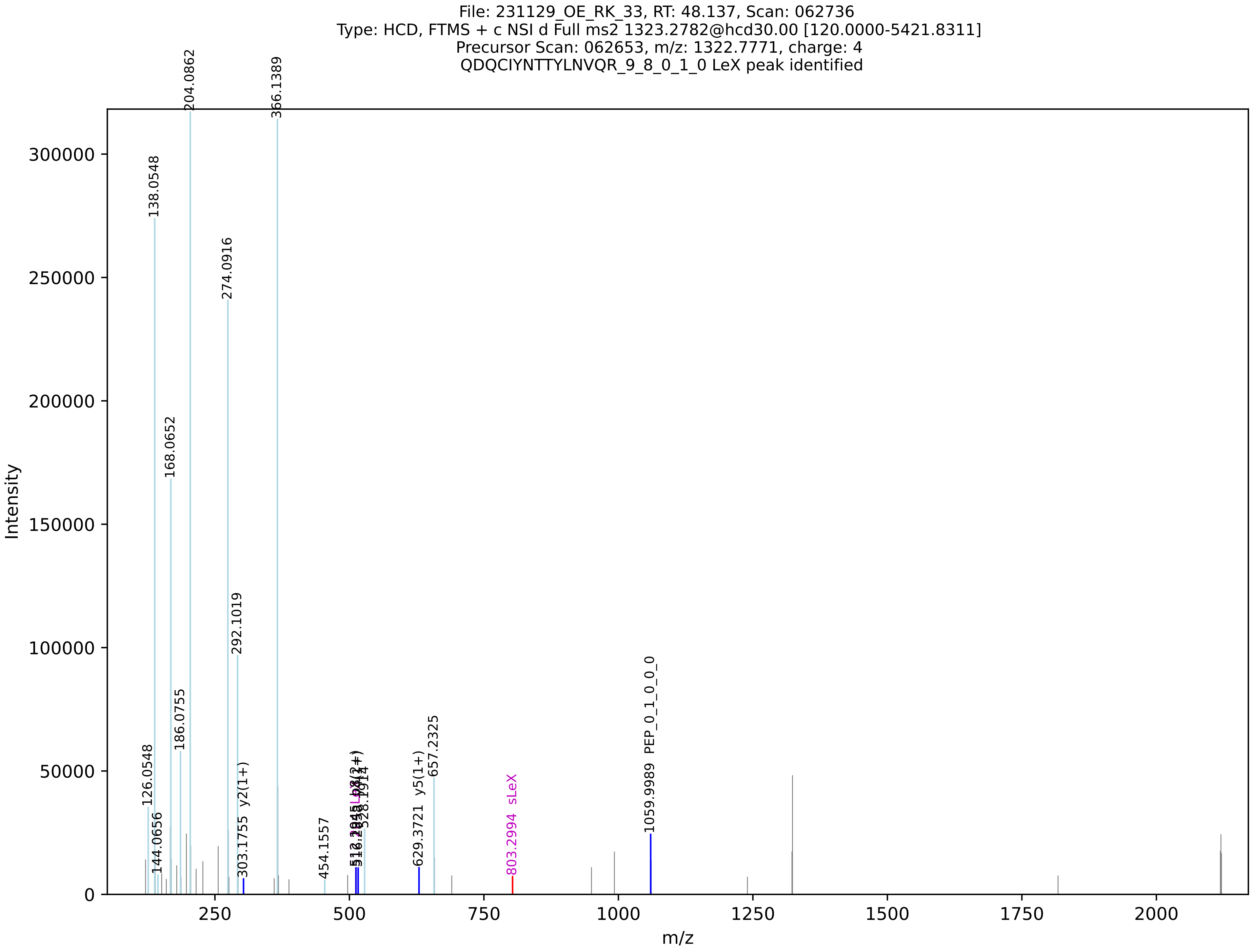
File: 231129_OE_RK_33, RT: 48.137, Scan: 062736
Type: HCD, FTMS + c NSI d Full ms2 1323.2782@hcd30.00 [120.0000-5421.8311]
Precursor Scan: 062653, m/z: 1322.7771, charge: 4
QDQCIYNTTYLNVQR_9_8_0_1_0 LeX peak identified
Intensity
m/z
204.0862
366.1389
138.0548
274.0916
168.0652
292.1019
186.0755
126.0548
144.0656
303.1755 y2(1+)
454.1557
528.1914
629.3721 y5(1+)
657.2325
803.2994 sLeX
1059.9989 PEP_0_1_0_0_0
0
50000
100000
150000
200000
250000
300000
250
500
750
1000
1250
1500
1750
2000

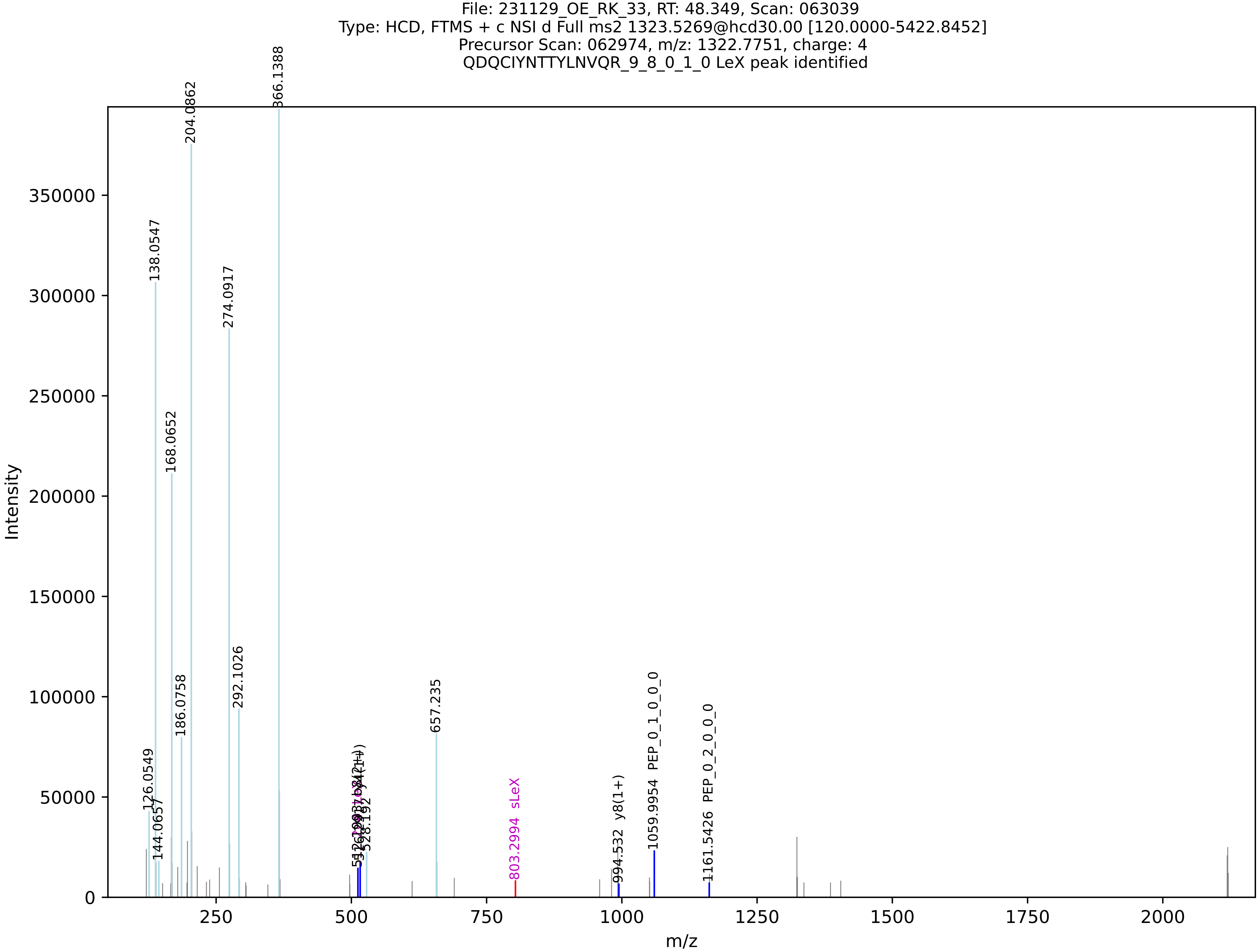
File: 231129_OE_RK_33, RT: 48.349, Scan: 063039
Type: HCD, FTMS + c NSI d Full ms2 1323.5269@hcd30.00 [120.0000-5422.8452]
Precursor Scan: 062974, m/z: 1322.7751, charge: 4
QDQCIYNTTYLNVQR_9_8_0_1_0 LeX peak identified
Intensity
m/z
366.1388
204.0862
138.0547
274.0917
168.0652
292.1026
186.0758
657.235
126.0549
144.0657
528.192
803.2994 sLeX
994.532 y8(1+)
1059.9954 PEP_0_1_0_0_0
1161.5426 PEP_0_2_0_0_0
0
50000
100000
150000
200000
250000
300000
350000
250
500
750
1000
1250
1500
1750
2000

File: 231129_OE_RK_33, RT: 48.352, Scan: 063044
Type: HCD, FTMS + c NSI d Full ms2 1376.5999@hcd30.00 [120.0000-4234.5376]
Precursor Scan: 062974, m/z: 1375.5981, charge: 3
ALLQKLASPNLSR_6_5_1_2_0 LeX peak identified

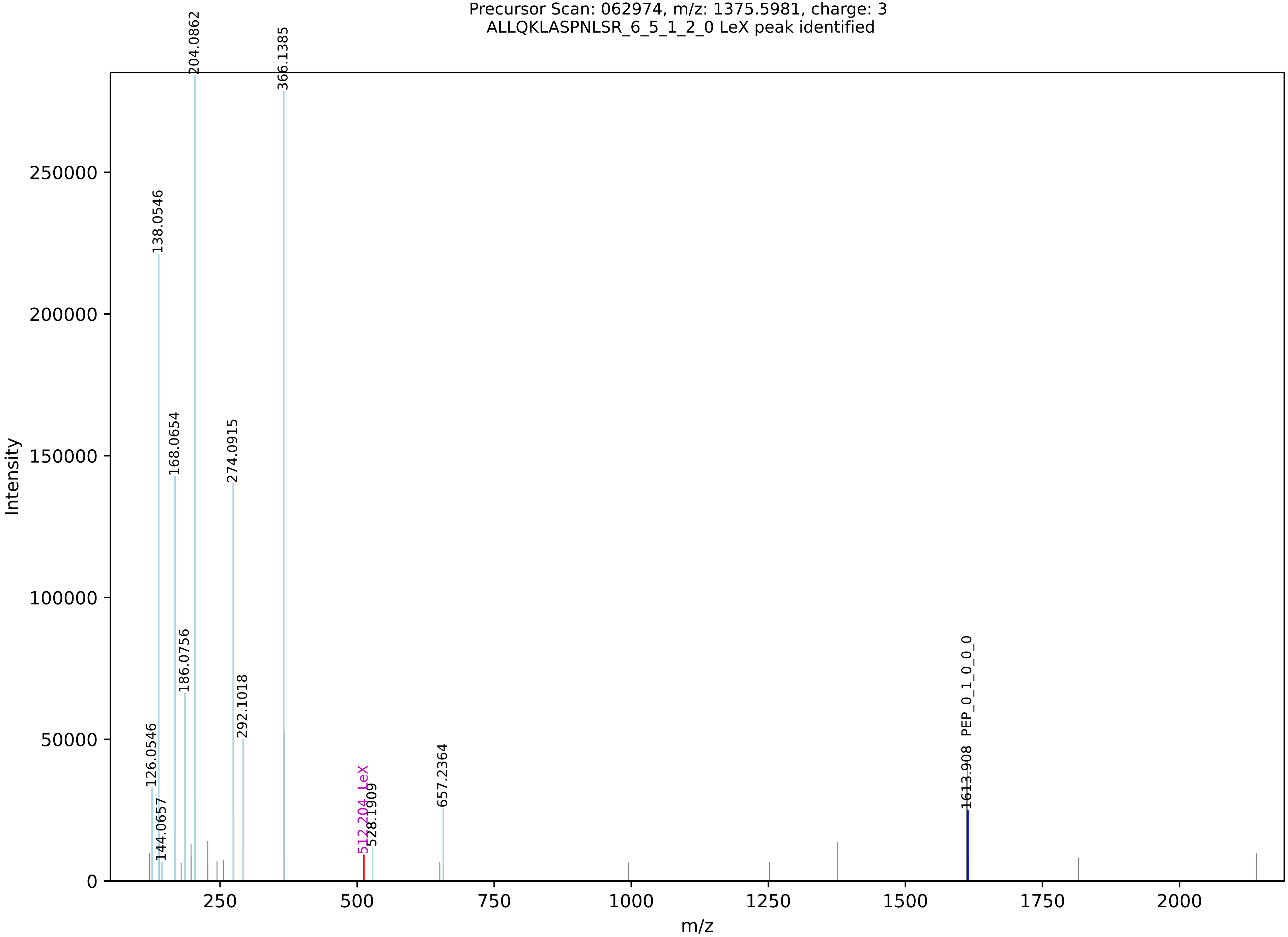

File: 231129_OE_RK_33, RT: 51.779, Scan: 068021
Type: HCD, FTMS + c NSI d Full ms2 1232.2432@hcd30.00 [120.0000-5050.4082]
Precursor Scan: 067930, m/z: 1231.4934, charge: 4
QDQCIYNTTYLNVQR_6_5_1_3_0 LeX peak identified

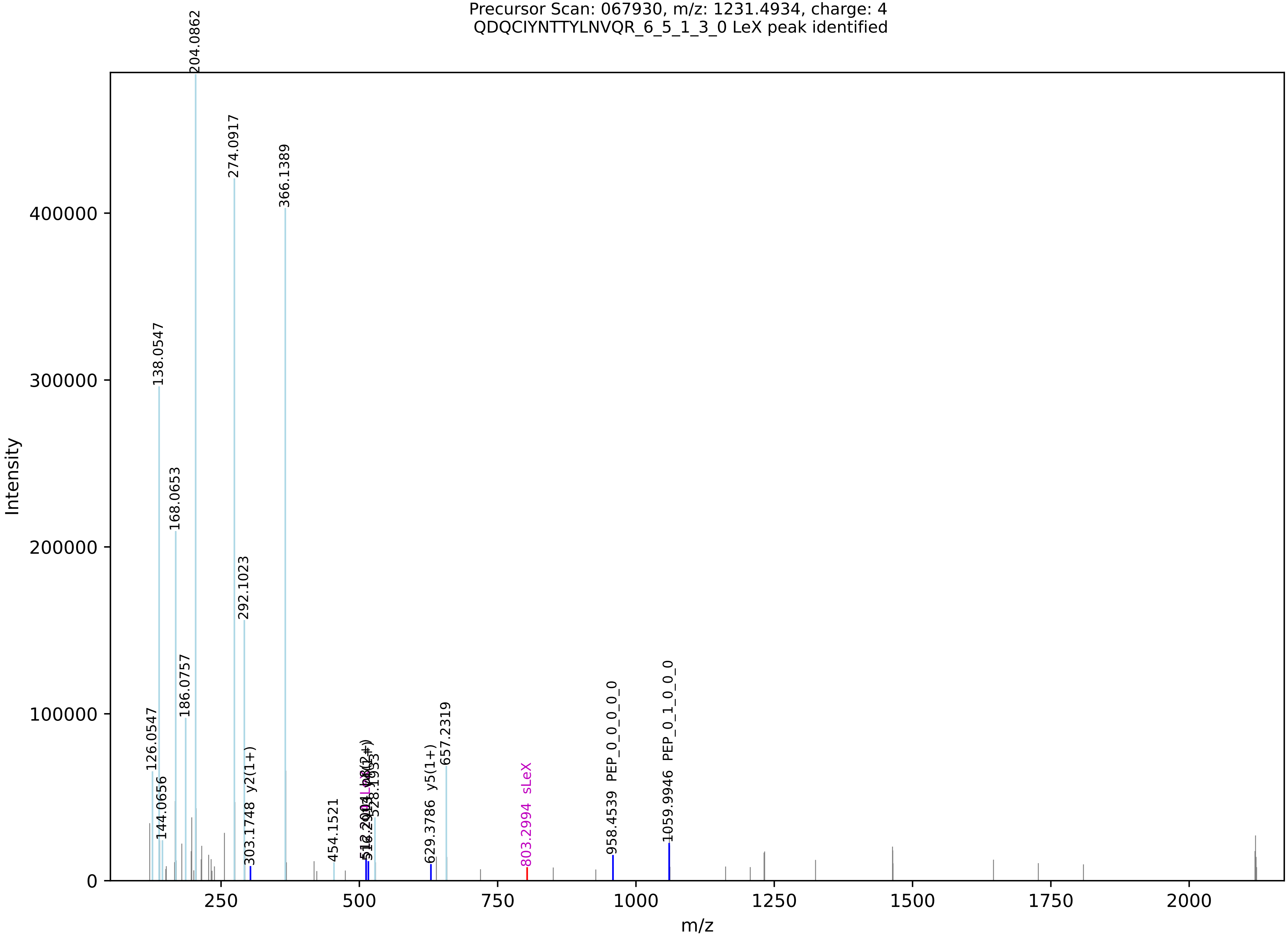

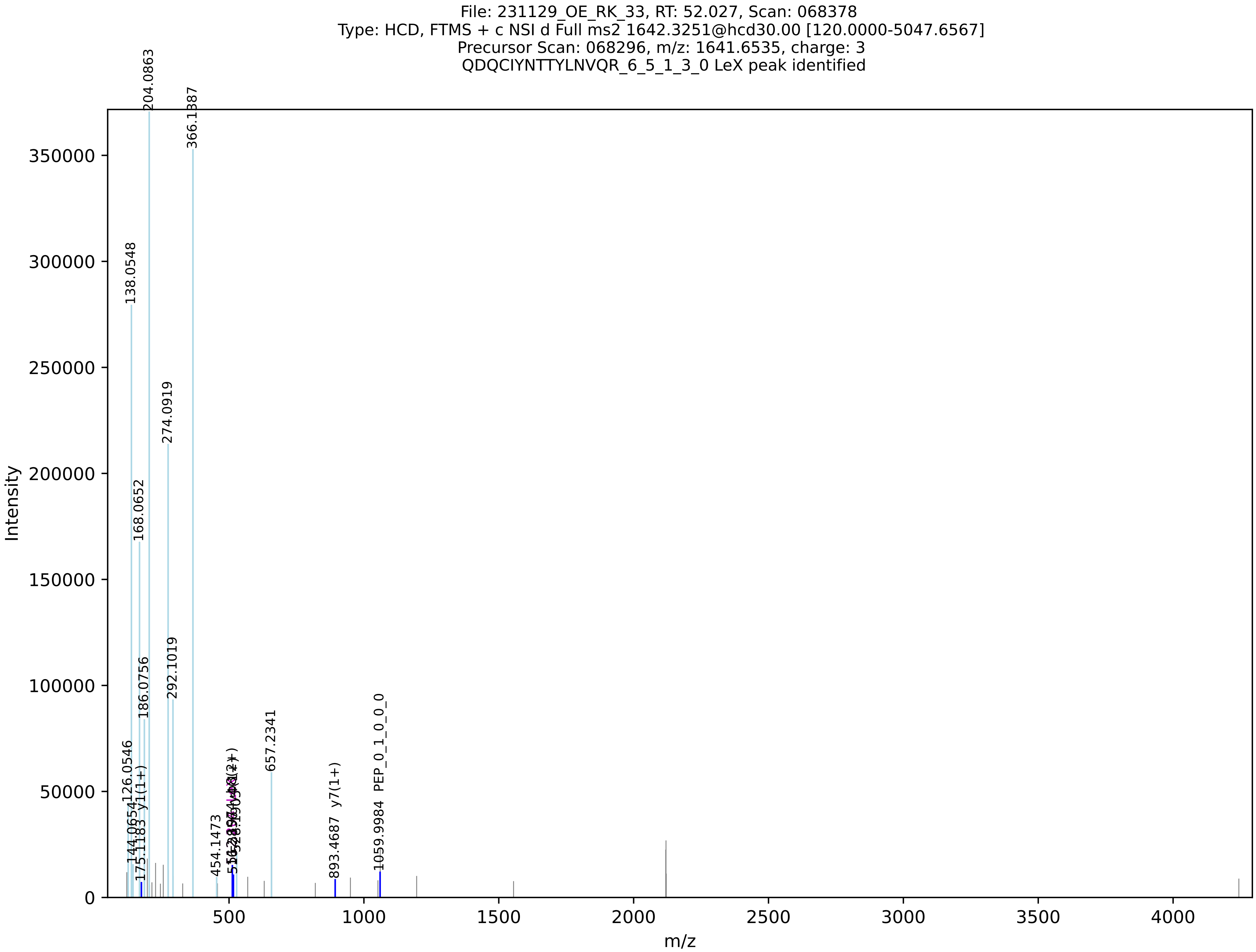
File: 231129_OE_RK_33, RT: 52.027, Scan: 068378
Type: HCD, FTMS + c NSI d Full ms2 1642.3251@hcd30.00 [120.0000-5047.6567]
Precursor Scan: 068296, m/z: 1641.6535, charge: 3
QDQCIYNTTYLNVQR_6_5_1_3_0 LeX peak identified
Intensity
m/z
204.0863
366.1387
138.0548
274.0919
168.0652
292.1019
186.0756
126.0546
y1(1+)
144.0654
175.1183
454.1473
657.2341
893.4687 y7(1+)
1059.9984 PEP_0_1_0_0_0_
0
50000
100000
150000
200000
250000
300000
350000
500
1000
1500
2000
2500
3000
3500
4000

File: 231129_OE_RK_33, RT: 52.861, Scan: 069582
Type: HCD, FTMS + c NSI d Full ms2 1266.5432@hcd30.00 [120.0000-3897.7642]
Precursor Scan: 069503, m/z: 1265.8749, charge: 3
ALPQPQNVTSLLGCTH_5_4_1_1_0 LeX peak identified

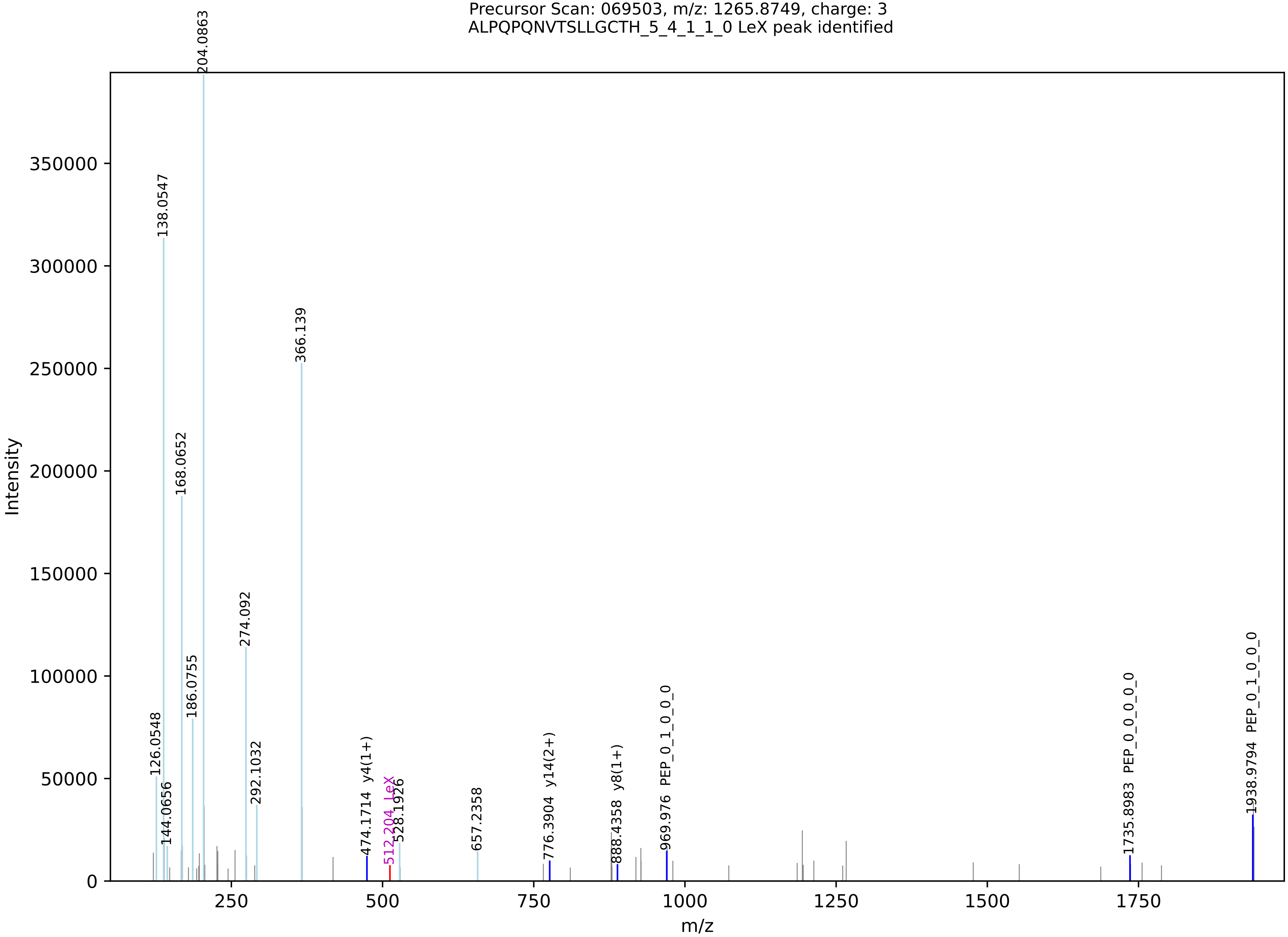

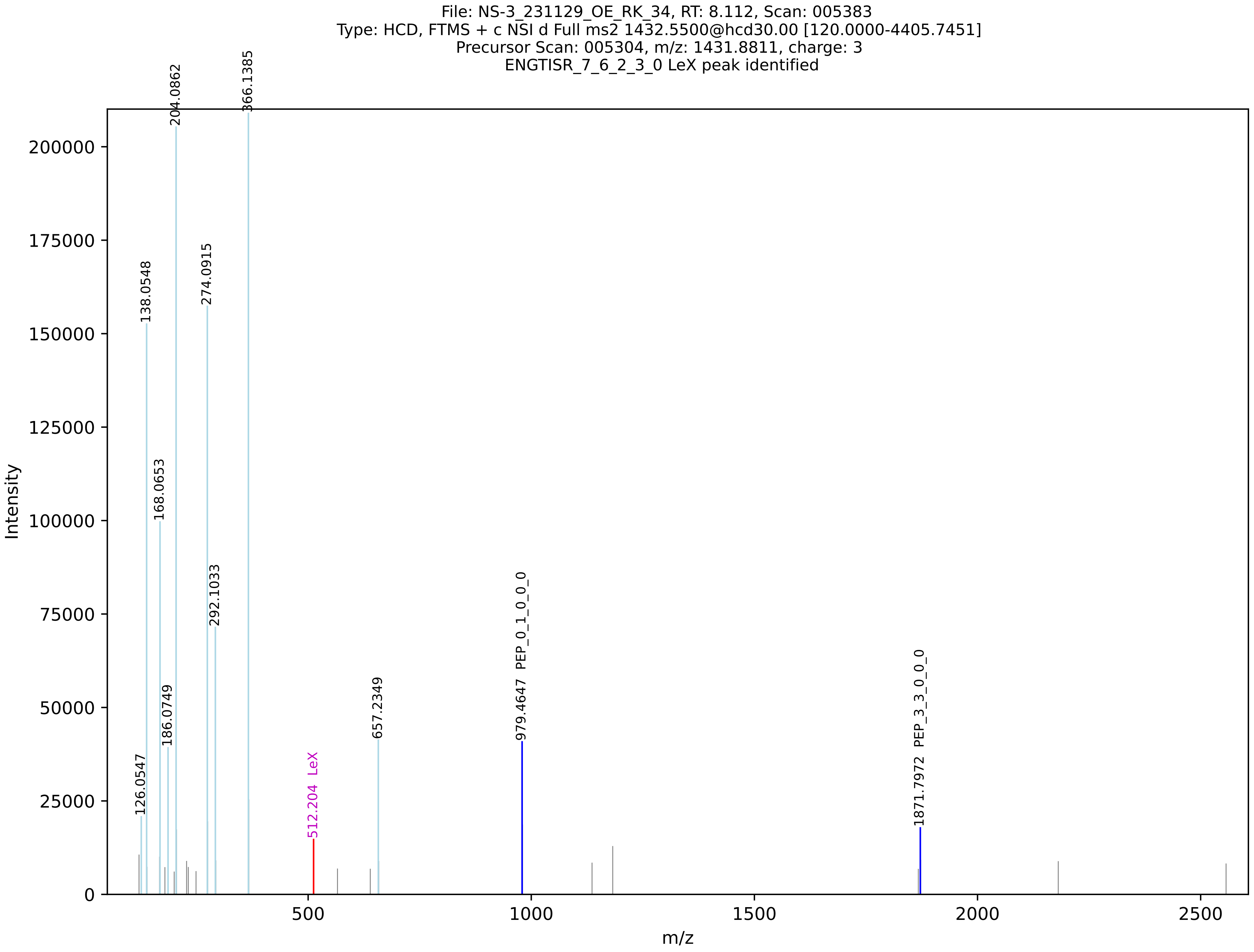
File: NS-3_231129_OE_RK_34, RT: 8.112, Scan: 005383
Type: HCD, FTMS + c NSI d Full ms2 1432.5500@hcd30.00 [120.0000-4405.7451]
Precursor Scan: 005304, m/z: 1431.8811, charge: 3
ENGTISR_7_6_2_3_0 LeX peak identified
Intensity
m/z
366.1385
204.0862
274.0915
138.0548
168.0653
292.1033
186.0749
126.0547
512.204 LeX
657.2349
979.4647 PEP_0_1_0_0_0
1871.7972 PEP_3_3_0_0_0
0
25000
50000
75000
100000
125000
150000
175000
200000
500
1000
1500
2000
2500

File: NS-3_231129_OE_RK_34, RT: 8.164, Scan: 005459
Type: HCD, FTMS + c NSI d Full ms2 1170.7970@hcd30.00 [120.0000-3604.7810]
Precursor Scan: 005378, m/z: 1170.1288, charge: 3
ENGTISR_7_5_0_2_0 LeX peak identified

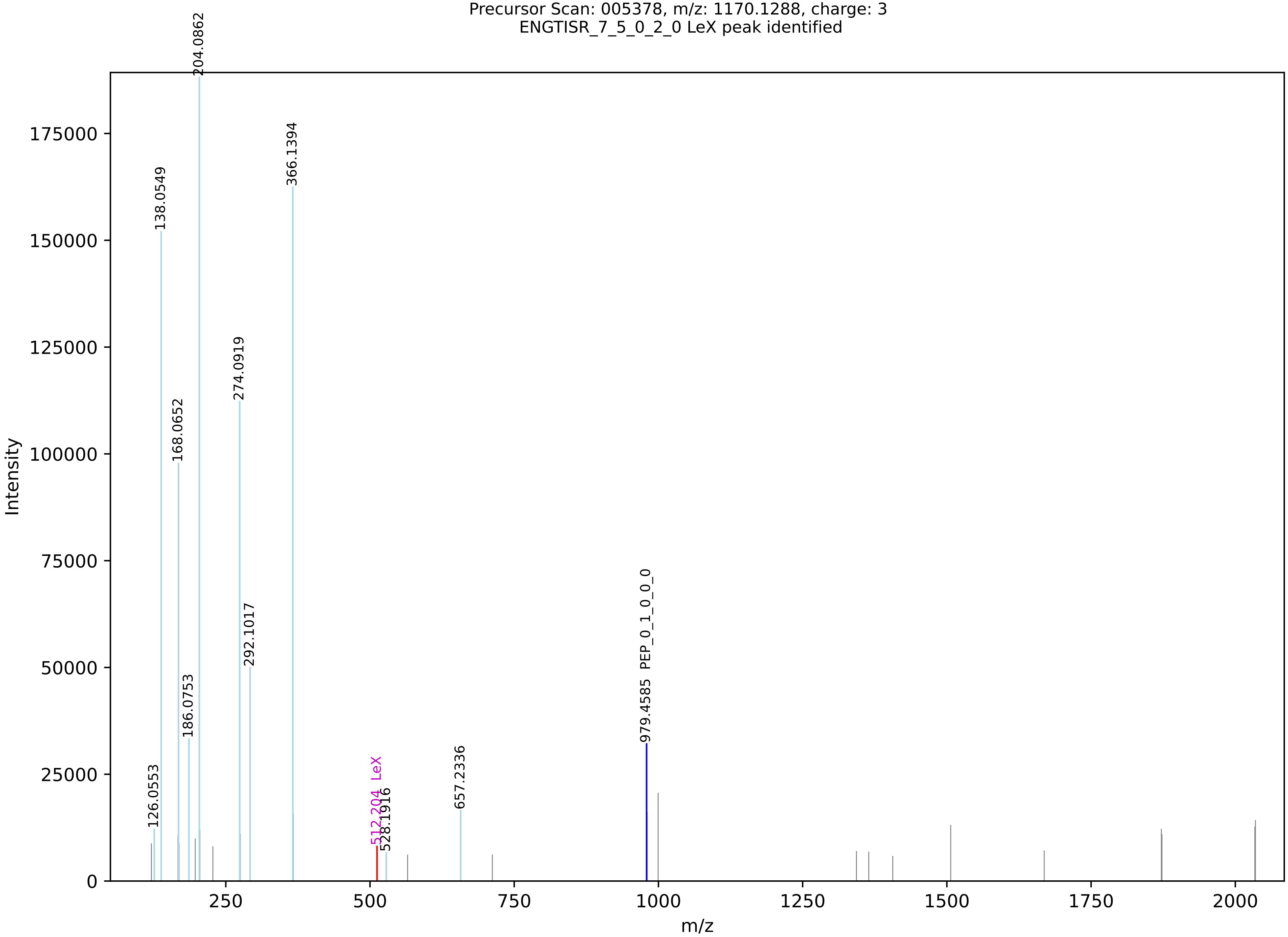

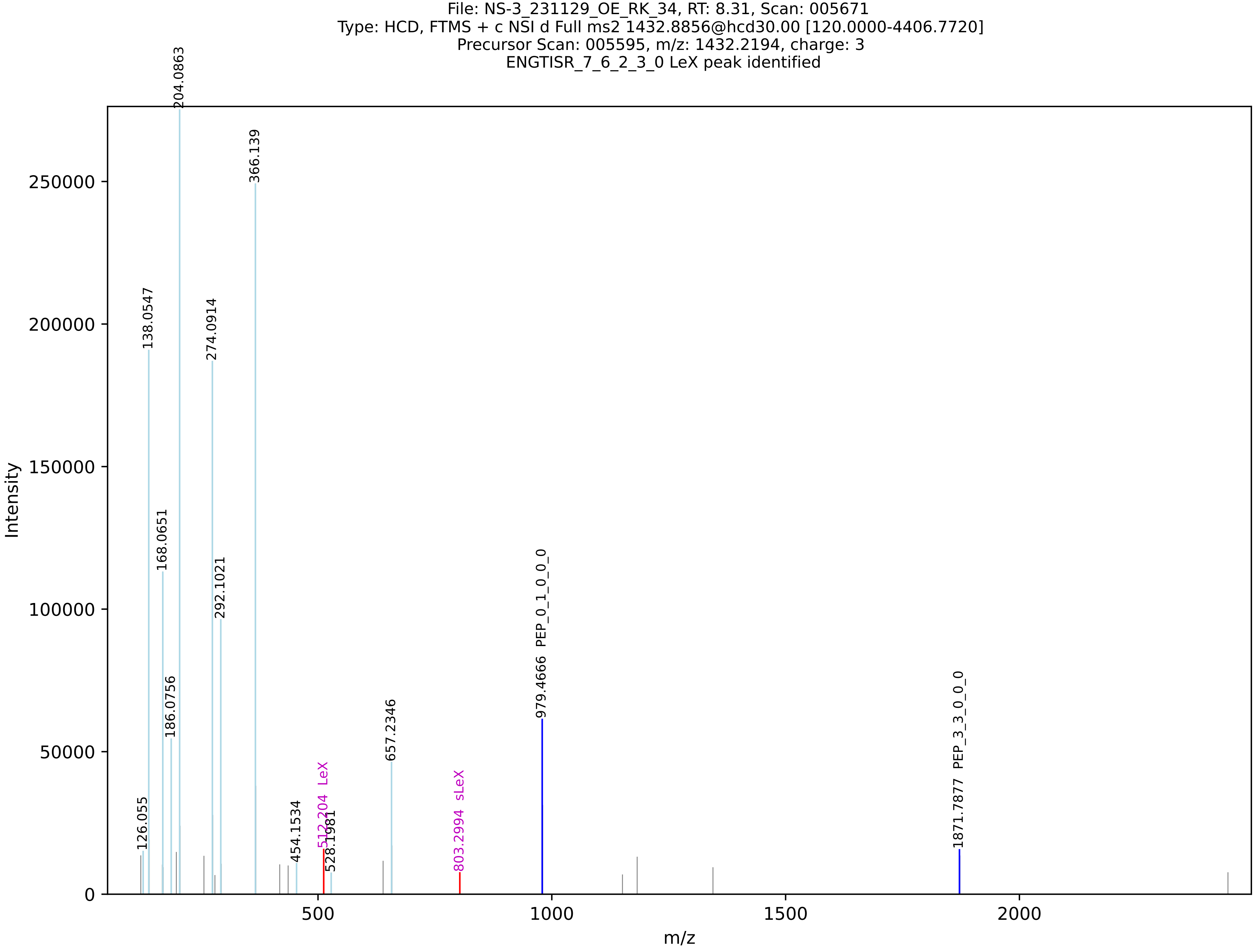
File: NS-3_231129_OE_RK_34, RT: 8.31, Scan: 005671
Type: HCD, FTMS + c NSI d Full ms2 1432.8856@hcd30.00 [120.0000-4406.7720]
Precursor Scan: 005595, m/z: 1432.2194, charge: 3
ENGTISR_7_6_2_3_0 LeX peak identified
Intensity
m/z
204.0863
366.139
138.0547
274.0914
168.0651
292.1021
186.0756
126.055
454.1534
512.204 LeX
528.1981
657.2346
803.2994 sLeX
979.4666 PEP_0_1_0_0_0
1871.7877 PEP_3_3_0_0_0
250000
200000
150000
100000
50000
0
500
1000
1500
2000

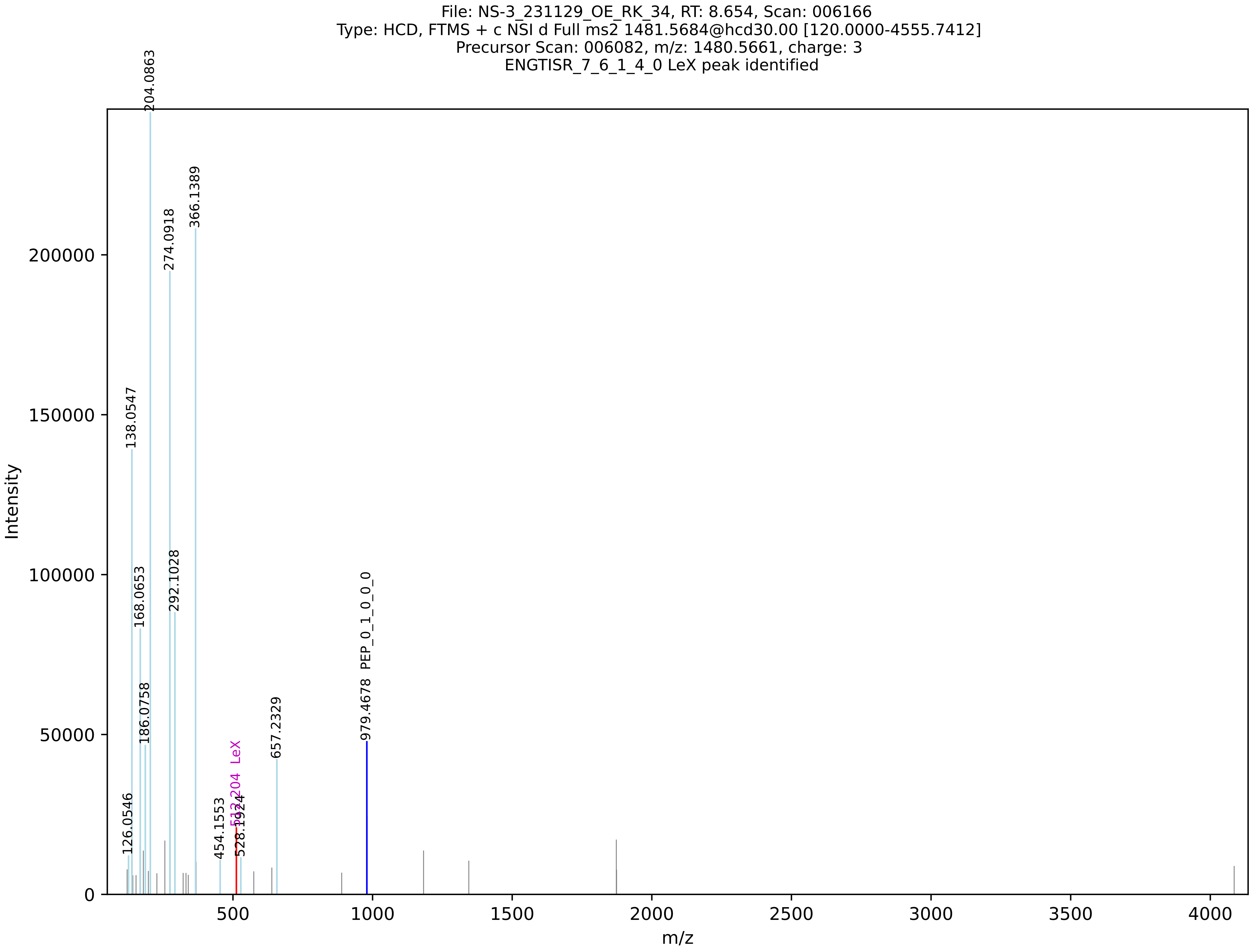

File: NS-3_231129_OE_RK_34, RT: 8.654, Scan: 006166
Type: HCD, FTMS + c NSI d Full ms2 1481.5684@hcd30.00 [120.0000-4555.7412]
Precursor Scan: 006082, m/z: 1480.5661, charge: 3
ENGTISR_7_6_1_4_0 LeX peak identified
204.0863
366.1389
274.0918
138.0547
292.1028
168.0653
186.0758
126.0546
454.1553
512.204 LeX
528.1924
657.2329
979.4678 PEP_0_1_0_0_0
Intensity
m/z
0
50000
100000
150000
200000
500
1000
1500
2000
2500
3000
3500
4000

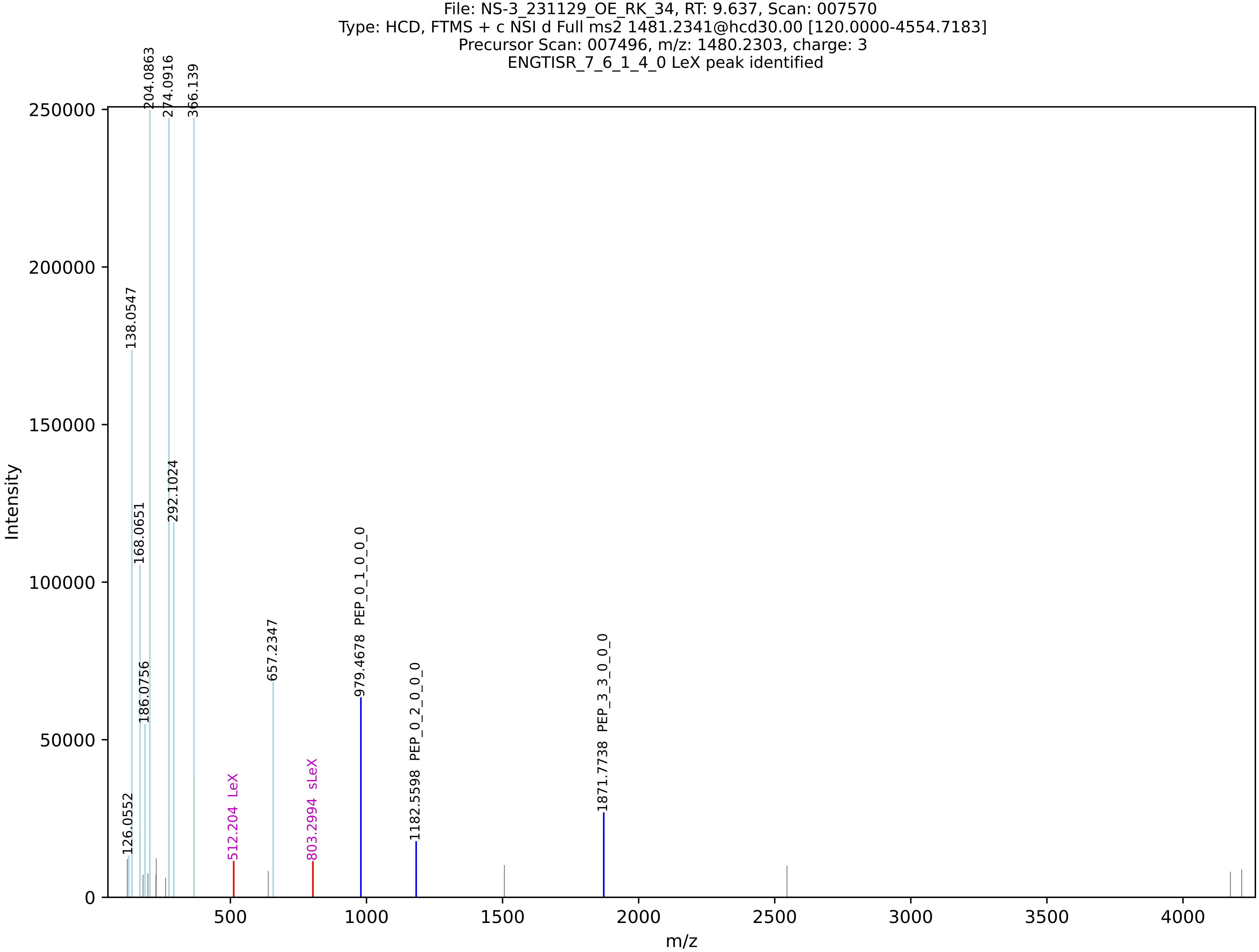

File: NS-3_231129_OE_RK_34, RT: 9.637, Scan: 007570
Type: HCD, FTMS + c NSI d Full ms2 1481.2341@hcd30.00 [120.0000-4554.7183]
Precursor Scan: 007496, m/z: 1480.2303, charge: 3
ENGTISR_7_6_1_4_0 LeX peak identified
Intensity
m/z
0
50000
100000
150000
200000
250000
500
1000
1500
2000
2500
3000
3500
4000
126.0552
138.0547
168.0651
186.0756
204.0863
274.0916
292.1024
366.139
512.204 LeX
657.2347
803.2994 sLeX
979.4678 PEP_0_1_0_0_
1182.5598 PEP_0_2_0_0_
1871.7738 PEP_3_3_0_0_

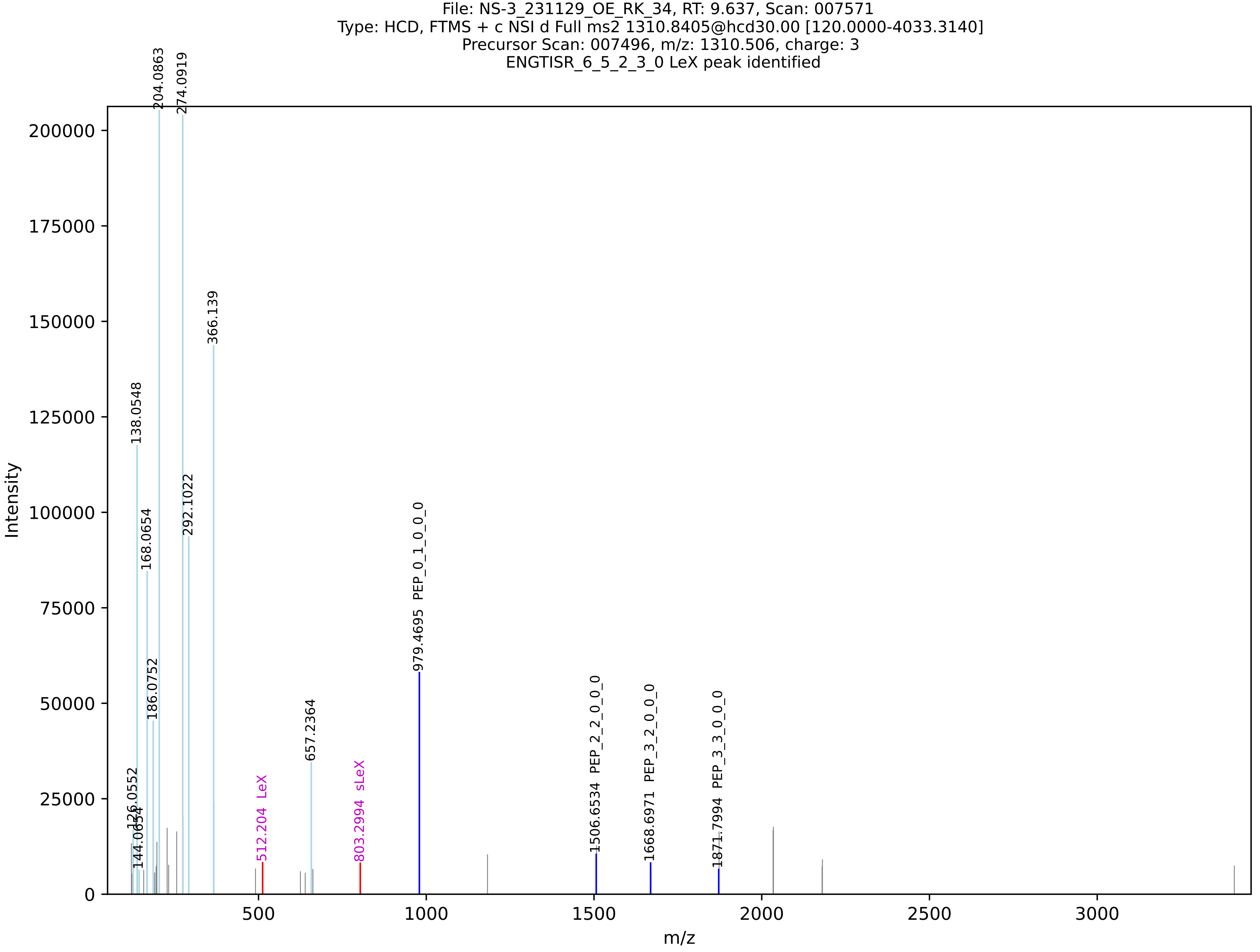

File: NS-3_231129_OE_RK_34, RT: 9.637, Scan: 007571
Type: HCD, FTMS + c NSI d Full ms2 1310.8405@hcd30.00 [120.0000-4033.3140]
Precursor Scan: 007496, m/z: 1310.506, charge: 3
ENGTISR_6_5_2_3_0 LeX peak identified
Intensity
m/z
204.0863
274.0919
366.139
138.0548
292.1022
168.0654
186.0752
126.0552
144.0654
512.204 LeX
657.2364
803.2994 sLeX
979.4695 PEP_0_1_0_0_0
1506.6534 PEP_2_2_0_0_0
1668.6971 PEP_3_2_0_0_0
1871.7994 PEP_3_3_0_0_0

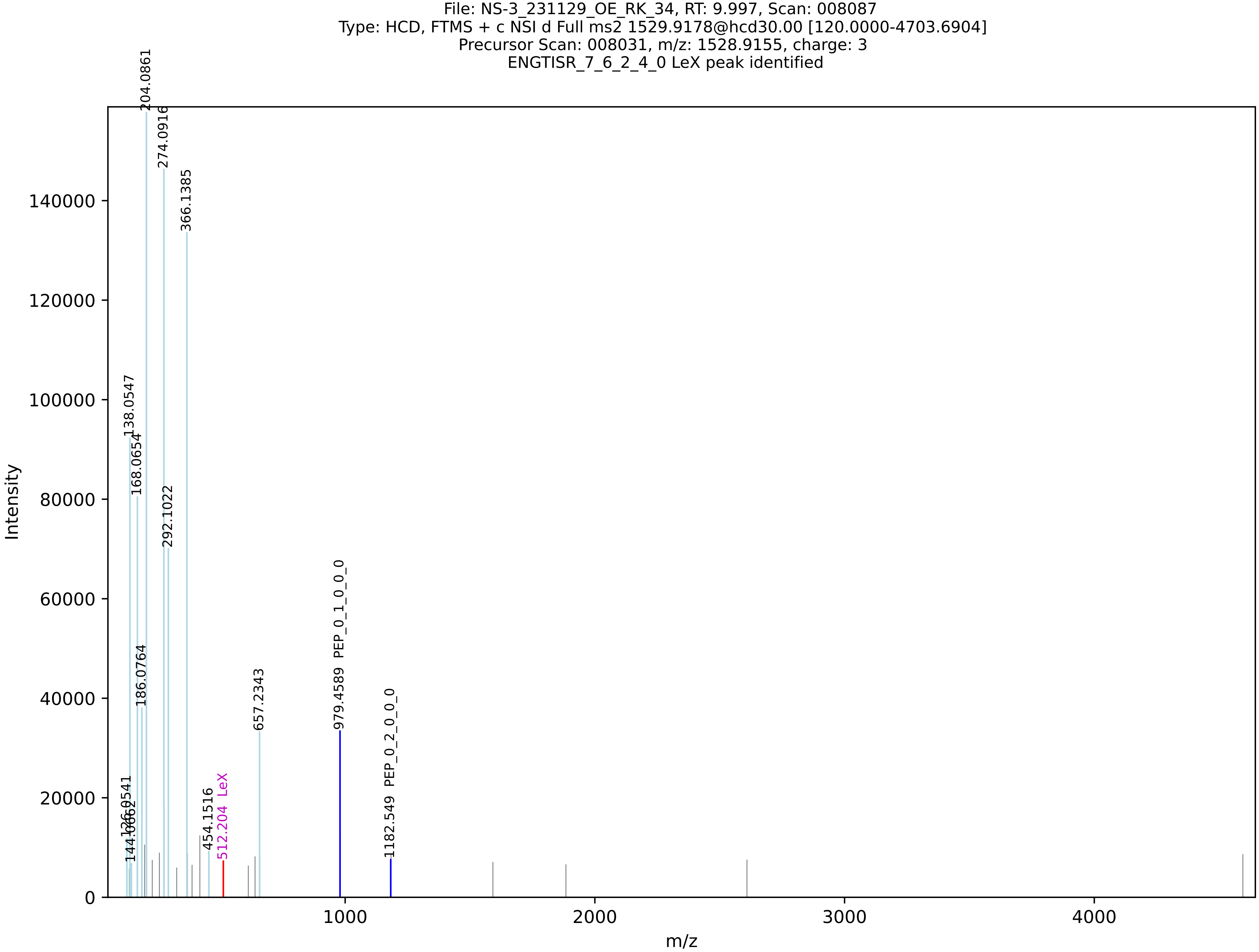

File: NS-3_231129_OE_RK_34, RT: 9.997, Scan: 008087
Type: HCD, FTMS + c NSI d Full ms2 1529.9178@hcd30.00 [120.0000-4703.6904]
Precursor Scan: 008031, m/z: 1528.9155, charge: 3
ENGTISR_7_6_2_4_0 LeX peak identified
204.0861
274.0916
366.1385
138.0547
168.0654
292.1022
186.0764
657.2343
979.4589 PEP_0_1_0_0_0
1182.549 PEP_0_2_0_0_0
126.0541
144.0662
454.1516
512.204 LeX
Intensity
0
20000
40000
60000
80000
100000
120000
140000
1000
2000
3000
4000
m/z

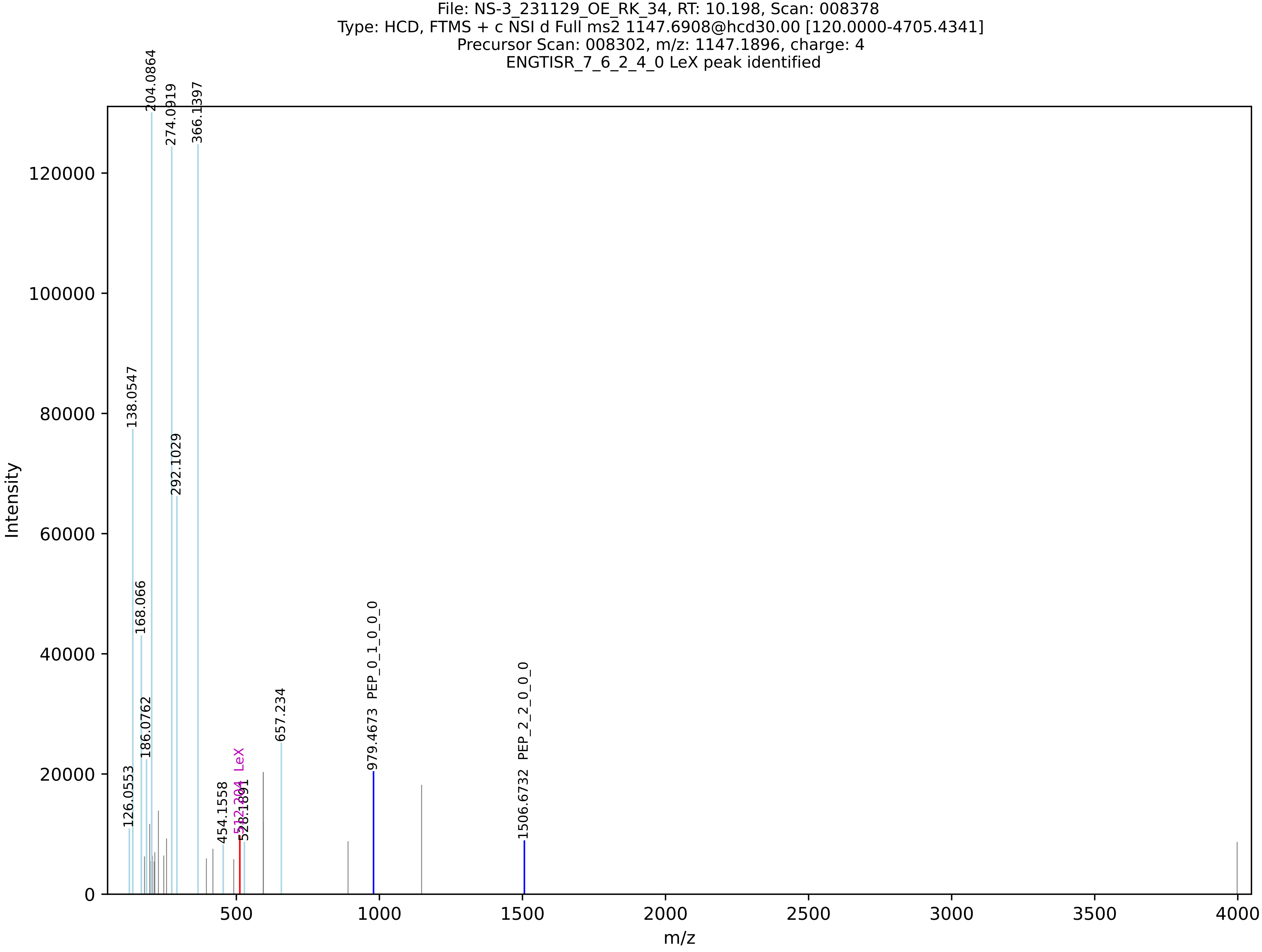

File: NS-3_231129_OE_RK_34, RT: 10.198, Scan: 008378
Type: HCD, FTMS + c NSI d Full ms2 1147.6908@hcd30.00 [120.0000-4705.4341]
Precursor Scan: 008302, m/z: 1147.1896, charge: 4
ENGTISR_7_6_2_4_0 LeX peak identified
Intensity
m/z
0
20000
40000
60000
80000
100000
120000
500
1000
1500
2000
2500
3000
3500
4000
204.0864
274.0919
366.1397
138.0547
292.1029
168.066
186.0762
126.0553
454.1558
512.204 LeX
528.1891
657.234
979.4673 PEP_0_1_0_0_0_
1506.6732 PEP_2_2_0_0_0_

File: NS-3_231129_OE_RK_34, RT: 10.843, Scan: 009312
Type: HCD, FTMS + c NSI d Full ms2 1529.5848@hcd30.00 [120.0000-4702.6714]
Precursor Scan: 009225, m/z: 1528.9167, charge: 3
ENGTISR_7_6_2_4_0 LeX peak identified

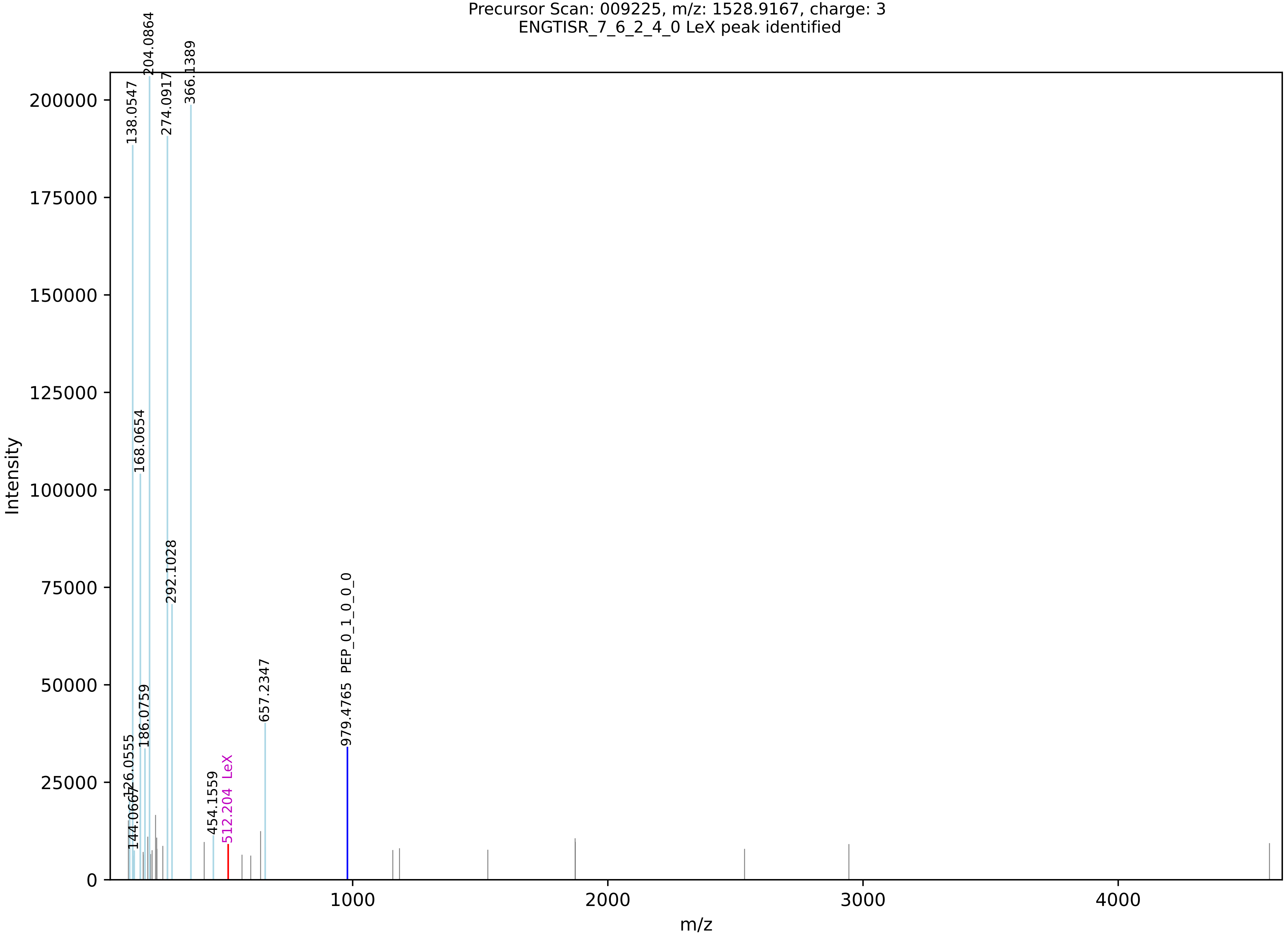

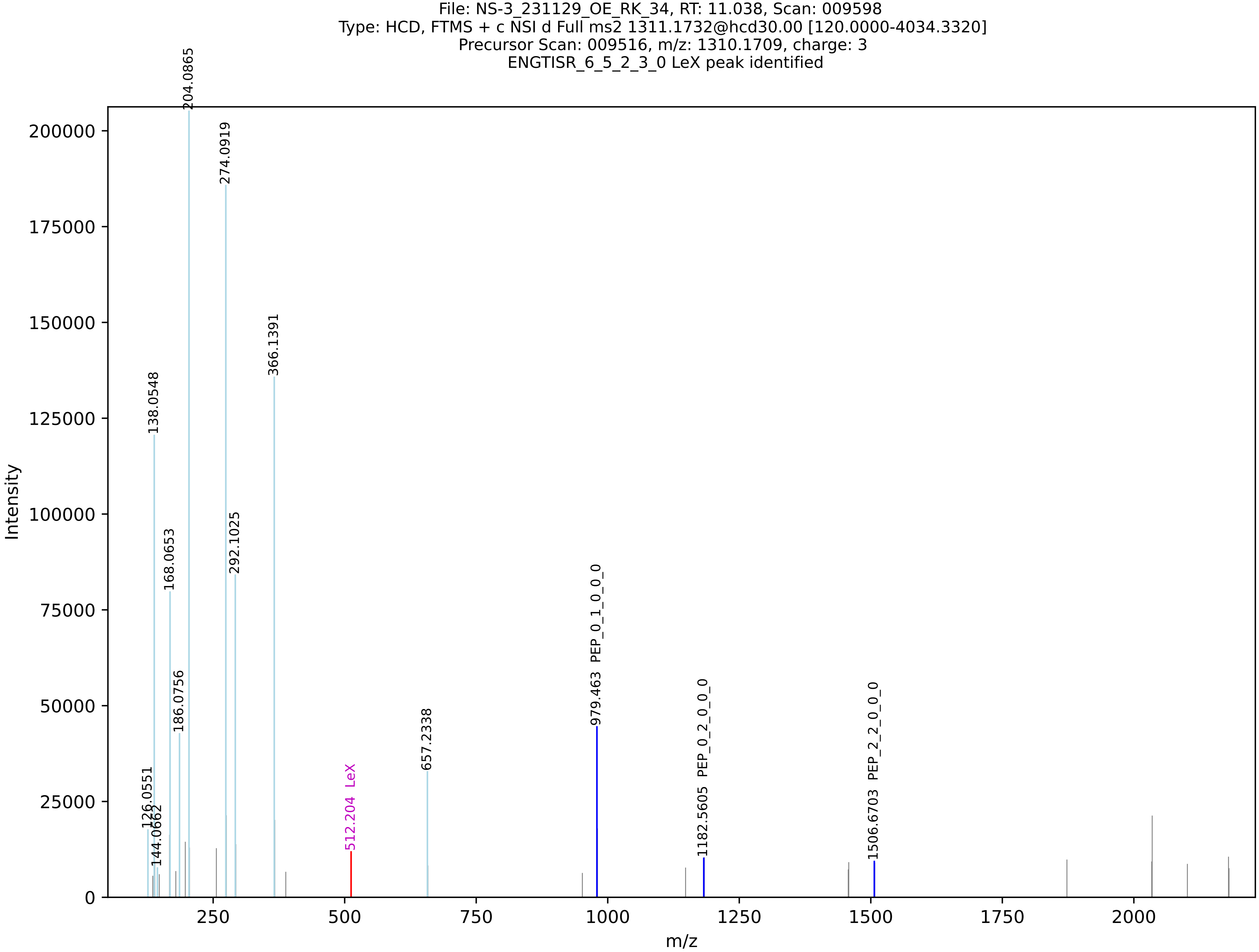

File: NS-3_231129_OE_RK_34, RT: 11.038, Scan: 009598
Type: HCD, FTMS + c NSI d Full ms2 1311.1732@hcd30.00 [120.0000-4034.3320]
Precursor Scan: 009516, m/z: 1310.1709, charge: 3
ENGTISR_6_5_2_3_0 LeX peak identified
Intensity
m/z
204.0865
274.0919
366.1391
138.0548
292.1025
168.0653
186.0756
126.0551
144.0662
512.204 LeX
657.2338
979.463 PEP_0_1_0_0_
1182.5605 PEP_0_2_0_0_
1506.6703 PEP_2_2_0_0_

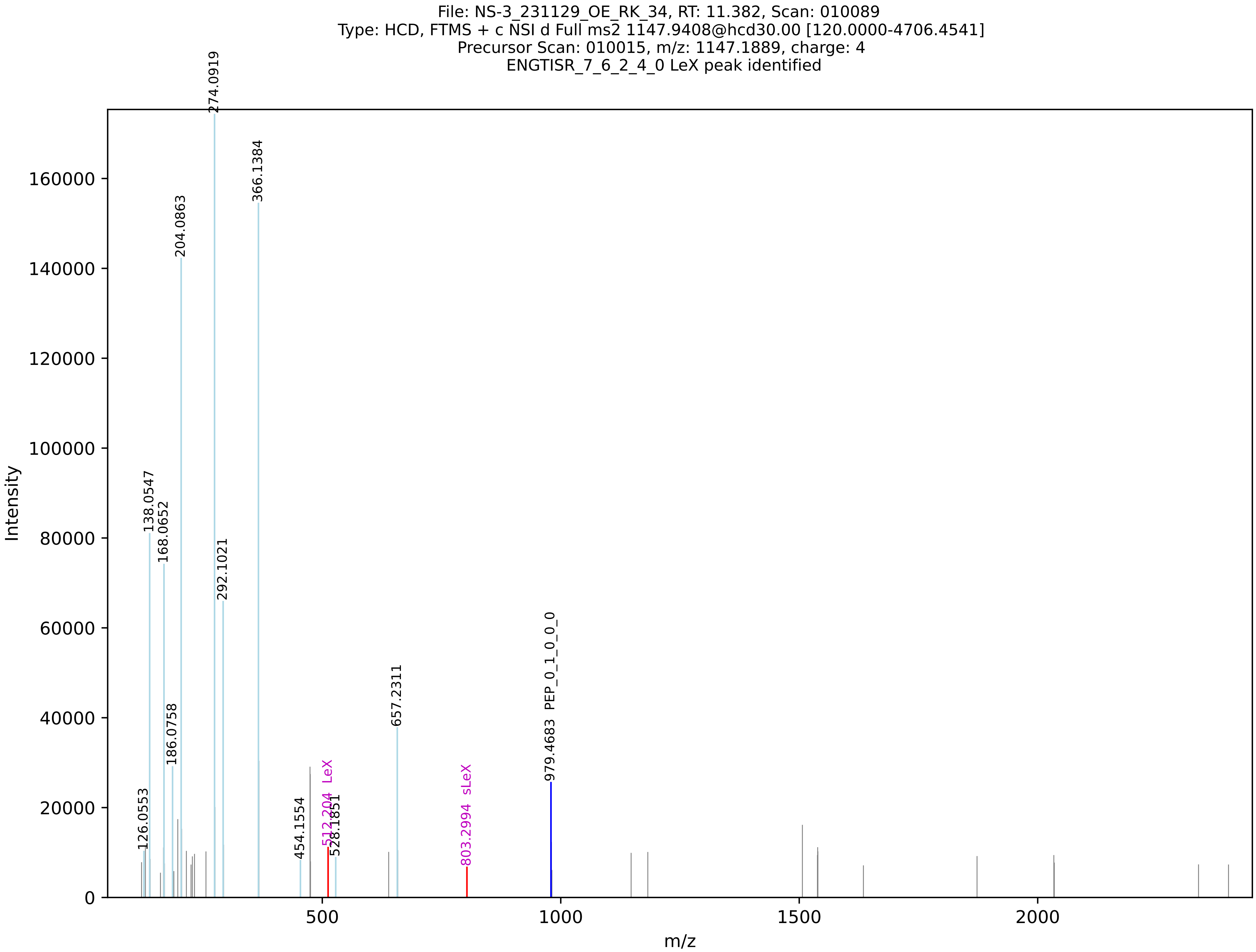
File: NS-3_231129_OE_RK_34, RT: 11.382, Scan: 010089
Type: HCD, FTMS + c NSI d Full ms2 1147.9408@hcd30.00 [120.0000-4706.4541]
Precursor Scan: 010015, m/z: 1147.1889, charge: 4
ENGTISR_7_6_2_4_0 LeX peak identified
Intensity
m/z
274.0919
366.1384
204.0863
138.0547
168.0652
292.1021
186.0758
126.0553
454.1554
512.204 LeX
528.1851
657.2311
803.2994 sLeX
979.4683 PEP_0_1_0_0_0

File: NS-3_231129_OE_RK_34, RT: 11.435, Scan: 010167
Type: HCD, FTMS + c NSI d Full ms2 1310.8392@hcd30.00 [120.0000-4033.3101]
Precursor Scan: 010085, m/z: 1310.508, charge: 3
ENGTISR_6_5_2_3_0 LeX peak identified

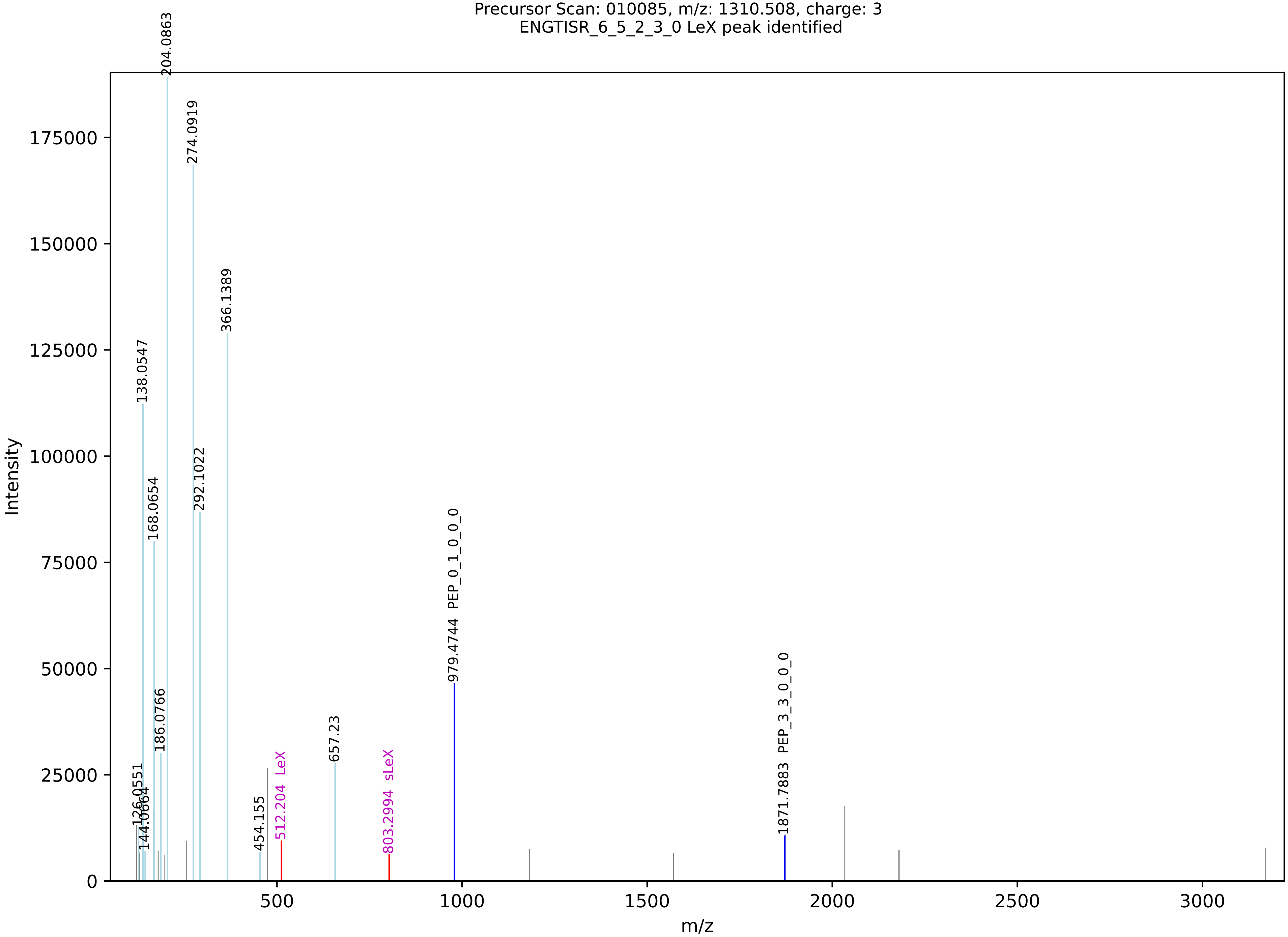

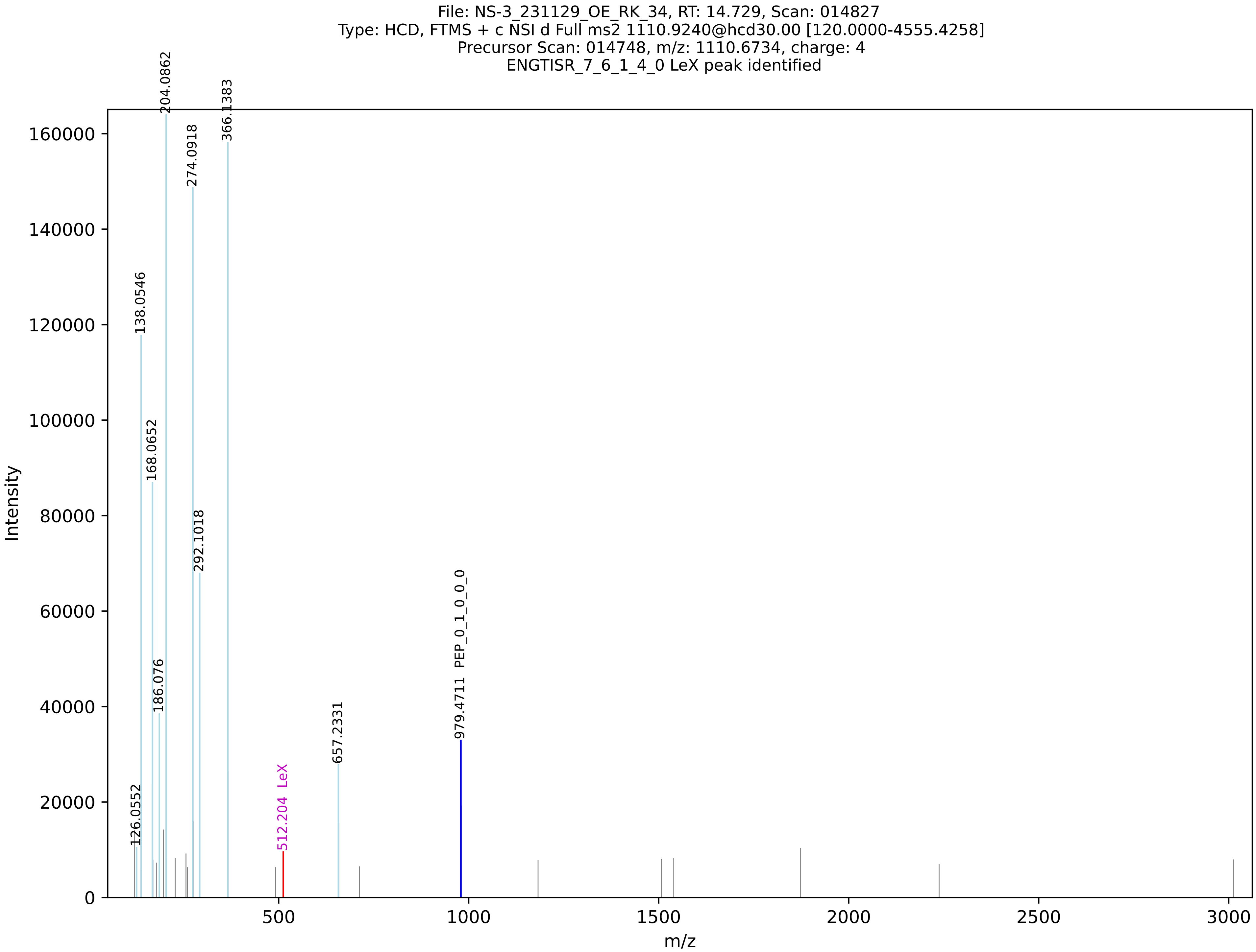
File: NS-3_231129_OE_RK_34, RT: 14.729, Scan: 014827
Type: HCD, FTMS + c NSI d Full ms2 1110.9240@hcd30.00 [120.0000-4555.4258]
Precursor Scan: 014748, m/z: 1110.6734, charge: 4
ENGTISR_7_6_1_4_0 LeX peak identified
Intensity
m/z
204.0862
366.1383
274.0918
138.0546
168.0652
292.1018
186.076
126.0552
512.204 LeX
657.2331
979.4711 PEP_0_1_0_0_0_
0
20000
40000
60000
80000
100000
120000
140000
160000
500
1000
1500
2000
2500
3000

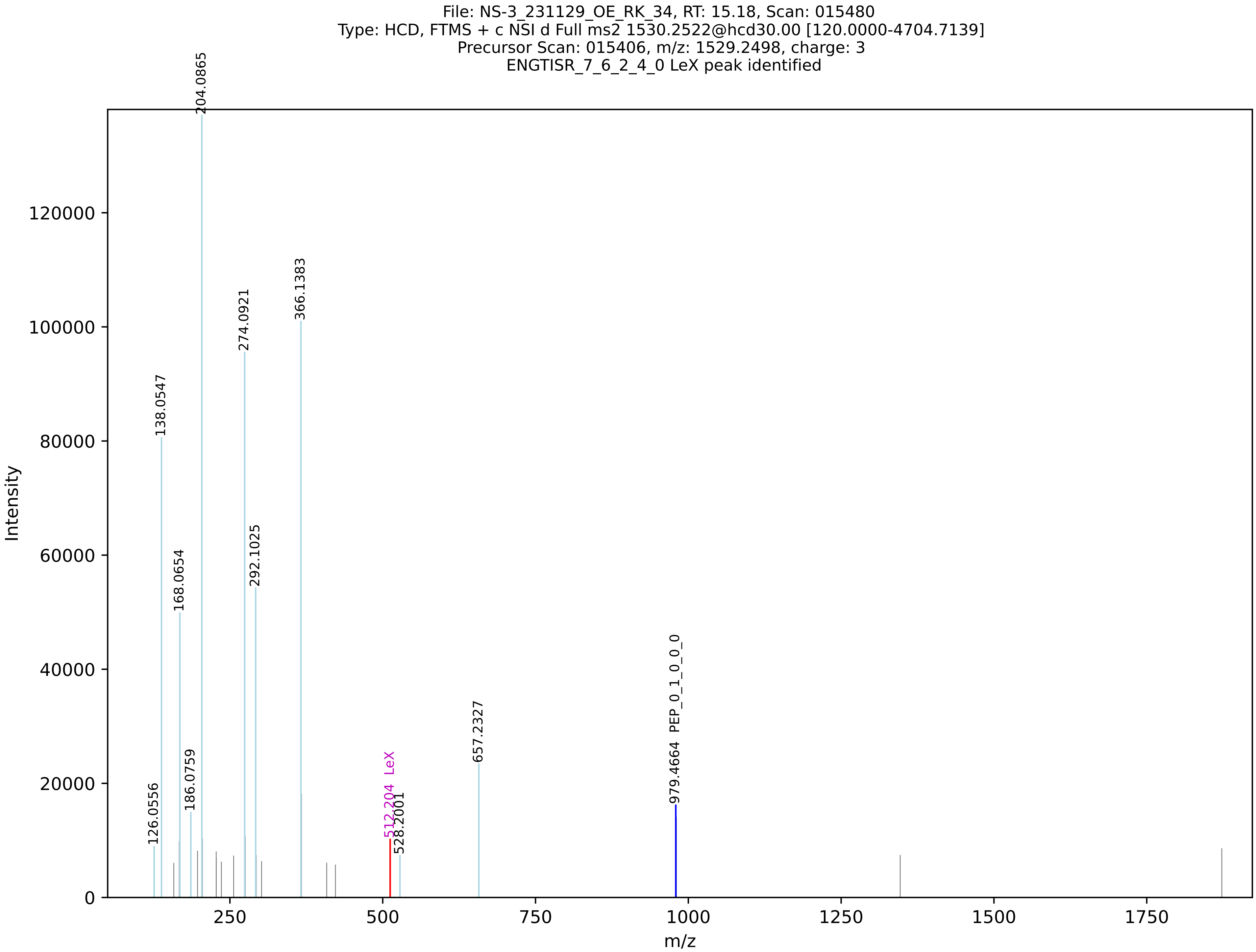
File: NS-3_231129_OE_RK_34, RT: 15.18, Scan: 015480
Type: HCD, FTMS + c NSI d Full ms2 1530.2522@hcd30.00 [120.0000-4704.7139]
Precursor Scan: 015406, m/z: 1529.2498, charge: 3
ENGTISR_7_6_2_4_0 LeX peak identified
Intensity
m/z
204.0865
366.1383
274.0921
138.0547
168.0654
292.1025
126.0556
186.0759
512.204 LeX
528.2001
657.2327
979.4664 PEP_0_1_0_0_0
0
20000
40000
60000
80000
100000
120000
250
500
750
1000
1250
1500
1750

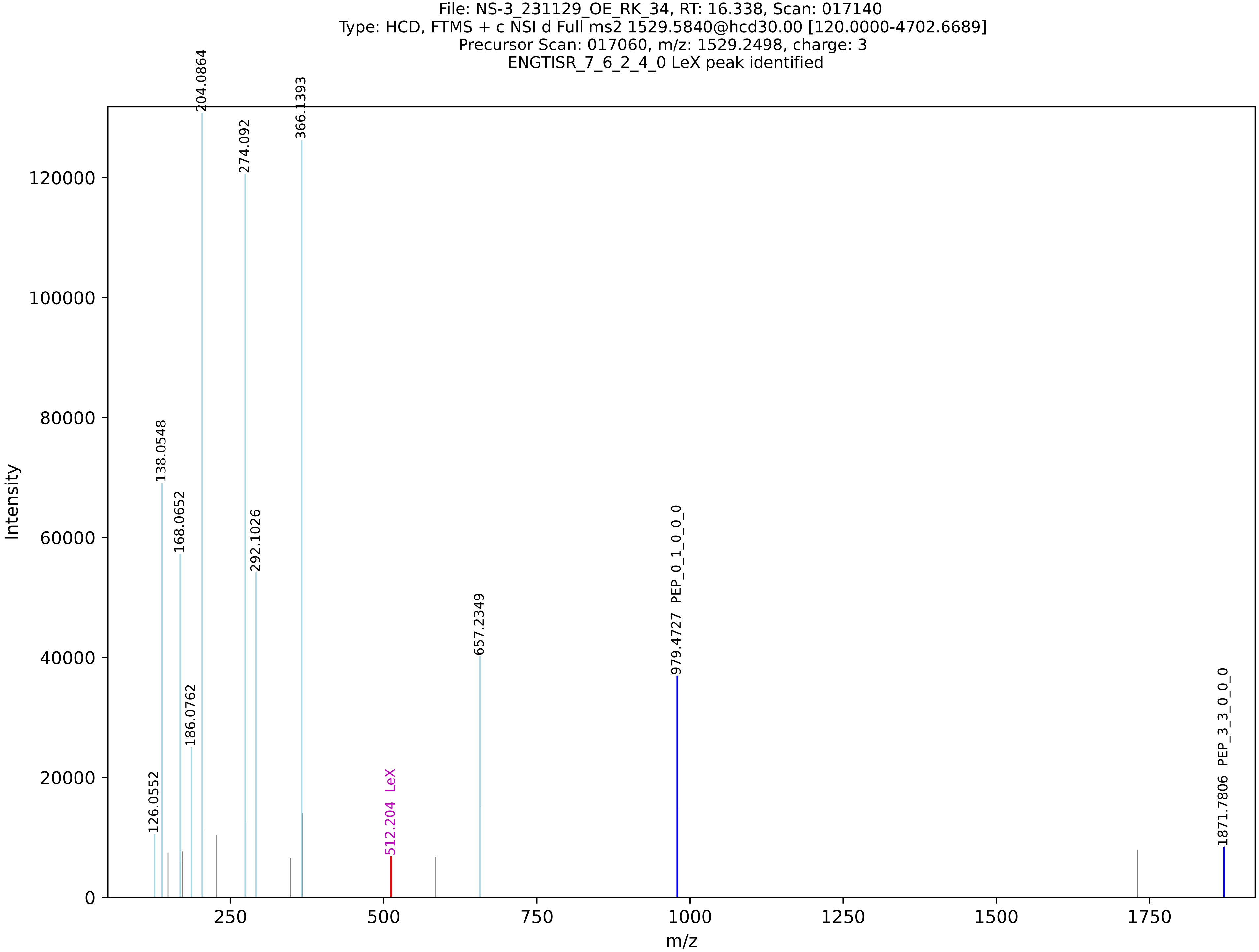

File: NS-3_231129_OE_RK_34, RT: 16.338, Scan: 017140
Type: HCD, FTMS + c NSI d Full ms2 1529.5840@hcd30.00 [120.0000-4702.6689]
Precursor Scan: 017060, m/z: 1529.2498, charge: 3
ENGTISR_7_6_2_4_0 LeX peak identified
Intensity
m/z
204.0864
274.092
366.1393
138.0548
168.0652
292.1026
186.0762
126.0552
512.204 LeX
657.2349
979.4727 PEP_0_1_0_0_0
1871.7806 PEP_3_3_0_0_0

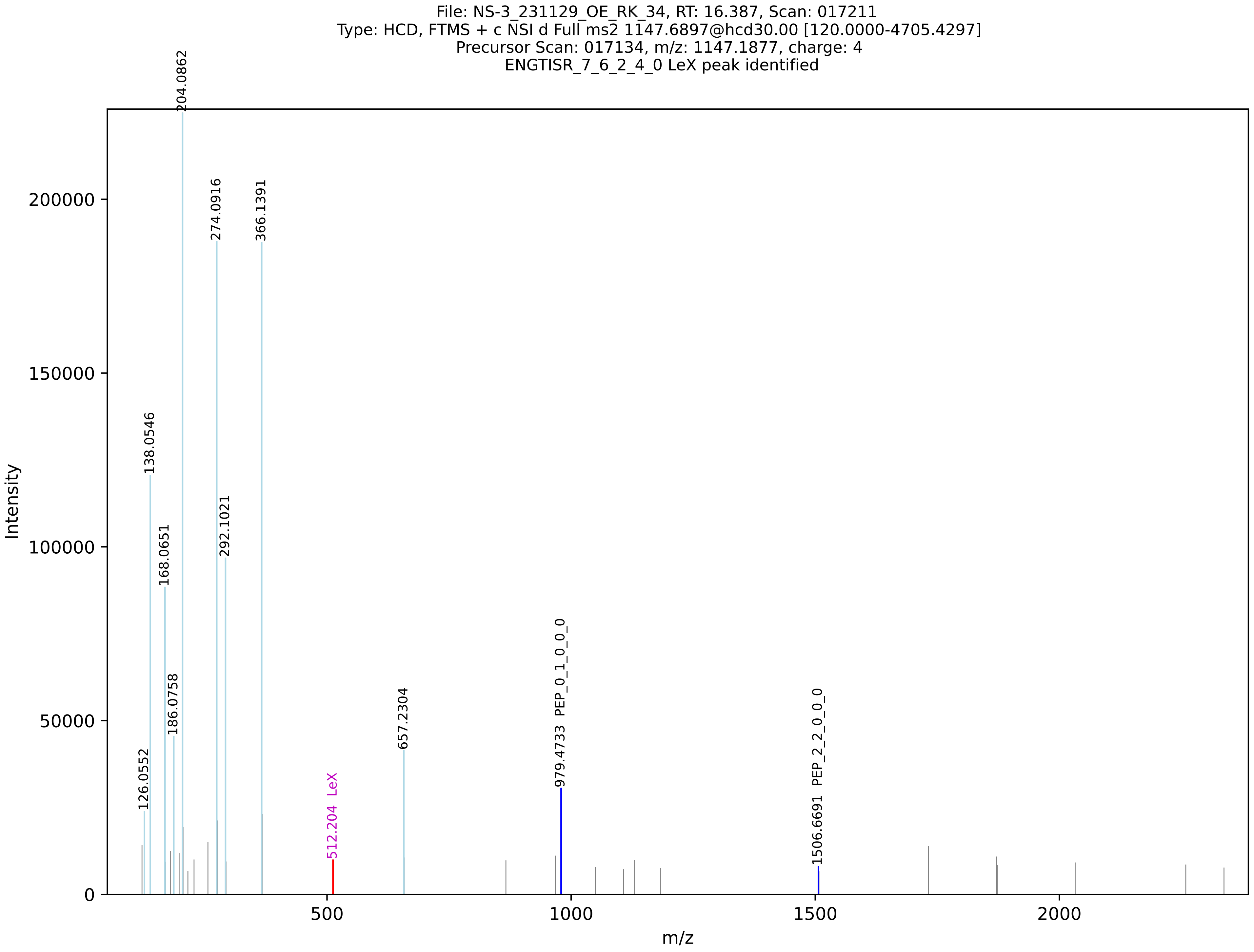
File: NS-3_231129_OE_RK_34, RT: 16.387, Scan: 017211
Type: HCD, FTMS + c NSI d Full ms2 1147.6897@hcd30.00 [120.0000-4705.4297]
Precursor Scan: 017134, m/z: 1147.1877, charge: 4
ENGTISR_7_6_2_4_0 LeX peak identified
Intensity
m/z
204.0862
274.0916
366.1391
138.0546
292.1021
168.0651
186.0758
126.0552
657.2304
512.204 LeX
979.4733 PEP_0_1_0_0_0
1506.6691 PEP_2_2_0_0_0
0
50000
100000
150000
200000
500
1000
1500
2000

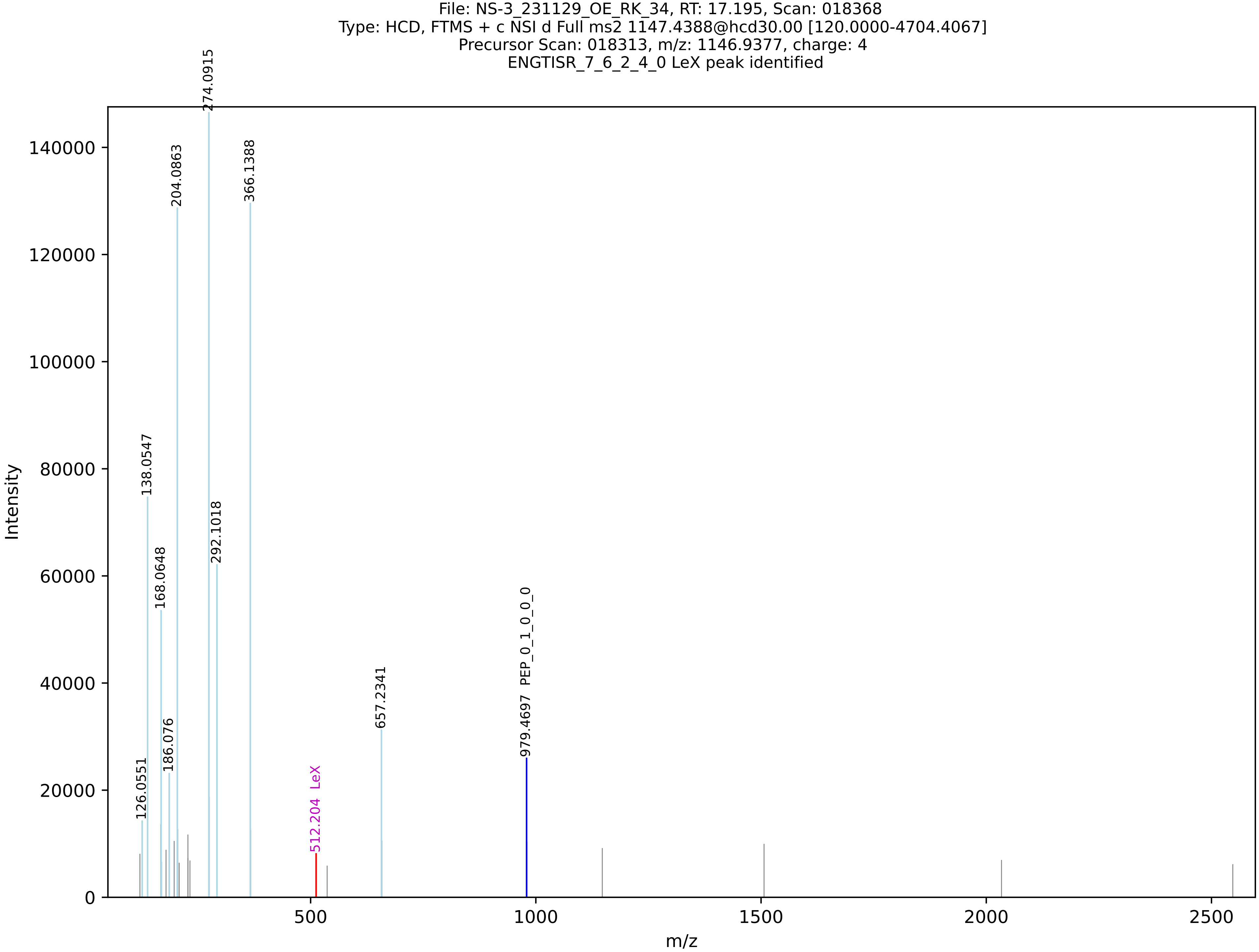
File: NS-3_231129_OE_RK_34, RT: 17.195, Scan: 018368
Type: HCD, FTMS + c NSI d Full ms2 1147.4388@hcd30.00 [120.0000-4704.4067]
Precursor Scan: 018313, m/z: 1146.9377, charge: 4
ENGTISR_7_6_2_4_0 LeX peak identified
274.0915
366.1388
204.0863
138.0547
292.1018
168.0648
PEP_0_1_0_0_0
979.4697
657.2341
186.076
126.0551
512.204 LeX
Intensity
m/z
0
20000
40000
60000
80000
100000
120000
140000
500
1000
1500
2000
2500

File: NS-3_231129_OE_RK_34, RT: 17.347, Scan: 018589
Type: HCD, FTMS + c NSI d Full ms2 1143.1530@hcd30.00 [120.0000-3520.1902]
Precursor Scan: 018506, m/z: 1142.8278, charge: 3
VVNSTTGPGEHLR_5_4_1_1_0 LeX peak identified

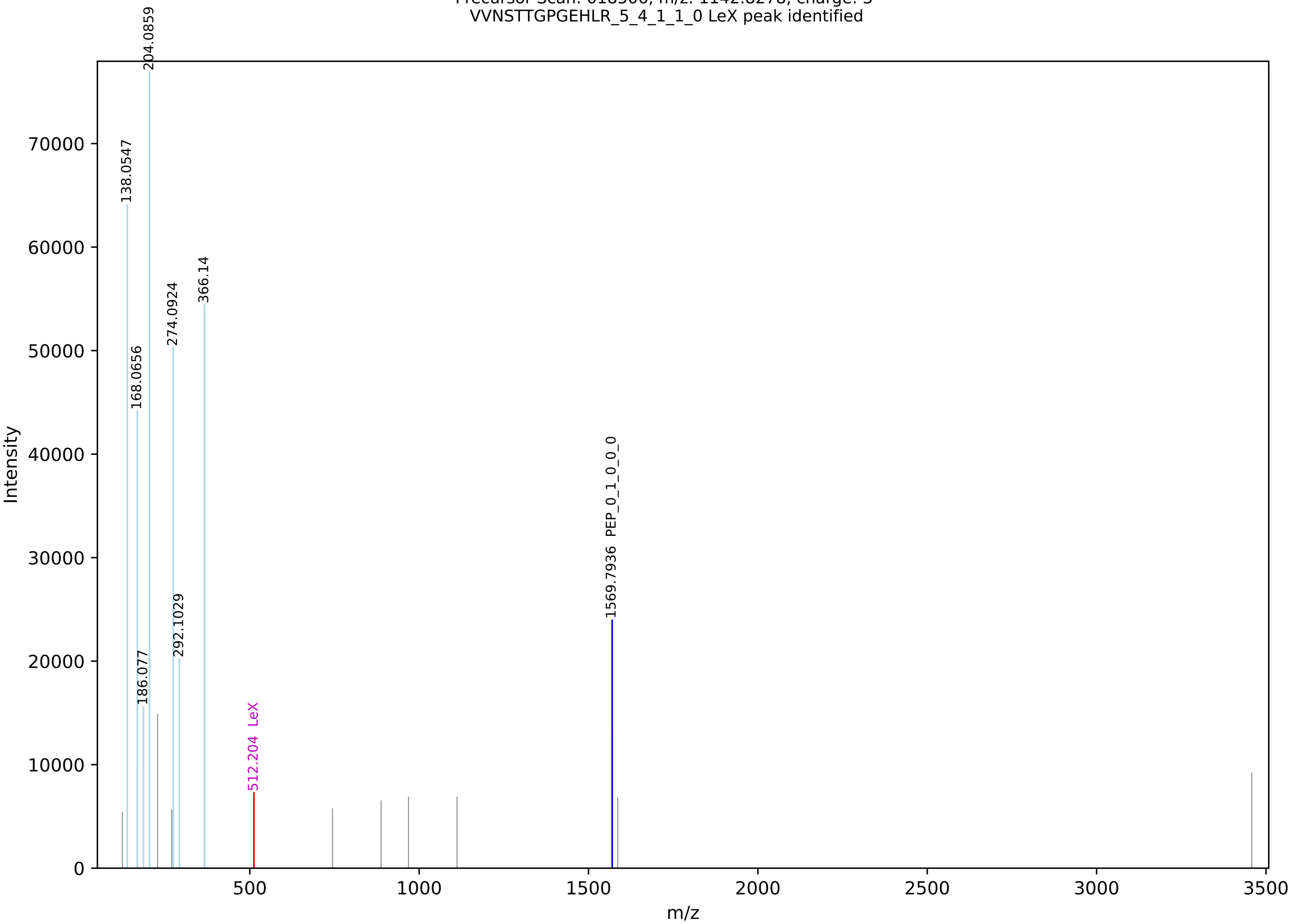

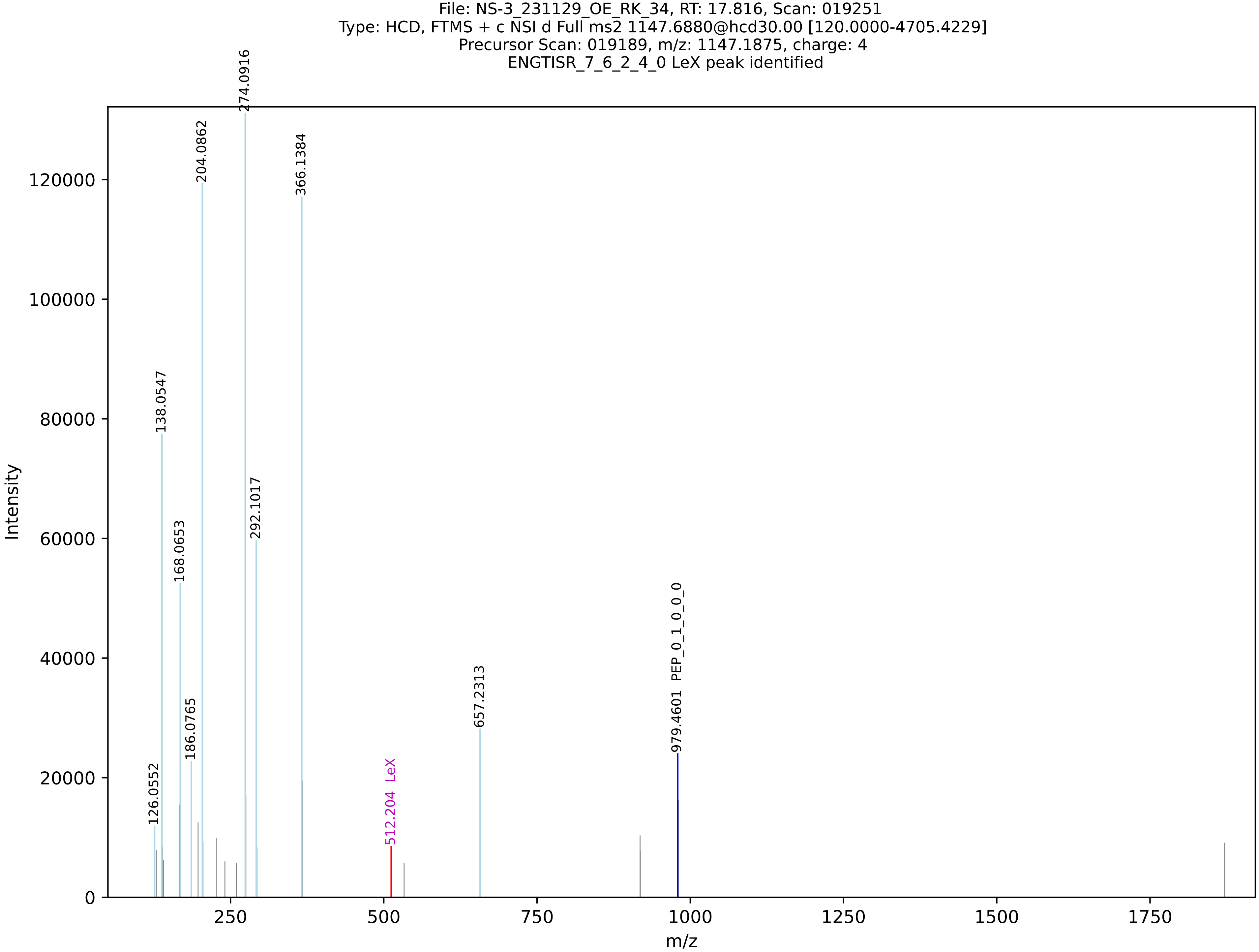
File: NS-3_231129_OE_RK_34, RT: 17.816, Scan: 019251
Type: HCD, FTMS + c NSI d Full ms2 1147.6880@hcd30.00 [120.0000-4705.4229]
Precursor Scan: 019189, m/z: 1147.1875, charge: 4
ENGTISR_7_6_2_4_0 LeX peak identified
274.0916
204.0862
366.1384
138.0547
292.1017
168.0653
PEP_0_1_0_0_0
657.2313
979.4601
186.0765
LeX
126.0552
512.204
Intensity
m/z
0
20000
40000
60000
80000
100000
120000
250
500
750
1000
1250
1500
1750

File: NS-3_231129_OE_RK_34, RT: 35.973, Scan: 045239
Type: HCD, FTMS + c NSI d Full ms2 1275.5417@hcd30.00 [120.0000-5227.0659]
Precursor Scan: 045159, m/z: 1274.7899, charge: 4
DVLWFRPTLLNDTGNYTCMLR_4_7_1_1_0 LeX peak identified

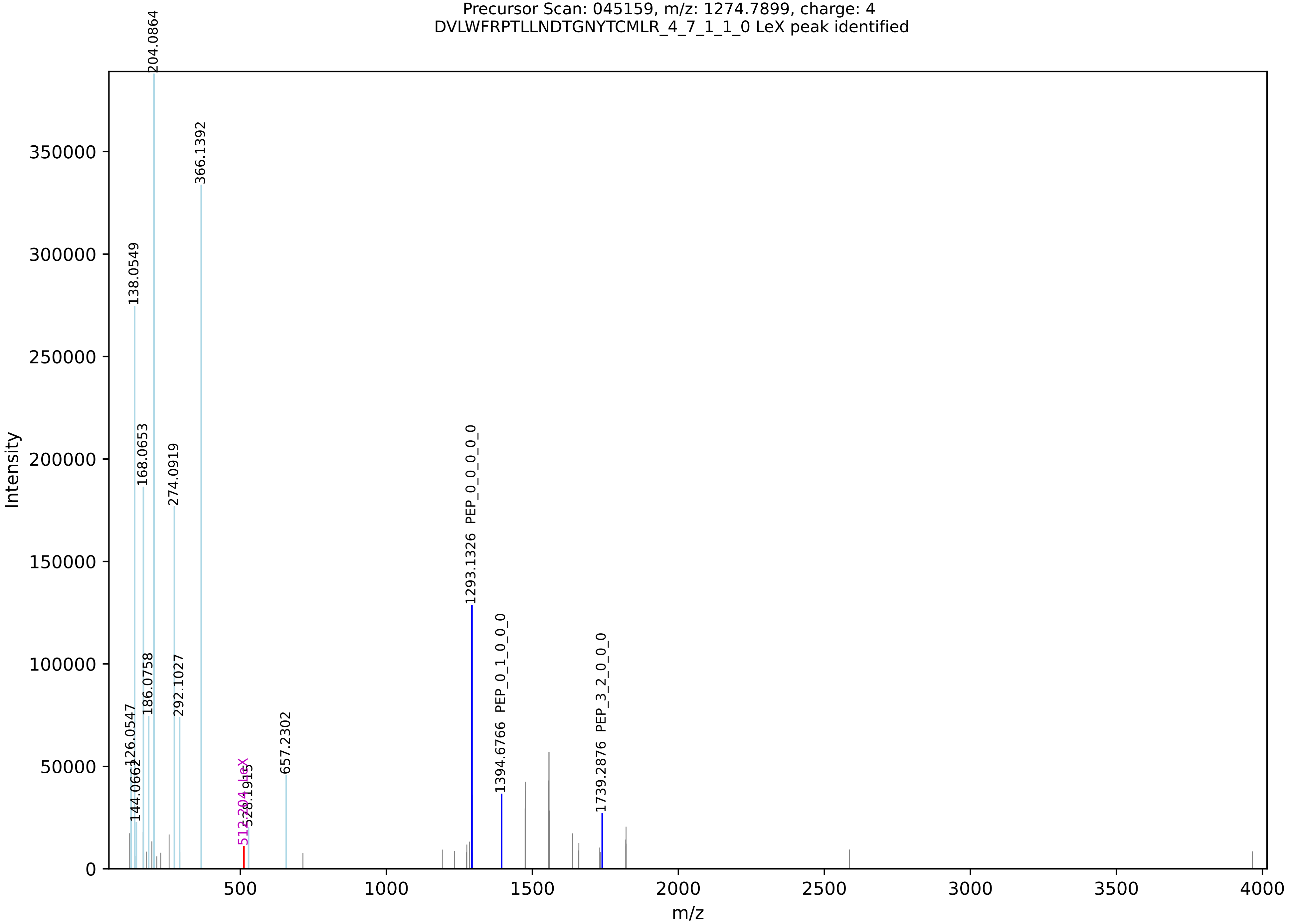

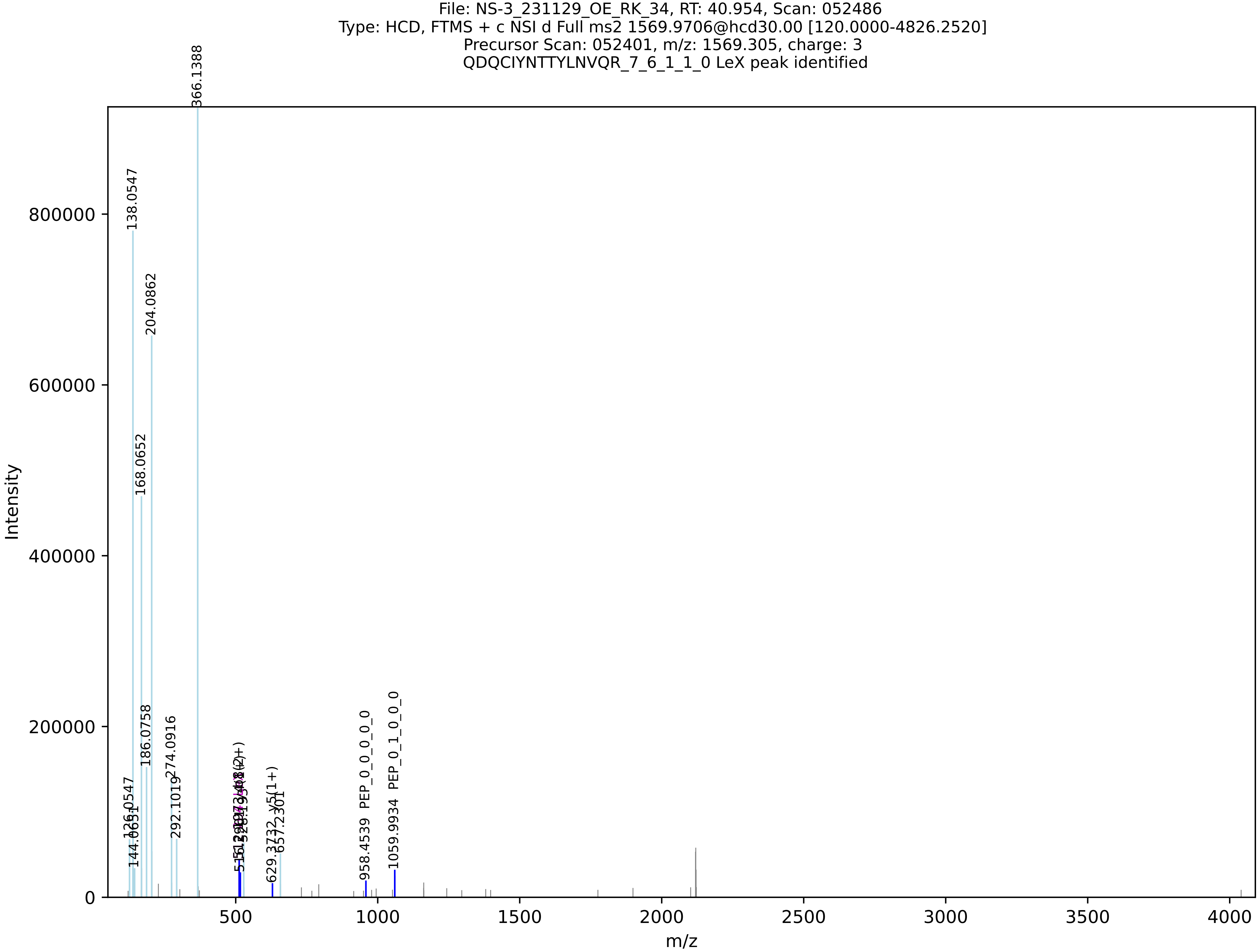
File: NS-3_231129_OE_RK_34, RT: 40.954, Scan: 052486
Type: HCD, FTMS + c NSI d Full ms2 1569.9706@hcd30.00 [120.0000-4826.2520]
Precursor Scan: 052401, m/z: 1569.305, charge: 3
QDQCIYNTTYLNVQR_7_6_1_1_0 LeX peak identified
366.1388
138.0547
204.0862
168.0652
186.0758
274.0916
292.1019
126.0547
144.0651
629.3732_y5(1+)
657.2301
958.4539 PEP_0_0_0_0_0
1059.9934 PEP_0_1_0_0_0
Intensity
0
200000
400000
600000
800000
500
1000
1500
2000
2500
3000
3500
4000
m/z

File: NS-3_231129_OE_RK_34, RT: 41.15, Scan: 052768
Type: HCD, FTMS + c NSI d Full ms2 1570.3026@hcd30.00 [120.0000-4827.2681]
Precursor Scan: 052695, m/z: 1569.3025, charge: 3
QDQCIYNTTYLNVQR_7_6_1_1_0 LeX peak identified

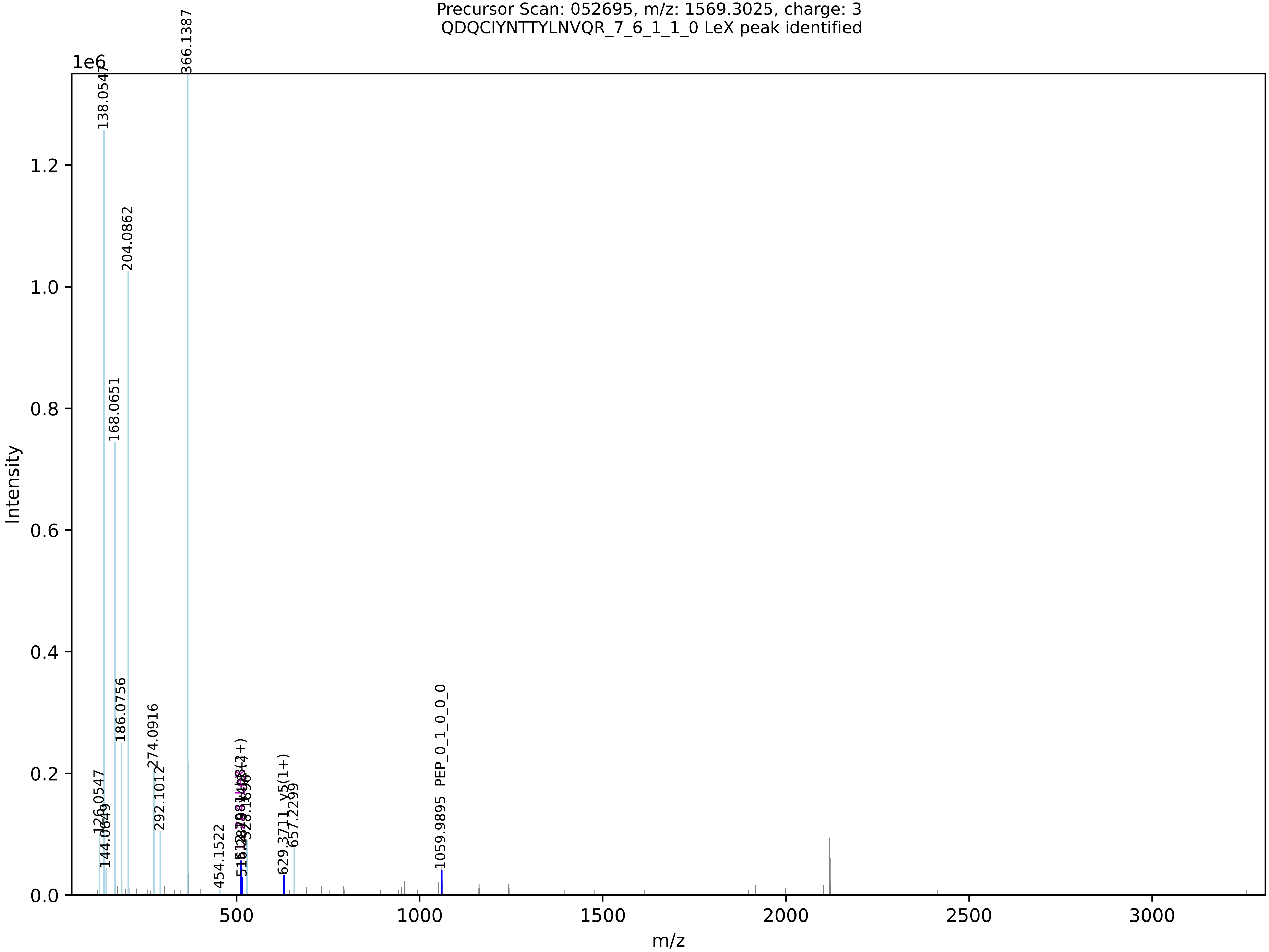

File: NS-3_231129_OE_RK_34, RT: 42.813, Scan: 055191
Type: HCD, FTMS + c NSI d Full ms2 1250.5050@hcd30.00 [120.0000-5124.9160]
Precursor Scan: 055116, m/z: 1250.004, charge: 4
QDQCIYNTTYLNVQR_7_6_1_2_0 LeX peak identified

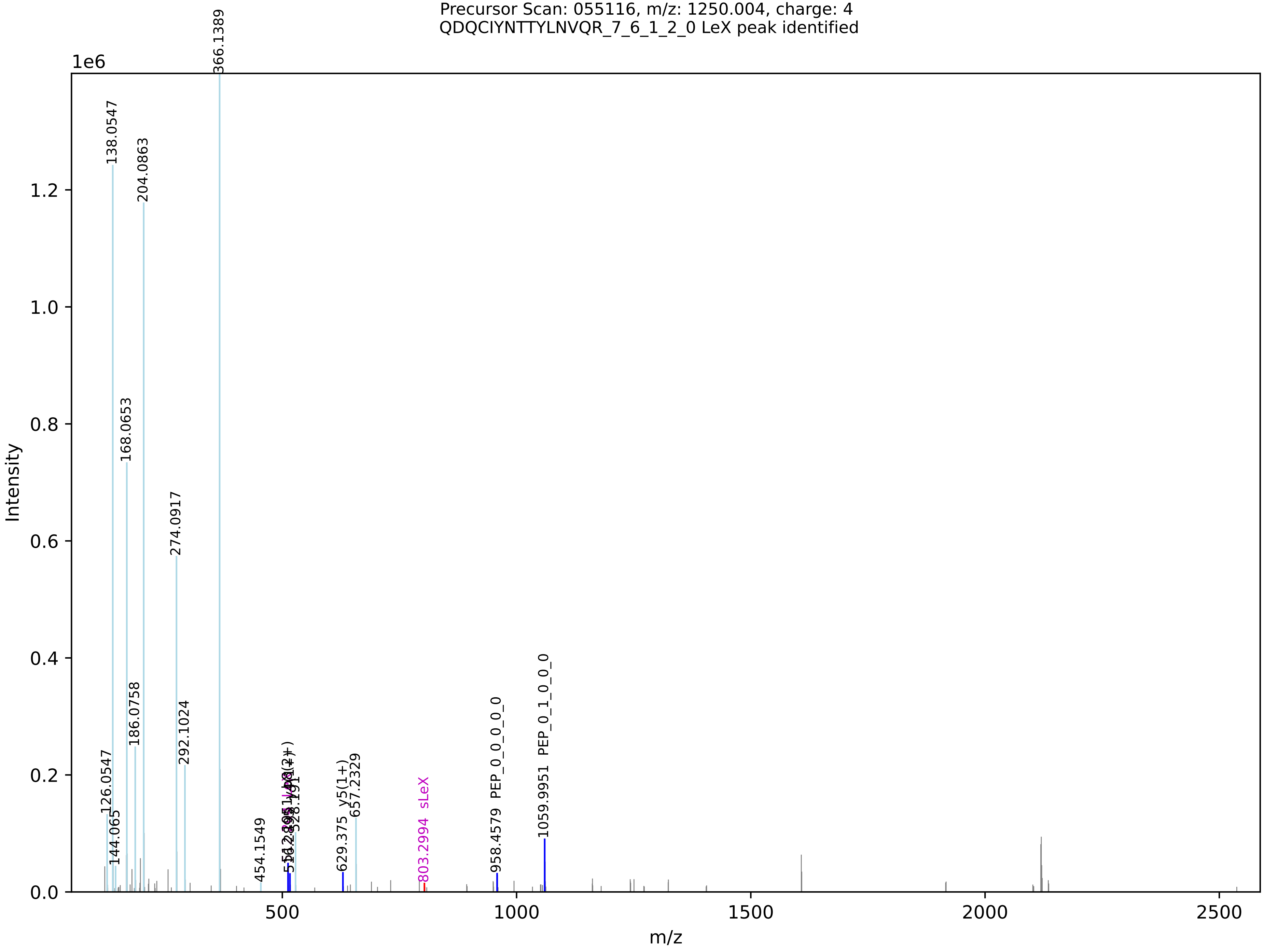

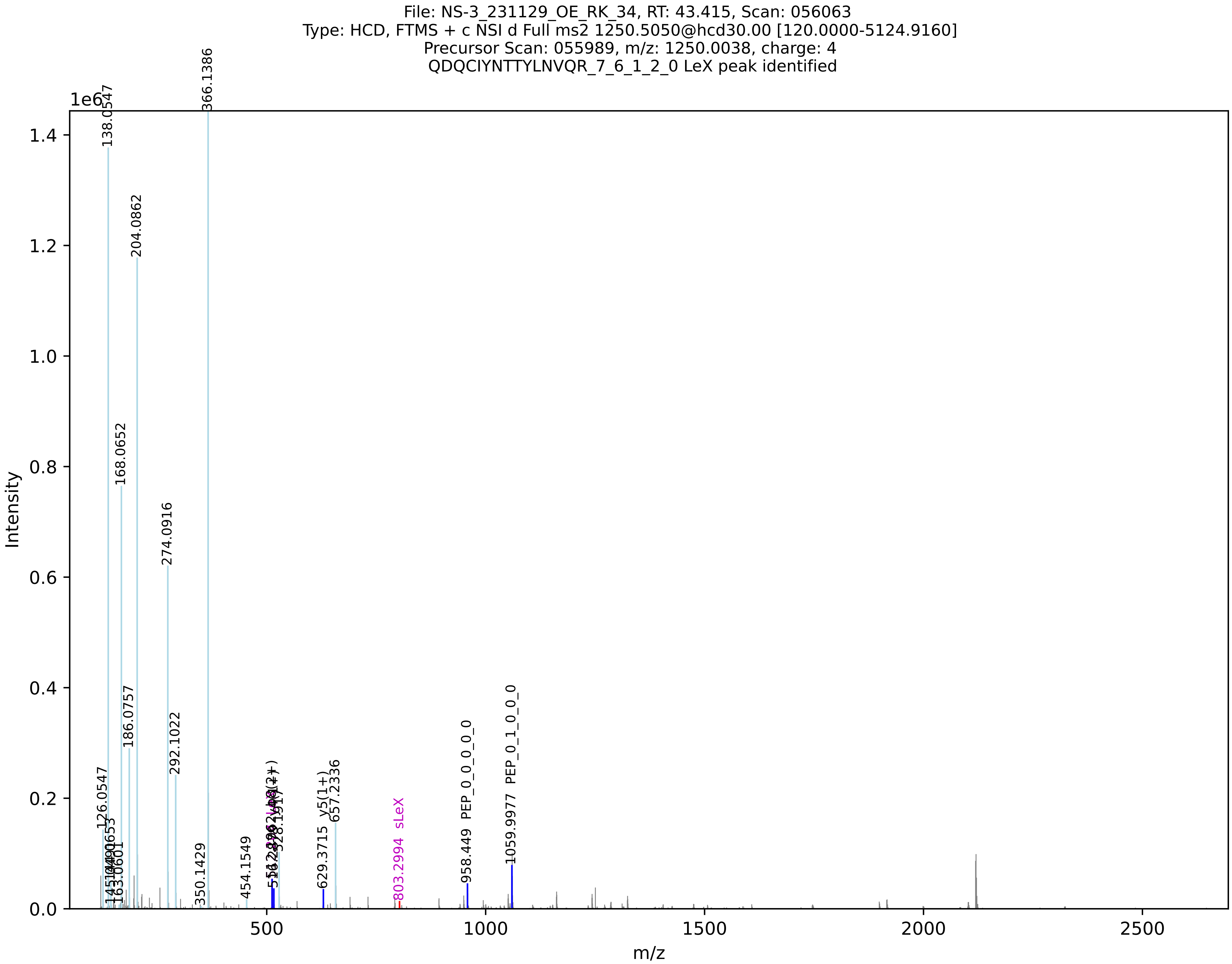
File: NS-3_231129_OE_RK_34, RT: 43.415, Scan: 056063
Type: HCD, FTMS + c NSI d Full ms2 1250.5050@hcd30.00 [120.0000-5124.9160]
Precursor Scan: 055989, m/z: 1250.0038, charge: 4
QDQCIYNTTYLNVQR_7_6_1_2_0 LeX peak identified
1e6
Intensity
m/z
138.0547
366.1386
204.0862
168.0652
274.0916
186.0757
292.1022
126.0547
163.0601
350.1429
454.1549
528.1917
629.3715 y5(1+)
657.2336
803.2994 sLeX
958.449 PEP_0_0_0_0_0
1059.9977 PEP_0_1_0_0_0

File: NS-3_231129_OE_RK_34, RT: 43.418, Scan: 056068
Type: HCD, FTMS + c NSI d Full ms2 1287.0188@hcd30.00 [120.0000-5273.8926]
Precursor Scan: 055989, m/z: 1286.5176, charge: 4
QDQCIYNTTYLNVQR_7_6_2_2_0 LeX peak identified

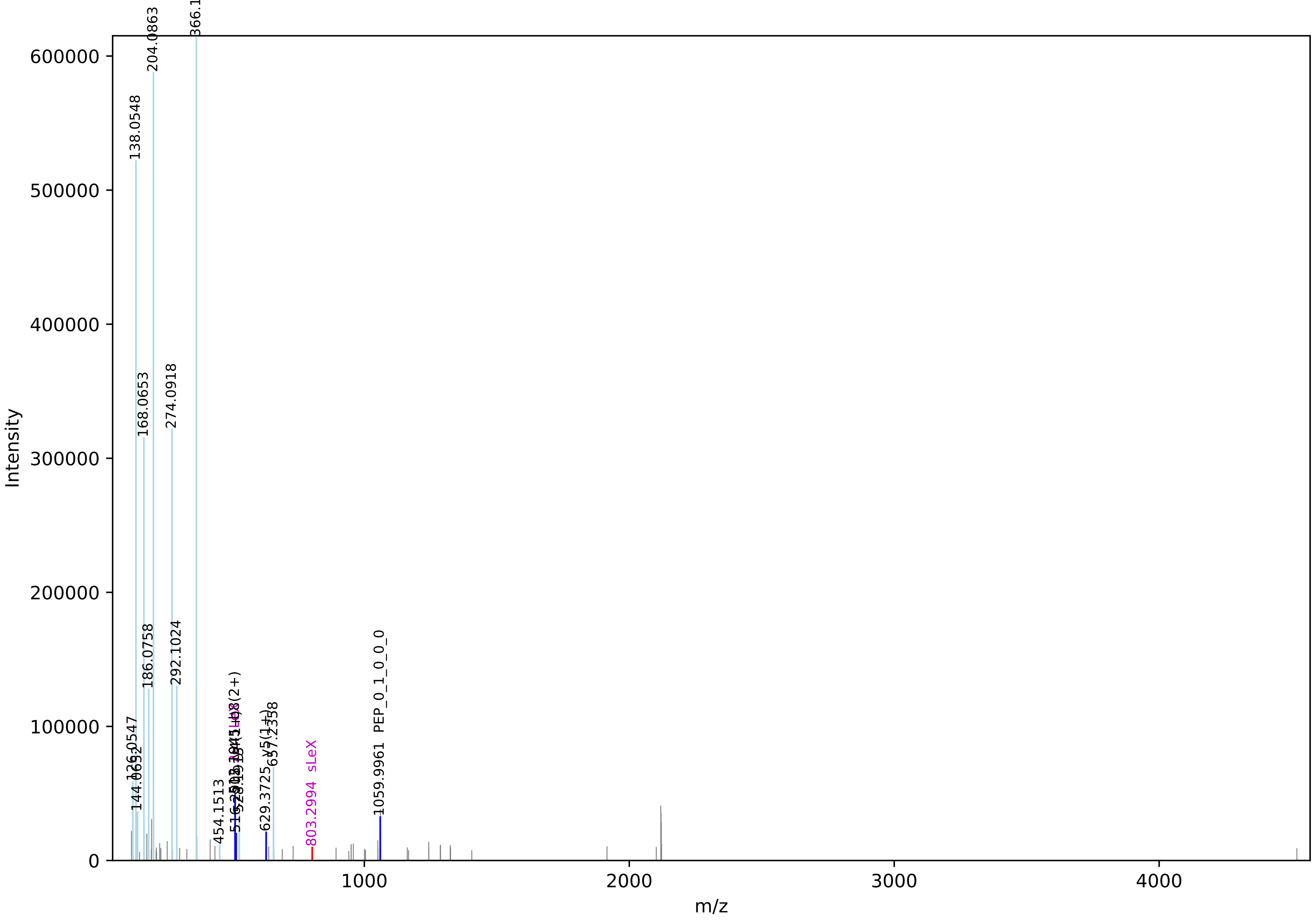

File: NS-3_231129_OE_RK_34, RT: 43.521, Scan: 056215
Type: HCD, FTMS + c NSI d Full ms2 1545.6266@hcd30.00 [120.0000-4751.7593]
Precursor Scan: 056136, m/z: 1544.6248, charge: 3
QDQCIYNTTYLNVQR_6_5_1_2_0 LeX peak identified

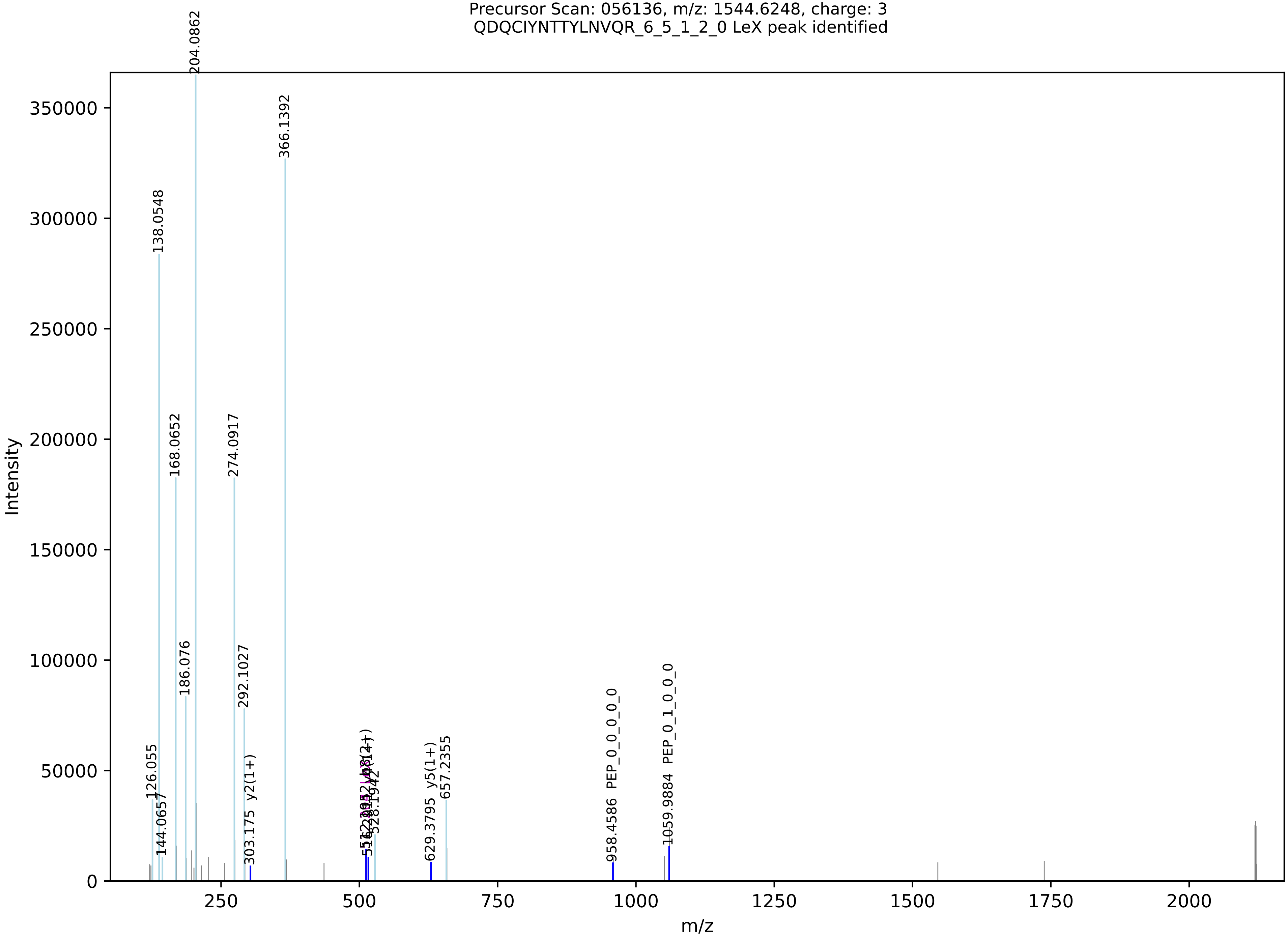

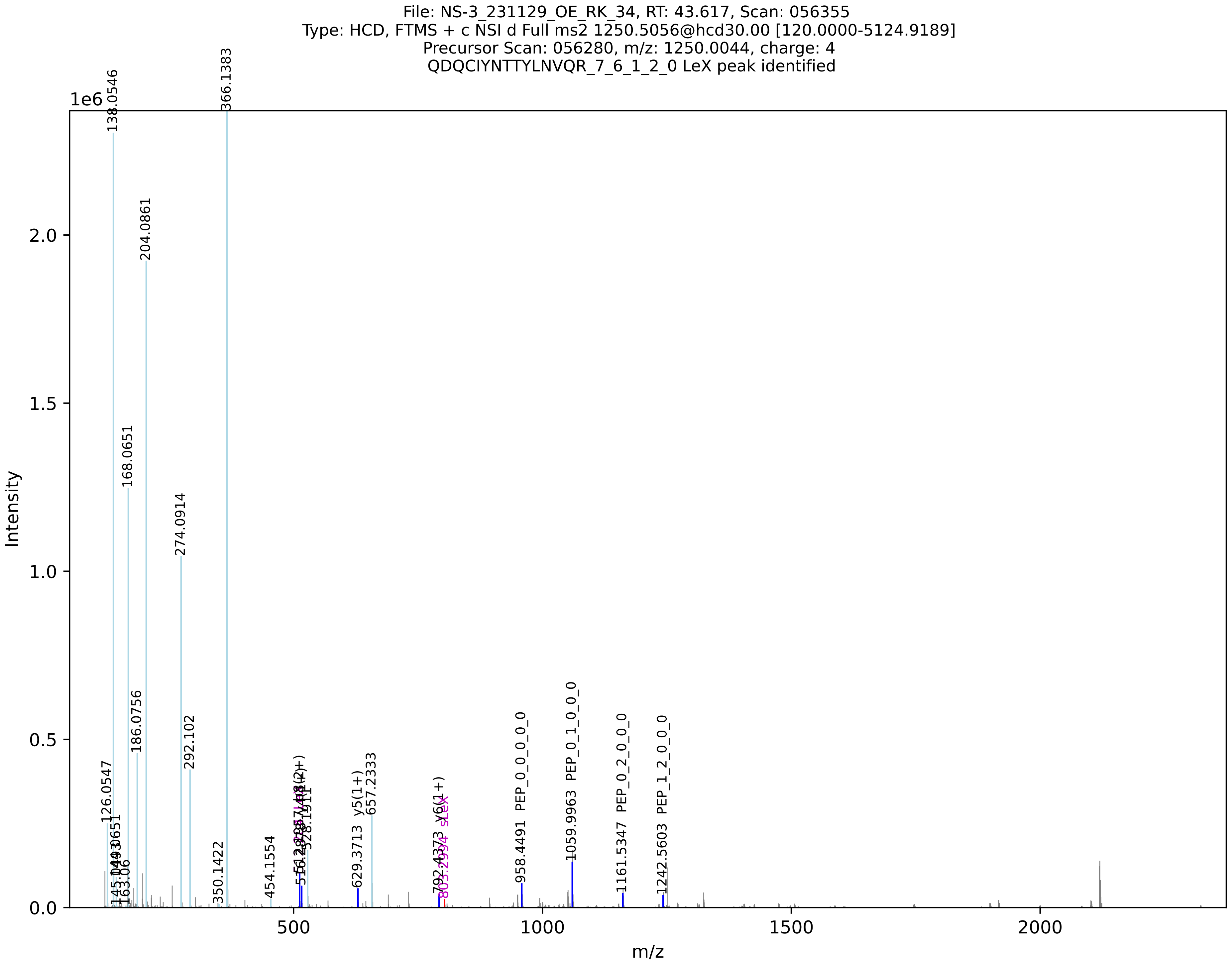
File: NS-3_231129_OE_RK_34, RT: 43.617, Scan: 056355
Type: HCD, FTMS + c NSI d Full ms2 1250.5056@hcd30.00 [120.0000-5124.9189]
Precursor Scan: 056280, m/z: 1250.0044, charge: 4
QDQCIYNTTYLNVQR_7_6_1_2_0 LeX peak identified
1e6
Intensity
m/z
0.0
0.5
1.0
1.5
2.0
500
1000
1500
2000
138.0546
366.1383
204.0861
168.0651
274.0914
186.0756
292.102
126.0547
163.06
350.1422
454.1554
528.1911
629.3713 y5(1+)
657.2333
792.4373 y6(1+)
803.2934 sLeX
958.4491 PEP_0_0_0_0_0
1059.9963 PEP_0_1_0_0_0
1161.5347 PEP_0_2_0_0_0
1242.5603 PEP_1_2_0_0_0

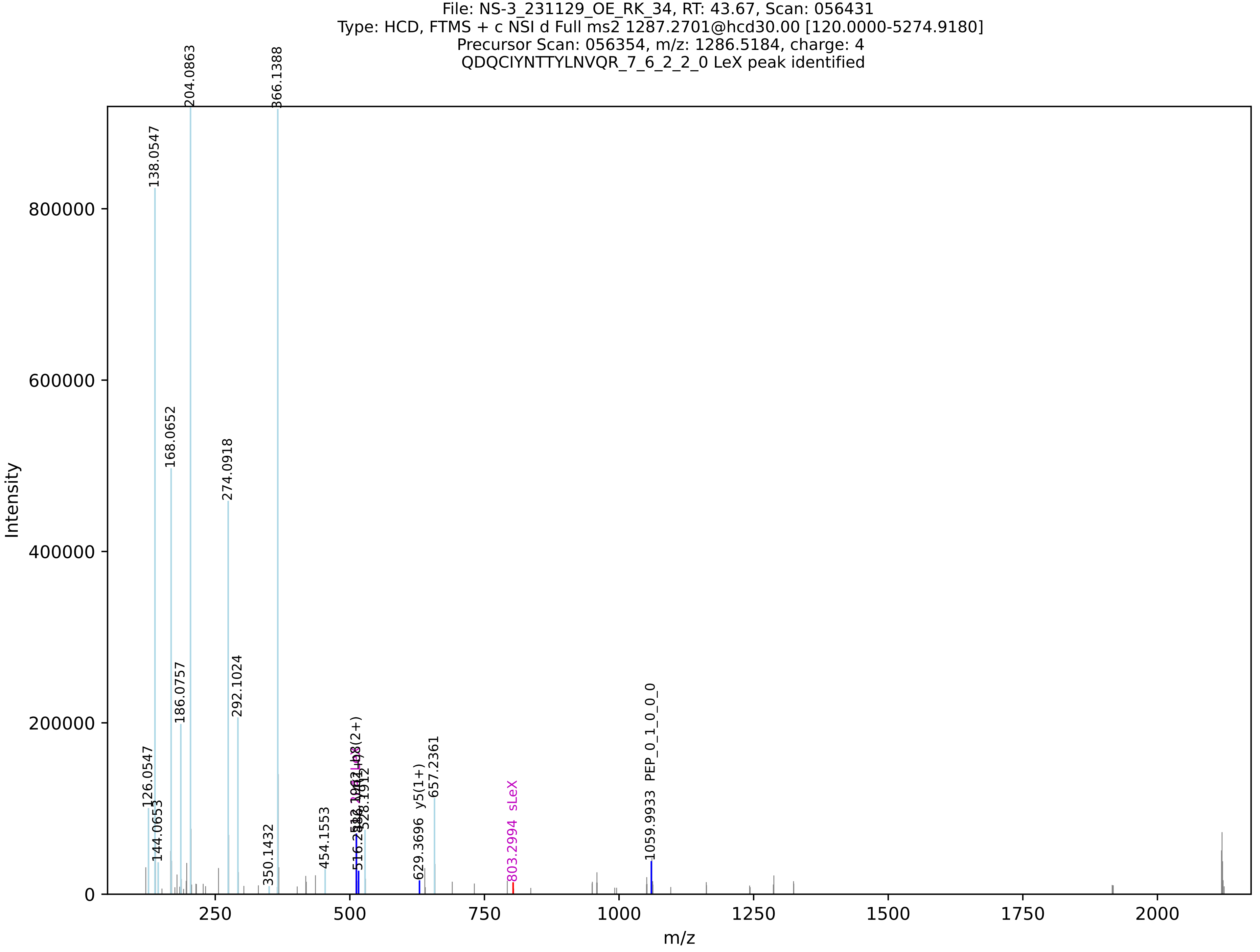

File: NS-3_231129_OE_RK_34, RT: 43.67, Scan: 056431
Type: HCD, FTMS + c NSI d Full ms2 1287.2701@hcd30.00 [120.0000-5274.9180]
Precursor Scan: 056354, m/z: 1286.5184, charge: 4
QDQCIYNTTYLNVQR_7_6_2_2_0 LeX peak identified
Intensity
m/z
204.0863
366.1388
138.0547
168.0652
274.0918
186.0757
292.1024
126.0547
144.0653
350.1432
454.1553
516.2896
528.1912
b3(2+)
629.3696 y5(1+)
657.2361
803.2994 sLeX
1059.9933 PEP_0_1_0_0_0

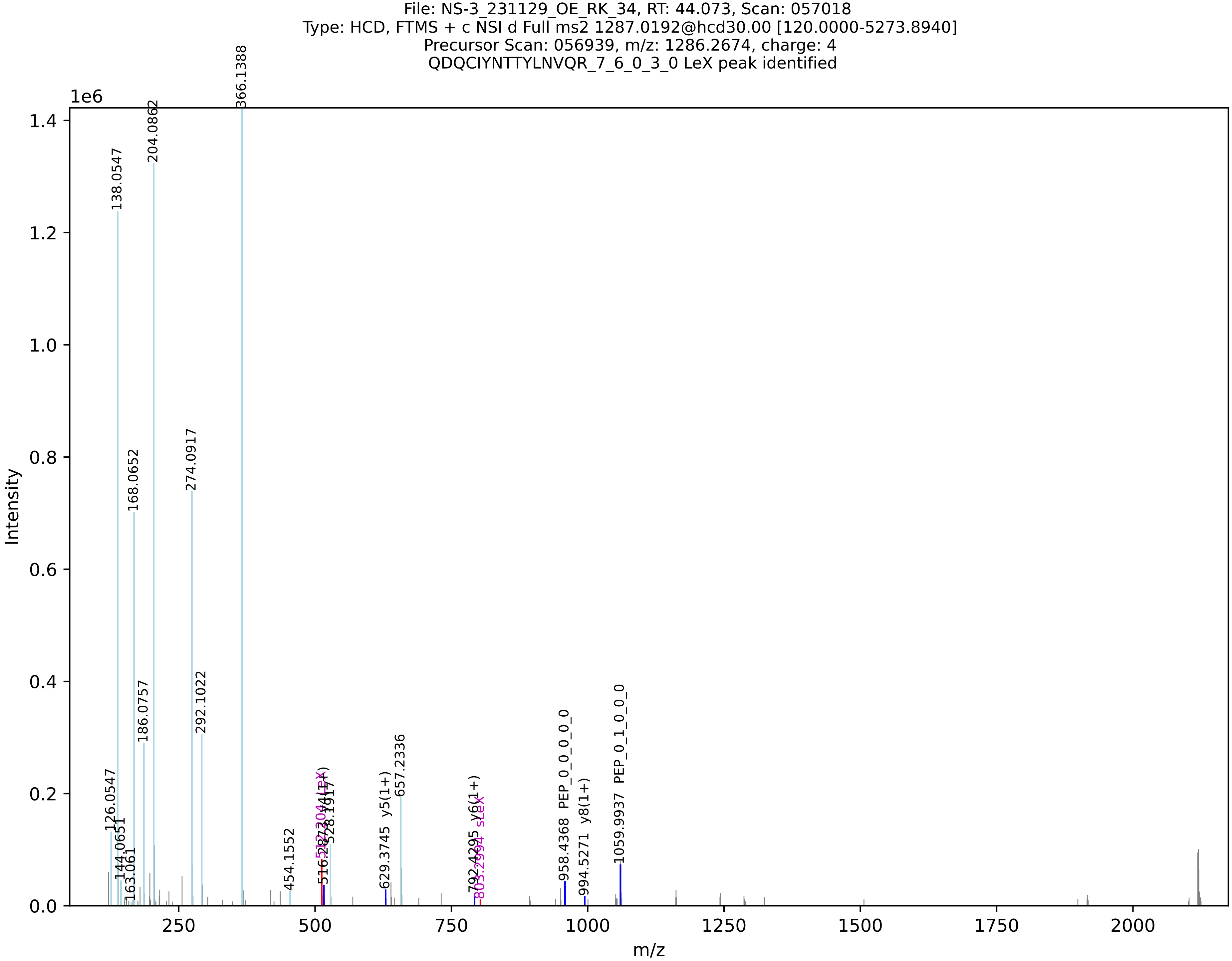
File: NS-3_231129_OE_RK_34, RT: 44.073, Scan: 057018
Type: HCD, FTMS + c NSI d Full ms2 1287.0192@hcd30.00 [120.0000-5273.8940]
Precursor Scan: 056939, m/z: 1286.2674, charge: 4
QDQCIYNTTYLNVQR_7_6_0_3_0 LeX peak identified
1e6
Intensity
m/z
366.1388
204.0862
138.0547
168.0652
274.0917
186.0757
292.1022
126.0547
144.0651
163.061
454.1552
516.2873 y4(1+)
528.1917
629.3745 y5(1+)
657.2336
792.4295 y6(1+)
803.2994 sLeX
958.4368 PEP_0_0_0_0_0
994.5271 y8(1+)
1059.9937 PEP_0_1_0_0_0

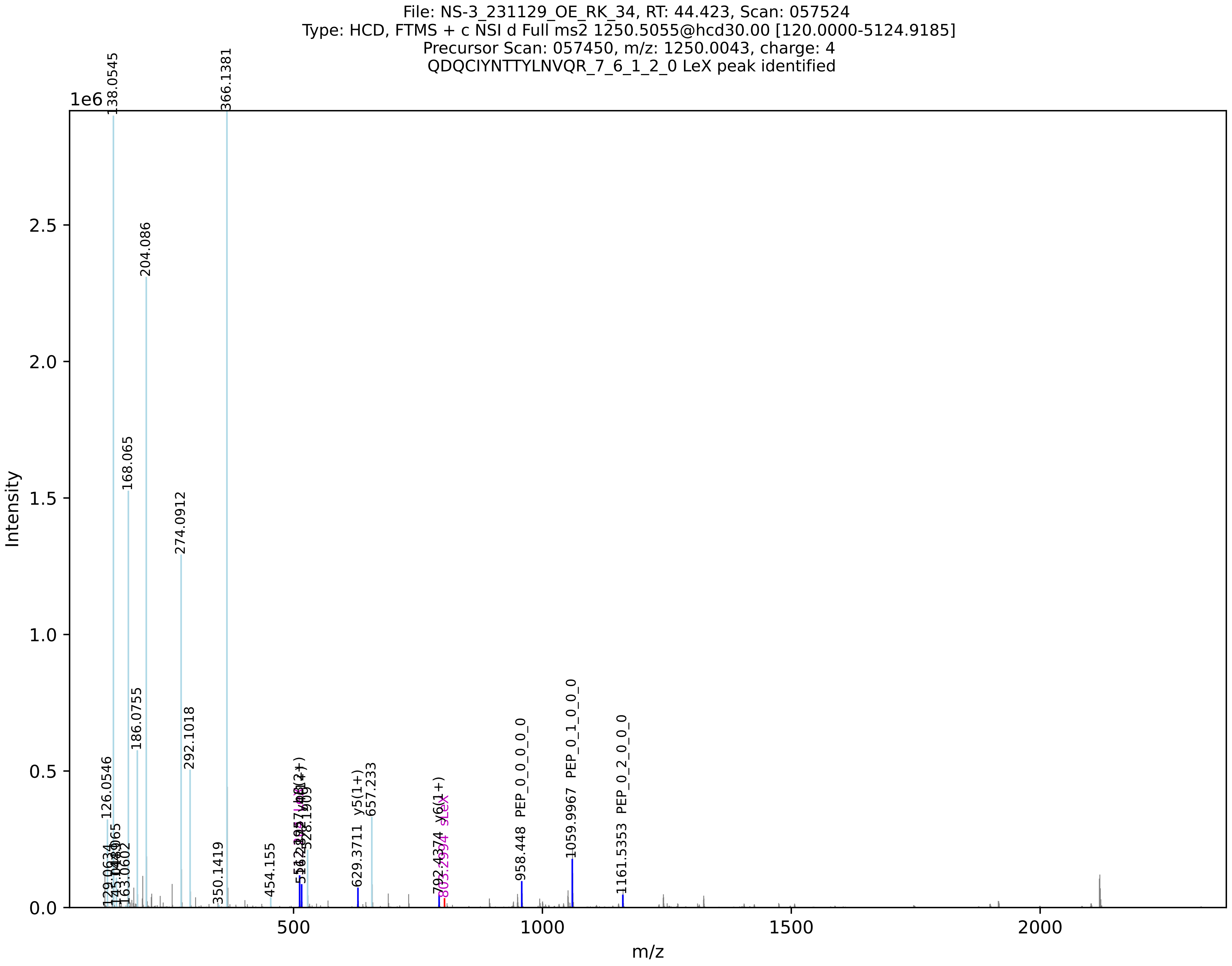

File: NS-3_231129_OE_RK_34, RT: 44.423, Scan: 057524
Type: HCD, FTMS + c NSI d Full ms2 1250.5055@hcd30.00 [120.0000-5124.9185]
Precursor Scan: 057450, m/z: 1250.0043, charge: 4
QDQCIYNTTYLNVQR_7_6_1_2_0 LeX peak identified
1e6
Intensity
m/z
138.0545
366.1381
204.086
168.065
274.0912
186.0755
292.1018
126.0546
350.1419
454.155
629.3711 y5(1+)
657.233
792.4374 y6(1+)
958.448 PEP_0_0_0_0_0
1059.9967 PEP_0_1_0_0_0
1161.5353 PEP_0_2_0_0_0

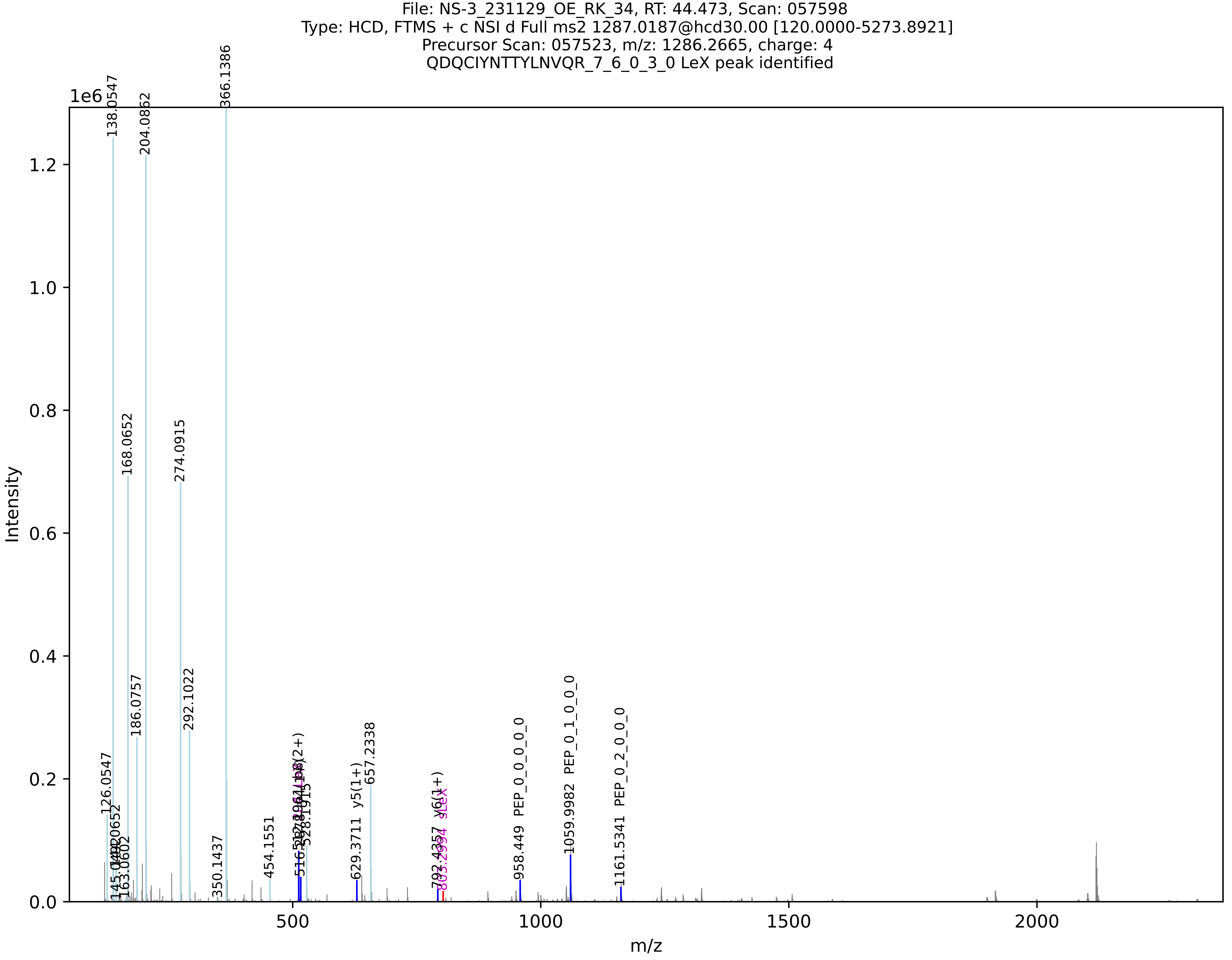

File: NS-3_231129_OE_RK_34, RT: 44.473, Scan: 057598
Type: HCD, FTMS + c NSI d Full ms2 1287.0187@hcd30.00 [120.0000-5273.8921]
Precursor Scan: 057523, m/z: 1286.2665, charge: 4
QDQCIYNTTYLNVQR_7_6_0_3_0 LeX peak identified
1e6
Intensity
m/z
138.0547
204.0862
366.1386
168.0652
274.0915
186.0757
292.1022
126.0547
163.0602
350.1437
454.1551
528.1915
y5(1+)
629.3711
657.2338
y6(1+)
792.4357
sLeX
PEP_0_0_0_0_0
958.449
PEP_0_1_0_0_0
1059.9982
PEP_0_2_0_0_0
1161.5341

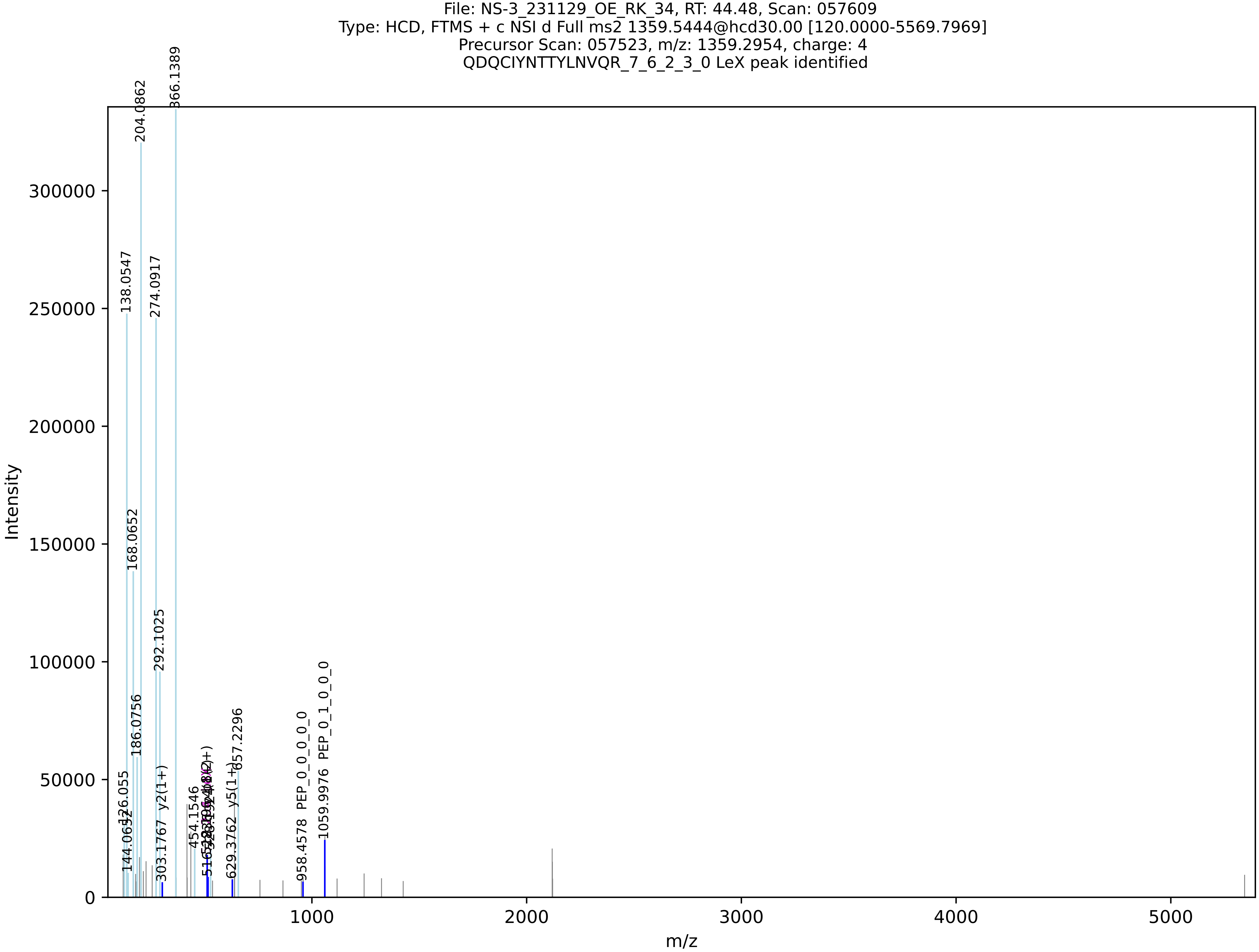
File: NS-3_231129_OE_RK_34, RT: 44.48, Scan: 057609
Type: HCD, FTMS + c NSI d Full ms2 1359.5444@hcd30.00 [120.0000-5569.7969]
Precursor Scan: 057523, m/z: 1359.2954, charge: 4
QDQCIYNTTYLNVQR_7_6_2_3_0 LeX peak identified
Intensity
m/z
366.1389
204.0862
138.0547
274.0917
168.0652
292.1025
186.0756
126.055
144.0652
303.1767 y2(1+)
454.1546
629.3762 y5(1+)
657.2296
958.4578 PEP_0_0_0_0_0
1059.9976 PEP_0_1_0_0_0
0
50000
100000
150000
200000
250000
300000
1000
2000
3000
4000
5000

File: NS-3_231129_OE_RK_34, RT: 44.825, Scan: 058110
Type: HCD, FTMS + c NSI d Full ms2 1250.5040@hcd30.00 [120.0000-5124.9126]
Precursor Scan: 058036, m/z: 1250.0026, charge: 4
QDQCIYNTTYLNVQR_7_6_1_2_0 LeX peak identified

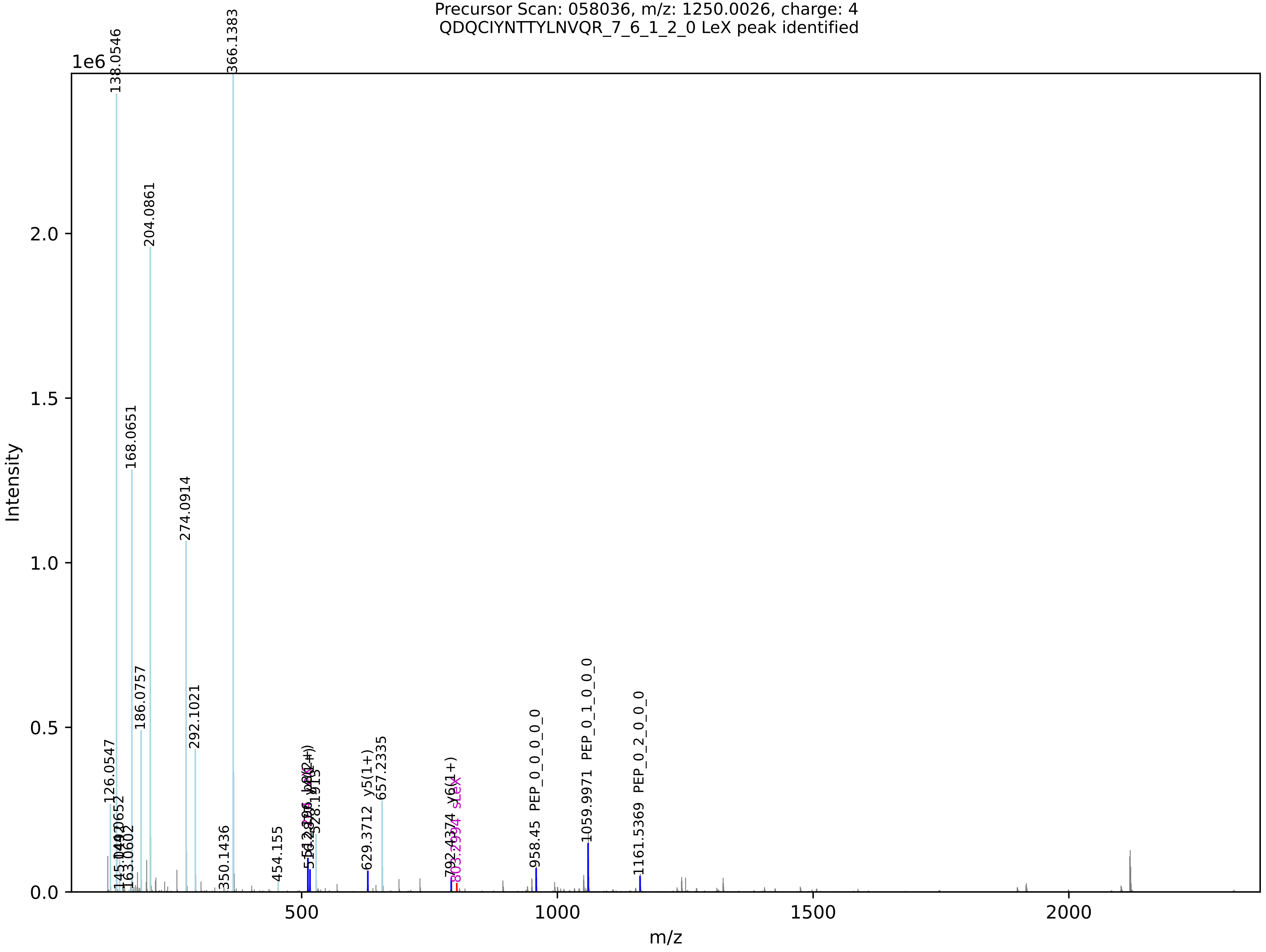

File: NS-3_231129_OE_RK_34, RT: 45.027, Scan: 058406
Type: HCD, FTMS + c NSI d Full ms2 1250.7550@hcd30.00 [120.0000-5125.9365]
Precursor Scan: 058330, m/z: 1250.0033, charge: 4
QDQCIYNTTYLNVQR_7_6_1_2_0 LeX peak identified

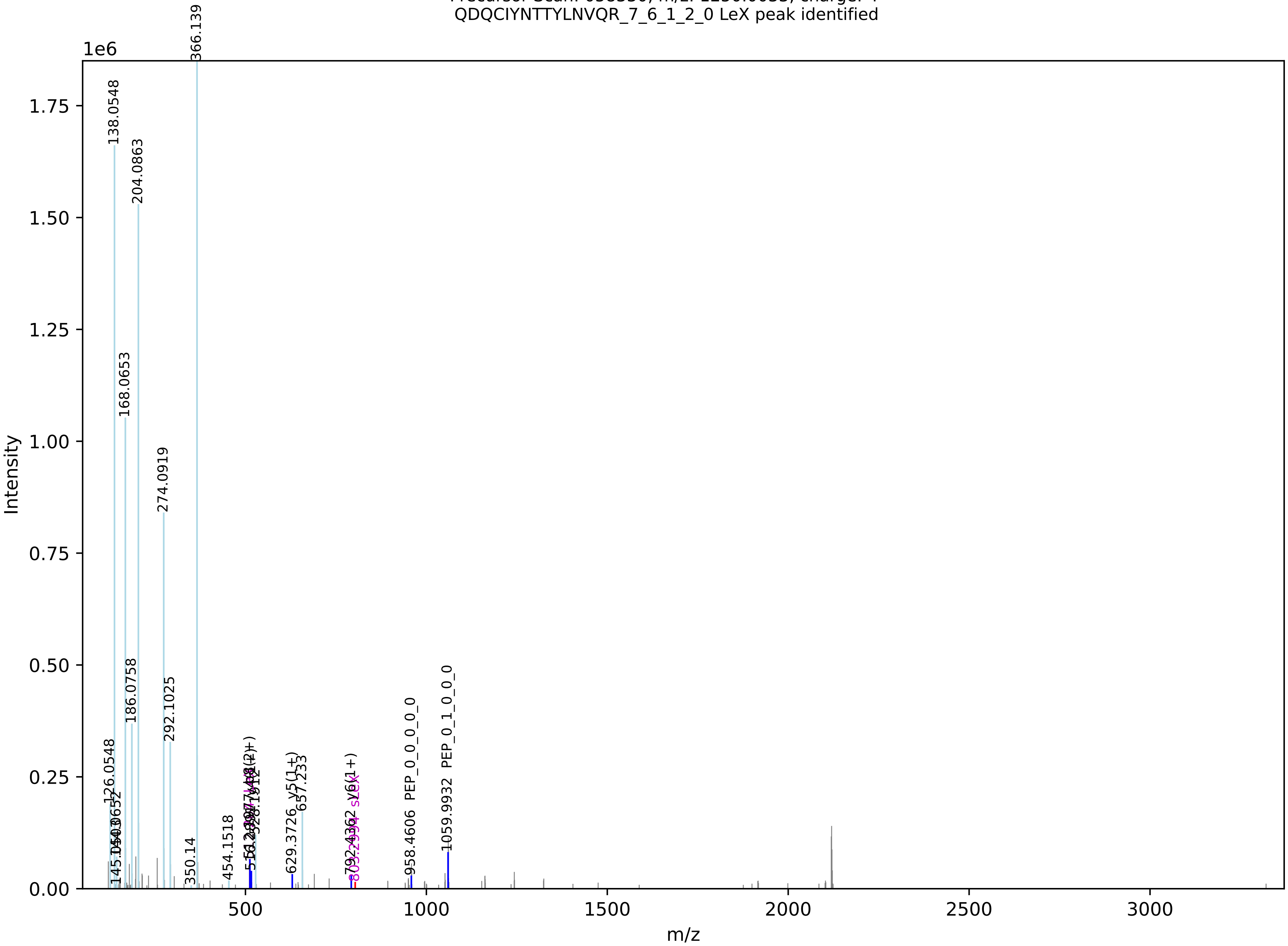

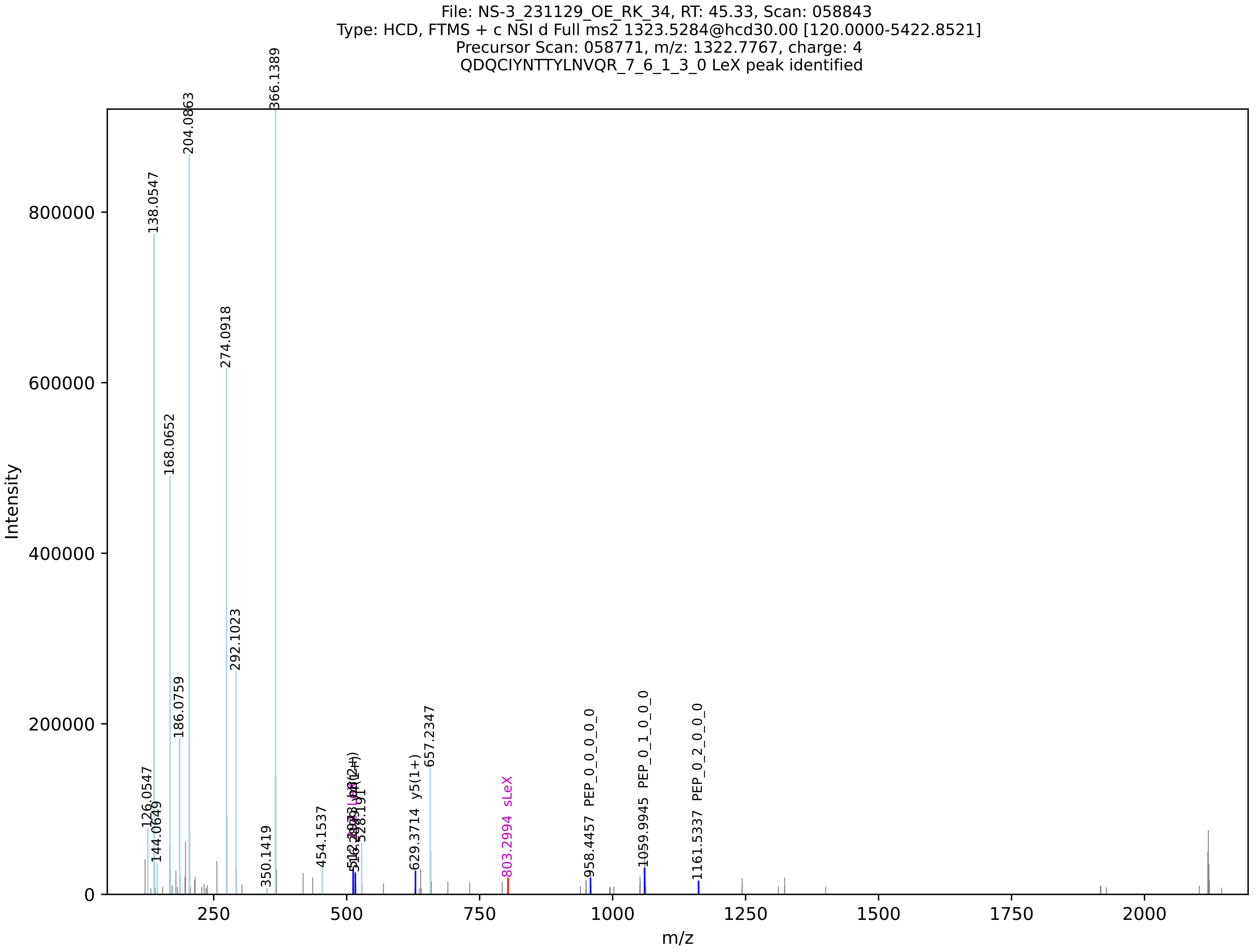
File: NS-3_231129_OE_RK_34, RT: 45.33, Scan: 058843
Type: HCD, FTMS + c NSI d Full ms2 1323.5284@hcd30.00 [120.0000-5422.8521]
Precursor Scan: 058771, m/z: 1322.7767, charge: 4
QDQCIYNTTYLNVQR_7_6_1_3_0 LeX peak identified
Intensity
m/z
366.1389
204.0863
138.0547
274.0918
168.0652
292.1023
186.0759
126.0547
144.0649
350.1419
454.1537
528.191
629.3714 y5(1+)
657.2347
803.2994 sLeX
958.4457 PEP_0_0_0_0_0
1059.9945 PEP_0_1_0_0_0
1161.5337 PEP_0_2_0_0_0

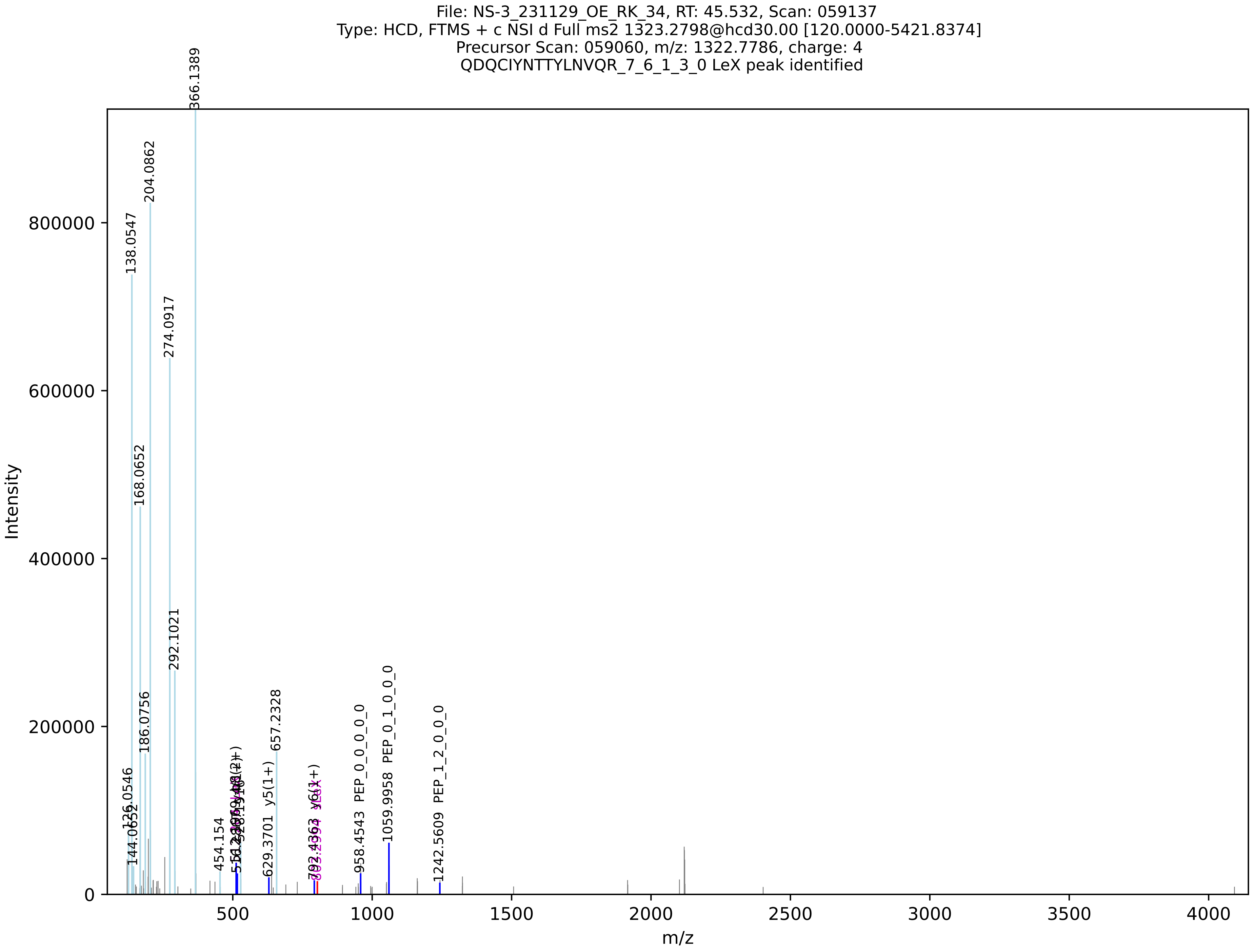
File: NS-3_231129_OE_RK_34, RT: 45.532, Scan: 059137
Type: HCD, FTMS + c NSI d Full ms2 1323.2798@hcd30.00 [120.0000-5421.8374]
Precursor Scan: 059060, m/z: 1322.7786, charge: 4
QDQCIYNTTYLNVQR_7_6_1_3_0 LeX peak identified
Intensity
m/z
366.1389
204.0862
138.0547
274.0917
168.0652
292.1021
186.0756
126.0546
144.0652
454.154
657.2328
629.3701 y5(1+)
958.4543 PEP_0_0_0_0_0_
1059.9958 PEP_0_1_0_0_0_
1242.5609 PEP_1_2_0_0_0_
0
200000
400000
600000
800000
500
1000
1500
2000
2500
3000
3500
4000

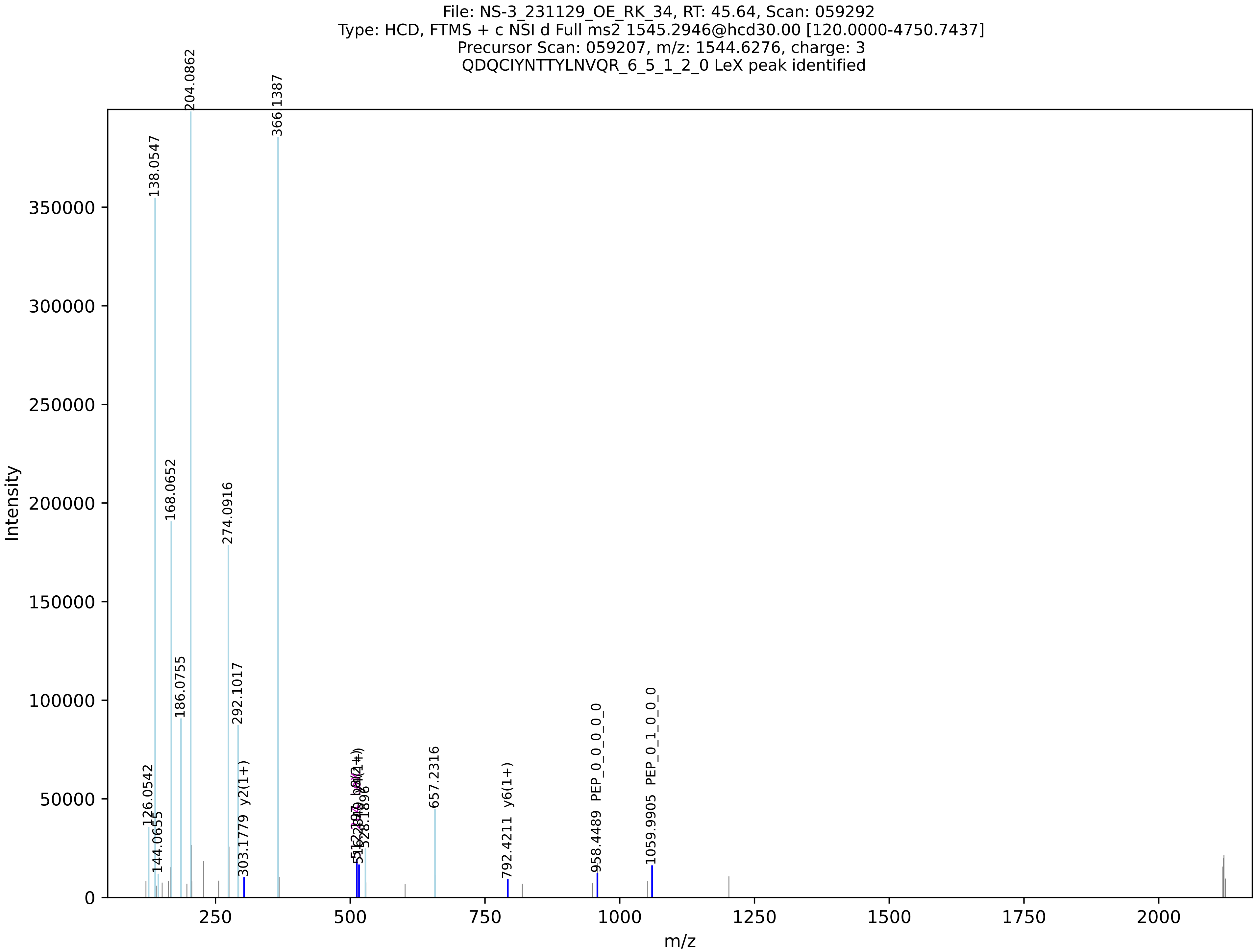
File: NS-3_231129_OE_RK_34, RT: 45.64, Scan: 059292
Type: HCD, FTMS + c NSI d Full ms2 1545.2946@hcd30.00 [120.0000-4750.7437]
Precursor Scan: 059207, m/z: 1544.6276, charge: 3
QDQCIYNTTYLNVQR_6_5_1_2_0 LeX peak identified
204.0862
366.1387
138.0547
168.0652
274.0916
186.0755
292.1017
126.0542
144.0655
303.1779 y2(1+)
528.1896
657.2316
792.4211 y6(1+)
958.4489 PEP_0_0_0_0_0
1059.9905 PEP_0_1_0_0_0
Intensity
m/z
0
50000
100000
150000
200000
250000
300000
350000
250
500
750
1000
1250
1500
1750
2000

File: NS-3_231129_OE_RK_34, RT: 45.783, Scan: 059497
Type: HCD, FTMS + c NSI d Full ms2 1323.2800@hcd30.00 [120.0000-5421.8389]
Precursor Scan: 059425, m/z: 1322.7784, charge: 4
QDQCIYNTTYLNVQR_7_6_1_3_0 LeX peak identified

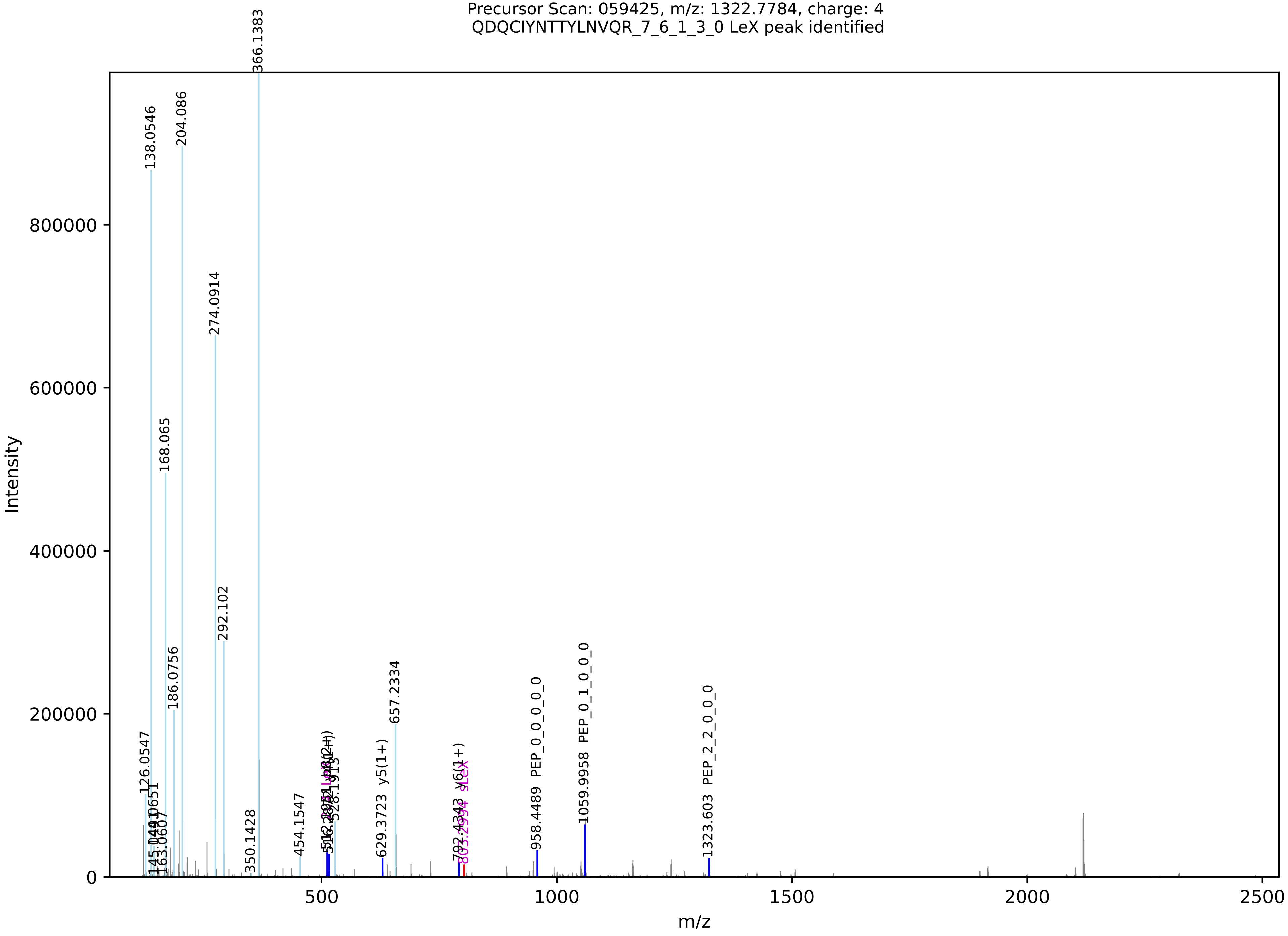

File: NS-3_231129_OE_RK_34, RT: 45.987, Scan: 059789
Type: HCD, FTMS + c NSI d Full ms2 1323.2800@hcd30.00 [120.0000-5421.8384]
Precursor Scan: 059713, m/z: 1322.7788, charge: 4
QDQCIYNTTYLNVQR_7_6_1_3_0 LeX peak identified

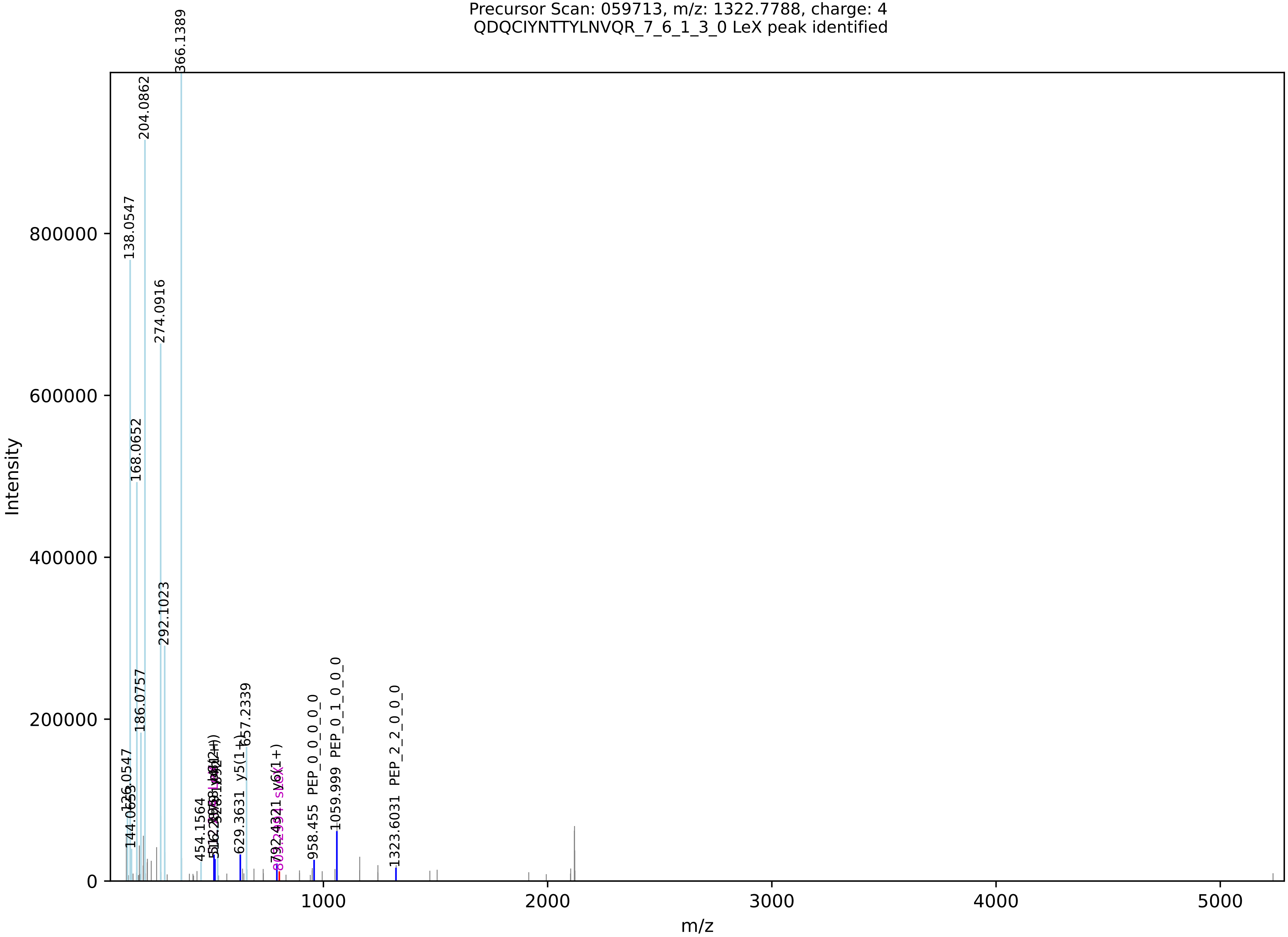

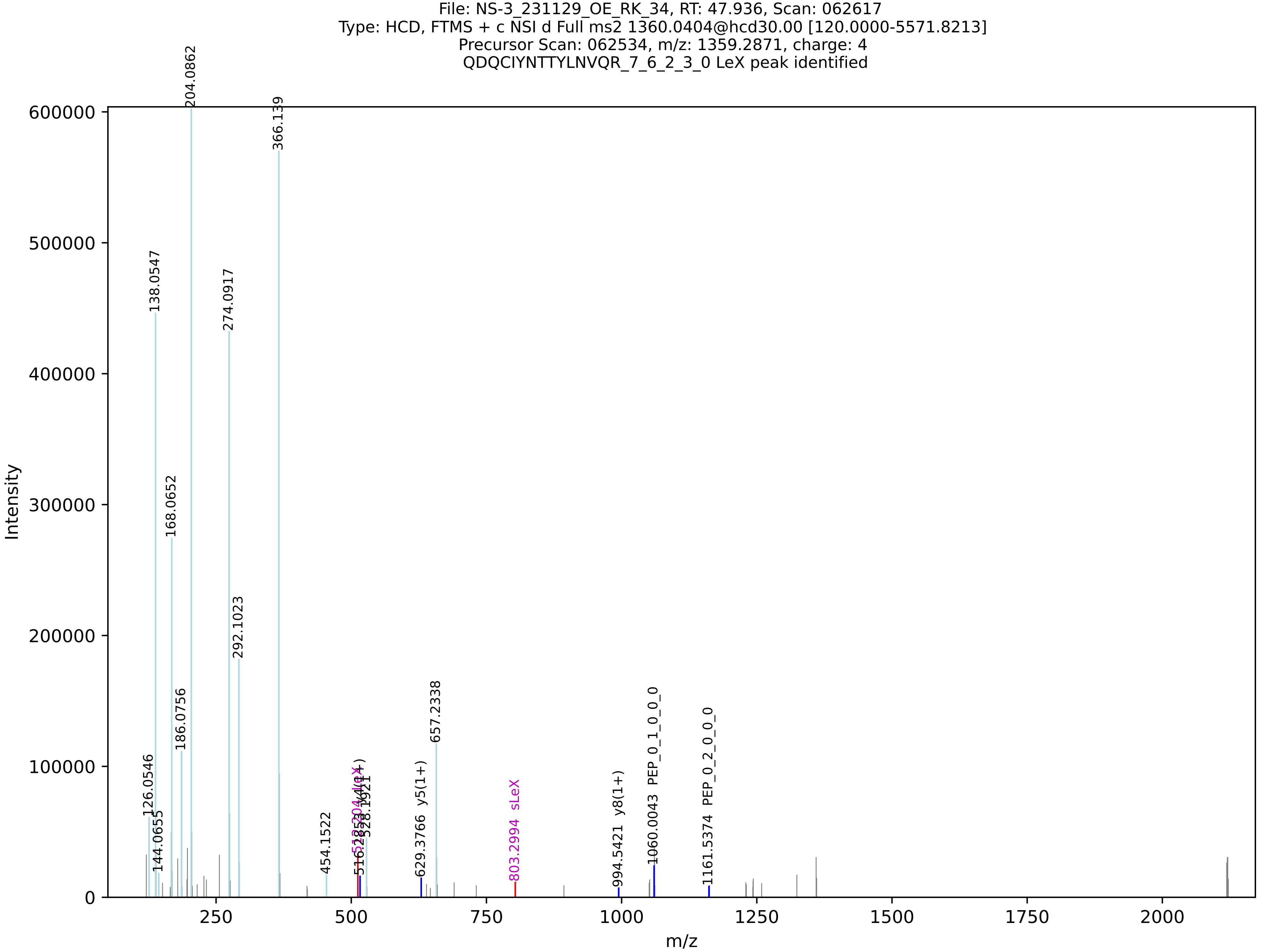
File: NS-3_231129_OE_RK_34, RT: 47.936, Scan: 062617
Type: HCD, FTMS + c NSI d Full ms2 1360.0404@hcd30.00 [120.0000-5571.8213]
Precursor Scan: 062534, m/z: 1359.2871, charge: 4
QDQCIYNTTYLNVQR_7_6_2_3_0 LeX peak identified
Intensity
m/z
204.0862
366.139
138.0547
274.0917
168.0652
292.1023
186.0756
126.0546
144.0655
454.1522
LeX
516.2853 y4(1+)
528.1921
629.3766 y5(1+)
657.2338
803.2994 sLeX
994.5421 y8(1+)
1060.0043 PEP_0_1_0_0_0
1161.5374 PEP_0_2_0_0_0
0
100000
200000
300000
400000
500000
600000
250
500
750
1000
1250
1500
1750
2000

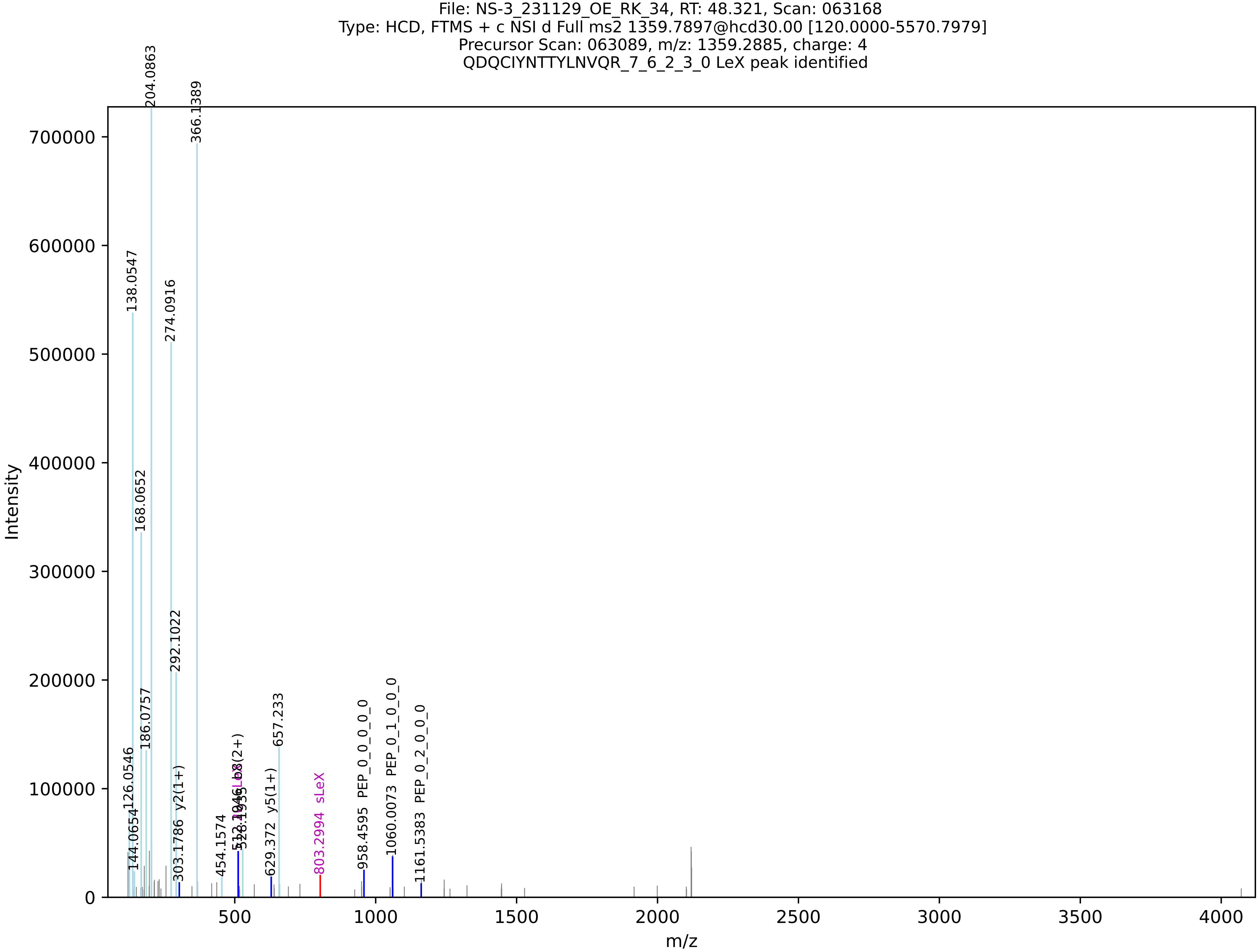
File: NS-3_231129_OE_RK_34, RT: 48.321, Scan: 063168
Type: HCD, FTMS + c NSI d Full ms2 1359.7897@hcd30.00 [120.0000-5570.7979]
Precursor Scan: 063089, m/z: 1359.2885, charge: 4
QDQCIYNTTYLNVQR_7_6_2_3_0 LeX peak identified
Intensity
m/z
204.0863
366.1389
138.0547
274.0916
168.0652
292.1022
186.0757
126.0546
144.0654
303.1786 y2(1+)
454.1574
657.233
629.372 y5(1+)
803.2994 sLeX
958.4595 PEP_0_0_0_0_0
1060.0073 PEP_0_1_0_0_0
1161.5383 PEP_0_2_0_0_0
0
100000
200000
300000
400000
500000
600000
700000
500
1000
1500
2000
2500
3000
3500
4000

File: NS-3_231129_OE_RK_34, RT: 48.38, Scan: 063256
Type: HCD, FTMS + c NSI d Full ms2 1396.0538@hcd30.00 [120.0000-5718.7554]
Precursor Scan: 063163, m/z: 1395.5527, charge: 4
QDQCIYNTTYLNVQR_7_6_1_4_0 LeX peak identified

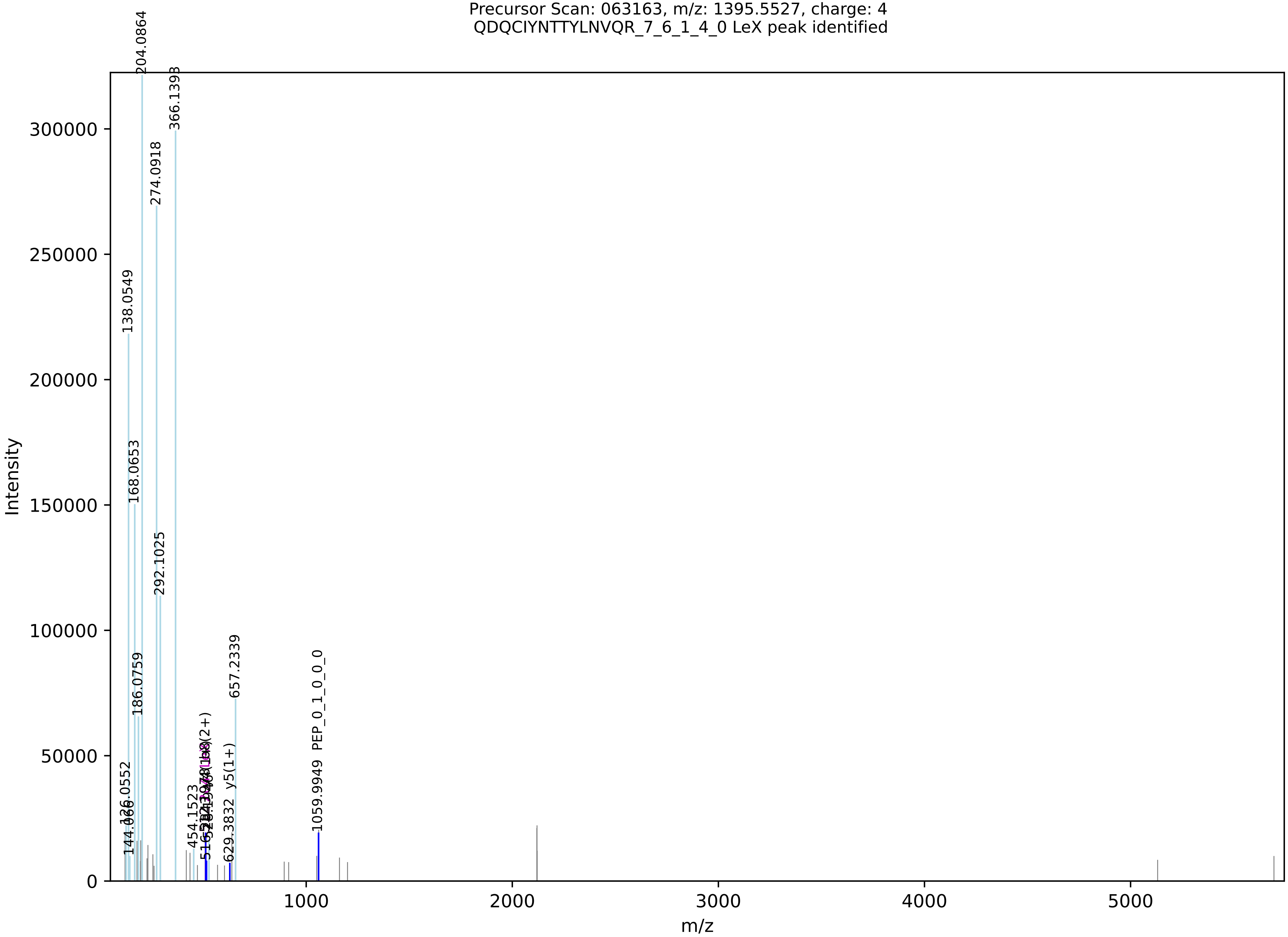

File: NS-3_231129_OE_RK_34, RT: 48.985, Scan: 064142
Type: HCD, FTMS + c NSI d Full ms2 1396.3027@hcd30.00 [120.0000-5719.7715]
Precursor Scan: 064049, m/z: 1395.551, charge: 4
QDQCIYNTTYLNVQR_7_6_1_4_0 LeX peak identified

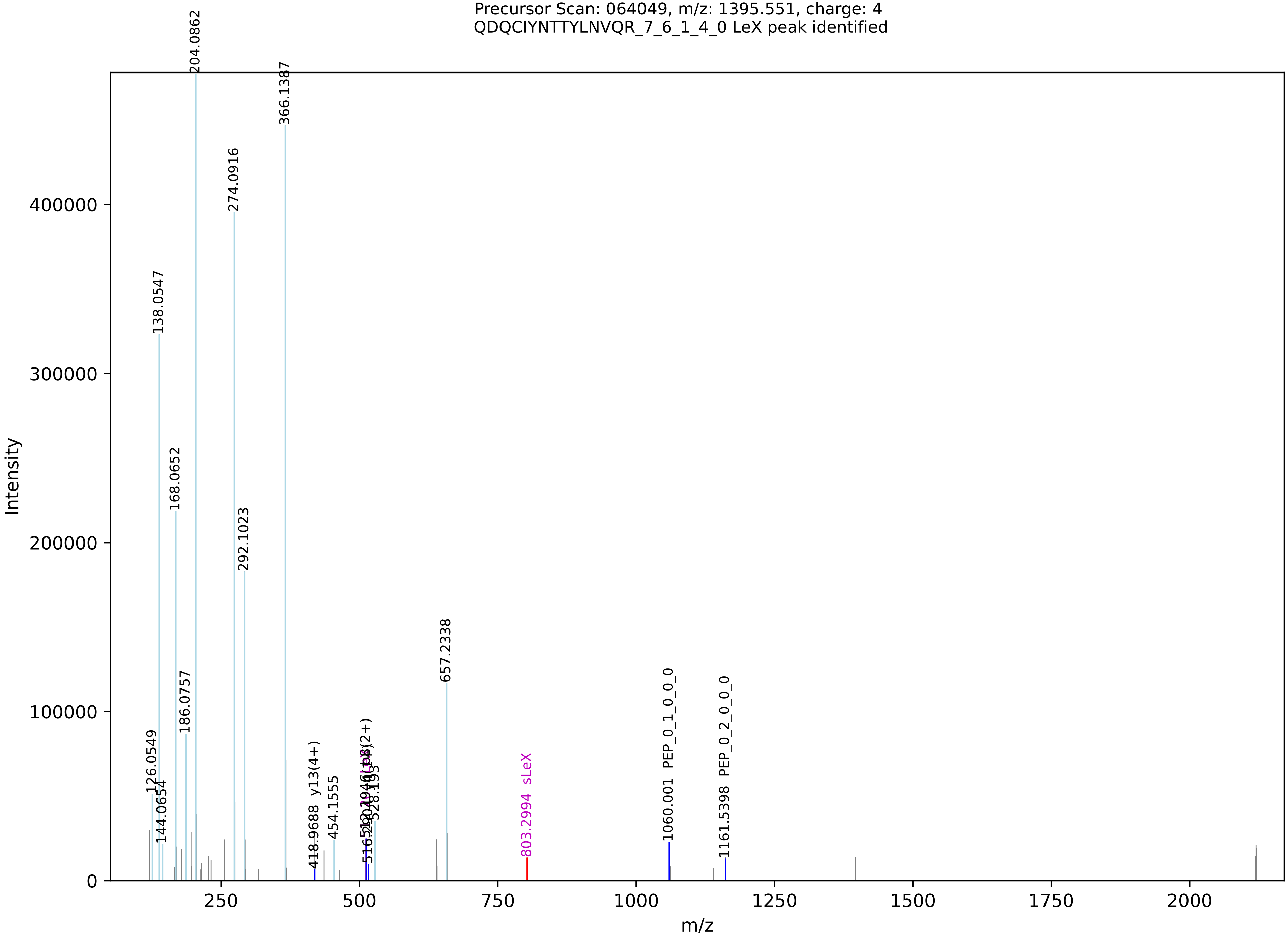

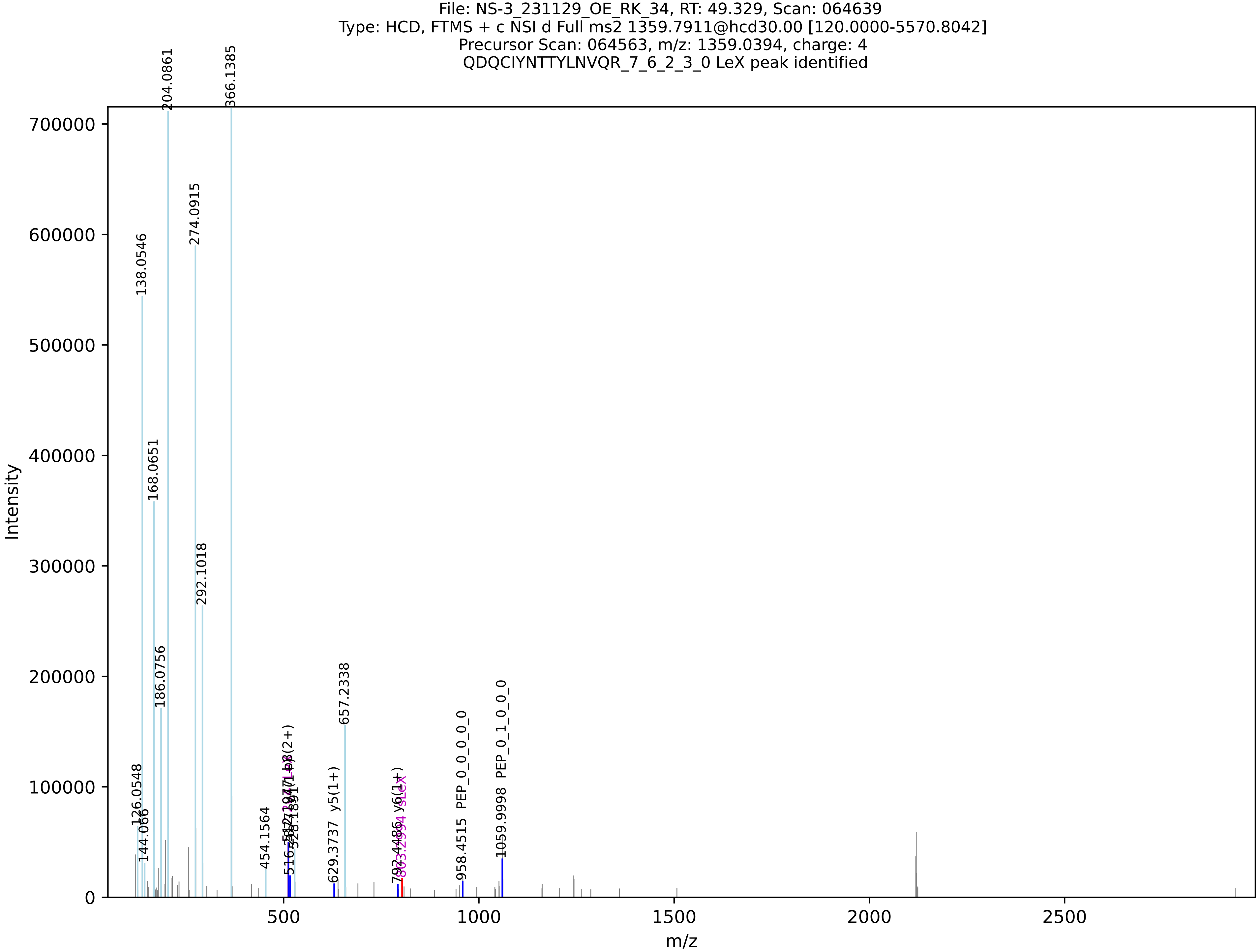
File: NS-3_231129_OE_RK_34, RT: 49.329, Scan: 064639
Type: HCD, FTMS + c NSI d Full ms2 1359.7911@hcd30.00 [120.0000-5570.8042]
Precursor Scan: 064563, m/z: 1359.0394, charge: 4
QDQCIYNTTYLNVQR_7_6_2_3_0 LeX peak identified
Intensity
m/z
204.0861
366.1385
274.0915
138.0546
168.0651
292.1018
186.0756
657.2338
126.0548
144.066
454.1564
528.1891
629.3737 y5(1+)
y6(1+)
sLeX
958.4515 PEP_0_0_0_0_0_
1059.9998 PEP_0_1_0_0_0_

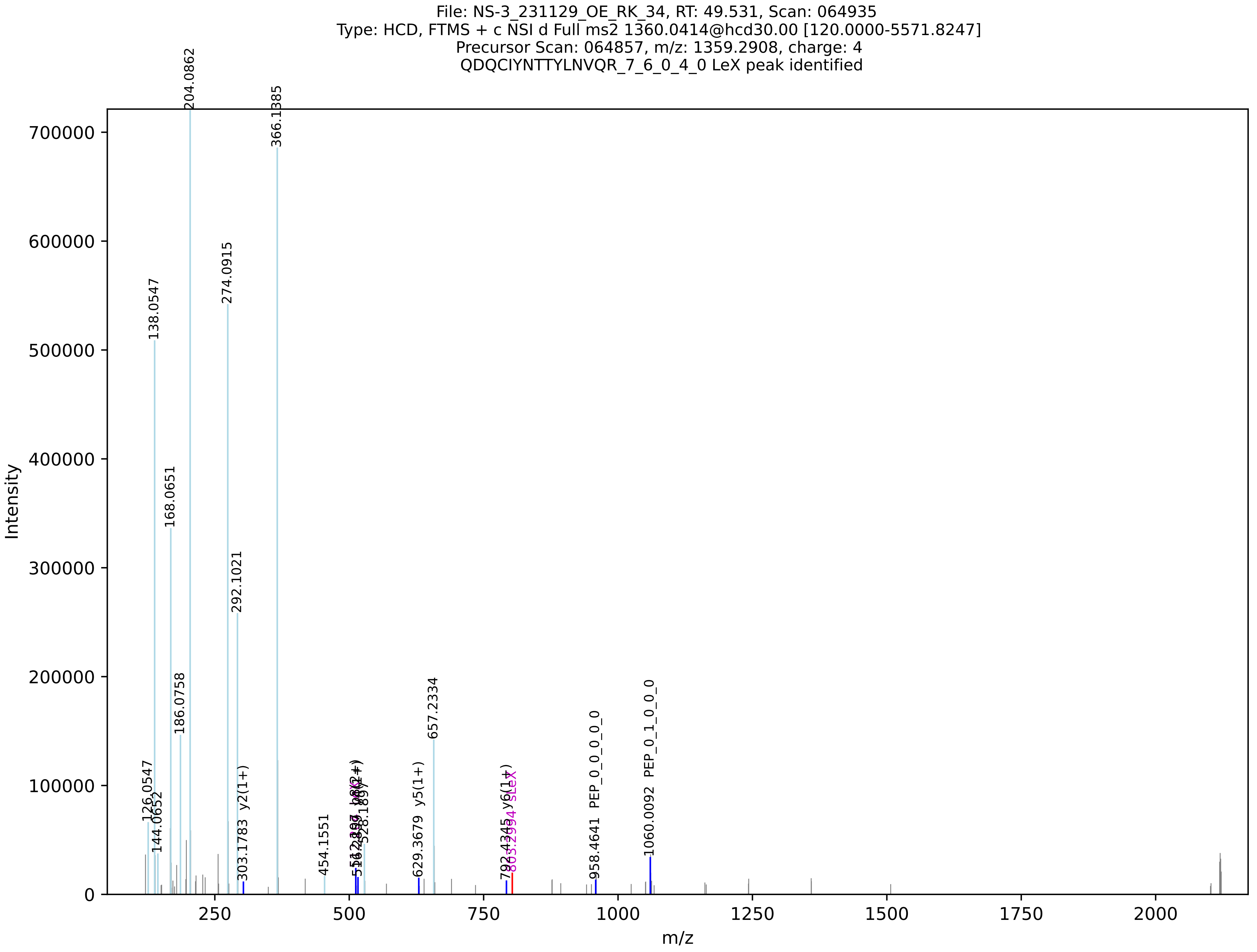

File: NS-3_231129_OE_RK_34, RT: 49.531, Scan: 064935
Type: HCD, FTMS + c NSI d Full ms2 1360.0414@hcd30.00 [120.0000-5571.8247]
Precursor Scan: 064857, m/z: 1359.2908, charge: 4
QDQCIYNTTYLNVQR_7_6_0_4_0 LeX peak identified
Intensity
m/z
204.0862
366.1385
274.0915
138.0547
168.0651
292.1021
186.0758
657.2334
126.0547
144.0652
303.1783 y2(1+)
454.1551
528.1897
629.3679 y5(1+)
792.4345 y6(1+)
803.2994 sLeX
958.4641 PEP_0_0_0_0_0
1060.0092 PEP_0_1_0_0_0

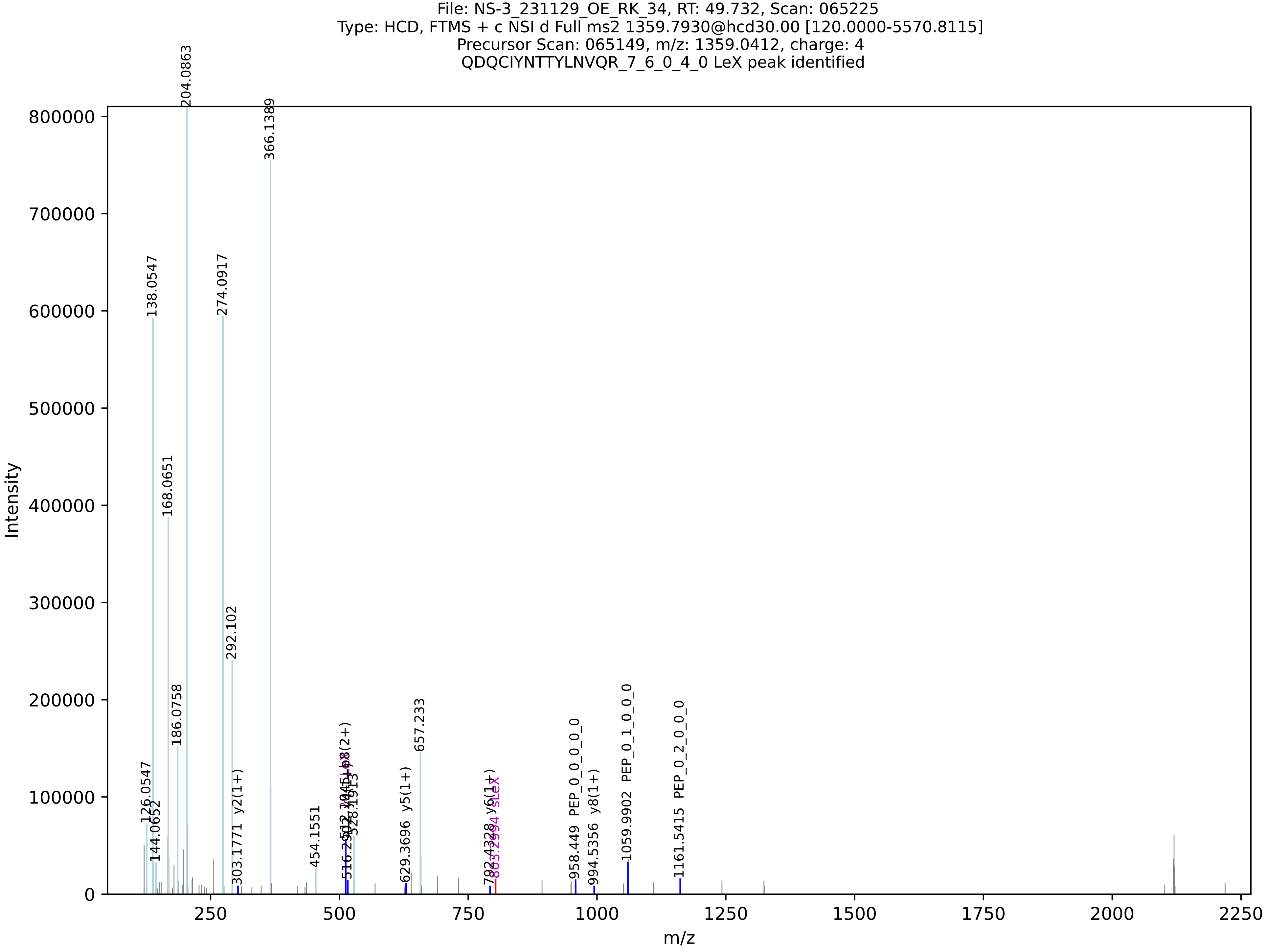
File: NS-3_231129_OE_RK_34, RT: 49.732, Scan: 065225
Type: HCD, FTMS + c NSI d Full ms2 1359.7930@hcd30.00 [120.0000-5570.8115]
Precursor Scan: 065149, m/z: 1359.0412, charge: 4
QDQCIYNTTYLNVQR_7_6_0_4_0 LeX peak identified
Intensity
m/z
204.0863
366.1389
138.0547
274.0917
168.0651
292.102
186.0758
126.0547
144.0652
303.1771 y2(1+)
454.1551
516.2903 b8(2+)
528.1913
657.233
629.3696 y5(1+)
792.4328 y6(1+)
sLeX
958.449 PEP_0_0_0_0_0
994.5356 y8(1+)
1059.9902 PEP_0_1_0_0_0
1161.5415 PEP_0_2_0_0_0

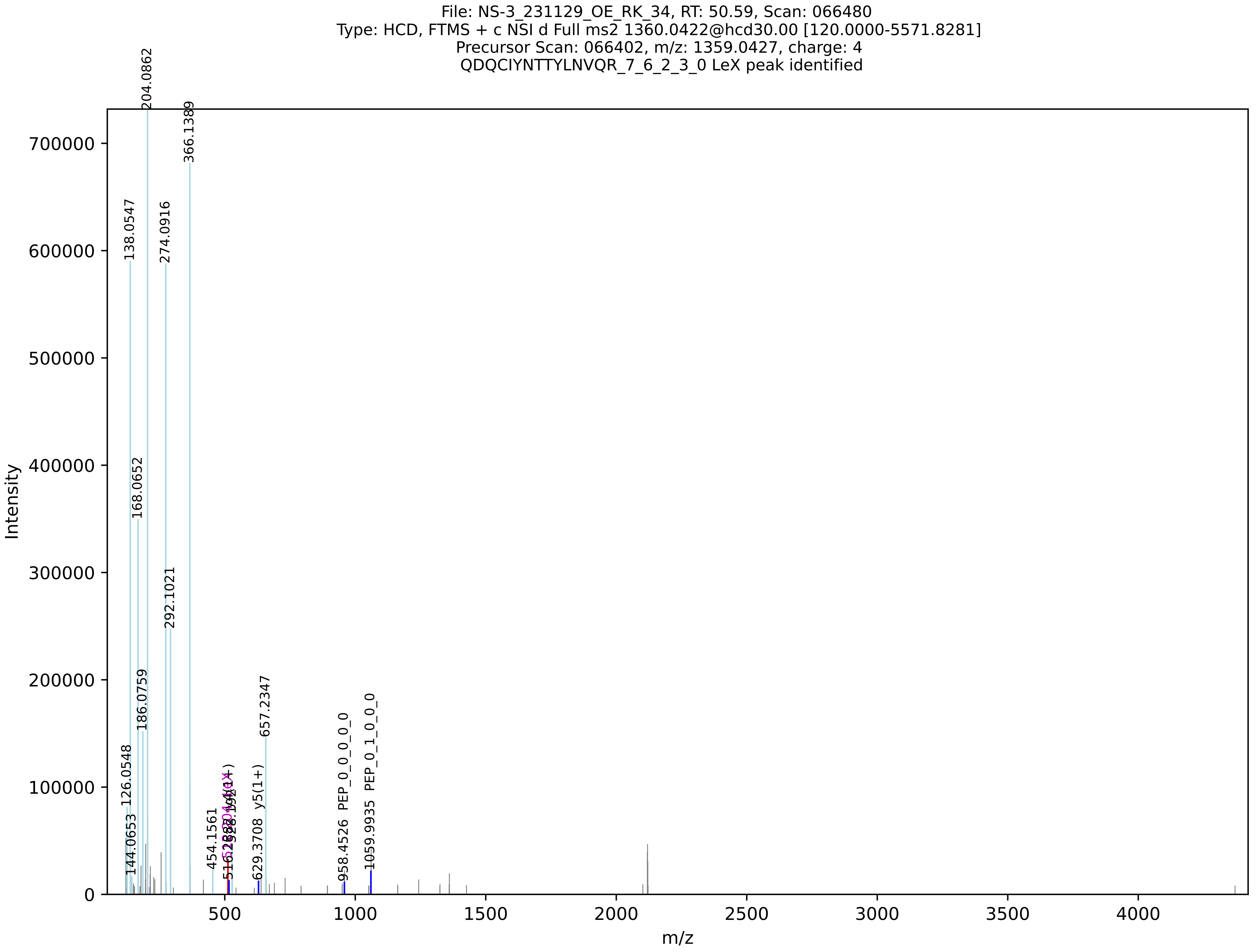
File: NS-3_231129_OE_RK_34, RT: 50.59, Scan: 066480
Type: HCD, FTMS + c NSI d Full ms2 1360.0422@hcd30.00 [120.0000-5571.8281]
Precursor Scan: 066402, m/z: 1359.0427, charge: 4
QDQCIYNTTYLNVQR_7_6_2_3_0 LeX peak identified
Intensity
m/z
204.0862
366.1389
138.0547
274.0916
168.0652
292.1021
186.0759
126.0548
144.0653
454.1561
657.2347
629.3708 y5(1+)
958.4526 PEP_0_0_0_0_0
1059.9935 PEP_0_1_0_0_0

File: NS-3_231129_OE_RK_34, RT: 50.696, Scan: 066637
Type: HCD, FTMS + c NSI d Full ms2 1456.6512@hcd30.00 [120.0000-4479.4951]
Precursor Scan: 066550, m/z: 1455.9851, charge: 3
VVLHPNYSQVDIGLIK_6_5_0_2_0 LeX peak identified

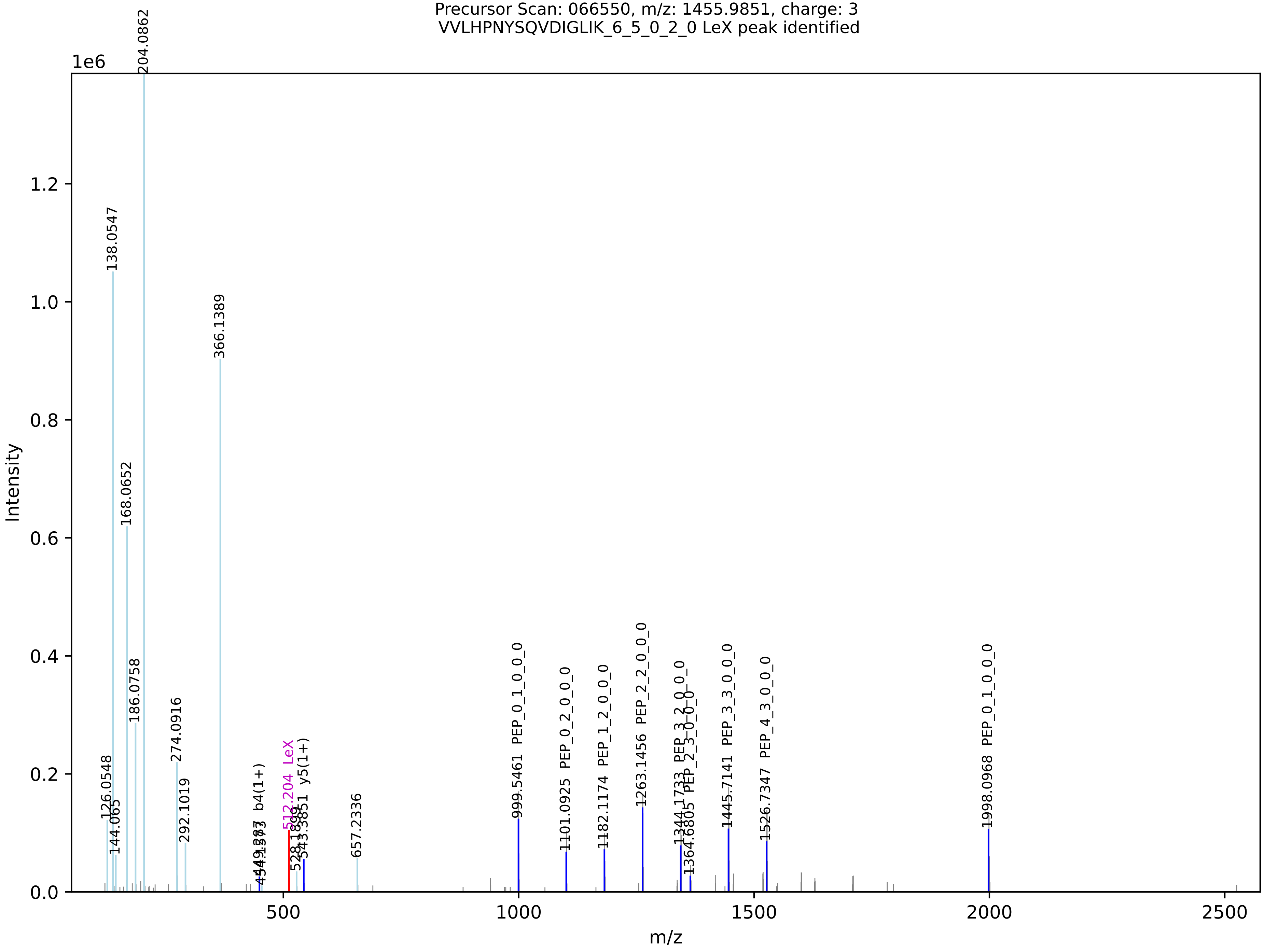

File: NS-3_231129_OE_RK_34, RT: 50.703, Scan: 066648
Type: HCD, FTMS + c NSI d Full ms2 1092.7388@hcd30.00 [120.0000-4481.2300]
Precursor Scan: 066550, m/z: 1092.49, charge: 4
VVLHPNYSQVDIGLIK_6_5_0_2_0 LeX peak identified

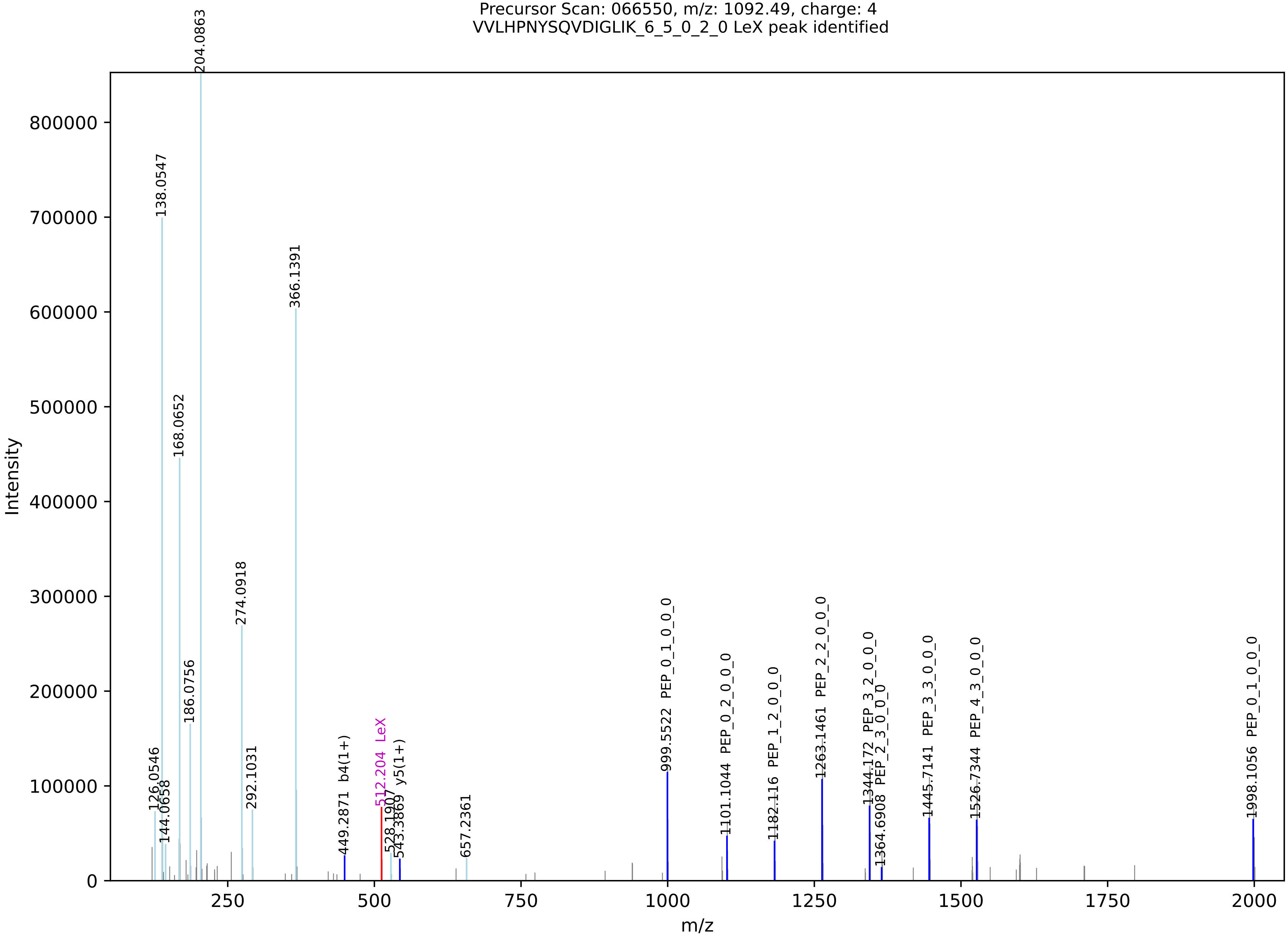

File: NS-3_231129_OE_RK_34, RT: 68.936, Scan: 093124
Type: HCD, FTMS + c NSI d Full ms2 1369.9186@hcd30.00 [120.0000-4214.0928]
Precursor Scan: 093045, m/z: 1369.5807, charge: 3
YLGNATAIFFLPDEGK_5_4_1_2_0 LeX peak identified

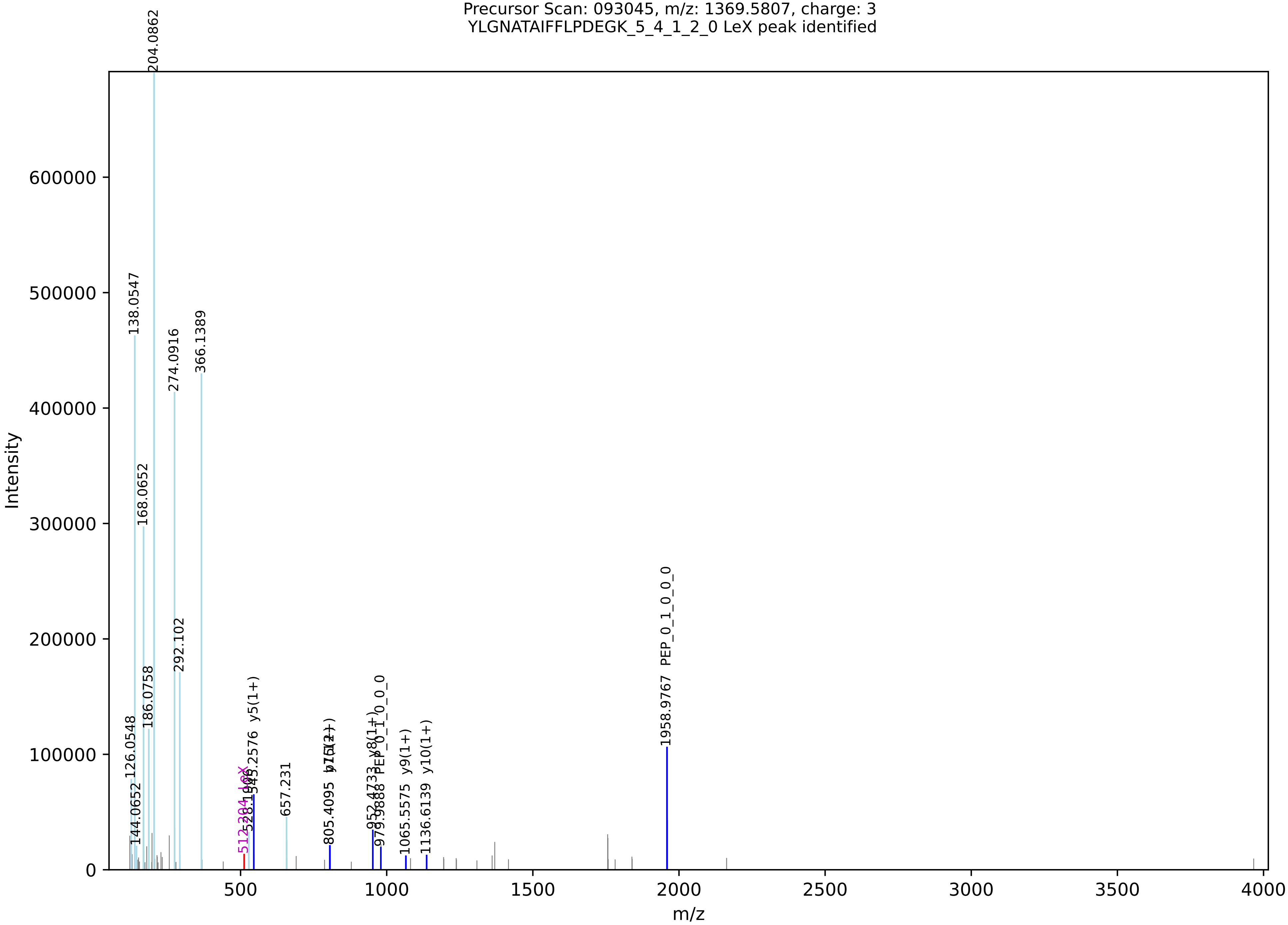

File: NS-3_231129_OE_RK_34, RT: 69.094, Scan: 093351
Type: HCD, FTMS + c NSI d Full ms2 1349.8479@hcd30.00 [120.0000-5530.2354]
Precursor Scan: 093264, m/z: 1349.6015, charge: 4
VVNSTTGPGEHLRNALWHTGNTPGQVR_5_4_2_2_0 LeX peak identified

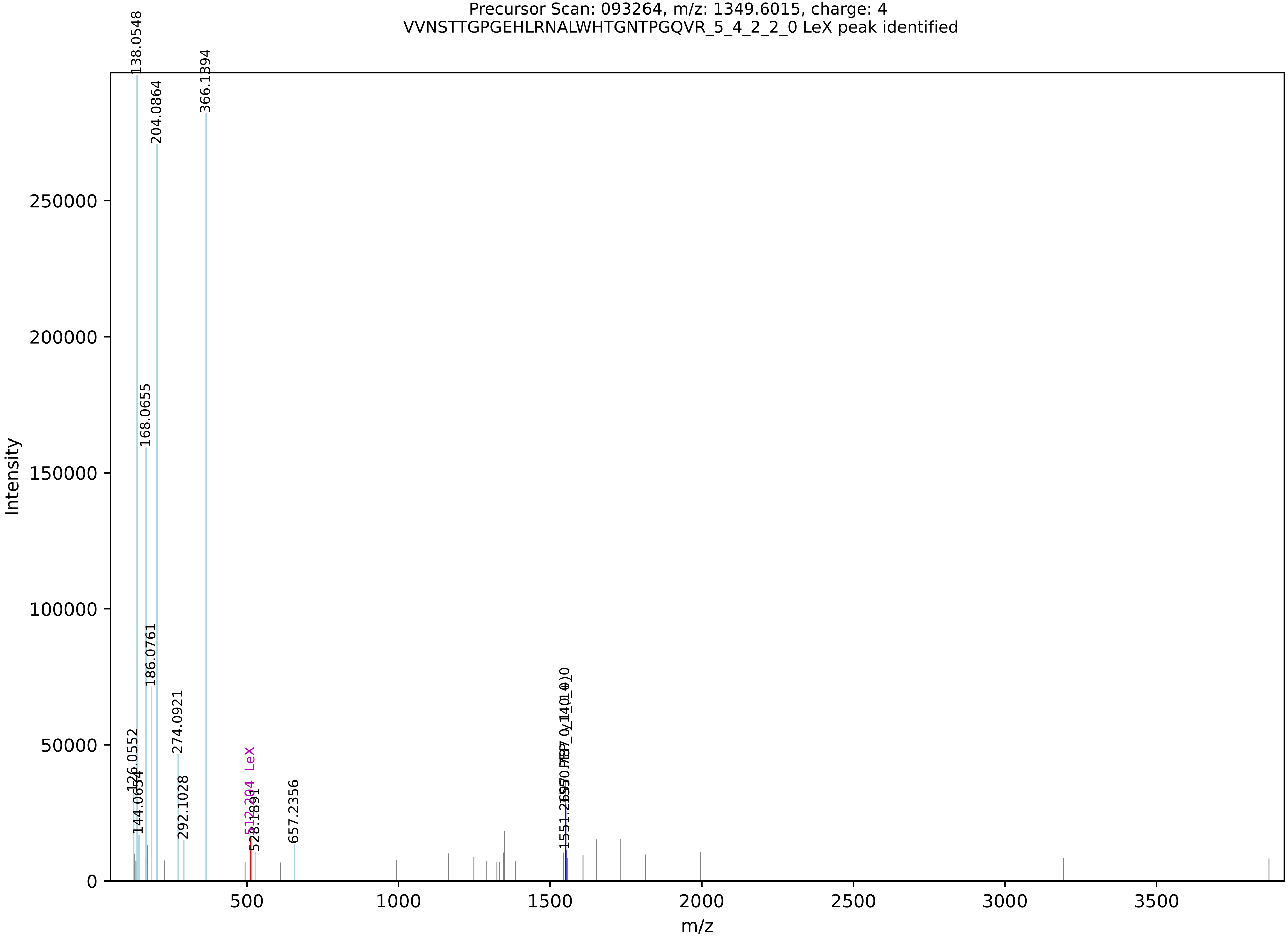

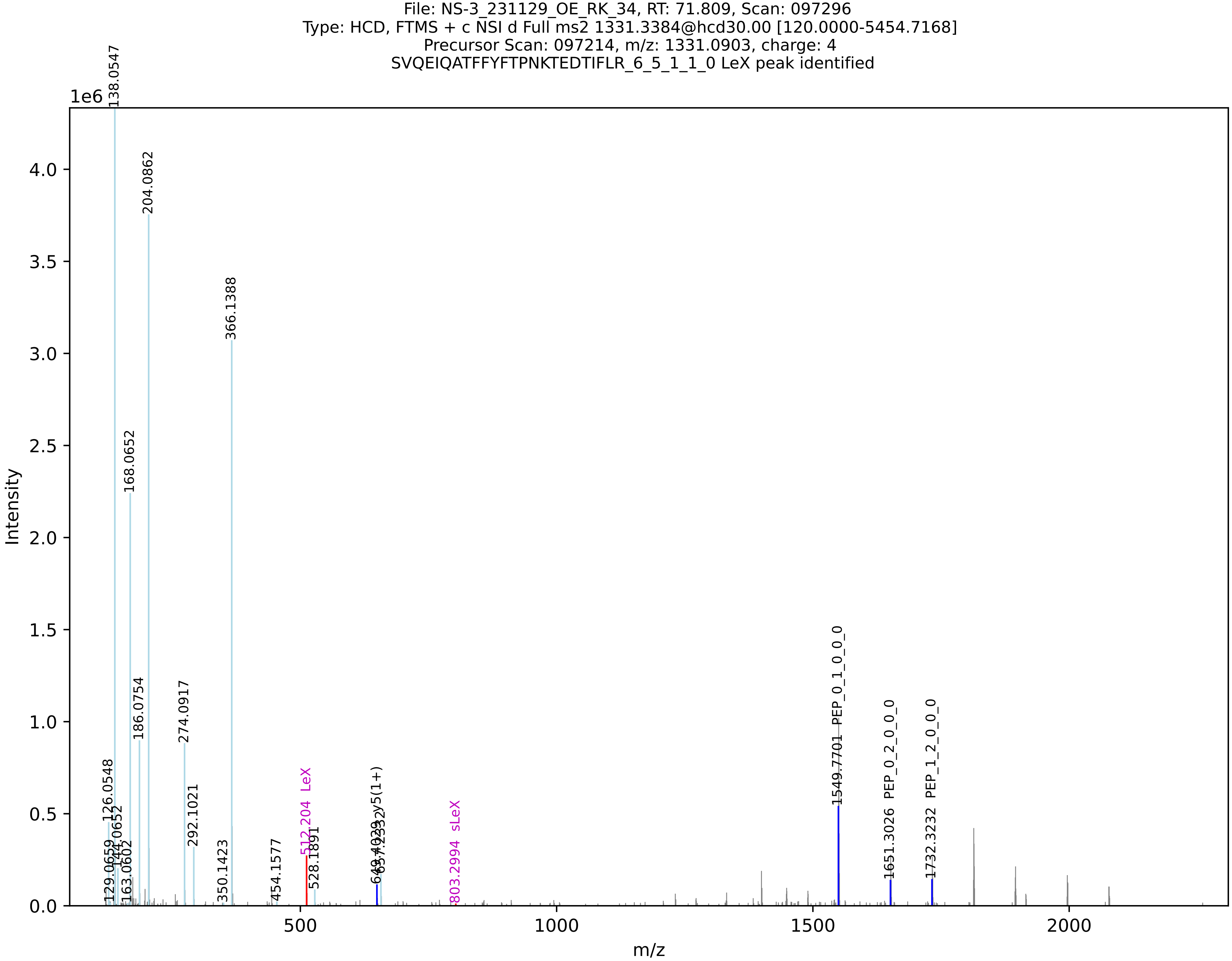

File: NS-3_231129_OE_RK_34, RT: 71.809, Scan: 097296
Type: HCD, FTMS + c NSI d Full ms2 1331.3384@hcd30.00 [120.0000-5454.7168]
Precursor Scan: 097214, m/z: 1331.0903, charge: 4
SVQEIQATFFYFTPNKTEDTIFLR_6_5_1_1_0 LeX peak identified
1e6
Intensity
m/z
138.0547
204.0862
366.1388
168.0652
186.0754
274.0917
126.0548
144.0652
129.0659
163.0602
292.1021
350.1423
454.1577
512.204 LeX
528.1891
649.4039 y5(1+)
657.2332
803.2994 sLeX
1549.7701 PEP_0_1_0_0_0
1651.3026 PEP_0_2_0_0_0
1732.3232 PEP_1_2_0_0_0

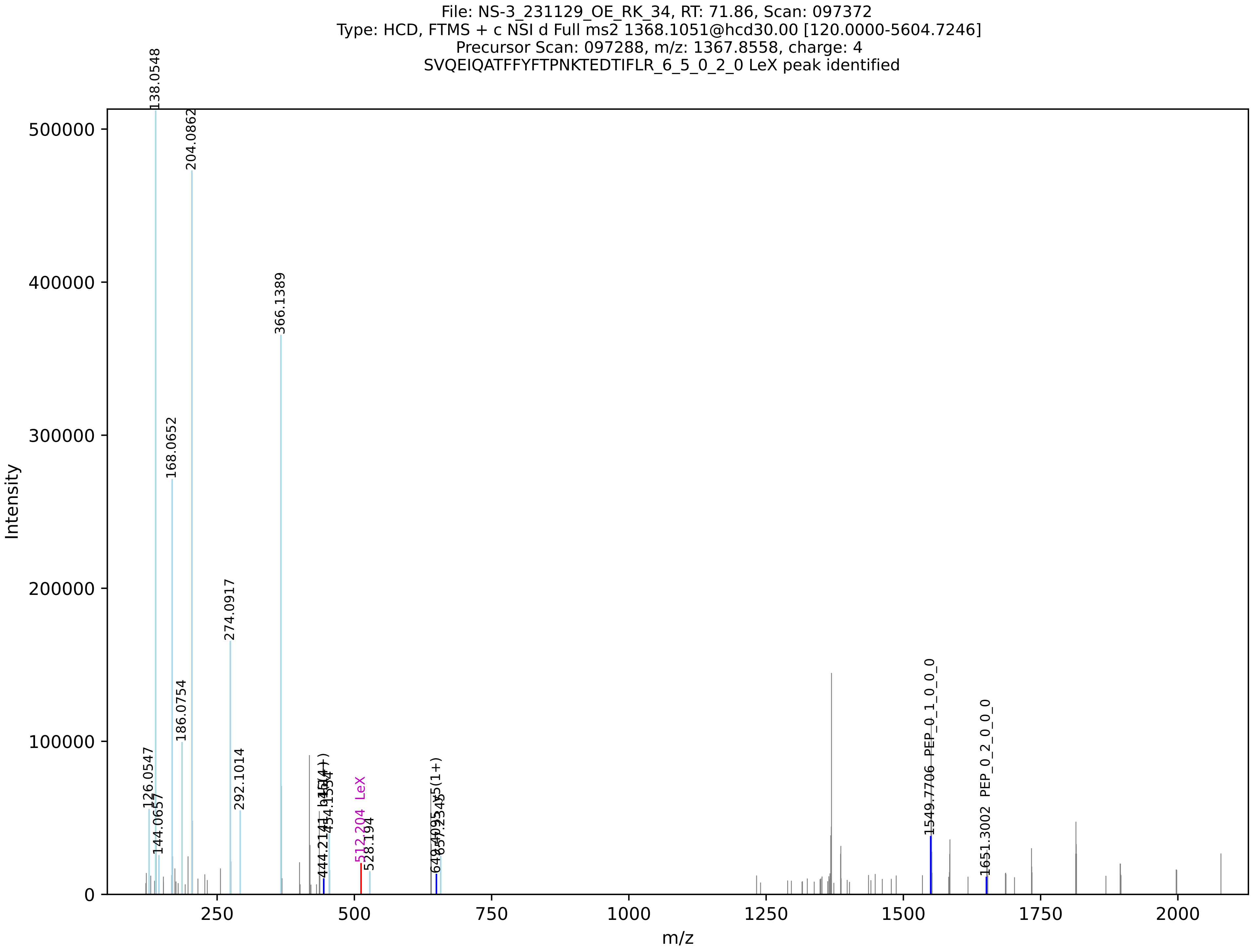
File: NS-3_231129_OE_RK_34, RT: 71.86, Scan: 097372
Type: HCD, FTMS + c NSI d Full ms2 1368.1051@hcd30.00 [120.0000-5604.7246]
Precursor Scan: 097288, m/z: 1367.8558, charge: 4
SVQEIQATFFYFTPNKTEDTIFLR_6_5_0_2_0 LeX peak identified
Intensity
m/z
138.0548
204.0862
366.1389
168.0652
274.0917
186.0754
126.0547
144.0657
292.1014
512.204 LeX
528.194
1549.7706 PEP_0_1_0_0_0
1651.3002 PEP_0_2_0_0_0

File: NS-3_231129_OE_RK_34, RT: 72.008, Scan: 097588
Type: HCD, FTMS + c NSI d Full ms2 1331.3389@hcd30.00 [120.0000-5454.7188]
Precursor Scan: 097510, m/z: 1331.0879, charge: 4
SVQEIQATFFYFTPNKTEDTIFLR_6_5_1_1_0 LeX peak identified

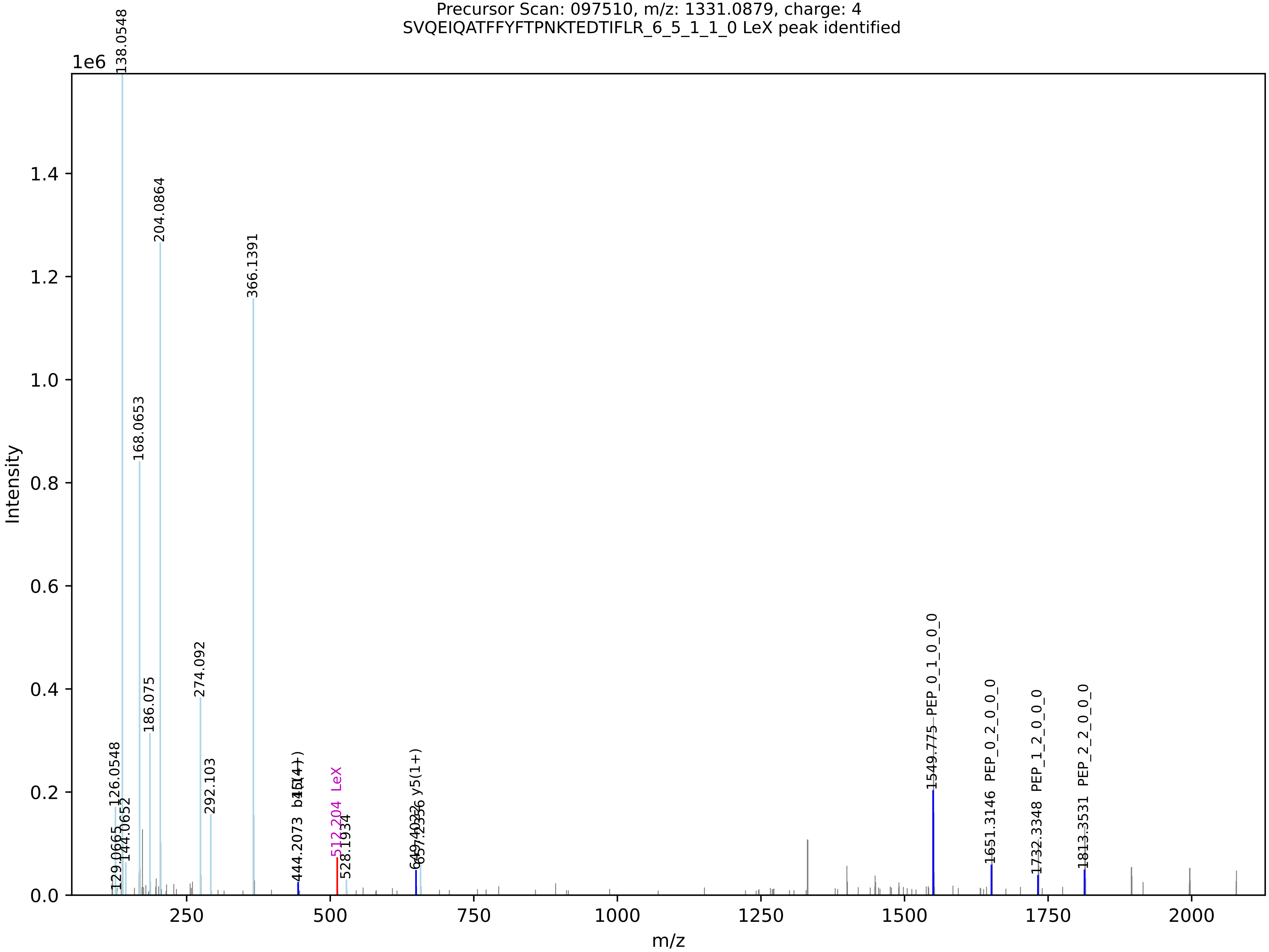

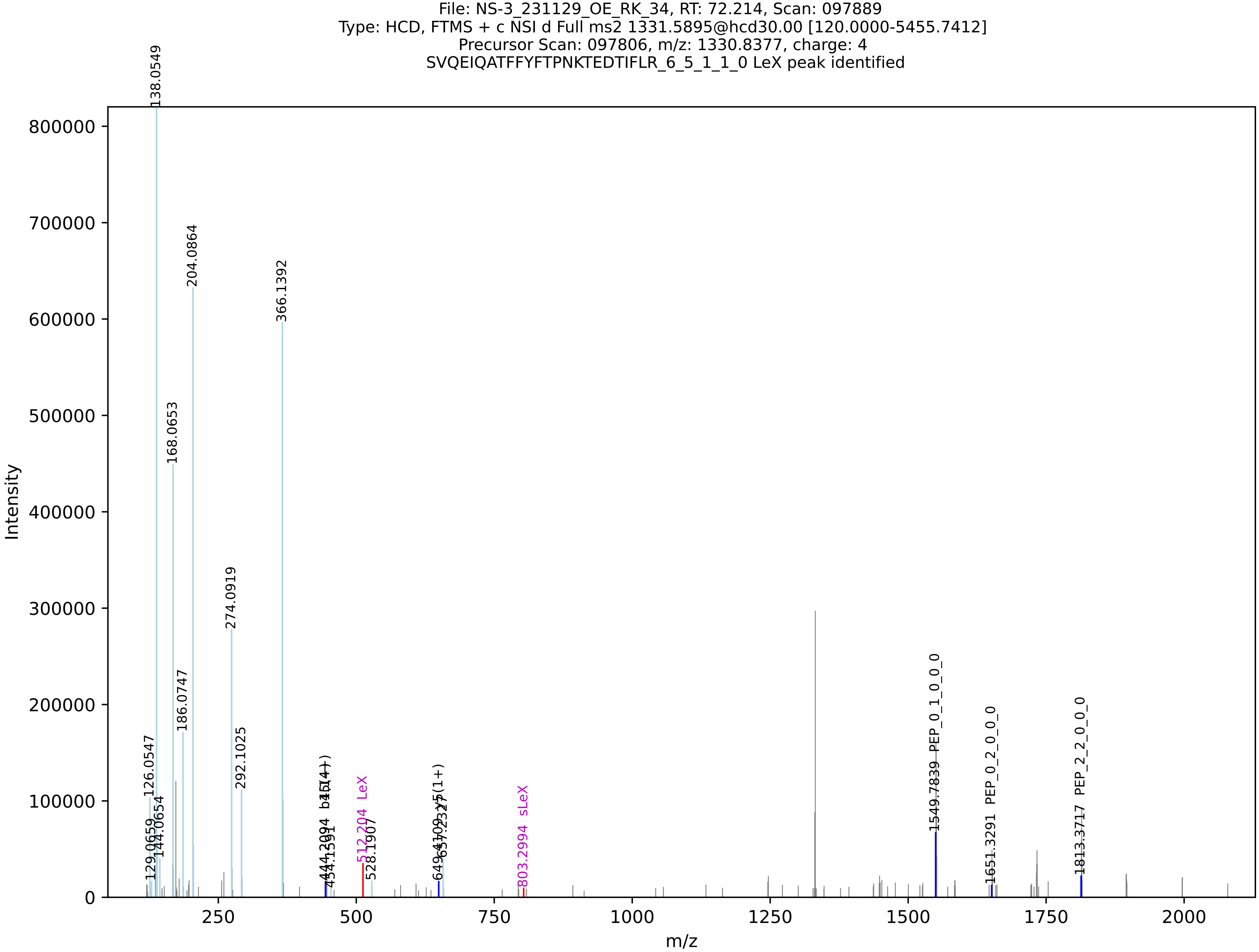
File: NS-3_231129_OE_RK_34, RT: 72.214, Scan: 097889
Type: HCD, FTMS + c NSI d Full ms2 1331.5895@hcd30.00 [120.0000-5455.7412]
Precursor Scan: 097806, m/z: 1330.8377, charge: 4
SVQEIQATFFYFTPNKTEDTIFLR_6_5_1_1_0 LeX peak identified
Intensity
m/z
138.0549
204.0864
366.1392
168.0653
274.0919
186.0747
292.1025
126.0547
144.0654
129.0659
512.204 LeX
528.1907
803.2994 sLeX
1549.7839 PEP_0_1_0_0_0
1651.3291 PEP_0_2_0_0_0
1813.3717 PEP_2_2_0_0_0

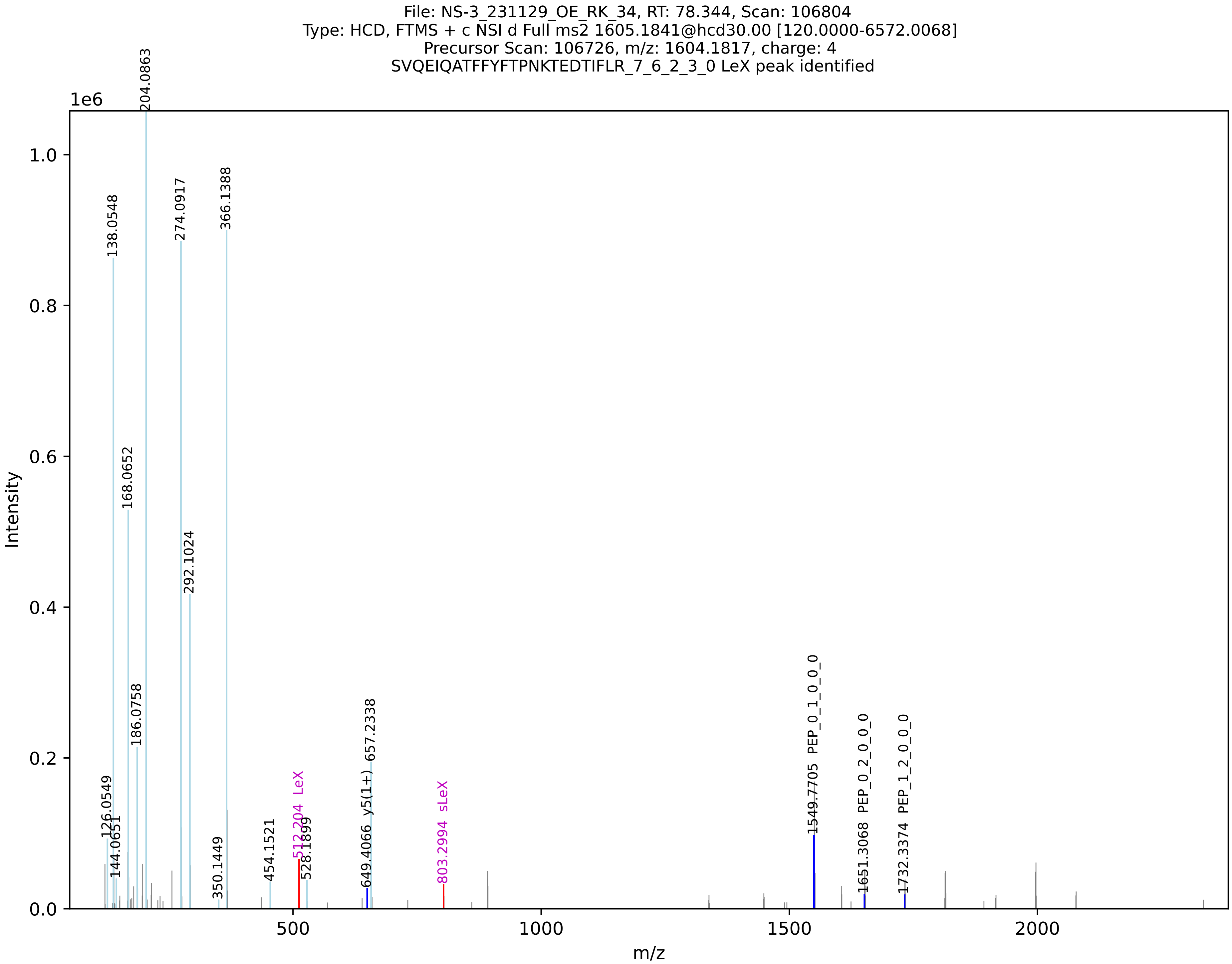
File: NS-3_231129_OE_RK_34, RT: 78.344, Scan: 106804
Type: HCD, FTMS + c NSI d Full ms2 1605.1841@hcd30.00 [120.0000-6572.0068]
Precursor Scan: 106726, m/z: 1604.1817, charge: 4
SVQEIQATFFYFTPNKTEDTIFLR_7_6_2_3_0 LeX peak identified
1e6
Intensity
m/z
204.0863
138.0548
274.0917
366.1388
168.0652
292.1024
186.0758
126.0549
144.0651
350.1449
454.1521
512.204 LeX
528.1899
649.4066 y5(1+)
657.2338
803.2994 sLeX
1549.7705 PEP_0_1_0_0_0
1651.3068 PEP_0_2_0_0_0
1732.3374 PEP_1_2_0_0_0

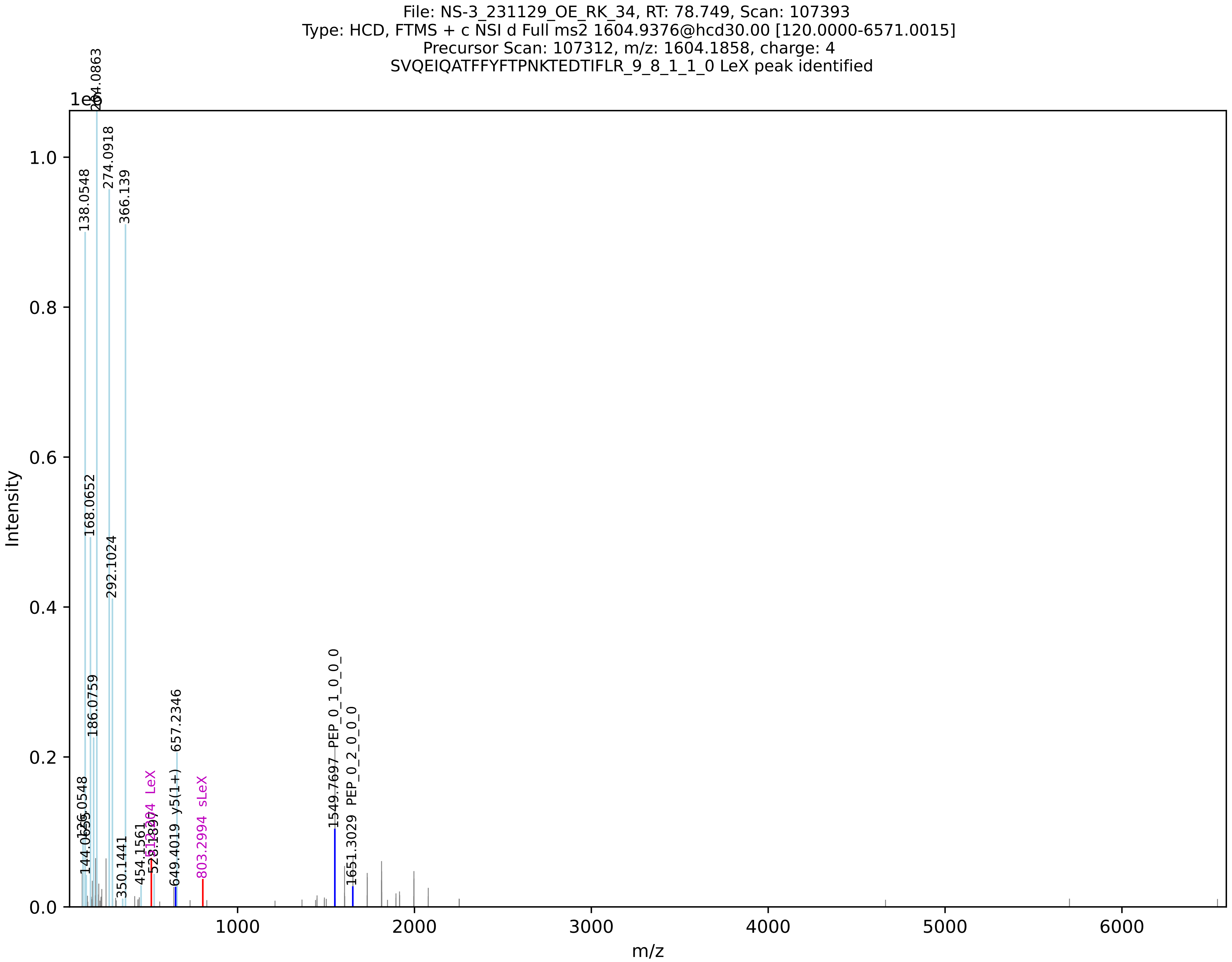
File: NS-3_231129_OE_RK_34, RT: 78.749, Scan: 107393
Type: HCD, FTMS + c NSI d Full ms2 1604.9376@hcd30.00 [120.0000-6571.0015]
Precursor Scan: 107312, m/z: 1604.1858, charge: 4
SVQEIQATFFYFTPNKTEDTIFLR_9_8_1_1_0 LeX peak identified
1e6
Intensity
m/z
138.0548
274.0918
366.139
168.0652
292.1024
186.0759
126.0548
144.0655
350.1441
454.1561
528.1897
LeX
649.4019 y5(1+)
657.2346
803.2994 sLeX
1549.7697 PEP_0_1_0_0_0
1651.3029 PEP_0_2_0_0_0
0.0
0.2
0.4
0.6
0.8
1.0
1000
2000
3000
4000
5000
6000

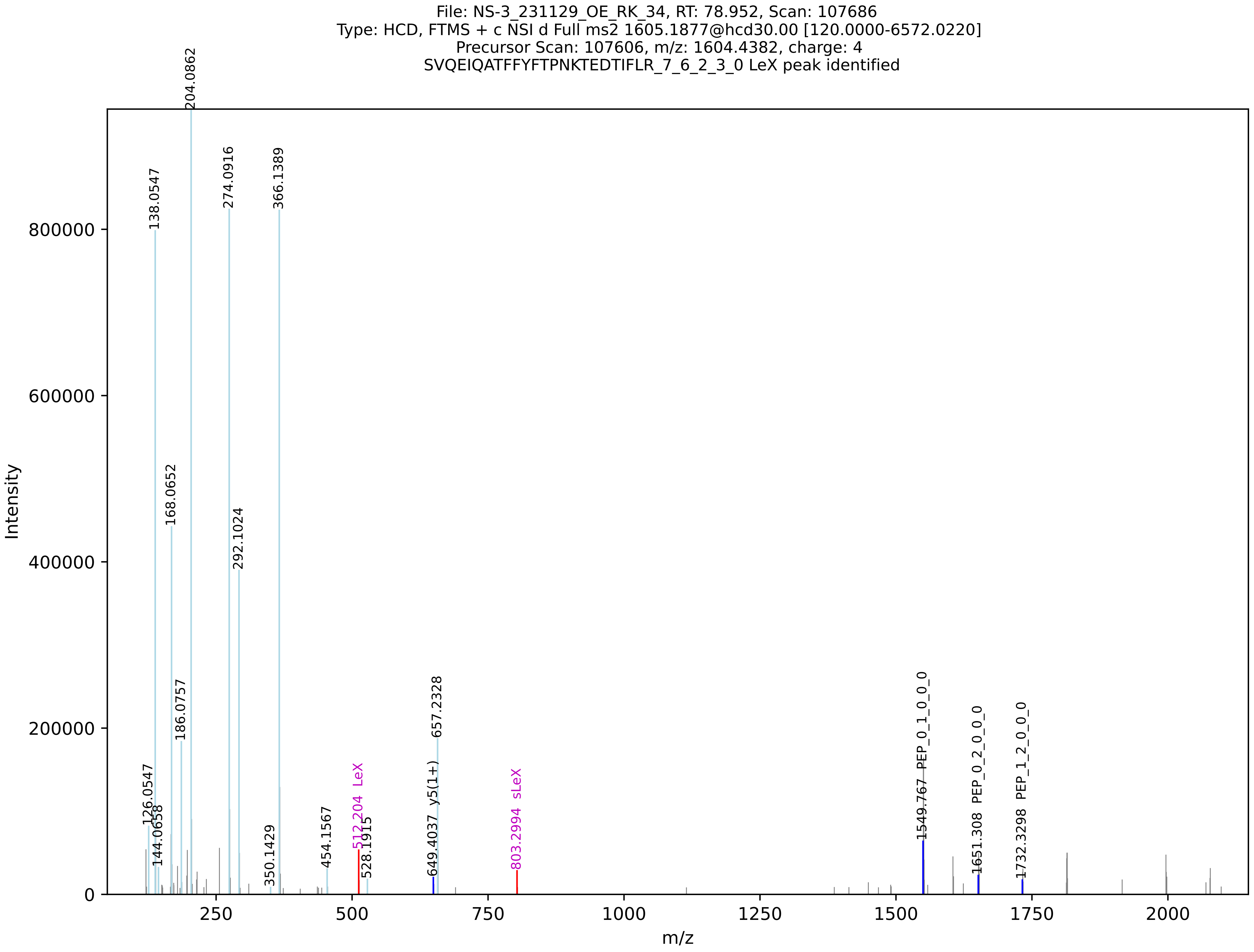

File: NS-3_231129_OE_RK_34, RT: 78.952, Scan: 107686
Type: HCD, FTMS + c NSI d Full ms2 1605.1877@hcd30.00 [120.0000-6572.0220]
Precursor Scan: 107606, m/z: 1604.4382, charge: 4
SVQEIQATFFYFTPNKTEDTIFLR_7_6_2_3_0 LeX peak identified
Intensity
m/z
204.0862
274.0916
366.1389
138.0547
168.0652
292.1024
186.0757
126.0547
144.0658
350.1429
454.1567
512.204 LeX
528.1915
649.4037 y5(1+)
657.2328
803.2994 sLeX
1549.767 PEP_0_1_0_0_0
1651.308 PEP_0_2_0_0_0
1732.3298 PEP_1_2_0_0_0

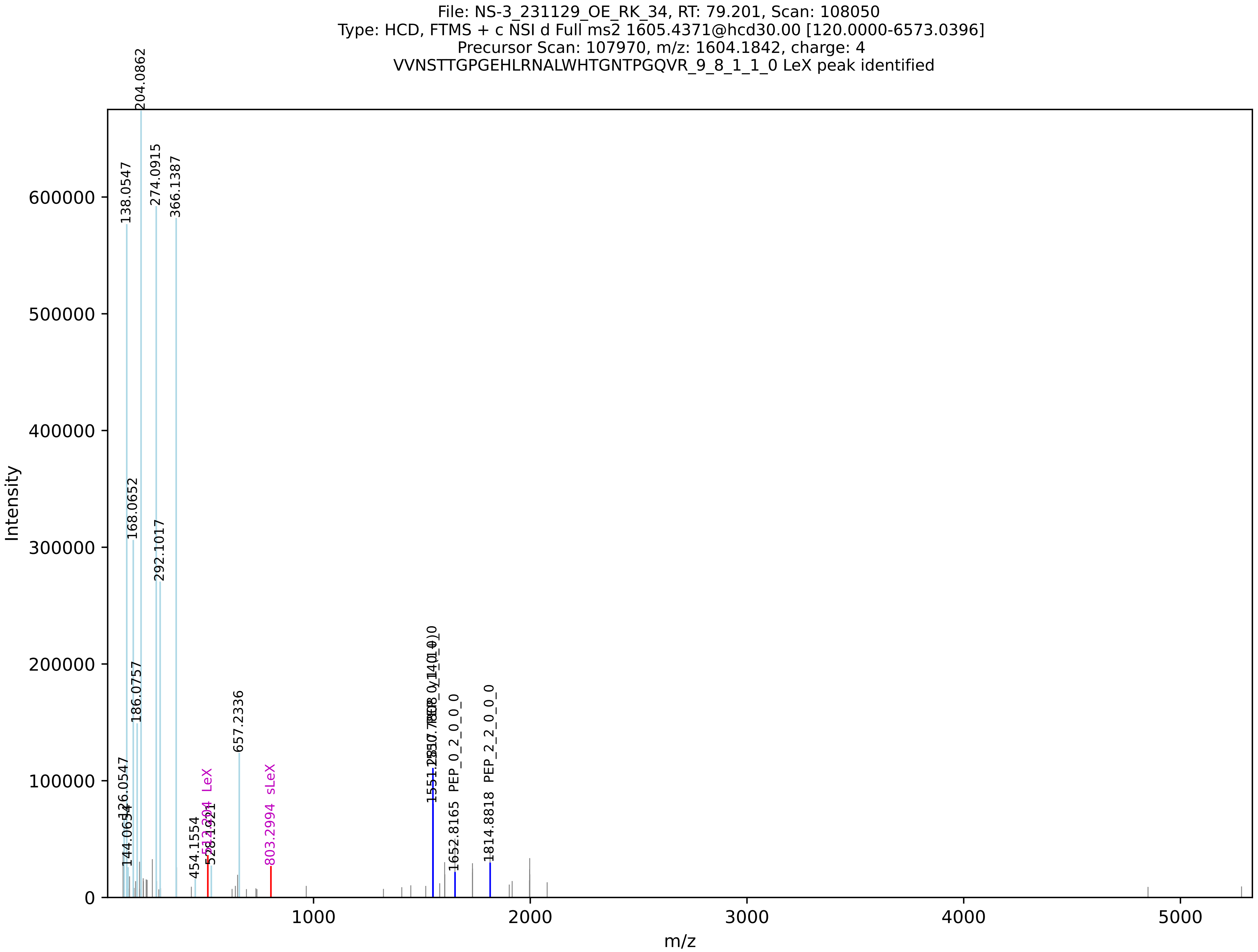
File: NS-3_231129_OE_RK_34, RT: 79.201, Scan: 108050
Type: HCD, FTMS + c NSI d Full ms2 1605.4371@hcd30.00 [120.0000-6573.0396]
Precursor Scan: 107970, m/z: 1604.1842, charge: 4
VVNSTTGPGEHLRNALWHTGNTPGQVR_9_8_1_1_0 LeX peak identified
Intensity
m/z
0
100000
200000
300000
400000
500000
600000
1000
2000
3000
4000
5000
204.0862
138.0547
274.0915
366.1387
168.0652
292.1017
186.0757
126.0547
144.0654
454.1554
528.1921
LeX
657.2336
803.2994 sLeX
PEP_0_2_0_0_0
1652.8165
PEP_2_2_0_0_0
1814.8818

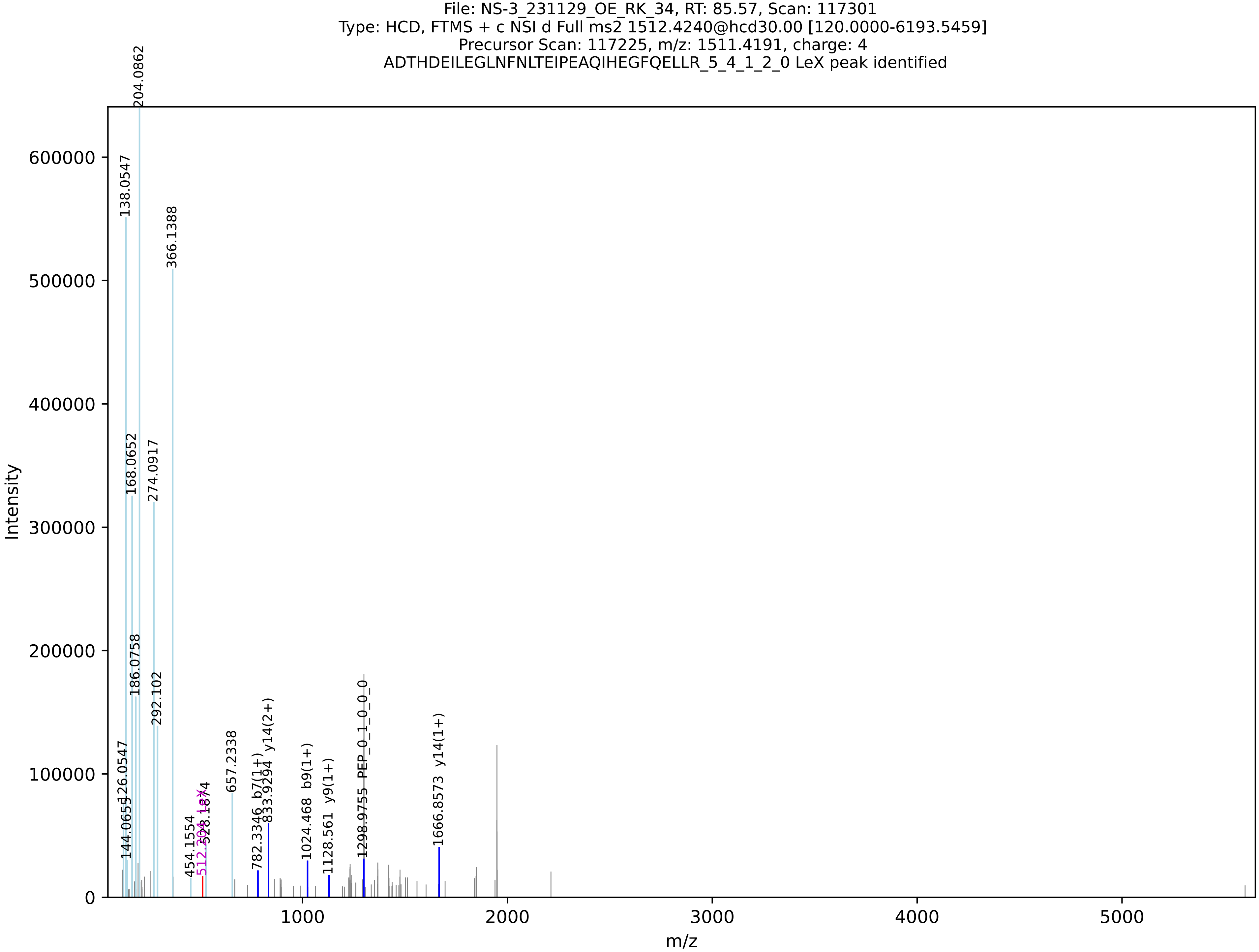

File: NS-3_231129_OE_RK_34, RT: 85.57, Scan: 117301
Type: HCD, FTMS + c NSI d Full ms2 1512.4240@hcd30.00 [120.0000-6193.5459]
Precursor Scan: 117225, m/z: 1511.4191, charge: 4
ADTHDEILEGLNFNLTEIPEAQIHEGFQELLR_5_4_1_2_0 LeX peak identified
Intensity
m/z
204.0862
138.0547
366.1388
168.0652
274.0917
186.0758
292.102
126.0547
144.0655
454.1554
512.204 LeX
528.1874
657.2338
782.3346 b7(1+)
833.9294 y14(2+)
1024.468 b9(1+)
1128.561 y9(1+)
1298.9755 PEP_0_1_0_0_0
1666.8573 y14(1+)

File: NS-3_231129_OE_RK_34, RT: 88.22, Scan: 121123
Type: HCD, FTMS + c NSI d Full ms2 1341.1847@hcd30.00 [120.0000-6863.6118]
Precursor Scan: 121048, m/z: 1340.7887, charge: 5
ADTHDEILEGLNFNLTEIPEAQIHEGFQELLR_6_5_1_3_0 LeX peak identified

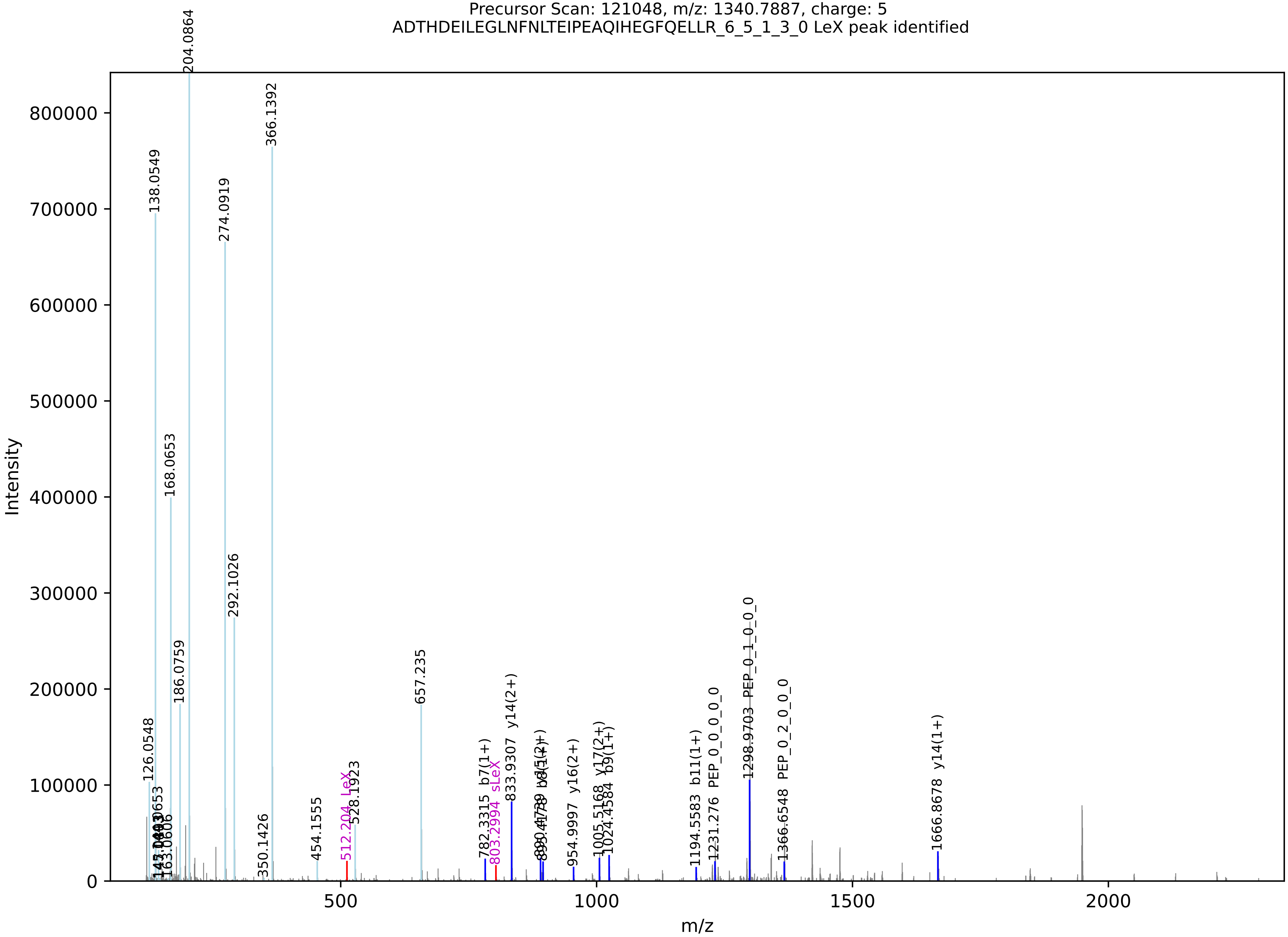

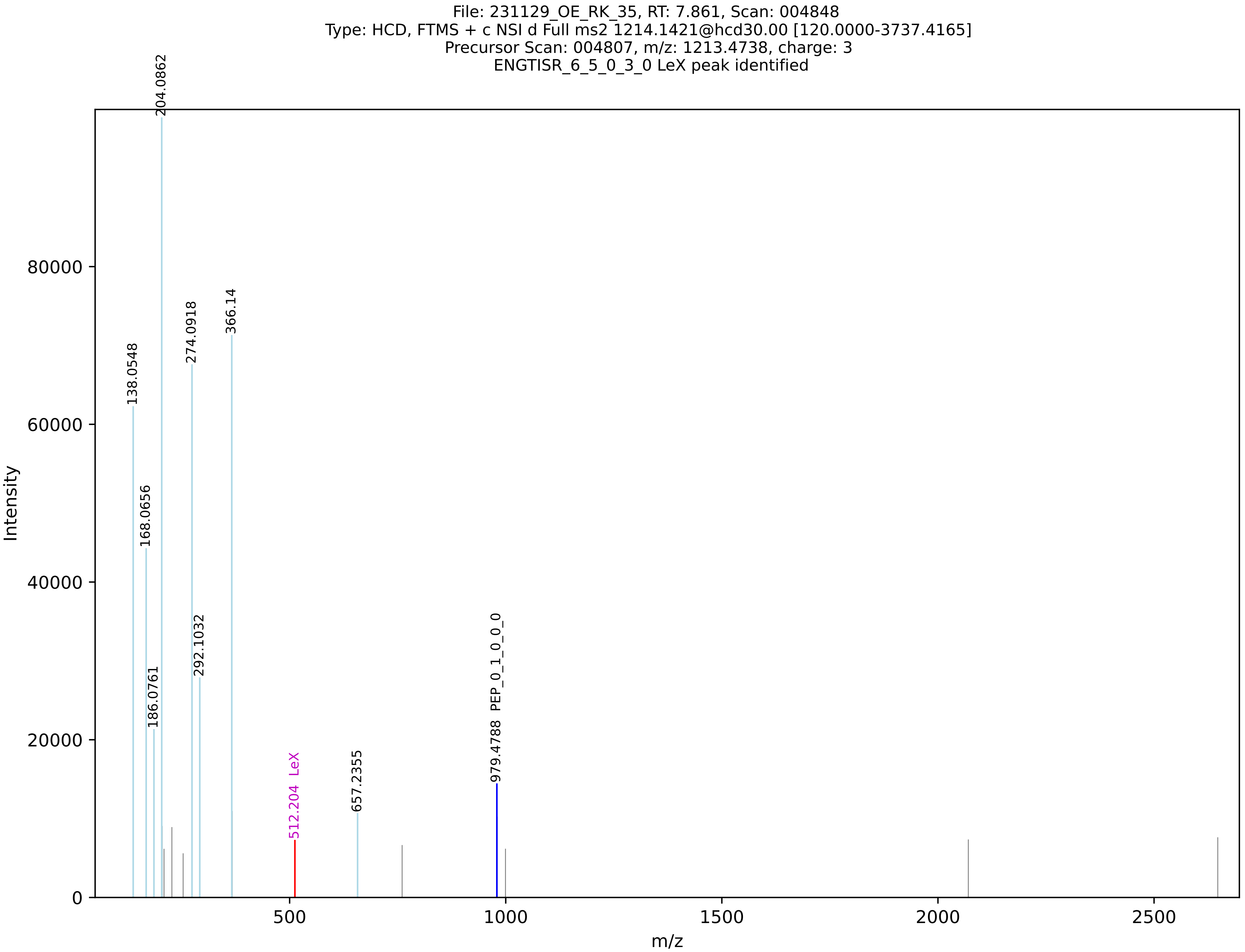
File: 231129_OE_RK_35, RT: 7.861, Scan: 004848
Type: HCD, FTMS + c NSI d Full ms2 1214.1421@hcd30.00 [120.0000-3737.4165]
Precursor Scan: 004807, m/z: 1213.4738, charge: 3
ENGTISR_6_5_0_3_0 LeX peak identified
Intensity
m/z
0
20000
40000
60000
80000
500
1000
1500
2000
2500
204.0862
366.14
274.0918
138.0548
168.0656
292.1032
186.0761
512.204 LeX
657.2355
979.4788 PEP_0_1_0_0_0

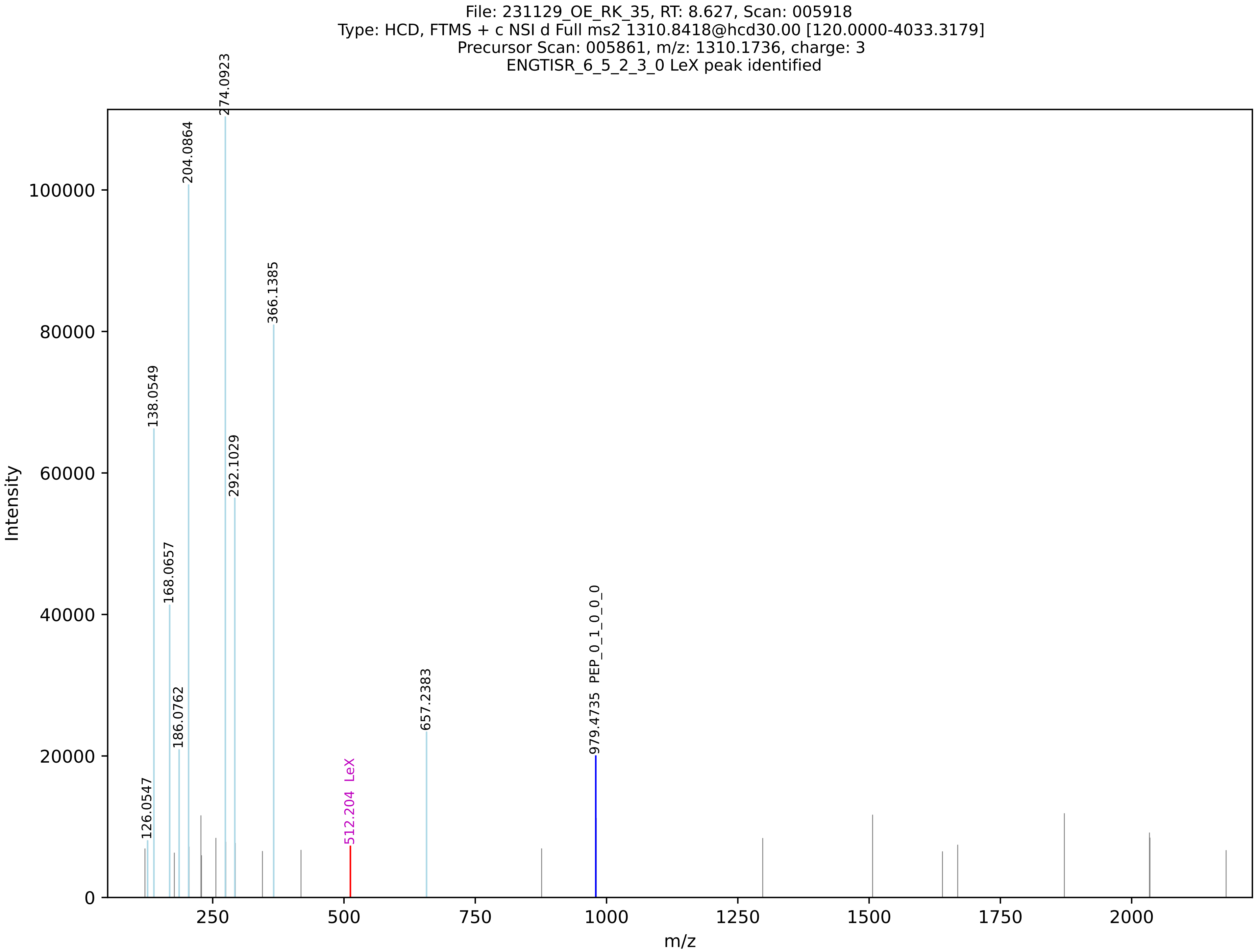
File: 231129_OE_RK_35, RT: 8.627, Scan: 005918
Type: HCD, FTMS + c NSI d Full ms2 1310.8418@hcd30.00 [120.0000-4033.3179]
Precursor Scan: 005861, m/z: 1310.1736, charge: 3
ENGTISR_6_5_2_3_0 LeX peak identified
274.0923
204.0864
366.1385
138.0549
292.1029
168.0657
186.0762
126.0547
512.204 LeX
657.2383
979.4735 PEP_0_1_0_0_0
Intensity
m/z
0
20000
40000
60000
80000
100000
250
500
750
1000
1250
1500
1750
2000

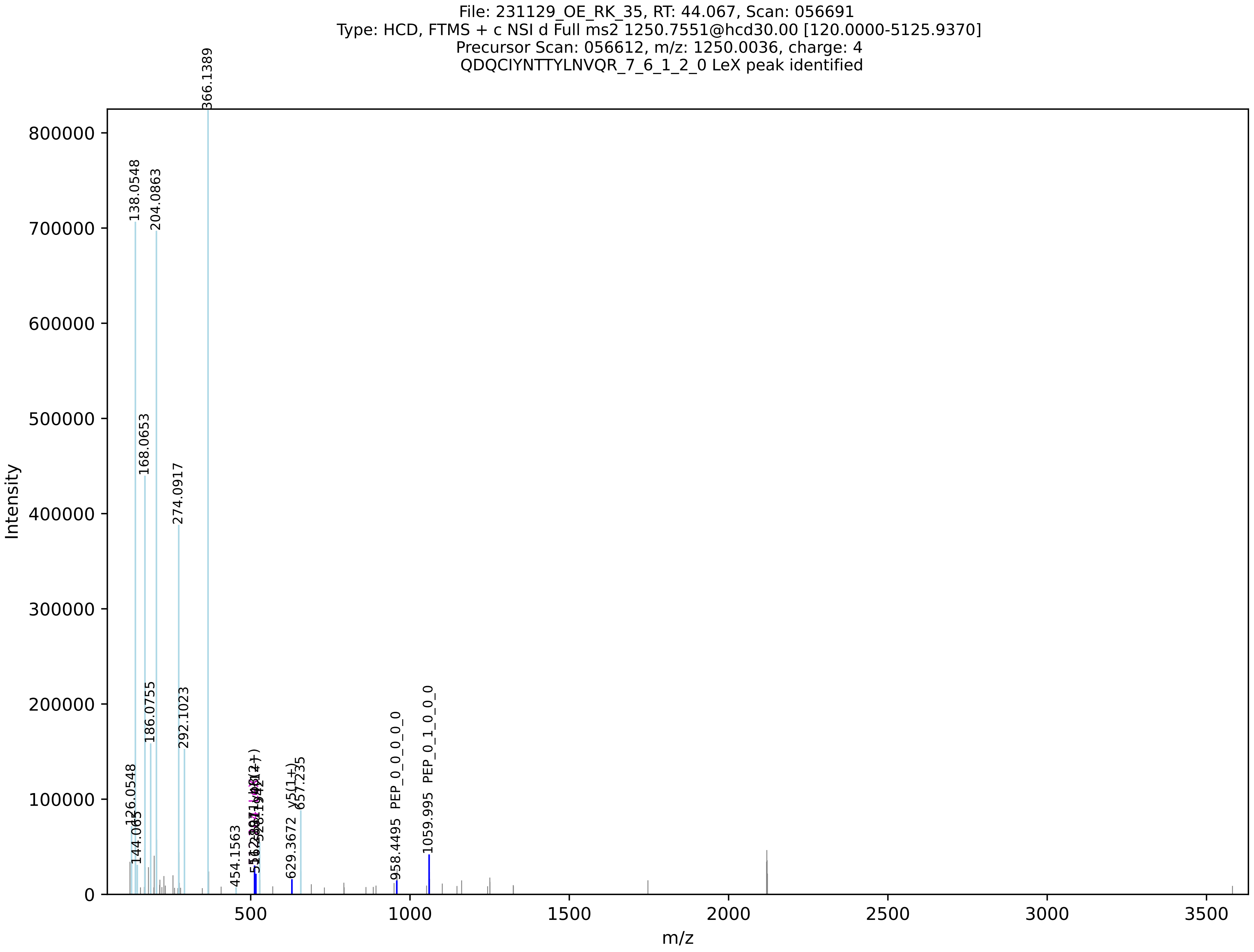
File: 231129_OE_RK_35, RT: 44.067, Scan: 056691
Type: HCD, FTMS + c NSI d Full ms2 1250.7551@hcd30.00 [120.0000-5125.9370]
Precursor Scan: 056612, m/z: 1250.0036, charge: 4
QDQCIYNTTYLNVQR_7_6_1_2_0 LeX peak identified
366.1389
138.0548
204.0863
168.0653
274.0917
186.0755
292.1023
126.0548
144.065
454.1563
629.3672
y5(1+)
657.235
958.4495
PEP_0_0_0_0_0
1059.995
PEP_0_1_0_0_0
Intensity
m/z
0
100000
200000
300000
400000
500000
600000
700000
800000
500
1000
1500
2000
2500
3000
3500

File: 231129_OE_RK_35, RT: 44.269, Scan: 056986
Type: HCD, FTMS + c NSI d Full ms2 1250.7555@hcd30.00 [120.0000-5125.9385]
Precursor Scan: 056905, m/z: 1250.0036, charge: 4
QDQCIYNTTYLNVQR_7_6_1_2_0 LeX peak identified

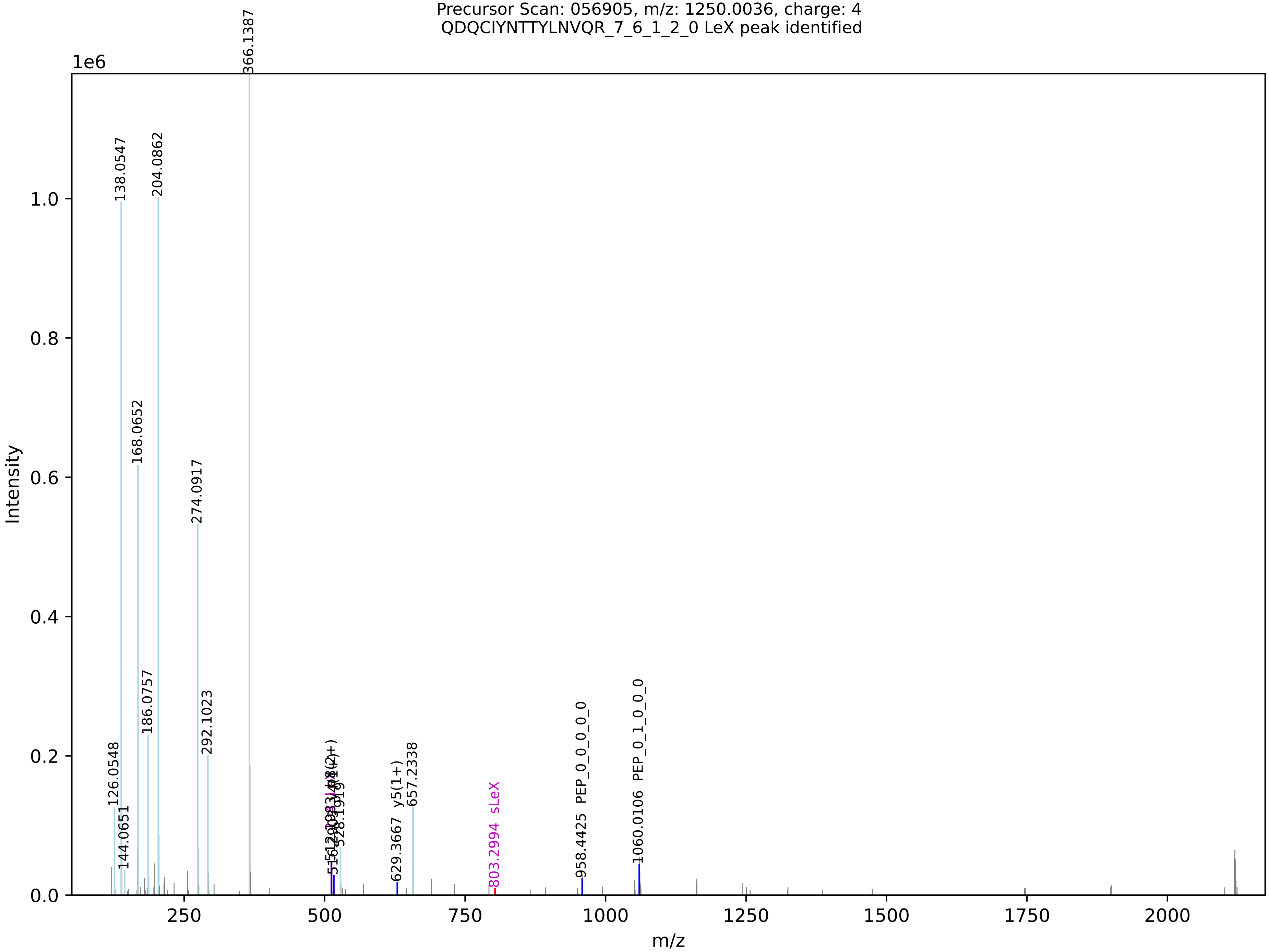

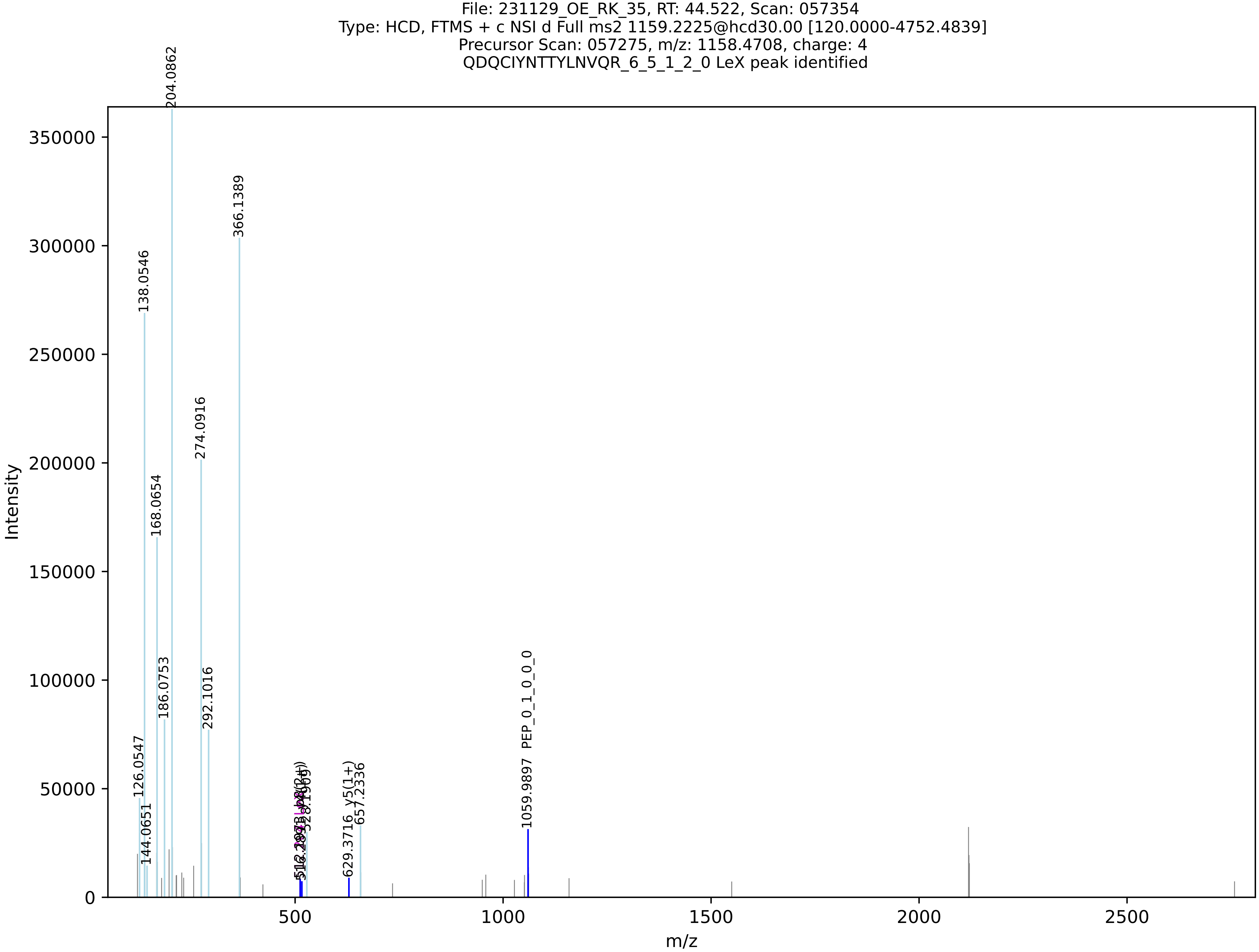
File: 231129_OE_RK_35, RT: 44.522, Scan: 057354
Type: HCD, FTMS + c NSI d Full ms2 1159.2225@hcd30.00 [120.0000-4752.4839]
Precursor Scan: 057275, m/z: 1158.4708, charge: 4
QDQCIYNTTYLNVQR_6_5_1_2_0 LeX peak identified
204.0862
366.1389
138.0546
274.0916
168.0654
186.0753
292.1016
126.0547
144.0651
629.3716_y5(1+)
657.2336
PEP_0_1_0_0_0
1059.9897
Intensity
m/z
0
50000
100000
150000
200000
250000
300000
350000
500
1000
1500
2000
2500

File: 231129_OE_RK_35, RT: 44.57, Scan: 057425
Type: HCD, FTMS + c NSI d Full ms2 1286.7661@hcd30.00 [120.0000-5272.8613]
Precursor Scan: 057346, m/z: 1286.2653, charge: 4
QDQCIYNTTYLNVQR_7_6_0_3_0 LeX peak identified

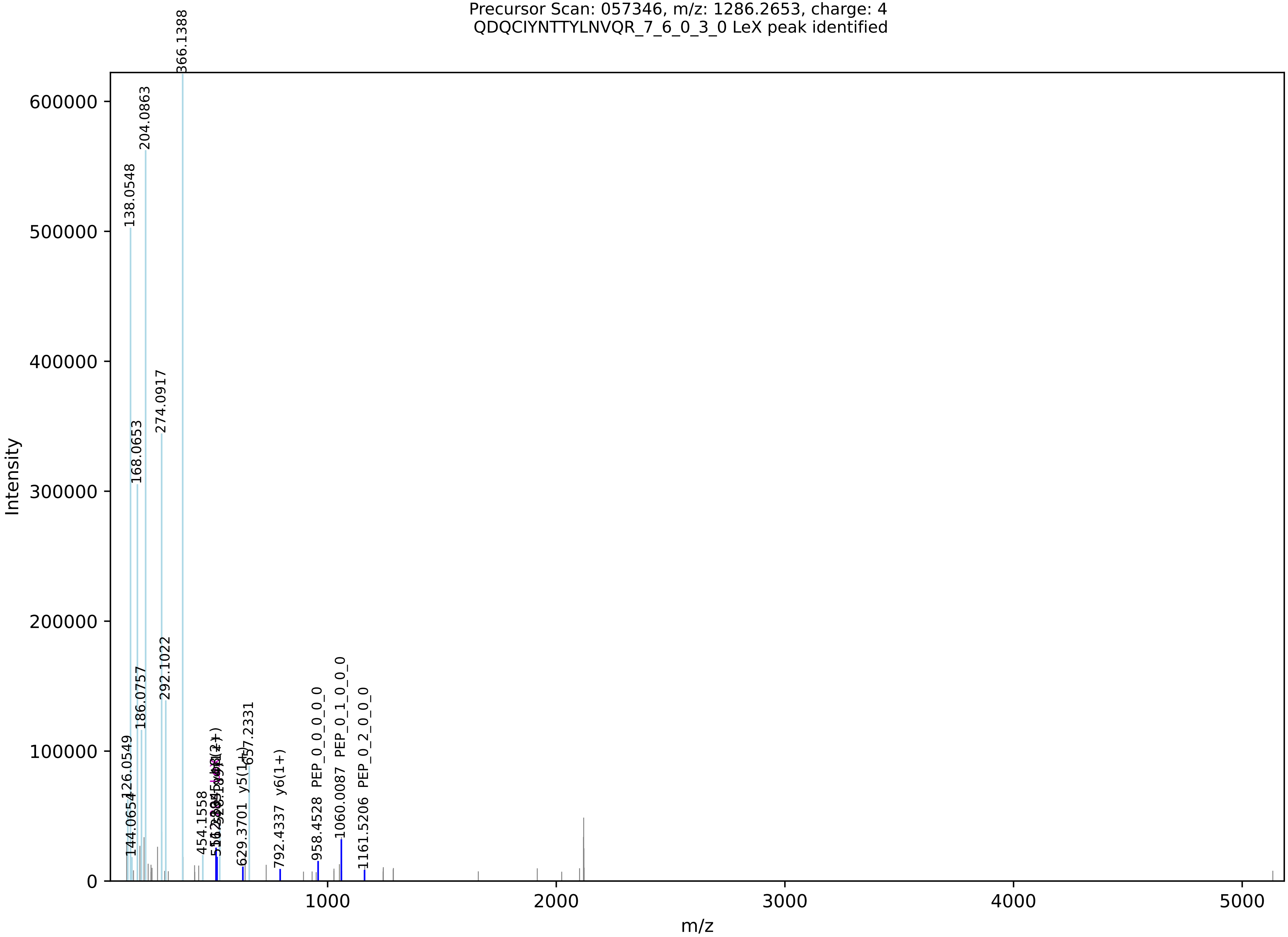

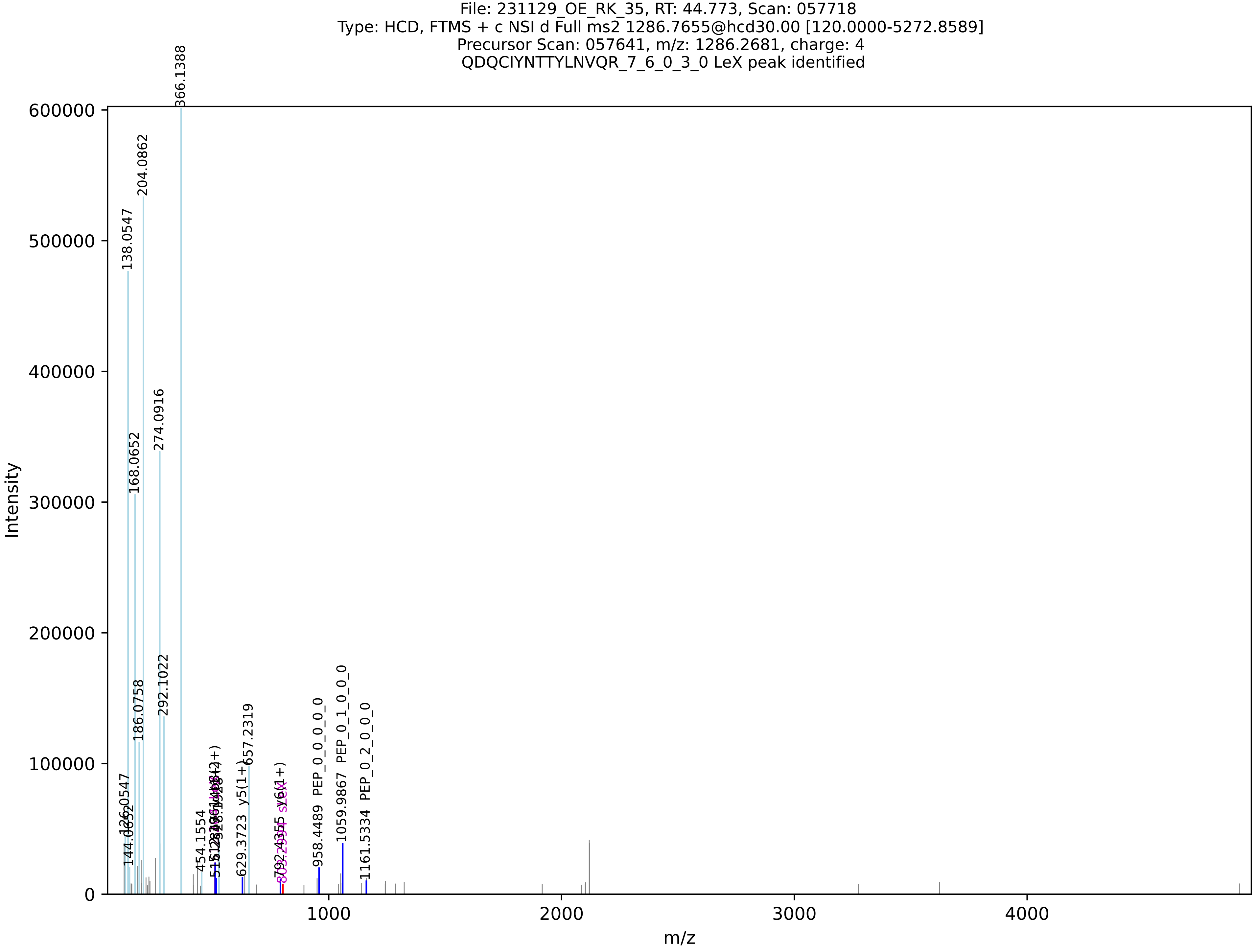
File: 231129_OE_RK_35, RT: 44.773, Scan: 057718
Type: HCD, FTMS + c NSI d Full ms2 1286.7655@hcd30.00 [120.0000-5272.8589]
Precursor Scan: 057641, m/z: 1286.2681, charge: 4
QDQCIYNTTYLNVQR_7_6_0_3_0 LeX peak identified
366.1388
204.0862
138.0547
274.0916
168.0652
186.0758
292.1022
126.0547
144.0652
454.1554
657.2319
629.3723 y5(1+)
958.4489 PEP_0_0_0_0_0
1059.9867 PEP_0_1_0_0_0
1161.5334 PEP_0_2_0_0_0
Intensity
m/z
0
100000
200000
300000
400000
500000
600000
1000
2000
3000
4000

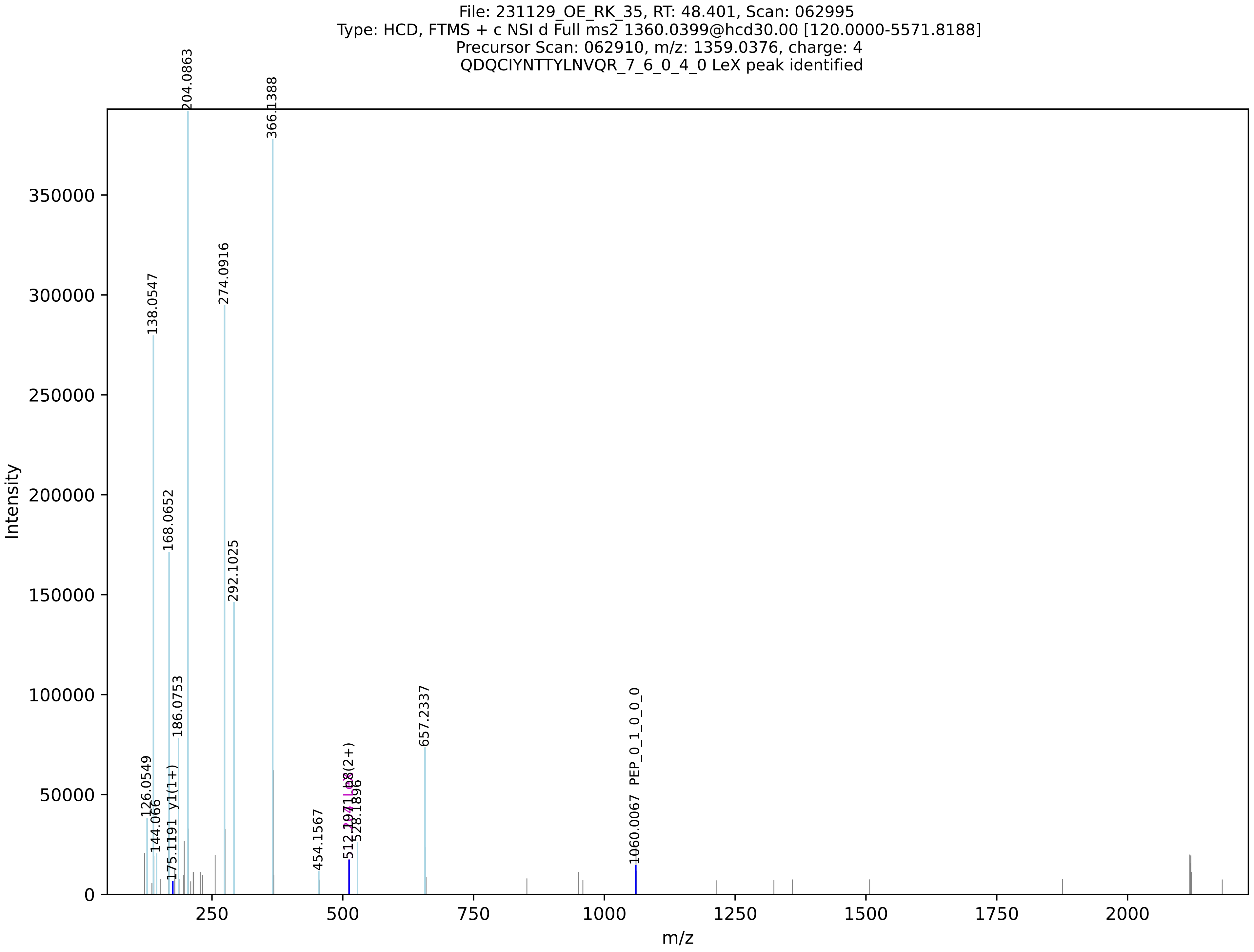
File: 231129_OE_RK_35, RT: 48.401, Scan: 062995
Type: HCD, FTMS + c NSI d Full ms2 1360.0399@hcd30.00 [120.0000-5571.8188]
Precursor Scan: 062910, m/z: 1359.0376, charge: 4
QDQCIYNTTYLNVQR_7_6_0_4_0 LeX peak identified
204.0863
366.1388
138.0547
274.0916
168.0652
292.1025
186.0753
657.2337
1060.0067 PEP_0_1_0_0_0
126.0549
144.066
175.1191 y1(1+)
454.1567
512.1971 b8(2+)
528.1896
Intensity
m/z

File: 231129_OE_RK_35, RT: 50.865, Scan: 066580
Type: HCD, FTMS + c NSI d Full ms2 1092.7405@hcd30.00 [120.0000-4481.2373]
Precursor Scan: 066495, m/z: 1092.2396, charge: 4
VVLHPNYSQVDIGLIK_6_5_0_2_0 LeX peak identified

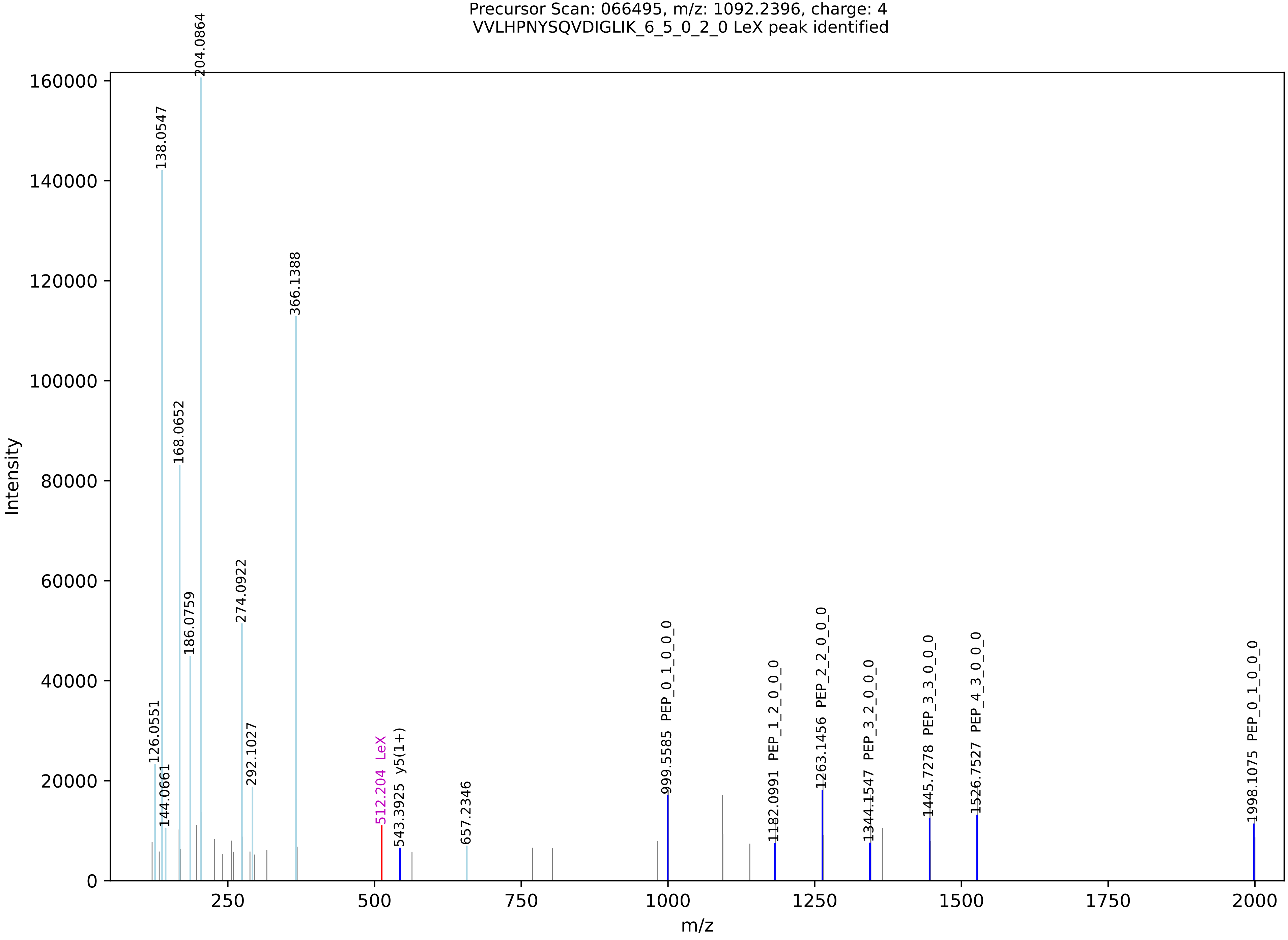

File: 231129_OE_RK_35, RT: 65.222, Scan: 087470
Type: HCD, FTMS + c NSI d Full ms2 1273.2174@hcd30.00 [120.0000-3918.1873]
Precursor Scan: 087386, m/z: 1272.5492, charge: 3
YLGNATAIFFLPDEGK_5_4_1_1_0 LeX peak identified

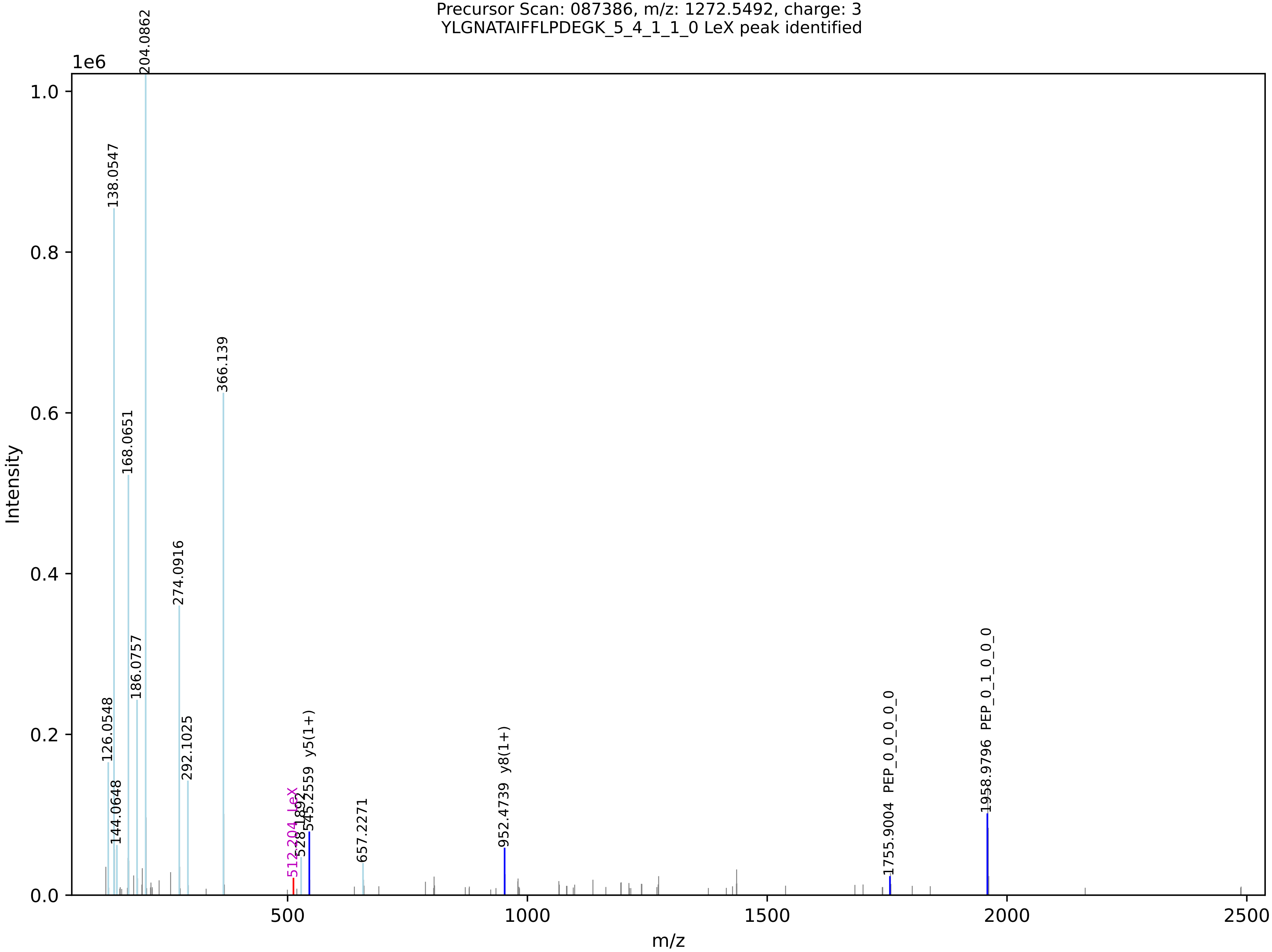

File: 231129_OE_RK_35, RT: 78.535, Scan: 106841
Type: HCD, FTMS + c NSI d Full ms2 1568.4186@hcd30.00 [120.0000-6422.0039]
Precursor Scan: 106760, m/z: 1567.6669, charge: 4
SVQEIQATFFYFTPNKTEDTIFLR_7_6_1_3_0 LeX peak identified

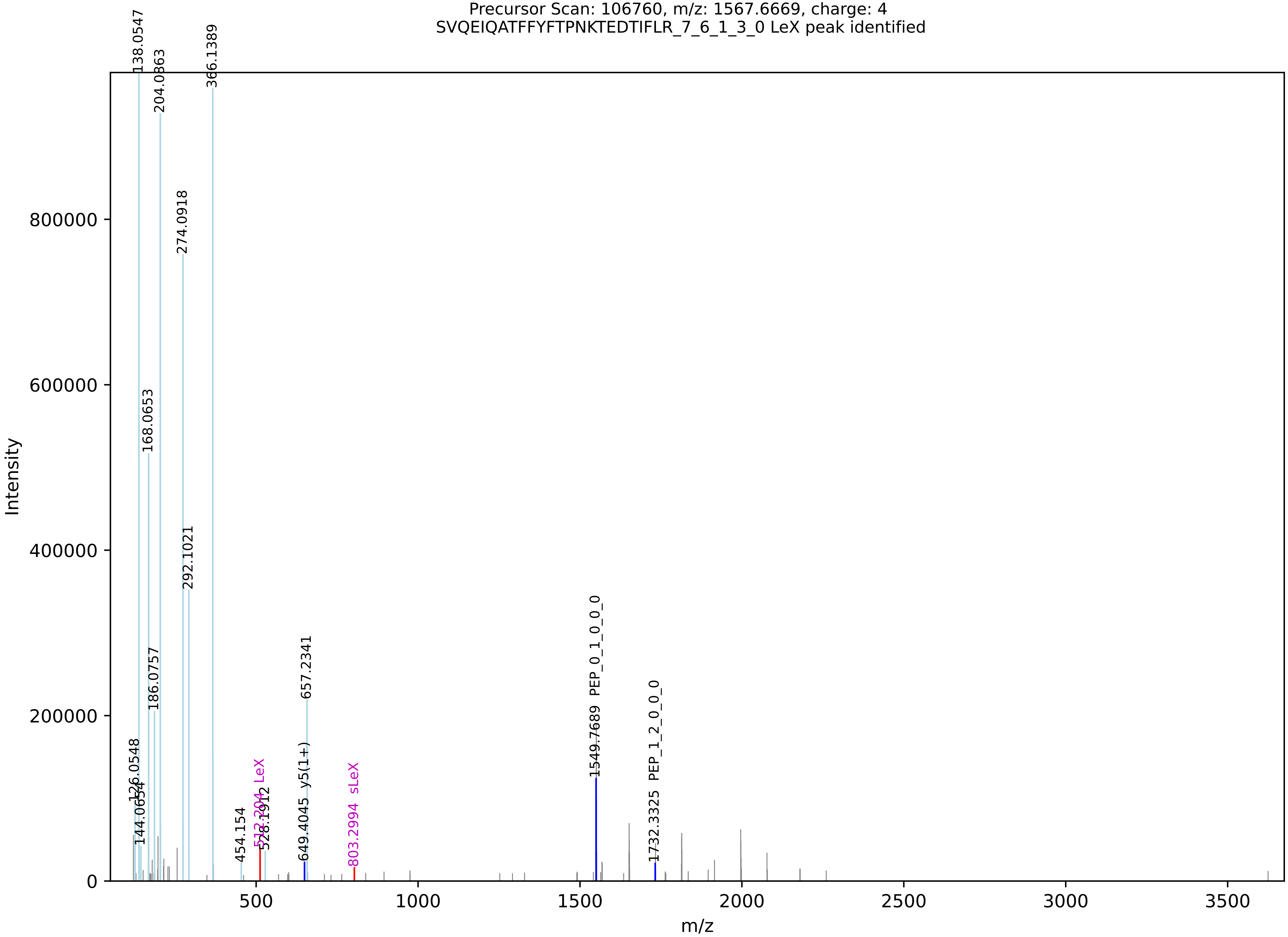

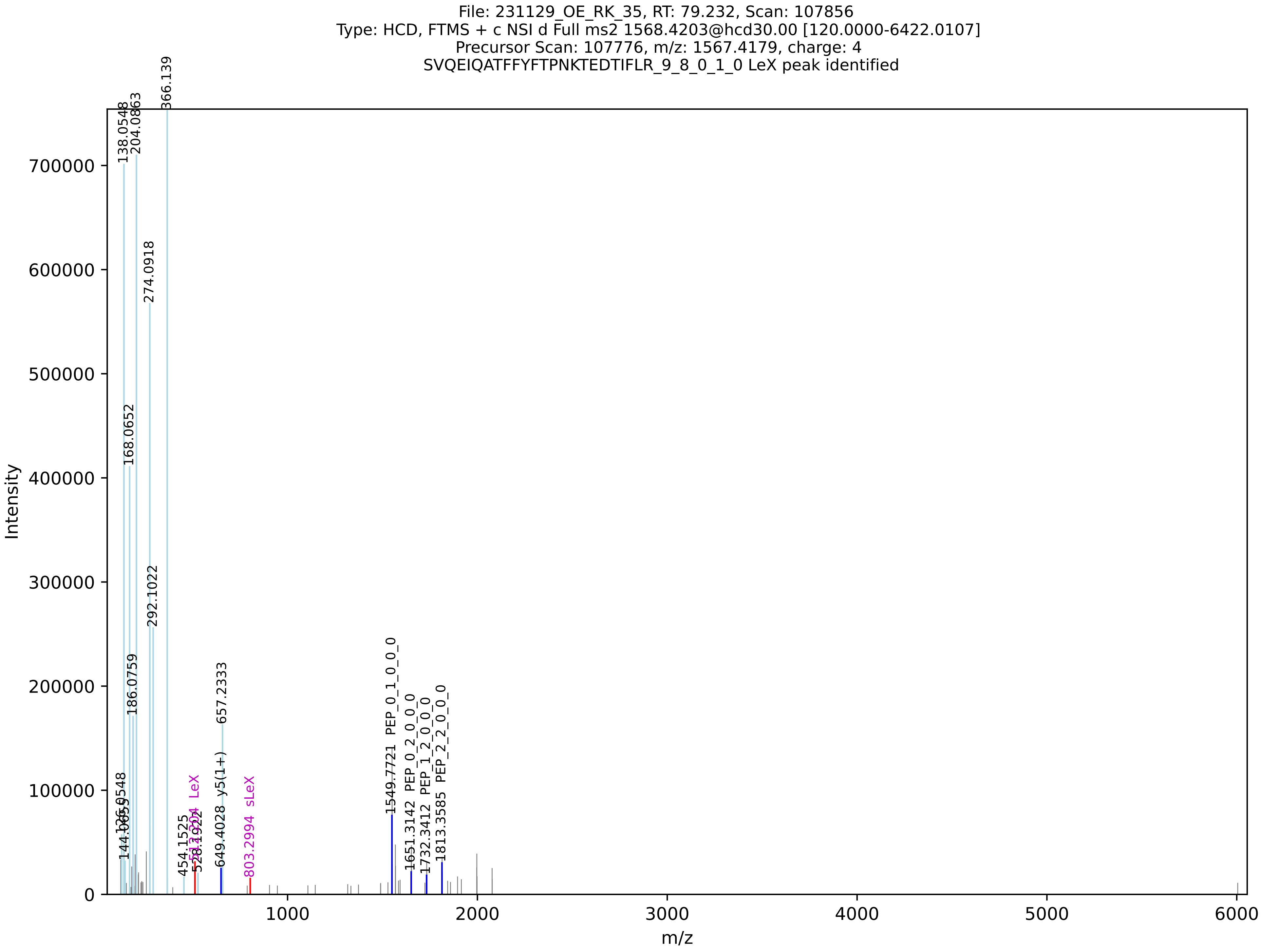
File: 231129_OE_RK_35, RT: 79.232, Scan: 107856
Type: HCD, FTMS + c NSI d Full ms2 1568.4203@hcd30.00 [120.0000-6422.0107]
Precursor Scan: 107776, m/z: 1567.4179, charge: 4
SVQEIQATFFYFTPNKTEDTIFLR_9_8_0_1_0 LeX peak identified
366.139
138.0548
204.0863
274.0918
168.0652
292.1022
186.0759
657.2333
126.0548
144.0655
454.1525
528.1922
649.4028 y5(1+)
803.2994 sLeX
1549.7721 PEP_0_1_0_0_0
1651.3142 PEP_0_2_0_0_0
1732.3412 PEP_1_2_0_0_0
1813.3585 PEP_2_2_0_0_0
Intensity
m/z

File: 231129_OE_RK_36, RT: 6.861, Scan: 002775
Type: HCD, FTMS + c NSI d Full ms2 1050.0261@hcd30.00 [120.0000-3235.2217]
Precursor Scan: 002758, m/z: 1050.0261, charge: 3
NEEYNK_5_4_1_2_0 LeX peak identified

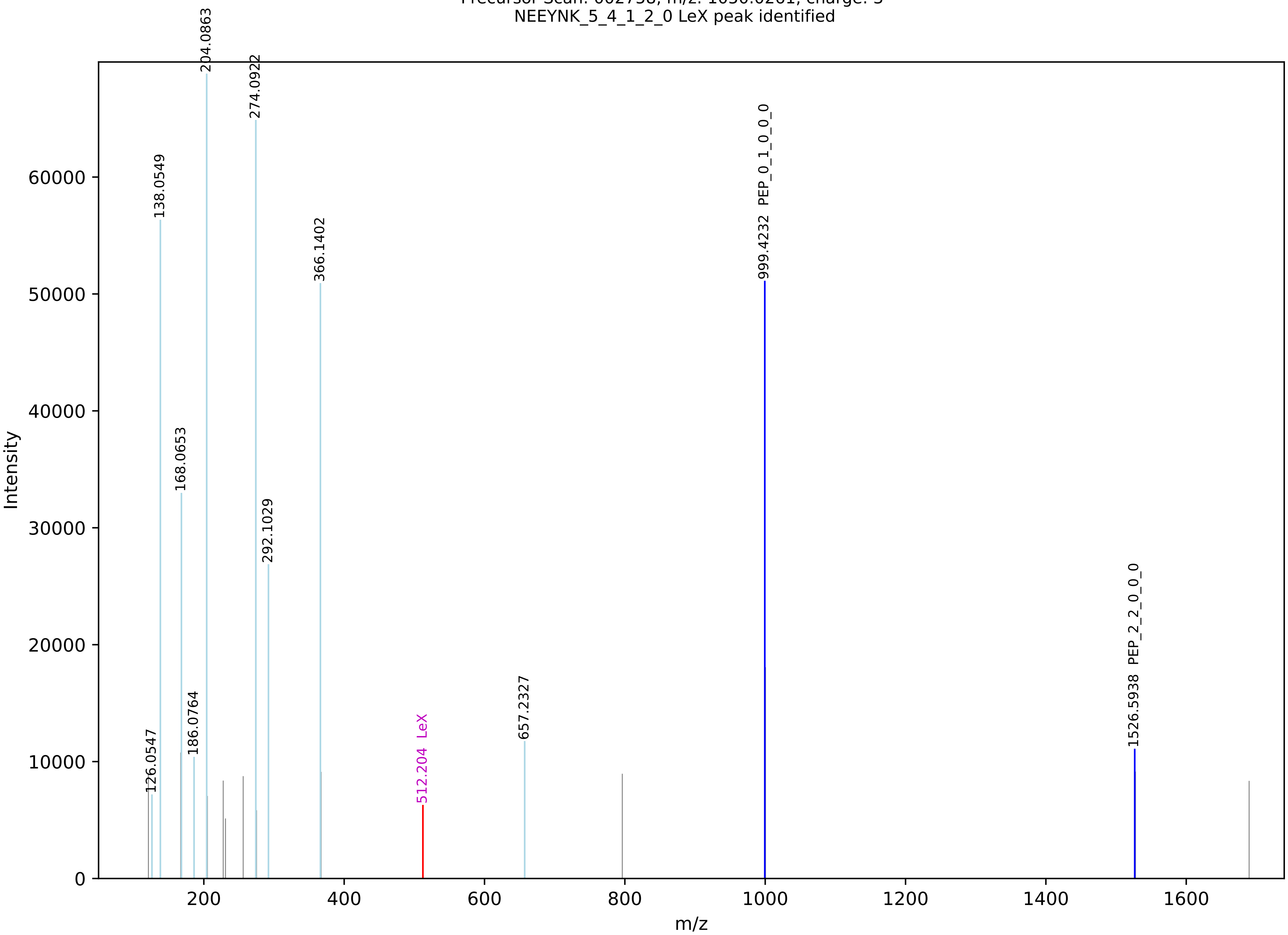

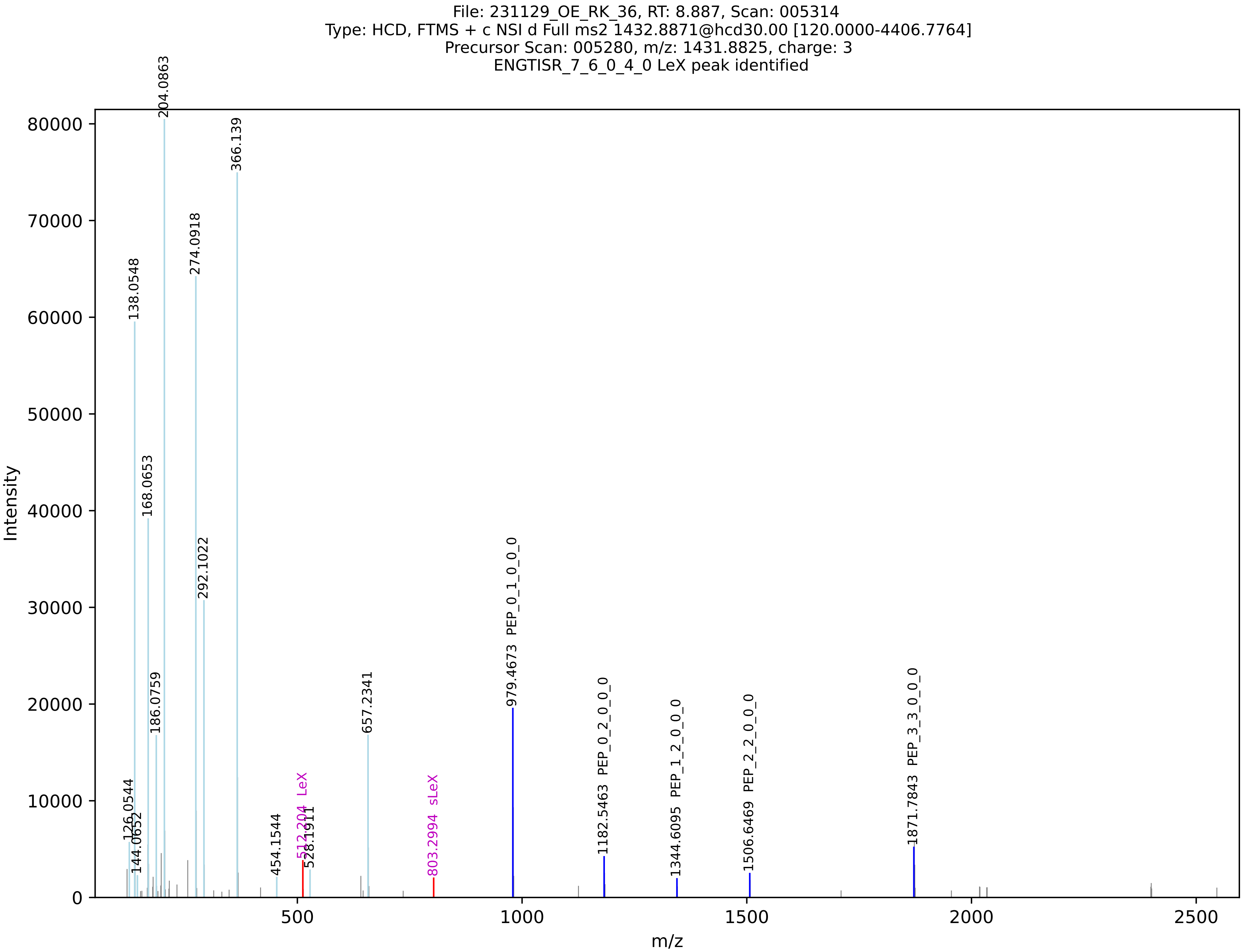
File: 231129_OE_RK_36, RT: 8.887, Scan: 005314
Type: HCD, FTMS + c NSI d Full ms2 1432.8871@hcd30.00 [120.0000-4406.7764]
Precursor Scan: 005280, m/z: 1431.8825, charge: 3
ENGTISR_7_6_0_4_0 LeX peak identified
Intensity
m/z
204.0863
366.139
274.0918
138.0548
168.0653
292.1022
186.0759
126.0544
144.0652
454.1544
512.204 LeX
528.1911
657.2341
803.2994 sLeX
979.4673 PEP_0_1_0_0_0
1182.5463 PEP_0_2_0_0_0
1344.6095 PEP_1_2_0_0_0
1506.6469 PEP_2_2_0_0_0
1871.7843 PEP_3_3_0_0_0

File: 231129_OE_RK_36, RT: 38.834, Scan: 046663
Type: HCD, FTMS + c NSI d Full ms2 1156.1405@hcd30.00 [120.0000-3559.9319]
Precursor Scan: 046624, m/z: 1155.4722, charge: 3
SWPAVGNCSSALR_5_4_1_1_0 LeX peak identified

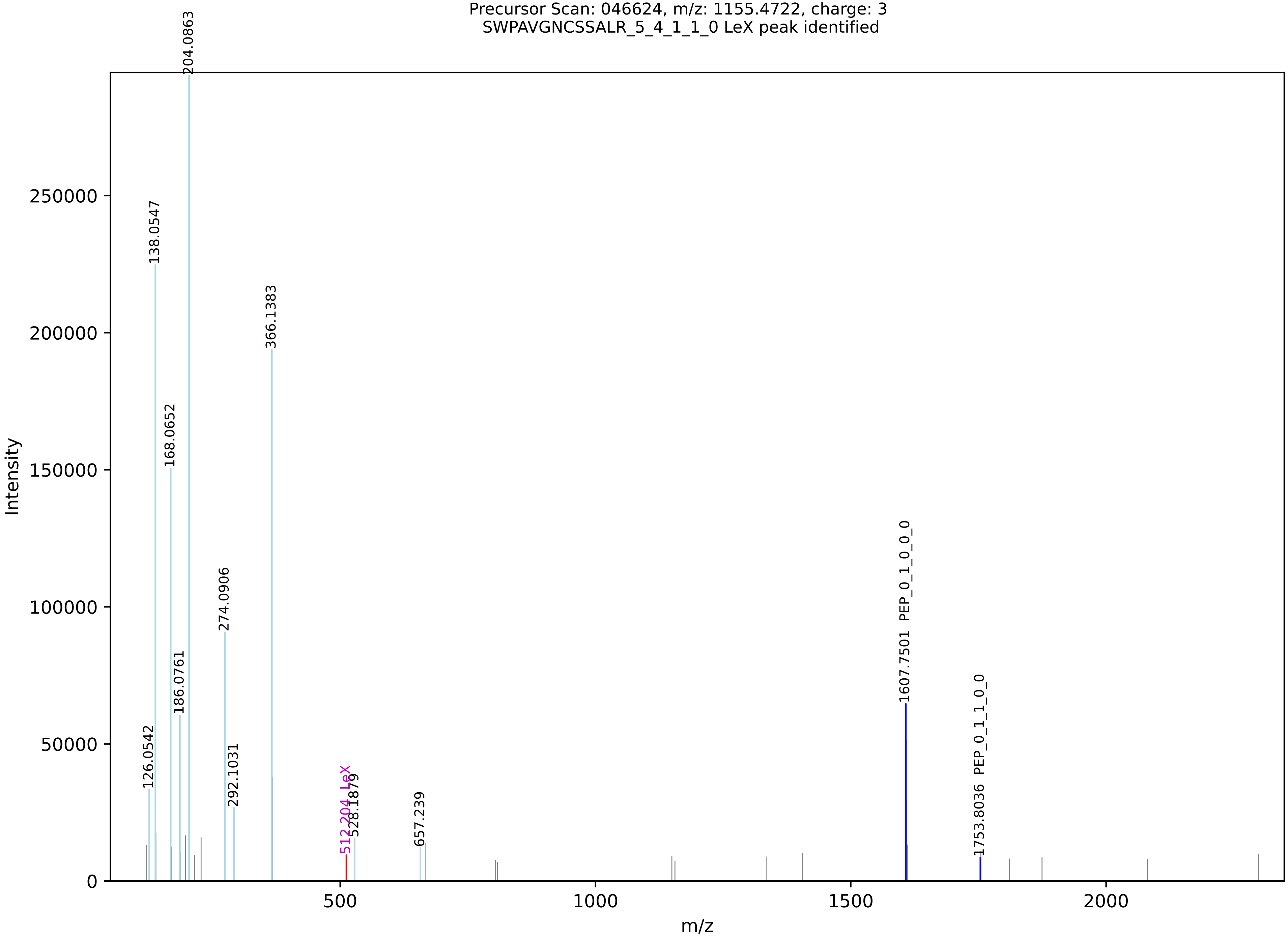

File: 231129_OE_RK_36, RT: 43.182, Scan: 052889
Type: HCD, FTMS + c NSI d Full ms2 1159.2214@hcd30.00 [120.0000-4752.4795]
Precursor Scan: 052809, m/z: 1158.7203, charge: 4
QDQCIYNTTYLNVQR_6_5_1_2_0 LeX peak identified

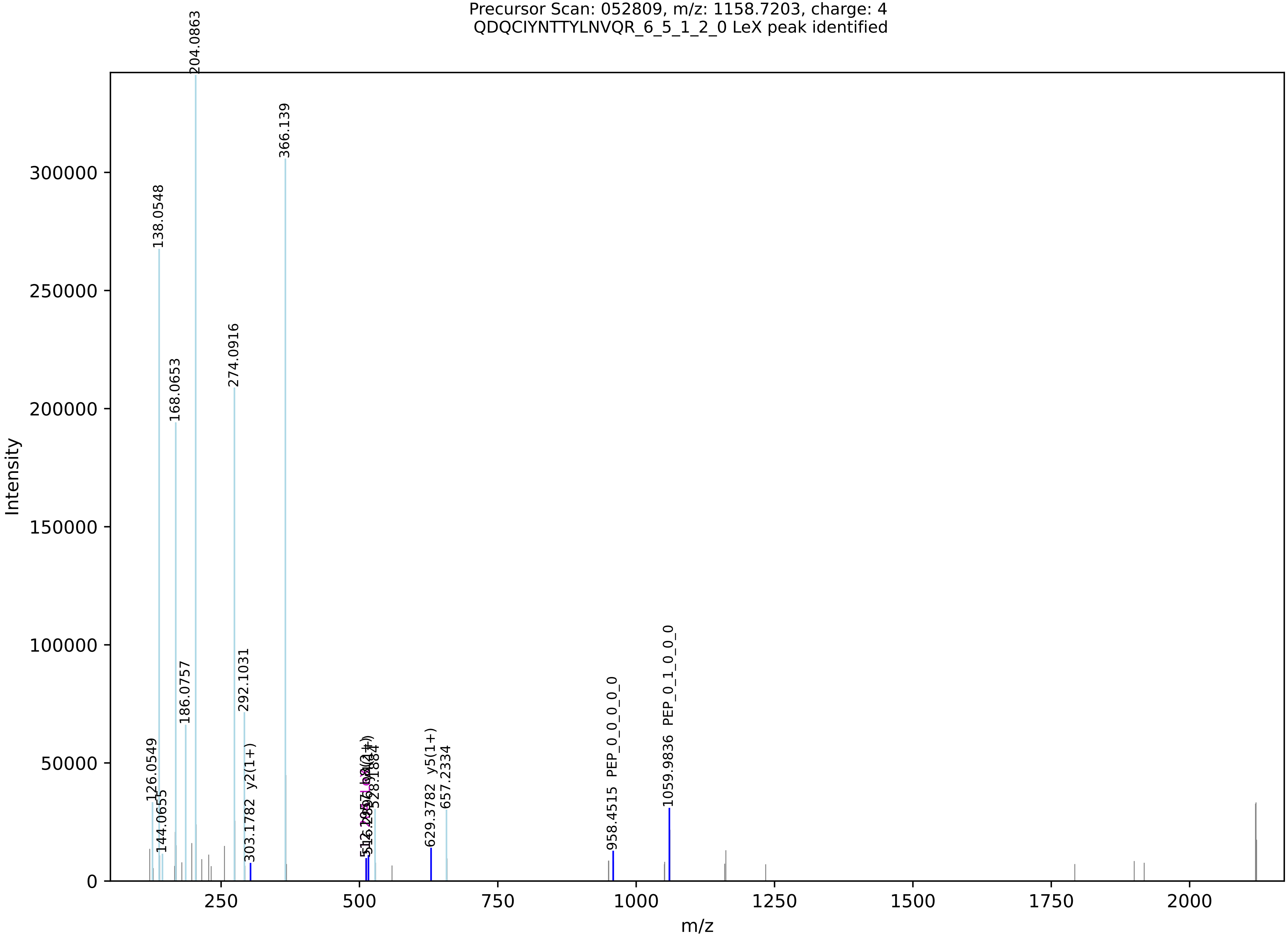

File: 231129_OE_RK_36, RT: 44.472, Scan: 054757
Type: HCD, FTMS + c NSI d Full ms2 1360.0443@hcd30.00 [120.0000-5571.8369]
Precursor Scan: 054678, m/z: 1359.2926, charge: 4
QDQCIYNTTYLNVQR_7_6_2_3_0 LeX peak identified

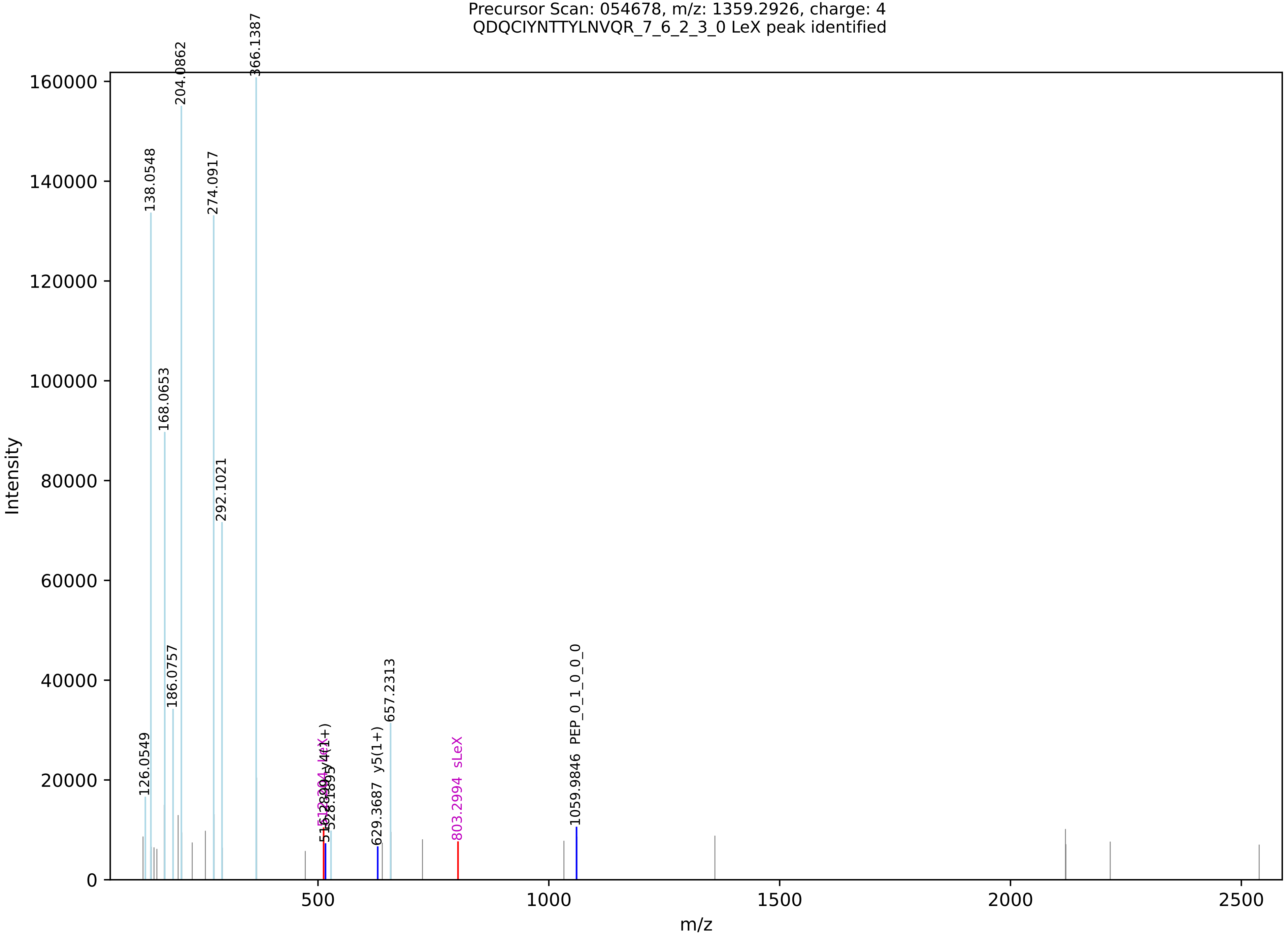

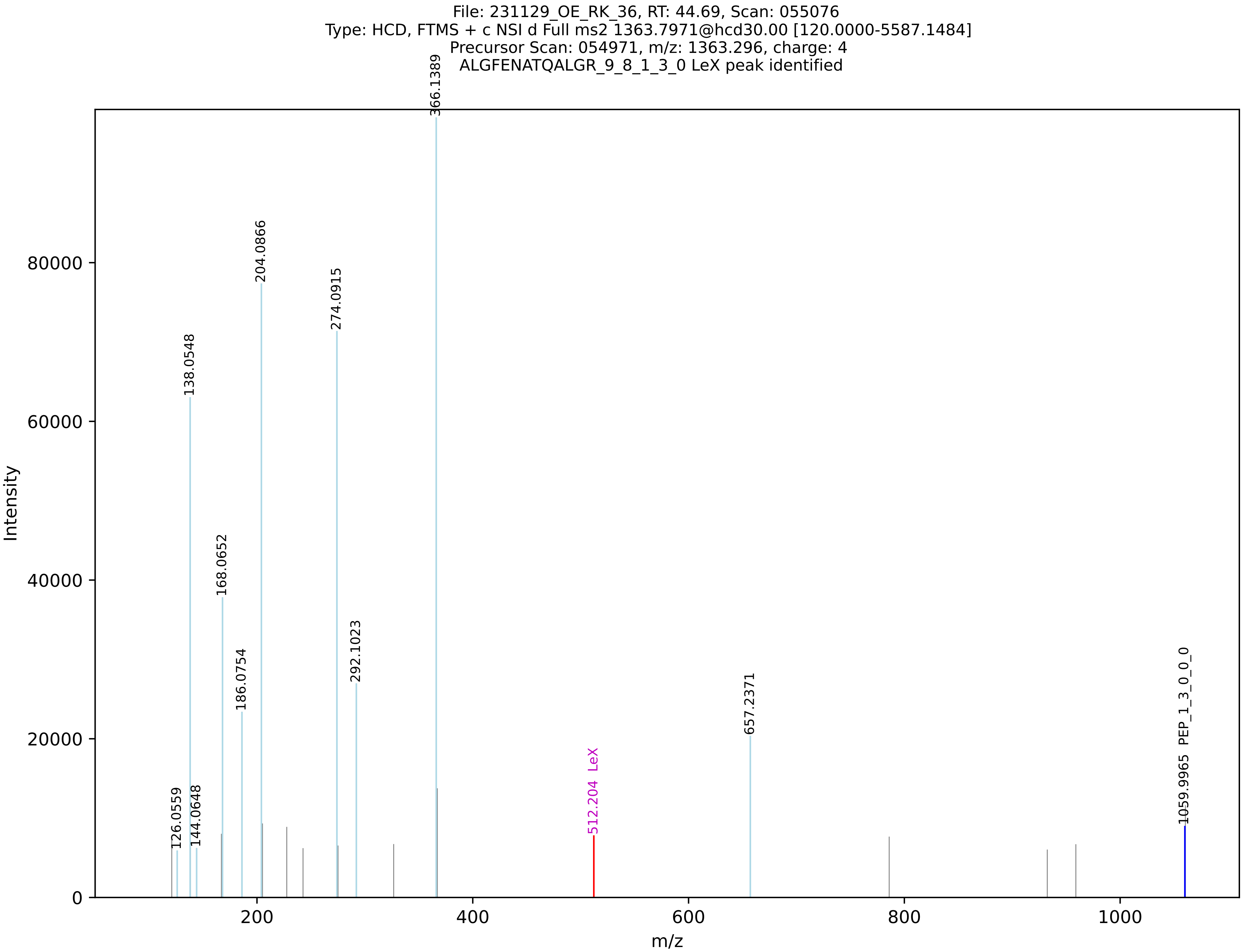
File: 231129_OE_RK_36, RT: 44.69, Scan: 055076
Type: HCD, FTMS + c NSI d Full ms2 1363.7971@hcd30.00 [120.0000-5587.1484]
Precursor Scan: 054971, m/z: 1363.296, charge: 4
ALGFENATQALGR_9_8_1_3_0 LeX peak identified
Intensity
m/z
0
20000
40000
60000
80000
200
400
600
800
1000
366.1389
204.0866
274.0915
138.0548
168.0652
292.1023
186.0754
657.2371
PEP_1_3_0_0_0
1059.9965
512.204 LeX
126.0559
144.0648

File: 231129_OE_RK_36, RT: 45.22, Scan: 055843
Type: HCD, FTMS + c NSI d Full ms2 1359.7931@hcd30.00 [120.0000-5570.8120]
Precursor Scan: 055781, m/z: 1359.288, charge: 4
QDQCIYNTTYLNVQR_7_6_0_4_0 LeX peak identified

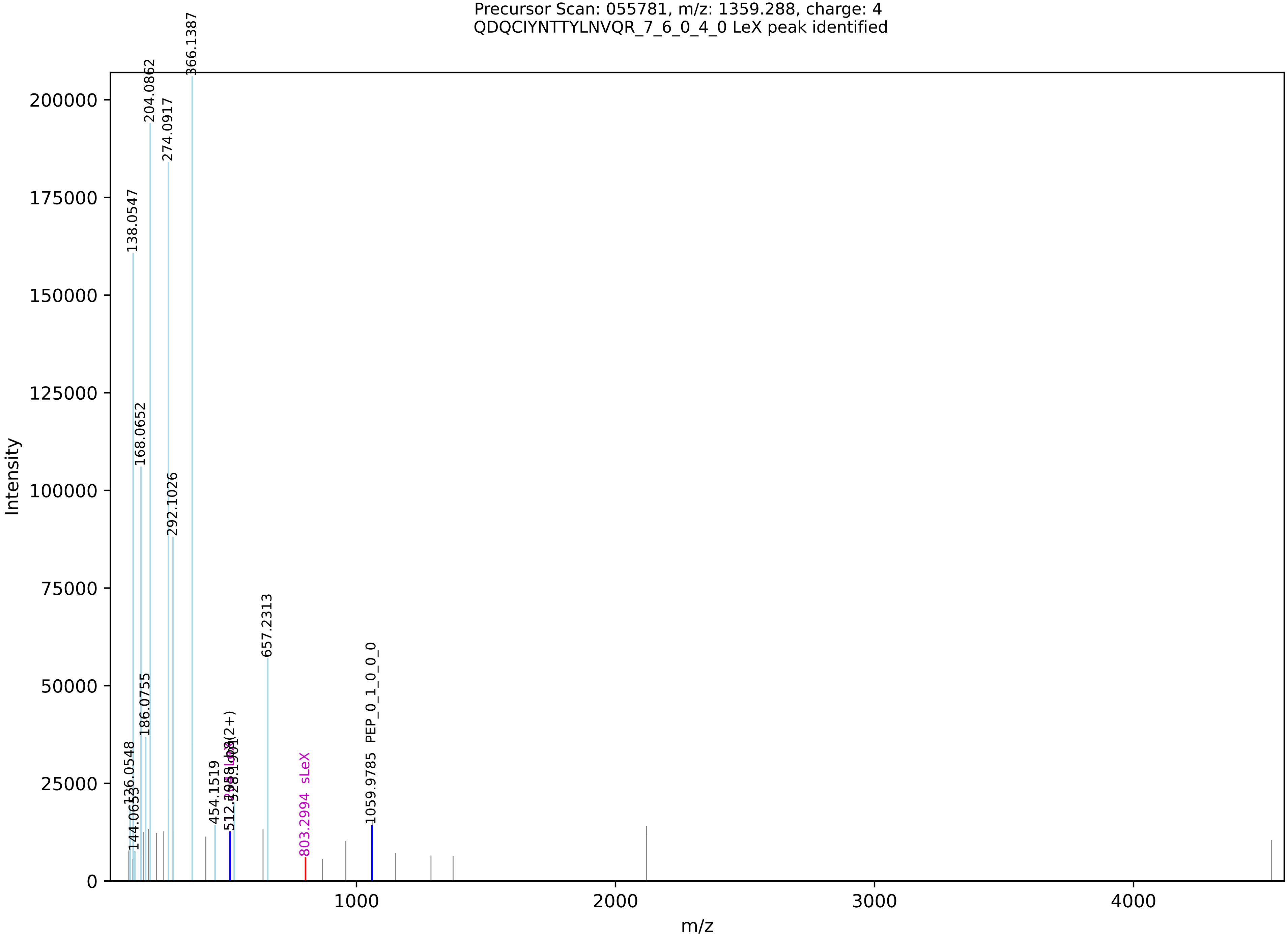

File: 231129_OE_RK_36, RT: 46.941, Scan: 058331
Type: HCD, FTMS + c NSI d Full ms2 1396.3048@hcd30.00 [120.0000-5719.7798]
Precursor Scan: 058250, m/z: 1395.5517, charge: 4
QDQCIYNTTYLNVQR_7_6_1_4_0 LeX peak identified

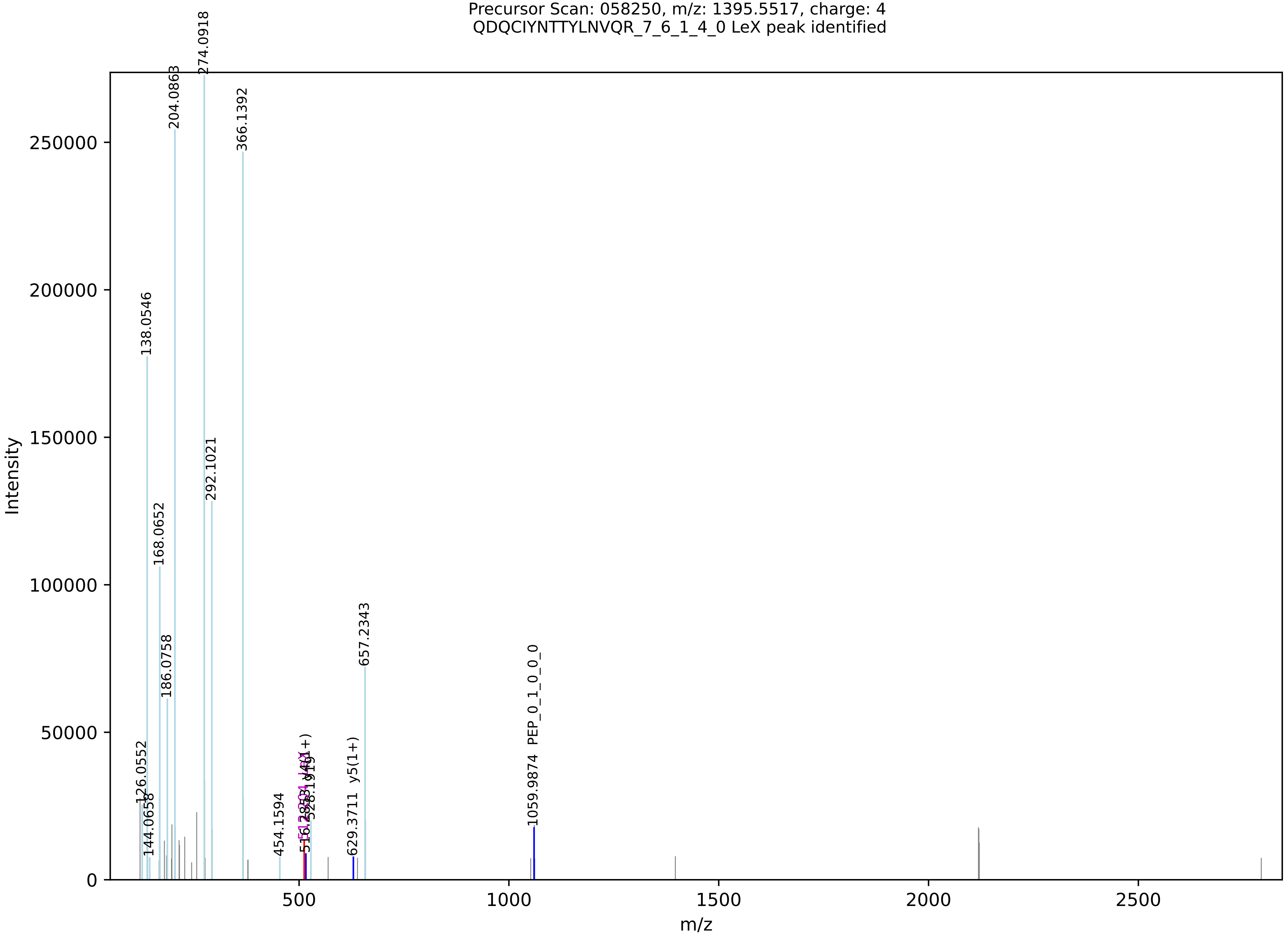

File: 231129_OE_RK_36, RT: 48.76, Scan: 060916
Type: HCD, FTMS + c NSI d Full ms2 1396.0519@hcd30.00 [120.0000-5718.7476]
Precursor Scan: 060835, m/z: 1395.5509, charge: 4
QDQCIYNTTYLNVQR_7_6_1_4_0 LeX peak identified

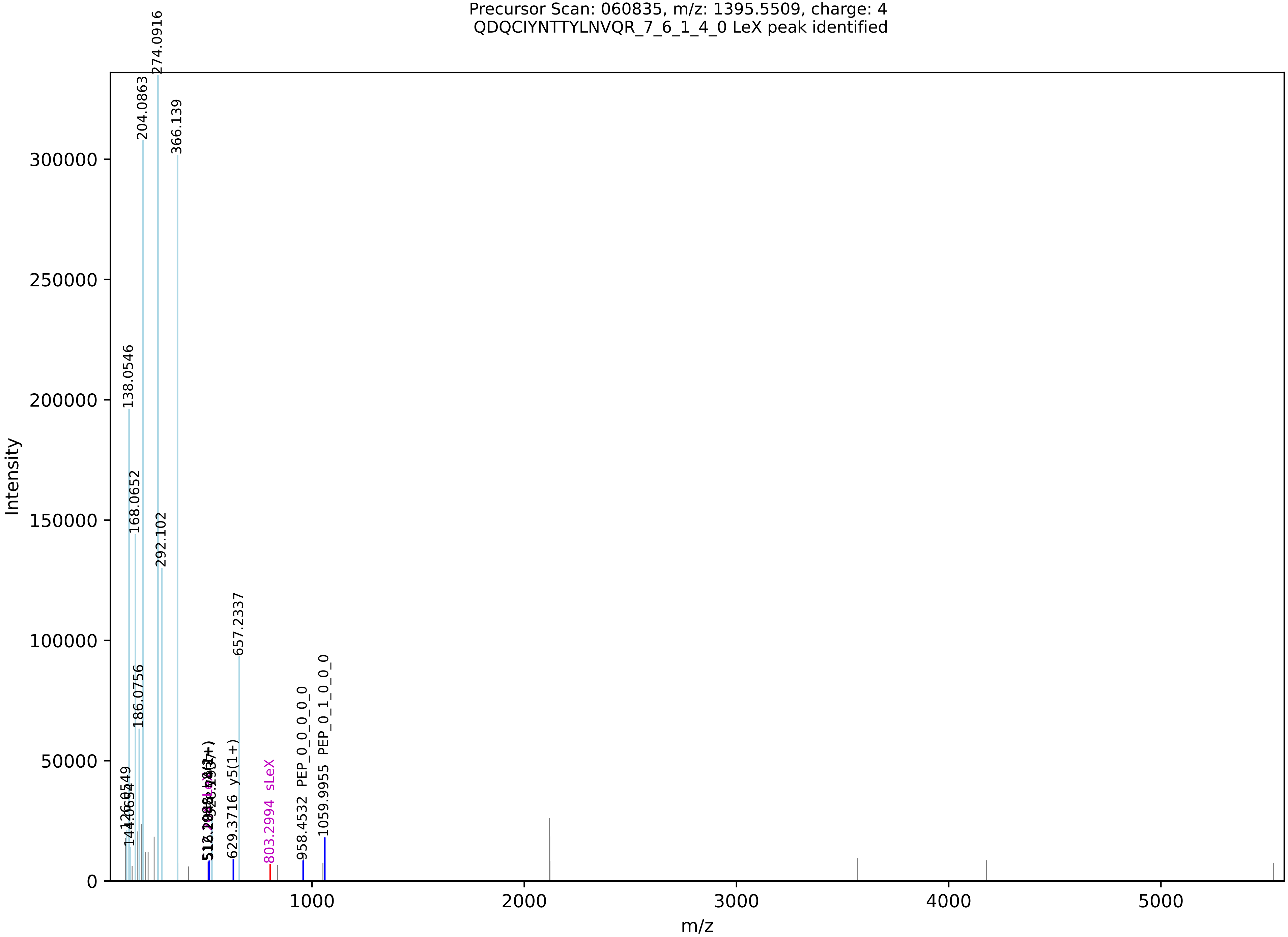

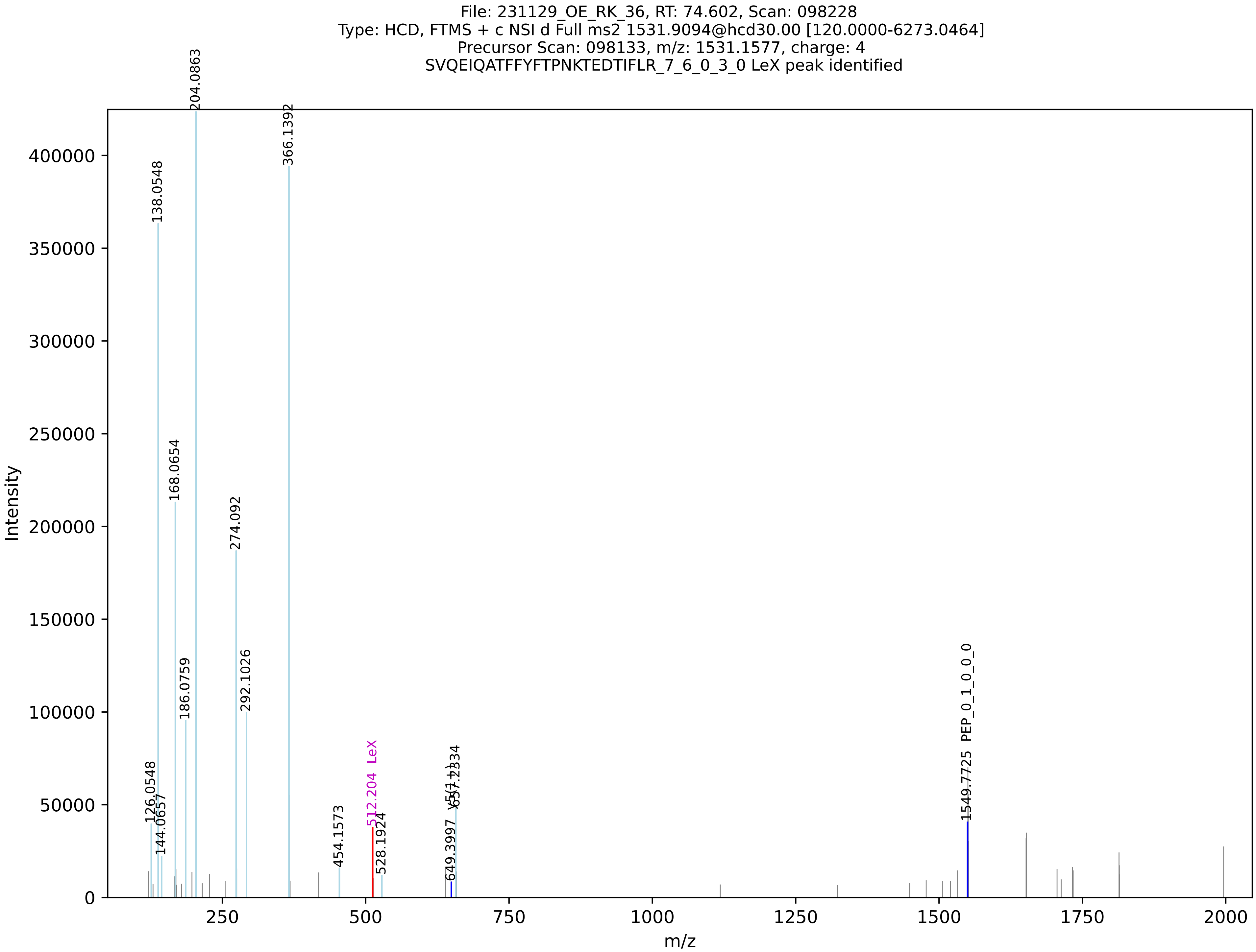
File: 231129_OE_RK_36, RT: 74.602, Scan: 098228
Type: HCD, FTMS + c NSI d Full ms2 1531.9094@hcd30.00 [120.0000-6273.0464]
Precursor Scan: 098133, m/z: 1531.1577, charge: 4
SVQEIQATFFYFTPNKTEDTIFLR_7_6_0_3_0 LeX peak identified
Intensity
m/z
0
50000
100000
150000
200000
250000
300000
350000
400000
250
500
750
1000
1250
1500
1750
2000
204.0863
366.1392
138.0548
168.0654
274.092
186.0759
292.1026
126.0548
144.0657
454.1573
512.204 LeX
528.1924
649.3997 y5(1+)
657.2334
1549.7725 PEP_0_1_0_0_0_

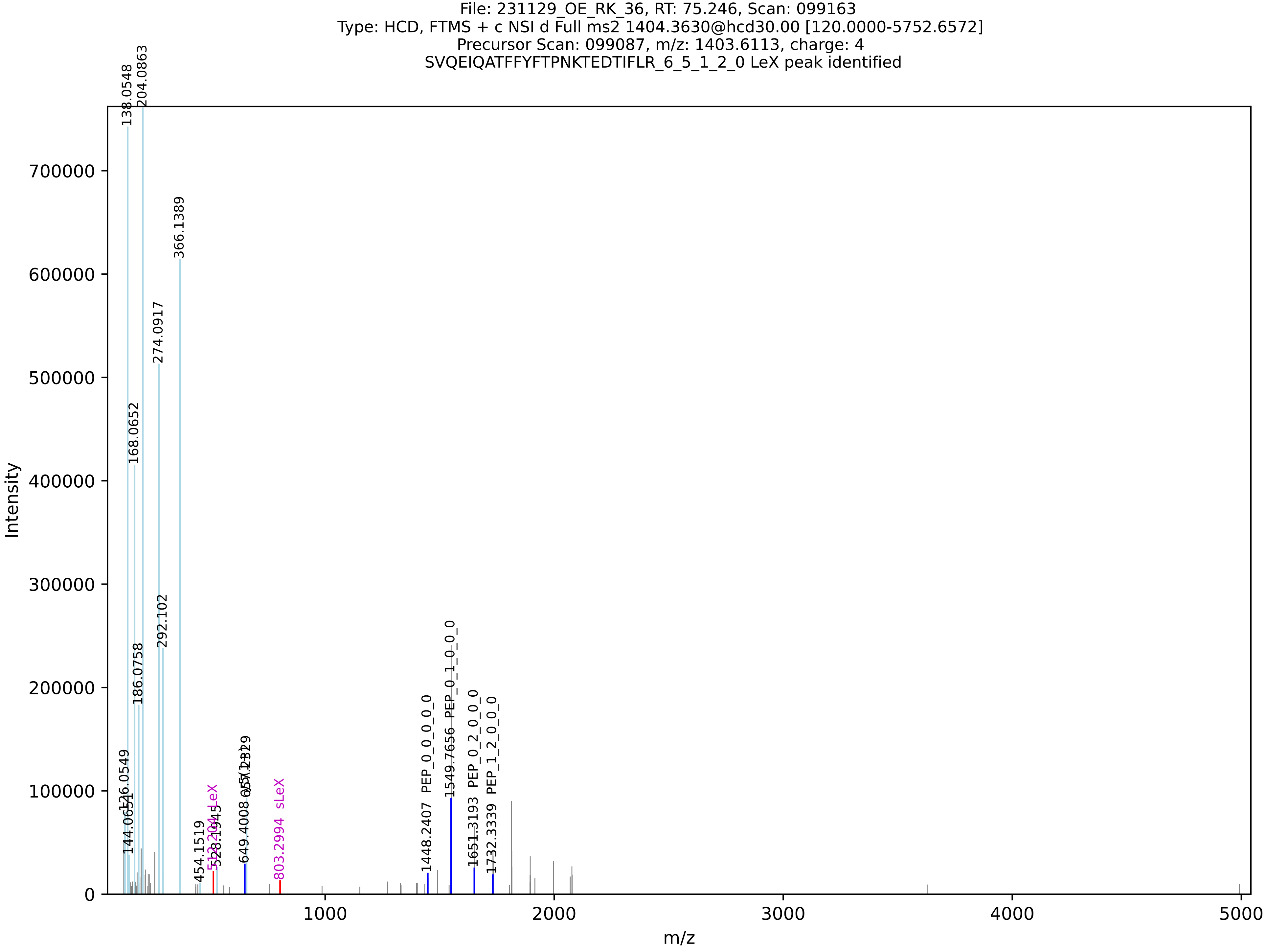

File: 231129_OE_RK_36, RT: 75.246, Scan: 099163
Type: HCD, FTMS + c NSI d Full ms2 1404.3630@hcd30.00 [120.0000-5752.6572]
Precursor Scan: 099087, m/z: 1403.6113, charge: 4
SVQEIQATFFYFTPNKTEDTIFLR_6_5_1_2_0 LeX peak identified
Intensity
m/z
138.0548
204.0863
366.1389
274.0917
168.0652
292.102
186.0758
126.0549
144.0651
454.1519
512.2004 LeX
528.1945
649.4008
657.2329
803.2994 sLeX
1448.2407 PEP_0_0_0_0_0_
1549.7656 PEP_0_1_0_0_0_
1651.3193 PEP_0_2_0_0_0_
1732.3339 PEP_1_2_0_0_0_

File: 231129_OE_RK_37, RT: 8.874, Scan: 006913
Type: HCD, FTMS + c NSI d Full ms2 1384.9227@hcd30.00 [120.0000-4260.0059]
Precursor Scan: 006767, m/z: 1384.9228, charge: 3
ENGTISR_9_8_0_1_0 LeX peak identified

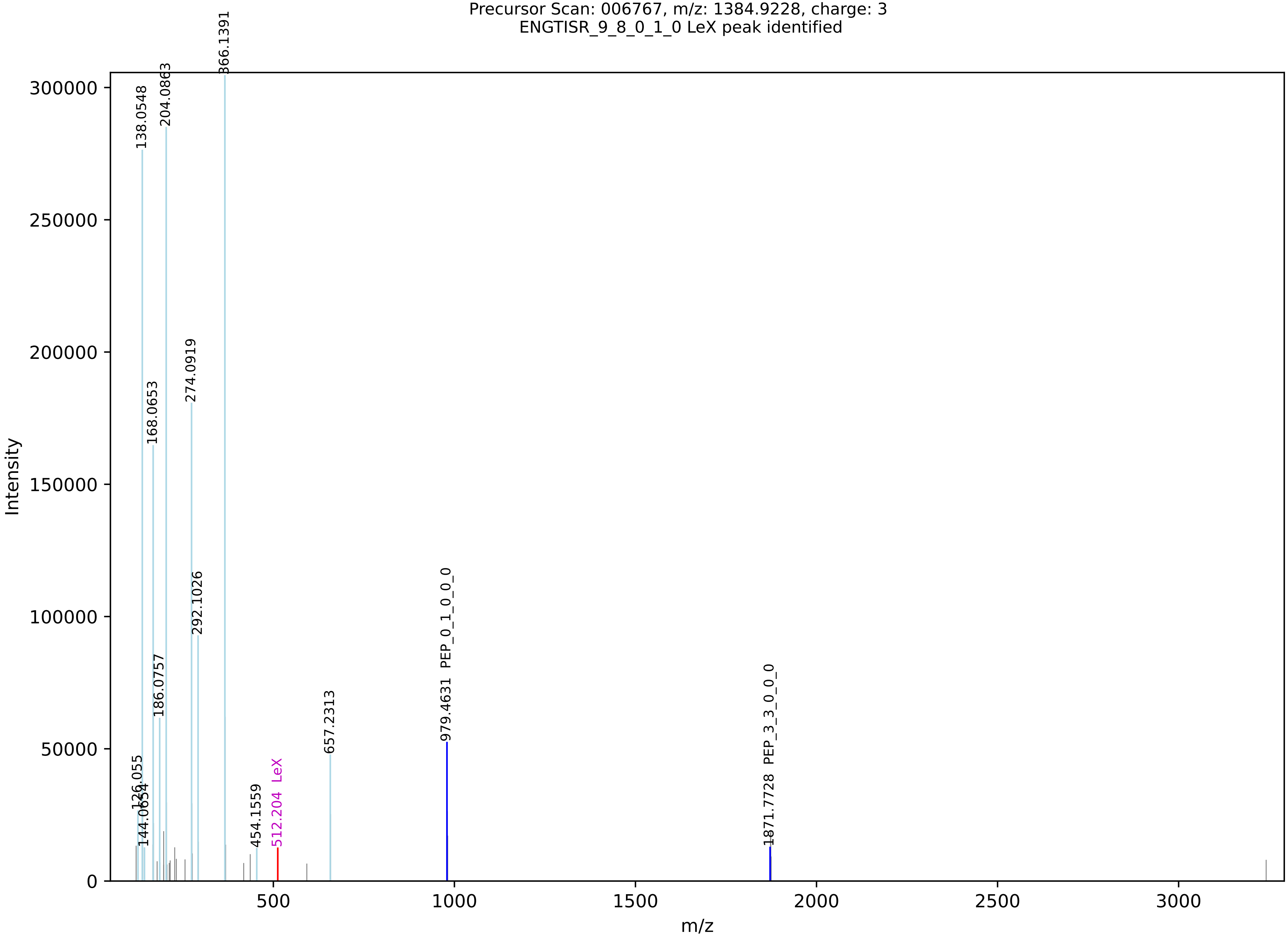

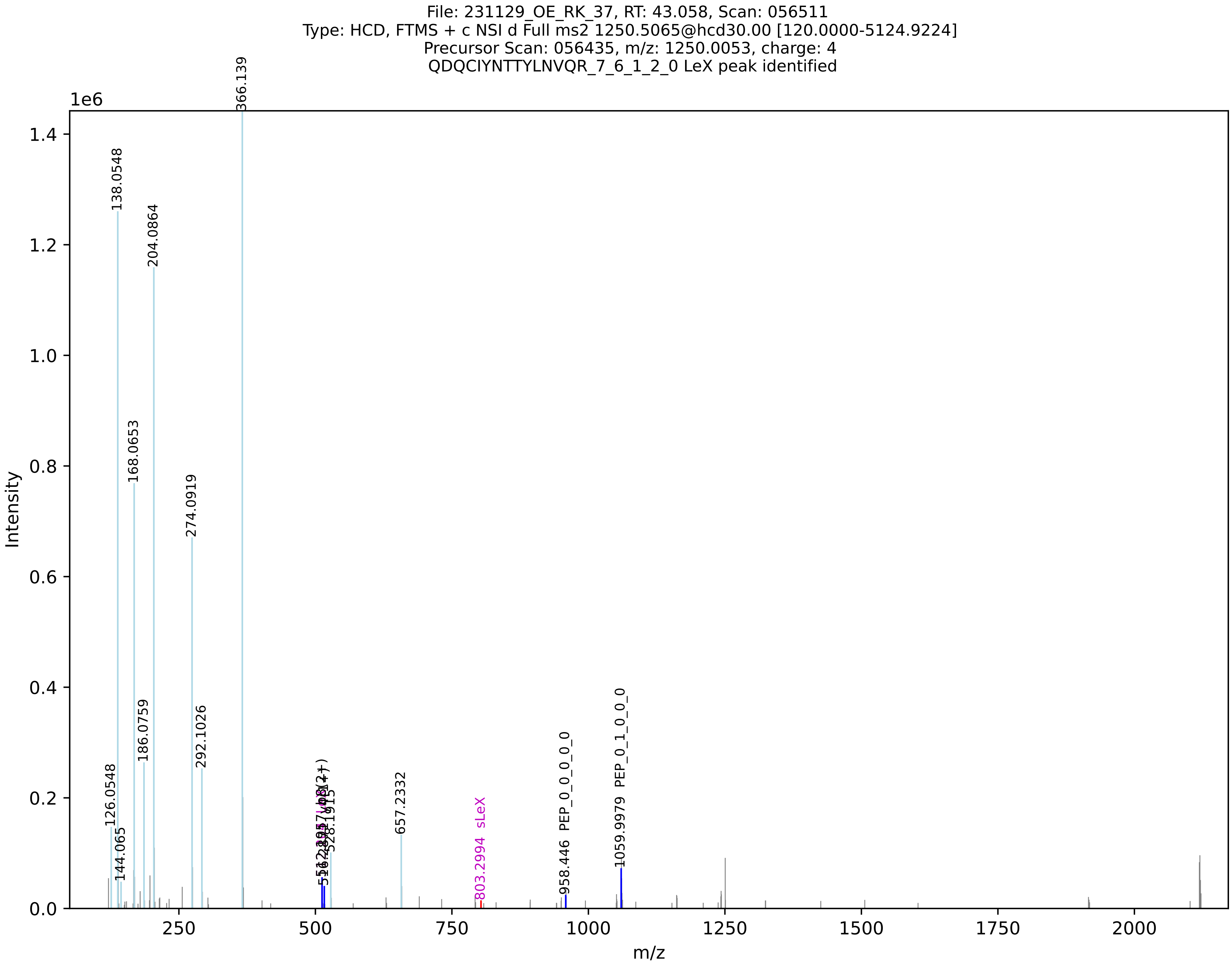
File: 231129_OE_RK_37, RT: 43.058, Scan: 056511
Type: HCD, FTMS + c NSI d Full ms2 1250.5065@hcd30.00 [120.0000-5124.9224]
Precursor Scan: 056435, m/z: 1250.0053, charge: 4
QDQCIYNTTYLNVQR_7_6_1_2_0 LeX peak identified
1e6
Intensity
m/z
366.139
138.0548
204.0864
168.0653
274.0919
186.0759
292.1026
126.0548
144.065
657.2332
803.2994 sLeX
958.446 PEP_0_0_0_0_0_
1059.9979 PEP_0_1_0_0_0_
1.4
1.2
1.0
0.8
0.6
0.4
0.2
0.0
250
500
750
1000
1250
1500
1750
2000

File: 231129_OE_RK_37, RT: 43.219, Scan: 056748
Type: HCD, FTMS + c NSI d Full ms2 1287.2732@hcd30.00 [120.0000-5274.9302]
Precursor Scan: 056654, m/z: 1286.5214, charge: 4
QDQCIYNTTYLNVQR_7_6_0_3_0 LeX peak identified

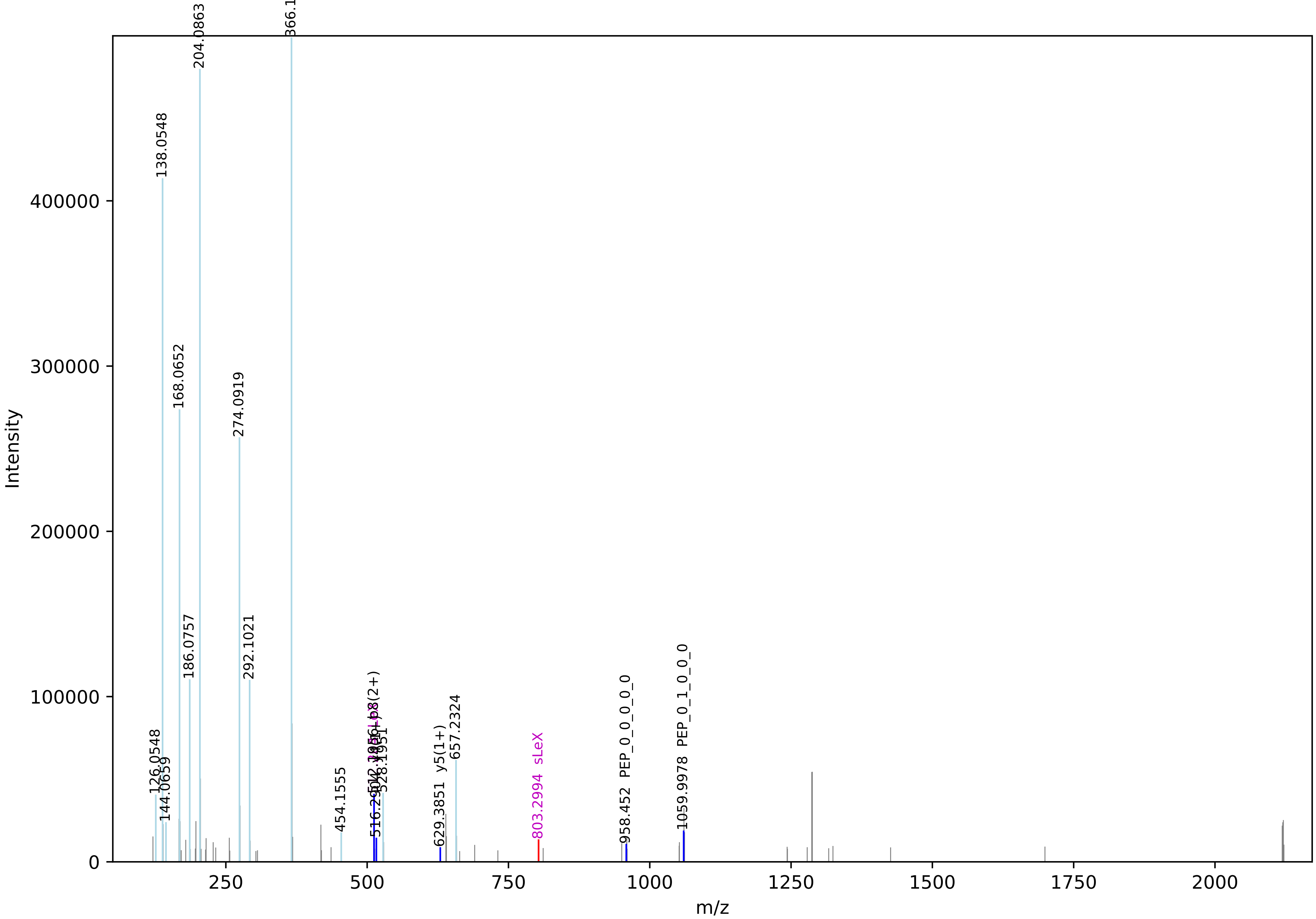

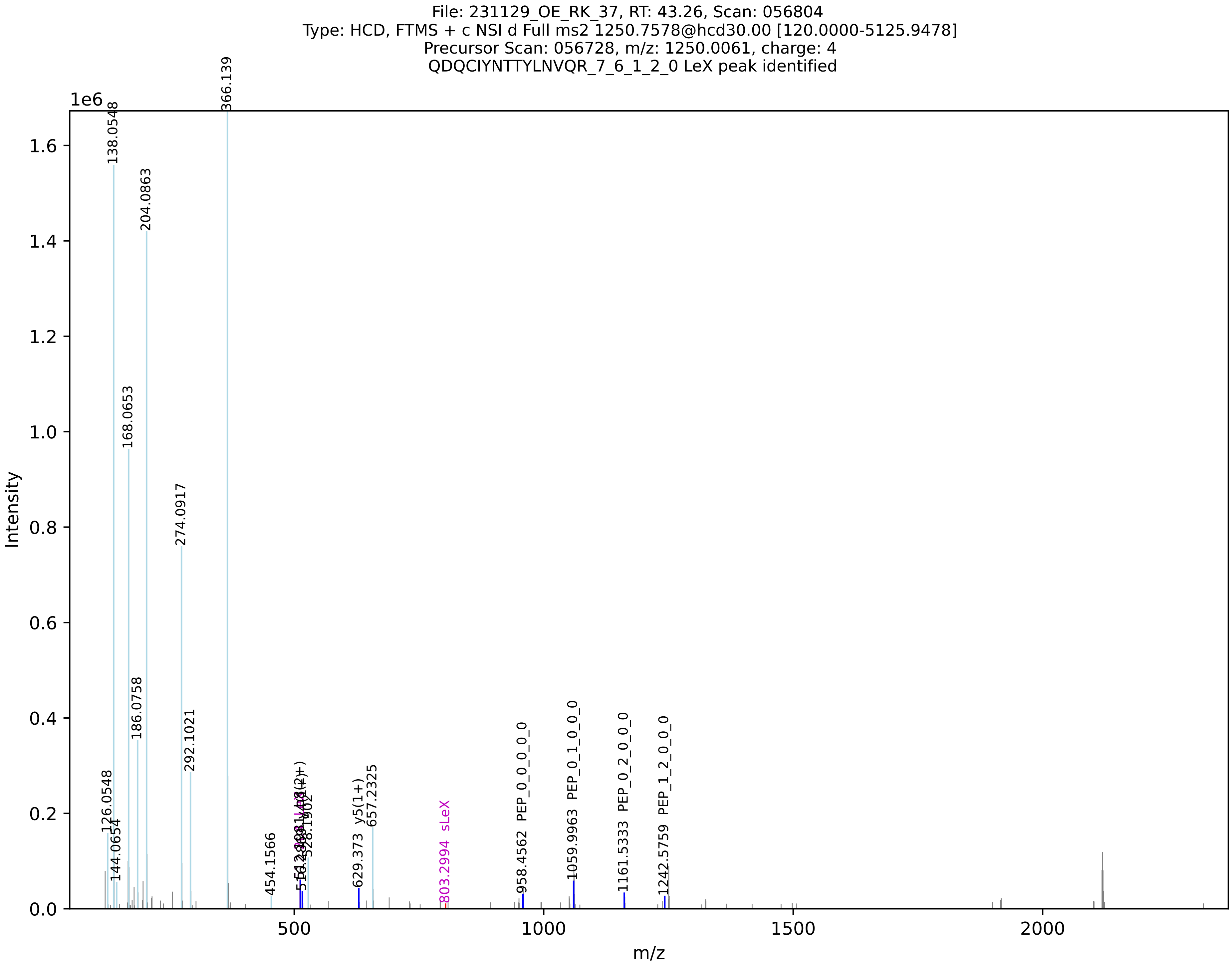
File: 231129_OE_RK_37, RT: 43.26, Scan: 056804
Type: HCD, FTMS + c NSI d Full ms2 1250.7578@hcd30.00 [120.0000-5125.9478]
Precursor Scan: 056728, m/z: 1250.0061, charge: 4
QDQCIYNTTYLNVQR_7_6_1_2_0 LeX peak identified
1e6
Intensity
m/z
366.139
138.0548
204.0863
168.0653
274.0917
186.0758
292.1021
126.0548
144.0654
454.1566
629.373 y5(1+)
657.2325
803.2994 sLeX
958.4562 PEP_0_0_0_0_0
1059.9963 PEP_0_1_0_0_0
1161.5333 PEP_0_2_0_0_0
1242.5759 PEP_1_2_0_0_0

File: 231129_OE_RK_37, RT: 43.46, Scan: 057096
Type: HCD, FTMS + c NSI d Full ms2 1250.7571@hcd30.00 [120.0000-5125.9448]
Precursor Scan: 057021, m/z: 1250.0055, charge: 4
QDQCIYNTTYLNVQR_7_6_1_2_0 LeX peak identified

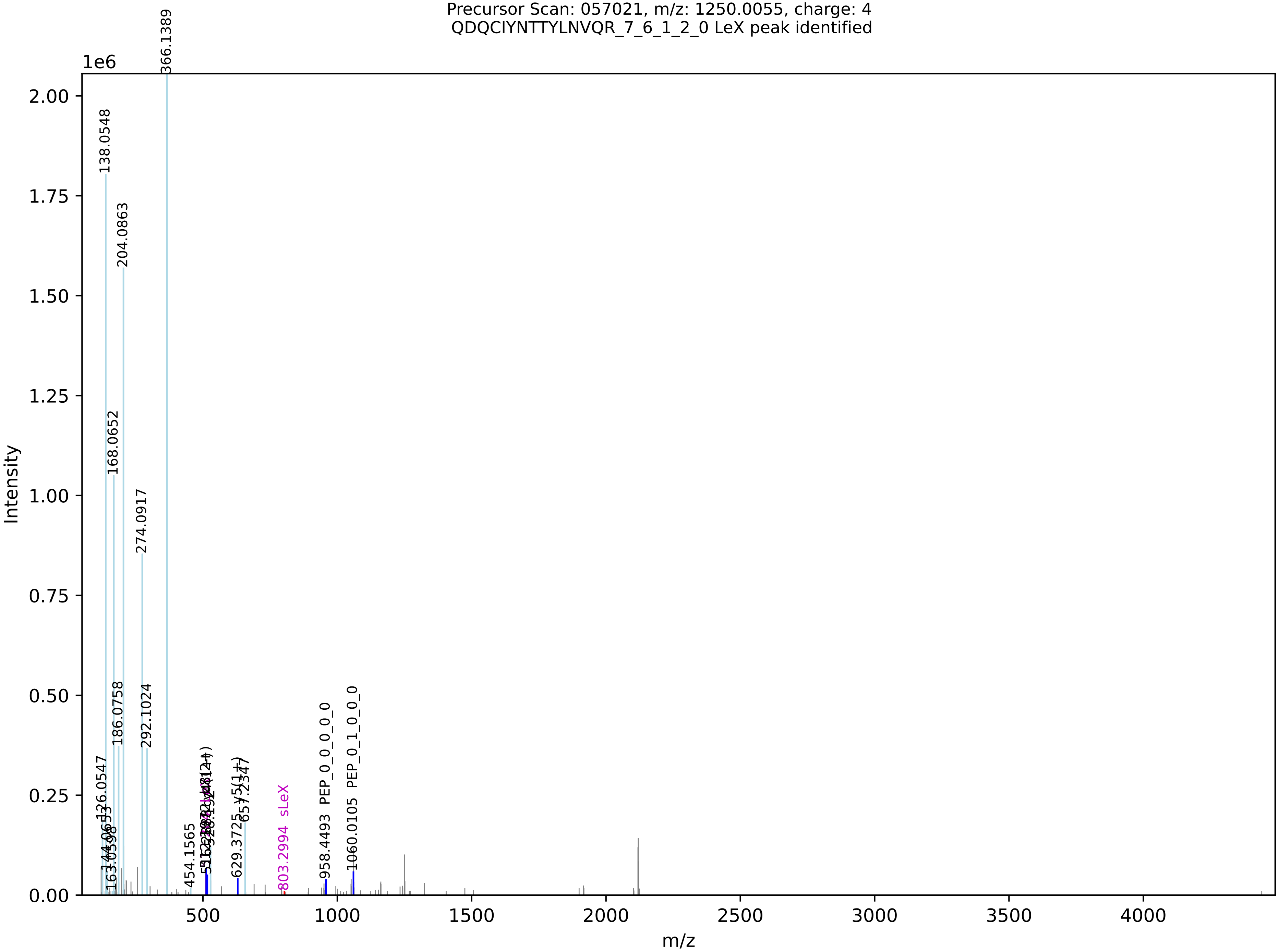

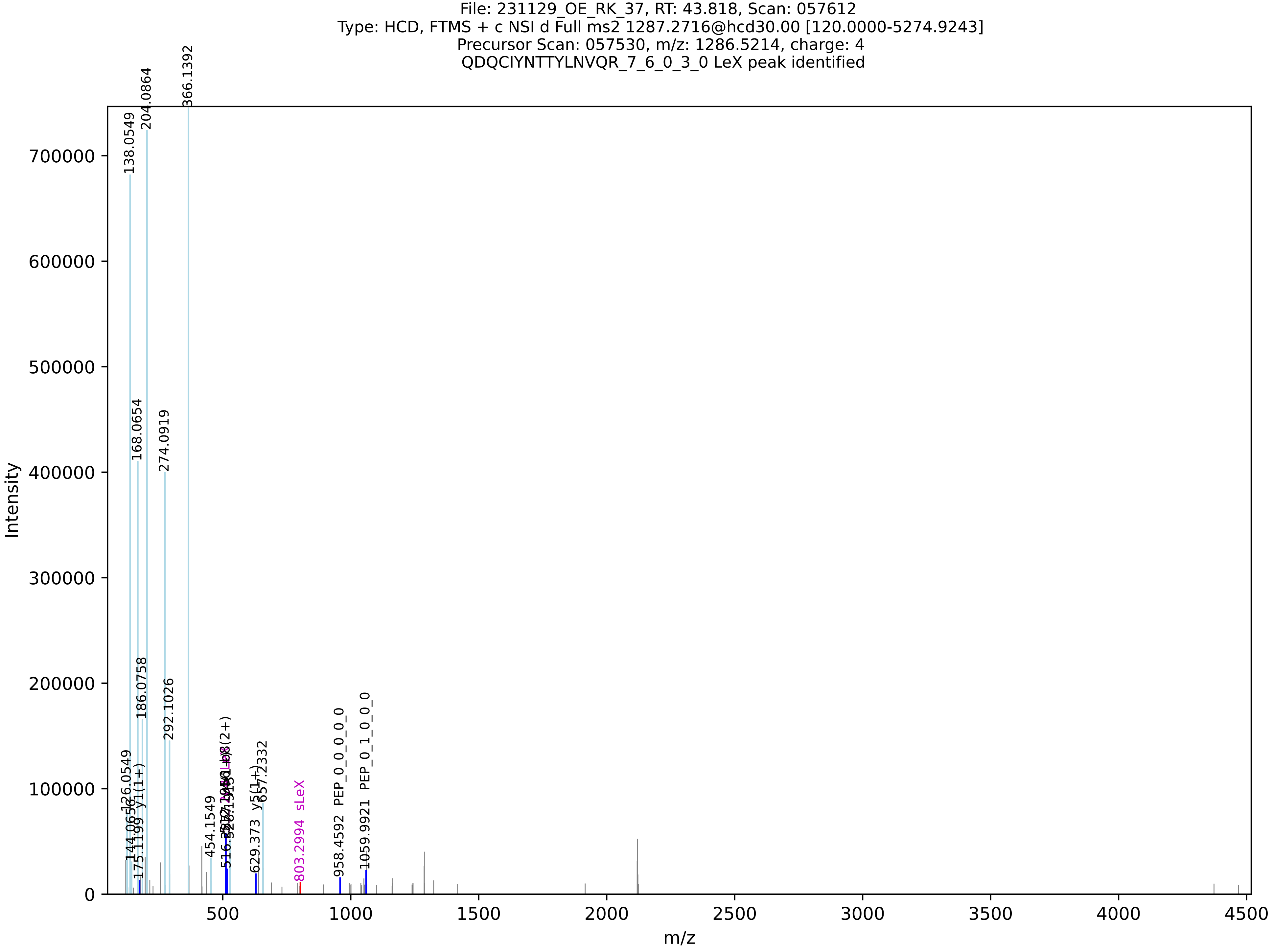
File: 231129_OE_RK_37, RT: 43.818, Scan: 057612
Type: HCD, FTMS + c NSI d Full ms2 1287.2716@hcd30.00 [120.0000-5274.9243]
Precursor Scan: 057530, m/z: 1286.5214, charge: 4
QDQCIYNTTYLNVQR_7_6_0_3_0 LeX peak identified
Intensity
m/z
366.1392
204.0864
138.0549
168.0654
274.0919
186.0758
292.1026
126.0549
144.0656
175.1199 y1(1+)
454.1549
629.373 y5(1+)
657.2332
803.2994 sLeX
958.4592 PEP_0_0_0_0_0_
1059.9921 PEP_0_1_0_0_0_

File: 231129_OE_RK_37, RT: 43.863, Scan: 057677
Type: HCD, FTMS + c NSI d Full ms2 1250.5065@hcd30.00 [120.0000-5124.9224]
Precursor Scan: 057604, m/z: 1250.0053, charge: 4
QDQCIYNTTYLNVQR_7_6_1_2_0 LeX peak identified

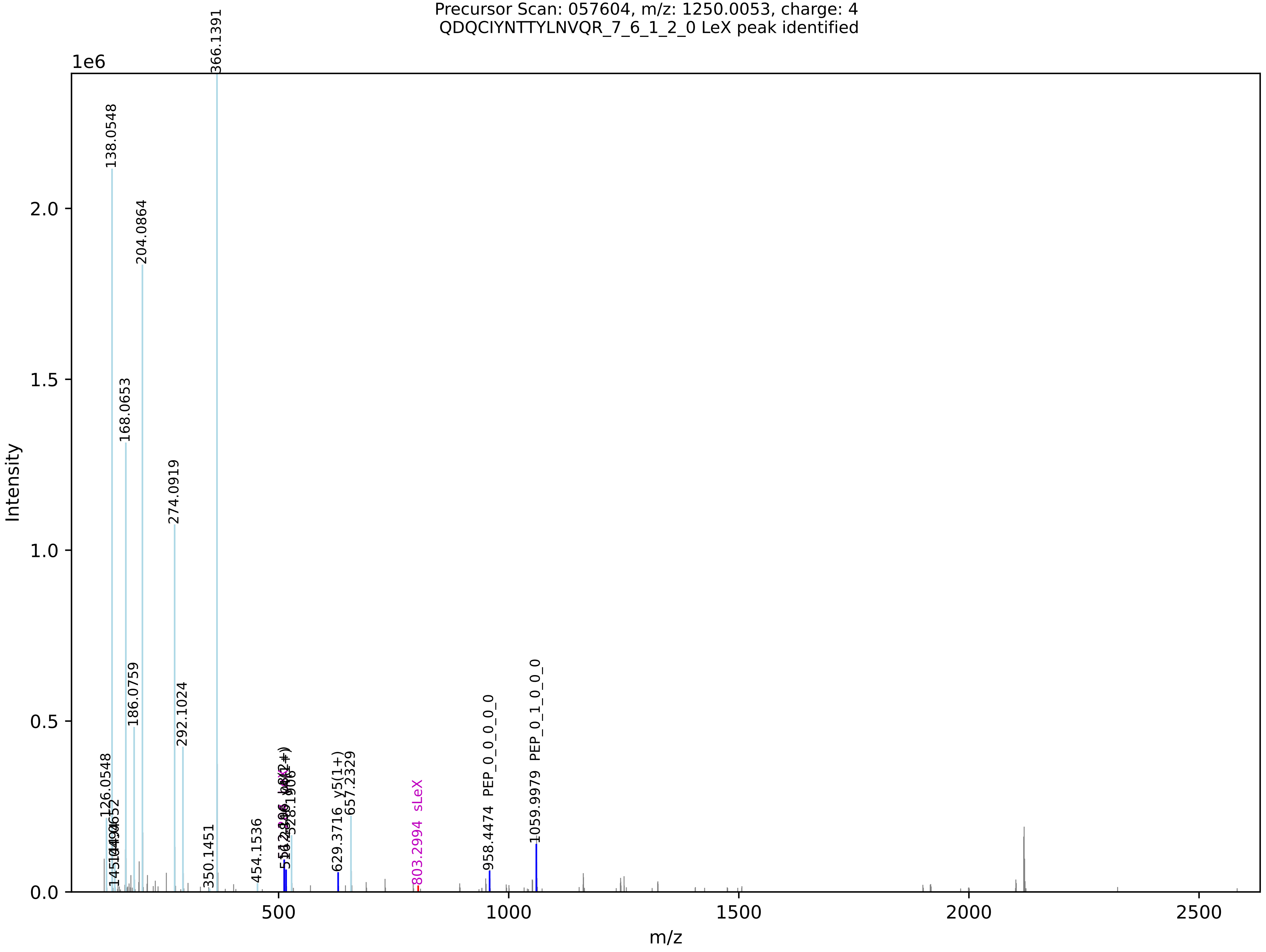

File: 231129_OE_RK_37, RT: 44.267, Scan: 058262
Type: HCD, FTMS + c NSI d Full ms2 1250.5062@hcd30.00 [120.0000-5124.9214]
Precursor Scan: 058190, m/z: 1250.0032, charge: 4
QDQCIYNTTYLNVQR_7_6_1_2_0 LeX peak identified

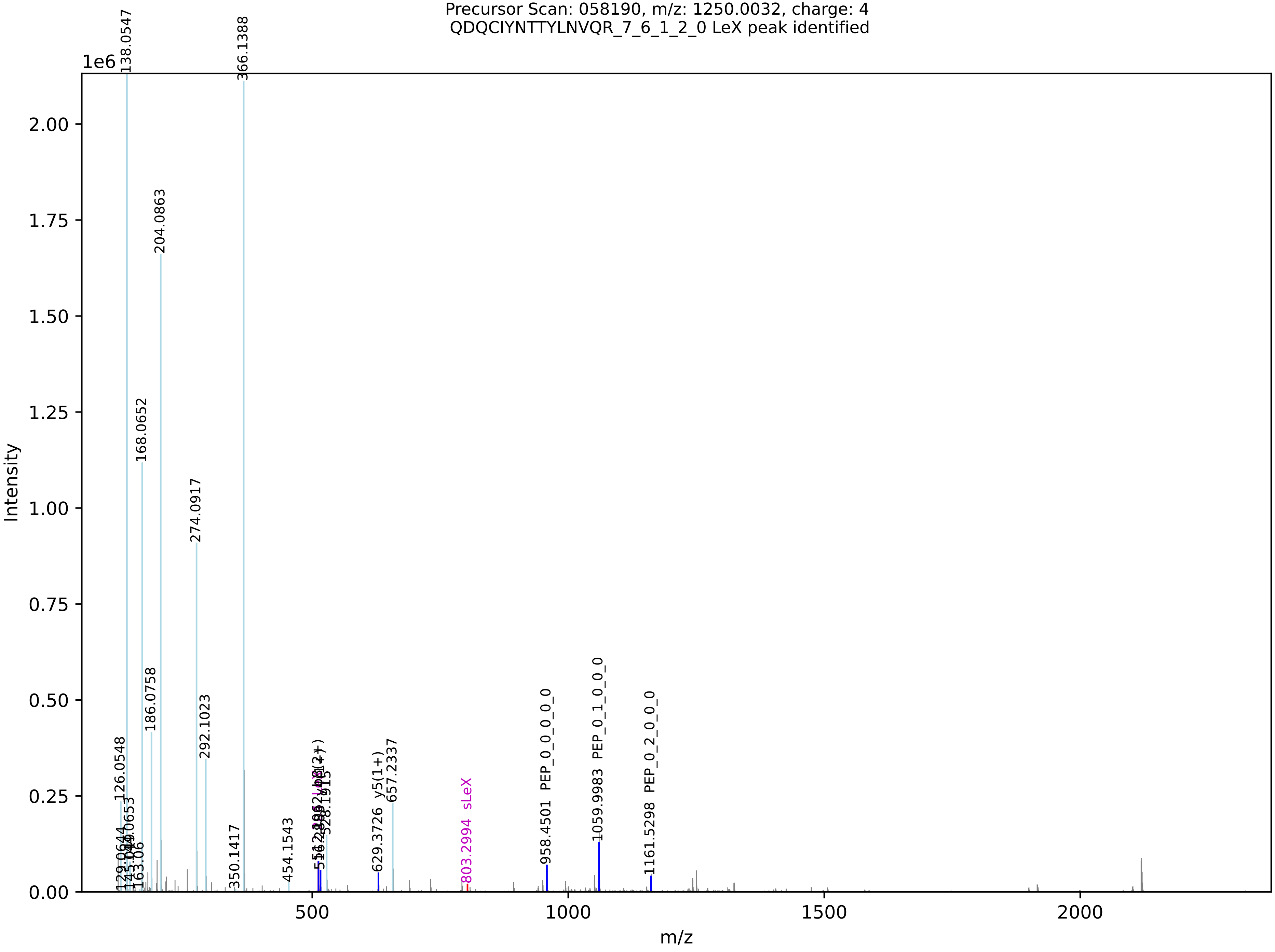

File: 231129_OE_RK_37, RT: 49.501, Scan: 065819
Type: HCD, FTMS + c NSI d Full ms2 1359.7942@hcd30.00 [120.0000-5570.8164]
Precursor Scan: 065739, m/z: 1359.0424, charge: 4
QDQCIYNTTYLNVQR_7_6_0_4_0 LeX peak identified

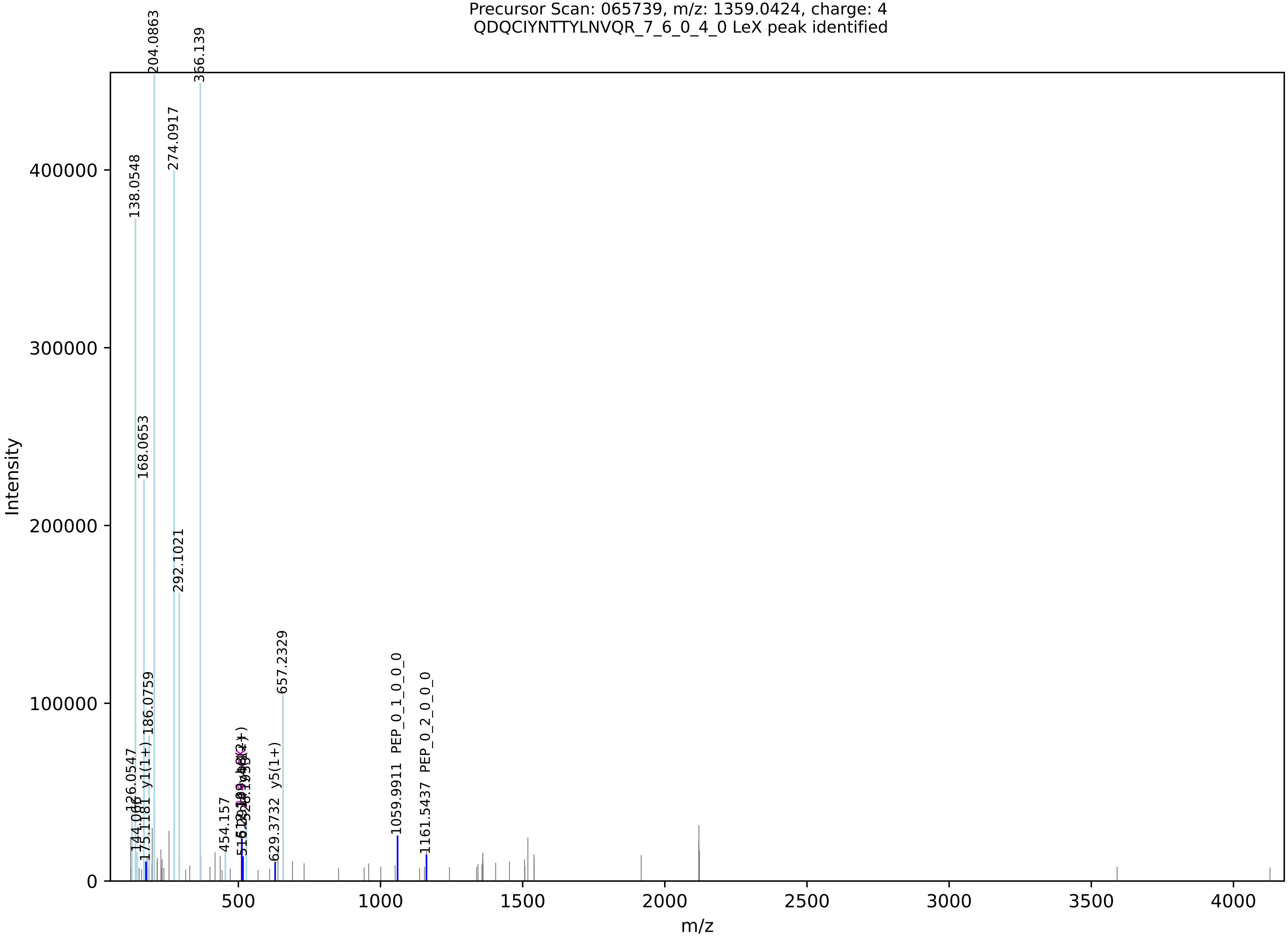

File: 231129_OE_RK_37, RT: 49.905, Scan: 066402
Type: HCD, FTMS + c NSI d Full ms2 1360.0419@hcd30.00 [120.0000-5571.8267]
Precursor Scan: 066322, m/z: 1359.0395, charge: 4
QDQCIYNTTYLNVQR_7_6_0_4_0 LeX peak identified

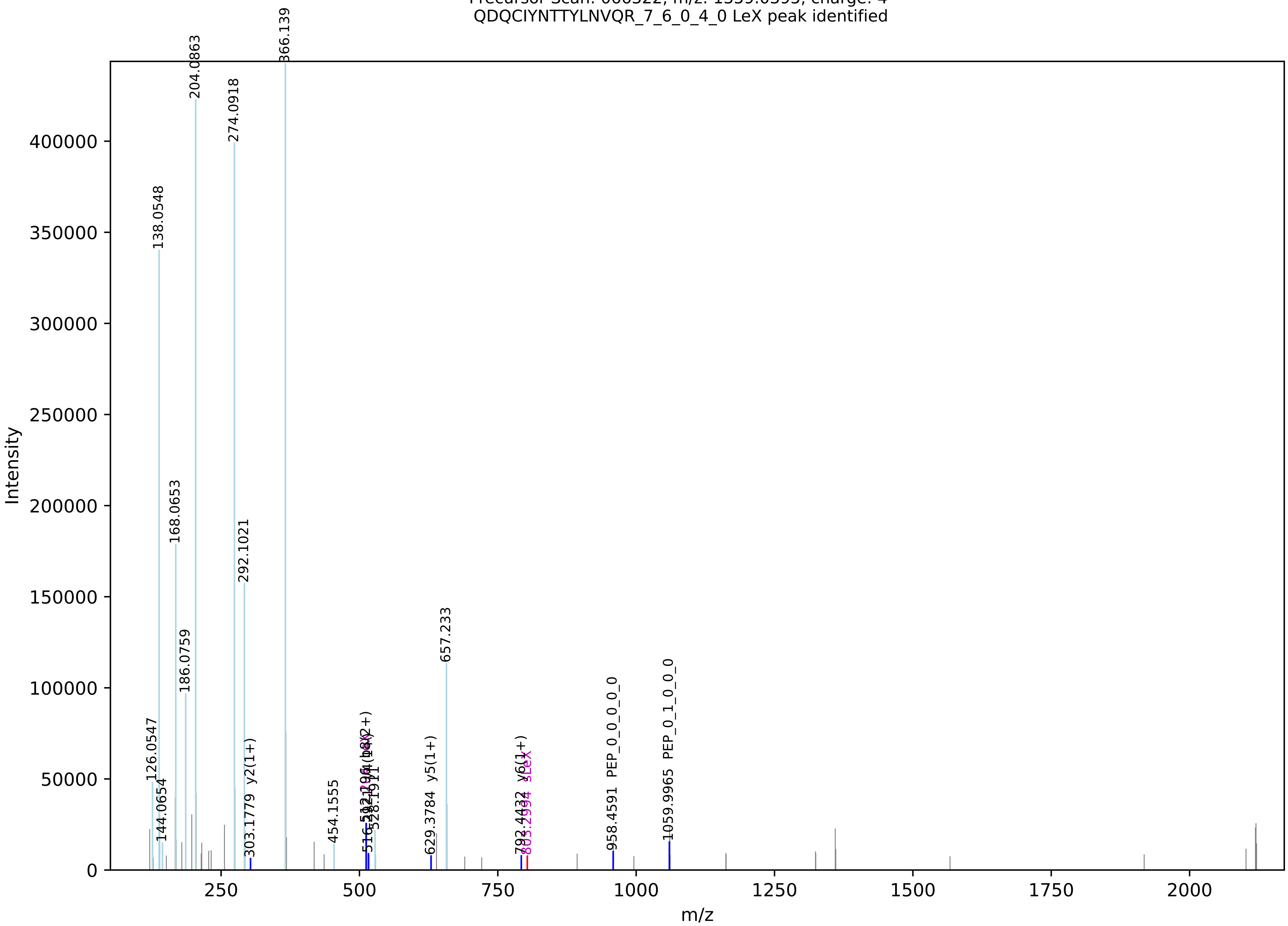

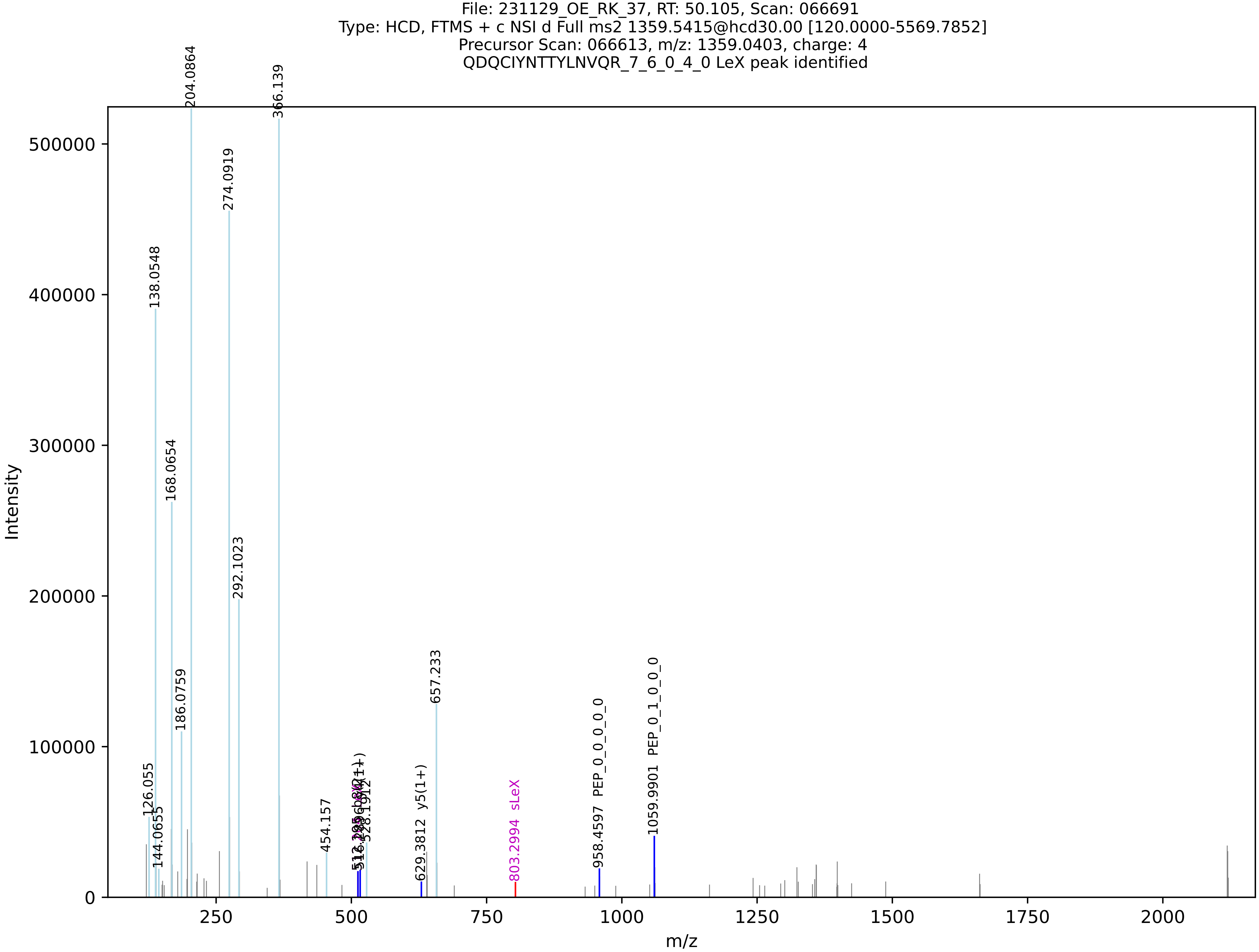
File: 231129_OE_RK_37, RT: 50.105, Scan: 066691
Type: HCD, FTMS + c NSI d Full ms2 1359.5415@hcd30.00 [120.0000-5569.7852]
Precursor Scan: 066613, m/z: 1359.0403, charge: 4
QDQCIYNTTYLNVQR_7_6_0_4_0 LeX peak identified
204.0864
366.139
274.0919
138.0548
168.0654
292.1023
186.0759
657.233
126.055
144.0655
454.157
528.1912
629.3812 y5(1+)
803.2994 sLeX
958.4597 PEP_0_0_0_0_0
1059.9901 PEP_0_1_0_0_0
Intensity
m/z
0
100000
200000
300000
400000
500000
250
500
750
1000
1250
1500
1750
2000

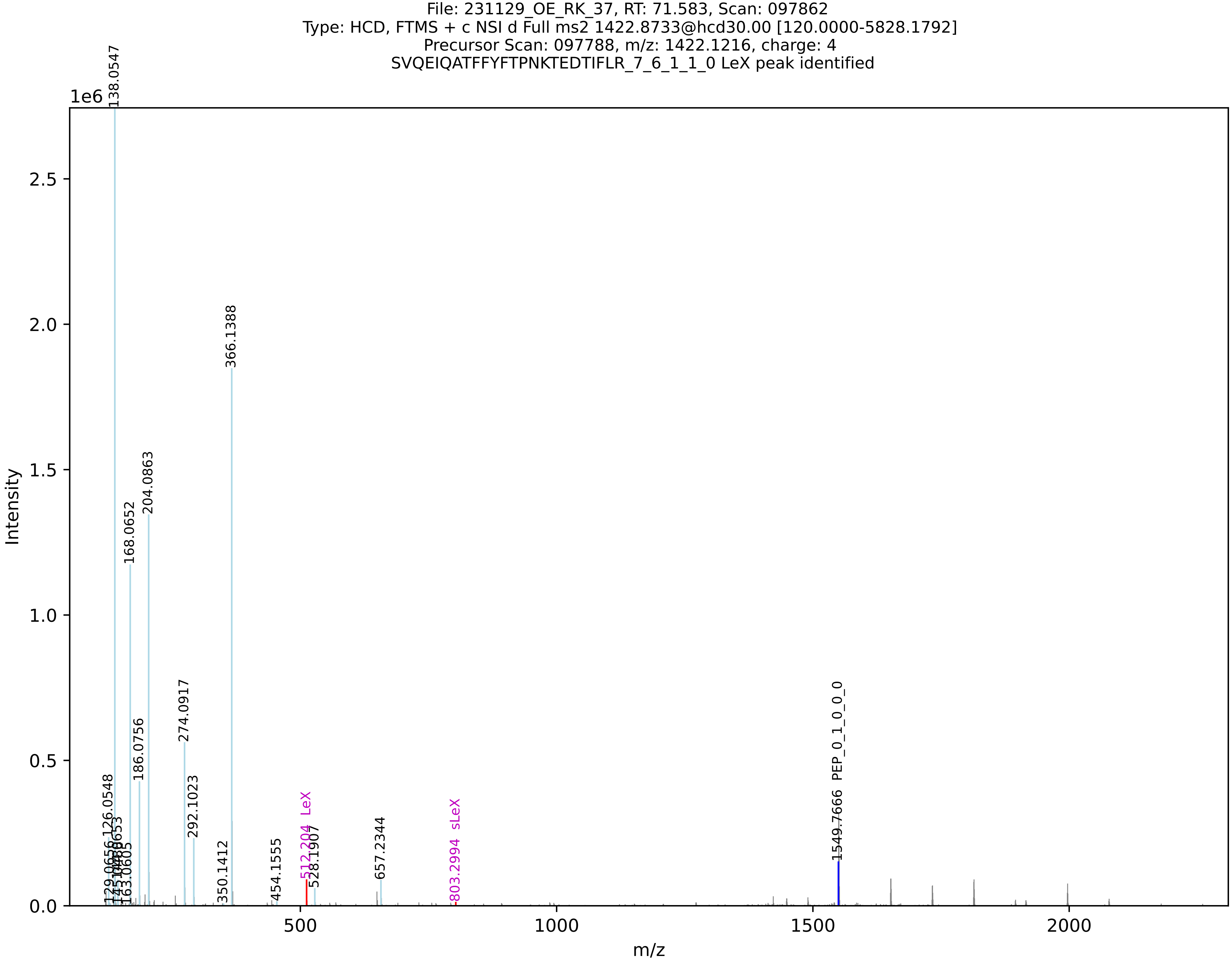

File: 231129_OE_RK_37, RT: 71.583, Scan: 097862
Type: HCD, FTMS + c NSI d Full ms2 1422.8733@hcd30.00 [120.0000-5828.1792]
Precursor Scan: 097788, m/z: 1422.1216, charge: 4
SVQEIQATFFYFTPNKTEDTIFLR_7_6_1_1_0 LeX peak identified
1e6
Intensity
m/z
138.0547
366.1388
204.0863
168.0652
186.0756
274.0917
292.1023
126.0548
163.0605
350.1412
454.1555
512.204 LeX
528.1907
657.2344
803.2994 sLeX
1549.7666 PEP_0_1_0_0_0

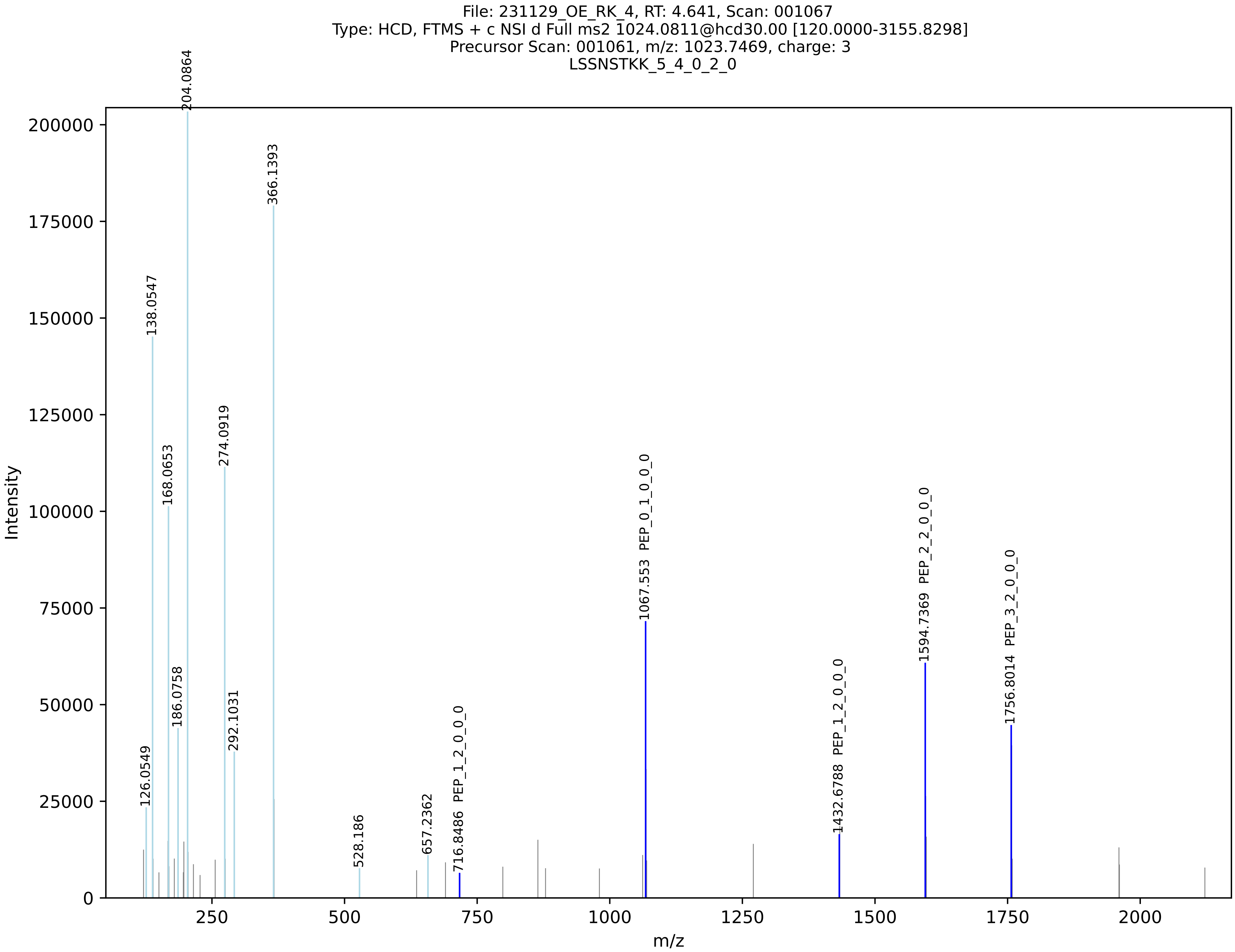
File: 231129_OE_RK_4, RT: 4.641, Scan: 001067
Type: HCD, FTMS + c NSI d Full ms2 1024.0811@hcd30.00 [120.0000-3155.8298]
Precursor Scan: 001061, m/z: 1023.7469, charge: 3
LSSNSTKK_5_4_0_2_0
Intensity
m/z
204.0864
366.1393
138.0547
274.0919
168.0653
186.0758
292.1031
126.0549
528.186
657.2362
716.8486 PEP_1_2_0_0_0
1067.553 PEP_0_1_0_0_0
1432.6788 PEP_1_2_0_0_0
1594.7369 PEP_2_2_0_0_0
1756.8014 PEP_3_2_0_0_0

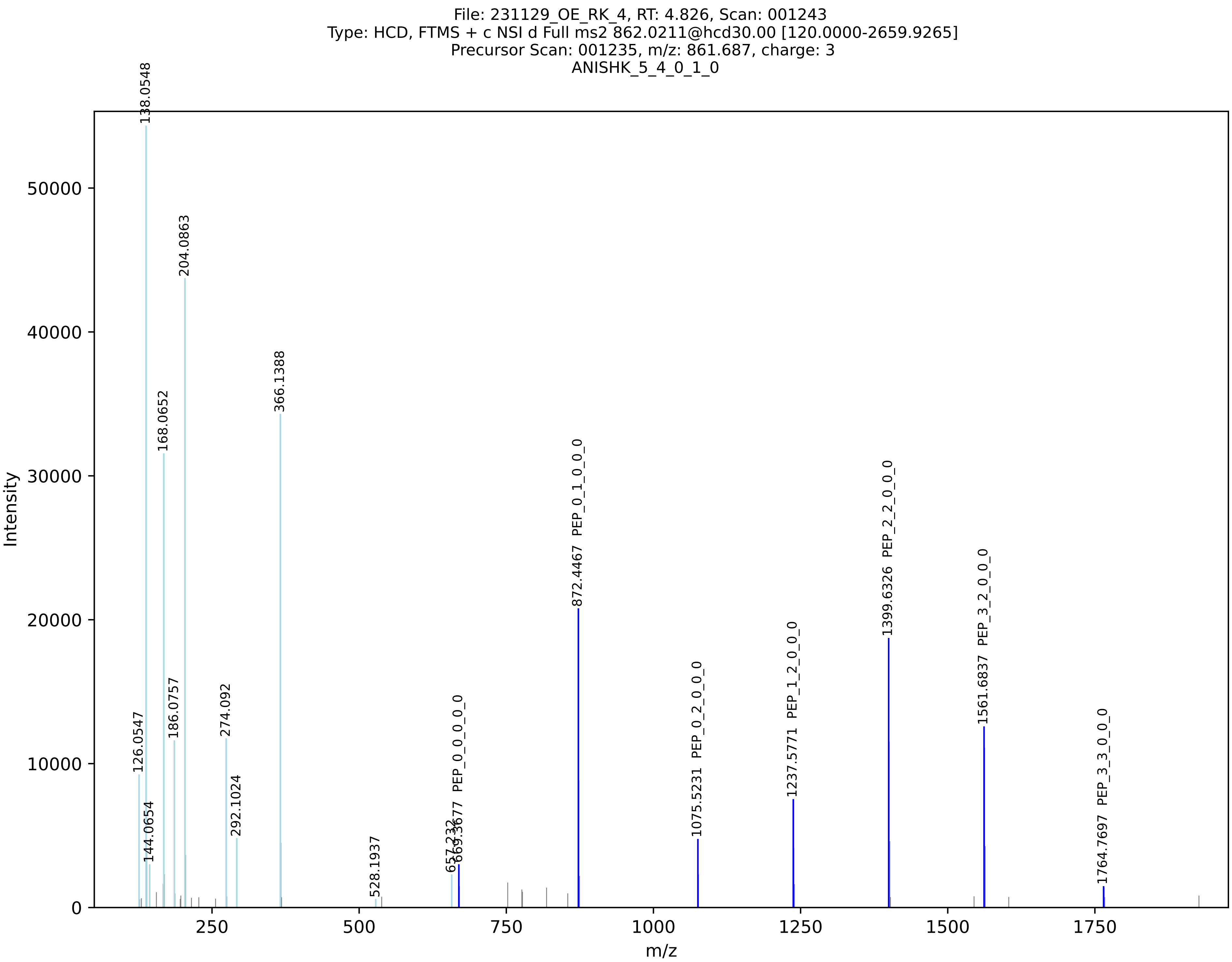

File: 231129_OE_RK_4, RT: 4.826, Scan: 001243
Type: HCD, FTMS + c NSI d Full ms2 862.0211@hcd30.00 [120.0000-2659.9265]
Precursor Scan: 001235, m/z: 861.687, charge: 3
ANISHK_5_4_0_1_0
Intensity
m/z
138.0548
204.0863
366.1388
168.0652
186.0757
274.092
126.0547
292.1024
144.0654
528.1937
657.232
669.3677 PEP_0_0_0_0_0
872.4467 PEP_0_1_0_0_0
1075.5231 PEP_0_2_0_0_0
1237.5771 PEP_1_2_0_0_0
1399.6326 PEP_2_2_0_0_0
1561.6837 PEP_3_2_0_0_0
1764.7697 PEP_3_3_0_0_0

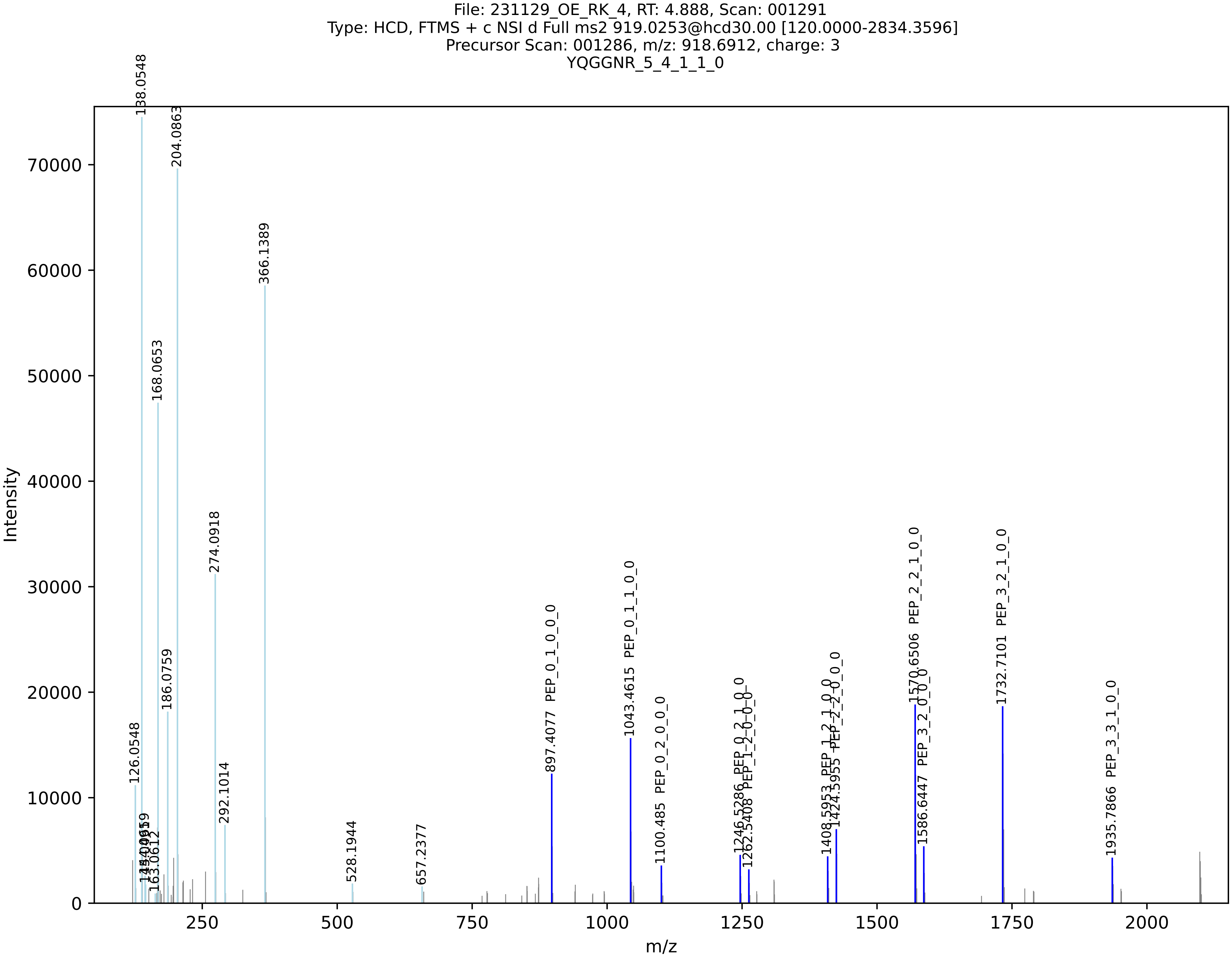
File: 231129_OE_RK_4, RT: 4.888, Scan: 001291
Type: HCD, FTMS + c NSI d Full ms2 919.0253@hcd30.00 [120.0000-2834.3596]
Precursor Scan: 001286, m/z: 918.6912, charge: 3
YQGGNR_5_4_1_1_0
138.0548
204.0863
366.1389
168.0653
274.0918
186.0759
126.0548
292.1014
163.0612
528.1944
657.2377
897.4077 PEP_0_1_0_0_0
1043.4615 PEP_0_1_1_0_0
1100.485 PEP_0_2_0_0_0
1246.5286 PEP_0_2_1_0_0
1262.5408 PEP_1_2_0_0_0
1408.5953 PEP_1_2_1_0_0
1424.5955 PEP_2_2_0_0_0
1570.6506 PEP_2_2_1_0_0
1586.6447 PEP_3_2_0_0_0
1732.7101 PEP_3_2_1_0_0
1935.7866 PEP_3_3_1_0_0
Intensity
m/z
0
10000
20000
30000
40000
50000
60000
70000
250
500
750
1000
1250
1500
1750
2000

File: 231129_OE_RK_4, RT: 4.979, Scan: 001373
Type: HCD, FTMS + c NSI d Full ms2 959.0519@hcd30.00 [120.0000-2956.8408]
Precursor Scan: 001363, m/z: 958.7178, charge: 3
ANISHK_5_4_0_2_0

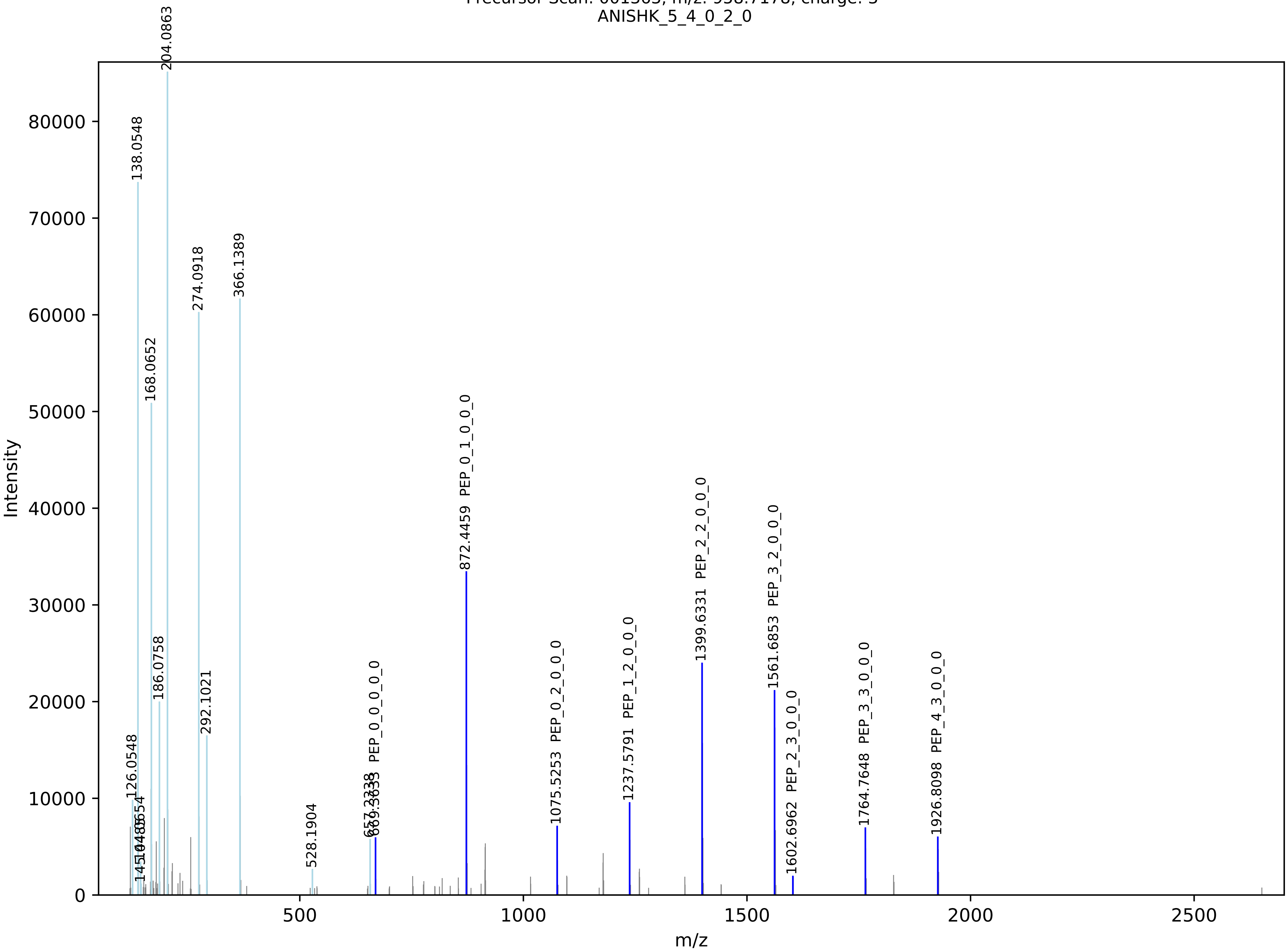

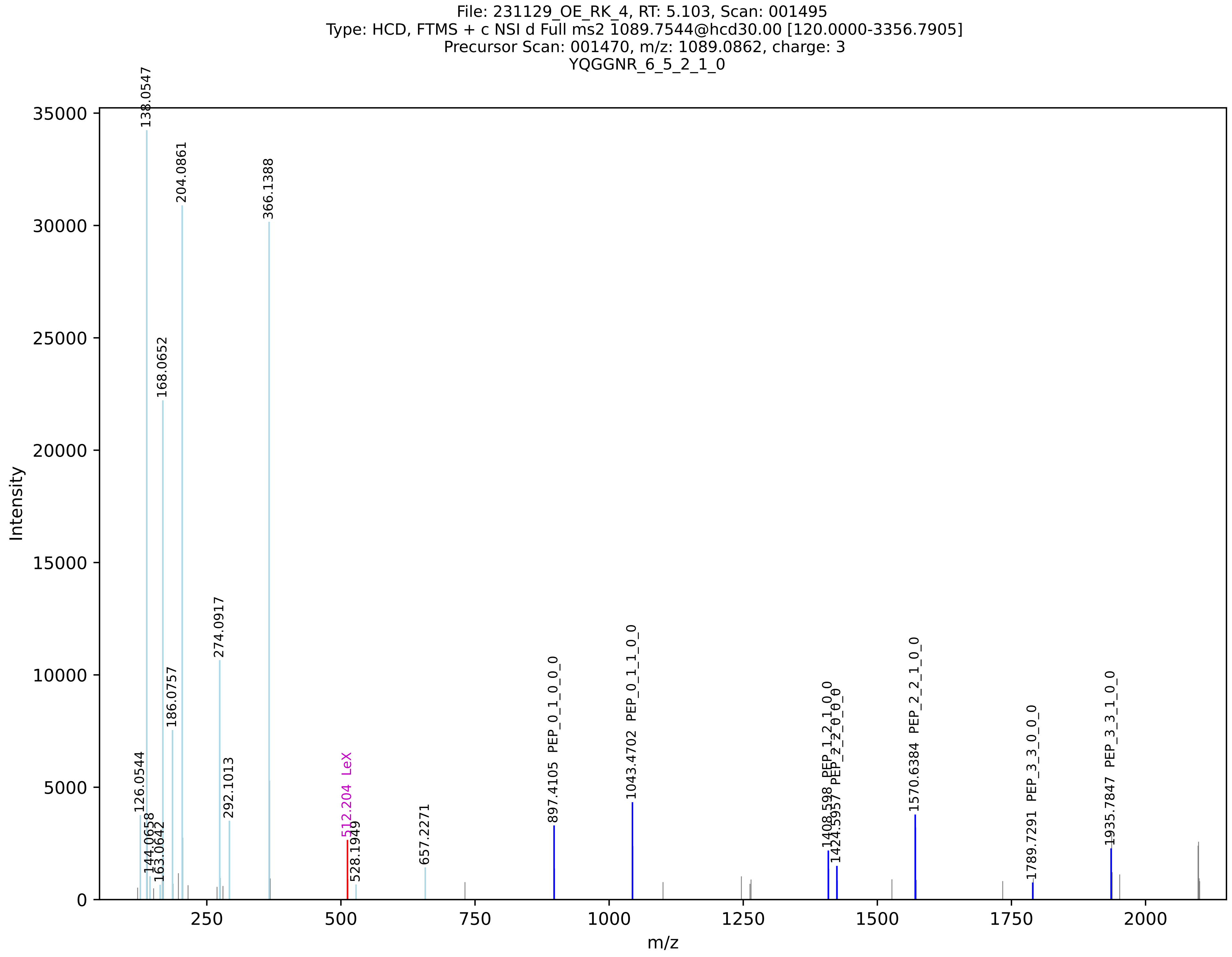
File: 231129_OE_RK_4, RT: 5.103, Scan: 001495
Type: HCD, FTMS + c NSI d Full ms2 1089.7544@hcd30.00 [120.0000-3356.7905]
Precursor Scan: 001470, m/z: 1089.0862, charge: 3
YQGGNR_6_5_2_1_0
Intensity
m/z
0
5000
10000
15000
20000
25000
30000
35000
250
500
750
1000
1250
1500
1750
2000
138.0547
204.0861
366.1388
168.0652
274.0917
186.0757
126.0544
144.0658
163.0642
292.1013
512.204 LeX
528.1949
657.2271
897.4105 PEP_0_1_0_0_0
1043.4702 PEP_0_1_1_0_0
1408.598 PEP_1_2_1_0_0
1424.5957 PEP_2_2_0_0_0
1570.6384 PEP_2_2_1_0_0
1789.7291 PEP_3_3_0_0_0
1935.7847 PEP_3_3_1_0_0

File: 231129_OE_RK_4, RT: 5.149, Scan: 001546
Type: HCD, FTMS + c NSI d Full ms2 958.3821@hcd30.00 [120.0000-3933.0547]
Precursor Scan: 001526, m/z: 957.881, charge: 4
HSHNNNSSDLHPHK_5_4_0_2_0

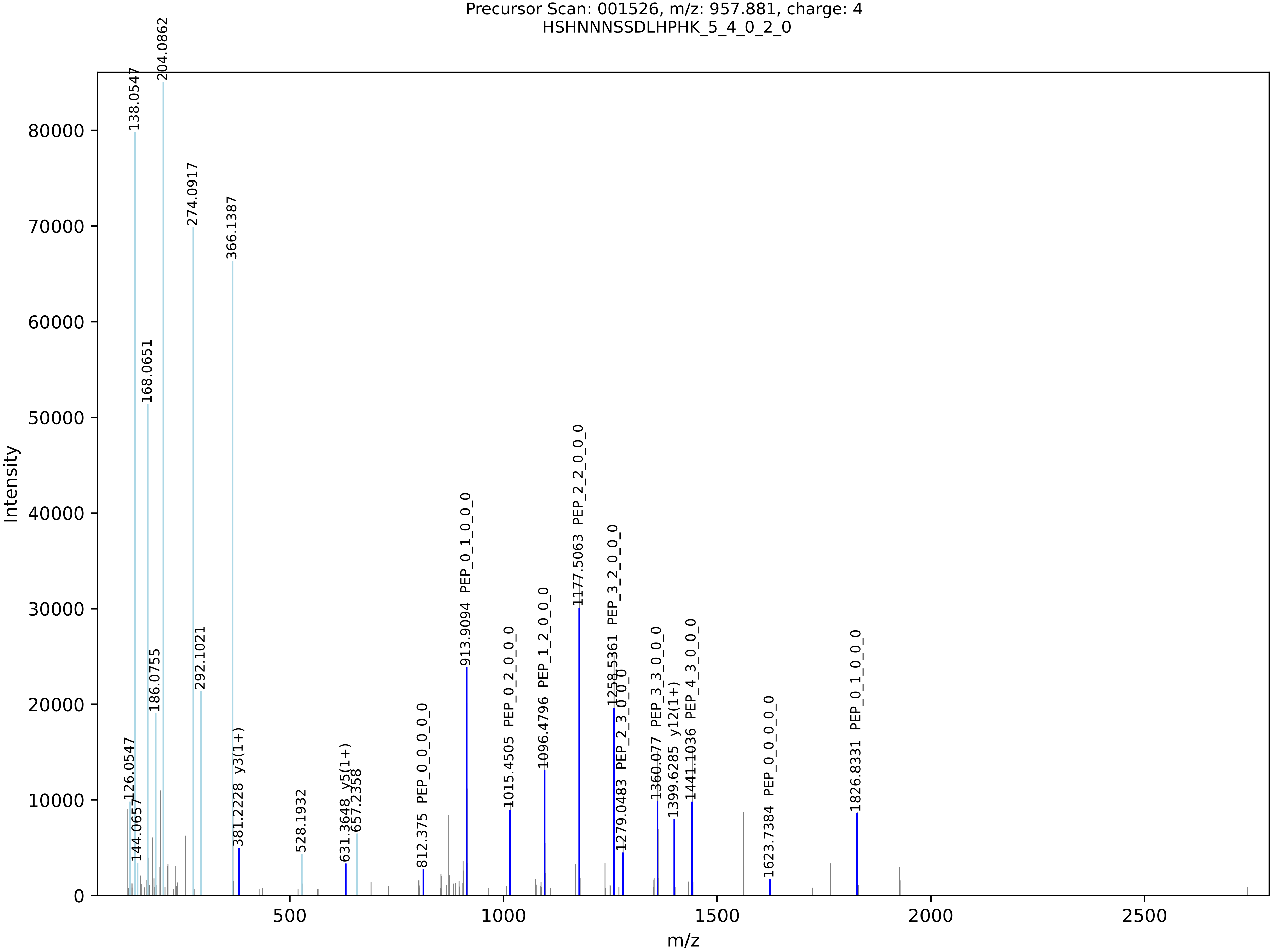

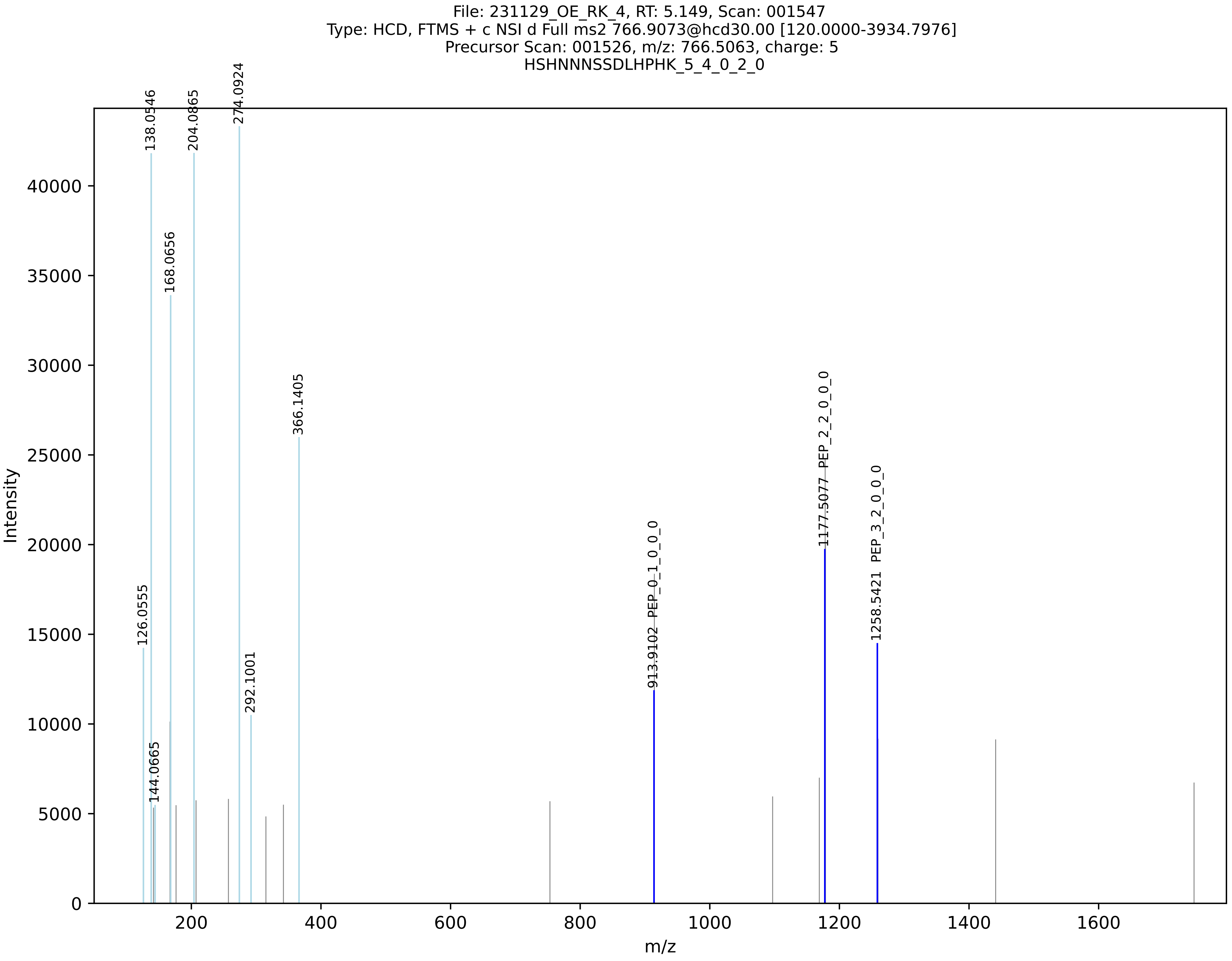
File: 231129_OE_RK_4, RT: 5.149, Scan: 001547
Type: HCD, FTMS + c NSI d Full ms2 766.9073@hcd30.00 [120.0000-3934.7976]
Precursor Scan: 001526, m/z: 766.5063, charge: 5
HSHNNNSSDLHPHK_5_4_0_2_0
138.0546
204.0865
274.0924
168.0656
366.1405
126.0555
144.0665
292.1001
913.9102_PEP_0_1_0_0_0
1177.5077_PEP_2_2_0_0_0
1258.5421_PEP_3_2_0_0_0
Intensity
m/z
0
5000
10000
15000
20000
25000
30000
35000
40000
200
400
600
800
1000
1200
1400
1600

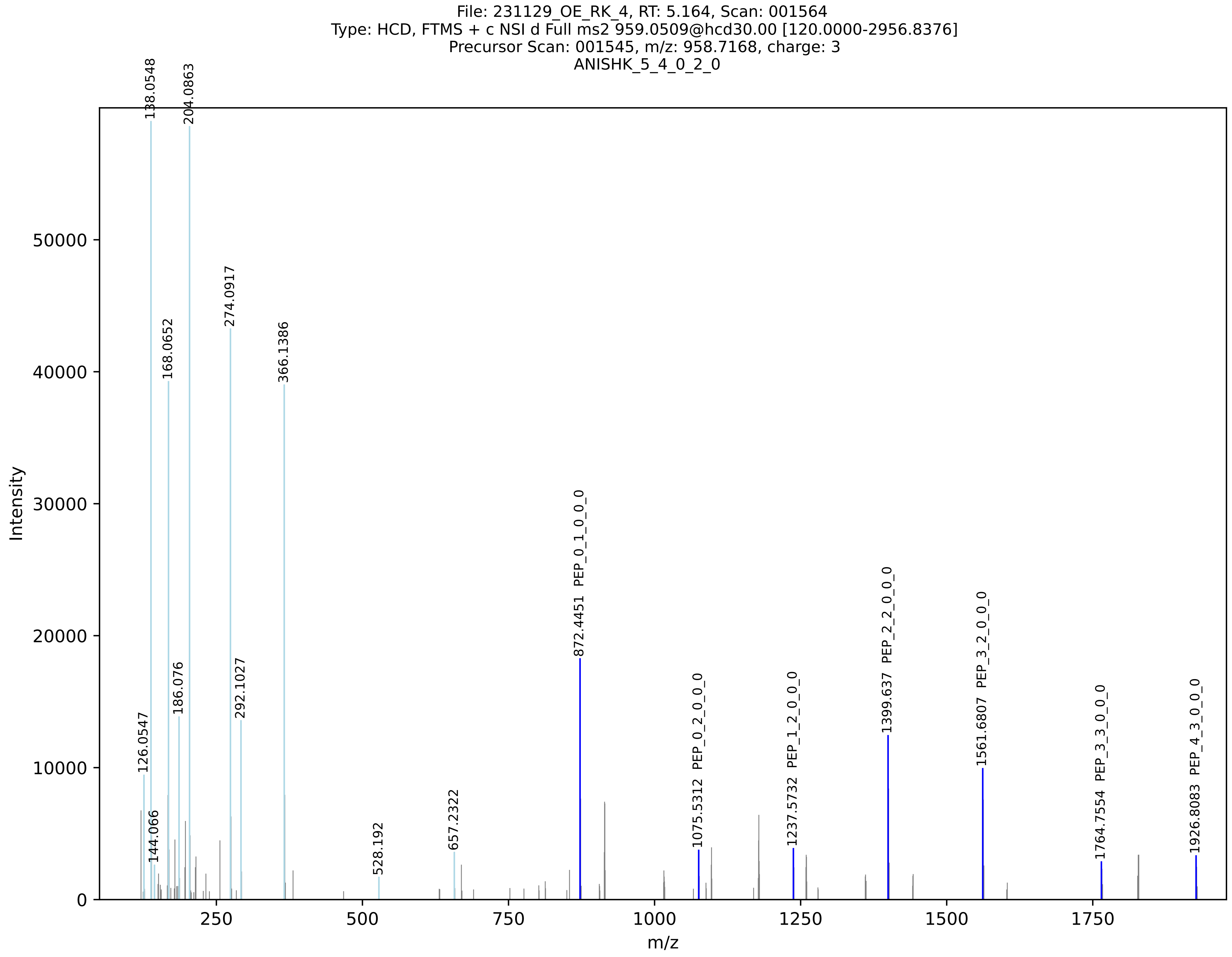
File: 231129_OE_RK_4, RT: 5.164, Scan: 001564
Type: HCD, FTMS + c NSI d Full ms2 959.0509@hcd30.00 [120.0000-2956.8376]
Precursor Scan: 001545, m/z: 958.7168, charge: 3
ANISHK_5_4_0_2_0
Intensity
m/z
138.0548
204.0863
168.0652
274.0917
366.1386
186.076
292.1027
126.0547
144.066
528.192
657.2322
872.4451 PEP_0_1_0_0_0
1075.5312 PEP_0_2_0_0_0
1237.5732 PEP_1_2_0_0_0
1399.637 PEP_2_2_0_0_0
1561.6807 PEP_3_2_0_0_0
1764.7554 PEP_3_3_0_0_0
1926.8083 PEP_4_3_0_0_0

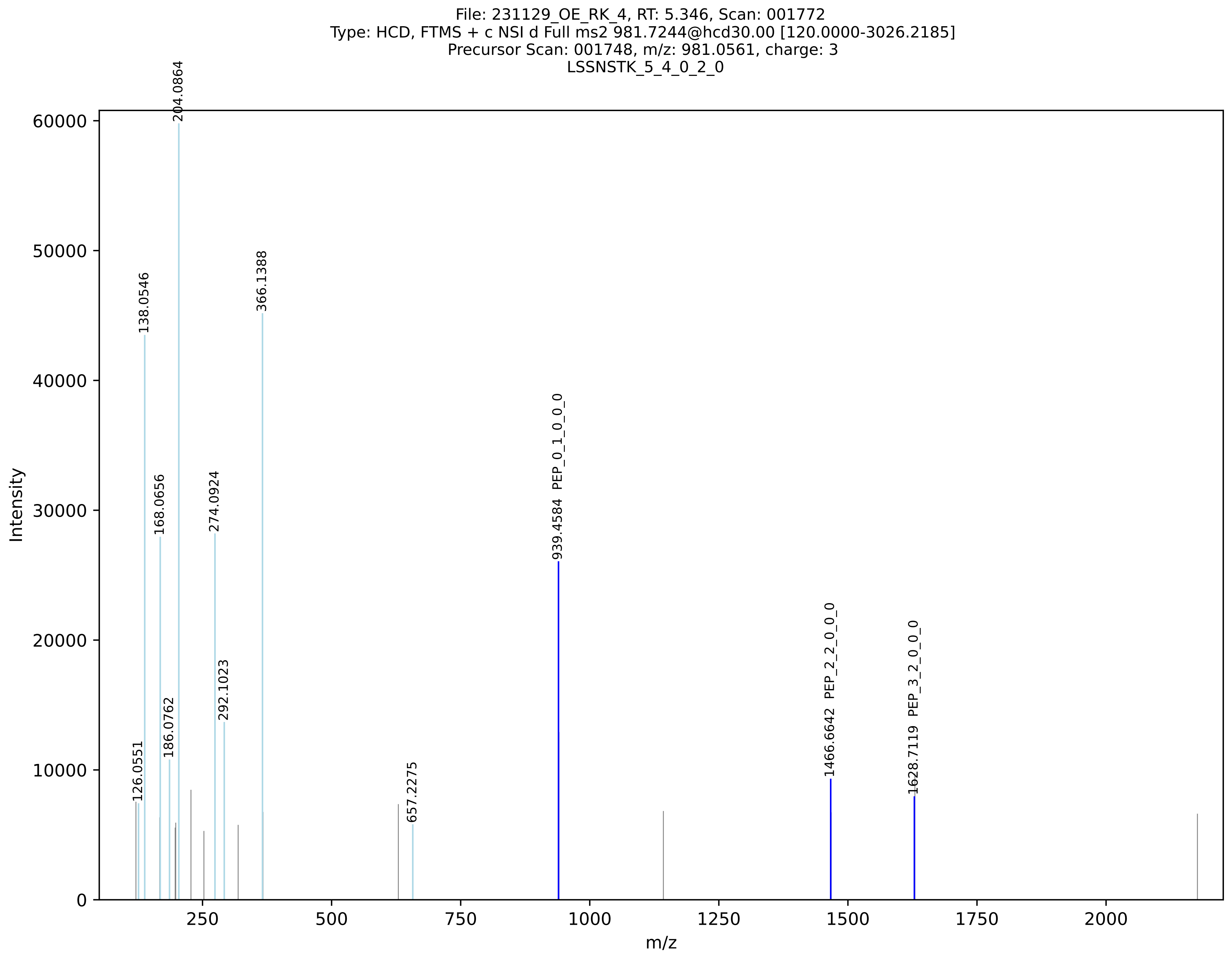
File: 231129_OE_RK_4, RT: 5.346, Scan: 001772
Type: HCD, FTMS + c NSI d Full ms2 981.7244@hcd30.00 [120.0000-3026.2185]
Precursor Scan: 001748, m/z: 981.0561, charge: 3
LSSNSTK_5_4_0_2_0
Intensity
m/z
204.0864
138.0546
366.1388
168.0656
274.0924
292.1023
186.0762
126.0551
657.2275
939.4584 PEP_0_1_0_0_0_
1466.6642 PEP_2_2_0_0_0_
1628.7119 PEP_3_2_0_0_0_
0
10000
20000
30000
40000
50000
60000
250
500
750
1000
1250
1500
1750
2000

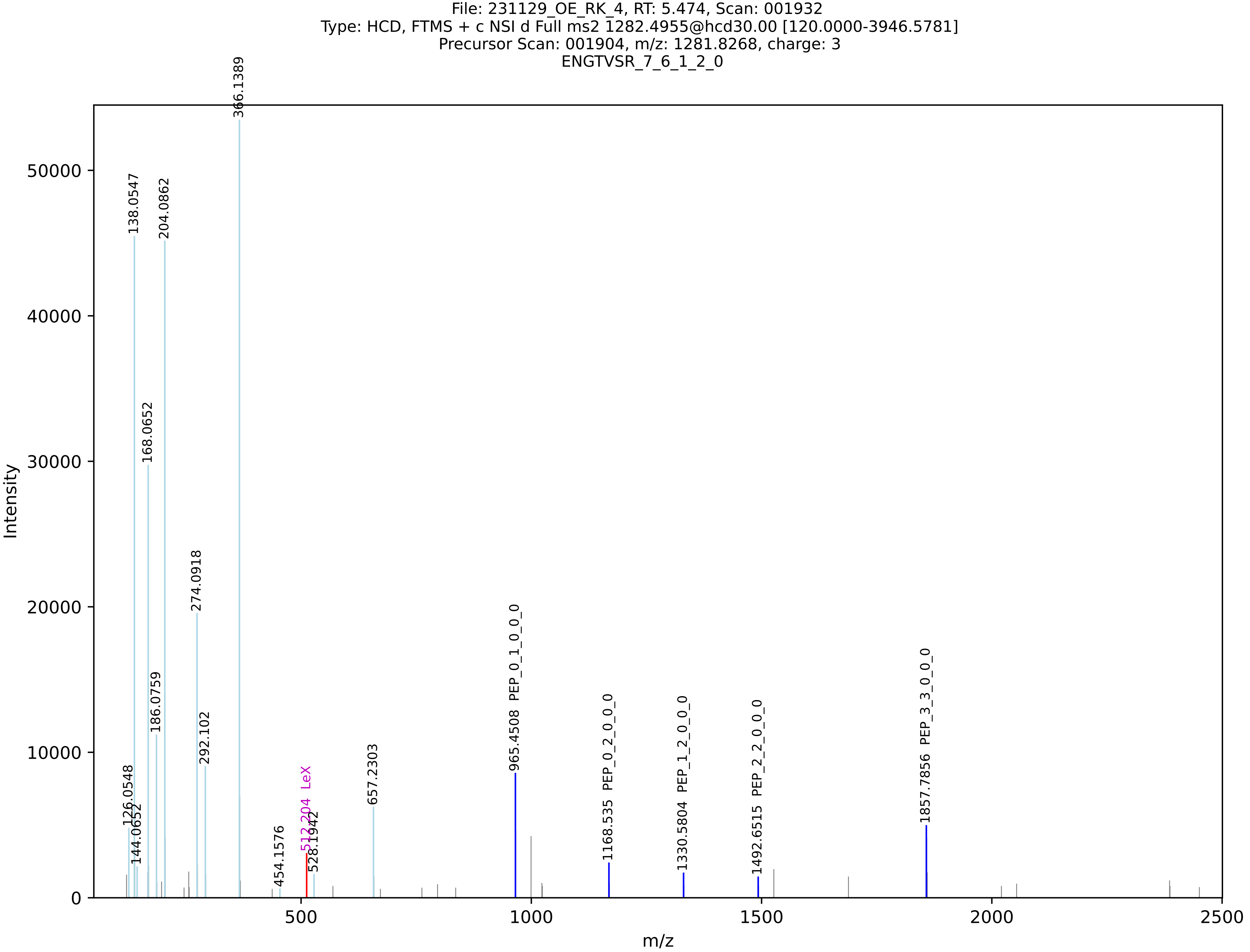

File: 231129_OE_RK_4, RT: 5.474, Scan: 001932
Type: HCD, FTMS + c NSI d Full ms2 1282.4955@hcd30.00 [120.0000-3946.5781]
Precursor Scan: 001904, m/z: 1281.8268, charge: 3
ENGTVSR_7_6_1_2_0
Intensity
m/z
366.1389
138.0547
204.0862
168.0652
274.0918
186.0759
292.102
126.0548
144.0652
454.1576
512.204 LeX
528.1942
657.2303
965.4508 PEP_0_1_0_0_0_
1168.535 PEP_0_2_0_0_0_
1330.5804 PEP_1_2_0_0_0_
1492.6515 PEP_2_2_0_0_0_
1857.7856 PEP_3_3_0_0_0_
0
10000
20000
30000
40000
50000
500
1000
1500
2000
2500

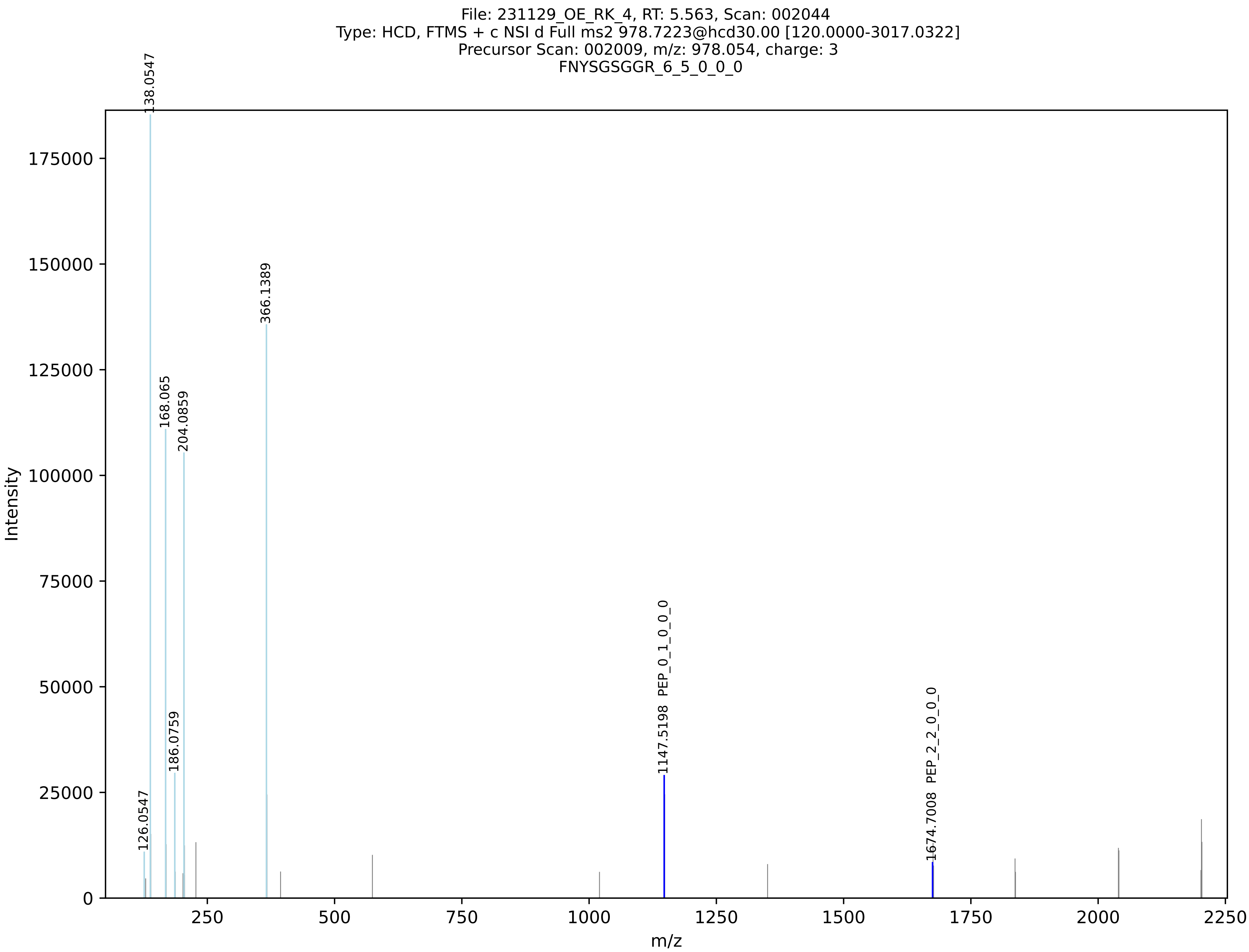

File: 231129_OE_RK_4, RT: 5.563, Scan: 002044
Type: HCD, FTMS + c NSI d Full ms2 978.7223@hcd30.00 [120.0000-3017.0322]
Precursor Scan: 002009, m/z: 978.054, charge: 3
FNYSGSGGR_6_5_0_0_0
Intensity
m/z
138.0547
366.1389
168.065
204.0859
186.0759
126.0547
1147.5198 PEP_0_1_0_0_0
1674.7008 PEP_2_2_0_0_0
0
25000
50000
75000
100000
125000
150000
175000
250
500
750
1000
1250
1500
1750
2000
2250

File: 231129_OE_RK_4, RT: 5.594, Scan: 002085
Type: HCD, FTMS + c NSI d Full ms2 1111.7643@hcd30.00 [120.0000-3424.1409]
Precursor Scan: 002040, m/z: 1111.4309, charge: 3
ENGTVSR_6_5_0_2_0

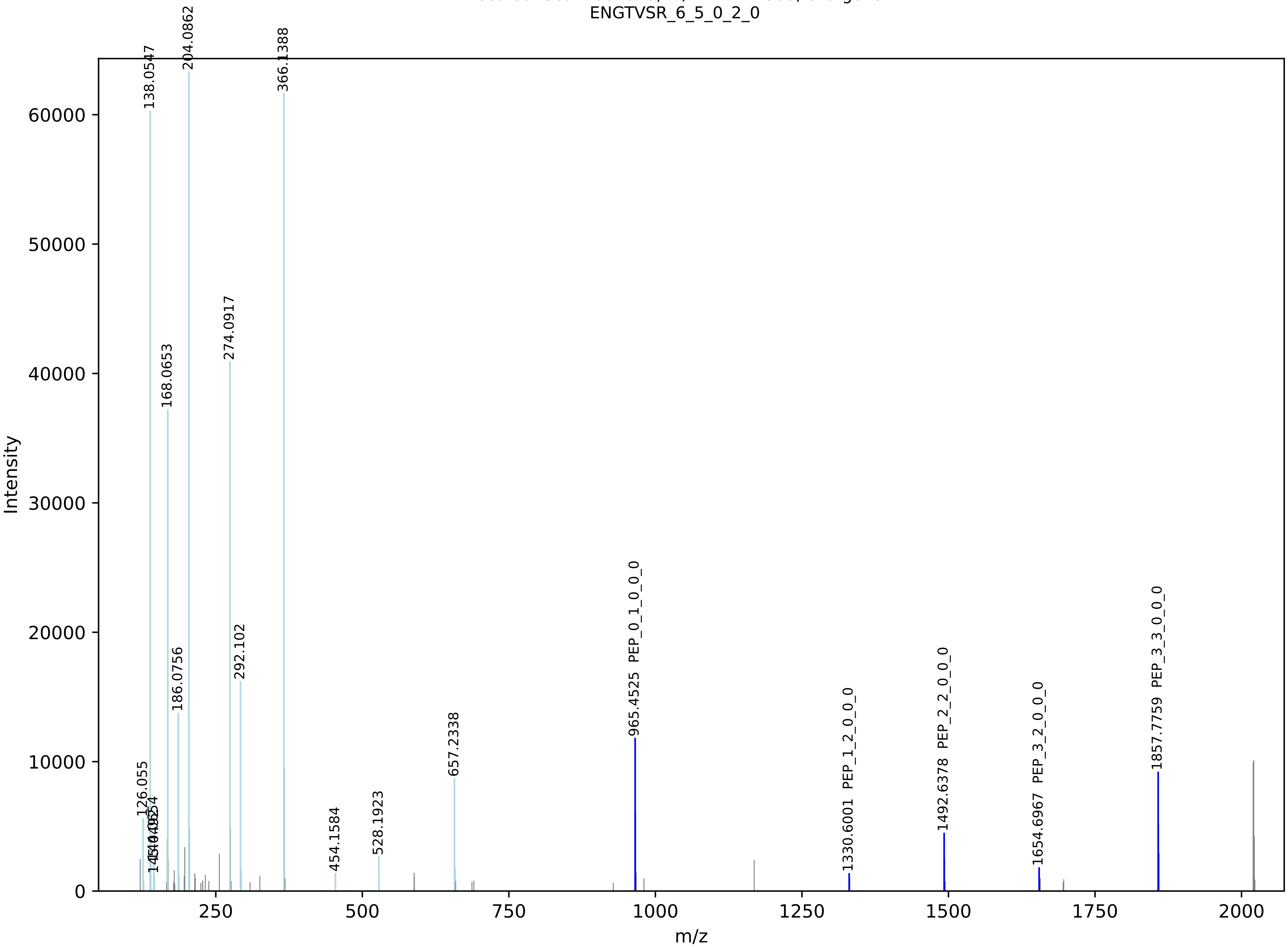

File: 231129_OE_RK_4, RT: 5.646, Scan: 002154
Type: HCD, FTMS + c NSI d Full ms2 990.0536@hcd30.00 [120.0000-3051.7061]
Precursor Scan: 002113, m/z: 989.7195, charge: 3
ENGTVSR_5_4_0_2_0

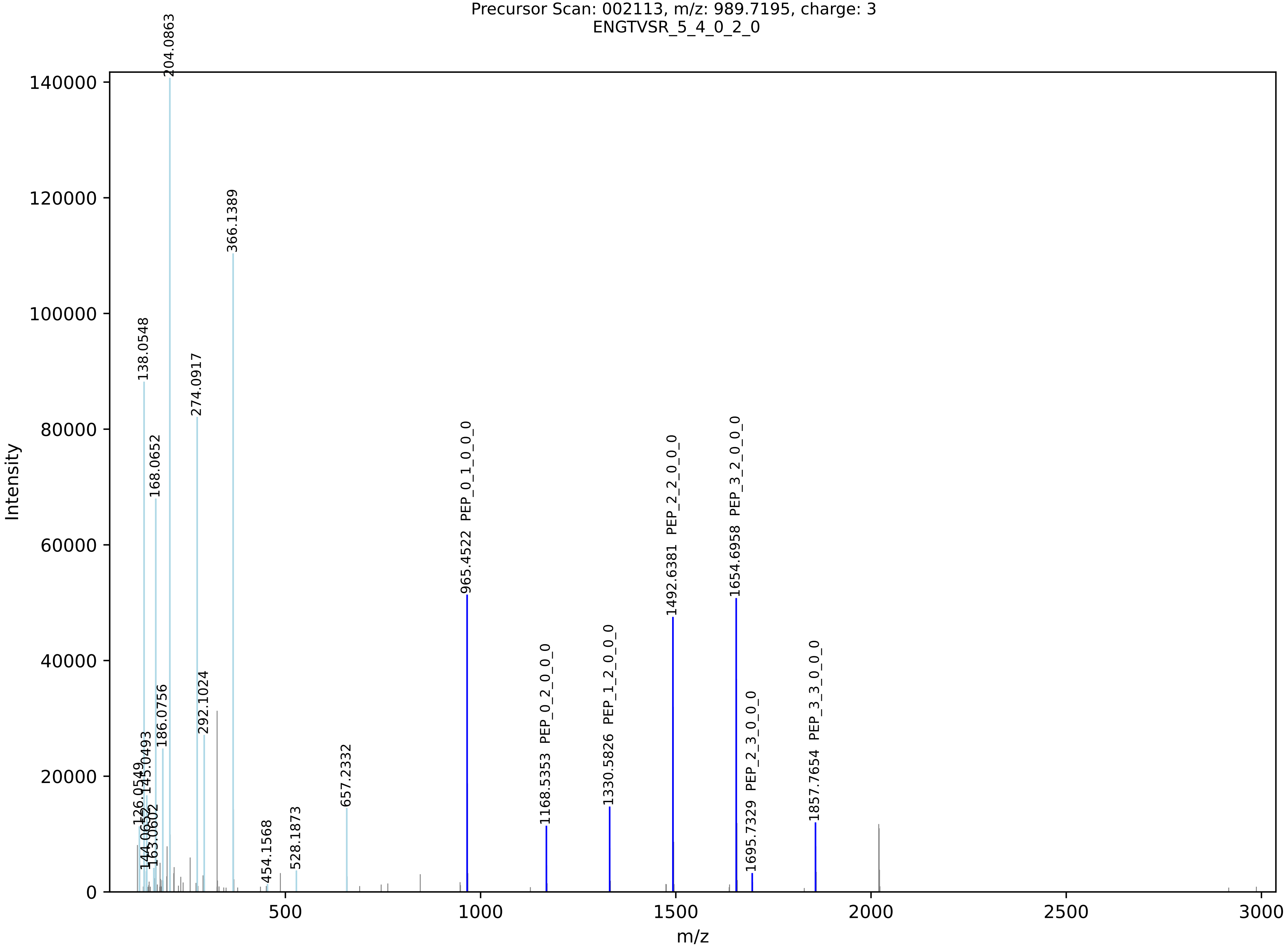

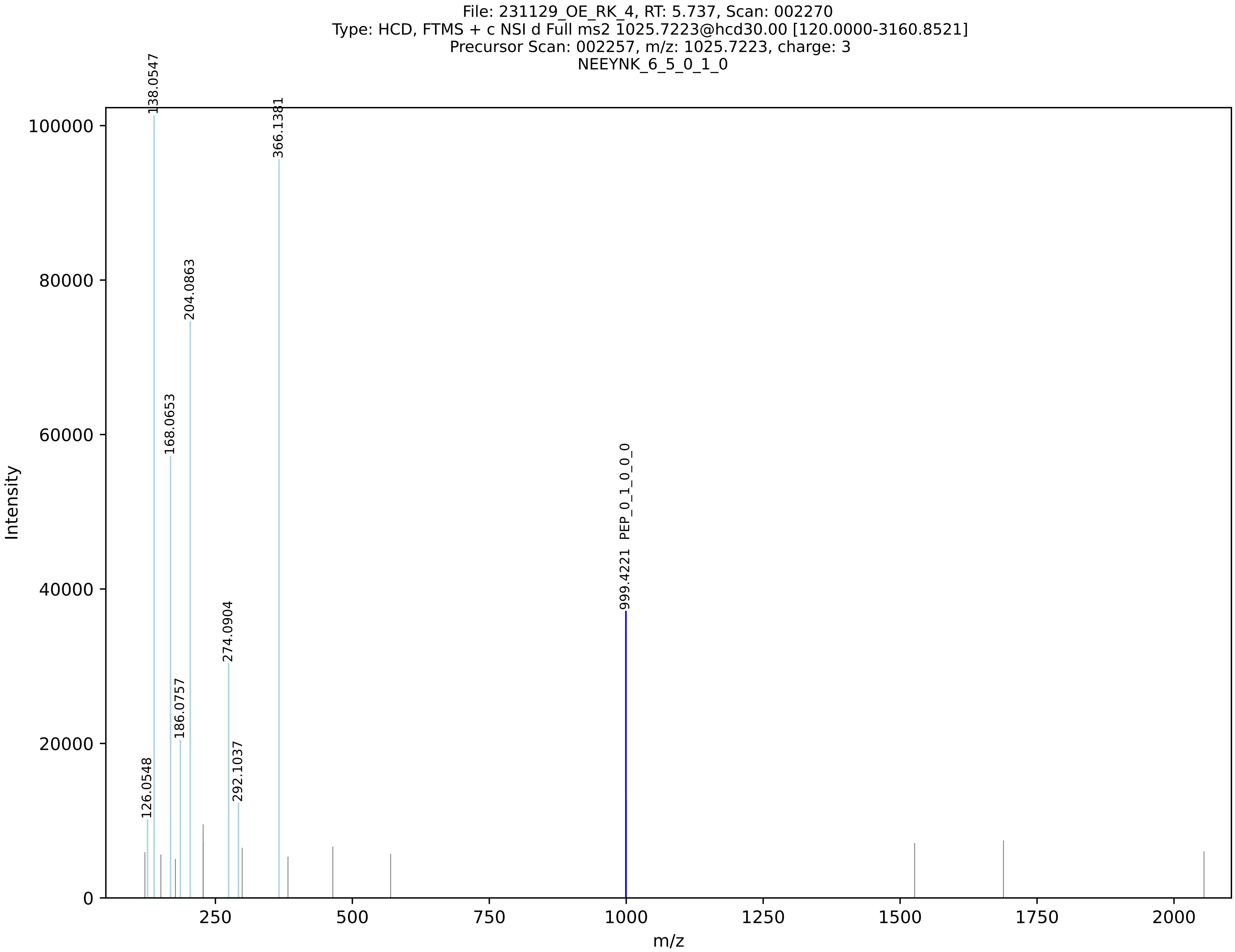
File: 231129_OE_RK_4, RT: 5.737, Scan: 002270
Type: HCD, FTMS + c NSI d Full ms2 1025.7223@hcd30.00 [120.0000-3160.8521]
Precursor Scan: 002257, m/z: 1025.7223, charge: 3
NEEYNK_6_5_0_1_0
138.0547
366.1381
204.0863
168.0653
999.4221 PEP_0_1_0_0_0
274.0904
186.0757
292.1037
126.0548
Intensity
m/z
0
20000
40000
60000
80000
100000
250
500
750
1000
1250
1500
1750
2000

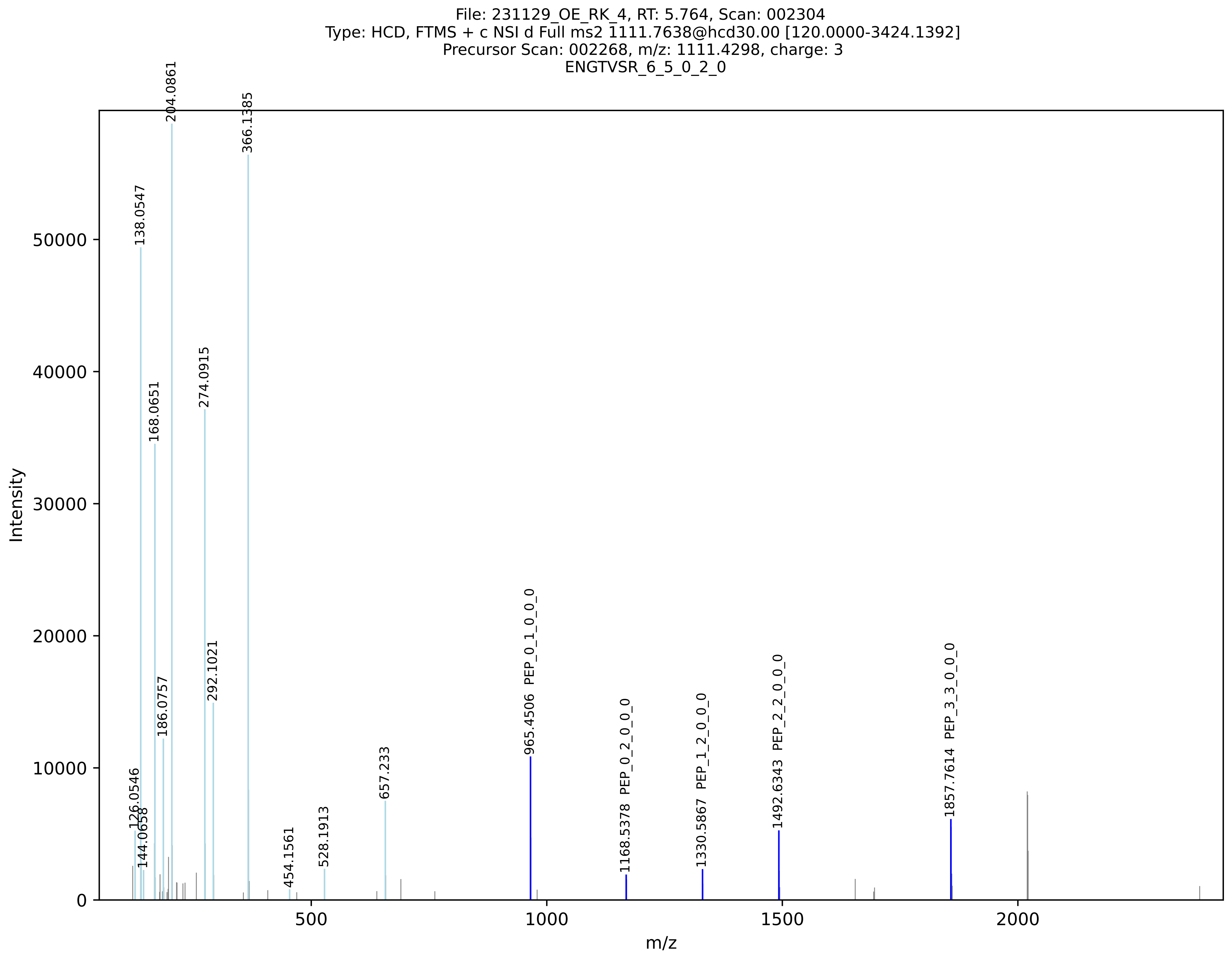
File: 231129_OE_RK_4, RT: 5.764, Scan: 002304
Type: HCD, FTMS + c NSI d Full ms2 1111.7638@hcd30.00 [120.0000-3424.1392]
Precursor Scan: 002268, m/z: 1111.4298, charge: 3
ENGTVSR_6_5_0_2_0
204.0861
366.1385
138.0547
274.0915
168.0651
292.1021
186.0757
126.0546
144.0658
454.1561
528.1913
657.233
965.4506 PEP_0_1_0_0_0
1168.5378 PEP_0_2_0_0_0
1330.5867 PEP_1_2_0_0_0
1492.6343 PEP_2_2_0_0_0
1857.7614 PEP_3_3_0_0_0
Intensity
m/z
0
10000
20000
30000
40000
50000
500
1000
1500
2000

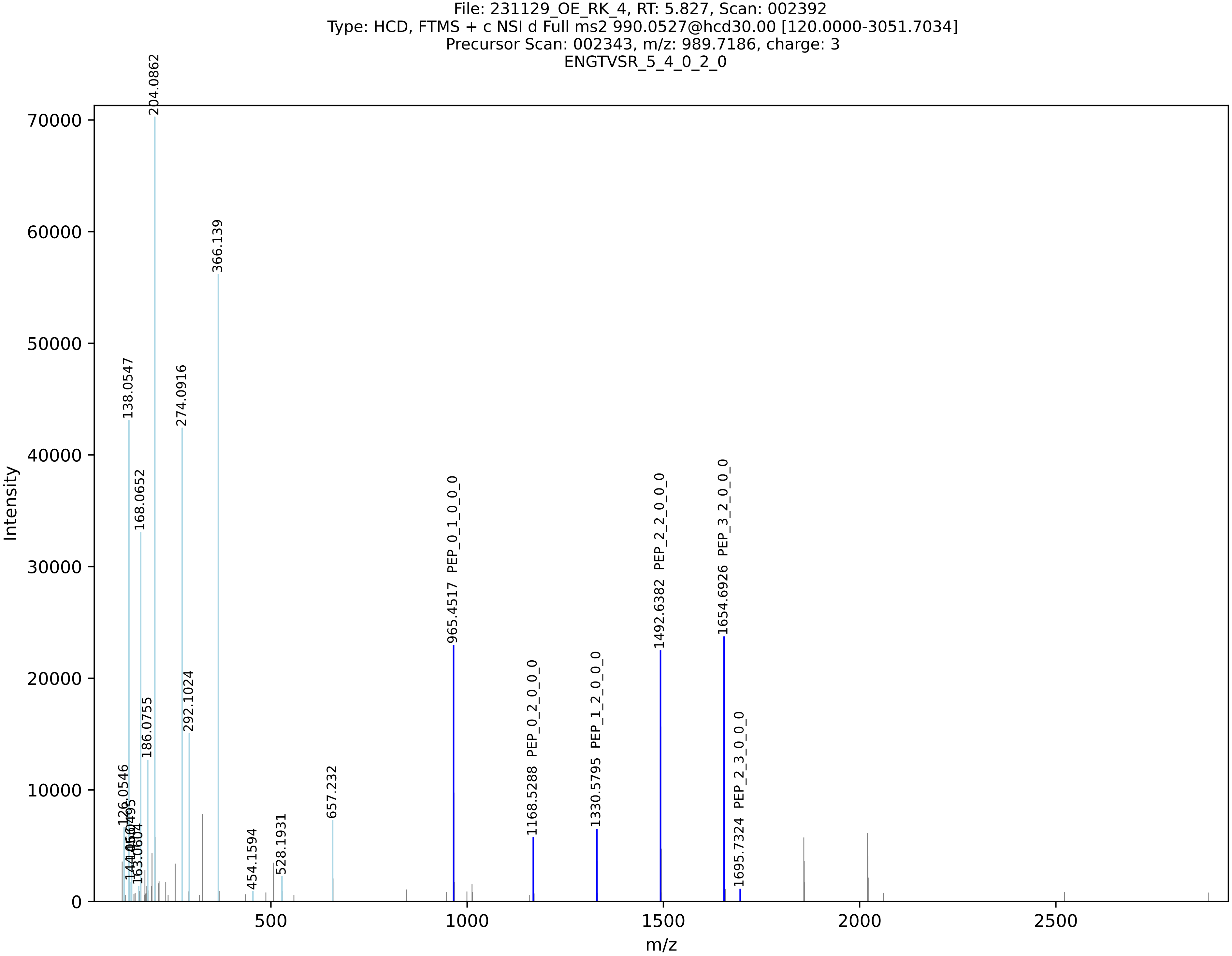
File: 231129_OE_RK_4, RT: 5.827, Scan: 002392
Type: HCD, FTMS + c NSI d Full ms2 990.0527@hcd30.00 [120.0000-3051.7034]
Precursor Scan: 002343, m/z: 989.7186, charge: 3
ENGTVSR_5_4_0_2_0
Intensity
m/z
204.0862
366.139
138.0547
274.0916
168.0652
186.0755
292.1024
126.0546
163.0604
454.1594
528.1931
657.232
965.4517 PEP_0_1_0_0_0
1168.5288 PEP_0_2_0_0_0
1330.5795 PEP_1_2_0_0_0
1492.6382 PEP_2_2_0_0_0
1654.6926 PEP_3_2_0_0_0
1695.7324 PEP_2_3_0_0_0

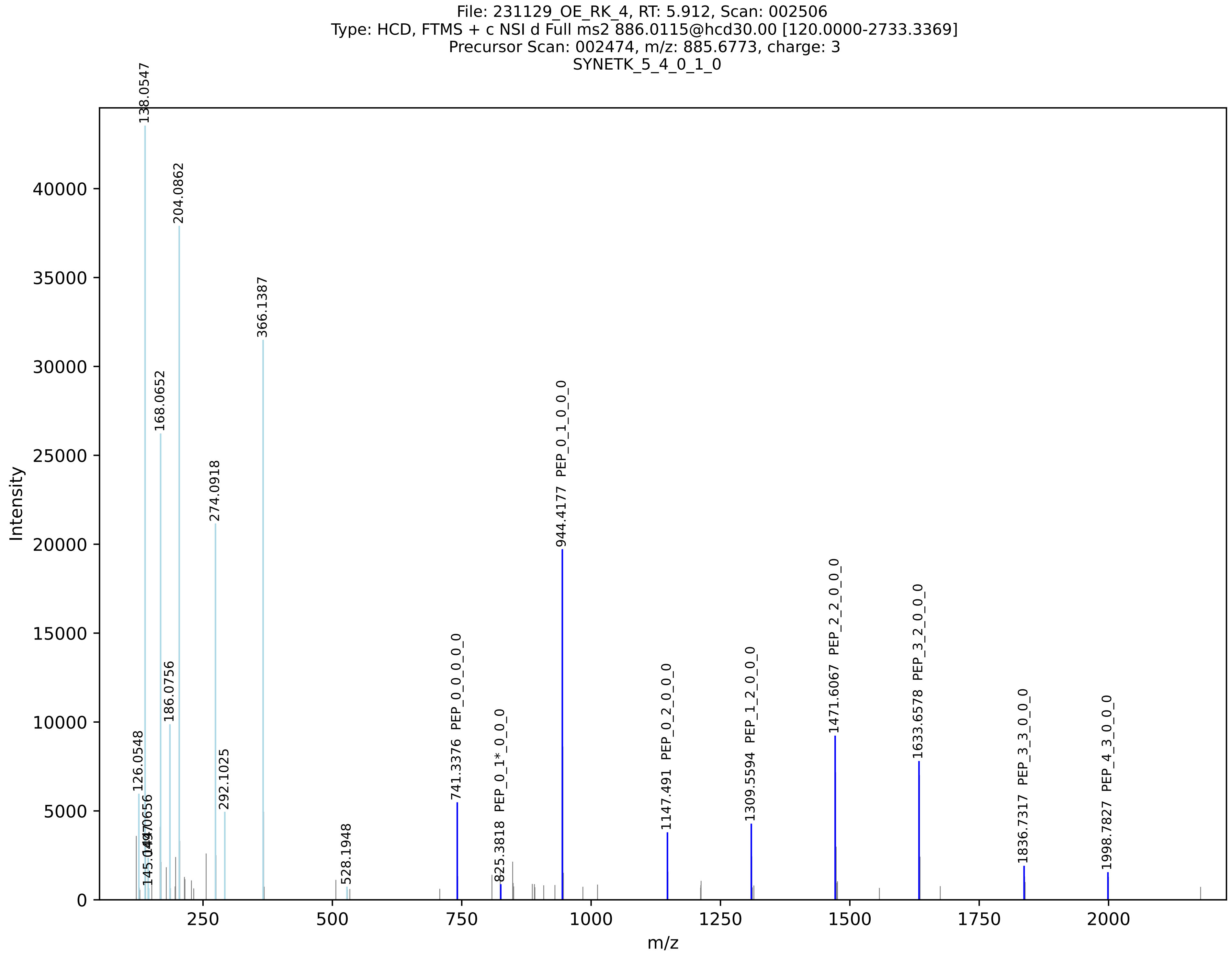

File: 231129_OE_RK_4, RT: 5.912, Scan: 002506
Type: HCD, FTMS + c NSI d Full ms2 886.0115@hcd30.00 [120.0000-2733.3369]
Precursor Scan: 002474, m/z: 885.6773, charge: 3
SYNETK_5_4_0_1_0
Intensity
m/z
138.0547
204.0862
366.1387
168.0652
274.0918
186.0756
126.0548
292.1025
145.0496
147.0656
528.1948
741.3376 PEP_0_0_0_0_0
825.3818 PEP_0_1*_0_0_0
944.4177 PEP_0_1_0_0_0
1147.491 PEP_0_2_0_0_0
1309.5594 PEP_1_2_0_0_0
1471.6067 PEP_2_2_0_0_0
1633.6578 PEP_3_2_0_0_0
1836.7317 PEP_3_3_0_0_0
1998.7827 PEP_4_3_0_0_0

File: 231129_OE_RK_4, RT: 5.94, Scan: 002540
Type: HCD, FTMS + c NSI d Full ms2 938.6899@hcd30.00 [120.0000-2894.5332]
Precursor Scan: 002505, m/z: 938.3558, charge: 3
SMTNR_5_4_0_2_0

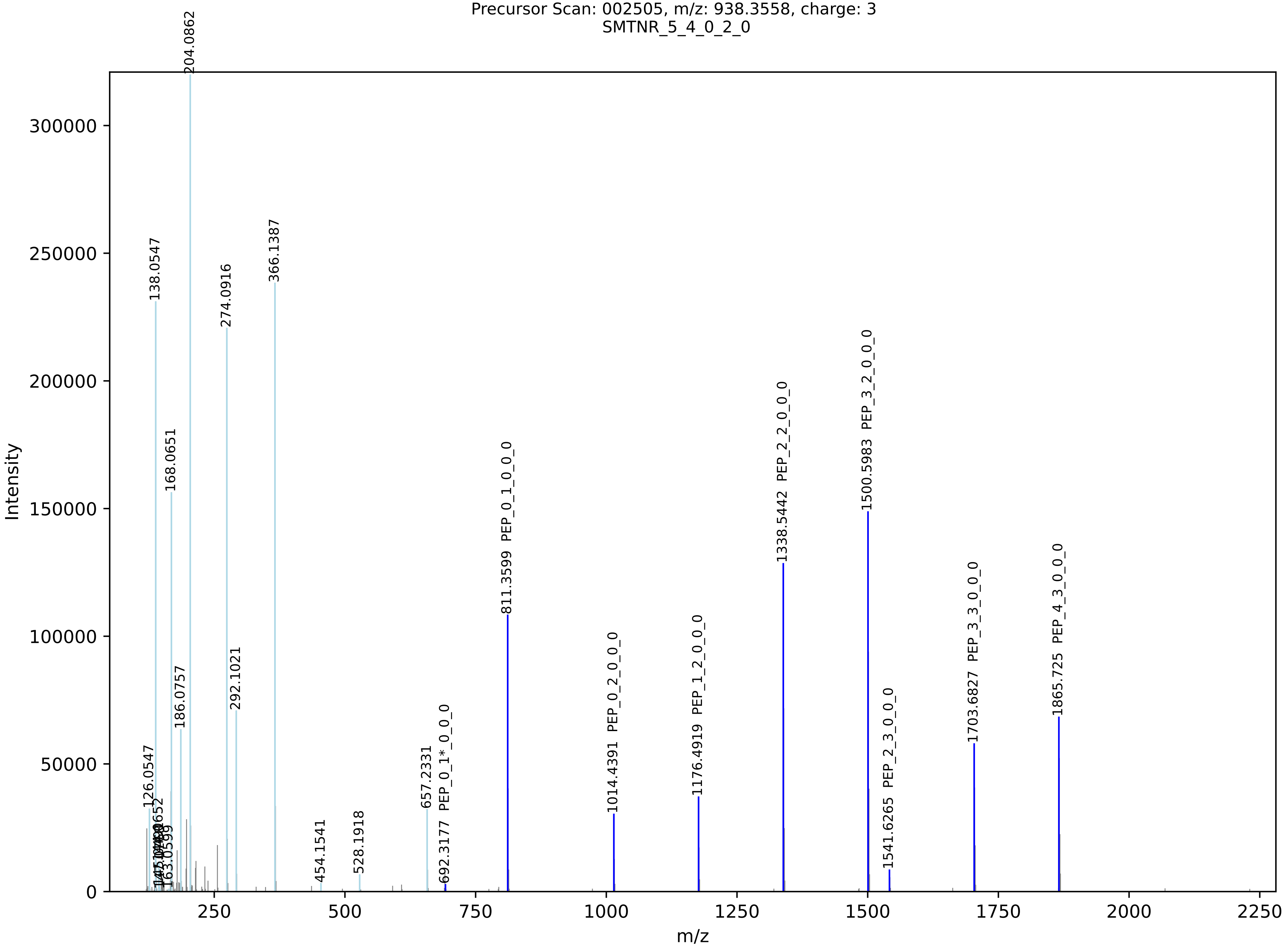

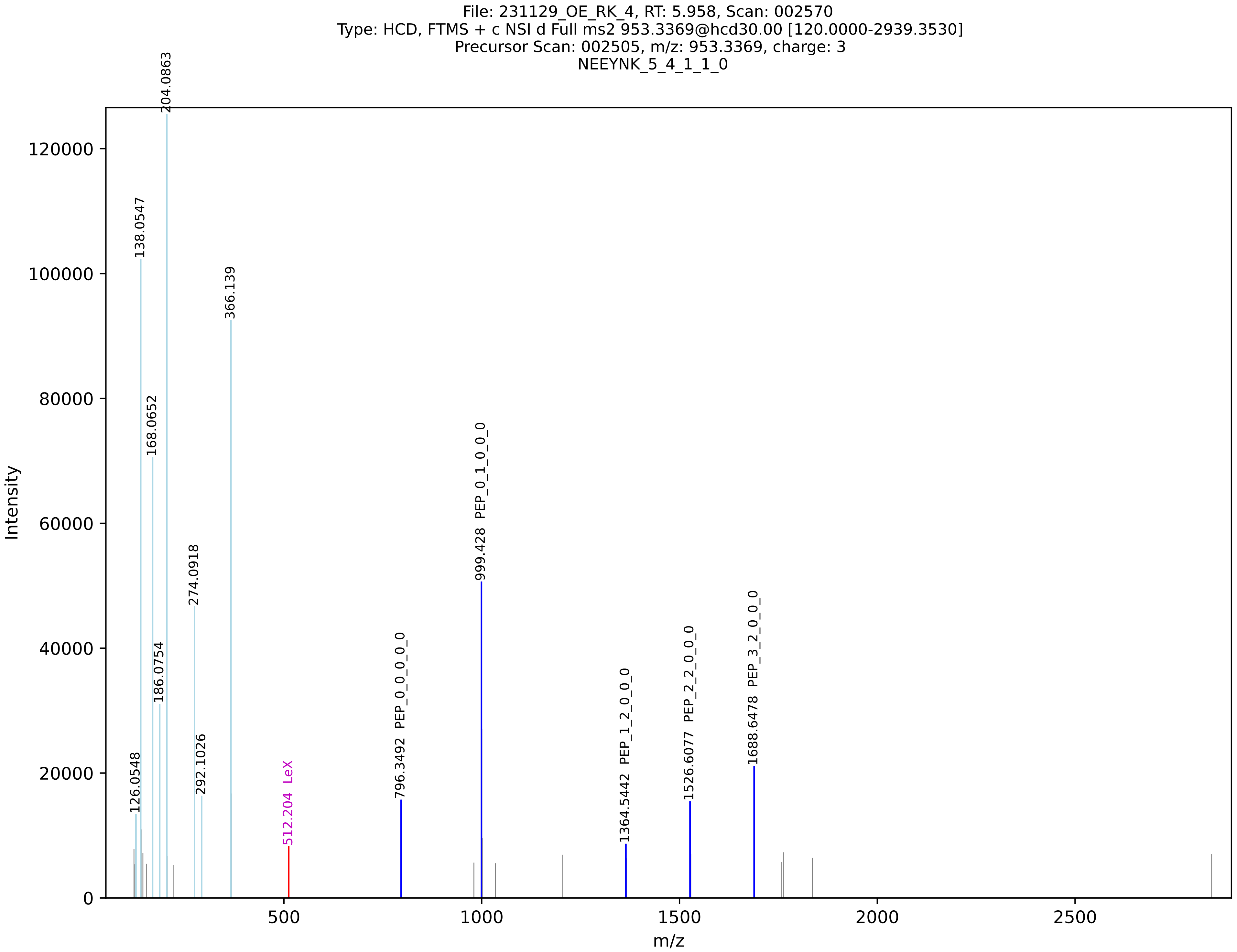
File: 231129_OE_RK_4, RT: 5.958, Scan: 002570
Type: HCD, FTMS + c NSI d Full ms2 953.3369@hcd30.00 [120.0000-2939.3530]
Precursor Scan: 002505, m/z: 953.3369, charge: 3
NEEYNK_5_4_1_1_0
Intensity
m/z
204.0863
138.0547
366.139
168.0652
274.0918
186.0754
292.1026
126.0548
512.204 LeX
796.3492 PEP_0_0_0_0_0
999.428 PEP_0_1_0_0_0
1364.5442 PEP_1_2_0_0_0
1526.6077 PEP_2_2_0_0_0
1688.6478 PEP_3_2_0_0_0

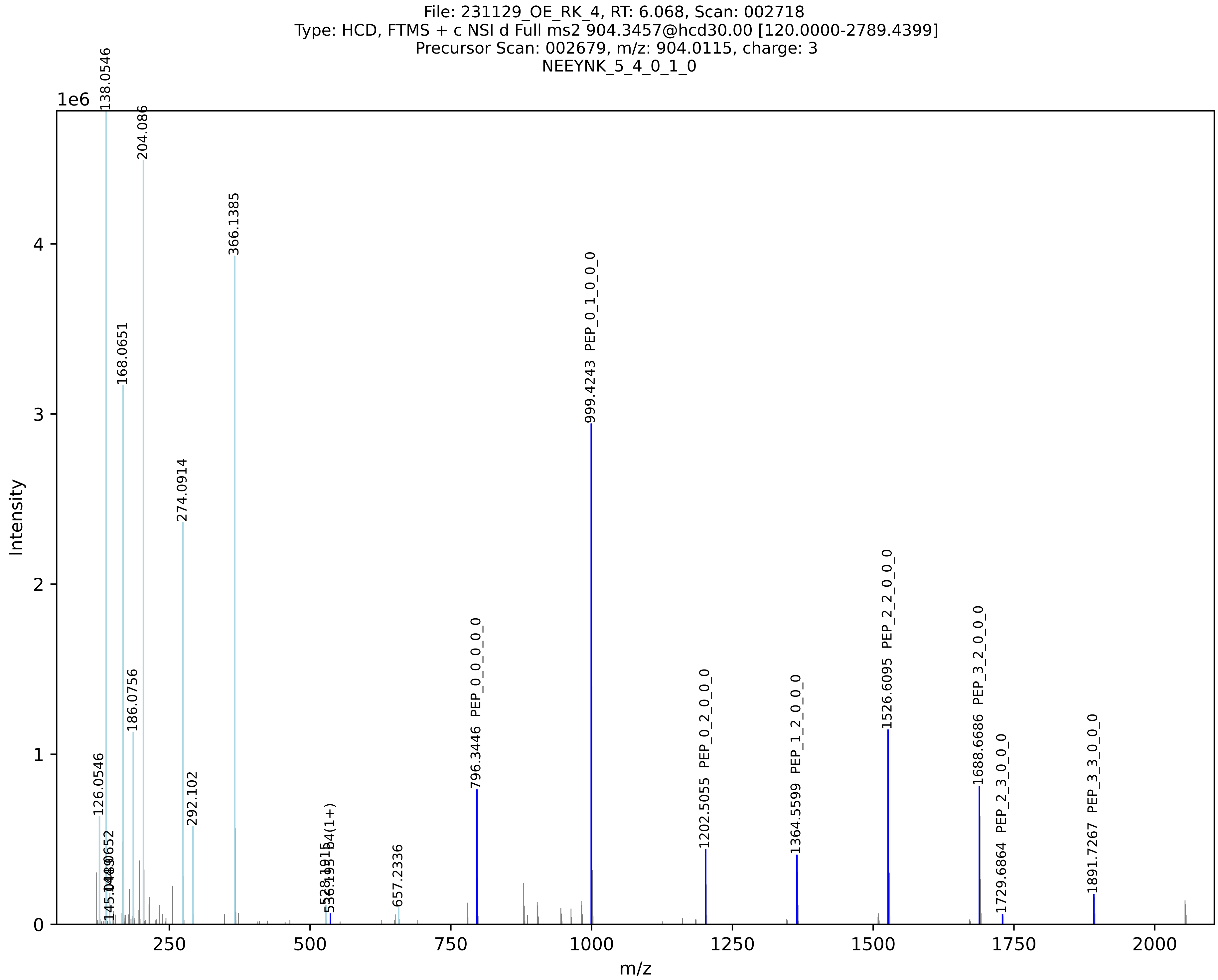
File: 231129_OE_RK_4, RT: 6.068, Scan: 002718
Type: HCD, FTMS + c NSI d Full ms2 904.3457@hcd30.00 [120.0000-2789.4399]
Precursor Scan: 002679, m/z: 904.0115, charge: 3
NEEYNK_5_4_0_1_0
1e6
Intensity
m/z
138.0546
204.086
366.1385
168.0651
274.0914
186.0756
126.0546
292.102
657.2336
796.3446 PEP_0_0_0_0_0
999.4243 PEP_0_1_0_0_0
1202.5055 PEP_0_2_0_0_0
1364.5599 PEP_1_2_0_0_0
1526.6095 PEP_2_2_0_0_0
1688.6686 PEP_3_2_0_0_0
1729.6864 PEP_2_3_0_0_0
1891.7267 PEP_3_3_0_0_0
0
1
2
3
4
250
500
750
1000
1250
1500
1750
2000

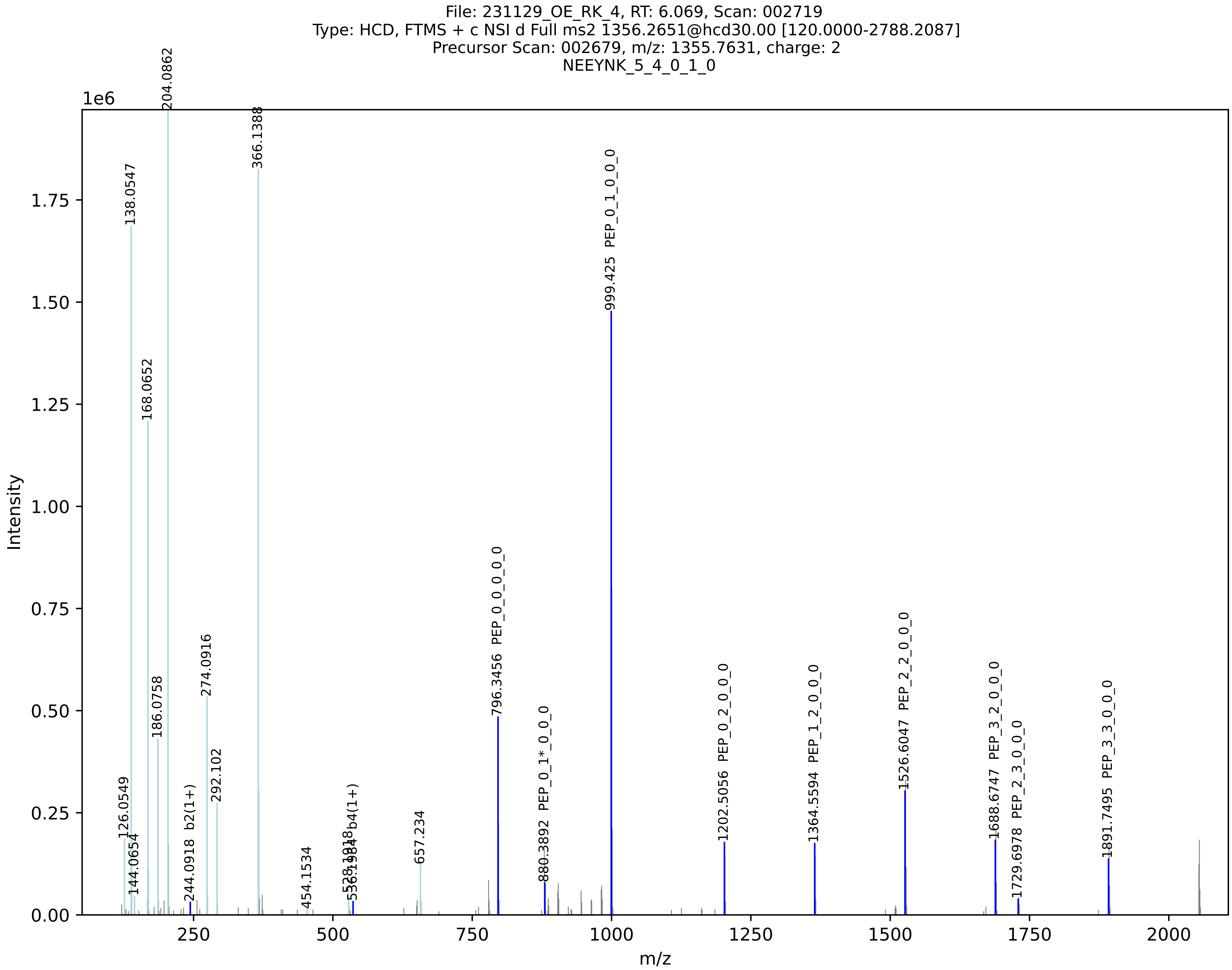
File: 231129_OE_RK_4, RT: 6.069, Scan: 002719
Type: HCD, FTMS + c NSI d Full ms2 1356.2651@hcd30.00 [120.0000-2788.2087]
Precursor Scan: 002679, m/z: 1355.7631, charge: 2
NEEYNK_5_4_0_1_0
1e6
Intensity
m/z
0.00
0.25
0.50
0.75
1.00
1.25
1.50
1.75
250
500
750
1000
1250
1500
1750
2000
204.0862
366.1388
138.0547
168.0652
186.0758
274.0916
292.102
126.0549
144.0654
244.0918 b2(1+)
454.1534
528.1918
536.1984 b4(1+)
657.234
796.3456 PEP_0_0_0_0_0
880.3892 PEP_0_1*_0_0_0
999.425 PEP_0_1_0_0_0
1202.5056 PEP_0_2_0_0_0
1364.5594 PEP_1_2_0_0_0
1526.6047 PEP_2_2_0_0_0
1688.6747 PEP_3_2_0_0_0
1729.6978 PEP_2_3_0_0_0
1891.7495 PEP_3_3_0_0_0

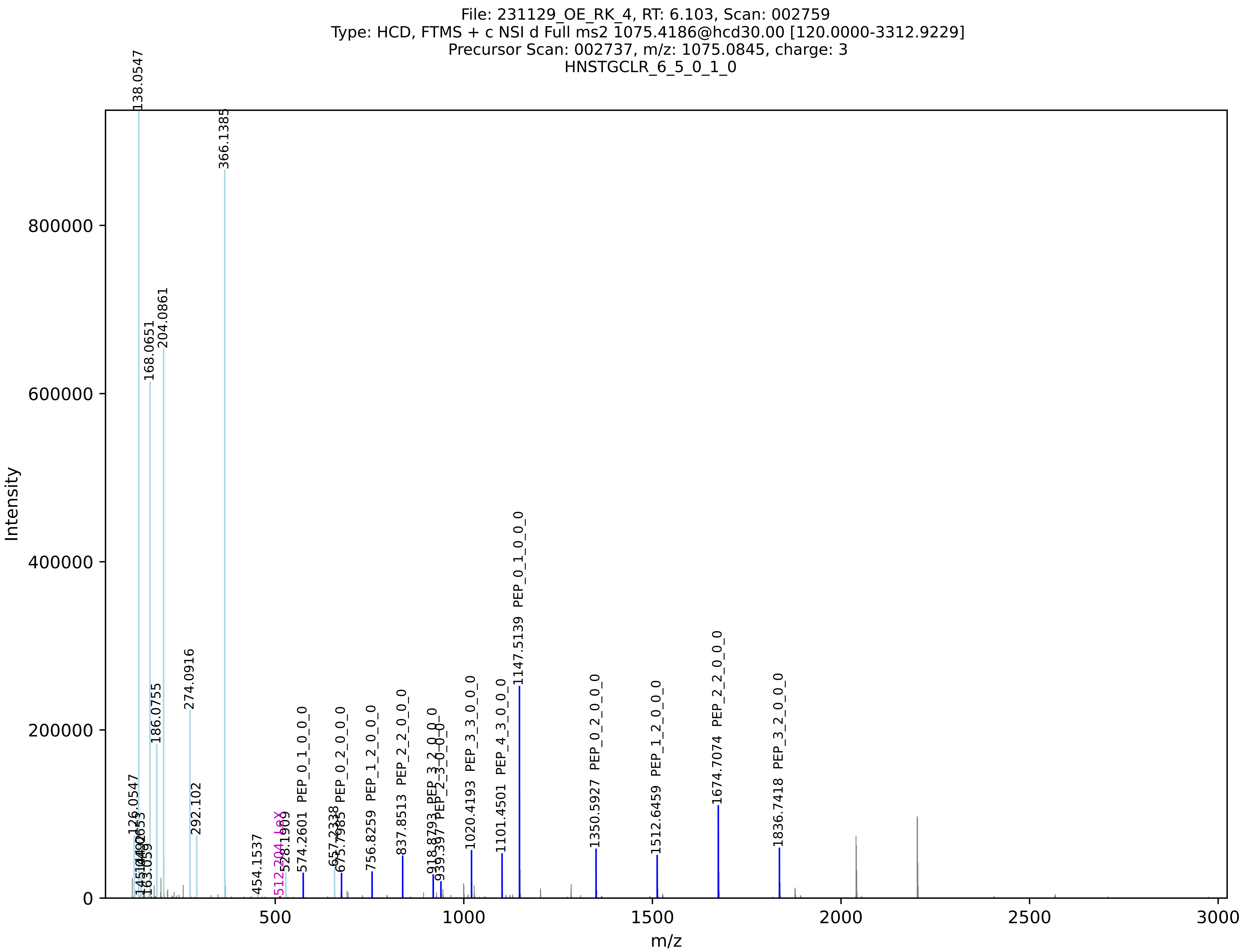
File: 231129_OE_RK_4, RT: 6.103, Scan: 002759
Type: HCD, FTMS + c NSI d Full ms2 1075.4186@hcd30.00 [120.0000-3312.9229]
Precursor Scan: 002737, m/z: 1075.0845, charge: 3
HNSTGCLR_6_5_0_1_0
Intensity
m/z
138.0547
366.1385
204.0861
168.0651
274.0916
186.0755
292.102
126.0547
163.059
454.1537
512.204_LeX
528.1909
574.2601 PEP_0_1_0_0_0
657.2338
675.7985 PEP_0_2_0_0_0
756.8259 PEP_1_2_0_0_0
837.8513 PEP_2_2_0_0_0
918.8793 PEP_3_2_0_0_0
939.397 PEP_2_3_0_0_0
1020.4193 PEP_3_3_0_0_0
1101.4501 PEP_4_3_0_0_0
1147.5139 PEP_0_1_0_0_0
1350.5927 PEP_0_2_0_0_0
1512.6459 PEP_1_2_0_0_0
1674.7074 PEP_2_2_0_0_0
1836.7418 PEP_3_2_0_0_0

File: 231129_OE_RK_4, RT: 6.103, Scan: 002760
Type: HCD, FTMS + c NSI d Full ms2 1274.9869@hcd30.00 [120.0000-2622.4014]
Precursor Scan: 002737, m/z: 1274.4858, charge: 2
NEEYNK_4_4_0_1_0

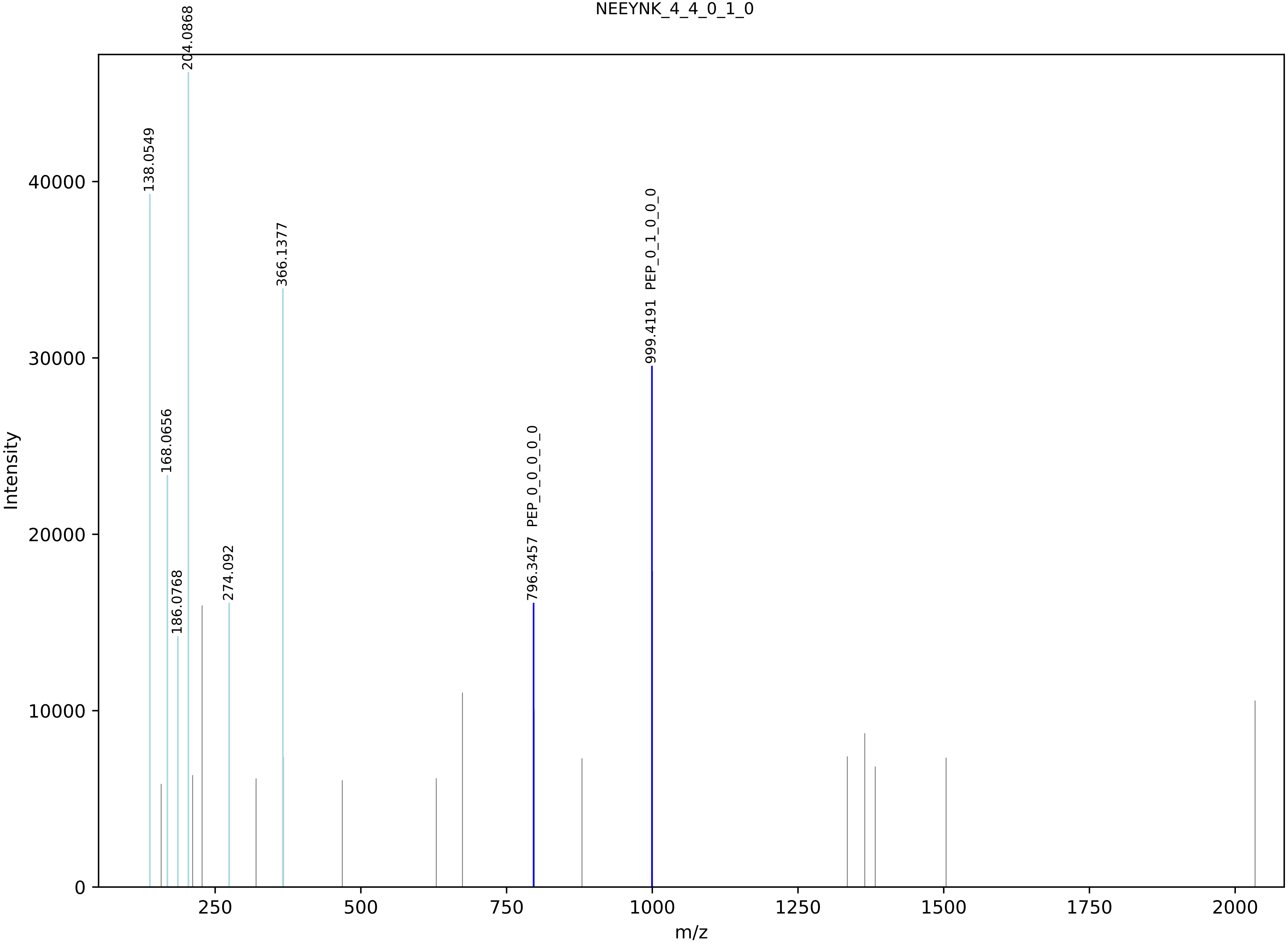

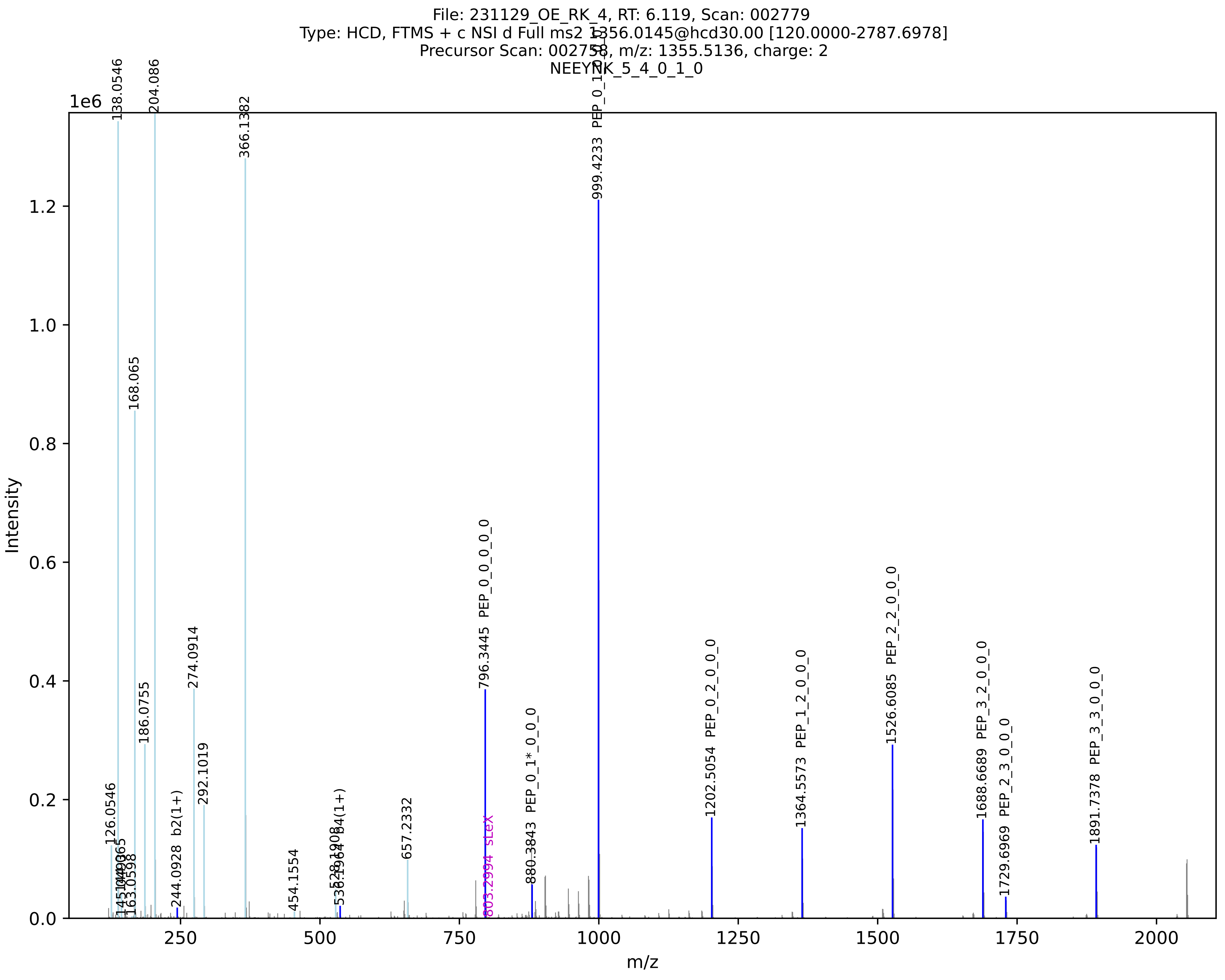

File: 231129_OE_RK_4, RT: 6.119, Scan: 002779
Type: HCD, FTMS + c NSI d Full ms2 1356.0145@hcd30.00 [120.0000-2787.6978]
Precursor Scan: 002758, m/z: 1355.5136, charge: 2
NEEYNK_5_4_0_1_0
1e6
Intensity
m/z
138.0546
204.086
366.1382
999.4233 PEP_0_1_0_0_0
168.065
186.0755
274.0914
292.1019
126.0546
163.0598
244.0928 b2(1+)
454.1554
528.1908
538.1964 b4(1+)
657.2332
796.3445 PEP_0_0_0_0_0
803.2994 sLeX
880.3843 PEP_0_1*_0_0_0
1202.5054 PEP_0_2_0_0_0
1364.5573 PEP_1_2_0_0_0
1526.6085 PEP_2_2_0_0_0
1688.6689 PEP_3_2_0_0_0
1729.6969 PEP_2_3_0_0_0
1891.7378 PEP_3_3_0_0_0

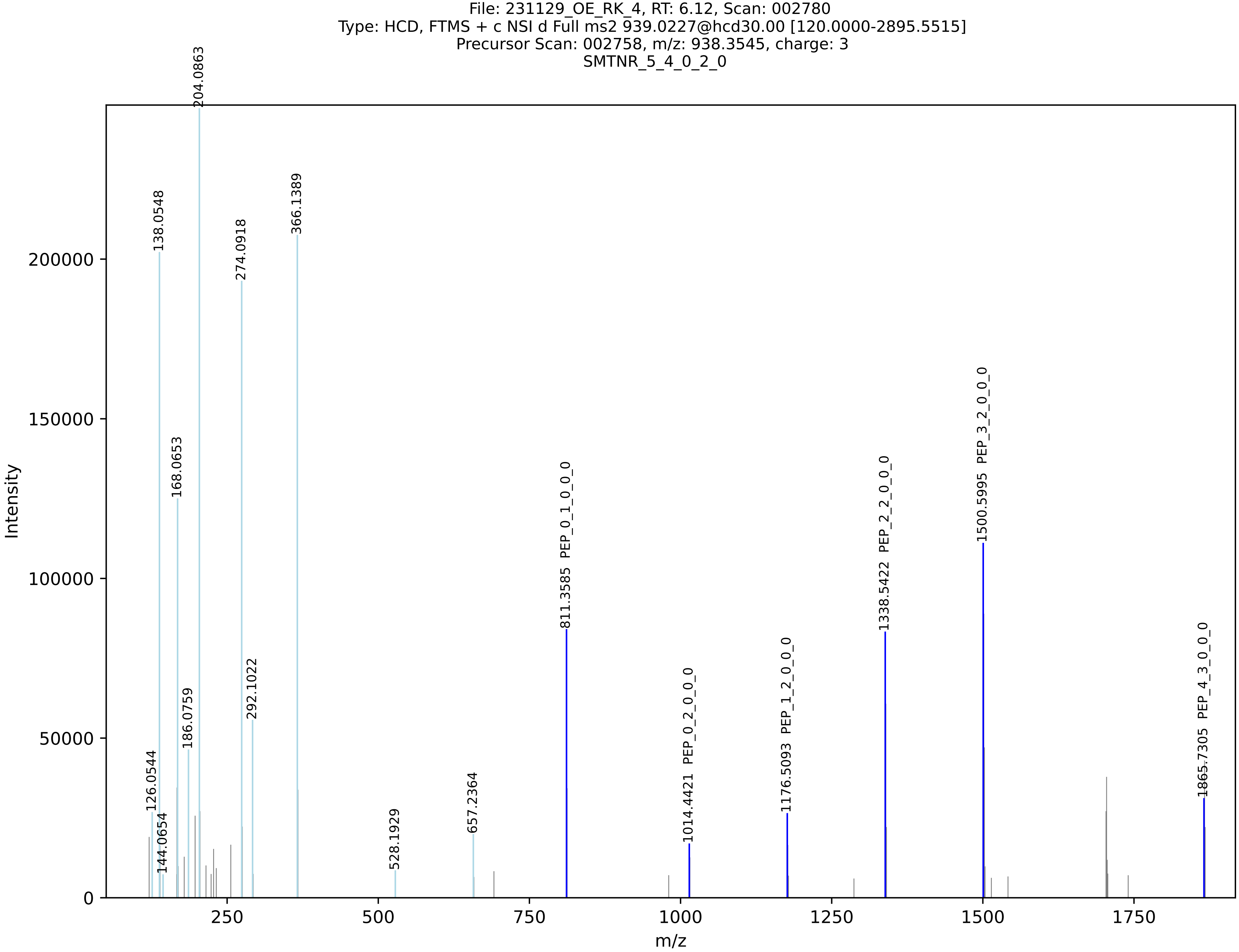
File: 231129_OE_RK_4, RT: 6.12, Scan: 002780
Type: HCD, FTMS + c NSI d Full ms2 939.0227@hcd30.00 [120.0000-2895.5515]
Precursor Scan: 002758, m/z: 938.3545, charge: 3
SMTNR_5_4_0_2_0
Intensity
m/z
204.0863
138.0548
366.1389
274.0918
168.0653
186.0759
292.1022
126.0544
144.0654
528.1929
657.2364
811.3585 PEP_0_1_0_0_0
1014.4421 PEP_0_2_0_0_0
1176.5093 PEP_1_2_0_0_0
1338.5422 PEP_2_2_0_0_0
1500.5995 PEP_3_2_0_0_0
1865.7305 PEP_4_3_0_0_0

File: 231129_OE_RK_4, RT: 6.144, Scan: 002812
Type: HCD, FTMS + c NSI d Full ms2 1067.1039@hcd30.00 [120.0000-3287.4800]
Precursor Scan: 002778, m/z: 1067.1039, charge: 3
TRLSSNSTK_5_4_0_2_0

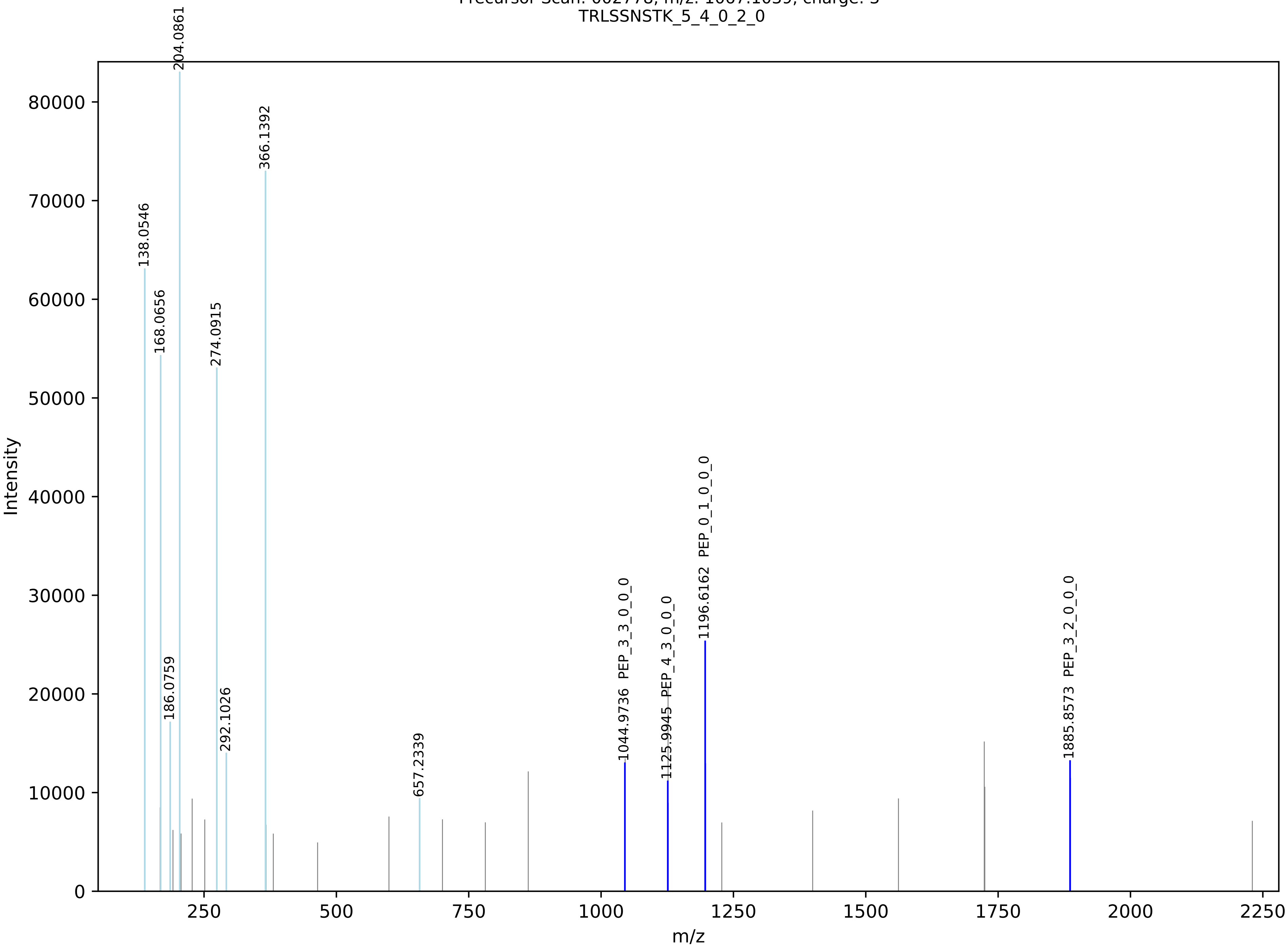

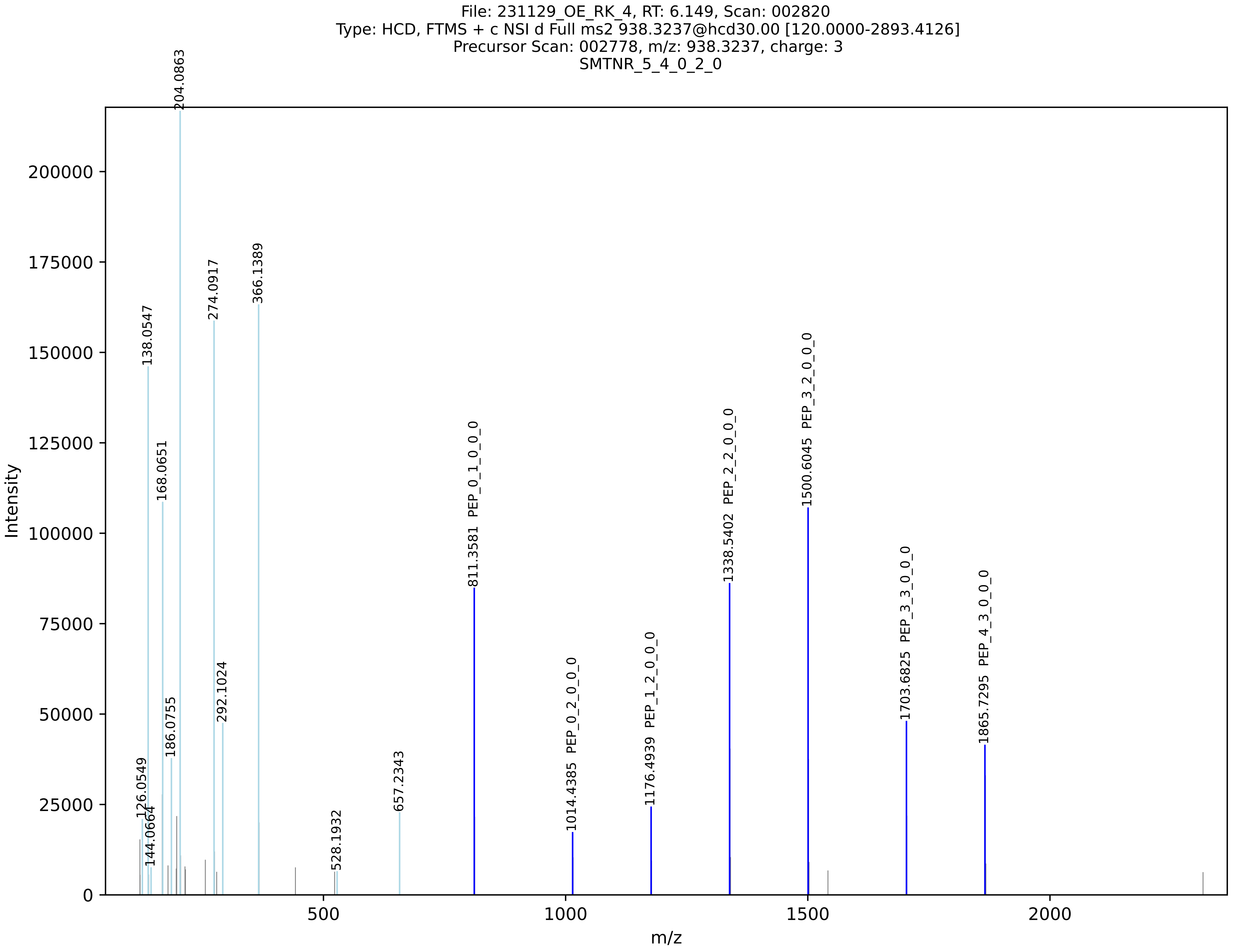
File: 231129_OE_RK_4, RT: 6.149, Scan: 002820
Type: HCD, FTMS + c NSI d Full ms2 938.3237@hcd30.00 [120.0000-2893.4126]
Precursor Scan: 002778, m/z: 938.3237, charge: 3
SMTNR_5_4_0_2_0
Intensity
m/z
204.0863
138.0547
274.0917
366.1389
168.0651
186.0755
292.1024
126.0549
144.0664
528.1932
657.2343
811.3581 PEP_0_1_0_0_0
1014.4385 PEP_0_2_0_0_0
1176.4939 PEP_1_2_0_0_0
1338.5402 PEP_2_2_0_0_0
1500.6045 PEP_3_2_0_0_0
1703.6825 PEP_3_3_0_0_0
1865.7295 PEP_4_3_0_0_0

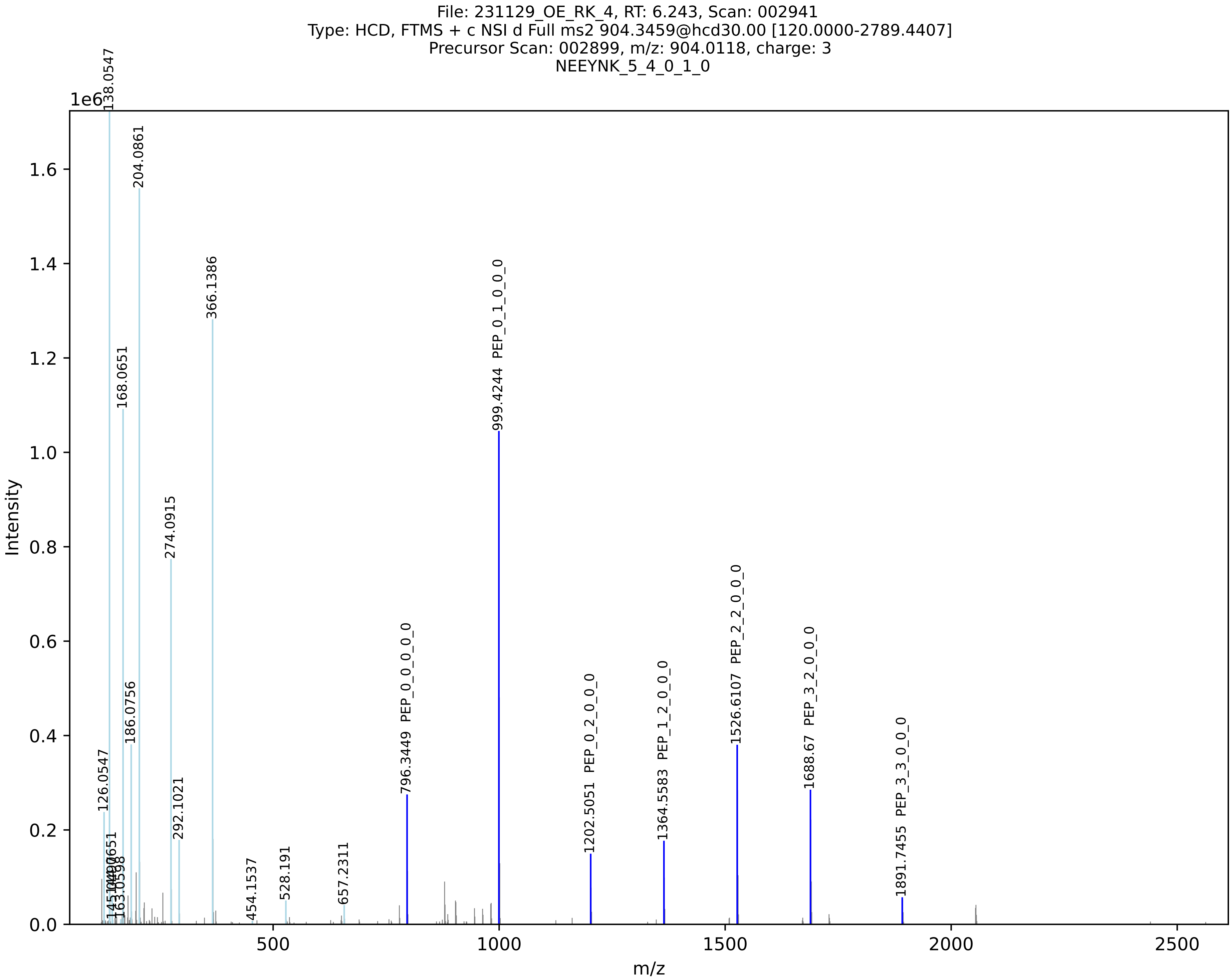

File: 231129_OE_RK_4, RT: 6.243, Scan: 002941
Type: HCD, FTMS + c NSI d Full ms2 904.3459@hcd30.00 [120.0000-2789.4407]
Precursor Scan: 002899, m/z: 904.0118, charge: 3
NEEYNK_5_4_0_1_0
1e6
Intensity
m/z
138.0547
204.0861
366.1386
168.0651
274.0915
186.0756
126.0547
292.1021
454.1537
528.191
657.2311
796.3449 PEP_0_0_0_0_0
999.4244 PEP_0_1_0_0_0
1202.5051 PEP_0_2_0_0_0
1364.5583 PEP_1_2_0_0_0
1526.6107 PEP_2_2_0_0_0
1688.67 PEP_3_2_0_0_0
1891.7455 PEP_3_3_0_0_0

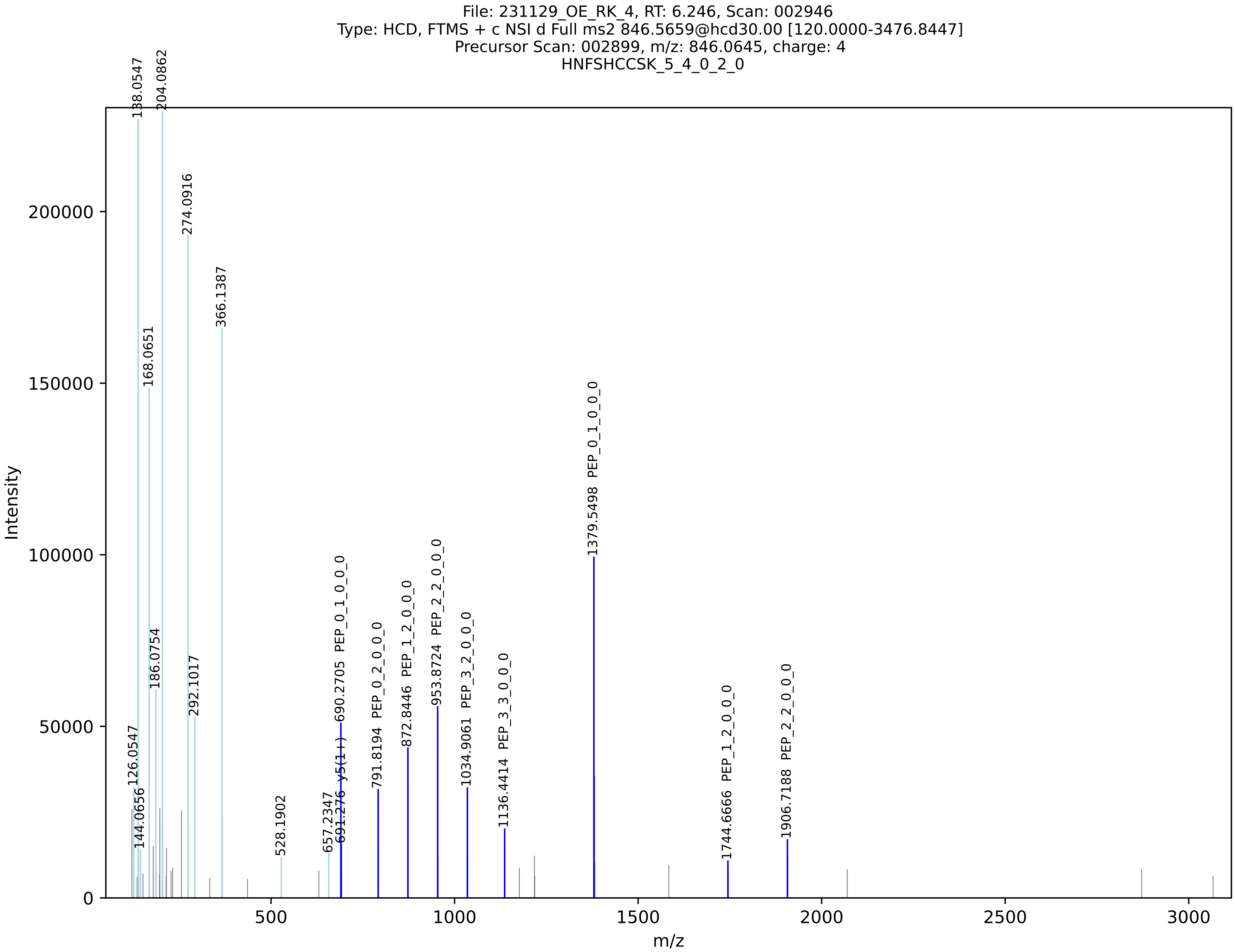
File: 231129_OE_RK_4, RT: 6.246, Scan: 002946
Type: HCD, FTMS + c NSI d Full ms2 846.5659@hcd30.00 [120.0000-3476.8447]
Precursor Scan: 002899, m/z: 846.0645, charge: 4
HNFSHCCSK_5_4_0_2_0
Intensity
m/z
0
50000
100000
150000
200000
500
1000
1500
2000
2500
3000
138.0547
204.0862
274.0916
366.1387
168.0651
186.0754
292.1017
126.0547
144.0656
528.1902
657.2347
691.276 y5(1+)
690.2705 PEP_0_1_0_0_0
791.8194 PEP_0_2_0_0_0
872.8446 PEP_1_2_0_0_0
953.8724 PEP_2_2_0_0_0
1034.9061 PEP_3_2_0_0_0
1136.4414 PEP_3_3_0_0_0
1379.5498 PEP_0_1_0_0_0
1744.6666 PEP_1_2_0_0_0
1906.7188 PEP_2_2_0_0_0

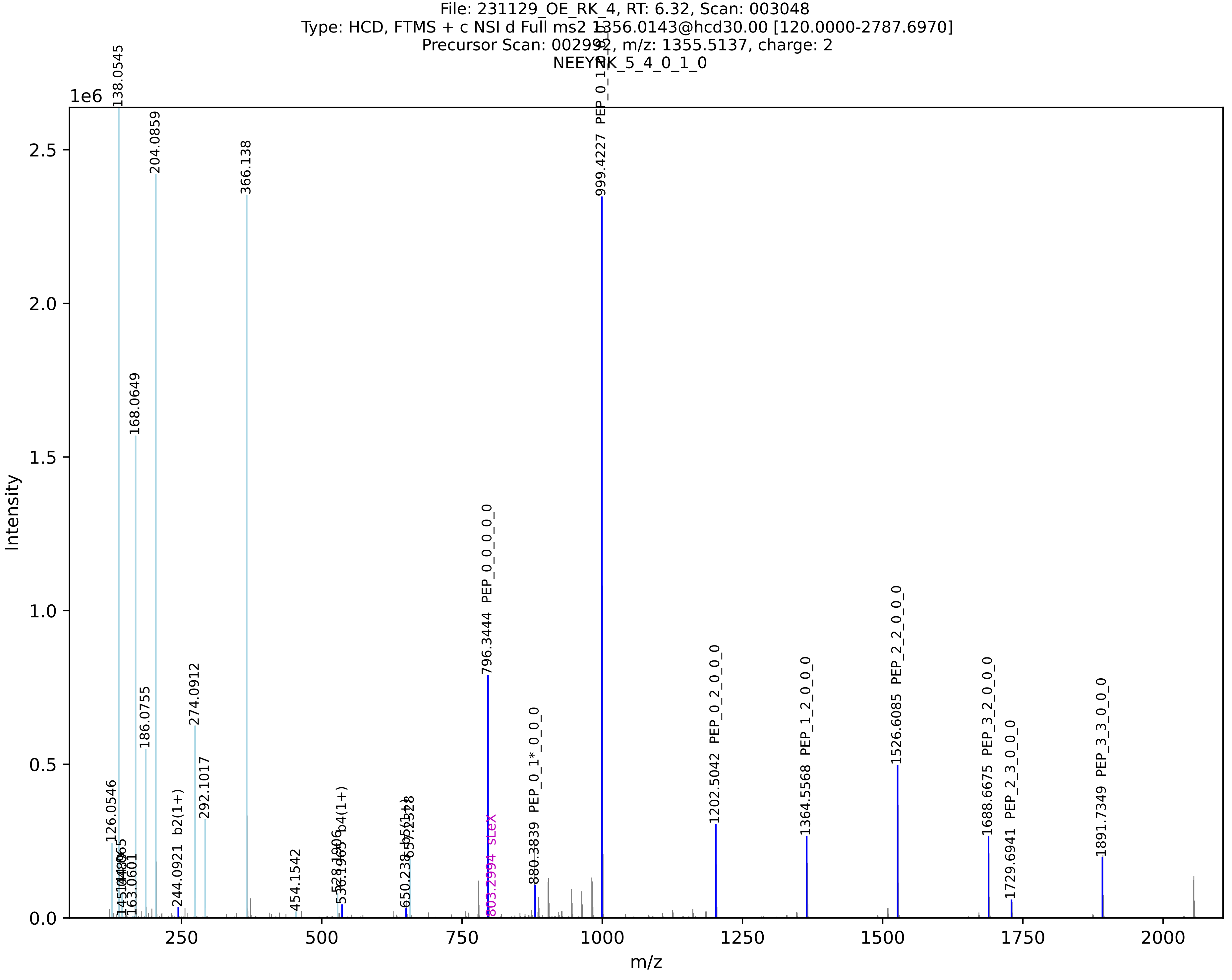
File: 231129_OE_RK_4, RT: 6.32, Scan: 003048
Type: HCD, FTMS + c NSI d Full ms2 1356.0143@hcd30.00 [120.0000-2787.6970]
Precursor Scan: 002992, m/z: 1355.5137, charge: 2
NEEYNK_5_4_0_1_0
1e6
Intensity
m/z
138.0545
204.0859
366.138
168.0649
186.0755
274.0912
292.1017
126.0546
163.0601
244.0921 b2(1+)
454.1542
536.1965 b4(1+)
796.3444 PEP_0_0_0_0_0
803.2994 sLeX
880.3839 PEP_0_1*_0_0_0
999.4227 PEP_0_1_0
1202.5042 PEP_0_2_0_0_0
1364.5568 PEP_1_2_0_0_0
1526.6085 PEP_2_2_0_0_0
1688.6675 PEP_3_2_0_0_0
1729.6941 PEP_2_3_0_0_0
1891.7349 PEP_3_3_0_0_0
657.2528

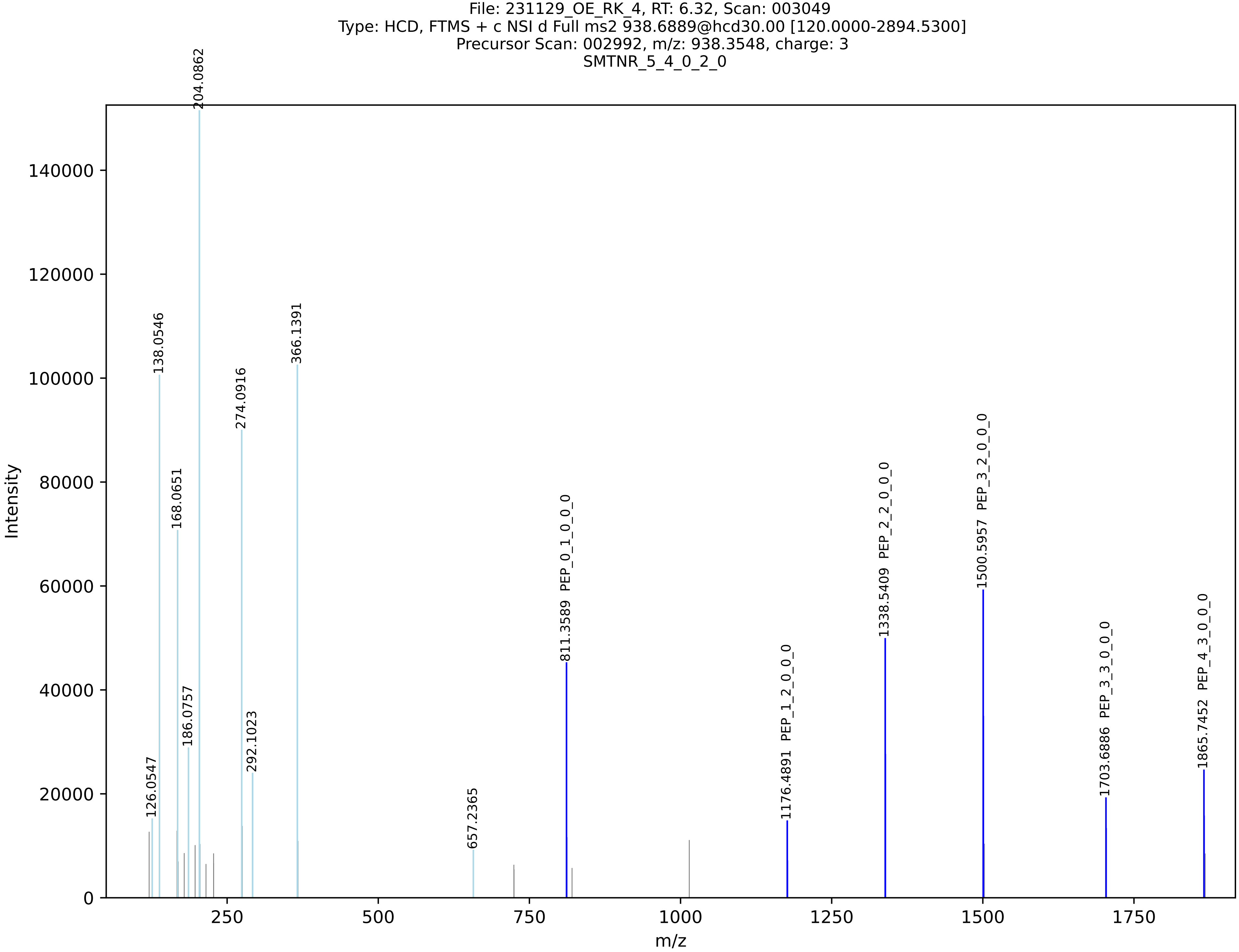
File: 231129_OE_RK_4, RT: 6.32, Scan: 003049
Type: HCD, FTMS + c NSI d Full ms2 938.6889@hcd30.00 [120.0000-2894.5300]
Precursor Scan: 002992, m/z: 938.3548, charge: 3
SMTNR_5_4_0_2_0
Intensity
m/z
204.0862
138.0546
366.1391
274.0916
168.0651
186.0757
292.1023
126.0547
657.2365
811.3589 PEP_0_1_0_0_0
1176.4891 PEP_1_2_0_0_0
1338.5409 PEP_2_2_0_0_0
1500.5957 PEP_3_2_0_0_0
1703.6886 PEP_3_3_0_0_0
1865.7452 PEP_4_3_0_0_0

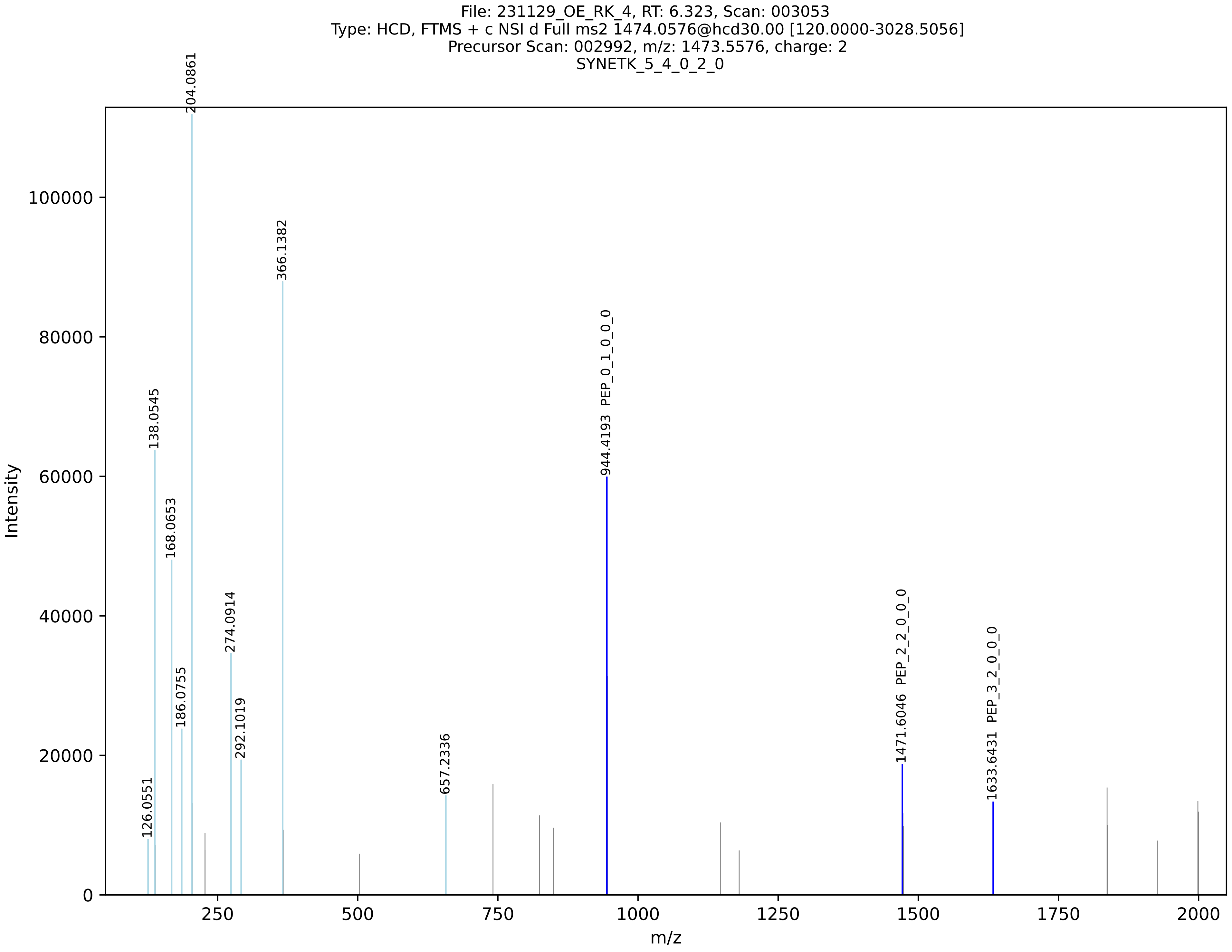
File: 231129_OE_RK_4, RT: 6.323, Scan: 003053
Type: HCD, FTMS + c NSI d Full ms2 1474.0576@hcd30.00 [120.0000-3028.5056]
Precursor Scan: 002992, m/z: 1473.5576, charge: 2
SYNETK_5_4_0_2_0
204.0861
366.1382
944.4193 PEP_0_1_0_0_0_
138.0545
168.0653
274.0914
186.0755
292.1019
1471.6046 PEP_2_2_0_0_0_
1633.6431 PEP_3_2_0_0_0_
657.2336
126.0551
Intensity
m/z
0
20000
40000
60000
80000
100000
250
500
750
1000
1250
1500
1750
2000

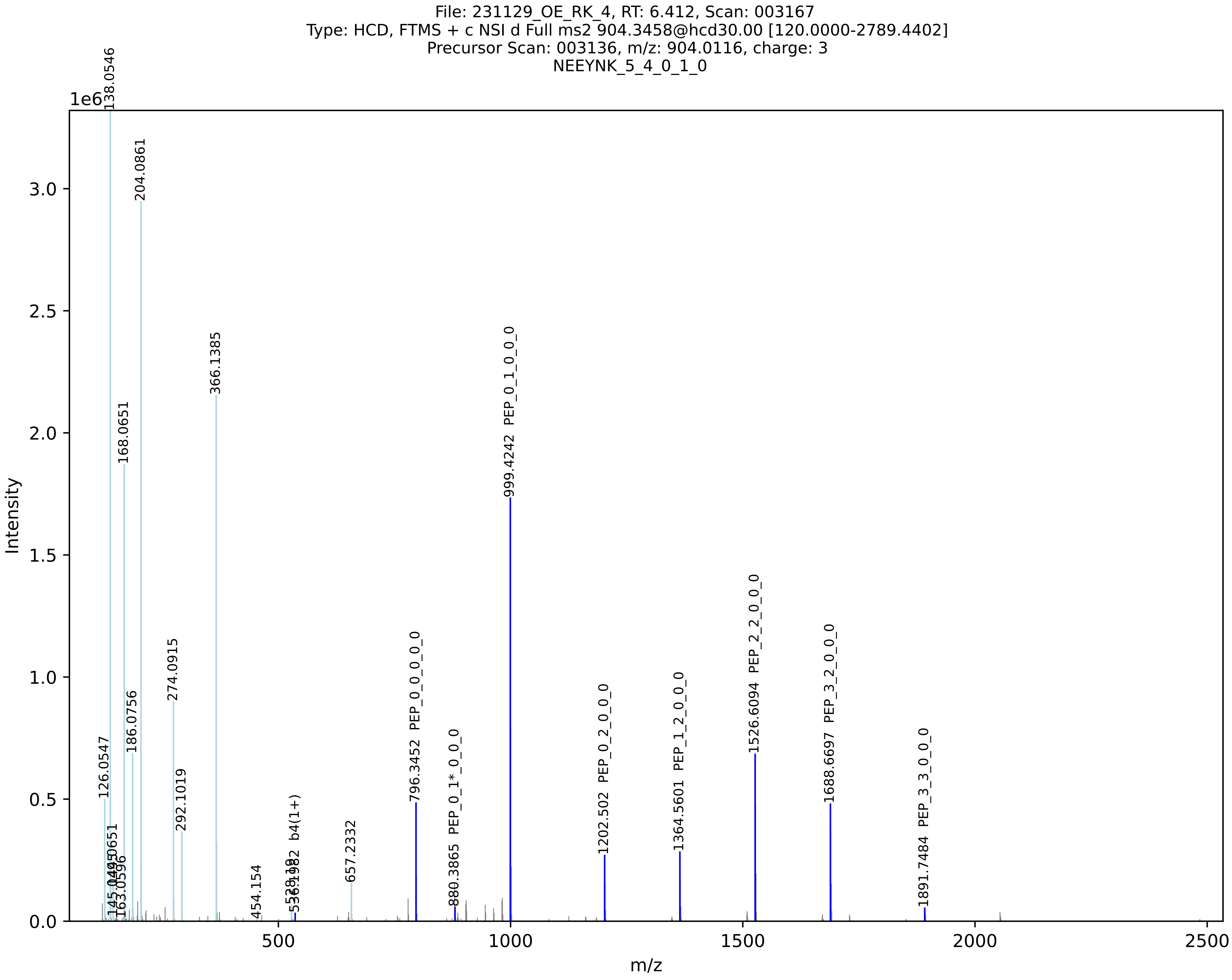
File: 231129_OE_RK_4, RT: 6.412, Scan: 003167
Type: HCD, FTMS + c NSI d Full ms2 904.3458@hcd30.00 [120.0000-2789.4402]
Precursor Scan: 003136, m/z: 904.0116, charge: 3
NEEYNK_5_4_0_1_0
1e6
Intensity
m/z
138.0546
204.0861
366.1385
168.0651
274.0915
186.0756
126.0547
292.1019
145.0495
150.0651
163.0596
454.154
528.19
536.1982 b4(1+)
657.2332
796.3452 PEP_0_0_0_0_0_
880.3865 PEP_0_1*_0_0_0_
999.4242 PEP_0_1_0_0_0_
1202.502 PEP_0_2_0_0_0_
1364.5601 PEP_1_2_0_0_0_
1526.6094 PEP_2_2_0_0_0_
1688.6697 PEP_3_2_0_0_0_
1891.7484 PEP_3_3_0_0_0_

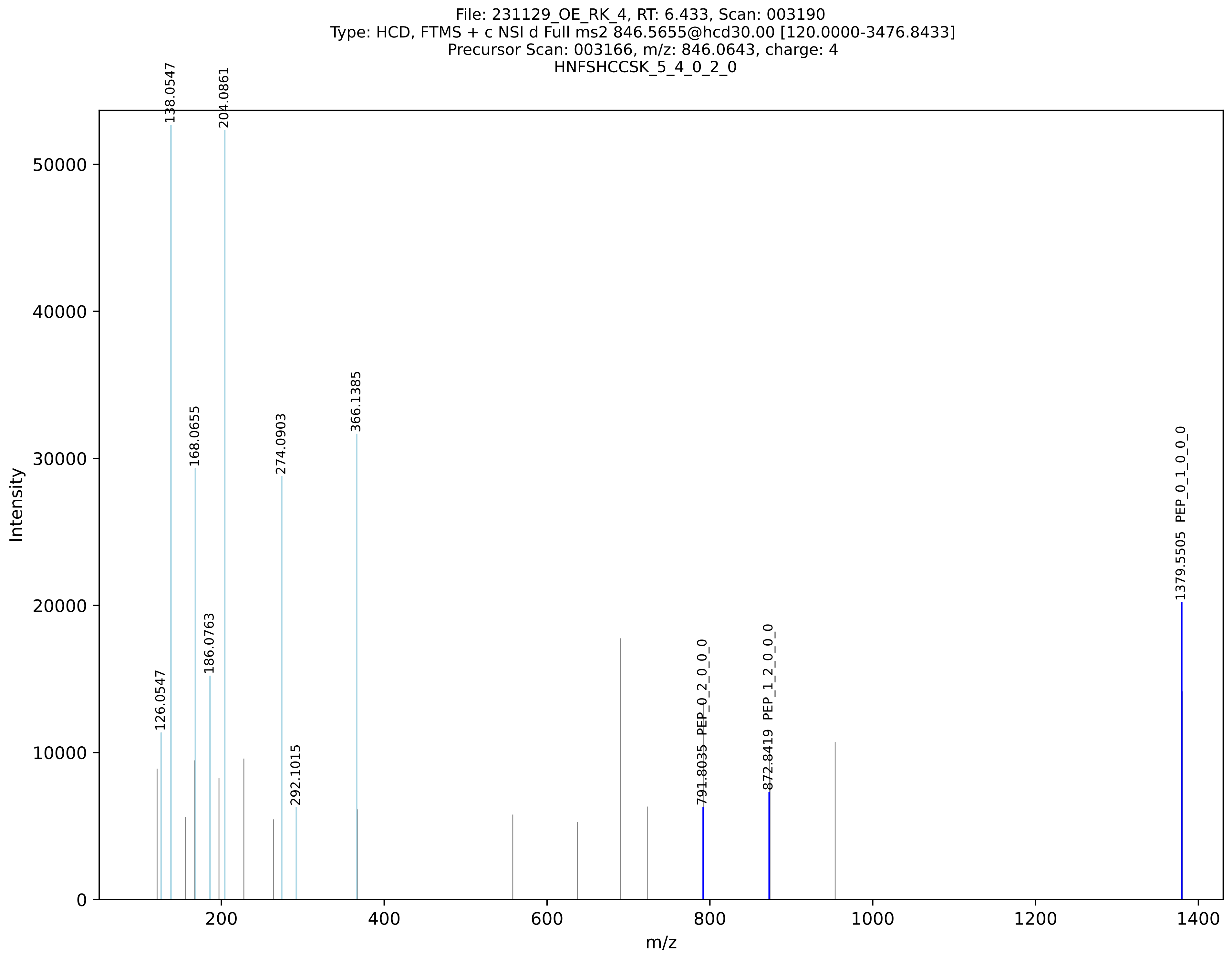
File: 231129_OE_RK_4, RT: 6.433, Scan: 003190
Type: HCD, FTMS + c NSI d Full ms2 846.5655@hcd30.00 [120.0000-3476.8433]
Precursor Scan: 003166, m/z: 846.0643, charge: 4
HNFSHCCSK_5_4_0_2_0
Intensity
m/z
126.0547
138.0547
168.0655
186.0763
204.0861
274.0903
292.1015
366.1385
791.8035 PEP_0_2_0_0_0
872.8419 PEP_1_2_0_0_0
1379.5505 PEP_0_1_0_0_0
0
10000
20000
30000
40000
50000
200
400
600
800
1000
1200
1400

File: 231129_OE_RK_4, RT: 6.434, Scan: 003191
Type: HCD, FTMS + c NSI d Full ms2 1122.7534@hcd30.00 [120.0000-3457.7673]
Precursor Scan: 003166, m/z: 1122.7534, charge: 3
NEEYNK_6_5_0_2_0

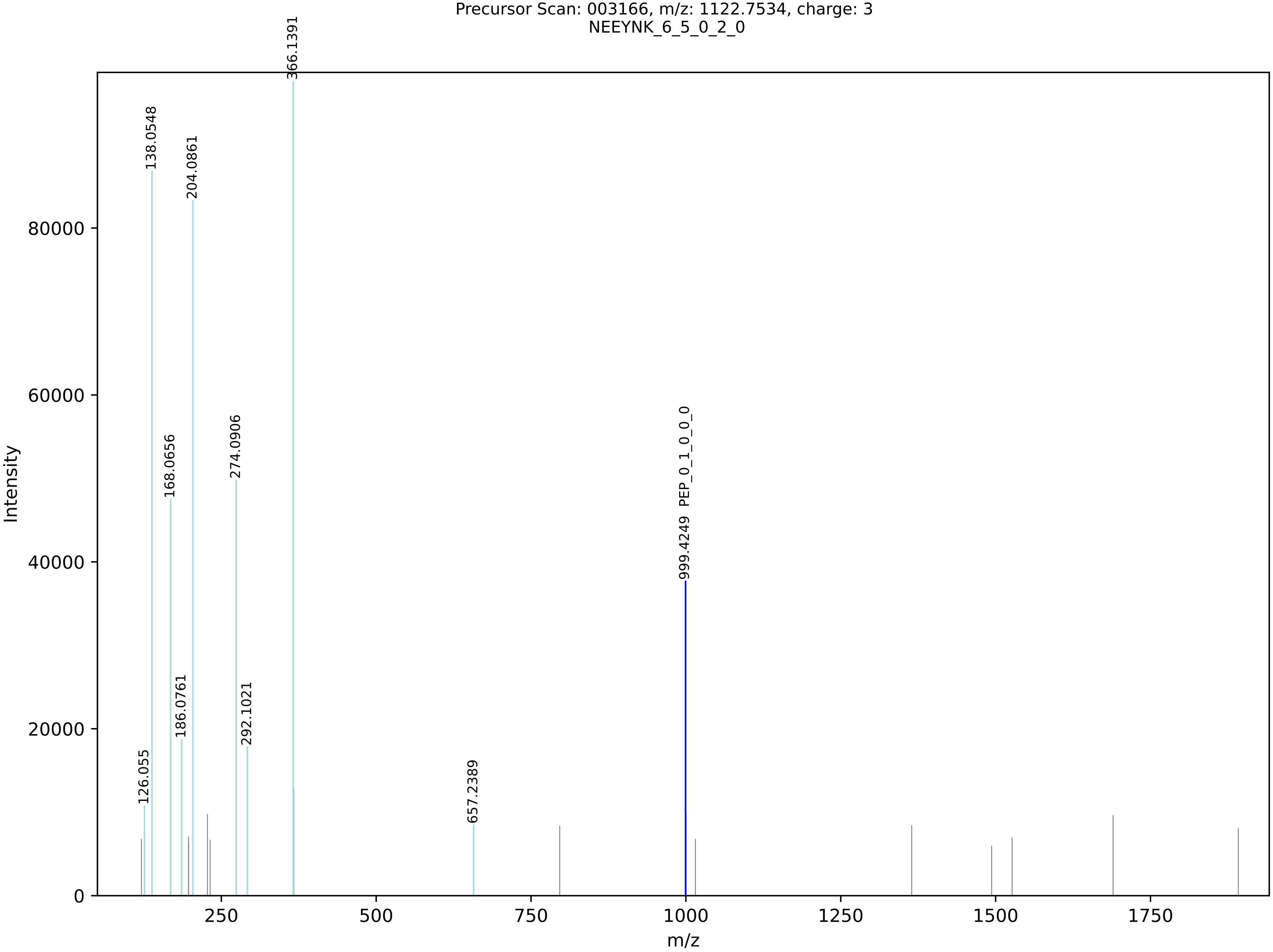

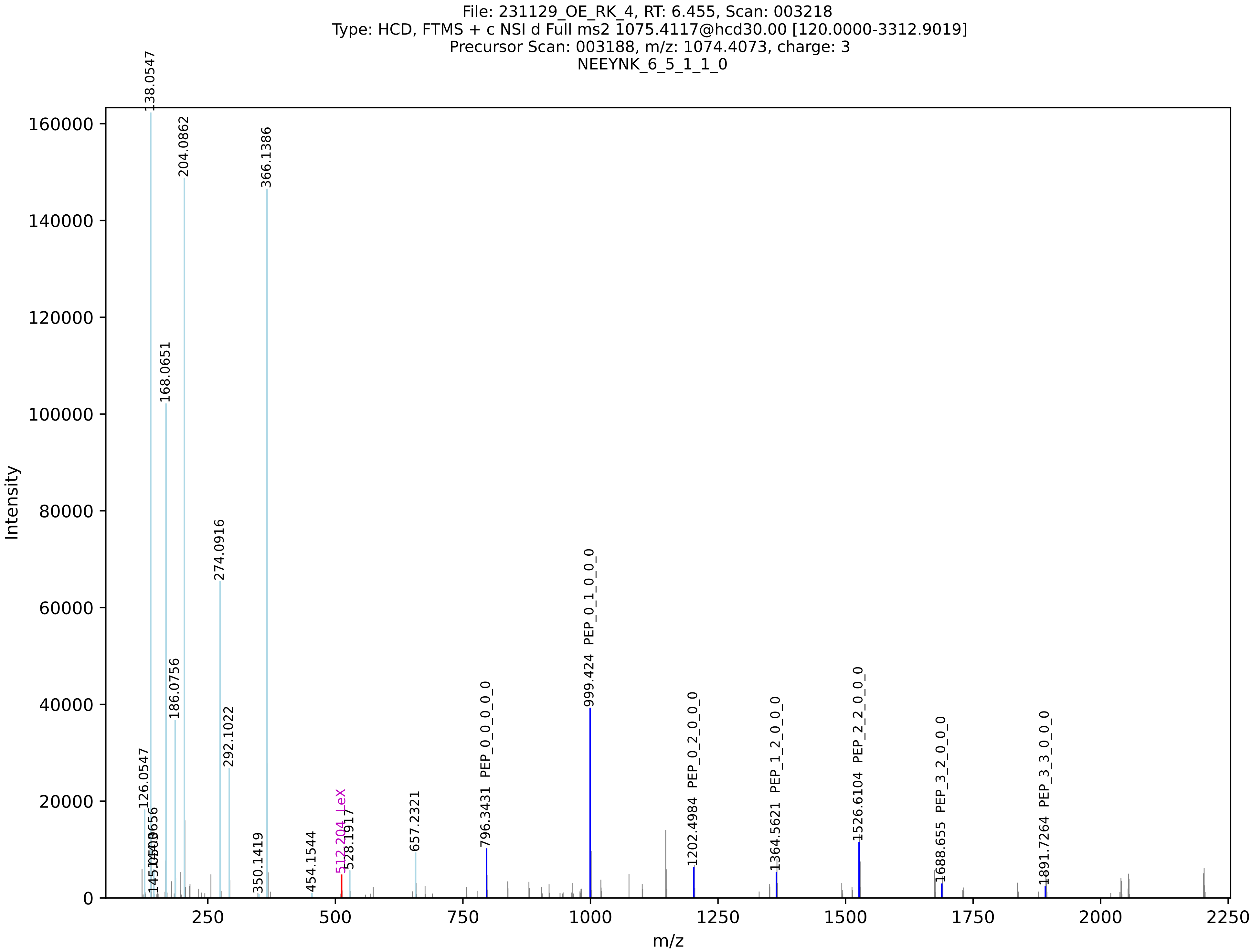
File: 231129_OE_RK_4, RT: 6.455, Scan: 003218
Type: HCD, FTMS + c NSI d Full ms2 1075.4117@hcd30.00 [120.0000-3312.9019]
Precursor Scan: 003188, m/z: 1074.4073, charge: 3
NEEYNK_6_5_1_1_0
Intensity
m/z
138.0547
204.0862
366.1386
168.0651
274.0916
186.0756
292.1022
126.0547
350.1419
454.1544
512.204 LeX
528.1917
657.2321
796.3431 PEP_0_0_0_0_0
999.424 PEP_0_1_0_0_0
1202.4984 PEP_0_2_0_0_0
1364.5621 PEP_1_2_0_0_0
1526.6104 PEP_2_2_0_0_0
1688.655 PEP_3_2_0_0_0
1891.7264 PEP_3_3_0_0_0

File: 231129_OE_RK_4, RT: 6.484, Scan: 003258
Type: HCD, FTMS + c NSI d Full ms2 1177.4474@hcd30.00 [120.0000-3625.1311]
Precursor Scan: 003217, m/z: 1177.1172, charge: 3
NEEYNK_7_5_2_1_0

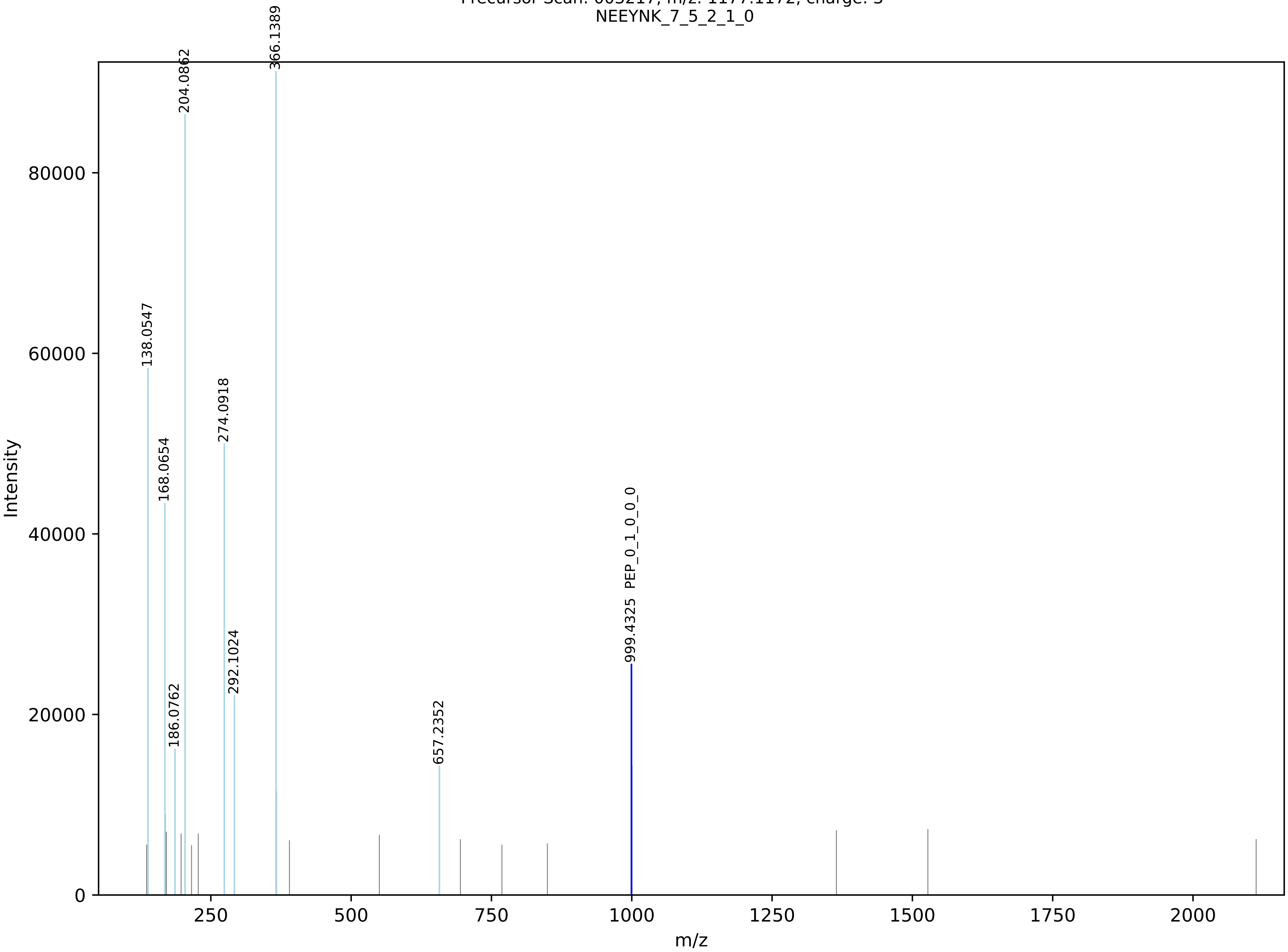

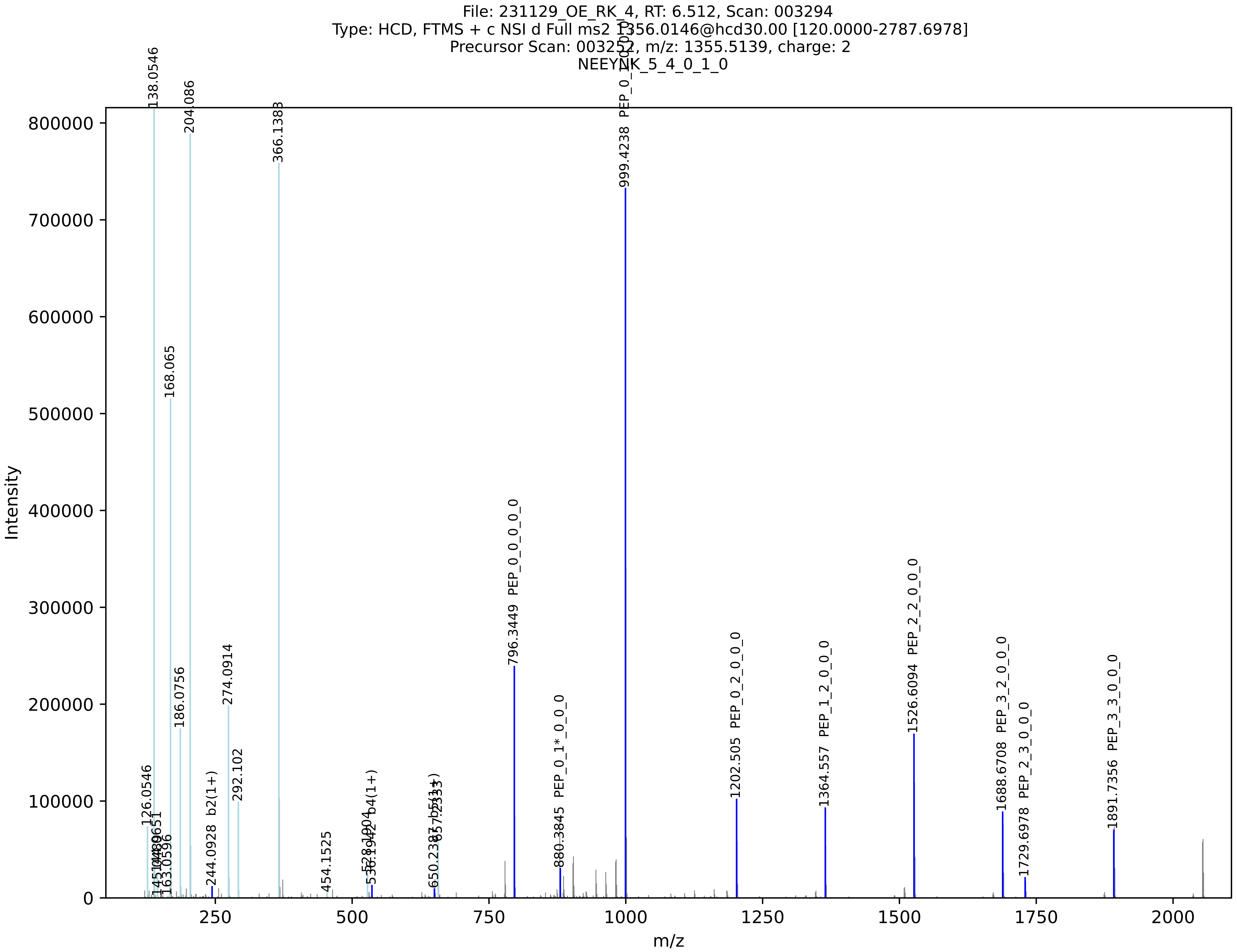

File: 231129_OE_RK_4, RT: 6.512, Scan: 003294
Type: HCD, FTMS + c NSI d Full ms2 1356.0146@hcd30.00 [120.0000-2787.6978]
Precursor Scan: 003252, m/z: 1355.5139, charge: 2
NEEYNK_5_4_0_1_0
Intensity
m/z
138.0546
204.086
366.1383
168.065
186.0756
274.0914
292.102
126.0546
163.0596
244.0928 b2(1+)
454.1525
b4(1+)
657.2333
796.3449 PEP_0_0_0_0_0
880.3845 PEP_0_1*_0_0_0
999.4238
1202.505 PEP_0_2_0_0_0
1364.557 PEP_1_2_0_0_0
1526.6094 PEP_2_2_0_0_0
1688.6708 PEP_3_2_0_0_0
1729.6978 PEP_2_3_0_0_0
1891.7356 PEP_3_3_0_0_0

File: 231129_OE_RK_4, RT: 6.515, Scan: 003298
Type: HCD, FTMS + c NSI d Full ms2 1474.0609@hcd30.00 [120.0000-3028.5122]
Precursor Scan: 003252, m/z: 1473.5583, charge: 2
SYNETK_5_4_0_2_0

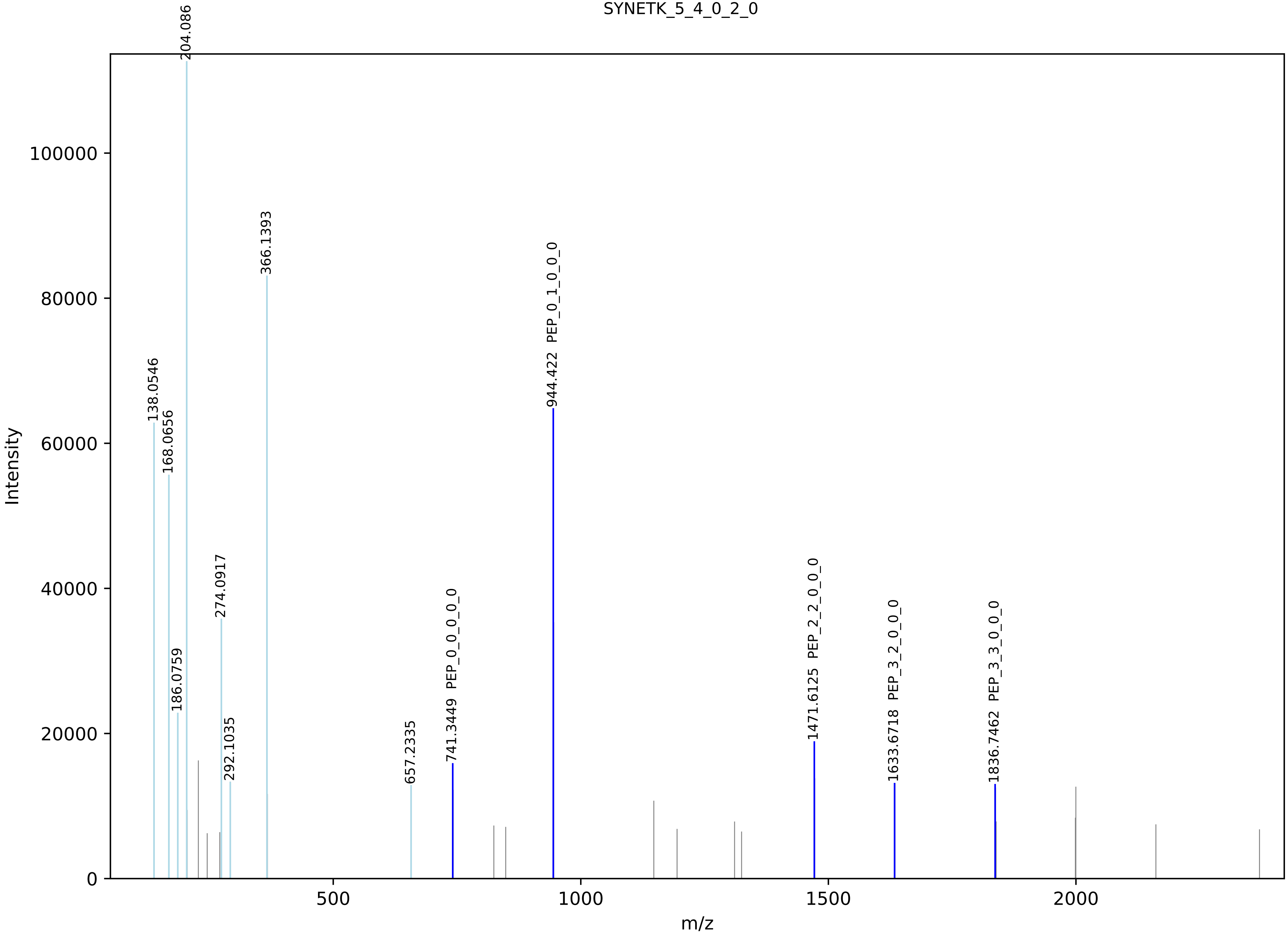

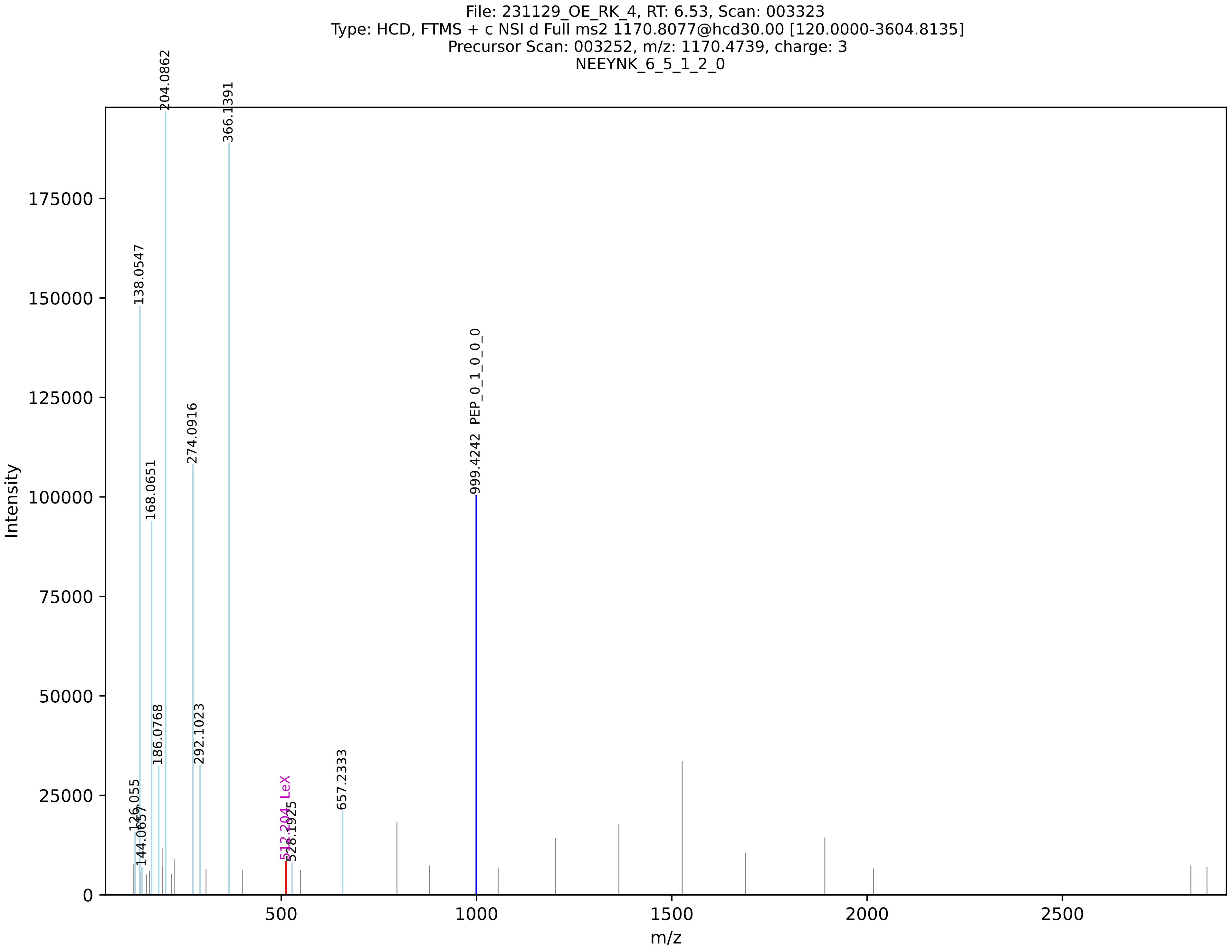
File: 231129_OE_RK_4, RT: 6.53, Scan: 003323
Type: HCD, FTMS + c NSI d Full ms2 1170.8077@hcd30.00 [120.0000-3604.8135]
Precursor Scan: 003252, m/z: 1170.4739, charge: 3
NEEYNK_6_5_1_2_0
Intensity
m/z
204.0862
366.1391
138.0547
999.4242 PEP_0_1_0_0_0
274.0916
168.0651
186.0768
292.1023
126.055
144.0657
512.204_LeX
528.1925
657.2333
0
25000
50000
75000
100000
125000
150000
175000
500
1000
1500
2000
2500

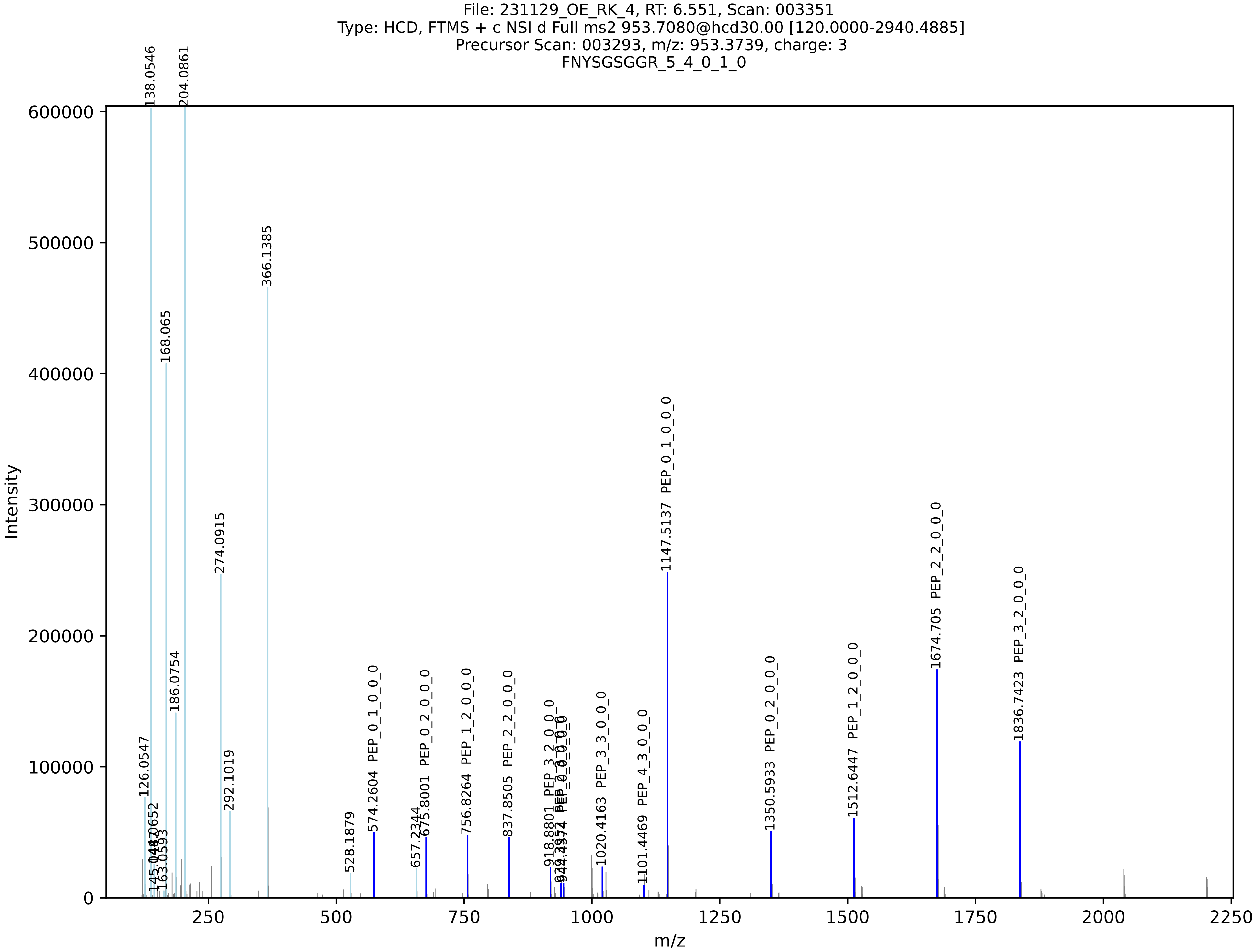
File: 231129_OE_RK_4, RT: 6.551, Scan: 003351
Type: HCD, FTMS + c NSI d Full ms2 953.7080@hcd30.00 [120.0000-2940.4885]
Precursor Scan: 003293, m/z: 953.3739, charge: 3
FNYSGSGGR_5_4_0_1_0
Intensity
m/z
138.0546
204.0861
366.1385
168.065
274.0915
186.0754
126.0547
292.1019
528.1879
574.2604 PEP_0_1_0_0_0
657.2344
675.8001 PEP_0_2_0_0_0
756.8264 PEP_1_2_0_0_0
837.8505 PEP_2_2_0_0_0
918.8801 PEP_3_2_0_0_0
1020.4163 PEP_3_3_0_0_0
1101.4469 PEP_4_3_0_0_0
1147.5137 PEP_0_1_0_0_0
1350.5933 PEP_0_2_0_0_0
1512.6447 PEP_1_2_0_0_0
1674.705 PEP_2_2_0_0_0
1836.7423 PEP_3_2_0_0_0

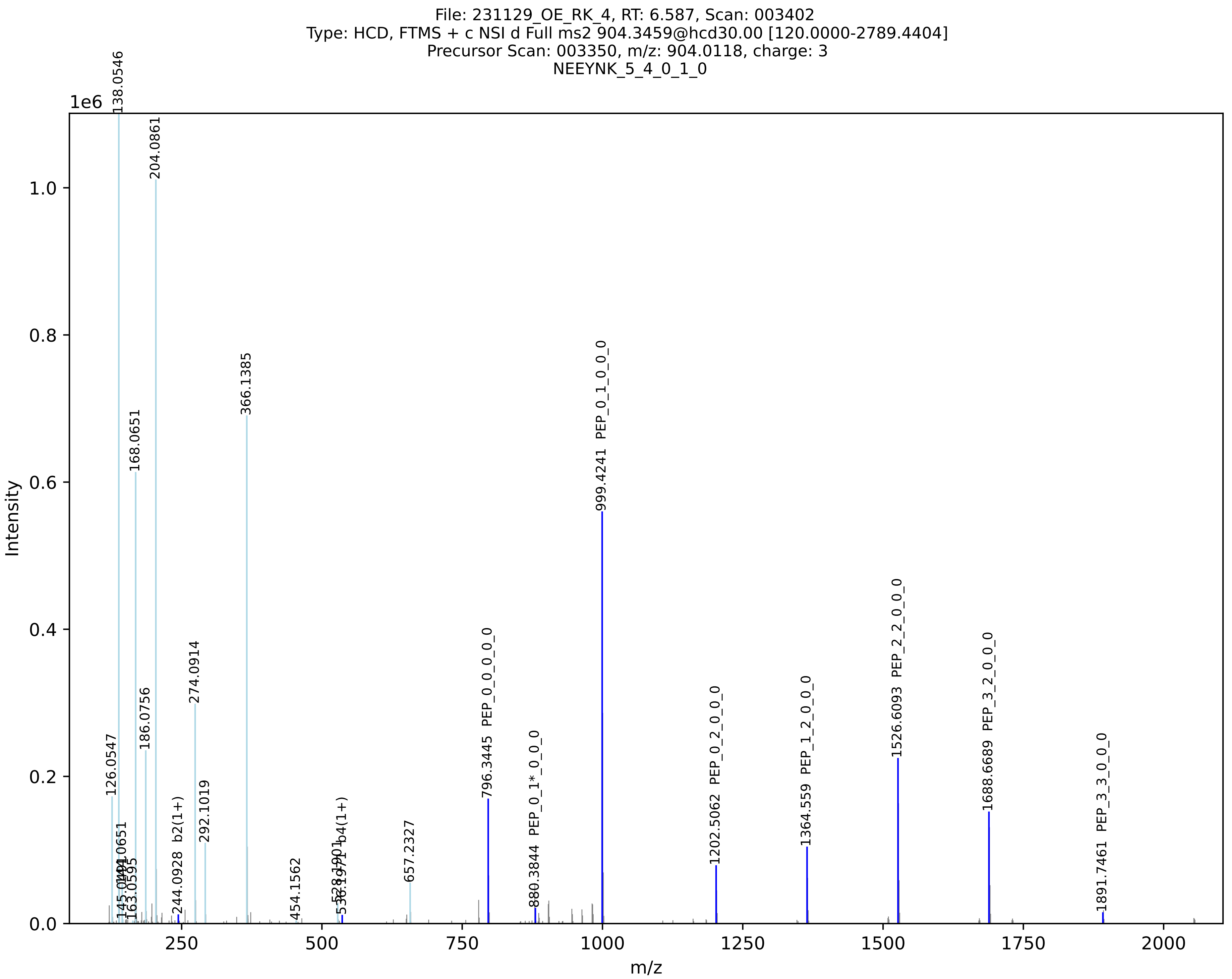
File: 231129_OE_RK_4, RT: 6.587, Scan: 003402
Type: HCD, FTMS + c NSI d Full ms2 904.3459@hcd30.00 [120.0000-2789.4404]
Precursor Scan: 003350, m/z: 904.0118, charge: 3
NEEYNK_5_4_0_1_0
1e6
Intensity
m/z
138.0546
204.0861
168.0651
366.1385
186.0756
274.0914
126.0547
292.1019
244.0928 b2(1+)
163.0595
454.1562
657.2327
796.3445 PEP_0_0_0_0_0
880.3844 PEP_0_1*_0_0_0
999.4241 PEP_0_1_0_0_0
1202.5062 PEP_0_2_0_0_0
1364.559 PEP_1_2_0_0_0
1526.6093 PEP_2_2_0_0_0
1688.6689 PEP_3_2_0_0_0
1891.7461 PEP_3_3_0_0_0

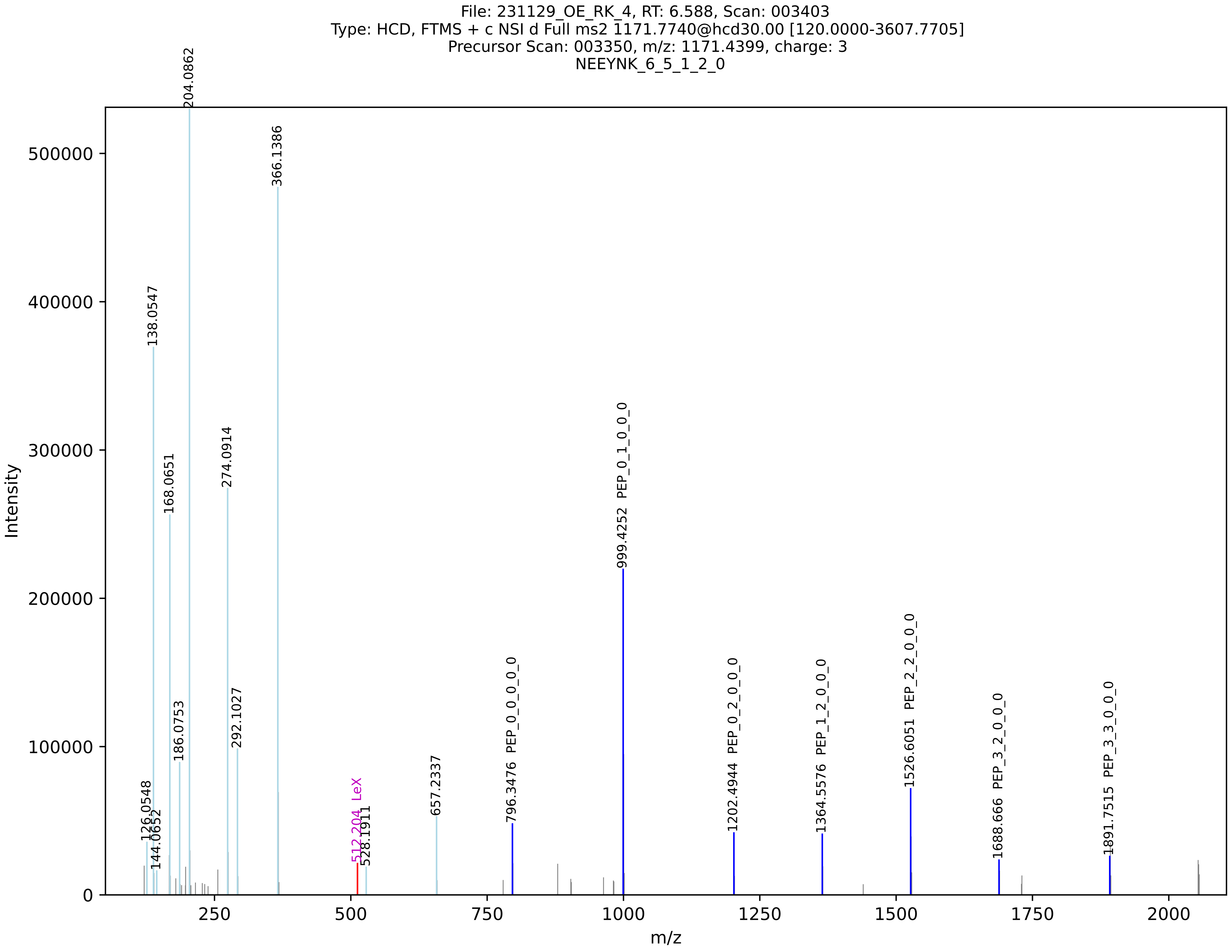

File: 231129_OE_RK_4, RT: 6.588, Scan: 003403
Type: HCD, FTMS + c NSI d Full ms2 1171.7740@hcd30.00 [120.0000-3607.7705]
Precursor Scan: 003350, m/z: 1171.4399, charge: 3
NEEYNK_6_5_1_2_0
Intensity
m/z
204.0862
366.1386
138.0547
274.0914
168.0651
292.1027
186.0753
126.0548
144.0652
512.204_LeX
528.1911
657.2337
796.3476 PEP_0_0_0_0_0
999.4252 PEP_0_1_0_0_0
1202.4944 PEP_0_2_0_0_0
1364.5576 PEP_1_2_0_0_0
1526.6051 PEP_2_2_0_0_0
1688.666 PEP_3_2_0_0_0
1891.7515 PEP_3_3_0_0_0

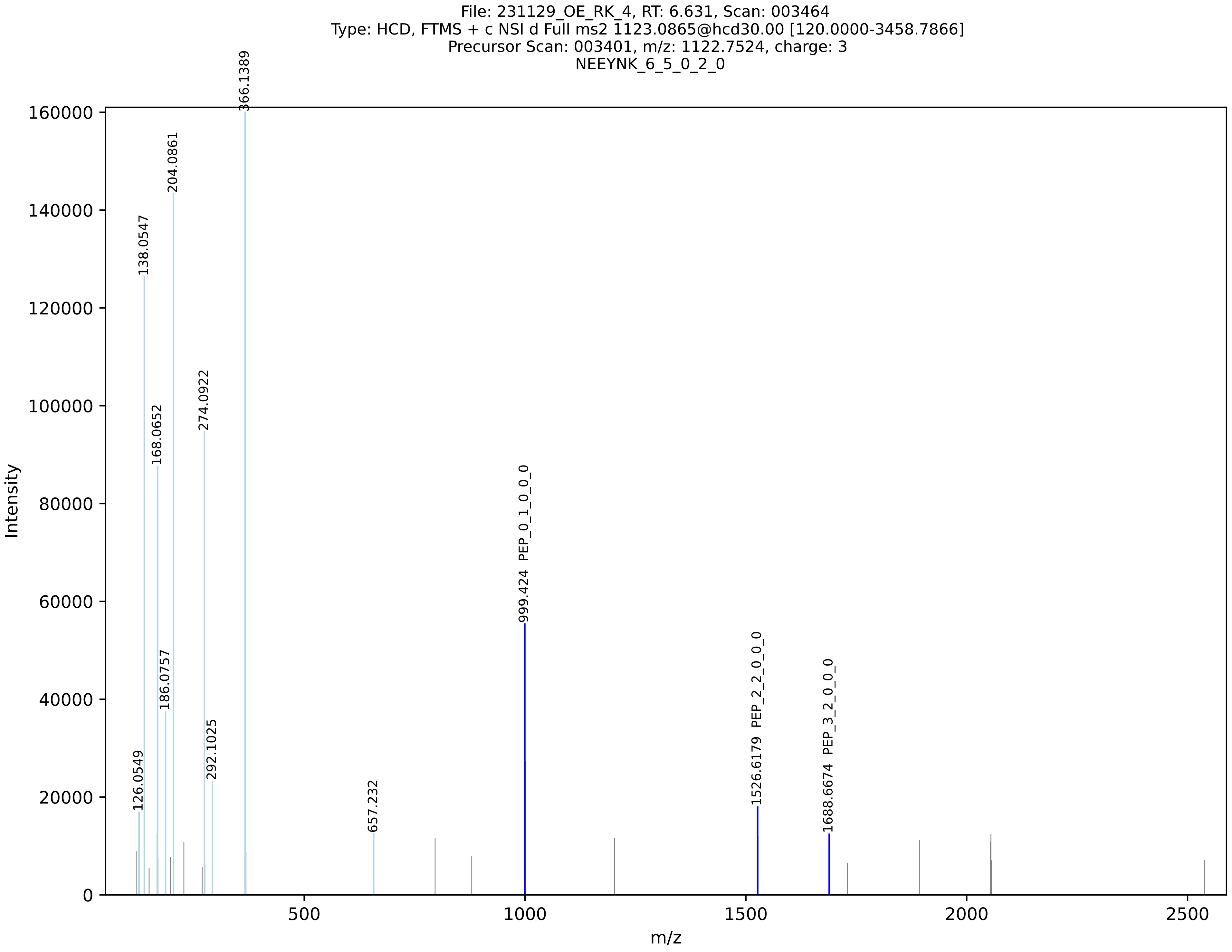

File: 231129_OE_RK_4, RT: 6.631, Scan: 003464
Type: HCD, FTMS + c NSI d Full ms2 1123.0865@hcd30.00 [120.0000-3458.7866]
Precursor Scan: 003401, m/z: 1122.7524, charge: 3
NEEYNK_6_5_0_2_0
Intensity
m/z
366.1389
204.0861
138.0547
274.0922
168.0652
186.0757
292.1025
126.0549
657.232
999.424 PEP_0_1_0_0_0
1526.6179 PEP_2_2_0_0_0
1688.6674 PEP_3_2_0_0_0

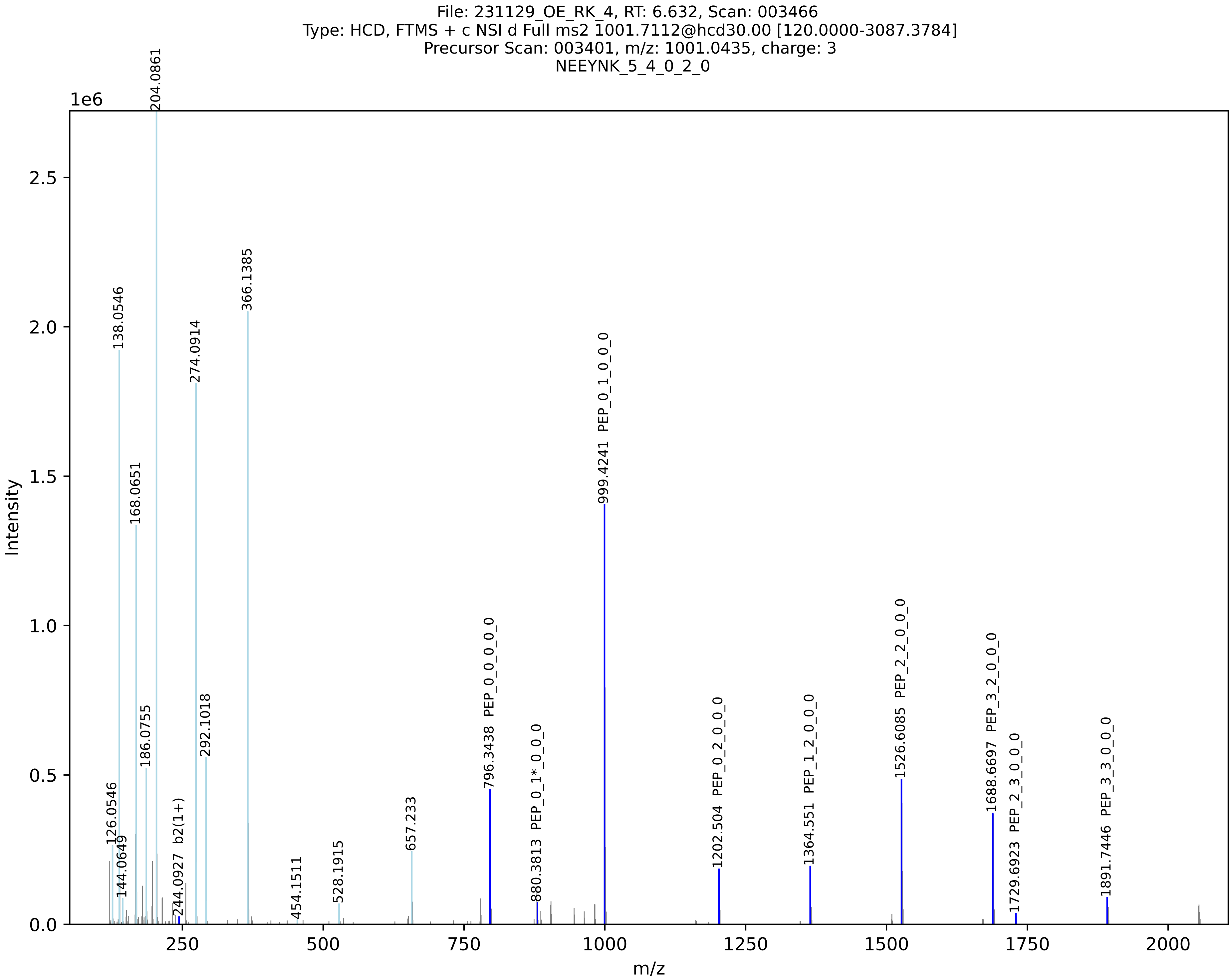
File: 231129_OE_RK_4, RT: 6.632, Scan: 003466
Type: HCD, FTMS + c NSI d Full ms2 1001.7112@hcd30.00 [120.0000-3087.3784]
Precursor Scan: 003401, m/z: 1001.0435, charge: 3
NEEYNK_5_4_0_2_0
1e6
Intensity
m/z
204.0861
138.0546
366.1385
274.0914
168.0651
186.0755
292.1018
126.0546
144.0649
244.0927 b2(1+)
454.1511
528.1915
657.233
796.3438 PEP_0_0_0_0_0
880.3813 PEP_0_1*_0_0_0
999.4241 PEP_0_1_0_0_0
1202.504 PEP_0_2_0_0_0
1364.551 PEP_1_2_0_0_0
1526.6085 PEP_2_2_0_0_0
1688.6697 PEP_3_2_0_0_0
1729.6923 PEP_2_3_0_0_0
1891.7446 PEP_3_3_0_0_0

File: 231129_OE_RK_4, RT: 6.652, Scan: 003499
Type: HCD, FTMS + c NSI d Full ms2 1501.5627@hcd30.00 [120.0000-3084.6160]
Precursor Scan: 003401, m/z: 1501.0616, charge: 2
NEEYNK_5_4_0_2_0

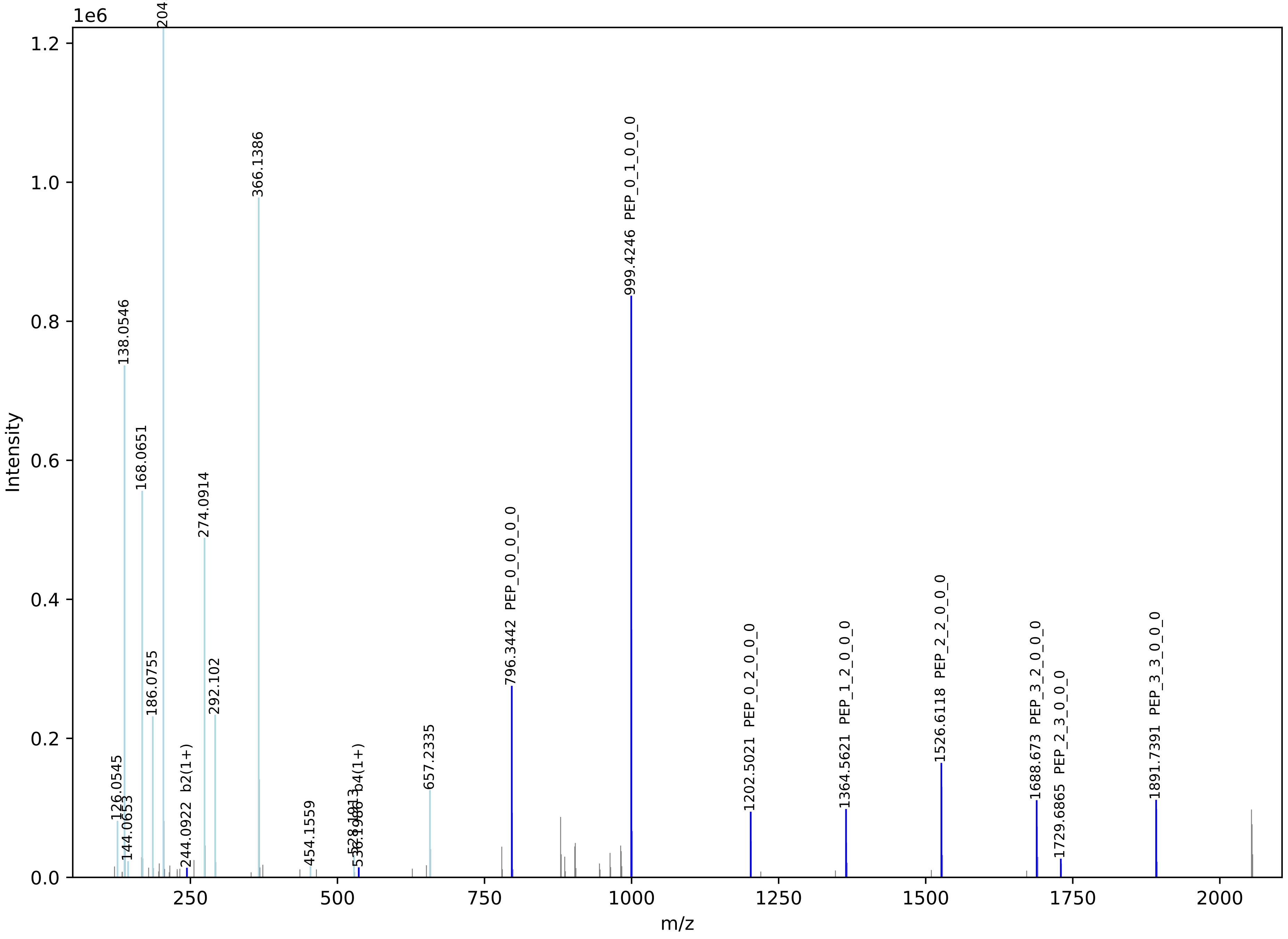

File: 231129_OE_RK_4, RT: 6.681, Scan: 003534
Type: HCD, FTMS + c NSI d Full ms2 1026.3893@hcd30.00 [120.0000-3162.8931]
Precursor Scan: 003462, m/z: 1025.721, charge: 3
NEEYNK_6_5_0_1_0

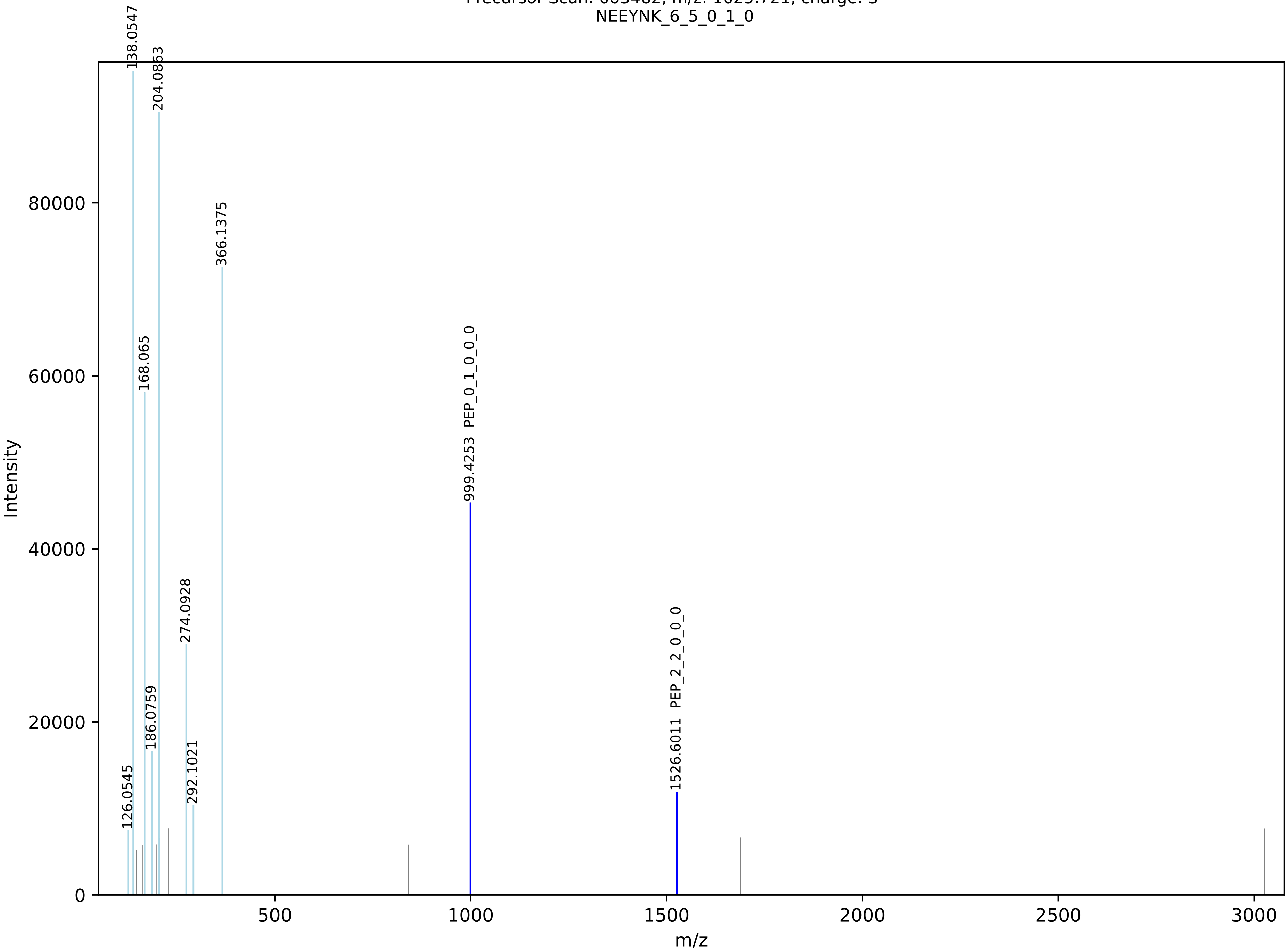

File: 231129_OE_RK_4, RT: 6.689, Scan: 003547
Type: HCD, FTMS + c NSI d Full ms2 988.7173@hcd30.00 [120.0000-3047.6172]
Precursor Scan: 003462, m/z: 988.0491, charge: 3
SYNETK_6_4_1_1_0

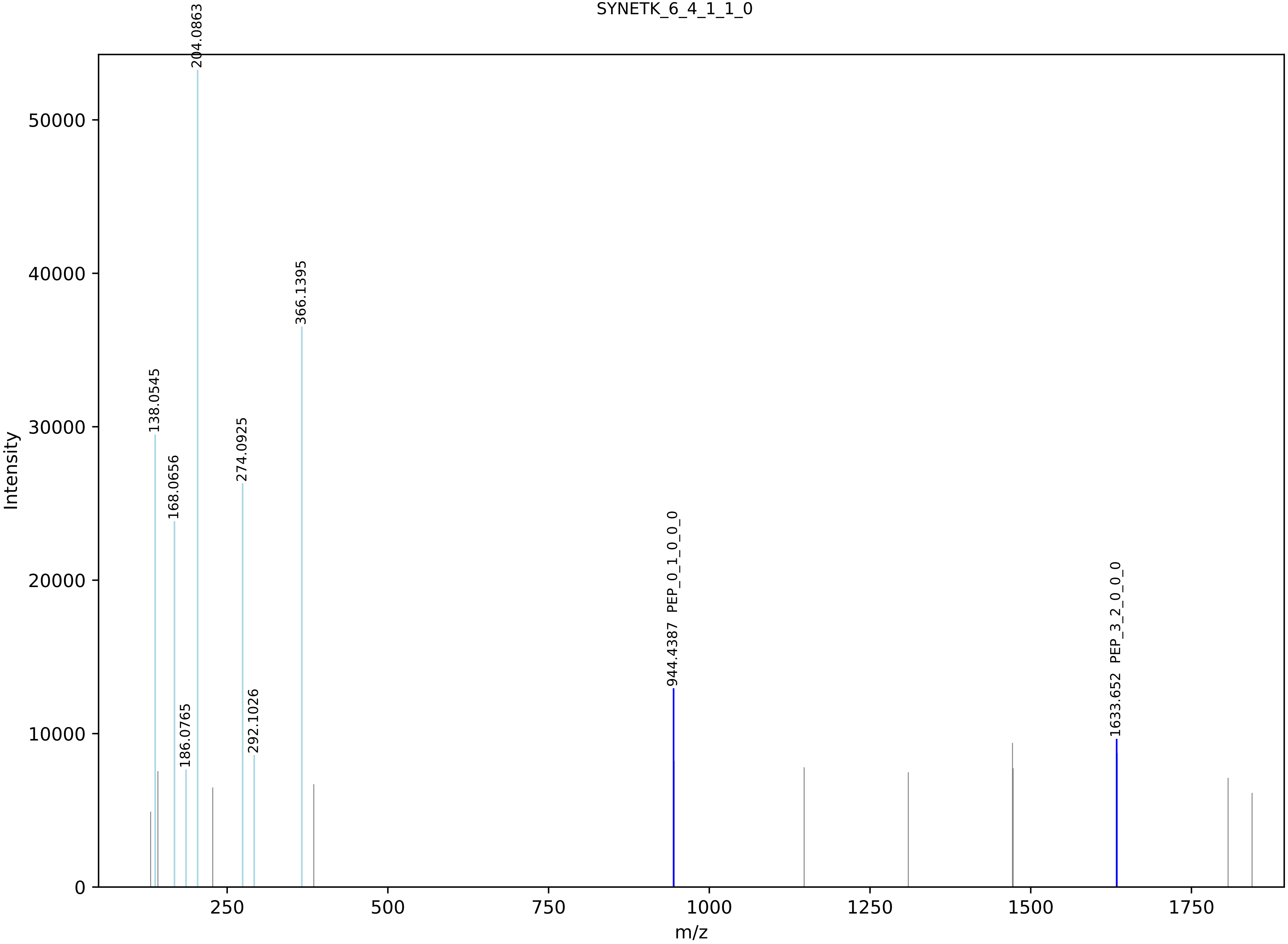

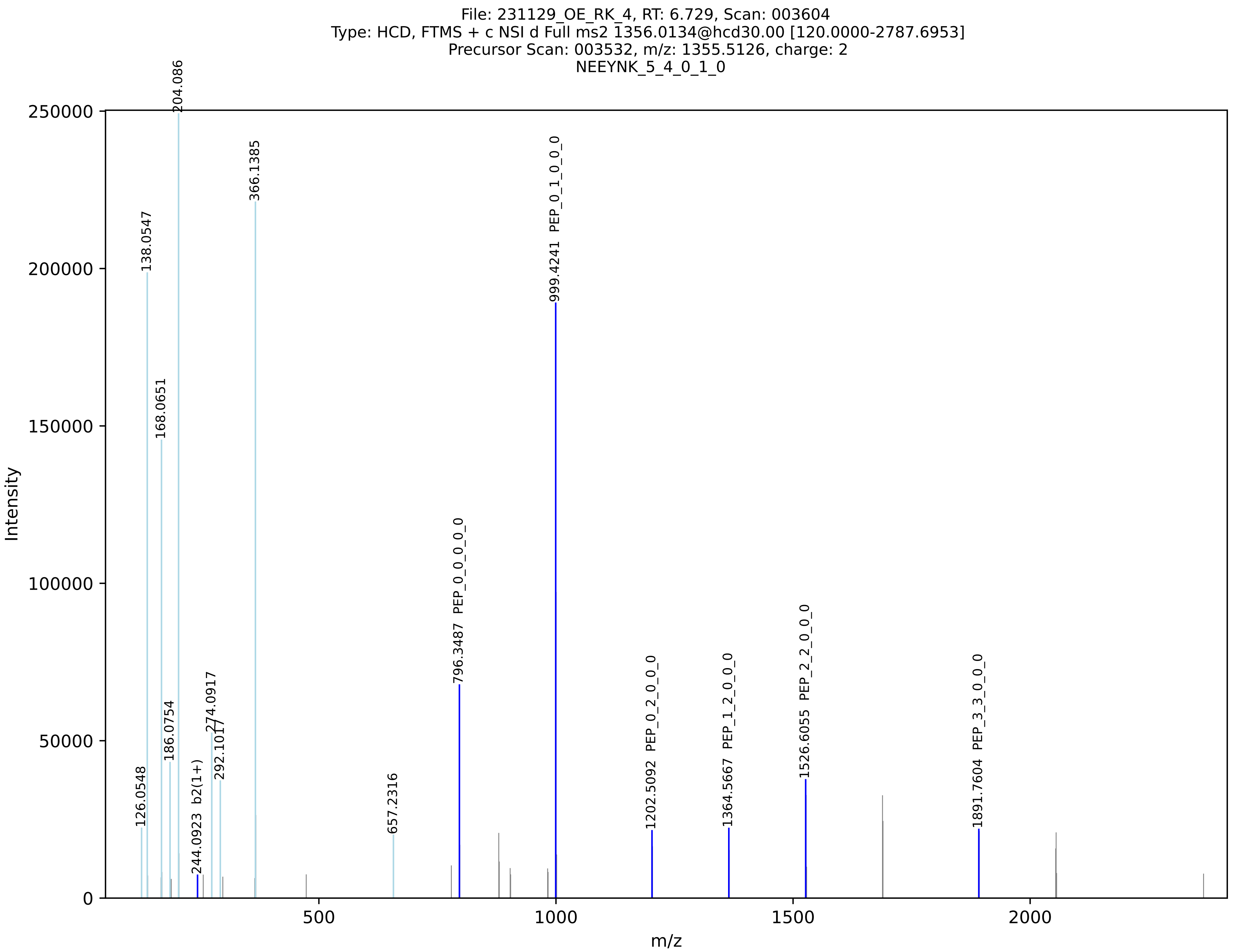
File: 231129_OE_RK_4, RT: 6.729, Scan: 003604
Type: HCD, FTMS + c NSI d Full ms2 1356.0134@hcd30.00 [120.0000-2787.6953]
Precursor Scan: 003532, m/z: 1355.5126, charge: 2
NEEYNK_5_4_0_1_0
Intensity
m/z
204.086
366.1385
138.0547
168.0651
186.0754
274.0917
292.1017
126.0548
244.0923 b2(1+)
657.2316
796.3487 PEP_0_0_0_0_0
999.4241 PEP_0_1_0_0_0
1202.5092 PEP_0_2_0_0_0
1364.5667 PEP_1_2_0_0_0
1526.6055 PEP_2_2_0_0_0
1891.7604 PEP_3_3_0_0_0

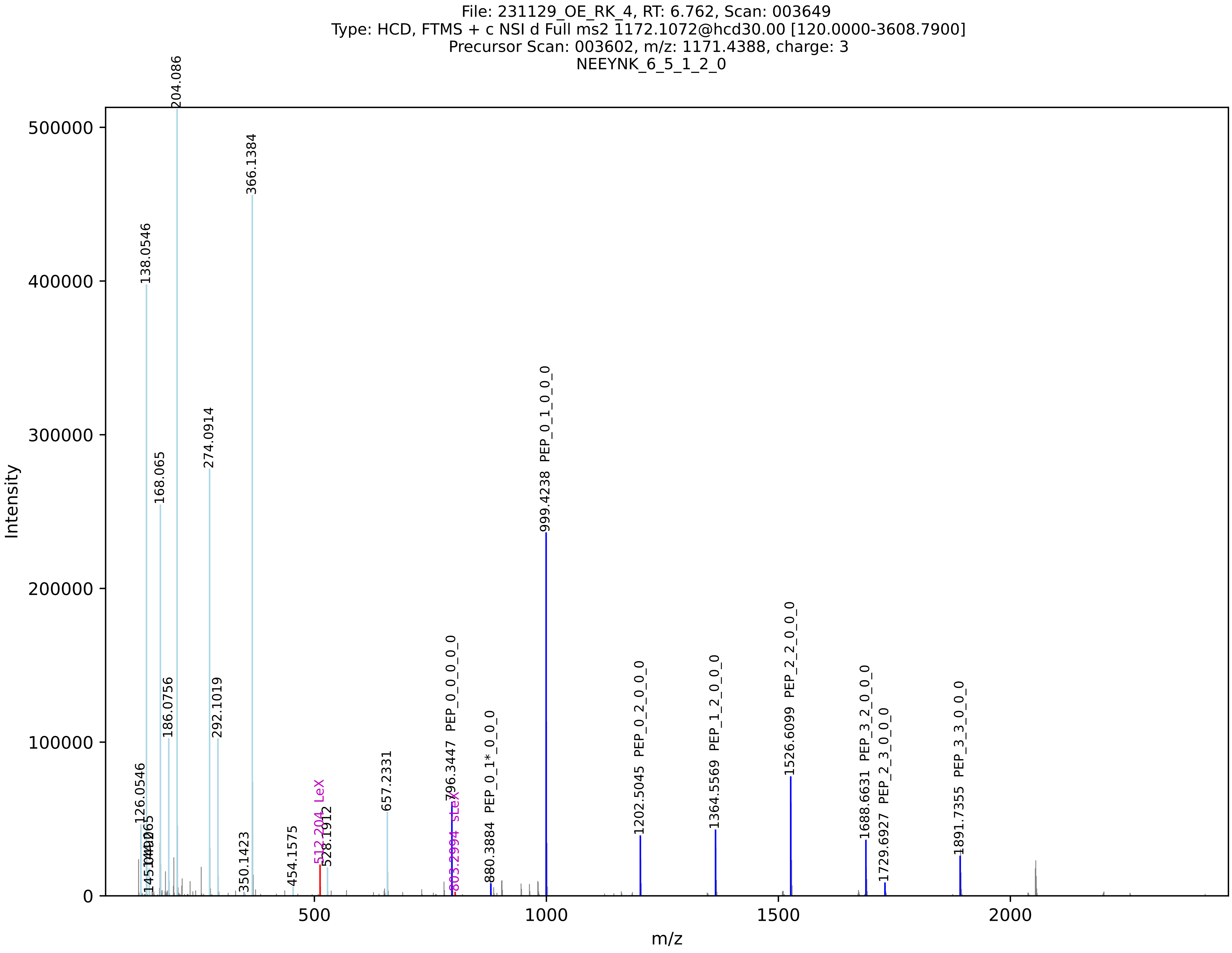
File: 231129_OE_RK_4, RT: 6.762, Scan: 003649
Type: HCD, FTMS + c NSI d Full ms2 1172.1072@hcd30.00 [120.0000-3608.7900]
Precursor Scan: 003602, m/z: 1171.4388, charge: 3
NEEYNK_6_5_1_2_0
Intensity
m/z
0
100000
200000
300000
400000
500000
500
1000
1500
2000
204.086
366.1384
138.0546
168.065
274.0914
186.0756
292.1019
126.0546
350.1423
454.1575
512.204 LeX
528.1912
657.2331
796.3447 PEP_0_0_0_0_0_
803.2994 sLeX
880.3884 PEP_0_1*_0_0_0
999.4238 PEP_0_1_0_0_0_
1202.5045 PEP_0_2_0_0_0_
1364.5569 PEP_1_2_0_0_0_
1526.6099 PEP_2_2_0_0_0_
1688.6631 PEP_3_2_0_0_0_
1729.6927 PEP_2_3_0_0_0_
1891.7355 PEP_3_3_0_0_0_

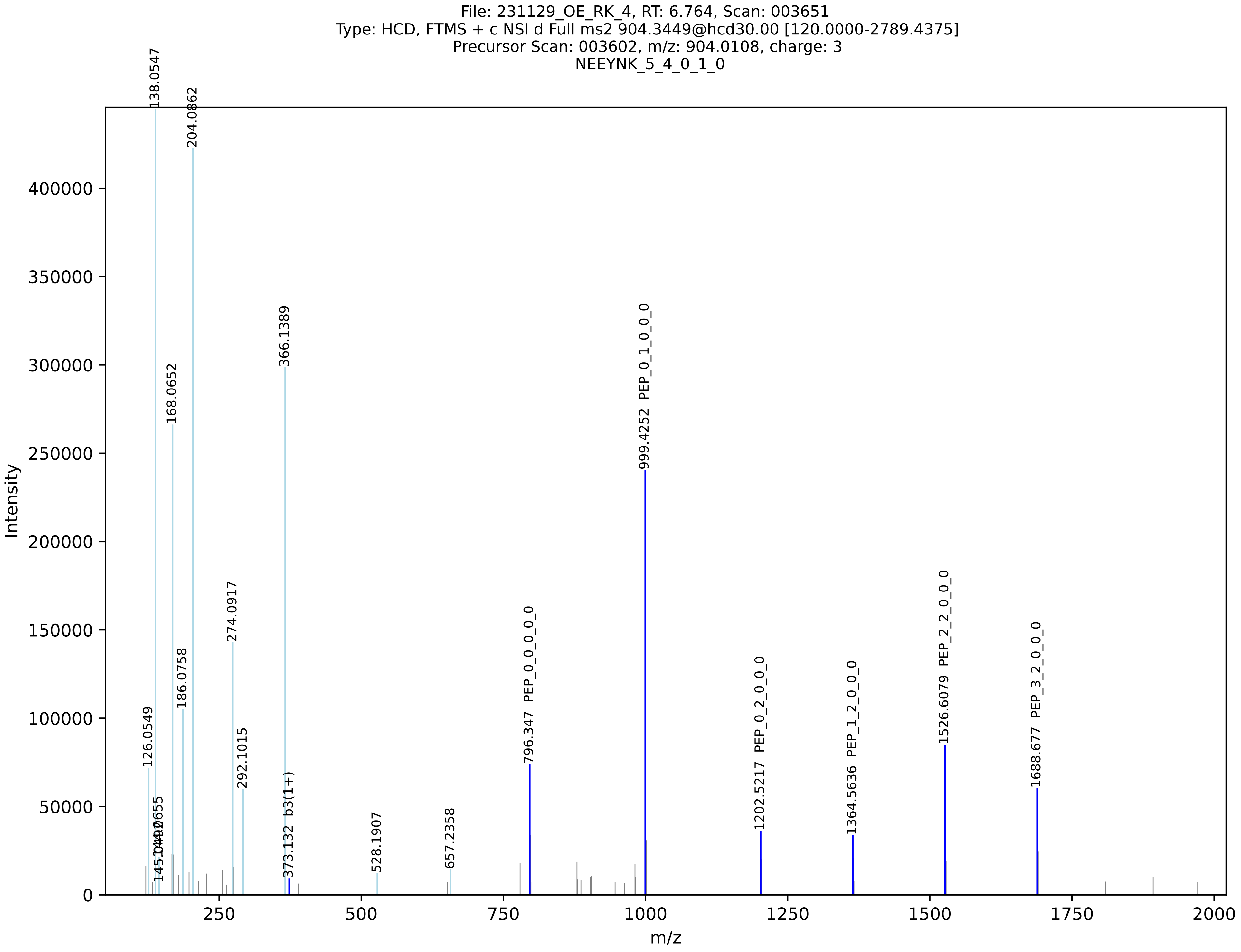
File: 231129_OE_RK_4, RT: 6.764, Scan: 003651
Type: HCD, FTMS + c NSI d Full ms2 904.3449@hcd30.00 [120.0000-2789.4375]
Precursor Scan: 003602, m/z: 904.0108, charge: 3
NEEYNK_5_4_0_1_0
Intensity
m/z
138.0547
204.0862
168.0652
366.1389
186.0758
274.0917
126.0549
292.1015
373.132 b3(1+)
528.1907
657.2358
796.347 PEP_0_0_0_0_0
999.4252 PEP_0_1_0_0_0
1202.5217 PEP_0_2_0_0_0
1364.5636 PEP_1_2_0_0_0
1526.6079 PEP_2_2_0_0_0
1688.677 PEP_3_2_0_0_0

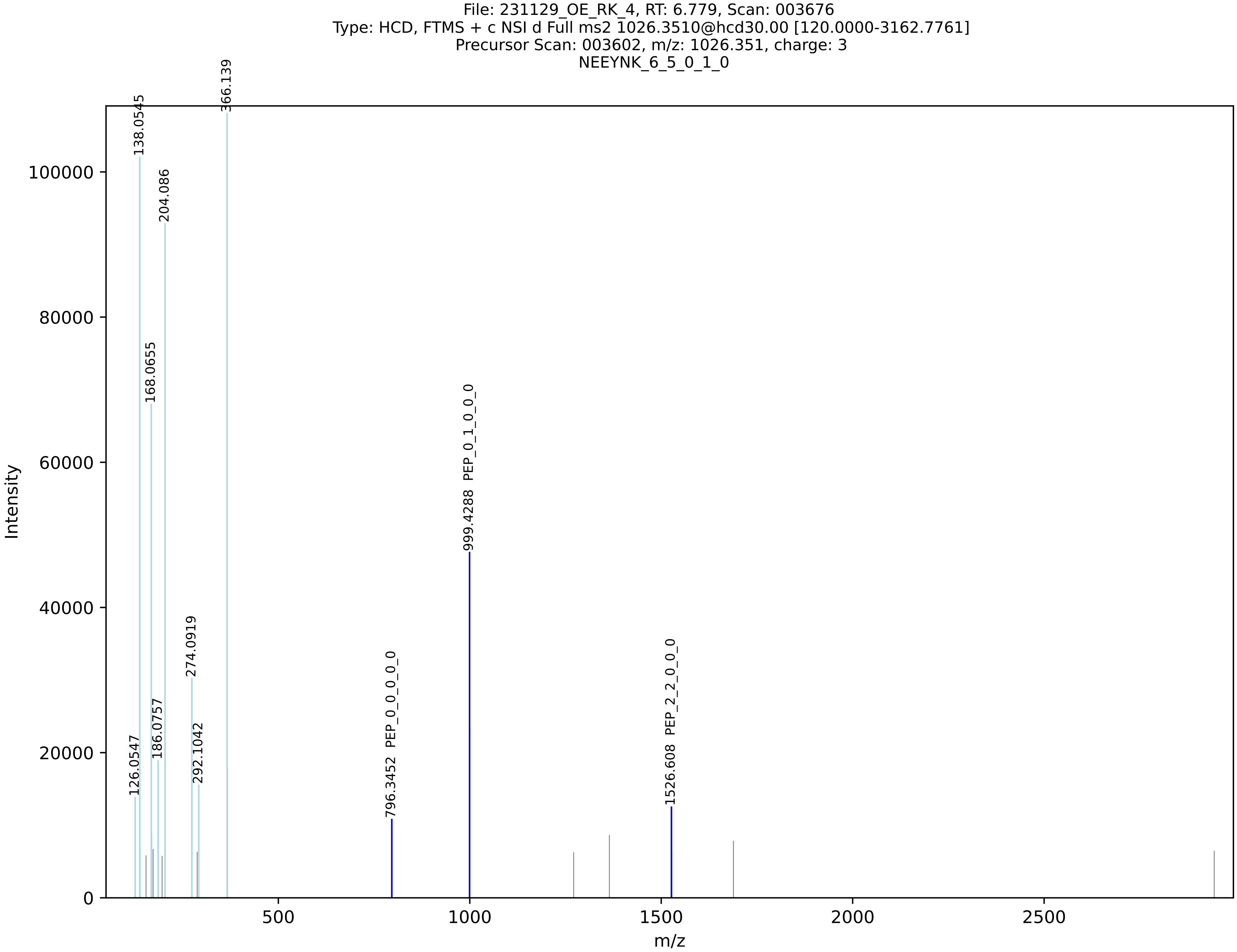
File: 231129_OE_RK_4, RT: 6.779, Scan: 003676
Type: HCD, FTMS + c NSI d Full ms2 1026.3510@hcd30.00 [120.0000-3162.7761]
Precursor Scan: 003602, m/z: 1026.351, charge: 3
NEEYNK_6_5_0_1_0
Intensity
m/z
0
20000
40000
60000
80000
100000
500
1000
1500
2000
2500
126.0547
138.0545
168.0655
186.0757
204.086
274.0919
292.1042
366.139
796.3452 PEP_0_0_0_0_0_
999.4288 PEP_0_1_0_0_0_
1526.608 PEP_2_2_0_0_0_

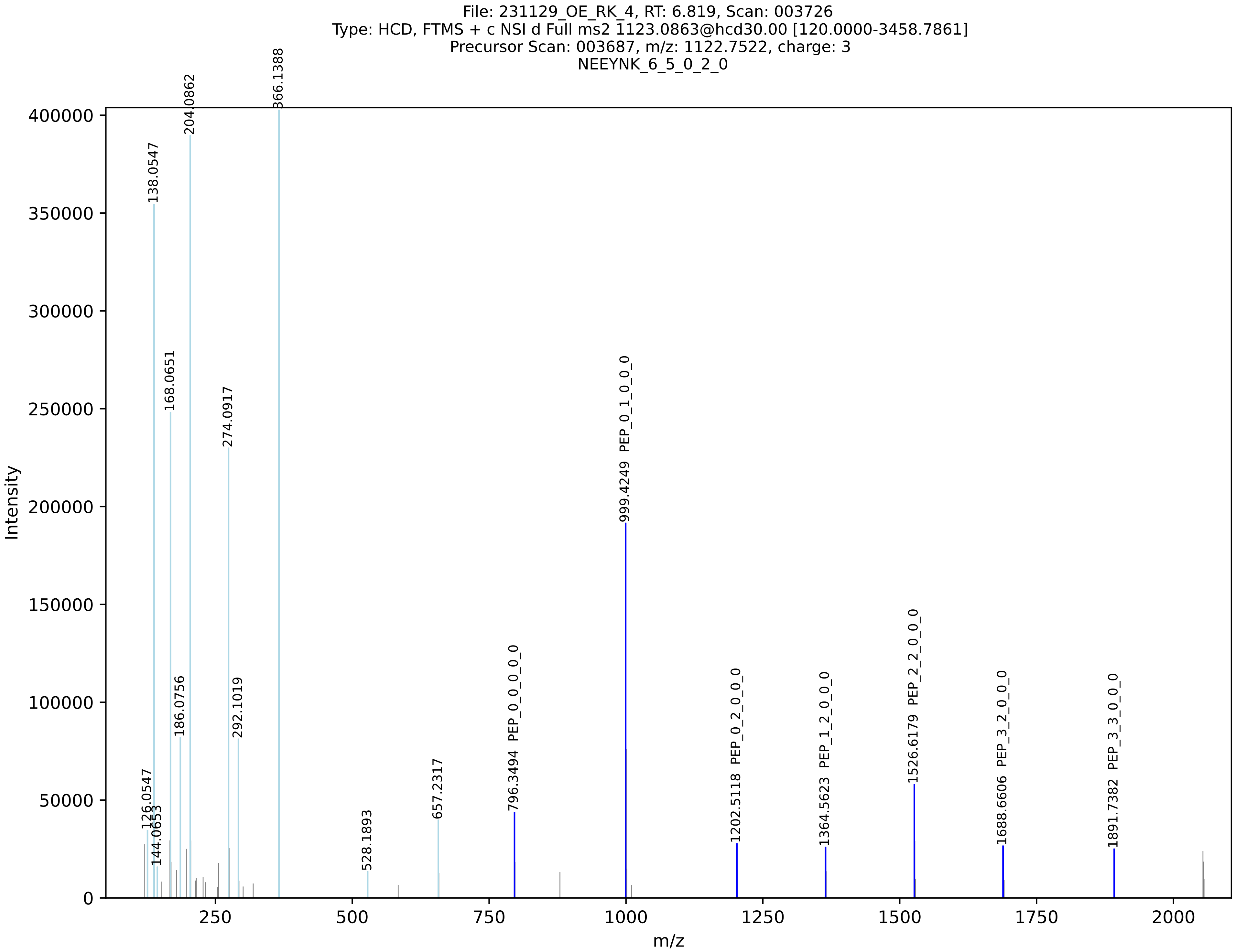

File: 231129_OE_RK_4, RT: 6.819, Scan: 003726
Type: HCD, FTMS + c NSI d Full ms2 1123.0863@hcd30.00 [120.0000-3458.7861]
Precursor Scan: 003687, m/z: 1122.7522, charge: 3
NEEYNK_6_5_0_2_0
366.1388
204.0862
138.0547
168.0651
274.0917
186.0756
292.1019
126.0547
144.0653
528.1893
657.2317
796.3494 PEP_0_0_0_0_0
999.4249 PEP_0_1_0_0_0
1202.5118 PEP_0_2_0_0_0
1364.5623 PEP_1_2_0_0_0
1526.6179 PEP_2_2_0_0_0
1688.6606 PEP_3_2_0_0_0
1891.7382 PEP_3_3_0_0_0
Intensity
m/z
0
50000
100000
150000
200000
250000
300000
350000
400000
250
500
750
1000
1250
1500
1750
2000

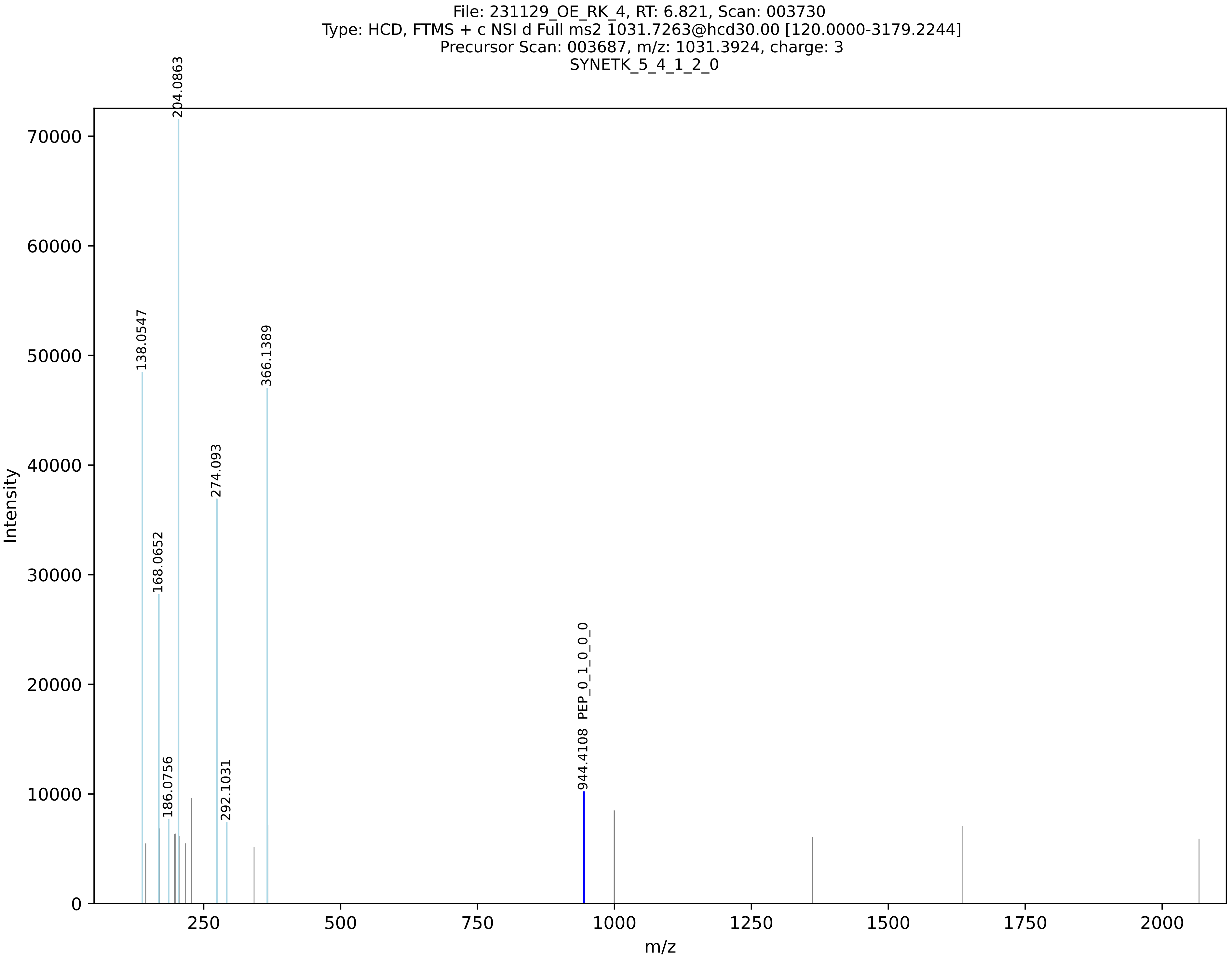
File: 231129_OE_RK_4, RT: 6.821, Scan: 003730
Type: HCD, FTMS + c NSI d Full ms2 1031.7263@hcd30.00 [120.0000-3179.2244]
Precursor Scan: 003687, m/z: 1031.3924, charge: 3
SYNETK_5_4_1_2_0
204.0863
138.0547
366.1389
274.093
168.0652
186.0756
292.1031
944.4108 PEP_0_1_0_0_0
Intensity
m/z
0
10000
20000
30000
40000
50000
60000
70000
250
500
750
1000
1250
1500
1750
2000

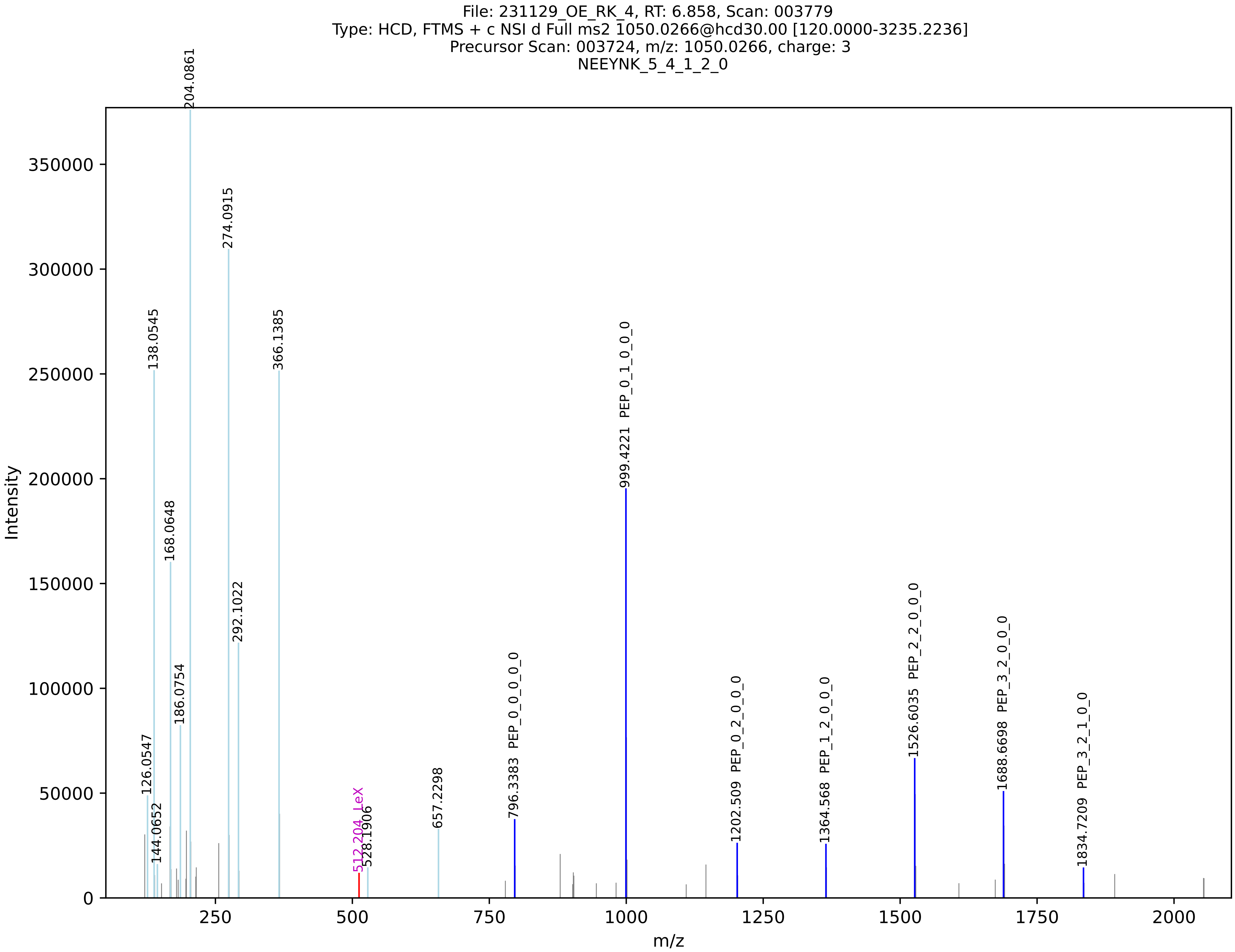

File: 231129_OE_RK_4, RT: 6.858, Scan: 003779
Type: HCD, FTMS + c NSI d Full ms2 1050.0266@hcd30.00 [120.0000-3235.2236]
Precursor Scan: 003724, m/z: 1050.0266, charge: 3
NEEYNK_5_4_1_2_0
Intensity
m/z
204.0861
274.0915
138.0545
366.1385
168.0648
292.1022
186.0754
126.0547
144.0652
512.204_LeX
528.1906
657.2298
796.3383 PEP_0_0_0_0_0
999.4221 PEP_0_1_0_0_0
1202.509 PEP_0_2_0_0_0
1364.568 PEP_1_2_0_0_0
1526.6035 PEP_2_2_0_0_0
1688.6698 PEP_3_2_0_0_0
1834.7209 PEP_3_2_1_0_0

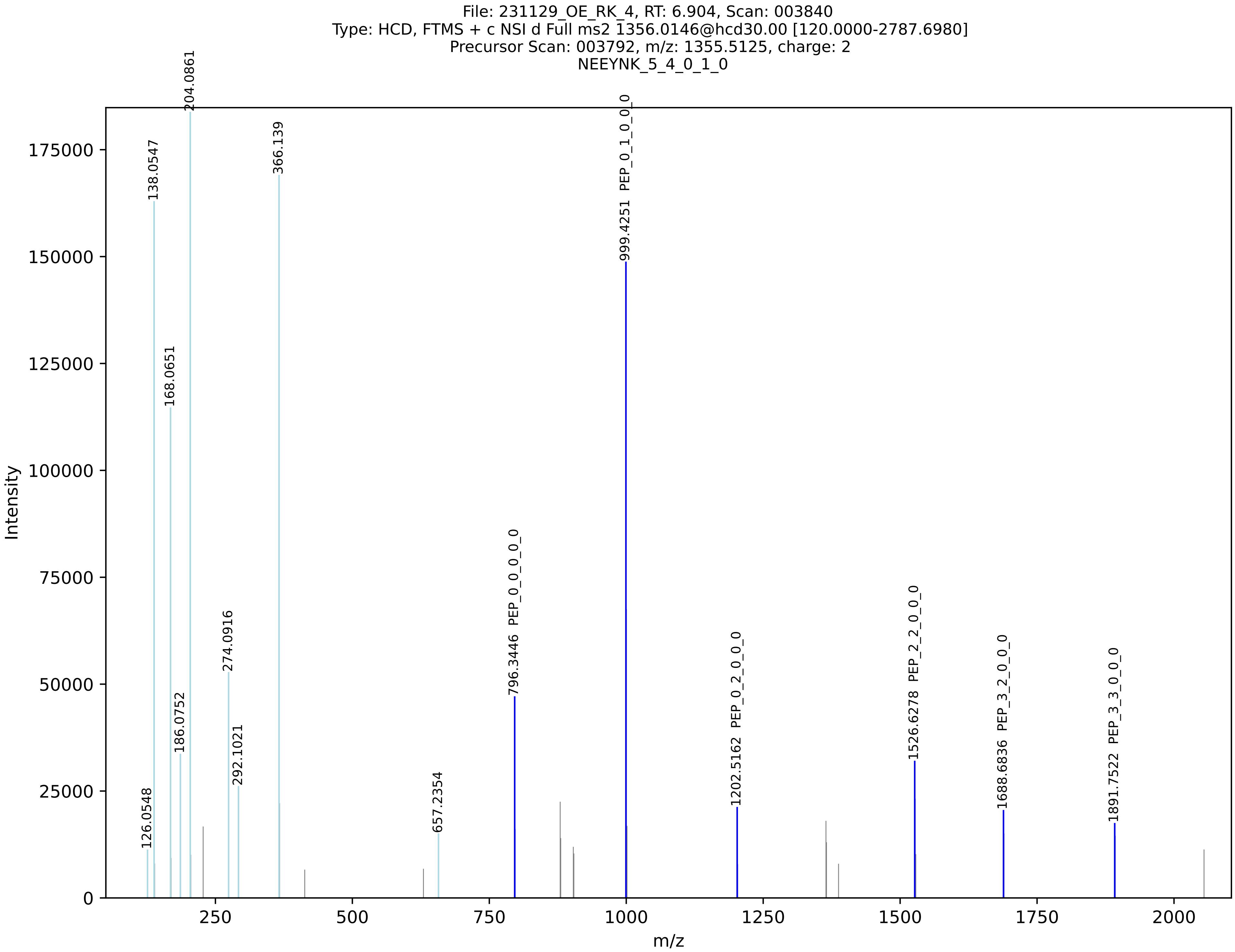
File: 231129_OE_RK_4, RT: 6.904, Scan: 003840
Type: HCD, FTMS + c NSI d Full ms2 1356.0146@hcd30.00 [120.0000-2787.6980]
Precursor Scan: 003792, m/z: 1355.5125, charge: 2
NEEYNK_5_4_0_1_0
Intensity
m/z
0
25000
50000
75000
100000
125000
150000
175000
250
500
750
1000
1250
1500
1750
2000
126.0548
138.0547
168.0651
186.0752
204.0861
274.0916
292.1021
366.139
657.2354
796.3446 PEP_0_0_0_0_0
999.4251 PEP_0_1_0_0_0
1202.5162 PEP_0_2_0_0_0
1526.6278 PEP_2_2_0_0_0
1688.6836 PEP_3_2_0_0_0
1891.7522 PEP_3_3_0_0_0

File: 231129_OE_RK_4, RT: 6.906, Scan: 003844
Type: HCD, FTMS + c NSI d Full ms2 1336.1829@hcd30.00 [120.0000-4110.8613]
Precursor Scan: 003792, m/z: 1335.1805, charge: 3
ENGTISR_7_6_2_2_0

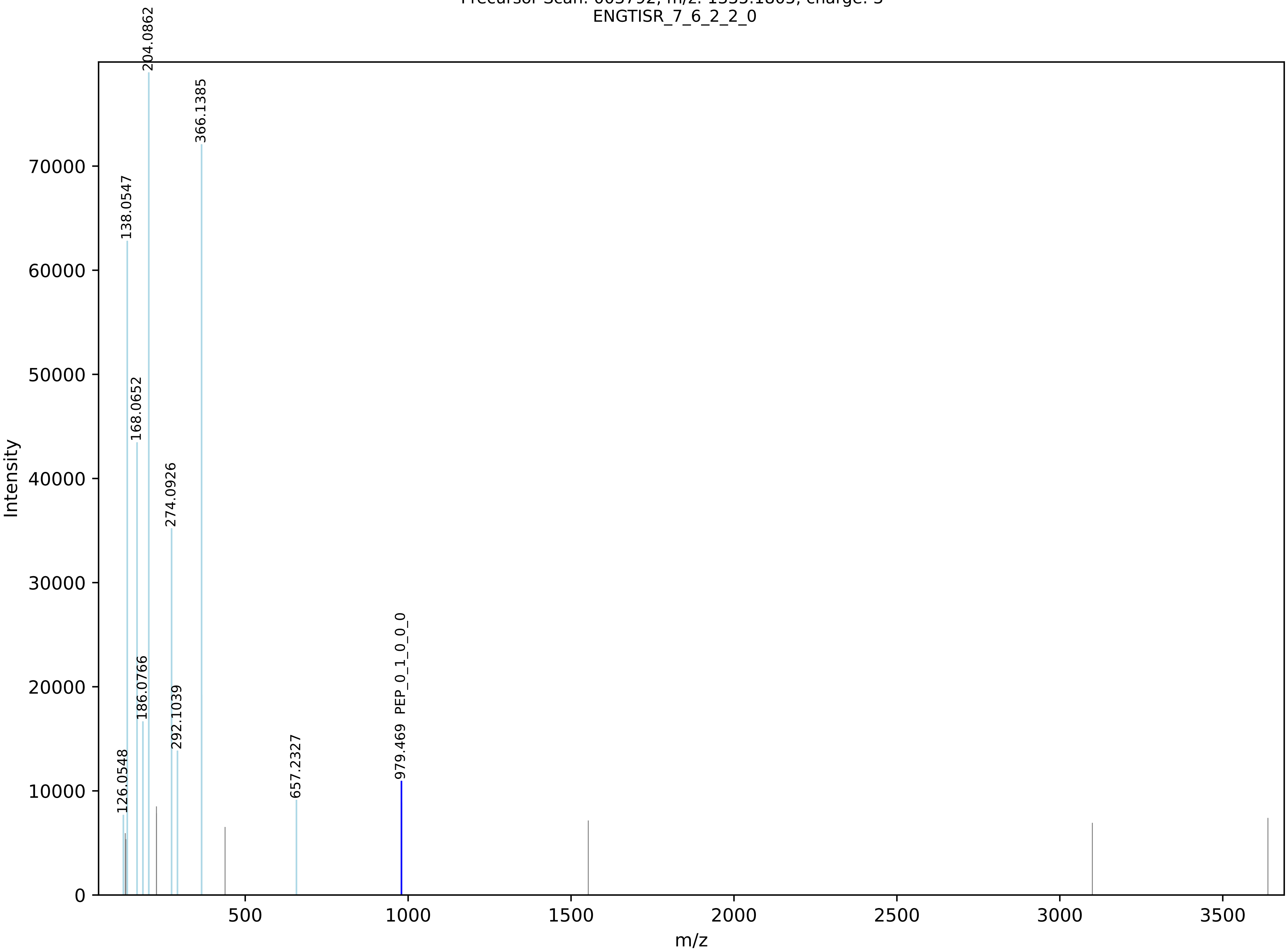

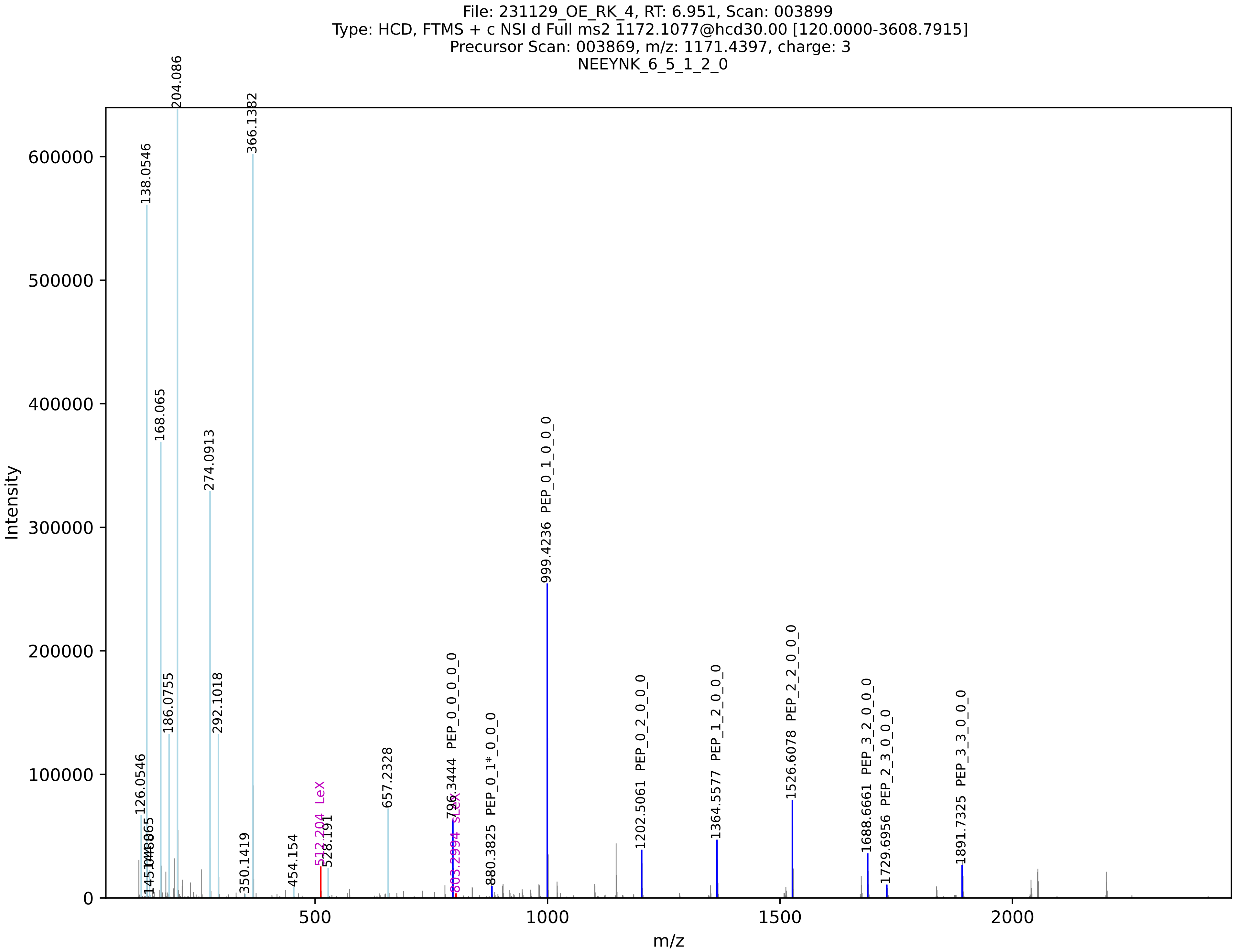

File: 231129_OE_RK_4, RT: 6.951, Scan: 003899
Type: HCD, FTMS + c NSI d Full ms2 1172.1077@hcd30.00 [120.0000-3608.7915]
Precursor Scan: 003869, m/z: 1171.4397, charge: 3
NEEYNK_6_5_1_2_0
Intensity
m/z
204.086
366.1382
138.0546
168.065
274.0913
186.0755
292.1018
126.0546
350.1419
454.154
512.204 LeX
528.191
657.2328
796.3444 PEP_0_0_0_0_0
803.2994 sLeX
880.3825 PEP_0_1*_0_0_0
999.4236 PEP_0_1_0_0_0
1202.5061 PEP_0_2_0_0_0
1364.5577 PEP_1_2_0_0_0
1526.6078 PEP_2_2_0_0_0
1688.6661 PEP_3_2_0_0_0
1729.6956 PEP_2_3_0_0_0
1891.7325 PEP_3_3_0_0_0

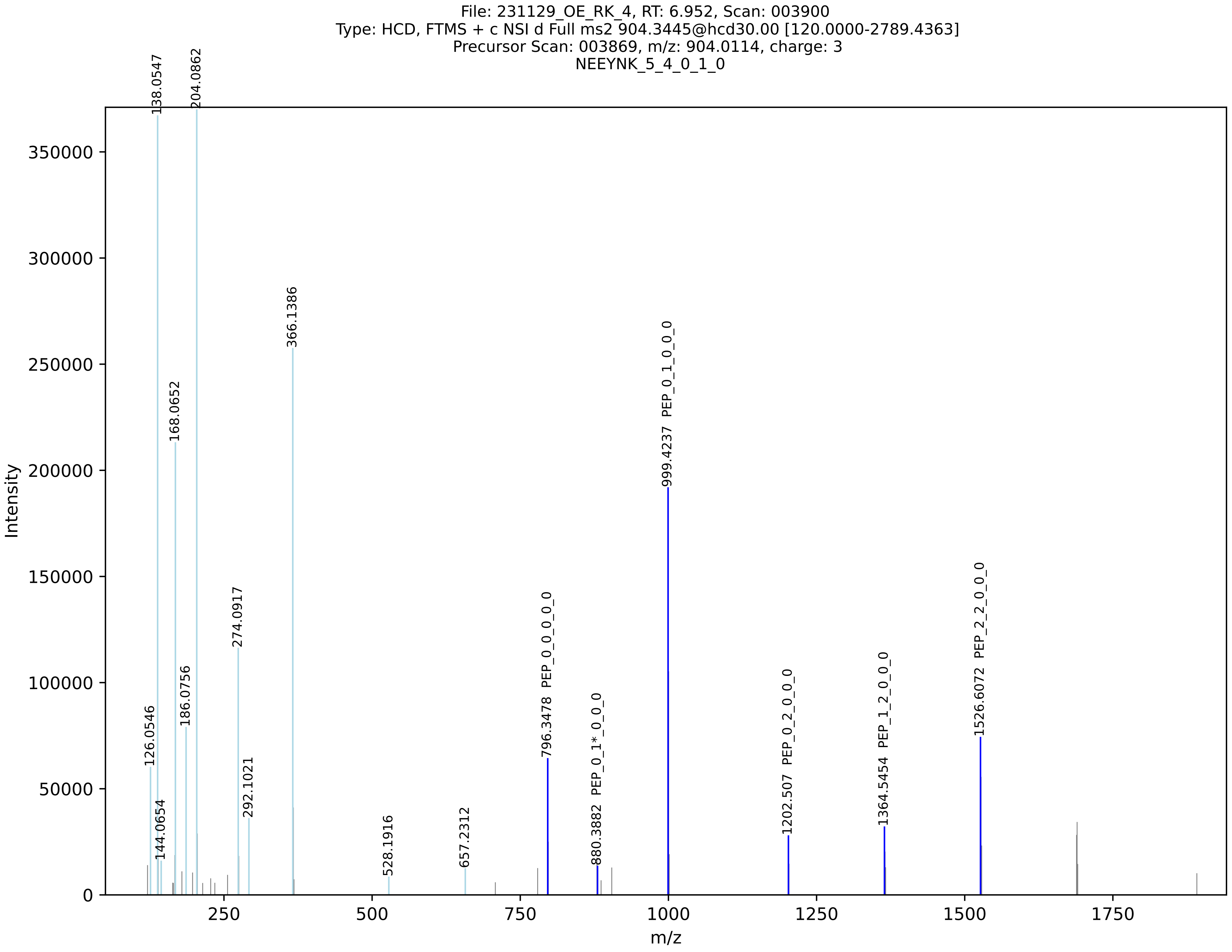
File: 231129_OE_RK_4, RT: 6.952, Scan: 003900
Type: HCD, FTMS + c NSI d Full ms2 904.3445@hcd30.00 [120.0000-2789.4363]
Precursor Scan: 003869, m/z: 904.0114, charge: 3
NEEYNK_5_4_0_1_0
138.0547
204.0862
366.1386
168.0652
999.4237 PEP_0_1_0_0_
186.0756
126.0546
274.0917
796.3478 PEP_0_0_0_0_0
880.3882 PEP_0_1*_0_0_0
1202.507 PEP_0_2_0_0_0
1364.5454 PEP_1_2_0_0_0
1526.6072 PEP_2_2_0_0_0
292.1021
144.0654
528.1916
657.2312
Intensity
m/z

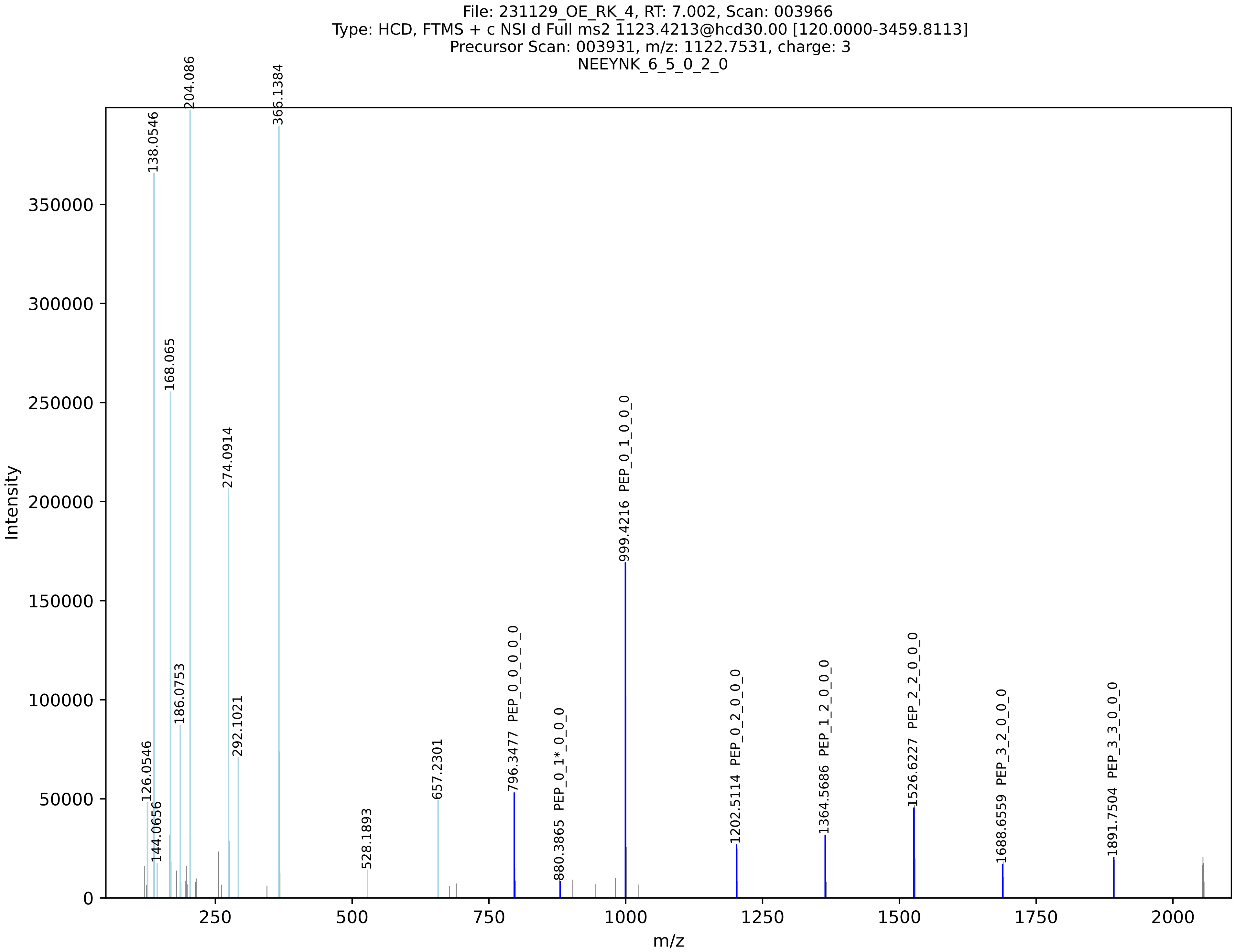
File: 231129_OE_RK_4, RT: 7.002, Scan: 003966
Type: HCD, FTMS + c NSI d Full ms2 1123.4213@hcd30.00 [120.0000-3459.8113]
Precursor Scan: 003931, m/z: 1122.7531, charge: 3
NEEYNK_6_5_0_2_0
Intensity
m/z
0
50000
100000
150000
200000
250000
300000
350000
250
500
750
1000
1250
1500
1750
2000
126.0546
138.0546
144.0656
168.065
186.0753
204.086
274.0914
292.1021
366.1384
528.1893
657.2301
796.3477 PEP_0_0_0_0_0
880.3865 PEP_0_1*_0_0_0
999.4216 PEP_0_1_0_0_0
1202.5114 PEP_0_2_0_0_0
1364.5686 PEP_1_2_0_0_0
1526.6227 PEP_2_2_0_0_0
1688.6559 PEP_3_2_0_0_0
1891.7504 PEP_3_3_0_0_0

File: 231129_OE_RK_4, RT: 7.018, Scan: 003984
Type: HCD, FTMS + c NSI d Full ms2 1501.5616@hcd30.00 [120.0000-3084.6138]
Precursor Scan: 003962, m/z: 1501.0598, charge: 2
NEEYNK_5_4_0_2_0

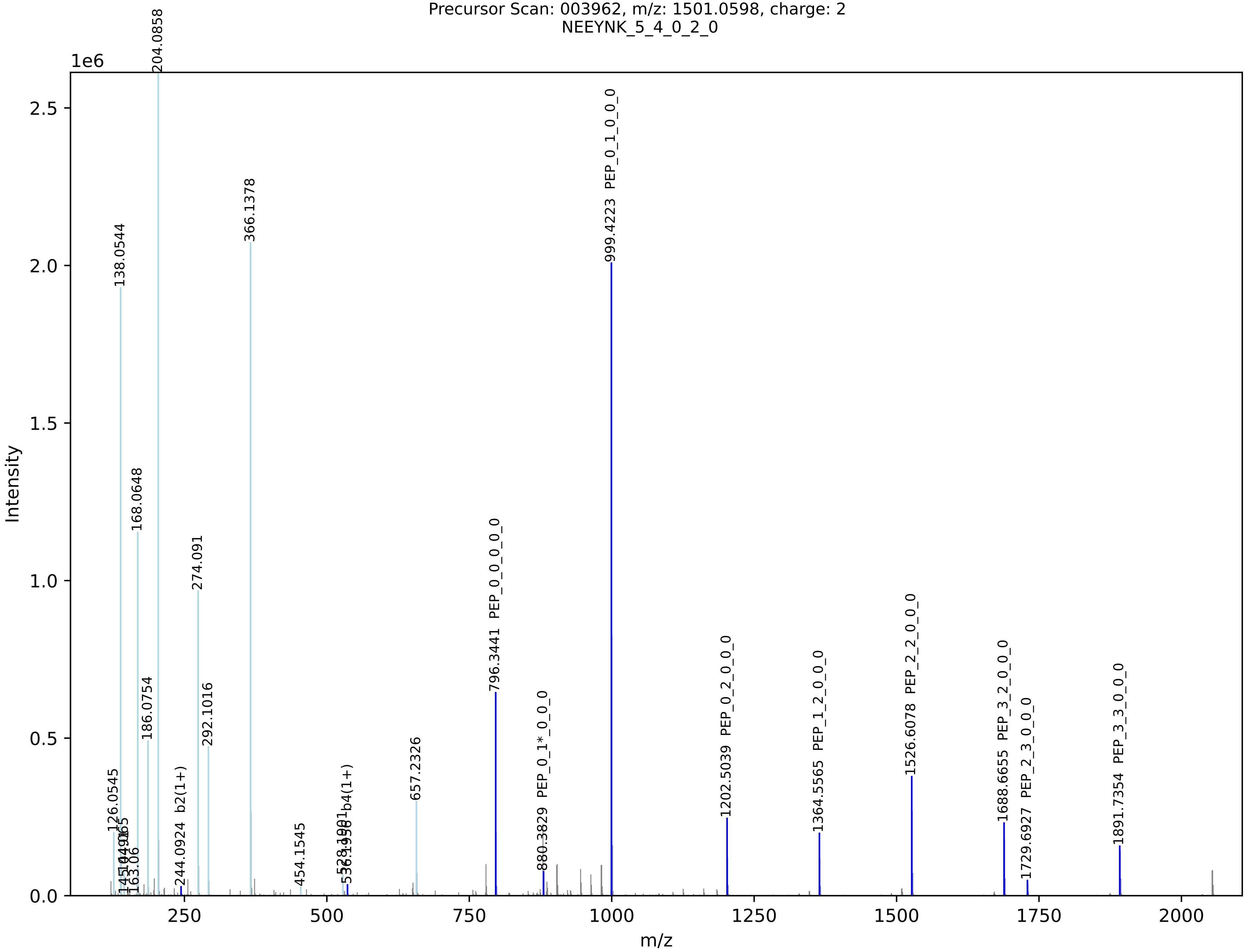

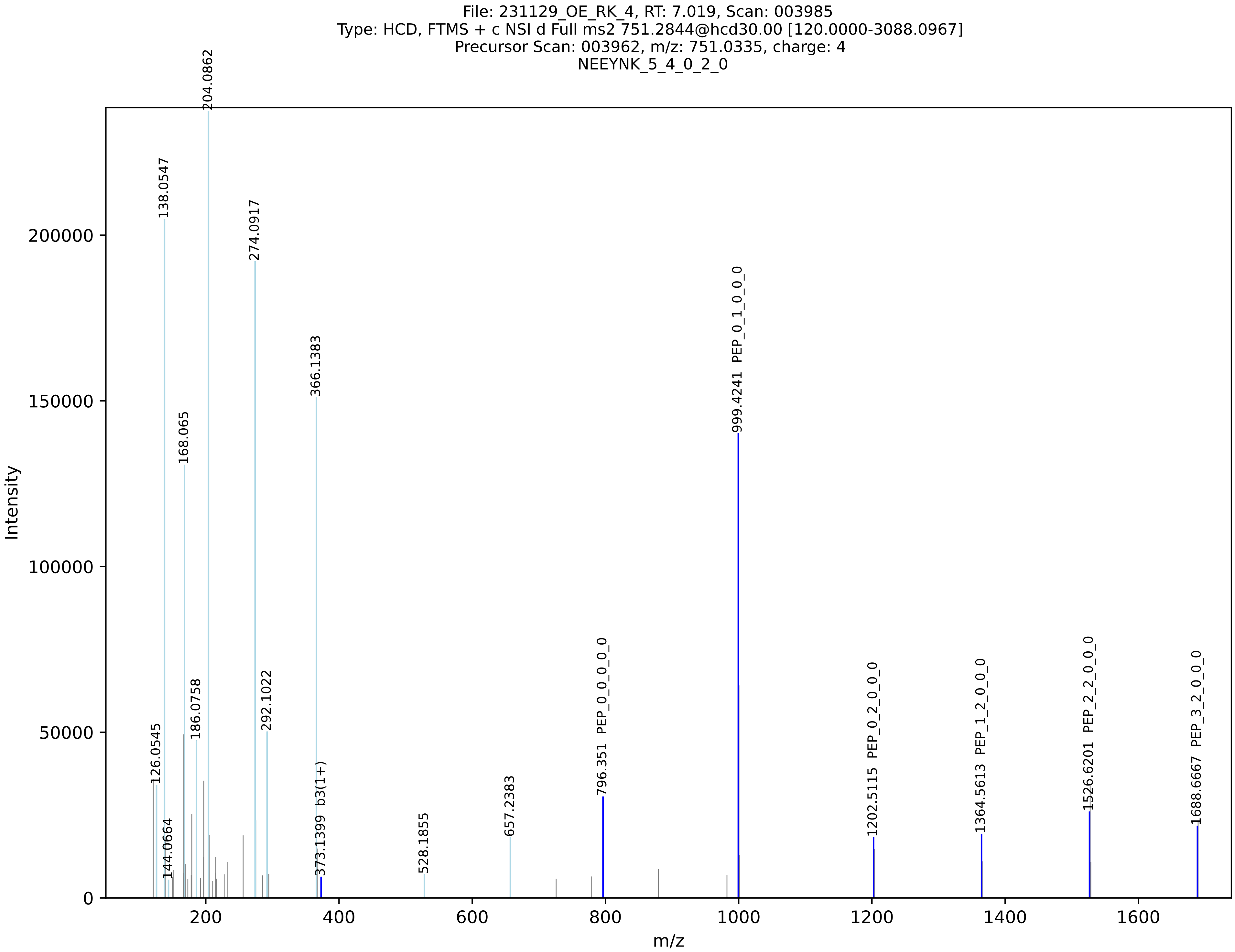
File: 231129_OE_RK_4, RT: 7.019, Scan: 003985
Type: HCD, FTMS + c NSI d Full ms2 751.2844@hcd30.00 [120.0000-3088.0967]
Precursor Scan: 003962, m/z: 751.0335, charge: 4
NEEYNK_5_4_0_2_0
204.0862
138.0547
274.0917
366.1383
168.065
186.0758
292.1022
126.0545
144.0664
373.1399 b3(1+)
528.1855
657.2383
796.351 PEP_0_0_0_0_0
999.4241 PEP_0_1_0_0_0
1202.5115 PEP_0_2_0_0_0
1364.5613 PEP_1_2_0_0_0
1526.6201 PEP_2_2_0_0_0
1688.6667 PEP_3_2_0_0_0
Intensity
m/z
0
50000
100000
150000
200000
200
400
600
800
1000
1200
1400
1600

File: 231129_OE_RK_4, RT: 7.023, Scan: 003991
Type: HCD, FTMS + c NSI d Full ms2 1269.1427@hcd30.00 [120.0000-3905.7188]
Precursor Scan: 003962, m/z: 1268.1403, charge: 3
NEEYNK_6_5_1_3_0

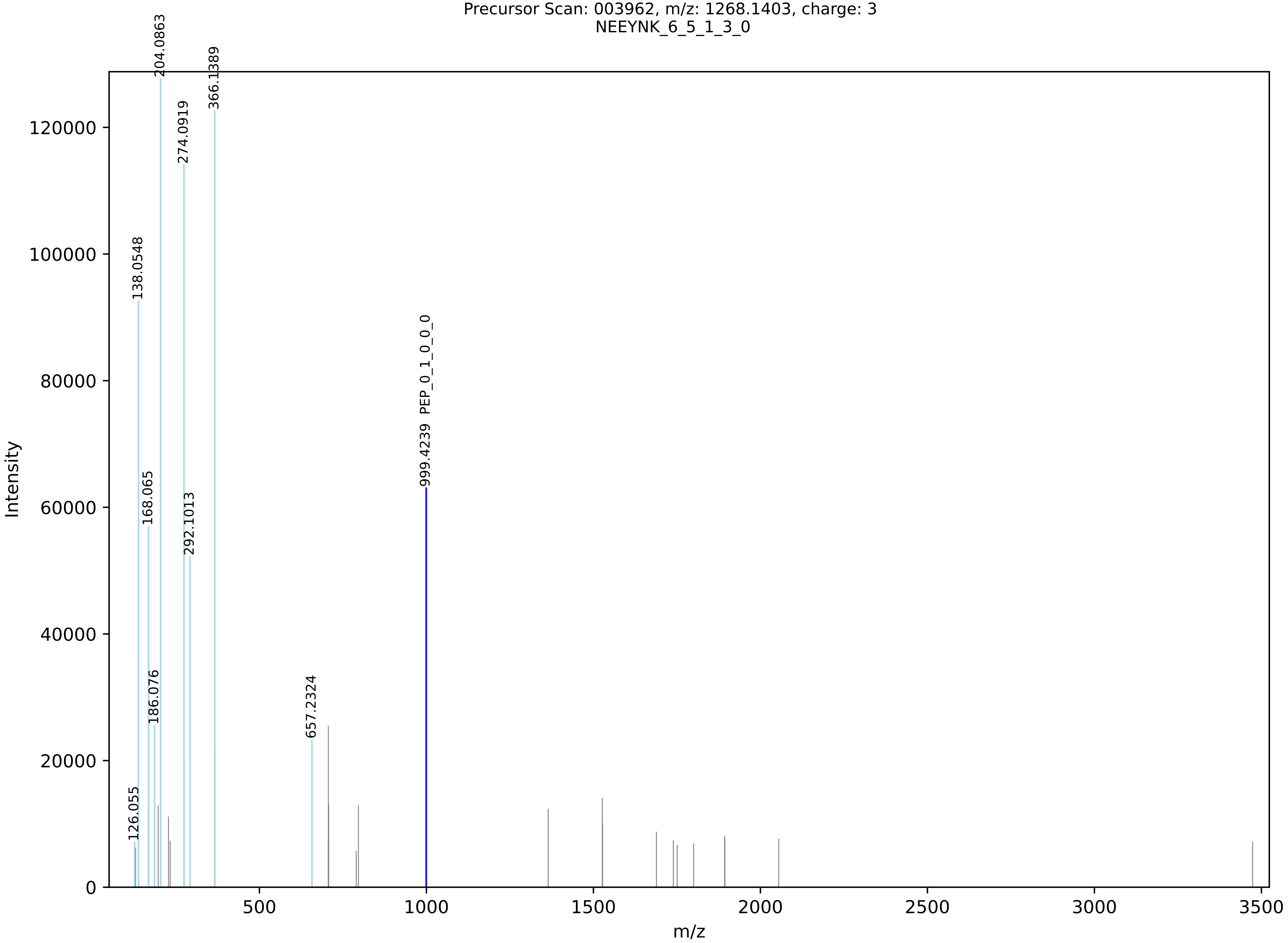

File: 231129_OE_RK_4, RT: 7.07, Scan: 004054
Type: HCD, FTMS + c NSI d Full ms2 1177.7820@hcd30.00 [120.0000-3626.1548]
Precursor Scan: 004017, m/z: 1177.1137, charge: 3
NEEYNK_7_5_2_1_0

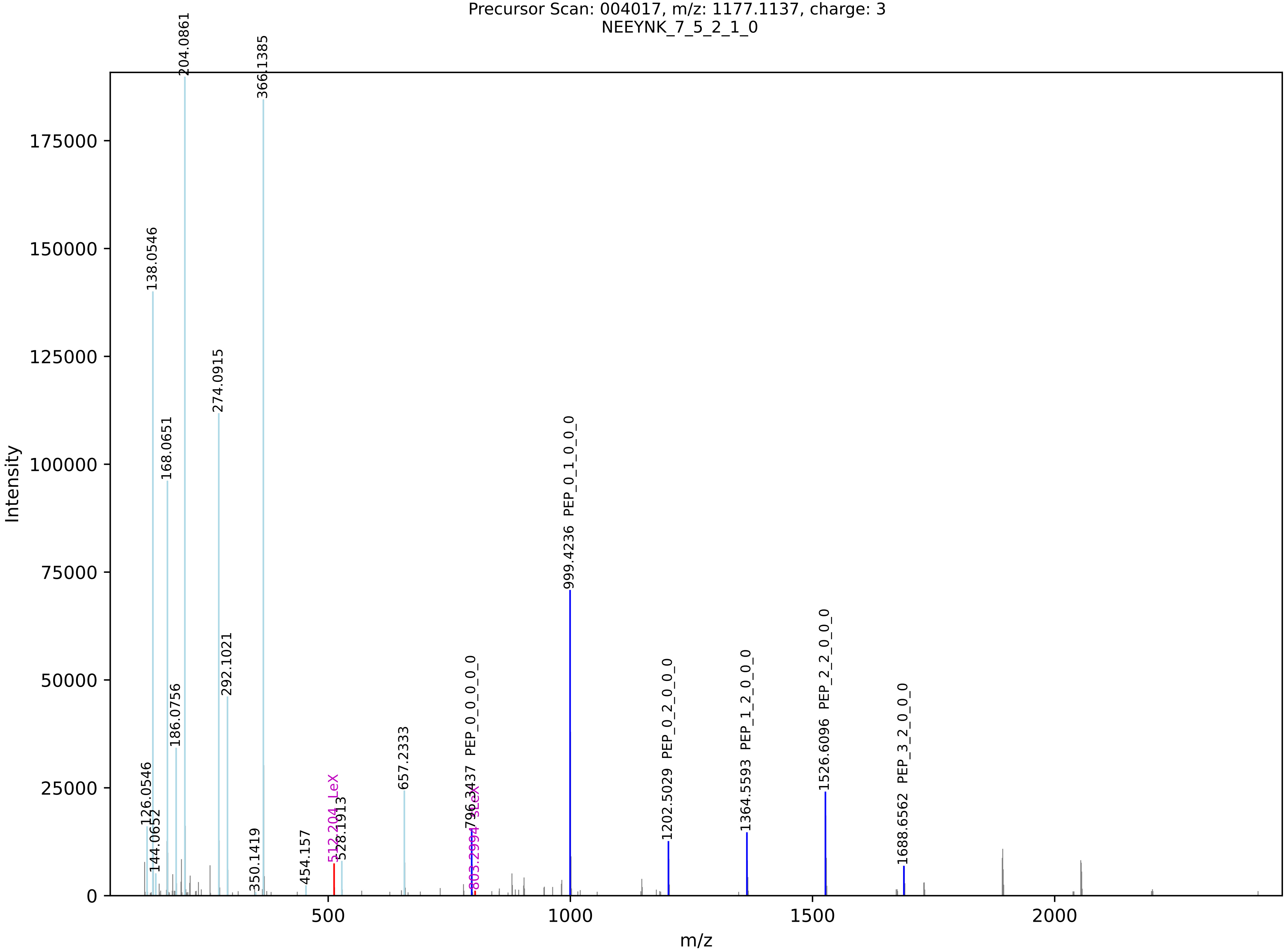

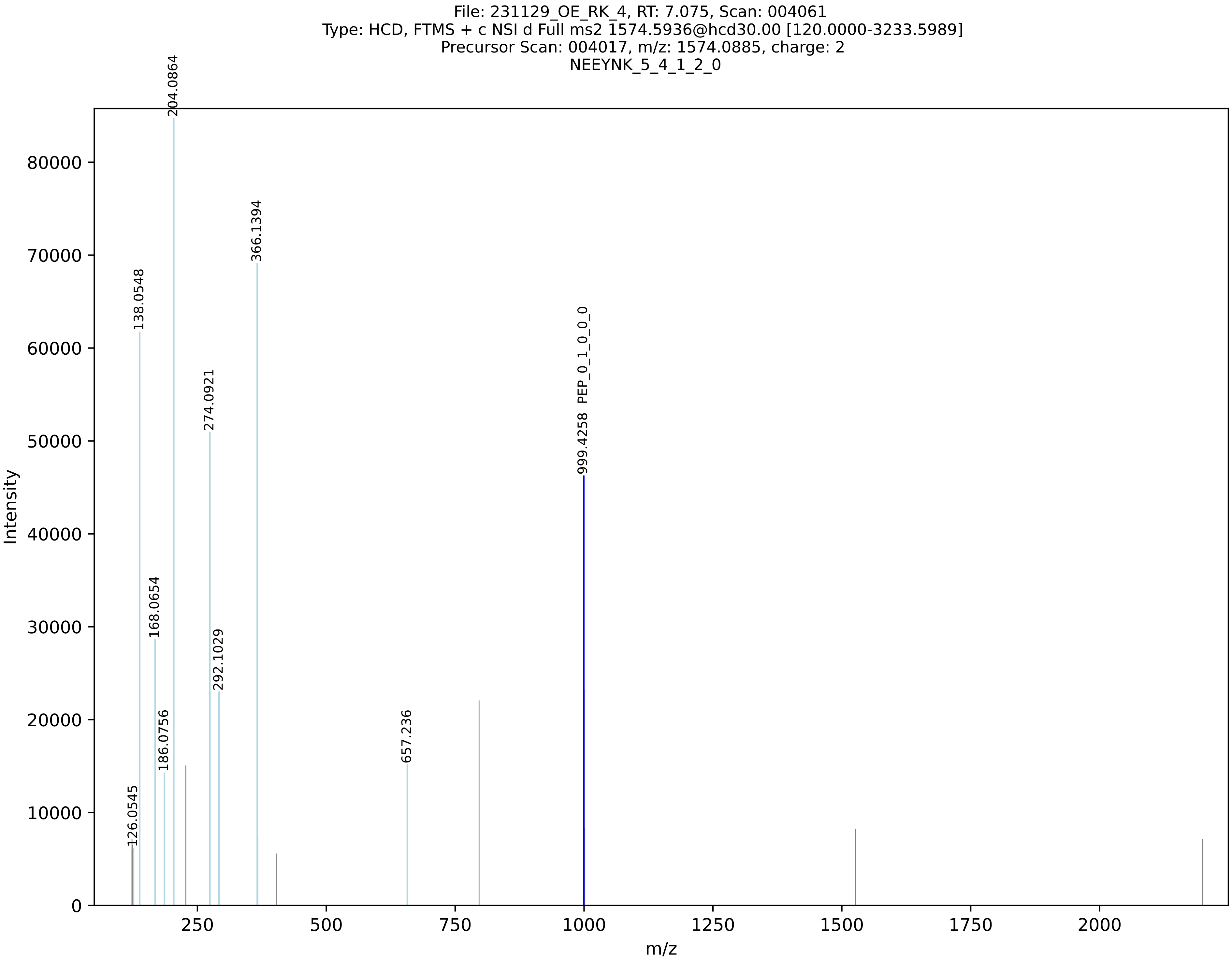

File: 231129_OE_RK_4, RT: 7.075, Scan: 004061
Type: HCD, FTMS + c NSI d Full ms2 1574.5936@hcd30.00 [120.0000-3233.5989]
Precursor Scan: 004017, m/z: 1574.0885, charge: 2
NEEYNK_5_4_1_2_0
204.0864
366.1394
138.0548
274.0921
999.4258 PEP_0_1_0_0_0
168.0654
292.1029
186.0756
657.236
126.0545
Intensity
m/z
0
10000
20000
30000
40000
50000
60000
70000
80000
250
500
750
1000
1250
1500
1750
2000

File: 231129_OE_RK_4, RT: 7.123, Scan: 004122
Type: HCD, FTMS + c NSI d Full ms2 953.7083@hcd30.00 [120.0000-2940.4895]
Precursor Scan: 004082, m/z: 953.3739, charge: 3
HNSTGCLR_5_4_0_1_0

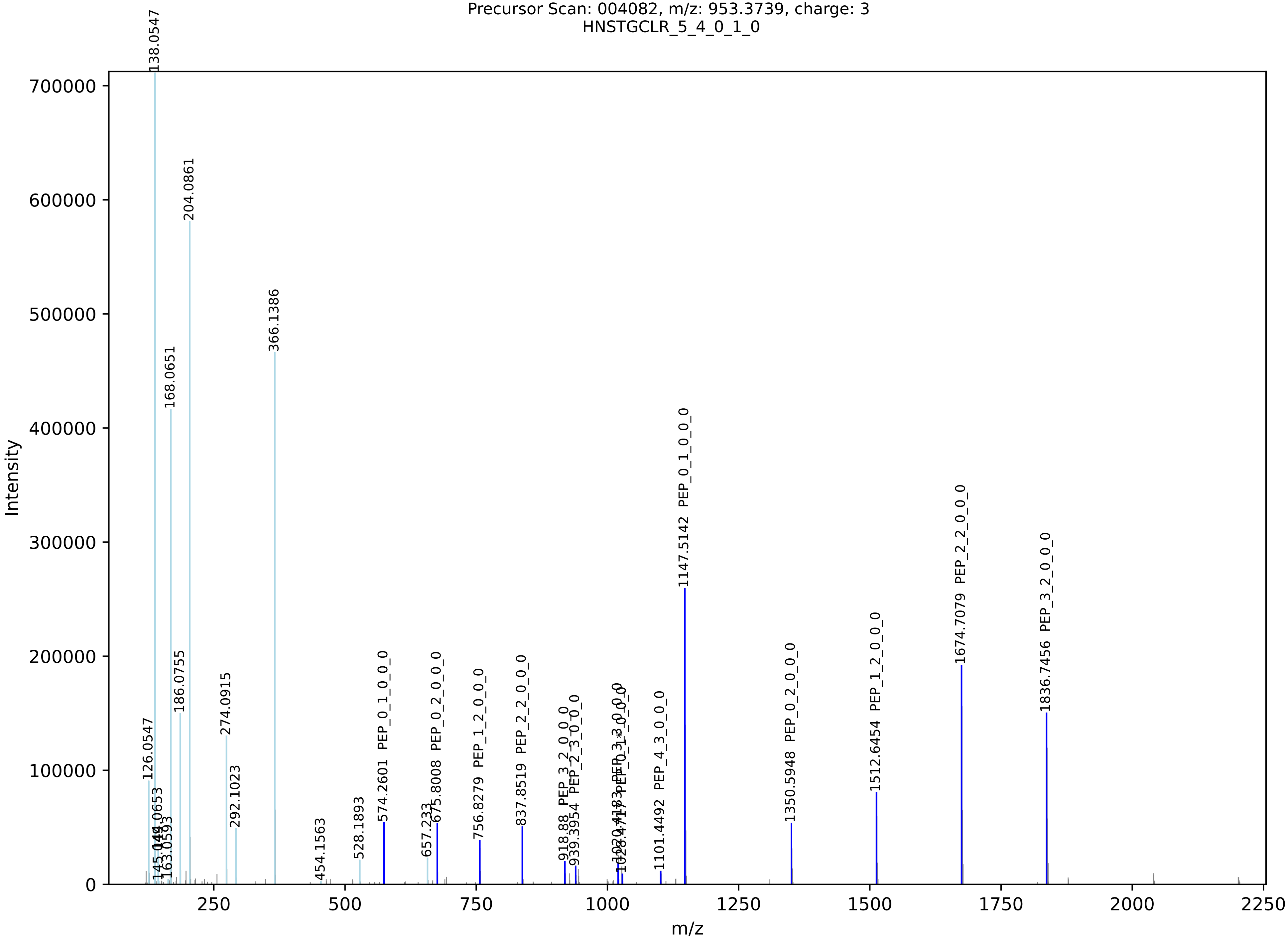

File: 231129_OE_RK_4, RT: 7.181, Scan: 004203
Type: HCD, FTMS + c NSI d Full ms2 1049.6890@hcd30.00 [120.0000-3234.1902]
Precursor Scan: 004151, m/z: 1048.6851, charge: 3
NEEYNK_5_4_1_2_0

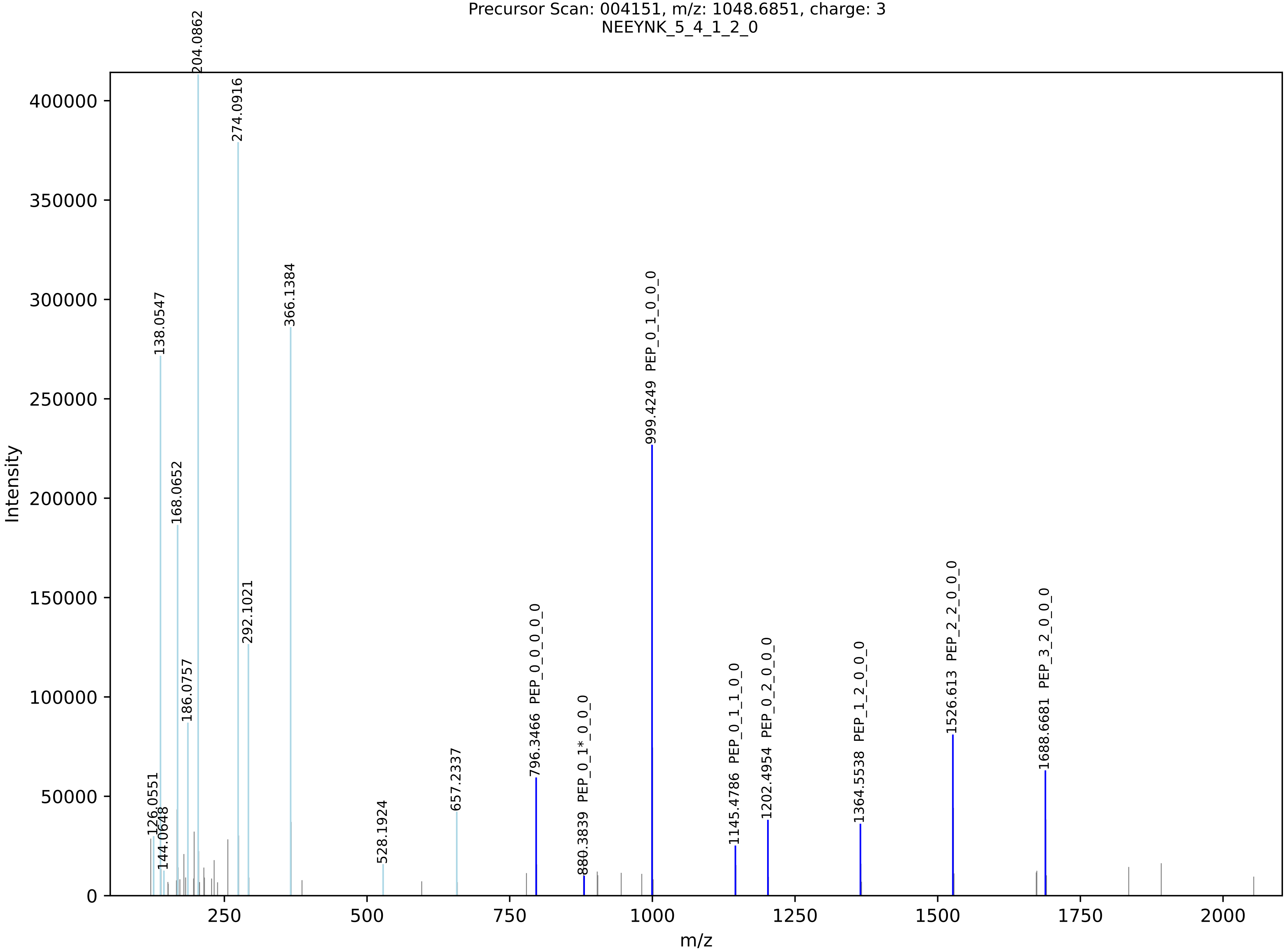

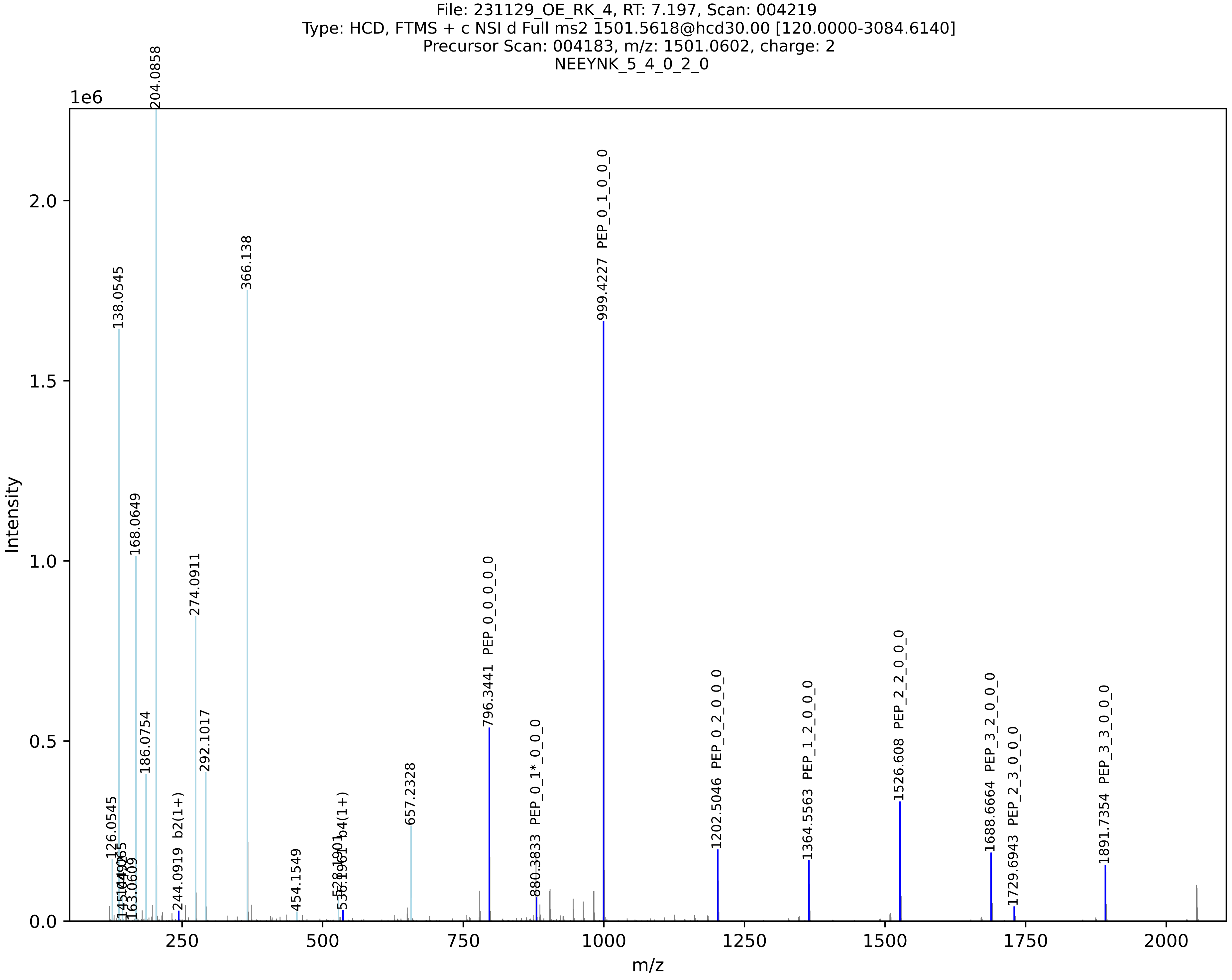
File: 231129_OE_RK_4, RT: 7.197, Scan: 004219
Type: HCD, FTMS + c NSI d Full ms2 1501.5618@hcd30.00 [120.0000-3084.6140]
Precursor Scan: 004183, m/z: 1501.0602, charge: 2
NEEYNK_5_4_0_2_0
1e6
Intensity
m/z
204.0858
138.0545
366.138
168.0649
274.0911
186.0754
292.1017
126.0545
163.0609
244.0919 b2(1+)
454.1549
b4(1+)
657.2328
796.3441 PEP_0_0_0_0_0
880.3833 PEP_0_1*_0_0_0
999.4227 PEP_0_1_0_0_0
1202.5046 PEP_0_2_0_0_0
1364.5563 PEP_1_2_0_0_0
1526.608 PEP_2_2_0_0_0
1688.6664 PEP_3_2_0_0_0
1729.6943 PEP_2_3_0_0_0
1891.7354 PEP_3_3_0_0_0

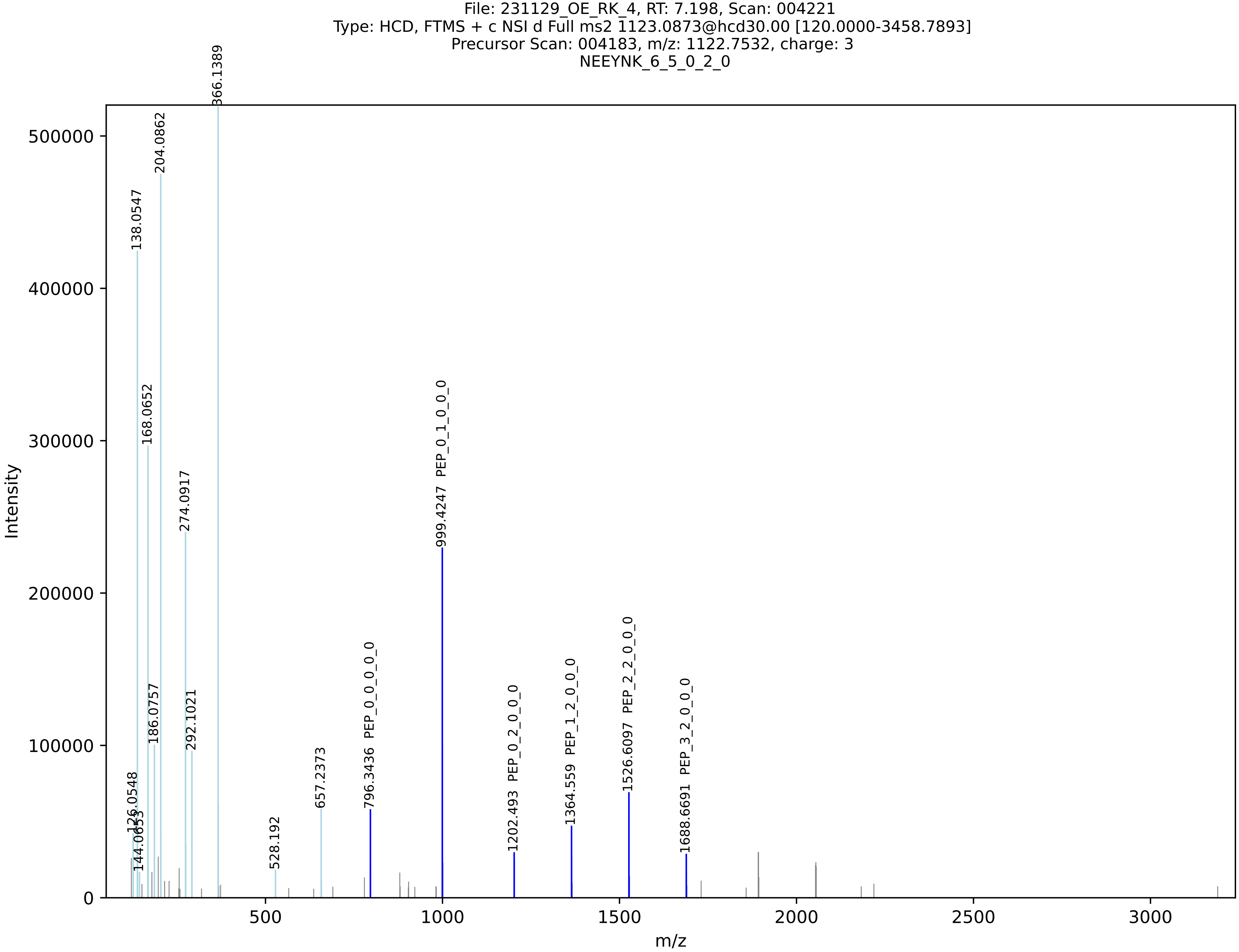
File: 231129_OE_RK_4, RT: 7.198, Scan: 004221
Type: HCD, FTMS + c NSI d Full ms2 1123.0873@hcd30.00 [120.0000-3458.7893]
Precursor Scan: 004183, m/z: 1122.7532, charge: 3
NEEYNK_6_5_0_2_0
366.1389
204.0862
138.0547
168.0652
274.0917
186.0757
292.1021
126.0548
144.0653
528.192
657.2373
796.3436 PEP_0_0_0_0_0
999.4247 PEP_0_1_0_0_0
1202.493 PEP_0_2_0_0_0
1364.559 PEP_1_2_0_0_0
1526.6097 PEP_2_2_0_0_0
1688.6691 PEP_3_2_0_0_0
Intensity
m/z
0
100000
200000
300000
400000
500000
500
1000
1500
2000
2500
3000

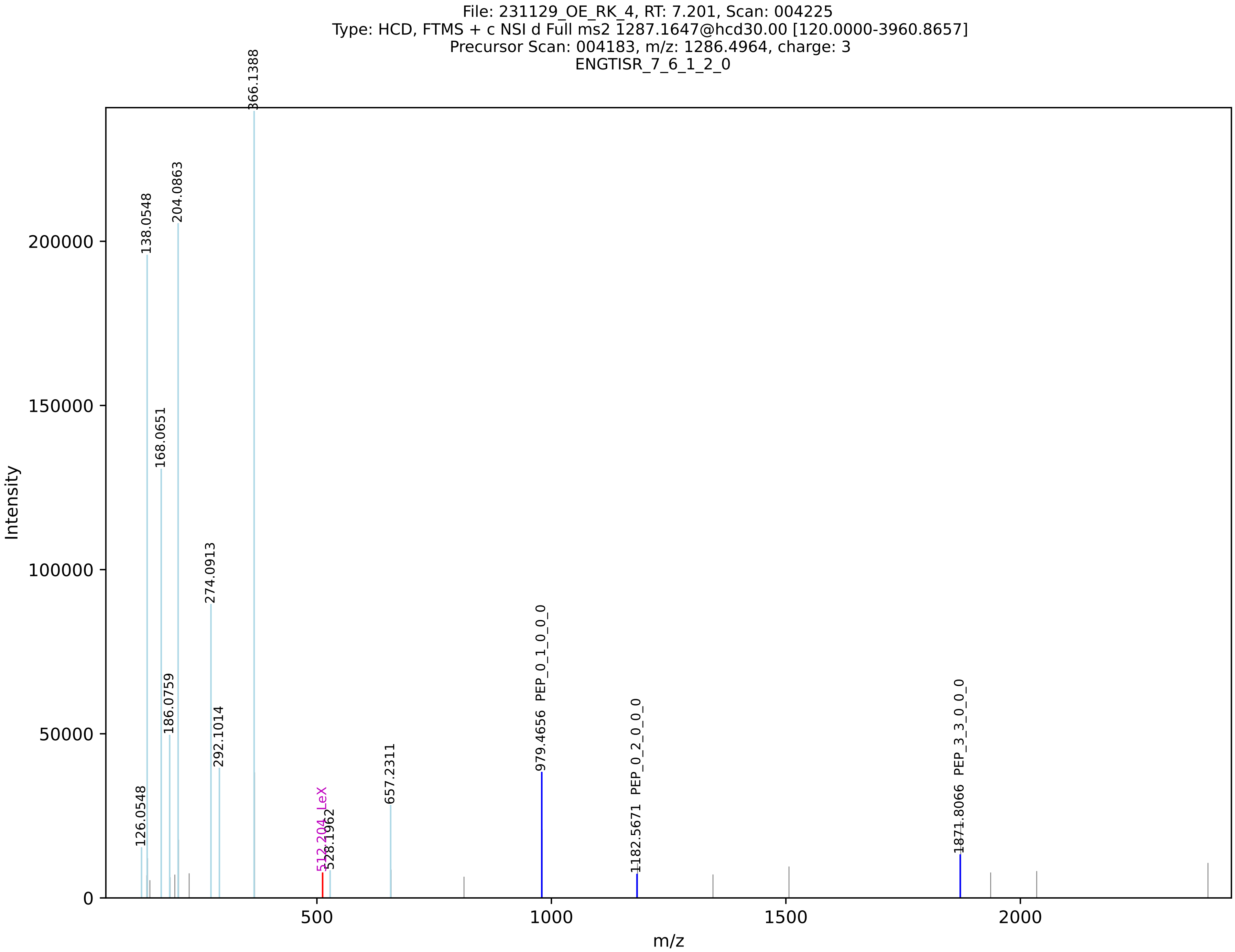
File: 231129_OE_RK_4, RT: 7.201, Scan: 004225
Type: HCD, FTMS + c NSI d Full ms2 1287.1647@hcd30.00 [120.0000-3960.8657]
Precursor Scan: 004183, m/z: 1286.4964, charge: 3
ENGTISR_7_6_1_2_0
366.1388
204.0863
138.0548
168.0651
274.0913
186.0759
292.1014
126.0548
512.204_LeX
528.1962
657.2311
979.4656 PEP_0_1_0_0_0
1182.5671 PEP_0_2_0_0_0
1871.8066 PEP_3_3_0_0_0
Intensity
0
50000
100000
150000
200000
500
1000
1500
2000
m/z

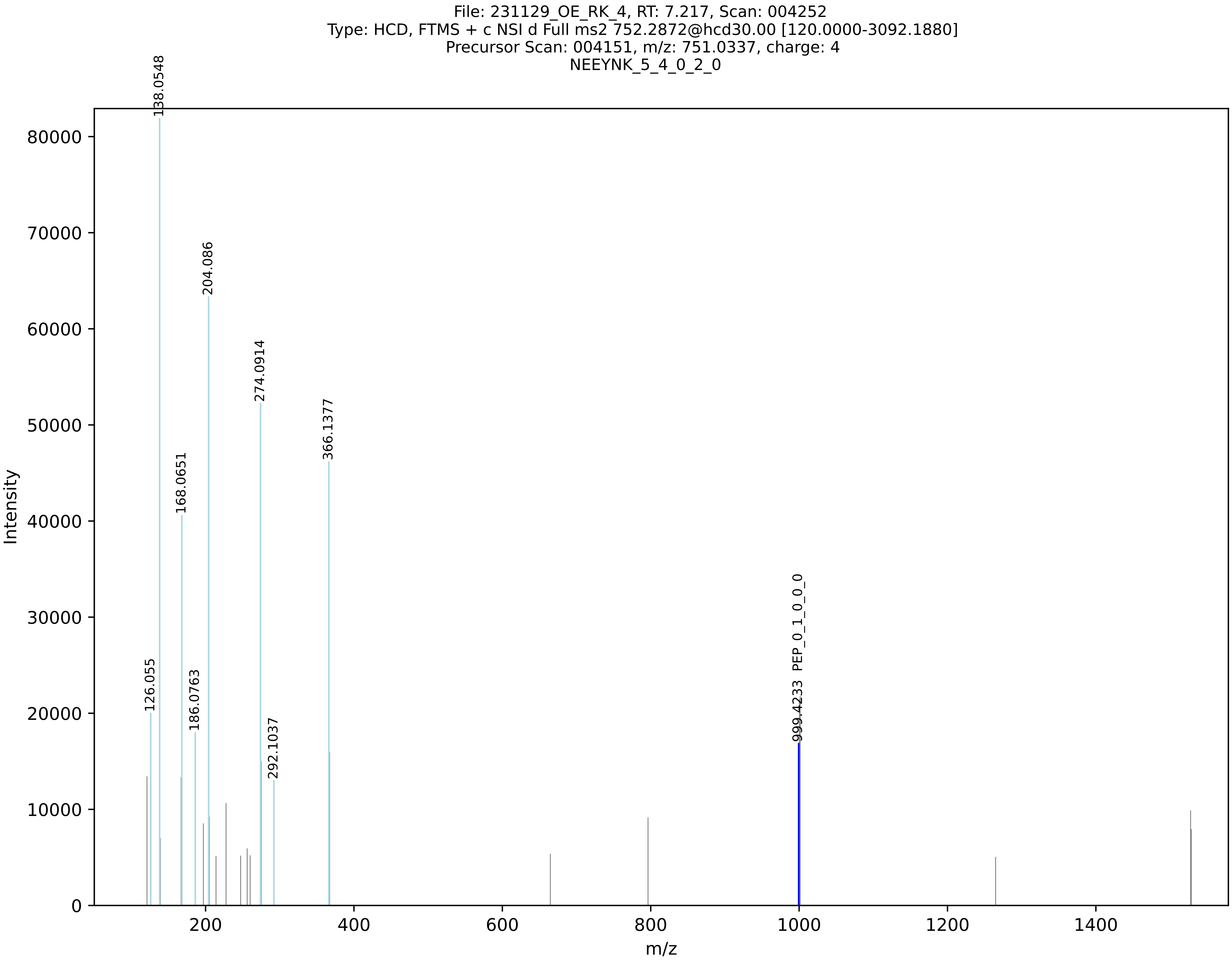
File: 231129_OE_RK_4, RT: 7.217, Scan: 004252
Type: HCD, FTMS + c NSI d Full ms2 752.2872@hcd30.00 [120.0000-3092.1880]
Precursor Scan: 004151, m/z: 751.0337, charge: 4
NEEYNK_5_4_0_2_0
Intensity
m/z
138.0548
204.086
274.0914
366.1377
168.0651
126.055
186.0763
292.1037
999.4233 PEP_0_1_0_0_0
0
10000
20000
30000
40000
50000
60000
70000
80000
200
400
600
800
1000
1200
1400

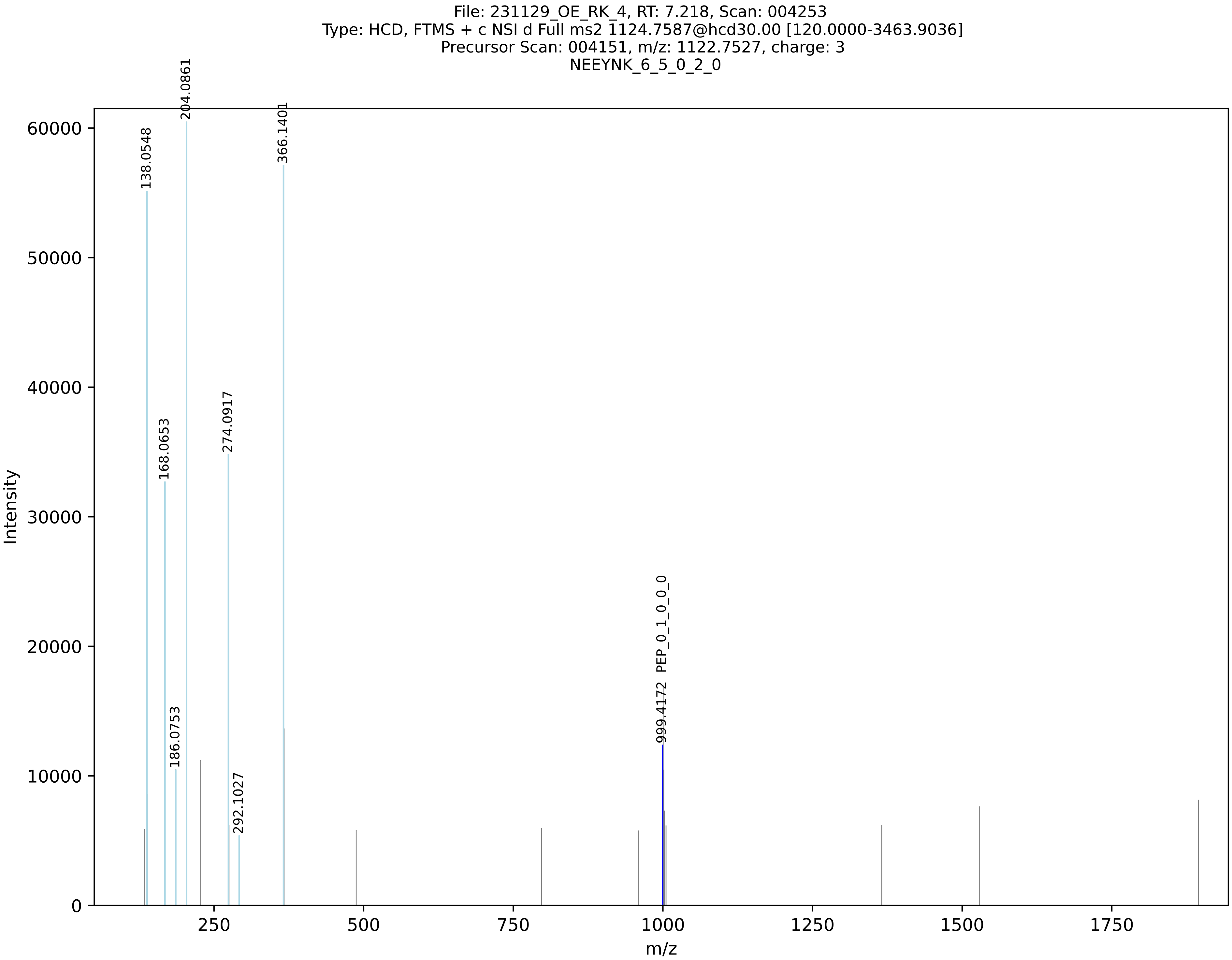

File: 231129_OE_RK_4, RT: 7.218, Scan: 004253
Type: HCD, FTMS + c NSI d Full ms2 1124.7587@hcd30.00 [120.0000-3463.9036]
Precursor Scan: 004151, m/z: 1122.7527, charge: 3
NEEYNK_6_5_0_2_0
204.0861
366.1401
138.0548
168.0653
274.0917
186.0753
292.1027
PEP_0_1_0_0_
999.4172
Intensity
0
10000
20000
30000
40000
50000
60000
250
500
750
1000
1250
1500
1750
m/z

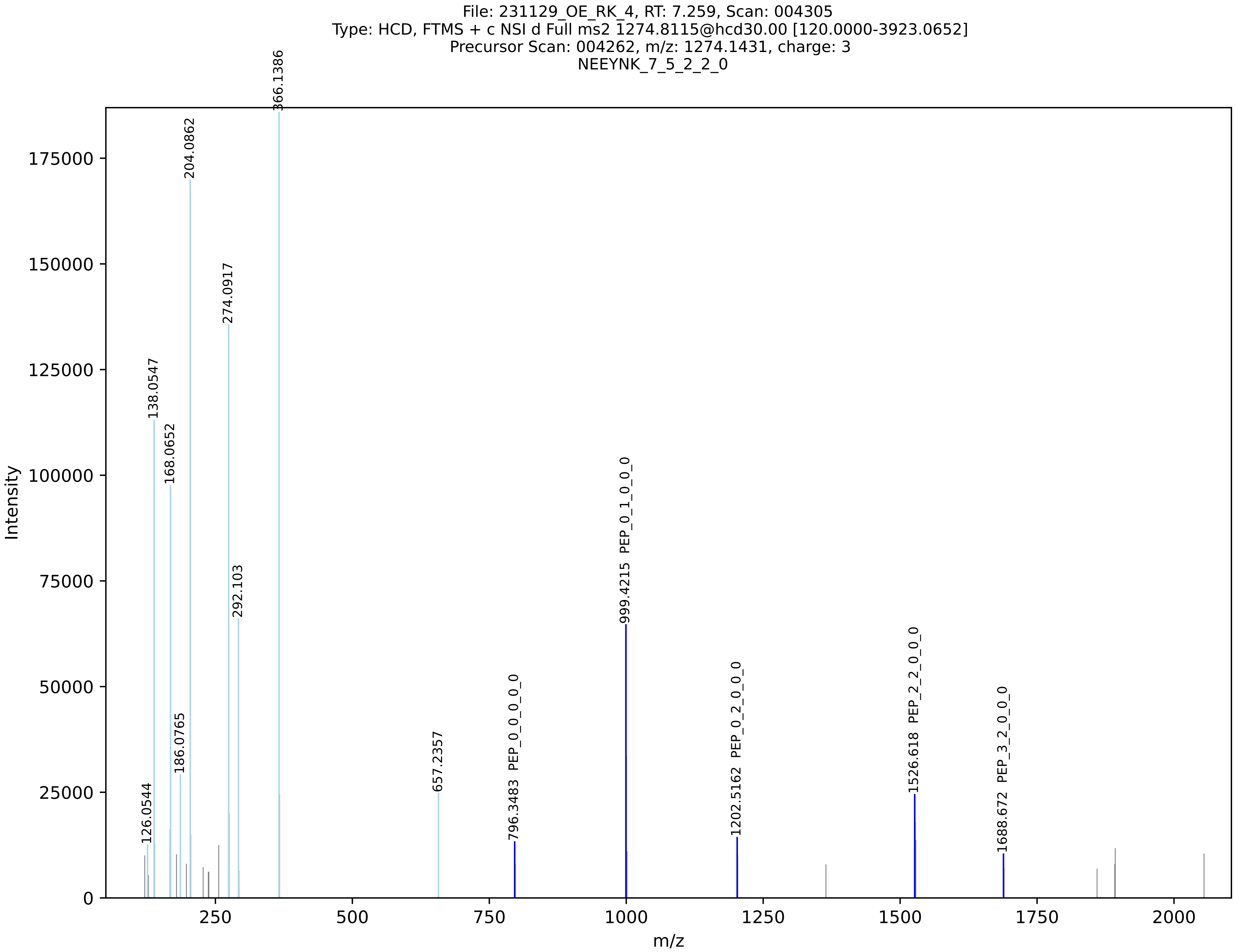
File: 231129_OE_RK_4, RT: 7.259, Scan: 004305
Type: HCD, FTMS + c NSI d Full ms2 1274.8115@hcd30.00 [120.0000-3923.0652]
Precursor Scan: 004262, m/z: 1274.1431, charge: 3
NEEYNK_7_5_2_2_0
Intensity
m/z
366.1386
204.0862
274.0917
138.0547
168.0652
292.103
186.0765
126.0544
657.2357
796.3483 PEP_0_0_0_0_0
999.4215 PEP_0_1_0_0_0
1202.5162 PEP_0_2_0_0_0
1526.618 PEP_2_2_0_0_0
1688.672 PEP_3_2_0_0_0

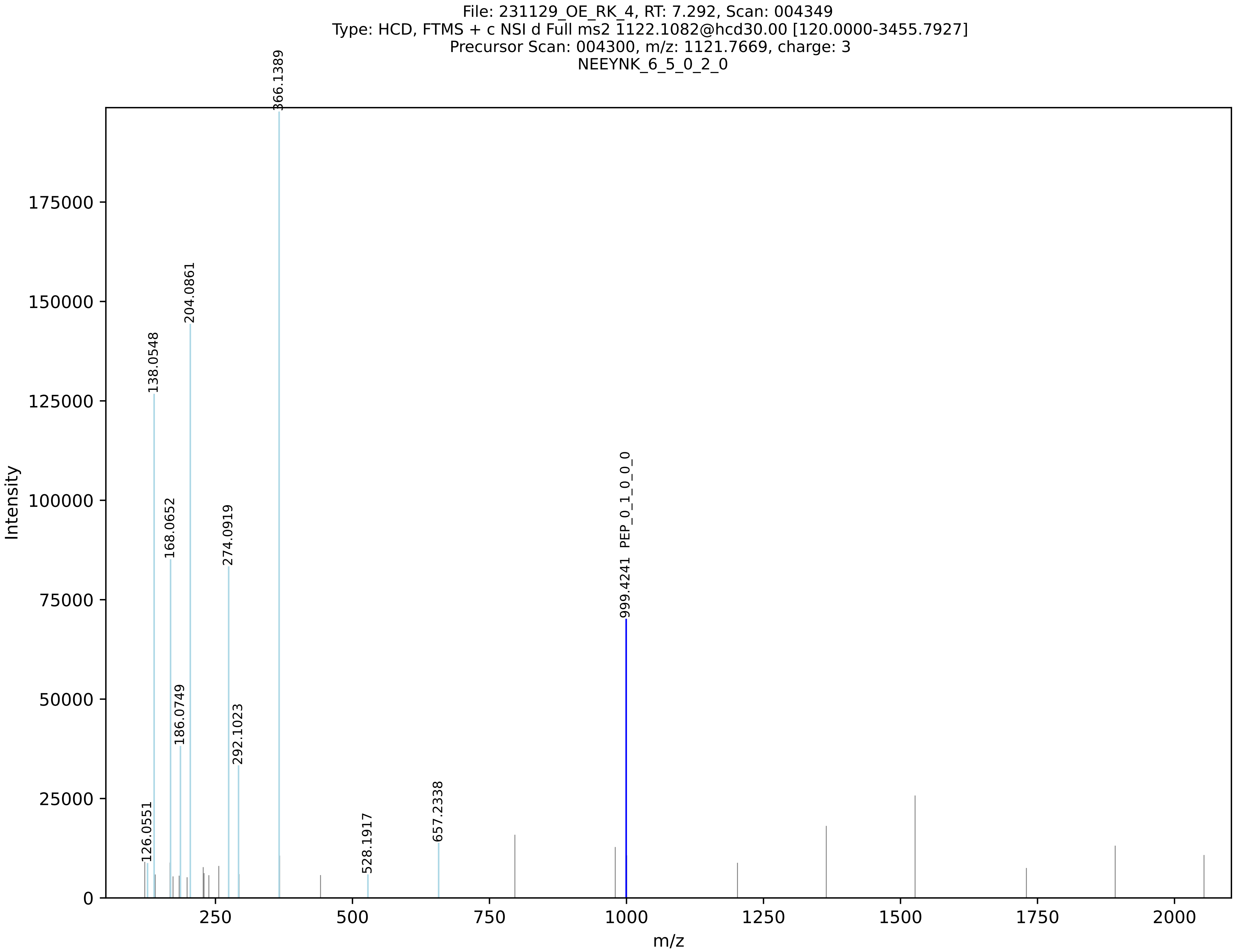
File: 231129_OE_RK_4, RT: 7.292, Scan: 004349
Type: HCD, FTMS + c NSI d Full ms2 1122.1082@hcd30.00 [120.0000-3455.7927]
Precursor Scan: 004300, m/z: 1121.7669, charge: 3
NEEYNK_6_5_0_2_0
366.1389
204.0861
138.0548
168.0652
274.0919
186.0749
292.1023
126.0551
528.1917
657.2338
999.4241 PEP_0_1_0_0_0
Intensity
m/z
0
25000
50000
75000
100000
125000
150000
175000
250
500
750
1000
1250
1500
1750
2000

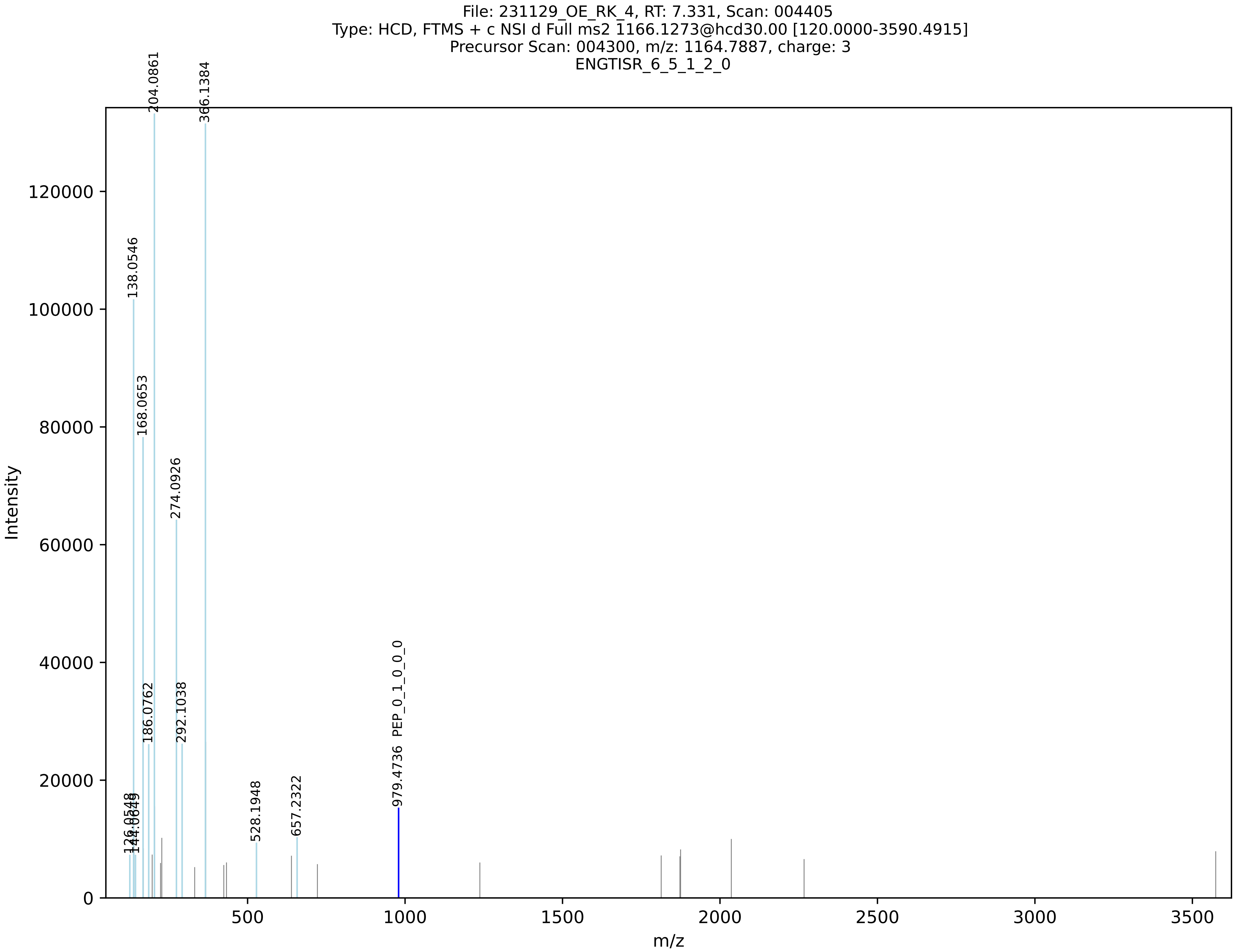
File: 231129_OE_RK_4, RT: 7.331, Scan: 004405
Type: HCD, FTMS + c NSI d Full ms2 1166.1273@hcd30.00 [120.0000-3590.4915]
Precursor Scan: 004300, m/z: 1164.7887, charge: 3
ENGTISR_6_5_1_2_0
204.0861
366.1384
138.0546
168.0653
274.0926
186.0762
292.1038
528.1948
657.2322
979.4736
PEP_0_1_0_0_0
Intensity
m/z
0
20000
40000
60000
80000
100000
120000
500
1000
1500
2000
2500
3000
3500

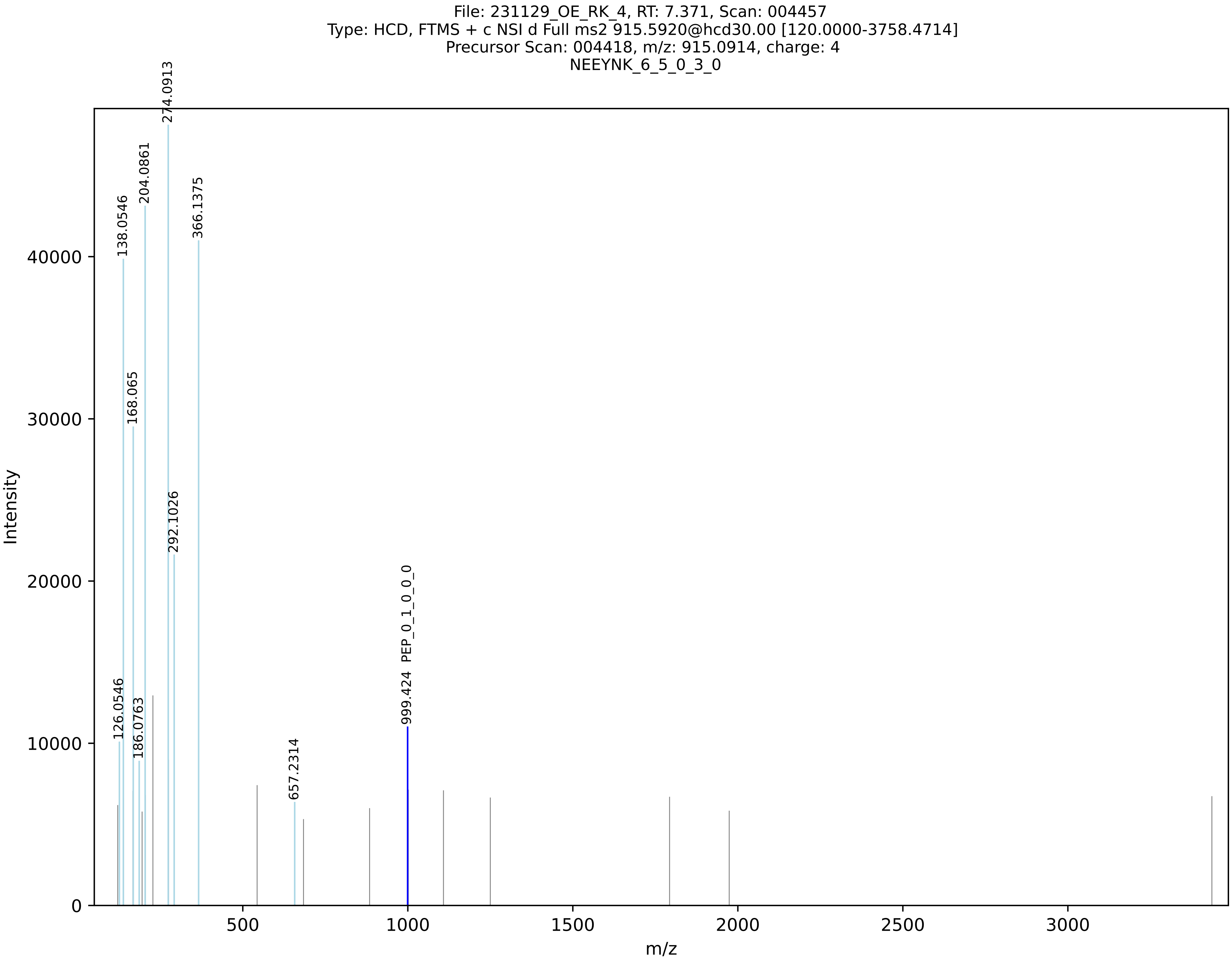
File: 231129_OE_RK_4, RT: 7.371, Scan: 004457
Type: HCD, FTMS + c NSI d Full ms2 915.5920@hcd30.00 [120.0000-3758.4714]
Precursor Scan: 004418, m/z: 915.0914, charge: 4
NEEYNK_6_5_0_3_0
274.0913
204.0861
366.1375
138.0546
168.065
292.1026
PEP_0_1_0_0_0
999.424
126.0546
186.0763
657.2314
Intensity
m/z
0
10000
20000
30000
40000
500
1000
1500
2000
2500
3000

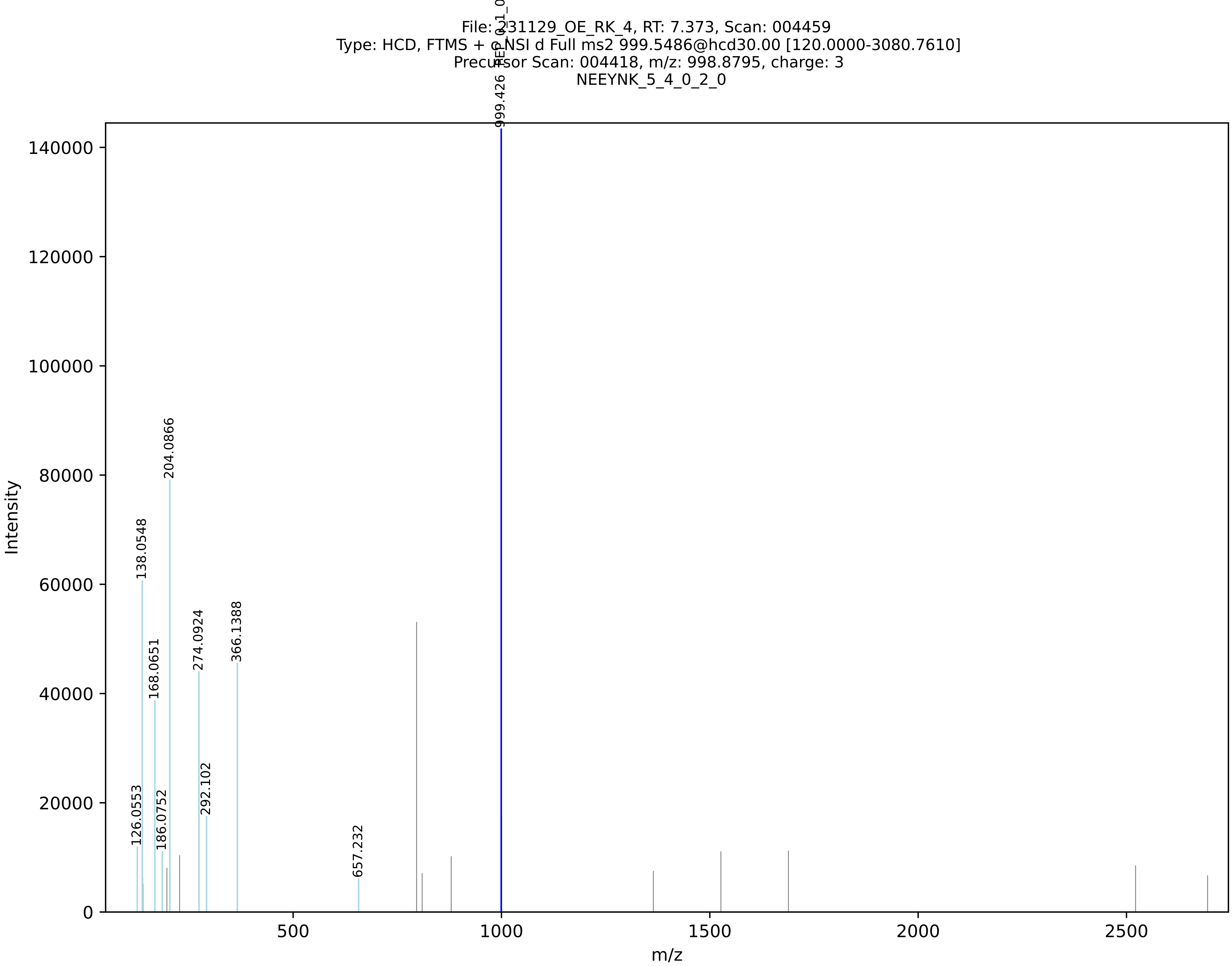
File: 231129_OE_RK_4, RT: 7.373, Scan: 004459
Type: HCD, FTMS + c NSI d Full ms2 999.5486@hcd30.00 [120.0000-3080.7610]
Precursor Scan: 004418, m/z: 998.8795, charge: 3
NEEYNK_5_4_0_2_0
999.426
204.0866
138.0548
168.0651
274.0924
366.1388
126.0553
186.0752
292.102
657.232
Intensity
m/z
0
20000
40000
60000
80000
100000
120000
140000
500
1000
1500
2000
2500

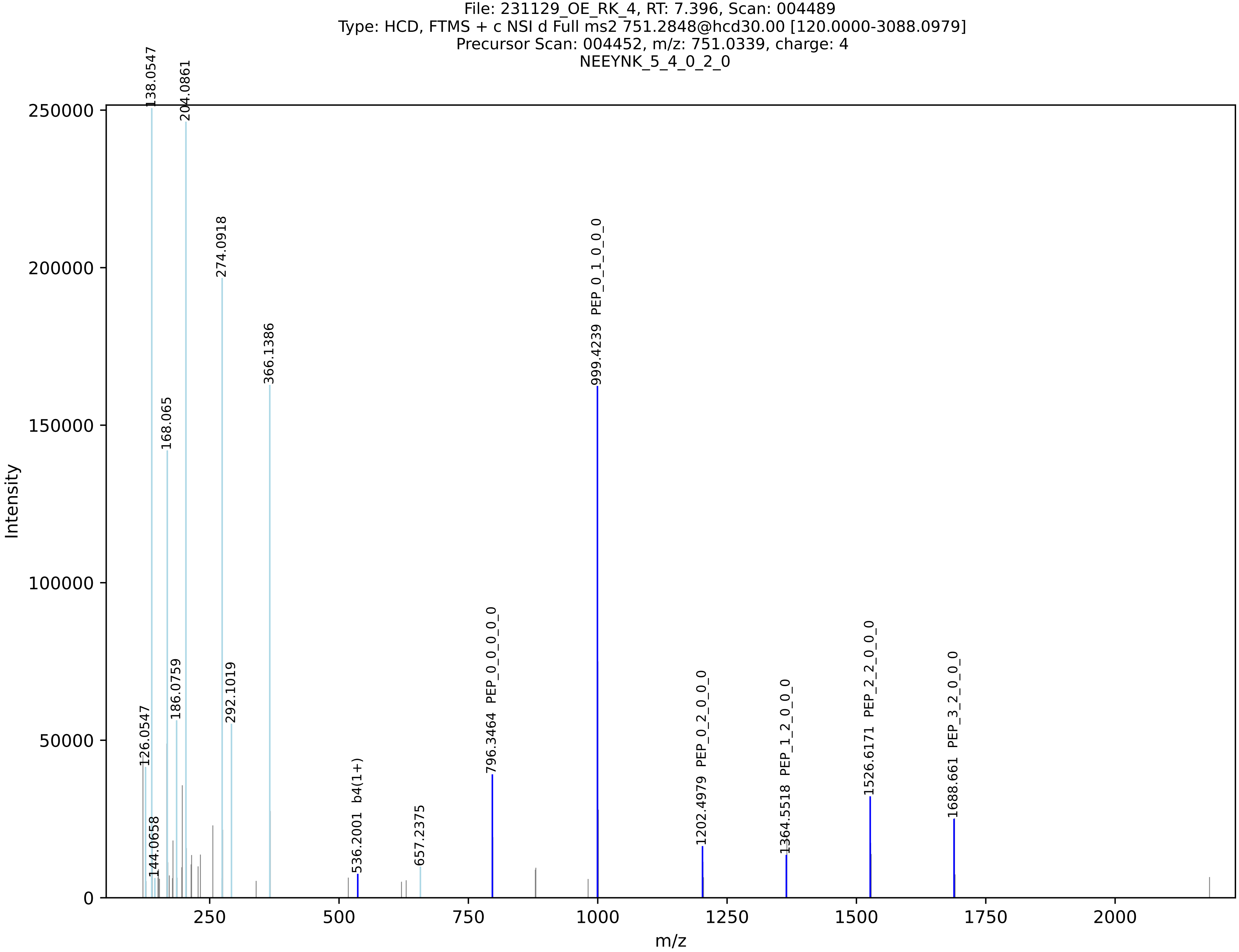

File: 231129_OE_RK_4, RT: 7.396, Scan: 004489
Type: HCD, FTMS + c NSI d Full ms2 751.2848@hcd30.00 [120.0000-3088.0979]
Precursor Scan: 004452, m/z: 751.0339, charge: 4
NEEYNK_5_4_0_2_0
Intensity
m/z
0
50000
100000
150000
200000
250000
250
500
750
1000
1250
1500
1750
2000
138.0547
204.0861
274.0918
366.1386
168.065
186.0759
292.1019
126.0547
144.0658
536.2001 b4(1+)
657.2375
796.3464 PEP_0_0_0_0_0
999.4239 PEP_0_1_0_0_0
1202.4979 PEP_0_2_0_0_0
1364.5518 PEP_1_2_0_0_0
1526.6171 PEP_2_2_0_0_0
1688.661 PEP_3_2_0_0_0

File: 231129_OE_RK_4, RT: 7.396, Scan: 004490
Type: HCD, FTMS + c NSI d Full ms2 1123.0867@hcd30.00 [120.0000-3458.7871]
Precursor Scan: 004452, m/z: 1122.7525, charge: 3
NEEYNK_6_5_0_2_0

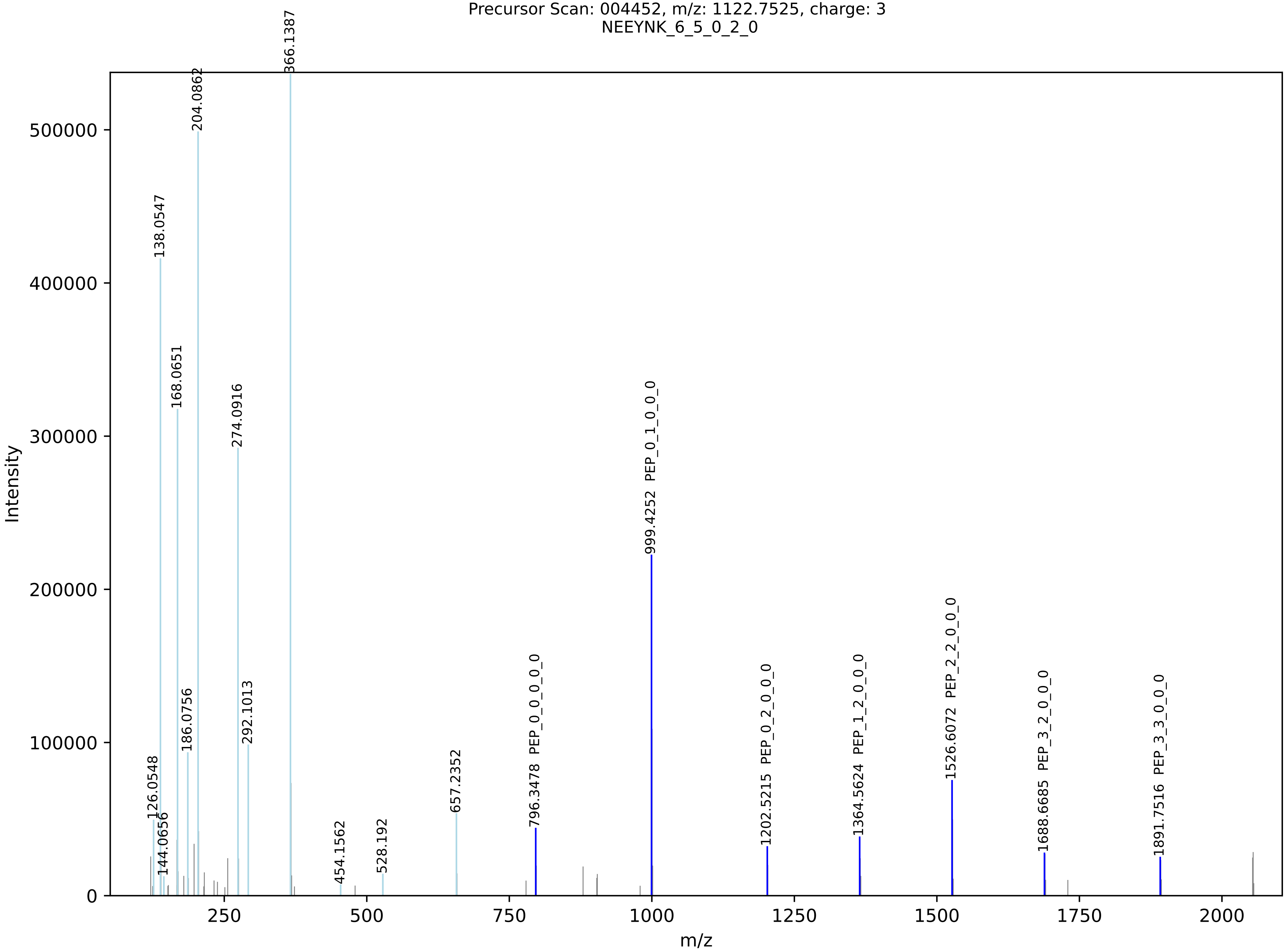

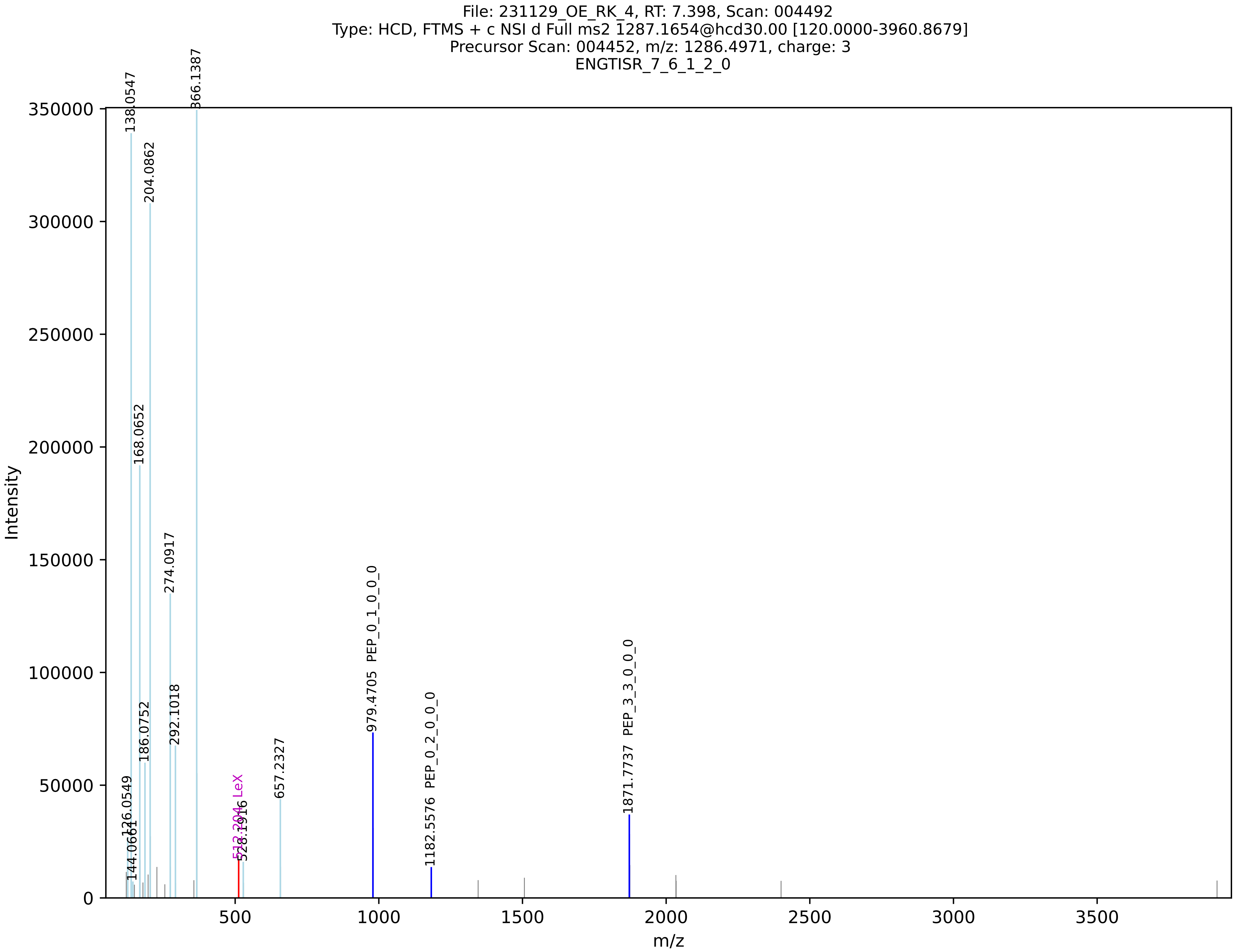
File: 231129_OE_RK_4, RT: 7.398, Scan: 004492
Type: HCD, FTMS + c NSI d Full ms2 1287.1654@hcd30.00 [120.0000-3960.8679]
Precursor Scan: 004452, m/z: 1286.4971, charge: 3
ENGTISR_7_6_1_2_0
Intensity
m/z
138.0547
366.1387
204.0862
168.0652
274.0917
186.0752
292.1018
126.0549
144.0661
512.204 LeX
528.1916
657.2327
979.4705 PEP_0_1_0_0_0
1182.5576 PEP_0_2_0_0_0
1871.7737 PEP_3_3_0_0_0
0
50000
100000
150000
200000
250000
300000
350000
500
1000
1500
2000
2500
3000
3500

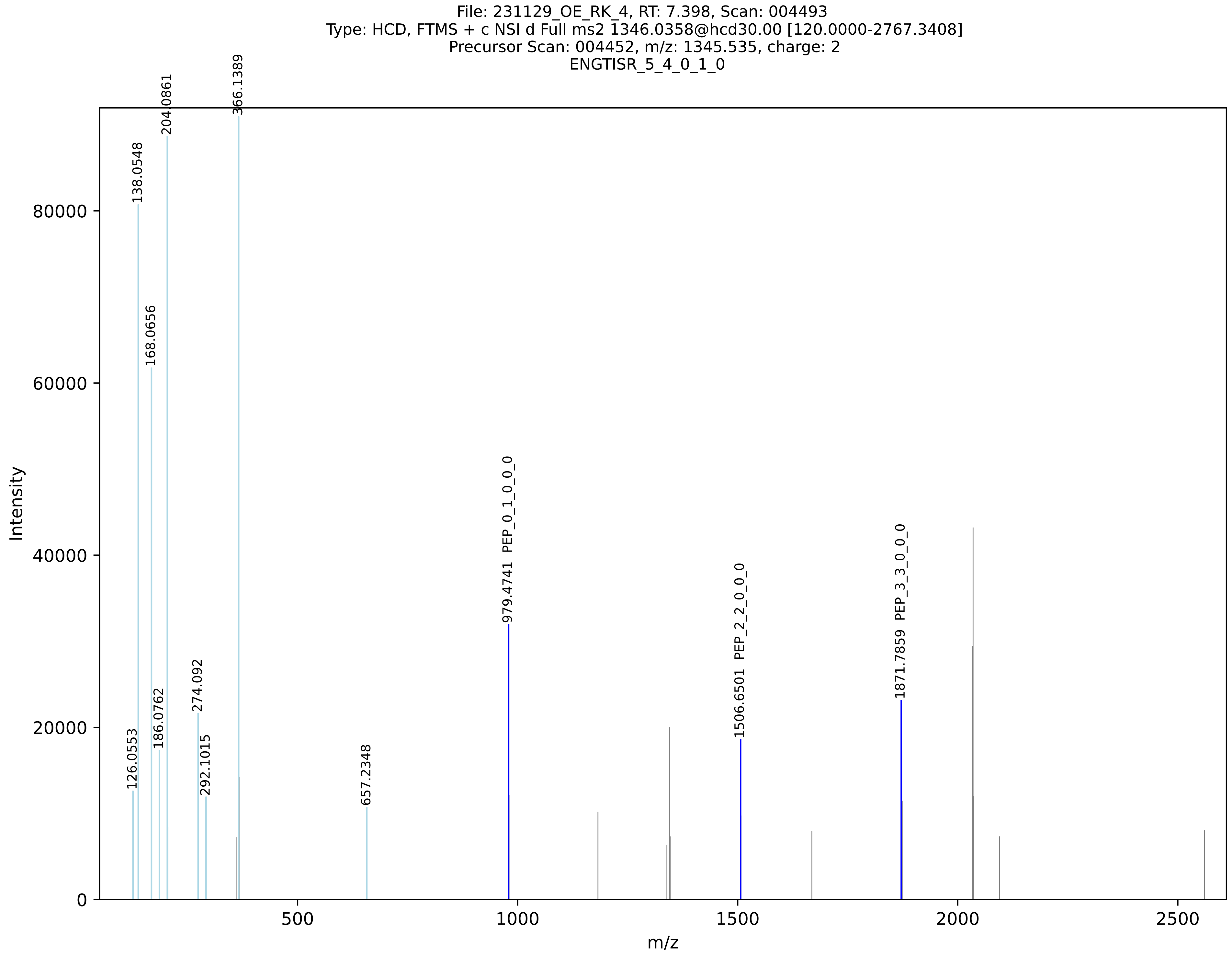
File: 231129_OE_RK_4, RT: 7.398, Scan: 004493
Type: HCD, FTMS + c NSI d Full ms2 1346.0358@hcd30.00 [120.0000-2767.3408]
Precursor Scan: 004452, m/z: 1345.535, charge: 2
ENGTISR_5_4_0_1_0
Intensity
m/z
126.0553
138.0548
168.0656
186.0762
204.0861
274.092
292.1015
366.1389
657.2348
979.4741 PEP_0_1_0_0_0
1506.6501 PEP_2_2_0_0_0
1871.7859 PEP_3_3_0_0_0
0
20000
40000
60000
80000
500
1000
1500
2000
2500

File: 231129_OE_RK_4, RT: 7.417, Scan: 004523
Type: HCD, FTMS + c NSI d Full ms2 1051.3623@hcd30.00 [120.0000-3239.3108]
Precursor Scan: 004452, m/z: 1050.6961, charge: 3
NEEYNK_5_4_1_2_0

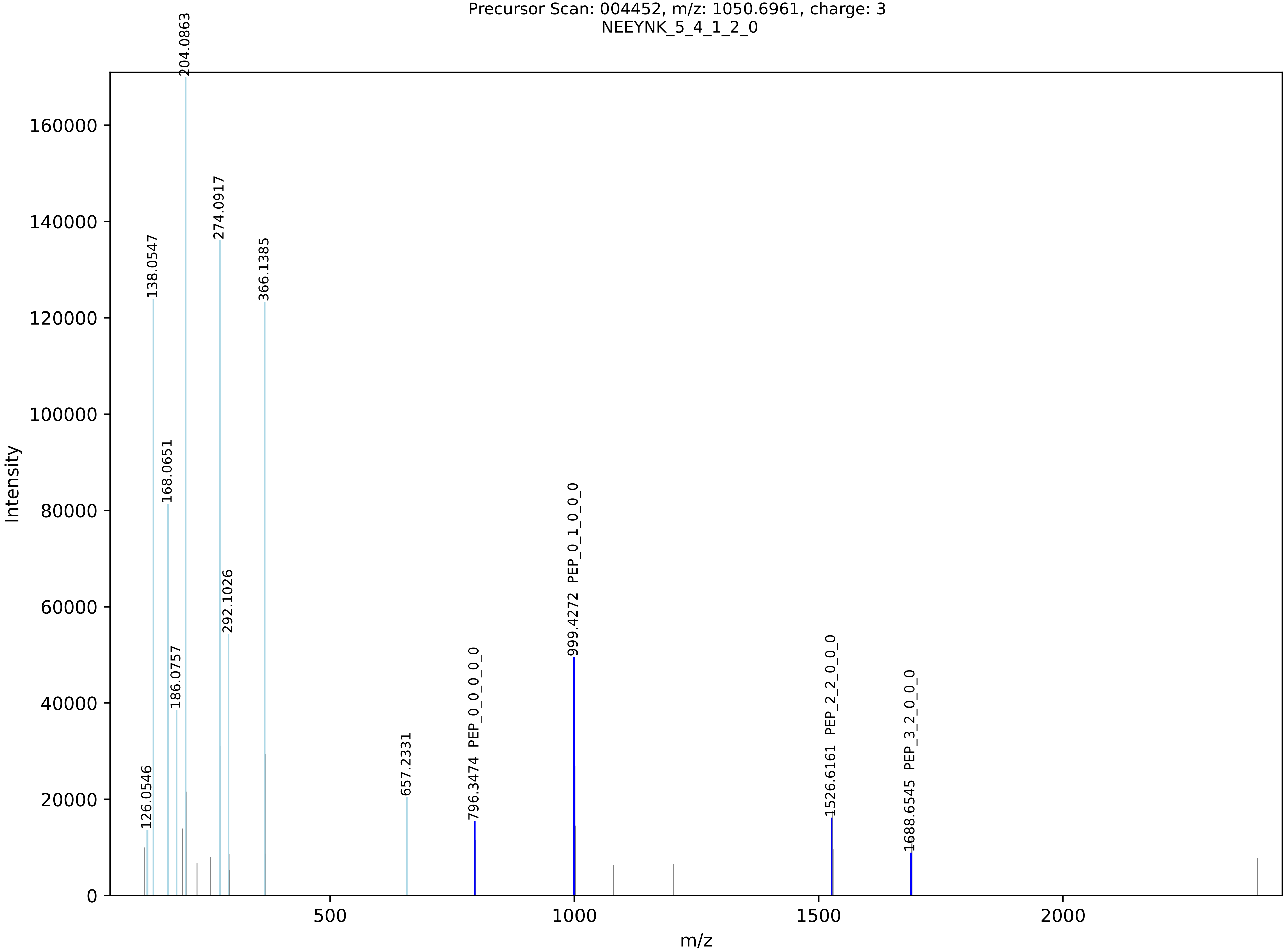

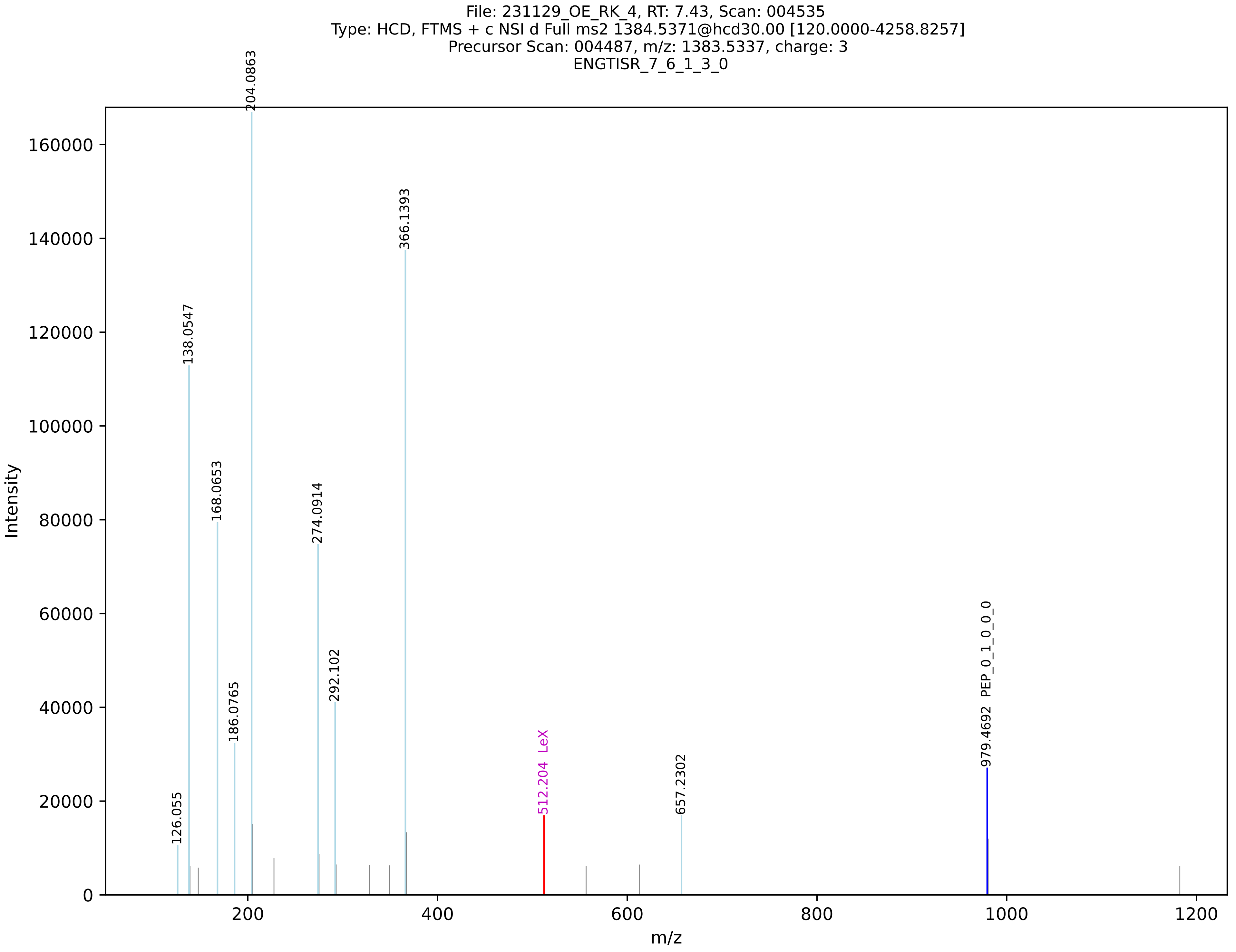

File: 231129_OE_RK_4, RT: 7.43, Scan: 004535
Type: HCD, FTMS + c NSI d Full ms2 1384.5371@hcd30.00 [120.0000-4258.8257]
Precursor Scan: 004487, m/z: 1383.5337, charge: 3
ENGTISR_7_6_1_3_0
Intensity
m/z
126.055
138.0547
168.0653
186.0765
204.0863
274.0914
292.102
366.1393
512.204 LeX
657.2302
979.4692 PEP_0_1_0_0_0

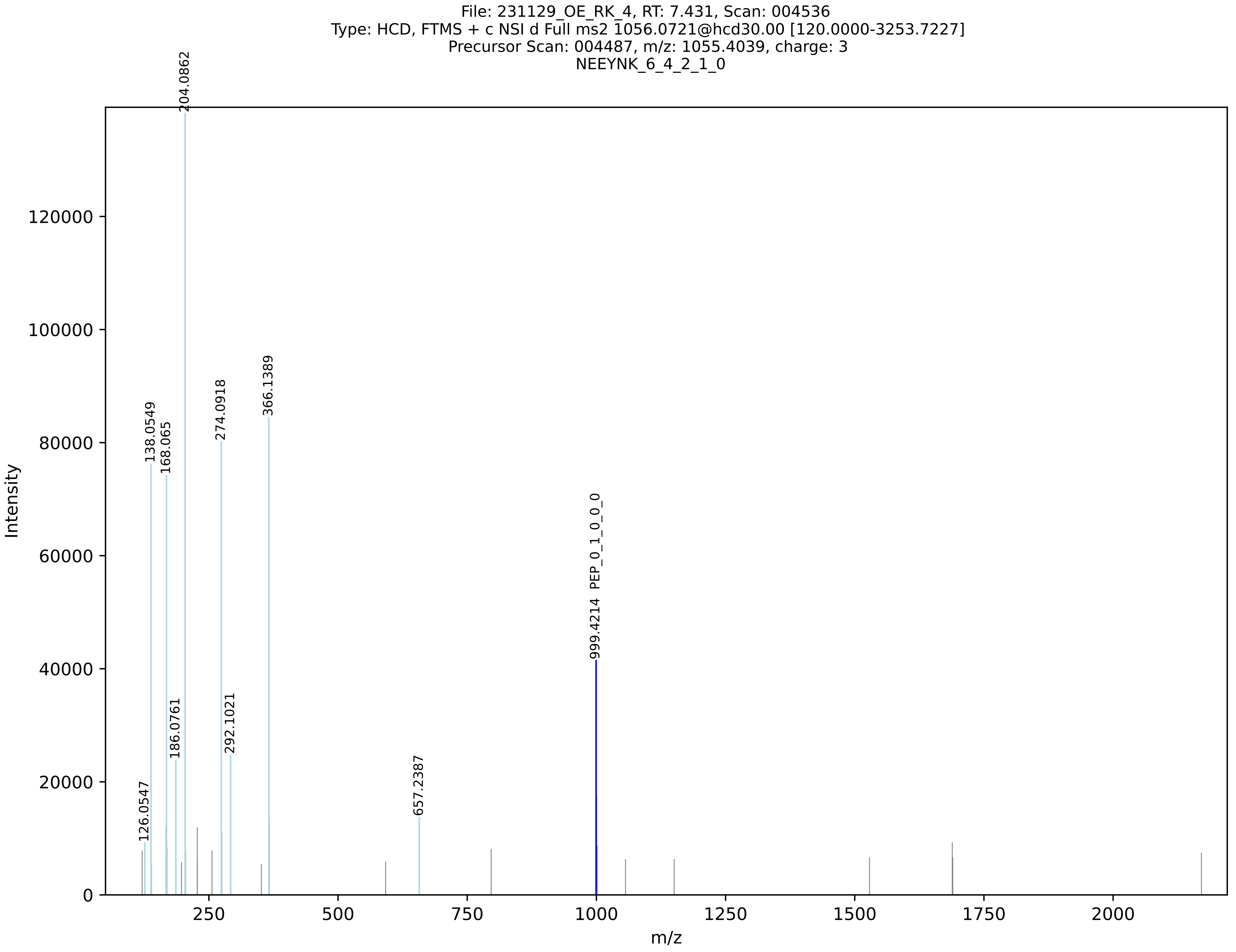
File: 231129_OE_RK_4, RT: 7.431, Scan: 004536
Type: HCD, FTMS + c NSI d Full ms2 1056.0721@hcd30.00 [120.0000-3253.7227]
Precursor Scan: 004487, m/z: 1055.4039, charge: 3
NEEYNK_6_4_2_1_0
204.0862
366.1389
274.0918
138.0549
168.065
999.4214 PEP_0_1_0_0_0
186.0761
292.1021
657.2387
126.0547
Intensity
m/z
0
20000
40000
60000
80000
100000
120000
250
500
750
1000
1250
1500
1750
2000

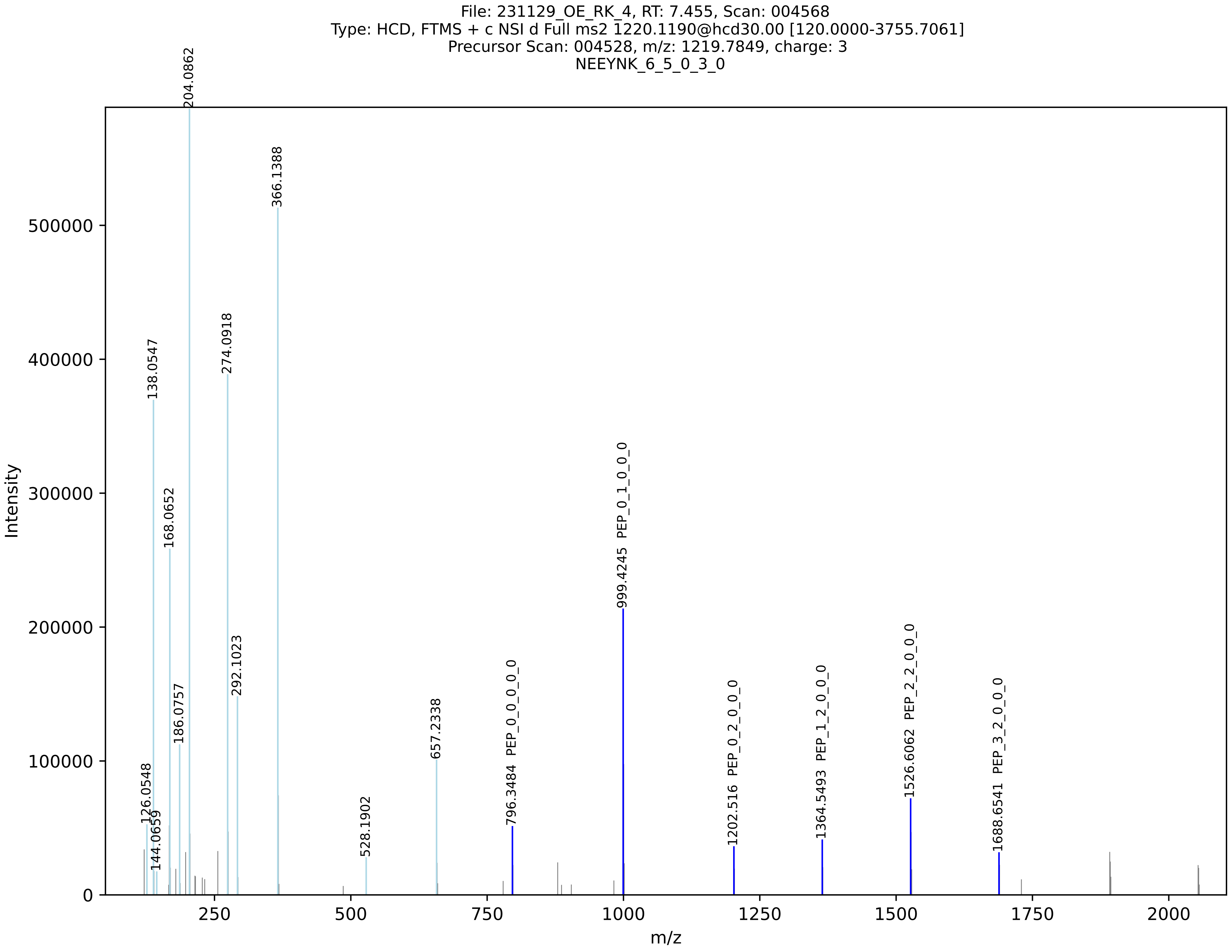
File: 231129_OE_RK_4, RT: 7.455, Scan: 004568
Type: HCD, FTMS + c NSI d Full ms2 1220.1190@hcd30.00 [120.0000-3755.7061]
Precursor Scan: 004528, m/z: 1219.7849, charge: 3
NEEYNK_6_5_0_3_0
Intensity
m/z
204.0862
366.1388
274.0918
138.0547
168.0652
186.0757
292.1023
126.0548
144.0659
528.1902
657.2338
796.3484 PEP_0_0_0_0_0
999.4245 PEP_0_1_0_0_0
1202.516 PEP_0_2_0_0_0
1364.5493 PEP_1_2_0_0_0
1526.6062 PEP_2_2_0_0_0
1688.6541 PEP_3_2_0_0_0

File: 231129_OE_RK_4, RT: 7.457, Scan: 004571
Type: HCD, FTMS + c NSI d Full ms2 1177.7825@hcd30.00 [120.0000-3626.1563]
Precursor Scan: 004528, m/z: 1177.1142, charge: 3
NEEYNK_7_5_2_1_0

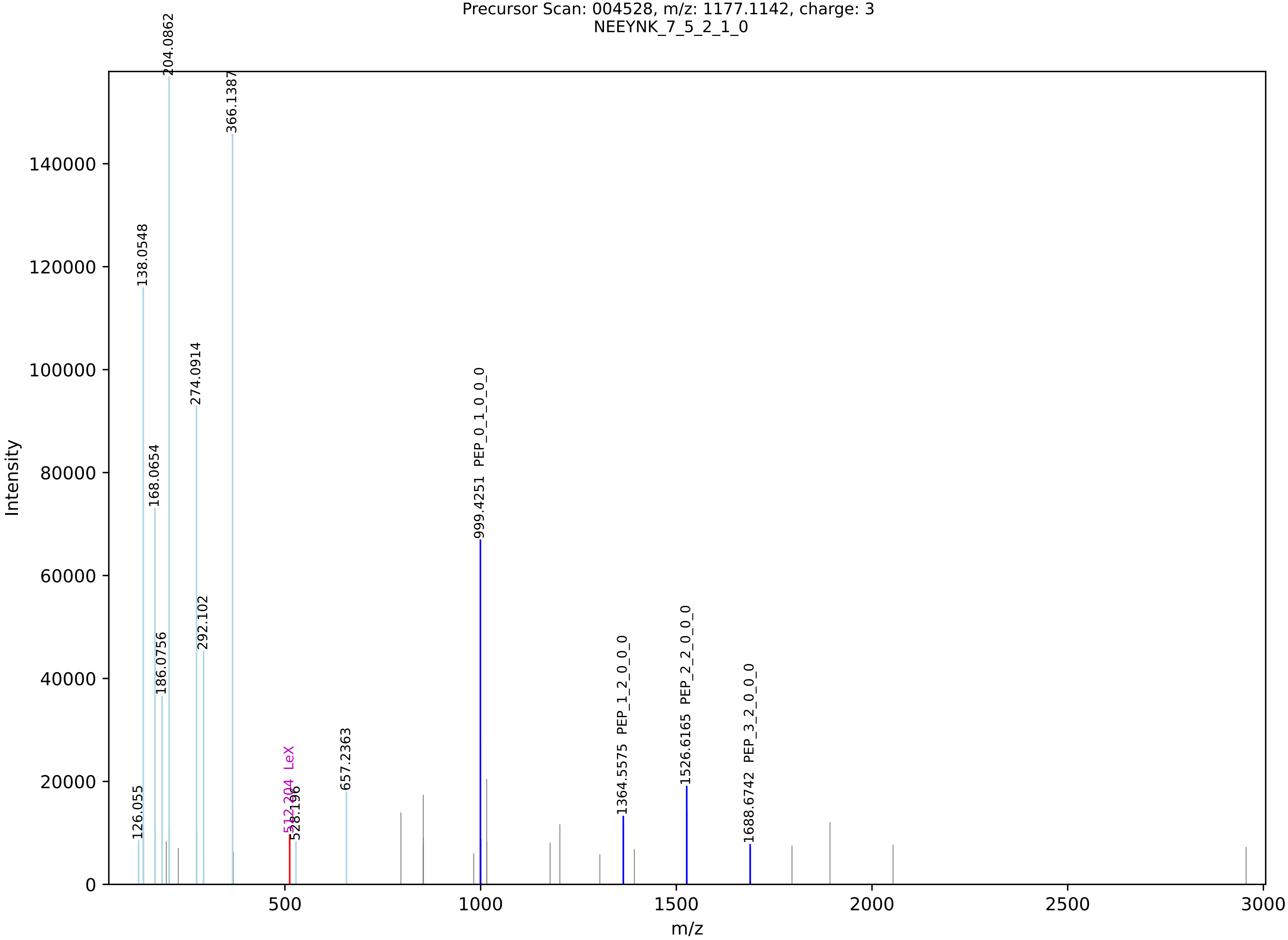

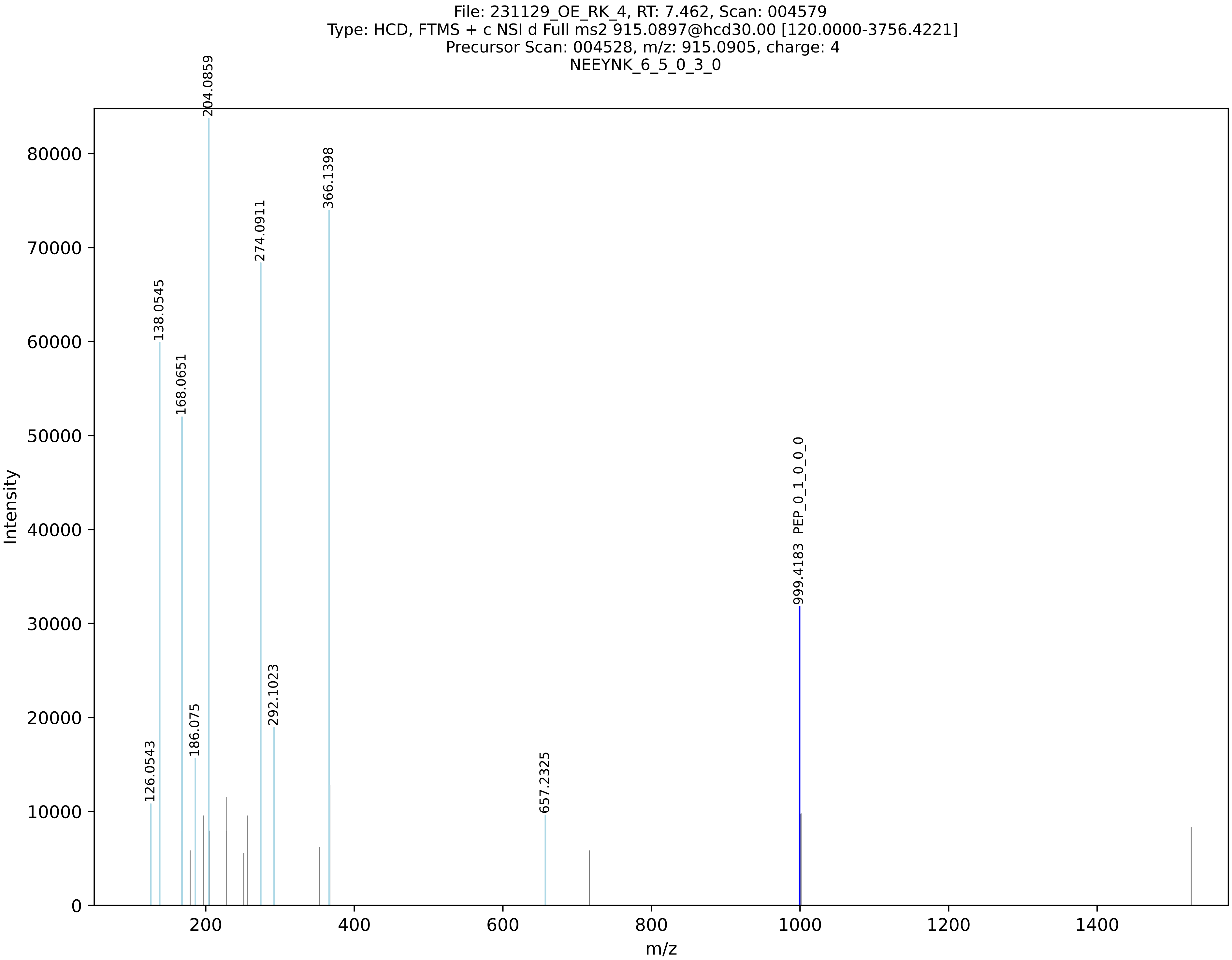

File: 231129_OE_RK_4, RT: 7.462, Scan: 004579
Type: HCD, FTMS + c NSI d Full ms2 915.0897@hcd30.00 [120.0000-3756.4221]
Precursor Scan: 004528, m/z: 915.0905, charge: 4
NEEYNK_6_5_0_3_0
Intensity
m/z
204.0859
366.1398
274.0911
138.0545
168.0651
292.1023
186.075
126.0543
657.2325
999.4183 PEP_0_1_0_0_0
0
10000
20000
30000
40000
50000
60000
70000
80000
200
400
600
800
1000
1200
1400

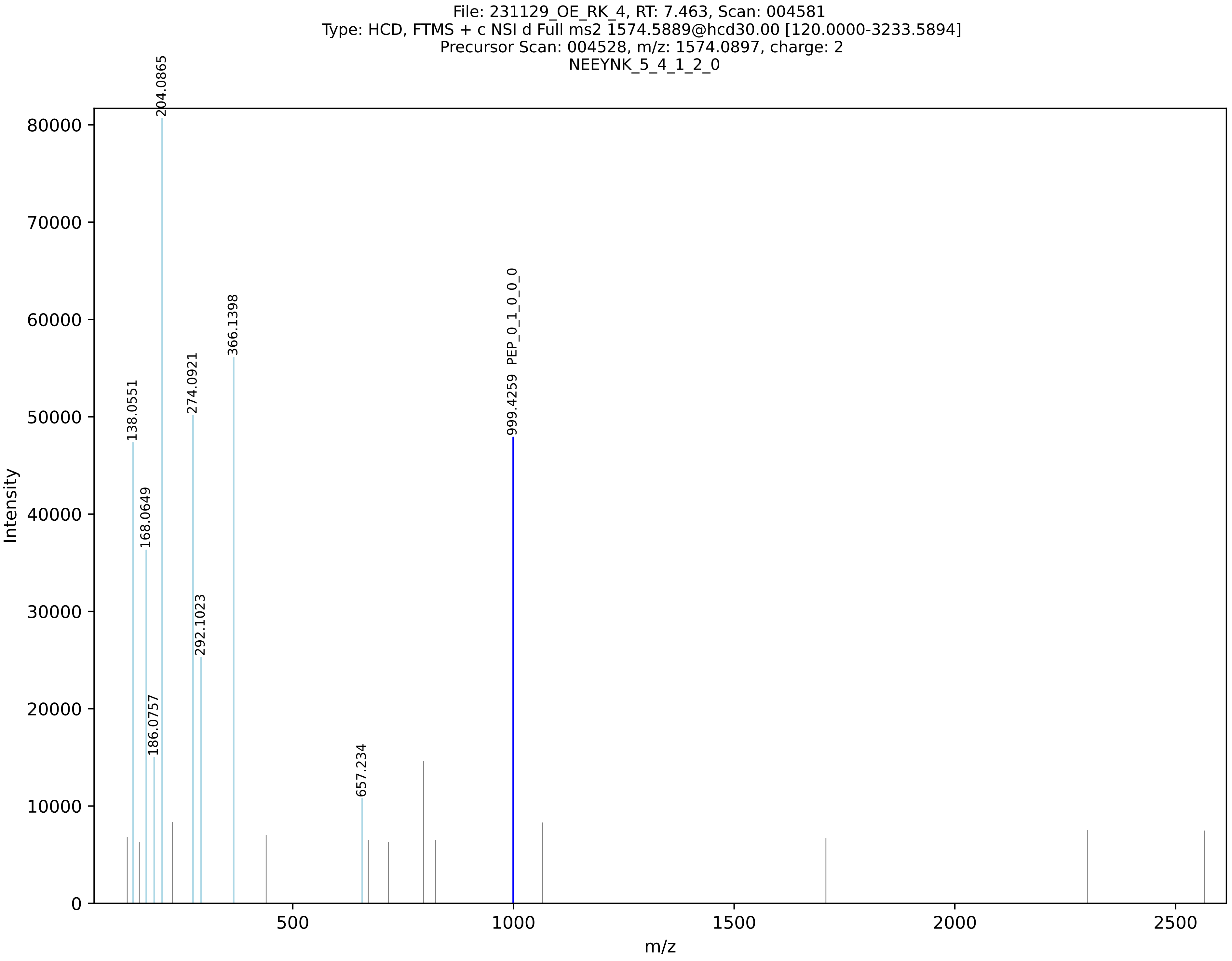
File: 231129_OE_RK_4, RT: 7.463, Scan: 004581
Type: HCD, FTMS + c NSI d Full ms2 1574.5889@hcd30.00 [120.0000-3233.5894]
Precursor Scan: 004528, m/z: 1574.0897, charge: 2
NEEYNK_5_4_1_2_0
204.0865
138.0551
168.0649
186.0757
274.0921
292.1023
366.1398
657.234
999.4259 PEP_0_1_0_0_0
Intensity
m/z
0
10000
20000
30000
40000
50000
60000
70000
80000
500
1000
1500
2000
2500

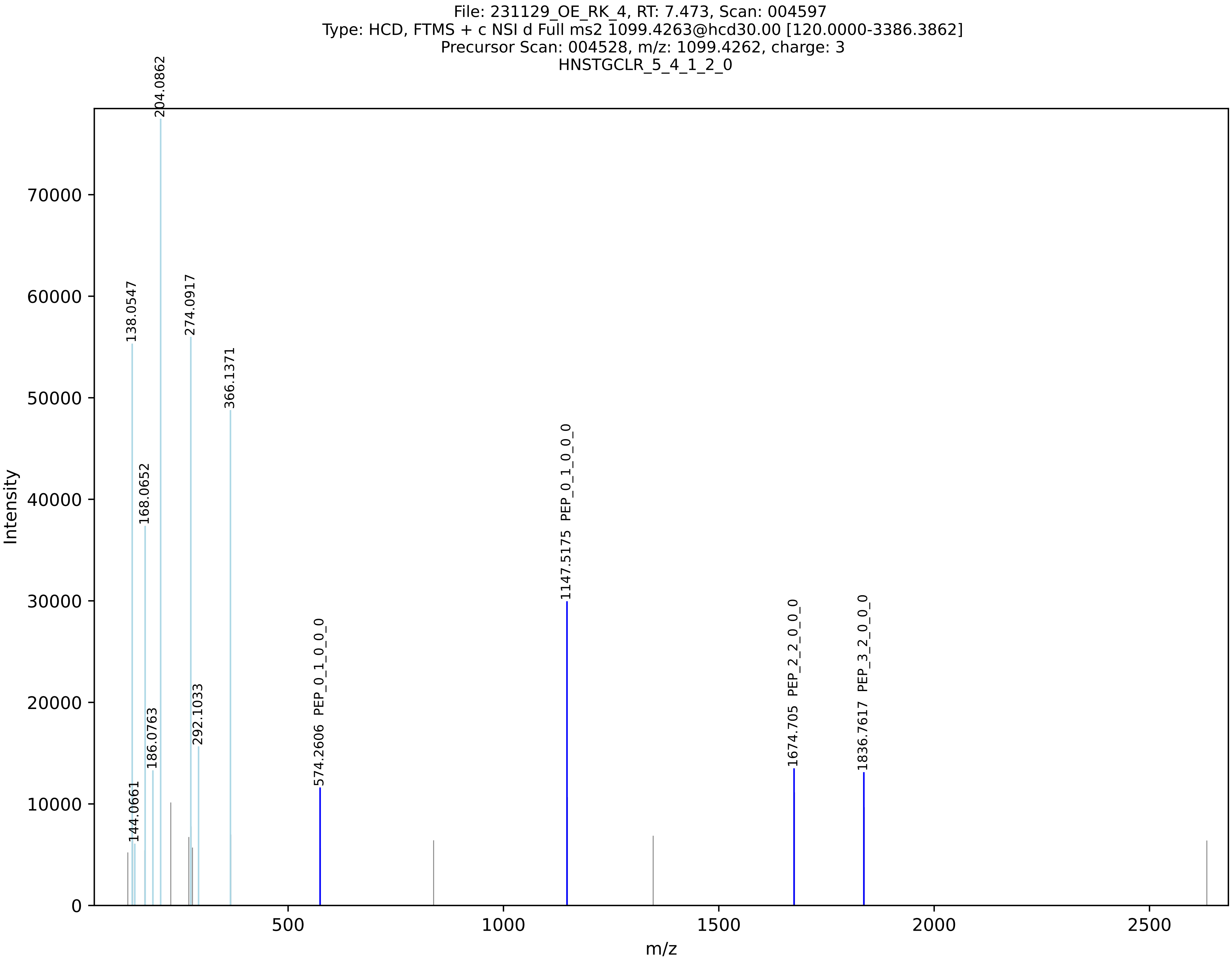

File: 231129_OE_RK_4, RT: 7.473, Scan: 004597
Type: HCD, FTMS + c NSI d Full ms2 1099.4263@hcd30.00 [120.0000-3386.3862]
Precursor Scan: 004528, m/z: 1099.4262, charge: 3
HNSTGCLR_5_4_1_2_0
Intensity
m/z
0
10000
20000
30000
40000
50000
60000
70000
500
1000
1500
2000
2500
204.0862
138.0547
274.0917
366.1371
168.0652
186.0763
292.1033
144.0661
574.2606 PEP_0_1_0_0_0_
1147.5175 PEP_0_1_0_0_0_
1674.705 PEP_2_2_0_0_0
1836.7617 PEP_3_2_0_0_0

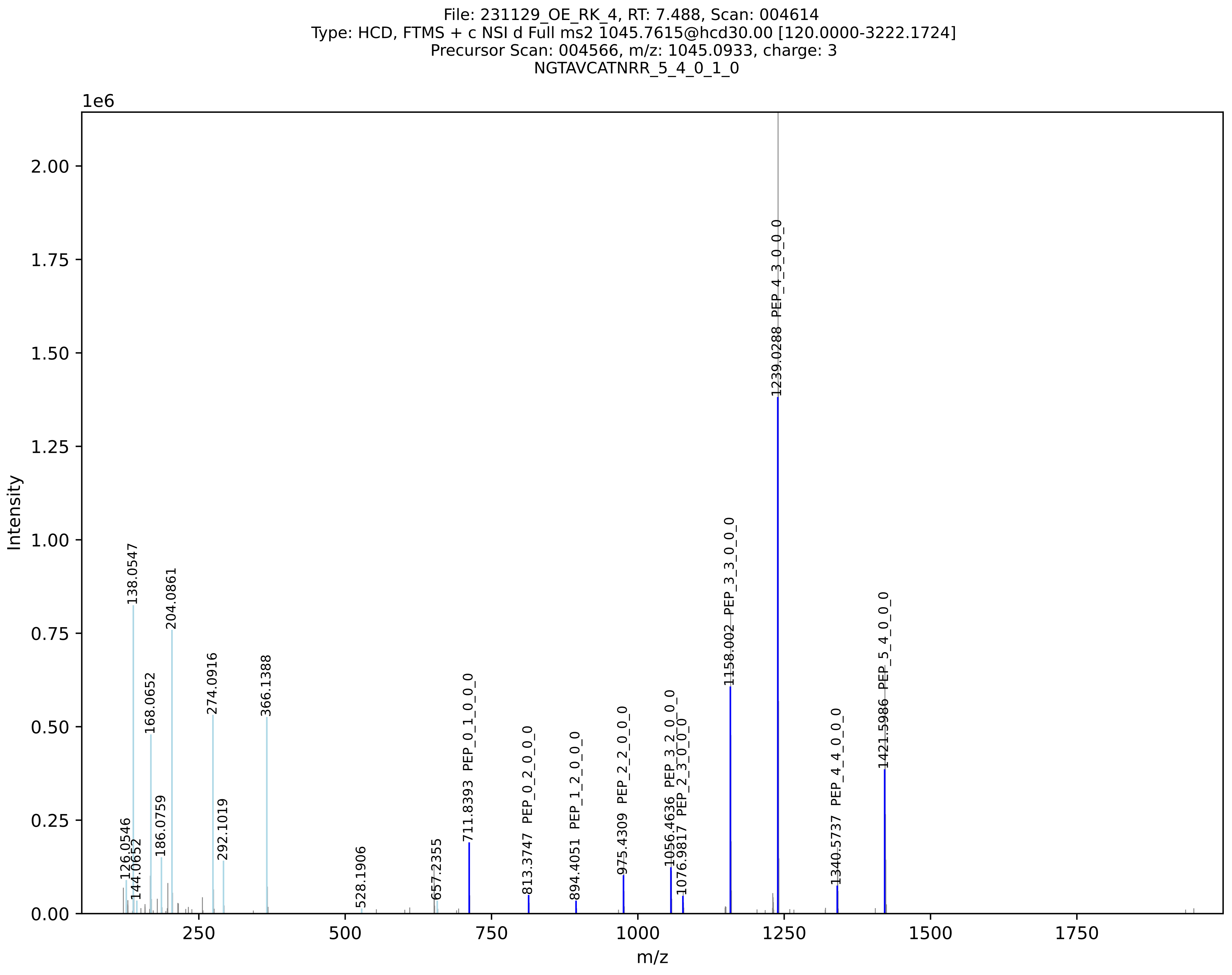
File: 231129_OE_RK_4, RT: 7.488, Scan: 004614
Type: HCD, FTMS + c NSI d Full ms2 1045.7615@hcd30.00 [120.0000-3222.1724]
Precursor Scan: 004566, m/z: 1045.0933, charge: 3
NGTAVCATNRR_5_4_0_1_0
1e6
Intensity
m/z
138.0547
204.0861
168.0652
274.0916
366.1388
186.0759
292.1019
126.0546
144.0652
528.1906
657.2355
711.8393 PEP_0_1_0_0_0
813.3747 PEP_0_2_0_0_0
894.4051 PEP_1_2_0_0_0
975.4309 PEP_2_2_0_0_0
1056.4636 PEP_3_2_0_0_0
1076.9817 PEP_2_3_0_0_0
1158.002 PEP_3_3_0_0_0
1239.0288 PEP_4_3_0_0_0
1340.5737 PEP_4_4_0_0_0
1421.5986 PEP_5_4_0_0_0

File: 231129_OE_RK_4, RT: 7.49, Scan: 004617
Type: HCD, FTMS + c NSI d Full ms2 952.1061@hcd30.00 [120.0000-3907.4487]
Precursor Scan: 004566, m/z: 951.6046, charge: 4
NEEYNK_6_5_1_3_0

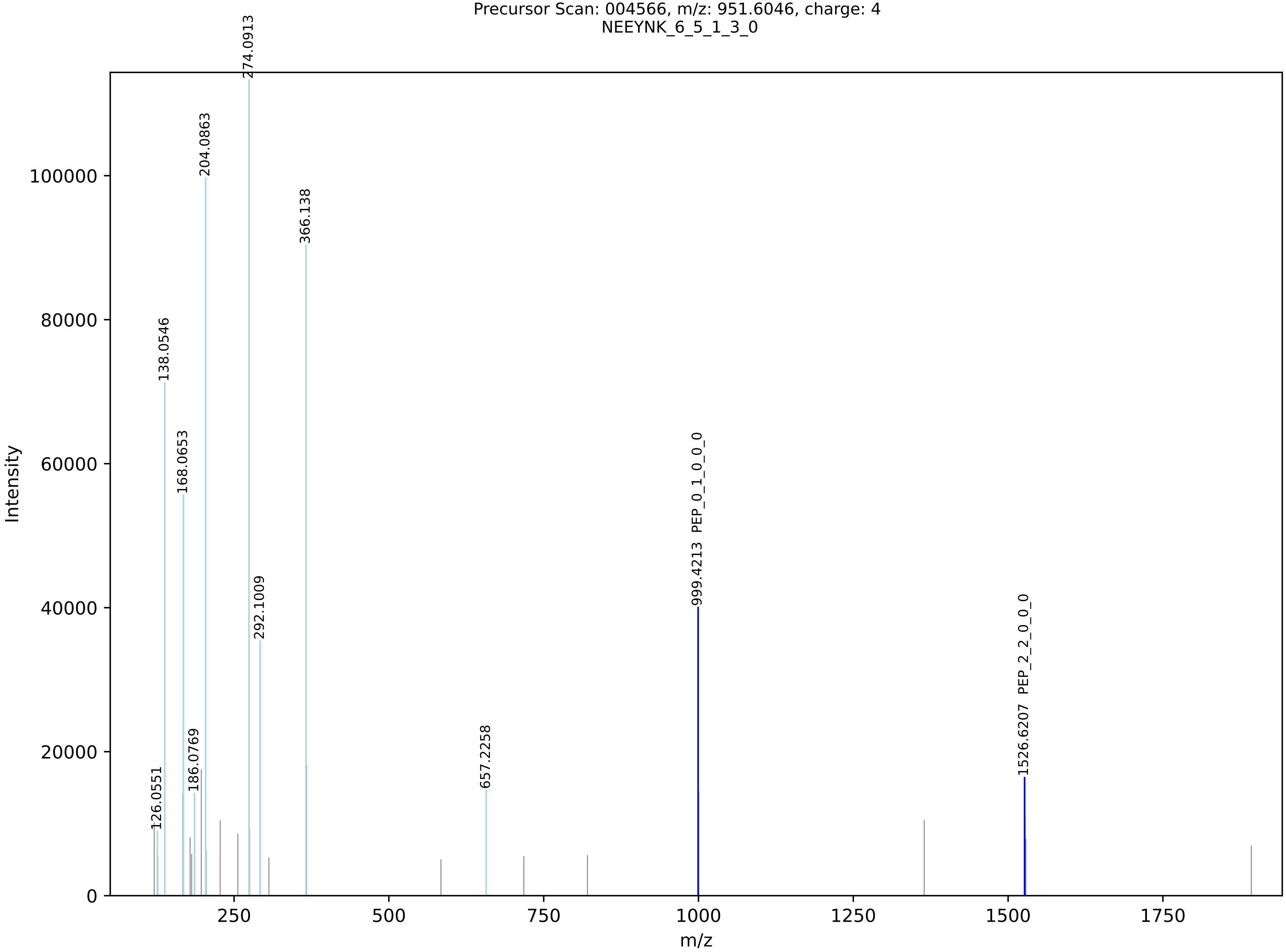

File: 231129_OE_RK_4, RT: 7.493, Scan: 004622
Type: HCD, FTMS + c NSI d Full ms2 1099.7596@hcd30.00 [120.0000-3387.4065]
Precursor Scan: 004566, m/z: 1099.0914, charge: 3
HNSTGCLR_5_4_1_2_0

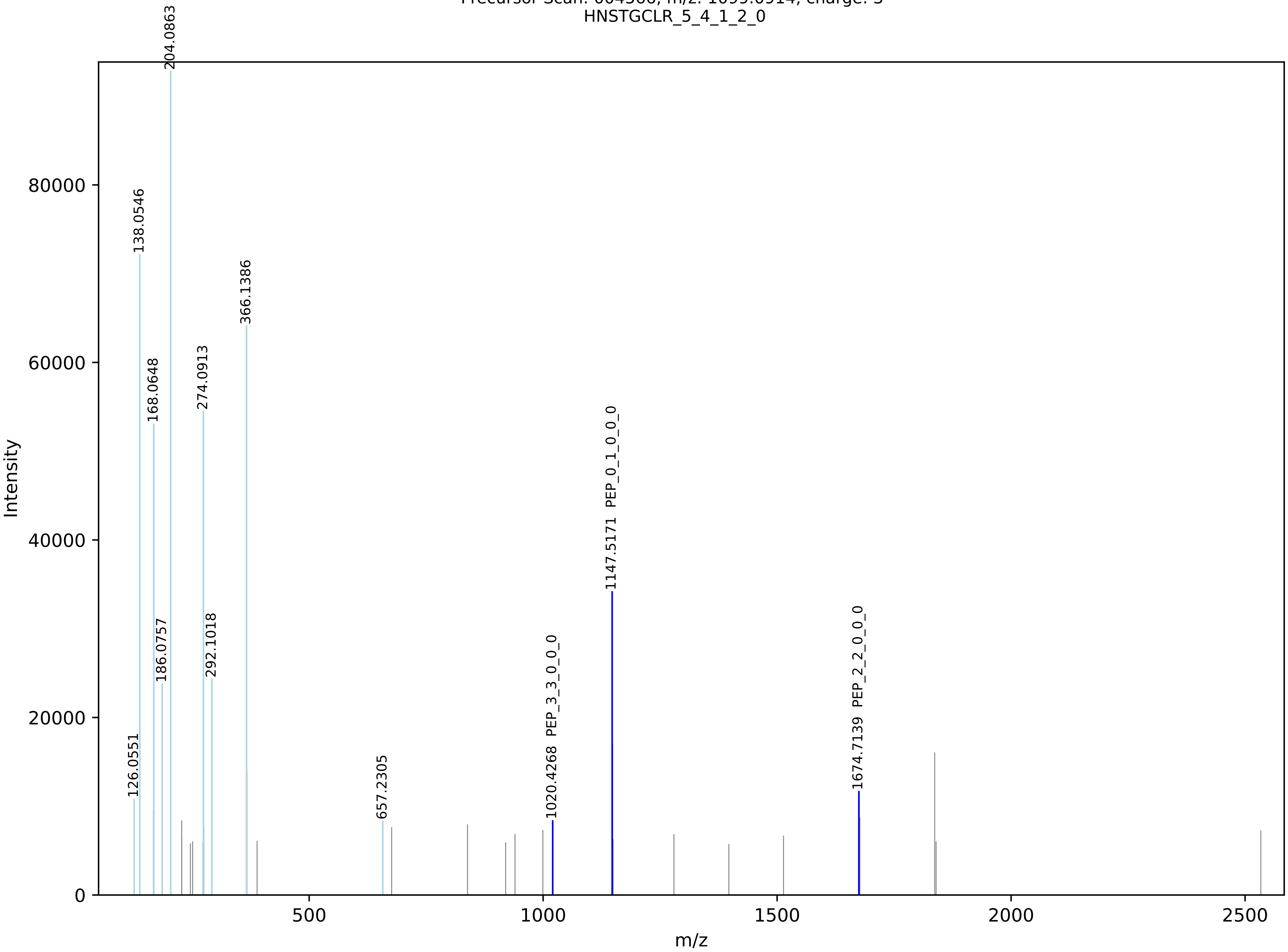

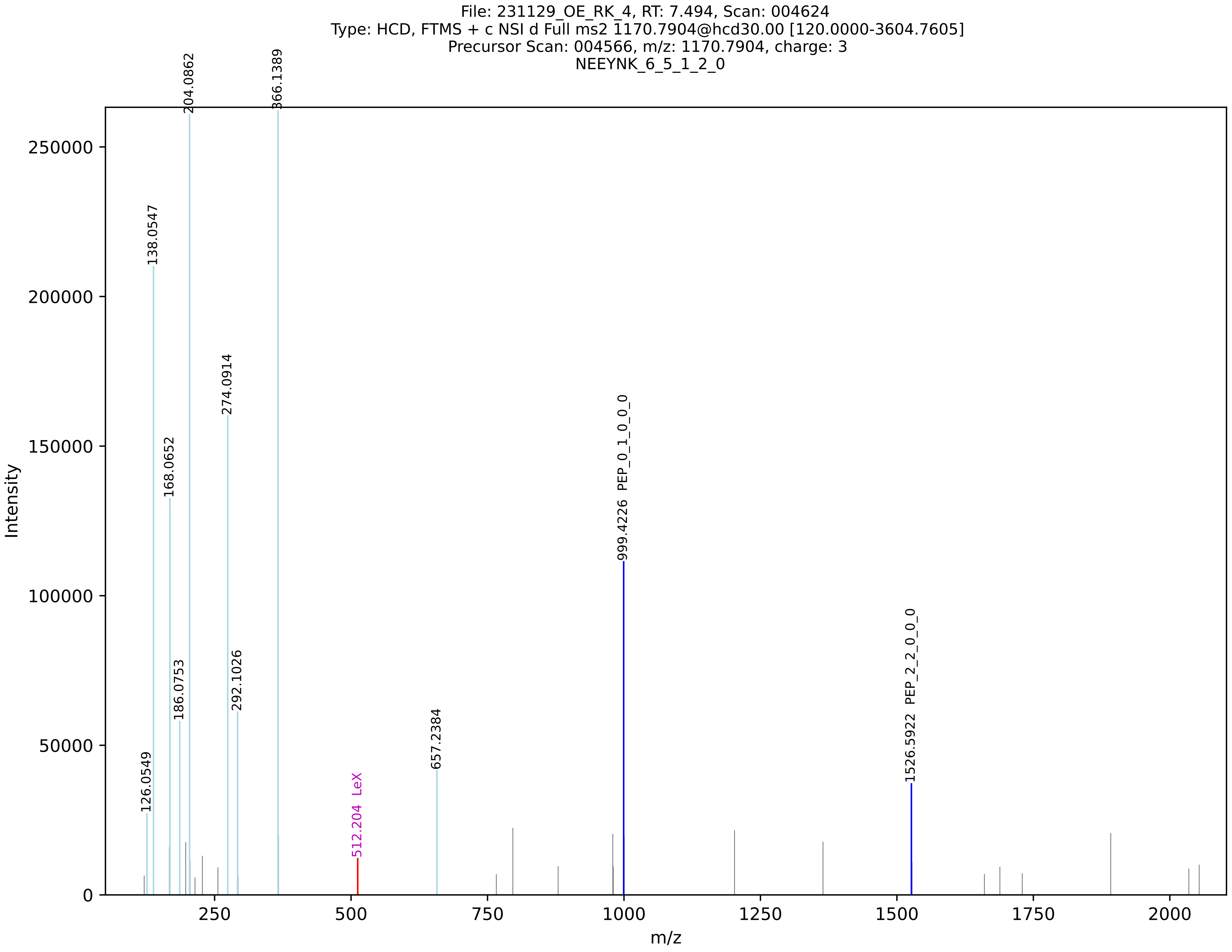
File: 231129_OE_RK_4, RT: 7.494, Scan: 004624
Type: HCD, FTMS + c NSI d Full ms2 1170.7904@hcd30.00 [120.0000-3604.7605]
Precursor Scan: 004566, m/z: 1170.7904, charge: 3
NEEYNK_6_5_1_2_0
204.0862
366.1389
138.0547
274.0914
168.0652
186.0753
292.1026
126.0549
512.204 LeX
657.2384
999.4226 PEP_0_1_0_0_0
1526.5922 PEP_2_2_0_0_0
Intensity
m/z
0
50000
100000
150000
200000
250000
250
500
750
1000
1250
1500
1750
2000

File: 231129_OE_RK_4, RT: 7.498, Scan: 004630
Type: HCD, FTMS + c NSI d Full ms2 1219.1473@hcd30.00 [120.0000-3752.7327]
Precursor Scan: 004566, m/z: 1219.1473, charge: 3
NEEYNK_6_5_0_3_0

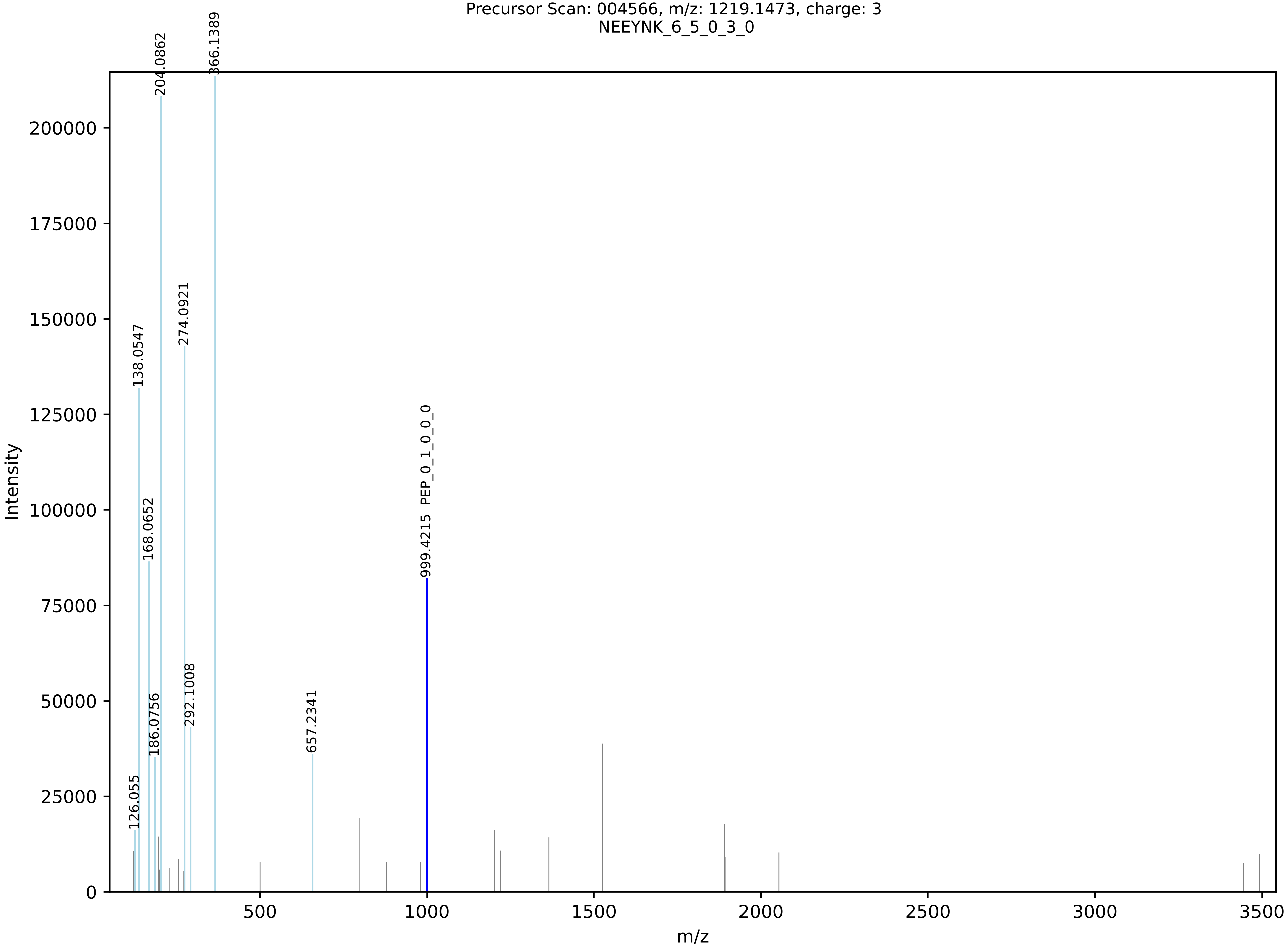

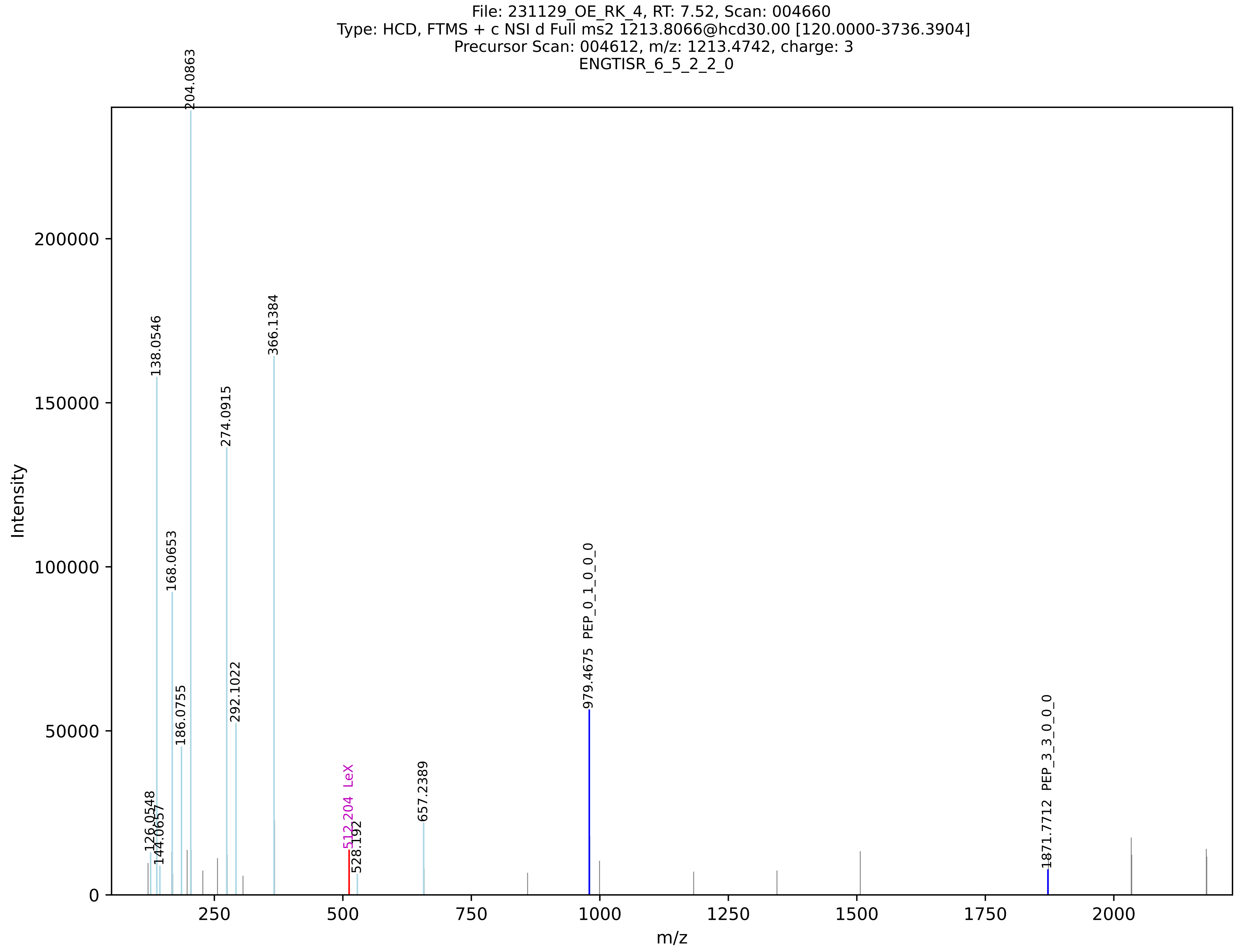
File: 231129_OE_RK_4, RT: 7.52, Scan: 004660
Type: HCD, FTMS + c NSI d Full ms2 1213.8066@hcd30.00 [120.0000-3736.3904]
Precursor Scan: 004612, m/z: 1213.4742, charge: 3
ENGTISR_6_5_2_2_0
Intensity
m/z
0
50000
100000
150000
200000
250
500
750
1000
1250
1500
1750
2000
204.0863
138.0546
366.1384
274.0915
168.0653
186.0755
292.1022
126.0548
144.0657
512.204 LeX
528.192
657.2389
979.4675 PEP_0_1_0_0_0
1871.7712 PEP_3_3_0_0_0

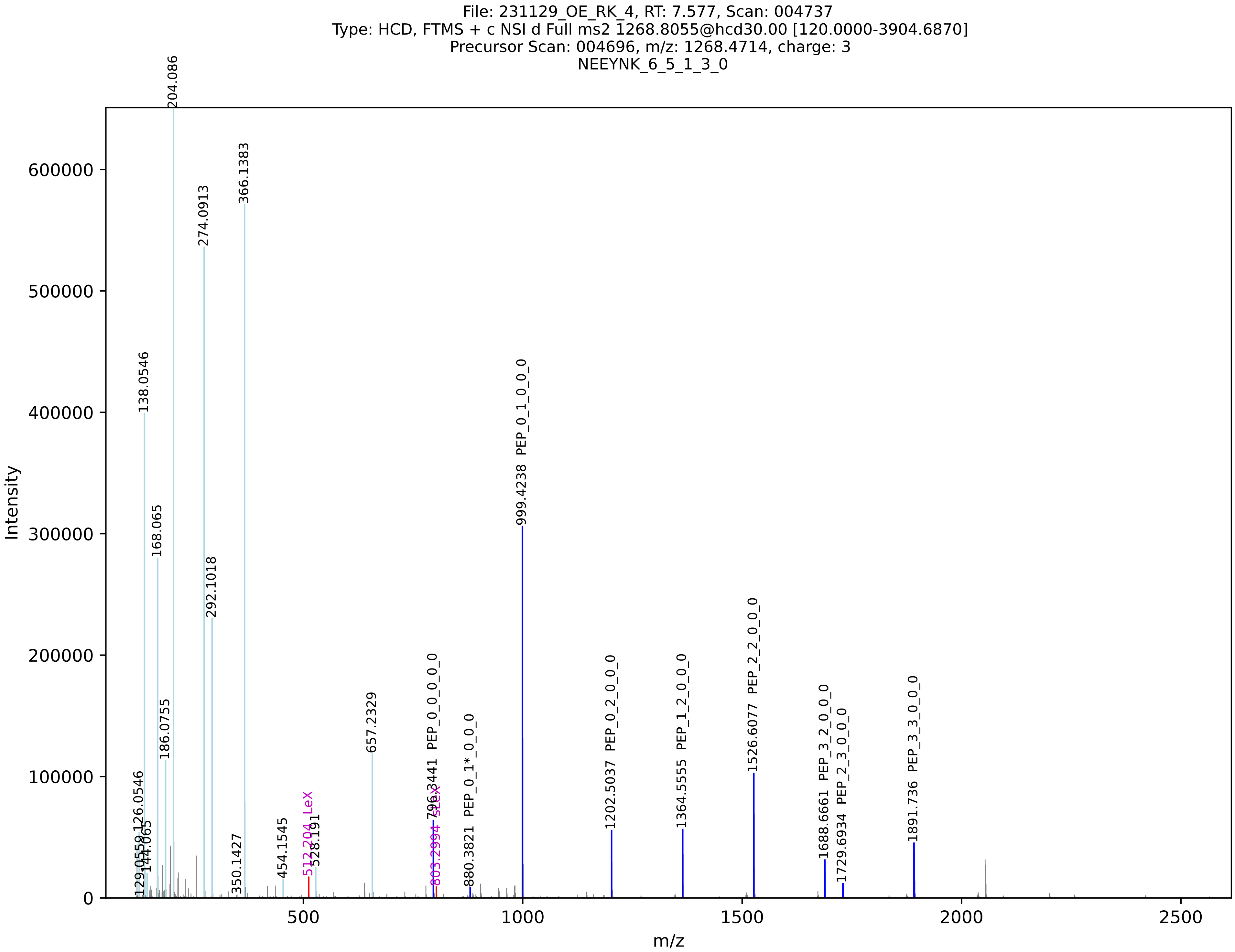
File: 231129_OE_RK_4, RT: 7.577, Scan: 004737
Type: HCD, FTMS + c NSI d Full ms2 1268.8055@hcd30.00 [120.0000-3904.6870]
Precursor Scan: 004696, m/z: 1268.4714, charge: 3
NEEYNK_6_5_1_3_0
Intensity
m/z
204.086
366.1383
274.0913
138.0546
168.065
292.1018
186.0755
126.0546
129.0559
144.065
350.1427
454.1545
512.204 LeX
528.191
657.2329
796.3441 PEP_0_0_0_0_0
803.2994 SLeX
880.3821 PEP_0_1*_0_0_0
999.4238 PEP_0_1_0_0_0
1202.5037 PEP_0_2_0_0_0
1364.5555 PEP_1_2_0_0_0
1526.6077 PEP_2_2_0_0_0
1688.6661 PEP_3_2_0_0_0
1729.6934 PEP_2_3_0_0_0
1891.736 PEP_3_3_0_0_0

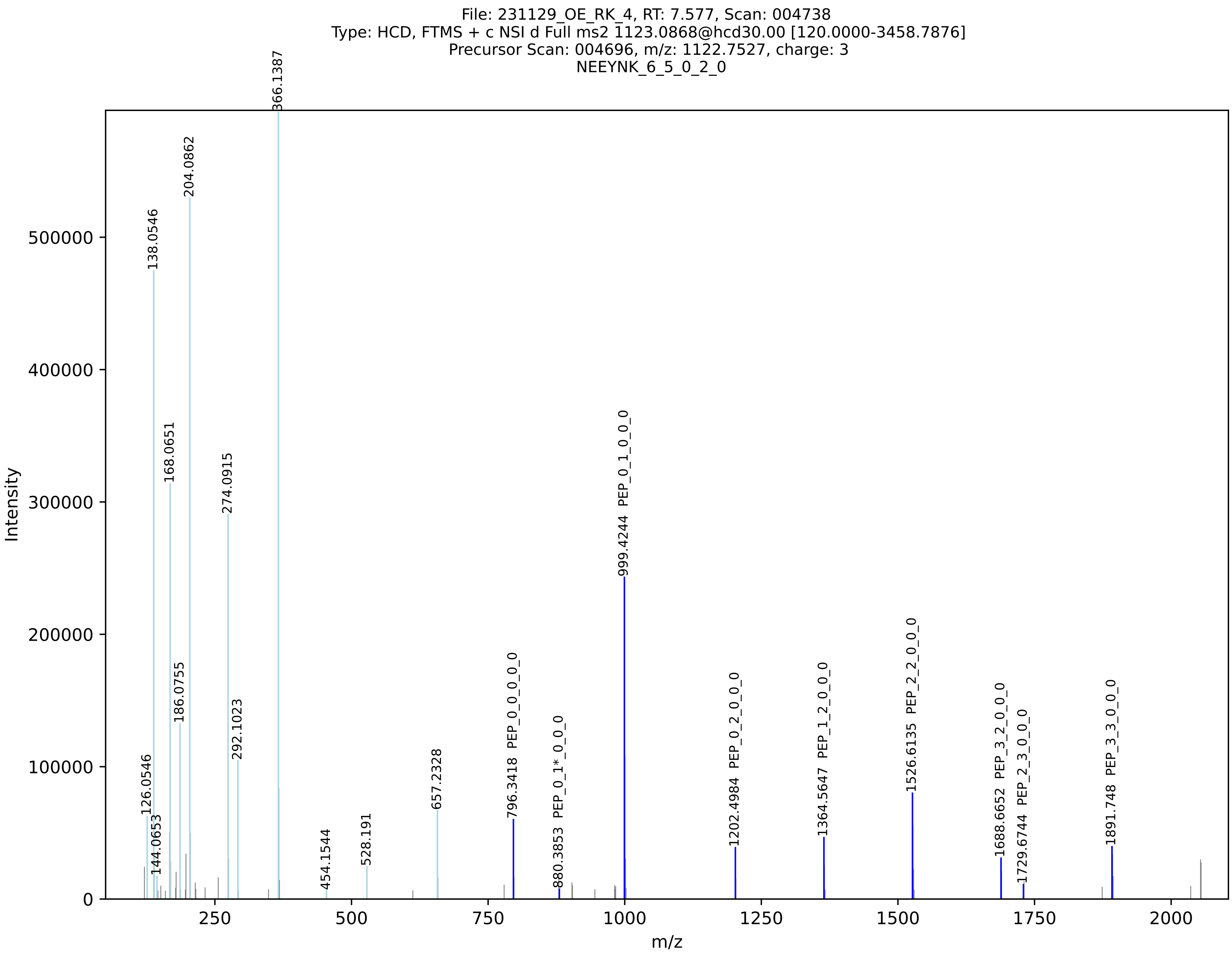
File: 231129_OE_RK_4, RT: 7.577, Scan: 004738
Type: HCD, FTMS + c NSI d Full ms2 1123.0868@hcd30.00 [120.0000-3458.7876]
Precursor Scan: 004696, m/z: 1122.7527, charge: 3
NEEYNK_6_5_0_2_0
Intensity
m/z
366.1387
204.0862
138.0546
168.0651
274.0915
186.0755
292.1023
126.0546
144.0653
454.1544
528.191
657.2328
796.3418 PEP_0_0_0_0_0
880.3853 PEP_0_1*_0_0_0
999.4244 PEP_0_1_0_0_0
1202.4984 PEP_0_2_0_0_0
1364.5647 PEP_1_2_0_0_0
1526.6135 PEP_2_2_0_0_0
1688.6652 PEP_3_2_0_0_0
1729.6744 PEP_2_3_0_0_0
1891.748 PEP_3_3_0_0_0

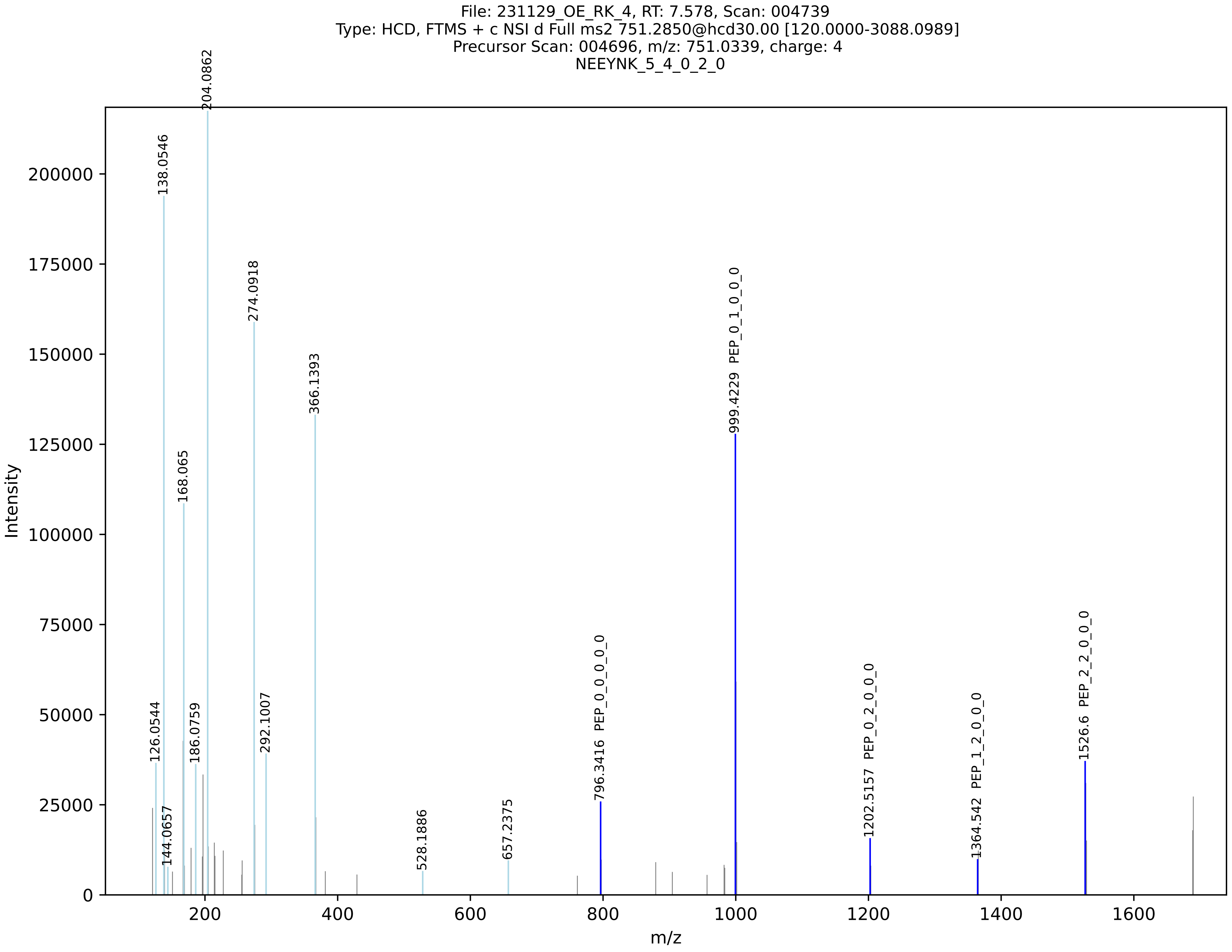

File: 231129_OE_RK_4, RT: 7.578, Scan: 004739
Type: HCD, FTMS + c NSI d Full ms2 751.2850@hcd30.00 [120.0000-3088.0989]
Precursor Scan: 004696, m/z: 751.0339, charge: 4
NEEYNK_5_4_0_2_0
204.0862
138.0546
274.0918
366.1393
168.065
999.4229 PEP_0_1_0_0_0
126.0544
186.0759
292.1007
796.3416 PEP_0_0_0_0_0
1202.5157 PEP_0_2_0_0_0
1364.542 PEP_1_2_0_0_0
1526.6 PEP_2_2_0_0_0
144.0657
528.1886
657.2375
Intensity
m/z
0
25000
50000
75000
100000
125000
150000
175000
200000
200
400
600
800
1000
1200
1400
1600

File: 231129_OE_RK_4, RT: 7.578, Scan: 004740
Type: HCD, FTMS + c NSI d Full ms2 1286.8311@hcd30.00 [120.0000-3959.8450]
Precursor Scan: 004696, m/z: 1286.4976, charge: 3
ENGTISR_7_6_1_2_0

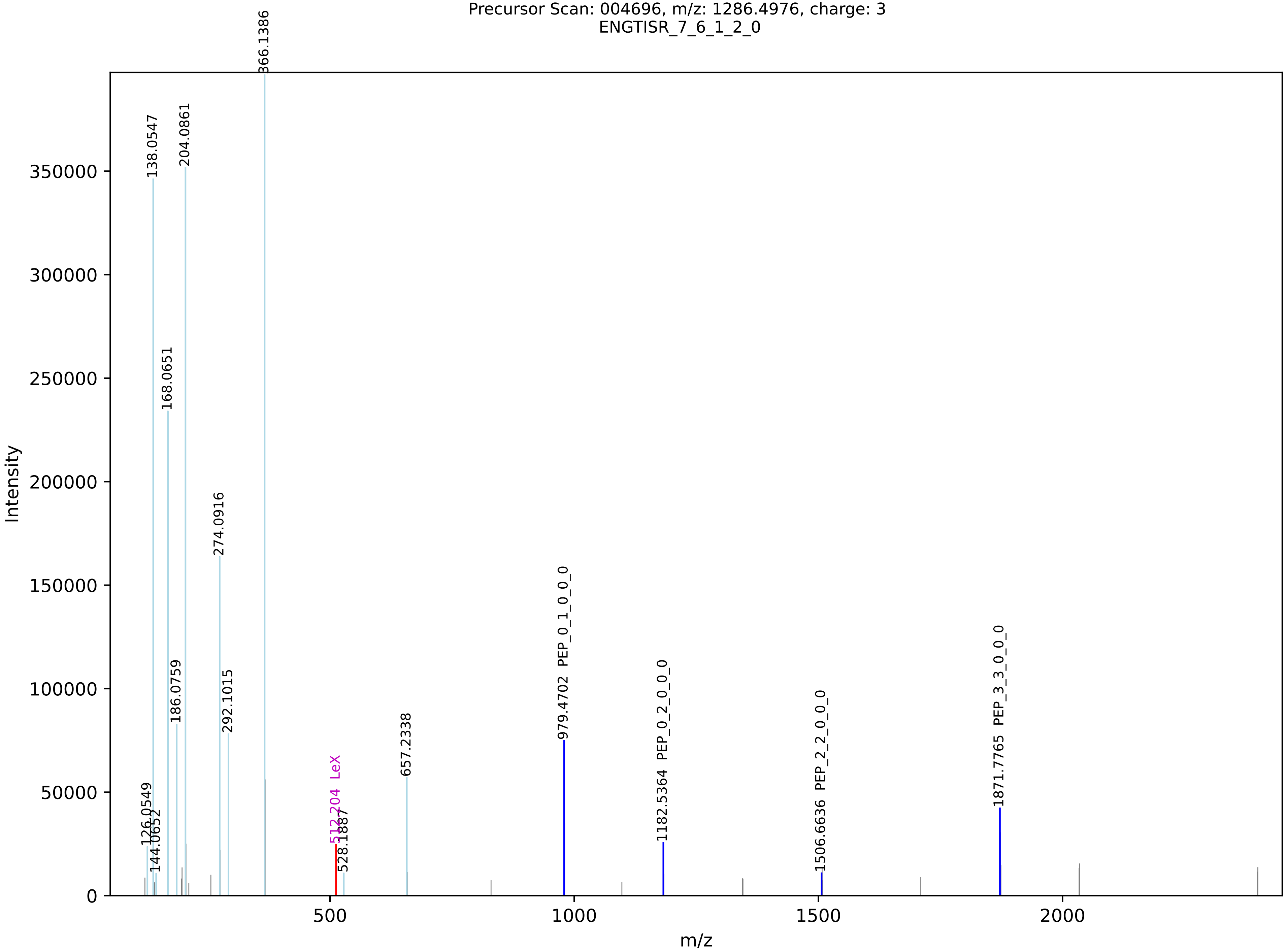

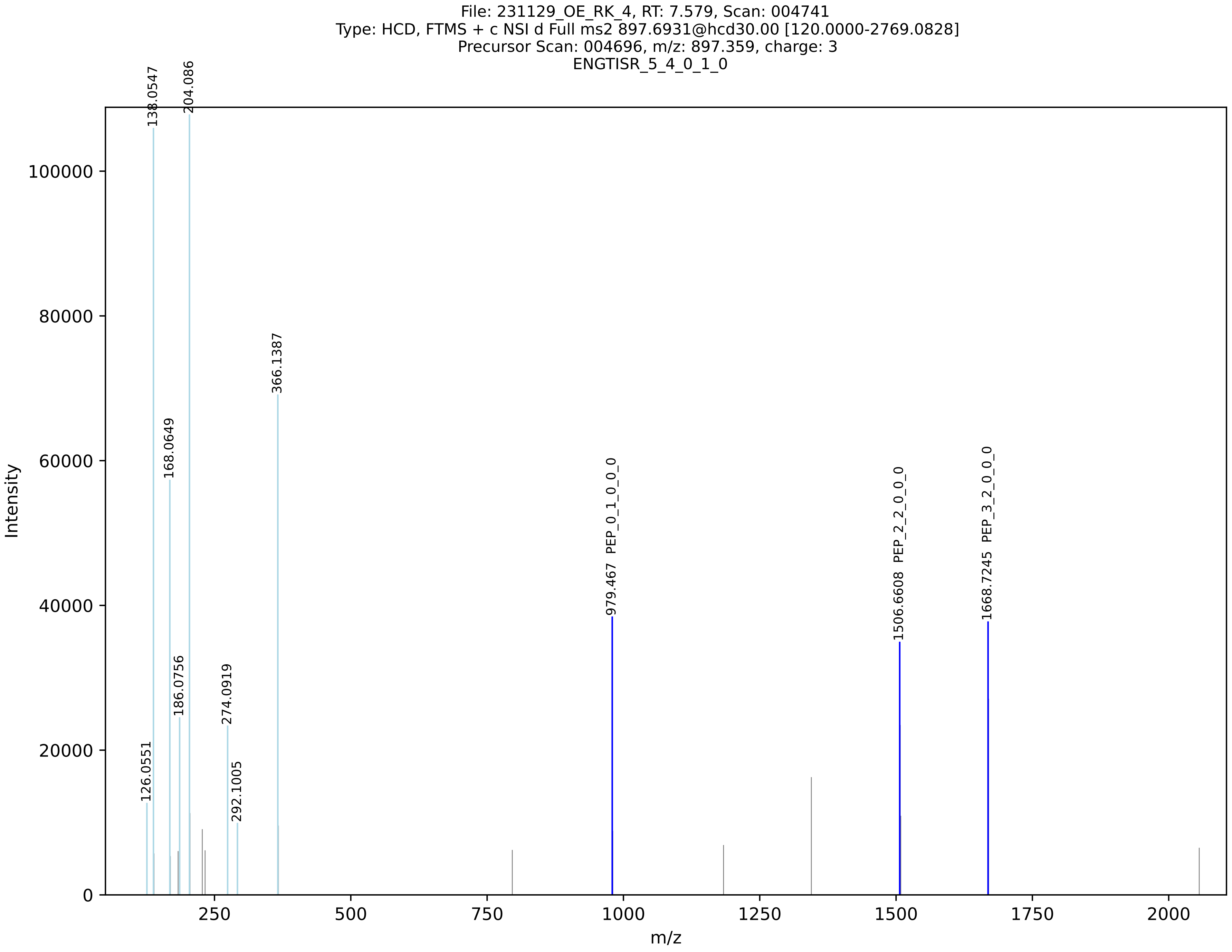
File: 231129_OE_RK_4, RT: 7.579, Scan: 004741
Type: HCD, FTMS + c NSI d Full ms2 897.6931@hcd30.00 [120.0000-2769.0828]
Precursor Scan: 004696, m/z: 897.359, charge: 3
ENGTISR_5_4_0_1_0
138.0547
204.086
366.1387
168.0649
186.0756
274.0919
126.0551
292.1005
979.467 PEP_0_1_0_0_0
1506.6608 PEP_2_2_0_0_0
1668.7245 PEP_3_2_0_0_0
Intensity
m/z
0
20000
40000
60000
80000
100000
250
500
750
1000
1250
1500
1750
2000

File: 231129_OE_RK_4, RT: 7.58, Scan: 004742
Type: HCD, FTMS + c NSI d Full ms2 1000.7068@hcd30.00 [120.0000-3084.3049]
Precursor Scan: 004696, m/z: 1000.3728, charge: 3
NEEYNK_5_4_0_2_0

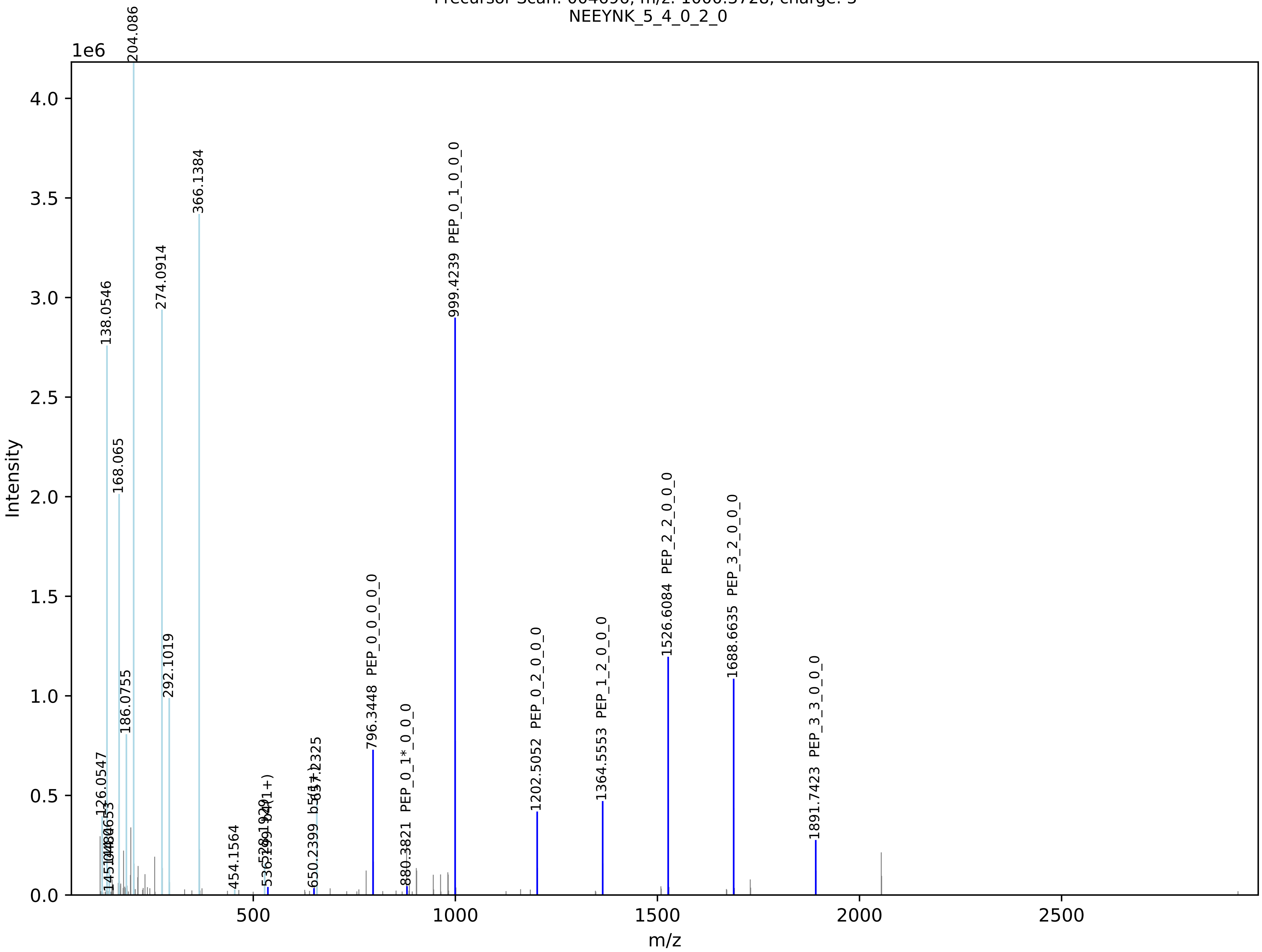

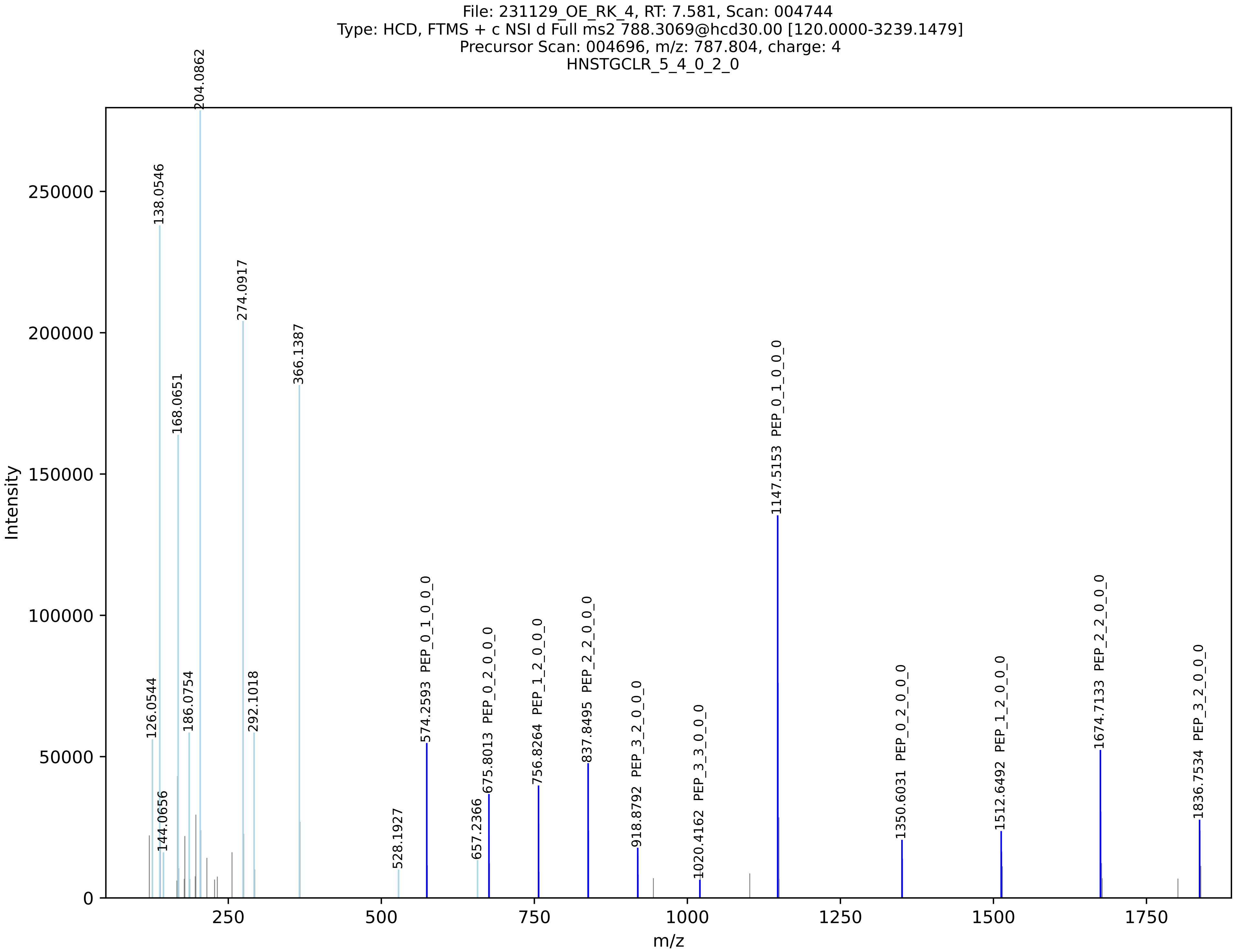

File: 231129_OE_RK_4, RT: 7.581, Scan: 004744
Type: HCD, FTMS + c NSI d Full ms2 788.3069@hcd30.00 [120.0000-3239.1479]
Precursor Scan: 004696, m/z: 787.804, charge: 4
HNSTGCLR_5_4_0_2_0
Intensity
m/z
204.0862
138.0546
274.0917
366.1387
168.0651
126.0544
186.0754
292.1018
144.0656
528.1927
574.2593 PEP_0_1_0_0_0
657.2366
675.8013 PEP_0_2_0_0_0
756.8264 PEP_1_2_0_0_0
837.8495 PEP_2_2_0_0_0
918.8792 PEP_3_2_0_0_0
1020.4162 PEP_3_3_0_0_0
1147.5153 PEP_0_1_0_0_0
1350.6031 PEP_0_2_0_0_0
1512.6492 PEP_1_2_0_0_0
1674.7133 PEP_2_2_0_0_0
1836.7534 PEP_3_2_0_0_0

File: 231129_OE_RK_4, RT: 7.594, Scan: 004765
Type: HCD, FTMS + c NSI d Full ms2 1049.0138@hcd30.00 [120.0000-3232.1240]
Precursor Scan: 004696, m/z: 1049.0138, charge: 3
NEEYNK_5_4_1_2_0

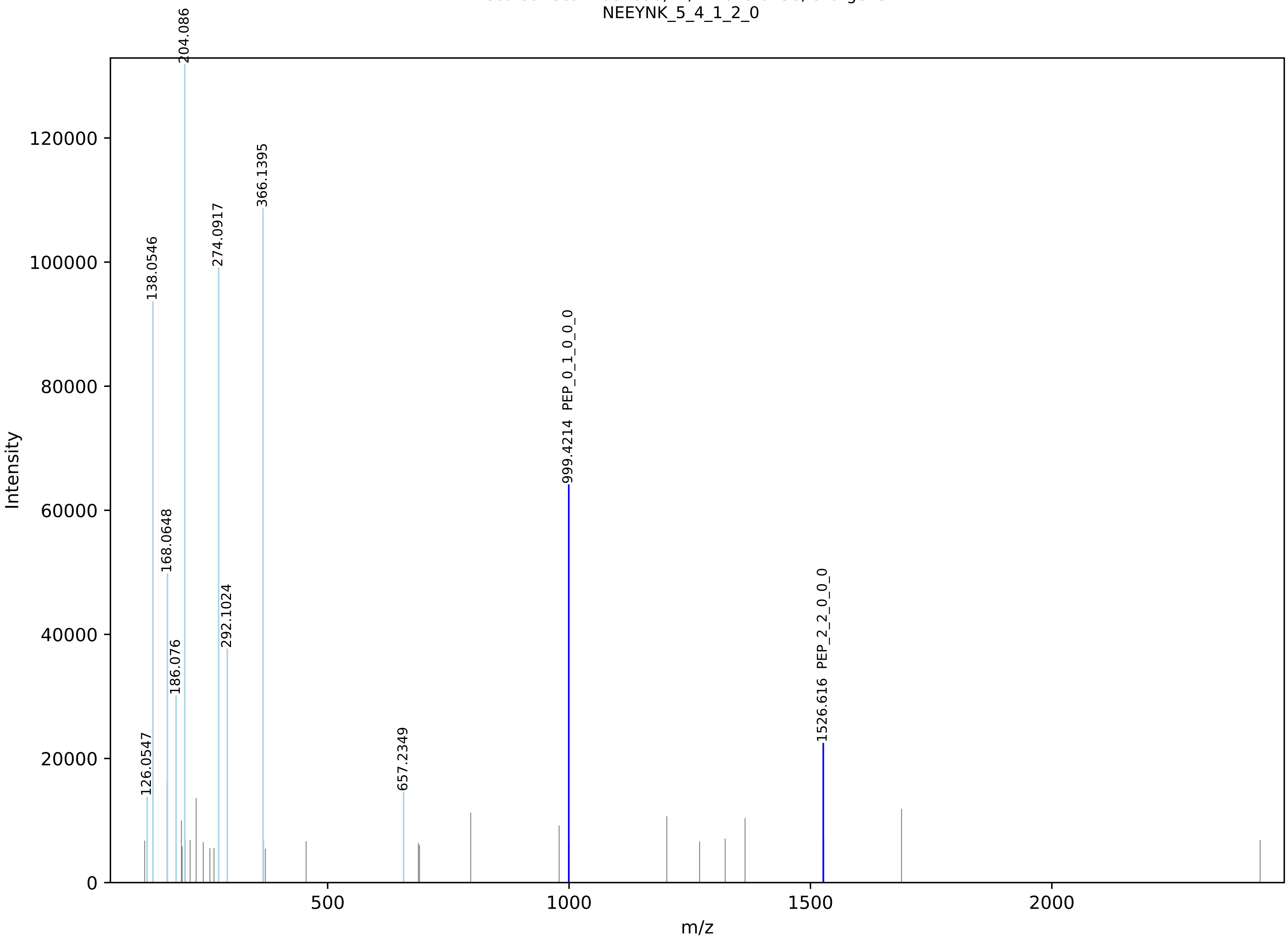

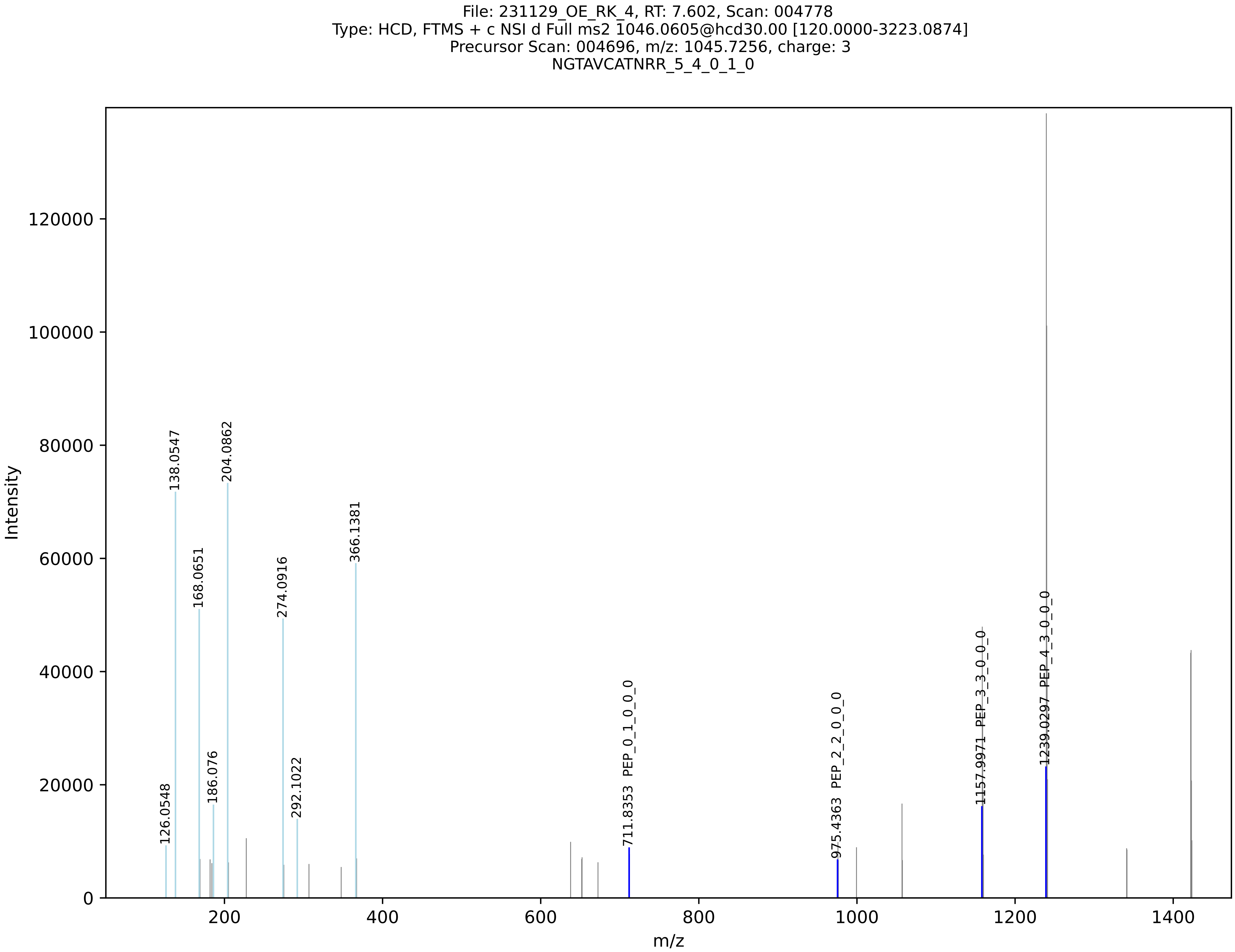
File: 231129_OE_RK_4, RT: 7.602, Scan: 004778
Type: HCD, FTMS + c NSI d Full ms2 1046.0605@hcd30.00 [120.0000-3223.0874]
Precursor Scan: 004696, m/z: 1045.7256, charge: 3
NGTAVCATNRR_5_4_0_1_0
Intensity
m/z
126.0548
138.0547
168.0651
186.076
204.0862
274.0916
292.1022
366.1381
711.8353 PEP_0_1_0_0_0
975.4363 PEP_2_2_0_0_0
1157.9971 PEP_3_3_0_0_0
1239.0297 PEP_4_3_0_0_0
0
20000
40000
60000
80000
100000
120000
200
400
600
800
1000
1200
1400

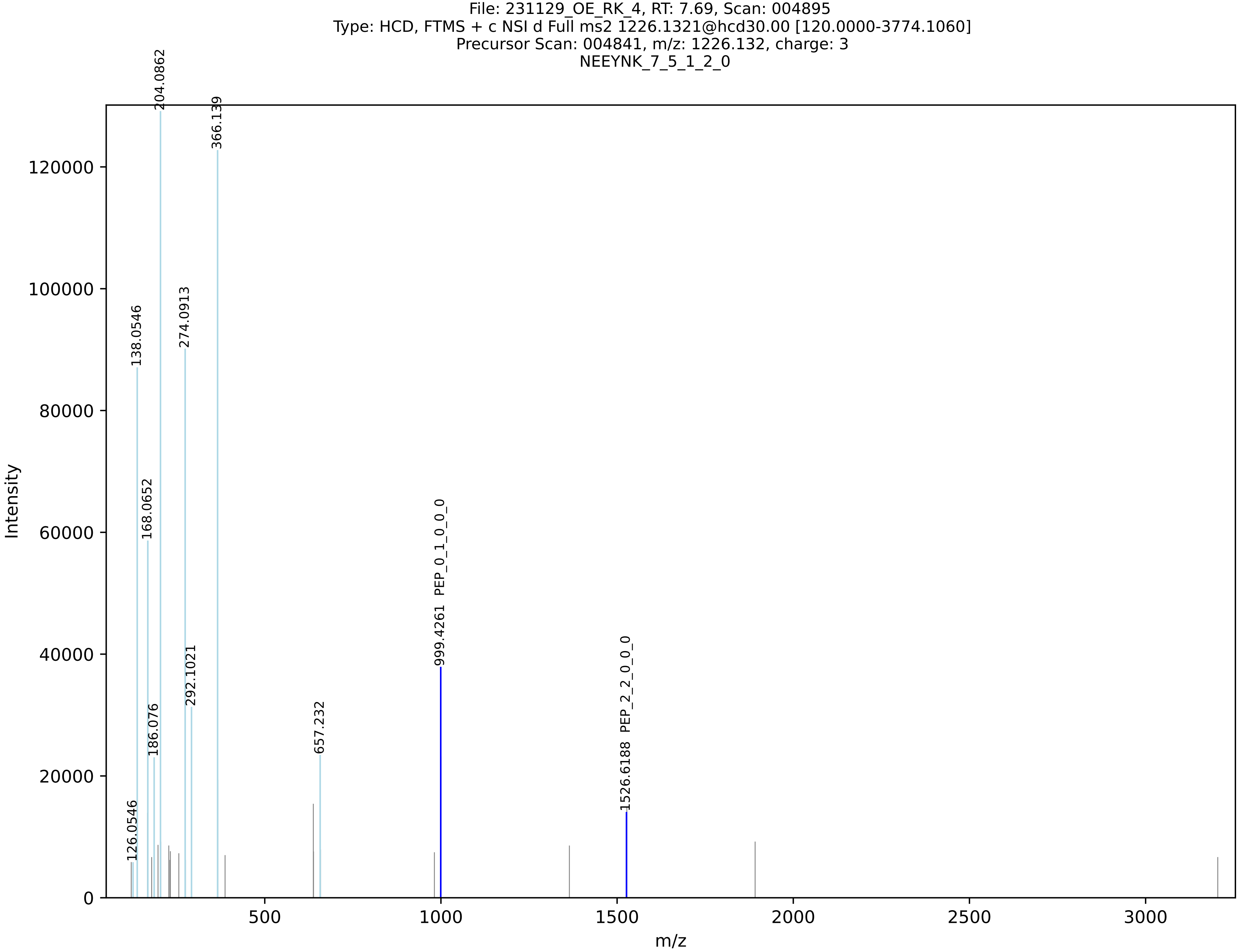
File: 231129_OE_RK_4, RT: 7.69, Scan: 004895
Type: HCD, FTMS + c NSI d Full ms2 1226.1321@hcd30.00 [120.0000-3774.1060]
Precursor Scan: 004841, m/z: 1226.132, charge: 3
NEEYNK_7_5_1_2_0
Intensity
m/z
204.0862
366.139
138.0546
274.0913
168.0652
999.4261 PEP_0_1_0_0_0
1526.6188 PEP_2_2_0_0_0
292.1021
186.076
657.232
126.0546
0
20000
40000
60000
80000
100000
120000
500
1000
1500
2000
2500
3000

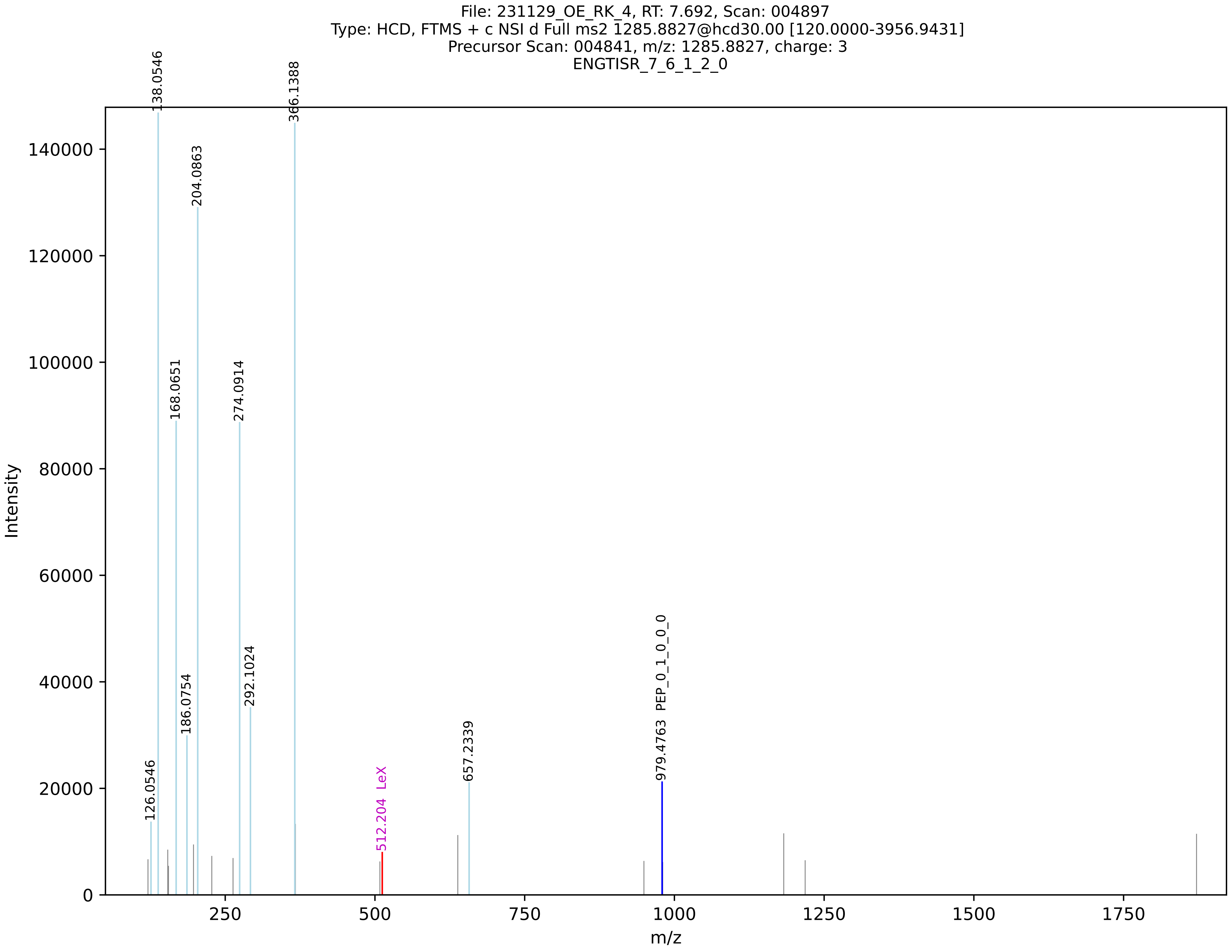

File: 231129_OE_RK_4, RT: 7.692, Scan: 004897
Type: HCD, FTMS + c NSI d Full ms2 1285.8827@hcd30.00 [120.0000-3956.9431]
Precursor Scan: 004841, m/z: 1285.8827, charge: 3
ENGTISR_7_6_1_2_0
138.0546
366.1388
204.0863
168.0651
274.0914
292.1024
186.0754
126.0546
512.204 LeX
657.2339
979.4763 PEP_0_1_0_0_0
Intensity
m/z
0
20000
40000
60000
80000
100000
120000
140000
250
500
750
1000
1250
1500
1750

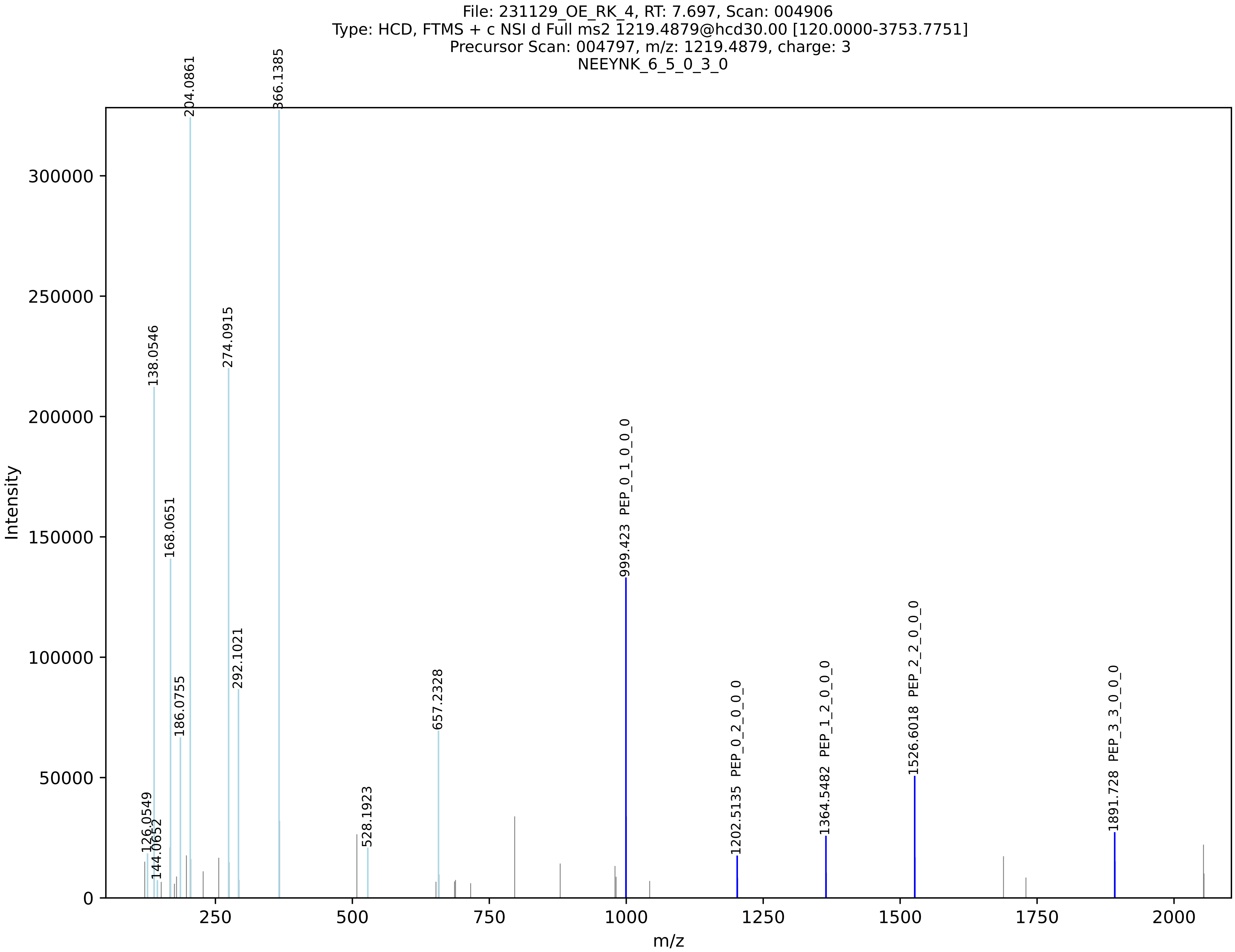

File: 231129_OE_RK_4, RT: 7.697, Scan: 004906
Type: HCD, FTMS + c NSI d Full ms2 1219.4879@hcd30.00 [120.0000-3753.7751]
Precursor Scan: 004797, m/z: 1219.4879, charge: 3
NEEYNK_6_5_0_3_0
Intensity
m/z
0
50000
100000
150000
200000
250000
300000
250
500
750
1000
1250
1500
1750
2000
126.0549
144.0652
138.0546
168.0651
186.0755
204.0861
274.0915
292.1021
366.1385
528.1923
657.2328
999.423 PEP_0_1_0_0_0
1202.5135 PEP_0_2_0_0_0
1364.5482 PEP_1_2_0_0_0
1526.6018 PEP_2_2_0_0_0
1891.728 PEP_3_3_0_0_0

File: 231129_OE_RK_4, RT: 7.71, Scan: 004920
Type: HCD, FTMS + c NSI d Full ms2 1213.8075@hcd30.00 [120.0000-3736.3928]
Precursor Scan: 004885, m/z: 1213.4721, charge: 3
ENGTISR_6_5_2_2_0

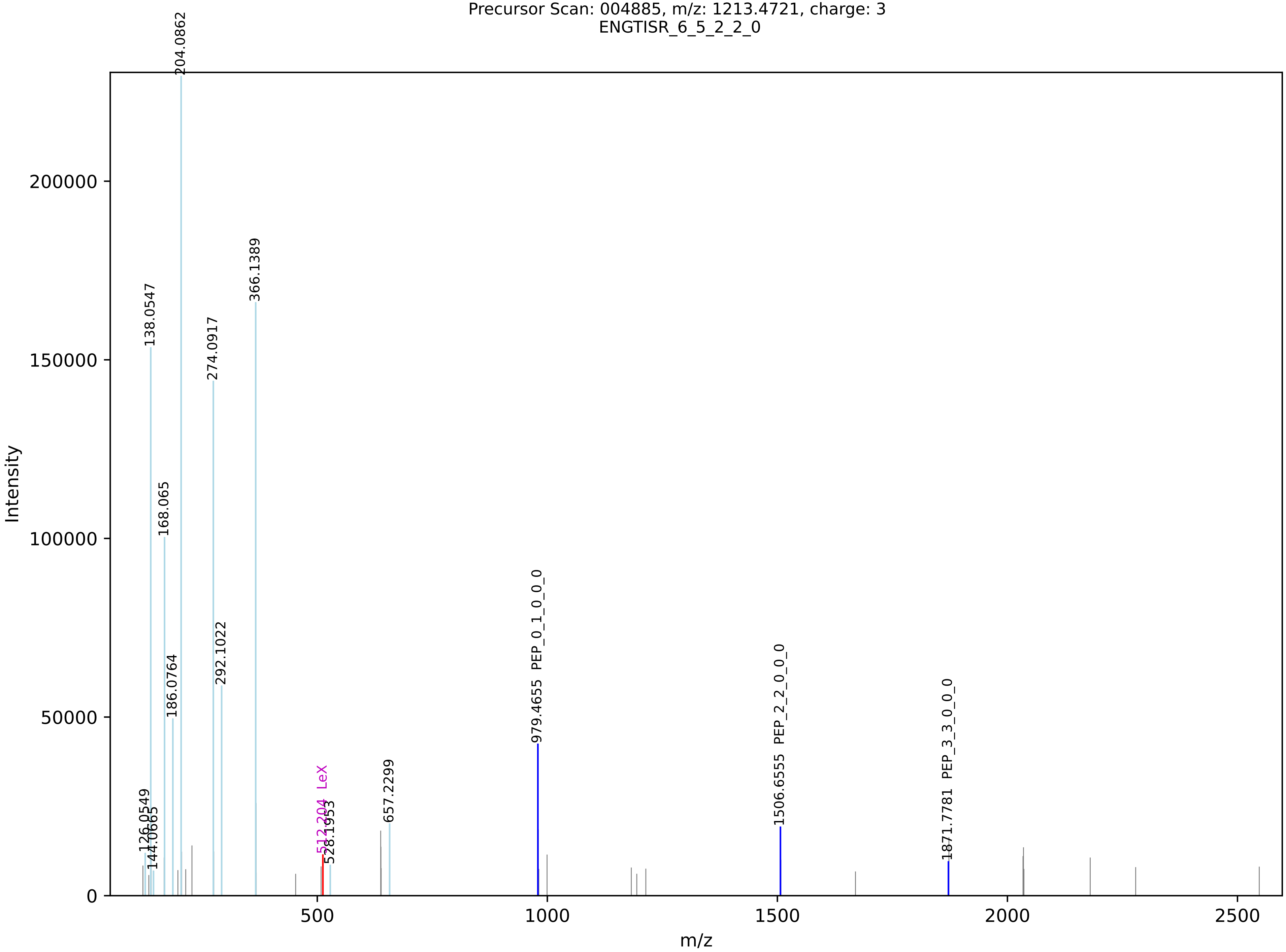

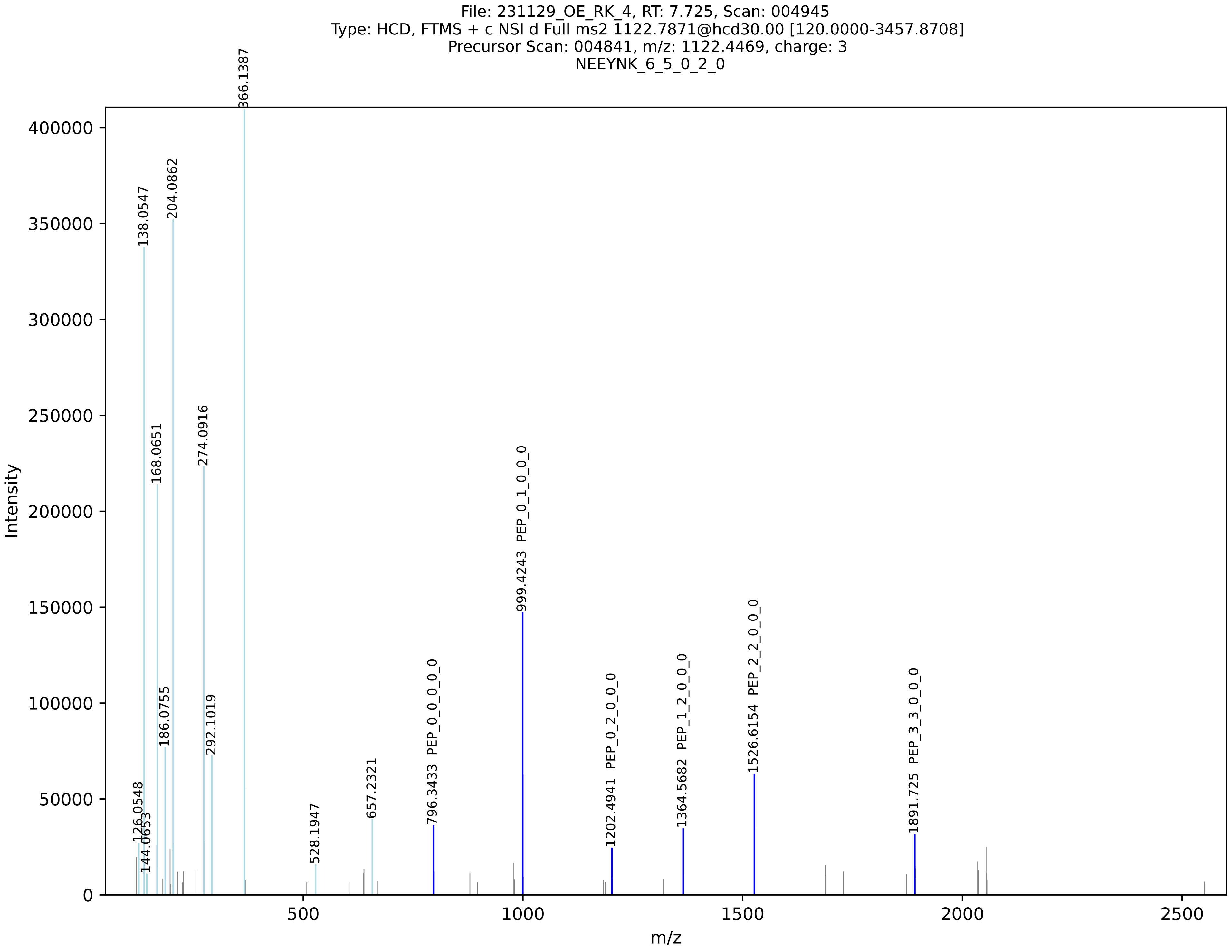

File: 231129_OE_RK_4, RT: 7.725, Scan: 004945
Type: HCD, FTMS + c NSI d Full ms2 1122.7871@hcd30.00 [120.0000-3457.8708]
Precursor Scan: 004841, m/z: 1122.4469, charge: 3
NEEYNK_6_5_0_2_0
Intensity
m/z
366.1387
138.0547
204.0862
168.0651
274.0916
186.0755
292.1019
126.0548
144.0653
528.1947
657.2321
796.3433 PEP_0_0_0_0_0_
999.4243 PEP_0_1_0_0_0_
1202.4941 PEP_0_2_0_0_0_
1364.5682 PEP_1_2_0_0_0_
1526.6154 PEP_2_2_0_0_0_
1891.725 PEP_3_3_0_0_0_

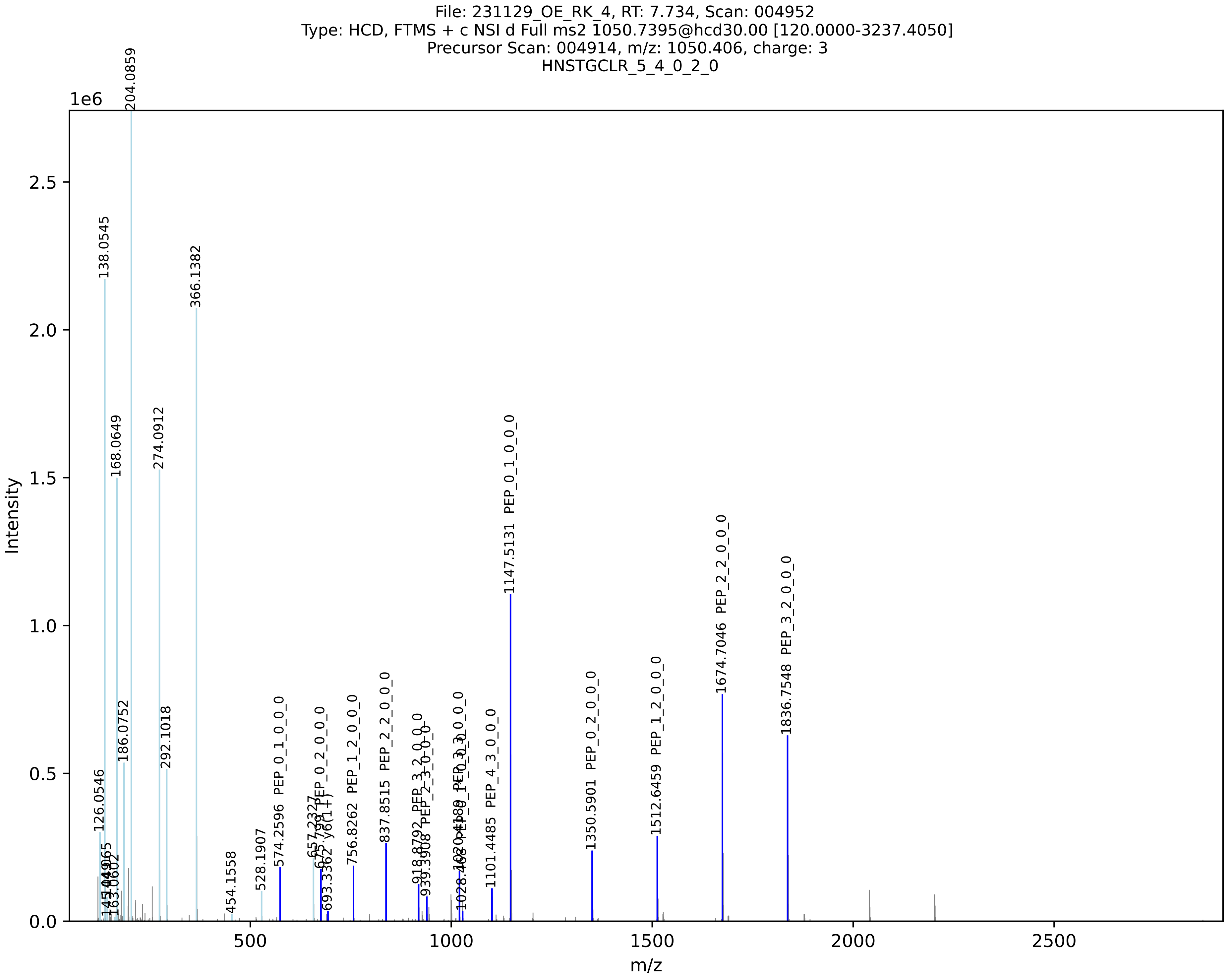
File: 231129_OE_RK_4, RT: 7.734, Scan: 004952
Type: HCD, FTMS + c NSI d Full ms2 1050.7395@hcd30.00 [120.0000-3237.4050]
Precursor Scan: 004914, m/z: 1050.406, charge: 3
HNSTGCLR_5_4_0_2_0
1e6
Intensity
m/z
204.0859
138.0545
366.1382
274.0912
168.0649
186.0752
292.1018
126.0546
454.1558
528.1907
574.2596 PEP_0_1_0_0_0
657.2327
756.8262 PEP_1_2_0_0_0
837.8515 PEP_2_2_0_0_0
1101.4485 PEP_4_3_0_0_0
1147.5131 PEP_0_1_0_0_0
1350.5901 PEP_0_2_0_0_0
1512.6459 PEP_1_2_0_0_0
1674.7046 PEP_2_2_0_0_0
1836.7548 PEP_3_2_0_0_0

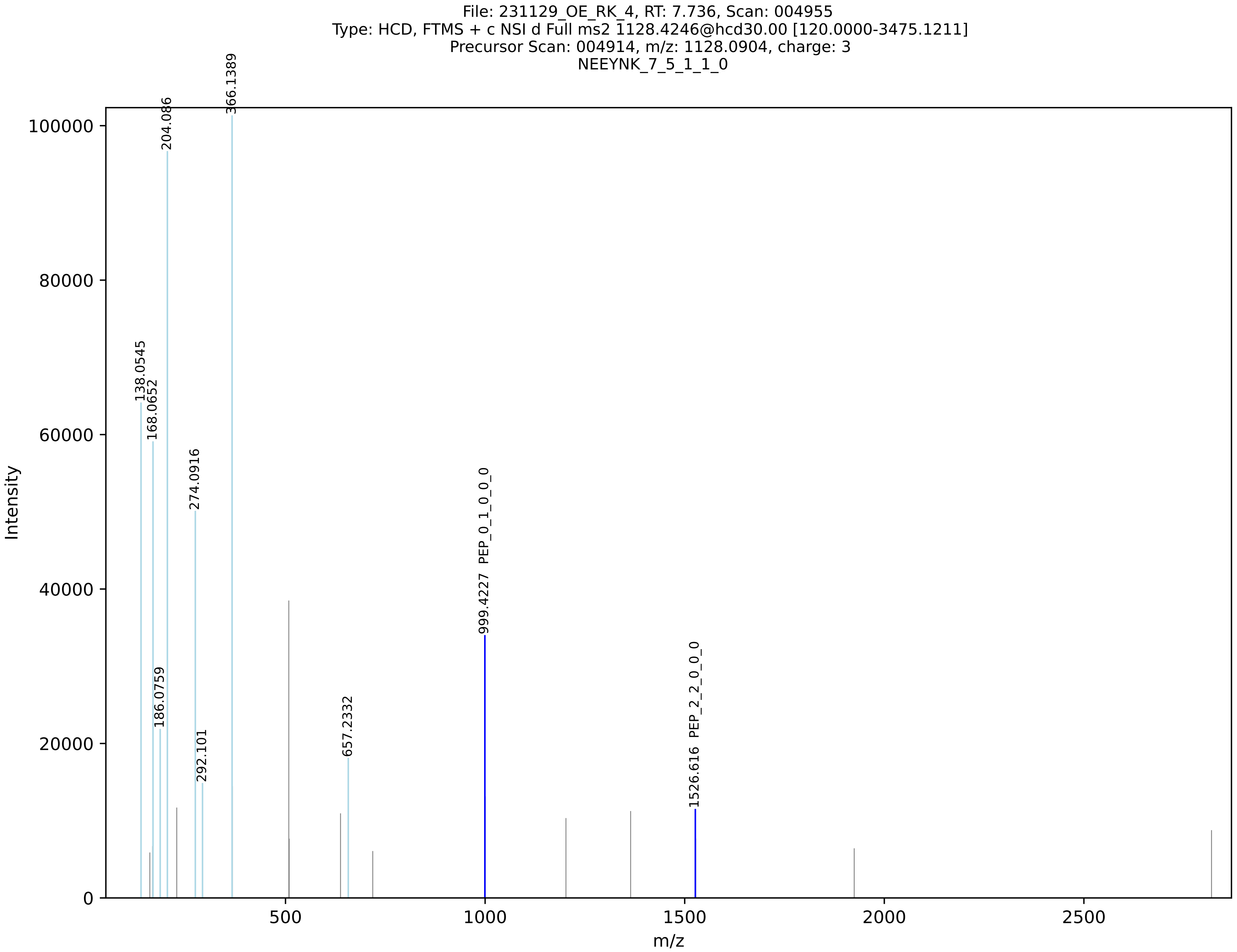
File: 231129_OE_RK_4, RT: 7.736, Scan: 004955
Type: HCD, FTMS + c NSI d Full ms2 1128.4246@hcd30.00 [120.0000-3475.1211]
Precursor Scan: 004914, m/z: 1128.0904, charge: 3
NEEYNK_7_5_1_1_0
366.1389
204.086
138.0545
168.0652
274.0916
999.4227 PEP_0_1_0_0_0
186.0759
292.101
657.2332
1526.616 PEP_2_2_0_0_0
Intensity
0
20000
40000
60000
80000
100000
500
1000
1500
2000
2500
m/z

File: 231129_OE_RK_4, RT: 7.737, Scan: 004957
Type: HCD, FTMS + c NSI d Full ms2 965.3740@hcd30.00 [120.0000-3961.5818]
Precursor Scan: 004914, m/z: 965.1246, charge: 4
ENGTISR_7_6_1_2_0

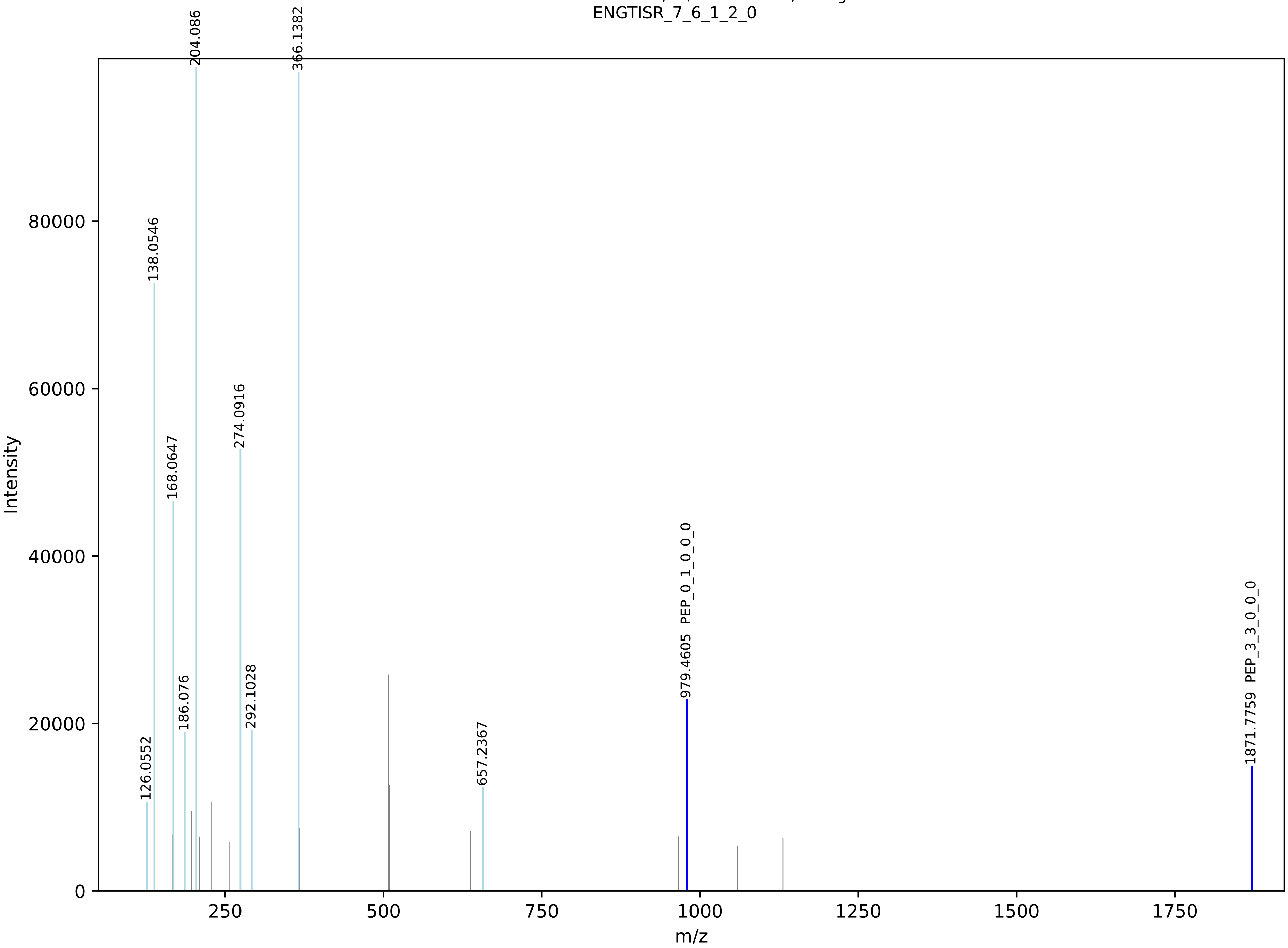

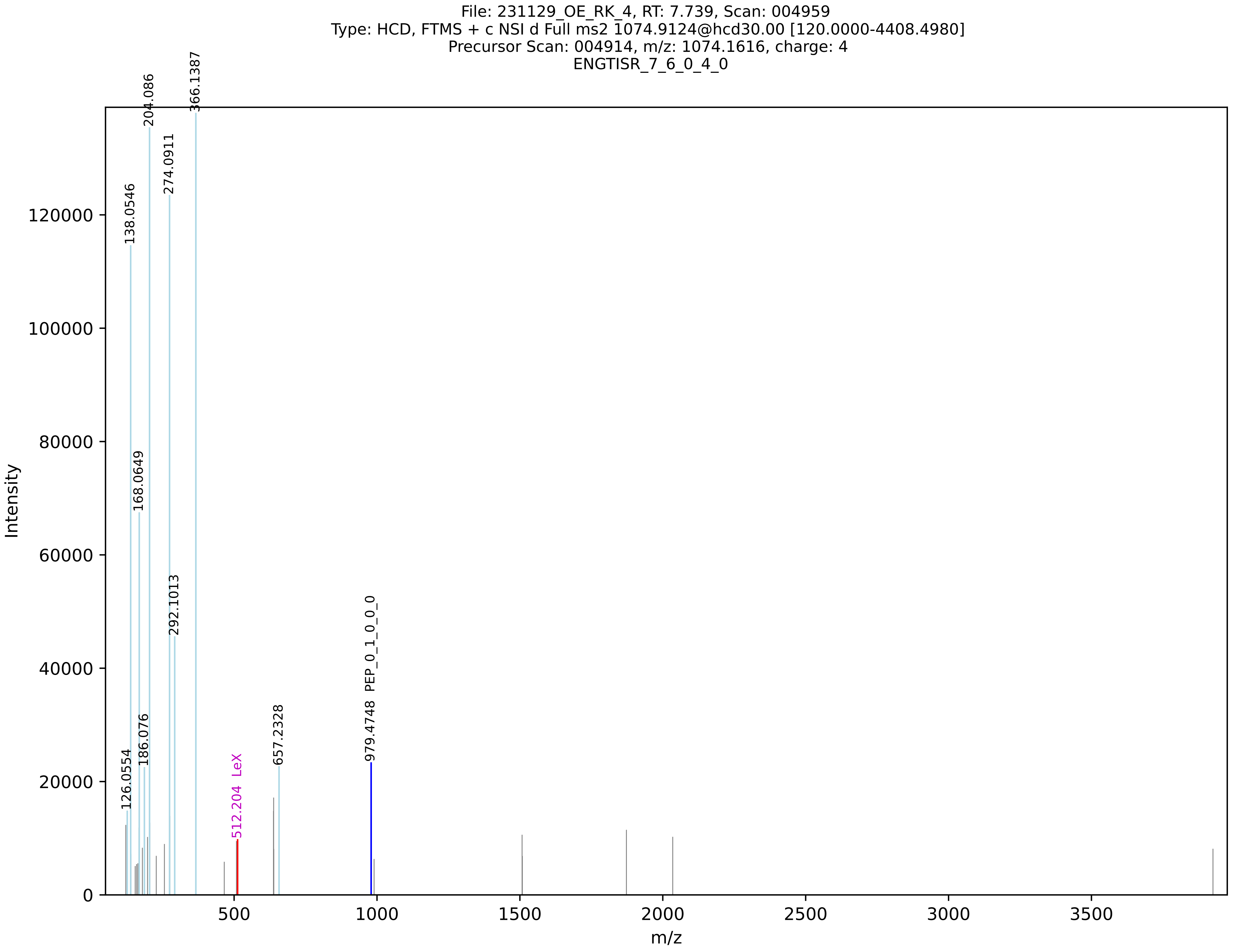
File: 231129_OE_RK_4, RT: 7.739, Scan: 004959
Type: HCD, FTMS + c NSI d Full ms2 1074.9124@hcd30.00 [120.0000-4408.4980]
Precursor Scan: 004914, m/z: 1074.1616, charge: 4
ENGTISR_7_6_0_4_0
Intensity
m/z
366.1387
204.086
274.0911
138.0546
168.0649
292.1013
979.4748 PEP_0_1_0_0_0_
657.2328
186.076
126.0554
512.204 LeX
0
20000
40000
60000
80000
100000
120000
500
1000
1500
2000
2500
3000
3500

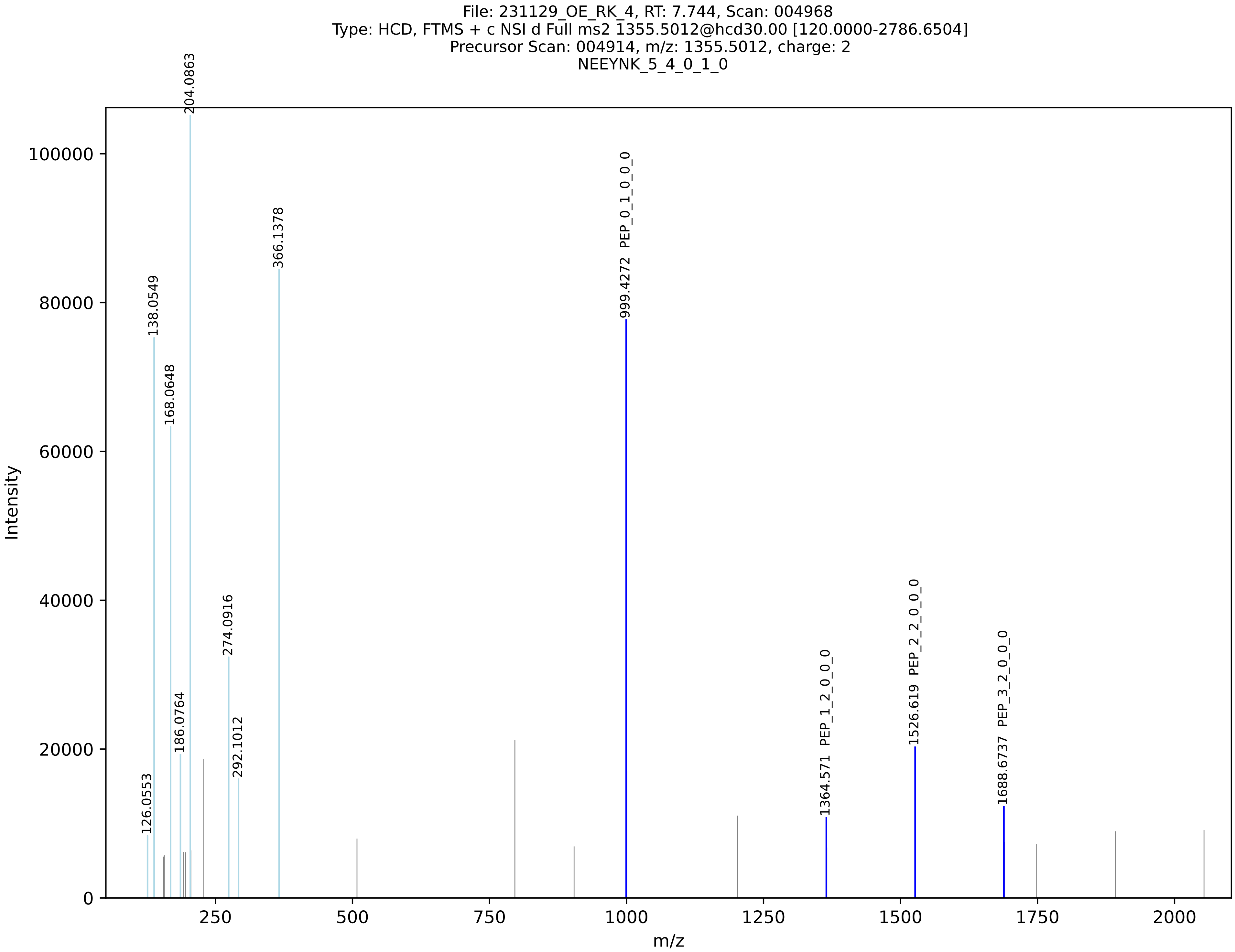

File: 231129_OE_RK_4, RT: 7.744, Scan: 004968
Type: HCD, FTMS + c NSI d Full ms2 1355.5012@hcd30.00 [120.0000-2786.6504]
Precursor Scan: 004914, m/z: 1355.5012, charge: 2
NEEYNK_5_4_0_1_0
204.0863
999.4272 PEP_0_1_0_0_0
366.1378
138.0549
168.0648
274.0916
186.0764
292.1012
126.0553
1364.571 PEP_1_2_0_0_0
1526.619 PEP_2_2_0_0_0
1688.6737 PEP_3_2_0_0_0
Intensity
m/z
0
20000
40000
60000
80000
100000
250
500
750
1000
1250
1500
1750
2000

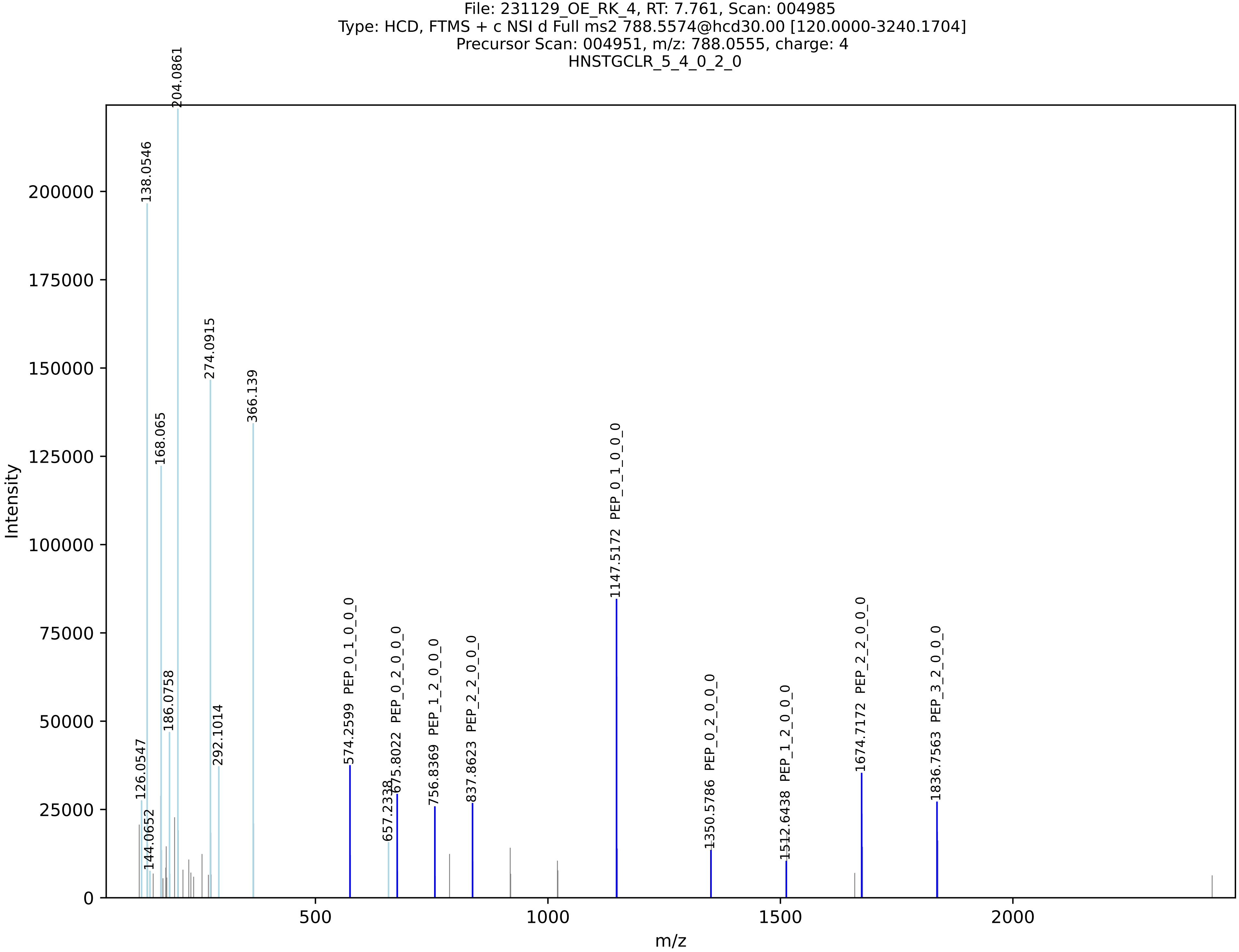
File: 231129_OE_RK_4, RT: 7.761, Scan: 004985
Type: HCD, FTMS + c NSI d Full ms2 788.5574@hcd30.00 [120.0000-3240.1704]
Precursor Scan: 004951, m/z: 788.0555, charge: 4
HNSTGCLR_5_4_0_2_0
Intensity
m/z
204.0861
138.0546
168.065
274.0915
366.139
186.0758
292.1014
126.0547
144.0652
574.2599 PEP_0_1_0_0_0
657.2338
675.8022 PEP_0_2_0_0_0
756.8369 PEP_1_2_0_0_0
837.8623 PEP_2_2_0_0_0
1147.5172 PEP_0_1_0_0_0
1350.5786 PEP_0_2_0_0_0
1512.6438 PEP_1_2_0_0_0
1674.7172 PEP_2_2_0_0_0
1836.7563 PEP_3_2_0_0_0

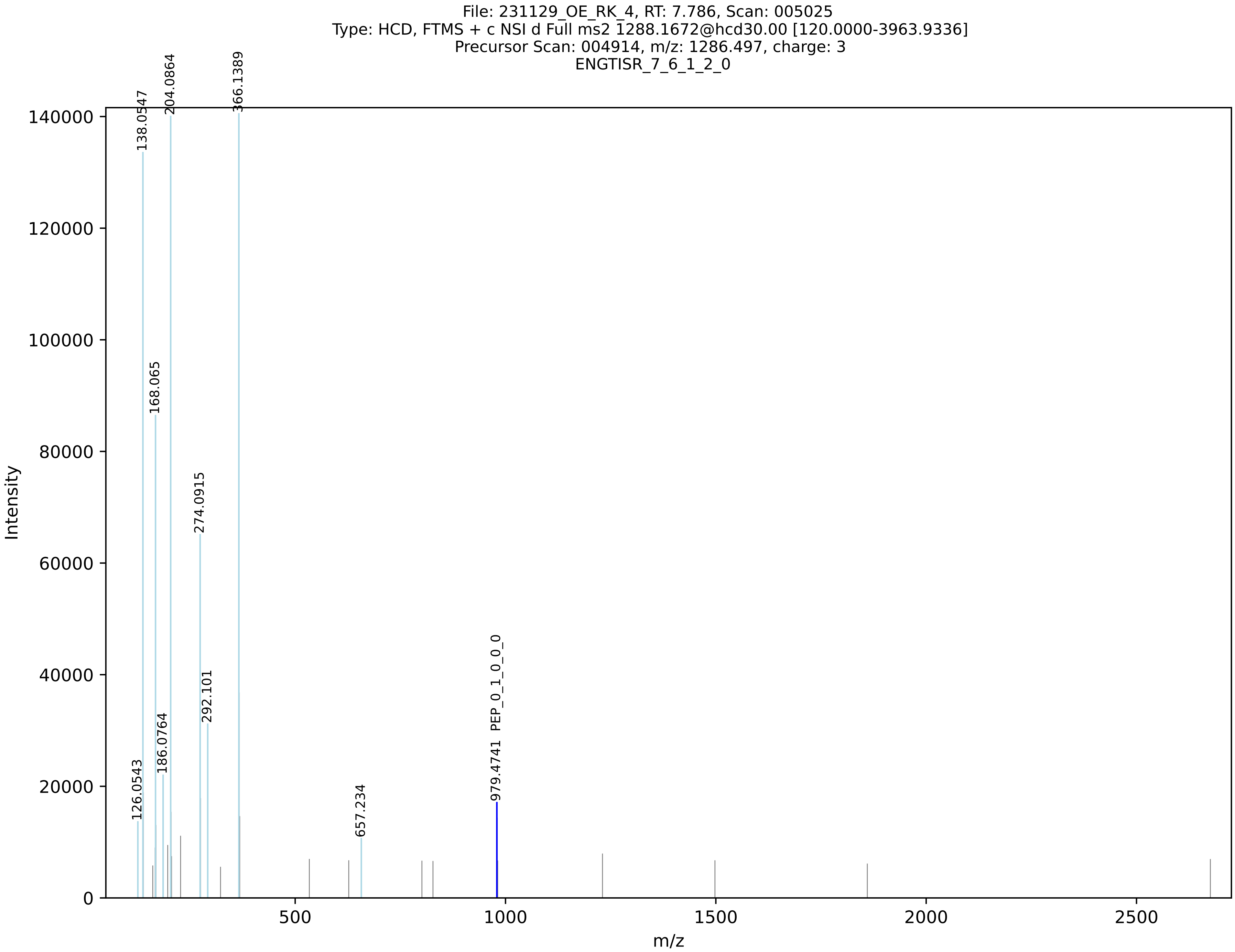
File: 231129_OE_RK_4, RT: 7.786, Scan: 005025
Type: HCD, FTMS + c NSI d Full ms2 1288.1672@hcd30.00 [120.0000-3963.9336]
Precursor Scan: 004914, m/z: 1286.497, charge: 3
ENGTISR_7_6_1_2_0
Intensity
m/z
0
20000
40000
60000
80000
100000
120000
140000
500
1000
1500
2000
2500
126.0543
138.0547
168.065
186.0764
204.0864
274.0915
292.101
366.1389
657.234
979.4741 PEP_0_1_0_0_0

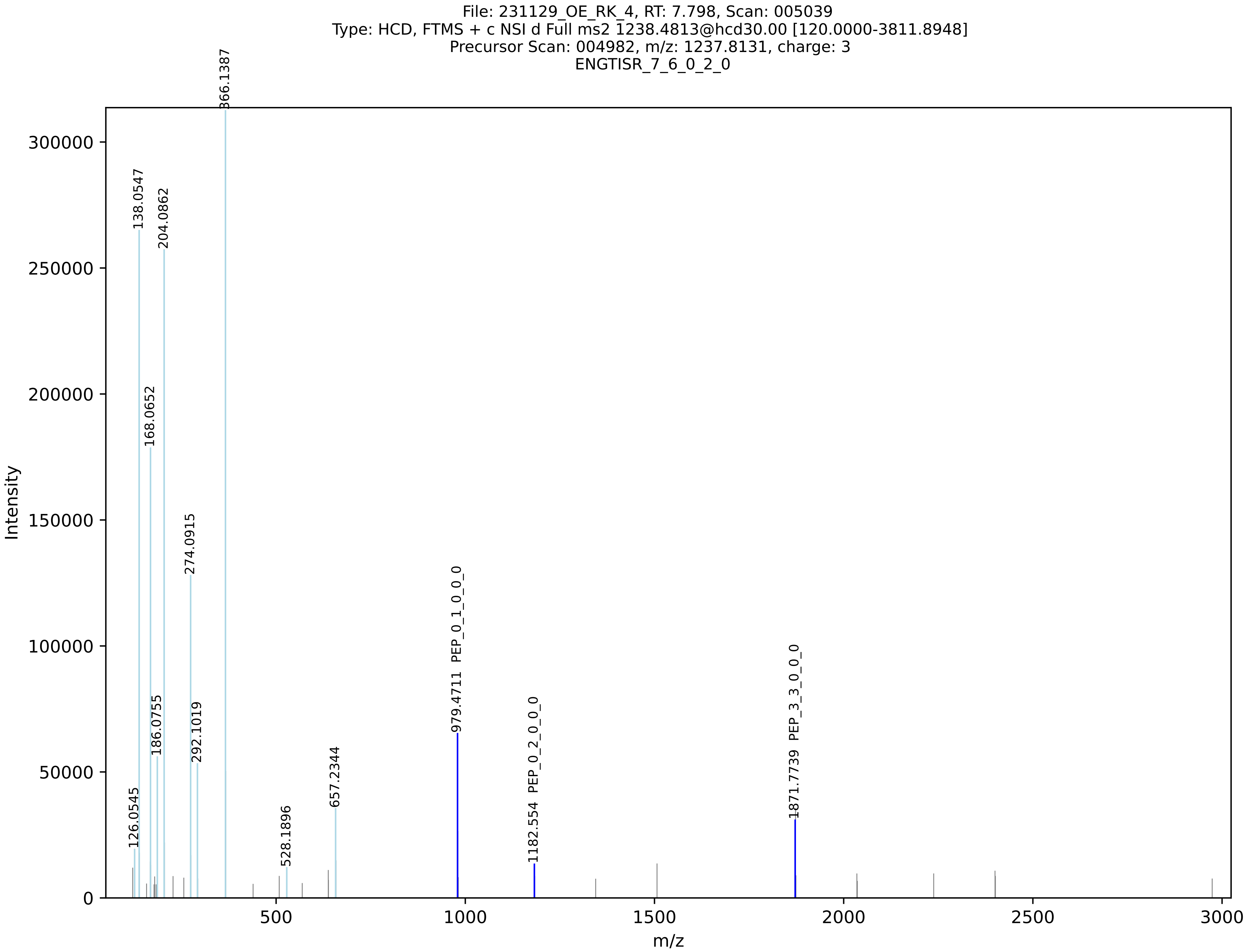
File: 231129_OE_RK_4, RT: 7.798, Scan: 005039
Type: HCD, FTMS + c NSI d Full ms2 1238.4813@hcd30.00 [120.0000-3811.8948]
Precursor Scan: 004982, m/z: 1237.8131, charge: 3
ENGTISR_7_6_0_2_0
Intensity
m/z
366.1387
138.0547
204.0862
168.0652
274.0915
186.0755
292.1019
126.0545
528.1896
657.2344
979.4711 PEP_0_1_0_0_0_
1182.554 PEP_0_2_0_0_0_
1871.7739 PEP_3_3_0_0_0_
0
50000
100000
150000
200000
250000
300000
500
1000
1500
2000
2500
3000

File: 231129_OE_RK_4, RT: 7.825, Scan: 005081
Type: HCD, FTMS + c NSI d Full ms2 1220.1600@hcd30.00 [120.0000-3755.8318]
Precursor Scan: 004951, m/z: 1219.1465, charge: 3
NEEYNK_6_5_0_3_0

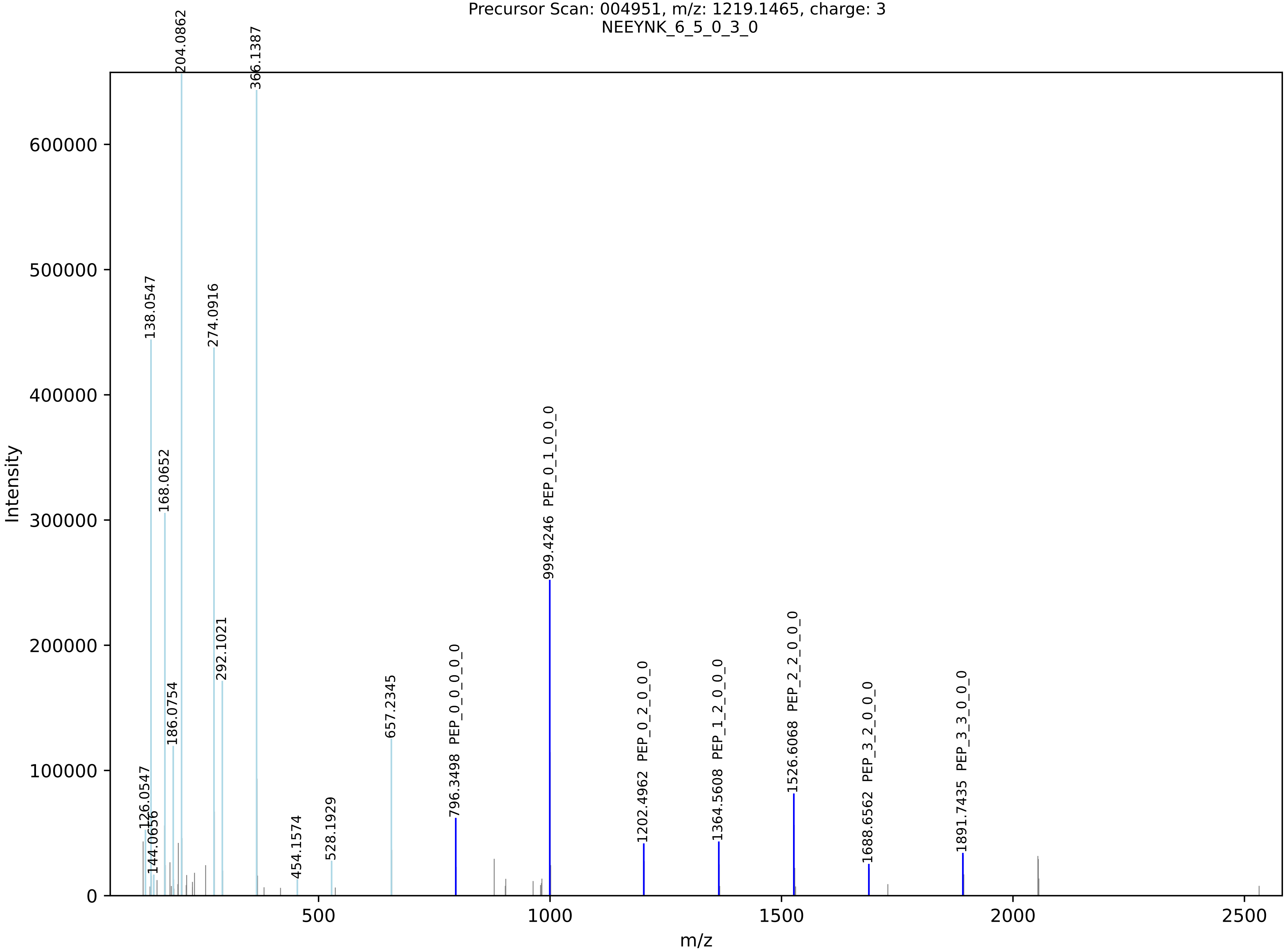

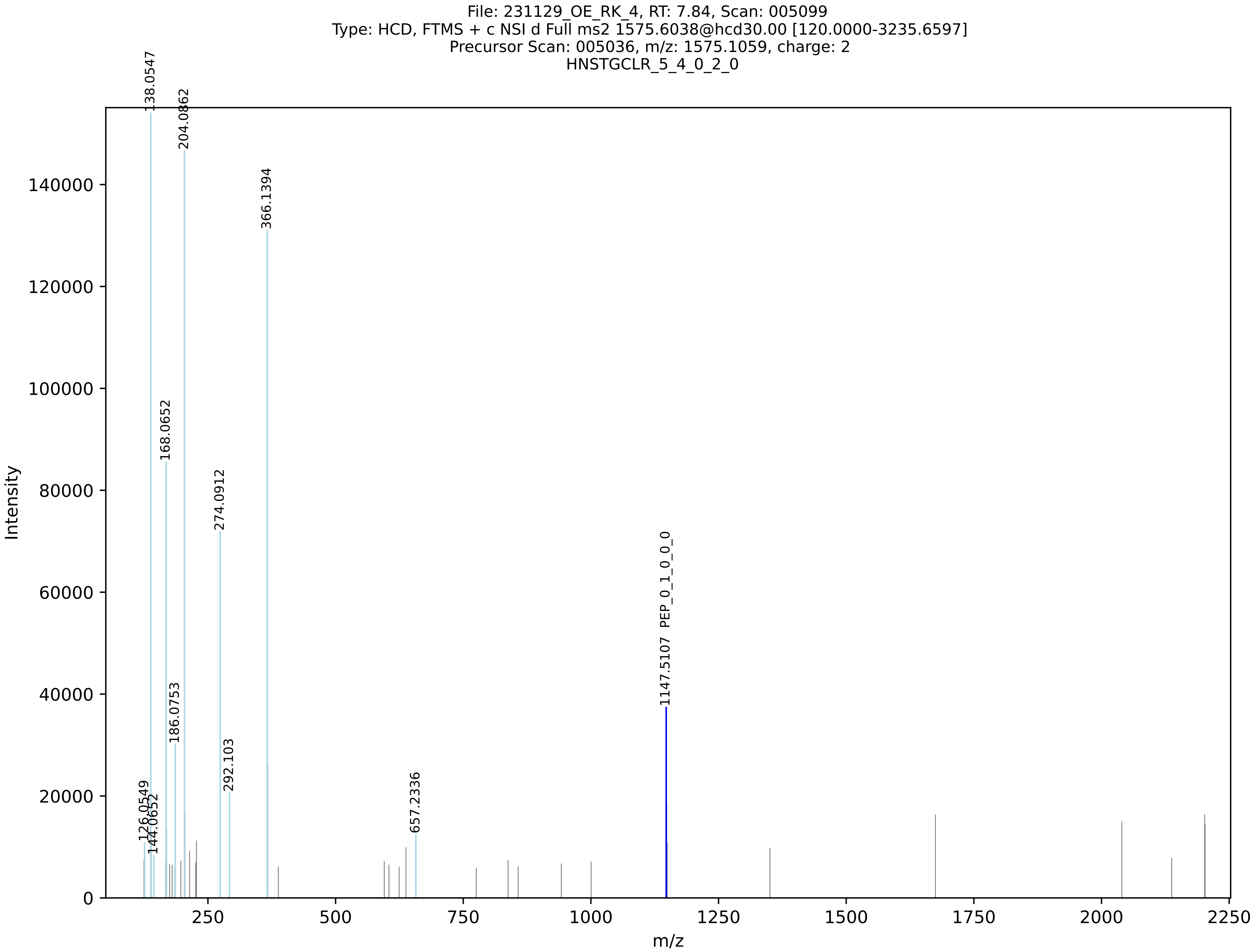
File: 231129_OE_RK_4, RT: 7.84, Scan: 005099
Type: HCD, FTMS + c NSI d Full ms2 1575.6038@hcd30.00 [120.0000-3235.6597]
Precursor Scan: 005036, m/z: 1575.1059, charge: 2
HNSTGCLR_5_4_0_2_0
138.0547
204.0862
366.1394
168.0652
274.0912
186.0753
292.103
126.0549
144.0652
657.2336
1147.5107 PEP_0_1_0_0_0
Intensity
m/z
0
20000
40000
60000
80000
100000
120000
140000
250
500
750
1000
1250
1500
1750
2000
2250

File: 231129_OE_RK_4, RT: 7.841, Scan: 005102
Type: HCD, FTMS + c NSI d Full ms2 1043.4081@hcd30.00 [120.0000-3214.9707]
Precursor Scan: 005036, m/z: 1043.076, charge: 3
ENGTISR_5_4_1_2_0

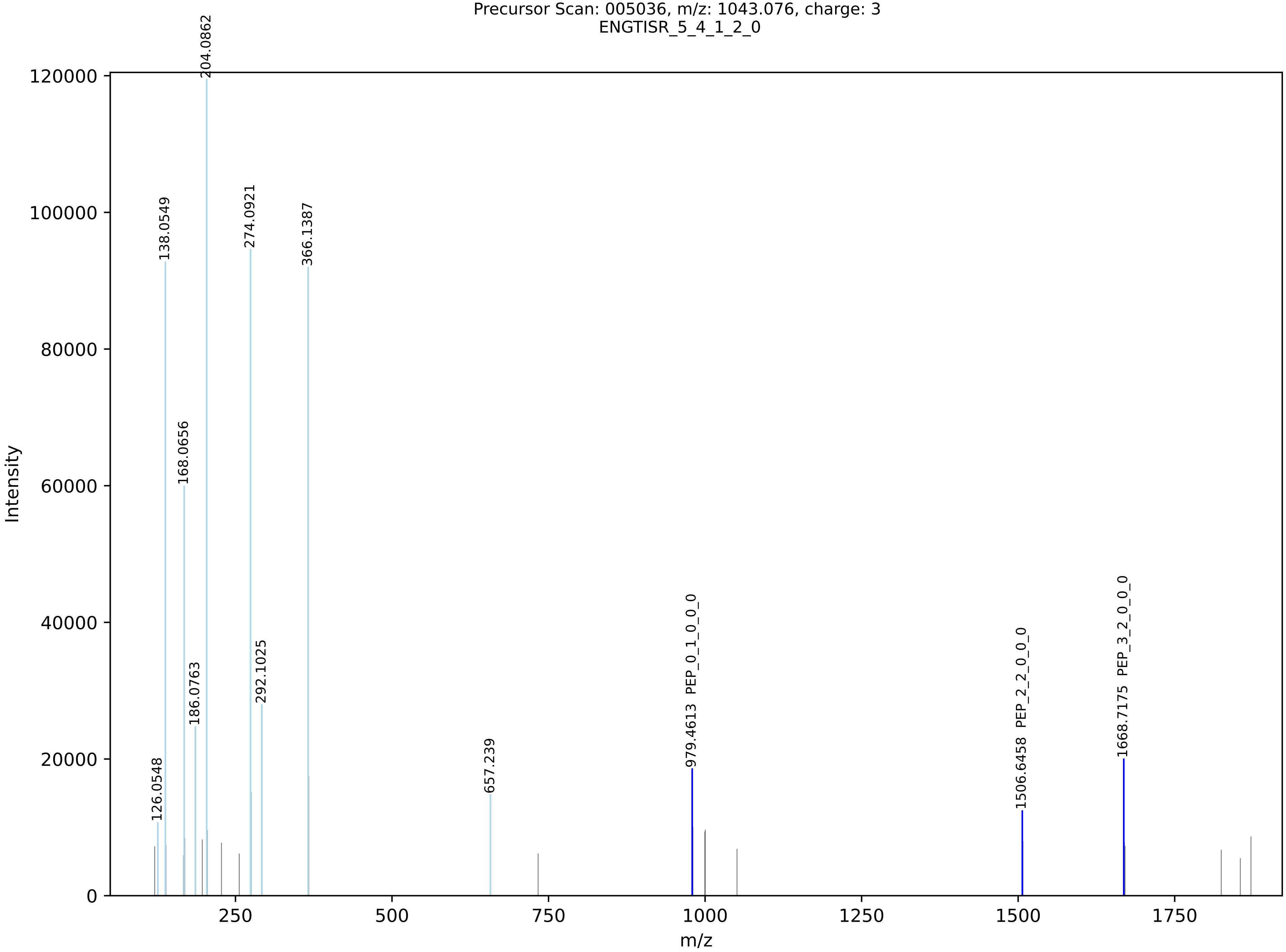

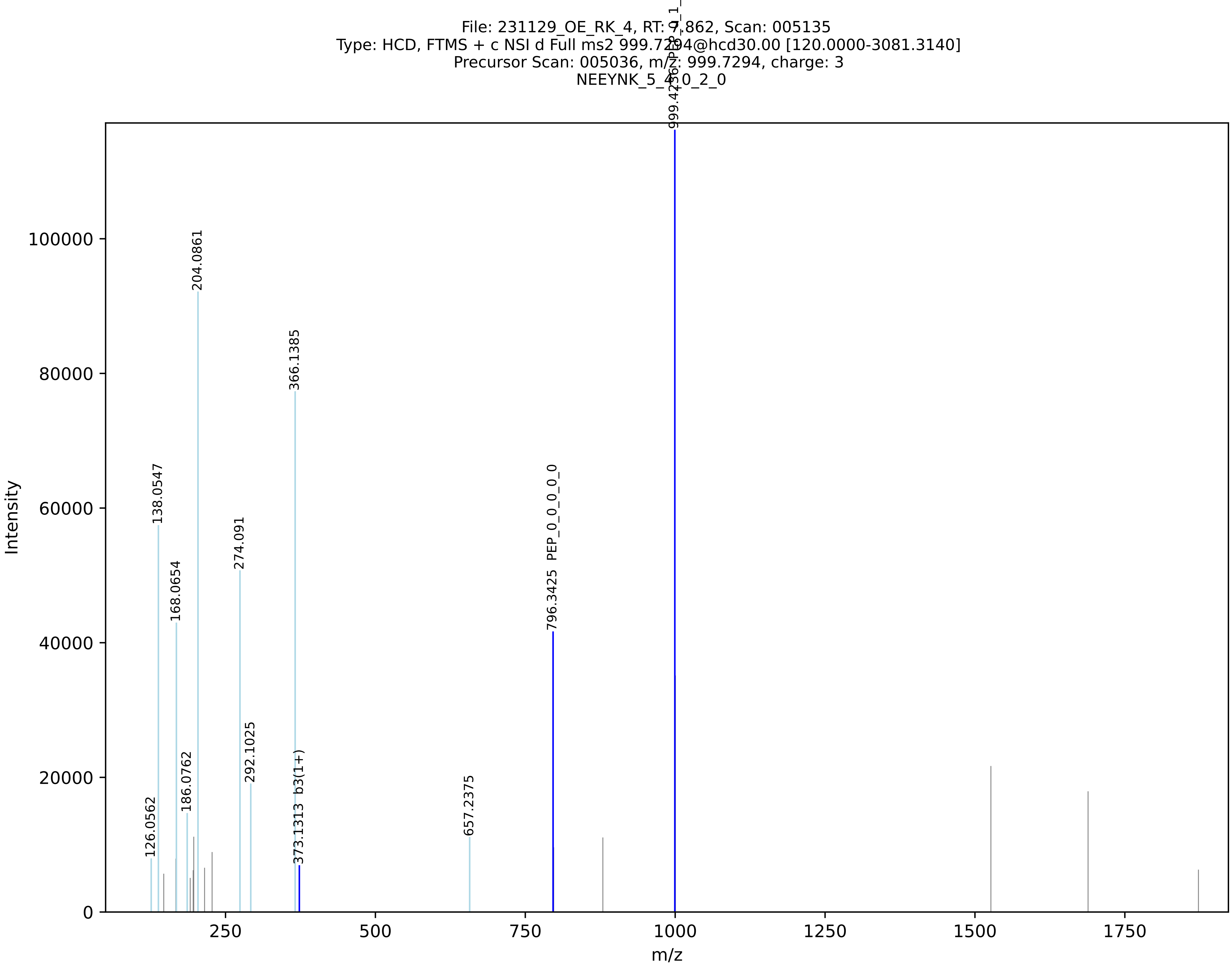

File: 231129_OE_RK_4, RT: 7.862, Scan: 005135
Type: HCD, FTMS + c NSI d Full ms2 999.7294@hcd30.00 [120.0000-3081.3140]
Precursor Scan: 005036, m/z: 999.7294, charge: 3
NEEYNK_5_4_0_2_0
204.0861
366.1385
138.0547
274.091
168.0654
796.3425 PEP_0_0_0_0_0
292.1025
186.0762
373.1313 b3(1+)
657.2375
126.0562
Intensity
m/z
0
20000
40000
60000
80000
100000
250
500
750
1000
1250
1500
1750

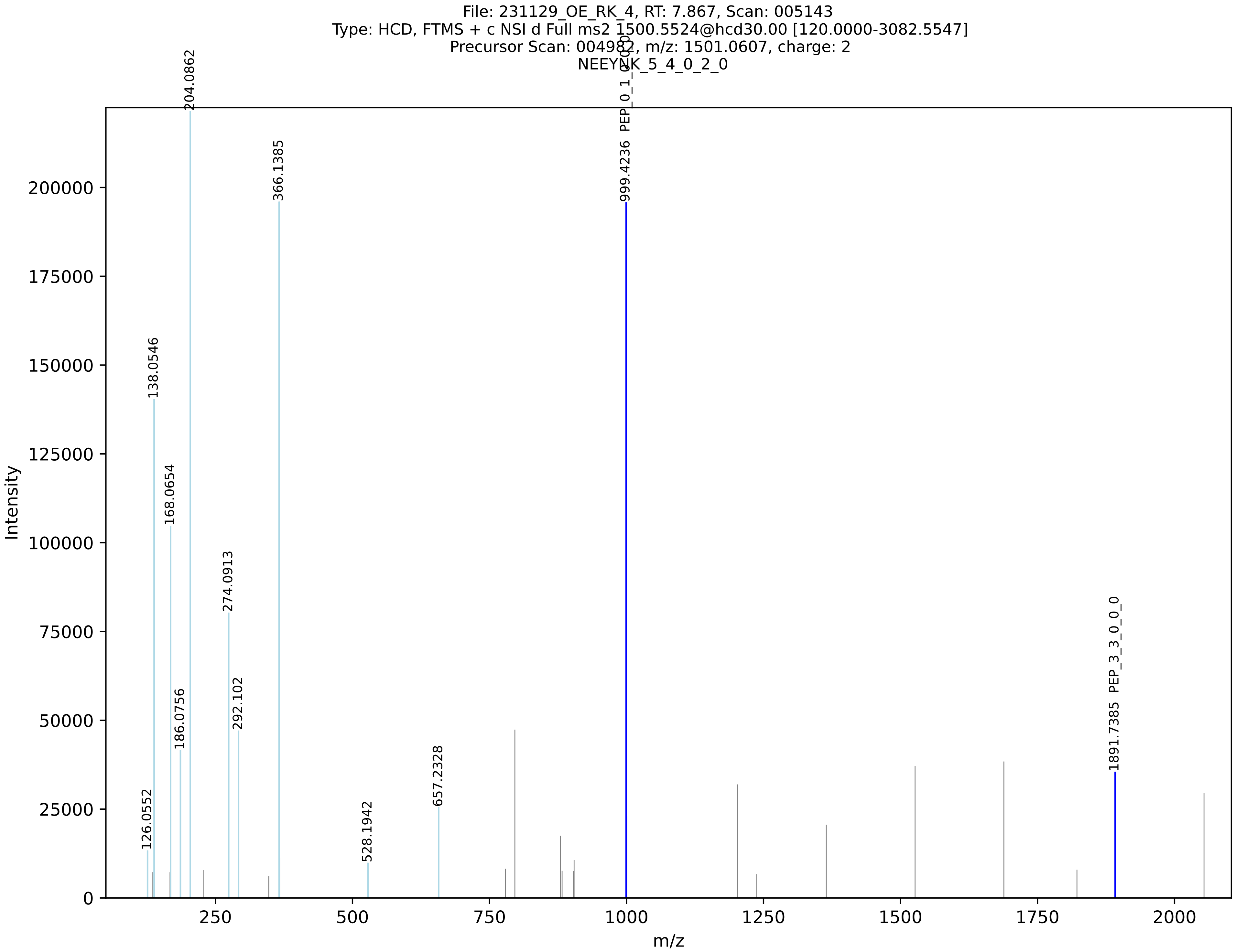
File: 231129_OE_RK_4, RT: 7.867, Scan: 005143
Type: HCD, FTMS + c NSI d Full ms2 1500.5524@hcd30.00 [120.0000-3082.5547]
Precursor Scan: 004982, m/z: 1501.0607, charge: 2
NEEYNK_5_4_0_2_0
Intensity
m/z
0
25000
50000
75000
100000
125000
150000
175000
200000
250
500
750
1000
1250
1500
1750
2000
204.0862
366.1385
999.4236 PEP_0_1_[illegible]
138.0546
168.0654
274.0913
292.102
186.0756
657.2328
528.1942
126.0552
1891.7385 PEP_3_3_0_0_0

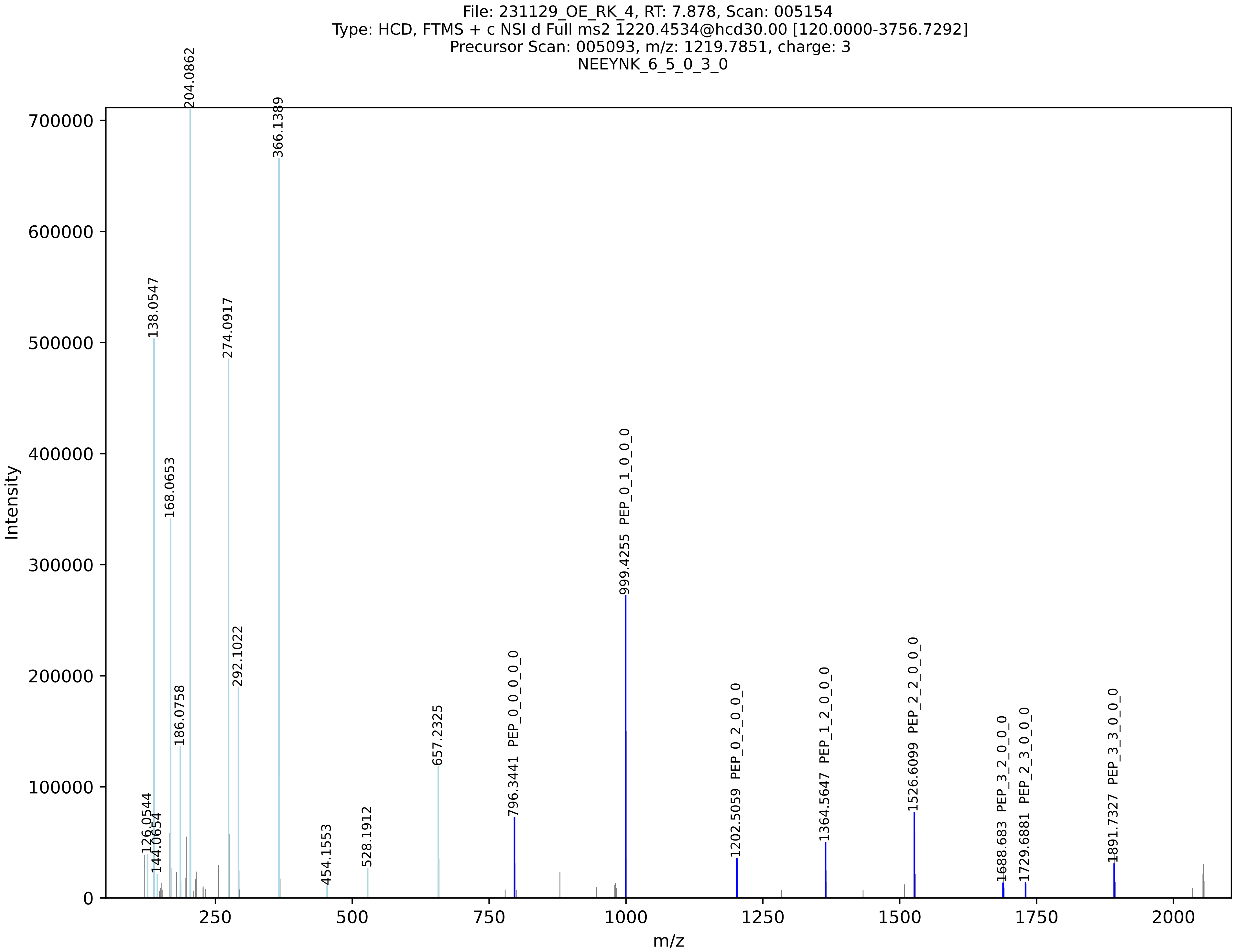
File: 231129_OE_RK_4, RT: 7.878, Scan: 005154
Type: HCD, FTMS + c NSI d Full ms2 1220.4534@hcd30.00 [120.0000-3756.7292]
Precursor Scan: 005093, m/z: 1219.7851, charge: 3
NEEYNK_6_5_0_3_0
Intensity
m/z
204.0862
366.1389
138.0547
274.0917
168.0653
292.1022
186.0758
126.0544
144.0654
454.1553
528.1912
657.2325
796.3441 PEP_0_0_0_0_0
999.4255 PEP_0_1_0_0_0
1202.5059 PEP_0_2_0_0_0
1364.5647 PEP_1_2_0_0_0
1526.6099 PEP_2_2_0_0_0
1688.683 PEP_3_2_0_0_0
1729.6881 PEP_2_3_0_0_0
1891.7327 PEP_3_3_0_0_0

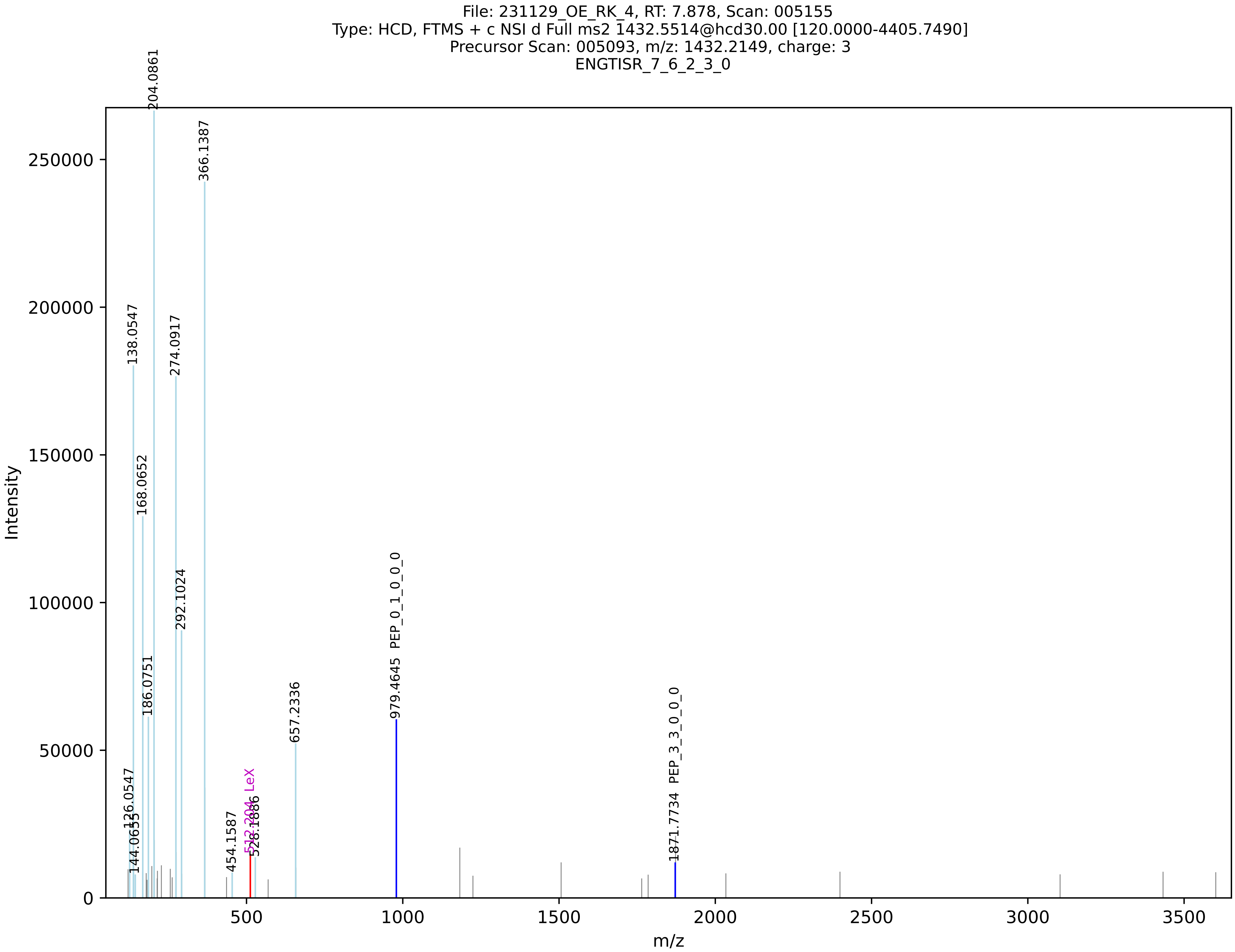
File: 231129_OE_RK_4, RT: 7.878, Scan: 005155
Type: HCD, FTMS + c NSI d Full ms2 1432.5514@hcd30.00 [120.0000-4405.7490]
Precursor Scan: 005093, m/z: 1432.2149, charge: 3
ENGTISR_7_6_2_3_0
Intensity
m/z
204.0861
366.1387
138.0547
274.0917
168.0652
292.1024
186.0751
126.0547
144.0655
454.1587
512.204 LeX
528.1886
657.2336
979.4645 PEP_0_1_0_0_0
1871.7734 PEP_3_3_0_0_0

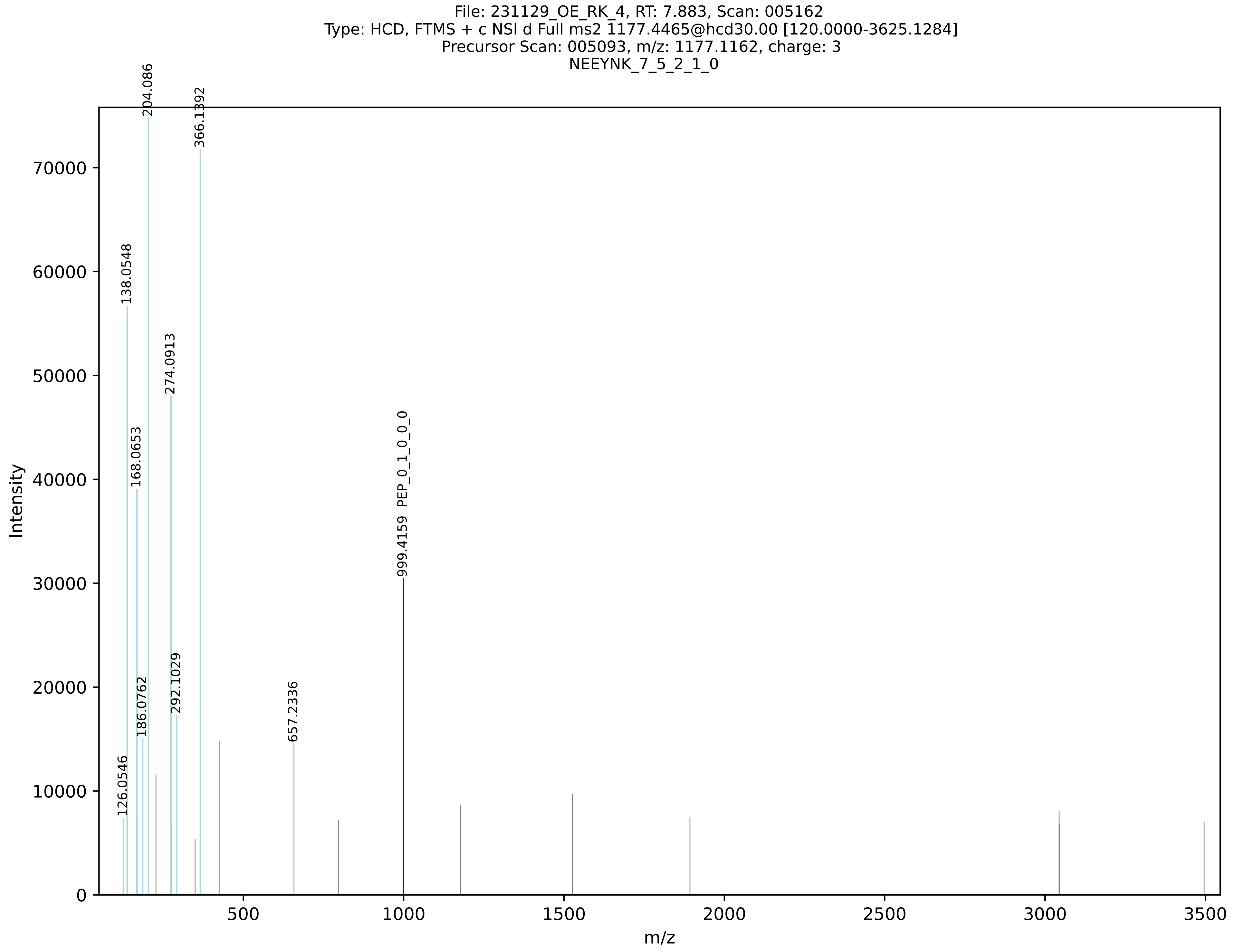

File: 231129_OE_RK_4, RT: 7.883, Scan: 005162
Type: HCD, FTMS + c NSI d Full ms2 1177.4465@hcd30.00 [120.0000-3625.1284]
Precursor Scan: 005093, m/z: 1177.1162, charge: 3
NEEYNK_7_5_2_1_0
Intensity
m/z
204.086
366.1392
138.0548
274.0913
168.0653
PEP_0_1_0_0_0
999.4159
292.1029
186.0762
657.2336
126.0546

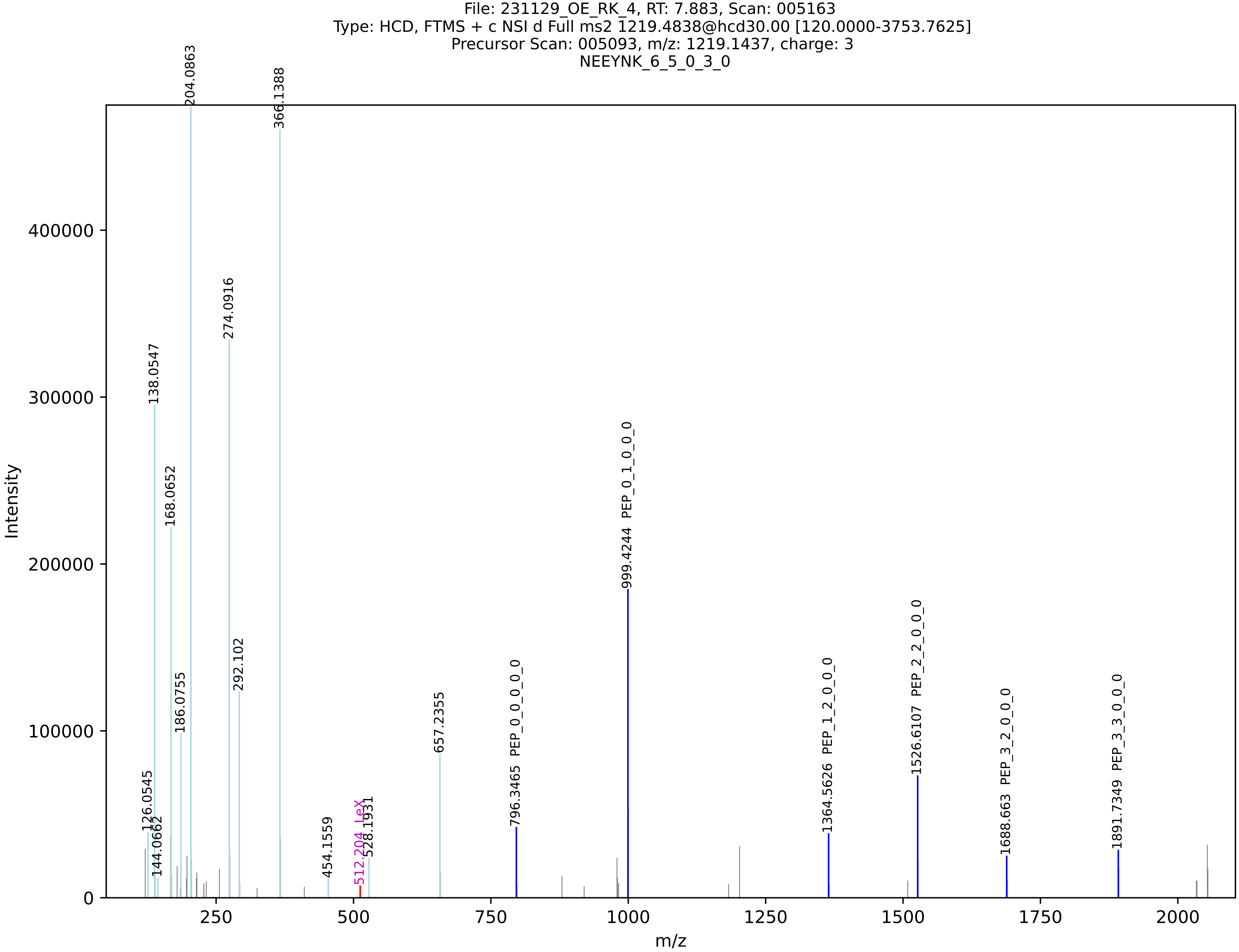

File: 231129_OE_RK_4, RT: 7.883, Scan: 005163
Type: HCD, FTMS + c NSI d Full ms2 1219.4838@hcd30.00 [120.0000-3753.7625]
Precursor Scan: 005093, m/z: 1219.1437, charge: 3
NEEYNK_6_5_0_3_0
Intensity
m/z
204.0863
366.1388
274.0916
138.0547
168.0652
292.102
186.0755
126.0545
144.0662
454.1559
512.204_LeX
528.1931
657.2355
796.3465 PEP_0_0_0_0_0
999.4244 PEP_0_1_0_0_0
1364.5626 PEP_1_2_0_0_0
1526.6107 PEP_2_2_0_0_0
1688.663 PEP_3_2_0_0_0
1891.7349 PEP_3_3_0_0_0
0
100000
200000
300000
400000
250
500
750
1000
1250
1500
1750
2000

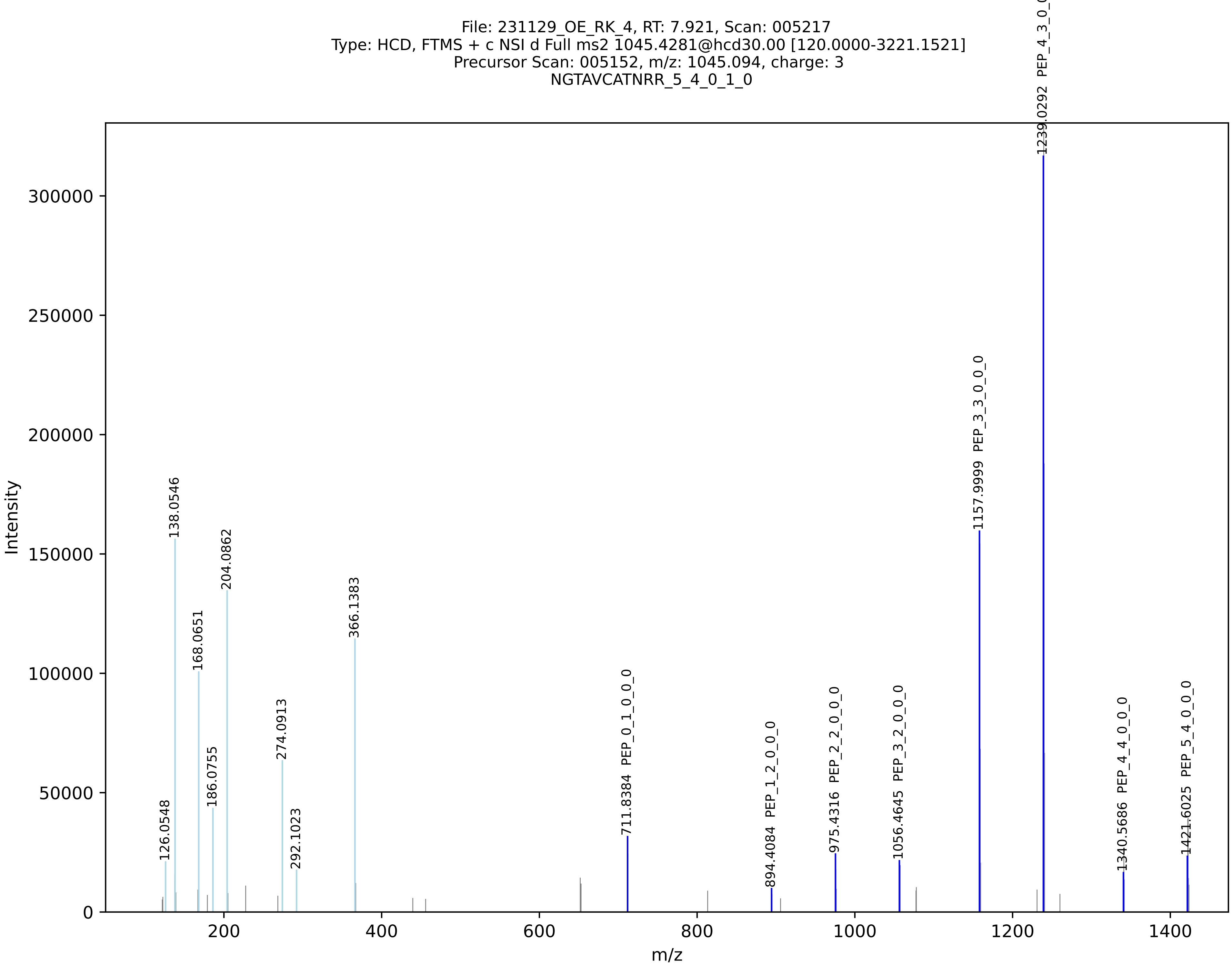
File: 231129_OE_RK_4, RT: 7.921, Scan: 005217
Type: HCD, FTMS + c NSI d Full ms2 1045.4281@hcd30.00 [120.0000-3221.1521]
Precursor Scan: 005152, m/z: 1045.094, charge: 3
NGTAVCATNRR_5_4_0_1_0
Intensity
m/z
0
50000
100000
150000
200000
250000
300000
200
400
600
800
1000
1200
1400
126.0548
138.0546
168.0651
186.0755
204.0862
274.0913
292.1023
366.1383
711.8384 PEP_0_1_0_0_0
894.4084 PEP_1_2_0_0_0
975.4316 PEP_2_2_0_0_0
1056.4645 PEP_3_2_0_0_0
1157.9999 PEP_3_3_0_0_0
1239.0292 PEP_4_3_0_0
1340.5686 PEP_4_4_0_0_0
1421.6025 PEP_5_4_0_0_0

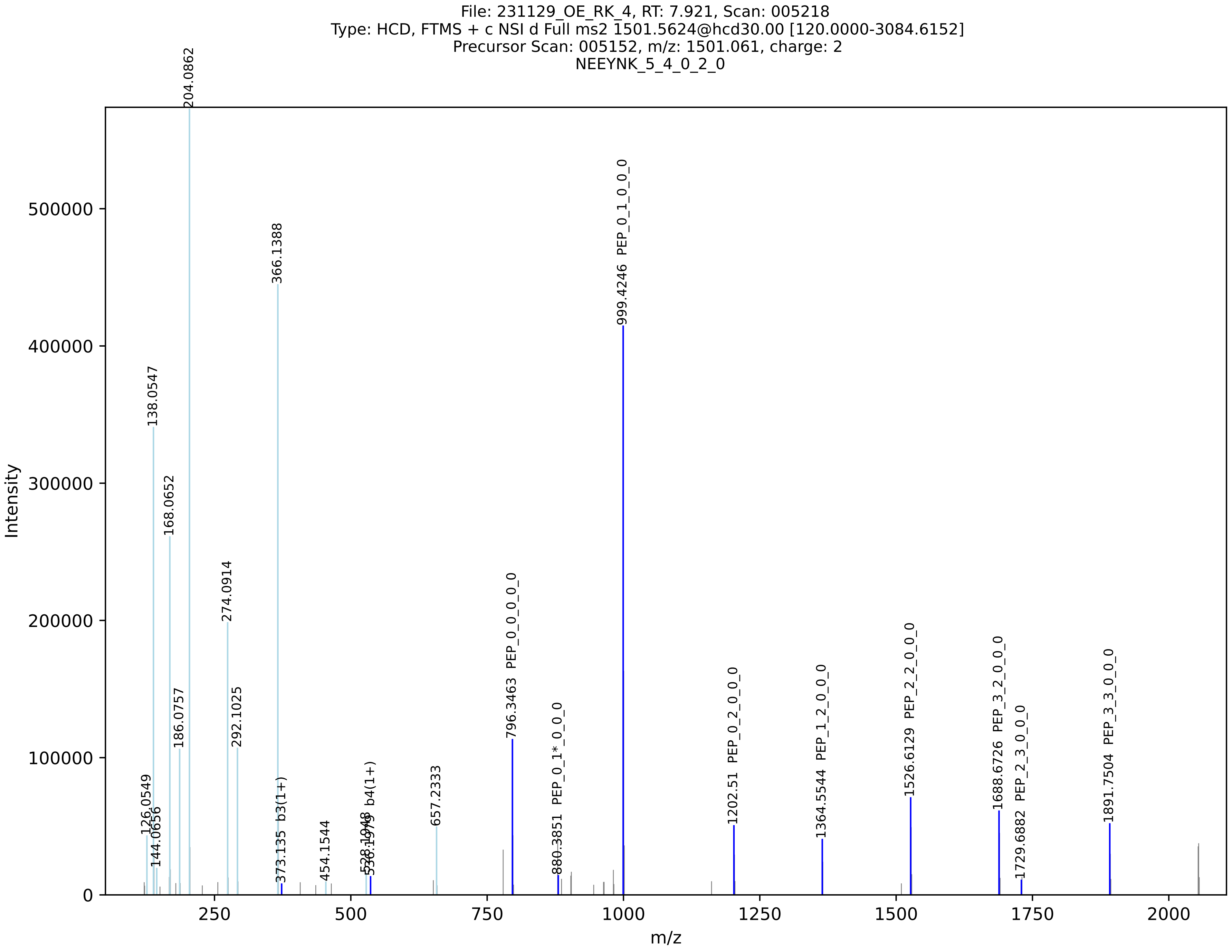

File: 231129_OE_RK_4, RT: 7.921, Scan: 005218
Type: HCD, FTMS + c NSI d Full ms2 1501.5624@hcd30.00 [120.0000-3084.6152]
Precursor Scan: 005152, m/z: 1501.061, charge: 2
NEEYNK_5_4_0_2_0
Intensity
m/z
204.0862
366.1388
138.0547
168.0652
274.0914
292.1025
186.0757
126.0549
144.0656
373.135 b3(1+)
454.1544
528.1048
536.1979 b4(1+)
657.2333
796.3463 PEP_0_0_0_0_0
880.3851 PEP_0_1*_0_0_0
999.4246 PEP_0_1_0_0_0
1202.51 PEP_0_2_0_0_0
1364.5544 PEP_1_2_0_0_0
1526.6129 PEP_2_2_0_0_0
1688.6726 PEP_3_2_0_0_0
1729.6882 PEP_2_3_0_0_0
1891.7504 PEP_3_3_0_0_0

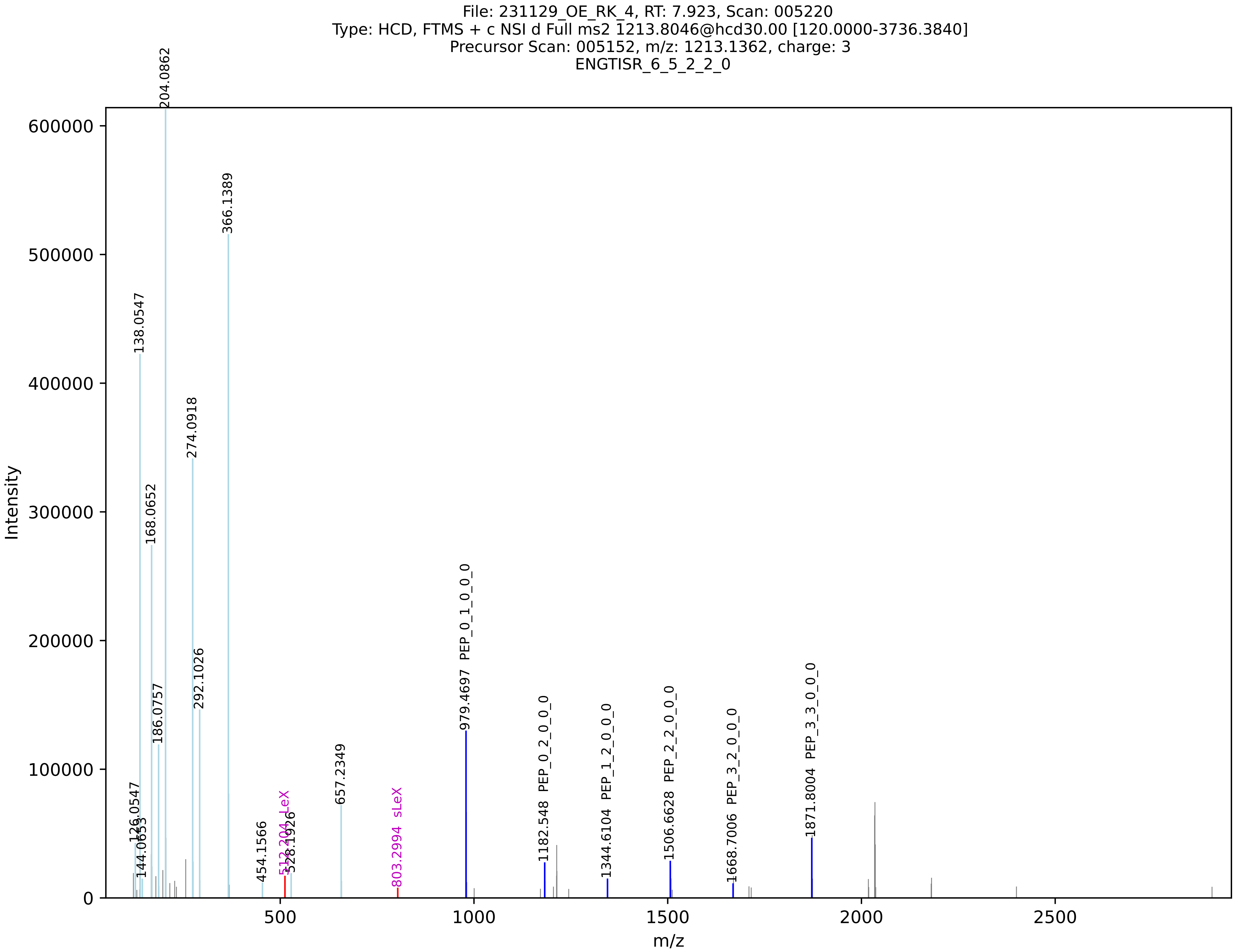
File: 231129_OE_RK_4, RT: 7.923, Scan: 005220
Type: HCD, FTMS + c NSI d Full ms2 1213.8046@hcd30.00 [120.0000-3736.3840]
Precursor Scan: 005152, m/z: 1213.1362, charge: 3
ENGTISR_6_5_2_2_0
Intensity
m/z
204.0862
366.1389
138.0547
274.0918
168.0652
292.1026
186.0757
126.0547
144.0653
454.1566
512.204 LeX
528.1926
657.2349
803.2994 sLeX
979.4697 PEP_0_1_0_0_0
1182.548 PEP_0_2_0_0_0
1344.6104 PEP_1_2_0_0_0
1506.6628 PEP_2_2_0_0_0
1668.7006 PEP_3_2_0_0_0
1871.8004 PEP_3_3_0_0_0

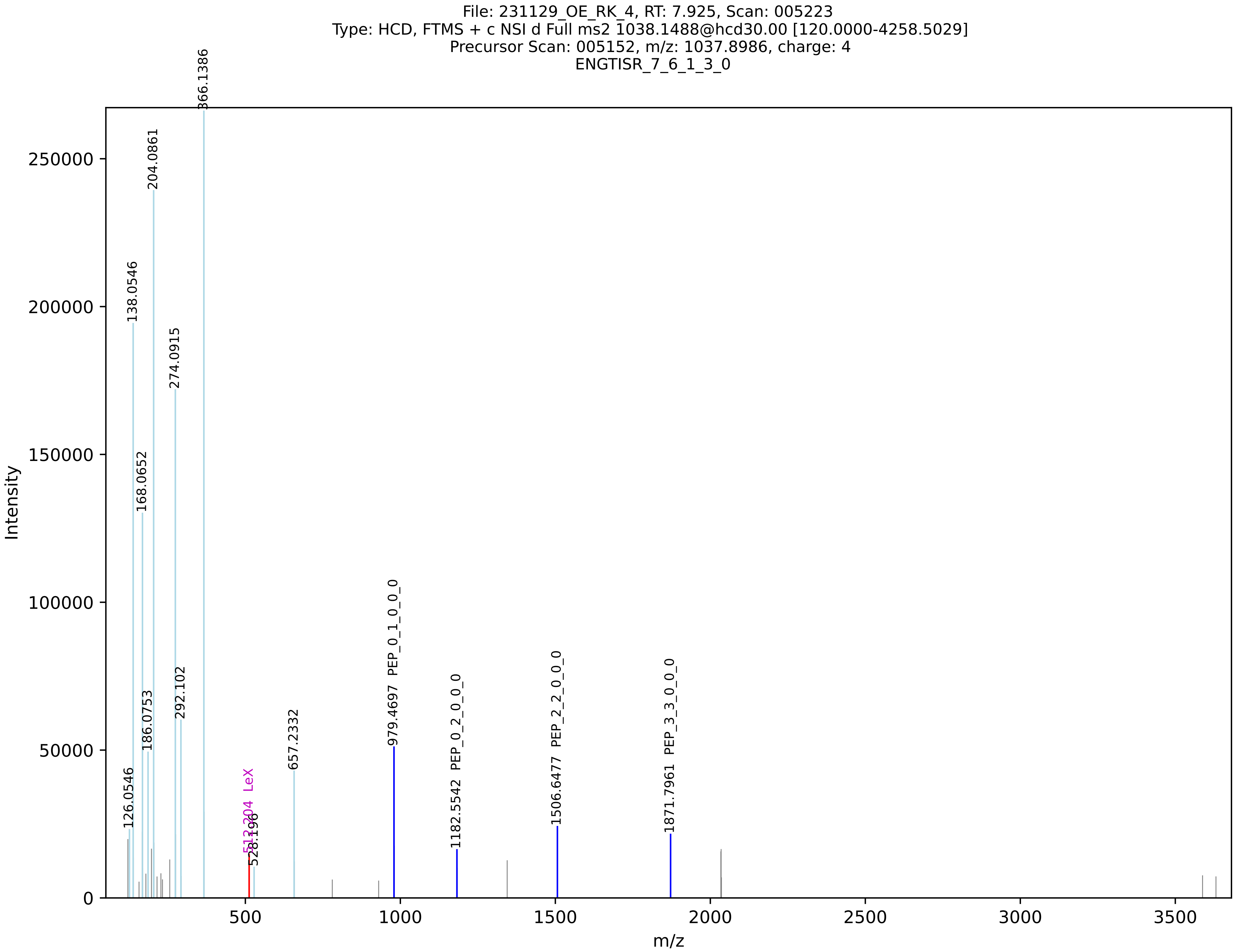
File: 231129_OE_RK_4, RT: 7.925, Scan: 005223
Type: HCD, FTMS + c NSI d Full ms2 1038.1488@hcd30.00 [120.0000-4258.5029]
Precursor Scan: 005152, m/z: 1037.8986, charge: 4
ENGTISR_7_6_1_3_0
Intensity
m/z
366.1386
204.0861
138.0546
274.0915
168.0652
186.0753
292.102
126.0546
512.204 LeX
528.196
657.2332
979.4697 PEP_0_1_0_0_0
1182.5542 PEP_0_2_0_0_0
1506.6477 PEP_2_2_0_0_0
1871.7961 PEP_3_3_0_0_0
0
50000
100000
150000
200000
250000
500
1000
1500
2000
2500
3000
3500

File: 231129_OE_RK_4, RT: 7.926, Scan: 005225
Type: HCD, FTMS + c NSI d Full ms2 1074.6630@hcd30.00 [120.0000-4407.4810]
Precursor Scan: 005152, m/z: 1074.4133, charge: 4
ENGTISR_7_6_2_3_0

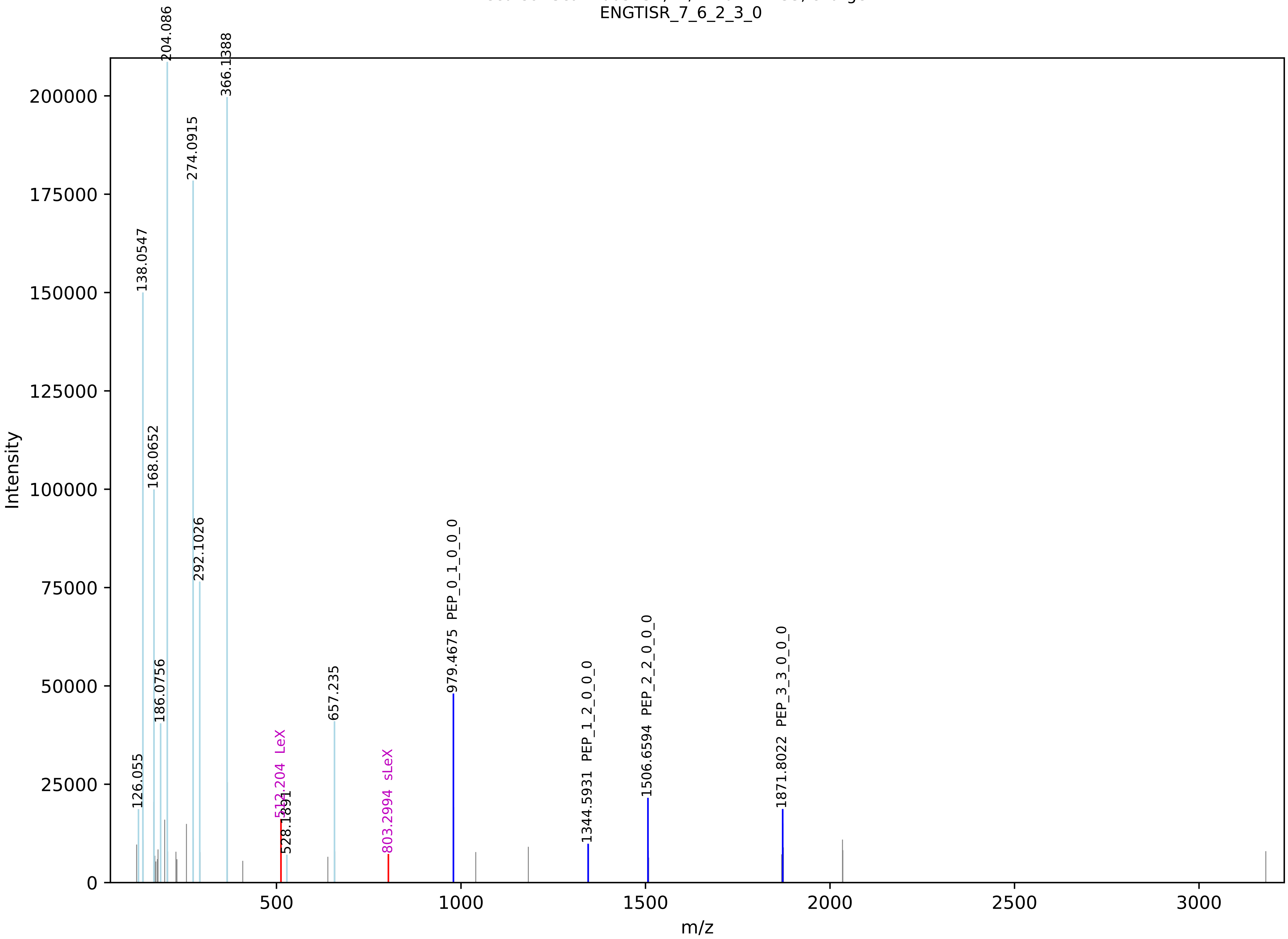

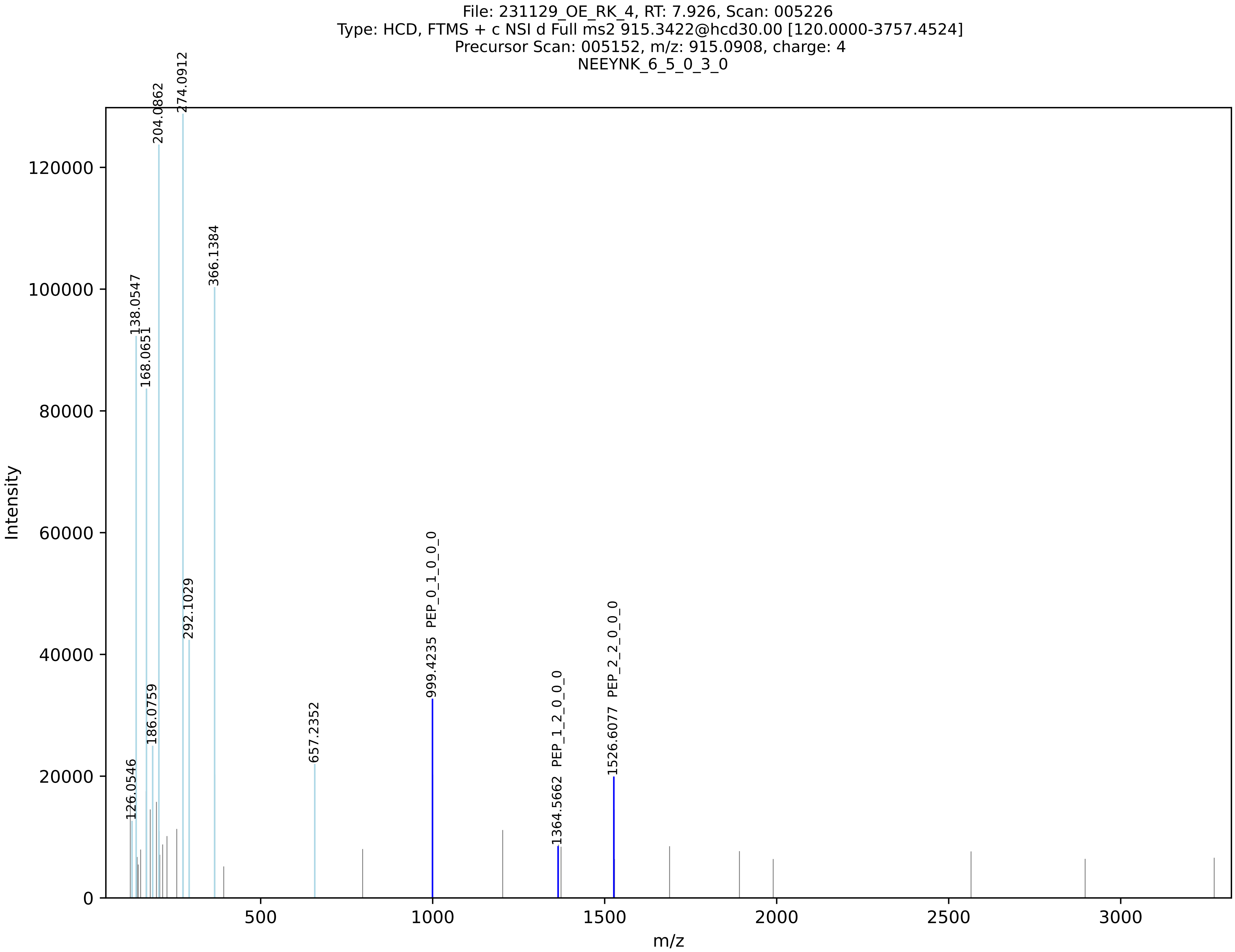
File: 231129_OE_RK_4, RT: 7.926, Scan: 005226
Type: HCD, FTMS + c NSI d Full ms2 915.3422@hcd30.00 [120.0000-3757.4524]
Precursor Scan: 005152, m/z: 915.0908, charge: 4
NEEYNK_6_5_0_3_0
Intensity
m/z
274.0912
204.0862
366.1384
138.0547
168.0651
292.1029
186.0759
126.0546
657.2352
999.4235 PEP_0_1_0_0_0
1364.5662 PEP_1_2_0_0_0
1526.6077 PEP_2_2_0_0_0
0
20000
40000
60000
80000
100000
120000
500
1000
1500
2000
2500
3000

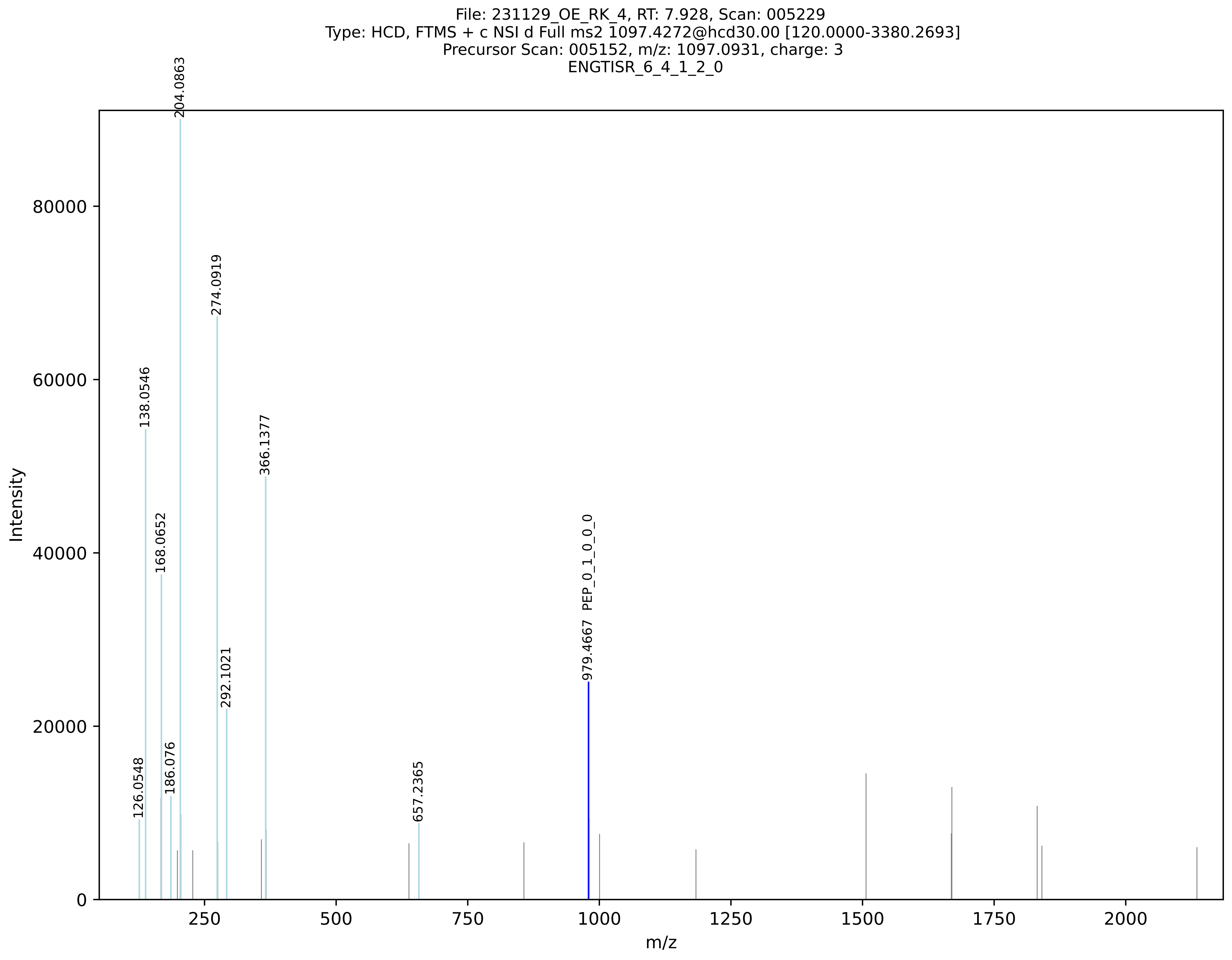
File: 231129_OE_RK_4, RT: 7.928, Scan: 005229
Type: HCD, FTMS + c NSI d Full ms2 1097.4272@hcd30.00 [120.0000-3380.2693]
Precursor Scan: 005152, m/z: 1097.0931, charge: 3
ENGTISR_6_4_1_2_0
204.0863
274.0919
138.0546
366.1377
168.0652
979.4667 PEP_0_1_0_0_0
292.1021
186.076
126.0548
657.2365
Intensity
80000
60000
40000
20000
0
250
500
750
1000
1250
1500
1750
2000
m/z

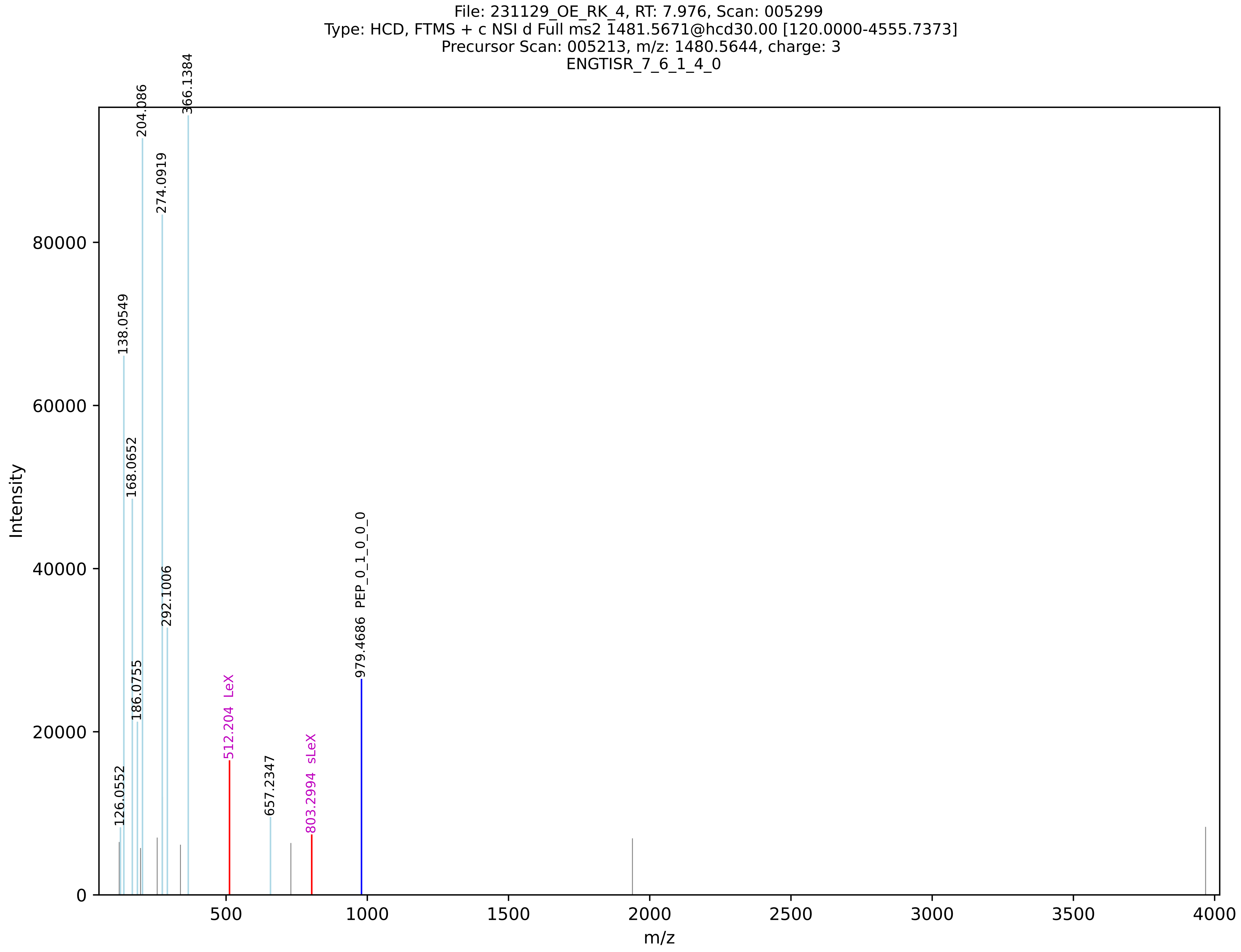
File: 231129_OE_RK_4, RT: 7.976, Scan: 005299
Type: HCD, FTMS + c NSI d Full ms2 1481.5671@hcd30.00 [120.0000-4555.7373]
Precursor Scan: 005213, m/z: 1480.5644, charge: 3
ENGTISR_7_6_1_4_0
Intensity
m/z
366.1384
204.086
274.0919
138.0549
168.0652
292.1006
186.0755
126.0552
512.204 LeX
657.2347
803.2994 sLeX
979.4686 PEP_0_1_0_0_0
0
20000
40000
60000
80000
500
1000
1500
2000
2500
3000
3500
4000

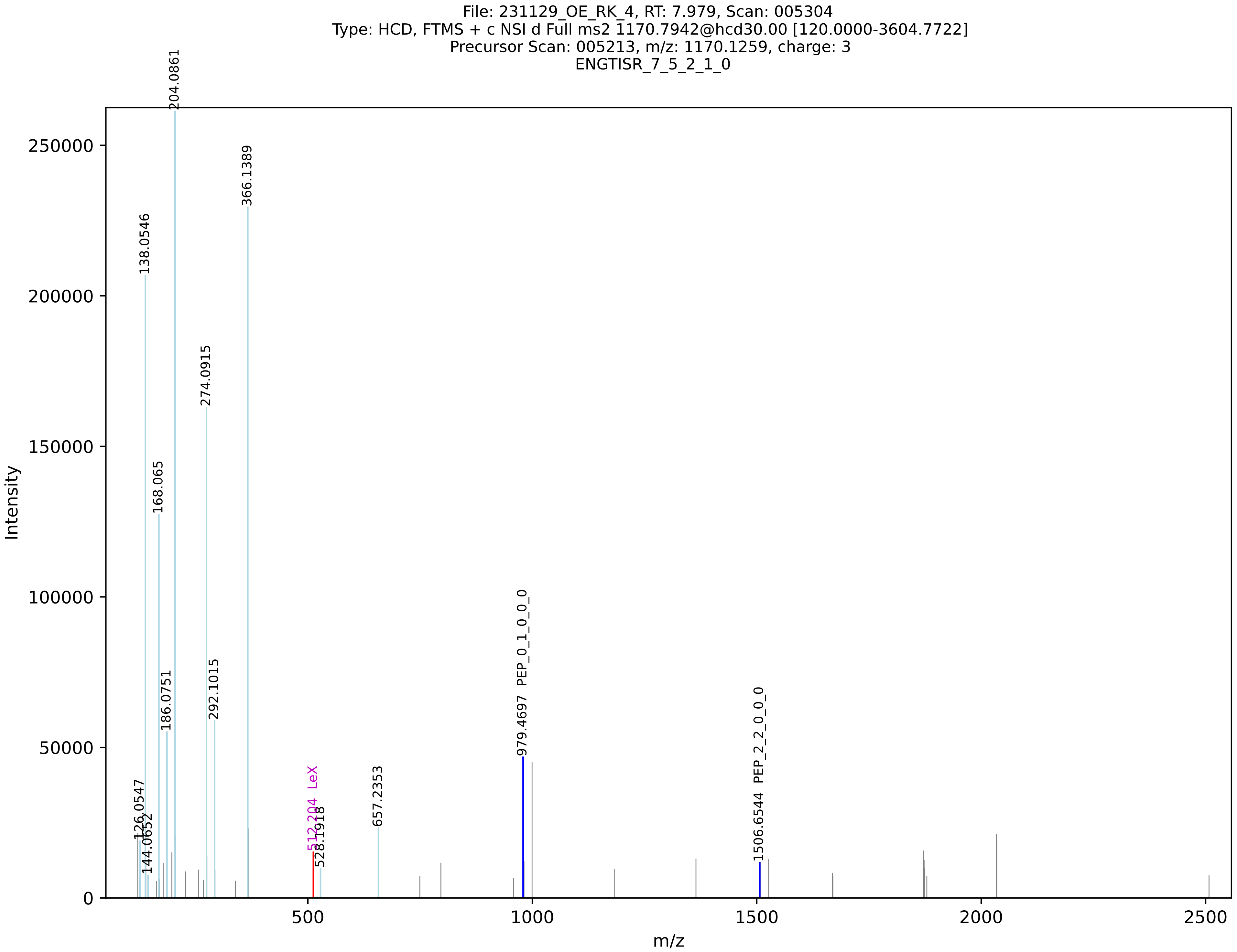
File: 231129_OE_RK_4, RT: 7.979, Scan: 005304
Type: HCD, FTMS + c NSI d Full ms2 1170.7942@hcd30.00 [120.0000-3604.7722]
Precursor Scan: 005213, m/z: 1170.1259, charge: 3
ENGTISR_7_5_2_1_0
Intensity
m/z
204.0861
366.1389
138.0546
274.0915
168.065
186.0751
292.1015
126.0547
144.0652
512.204 LeX
528.1918
657.2353
979.4697 PEP_0_1_0_0_0
1506.6544 PEP_2_2_0_0_0
0
50000
100000
150000
200000
250000
500
1000
1500
2000
2500

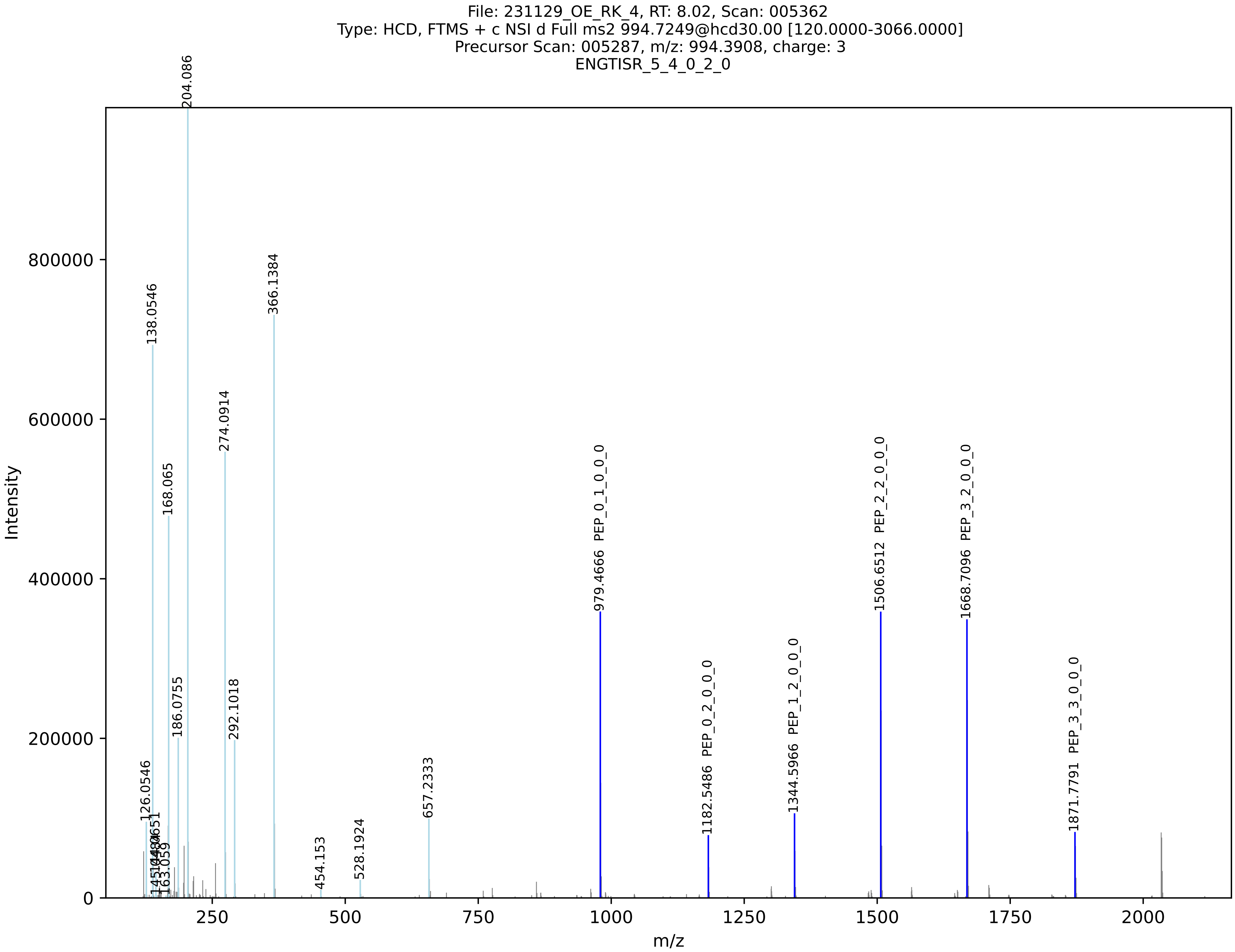
File: 231129_OE_RK_4, RT: 8.02, Scan: 005362
Type: HCD, FTMS + c NSI d Full ms2 994.7249@hcd30.00 [120.0000-3066.0000]
Precursor Scan: 005287, m/z: 994.3908, charge: 3
ENGTISR_5_4_0_2_0
Intensity
m/z
0
200000
400000
600000
800000
250
500
750
1000
1250
1500
1750
2000
204.086
138.0546
366.1384
274.0914
168.065
186.0755
292.1018
126.0546
163.059
454.153
528.1924
657.2333
979.4666 PEP_0_1_0_0_0
1182.5486 PEP_0_2_0_0_0
1344.5966 PEP_1_2_0_0_0
1506.6512 PEP_2_2_0_0_0
1668.7096 PEP_3_2_0_0_0
1871.7791 PEP_3_3_0_0_0

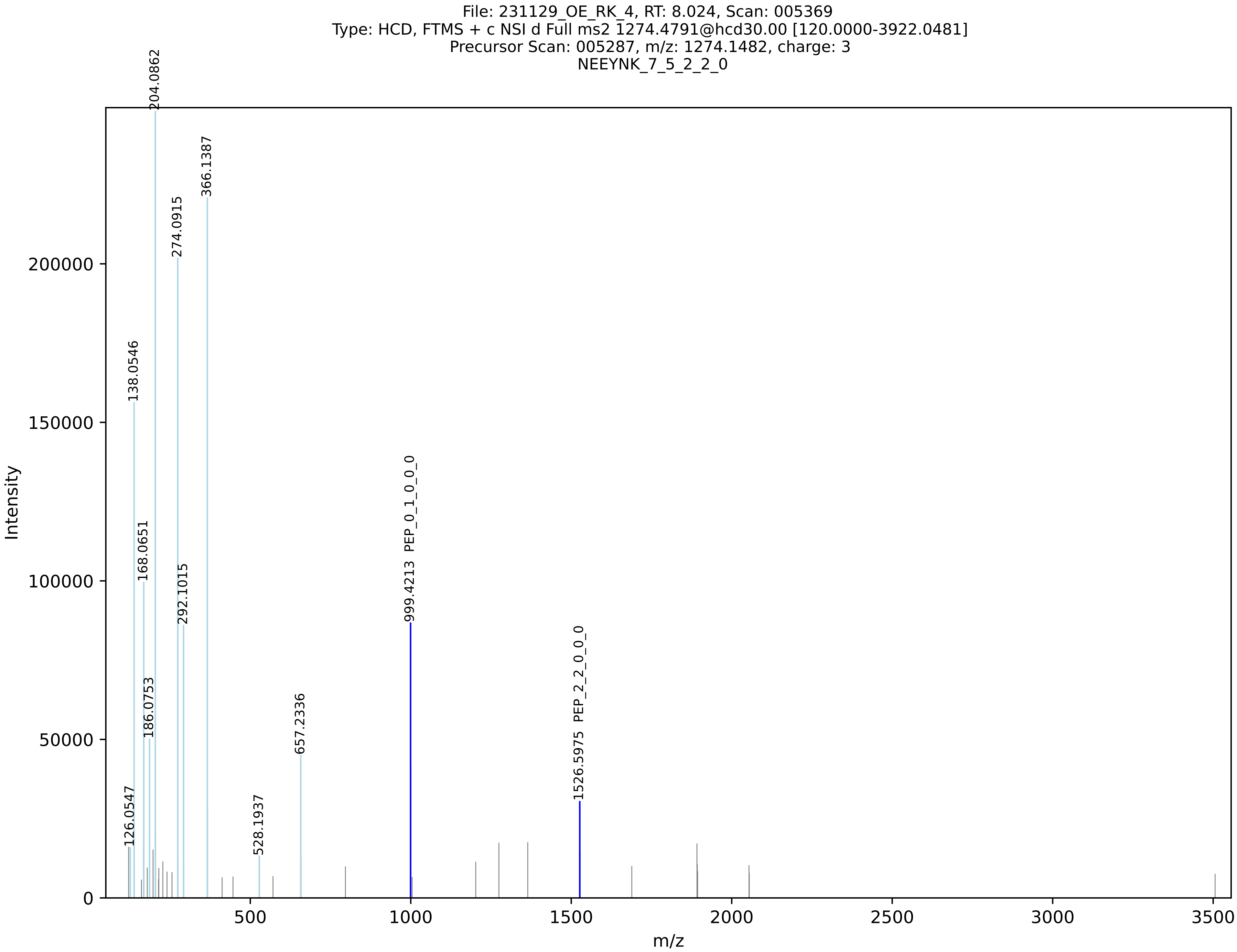
File: 231129_OE_RK_4, RT: 8.024, Scan: 005369
Type: HCD, FTMS + c NSI d Full ms2 1274.4791@hcd30.00 [120.0000-3922.0481]
Precursor Scan: 005287, m/z: 1274.1482, charge: 3
NEEYNK_7_5_2_2_0
Intensity
m/z
204.0862
366.1387
274.0915
138.0546
168.0651
292.1015
186.0753
126.0547
528.1937
657.2336
999.4213 PEP_0_1_0_0_0
1526.5975 PEP_2_2_0_0_0
0
50000
100000
150000
200000
500
1000
1500
2000
2500
3000
3500

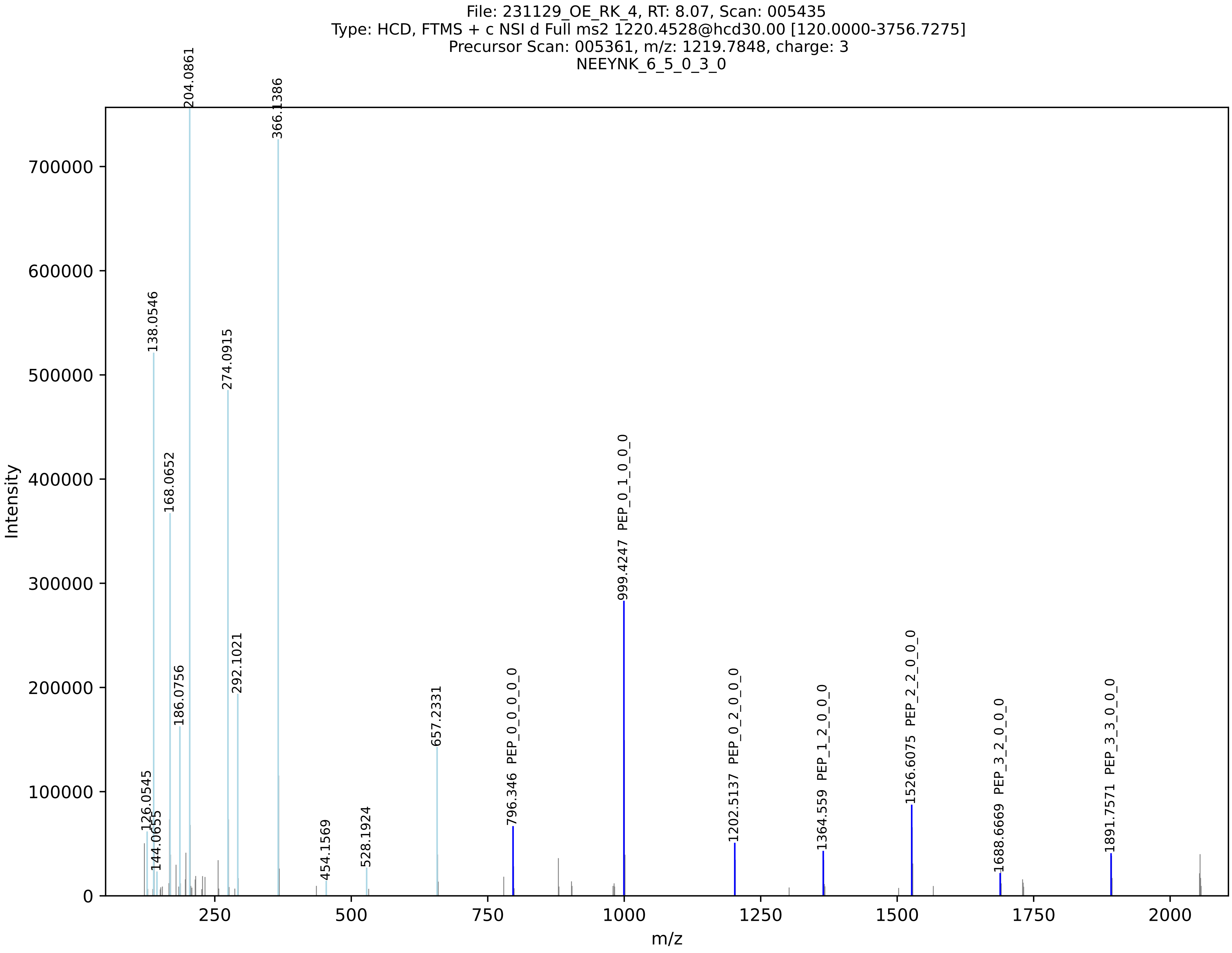
File: 231129_OE_RK_4, RT: 8.07, Scan: 005435
Type: HCD, FTMS + c NSI d Full ms2 1220.4528@hcd30.00 [120.0000-3756.7275]
Precursor Scan: 005361, m/z: 1219.7848, charge: 3
NEEYNK_6_5_0_3_0
Intensity
m/z
0
100000
200000
300000
400000
500000
600000
700000
250
500
750
1000
1250
1500
1750
2000
204.0861
366.1386
138.0546
274.0915
168.0652
292.1021
186.0756
126.0545
144.0655
454.1569
528.1924
657.2331
796.346 PEP_0_0_0_0_0
999.4247 PEP_0_1_0_0_0
1202.5137 PEP_0_2_0_0_0
1364.559 PEP_1_2_0_0_0
1526.6075 PEP_2_2_0_0_0
1688.6669 PEP_3_2_0_0_0
1891.7571 PEP_3_3_0_0_0

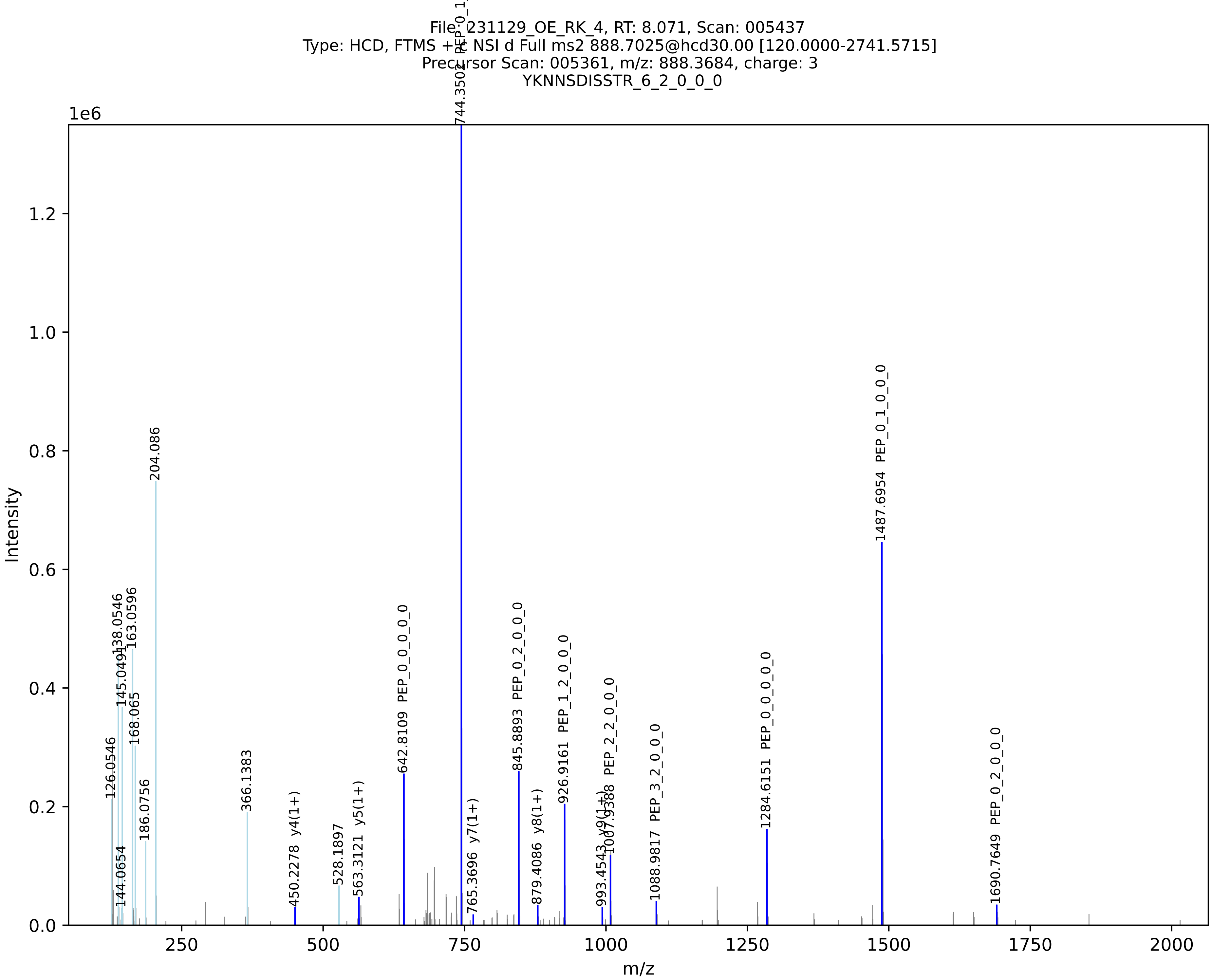

File: 231129_OE_RK_4, RT: 8.071, Scan: 005437
Type: HCD, FTMS + p NSI d Full ms2 888.7025@hcd30.00 [120.0000-2741.5715]
Precursor Scan: 005361, m/z: 888.3684, charge: 3
YKNNSDISSTR_6_2_0_0_0
1e6
Intensity
m/z
744.3502 PEP_0_1
1487.6954 PEP_0_1_0_0_0
204.086
138.0546
163.0596
145.0491
168.065
126.0546
186.0756
144.0654
366.1383
450.2278 y4(1+)
528.1897
563.3121 y5(1+)
642.8109 PEP_0_0_0_0_0
765.3696 y7(1+)
845.8893 PEP_0_2_0_0_0
879.4086 y8(1+)
926.9161 PEP_1_2_0_0_0
993.4543 y9(1+)
1007.9388 PEP_2_2_0_0_0
1088.9817 PEP_3_2_0_0_0
1284.6151 PEP_0_0_0_0_0
1690.7649 PEP_0_2_0_0_0

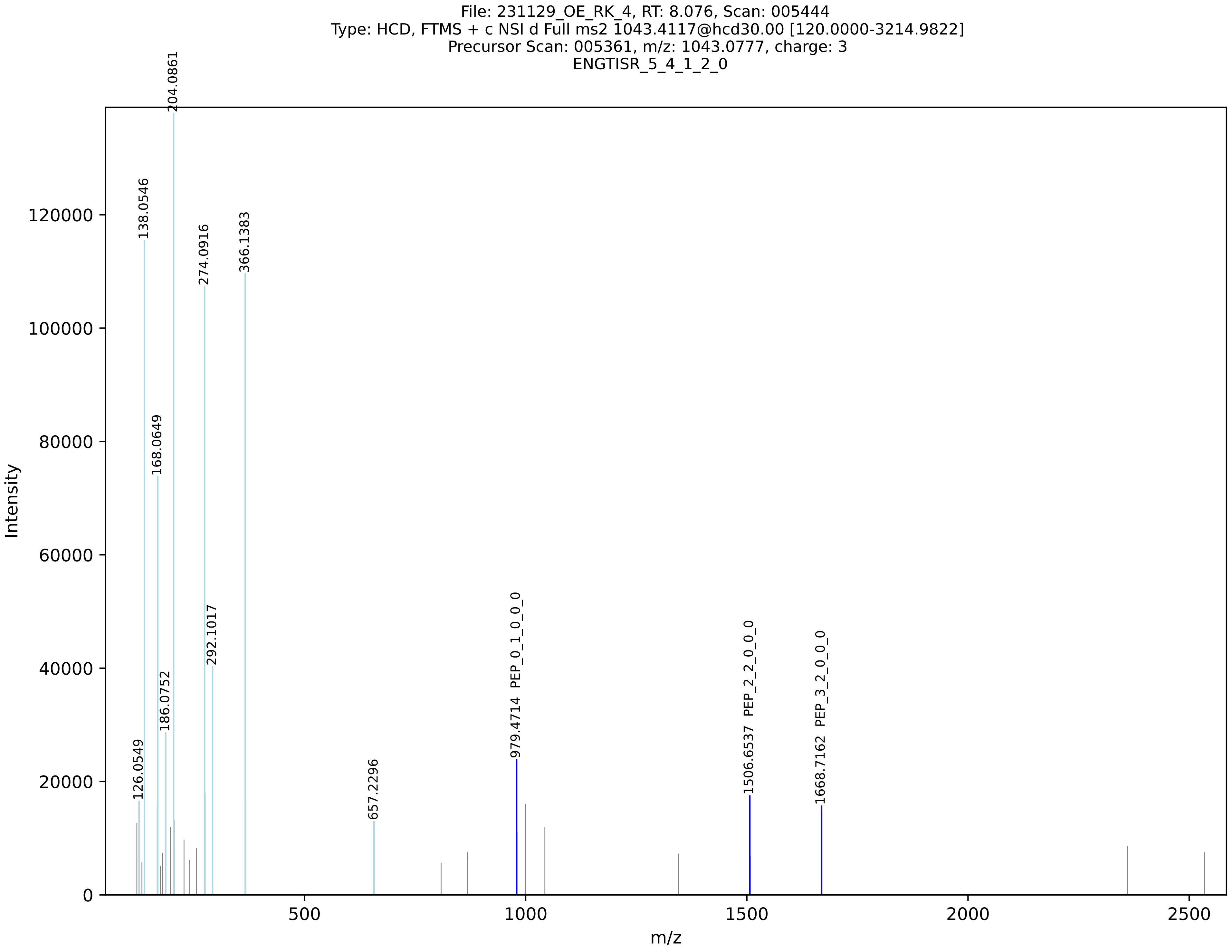

File: 231129_OE_RK_4, RT: 8.076, Scan: 005444
Type: HCD, FTMS + c NSI d Full ms2 1043.4117@hcd30.00 [120.0000-3214.9822]
Precursor Scan: 005361, m/z: 1043.0777, charge: 3
ENGTISR_5_4_1_2_0
204.0861
138.0546
274.0916
366.1383
168.0649
292.1017
186.0752
126.0549
657.2296
979.4714 PEP_0_1_0_0_0
1506.6537 PEP_2_2_0_0_0
1668.7162 PEP_3_2_0_0_0
Intensity
m/z
0
20000
40000
60000
80000
100000
120000
500
1000
1500
2000
2500

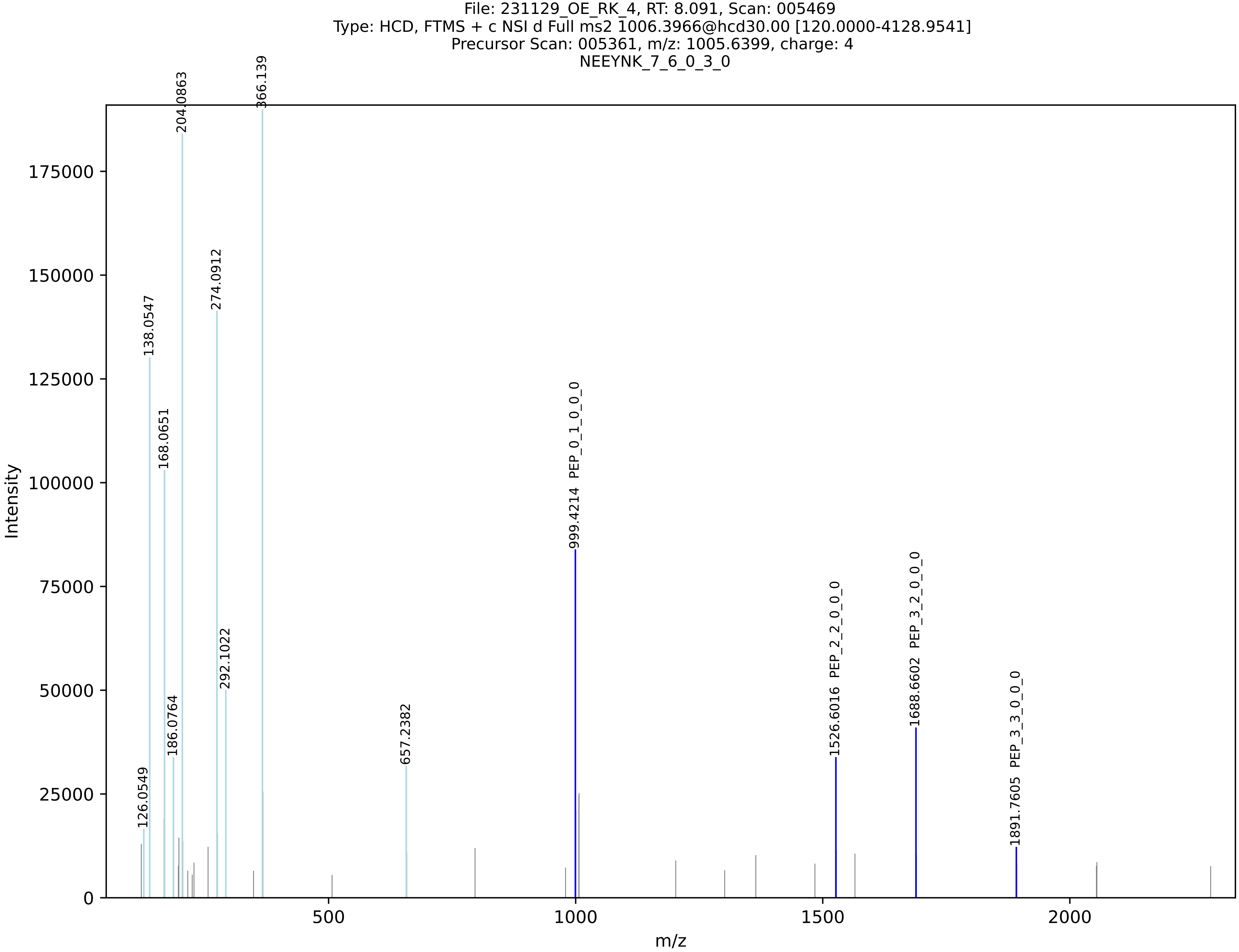

File: 231129_OE_RK_4, RT: 8.091, Scan: 005469
Type: HCD, FTMS + c NSI d Full ms2 1006.3966@hcd30.00 [120.0000-4128.9541]
Precursor Scan: 005361, m/z: 1005.6399, charge: 4
NEEYNK_7_6_0_3_0
Intensity
m/z
0
25000
50000
75000
100000
125000
150000
175000
500
1000
1500
2000
126.0549
138.0547
168.0651
186.0764
204.0863
274.0912
292.1022
366.139
657.2382
999.4214 PEP_0_1_0_0_0
1526.6016 PEP_2_2_0_0_0
1688.6602 PEP_3_2_0_0_0
1891.7605 PEP_3_3_0_0_0

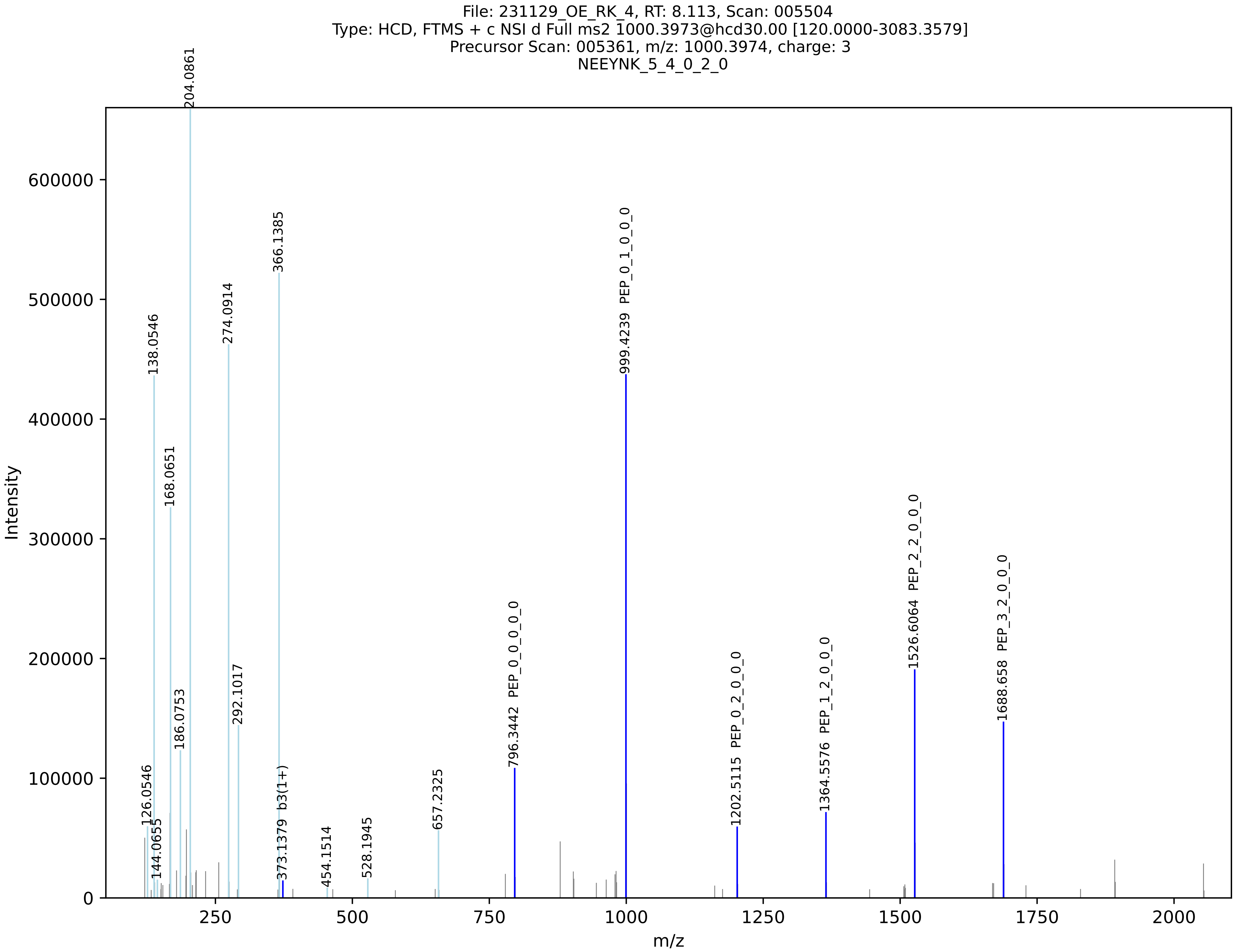

File: 231129_OE_RK_4, RT: 8.113, Scan: 005504
Type: HCD, FTMS + c NSI d Full ms2 1000.3973@hcd30.00 [120.0000-3083.3579]
Precursor Scan: 005361, m/z: 1000.3974, charge: 3
NEEYNK_5_4_0_2_0
Intensity
m/z
204.0861
366.1385
274.0914
138.0546
168.0651
186.0753
292.1017
126.0546
144.0655
373.1379 b3(1+)
454.1514
528.1945
657.2325
796.3442 PEP_0_0_0_0_0
999.4239 PEP_0_1_0_0_0
1202.5115 PEP_0_2_0_0_0
1364.5576 PEP_1_2_0_0_0
1526.6064 PEP_2_2_0_0_0
1688.658 PEP_3_2_0_0_0

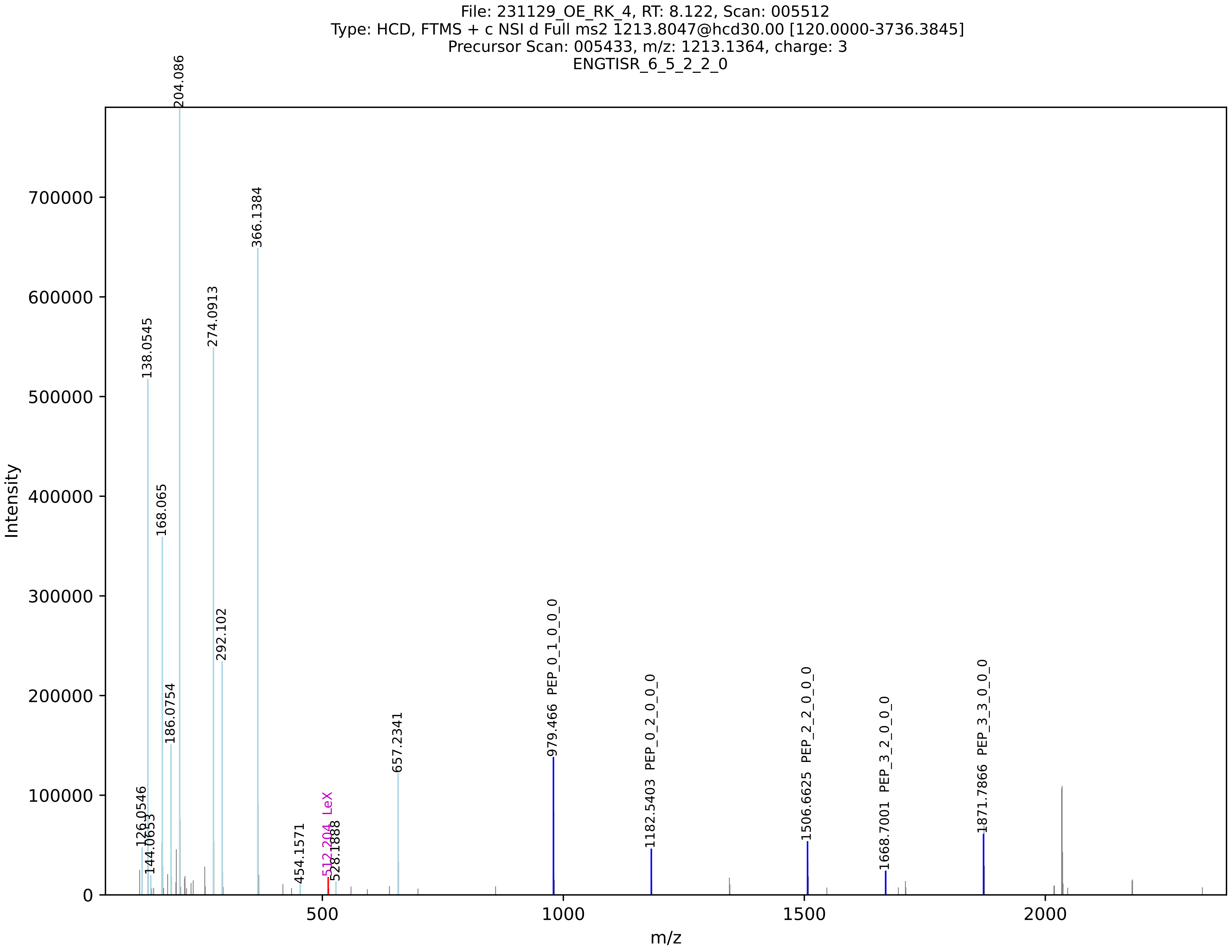
File: 231129_OE_RK_4, RT: 8.122, Scan: 005512
Type: HCD, FTMS + c NSI d Full ms2 1213.8047@hcd30.00 [120.0000-3736.3845]
Precursor Scan: 005433, m/z: 1213.1364, charge: 3
ENGTISR_6_5_2_2_0
Intensity
m/z
204.086
366.1384
274.0913
138.0545
168.065
292.102
186.0754
126.0546
144.0653
454.1571
512.204 LeX
528.1888
657.2341
979.466 PEP_0_1_0_0_0
1182.5403 PEP_0_2_0_0_0
1506.6625 PEP_2_2_0_0_0
1668.7001 PEP_3_2_0_0_0
1871.7866 PEP_3_3_0_0_0
0
100000
200000
300000
400000
500000
600000
700000
500
1000
1500
2000

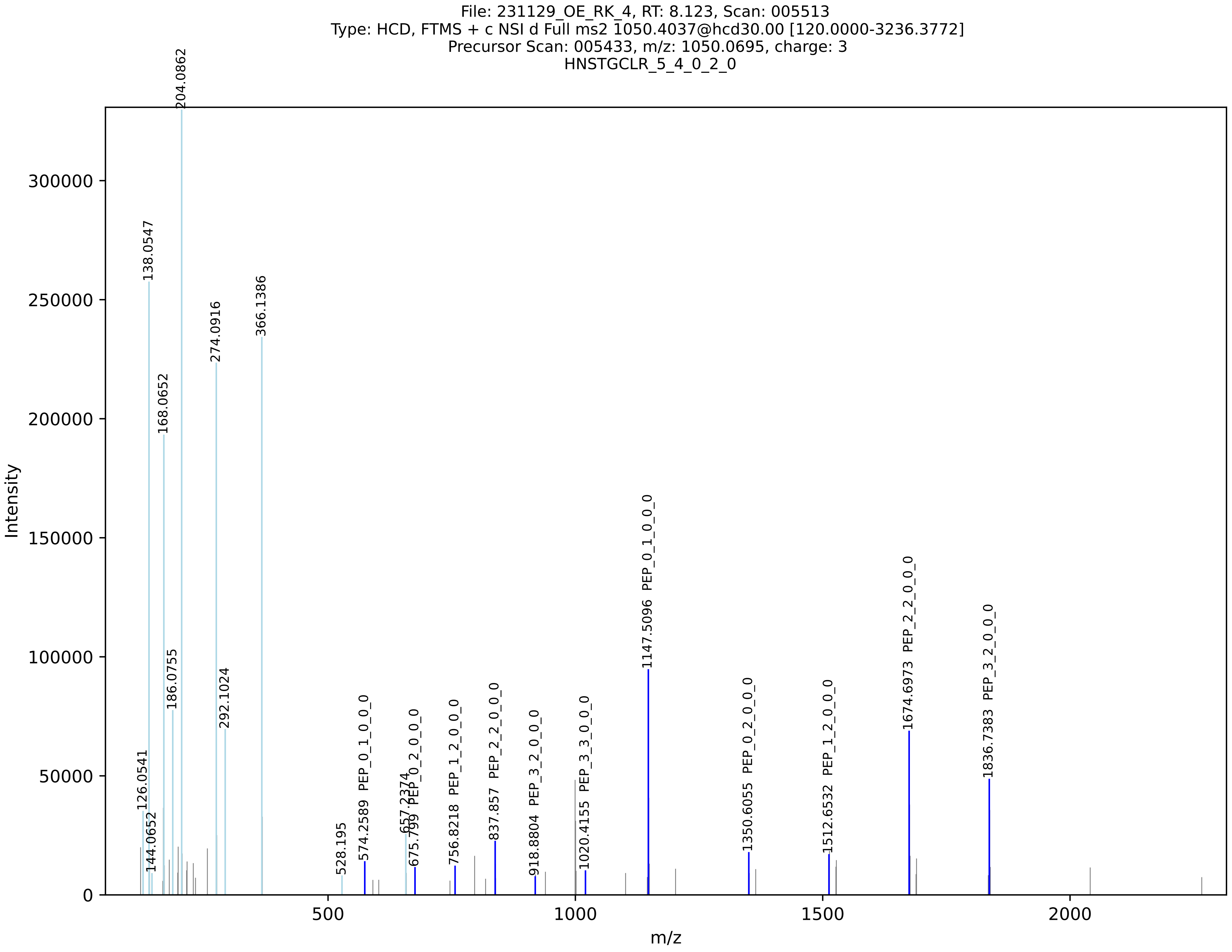
File: 231129_OE_RK_4, RT: 8.123, Scan: 005513
Type: HCD, FTMS + c NSI d Full ms2 1050.4037@hcd30.00 [120.0000-3236.3772]
Precursor Scan: 005433, m/z: 1050.0695, charge: 3
HNSTGCLR_5_4_0_2_0
204.0862
138.0547
366.1386
274.0916
168.0652
186.0755
292.1024
126.0541
144.0652
528.195
574.2589 PEP_0_1_0_0_0
657.2374
675.799 PEP_0_2_0_0_0
756.8218 PEP_1_2_0_0_0
837.857 PEP_2_2_0_0_0
918.8804 PEP_3_2_0_0_0
1020.4155 PEP_3_3_0_0_0
1147.5096 PEP_0_1_0_0_0
1350.6055 PEP_0_2_0_0_0
1512.6532 PEP_1_2_0_0_0
1674.6973 PEP_2_2_0_0_0
1836.7383 PEP_3_2_0_0_0
Intensity
m/z
0
50000
100000
150000
200000
250000
300000
500
1000
1500
2000

File: 231129_OE_RK_4, RT: 8.124, Scan: 005514
Type: HCD, FTMS + c NSI d Full ms2 1262.4885@hcd30.00 [120.0000-3885.3569]
Precursor Scan: 005433, m/z: 1261.8203, charge: 3
ENGTISR_6_5_1_3_0

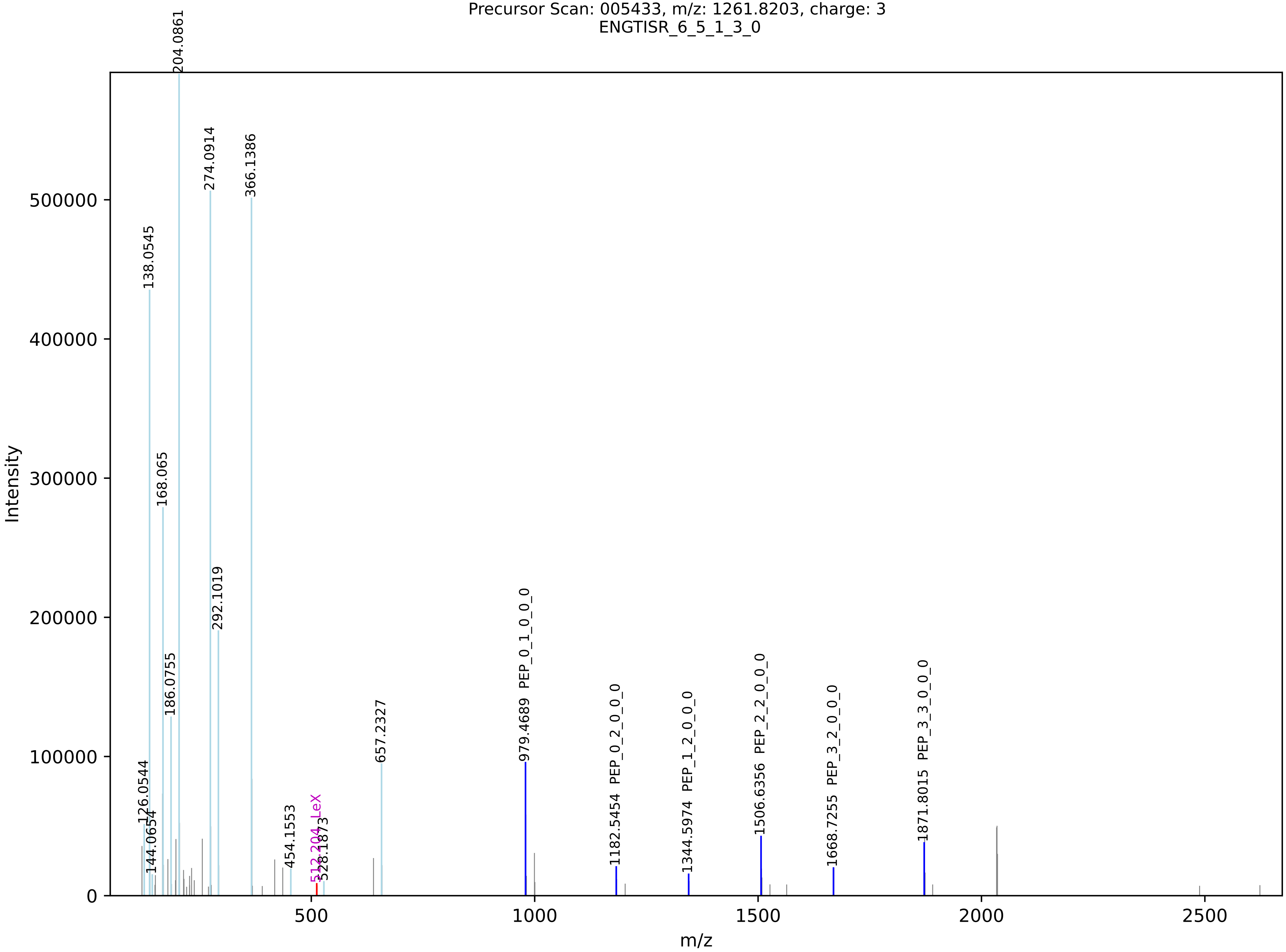

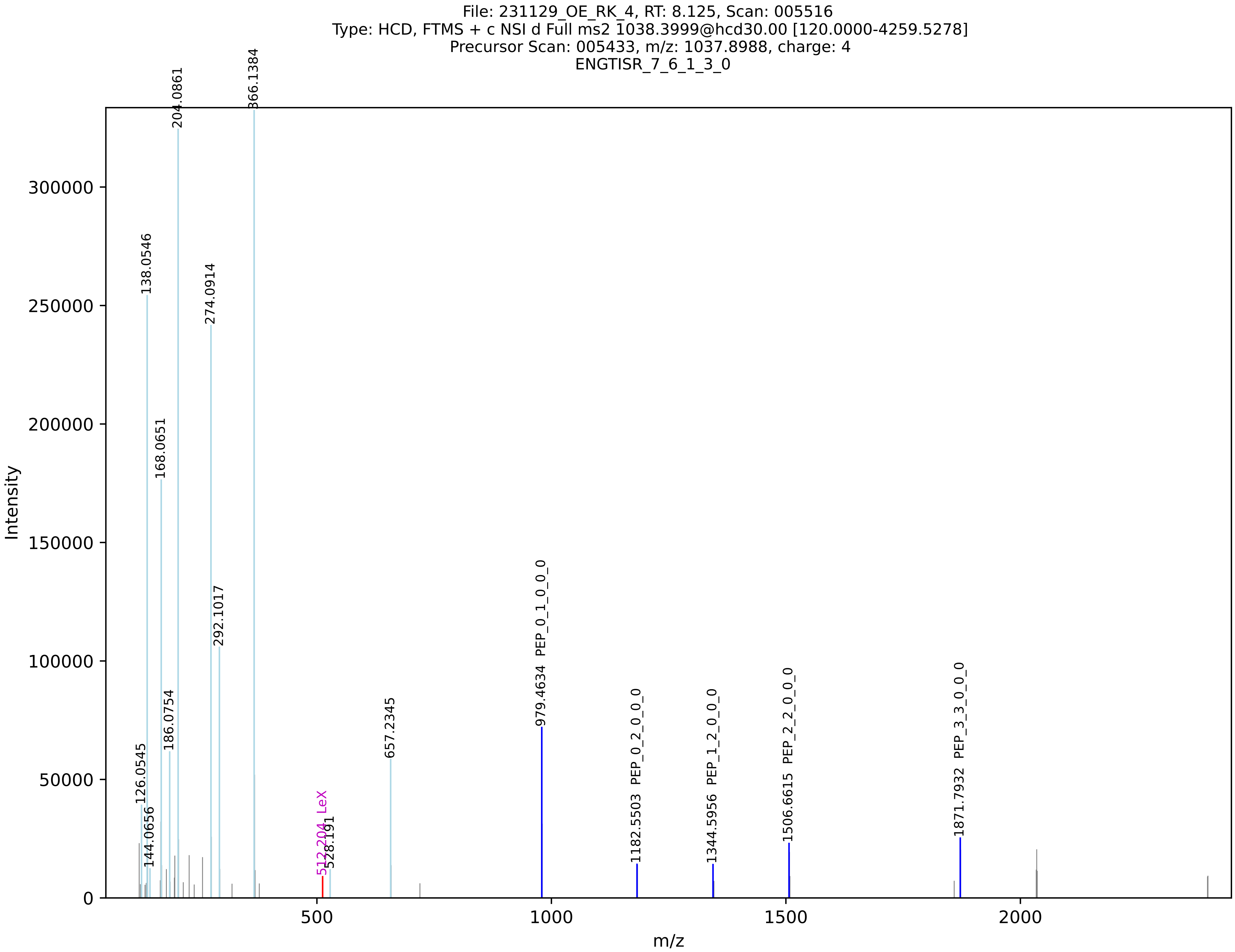
File: 231129_OE_RK_4, RT: 8.125, Scan: 005516
Type: HCD, FTMS + c NSI d Full ms2 1038.3999@hcd30.00 [120.0000-4259.5278]
Precursor Scan: 005433, m/z: 1037.8988, charge: 4
ENGTISR_7_6_1_3_0
Intensity
m/z
366.1384
204.0861
138.0546
274.0914
168.0651
292.1017
186.0754
126.0545
144.0656
512.204_LeX
528.191
657.2345
979.4634 PEP_0_1_0_0_0
1182.5503 PEP_0_2_0_0_0
1344.5956 PEP_1_2_0_0_0
1506.6615 PEP_2_2_0_0_0
1871.7932 PEP_3_3_0_0_0

File: 231129_OE_RK_4, RT: 8.125, Scan: 005517
Type: HCD, FTMS + c NSI d Full ms2 1501.5618@hcd30.00 [120.0000-3084.6140]
Precursor Scan: 005433, m/z: 1501.0609, charge: 2
NEEYNK_5_4_0_2_0

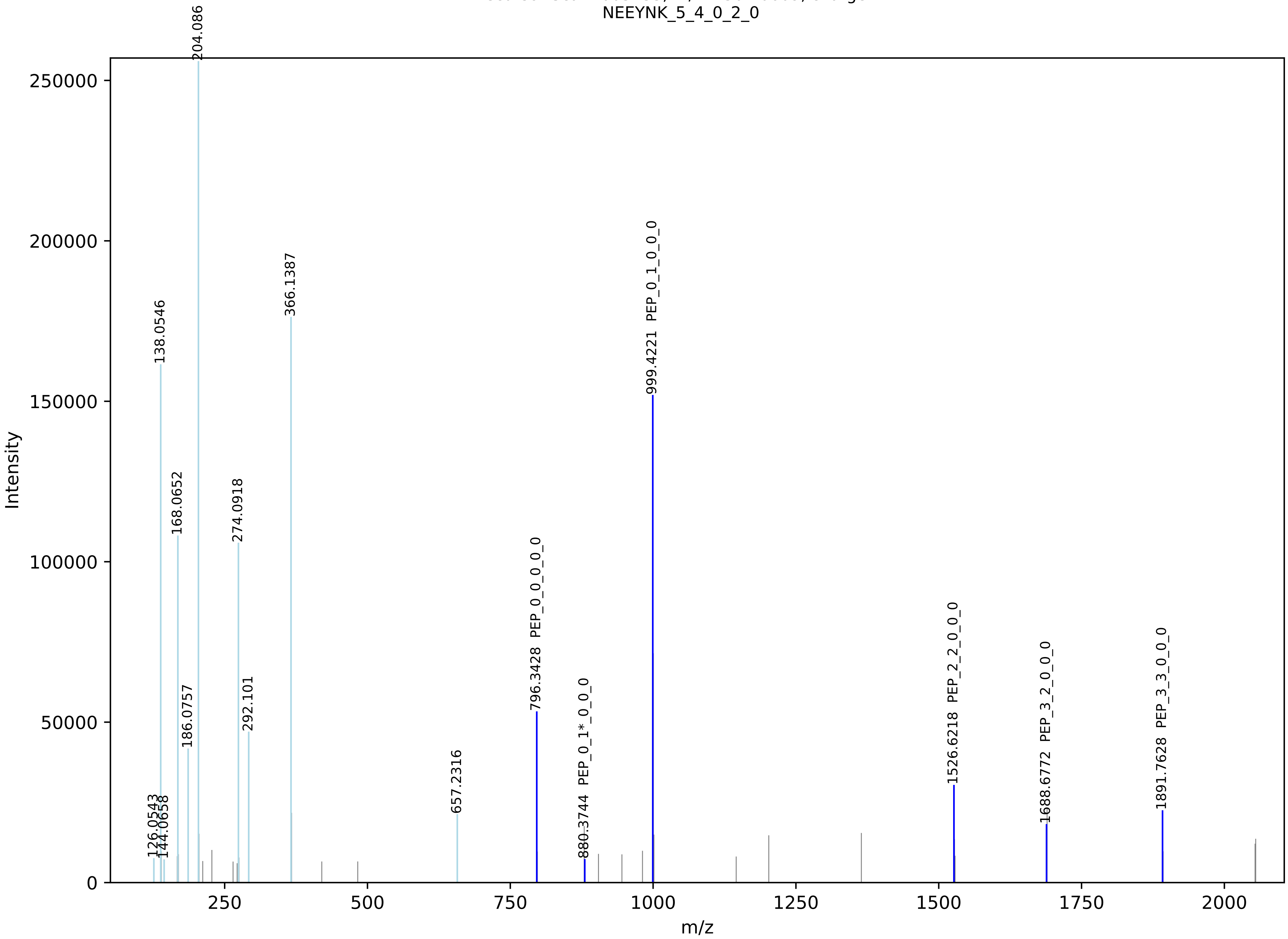

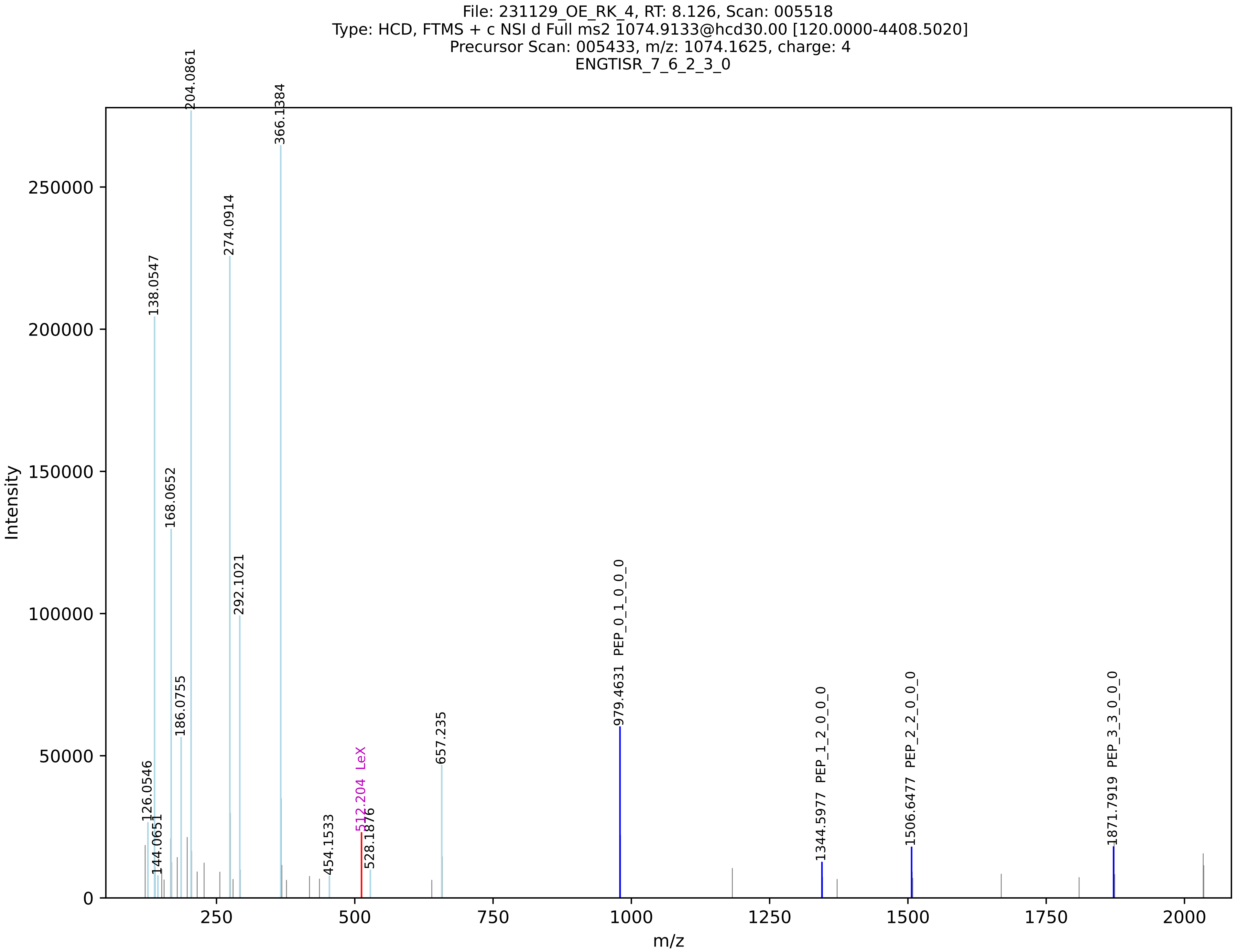

File: 231129_OE_RK_4, RT: 8.126, Scan: 005518
Type: HCD, FTMS + c NSI d Full ms2 1074.9133@hcd30.00 [120.0000-4408.5020]
Precursor Scan: 005433, m/z: 1074.1625, charge: 4
ENGTISR_7_6_2_3_0
Intensity
m/z
204.0861
366.1384
274.0914
138.0547
168.0652
292.1021
186.0755
126.0546
144.0651
454.1533
512.204 LeX
528.1876
657.235
979.4631 PEP_0_1_0_0_0_
1344.5977 PEP_1_2_0_0_0_
1506.6477 PEP_2_2_0_0_0_
1871.7919 PEP_3_3_0_0_0_

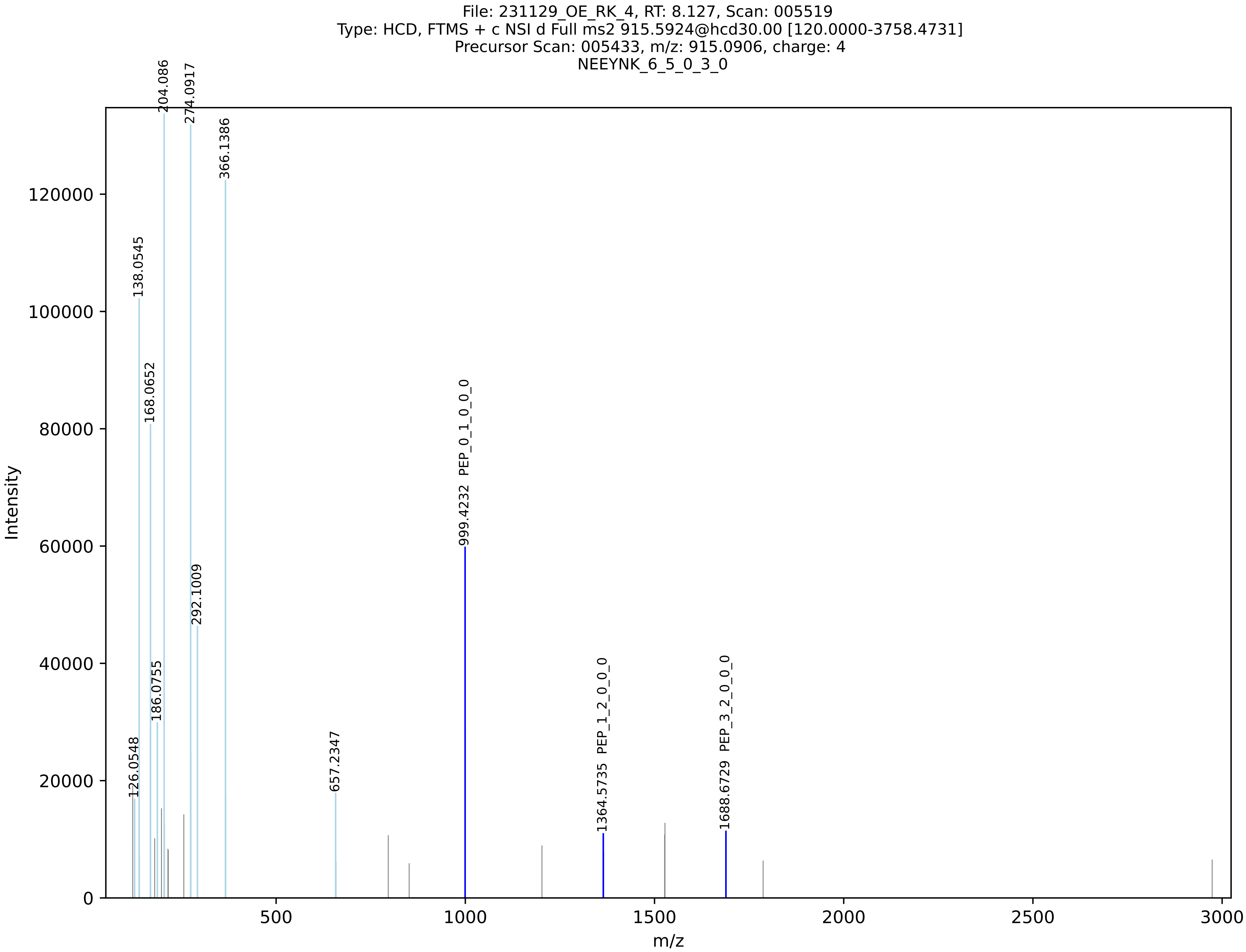
File: 231129_OE_RK_4, RT: 8.127, Scan: 005519
Type: HCD, FTMS + c NSI d Full ms2 915.5924@hcd30.00 [120.0000-3758.4731]
Precursor Scan: 005433, m/z: 915.0906, charge: 4
NEEYNK_6_5_0_3_0
204.086
274.0917
366.1386
138.0545
168.0652
999.4232 PEP_0_1_0_0_0
292.1009
186.0755
1364.5735 PEP_1_2_0_0_0
1688.6729 PEP_3_2_0_0_0
126.0548
657.2347
Intensity
m/z
0
20000
40000
60000
80000
100000
120000
500
1000
1500
2000
2500
3000

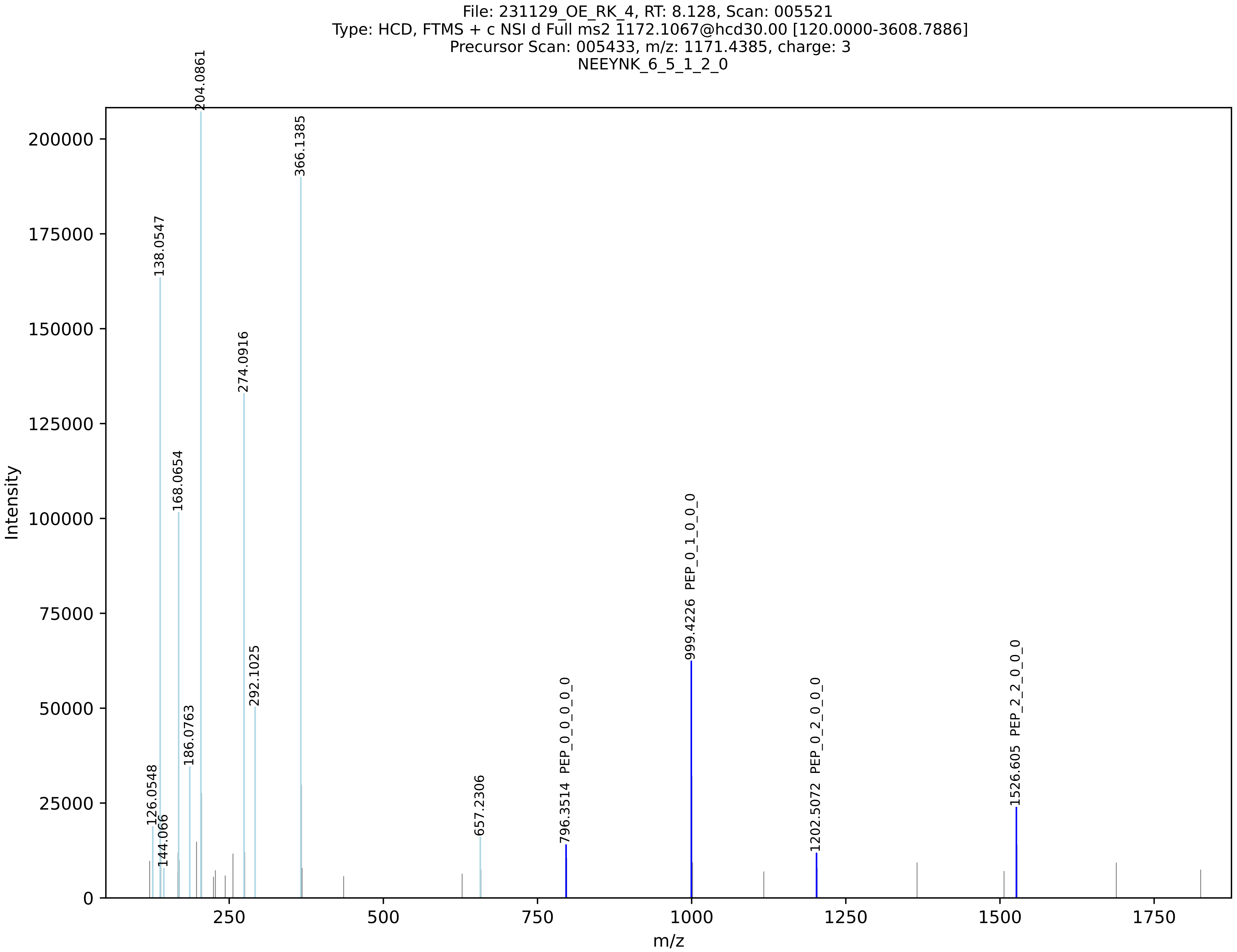
File: 231129_OE_RK_4, RT: 8.128, Scan: 005521
Type: HCD, FTMS + c NSI d Full ms2 1172.1067@hcd30.00 [120.0000-3608.7886]
Precursor Scan: 005433, m/z: 1171.4385, charge: 3
NEEYNK_6_5_1_2_0
Intensity
m/z
204.0861
366.1385
138.0547
274.0916
168.0654
292.1025
186.0763
126.0548
144.066
657.2306
796.3514 PEP_0_0_0_0_0
999.4226 PEP_0_1_0_0_0
1202.5072 PEP_0_2_0_0_0
1526.605 PEP_2_2_0_0_0
0
25000
50000
75000
100000
125000
150000
175000
200000
250
500
750
1000
1250
1500
1750

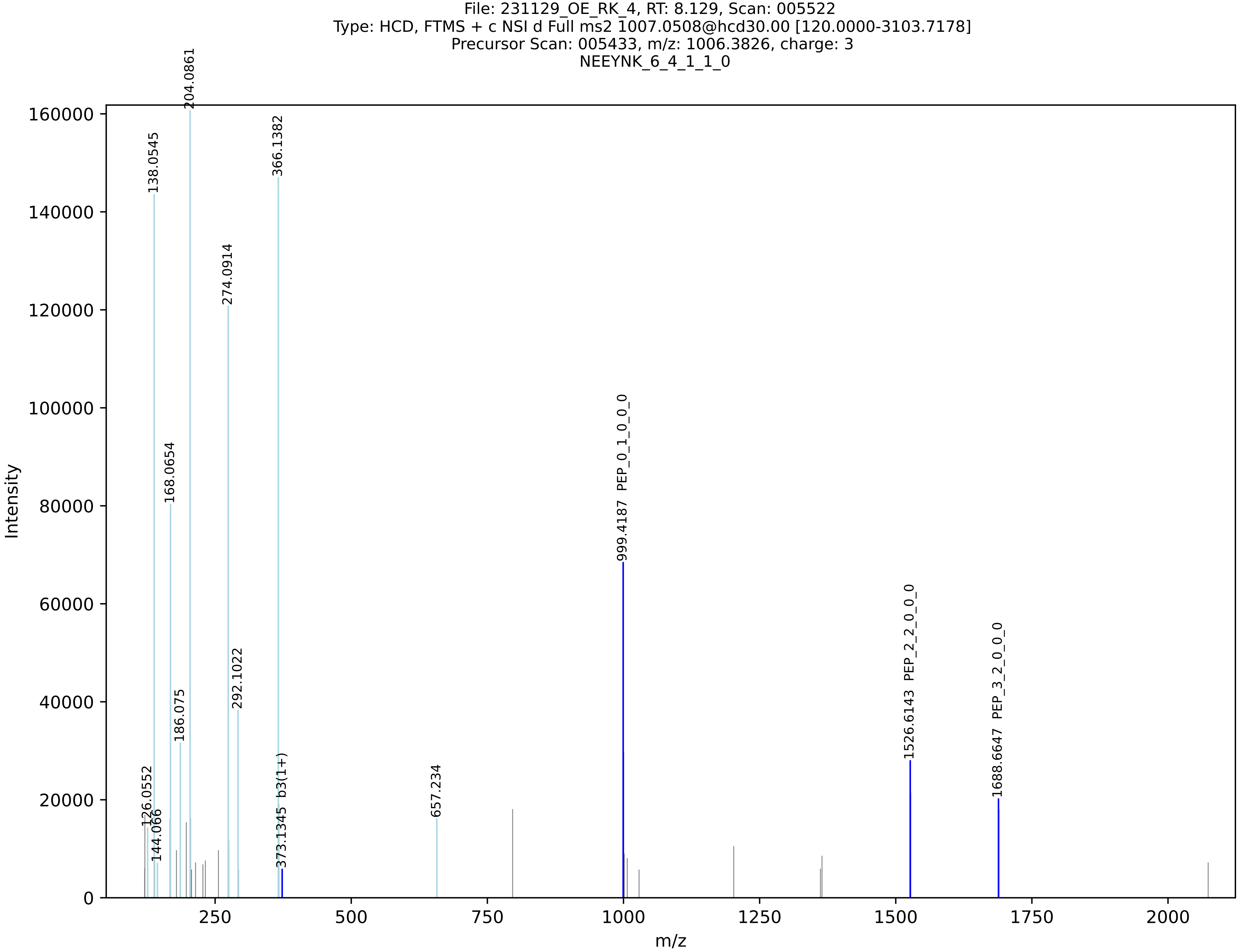
File: 231129_OE_RK_4, RT: 8.129, Scan: 005522
Type: HCD, FTMS + c NSI d Full ms2 1007.0508@hcd30.00 [120.0000-3103.7178]
Precursor Scan: 005433, m/z: 1006.3826, charge: 3
NEEYNK_6_4_1_1_0
Intensity
m/z
204.0861
138.0545
366.1382
274.0914
168.0654
186.075
292.1022
126.0552
144.066
373.1345 b3(1+)
657.234
999.4187 PEP_0_1_0_0_0
1526.6143 PEP_2_2_0_0_0
1688.6647 PEP_3_2_0_0_0

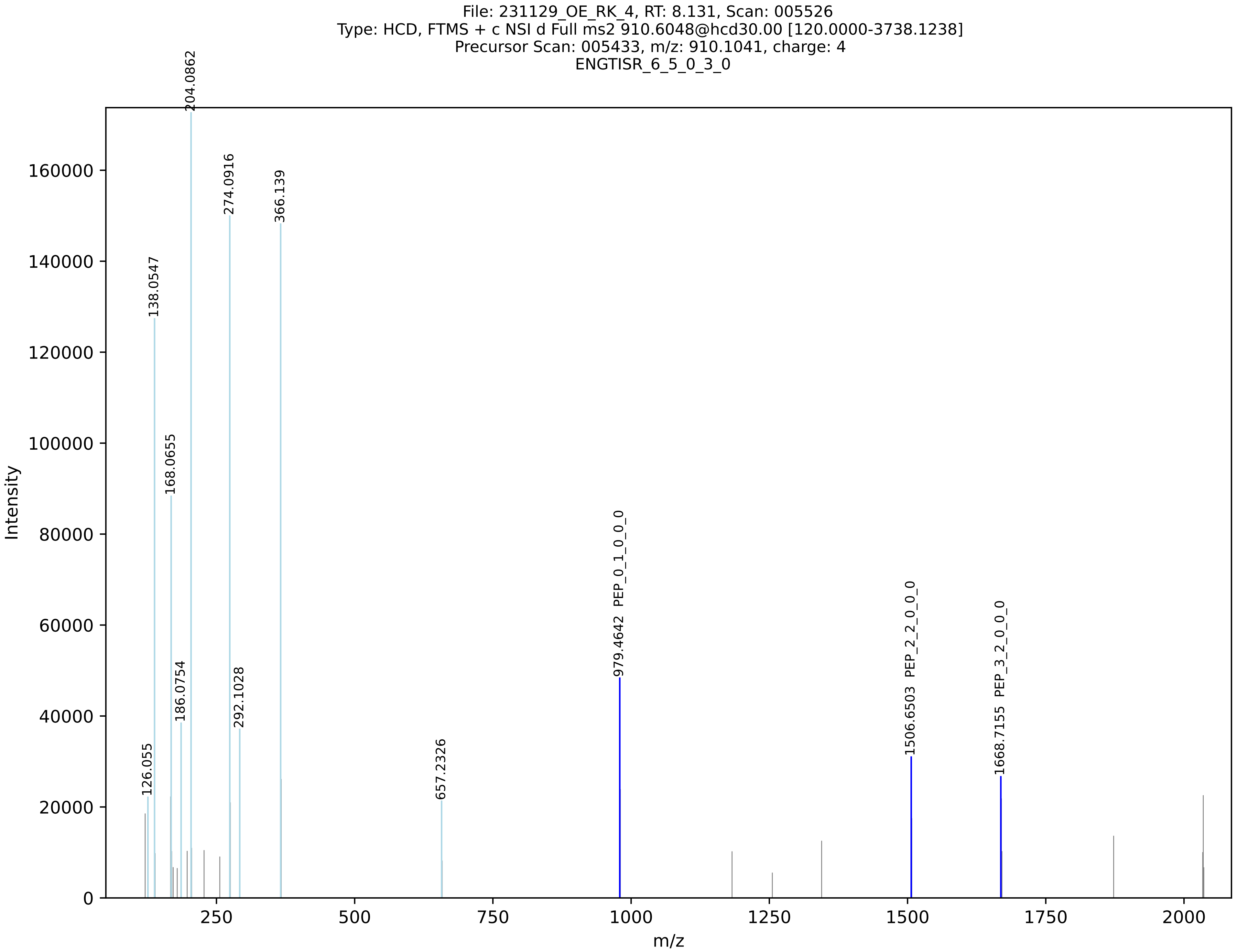

File: 231129_OE_RK_4, RT: 8.131, Scan: 005526
Type: HCD, FTMS + c NSI d Full ms2 910.6048@hcd30.00 [120.0000-3738.1238]
Precursor Scan: 005433, m/z: 910.1041, charge: 4
ENGTISR_6_5_0_3_0
Intensity
m/z
204.0862
274.0916
366.139
138.0547
168.0655
186.0754
292.1028
126.055
657.2326
979.4642 PEP_0_1_0_0_0_
1506.6503 PEP_2_2_0_0_0_
1668.7155 PEP_3_2_0_0_0

File: 231129_OE_RK_4, RT: 8.172, Scan: 005582
Type: HCD, FTMS + c NSI d Full ms2 1269.1392@hcd30.00 [120.0000-3905.7080]
Precursor Scan: 005507, m/z: 1268.4716, charge: 3
NEEYNK_6_5_1_3_0

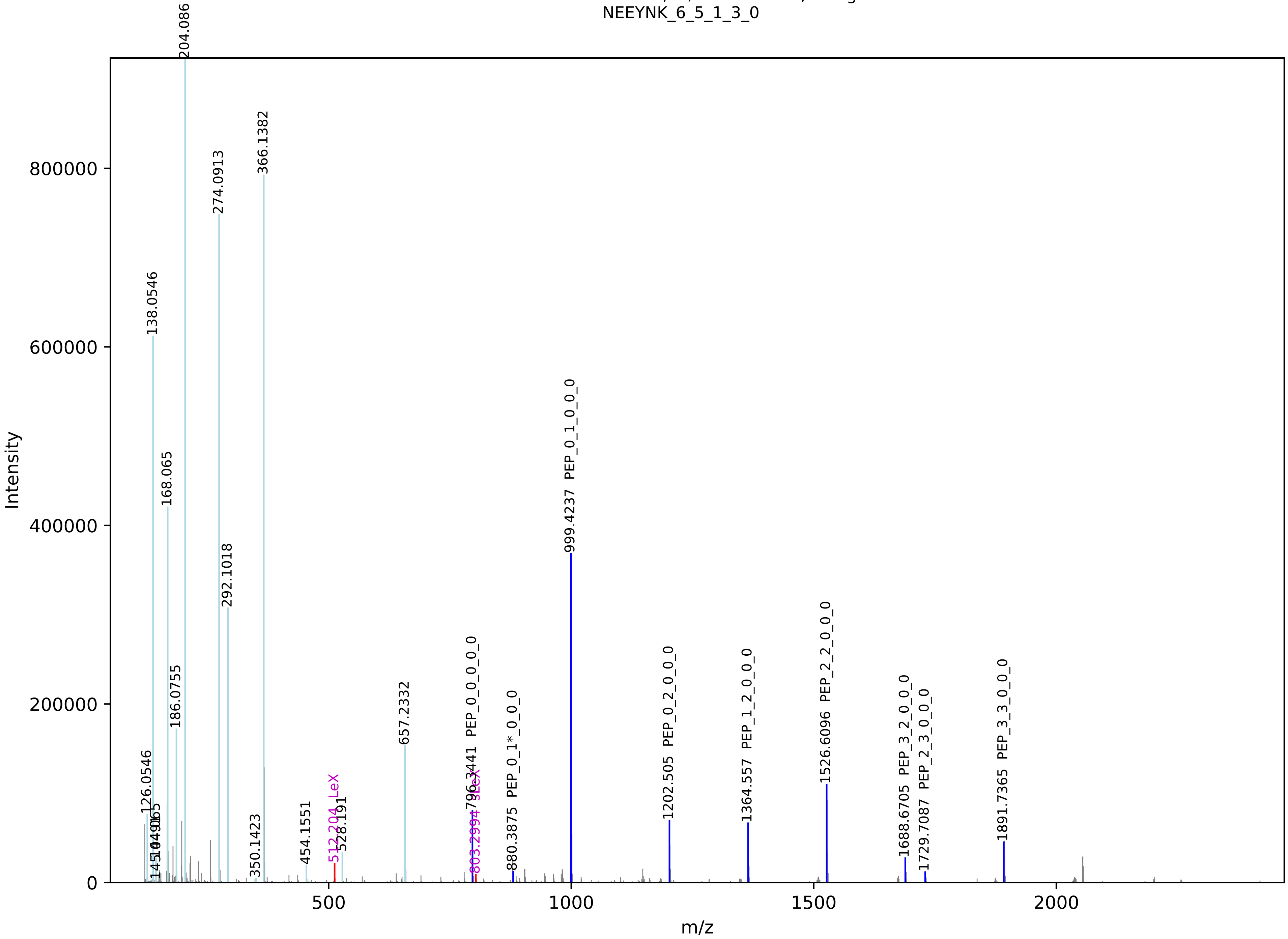

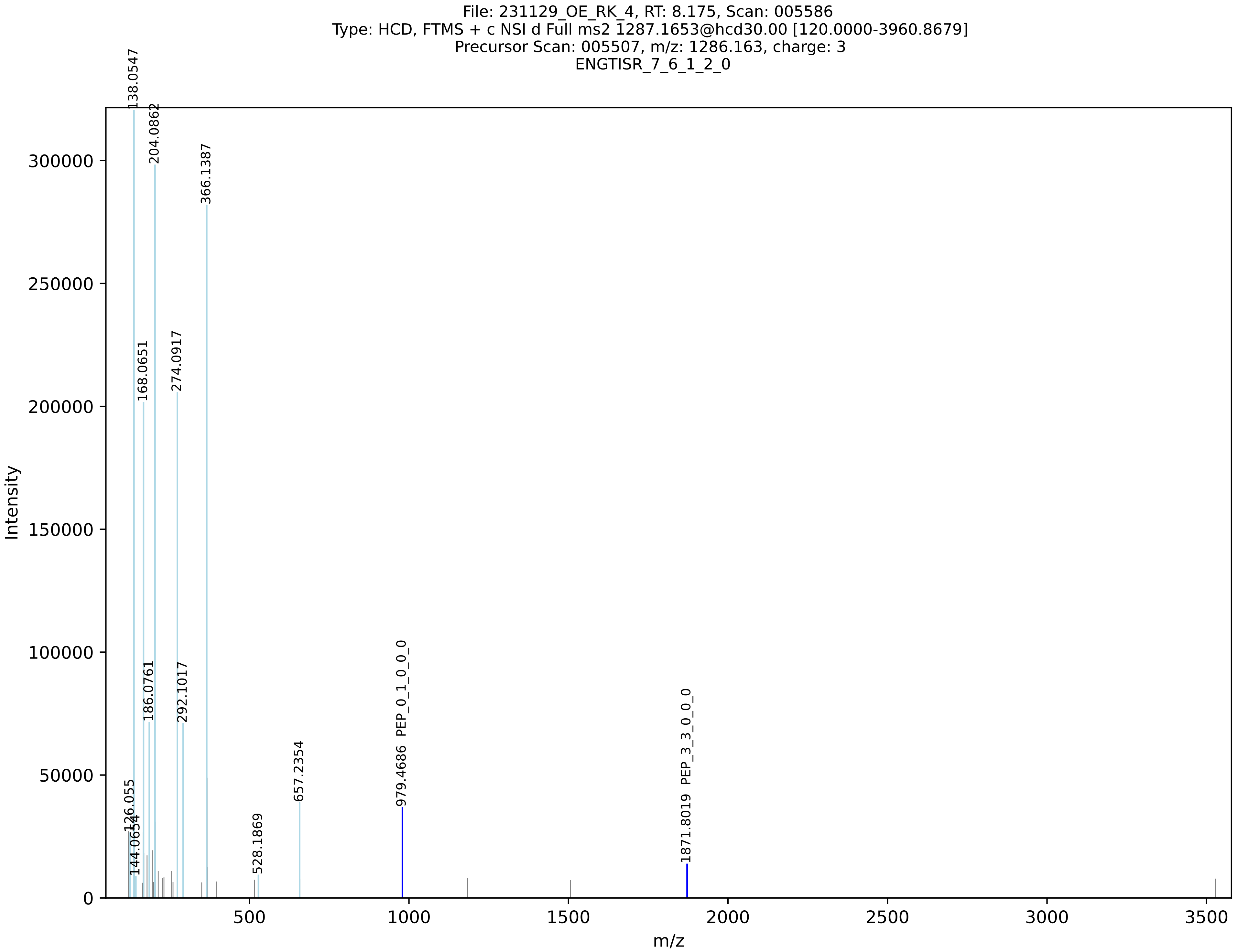
File: 231129_OE_RK_4, RT: 8.175, Scan: 005586
Type: HCD, FTMS + c NSI d Full ms2 1287.1653@hcd30.00 [120.0000-3960.8679]
Precursor Scan: 005507, m/z: 1286.163, charge: 3
ENGTISR_7_6_1_2_0
Intensity
m/z
138.0547
204.0862
366.1387
168.0651
274.0917
186.0761
292.1017
126.055
144.0654
528.1869
657.2354
979.4686 PEP_0_1_0_0_0
1871.8019 PEP_3_3_0_0_0

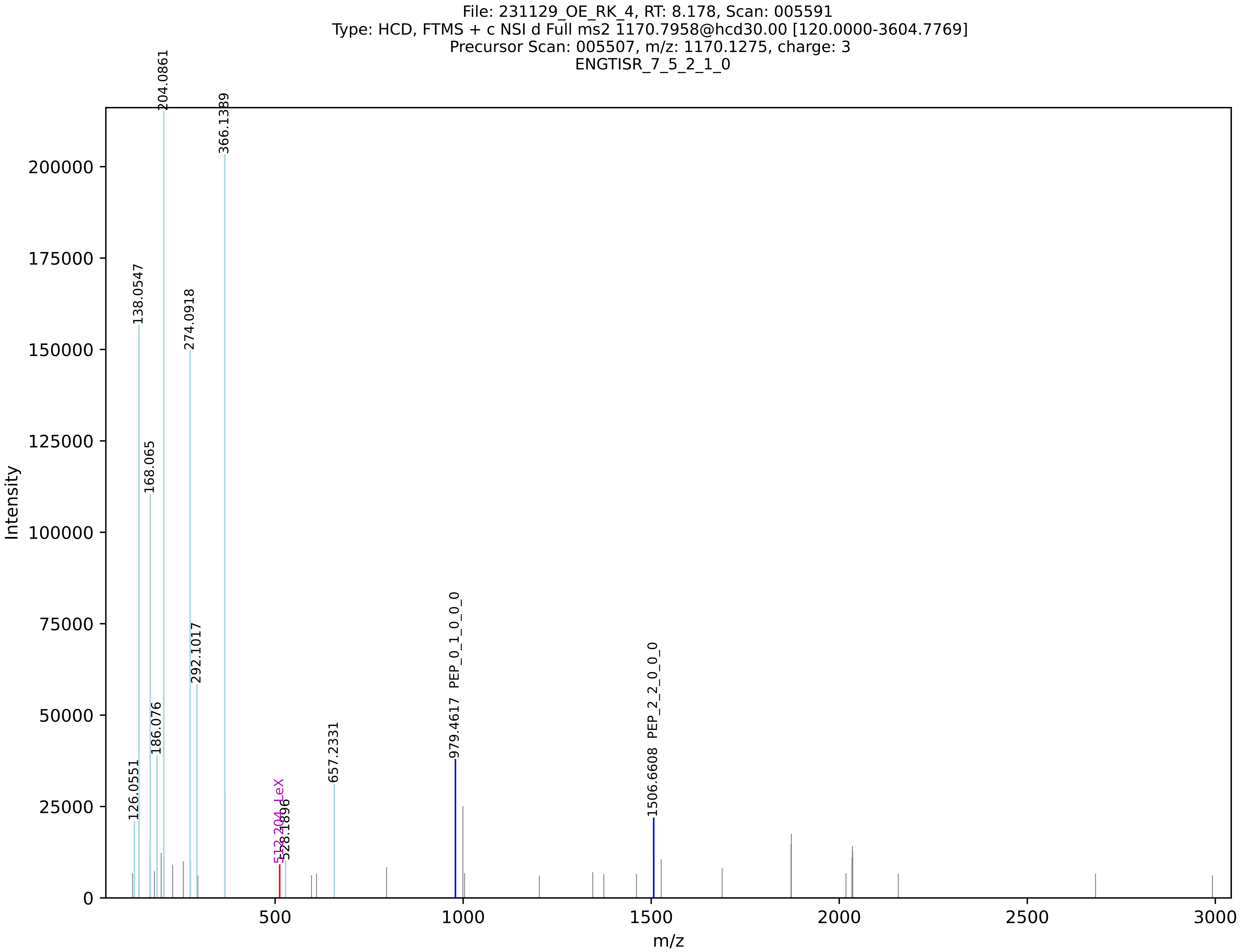
File: 231129_OE_RK_4, RT: 8.178, Scan: 005591
Type: HCD, FTMS + c NSI d Full ms2 1170.7958@hcd30.00 [120.0000-3604.7769]
Precursor Scan: 005507, m/z: 1170.1275, charge: 3
ENGTISR_7_5_2_1_0
204.0861
366.1389
138.0547
274.0918
168.065
292.1017
186.076
126.0551
512.204_LeX
528.1896
657.2331
979.4617 PEP_0_1_0_0_0
1506.6608 PEP_2_2_0_0_0
Intensity
m/z
0
25000
50000
75000
100000
125000
150000
175000
200000
500
1000
1500
2000
2500
3000

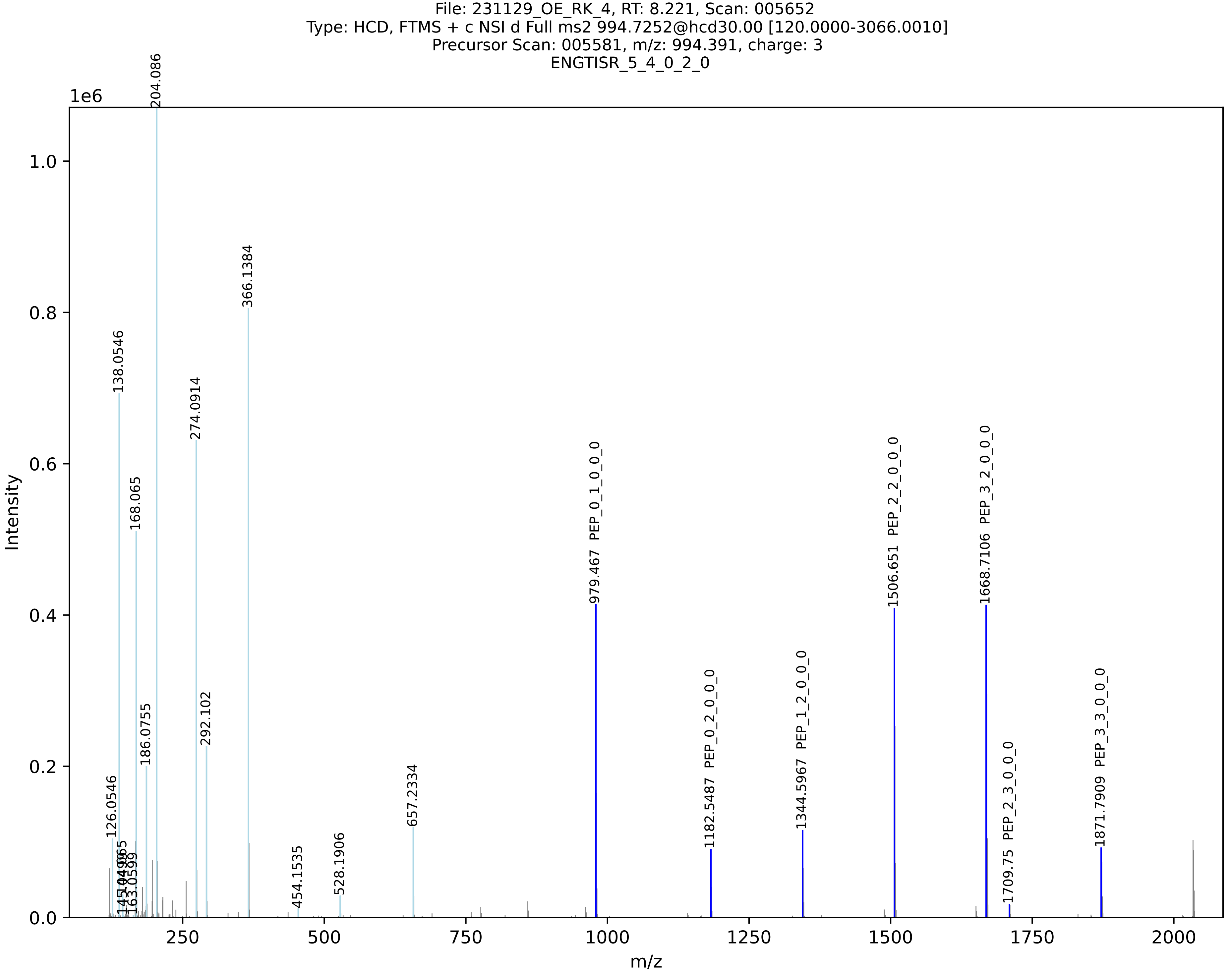
File: 231129_OE_RK_4, RT: 8.221, Scan: 005652
Type: HCD, FTMS + c NSI d Full ms2 994.7252@hcd30.00 [120.0000-3066.0010]
Precursor Scan: 005581, m/z: 994.391, charge: 3
ENGTISR_5_4_0_2_0
1e6
Intensity
m/z
204.086
366.1384
138.0546
274.0914
168.065
292.102
186.0755
126.0546
163.0599
454.1535
528.1906
657.2334
979.467 PEP_0_1_0_0_0
1182.5487 PEP_0_2_0_0_0
1344.5967 PEP_1_2_0_0_0
1506.651 PEP_2_2_0_0_0
1668.7106 PEP_3_2_0_0_0
1709.75 PEP_2_3_0_0_0
1871.7909 PEP_3_3_0_0_0

File: 231129_OE_RK_4, RT: 8.223, Scan: 005656
Type: HCD, FTMS + c NSI d Full ms2 1123.0867@hcd30.00 [120.0000-3458.7871]
Precursor Scan: 005581, m/z: 1122.7525, charge: 3
NEEYNK_6_5_0_2_0

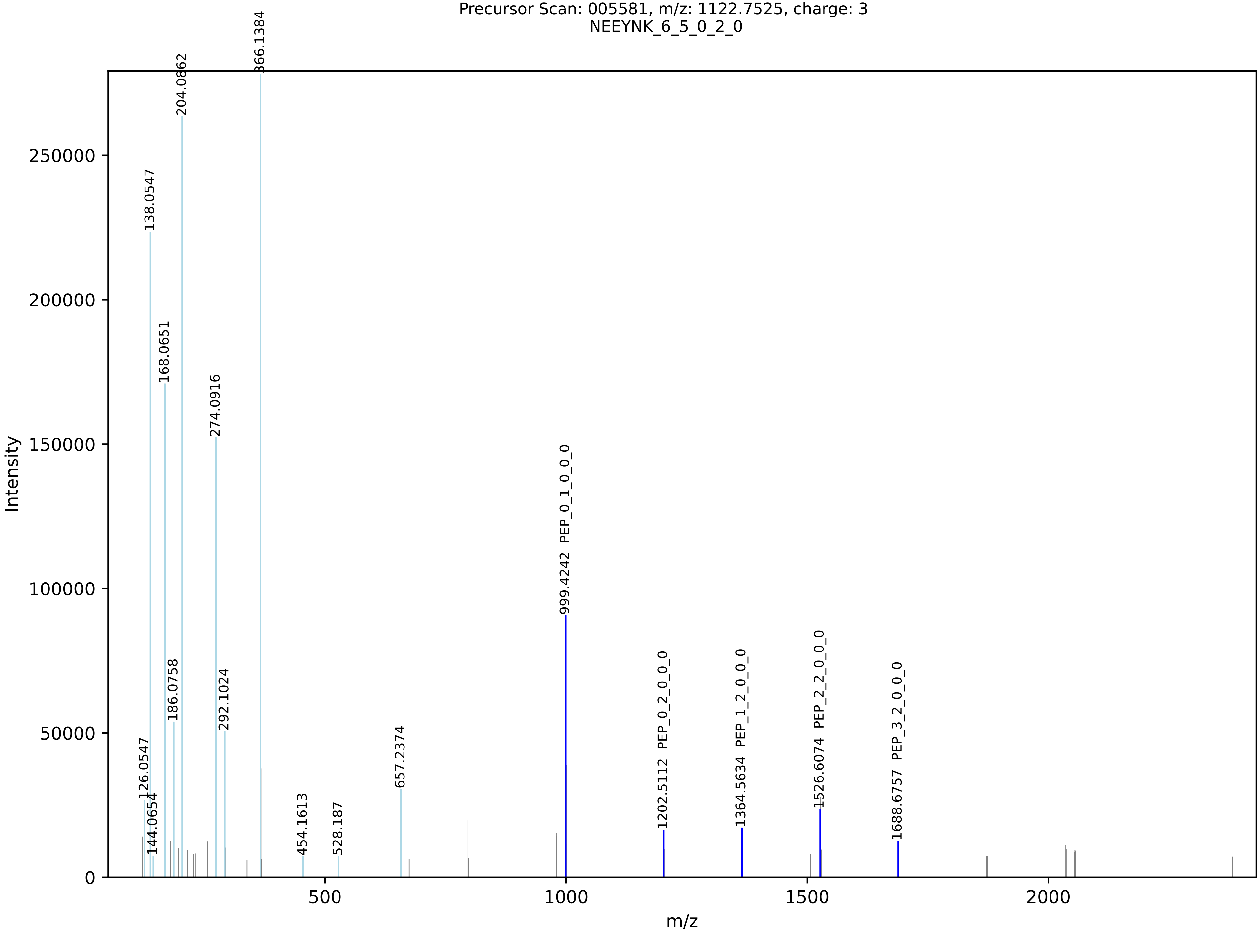

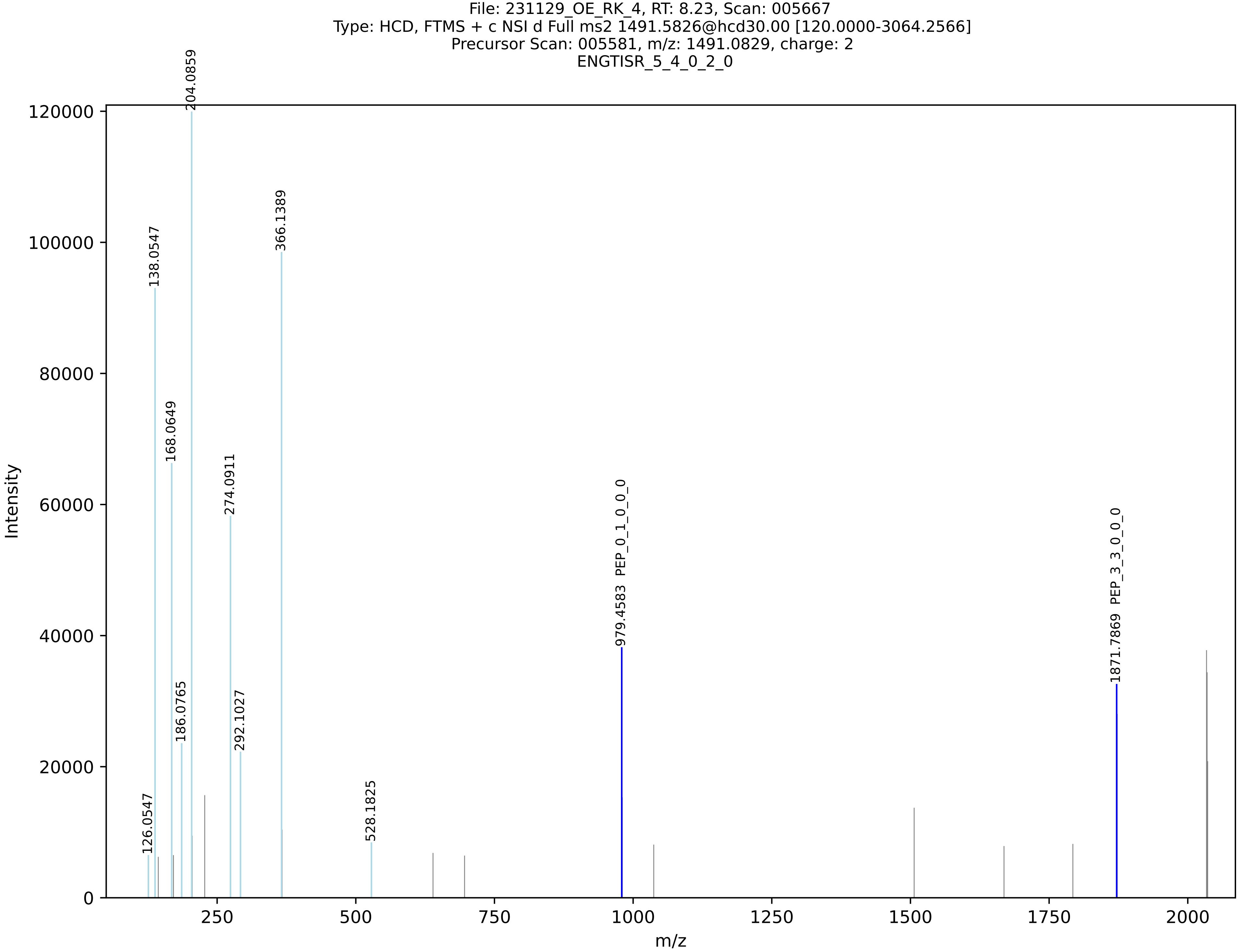
File: 231129_OE_RK_4, RT: 8.23, Scan: 005667
Type: HCD, FTMS + c NSI d Full ms2 1491.5826@hcd30.00 [120.0000-3064.2566]
Precursor Scan: 005581, m/z: 1491.0829, charge: 2
ENGTISR_5_4_0_2_0
Intensity
m/z
126.0547
138.0547
168.0649
186.0765
204.0859
274.0911
292.1027
366.1389
528.1825
979.4583 PEP_0_1_0_0_0
1871.7869 PEP_3_3_0_0_0

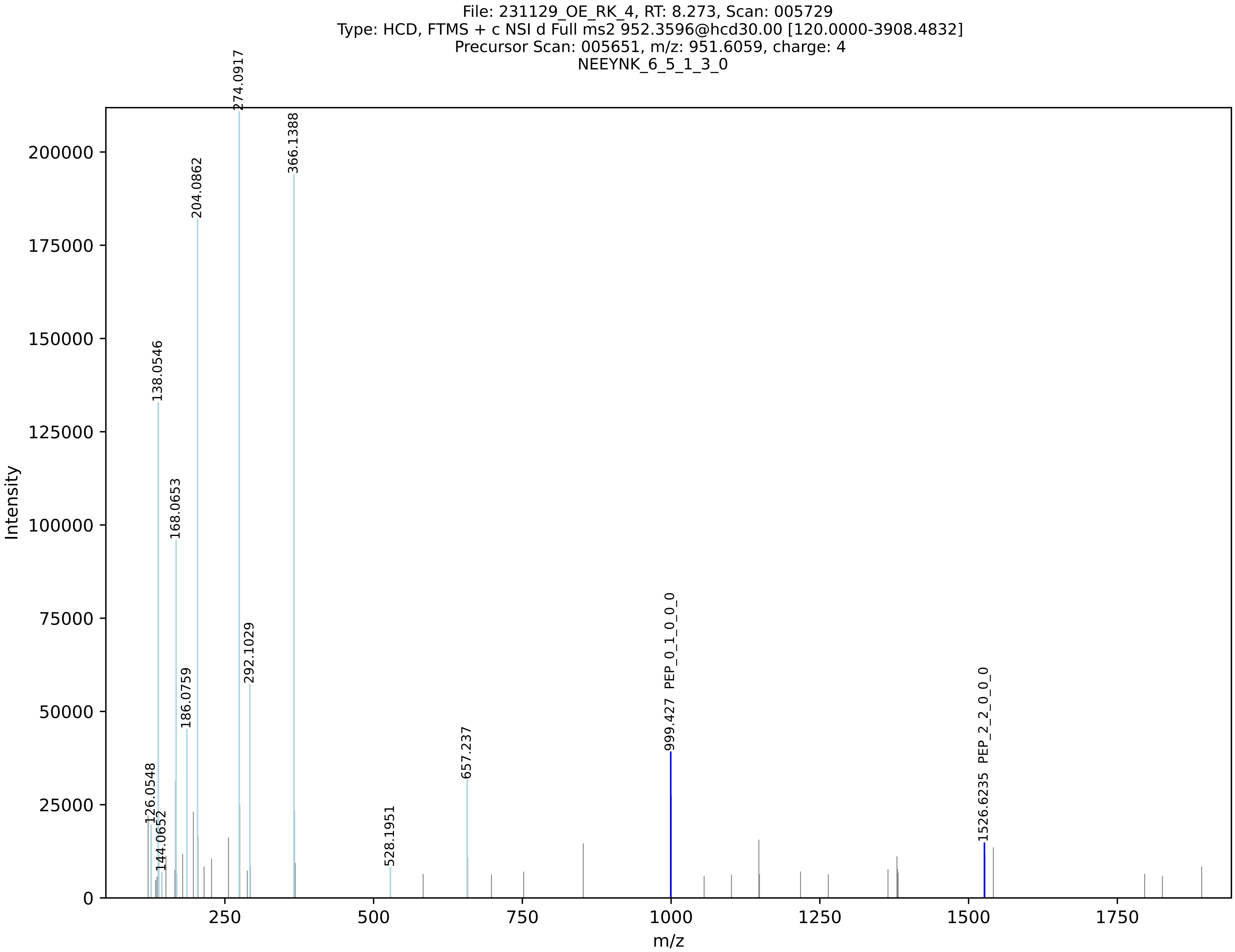
File: 231129_OE_RK_4, RT: 8.273, Scan: 005729
Type: HCD, FTMS + c NSI d Full ms2 952.3596@hcd30.00 [120.0000-3908.4832]
Precursor Scan: 005651, m/z: 951.6059, charge: 4
NEEYNK_6_5_1_3_0
Intensity
m/z
274.0917
366.1388
204.0862
138.0546
168.0653
292.1029
186.0759
126.0548
144.0652
528.1951
657.237
999.427 PEP_0_1_0_0_0
1526.6235 PEP_2_2_0_0_0
0
25000
50000
75000
100000
125000
150000
175000
200000
250
500
750
1000
1250
1500
1750

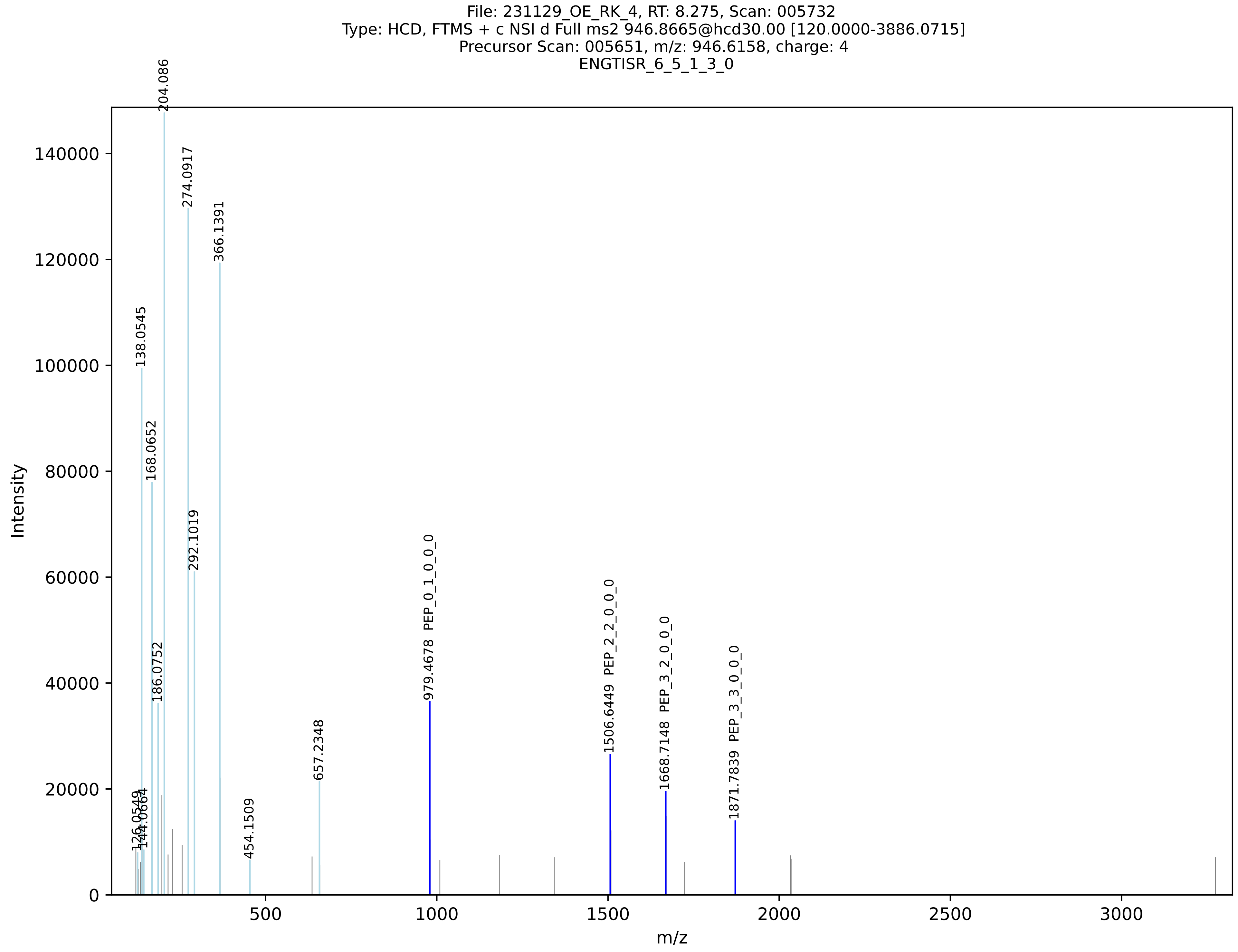
File: 231129_OE_RK_4, RT: 8.275, Scan: 005732
Type: HCD, FTMS + c NSI d Full ms2 946.8665@hcd30.00 [120.0000-3886.0715]
Precursor Scan: 005651, m/z: 946.6158, charge: 4
ENGTISR_6_5_1_3_0
204.086
274.0917
366.1391
138.0545
168.0652
292.1019
186.0752
126.0549
144.0664
454.1509
657.2348
979.4678 PEP_0_1_0_0_0
1506.6449 PEP_2_2_0_0_0
1668.7148 PEP_3_2_0_0_0
1871.7839 PEP_3_3_0_0_0
Intensity
m/z
0
20000
40000
60000
80000
100000
120000
140000
500
1000
1500
2000
2500
3000

File: 231129_OE_RK_4, RT: 8.289, Scan: 005755
Type: HCD, FTMS + c NSI d Full ms2 1006.1412@hcd30.00 [120.0000-4127.9121]
Precursor Scan: 005651, m/z: 1005.6418, charge: 4
NEEYNK_7_6_0_3_0

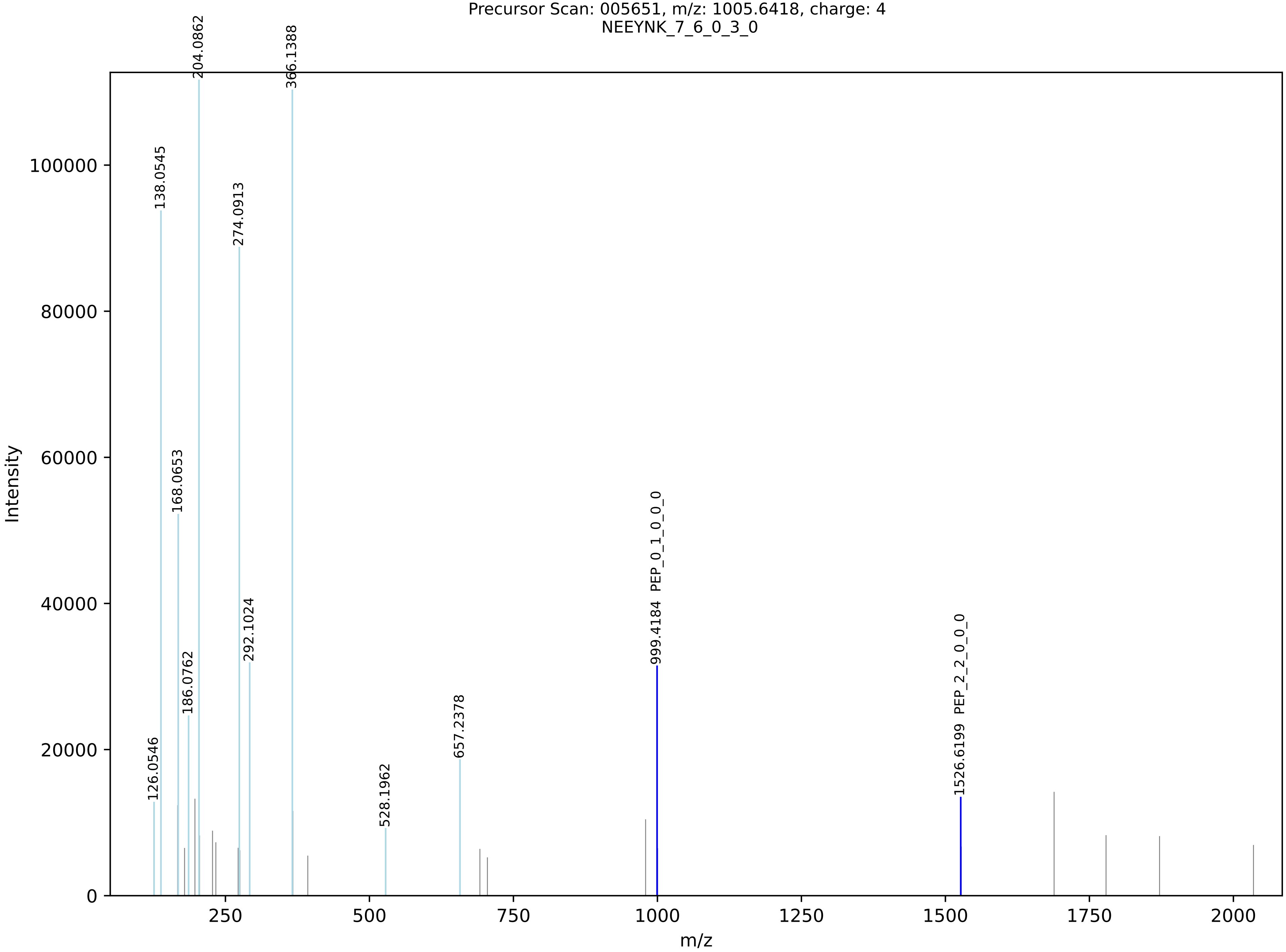

File: 231129_OE_RK_4, RT: 8.324, Scan: 005801
Type: HCD, FTMS + c NSI d Full ms2 1213.4684@hcd30.00 [120.0000-3735.3555]
Precursor Scan: 005725, m/z: 1213.134, charge: 3
ENGTISR_6_5_2_2_0

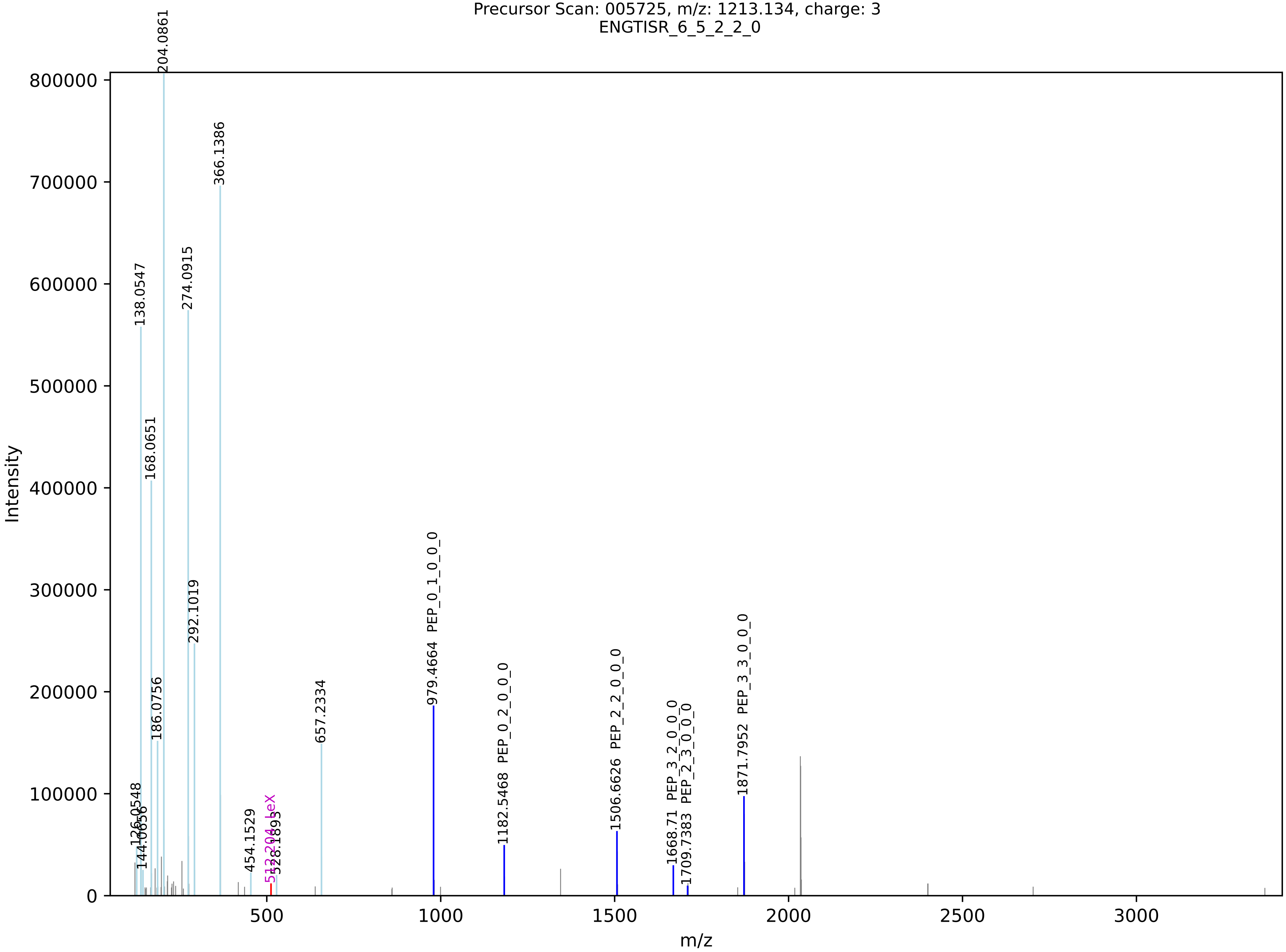

File: 231129_OE_RK_4, RT: 8.326, Scan: 005804
Type: HCD, FTMS + c NSI d Full ms2 1262.1533@hcd30.00 [120.0000-3884.3311]
Precursor Scan: 005725, m/z: 1261.8198, charge: 3
ENGTISR_6_5_1_3_0

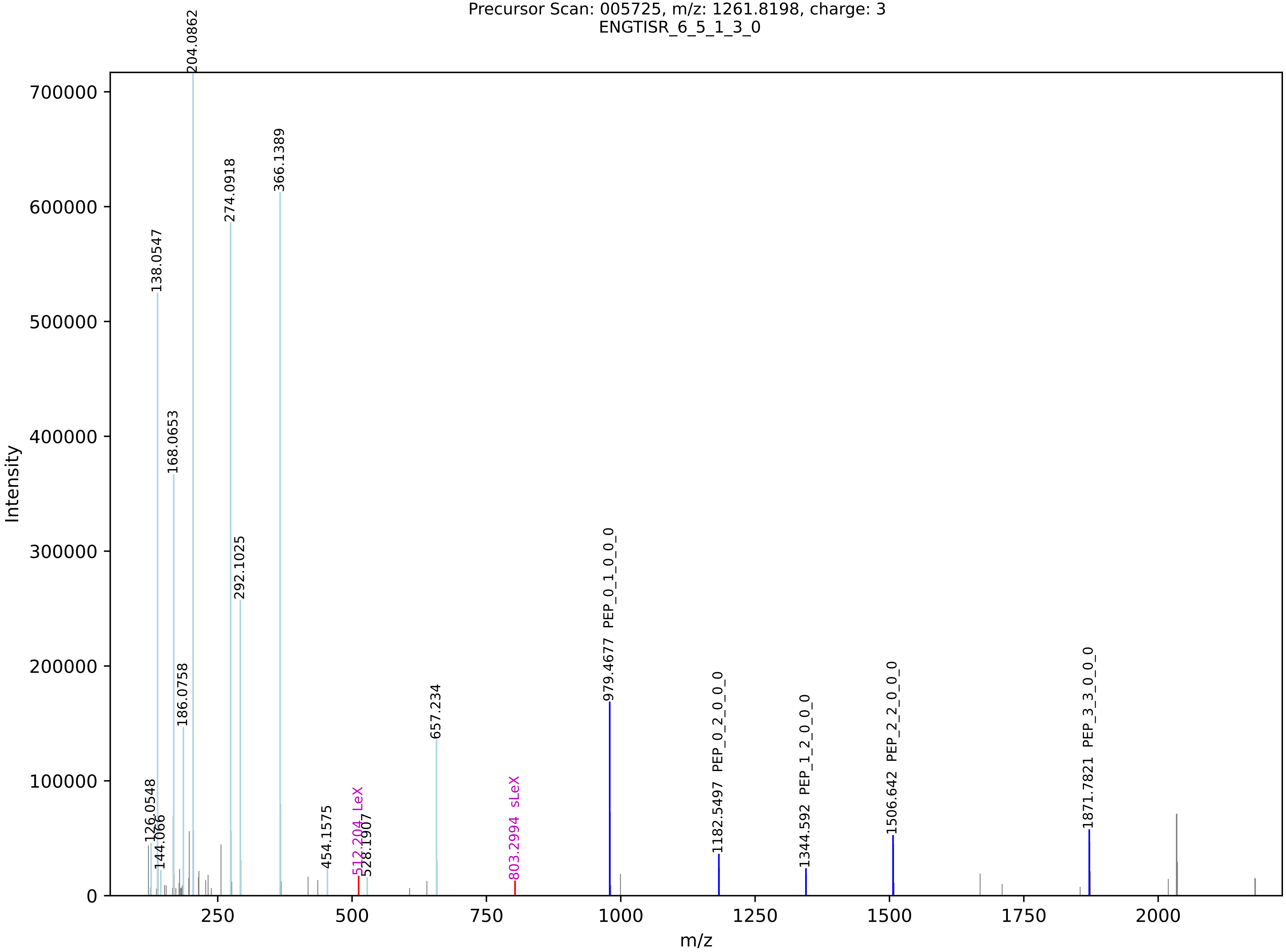

File: 231129_OE_RK_4, RT: 8.328, Scan: 005807
Type: HCD, FTMS + c NSI d Full ms2 1074.6637@hcd30.00 [120.0000-4407.4839]
Precursor Scan: 005725, m/z: 1074.4125, charge: 4
ENGTISR_7_6_2_3_0

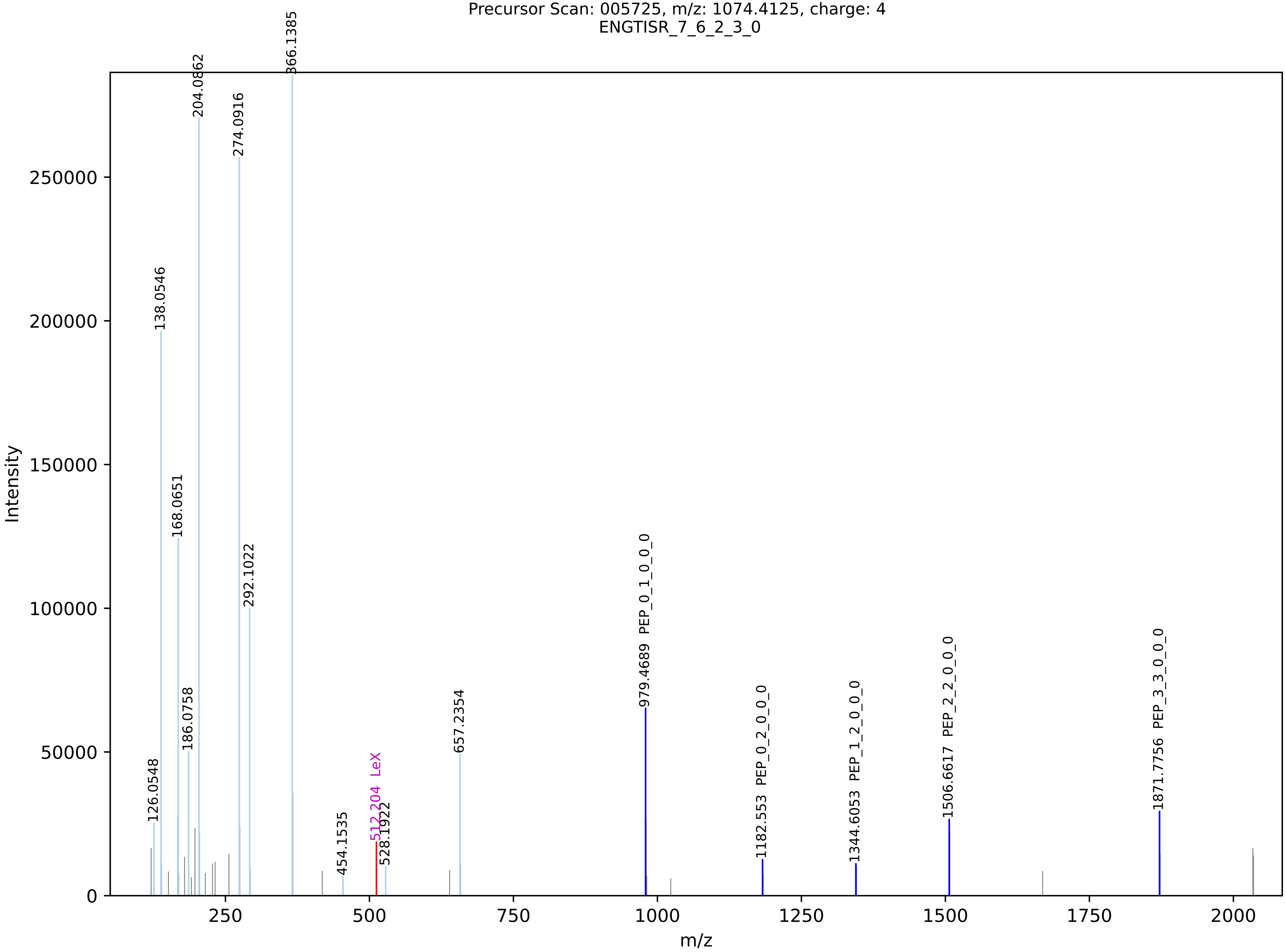

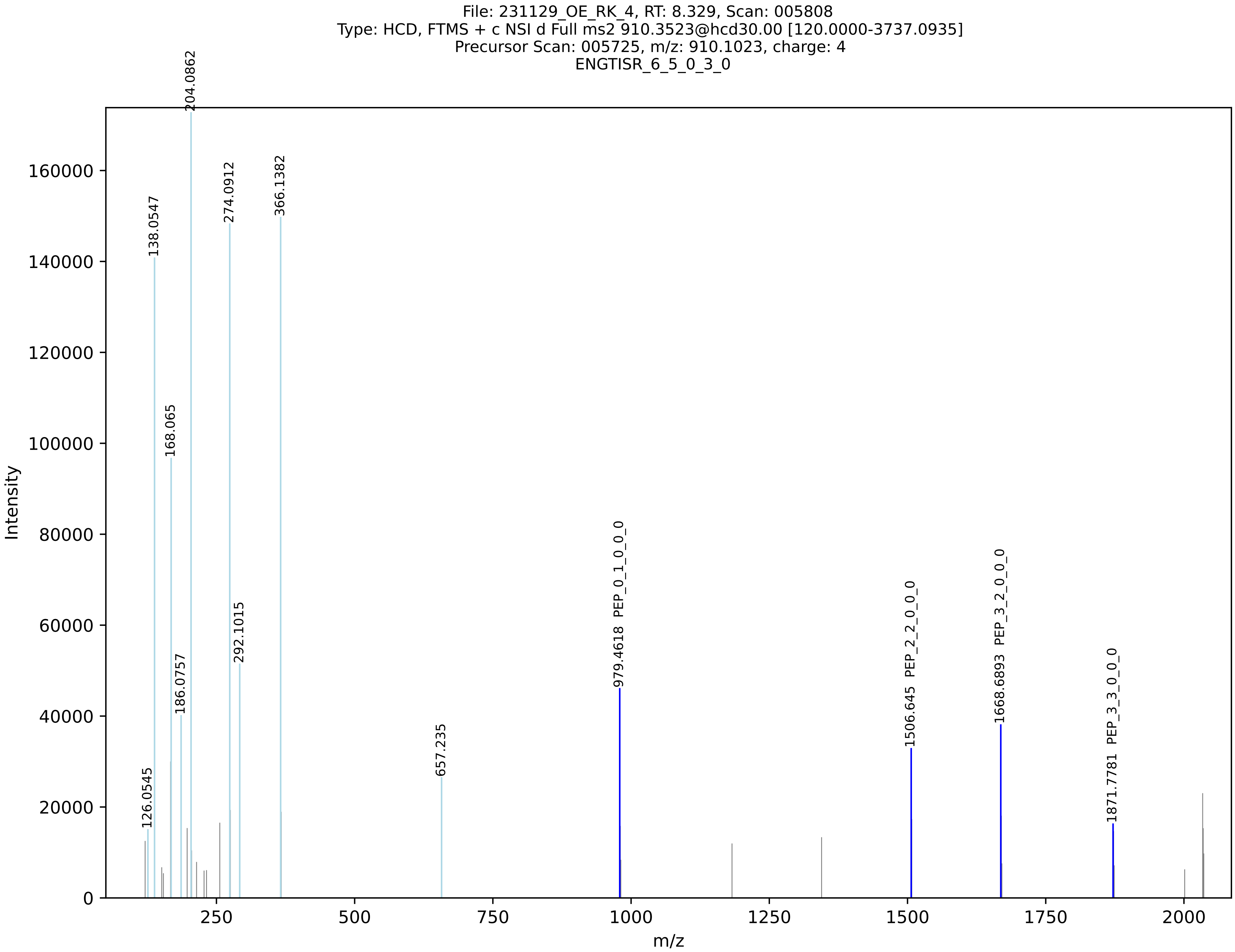
File: 231129_OE_RK_4, RT: 8.329, Scan: 005808
Type: HCD, FTMS + c NSI d Full ms2 910.3523@hcd30.00 [120.0000-3737.0935]
Precursor Scan: 005725, m/z: 910.1023, charge: 4
ENGTISR_6_5_0_3_0
Intensity
m/z
204.0862
138.0547
168.065
186.0757
126.0545
274.0912
292.1015
366.1382
657.235
979.4618 PEP_0_1_0_0_0
1506.645 PEP_2_2_0_0_0
1668.6893 PEP_3_2_0_0_0
1871.7781 PEP_3_3_0_0_0

File: 231129_OE_RK_4, RT: 8.331, Scan: 005812
Type: HCD, FTMS + c NSI d Full ms2 915.3415@hcd30.00 [120.0000-3757.4492]
Precursor Scan: 005725, m/z: 915.0905, charge: 4
NEEYNK_6_5_0_3_0

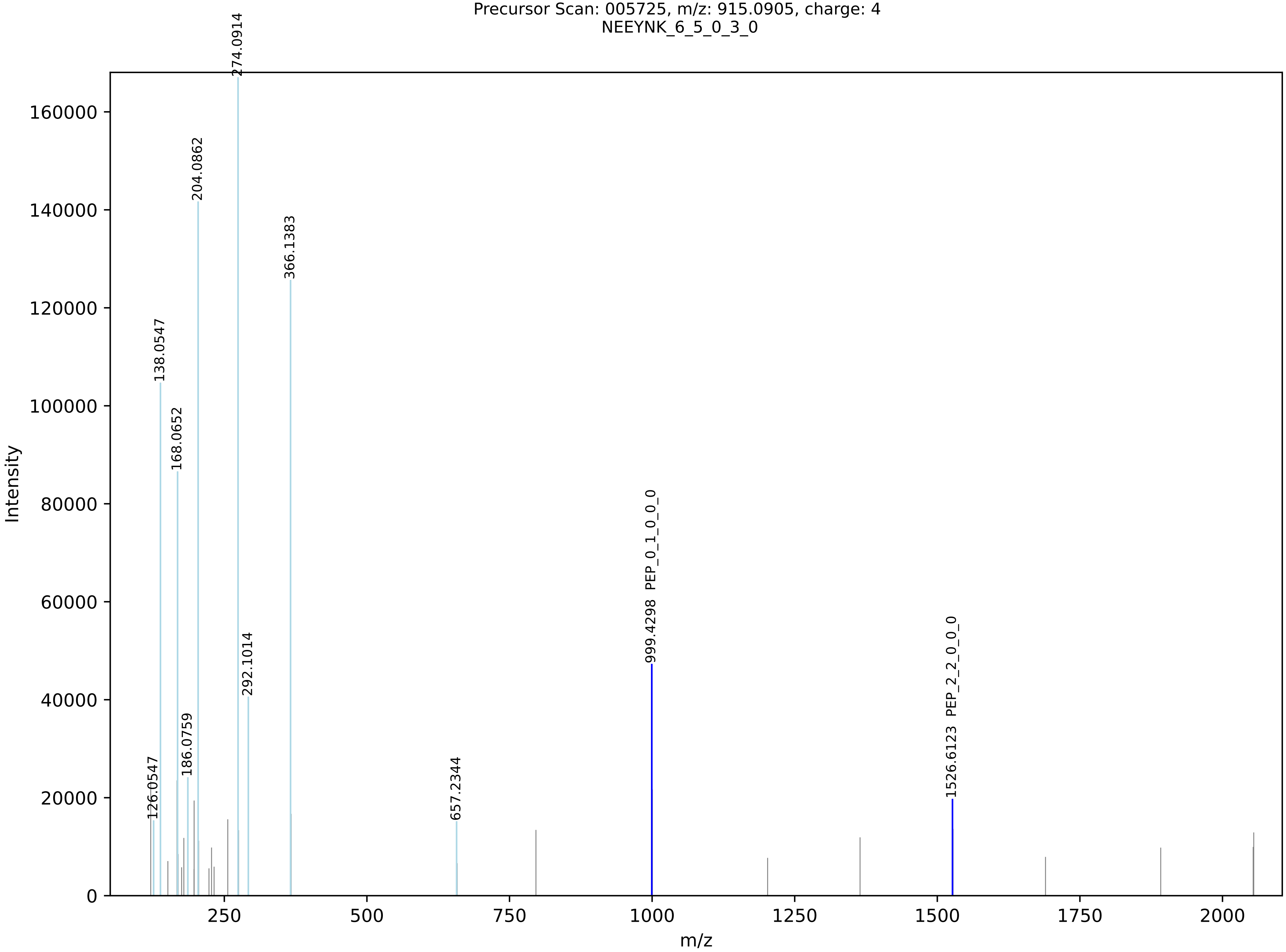

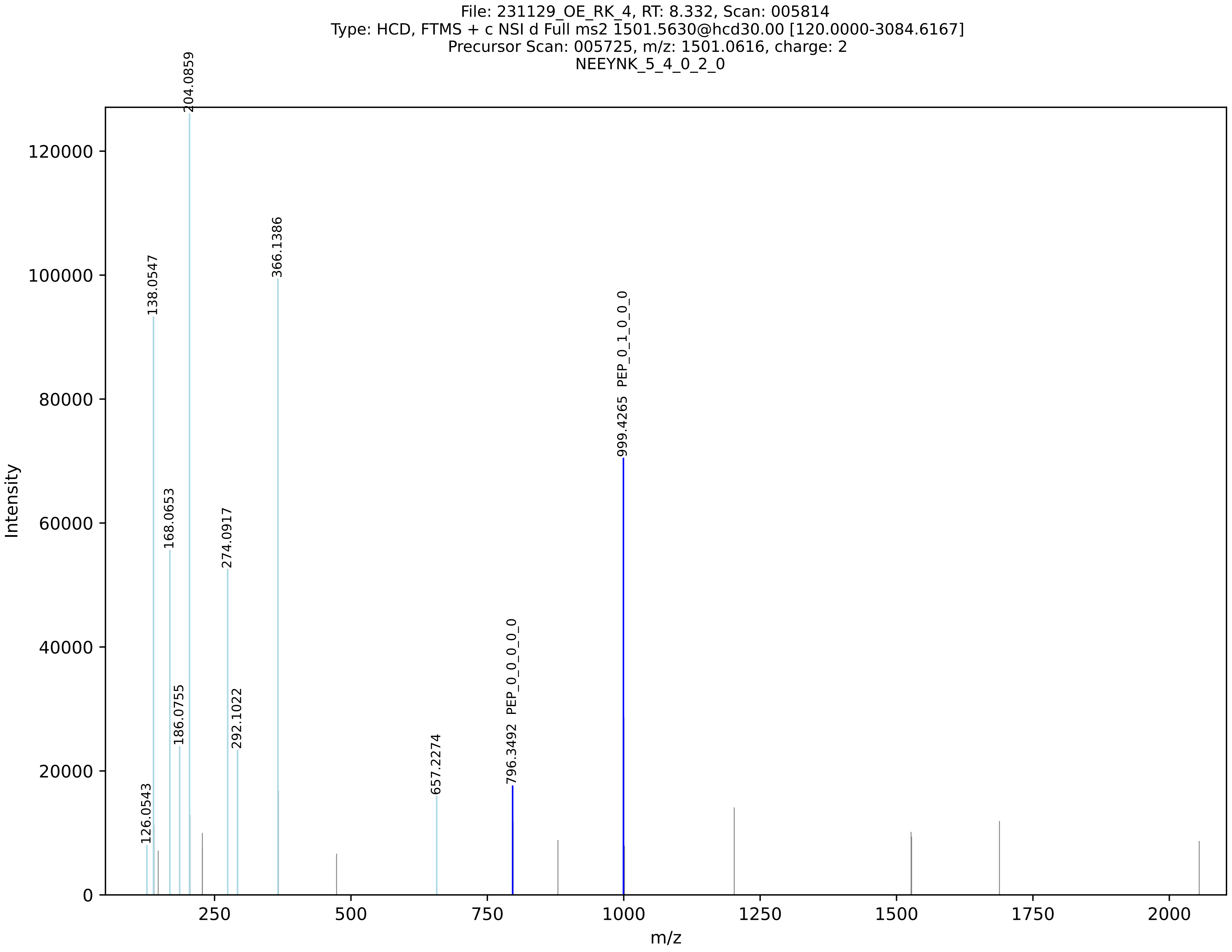
File: 231129_OE_RK_4, RT: 8.332, Scan: 005814
Type: HCD, FTMS + c NSI d Full ms2 1501.5630@hcd30.00 [120.0000-3084.6167]
Precursor Scan: 005725, m/z: 1501.0616, charge: 2
NEEYNK_5_4_0_2_0
204.0859
138.0547
366.1386
999.4265 PEP_0_1_0_0_0
168.0653
274.0917
186.0755
292.1022
657.2274
796.3492 PEP_0_0_0_0_0
126.0543
Intensity
m/z
0
20000
40000
60000
80000
100000
120000
250
500
750
1000
1250
1500
1750
2000

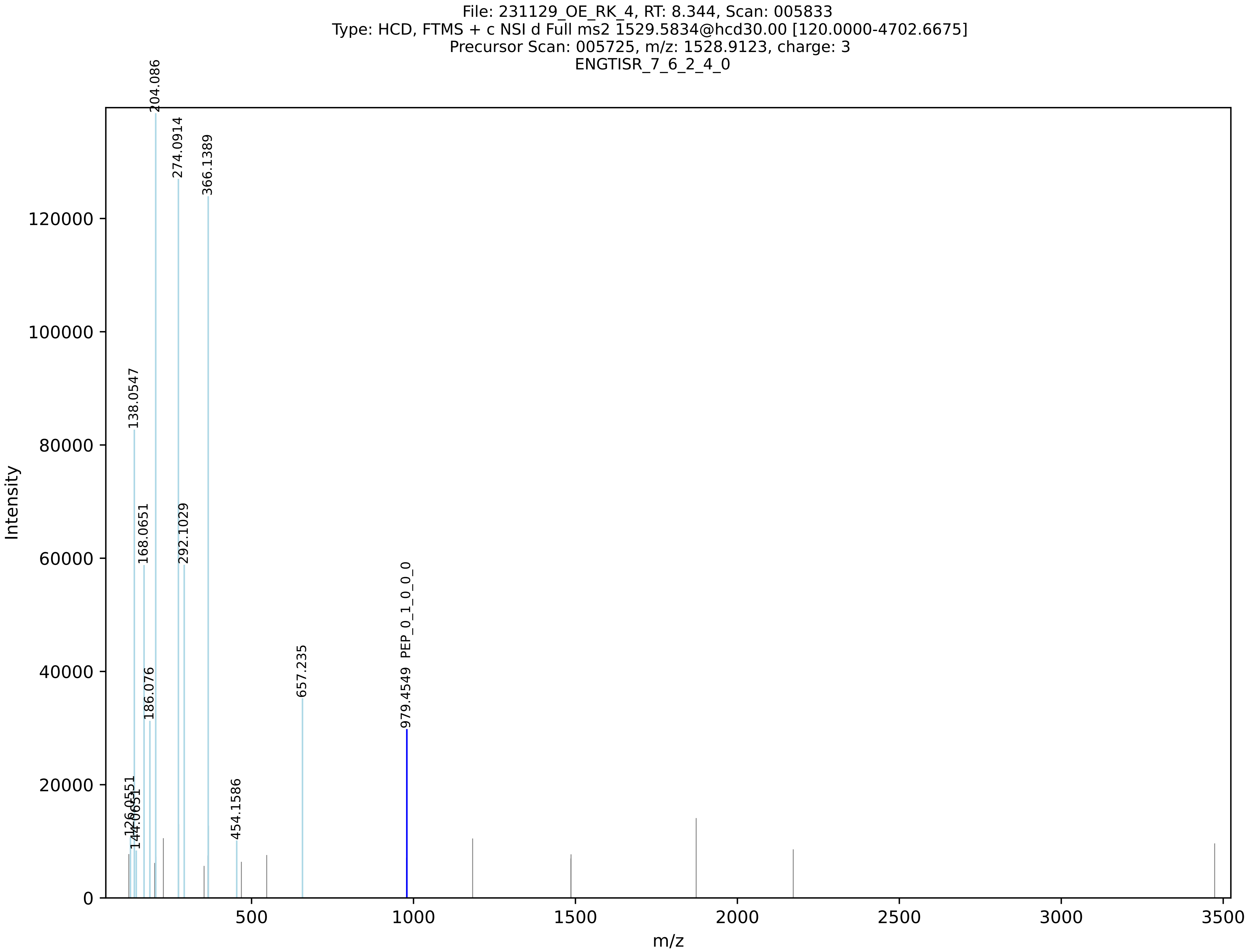
File: 231129_OE_RK_4, RT: 8.344, Scan: 005833
Type: HCD, FTMS + c NSI d Full ms2 1529.5834@hcd30.00 [120.0000-4702.6675]
Precursor Scan: 005725, m/z: 1528.9123, charge: 3
ENGTISR_7_6_2_4_0
Intensity
m/z
204.086
274.0914
366.1389
138.0547
168.0651
292.1029
186.076
657.235
979.4549 PEP_0_1_0_0_0
126.0551
144.0651
454.1586
0
20000
40000
60000
80000
100000
120000
500
1000
1500
2000
2500
3000
3500

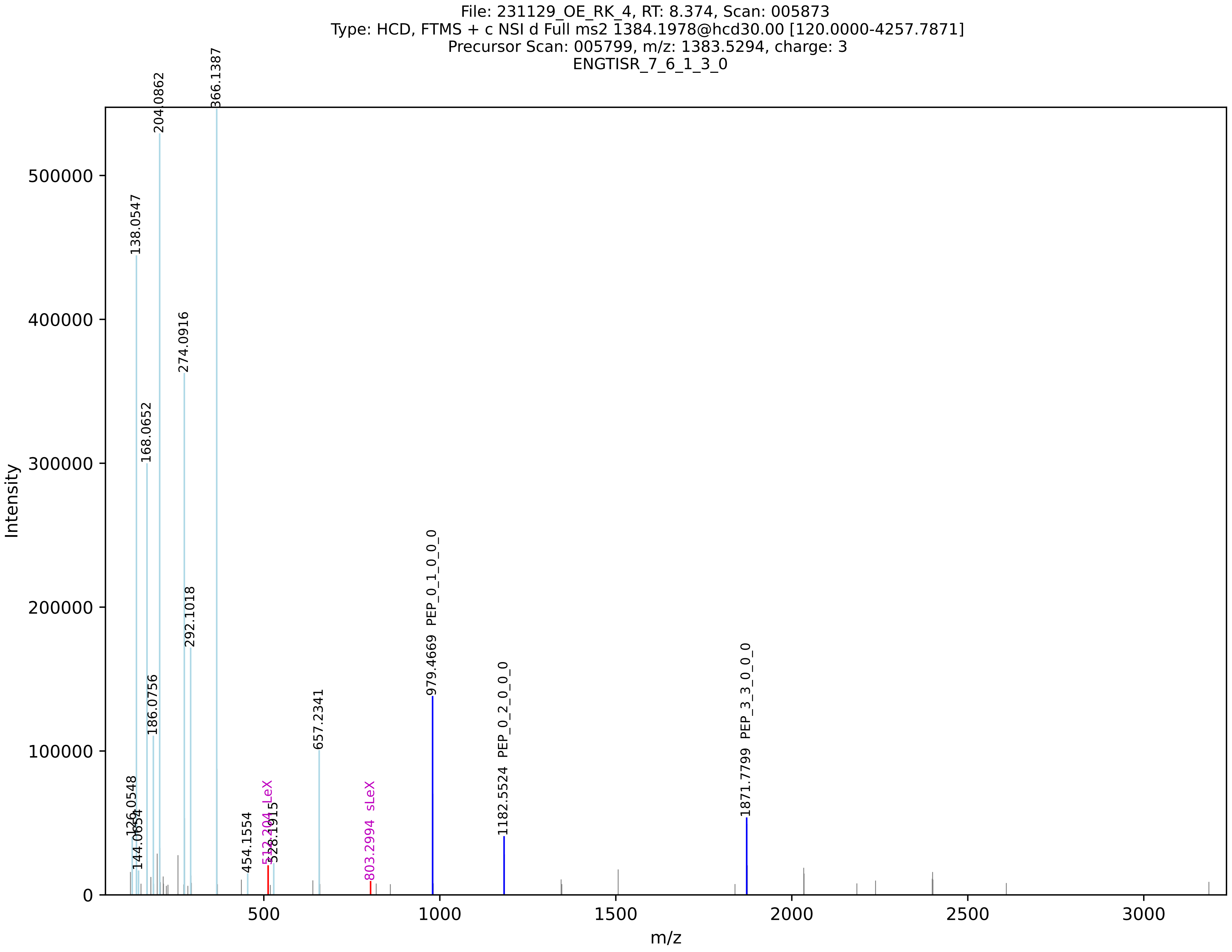
File: 231129_OE_RK_4, RT: 8.374, Scan: 005873
Type: HCD, FTMS + c NSI d Full ms2 1384.1978@hcd30.00 [120.0000-4257.7871]
Precursor Scan: 005799, m/z: 1383.5294, charge: 3
ENGTISR_7_6_1_3_0
Intensity
m/z
0
100000
200000
300000
400000
500000
500
1000
1500
2000
2500
3000
126.0548
144.0654
138.0547
168.0652
186.0756
204.0862
274.0916
292.1018
366.1387
454.1554
512.204 LeX
528.1915
657.2341
803.2994 sLeX
979.4669 PEP_0_1_0_0_0
1182.5524 PEP_0_2_0_0_0
1871.7799 PEP_3_3_0_0_0

File: 231129_OE_RK_4, RT: 8.376, Scan: 005876
Type: HCD, FTMS + c NSI d Full ms2 1481.5682@hcd30.00 [120.0000-4555.7412]
Precursor Scan: 005799, m/z: 1480.5664, charge: 3
ENGTISR_7_6_1_4_0

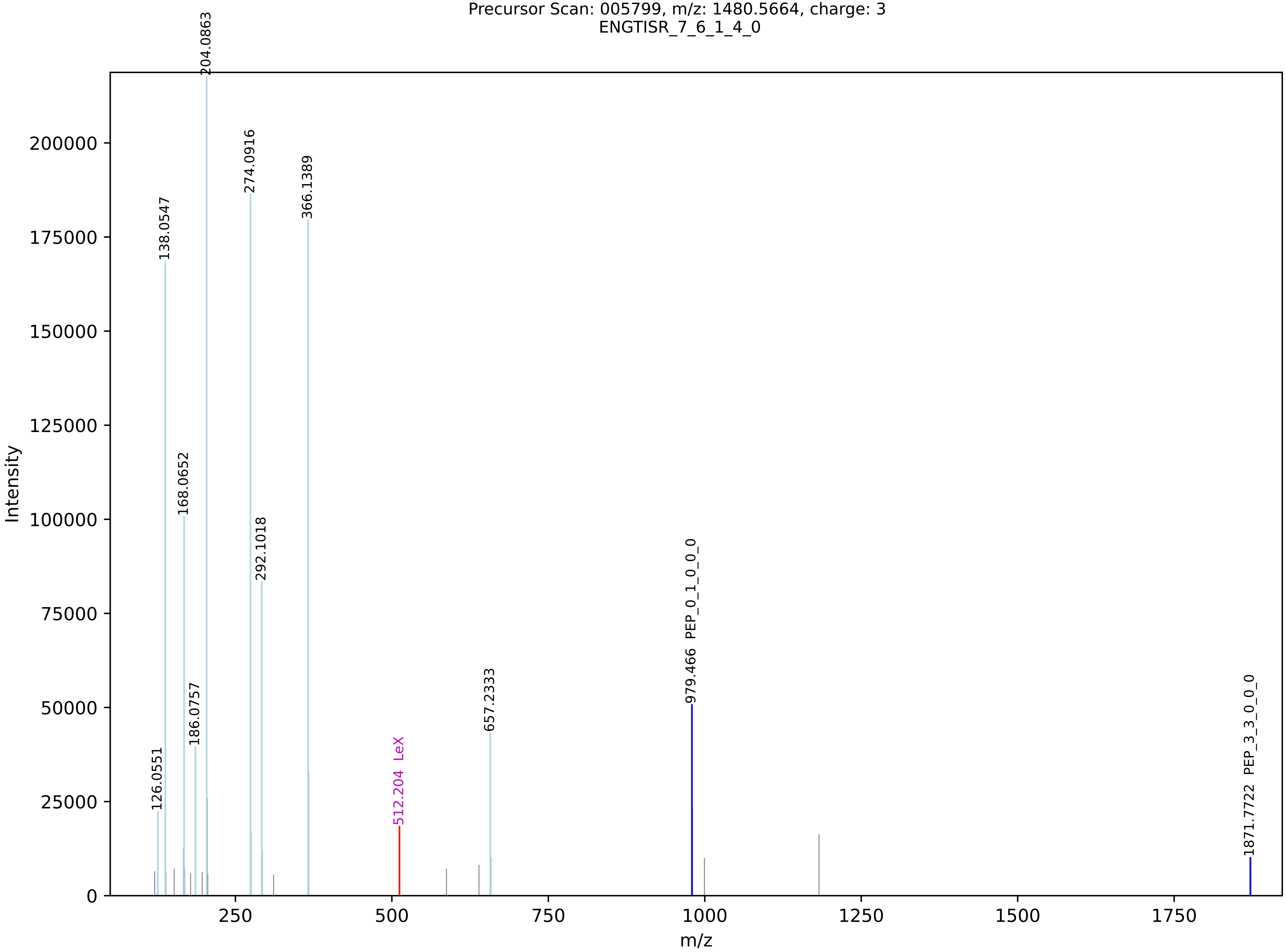

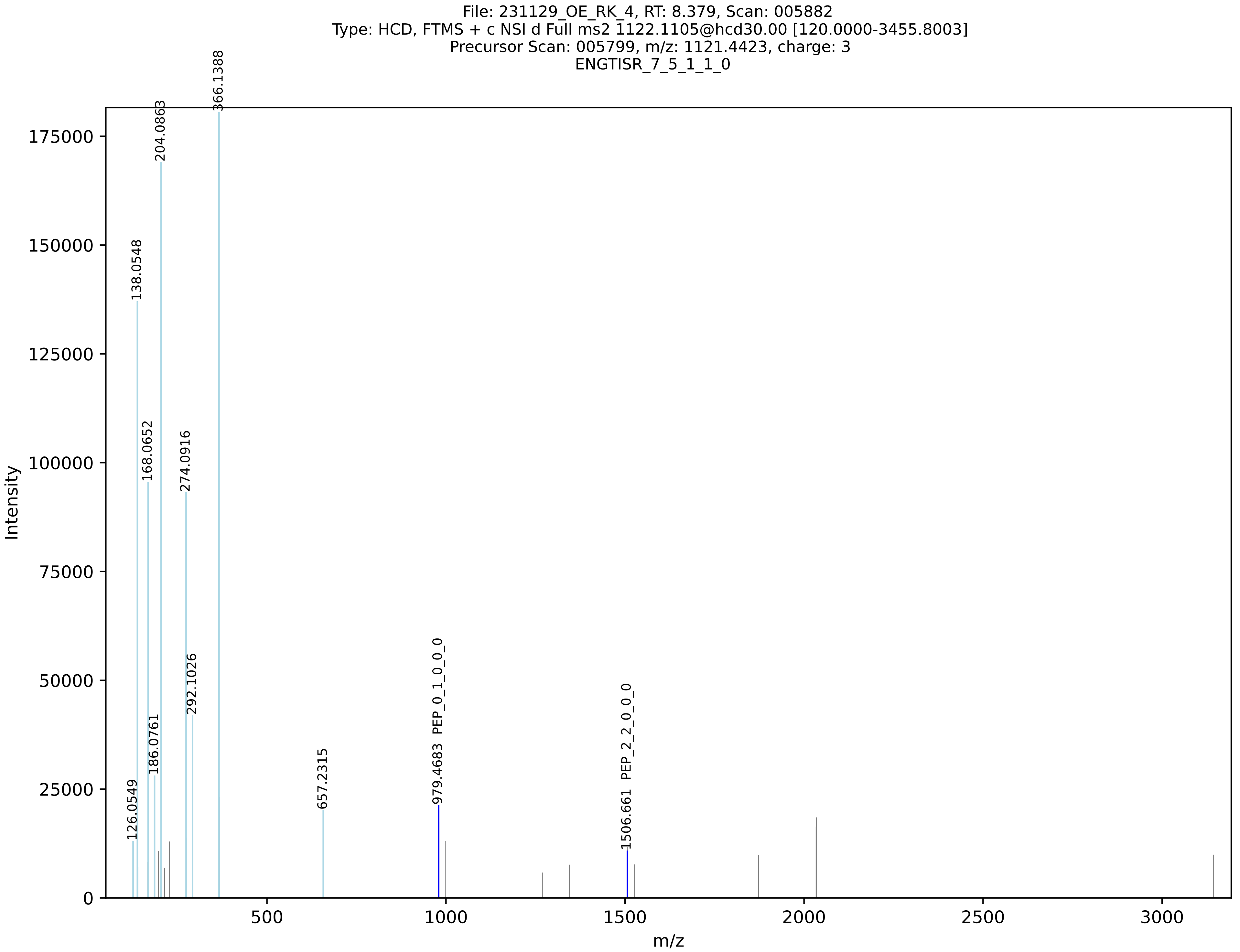

File: 231129_OE_RK_4, RT: 8.379, Scan: 005882
Type: HCD, FTMS + c NSI d Full ms2 1122.1105@hcd30.00 [120.0000-3455.8003]
Precursor Scan: 005799, m/z: 1121.4423, charge: 3
ENGTISR_7_5_1_1_0
Intensity
m/z
366.1388
204.0863
138.0548
168.0652
274.0916
292.1026
186.0761
126.0549
657.2315
979.4683 PEP_0_1_0_0_0
1506.661 PEP_2_2_0_0_0

File: 231129_OE_RK_4, RT: 8.423, Scan: 005945
Type: HCD, FTMS + c NSI d Full ms2 994.7255@hcd30.00 [120.0000-3066.0022]
Precursor Scan: 005870, m/z: 994.3914, charge: 3
ENGTISR_5_4_0_2_0

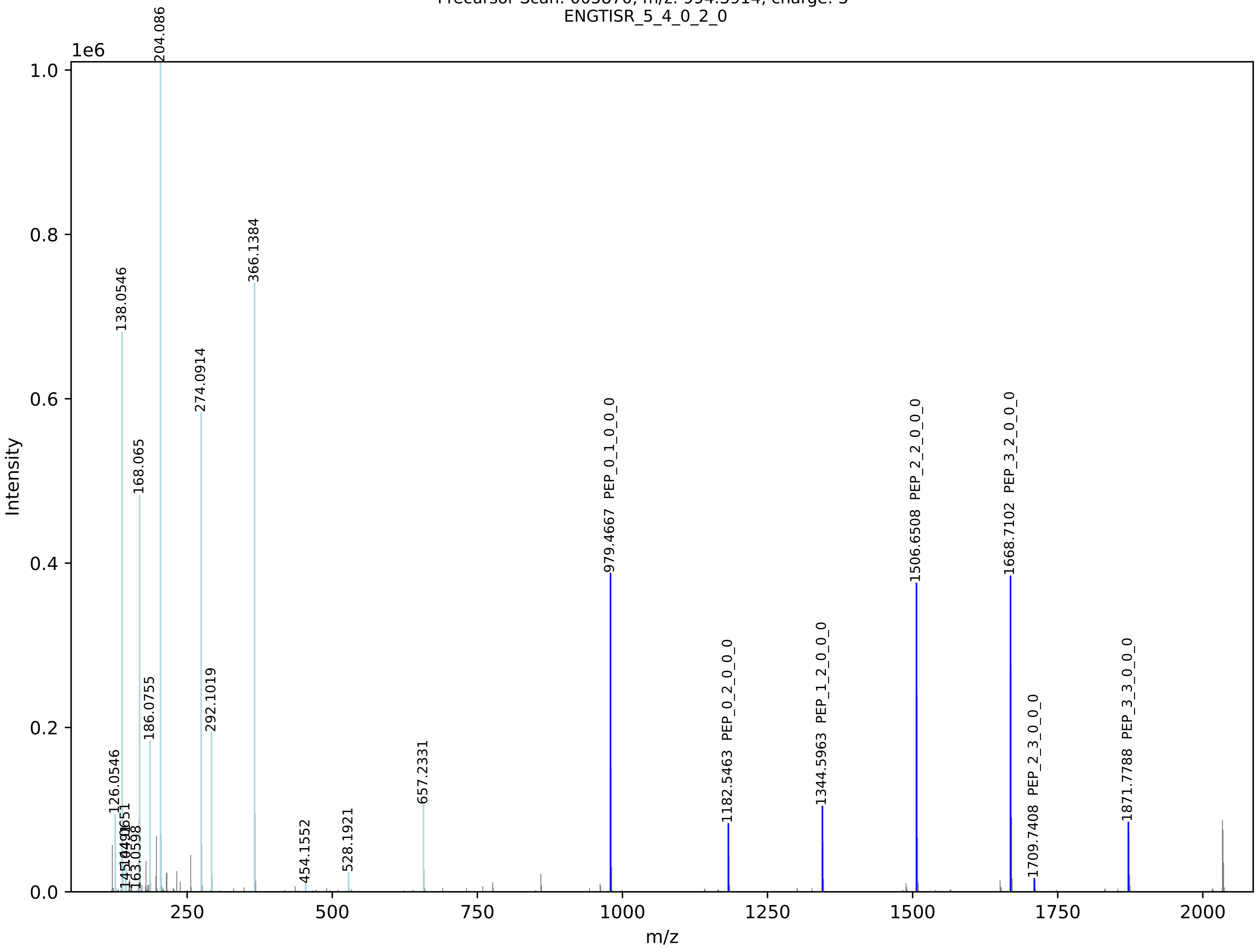

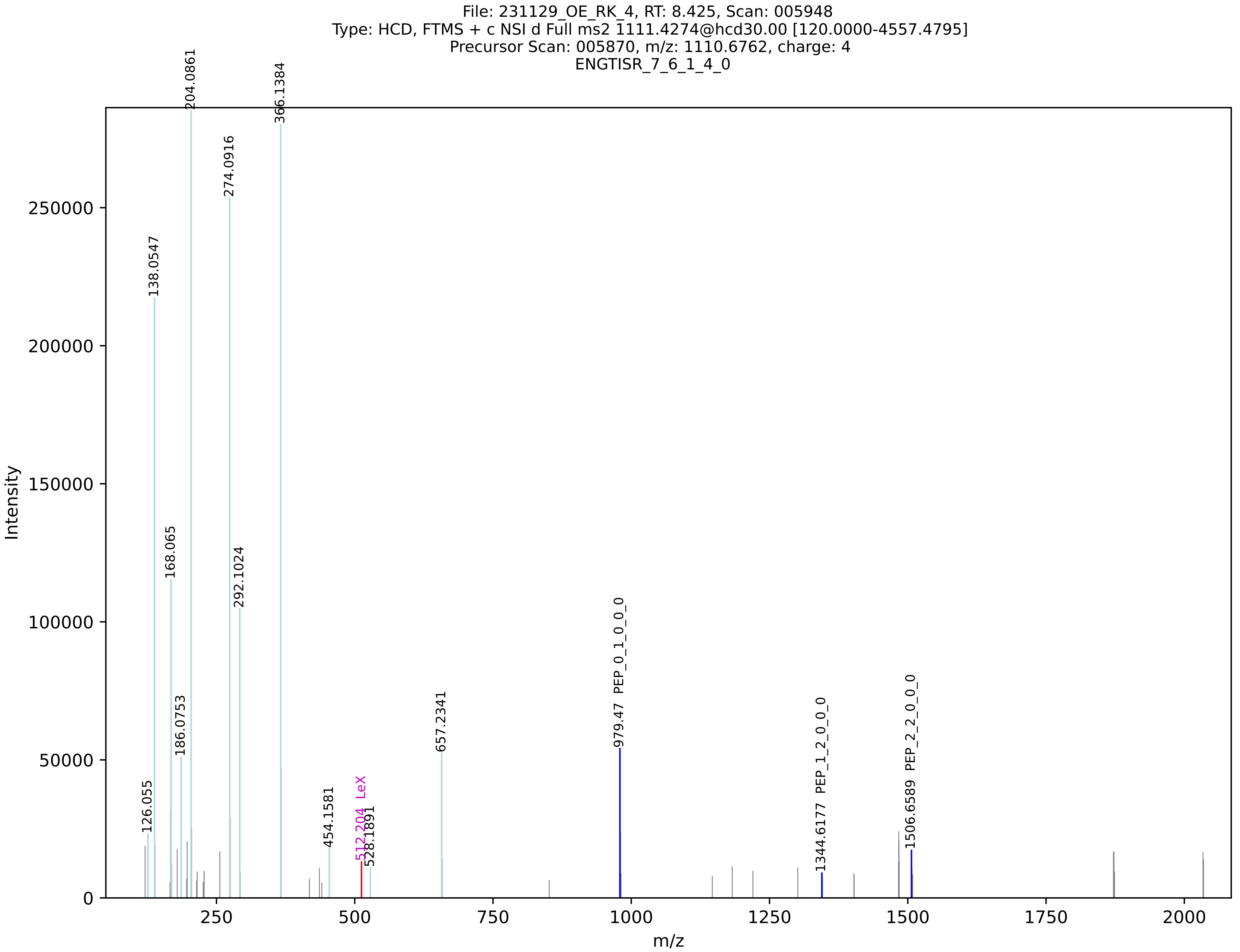
File: 231129_OE_RK_4, RT: 8.425, Scan: 005948
Type: HCD, FTMS + c NSI d Full ms2 1111.4274@hcd30.00 [120.0000-4557.4795]
Precursor Scan: 005870, m/z: 1110.6762, charge: 4
ENGTISR_7_6_1_4_0
Intensity
m/z
204.0861
366.1384
274.0916
138.0547
168.065
292.1024
186.0753
126.055
454.1581
512.204 LeX
528.1891
657.2341
979.47 PEP_0_1_0_0_0
1344.6177 PEP_1_2_0_0_0
1506.6589 PEP_2_2_0_0_0
0
50000
100000
150000
200000
250000
250
500
750
1000
1250
1500
1750
2000

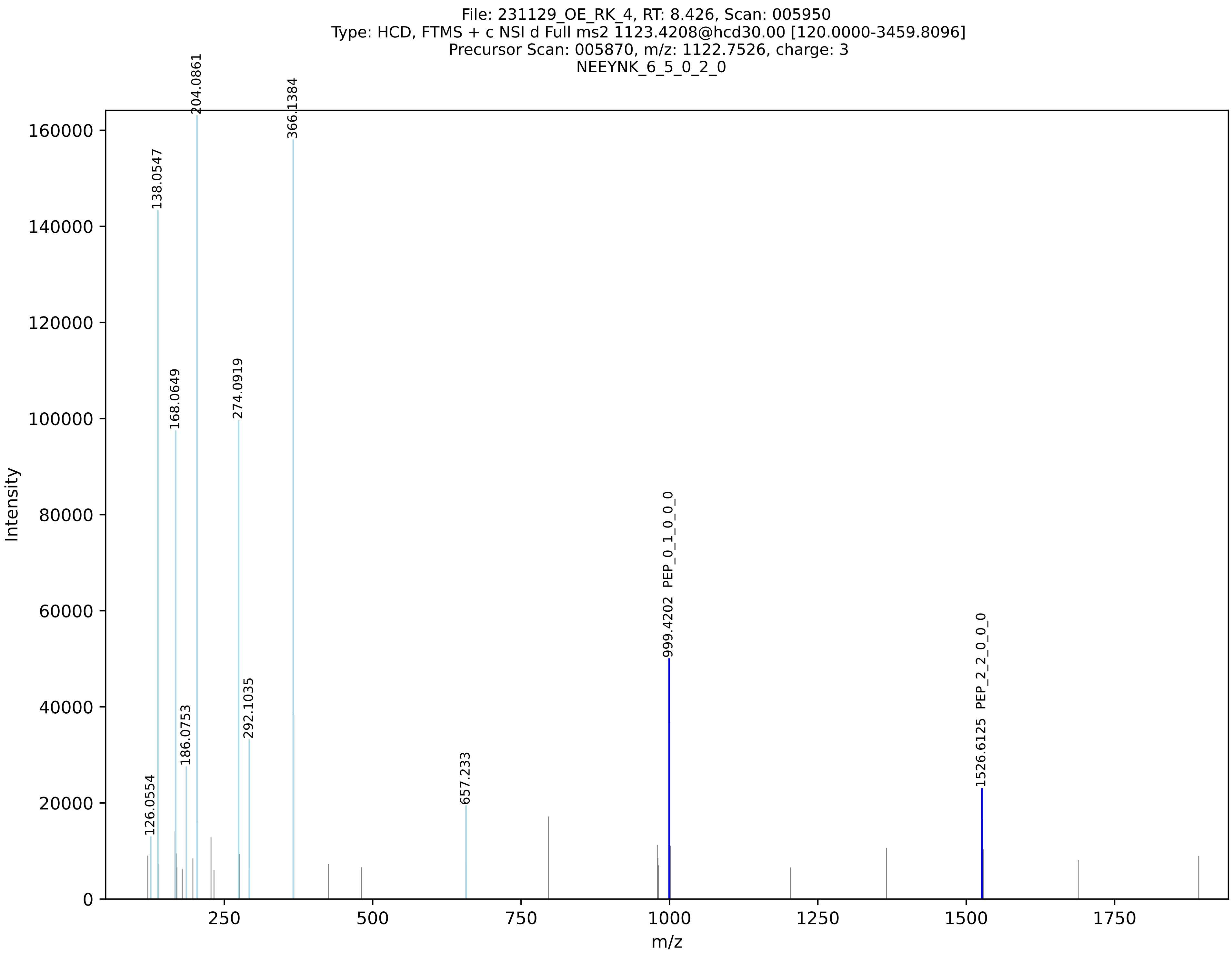

File: 231129_OE_RK_4, RT: 8.426, Scan: 005950
Type: HCD, FTMS + c NSI d Full ms2 1123.4208@hcd30.00 [120.0000-3459.8096]
Precursor Scan: 005870, m/z: 1122.7526, charge: 3
NEEYNK_6_5_0_2_0
204.0861
366.1384
138.0547
168.0649
274.0919
999.4202 PEP_0_1_0_0_0
1526.6125 PEP_2_2_0_0_0
292.1035
186.0753
657.233
126.0554
Intensity
m/z
0
20000
40000
60000
80000
100000
120000
140000
160000
250
500
750
1000
1250
1500
1750

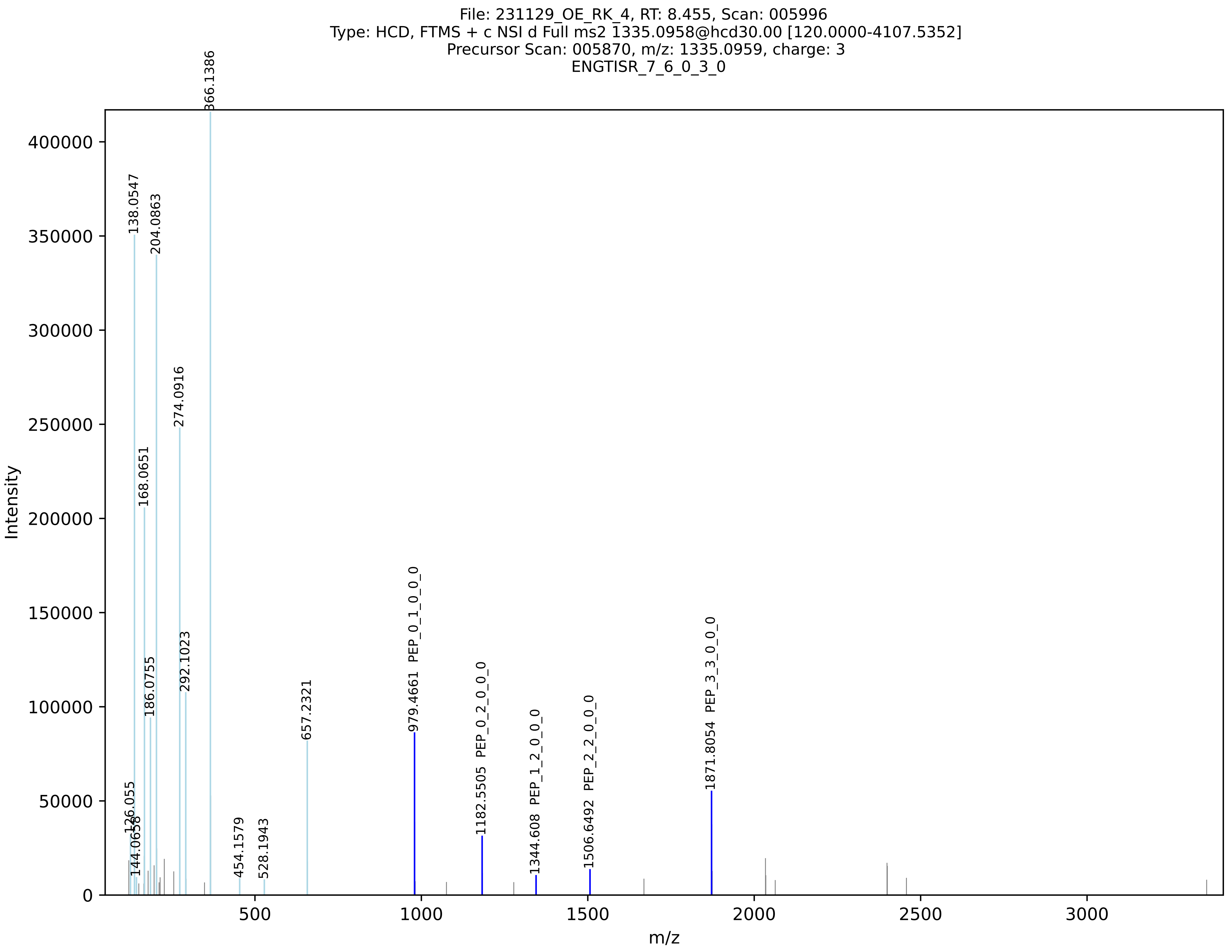
File: 231129_OE_RK_4, RT: 8.455, Scan: 005996
Type: HCD, FTMS + c NSI d Full ms2 1335.0958@hcd30.00 [120.0000-4107.5352]
Precursor Scan: 005870, m/z: 1335.0959, charge: 3
ENGTISR_7_6_0_3_0
Intensity
m/z
366.1386
138.0547
204.0863
274.0916
168.0651
292.1023
186.0755
126.055
144.0658
454.1579
528.1943
657.2321
979.4661 PEP_0_1_0_0_0
1182.5505 PEP_0_2_0_0_0
1344.608 PEP_1_2_0_0_0
1506.6492 PEP_2_2_0_0_0
1871.8054 PEP_3_3_0_0_0

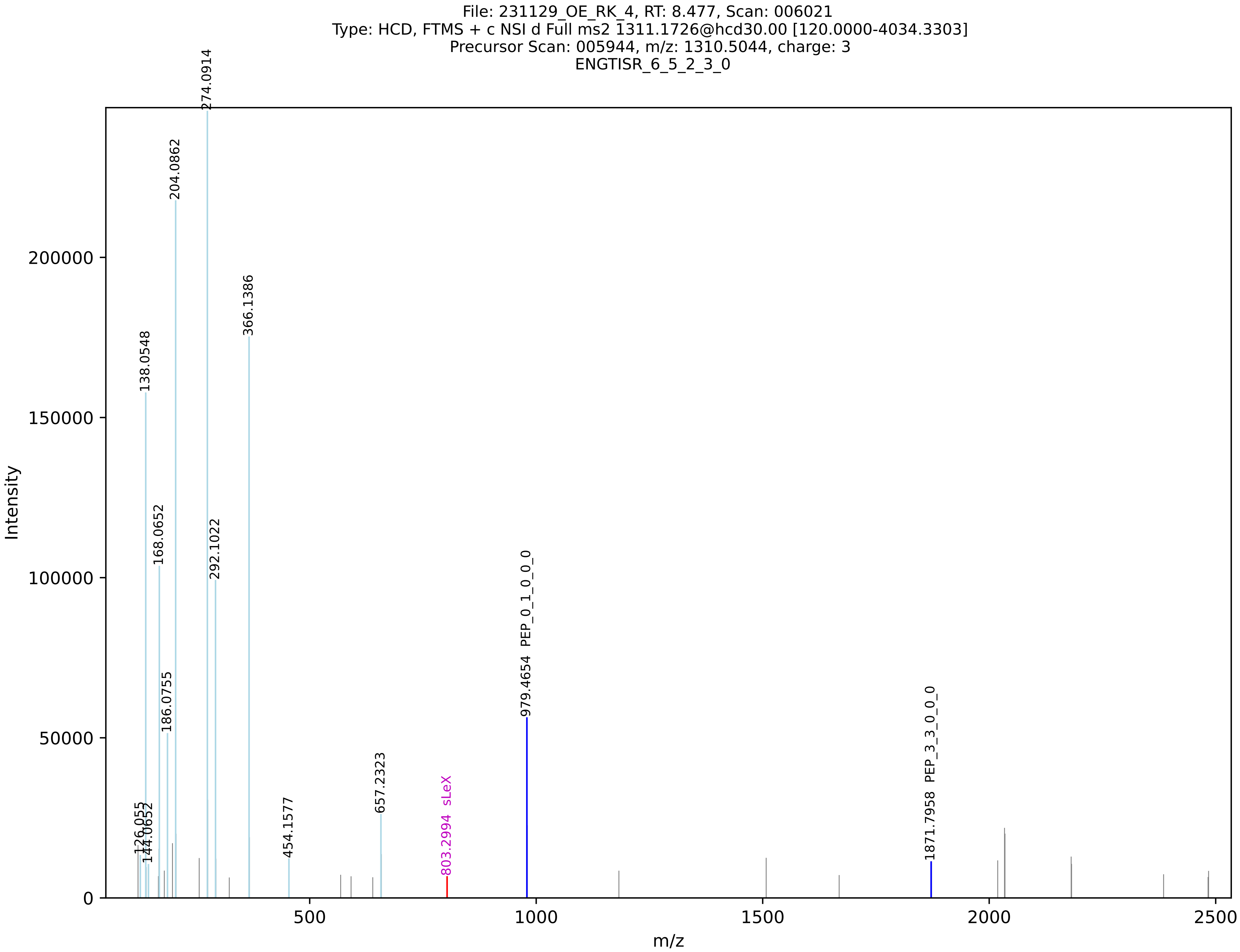

File: 231129_OE_RK_4, RT: 8.477, Scan: 006021
Type: HCD, FTMS + c NSI d Full ms2 1311.1726@hcd30.00 [120.0000-4034.3303]
Precursor Scan: 005944, m/z: 1310.5044, charge: 3
ENGTISR_6_5_2_3_0
Intensity
m/z
274.0914
204.0862
366.1386
138.0548
168.0652
292.1022
186.0755
126.055
144.0652
454.1577
657.2323
803.2994 sLeX
979.4654 PEP_0_1_0_0_0_
1871.7958 PEP_3_3_0_0_0_
0
50000
100000
150000
200000
500
1000
1500
2000
2500

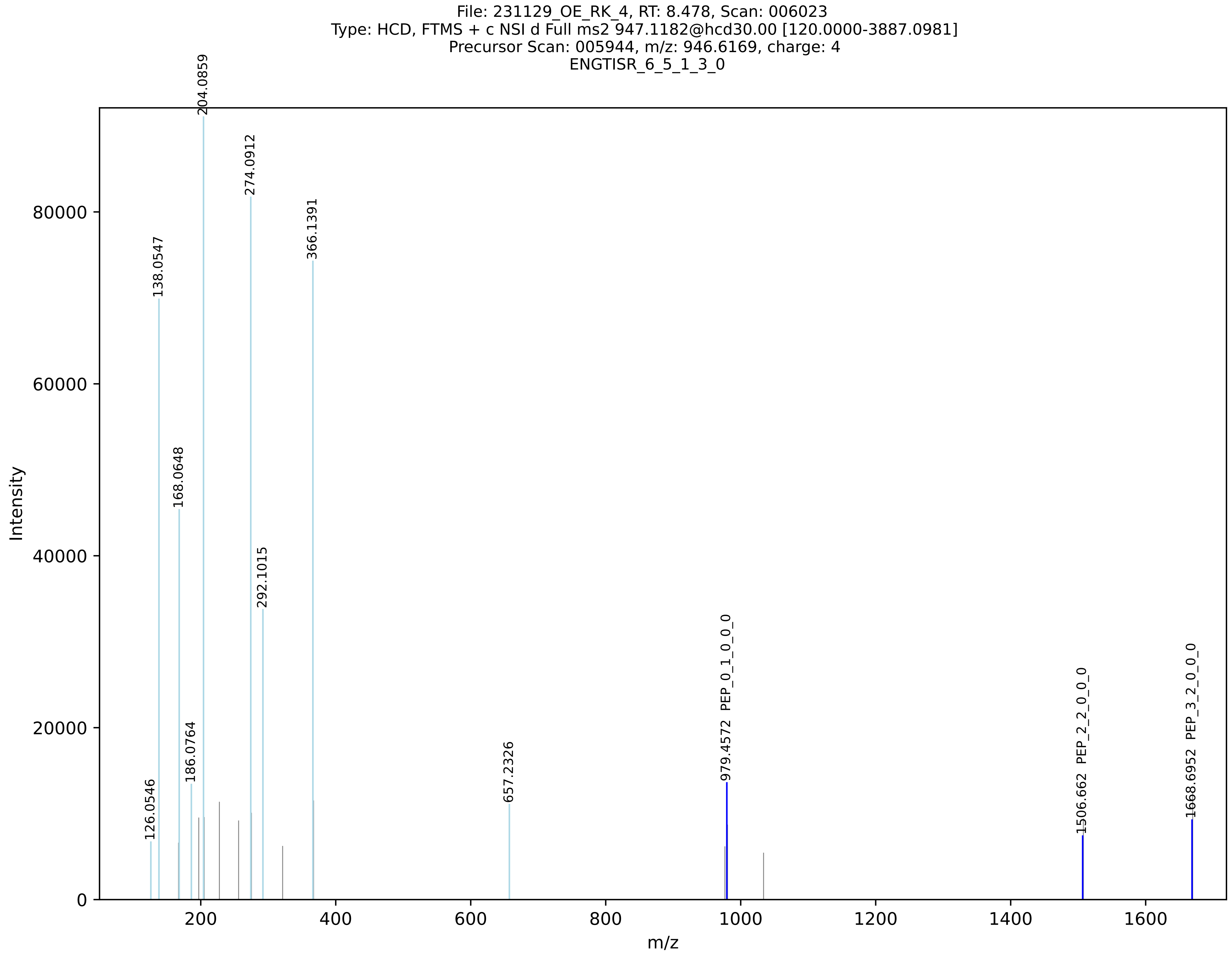

File: 231129_OE_RK_4, RT: 8.478, Scan: 006023
Type: HCD, FTMS + c NSI d Full ms2 947.1182@hcd30.00 [120.0000-3887.0981]
Precursor Scan: 005944, m/z: 946.6169, charge: 4
ENGTISR_6_5_1_3_0
Intensity
m/z
204.0859
274.0912
366.1391
138.0547
168.0648
292.1015
186.0764
126.0546
657.2326
979.4572 PEP_0_1_0_0_0
1506.662 PEP_2_2_0_0_0
1668.6952 PEP_3_2_0_0_0
0
20000
40000
60000
80000
200
400
600
800
1000
1200
1400
1600

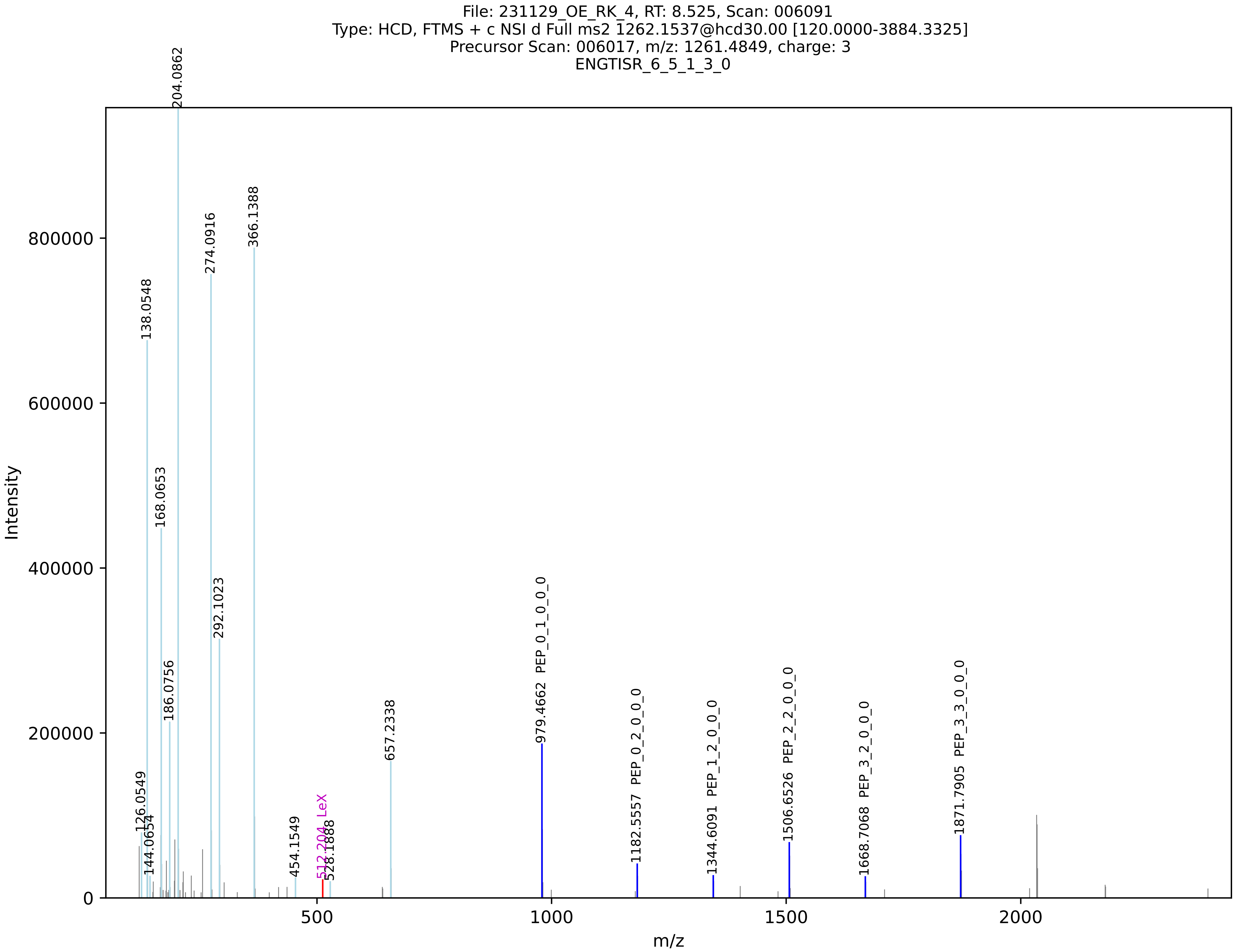
File: 231129_OE_RK_4, RT: 8.525, Scan: 006091
Type: HCD, FTMS + c NSI d Full ms2 1262.1537@hcd30.00 [120.0000-3884.3325]
Precursor Scan: 006017, m/z: 1261.4849, charge: 3
ENGTISR_6_5_1_3_0
Intensity
m/z
204.0862
366.1388
274.0916
138.0548
168.0653
292.1023
186.0756
126.0549
144.0654
454.1549
512.204 LeX
528.1888
657.2338
979.4662 PEP_0_1_0_0_0
1182.5557 PEP_0_2_0_0_0
1344.6091 PEP_1_2_0_0_0
1506.6526 PEP_2_2_0_0_0
1668.7068 PEP_3_2_0_0_0
1871.7905 PEP_3_3_0_0_0

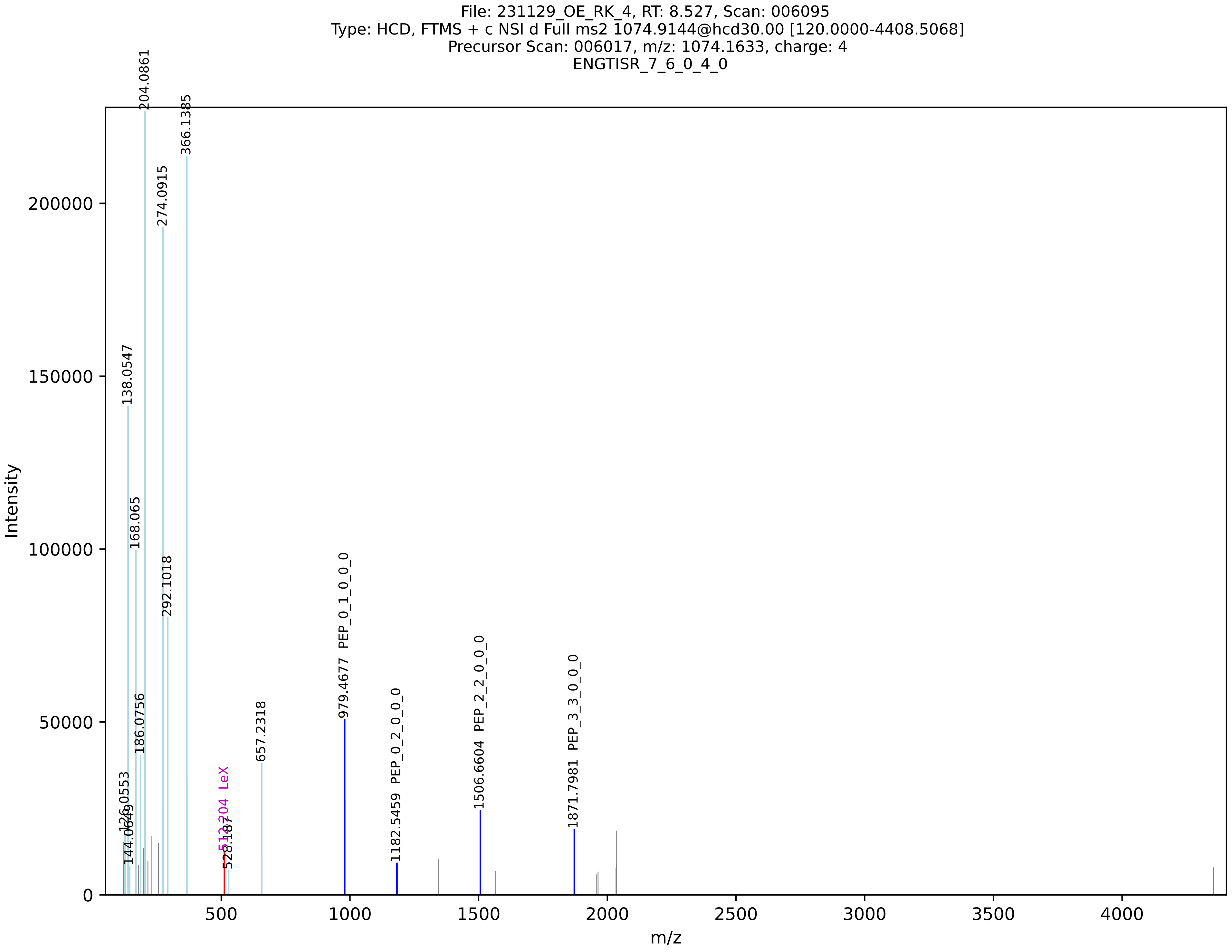
File: 231129_OE_RK_4, RT: 8.527, Scan: 006095
Type: HCD, FTMS + c NSI d Full ms2 1074.9144@hcd30.00 [120.0000-4408.5068]
Precursor Scan: 006017, m/z: 1074.1633, charge: 4
ENGTISR_7_6_0_4_0
204.0861
366.1385
274.0915
138.0547
168.065
292.1018
186.0756
126.0553
144.0649
512.204 LeX
528.187
657.2318
979.4677 PEP_0_1_0_0_0
1182.5459 PEP_0_2_0_0_0
1506.6604 PEP_2_2_0_0_0
1871.7981 PEP_3_3_0_0_0
Intensity
m/z
0
50000
100000
150000
200000
500
1000
1500
2000
2500
3000
3500
4000

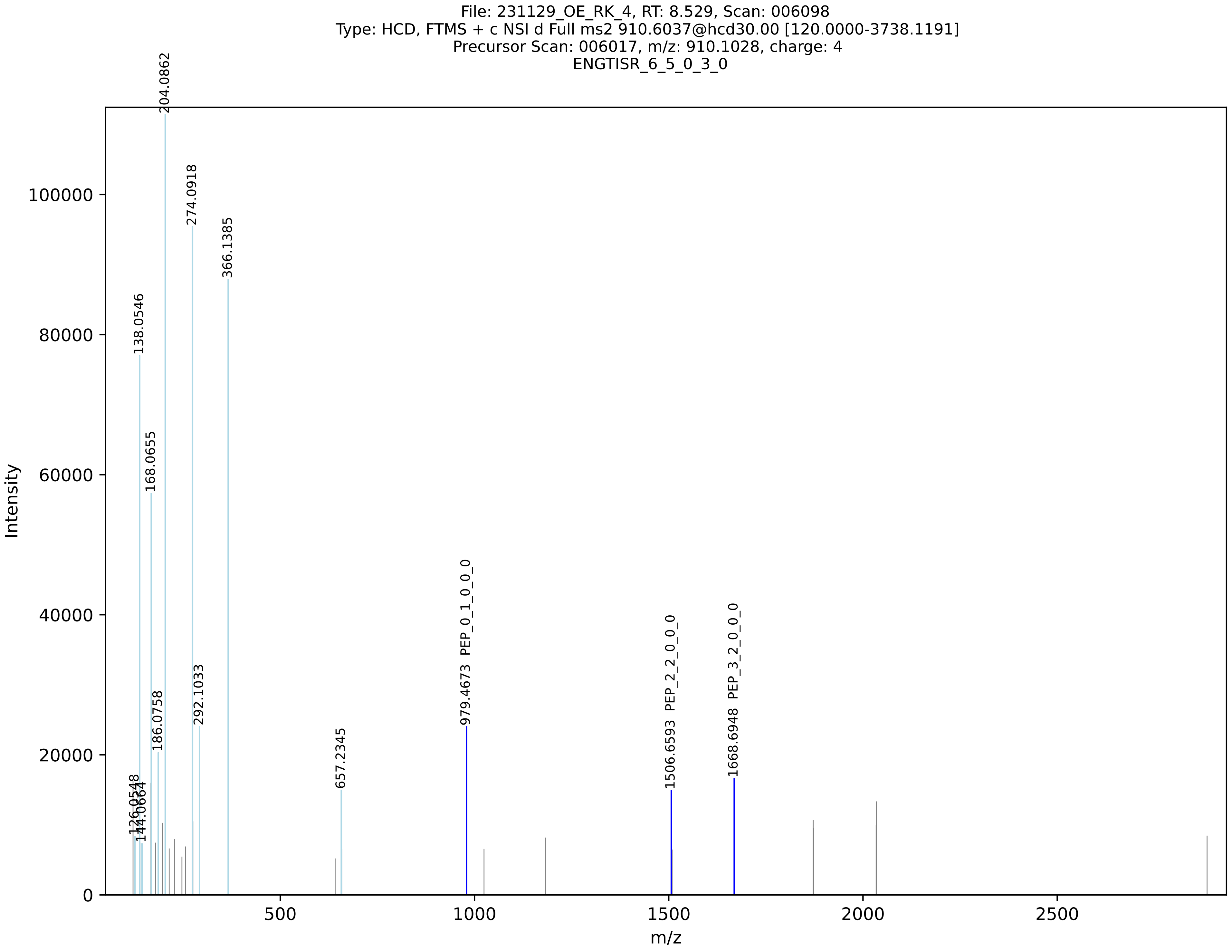
File: 231129_OE_RK_4, RT: 8.529, Scan: 006098
Type: HCD, FTMS + c NSI d Full ms2 910.6037@hcd30.00 [120.0000-3738.1191]
Precursor Scan: 006017, m/z: 910.1028, charge: 4
ENGTISR_6_5_0_3_0
204.0862
274.0918
366.1385
138.0546
168.0655
186.0758
292.1033
126.0548
144.0664
657.2345
979.4673 PEP_0_1_0_0_0
1506.6593 PEP_2_2_0_0_0
1668.6948 PEP_3_2_0_0_0
Intensity
m/z
0
20000
40000
60000
80000
100000
500
1000
1500
2000
2500

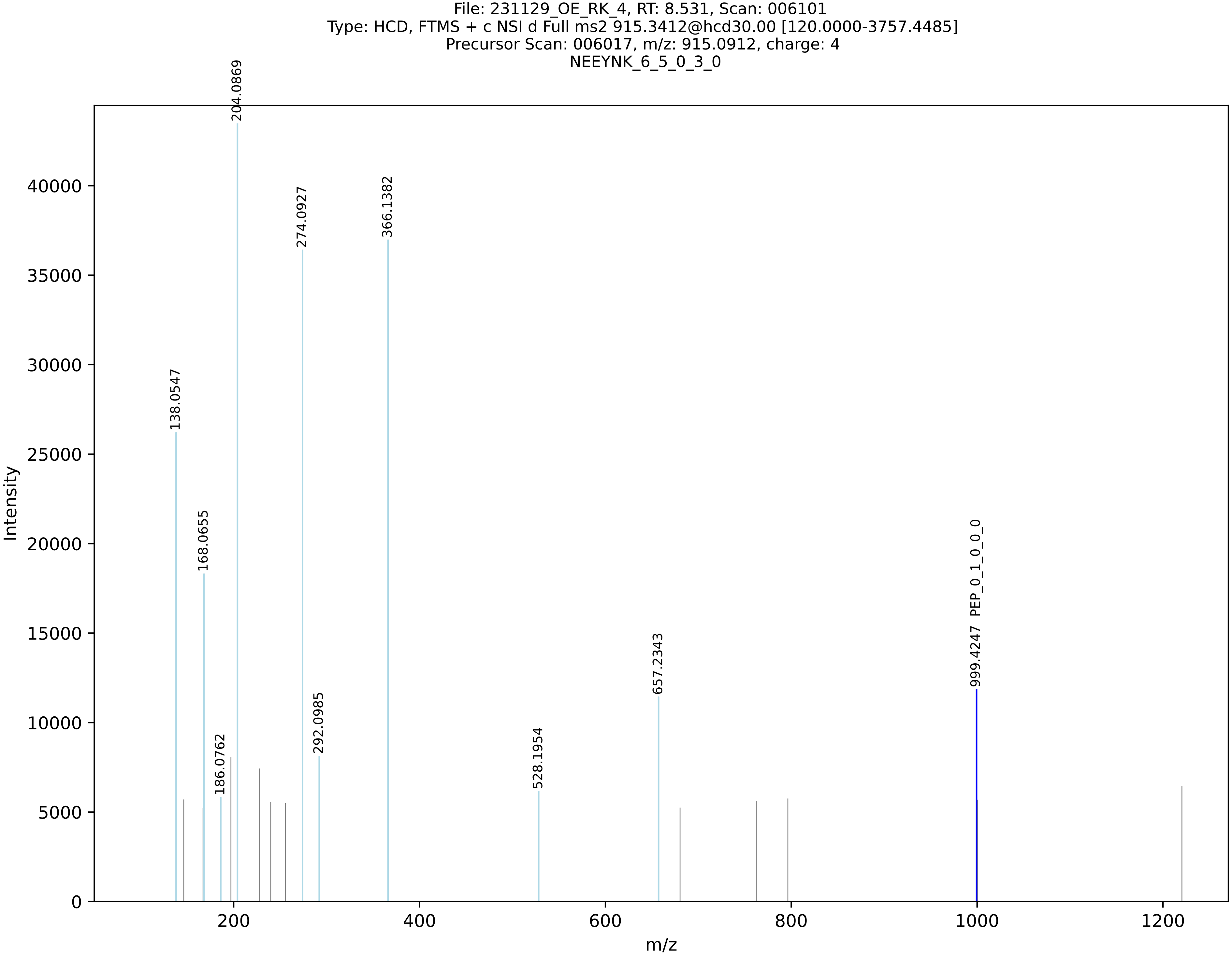
File: 231129_OE_RK_4, RT: 8.531, Scan: 006101
Type: HCD, FTMS + c NSI d Full ms2 915.3412@hcd30.00 [120.0000-3757.4485]
Precursor Scan: 006017, m/z: 915.0912, charge: 4
NEEYNK_6_5_0_3_0
204.0869
274.0927
366.1382
138.0547
168.0655
186.0762
292.0985
528.1954
657.2343
999.4247 PEP_0_1_0_0_
Intensity
m/z
0
5000
10000
15000
20000
25000
30000
35000
40000
200
400
600
800
1000
1200

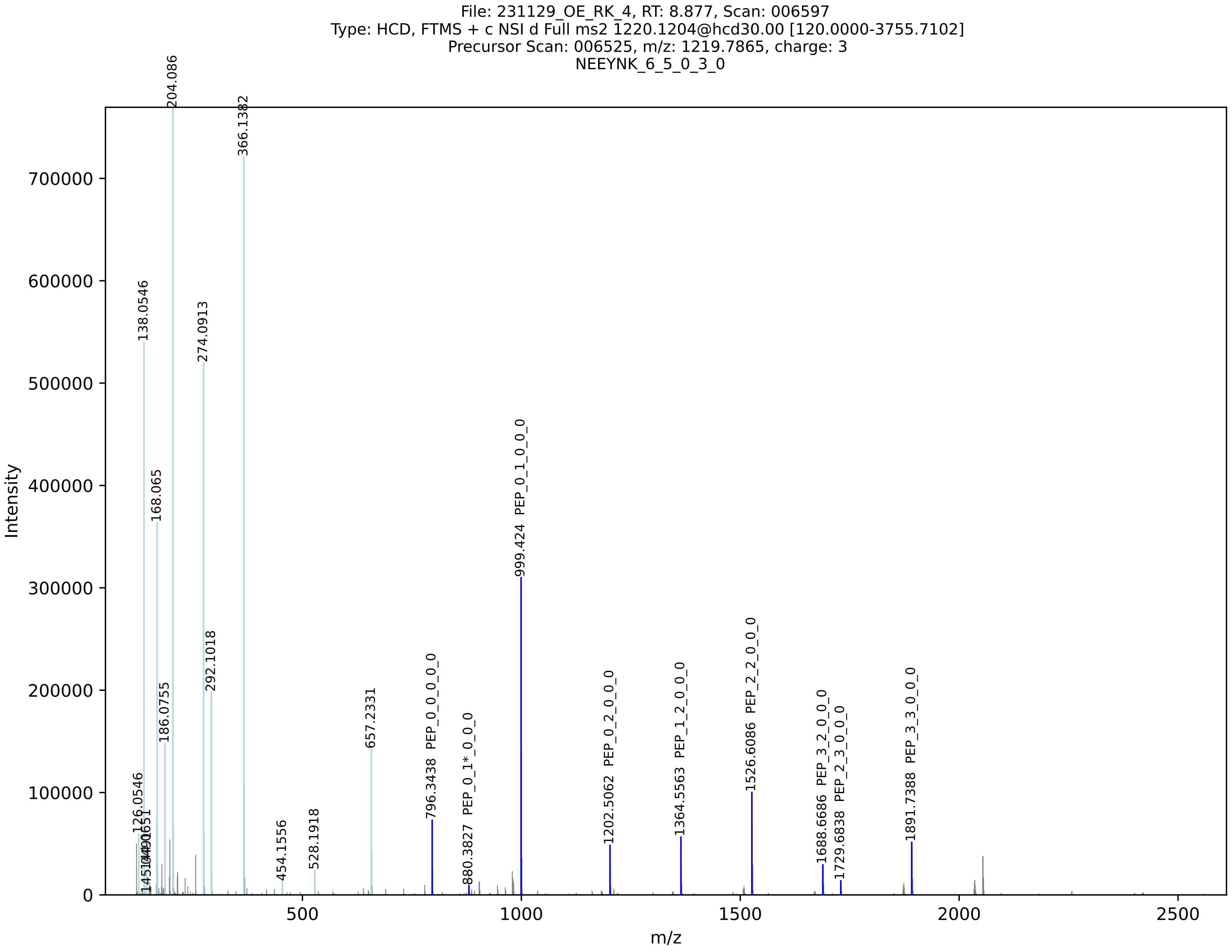
File: 231129_OE_RK_4, RT: 8.877, Scan: 006597
Type: HCD, FTMS + c NSI d Full ms2 1220.1204@hcd30.00 [120.0000-3755.7102]
Precursor Scan: 006525, m/z: 1219.7865, charge: 3
NEEYNK_6_5_0_3_0
Intensity
m/z
0
100000
200000
300000
400000
500000
600000
700000
500
1000
1500
2000
2500
204.086
366.1382
138.0546
274.0913
168.065
292.1018
186.0755
126.0546
454.1556
528.1918
657.2331
796.3438 PEP_0_0_0_0_0_
880.3827 PEP_0_1*_0_0_0
999.424 PEP_0_1_0_0_0
1202.5062 PEP_0_2_0_0_0
1364.5563 PEP_1_2_0_0_0
1526.6086 PEP_2_2_0_0_0
1688.6686 PEP_3_2_0_0_0
1729.6838 PEP_2_3_0_0_0
1891.7388 PEP_3_3_0_0_0

File: 231129_OE_RK_4, RT: 8.879, Scan: 006599
Type: HCD, FTMS + c NSI d Full ms2 1310.8401@hcd30.00 [120.0000-4033.3127]
Precursor Scan: 006525, m/z: 1310.5061, charge: 3
ENGTISR_6_5_2_3_0

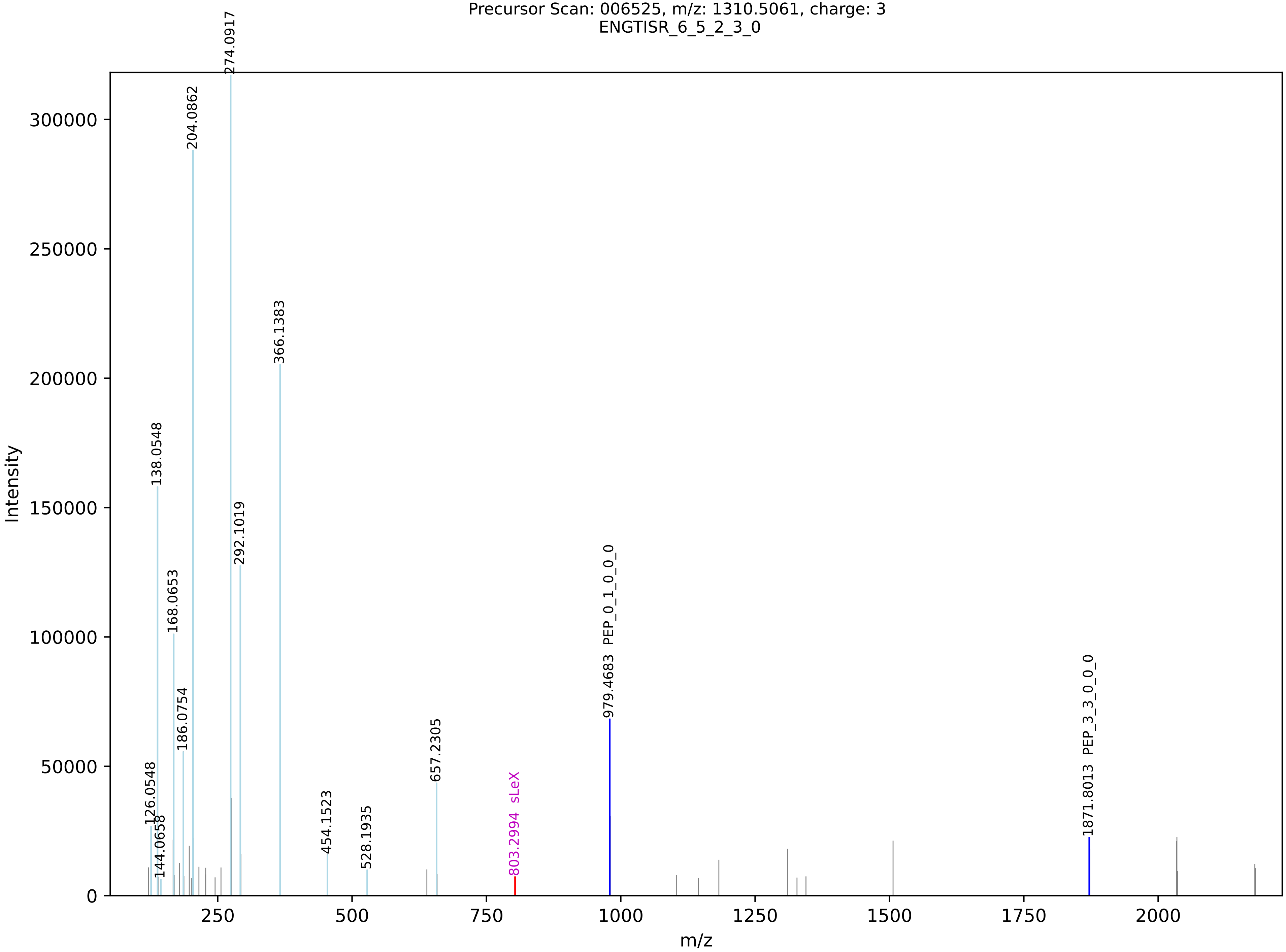

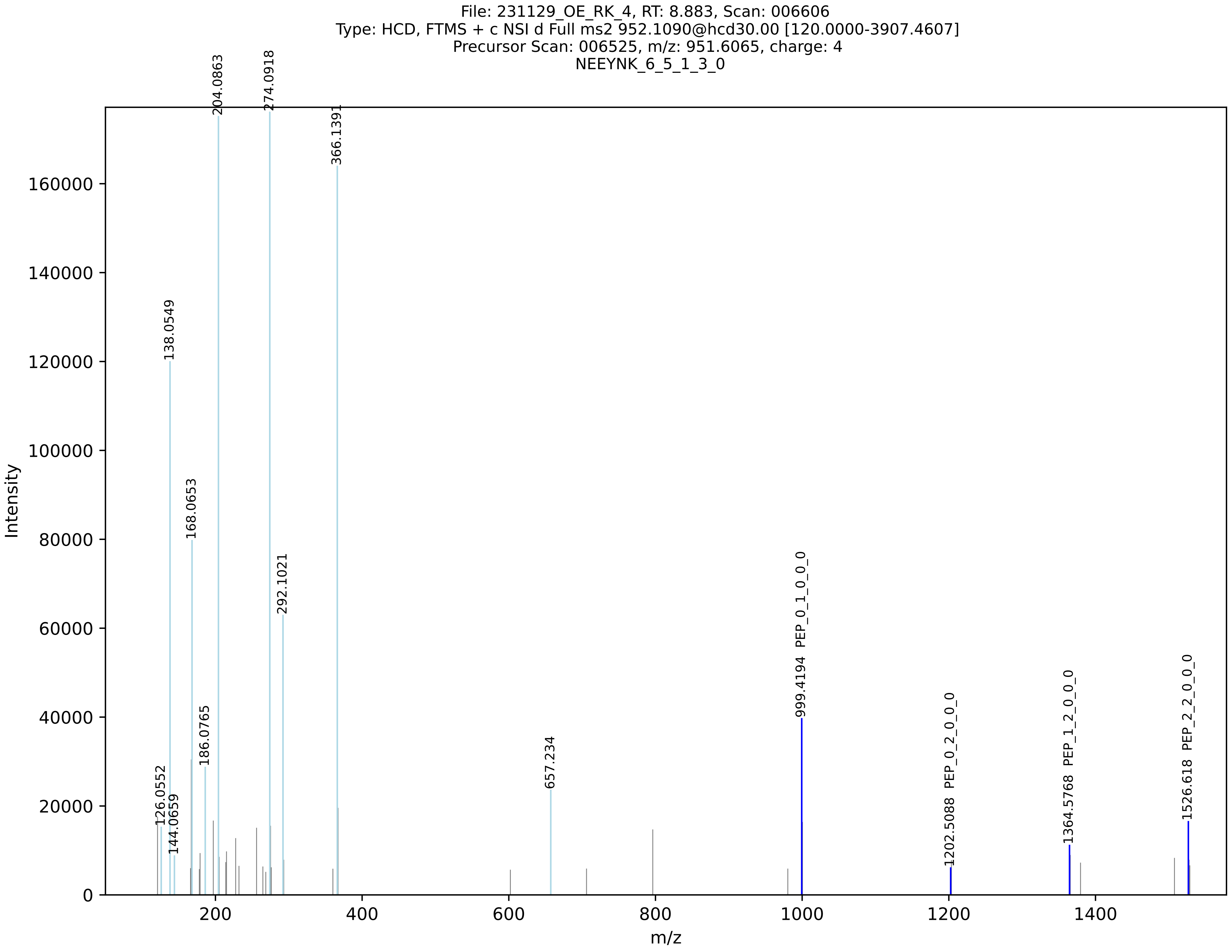
File: 231129_OE_RK_4, RT: 8.883, Scan: 006606
Type: HCD, FTMS + c NSI d Full ms2 952.1090@hcd30.00 [120.0000-3907.4607]
Precursor Scan: 006525, m/z: 951.6065, charge: 4
NEEYNK_6_5_1_3_0
Intensity
m/z
126.0552
138.0549
144.0659
168.0653
186.0765
204.0863
274.0918
292.1021
366.1391
657.234
999.4194 PEP_0_1_0_0_0
1202.5088 PEP_0_2_0_0_0
1364.5768 PEP_1_2_0_0_0
1526.618 PEP_2_2_0_0_0

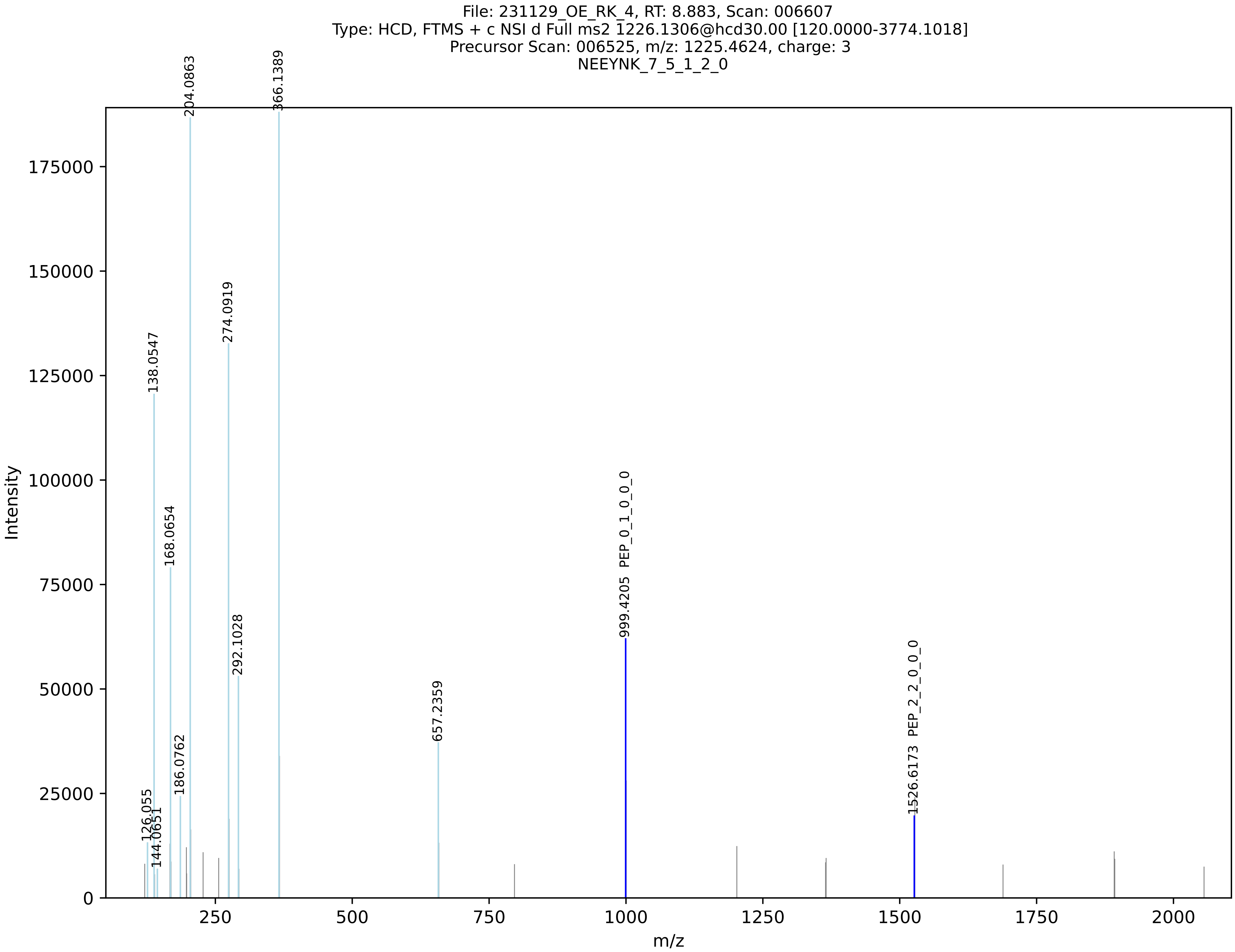
File: 231129_OE_RK_4, RT: 8.883, Scan: 006607
Type: HCD, FTMS + c NSI d Full ms2 1226.1306@hcd30.00 [120.0000-3774.1018]
Precursor Scan: 006525, m/z: 1225.4624, charge: 3
NEEYNK_7_5_1_2_0
Intensity
m/z
204.0863
366.1389
274.0919
138.0547
168.0654
292.1028
186.0762
126.055
144.0651
657.2359
999.4205 PEP_0_1_0_0_0
1526.6173 PEP_2_2_0_0_0

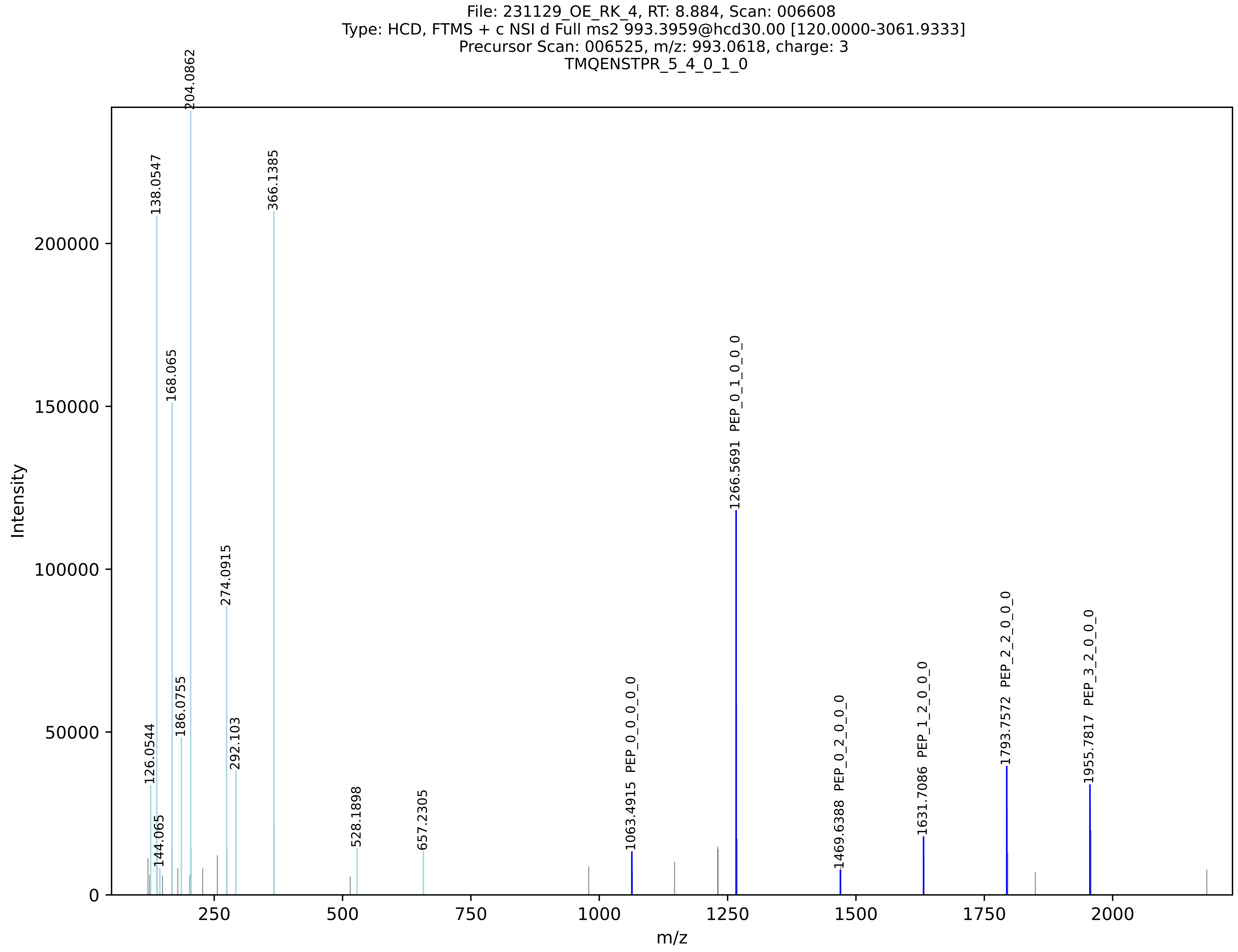
File: 231129_OE_RK_4, RT: 8.884, Scan: 006608
Type: HCD, FTMS + c NSI d Full ms2 993.3959@hcd30.00 [120.0000-3061.9333]
Precursor Scan: 006525, m/z: 993.0618, charge: 3
TMQENSTPR_5_4_0_1_0
Intensity
m/z
204.0862
138.0547
366.1385
168.065
274.0915
186.0755
126.0544
292.103
144.065
528.1898
657.2305
1063.4915 PEP_0_0_0_0_0
1266.5691 PEP_0_1_0_0_0
1469.6388 PEP_0_2_0_0_0
1631.7086 PEP_1_2_0_0_0
1793.7572 PEP_2_2_0_0_0
1955.7817 PEP_3_2_0_0_0
0
50000
100000
150000
200000
250
500
750
1000
1250
1500
1750
2000

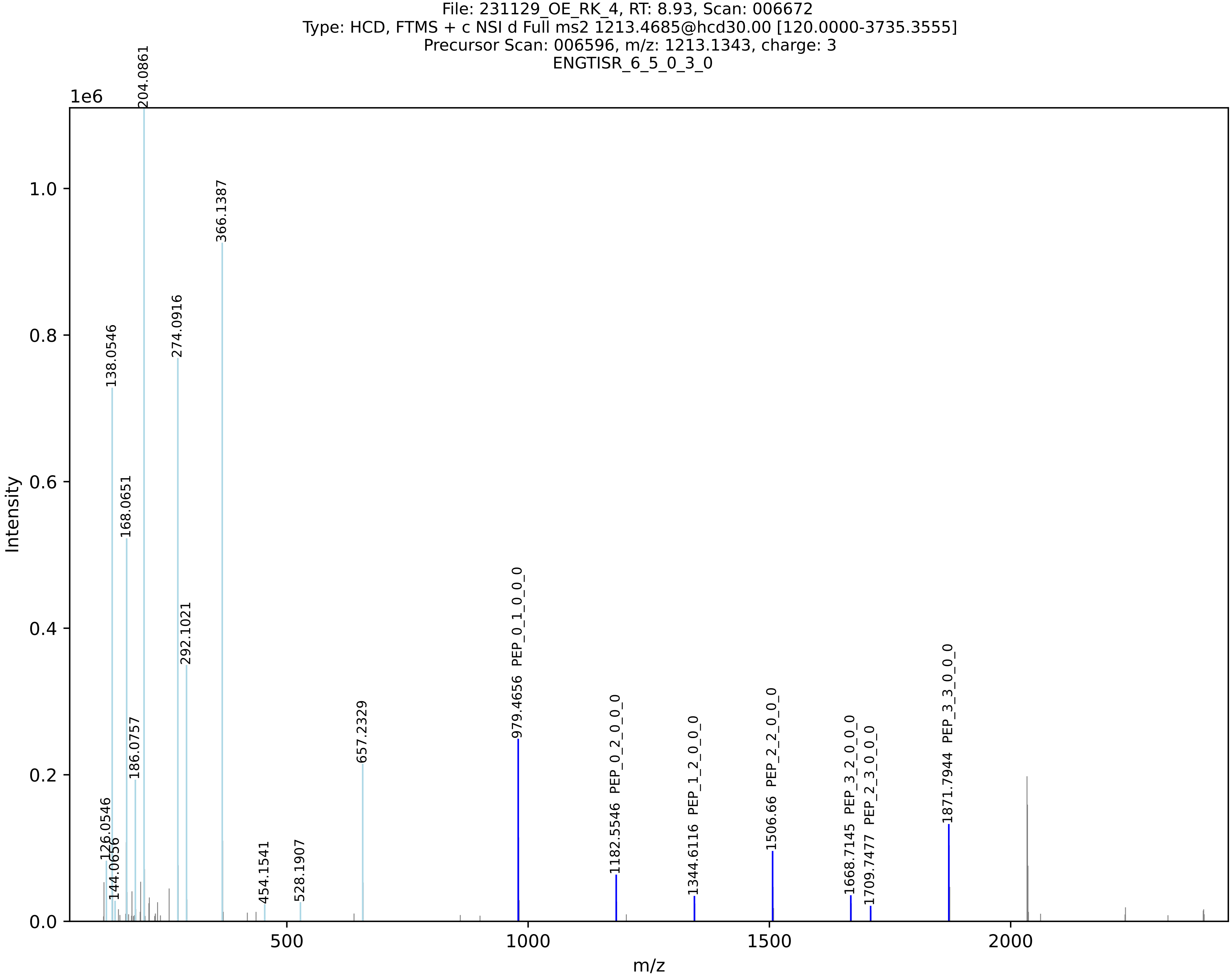
File: 231129_OE_RK_4, RT: 8.93, Scan: 006672
Type: HCD, FTMS + c NSI d Full ms2 1213.4685@hcd30.00 [120.0000-3735.3555]
Precursor Scan: 006596, m/z: 1213.1343, charge: 3
ENGTISR_6_5_0_3_0
1e6
Intensity
m/z
204.0861
366.1387
274.0916
138.0546
168.0651
292.1021
186.0757
126.0546
144.0656
454.1541
528.1907
657.2329
979.4656 PEP_0_1_0_0_0
1182.5546 PEP_0_2_0_0_0
1344.6116 PEP_1_2_0_0_0
1506.66 PEP_2_2_0_0_0
1668.7145 PEP_3_2_0_0_0
1709.7477 PEP_2_3_0_0_0
1871.7944 PEP_3_3_0_0_0

File: 231129_OE_RK_4, RT: 8.93, Scan: 006673
Type: HCD, FTMS + c NSI d Full ms2 1262.1542@hcd30.00 [120.0000-3884.3337]
Precursor Scan: 006596, m/z: 1261.8202, charge: 3
ENGTISR_6_5_1_3_0

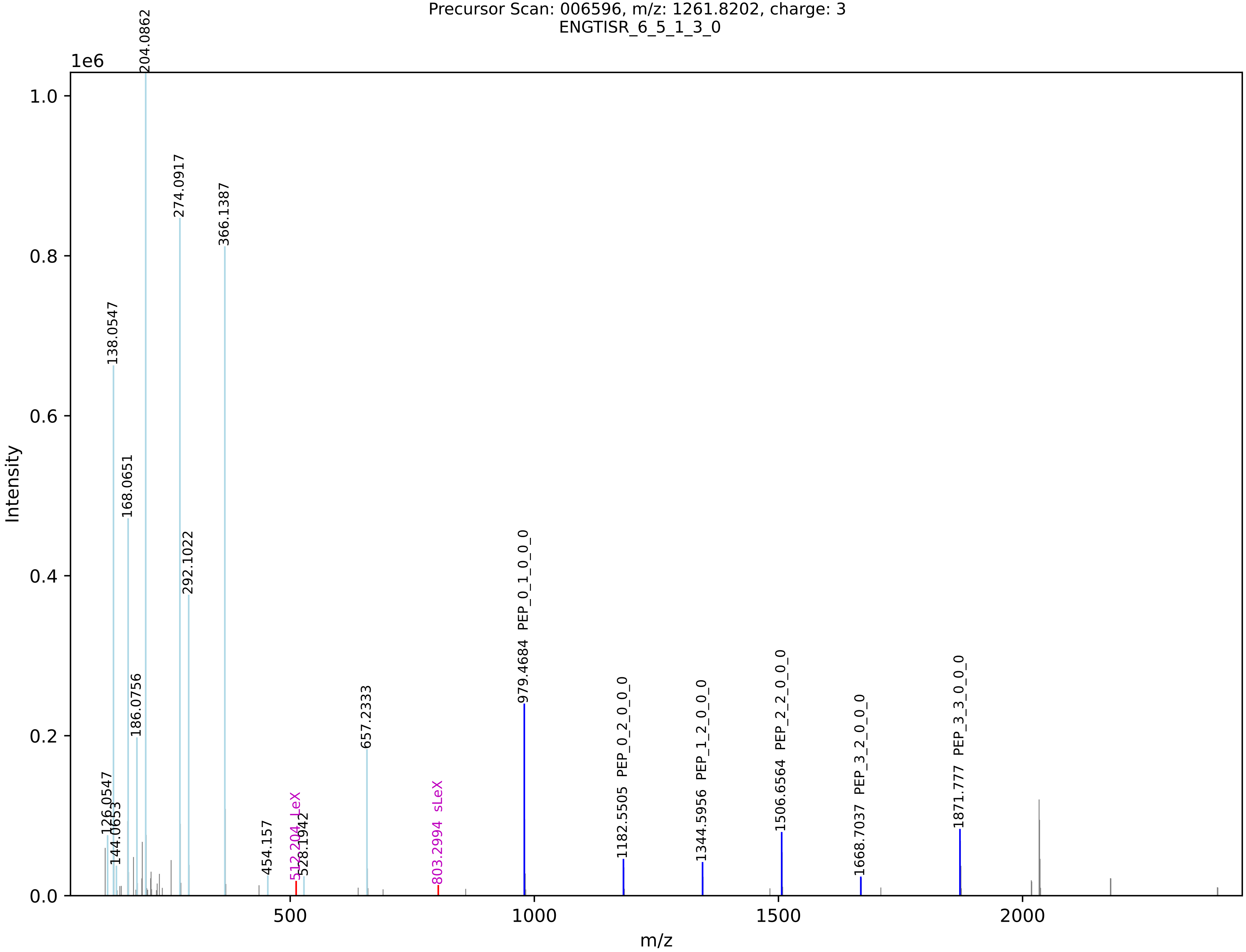

File: 231129_OE_RK_4, RT: 8.936, Scan: 006683
Type: HCD, FTMS + c NSI d Full ms2 915.5923@hcd30.00 [120.0000-3758.4729]
Precursor Scan: 006596, m/z: 915.0915, charge: 4
NEEYNK_6_5_0_3_0

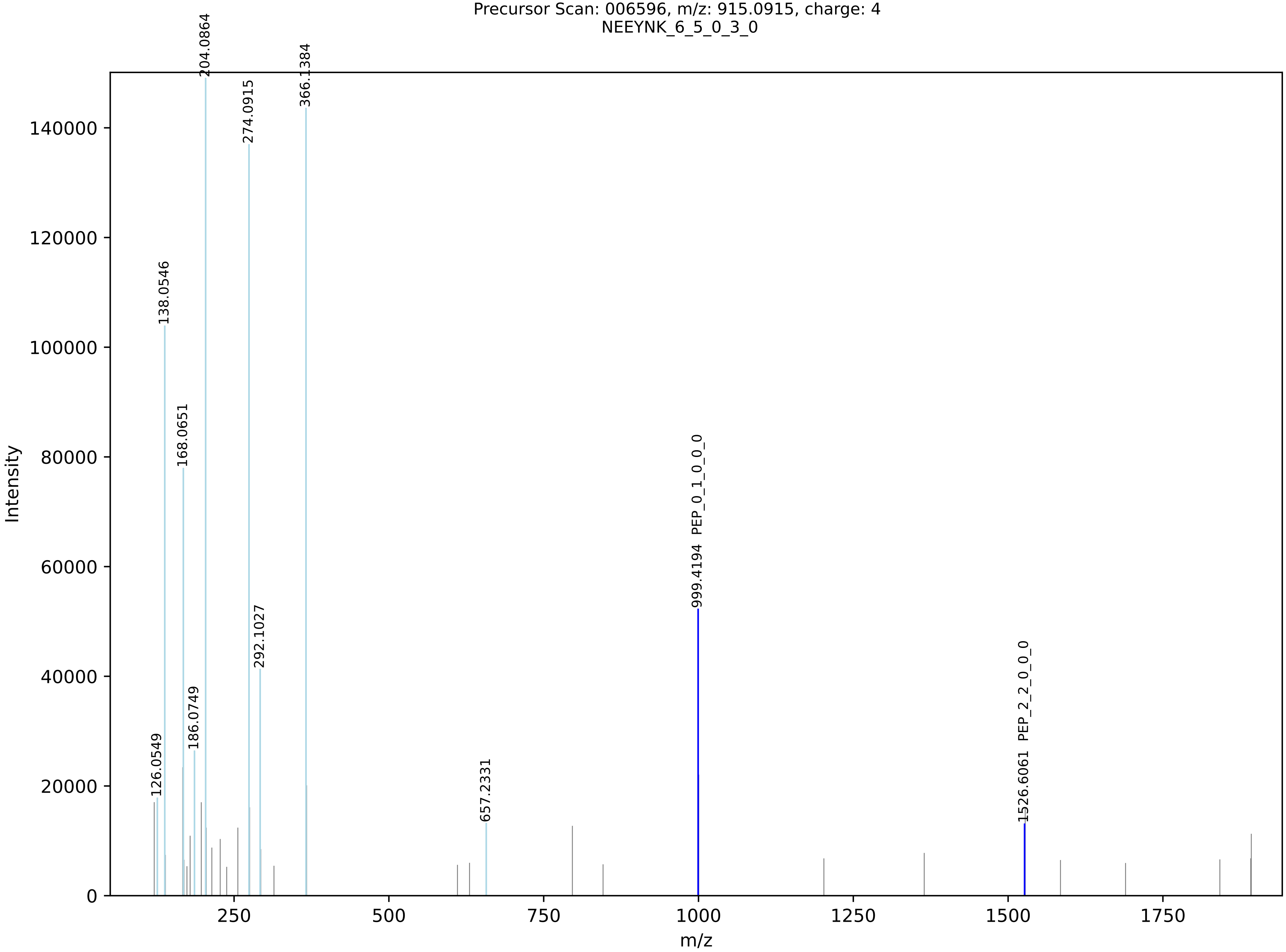

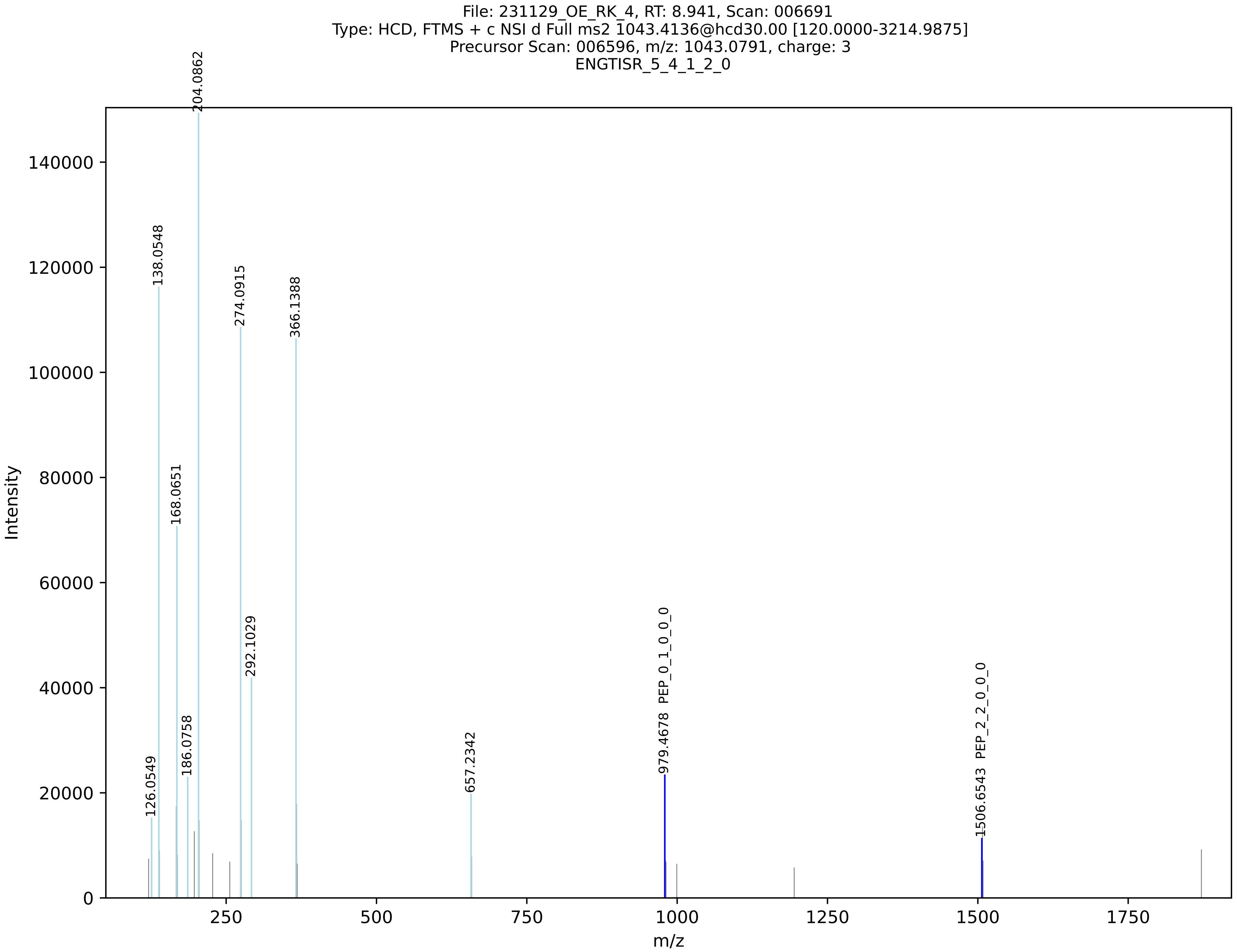
File: 231129_OE_RK_4, RT: 8.941, Scan: 006691
Type: HCD, FTMS + c NSI d Full ms2 1043.4136@hcd30.00 [120.0000-3214.9875]
Precursor Scan: 006596, m/z: 1043.0791, charge: 3
ENGTISR_5_4_1_2_0
204.0862
138.0548
274.0915
366.1388
168.0651
292.1029
186.0758
126.0549
657.2342
979.4678 PEP_0_1_0_0_0
1506.6543 PEP_2_2_0_0_0
Intensity
m/z
0
20000
40000
60000
80000
100000
120000
140000
250
500
750
1000
1250
1500
1750

File: 231129_OE_RK_4, RT: 8.978, Scan: 006743
Type: HCD, FTMS + c NSI d Full ms2 1147.6897@hcd30.00 [120.0000-4705.4302]
Precursor Scan: 006670, m/z: 1147.1881, charge: 4
ENGTISR_7_6_2_4_0

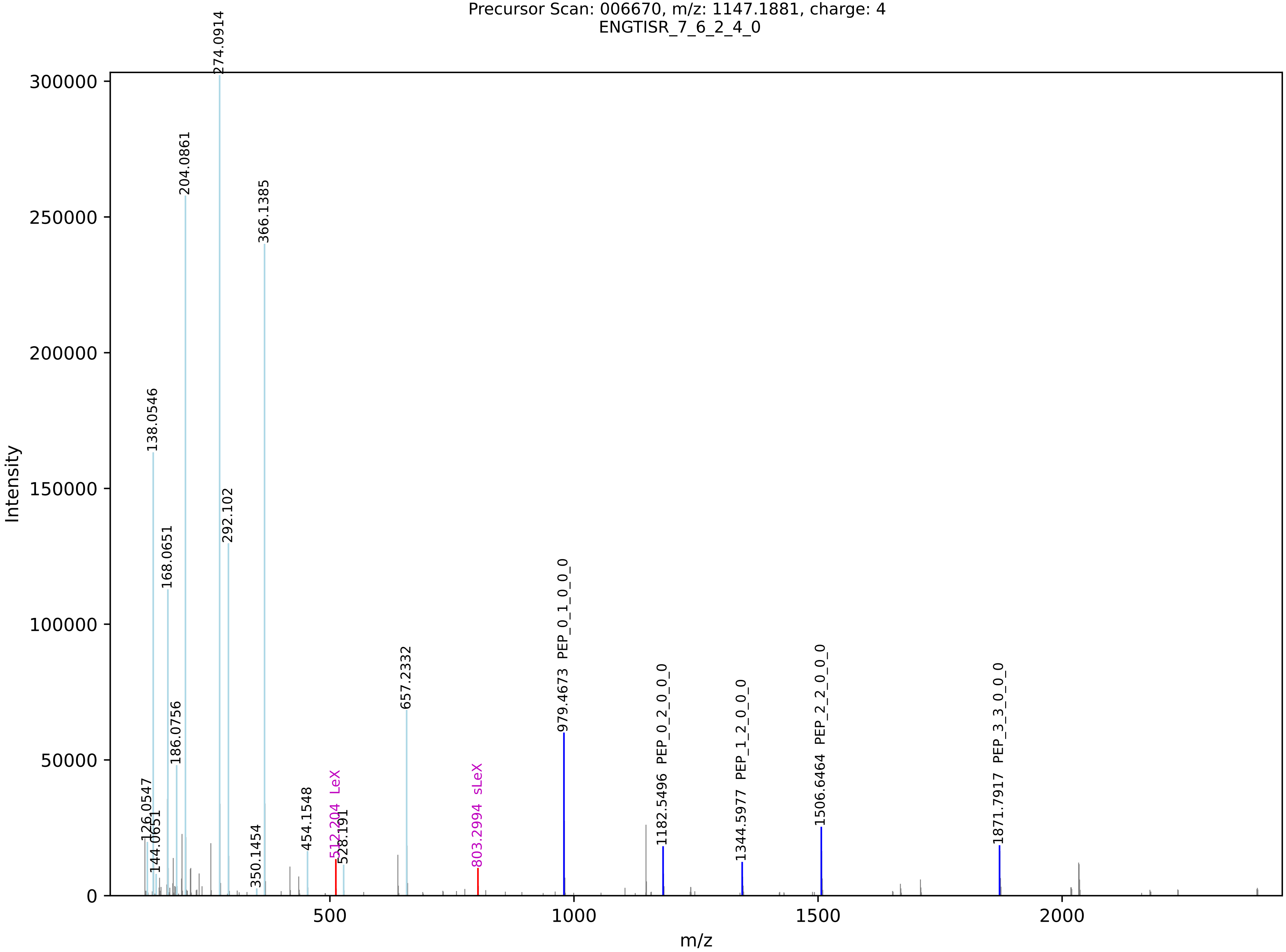

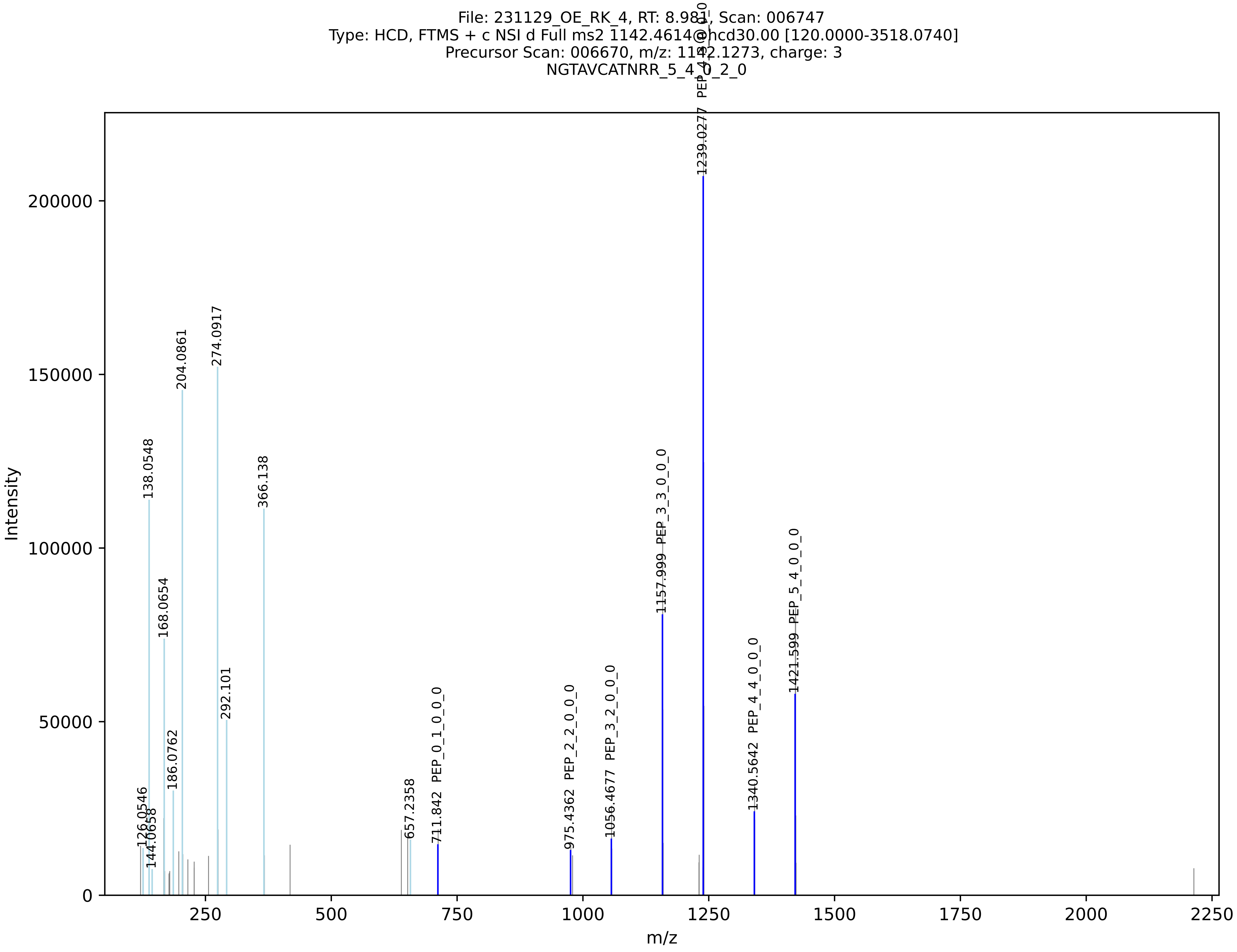
File: 231129_OE_RK_4, RT: 8.981, Scan: 006747
Type: HCD, FTMS + c NSI d Full ms2 1142.4614@hcd30.00 [120.0000-3518.0740]
Precursor Scan: 006670, m/z: 1142.1273, charge: 3
NGTAVCATNRR_5_4_0_2_0
Intensity
m/z
126.0546
144.0658
138.0548
168.0654
186.0762
204.0861
274.0917
292.101
366.138
657.2358
711.842 PEP_0_1_0_0_0
975.4362 PEP_2_2_0_0_0
1056.4677 PEP_3_2_0_0_0
1157.999 PEP_3_3_0_0_0
1239.0277 PEP_4_3_0_0_0
1340.5642 PEP_4_4_0_0_0
1421.599 PEP_5_4_0_0_0

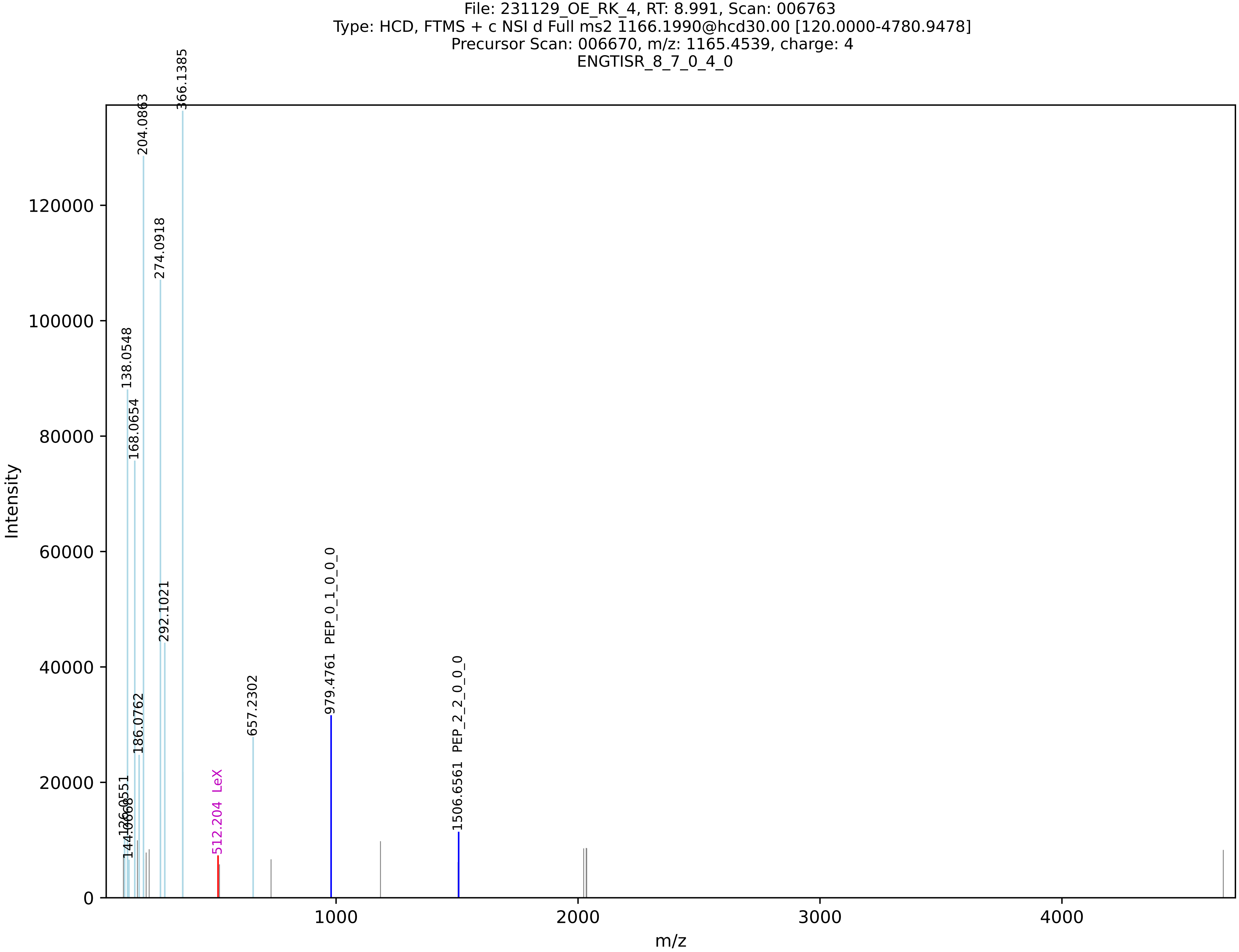
File: 231129_OE_RK_4, RT: 8.991, Scan: 006763
Type: HCD, FTMS + c NSI d Full ms2 1166.1990@hcd30.00 [120.0000-4780.9478]
Precursor Scan: 006670, m/z: 1165.4539, charge: 4
ENGTISR_8_7_0_4_0
Intensity
m/z
0
20000
40000
60000
80000
100000
120000
1000
2000
3000
4000
366.1385
204.0863
274.0918
138.0548
168.0654
292.1021
186.0762
126.0551
144.0668
512.204 LeX
657.2302
979.4761 PEP_0_1_0_0_0
1506.6561 PEP_2_2_0_0_0

File: 231129_OE_RK_4, RT: 9.029, Scan: 006817
Type: HCD, FTMS + c NSI d Full ms2 1269.1400@hcd30.00 [120.0000-3905.7104]
Precursor Scan: 006742, m/z: 1268.4724, charge: 3
NEEYNK_6_5_1_3_0

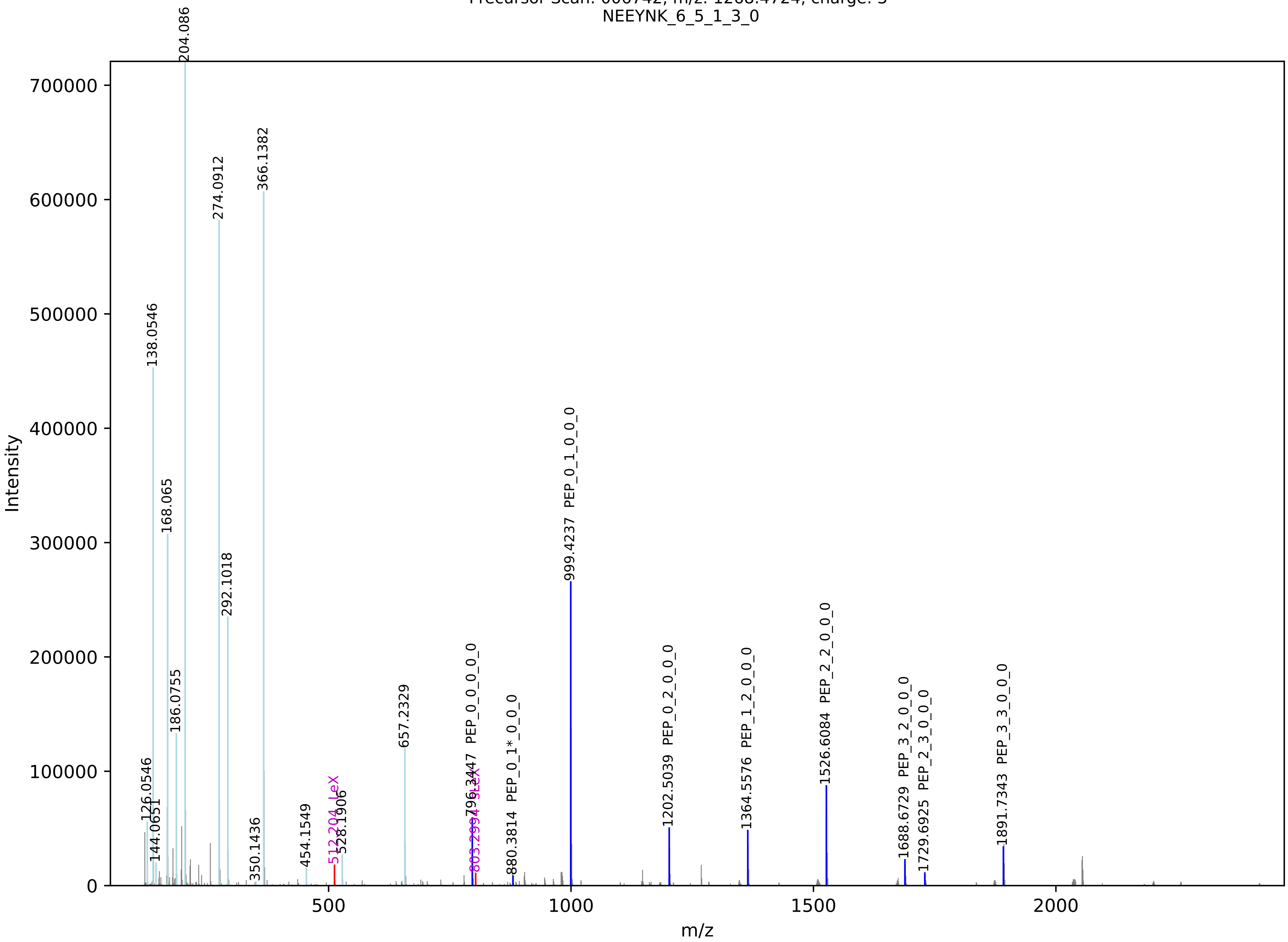

File: 231129_OE_RK_4, RT: 9.03, Scan: 006819
Type: HCD, FTMS + c NSI d Full ms2 1032.0804@hcd30.00 [120.0000-3180.3081]
Precursor Scan: 006742, m/z: 1031.7463, charge: 3
NVTAEQAR_5_4_0_2_0

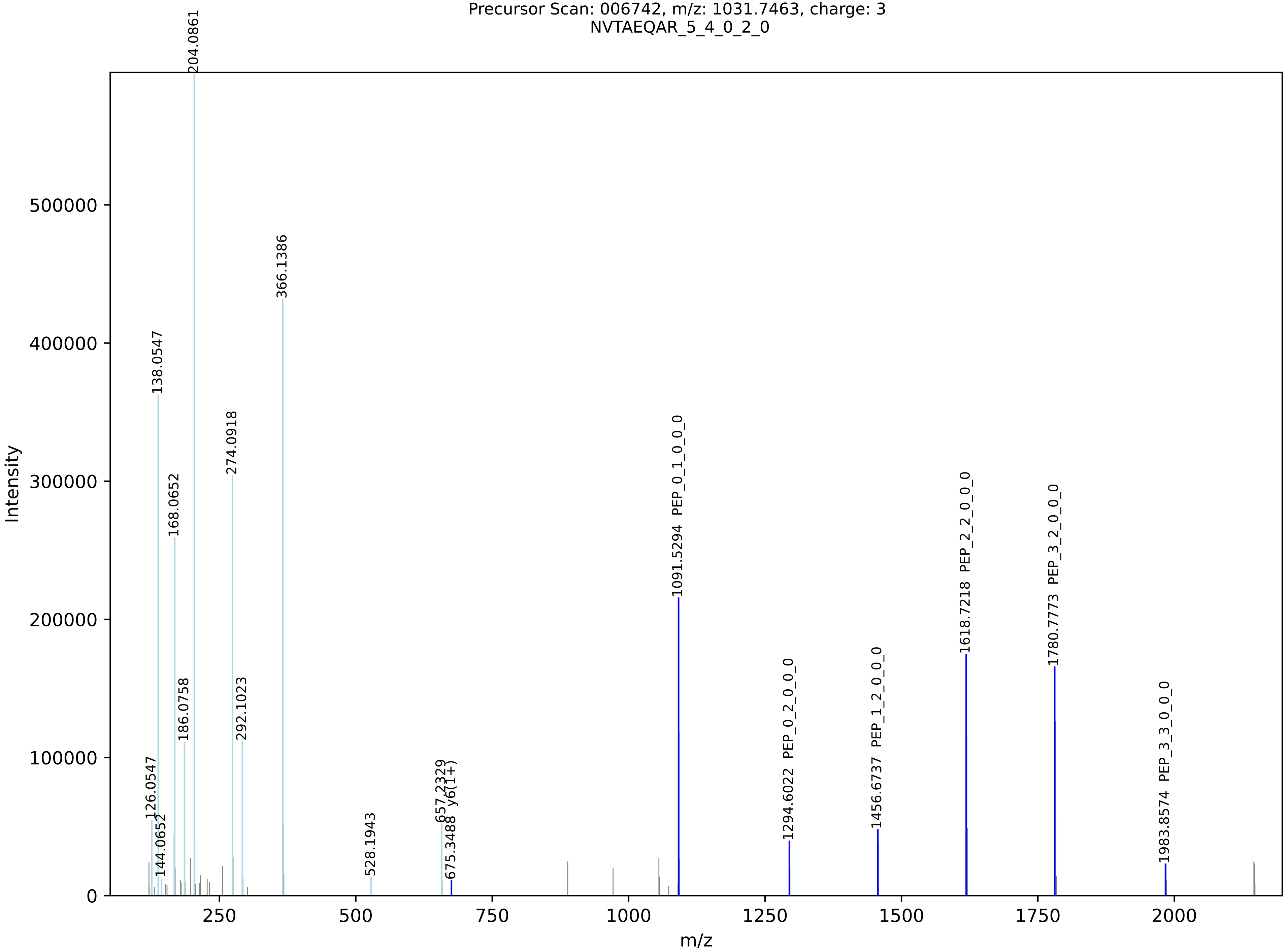

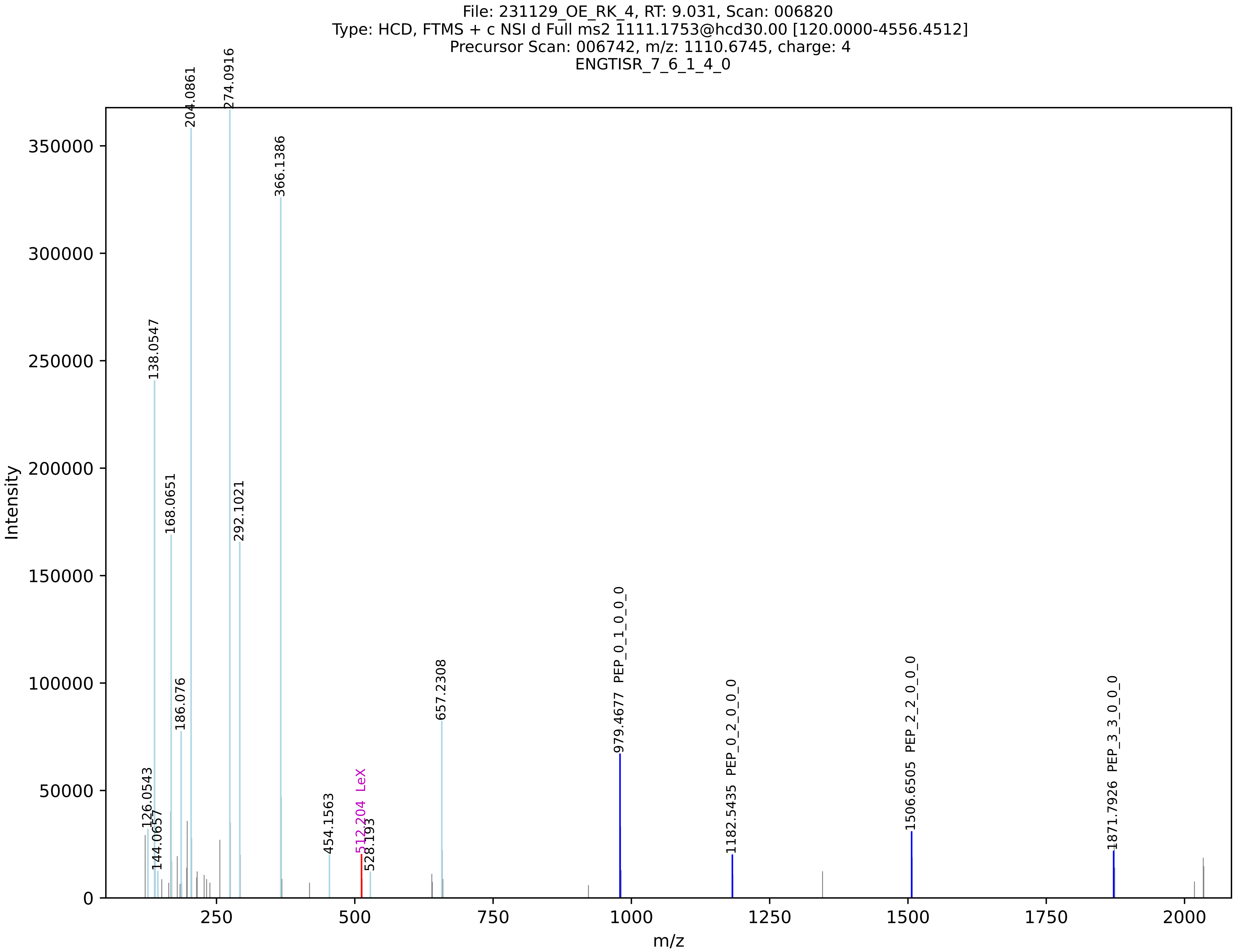
File: 231129_OE_RK_4, RT: 9.031, Scan: 006820
Type: HCD, FTMS + c NSI d Full ms2 1111.1753@hcd30.00 [120.0000-4556.4512]
Precursor Scan: 006742, m/z: 1110.6745, charge: 4
ENGTISR_7_6_1_4_0
Intensity
m/z
0
50000
100000
150000
200000
250000
300000
350000
250
500
750
1000
1250
1500
1750
2000
126.0543
138.0547
144.0657
168.0651
186.076
204.0861
274.0916
292.1021
366.1386
454.1563
512.204 LeX
528.193
657.2308
979.4677 PEP_0_1_0_0_0
1182.5435 PEP_0_2_0_0_0
1506.6505 PEP_2_2_0_0_0
1871.7926 PEP_3_3_0_0_0

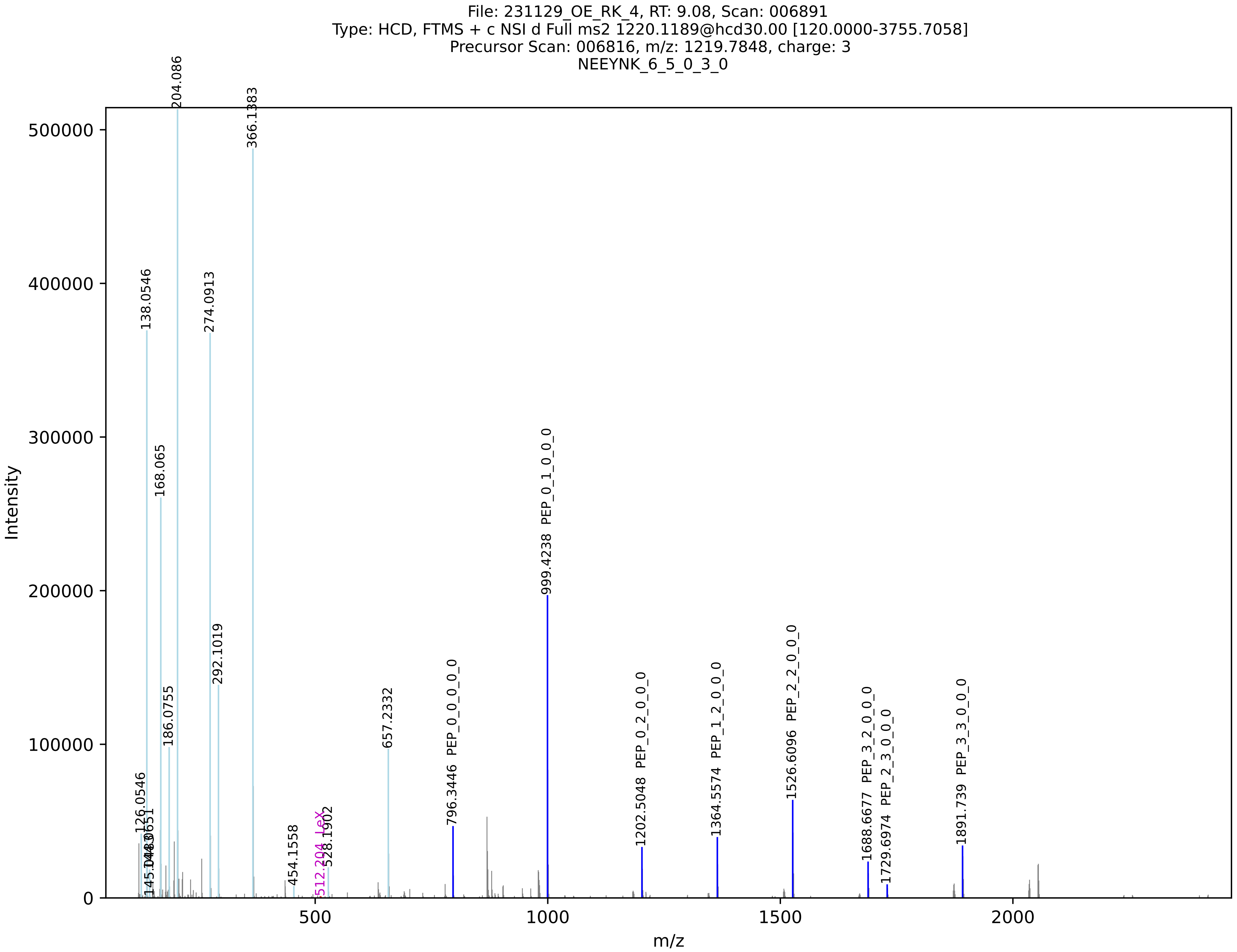

File: 231129_OE_RK_4, RT: 9.08, Scan: 006891
Type: HCD, FTMS + c NSI d Full ms2 1220.1189@hcd30.00 [120.0000-3755.7058]
Precursor Scan: 006816, m/z: 1219.7848, charge: 3
NEEYNK_6_5_0_3_0
Intensity
m/z
204.086
366.1383
138.0546
274.0913
168.065
292.1019
186.0755
126.0546
657.2332
454.1558
512.204_LeX
528.1902
796.3446 PEP_0_0_0_0_0
999.4238 PEP_0_1_0_0_0
1202.5048 PEP_0_2_0_0_0
1364.5574 PEP_1_2_0_0_0
1526.6096 PEP_2_2_0_0_0
1688.6677 PEP_3_2_0_0_0
1729.6974 PEP_2_3_0_0_0
1891.739 PEP_3_3_0_0_0

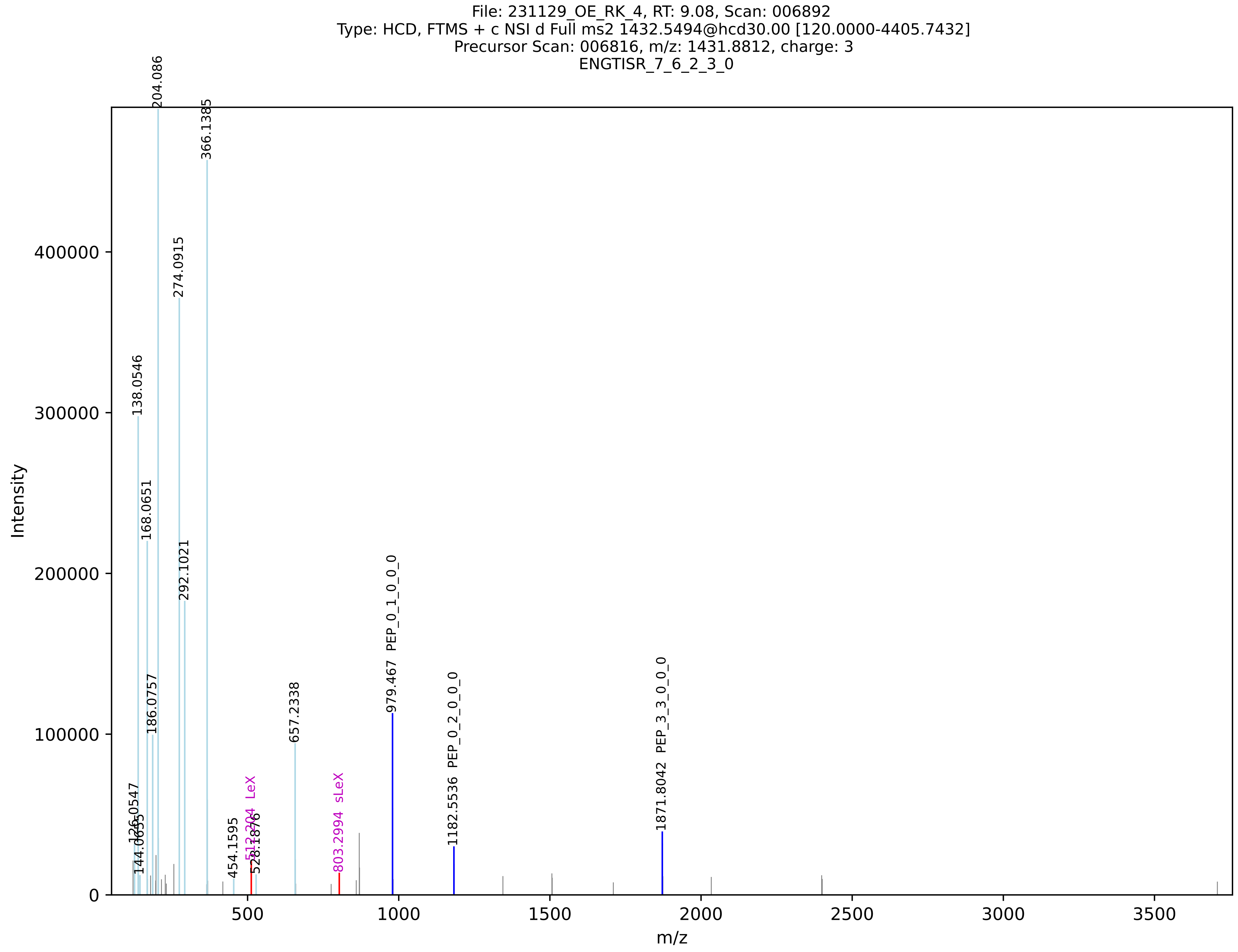

File: 231129_OE_RK_4, RT: 9.08, Scan: 006892
Type: HCD, FTMS + c NSI d Full ms2 1432.5494@hcd30.00 [120.0000-4405.7432]
Precursor Scan: 006816, m/z: 1431.8812, charge: 3
ENGTISR_7_6_2_3_0
Intensity
m/z
204.086
366.1385
274.0915
138.0546
168.0651
292.1021
186.0757
126.0547
144.0655
454.1595
512.204 LeX
528.1876
657.2338
803.2994 sLeX
979.467 PEP_0_1_0_0_0
1182.5536 PEP_0_2_0_0_0
1871.8042 PEP_3_3_0_0_0
0
100000
200000
300000
400000
500
1000
1500
2000
2500
3000
3500

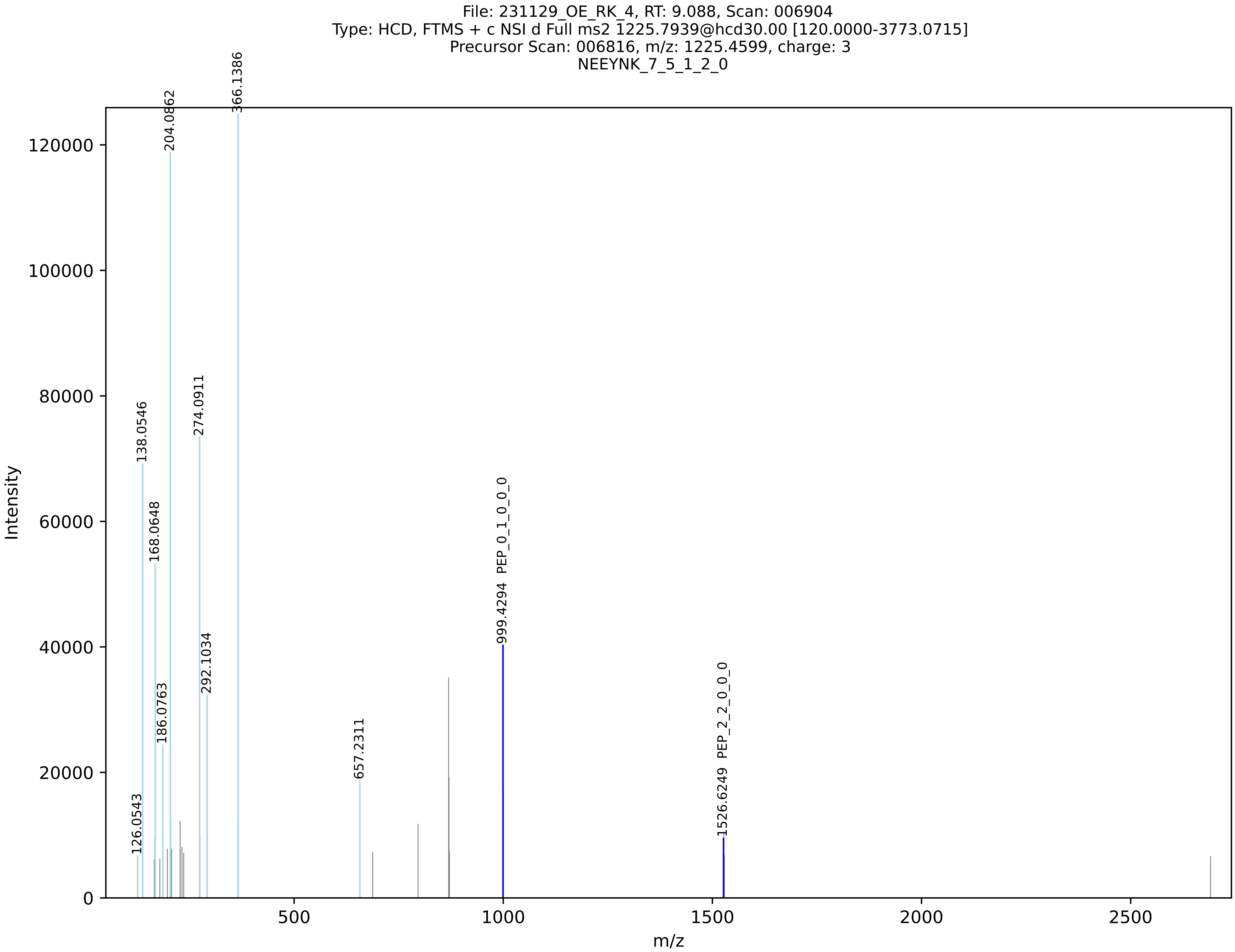
File: 231129_OE_RK_4, RT: 9.088, Scan: 006904
Type: HCD, FTMS + c NSI d Full ms2 1225.7939@hcd30.00 [120.0000-3773.0715]
Precursor Scan: 006816, m/z: 1225.4599, charge: 3
NEEYNK_7_5_1_2_0
Intensity
m/z
0
20000
40000
60000
80000
100000
120000
500
1000
1500
2000
2500
126.0543
138.0546
168.0648
186.0763
204.0862
274.0911
292.1034
366.1386
657.2311
999.4294 PEP_0_1_0_0_0
1526.6249 PEP_2_2_0_0_0

File: 231129_OE_RK_4, RT: 9.128, Scan: 006958
Type: HCD, FTMS + c NSI d Full ms2 1213.8011@hcd30.00 [120.0000-3736.3735]
Precursor Scan: 006890, m/z: 1213.1329, charge: 3
ENGTISR_6_5_0_3_0

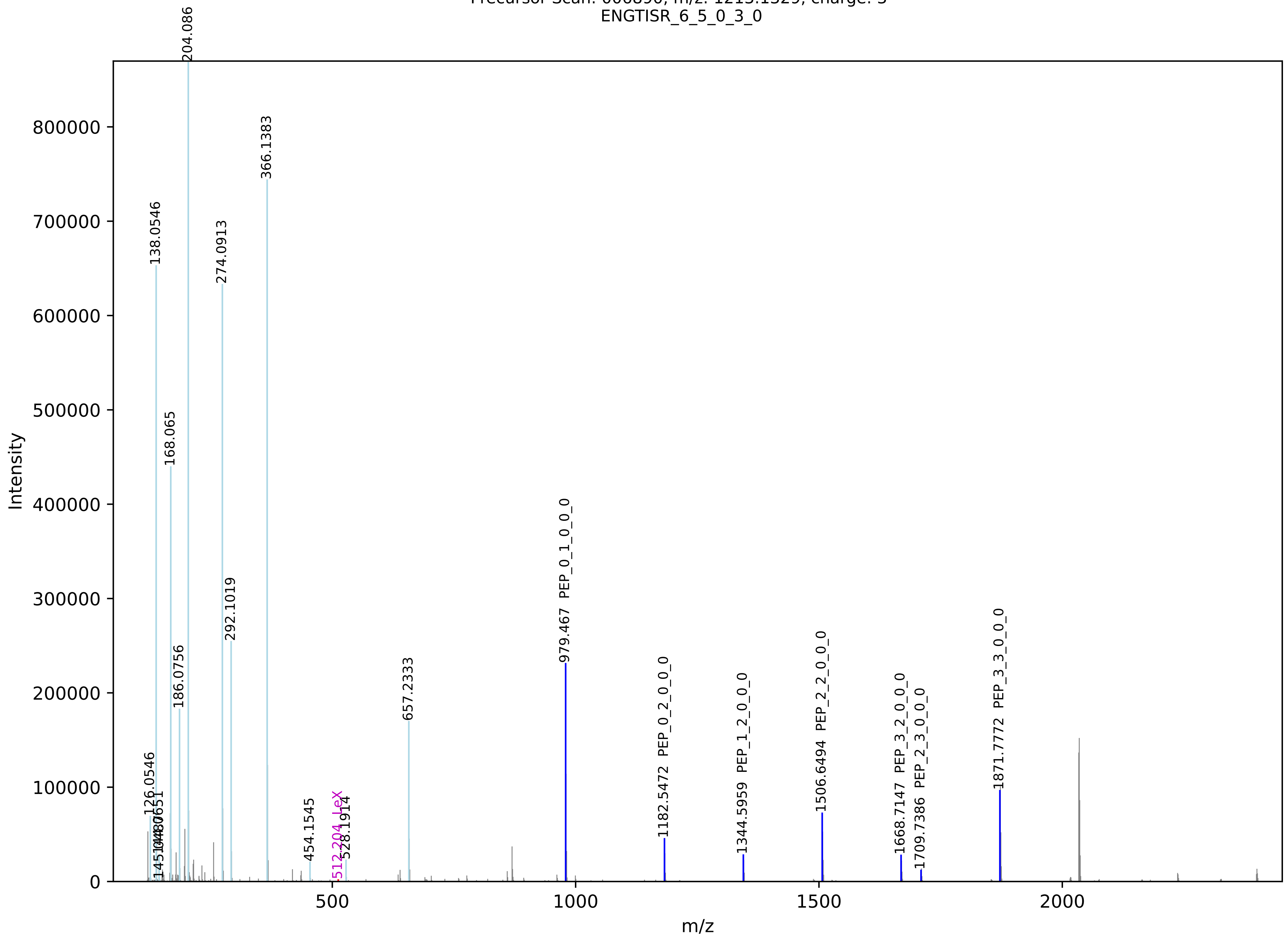

File: 231129_OE_RK_4, RT: 9.131, Scan: 006964
Type: HCD, FTMS + c NSI d Full ms2 1038.4009@hcd30.00 [120.0000-4259.5313]
Precursor Scan: 006890, m/z: 1037.6491, charge: 4
ENGTISR_7_6_1_3_0

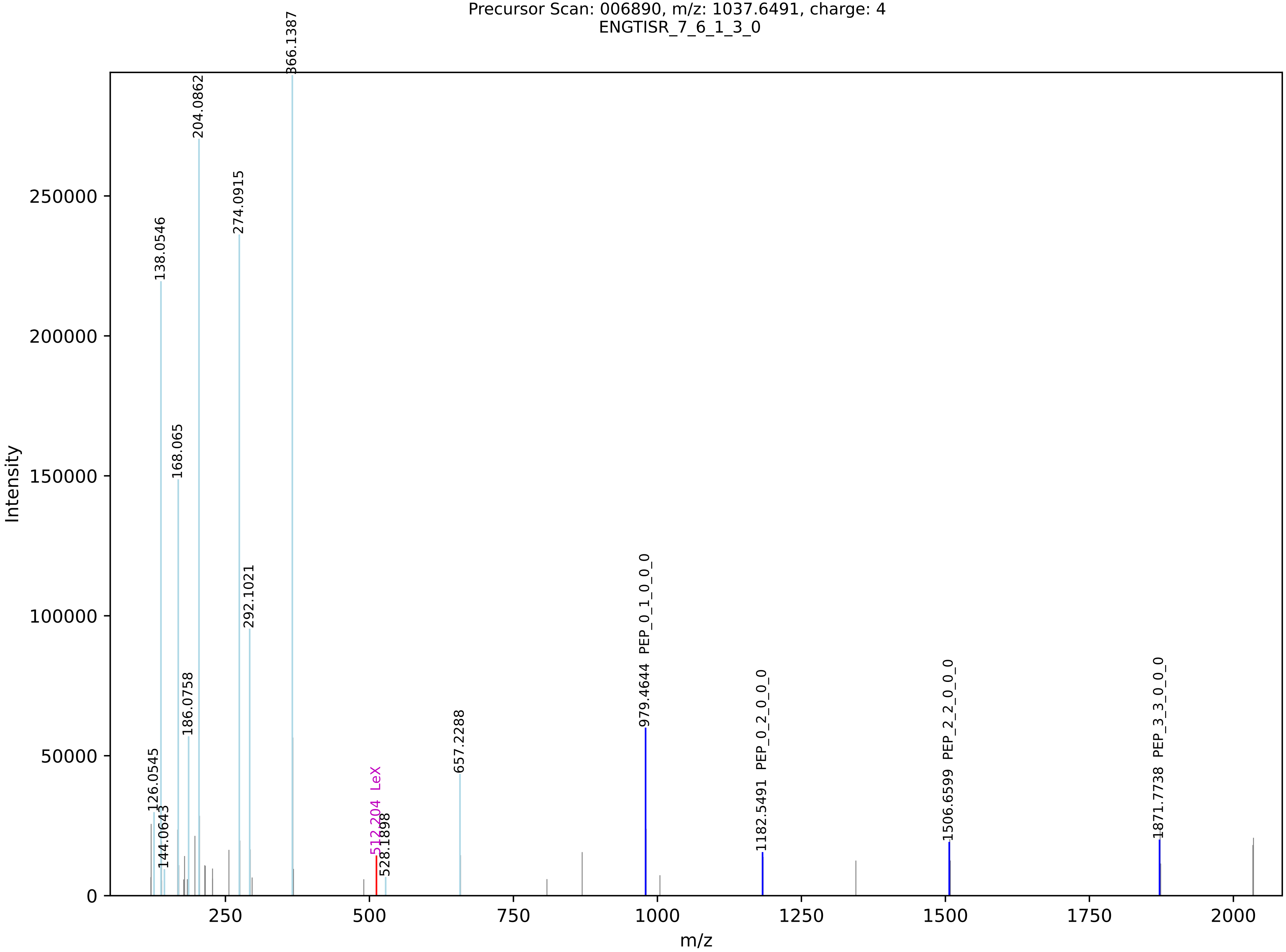

File: 231129_OE_RK_4, RT: 9.133, Scan: 006967
Type: HCD, FTMS + c NSI d Full ms2 1529.5846@hcd30.00 [120.0000-4702.6709]
Precursor Scan: 006890, m/z: 1528.916, charge: 3
ENGTISR_7_6_2_4_0

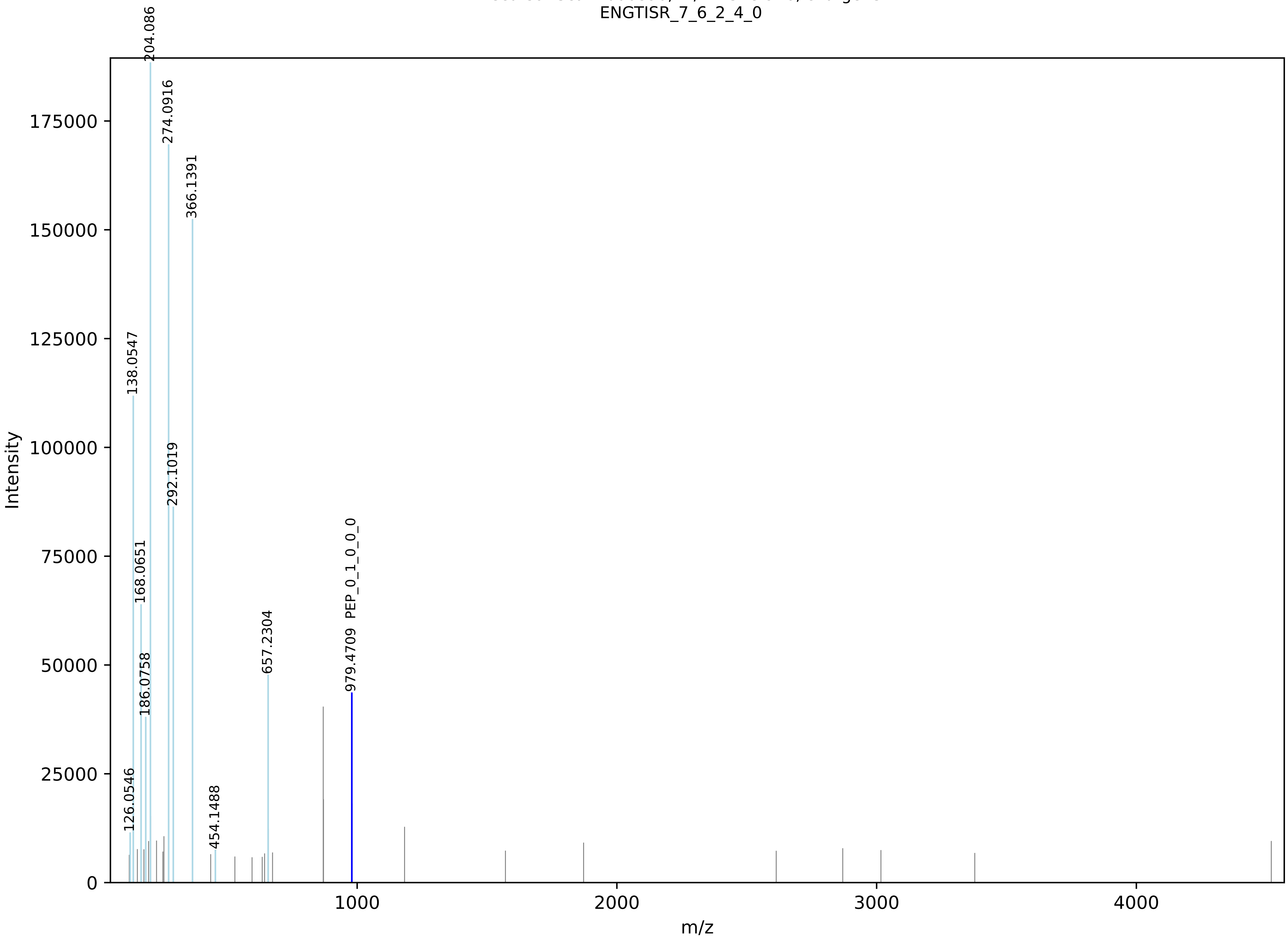

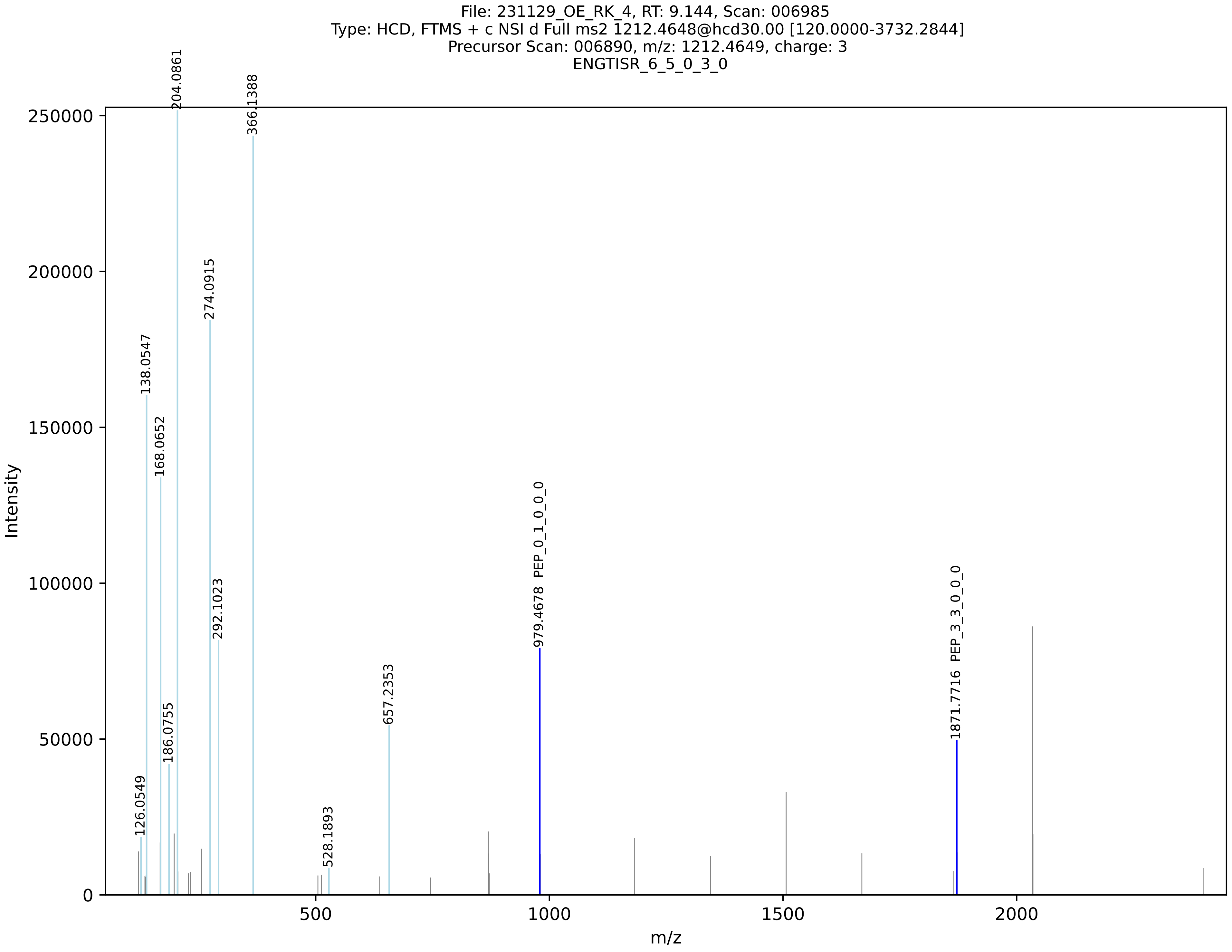
File: 231129_OE_RK_4, RT: 9.144, Scan: 006985
Type: HCD, FTMS + c NSI d Full ms2 1212.4648@hcd30.00 [120.0000-3732.2844]
Precursor Scan: 006890, m/z: 1212.4649, charge: 3
ENGTISR_6_5_0_3_0
Intensity
m/z
204.0861
366.1388
274.0915
138.0547
168.0652
979.4678 PEP_0_1_0_0_0_
1871.7716 PEP_3_3_0_0_0_
292.1023
657.2353
186.0755
126.0549
528.1893

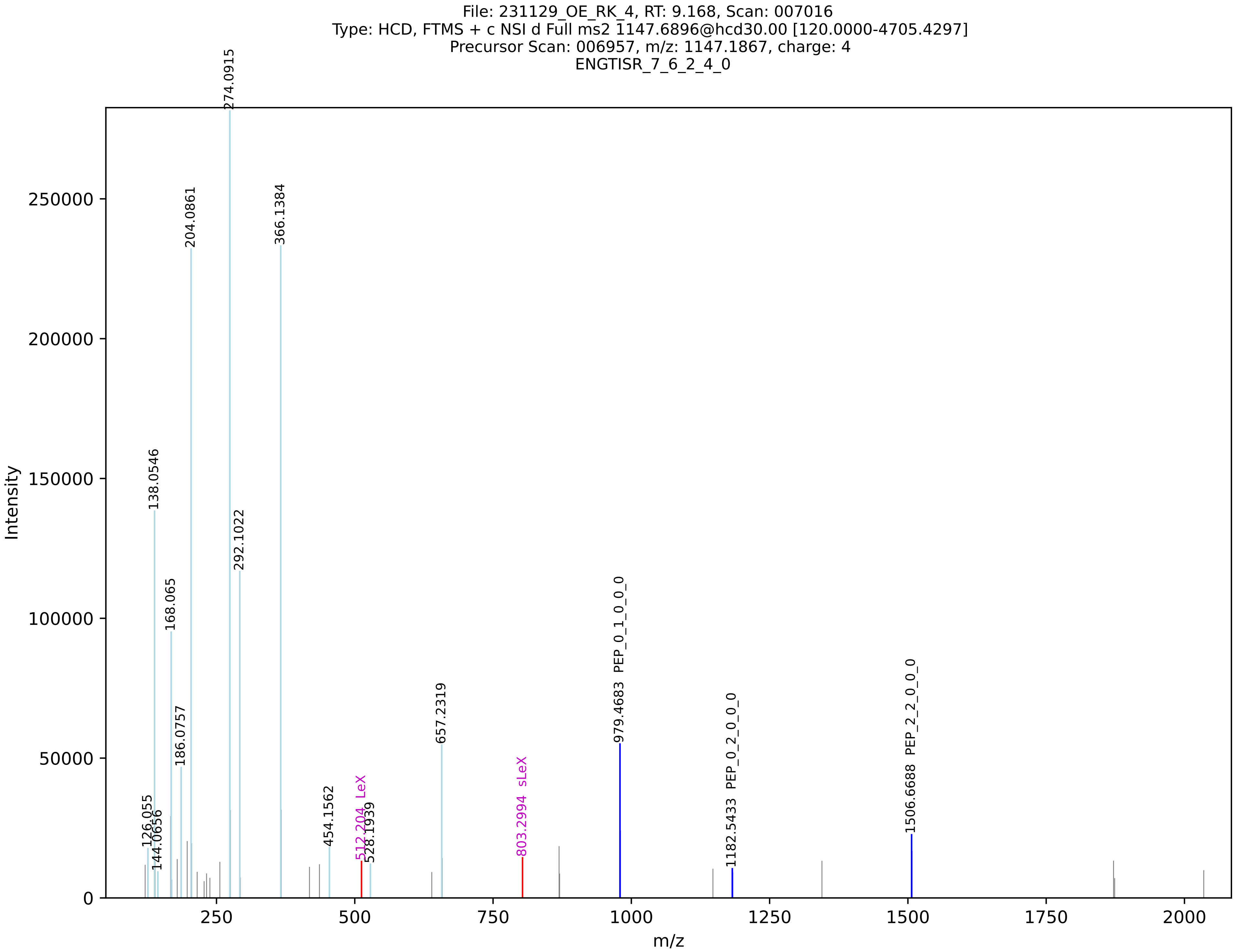
File: 231129_OE_RK_4, RT: 9.168, Scan: 007016
Type: HCD, FTMS + c NSI d Full ms2 1147.6896@hcd30.00 [120.0000-4705.4297]
Precursor Scan: 006957, m/z: 1147.1867, charge: 4
ENGTISR_7_6_2_4_0
Intensity
m/z
0
50000
100000
150000
200000
250000
250
500
750
1000
1250
1500
1750
2000
274.0915
204.0861
366.1384
138.0546
292.1022
168.065
186.0757
126.055
144.0656
454.1562
512.204 LeX
528.1939
657.2319
803.2994 sLeX
979.4683 PEP_0_1_0_0_0
1182.5433 PEP_0_2_0_0_0
1506.6688 PEP_2_2_0_0_0

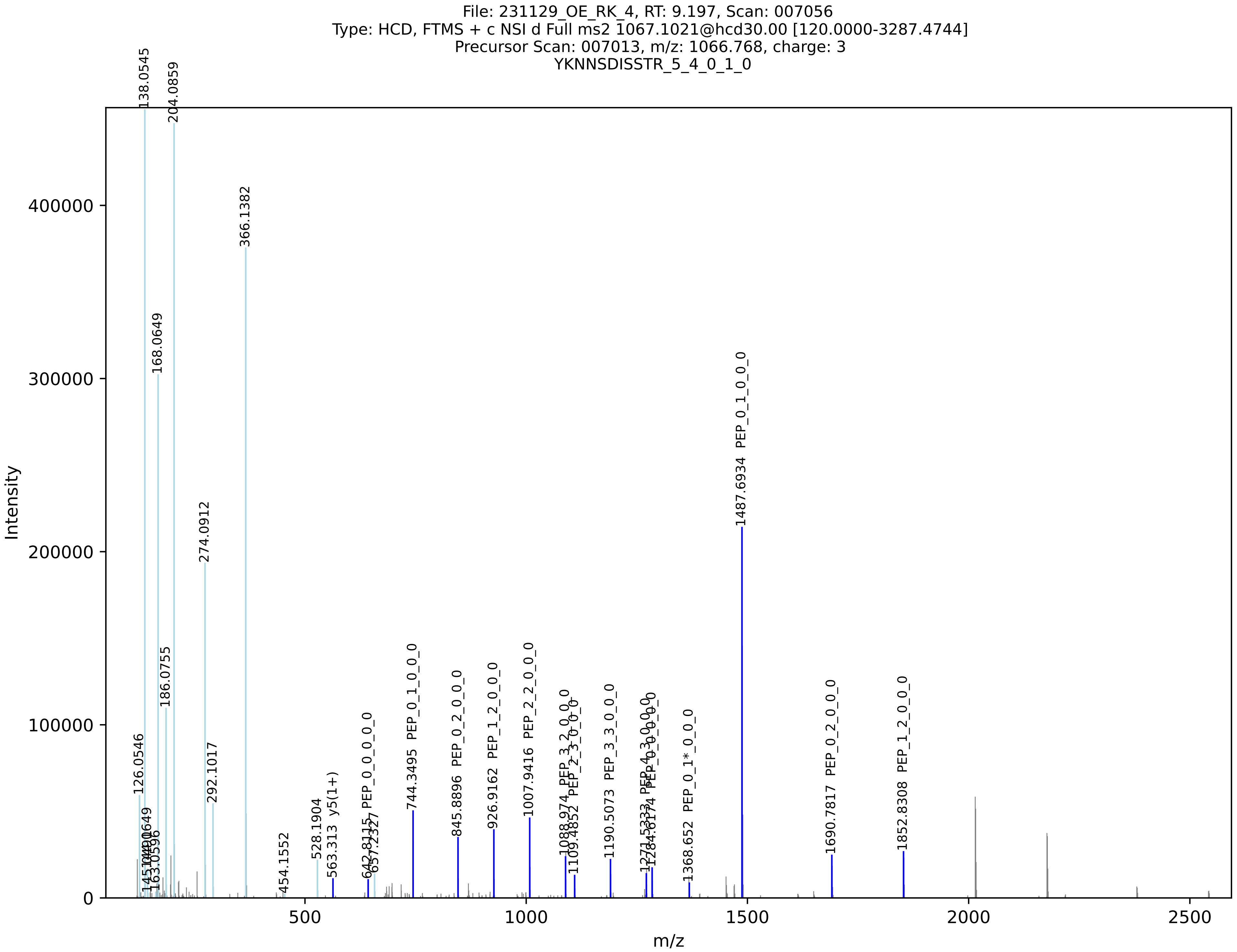

File: 231129_OE_RK_4, RT: 9.197, Scan: 007056
Type: HCD, FTMS + c NSI d Full ms2 1067.1021@hcd30.00 [120.0000-3287.4744]
Precursor Scan: 007013, m/z: 1066.768, charge: 3
YKNNSDISSTR_5_4_0_1_0
Intensity
m/z
138.0545
204.0859
366.1382
168.0649
274.0912
186.0755
126.0546
292.1017
163.0596
454.1552
528.1904
563.313 y5(1+)
642.8115 PEP_0_0_0_0_0
657.2327
744.3495 PEP_0_1_0_0_0
845.8896 PEP_0_2_0_0_0
926.9162 PEP_1_2_0_0_0
1007.9416 PEP_2_2_0_0_0
1088.974 PEP_3_2_0_0_0
1109.4852 PEP_2_3_0_0_0
1190.5073 PEP_3_3_0_0_0
1271.5333 PEP_4_3_0_0_0
1284.6174 PEP_0_0_0_0_0
1368.652 PEP_0_1* 0_0_0
1487.6934 PEP_0_1_0_0_0
1690.7817 PEP_0_2_0_0_0
1852.8308 PEP_1_2_0_0_0

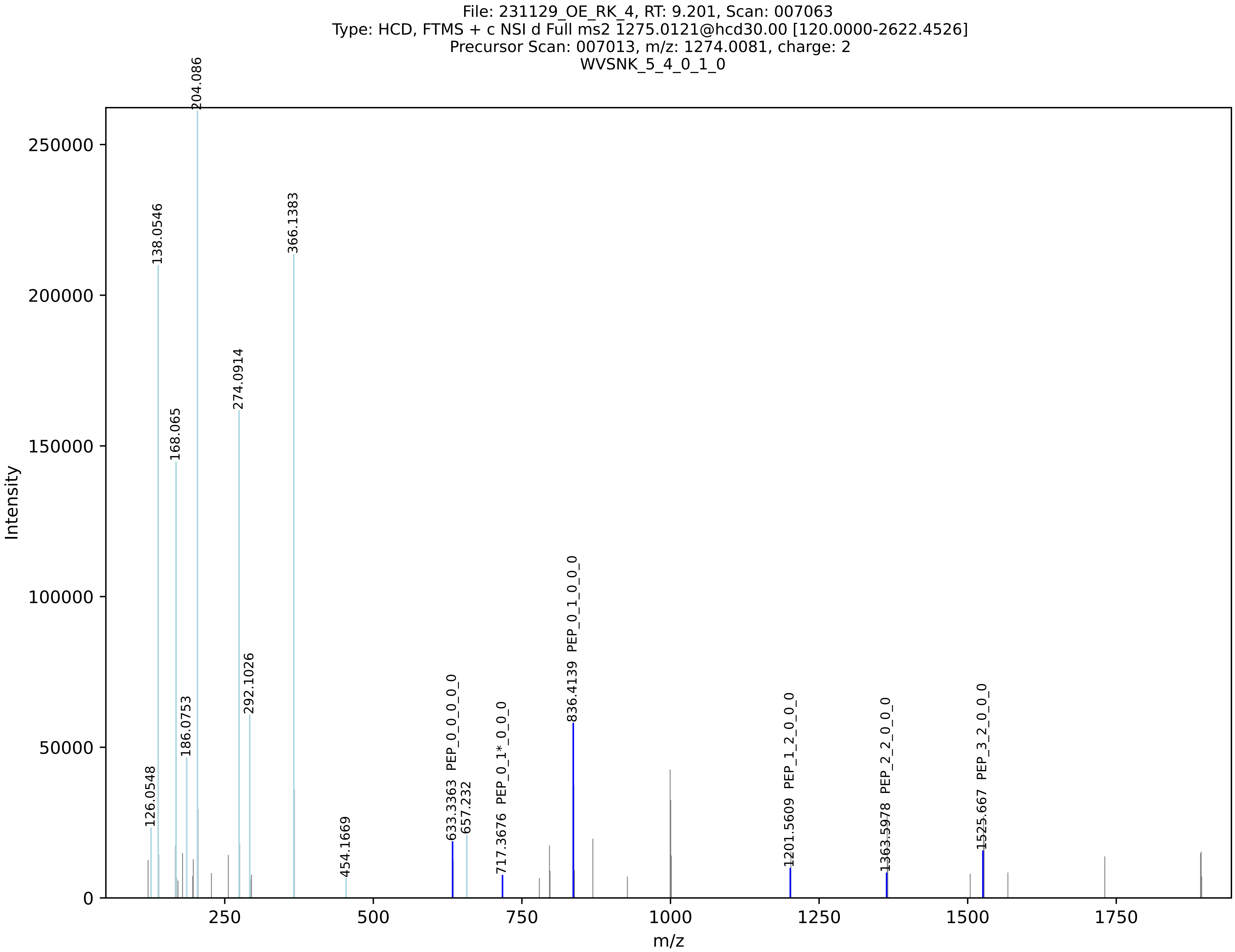

File: 231129_OE_RK_4, RT: 9.201, Scan: 007063
Type: HCD, FTMS + c NSI d Full ms2 1275.0121@hcd30.00 [120.0000-2622.4526]
Precursor Scan: 007013, m/z: 1274.0081, charge: 2
WVSNK_5_4_0_1_0
Intensity
m/z
204.086
138.0546
366.1383
274.0914
168.065
292.1026
186.0753
126.0548
454.1669
633.3363 PEP_0_0_0_0_0
657.232
717.3676 PEP_0_1*_0_0_0
836.4139 PEP_0_1_0_0_0
1201.5609 PEP_1_2_0_0_0
1363.5978 PEP_2_2_0_0_0
1525.667 PEP_3_2_0_0_0

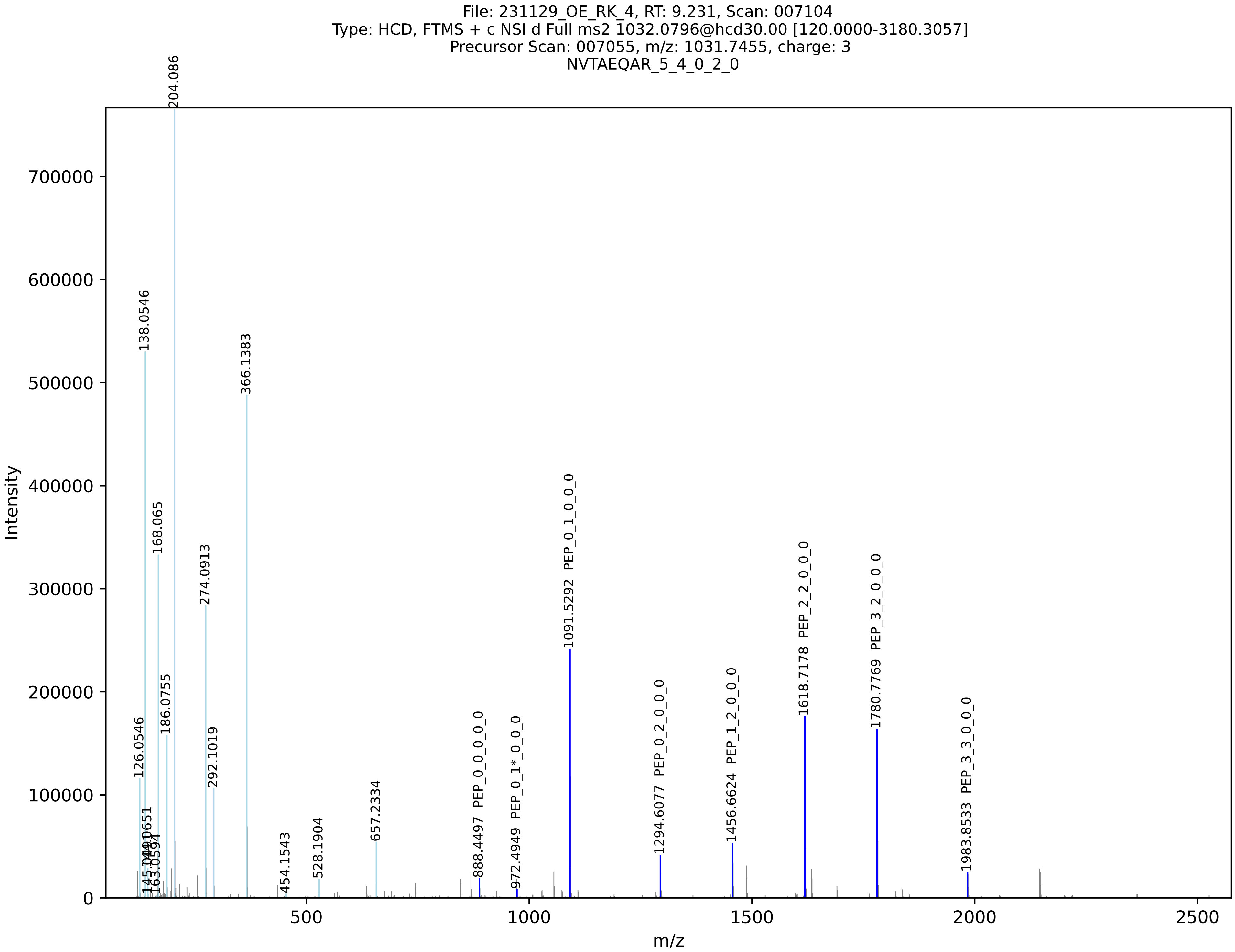
File: 231129_OE_RK_4, RT: 9.231, Scan: 007104
Type: HCD, FTMS + c NSI d Full ms2 1032.0796@hcd30.00 [120.0000-3180.3057]
Precursor Scan: 007055, m/z: 1031.7455, charge: 3
NVTAEQAR_5_4_0_2_0
Intensity
m/z
204.086
138.0546
366.1383
168.065
274.0913
186.0755
126.0546
292.1019
163.0594
454.1543
528.1904
657.2334
888.4497 PEP_0_0_0_0_0
972.4949 PEP_0_1*_0_0_0
1091.5292 PEP_0_1_0_0_0
1294.6077 PEP_0_2_0_0_0
1456.6624 PEP_1_2_0_0_0
1618.7178 PEP_2_2_0_0_0
1780.7769 PEP_3_2_0_0_0
1983.8533 PEP_3_3_0_0_0

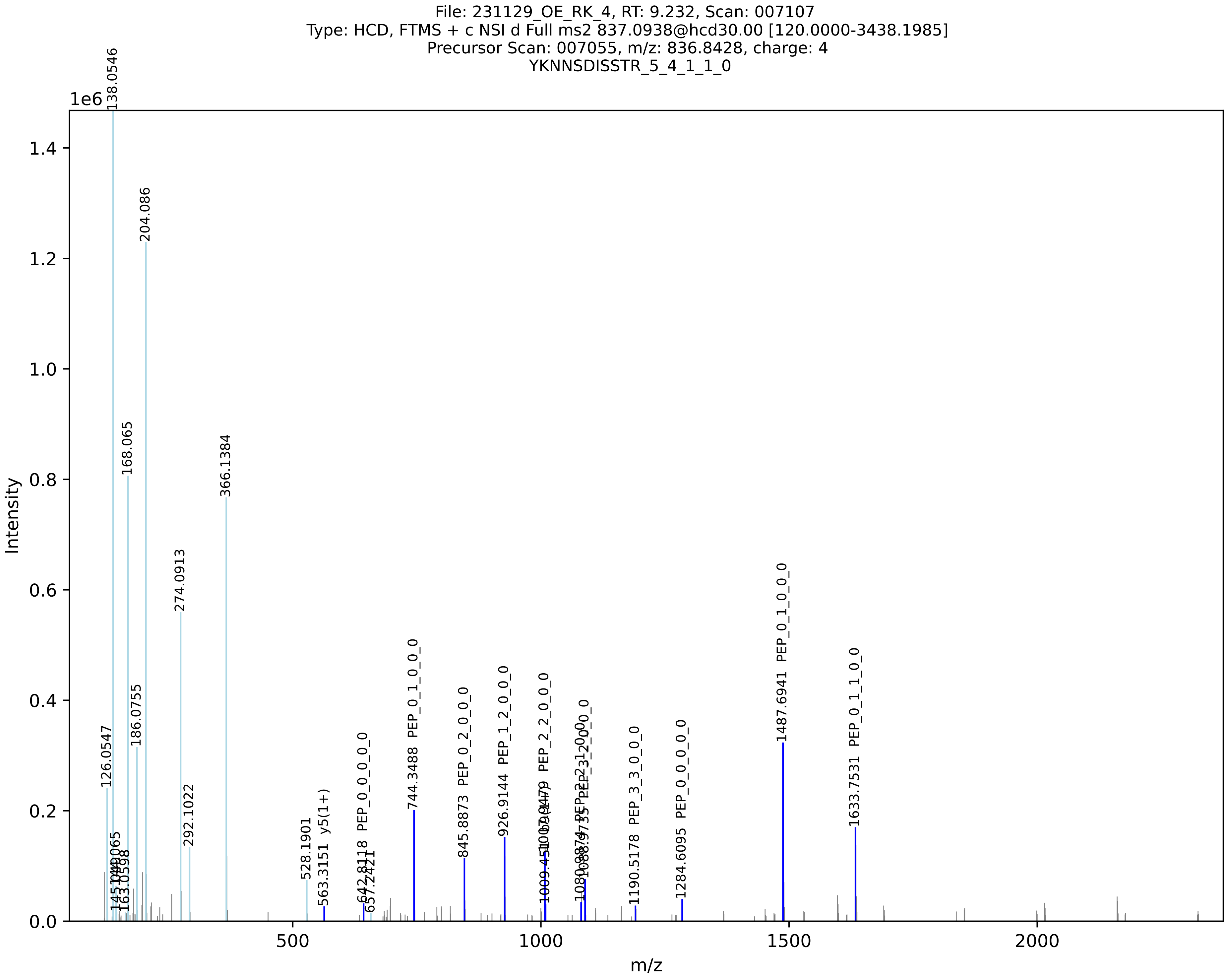

File: 231129_OE_RK_4, RT: 9.232, Scan: 007107
Type: HCD, FTMS + c NSI d Full ms2 837.0938@hcd30.00 [120.0000-3438.1985]
Precursor Scan: 007055, m/z: 836.8428, charge: 4
YKNNSDISSTR_5_4_1_1_0
1e6
Intensity
m/z
138.0546
204.086
168.065
366.1384
274.0913
186.0755
126.0547
292.1022
163.0598
528.1901
563.3151 y5(1+)
642.8118 PEP_0_0_0_0_0
657.2421
744.3488 PEP_0_1_0_0_0
845.8873 PEP_0_2_0_0_0
926.9144 PEP_1_2_0_0_0
PEP_2_2_0_0_0
1190.5178 PEP_3_3_0_0_0
1284.6095 PEP_0_0_0_0_0
1487.6941 PEP_0_1_0_0_0
1633.7531 PEP_0_1_1_0_0

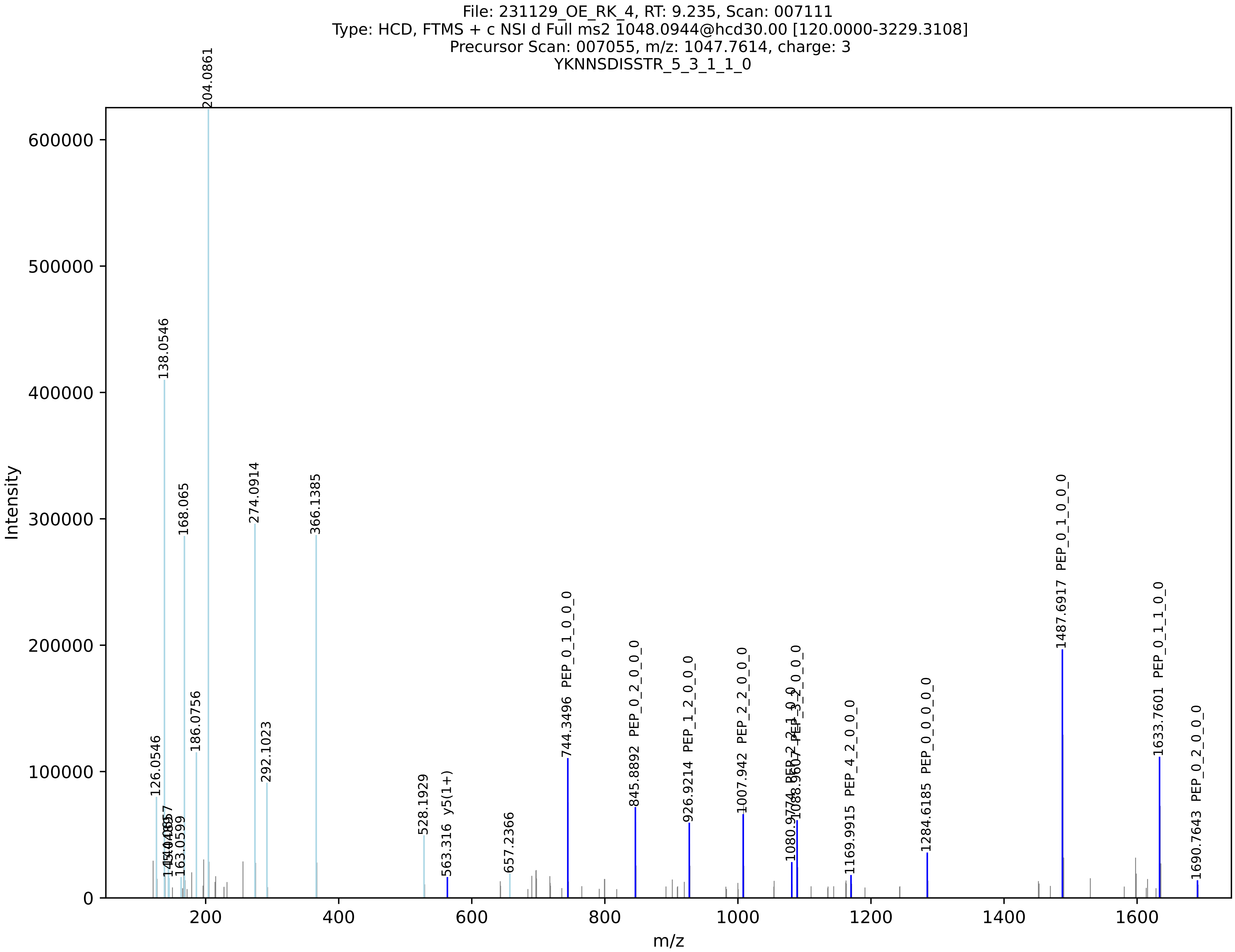
File: 231129_OE_RK_4, RT: 9.235, Scan: 007111
Type: HCD, FTMS + c NSI d Full ms2 1048.0944@hcd30.00 [120.0000-3229.3108]
Precursor Scan: 007055, m/z: 1047.7614, charge: 3
YKNNSDISSTR_5_3_1_1_0
Intensity
m/z
204.0861
138.0546
168.065
274.0914
366.1385
186.0756
292.1023
126.0546
163.0599
528.1929
563.316 y5(1+)
657.2366
744.3496 PEP_0_1_0_0_0
845.8892 PEP_0_2_0_0_0
926.9214 PEP_1_2_0_0_0
1007.942 PEP_2_2_0_0_0
1169.9915 PEP_4_2_0_0_0
1284.6185 PEP_0_0_0_0_0
1487.6917 PEP_0_1_0_0_0
1633.7601 PEP_0_1_1_0_0
1690.7643 PEP_0_2_0_0_0

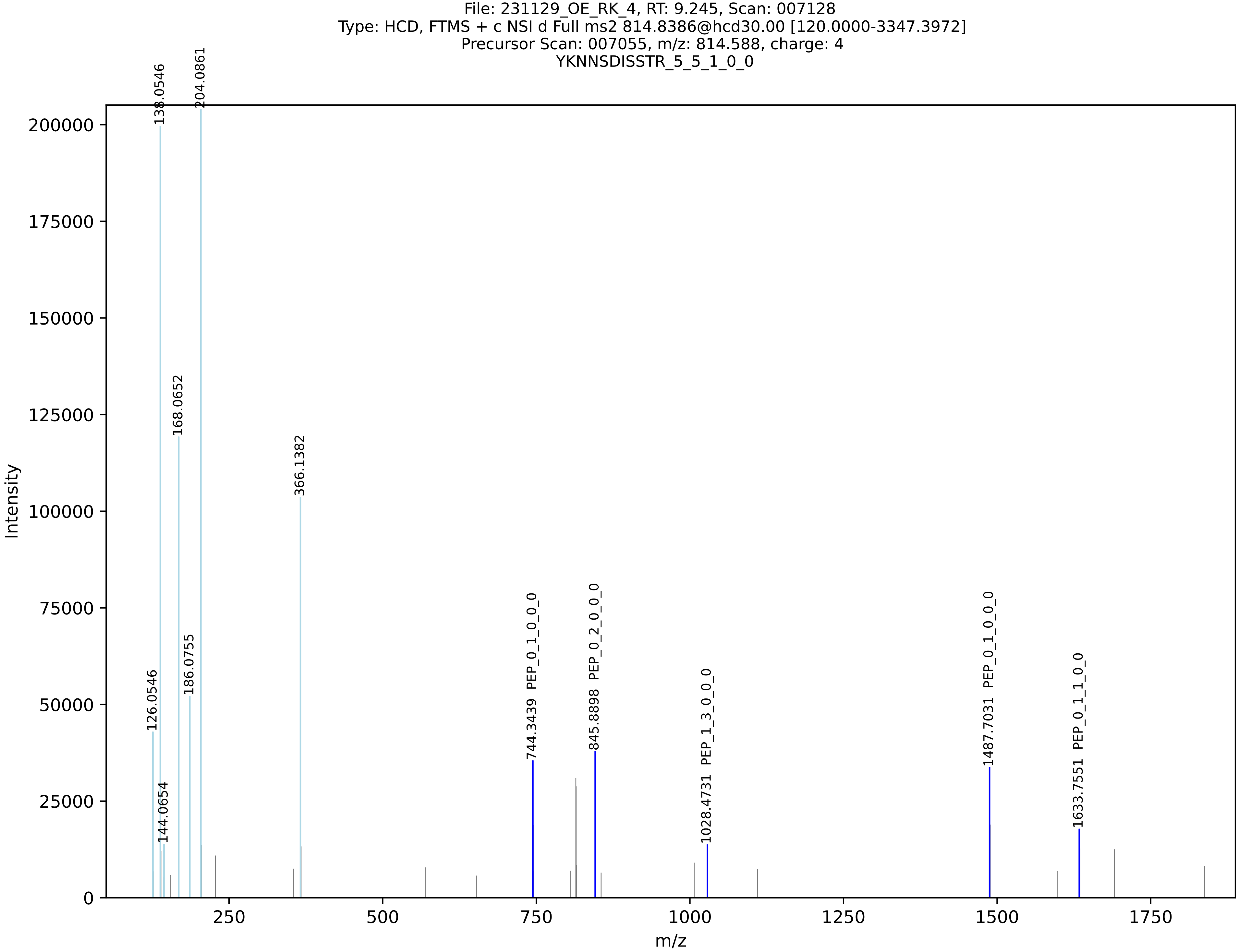
File: 231129_OE_RK_4, RT: 9.245, Scan: 007128
Type: HCD, FTMS + c NSI d Full ms2 814.8386@hcd30.00 [120.0000-3347.3972]
Precursor Scan: 007055, m/z: 814.588, charge: 4
YKNNSDISSTR_5_5_1_0_0
Intensity
m/z
0
25000
50000
75000
100000
125000
150000
175000
200000
250
500
750
1000
1250
1500
1750
126.0546
138.0546
144.0654
168.0652
186.0755
204.0861
366.1382
744.3439 PEP_0_1_0_0_0
845.8898 PEP_0_2_0_0_0
1028.4731 PEP_1_3_0_0_0
1487.7031 PEP_0_1_0_0_
1633.7551 PEP_0_1_1_0_

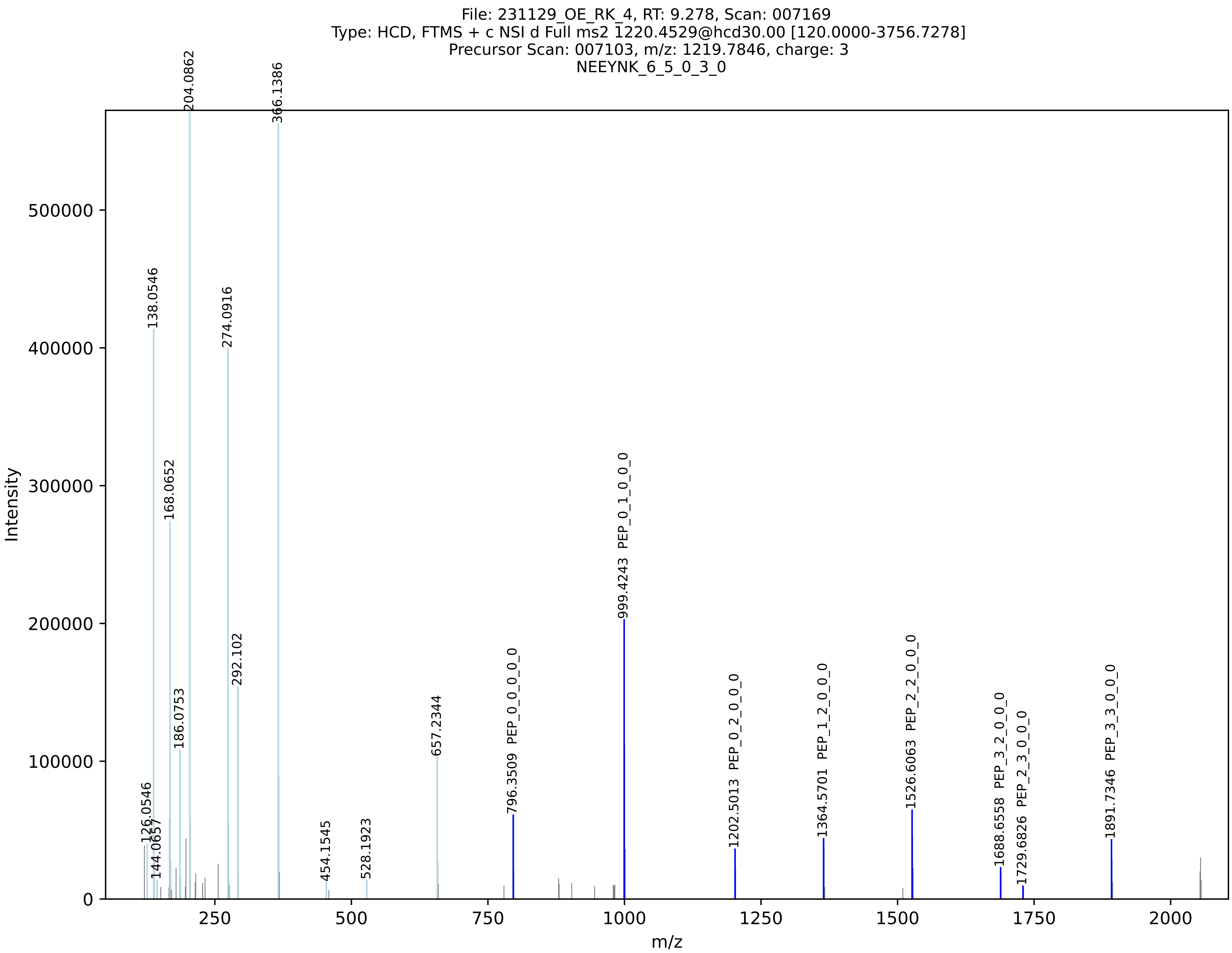
File: 231129_OE_RK_4, RT: 9.278, Scan: 007169
Type: HCD, FTMS + c NSI d Full ms2 1220.4529@hcd30.00 [120.0000-3756.7278]
Precursor Scan: 007103, m/z: 1219.7846, charge: 3
NEEYNK_6_5_0_3_0
Intensity
m/z
204.0862
366.1386
138.0546
274.0916
168.0652
292.102
186.0753
126.0546
144.0657
454.1545
528.1923
657.2344
796.3509 PEP_0_0_0_0_0
999.4243 PEP_0_1_0_0_0
1202.5013 PEP_0_2_0_0_0
1364.5701 PEP_1_2_0_0_0
1526.6063 PEP_2_2_0_0_0
1688.6558 PEP_3_2_0_0_0
1729.6826 PEP_2_3_0_0_0
1891.7346 PEP_3_3_0_0_0

File: 231129_OE_RK_4, RT: 9.279, Scan: 007172
Type: HCD, FTMS + c NSI d Full ms2 946.8664@hcd30.00 [120.0000-3886.0708]
Precursor Scan: 007103, m/z: 946.6161, charge: 4
ENGTISR_6_5_1_3_0

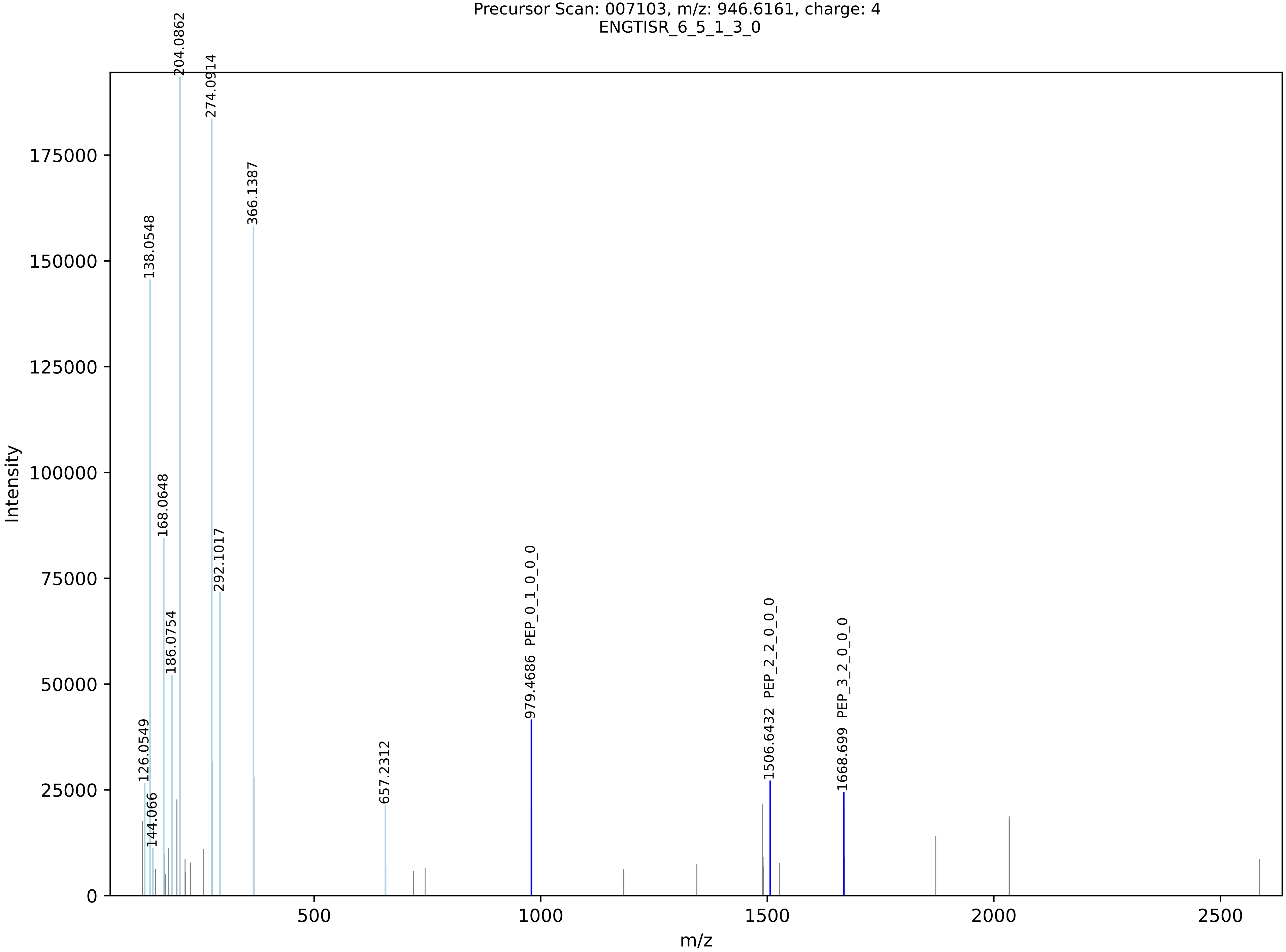

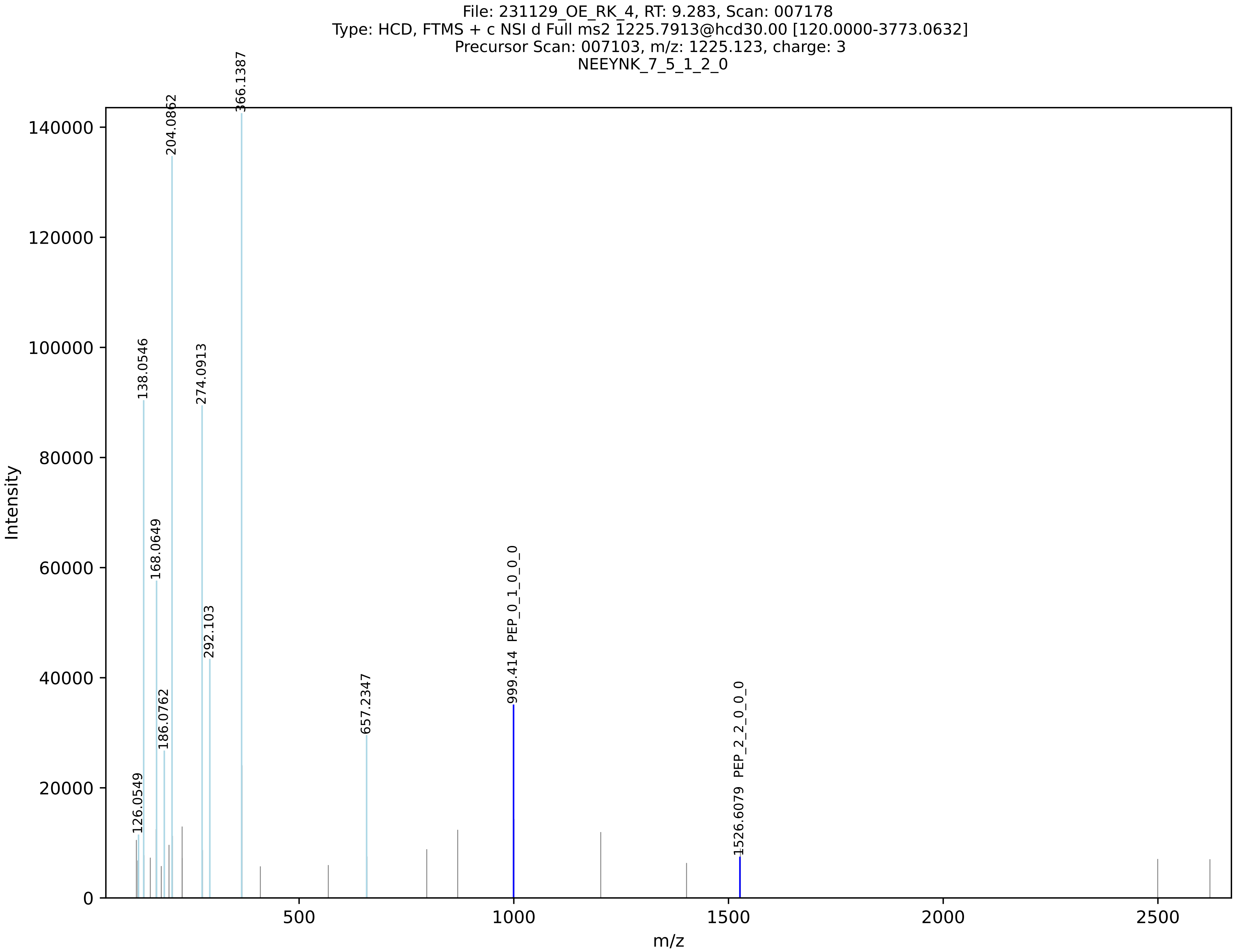
File: 231129_OE_RK_4, RT: 9.283, Scan: 007178
Type: HCD, FTMS + c NSI d Full ms2 1225.7913@hcd30.00 [120.0000-3773.0632]
Precursor Scan: 007103, m/z: 1225.123, charge: 3
NEEYNK_7_5_1_2_0
Intensity
m/z
0
20000
40000
60000
80000
100000
120000
140000
500
1000
1500
2000
2500
366.1387
204.0862
138.0546
274.0913
168.0649
292.103
186.0762
126.0549
657.2347
999.414 PEP_0_1_0_0_0
1526.6079 PEP_2_2_0_0_0

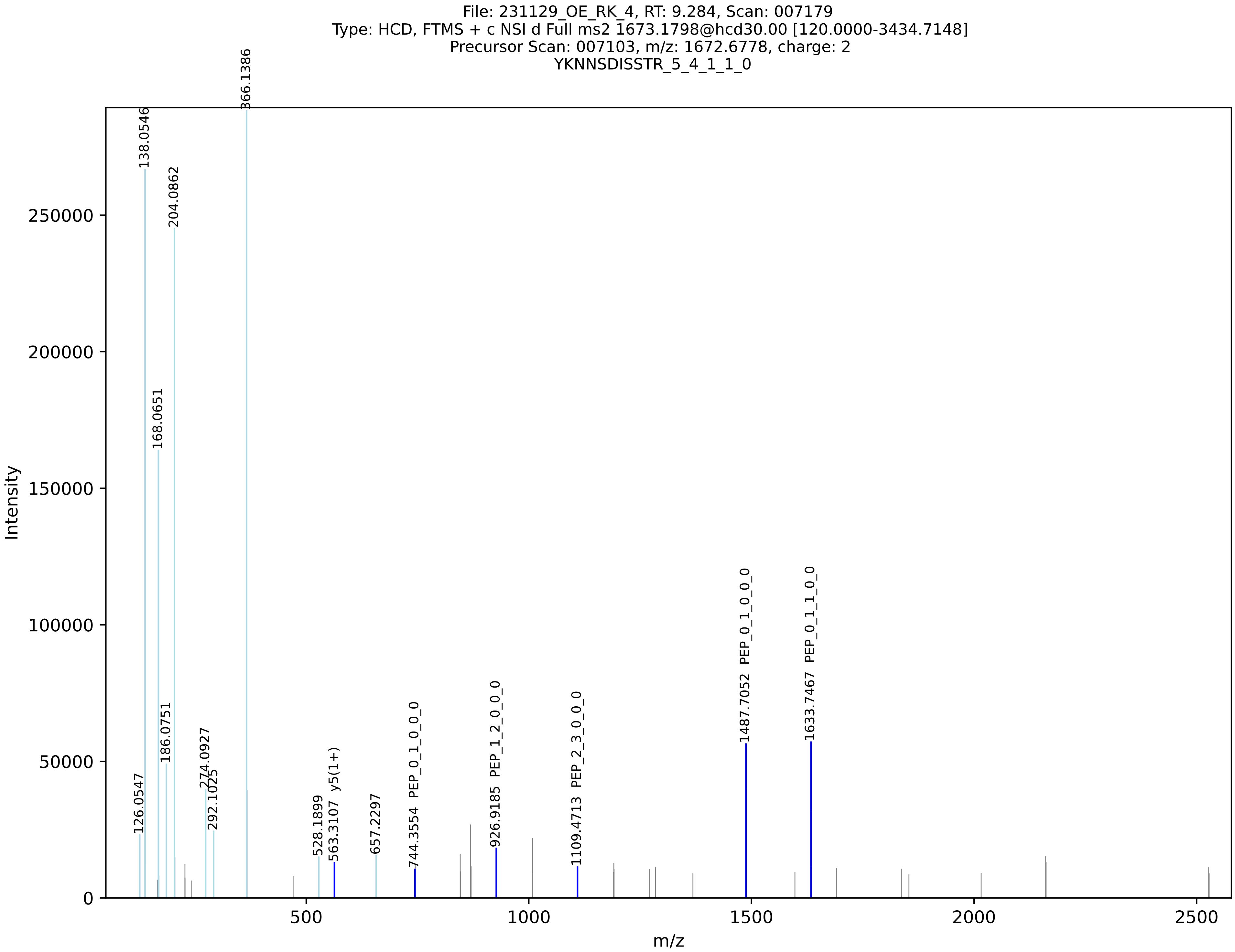

File: 231129_OE_RK_4, RT: 9.284, Scan: 007179
Type: HCD, FTMS + c NSI d Full ms2 1673.1798@hcd30.00 [120.0000-3434.7148]
Precursor Scan: 007103, m/z: 1672.6778, charge: 2
YKNNSDISSTR_5_4_1_1_0
Intensity
m/z
0
50000
100000
150000
200000
250000
500
1000
1500
2000
2500
126.0547
138.0546
168.0651
186.0751
204.0862
274.0927
292.1025
366.1386
528.1899
563.3107 y5(1+)
657.2297
744.3554 PEP_0_1_0_0_0_
926.9185 PEP_1_2_0_0_0_
1109.4713 PEP_2_3_0_0_0_
1487.7052 PEP_0_1_0_0_0_
1633.7467 PEP_0_1_1_0_0_

File: 231129_OE_RK_4, RT: 9.325, Scan: 007237
Type: HCD, FTMS + c NSI d Full ms2 1262.4868@hcd30.00 [120.0000-3885.3518]
Precursor Scan: 007167, m/z: 1261.8184, charge: 3
ENGTISR_6_5_1_3_0

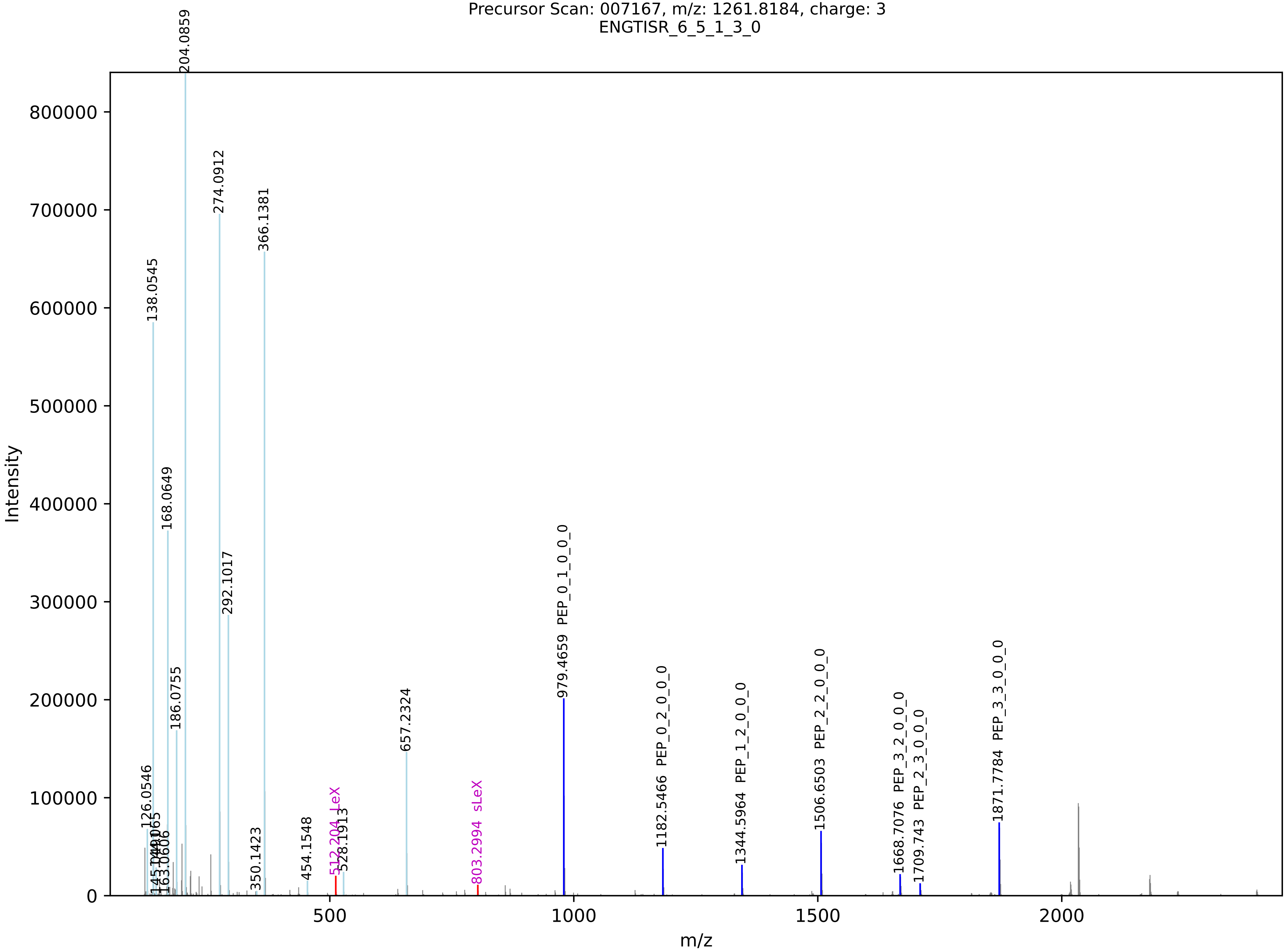

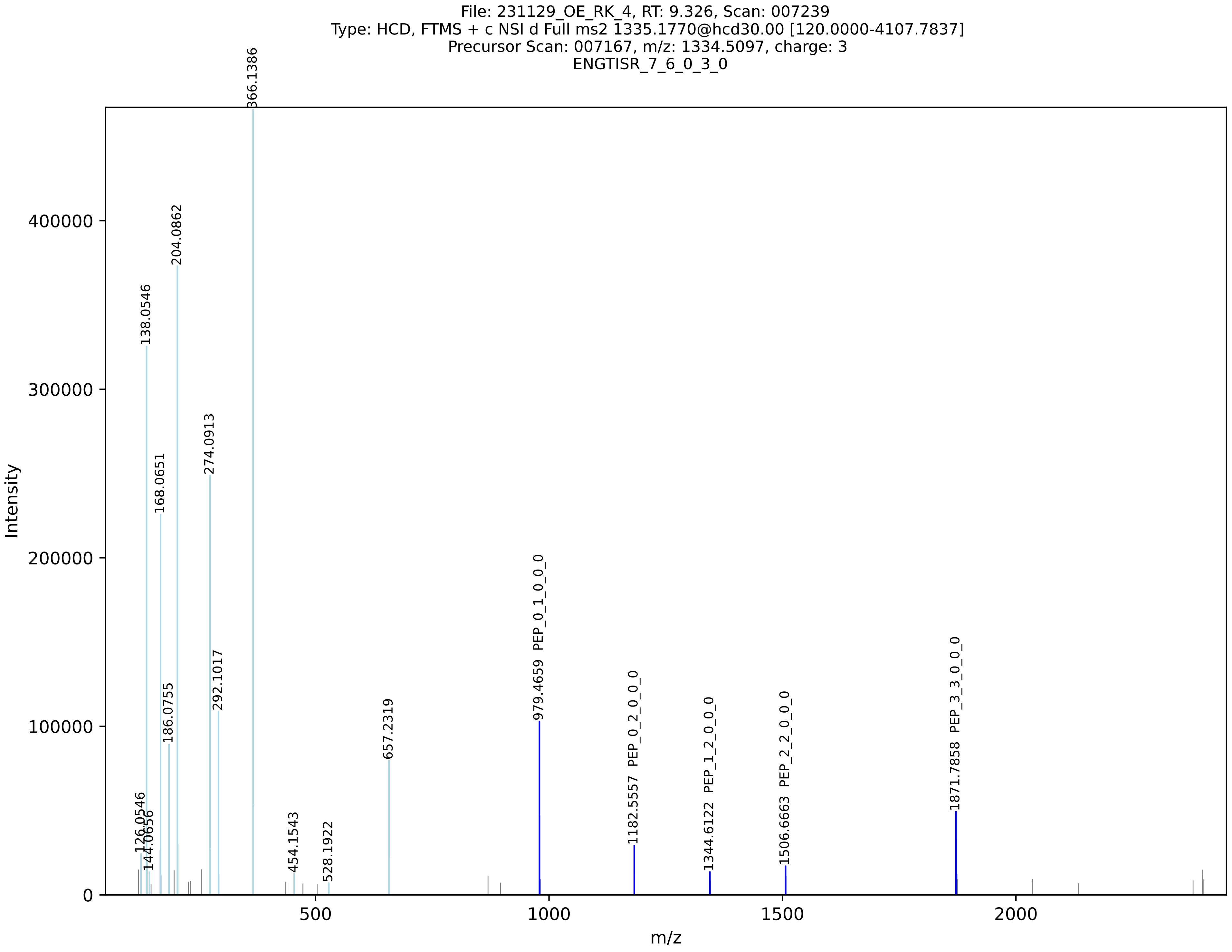
File: 231129_OE_RK_4, RT: 9.326, Scan: 007239
Type: HCD, FTMS + c NSI d Full ms2 1335.1770@hcd30.00 [120.0000-4107.7837]
Precursor Scan: 007167, m/z: 1334.5097, charge: 3
ENGTISR_7_6_0_3_0
366.1386
204.0862
138.0546
274.0913
168.0651
292.1017
186.0755
126.0546
144.0656
454.1543
528.1922
657.2319
979.4659 PEP_0_1_0_0_0
1182.5557 PEP_0_2_0_0_0
1344.6122 PEP_1_2_0_0_0
1506.6663 PEP_2_2_0_0_0
1871.7858 PEP_3_3_0_0_0
Intensity
0
100000
200000
300000
400000
500
1000
1500
2000
m/z

File: 231129_OE_RK_4, RT: 9.369, Scan: 007300
Type: HCD, FTMS + c NSI d Full ms2 1529.9178@hcd30.00 [120.0000-4703.6904]
Precursor Scan: 007236, m/z: 1529.2541, charge: 3
ENGTISR_7_6_2_4_0

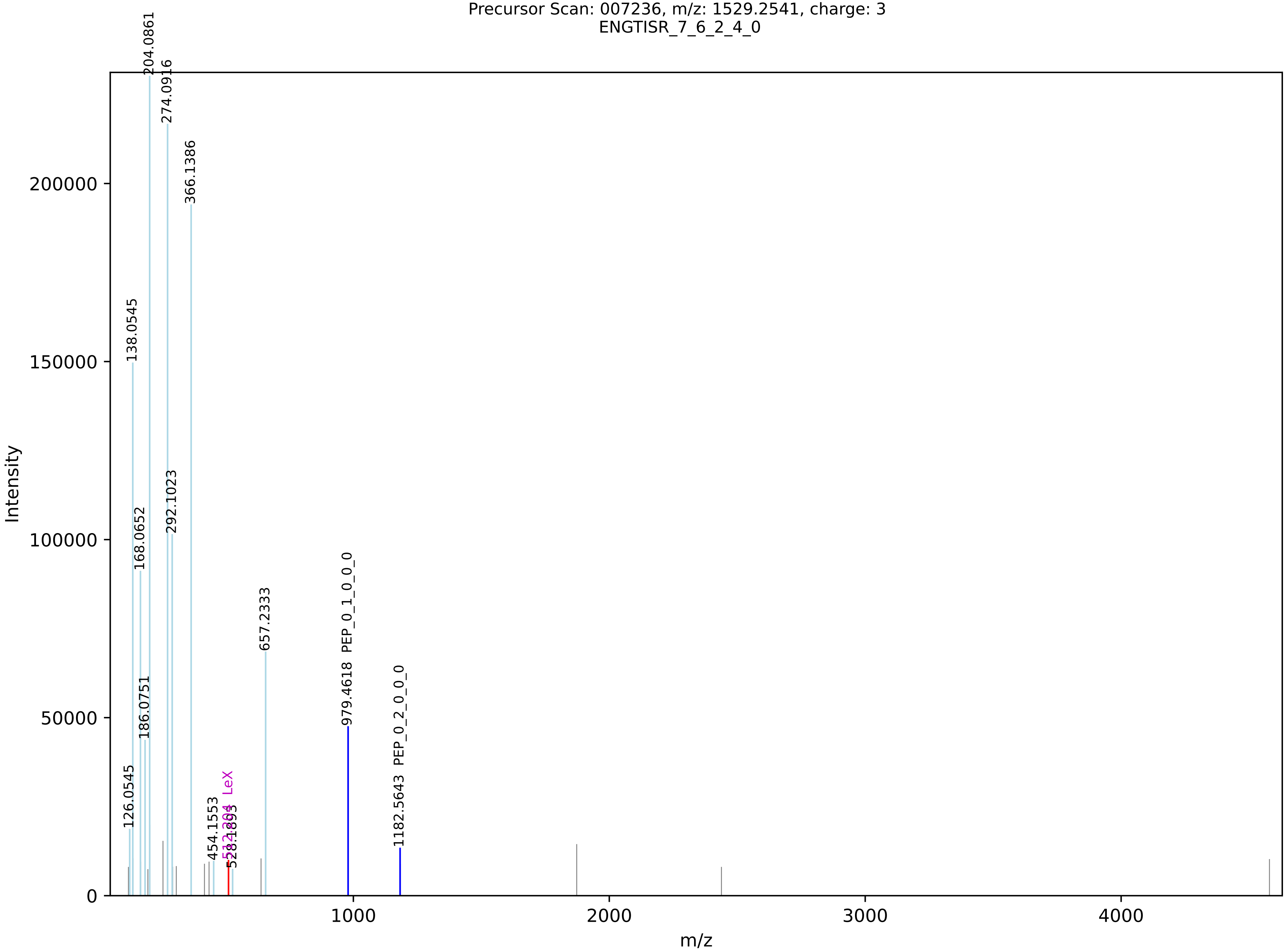

File: 231129_OE_RK_4, RT: 9.416, Scan: 007367
Type: HCD, FTMS + c NSI d Full ms2 1115.7882@hcd30.00 [120.0000-3436.4539]
Precursor Scan: 007295, m/z: 1115.4542, charge: 3
YKNNSDISSTR_5_4_1_1_0

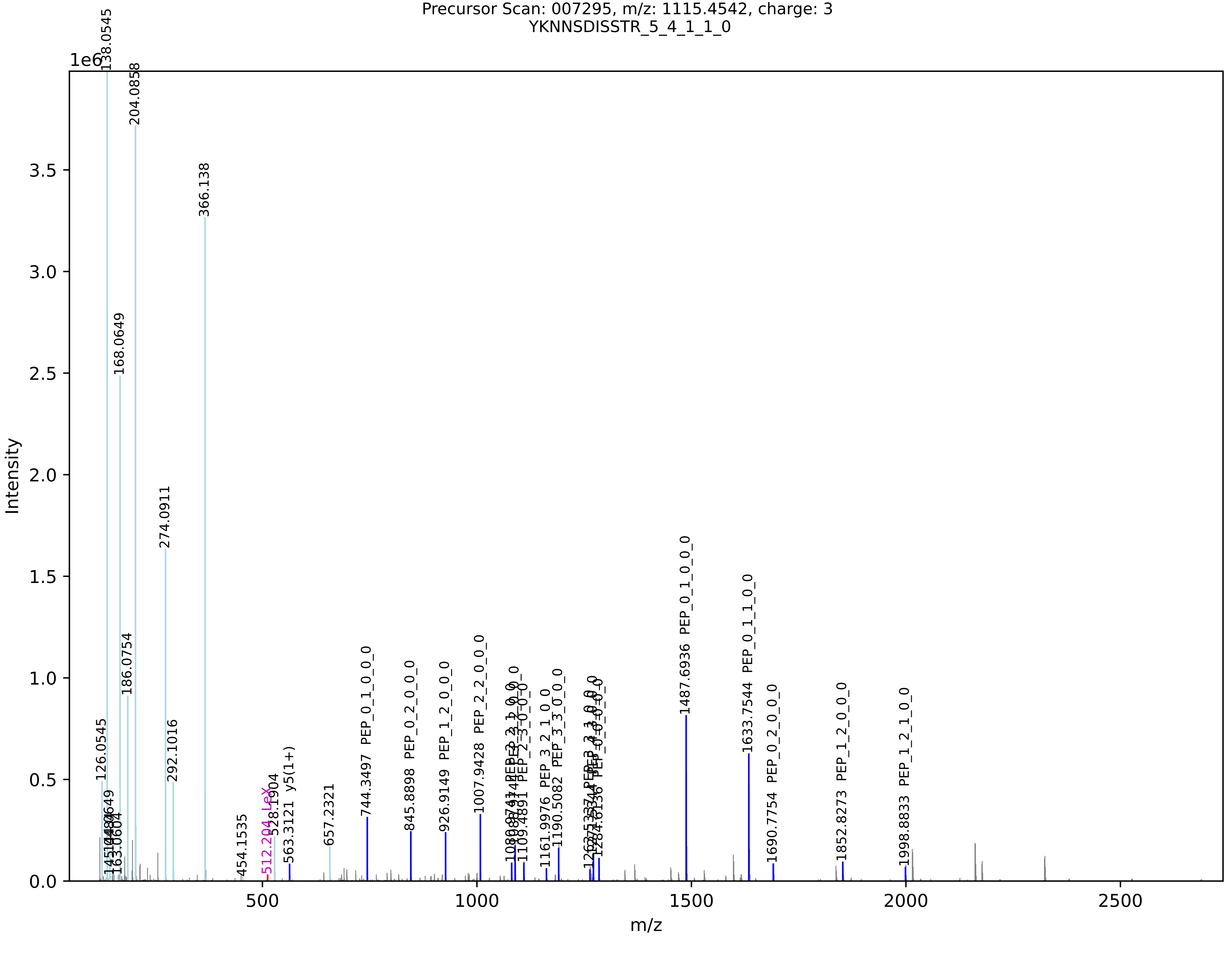

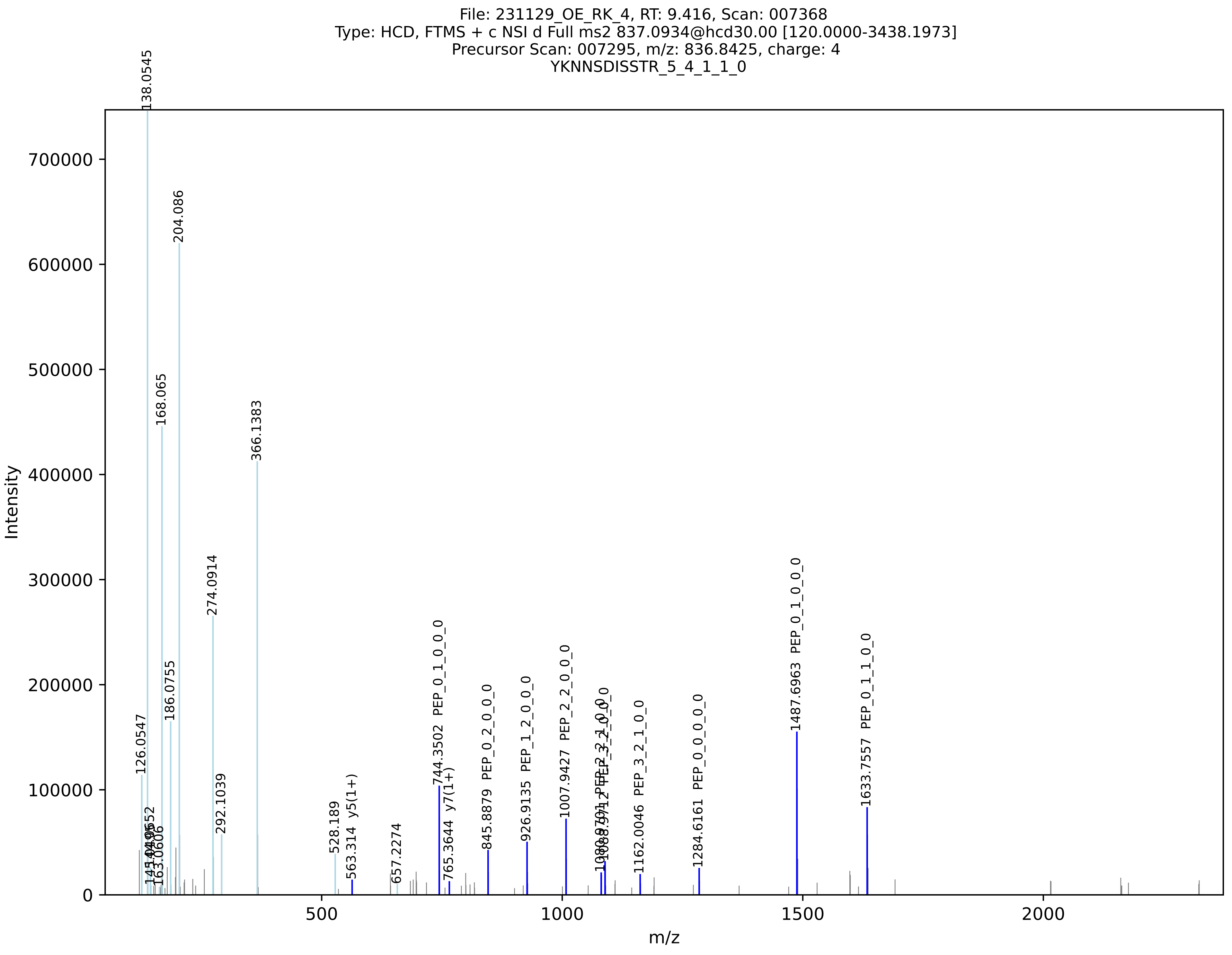

File: 231129_OE_RK_4, RT: 9.416, Scan: 007368
Type: HCD, FTMS + c NSI d Full ms2 837.0934@hcd30.00 [120.0000-3438.1973]
Precursor Scan: 007295, m/z: 836.8425, charge: 4
YKNNSDISSTR_5_4_1_1_0
Intensity
m/z
138.0545
204.086
168.065
366.1383
274.0914
186.0755
126.0547
292.1039
163.0606
528.189
563.314 y5(1+)
657.2274
744.3502 PEP_0_1_0_0_0_
765.3644 y7(1+)
845.8879 PEP_0_2_0_0_0_
926.9135 PEP_1_2_0_0_0_
1007.9427 PEP_2_2_0_0_0_
1162.0046 PEP_3_2_1_0_0_
1284.6161 PEP_0_0_0_0_0_
1487.6963 PEP_0_1_0_0_0_
1633.7557 PEP_0_1_1_0_0_

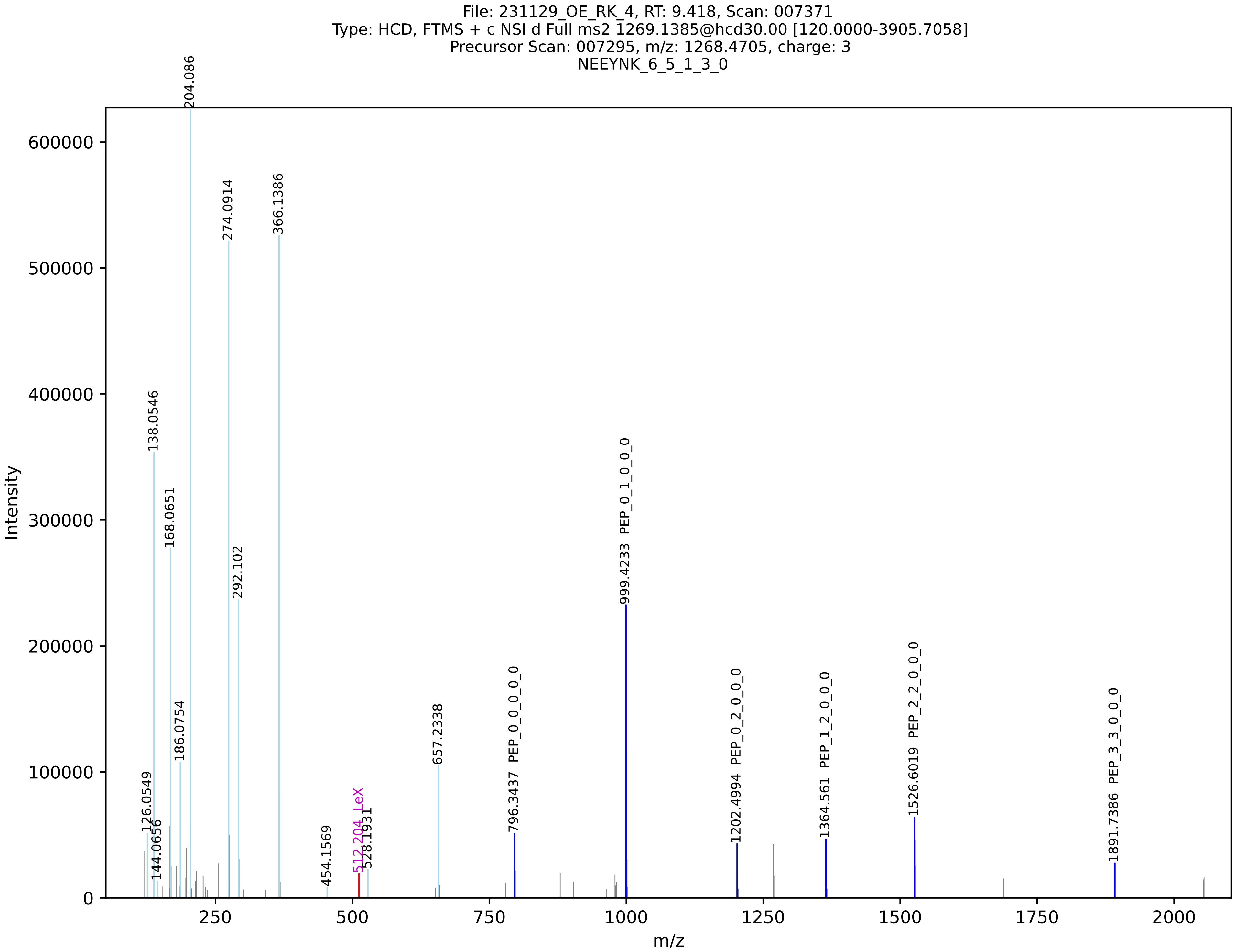

File: 231129_OE_RK_4, RT: 9.418, Scan: 007371
Type: HCD, FTMS + c NSI d Full ms2 1269.1385@hcd30.00 [120.0000-3905.7058]
Precursor Scan: 007295, m/z: 1268.4705, charge: 3
NEEYNK_6_5_1_3_0
Intensity
m/z
204.086
274.0914
366.1386
138.0546
168.0651
292.102
186.0754
126.0549
144.0656
454.1569
512.204_LeX
528.1931
657.2338
796.3437 PEP_0_0_0_0_0
999.4233 PEP_0_1_0_0_0
1202.4994 PEP_0_2_0_0_0
1364.561 PEP_1_2_0_0_0
1526.6019 PEP_2_2_0_0_0
1891.7386 PEP_3_3_0_0_0

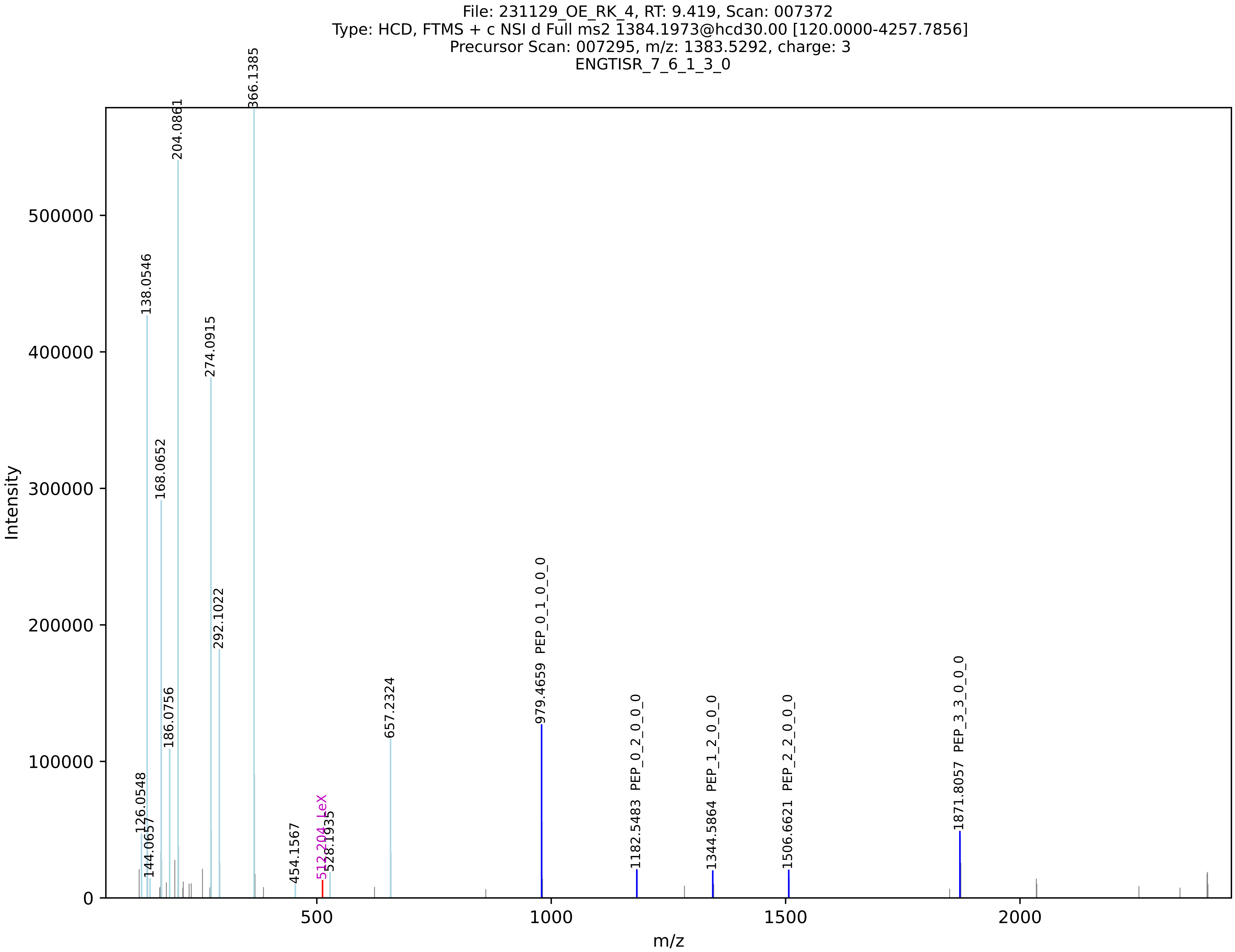
File: 231129_OE_RK_4, RT: 9.419, Scan: 007372
Type: HCD, FTMS + c NSI d Full ms2 1384.1973@hcd30.00 [120.0000-4257.7856]
Precursor Scan: 007295, m/z: 1383.5292, charge: 3
ENGTISR_7_6_1_3_0
366.1385
204.0861
138.0546
274.0915
168.0652
292.1022
186.0756
126.0548
144.0657
454.1567
512.204_LeX
528.1935
657.2324
979.4659 PEP_0_1_0_0_0
1182.5483 PEP_0_2_0_0_0
1344.5864 PEP_1_2_0_0_0
1506.6621 PEP_2_2_0_0_0
1871.8057 PEP_3_3_0_0_0
Intensity
m/z
0
100000
200000
300000
400000
500000
500
1000
1500
2000

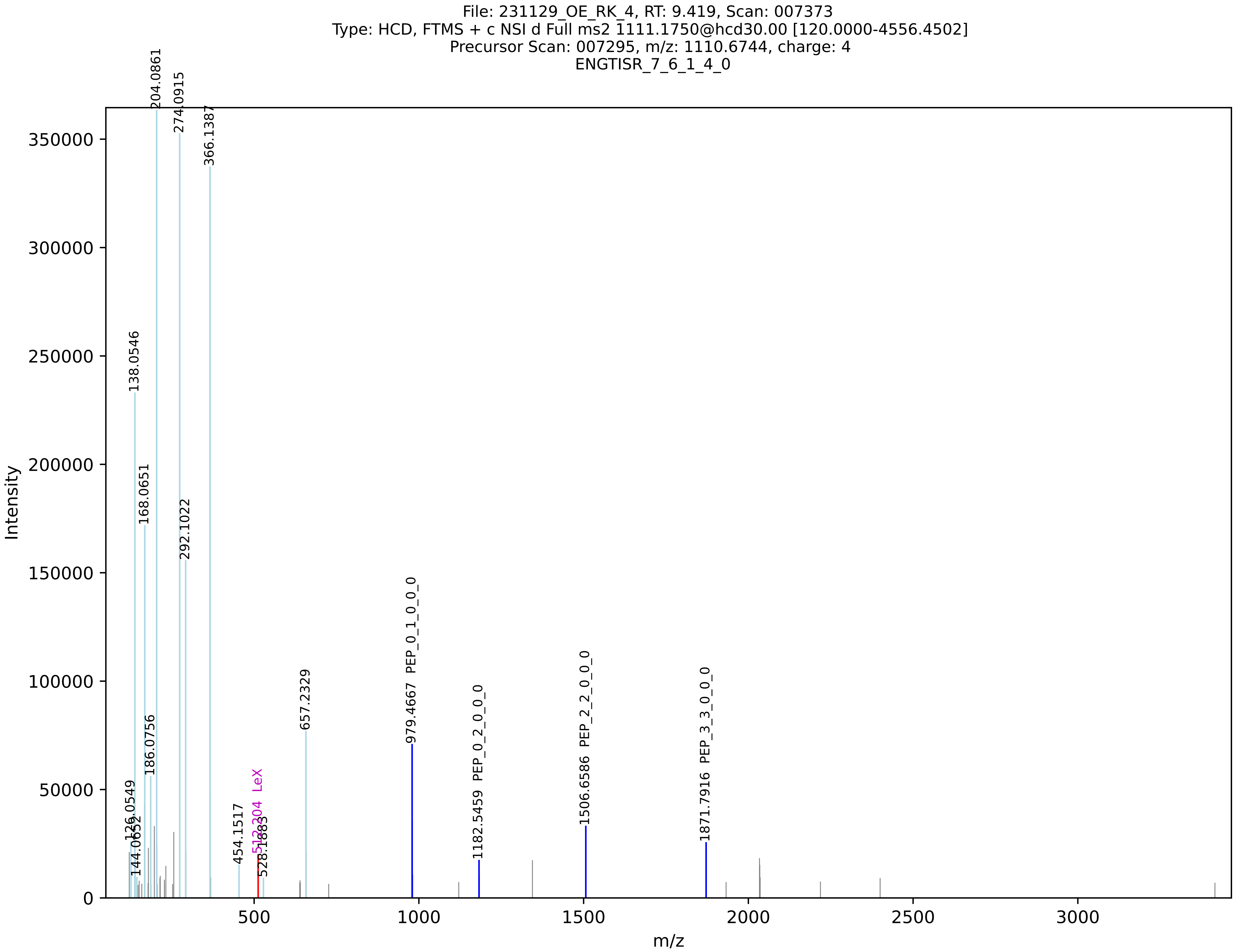

File: 231129_OE_RK_4, RT: 9.419, Scan: 007373
Type: HCD, FTMS + c NSI d Full ms2 1111.1750@hcd30.00 [120.0000-4556.4502]
Precursor Scan: 007295, m/z: 1110.6744, charge: 4
ENGTISR_7_6_1_4_0
Intensity
m/z
204.0861
274.0915
366.1387
138.0546
168.0651
292.1022
186.0756
126.0549
144.0652
454.1517
512.204 LeX
528.1883
657.2329
979.4667 PEP_0_1_0_0_0
1182.5459 PEP_0_2_0_0_0
1506.6586 PEP_2_2_0_0_0
1871.7916 PEP_3_3_0_0_0

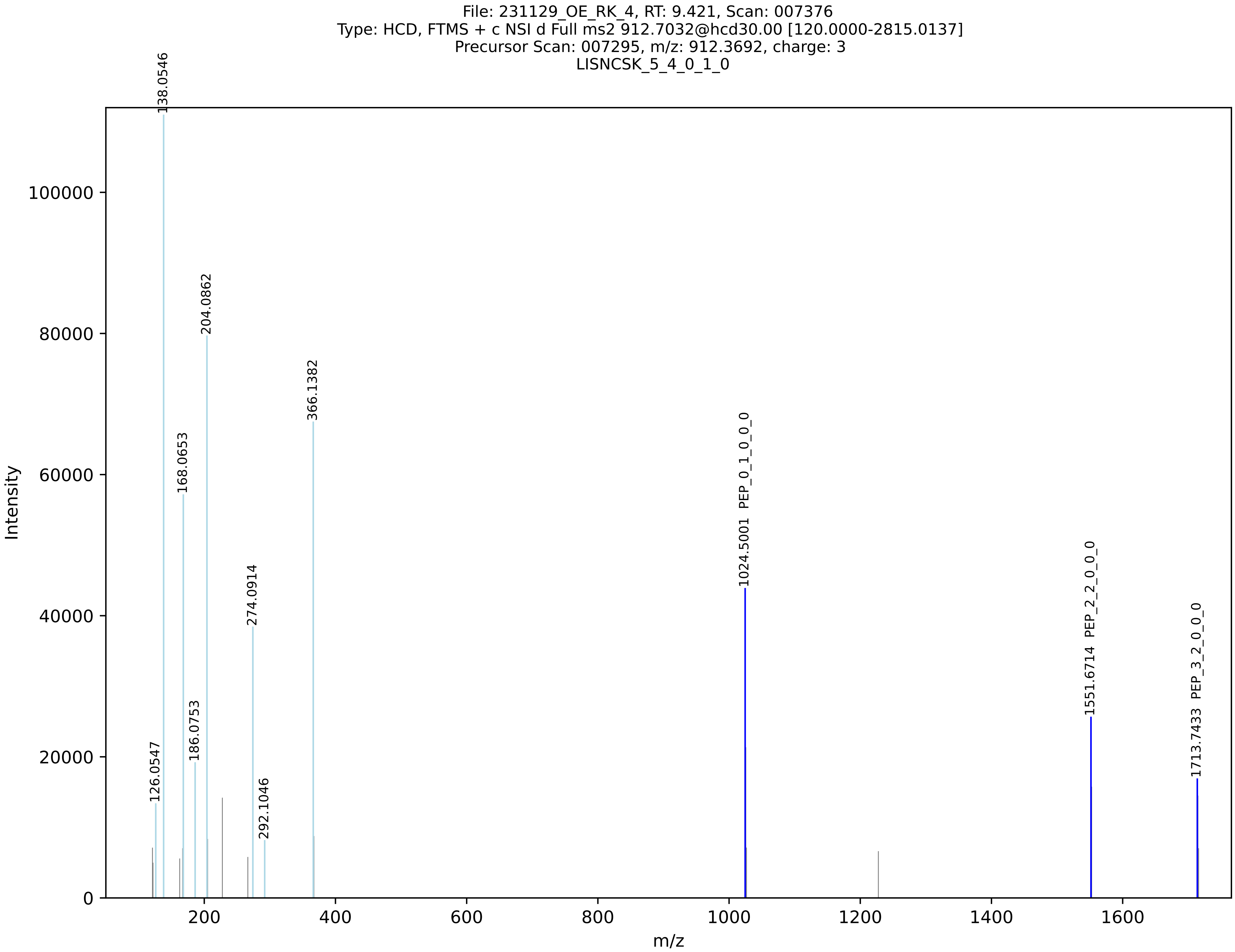

File: 231129_OE_RK_4, RT: 9.421, Scan: 007376
Type: HCD, FTMS + c NSI d Full ms2 912.7032@hcd30.00 [120.0000-2815.0137]
Precursor Scan: 007295, m/z: 912.3692, charge: 3
LISNCSK_5_4_0_1_0
Intensity
m/z
138.0546
204.0862
168.0653
366.1382
274.0914
186.0753
126.0547
292.1046
1024.5001 PEP_0_1_0_0_
1551.6714 PEP_2_2_0_0_0
1713.7433 PEP_3_2_0_0_0

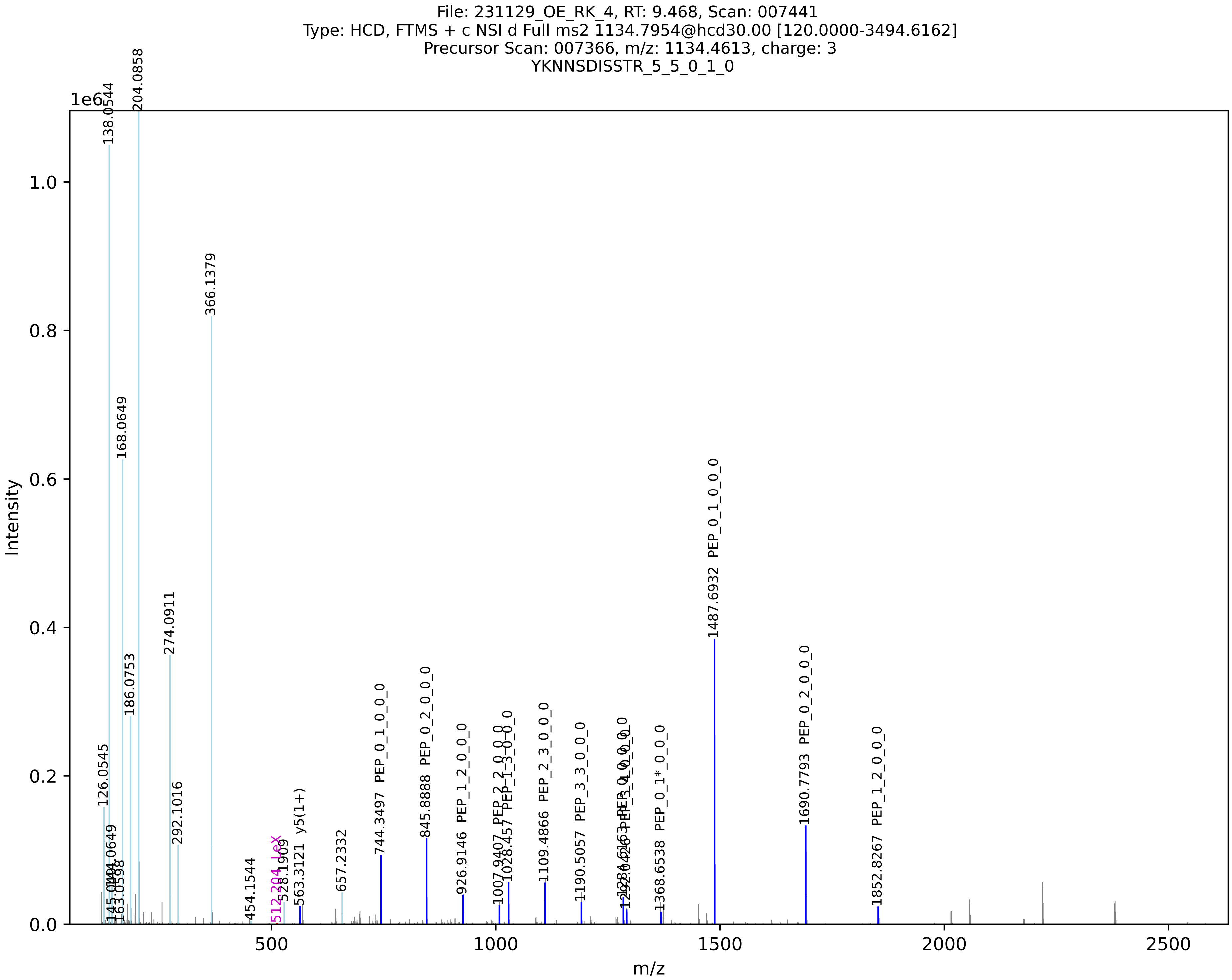

File: 231129_OE_RK_4, RT: 9.468, Scan: 007441
Type: HCD, FTMS + c NSI d Full ms2 1134.7954@hcd30.00 [120.0000-3494.6162]
Precursor Scan: 007366, m/z: 1134.4613, charge: 3
YKNNSDISSTR_5_5_0_1_0
1e6
Intensity
m/z
204.0858
138.0544
366.1379
168.0649
274.0911
186.0753
126.0545
292.1016
454.1544
512.204_LeX
528.1909
563.3121 y5(1+)
657.2332
744.3497 PEP_0_1_0_0_0
845.8888 PEP_0_2_0_0_0
926.9146 PEP_1_2_0_0_0
1007.9407 PEP_2_2_0_0_0
1028.457 PEP_1_3_0_0_0
1109.4866 PEP_2_3_0_0_0
1190.5057 PEP_3_3_0_0_0
1368.6538 PEP_0_1*_0_0_0
1487.6932 PEP_0_1_0_0_0
1690.7793 PEP_0_2_0_0_0
1852.8267 PEP_1_2_0_0_0

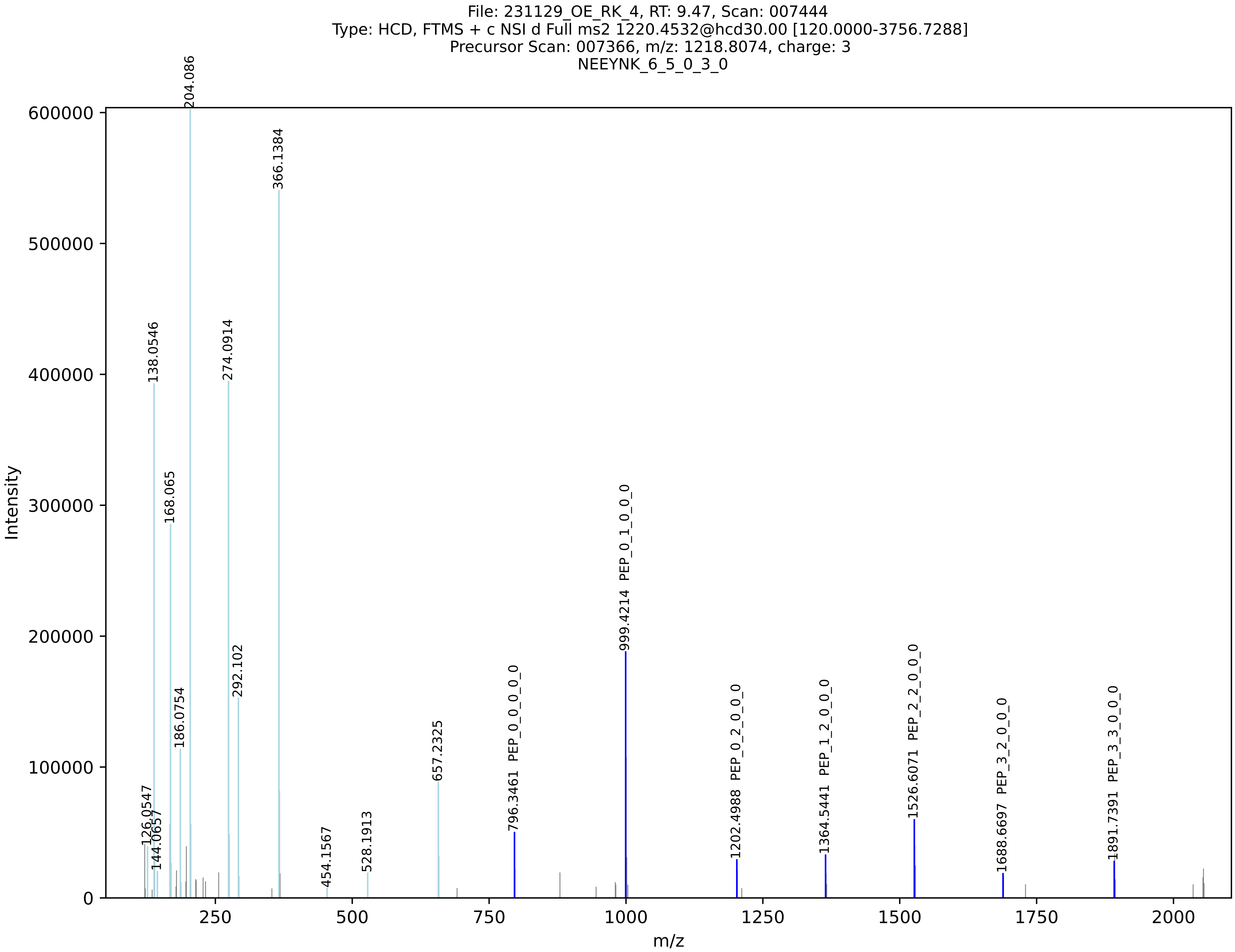

File: 231129_OE_RK_4, RT: 9.47, Scan: 007444
Type: HCD, FTMS + c NSI d Full ms2 1220.4532@hcd30.00 [120.0000-3756.7288]
Precursor Scan: 007366, m/z: 1218.8074, charge: 3
NEEYNK_6_5_0_3_0
Intensity
m/z
204.086
366.1384
138.0546
274.0914
168.065
186.0754
292.102
126.0547
144.0657
454.1567
528.1913
657.2325
796.3461 PEP_0_0_0_0_0
999.4214 PEP_0_1_0_0_0
1202.4988 PEP_0_2_0_0_0
1364.5441 PEP_1_2_0_0_0
1526.6071 PEP_2_2_0_0_0
1688.6697 PEP_3_2_0_0_0
1891.7391 PEP_3_3_0_0_0

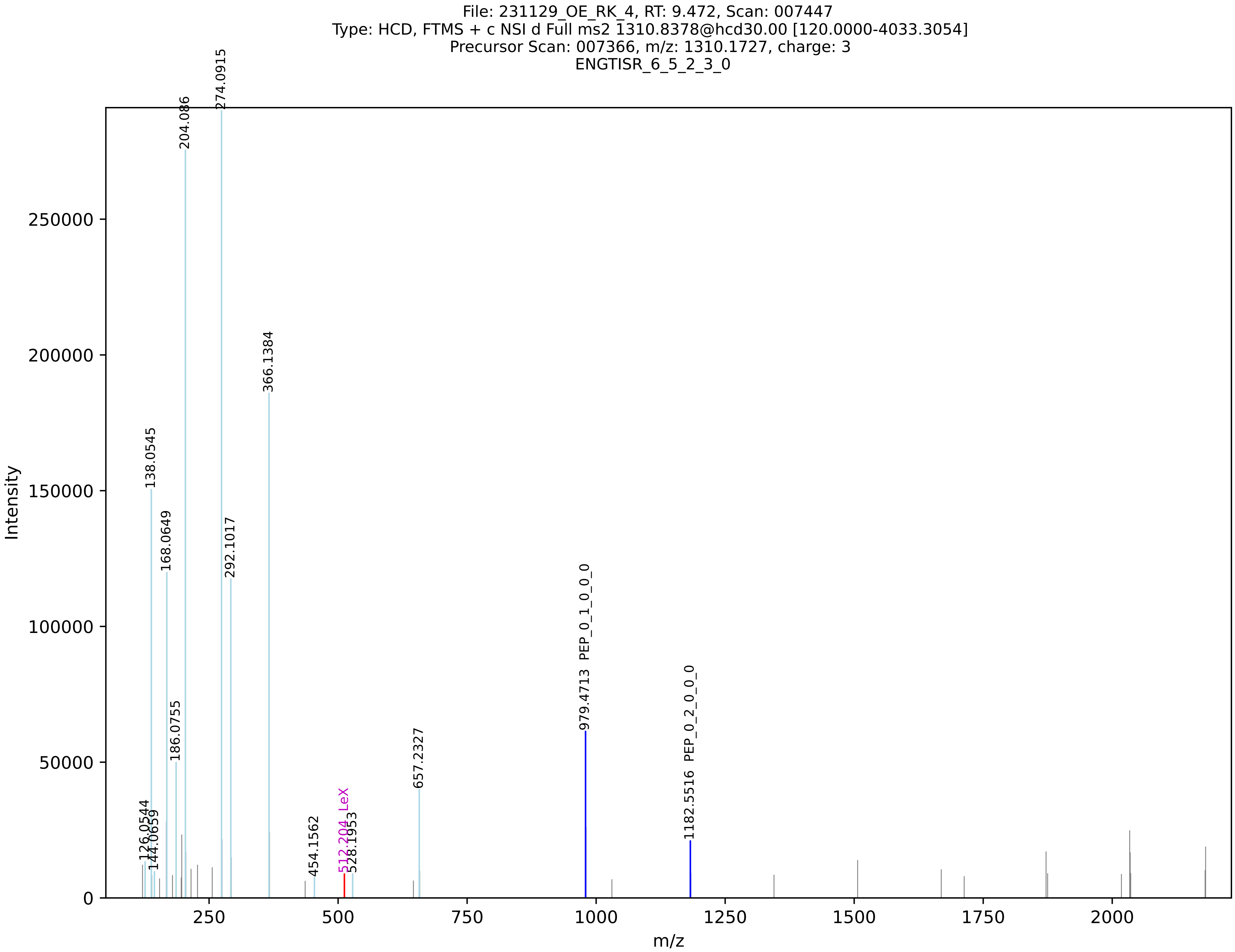
File: 231129_OE_RK_4, RT: 9.472, Scan: 007447
Type: HCD, FTMS + c NSI d Full ms2 1310.8378@hcd30.00 [120.0000-4033.3054]
Precursor Scan: 007366, m/z: 1310.1727, charge: 3
ENGTISR_6_5_2_3_0
Intensity
m/z
274.0915
204.086
366.1384
138.0545
168.0649
292.1017
186.0755
126.0544
144.0659
454.1562
512.204 LeX
528.1953
657.2327
979.4713 PEP_0_1_0_0_0
1182.5516 PEP_0_2_0_0_0

File: 231129_OE_RK_4, RT: 9.472, Scan: 007448
Type: HCD, FTMS + c NSI d Full ms2 946.8667@hcd30.00 [120.0000-3886.0723]
Precursor Scan: 007366, m/z: 946.6153, charge: 4
ENGTISR_6_5_1_3_0

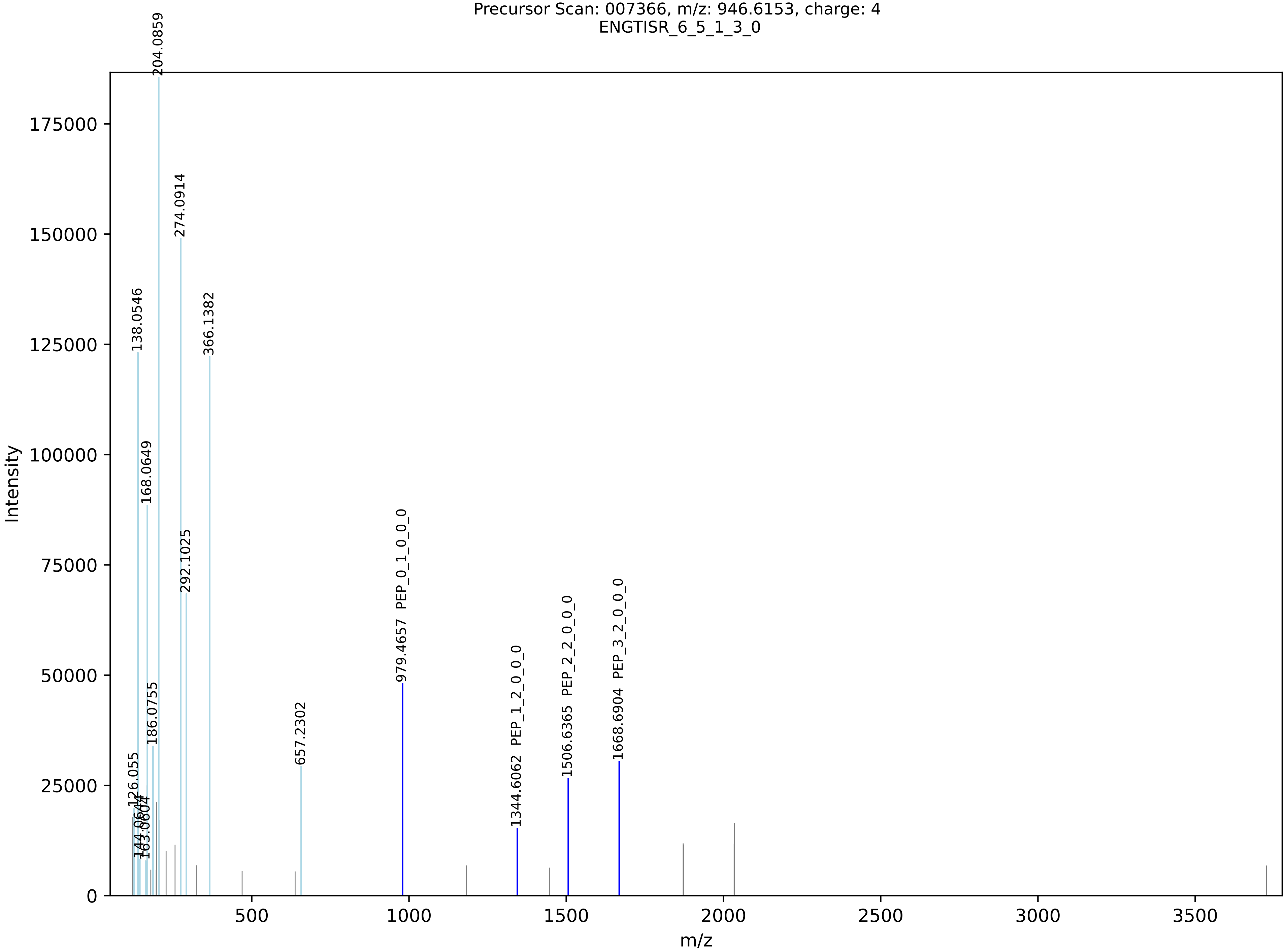

File: 231129_OE_RK_4, RT: 9.473, Scan: 007449
Type: HCD, FTMS + c NSI d Full ms2 1115.6855@hcd30.00 [120.0000-3436.1396]
Precursor Scan: 007366, m/z: 1115.6855, charge: 3
YKNNSDISSTR_5_4_1_1_0

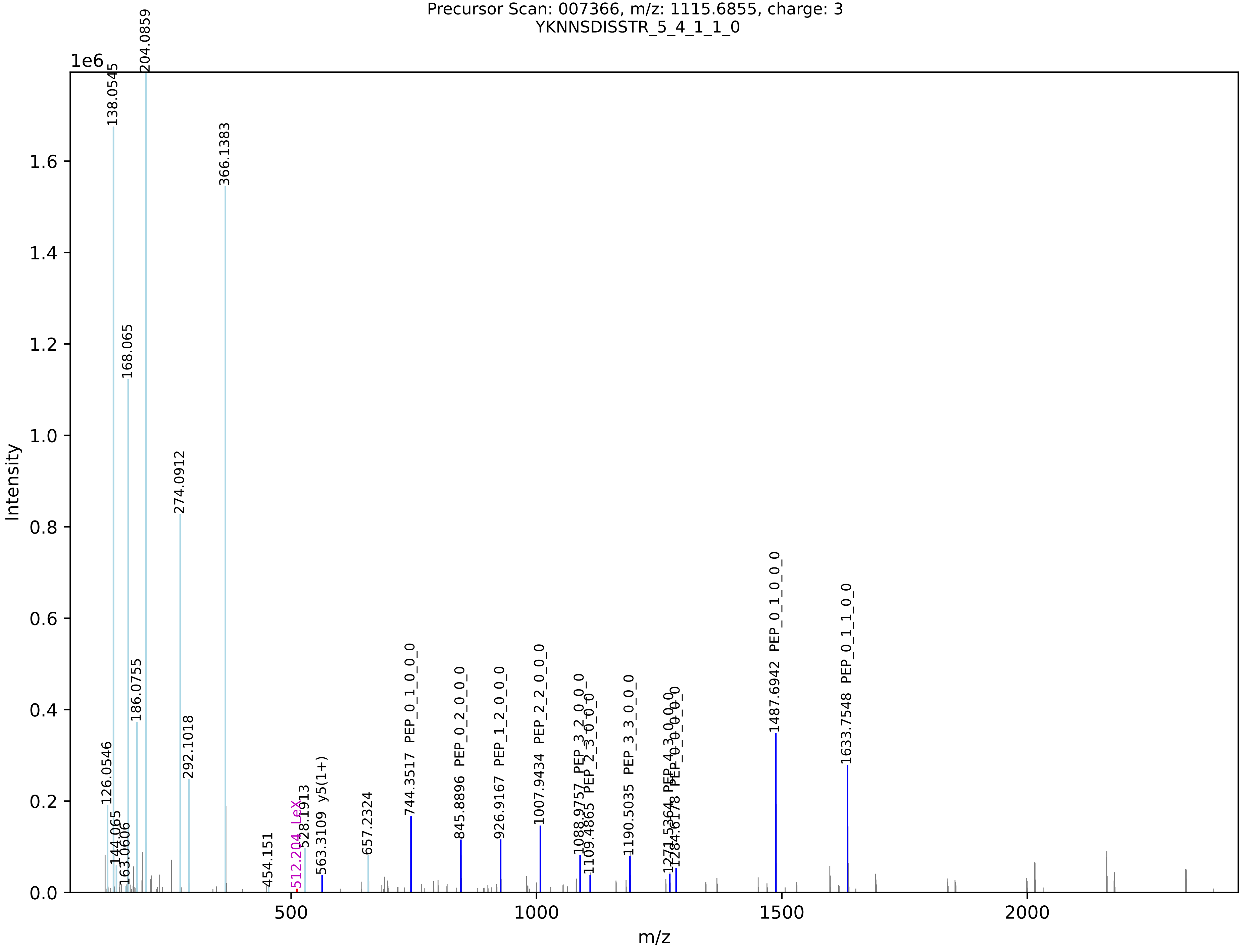

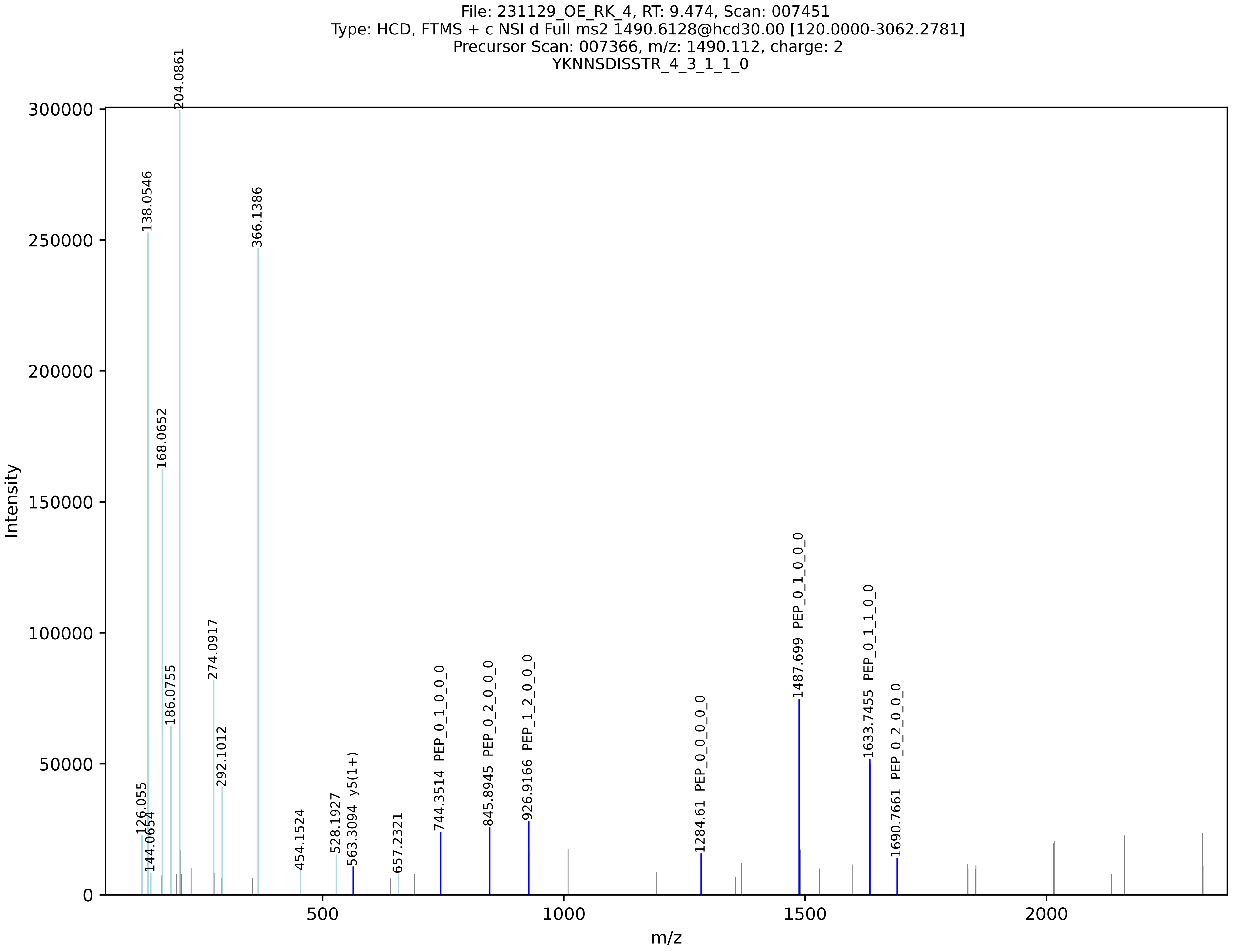

File: 231129_OE_RK_4, RT: 9.474, Scan: 007451
Type: HCD, FTMS + c NSI d Full ms2 1490.6128@hcd30.00 [120.0000-3062.2781]
Precursor Scan: 007366, m/z: 1490.112, charge: 2
YKNNSDISSTR_4_3_1_1_0
Intensity
m/z
204.0861
138.0546
366.1386
168.0652
186.0755
274.0917
292.1012
126.055
144.0654
454.1524
528.1927
563.3094 y5(1+)
657.2321
744.3514 PEP_0_1_0_0_0
845.8945 PEP_0_2_0_0_0
926.9166 PEP_1_2_0_0_0
1284.61 PEP_0_0_0_0_0
1487.699 PEP_0_1_0_0_0
1633.7455 PEP_0_1_1_0_0
1690.7661 PEP_0_2_0_0_0

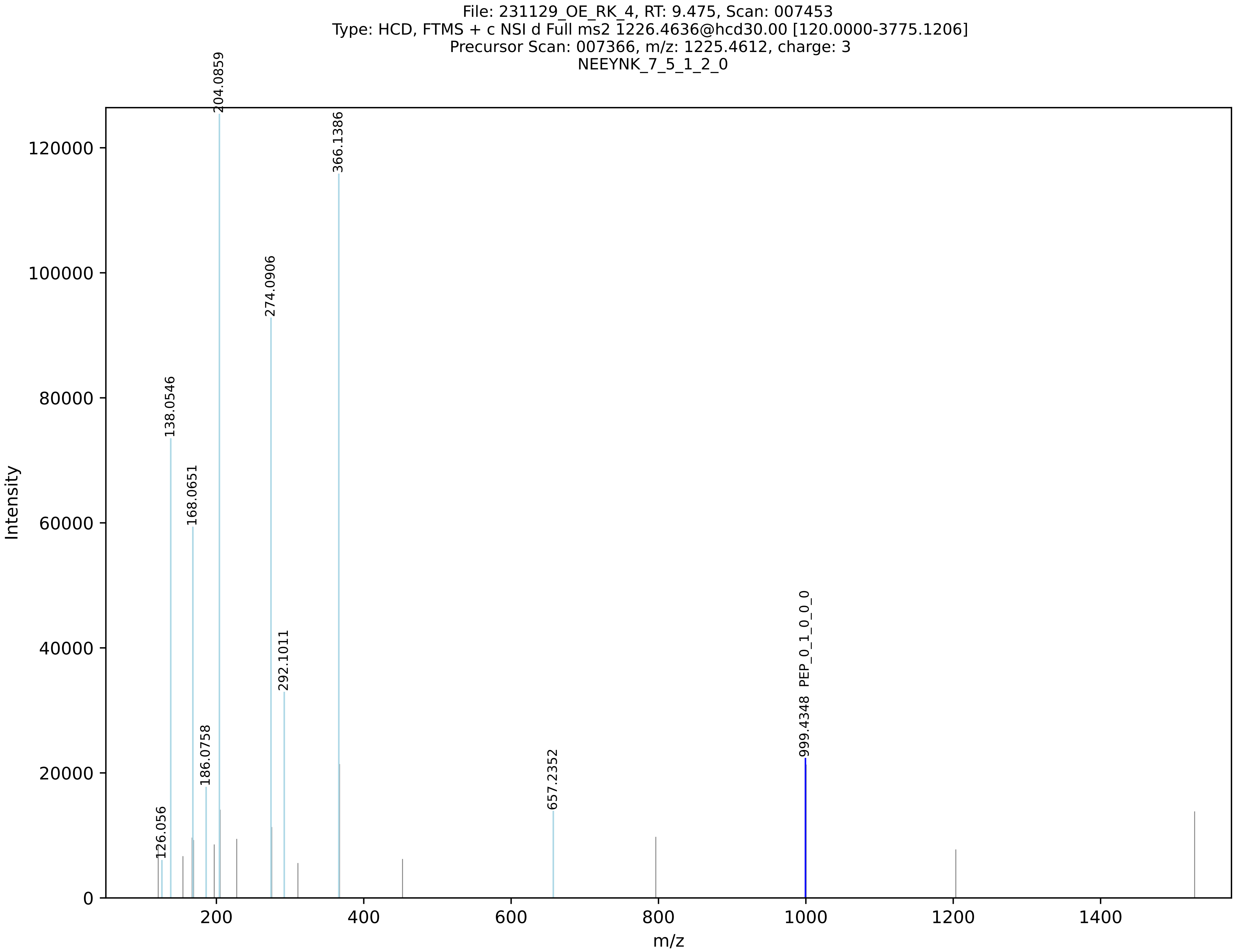

File: 231129_OE_RK_4, RT: 9.475, Scan: 007453
Type: HCD, FTMS + c NSI d Full ms2 1226.4636@hcd30.00 [120.0000-3775.1206]
Precursor Scan: 007366, m/z: 1225.4612, charge: 3
NEEYNK_7_5_1_2_0
Intensity
m/z
204.0859
366.1386
274.0906
138.0546
168.0651
292.1011
186.0758
126.056
657.2352
999.4348 PEP_0_1_0_0_0
0
20000
40000
60000
80000
100000
120000
200
400
600
800
1000
1200
1400

File: 231129_OE_RK_4, RT: 9.482, Scan: 007464
Type: HCD, FTMS + c NSI d Full ms2 994.4130@hcd30.00 [120.0000-3065.0457]
Precursor Scan: 007295, m/z: 993.7438, charge: 3
YKNNSDISSTR_4_3_1_1_0

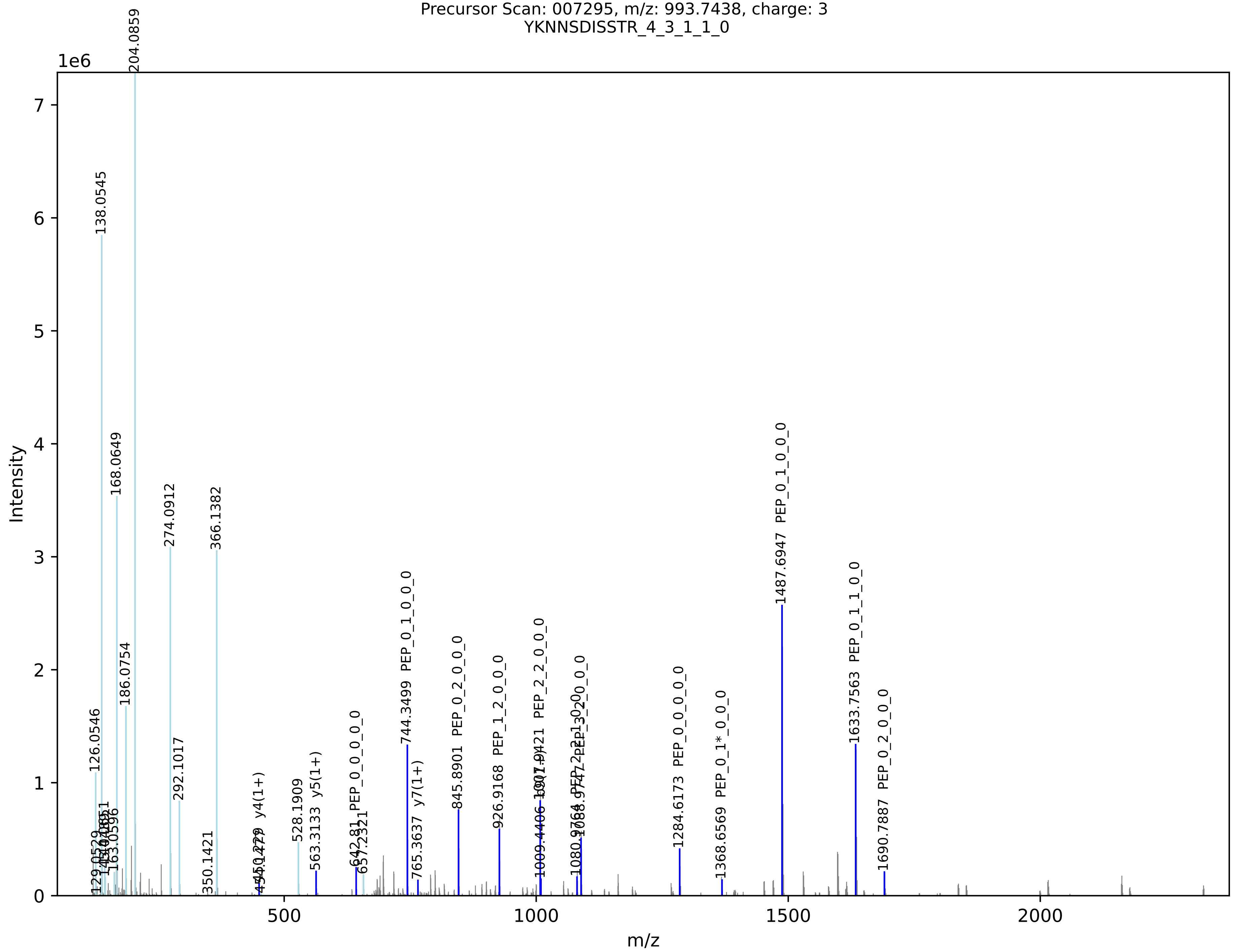

File: 231129_OE_RK_4, RT: 9.518, Scan: 007511
Type: HCD, FTMS + c NSI d Full ms2 1262.1526@hcd30.00 [120.0000-3884.3289]
Precursor Scan: 007440, m/z: 1261.4835, charge: 3
ENGTISR_6_5_1_3_0

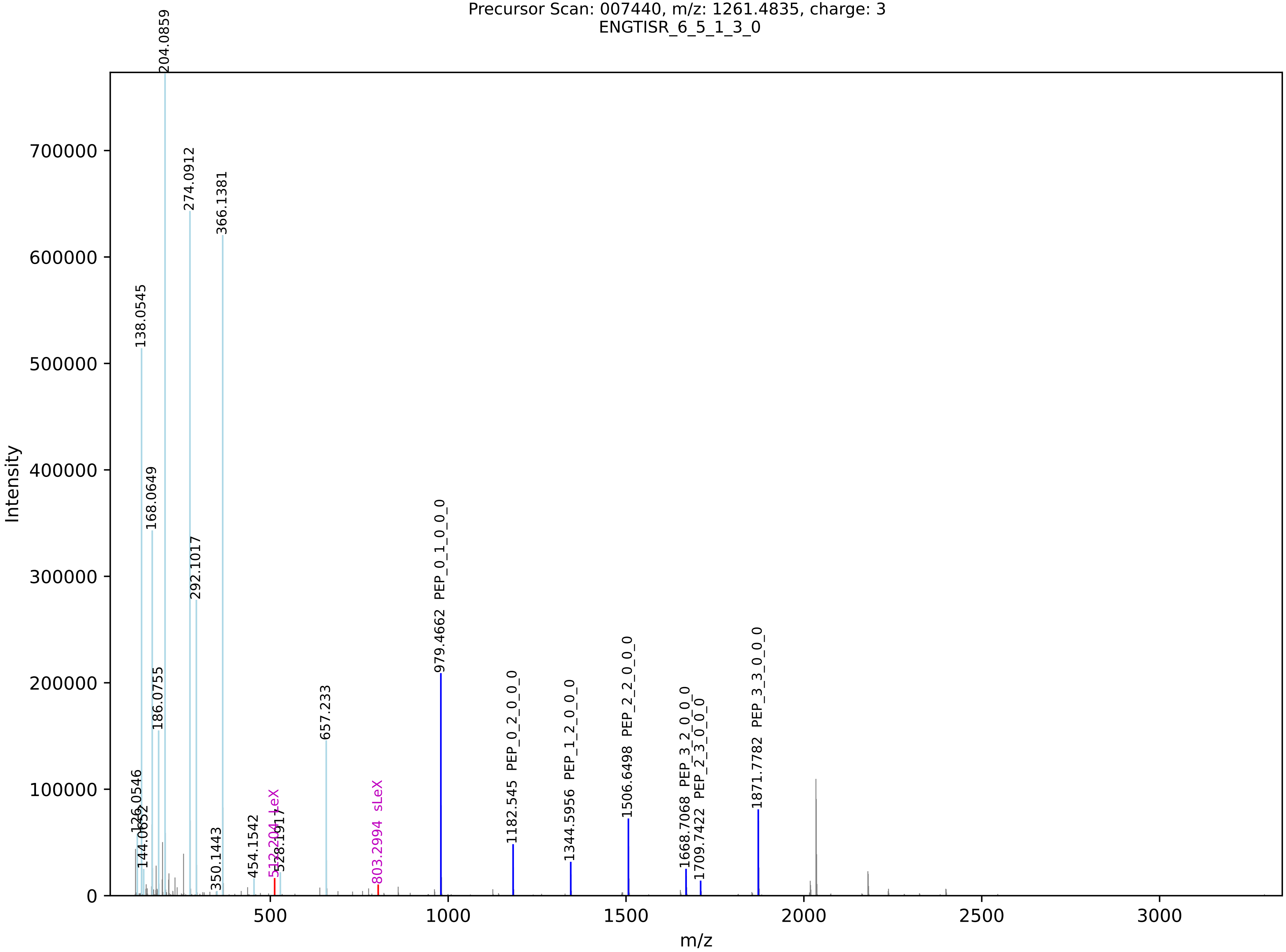

File: 231129_OE_RK_4, RT: 9.52, Scan: 007514
Type: HCD, FTMS + c NSI d Full ms2 1038.4003@hcd30.00 [120.0000-4259.5293]
Precursor Scan: 007440, m/z: 1037.8991, charge: 4
ENGTISR_7_6_1_3_0

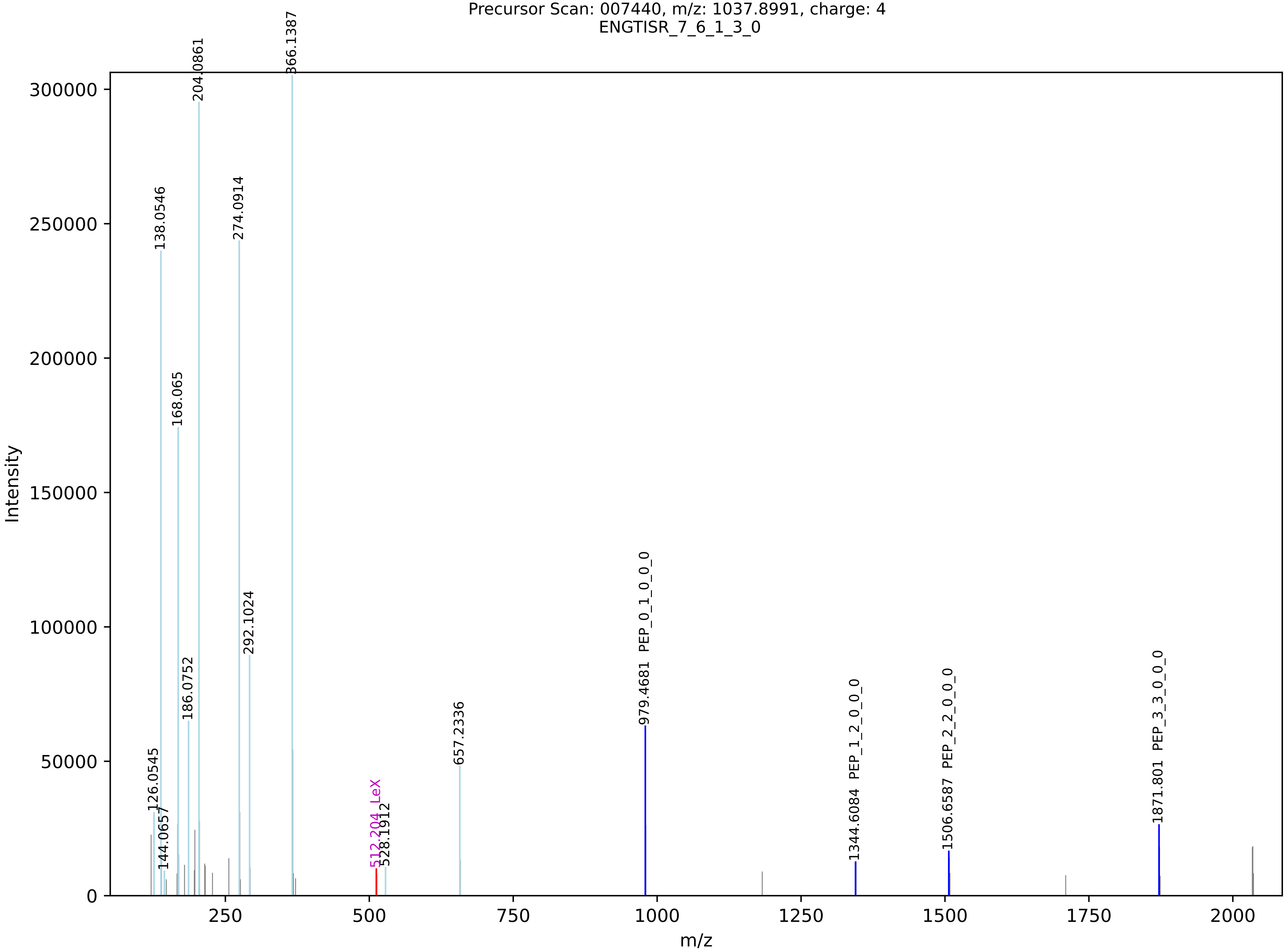

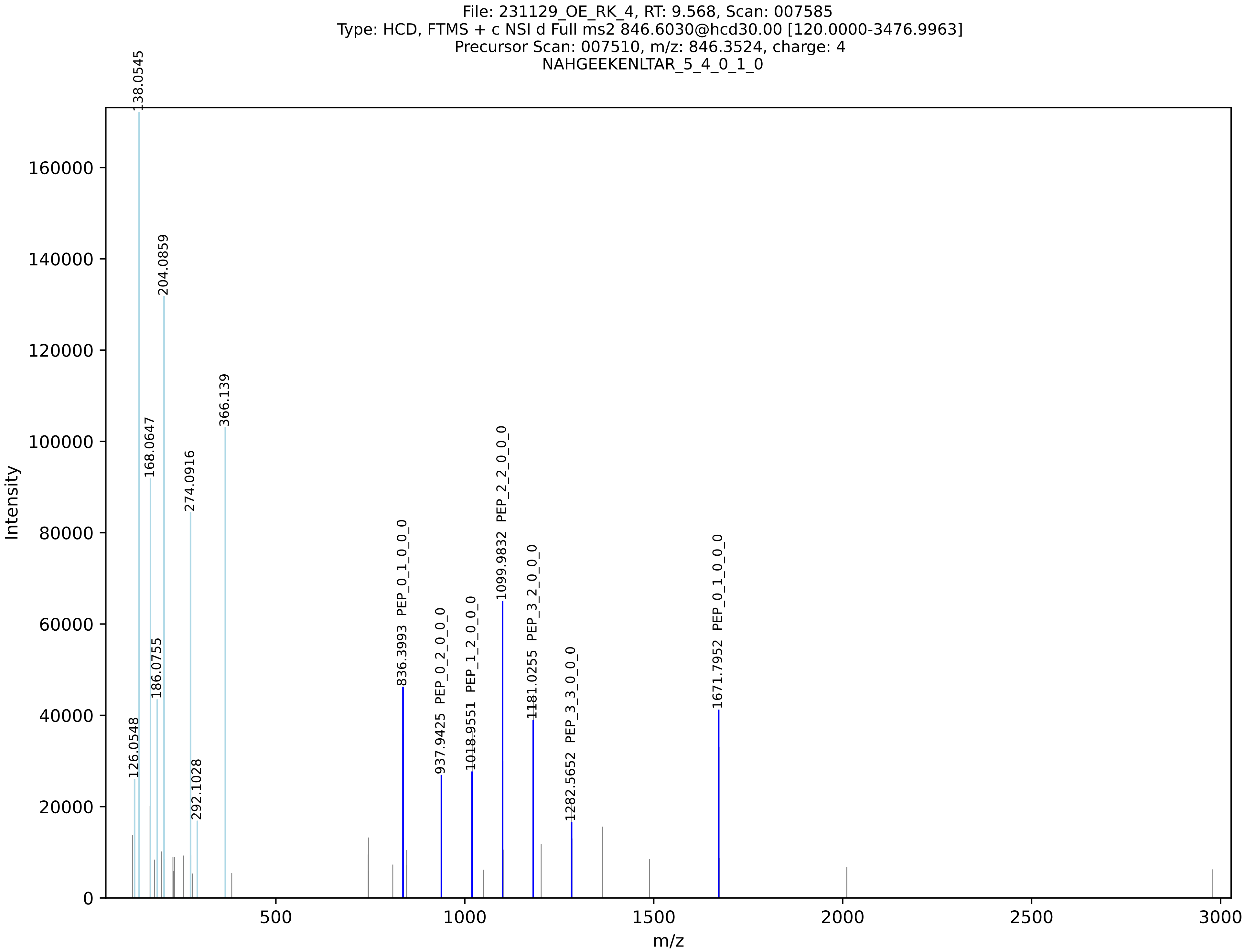

File: 231129_OE_RK_4, RT: 9.568, Scan: 007585
Type: HCD, FTMS + c NSI d Full ms2 846.6030@hcd30.00 [120.0000-3476.9963]
Precursor Scan: 007510, m/z: 846.3524, charge: 4
NAHGEEKENLTAR_5_4_0_1_0
Intensity
m/z
138.0545
204.0859
168.0647
366.139
274.0916
186.0755
126.0548
292.1028
836.3993 PEP_0_1_0_0_0
937.9425 PEP_0_2_0_0_0
1018.9551 PEP_1_2_0_0_0
1099.9832 PEP_2_2_0_0_0
1181.0255 PEP_3_2_0_0_0
1282.5652 PEP_3_3_0_0_0
1671.7952 PEP_0_1_0_0_0

File: 231129_OE_RK_4, RT: 9.57, Scan: 007588
Type: HCD, FTMS + c NSI d Full ms2 1529.5806@hcd30.00 [120.0000-4702.6587]
Precursor Scan: 007510, m/z: 1528.9119, charge: 3
ENGTISR_7_6_2_4_0

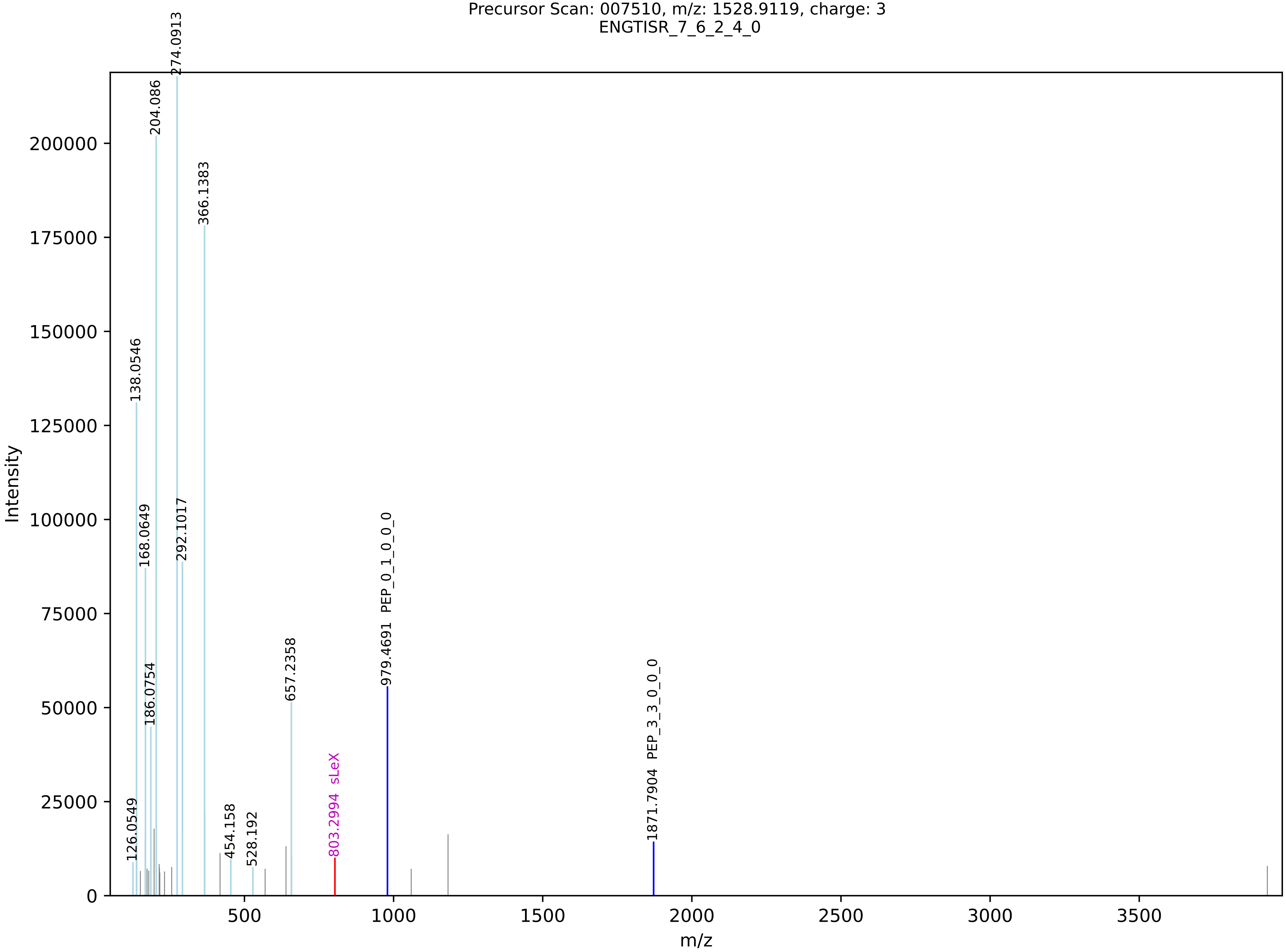

File: 231129_OE_RK_4, RT: 9.615, Scan: 007653
Type: HCD, FTMS + c NSI d Full ms2 994.0778@hcd30.00 [120.0000-3064.0200]
Precursor Scan: 007581, m/z: 993.7437, charge: 3
YKNNSDISSTR_4_3_1_1_0

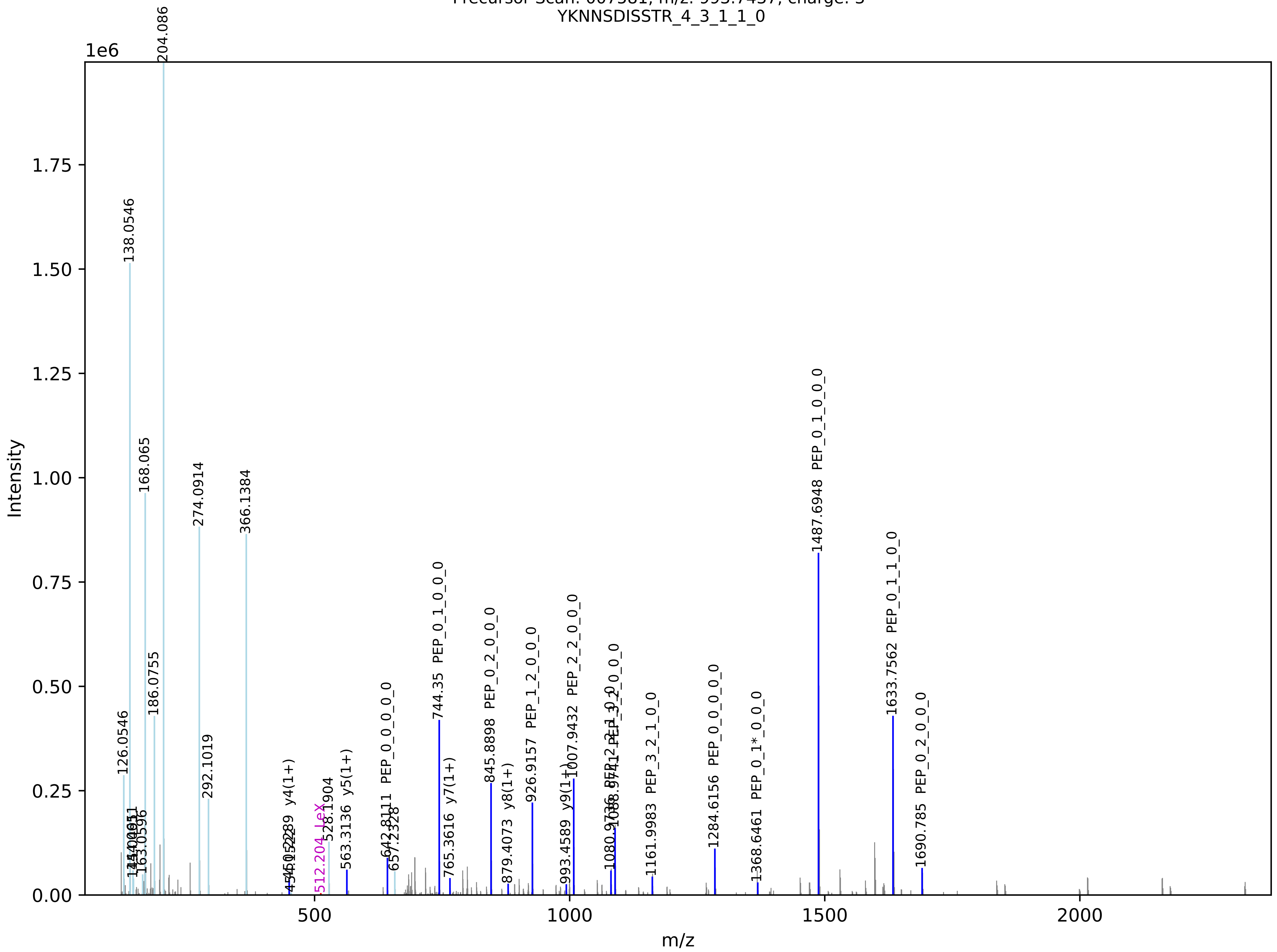

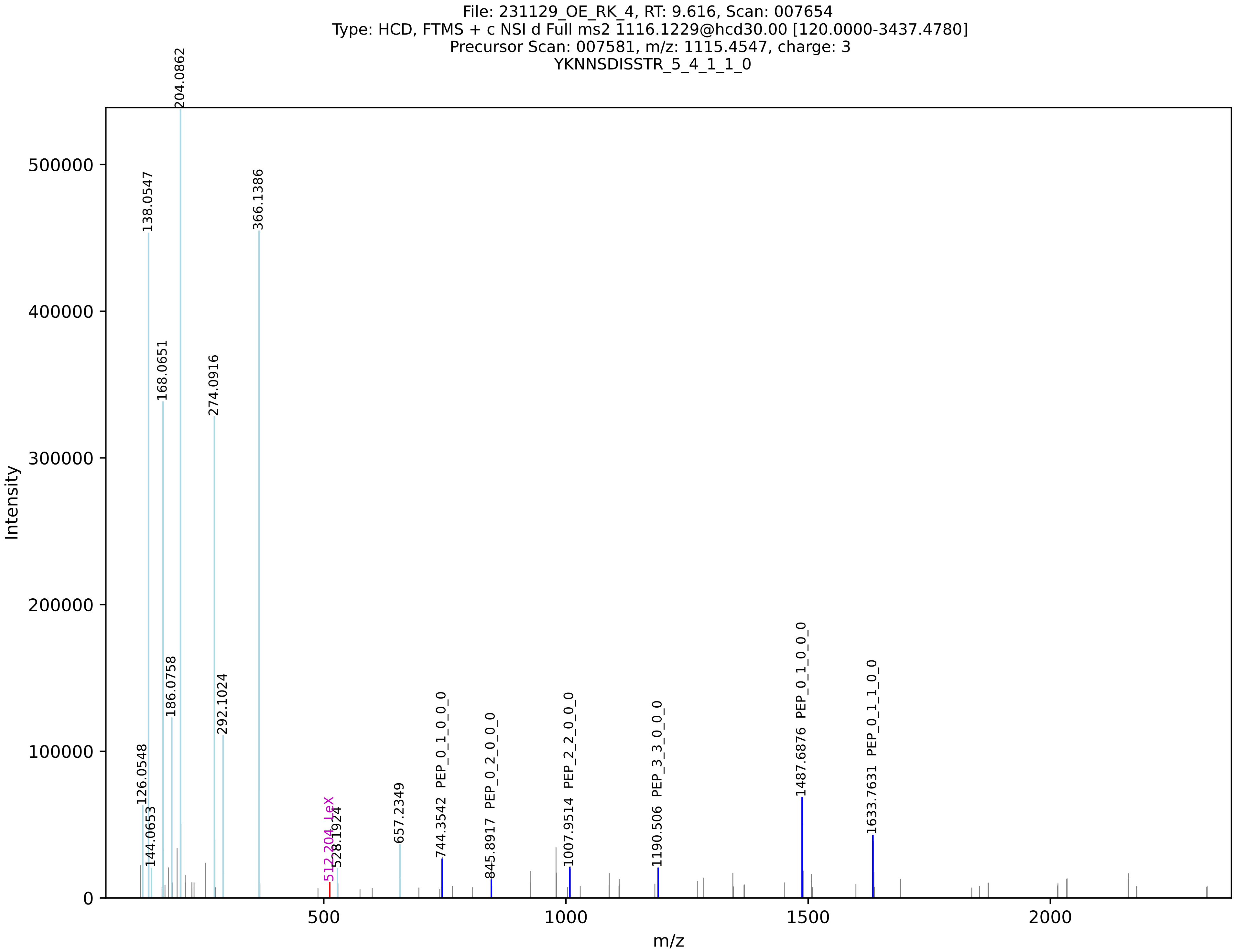

File: 231129_OE_RK_4, RT: 9.616, Scan: 007654
Type: HCD, FTMS + c NSI d Full ms2 1116.1229@hcd30.00 [120.0000-3437.4780]
Precursor Scan: 007581, m/z: 1115.4547, charge: 3
YKNNSDISSTR_5_4_1_1_0
Intensity
m/z
0
100000
200000
300000
400000
500000
500
1000
1500
2000
204.0862
138.0547
366.1386
168.0651
274.0916
186.0758
292.1024
126.0548
144.0653
512.204_LeX
528.1924
657.2349
744.3542 PEP_0_1_0_0_0
845.8917 PEP_0_2_0_0_0
1007.9514 PEP_2_2_0_0_0
1190.506 PEP_3_3_0_0_0
1487.6876 PEP_0_1_0_0_0
1633.7631 PEP_0_1_1_0_0

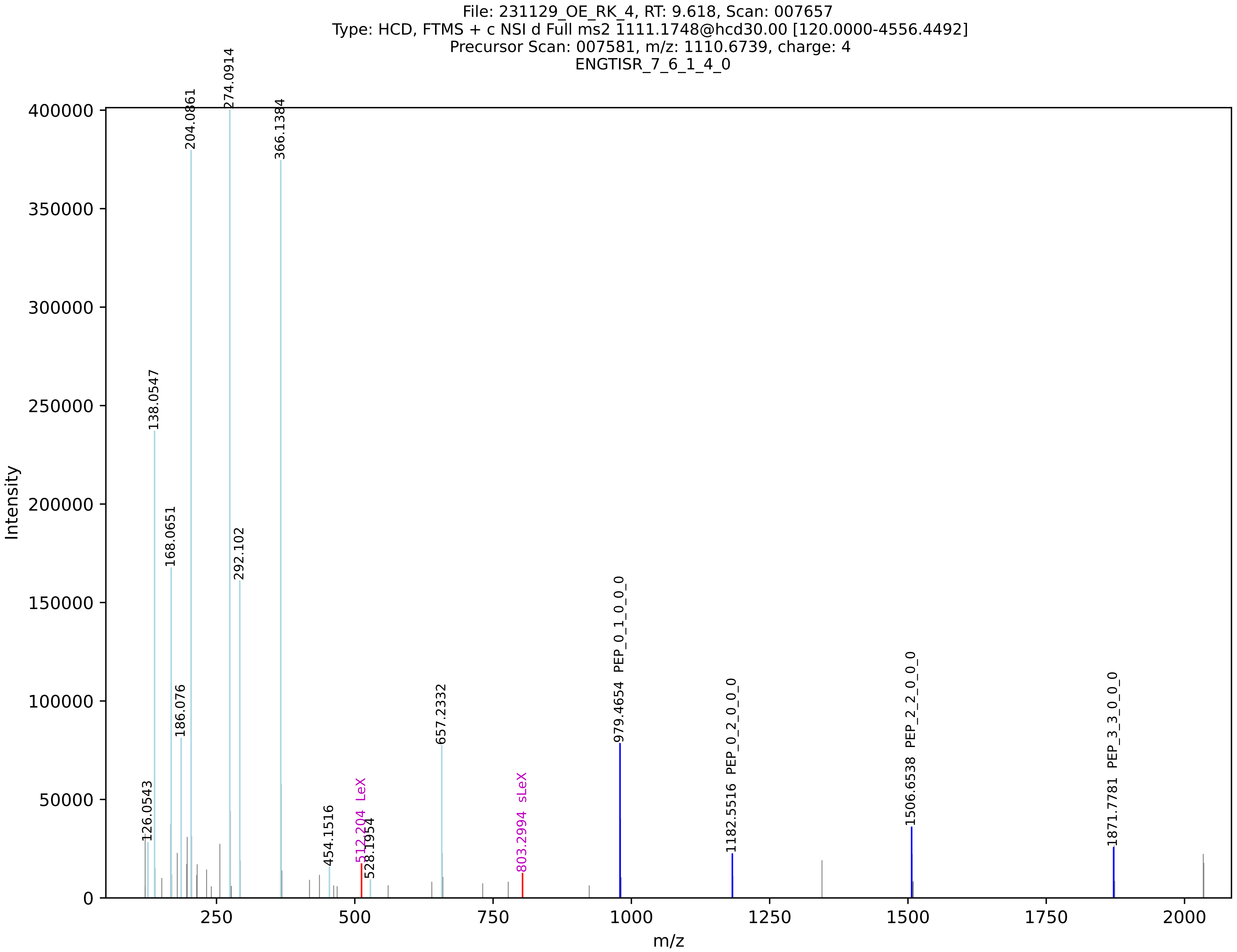
File: 231129_OE_RK_4, RT: 9.618, Scan: 007657
Type: HCD, FTMS + c NSI d Full ms2 1111.1748@hcd30.00 [120.0000-4556.4492]
Precursor Scan: 007581, m/z: 1110.6739, charge: 4
ENGTISR_7_6_1_4_0
Intensity
m/z
126.0543
138.0547
168.0651
186.076
204.0861
274.0914
292.102
366.1384
454.1516
512.204 LeX
528.1954
657.2332
803.2994 sLeX
979.4654 PEP_0_1_0_0_0
1182.5516 PEP_0_2_0_0_0
1506.6538 PEP_2_2_0_0_0
1871.7781 PEP_3_3_0_0_0

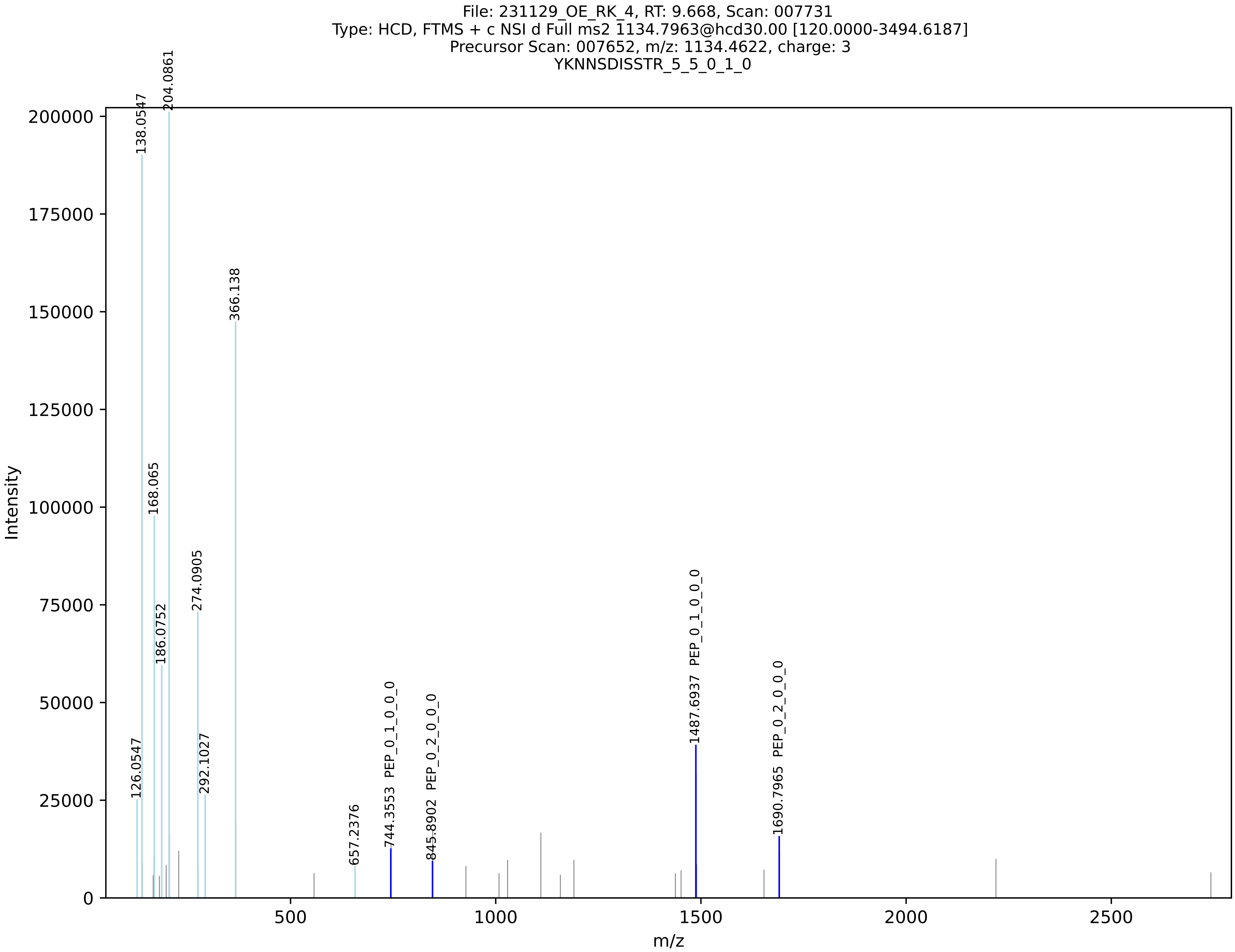

File: 231129_OE_RK_4, RT: 9.668, Scan: 007731
Type: HCD, FTMS + c NSI d Full ms2 1134.7963@hcd30.00 [120.0000-3494.6187]
Precursor Scan: 007652, m/z: 1134.4622, charge: 3
YKNNSDISSTR_5_5_0_1_0
Intensity
m/z
0
25000
50000
75000
100000
125000
150000
175000
200000
500
1000
1500
2000
2500
126.0547
138.0547
168.065
186.0752
204.0861
274.0905
292.1027
366.138
657.2376
744.3553 PEP_0_1_0_0_0
845.8902 PEP_0_2_0_0_0
1487.6937 PEP_0_1_0_0_0
1690.7965 PEP_0_2_0_0_0

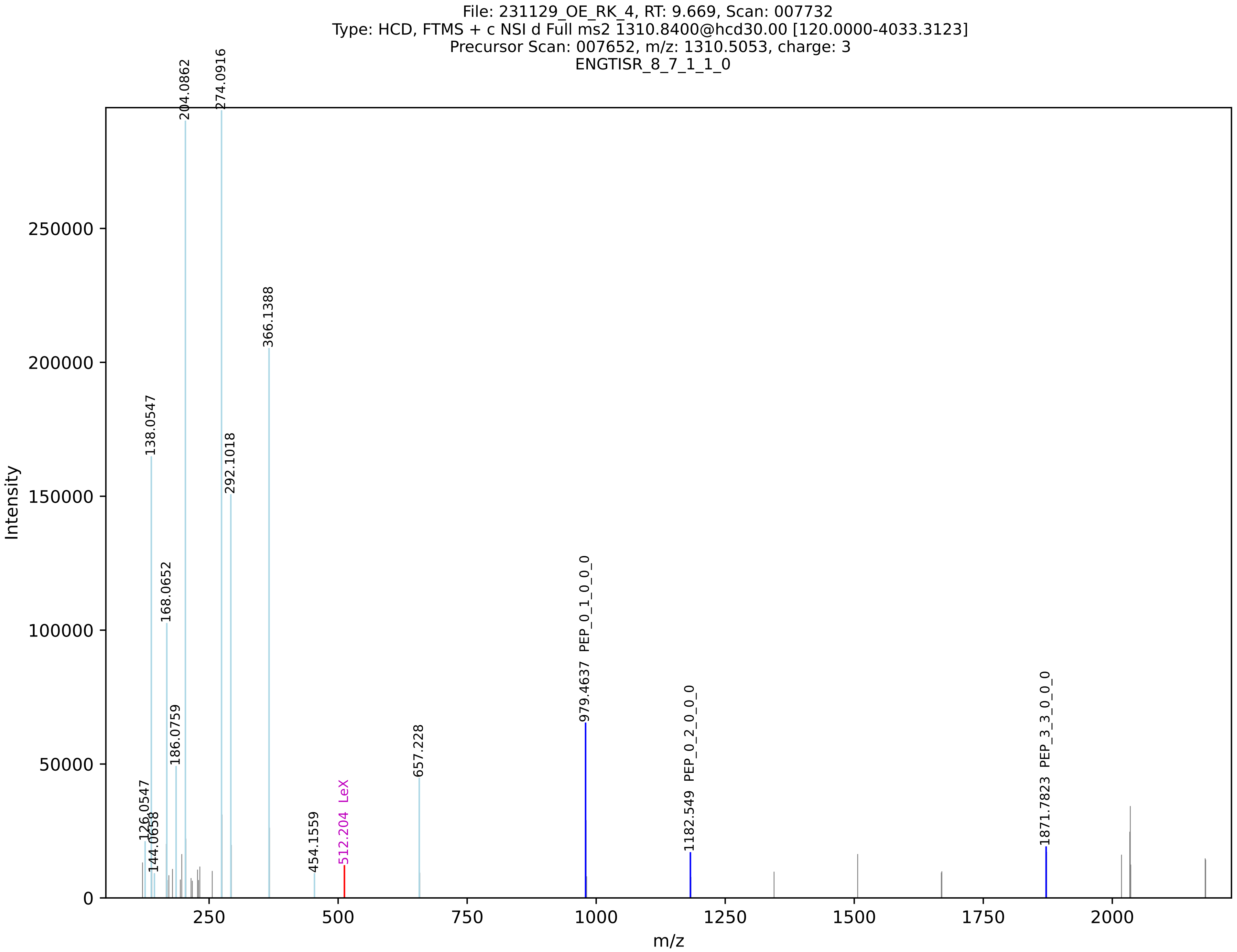

File: 231129_OE_RK_4, RT: 9.669, Scan: 007732
Type: HCD, FTMS + c NSI d Full ms2 1310.8400@hcd30.00 [120.0000-4033.3123]
Precursor Scan: 007652, m/z: 1310.5053, charge: 3
ENGTISR_8_7_1_1_0
Intensity
m/z
204.0862
274.0916
366.1388
138.0547
292.1018
168.0652
186.0759
126.0547
144.0658
454.1559
512.204 LeX
657.228
979.4637 PEP_0_1_0_0_0
1182.549 PEP_0_2_0_0_0
1871.7823 PEP_3_3_0_0_0

File: 231129_OE_RK_4, RT: 9.67, Scan: 007733
Type: HCD, FTMS + c NSI d Full ms2 947.1178@hcd30.00 [120.0000-3887.0964]
Precursor Scan: 007652, m/z: 946.6164, charge: 4
ENGTISR_6_5_1_3_0

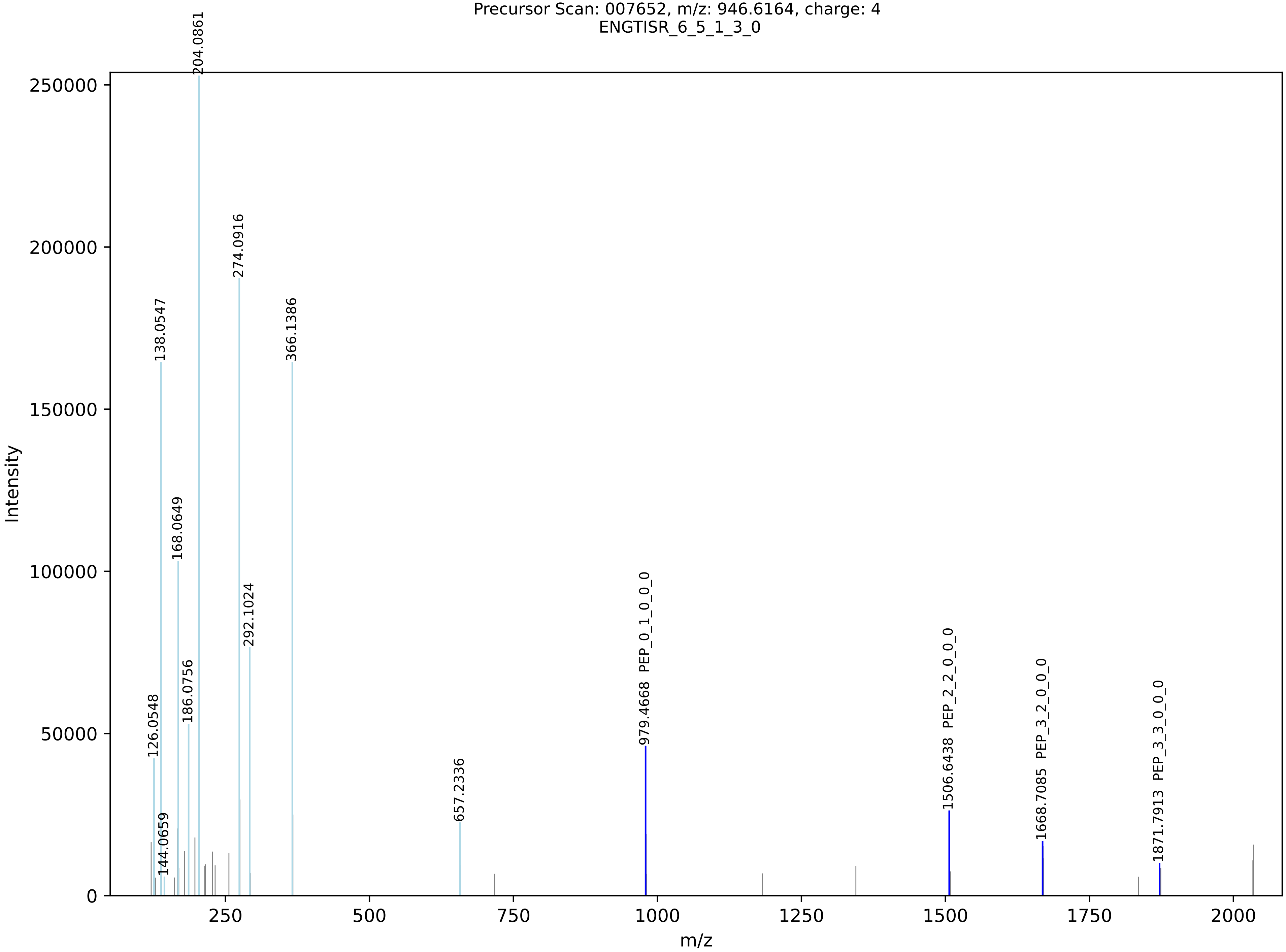

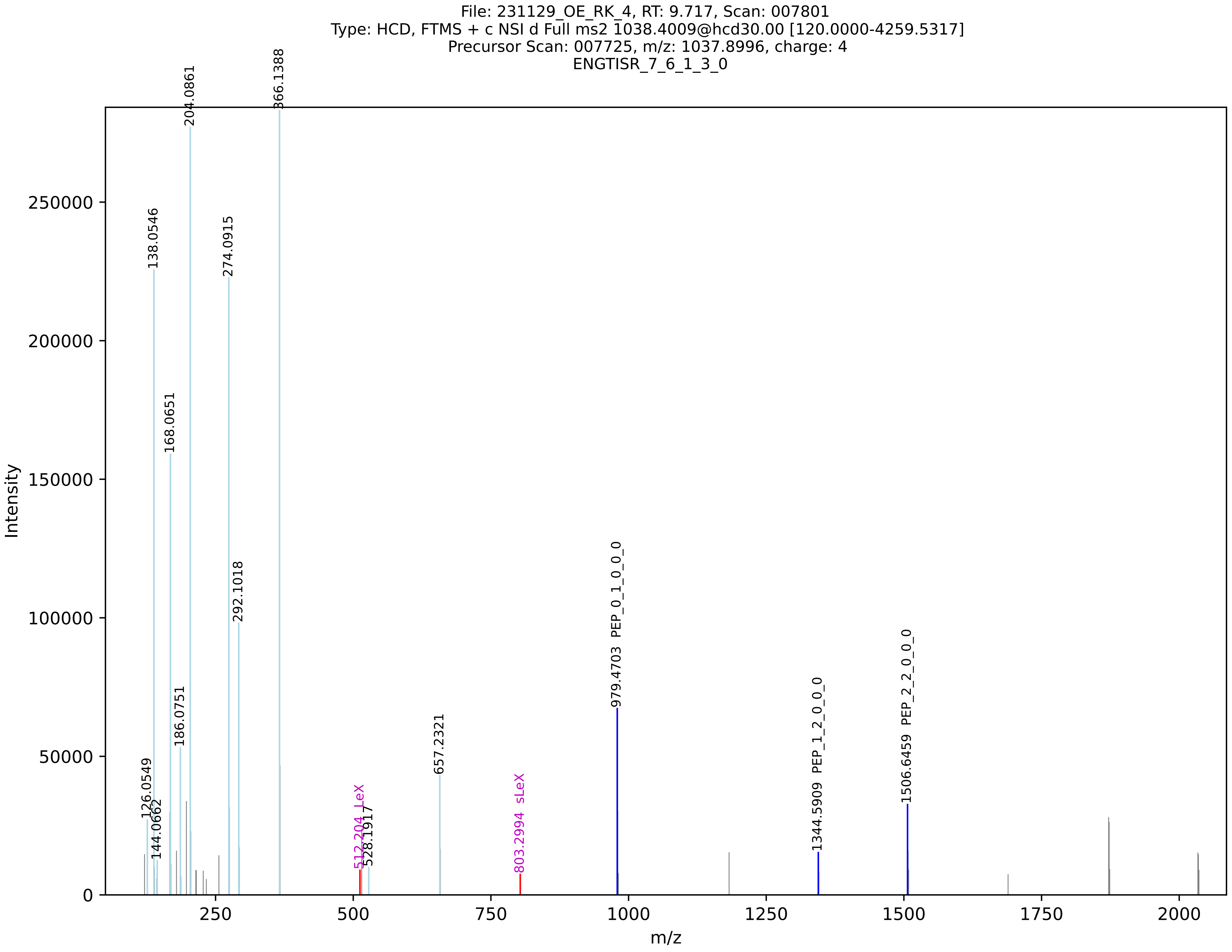
File: 231129_OE_RK_4, RT: 9.717, Scan: 007801
Type: HCD, FTMS + c NSI d Full ms2 1038.4009@hcd30.00 [120.0000-4259.5317]
Precursor Scan: 007725, m/z: 1037.8996, charge: 4
ENGTISR_7_6_1_3_0
Intensity
m/z
366.1388
204.0861
138.0546
274.0915
168.0651
292.1018
186.0751
126.0549
144.0662
512.204 LeX
528.1917
657.2321
803.2994 sLeX
979.4703 PEP_0_1_0_0_0
1344.5909 PEP_1_2_0_0_0
1506.6459 PEP_2_2_0_0_0
0
50000
100000
150000
200000
250000
250
500
750
1000
1250
1500
1750
2000

File: 231129_OE_RK_4, RT: 9.718, Scan: 007804
Type: HCD, FTMS + c NSI d Full ms2 915.3422@hcd30.00 [120.0000-3757.4519]
Precursor Scan: 007725, m/z: 915.0916, charge: 4
NEEYNK_6_5_0_3_0

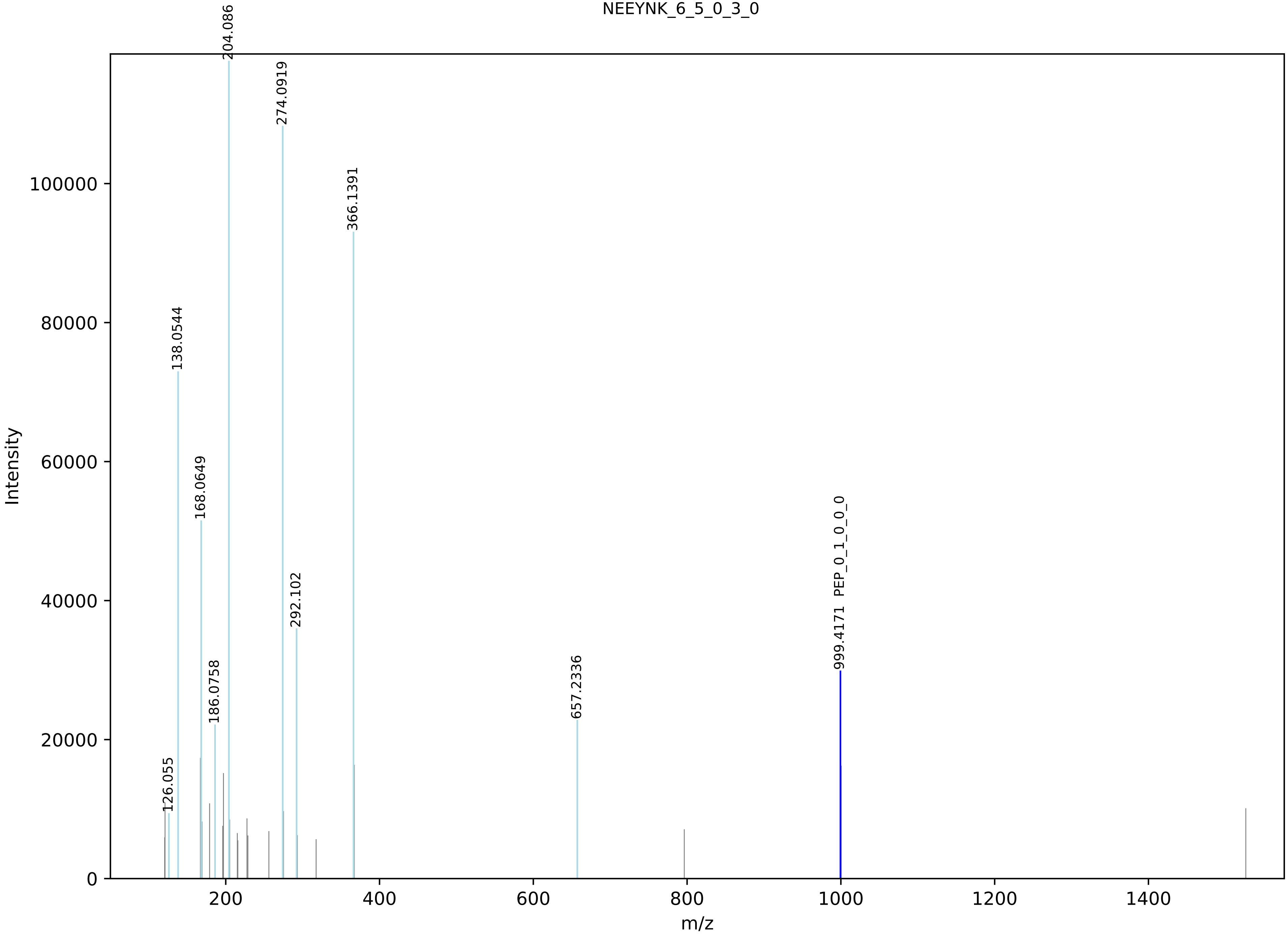

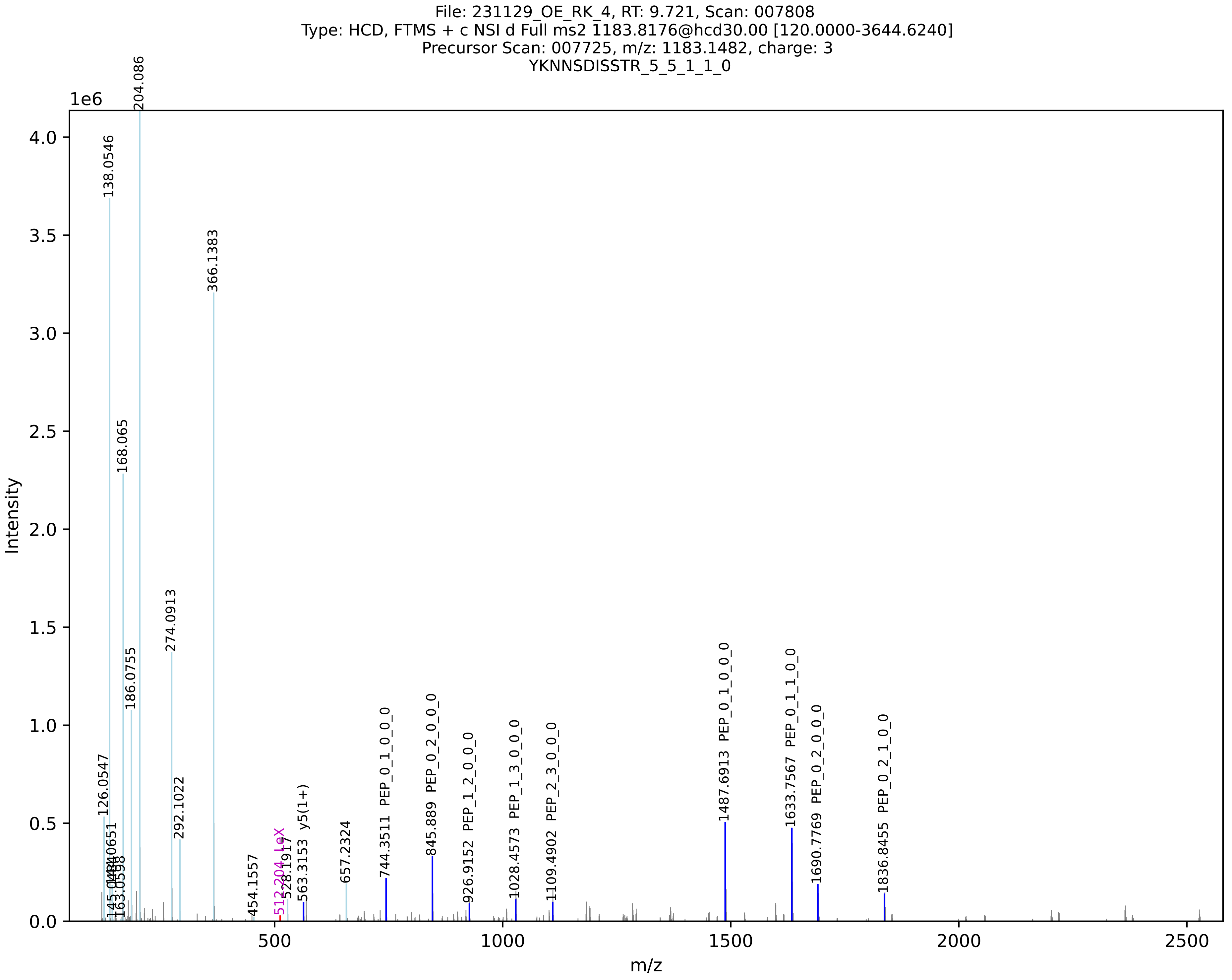
File: 231129_OE_RK_4, RT: 9.721, Scan: 007808
Type: HCD, FTMS + c NSI d Full ms2 1183.8176@hcd30.00 [120.0000-3644.6240]
Precursor Scan: 007725, m/z: 1183.1482, charge: 3
YKNNSDISSTR_5_5_1_1_0
1e6
Intensity
m/z
204.086
138.0546
366.1383
168.065
274.0913
186.0755
126.0547
292.1022
454.1557
512.204 LeX
528.1917
563.3153 y5(1+)
657.2324
744.3511 PEP_0_1_0_0_0
845.889 PEP_0_2_0_0_0
926.9152 PEP_1_2_0_0_0
1028.4573 PEP_1_3_0_0_0
1109.4902 PEP_2_3_0_0_0
1487.6913 PEP_0_1_0_0_0
1633.7567 PEP_0_1_1_0_0
1690.7769 PEP_0_2_0_0_0
1836.8455 PEP_0_2_1_0_0

File: 231129_OE_RK_4, RT: 9.724, Scan: 007813
Type: HCD, FTMS + c NSI d Full ms2 887.8638@hcd30.00 [120.0000-3645.3401]
Precursor Scan: 007725, m/z: 887.6127, charge: 4
YKNNSDISSTR_5_5_1_1_0

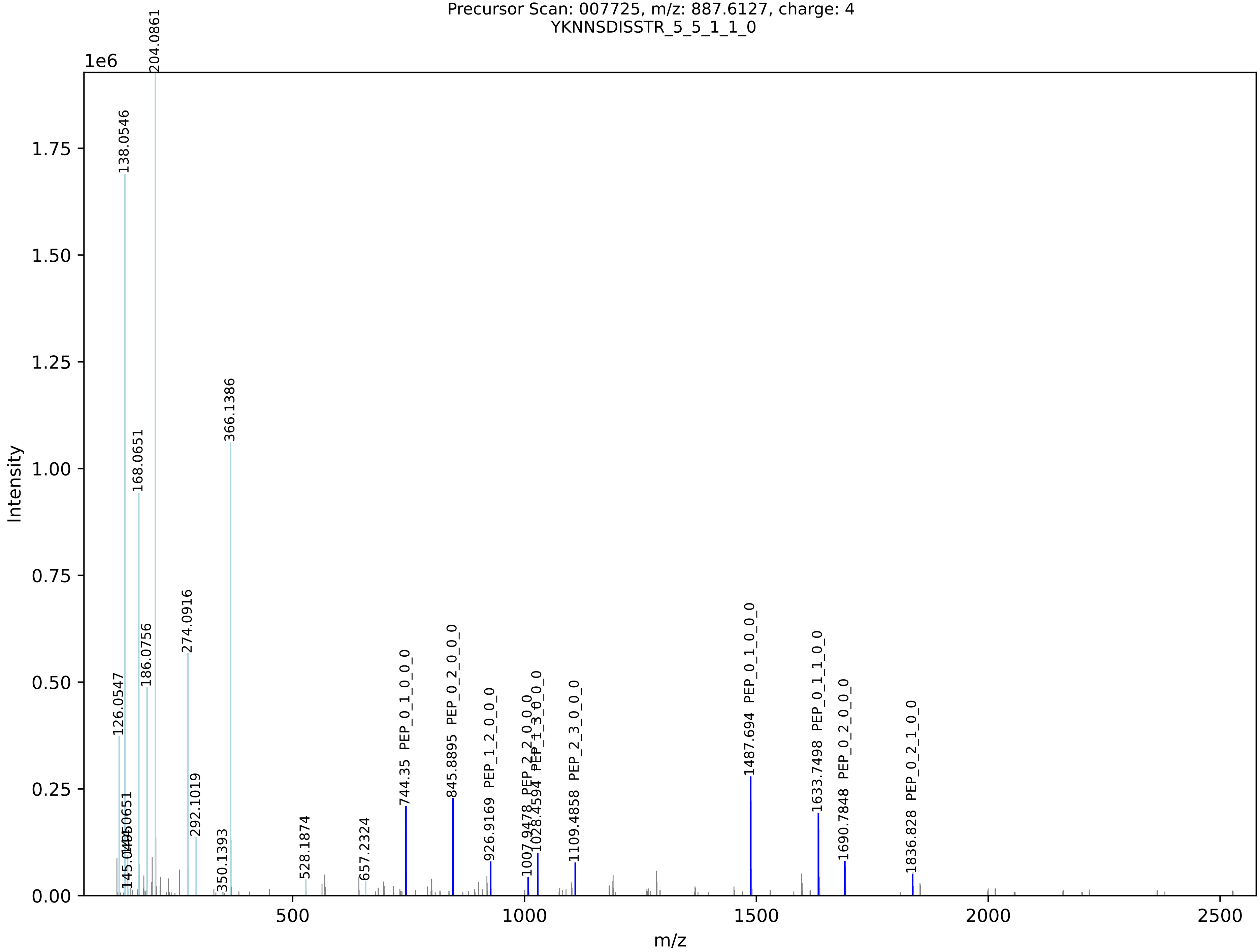

File: 231129_OE_RK_4, RT: 9.768, Scan: 007873
Type: HCD, FTMS + c NSI d Full ms2 1213.8029@hcd30.00 [120.0000-3736.3787]
Precursor Scan: 007798, m/z: 1213.1349, charge: 3
ENGTISR_6_5_0_3_0

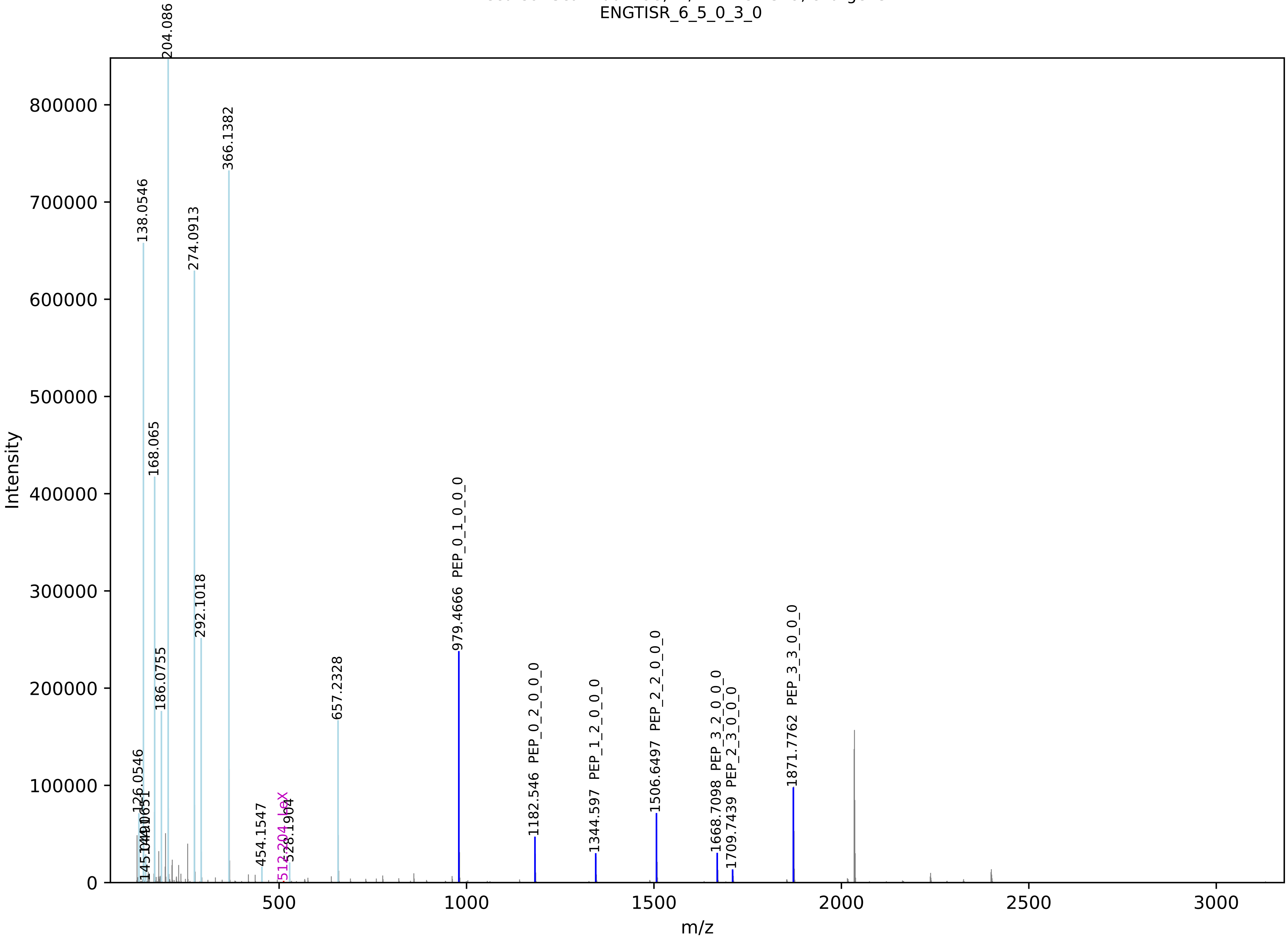

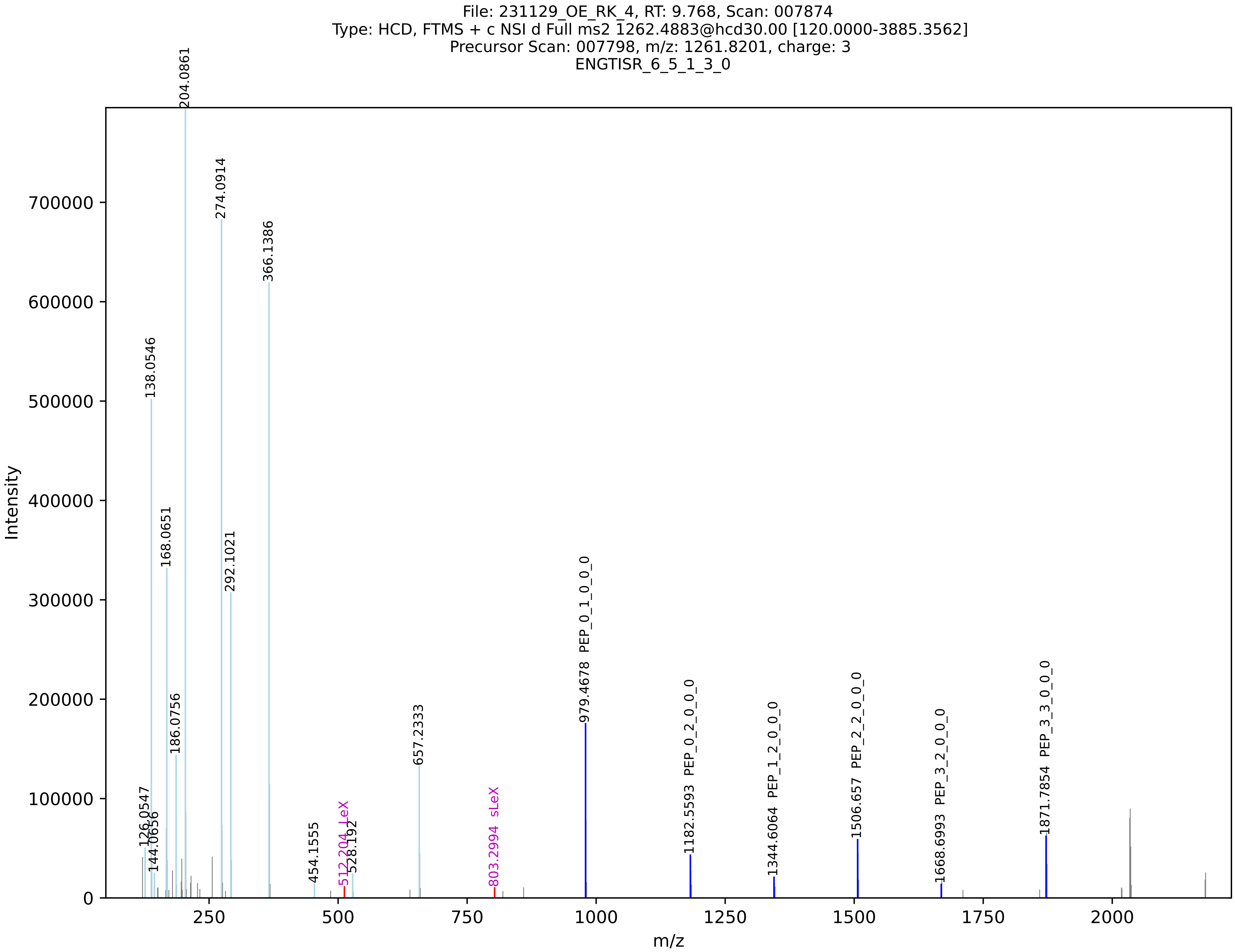
File: 231129_OE_RK_4, RT: 9.768, Scan: 007874
Type: HCD, FTMS + c NSI d Full ms2 1262.4883@hcd30.00 [120.0000-3885.3562]
Precursor Scan: 007798, m/z: 1261.8201, charge: 3
ENGTISR_6_5_1_3_0
Intensity
m/z
204.0861
274.0914
366.1386
138.0546
168.0651
292.1021
186.0756
126.0547
144.0656
454.1555
512.204 LeX
528.192
657.2333
803.2994 sLeX
979.4678 PEP_0_1_0_0_0
1182.5593 PEP_0_2_0_0_0
1344.6064 PEP_1_2_0_0_0
1506.657 PEP_2_2_0_0_0
1668.6993 PEP_3_2_0_0_0
1871.7854 PEP_3_3_0_0_0

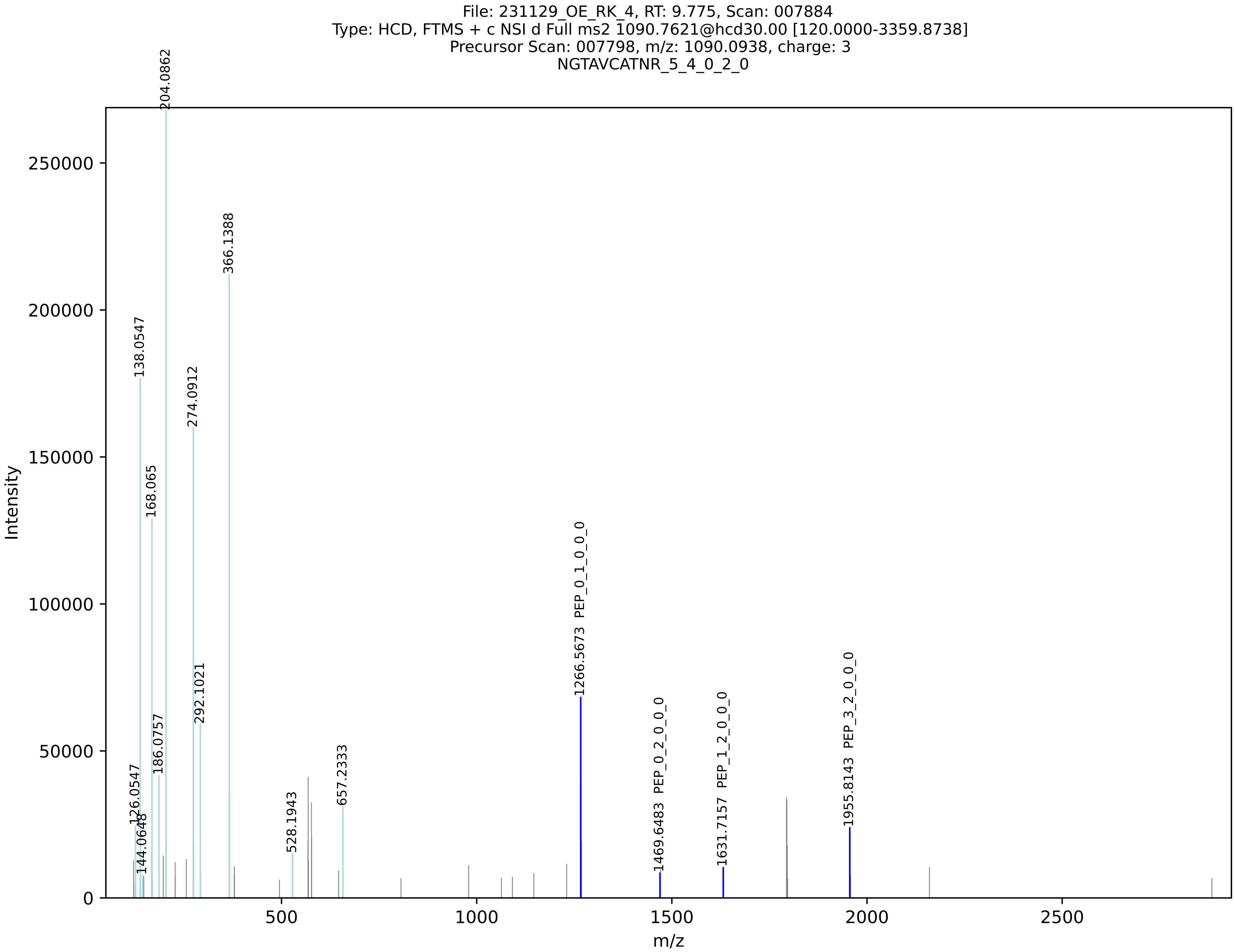
File: 231129_OE_RK_4, RT: 9.775, Scan: 007884
Type: HCD, FTMS + c NSI d Full ms2 1090.7621@hcd30.00 [120.0000-3359.8738]
Precursor Scan: 007798, m/z: 1090.0938, charge: 3
NGTAVCATNR_5_4_0_2_0
Intensity
m/z
204.0862
366.1388
138.0547
274.0912
168.065
292.1021
186.0757
126.0547
144.0648
528.1943
657.2333
1266.5673 PEP_0_1_0_0_0
1469.6483 PEP_0_2_0_0_0
1631.7157 PEP_1_2_0_0_0
1955.8143 PEP_3_2_0_0_0
0
50000
100000
150000
200000
250000
500
1000
1500
2000
2500

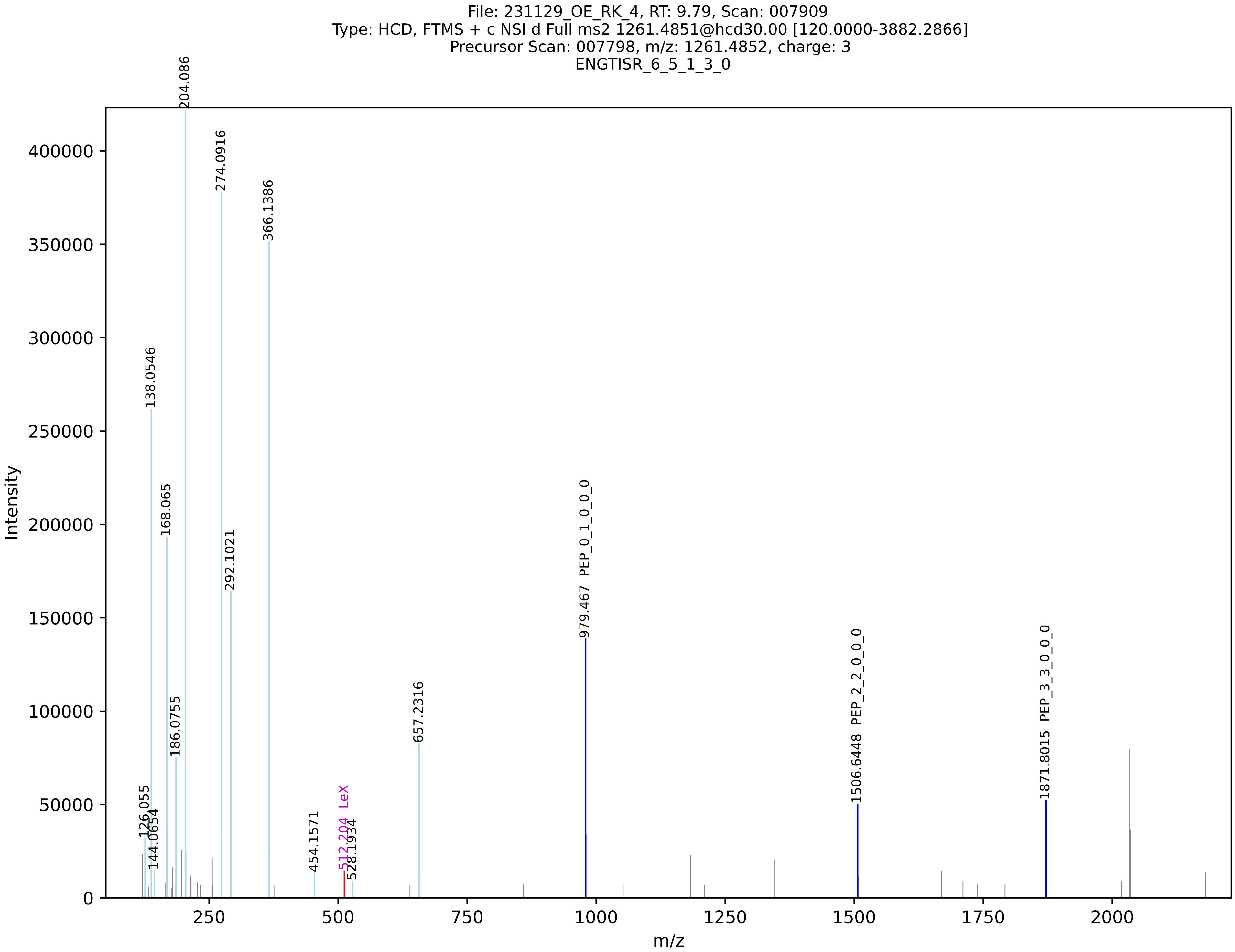
File: 231129_OE_RK_4, RT: 9.79, Scan: 007909
Type: HCD, FTMS + c NSI d Full ms2 1261.4851@hcd30.00 [120.0000-3882.2866]
Precursor Scan: 007798, m/z: 1261.4852, charge: 3
ENGTISR_6_5_1_3_0
Intensity
m/z
0
50000
100000
150000
200000
250000
300000
350000
400000
250
500
750
1000
1250
1500
1750
2000
204.086
274.0916
366.1386
138.0546
168.065
292.1021
186.0755
126.055
144.0654
454.1571
512.204 LeX
528.1934
657.2316
979.467 PEP_0_1_0_0_0
1506.6448 PEP_2_2_0_0_0
1871.8015 PEP_3_3_0_0_0

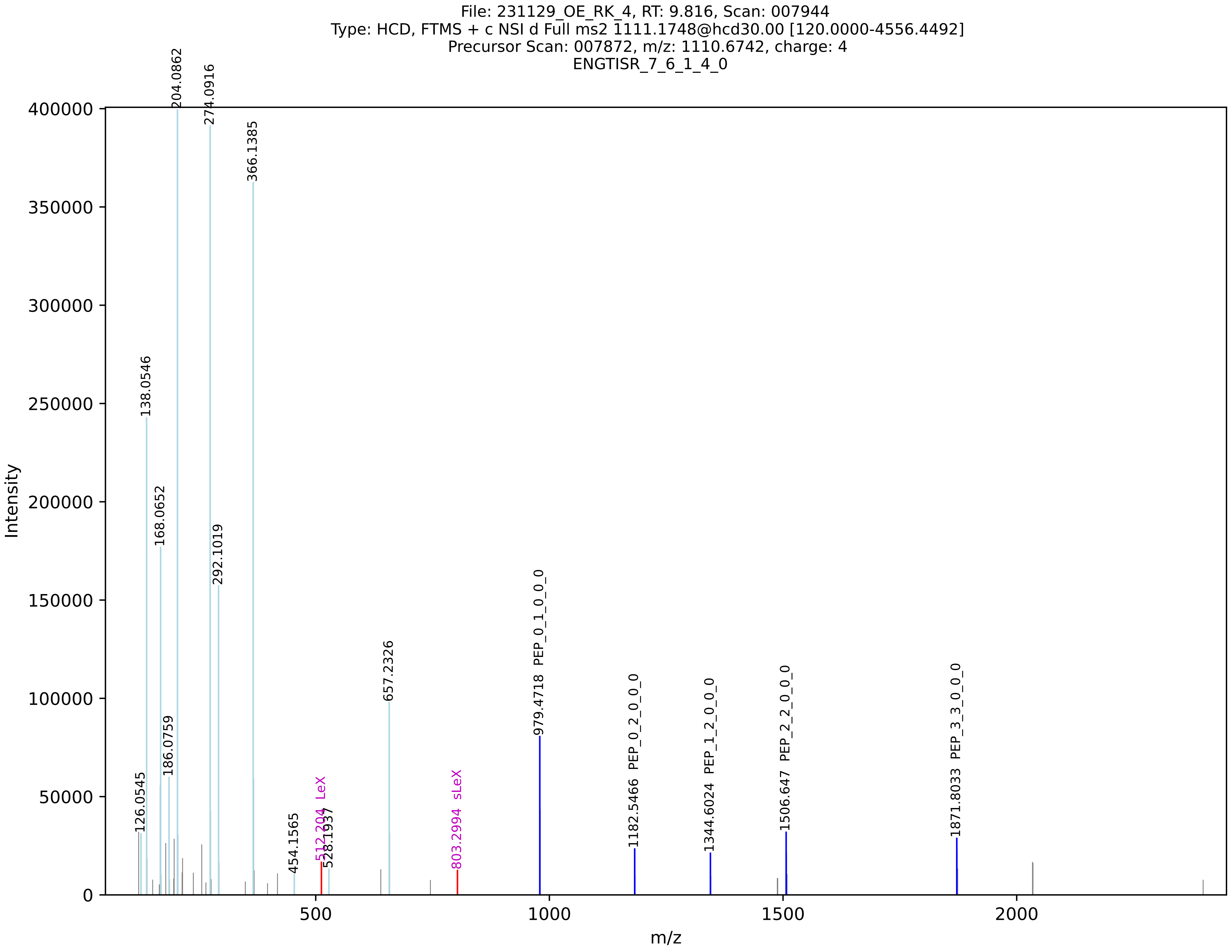
File: 231129_OE_RK_4, RT: 9.816, Scan: 007944
Type: HCD, FTMS + c NSI d Full ms2 1111.1748@hcd30.00 [120.0000-4556.4492]
Precursor Scan: 007872, m/z: 1110.6742, charge: 4
ENGTISR_7_6_1_4_0
Intensity
m/z
204.0862
274.0916
366.1385
138.0546
168.0652
292.1019
186.0759
126.0545
454.1565
512.204 LeX
528.1937
657.2326
803.2994 sLeX
979.4718 PEP_0_1_0_0_0
1182.5466 PEP_0_2_0_0_0
1344.6024 PEP_1_2_0_0_0
1506.647 PEP_2_2_0_0_0
1871.8033 PEP_3_3_0_0_0

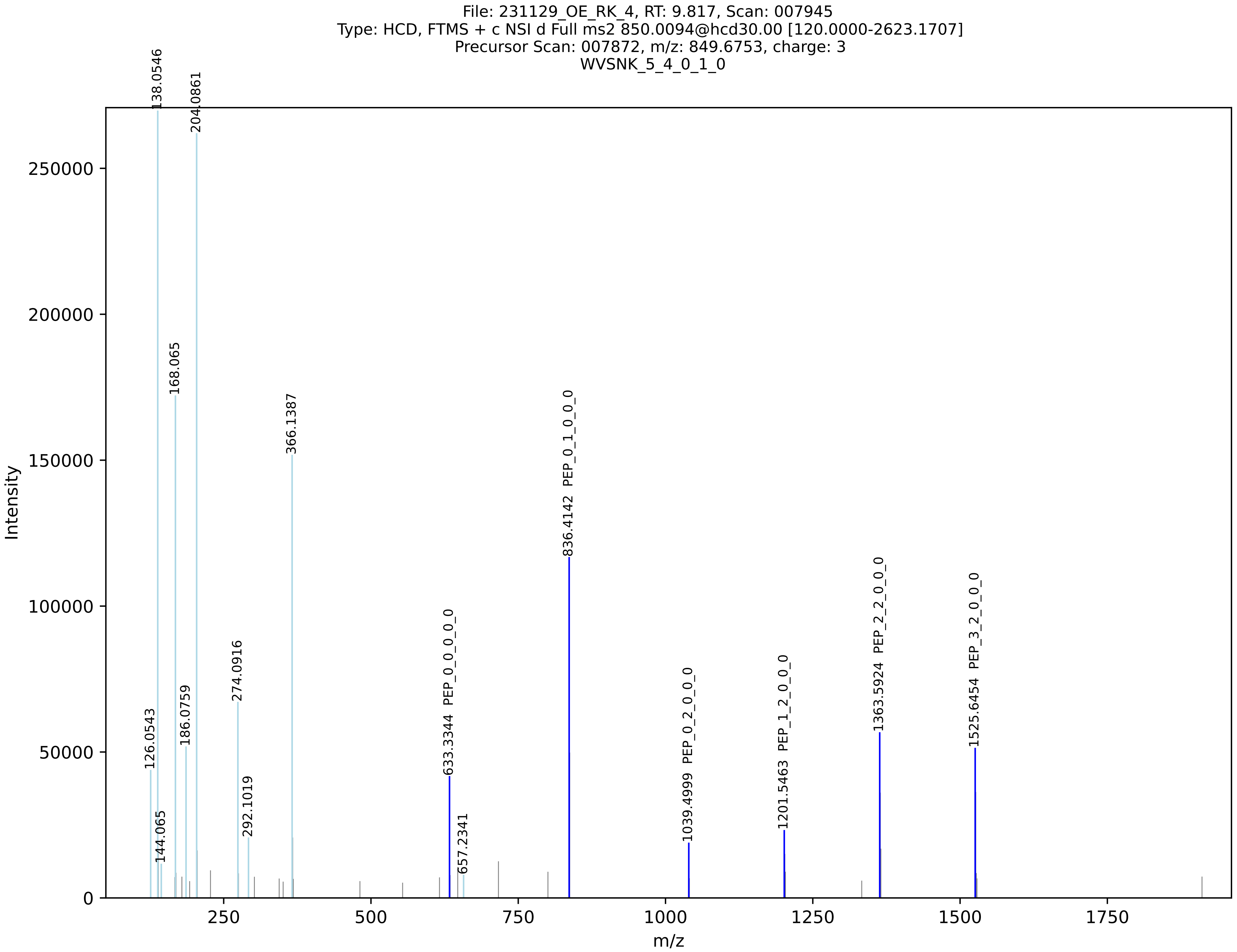
File: 231129_OE_RK_4, RT: 9.817, Scan: 007945
Type: HCD, FTMS + c NSI d Full ms2 850.0094@hcd30.00 [120.0000-2623.1707]
Precursor Scan: 007872, m/z: 849.6753, charge: 3
WVSNK_5_4_0_1_0
Intensity
m/z
138.0546
204.0861
168.065
366.1387
836.4142 PEP_0_1_0_0_0
633.3344 PEP_0_0_0_0_0
1039.4999 PEP_0_2_0_0_0
1201.5463 PEP_1_2_0_0_0
1363.5924 PEP_2_2_0_0_0
1525.6454 PEP_3_2_0_0_0
126.0543
186.0759
274.0916
292.1019
144.065
657.2341
0
50000
100000
150000
200000
250000
250
500
750
1000
1250
1500
1750

File: 231129_OE_RK_4, RT: 9.818, Scan: 007946
Type: HCD, FTMS + c NSI d Full ms2 994.0786@hcd30.00 [120.0000-3064.0225]
Precursor Scan: 007872, m/z: 993.7444, charge: 3
YKNNSDISSTR_4_3_1_1_0

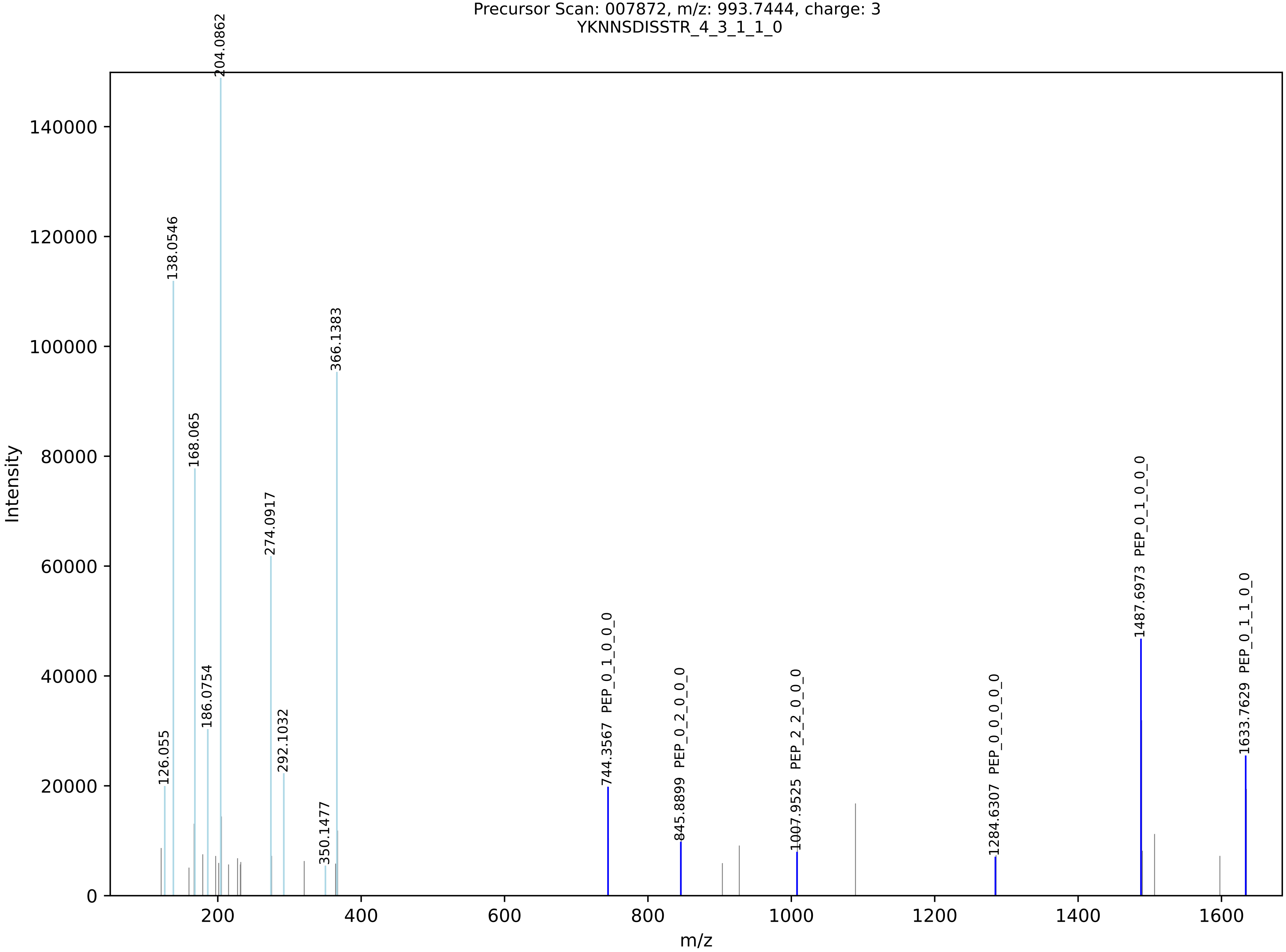

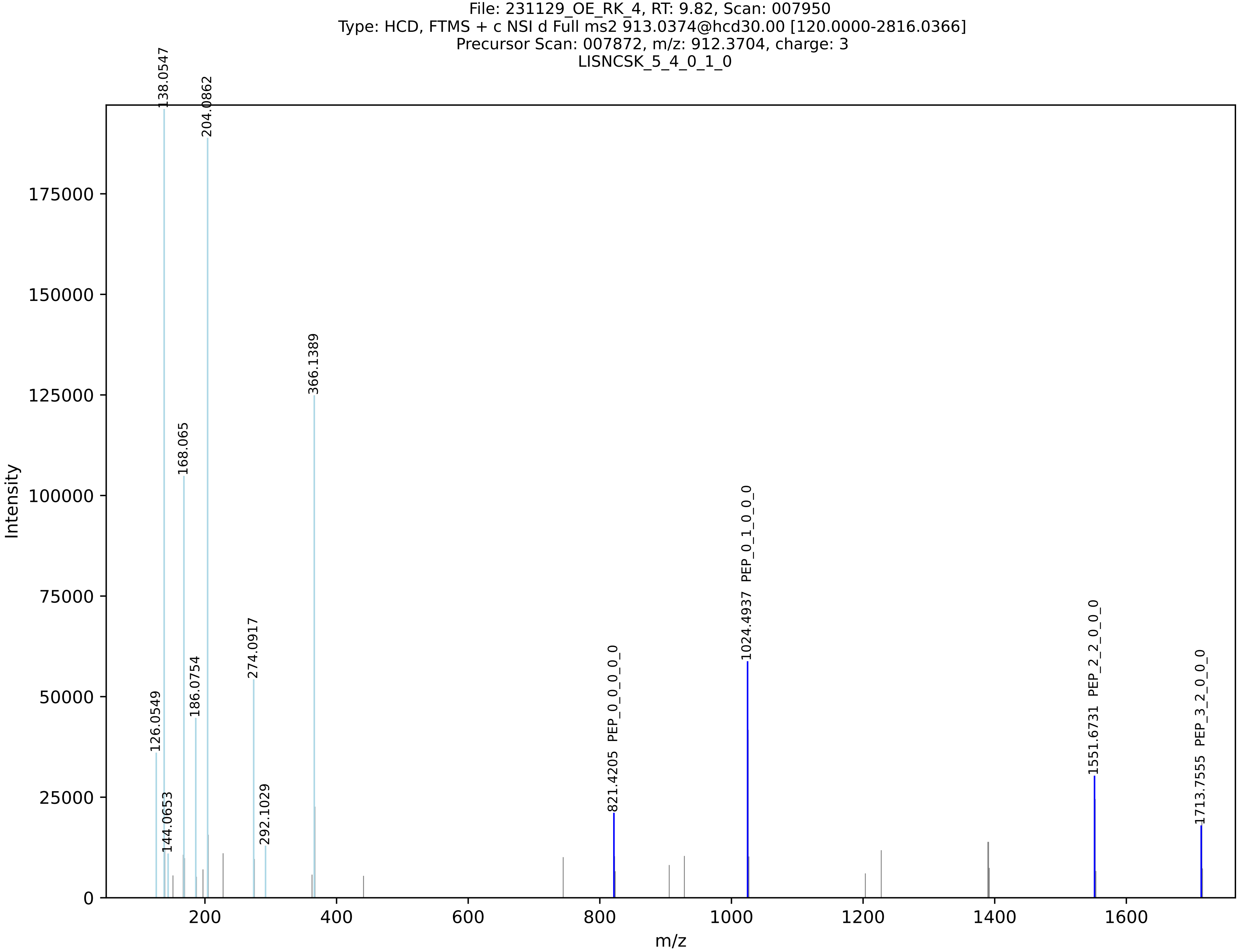

File: 231129_OE_RK_4, RT: 9.82, Scan: 007950
Type: HCD, FTMS + c NSI d Full ms2 913.0374@hcd30.00 [120.0000-2816.0366]
Precursor Scan: 007872, m/z: 912.3704, charge: 3
LISNCSK_5_4_0_1_0
138.0547
204.0862
366.1389
168.065
186.0754
274.0917
126.0549
144.0653
292.1029
821.4205 PEP_0_0_0_0_0_
1024.4937 PEP_0_1_0_0_0_
1551.6731 PEP_2_2_0_0_0_
1713.7555 PEP_3_2_0_0_0_
Intensity
m/z

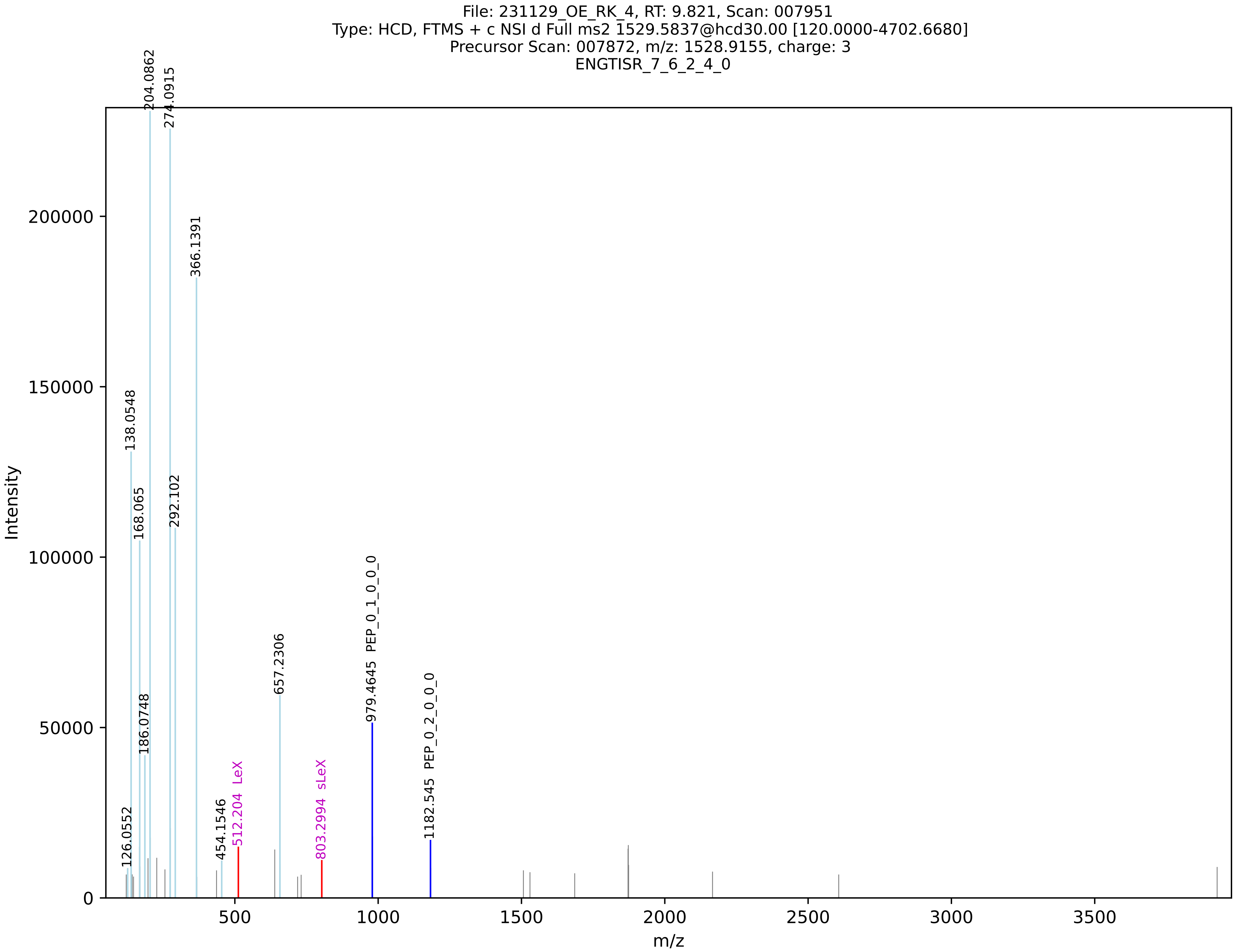
File: 231129_OE_RK_4, RT: 9.821, Scan: 007951
Type: HCD, FTMS + c NSI d Full ms2 1529.5837@hcd30.00 [120.0000-4702.6680]
Precursor Scan: 007872, m/z: 1528.9155, charge: 3
ENGTISR_7_6_2_4_0
204.0862
274.0915
366.1391
138.0548
168.065
292.102
186.0748
126.0552
454.1546
512.204 LeX
657.2306
803.2994 sLeX
979.4645 PEP_0_1_0_0_
1182.545 PEP_0_2_0_0_0
Intensity
0
50000
100000
150000
200000
500
1000
1500
2000
2500
3000
3500
m/z

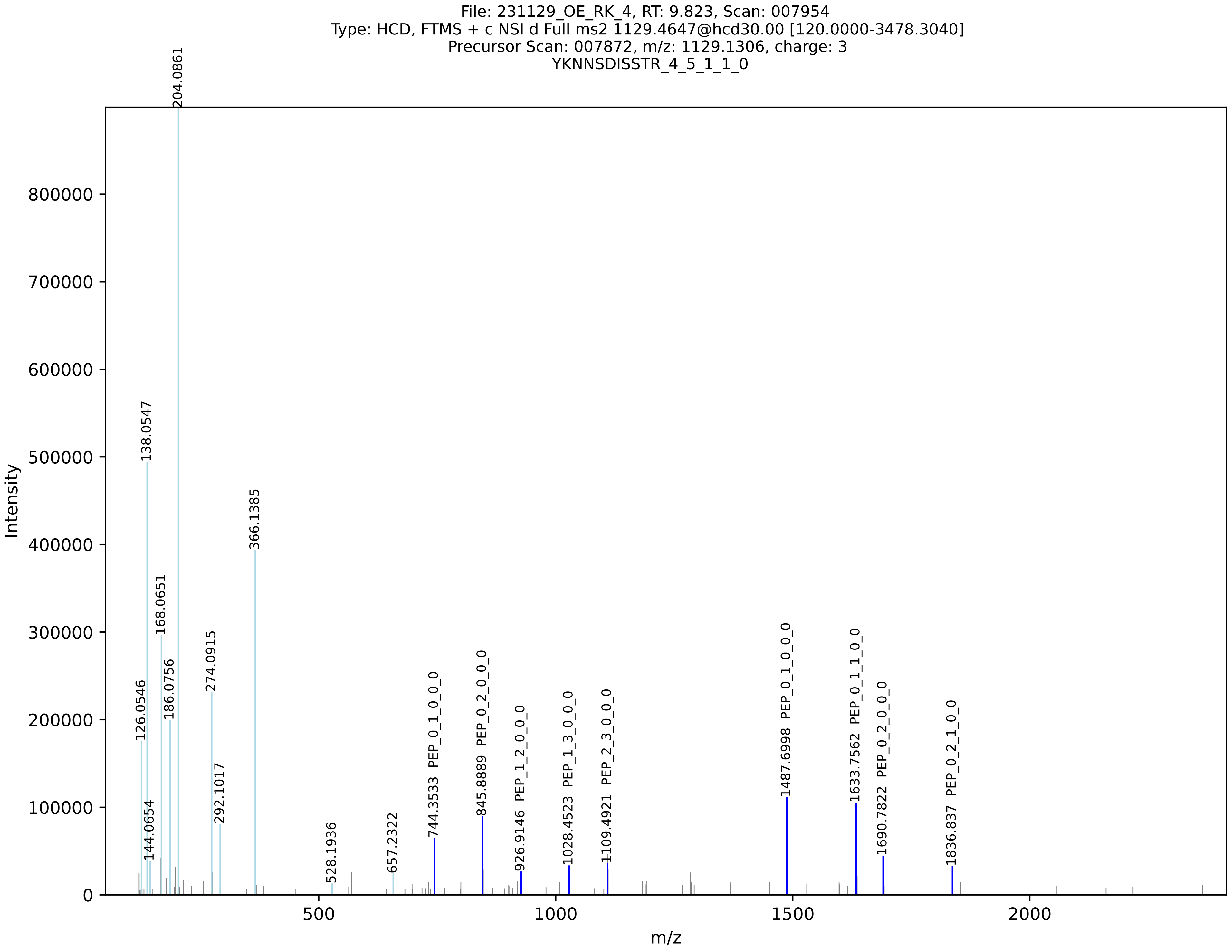

File: 231129_OE_RK_4, RT: 9.823, Scan: 007954
Type: HCD, FTMS + c NSI d Full ms2 1129.4647@hcd30.00 [120.0000-3478.3040]
Precursor Scan: 007872, m/z: 1129.1306, charge: 3
YKNNSDISSTR_4_5_1_1_0
Intensity
m/z
204.0861
138.0547
366.1385
168.0651
274.0915
186.0756
126.0546
292.1017
144.0654
528.1936
657.2322
744.3533 PEP_0_1_0_0_0
845.8889 PEP_0_2_0_0_0
926.9146 PEP_1_2_0_0_0
1028.4523 PEP_1_3_0_0_0
1109.4921 PEP_2_3_0_0_0
1487.6998 PEP_0_1_0_0_0
1633.7562 PEP_0_1_1_0_0
1690.7822 PEP_0_2_0_0_0
1836.837 PEP_0_2_1_0_0

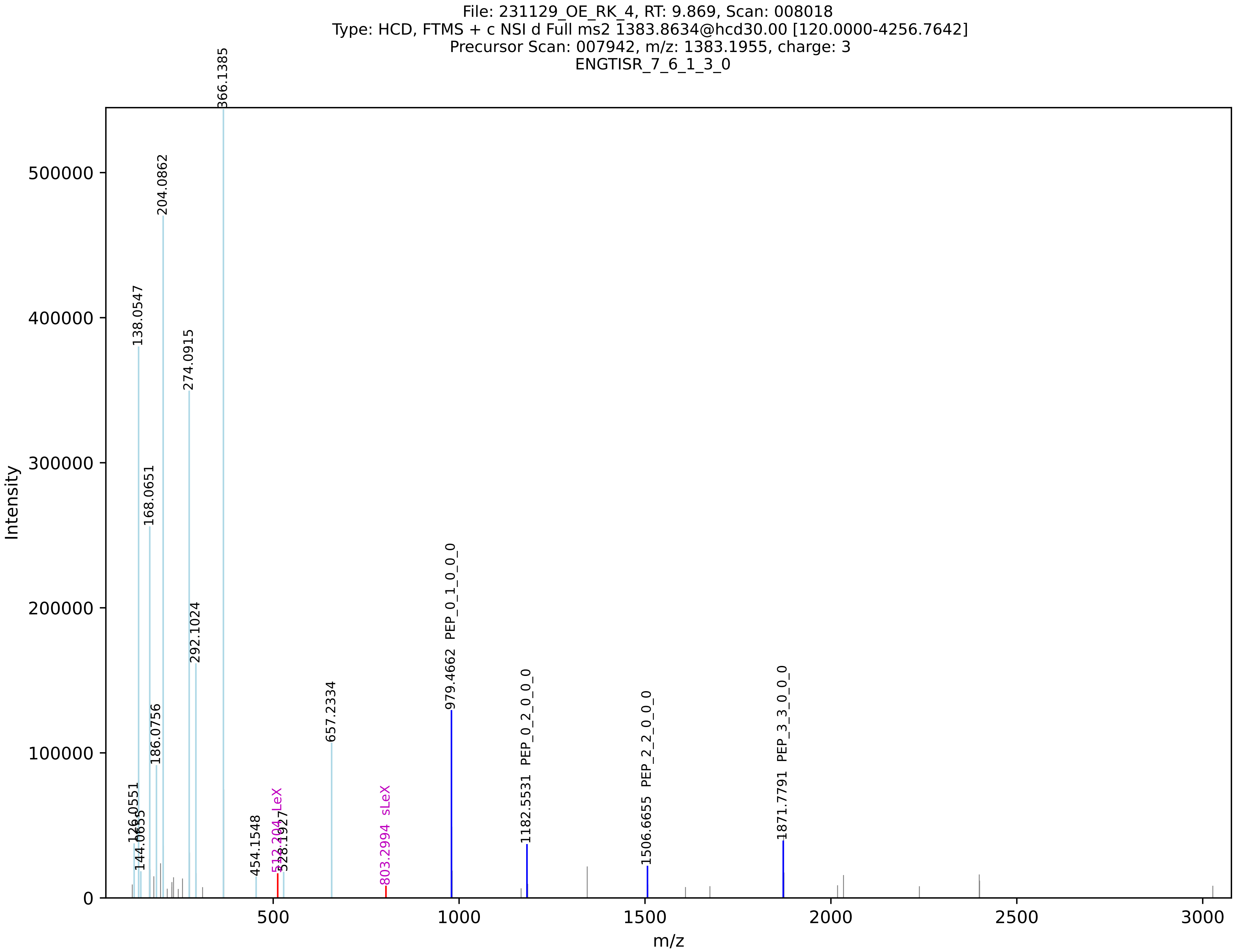

File: 231129_OE_RK_4, RT: 9.869, Scan: 008018
Type: HCD, FTMS + c NSI d Full ms2 1383.8634@hcd30.00 [120.0000-4256.7642]
Precursor Scan: 007942, m/z: 1383.1955, charge: 3
ENGTISR_7_6_1_3_0
Intensity
m/z
366.1385
204.0862
138.0547
274.0915
168.0651
292.1024
186.0756
126.0551
144.0655
454.1548
LeX
528.1927
657.2334
803.2994 sLeX
979.4662 PEP_0_1_0_0_0_
1182.5531 PEP_0_2_0_0_0_
1506.6655 PEP_2_2_0_0_0_
1871.7791 PEP_3_3_0_0_0_

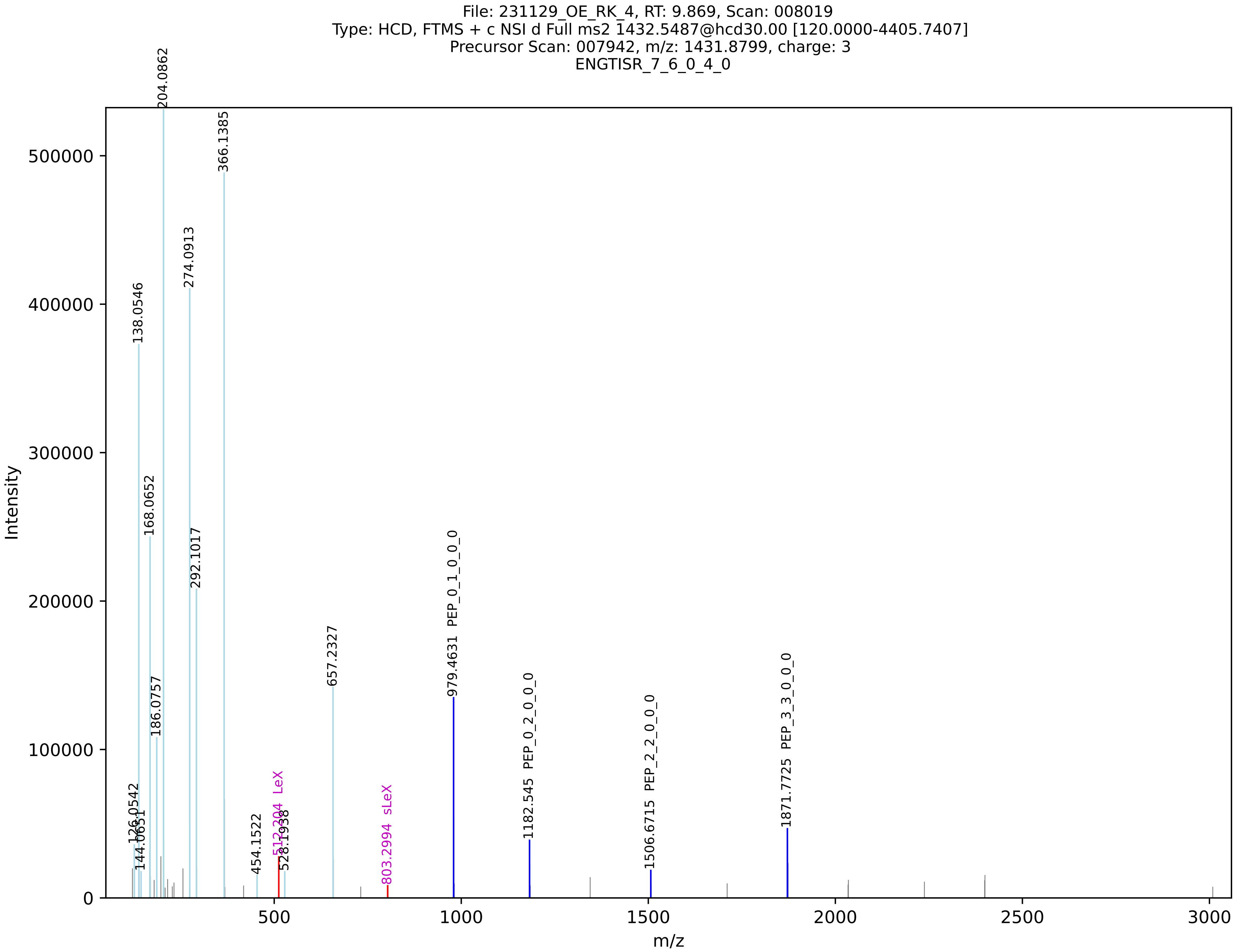
File: 231129_OE_RK_4, RT: 9.869, Scan: 008019
Type: HCD, FTMS + c NSI d Full ms2 1432.5487@hcd30.00 [120.0000-4405.7407]
Precursor Scan: 007942, m/z: 1431.8799, charge: 3
ENGTISR_7_6_0_4_0
204.0862
366.1385
274.0913
138.0546
168.0652
292.1017
186.0757
126.0542
144.0651
454.1522
512.204 LeX
528.1938
657.2327
803.2994 sLeX
979.4631 PEP_0_1_0_0_0
1182.545 PEP_0_2_0_0_0
1506.6715 PEP_2_2_0_0_0
1871.7725 PEP_3_3_0_0_0
Intensity
m/z
0
100000
200000
300000
400000
500000
500
1000
1500
2000
2500
3000

File: 231129_OE_RK_4, RT: 9.875, Scan: 008028
Type: HCD, FTMS + c NSI d Full ms2 952.1057@hcd30.00 [120.0000-3907.4470]
Precursor Scan: 007942, m/z: 951.6044, charge: 4
NEEYNK_6_5_1_3_0

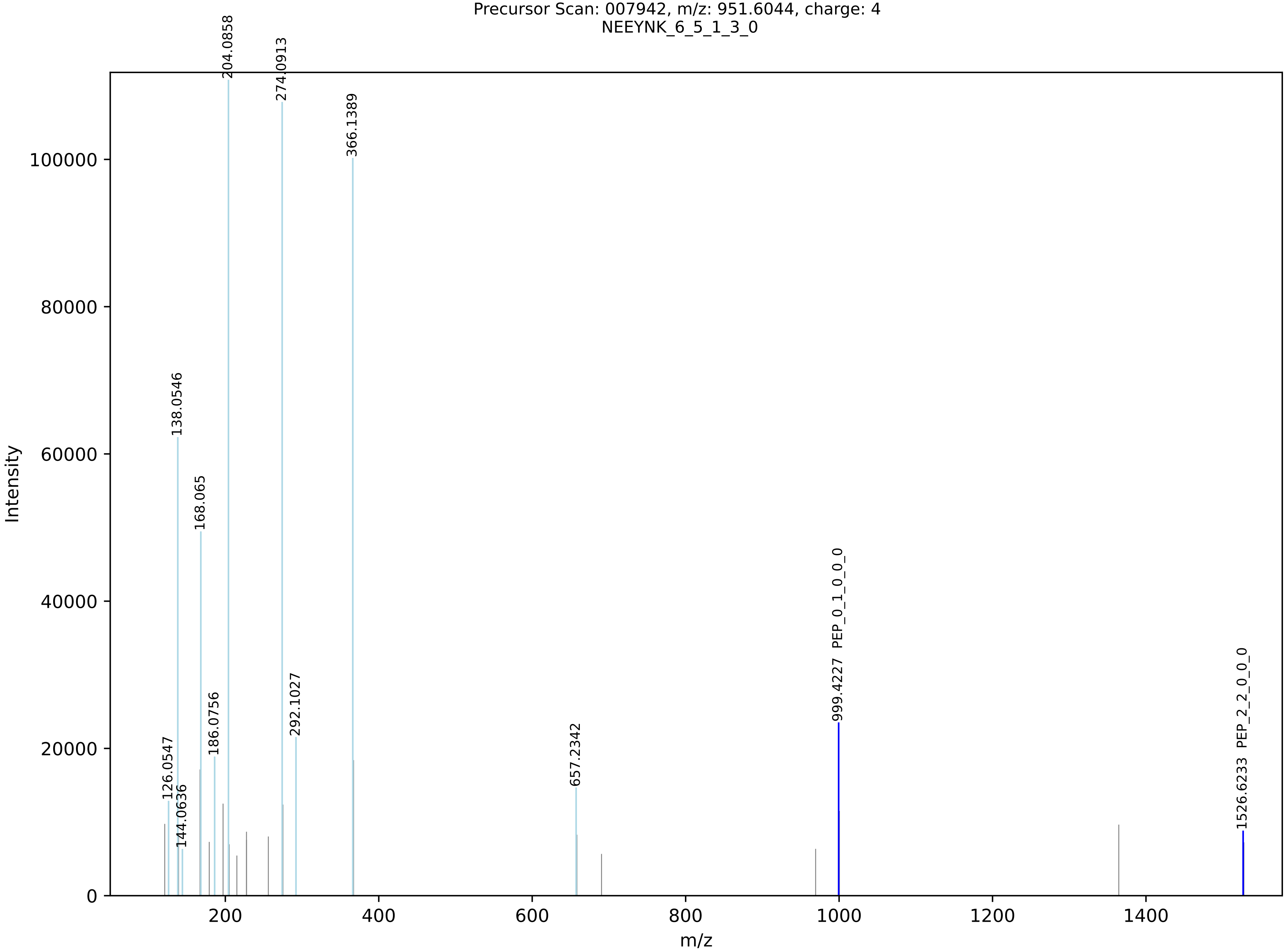

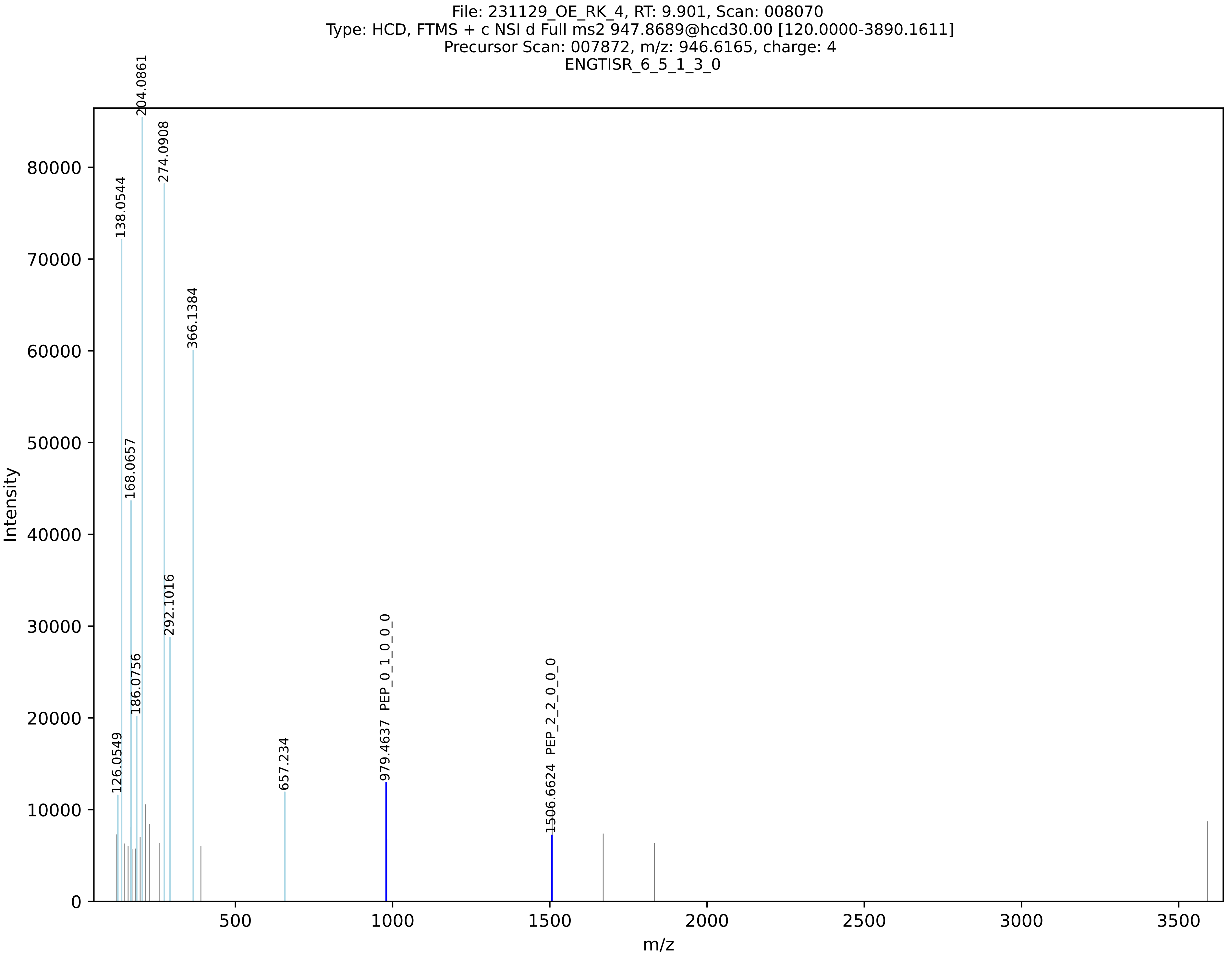
File: 231129_OE_RK_4, RT: 9.901, Scan: 008070
Type: HCD, FTMS + c NSI d Full ms2 947.8689@hcd30.00 [120.0000-3890.1611]
Precursor Scan: 007872, m/z: 946.6165, charge: 4
ENGTISR_6_5_1_3_0
204.0861
274.0908
138.0544
366.1384
168.0657
292.1016
186.0756
126.0549
657.234
979.4637 PEP_0_1_0_0_0
1506.6624 PEP_2_2_0_0_0
Intensity
m/z
0
10000
20000
30000
40000
50000
60000
70000
80000
500
1000
1500
2000
2500
3000
3500

File: 231129_OE_RK_4, RT: 9.913, Scan: 008082
Type: HCD, FTMS + c NSI d Full ms2 887.8631@hcd30.00 [120.0000-3645.3376]
Precursor Scan: 008016, m/z: 887.6126, charge: 4
YKNNSDISSTR_5_5_1_1_0

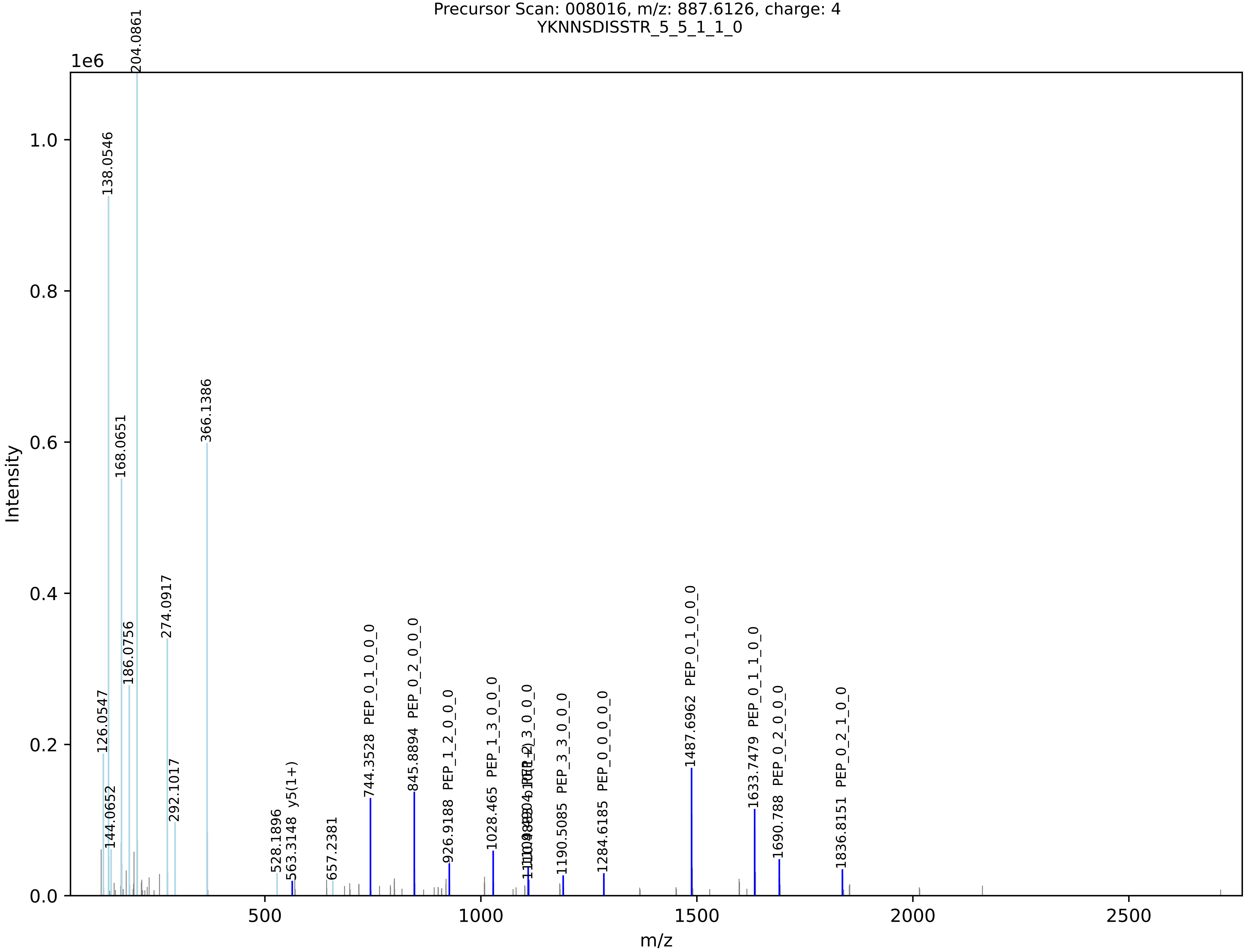

File: 231129_OE_RK_4, RT: 9.914, Scan: 008083
Type: HCD, FTMS + c NSI d Full ms2 1074.6620@hcd30.00 [120.0000-4407.4771]
Precursor Scan: 008016, m/z: 1074.1612, charge: 4
ENGTISR_7_6_0_4_0

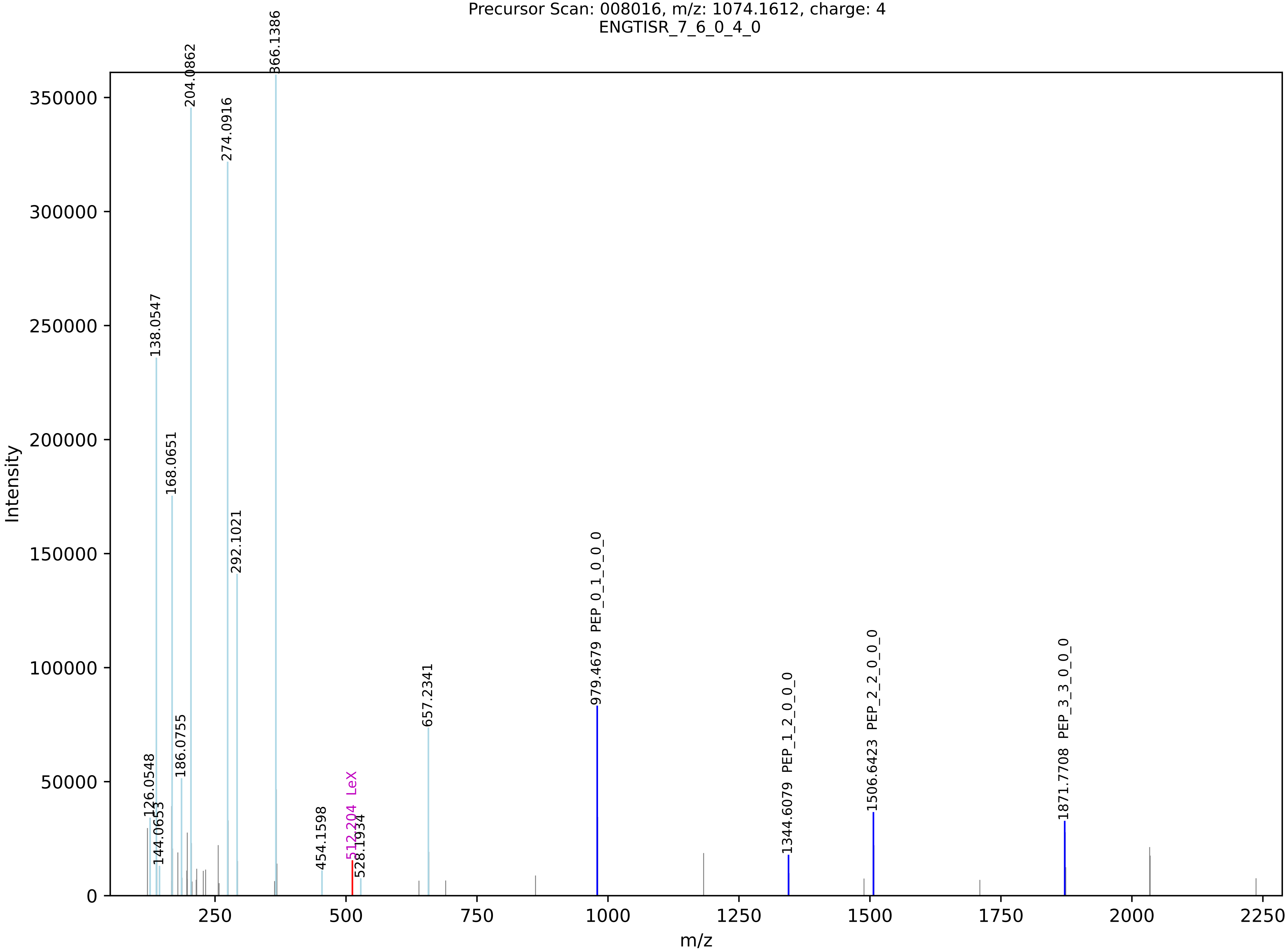

File: 231129_OE_RK_4, RT: 9.92, Scan: 008093
Type: HCD, FTMS + c NSI d Full ms2 983.6336@hcd30.00 [120.0000-4036.0811]
Precursor Scan: 008016, m/z: 983.1302, charge: 4
ENGTISR_6_5_2_3_0

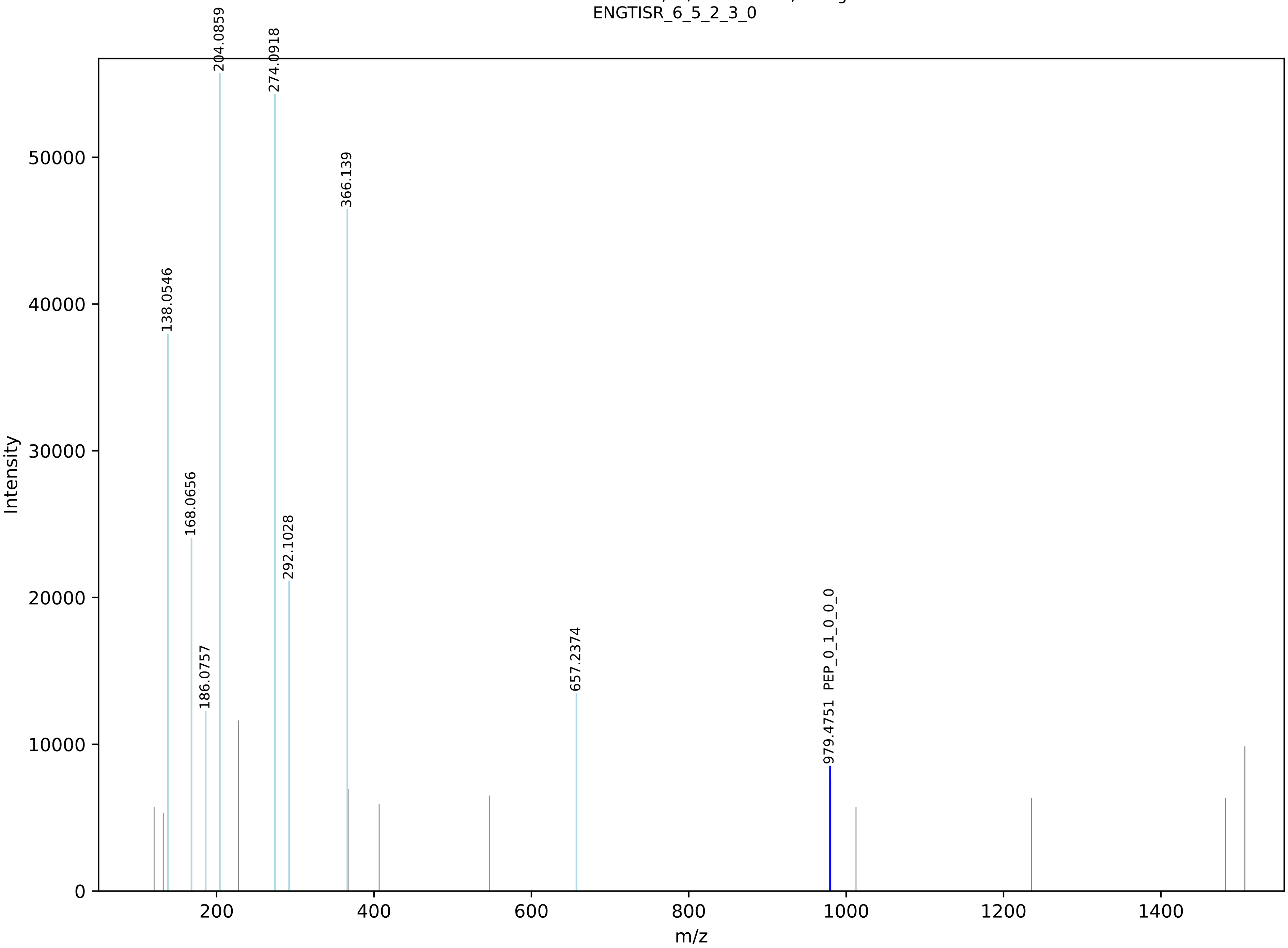

File: 231129_OE_RK_4, RT: 9.956, Scan: 008144
Type: HCD, FTMS + c NSI d Full ms2 1262.4877@hcd30.00 [120.0000-3885.3542]
Precursor Scan: 008080, m/z: 1261.8194, charge: 3
ENGTISR_6_5_1_3_0

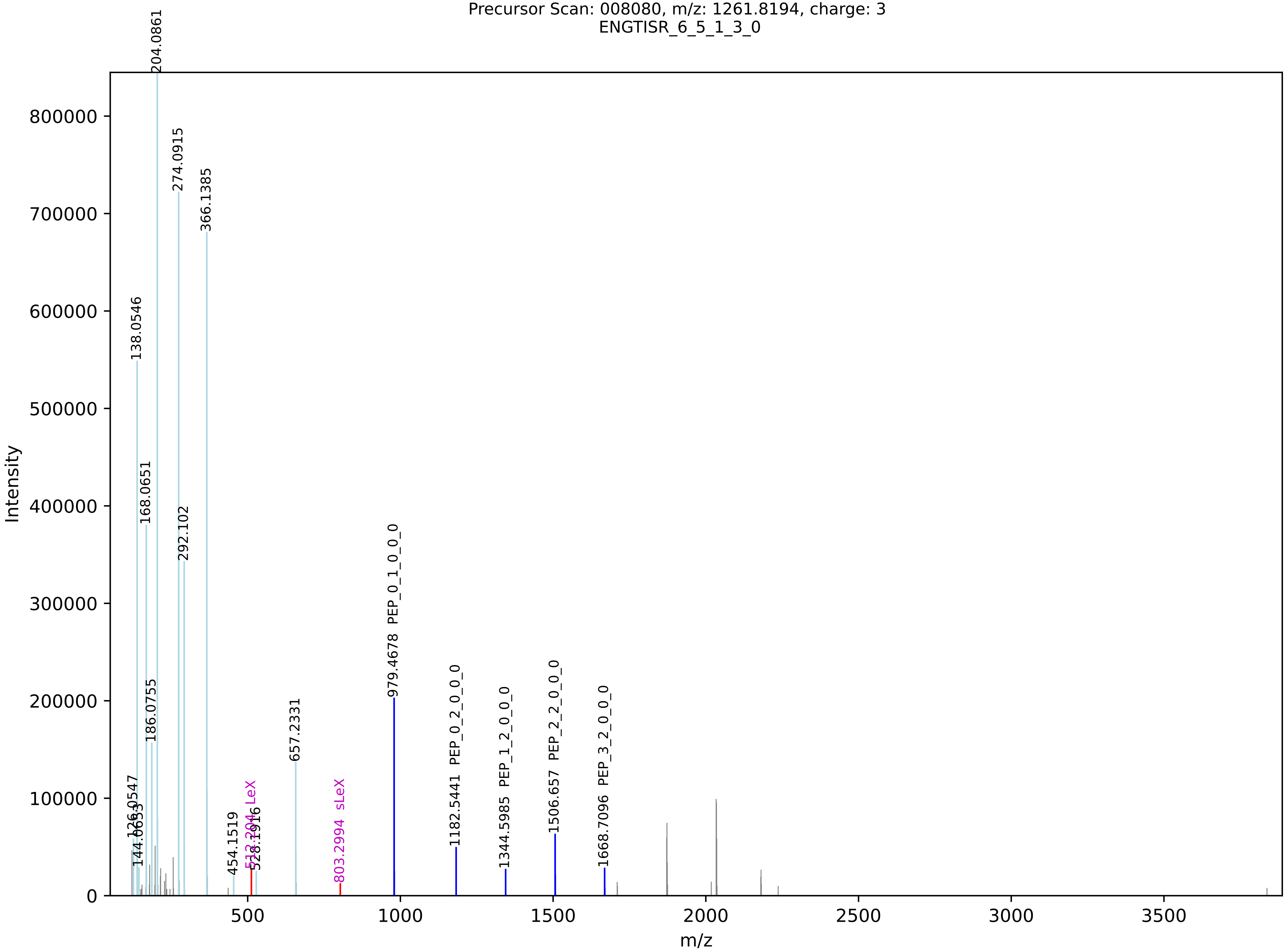

File: 231129_OE_RK_4, RT: 9.958, Scan: 008148
Type: HCD, FTMS + c NSI d Full ms2 910.3523@hcd30.00 [120.0000-3737.0933]
Precursor Scan: 008080, m/z: 910.1024, charge: 4
ENGTISR_6_5_0_3_0

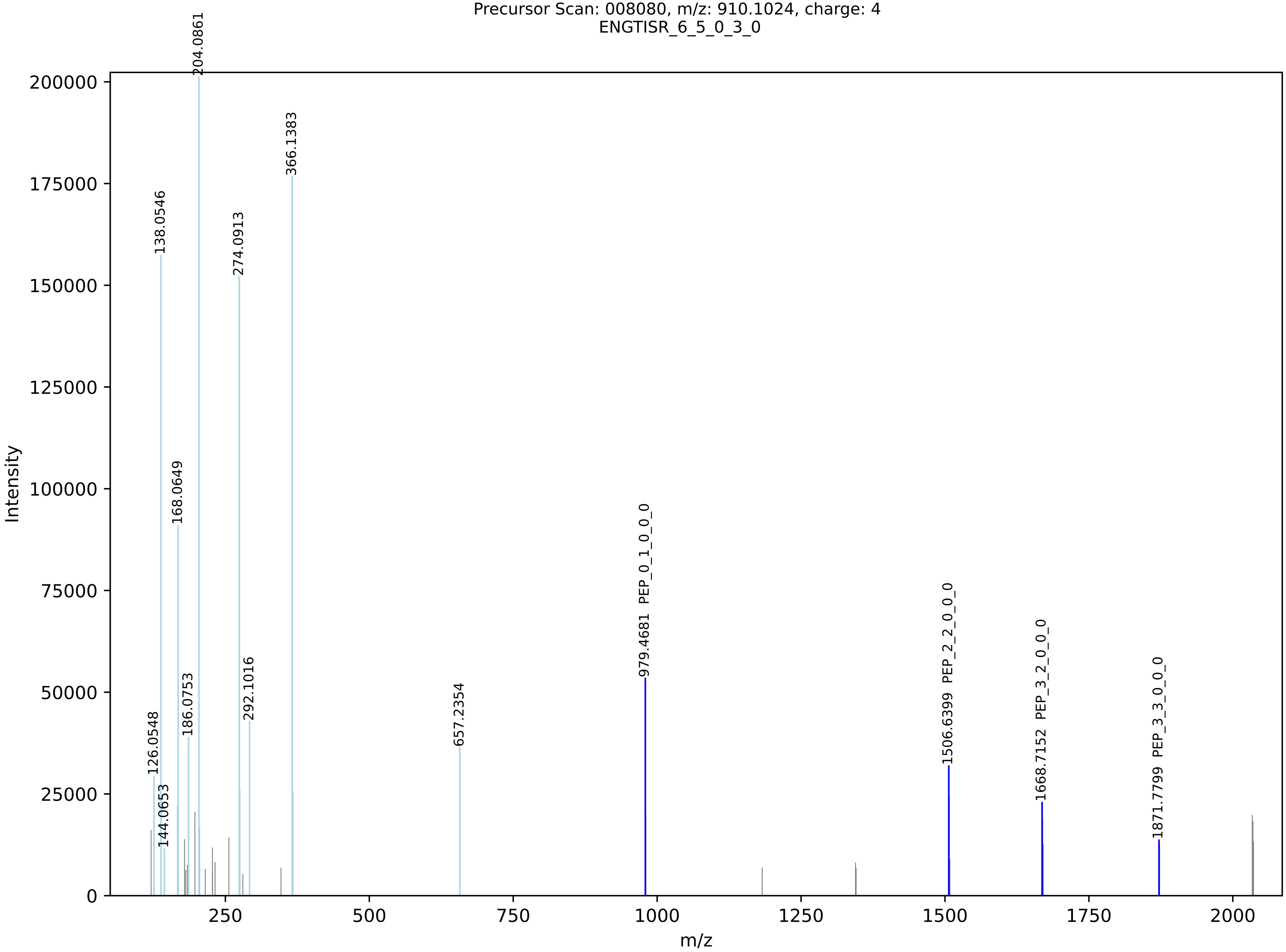

File: 231129_OE_RK_4, RT: 9.96, Scan: 008151
Type: HCD, FTMS + c NSI d Full ms2 1226.1289@hcd30.00 [120.0000-3774.0964]
Precursor Scan: 008080, m/z: 1225.4607, charge: 3
NEEYNK_7_5_1_2_0

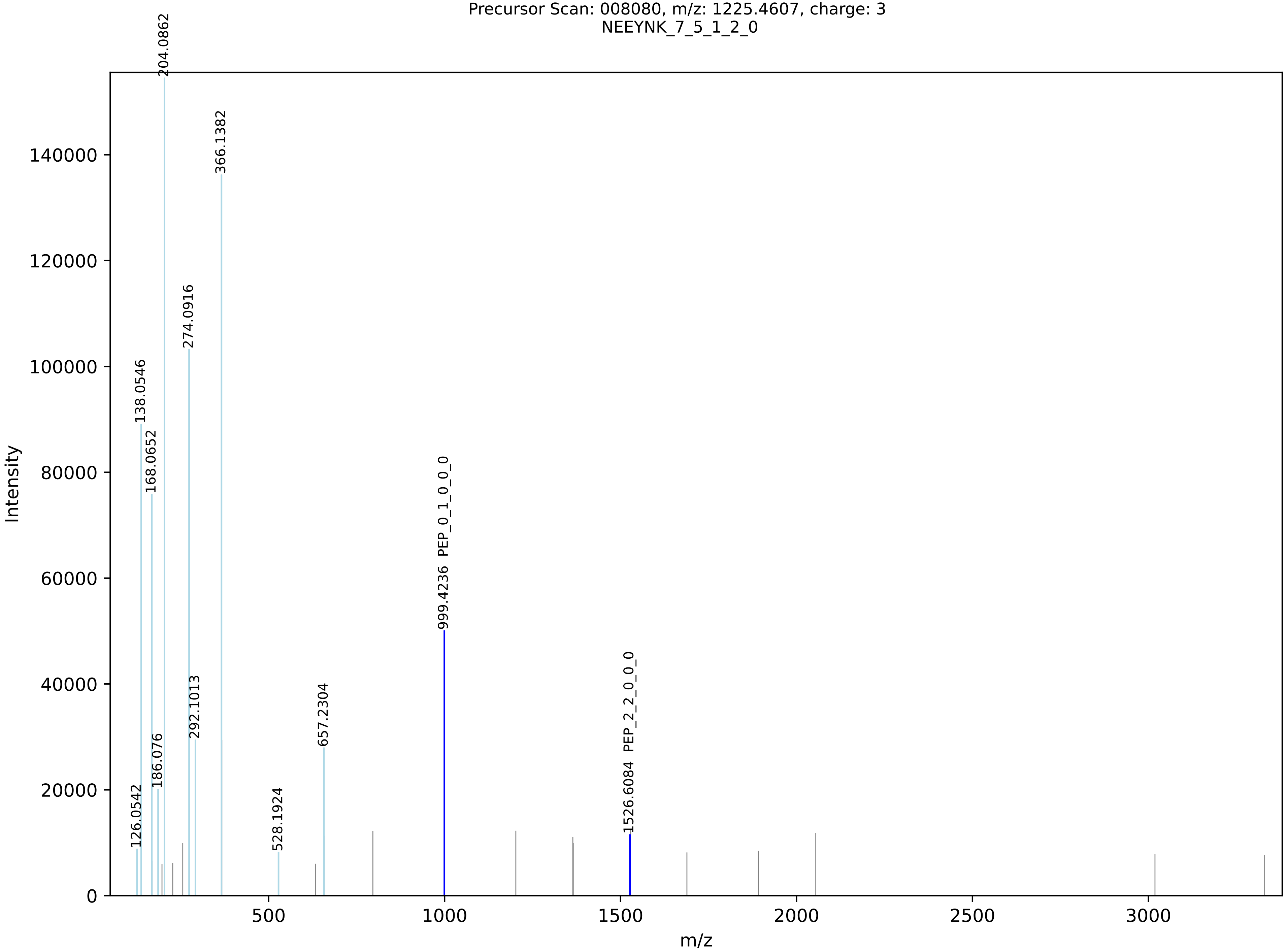

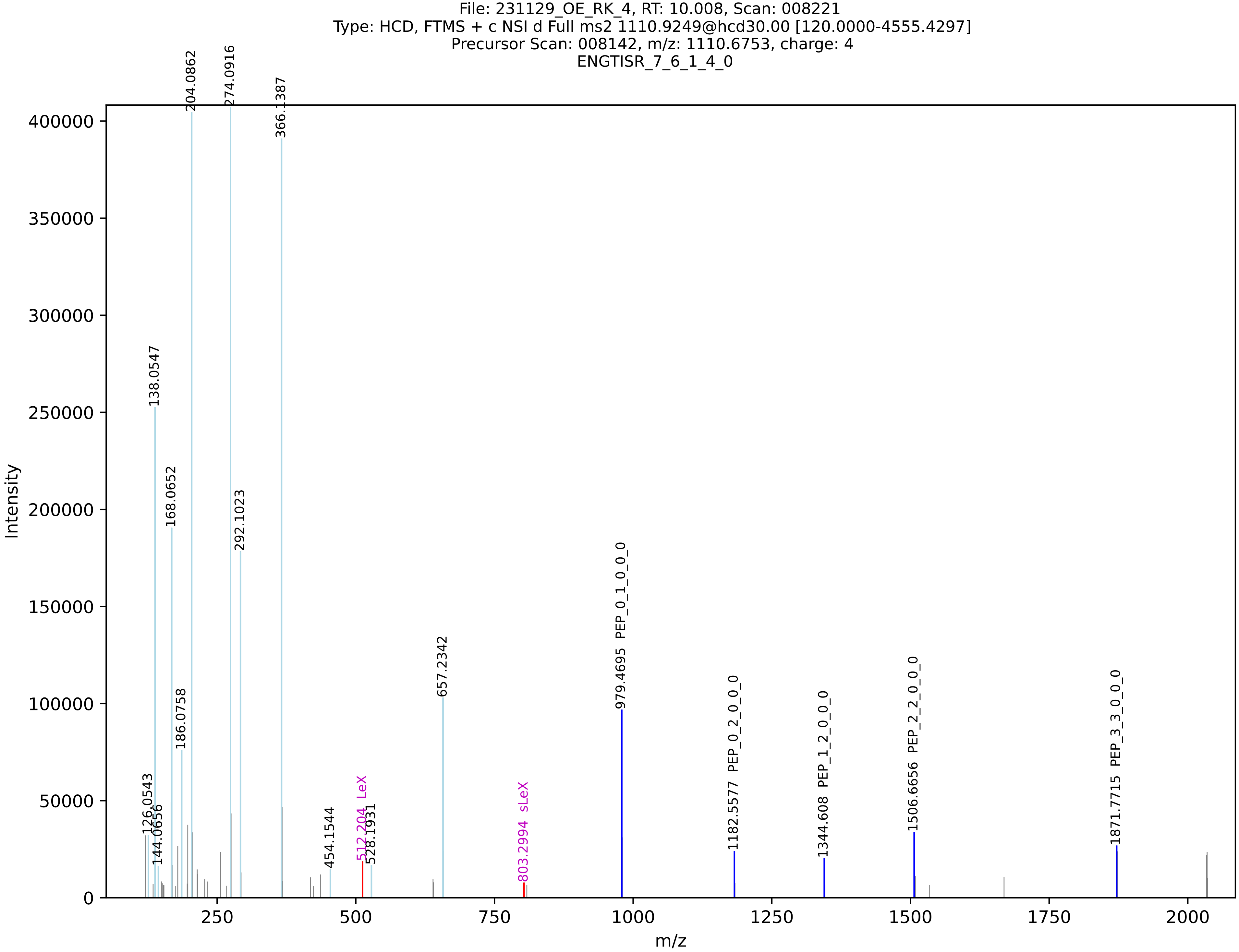
File: 231129_OE_RK_4, RT: 10.008, Scan: 008221
Type: HCD, FTMS + c NSI d Full ms2 1110.9249@hcd30.00 [120.0000-4555.4297]
Precursor Scan: 008142, m/z: 1110.6753, charge: 4
ENGTISR_7_6_1_4_0
Intensity
m/z
204.0862
274.0916
366.1387
138.0547
168.0652
292.1023
186.0758
126.0543
144.0656
454.1544
512.204 LeX
528.1931
657.2342
803.2994 sLeX
979.4695 PEP_0_1_0_0_0
1182.5577 PEP_0_2_0_0_0
1344.608 PEP_1_2_0_0_0
1506.6656 PEP_2_2_0_0_0
1871.7715 PEP_3_3_0_0_0

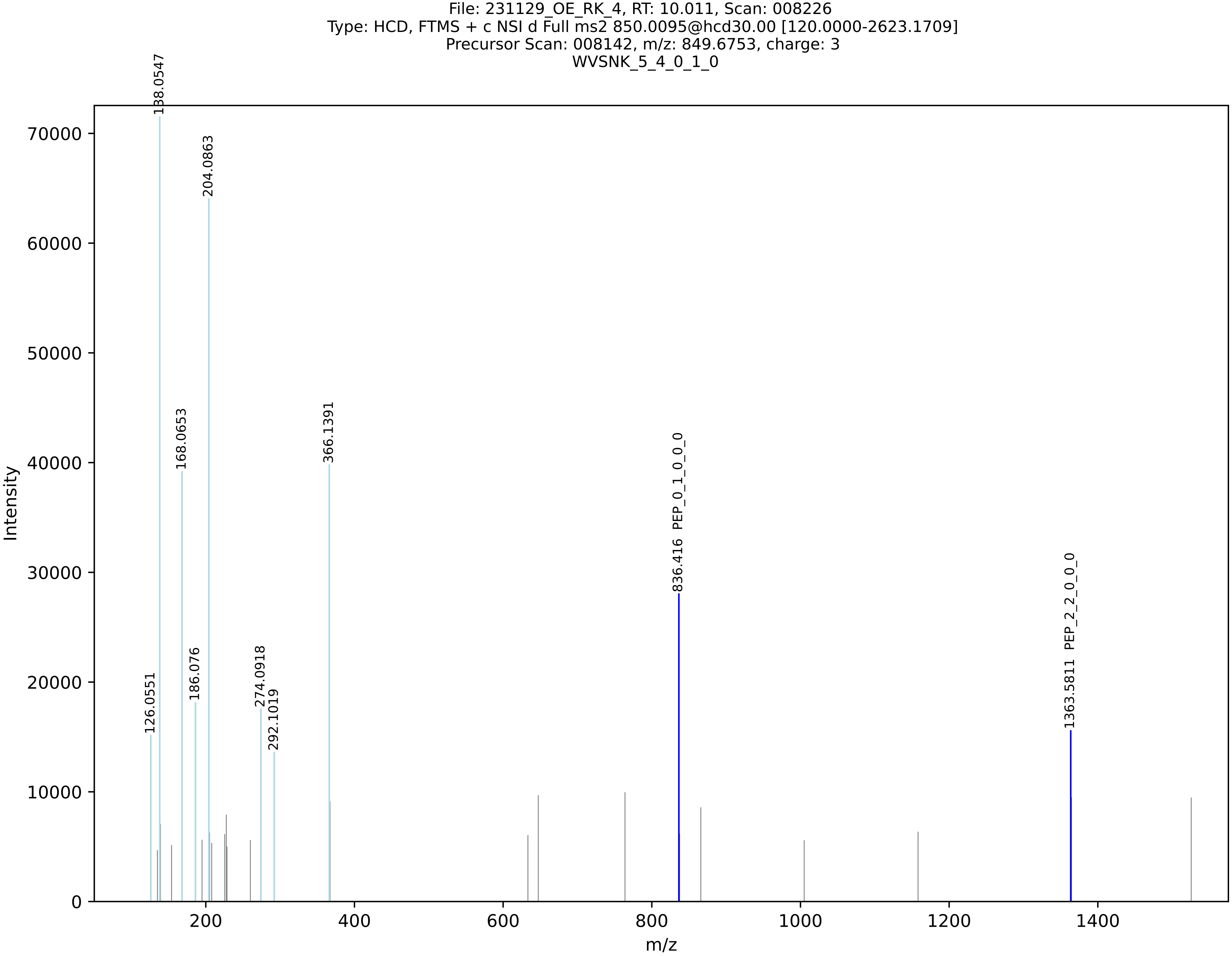

File: 231129_OE_RK_4, RT: 10.011, Scan: 008226
Type: HCD, FTMS + c NSI d Full ms2 850.0095@hcd30.00 [120.0000-2623.1709]
Precursor Scan: 008142, m/z: 849.6753, charge: 3
WVSNK_5_4_0_1_0
Intensity
m/z
138.0547
204.0863
168.0653
366.1391
836.416 PEP_0_1_0_0_
1363.5811 PEP_2_2_0_0_0
126.0551
186.076
274.0918
292.1019

File: 231129_OE_RK_4, RT: 10.014, Scan: 008230
Type: HCD, FTMS + c NSI d Full ms2 1274.4818@hcd30.00 [120.0000-3922.0564]
Precursor Scan: 008142, m/z: 1274.1441, charge: 3
NEEYNK_7_5_2_2_0

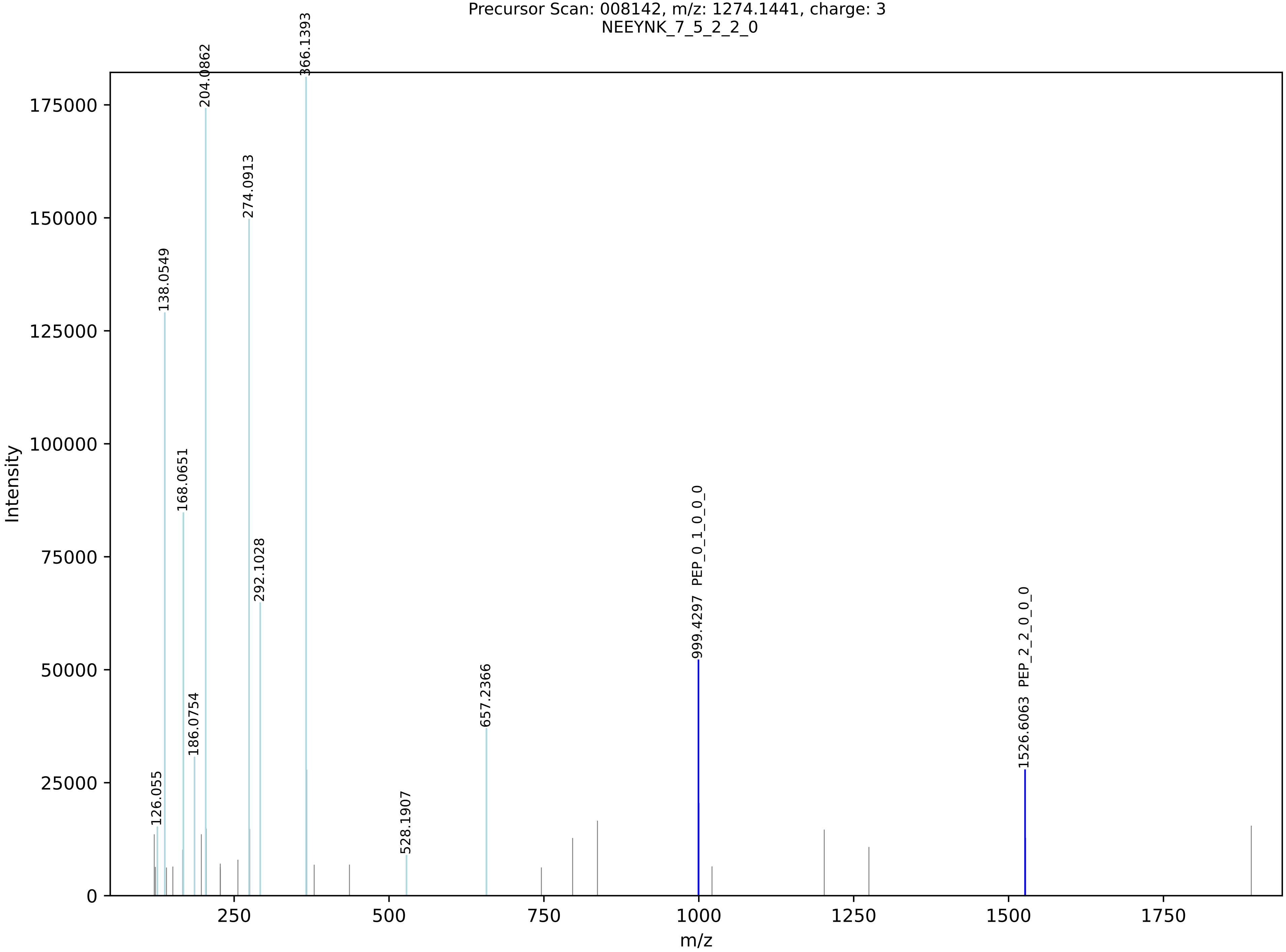

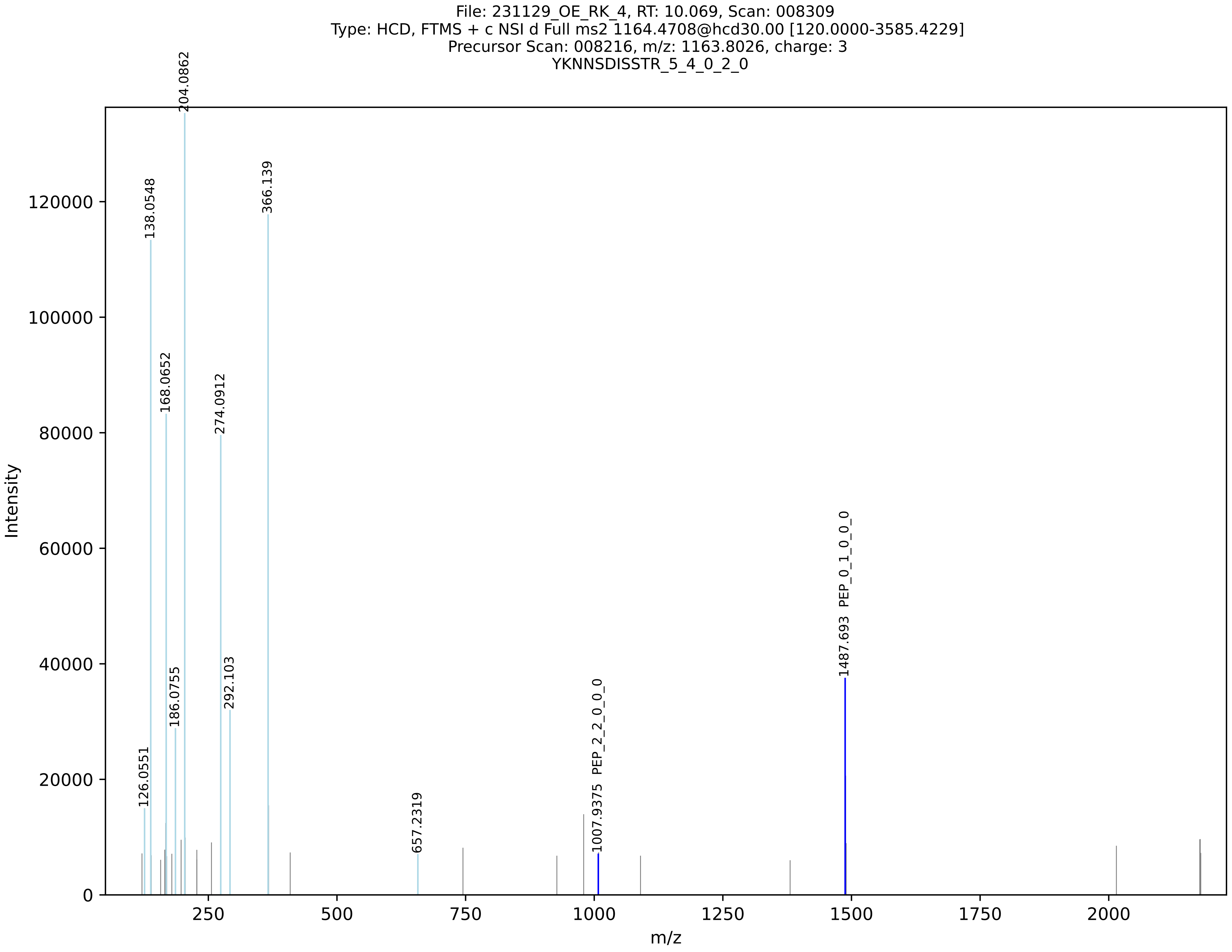
File: 231129_OE_RK_4, RT: 10.069, Scan: 008309
Type: HCD, FTMS + c NSI d Full ms2 1164.4708@hcd30.00 [120.0000-3585.4229]
Precursor Scan: 008216, m/z: 1163.8026, charge: 3
YKNNSDISSTR_5_4_0_2_0
Intensity
m/z
204.0862
366.139
138.0548
168.0652
274.0912
186.0755
292.103
126.0551
657.2319
1007.9375 PEP_2_2_0_0_0
1487.693 PEP_0_1_0_0_0

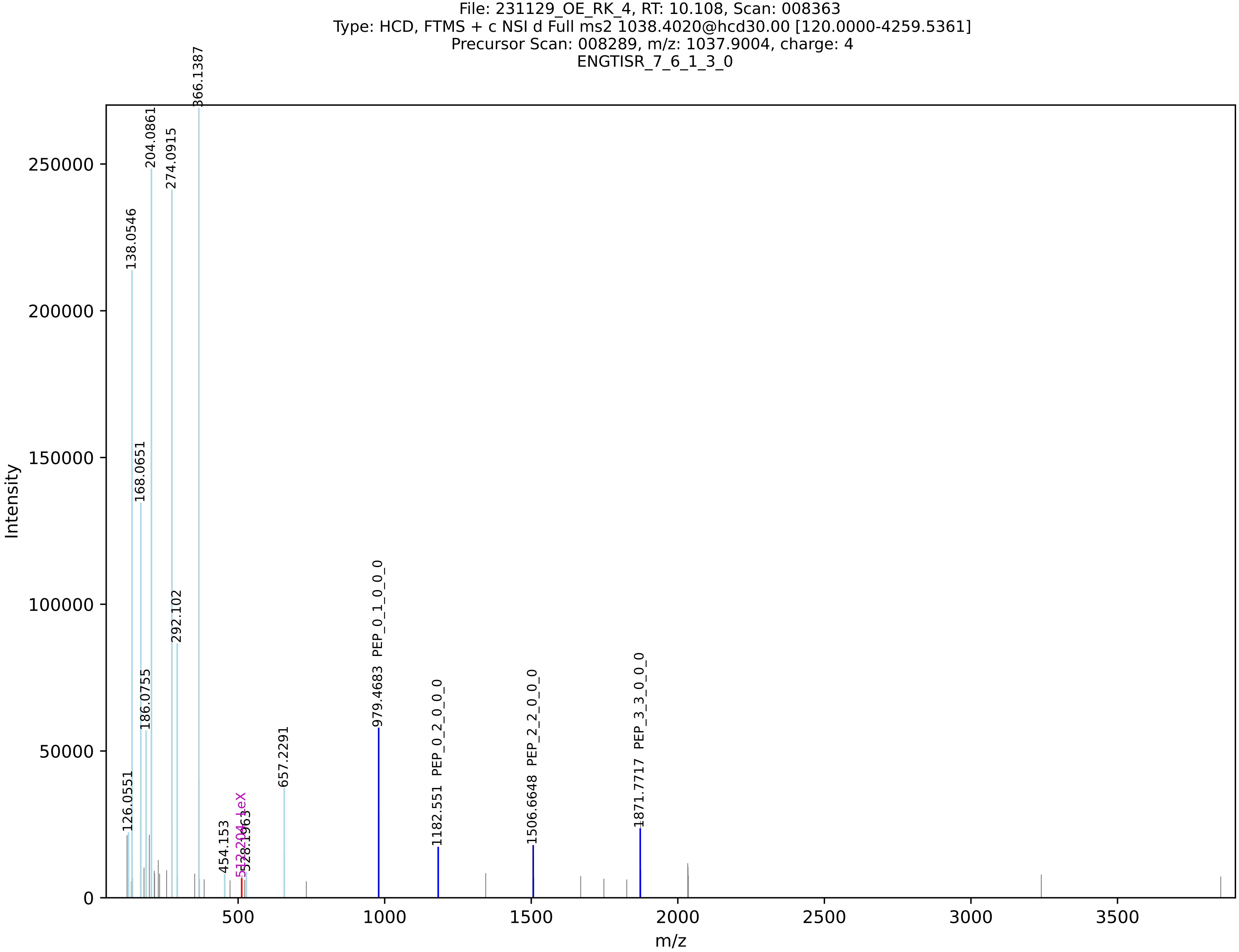

File: 231129_OE_RK_4, RT: 10.108, Scan: 008363
Type: HCD, FTMS + c NSI d Full ms2 1038.4020@hcd30.00 [120.0000-4259.5361]
Precursor Scan: 008289, m/z: 1037.9004, charge: 4
ENGTISR_7_6_1_3_0
366.1387
204.0861
274.0915
138.0546
168.0651
292.102
186.0755
126.0551
454.153
512.204 LeX
528.1963
657.2291
979.4683 PEP_0_1_0_0_0
1182.551 PEP_0_2_0_0_0
1506.6648 PEP_2_2_0_0_0
1871.7717 PEP_3_3_0_0_0
Intensity
m/z

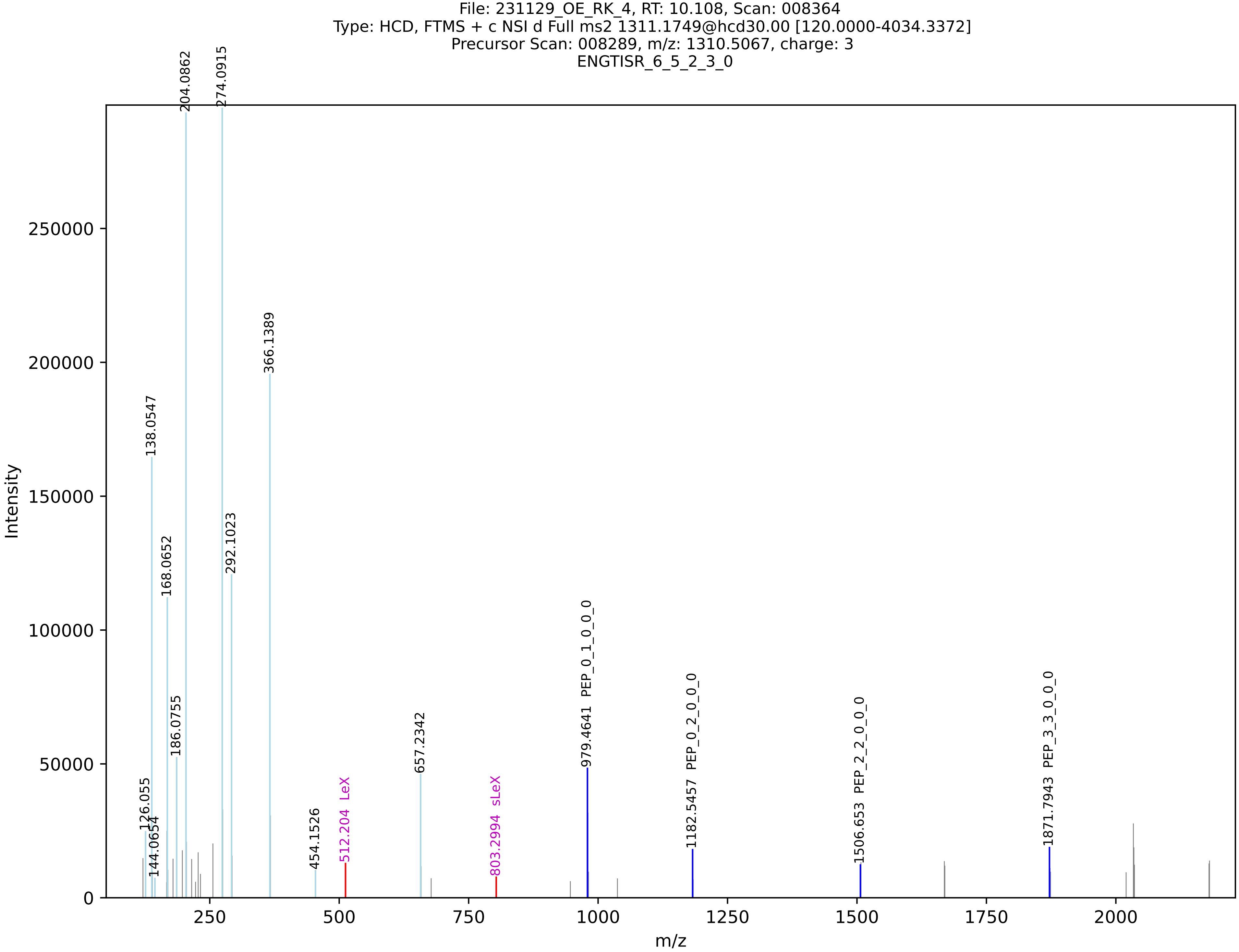

File: 231129_OE_RK_4, RT: 10.108, Scan: 008364
Type: HCD, FTMS + c NSI d Full ms2 1311.1749@hcd30.00 [120.0000-4034.3372]
Precursor Scan: 008289, m/z: 1310.5067, charge: 3
ENGTISR_6_5_2_3_0
Intensity
m/z
0
50000
100000
150000
200000
250000
250
500
750
1000
1250
1500
1750
2000
126.055
144.0654
138.0547
168.0652
186.0755
204.0862
274.0915
292.1023
366.1389
454.1526
512.204 LeX
657.2342
803.2994 sLeX
979.4641 PEP_0_1_0_0_0
1182.5457 PEP_0_2_0_0_0
1506.653 PEP_2_2_0_0_0
1871.7943 PEP_3_3_0_0_0

File: 231129_OE_RK_4, RT: 10.113, Scan: 008372
Type: HCD, FTMS + c NSI d Full ms2 915.3425@hcd30.00 [120.0000-3757.4531]
Precursor Scan: 008289, m/z: 915.0916, charge: 4
NEEYNK_6_5_0_3_0

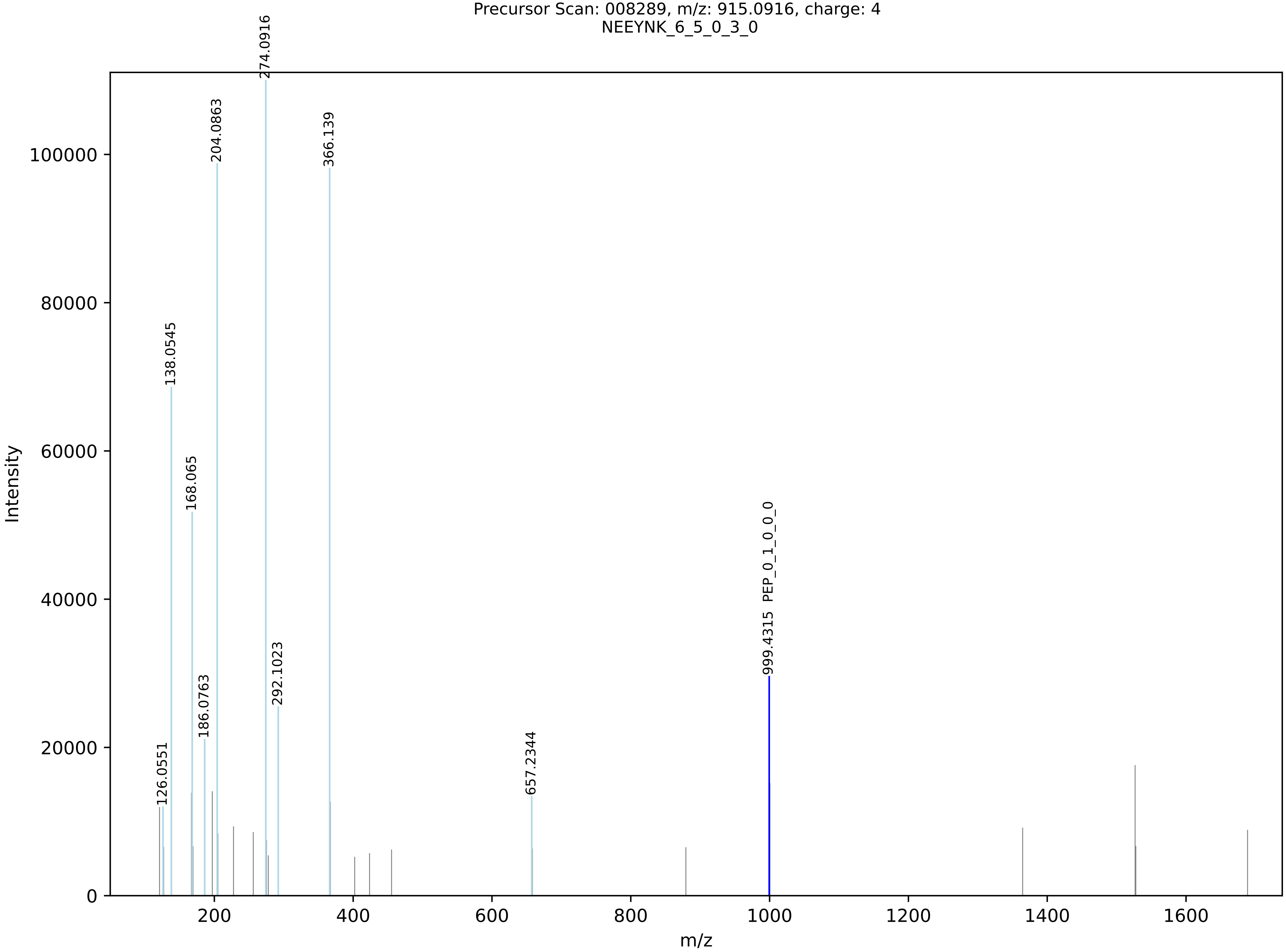

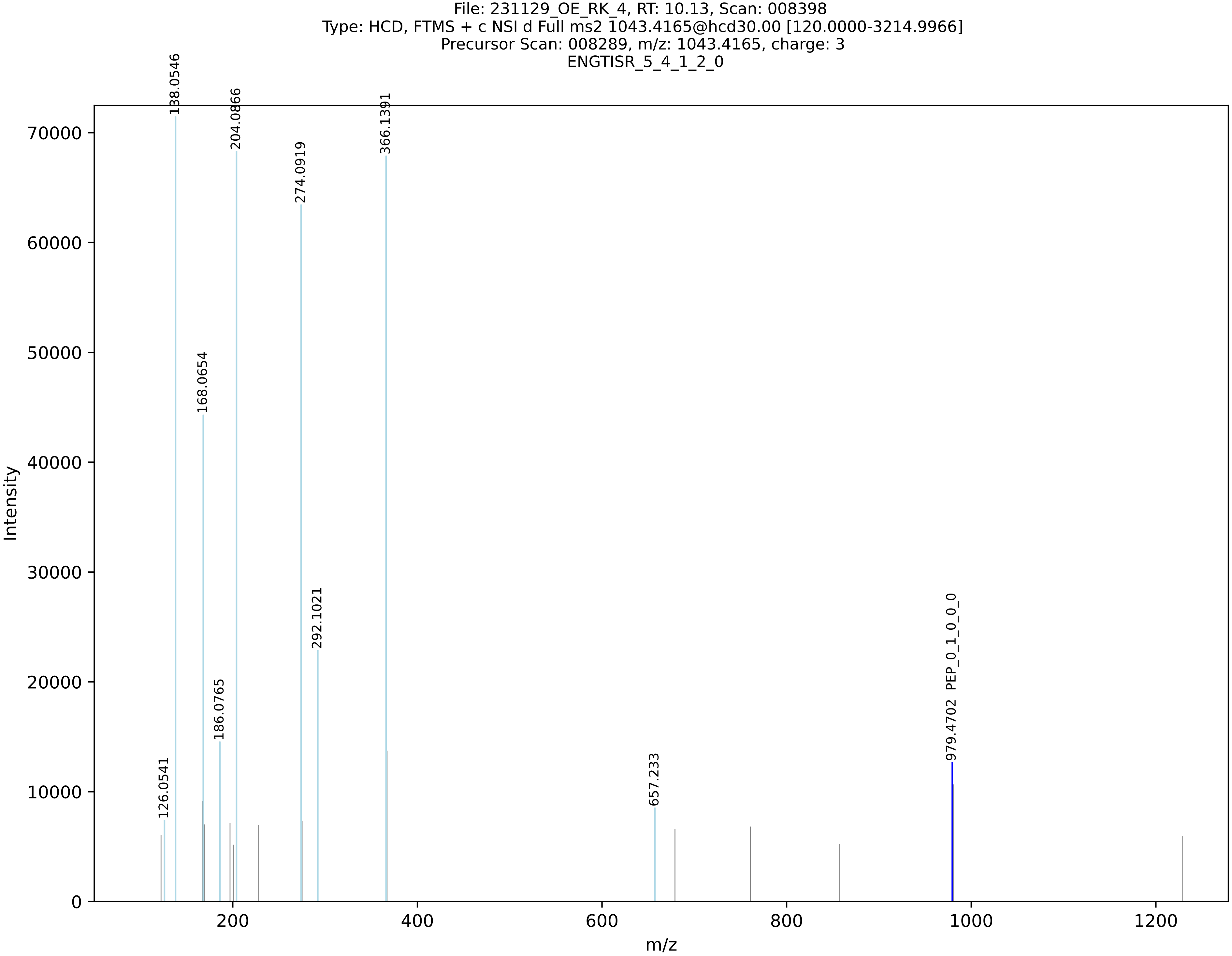
File: 231129_OE_RK_4, RT: 10.13, Scan: 008398
Type: HCD, FTMS + c NSI d Full ms2 1043.4165@hcd30.00 [120.0000-3214.9966]
Precursor Scan: 008289, m/z: 1043.4165, charge: 3
ENGTISR_5_4_1_2_0
138.0546
204.0866
366.1391
274.0919
168.0654
292.1021
186.0765
126.0541
657.233
979.4702 PEP_0_1_0_0_0
Intensity
m/z
0
10000
20000
30000
40000
50000
60000
70000
200
400
600
800
1000
1200

File: 231129_OE_RK_4, RT: 10.159, Scan: 008435
Type: HCD, FTMS + c NSI d Full ms2 1262.4885@hcd30.00 [120.0000-3885.3567]
Precursor Scan: 008359, m/z: 1261.8211, charge: 3
ENGTISR_6_5_1_3_0

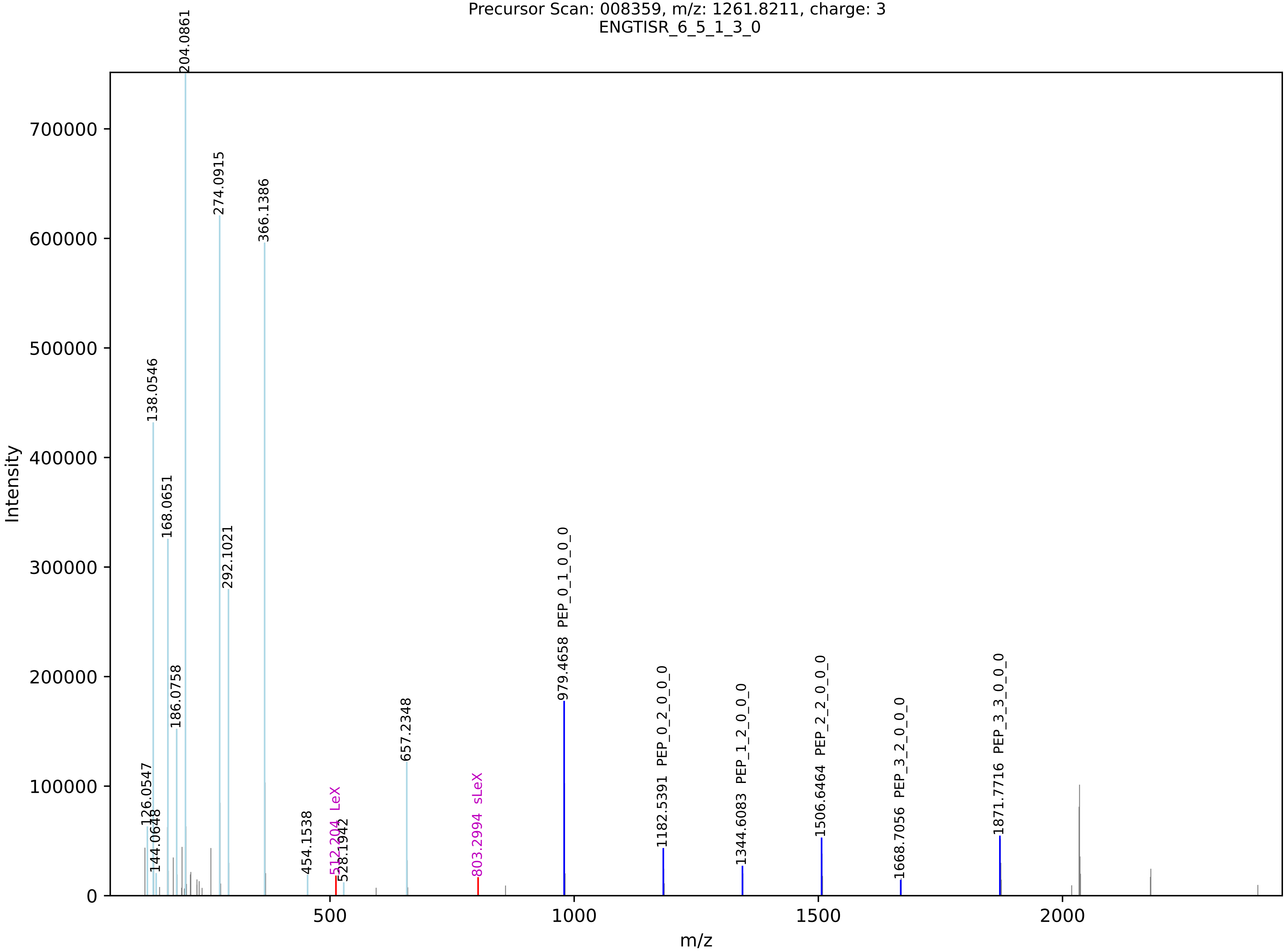

File: 231129_OE_RK_4, RT: 10.16, Scan: 008437
Type: HCD, FTMS + c NSI d Full ms2 1090.4279@hcd30.00 [120.0000-3358.8513]
Precursor Scan: 008359, m/z: 1090.0938, charge: 3
TMQENSTPR_5_4_0_2_0

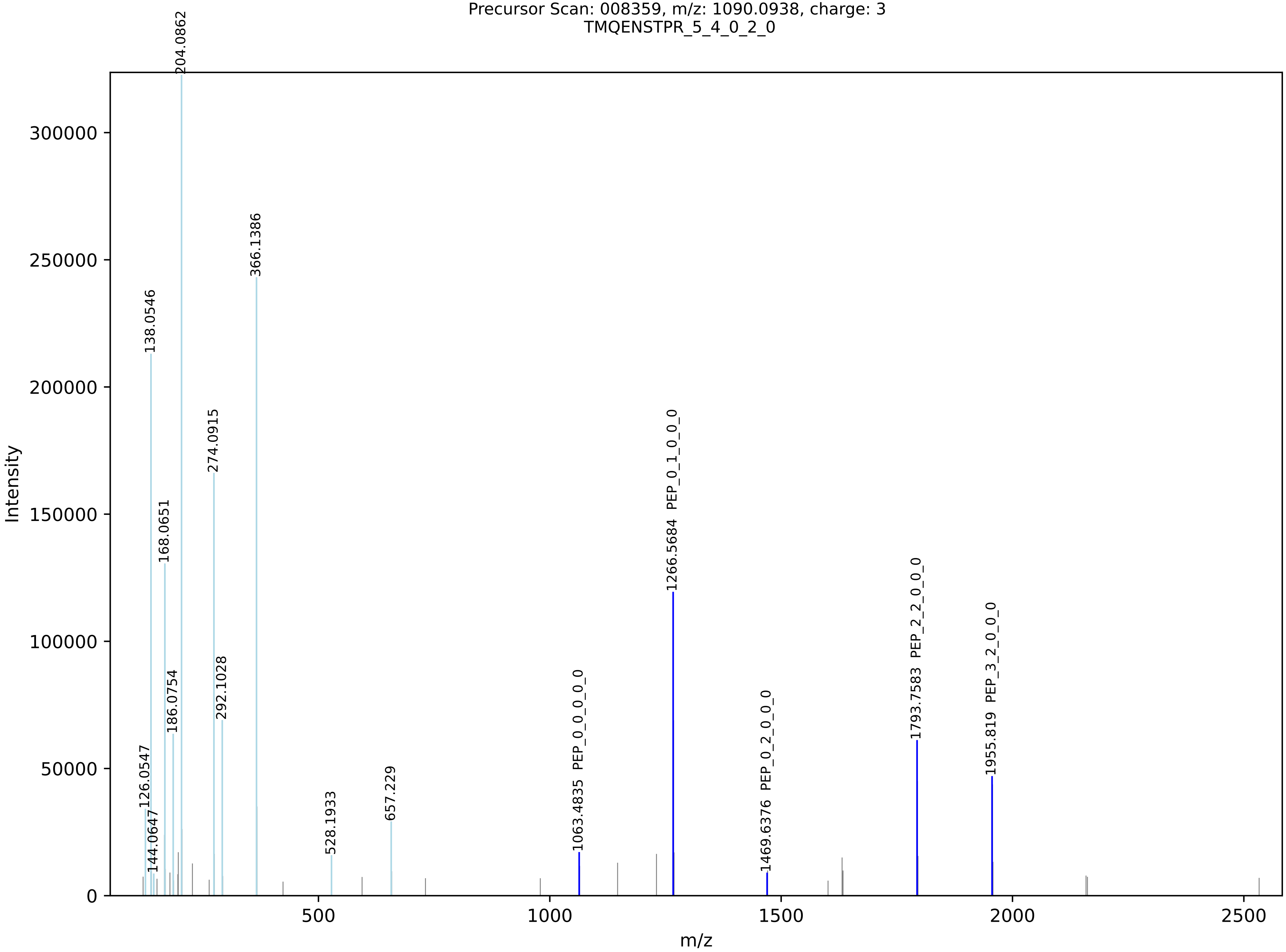

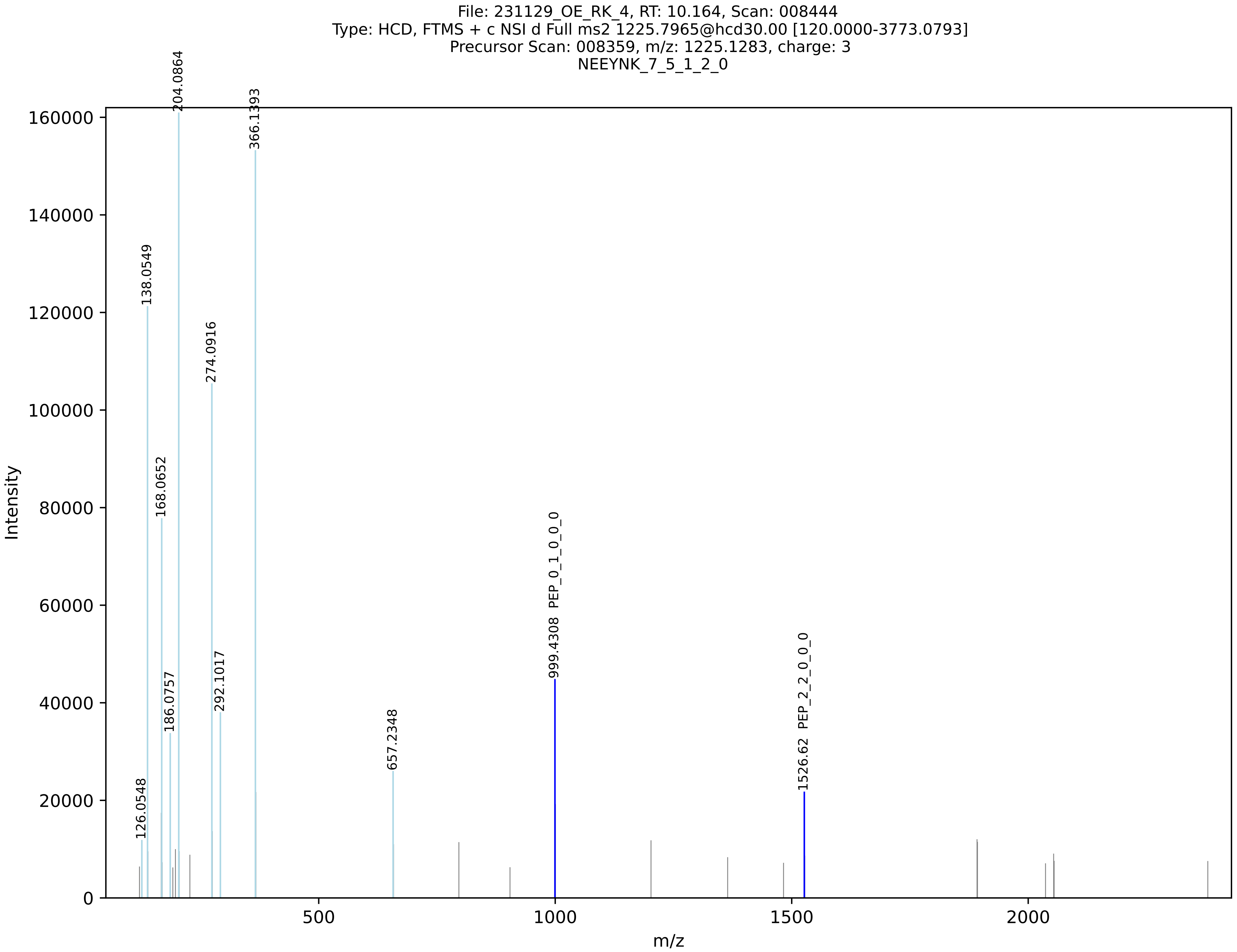
File: 231129_OE_RK_4, RT: 10.164, Scan: 008444
Type: HCD, FTMS + c NSI d Full ms2 1225.7965@hcd30.00 [120.0000-3773.0793]
Precursor Scan: 008359, m/z: 1225.1283, charge: 3
NEEYNK_7_5_1_2_0
Intensity
m/z
204.0864
366.1393
138.0549
274.0916
168.0652
186.0757
292.1017
126.0548
657.2348
999.4308 PEP_0_1_0_0_0
1526.62 PEP_2_2_0_0_0
0
20000
40000
60000
80000
100000
120000
140000
160000
500
1000
1500
2000

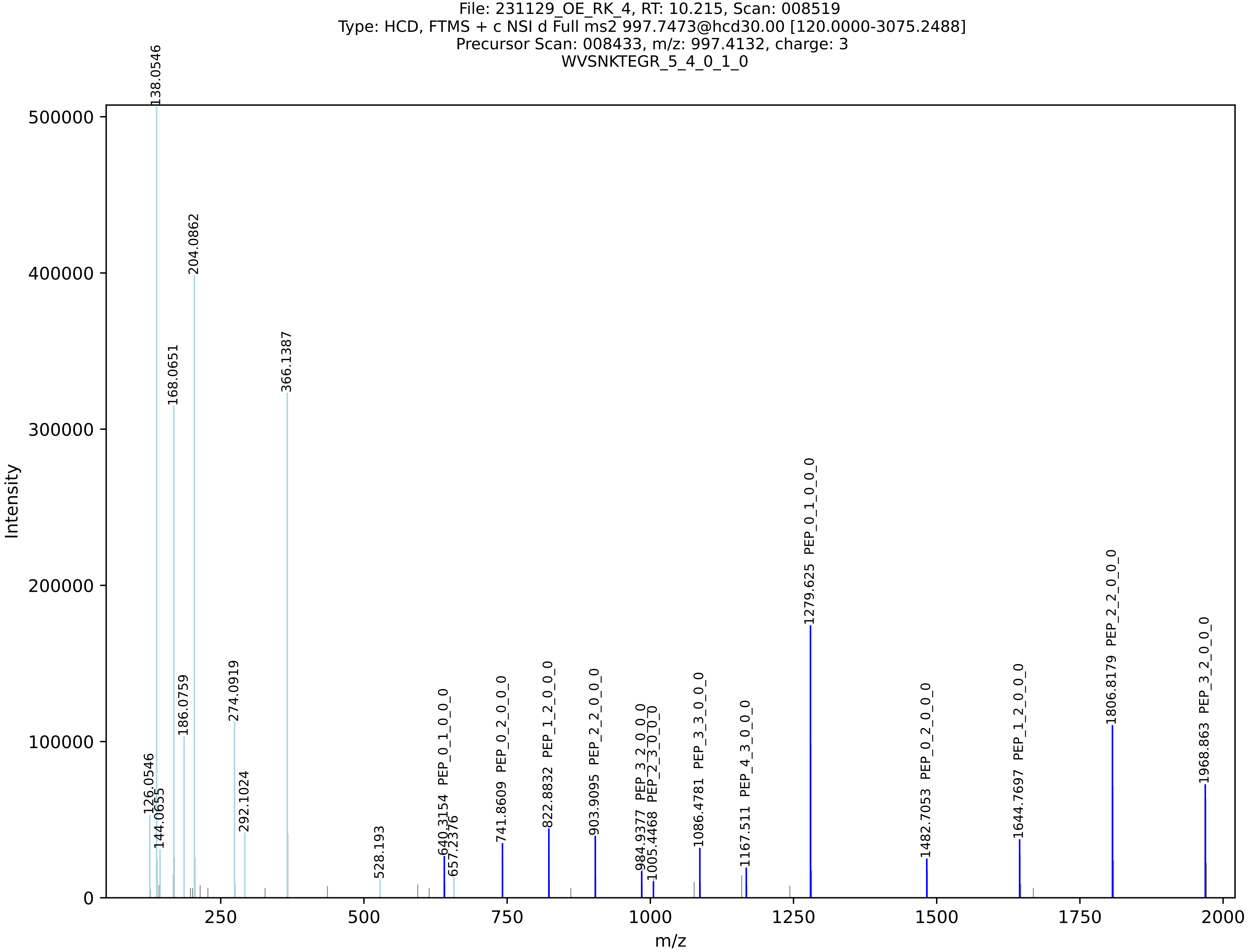

File: 231129_OE_RK_4, RT: 10.215, Scan: 008519
Type: HCD, FTMS + c NSI d Full ms2 997.7473@hcd30.00 [120.0000-3075.2488]
Precursor Scan: 008433, m/z: 997.4132, charge: 3
WVSNKTEGR_5_4_0_1_0
Intensity
m/z
138.0546
204.0862
366.1387
168.0651
186.0759
274.0919
126.0546
144.0655
292.1024
528.193
640.3154 PEP_0_1_0_0_0
657.2376
741.8609 PEP_0_2_0_0_0
822.8832 PEP_1_2_0_0_0
903.9095 PEP_2_2_0_0_0
984.9377 PEP_3_2_0_0_0
1005.4468 PEP_2_3_0_0_0
1086.4781 PEP_3_3_0_0_0
1167.511 PEP_4_3_0_0_0
1279.625 PEP_0_1_0_0_0
1482.7053 PEP_0_2_0_0_0
1644.7697 PEP_1_2_0_0_0
1806.8179 PEP_2_2_0_0_0
1968.863 PEP_3_2_0_0_0

File: 231129_OE_RK_4, RT: 10.231, Scan: 008543
Type: HCD, FTMS + c NSI d Full ms2 1232.7756@hcd30.00 [120.0000-3794.4353]
Precursor Scan: 008433, m/z: 1232.1074, charge: 3
MEACMLNGTVIGPGK_5_5_0_1_0

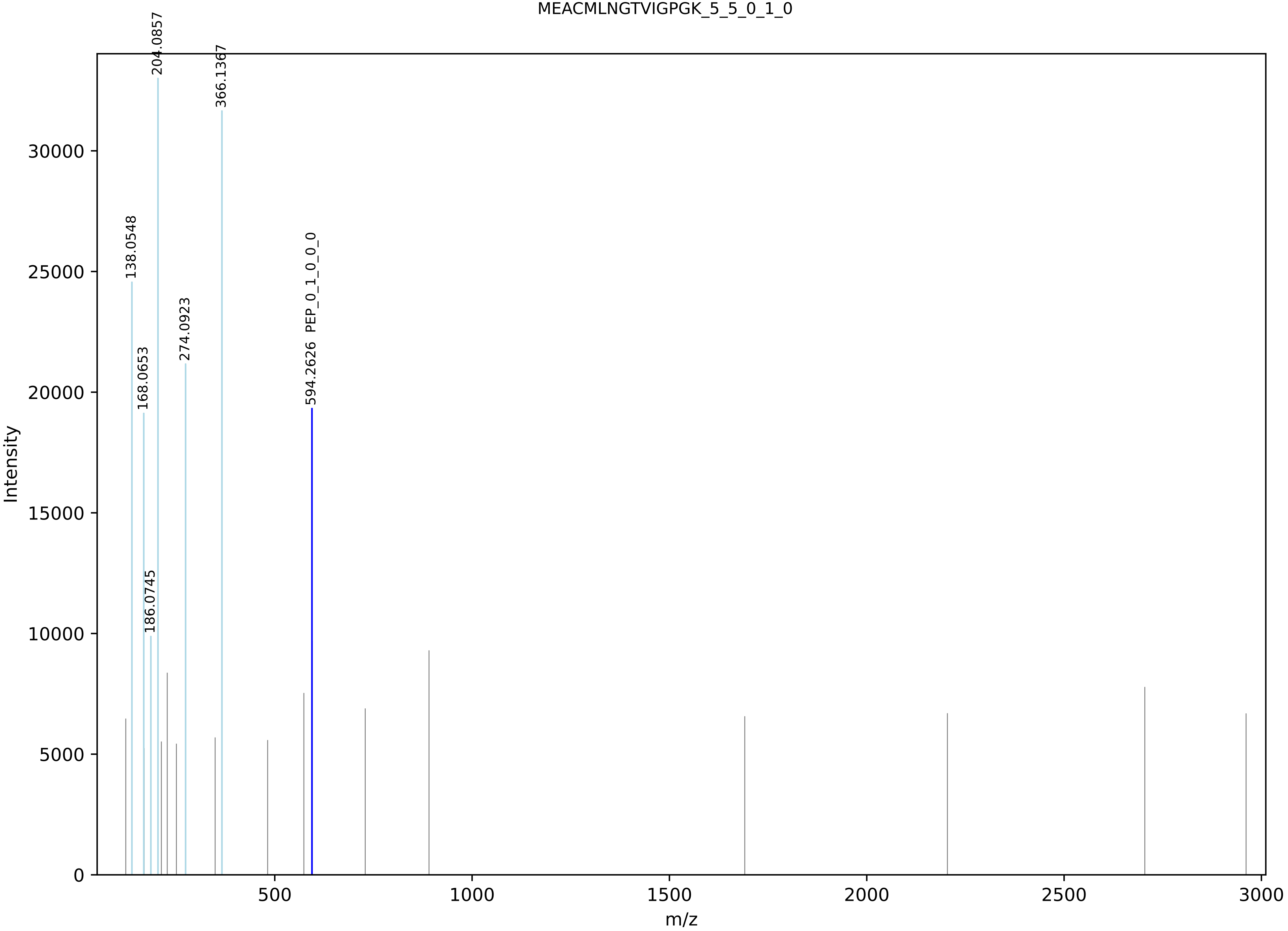

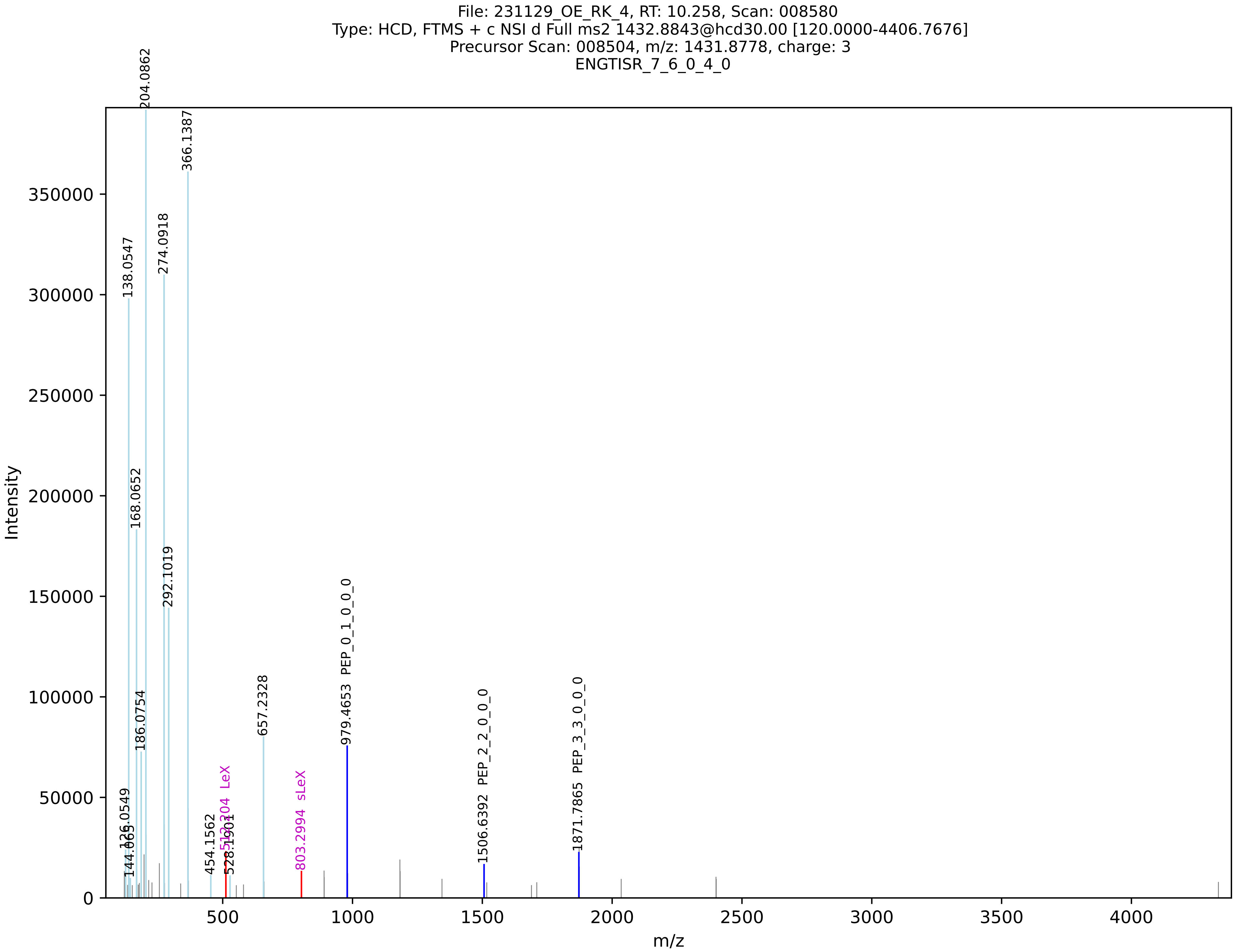

File: 231129_OE_RK_4, RT: 10.258, Scan: 008580
Type: HCD, FTMS + c NSI d Full ms2 1432.8843@hcd30.00 [120.0000-4406.7676]
Precursor Scan: 008504, m/z: 1431.8778, charge: 3
ENGTISR_7_6_0_4_0
Intensity
m/z
204.0862
366.1387
274.0918
138.0547
168.0652
292.1019
186.0754
657.2328
979.4653 PEP_0_1_0_0_0
1506.6392 PEP_2_2_0_0_0
1871.7865 PEP_3_3_0_0_0
454.1562
528.1901
LeX
803.2994 sLeX
126.0549
144.065

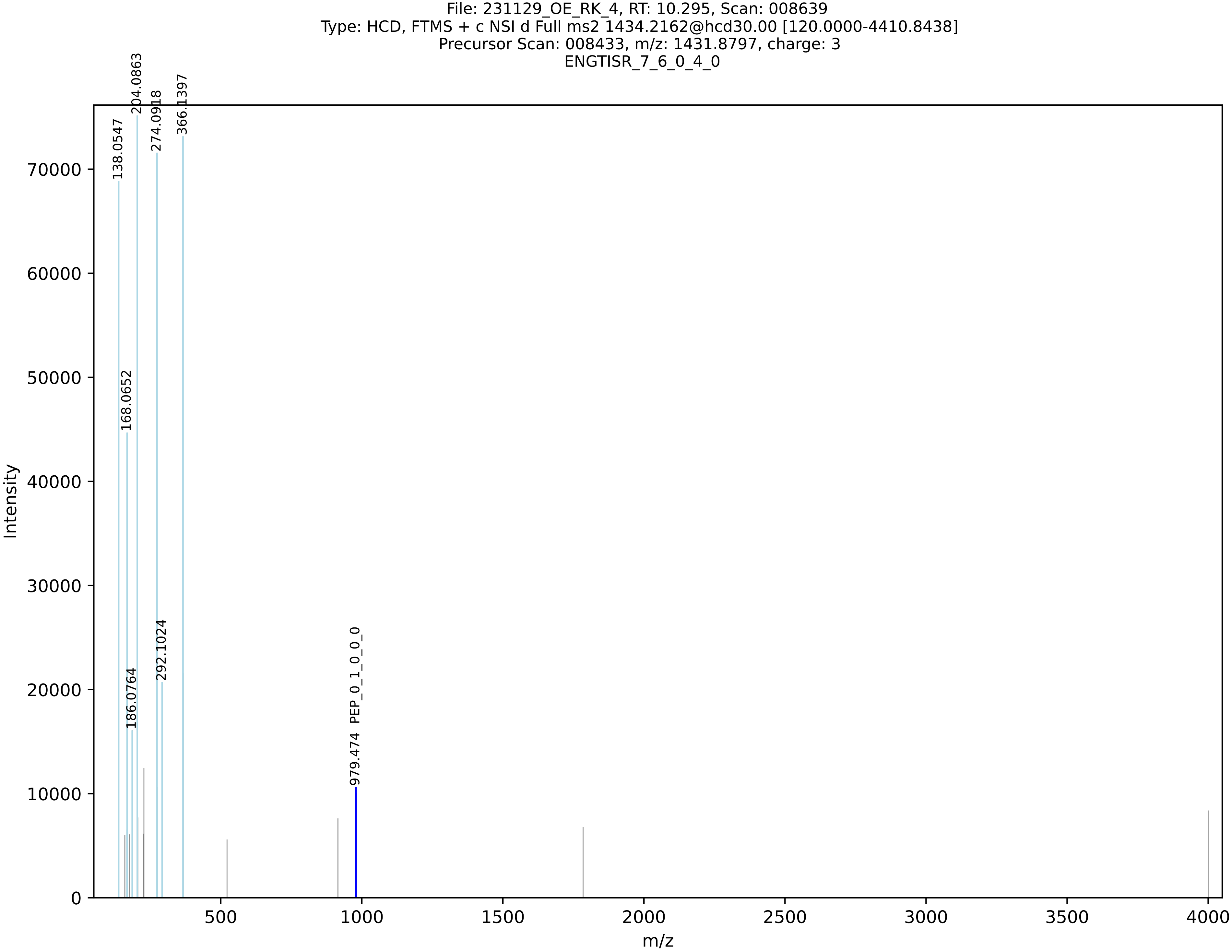

File: 231129_OE_RK_4, RT: 10.295, Scan: 008639
Type: HCD, FTMS + c NSI d Full ms2 1434.2162@hcd30.00 [120.0000-4410.8438]
Precursor Scan: 008433, m/z: 1431.8797, charge: 3
ENGTISR_7_6_0_4_0
138.0547
204.0863
274.0918
366.1397
168.0652
186.0764
292.1024
979.474 PEP_0_1_0_0_0_
Intensity
m/z
0
10000
20000
30000
40000
50000
60000
70000
500
1000
1500
2000
2500
3000
3500
4000

File: 231129_OE_RK_4, RT: 10.309, Scan: 008652
Type: HCD, FTMS + c NSI d Full ms2 1383.8638@hcd30.00 [120.0000-4256.7651]
Precursor Scan: 008578, m/z: 1383.5275, charge: 3
ENGTISR_7_6_1_3_0

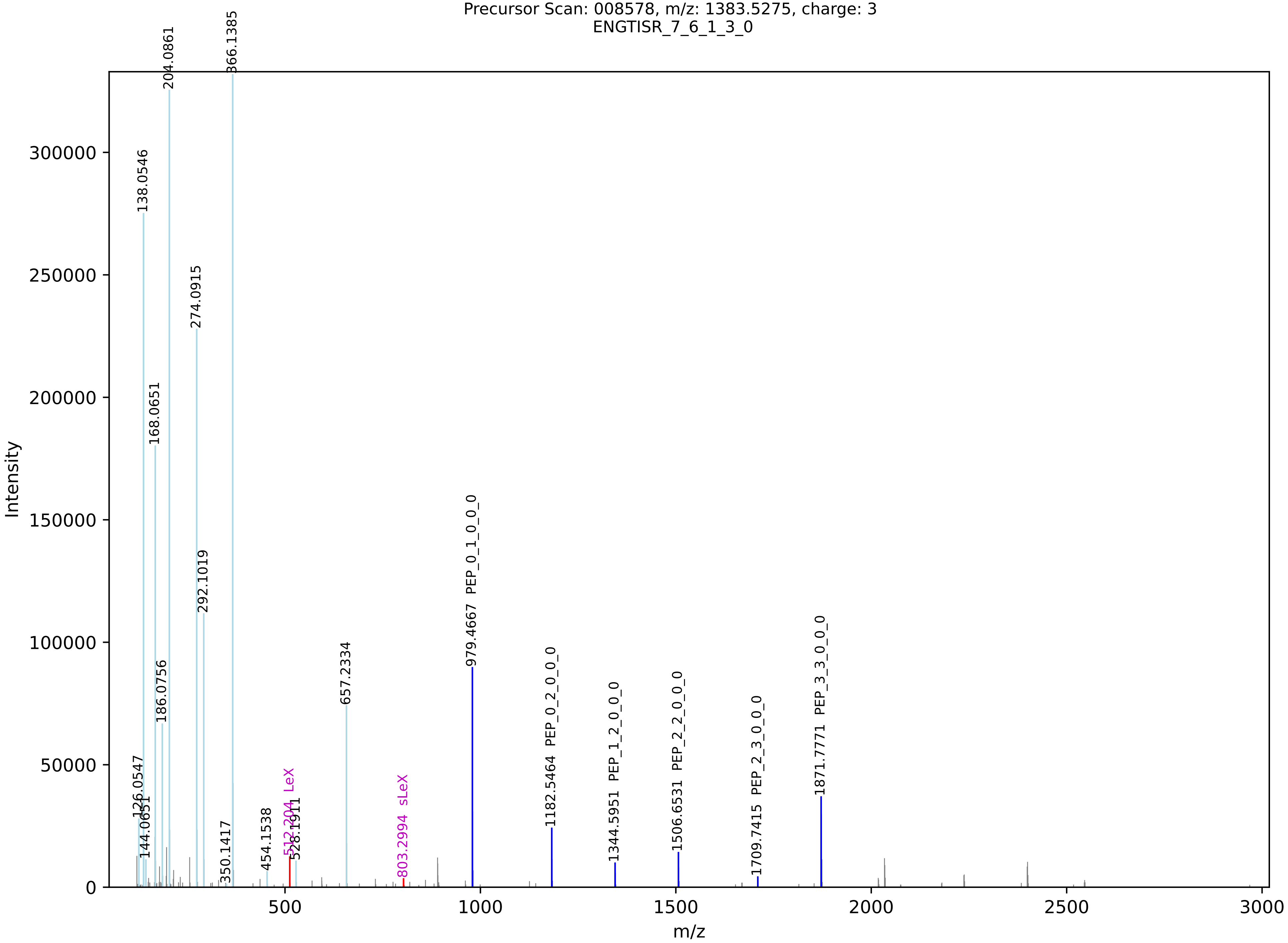

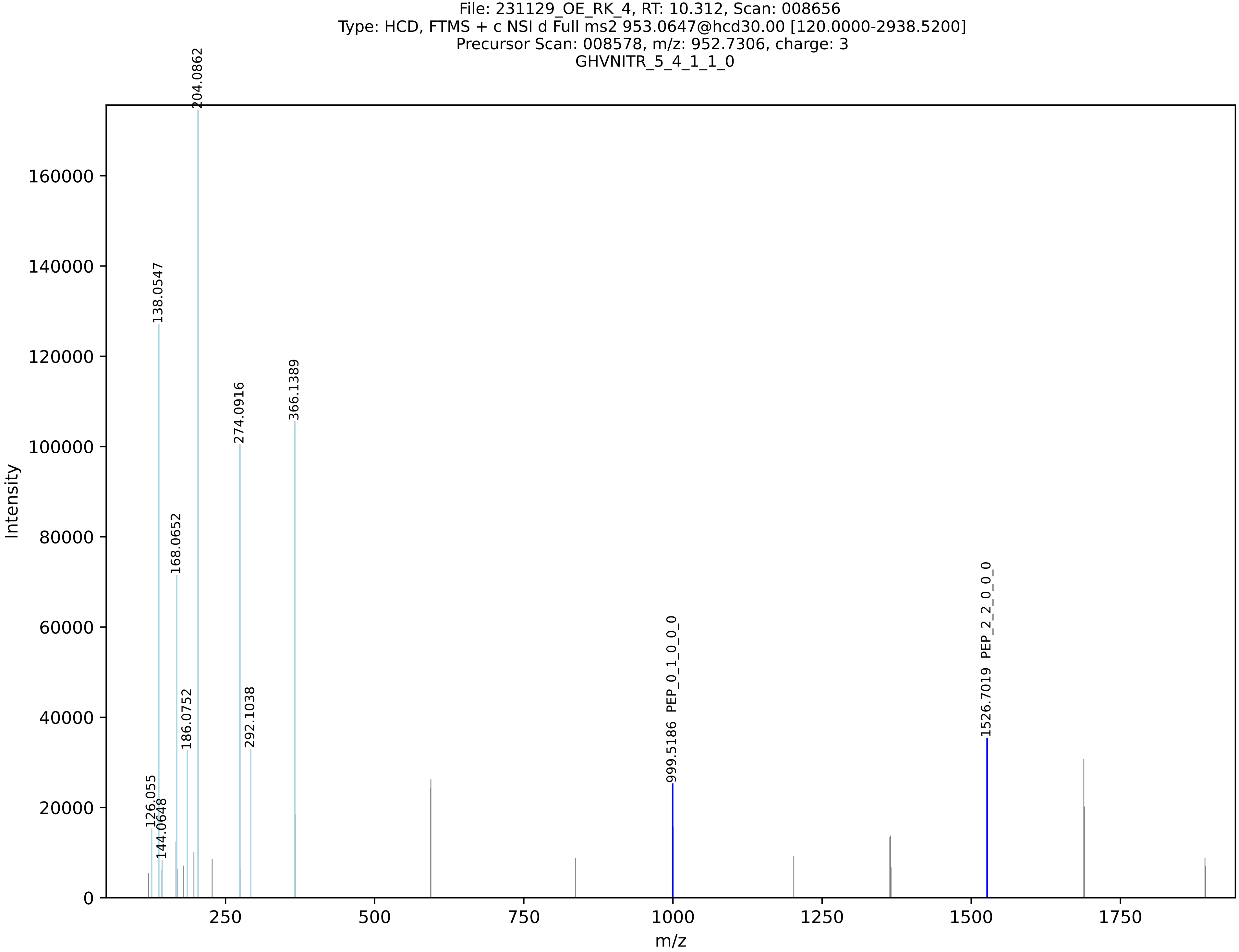

File: 231129_OE_RK_4, RT: 10.312, Scan: 008656
Type: HCD, FTMS + c NSI d Full ms2 953.0647@hcd30.00 [120.0000-2938.5200]
Precursor Scan: 008578, m/z: 952.7306, charge: 3
GHVNITR_5_4_1_1_0
Intensity
m/z
204.0862
138.0547
168.0652
274.0916
366.1389
186.0752
292.1038
126.055
144.0648
999.5186 PEP_0_1_0_0_0
1526.7019 PEP_2_2_0_0_0
0
20000
40000
60000
80000
100000
120000
140000
160000
250
500
750
1000
1250
1500
1750

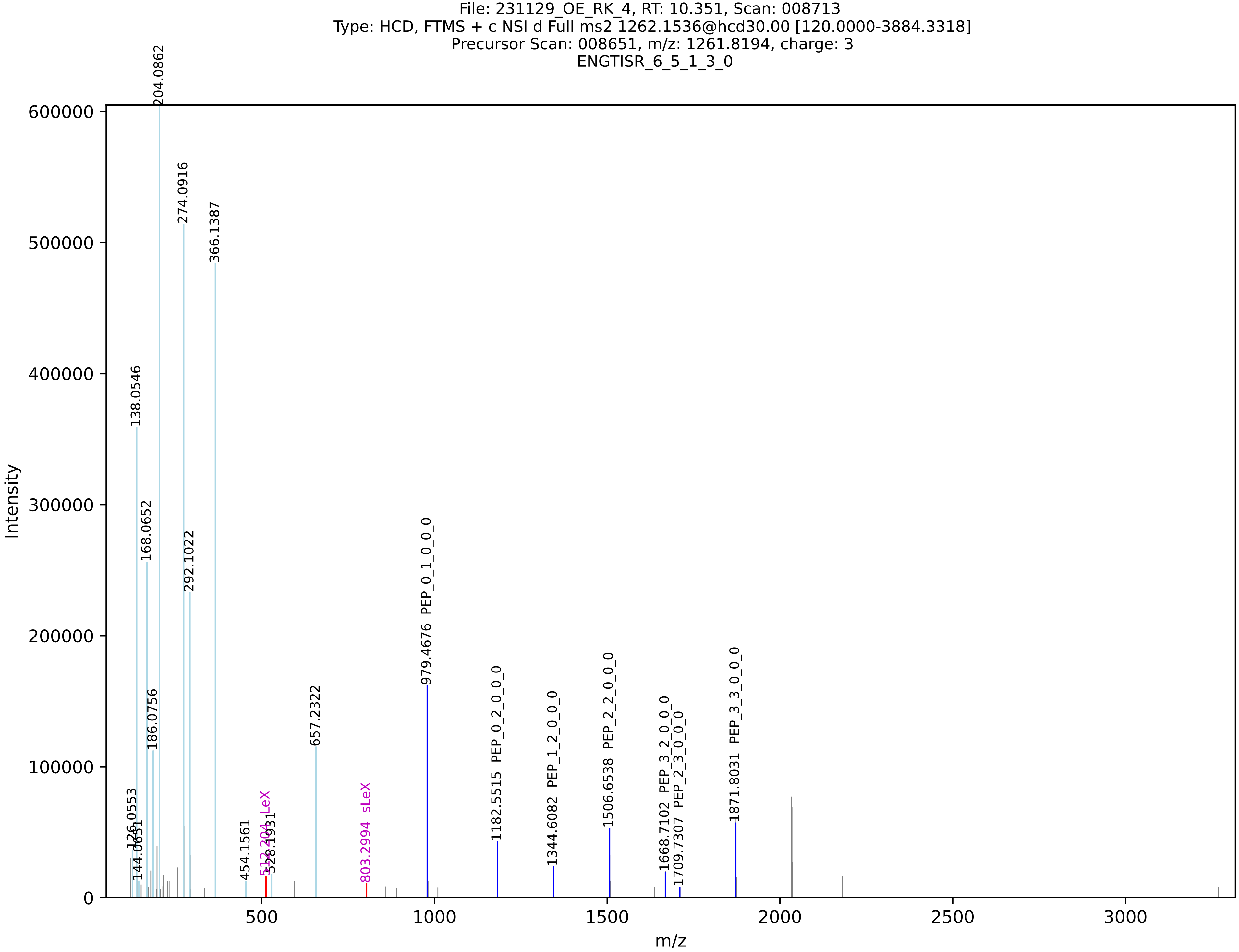
File: 231129_OE_RK_4, RT: 10.351, Scan: 008713
Type: HCD, FTMS + c NSI d Full ms2 1262.1536@hcd30.00 [120.0000-3884.3318]
Precursor Scan: 008651, m/z: 1261.8194, charge: 3
ENGTISR_6_5_1_3_0
Intensity
m/z
204.0862
274.0916
366.1387
138.0546
168.0652
292.1022
186.0756
126.0553
144.0651
454.1561
512.204 LeX
528.1931
657.2322
803.2994 sLeX
979.4676 PEP_0_1_0_0_
1182.5515 PEP_0_2_0_0_0
1344.6082 PEP_1_2_0_0_0
1506.6538 PEP_2_2_0_0_
1668.7102 PEP_3_2_0_0_0
1709.7307 PEP_2_3_0_0_
1871.8031 PEP_3_3_0_0_0

File: 231129_OE_RK_4, RT: 10.354, Scan: 008718
Type: HCD, FTMS + c NSI d Full ms2 1090.7606@hcd30.00 [120.0000-3359.8694]
Precursor Scan: 008651, m/z: 1090.0924, charge: 3
TMQENSTPR_5_4_0_2_0

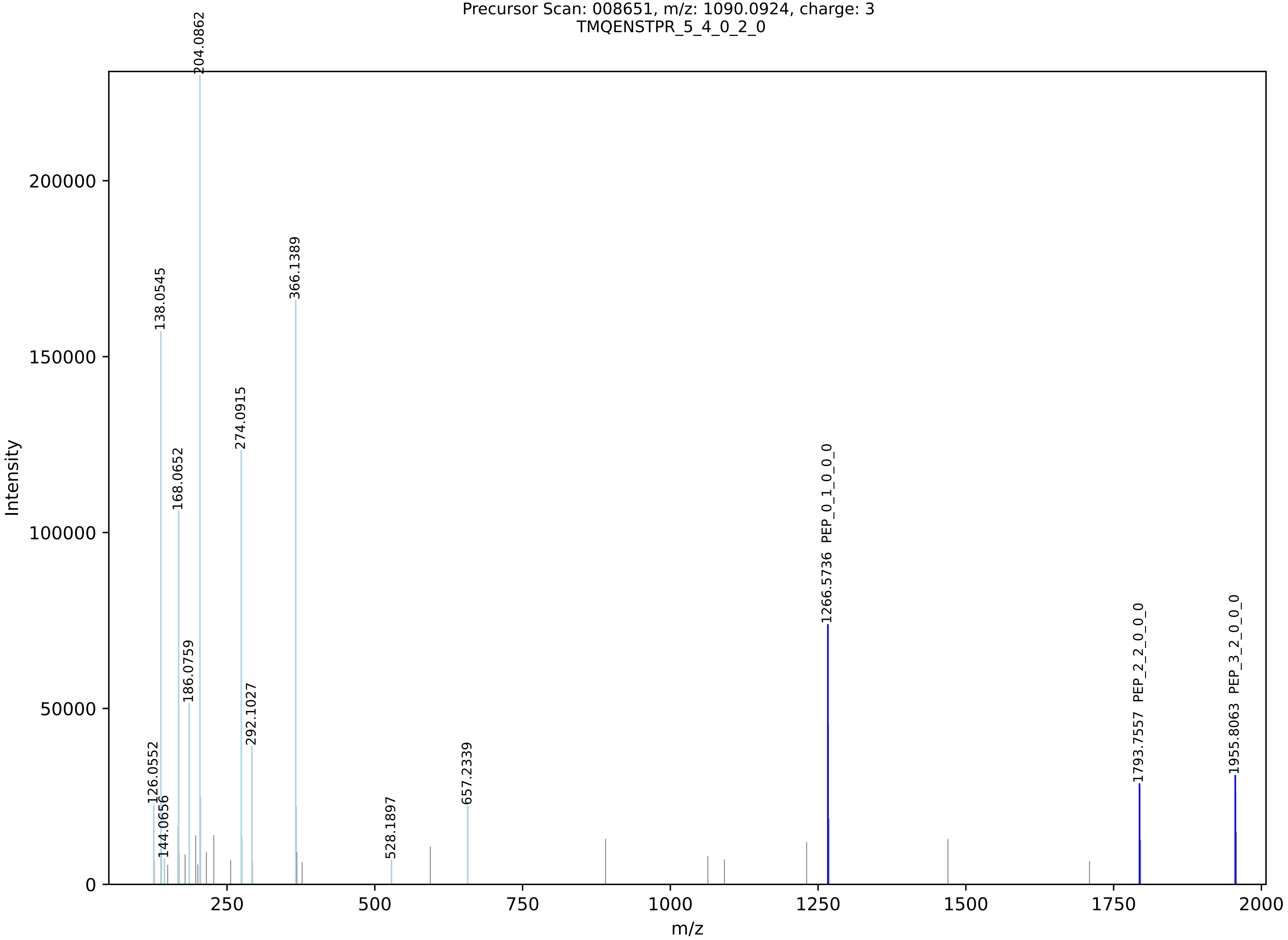

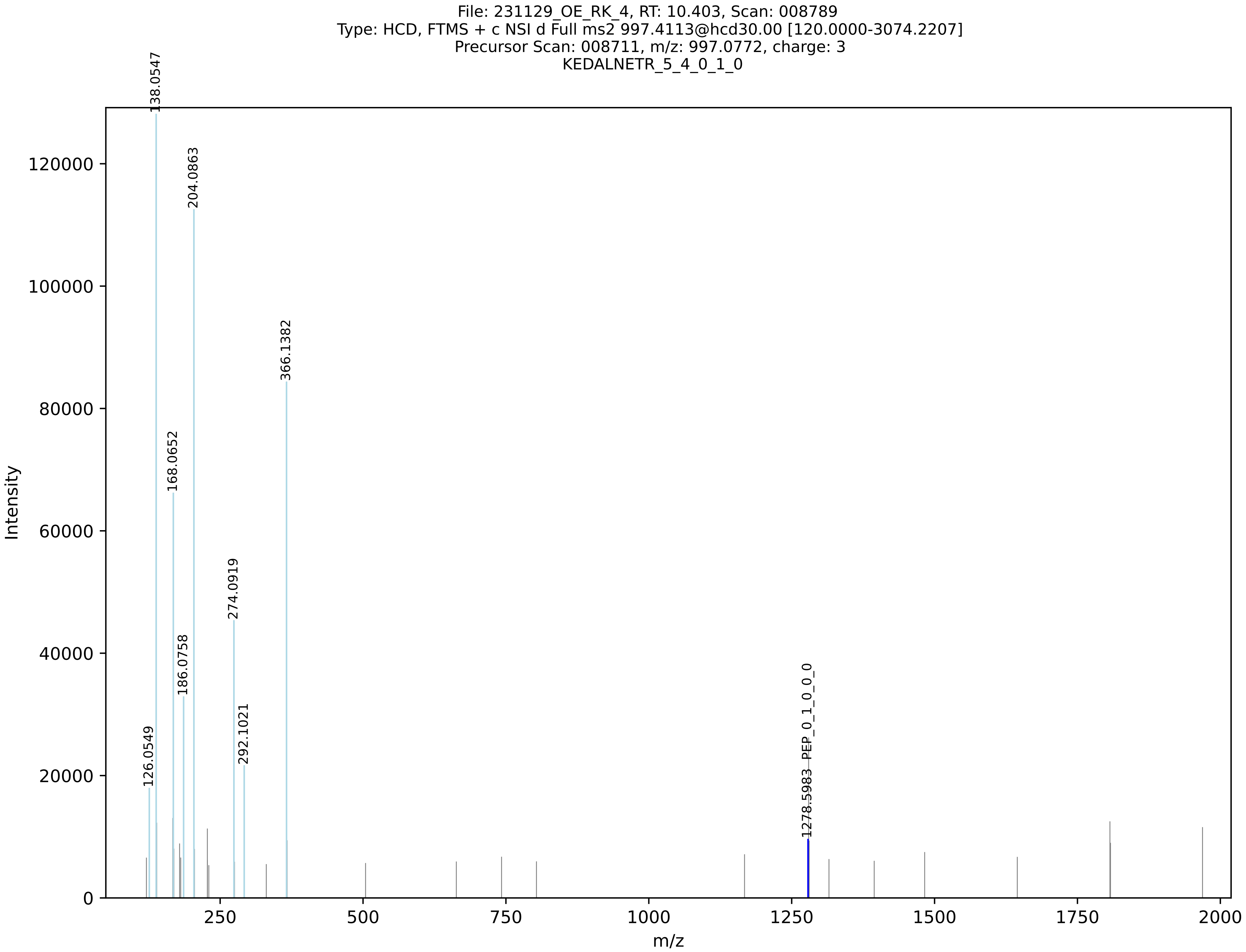
File: 231129_OE_RK_4, RT: 10.403, Scan: 008789
Type: HCD, FTMS + c NSI d Full ms2 997.4113@hcd30.00 [120.0000-3074.2207]
Precursor Scan: 008711, m/z: 997.0772, charge: 3
KEDALNETR_5_4_0_1_0
138.0547
204.0863
366.1382
168.0652
274.0919
186.0758
292.1021
126.0549
1278.5983_PEP_0_1_0_0_0
Intensity
m/z
0
20000
40000
60000
80000
100000
120000
250
500
750
1000
1250
1500
1750
2000

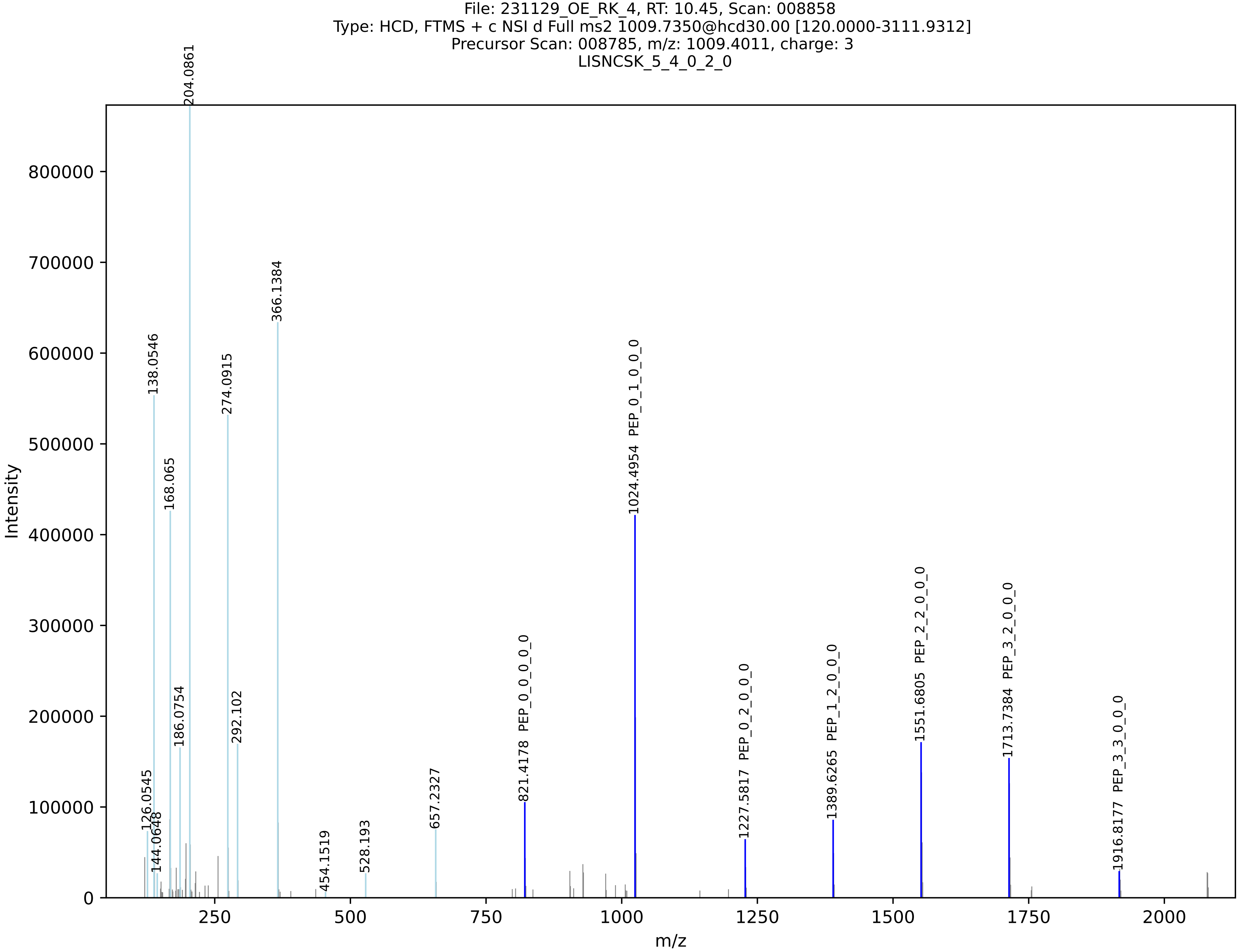
File: 231129_OE_RK_4, RT: 10.45, Scan: 008858
Type: HCD, FTMS + c NSI d Full ms2 1009.7350@hcd30.00 [120.0000-3111.9312]
Precursor Scan: 008785, m/z: 1009.4011, charge: 3
LISNCSK_5_4_0_2_0
Intensity
m/z
204.0861
366.1384
138.0546
274.0915
168.065
186.0754
292.102
126.0545
144.0648
454.1519
528.193
657.2327
821.4178 PEP_0_0_0_0_0
1024.4954 PEP_0_1_0_0_0
1227.5817 PEP_0_2_0_0_0
1389.6265 PEP_1_2_0_0_0
1551.6805 PEP_2_2_0_0_0
1713.7384 PEP_3_2_0_0_0
1916.8177 PEP_3_3_0_0_0

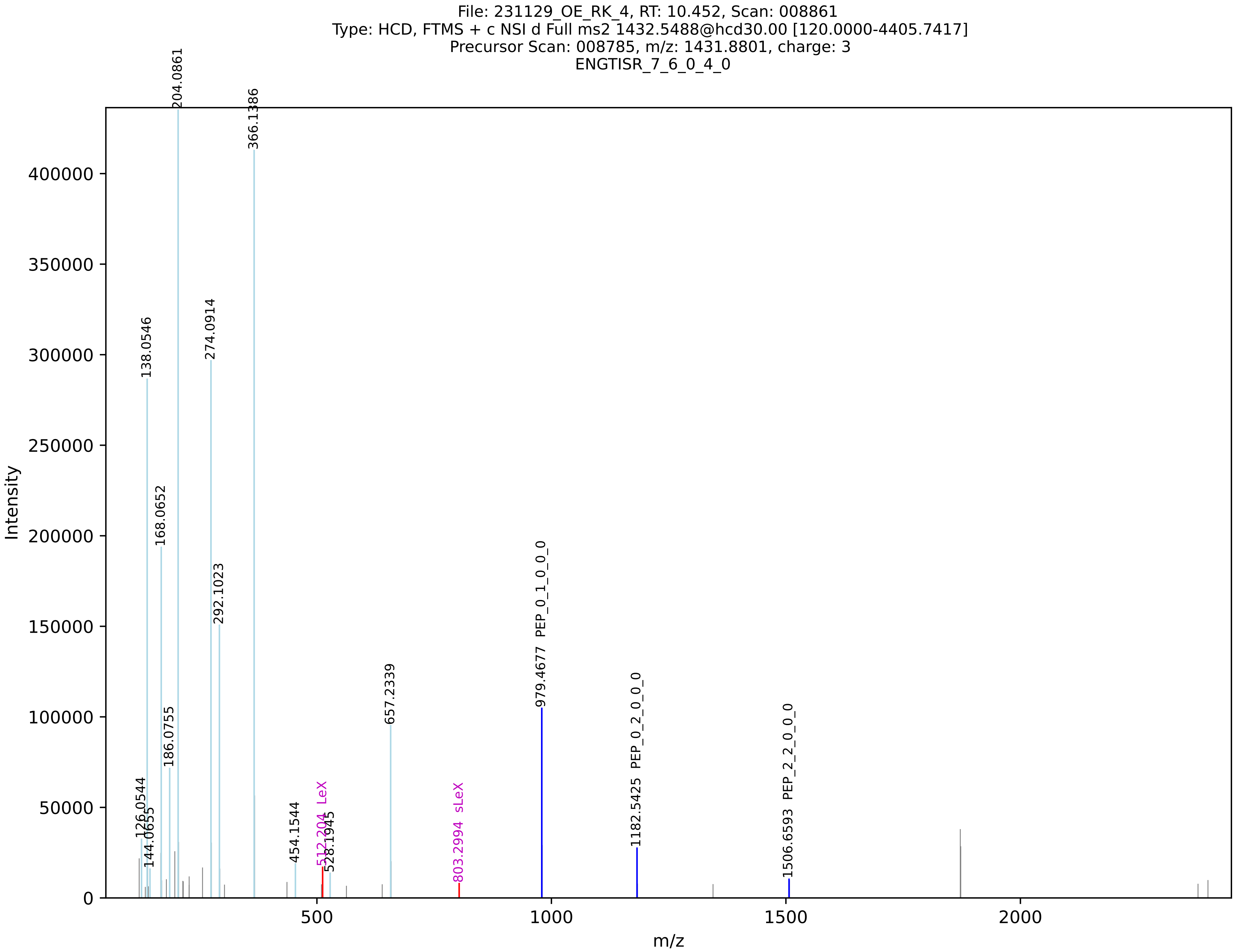
File: 231129_OE_RK_4, RT: 10.452, Scan: 008861
Type: HCD, FTMS + c NSI d Full ms2 1432.5488@hcd30.00 [120.0000-4405.7417]
Precursor Scan: 008785, m/z: 1431.8801, charge: 3
ENGTISR_7_6_0_4_0
Intensity
m/z
204.0861
366.1386
138.0546
274.0914
168.0652
292.1023
186.0755
126.0544
144.0655
454.1544
512.204 LeX
528.1945
657.2339
803.2994 sLeX
979.4677 PEP_0_1_0_0_0
1182.5425 PEP_0_2_0_0_0
1506.6593 PEP_2_2_0_0_0
0
50000
100000
150000
200000
250000
300000
350000
400000
500
1000
1500
2000

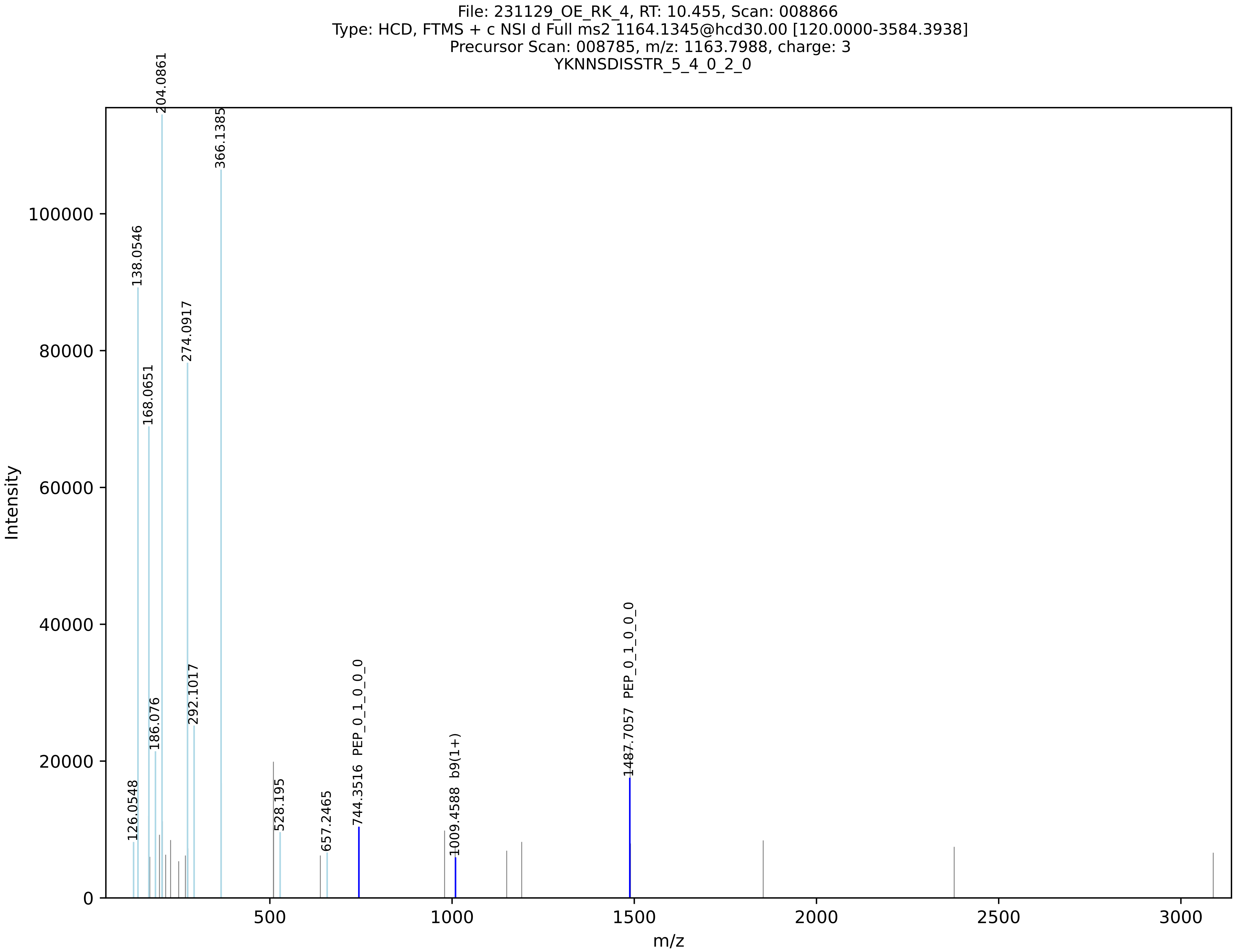
File: 231129_OE_RK_4, RT: 10.455, Scan: 008866
Type: HCD, FTMS + c NSI d Full ms2 1164.1345@hcd30.00 [120.0000-3584.3938]
Precursor Scan: 008785, m/z: 1163.7988, charge: 3
YKNNSDISSTR_5_4_0_2_0
204.0861
366.1385
138.0546
274.0917
168.0651
292.1017
186.076
126.0548
528.195
657.2465
744.3516 PEP_0_1_0_0_0
1009.4588 b9(1+)
1487.7057 PEP_0_1_0_0_0
Intensity
m/z
0
20000
40000
60000
80000
100000
500
1000
1500
2000
2500
3000

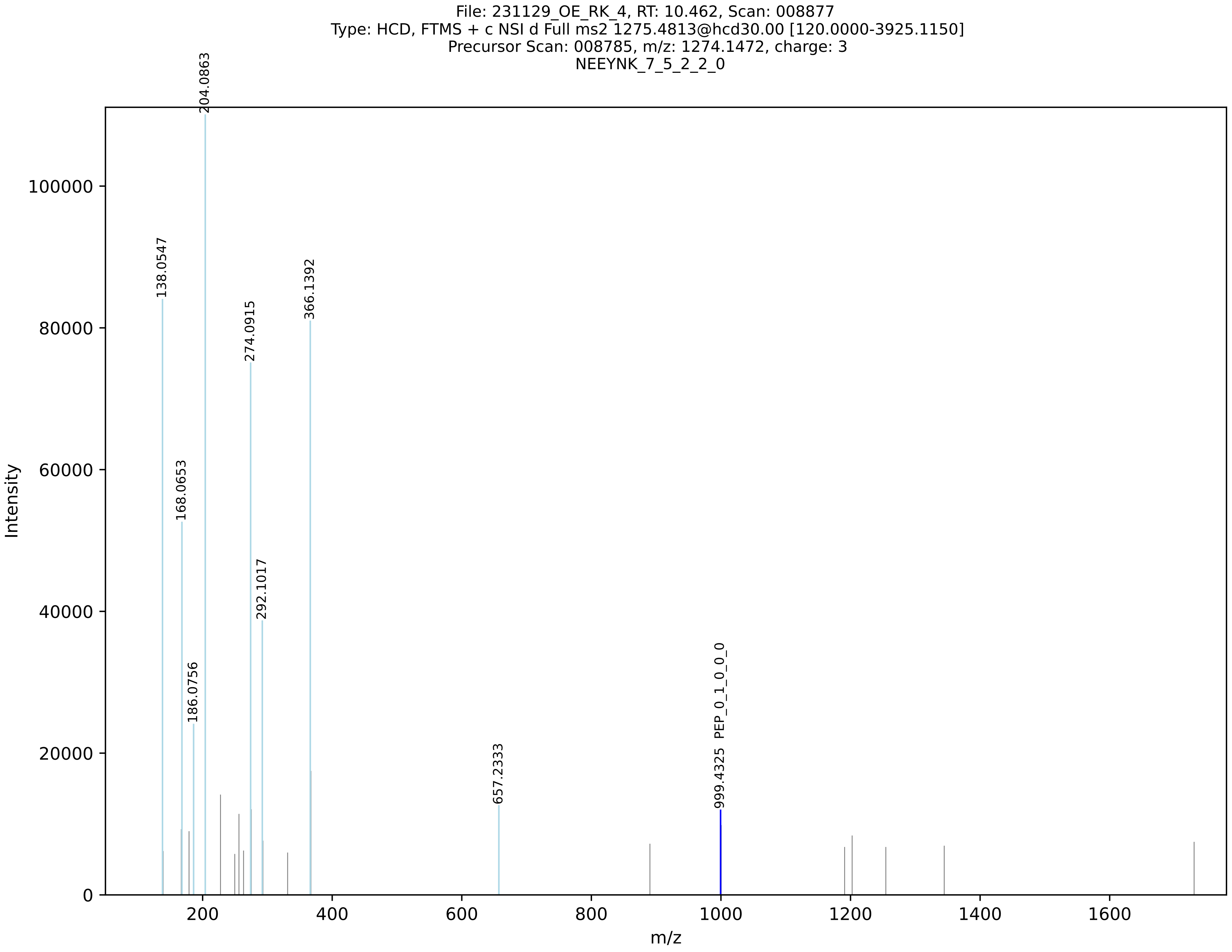
File: 231129_OE_RK_4, RT: 10.462, Scan: 008877
Type: HCD, FTMS + c NSI d Full ms2 1275.4813@hcd30.00 [120.0000-3925.1150]
Precursor Scan: 008785, m/z: 1274.1472, charge: 3
NEEYNK_7_5_2_2_0
Intensity
m/z
204.0863
138.0547
366.1392
274.0915
168.0653
292.1017
186.0756
657.2333
999.4325 PEP_0_1_0_0_0
0
20000
40000
60000
80000
100000
200
400
600
800
1000
1200
1400
1600

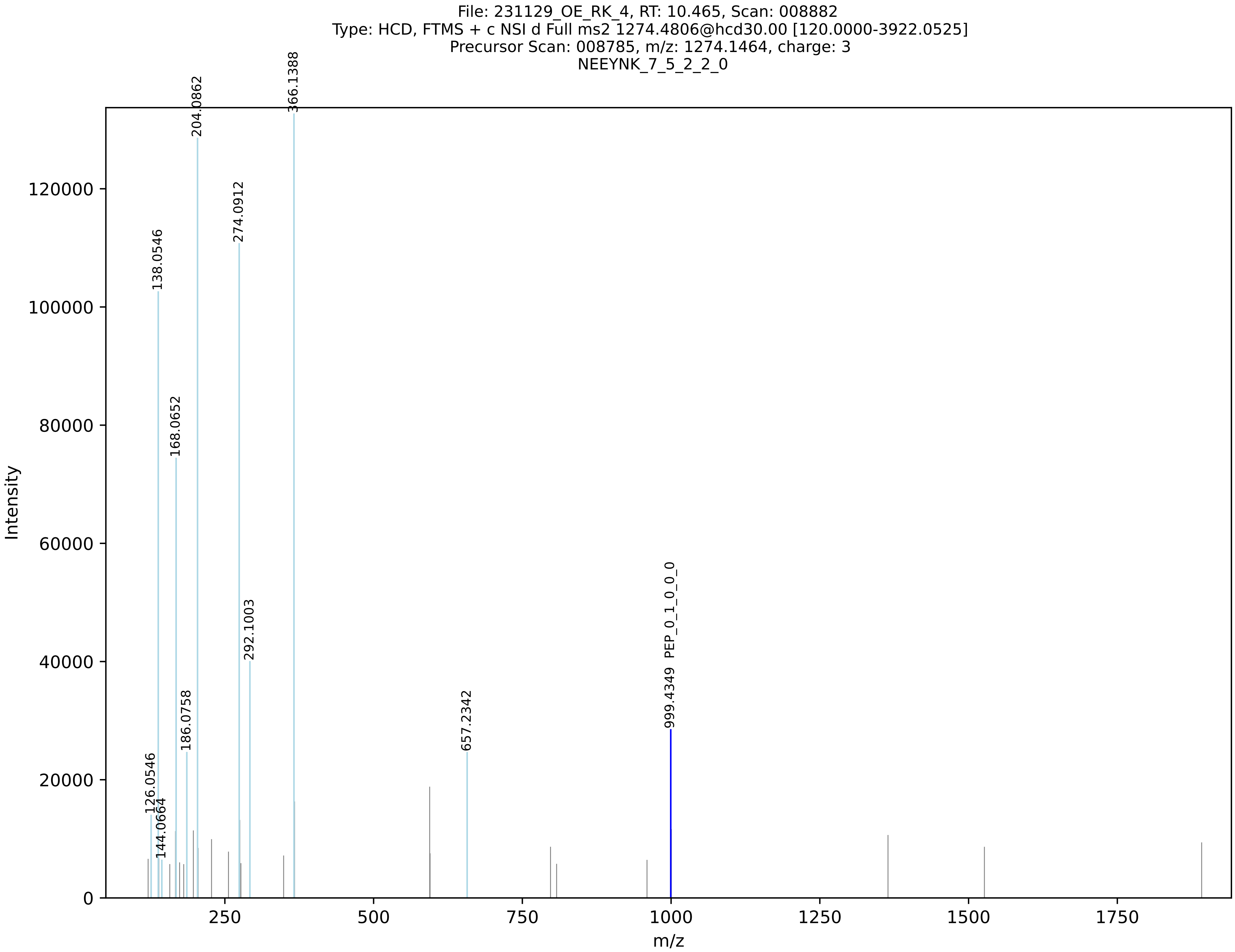

File: 231129_OE_RK_4, RT: 10.465, Scan: 008882
Type: HCD, FTMS + c NSI d Full ms2 1274.4806@hcd30.00 [120.0000-3922.0525]
Precursor Scan: 008785, m/z: 1274.1464, charge: 3
NEEYNK_7_5_2_2_0
366.1388
204.0862
274.0912
138.0546
168.0652
292.1003
186.0758
126.0546
144.0664
657.2342
999.4349 PEP_0_1_0_0_0
Intensity
m/z
0
20000
40000
60000
80000
100000
120000
250
500
750
1000
1250
1500
1750

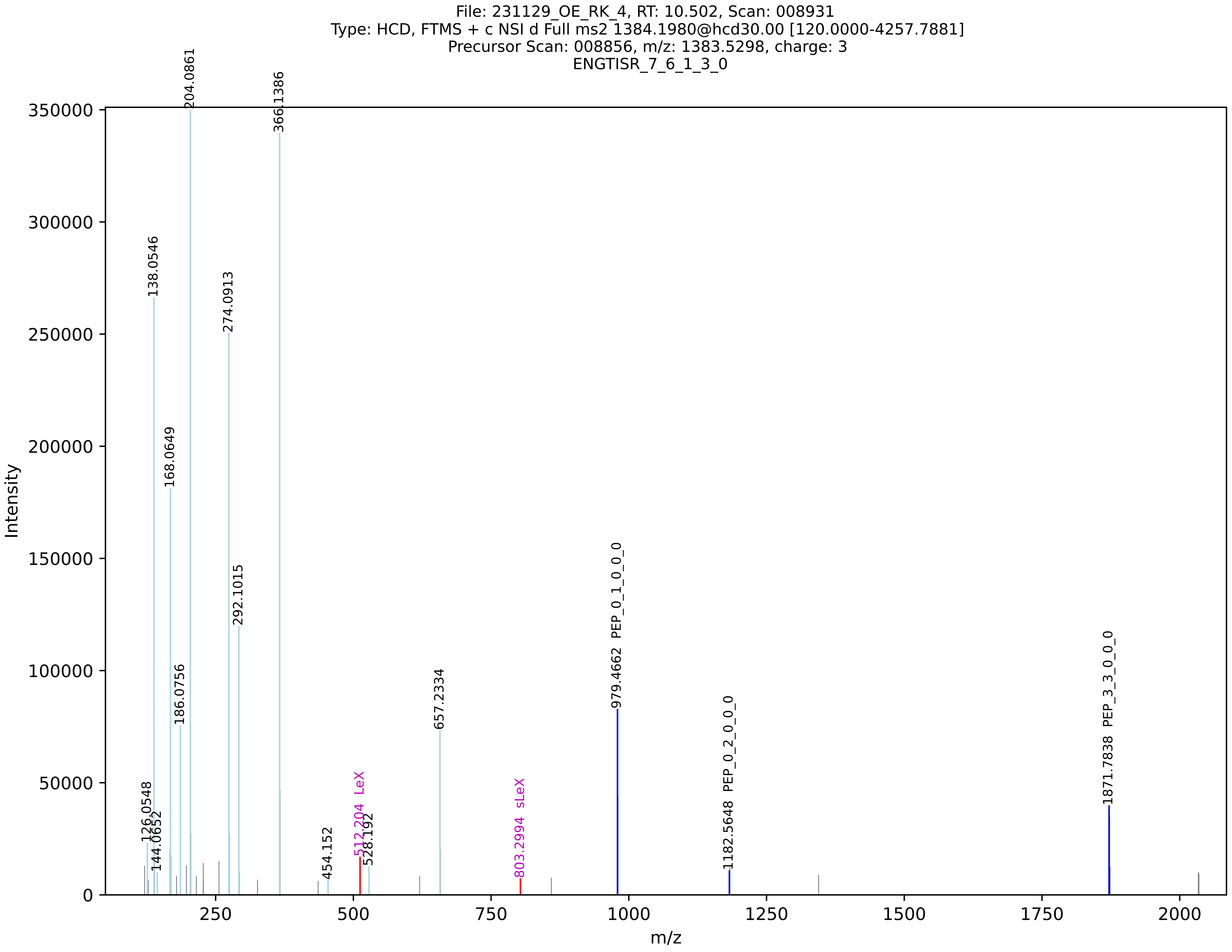
File: 231129_OE_RK_4, RT: 10.502, Scan: 008931
Type: HCD, FTMS + c NSI d Full ms2 1384.1980@hcd30.00 [120.0000-4257.7881]
Precursor Scan: 008856, m/z: 1383.5298, charge: 3
ENGTISR_7_6_1_3_0
Intensity
m/z
204.0861
366.1386
138.0546
274.0913
168.0649
292.1015
186.0756
126.0548
144.0652
454.152
512.204 LeX
528.192
657.2334
803.2994 sLeX
979.4662 PEP_0_1_0_0_0
1182.5648 PEP_0_2_0_0_0
1871.7838 PEP_3_3_0_0_0
350000
300000
250000
200000
150000
100000
50000
0
250
500
750
1000
1250
1500
1750
2000

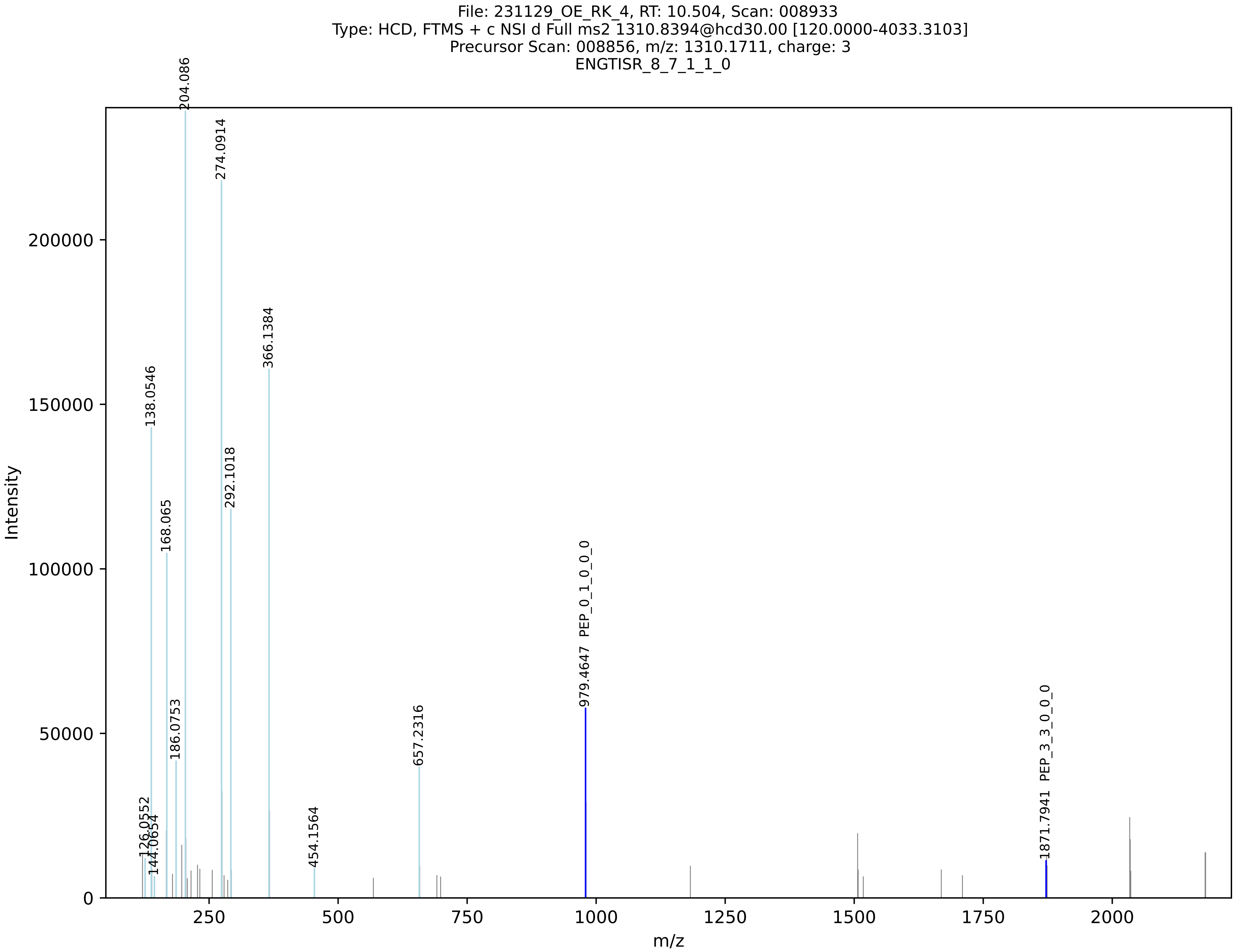
File: 231129_OE_RK_4, RT: 10.504, Scan: 008933
Type: HCD, FTMS + c NSI d Full ms2 1310.8394@hcd30.00 [120.0000-4033.3103]
Precursor Scan: 008856, m/z: 1310.1711, charge: 3
ENGTISR_8_7_1_1_0
Intensity
m/z
204.086
274.0914
366.1384
138.0546
292.1018
168.065
186.0753
126.0552
144.0654
454.1564
657.2316
979.4647 PEP_0_1_0_0_0
1871.7941 PEP_3_3_0_0_0

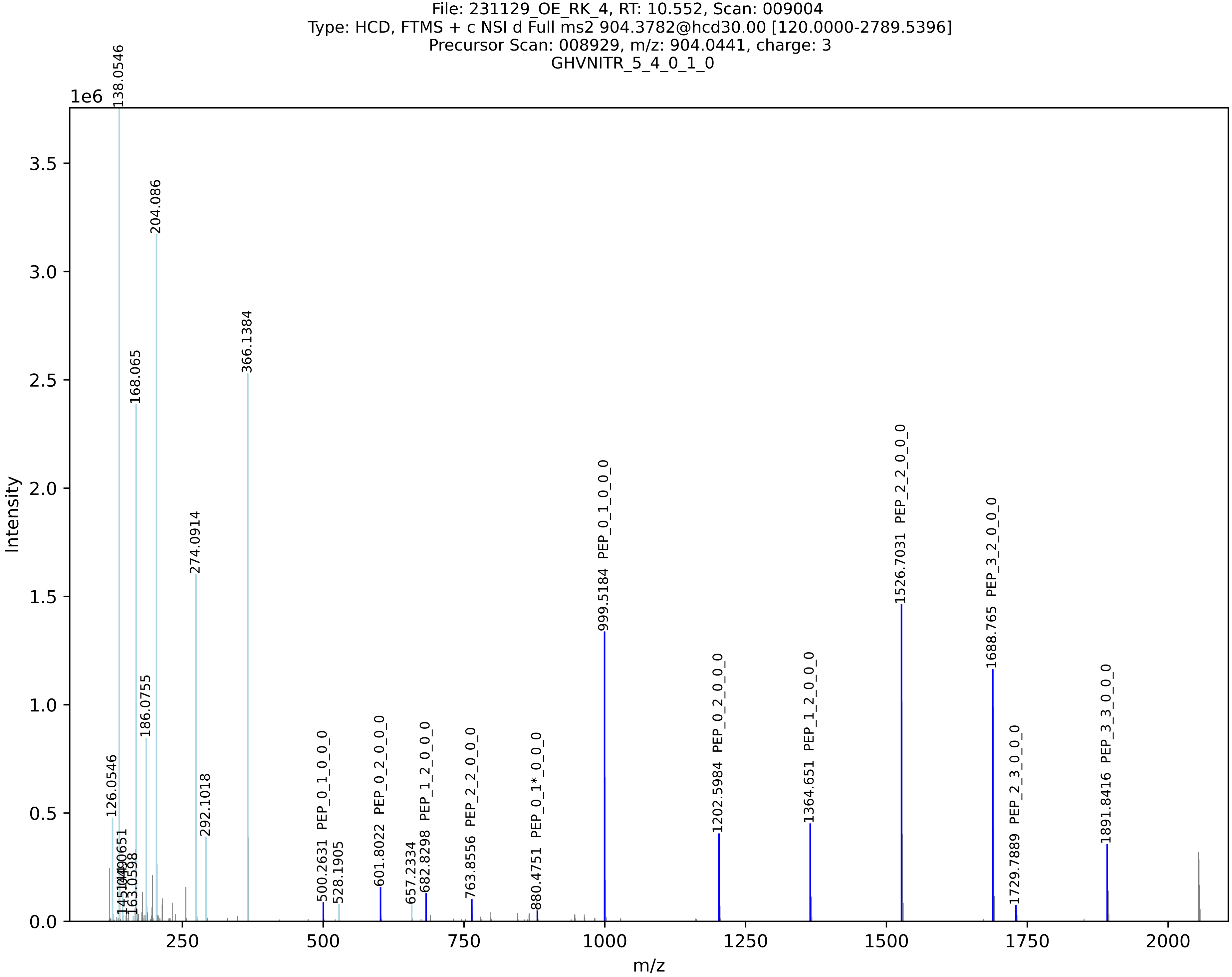
File: 231129_OE_RK_4, RT: 10.552, Scan: 009004
Type: HCD, FTMS + c NSI d Full ms2 904.3782@hcd30.00 [120.0000-2789.5396]
Precursor Scan: 008929, m/z: 904.0441, charge: 3
GHVNITR_5_4_0_1_0
1e6
Intensity
m/z
0.0
0.5
1.0
1.5
2.0
2.5
3.0
3.5
250
500
750
1000
1250
1500
1750
2000
138.0546
204.086
366.1384
168.065
274.0914
186.0755
126.0546
292.1018
163.0598
500.2631 PEP_0_1_0_0_0
528.1905
601.8022 PEP_0_2_0_0_0
657.2334
682.8298 PEP_1_2_0_0_0
763.8556 PEP_2_2_0_0_0
880.4751 PEP_0_1*_0_0_0
999.5184 PEP_0_1_0_0_0
1202.5984 PEP_0_2_0_0_0
1364.651 PEP_1_2_0_0_0
1526.7031 PEP_2_2_0_0_0
1688.765 PEP_3_2_0_0_0
1729.7889 PEP_2_3_0_0_0
1891.8416 PEP_3_3_0_0_0

File: 231129_OE_RK_4, RT: 10.553, Scan: 009005
Type: HCD, FTMS + c NSI d Full ms2 1262.1538@hcd30.00 [120.0000-3884.3325]
Precursor Scan: 008929, m/z: 1261.4863, charge: 3
ENGTISR_6_5_1_3_0

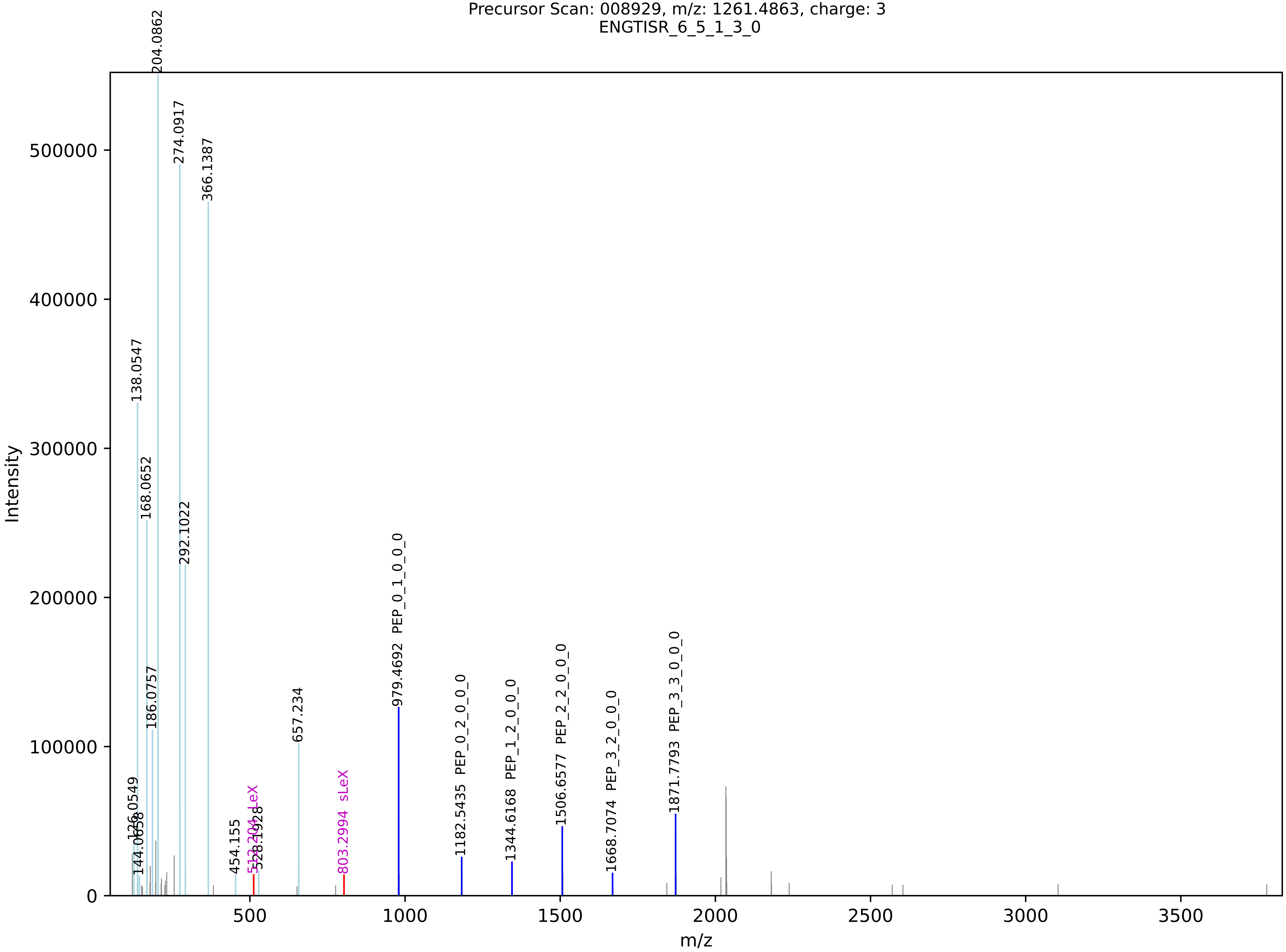

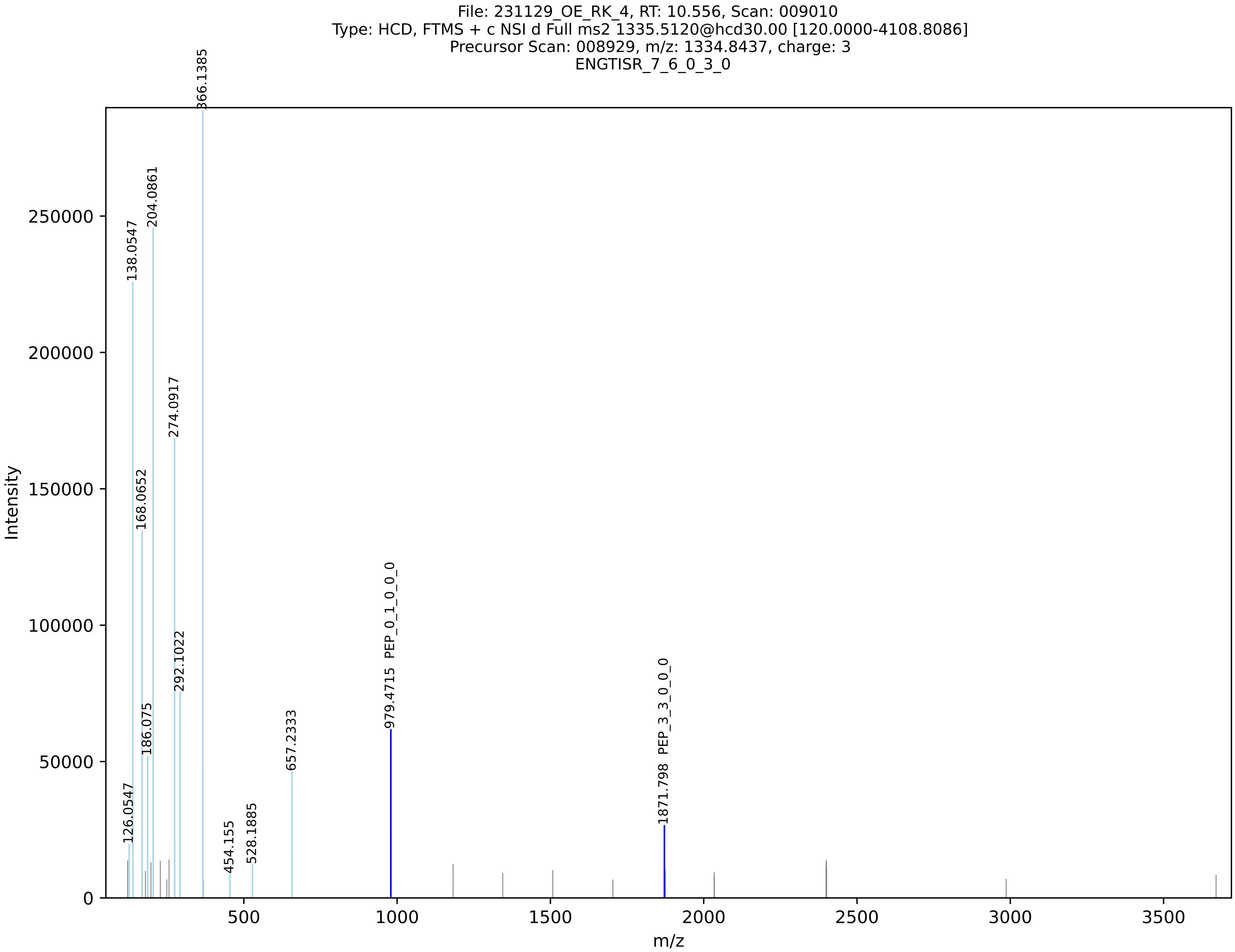

File: 231129_OE_RK_4, RT: 10.556, Scan: 009010
Type: HCD, FTMS + c NSI d Full ms2 1335.5120@hcd30.00 [120.0000-4108.8086]
Precursor Scan: 008929, m/z: 1334.8437, charge: 3
ENGTISR_7_6_0_3_0
366.1385
204.0861
138.0547
274.0917
168.0652
292.1022
186.075
126.0547
454.155
528.1885
657.2333
979.4715 PEP_0_1_0_0_0
1871.798 PEP_3_3_0_0_0
Intensity
m/z
0
50000
100000
150000
200000
250000
500
1000
1500
2000
2500
3000
3500

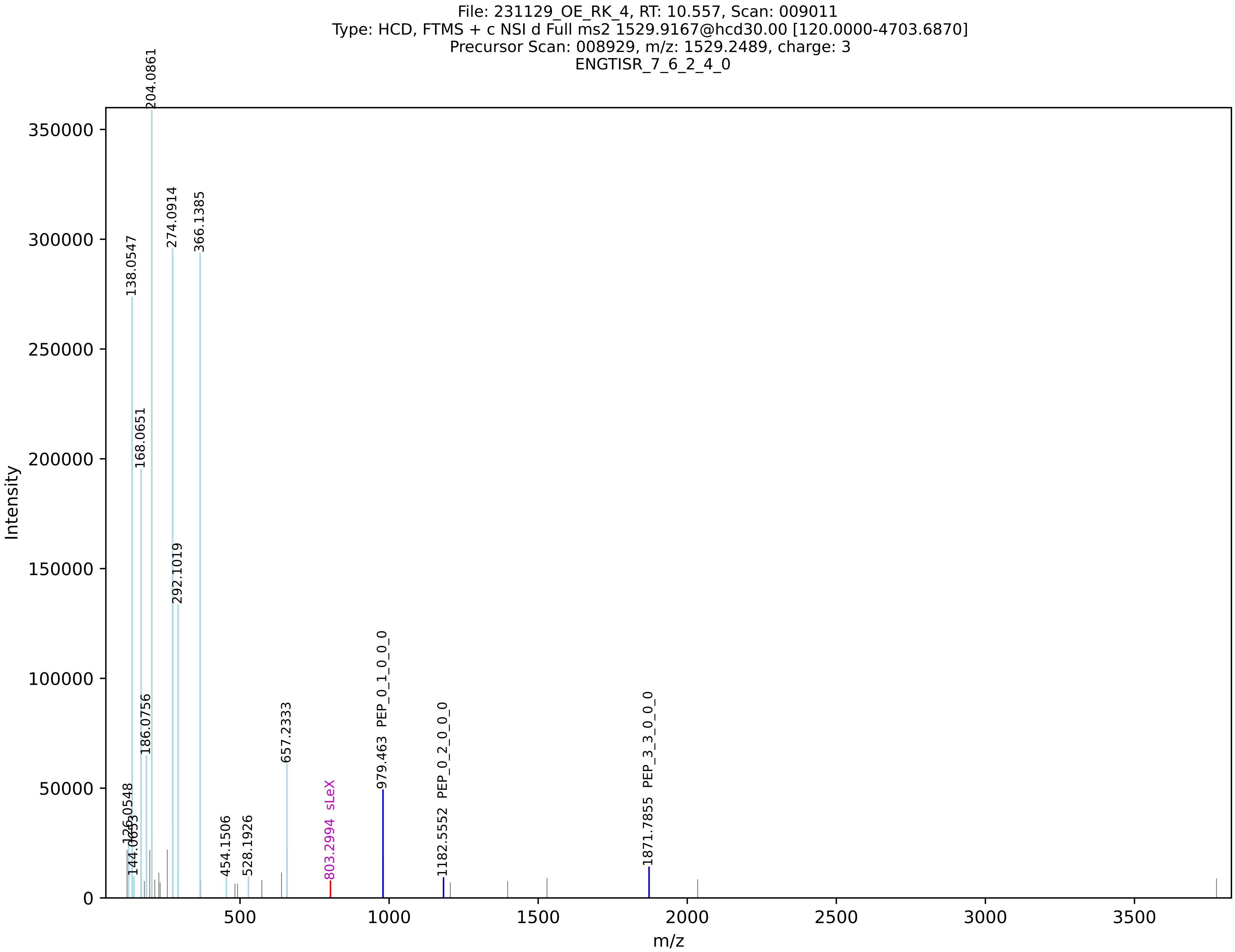
File: 231129_OE_RK_4, RT: 10.557, Scan: 009011
Type: HCD, FTMS + c NSI d Full ms2 1529.9167@hcd30.00 [120.0000-4703.6870]
Precursor Scan: 008929, m/z: 1529.2489, charge: 3
ENGTISR_7_6_2_4_0
Intensity
m/z
204.0861
274.0914
366.1385
138.0547
168.0651
292.1019
186.0756
657.2333
126.0548
144.0653
454.1506
528.1926
803.2994 sLeX
979.463 PEP_0_1_0_0_0
1182.5552 PEP_0_2_0_0_0
1871.7855 PEP_3_3_0_0_0

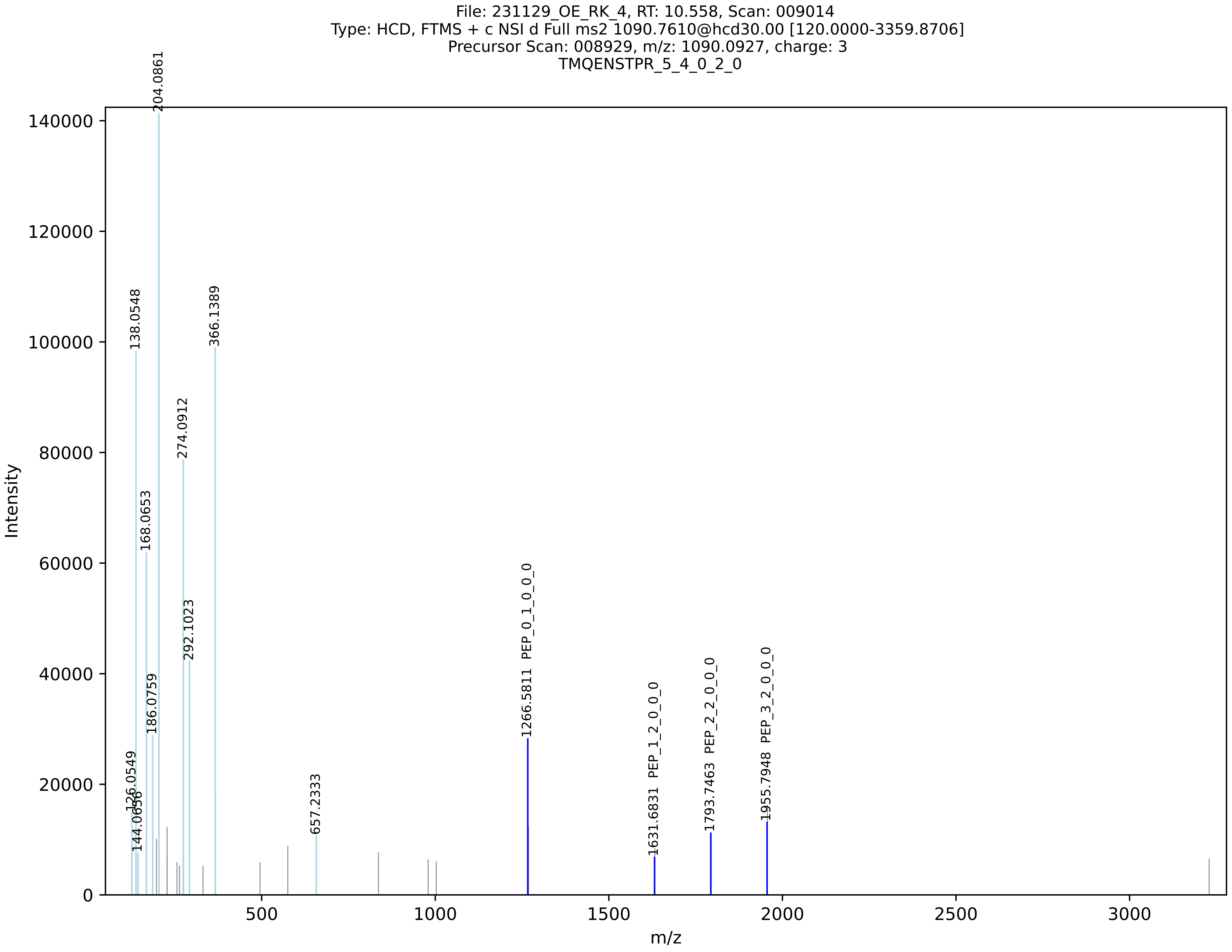

File: 231129_OE_RK_4, RT: 10.558, Scan: 009014
Type: HCD, FTMS + c NSI d Full ms2 1090.7610@hcd30.00 [120.0000-3359.8706]
Precursor Scan: 008929, m/z: 1090.0927, charge: 3
TMQENSTPR_5_4_0_2_0
Intensity
m/z
204.0861
138.0548
366.1389
274.0912
168.0653
292.1023
186.0759
126.0549
144.0656
657.2333
1266.5811 PEP_0_1_0_0_0
1631.6831 PEP_1_2_0_0_0
1793.7463 PEP_2_2_0_0_0
1955.7948 PEP_3_2_0_0_0

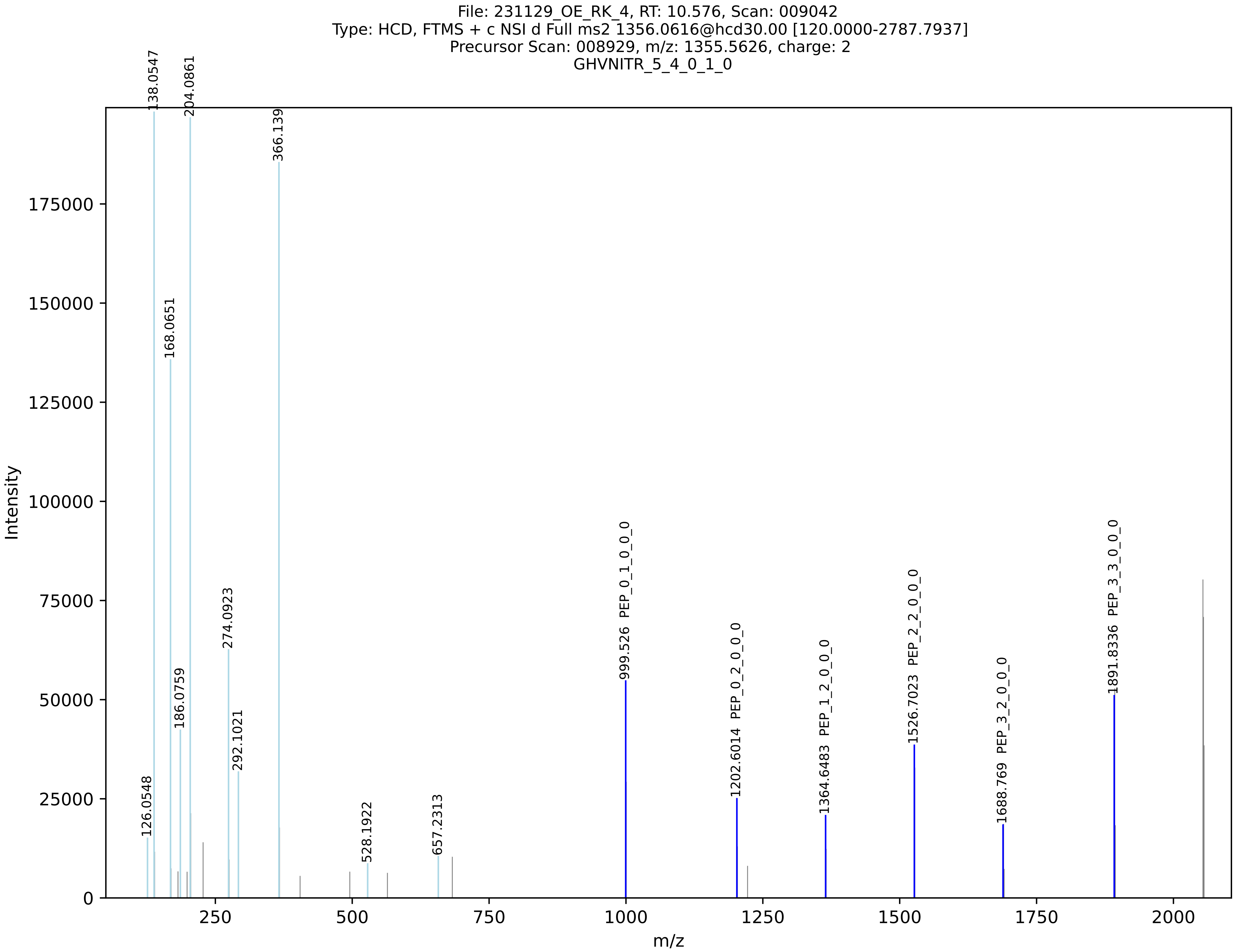

File: 231129_OE_RK_4, RT: 10.576, Scan: 009042
Type: HCD, FTMS + c NSI d Full ms2 1356.0616@hcd30.00 [120.0000-2787.7937]
Precursor Scan: 008929, m/z: 1355.5626, charge: 2
GHVNITR_5_4_0_1_0
Intensity
m/z
138.0547
204.0861
366.139
168.0651
186.0759
274.0923
292.1021
126.0548
528.1922
657.2313
999.526 PEP_0_1_0_0_0
1202.6014 PEP_0_2_0_0_0
1364.6483 PEP_1_2_0_0_0
1526.7023 PEP_2_2_0_0_0
1688.769 PEP_3_2_0_0_0
1891.8336 PEP_3_3_0_0_0

File: 231129_OE_RK_4, RT: 10.602, Scan: 009077
Type: HCD, FTMS + c NSI d Full ms2 1147.6896@hcd30.00 [120.0000-4705.4292]
Precursor Scan: 009000, m/z: 1147.1881, charge: 4
ENGTISR_7_6_2_4_0

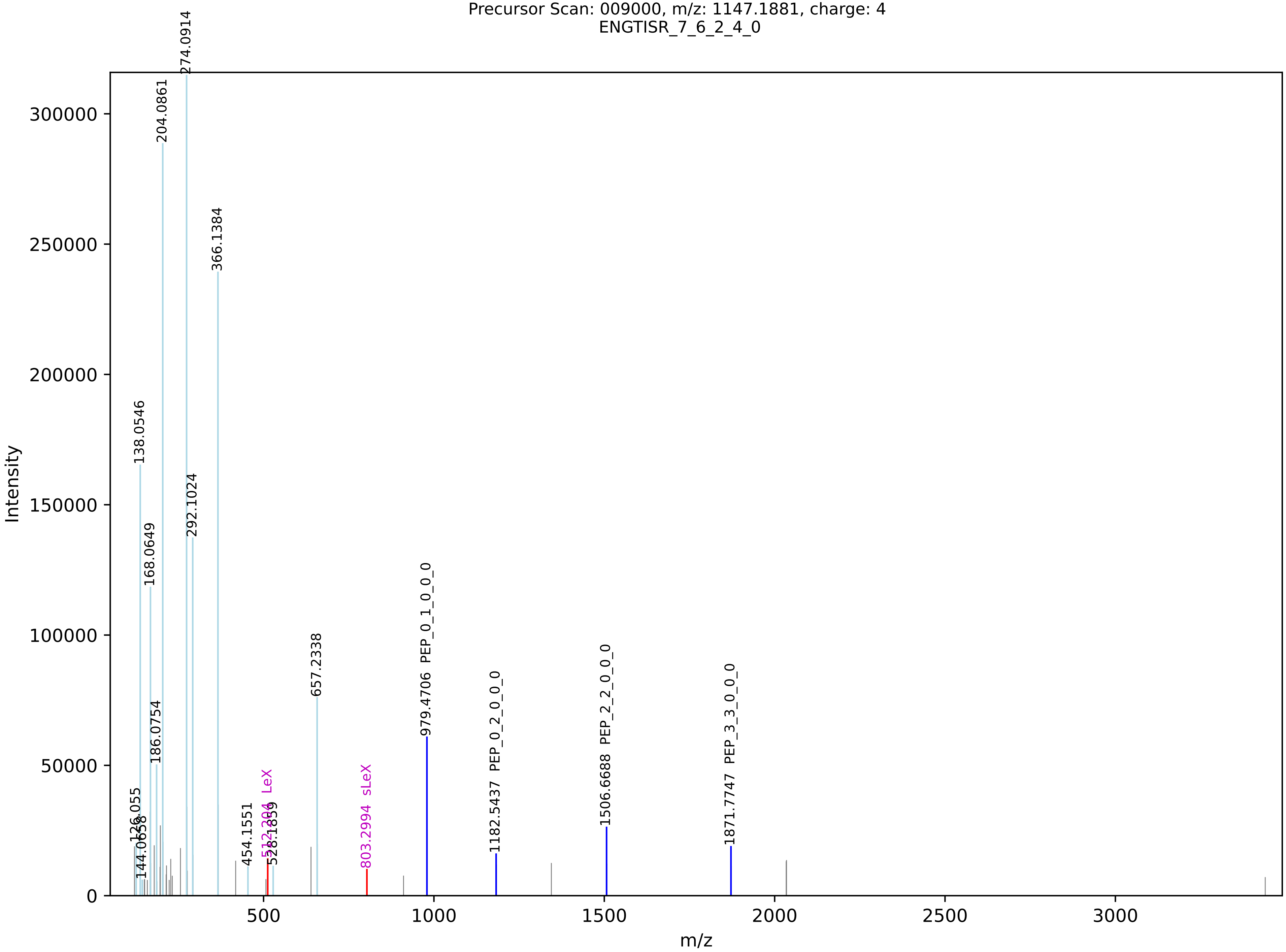

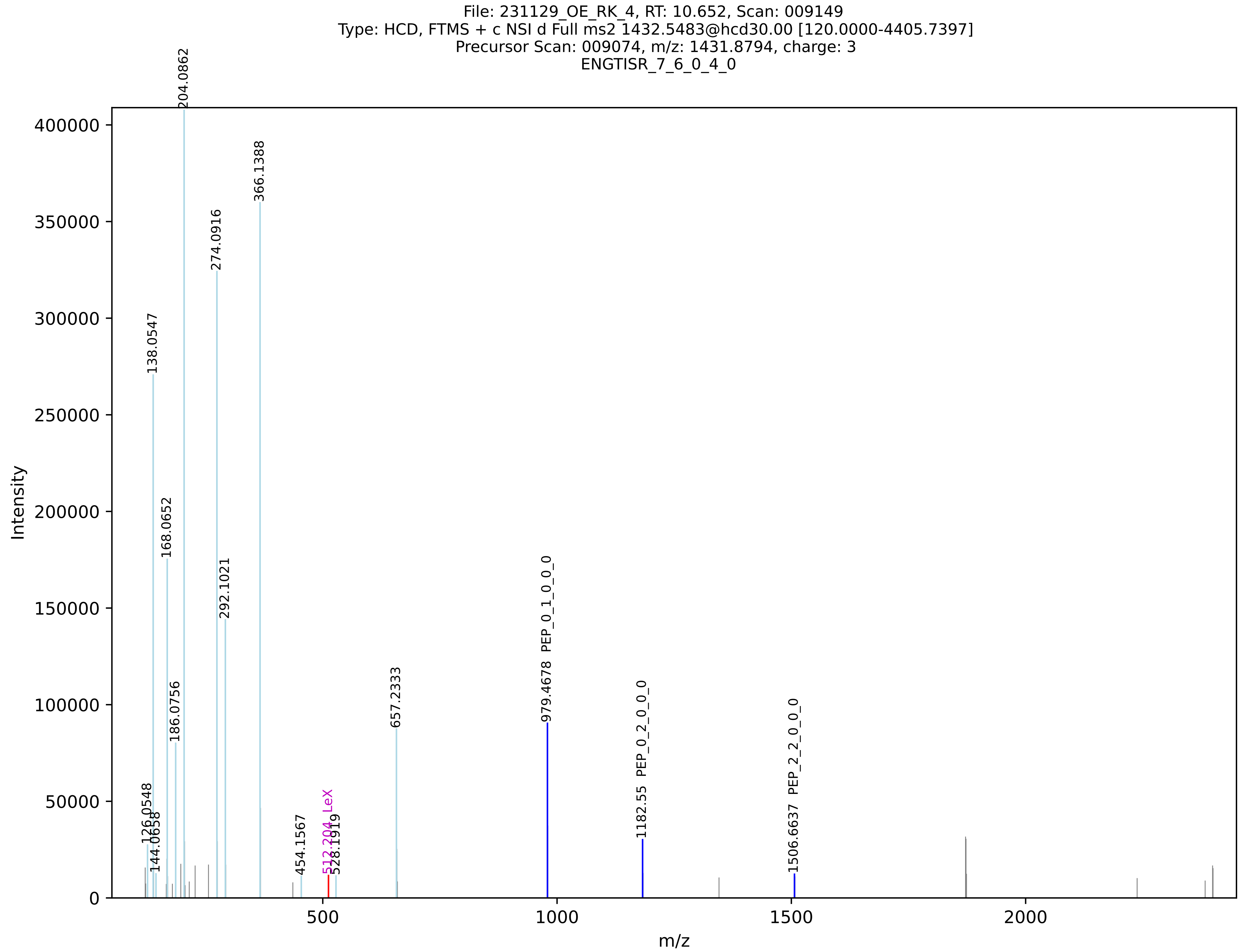
File: 231129_OE_RK_4, RT: 10.652, Scan: 009149
Type: HCD, FTMS + c NSI d Full ms2 1432.5483@hcd30.00 [120.0000-4405.7397]
Precursor Scan: 009074, m/z: 1431.8794, charge: 3
ENGTISR_7_6_0_4_0
Intensity
m/z
204.0862
366.1388
274.0916
138.0547
168.0652
292.1021
186.0756
126.0548
144.0658
454.1567
512.204 LeX
528.1919
657.2333
979.4678 PEP_0_1_0_0_0
1182.55 PEP_0_2_0_0_0
1506.6637 PEP_2_2_0_0_0
0
50000
100000
150000
200000
250000
300000
350000
400000
500
1000
1500
2000

File: 231129_OE_RK_4, RT: 10.703, Scan: 009224
Type: HCD, FTMS + c NSI d Full ms2 1038.1499@hcd30.00 [120.0000-4258.5073]
Precursor Scan: 009146, m/z: 1037.9007, charge: 4
ENGTISR_7_6_1_3_0

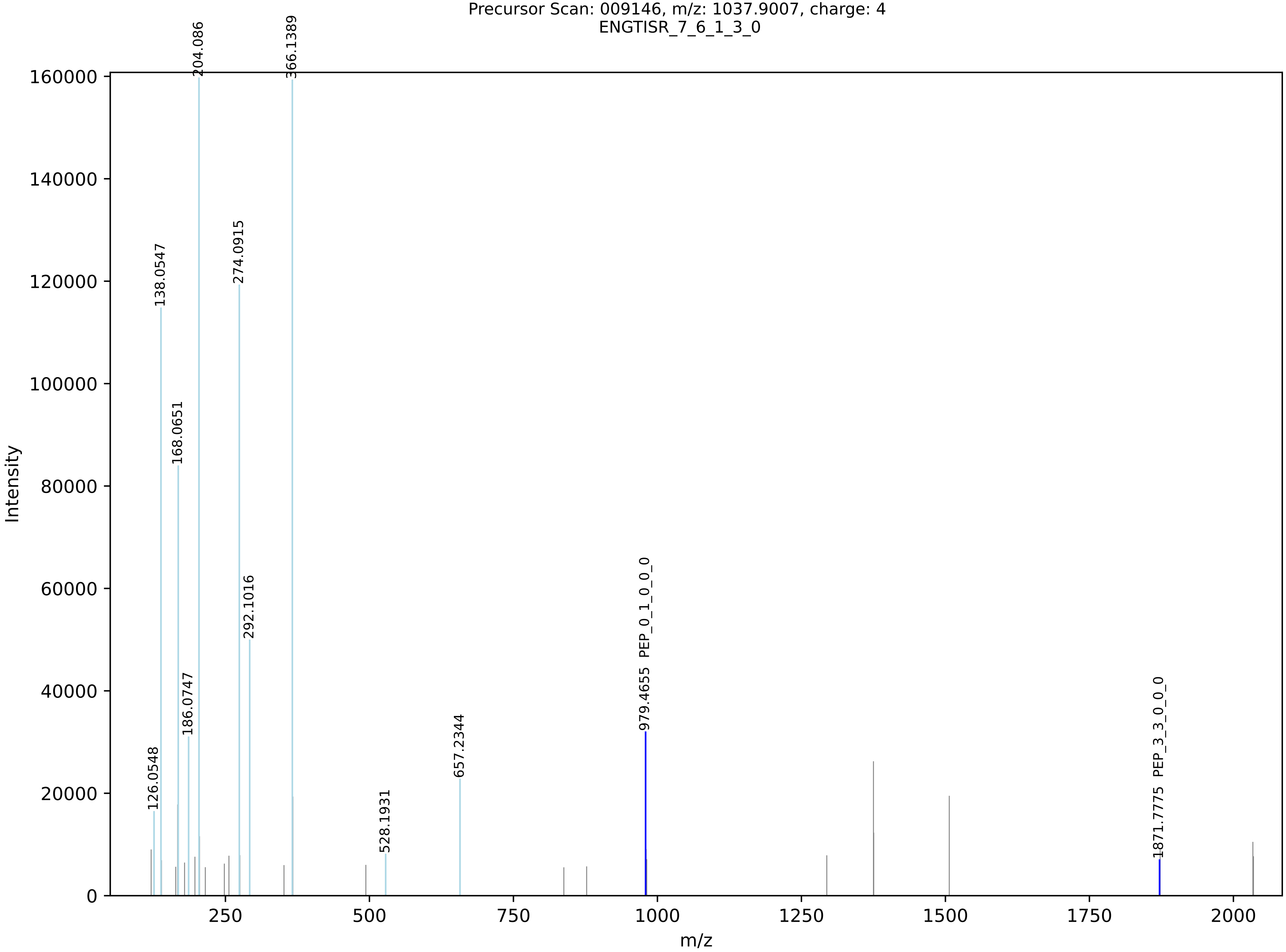

File: 231129_OE_RK_4, RT: 10.753, Scan: 009296
Type: HCD, FTMS + c NSI d Full ms2 904.3788@hcd30.00 [120.0000-2789.5413]
Precursor Scan: 009219, m/z: 904.0448, charge: 3
GHVNITR_5_4_0_1_0

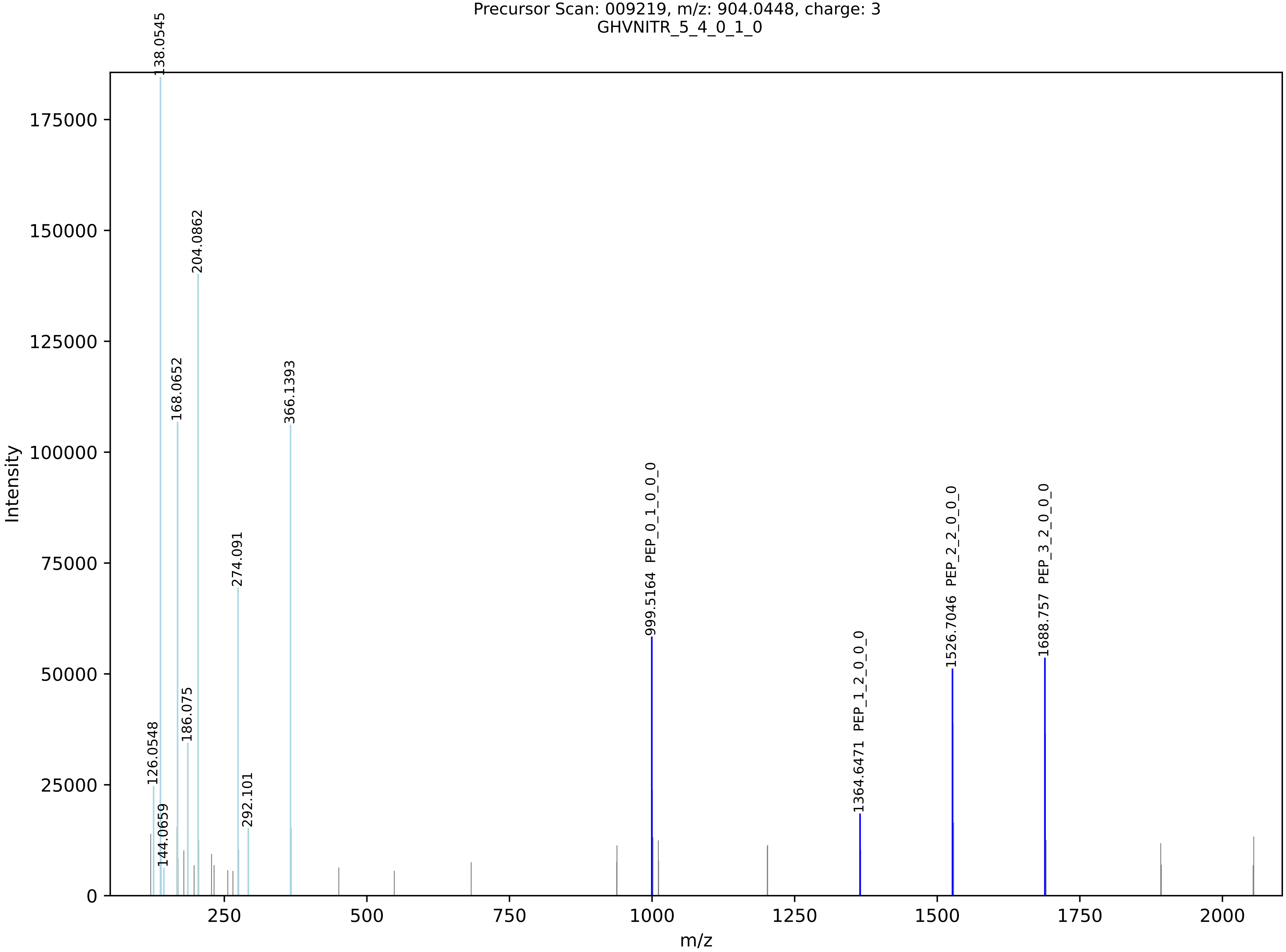

File: 231129_OE_RK_4, RT: 10.754, Scan: 009298
Type: HCD, FTMS + c NSI d Full ms2 1074.6625@hcd30.00 [120.0000-4407.4790]
Precursor Scan: 009219, m/z: 1074.1617, charge: 4
ENGTISR_7_6_0_4_0

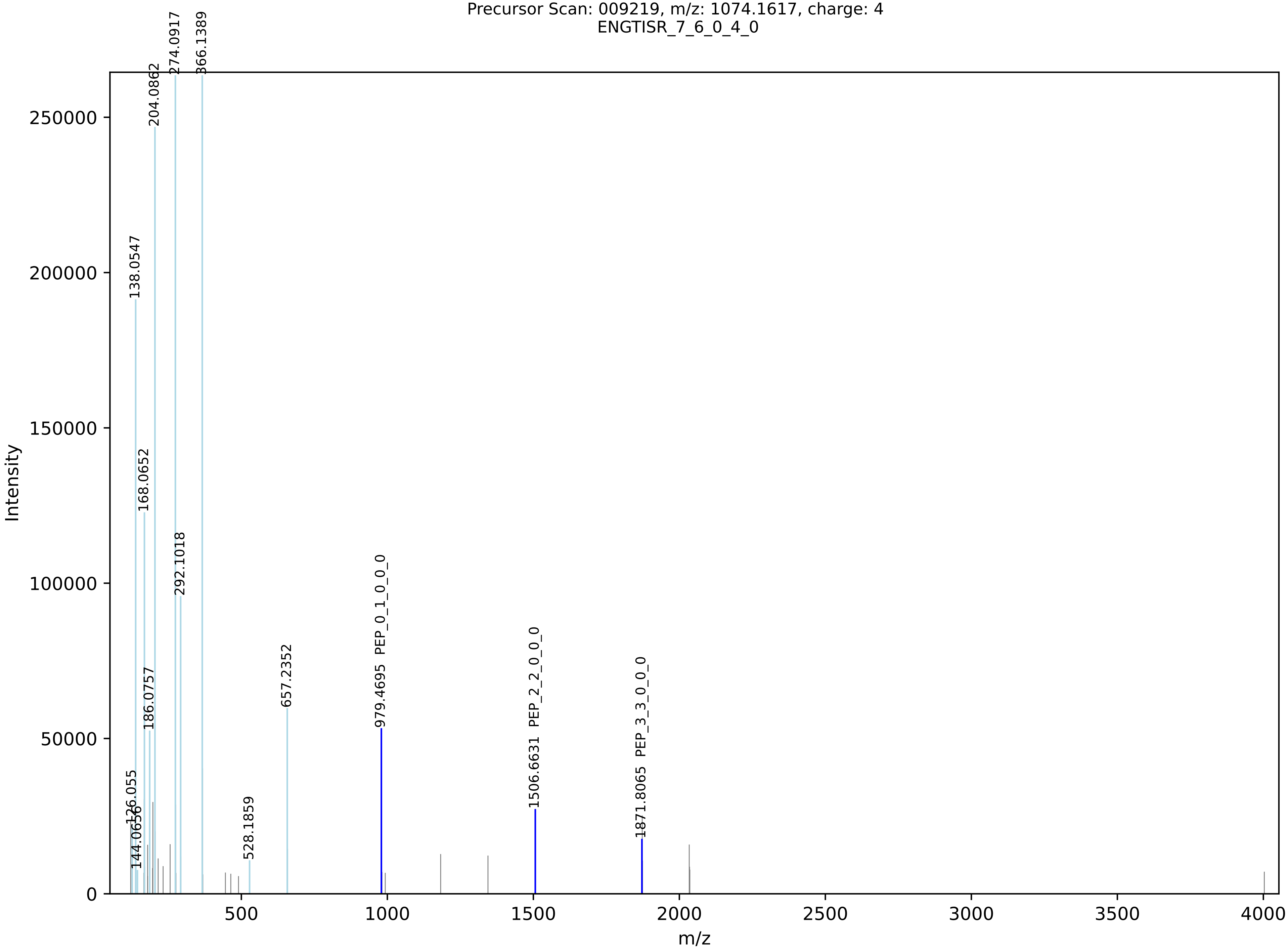

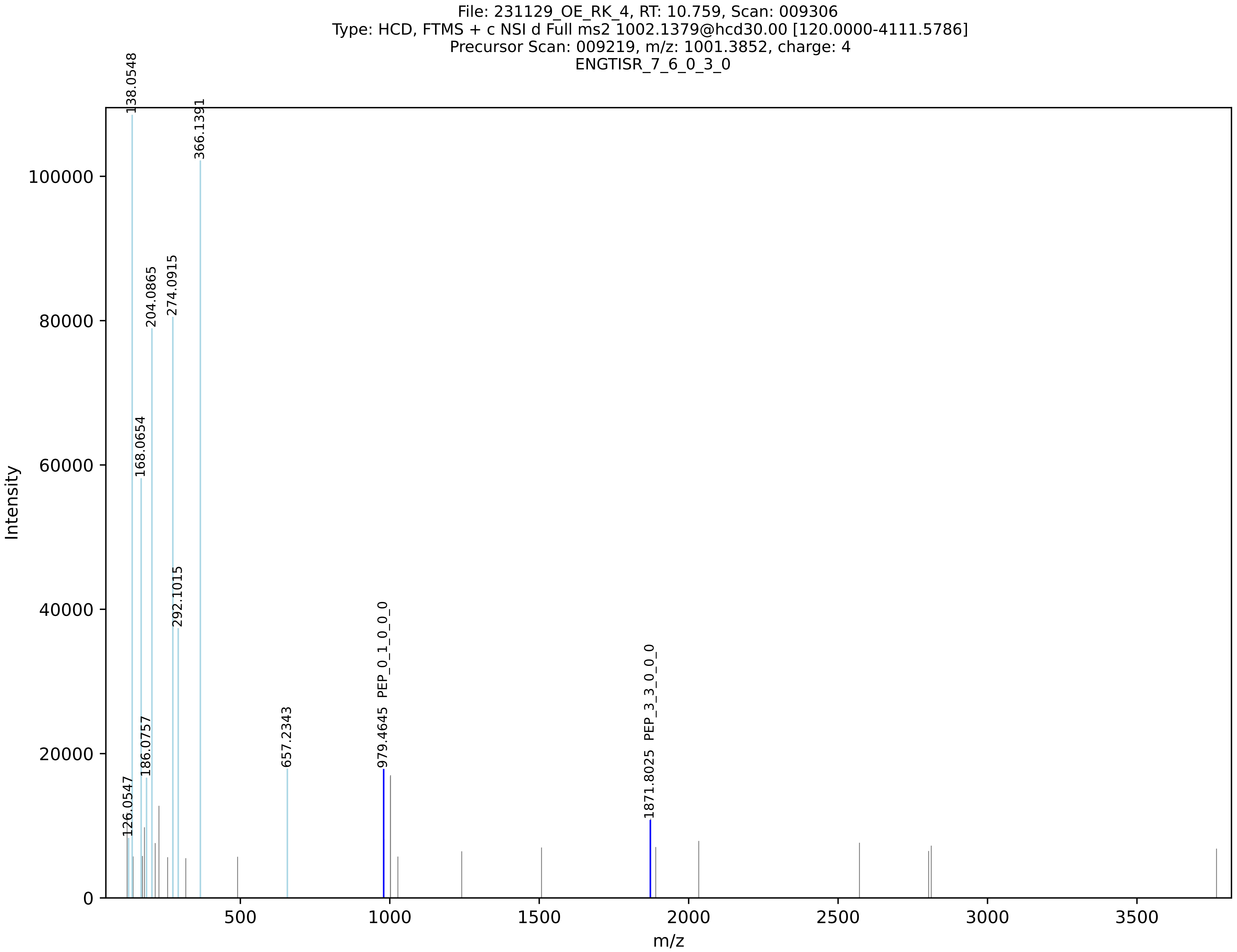

File: 231129_OE_RK_4, RT: 10.759, Scan: 009306
Type: HCD, FTMS + c NSI d Full ms2 1002.1379@hcd30.00 [120.0000-4111.5786]
Precursor Scan: 009219, m/z: 1001.3852, charge: 4
ENGTISR_7_6_0_3_0
138.0548
366.1391
204.0865
274.0915
168.0654
292.1015
979.4645 PEP_0_1_0_0_0
1871.8025 PEP_3_3_0_0_0
657.2343
186.0757
126.0547
Intensity
m/z
0
20000
40000
60000
80000
100000
500
1000
1500
2000
2500
3000
3500

File: 231129_OE_RK_4, RT: 10.778, Scan: 009337
Type: HCD, FTMS + c NSI d Full ms2 1094.1410@hcd30.00 [120.0000-3370.2136]
Precursor Scan: 009219, m/z: 1093.4701, charge: 3
TKPREEQYNSTYR_4_4_1_0_0

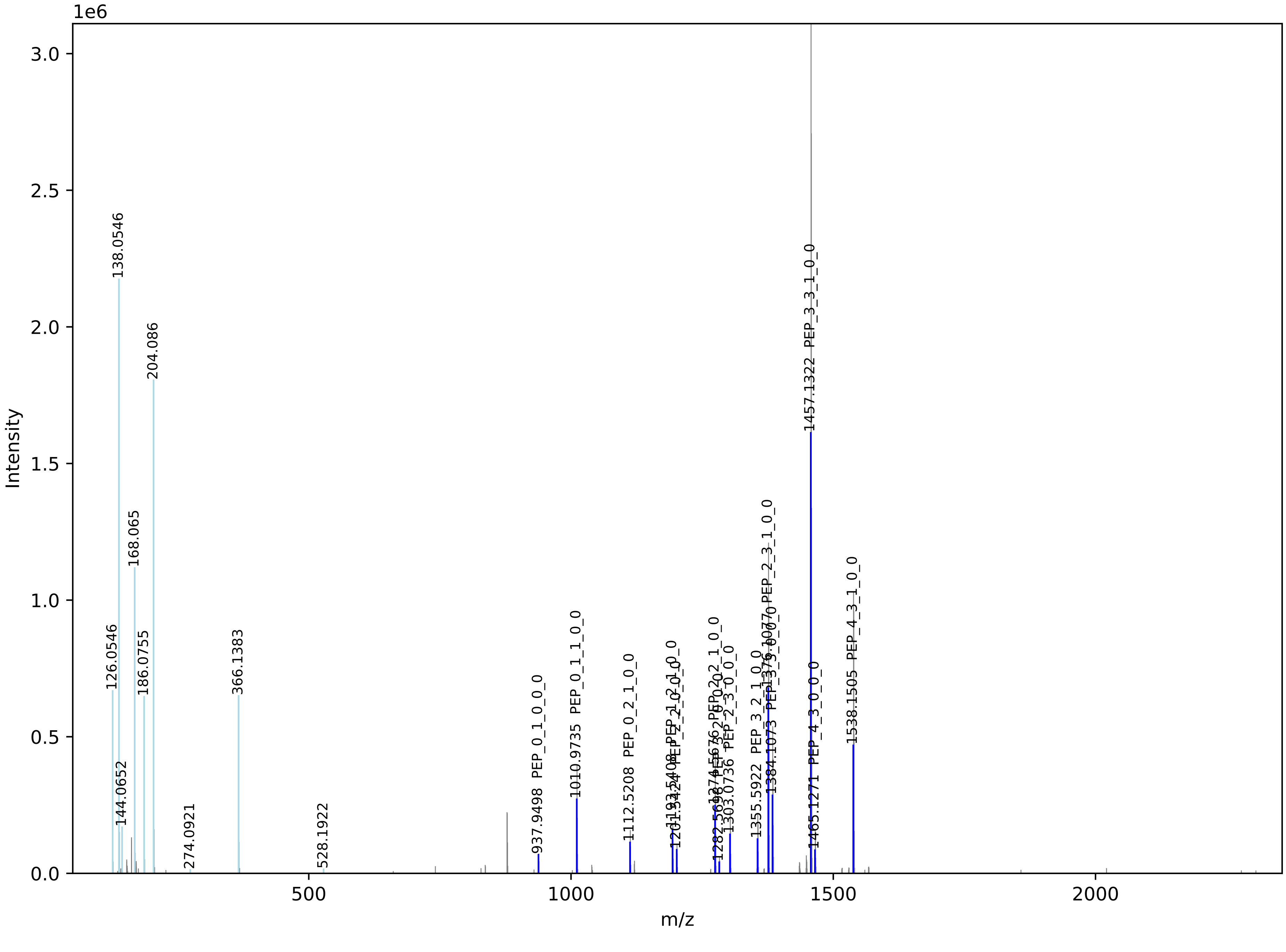

File: 231129_OE_RK_4, RT: 10.804, Scan: 009371
Type: HCD, FTMS + c NSI d Full ms2 1262.1537@hcd30.00 [120.0000-3884.3320]
Precursor Scan: 009292, m/z: 1261.819, charge: 3
ENGTISR_6_5_1_3_0

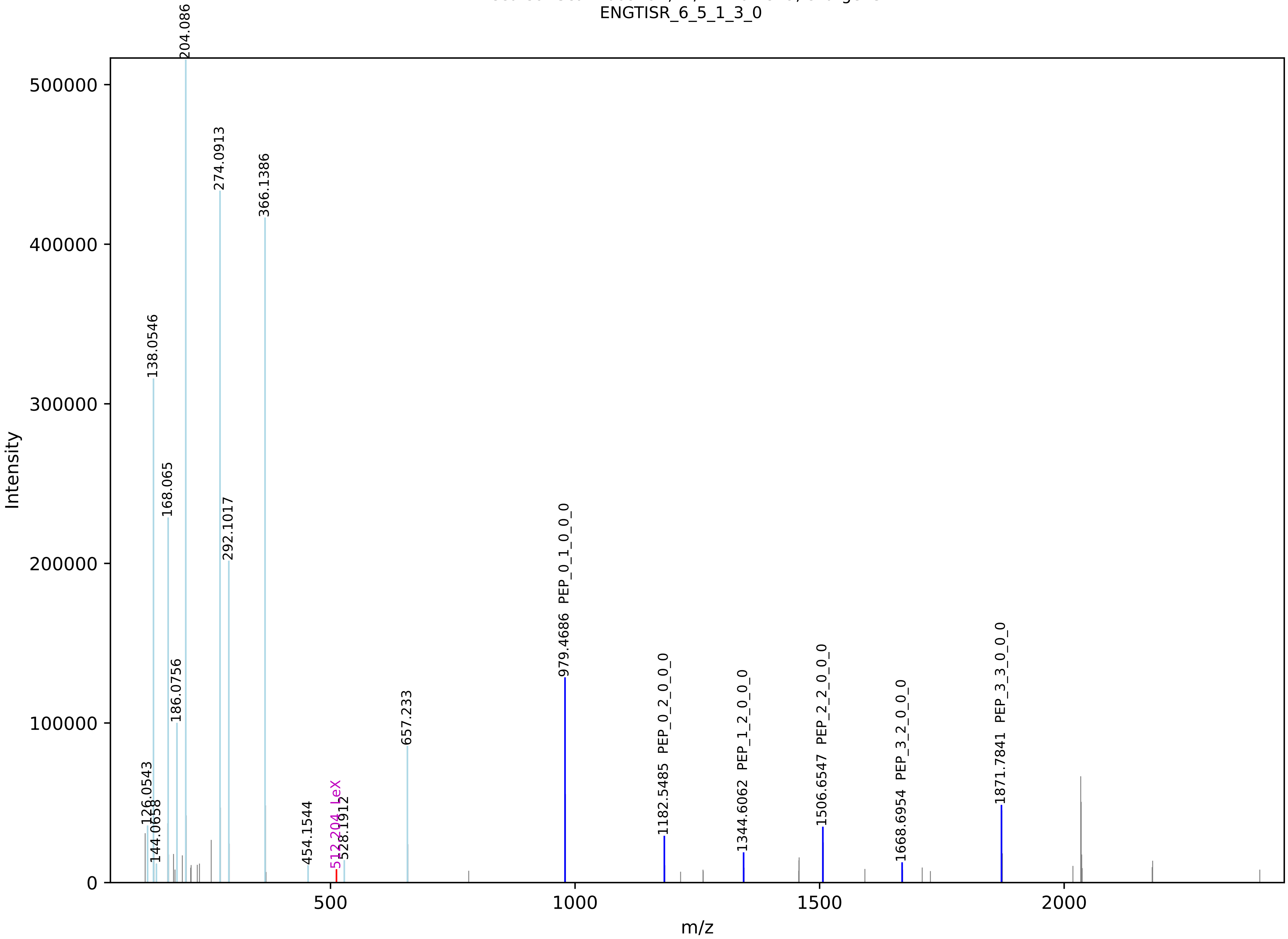

File: 231129_OE_RK_4, RT: 10.805, Scan: 009372
Type: HCD, FTMS + c NSI d Full ms2 1111.1746@hcd30.00 [120.0000-4556.4482]
Precursor Scan: 009292, m/z: 1110.674, charge: 4
ENGTISR_7_6_1_4_0

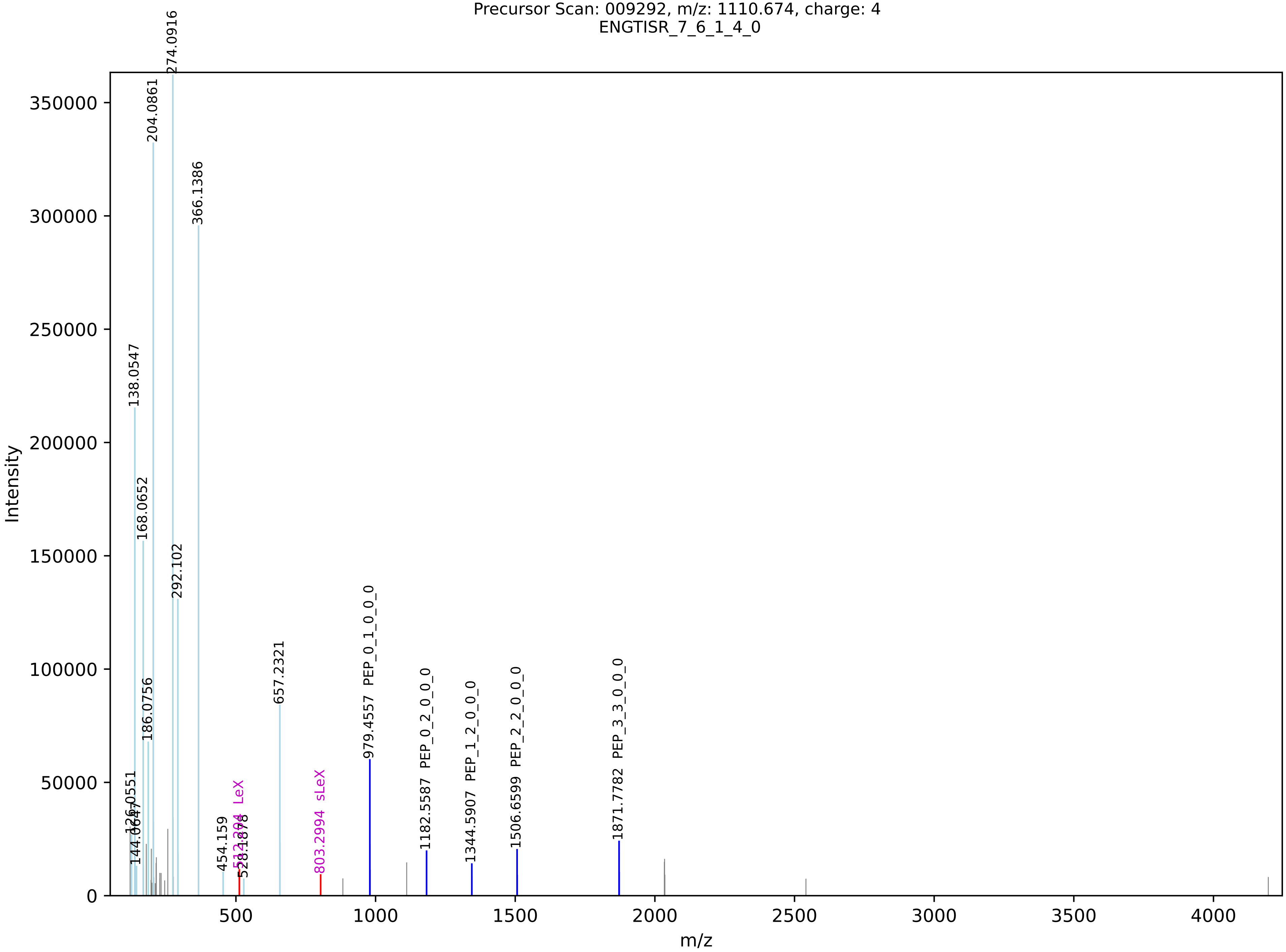

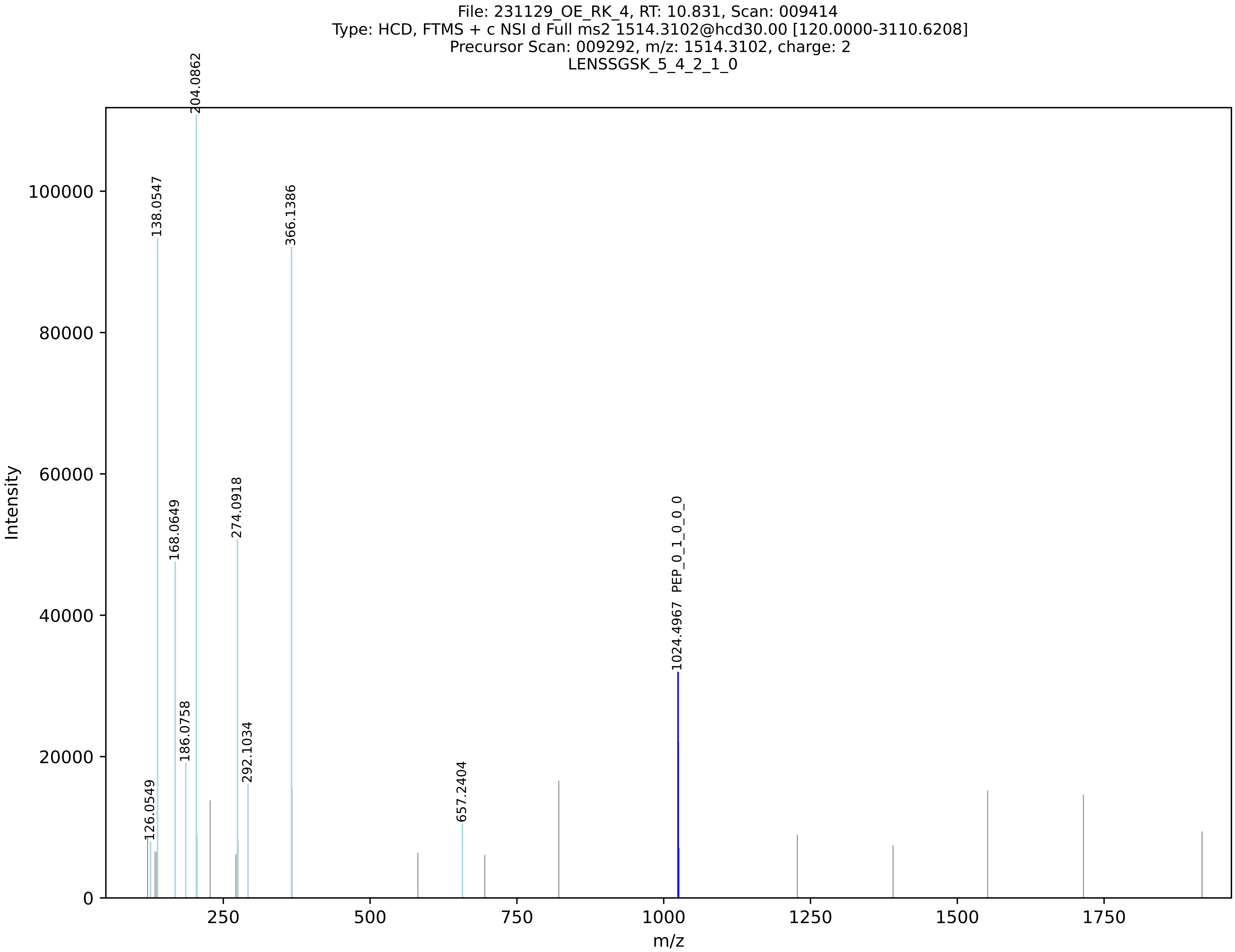
File: 231129_OE_RK_4, RT: 10.831, Scan: 009414
Type: HCD, FTMS + c NSI d Full ms2 1514.3102@hcd30.00 [120.0000-3110.6208]
Precursor Scan: 009292, m/z: 1514.3102, charge: 2
LENSSGSK_5_4_2_1_0
Intensity
m/z
204.0862
138.0547
366.1386
168.0649
274.0918
186.0758
292.1034
126.0549
657.2404
1024.4967 PEP_0_1_0_0_0
0
20000
40000
60000
80000
100000
250
500
750
1000
1250
1500
1750

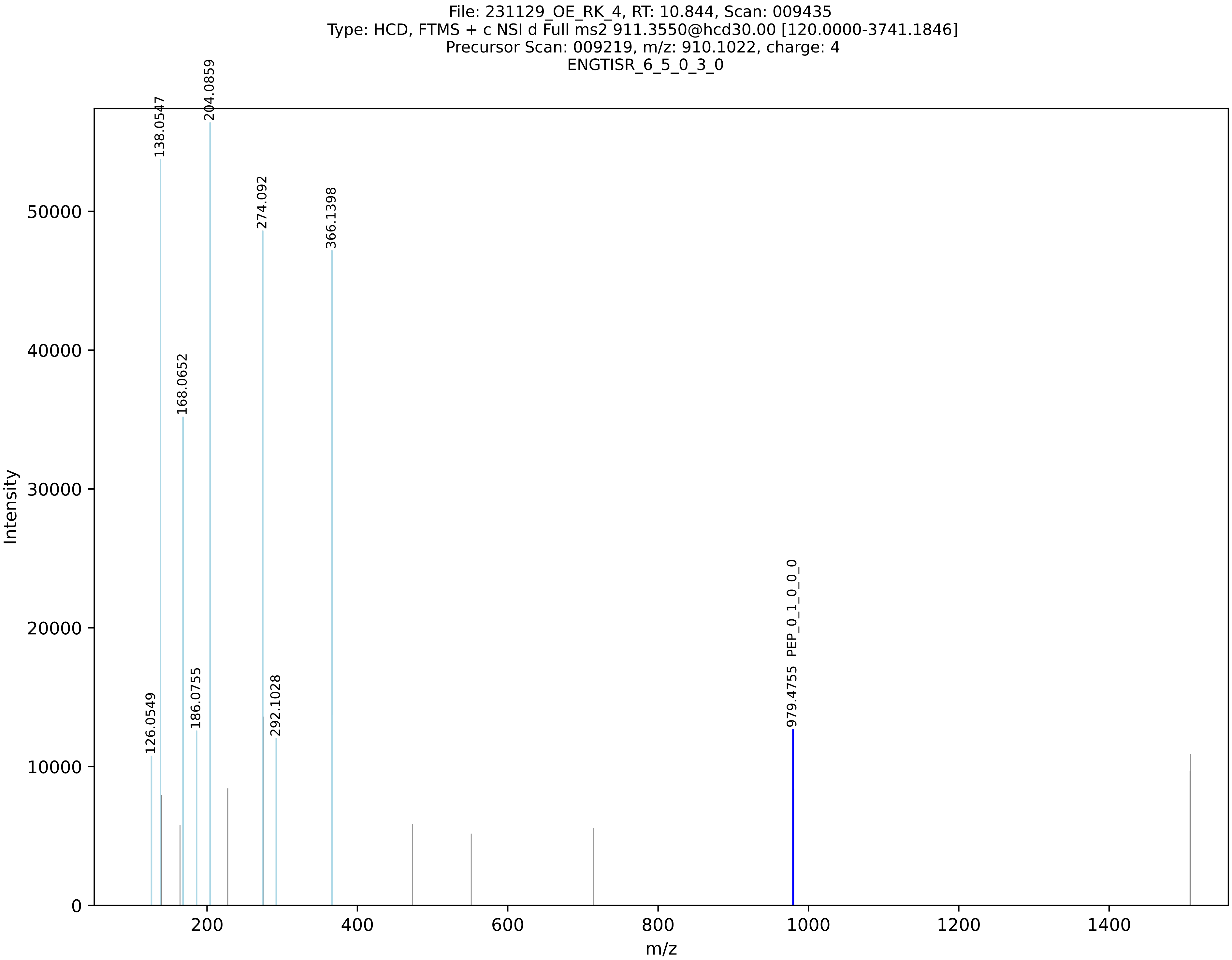
File: 231129_OE_RK_4, RT: 10.844, Scan: 009435
Type: HCD, FTMS + c NSI d Full ms2 911.3550@hcd30.00 [120.0000-3741.1846]
Precursor Scan: 009219, m/z: 910.1022, charge: 4
ENGTISR_6_5_0_3_0
138.0547
204.0859
274.092
366.1398
168.0652
126.0549
186.0755
292.1028
979.4755 PEP_0_1_0_0_0
Intensity
0
10000
20000
30000
40000
50000
200
400
600
800
1000
1200
1400
m/z

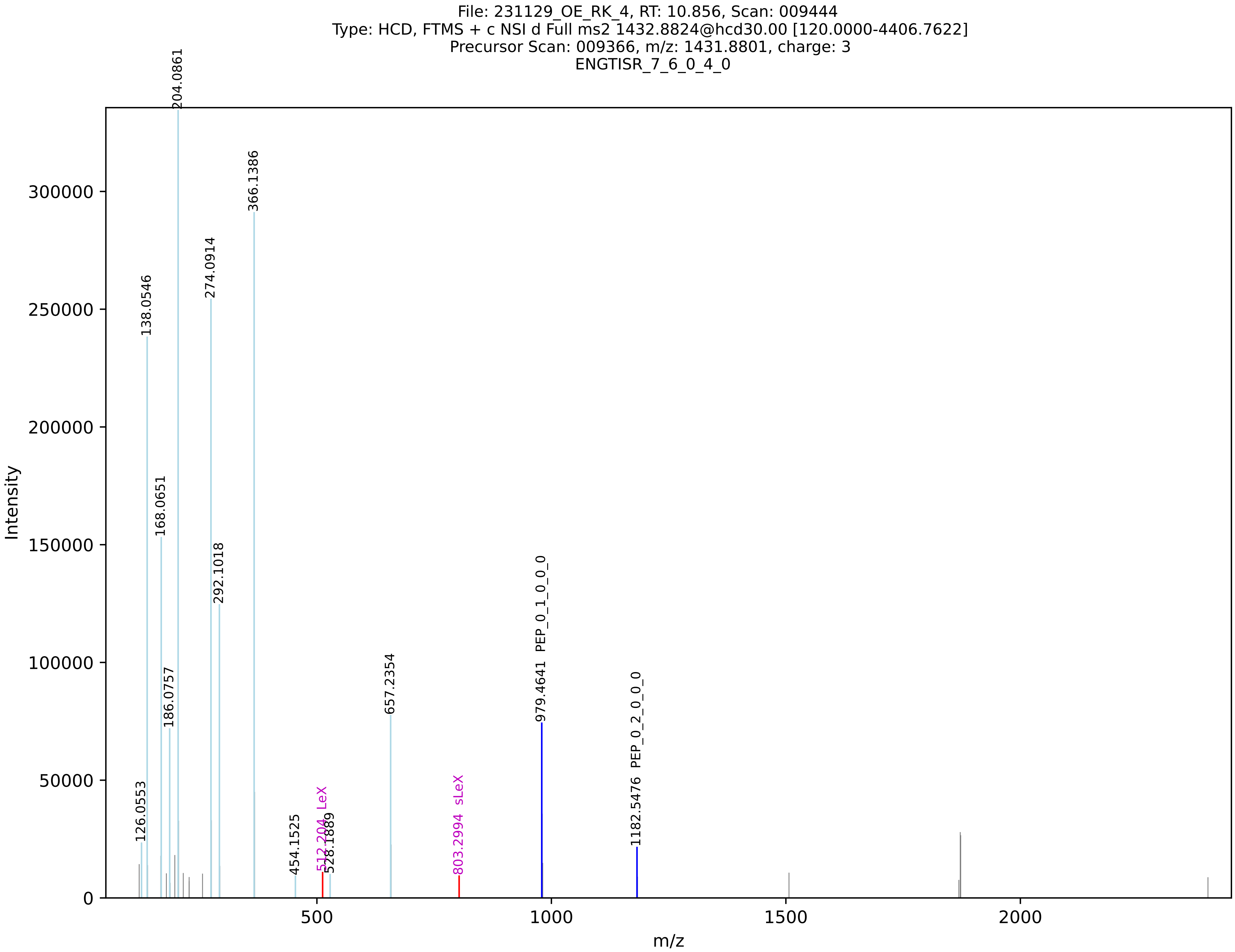
File: 231129_OE_RK_4, RT: 10.856, Scan: 009444
Type: HCD, FTMS + c NSI d Full ms2 1432.8824@hcd30.00 [120.0000-4406.7622]
Precursor Scan: 009366, m/z: 1431.8801, charge: 3
ENGTISR_7_6_0_4_0
Intensity
m/z
204.0861
366.1386
274.0914
138.0546
168.0651
292.1018
186.0757
126.0553
454.1525
512.204 LeX
528.1889
657.2354
803.2994 sLeX
979.4641 PEP_0_1_0_0_0
1182.5476 PEP_0_2_0_0_0

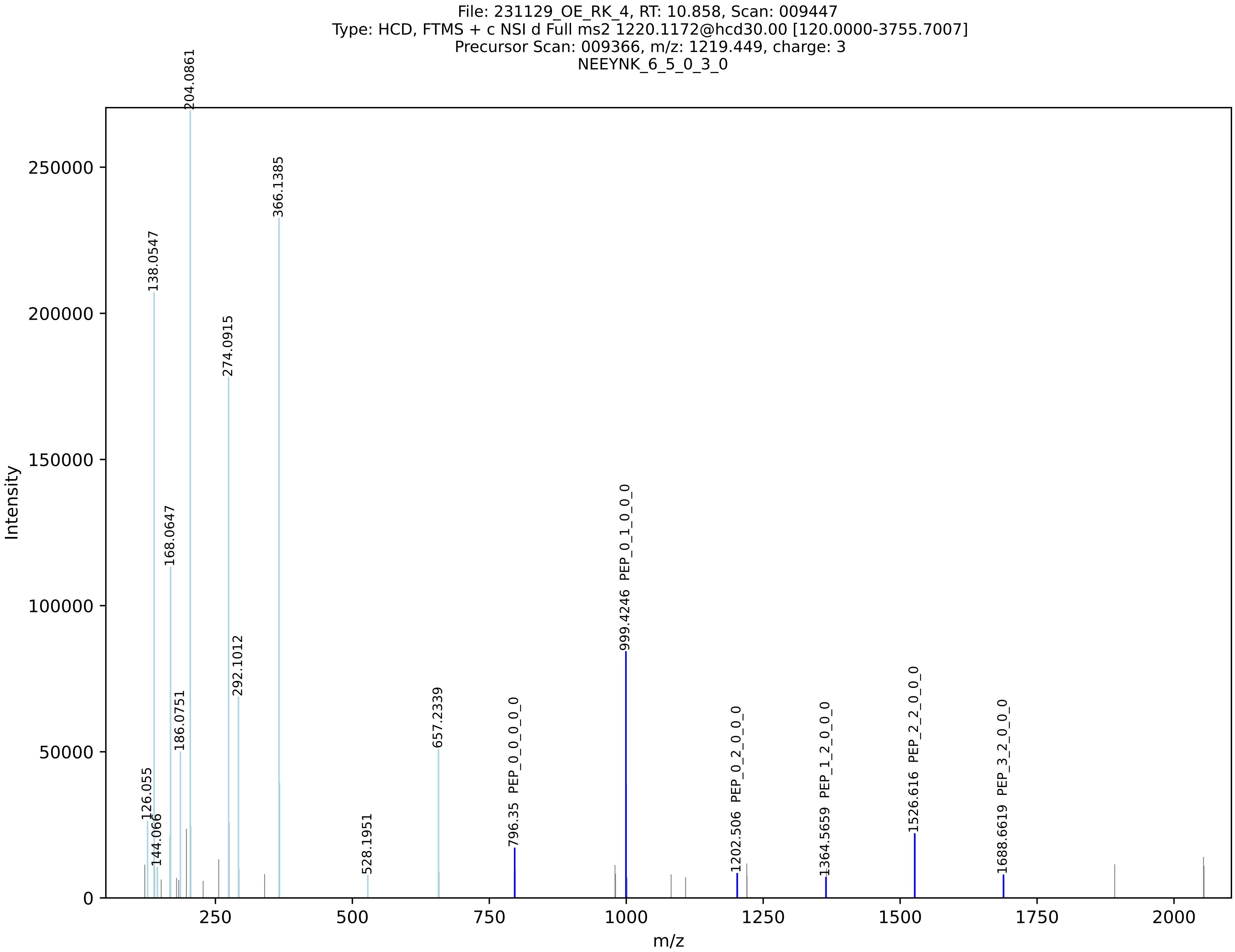

File: 231129_OE_RK_4, RT: 10.858, Scan: 009447
Type: HCD, FTMS + c NSI d Full ms2 1220.1172@hcd30.00 [120.0000-3755.7007]
Precursor Scan: 009366, m/z: 1219.449, charge: 3
NEEYNK_6_5_0_3_0
Intensity
m/z
204.0861
366.1385
138.0547
274.0915
168.0647
292.1012
186.0751
126.055
144.066
528.1951
657.2339
796.35 PEP_0_0_0_0_0
999.4246 PEP_0_1_0_0_0
1202.506 PEP_0_2_0_0_0
1364.5659 PEP_1_2_0_0_0
1526.616 PEP_2_2_0_0_0
1688.6619 PEP_3_2_0_0_0

File: 231129_OE_RK_4, RT: 10.898, Scan: 009504
Type: HCD, FTMS + c NSI d Full ms2 861.1172@hcd30.00 [120.0000-3536.2141]
Precursor Scan: 009440, m/z: 860.8666, charge: 4
CATPHGDNASLEATFVK_5_4_0_0_0

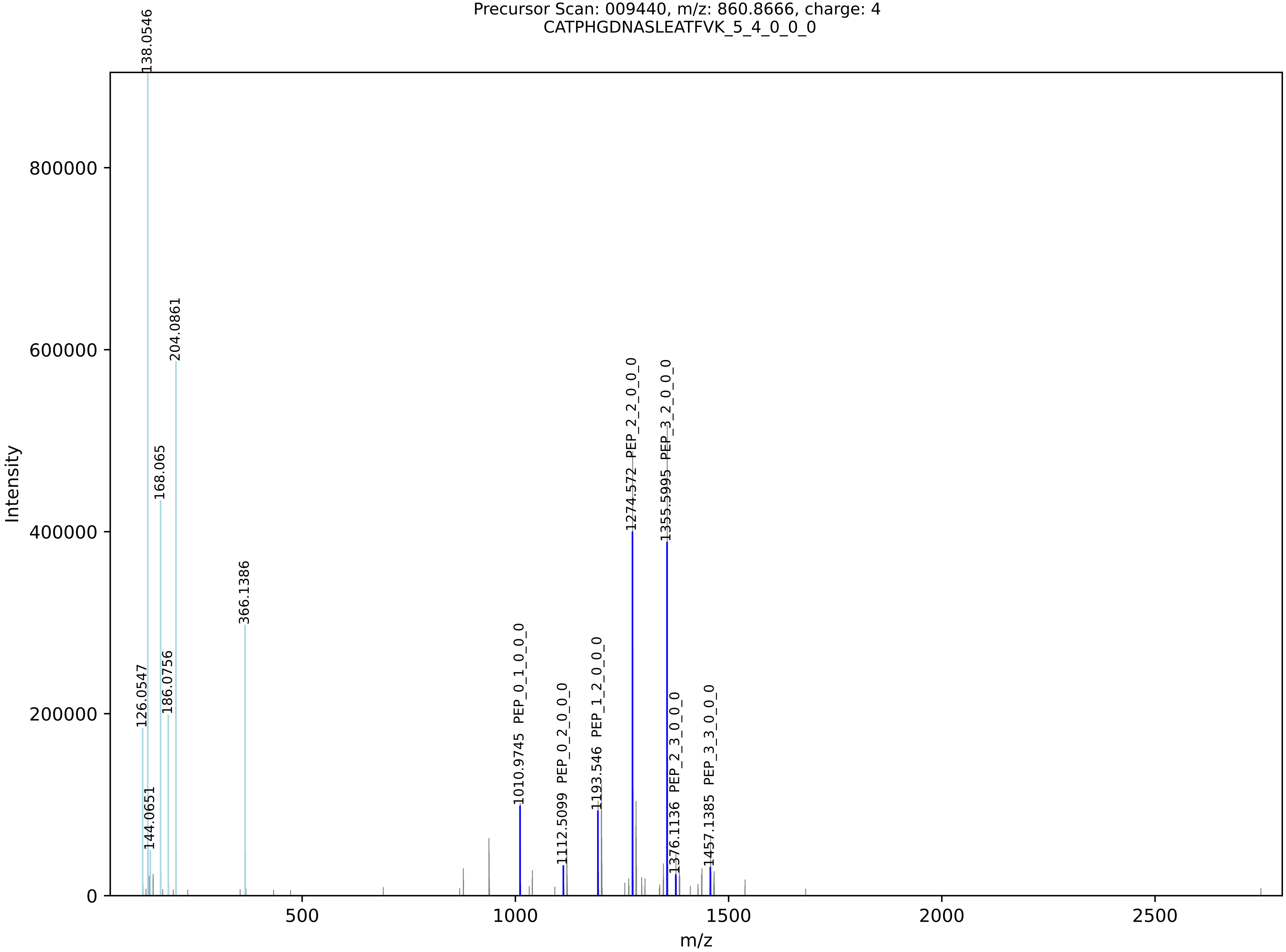

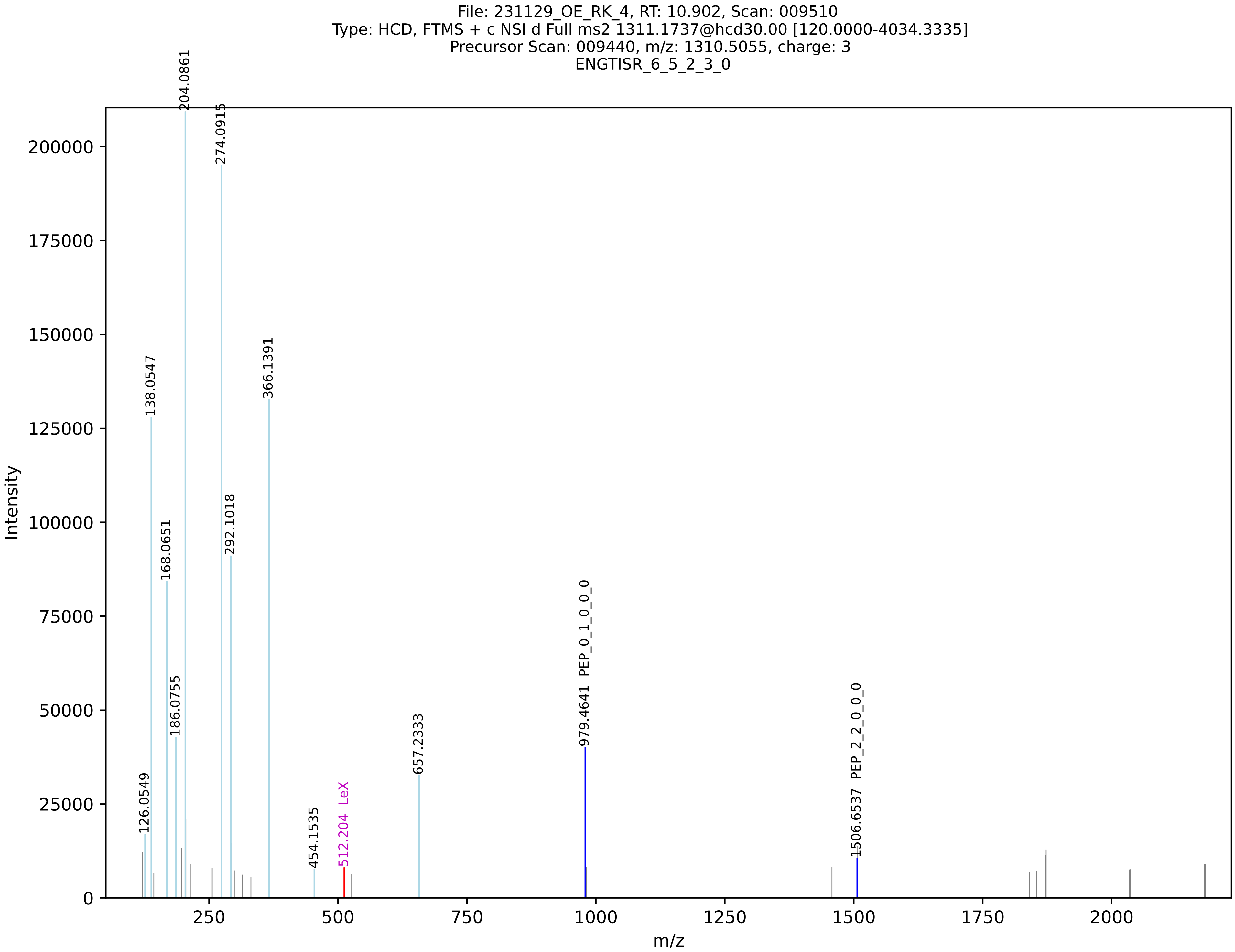
File: 231129_OE_RK_4, RT: 10.902, Scan: 009510
Type: HCD, FTMS + c NSI d Full ms2 1311.1737@hcd30.00 [120.0000-4034.3335]
Precursor Scan: 009440, m/z: 1310.5055, charge: 3
ENGTISR_6_5_2_3_0
Intensity
m/z
204.0861
274.0915
138.0547
366.1391
292.1018
168.0651
186.0755
126.0549
454.1535
512.204 LeX
657.2333
979.4641 PEP_0_1_0_0_0_
1506.6537 PEP_2_2_0_0_0_
0
25000
50000
75000
100000
125000
150000
175000
200000
250
500
750
1000
1250
1500
1750
2000

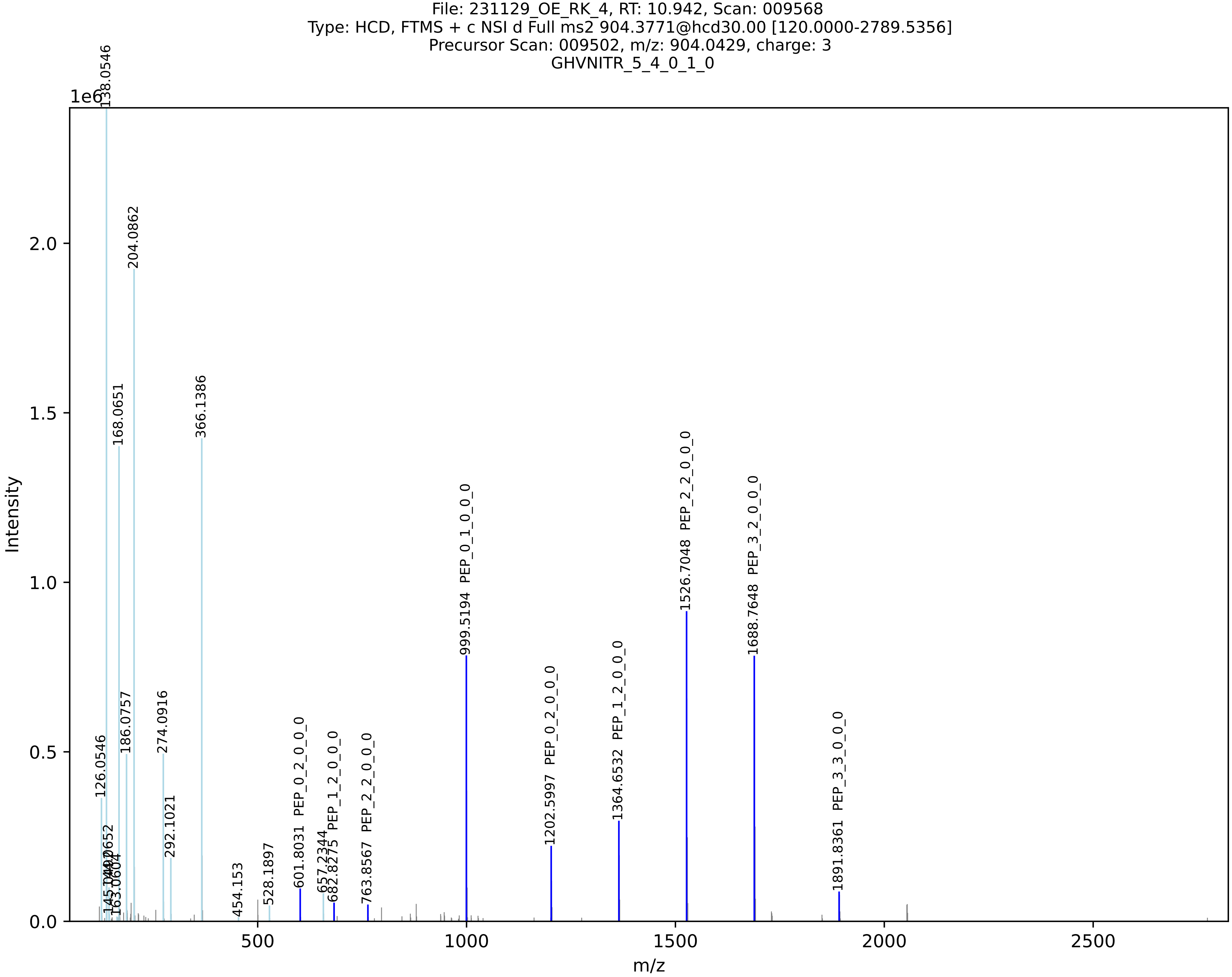
File: 231129_OE_RK_4, RT: 10.942, Scan: 009568
Type: HCD, FTMS + c NSI d Full ms2 904.3771@hcd30.00 [120.0000-2789.5356]
Precursor Scan: 009502, m/z: 904.0429, charge: 3
GHVNITR_5_4_0_1_0
1e6
Intensity
m/z
138.0546
204.0862
168.0651
366.1386
186.0757
274.0916
126.0546
292.1021
163.0604
454.153
528.1897
601.8031 PEP_0_2_0_0_
657.2344
682.8275 PEP_1_2_0_0_
763.8567 PEP_2_2_0_0_
999.5194 PEP_0_1_0_0_
1202.5997 PEP_0_2_0_0_
1364.6532 PEP_1_2_0_0_
1526.7048 PEP_2_2_0_0_
1688.7648 PEP_3_2_0_0_
1891.8361 PEP_3_3_0_0_

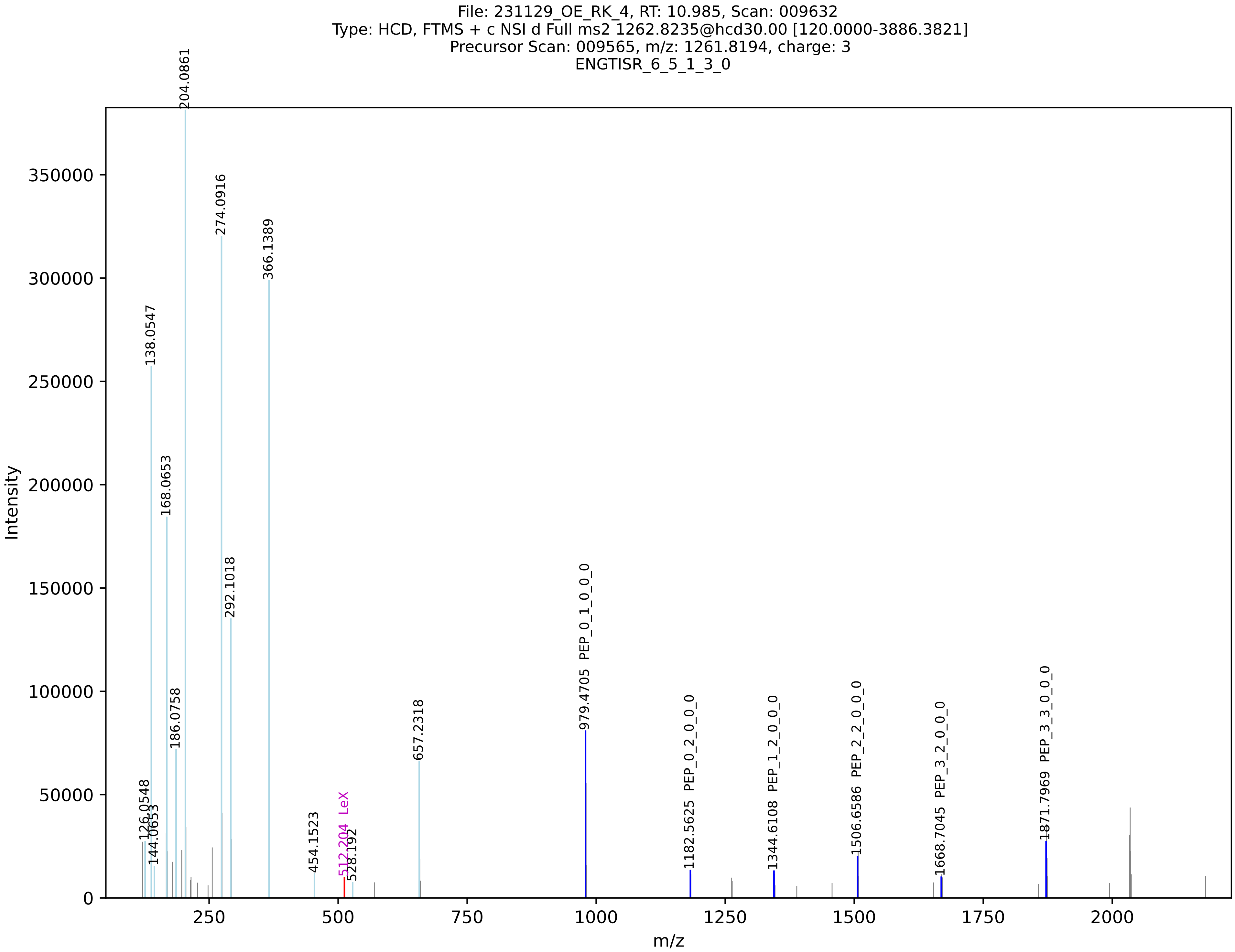
File: 231129_OE_RK_4, RT: 10.985, Scan: 009632
Type: HCD, FTMS + c NSI d Full ms2 1262.8235@hcd30.00 [120.0000-3886.3821]
Precursor Scan: 009565, m/z: 1261.8194, charge: 3
ENGTISR_6_5_1_3_0
Intensity
m/z
204.0861
274.0916
366.1389
138.0547
168.0653
292.1018
186.0758
126.0548
144.0653
454.1523
512.204 LeX
528.192
657.2318
979.4705 PEP_0_1_0_0_0
1182.5625 PEP_0_2_0_0_0
1344.6108 PEP_1_2_0_0_0
1506.6586 PEP_2_2_0_0_0
1668.7045 PEP_3_2_0_0_0
1871.7969 PEP_3_3_0_0_0

File: 231129_OE_RK_4, RT: 11.015, Scan: 009673
Type: HCD, FTMS + c NSI d Full ms2 1184.1582@hcd30.00 [120.0000-3645.6660]
Precursor Scan: 009627, m/z: 1183.8138, charge: 3
YEITAANSSGTTK_5_4_0_2_0

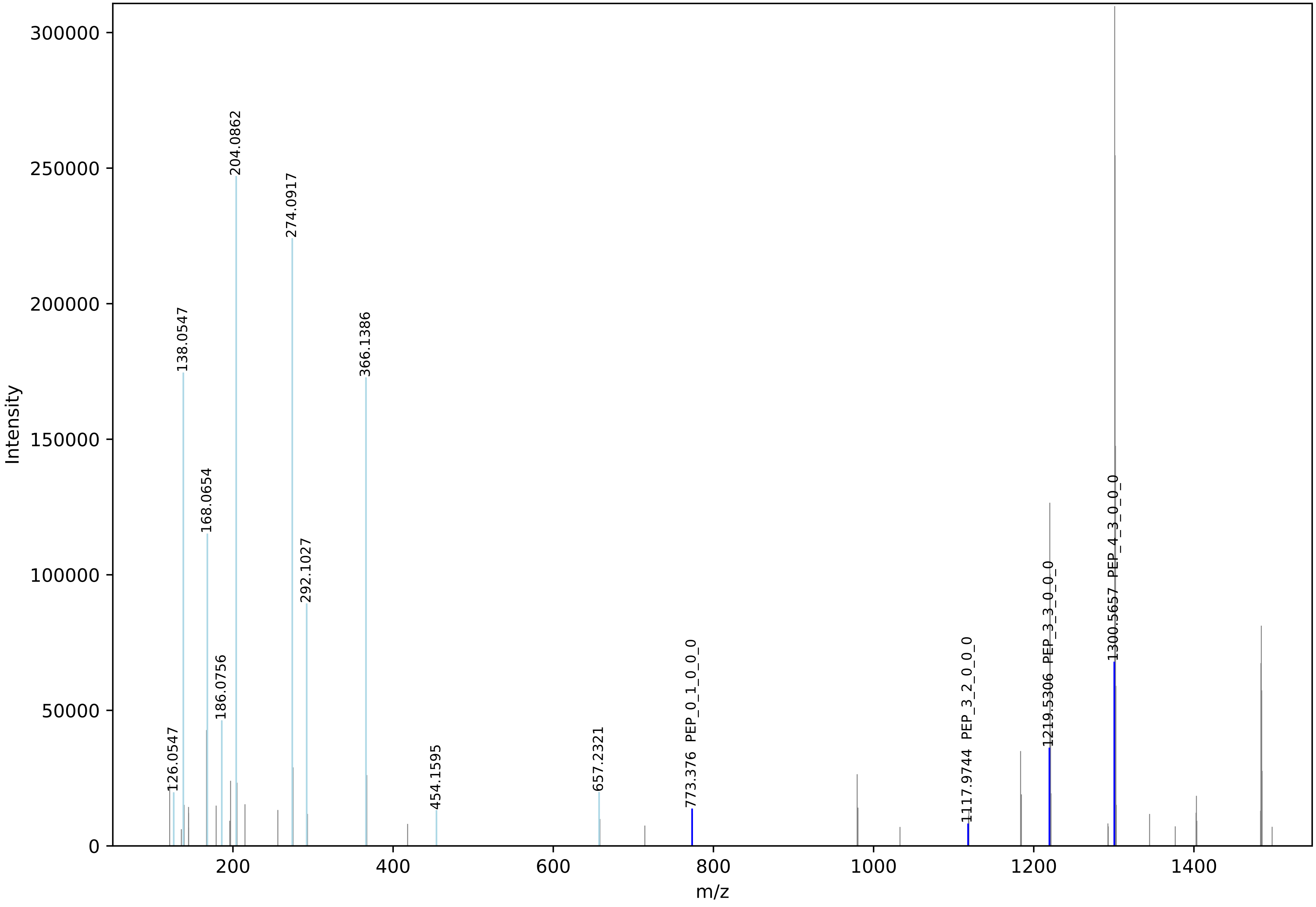

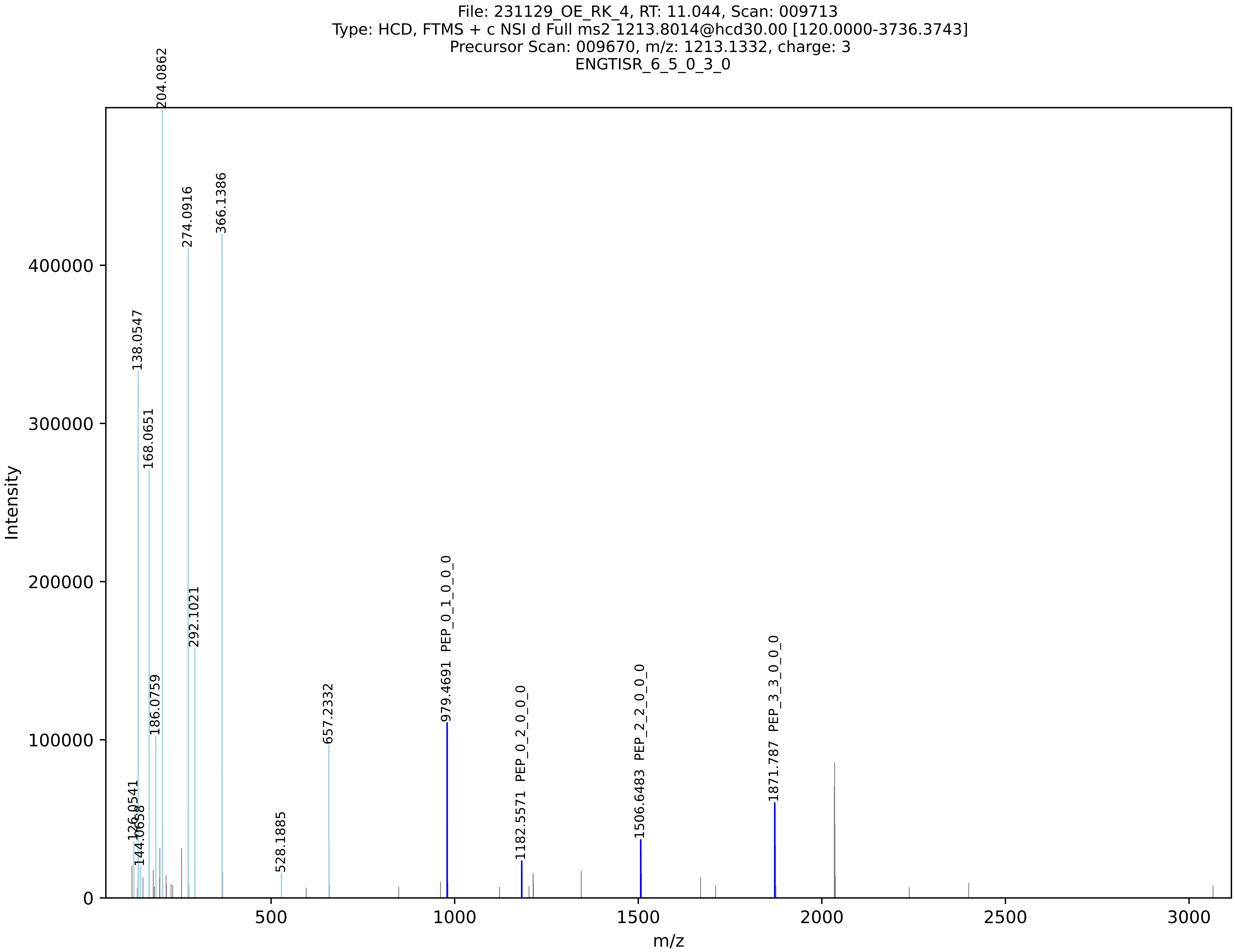
File: 231129_OE_RK_4, RT: 11.044, Scan: 009713
Type: HCD, FTMS + c NSI d Full ms2 1213.8014@hcd30.00 [120.0000-3736.3743]
Precursor Scan: 009670, m/z: 1213.1332, charge: 3
ENGTISR_6_5_0_3_0
Intensity
m/z
204.0862
366.1386
274.0916
138.0547
168.0651
292.1021
186.0759
126.0541
144.0658
528.1885
657.2332
979.4691 PEP_0_1_0_0_0
1182.5571 PEP_0_2_0_0_0
1506.6483 PEP_2_2_0_0_0
1871.787 PEP_3_3_0_0_0
0
100000
200000
300000
400000
500
1000
1500
2000
2500
3000

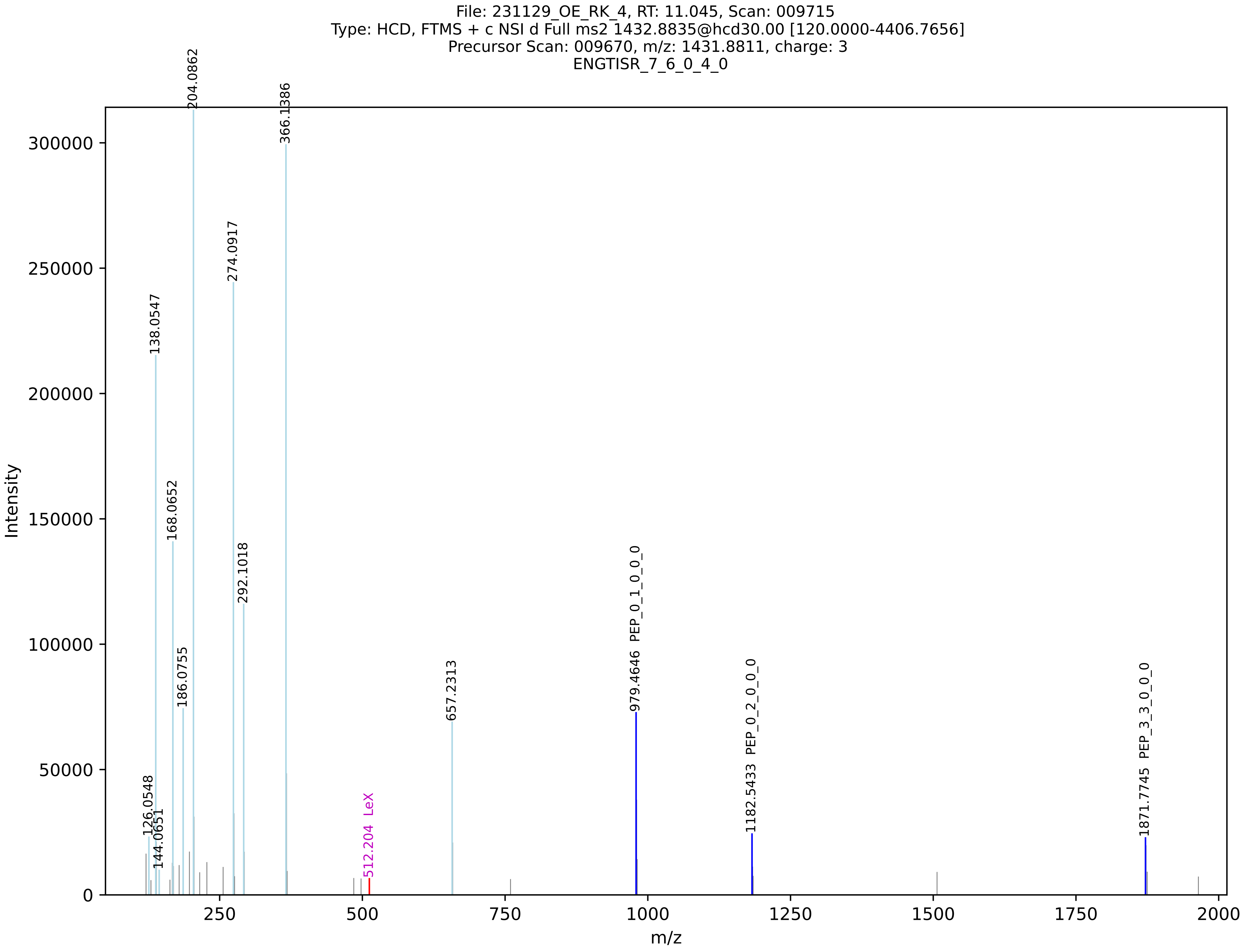
File: 231129_OE_RK_4, RT: 11.045, Scan: 009715
Type: HCD, FTMS + c NSI d Full ms2 1432.8835@hcd30.00 [120.0000-4406.7656]
Precursor Scan: 009670, m/z: 1431.8811, charge: 3
ENGTISR_7_6_0_4_0
Intensity
m/z
204.0862
366.1386
274.0917
138.0547
168.0652
292.1018
186.0755
657.2313
979.4646 PEP_0_1_0_0_0
1182.5433 PEP_0_2_0_0_0
1871.7745 PEP_3_3_0_0_0
126.0548
144.0651
512.204 LeX
0
50000
100000
150000
200000
250000
300000
250
500
750
1000
1250
1500
1750
2000

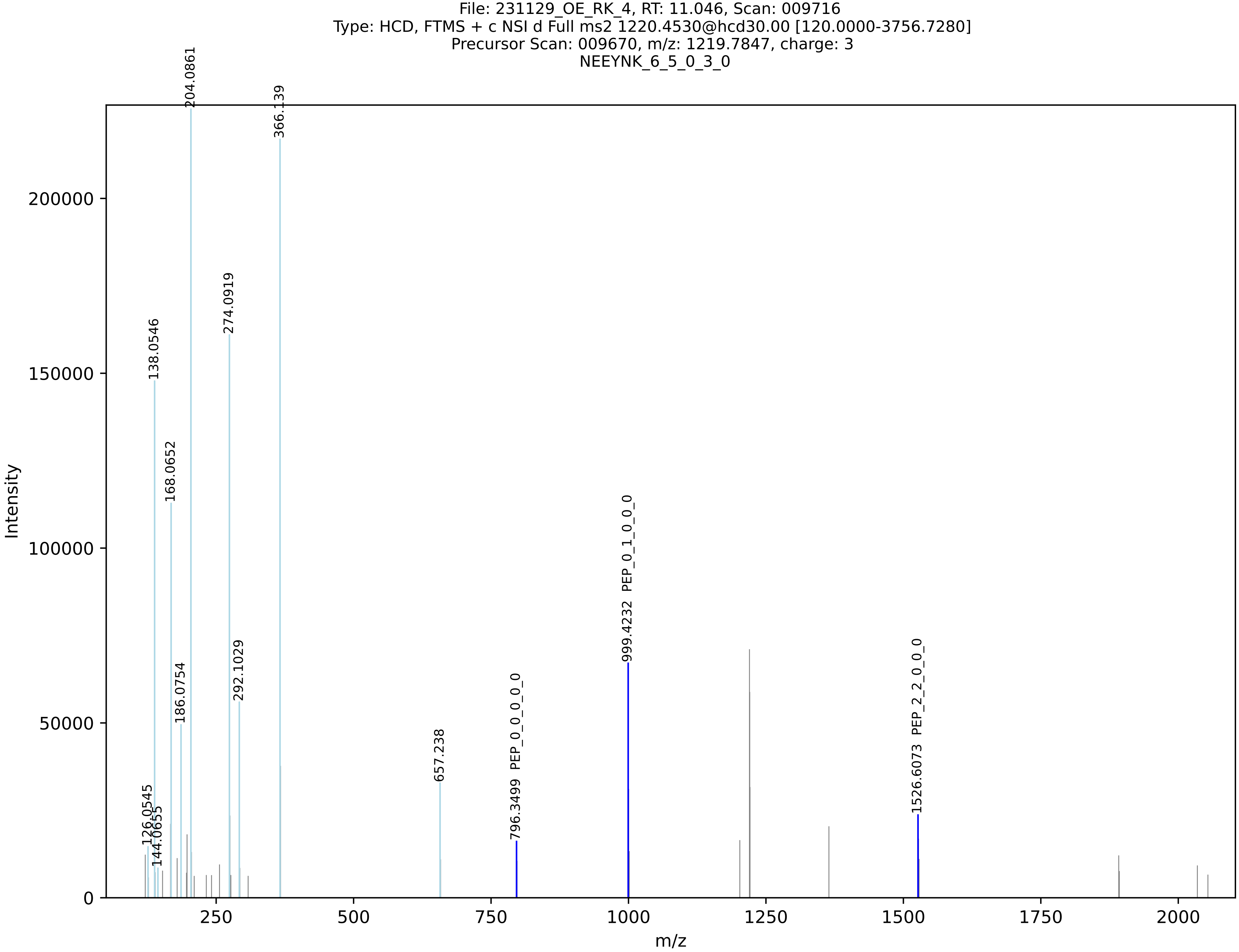
File: 231129_OE_RK_4, RT: 11.046, Scan: 009716
Type: HCD, FTMS + c NSI d Full ms2 1220.4530@hcd30.00 [120.0000-3756.7280]
Precursor Scan: 009670, m/z: 1219.7847, charge: 3
NEEYNK_6_5_0_3_0
Intensity
m/z
204.0861
366.139
274.0919
138.0546
168.0652
186.0754
292.1029
126.0545
144.0655
657.238
796.3499 PEP_0_0_0_0_0
999.4232 PEP_0_1_0_0_0
1526.6073 PEP_2_2_0_0_0
0
50000
100000
150000
200000
250
500
750
1000
1250
1500
1750
2000

File: 231129_OE_RK_4, RT: 11.048, Scan: 009720
Type: HCD, FTMS + c NSI d Full ms2 955.8903@hcd30.00 [120.0000-3922.8884]
Precursor Scan: 009670, m/z: 955.6397, charge: 4
NAHGEEKENLTAR_5_4_1_2_0

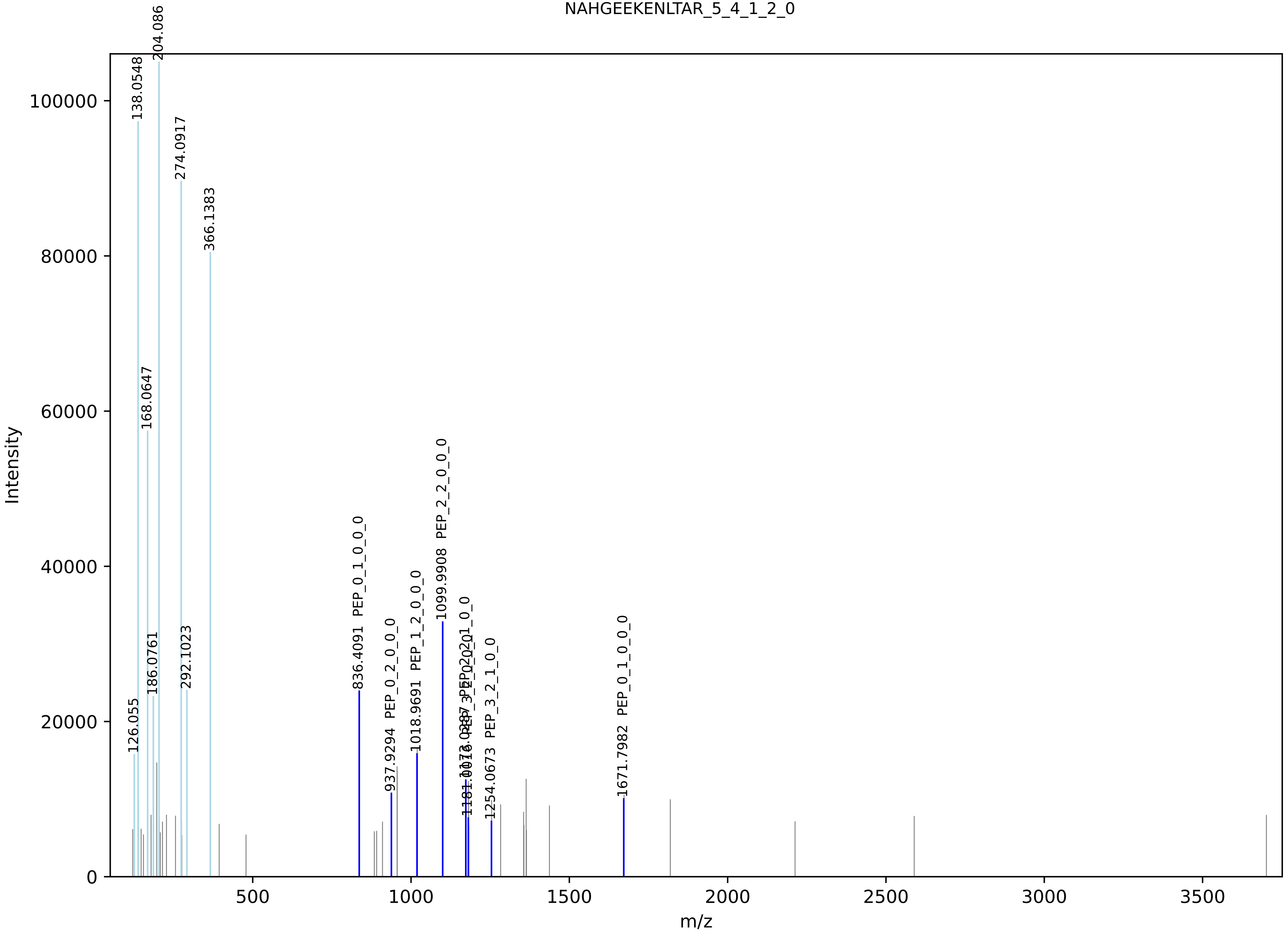

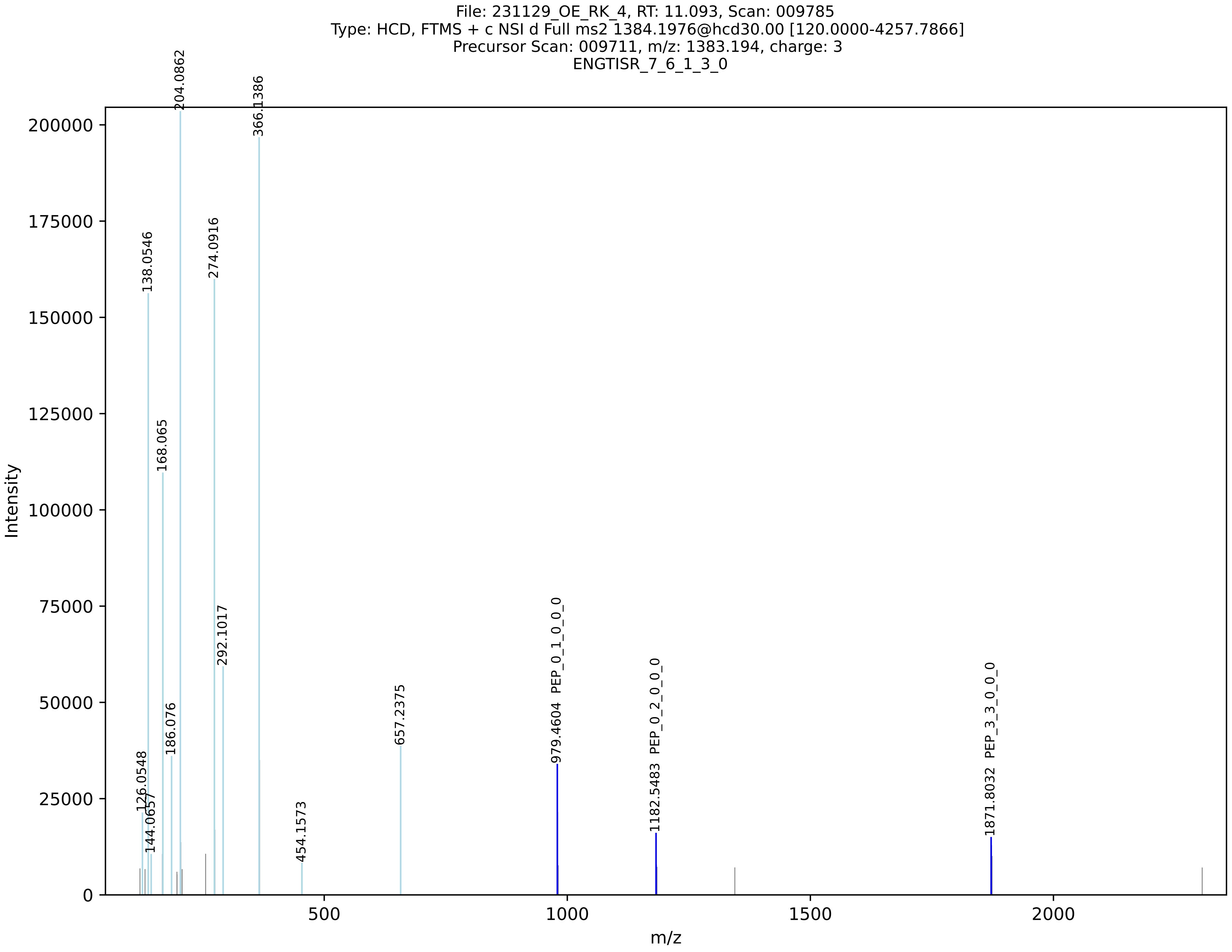
File: 231129_OE_RK_4, RT: 11.093, Scan: 009785
Type: HCD, FTMS + c NSI d Full ms2 1384.1976@hcd30.00 [120.0000-4257.7866]
Precursor Scan: 009711, m/z: 1383.194, charge: 3
ENGTISR_7_6_1_3_0
Intensity
m/z
204.0862
366.1386
138.0546
274.0916
168.065
292.1017
186.076
126.0548
144.0657
454.1573
657.2375
979.4604 PEP_0_1_0_0_0
1182.5483 PEP_0_2_0_0_0
1871.8032 PEP_3_3_0_0_0

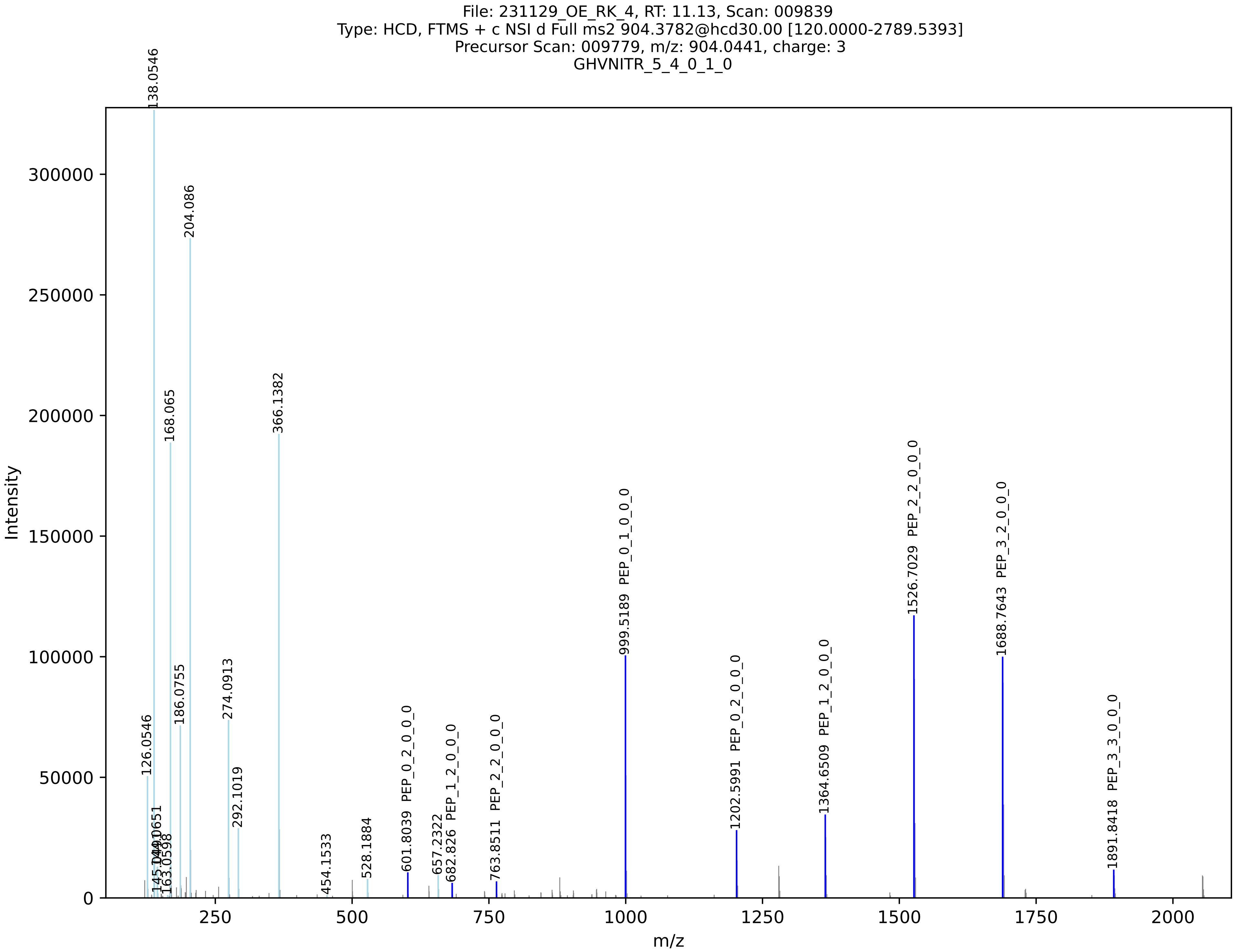
File: 231129_OE_RK_4, RT: 11.13, Scan: 009839
Type: HCD, FTMS + c NSI d Full ms2 904.3782@hcd30.00 [120.0000-2789.5393]
Precursor Scan: 009779, m/z: 904.0441, charge: 3
GHVNITR_5_4_0_1_0
Intensity
m/z
138.0546
204.086
366.1382
168.065
186.0755
274.0913
126.0546
292.1019
163.0598
454.1533
528.1884
601.8039 PEP_0_2_0_0_0
657.2322
682.826 PEP_1_2_0_0_0
763.8511 PEP_2_2_0_0_0
999.5189 PEP_0_1_0_0_0
1202.5991 PEP_0_2_0_0_0
1364.6509 PEP_1_2_0_0_0
1526.7029 PEP_2_2_0_0_0
1688.7643 PEP_3_2_0_0_0
1891.8418 PEP_3_3_0_0_0

File: 231129_OE_RK_4, RT: 11.131, Scan: 009840
Type: HCD, FTMS + c NSI d Full ms2 887.8645@hcd30.00 [120.0000-3645.3430]
Precursor Scan: 009779, m/z: 887.6142, charge: 4
EIRHNSTGCLR_5_4_0_2_0

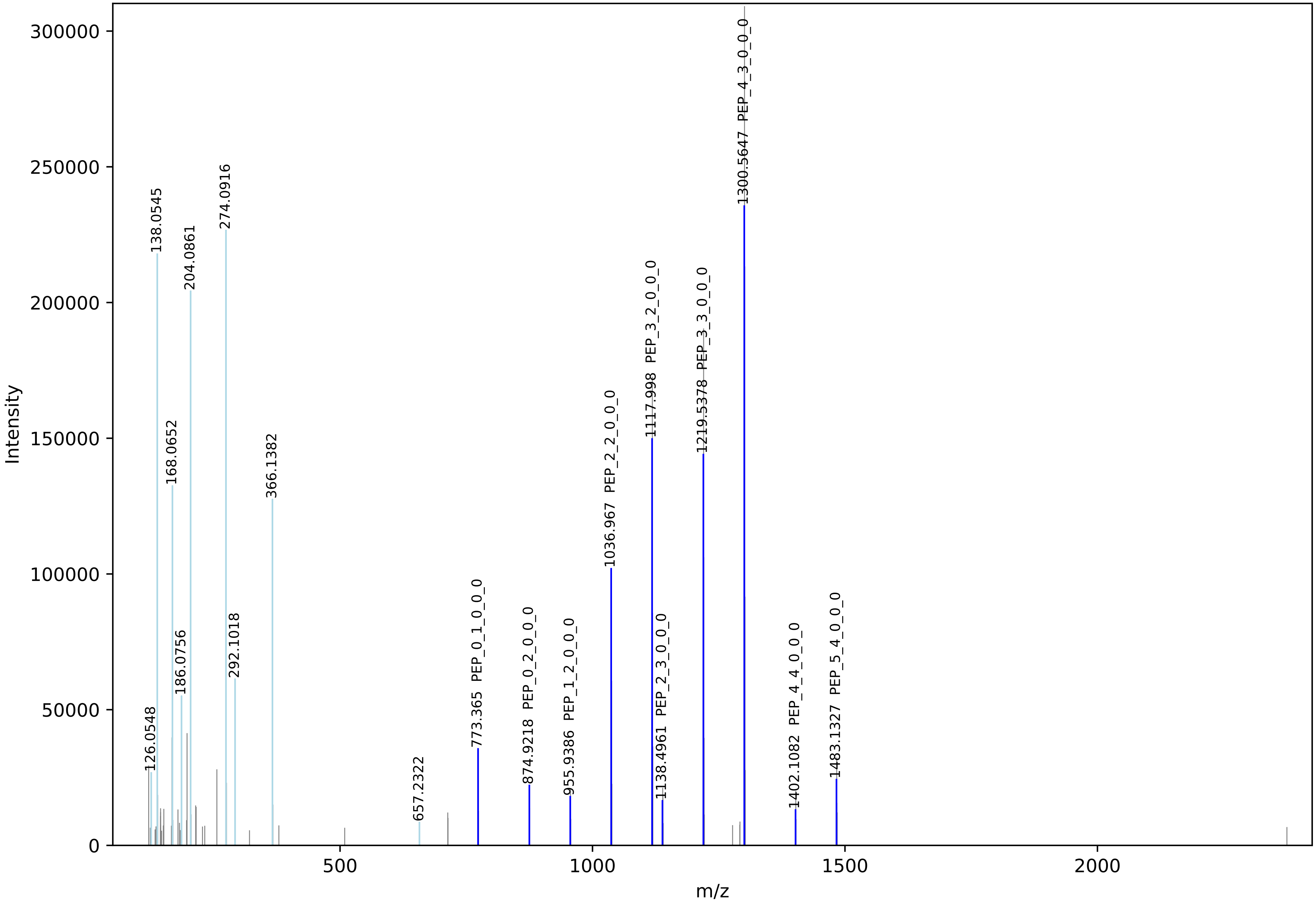

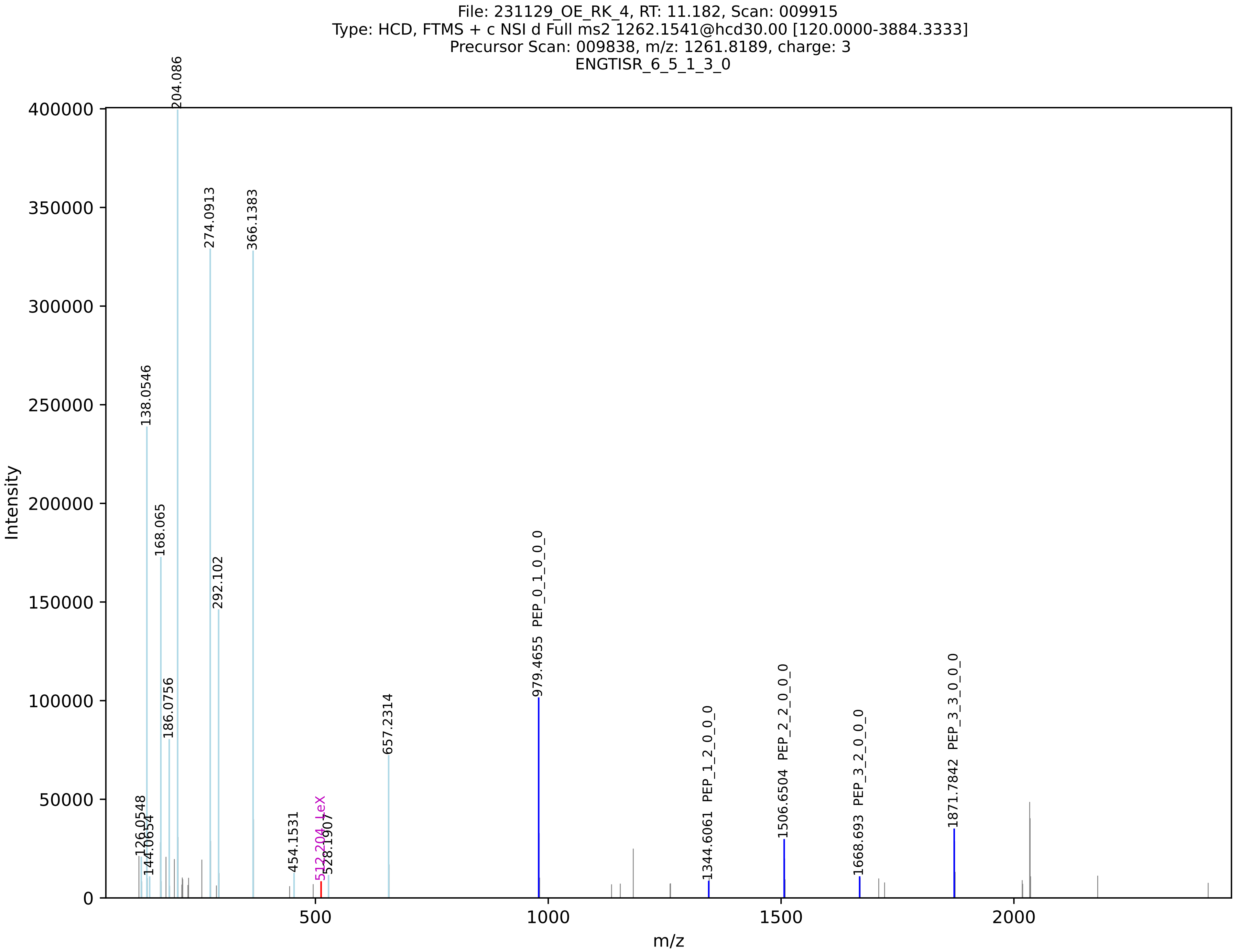
File: 231129_OE_RK_4, RT: 11.182, Scan: 009915
Type: HCD, FTMS + c NSI d Full ms2 1262.1541@hcd30.00 [120.0000-3884.3333]
Precursor Scan: 009838, m/z: 1261.8189, charge: 3
ENGTISR_6_5_1_3_0
Intensity
m/z
204.086
274.0913
366.1383
138.0546
168.065
292.102
186.0756
126.0548
144.0654
454.1531
512.204_LeX
528.1907
657.2314
979.4655 PEP_0_1_0_0_0
1344.6061 PEP_1_2_0_0_0
1506.6504 PEP_2_2_0_0_0
1668.693 PEP_3_2_0_0_0
1871.7842 PEP_3_3_0_0_0

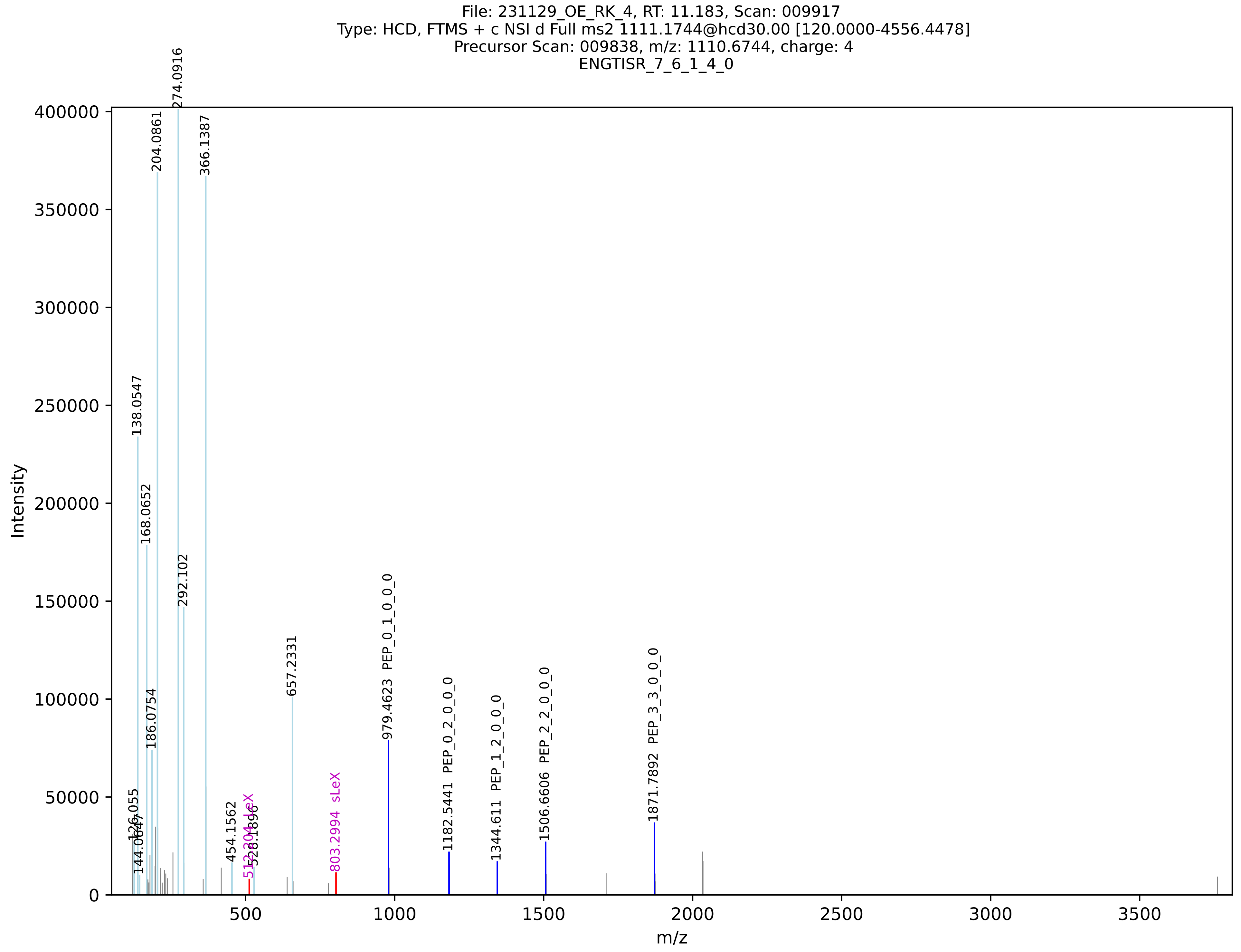
File: 231129_OE_RK_4, RT: 11.183, Scan: 009917
Type: HCD, FTMS + c NSI d Full ms2 1111.1744@hcd30.00 [120.0000-4556.4478]
Precursor Scan: 009838, m/z: 1110.6744, charge: 4
ENGTISR_7_6_1_4_0
Intensity
m/z
0
50000
100000
150000
200000
250000
300000
350000
400000
500
1000
1500
2000
2500
3000
3500
274.0916
204.0861
366.1387
138.0547
168.0652
292.102
186.0754
126.055
144.0647
454.1562
512.204 LeX
528.1896
657.2331
803.2994 sLeX
979.4623 PEP_0_1_0_0_0
1182.5441 PEP_0_2_0_0_0
1344.611 PEP_1_2_0_0_0
1506.6606 PEP_2_2_0_0_0
1871.7892 PEP_3_3_0_0_0

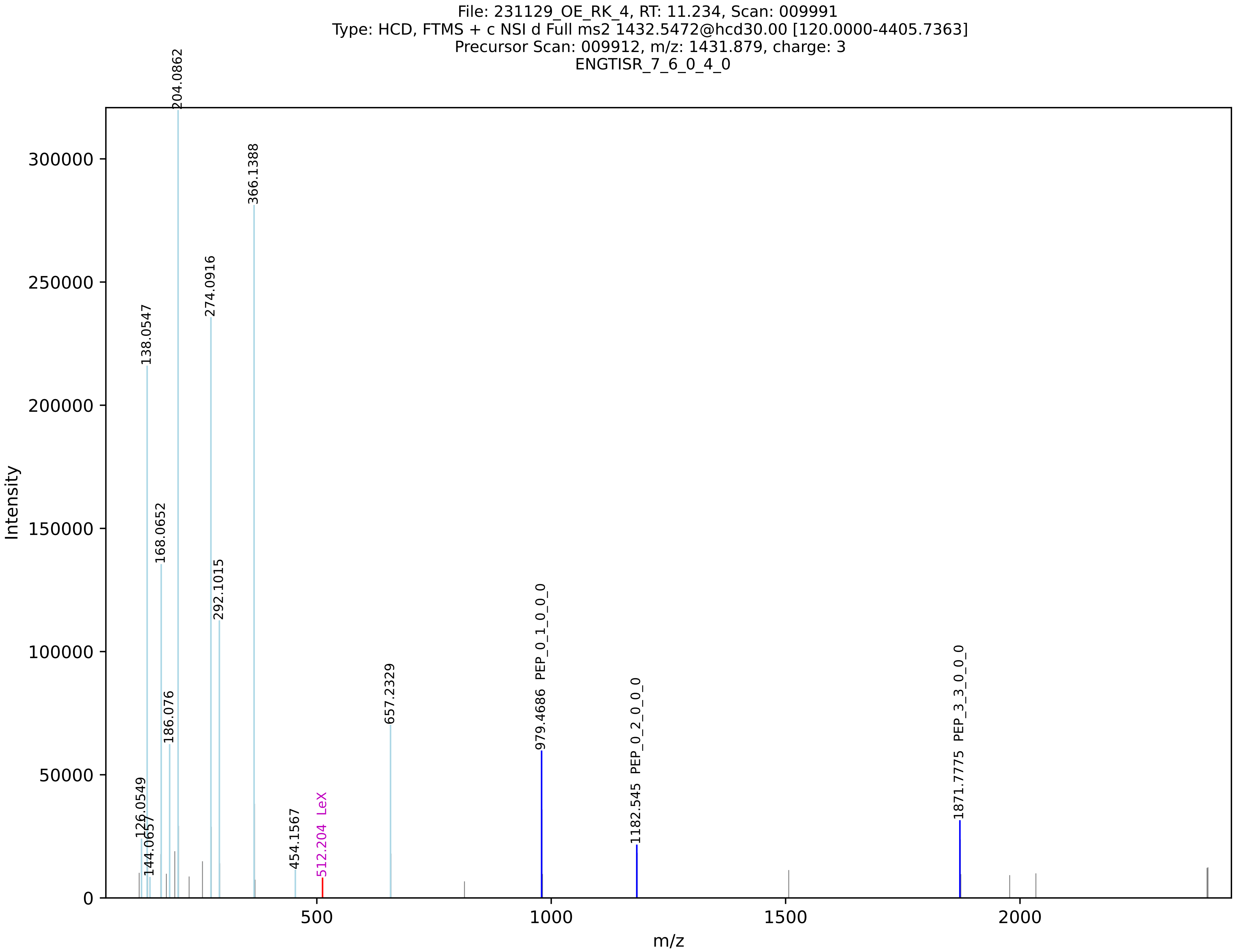

File: 231129_OE_RK_4, RT: 11.234, Scan: 009991
Type: HCD, FTMS + c NSI d Full ms2 1432.5472@hcd30.00 [120.0000-4405.7363]
Precursor Scan: 009912, m/z: 1431.879, charge: 3
ENGTISR_7_6_0_4_0
Intensity
m/z
204.0862
366.1388
274.0916
138.0547
168.0652
292.1015
186.076
126.0549
144.0657
454.1567
512.204 LeX
657.2329
979.4686 PEP_0_1_0_0_0
1182.545 PEP_0_2_0_0_0
1871.7775 PEP_3_3_0_0_0

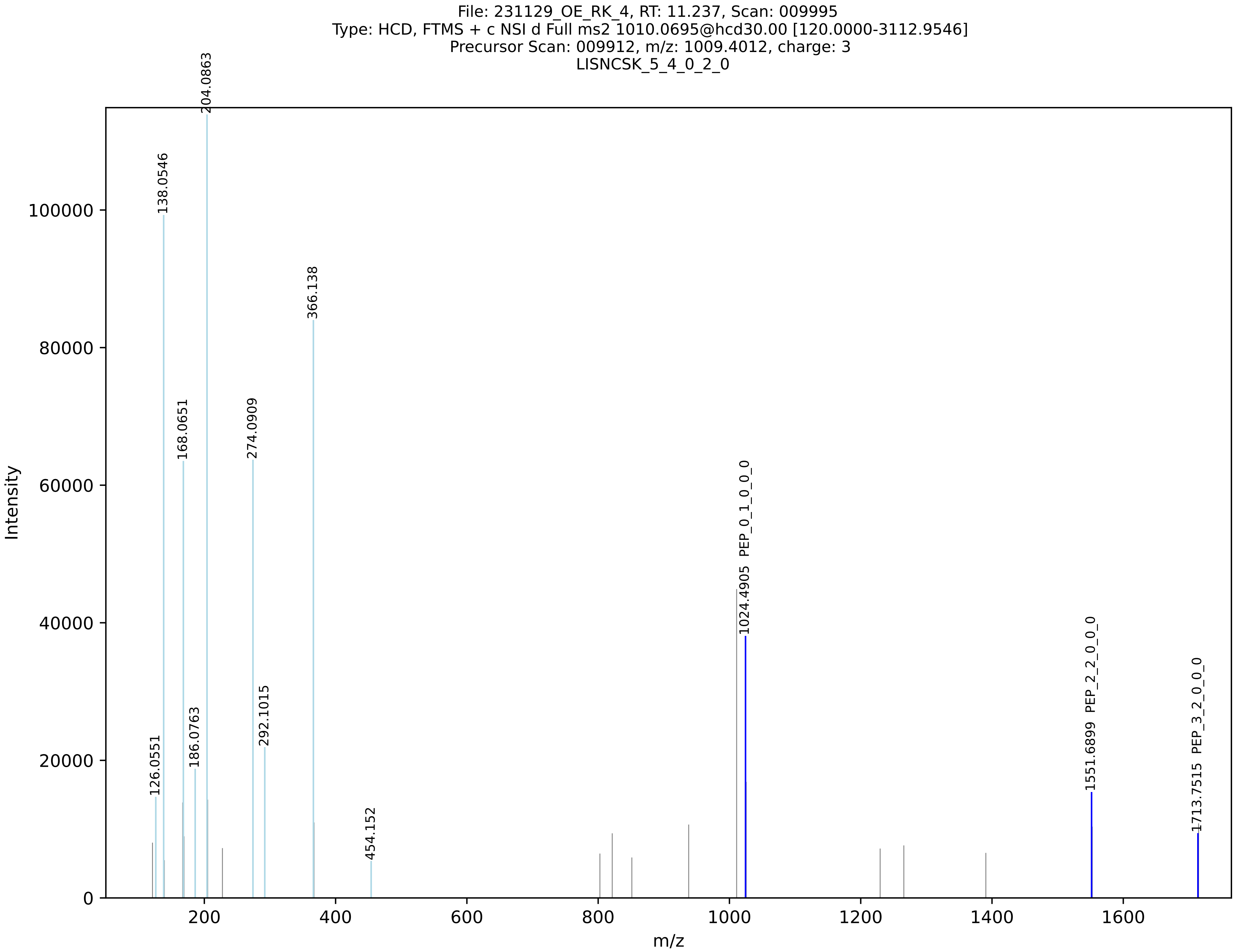
File: 231129_OE_RK_4, RT: 11.237, Scan: 009995
Type: HCD, FTMS + c NSI d Full ms2 1010.0695@hcd30.00 [120.0000-3112.9546]
Precursor Scan: 009912, m/z: 1009.4012, charge: 3
LISNCSK_5_4_0_2_0
Intensity
m/z
0
20000
40000
60000
80000
100000
200
400
600
800
1000
1200
1400
1600
204.0863
138.0546
366.138
168.0651
274.0909
292.1015
186.0763
126.0551
454.152
1024.4905 PEP_0_1_0_0_0
1551.6899 PEP_2_2_0_0_0
1713.7515 PEP_3_2_0_0_0

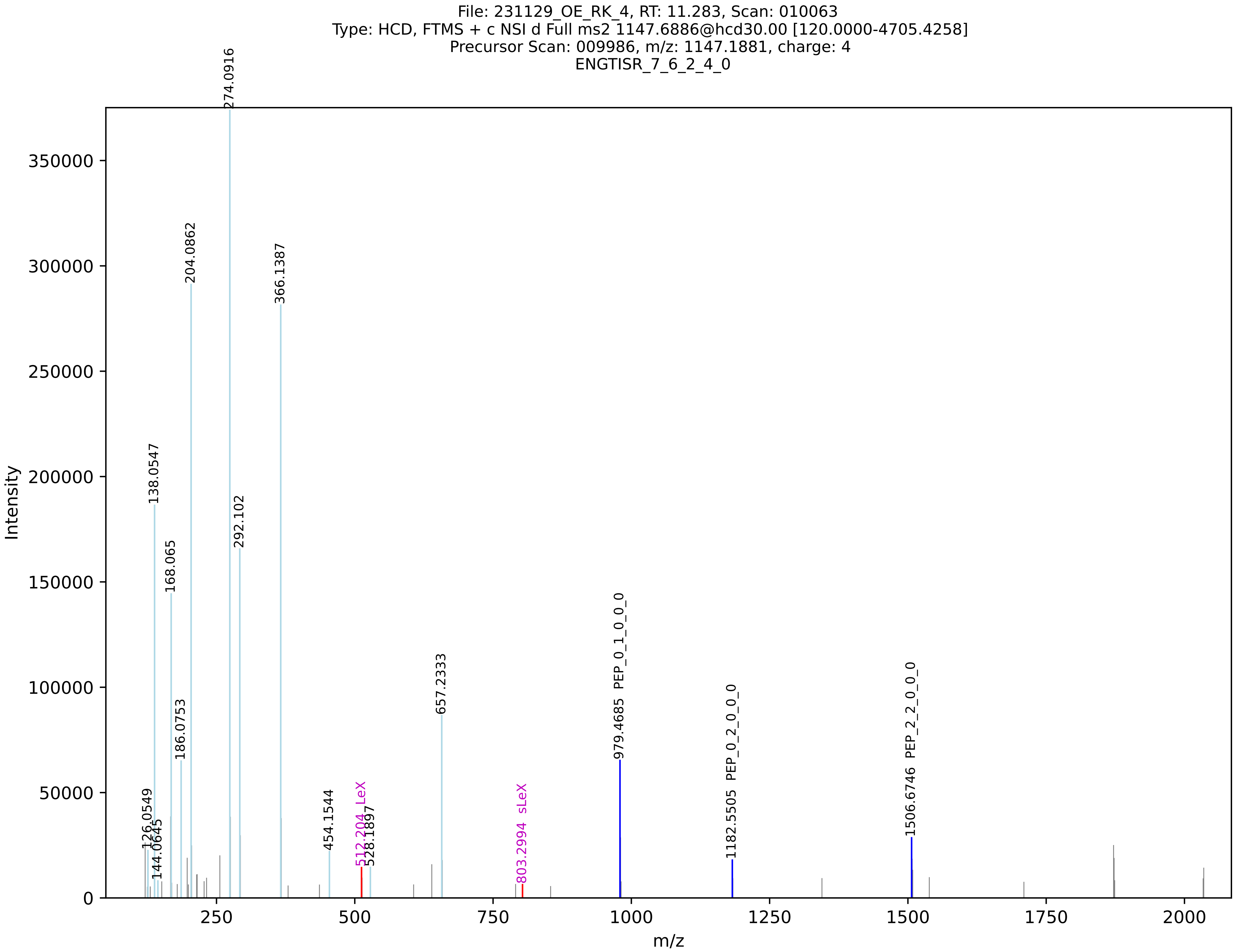
File: 231129_OE_RK_4, RT: 11.283, Scan: 010063
Type: HCD, FTMS + c NSI d Full ms2 1147.6886@hcd30.00 [120.0000-4705.4258]
Precursor Scan: 009986, m/z: 1147.1881, charge: 4
ENGTISR_7_6_2_4_0
274.0916
204.0862
366.1387
138.0547
292.102
168.065
186.0753
126.0549
144.0645
454.1544
512.204 LeX
528.1897
657.2333
803.2994 sLeX
979.4685 PEP_0_1_0_0_0
1182.5505 PEP_0_2_0_0_0
1506.6746 PEP_2_2_0_0_0
Intensity
m/z
0
50000
100000
150000
200000
250000
300000
350000
250
500
750
1000
1250
1500
1750
2000

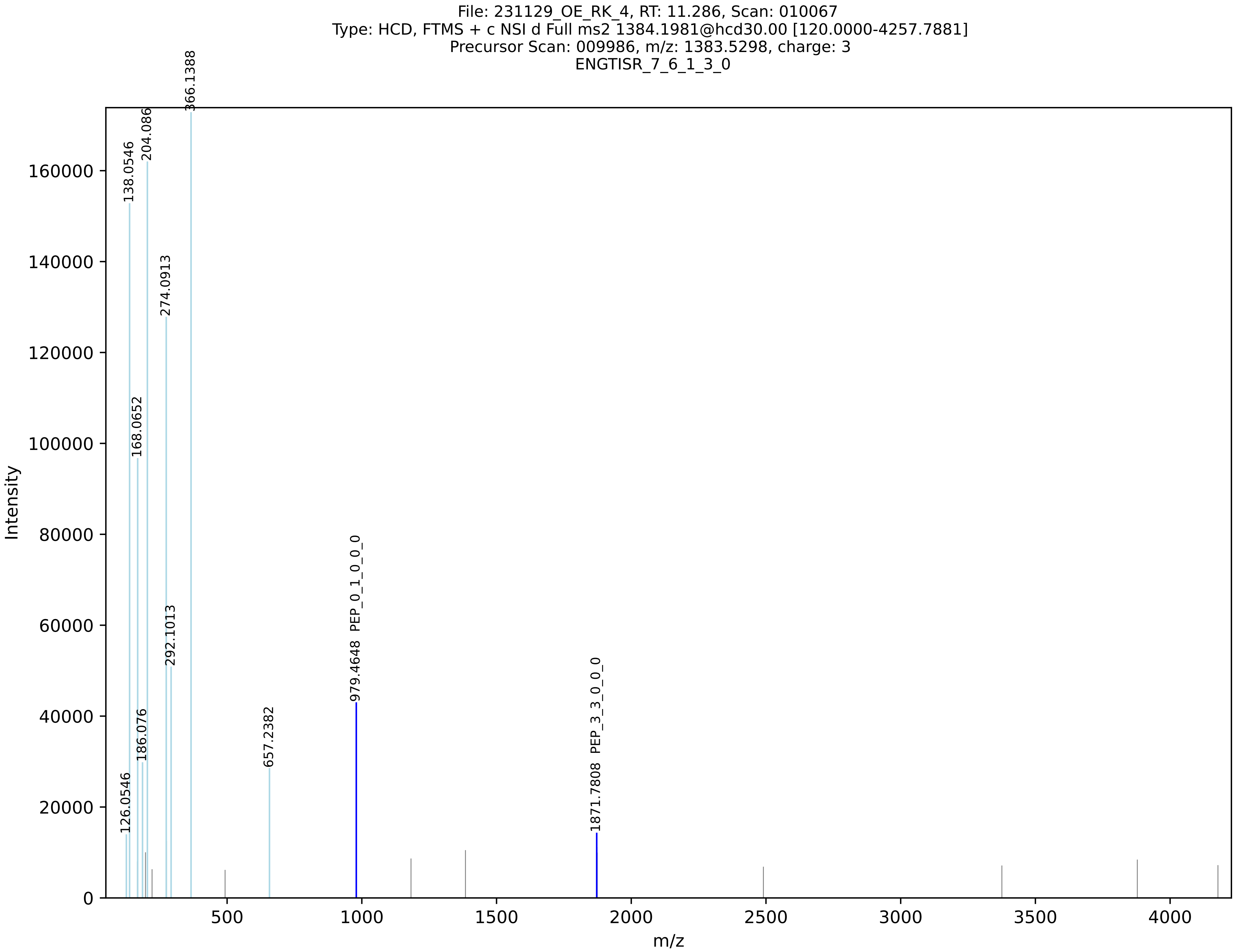
File: 231129_OE_RK_4, RT: 11.286, Scan: 010067
Type: HCD, FTMS + c NSI d Full ms2 1384.1981@hcd30.00 [120.0000-4257.7881]
Precursor Scan: 009986, m/z: 1383.5298, charge: 3
ENGTISR_7_6_1_3_0
366.1388
204.086
138.0546
274.0913
168.0652
292.1013
186.076
126.0546
657.2382
979.4648 PEP_0_1_0_0_0
1871.7808 PEP_3_3_0_0_0
Intensity
m/z
0
20000
40000
60000
80000
100000
120000
140000
160000
500
1000
1500
2000
2500
3000
3500
4000

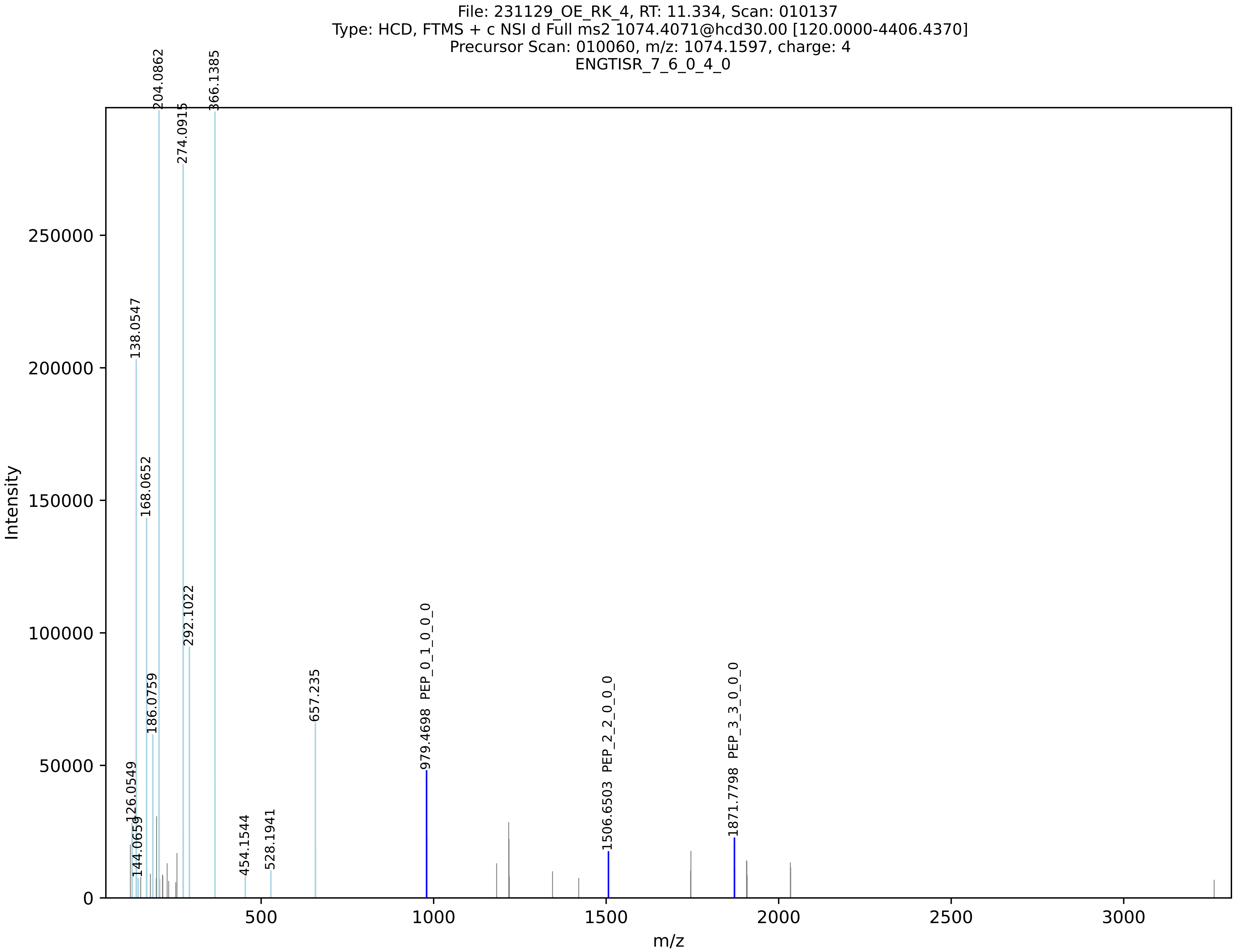
File: 231129_OE_RK_4, RT: 11.334, Scan: 010137
Type: HCD, FTMS + c NSI d Full ms2 1074.4071@hcd30.00 [120.0000-4406.4370]
Precursor Scan: 010060, m/z: 1074.1597, charge: 4
ENGTISR_7_6_0_4_0
Intensity
m/z
204.0862
366.1385
274.0915
138.0547
168.0652
292.1022
186.0759
126.0549
144.0659
454.1544
528.1941
657.235
979.4698 PEP_0_1_0_0_0
1506.6503 PEP_2_2_0_0_0
1871.7798 PEP_3_3_0_0_0
0
50000
100000
150000
200000
250000
500
1000
1500
2000
2500
3000

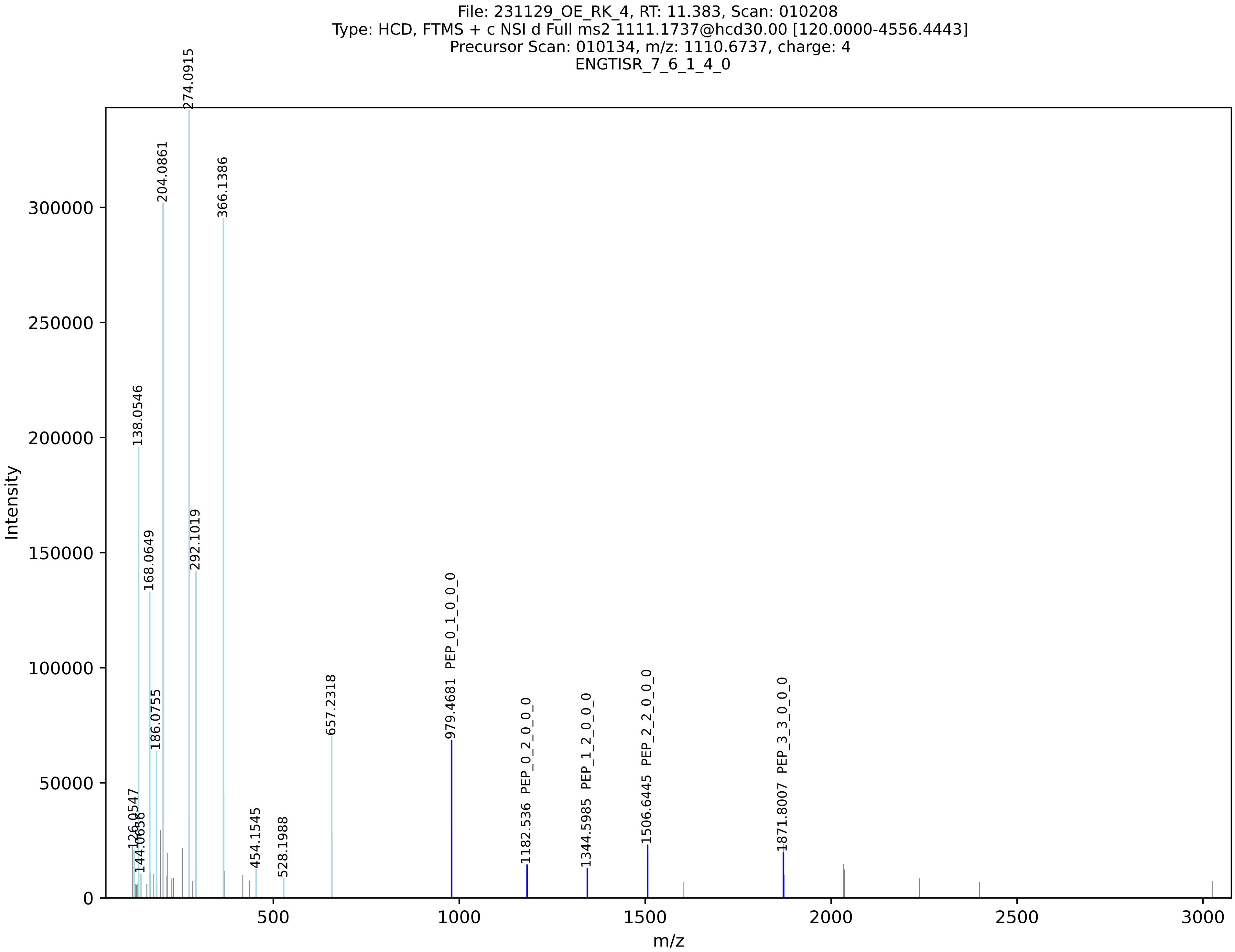
File: 231129_OE_RK_4, RT: 11.383, Scan: 010208
Type: HCD, FTMS + c NSI d Full ms2 1111.1737@hcd30.00 [120.0000-4556.4443]
Precursor Scan: 010134, m/z: 1110.6737, charge: 4
ENGTISR_7_6_1_4_0
Intensity
m/z
274.0915
204.0861
366.1386
138.0546
292.1019
168.0649
186.0755
126.0547
144.0656
454.1545
528.1988
657.2318
979.4681 PEP_0_1_0_0_
1182.536 PEP_0_2_0_0_
1344.5985 PEP_1_2_0_0_
1506.6445 PEP_2_2_0_0_
1871.8007 PEP_3_3_0_0_
0
50000
100000
150000
200000
250000
300000
500
1000
1500
2000
2500
3000

File: 231129_OE_RK_4, RT: 11.436, Scan: 010284
Type: HCD, FTMS + c NSI d Full ms2 1432.5458@hcd30.00 [120.0000-4405.7319]
Precursor Scan: 010206, m/z: 1431.8775, charge: 3
ENGTISR_7_6_0_4_0

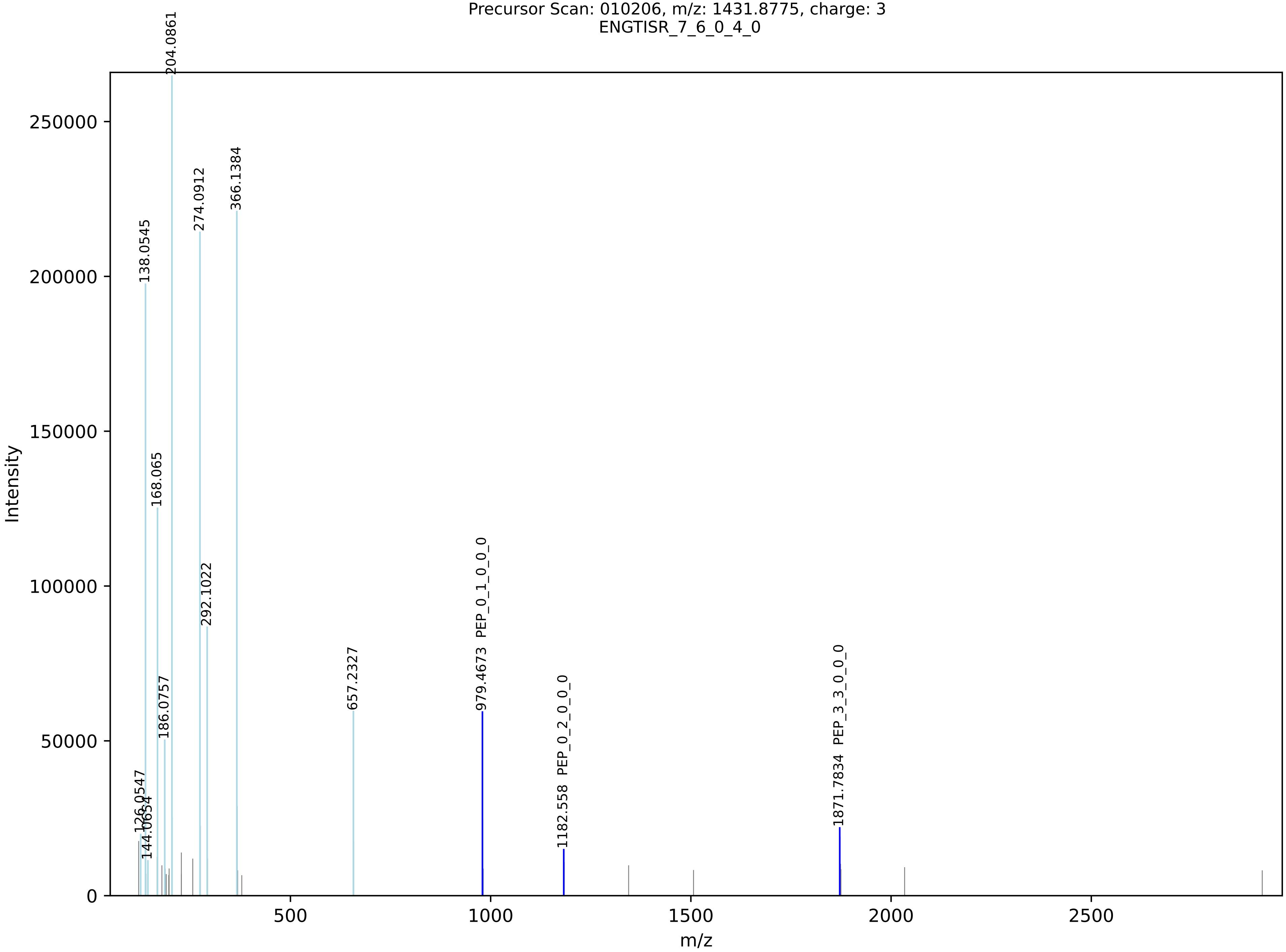

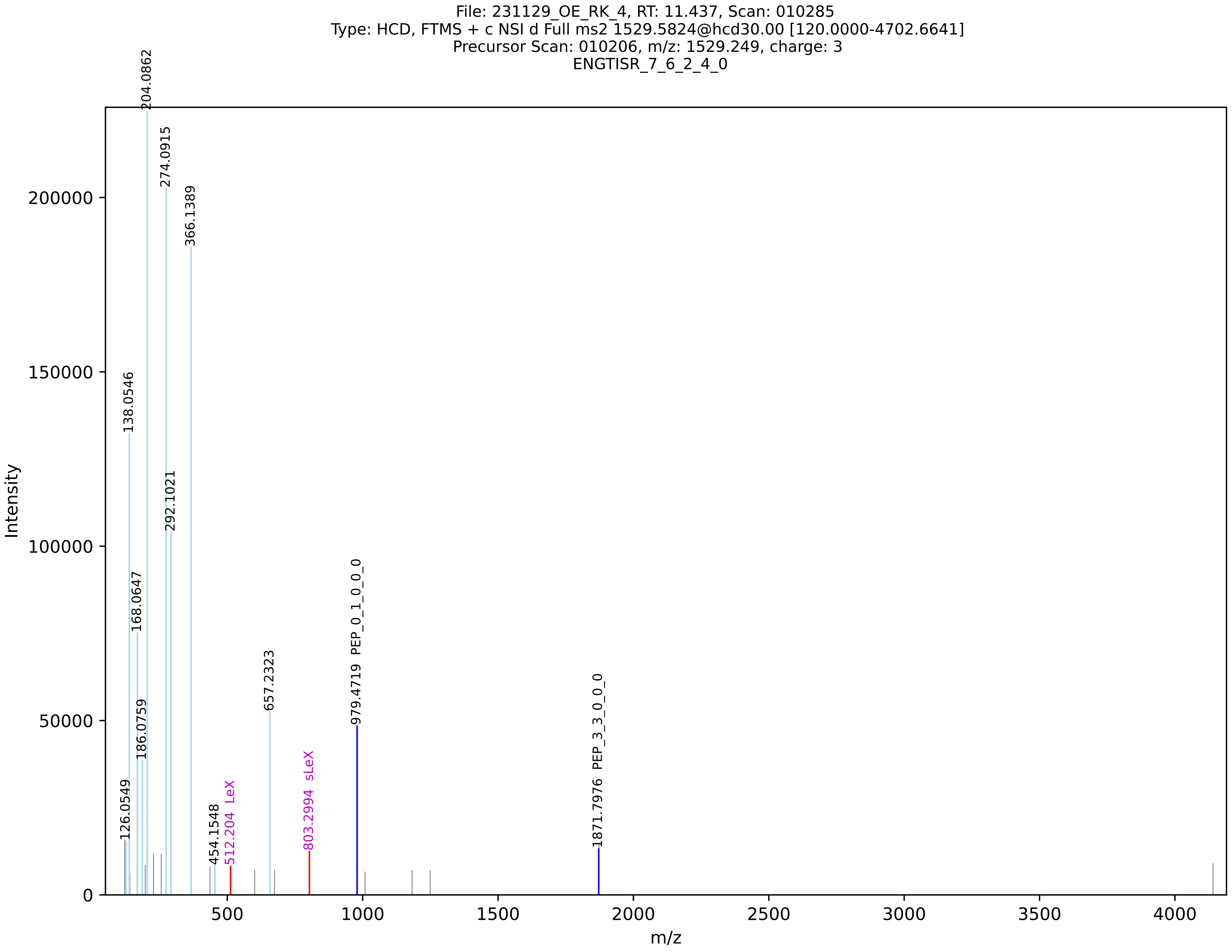

File: 231129_OE_RK_4, RT: 11.437, Scan: 010285
Type: HCD, FTMS + c NSI d Full ms2 1529.5824@hcd30.00 [120.0000-4702.6641]
Precursor Scan: 010206, m/z: 1529.249, charge: 3
ENGTISR_7_6_2_4_0
Intensity
m/z
204.0862
274.0915
366.1389
138.0546
292.1021
168.0647
186.0759
126.0549
454.1548
512.204 LeX
657.2323
803.2994 sLeX
979.4719 PEP_0_1_0_0_0
1871.7976 PEP_3_3_0_0_0
0
50000
100000
150000
200000
500
1000
1500
2000
2500
3000
3500
4000

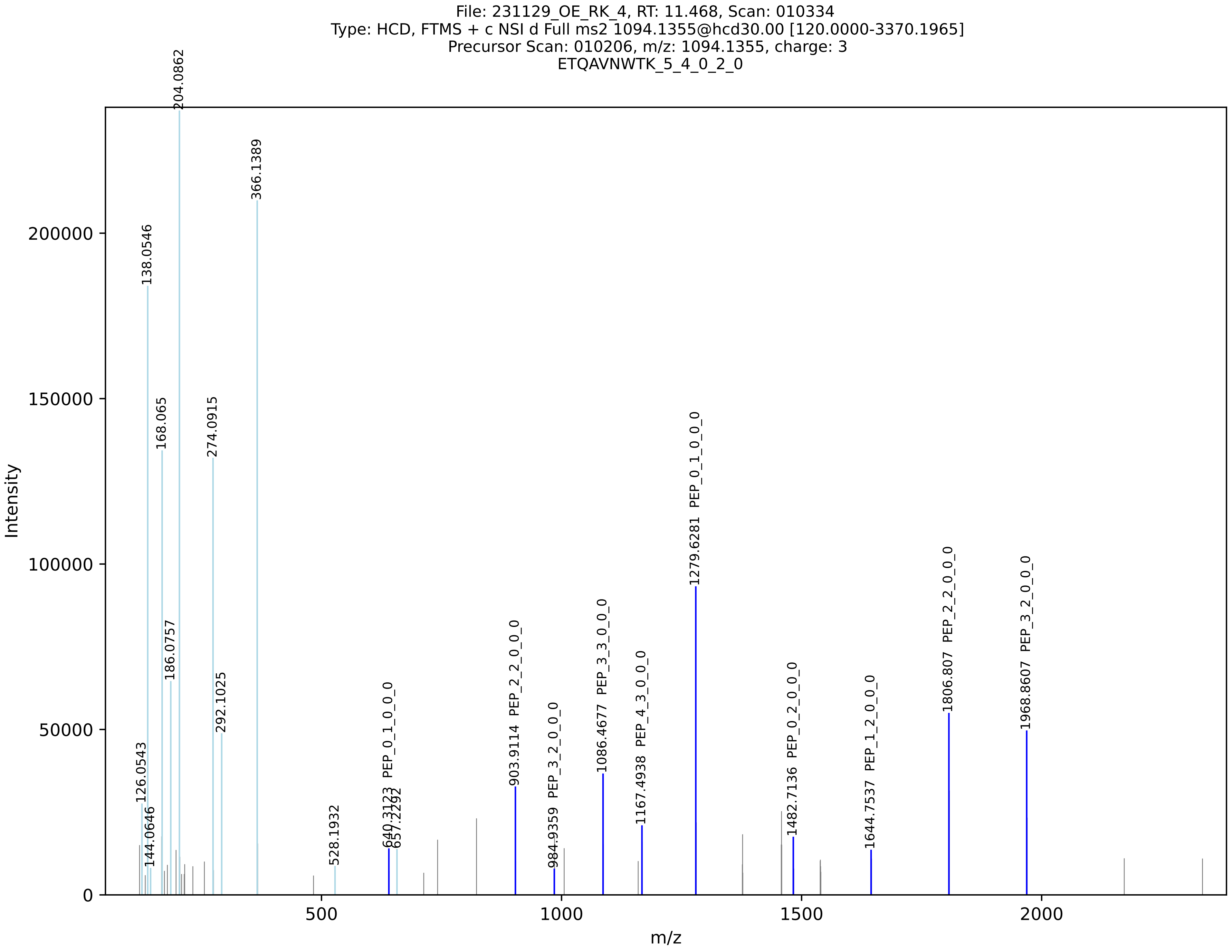

File: 231129_OE_RK_4, RT: 11.468, Scan: 010334
Type: HCD, FTMS + c NSI d Full ms2 1094.1355@hcd30.00 [120.0000-3370.1965]
Precursor Scan: 010206, m/z: 1094.1355, charge: 3
ETQAVNWTK_5_4_0_2_0
Intensity
m/z
0
50000
100000
150000
200000
500
1000
1500
2000
204.0862
366.1389
138.0546
168.065
274.0915
186.0757
292.1025
126.0543
144.0646
528.1932
640.3123 PEP_0_1_0_0_0
657.2292
903.9114 PEP_2_2_0_0_0
984.9359 PEP_3_2_0_0_0
1086.4677 PEP_3_3_0_0_0
1167.4938 PEP_4_3_0_0_0
1279.6281 PEP_0_1_0_0_0
1482.7136 PEP_0_2_0_0_0
1644.7537 PEP_1_2_0_0_0
1806.807 PEP_2_2_0_0_0
1968.8607 PEP_3_2_0_0_0

File: 231129_OE_RK_4, RT: 11.487, Scan: 010359
Type: HCD, FTMS + c NSI d Full ms2 1384.1991@hcd30.00 [120.0000-4257.7915]
Precursor Scan: 010279, m/z: 1383.5309, charge: 3
ENGTISR_7_6_1_3_0

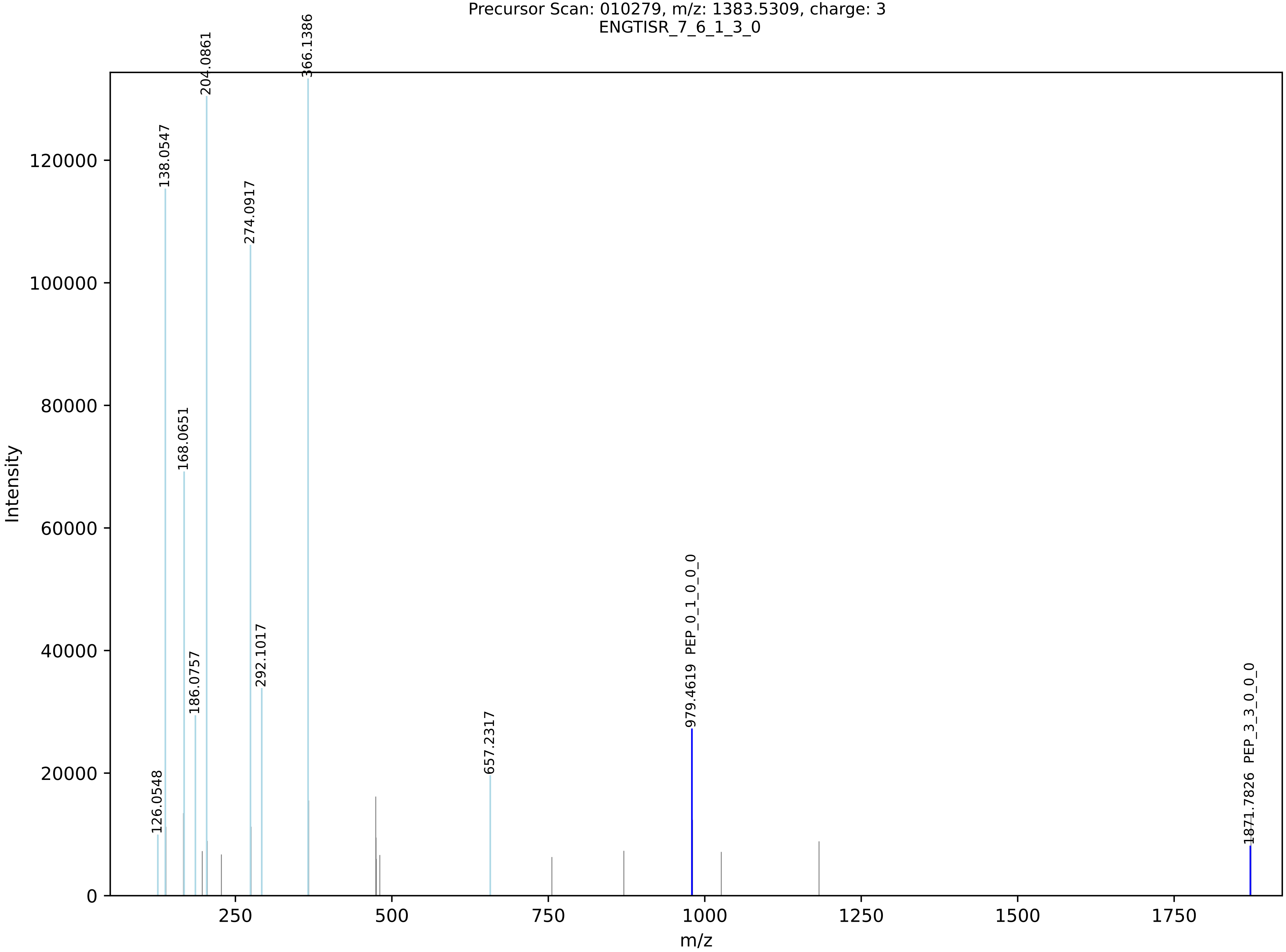

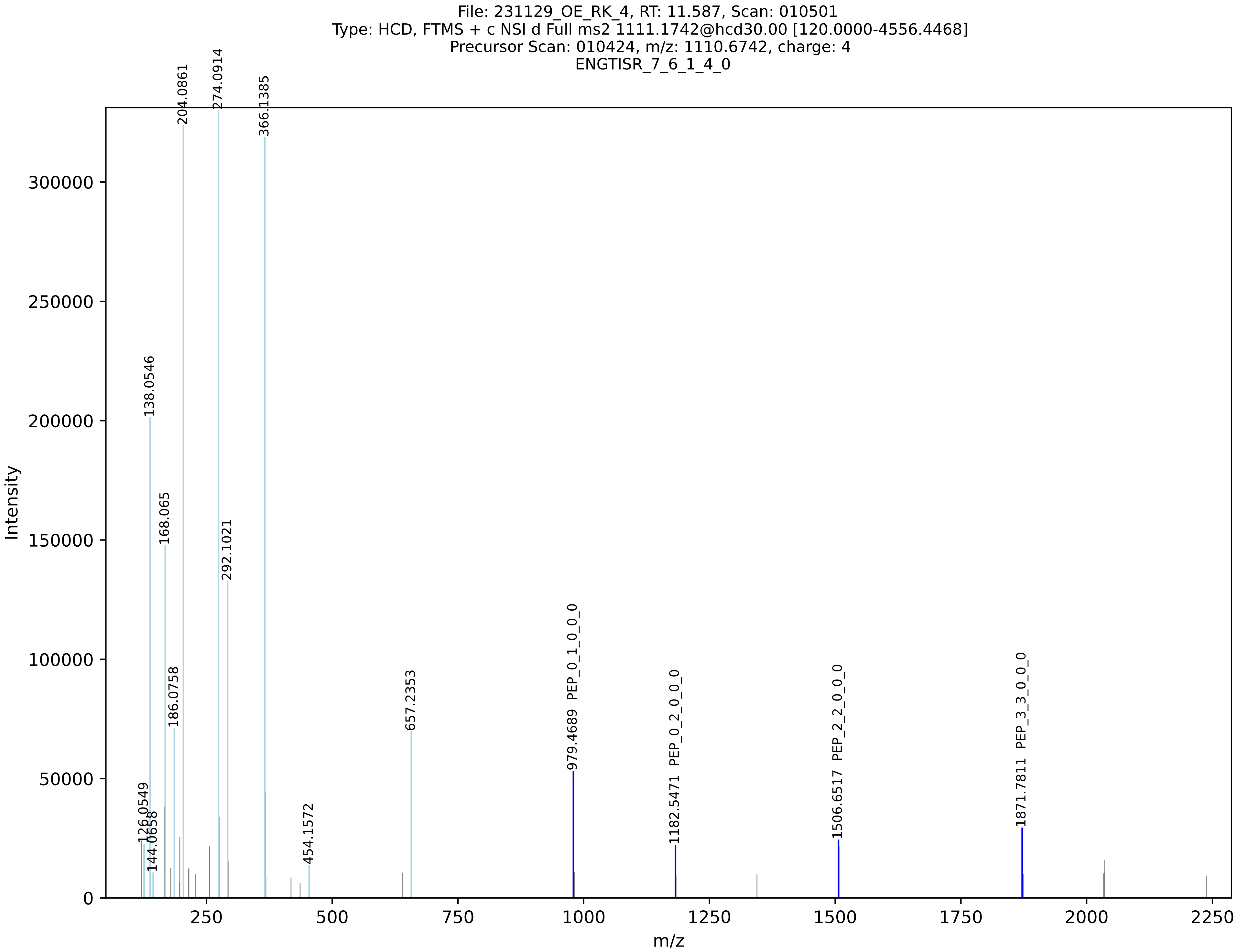
File: 231129_OE_RK_4, RT: 11.587, Scan: 010501
Type: HCD, FTMS + c NSI d Full ms2 1111.1742@hcd30.00 [120.0000-4556.4468]
Precursor Scan: 010424, m/z: 1110.6742, charge: 4
ENGTISR_7_6_1_4_0
Intensity
m/z
204.0861
274.0914
366.1385
138.0546
168.065
292.1021
186.0758
126.0549
144.0658
454.1572
657.2353
979.4689 PEP_0_1_0_0_0
1182.5471 PEP_0_2_0_0_0
1506.6517 PEP_2_2_0_0_0
1871.7811 PEP_3_3_0_0_0

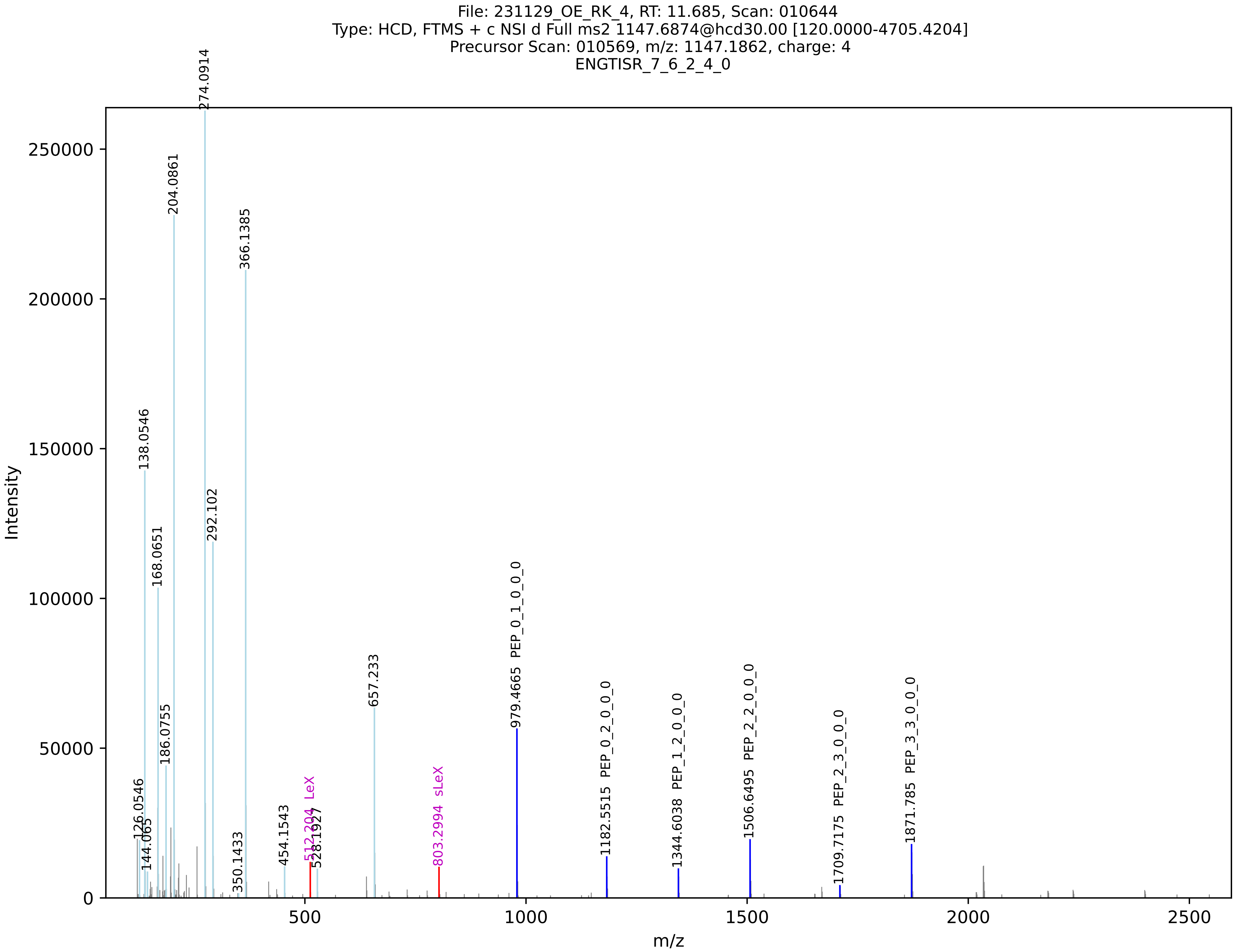

File: 231129_OE_RK_4, RT: 11.685, Scan: 010644
Type: HCD, FTMS + c NSI d Full ms2 1147.6874@hcd30.00 [120.0000-4705.4204]
Precursor Scan: 010569, m/z: 1147.1862, charge: 4
ENGTISR_7_6_2_4_0
Intensity
m/z
274.0914
204.0861
366.1385
138.0546
292.102
168.0651
186.0755
126.0546
144.065
350.1433
454.1543
512.204 LeX
528.1927
657.233
803.2994 sLeX
979.4665 PEP_0_1_0_0_0
1182.5515 PEP_0_2_0_0_0
1344.6038 PEP_1_2_0_0_0
1506.6495 PEP_2_2_0_0_0
1709.7175 PEP_2_3_0_0_0
1871.785 PEP_3_3_0_0_0

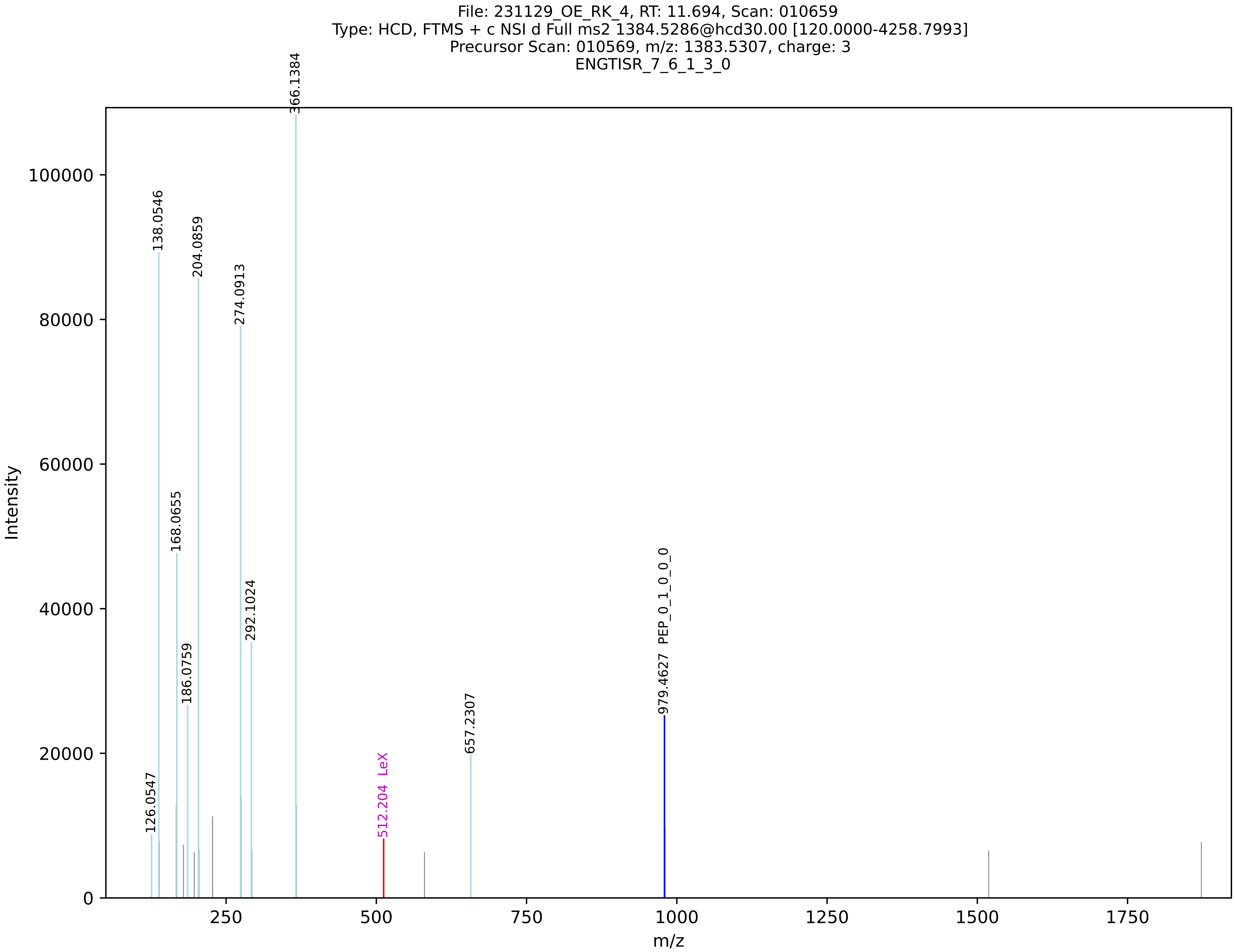
File: 231129_OE_RK_4, RT: 11.694, Scan: 010659
Type: HCD, FTMS + c NSI d Full ms2 1384.5286@hcd30.00 [120.0000-4258.7993]
Precursor Scan: 010569, m/z: 1383.5307, charge: 3
ENGTISR_7_6_1_3_0
366.1384
138.0546
204.0859
274.0913
168.0655
292.1024
186.0759
126.0547
512.204 LeX
657.2307
979.4627 PEP_0_1_0_0_0
Intensity
m/z
0
20000
40000
60000
80000
100000
250
500
750
1000
1250
1500
1750

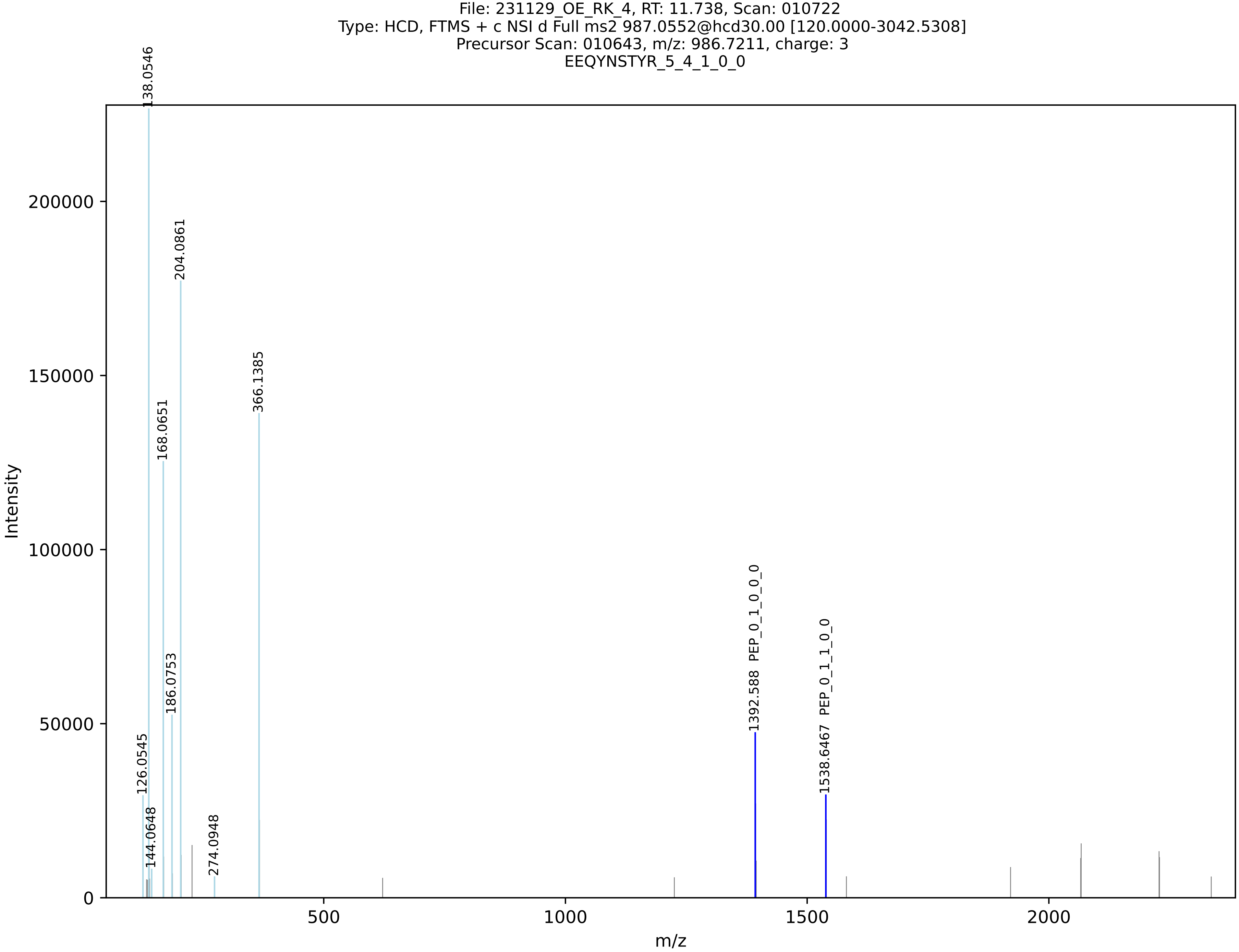
File: 231129_OE_RK_4, RT: 11.738, Scan: 010722
Type: HCD, FTMS + c NSI d Full ms2 987.0552@hcd30.00 [120.0000-3042.5308]
Precursor Scan: 010643, m/z: 986.7211, charge: 3
EEQYNSTYR_5_4_1_0_0
Intensity
m/z
138.0546
204.0861
366.1385
168.0651
186.0753
126.0545
144.0648
274.0948
1392.588 PEP_0_1_0_0_0
1538.6467 PEP_0_1_1_0_0
0
50000
100000
150000
200000
500
1000
1500
2000

File: 231129_OE_RK_4, RT: 11.743, Scan: 010730
Type: HCD, FTMS + c NSI d Full ms2 1335.1770@hcd30.00 [120.0000-4107.7837]
Precursor Scan: 010643, m/z: 1334.8435, charge: 3
ENGTISR_7_6_0_3_0

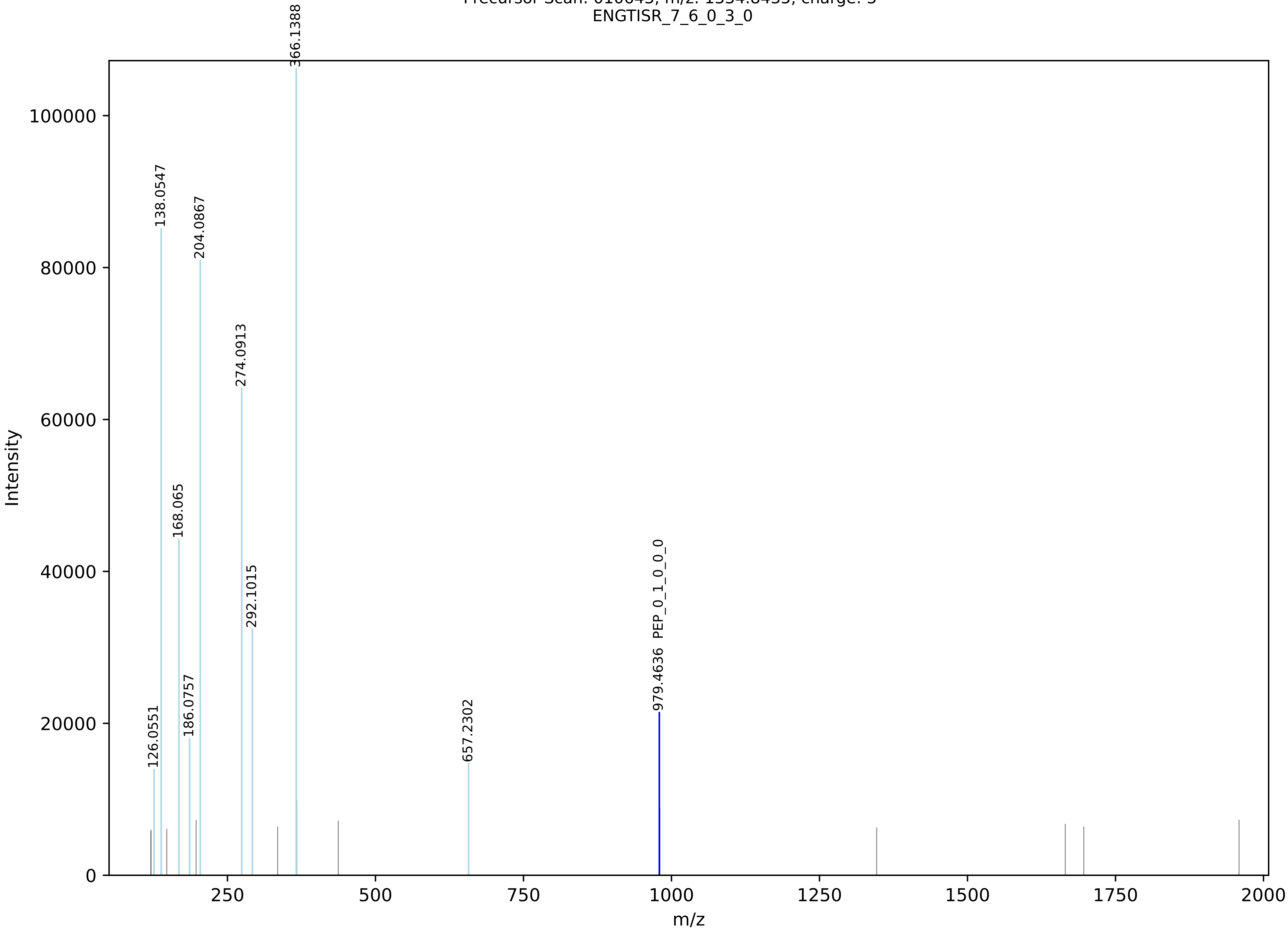

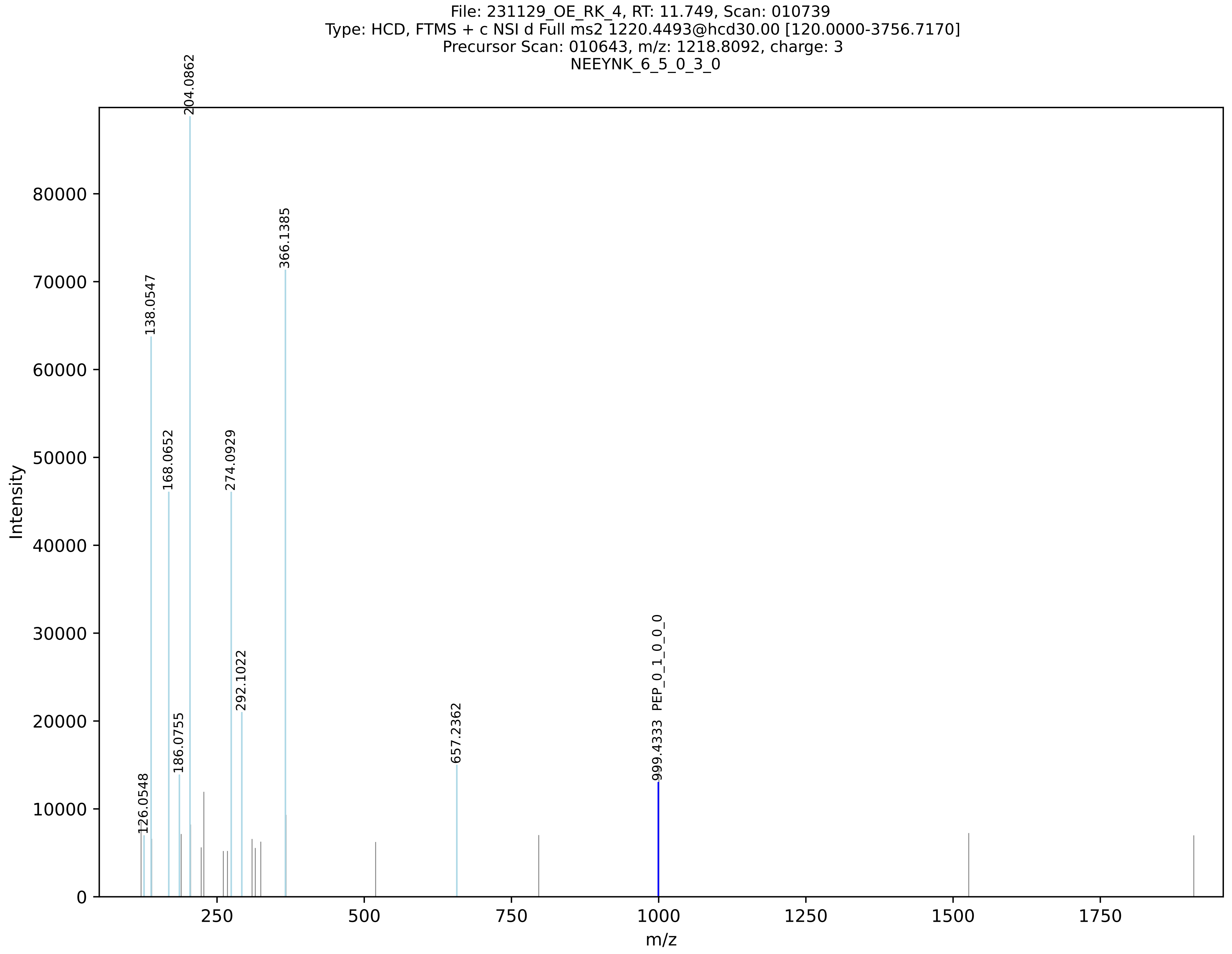
File: 231129_OE_RK_4, RT: 11.749, Scan: 010739
Type: HCD, FTMS + c NSI d Full ms2 1220.4493@hcd30.00 [120.0000-3756.7170]
Precursor Scan: 010643, m/z: 1218.8092, charge: 3
NEEYNK_6_5_0_3_0
Intensity
m/z
204.0862
366.1385
138.0547
168.0652
274.0929
292.1022
186.0755
126.0548
657.2362
999.4333 PEP_0_1_0_0_0
0
10000
20000
30000
40000
50000
60000
70000
80000
250
500
750
1000
1250
1500
1750

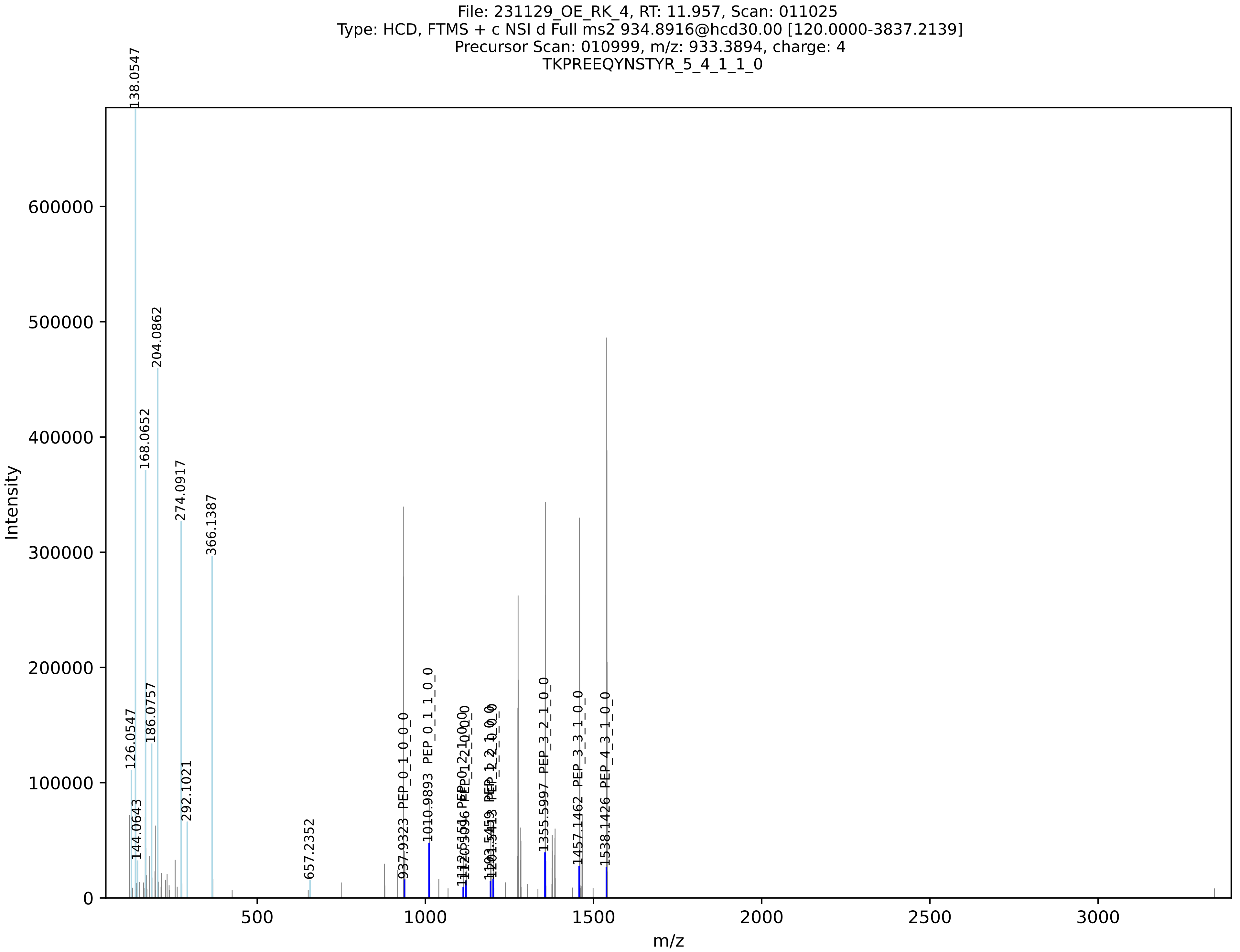
File: 231129_OE_RK_4, RT: 11.957, Scan: 011025
Type: HCD, FTMS + c NSI d Full ms2 934.8916@hcd30.00 [120.0000-3837.2139]
Precursor Scan: 010999, m/z: 933.3894, charge: 4
TKPREEQYNSTYR_5_4_1_1_0
Intensity
m/z
138.0547
204.0862
168.0652
274.0917
366.1387
186.0757
126.0547
292.1021
144.0643
657.2352
937.9323 PEP_0_1_0_0_0
1010.9893 PEP_0_1_1_0_0
1355.5997 PEP_3_2_1_0_0
1457.1462 PEP_3_3_1_0_0
1538.1426 PEP_4_3_1_0_0
0
100000
200000
300000
400000
500000
600000
500
1000
1500
2000
2500
3000

File: 231129_OE_RK_4, RT: 12.009, Scan: 011072
Type: HCD, FTMS + c NSI d Full ms2 1245.5205@hcd30.00 [120.0000-3833.4346]
Precursor Scan: 011058, m/z: 1244.5181, charge: 3
CATPHGDNASLEATFVK_5_4_0_1_0

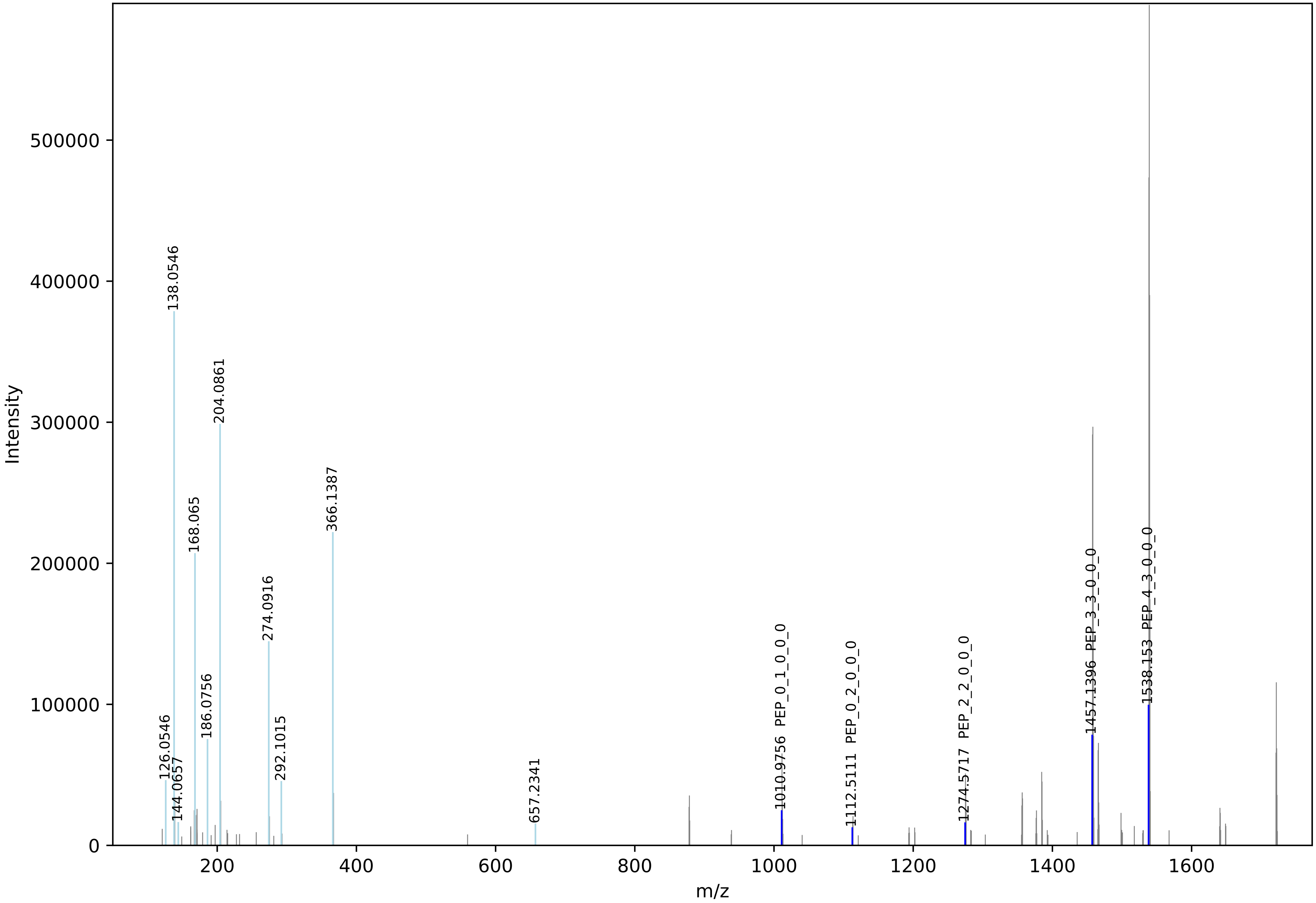

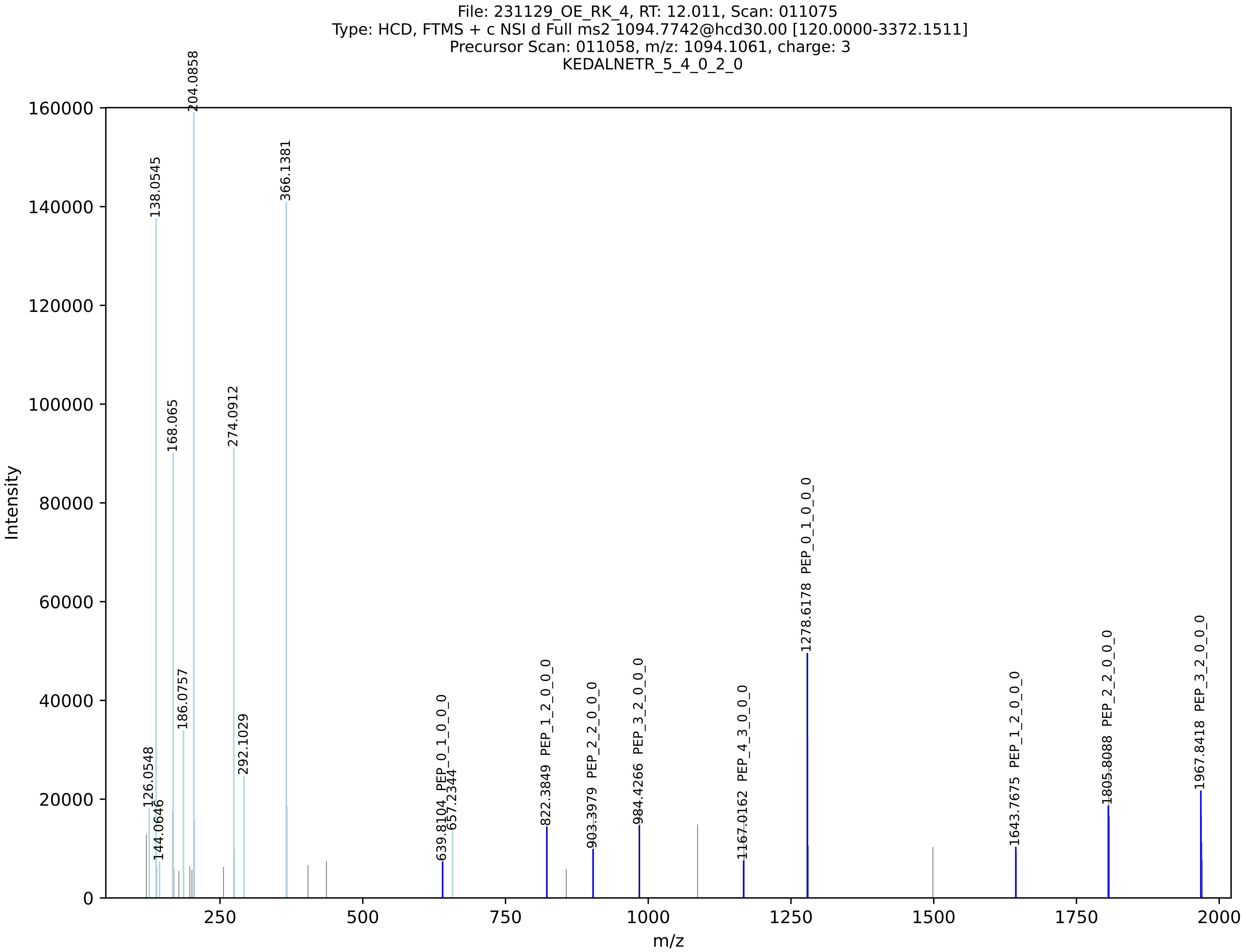

File: 231129_OE_RK_4, RT: 12.011, Scan: 011075
Type: HCD, FTMS + c NSI d Full ms2 1094.7742@hcd30.00 [120.0000-3372.1511]
Precursor Scan: 011058, m/z: 1094.1061, charge: 3
KEDALNETR_5_4_0_2_0
Intensity
m/z
204.0858
138.0545
366.1381
168.065
274.0912
186.0757
292.1029
126.0548
144.0646
639.8104 PEP_0_1_0_0_0
657.2344
822.3849 PEP_1_2_0_0_0
903.3979 PEP_2_2_0_0_0
984.4266 PEP_3_2_0_0_0
1167.0162 PEP_4_3_0_0_0
1278.6178 PEP_0_1_0_0_0
1643.7675 PEP_1_2_0_0_0
1805.8088 PEP_2_2_0_0_0
1967.8418 PEP_3_2_0_0_0

File: 231129_OE_RK_4, RT: 12.112, Scan: 011197
Type: HCD, FTMS + c NSI d Full ms2 1190.8344@hcd30.00 [120.0000-3666.0952]
Precursor Scan: 011166, m/z: 1190.4989, charge: 3
CATPHGDNASLEATFVK_4_4_0_1_0

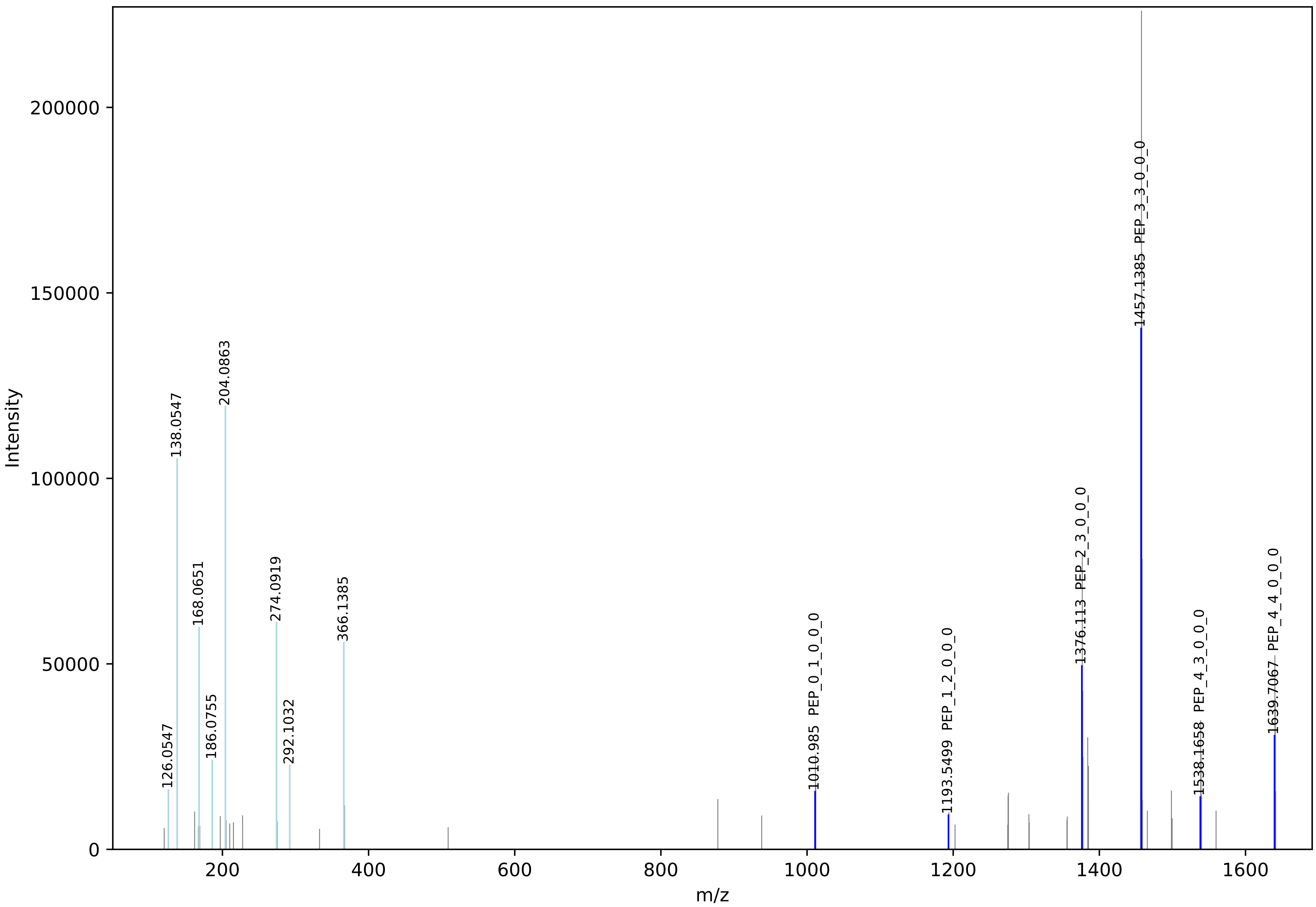

File: 231129_OE_RK_4, RT: 12.144, Scan: 011242
Type: HCD, FTMS + c NSI d Full ms2 1111.1727@hcd30.00 [120.0000-4556.4409]
Precursor Scan: 011191, m/z: 1110.6716, charge: 4
ENGTISR_7_6_1_4_0

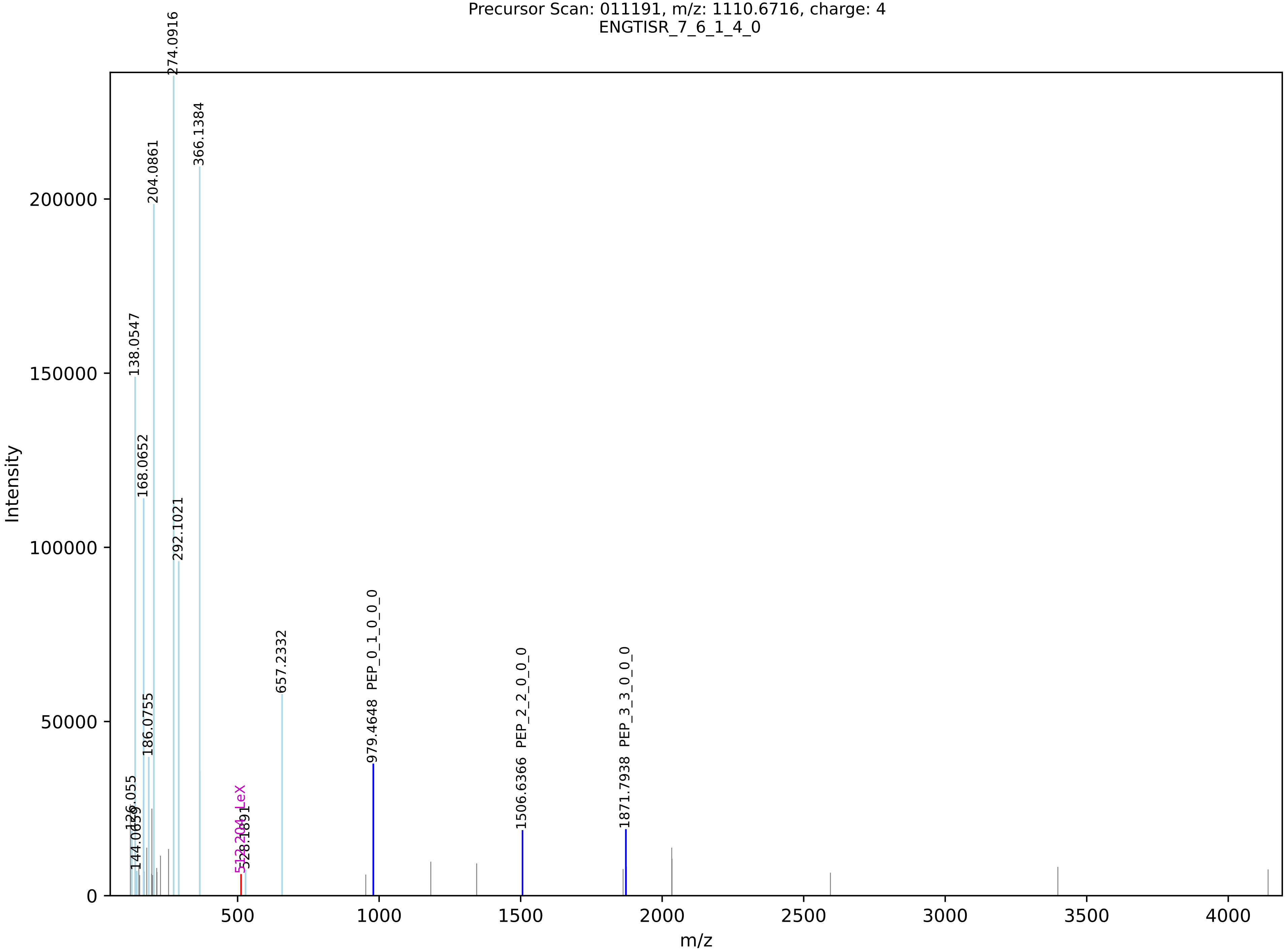

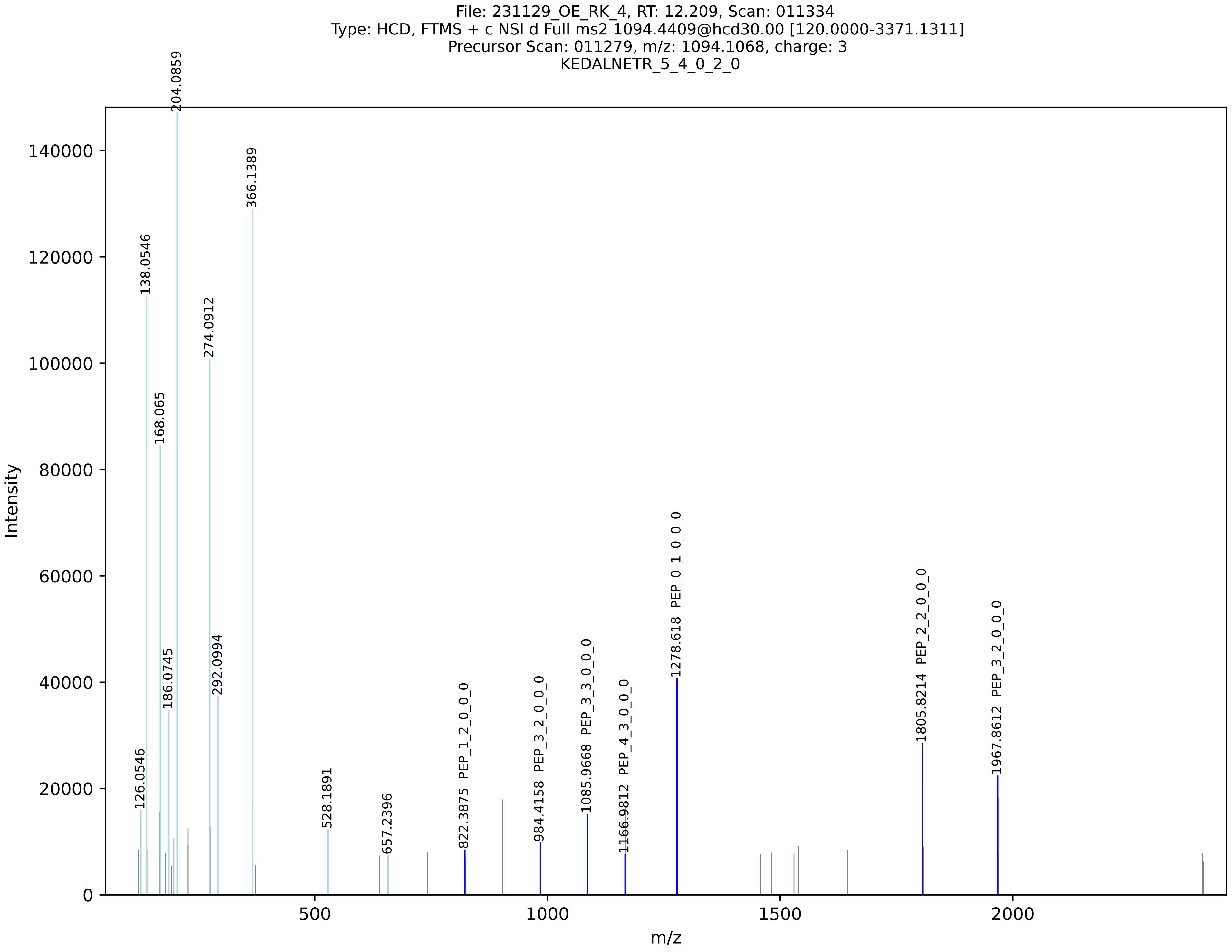
File: 231129_OE_RK_4, RT: 12.209, Scan: 011334
Type: HCD, FTMS + c NSI d Full ms2 1094.4409@hcd30.00 [120.0000-3371.1311]
Precursor Scan: 011279, m/z: 1094.1068, charge: 3
KEDALNETR_5_4_0_2_0
Intensity
m/z
204.0859
366.1389
138.0546
274.0912
168.065
186.0745
292.0994
126.0546
528.1891
657.2396
822.3875 PEP_1_2_0_0_0
984.4158 PEP_3_2_0_0_0
1085.9668 PEP_3_3_0_0_0
1166.9812 PEP_4_3_0_0_0
1278.618 PEP_0_1_0_0_0
1805.8214 PEP_2_2_0_0_0
1967.8612 PEP_3_2_0_0_0

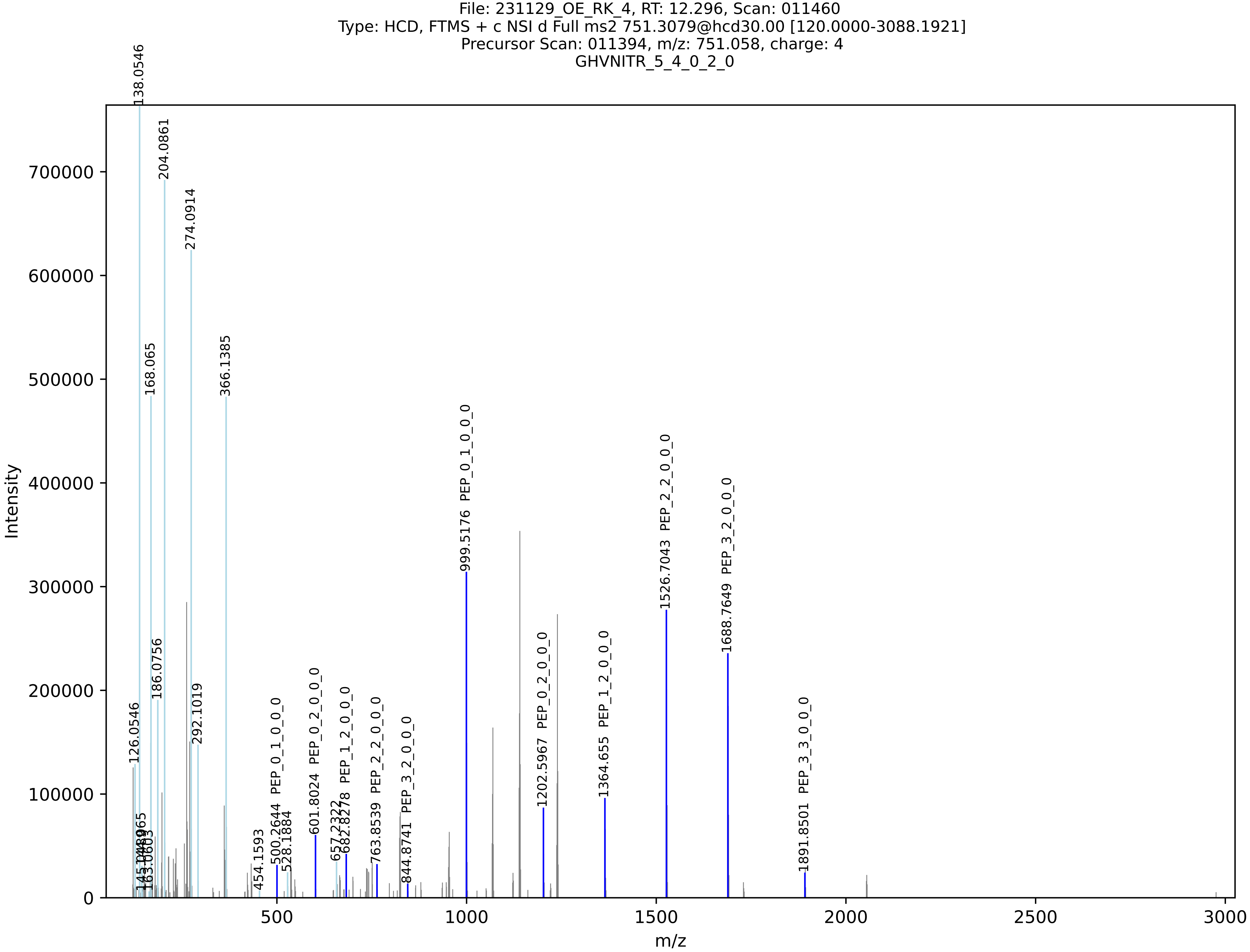

File: 231129_OE_RK_4, RT: 12.296, Scan: 011460
Type: HCD, FTMS + c NSI d Full ms2 751.3079@hcd30.00 [120.0000-3088.1921]
Precursor Scan: 011394, m/z: 751.058, charge: 4
GHVNITR_5_4_0_2_0
Intensity
m/z
138.0546
204.0861
274.0914
168.065
366.1385
186.0756
126.0546
292.1019
163.0603
454.1593
500.2644 PEP_0_1_0_0_0
528.1884
601.8024 PEP_0_2_0_0_0
657.2322
682.8278 PEP_1_2_0_0_0
763.8539 PEP_2_2_0_0_0
844.8741 PEP_3_2_0_0_0
999.5176 PEP_0_1_0_0_0
1202.5967 PEP_0_2_0_0_0
1364.655 PEP_1_2_0_0_0
1526.7043 PEP_2_2_0_0_0
1688.7649 PEP_3_2_0_0_0
1891.8501 PEP_3_3_0_0_0

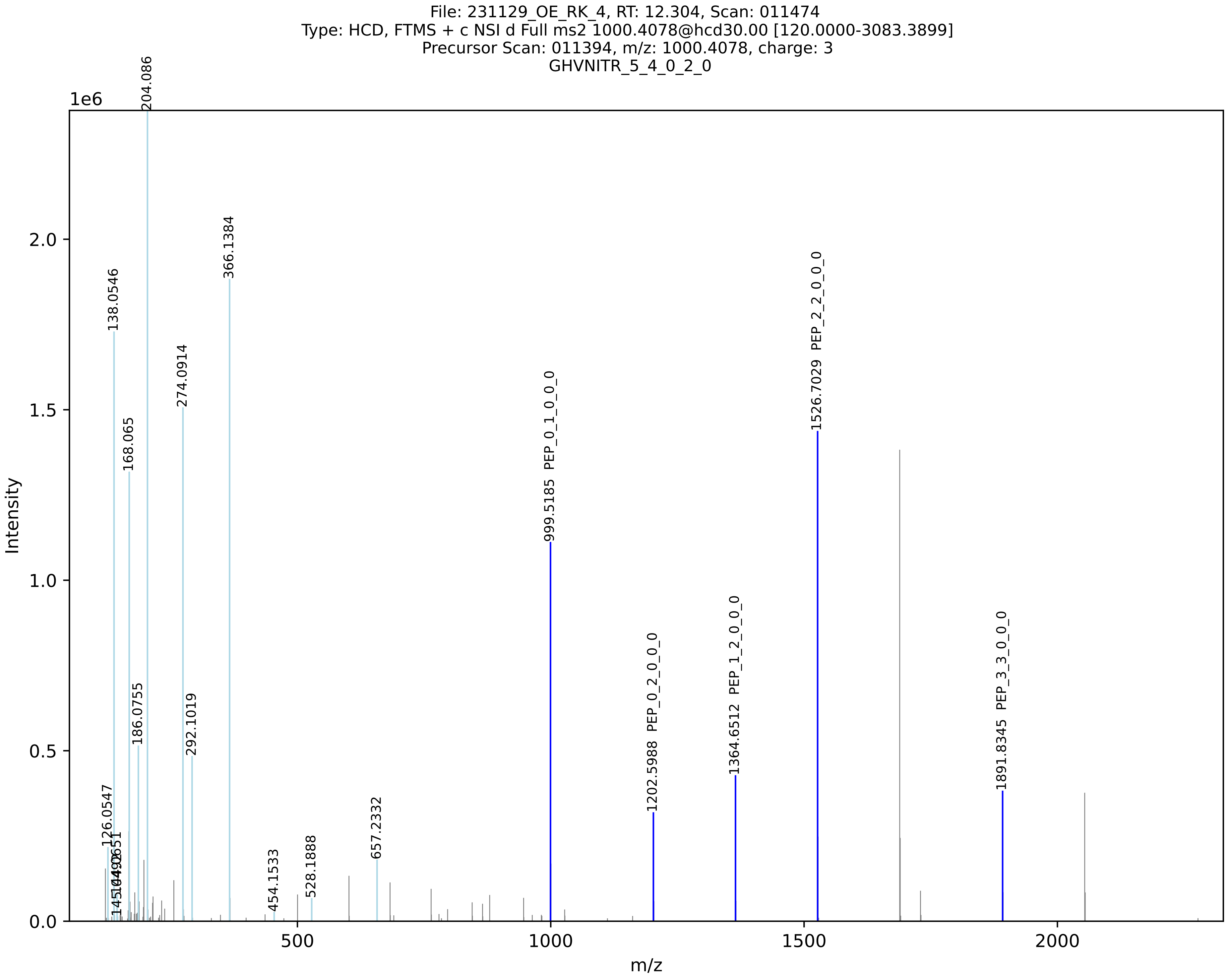
File: 231129_OE_RK_4, RT: 12.304, Scan: 011474
Type: HCD, FTMS + c NSI d Full ms2 1000.4078@hcd30.00 [120.0000-3083.3899]
Precursor Scan: 011394, m/z: 1000.4078, charge: 3
GHVNITR_5_4_0_2_0
1e6
Intensity
m/z
0.0
0.5
1.0
1.5
2.0
500
1000
1500
2000
204.086
138.0546
168.065
186.0755
126.0547
274.0914
292.1019
366.1384
454.1533
528.1888
657.2332
999.5185 PEP_0_1_0_0_0
1202.5988 PEP_0_2_0_0_0
1364.6512 PEP_1_2_0_0_0
1526.7029 PEP_2_2_0_0_0
1891.8345 PEP_3_3_0_0_0

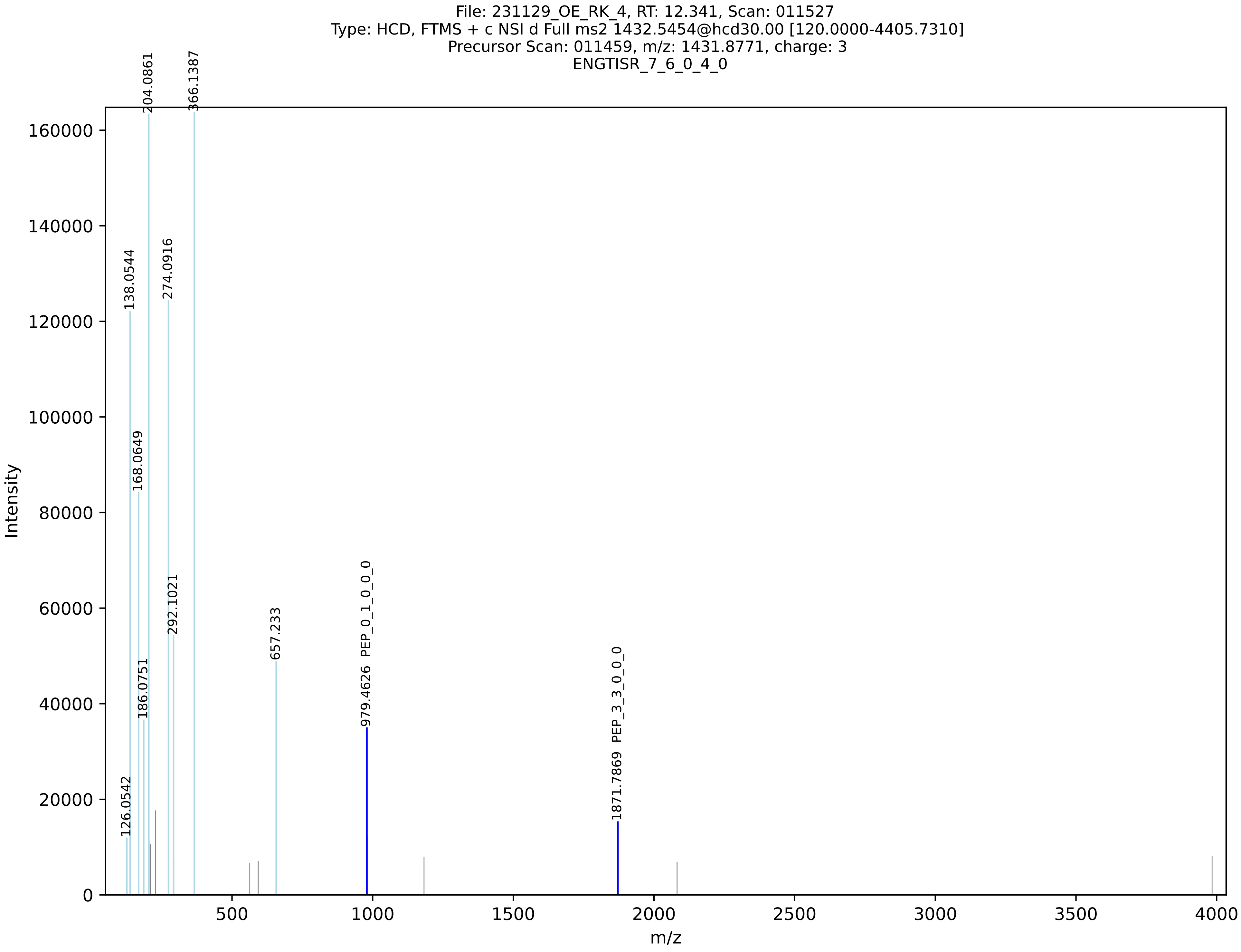
File: 231129_OE_RK_4, RT: 12.341, Scan: 011527
Type: HCD, FTMS + c NSI d Full ms2 1432.5454@hcd30.00 [120.0000-4405.7310]
Precursor Scan: 011459, m/z: 1431.8771, charge: 3
ENGTISR_7_6_0_4_0
204.0861
366.1387
138.0544
274.0916
168.0649
292.1021
657.233
979.4626 PEP_0_1_0_0_0
186.0751
1871.7869 PEP_3_3_0_0_0
126.0542
Intensity
m/z
0
20000
40000
60000
80000
100000
120000
140000
160000
500
1000
1500
2000
2500
3000
3500
4000

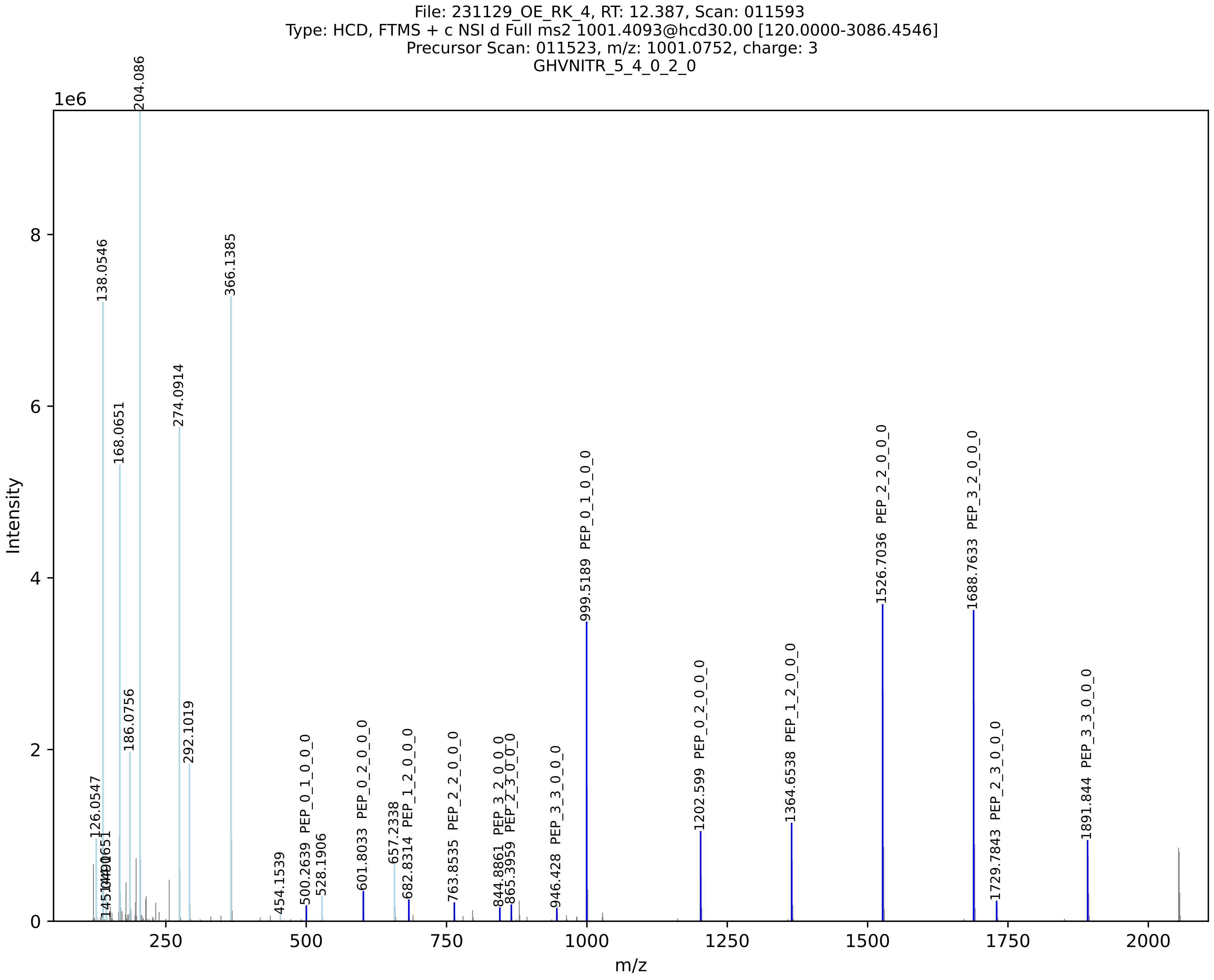

File: 231129_OE_RK_4, RT: 12.387, Scan: 011593
Type: HCD, FTMS + c NSI d Full ms2 1001.4093@hcd30.00 [120.0000-3086.4546]
Precursor Scan: 011523, m/z: 1001.0752, charge: 3
GHVNITR_5_4_0_2_0
1e6
Intensity
m/z
204.086
138.0546
366.1385
274.0914
168.0651
186.0756
292.1019
126.0547
454.1539
500.2639 PEP_0_1_0_0_0
528.1906
601.8033 PEP_0_2_0_0_0
657.2338
682.8314 PEP_1_2_0_0_0
763.8535 PEP_2_2_0_0_0
844.8861 PEP_3_2_0_0_0
865.3959 PEP_2_3_0_0_0
946.428 PEP_3_3_0_0_0
999.5189 PEP_0_1_0_0_0
1202.599 PEP_0_2_0_0_0
1364.6538 PEP_1_2_0_0_0
1526.7036 PEP_2_2_0_0_0
1688.7633 PEP_3_2_0_0_0
1729.7843 PEP_2_3_0_0_0
1891.844 PEP_3_3_0_0_0

File: 231129_OE_RK_4, RT: 12.393, Scan: 011603
Type: HCD, FTMS + c NSI d Full ms2 970.7205@hcd30.00 [120.0000-2992.5469]
Precursor Scan: 011523, m/z: 970.0523, charge: 3
ENLTAR_5_4_0_2_0

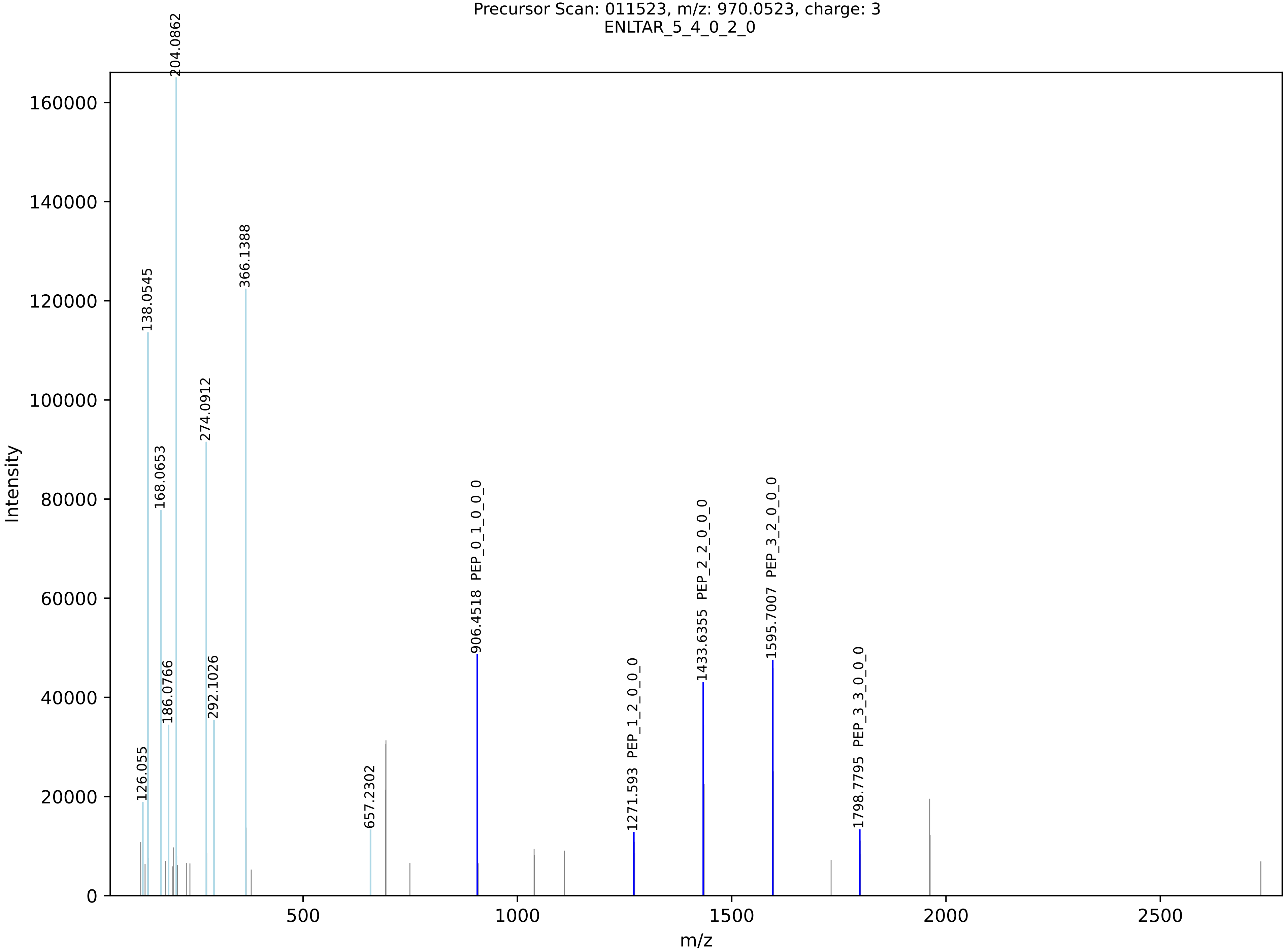

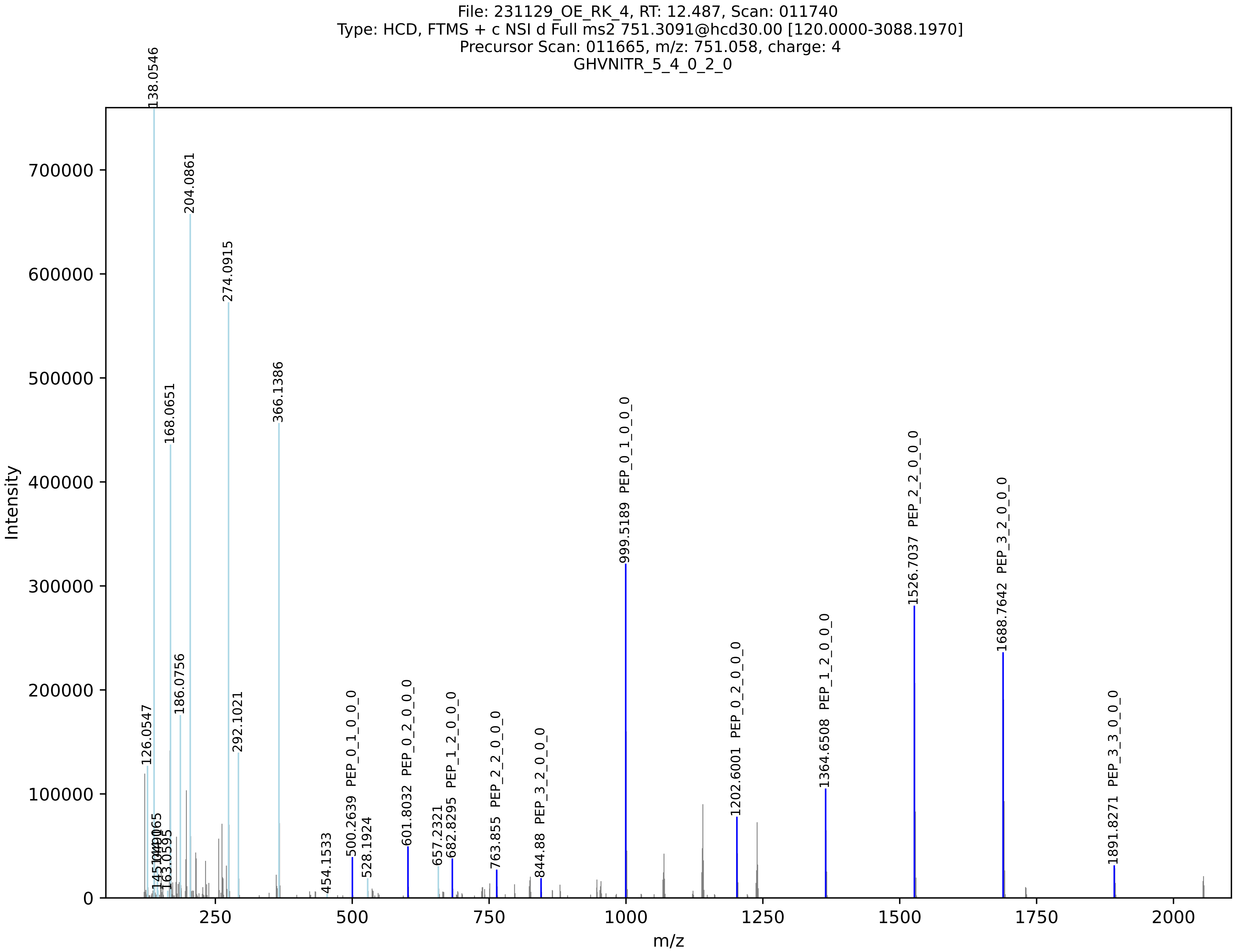
File: 231129_OE_RK_4, RT: 12.487, Scan: 011740
Type: HCD, FTMS + c NSI d Full ms2 751.3091@hcd30.00 [120.0000-3088.1970]
Precursor Scan: 011665, m/z: 751.058, charge: 4
GHVNITR_5_4_0_2_0
Intensity
m/z
138.0546
204.0861
274.0915
366.1386
168.0651
186.0756
126.0547
292.1021
163.0595
454.1533
500.2639 PEP_0_1_0_0_0
528.1924
601.8032 PEP_0_2_0_0_0
657.2321
682.8295 PEP_1_2_0_0_0
763.855 PEP_2_2_0_0_0
844.88 PEP_3_2_0_0_0
999.5189 PEP_0_1_0_0_0
1202.6001 PEP_0_2_0_0_0
1364.6508 PEP_1_2_0_0_0
1526.7037 PEP_2_2_0_0_0
1688.7642 PEP_3_2_0_0_0
1891.8271 PEP_3_3_0_0_0

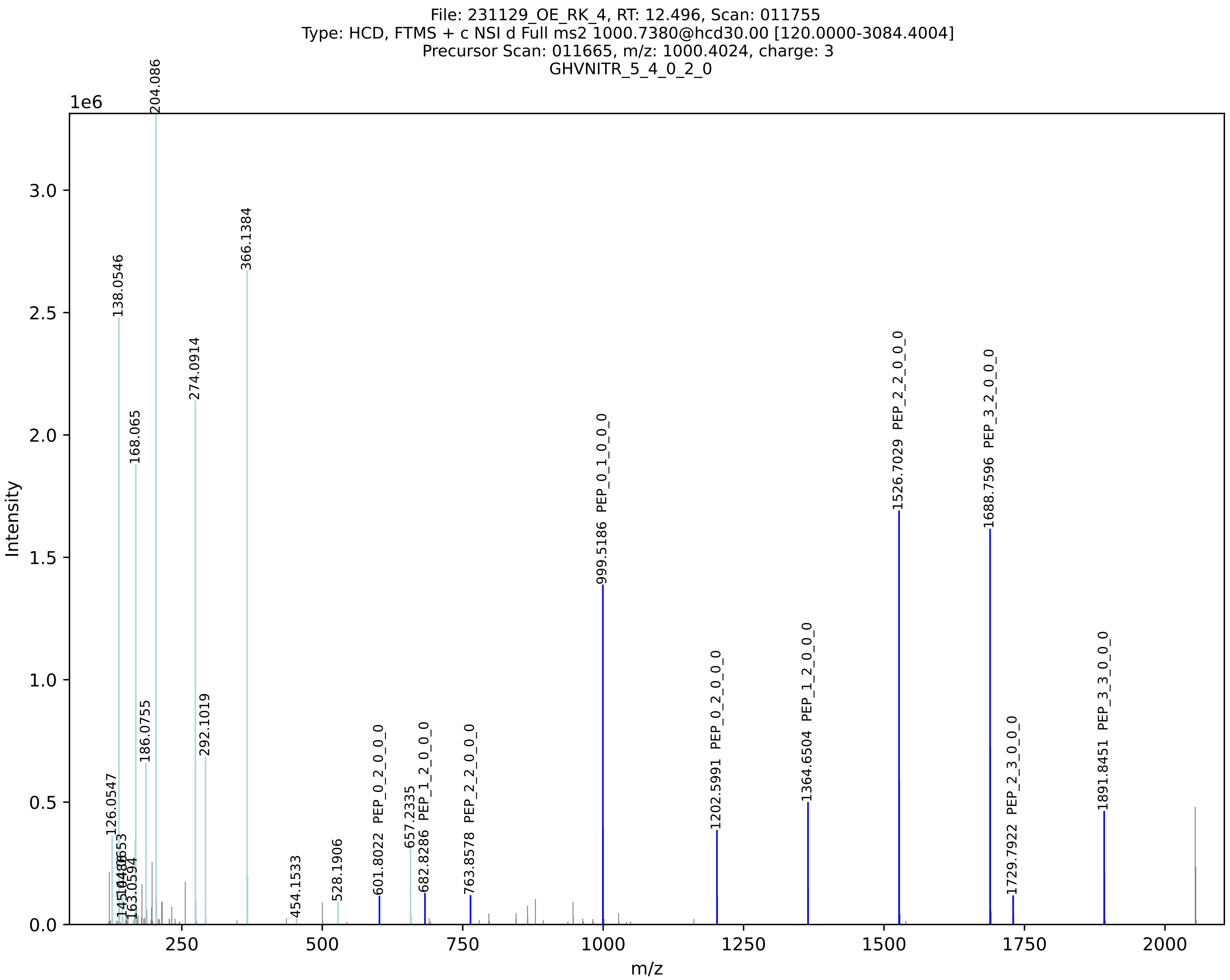
File: 231129_OE_RK_4, RT: 12.496, Scan: 011755
Type: HCD, FTMS + c NSI d Full ms2 1000.7380@hcd30.00 [120.0000-3084.4004]
Precursor Scan: 011665, m/z: 1000.4024, charge: 3
GHVNITR_5_4_0_2_0
1e6
Intensity
m/z
204.086
138.0546
366.1384
274.0914
168.065
186.0755
292.1019
126.0547
163.0594
454.1533
528.1906
601.8022 PEP_0_2_0_0_0
657.2335
682.8286 PEP_1_2_0_0_0
763.8578 PEP_2_2_0_0_0
999.5186 PEP_0_1_0_0_0
1202.5991 PEP_0_2_0_0_0
1364.6504 PEP_1_2_0_0_0
1526.7029 PEP_2_2_0_0_0
1688.7596 PEP_3_2_0_0_0
1729.7922 PEP_2_3_0_0_0
1891.8451 PEP_3_3_0_0_0

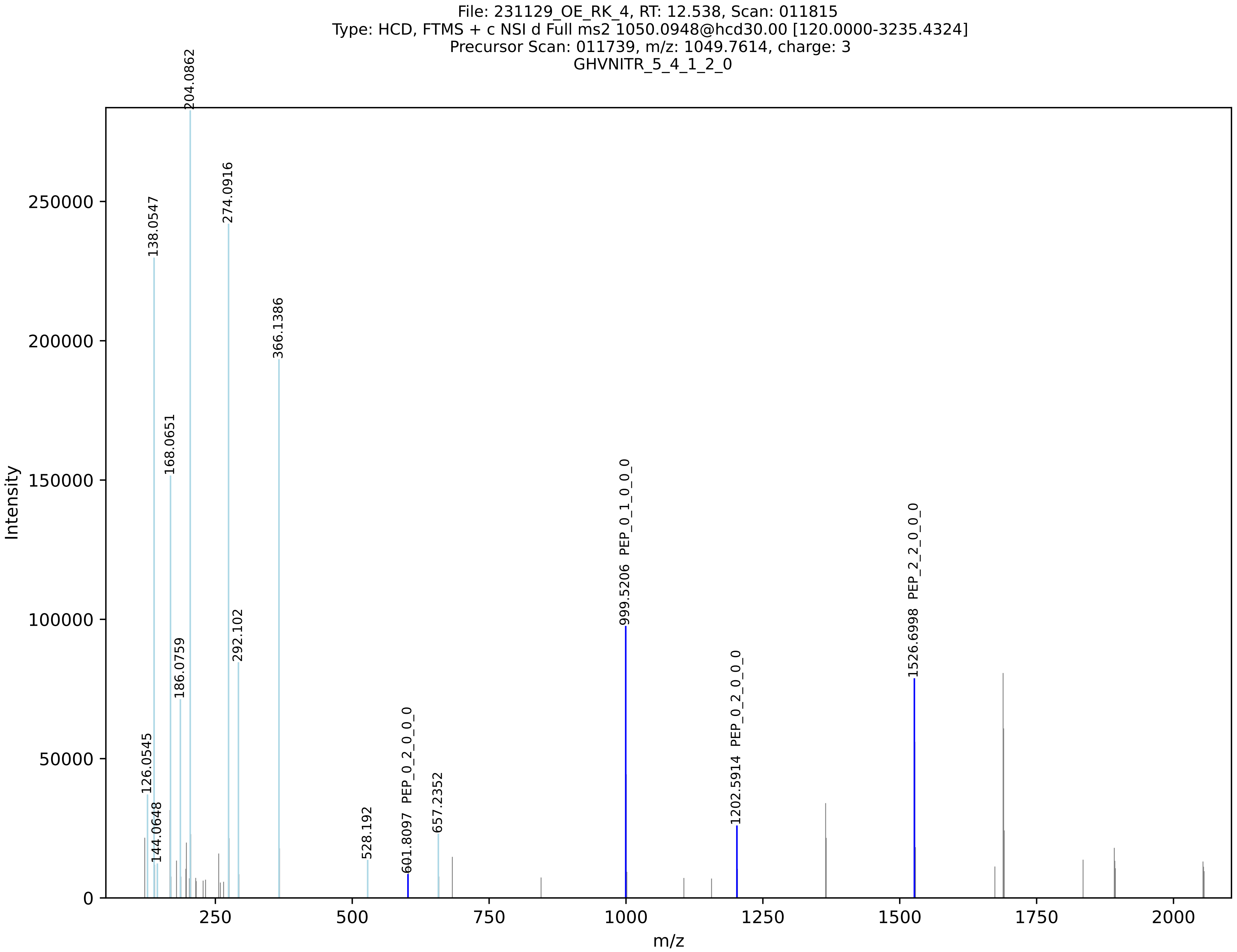

File: 231129_OE_RK_4, RT: 12.538, Scan: 011815
Type: HCD, FTMS + c NSI d Full ms2 1050.0948@hcd30.00 [120.0000-3235.4324]
Precursor Scan: 011739, m/z: 1049.7614, charge: 3
GHVNITR_5_4_1_2_0
Intensity
m/z
204.0862
274.0916
138.0547
366.1386
168.0651
186.0759
292.102
126.0545
144.0648
528.192
601.8097 PEP_0_2_0_0_0
657.2352
999.5206 PEP_0_1_0_0_0
1202.5914 PEP_0_2_0_0_0
1526.6998 PEP_2_2_0_0_0

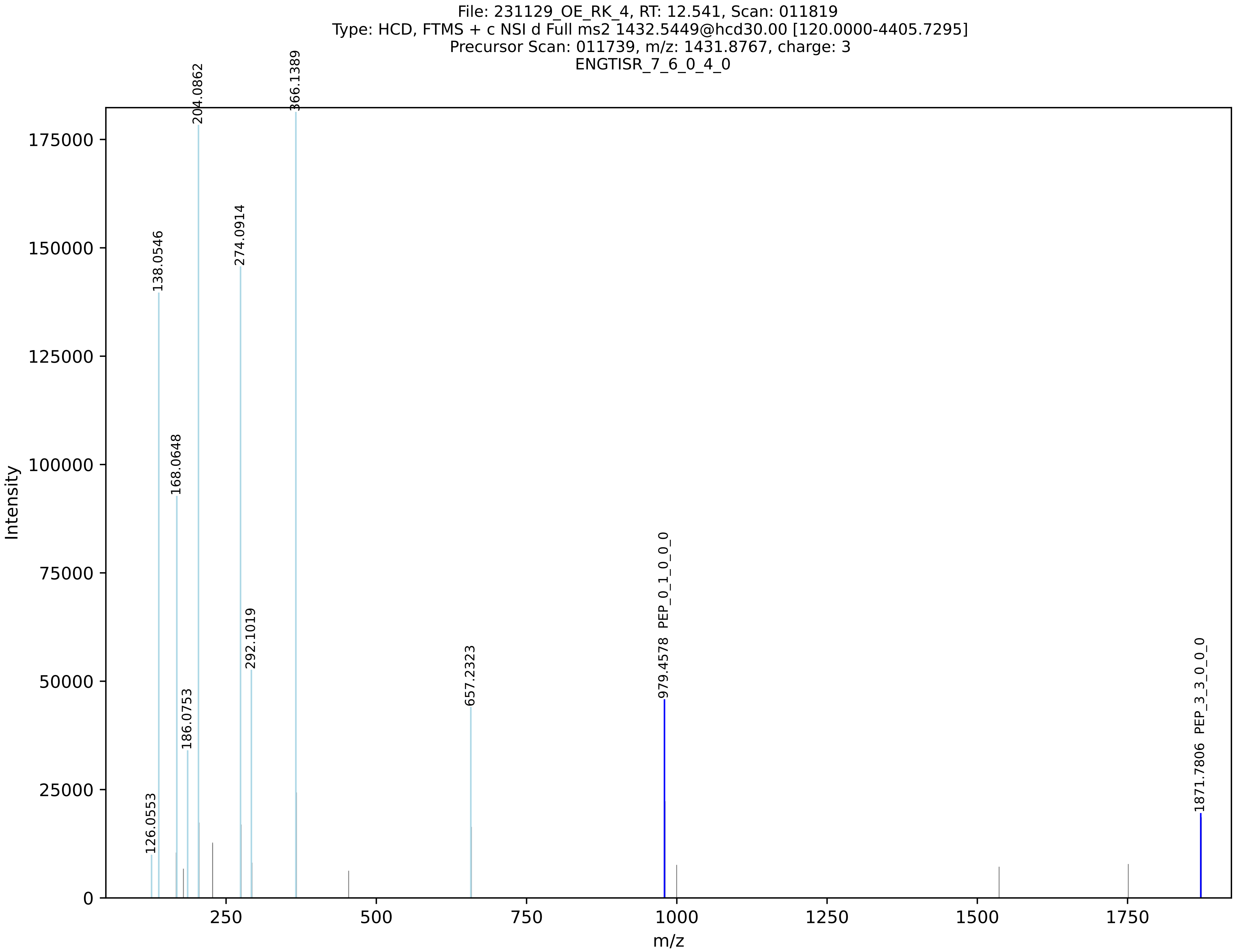
File: 231129_OE_RK_4, RT: 12.541, Scan: 011819
Type: HCD, FTMS + c NSI d Full ms2 1432.5449@hcd30.00 [120.0000-4405.7295]
Precursor Scan: 011739, m/z: 1431.8767, charge: 3
ENGTISR_7_6_0_4_0
Intensity
m/z
126.0553
138.0546
168.0648
186.0753
204.0862
274.0914
292.1019
366.1389
657.2323
979.4578 PEP_0_1_0_0_0
1871.7806 PEP_3_3_0_0_0
0
25000
50000
75000
100000
125000
150000
175000
250
500
750
1000
1250
1500
1750

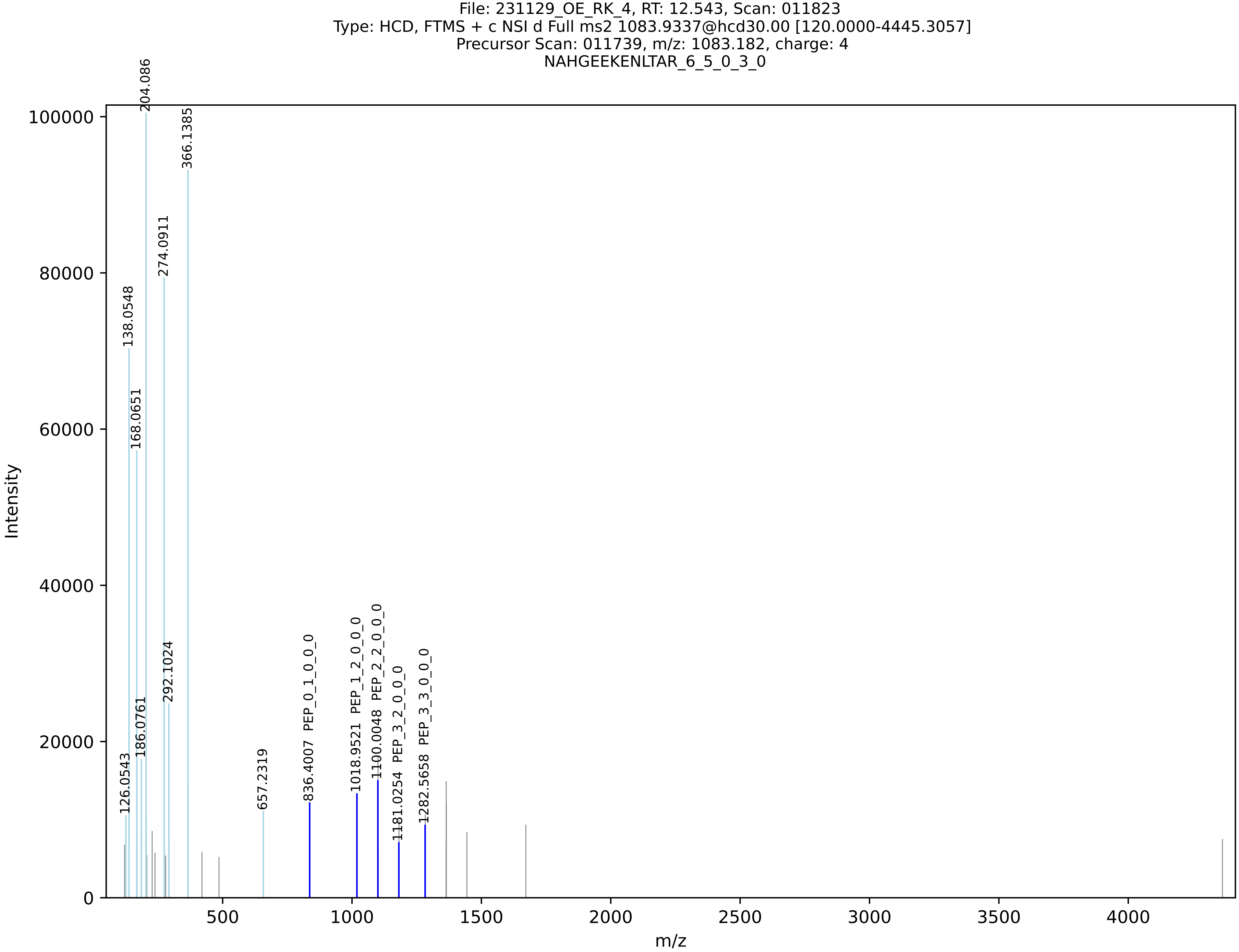

File: 231129_OE_RK_4, RT: 12.543, Scan: 011823
Type: HCD, FTMS + c NSI d Full ms2 1083.9337@hcd30.00 [120.0000-4445.3057]
Precursor Scan: 011739, m/z: 1083.182, charge: 4
NAHGEEKENLTAR_6_5_0_3_0
Intensity
m/z
204.086
366.1385
274.0911
138.0548
168.0651
292.1024
186.0761
126.0543
657.2319
836.4007 PEP_0_1_0_0_0
1018.9521 PEP_1_2_0_0_0
1100.0048 PEP_2_2_0_0_0
1181.0254 PEP_3_2_0_0_0
1282.5658 PEP_3_3_0_0_0

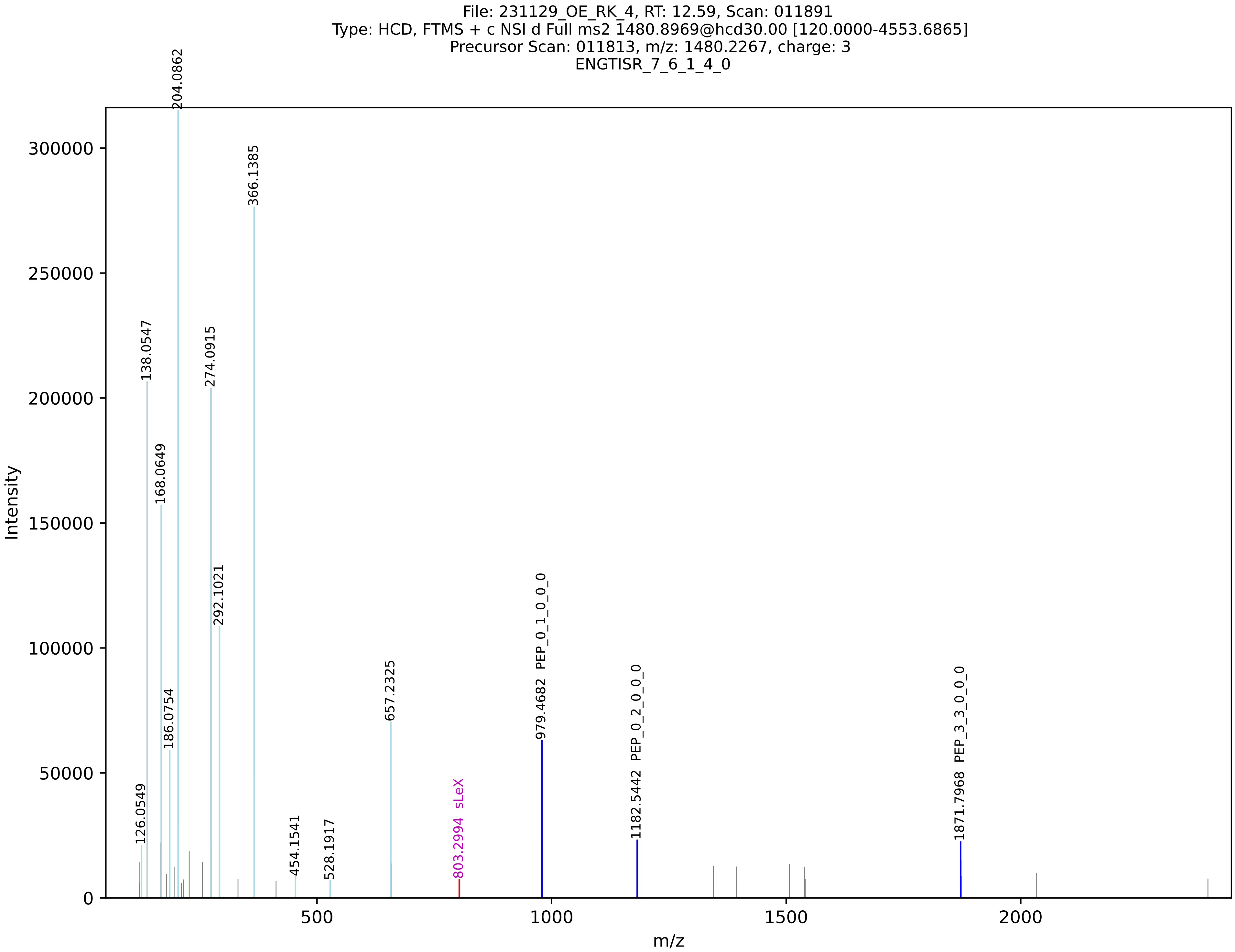

File: 231129_OE_RK_4, RT: 12.59, Scan: 011891
Type: HCD, FTMS + c NSI d Full ms2 1480.8969@hcd30.00 [120.0000-4553.6865]
Precursor Scan: 011813, m/z: 1480.2267, charge: 3
ENGTISR_7_6_1_4_0
Intensity
m/z
204.0862
366.1385
138.0547
274.0915
168.0649
292.1021
186.0754
126.0549
454.1541
528.1917
657.2325
803.2994 sLeX
979.4682 PEP_0_1_0_0_0
1182.5442 PEP_0_2_0_0_0
1871.7968 PEP_3_3_0_0_0

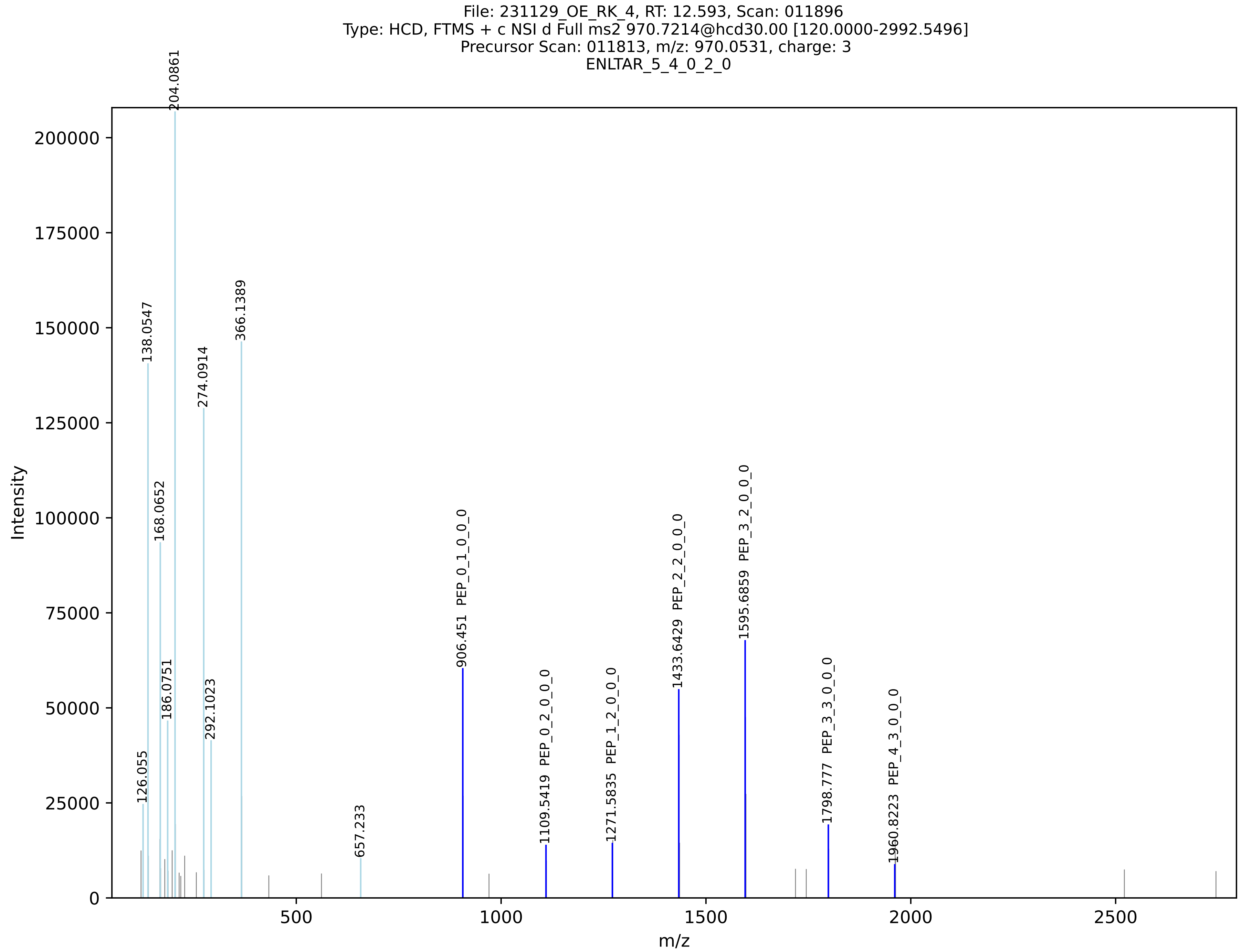
File: 231129_OE_RK_4, RT: 12.593, Scan: 011896
Type: HCD, FTMS + c NSI d Full ms2 970.7214@hcd30.00 [120.0000-2992.5496]
Precursor Scan: 011813, m/z: 970.0531, charge: 3
ENLTAR_5_4_0_2_0
Intensity
m/z
204.0861
138.0547
366.1389
274.0914
168.0652
186.0751
292.1023
126.055
657.233
906.451 PEP_0_1_0_0_0_
1109.5419 PEP_0_2_0_0_0_
1271.5835 PEP_1_2_0_0_0_
1433.6429 PEP_2_2_0_0_0_
1595.6859 PEP_3_2_0_0_0_
1798.777 PEP_3_3_0_0_0_
1960.8223 PEP_4_3_0_0_0_

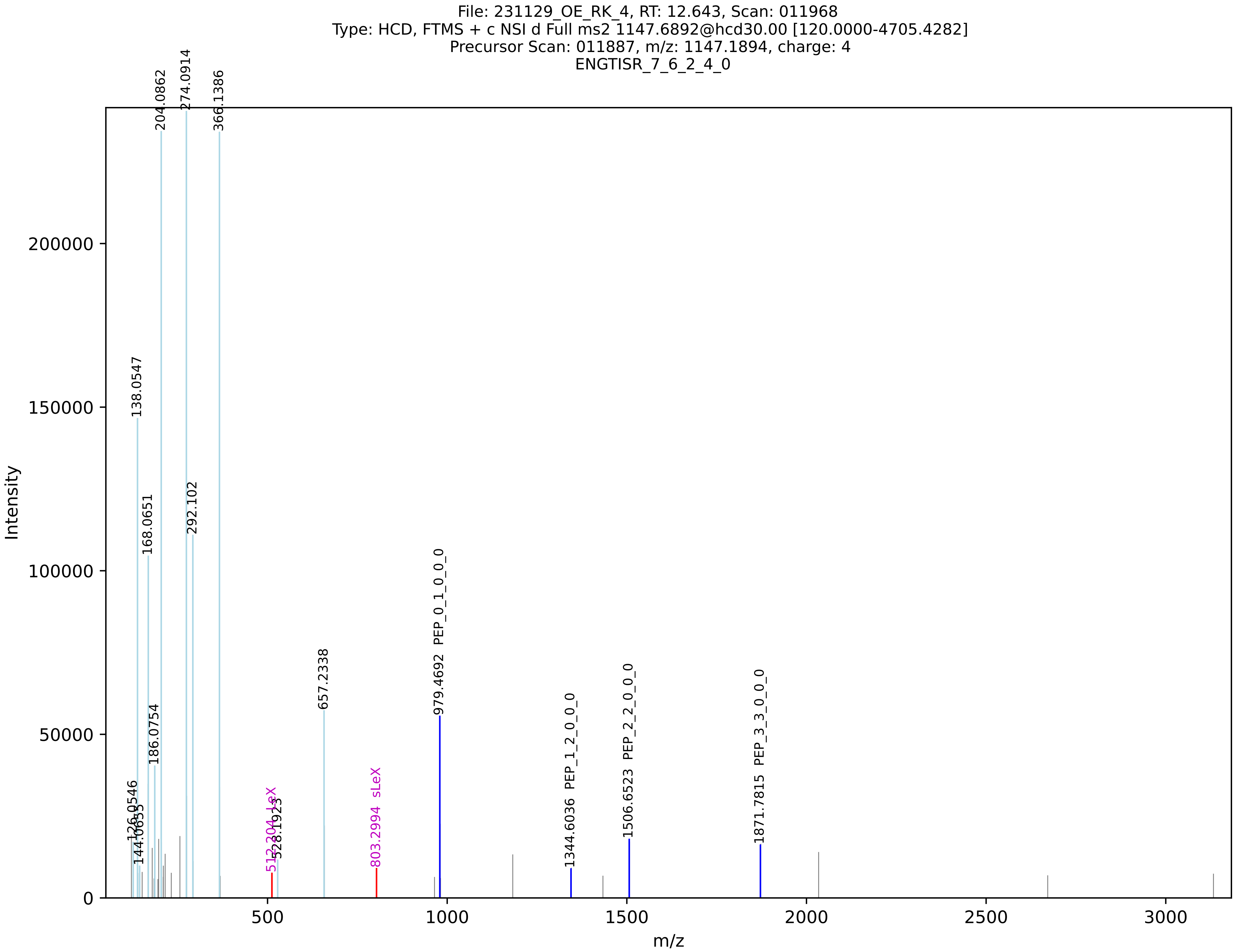
File: 231129_OE_RK_4, RT: 12.643, Scan: 011968
Type: HCD, FTMS + c NSI d Full ms2 1147.6892@hcd30.00 [120.0000-4705.4282]
Precursor Scan: 011887, m/z: 1147.1894, charge: 4
ENGTISR_7_6_2_4_0
Intensity
m/z
204.0862
274.0914
366.1386
138.0547
168.0651
292.102
186.0754
126.0546
144.0655
512.204 LeX
528.1923
657.2338
803.2994 sLeX
979.4692 PEP_0_1_0_0_0
1344.6036 PEP_1_2_0_0_0
1506.6523 PEP_2_2_0_0_0
1871.7815 PEP_3_3_0_0_0
0
50000
100000
150000
200000
500
1000
1500
2000
2500
3000

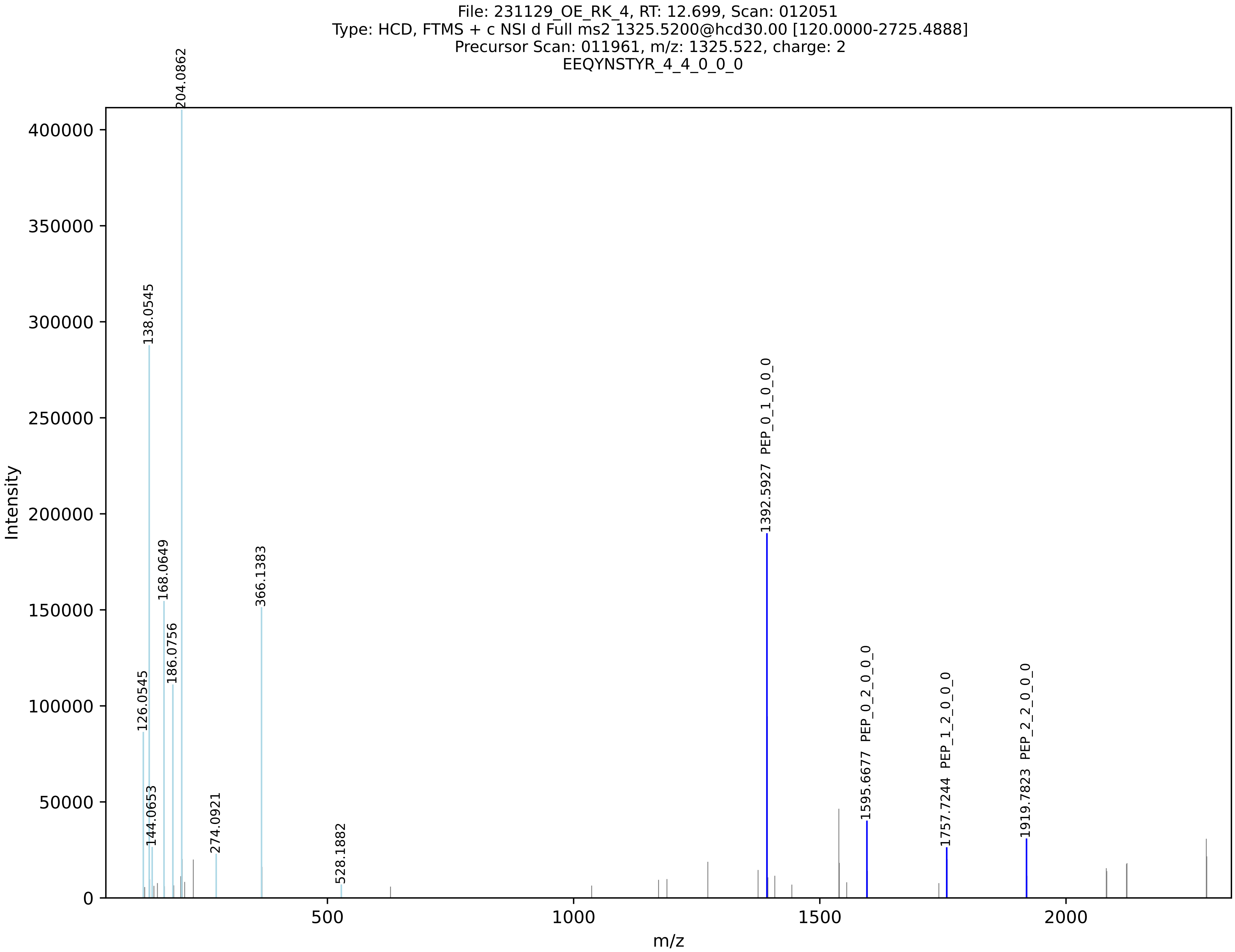
File: 231129_OE_RK_4, RT: 12.699, Scan: 012051
Type: HCD, FTMS + c NSI d Full ms2 1325.5200@hcd30.00 [120.0000-2725.4888]
Precursor Scan: 011961, m/z: 1325.522, charge: 2
EEQYNSTYR_4_4_0_0_0
Intensity
m/z
204.0862
138.0545
168.0649
186.0756
126.0545
144.0653
274.0921
366.1383
528.1882
1392.5927 PEP_0_1_0_0_0
1595.6677 PEP_0_2_0_0_0
1757.7244 PEP_1_2_0_0_0
1919.7823 PEP_2_2_0_0_0

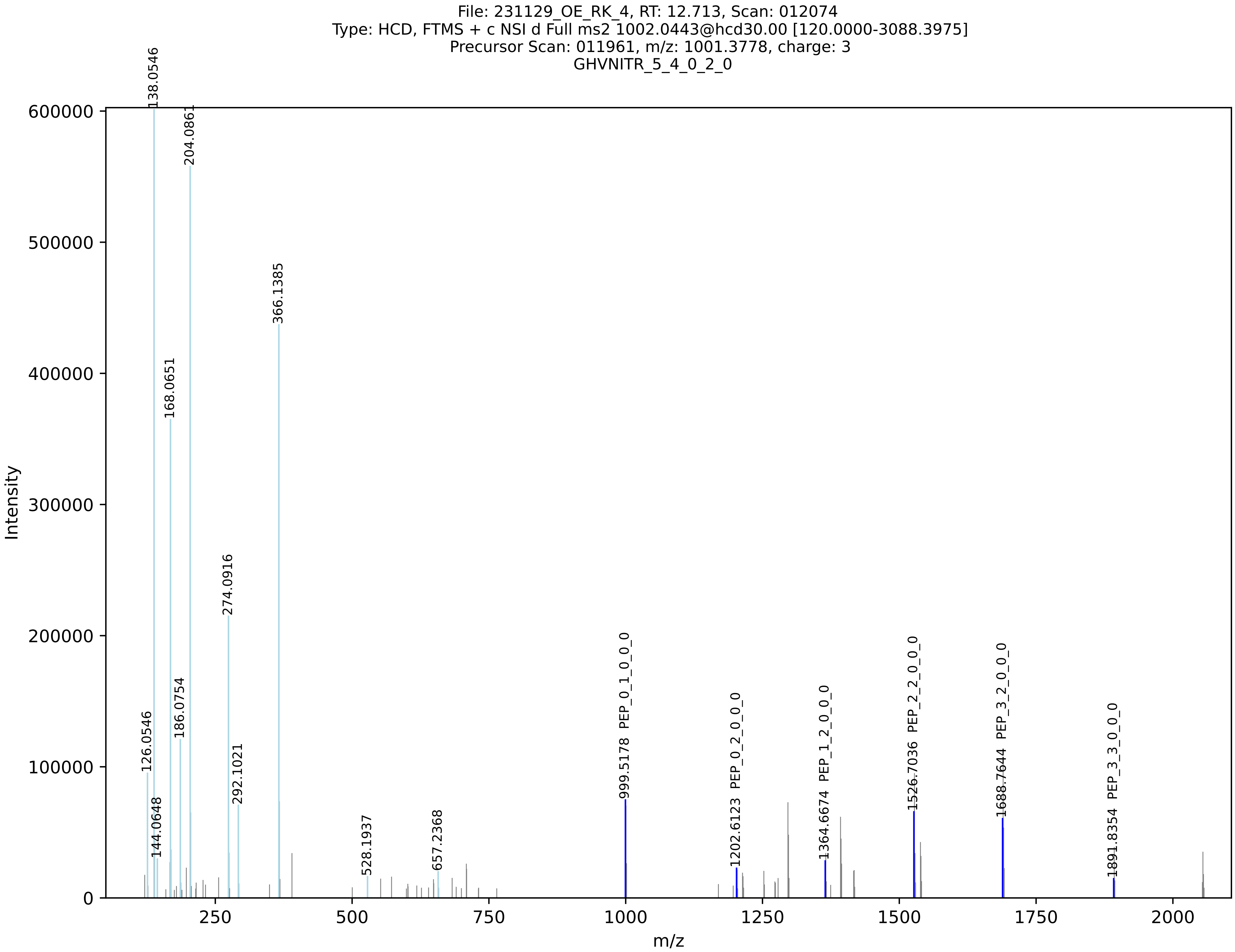
File: 231129_OE_RK_4, RT: 12.713, Scan: 012074
Type: HCD, FTMS + c NSI d Full ms2 1002.0443@hcd30.00 [120.0000-3088.3975]
Precursor Scan: 011961, m/z: 1001.3778, charge: 3
GHVNITR_5_4_0_2_0
Intensity
m/z
138.0546
204.0861
366.1385
168.0651
274.0916
186.0754
126.0546
292.1021
144.0648
528.1937
657.2368
999.5178 PEP_0_1_0_0_0
1202.6123 PEP_0_2_0_0_0
1364.6674 PEP_1_2_0_0_0
1526.7036 PEP_2_2_0_0_0
1688.7644 PEP_3_2_0_0_0
1891.8354 PEP_3_3_0_0_0

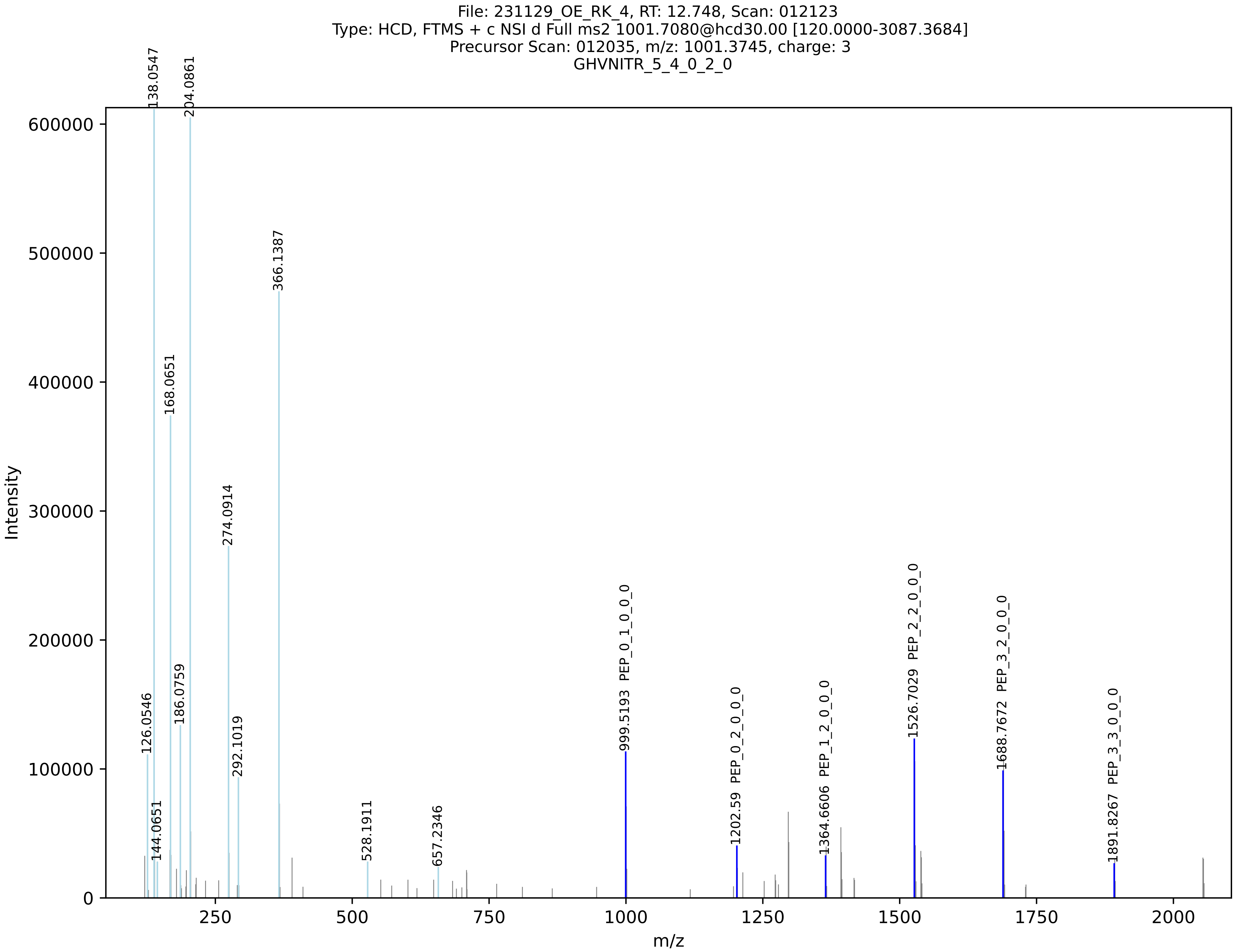
File: 231129_OE_RK_4, RT: 12.748, Scan: 012123
Type: HCD, FTMS + c NSI d Full ms2 1001.7080@hcd30.00 [120.0000-3087.3684]
Precursor Scan: 012035, m/z: 1001.3745, charge: 3
GHVNITR_5_4_0_2_0
Intensity
m/z
138.0547
204.0861
366.1387
168.0651
274.0914
186.0759
126.0546
292.1019
144.0651
528.1911
657.2346
999.5193 PEP_0_1_0_0_0
1202.59 PEP_0_2_0_0_0
1364.6606 PEP_1_2_0_0_0
1526.7029 PEP_2_2_0_0_0
1688.7672 PEP_3_2_0_0_0
1891.8267 PEP_3_3_0_0_0

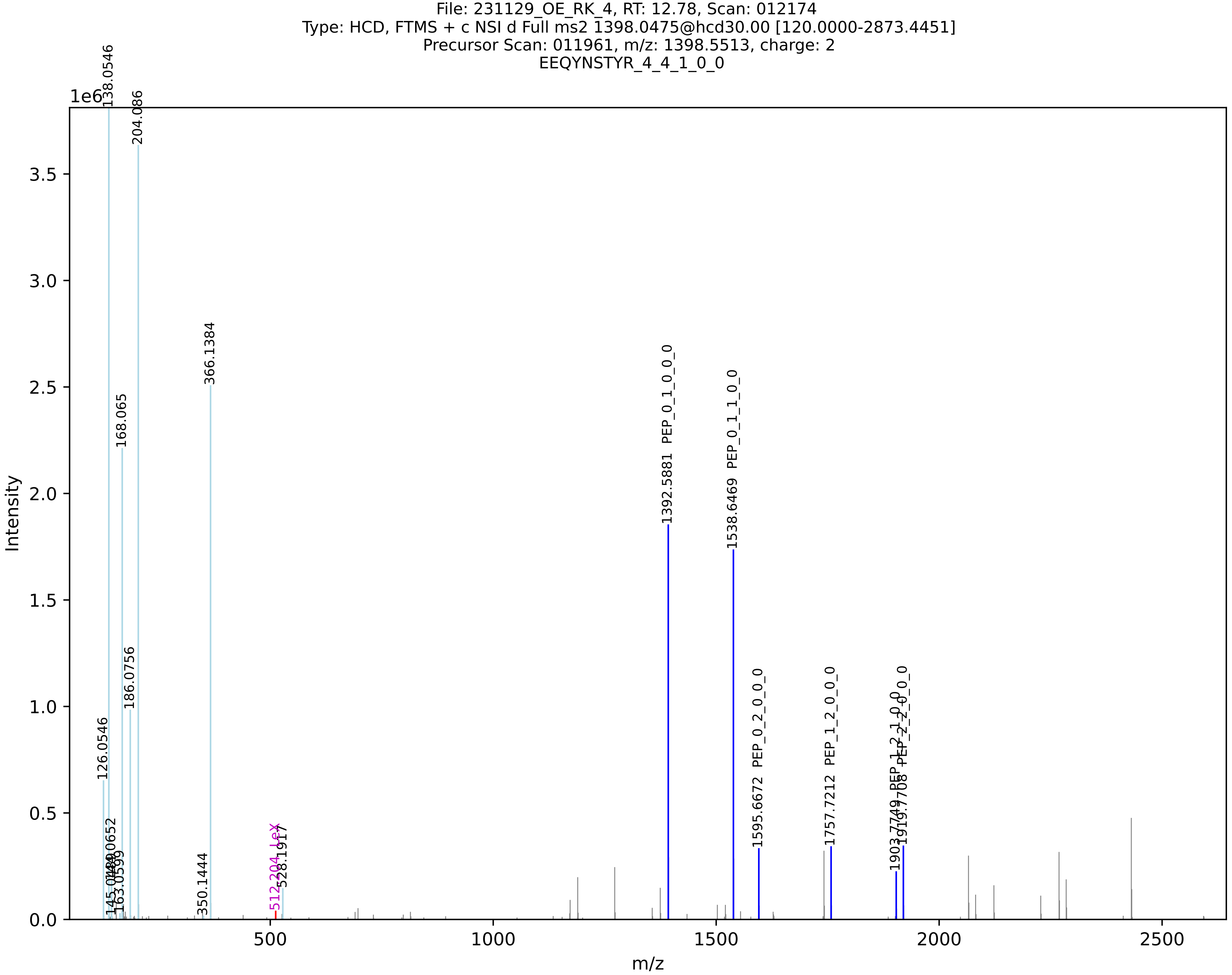

File: 231129_OE_RK_4, RT: 12.78, Scan: 012174
Type: HCD, FTMS + c NSI d Full ms2 1398.0475@hcd30.00 [120.0000-2873.4451]
Precursor Scan: 011961, m/z: 1398.5513, charge: 2
EEQYNSTYR_4_4_1_0_0
1e6
Intensity
m/z
0.0
0.5
1.0
1.5
2.0
2.5
3.0
3.5
500
1000
1500
2000
2500
138.0546
204.086
366.1384
168.065
186.0756
126.0546
350.1444
512.204_LeX
528.1917
1392.5881 PEP_0_1_0_0_0
1538.6469 PEP_0_1_1_0_0
1595.6672 PEP_0_2_0_0_0
1757.7212 PEP_1_2_0_0_0

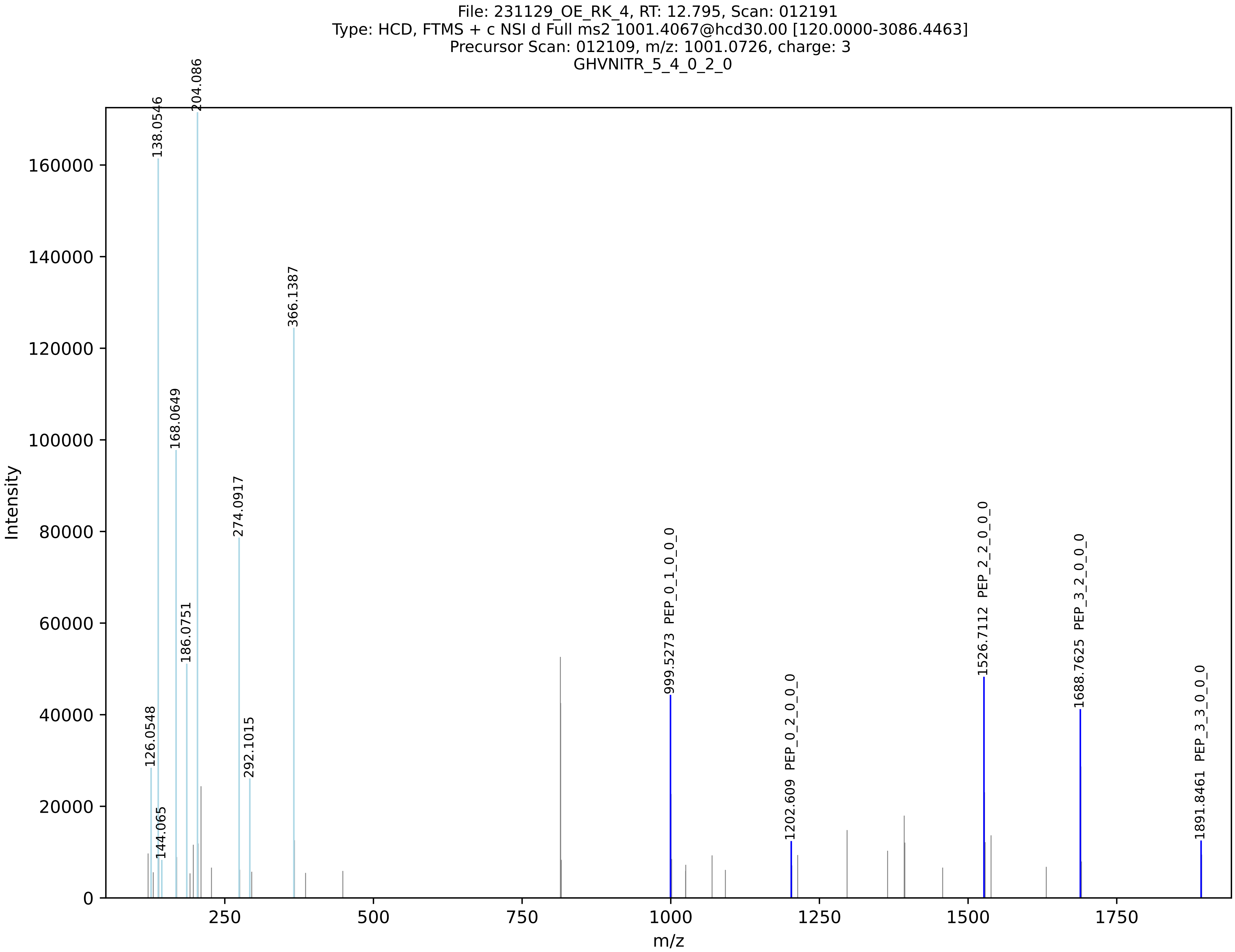
File: 231129_OE_RK_4, RT: 12.795, Scan: 012191
Type: HCD, FTMS + c NSI d Full ms2 1001.4067@hcd30.00 [120.0000-3086.4463]
Precursor Scan: 012109, m/z: 1001.0726, charge: 3
GHVNITR_5_4_0_2_0
Intensity
m/z
204.086
138.0546
366.1387
168.0649
274.0917
186.0751
126.0548
292.1015
144.065
999.5273 PEP_0_1_0_0_
1202.609 PEP_0_2_0_0_0
1526.7112 PEP_2_2_0_0_0
1688.7625 PEP_3_2_0_0_0
1891.8461 PEP_3_3_0_0_0

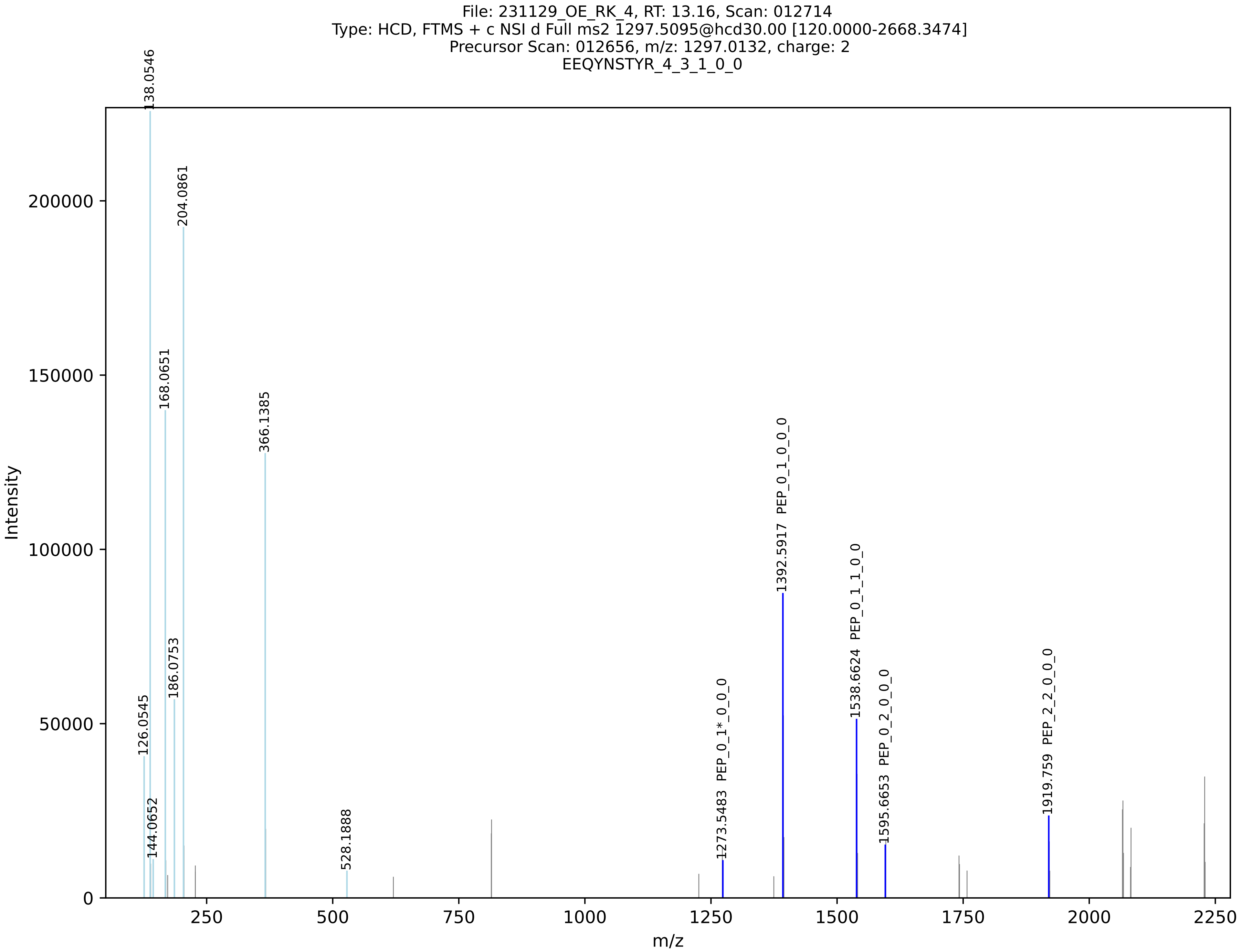
File: 231129_OE_RK_4, RT: 13.16, Scan: 012714
Type: HCD, FTMS + c NSI d Full ms2 1297.5095@hcd30.00 [120.0000-2668.3474]
Precursor Scan: 012656, m/z: 1297.0132, charge: 2
EEQYNSTYR_4_3_1_0_0
Intensity
m/z
138.0546
204.0861
168.0651
366.1385
186.0753
126.0545
144.0652
528.1888
1273.5483 PEP_0_1*_0_0_0
1392.5917 PEP_0_1_0_0_0
1538.6624 PEP_0_1_1_0_0
1595.6653 PEP_0_2_0_0_0
1919.759 PEP_2_2_0_0_0
0
50000
100000
150000
200000
250
500
750
1000
1250
1500
1750
2000
2250

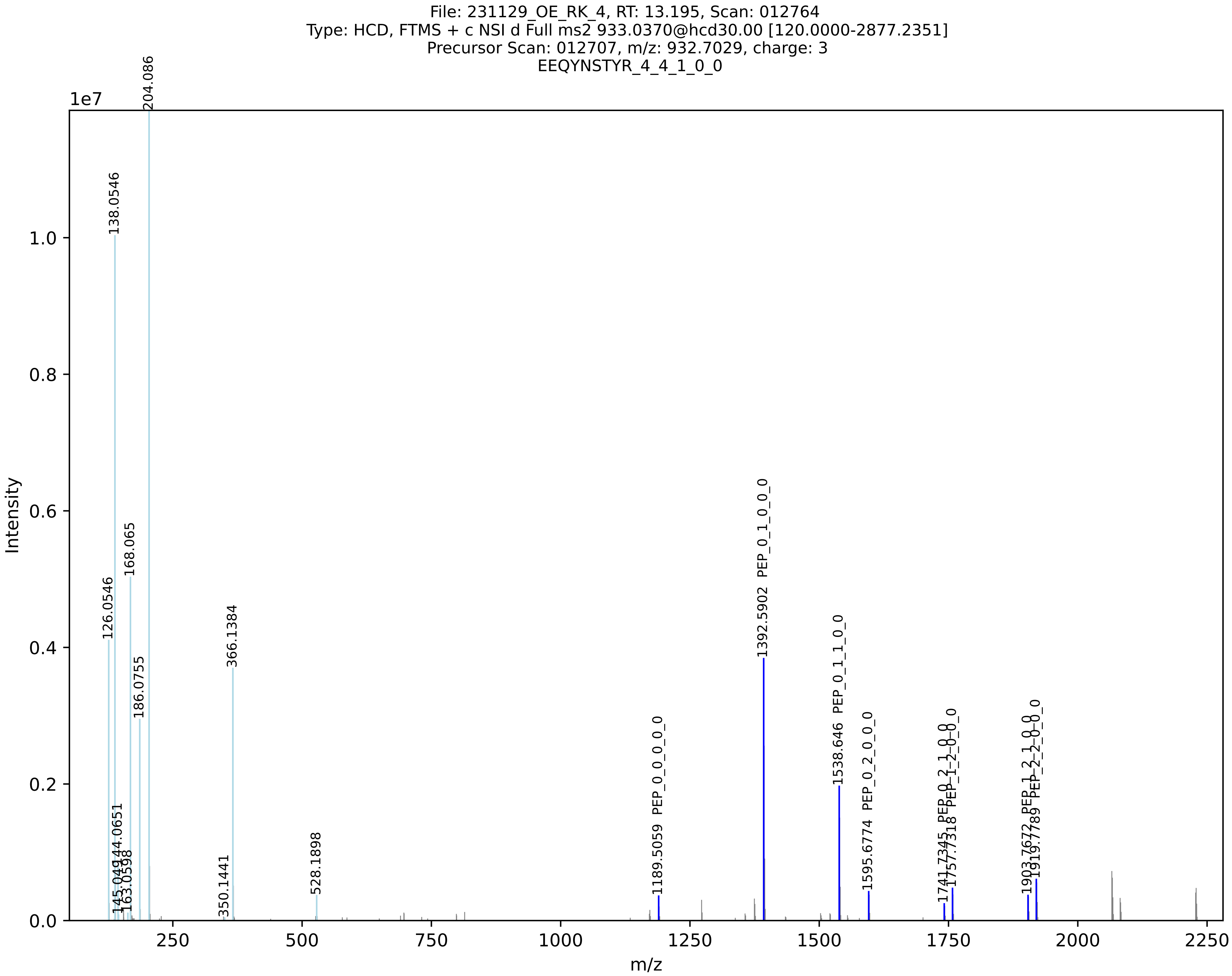
File: 231129_OE_RK_4, RT: 13.195, Scan: 012764
Type: HCD, FTMS + c NSI d Full ms2 933.0370@hcd30.00 [120.0000-2877.2351]
Precursor Scan: 012707, m/z: 932.7029, charge: 3
EEQYNSTYR_4_4_1_0_0
1e7
Intensity
m/z
204.086
138.0546
168.065
126.0546
186.0755
366.1384
144.0651
163.0598
350.1441
528.1898
1189.5059 PEP_0_0_0_0_0
1392.5902 PEP_0_1_0_0_0
1538.646 PEP_0_1_1_0_0
1595.6774 PEP_0_2_0_0_0
1741.7345 PEP_0_2_1_0_0
1757.7318 PEP_1_2_0_0_0
1903.7672 PEP_1_2_1_0_0
1919.7789 PEP_2_2_0_0_0

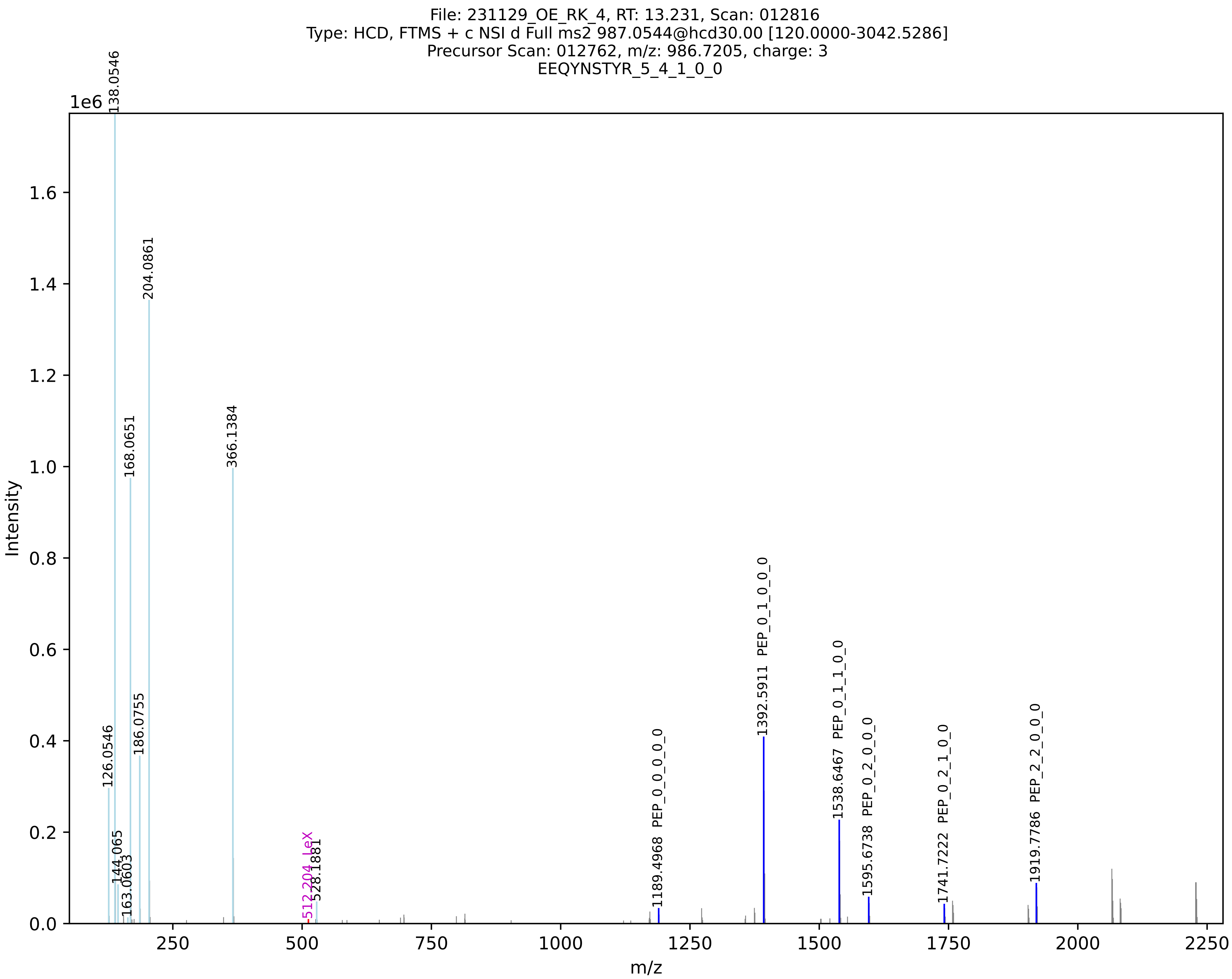
File: 231129_OE_RK_4, RT: 13.231, Scan: 012816
Type: HCD, FTMS + c NSI d Full ms2 987.0544@hcd30.00 [120.0000-3042.5286]
Precursor Scan: 012762, m/z: 986.7205, charge: 3
EEQYNSTYR_5_4_1_0_0
1e6
Intensity
m/z
138.0546
204.0861
168.0651
366.1384
186.0755
126.0546
144.065
163.0603
512.204_LeX
528.1881
1189.4968 PEP_0_0_0_0_0
1392.5911 PEP_0_1_0_0_0
1538.6467 PEP_0_1_1_0_0
1595.6738 PEP_0_2_0_0_0
1741.7222 PEP_0_2_1_0_0
1919.7786 PEP_2_2_0_0_0

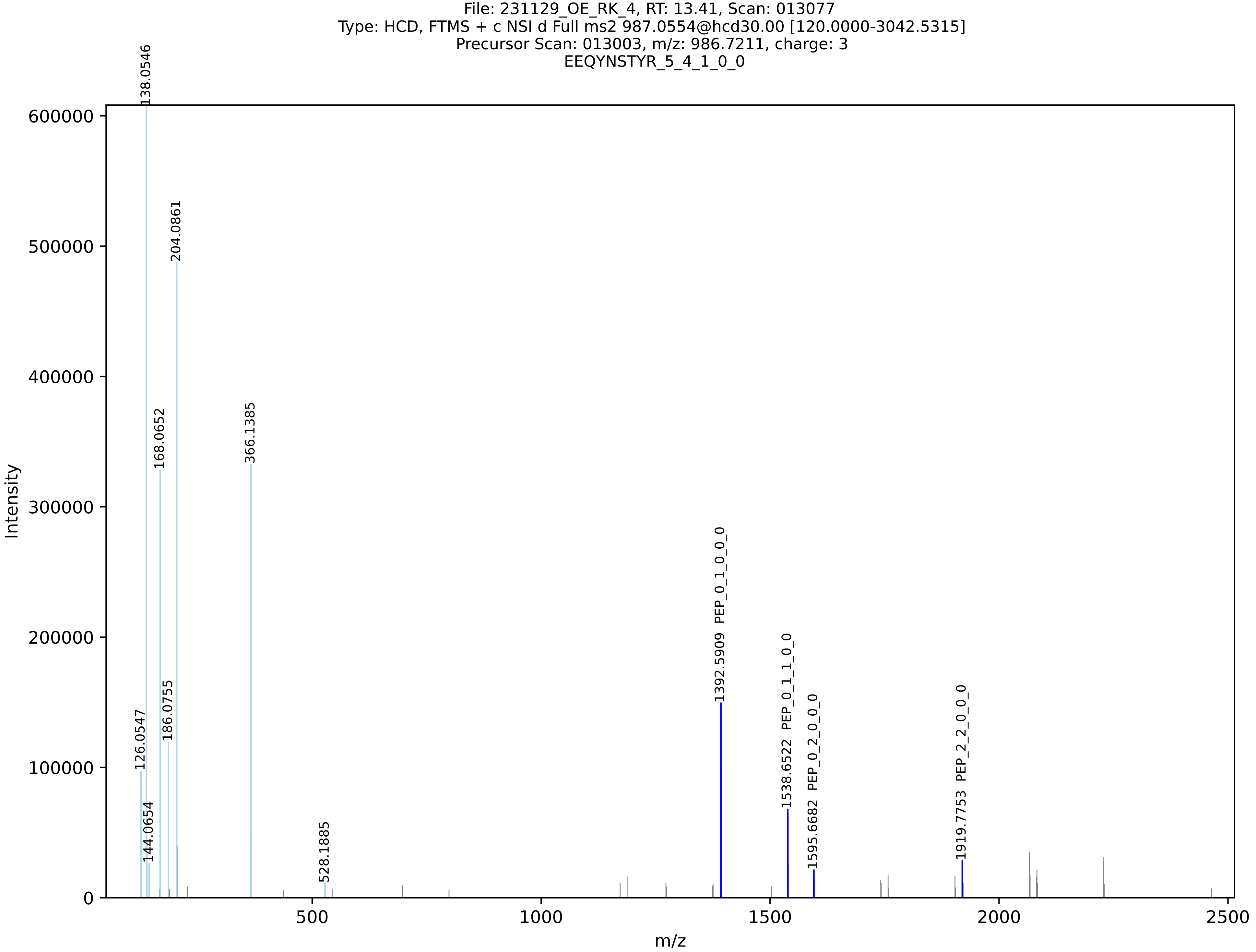
File: 231129_OE_RK_4, RT: 13.41, Scan: 013077
Type: HCD, FTMS + c NSI d Full ms2 987.0554@hcd30.00 [120.0000-3042.5315]
Precursor Scan: 013003, m/z: 986.7211, charge: 3
EEQYNSTYR_5_4_1_0_0
Intensity
m/z
0
100000
200000
300000
400000
500000
600000
500
1000
1500
2000
2500
138.0546
204.0861
168.0652
366.1385
126.0547
186.0755
144.0654
528.1885
1392.5909 PEP_0_1_0_0_0
1538.6522 PEP_0_1_1_0_0
1595.6682 PEP_0_2_0_0_0
1919.7753 PEP_2_2_0_0_0

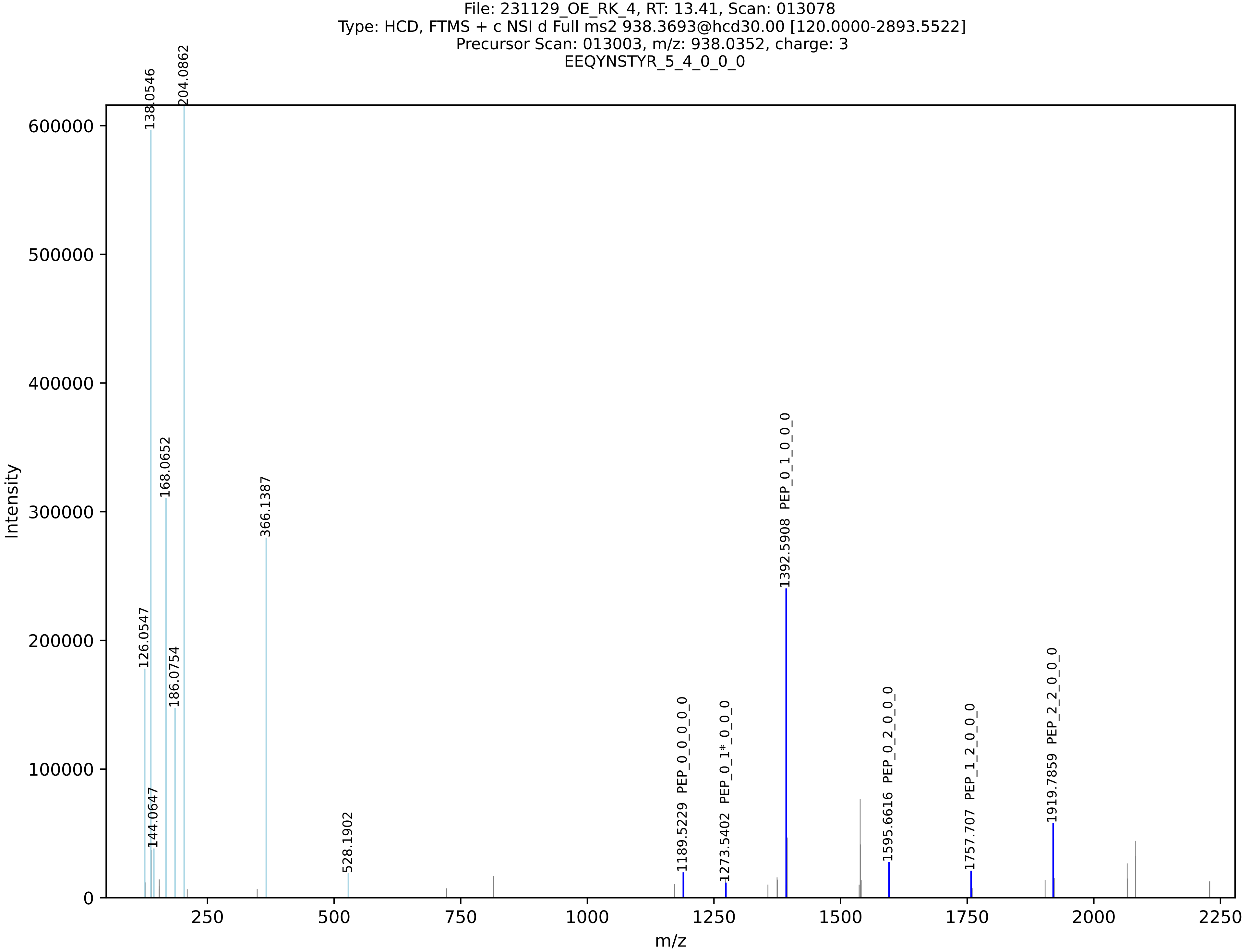
File: 231129_OE_RK_4, RT: 13.41, Scan: 013078
Type: HCD, FTMS + c NSI d Full ms2 938.3693@hcd30.00 [120.0000-2893.5522]
Precursor Scan: 013003, m/z: 938.0352, charge: 3
EEQYNSTYR_5_4_0_0_0
Intensity
m/z
138.0546
204.0862
168.0652
366.1387
126.0547
186.0754
144.0647
528.1902
1189.5229 PEP_0_0_0_0_0
1273.5402 PEP_0_1*_0_0_0
1392.5908 PEP_0_1_0_0_0
1595.6616 PEP_0_2_0_0_0
1757.707 PEP_1_2_0_0_0
1919.7859 PEP_2_2_0_0_0

File: 231129_OE_RK_4, RT: 13.414, Scan: 013084
Type: HCD, FTMS + c NSI d Full ms2 1481.2284@hcd30.00 [120.0000-4554.7007]
Precursor Scan: 013003, m/z: 1480.226, charge: 3
ENGTISR_7_6_1_4_0

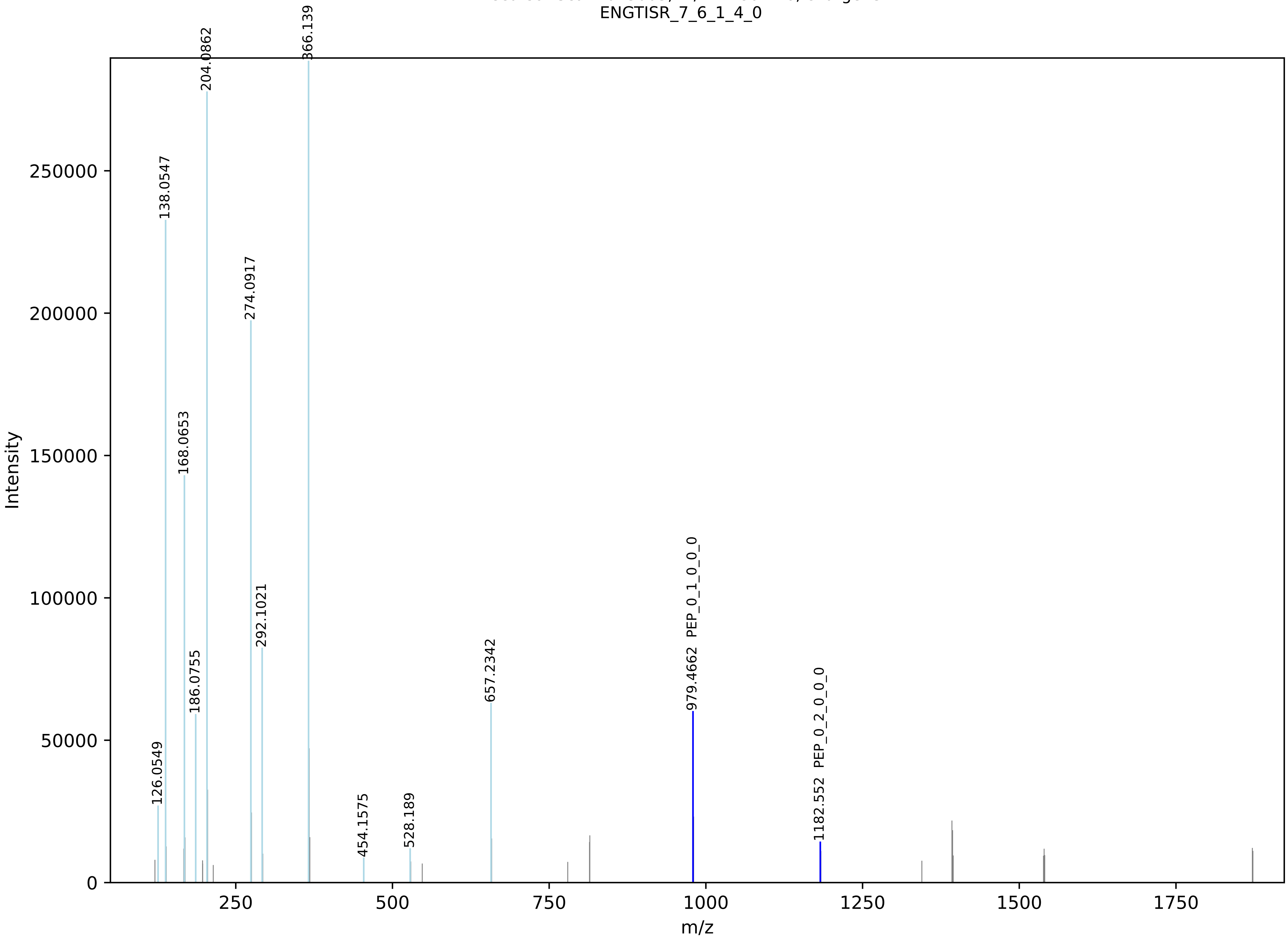

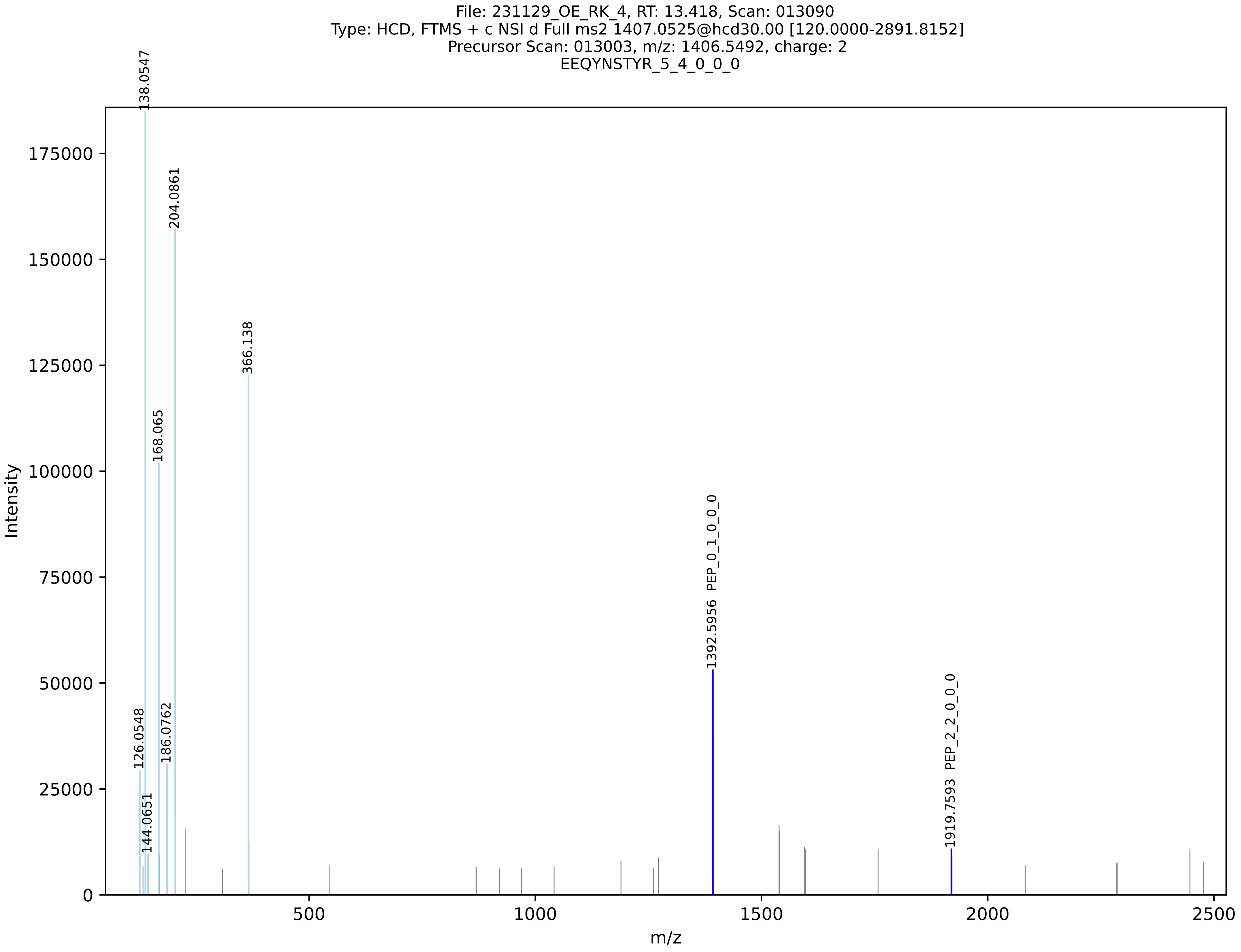
File: 231129_OE_RK_4, RT: 13.418, Scan: 013090
Type: HCD, FTMS + c NSI d Full ms2 1407.0525@hcd30.00 [120.0000-2891.8152]
Precursor Scan: 013003, m/z: 1406.5492, charge: 2
EEQYNSTYR_5_4_0_0_0
138.0547
204.0861
366.138
168.065
126.0548
186.0762
144.0651
1392.5956 PEP_0_1_0_0_0
1919.7593 PEP_2_2_0_0_0
Intensity
m/z
0
25000
50000
75000
100000
125000
150000
175000
500
1000
1500
2000
2500

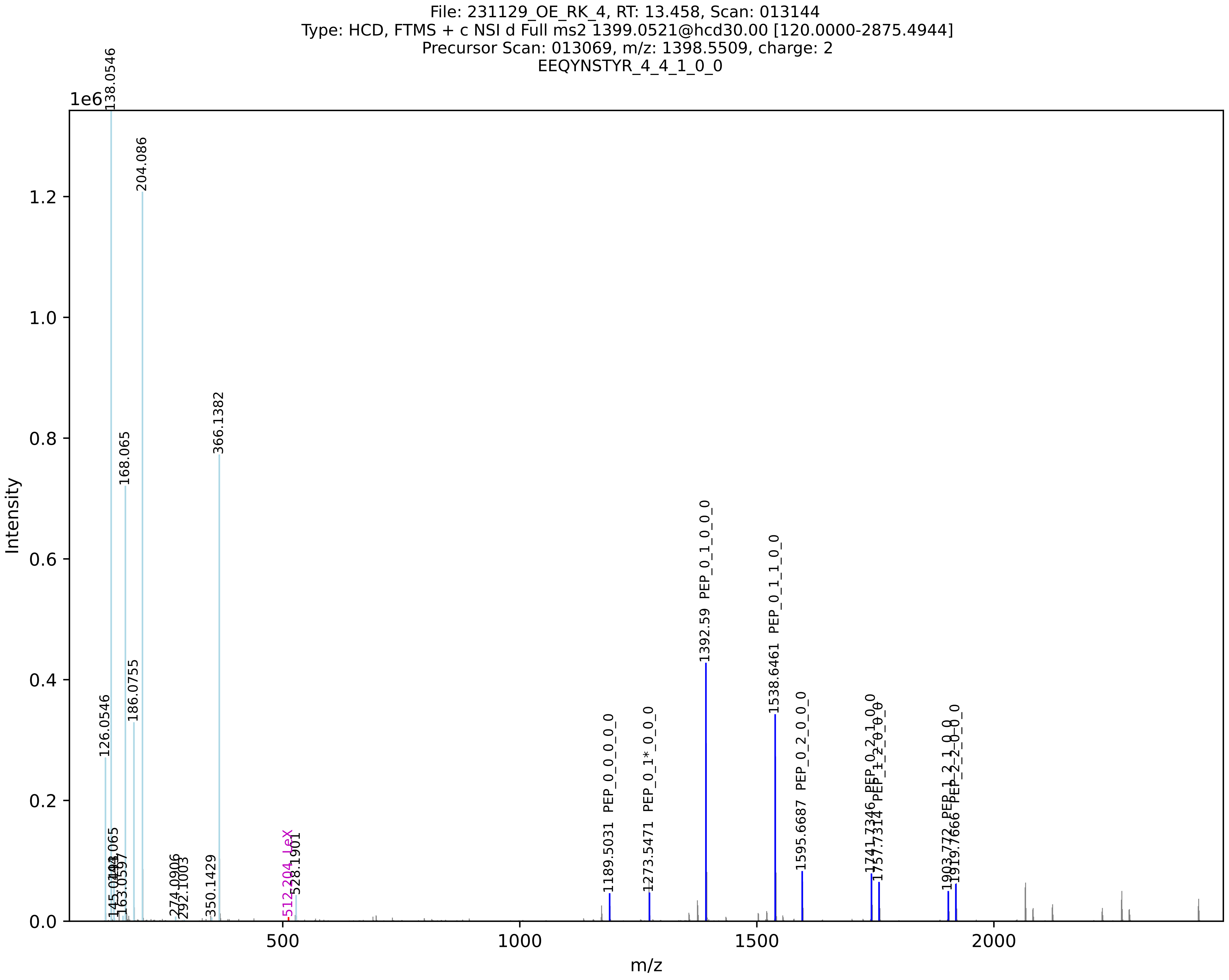
File: 231129_OE_RK_4, RT: 13.458, Scan: 013144
Type: HCD, FTMS + c NSI d Full ms2 1399.0521@hcd30.00 [120.0000-2875.4944]
Precursor Scan: 013069, m/z: 1398.5509, charge: 2
EEQYNSTYR_4_4_1_0_0
1e6
Intensity
m/z
138.0546
204.086
366.1382
168.065
186.0755
126.0546
274.0906
292.1003
350.1429
512.204_LeX
528.1901
1189.5031 PEP_0_0_0_0_0
1273.5471 PEP_0_1*_0_0_0
1392.59 PEP_0_1_0_0_0
1538.6461 PEP_0_1_1_0_0
1595.6687 PEP_0_2_0_0_0

File: 231129_OE_RK_4, RT: 13.508, Scan: 013217
Type: HCD, FTMS + c NSI d Full ms2 1326.0233@hcd30.00 [120.0000-2726.5156]
Precursor Scan: 013143, m/z: 1325.5218, charge: 2
EEQYNSTYR_4_4_0_0_0

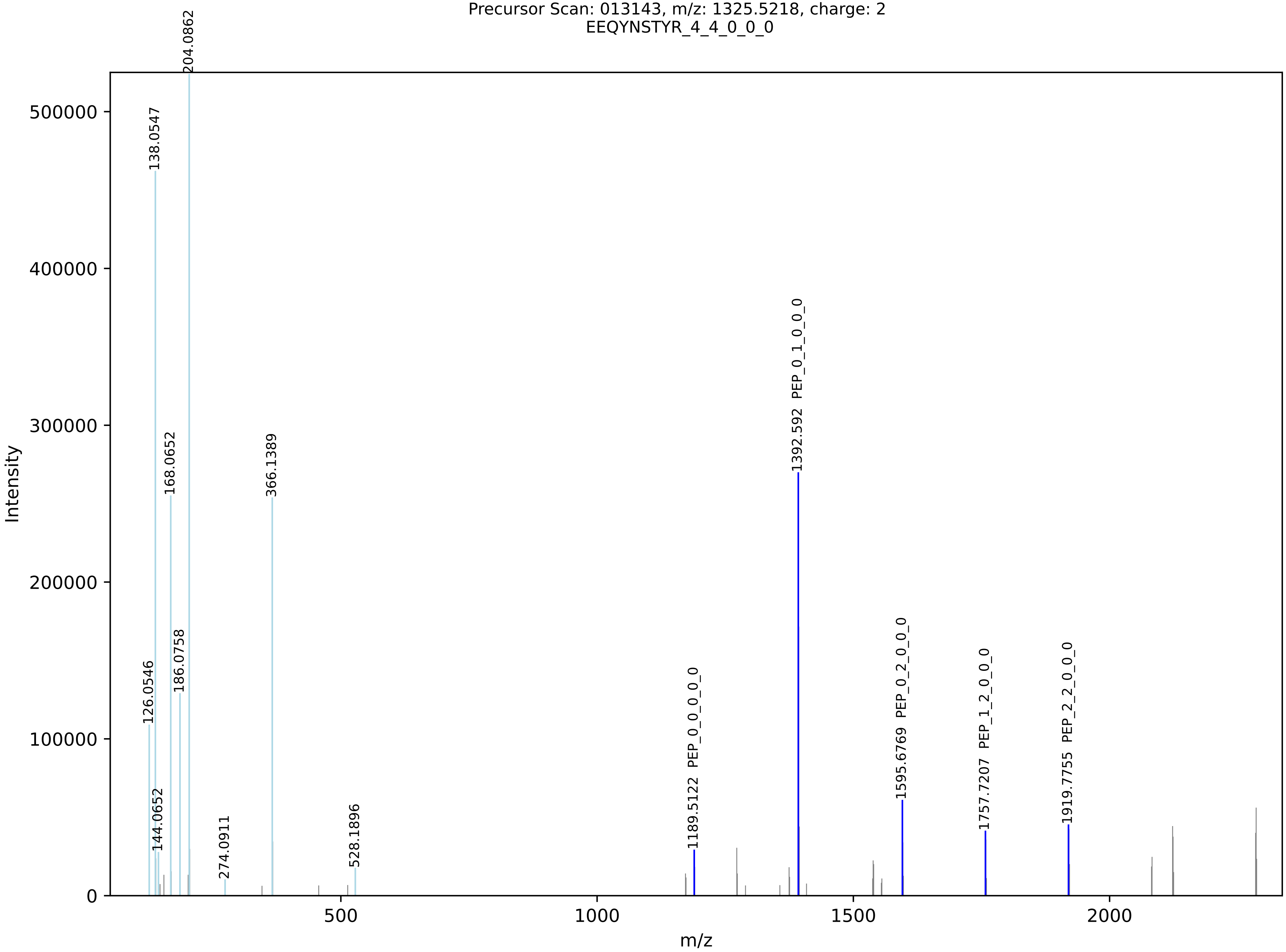

File: 231129_OE_RK_4, RT: 13.613, Scan: 013371
Type: HCD, FTMS + c NSI d Full ms2 1581.6204@hcd30.00 [120.0000-3247.9336]
Precursor Scan: 013287, m/z: 1581.1173, charge: 2
EEQYNSTYR_5_5_1_0_0

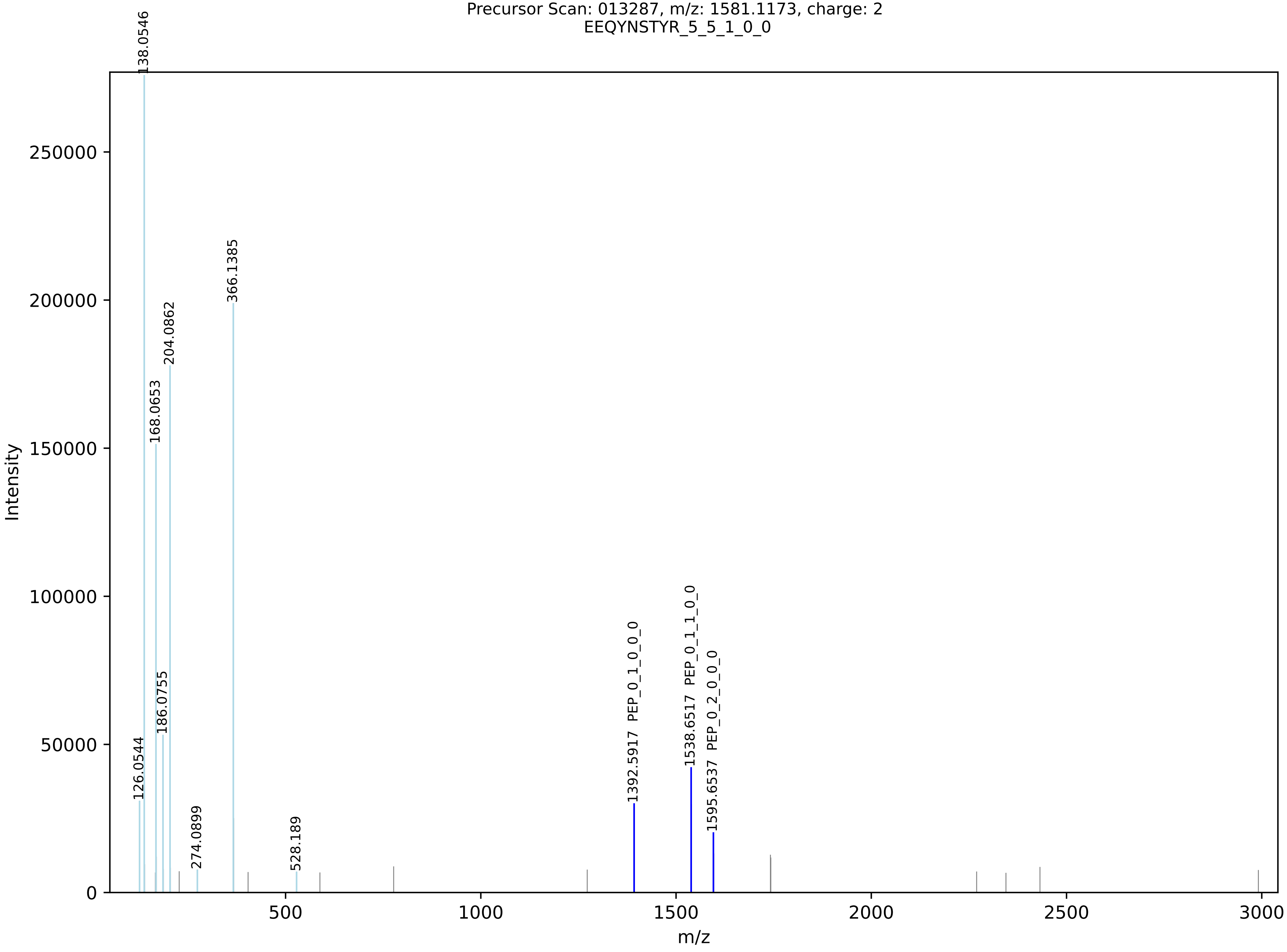

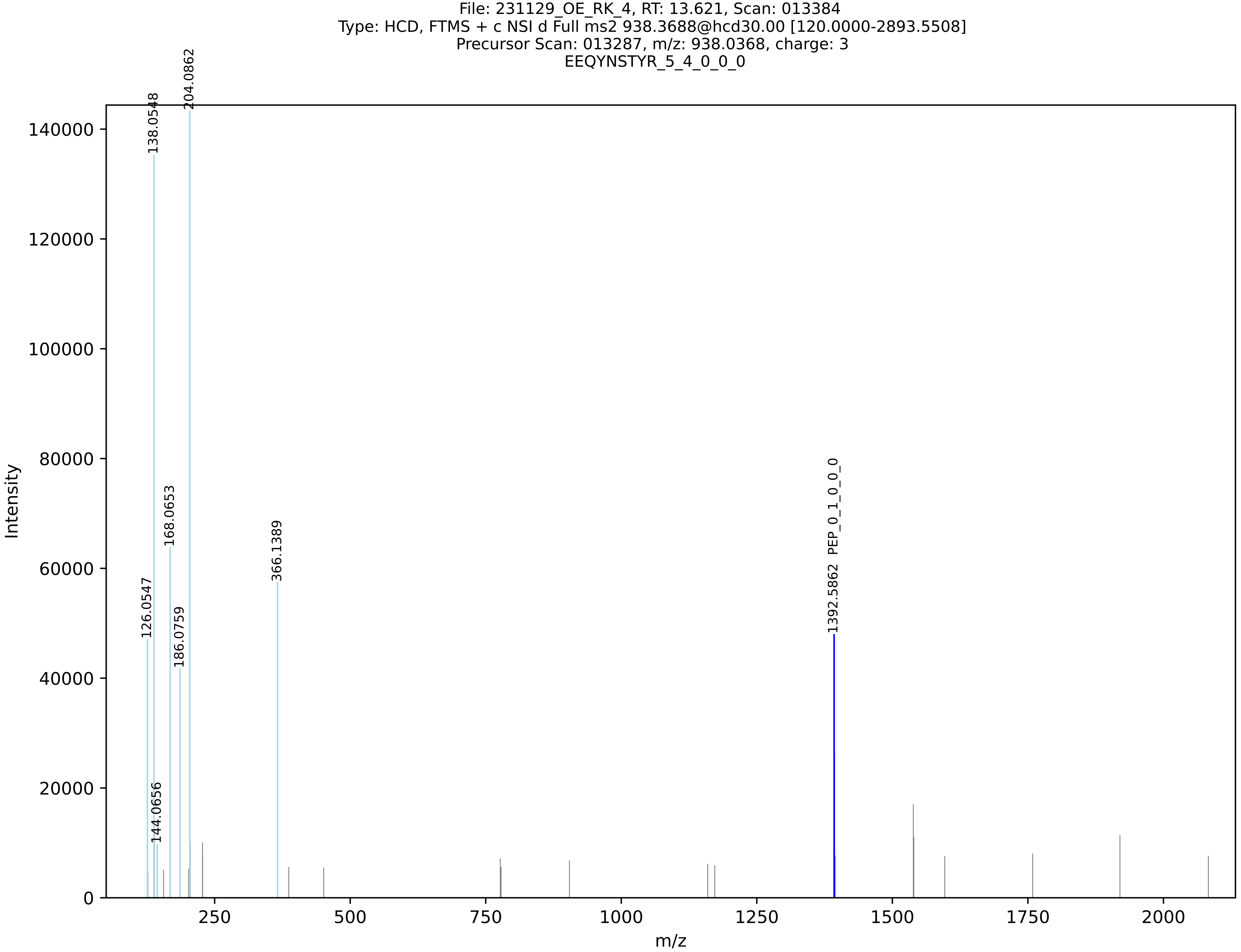
File: 231129_OE_RK_4, RT: 13.621, Scan: 013384
Type: HCD, FTMS + c NSI d Full ms2 938.3688@hcd30.00 [120.0000-2893.5508]
Precursor Scan: 013287, m/z: 938.0368, charge: 3
EEQYNSTYR_5_4_0_0_0
Intensity
m/z
138.0548
204.0862
168.0653
366.1389
126.0547
186.0759
144.0656
1392.5862 PEP_0_1_0_0_0
0
20000
40000
60000
80000
100000
120000
140000
250
500
750
1000
1250
1500
1750
2000

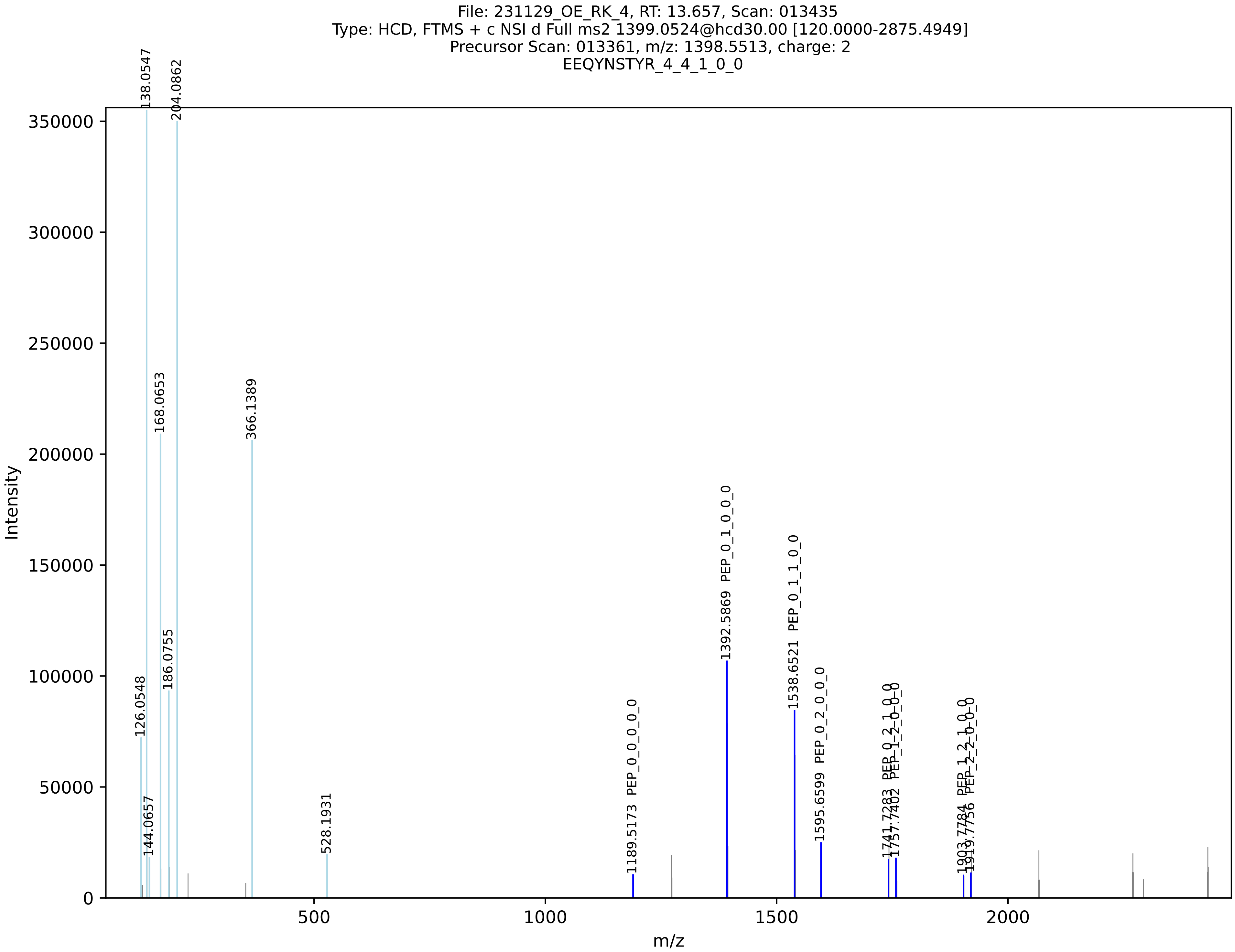
File: 231129_OE_RK_4, RT: 13.657, Scan: 013435
Type: HCD, FTMS + c NSI d Full ms2 1399.0524@hcd30.00 [120.0000-2875.4949]
Precursor Scan: 013361, m/z: 1398.5513, charge: 2
EEQYNSTYR_4_4_1_0_0
Intensity
m/z
138.0547
204.0862
168.0653
366.1389
186.0755
126.0548
144.0657
528.1931
1189.5173 PEP_0_0_0_0_0
1392.5869 PEP_0_1_0_0_0
1538.6521 PEP_0_1_1_0_0
1595.6599 PEP_0_2_0_0_0
1741.7283 PEP_0_2_1_0_0
1757.7402 PEP_1_2_0_0_0
1903.7784 PEP_1_2_1_0_0
1919.7756 PEP_2_2_0_0_0

File: 231129_OE_RK_4, RT: 13.712, Scan: 013518
Type: HCD, FTMS + c NSI d Full ms2 1056.0831@hcd30.00 [120.0000-3253.7561]
Precursor Scan: 013361, m/z: 1054.4131, charge: 3
EEQYNSTYR_5_5_1_0_0

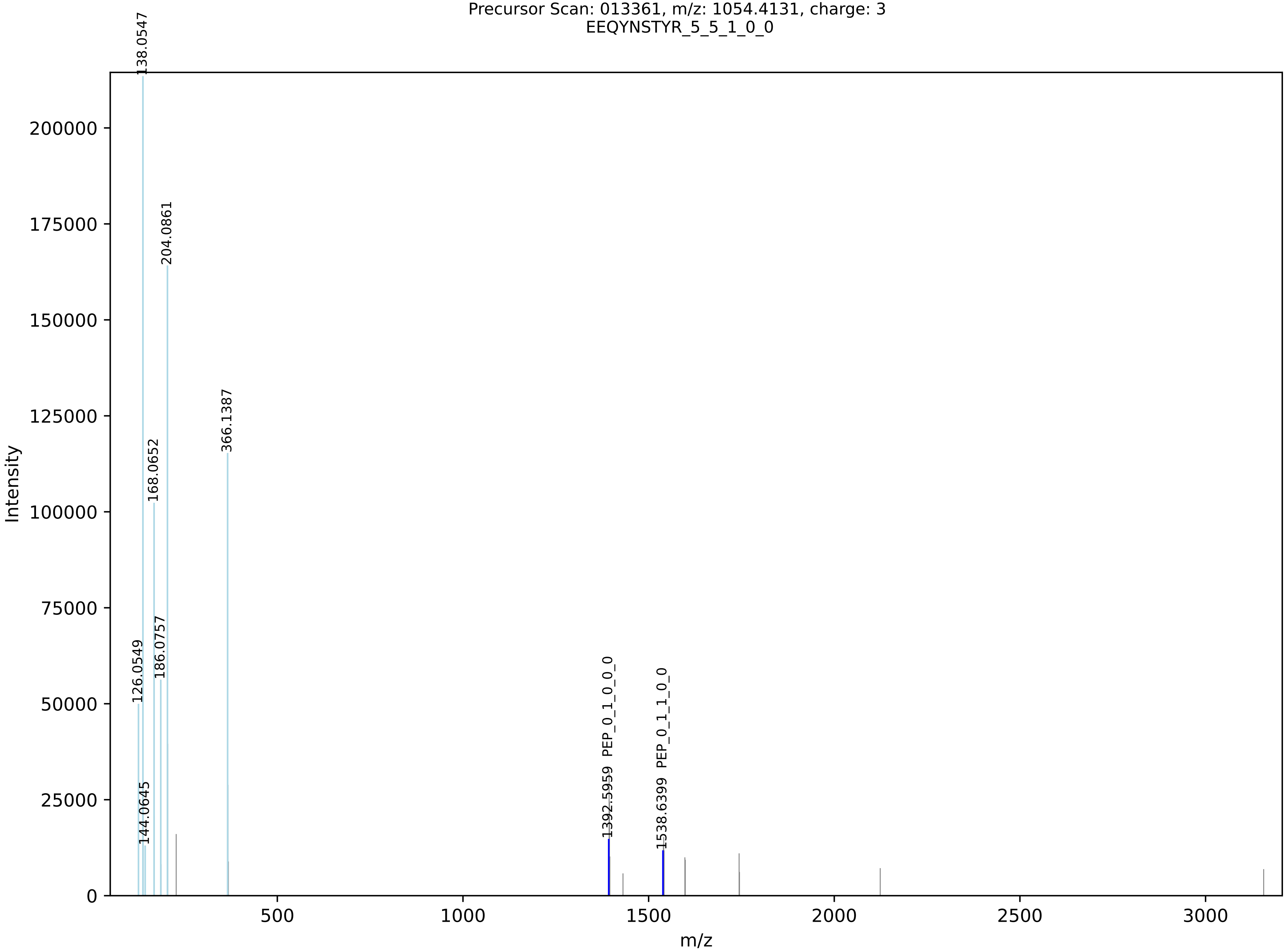

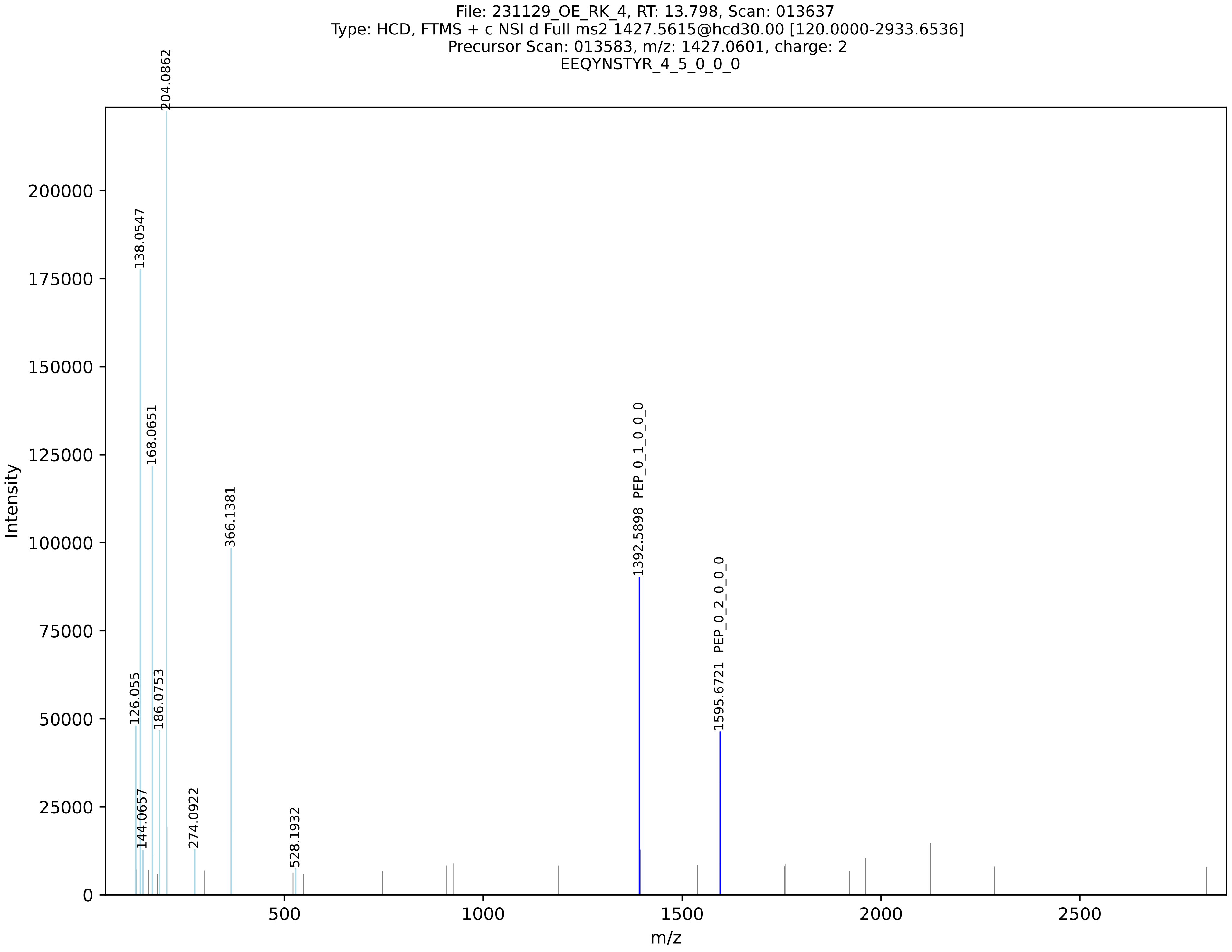
File: 231129_OE_RK_4, RT: 13.798, Scan: 013637
Type: HCD, FTMS + c NSI d Full ms2 1427.5615@hcd30.00 [120.0000-2933.6536]
Precursor Scan: 013583, m/z: 1427.0601, charge: 2
EEQYNSTYR_4_5_0_0_0
204.0862
138.0547
168.0651
366.1381
1392.5898 PEP_0_1_0_0_0
1595.6721 PEP_0_2_0_0_0
126.055
186.0753
144.0657
274.0922
528.1932
Intensity
m/z
0
25000
50000
75000
100000
125000
150000
175000
200000
500
1000
1500
2000
2500

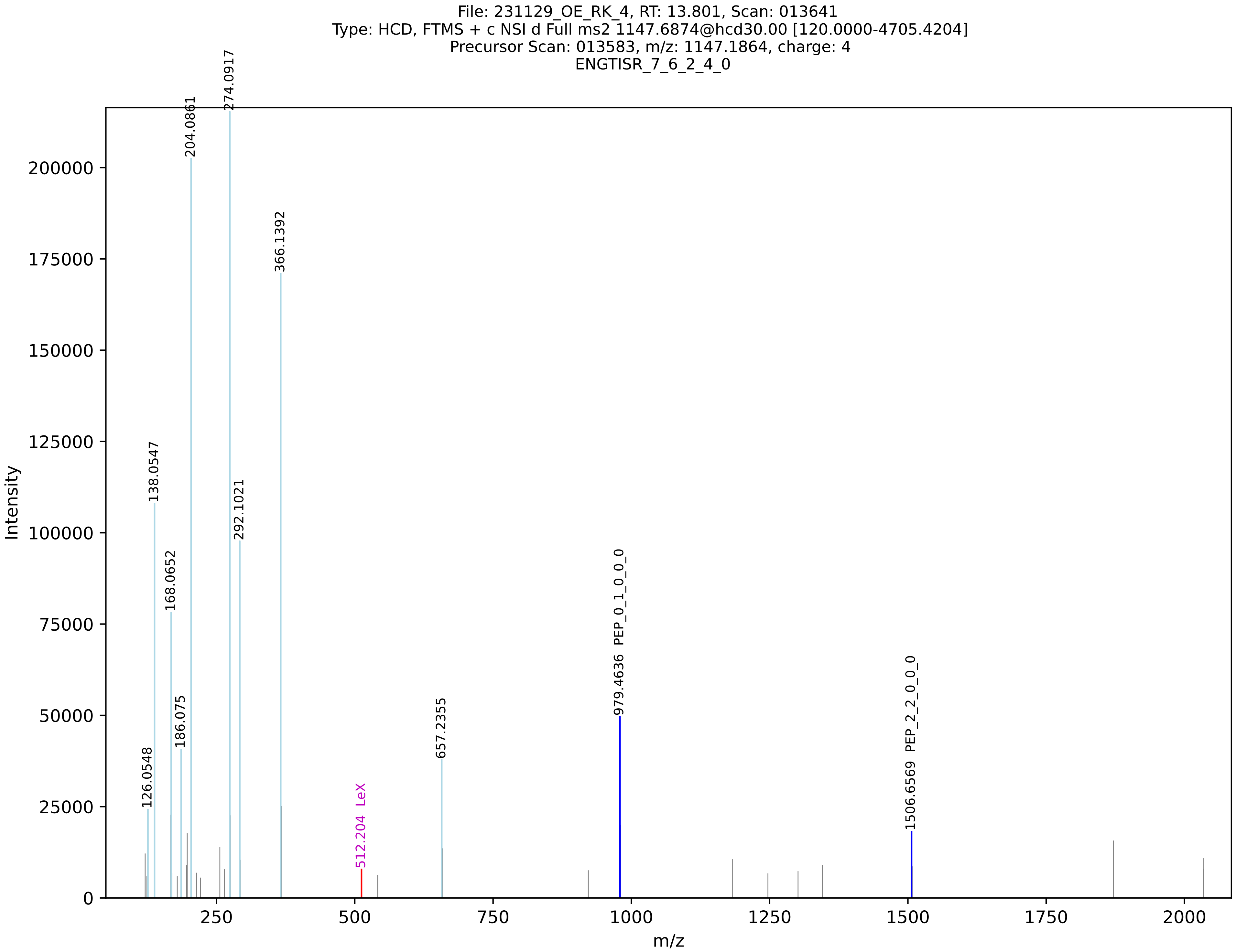

File: 231129_OE_RK_4, RT: 13.801, Scan: 013641
Type: HCD, FTMS + c NSI d Full ms2 1147.6874@hcd30.00 [120.0000-4705.4204]
Precursor Scan: 013583, m/z: 1147.1864, charge: 4
ENGTISR_7_6_2_4_0
Intensity
m/z
0
25000
50000
75000
100000
125000
150000
175000
200000
250
500
750
1000
1250
1500
1750
2000
126.0548
138.0547
168.0652
186.075
204.0861
274.0917
292.1021
366.1392
512.204 LeX
657.2355
979.4636 PEP_0_1_0_0_0
1506.6569 PEP_2_2_0_0_0

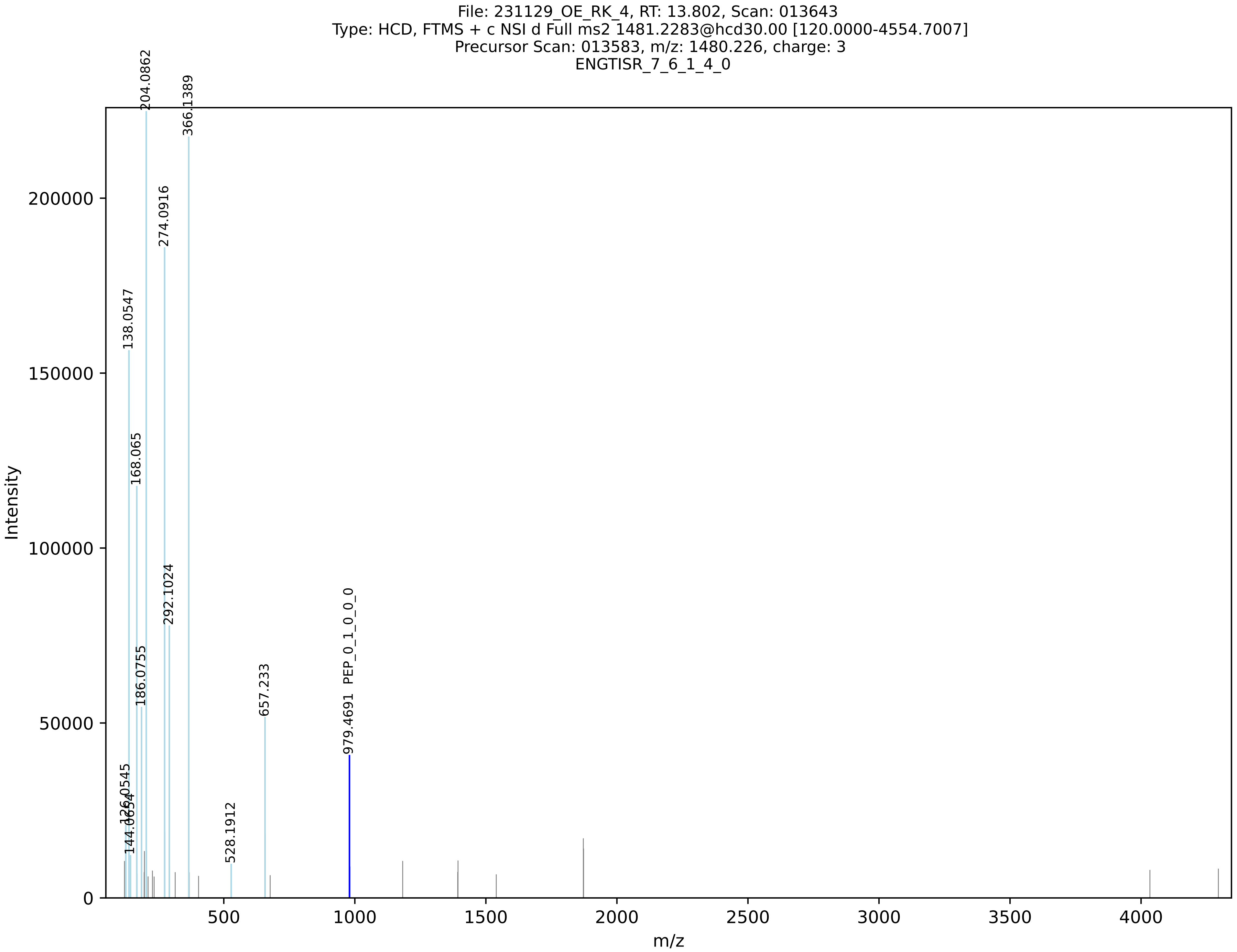

File: 231129_OE_RK_4, RT: 13.802, Scan: 013643
Type: HCD, FTMS + c NSI d Full ms2 1481.2283@hcd30.00 [120.0000-4554.7007]
Precursor Scan: 013583, m/z: 1480.226, charge: 3
ENGTISR_7_6_1_4_0
204.0862
366.1389
274.0916
138.0547
168.065
292.1024
186.0755
657.233
979.4691 PEP_0_1_0_0_0_
126.0545
144.0654
528.1912
Intensity
0
50000
100000
150000
200000
500
1000
1500
2000
2500
3000
3500
4000
m/z

File: 231129_OE_RK_4, RT: 13.837, Scan: 013692
Type: HCD, FTMS + c NSI d Full ms2 1399.5518@hcd30.00 [120.0000-2876.5137]
Precursor Scan: 013632, m/z: 1398.5497, charge: 2
EEQYNSTYR_4_4_1_0_0

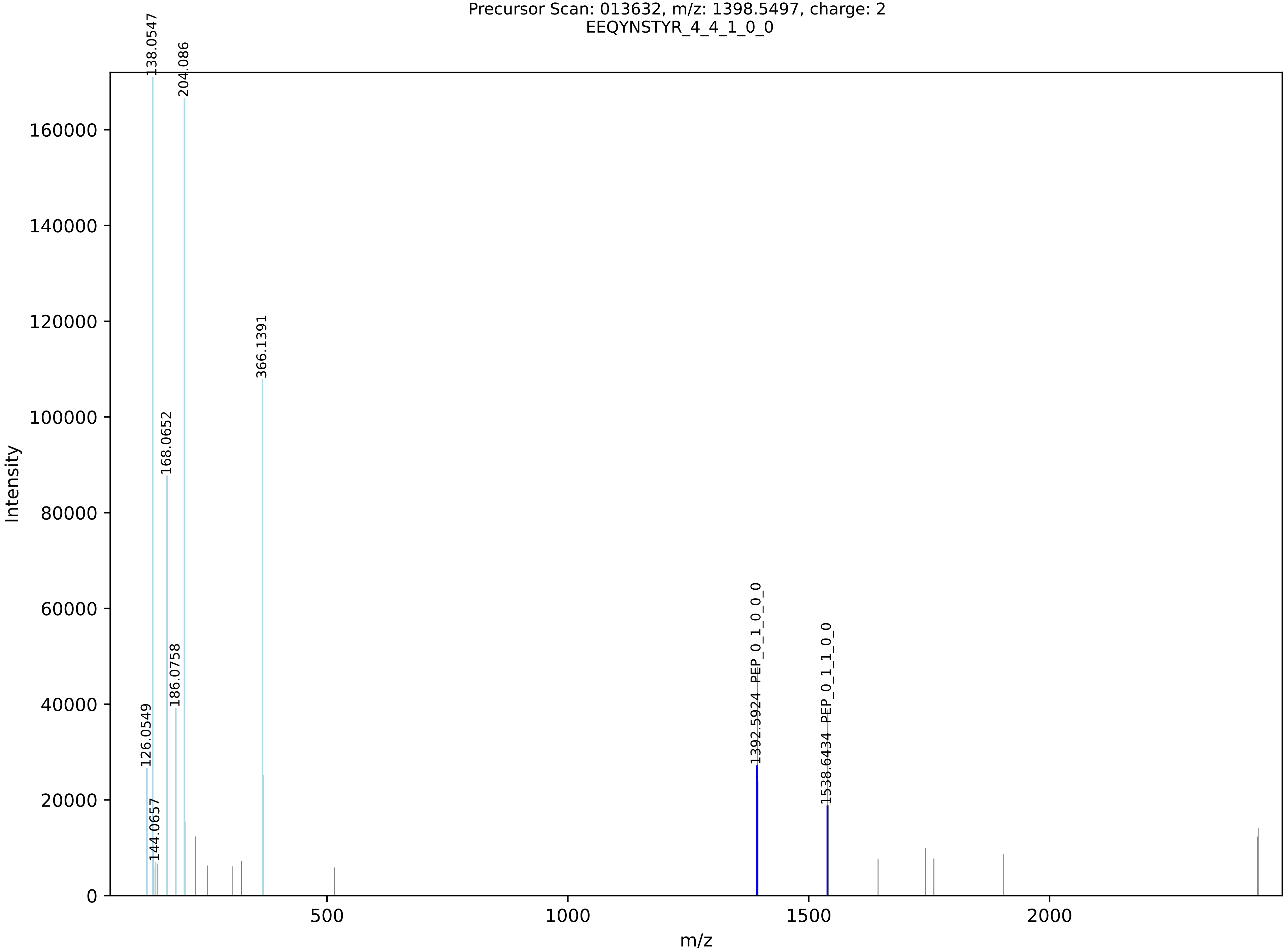

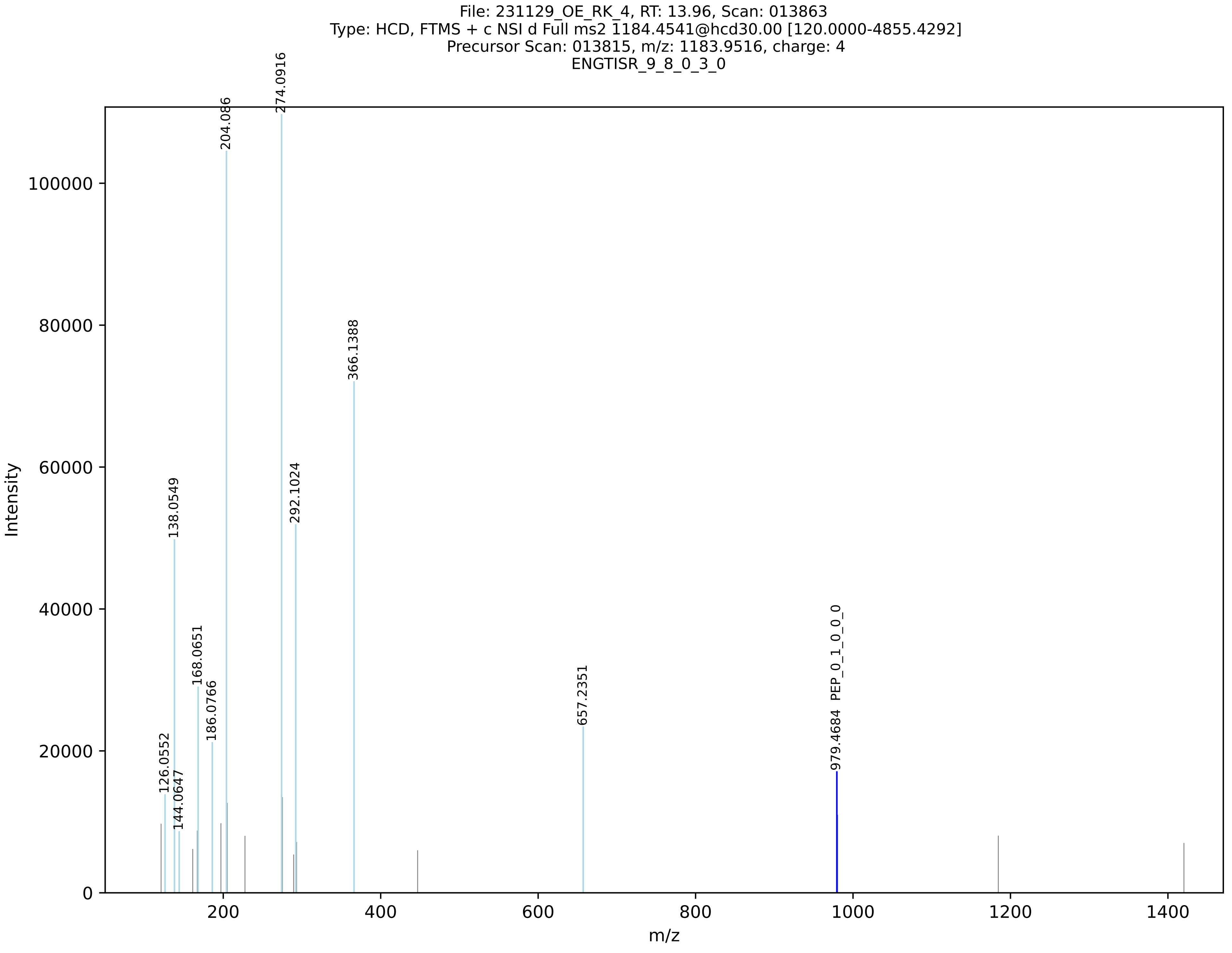
File: 231129_OE_RK_4, RT: 13.96, Scan: 013863
Type: HCD, FTMS + c NSI d Full ms2 1184.4541@hcd30.00 [120.0000-4855.4292]
Precursor Scan: 013815, m/z: 1183.9516, charge: 4
ENGTISR_9_8_0_3_0
274.0916
204.086
366.1388
292.1024
138.0549
168.0651
186.0766
126.0552
144.0647
657.2351
979.4684 PEP_0_1_0_0_0
Intensity
m/z
0
20000
40000
60000
80000
100000
200
400
600
800
1000
1200
1400

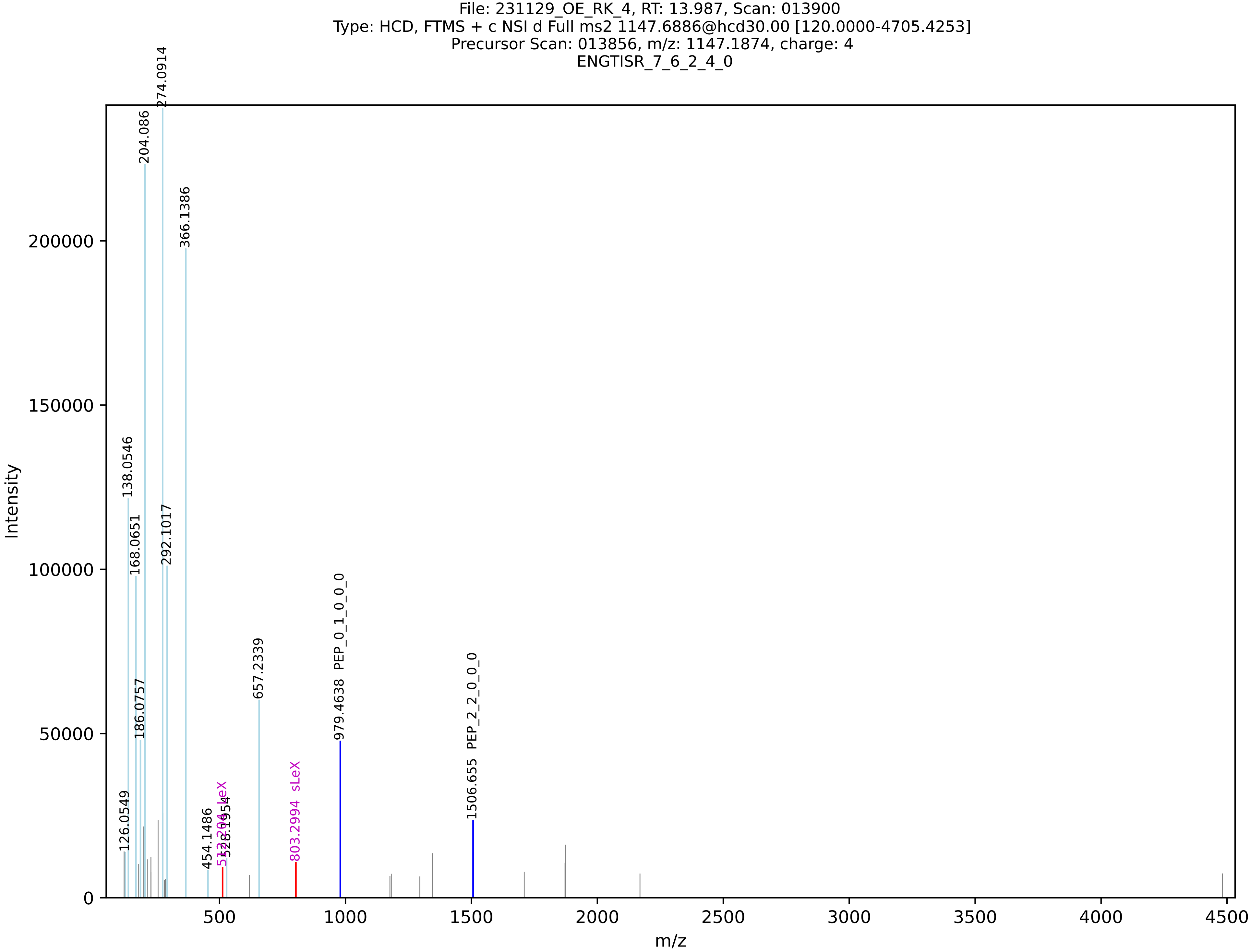
File: 231129_OE_RK_4, RT: 13.987, Scan: 013900
Type: HCD, FTMS + c NSI d Full ms2 1147.6886@hcd30.00 [120.0000-4705.4253]
Precursor Scan: 013856, m/z: 1147.1874, charge: 4
ENGTISR_7_6_2_4_0
Intensity
m/z
274.0914
204.086
366.1386
138.0546
168.0651
292.1017
186.0757
126.0549
454.1486
528.1954
657.2339
979.4638 PEP_0_1_0_0_0
803.2994 sLeX
1506.655 PEP_2_2_0_0_0
0
50000
100000
150000
200000
500
1000
1500
2000
2500
3000
3500
4000
4500

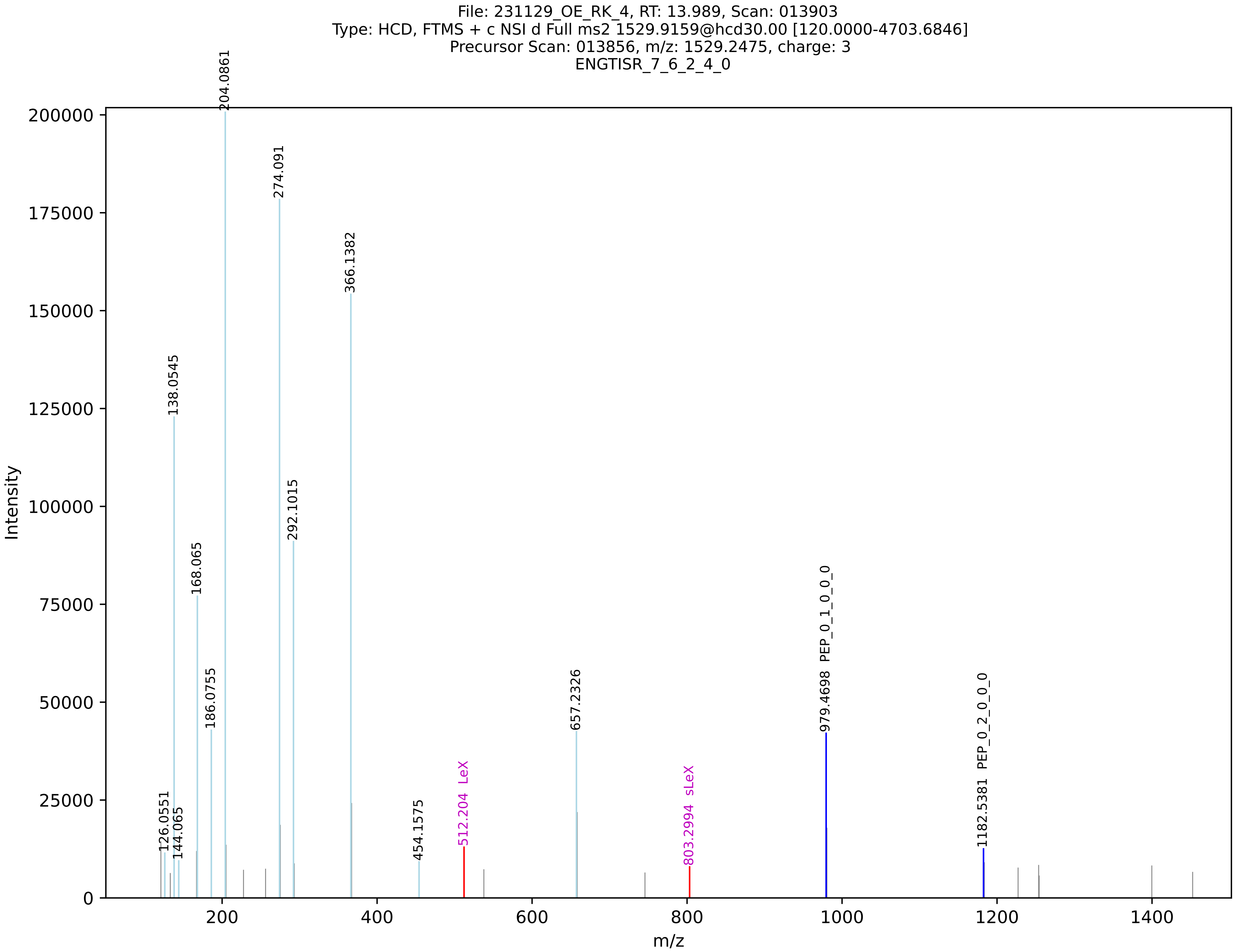
File: 231129_OE_RK_4, RT: 13.989, Scan: 013903
Type: HCD, FTMS + c NSI d Full ms2 1529.9159@hcd30.00 [120.0000-4703.6846]
Precursor Scan: 013856, m/z: 1529.2475, charge: 3
ENGTISR_7_6_2_4_0
Intensity
m/z
204.0861
274.091
366.1382
138.0545
292.1015
168.065
186.0755
126.0551
144.065
454.1575
512.204 LeX
657.2326
803.2994 sLeX
979.4698 PEP_0_1_0_0_0
1182.5381 PEP_0_2_0_0_0
0
25000
50000
75000
100000
125000
150000
175000
200000
200
400
600
800
1000
1200
1400

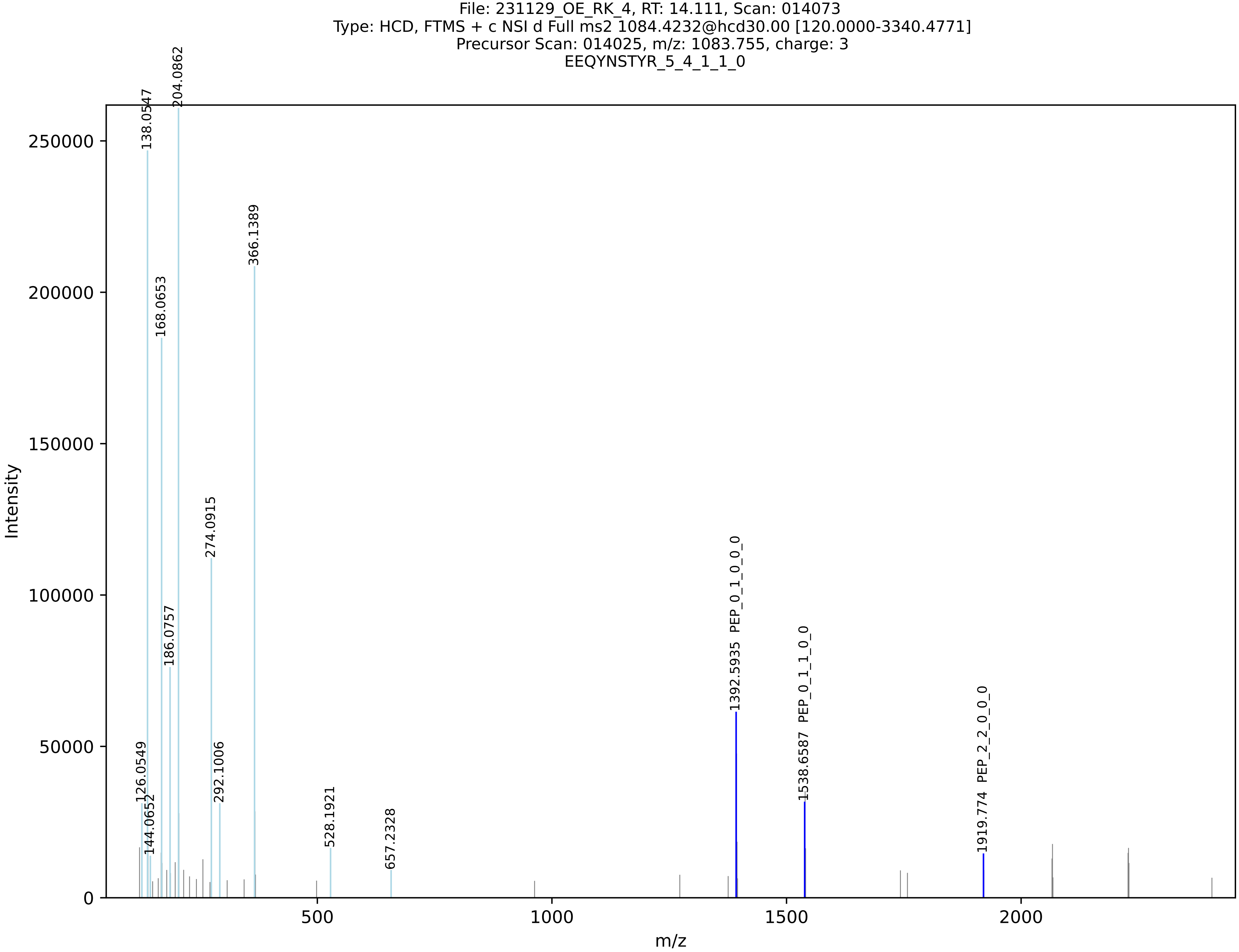

File: 231129_OE_RK_4, RT: 14.111, Scan: 014073
Type: HCD, FTMS + c NSI d Full ms2 1084.4232@hcd30.00 [120.0000-3340.4771]
Precursor Scan: 014025, m/z: 1083.755, charge: 3
EEQYNSTYR_5_4_1_1_0
Intensity
m/z
204.0862
138.0547
366.1389
168.0653
274.0915
186.0757
126.0549
144.0652
292.1006
528.1921
657.2328
1392.5935 PEP_0_1_0_0_
1538.6587 PEP_0_1_1_0_0
1919.774 PEP_2_2_0_0_0

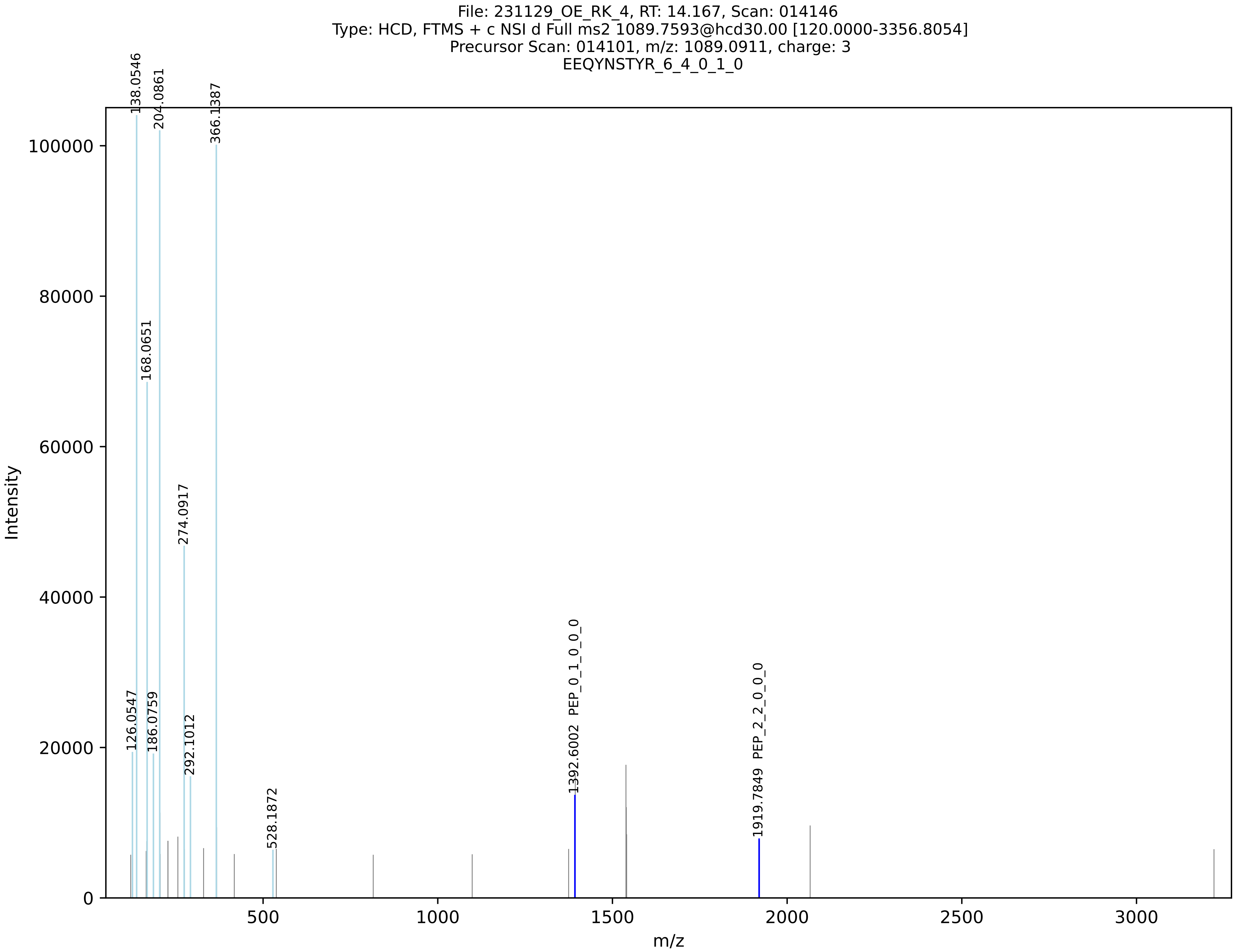
File: 231129_OE_RK_4, RT: 14.167, Scan: 014146
Type: HCD, FTMS + c NSI d Full ms2 1089.7593@hcd30.00 [120.0000-3356.8054]
Precursor Scan: 014101, m/z: 1089.0911, charge: 3
EEQYNSTYR_6_4_0_1_0
138.0546
204.0861
366.1387
168.0651
274.0917
126.0547
186.0759
292.1012
528.1872
1392.6002 PEP_0_1_0_0_0
1919.7849 PEP_2_2_0_0_0
Intensity
m/z
0
20000
40000
60000
80000
100000
500
1000
1500
2000
2500
3000

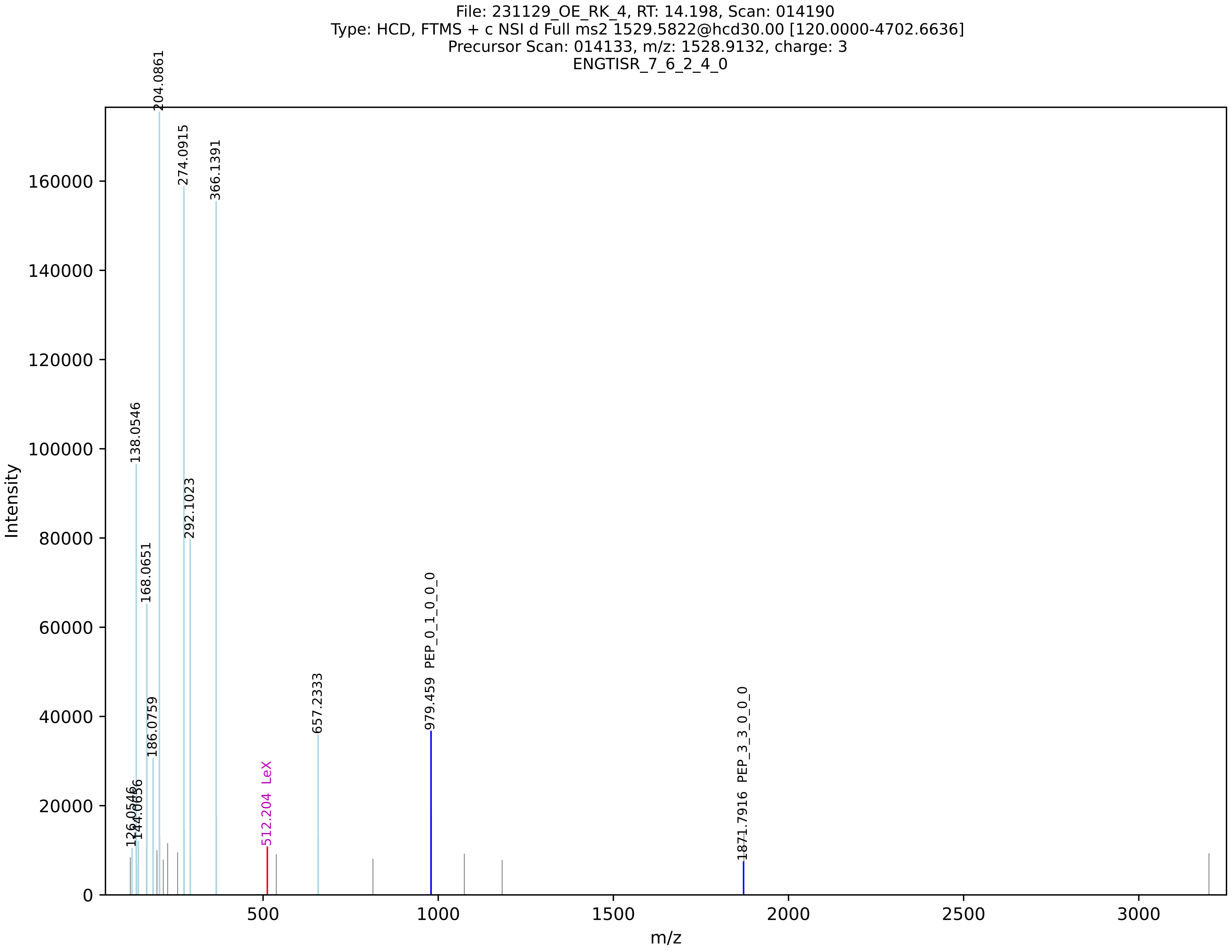

File: 231129_OE_RK_4, RT: 14.198, Scan: 014190
Type: HCD, FTMS + c NSI d Full ms2 1529.5822@hcd30.00 [120.0000-4702.6636]
Precursor Scan: 014133, m/z: 1528.9132, charge: 3
ENGTISR_7_6_2_4_0
Intensity
m/z
204.0861
274.0915
366.1391
138.0546
292.1023
168.0651
186.0759
126.0546
144.0656
512.204 LeX
657.2333
979.459 PEP_0_1_0_0_0
1871.7916 PEP_3_3_0_0_0

File: 231129_OE_RK_4, RT: 14.318, Scan: 014363
Type: HCD, FTMS + c NSI d Full ms2 1084.0874@hcd30.00 [120.0000-3339.4495]
Precursor Scan: 014301, m/z: 1083.7533, charge: 3
EEQYNSTYR_5_4_1_1_0

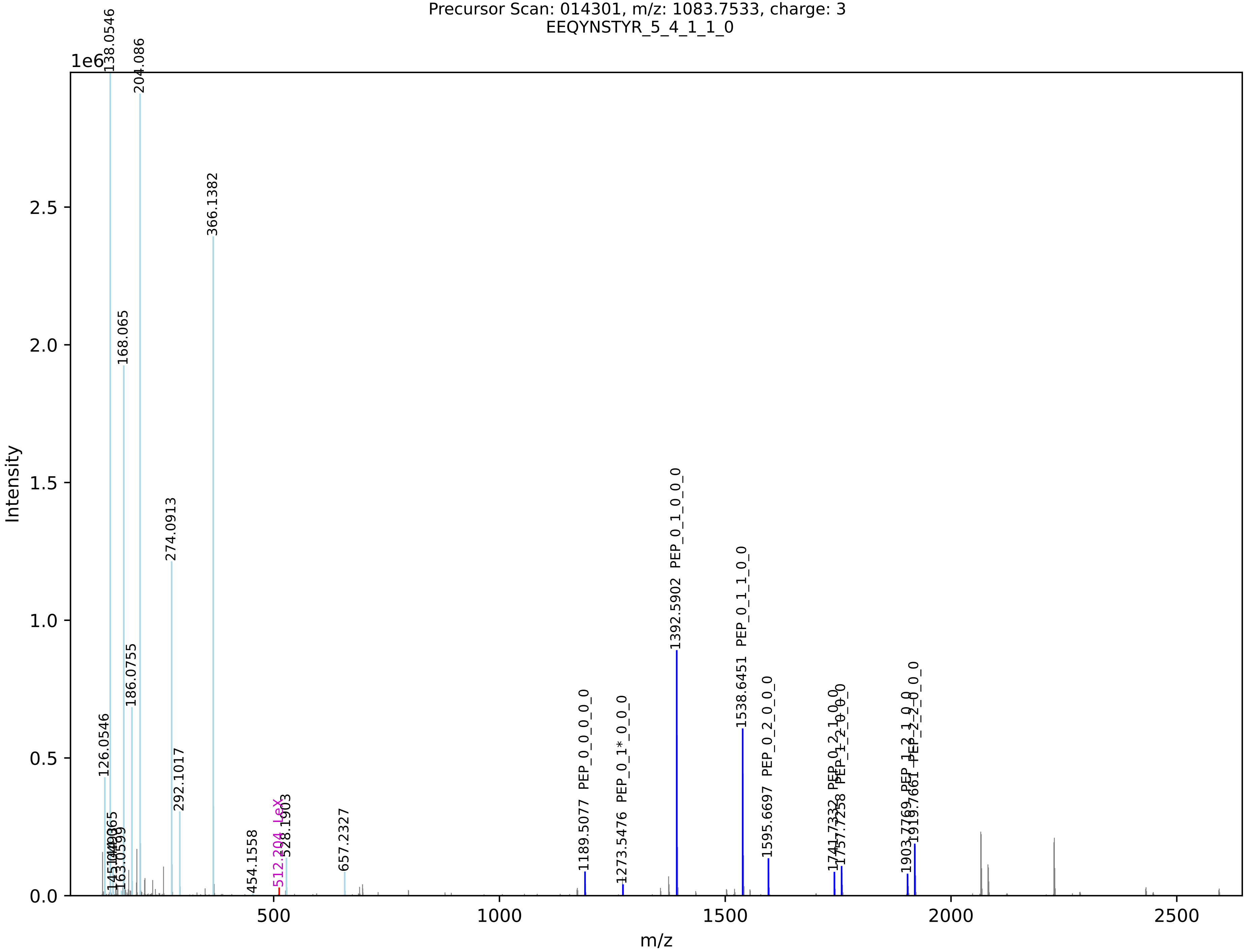

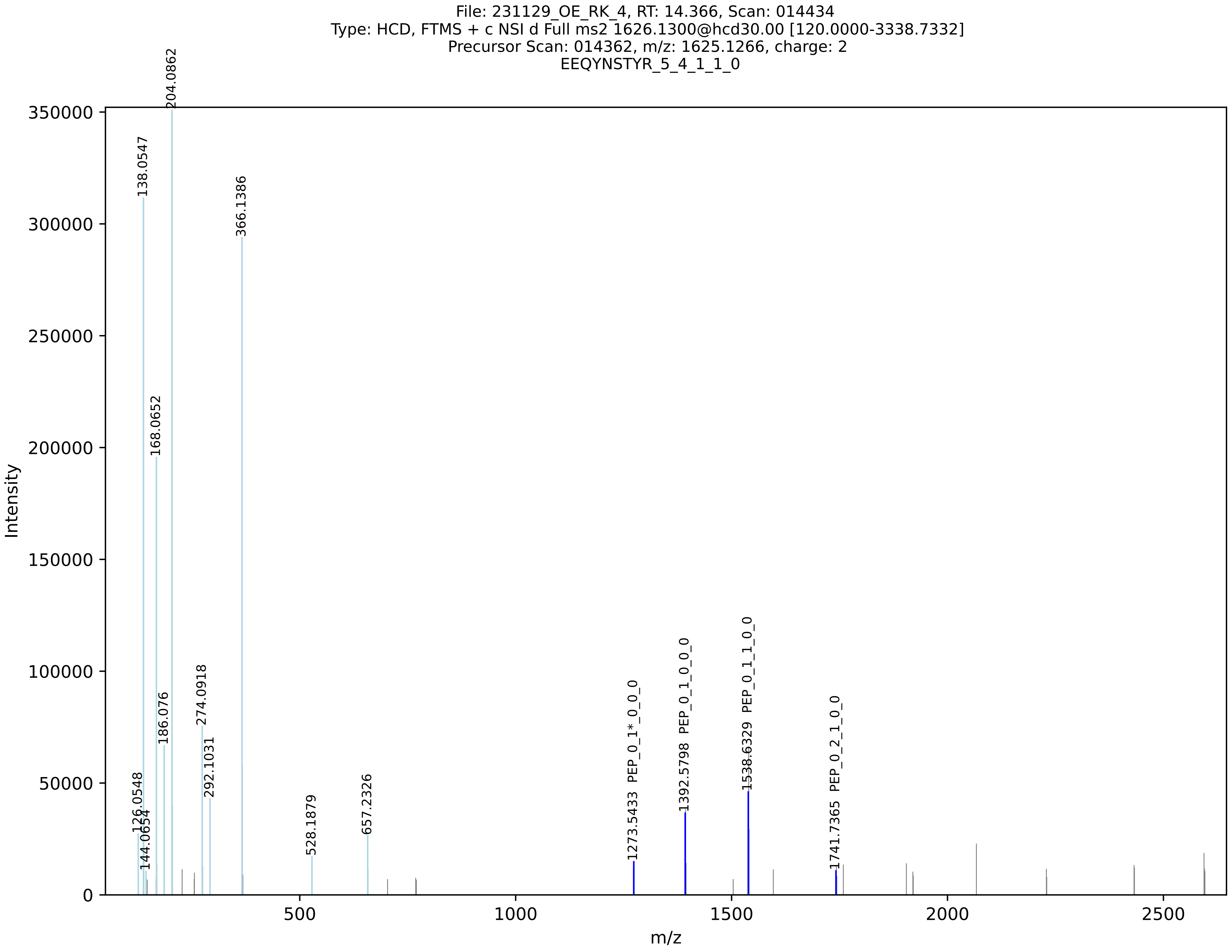
File: 231129_OE_RK_4, RT: 14.366, Scan: 014434
Type: HCD, FTMS + c NSI d Full ms2 1626.1300@hcd30.00 [120.0000-3338.7332]
Precursor Scan: 014362, m/z: 1625.1266, charge: 2
EEQYNSTYR_5_4_1_1_0
Intensity
m/z
204.0862
138.0547
366.1386
168.0652
186.076
274.0918
292.1031
126.0548
144.0654
528.1879
657.2326
1273.5433 PEP_0_1*_0_0_0
1392.5798 PEP_0_1_0_0_0
1538.6329 PEP_0_1_1_0_0
1741.7365 PEP_0_2_1_0_0

File: 231129_OE_RK_4, RT: 14.419, Scan: 014512
Type: HCD, FTMS + c NSI d Full ms2 1147.4375@hcd30.00 [120.0000-4704.4014]
Precursor Scan: 014432, m/z: 1147.1884, charge: 4
ENGTISR_7_6_2_4_0

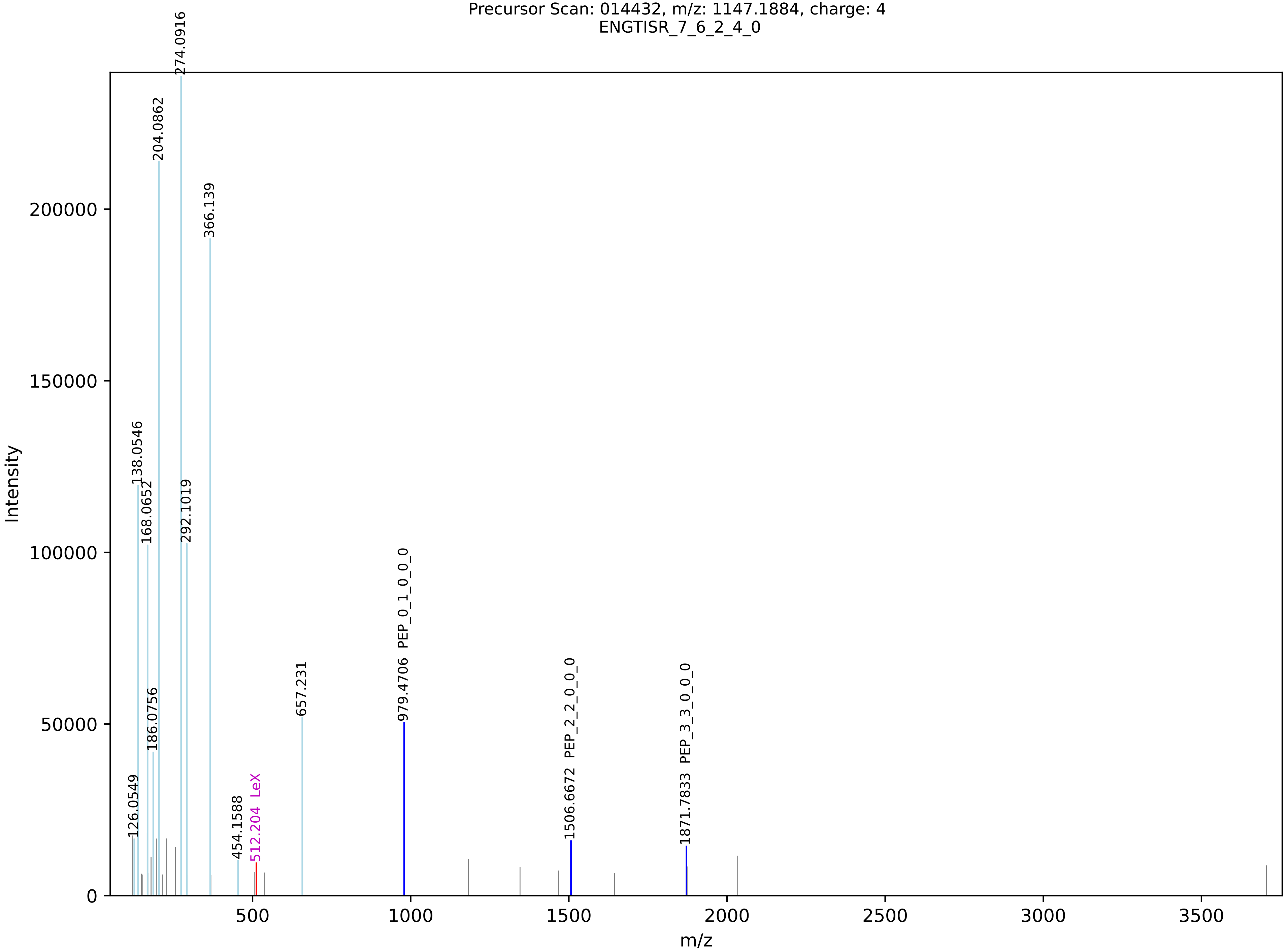

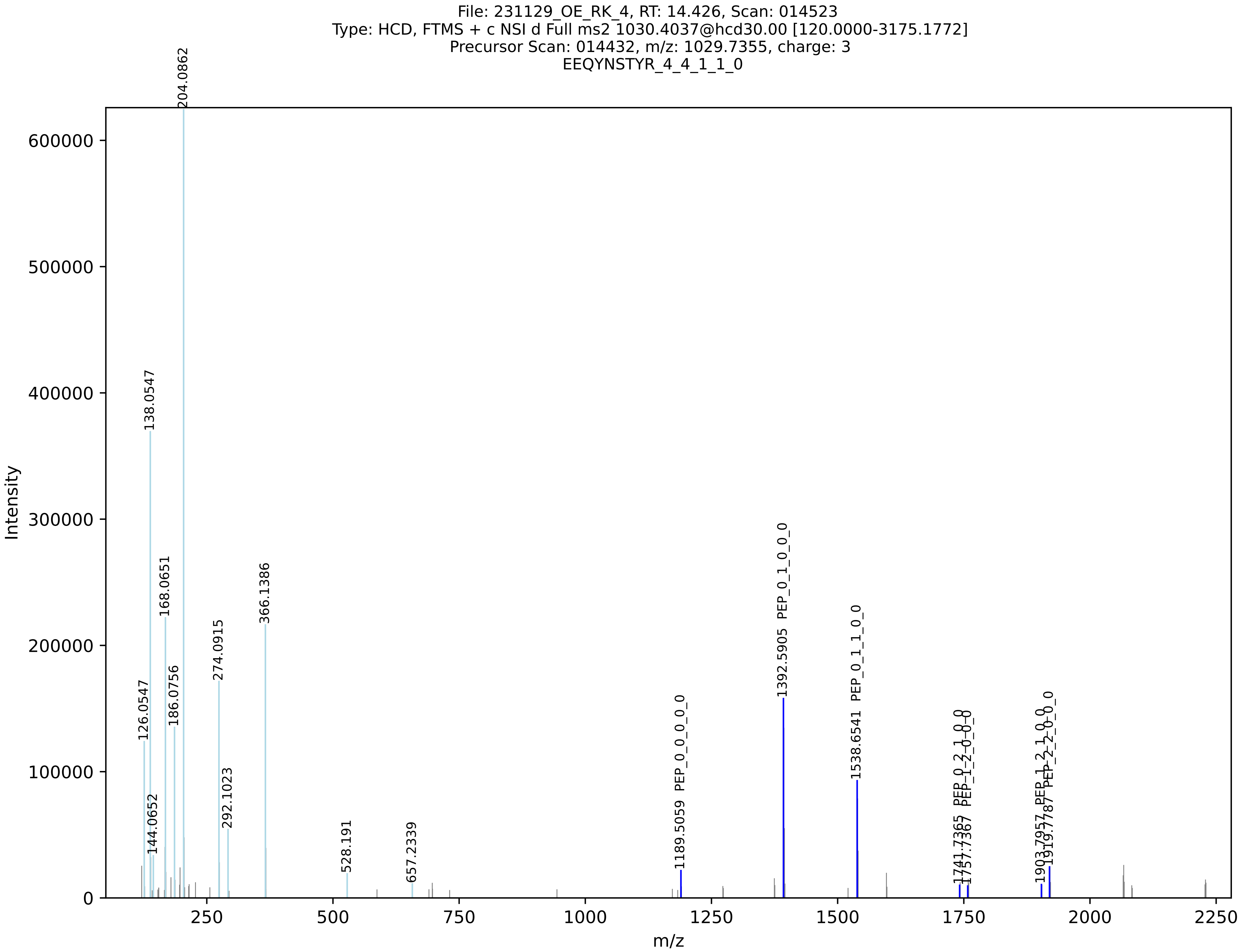
File: 231129_OE_RK_4, RT: 14.426, Scan: 014523
Type: HCD, FTMS + c NSI d Full ms2 1030.4037@hcd30.00 [120.0000-3175.1772]
Precursor Scan: 014432, m/z: 1029.7355, charge: 3
EEQYNSTYR_4_4_1_1_0
Intensity
m/z
204.0862
138.0547
168.0651
366.1386
274.0915
186.0756
126.0547
144.0652
292.1023
528.191
657.2339
1189.5059 PEP_0_0_0_0_0_
1392.5905 PEP_0_1_0_0_0_
1538.6541 PEP_0_1_1_0_0_
1741.7365 PEP_0_2_1_0_0_
1757.7367 PEP_1_2_0_0_0_
1903.7957 PEP_1_2_1_0_0_
1919.7787 PEP_2_2_0_0_0_

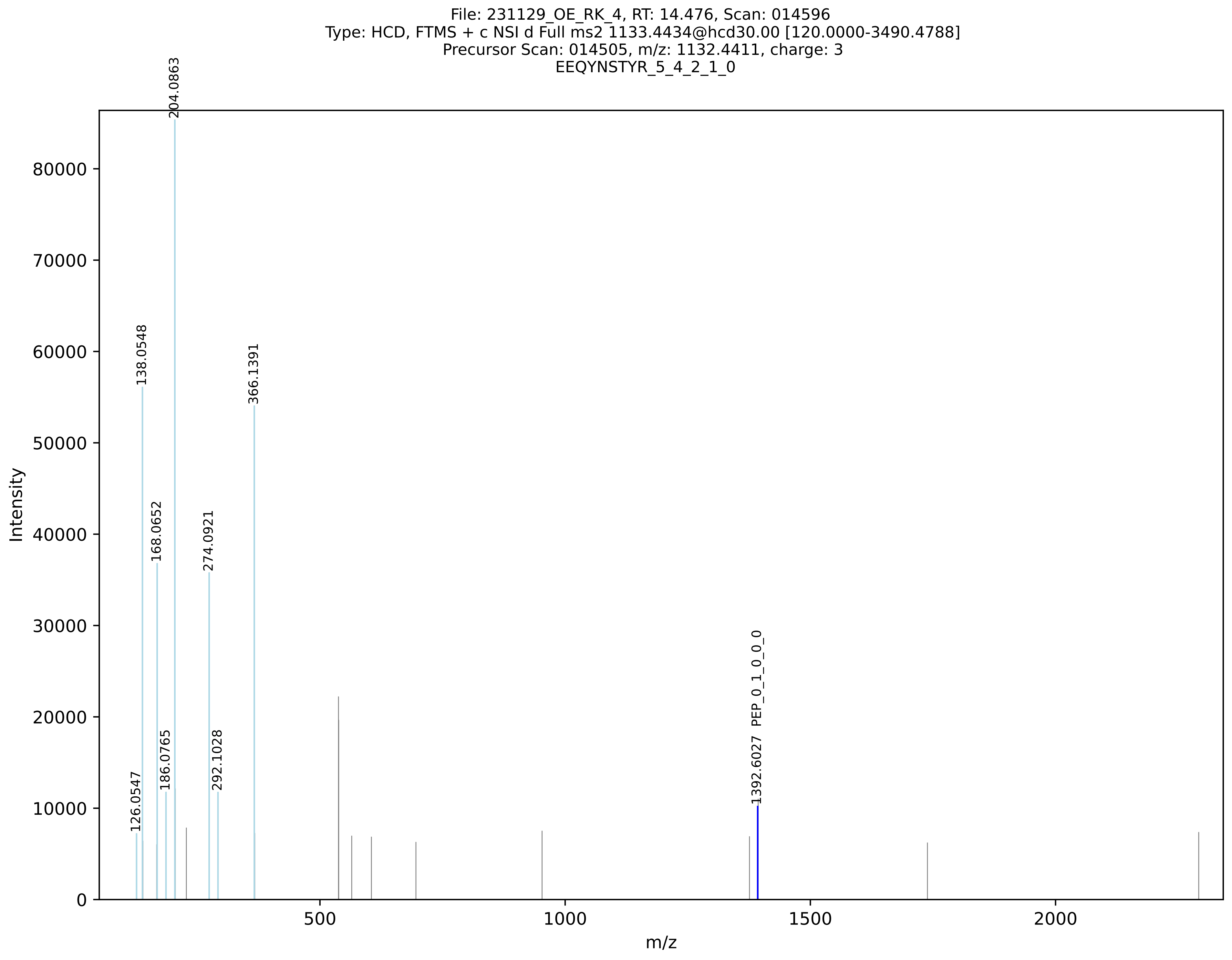
File: 231129_OE_RK_4, RT: 14.476, Scan: 014596
Type: HCD, FTMS + c NSI d Full ms2 1133.4434@hcd30.00 [120.0000-3490.4788]
Precursor Scan: 014505, m/z: 1132.4411, charge: 3
EEQYNSTYR_5_4_2_1_0
204.0863
138.0548
366.1391
168.0652
274.0921
PEP_0_1_0_0_0
186.0765
292.1028
126.0547
1392.6027
Intensity
m/z
0
10000
20000
30000
40000
50000
60000
70000
80000
500
1000
1500
2000

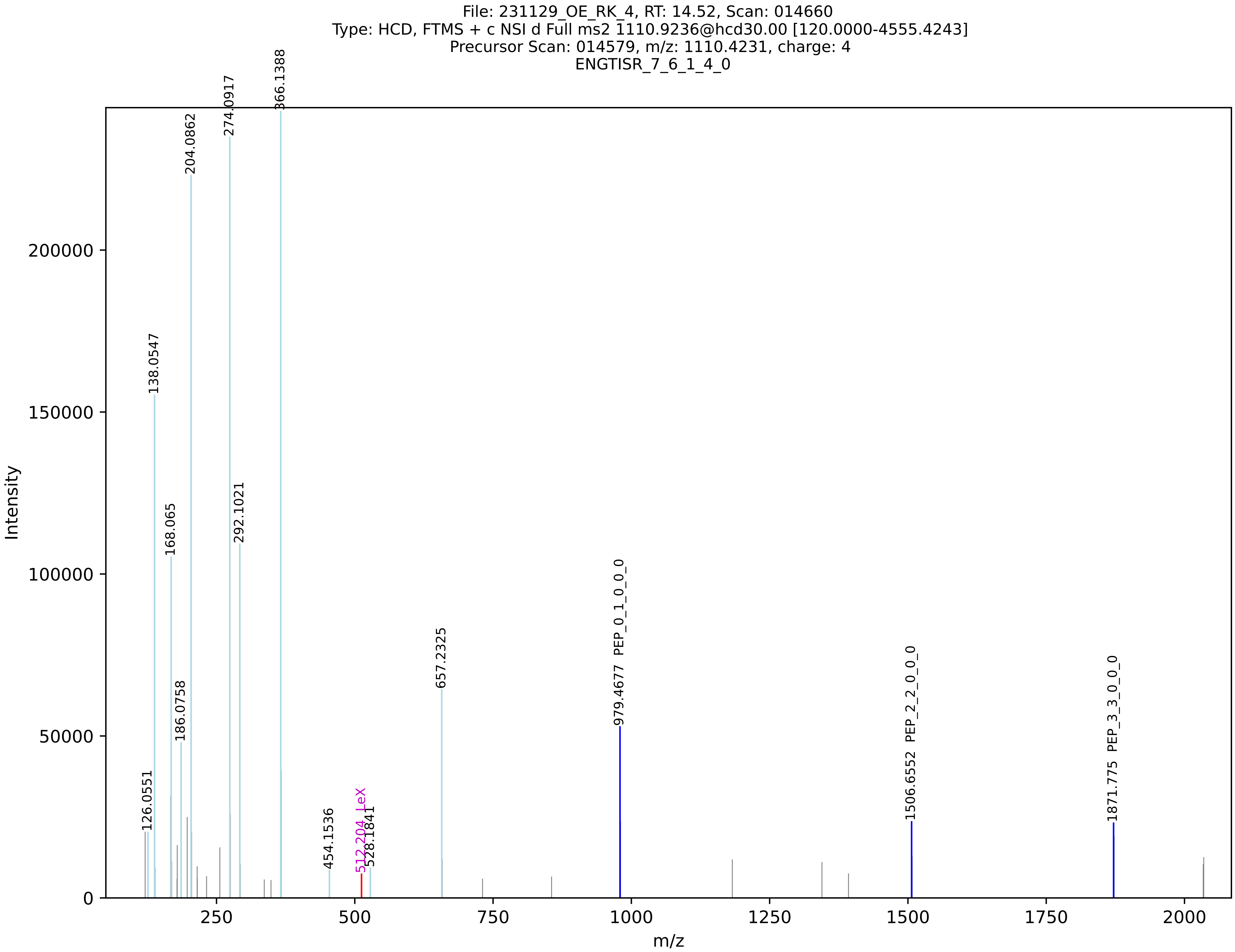
File: 231129_OE_RK_4, RT: 14.52, Scan: 014660
Type: HCD, FTMS + c NSI d Full ms2 1110.9236@hcd30.00 [120.0000-4555.4243]
Precursor Scan: 014579, m/z: 1110.4231, charge: 4
ENGTISR_7_6_1_4_0
Intensity
m/z
0
50000
100000
150000
200000
250
500
750
1000
1250
1500
1750
2000
366.1388
274.0917
204.0862
138.0547
168.065
292.1021
186.0758
126.0551
454.1536
512.204_LeX
528.1841
657.2325
979.4677 PEP_0_1_0_0_0
1506.6552 PEP_2_2_0_0_0
1871.775 PEP_3_3_0_0_0

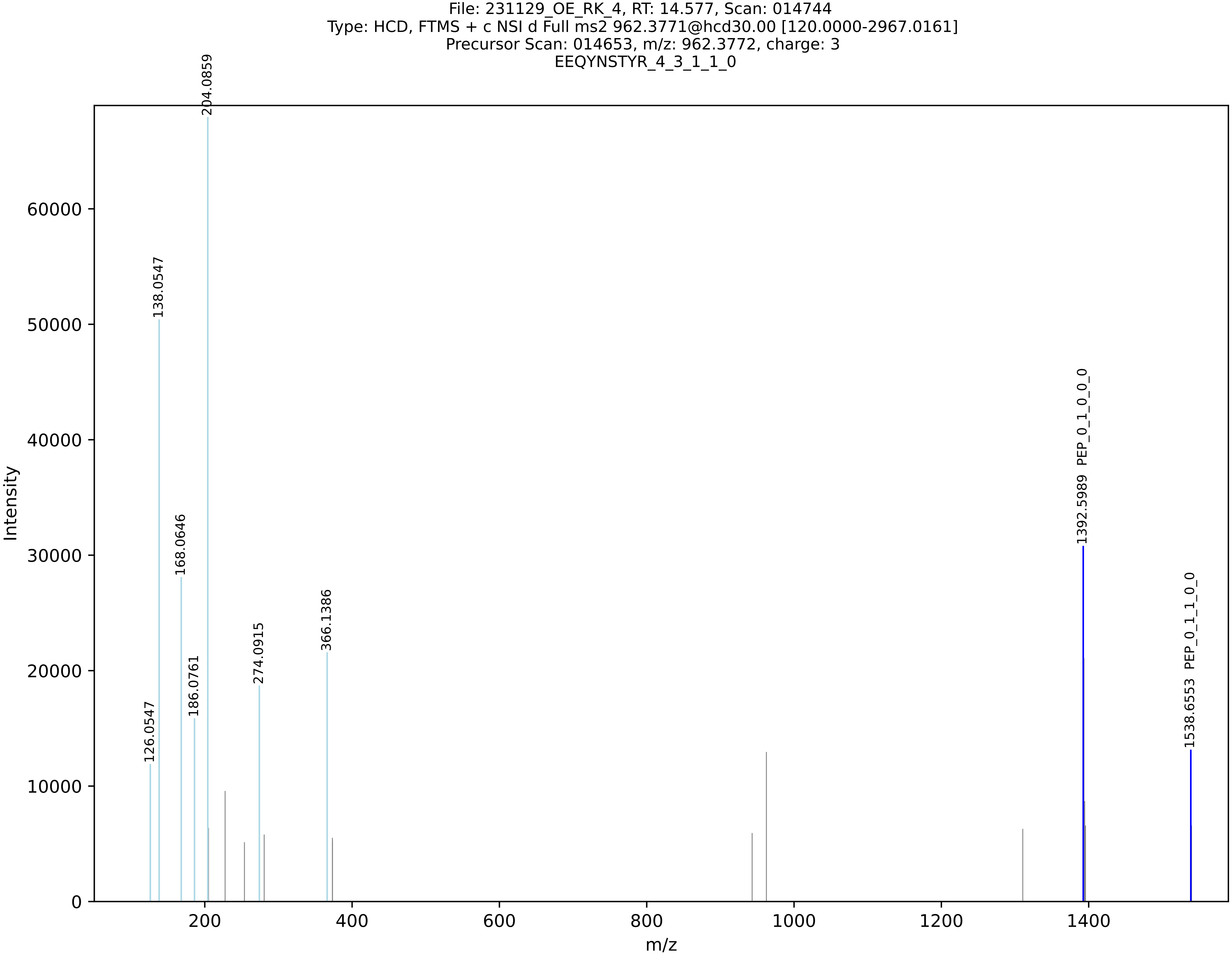
File: 231129_OE_RK_4, RT: 14.577, Scan: 014744
Type: HCD, FTMS + c NSI d Full ms2 962.3771@hcd30.00 [120.0000-2967.0161]
Precursor Scan: 014653, m/z: 962.3772, charge: 3
EEQYNSTYR_4_3_1_1_0
204.0859
138.0547
168.0646
186.0761
126.0547
274.0915
366.1386
1392.5989 PEP_0_1_0_0_
1538.6553 PEP_0_1_1_0_0
Intensity
m/z
0
10000
20000
30000
40000
50000
60000
200
400
600
800
1000
1200
1400

File: 231129_OE_RK_4, RT: 14.618, Scan: 014802
Type: HCD, FTMS + c NSI d Full ms2 1030.0697@hcd30.00 [120.0000-3174.1550]
Precursor Scan: 014727, m/z: 1029.7356, charge: 3
EEQYNSTYR_4_4_1_1_0

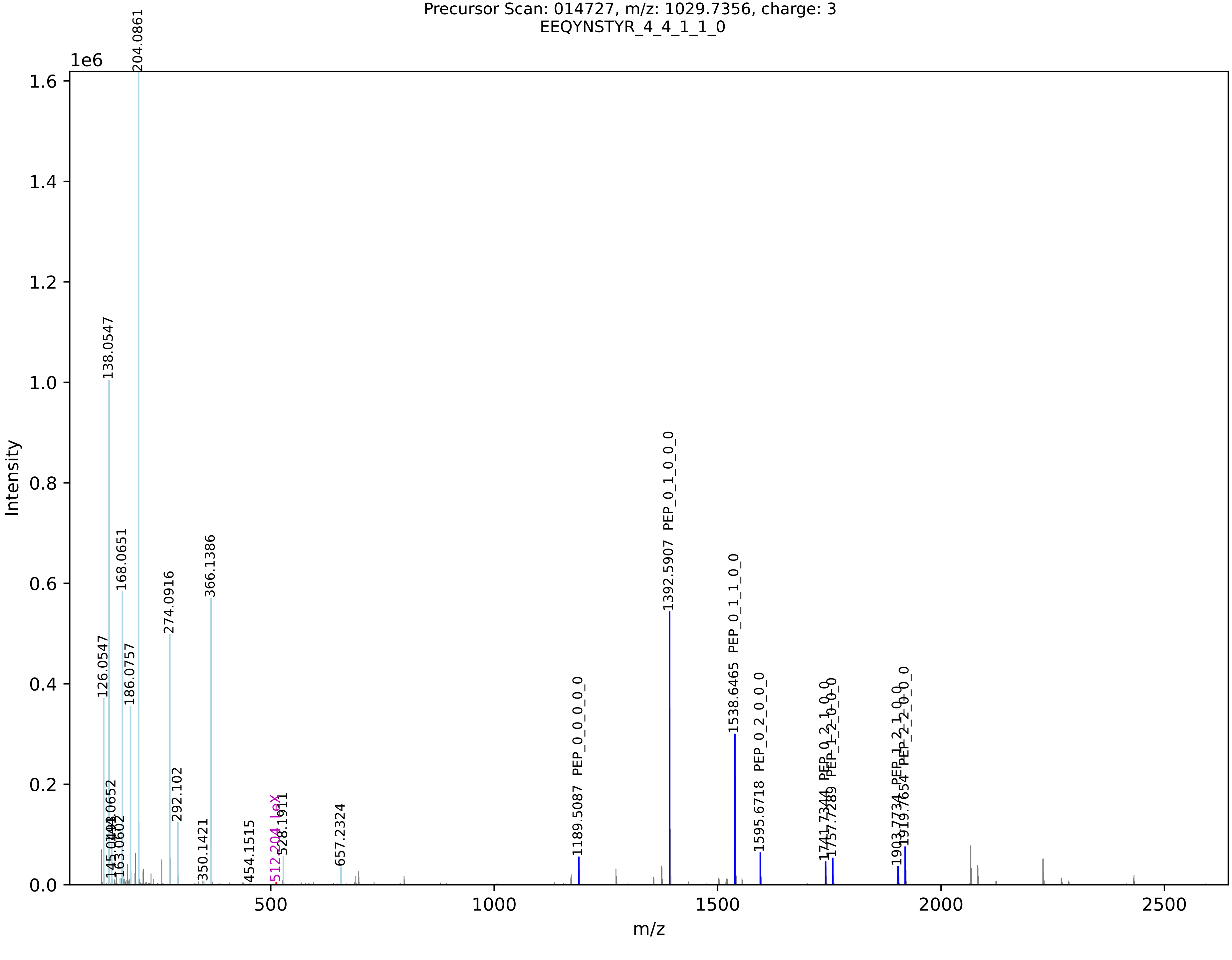

File: 231129_OE_RK_4, RT: 14.622, Scan: 014809
Type: HCD, FTMS + c NSI d Full ms2 1147.9395@hcd30.00 [120.0000-4706.4487]
Precursor Scan: 014727, m/z: 1147.1884, charge: 4
ENGTISR_7_6_2_4_0

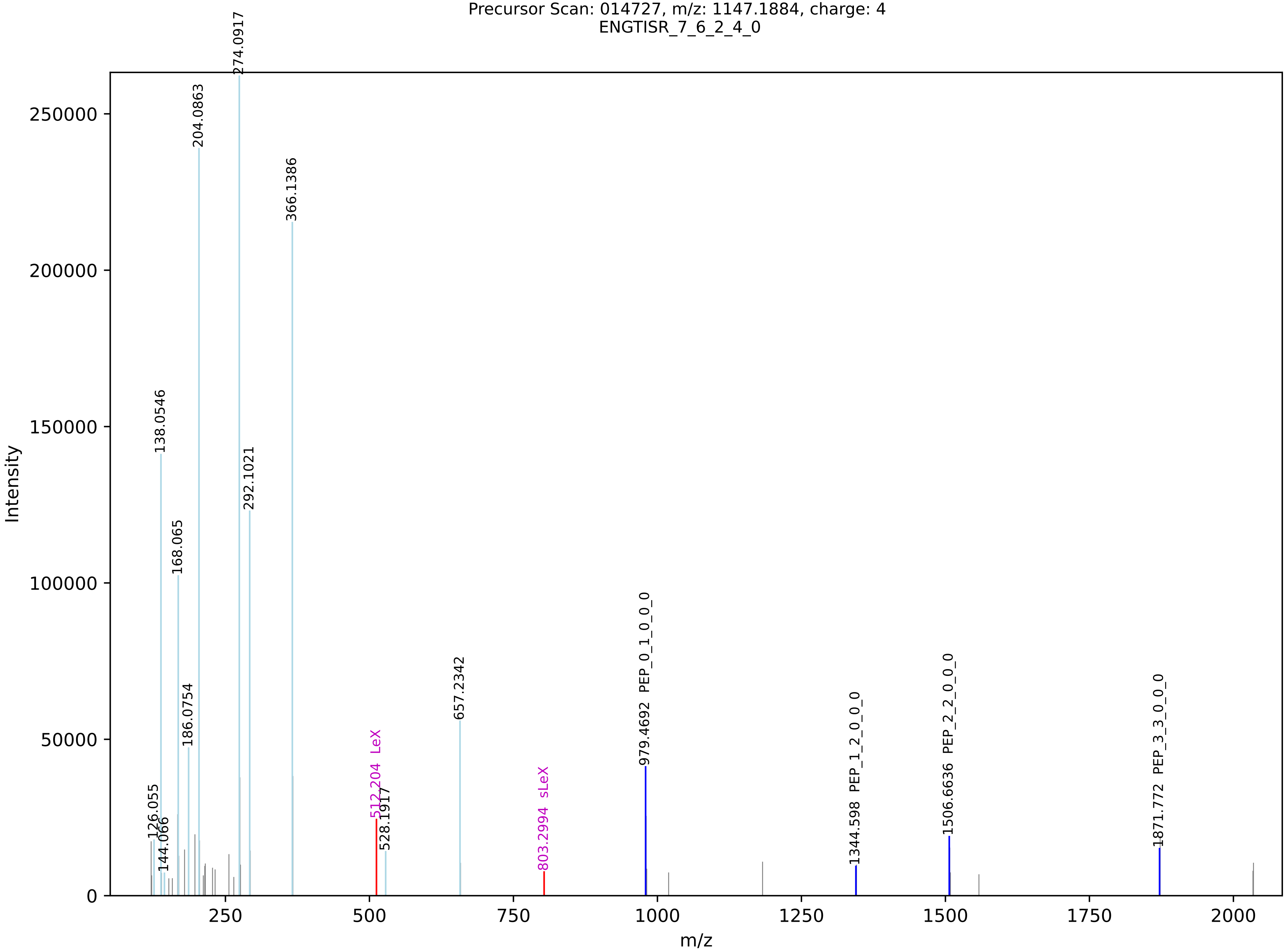

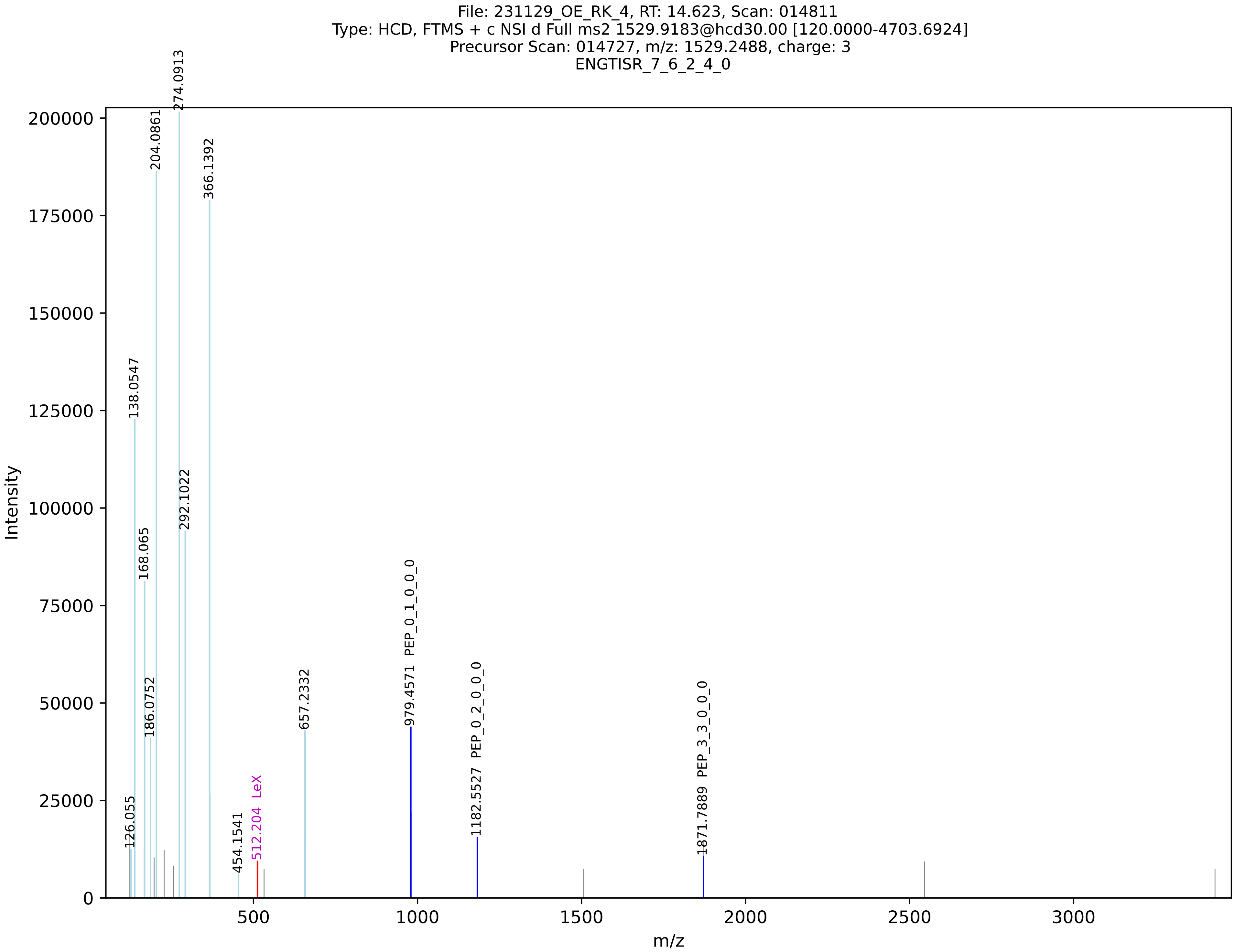

File: 231129_OE_RK_4, RT: 14.623, Scan: 014811
Type: HCD, FTMS + c NSI d Full ms2 1529.9183@hcd30.00 [120.0000-4703.6924]
Precursor Scan: 014727, m/z: 1529.2488, charge: 3
ENGTISR_7_6_2_4_0
Intensity
m/z
274.0913
204.0861
366.1392
138.0547
292.1022
168.065
186.0752
126.055
454.1541
512.204 LeX
657.2332
979.4571 PEP_0_1_0_0_0
1182.5527 PEP_0_2_0_0_0
1871.7889 PEP_3_3_0_0_0

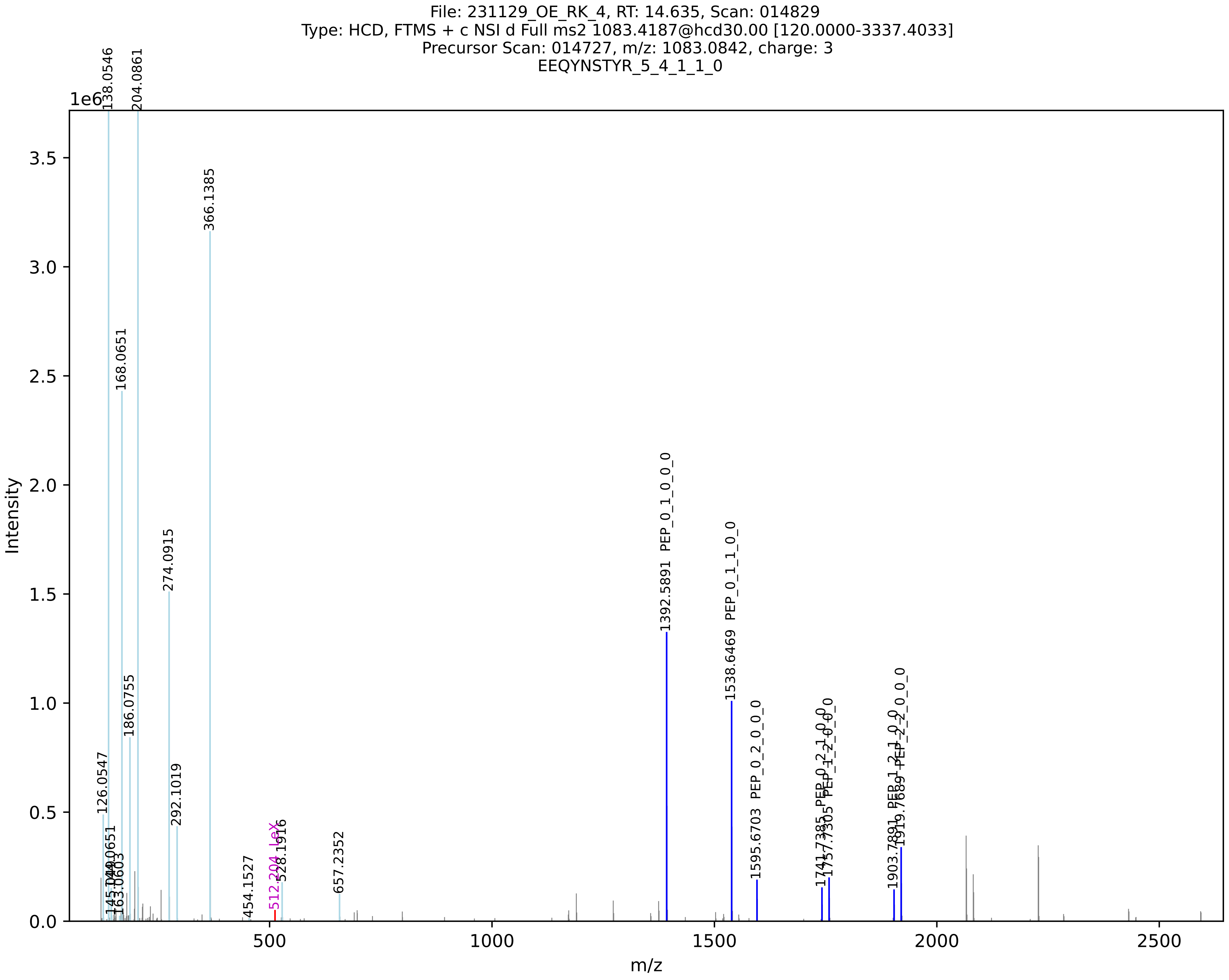

File: 231129_OE_RK_4, RT: 14.635, Scan: 014829
Type: HCD, FTMS + c NSI d Full ms2 1083.4187@hcd30.00 [120.0000-3337.4033]
Precursor Scan: 014727, m/z: 1083.0842, charge: 3
EEQYNSTYR_5_4_1_1_0
1e6
Intensity
m/z
138.0546
204.0861
366.1385
168.0651
274.0915
186.0755
292.1019
126.0547
163.0603
454.1527
512.204_LeX
528.1916
657.2352
1392.5891 PEP_0_1_0_0_0
1538.6469 PEP_0_1_1_0_0
1595.6703 PEP_0_2_0_0_0
1741.7385 PEP_0_2_1_0_0
1757.7305 PEP_1_2_0_0_0
1903.7891 PEP_1_2_1_0_0
1919.7689 PEP_2_2_0_0_0

File: 231129_OE_RK_4, RT: 14.717, Scan: 014946
Type: HCD, FTMS + c NSI d Full ms2 1111.4253@hcd30.00 [120.0000-4557.4712]
Precursor Scan: 014869, m/z: 1110.6736, charge: 4
ENGTISR_7_6_1_4_0

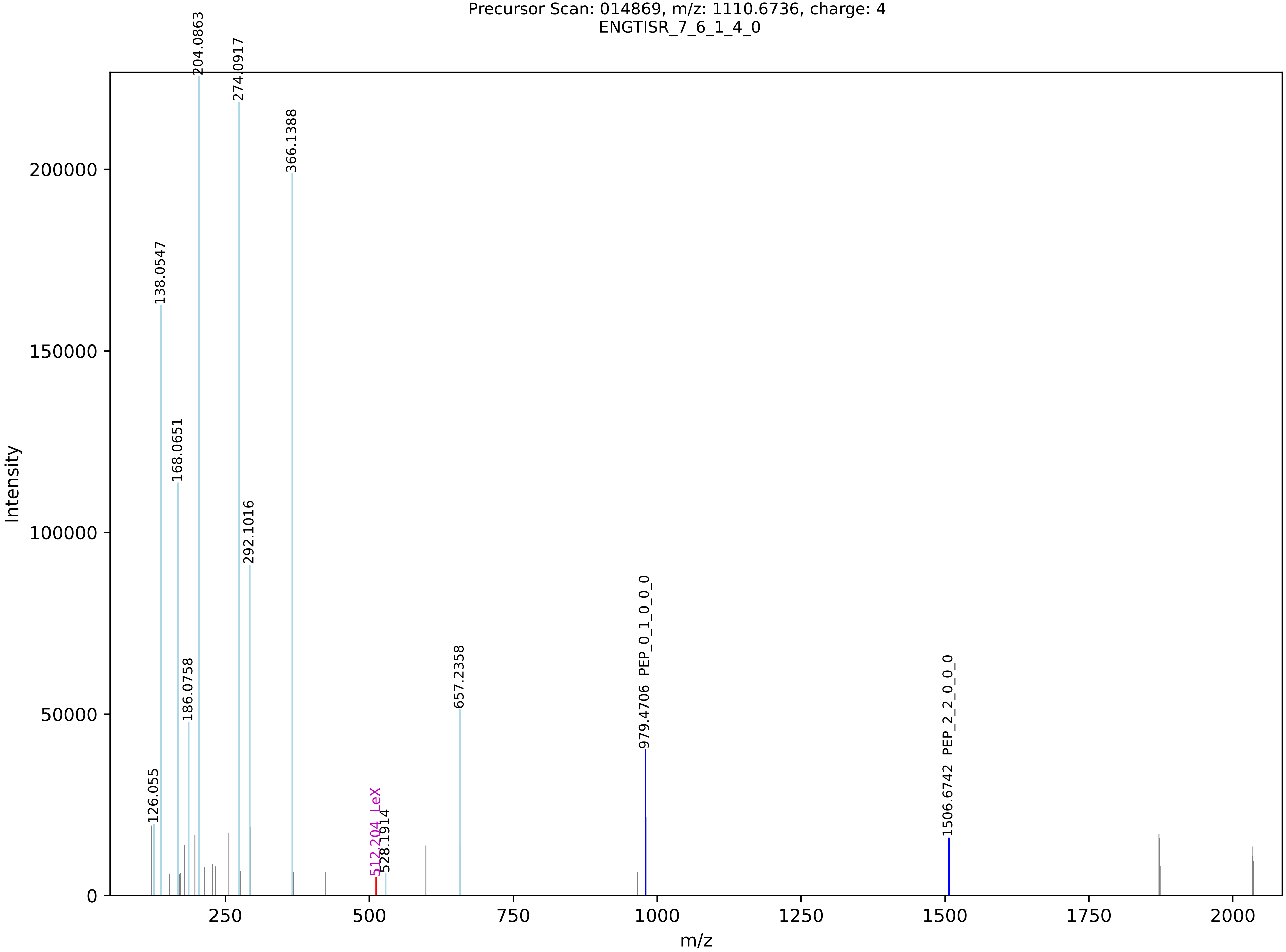

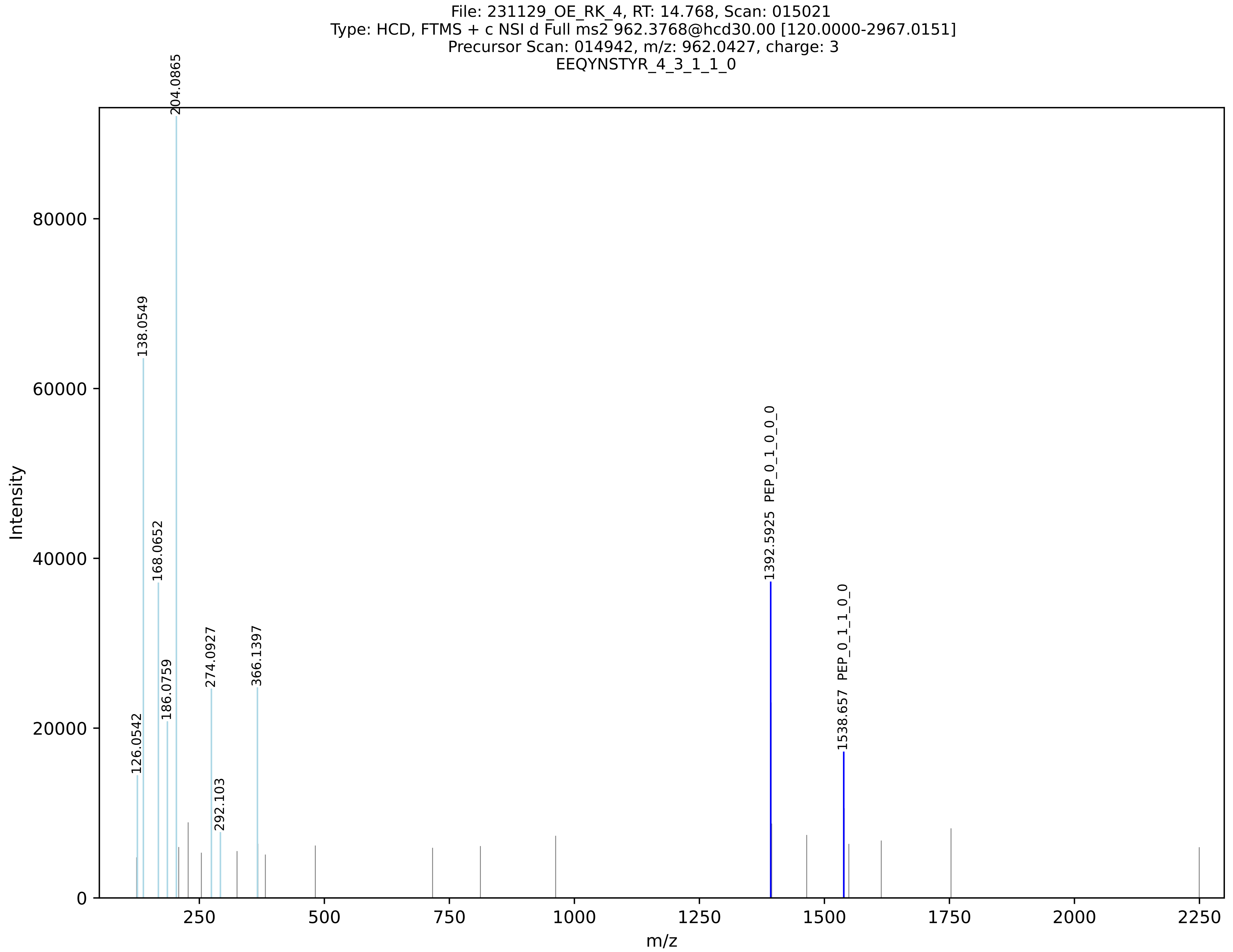
File: 231129_OE_RK_4, RT: 14.768, Scan: 015021
Type: HCD, FTMS + c NSI d Full ms2 962.3768@hcd30.00 [120.0000-2967.0151]
Precursor Scan: 014942, m/z: 962.0427, charge: 3
EEQYNSTYR_4_3_1_1_0
204.0865
138.0549
168.0652
186.0759
126.0542
274.0927
292.103
366.1397
1392.5925 PEP_0_1_0_0_0
1538.657 PEP_0_1_1_0_0
Intensity
m/z
0
20000
40000
60000
80000
250
500
750
1000
1250
1500
1750
2000
2250

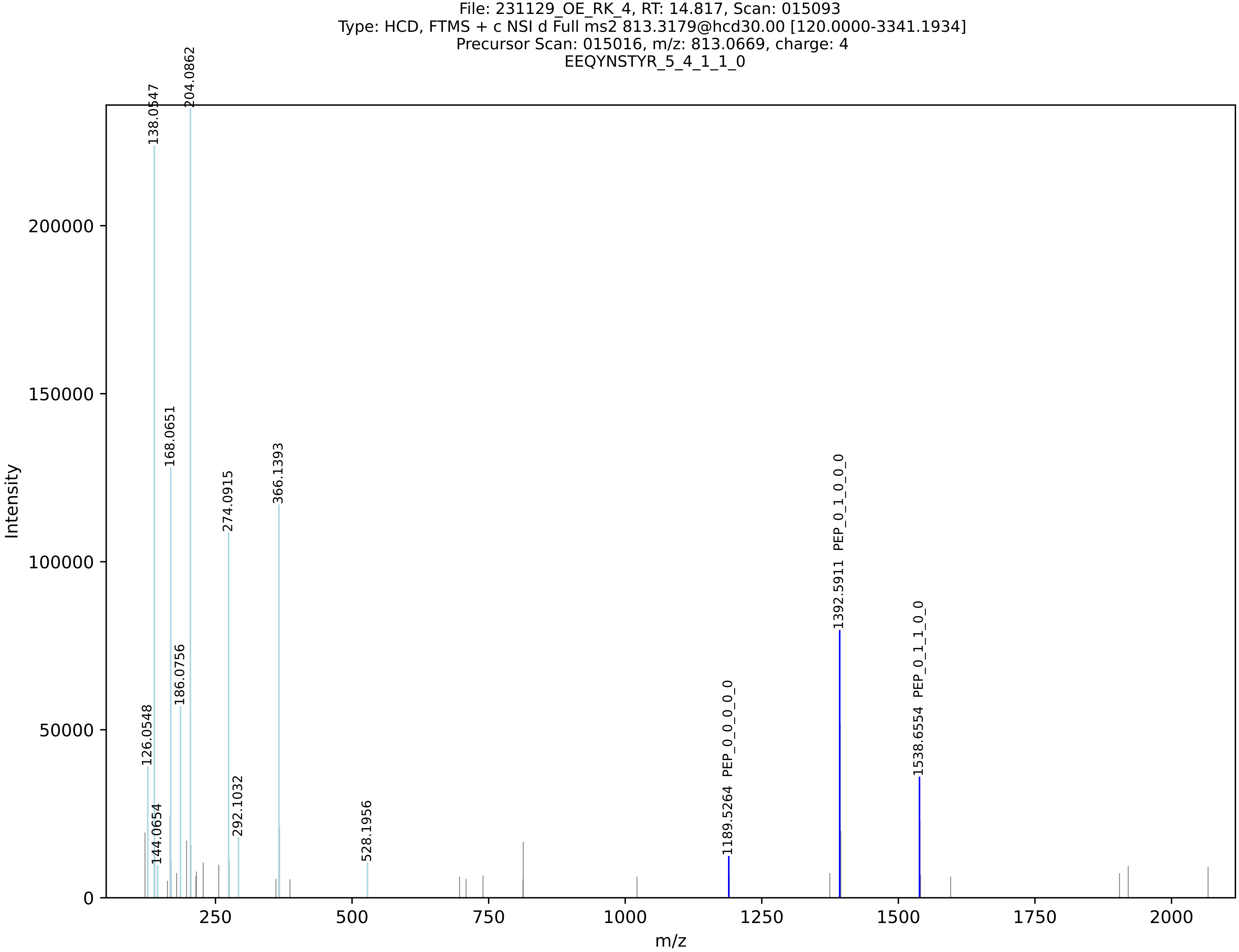
File: 231129_OE_RK_4, RT: 14.817, Scan: 015093
Type: HCD, FTMS + c NSI d Full ms2 813.3179@hcd30.00 [120.0000-3341.1934]
Precursor Scan: 015016, m/z: 813.0669, charge: 4
EEQYNSTYR_5_4_1_1_0
Intensity
m/z
0
50000
100000
150000
200000
250
500
750
1000
1250
1500
1750
2000
204.0862
138.0547
168.0651
366.1393
274.0915
186.0756
126.0548
292.1032
144.0654
528.1956
1189.5264 PEP_0_0_0_0_0
1392.5911 PEP_0_1_0_0_0
1538.6554 PEP_0_1_1_0_0

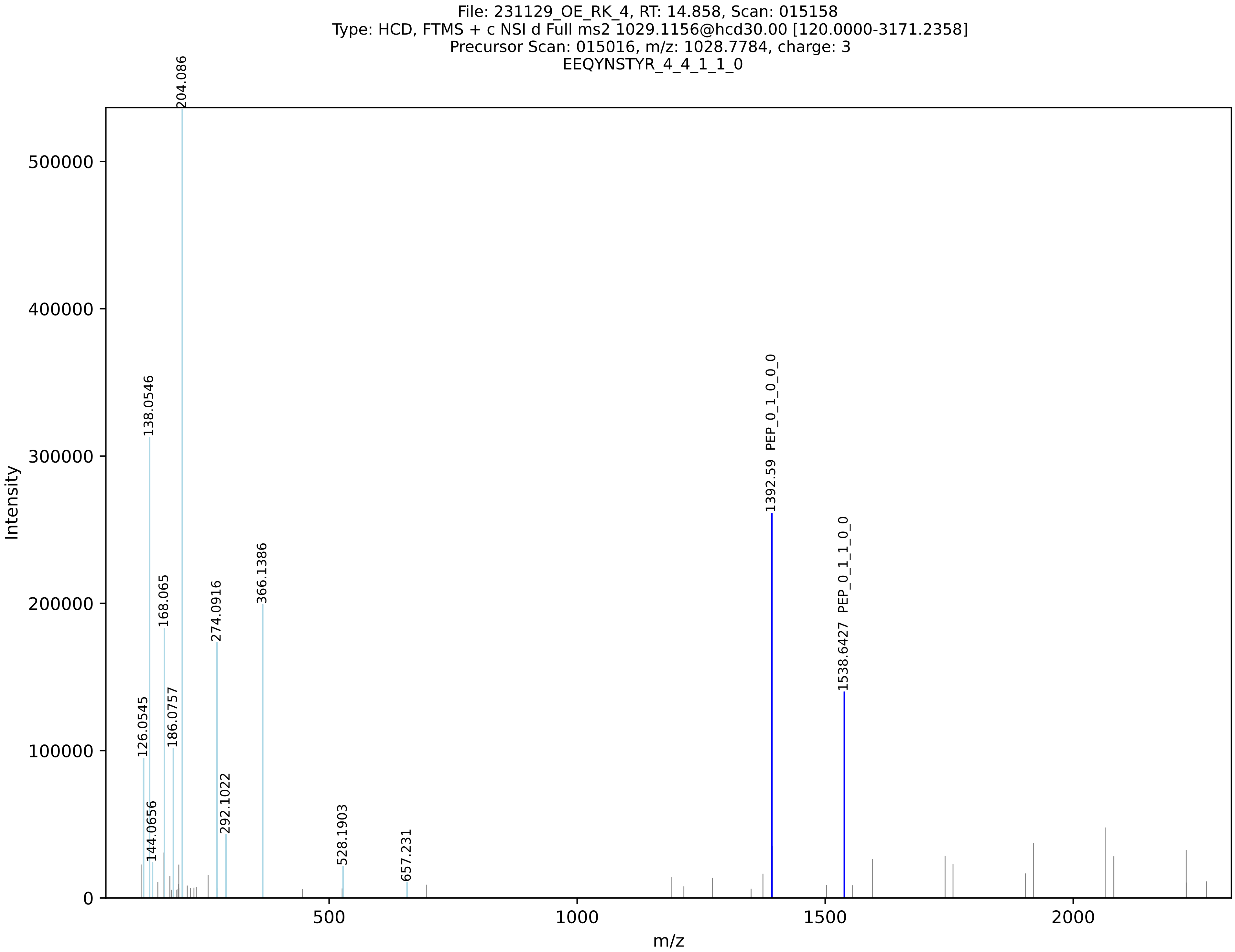
File: 231129_OE_RK_4, RT: 14.858, Scan: 015158
Type: HCD, FTMS + c NSI d Full ms2 1029.1156@hcd30.00 [120.0000-3171.2358]
Precursor Scan: 015016, m/z: 1028.7784, charge: 3
EEQYNSTYR_4_4_1_1_0
Intensity
m/z
204.086
138.0546
168.065
186.0757
126.0545
144.0656
274.0916
292.1022
366.1386
528.1903
657.231
1392.59 PEP_0_1_0_0_0
1538.6427 PEP_0_1_1_0_0
0
100000
200000
300000
400000
500000
500
1000
1500
2000

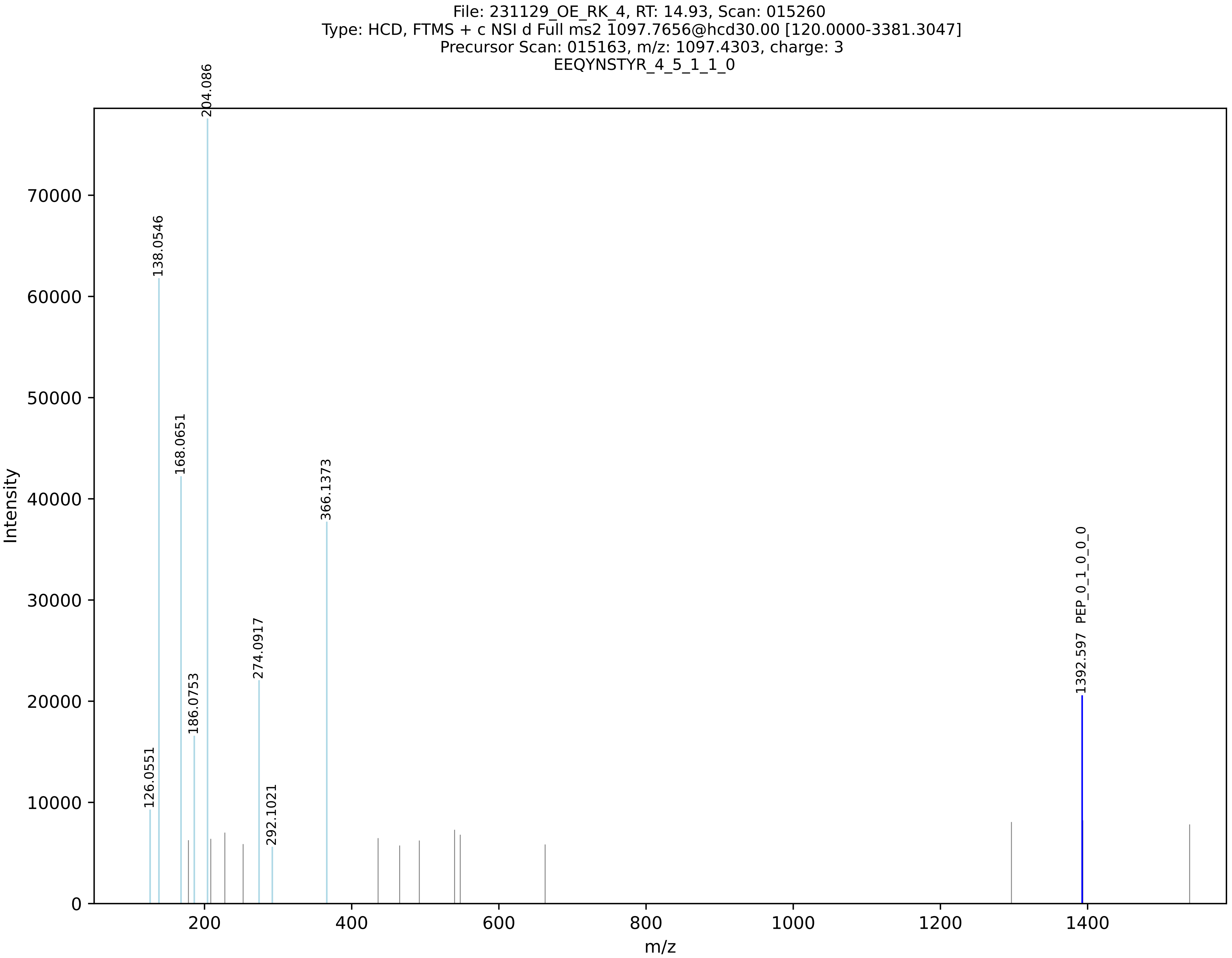

File: 231129_OE_RK_4, RT: 14.93, Scan: 015260
Type: HCD, FTMS + c NSI d Full ms2 1097.7656@hcd30.00 [120.0000-3381.3047]
Precursor Scan: 015163, m/z: 1097.4303, charge: 3
EEQYNSTYR_4_5_1_1_0
Intensity
m/z
0
10000
20000
30000
40000
50000
60000
70000
200
400
600
800
1000
1200
1400
126.0551
138.0546
168.0651
186.0753
204.086
274.0917
292.1021
366.1373
1392.597 PEP_0_1_0_0_0_

File: 231129_OE_RK_4, RT: 14.97, Scan: 015316
Type: HCD, FTMS + c NSI d Full ms2 1432.5444@hcd30.00 [120.0000-4405.7280]
Precursor Scan: 015237, m/z: 1431.877, charge: 3
ENGTISR_7_6_0_4_0

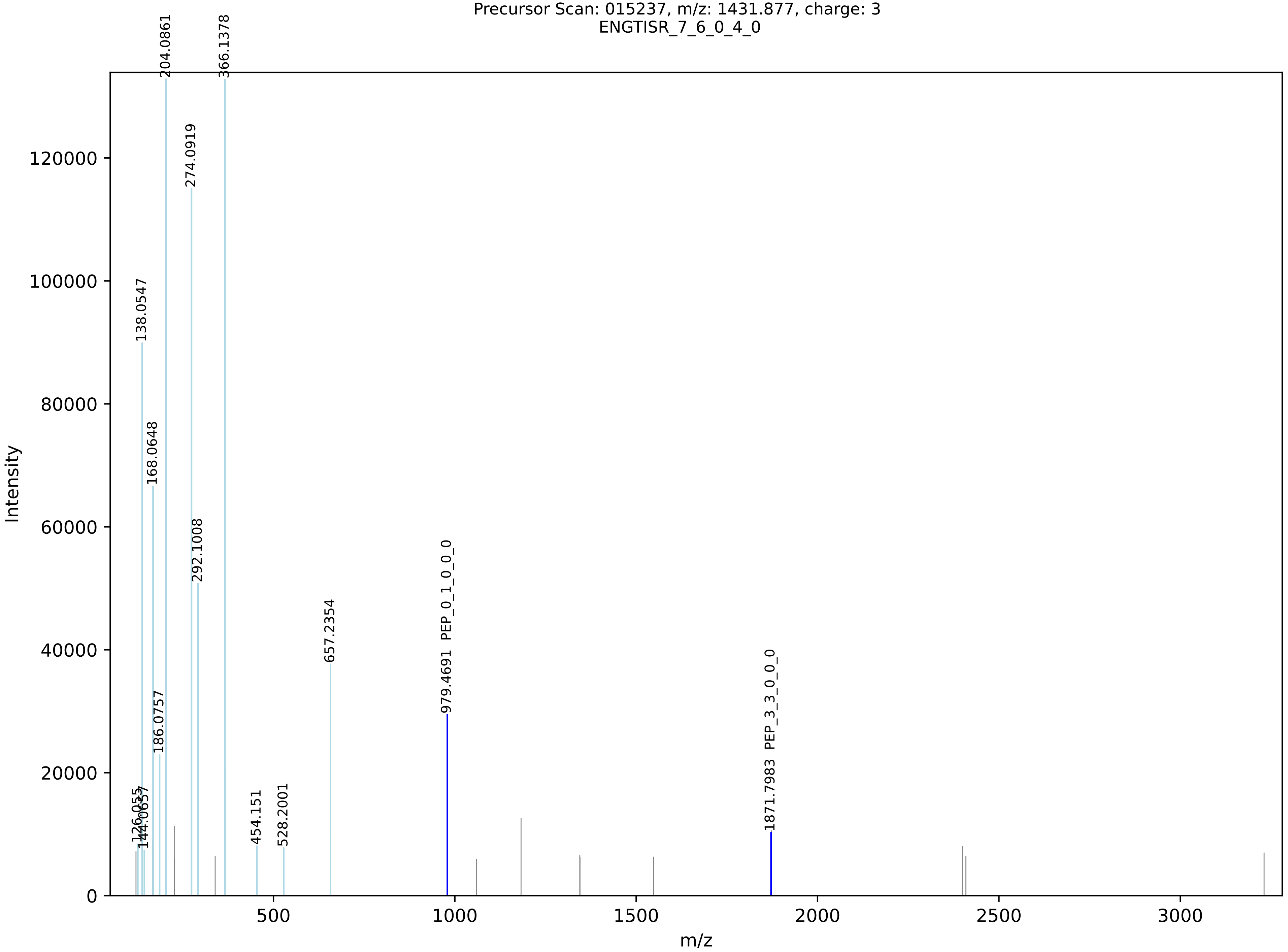

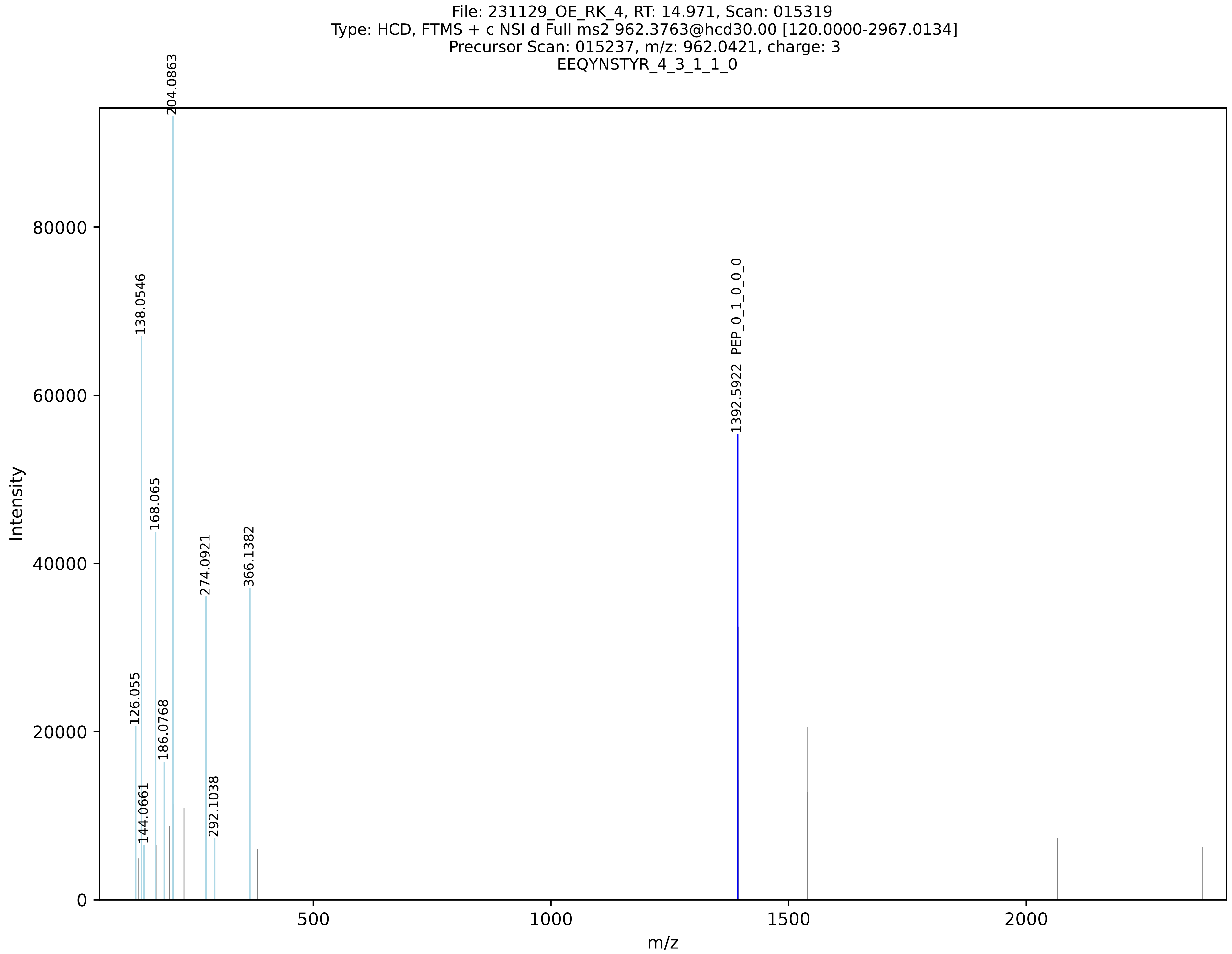
File: 231129_OE_RK_4, RT: 14.971, Scan: 015319
Type: HCD, FTMS + c NSI d Full ms2 962.3763@hcd30.00 [120.0000-2967.0134]
Precursor Scan: 015237, m/z: 962.0421, charge: 3
EEQYNSTYR_4_3_1_1_0
Intensity
m/z
0
20000
40000
60000
80000
500
1000
1500
2000
204.0863
138.0546
168.065
274.0921
366.1382
126.055
186.0768
144.0661
292.1038
1392.5922 PEP_0_1_0_0_0

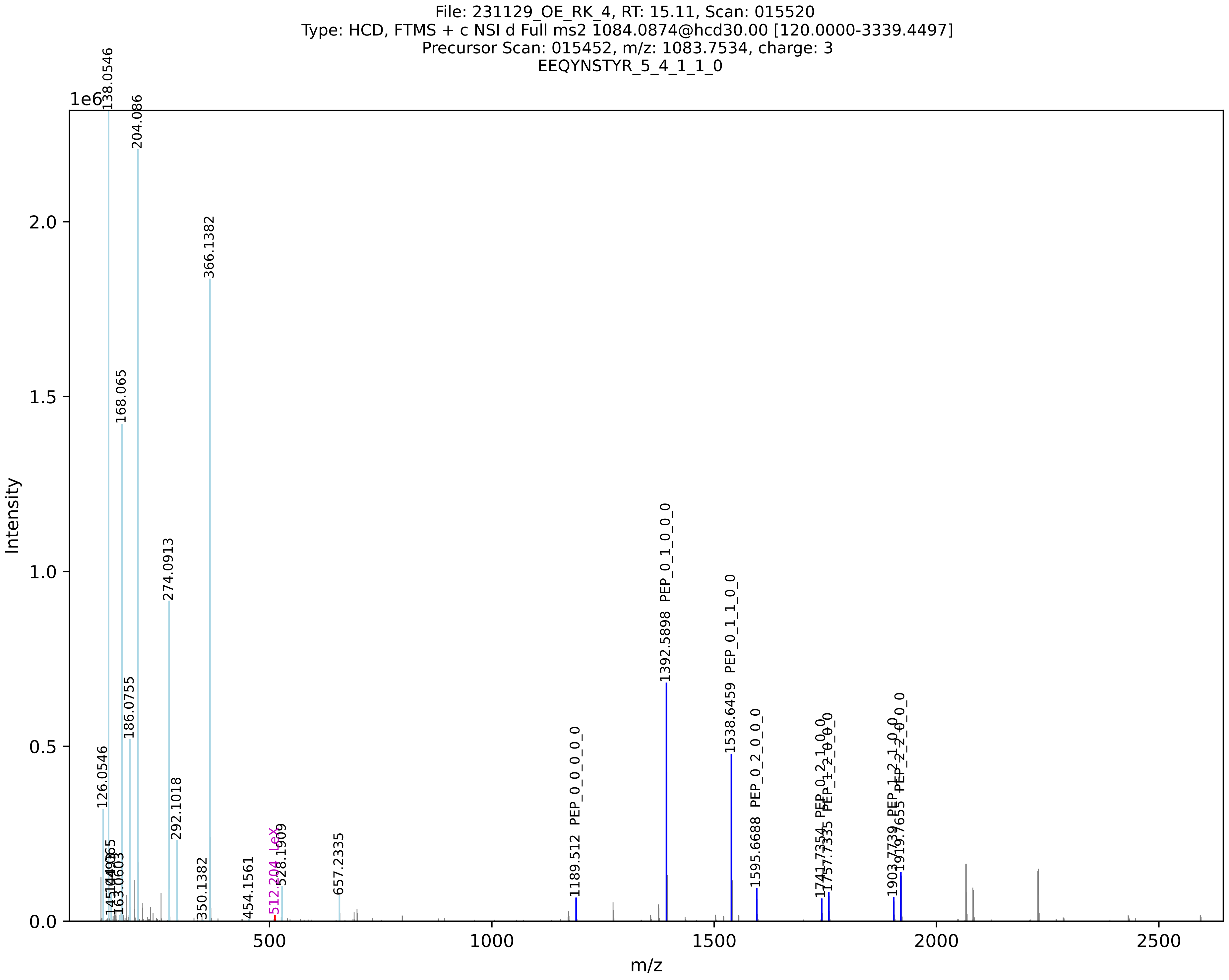

File: 231129_OE_RK_4, RT: 15.11, Scan: 015520
Type: HCD, FTMS + c NSI d Full ms2 1084.0874@hcd30.00 [120.0000-3339.4497]
Precursor Scan: 015452, m/z: 1083.7534, charge: 3
EEQYNSTYR_5_4_1_1_0
1e6
Intensity
m/z
138.0546
204.086
366.1382
168.065
274.0913
186.0755
126.0546
292.1018
350.1382
454.1561
512.204_LeX
528.1909
657.2335
1189.512 PEP_0_0_0_0_0_
1392.5898 PEP_0_1_0_0_0_
1538.6459 PEP_0_1_1_0_0_
1595.6688 PEP_0_2_0_0_0_
1741.7254 PEP_0_2_1_0_0_
1757.7335 PEP_1_2_0_0_
1903.7739 PEP_1_2_1_0_0_
1919.7655 PEP_2_2_0_0_0_
0.0
0.5
1.0
1.5
2.0
500
1000
1500
2000
2500

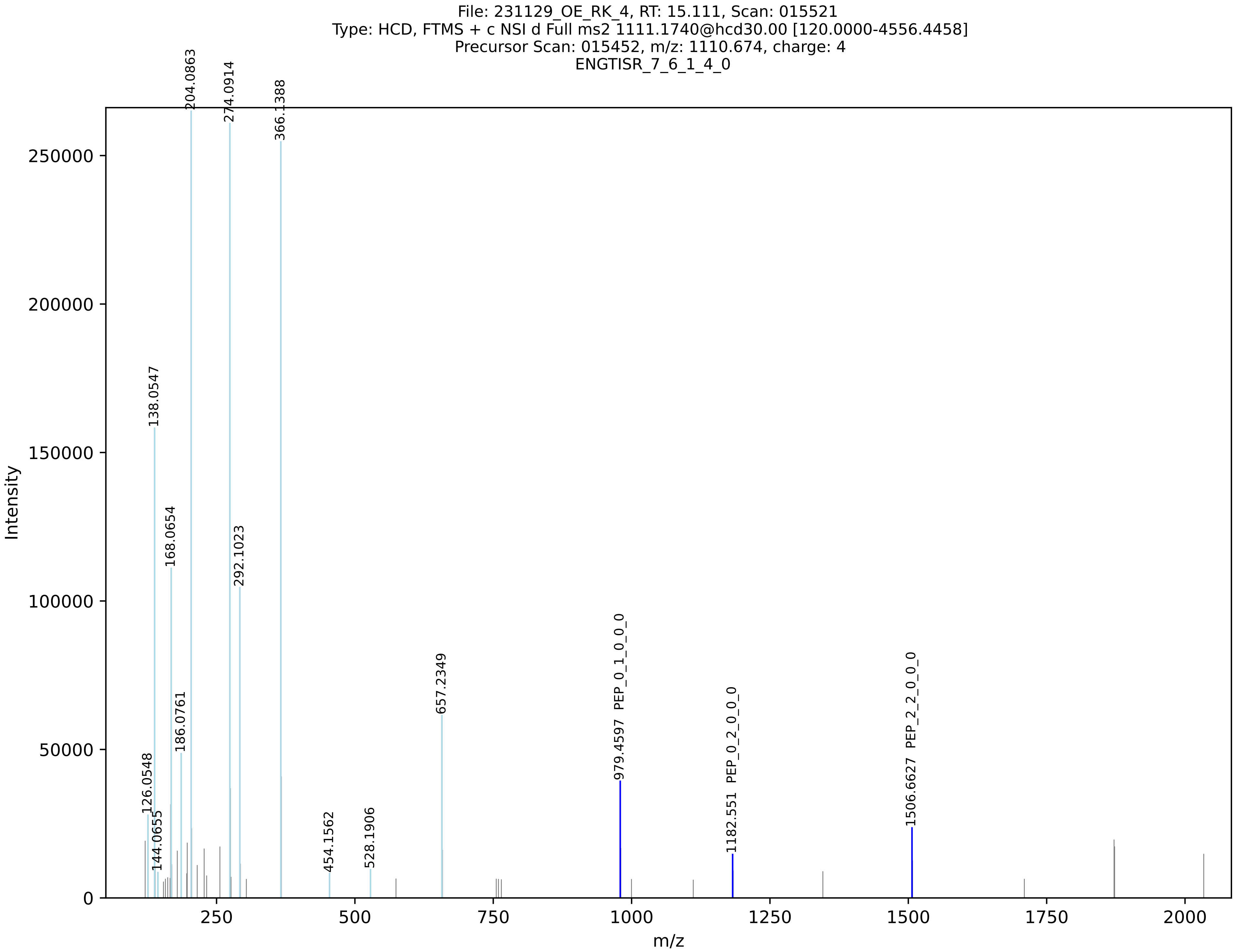
File: 231129_OE_RK_4, RT: 15.111, Scan: 015521
Type: HCD, FTMS + c NSI d Full ms2 1111.1740@hcd30.00 [120.0000-4556.4458]
Precursor Scan: 015452, m/z: 1110.674, charge: 4
ENGTISR_7_6_1_4_0
204.0863
274.0914
366.1388
138.0547
168.0654
292.1023
186.0761
126.0548
144.0655
454.1562
528.1906
657.2349
979.4597 PEP_0_1_0_0_0
1182.551 PEP_0_2_0_0_0
1506.6627 PEP_2_2_0_0_0
Intensity
m/z
0
50000
100000
150000
200000
250000
250
500
750
1000
1250
1500
1750
2000

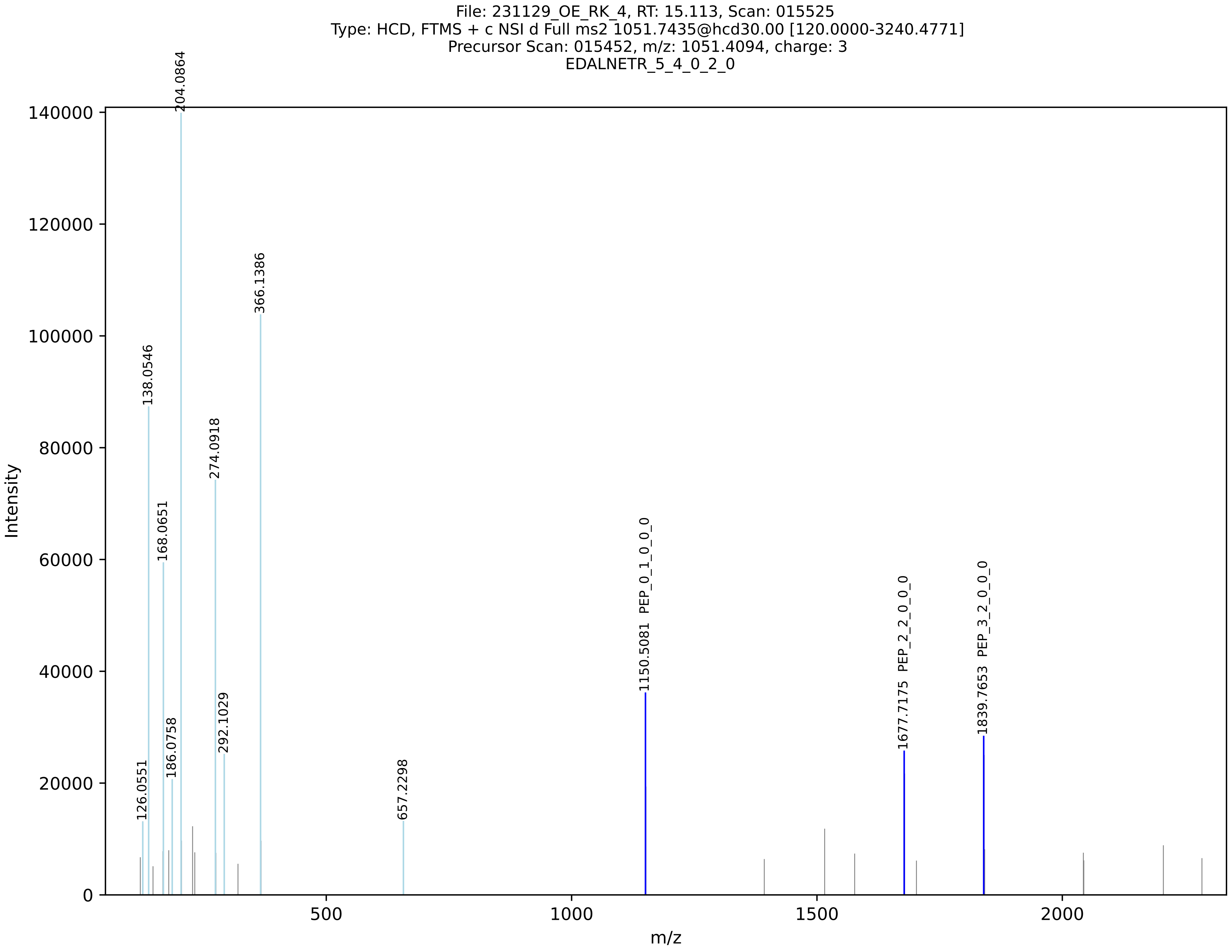
File: 231129_OE_RK_4, RT: 15.113, Scan: 015525
Type: HCD, FTMS + c NSI d Full ms2 1051.7435@hcd30.00 [120.0000-3240.4771]
Precursor Scan: 015452, m/z: 1051.4094, charge: 3
EDALNETR_5_4_0_2_0
Intensity
m/z
204.0864
366.1386
138.0546
274.0918
168.0651
1150.5081 PEP_0_1_0_0_0
1677.7175 PEP_2_2_0_0_0
1839.7653 PEP_3_2_0_0_0
292.1029
186.0758
126.0551
657.2298

File: 231129_OE_RK_4, RT: 15.153, Scan: 015582
Type: HCD, FTMS + c NSI d Full ms2 962.7112@hcd30.00 [120.0000-2968.0383]
Precursor Scan: 015519, m/z: 962.043, charge: 3
EEQYNSTYR_4_3_1_1_0

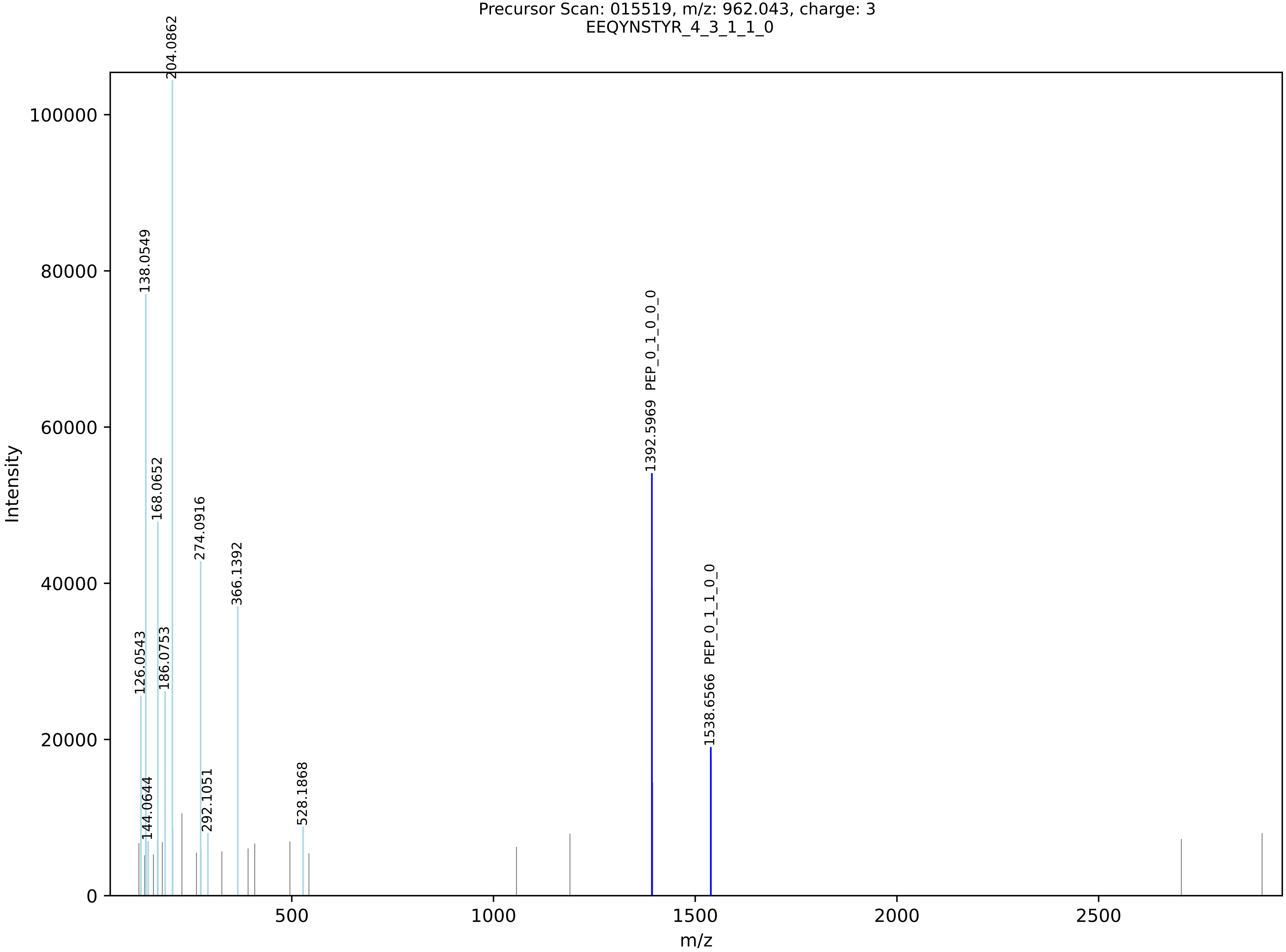

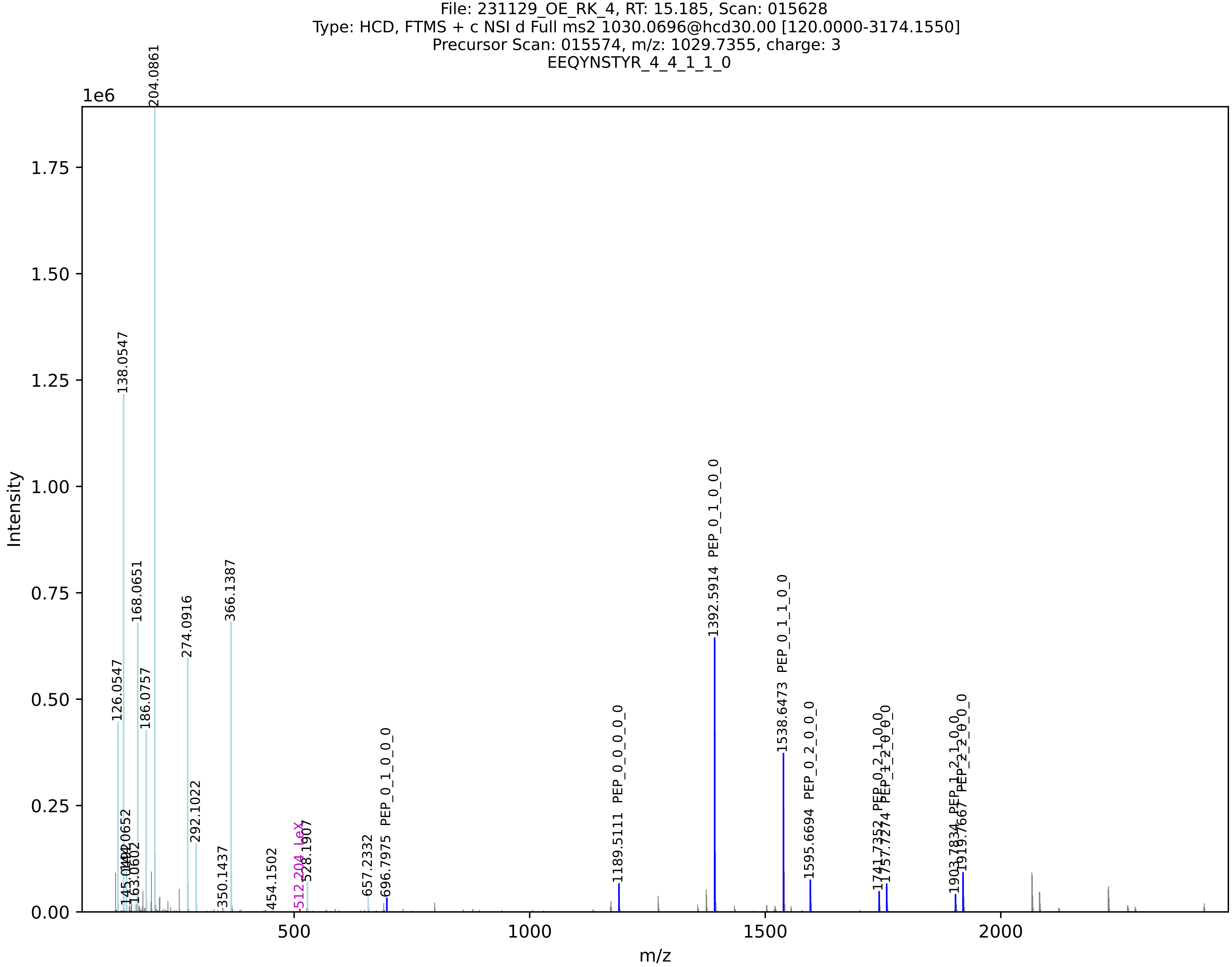
File: 231129_OE_RK_4, RT: 15.185, Scan: 015628
Type: HCD, FTMS + c NSI d Full ms2 1030.0696@hcd30.00 [120.0000-3174.1550]
Precursor Scan: 015574, m/z: 1029.7355, charge: 3
EEQYNSTYR_4_4_1_1_0
1e6
Intensity
m/z
204.0861
138.0547
168.0651
366.1387
274.0916
126.0547
186.0757
292.1022
163.0602
350.1437
454.1502
512.204 LeX
528.1907
657.2332
696.7975 PEP_0_1_0_0_0
1189.5111 PEP_0_0_0_0_0
1392.5914 PEP_0_1_0_0_
1538.6473 PEP_0_1_1_0_0
1595.6694 PEP_0_2_0_0_0

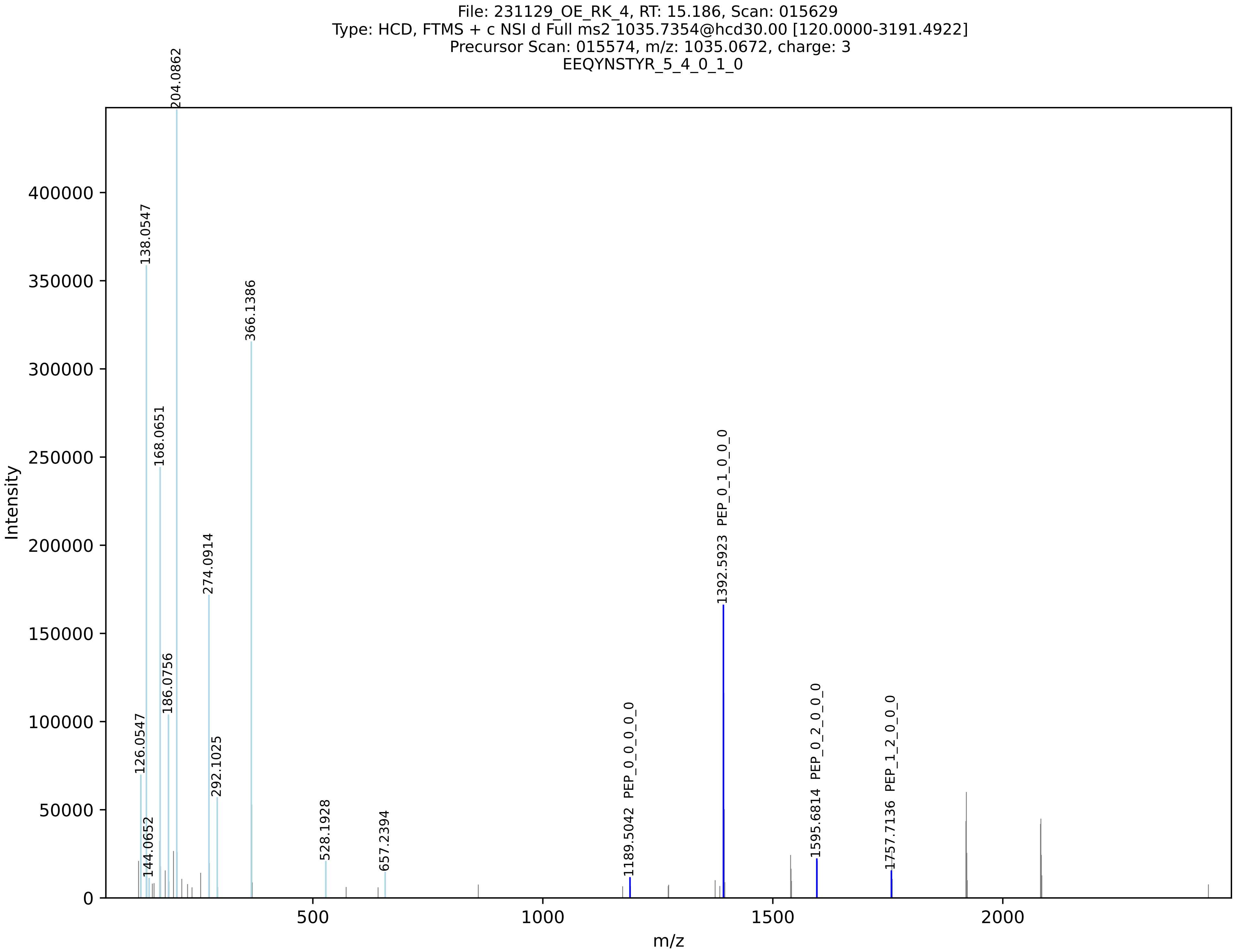
File: 231129_OE_RK_4, RT: 15.186, Scan: 015629
Type: HCD, FTMS + c NSI d Full ms2 1035.7354@hcd30.00 [120.0000-3191.4922]
Precursor Scan: 015574, m/z: 1035.0672, charge: 3
EEQYNSTYR_5_4_0_1_0
204.0862
138.0547
366.1386
168.0651
274.0914
186.0756
126.0547
292.1025
144.0652
528.1928
657.2394
1189.5042 PEP_0_0_0_0_0
1392.5923 PEP_0_1_0_0_0
1595.6814 PEP_0_2_0_0_0
1757.7136 PEP_1_2_0_0_0
Intensity
m/z
0
50000
100000
150000
200000
250000
300000
350000
400000
500
1000
1500
2000

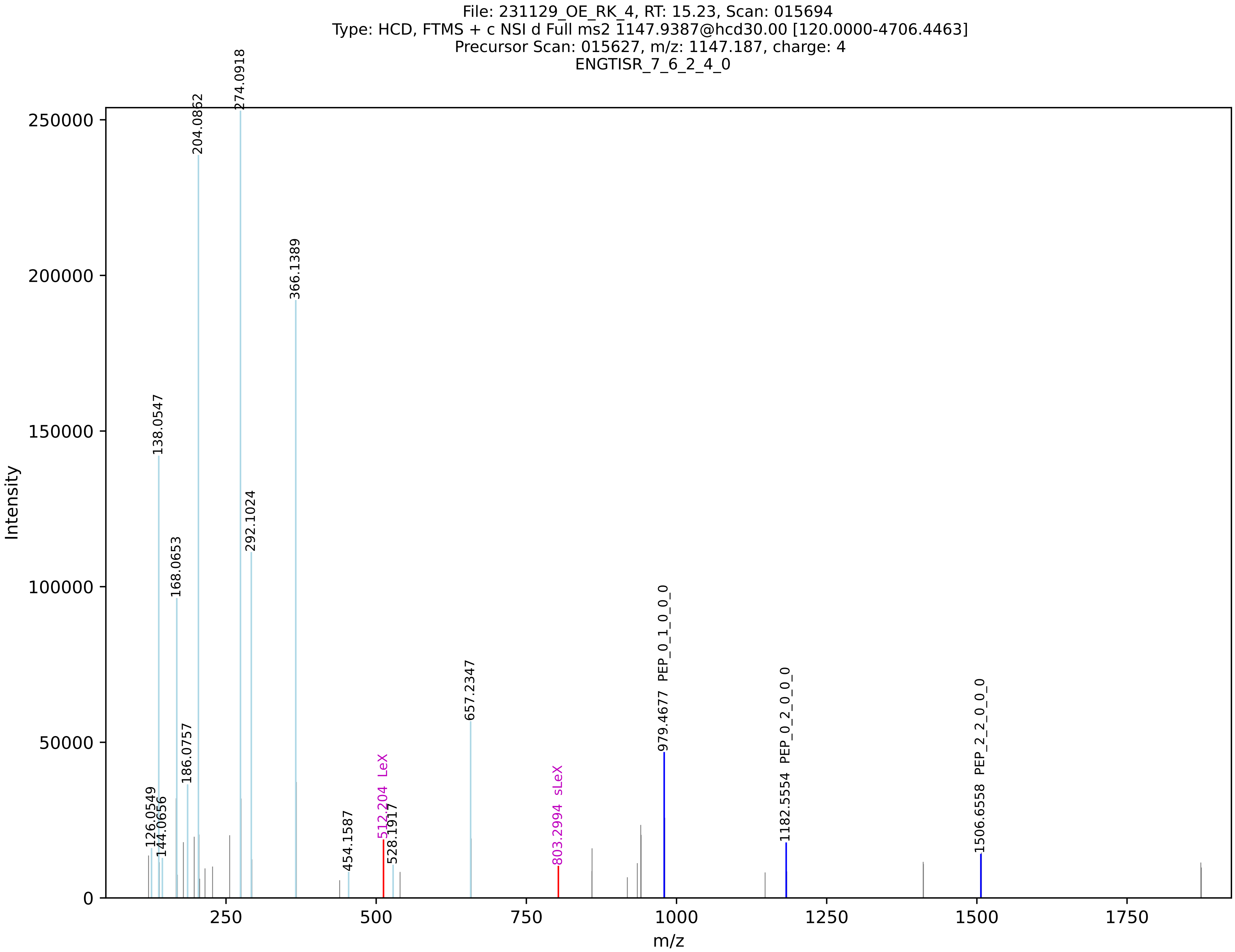
File: 231129_OE_RK_4, RT: 15.23, Scan: 015694
Type: HCD, FTMS + c NSI d Full ms2 1147.9387@hcd30.00 [120.0000-4706.4463]
Precursor Scan: 015627, m/z: 1147.187, charge: 4
ENGTISR_7_6_2_4_0
Intensity
m/z
274.0918
204.0862
366.1389
138.0547
292.1024
168.0653
186.0757
126.0549
144.0656
454.1587
512.204 LeX
528.1917
657.2347
803.2994 sLeX
979.4677 PEP_0_1_0_0_0
1182.5554 PEP_0_2_0_0_0
1506.6558 PEP_2_2_0_0_0
0
50000
100000
150000
200000
250000
250
500
750
1000
1250
1500
1750

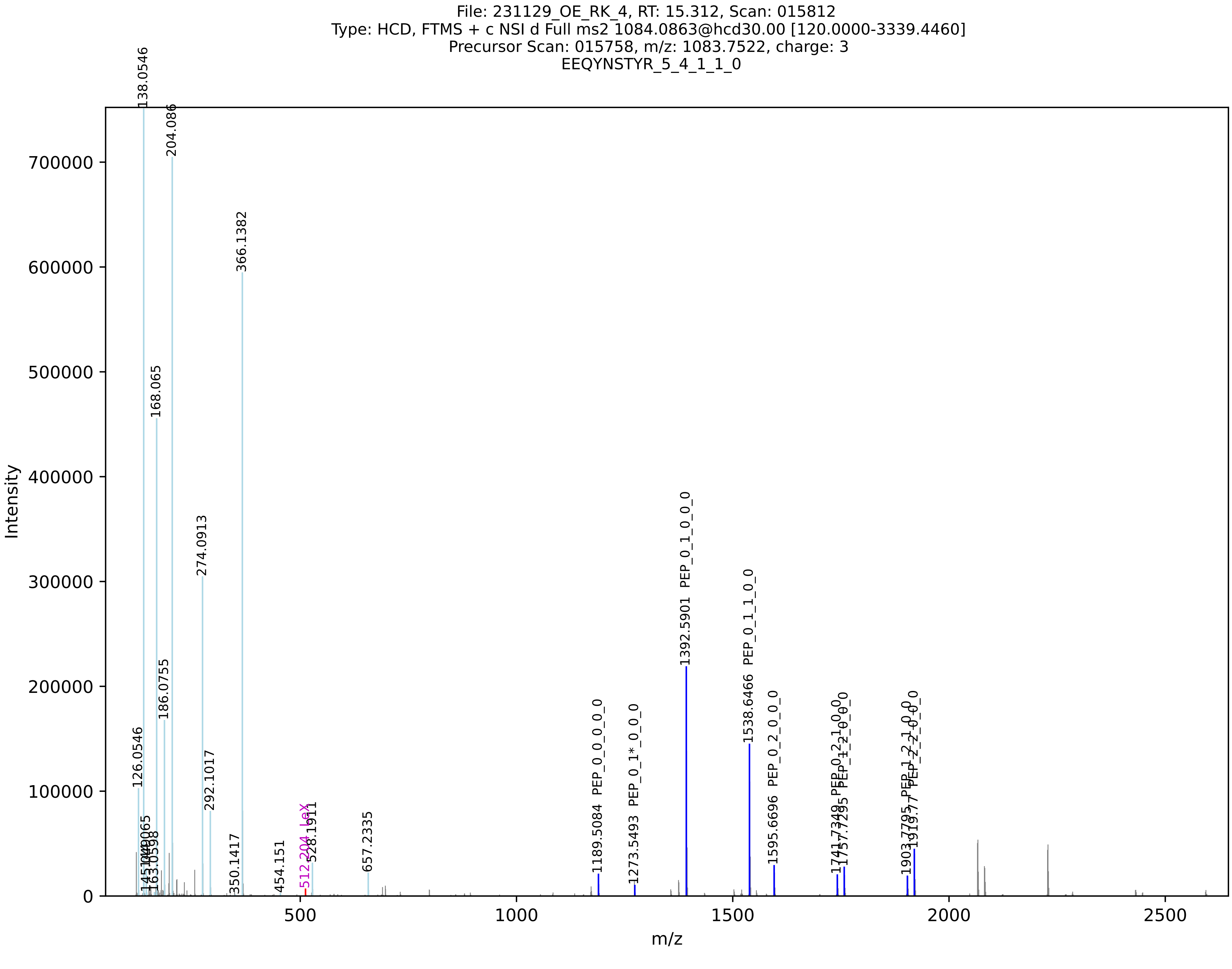
File: 231129_OE_RK_4, RT: 15.312, Scan: 015812
Type: HCD, FTMS + c NSI d Full ms2 1084.0863@hcd30.00 [120.0000-3339.4460]
Precursor Scan: 015758, m/z: 1083.7522, charge: 3
EEQYNSTYR_5_4_1_1_0
Intensity
m/z
138.0546
204.086
366.1382
168.065
274.0913
186.0755
126.0546
292.1017
350.1417
454.151
512.204 LeX
528.1911
657.2335
1189.5084 PEP_0_0_0_0_0
1273.5493 PEP_0_1*_0_0_0
1392.5901 PEP_0_1_0_0_0
1538.6466 PEP_0_1_1_0_0
1595.6696 PEP_0_2_0_0_0
1741.7349 PEP_0_2_1_0_0
1757.7295 PEP_1_2_0_0_0
1903.7795 PEP_1_2_1_0_0
1919.77 PEP_2_2_0_0_0

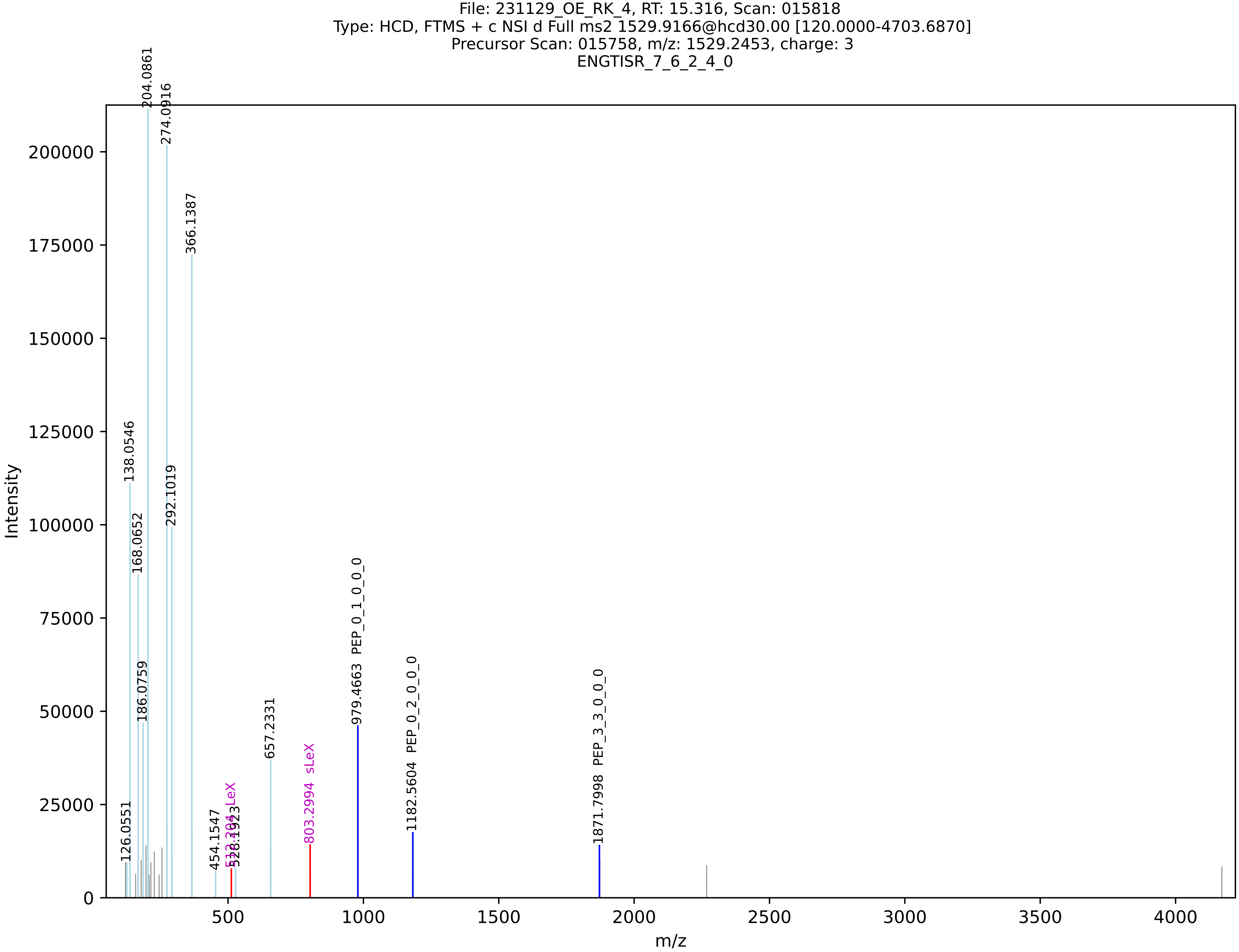
File: 231129_OE_RK_4, RT: 15.316, Scan: 015818
Type: HCD, FTMS + c NSI d Full ms2 1529.9166@hcd30.00 [120.0000-4703.6870]
Precursor Scan: 015758, m/z: 1529.2453, charge: 3
ENGTISR_7_6_2_4_0
204.0861
274.0916
366.1387
138.0546
292.1019
168.0652
186.0759
126.0551
454.1547
sLeX
528.1923
657.2331
803.2994 sLeX
979.4663 PEP_0_1_0_0_0
1182.5604 PEP_0_2_0_0_0
1871.7998 PEP_3_3_0_0_0
Intensity
m/z
0
25000
50000
75000
100000
125000
150000
175000
200000
500
1000
1500
2000
2500
3000
3500
4000

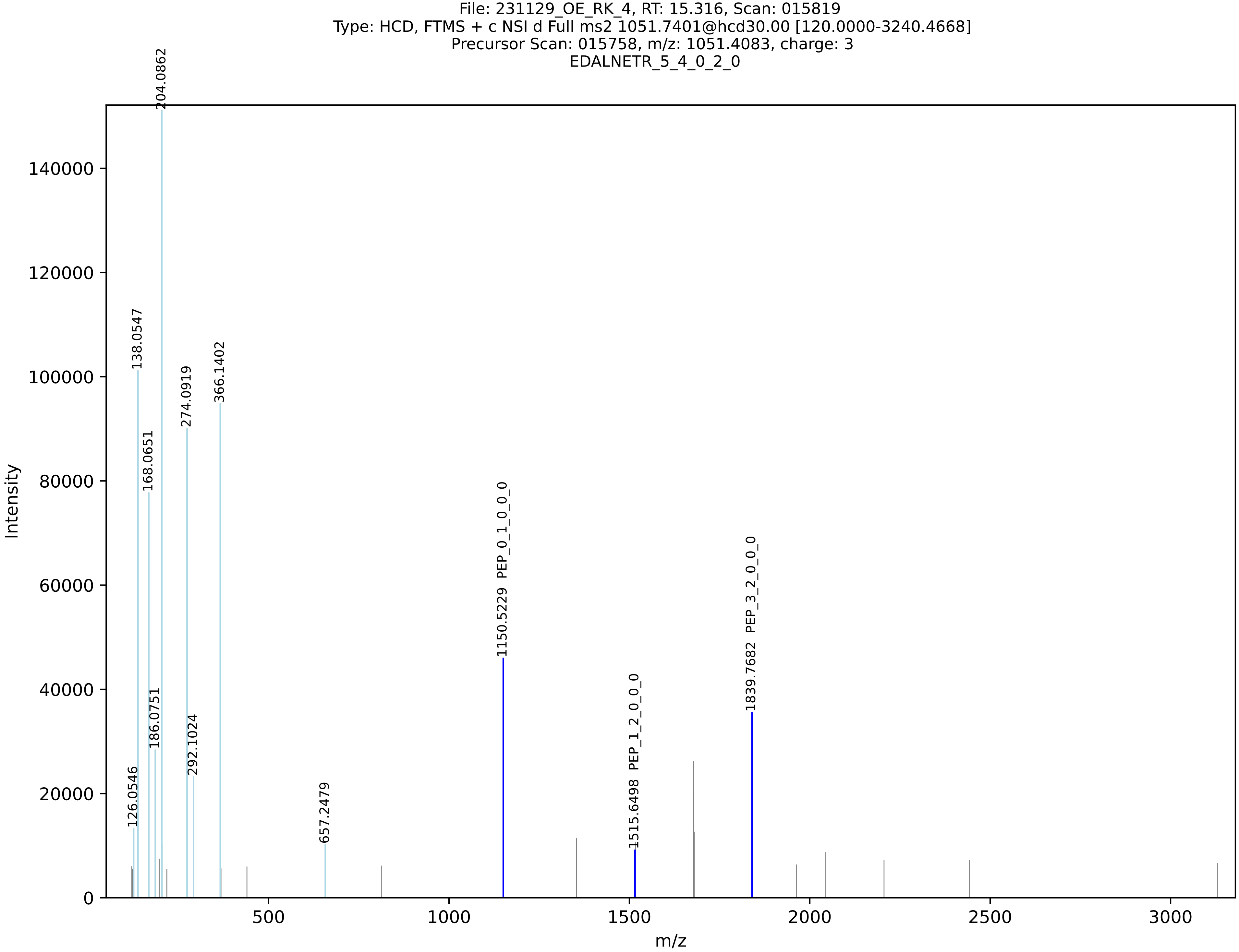
File: 231129_OE_RK_4, RT: 15.316, Scan: 015819
Type: HCD, FTMS + c NSI d Full ms2 1051.7401@hcd30.00 [120.0000-3240.4668]
Precursor Scan: 015758, m/z: 1051.4083, charge: 3
EDALNETR_5_4_0_2_0
Intensity
m/z
204.0862
138.0547
168.0651
274.0919
366.1402
186.0751
292.1024
126.0546
657.2479
1150.5229 PEP_0_1_0_0_0
1515.6498 PEP_1_2_0_0_0
1839.7682 PEP_3_2_0_0_0

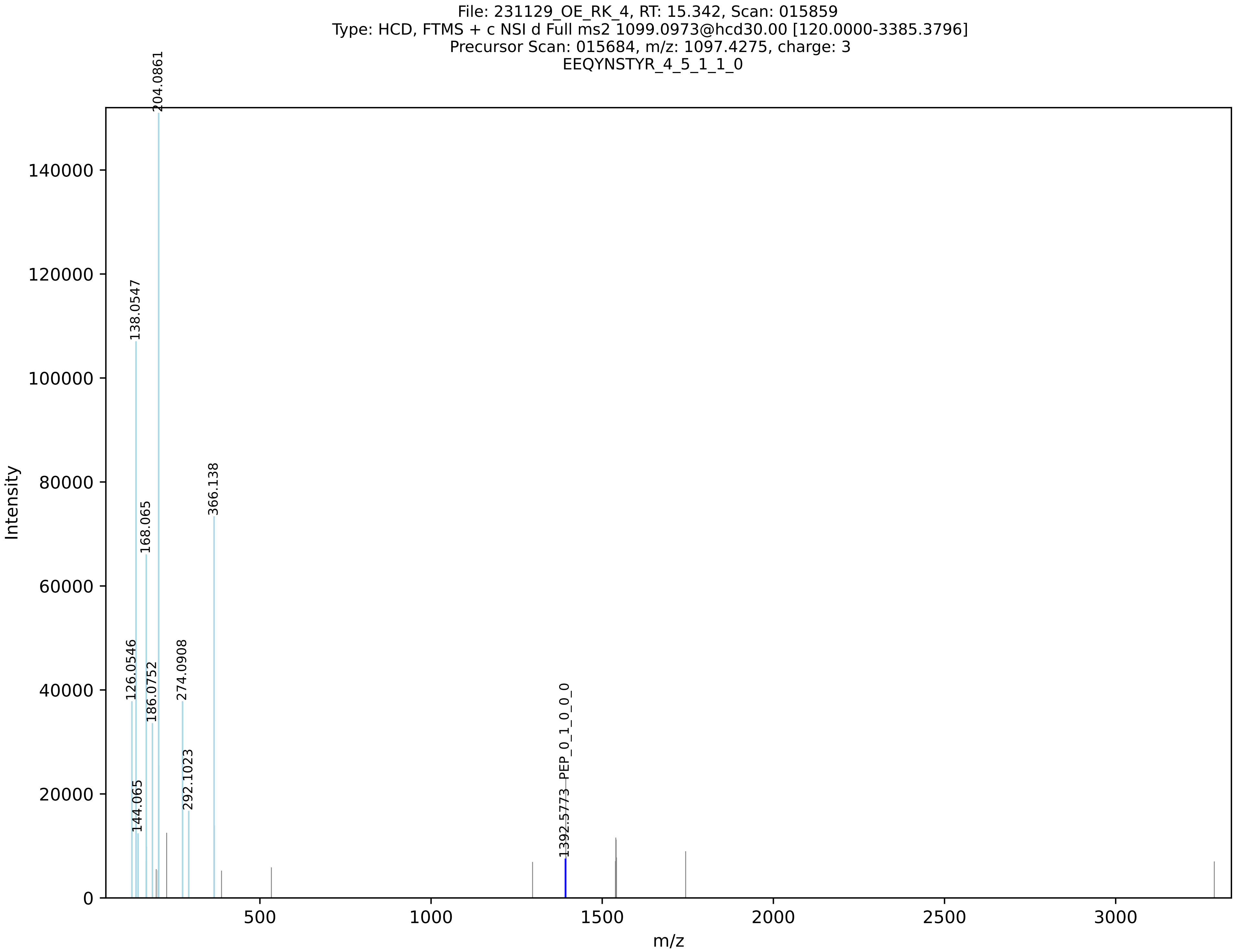
File: 231129_OE_RK_4, RT: 15.342, Scan: 015859
Type: HCD, FTMS + c NSI d Full ms2 1099.0973@hcd30.00 [120.0000-3385.3796]
Precursor Scan: 015684, m/z: 1097.4275, charge: 3
EEQYNSTYR_4_5_1_1_0
204.0861
138.0547
366.138
168.065
126.0546
186.0752
274.0908
292.1023
144.065
1392.5773_PEP_0_1_0_0_0
Intensity
m/z
0
20000
40000
60000
80000
100000
120000
140000
500
1000
1500
2000
2500
3000

File: 231129_OE_RK_4, RT: 15.411, Scan: 015958
Type: HCD, FTMS + c NSI d Full ms2 972.0431@hcd30.00 [120.0000-2996.5938]
Precursor Scan: 015871, m/z: 971.3748, charge: 3
MFSQNDTR_5_4_0_1_0

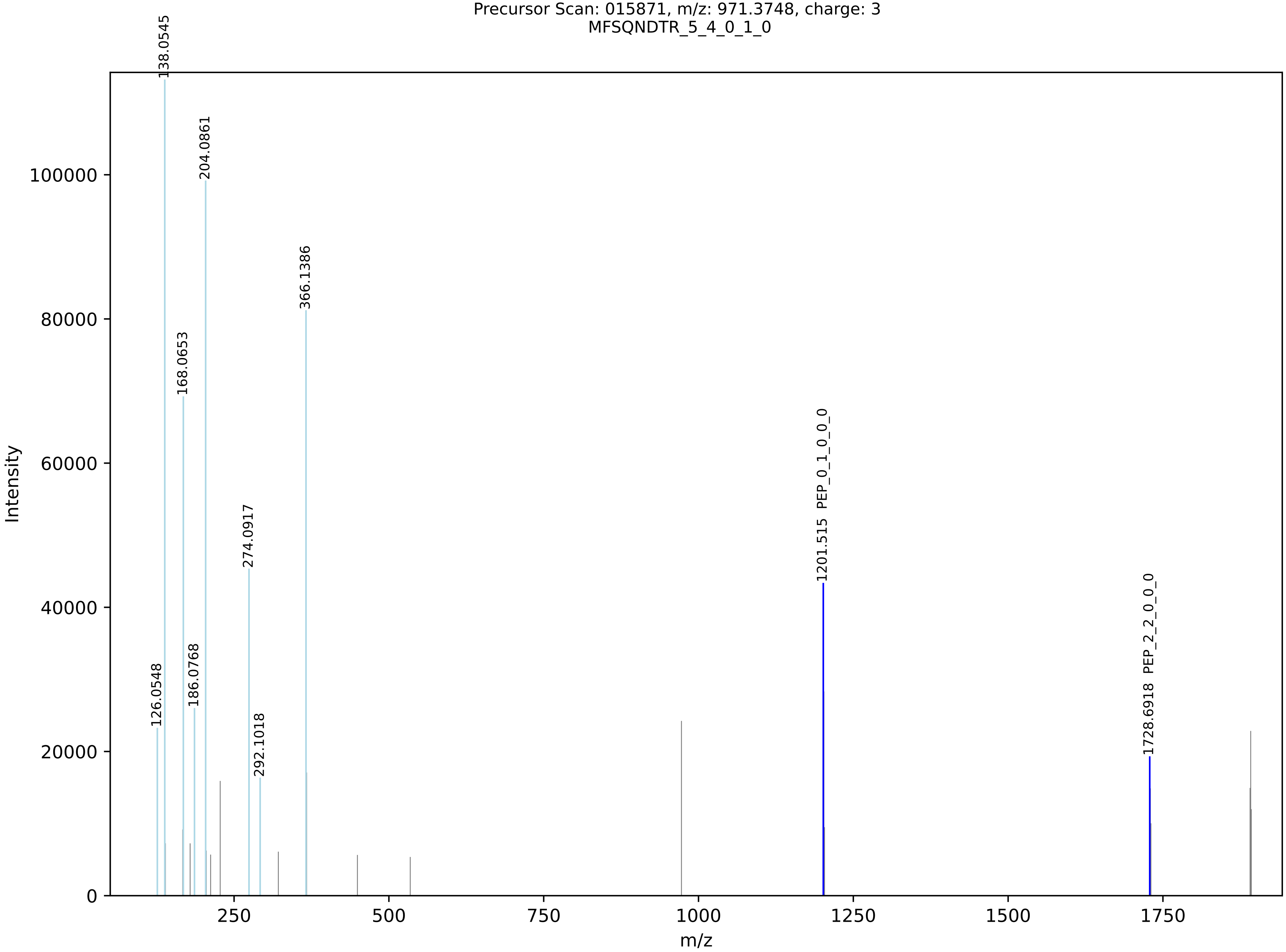

File: 231129_OE_RK_4, RT: 15.497, Scan: 016082
Type: HCD, FTMS + c NSI d Full ms2 1084.4205@hcd30.00 [120.0000-3340.4688]
Precursor Scan: 016019, m/z: 1083.7523, charge: 3
EEQYNSTYR_5_4_1_1_0

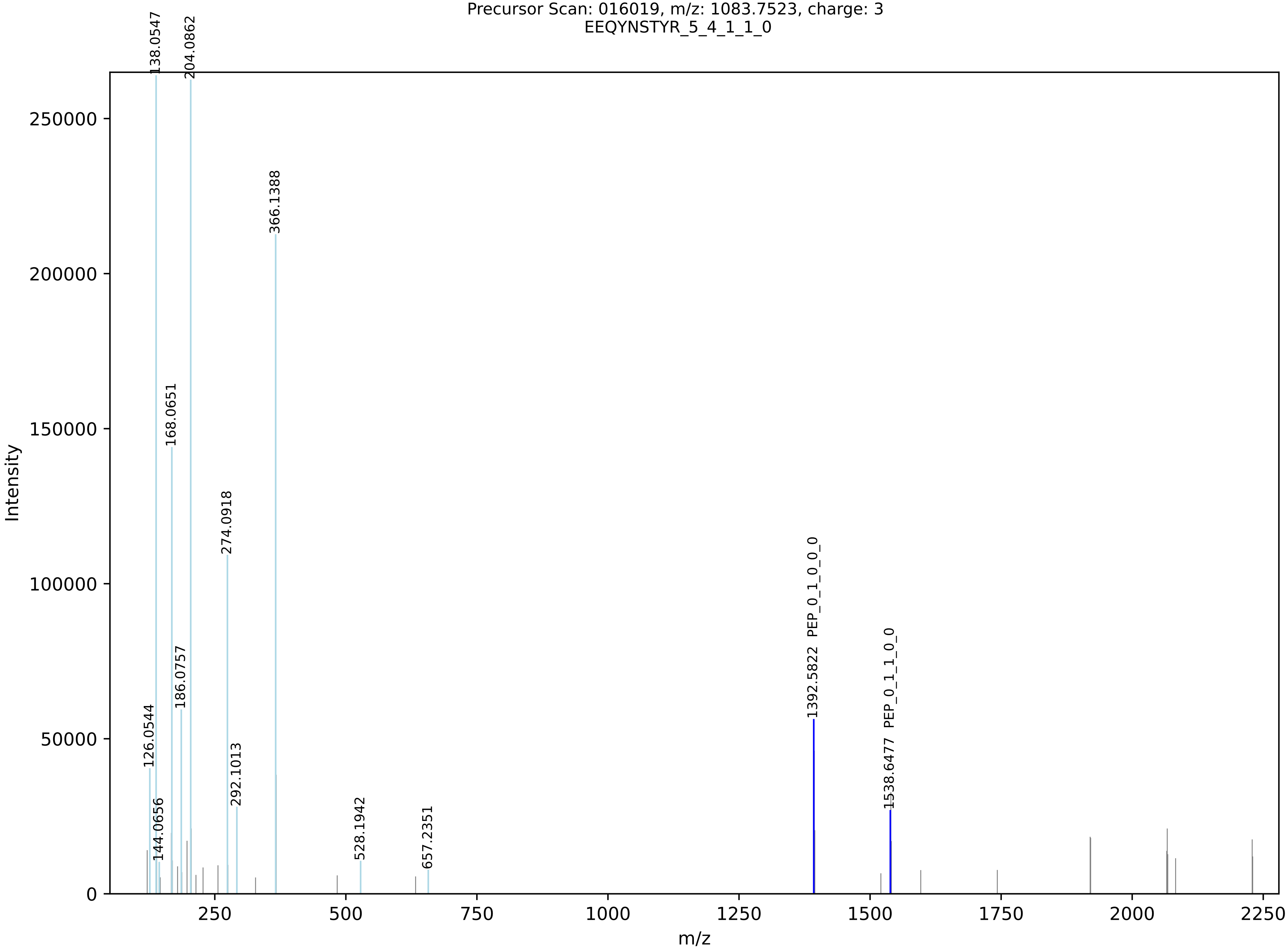

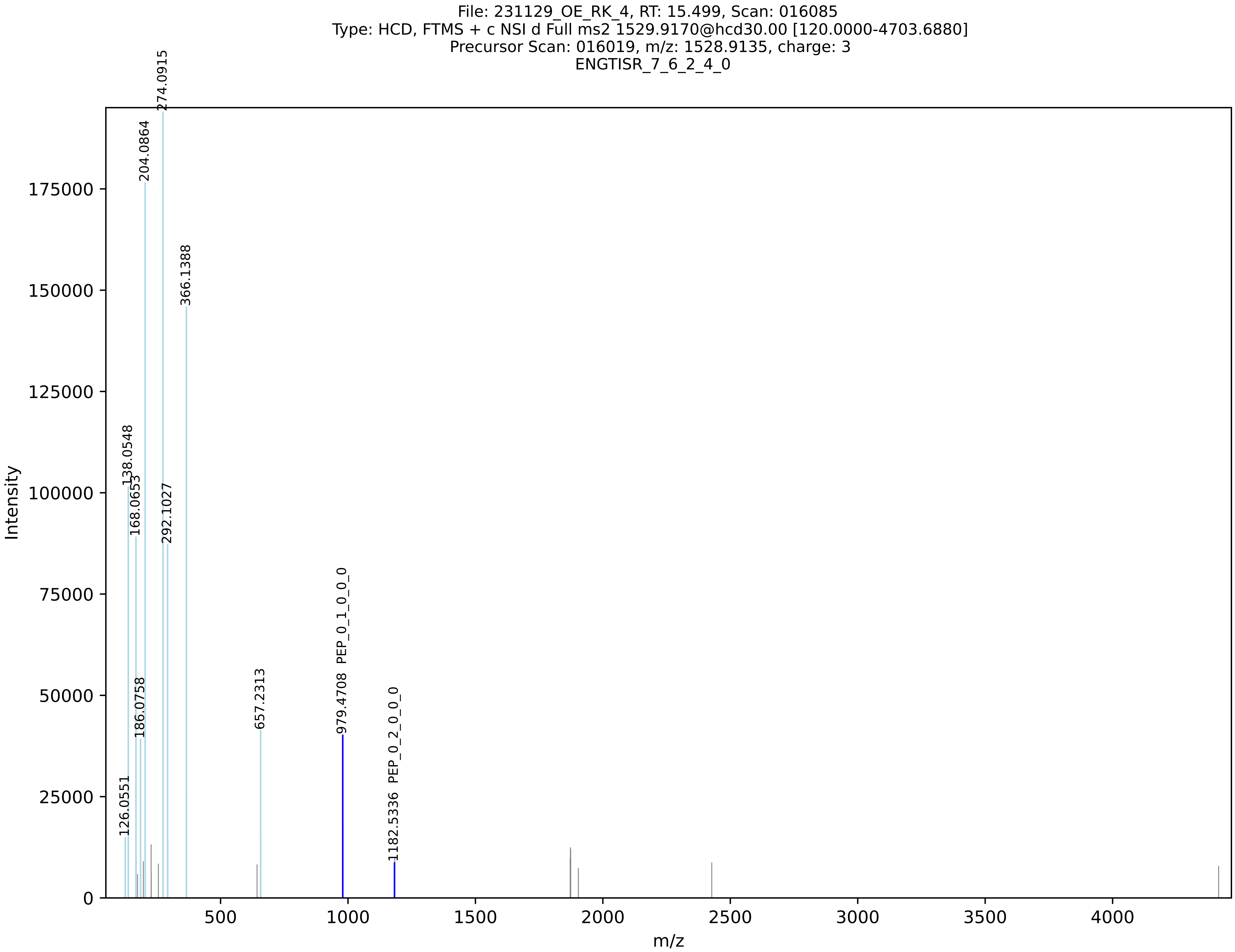
File: 231129_OE_RK_4, RT: 15.499, Scan: 016085
Type: HCD, FTMS + c NSI d Full ms2 1529.9170@hcd30.00 [120.0000-4703.6880]
Precursor Scan: 016019, m/z: 1528.9135, charge: 3
ENGTISR_7_6_2_4_0
274.0915
204.0864
366.1388
138.0548
168.0653
292.1027
979.4708 PEP_0_1_0_0_0
657.2313
186.0758
1182.5336 PEP_0_2_0_0_0
126.0551
Intensity
m/z
175000
150000
125000
100000
75000
50000
25000
0
500
1000
1500
2000
2500
3000
3500
4000

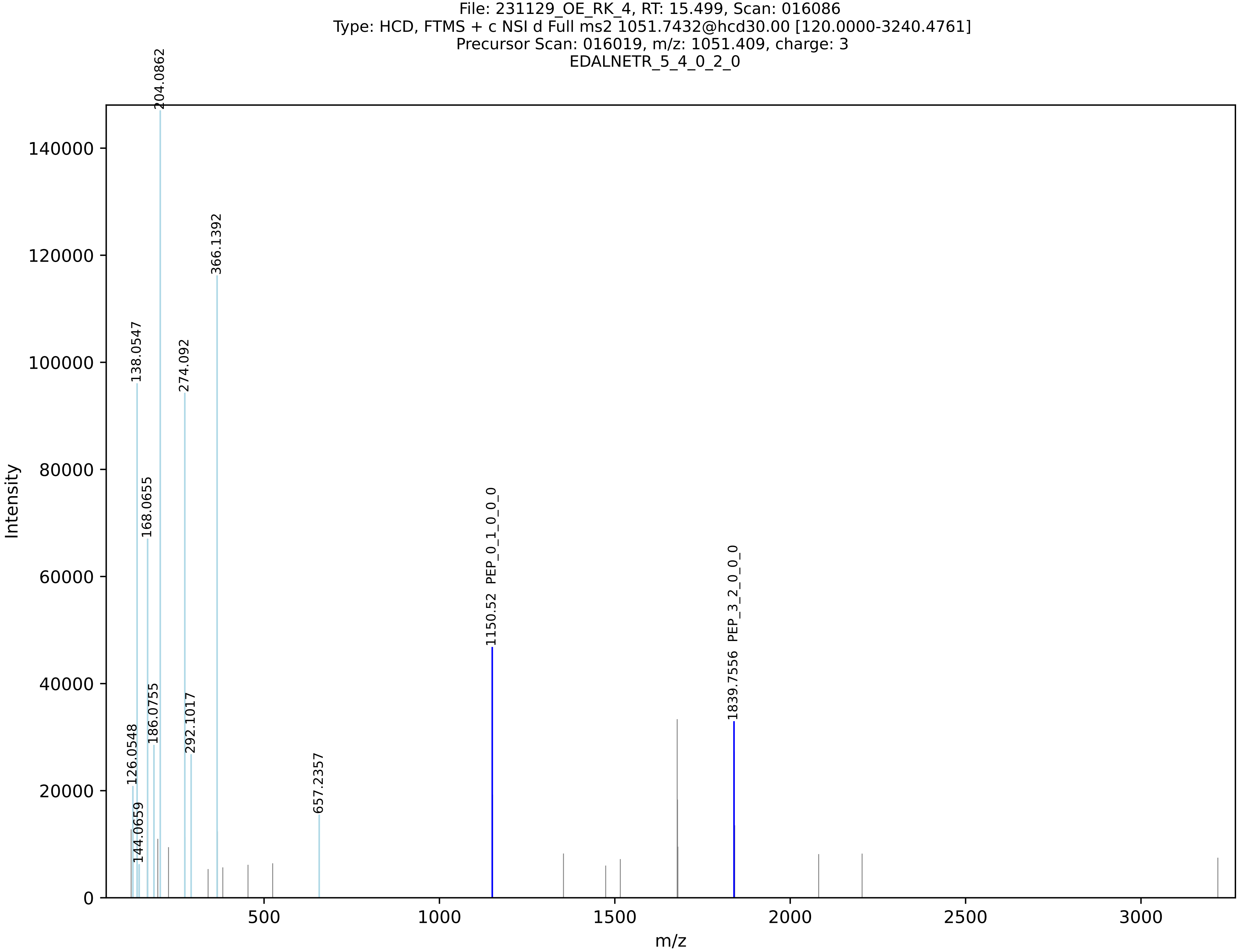
File: 231129_OE_RK_4, RT: 15.499, Scan: 016086
Type: HCD, FTMS + c NSI d Full ms2 1051.7432@hcd30.00 [120.0000-3240.4761]
Precursor Scan: 016019, m/z: 1051.409, charge: 3
EDALNETR_5_4_0_2_0
204.0862
366.1392
138.0547
274.092
168.0655
1150.52 PEP_0_1_0_0_0_
1839.7556 PEP_3_2_0_0_0
186.0755
292.1017
126.0548
657.2357
144.0659
Intensity
m/z
0
20000
40000
60000
80000
100000
120000
140000
500
1000
1500
2000
2500
3000

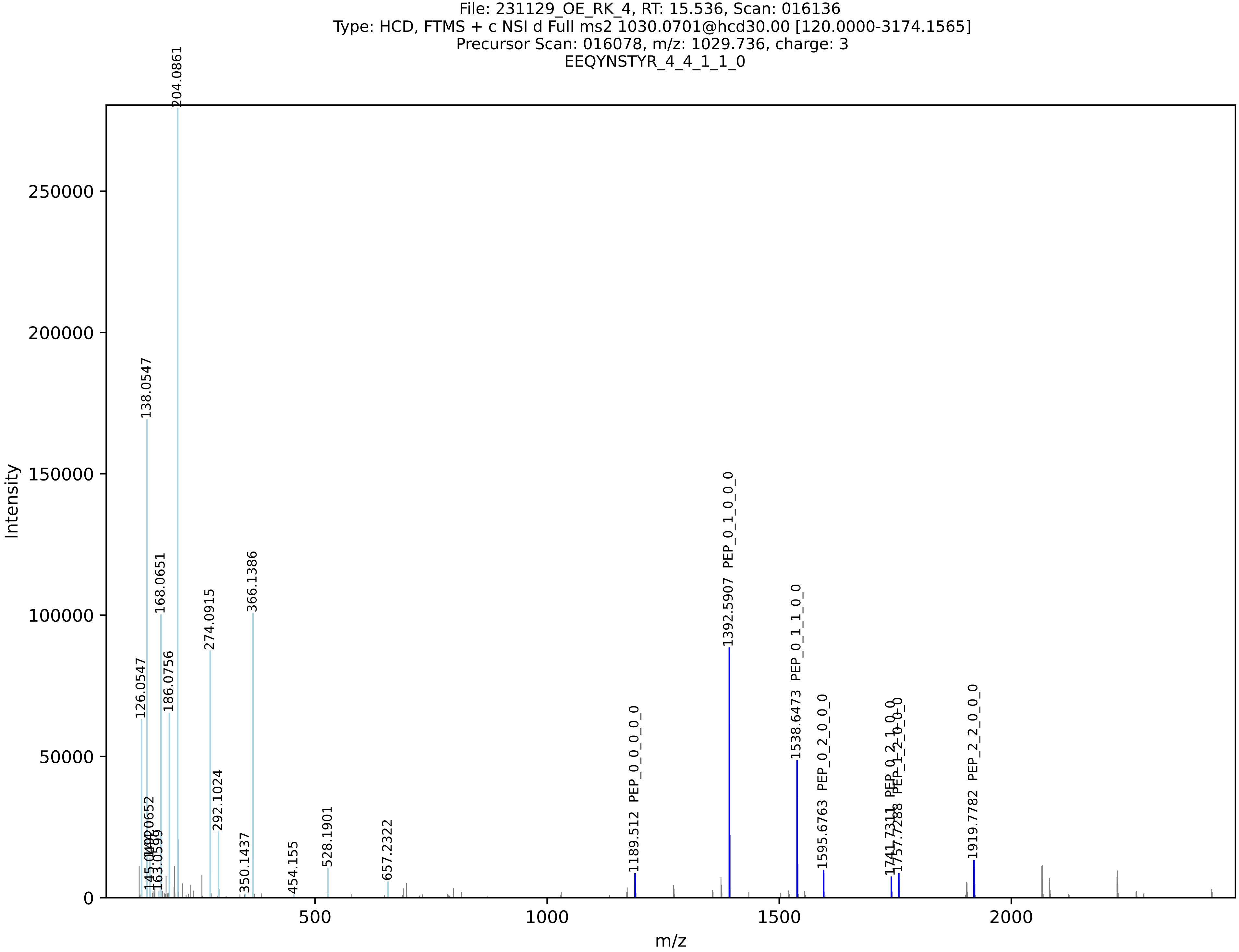
File: 231129_OE_RK_4, RT: 15.536, Scan: 016136
Type: HCD, FTMS + c NSI d Full ms2 1030.0701@hcd30.00 [120.0000-3174.1565]
Precursor Scan: 016078, m/z: 1029.736, charge: 3
EEQYNSTYR_4_4_1_1_0
Intensity
m/z
204.0861
138.0547
168.0651
126.0547
186.0756
274.0915
292.1024
366.1386
350.1437
454.155
528.1901
657.2322
1189.512 PEP_0_0_0_0_0
1392.5907 PEP_0_1_0_0_0
1538.6473 PEP_0_1_1_0_0
1595.6763 PEP_0_2_0_0_0
1741.7311 PEP_0_2_1_0_0
1757.7288 PEP_1_2_0_0_0
1919.7782 PEP_2_2_0_0_0
0
50000
100000
150000
200000
250000
500
1000
1500
2000

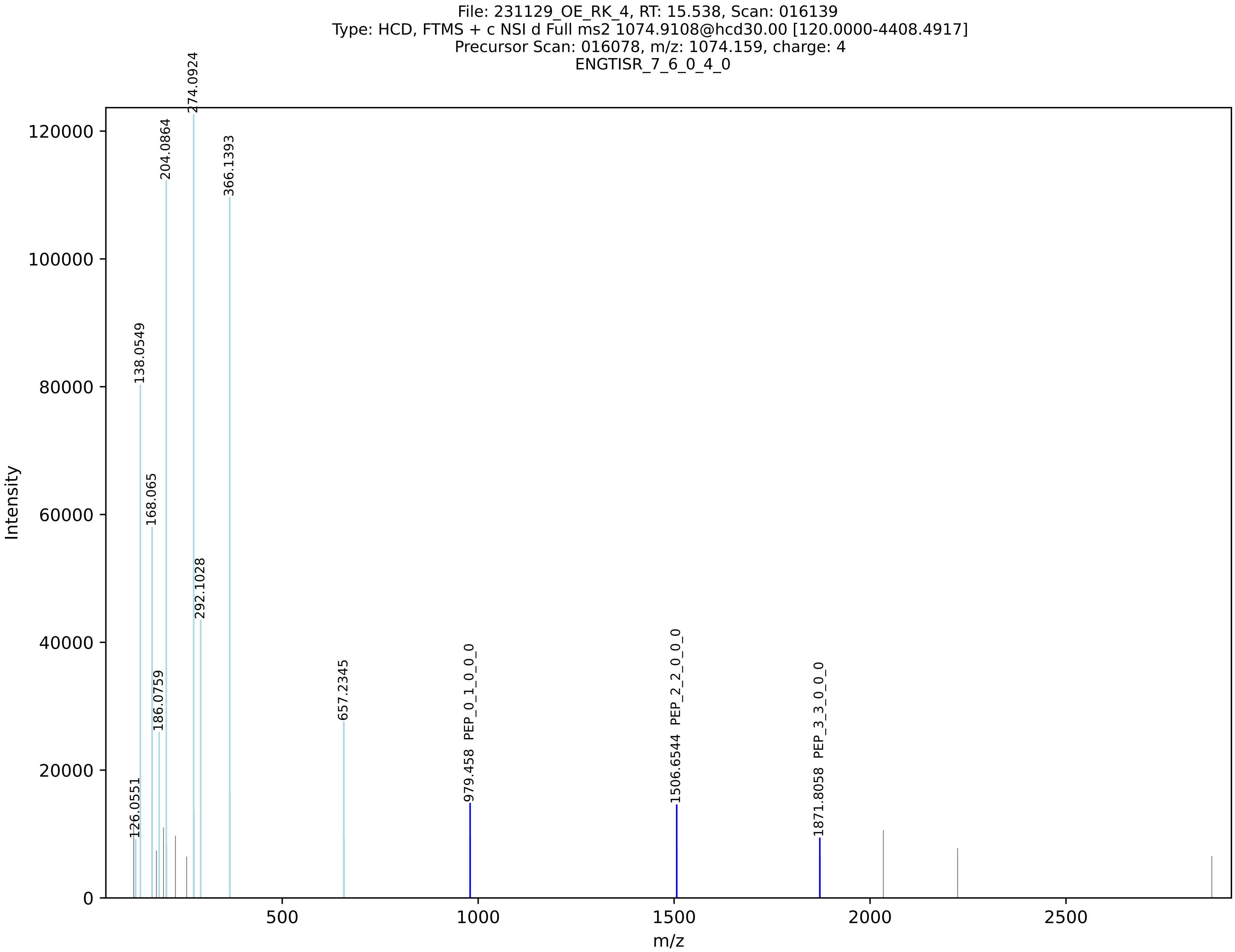
File: 231129_OE_RK_4, RT: 15.538, Scan: 016139
Type: HCD, FTMS + c NSI d Full ms2 1074.9108@hcd30.00 [120.0000-4408.4917]
Precursor Scan: 016078, m/z: 1074.159, charge: 4
ENGTISR_7_6_0_4_0
Intensity
m/z
0
20000
40000
60000
80000
100000
120000
500
1000
1500
2000
2500
274.0924
204.0864
366.1393
138.0549
168.065
292.1028
186.0759
126.0551
657.2345
979.458 PEP_0_1_0_0_0
1506.6544 PEP_2_2_0_0_0
1871.8058 PEP_3_3_0_0_0

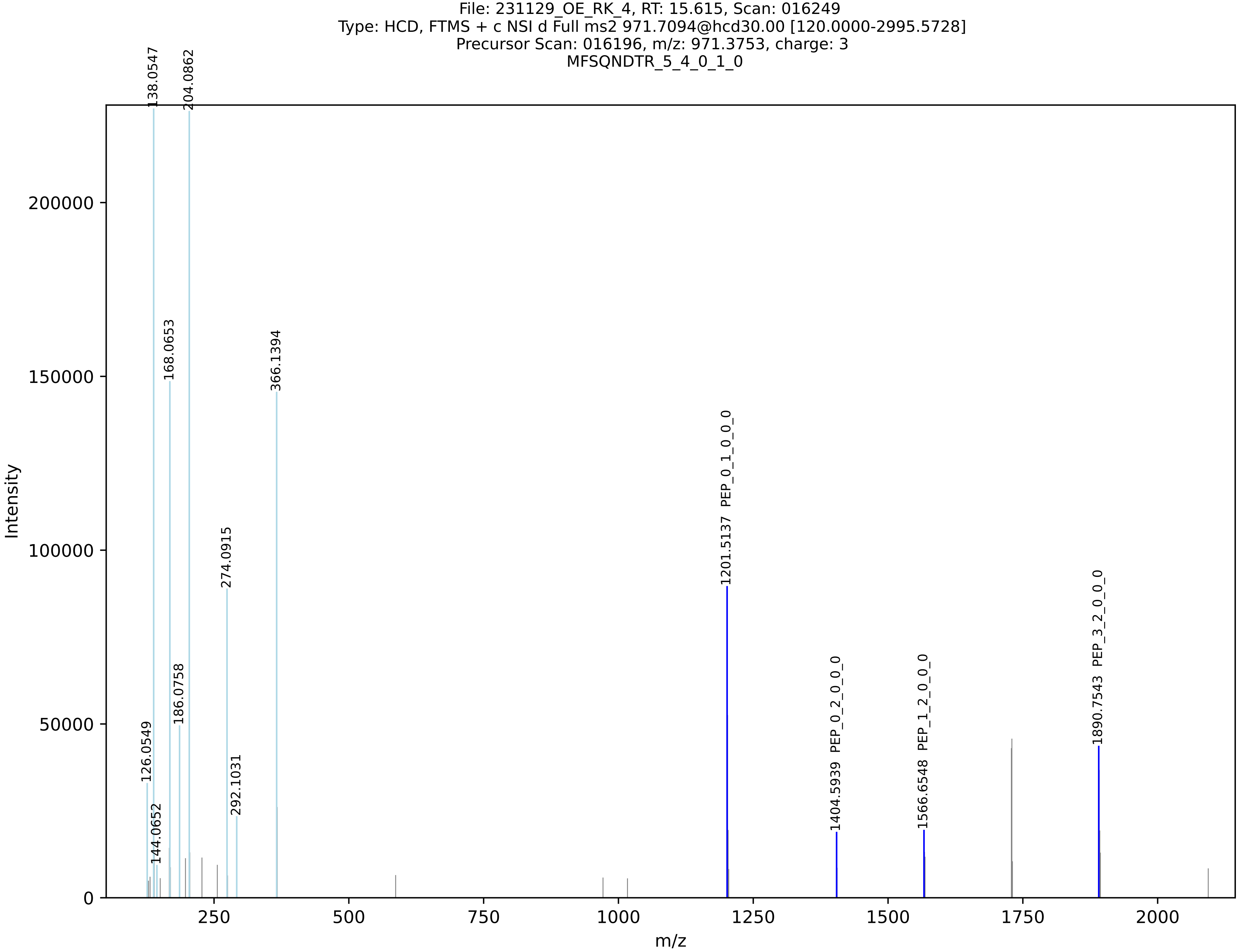
File: 231129_OE_RK_4, RT: 15.615, Scan: 016249
Type: HCD, FTMS + c NSI d Full ms2 971.7094@hcd30.00 [120.0000-2995.5728]
Precursor Scan: 016196, m/z: 971.3753, charge: 3
MFSQNDTR_5_4_0_1_0
138.0547
204.0862
168.0653
366.1394
274.0915
186.0758
126.0549
292.1031
144.0652
1201.5137 PEP_0_1_0_0_0
1404.5939 PEP_0_2_0_0_0
1566.6548 PEP_1_2_0_0_0
1890.7543 PEP_3_2_0_0_0
Intensity
m/z
0
50000
100000
150000
200000
250
500
750
1000
1250
1500
1750
2000

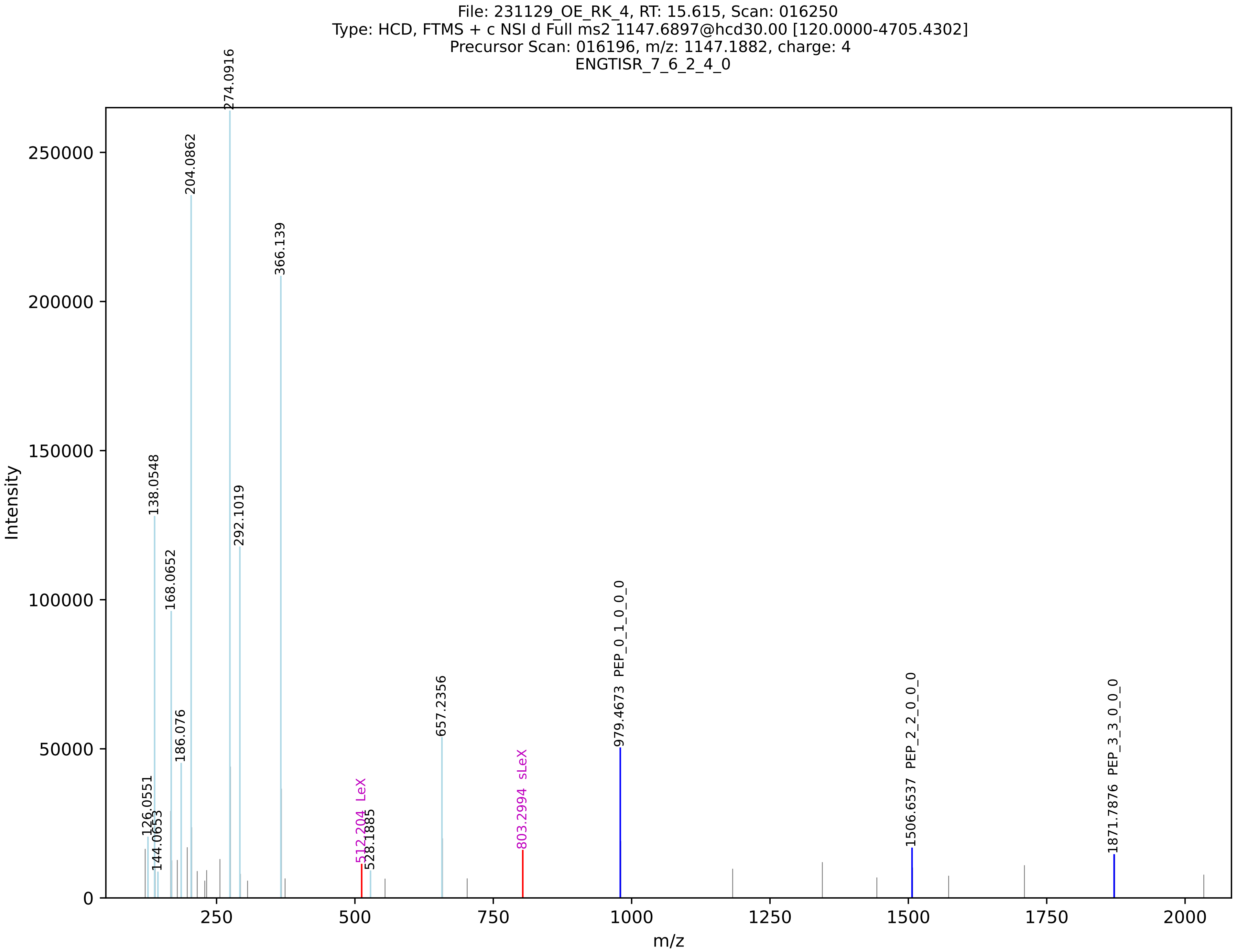
File: 231129_OE_RK_4, RT: 15.615, Scan: 016250
Type: HCD, FTMS + c NSI d Full ms2 1147.6897@hcd30.00 [120.0000-4705.4302]
Precursor Scan: 016196, m/z: 1147.1882, charge: 4
ENGTISR_7_6_2_4_0
Intensity
m/z
274.0916
204.0862
366.139
138.0548
292.1019
168.0652
186.076
126.0551
144.0653
512.204 LeX
528.1885
657.2356
803.2994 sLeX
979.4673 PEP_0_1_0_0_0_
1506.6537 PEP_2_2_0_0_0_
1871.7876 PEP_3_3_0_0_0_

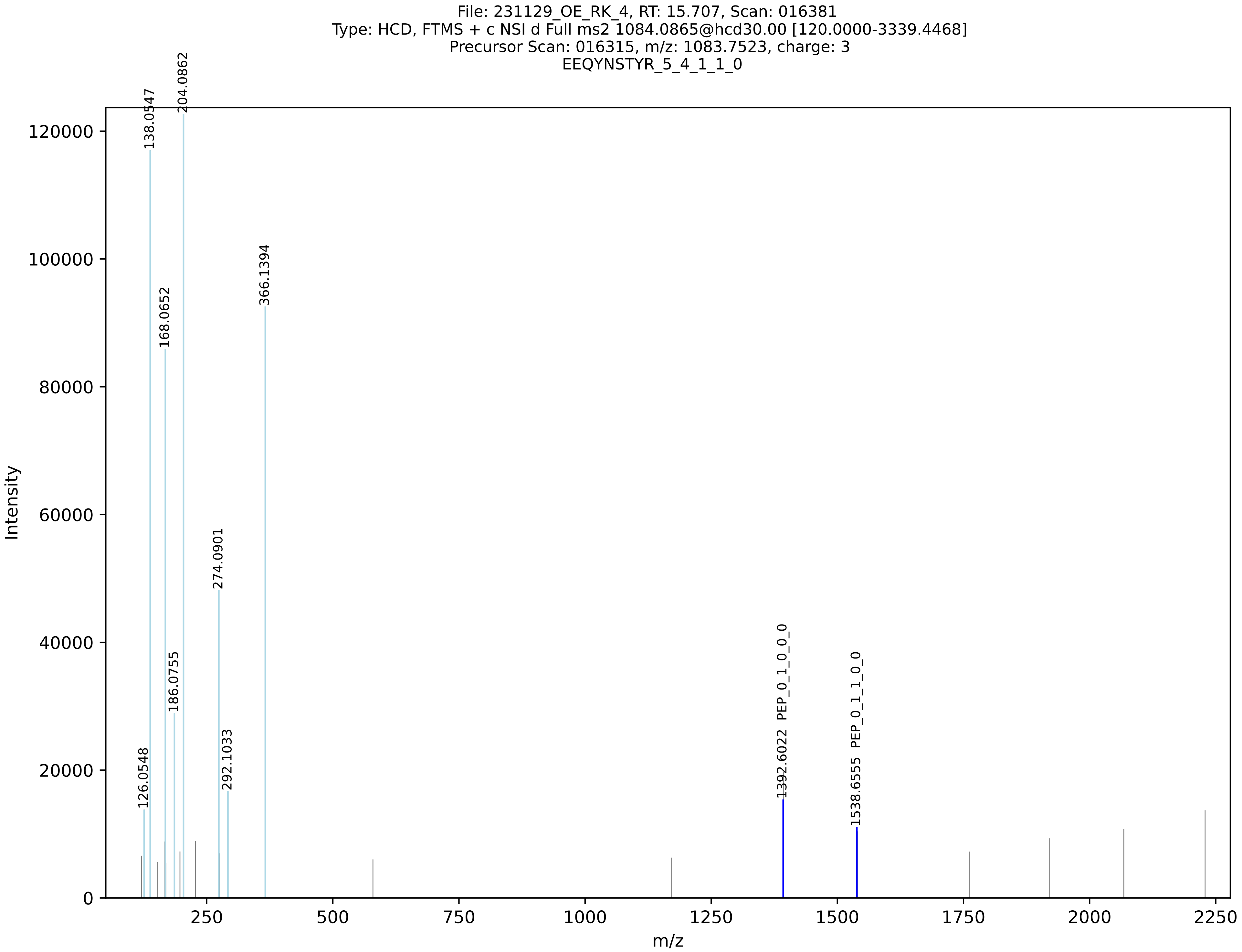
File: 231129_OE_RK_4, RT: 15.707, Scan: 016381
Type: HCD, FTMS + c NSI d Full ms2 1084.0865@hcd30.00 [120.0000-3339.4468]
Precursor Scan: 016315, m/z: 1083.7523, charge: 3
EEQYNSTYR_5_4_1_1_0
204.0862
138.0547
366.1394
168.0652
274.0901
186.0755
126.0548
292.1033
1392.6022 PEP_0_1_0_0_0
1538.6555 PEP_0_1_1_0_0
Intensity
m/z
0
20000
40000
60000
80000
100000
120000
250
500
750
1000
1250
1500
1750
2000
2250

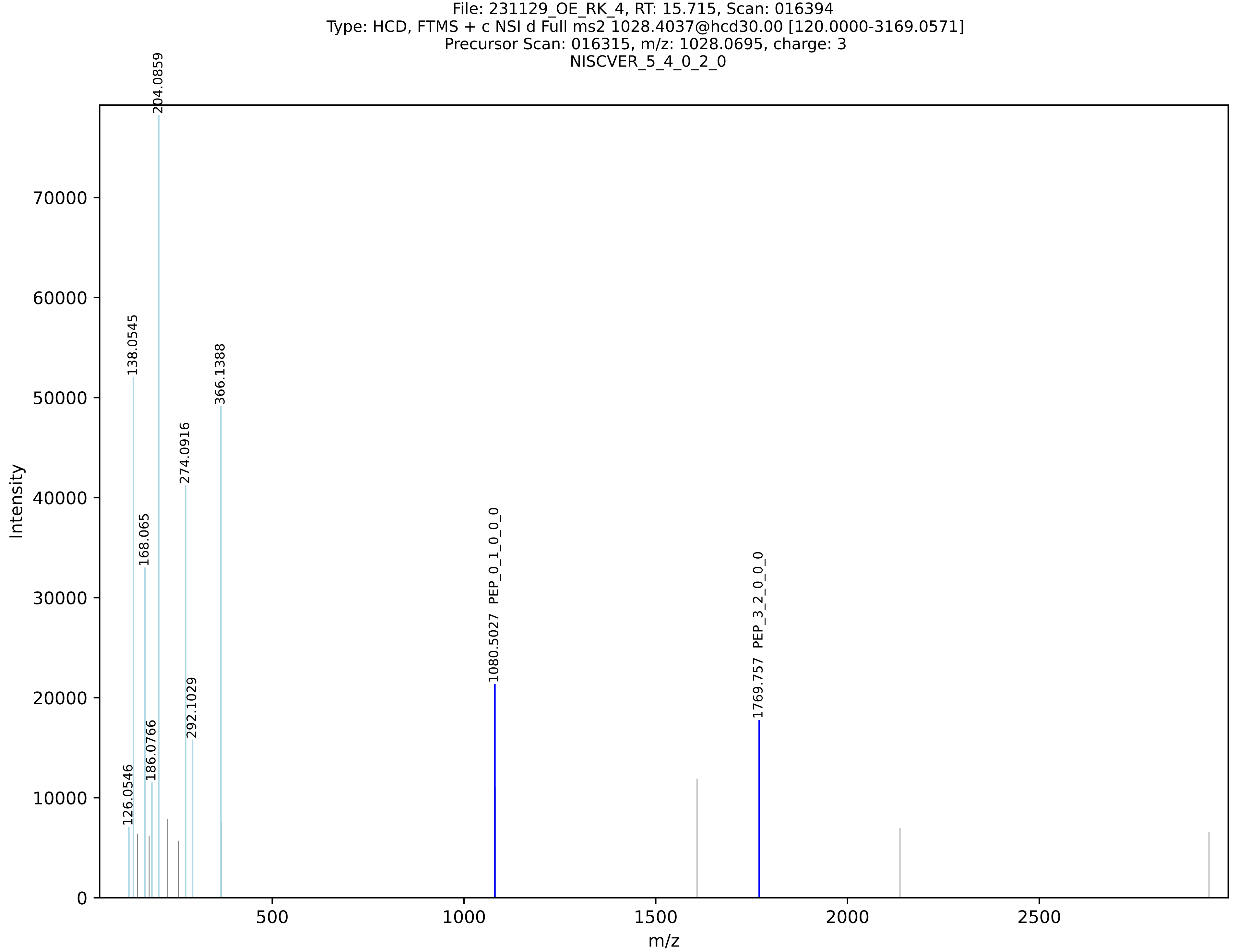

File: 231129_OE_RK_4, RT: 15.715, Scan: 016394
Type: HCD, FTMS + c NSI d Full ms2 1028.4037@hcd30.00 [120.0000-3169.0571]
Precursor Scan: 016315, m/z: 1028.0695, charge: 3
NISCVER_5_4_0_2_0
204.0859
138.0545
366.1388
274.0916
168.065
PEP_0_1_0_0_0
1080.5027
PEP_3_2_0_0_0
1769.757
292.1029
186.0766
126.0546
Intensity
m/z
70000
60000
50000
40000
30000
20000
10000
0
500
1000
1500
2000
2500

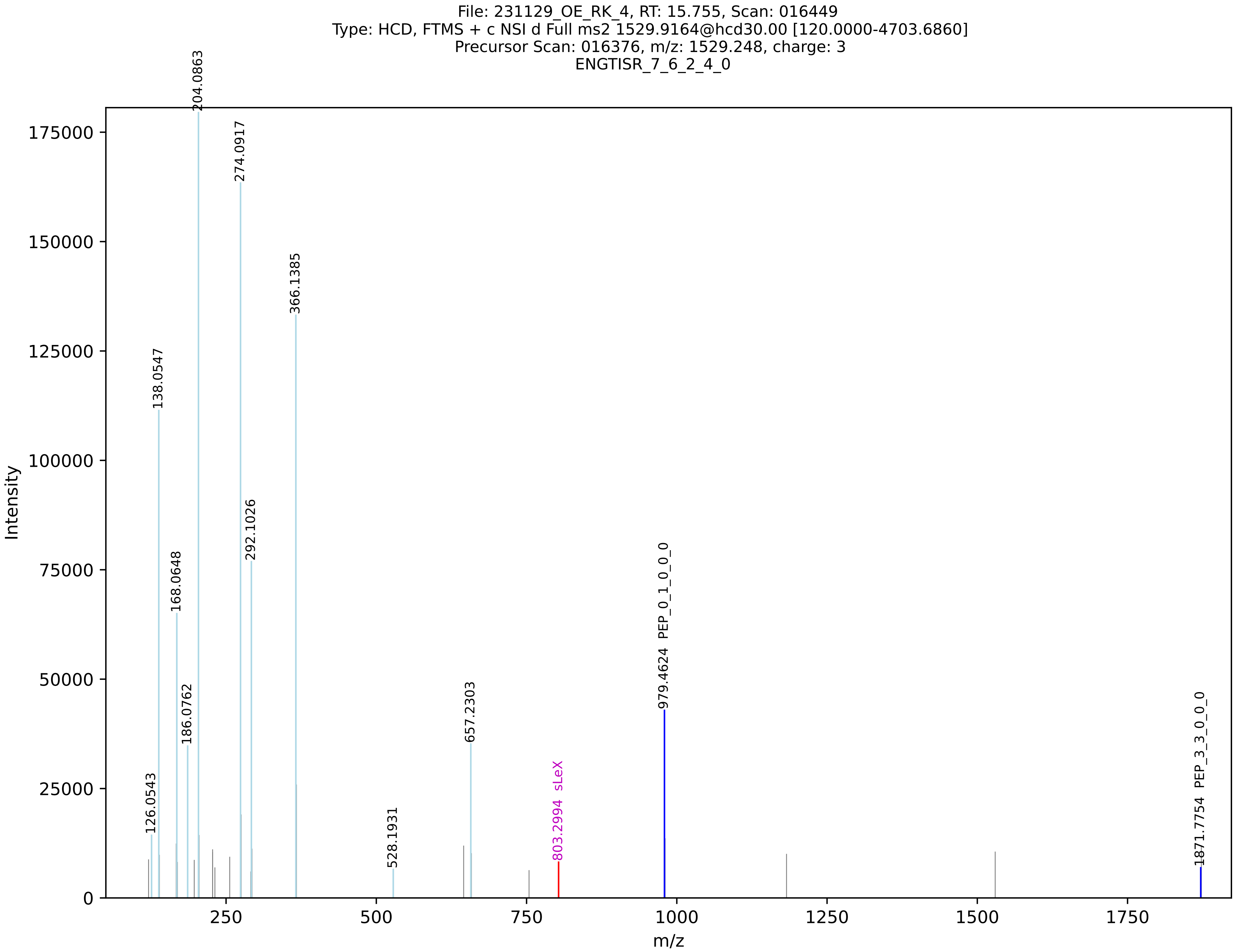

File: 231129_OE_RK_4, RT: 15.755, Scan: 016449
Type: HCD, FTMS + c NSI d Full ms2 1529.9164@hcd30.00 [120.0000-4703.6860]
Precursor Scan: 016376, m/z: 1529.248, charge: 3
ENGTISR_7_6_2_4_0
Intensity
m/z
204.0863
274.0917
366.1385
138.0547
292.1026
168.0648
186.0762
126.0543
528.1931
657.2303
803.2994 sLeX
979.4624 PEP_0_1_0_0_0
1871.7754 PEP_3_3_0_0_0

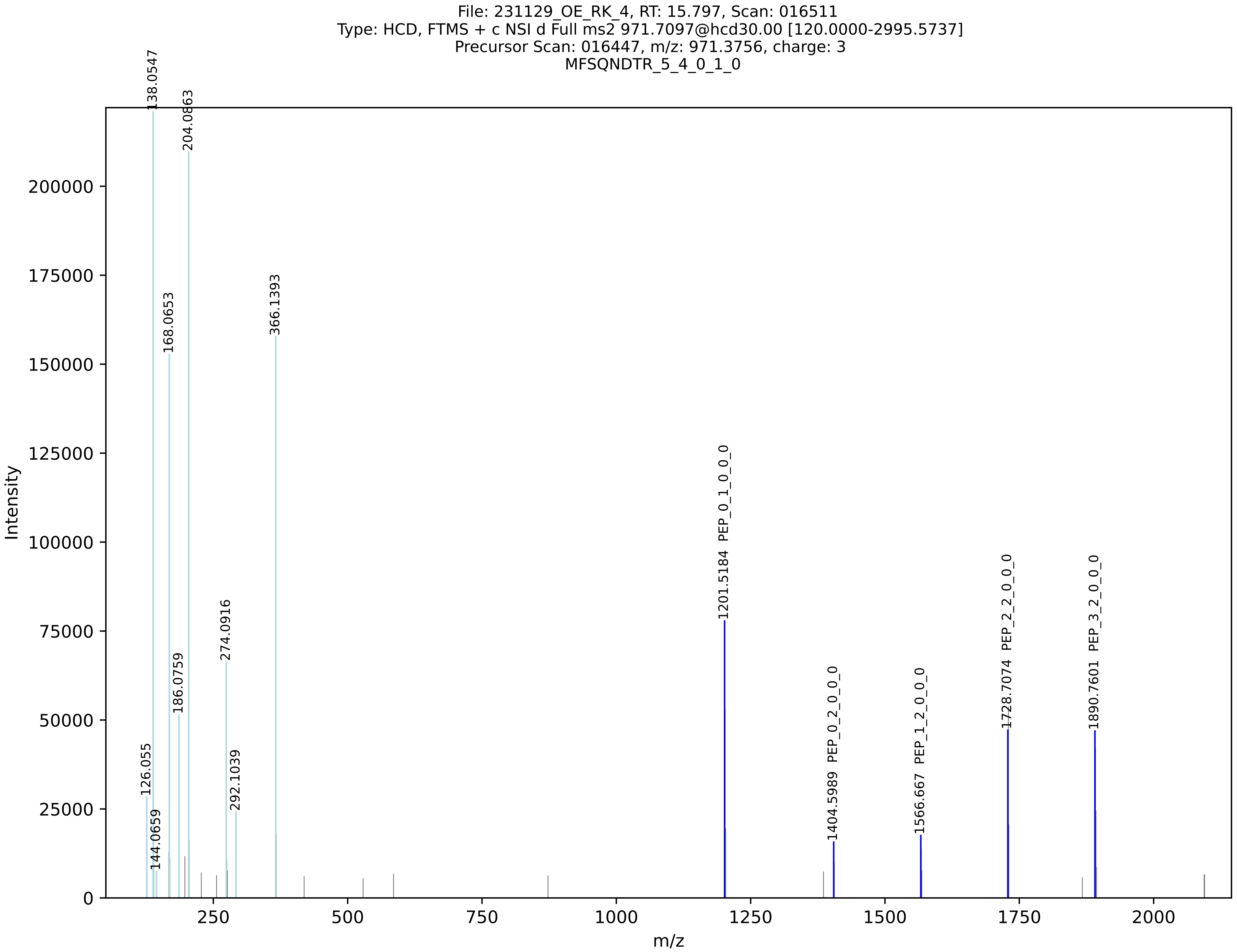
File: 231129_OE_RK_4, RT: 15.797, Scan: 016511
Type: HCD, FTMS + c NSI d Full ms2 971.7097@hcd30.00 [120.0000-2995.5737]
Precursor Scan: 016447, m/z: 971.3756, charge: 3
MFSQNDTR_5_4_0_1_0
Intensity
m/z
138.0547
204.0863
168.0653
366.1393
186.0759
274.0916
126.055
144.0659
292.1039
1201.5184 PEP_0_1_0_0_0
1404.5989 PEP_0_2_0_0_0
1566.667 PEP_1_2_0_0_0
1728.7074 PEP_2_2_0_0_0
1890.7601 PEP_3_2_0_0_0

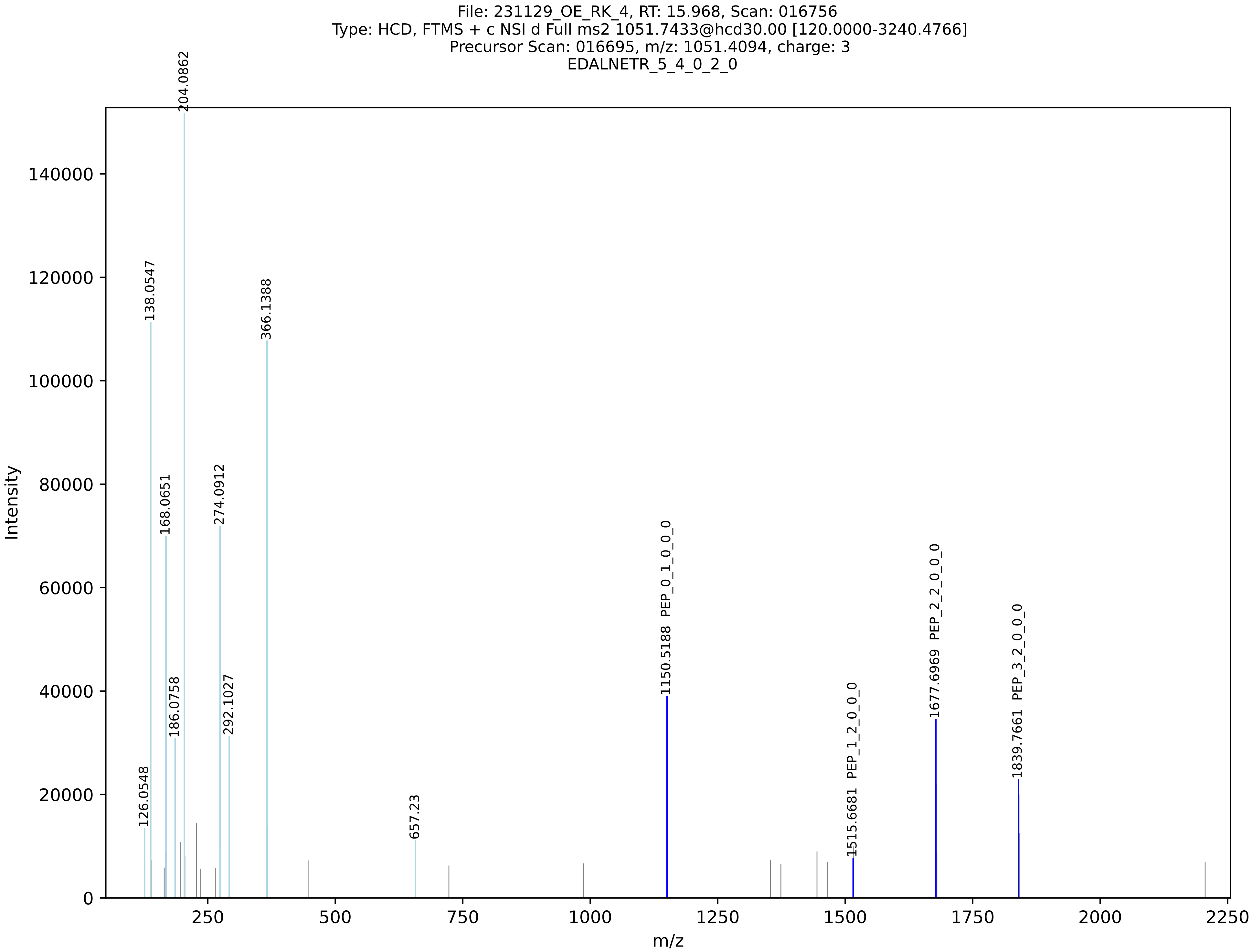
File: 231129_OE_RK_4, RT: 15.968, Scan: 016756
Type: HCD, FTMS + c NSI d Full ms2 1051.7433@hcd30.00 [120.0000-3240.4766]
Precursor Scan: 016695, m/z: 1051.4094, charge: 3
EDALNETR_5_4_0_2_0
204.0862
138.0547
366.1388
168.0651
274.0912
186.0758
292.1027
126.0548
657.23
1150.5188 PEP_0_1_0_0_0
1515.6681 PEP_1_2_0_0_0
1677.6969 PEP_2_2_0_0_0
1839.7661 PEP_3_2_0_0_0
Intensity
m/z
0
20000
40000
60000
80000
100000
120000
140000
250
500
750
1000
1250
1500
1750
2000
2250

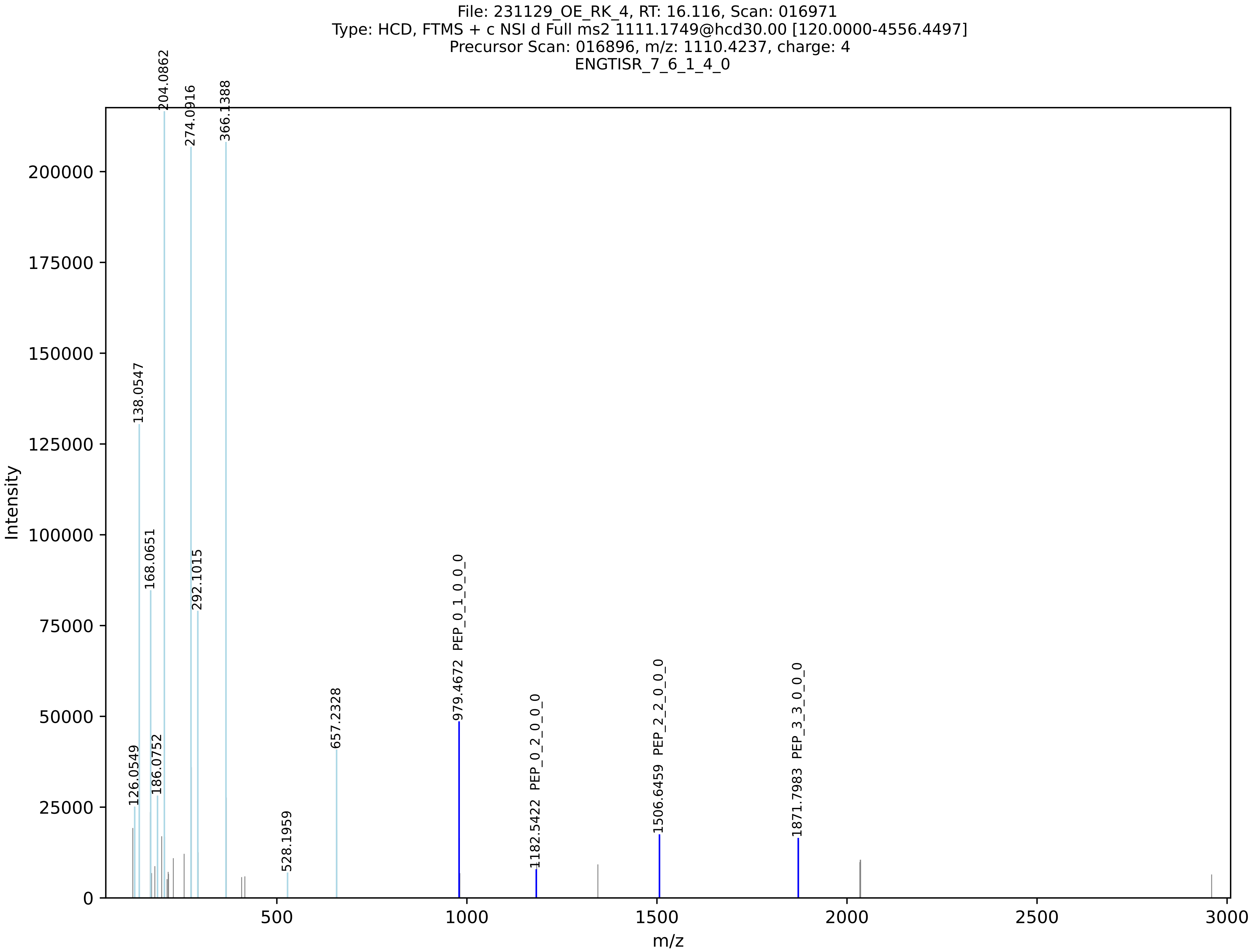
File: 231129_OE_RK_4, RT: 16.116, Scan: 016971
Type: HCD, FTMS + c NSI d Full ms2 1111.1749@hcd30.00 [120.0000-4556.4497]
Precursor Scan: 016896, m/z: 1110.4237, charge: 4
ENGTISR_7_6_1_4_0
204.0862
274.0916
366.1388
138.0547
168.0651
292.1015
126.0549
186.0752
528.1959
657.2328
979.4672 PEP_0_1_0_0_0
1182.5422 PEP_0_2_0_0_0
1506.6459 PEP_2_2_0_0_0
1871.7983 PEP_3_3_0_0_0
Intensity
m/z
200000
175000
150000
125000
100000
75000
50000
25000
0
500
1000
1500
2000
2500
3000

File: 231129_OE_RK_4, RT: 16.167, Scan: 017046
Type: HCD, FTMS + c NSI d Full ms2 1432.5461@hcd30.00 [120.0000-4405.7334]
Precursor Scan: 016969, m/z: 1431.8779, charge: 3
ENGTISR_7_6_0_4_0

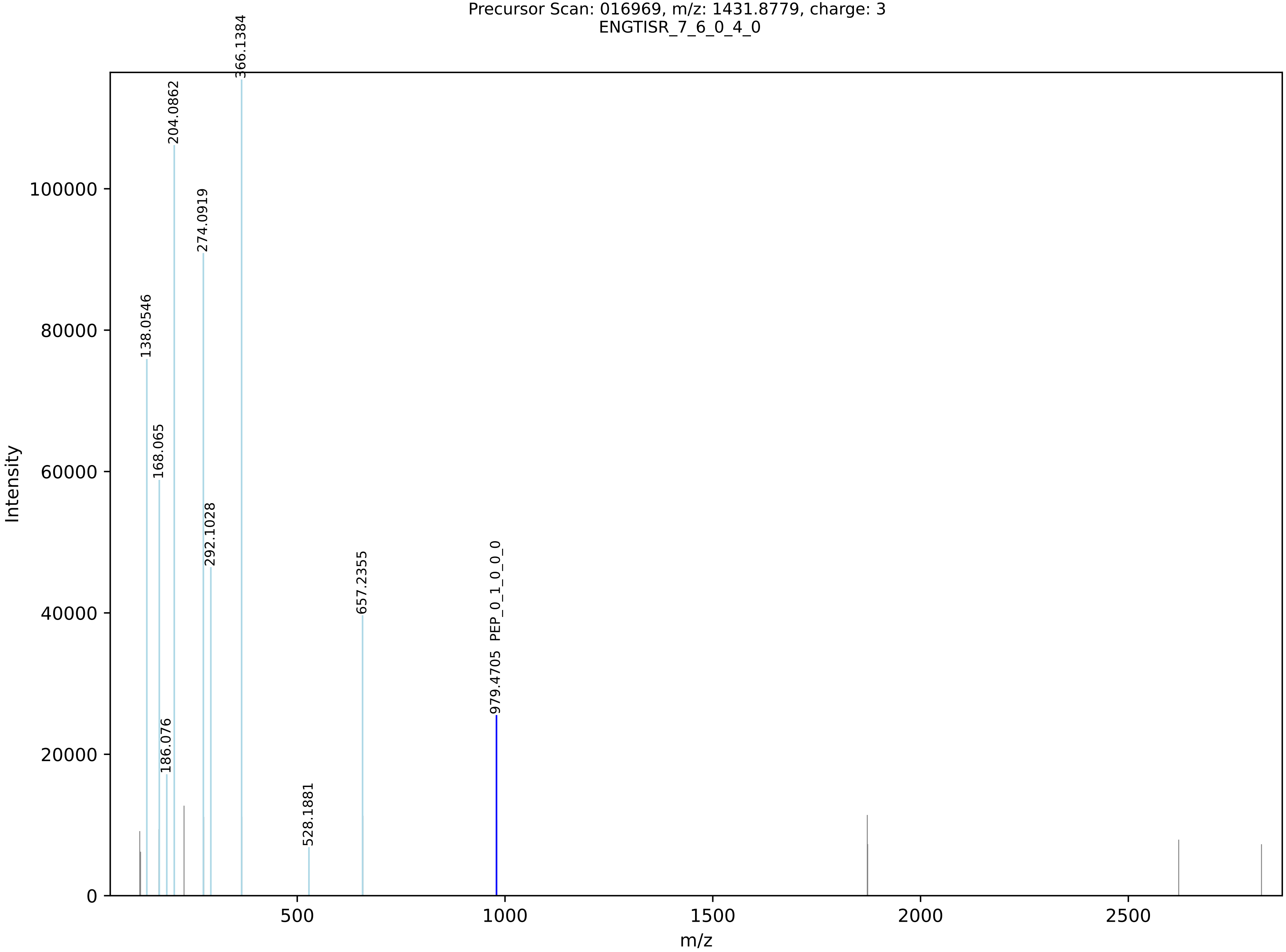

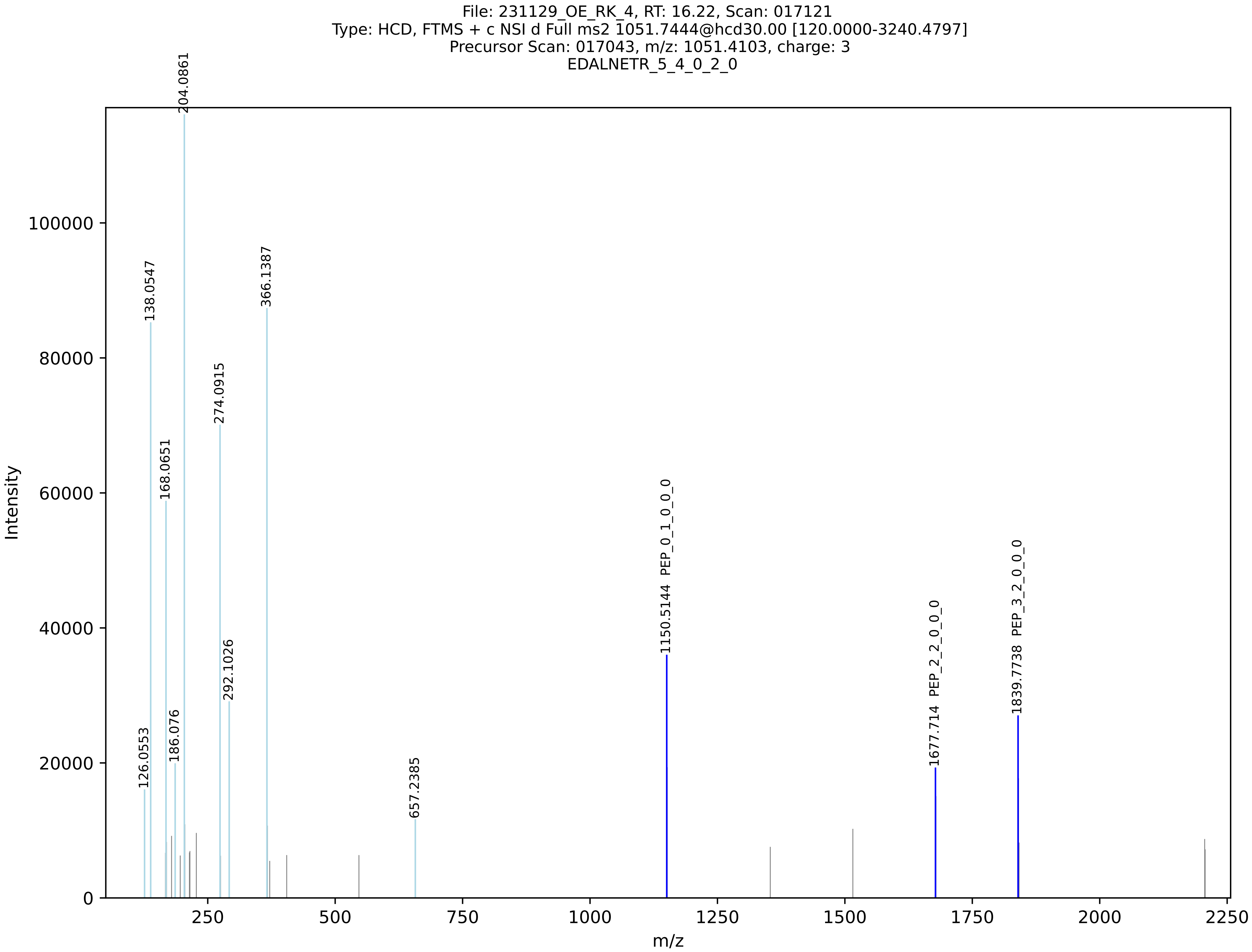
File: 231129_OE_RK_4, RT: 16.22, Scan: 017121
Type: HCD, FTMS + c NSI d Full ms2 1051.7444@hcd30.00 [120.0000-3240.4797]
Precursor Scan: 017043, m/z: 1051.4103, charge: 3
EDALNETR_5_4_0_2_0
204.0861
138.0547
366.1387
274.0915
168.0651
292.1026
186.076
126.0553
657.2385
1150.5144 PEP_0_1_0_0_0_
1677.714 PEP_2_2_0_0_0_
1839.7738 PEP_3_2_0_0_0_
Intensity
m/z
0
20000
40000
60000
80000
100000
250
500
750
1000
1250
1500
1750
2000
2250

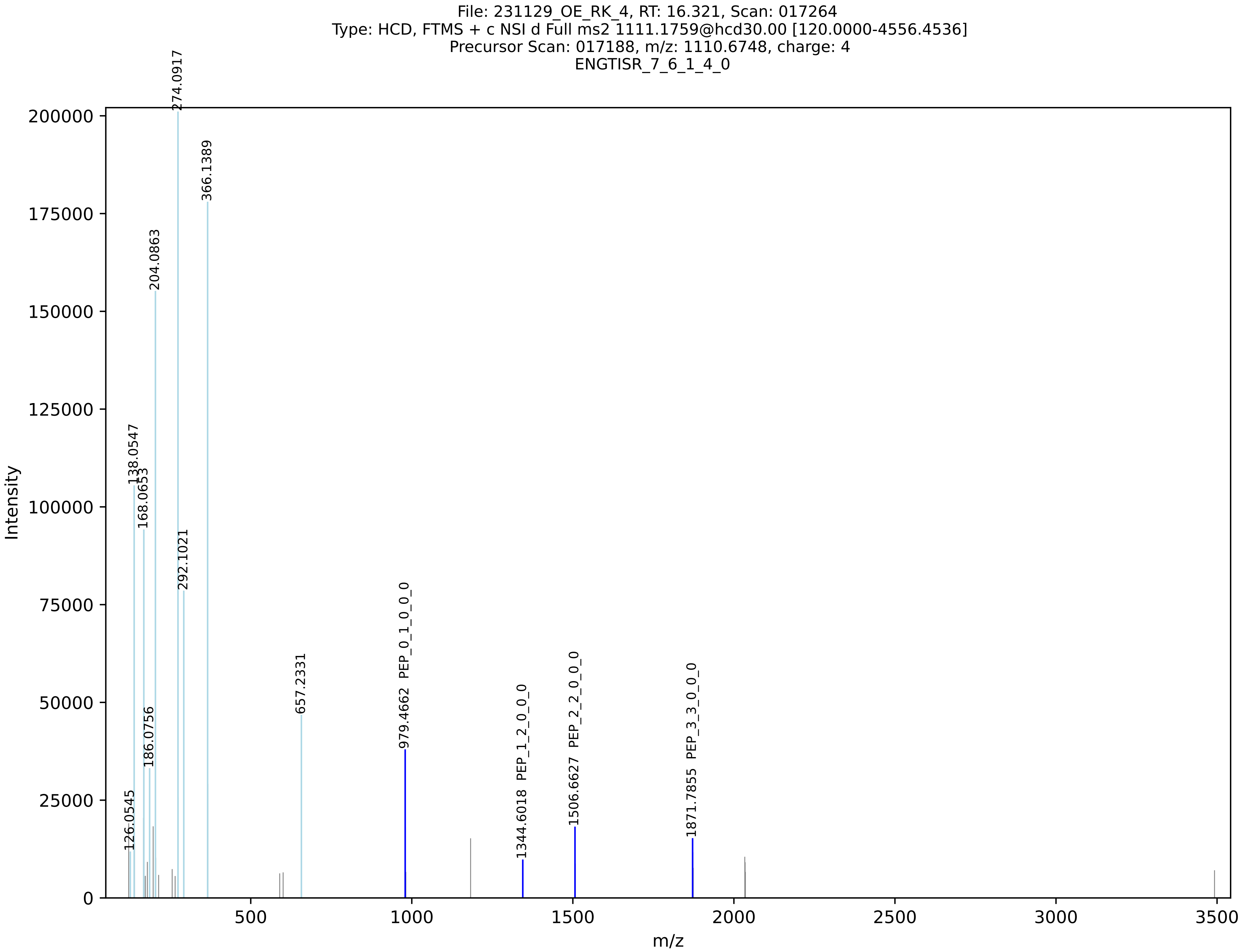
File: 231129_OE_RK_4, RT: 16.321, Scan: 017264
Type: HCD, FTMS + c NSI d Full ms2 1111.1759@hcd30.00 [120.0000-4556.4536]
Precursor Scan: 017188, m/z: 1110.6748, charge: 4
ENGTISR_7_6_1_4_0
274.0917
366.1389
204.0863
138.0547
168.0653
292.1021
657.2331
979.4662 PEP_0_1_0_0_0
186.0756
126.0545
1344.6018 PEP_1_2_0_0_0
1506.6627 PEP_2_2_0_0_0
1871.7855 PEP_3_3_0_0_0
Intensity
m/z
0
25000
50000
75000
100000
125000
150000
175000
200000
500
1000
1500
2000
2500
3000
3500

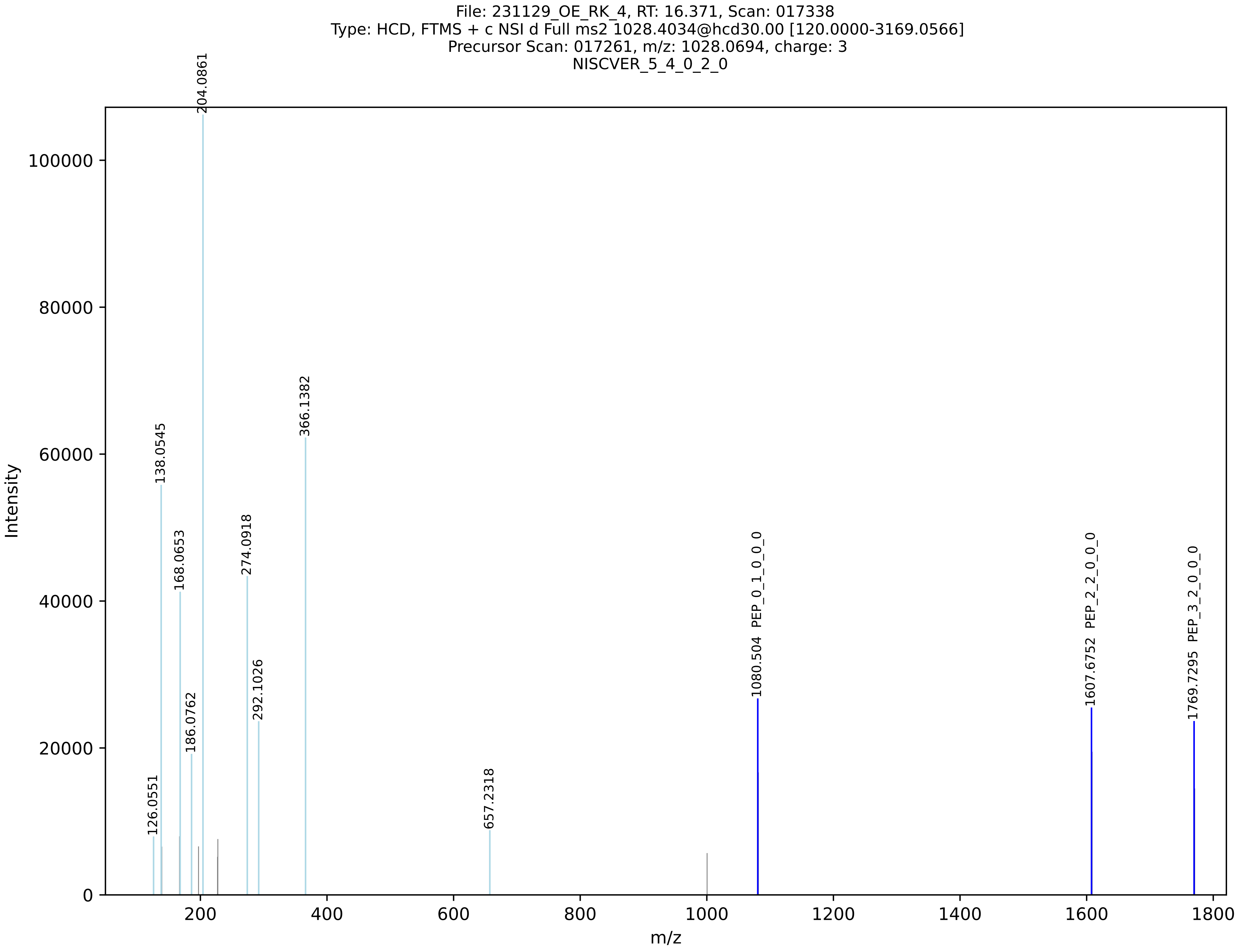
File: 231129_OE_RK_4, RT: 16.371, Scan: 017338
Type: HCD, FTMS + c NSI d Full ms2 1028.4034@hcd30.00 [120.0000-3169.0566]
Precursor Scan: 017261, m/z: 1028.0694, charge: 3
NISCVER_5_4_0_2_0
204.0861
138.0545
366.1382
168.0653
274.0918
292.1026
186.0762
126.0551
657.2318
1080.504 PEP_0_1_0_0_0
1607.6752 PEP_2_2_0_0_0
1769.7295 PEP_3_2_0_0_0
Intensity
m/z
0
20000
40000
60000
80000
100000
200
400
600
800
1000
1200
1400
1600
1800

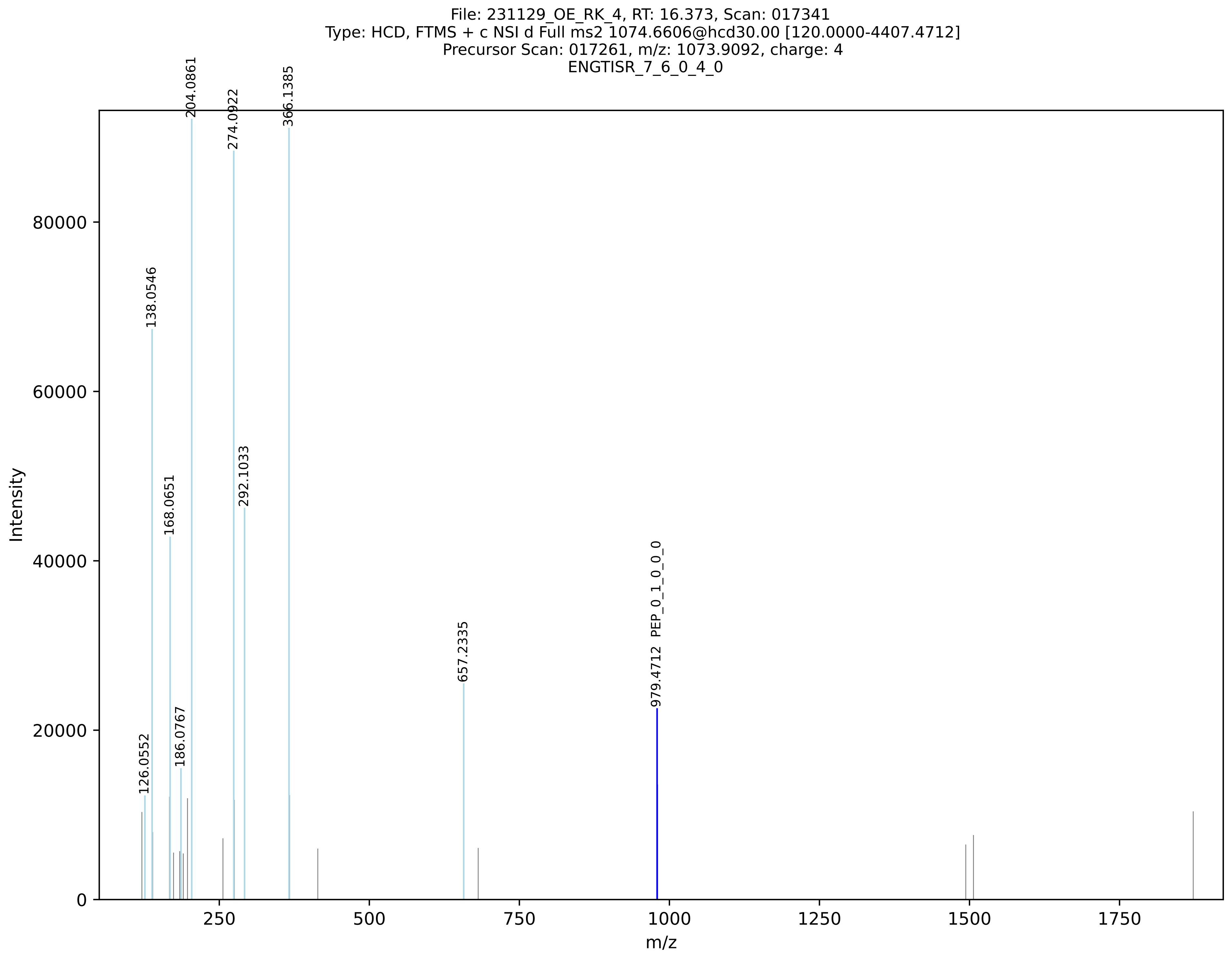

File: 231129_OE_RK_4, RT: 16.373, Scan: 017341
Type: HCD, FTMS + c NSI d Full ms2 1074.6606@hcd30.00 [120.0000-4407.4712]
Precursor Scan: 017261, m/z: 1073.9092, charge: 4
ENGTISR_7_6_0_4_0
204.0861
274.0922
366.1385
138.0546
168.0651
292.1033
186.0767
126.0552
657.2335
979.4712 PEP_0_1_0_0_0
Intensity
m/z
0
20000
40000
60000
80000
250
500
750
1000
1250
1500
1750

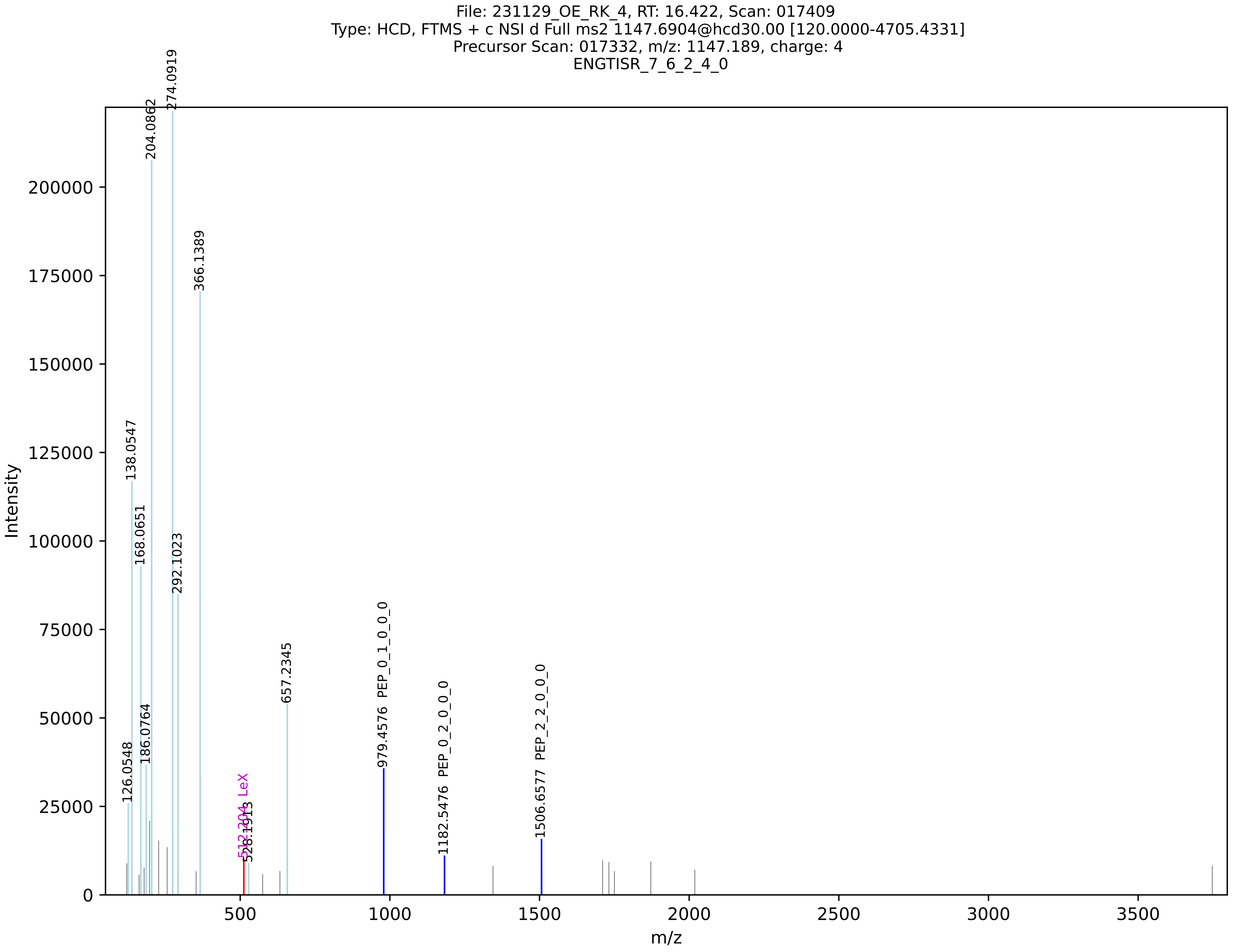
File: 231129_OE_RK_4, RT: 16.422, Scan: 017409
Type: HCD, FTMS + c NSI d Full ms2 1147.6904@hcd30.00 [120.0000-4705.4331]
Precursor Scan: 017332, m/z: 1147.189, charge: 4
ENGTISR_7_6_2_4_0
274.0919
204.0862
366.1389
138.0547
168.0651
292.1023
657.2345
979.4576 PEP_0_1_0_0_0
1182.5476 PEP_0_2_0_0_0
1506.6577 PEP_2_2_0_0_0
186.0764
126.0548
512.204 LeX
528.1913
Intensity
m/z
0
25000
50000
75000
100000
125000
150000
175000
200000
500
1000
1500
2000
2500
3000
3500

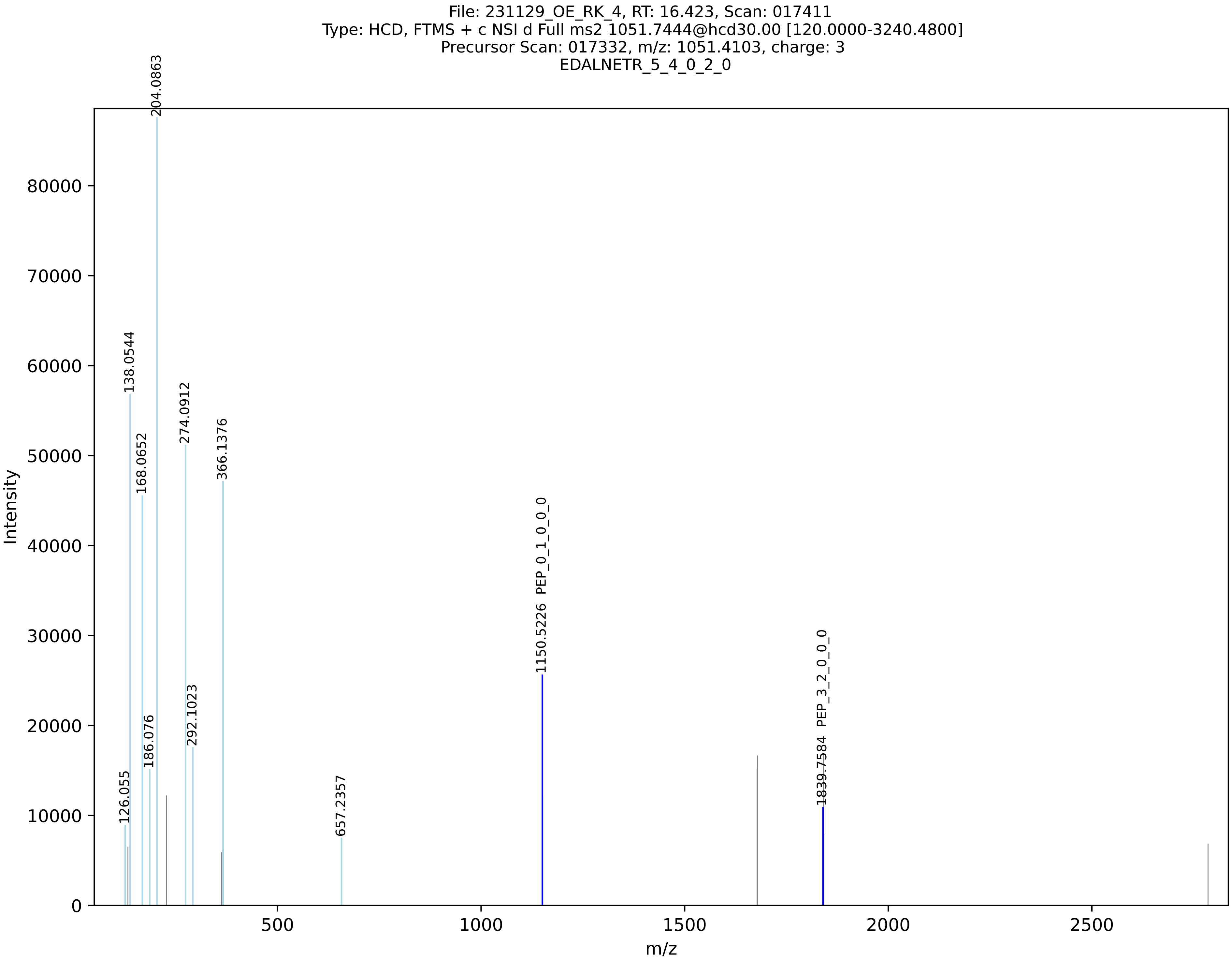

File: 231129_OE_RK_4, RT: 16.423, Scan: 017411
Type: HCD, FTMS + c NSI d Full ms2 1051.7444@hcd30.00 [120.0000-3240.4800]
Precursor Scan: 017332, m/z: 1051.4103, charge: 3
EDALNETR_5_4_0_2_0
Intensity
m/z
204.0863
138.0544
168.0652
274.0912
366.1376
1150.5226 PEP_0_1_0_0_0
1839.7584 PEP_3_2_0_0_0
292.1023
186.076
126.055
657.2357
0
10000
20000
30000
40000
50000
60000
70000
80000
500
1000
1500
2000
2500

File: 231129_OE_RK_4, RT: 16.52, Scan: 017549
Type: HCD, FTMS + c NSI d Full ms2 1111.1749@hcd30.00 [120.0000-4556.4497]
Precursor Scan: 017477, m/z: 1110.6736, charge: 4
ENGTISR_7_6_1_4_0

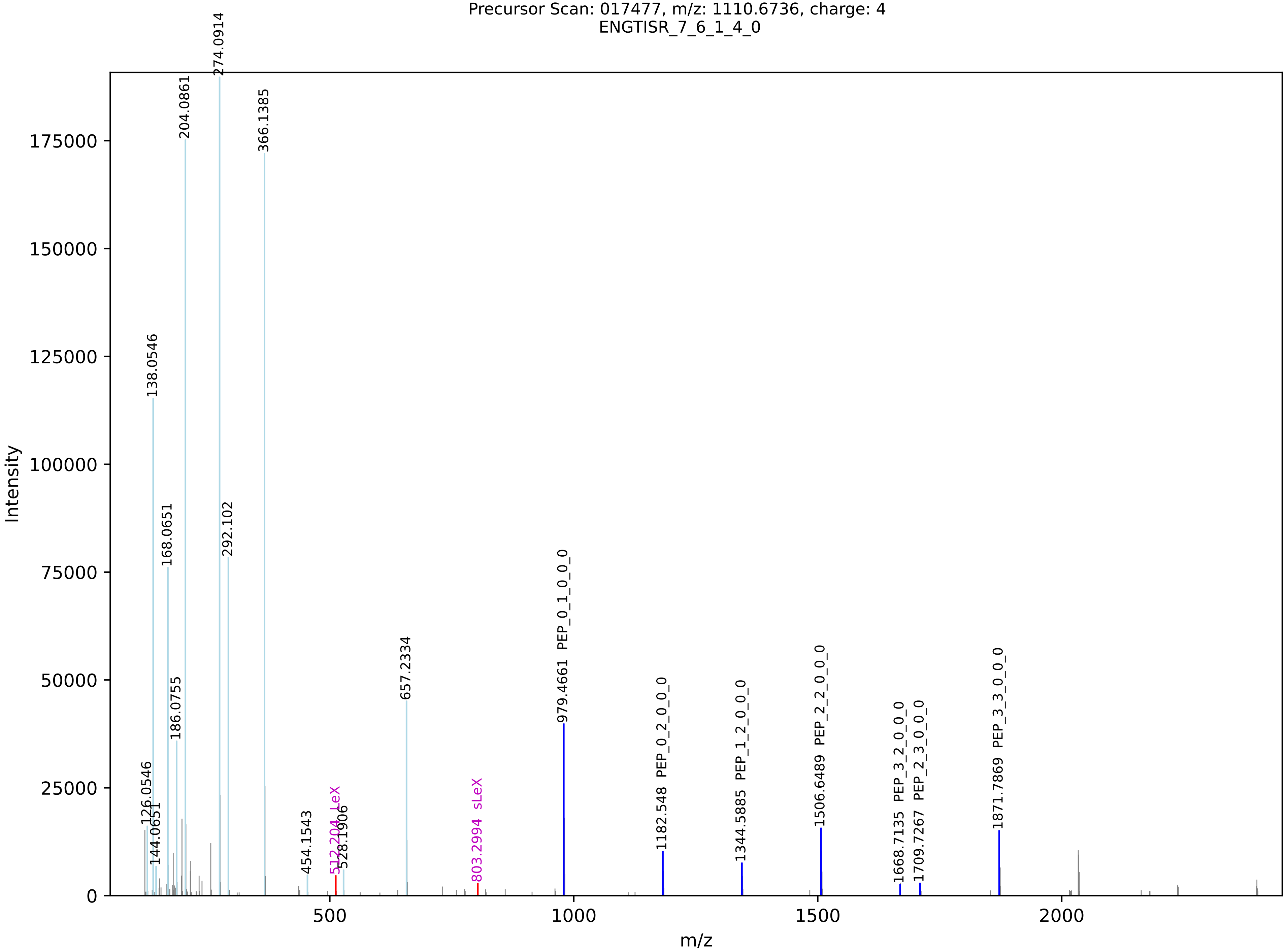

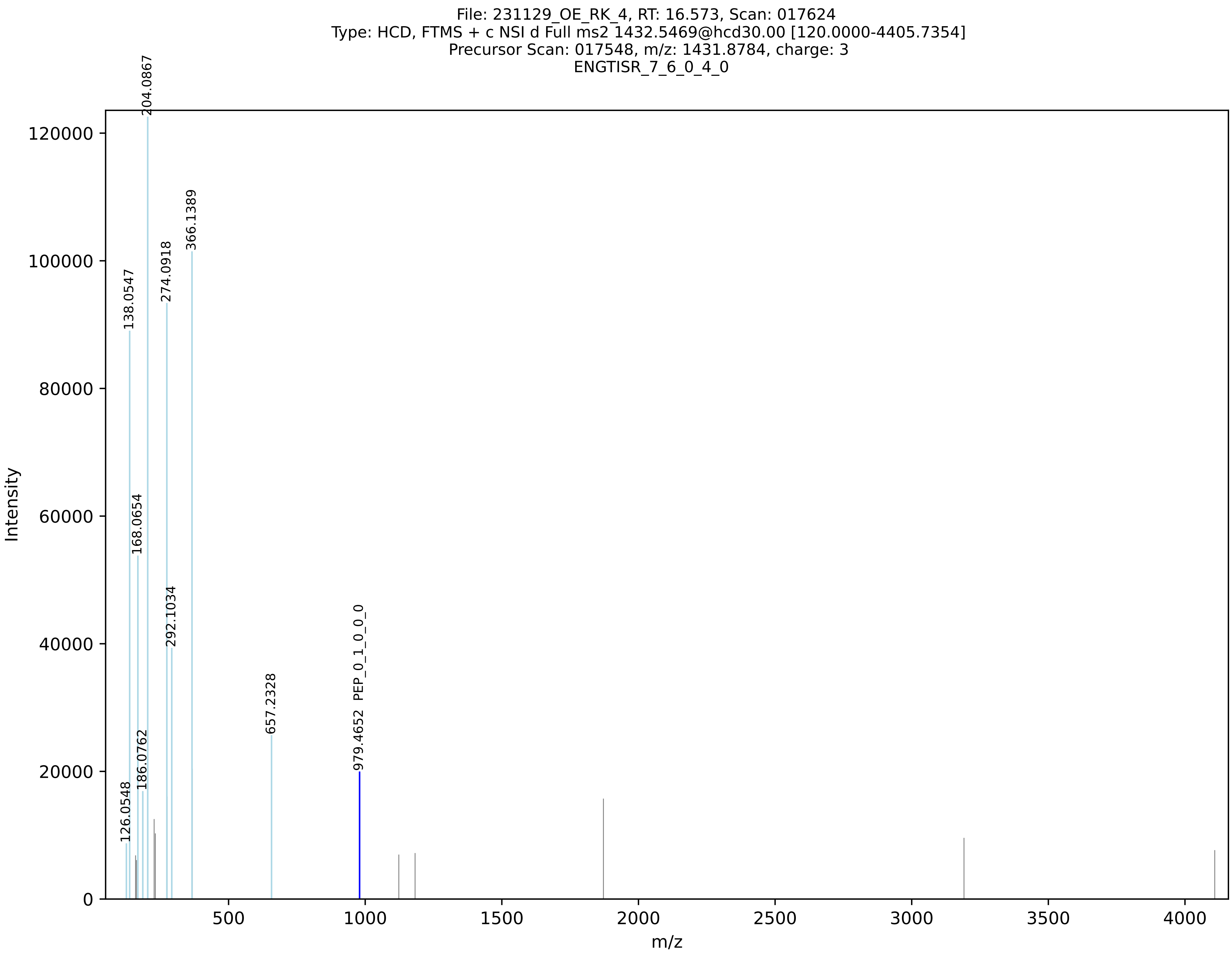

File: 231129_OE_RK_4, RT: 16.573, Scan: 017624
Type: HCD, FTMS + c NSI d Full ms2 1432.5469@hcd30.00 [120.0000-4405.7354]
Precursor Scan: 017548, m/z: 1431.8784, charge: 3
ENGTISR_7_6_0_4_0
Intensity
m/z
204.0867
366.1389
274.0918
138.0547
168.0654
292.1034
657.2328
979.4652 PEP_0_1_0_0_0
186.0762
126.0548
0
20000
40000
60000
80000
100000
120000
500
1000
1500
2000
2500
3000
3500
4000

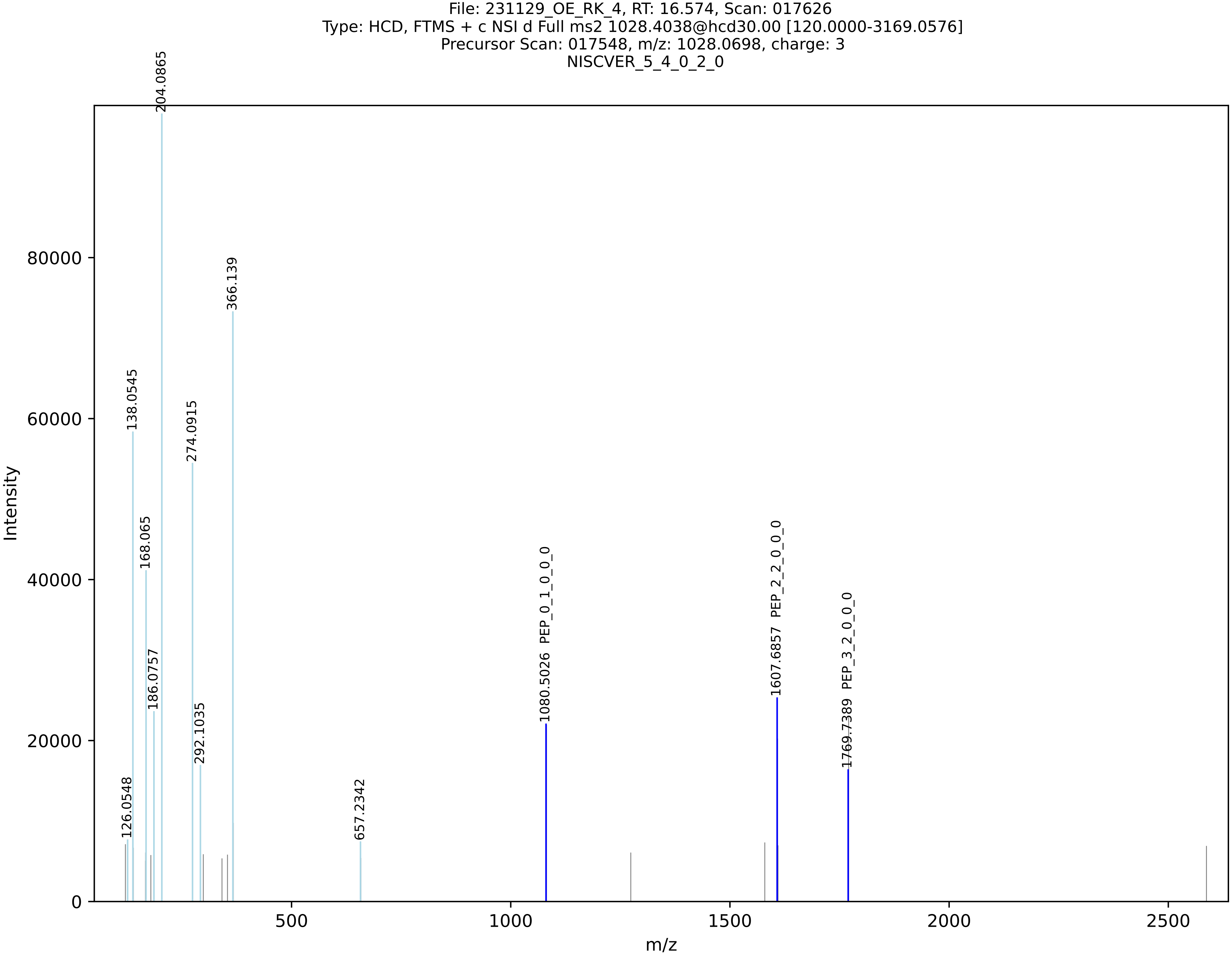
File: 231129_OE_RK_4, RT: 16.574, Scan: 017626
Type: HCD, FTMS + c NSI d Full ms2 1028.4038@hcd30.00 [120.0000-3169.0576]
Precursor Scan: 017548, m/z: 1028.0698, charge: 3
NISCVER_5_4_0_2_0
204.0865
366.139
138.0545
274.0915
168.065
186.0757
292.1035
126.0548
657.2342
1080.5026 PEP_0_1_0_0_0
1607.6857 PEP_2_2_0_0_0
1769.7389 PEP_3_2_0_0_0
Intensity
m/z
80000
60000
40000
20000
0
500
1000
1500
2000
2500

File: 231129_OE_RK_4, RT: 16.624, Scan: 017697
Type: HCD, FTMS + c NSI d Full ms2 1051.7449@hcd30.00 [120.0000-3240.4812]
Precursor Scan: 017621, m/z: 1051.4107, charge: 3
EDALNETR_5_4_0_2_0

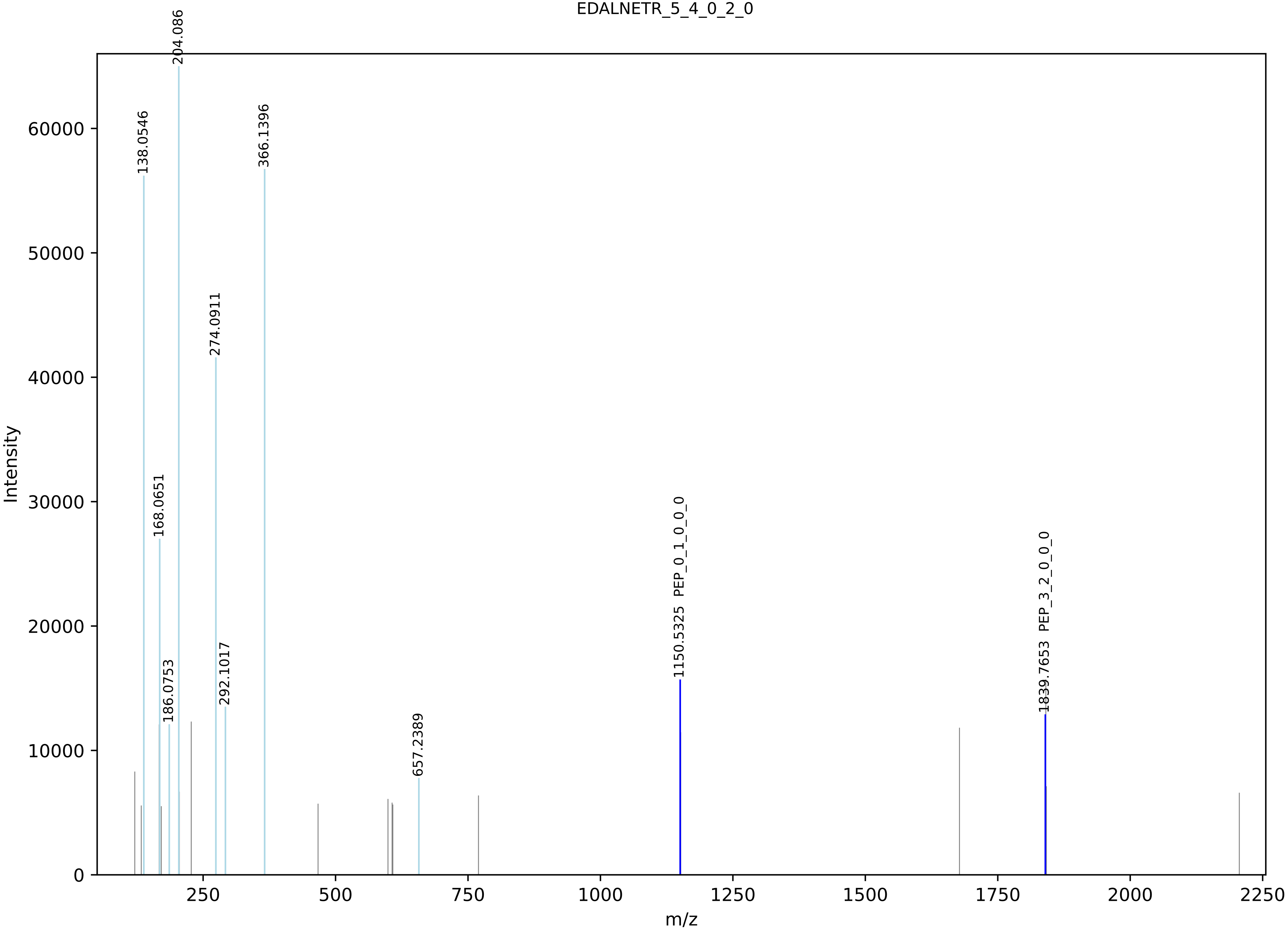

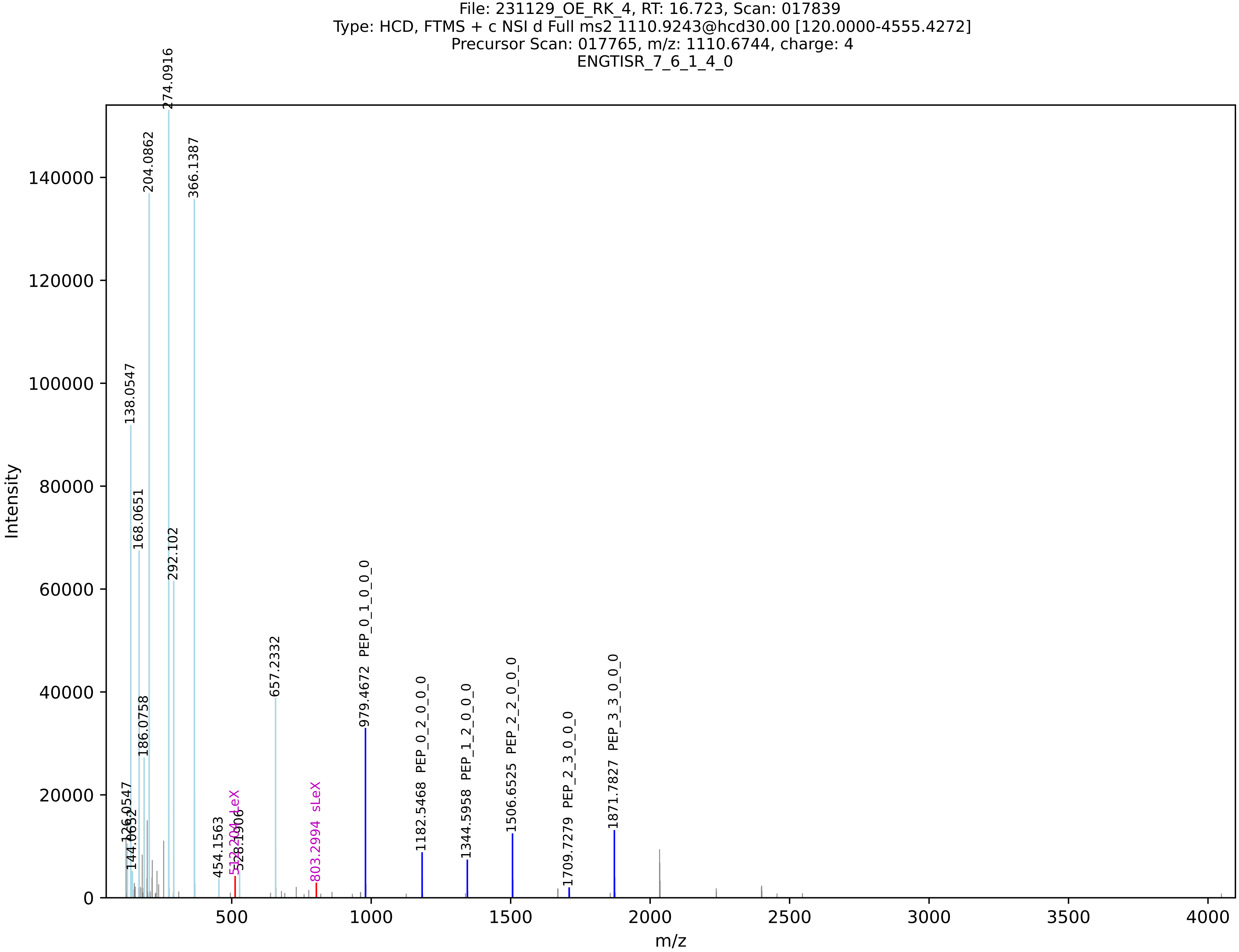

File: 231129_OE_RK_4, RT: 16.723, Scan: 017839
Type: HCD, FTMS + c NSI d Full ms2 1110.9243@hcd30.00 [120.0000-4555.4272]
Precursor Scan: 017765, m/z: 1110.6744, charge: 4
ENGTISR_7_6_1_4_0
Intensity
m/z
274.0916
204.0862
366.1387
138.0547
168.0651
292.102
186.0758
126.0547
144.0652
454.1563
512.204 LeX
528.1906
657.2332
803.2994 sLeX
979.4672 PEP_0_1_0_0_0
1182.5468 PEP_0_2_0_0_0
1344.5958 PEP_1_2_0_0_0
1506.6525 PEP_2_2_0_0_0
1709.7279 PEP_2_3_0_0_0
1871.7827 PEP_3_3_0_0_0

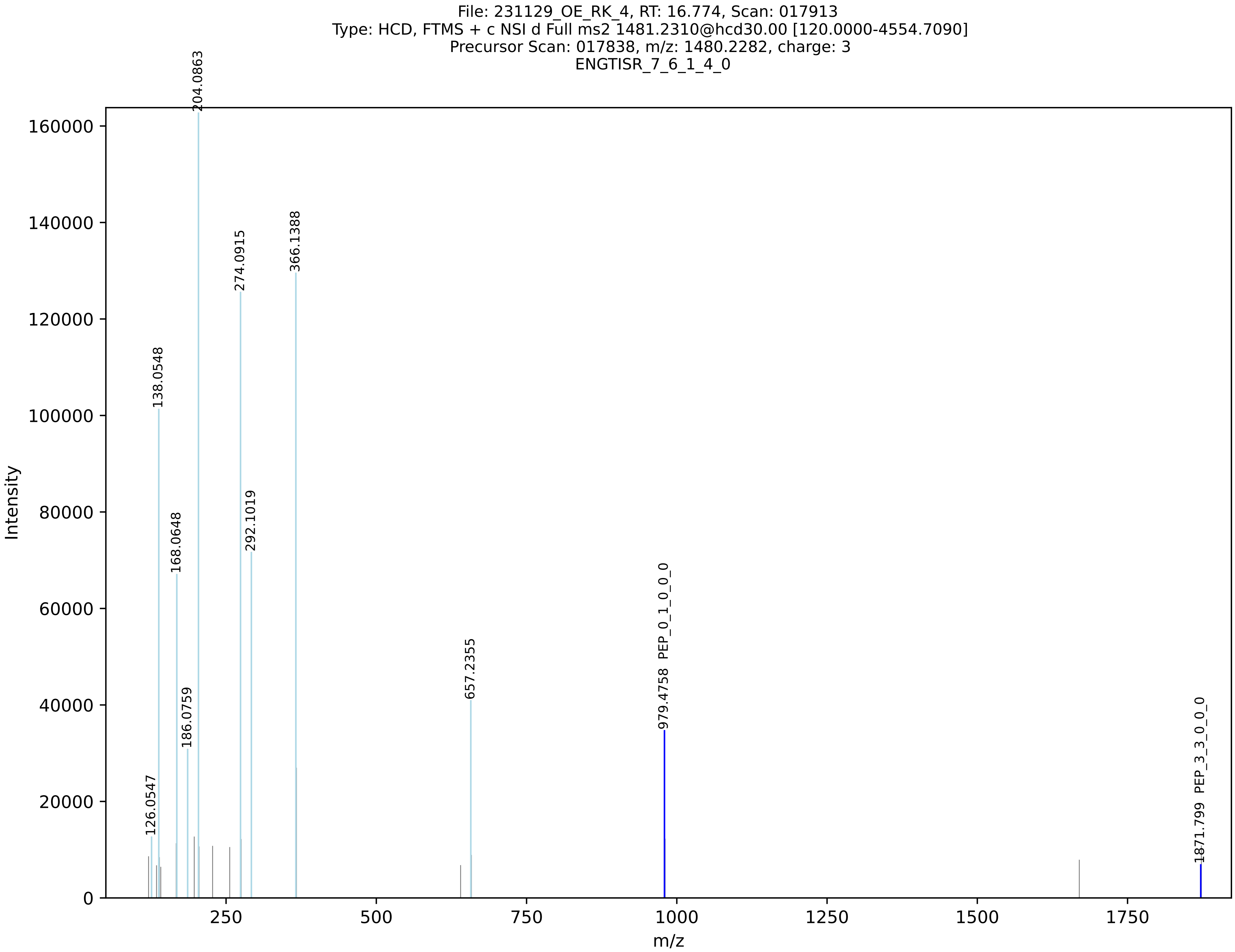

File: 231129_OE_RK_4, RT: 16.774, Scan: 017913
Type: HCD, FTMS + c NSI d Full ms2 1481.2310@hcd30.00 [120.0000-4554.7090]
Precursor Scan: 017838, m/z: 1480.2282, charge: 3
ENGTISR_7_6_1_4_0
Intensity
m/z
204.0863
366.1388
274.0915
138.0548
292.1019
168.0648
186.0759
126.0547
657.2355
979.4758 PEP_0_1_0_0_0
1871.799 PEP_3_3_0_0_0

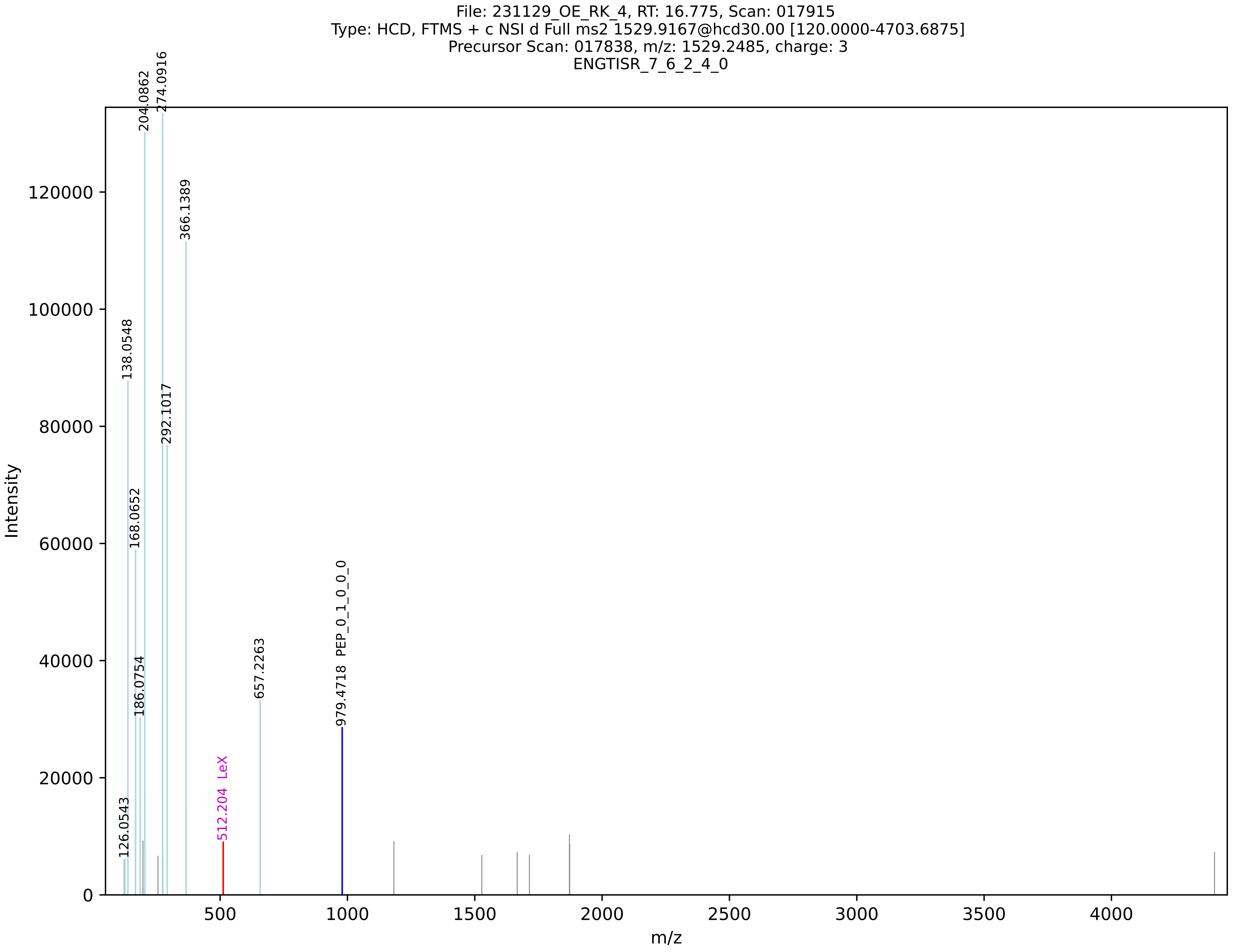
File: 231129_OE_RK_4, RT: 16.775, Scan: 017915
Type: HCD, FTMS + c NSI d Full ms2 1529.9167@hcd30.00 [120.0000-4703.6875]
Precursor Scan: 017838, m/z: 1529.2485, charge: 3
ENGTISR_7_6_2_4_0
Intensity
m/z
204.0862
274.0916
366.1389
138.0548
292.1017
168.0652
186.0754
126.0543
512.204 LeX
657.2263
979.4718 PEP_0_1_0_0_0
0
20000
40000
60000
80000
100000
120000
500
1000
1500
2000
2500
3000
3500
4000

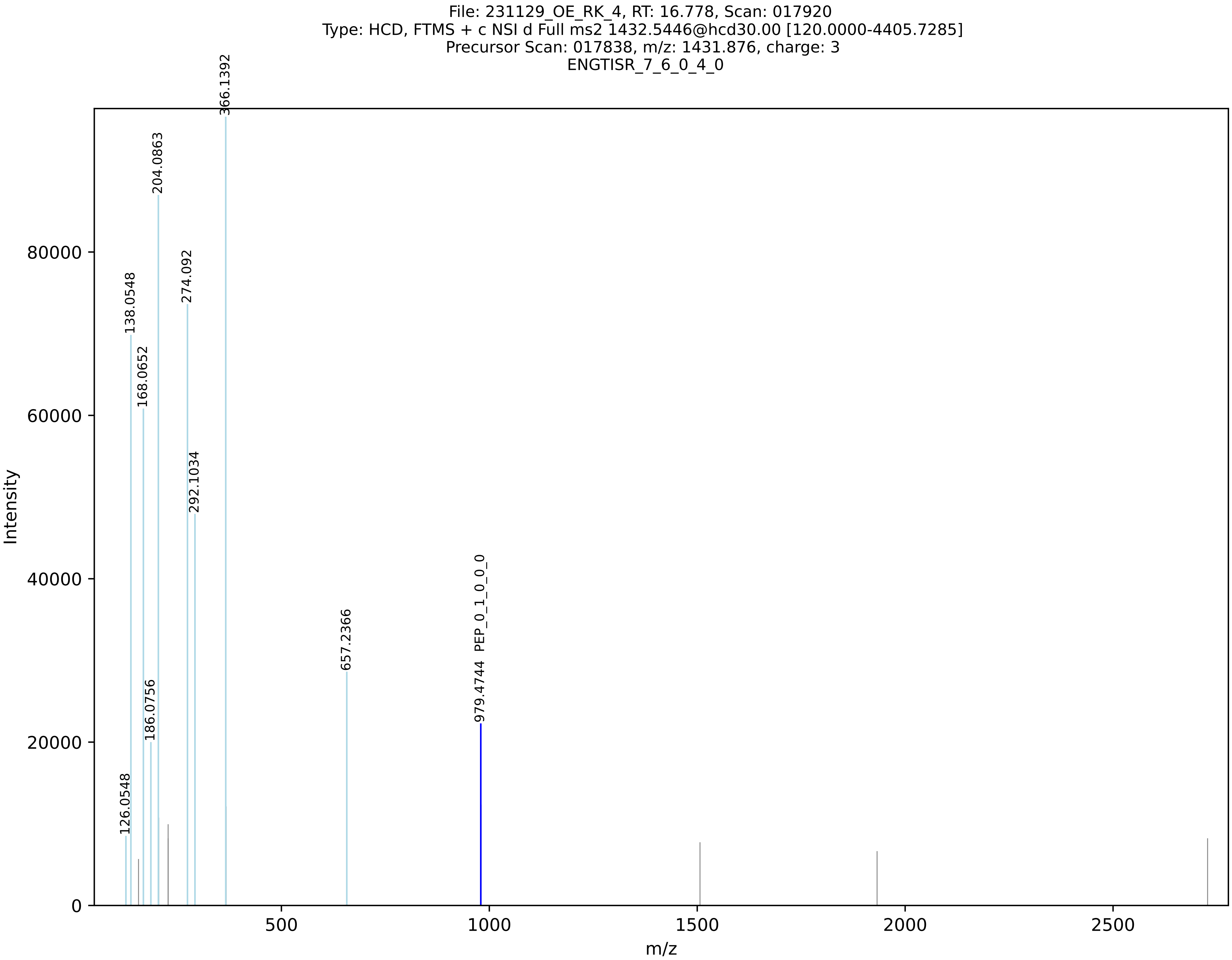
File: 231129_OE_RK_4, RT: 16.778, Scan: 017920
Type: HCD, FTMS + c NSI d Full ms2 1432.5446@hcd30.00 [120.0000-4405.7285]
Precursor Scan: 017838, m/z: 1431.876, charge: 3
ENGTISR_7_6_0_4_0
366.1392
204.0863
274.092
138.0548
168.0652
292.1034
657.2366
979.4744 PEP_0_1_0_0_0
186.0756
126.0548
Intensity
m/z
0
20000
40000
60000
80000
500
1000
1500
2000
2500

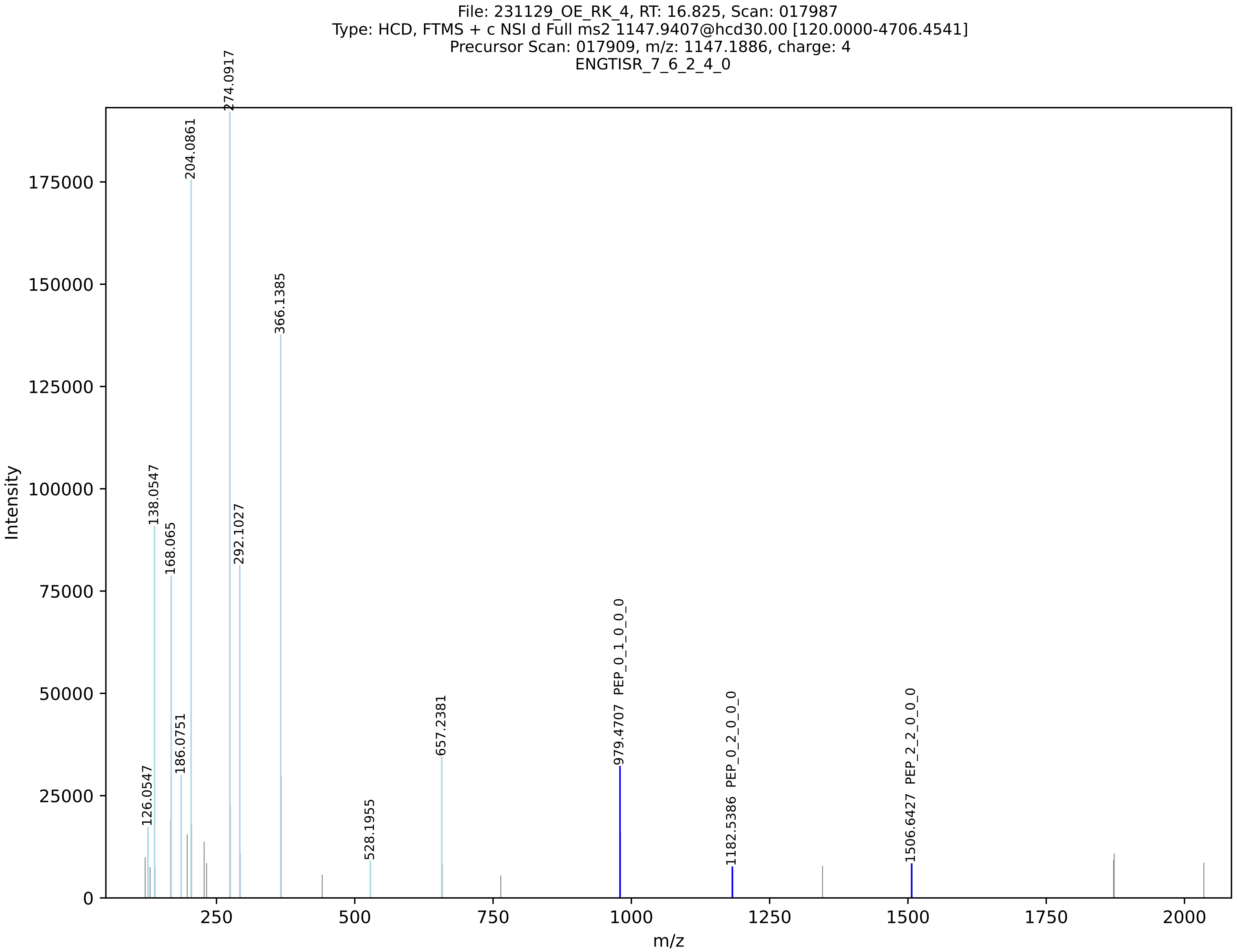
File: 231129_OE_RK_4, RT: 16.825, Scan: 017987
Type: HCD, FTMS + c NSI d Full ms2 1147.9407@hcd30.00 [120.0000-4706.4541]
Precursor Scan: 017909, m/z: 1147.1886, charge: 4
ENGTISR_7_6_2_4_0
274.0917
204.0861
366.1385
138.0547
168.065
292.1027
979.4707 PEP_0_1_0_0_0_
186.0751
126.0547
657.2381
528.1955
1182.5386 PEP_0_2_0_0_0
1506.6427 PEP_2_2_0_0_0_
Intensity
m/z
0
25000
50000
75000
100000
125000
150000
175000
250
500
750
1000
1250
1500
1750
2000

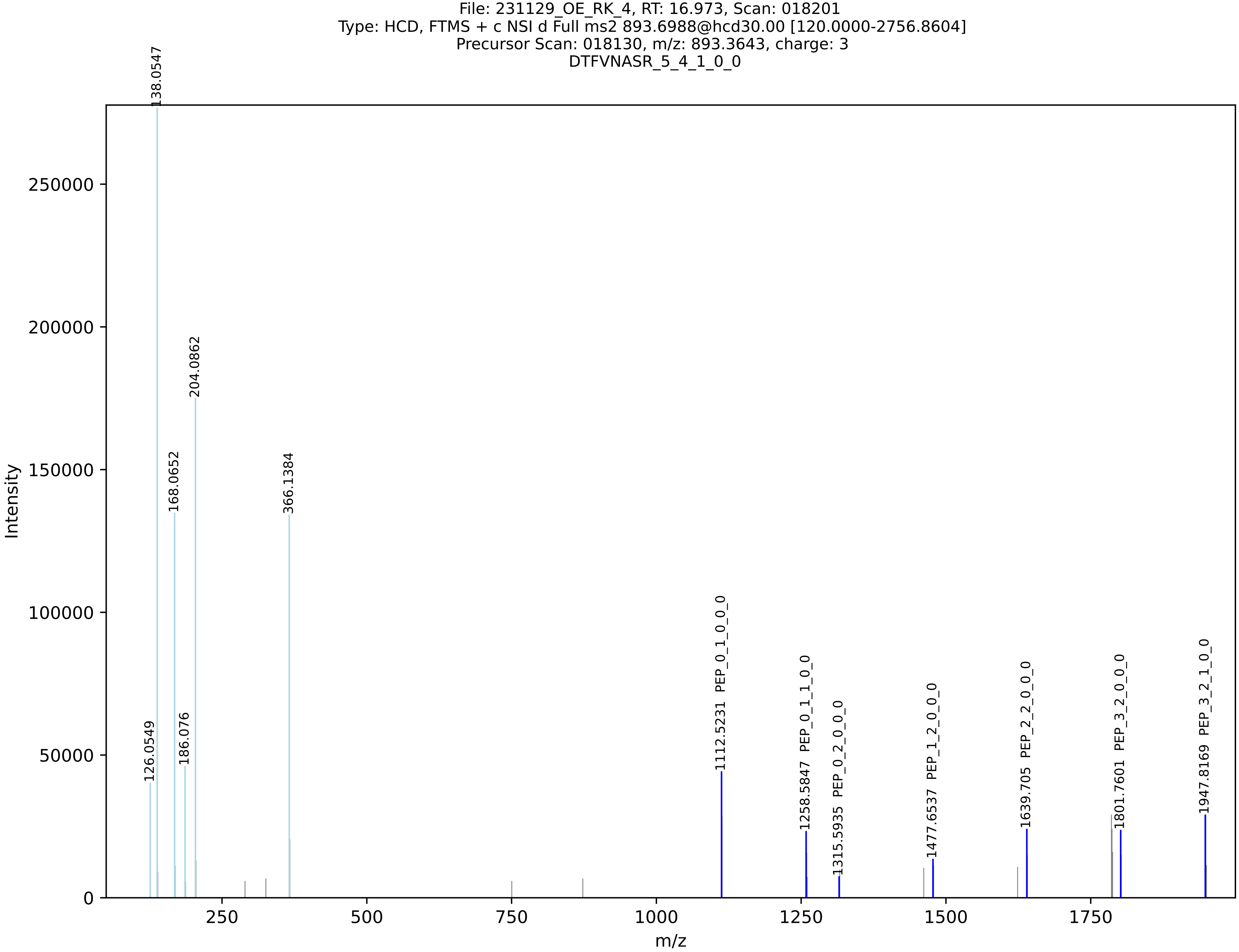

File: 231129_OE_RK_4, RT: 16.973, Scan: 018201
Type: HCD, FTMS + c NSI d Full ms2 893.6988@hcd30.00 [120.0000-2756.8604]
Precursor Scan: 018130, m/z: 893.3643, charge: 3
DTFVNASR_5_4_1_0_0
Intensity
m/z
0
50000
100000
150000
200000
250000
250
500
750
1000
1250
1500
1750
138.0547
204.0862
168.0652
366.1384
126.0549
186.076
1112.5231 PEP_0_1_0_0_0
1258.5847 PEP_0_1_1_0_0
1315.5935 PEP_0_2_0_0_0
1477.6537 PEP_1_2_0_0_0
1639.705 PEP_2_2_0_0_0
1801.7601 PEP_3_2_0_0_0
1947.8169 PEP_3_2_1_0_0

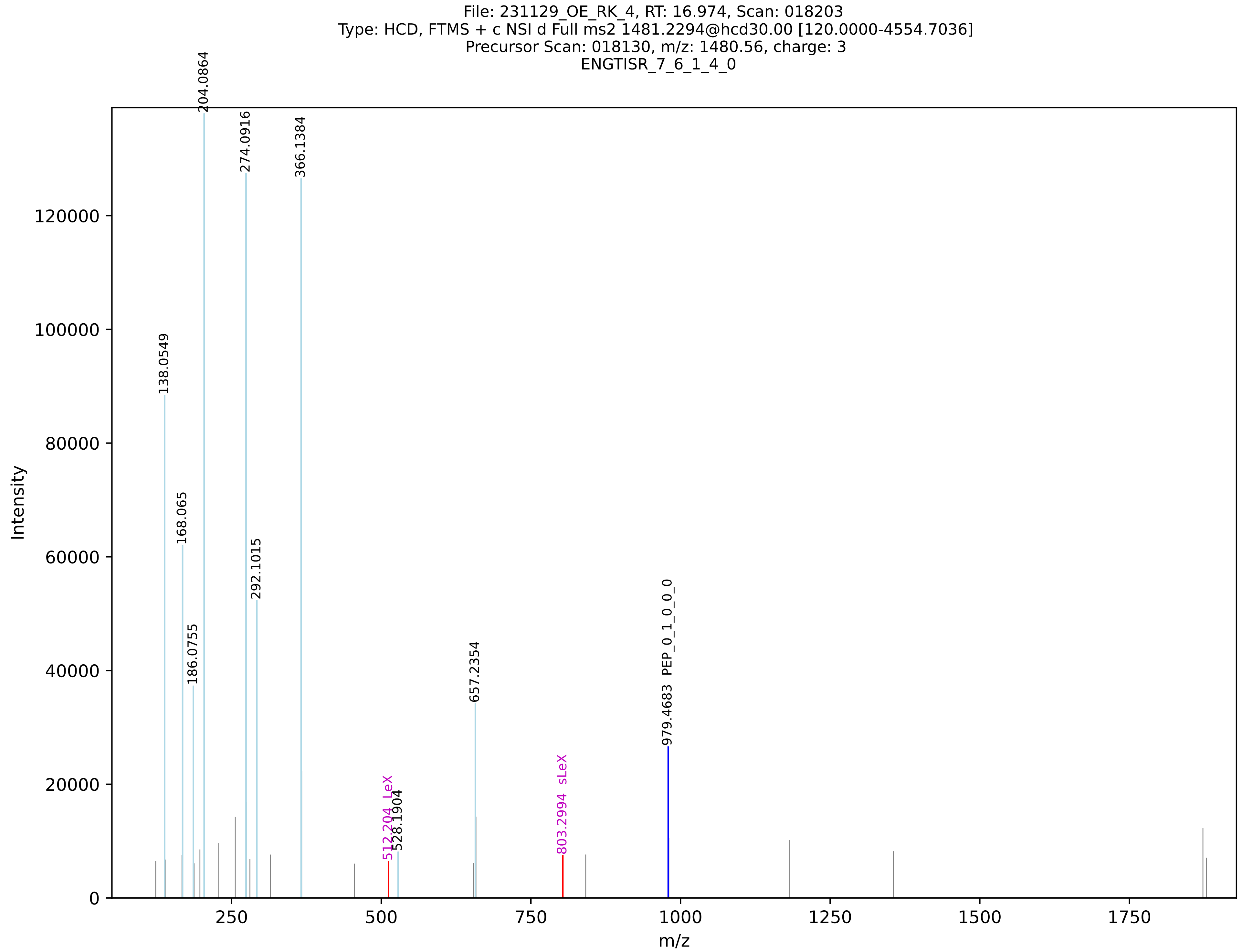

File: 231129_OE_RK_4, RT: 16.974, Scan: 018203
Type: HCD, FTMS + c NSI d Full ms2 1481.2294@hcd30.00 [120.0000-4554.7036]
Precursor Scan: 018130, m/z: 1480.56, charge: 3
ENGTISR_7_6_1_4_0
Intensity
m/z
204.0864
274.0916
366.1384
138.0549
168.065
292.1015
186.0755
657.2354
979.4683 PEP_0_1_0_0_0
512.204 LeX
528.1904
803.2994 sLeX
0
20000
40000
60000
80000
100000
120000
250
500
750
1000
1250
1500
1750

File: 231129_OE_RK_4, RT: 16.976, Scan: 018205
Type: HCD, FTMS + c NSI d Full ms2 1530.2493@hcd30.00 [120.0000-4704.7046]
Precursor Scan: 018130, m/z: 1528.9137, charge: 3
ENGTISR_7_6_2_4_0

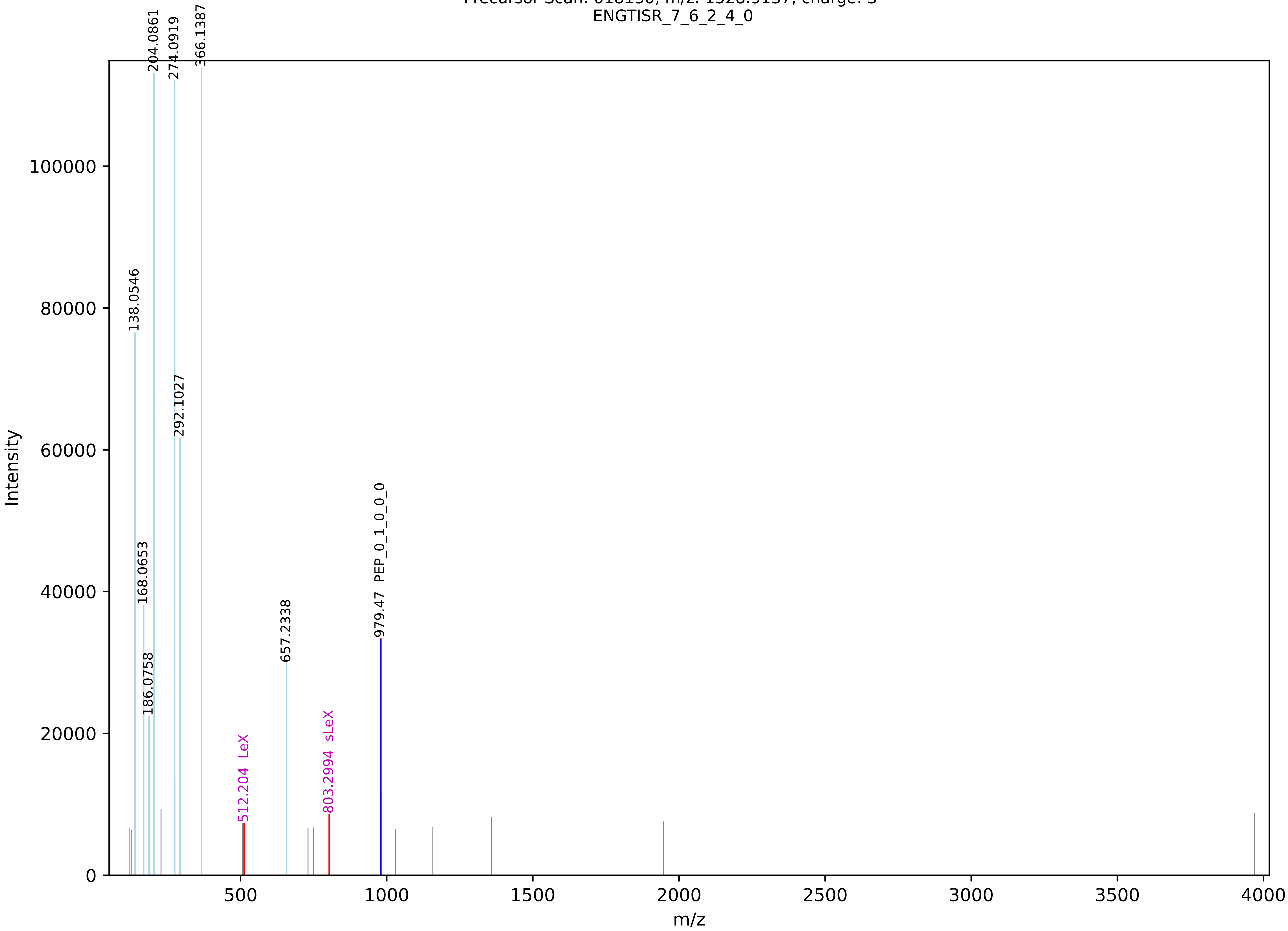

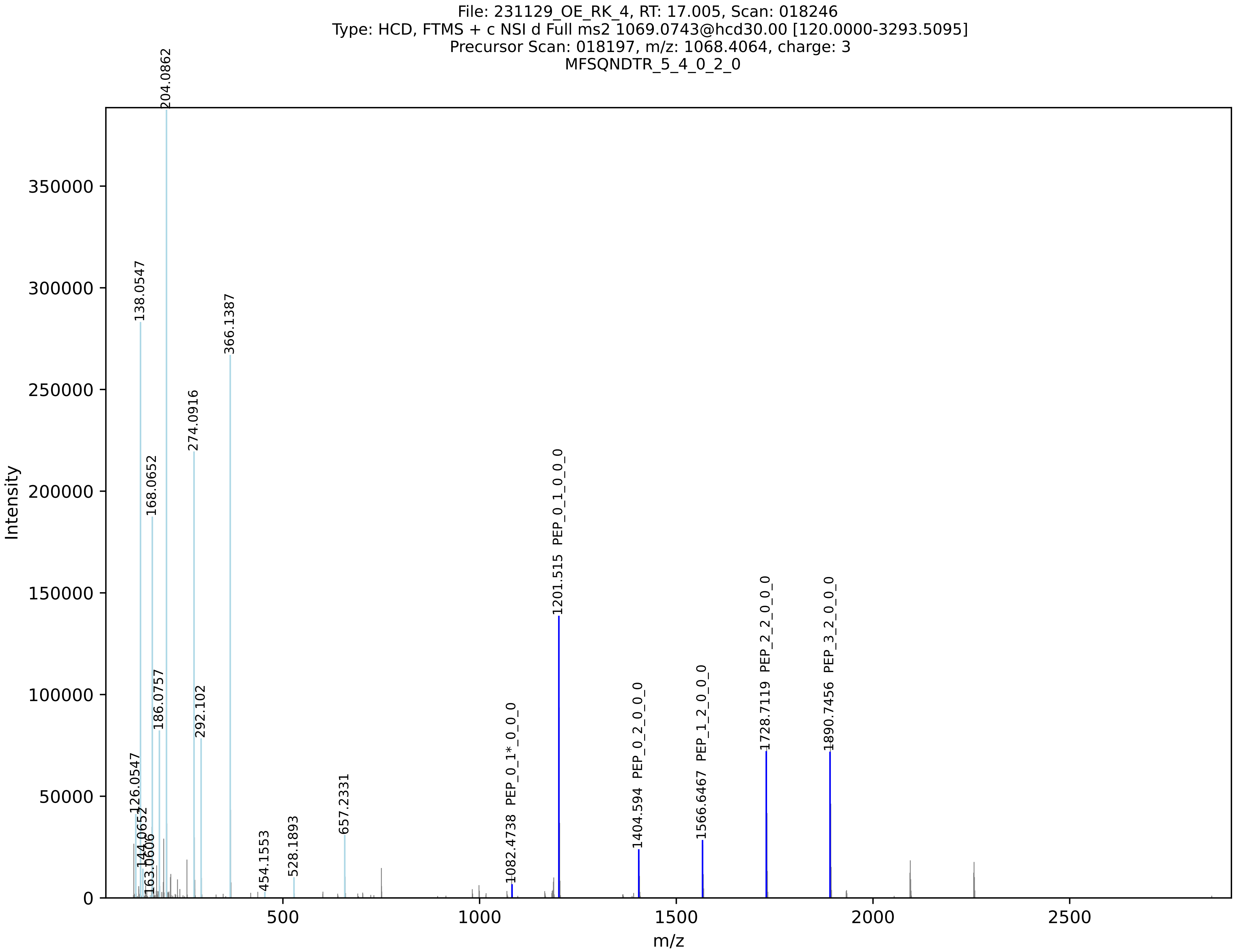

File: 231129_OE_RK_4, RT: 17.005, Scan: 018246
Type: HCD, FTMS + c NSI d Full ms2 1069.0743@hcd30.00 [120.0000-3293.5095]
Precursor Scan: 018197, m/z: 1068.4064, charge: 3
MFSQNDTR_5_4_0_2_0
Intensity
m/z
204.0862
138.0547
366.1387
274.0916
168.0652
186.0757
292.102
126.0547
144.0652
163.0606
454.1553
528.1893
657.2331
1082.4738 PEP_0_1*_0_0_0
1201.515 PEP_0_1_0_0_0
1404.594 PEP_0_2_0_0_0
1566.6467 PEP_1_2_0_0_0
1728.7119 PEP_2_2_0_0_0
1890.7456 PEP_3_2_0_0_0

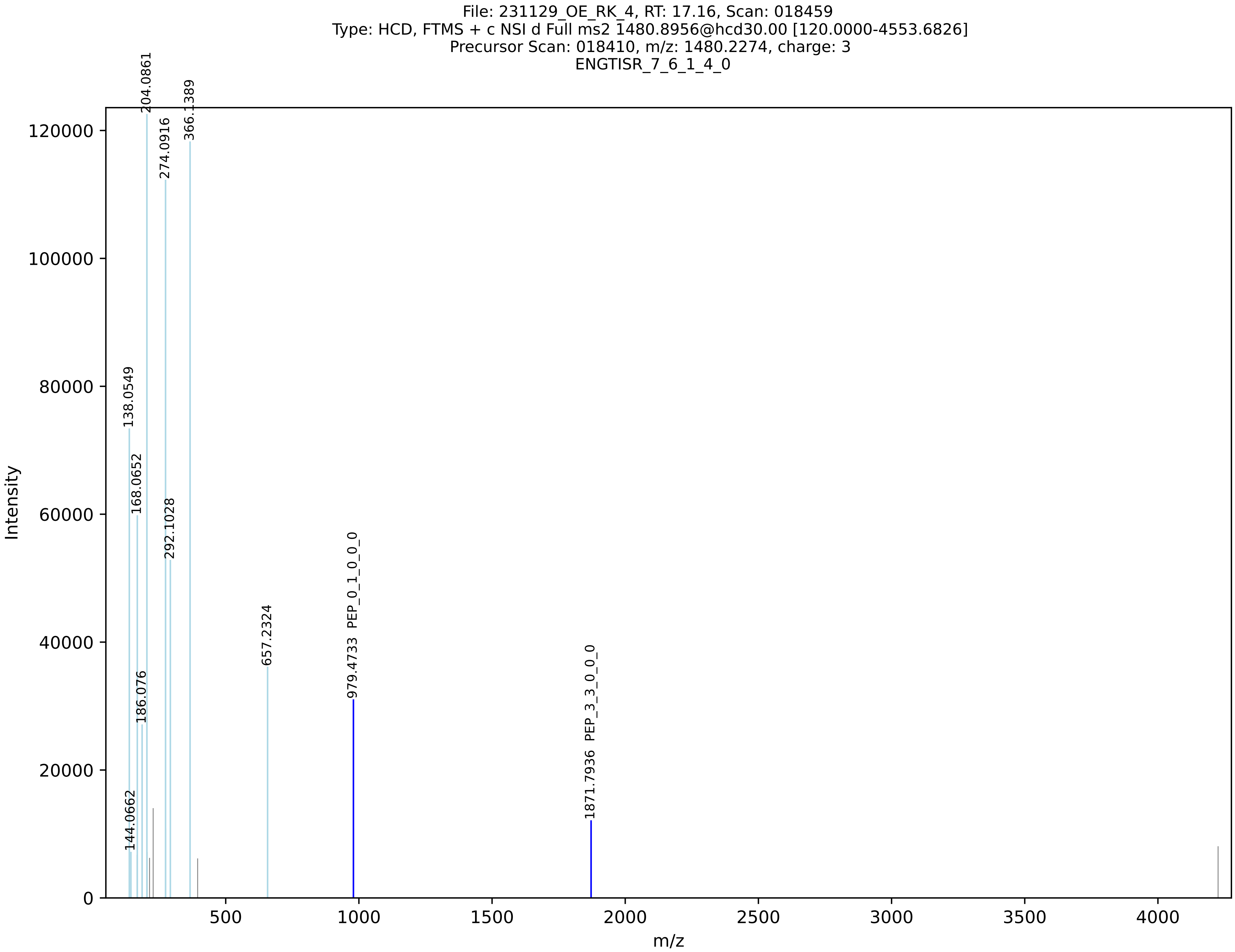
File: 231129_OE_RK_4, RT: 17.16, Scan: 018459
Type: HCD, FTMS + c NSI d Full ms2 1480.8956@hcd30.00 [120.0000-4553.6826]
Precursor Scan: 018410, m/z: 1480.2274, charge: 3
ENGTISR_7_6_1_4_0
Intensity
m/z
204.0861
366.1389
274.0916
138.0549
168.0652
292.1028
657.2324
979.4733 PEP_0_1_0_0_0
1871.7936 PEP_3_3_0_0_0
186.076
144.0662
0
20000
40000
60000
80000
100000
120000
500
1000
1500
2000
2500
3000
3500
4000

File: 231129_OE_RK_4, RT: 17.202, Scan: 018521
Type: HCD, FTMS + c NSI d Full ms2 845.0132@hcd30.00 [120.0000-2607.8823]
Precursor Scan: 018456, m/z: 844.6789, charge: 3
DTFVNASR_5_4_0_0_0

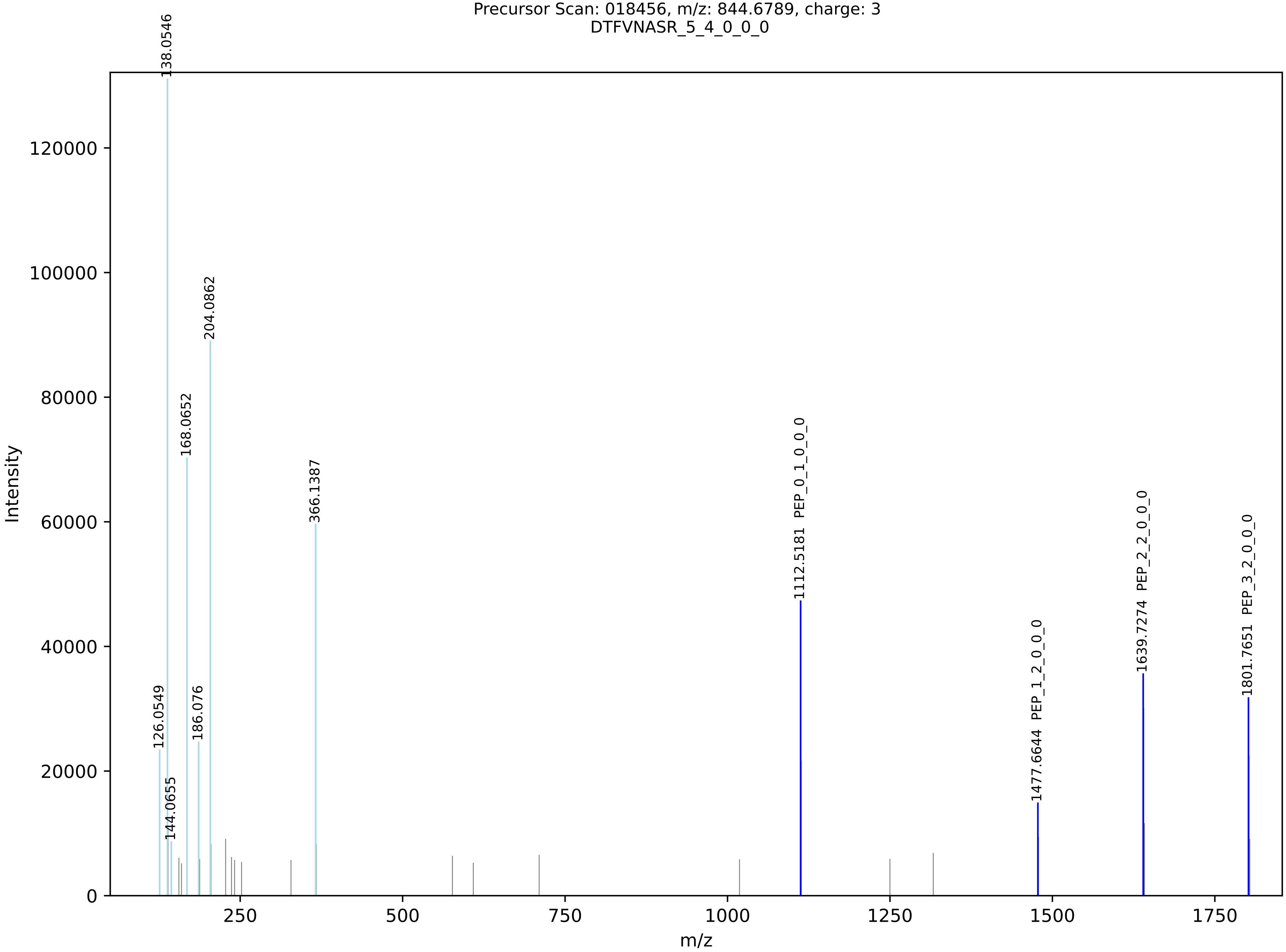

File: 231129_OE_RK_4, RT: 17.203, Scan: 018523
Type: HCD, FTMS + c NSI d Full ms2 1147.6887@hcd30.00 [120.0000-4705.4263]
Precursor Scan: 018456, m/z: 1147.1879, charge: 4
ENGTISR_7_6_2_4_0

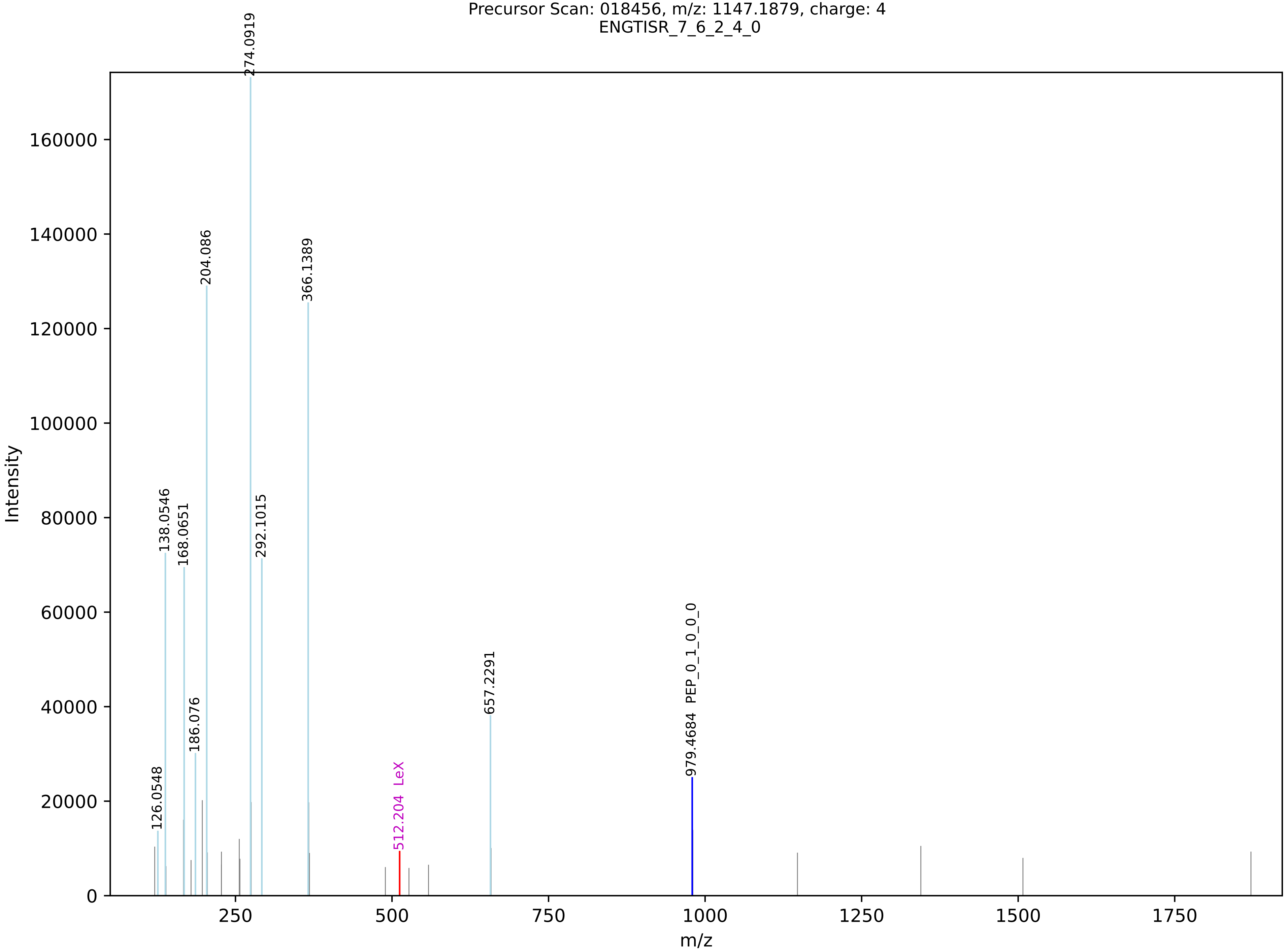

File: 231129_OE_RK_4, RT: 17.21, Scan: 018534
Type: HCD, FTMS + c NSI d Full ms2 1074.0784@hcd30.00 [120.0000-3308.8218]
Precursor Scan: 018456, m/z: 1074.0784, charge: 3
MFSQNDTR_6_4_1_1_0

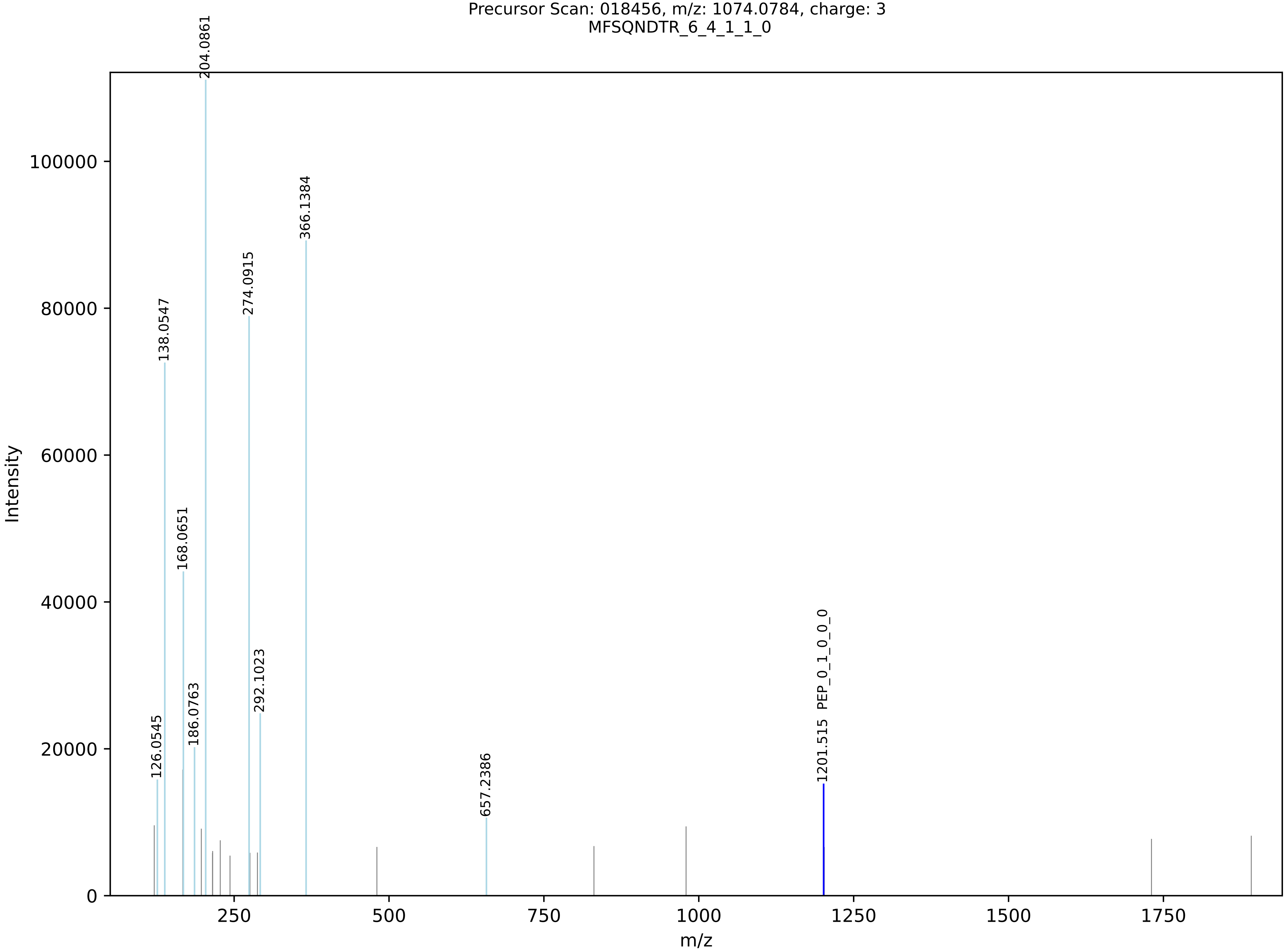

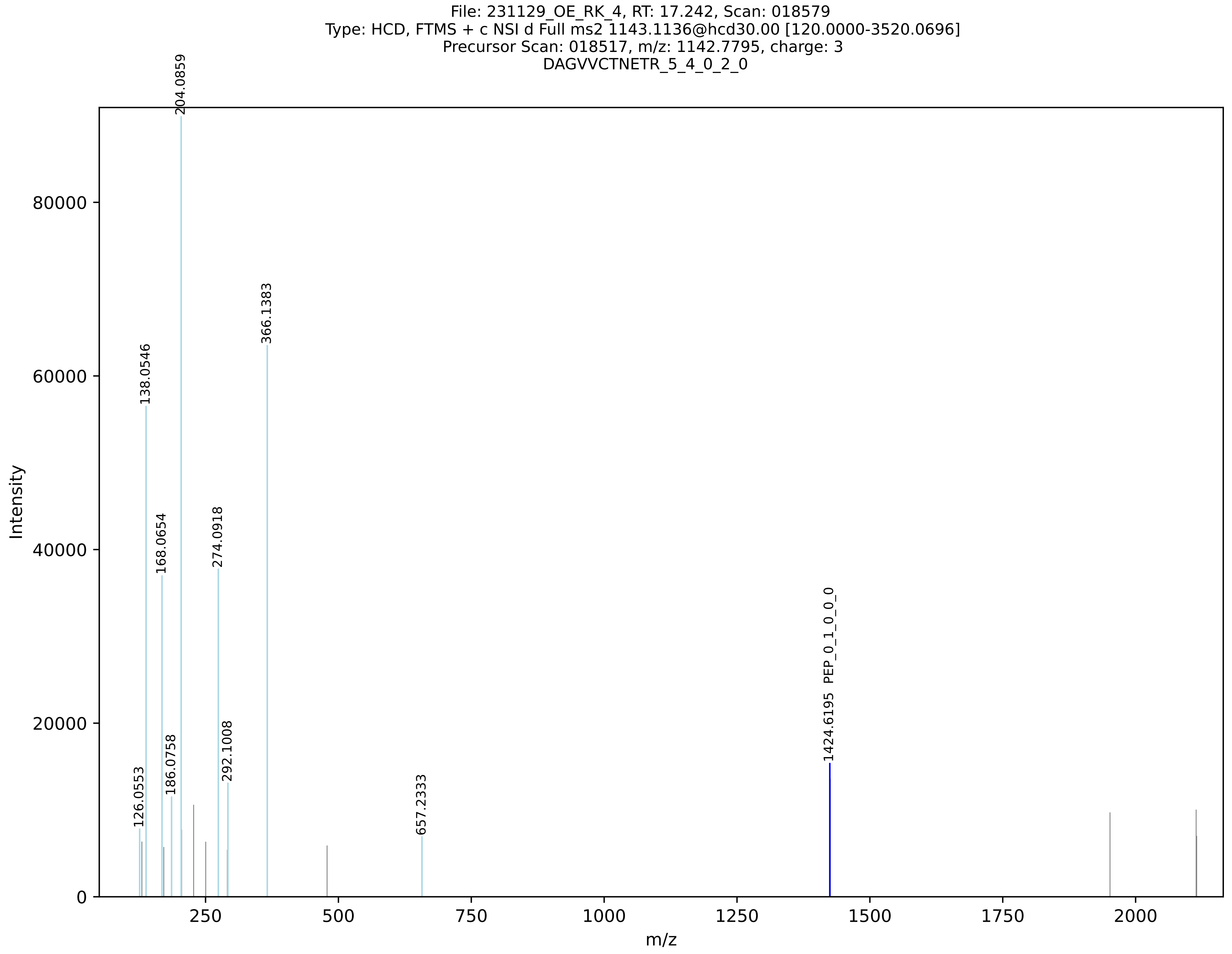
File: 231129_OE_RK_4, RT: 17.242, Scan: 018579
Type: HCD, FTMS + c NSI d Full ms2 1143.1136@hcd30.00 [120.0000-3520.0696]
Precursor Scan: 018517, m/z: 1142.7795, charge: 3
DAGVVCTNETR_5_4_0_2_0
204.0859
366.1383
138.0546
168.0654
274.0918
186.0758
292.1008
126.0553
657.2333
1424.6195 PEP_0_1_0_0_0
Intensity
m/z
0
20000
40000
60000
80000
250
500
750
1000
1250
1500
1750
2000

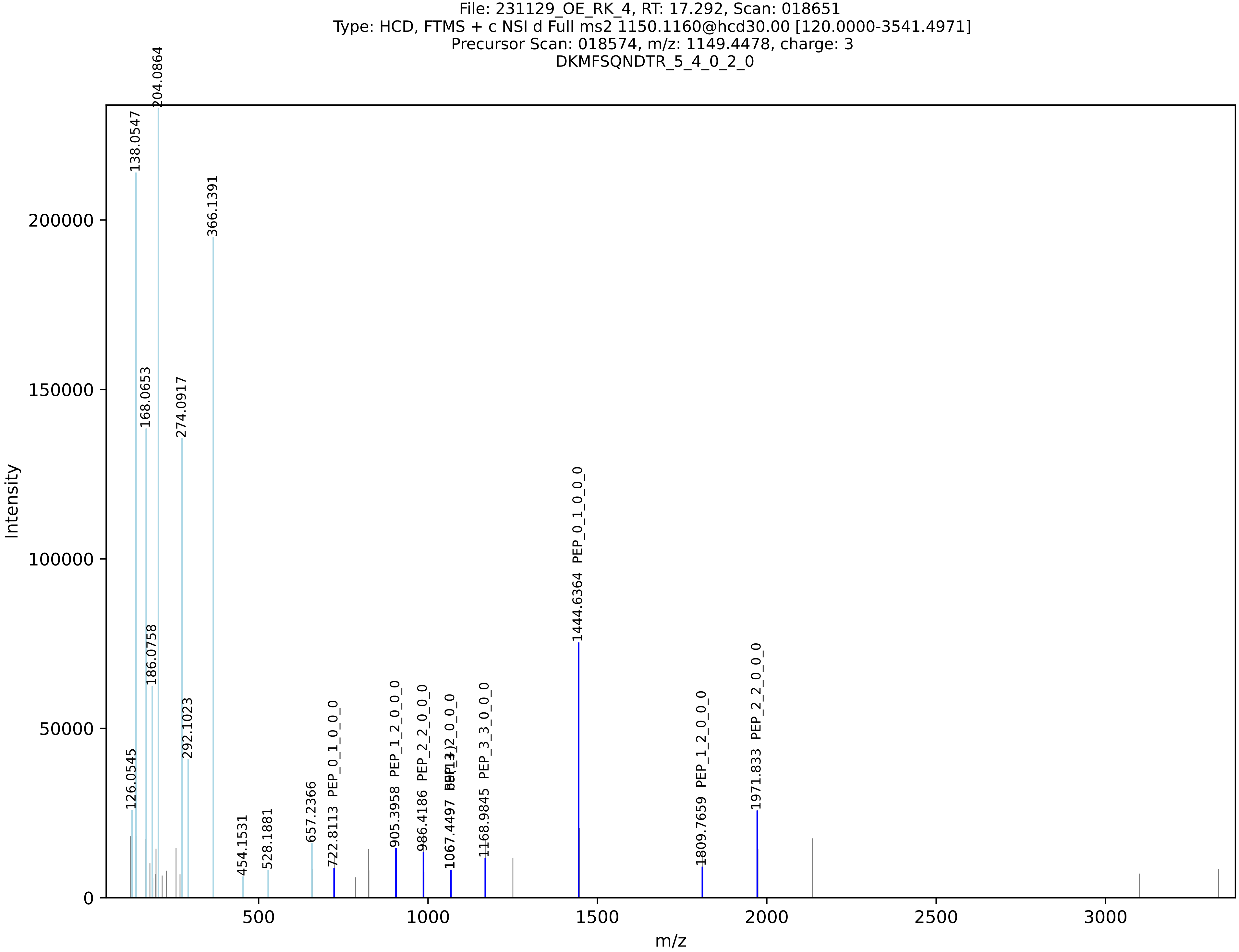
File: 231129_OE_RK_4, RT: 17.292, Scan: 018651
Type: HCD, FTMS + c NSI d Full ms2 1150.1160@hcd30.00 [120.0000-3541.4971]
Precursor Scan: 018574, m/z: 1149.4478, charge: 3
DKMFSQNDTR_5_4_0_2_0
Intensity
m/z
0
50000
100000
150000
200000
500
1000
1500
2000
2500
3000
204.0864
138.0547
366.1391
168.0653
274.0917
186.0758
292.1023
126.0545
454.1531
528.1881
657.2366
722.8113 PEP_0_1_0_0_0
905.3958 PEP_1_2_0_0_0
986.4186 PEP_2_2_0_0_0
1067.4497
1168.9845 PEP_3_3_0_0_0
1444.6364 PEP_0_1_0_0_0
1809.7659 PEP_1_2_0_0_0
1971.833 PEP_2_2_0_0_0

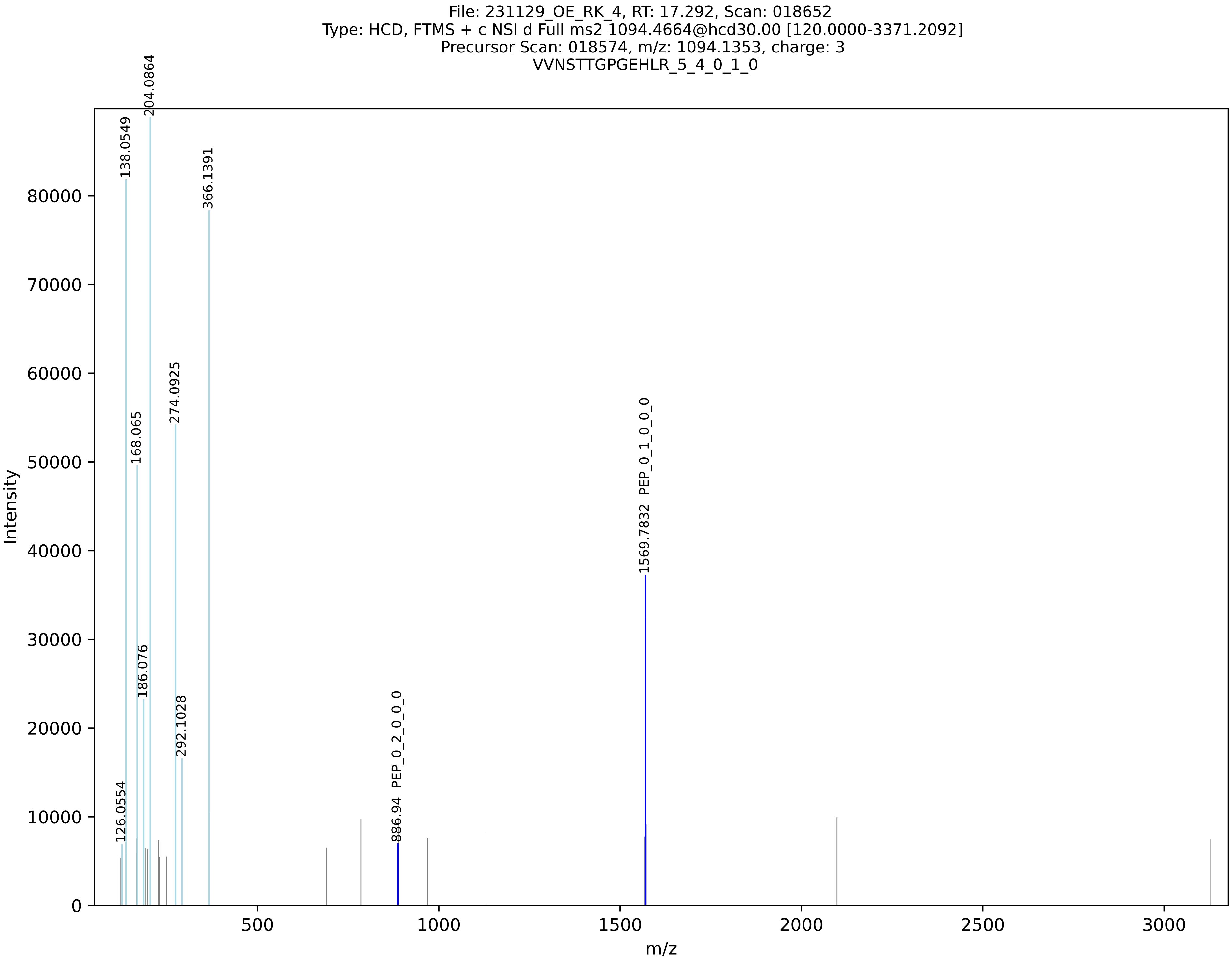
File: 231129_OE_RK_4, RT: 17.292, Scan: 018652
Type: HCD, FTMS + c NSI d Full ms2 1094.4664@hcd30.00 [120.0000-3371.2092]
Precursor Scan: 018574, m/z: 1094.1353, charge: 3
VVNSTTGPGEHLR_5_4_0_1_0
Intensity
m/z
204.0864
138.0549
366.1391
274.0925
168.065
186.076
292.1028
126.0554
886.94 PEP_0_2_0_0_0
1569.7832 PEP_0_1_0_0_0
0
10000
20000
30000
40000
50000
60000
70000
80000
500
1000
1500
2000
2500
3000

File: 231129_OE_RK_4, RT: 17.344, Scan: 018728
Type: HCD, FTMS + c NSI d Full ms2 1481.5667@hcd30.00 [120.0000-4555.7358]
Precursor Scan: 018648, m/z: 1480.5632, charge: 3
ENGTISR_7_6_1_4_0

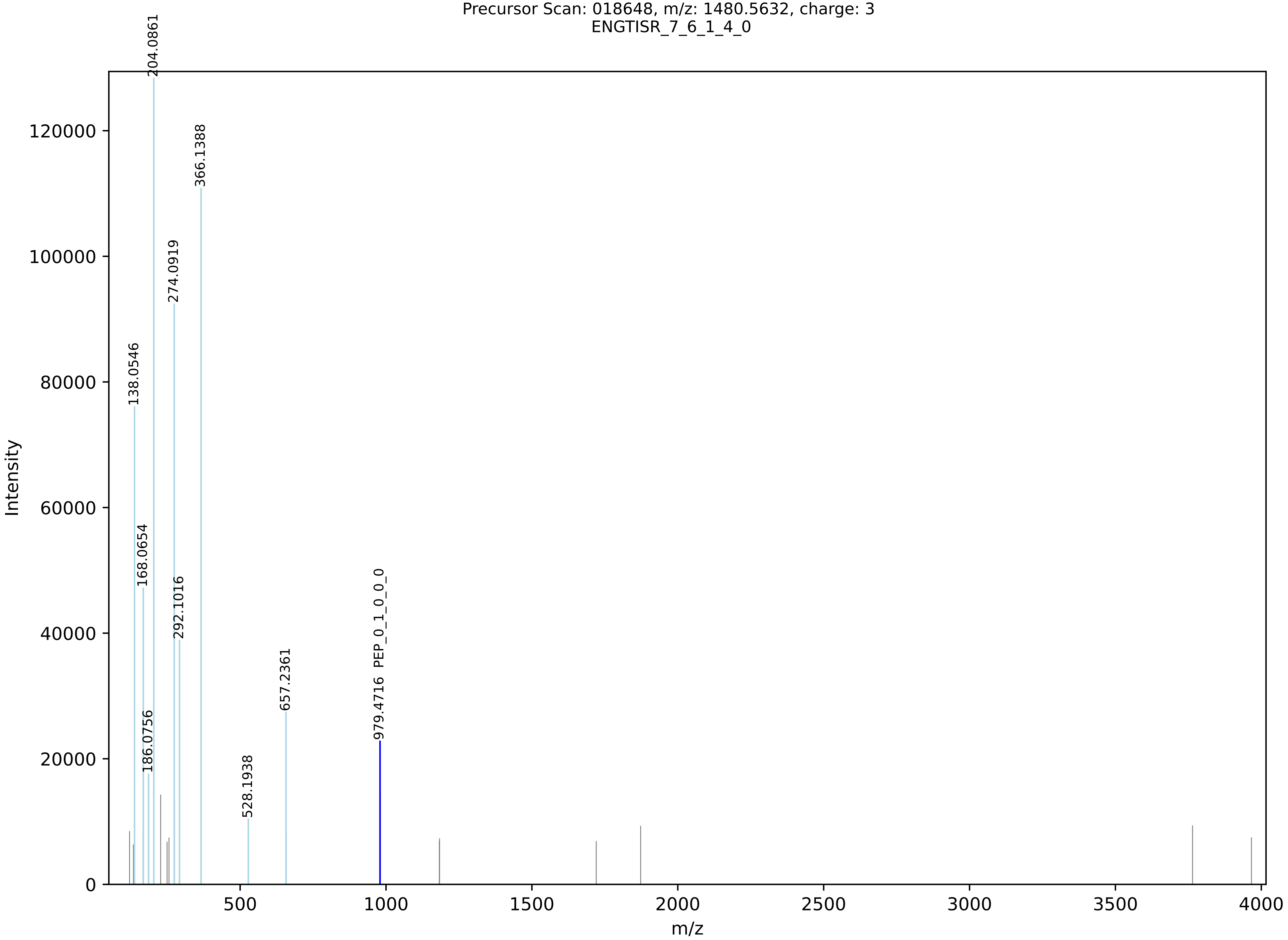

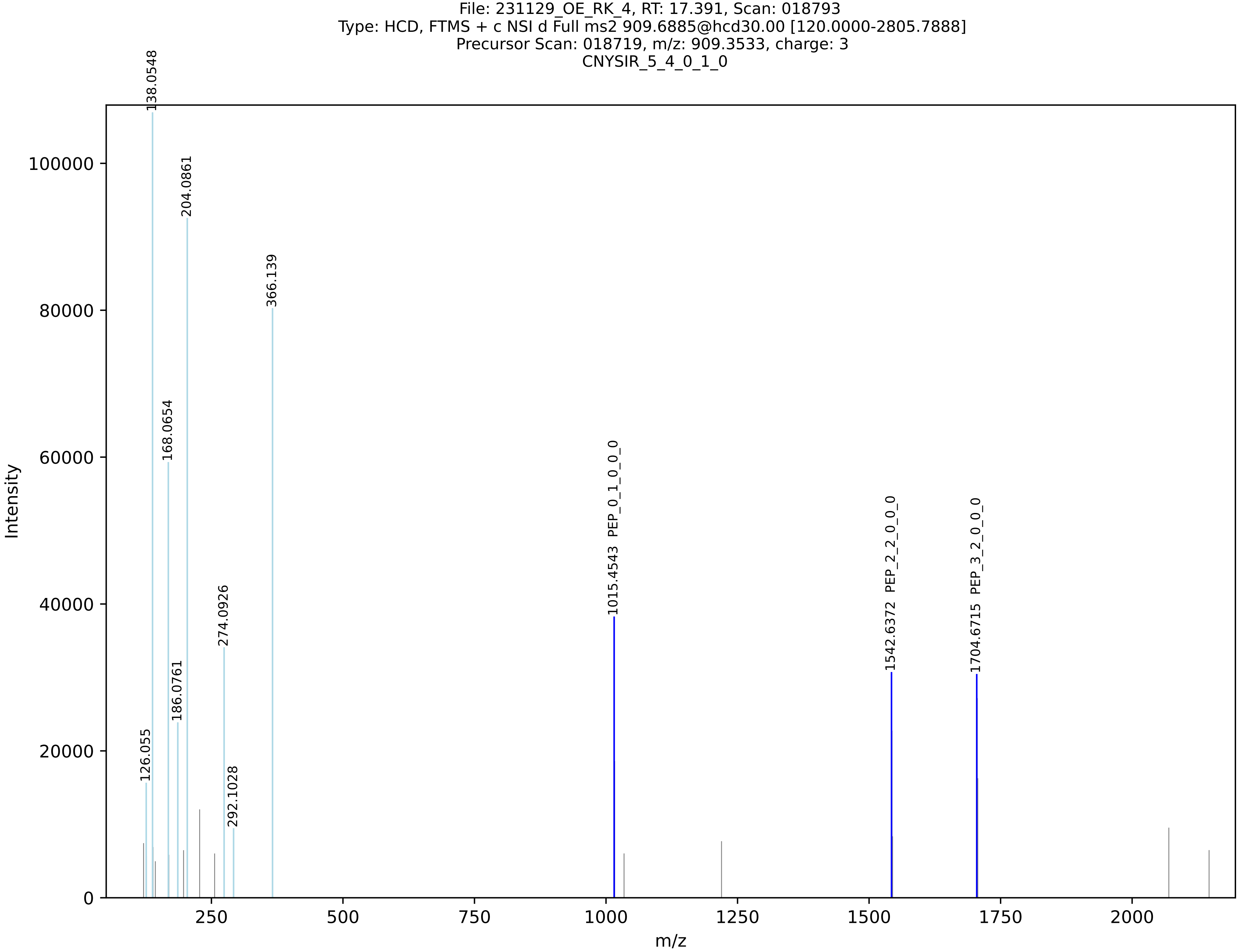

File: 231129_OE_RK_4, RT: 17.391, Scan: 018793
Type: HCD, FTMS + c NSI d Full ms2 909.6885@hcd30.00 [120.0000-2805.7888]
Precursor Scan: 018719, m/z: 909.3533, charge: 3
CNYSIR_5_4_0_1_0
138.0548
204.0861
366.139
168.0654
186.0761
274.0926
126.055
292.1028
1015.4543 PEP_0_1_0_0_0
1542.6372 PEP_2_2_0_0_0
1704.6715 PEP_3_2_0_0_0
Intensity
m/z
0
20000
40000
60000
80000
100000
250
500
750
1000
1250
1500
1750
2000

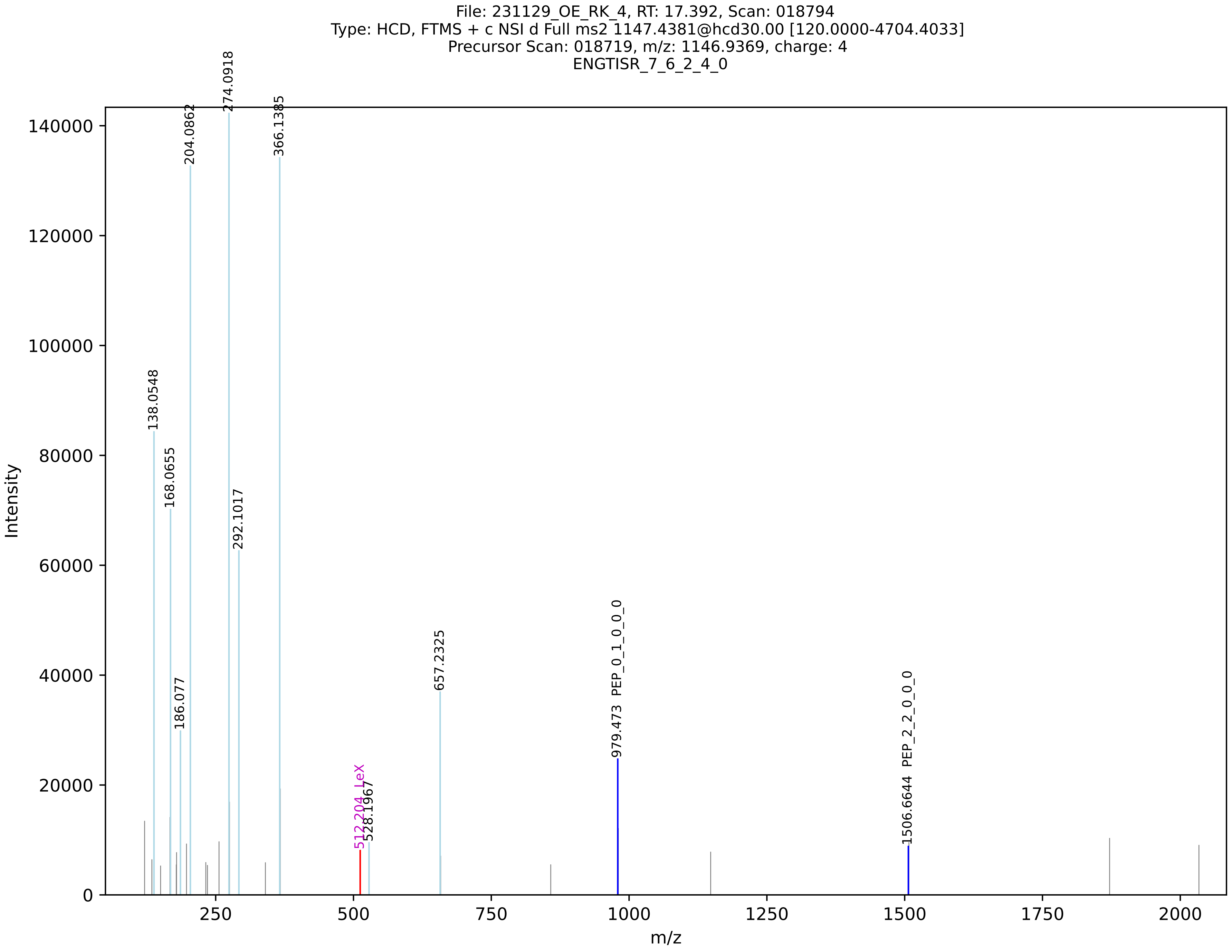
File: 231129_OE_RK_4, RT: 17.392, Scan: 018794
Type: HCD, FTMS + c NSI d Full ms2 1147.4381@hcd30.00 [120.0000-4704.4033]
Precursor Scan: 018719, m/z: 1146.9369, charge: 4
ENGTISR_7_6_2_4_0
274.0918
366.1385
204.0862
138.0548
168.0655
292.1017
186.077
512.204_LeX
528.1967
657.2325
979.473 PEP_0_1_0_0_0
1506.6644 PEP_2_2_0_0_0
Intensity
m/z
0
20000
40000
60000
80000
100000
120000
140000
250
500
750
1000
1250
1500
1750
2000

File: 231129_OE_RK_4, RT: 17.394, Scan: 018797
Type: HCD, FTMS + c NSI d Full ms2 1181.4536@hcd30.00 [120.0000-3637.3899]
Precursor Scan: 018719, m/z: 1180.7854, charge: 3
EEQYNSTYR_5_4_1_2_0

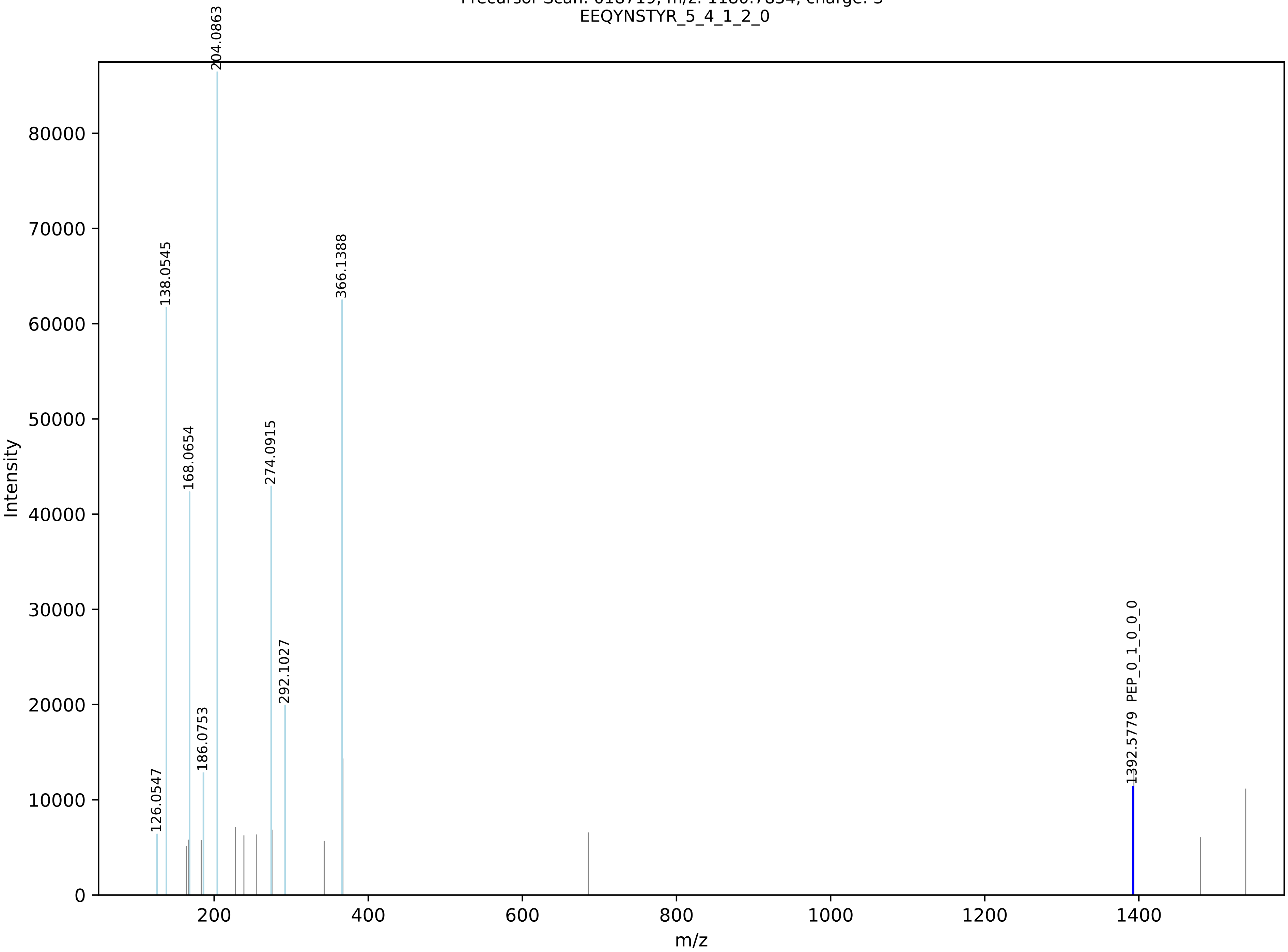

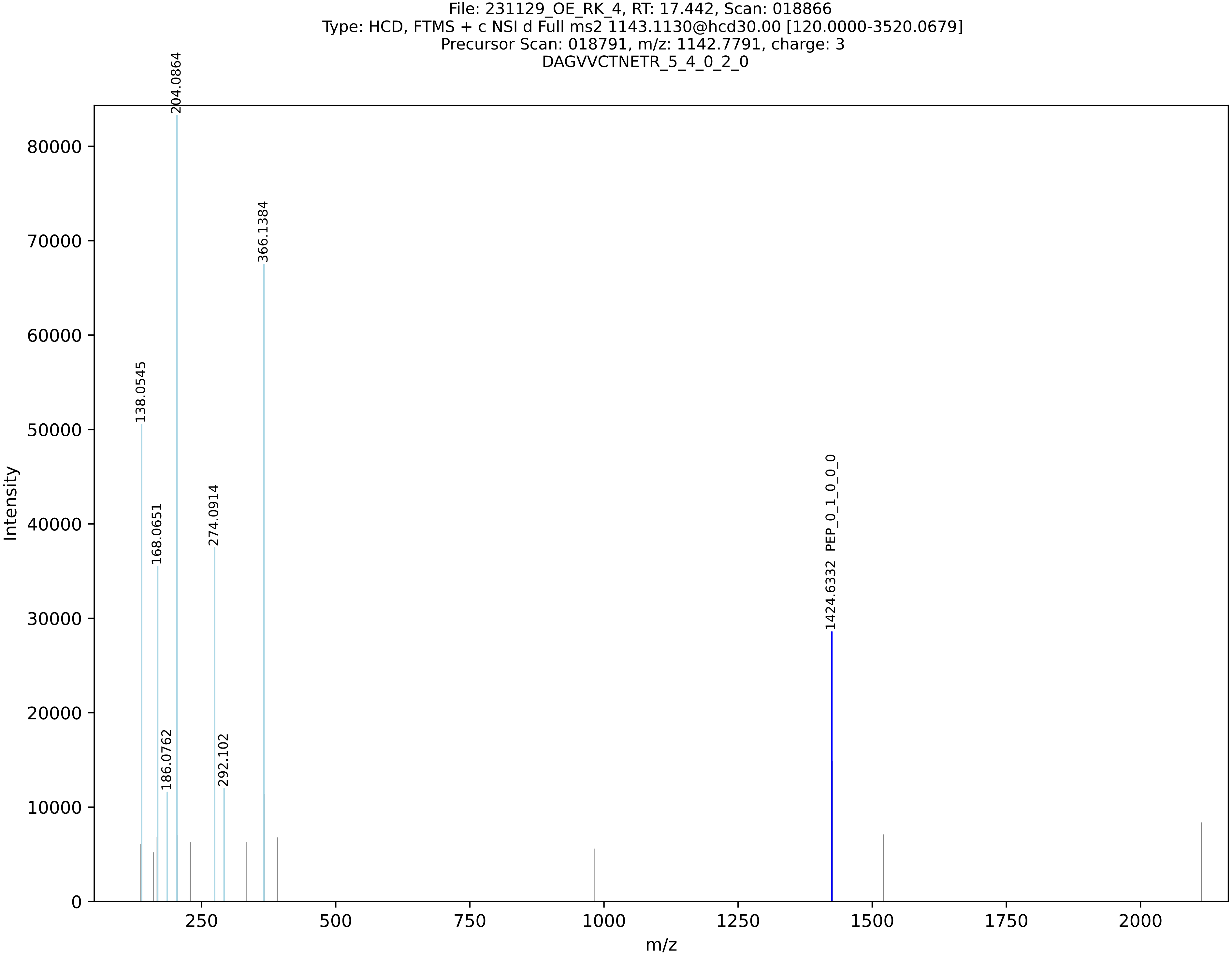

File: 231129_OE_RK_4, RT: 17.442, Scan: 018866
Type: HCD, FTMS + c NSI d Full ms2 1143.1130@hcd30.00 [120.0000-3520.0679]
Precursor Scan: 018791, m/z: 1142.7791, charge: 3
DAGVVCTNETR_5_4_0_2_0
204.0864
366.1384
138.0545
168.0651
274.0914
186.0762
292.102
1424.6332 PEP_0_1_0_0_0
Intensity
m/z

File: 231129_OE_RK_4, RT: 17.494, Scan: 018939
Type: HCD, FTMS + c NSI d Full ms2 1117.4280@hcd30.00 [120.0000-3441.4714]
Precursor Scan: 018861, m/z: 1117.0938, charge: 3
MFSQNDTR_5_4_1_2_0

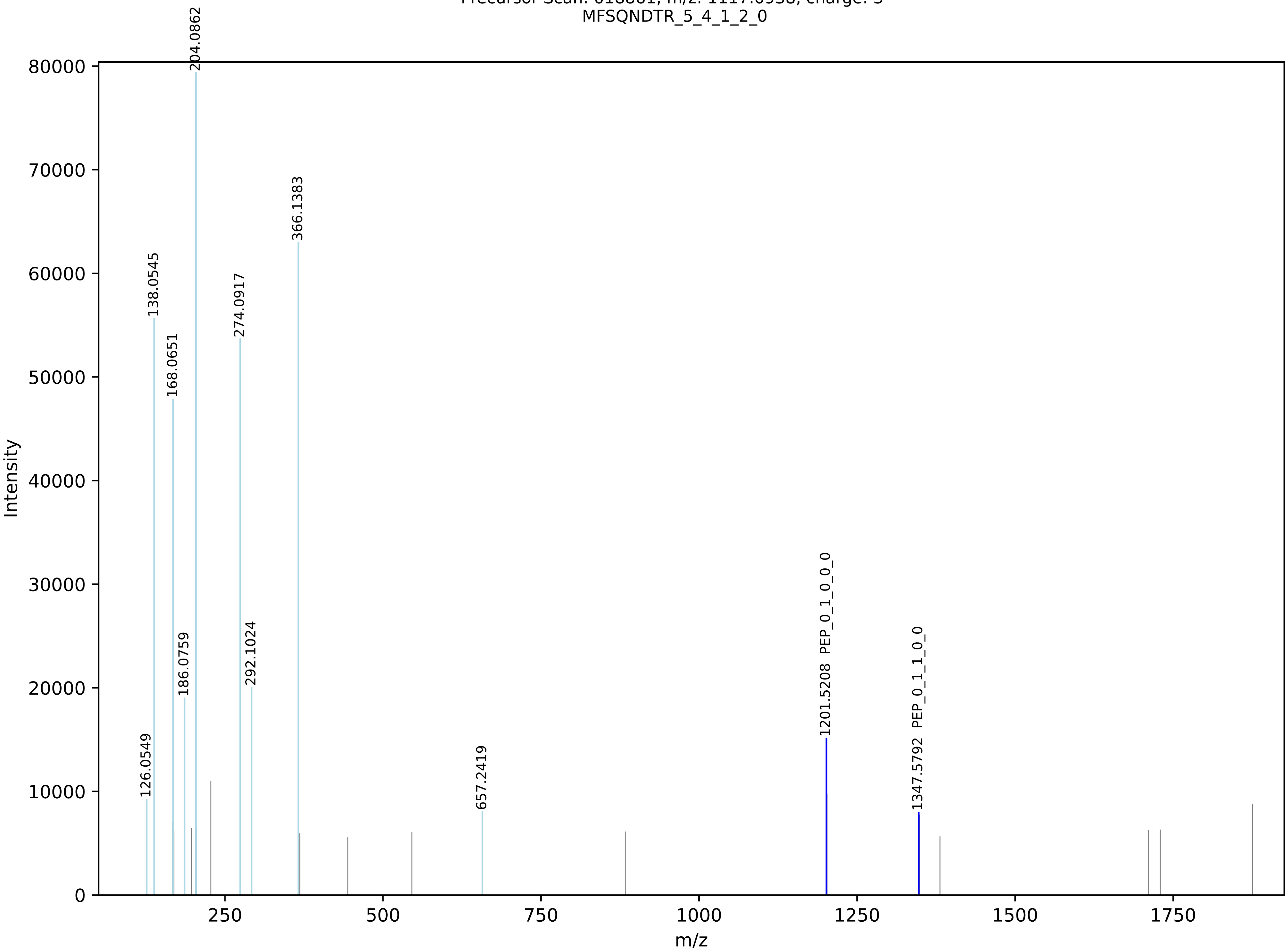

File: 231129_OE_RK_4, RT: 17.593, Scan: 019084
Type: HCD, FTMS + c NSI d Full ms2 1068.7397@hcd30.00 [120.0000-3292.4856]
Precursor Scan: 019005, m/z: 1068.4054, charge: 3
MFSQNDTR_5_4_0_2_0

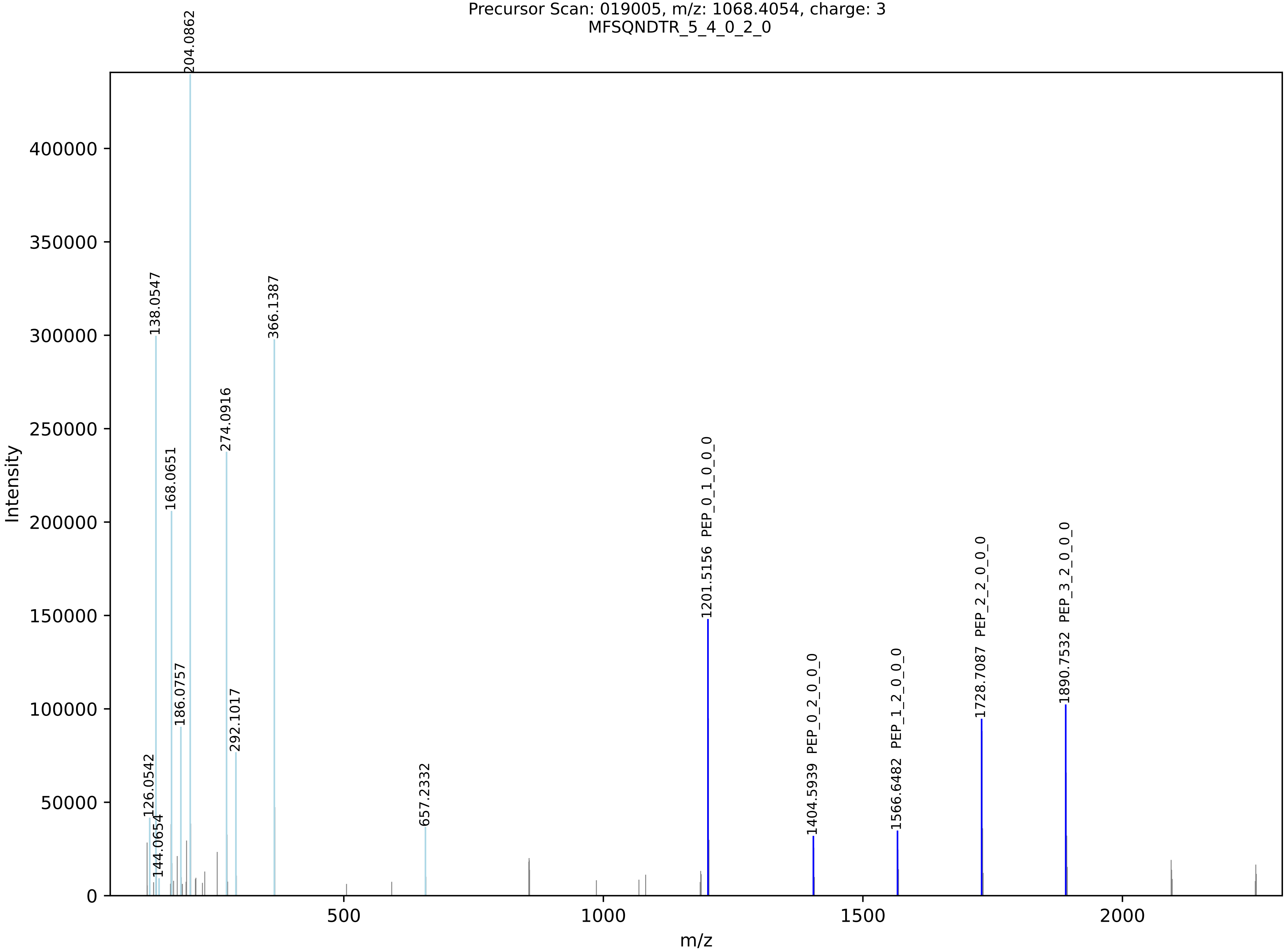

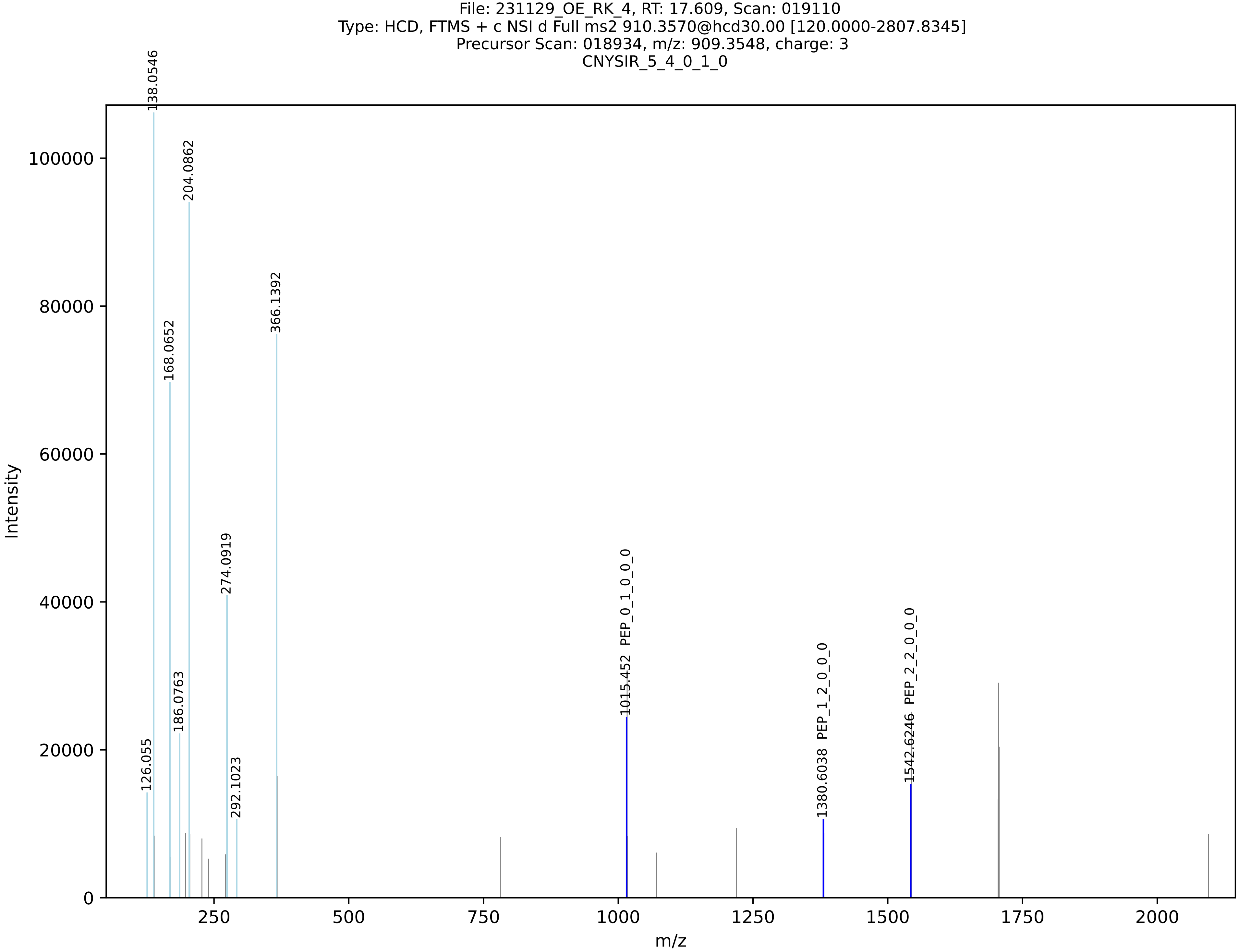
File: 231129_OE_RK_4, RT: 17.609, Scan: 019110
Type: HCD, FTMS + c NSI d Full ms2 910.3570@hcd30.00 [120.0000-2807.8345]
Precursor Scan: 018934, m/z: 909.3548, charge: 3
CNYSIR_5_4_0_1_0
Intensity
m/z
138.0546
204.0862
366.1392
168.0652
274.0919
186.0763
126.055
292.1023
1015.452 PEP_0_1_0_0_0
1380.6038 PEP_1_2_0_0_0
1542.6246 PEP_2_2_0_0_0

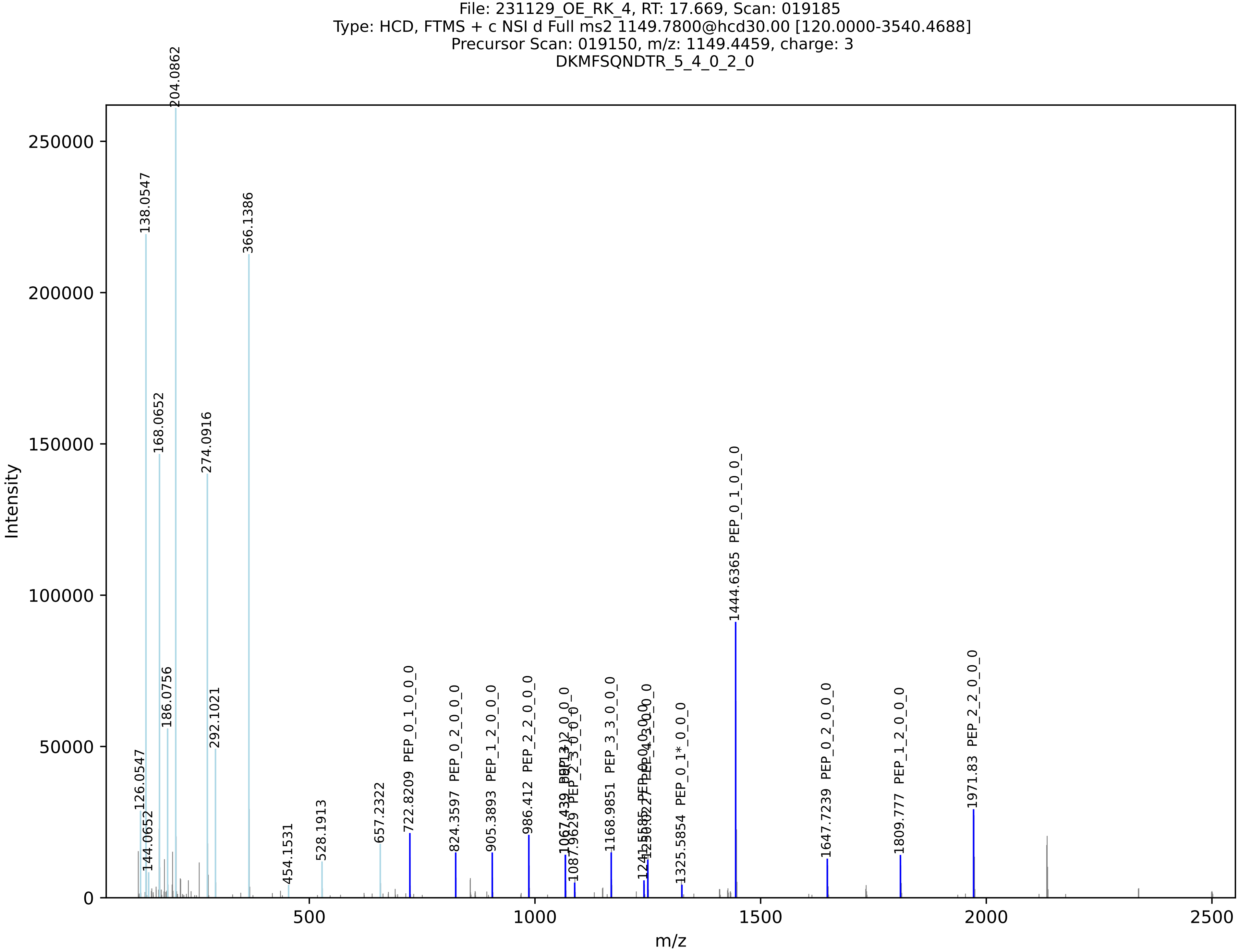
File: 231129_OE_RK_4, RT: 17.669, Scan: 019185
Type: HCD, FTMS + c NSI d Full ms2 1149.7800@hcd30.00 [120.0000-3540.4688]
Precursor Scan: 019150, m/z: 1149.4459, charge: 3
DKMFSQNDTR_5_4_0_2_0
Intensity
m/z
204.0862
138.0547
366.1386
168.0652
274.0916
186.0756
292.1021
126.0547
144.0652
454.1531
528.1913
657.2322
722.8209 PEP_0_1_0_0_0
824.3597 PEP_0_2_0_0_0
905.3893 PEP_1_2_0_0_0
986.412 PEP_2_2_0_0_0
1168.9851 PEP_3_3_0_0_0
1325.5854 PEP_0_1*_0_0_0
1444.6365 PEP_0_1_0_0_0
1647.7239 PEP_0_2_0_0_0
1809.777 PEP_1_2_0_0_0
1971.83 PEP_2_2_0_0_0

File: 231129_OE_RK_4, RT: 17.67, Scan: 019186
Type: HCD, FTMS + c NSI d Full ms2 1110.9222@hcd30.00 [120.0000-4555.4185]
Precursor Scan: 019150, m/z: 1110.4214, charge: 4
ENGTISR_7_6_1_4_0

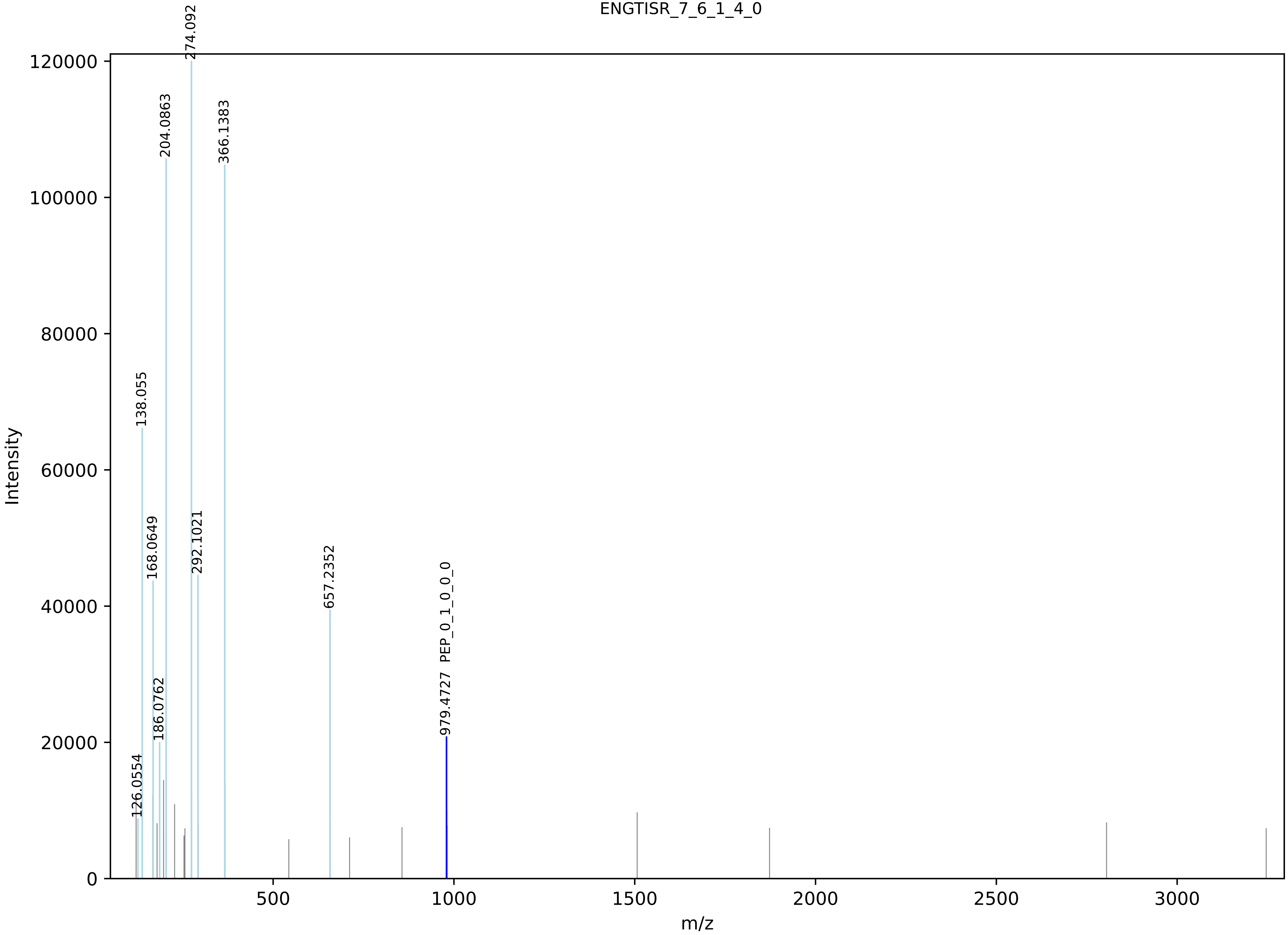

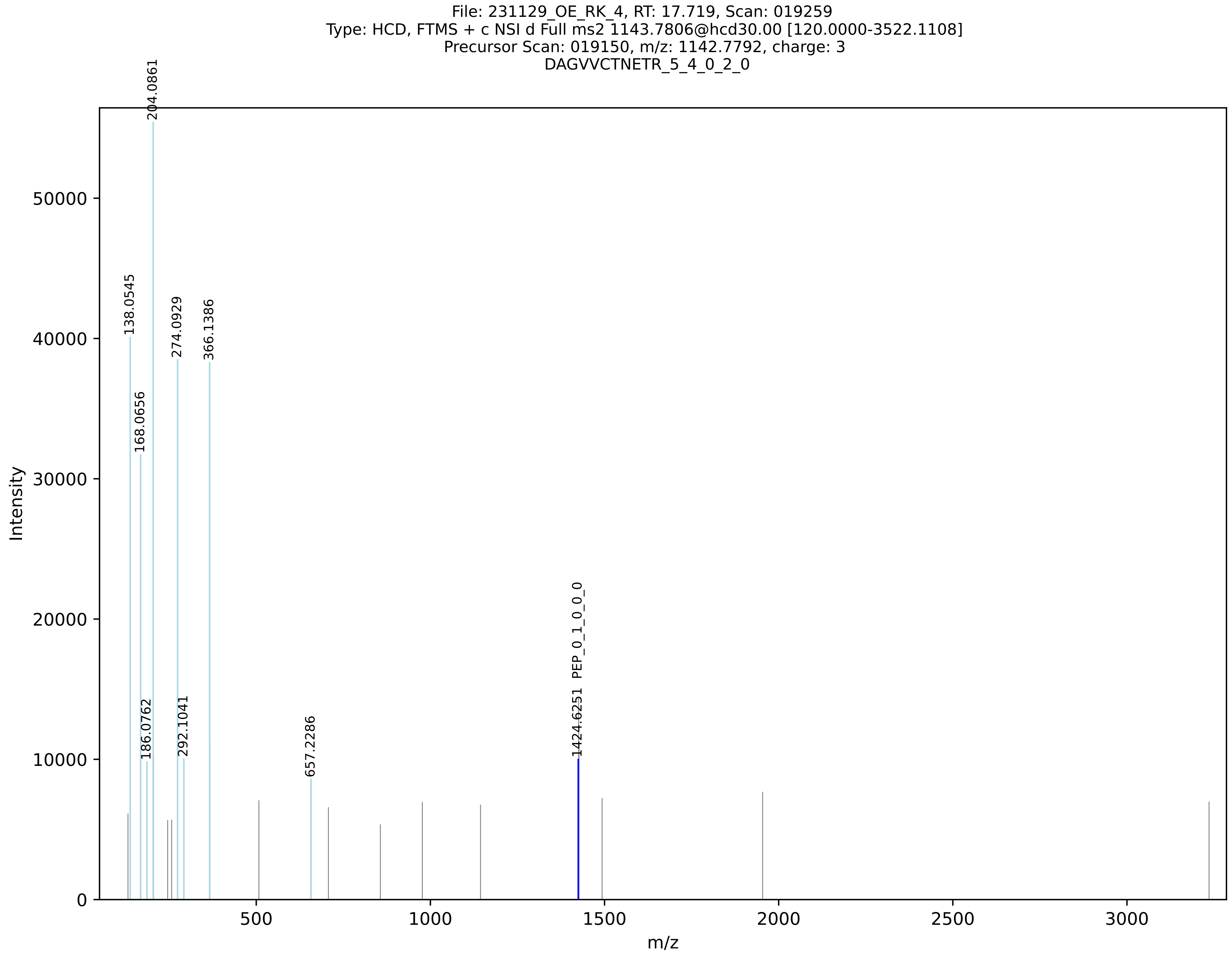

File: 231129_OE_RK_4, RT: 17.719, Scan: 019259
Type: HCD, FTMS + c NSI d Full ms2 1143.7806@hcd30.00 [120.0000-3522.1108]
Precursor Scan: 019150, m/z: 1142.7792, charge: 3
DAGVVCTNETR_5_4_0_2_0
204.0861
138.0545
274.0929
366.1386
168.0656
186.0762
292.1041
657.2286
1424.6251 PEP_0_1_0_0_0
Intensity
m/z

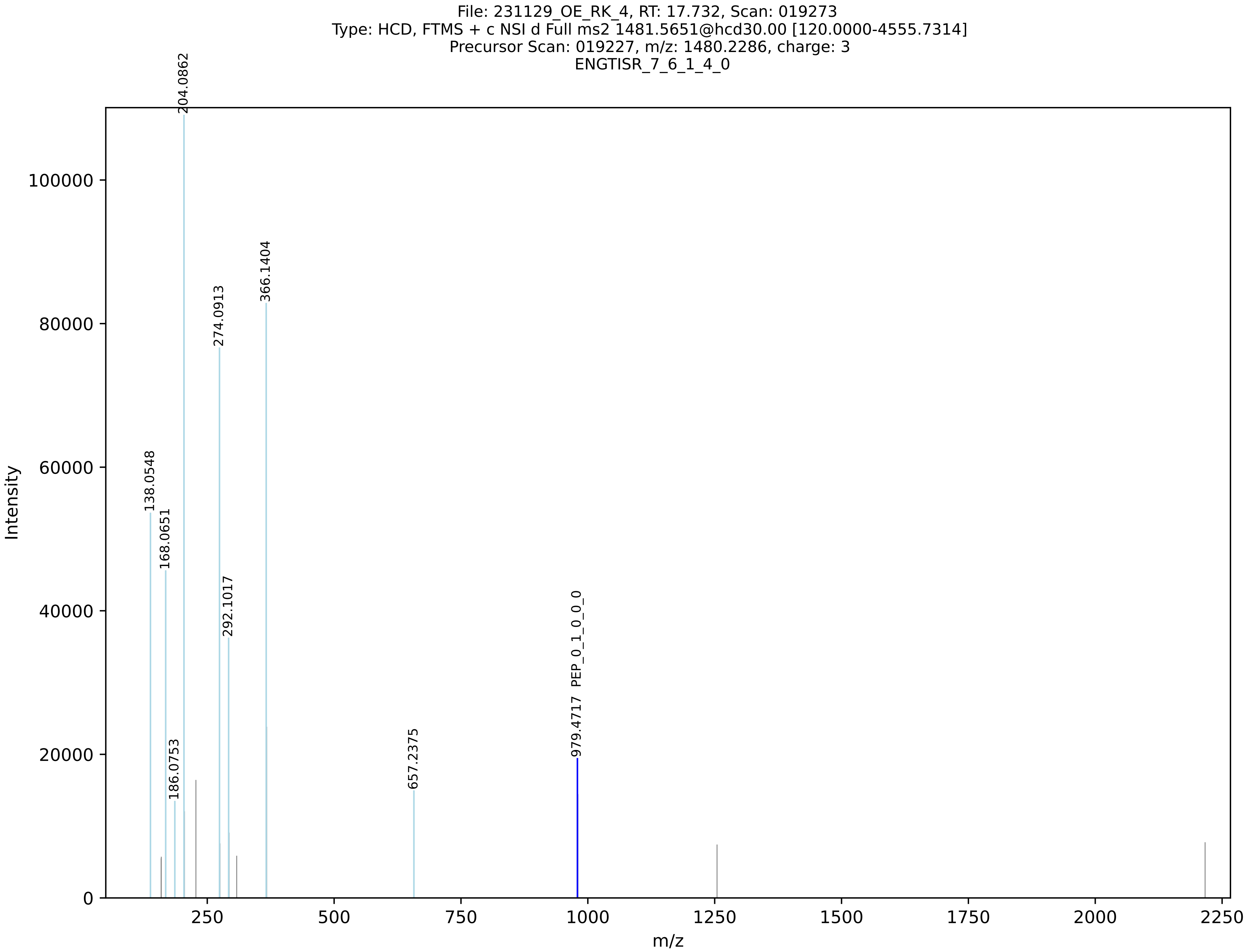
File: 231129_OE_RK_4, RT: 17.732, Scan: 019273
Type: HCD, FTMS + c NSI d Full ms2 1481.5651@hcd30.00 [120.0000-4555.7314]
Precursor Scan: 019227, m/z: 1480.2286, charge: 3
ENGTISR_7_6_1_4_0
204.0862
366.1404
274.0913
138.0548
168.0651
292.1017
PEP_0_1_0_0_0
979.4717
657.2375
186.0753
Intensity
m/z
0
20000
40000
60000
80000
100000
250
500
750
1000
1250
1500
1750
2000
2250

File: 231129_OE_RK_4, RT: 17.849, Scan: 019442
Type: HCD, FTMS + c NSI d Full ms2 1110.9221@hcd30.00 [120.0000-4555.4180]
Precursor Scan: 019372, m/z: 1110.4229, charge: 4
ENGTISR_7_6_1_4_0

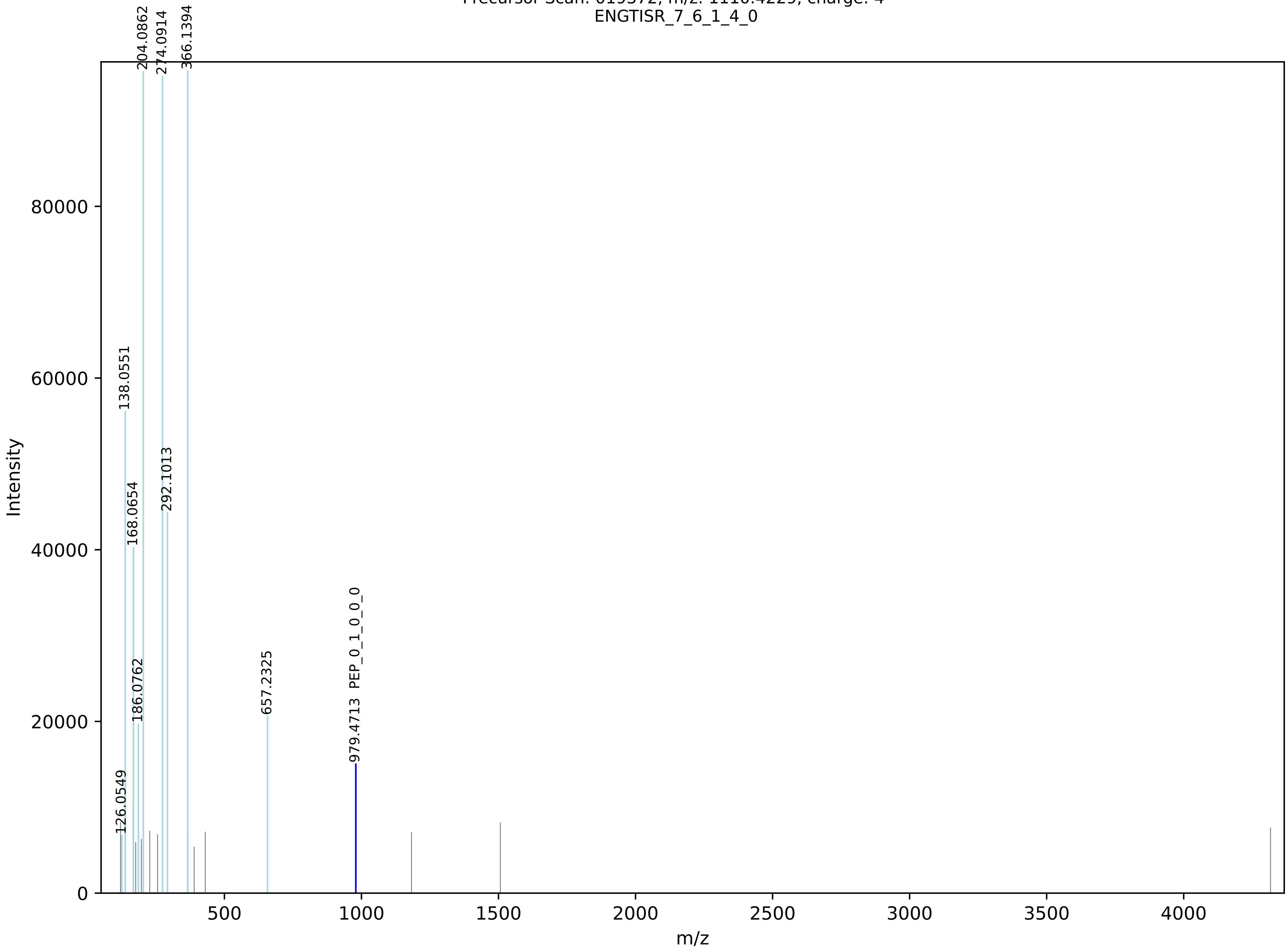

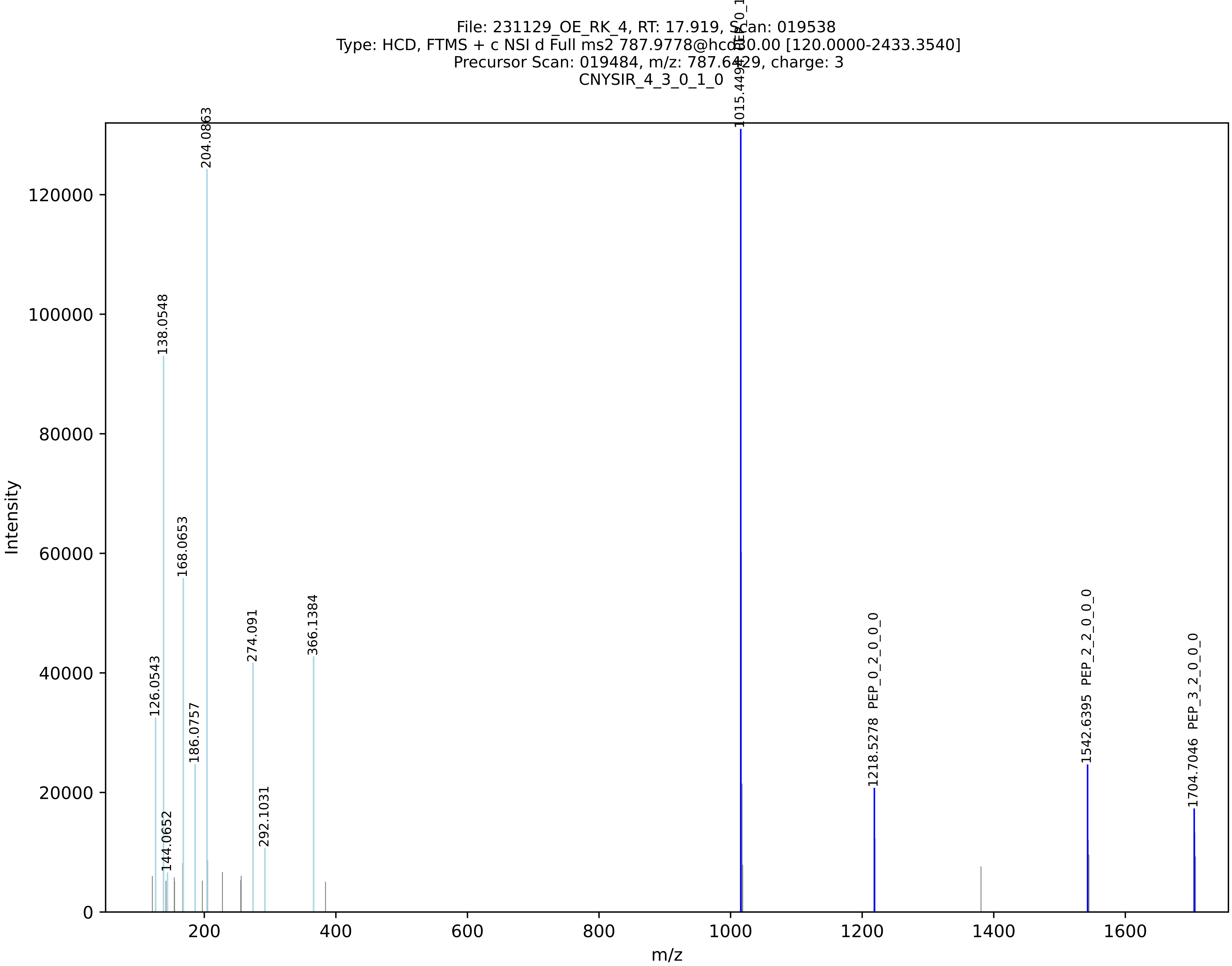
File: 231129_OE_RK_4, RT: 17.919, Scan: 019538
Type: HCD, FTMS + c NSI d Full ms2 787.9778@hcd30.00 [120.0000-2433.3540]
Precursor Scan: 019484, m/z: 787.6429, charge: 3
CNYSIR_4_3_0_1_0
Intensity
m/z
204.0863
138.0548
168.0653
126.0543
186.0757
144.0652
274.091
292.1031
366.1384
1015.4494
1218.5278 PEP_0_2_0_0_0
1542.6395 PEP_2_2_0_0_0
1704.7046 PEP_3_2_0_0_0

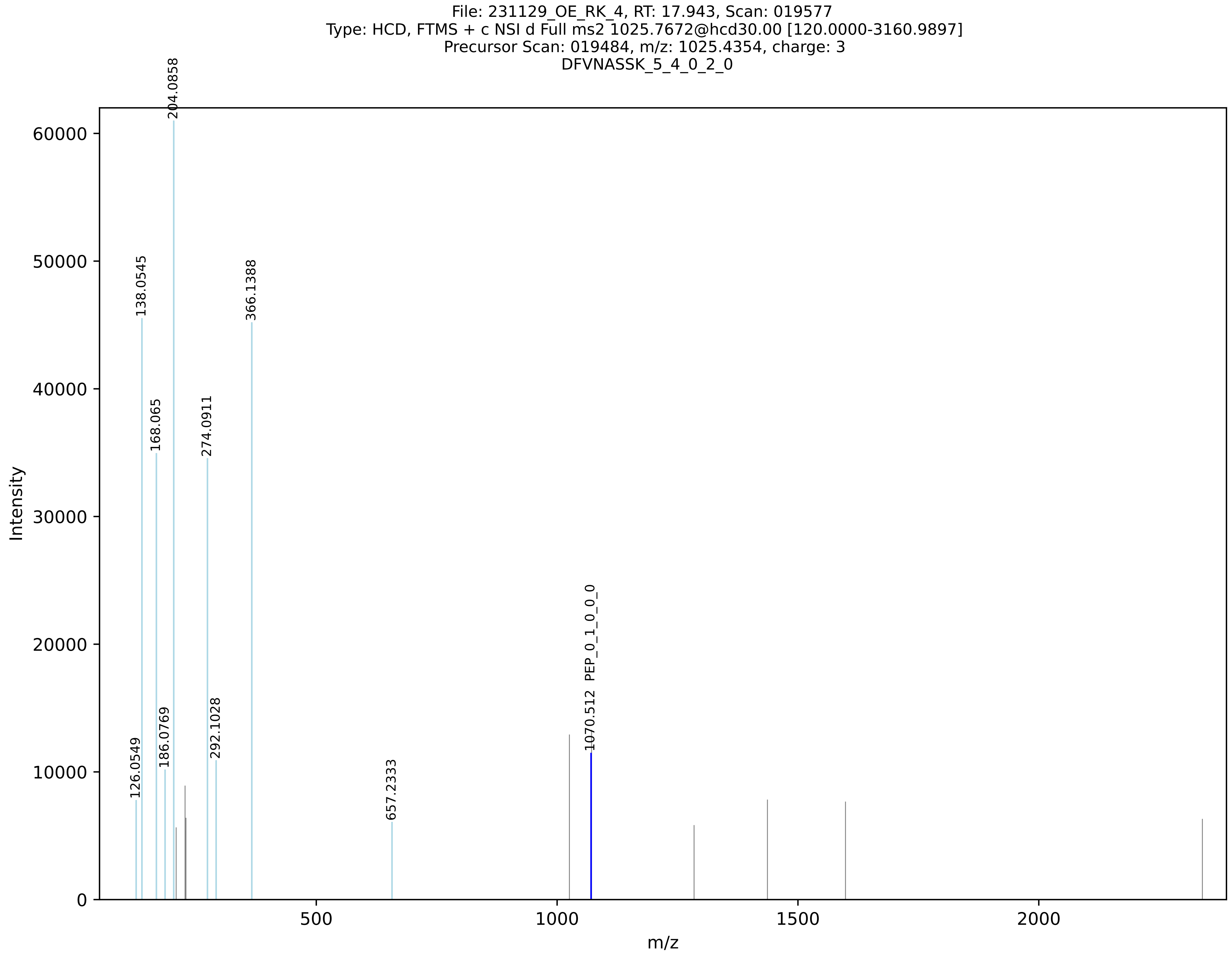

File: 231129_OE_RK_4, RT: 17.943, Scan: 019577
Type: HCD, FTMS + c NSI d Full ms2 1025.7672@hcd30.00 [120.0000-3160.9897]
Precursor Scan: 019484, m/z: 1025.4354, charge: 3
DFVNASSK_5_4_0_2_0
204.0858
138.0545
366.1388
168.065
274.0911
PEP_0_1_0_0_0_
1070.512
292.1028
186.0769
126.0549
657.2333
Intensity
60000
50000
40000
30000
20000
10000
0
500
1000
1500
2000
m/z

File: 231129_OE_RK_4, RT: 18.019, Scan: 019684
Type: HCD, FTMS + c NSI d Full ms2 1181.4634@hcd30.00 [120.0000-2431.6133]
Precursor Scan: 019605, m/z: 1180.9628, charge: 2
CNYSIR_4_3_0_1_0

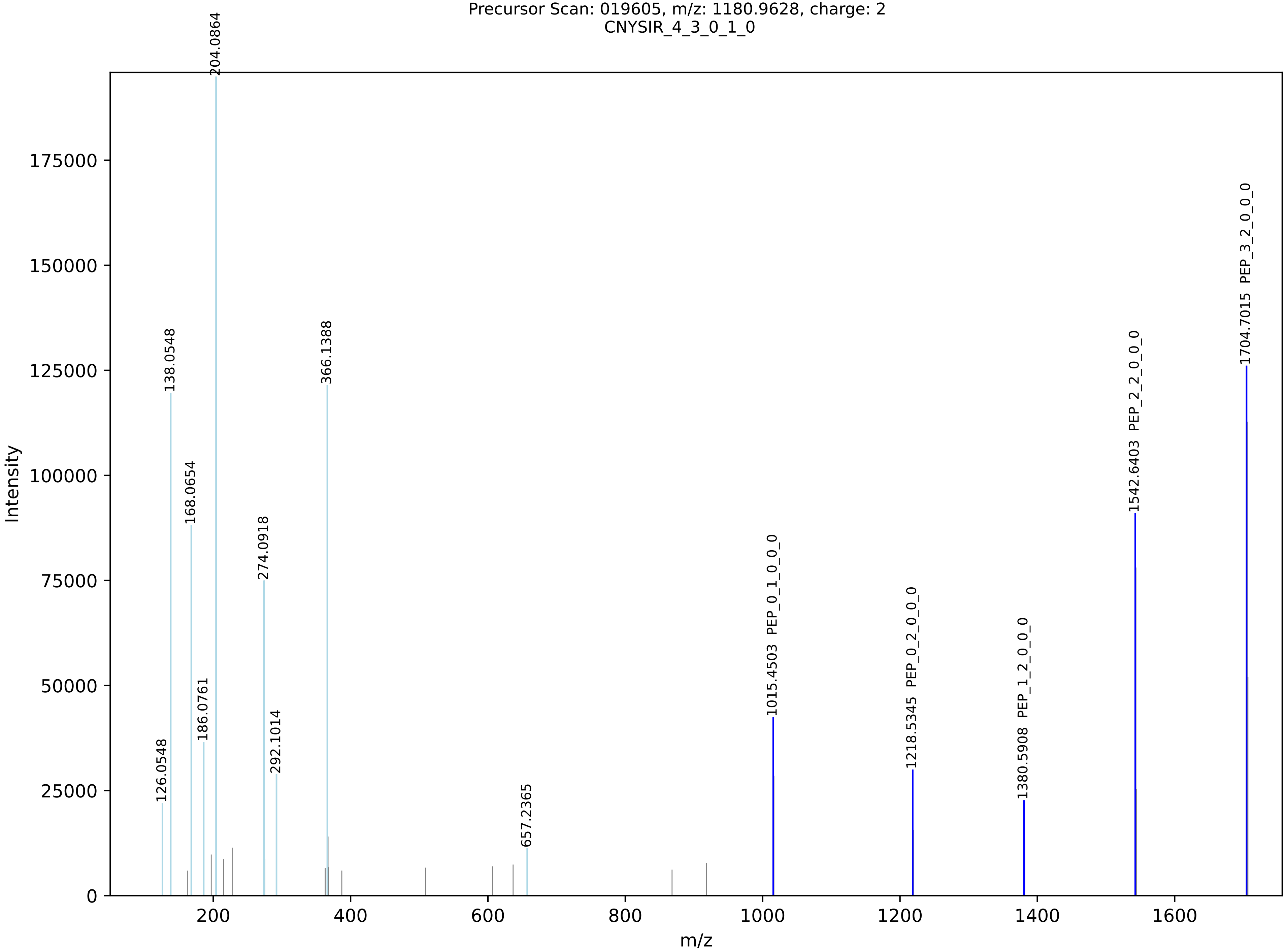

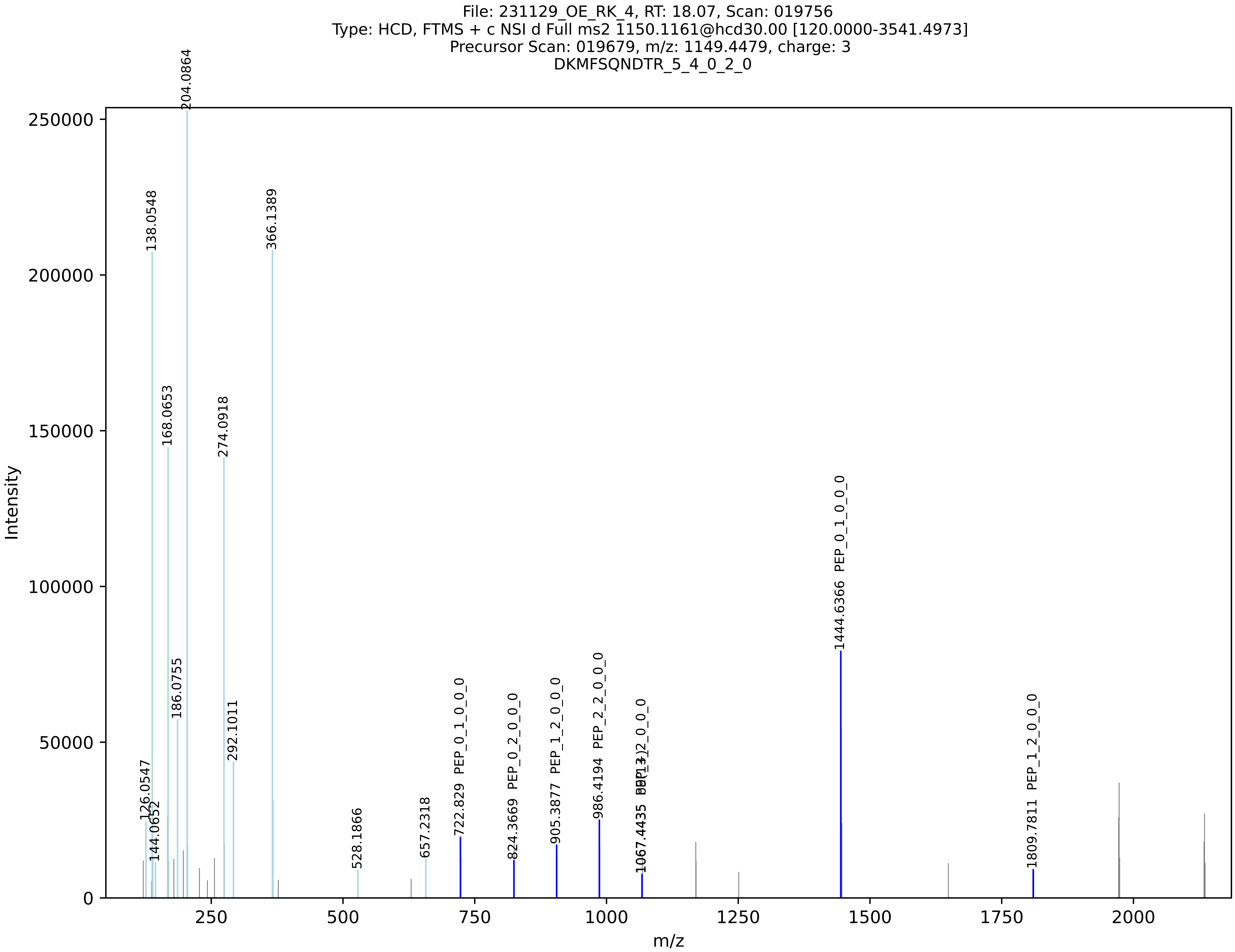
File: 231129_OE_RK_4, RT: 18.07, Scan: 019756
Type: HCD, FTMS + c NSI d Full ms2 1150.1161@hcd30.00 [120.0000-3541.4973]
Precursor Scan: 019679, m/z: 1149.4479, charge: 3
DKMFSQNDTR_5_4_0_2_0
Intensity
m/z
204.0864
138.0548
366.1389
168.0653
274.0918
186.0755
292.1011
126.0547
144.0652
528.1866
657.2318
722.829 PEP_0_1_0_0_0
824.3669 PEP_0_2_0_0_0
905.3877 PEP_1_2_0_0_0
986.4194 PEP_2_2_0_0_0
1067.4435 PEP_3_2_0_0_0
1444.6366 PEP_0_1_0_0_0
1809.7811 PEP_1_2_0_0_0

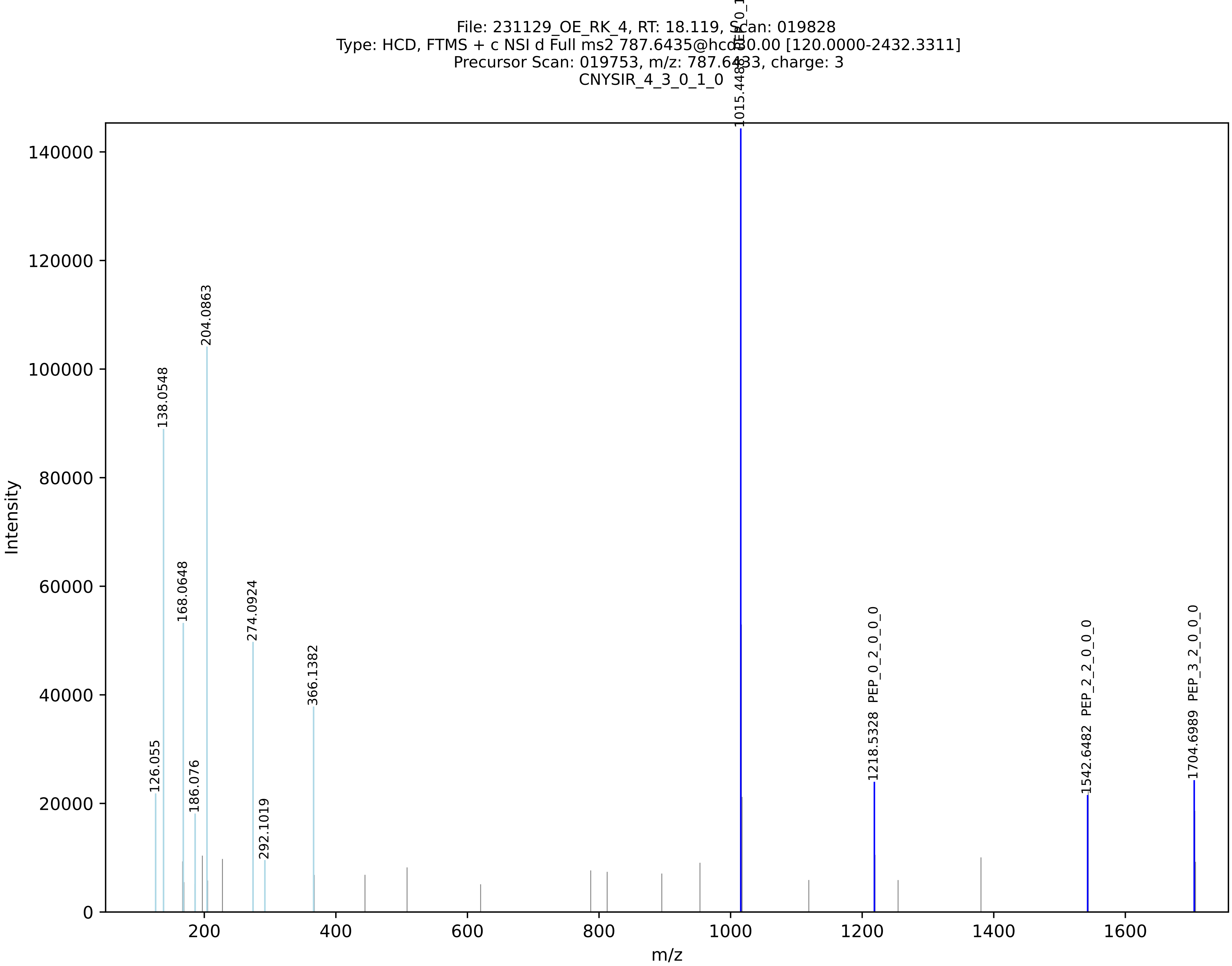
File: 231129_OE_RK_4, RT: 18.119, Scan: 019828
Type: HCD, FTMS + c NSI d Full ms2 787.6435@hcd30.00 [120.0000-2432.3311]
Precursor Scan: 019753, m/z: 787.6433, charge: 3
CNYSIR_4_3_0_1_0
Intensity
m/z
0
20000
40000
60000
80000
100000
120000
140000
200
400
600
800
1000
1200
1400
1600
126.055
138.0548
168.0648
186.076
204.0863
274.0924
292.1019
366.1382
1218.5328 PEP_0_2_0_0_0
1542.6482 PEP_2_2_0_0_0
1704.6989 PEP_3_2_0_0_0

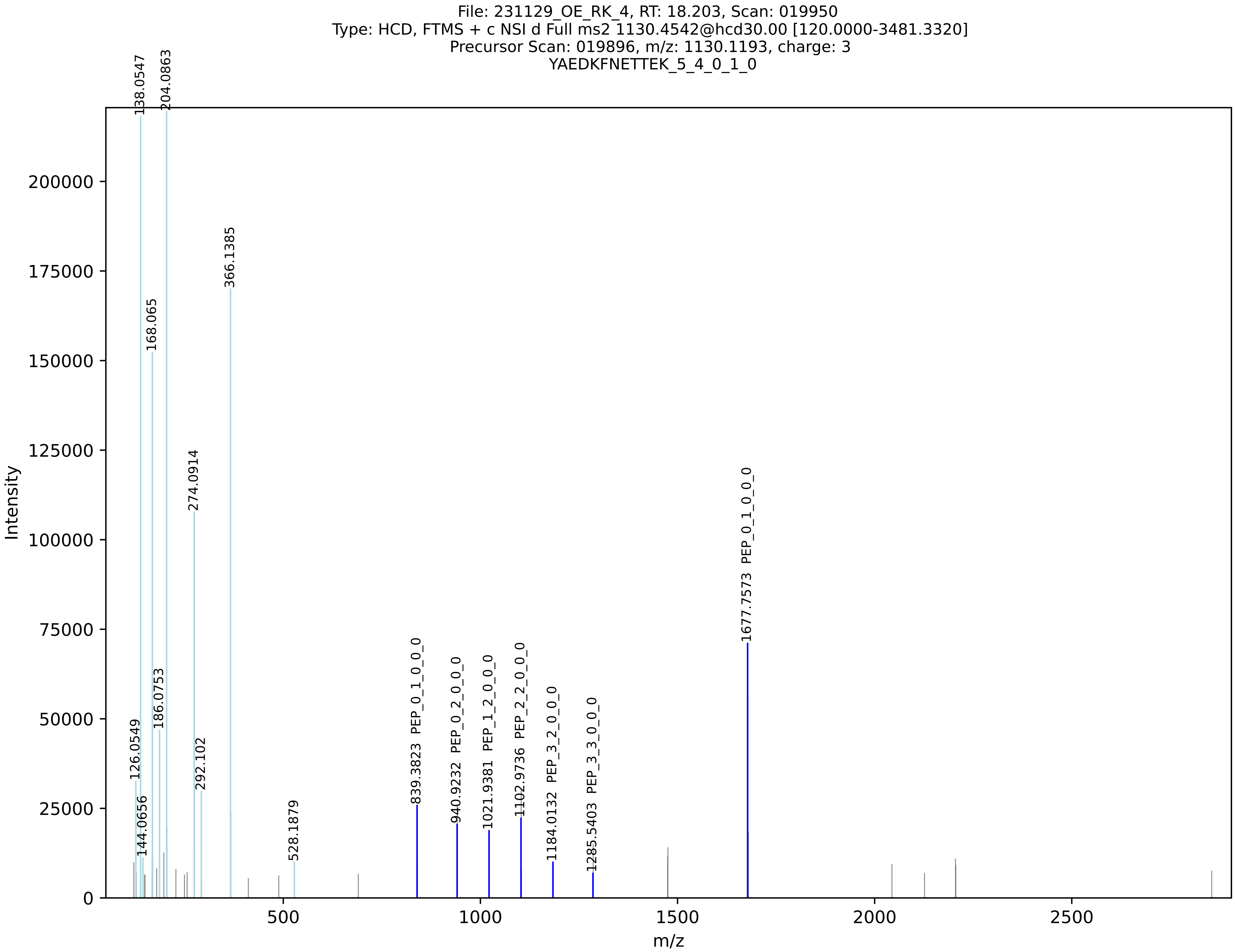
File: 231129_OE_RK_4, RT: 18.203, Scan: 019950
Type: HCD, FTMS + c NSI d Full ms2 1130.4542@hcd30.00 [120.0000-3481.3320]
Precursor Scan: 019896, m/z: 1130.1193, charge: 3
YAEDKFNETTEK_5_4_0_1_0
138.0547
204.0863
366.1385
168.065
274.0914
186.0753
126.0549
292.102
144.0656
528.1879
839.3823 PEP_0_1_0_0_0
940.9232 PEP_0_2_0_0_0
1021.9381 PEP_1_2_0_0_0
1102.9736 PEP_2_2_0_0_0
1184.0132 PEP_3_2_0_0_0
1285.5403 PEP_3_3_0_0_0
1677.7573 PEP_0_1_0_0_0
Intensity
m/z

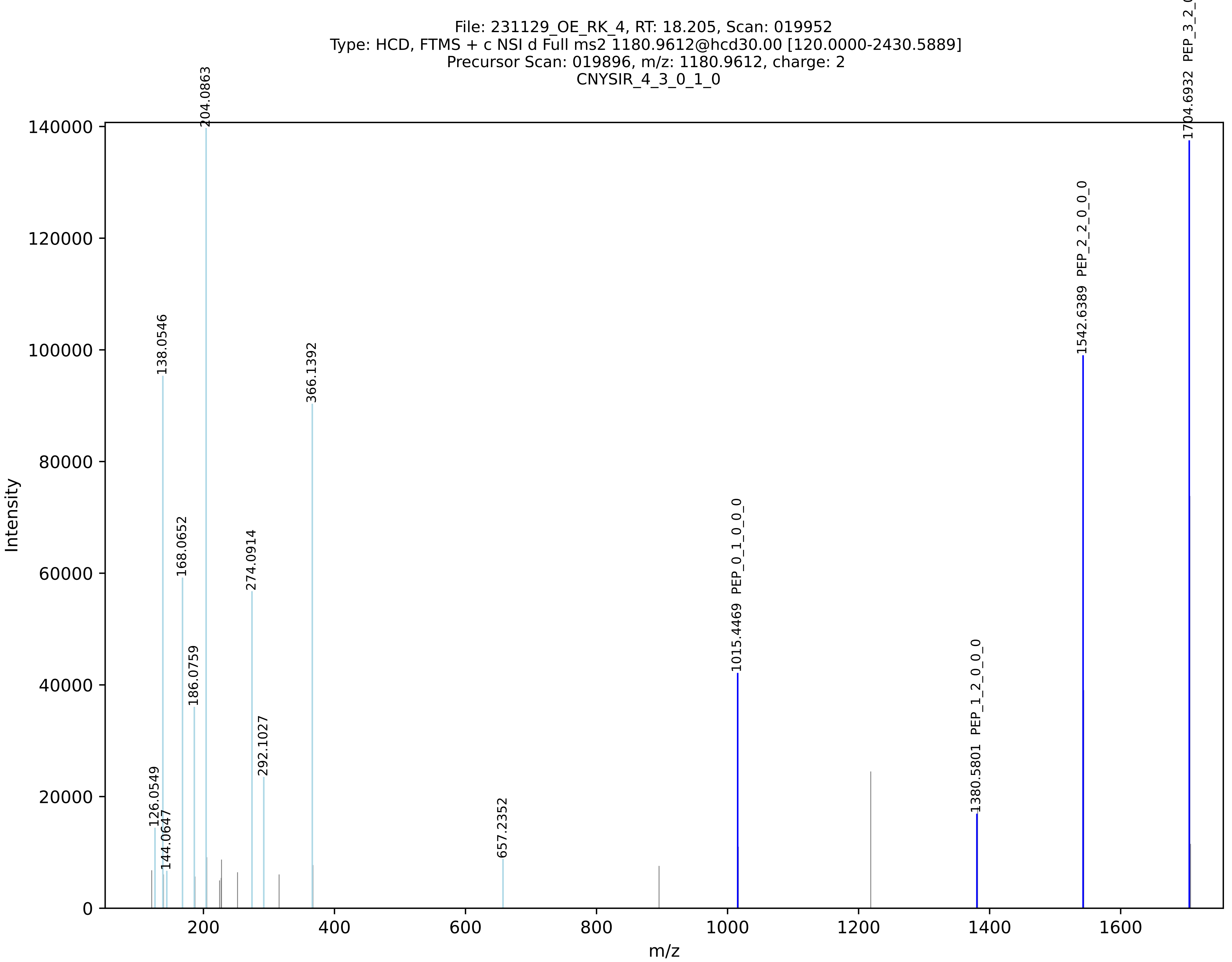

File: 231129_OE_RK_4, RT: 18.205, Scan: 019952
Type: HCD, FTMS + c NSI d Full ms2 1180.9612@hcd30.00 [120.0000-2430.5889]
Precursor Scan: 019896, m/z: 1180.9612, charge: 2
CNYSIR_4_3_0_1_0
Intensity
m/z
204.0863
138.0546
366.1392
168.0652
274.0914
186.0759
292.1027
126.0549
144.0647
657.2352
1015.4469 PEP_0_1_0_0_0
1380.5801 PEP_1_2_0_0_0
1542.6389 PEP_2_2_0_0_0
1704.6932 PEP_3_2_0

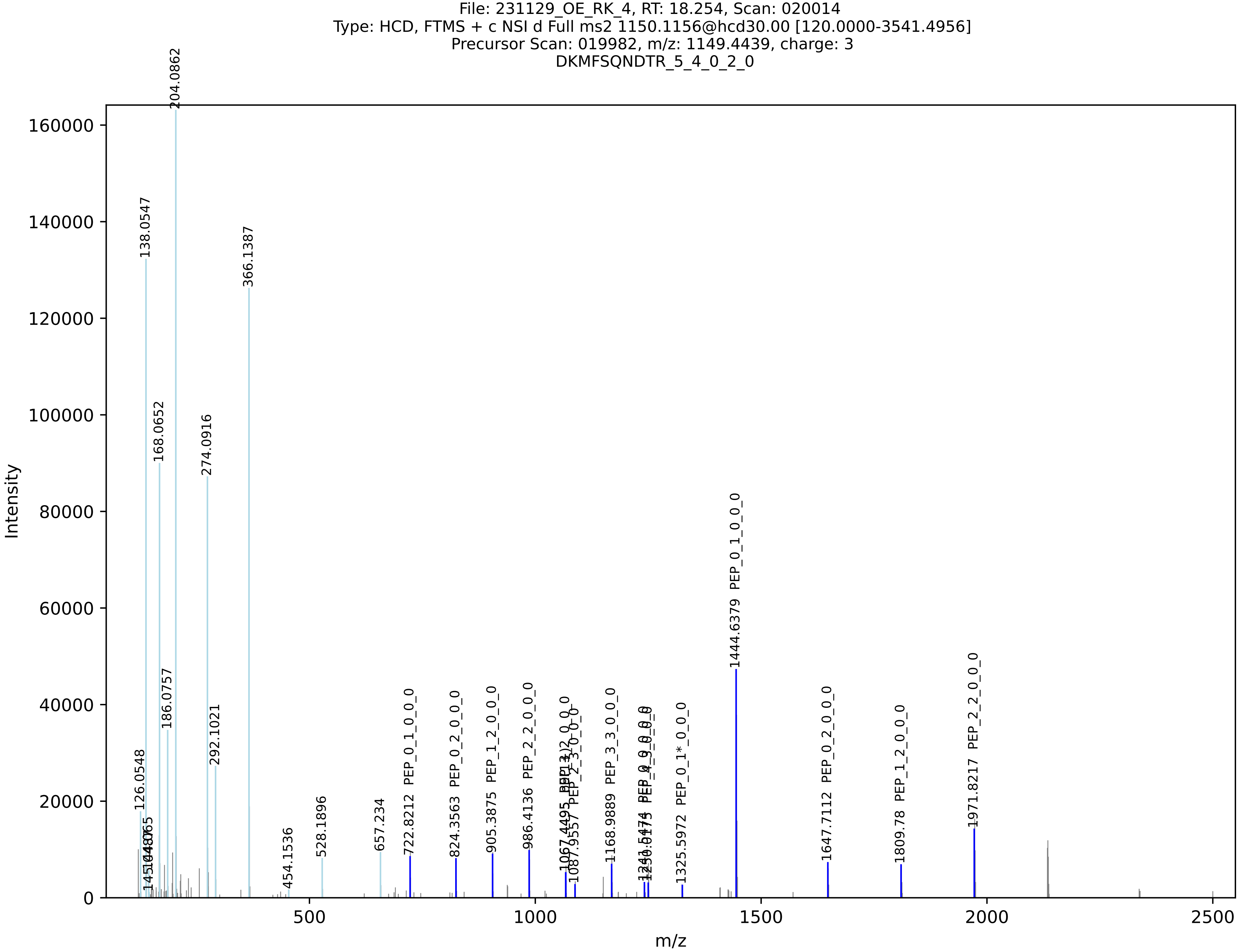
File: 231129_OE_RK_4, RT: 18.254, Scan: 020014
Type: HCD, FTMS + c NSI d Full ms2 1150.1156@hcd30.00 [120.0000-3541.4956]
Precursor Scan: 019982, m/z: 1149.4439, charge: 3
DKMFSQNDTR_5_4_0_2_0
Intensity
m/z
204.0862
138.0547
366.1387
168.0652
274.0916
186.0757
292.1021
126.0548
454.1536
528.1896
657.234
722.8212 PEP_0_1_0_0_
824.3563 PEP_0_2_0_0_
905.3875 PEP_1_2_0_0_
986.4136 PEP_2_2_0_0_
1087.9557 PEP_2_3_0_0_
1168.9889 PEP_3_3_0_0_
1325.5972 PEP_0_1*_0_0_
1444.6379 PEP_0_1_0_0_
1647.7112 PEP_0_2_0_0_
1809.78 PEP_1_2_0_0_
1971.8217 PEP_2_2_0_0_

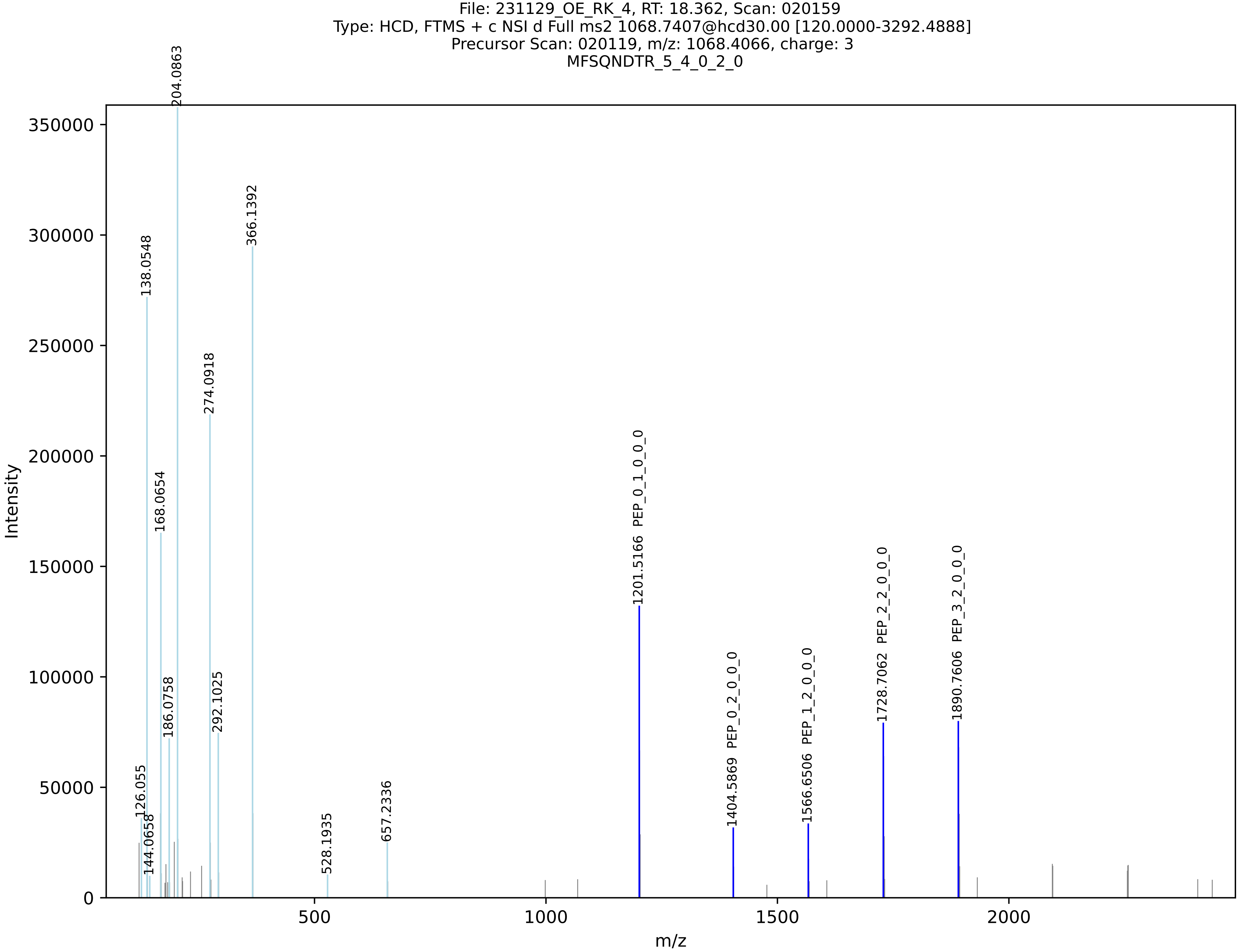
File: 231129_OE_RK_4, RT: 18.362, Scan: 020159
Type: HCD, FTMS + c NSI d Full ms2 1068.7407@hcd30.00 [120.0000-3292.4888]
Precursor Scan: 020119, m/z: 1068.4066, charge: 3
MFSQNDTR_5_4_0_2_0
Intensity
m/z
204.0863
366.1392
138.0548
274.0918
168.0654
186.0758
292.1025
126.055
144.0658
528.1935
657.2336
1201.5166 PEP_0_1_0_0_0
1404.5869 PEP_0_2_0_0_0
1566.6506 PEP_1_2_0_0_0
1728.7062 PEP_2_2_0_0_0
1890.7606 PEP_3_2_0_0_0
0
50000
100000
150000
200000
250000
300000
350000
500
1000
1500
2000

File: 231129_OE_RK_4, RT: 18.365, Scan: 020164
Type: HCD, FTMS + c NSI d Full ms2 1025.0690@hcd30.00 [120.0000-3158.8530]
Precursor Scan: 020119, m/z: 1024.7349, charge: 3
DFVNASSK_5_4_0_2_0

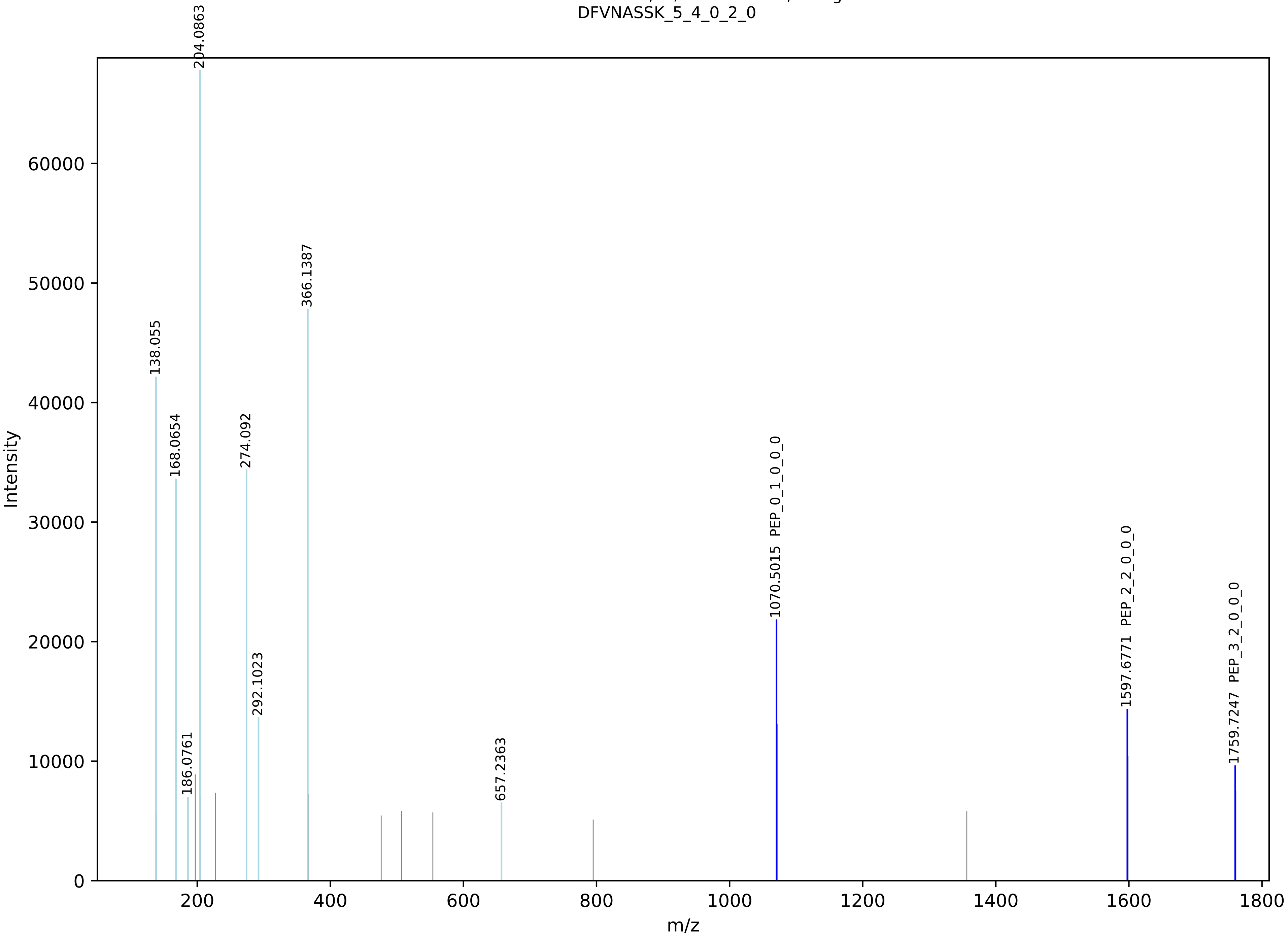

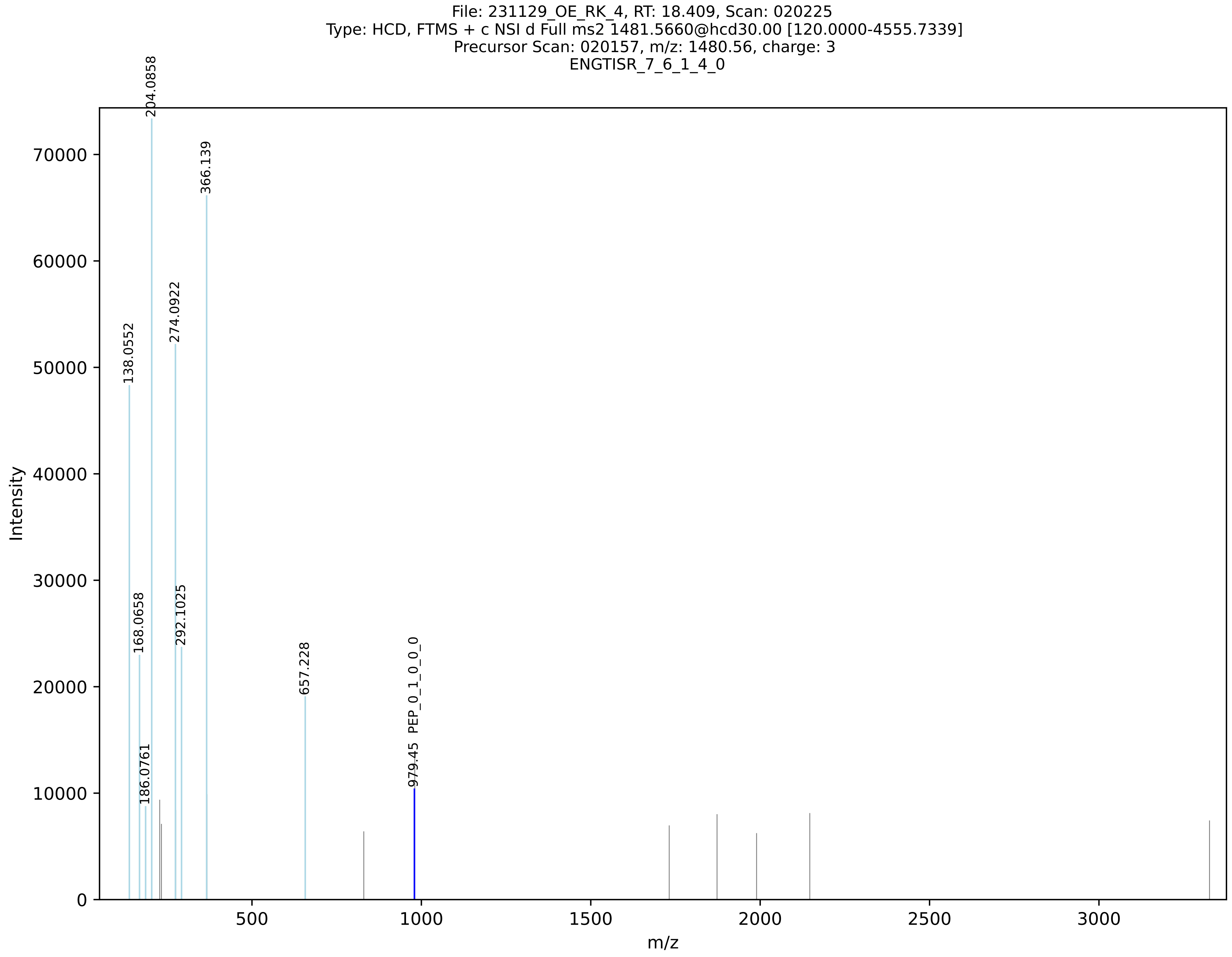
File: 231129_OE_RK_4, RT: 18.409, Scan: 020225
Type: HCD, FTMS + c NSI d Full ms2 1481.5660@hcd30.00 [120.0000-4555.7339]
Precursor Scan: 020157, m/z: 1480.56, charge: 3
ENGTISR_7_6_1_4_0
204.0858
366.139
274.0922
138.0552
168.0658
292.1025
657.228
PEP_0_1_0_0_0_
979.45
186.0761
Intensity
m/z
0
10000
20000
30000
40000
50000
60000
70000
500
1000
1500
2000
2500
3000

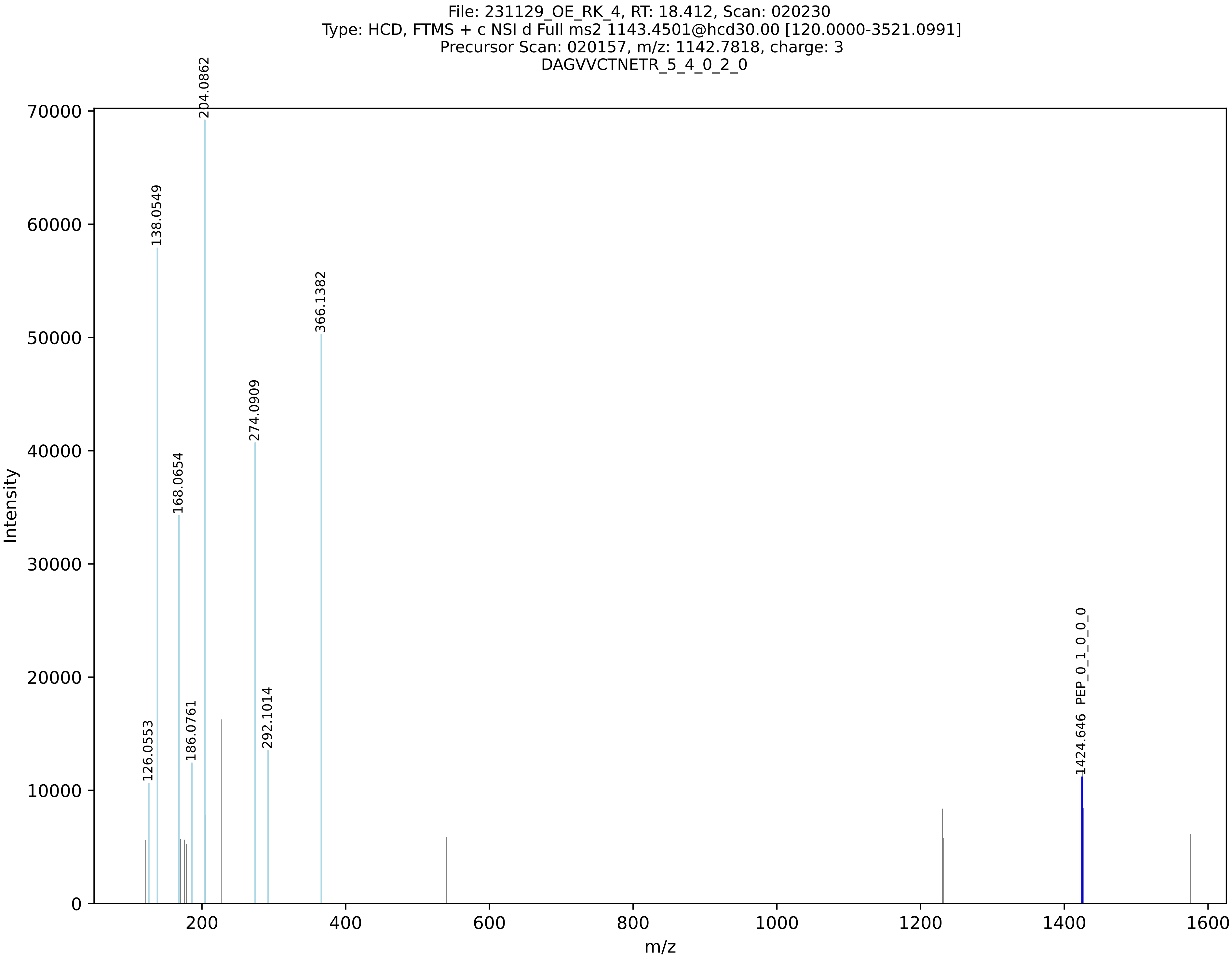
File: 231129_OE_RK_4, RT: 18.412, Scan: 020230
Type: HCD, FTMS + c NSI d Full ms2 1143.4501@hcd30.00 [120.0000-3521.0991]
Precursor Scan: 020157, m/z: 1142.7818, charge: 3
DAGVVCTNETR_5_4_0_2_0
Intensity
m/z
204.0862
138.0549
366.1382
274.0909
168.0654
292.1014
186.0761
126.0553
1424.646 PEP_0_1_0_0_0
0
10000
20000
30000
40000
50000
60000
70000
200
400
600
800
1000
1200
1400
1600

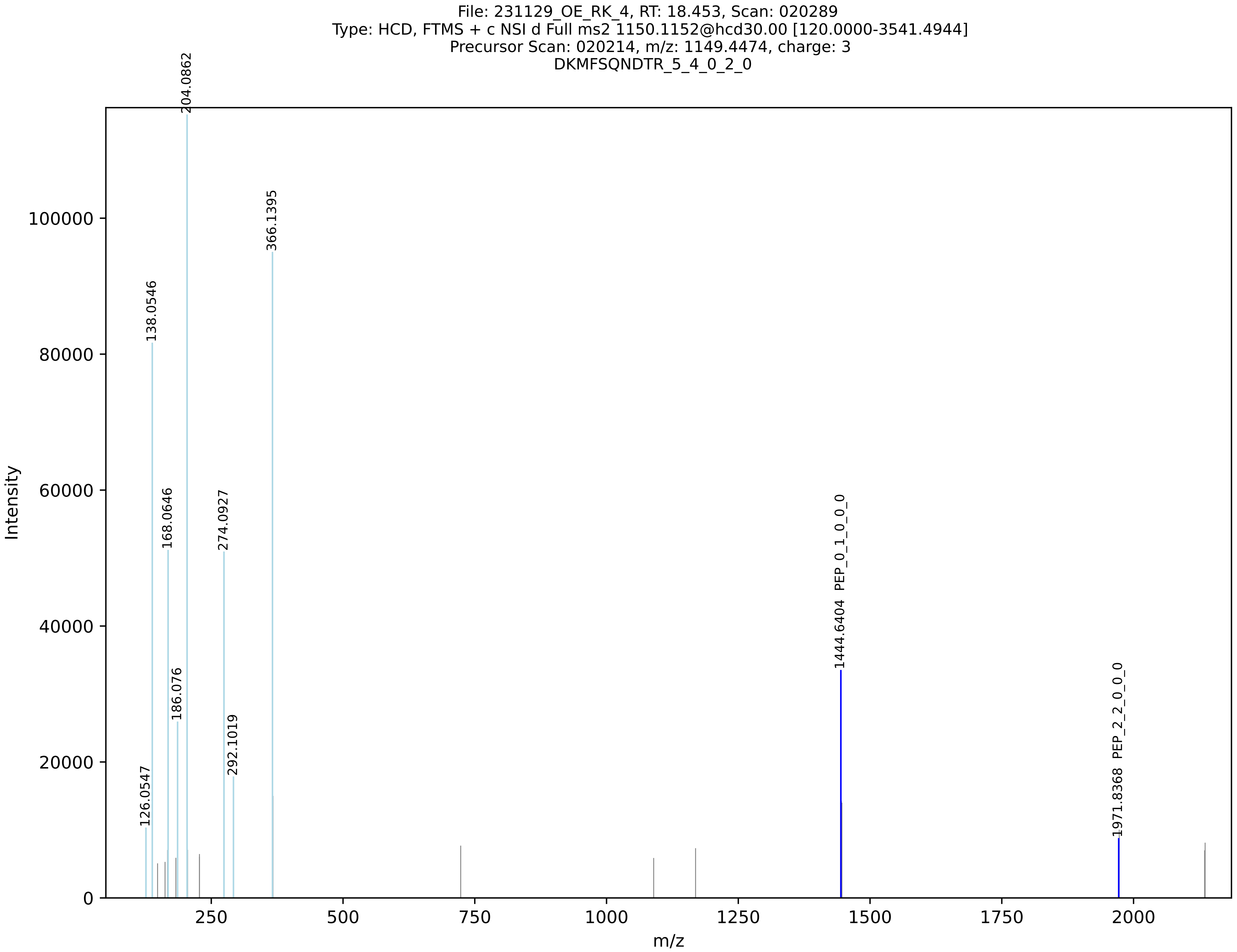
File: 231129_OE_RK_4, RT: 18.453, Scan: 020289
Type: HCD, FTMS + c NSI d Full ms2 1150.1152@hcd30.00 [120.0000-3541.4944]
Precursor Scan: 020214, m/z: 1149.4474, charge: 3
DKMFSQNDTR_5_4_0_2_0
204.0862
366.1395
138.0546
168.0646
274.0927
186.076
292.1019
126.0547
1444.6404 PEP_0_1_0_0_0
1971.8368 PEP_2_2_0_0_0
Intensity
m/z
0
20000
40000
60000
80000
100000
250
500
750
1000
1250
1500
1750
2000

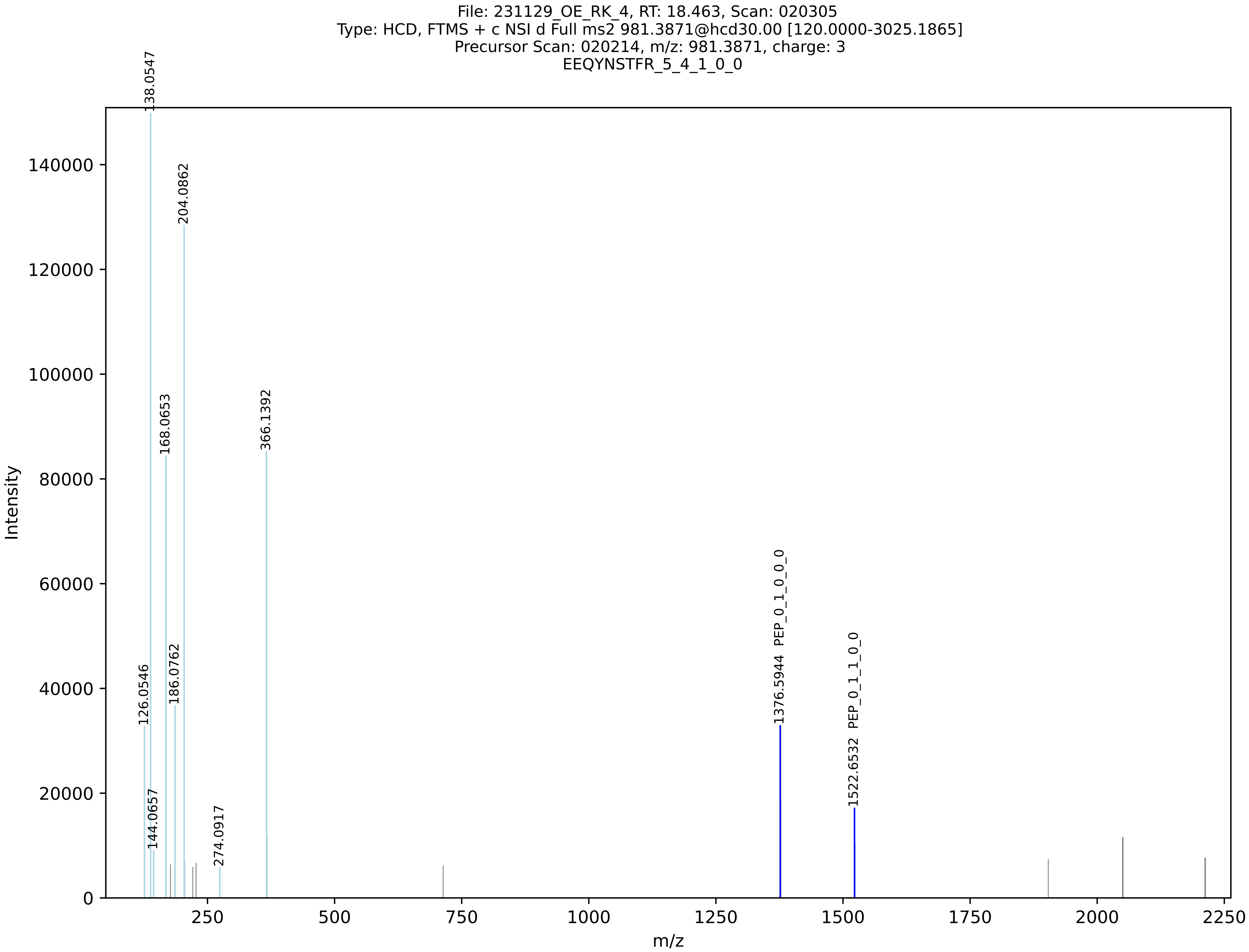
File: 231129_OE_RK_4, RT: 18.463, Scan: 020305
Type: HCD, FTMS + c NSI d Full ms2 981.3871@hcd30.00 [120.0000-3025.1865]
Precursor Scan: 020214, m/z: 981.3871, charge: 3
EEQYNSTFR_5_4_1_0_0
138.0547
204.0862
168.0653
366.1392
126.0546
186.0762
144.0657
274.0917
1376.5944 PEP_0_1_0_0_0
1522.6532 PEP_0_1_1_0_0
Intensity
m/z
0
20000
40000
60000
80000
100000
120000
140000
250
500
750
1000
1250
1500
1750
2000
2250

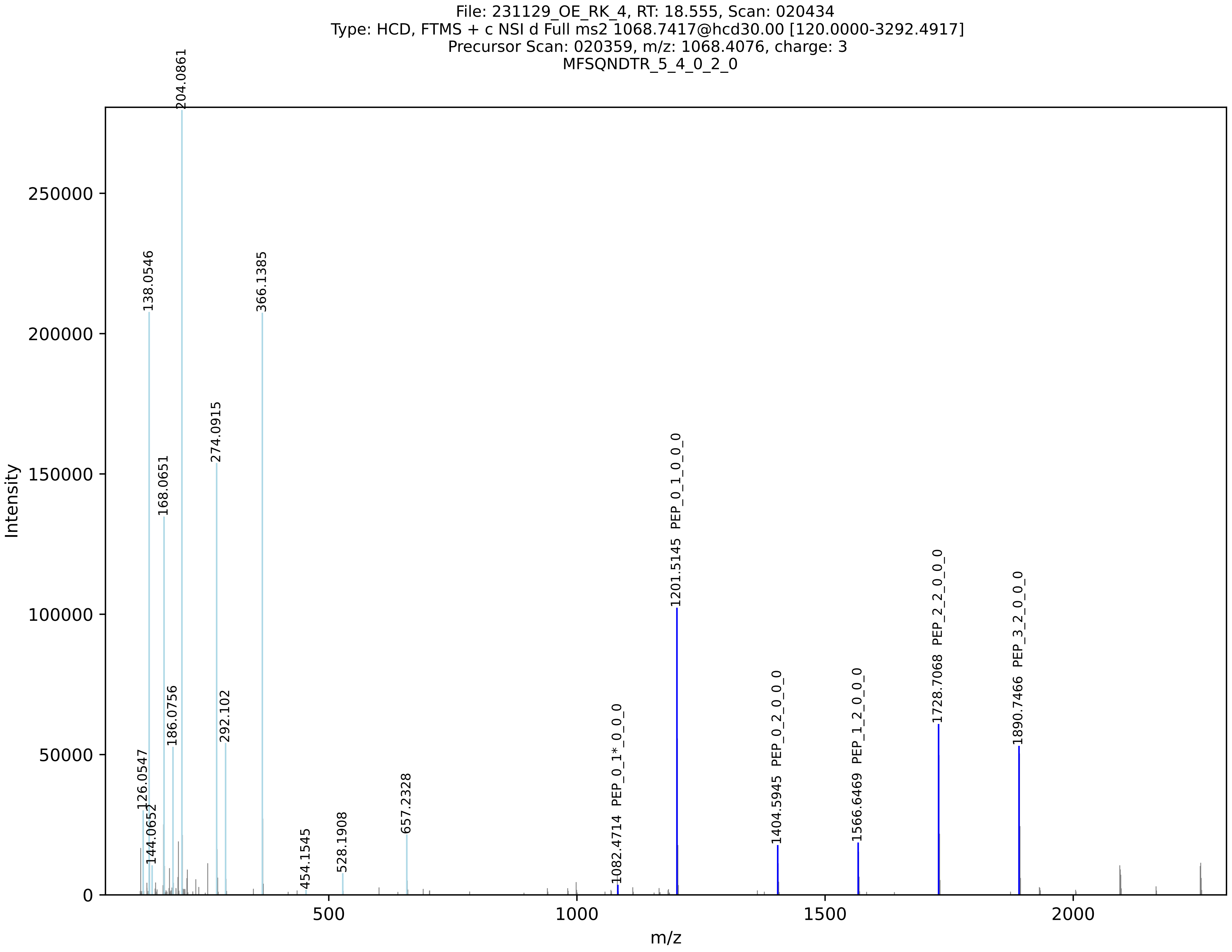
File: 231129_OE_RK_4, RT: 18.555, Scan: 020434
Type: HCD, FTMS + c NSI d Full ms2 1068.7417@hcd30.00 [120.0000-3292.4917]
Precursor Scan: 020359, m/z: 1068.4076, charge: 3
MFSQNDTR_5_4_0_2_0
Intensity
m/z
204.0861
138.0546
366.1385
274.0915
168.0651
186.0756
292.102
126.0547
144.0652
454.1545
528.1908
657.2328
1082.4714 PEP_0_1*_0_0_0
1201.5145 PEP_0_1_0_0_0
1404.5945 PEP_0_2_0_0_0
1566.6469 PEP_1_2_0_0_0
1728.7068 PEP_2_2_0_0_0
1890.7466 PEP_3_2_0_0_0

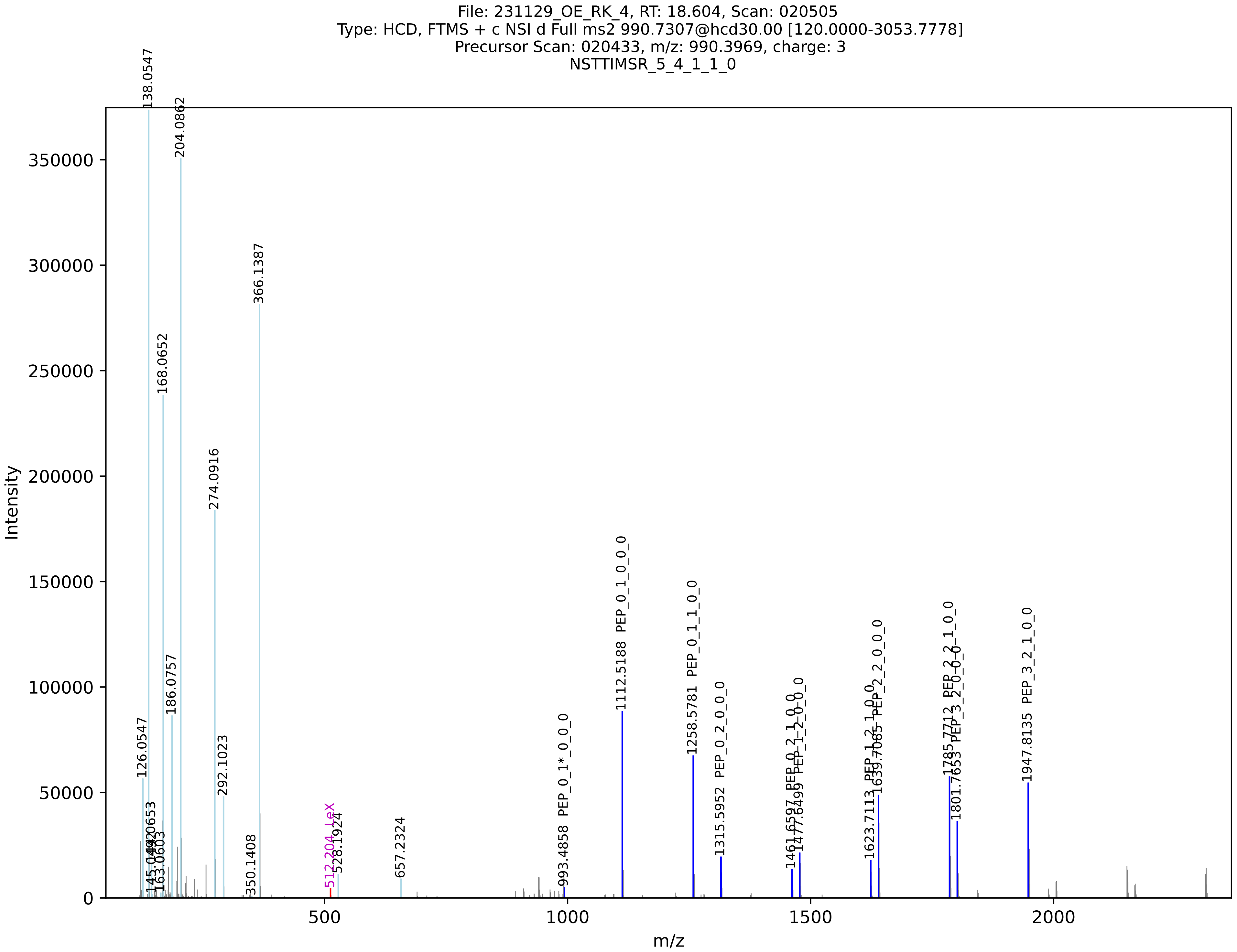
File: 231129_OE_RK_4, RT: 18.604, Scan: 020505
Type: HCD, FTMS + c NSI d Full ms2 990.7307@hcd30.00 [120.0000-3053.7778]
Precursor Scan: 020433, m/z: 990.3969, charge: 3
NSTTIMSR_5_4_1_1_0
Intensity
m/z
138.0547
204.0862
366.1387
168.0652
274.0916
186.0757
126.0547
292.1023
350.1408
512.204_LeX
528.1924
657.2324
993.4858 PEP_0_1*_0_0_
1112.5188 PEP_0_1_0_0_
1258.5781 PEP_0_1_1_0_
1315.5952 PEP_0_2_0_0_
1461.6597 PEP_0_2_1_0_
1477.6499 PEP_1_2_0_0_
1623.7113 PEP_1_2_1_0_
1639.7085 PEP_2_2_0_0_
1785.7712 PEP_2_2_1_0_
1801.7653 PEP_3_2_0_0_
1947.8135 PEP_3_2_1_0_

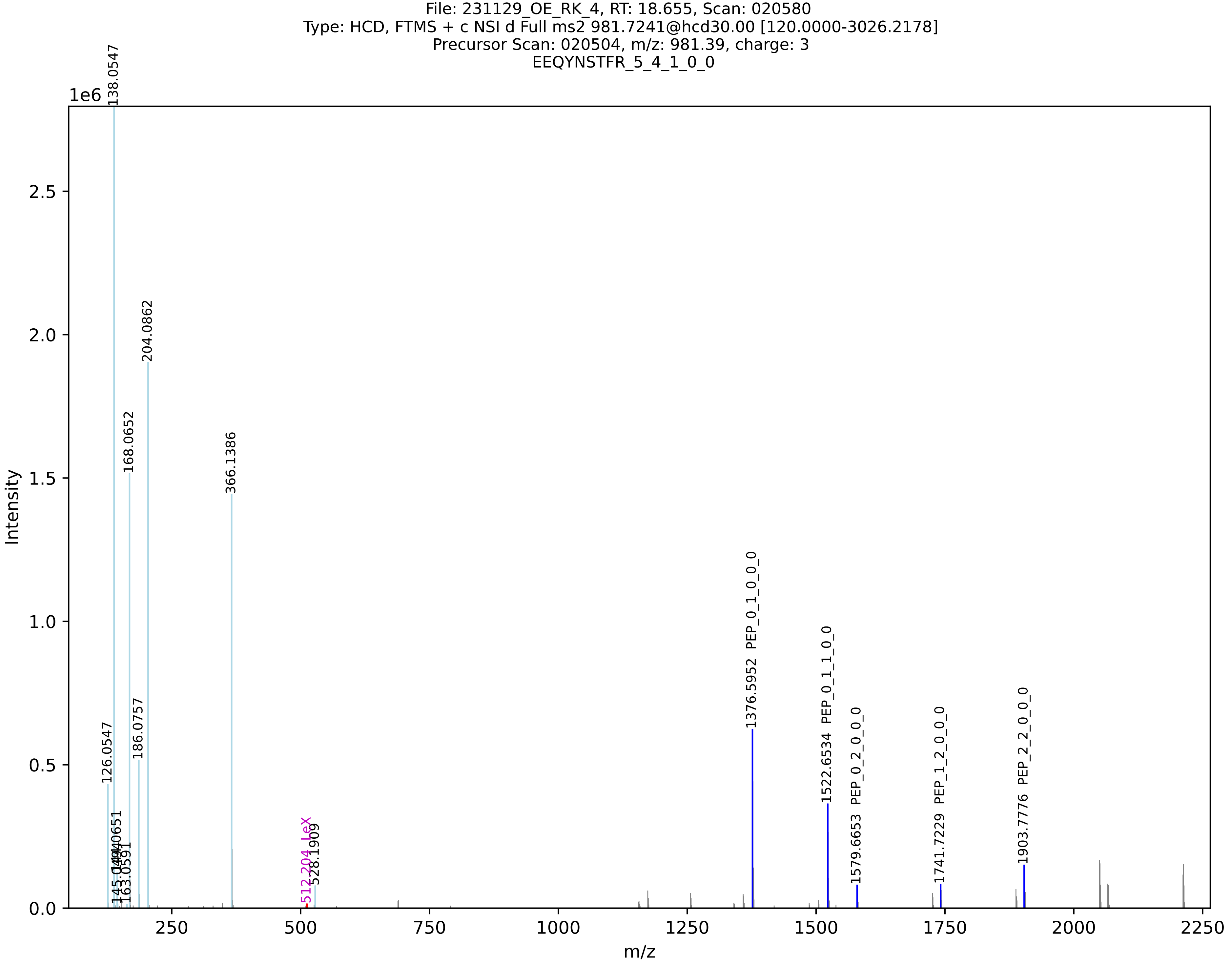

File: 231129_OE_RK_4, RT: 18.655, Scan: 020580
Type: HCD, FTMS + c NSI d Full ms2 981.7241@hcd30.00 [120.0000-3026.2178]
Precursor Scan: 020504, m/z: 981.39, charge: 3
EEQYNSTFR_5_4_1_0_0
1e6
Intensity
m/z
138.0547
204.0862
168.0652
366.1386
186.0757
126.0547
512.204_LeX
528.1909
1376.5952 PEP_0_1_0_0_0
1522.6534 PEP_0_1_1_0_0
1579.6653 PEP_0_2_0_0_0
1741.7229 PEP_1_2_0_0_0
1903.7776 PEP_2_2_0_0_0

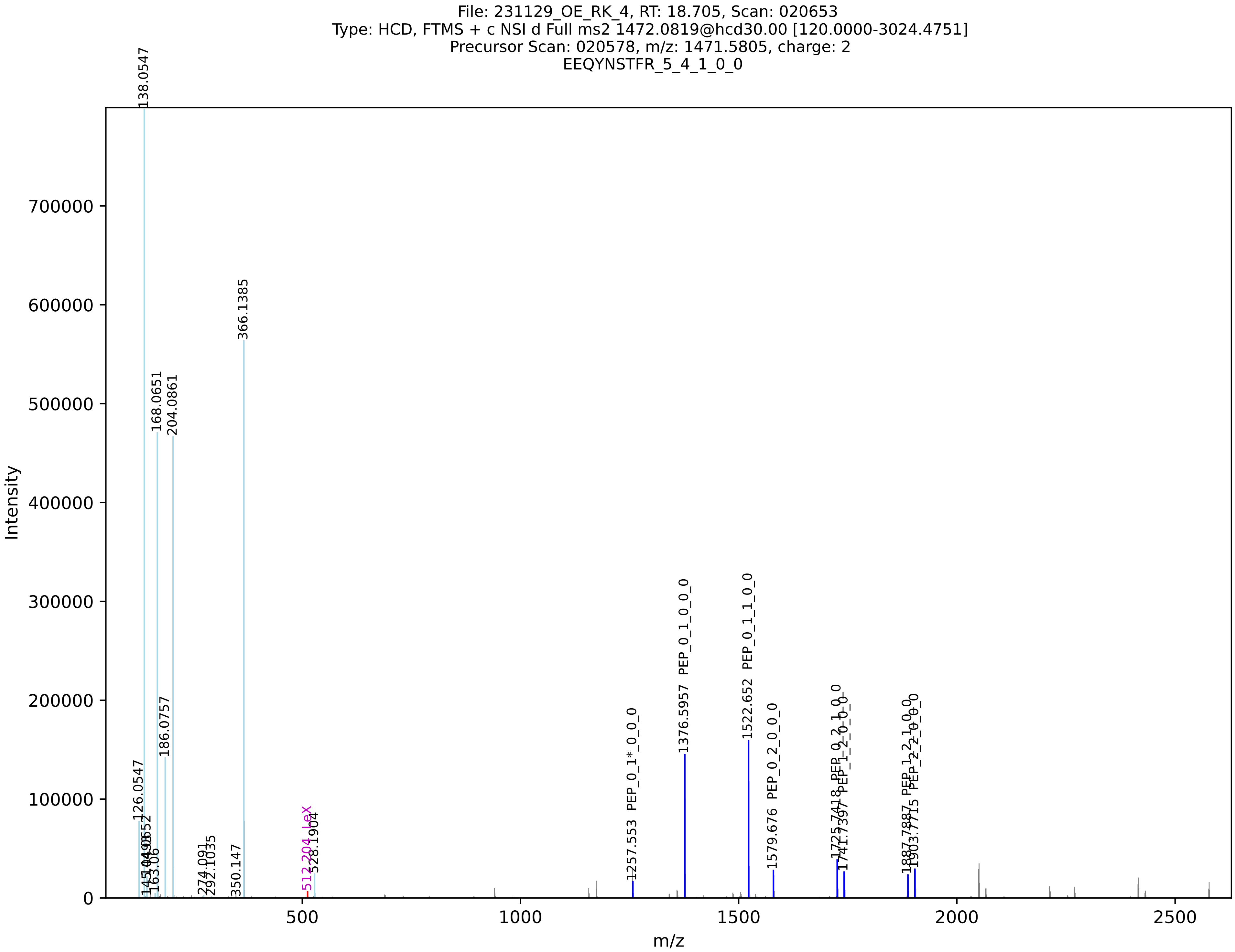

File: 231129_OE_RK_4, RT: 18.705, Scan: 020653
Type: HCD, FTMS + c NSI d Full ms2 1472.0819@hcd30.00 [120.0000-3024.4751]
Precursor Scan: 020578, m/z: 1471.5805, charge: 2
EEQYNSTFR_5_4_1_0_0
Intensity
m/z
138.0547
366.1385
168.0651
204.0861
186.0757
126.0547
512.204 LeX
528.1904
274.091
292.1035
350.147
1257.553 PEP_0_1*_0_0_0
1376.5957 PEP_0_1_0_0_0
1522.652 PEP_0_1_1_0_0
1579.676 PEP_0_2_0_0_0
1725.7418 PEP_0_2_1_0_0
1741.7397 PEP_1_2_0_0_0
1887.7887 PEP_1_2_1_0_0
1903.7715 PEP_2_2_0_0_0

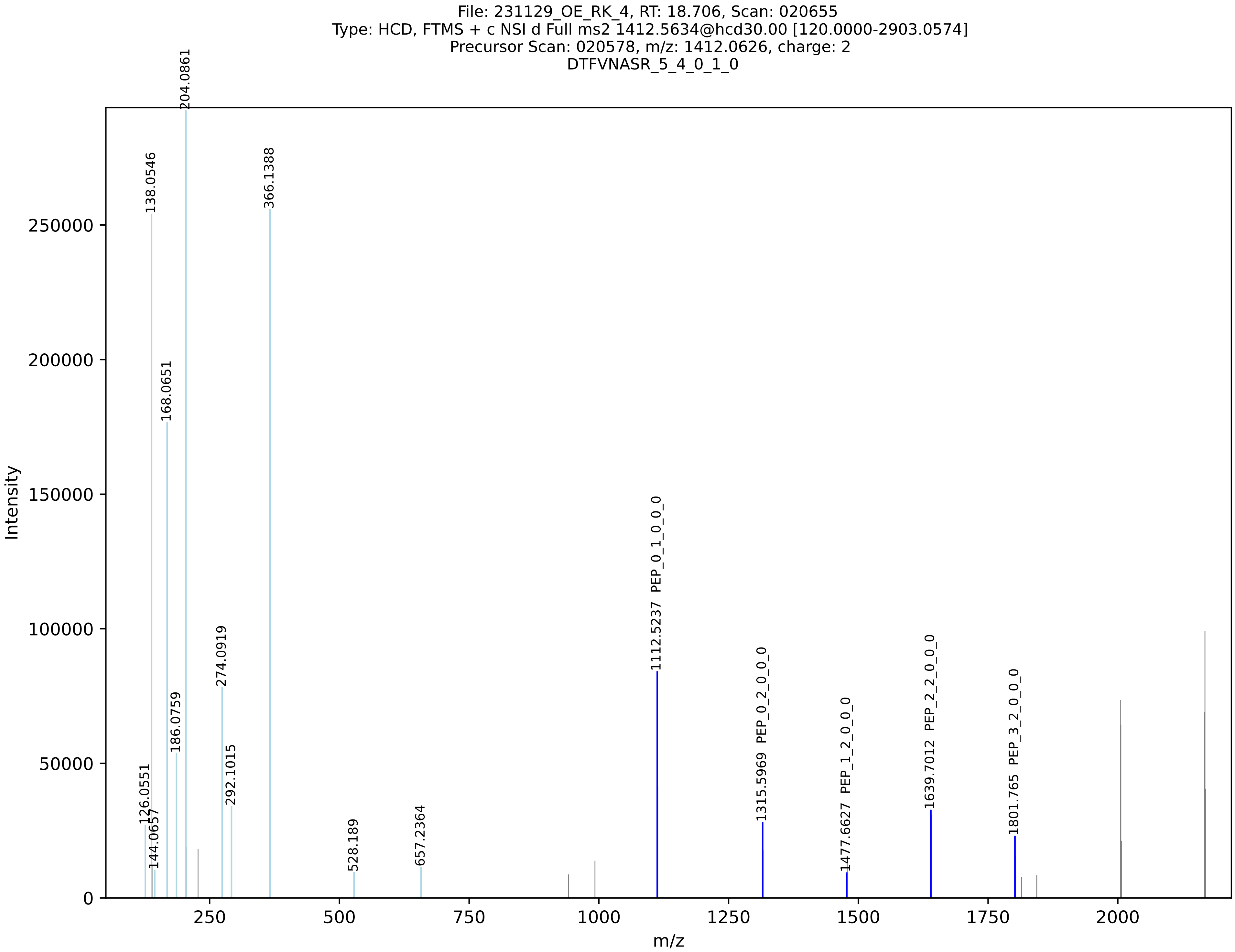

File: 231129_OE_RK_4, RT: 18.706, Scan: 020655
Type: HCD, FTMS + c NSI d Full ms2 1412.5634@hcd30.00 [120.0000-2903.0574]
Precursor Scan: 020578, m/z: 1412.0626, charge: 2
DTFVNASR_5_4_0_1_0
204.0861
138.0546
366.1388
168.0651
186.0759
274.0919
292.1015
126.0551
144.0657
528.189
657.2364
1112.5237 PEP_0_1_0_0_0
1315.5969 PEP_0_2_0_0_0
1477.6627 PEP_1_2_0_0_0
1639.7012 PEP_2_2_0_0_0
1801.765 PEP_3_2_0_0_0
Intensity
m/z

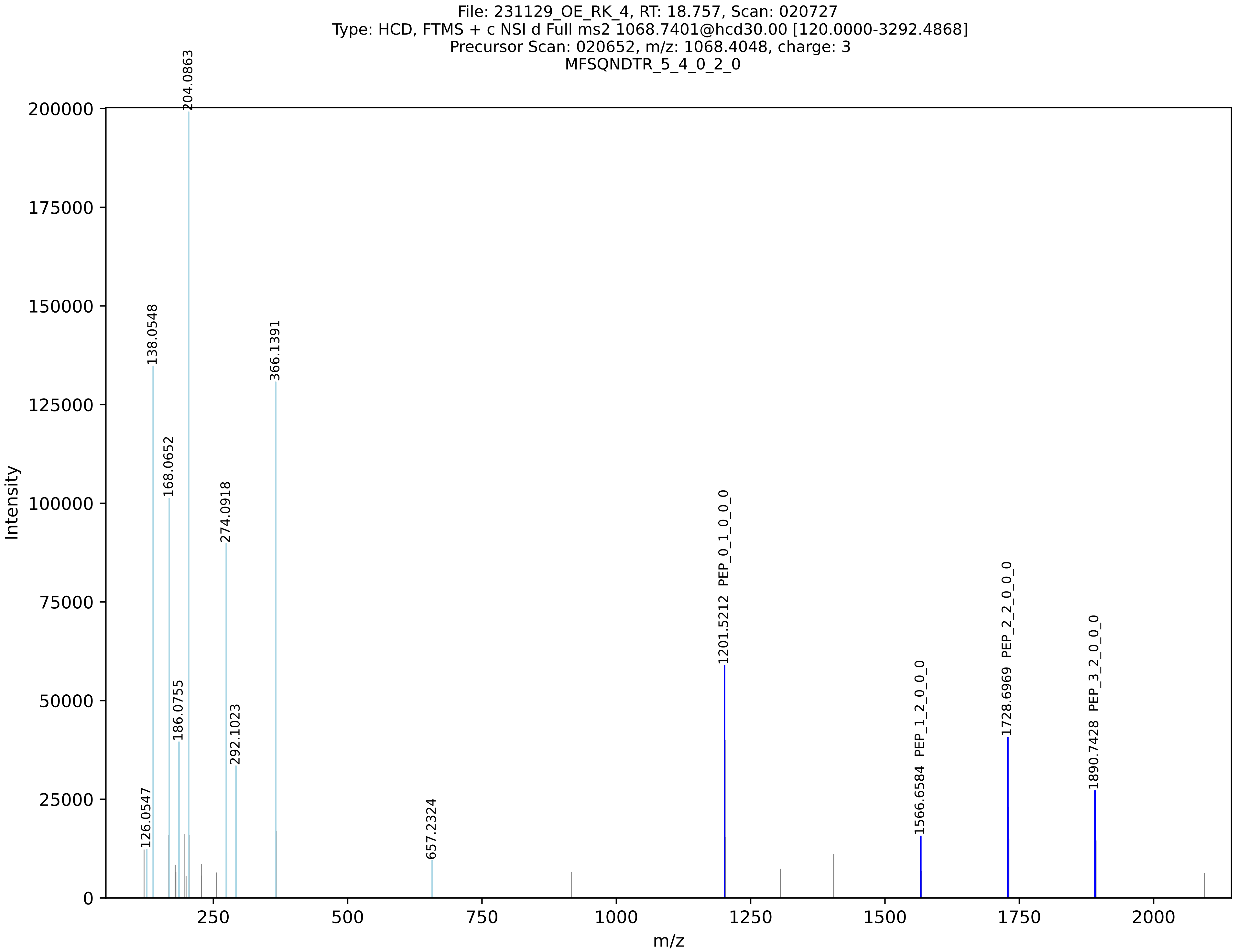
File: 231129_OE_RK_4, RT: 18.757, Scan: 020727
Type: HCD, FTMS + c NSI d Full ms2 1068.7401@hcd30.00 [120.0000-3292.4868]
Precursor Scan: 020652, m/z: 1068.4048, charge: 3
MFSQNDTR_5_4_0_2_0
Intensity
m/z
204.0863
138.0548
366.1391
168.0652
274.0918
186.0755
292.1023
126.0547
657.2324
1201.5212 PEP_0_1_0_0_0_
1566.6584 PEP_1_2_0_0_0_
1728.6969 PEP_2_2_0_0_0_
1890.7428 PEP_3_2_0_0_0_

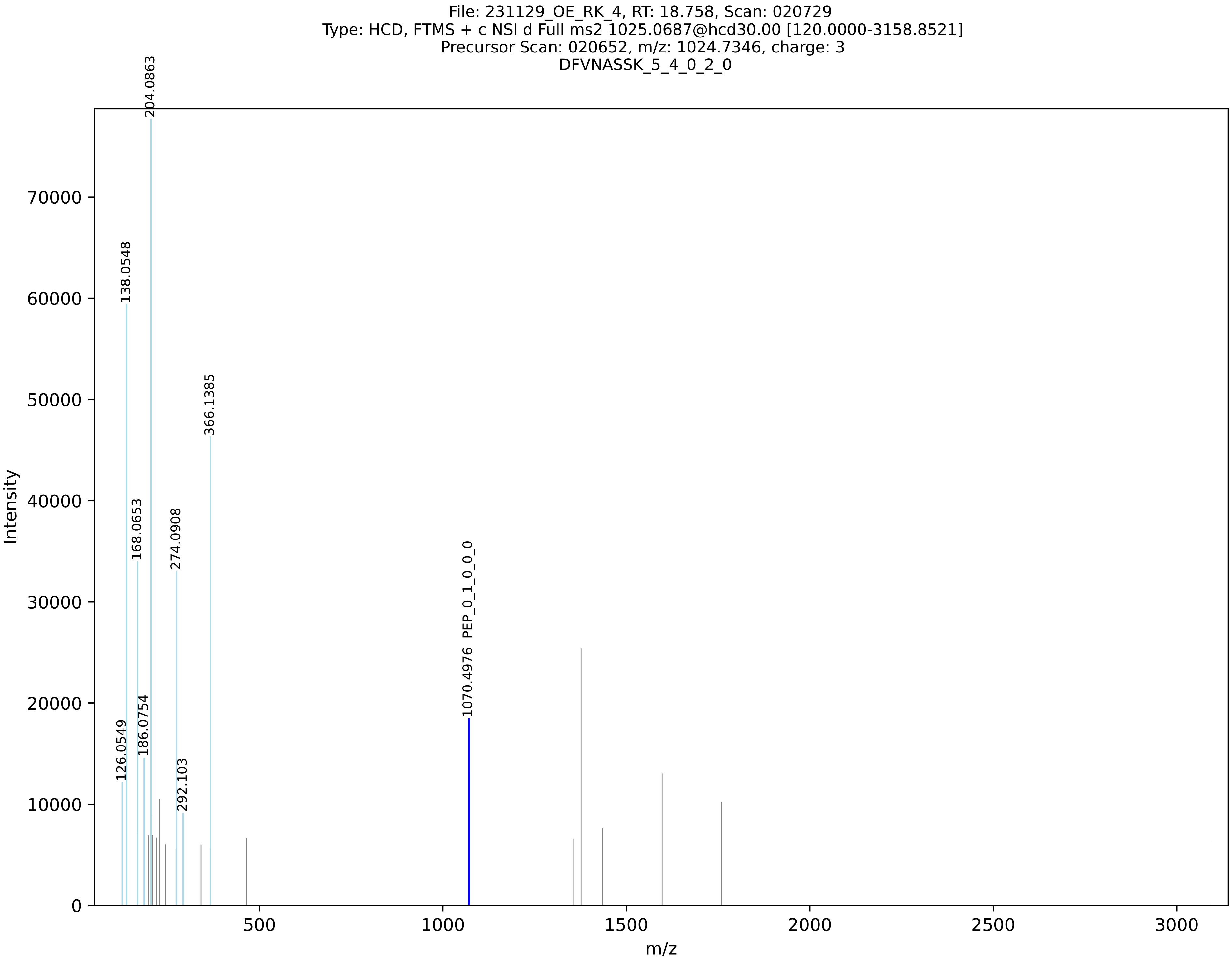
File: 231129_OE_RK_4, RT: 18.758, Scan: 020729
Type: HCD, FTMS + c NSI d Full ms2 1025.0687@hcd30.00 [120.0000-3158.8521]
Precursor Scan: 020652, m/z: 1024.7346, charge: 3
DFVNASSK_5_4_0_2_0
204.0863
138.0548
366.1385
168.0653
274.0908
1070.4976 PEP_0_1_0_0_
126.0549
186.0754
292.103
Intensity
m/z
0
10000
20000
30000
40000
50000
60000
70000
500
1000
1500
2000
2500
3000

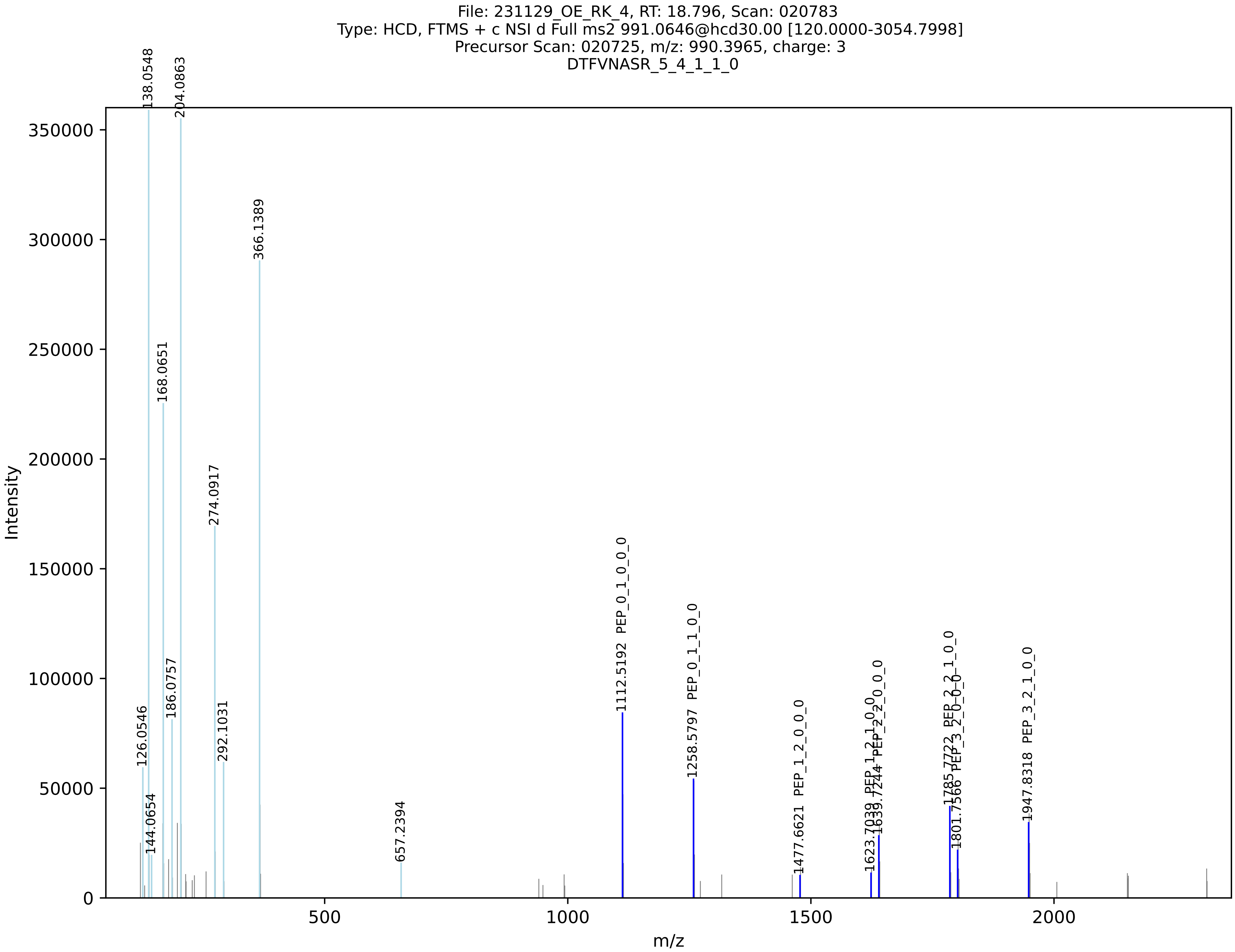
File: 231129_OE_RK_4, RT: 18.796, Scan: 020783
Type: HCD, FTMS + c NSI d Full ms2 991.0646@hcd30.00 [120.0000-3054.7998]
Precursor Scan: 020725, m/z: 990.3965, charge: 3
DTFVNASR_5_4_1_1_0
Intensity
m/z
138.0548
204.0863
366.1389
168.0651
274.0917
126.0546
144.0654
186.0757
292.1031
657.2394
1112.5192 PEP_0_1_0_0_0
1258.5797 PEP_0_1_1_0_0
1477.6621 PEP_1_2_0_0_0
1623.7039 PEP_1_2_1_0_0
1639.7244 PEP_2_2_0_0_0
1785.7722 PEP_2_2_1_0_0
1801.7566 PEP_3_2_0_0_0
1947.8318 PEP_3_2_1_0_0

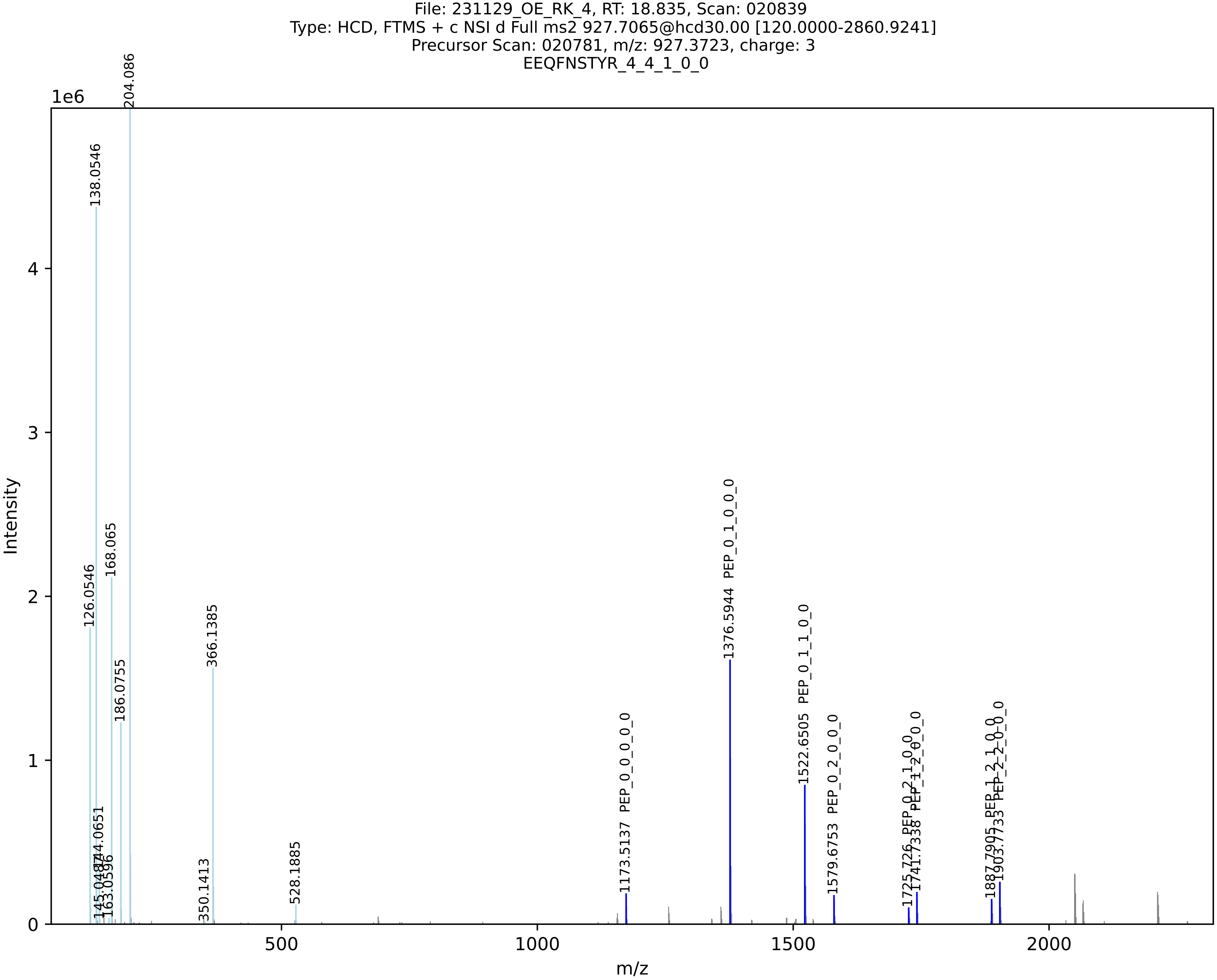

File: 231129_OE_RK_4, RT: 18.835, Scan: 020839
Type: HCD, FTMS + c NSI d Full ms2 927.7065@hcd30.00 [120.0000-2860.9241]
Precursor Scan: 020781, m/z: 927.3723, charge: 3
EEQFNSTYR_4_4_1_0_0
1e6
Intensity
m/z
204.086
138.0546
168.065
126.0546
366.1385
186.0755
144.0651
145.0487
163.0596
350.1413
528.1885
1173.5137 PEP_0_0_0_0_0
1376.5944 PEP_0_1_0_0_0
1522.6505 PEP_0_1_1_0_0
1579.6753 PEP_0_2_0_0_0
1725.726 PEP_0_2_1_0_0
1741.7338 PEP_1_2_0_0_0
1887.7905 PEP_1_2_1_0_0
1903.7733 PEP_2_2_0_0_0

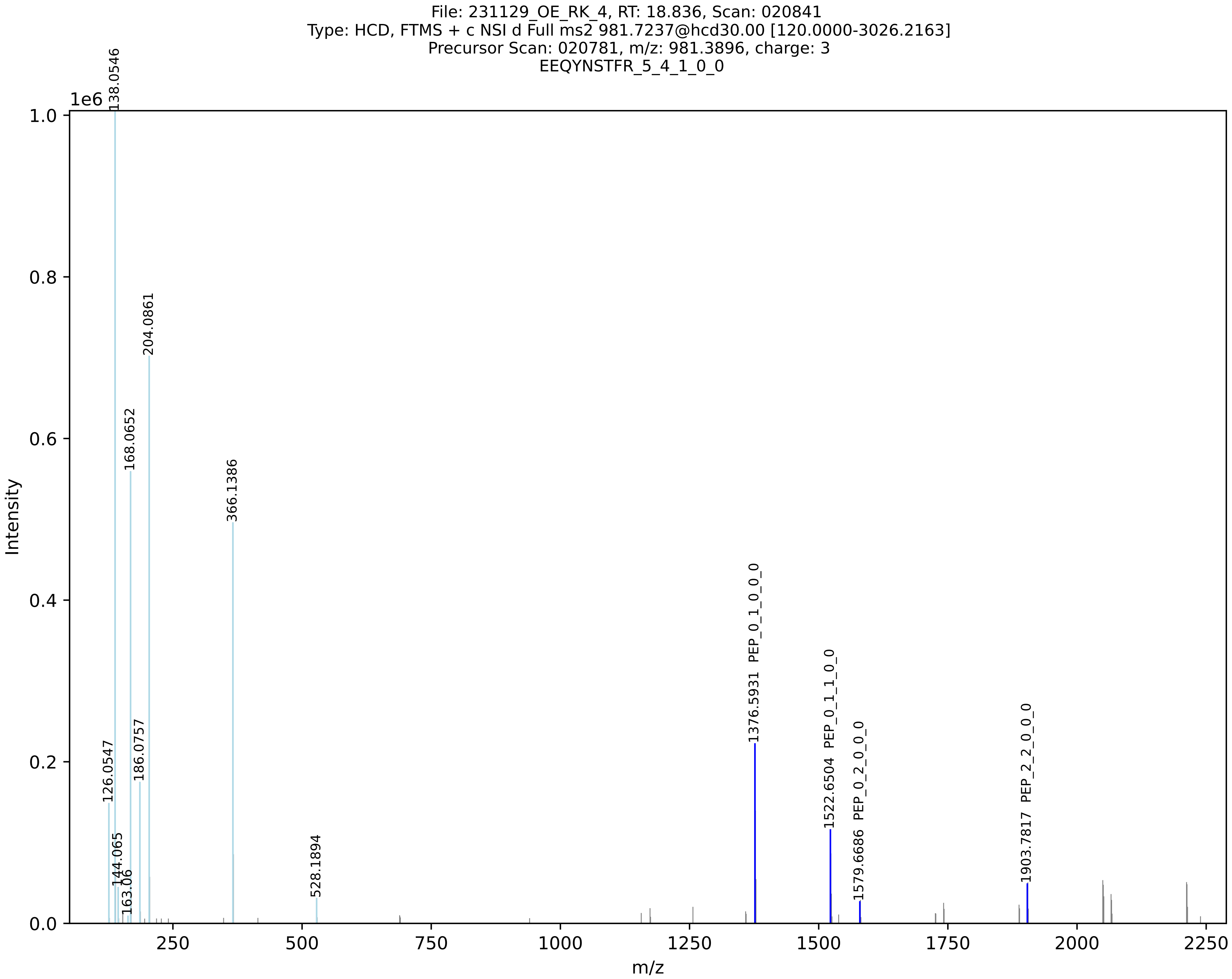
File: 231129_OE_RK_4, RT: 18.836, Scan: 020841
Type: HCD, FTMS + c NSI d Full ms2 981.7237@hcd30.00 [120.0000-3026.2163]
Precursor Scan: 020781, m/z: 981.3896, charge: 3
EEQYNSTFR_5_4_1_0_0
1e6
Intensity
m/z
138.0546
204.0861
168.0652
366.1386
126.0547
186.0757
144.065
163.06
528.1894
1376.5931 PEP_0_1_0_0_0
1522.6504 PEP_0_1_1_0_0
1579.6686 PEP_0_2_0_0_0
1903.7817 PEP_2_2_0_0_0

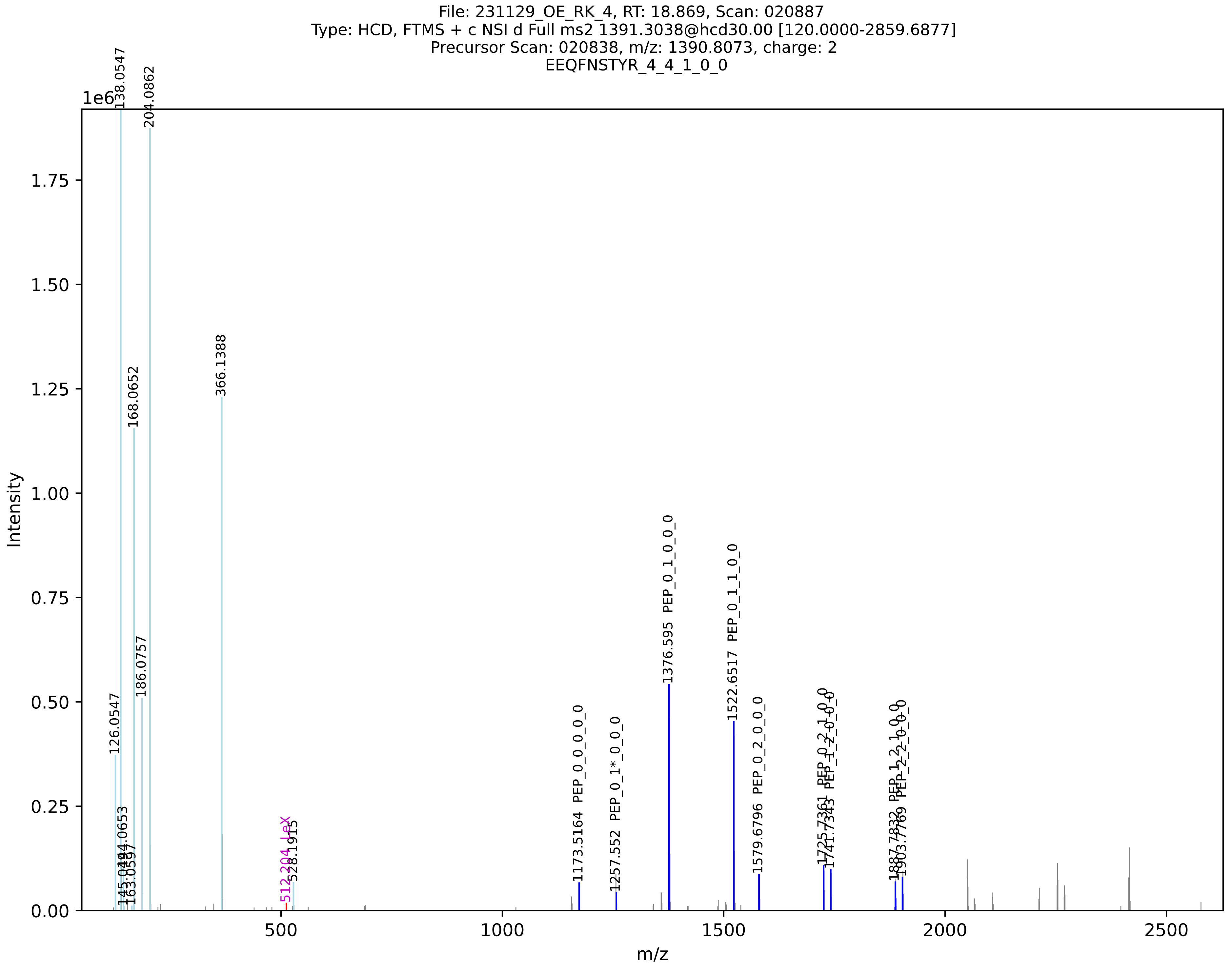
File: 231129_OE_RK_4, RT: 18.869, Scan: 020887
Type: HCD, FTMS + c NSI d Full ms2 1391.3038@hcd30.00 [120.0000-2859.6877]
Precursor Scan: 020838, m/z: 1390.8073, charge: 2
EEQFNSTYR_4_4_1_0_0
1e6
Intensity
m/z
138.0547
204.0862
366.1388
168.0652
186.0757
126.0547
144.0653
528.1915
512.204_LeX
1173.5164 PEP_0_0_0_0_0
1257.552 PEP_0_1*_0_0_0
1376.595 PEP_0_1_0_0_0
1522.6517 PEP_0_1_1_0_0
1579.6796 PEP_0_2_0_0_0
1725.7361 PEP_0_2_1_0_0
1741.7343 PEP_1_2_0_0_0
1887.7832 PEP_1_2_1_0_0
1903.7769 PEP_2_2_0_0_0

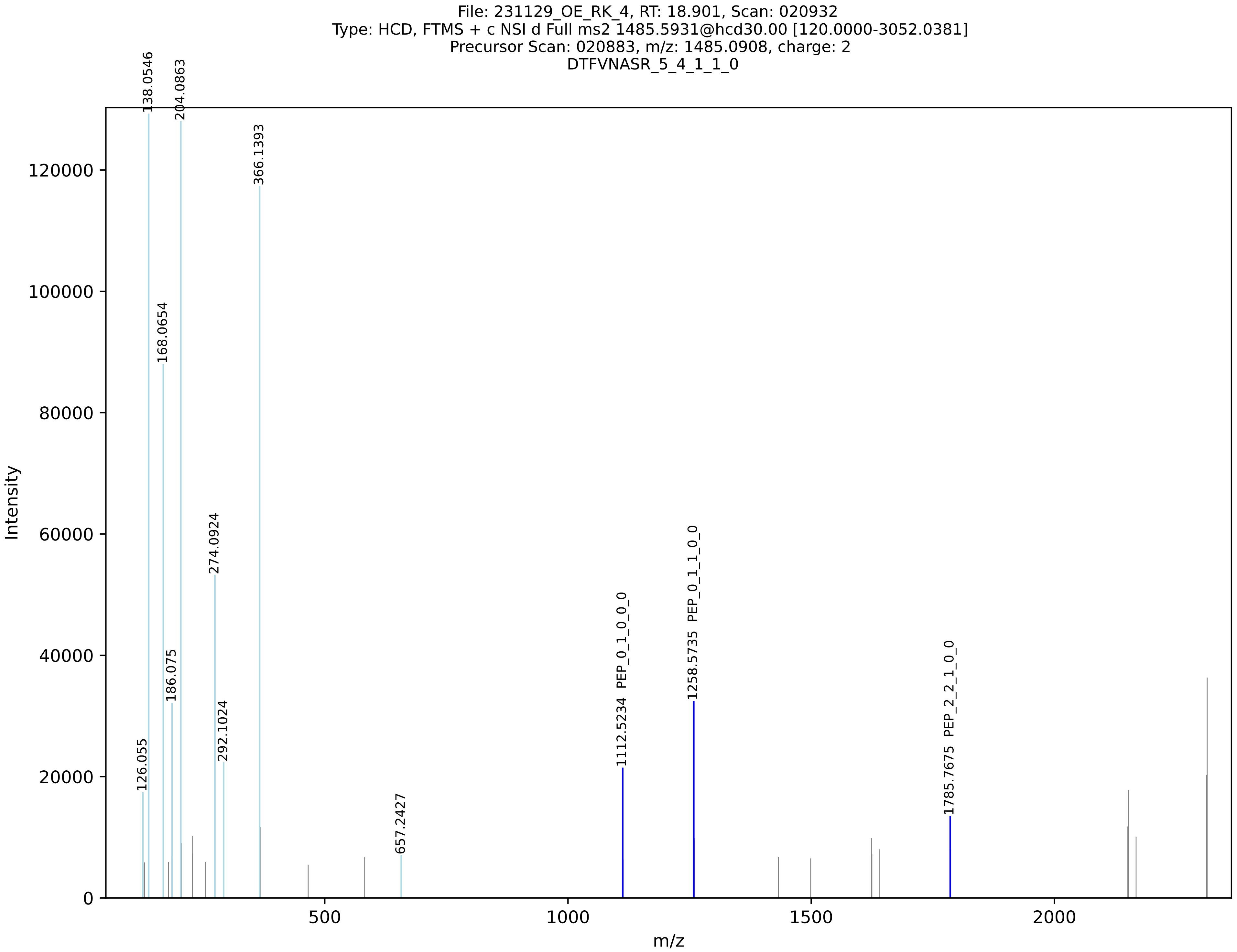

File: 231129_OE_RK_4, RT: 18.901, Scan: 020932
Type: HCD, FTMS + c NSI d Full ms2 1485.5931@hcd30.00 [120.0000-3052.0381]
Precursor Scan: 020883, m/z: 1485.0908, charge: 2
DTFVNASR_5_4_1_1_0
Intensity
m/z
0
20000
40000
60000
80000
100000
120000
500
1000
1500
2000
138.0546
204.0863
366.1393
168.0654
274.0924
186.075
292.1024
126.055
657.2427
1112.5234 PEP_0_1_0_0_0
1258.5735 PEP_0_1_1_0_0
1785.7675 PEP_2_2_1_0_0

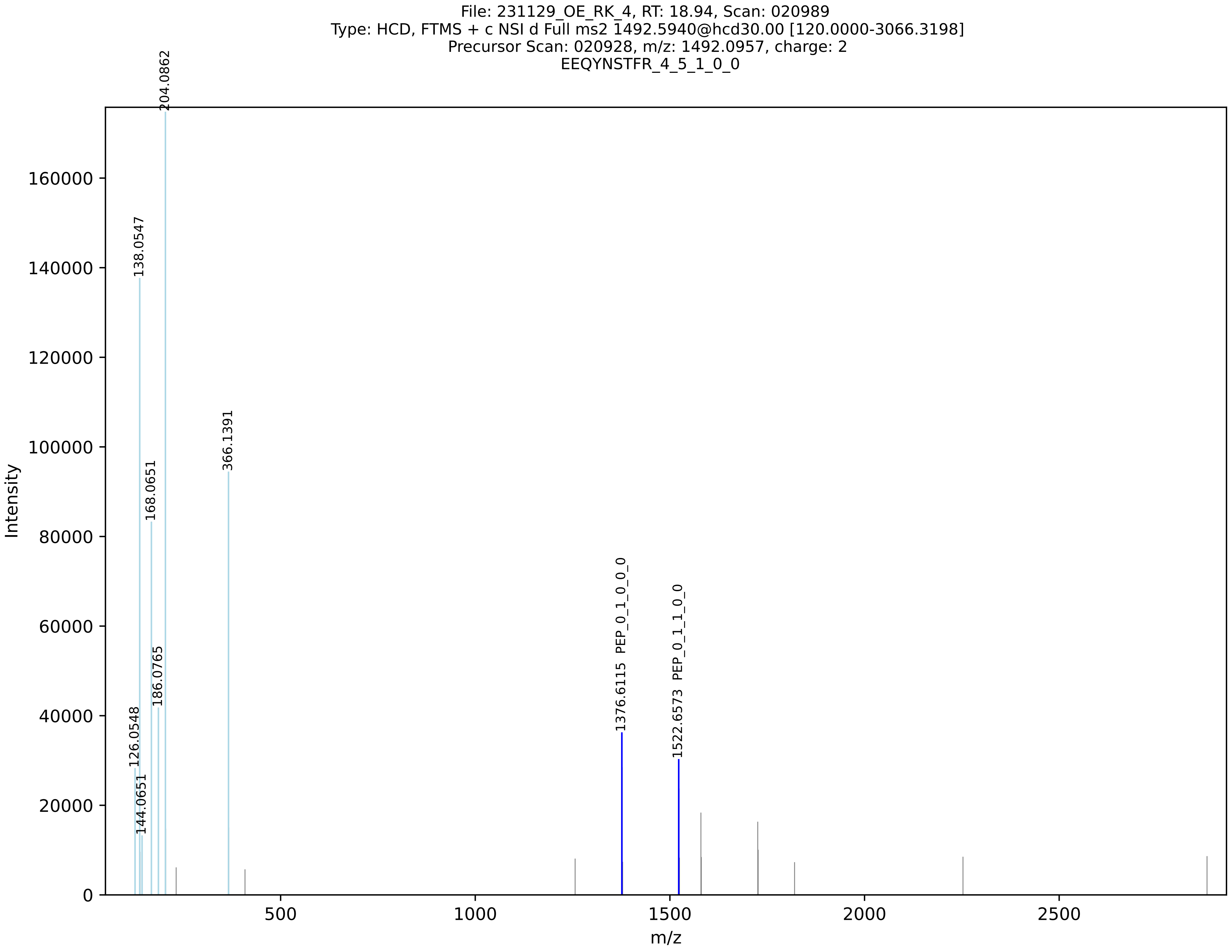
File: 231129_OE_RK_4, RT: 18.94, Scan: 020989
Type: HCD, FTMS + c NSI d Full ms2 1492.5940@hcd30.00 [120.0000-3066.3198]
Precursor Scan: 020928, m/z: 1492.0957, charge: 2
EEQYNSTFR_4_5_1_0_0
204.0862
138.0547
366.1391
168.0651
186.0765
126.0548
144.0651
1376.6115 PEP_0_1_0_0_0
1522.6573 PEP_0_1_1_0_0
Intensity
m/z
0
20000
40000
60000
80000
100000
120000
140000
160000
500
1000
1500
2000
2500

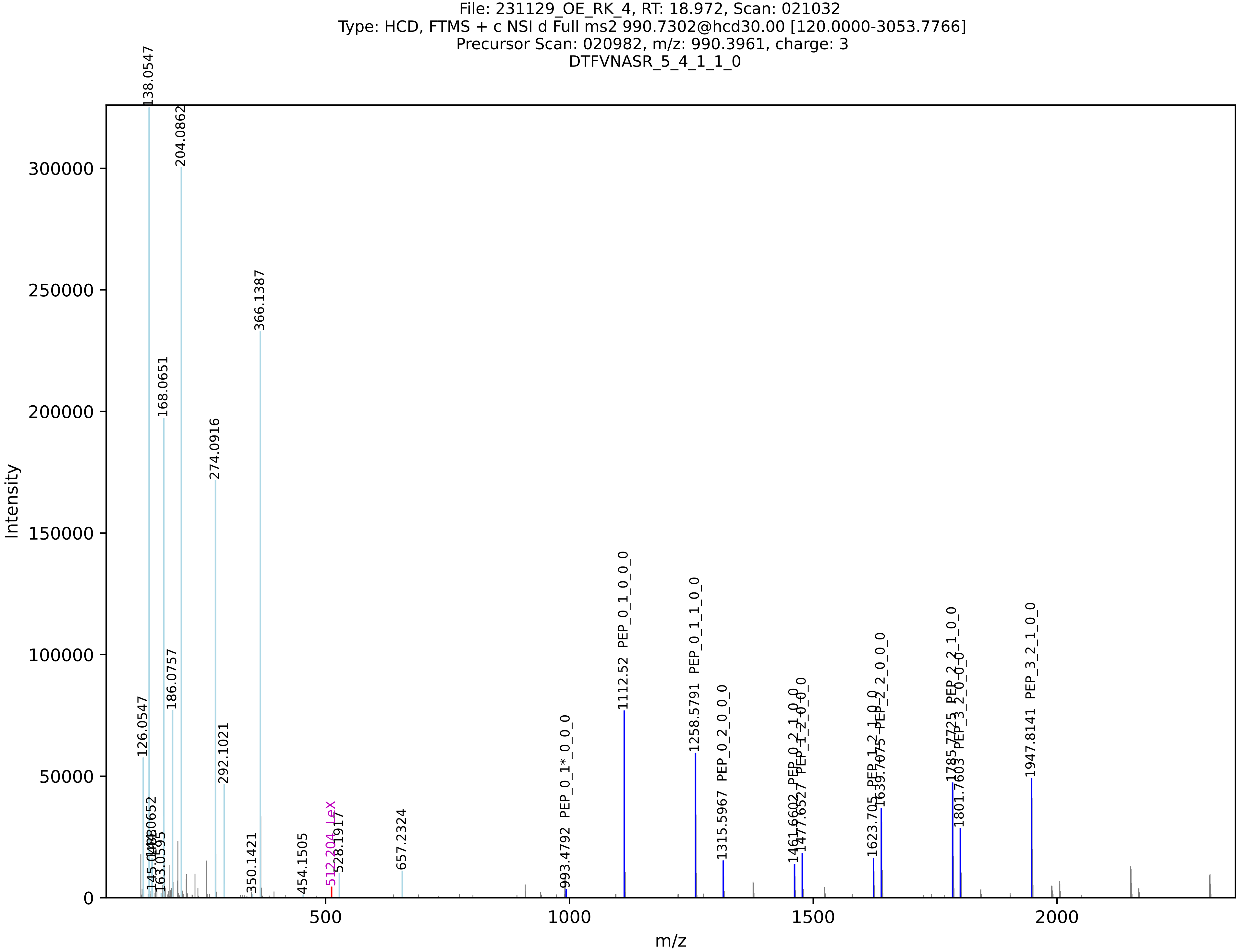
File: 231129_OE_RK_4, RT: 18.972, Scan: 021032
Type: HCD, FTMS + c NSI d Full ms2 990.7302@hcd30.00 [120.0000-3053.7766]
Precursor Scan: 020982, m/z: 990.3961, charge: 3
DTFVNASR_5_4_1_1_0
Intensity
m/z
138.0547
204.0862
366.1387
168.0651
274.0916
186.0757
126.0547
292.1021
350.1421
454.1505
512.204_LeX
528.1917
657.2324
993.4792 PEP_0_1*_0_0_
1112.52 PEP_0_1_0_0_0
1258.5791 PEP_0_1_1_0_0
1315.5967 PEP_0_2_0_0_0
1461.6602 PEP_0_2_1_0_0
1477.6527 PEP_1_2_0_0_0
1623.705 PEP_1_2_1_0_0
1639.7075 PEP_2_2_0_0_0
1785.7725 PEP_2_2_1_0_0
1801.7603 PEP_3_2_0_0_0
1947.8141 PEP_3_2_1_0_0

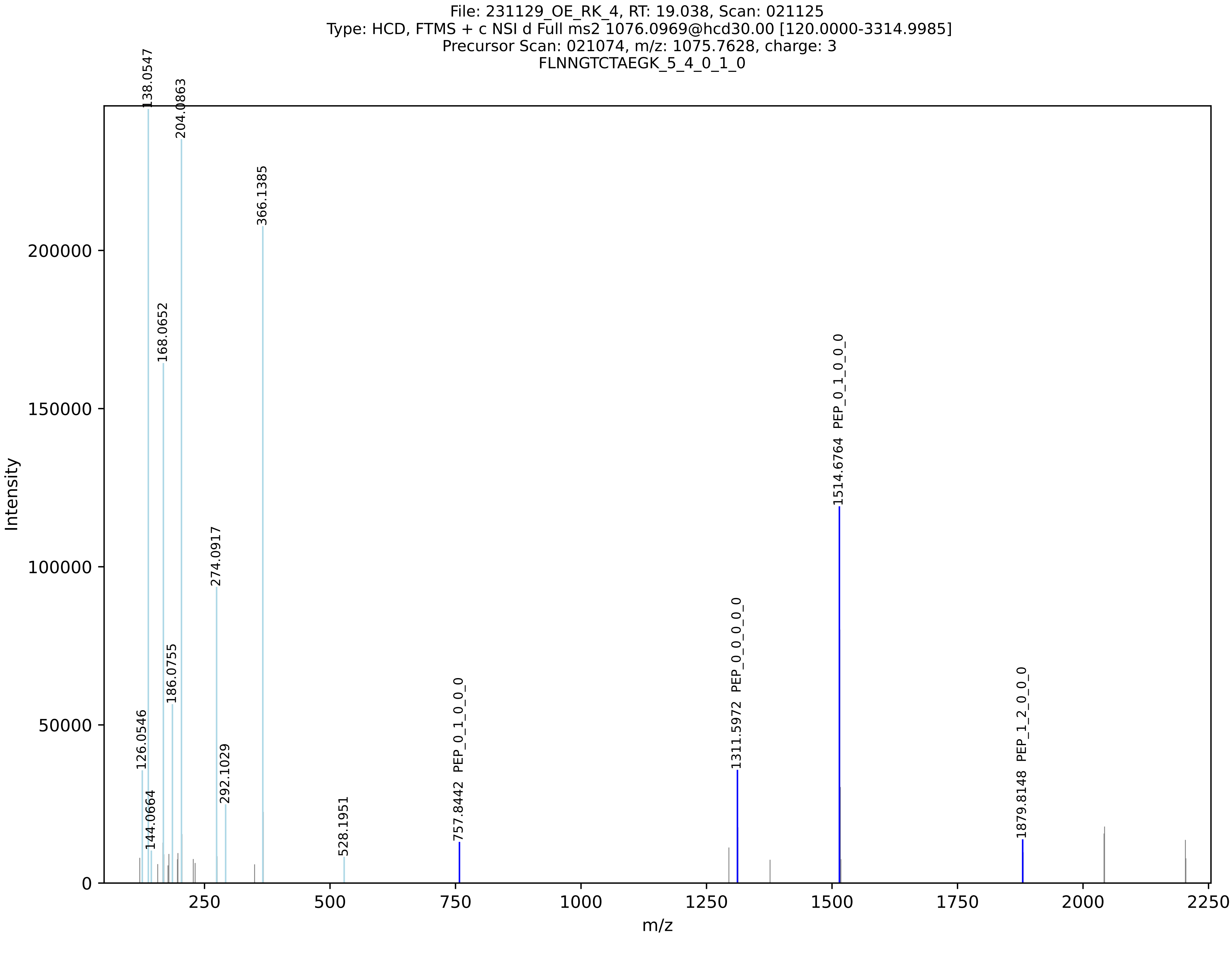
File: 231129_OE_RK_4, RT: 19.038, Scan: 021125
Type: HCD, FTMS + c NSI d Full ms2 1076.0969@hcd30.00 [120.0000-3314.9985]
Precursor Scan: 021074, m/z: 1075.7628, charge: 3
FLNNGTCTAEGK_5_4_0_1_0
138.0547
204.0863
366.1385
168.0652
274.0917
186.0755
126.0546
292.1029
144.0664
528.1951
757.8442 PEP_0_1_0_0_0_
1311.5972 PEP_0_0_0_0_0_
1514.6764 PEP_0_1_0_0_0_
1879.8148 PEP_1_2_0_0_0_
Intensity
0
50000
100000
150000
200000
250
500
750
1000
1250
1500
1750
2000
2250
m/z

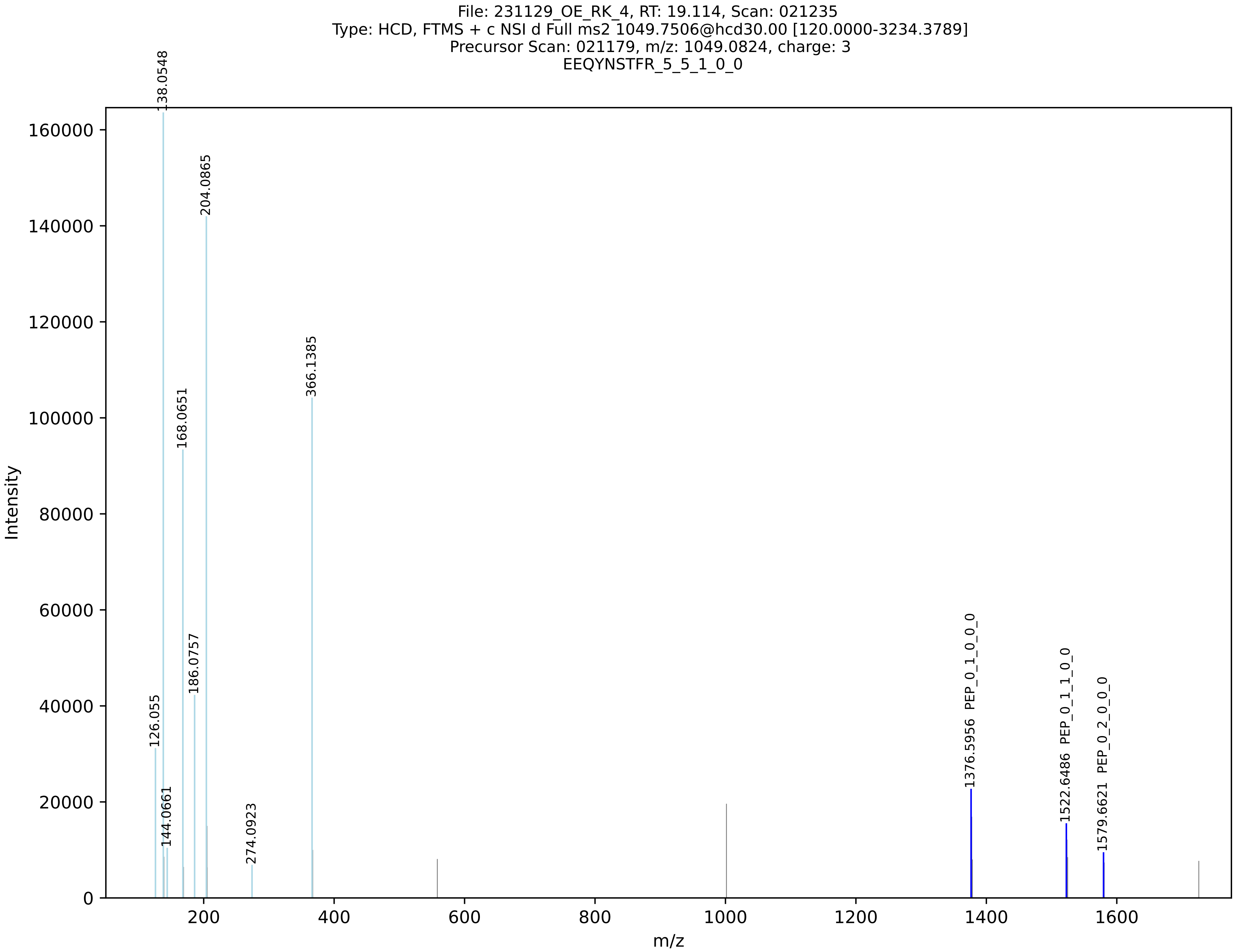
File: 231129_OE_RK_4, RT: 19.114, Scan: 021235
Type: HCD, FTMS + c NSI d Full ms2 1049.7506@hcd30.00 [120.0000-3234.3789]
Precursor Scan: 021179, m/z: 1049.0824, charge: 3
EEQYNSTFR_5_5_1_0_0
138.0548
204.0865
366.1385
168.0651
186.0757
126.055
144.0661
274.0923
1376.5956 PEP_0_1_0_0_0
1522.6486 PEP_0_1_1_0_0
1579.6621 PEP_0_2_0_0_0
Intensity
m/z
0
20000
40000
60000
80000
100000
120000
140000
160000
200
400
600
800
1000
1200
1400
1600

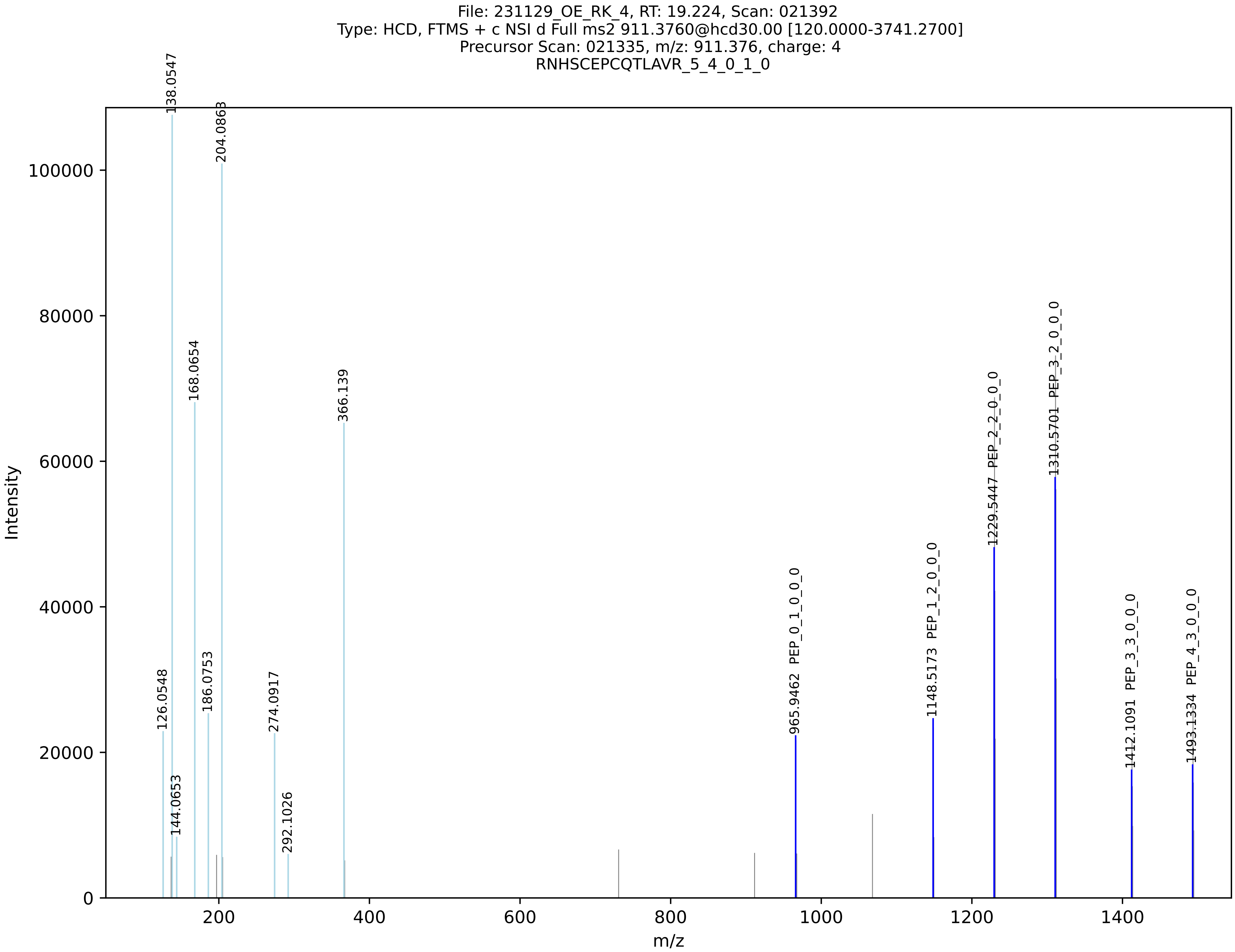

File: 231129_OE_RK_4, RT: 19.224, Scan: 021392
Type: HCD, FTMS + c NSI d Full ms2 911.3760@hcd30.00 [120.0000-3741.2700]
Precursor Scan: 021335, m/z: 911.376, charge: 4
RNHSCEPCQTLAVR_5_4_0_1_0
Intensity
m/z
138.0547
204.0863
168.0654
366.139
126.0548
186.0753
274.0917
144.0653
292.1026
965.9462 PEP_0_1_0_0_0
1148.5173 PEP_1_2_0_0_0
1229.5447 PEP_2_2_0_0_0
1310.5701 PEP_3_2_0_0_0
1412.1091 PEP_3_3_0_0_0
1493.1334 PEP_4_3_0_0_0

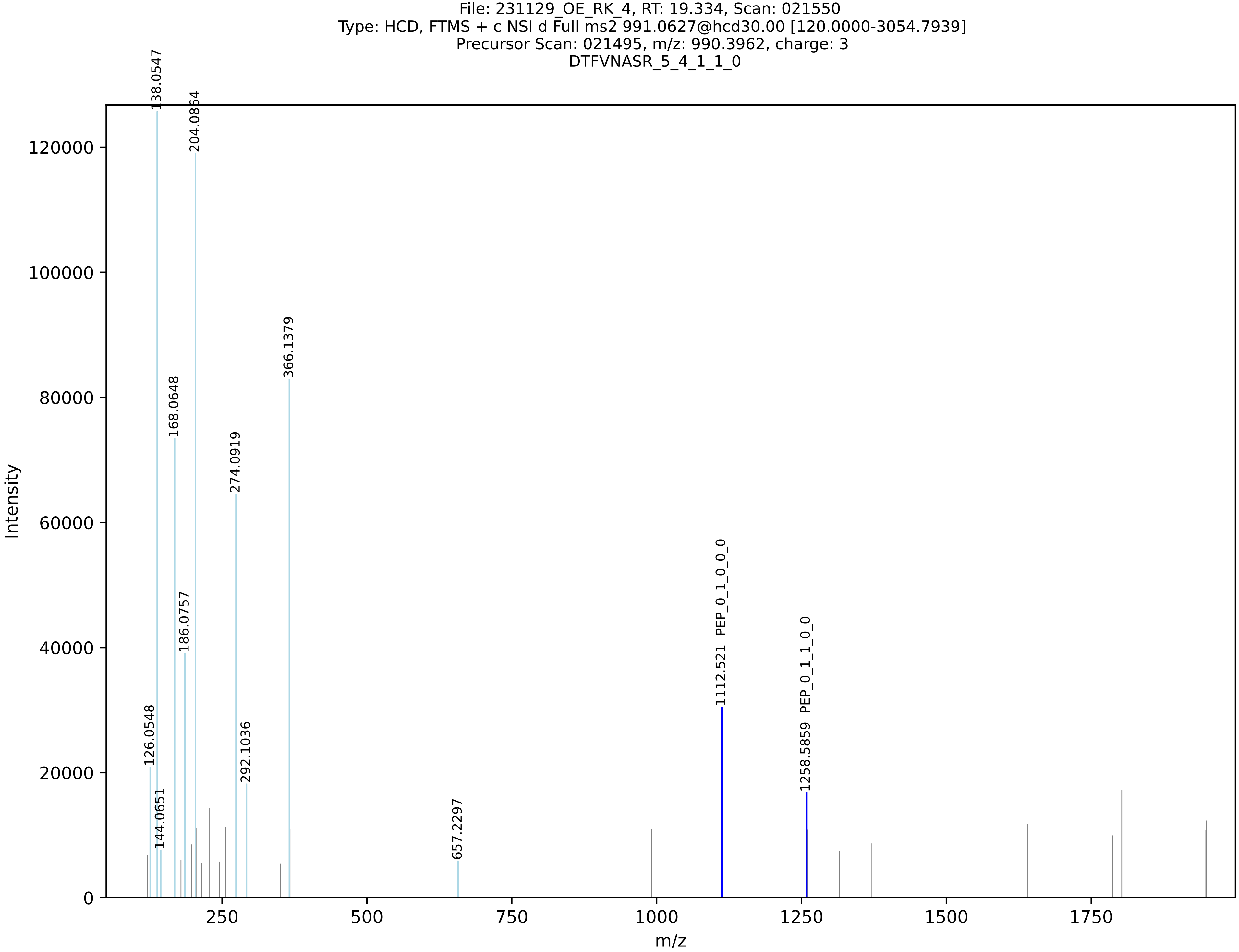
File: 231129_OE_RK_4, RT: 19.334, Scan: 021550
Type: HCD, FTMS + c NSI d Full ms2 991.0627@hcd30.00 [120.0000-3054.7939]
Precursor Scan: 021495, m/z: 990.3962, charge: 3
DTFVNASR_5_4_1_1_0
138.0547
204.0864
366.1379
168.0648
274.0919
186.0757
126.0548
292.1036
144.0651
657.2297
1112.521 PEP_0_1_0_0_0
1258.5859 PEP_0_1_1_0_0
Intensity
m/z
0
20000
40000
60000
80000
100000
120000
250
500
750
1000
1250
1500
1750

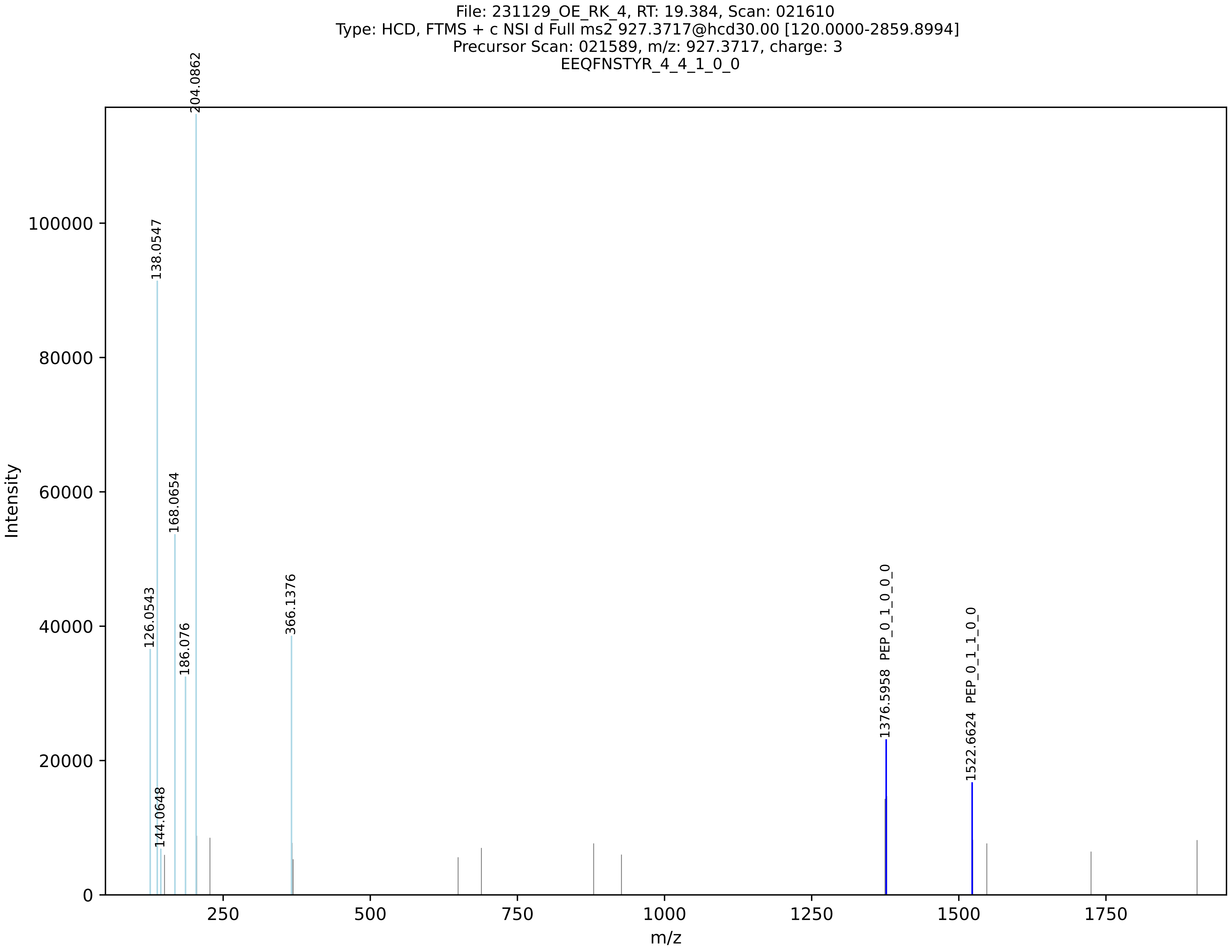
File: 231129_OE_RK_4, RT: 19.384, Scan: 021610
Type: HCD, FTMS + c NSI d Full ms2 927.3717@hcd30.00 [120.0000-2859.8994]
Precursor Scan: 021589, m/z: 927.3717, charge: 3
EEQFNSTYR_4_4_1_0_0
Intensity
m/z
204.0862
138.0547
168.0654
126.0543
186.076
144.0648
366.1376
1376.5958 PEP_0_1_0_0_0
1522.6624 PEP_0_1_1_0_0
0
20000
40000
60000
80000
100000
250
500
750
1000
1250
1500
1750

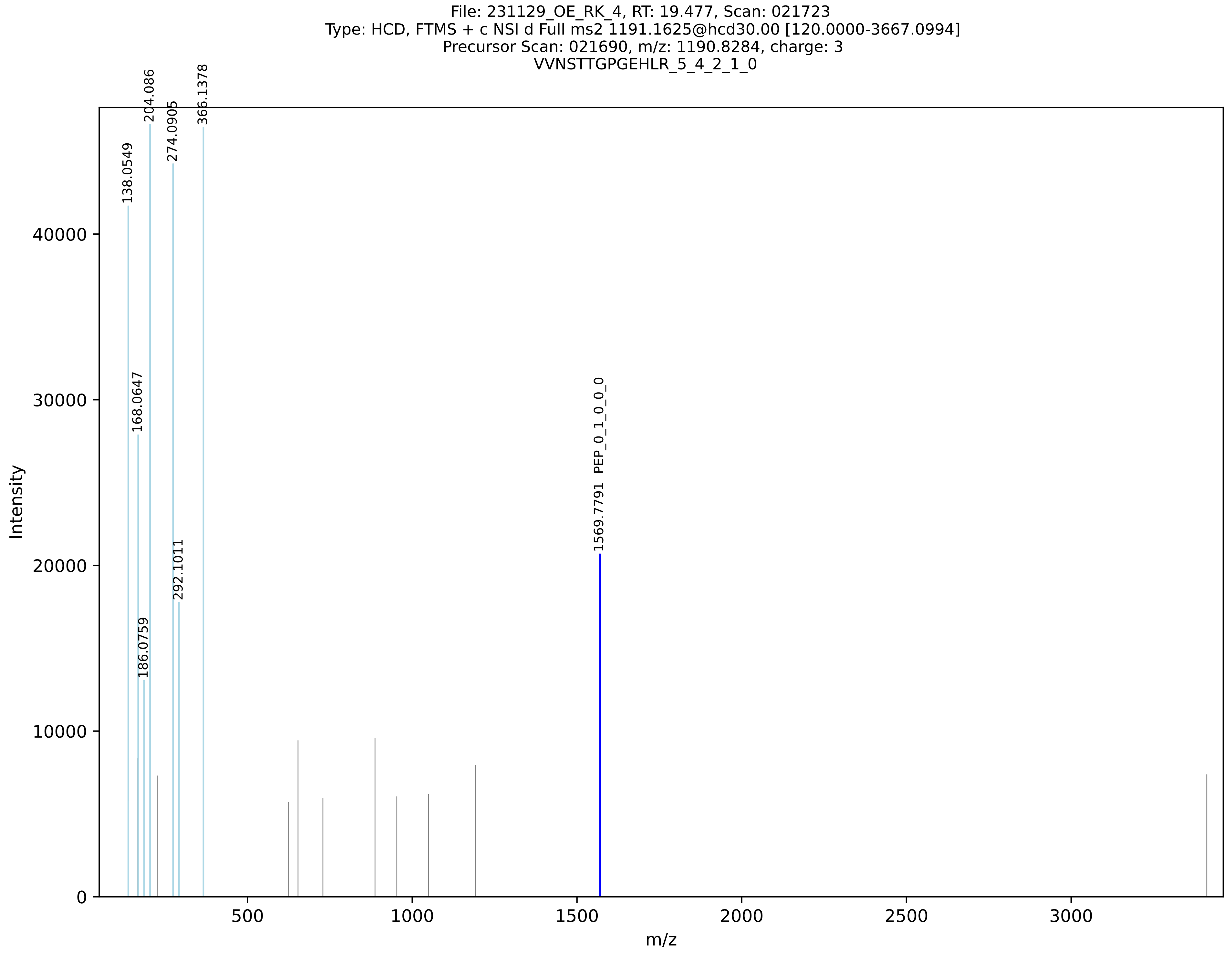
File: 231129_OE_RK_4, RT: 19.477, Scan: 021723
Type: HCD, FTMS + c NSI d Full ms2 1191.1625@hcd30.00 [120.0000-3667.0994]
Precursor Scan: 021690, m/z: 1190.8284, charge: 3
VVNSTTGPGEHLR_5_4_2_1_0
204.086
366.1378
274.0905
138.0549
168.0647
1569.7791 PEP_0_1_0_0_0
292.1011
186.0759
Intensity
m/z
0
10000
20000
30000
40000
500
1000
1500
2000
2500
3000

File: 231129_OE_RK_4, RT: 19.534, Scan: 021794
Type: HCD, FTMS + c NSI d Full ms2 962.3915@hcd30.00 [120.0000-2967.0603]
Precursor Scan: 021759, m/z: 962.0558, charge: 3
NYTLTGR_5_4_1_1_0

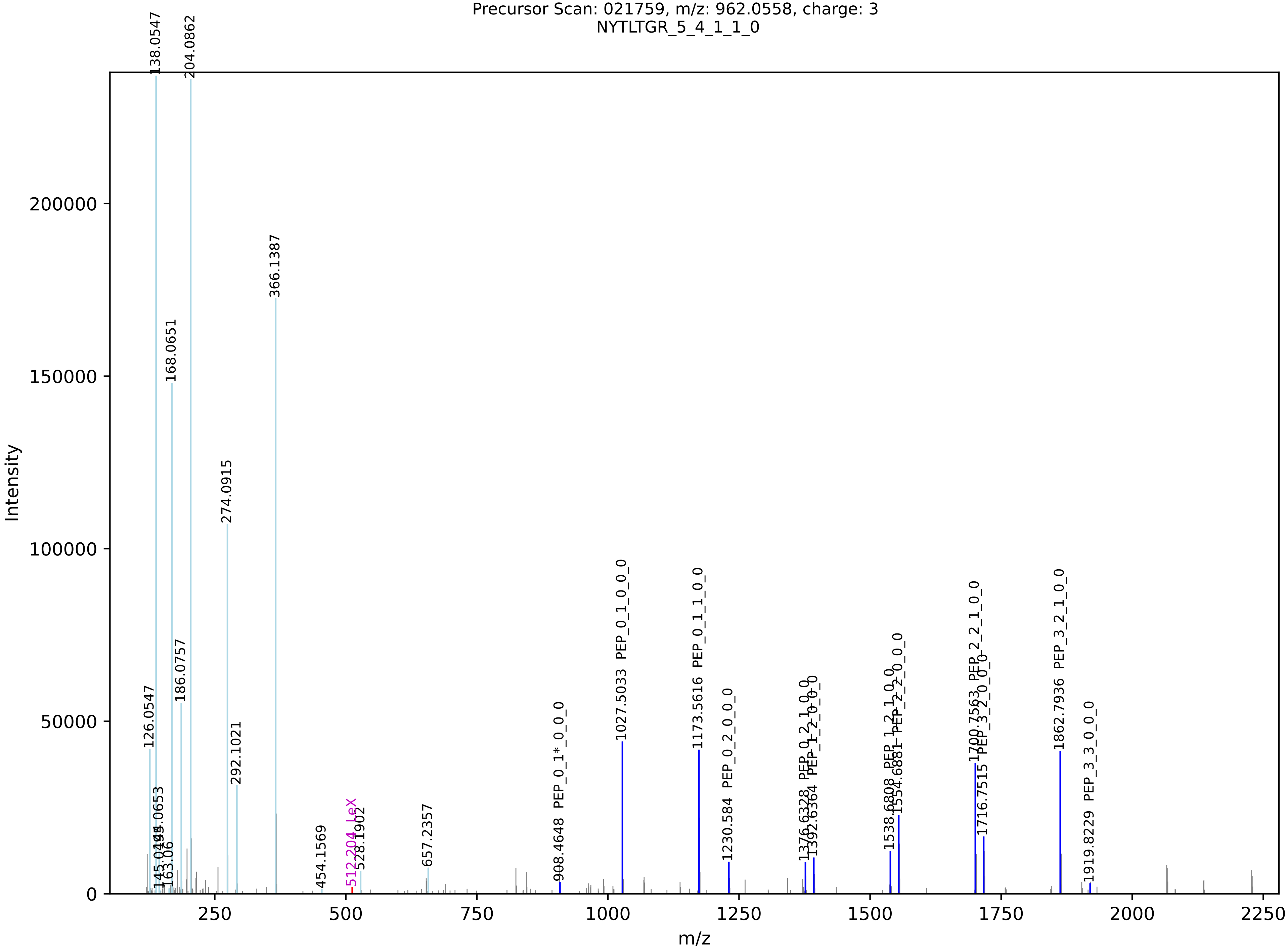

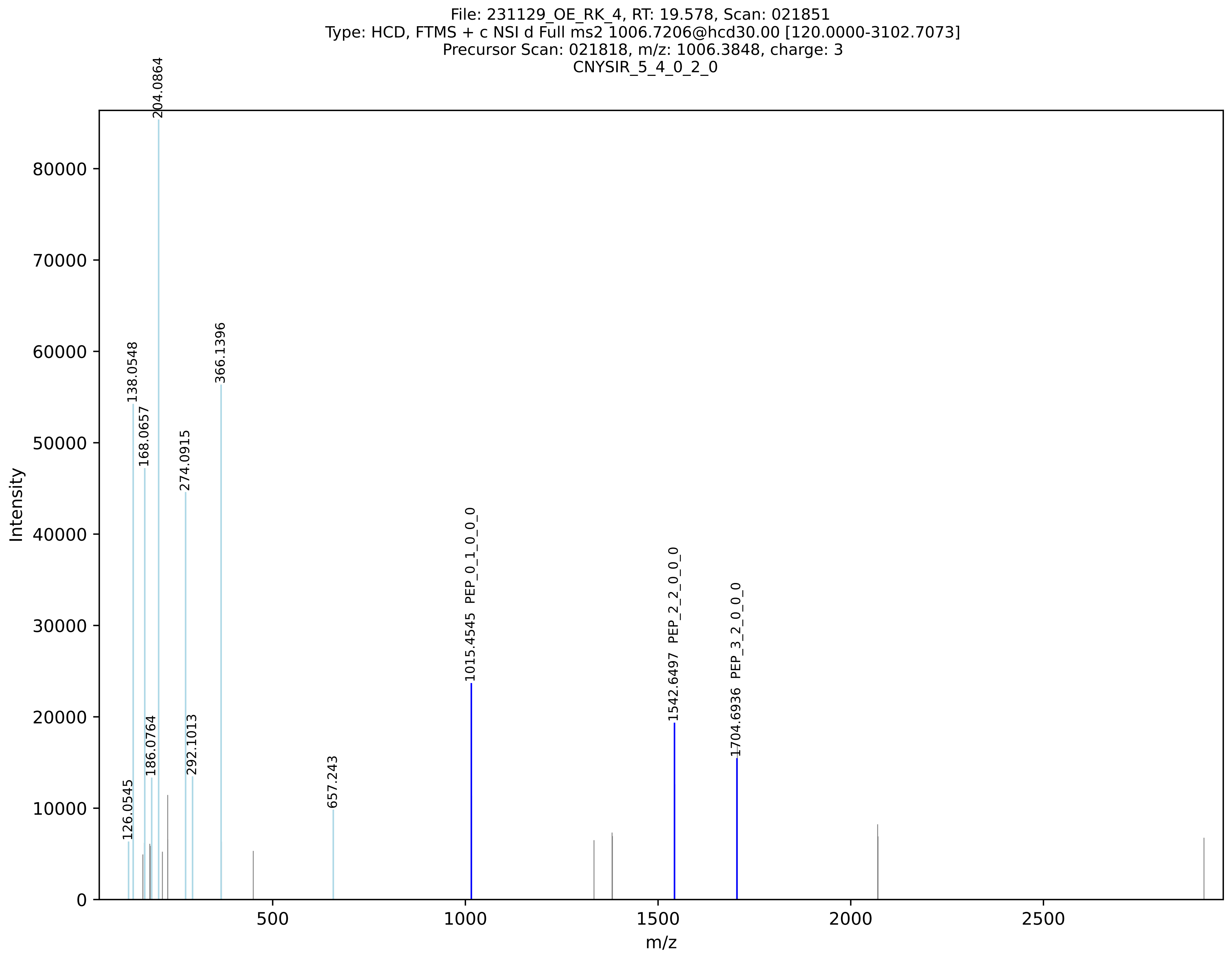

File: 231129_OE_RK_4, RT: 19.578, Scan: 021851
Type: HCD, FTMS + c NSI d Full ms2 1006.7206@hcd30.00 [120.0000-3102.7073]
Precursor Scan: 021818, m/z: 1006.3848, charge: 3
CNYSIR_5_4_0_2_0
Intensity
m/z
204.0864
138.0548
168.0657
274.0915
366.1396
126.0545
186.0764
292.1013
657.243
1015.4545 PEP_0_1_0_0_0
1542.6497 PEP_2_2_0_0_0
1704.6936 PEP_3_2_0_0_0

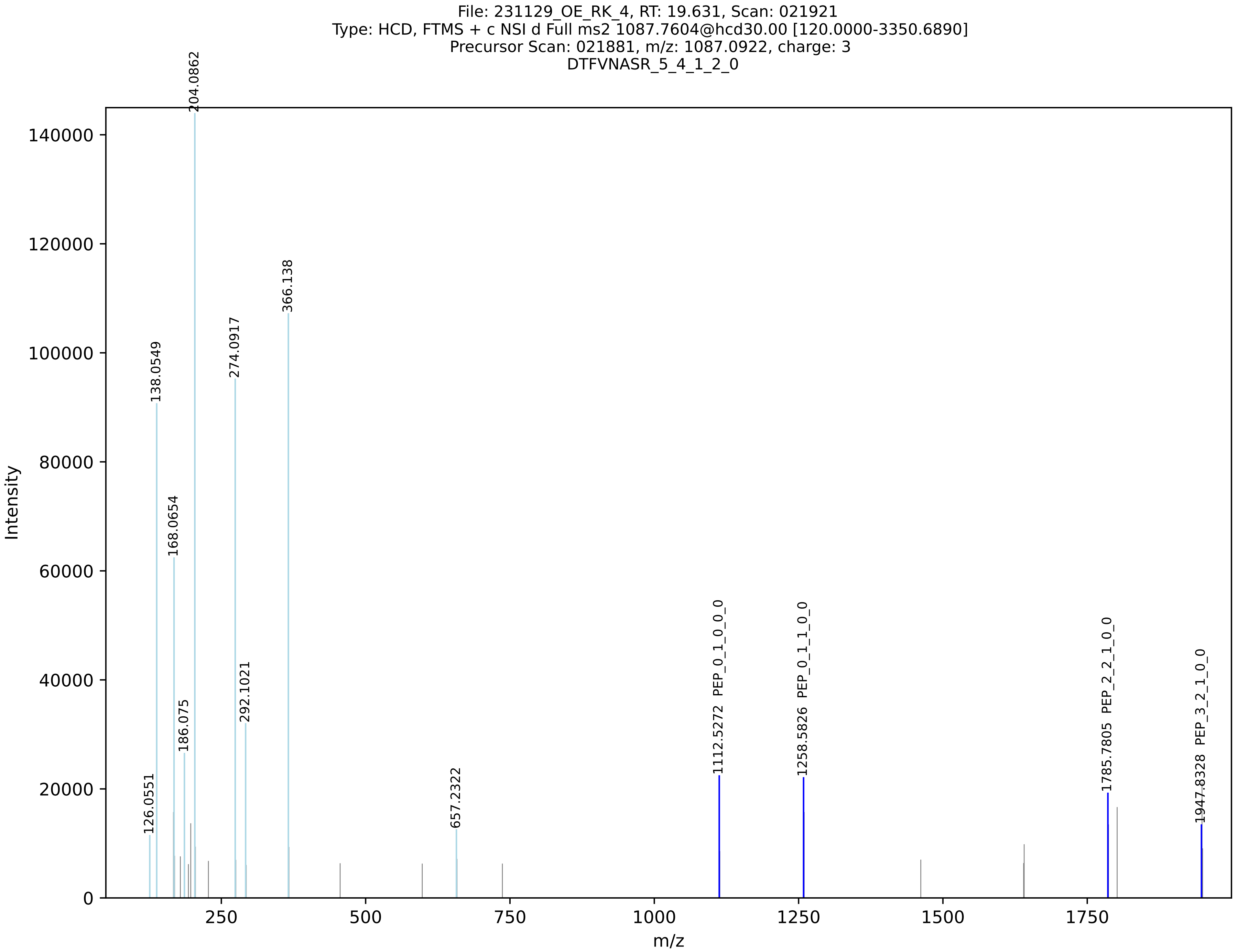
File: 231129_OE_RK_4, RT: 19.631, Scan: 021921
Type: HCD, FTMS + c NSI d Full ms2 1087.7604@hcd30.00 [120.0000-3350.6890]
Precursor Scan: 021881, m/z: 1087.0922, charge: 3
DTFVNASR_5_4_1_2_0
Intensity
m/z
204.0862
366.138
138.0549
274.0917
168.0654
292.1021
186.075
126.0551
657.2322
1112.5272 PEP_0_1_0_0_0
1258.5826 PEP_0_1_1_0_0
1785.7805 PEP_2_2_1_0_0
1947.8328 PEP_3_2_1_0_0

File: 231129_OE_RK_4, RT: 19.739, Scan: 022072
Type: HCD, FTMS + c NSI d Full ms2 962.3920@hcd30.00 [120.0000-2967.0615]
Precursor Scan: 022008, m/z: 962.0579, charge: 3
NYTLTGR_5_4_1_1_0

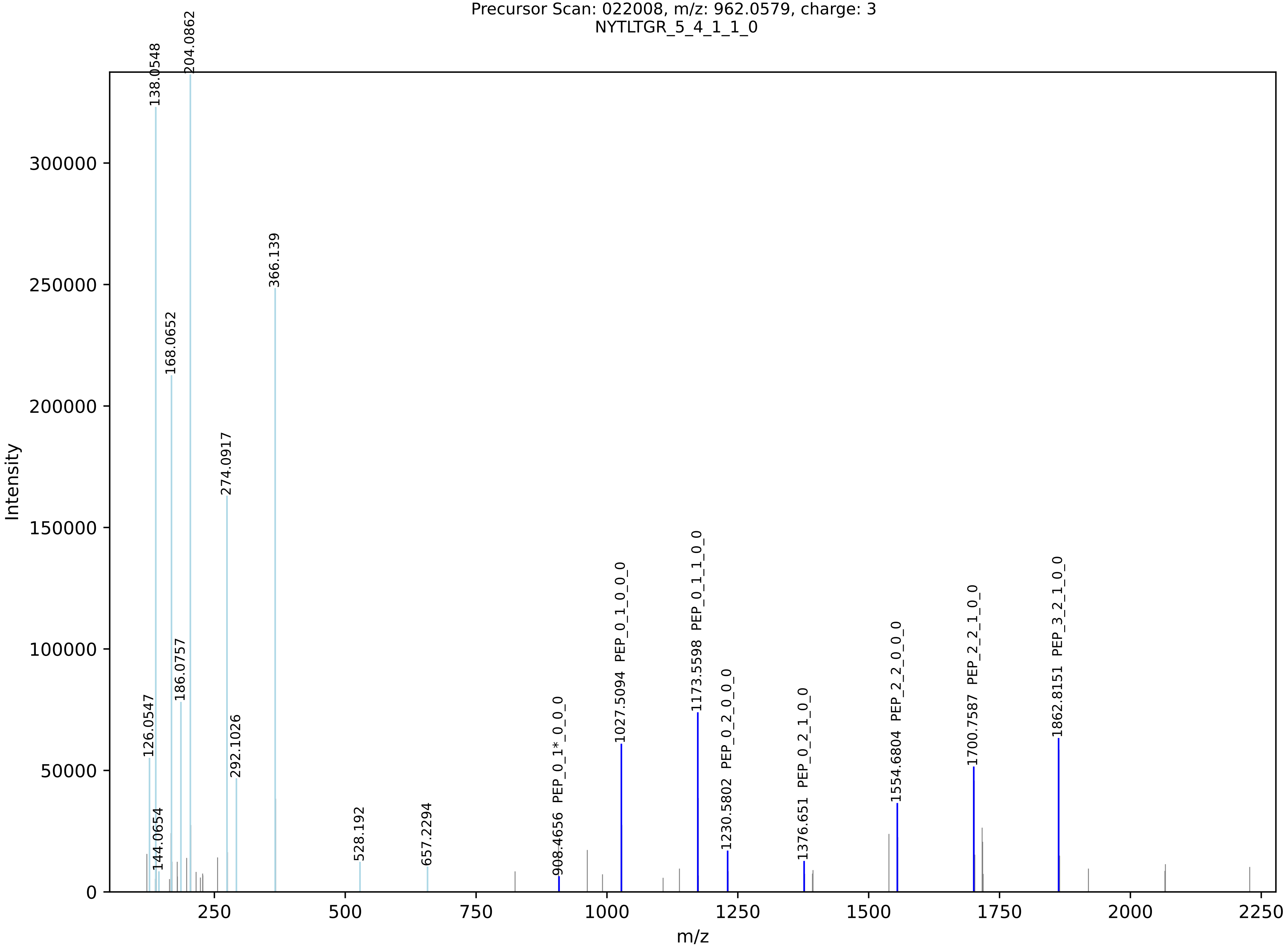

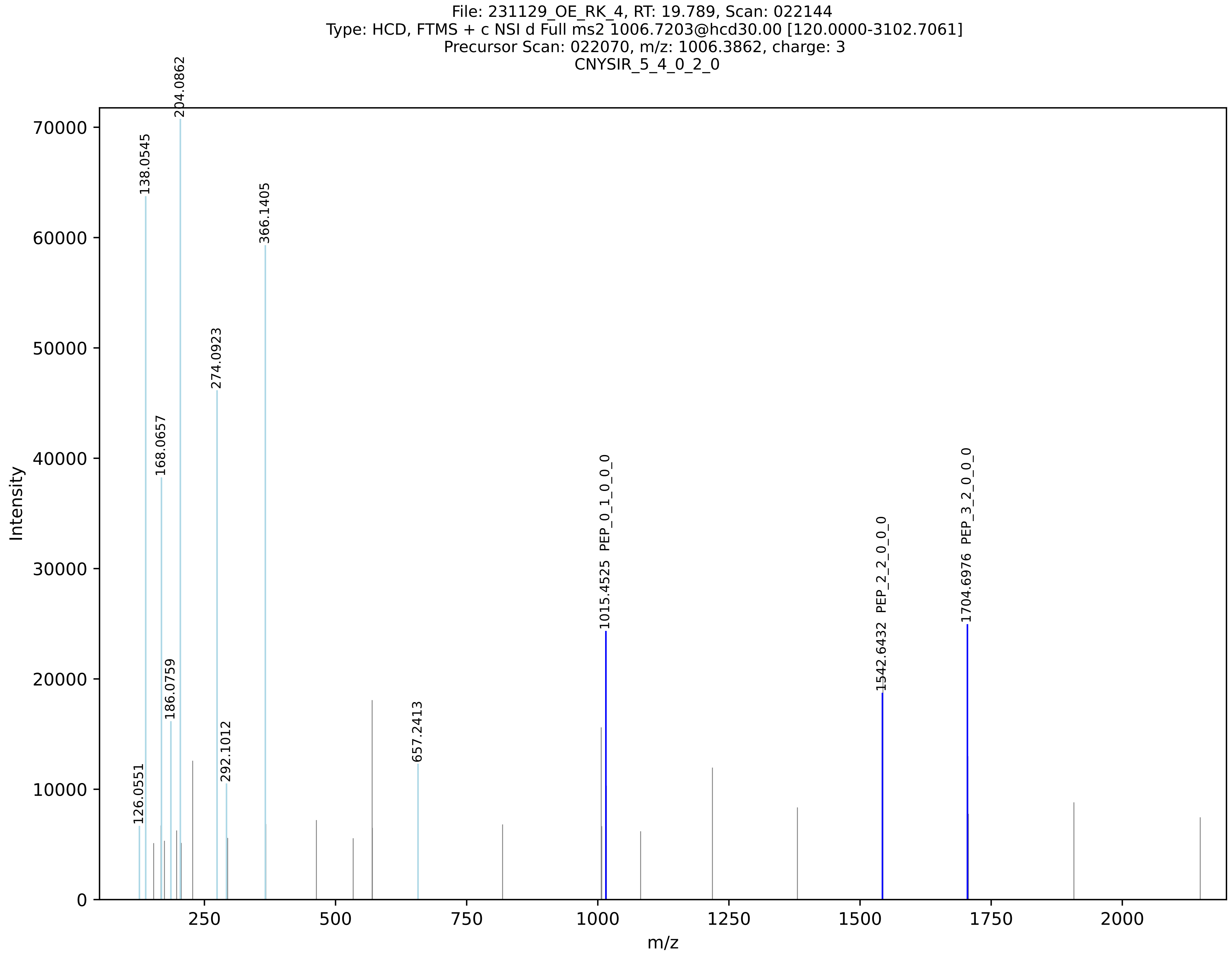
File: 231129_OE_RK_4, RT: 19.789, Scan: 022144
Type: HCD, FTMS + c NSI d Full ms2 1006.7203@hcd30.00 [120.0000-3102.7061]
Precursor Scan: 022070, m/z: 1006.3862, charge: 3
CNYSIR_5_4_0_2_0
Intensity
m/z
204.0862
138.0545
366.1405
274.0923
168.0657
186.0759
126.0551
292.1012
657.2413
1015.4525 PEP_0_1_0_0_0
1542.6432 PEP_2_2_0_0_0
1704.6976 PEP_3_2_0_0_0

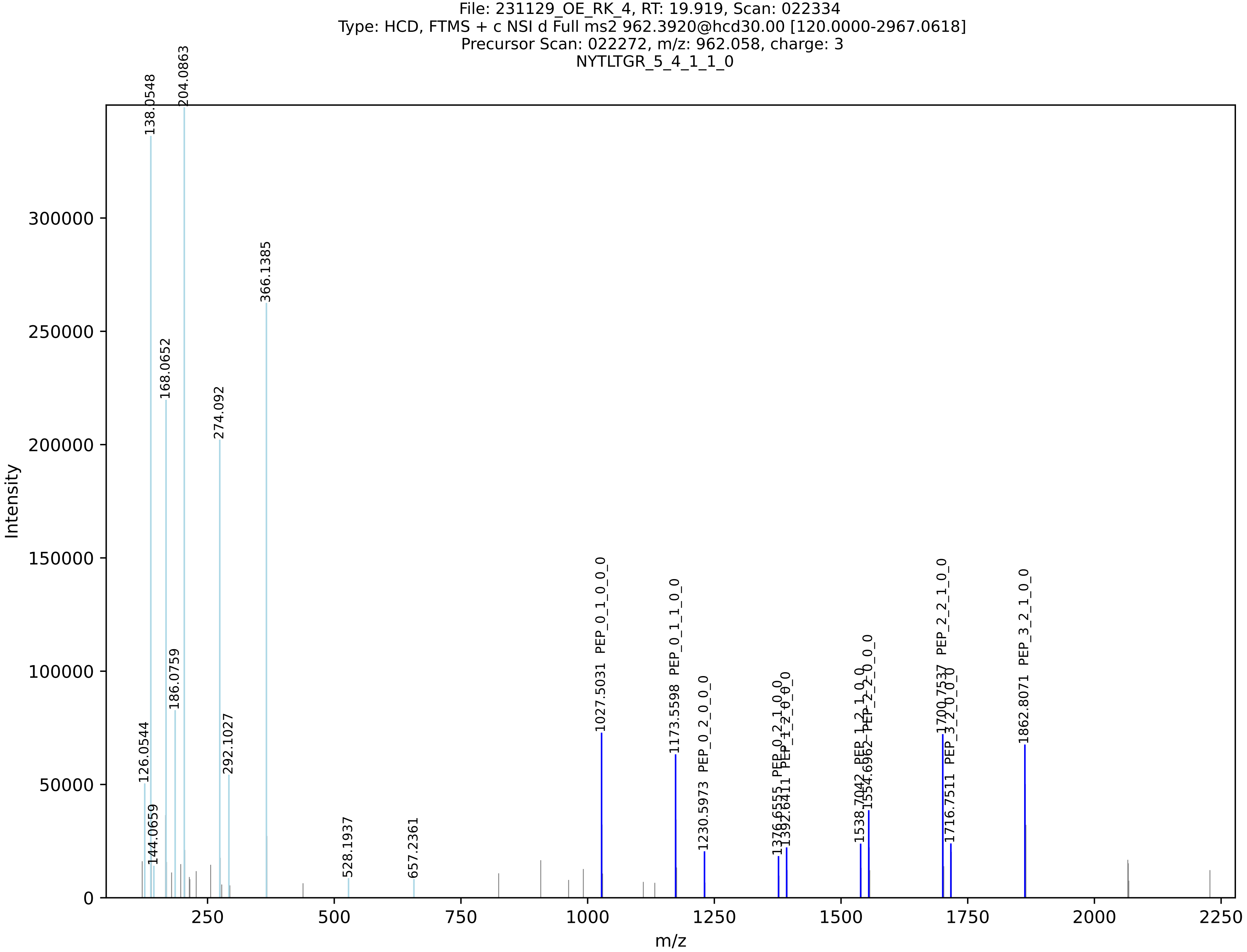
File: 231129_OE_RK_4, RT: 19.919, Scan: 022334
Type: HCD, FTMS + c NSI d Full ms2 962.3920@hcd30.00 [120.0000-2967.0618]
Precursor Scan: 022272, m/z: 962.058, charge: 3
NYTLTGR_5_4_1_1_0
138.0548
204.0863
366.1385
168.0652
274.092
186.0759
292.1027
126.0544
144.0659
528.1937
657.2361
1027.5031 PEP_0_1_0_0_0
1173.5598 PEP_0_1_1_0_0
1230.5973 PEP_0_2_0_0_0
1376.6555 PEP_0_2_1_0_0
1392.6411 PEP_1_2_0_0_0
1538.7042 PEP_1_2_1_0_0
1554.6962 PEP_2_2_0_0_0
1700.7537 PEP_2_2_1_0_0
1716.7511 PEP_3_2_0_0_0
1862.8071 PEP_3_2_1_0_0
Intensity
m/z

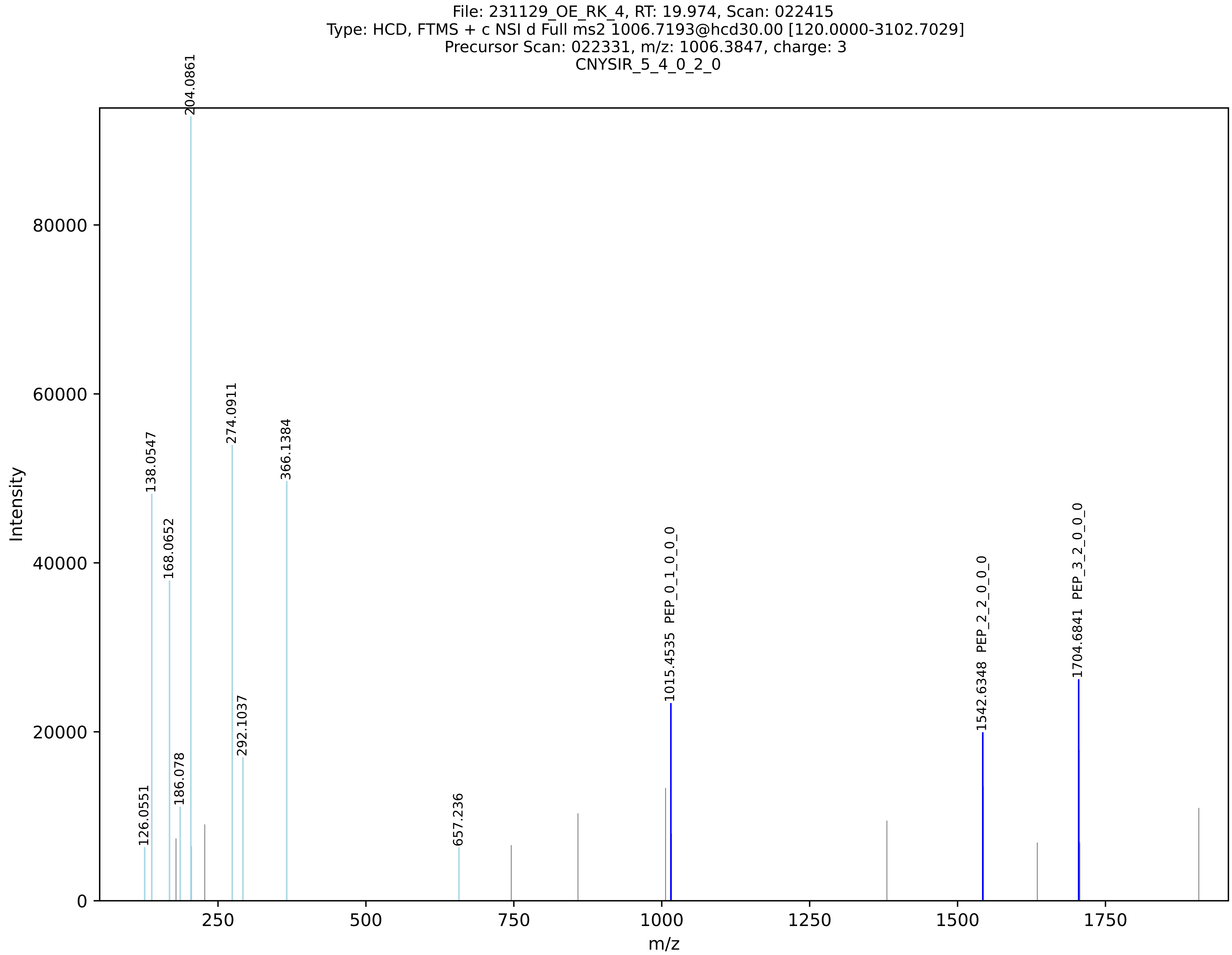
File: 231129_OE_RK_4, RT: 19.974, Scan: 022415
Type: HCD, FTMS + c NSI d Full ms2 1006.7193@hcd30.00 [120.0000-3102.7029]
Precursor Scan: 022331, m/z: 1006.3847, charge: 3
CNYSIR_5_4_0_2_0
204.0861
274.0911
366.1384
138.0547
168.0652
292.1037
186.078
126.0551
657.236
1015.4535 PEP_0_1_0_0_0
1542.6348 PEP_2_2_0_0_0
1704.6841 PEP_3_2_0_0_0
Intensity
m/z
0
20000
40000
60000
80000
250
500
750
1000
1250
1500
1750

File: 231129_OE_RK_4, RT: 19.984, Scan: 022431
Type: HCD, FTMS + c NSI d Full ms2 1038.7408@hcd30.00 [120.0000-3200.6890]
Precursor Scan: 022331, m/z: 1038.4067, charge: 3
DTFVNASR_5_4_0_2_0

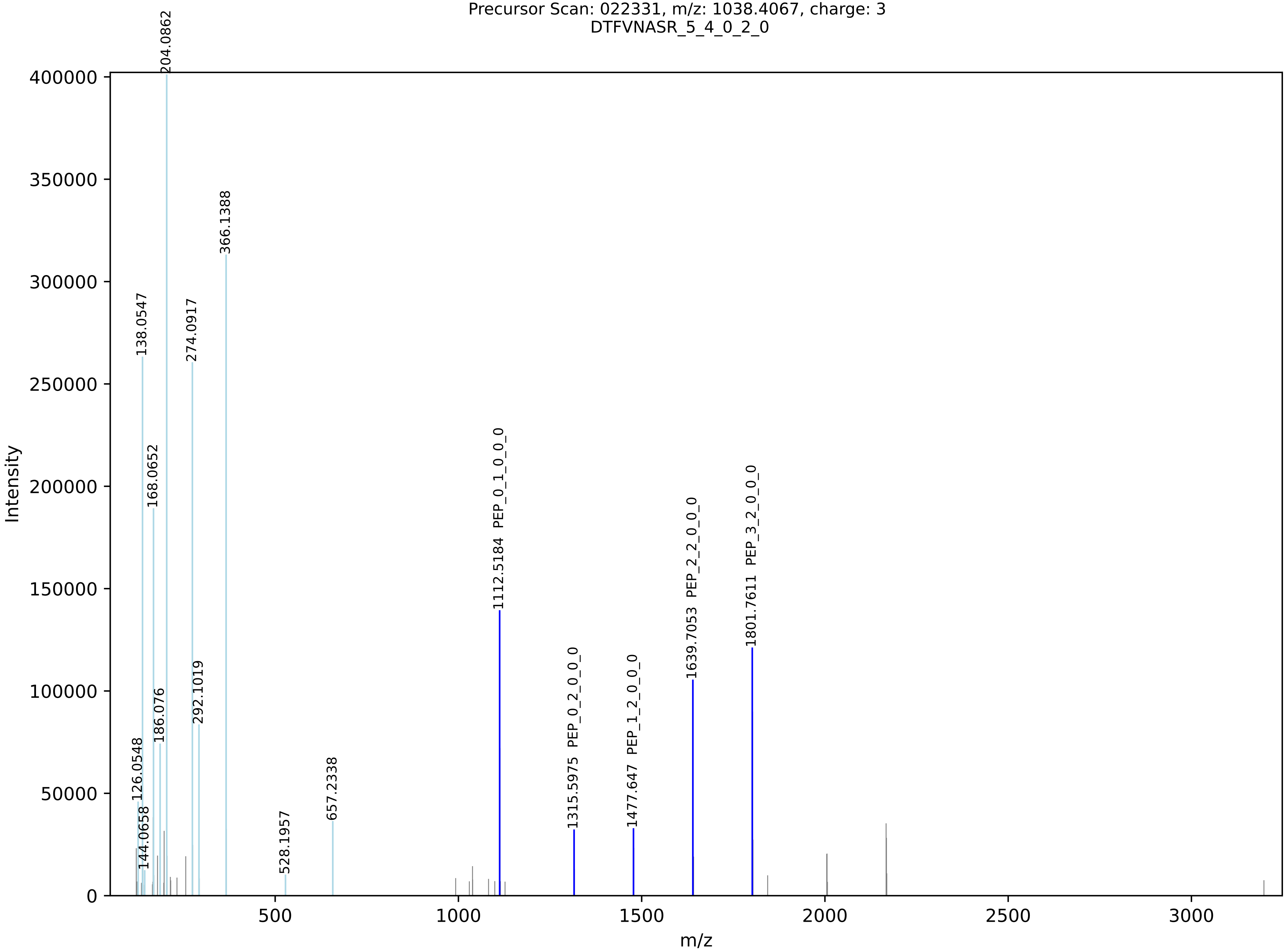

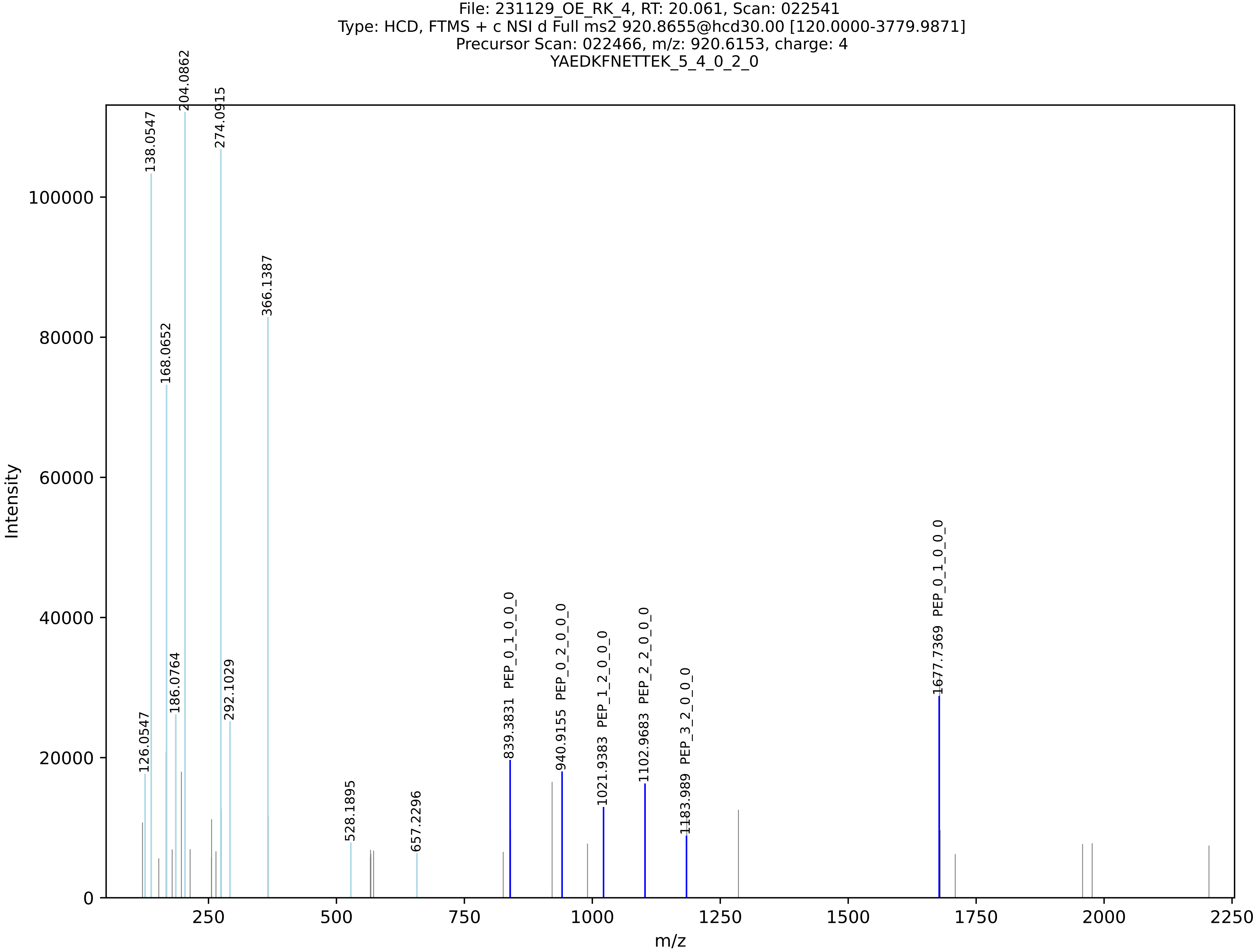

File: 231129_OE_RK_4, RT: 20.061, Scan: 022541
Type: HCD, FTMS + c NSI d Full ms2 920.8655@hcd30.00 [120.0000-3779.9871]
Precursor Scan: 022466, m/z: 920.6153, charge: 4
YAEDKFNETTEK_5_4_0_2_0
Intensity
m/z
126.0547
138.0547
168.0652
186.0764
204.0862
274.0915
292.1029
366.1387
528.1895
657.2296
839.3831 PEP_0_1_0_0_0_
940.9155 PEP_0_2_0_0_0_
1021.9383 PEP_1_2_0_0_0_
1102.9683 PEP_2_2_0_0_0_
1183.989 PEP_3_2_0_0_0_
1677.7369 PEP_0_1_0_0_0_

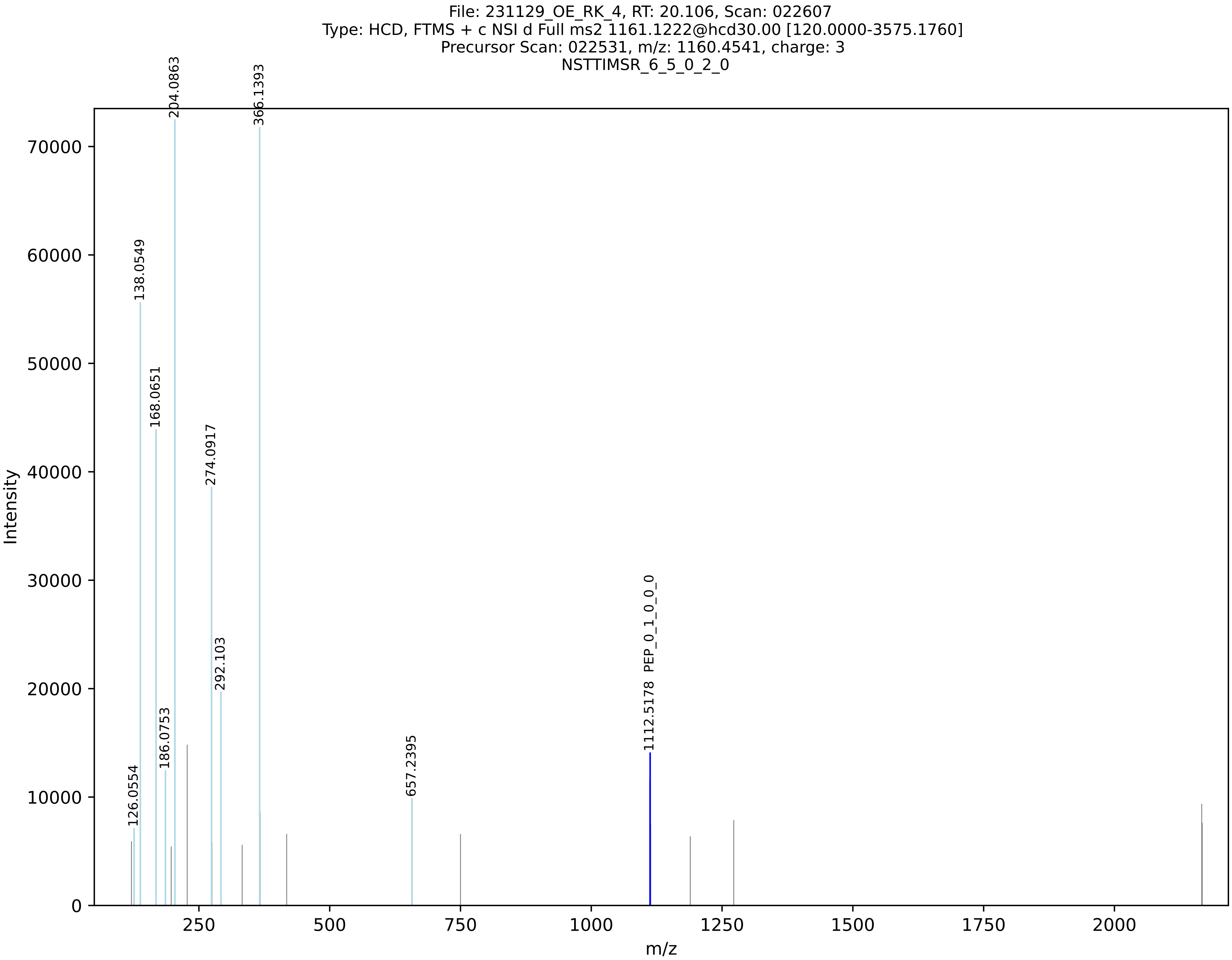
File: 231129_OE_RK_4, RT: 20.106, Scan: 022607
Type: HCD, FTMS + c NSI d Full ms2 1161.1222@hcd30.00 [120.0000-3575.1760]
Precursor Scan: 022531, m/z: 1160.4541, charge: 3
NSTTIMSR_6_5_0_2_0
204.0863
366.1393
138.0549
168.0651
274.0917
292.103
186.0753
126.0554
657.2395
1112.5178 PEP_0_1_0_0_0
Intensity
m/z
0
10000
20000
30000
40000
50000
60000
70000
250
500
750
1000
1250
1500
1750
2000

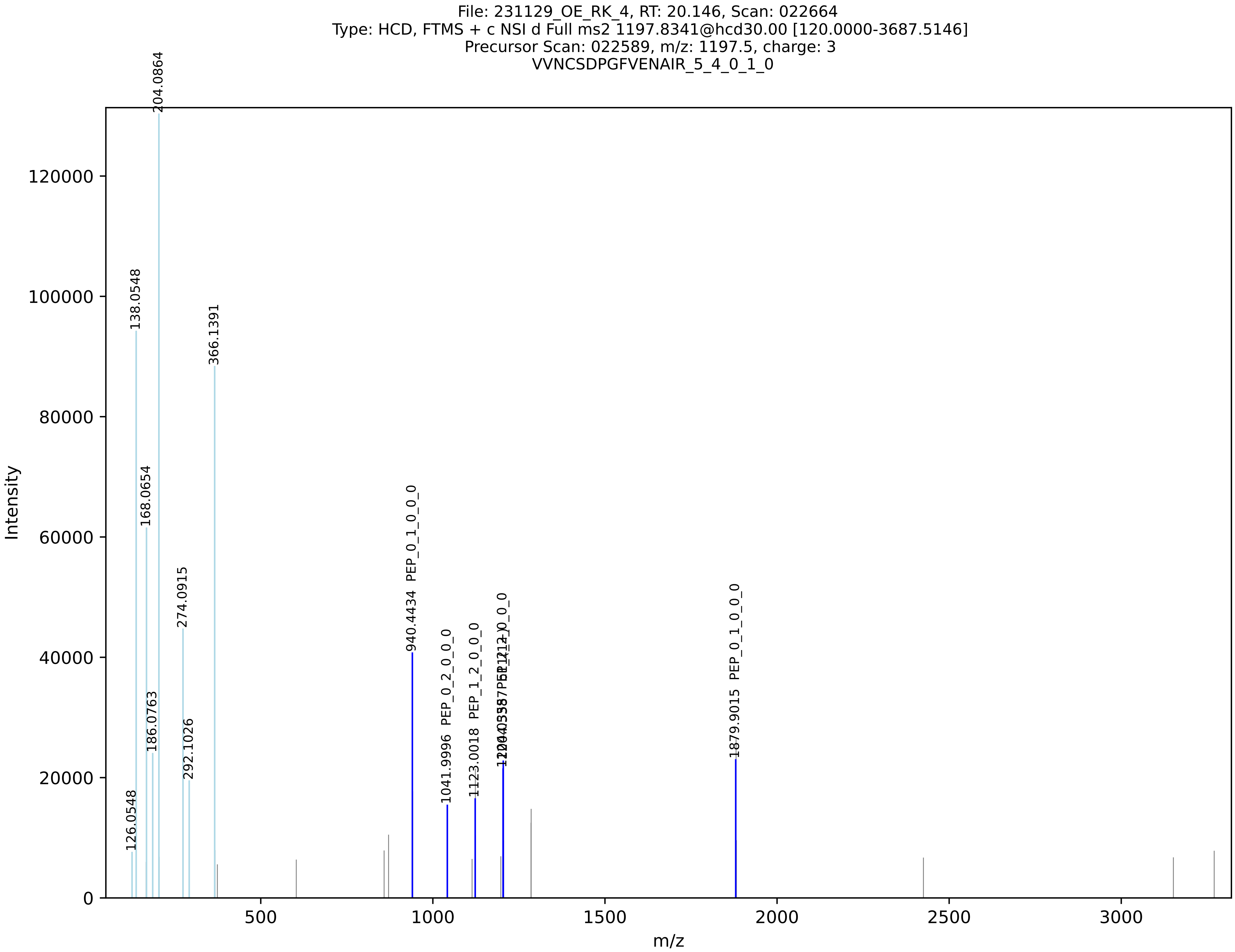
File: 231129_OE_RK_4, RT: 20.146, Scan: 022664
Type: HCD, FTMS + c NSI d Full ms2 1197.8341@hcd30.00 [120.0000-3687.5146]
Precursor Scan: 022589, m/z: 1197.5, charge: 3
VVNCSDPGFVENAIR_5_4_0_1_0
Intensity
m/z
0
20000
40000
60000
80000
100000
120000
500
1000
1500
2000
2500
3000
204.0864
138.0548
366.1391
168.0654
274.0915
186.0763
292.1026
126.0548
940.4434 PEP_0_1_0_0_0
1041.9996 PEP_0_2_0_0_0
1123.0018 PEP_1_2_0_0_0
1879.9015 PEP_0_1_0_0_0

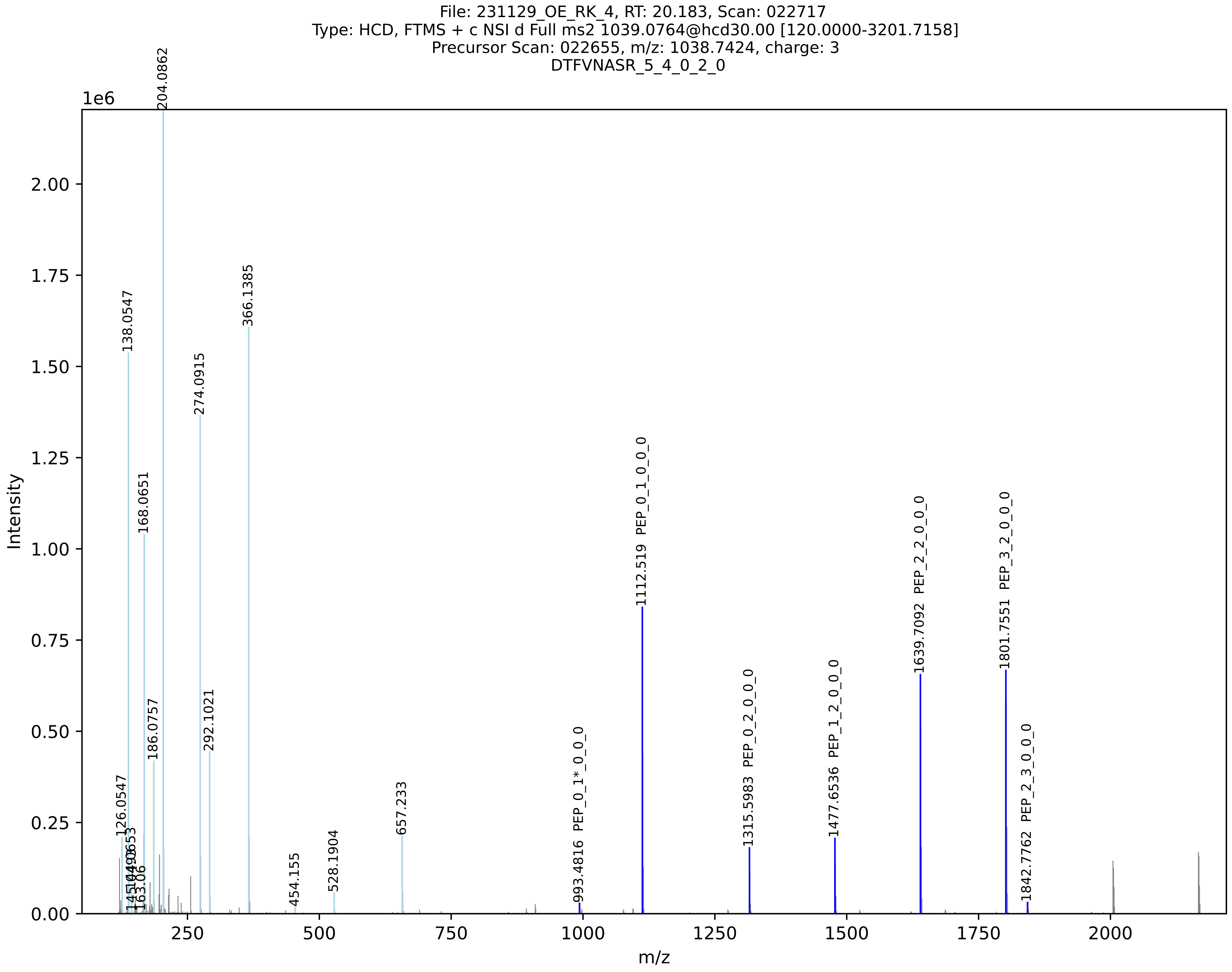

File: 231129_OE_RK_4, RT: 20.183, Scan: 022717
Type: HCD, FTMS + c NSI d Full ms2 1039.0764@hcd30.00 [120.0000-3201.7158]
Precursor Scan: 022655, m/z: 1038.7424, charge: 3
DTFVNASR_5_4_0_2_0
1e6
Intensity
m/z
204.0862
138.0547
366.1385
274.0915
168.0651
186.0757
292.1021
126.0547
163.06
454.155
528.1904
657.233
993.4816 PEP_0_1*_0_0_0
1112.519 PEP_0_1_0_0_0
1315.5983 PEP_0_2_0_0_0
1477.6536 PEP_1_2_0_0_0
1639.7092 PEP_2_2_0_0_0
1801.7551 PEP_3_2_0_0_0
1842.7762 PEP_2_3_0_0_0

File: 231129_OE_RK_4, RT: 20.196, Scan: 022739
Type: HCD, FTMS + c NSI d Full ms2 1083.4731@hcd30.00 [120.0000-3337.5698]
Precursor Scan: 022655, m/z: 1082.8066, charge: 3
TKPREEQFNSTFR_4_4_1_0_0

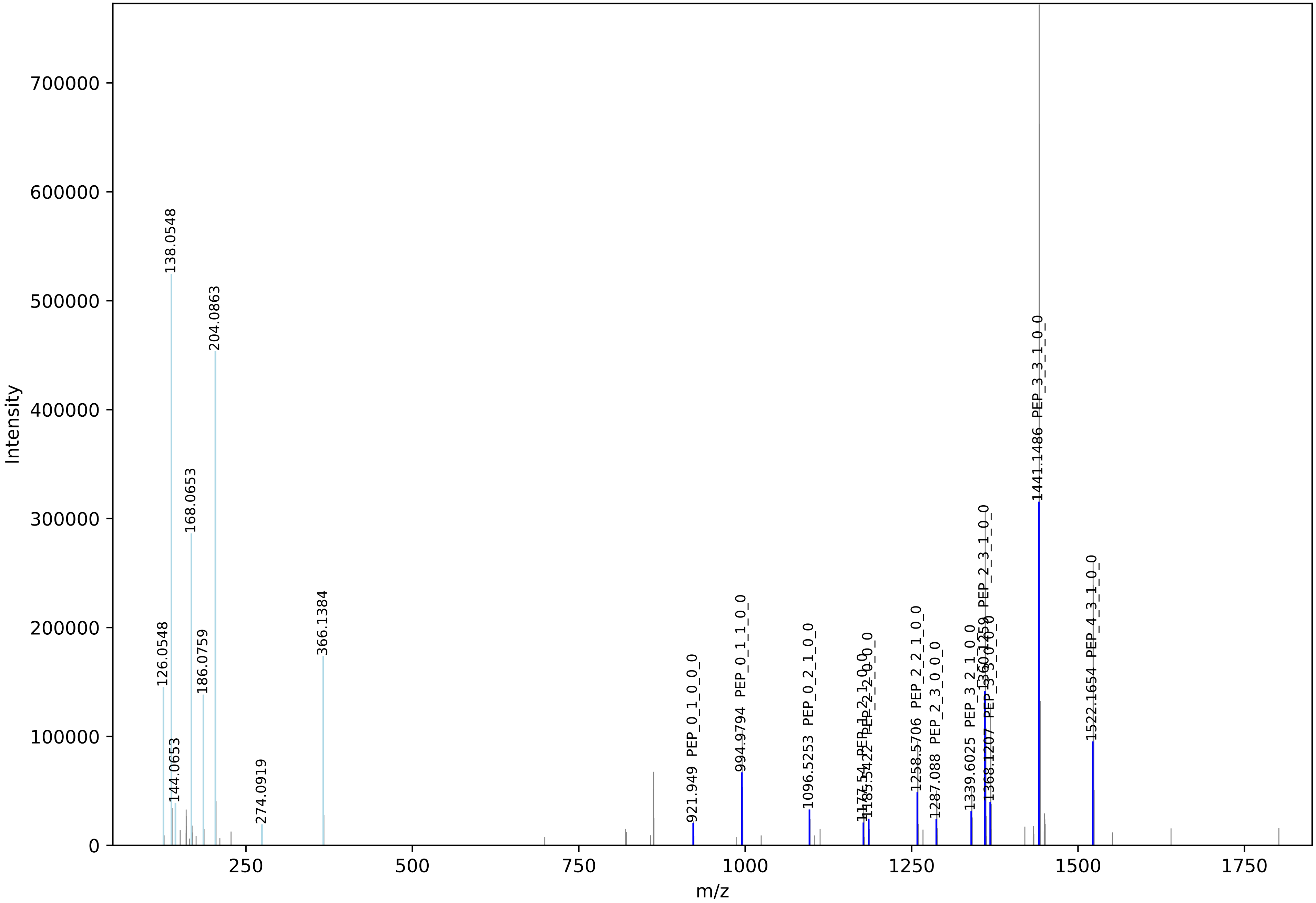

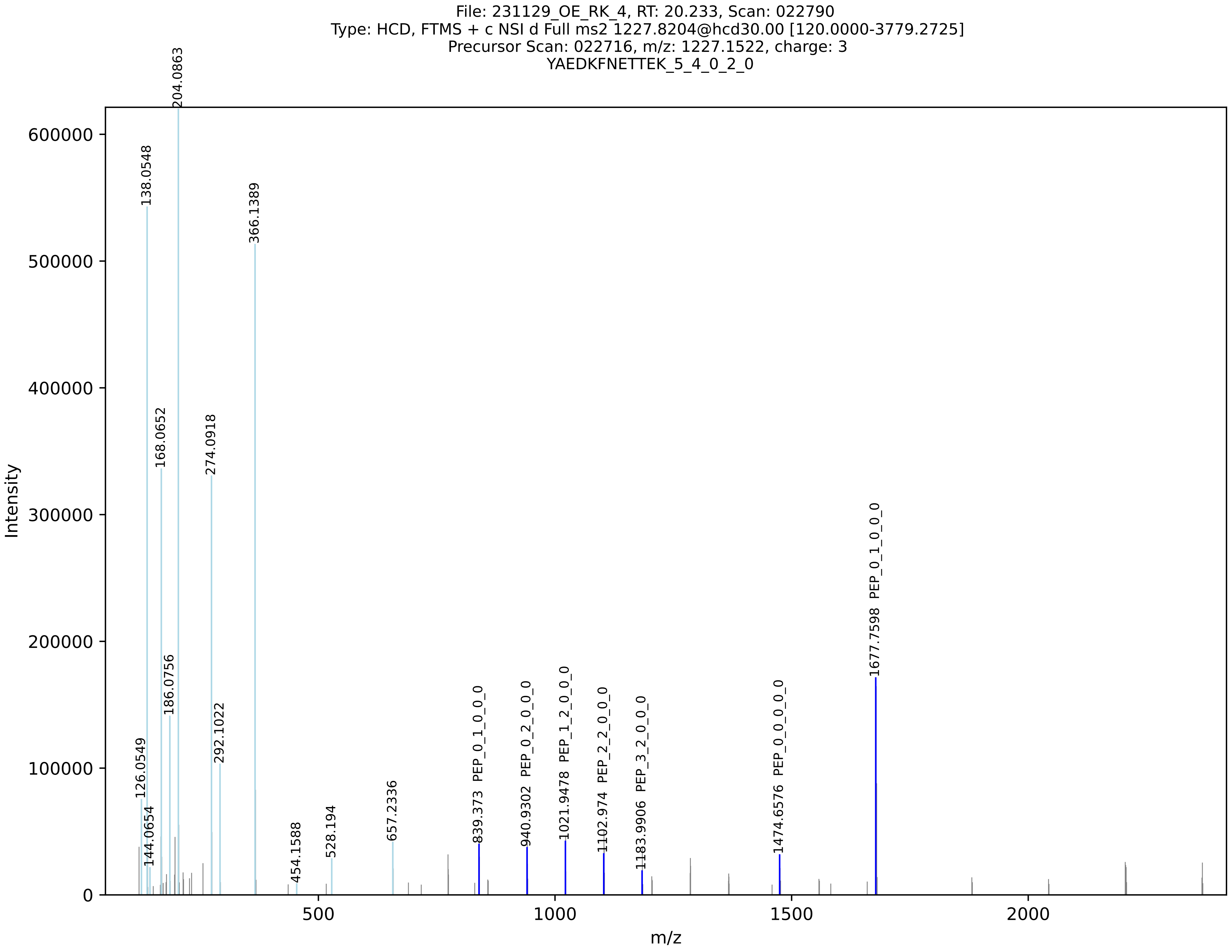

File: 231129_OE_RK_4, RT: 20.233, Scan: 022790
Type: HCD, FTMS + c NSI d Full ms2 1227.8204@hcd30.00 [120.0000-3779.2725]
Precursor Scan: 022716, m/z: 1227.1522, charge: 3
YAEDKFNETTEK_5_4_0_2_0
Intensity
m/z
0
100000
200000
300000
400000
500000
600000
500
1000
1500
2000
204.0863
138.0548
366.1389
168.0652
274.0918
186.0756
292.1022
126.0549
144.0654
454.1588
528.194
657.2336
839.373 PEP_0_1_0_0_0
940.9302 PEP_0_2_0_0_0
1021.9478 PEP_1_2_0_0_0
1102.974 PEP_2_2_0_0_0
1183.9906 PEP_3_2_0_0_0
1474.6576 PEP_0_0_0_0_0
1677.7598 PEP_0_1_0_0_0

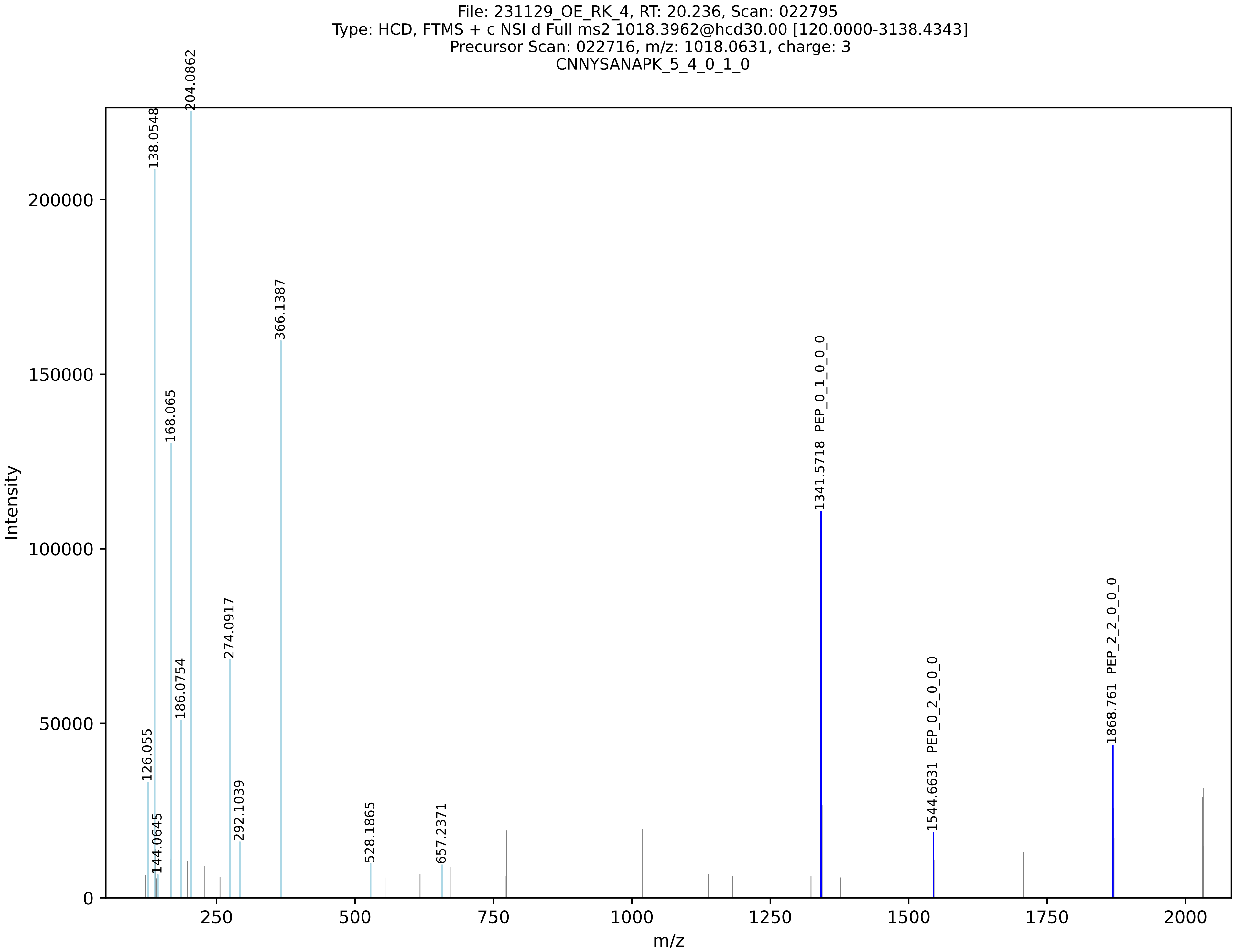
File: 231129_OE_RK_4, RT: 20.236, Scan: 022795
Type: HCD, FTMS + c NSI d Full ms2 1018.3962@hcd30.00 [120.0000-3138.4343]
Precursor Scan: 022716, m/z: 1018.0631, charge: 3
CNNYSANAPK_5_4_0_1_0
204.0862
138.0548
168.065
366.1387
1341.5718 PEP_0_1_0_0_0
274.0917
186.0754
1868.761 PEP_2_2_0_0_0
1544.6631 PEP_0_2_0_0_0
126.055
144.0645
292.1039
528.1865
657.2371
Intensity
m/z
0
50000
100000
150000
200000
250
500
750
1000
1250
1500
1750
2000

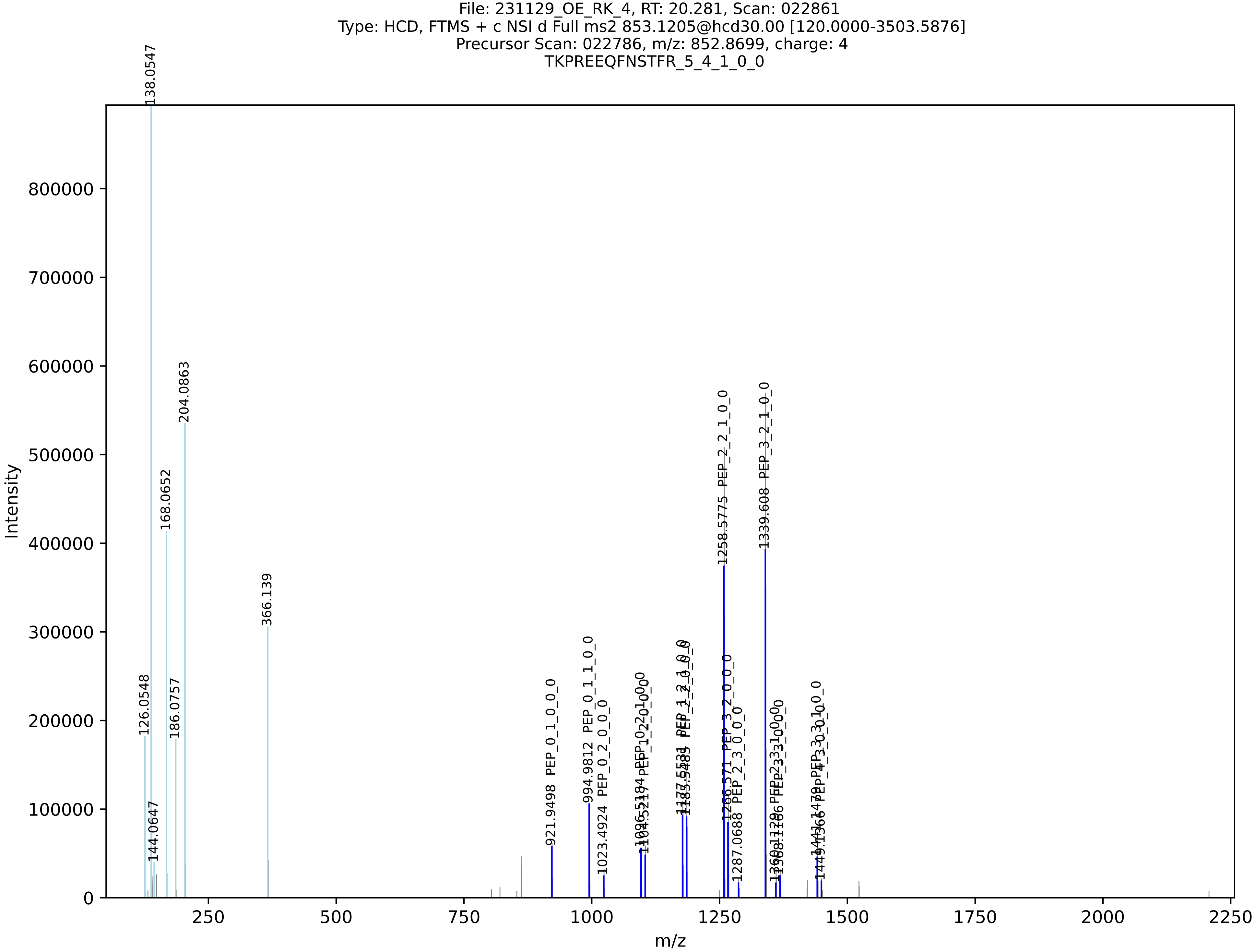
File: 231129_OE_RK_4, RT: 20.281, Scan: 022861
Type: HCD, FTMS + c NSI d Full ms2 853.1205@hcd30.00 [120.0000-3503.5876]
Precursor Scan: 022786, m/z: 852.8699, charge: 4
TKPREEQFNSTFR_5_4_1_0_0
Intensity
m/z
138.0547
204.0863
168.0652
366.139
126.0548
186.0757
144.0647
921.9498 PEP_0_1_0_0_0
994.9812 PEP_0_1_1_0_0
1023.4924 PEP_0_2_0_0_0
1258.5775 PEP_2_2_1_0_0
1339.608 PEP_3_2_1_0_0
1287.0688 PEP_2_3_0_0_0

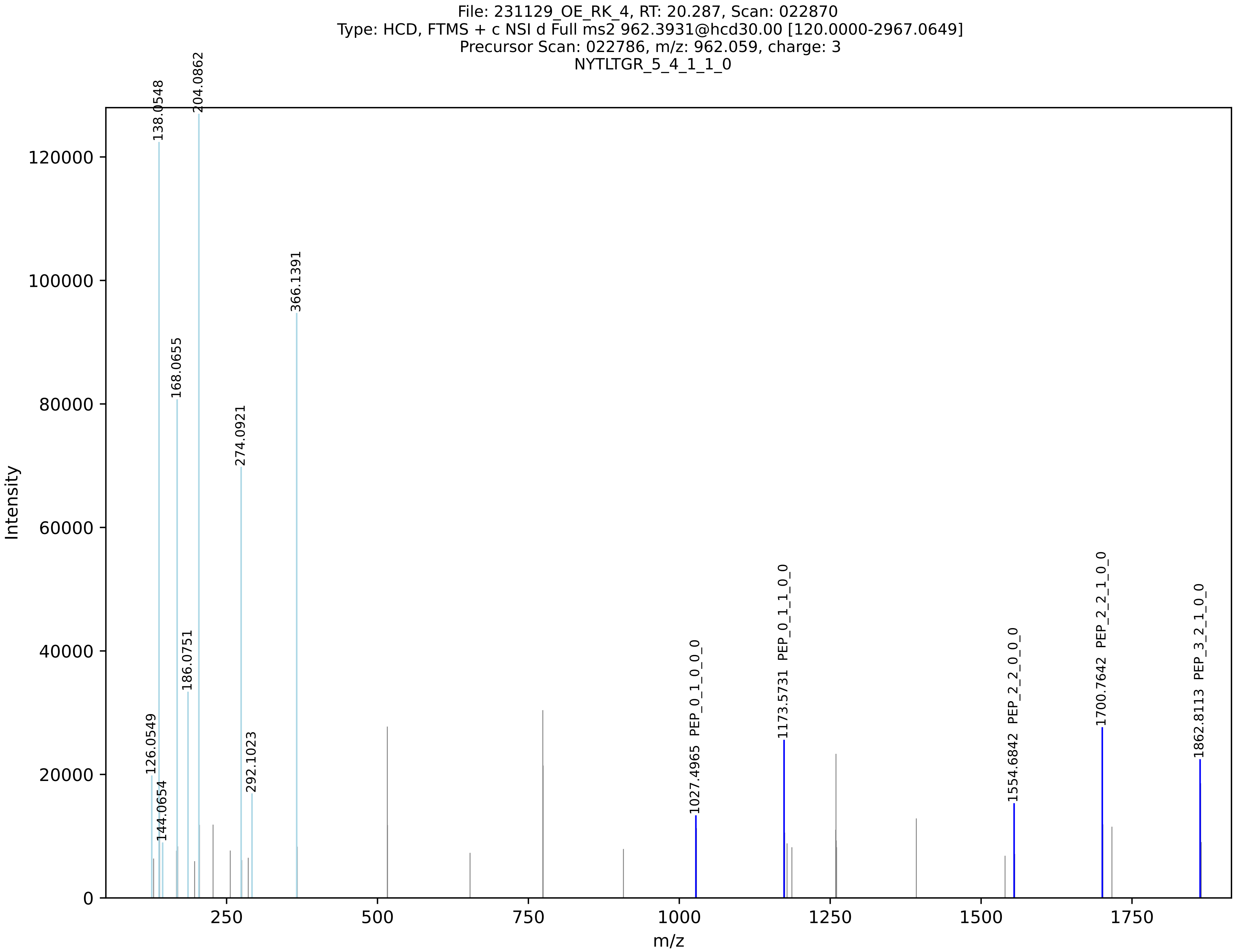
File: 231129_OE_RK_4, RT: 20.287, Scan: 022870
Type: HCD, FTMS + c NSI d Full ms2 962.3931@hcd30.00 [120.0000-2967.0649]
Precursor Scan: 022786, m/z: 962.059, charge: 3
NYTLTGR_5_4_1_1_0
Intensity
m/z
138.0548
204.0862
366.1391
168.0655
274.0921
186.0751
126.0549
144.0654
292.1023
1027.4965 PEP_0_1_0_0_0
1173.5731 PEP_0_1_1_0_0
1554.6842 PEP_2_2_0_0_0
1700.7642 PEP_2_2_1_0_0
1862.8113 PEP_3_2_1_0_0

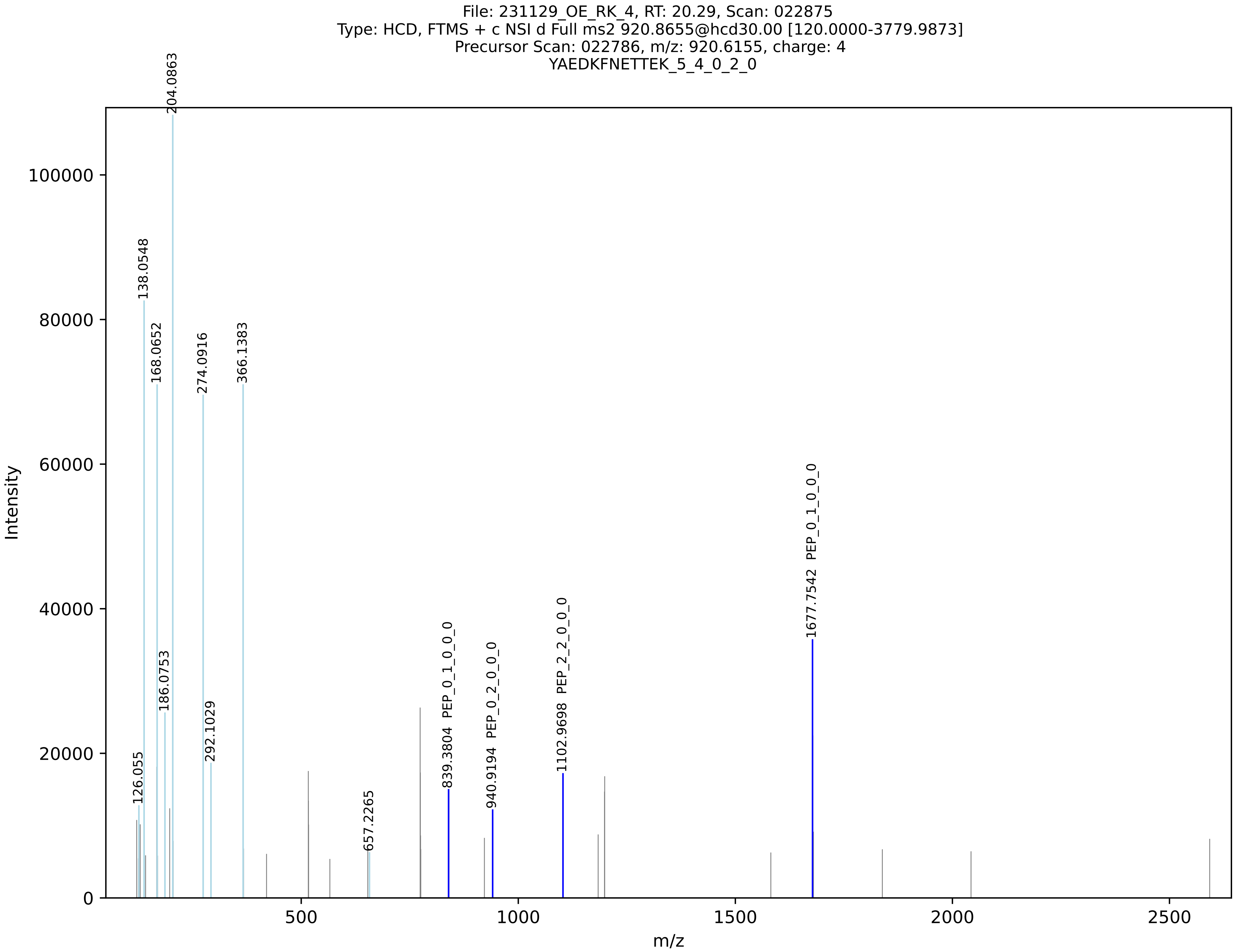
File: 231129_OE_RK_4, RT: 20.29, Scan: 022875
Type: HCD, FTMS + c NSI d Full ms2 920.8655@hcd30.00 [120.0000-3779.9873]
Precursor Scan: 022786, m/z: 920.6155, charge: 4
YAEDKFNETTEK_5_4_0_2_0
204.0863
138.0548
168.0652
274.0916
366.1383
186.0753
292.1029
126.055
657.2265
839.3804 PEP_0_1_0_0_0
940.9194 PEP_0_2_0_0_0
1102.9698 PEP_2_2_0_0_0
1677.7542 PEP_0_1_0_0_
Intensity
m/z
0
20000
40000
60000
80000
100000
500
1000
1500
2000
2500

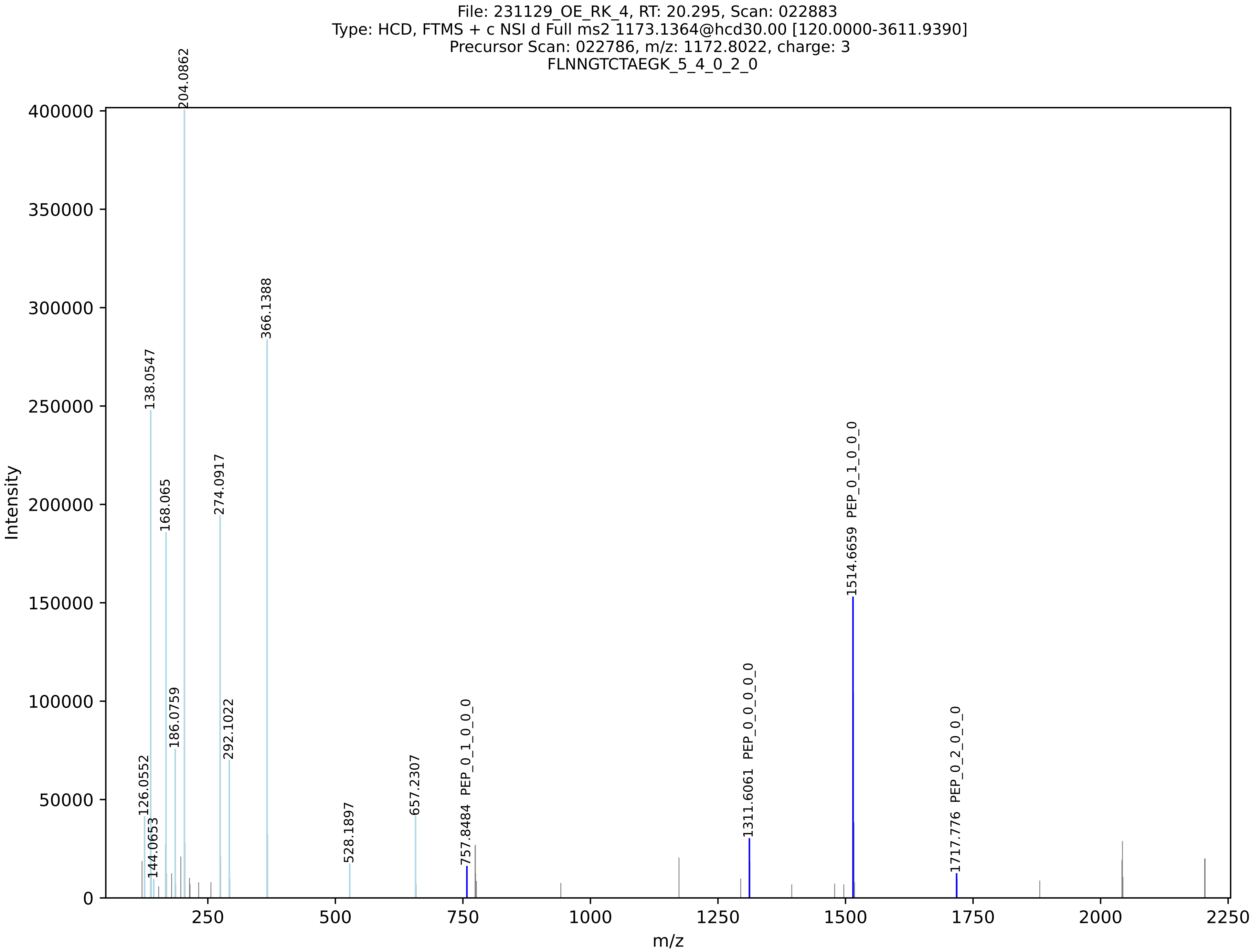
File: 231129_OE_RK_4, RT: 20.295, Scan: 022883
Type: HCD, FTMS + c NSI d Full ms2 1173.1364@hcd30.00 [120.0000-3611.9390]
Precursor Scan: 022786, m/z: 1172.8022, charge: 3
FLNNGTCTAEGK_5_4_0_2_0
Intensity
m/z
204.0862
366.1388
138.0547
274.0917
168.065
186.0759
292.1022
126.0552
144.0653
528.1897
657.2307
757.8484 PEP_0_1_0_0_0
1311.6061 PEP_0_0_0_0_0
1514.6659 PEP_0_1_0_0_0
1717.776 PEP_0_2_0_0_0

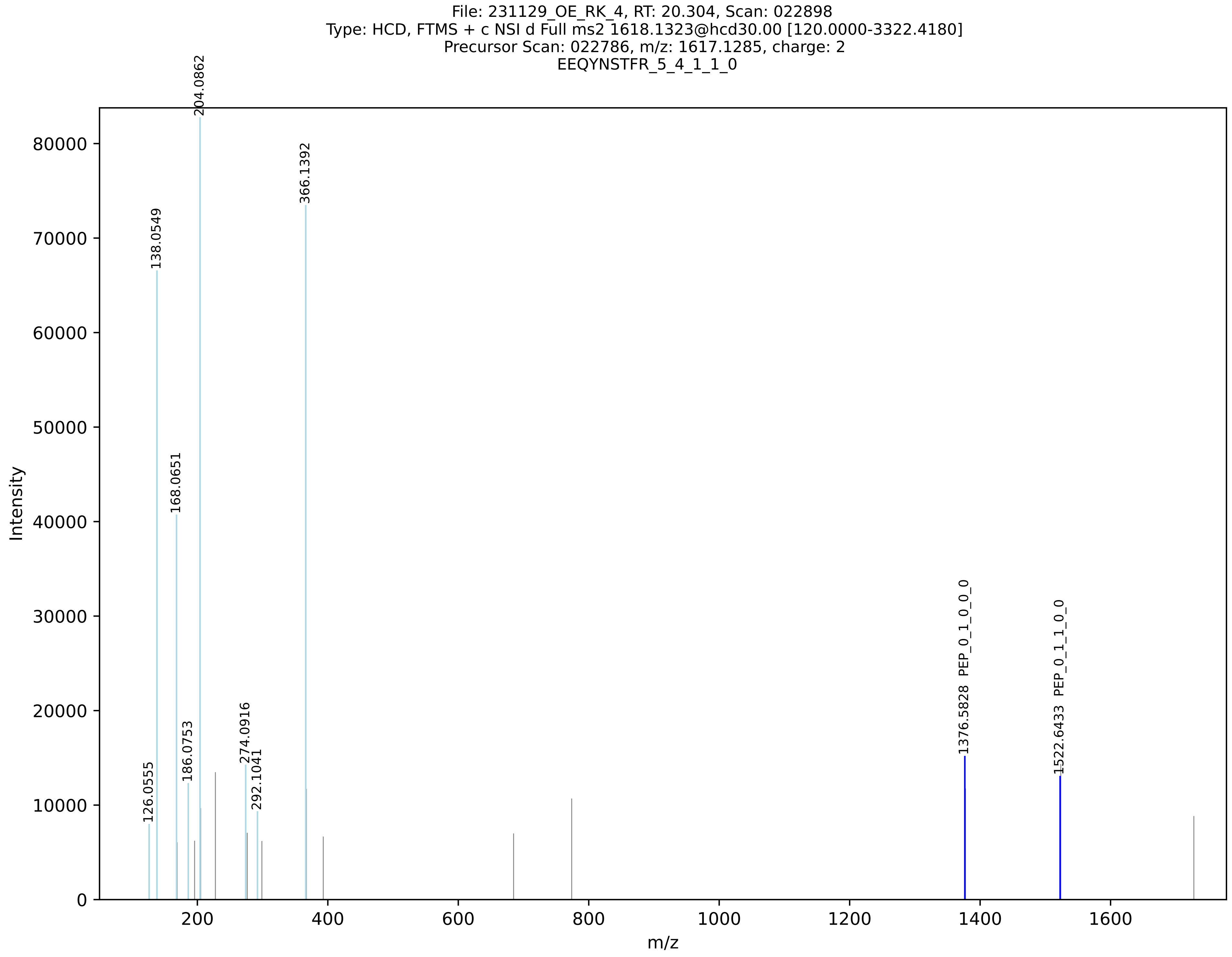

File: 231129_OE_RK_4, RT: 20.304, Scan: 022898
Type: HCD, FTMS + c NSI d Full ms2 1618.1323@hcd30.00 [120.0000-3322.4180]
Precursor Scan: 022786, m/z: 1617.1285, charge: 2
EEQYNSTFR_5_4_1_1_0
Intensity
m/z
0
10000
20000
30000
40000
50000
60000
70000
80000
200
400
600
800
1000
1200
1400
1600
126.0555
138.0549
168.0651
186.0753
204.0862
274.0916
292.1041
366.1392
1376.5828 PEP_0_1_0_0_0
1522.6433 PEP_0_1_1_0_0

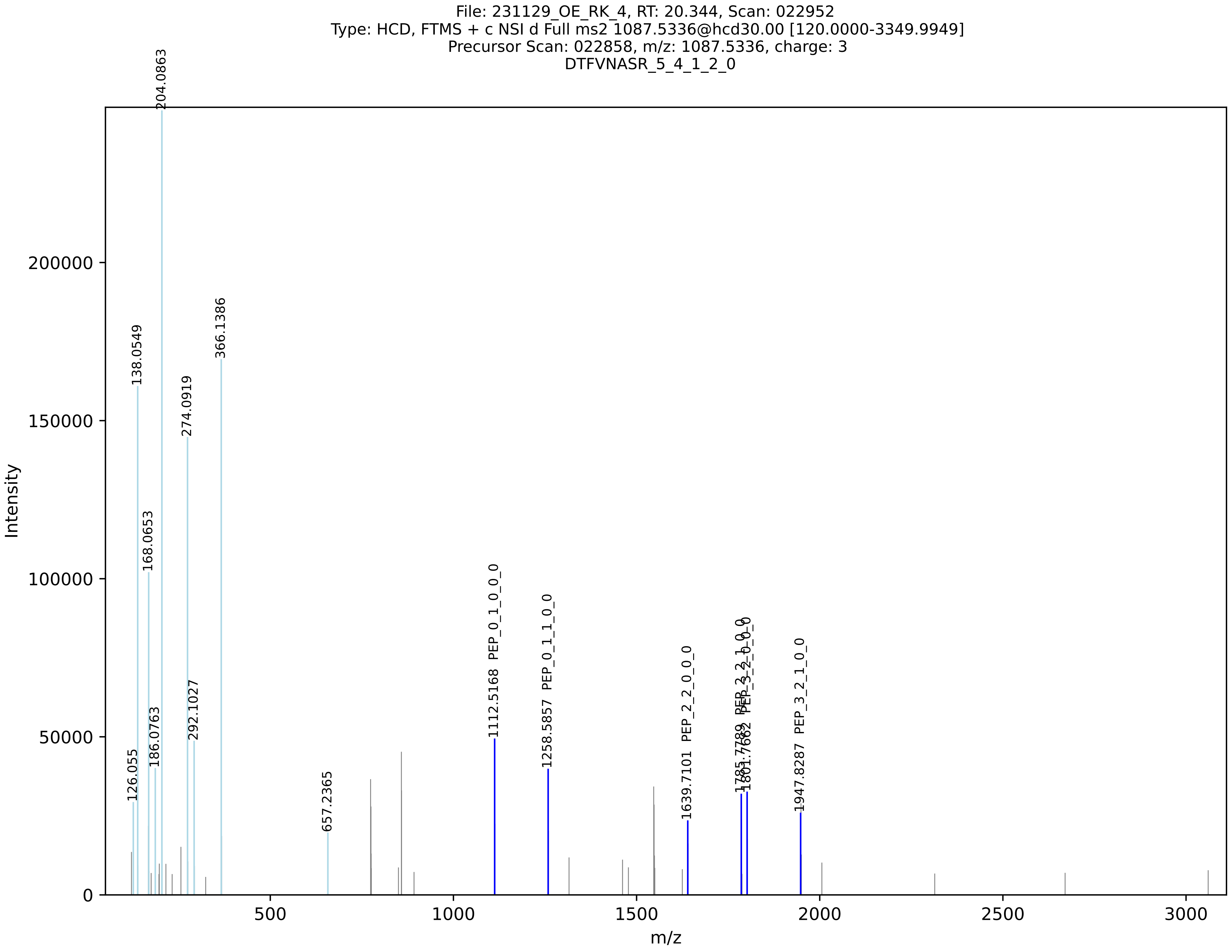
File: 231129_OE_RK_4, RT: 20.344, Scan: 022952
Type: HCD, FTMS + c NSI d Full ms2 1087.5336@hcd30.00 [120.0000-3349.9949]
Precursor Scan: 022858, m/z: 1087.5336, charge: 3
DTFVNASR_5_4_1_2_0
204.0863
138.0549
366.1386
274.0919
168.0653
186.0763
292.1027
126.055
657.2365
1112.5168 PEP_0_1_0_0_0
1258.5857 PEP_0_1_1_0_0
1639.7101 PEP_2_2_0_0_0
1785.7789 PEP_3_2_1_0_0
1801.7662 PEP_3_2_0_0_0
1947.8287 PEP_3_2_1_0_0
Intensity
m/z
0
50000
100000
150000
200000
500
1000
1500
2000
2500
3000

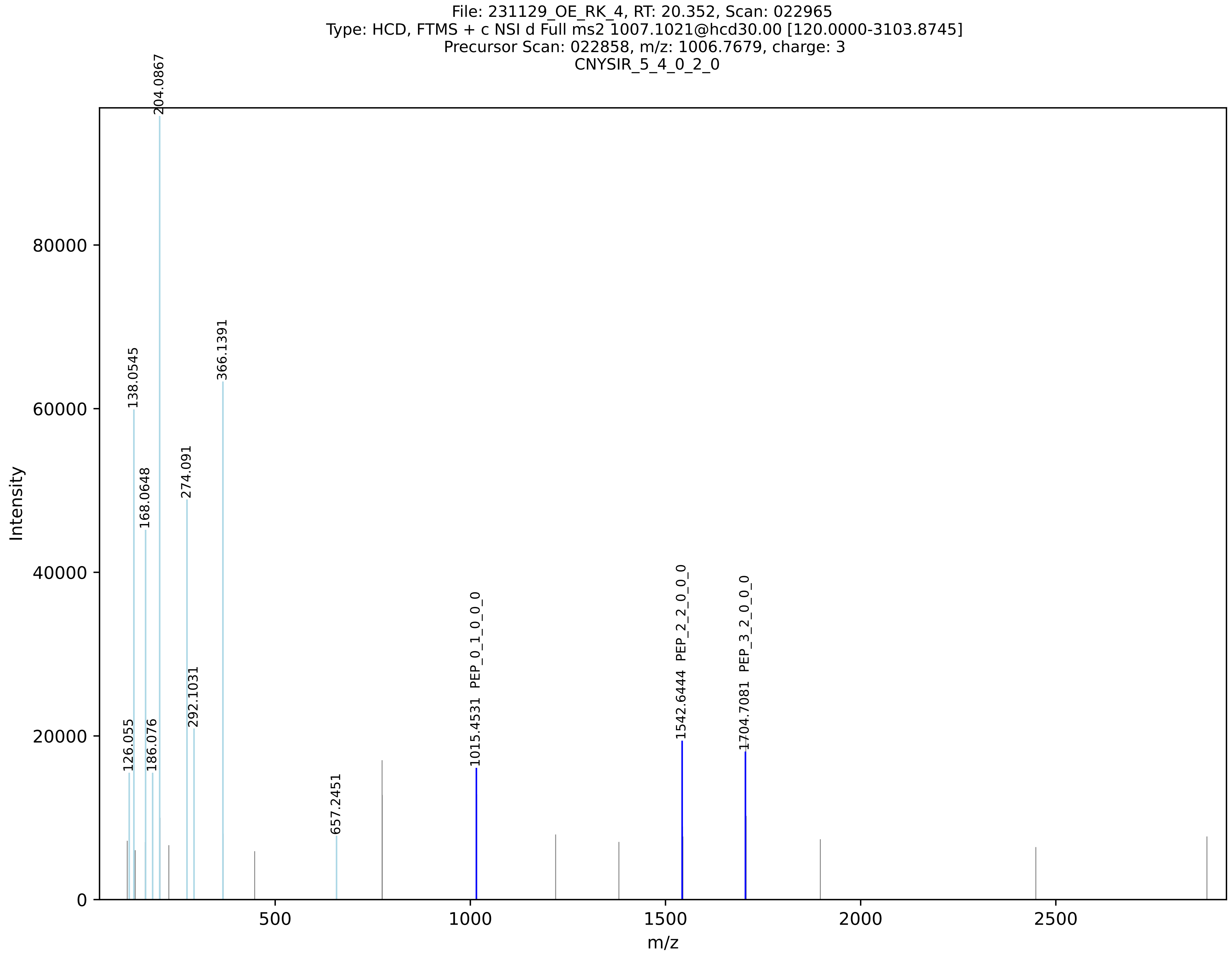
File: 231129_OE_RK_4, RT: 20.352, Scan: 022965
Type: HCD, FTMS + c NSI d Full ms2 1007.1021@hcd30.00 [120.0000-3103.8745]
Precursor Scan: 022858, m/z: 1006.7679, charge: 3
CNYSIR_5_4_0_2_0
Intensity
m/z
204.0867
138.0545
366.1391
168.0648
274.091
292.1031
126.055
186.076
657.2451
1015.4531 PEP_0_1_0_0_0
1542.6444 PEP_2_2_0_0_0
1704.7081 PEP_3_2_0_0_0
0
20000
40000
60000
80000
500
1000
1500
2000
2500

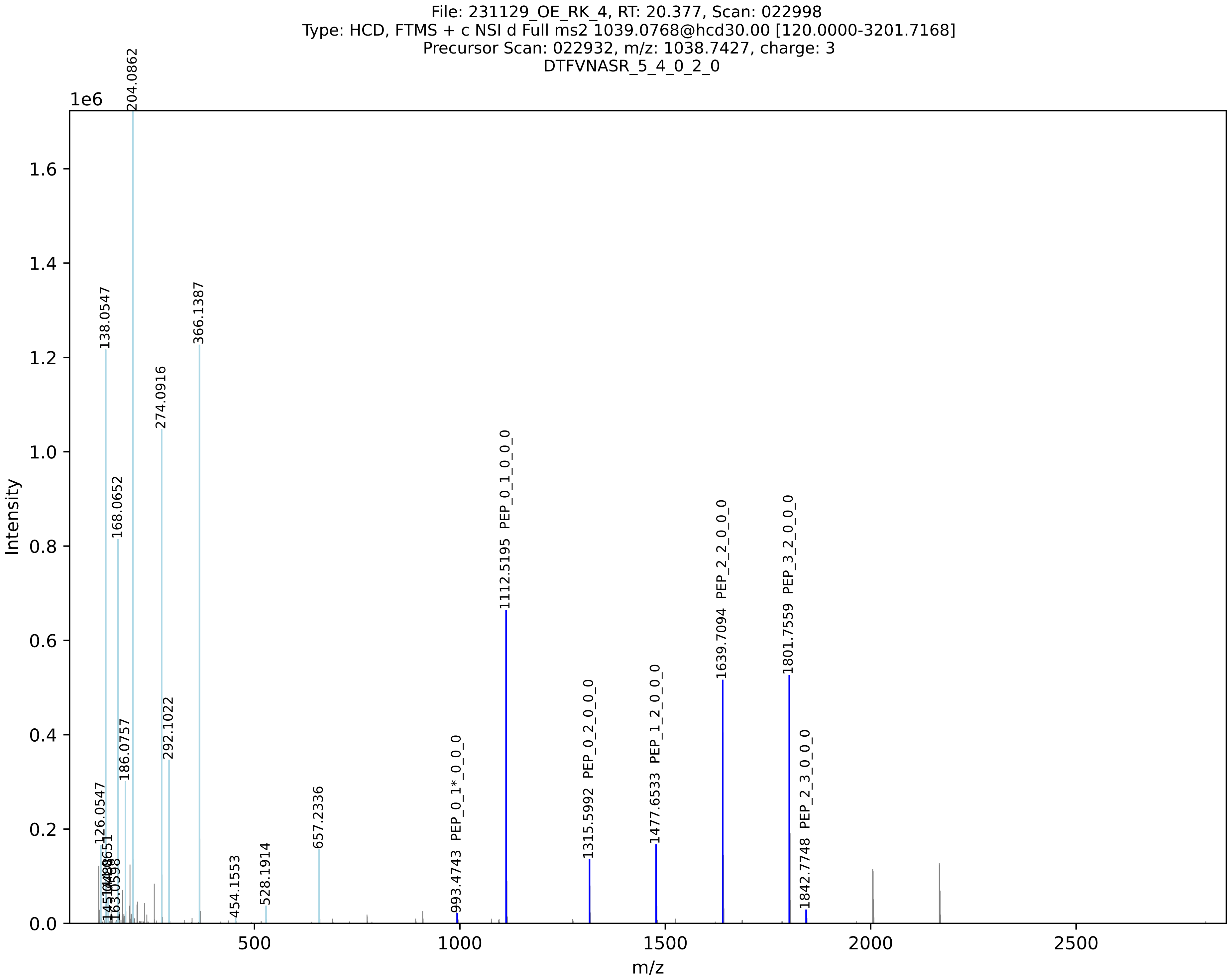
File: 231129_OE_RK_4, RT: 20.377, Scan: 022998
Type: HCD, FTMS + c NSI d Full ms2 1039.0768@hcd30.00 [120.0000-3201.7168]
Precursor Scan: 022932, m/z: 1038.7427, charge: 3
DTFVNASR_5_4_0_2_0
1e6
Intensity
m/z
204.0862
138.0547
366.1387
274.0916
168.0652
186.0757
292.1022
126.0547
163.0598
657.2336
454.1553
528.1914
993.4743 PEP_0_1*_0_0_0
1112.5195 PEP_0_1_0_0_0
1315.5992 PEP_0_2_0_0_0
1477.6533 PEP_1_2_0_0_0
1639.7094 PEP_2_2_0_0_0
1801.7559 PEP_3_2_0_0_0
1842.7748 PEP_2_3_0_0_0

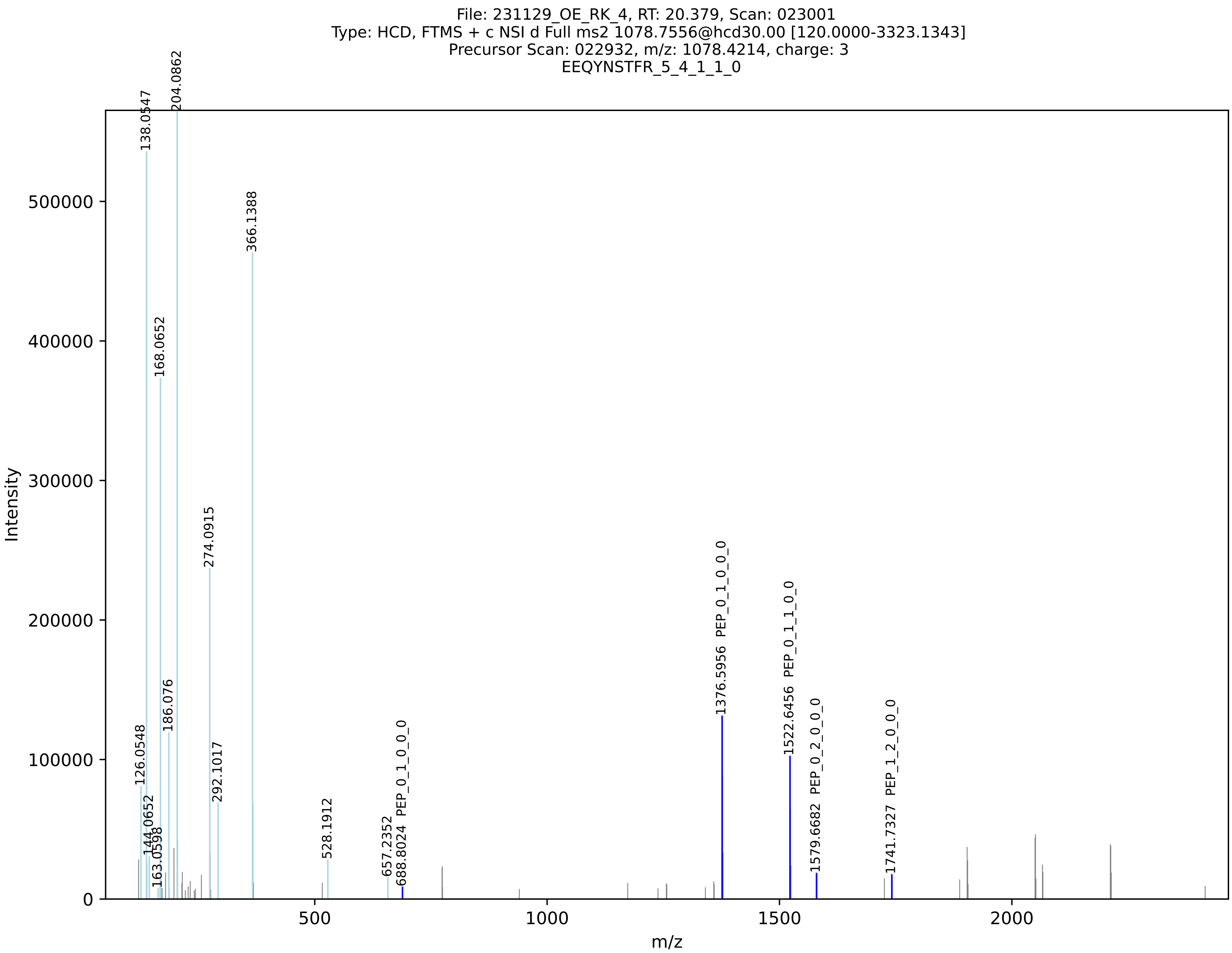
File: 231129_OE_RK_4, RT: 20.379, Scan: 023001
Type: HCD, FTMS + c NSI d Full ms2 1078.7556@hcd30.00 [120.0000-3323.1343]
Precursor Scan: 022932, m/z: 1078.4214, charge: 3
EEQYNSTFR_5_4_1_1_0
Intensity
m/z
204.0862
138.0547
366.1388
168.0652
274.0915
186.076
126.0548
292.1017
144.0652
163.0598
528.1912
657.2352
688.8024 PEP_0_1_0_0_0
1376.5956 PEP_0_1_0_0_0
1522.6456 PEP_0_1_1_0_0
1579.6682 PEP_0_2_0_0_0
1741.7327 PEP_1_2_0_0_0
0
100000
200000
300000
400000
500000
500
1000
1500
2000

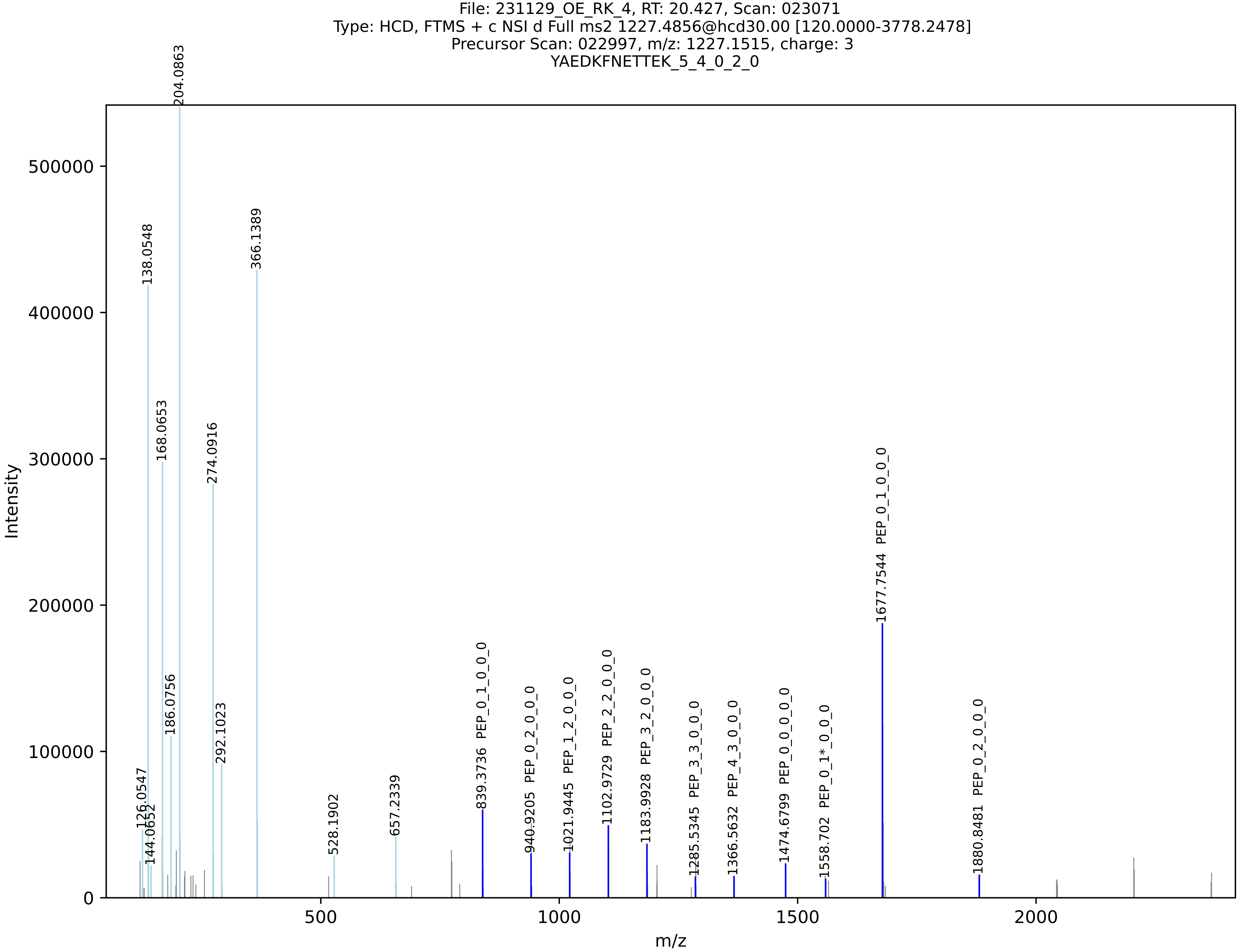
File: 231129_OE_RK_4, RT: 20.427, Scan: 023071
Type: HCD, FTMS + c NSI d Full ms2 1227.4856@hcd30.00 [120.0000-3778.2478]
Precursor Scan: 022997, m/z: 1227.1515, charge: 3
YAEDKFNETTEK_5_4_0_2_0
Intensity
m/z
204.0863
138.0548
366.1389
168.0653
274.0916
186.0756
292.1023
126.0547
144.0652
528.1902
657.2339
839.3736 PEP_0_1_0_0_0
940.9205 PEP_0_2_0_0_0
1021.9445 PEP_1_2_0_0_0
1102.9729 PEP_2_2_0_0_0
1183.9928 PEP_3_2_0_0_0
1285.5345 PEP_3_3_0_0_0
1366.5632 PEP_4_3_0_0_0
1474.6799 PEP_0_0_0_0_0
1558.702 PEP_0_1*_0_0_0
1677.7544 PEP_0_1_0_0_0
1880.8481 PEP_0_2_0_0_0

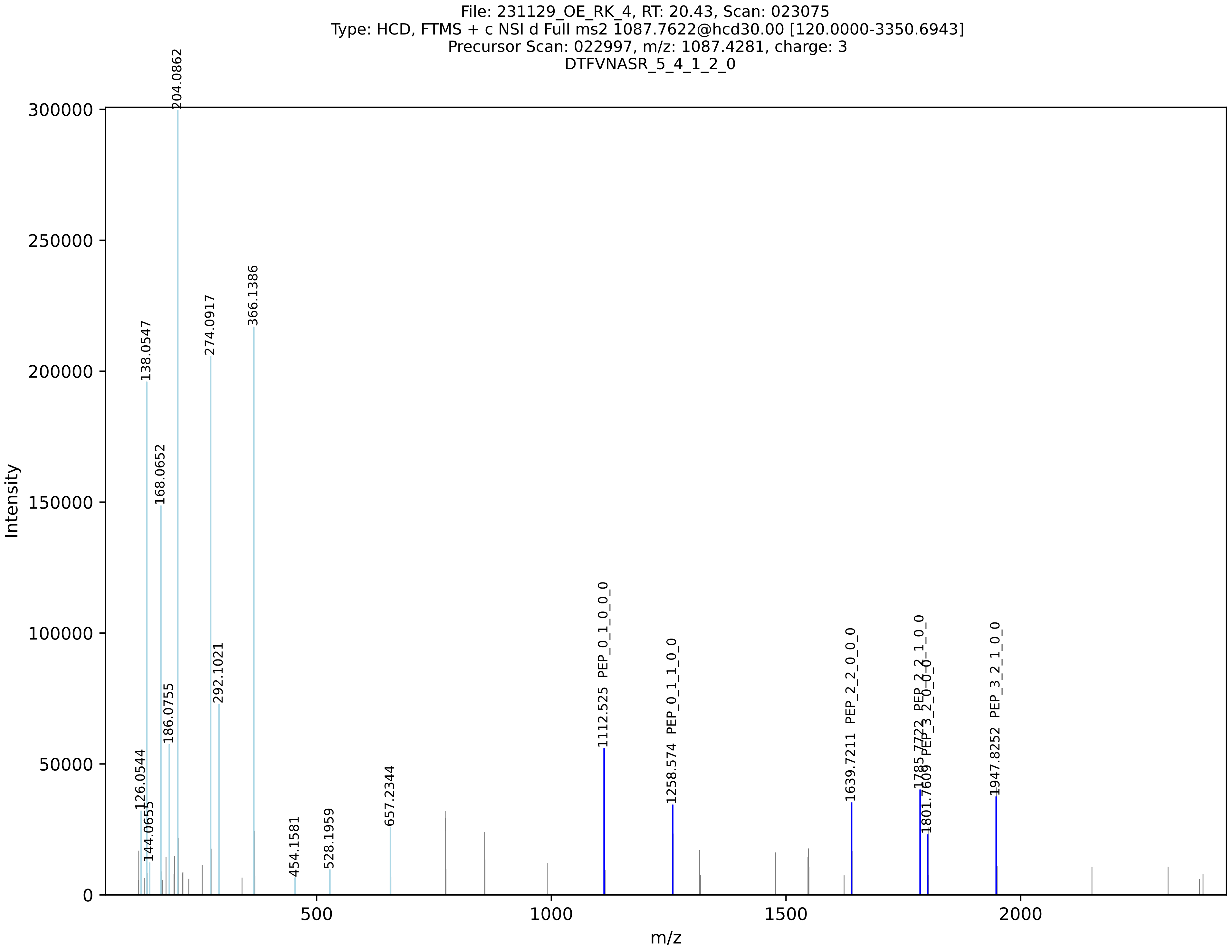
File: 231129_OE_RK_4, RT: 20.43, Scan: 023075
Type: HCD, FTMS + c NSI d Full ms2 1087.7622@hcd30.00 [120.0000-3350.6943]
Precursor Scan: 022997, m/z: 1087.4281, charge: 3
DTFVNASR_5_4_1_2_0
Intensity
m/z
0
50000
100000
150000
200000
250000
300000
500
1000
1500
2000
204.0862
138.0547
168.0652
274.0917
366.1386
292.1021
186.0755
126.0544
144.0655
454.1581
528.1959
657.2344
1112.525 PEP_0_1_0_0_0
1258.574 PEP_0_1_1_0_0
1639.7211 PEP_2_2_0_0_0
1785.7722 PEP_2_2_1_0_0
1801.7609 PEP_3_2_0_0_0
1947.8252 PEP_3_2_1_0_0

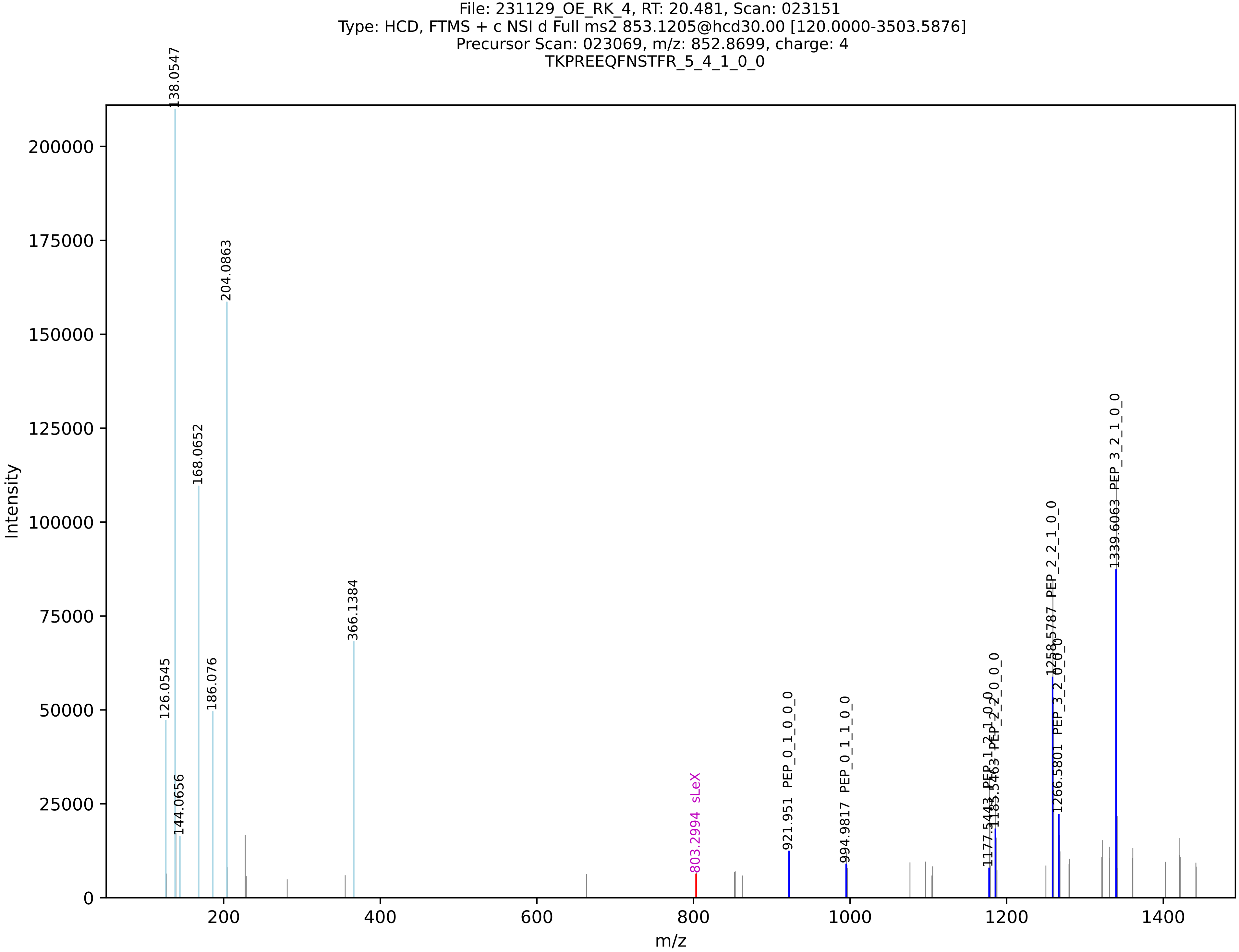
File: 231129_OE_RK_4, RT: 20.481, Scan: 023151
Type: HCD, FTMS + c NSI d Full ms2 853.1205@hcd30.00 [120.0000-3503.5876]
Precursor Scan: 023069, m/z: 852.8699, charge: 4
TKPREEQFNSTFR_5_4_1_0_0
Intensity
m/z
138.0547
204.0863
168.0652
366.1384
126.0545
186.076
144.0656
803.2994 sLeX
921.951 PEP_0_1_0_0_0
994.9817 PEP_0_1_1_0_0
1258.5787 PEP_2_2_1_0_0
1339.6063 PEP_3_2_1_0_0
1266.5801 PEP_3_2_0_0_0
0
25000
50000
75000
100000
125000
150000
175000
200000
200
400
600
800
1000
1200
1400

File: 231129_OE_RK_4, RT: 20.568, Scan: 023276
Type: HCD, FTMS + c NSI d Full ms2 1078.7545@hcd30.00 [120.0000-3323.1306]
Precursor Scan: 023201, m/z: 1078.4206, charge: 3
EEQYNSTFR_5_4_1_1_0

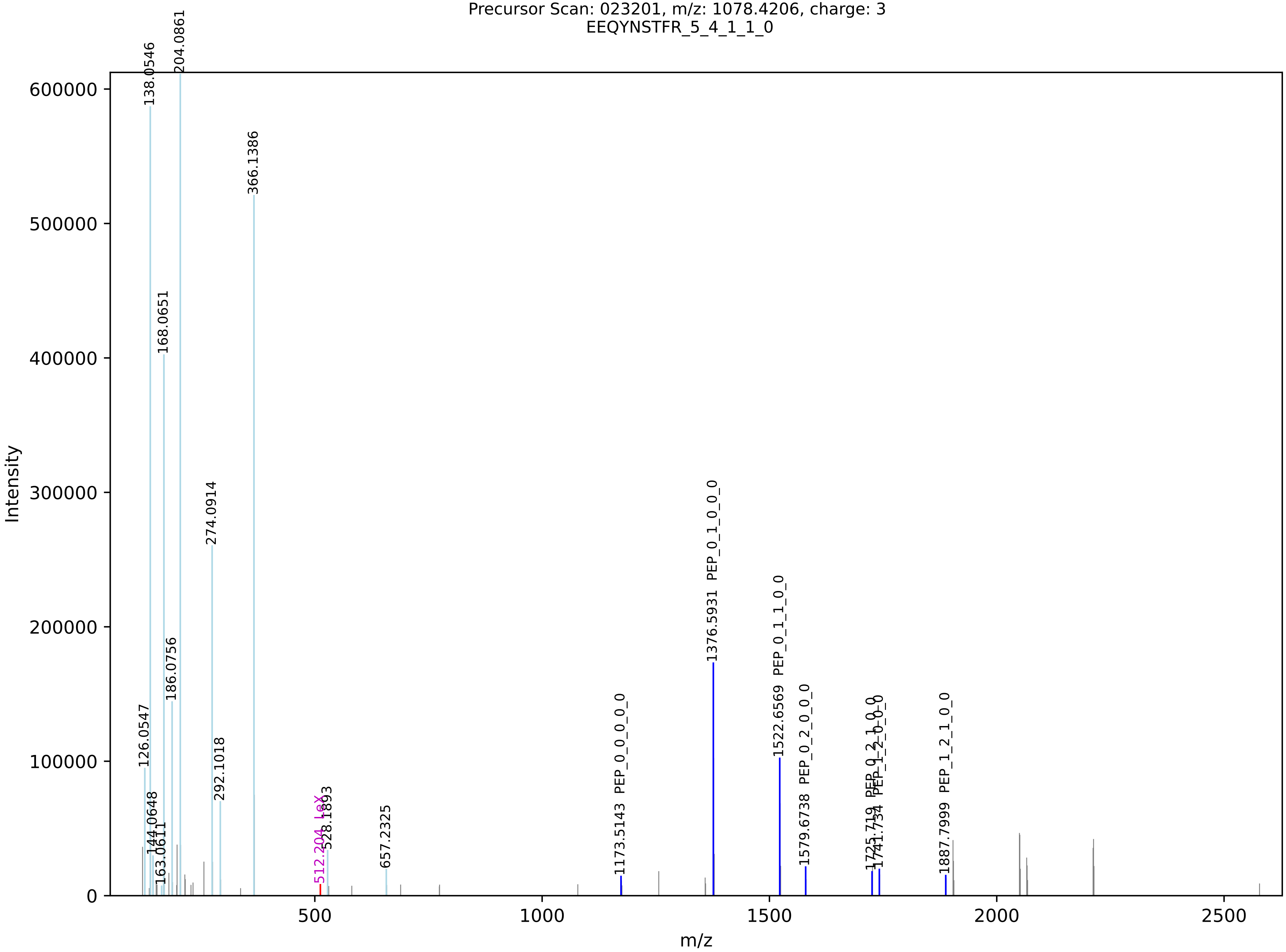

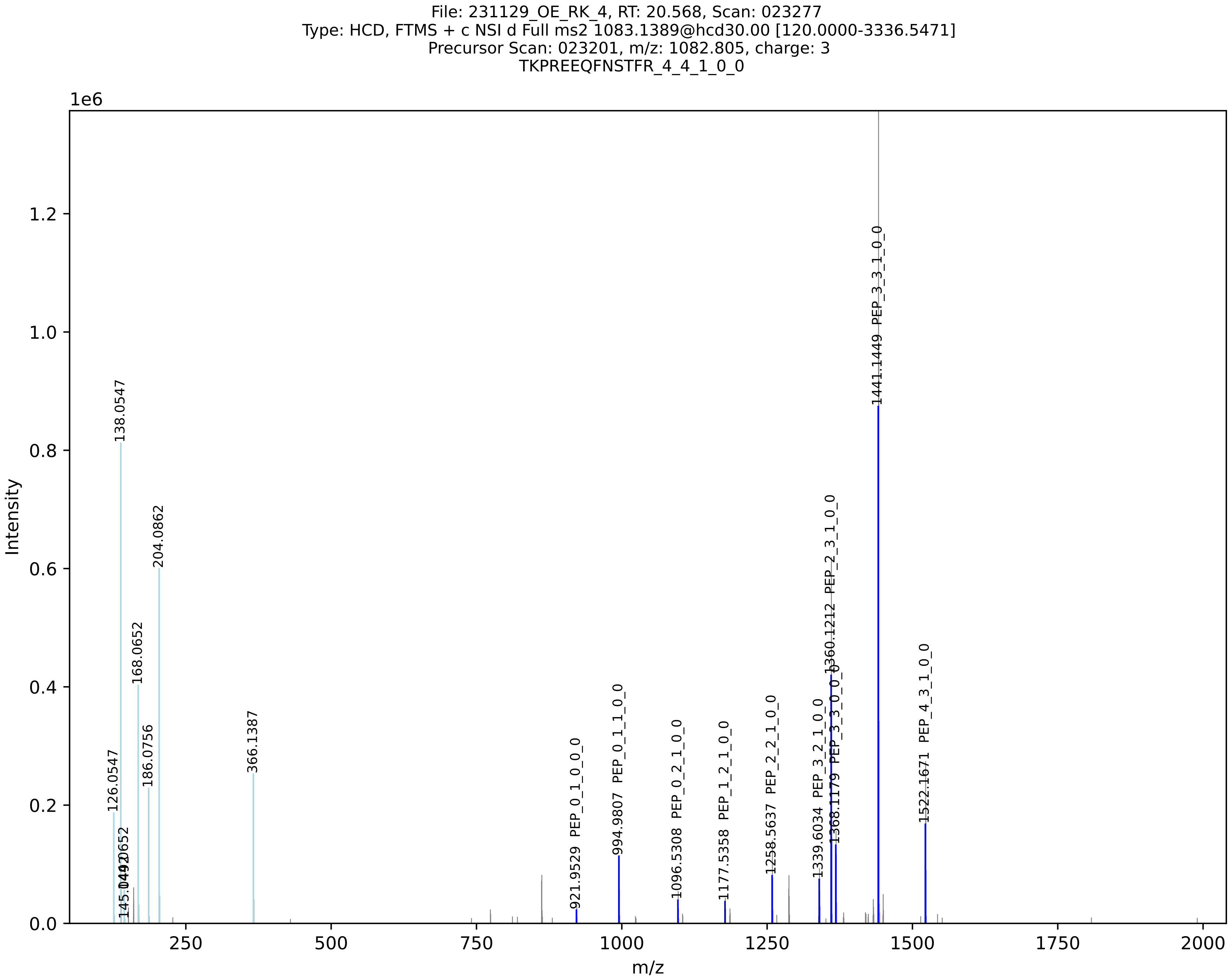

File: 231129_OE_RK_4, RT: 20.568, Scan: 023277
Type: HCD, FTMS + c NSI d Full ms2 1083.1389@hcd30.00 [120.0000-3336.5471]
Precursor Scan: 023201, m/z: 1082.805, charge: 3
TKPREEQFNSTFR_4_4_1_0_0
1e6
Intensity
m/z
138.0547
204.0862
168.0652
186.0756
126.0547
366.1387
921.9529 PEP_0_1_0_0_0
994.9807 PEP_0_1_1_0_0
1096.5308 PEP_0_2_1_0_0
1177.5358 PEP_1_2_1_0_0
1258.5637 PEP_2_2_1_0_0
1339.6034 PEP_3_2_1_0_0
1360.1212 PEP_2_3_1_0_0
1368.1179 PEP_3_3_0_0_0
1441.1449 PEP_3_3_1_0_0
1522.1671 PEP_4_3_1_0_0

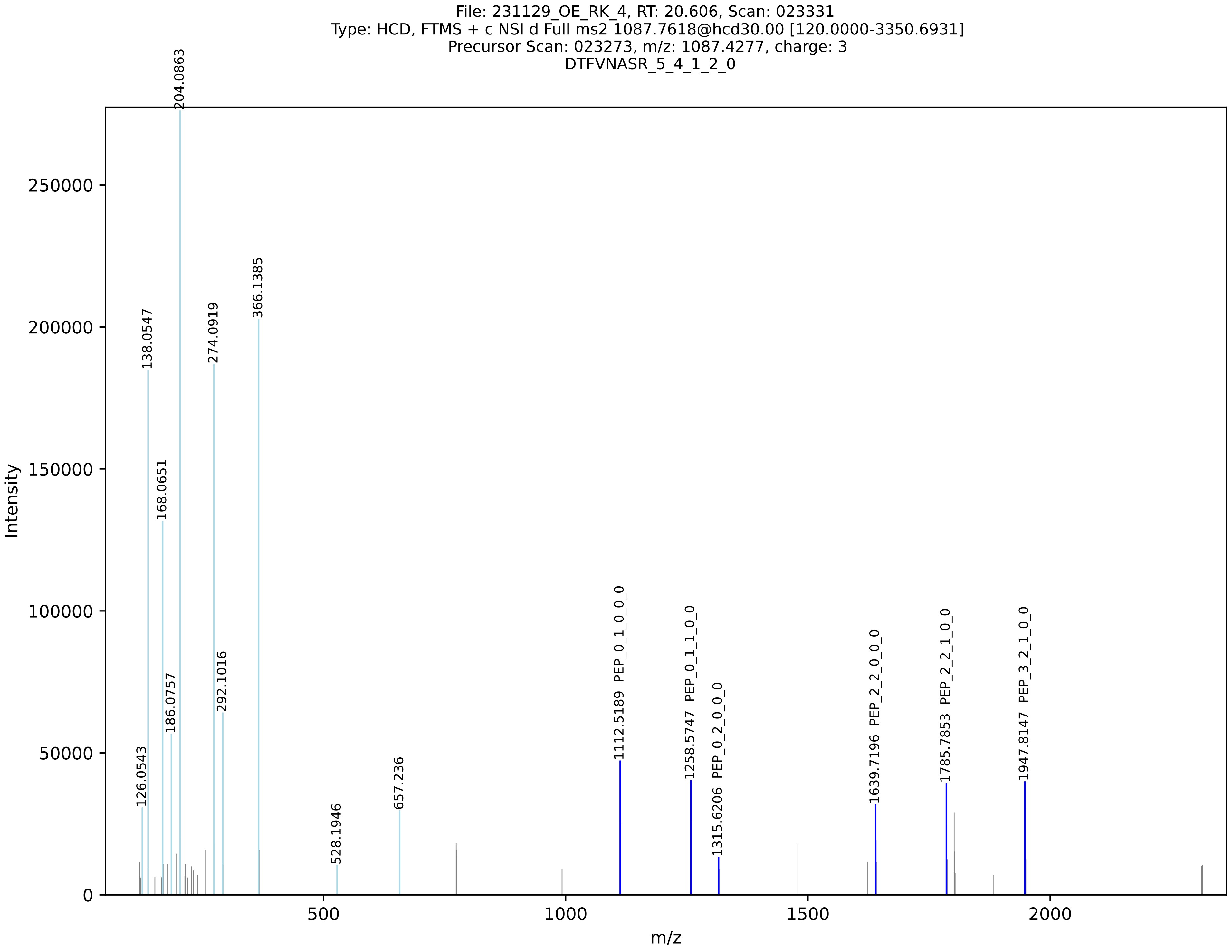
File: 231129_OE_RK_4, RT: 20.606, Scan: 023331
Type: HCD, FTMS + c NSI d Full ms2 1087.7618@hcd30.00 [120.0000-3350.6931]
Precursor Scan: 023273, m/z: 1087.4277, charge: 3
DTFVNASR_5_4_1_2_0
Intensity
m/z
0
50000
100000
150000
200000
250000
500
1000
1500
2000
204.0863
366.1385
138.0547
274.0919
168.0651
292.1016
186.0757
126.0543
528.1946
657.236
1112.5189 PEP_0_1_0_0_0
1258.5747 PEP_0_1_1_0_0
1315.6206 PEP_0_2_0_0_0
1639.7196 PEP_2_2_0_0_0
1785.7853 PEP_2_2_1_0_0
1947.8147 PEP_3_2_1_0_0

File: 231129_OE_RK_4, RT: 21.77, Scan: 024938
Type: HCD, FTMS + c NSI d Full ms2 1087.7625@hcd30.00 [120.0000-3350.6953]
Precursor Scan: 024860, m/z: 1087.4284, charge: 3
DTFVNASR_5_4_1_2_0

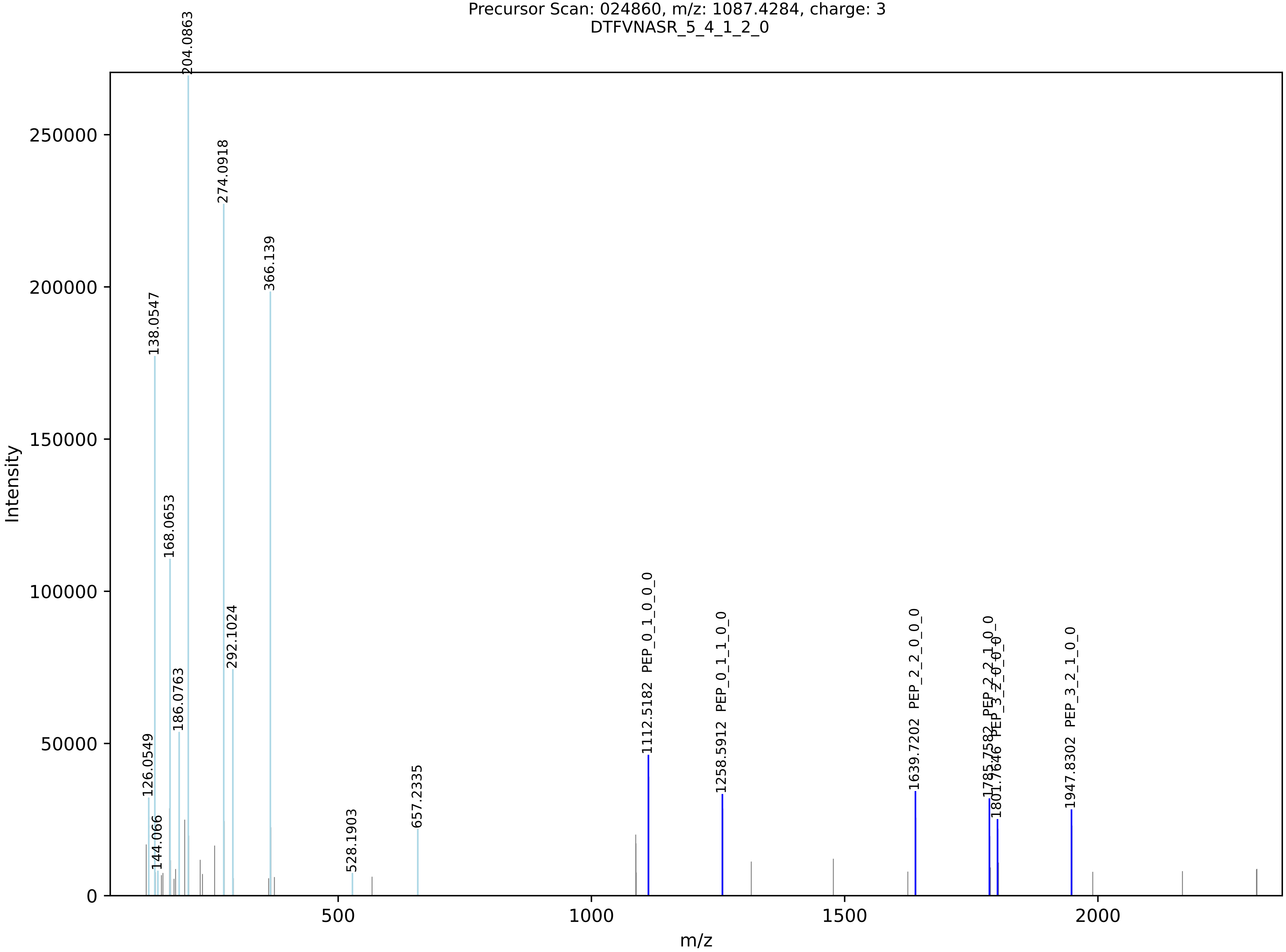

File: 231129_OE_RK_4, RT: 21.82, Scan: 025011
Type: HCD, FTMS + c NSI d Full ms2 1173.1300@hcd30.00 [120.0000-3611.9199]
Precursor Scan: 024934, m/z: 1172.796, charge: 3
FLNNGTCTAEGK_5_4_0_2_0

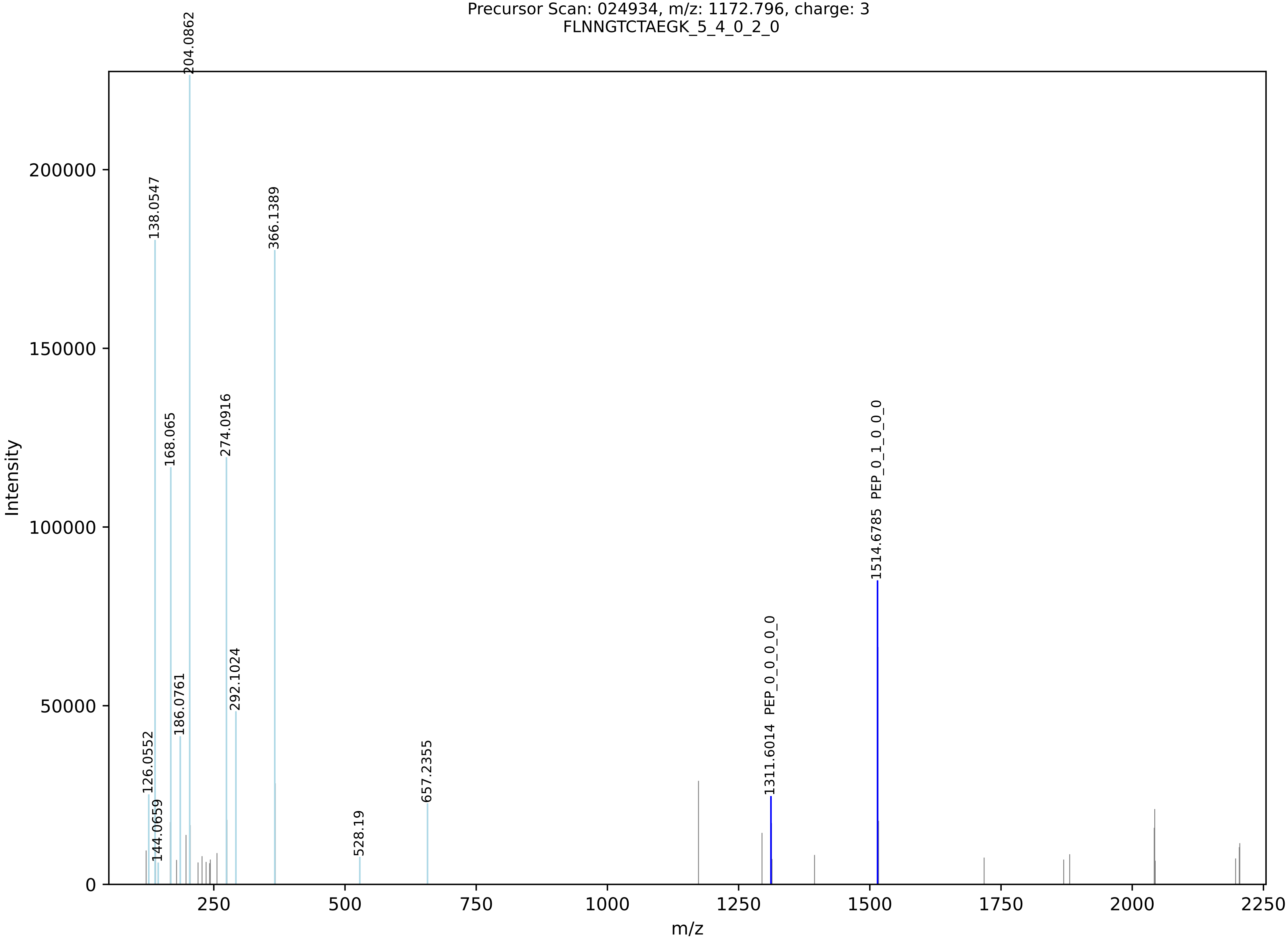

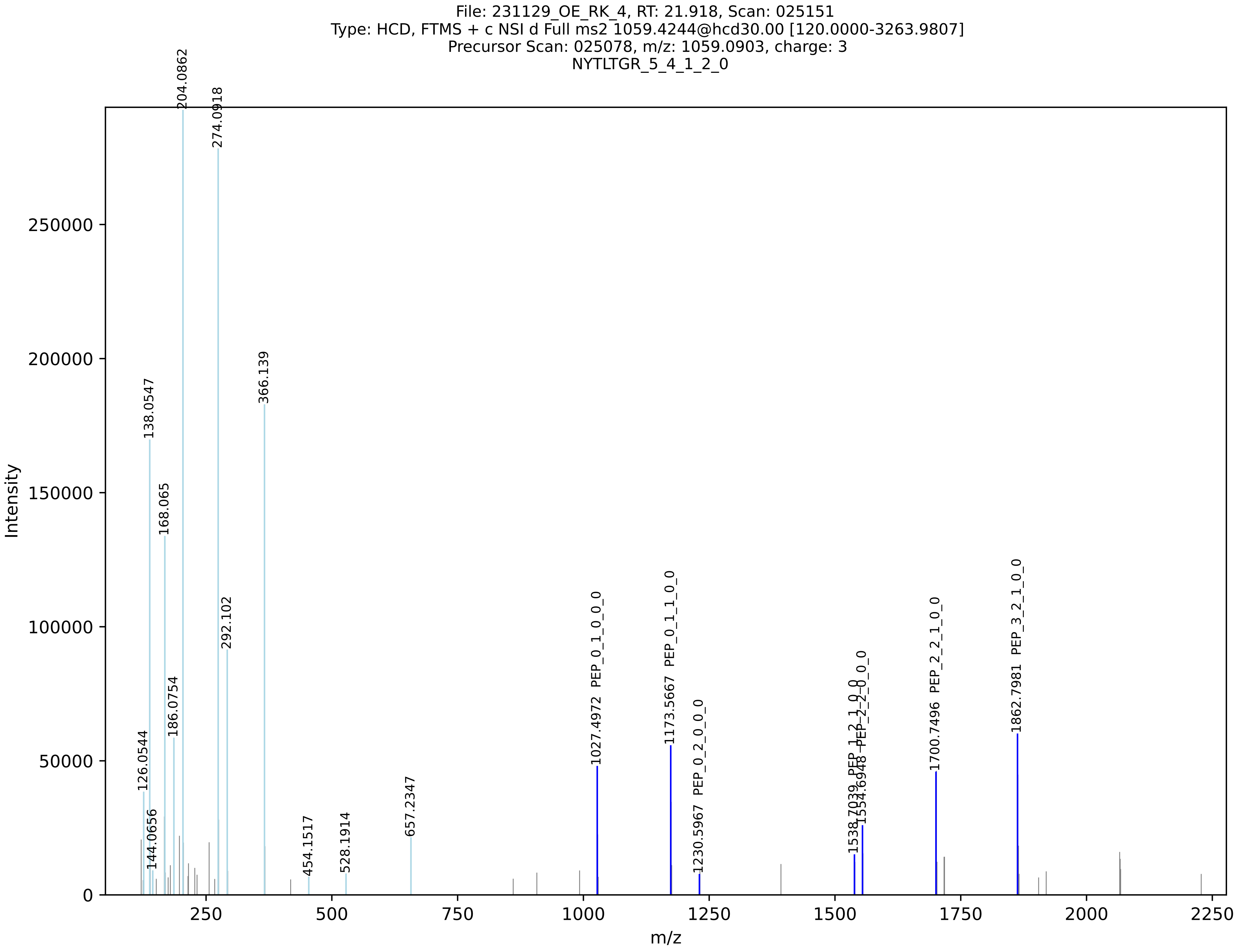
File: 231129_OE_RK_4, RT: 21.918, Scan: 025151
Type: HCD, FTMS + c NSI d Full ms2 1059.4244@hcd30.00 [120.0000-3263.9807]
Precursor Scan: 025078, m/z: 1059.0903, charge: 3
NYTLTGR_5_4_1_2_0
Intensity
m/z
204.0862
274.0918
366.139
138.0547
168.065
292.102
186.0754
126.0544
144.0656
454.1517
528.1914
657.2347
1027.4972 PEP_0_1_0_0_0
1173.5667 PEP_0_1_1_0_0
1230.5967 PEP_0_2_0_0_0
1538.7039 PEP_1_2_1_0_0
1554.6948 PEP_2_2_0_0_0
1700.7496 PEP_2_2_1_0_0
1862.7981 PEP_3_2_1_0_0
0
50000
100000
150000
200000
250000
250
500
750
1000
1250
1500
1750
2000
2250

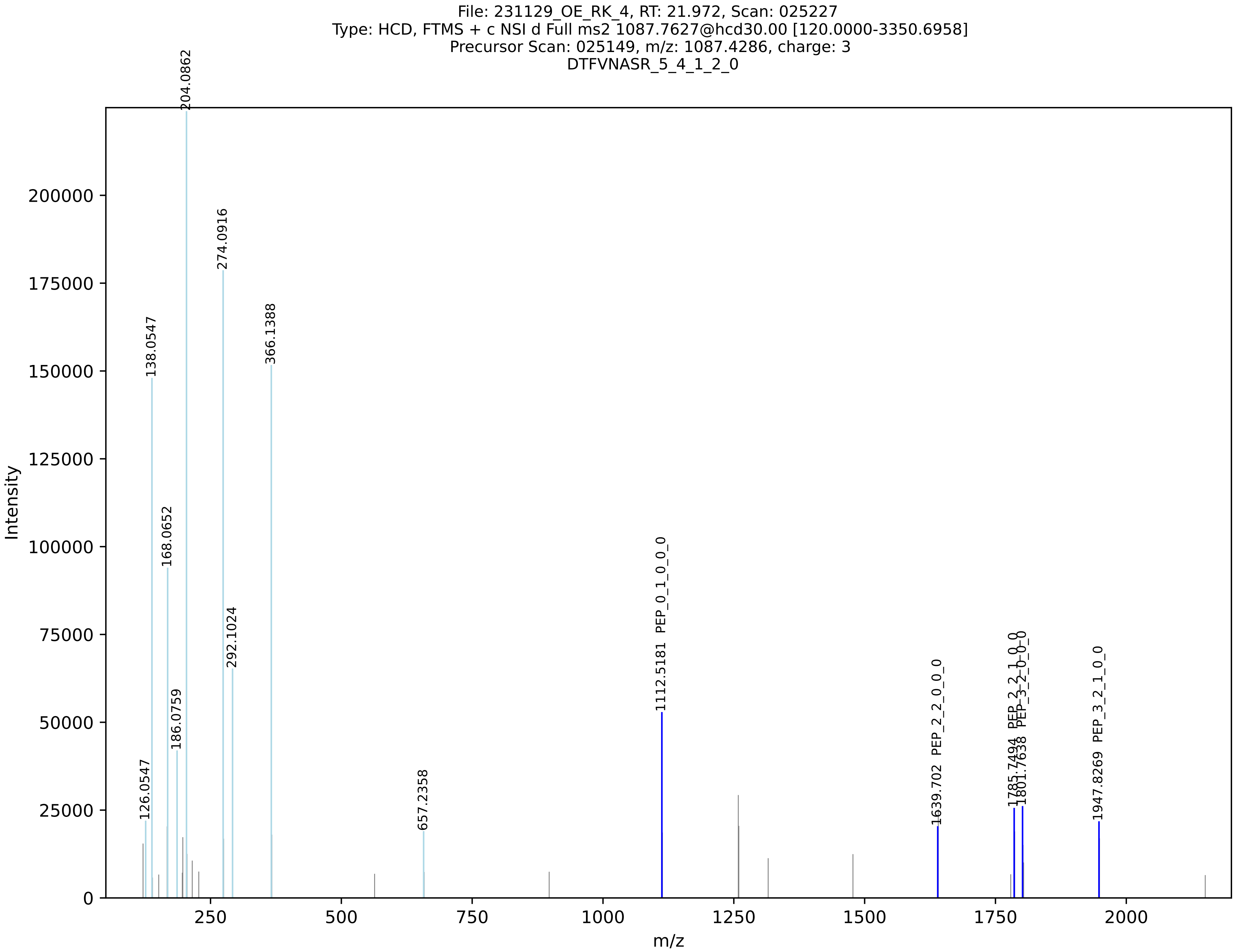
File: 231129_OE_RK_4, RT: 21.972, Scan: 025227
Type: HCD, FTMS + c NSI d Full ms2 1087.7627@hcd30.00 [120.0000-3350.6958]
Precursor Scan: 025149, m/z: 1087.4286, charge: 3
DTFVNASR_5_4_1_2_0
Intensity
m/z
0
25000
50000
75000
100000
125000
150000
175000
200000
250
500
750
1000
1250
1500
1750
2000
126.0547
138.0547
168.0652
186.0759
204.0862
274.0916
292.1024
366.1388
657.2358
1112.5181 PEP_0_1_0_0_0
1639.702 PEP_2_2_0_0_0
1785.7494 PEP_2_2_1_0_0
1801.7638 PEP_3_2_0_0_0
1947.8269 PEP_3_2_1_0_0

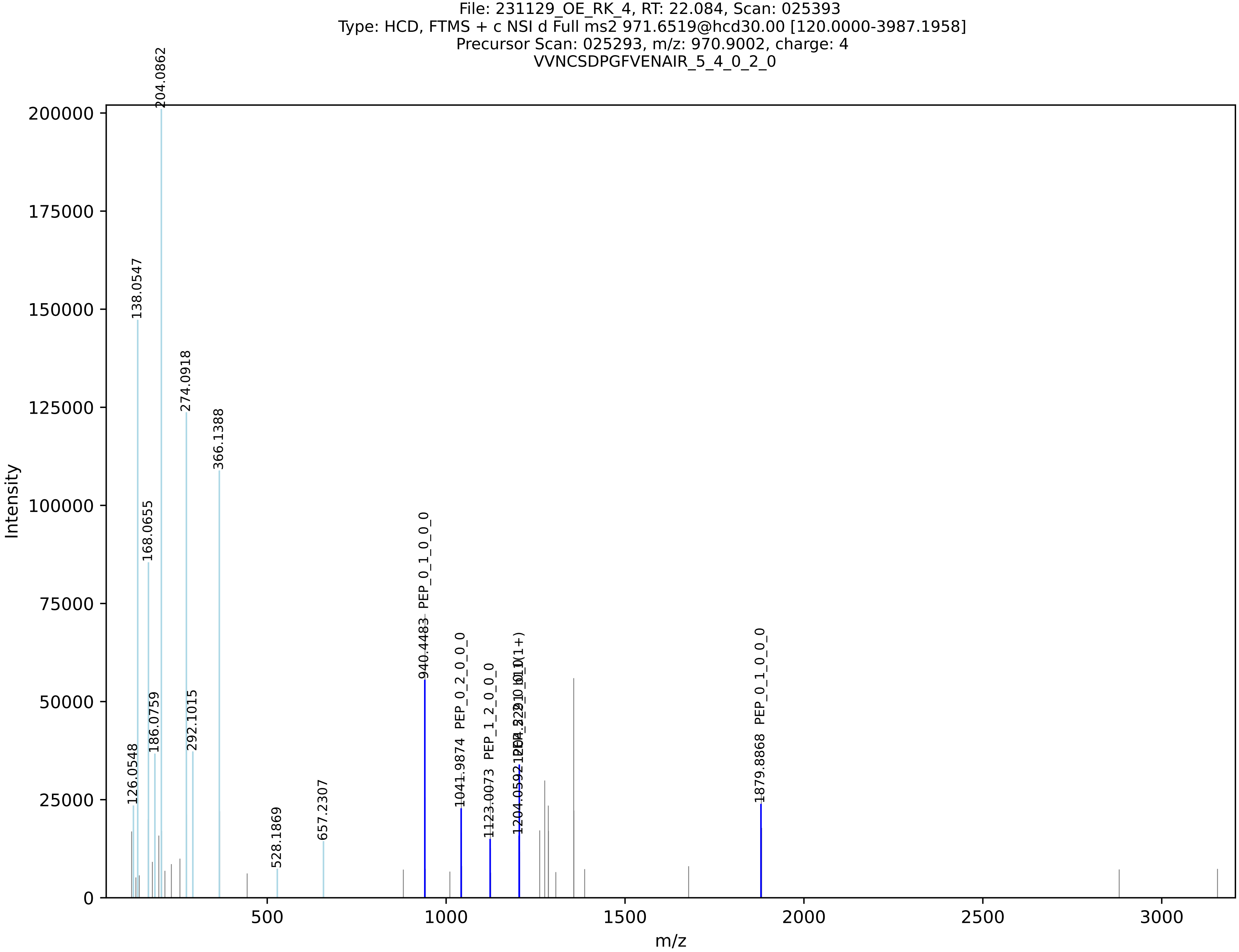
File: 231129_OE_RK_4, RT: 22.084, Scan: 025393
Type: HCD, FTMS + c NSI d Full ms2 971.6519@hcd30.00 [120.0000-3987.1958]
Precursor Scan: 025293, m/z: 970.9002, charge: 4
VVNCSDPGFVENAIR_5_4_0_2_0
Intensity
m/z
204.0862
138.0547
274.0918
366.1388
168.0655
186.0759
292.1015
126.0548
528.1869
657.2307
940.4483 PEP_0_1_0_0_0
1041.9874 PEP_0_2_0_0_0
1123.0073 PEP_1_2_0_0_0
1204.0592
1879.8868 PEP_0_1_0_0_0
0
25000
50000
75000
100000
125000
150000
175000
200000
500
1000
1500
2000
2500
3000

File: 231129_OE_RK_4, RT: 22.12, Scan: 025443
Type: HCD, FTMS + c NSI d Full ms2 1059.4237@hcd30.00 [120.0000-3263.9788]
Precursor Scan: 025367, m/z: 1059.0895, charge: 3
NYTLTGR_5_4_1_2_0

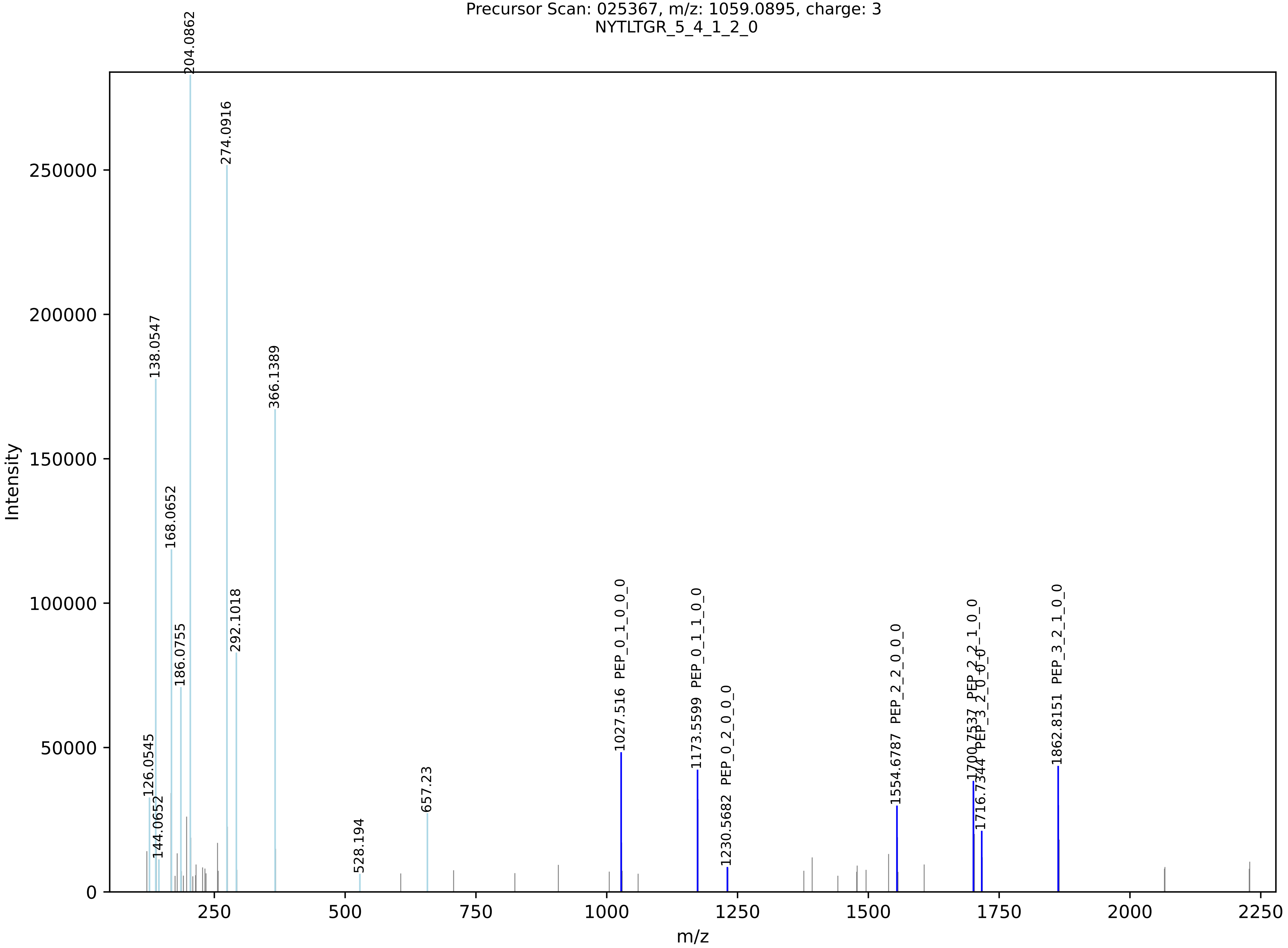

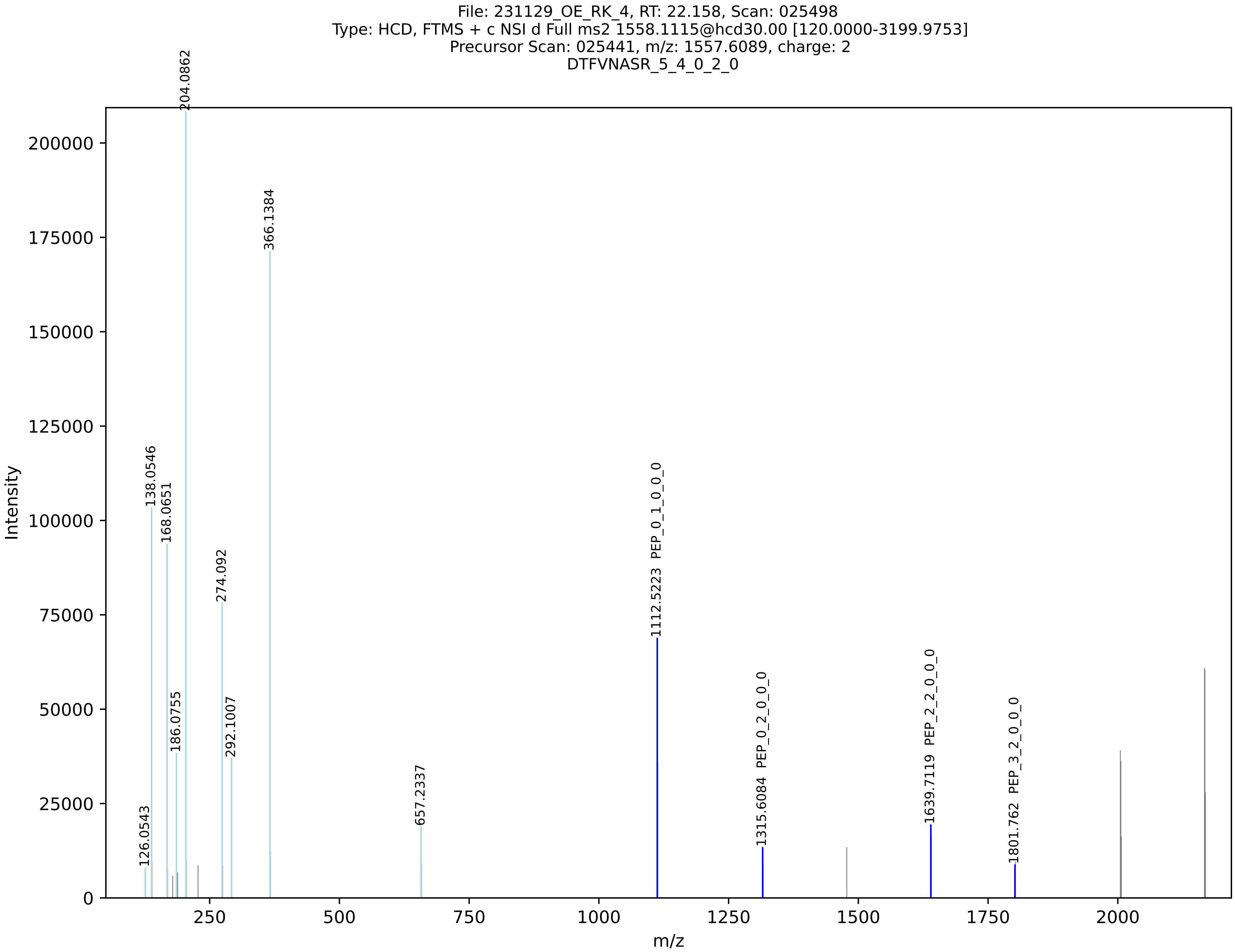

File: 231129_OE_RK_4, RT: 22.158, Scan: 025498
Type: HCD, FTMS + c NSI d Full ms2 1558.1115@hcd30.00 [120.0000-3199.9753]
Precursor Scan: 025441, m/z: 1557.6089, charge: 2
DTFVNASR_5_4_0_2_0
Intensity
m/z
204.0862
366.1384
138.0546
168.0651
274.092
186.0755
292.1007
126.0543
657.2337
1112.5223 PEP_0_1_0_0_0
1315.6084 PEP_0_2_0_0_0
1639.7119 PEP_2_2_0_0_0
1801.762 PEP_3_2_0_0_0

File: 231129_OE_RK_4, RT: 22.158, Scan: 025499
Type: HCD, FTMS + c NSI d Full ms2 1087.7579@hcd30.00 [120.0000-3350.6812]
Precursor Scan: 025441, m/z: 1087.4273, charge: 3
DTFVNASR_5_4_1_2_0

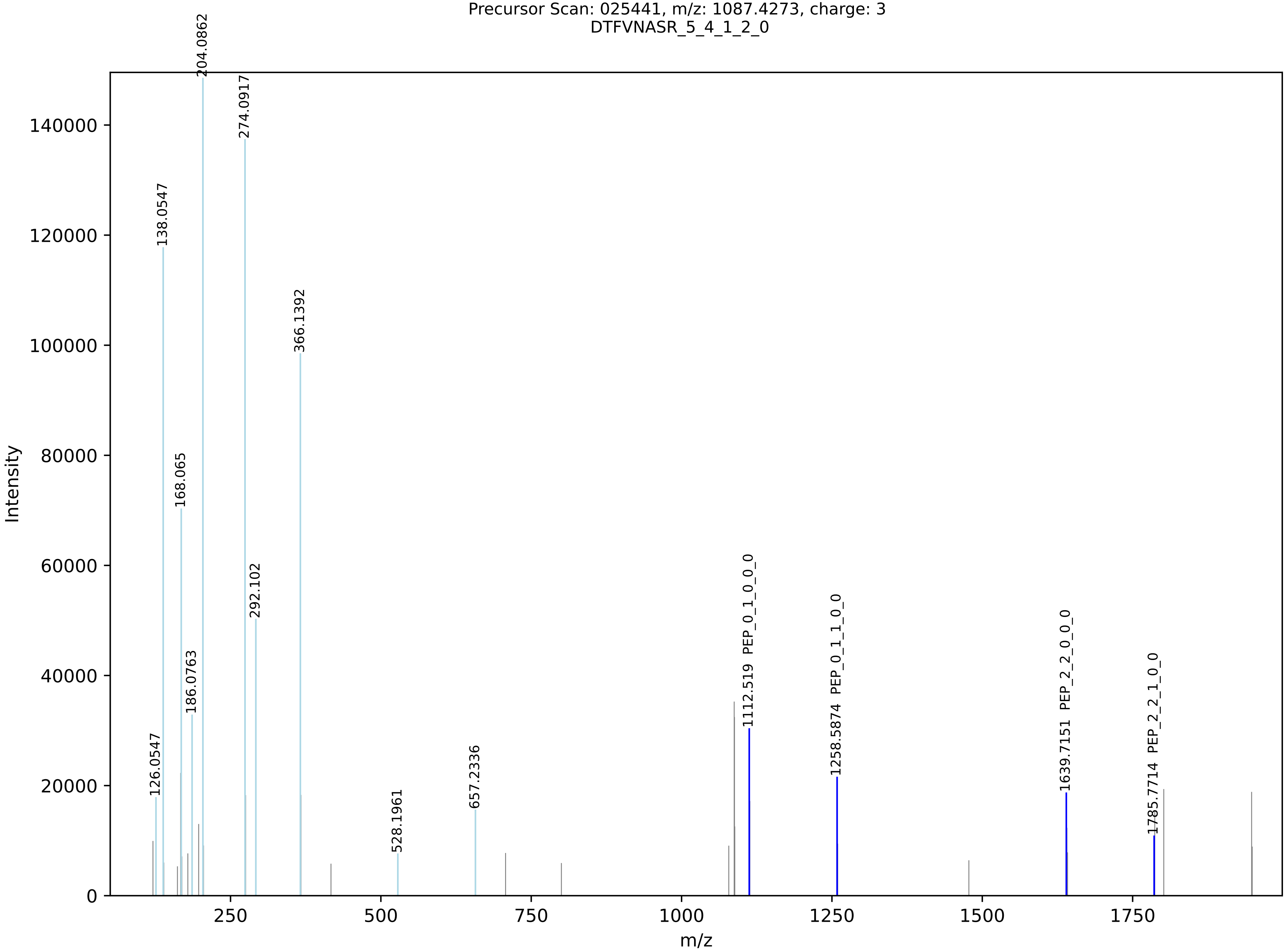

File: 231129_OE_RK_4, RT: 22.414, Scan: 025836
Type: HCD, FTMS + c NSI d Full ms2 1294.8640@hcd30.00 [120.0000-3984.4258]
Precursor Scan: 025763, m/z: 1294.5302, charge: 3
VVNCSDPGFVENAIR_5_4_0_2_0

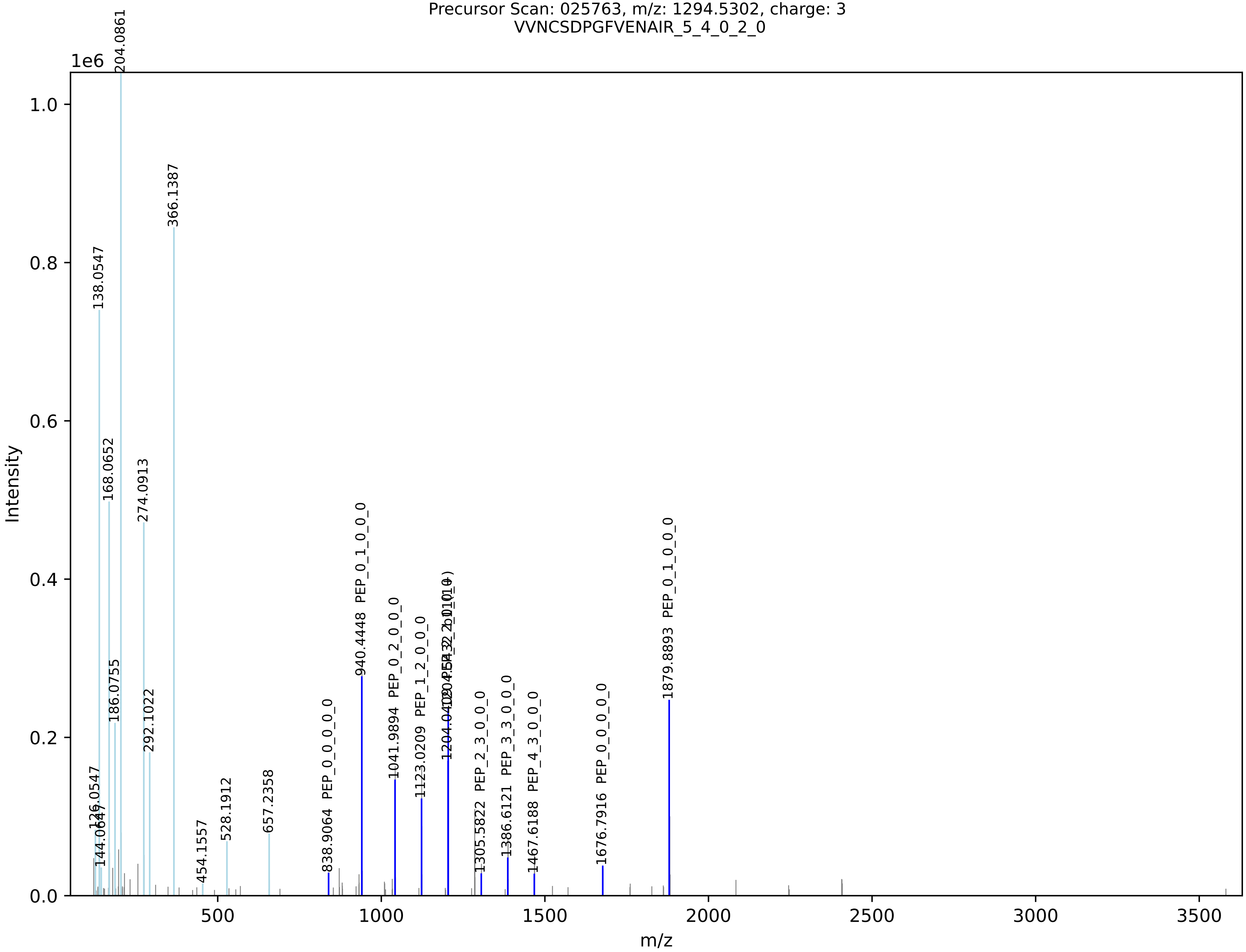

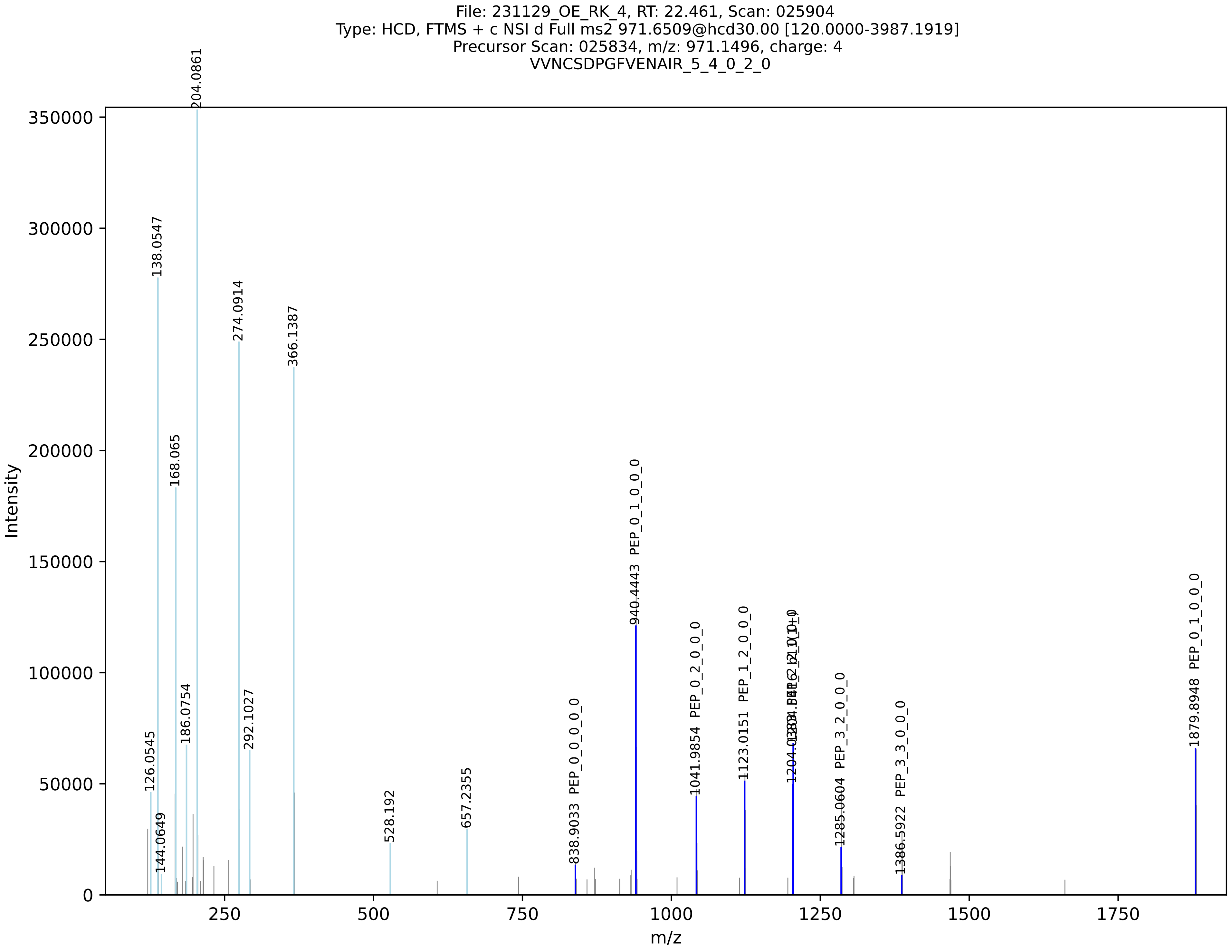
File: 231129_OE_RK_4, RT: 22.461, Scan: 025904
Type: HCD, FTMS + c NSI d Full ms2 971.6509@hcd30.00 [120.0000-3987.1919]
Precursor Scan: 025834, m/z: 971.1496, charge: 4
VVNCSDPGFVENAIR_5_4_0_2_0
Intensity
m/z
0
50000
100000
150000
200000
250000
300000
350000
250
500
750
1000
1250
1500
1750
126.0545
138.0547
144.0649
168.065
186.0754
204.0861
274.0914
292.1027
366.1387
528.192
657.2355
838.9033 PEP_0_0_0_0_0
940.4443 PEP_0_1_0_0_0
1041.9854 PEP_0_2_0_0_0
1123.0151 PEP_1_2_0_0_0
1285.0604 PEP_3_2_0_0_0
1386.5922 PEP_3_3_0_0_0
1879.8948 PEP_0_1_0_0_0

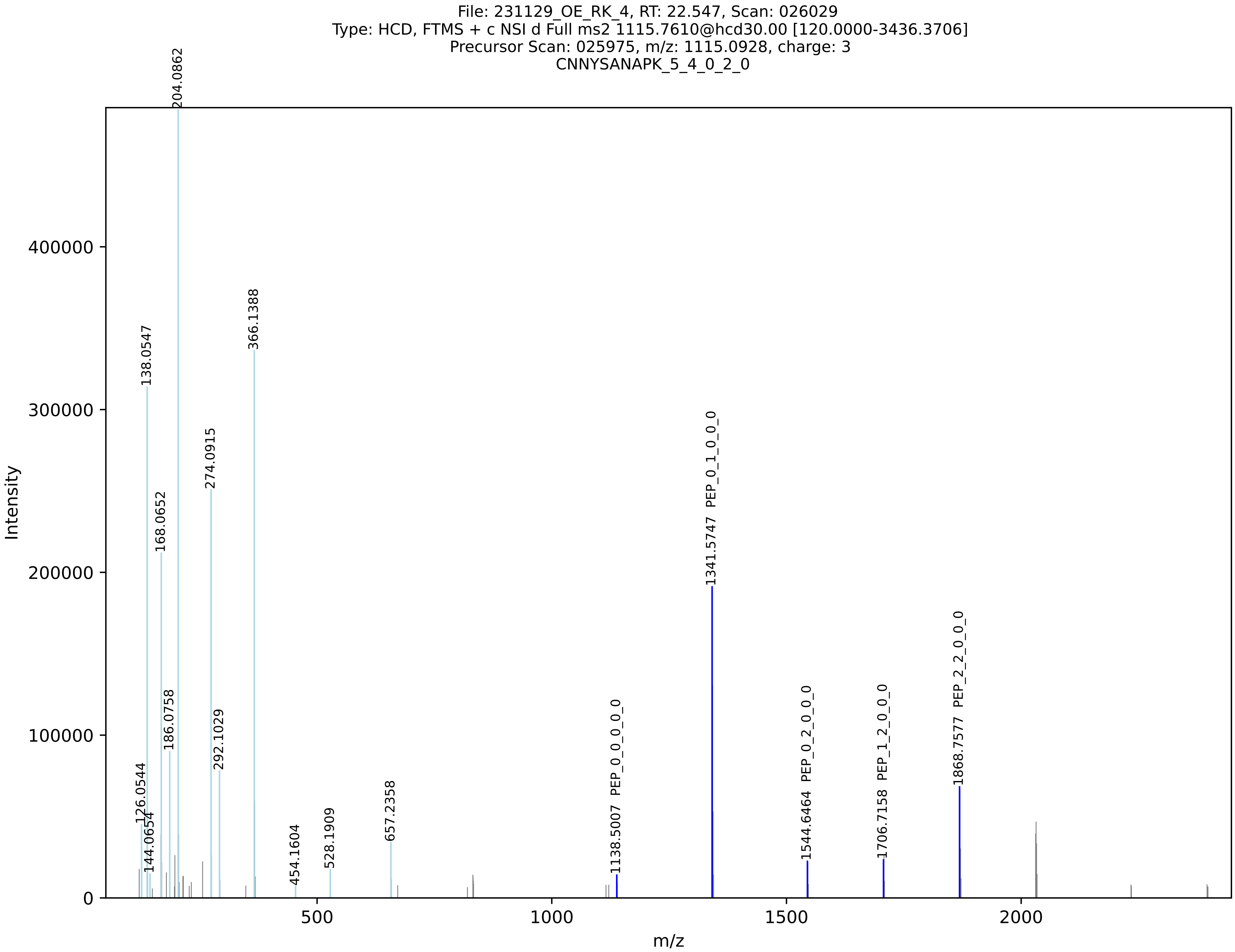
File: 231129_OE_RK_4, RT: 22.547, Scan: 026029
Type: HCD, FTMS + c NSI d Full ms2 1115.7610@hcd30.00 [120.0000-3436.3706]
Precursor Scan: 025975, m/z: 1115.0928, charge: 3
CNNYSANAPK_5_4_0_2_0
Intensity
m/z
204.0862
366.1388
138.0547
274.0915
168.0652
186.0758
292.1029
126.0544
144.0654
454.1604
528.1909
657.2358
1138.5007 PEP_0_0_0_0_0_
1341.5747 PEP_0_1_0_0_0_
1544.6464 PEP_0_2_0_0_0_
1706.7158 PEP_1_2_0_0_0_
1868.7577 PEP_2_2_0_0_0_

File: 231129_OE_RK_4, RT: 22.61, Scan: 026115
Type: HCD, FTMS + c NSI d Full ms2 925.8928@hcd30.00 [120.0000-3800.4985]
Precursor Scan: 026080, m/z: 925.6424, charge: 4
TKPREEQFNSTFR_5_4_1_1_0

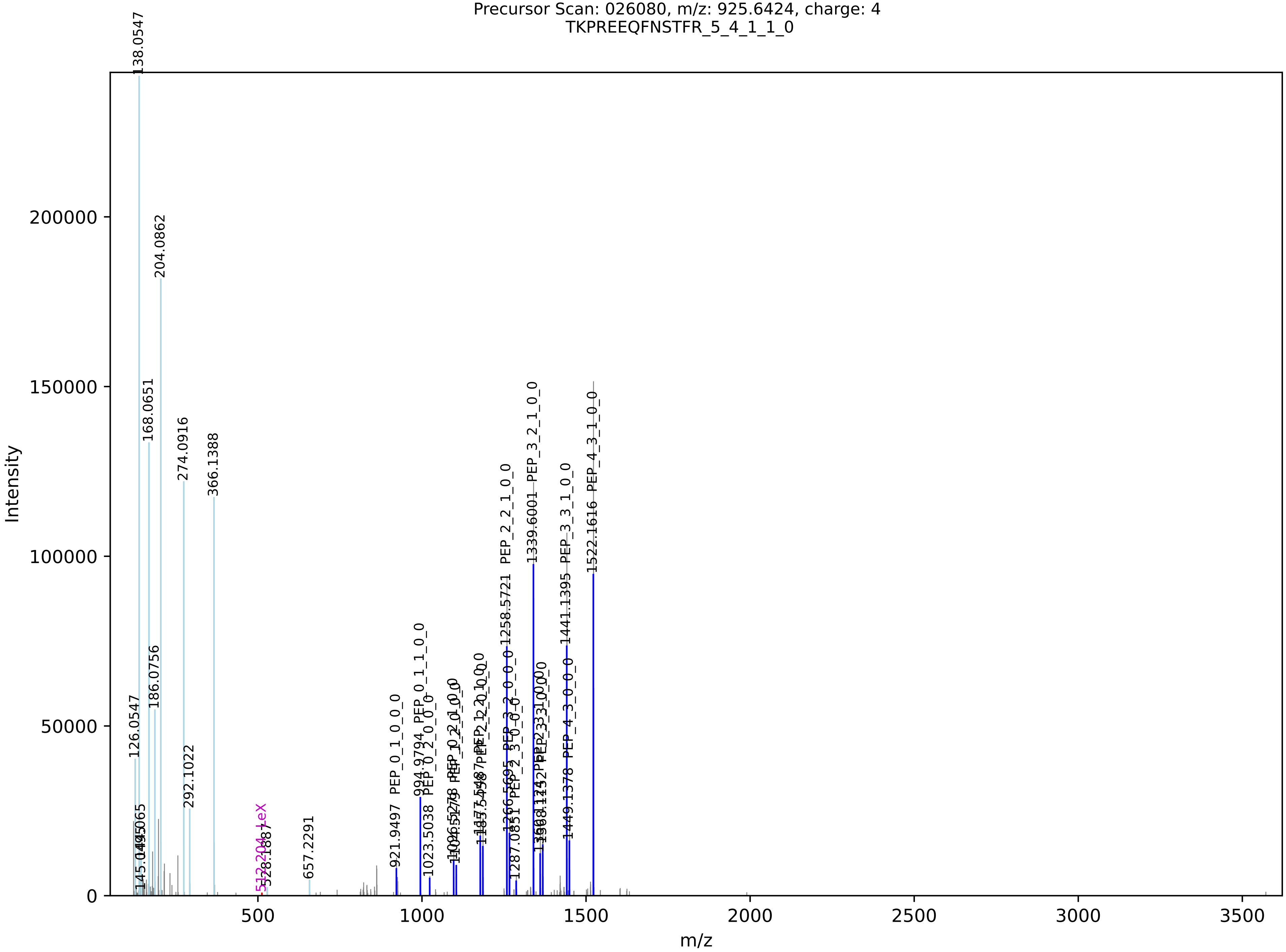

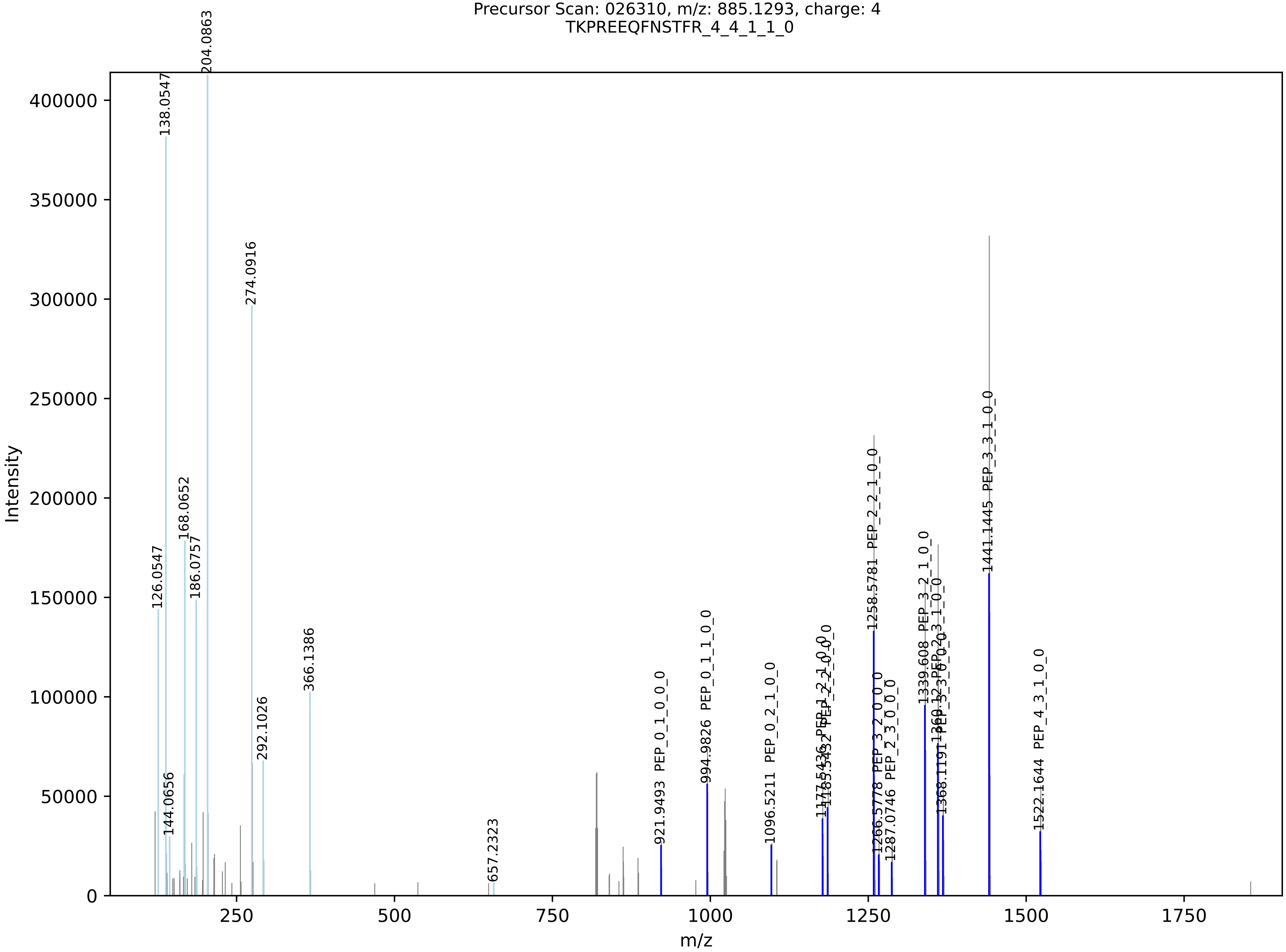
File: 231129_OE_RK_4, RT: 22.795, Scan: 026354
Type: HCD, FTMS + c NSI d Full ms2 885.6307@hcd30.00 [120.0000-3636.2290]
Precursor Scan: 026310, m/z: 885.1293, charge: 4
TKPREEQFNSTFR_4_4_1_1_0
Intensity
m/z
204.0863
138.0547
274.0916
168.0652
186.0757
126.0547
366.1386
292.1026
144.0656
657.2323
921.9493 PEP_0_1_0_0_0
994.9826 PEP_0_1_1_0_0
1096.5211 PEP_0_2_1_0_0
1258.5781 PEP_2_2_1_0_0
1266.5778 PEP_3_2_0_0_0
1287.0746 PEP_2_3_0_0_0
1339.608 PEP_3_2_1_0_0
1368.1191 PEP_3_3_0_0_0
1441.1445 PEP_3_3_1_0_0
1522.1644 PEP_4_3_1_0_0

File: 231129_OE_RK_4, RT: 22.915, Scan: 026513
Type: HCD, FTMS + c NSI d Full ms2 1115.7594@hcd30.00 [120.0000-3436.3660]
Precursor Scan: 026479, m/z: 1115.0912, charge: 3
CNNYSANAPK_5_4_0_2_0

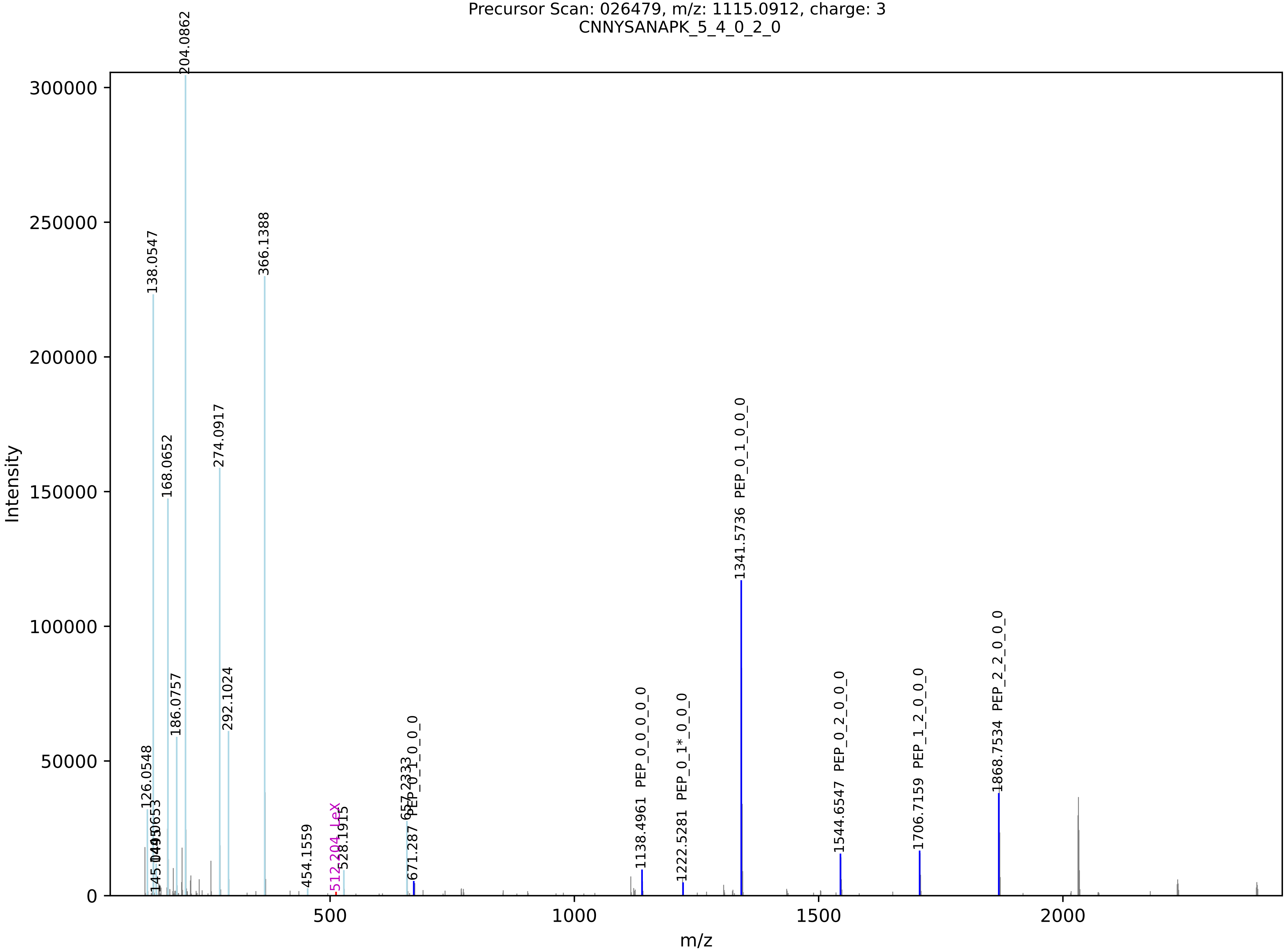

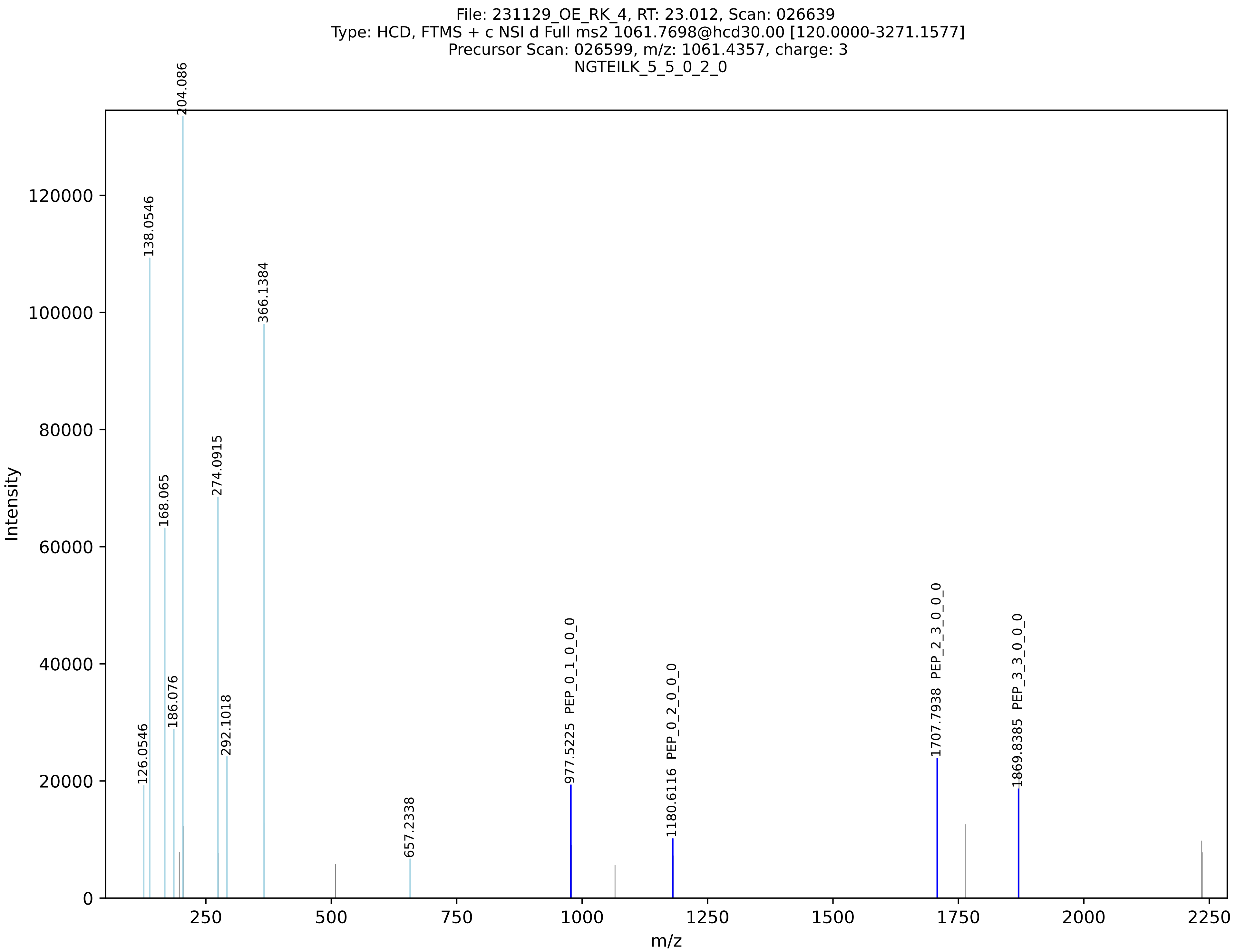
File: 231129_OE_RK_4, RT: 23.012, Scan: 026639
Type: HCD, FTMS + c NSI d Full ms2 1061.7698@hcd30.00 [120.0000-3271.1577]
Precursor Scan: 026599, m/z: 1061.4357, charge: 3
NGTEILK_5_5_0_2_0
204.086
138.0546
366.1384
274.0915
168.065
186.076
292.1018
126.0546
657.2338
977.5225 PEP_0_1_0_0_0
1180.6116 PEP_0_2_0_0_0
1707.7938 PEP_2_3_0_0_0
1869.8385 PEP_3_3_0_0_0
Intensity
m/z
0
20000
40000
60000
80000
100000
120000
250
500
750
1000
1250
1500
1750
2000
2250

File: 231129_OE_RK_4, RT: 23.157, Scan: 026839
Type: HCD, FTMS + c NSI d Full ms2 949.8831@hcd30.00 [120.0000-3898.3789]
Precursor Scan: 026777, m/z: 949.3811, charge: 4
LQNNENNISCVER_5_4_0_2_0

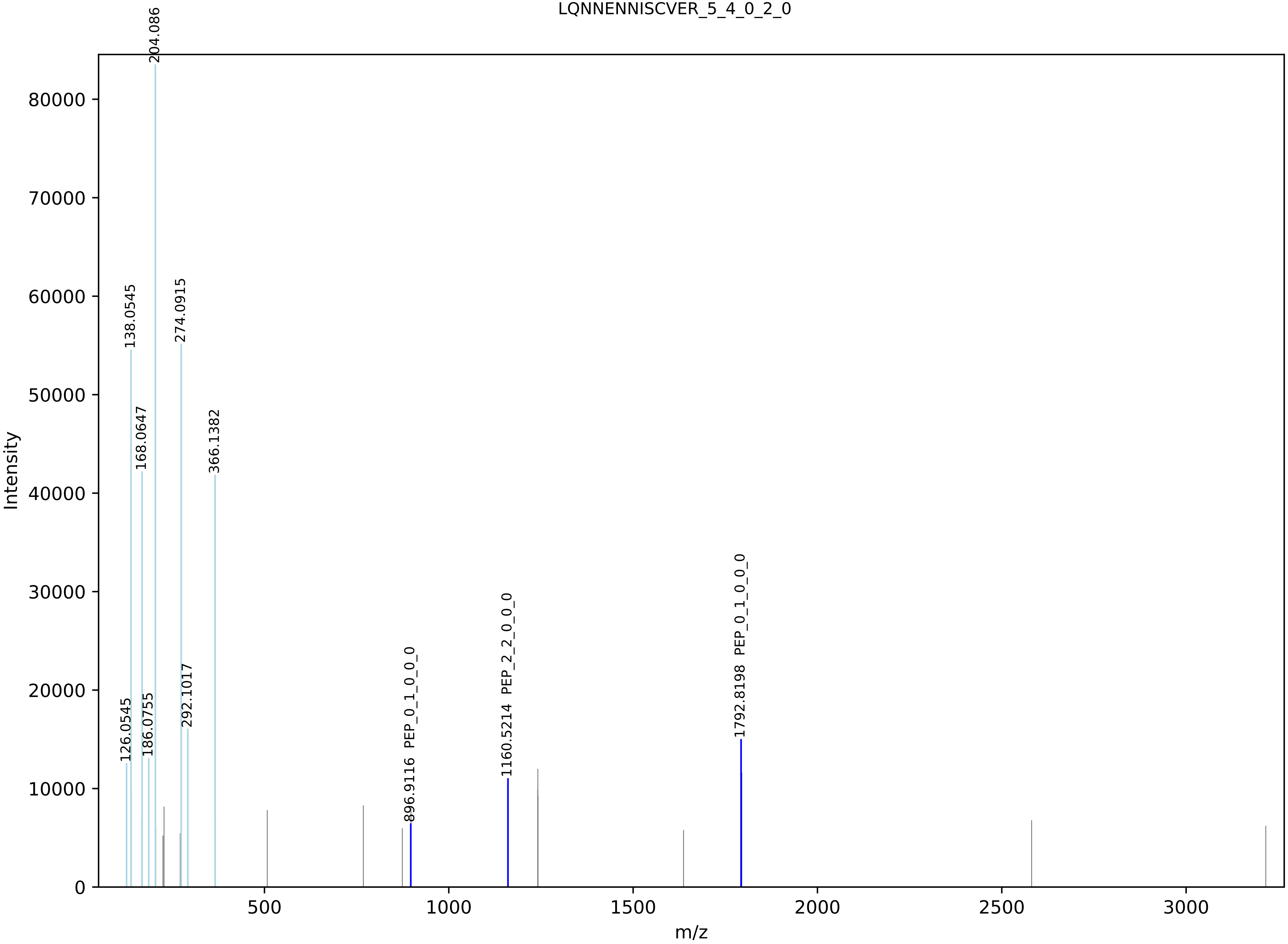

File: 231129_OE_RK_4, RT: 23.342, Scan: 027101
Type: HCD, FTMS + c NSI d Full ms2 949.8835@hcd30.00 [120.0000-3898.3809]
Precursor Scan: 027020, m/z: 949.3826, charge: 4
LQNNENNISCVER_5_4_0_2_0

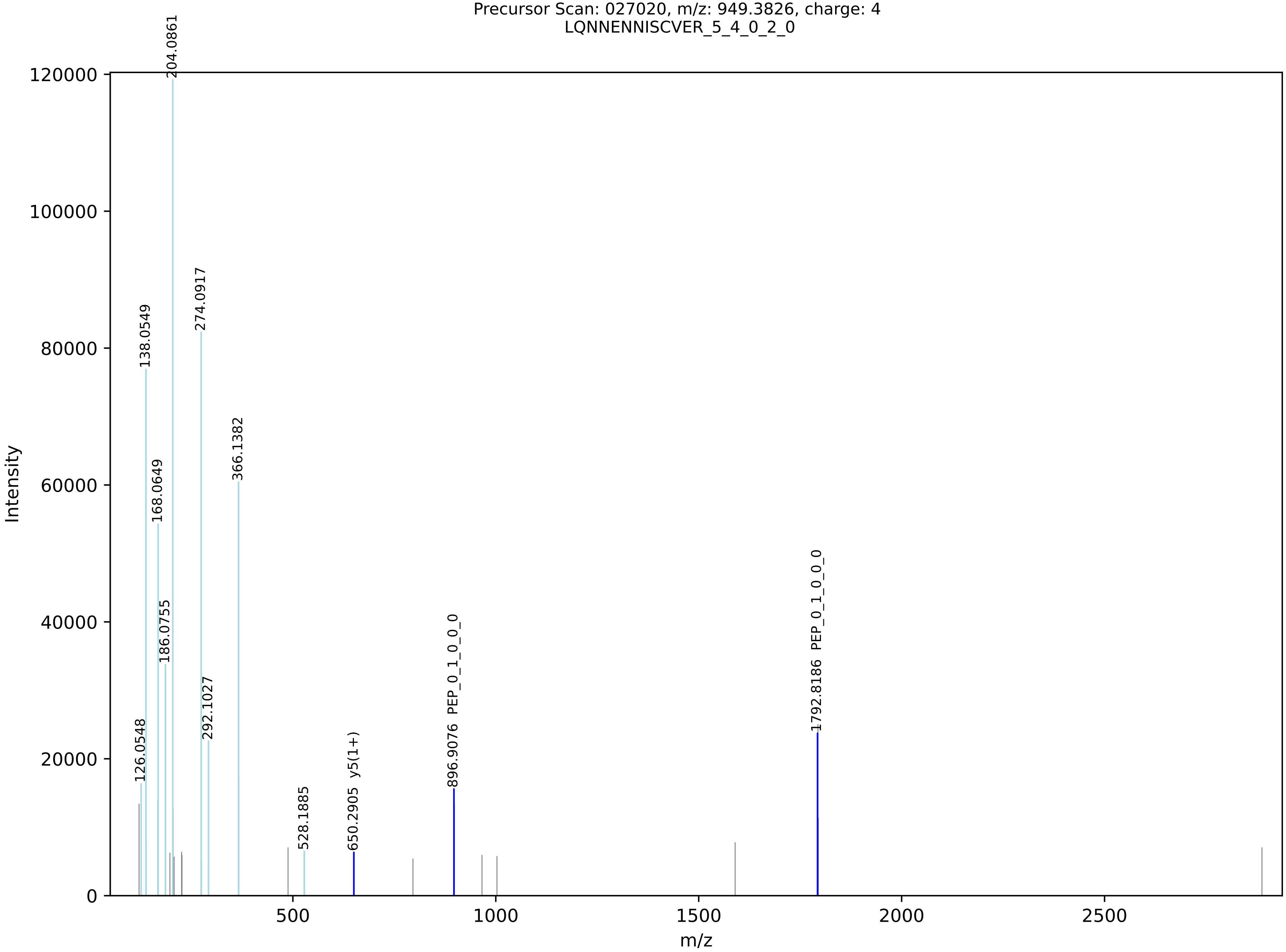

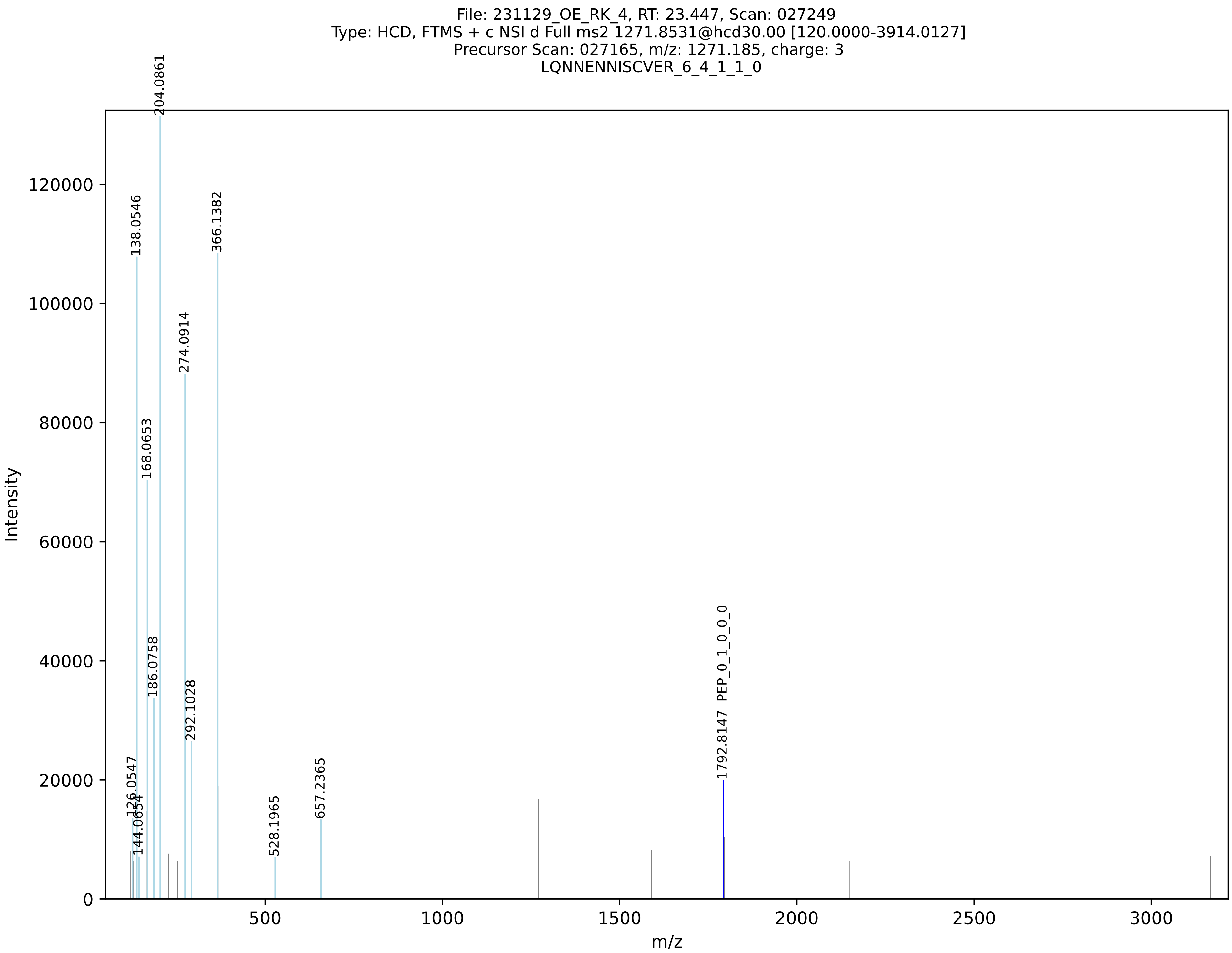
File: 231129_OE_RK_4, RT: 23.447, Scan: 027249
Type: HCD, FTMS + c NSI d Full ms2 1271.8531@hcd30.00 [120.0000-3914.0127]
Precursor Scan: 027165, m/z: 1271.185, charge: 3
LQNNENNISCVER_6_4_1_1_0
Intensity
m/z
204.0861
138.0546
366.1382
274.0914
168.0653
186.0758
292.1028
126.0547
144.0654
528.1965
657.2365
1792.8147 PEP_0_1_0_0_0
0
20000
40000
60000
80000
100000
120000
500
1000
1500
2000
2500
3000

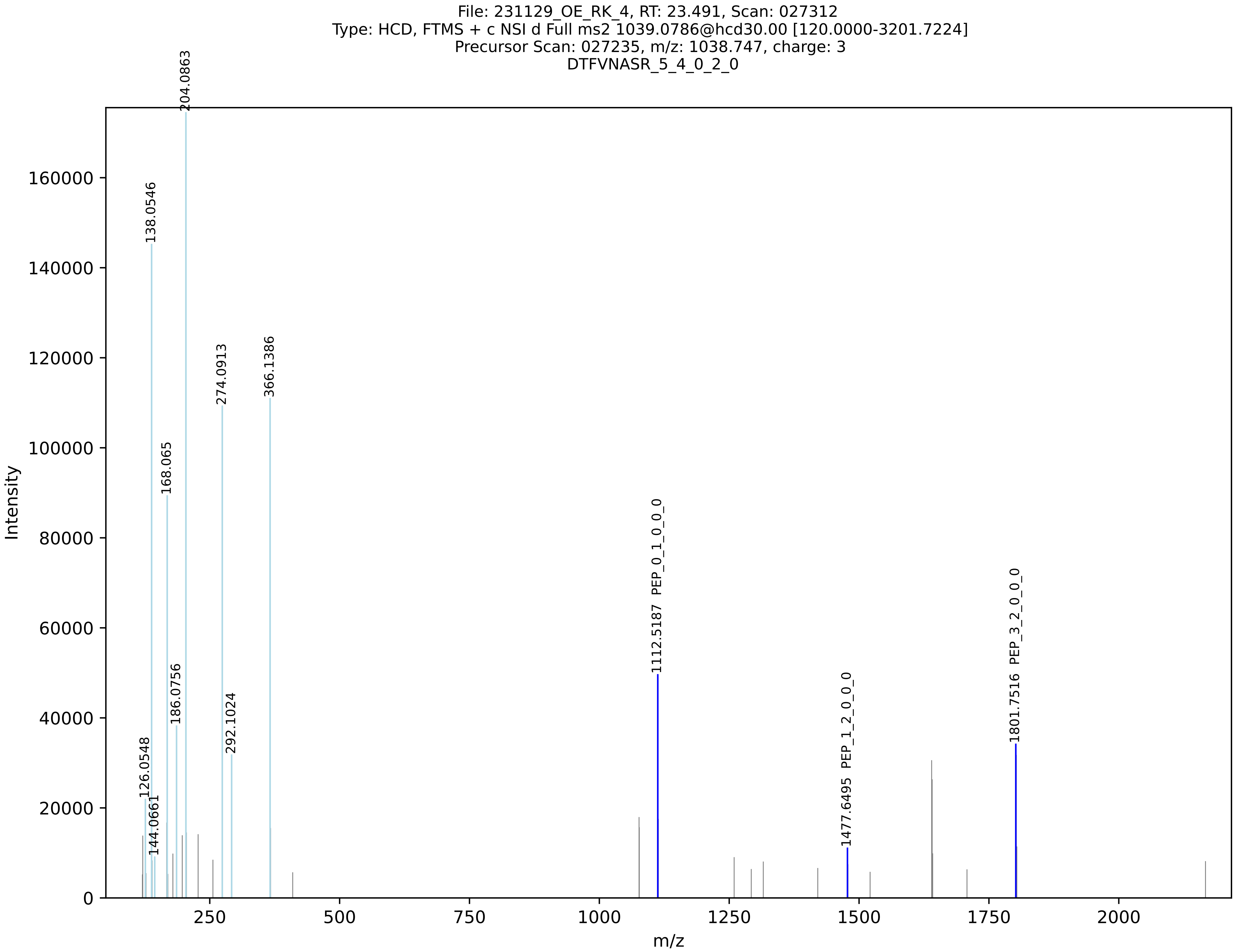

File: 231129_OE_RK_4, RT: 23.491, Scan: 027312
Type: HCD, FTMS + c NSI d Full ms2 1039.0786@hcd30.00 [120.0000-3201.7224]
Precursor Scan: 027235, m/z: 1038.747, charge: 3
DTFVNASR_5_4_0_2_0
Intensity
m/z
204.0863
138.0546
168.065
274.0913
366.1386
186.0756
292.1024
126.0548
144.0661
1112.5187 PEP_0_1_0_0_0
1477.6495 PEP_1_2_0_0_0
1801.7516 PEP_3_2_0_0_0

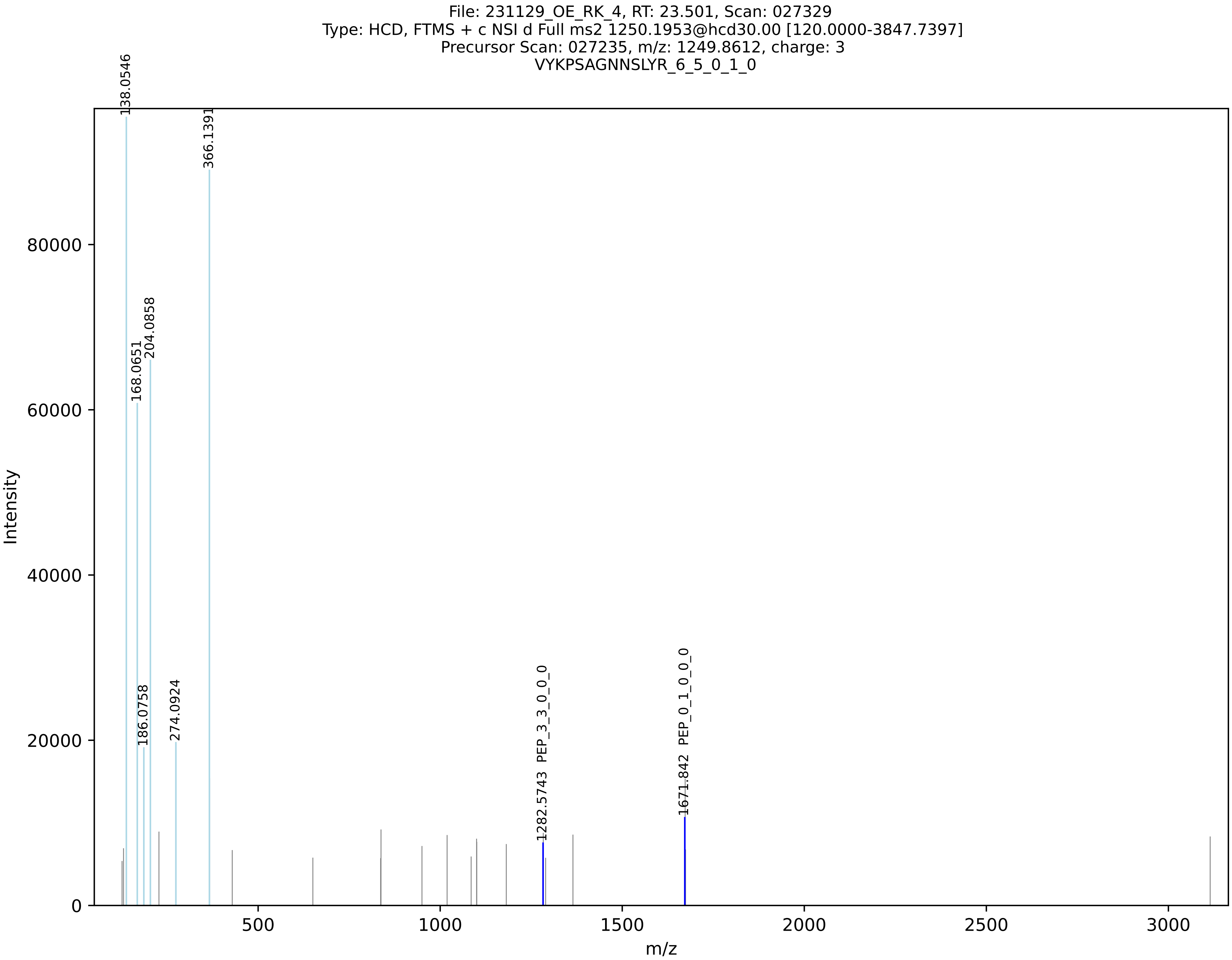
File: 231129_OE_RK_4, RT: 23.501, Scan: 027329
Type: HCD, FTMS + c NSI d Full ms2 1250.1953@hcd30.00 [120.0000-3847.7397]
Precursor Scan: 027235, m/z: 1249.8612, charge: 3
VYKPSAGNNSLYR_6_5_0_1_0
138.0546
366.1391
204.0858
168.0651
186.0758
274.0924
1282.5743 PEP_3_3_0_0_0
1671.842 PEP_0_1_0_0_0
Intensity
m/z
0
20000
40000
60000
80000
500
1000
1500
2000
2500
3000

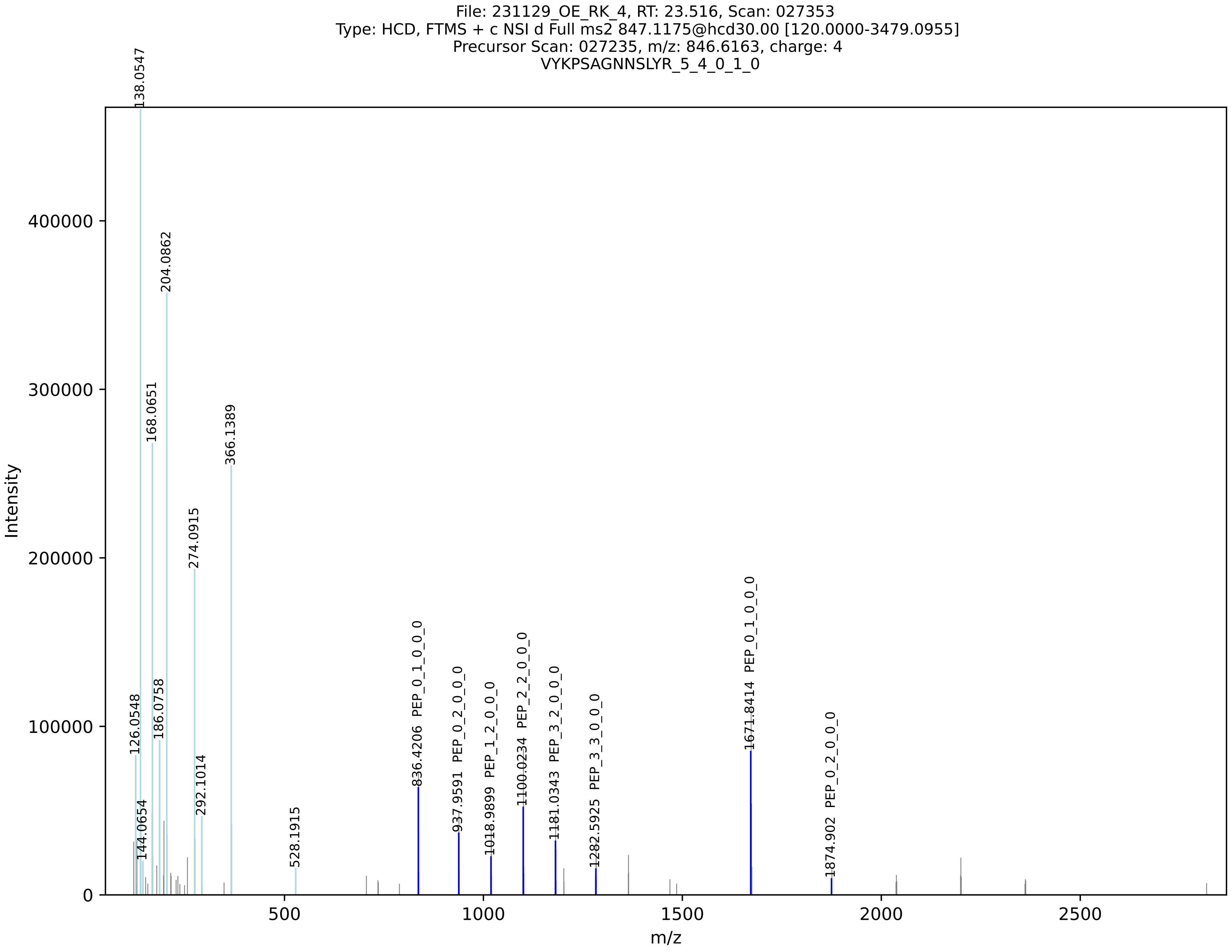

File: 231129_OE_RK_4, RT: 23.516, Scan: 027353
Type: HCD, FTMS + c NSI d Full ms2 847.1175@hcd30.00 [120.0000-3479.0955]
Precursor Scan: 027235, m/z: 846.6163, charge: 4
VYKPSAGNNSLYR_5_4_0_1_0
Intensity
m/z
0
100000
200000
300000
400000
500
1000
1500
2000
2500
138.0547
204.0862
168.0651
366.1389
274.0915
126.0548
186.0758
292.1014
144.0654
528.1915
836.4206 PEP_0_1_0_0_0
937.9591 PEP_0_2_0_0_0
1018.9899 PEP_1_2_0_0_0
1100.0234 PEP_2_2_0_0_0
1181.0343 PEP_3_2_0_0_0
1282.5925 PEP_3_3_0_0_0
1671.8414 PEP_0_1_0_0_0
1874.902 PEP_0_2_0_0_0

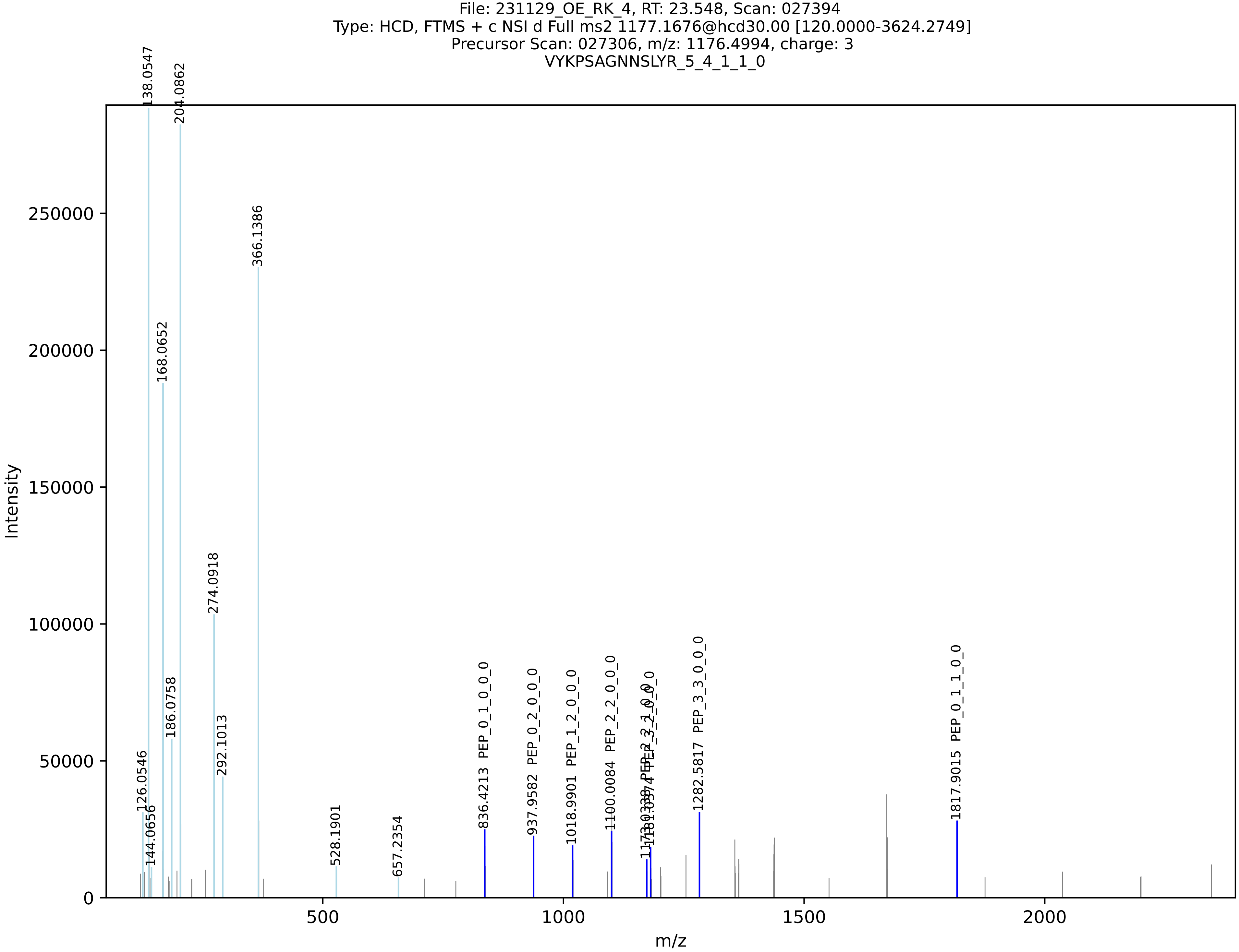

File: 231129_OE_RK_4, RT: 23.548, Scan: 027394
Type: HCD, FTMS + c NSI d Full ms2 1177.1676@hcd30.00 [120.0000-3624.2749]
Precursor Scan: 027306, m/z: 1176.4994, charge: 3
VYKPSAGNNSLYR_5_4_1_1_0
Intensity
m/z
138.0547
204.0862
366.1386
168.0652
274.0918
186.0758
292.1013
126.0546
144.0656
528.1901
657.2354
836.4213 PEP_0_1_0_0_0
937.9582 PEP_0_2_0_0_0
1018.9901 PEP_1_2_0_0_0
1100.0084 PEP_2_2_0_0_0
1282.5817 PEP_3_3_0_0_0
1817.9015 PEP_0_1_1_0_0

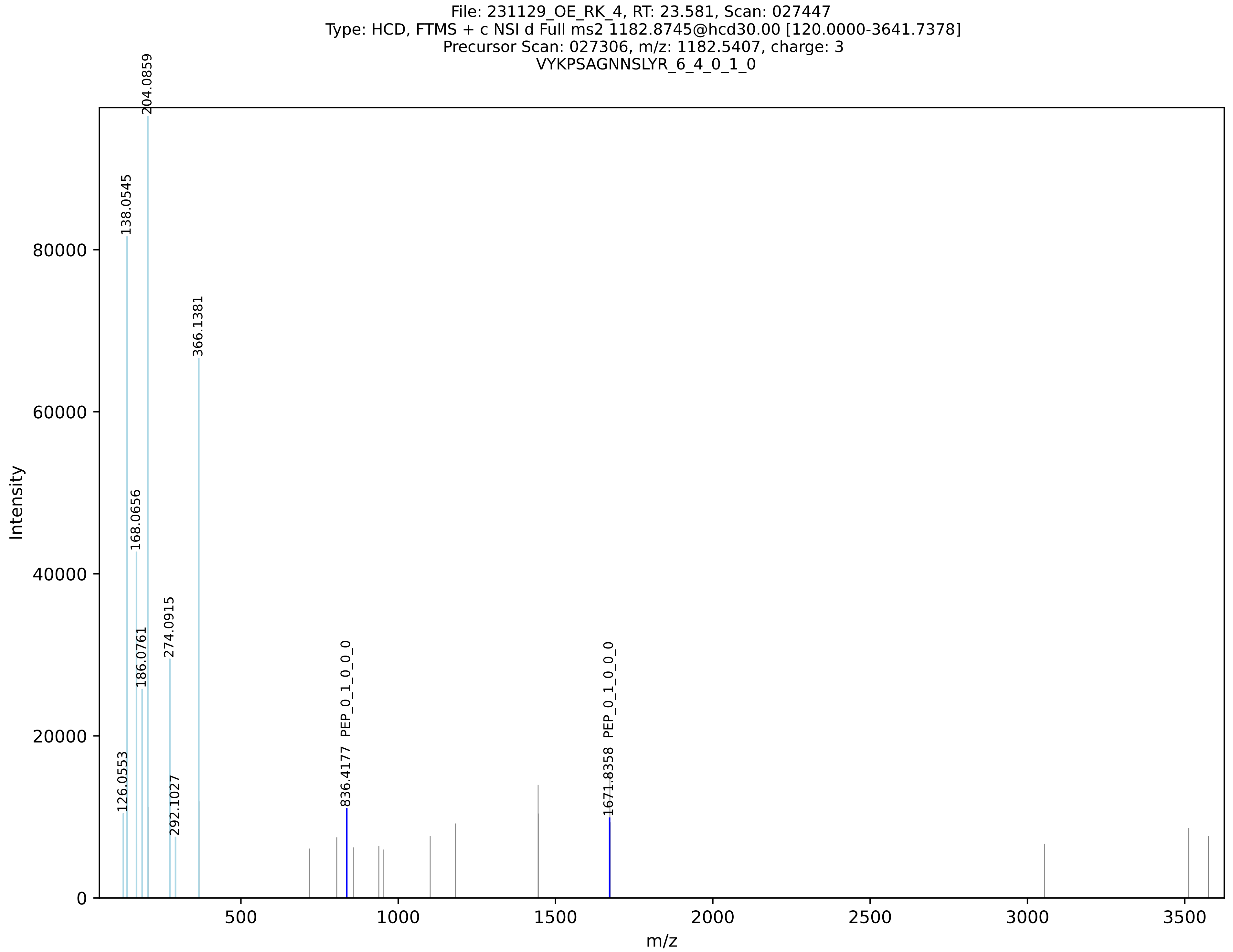
File: 231129_OE_RK_4, RT: 23.581, Scan: 027447
Type: HCD, FTMS + c NSI d Full ms2 1182.8745@hcd30.00 [120.0000-3641.7378]
Precursor Scan: 027306, m/z: 1182.5407, charge: 3
VYKPSAGNNSLYR_6_4_0_1_0
204.0859
138.0545
366.1381
168.0656
274.0915
186.0761
126.0553
292.1027
836.4177 PEP_0_1_0_0_0
1671.8358 PEP_0_1_0_0_0
Intensity
m/z
0
20000
40000
60000
80000
500
1000
1500
2000
2500
3000
3500

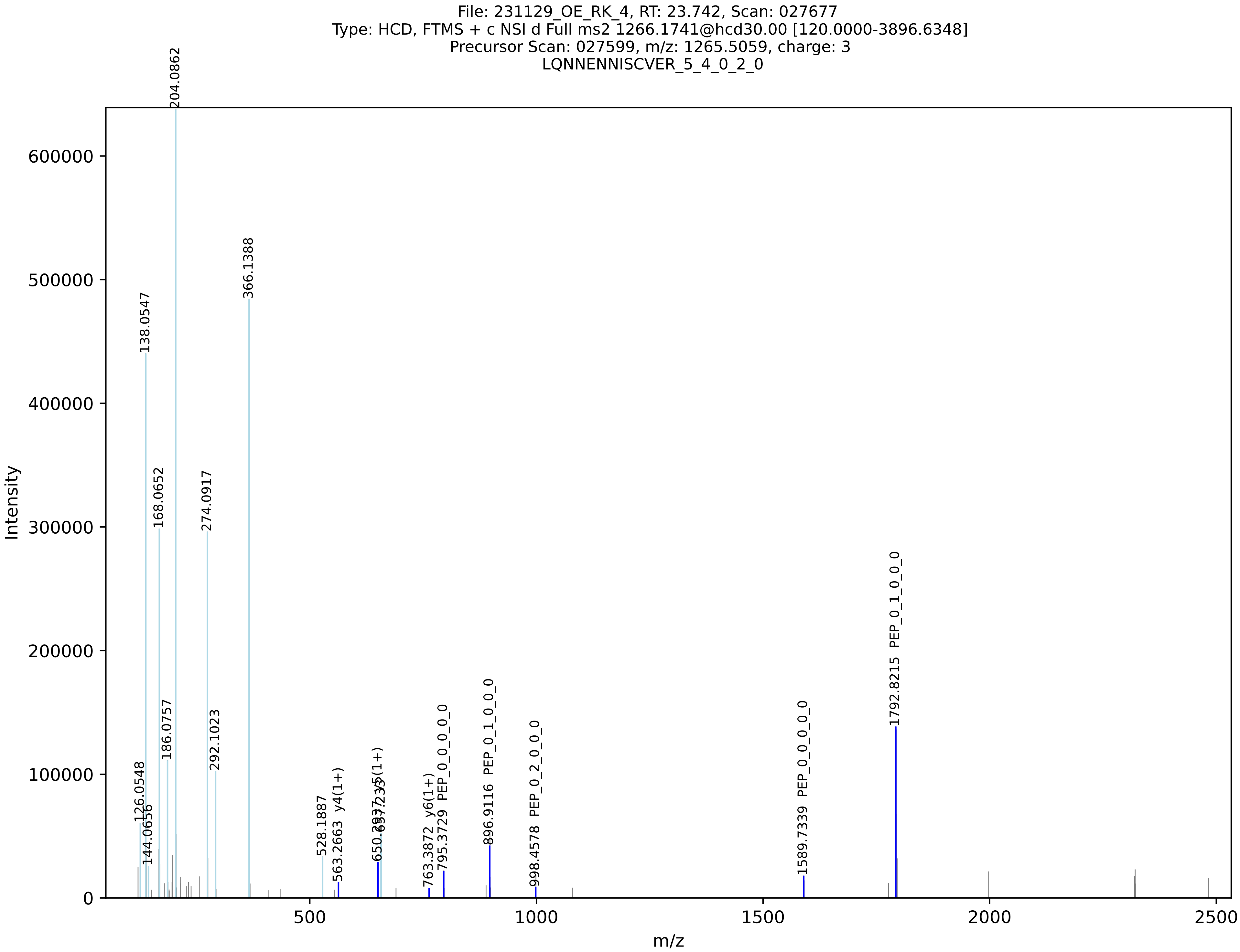

File: 231129_OE_RK_4, RT: 23.742, Scan: 027677
Type: HCD, FTMS + c NSI d Full ms2 1266.1741@hcd30.00 [120.0000-3896.6348]
Precursor Scan: 027599, m/z: 1265.5059, charge: 3
LQNNENNISCVER_5_4_0_2_0
Intensity
m/z
204.0862
366.1388
138.0547
168.0652
274.0917
186.0757
292.1023
126.0548
144.0656
528.1887
563.2663 y4(1+)
650.2937 y5(1+)
657.233
763.3872 y6(1+)
795.3729 PEP_0_0_0_0_0_
896.9116 PEP_0_1_0_0_0_
998.4578 PEP_0_2_0_0_0_
1589.7339 PEP_0_0_0_0_0_
1792.8215 PEP_0_1_0_0_0_

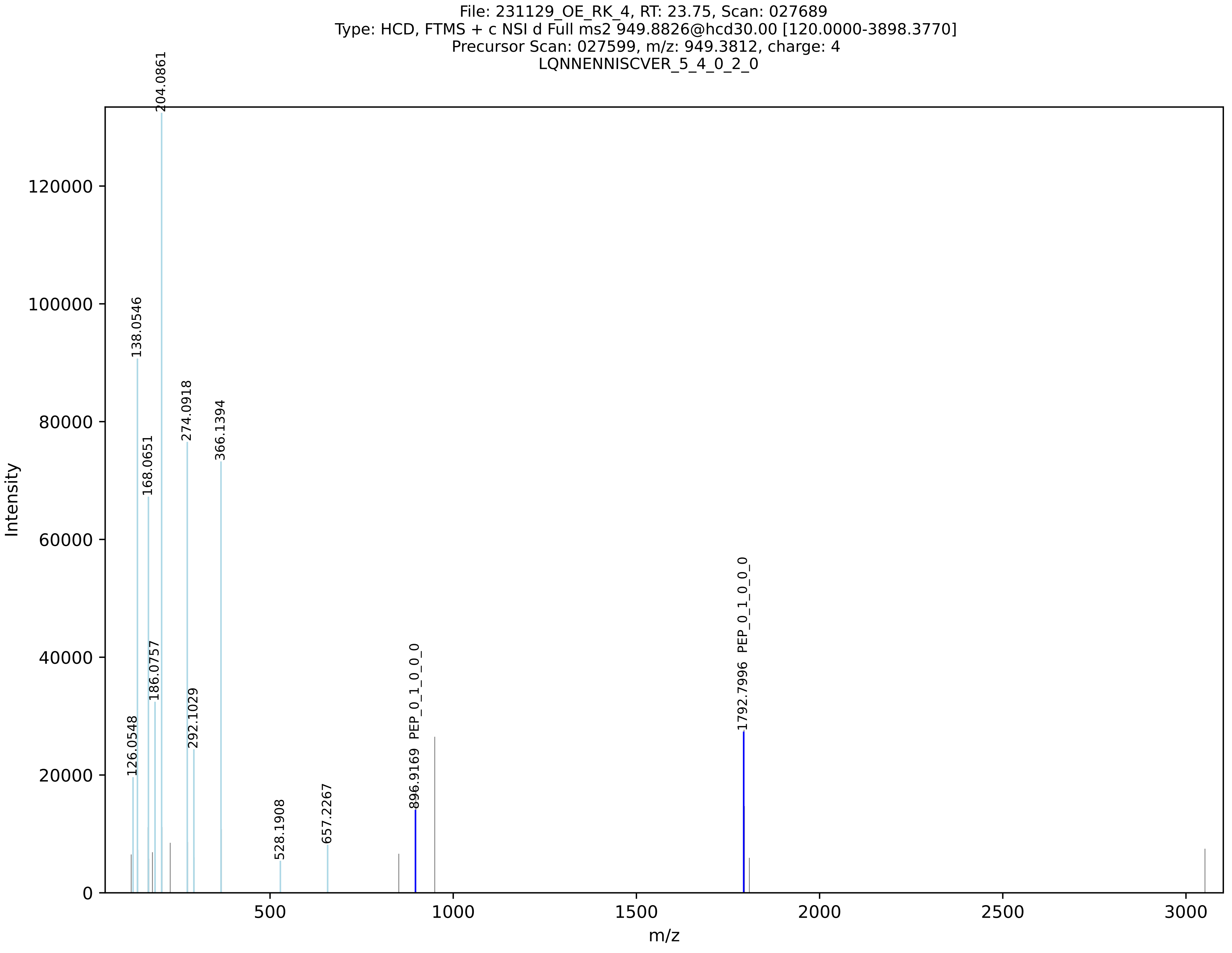

File: 231129_OE_RK_4, RT: 23.75, Scan: 027689
Type: HCD, FTMS + c NSI d Full ms2 949.8826@hcd30.00 [120.0000-3898.3770]
Precursor Scan: 027599, m/z: 949.3812, charge: 4
LQNNENNISCVER_5_4_0_2_0
Intensity
m/z
204.0861
138.0546
168.0651
274.0918
366.1394
186.0757
292.1029
126.0548
528.1908
657.2267
896.9169 PEP_0_1_0_0_0
1792.7996 PEP_0_1_0_0_0

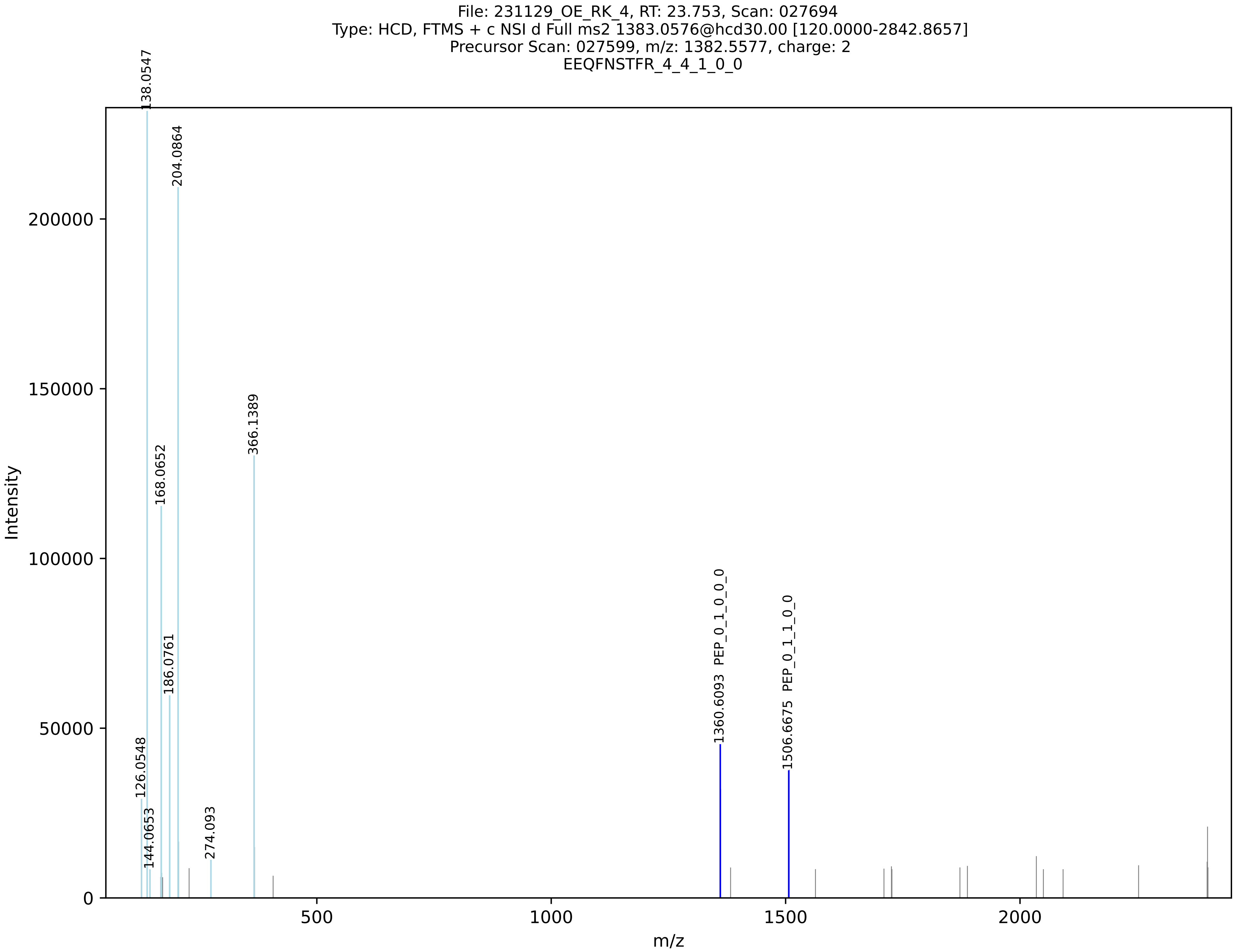
File: 231129_OE_RK_4, RT: 23.753, Scan: 027694
Type: HCD, FTMS + c NSI d Full ms2 1383.0576@hcd30.00 [120.0000-2842.8657]
Precursor Scan: 027599, m/z: 1382.5577, charge: 2
EEQFNSTFR_4_4_1_0_0
138.0547
204.0864
366.1389
168.0652
186.0761
126.0548
144.0653
274.093
1360.6093 PEP_0_1_0_0_0
1506.6675 PEP_0_1_1_0_0
Intensity
200000
150000
100000
50000
0
500
1000
1500
2000
m/z

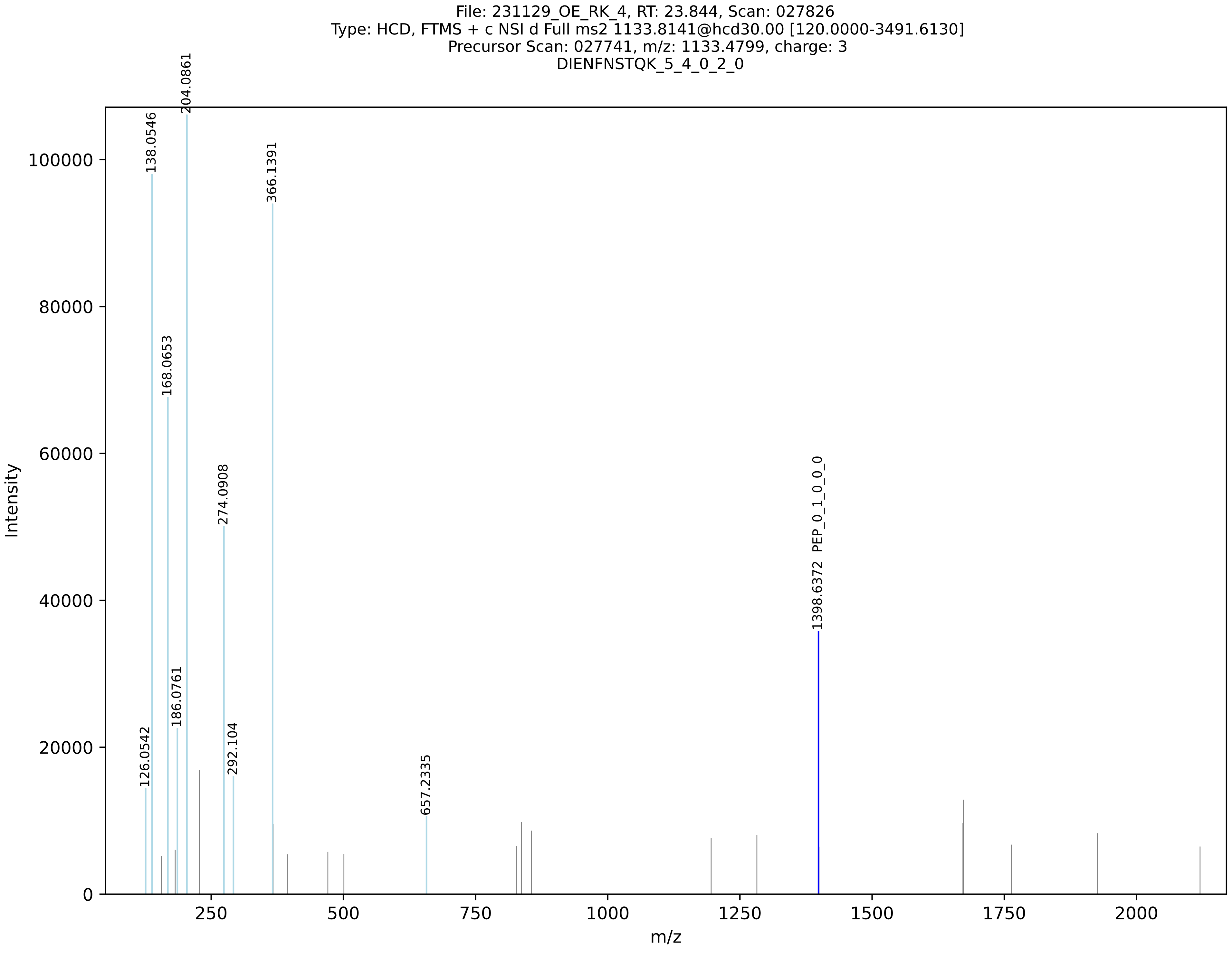
File: 231129_OE_RK_4, RT: 23.844, Scan: 027826
Type: HCD, FTMS + c NSI d Full ms2 1133.8141@hcd30.00 [120.0000-3491.6130]
Precursor Scan: 027741, m/z: 1133.4799, charge: 3
DIENFNSTQK_5_4_0_2_0
204.0861
138.0546
366.1391
168.0653
274.0908
1398.6372 PEP_0_1_0_0_0
186.0761
126.0542
292.104
657.2335
Intensity
m/z
0
20000
40000
60000
80000
100000
250
500
750
1000
1250
1500
1750
2000

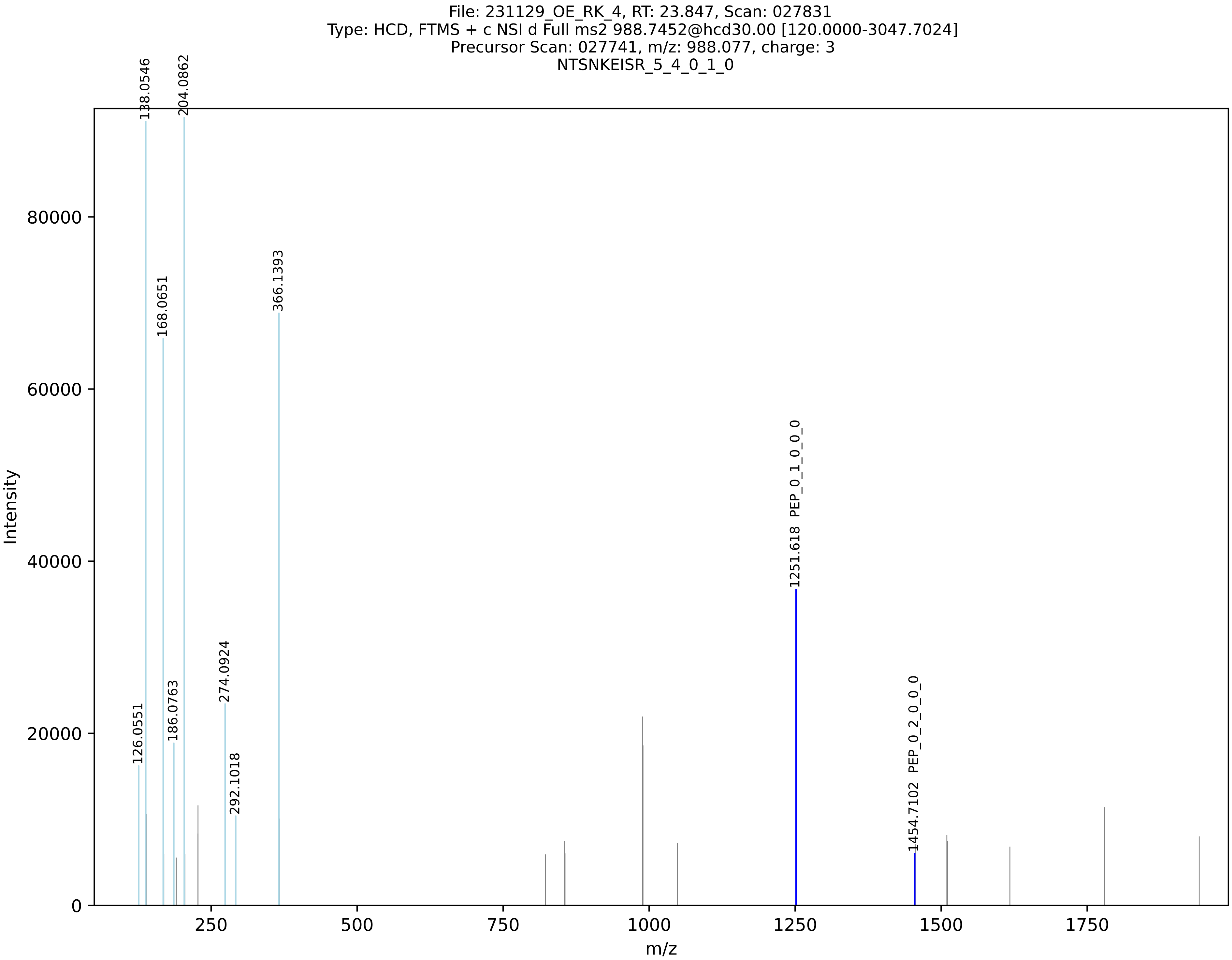
File: 231129_OE_RK_4, RT: 23.847, Scan: 027831
Type: HCD, FTMS + c NSI d Full ms2 988.7452@hcd30.00 [120.0000-3047.7024]
Precursor Scan: 027741, m/z: 988.077, charge: 3
NTSNKEISR_5_4_0_1_0
138.0546
204.0862
168.0651
366.1393
1251.618 PEP_0_1_0_0_0
274.0924
186.0763
126.0551
292.1018
1454.7102 PEP_0_2_0_0_0
Intensity
m/z
0
20000
40000
60000
80000
250
500
750
1000
1250
1500
1750

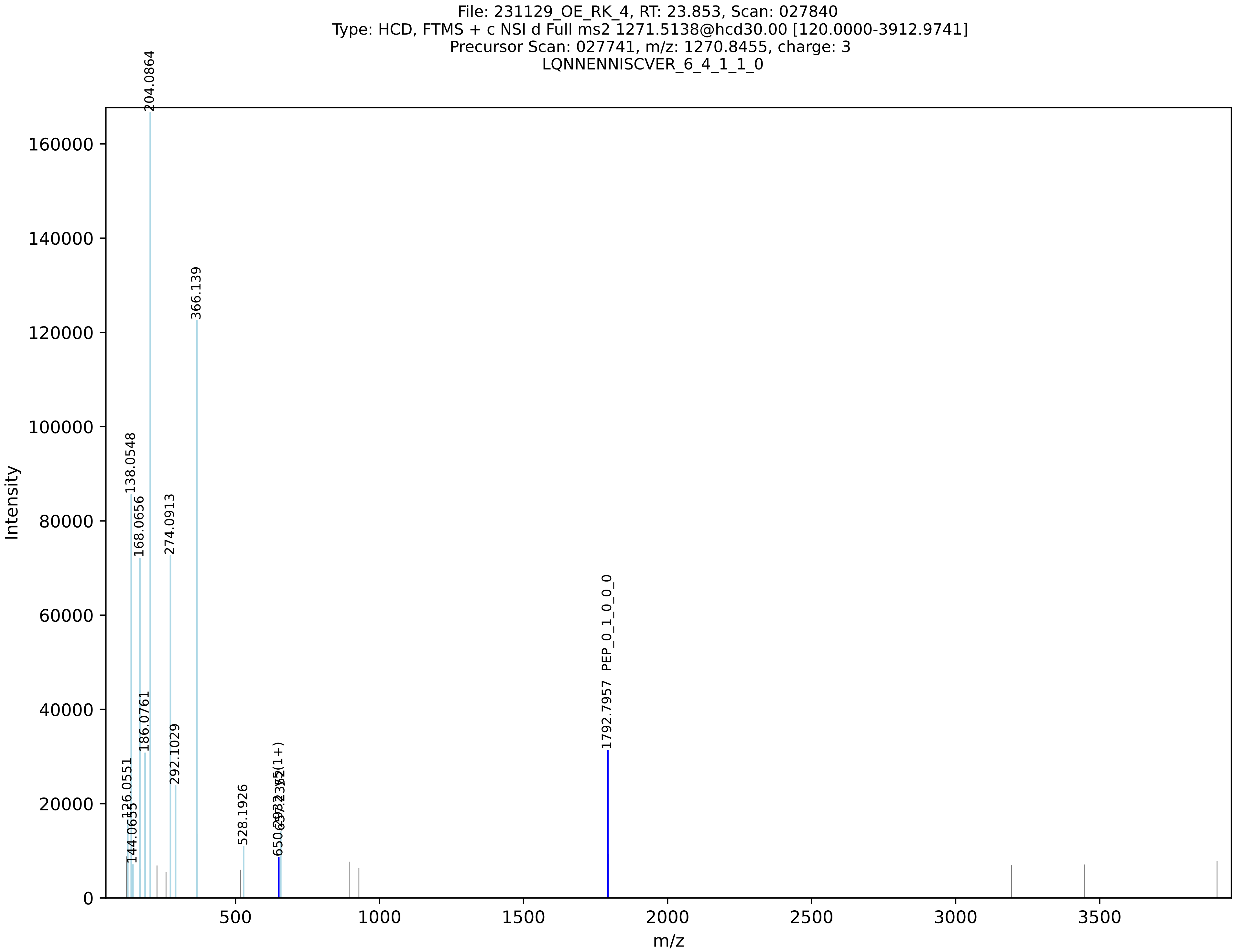
File: 231129_OE_RK_4, RT: 23.853, Scan: 027840
Type: HCD, FTMS + c NSI d Full ms2 1271.5138@hcd30.00 [120.0000-3912.9741]
Precursor Scan: 027741, m/z: 1270.8455, charge: 3
LQNNENNISCVER_6_4_1_1_0
204.0864
366.139
138.0548
168.0656
274.0913
1792.7957 PEP_0_1_0_0_0
186.0761
292.1029
126.0551
144.0655
528.1926
Intensity
m/z
160000
140000
120000
100000
80000
60000
40000
20000
0
500
1000
1500
2000
2500
3000
3500

File: 231129_OE_RK_4, RT: 23.872, Scan: 027871
Type: HCD, FTMS + c NSI d Full ms2 1127.4746@hcd30.00 [120.0000-3472.2144]
Precursor Scan: 027741, m/z: 1127.8152, charge: 3
VYKPSAGNNSLYR_5_4_0_1_0

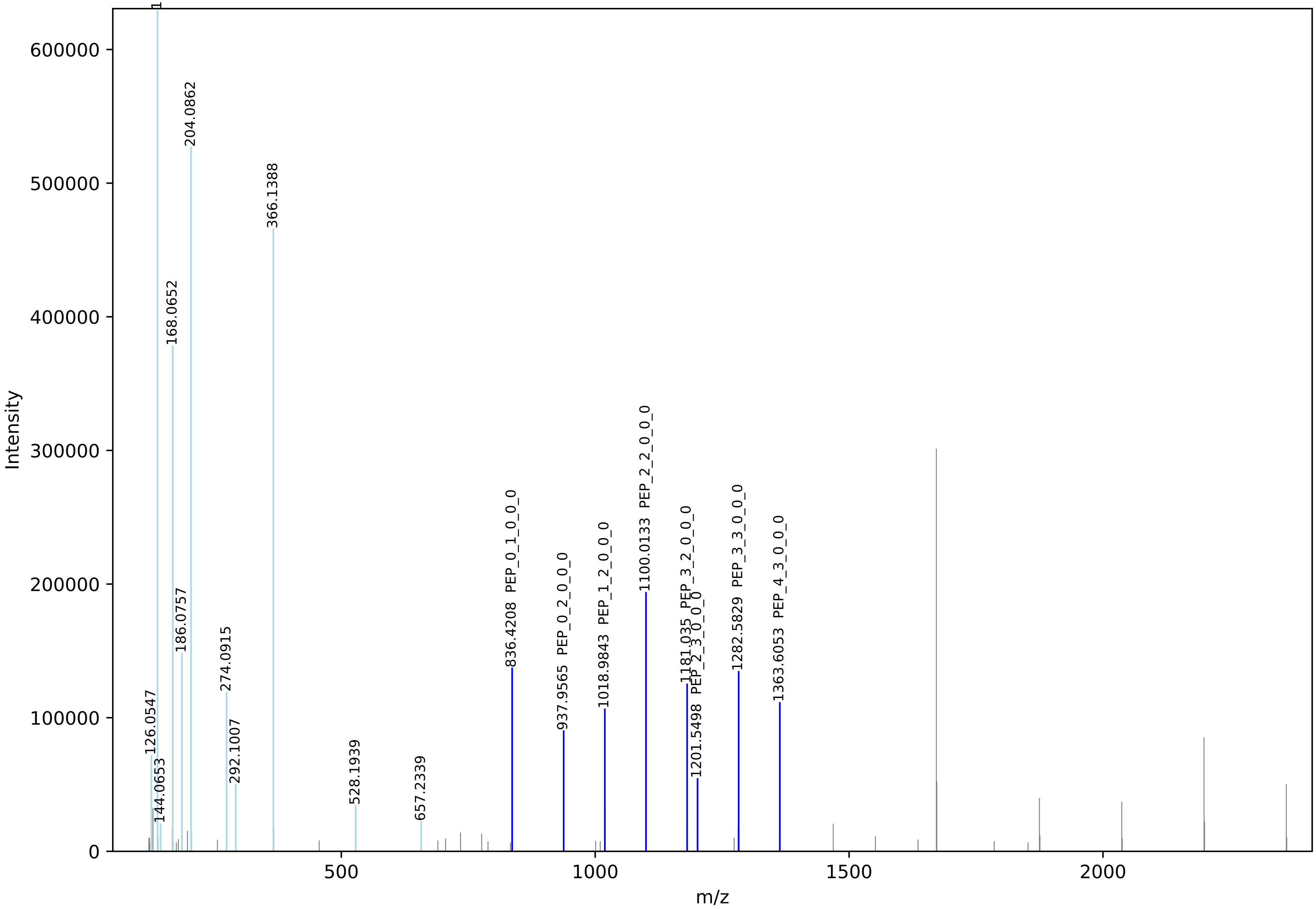

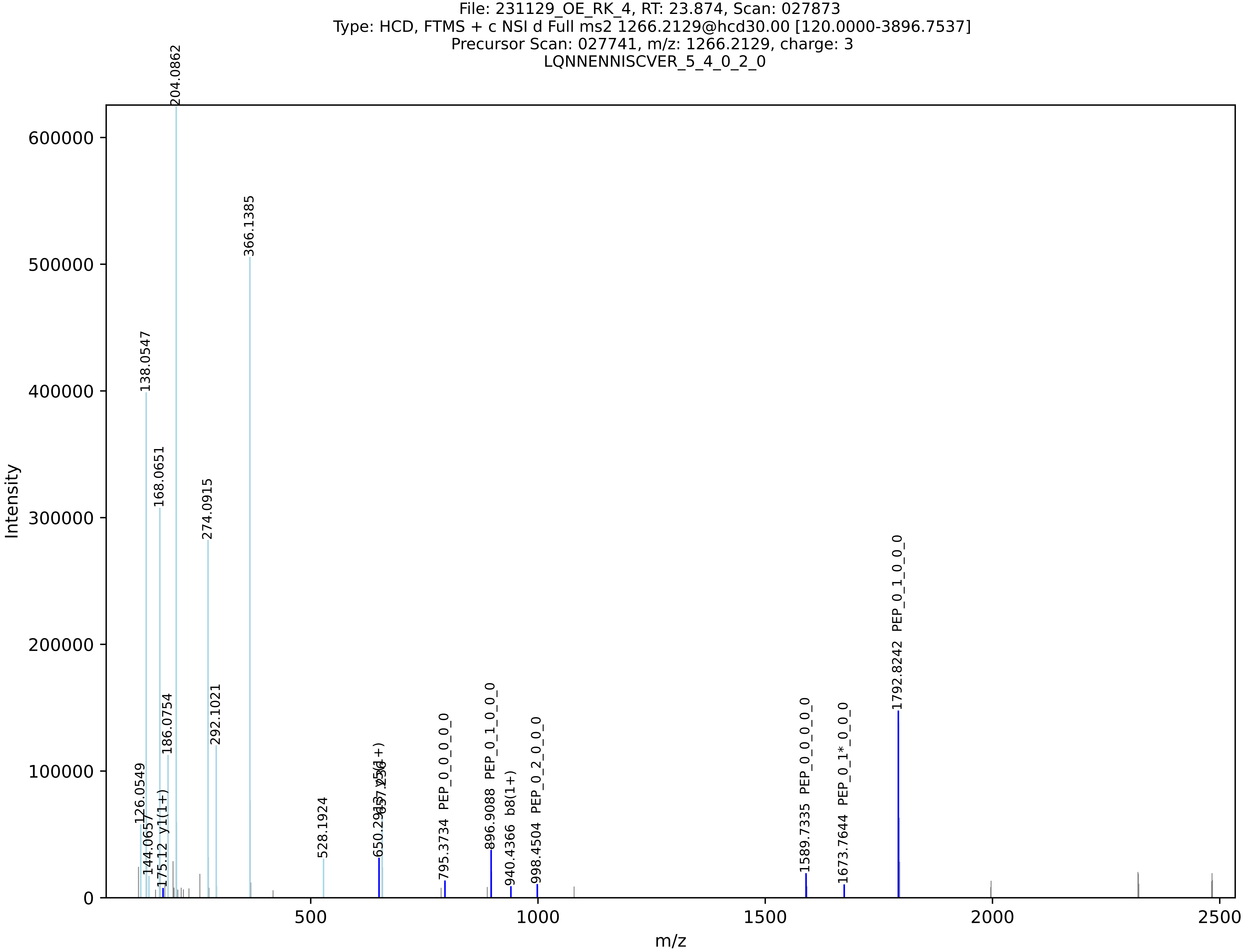
File: 231129_OE_RK_4, RT: 23.874, Scan: 027873
Type: HCD, FTMS + c NSI d Full ms2 1266.2129@hcd30.00 [120.0000-3896.7537]
Precursor Scan: 027741, m/z: 1266.2129, charge: 3
LQNNENNISCVER_5_4_0_2_0
204.0862
366.1385
138.0547
168.0651
274.0915
186.0754
292.1021
126.0549
144.0657
175.12 y1(1+)
528.1924
650.2913 y5(1+)
657.236
795.3734 PEP_0_0_0_0_0
896.9088 PEP_0_1_0_0_0
940.4366 b8(1+)
998.4504 PEP_0_2_0_0_0
1589.7335 PEP_0_0_0_0_0
1673.7644 PEP_0_1*_0_0_0
1792.8242 PEP_0_1_0_0_0
Intensity
0
100000
200000
300000
400000
500000
600000
500
1000
1500
2000
2500
m/z

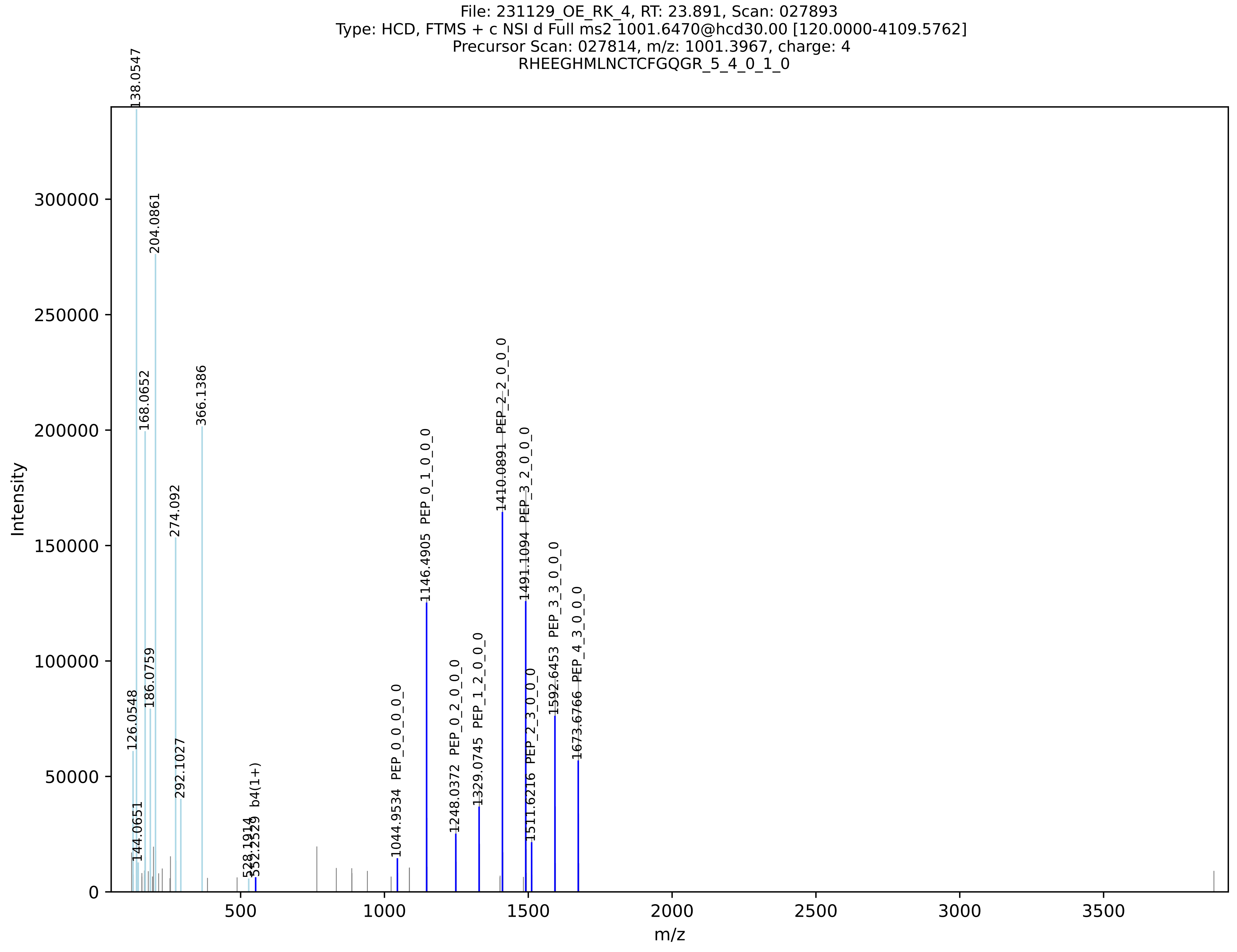
File: 231129_OE_RK_4, RT: 23.891, Scan: 027893
Type: HCD, FTMS + c NSI d Full ms2 1001.6470@hcd30.00 [120.0000-4109.5762]
Precursor Scan: 027814, m/z: 1001.3967, charge: 4
RHEEGHMLNCTCFGQGR_5_4_0_1_0
Intensity
m/z
138.0547
204.0861
168.0652
366.1386
274.092
186.0759
126.0548
292.1027
144.0651
528.1914
552.2529 b4(1+)
1044.9534 PEP_0_0_0_0_0
1146.4905 PEP_0_1_0_0_0
1248.0372 PEP_0_2_0_0_0
1329.0745 PEP_1_2_0_0_0
1410.0891 PEP_2_2_0_0_0
1491.1094 PEP_3_2_0_0_0
1511.6216 PEP_2_3_0_0_0
1592.6453 PEP_3_3_0_0_0
1673.6766 PEP_4_3_0_0_0

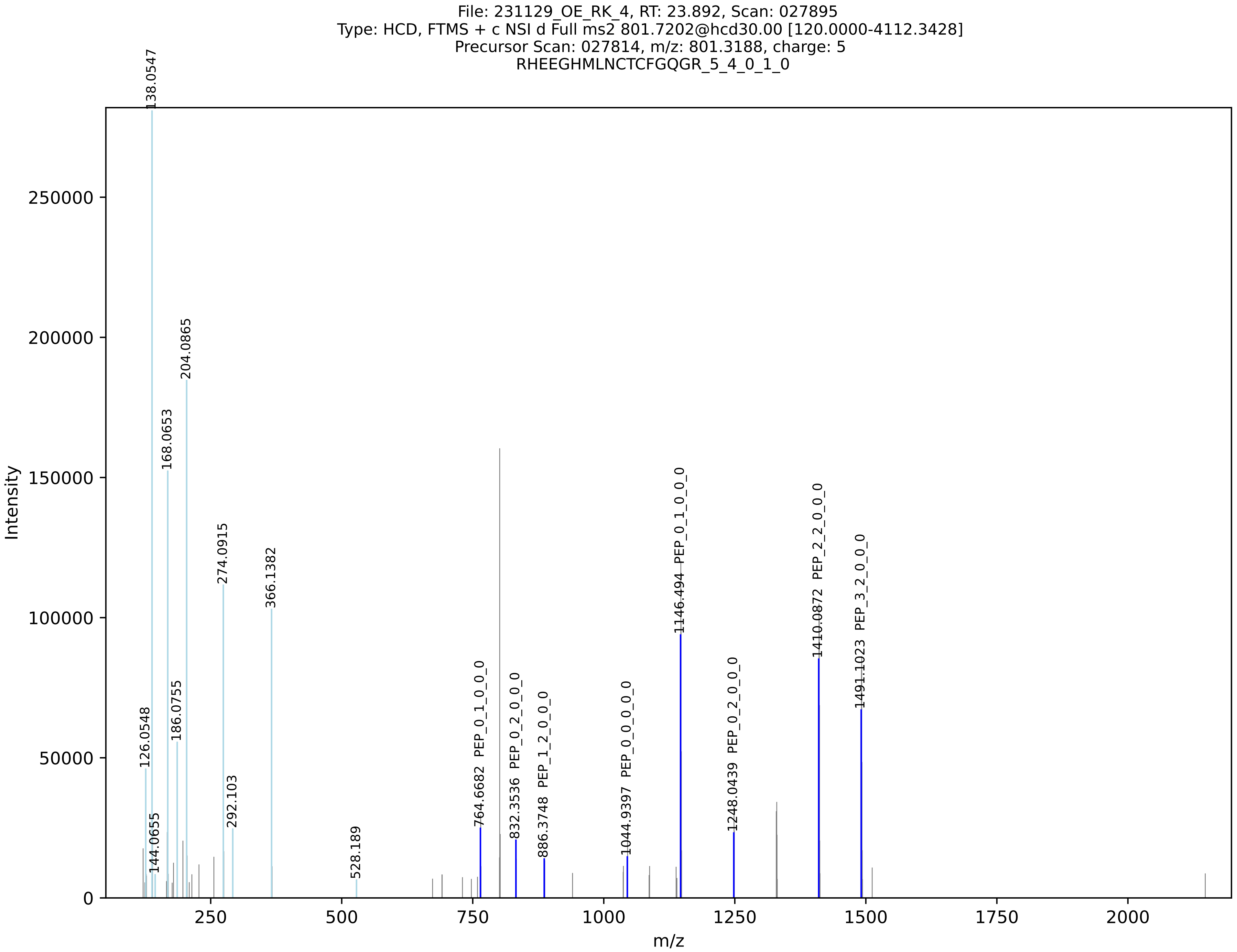
File: 231129_OE_RK_4, RT: 23.892, Scan: 027895
Type: HCD, FTMS + c NSI d Full ms2 801.7202@hcd30.00 [120.0000-4112.3428]
Precursor Scan: 027814, m/z: 801.3188, charge: 5
RHEEGHMLNCTCFGQGR_5_4_0_1_0
Intensity
m/z
138.0547
204.0865
168.0653
274.0915
366.1382
186.0755
126.0548
292.103
144.0655
528.189
764.6682 PEP_0_1_0_0_0
832.3536 PEP_0_2_0_0_0
886.3748 PEP_1_2_0_0_0
1044.9397 PEP_0_0_0_0_0
1146.494 PEP_0_1_0_0_0
1248.0439 PEP_0_2_0_0_0
1410.0872 PEP_2_2_0_0_0
1491.1023 PEP_3_2_0_0_0

File: 231129_OE_RK_4, RT: 23.897, Scan: 027903
Type: HCD, FTMS + c NSI d Full ms2 1335.5271@hcd30.00 [120.0000-4108.8550]
Precursor Scan: 027814, m/z: 1334.8598, charge: 3
RHEEGHMLNCTCFGQGR_5_4_0_1_0

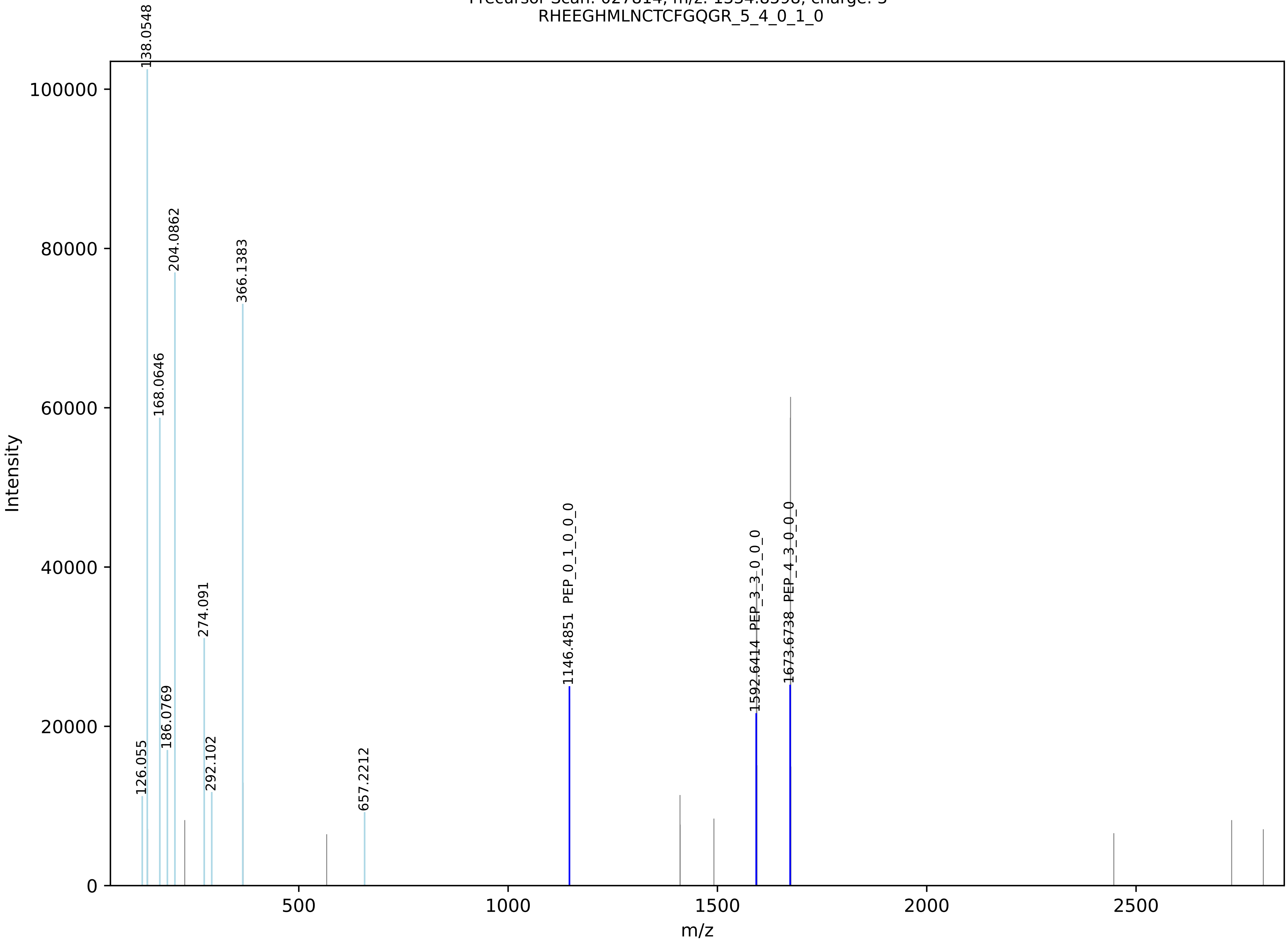

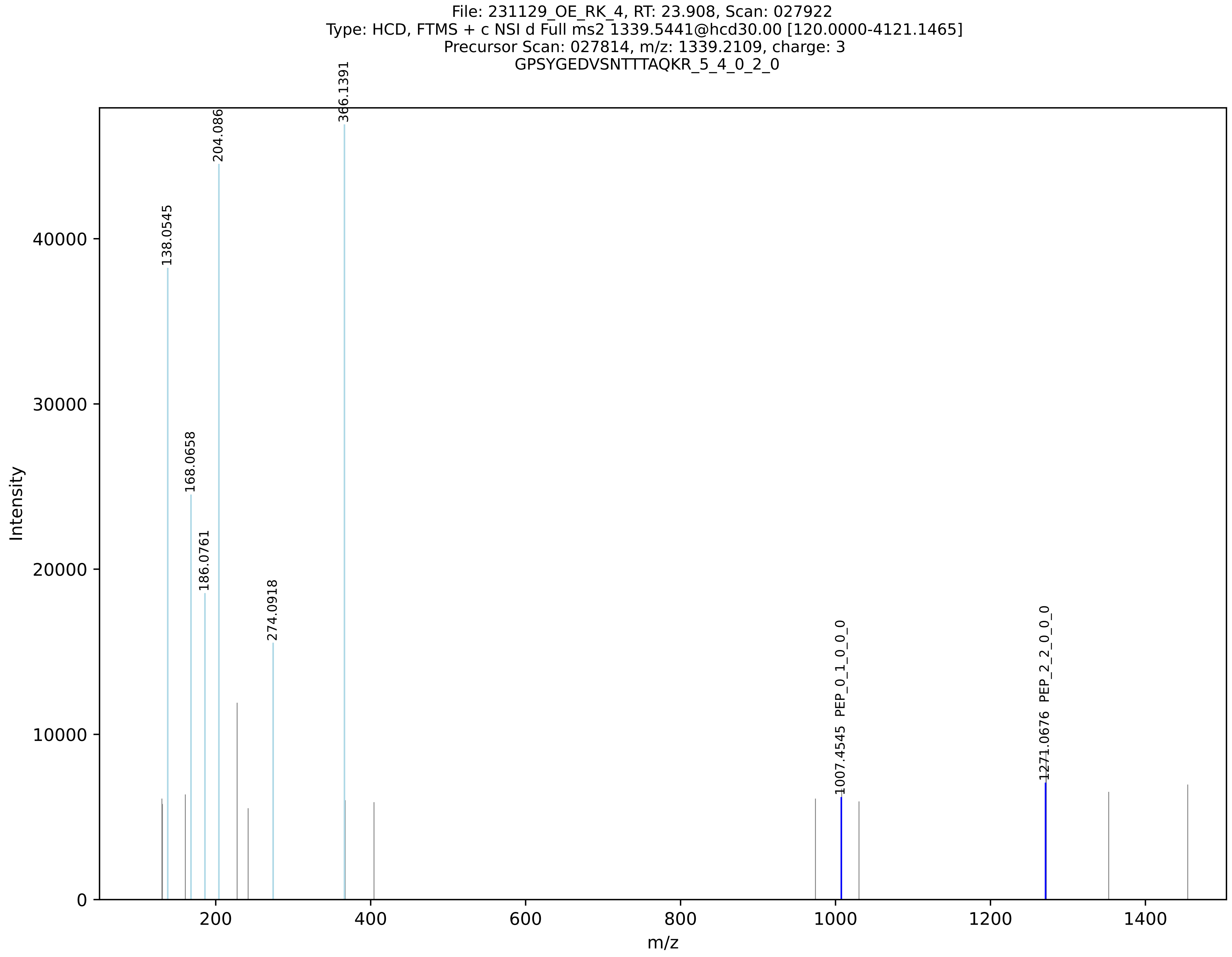

File: 231129_OE_RK_4, RT: 23.908, Scan: 027922
Type: HCD, FTMS + c NSI d Full ms2 1339.5441@hcd30.00 [120.0000-4121.1465]
Precursor Scan: 027814, m/z: 1339.2109, charge: 3
GPSYGEDVSNTTTAQKR_5_4_0_2_0
Intensity
m/z
138.0545
168.0658
186.0761
204.086
274.0918
366.1391
1007.4545 PEP_0_1_0_0_0
1271.0676 PEP_2_2_0_0_0

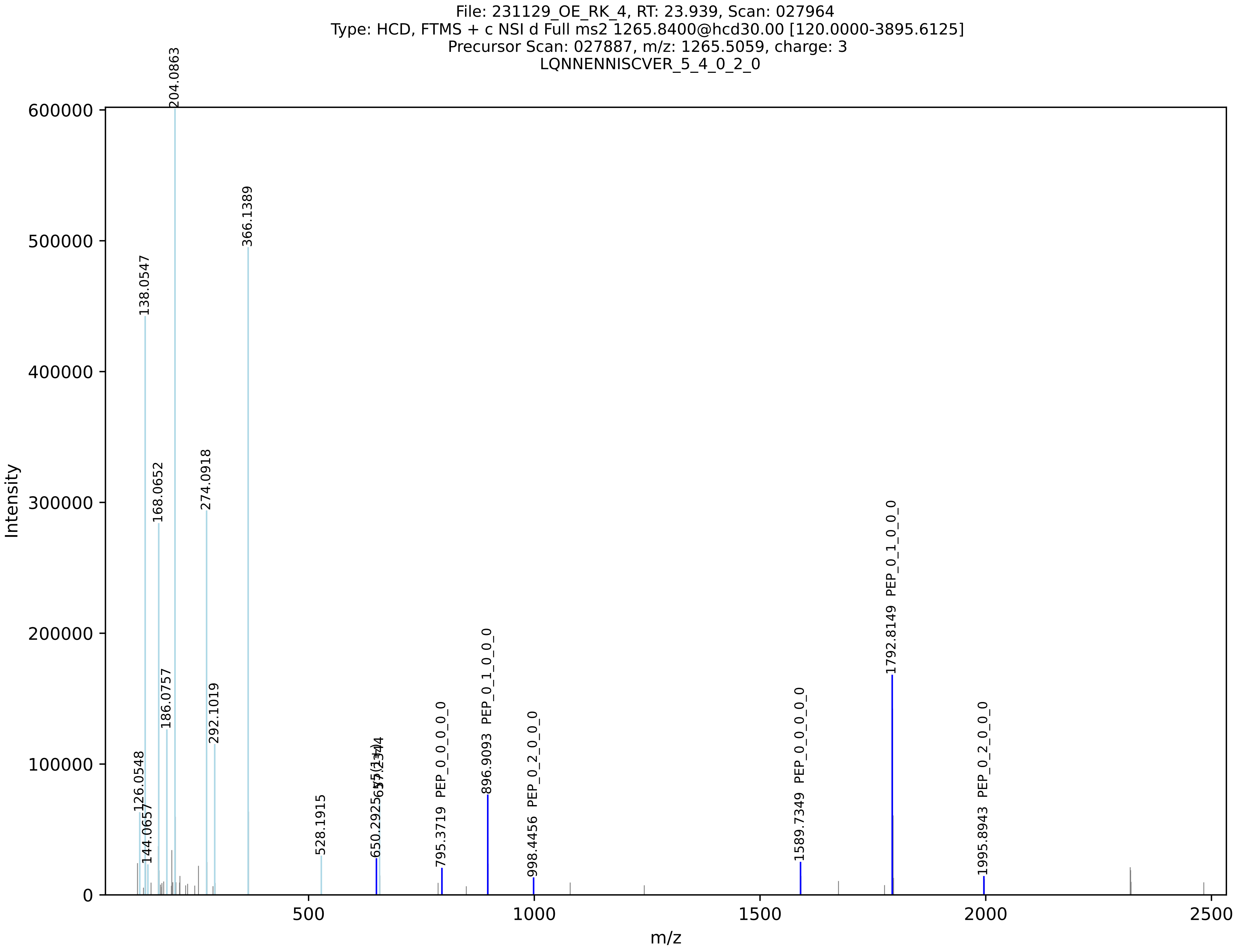
File: 231129_OE_RK_4, RT: 23.939, Scan: 027964
Type: HCD, FTMS + c NSI d Full ms2 1265.8400@hcd30.00 [120.0000-3895.6125]
Precursor Scan: 027887, m/z: 1265.5059, charge: 3
LQNNENNISCVER_5_4_0_2_0
Intensity
m/z
0
100000
200000
300000
400000
500000
600000
500
1000
1500
2000
2500
204.0863
366.1389
138.0547
168.0652
274.0918
186.0757
292.1019
126.0548
144.0657
528.1915
650.2925
657.2344
795.3719 PEP_0_0_0_0_0
896.9093 PEP_0_1_0_0_0
998.4456 PEP_0_2_0_0_0
1589.7349 PEP_0_0_0_0_0
1792.8149 PEP_0_1_0_0_0
1995.8943 PEP_0_2_0_0_0

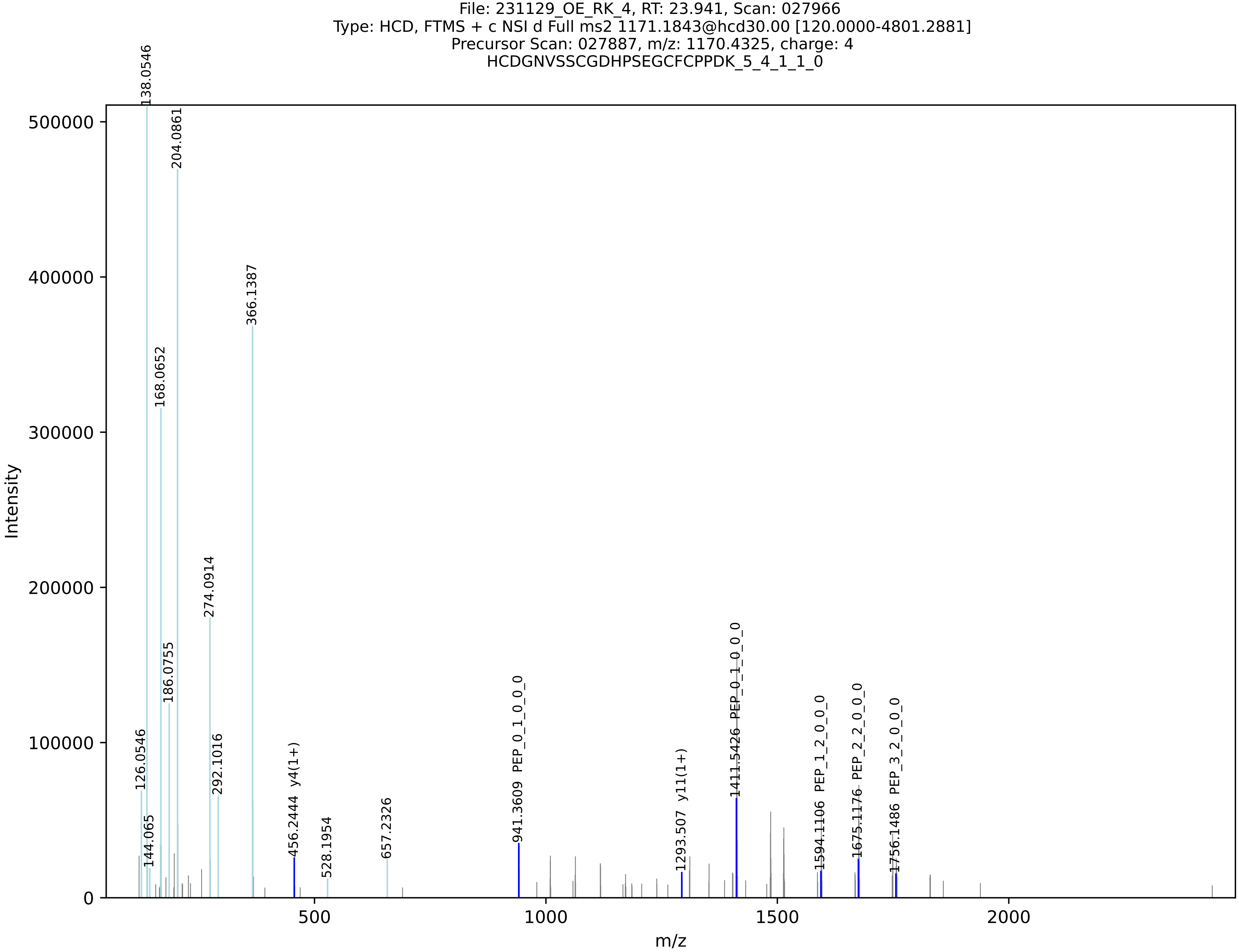
File: 231129_OE_RK_4, RT: 23.941, Scan: 027966
Type: HCD, FTMS + c NSI d Full ms2 1171.1843@hcd30.00 [120.0000-4801.2881]
Precursor Scan: 027887, m/z: 1170.4325, charge: 4
HCDGNVSSCGDHPSEGCFCPPDK_5_4_1_1_0
Intensity
m/z
138.0546
204.0861
366.1387
168.0652
274.0914
186.0755
126.0546
292.1016
144.065
456.2444 y4(1+)
528.1954
657.2326
941.3609 PEP_0_1_0_0_0
1293.507 y11(1+)
1411.5426 PEP_0_1_0_0_0
1594.1106 PEP_1_2_0_0_0
1675.1176 PEP_2_2_0_0_0
1756.1486 PEP_3_2_0_0_0

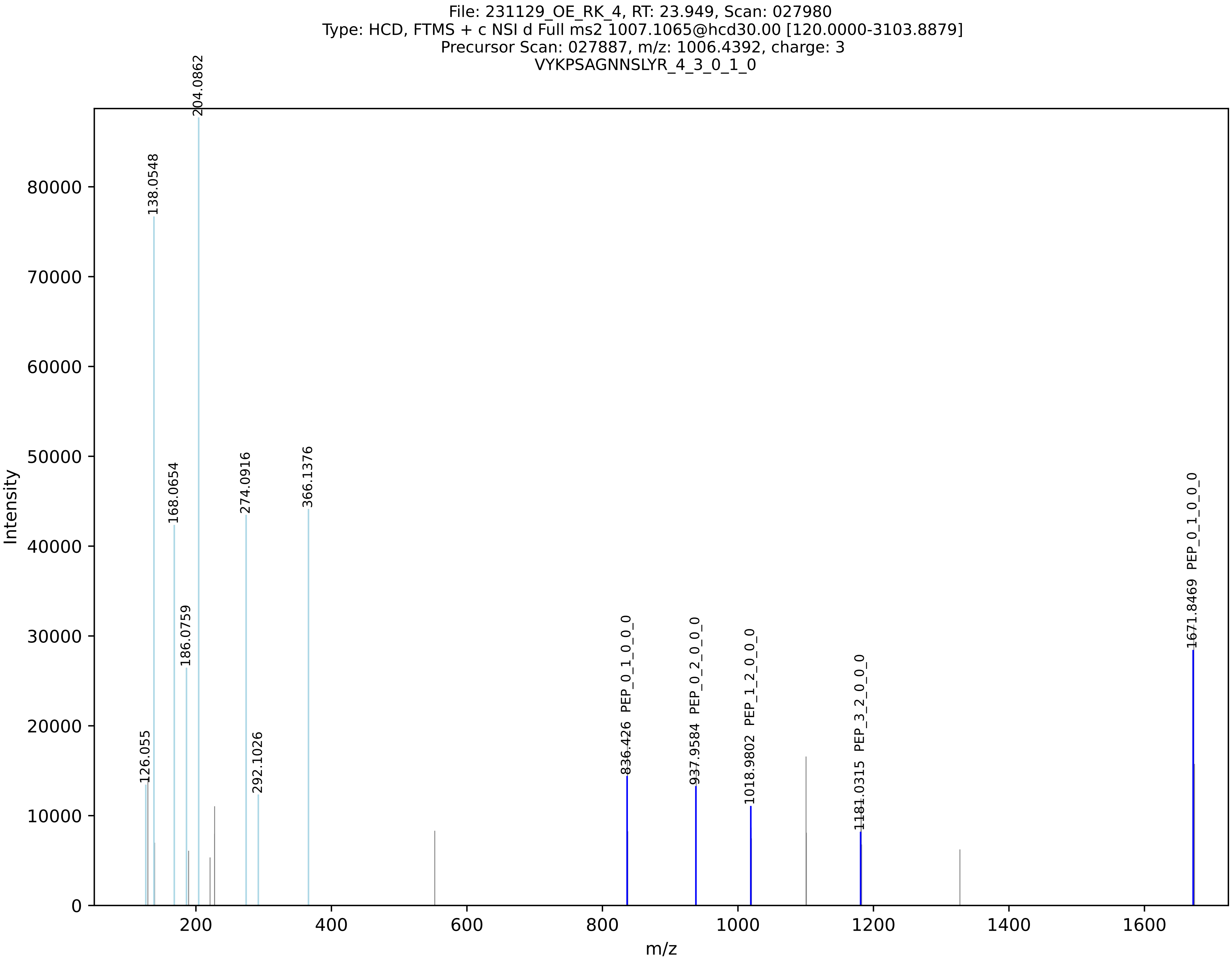
File: 231129_OE_RK_4, RT: 23.949, Scan: 027980
Type: HCD, FTMS + c NSI d Full ms2 1007.1065@hcd30.00 [120.0000-3103.8879]
Precursor Scan: 027887, m/z: 1006.4392, charge: 3
VYKPSAGNNSLYR_4_3_0_1_0
Intensity
m/z
126.055
138.0548
168.0654
186.0759
204.0862
274.0916
292.1026
366.1376
836.426 PEP_0_1_0_0_0
937.9584 PEP_0_2_0_0_0
1018.9802 PEP_1_2_0_0_0
1181.0315 PEP_3_2_0_0_0
1671.8469 PEP_0_1_0_0_0

File: 231129_OE_RK_4, RT: 23.988, Scan: 028035
Type: HCD, FTMS + c NSI d Full ms2 1114.1071@hcd30.00 [120.0000-3431.3096]
Precursor Scan: 027960, m/z: 1113.439, charge: 3
MDGASNVTCINSR_5_4_0_1_0

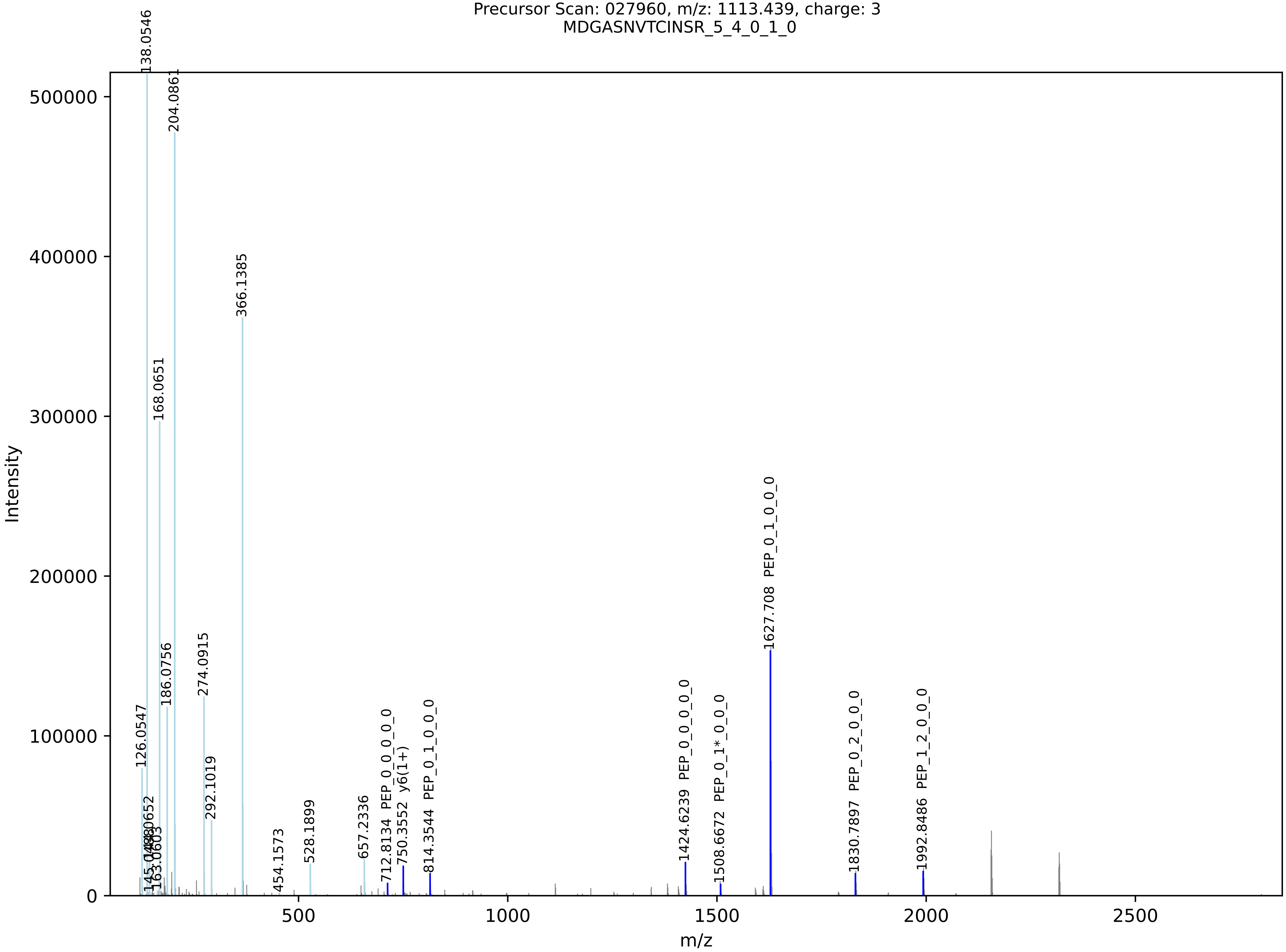

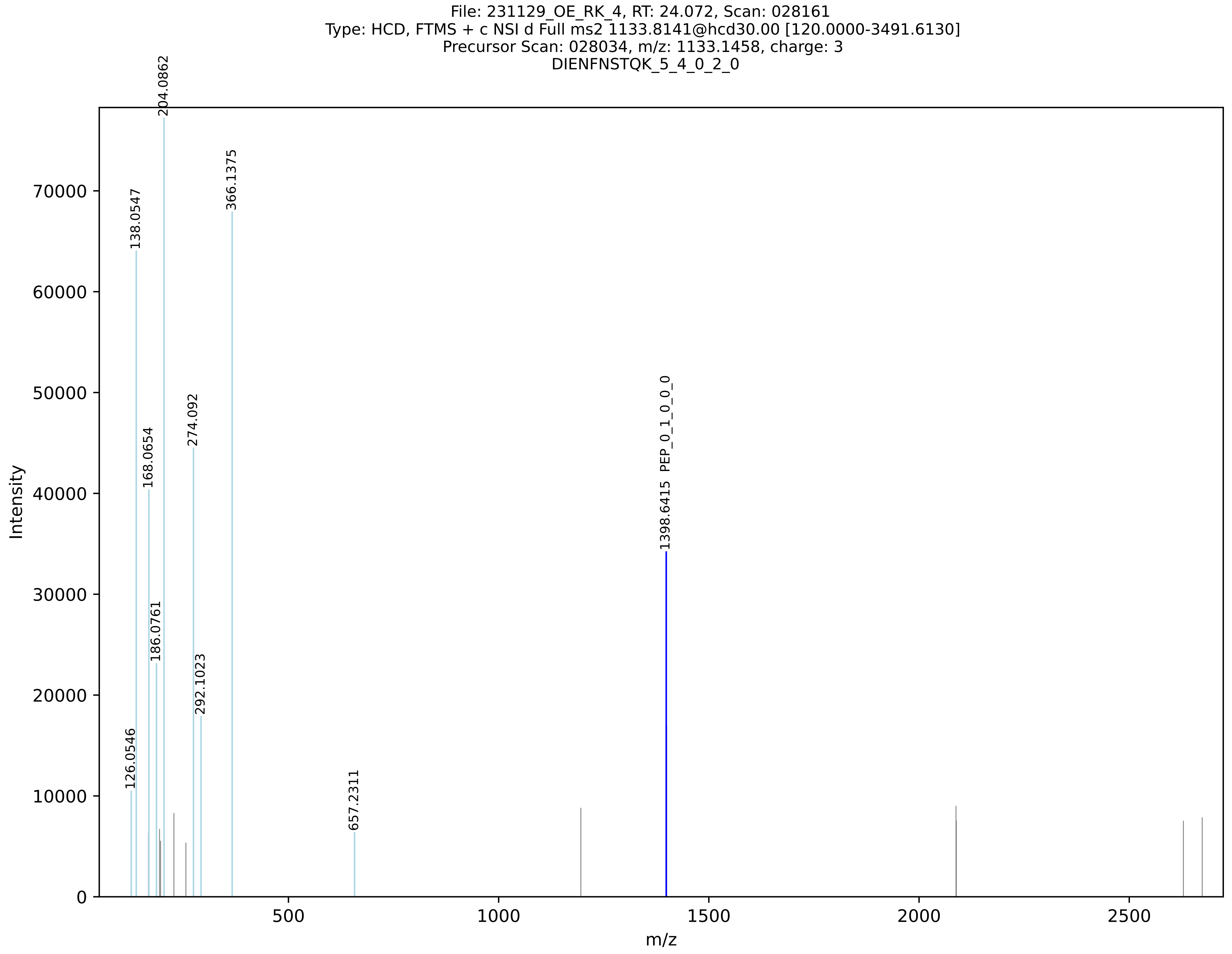
File: 231129_OE_RK_4, RT: 24.072, Scan: 028161
Type: HCD, FTMS + c NSI d Full ms2 1133.8141@hcd30.00 [120.0000-3491.6130]
Precursor Scan: 028034, m/z: 1133.1458, charge: 3
DIENFNSTQK_5_4_0_2_0
204.0862
366.1375
138.0547
274.092
168.0654
186.0761
292.1023
126.0546
657.2311
1398.6415 PEP_0_1_0_0_0
Intensity
m/z
0
10000
20000
30000
40000
50000
60000
70000
500
1000
1500
2000
2500

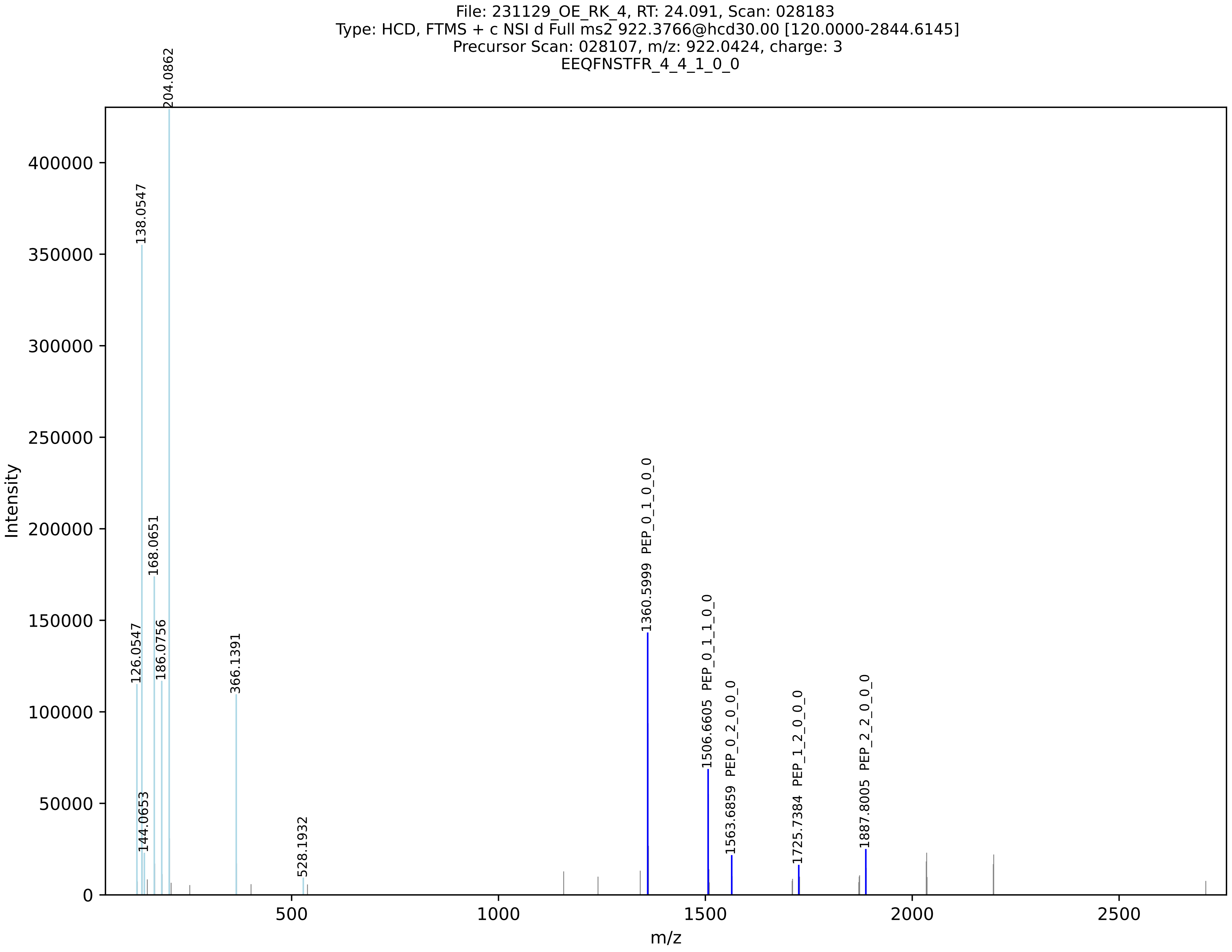
File: 231129_OE_RK_4, RT: 24.091, Scan: 028183
Type: HCD, FTMS + c NSI d Full ms2 922.3766@hcd30.00 [120.0000-2844.6145]
Precursor Scan: 028107, m/z: 922.0424, charge: 3
EEQFNSTFR_4_4_1_0_0
204.0862
138.0547
168.0651
126.0547
186.0756
366.1391
144.0653
528.1932
1360.5999 PEP_0_1_0_0_0
1506.6605 PEP_0_1_1_0_0
1563.6859 PEP_0_2_0_0_0
1725.7384 PEP_1_2_0_0_0
1887.8005 PEP_2_2_0_0_0
Intensity
m/z
0
50000
100000
150000
200000
250000
300000
350000
400000
500
1000
1500
2000
2500

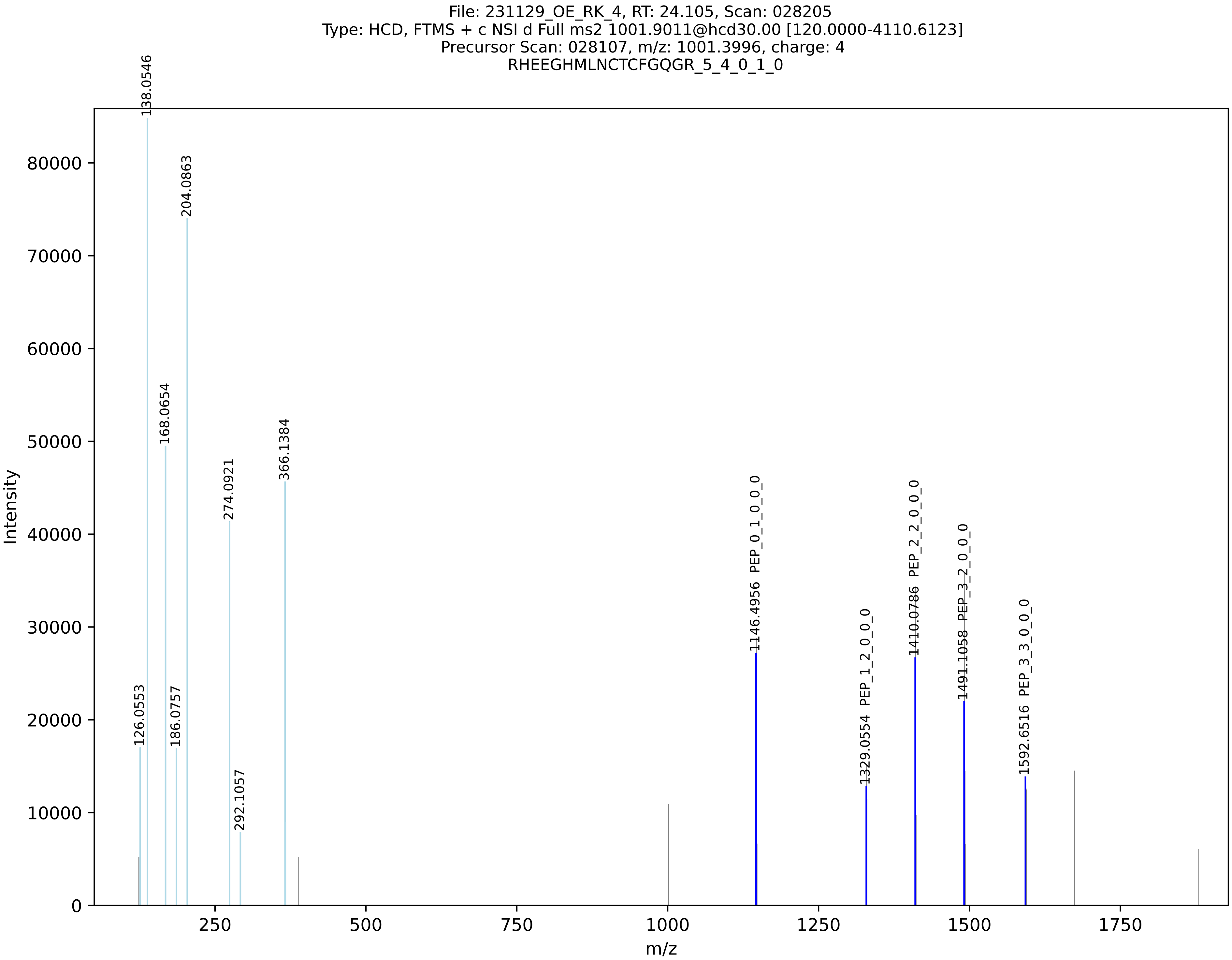
File: 231129_OE_RK_4, RT: 24.105, Scan: 028205
Type: HCD, FTMS + c NSI d Full ms2 1001.9011@hcd30.00 [120.0000-4110.6123]
Precursor Scan: 028107, m/z: 1001.3996, charge: 4
RHEEGHMLNCTCFGQGR_5_4_0_1_0
Intensity
m/z
138.0546
204.0863
168.0654
366.1384
274.0921
126.0553
186.0757
292.1057
1146.4956 PEP_0_1_0_0_0
1329.0554 PEP_1_2_0_0_0
1410.0786 PEP_2_2_0_0_0
1491.1058 PEP_3_2_0_0_0
1592.6516 PEP_3_3_0_0_0

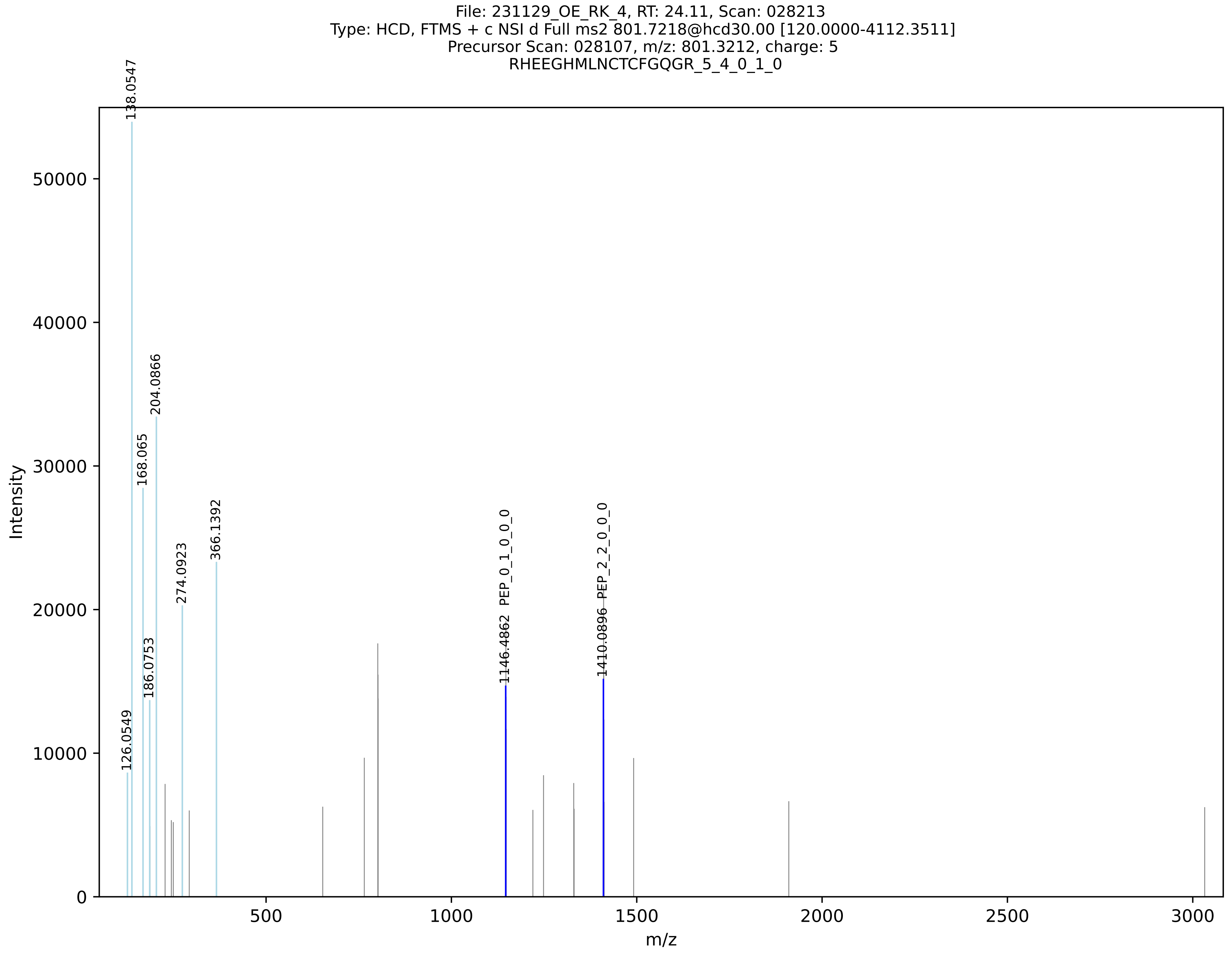

File: 231129_OE_RK_4, RT: 24.11, Scan: 028213
Type: HCD, FTMS + c NSI d Full ms2 801.7218@hcd30.00 [120.0000-4112.3511]
Precursor Scan: 028107, m/z: 801.3212, charge: 5
RHEEGHMLNCTCFGQGR_5_4_0_1_0
138.0547
204.0866
168.065
186.0753
126.0549
274.0923
366.1392
1146.4862 PEP_0_1_0_0_0
1410.0896 PEP_2_2_0_0_0
Intensity
m/z
0
10000
20000
30000
40000
50000
500
1000
1500
2000
2500
3000

File: 231129_OE_RK_4, RT: 24.141, Scan: 028253
Type: HCD, FTMS + c NSI d Full ms2 1170.9357@hcd30.00 [120.0000-4800.2734]
Precursor Scan: 028181, m/z: 1170.4344, charge: 4
HCDGNVSSCGDHPSEGCFCPPDK_5_4_1_1_0

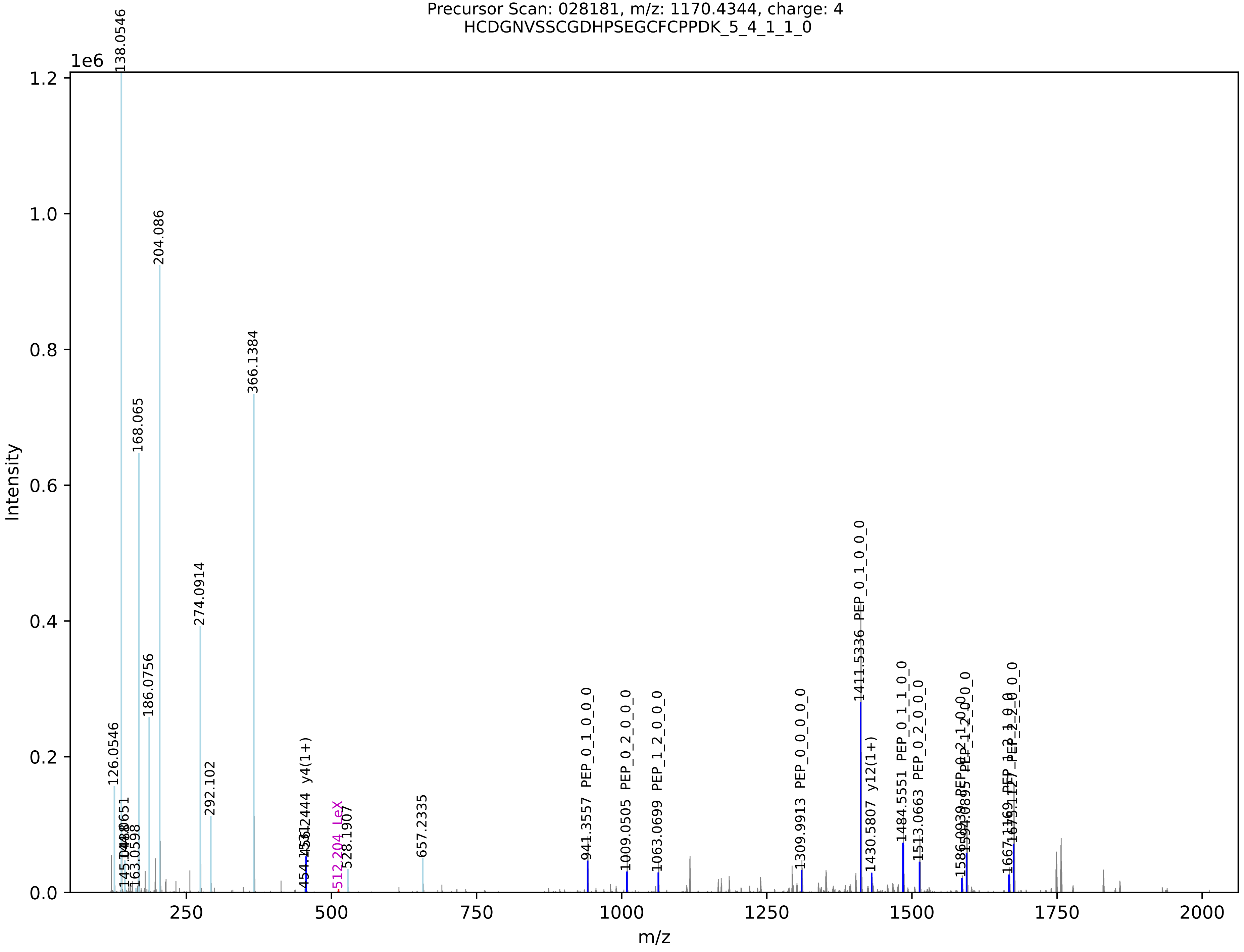

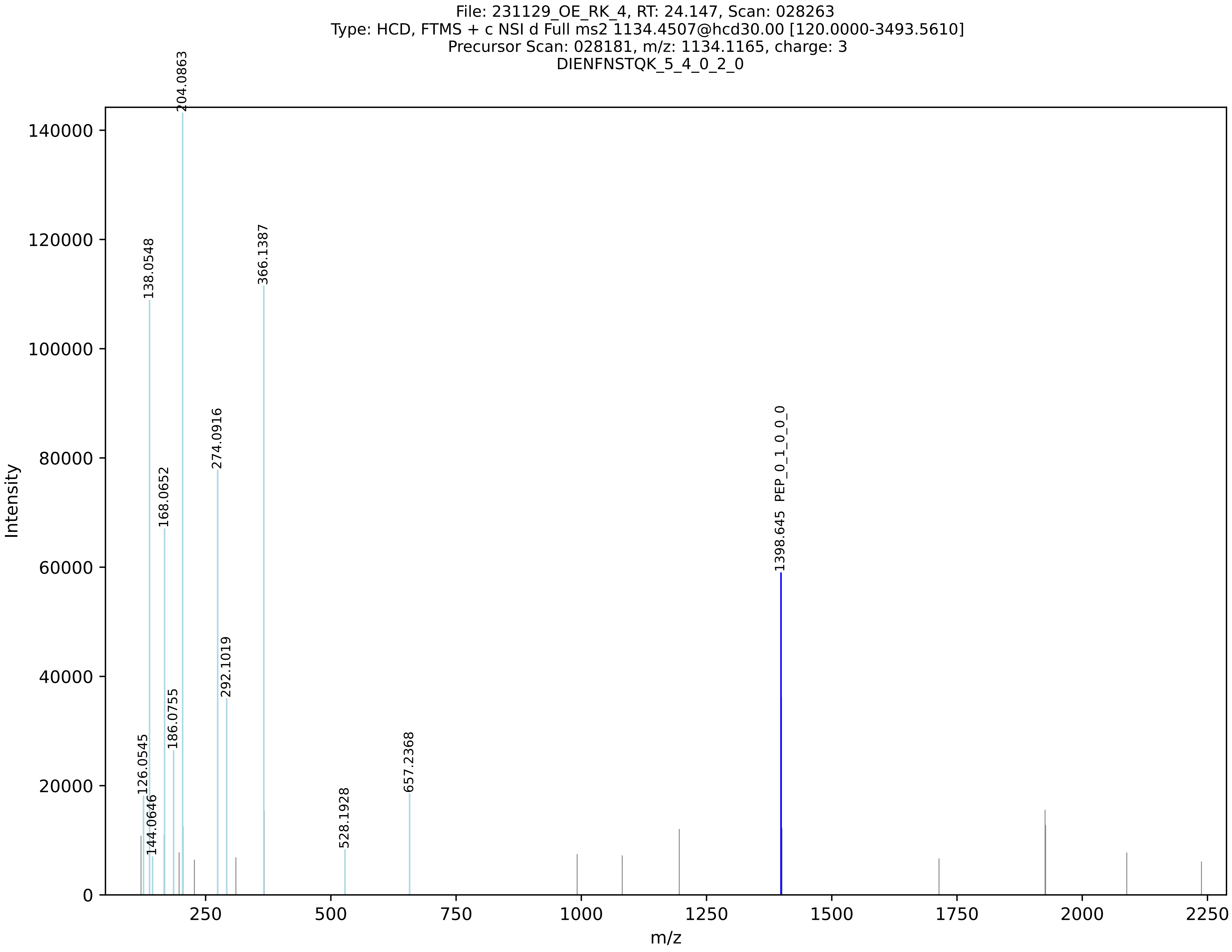
File: 231129_OE_RK_4, RT: 24.147, Scan: 028263
Type: HCD, FTMS + c NSI d Full ms2 1134.4507@hcd30.00 [120.0000-3493.5610]
Precursor Scan: 028181, m/z: 1134.1165, charge: 3
DIENFNSTQK_5_4_0_2_0
Intensity
m/z
204.0863
138.0548
366.1387
274.0916
168.0652
292.1019
186.0755
126.0545
144.0646
528.1928
657.2368
1398.645 PEP_0_1_0_0_0
0
20000
40000
60000
80000
100000
120000
140000
250
500
750
1000
1250
1500
1750
2000
2250

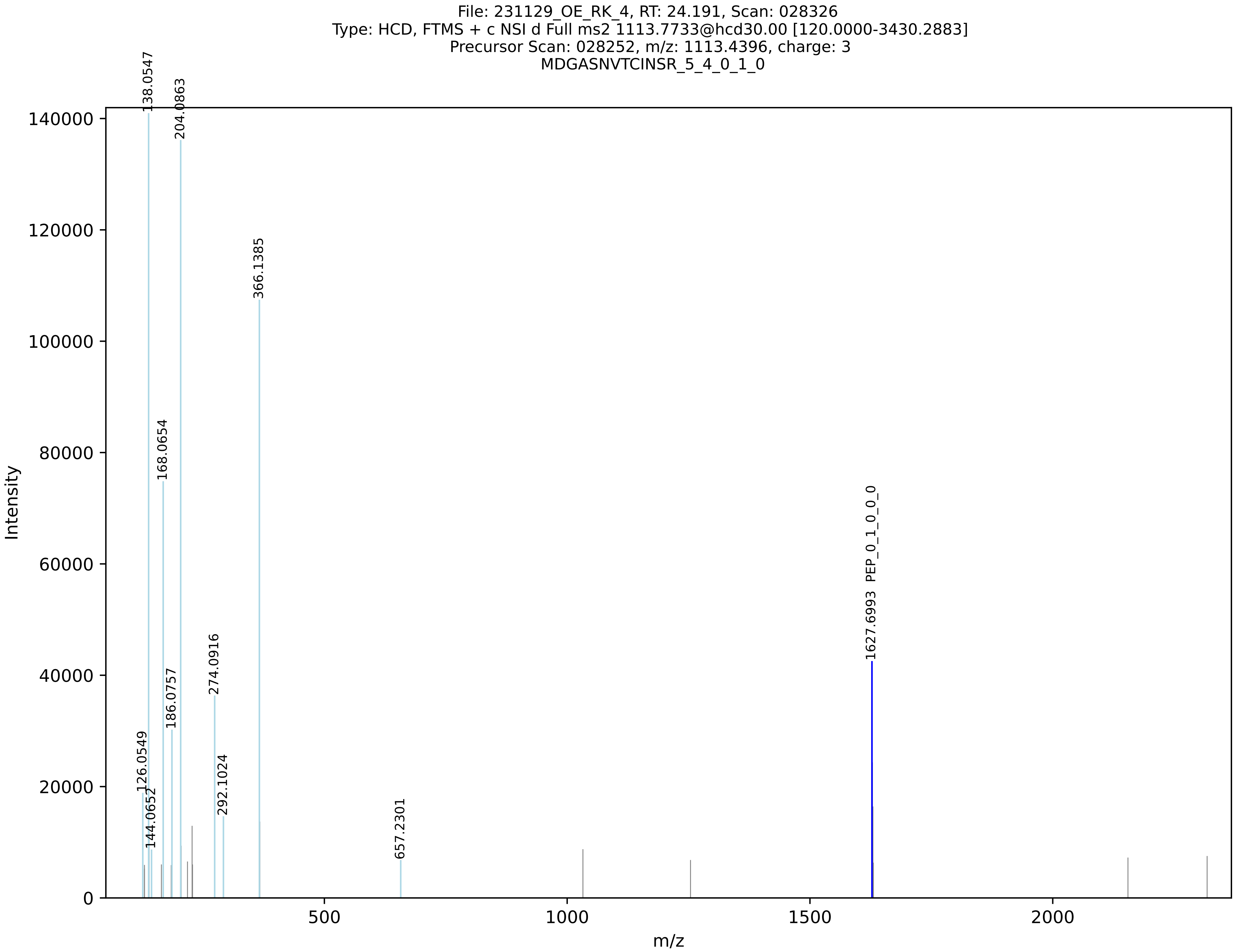

File: 231129_OE_RK_4, RT: 24.191, Scan: 028326
Type: HCD, FTMS + c NSI d Full ms2 1113.7733@hcd30.00 [120.0000-3430.2883]
Precursor Scan: 028252, m/z: 1113.4396, charge: 3
MDGASNVTCINSR_5_4_0_1_0
138.0547
204.0863
366.1385
168.0654
1627.6993 PEP_0_1_0_0_0
274.0916
186.0757
126.0549
144.0652
292.1024
657.2301
Intensity
m/z
0
20000
40000
60000
80000
100000
120000
140000
500
1000
1500
2000

File: 231129_OE_RK_4, RT: 24.241, Scan: 028399
Type: HCD, FTMS + c NSI d Full ms2 1364.6028@hcd30.00 [120.0000-2805.2178]
Precursor Scan: 028323, m/z: 1364.1026, charge: 2
TPLTANITK_5_4_1_0_0

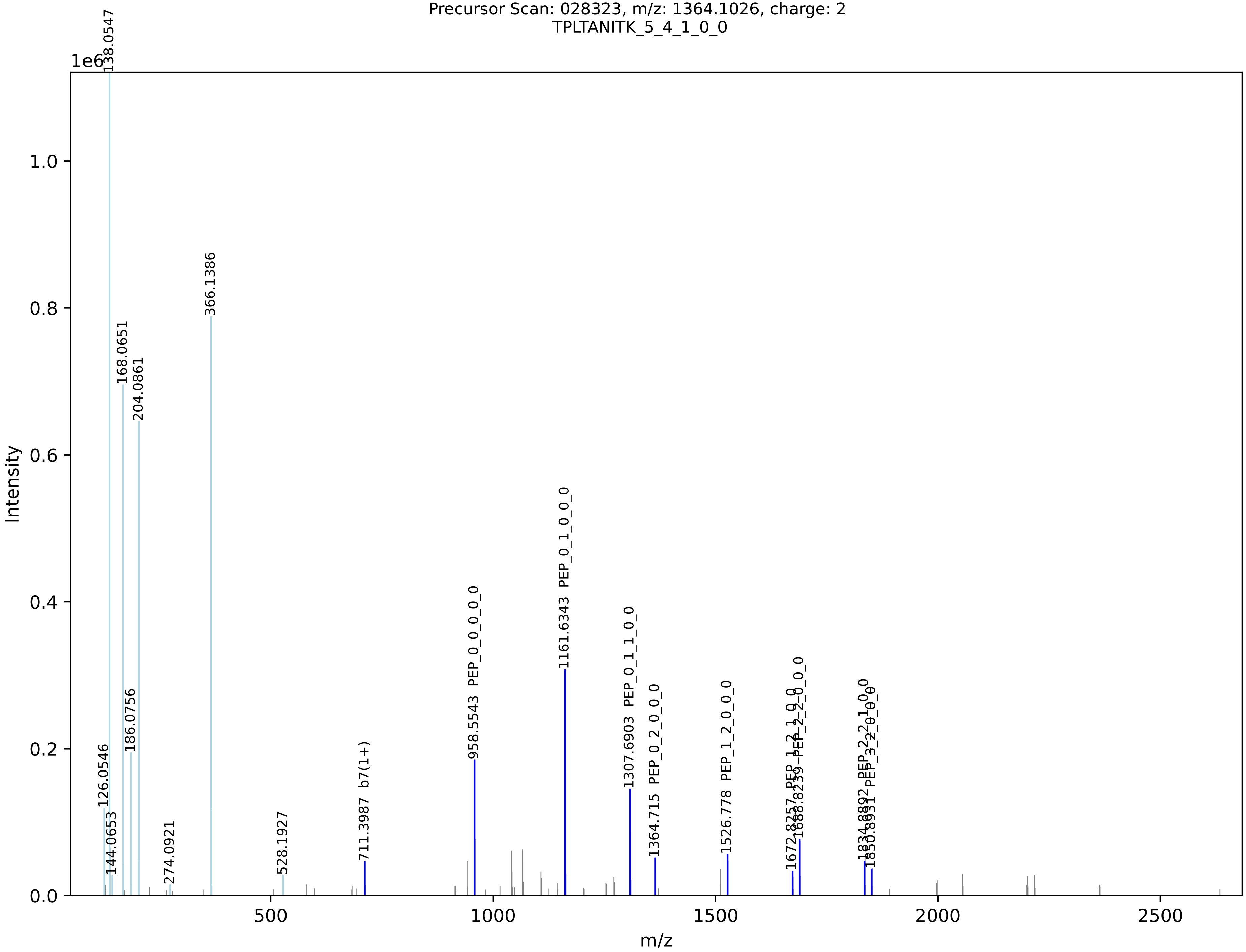

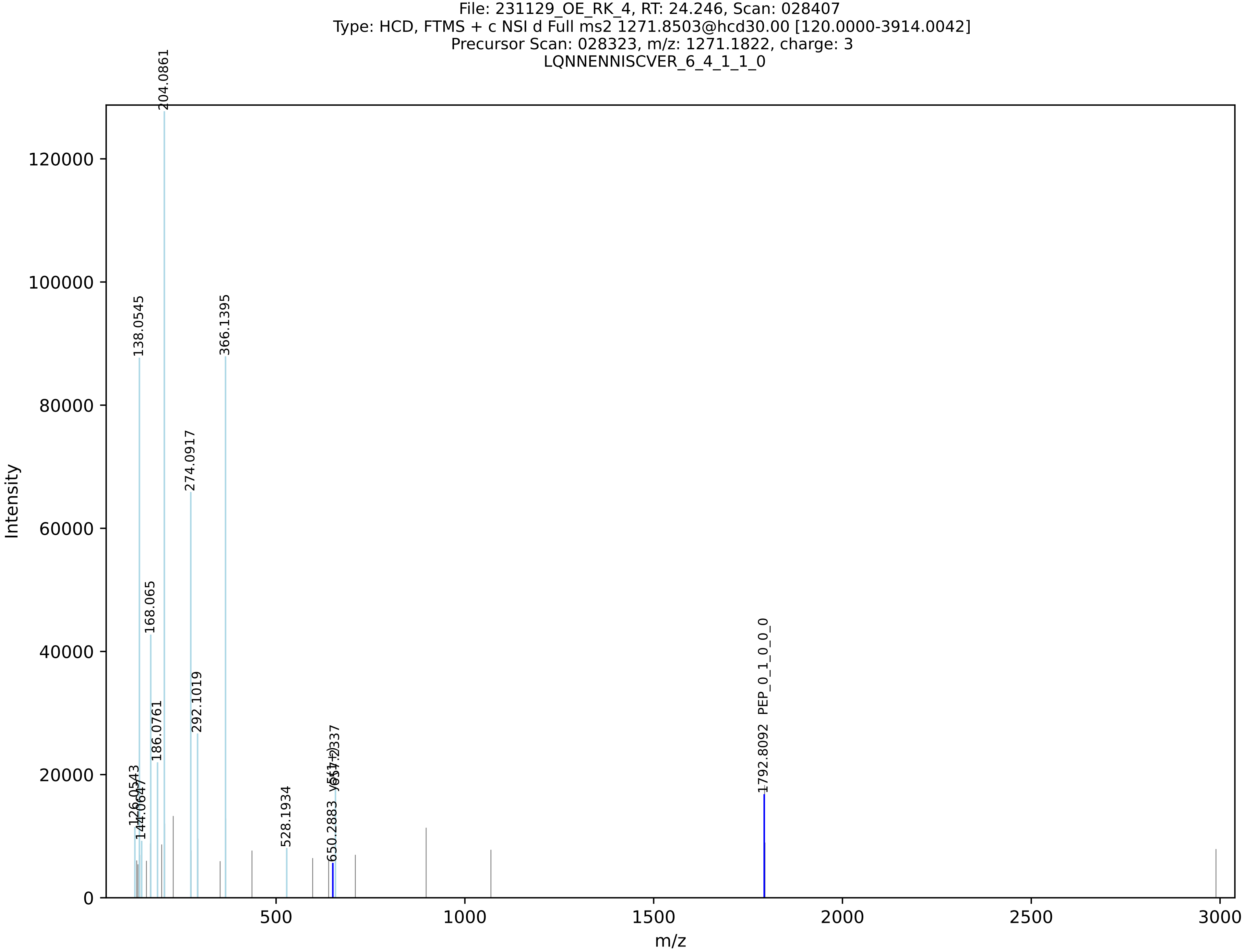

File: 231129_OE_RK_4, RT: 24.246, Scan: 028407
Type: HCD, FTMS + c NSI d Full ms2 1271.8503@hcd30.00 [120.0000-3914.0042]
Precursor Scan: 028323, m/z: 1271.1822, charge: 3
LQNNENNISCVER_6_4_1_1_0
Intensity
m/z
0
20000
40000
60000
80000
100000
120000
500
1000
1500
2000
2500
3000
204.0861
138.0545
366.1395
274.0917
168.065
292.1019
186.0761
126.0543
144.0647
528.1934
650.2883
y5(1+)
657.2337
1792.8092
PEP_0_1_0_0_0

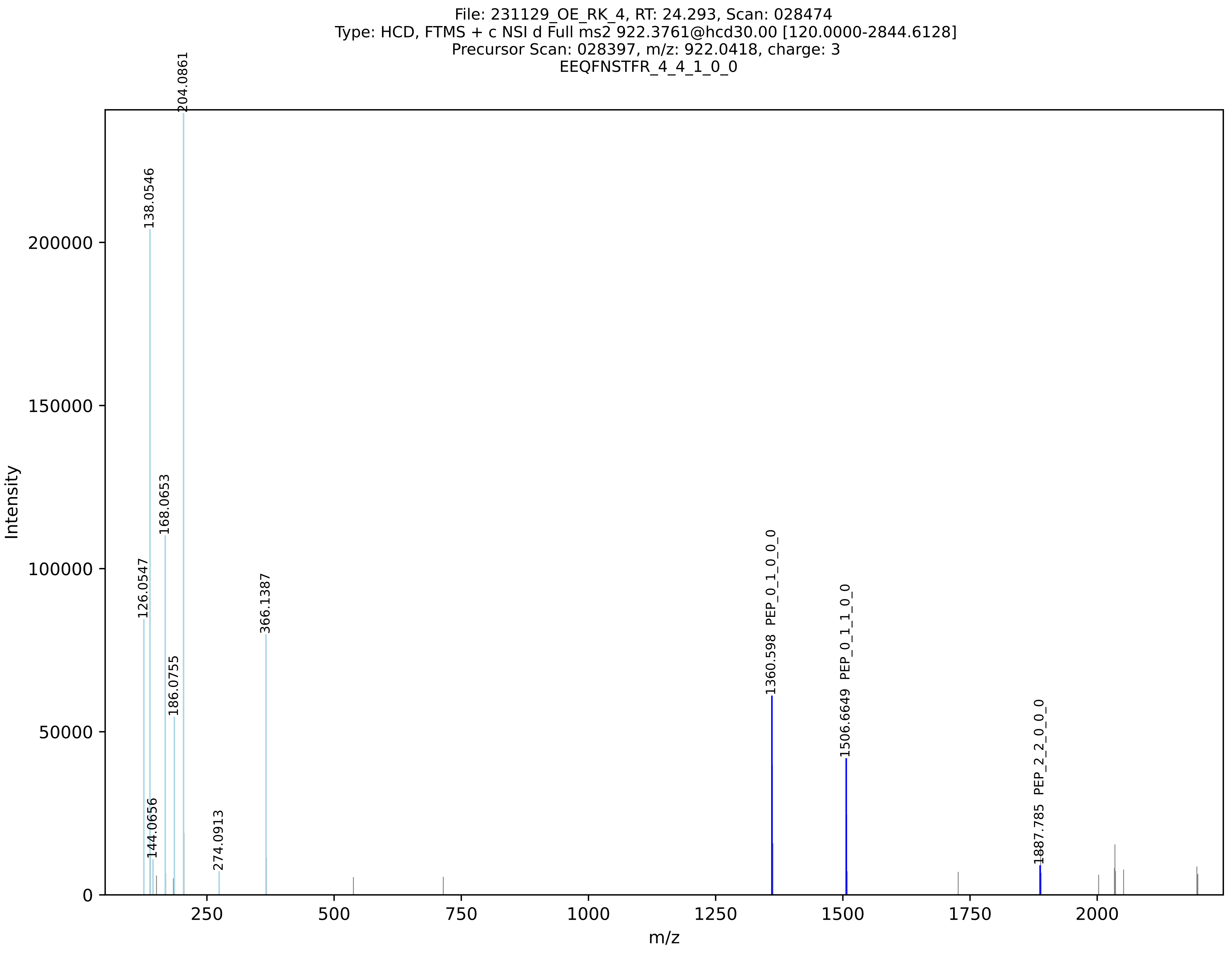
File: 231129_OE_RK_4, RT: 24.293, Scan: 028474
Type: HCD, FTMS + c NSI d Full ms2 922.3761@hcd30.00 [120.0000-2844.6128]
Precursor Scan: 028397, m/z: 922.0418, charge: 3
EEQFNSTFR_4_4_1_0_0
204.0861
138.0546
168.0653
126.0547
366.1387
186.0755
144.0656
274.0913
1360.598 PEP_0_1_0_0_0
1506.6649 PEP_0_1_1_0_0
1887.785 PEP_2_2_0_0_0
Intensity
m/z
0
50000
100000
150000
200000
250
500
750
1000
1250
1500
1750
2000

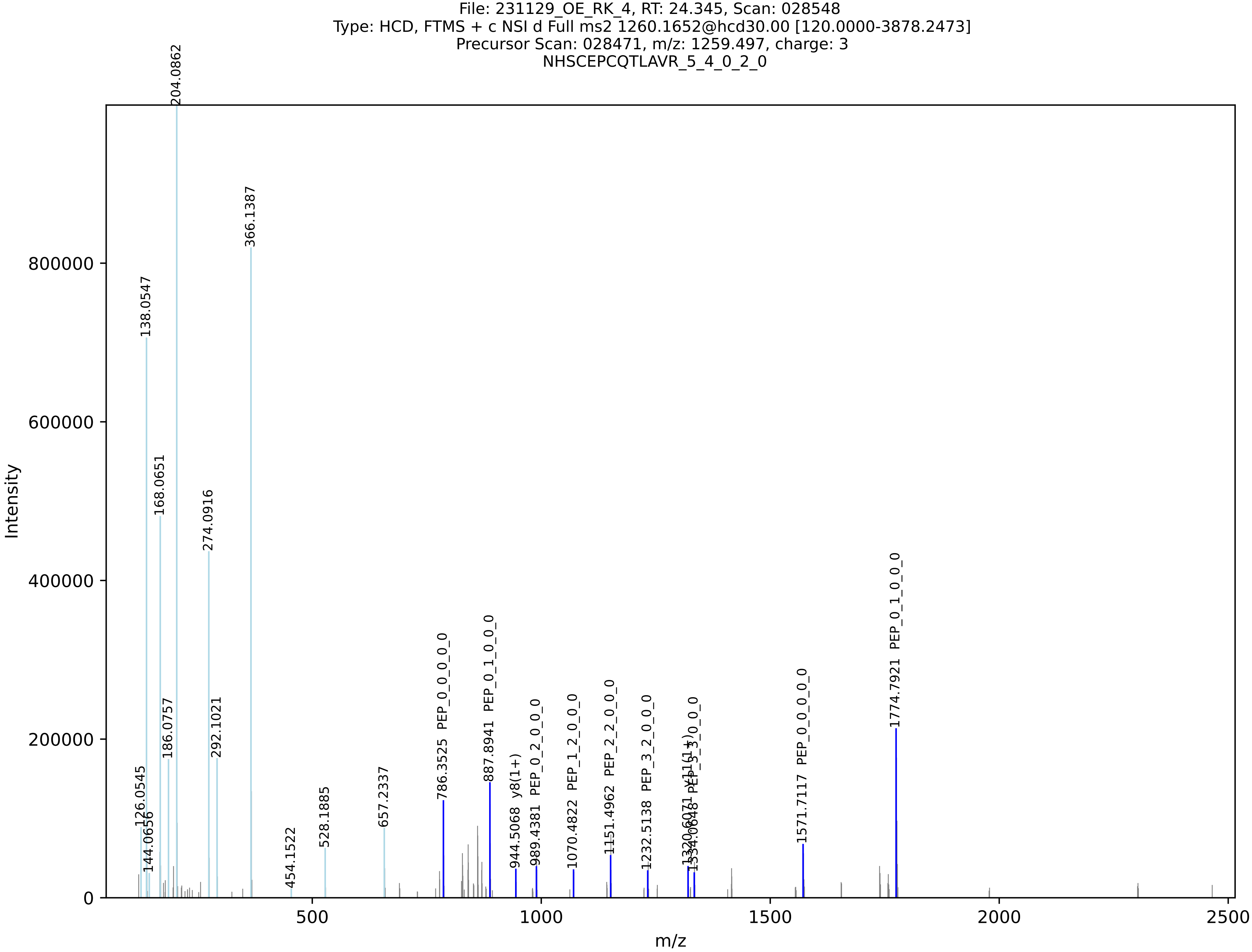
File: 231129_OE_RK_4, RT: 24.345, Scan: 028548
Type: HCD, FTMS + c NSI d Full ms2 1260.1652@hcd30.00 [120.0000-3878.2473]
Precursor Scan: 028471, m/z: 1259.497, charge: 3
NHSCEPCQTLAVR_5_4_0_2_0
204.0862
366.1387
138.0547
168.0651
274.0916
186.0757
292.1021
126.0545
144.0656
454.1522
528.1885
657.2337
786.3525 PEP_0_0_0_0_0
887.8941 PEP_0_1_0_0_0
944.5068 y8(1+)
989.4381 PEP_0_2_0_0_0
1070.4822 PEP_1_2_0_0_0
1151.4962 PEP_2_2_0_0_0
1232.5138 PEP_3_2_0_0_0
1320.6071 y11(1+)
1334.0648 PEP_5_3_0_0_0
1571.7117 PEP_0_0_0_0_0
1774.7921 PEP_0_1_0_0_0
Intensity
m/z
0
200000
400000
600000
800000
500
1000
1500
2000
2500

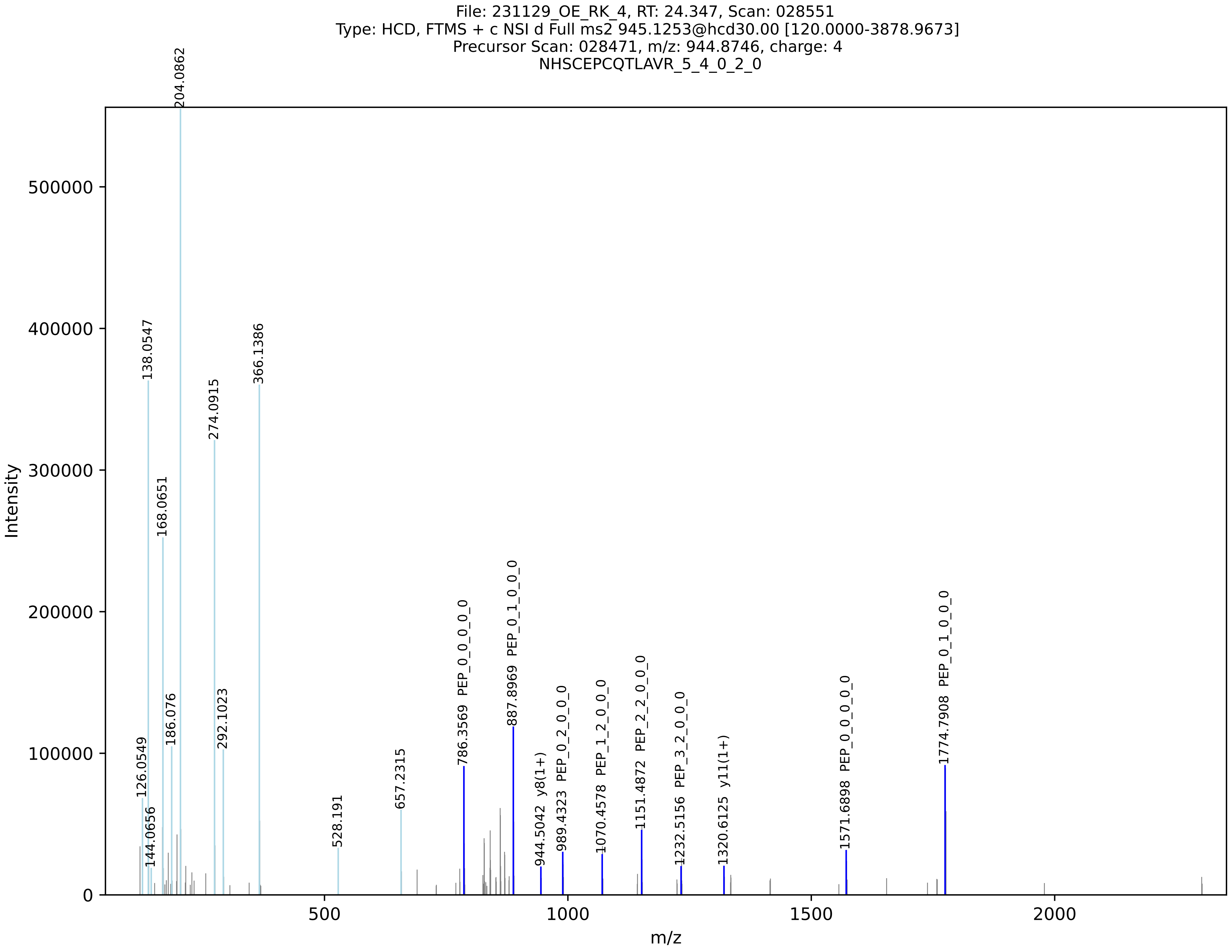
File: 231129_OE_RK_4, RT: 24.347, Scan: 028551
Type: HCD, FTMS + c NSI d Full ms2 945.1253@hcd30.00 [120.0000-3878.9673]
Precursor Scan: 028471, m/z: 944.8746, charge: 4
NHSCEPCQTLAVR_5_4_0_2_0
Intensity
m/z
204.0862
138.0547
366.1386
274.0915
168.0651
186.076
292.1023
126.0549
144.0656
528.191
657.2315
786.3569 PEP_0_0_0_0_0
887.8969 PEP_0_1_0_0_0
944.5042 y8(1+)
989.4323 PEP_0_2_0_0_0
1070.4578 PEP_1_2_0_0_0
1151.4872 PEP_2_2_0_0_0
1232.5156 PEP_3_2_0_0_0
1320.6125 y11(1+)
1571.6898 PEP_0_0_0_0_0
1774.7908 PEP_0_1_0_0_0
0
100000
200000
300000
400000
500000
500
1000
1500
2000

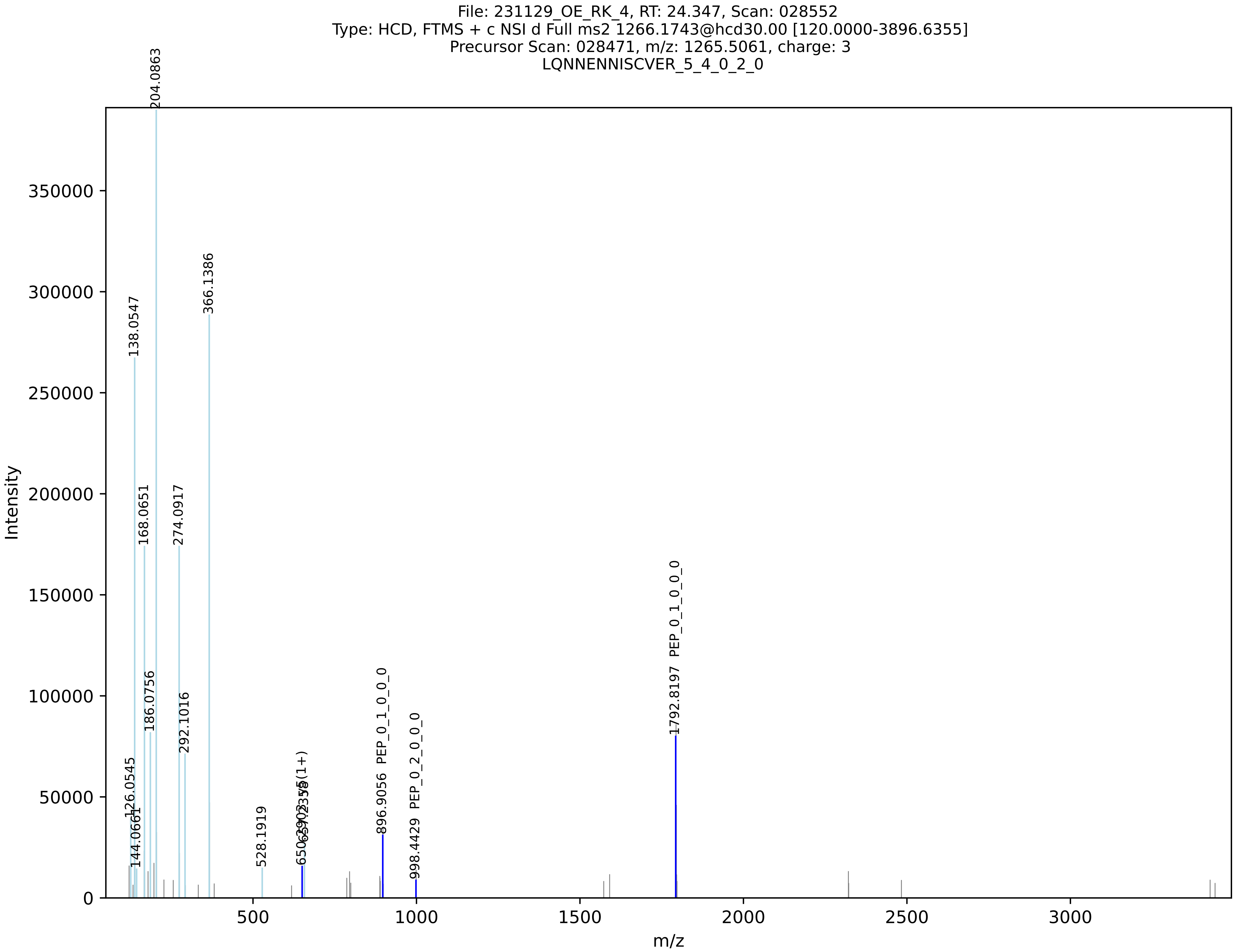
File: 231129_OE_RK_4, RT: 24.347, Scan: 028552
Type: HCD, FTMS + c NSI d Full ms2 1266.1743@hcd30.00 [120.0000-3896.6355]
Precursor Scan: 028471, m/z: 1265.5061, charge: 3
LQNNENNISCVER_5_4_0_2_0
Intensity
m/z
0
50000
100000
150000
200000
250000
300000
350000
500
1000
1500
2000
2500
3000
204.0863
366.1386
138.0547
168.0651
274.0917
186.0756
292.1016
126.0545
144.0661
528.1919
657.2358
896.9056 PEP_0_1_0_0_0
998.4429 PEP_0_2_0_0_0
1792.8197 PEP_0_1_0_0_0

File: 231129_OE_RK_4, RT: 24.35, Scan: 028556
Type: HCD, FTMS + c NSI d Full ms2 1134.7845@hcd30.00 [120.0000-3494.5830]
Precursor Scan: 028471, m/z: 1134.1164, charge: 3
DIENFNSTQK_5_4_0_2_0

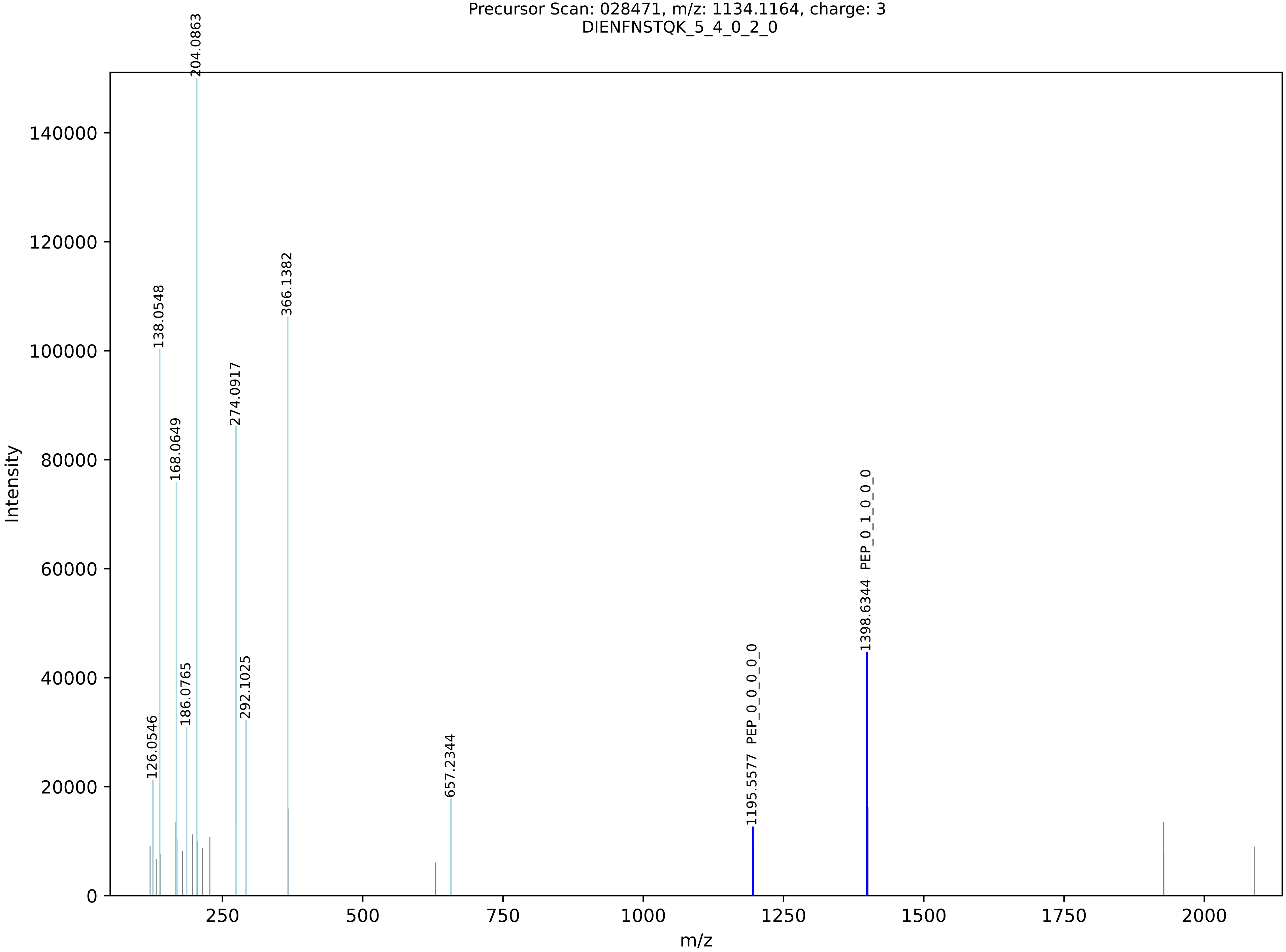

File: 231129_OE_RK_4, RT: 24.393, Scan: 028617
Type: HCD, FTMS + c NSI d Full ms2 910.0713@hcd30.00 [120.0000-2806.9600]
Precursor Scan: 028545, m/z: 909.7369, charge: 3
TPLTANITK_5_4_1_0_0

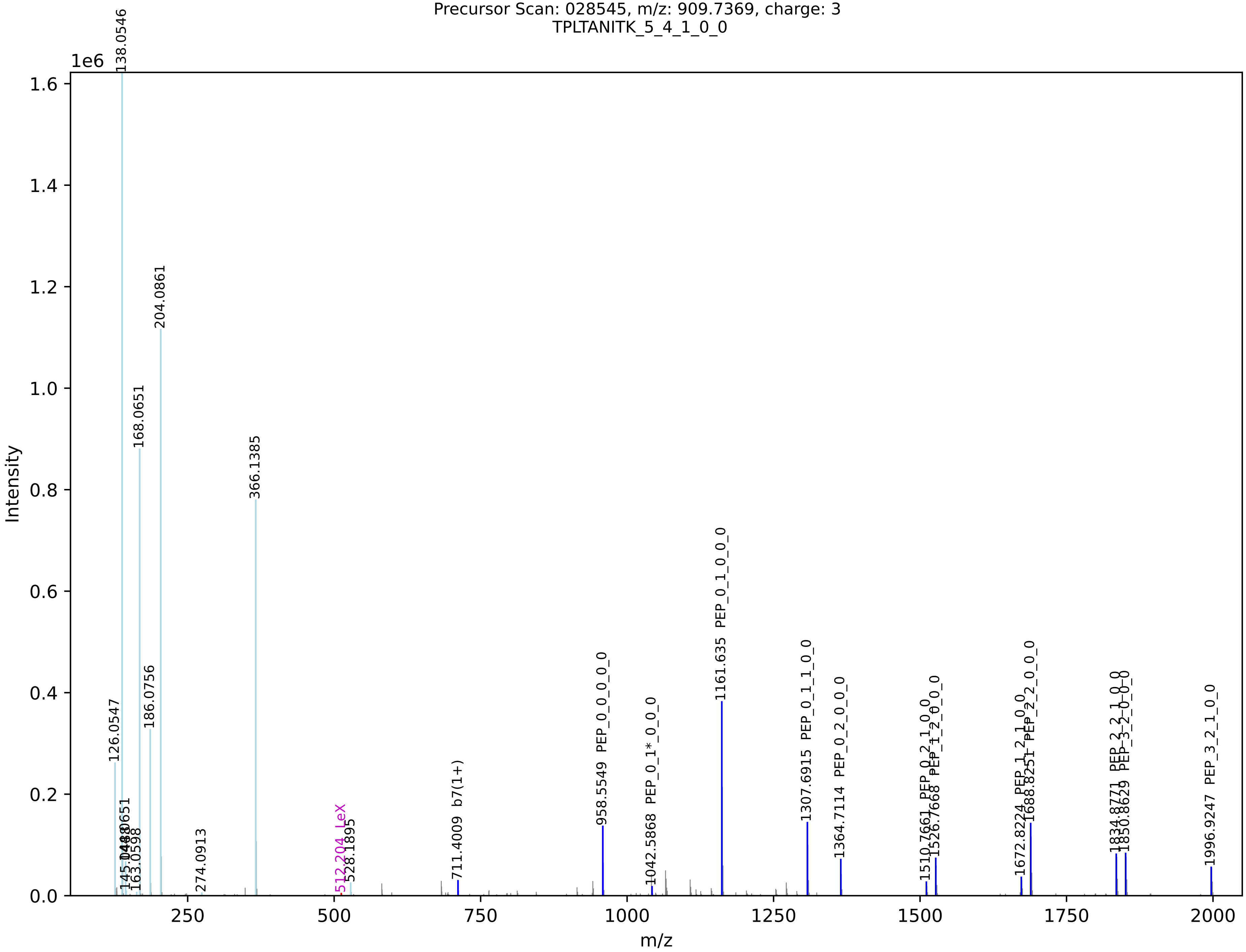

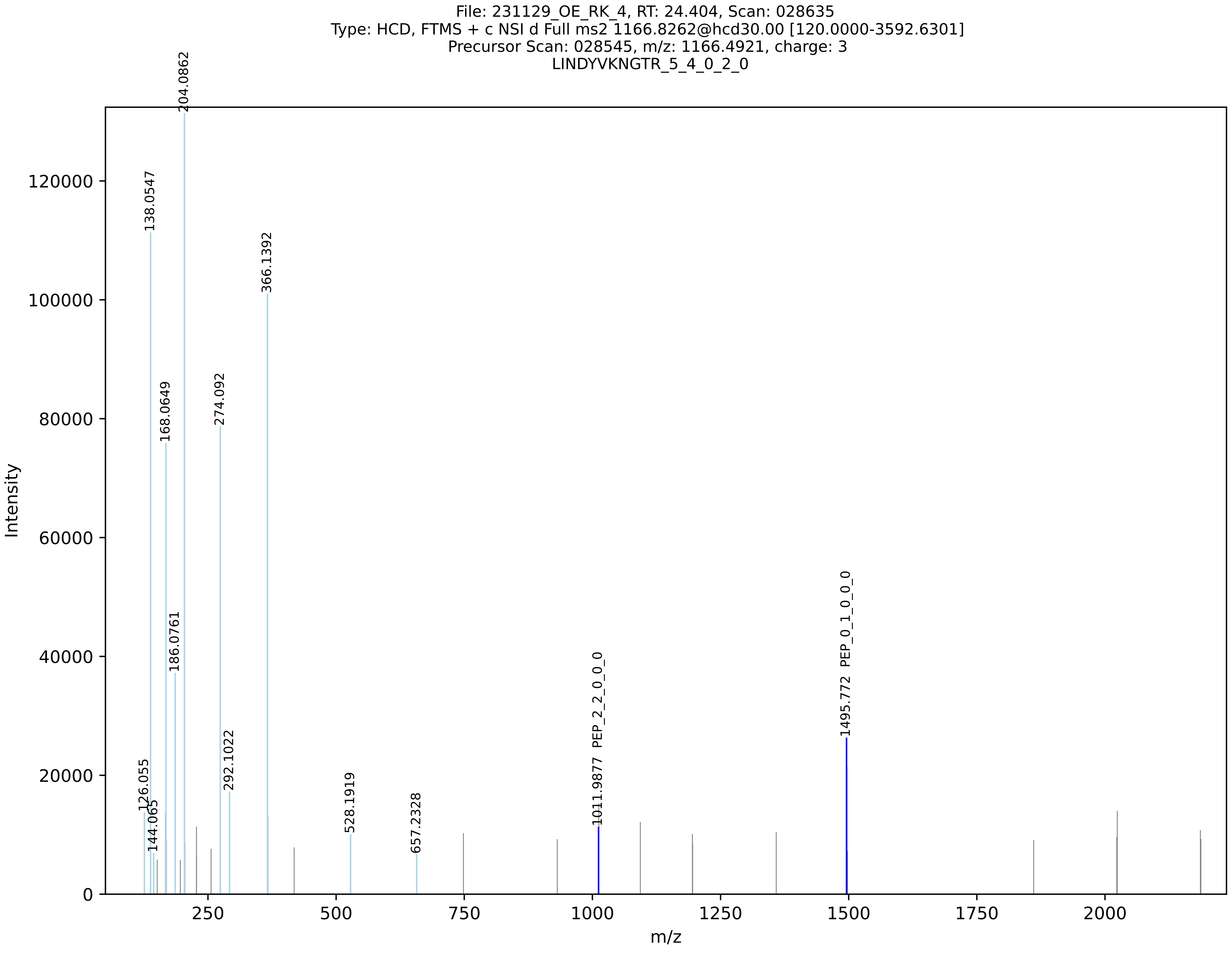

File: 231129_OE_RK_4, RT: 24.404, Scan: 028635
Type: HCD, FTMS + c NSI d Full ms2 1166.8262@hcd30.00 [120.0000-3592.6301]
Precursor Scan: 028545, m/z: 1166.4921, charge: 3
LINDYVKNGTR_5_4_0_2_0
Intensity
m/z
0
20000
40000
60000
80000
100000
120000
250
500
750
1000
1250
1500
1750
2000
204.0862
138.0547
366.1392
168.0649
274.092
186.0761
126.055
144.065
292.1022
528.1919
657.2328
1011.9877 PEP_2_2_0_0_0
1495.772 PEP_0_1_0_0_0

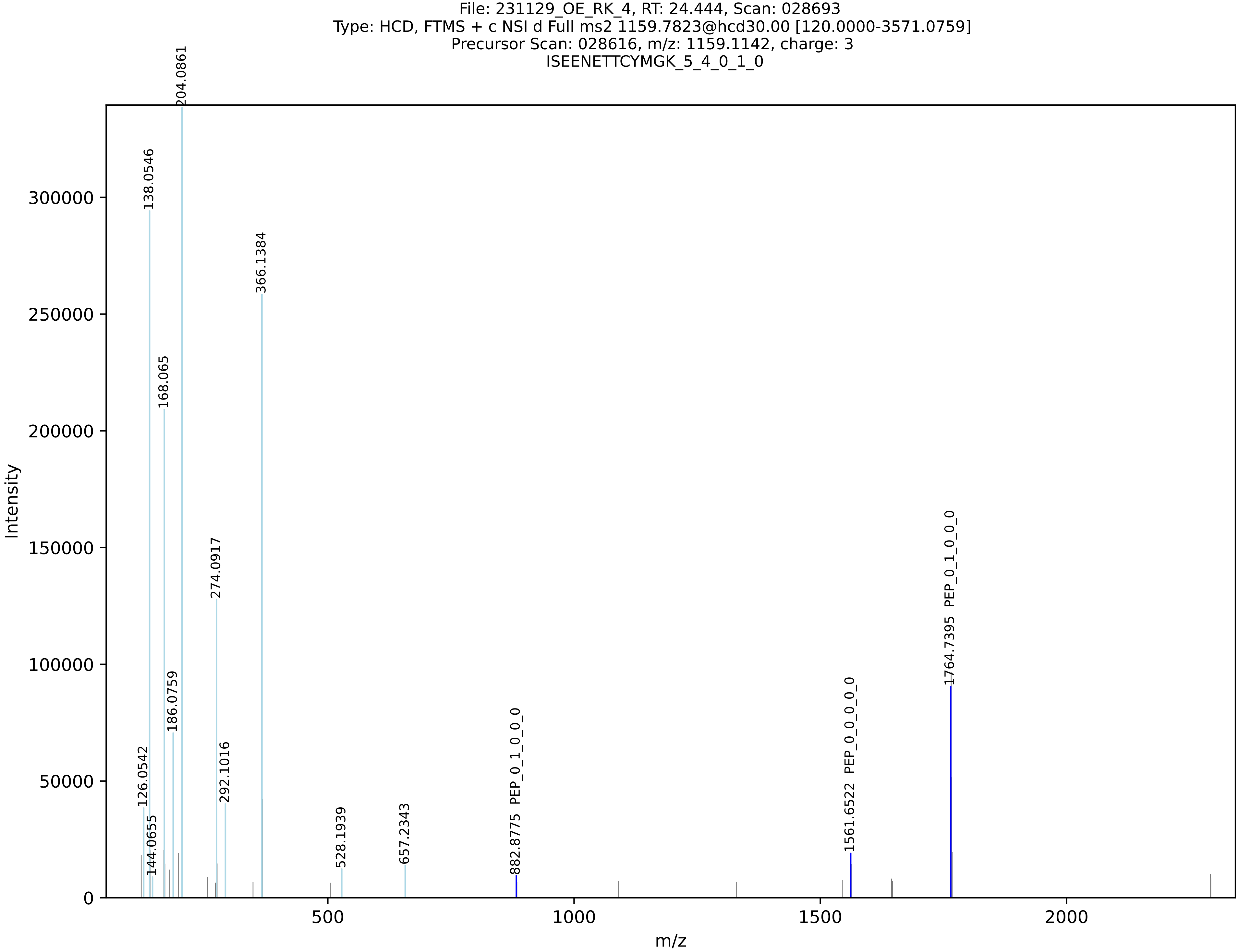

File: 231129_OE_RK_4, RT: 24.444, Scan: 028693
Type: HCD, FTMS + c NSI d Full ms2 1159.7823@hcd30.00 [120.0000-3571.0759]
Precursor Scan: 028616, m/z: 1159.1142, charge: 3
ISEENETTCYMGK_5_4_0_1_0
Intensity
m/z
204.0861
138.0546
366.1384
168.065
274.0917
186.0759
126.0542
292.1016
144.0655
528.1939
657.2343
882.8775 PEP_0_1_0_0_0
1561.6522 PEP_0_0_0_0_0
1764.7395 PEP_0_1_0_0_
0
50000
100000
150000
200000
250000
300000
500
1000
1500
2000

File: 231129_OE_RK_4, RT: 24.546, Scan: 028839
Type: HCD, FTMS + c NSI d Full ms2 1260.1642@hcd30.00 [120.0000-3878.2444]
Precursor Scan: 028764, m/z: 1259.4961, charge: 3
NHSCEPCQTLAVR_5_4_0_2_0

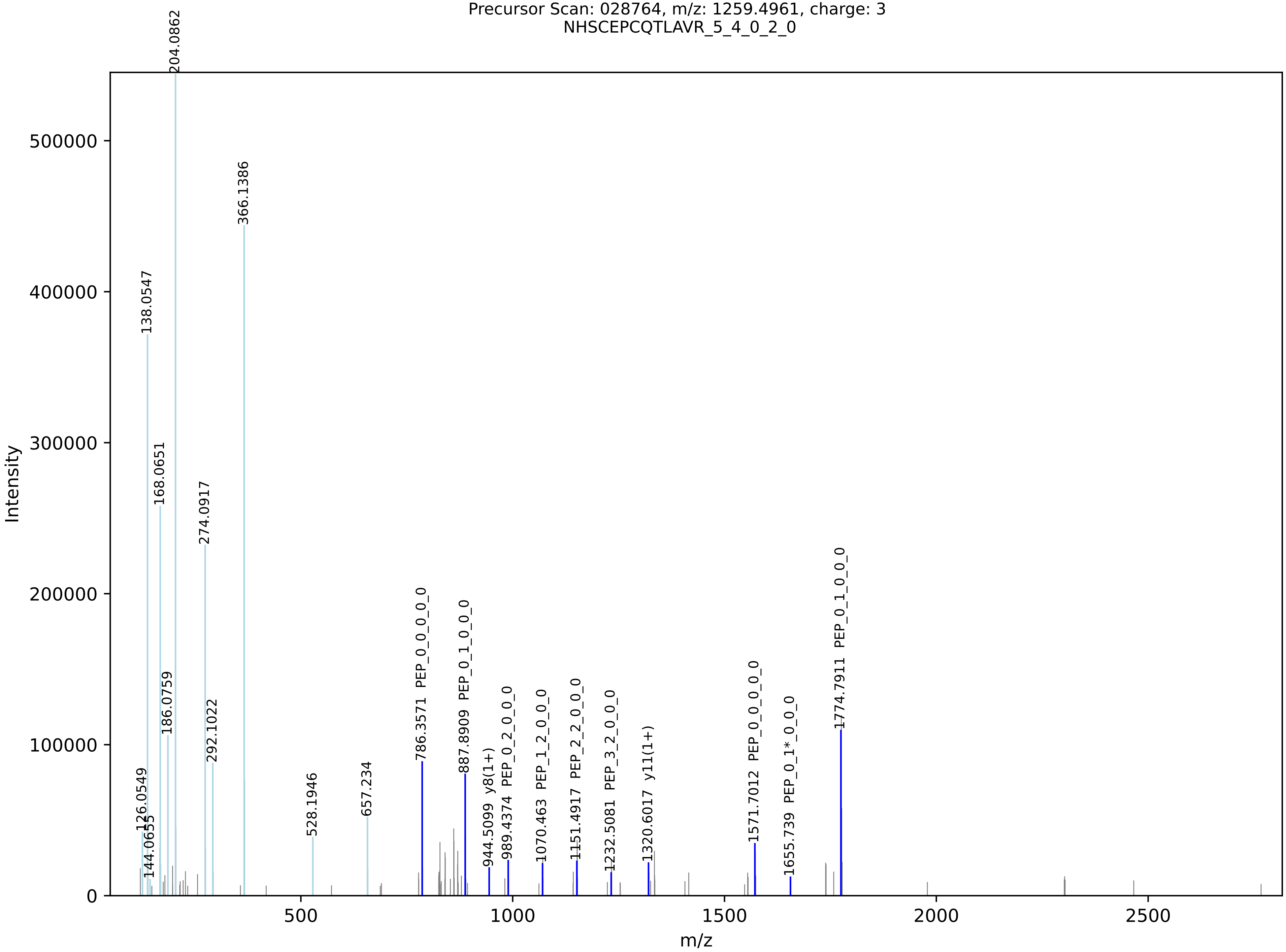

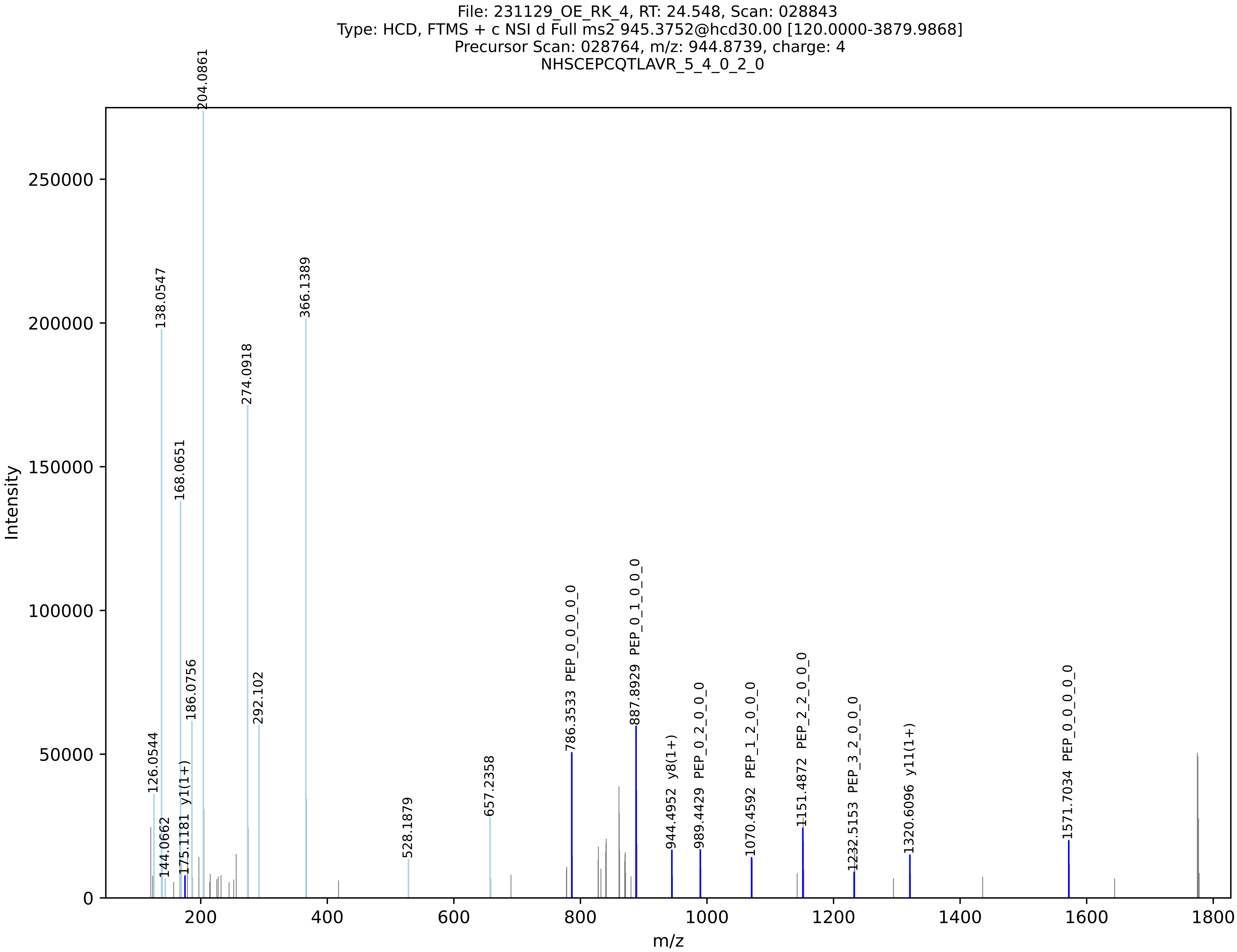

File: 231129_OE_RK_4, RT: 24.548, Scan: 028843
Type: HCD, FTMS + c NSI d Full ms2 945.3752@hcd30.00 [120.0000-3879.9868]
Precursor Scan: 028764, m/z: 944.8739, charge: 4
NHSCEPCQTLAVR_5_4_0_2_0
Intensity
m/z
204.0861
138.0547
366.1389
274.0918
168.0651
186.0756
292.102
126.0544
144.0662
175.1181 y1(1+)
528.1879
657.2358
786.3533 PEP_0_0_0_0_0
887.8929 PEP_0_1_0_0_0
944.4952 y8(1+)
989.4429 PEP_0_2_0_0_0
1070.4592 PEP_1_2_0_0_0
1151.4872 PEP_2_2_0_0_0
1232.5153 PEP_3_2_0_0_0
1320.6096 y11(1+)
1571.7034 PEP_0_0_0_0_0

File: 231129_OE_RK_4, RT: 24.643, Scan: 028981
Type: HCD, FTMS + c NSI d Full ms2 977.7634@hcd30.00 [120.0000-3014.0979]
Precursor Scan: 028909, m/z: 977.4292, charge: 3
TPLTANITK_5_5_1_0_0

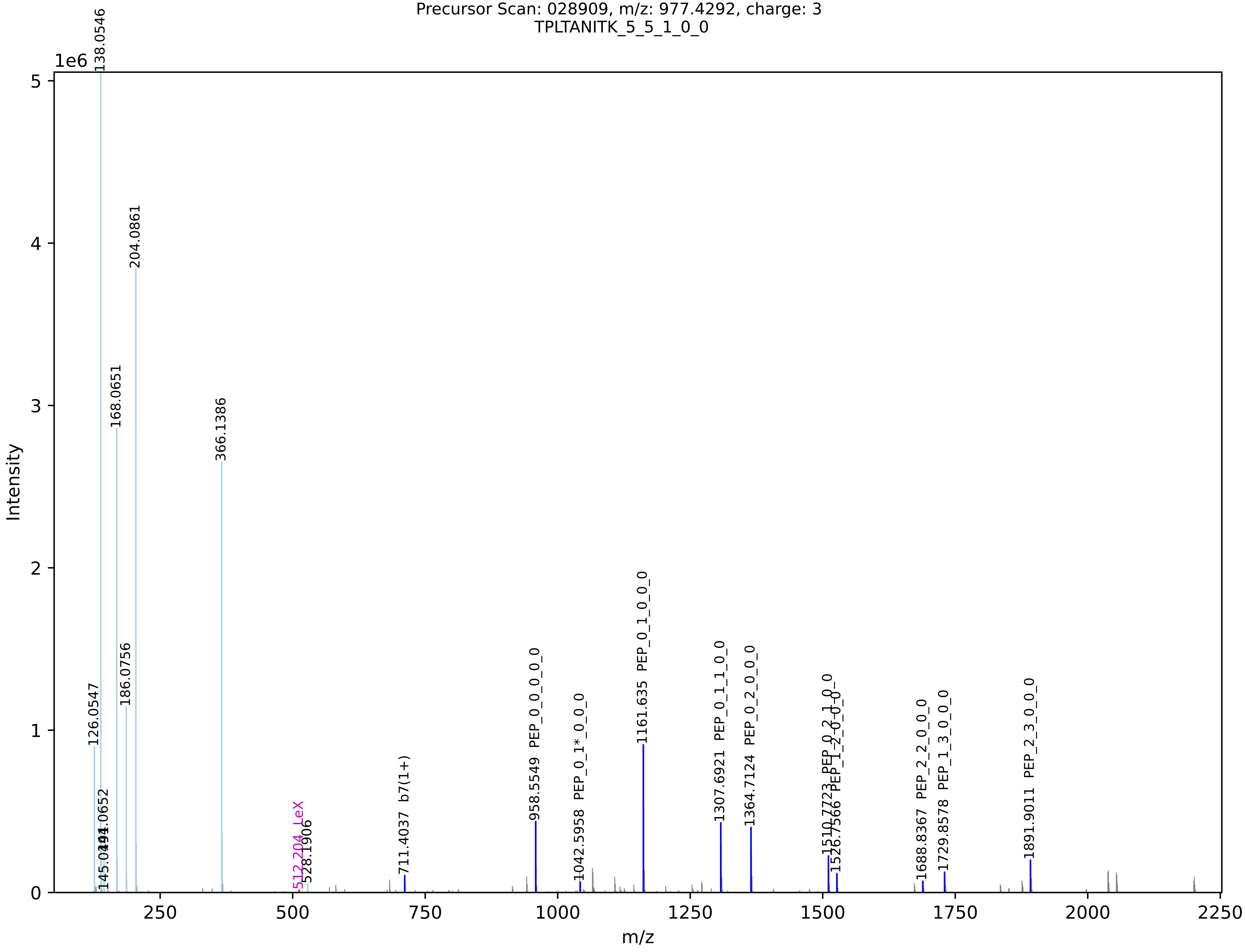

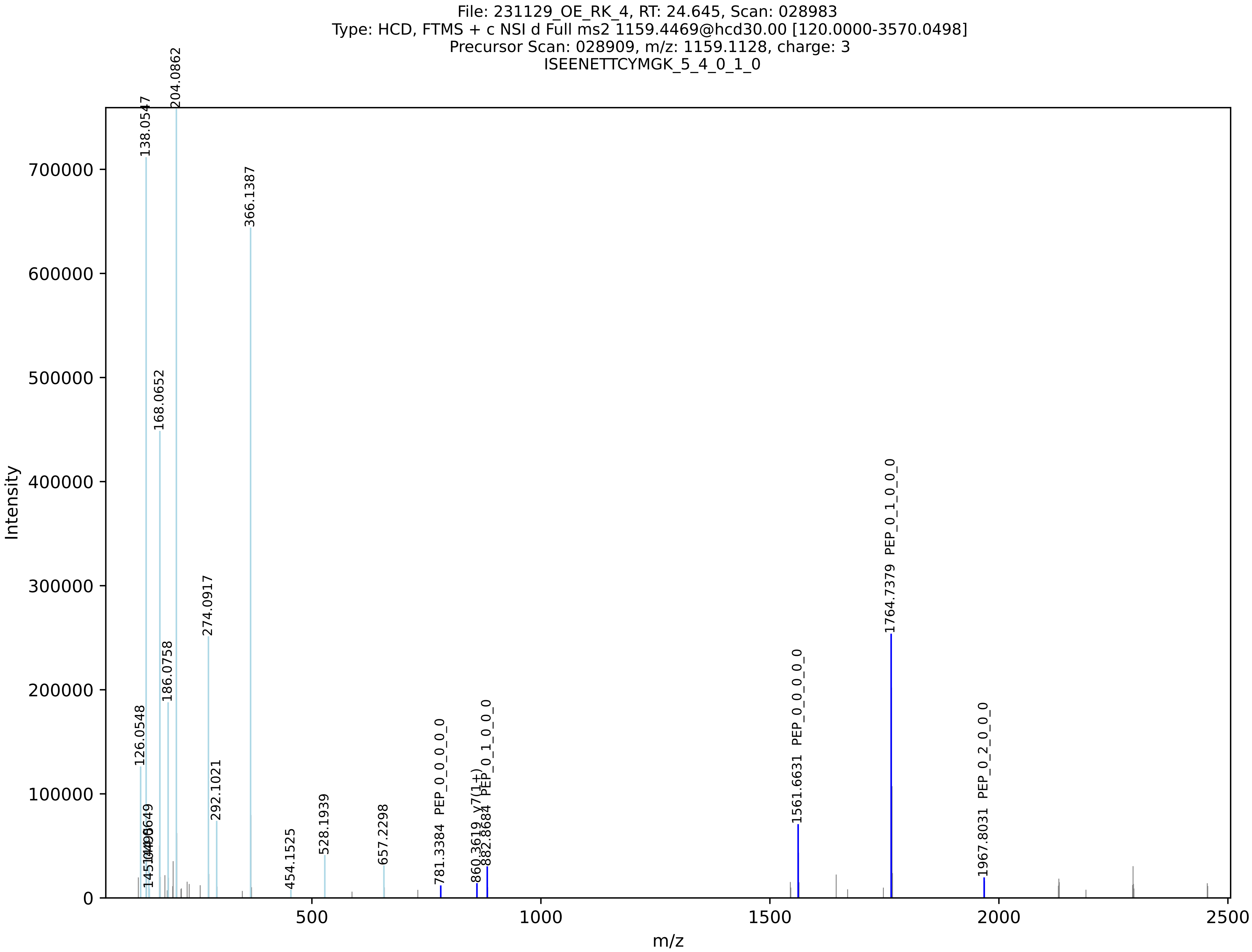
File: 231129_OE_RK_4, RT: 24.645, Scan: 028983
Type: HCD, FTMS + c NSI d Full ms2 1159.4469@hcd30.00 [120.0000-3570.0498]
Precursor Scan: 028909, m/z: 1159.1128, charge: 3
ISEENETTCYMGK_5_4_0_1_0
Intensity
m/z
204.0862
138.0547
366.1387
168.0652
274.0917
186.0758
126.0548
292.1021
454.1525
528.1939
657.2298
781.3384 PEP_0_0_0_0_0
860.3619 y7(1+)
882.8684 PEP_0_1_0_0_0
1561.6631 PEP_0_0_0_0_0
1764.7379 PEP_0_1_0_0_0
1967.8031 PEP_0_2_0_0_0

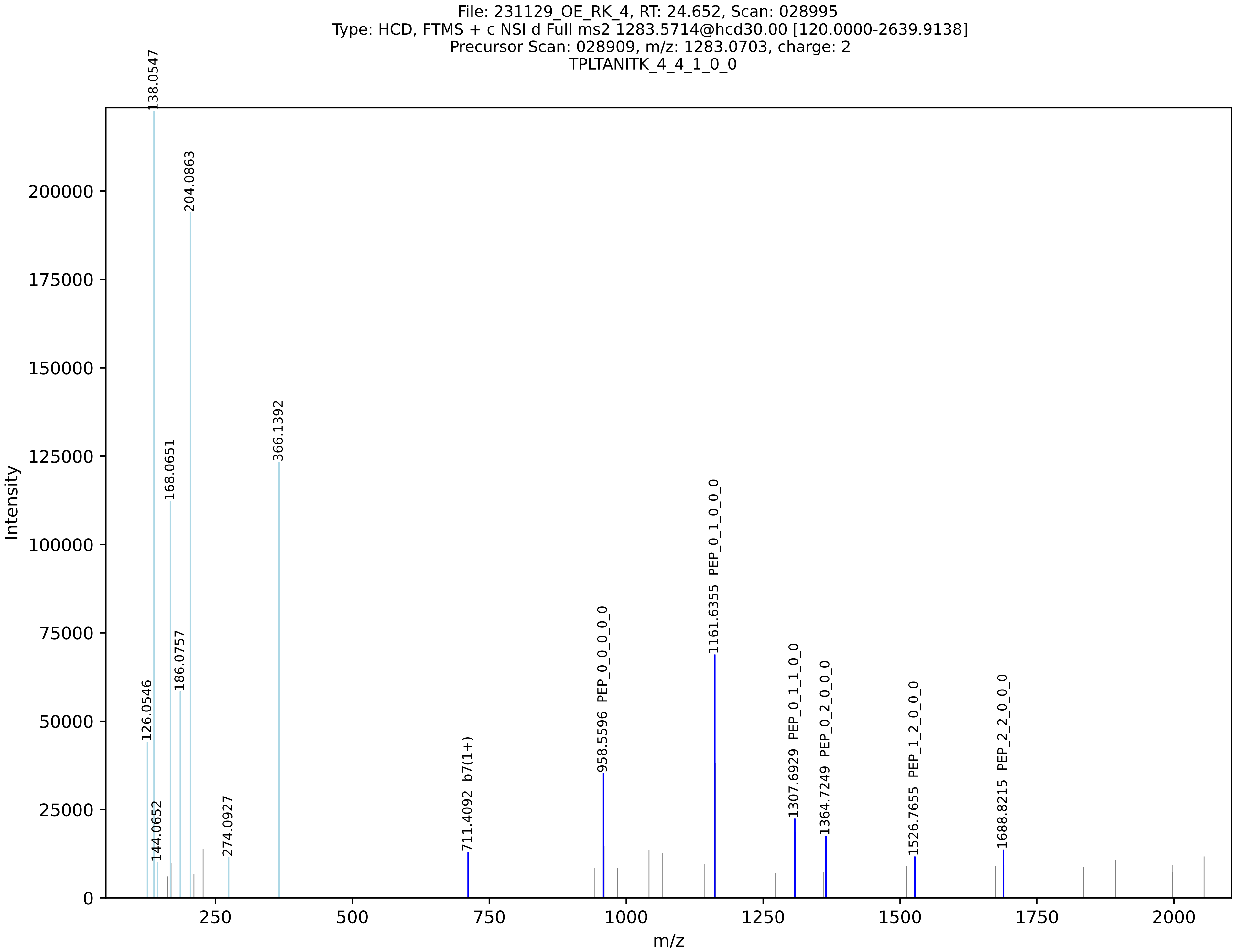

File: 231129_OE_RK_4, RT: 24.652, Scan: 028995
Type: HCD, FTMS + c NSI d Full ms2 1283.5714@hcd30.00 [120.0000-2639.9138]
Precursor Scan: 028909, m/z: 1283.0703, charge: 2
TPLTANITK_4_4_1_0_0
Intensity
m/z
138.0547
204.0863
366.1392
168.0651
186.0757
126.0546
144.0652
274.0927
711.4092 b7(1+)
958.5596 PEP_0_0_0_0_0
1161.6355 PEP_0_1_0_0_0
1307.6929 PEP_0_1_1_0_0
1364.7249 PEP_0_2_0_0_0
1526.7655 PEP_1_2_0_0_0
1688.8215 PEP_2_2_0_0_0

File: 231129_OE_RK_4, RT: 24.663, Scan: 029012
Type: HCD, FTMS + c NSI d Full ms2 976.7603@hcd30.00 [120.0000-3011.0286]
Precursor Scan: 028909, m/z: 976.7603, charge: 3
TPLTANITK_5_5_1_0_0

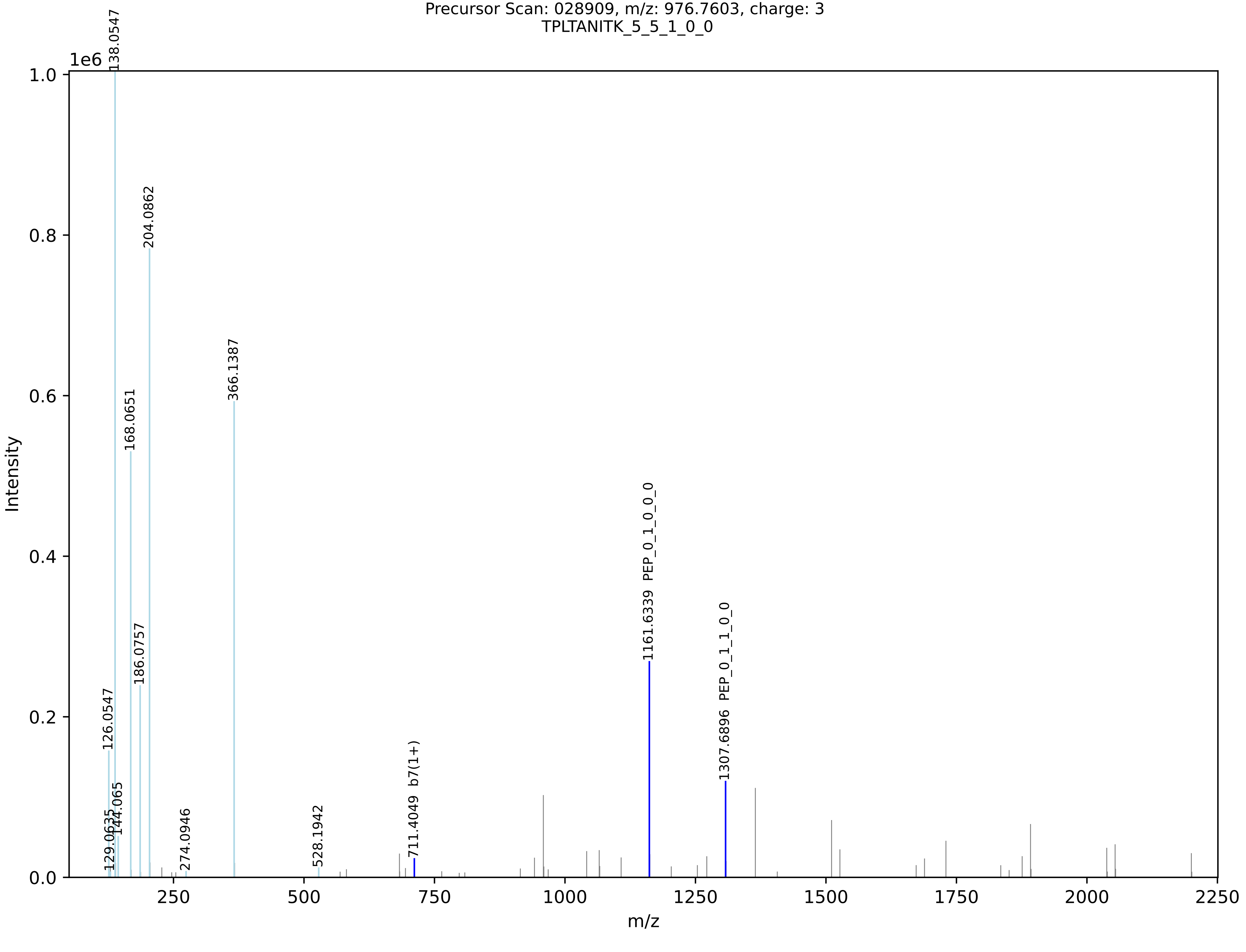

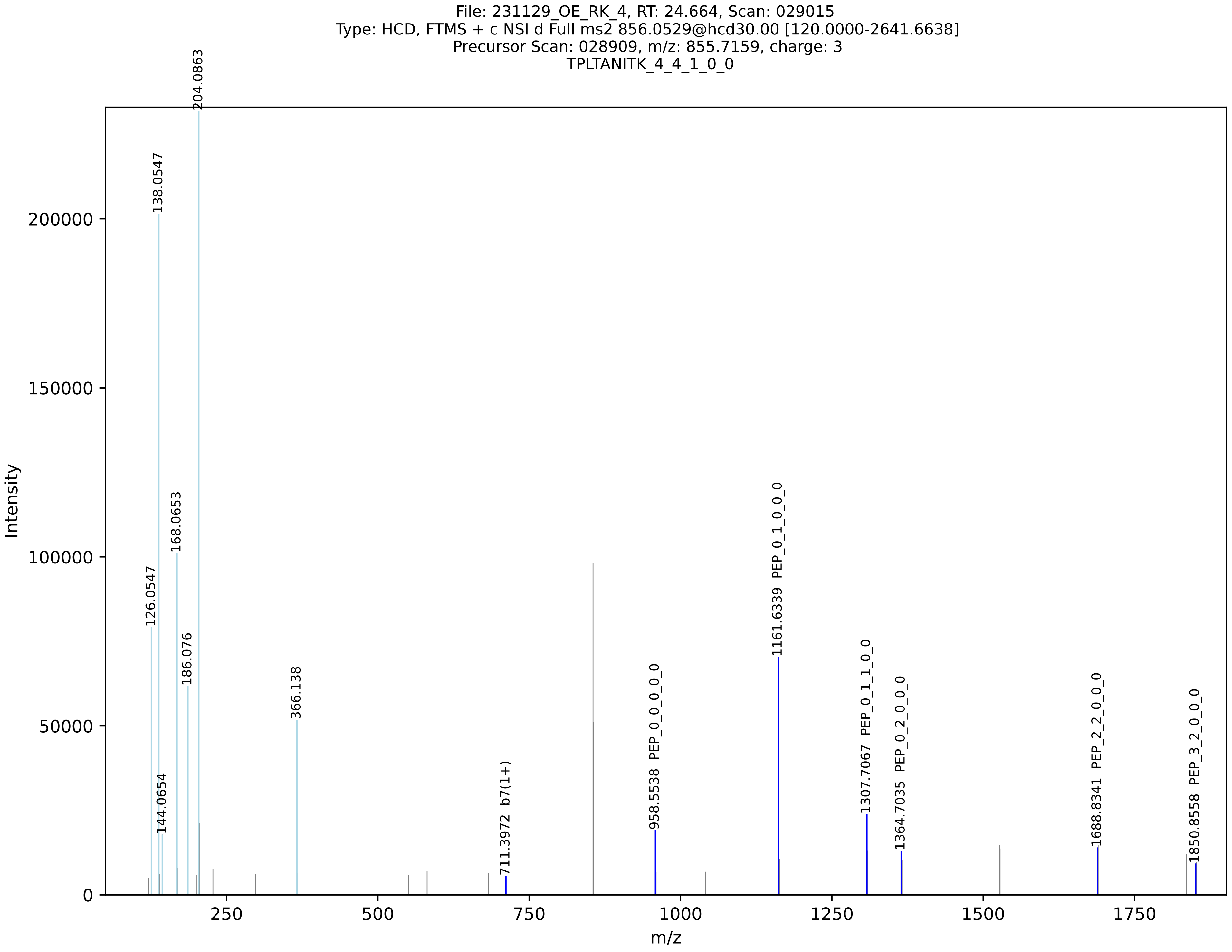
File: 231129_OE_RK_4, RT: 24.664, Scan: 029015
Type: HCD, FTMS + c NSI d Full ms2 856.0529@hcd30.00 [120.0000-2641.6638]
Precursor Scan: 028909, m/z: 855.7159, charge: 3
TPLTANITK_4_4_1_0_0
Intensity
m/z
204.0863
138.0547
168.0653
126.0547
186.076
366.138
144.0654
711.3972 b7(1+)
958.5538 PEP_0_0_0_0_0
1161.6339 PEP_0_1_0_0_0
1307.7067 PEP_0_1_1_0_0
1364.7035 PEP_0_2_0_0_0
1688.8341 PEP_2_2_0_0_0
1850.8558 PEP_3_2_0_0_0

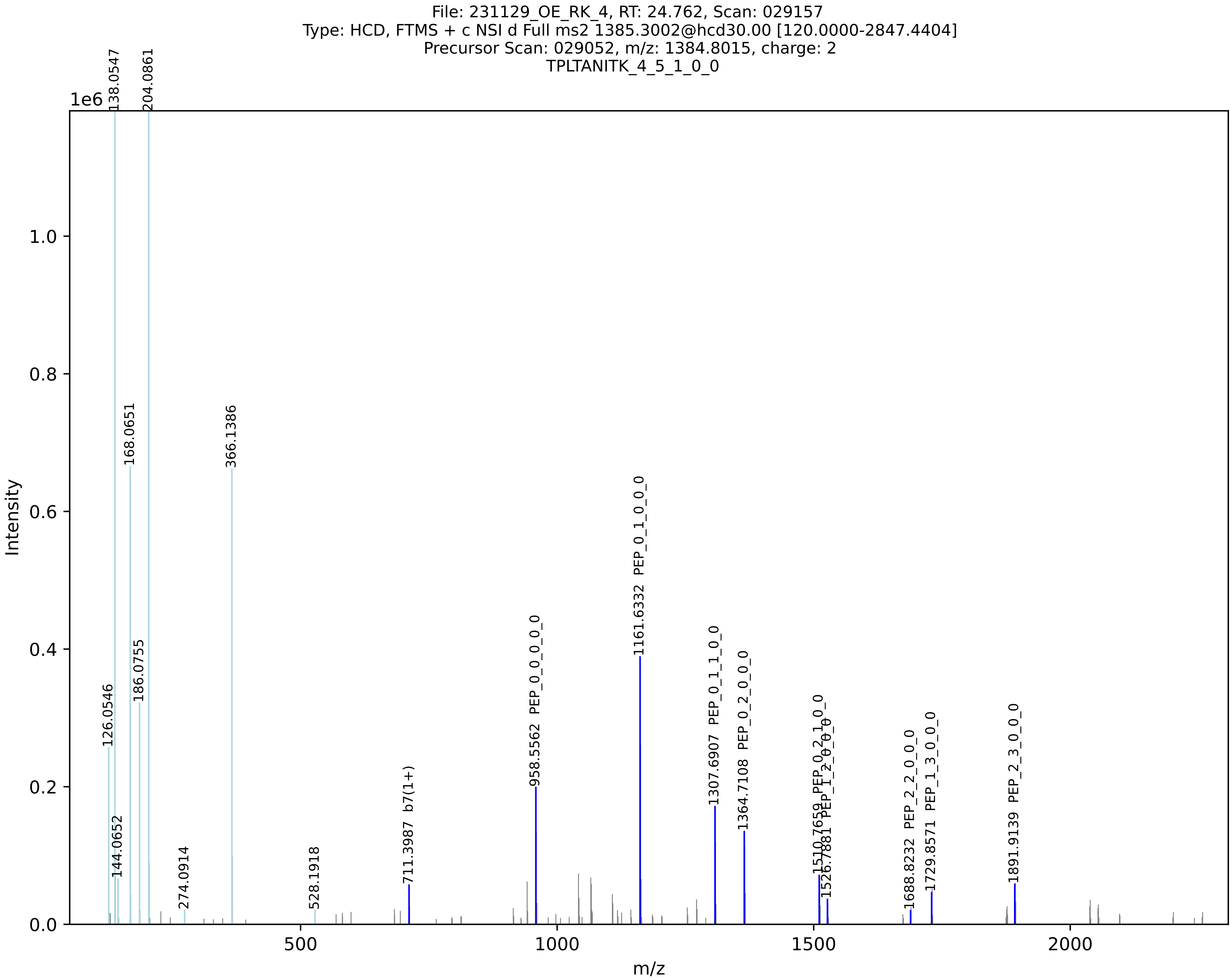

File: 231129_OE_RK_4, RT: 24.762, Scan: 029157
Type: HCD, FTMS + c NSI d Full ms2 1385.3002@hcd30.00 [120.0000-2847.4404]
Precursor Scan: 029052, m/z: 1384.8015, charge: 2
TPLTANITK_4_5_1_0_0
1e6
Intensity
m/z
138.0547
204.0861
168.0651
366.1386
186.0755
126.0546
144.0652
274.0914
528.1918
711.3987 b7(1+)
958.5562 PEP_0_0_0_0_0
1161.6332 PEP_0_1_0_0_0
1307.6907 PEP_0_1_1_0_0
1364.7108 PEP_0_2_0_0_0
1510.7659 PEP_0_2_1_0_0
1526.7881 PEP_1_2_0_0_0
1688.8232 PEP_2_2_0_0_0
1729.8571 PEP_1_3_0_0_0
1891.9139 PEP_2_3_0_0_0

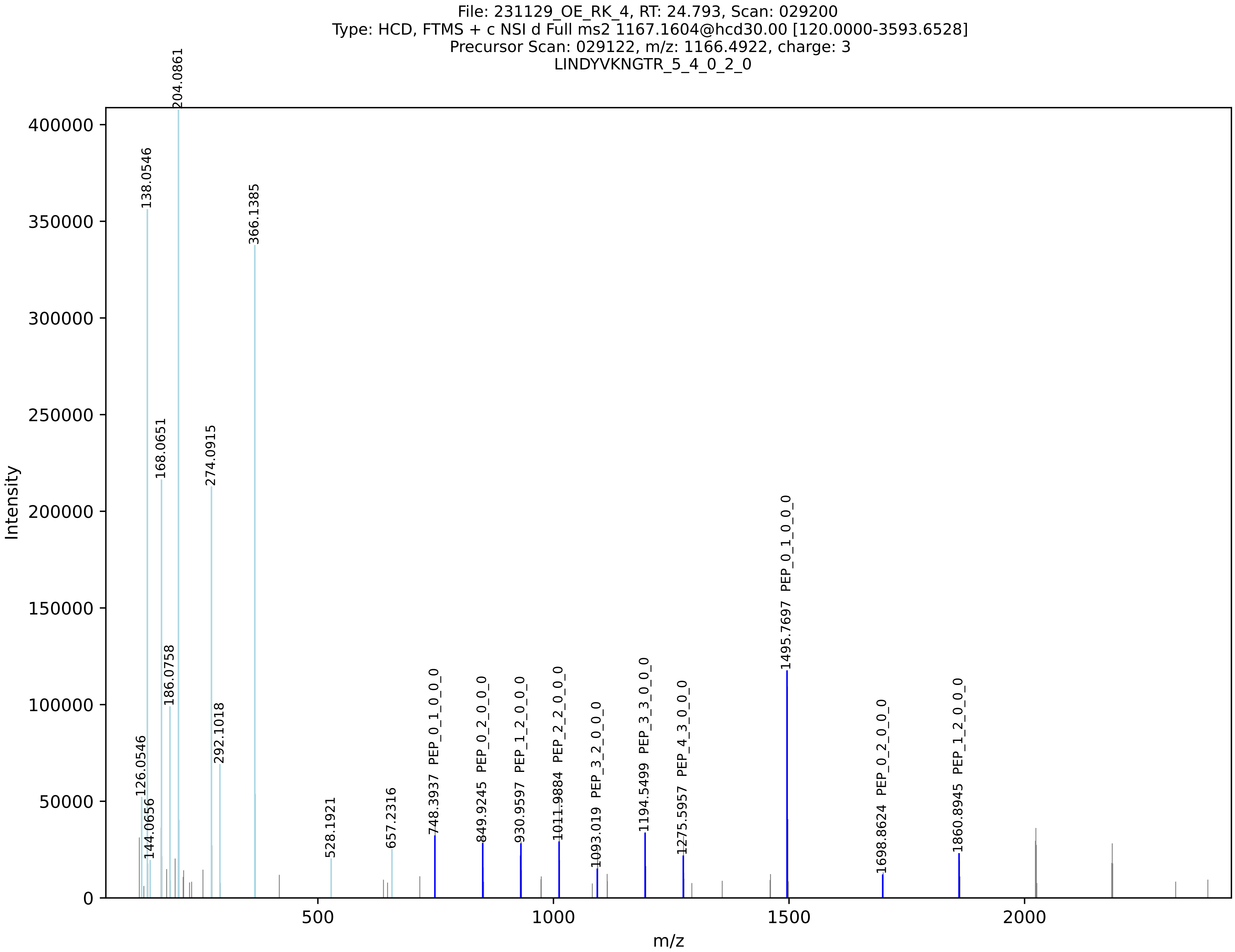
File: 231129_OE_RK_4, RT: 24.793, Scan: 029200
Type: HCD, FTMS + c NSI d Full ms2 1167.1604@hcd30.00 [120.0000-3593.6528]
Precursor Scan: 029122, m/z: 1166.4922, charge: 3
LINDYVKNGTR_5_4_0_2_0
204.0861
138.0546
366.1385
168.0651
274.0915
186.0758
292.1018
126.0546
144.0656
528.1921
657.2316
748.3937 PEP_0_1_0_0_0
849.9245 PEP_0_2_0_0_0
930.9597 PEP_1_2_0_0_0
1011.9884 PEP_2_2_0_0_0
1093.019 PEP_3_2_0_0_0
1194.5499 PEP_3_3_0_0_0
1275.5957 PEP_4_3_0_0_0
1495.7697 PEP_0_1_0_0_0
1698.8624 PEP_0_2_0_0_0
1860.8945 PEP_1_2_0_0_0
Intensity
m/z
0
50000
100000
150000
200000
250000
300000
350000
400000
500
1000
1500
2000

File: 231129_OE_RK_4, RT: 24.879, Scan: 029318
Type: HCD, FTMS + c NSI d Full ms2 1385.1136@hcd30.00 [120.0000-2847.0601]
Precursor Scan: 029284, m/z: 1384.6128, charge: 2
TPLTANITK_4_5_1_0_0

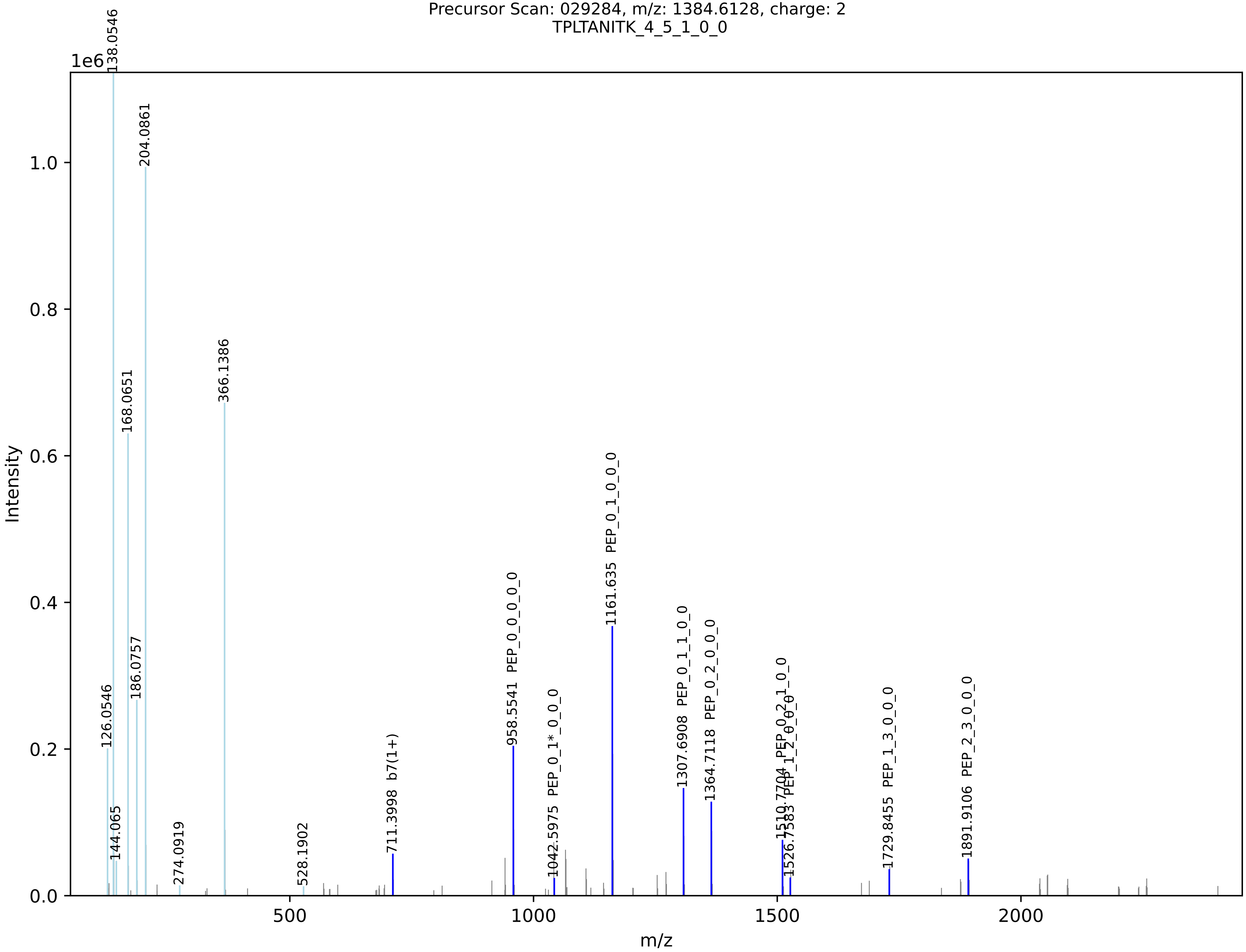

File: 231129_OE_RK_4, RT: 24.888, Scan: 029332
Type: HCD, FTMS + c NSI d Full ms2 1210.8051@hcd30.00 [120.0000-3727.2056]
Precursor Scan: 029284, m/z: 1210.4709, charge: 3
MDGASNVTCINSR_5_4_0_2_0

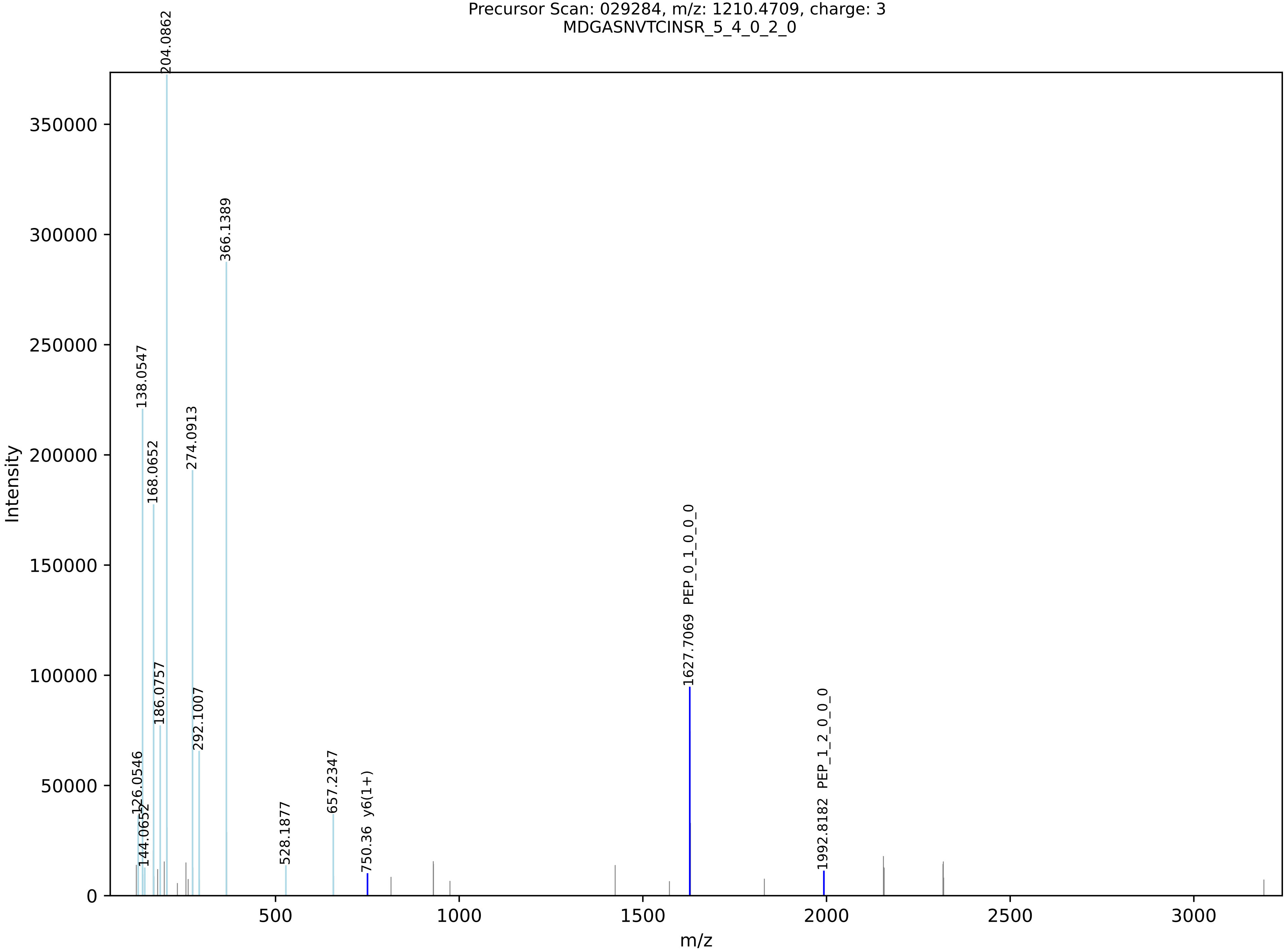

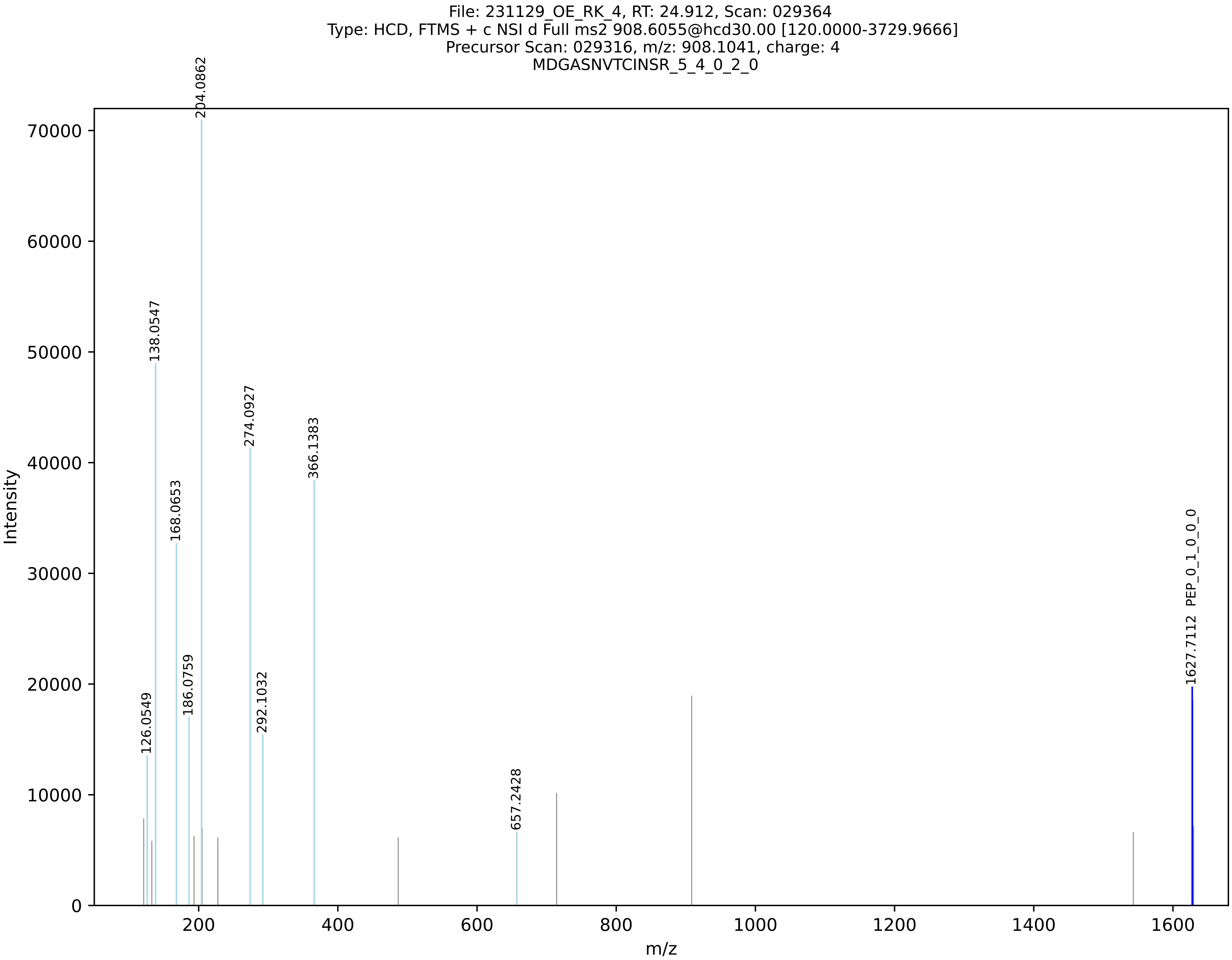
File: 231129_OE_RK_4, RT: 24.912, Scan: 029364
Type: HCD, FTMS + c NSI d Full ms2 908.6055@hcd30.00 [120.0000-3729.9666]
Precursor Scan: 029316, m/z: 908.1041, charge: 4
MDGASNVTCINSR_5_4_0_2_0
Intensity
m/z
204.0862
138.0547
274.0927
366.1383
168.0653
186.0759
292.1032
126.0549
657.2428
1627.7112 PEP_0_1_0_0_0

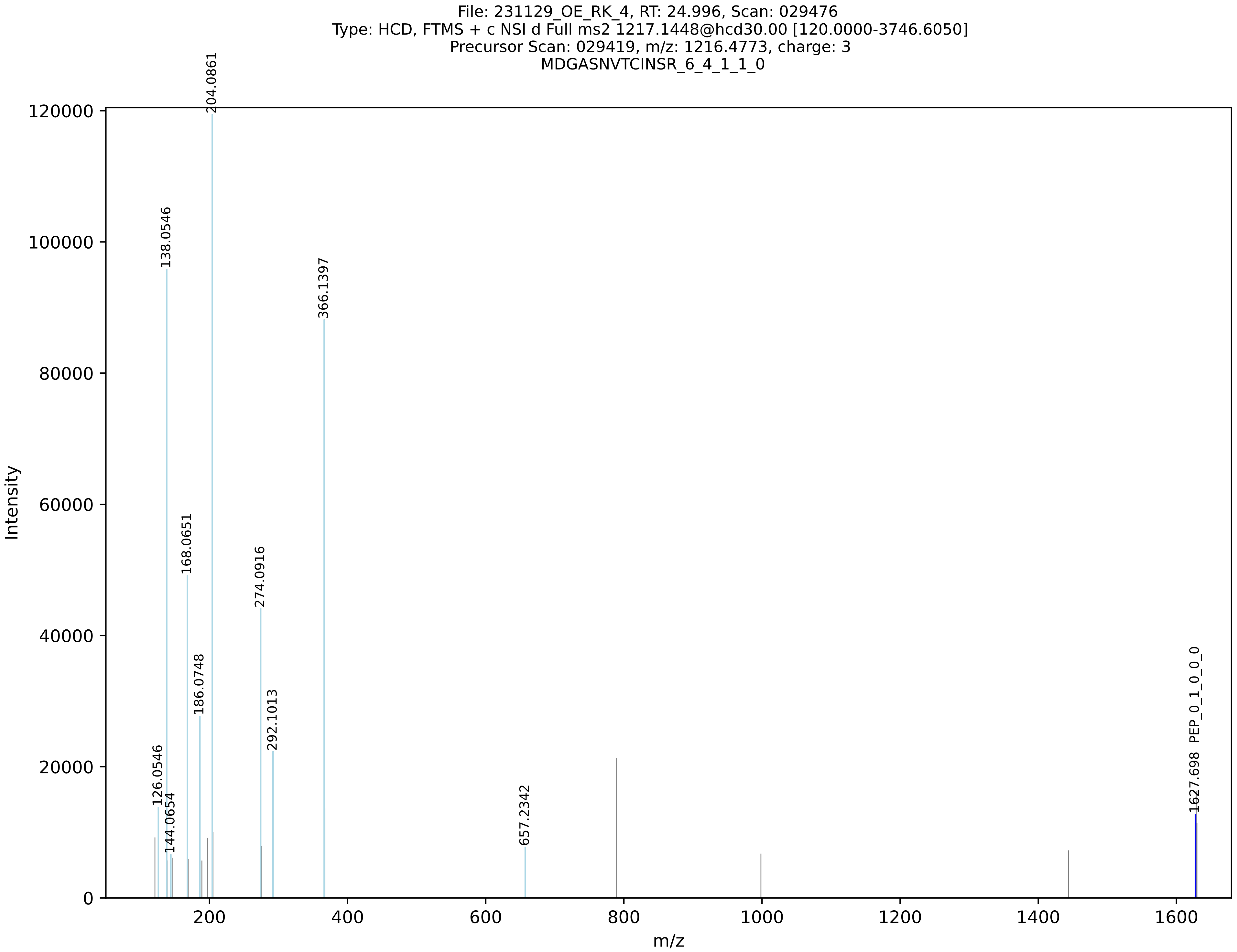

File: 231129_OE_RK_4, RT: 24.996, Scan: 029476
Type: HCD, FTMS + c NSI d Full ms2 1217.1448@hcd30.00 [120.0000-3746.6050]
Precursor Scan: 029419, m/z: 1216.4773, charge: 3
MDGASNVTCINSR_6_4_1_1_0
Intensity
m/z
204.0861
138.0546
366.1397
168.0651
274.0916
186.0748
292.1013
126.0546
144.0654
657.2342
1627.698 PEP_0_1_0_0_0
0
20000
40000
60000
80000
100000
120000
200
400
600
800
1000
1200
1400
1600

File: 231129_OE_RK_4, RT: 25.066, Scan: 029575
Type: HCD, FTMS + c NSI d Full ms2 1210.8033@hcd30.00 [120.0000-3727.2004]
Precursor Scan: 029515, m/z: 1210.4693, charge: 3
MDGASNVTCINSR_5_4_0_2_0

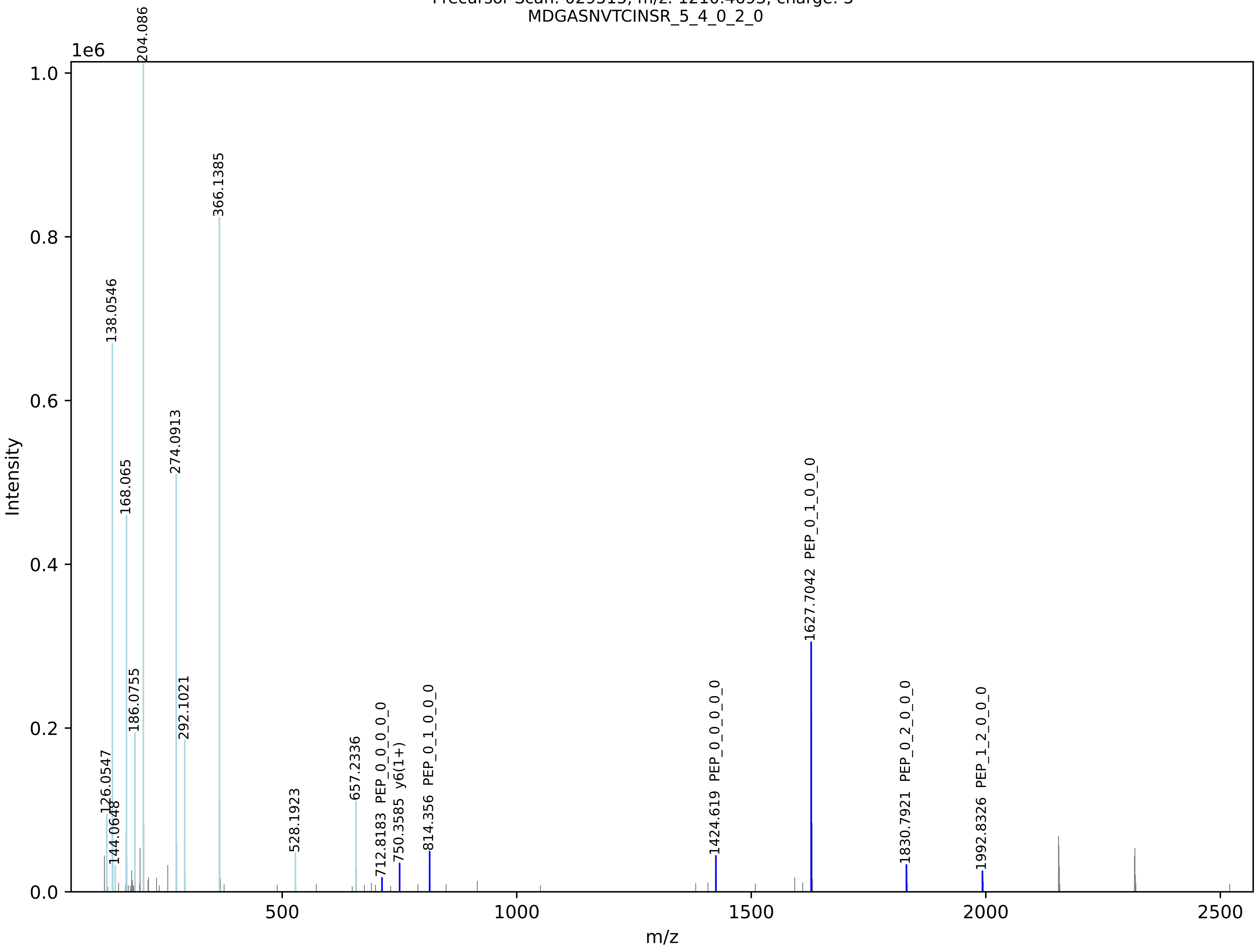

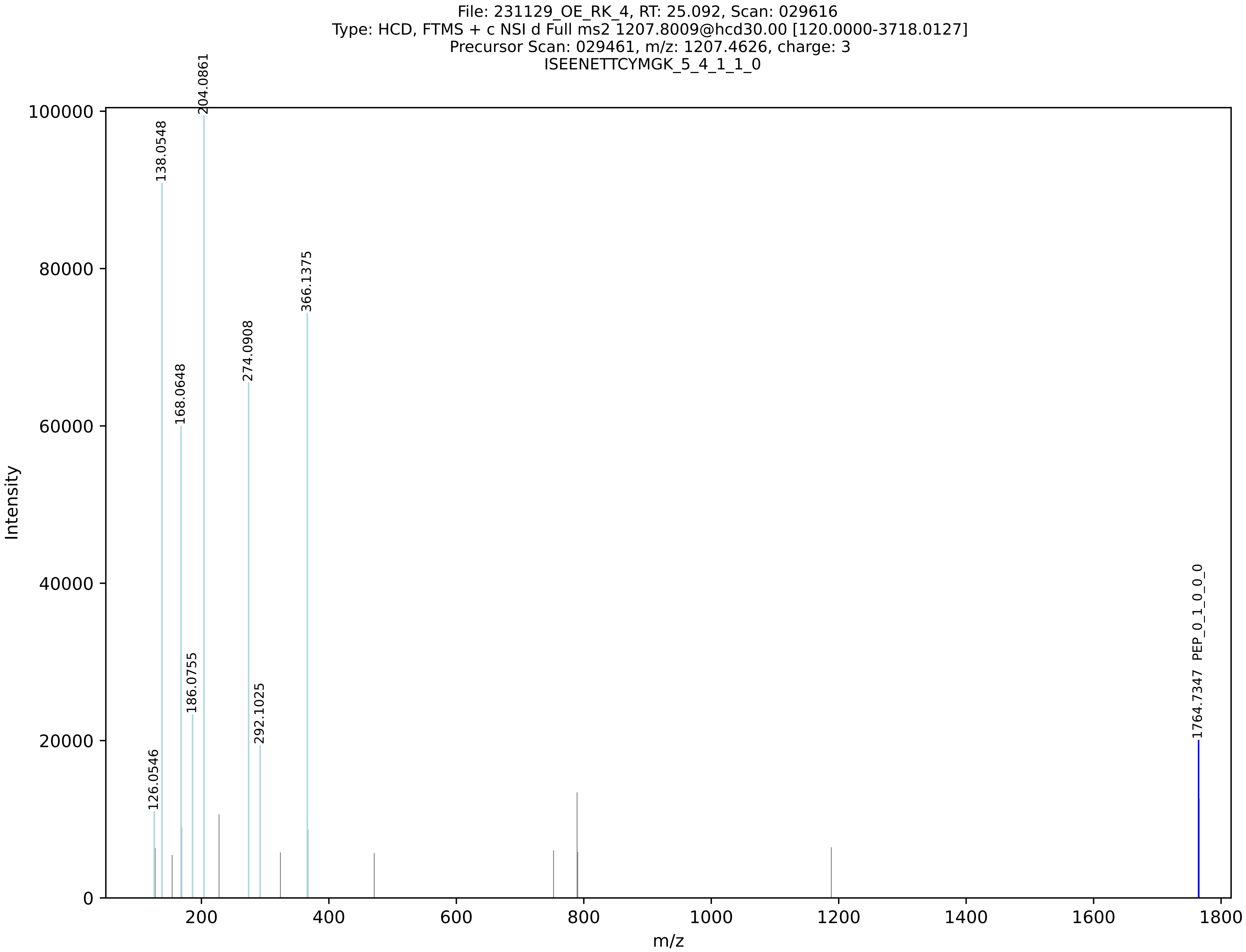
File: 231129_OE_RK_4, RT: 25.092, Scan: 029616
Type: HCD, FTMS + c NSI d Full ms2 1207.8009@hcd30.00 [120.0000-3718.0127]
Precursor Scan: 029461, m/z: 1207.4626, charge: 3
ISEENETTCYMGK_5_4_1_1_0
Intensity
m/z
204.0861
138.0548
168.0648
366.1375
274.0908
186.0755
292.1025
126.0546
1764.7347 PEP_0_1_0_0_0
0
20000
40000
60000
80000
100000
200
400
600
800
1000
1200
1400
1600
1800

File: 231129_OE_RK_4, RT: 25.157, Scan: 029707
Type: HCD, FTMS + c NSI d Full ms2 1174.4988@hcd30.00 [120.0000-3616.1084]
Precursor Scan: 029638, m/z: 1174.1649, charge: 3
ESGTTAWQLVNSSVK_5_4_0_1_0

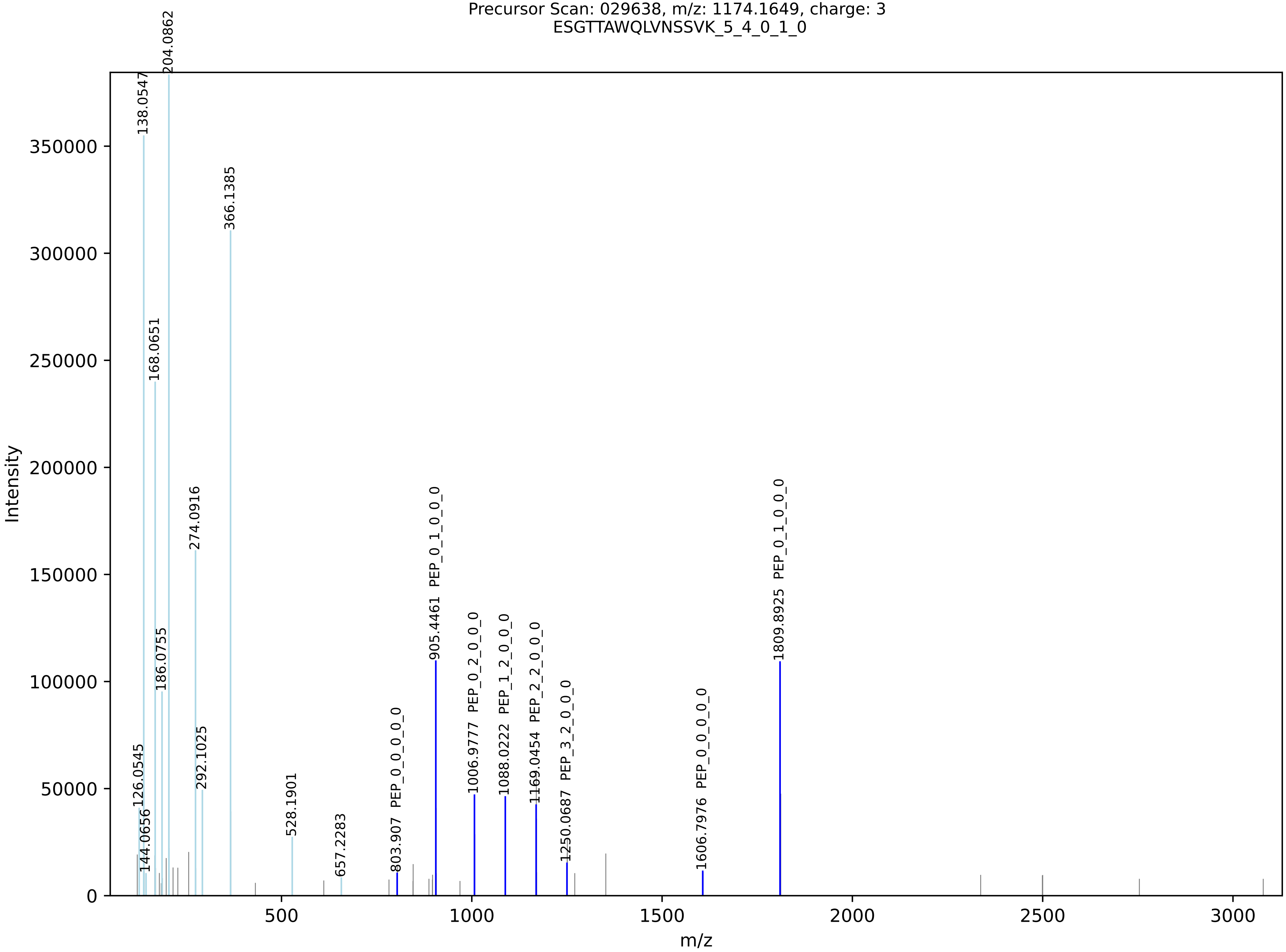

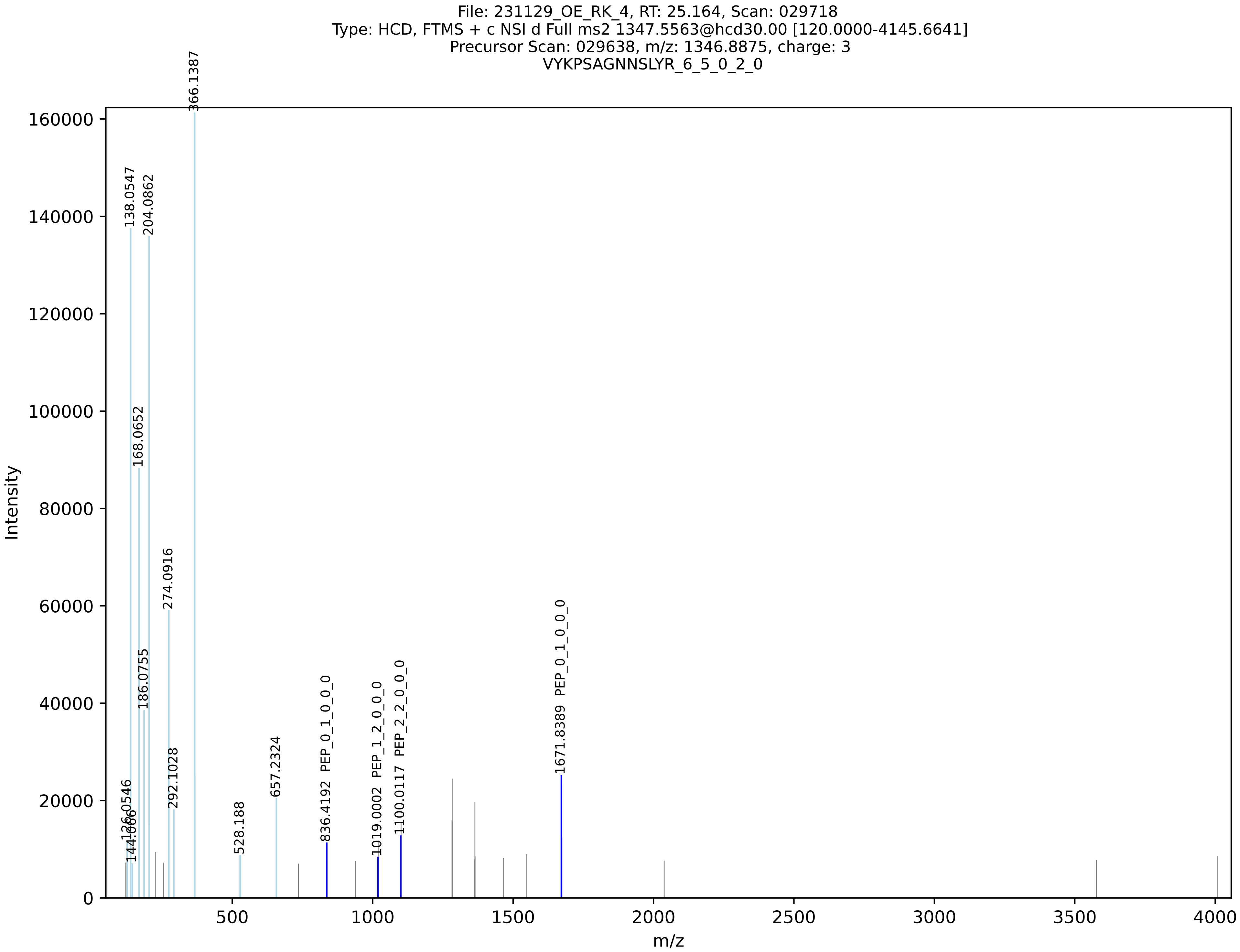

File: 231129_OE_RK_4, RT: 25.164, Scan: 029718
Type: HCD, FTMS + c NSI d Full ms2 1347.5563@hcd30.00 [120.0000-4145.6641]
Precursor Scan: 029638, m/z: 1346.8875, charge: 3
VYKPSAGNNSLYR_6_5_0_2_0
366.1387
138.0547
204.0862
168.0652
274.0916
186.0755
292.1028
126.0546
144.066
528.188
657.2324
836.4192 PEP_0_1_0_0_0
1019.0002 PEP_1_2_0_0_0
1100.0117 PEP_2_2_0_0_0
1671.8389 PEP_0_1_0_0_0
Intensity
m/z
0
20000
40000
60000
80000
100000
120000
140000
160000
500
1000
1500
2000
2500
3000
3500
4000

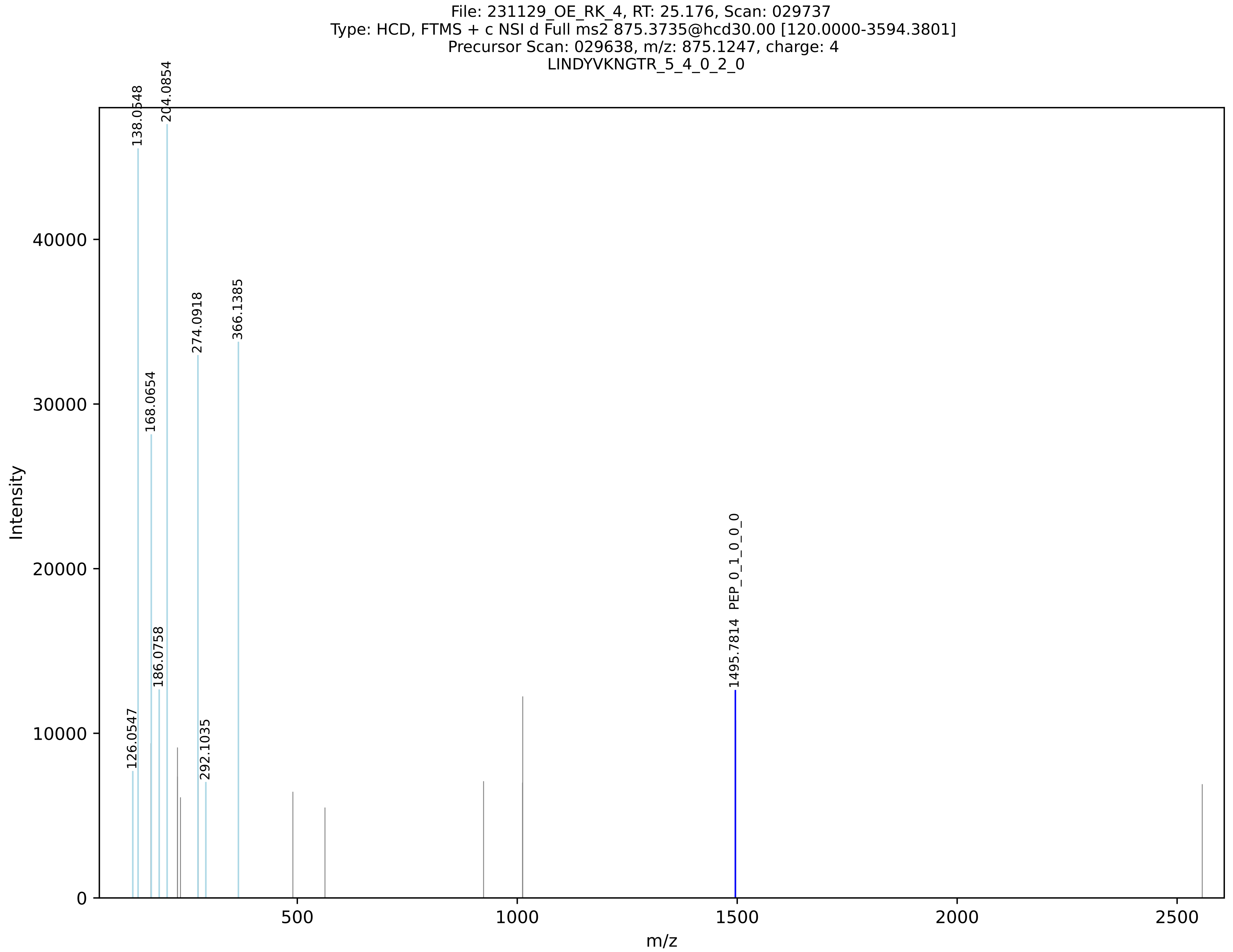
File: 231129_OE_RK_4, RT: 25.176, Scan: 029737
Type: HCD, FTMS + c NSI d Full ms2 875.3735@hcd30.00 [120.0000-3594.3801]
Precursor Scan: 029638, m/z: 875.1247, charge: 4
LINDYVKNGTR_5_4_0_2_0
Intensity
m/z
0
10000
20000
30000
40000
500
1000
1500
2000
2500
126.0547
138.0548
168.0654
186.0758
204.0854
274.0918
292.1035
366.1385
1495.7814 PEP_0_1_0_0_0

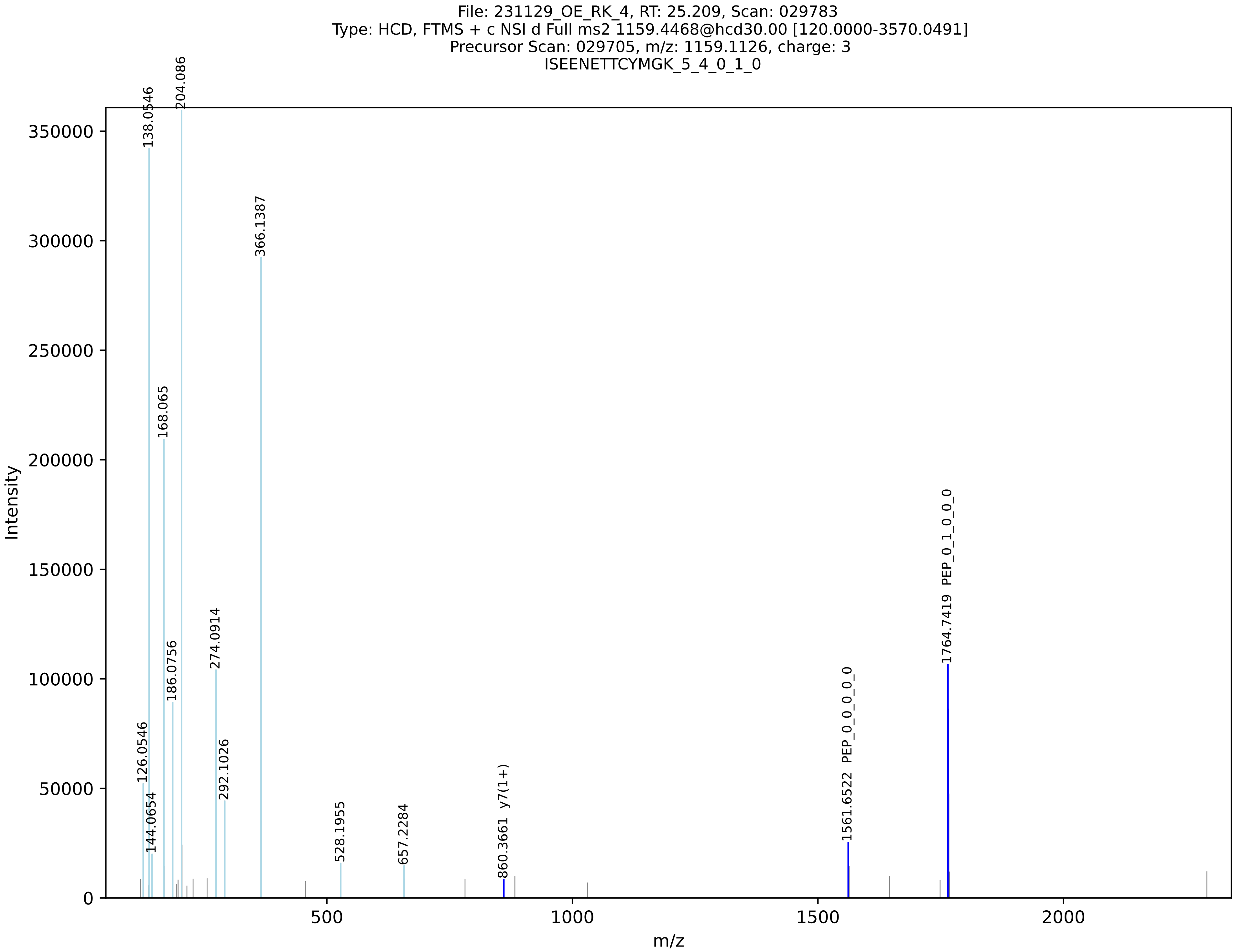
File: 231129_OE_RK_4, RT: 25.209, Scan: 029783
Type: HCD, FTMS + c NSI d Full ms2 1159.4468@hcd30.00 [120.0000-3570.0491]
Precursor Scan: 029705, m/z: 1159.1126, charge: 3
ISEENETTCYMGK_5_4_0_1_0
Intensity
m/z
204.086
138.0546
366.1387
168.065
274.0914
186.0756
126.0546
292.1026
144.0654
528.1955
657.2284
860.3661 y7(1+)
1561.6522 PEP_0_0_0_0_0
1764.7419 PEP_0_1_0_0_0
0
50000
100000
150000
200000
250000
300000
350000
500
1000
1500
2000

File: 231129_OE_RK_4, RT: 25.241, Scan: 029834
Type: HCD, FTMS + c NSI d Full ms2 1030.0762@hcd30.00 [120.0000-3174.1750]
Precursor Scan: 029705, m/z: 1030.0762, charge: 3
EEQFNSTFR_6_4_1_0_0

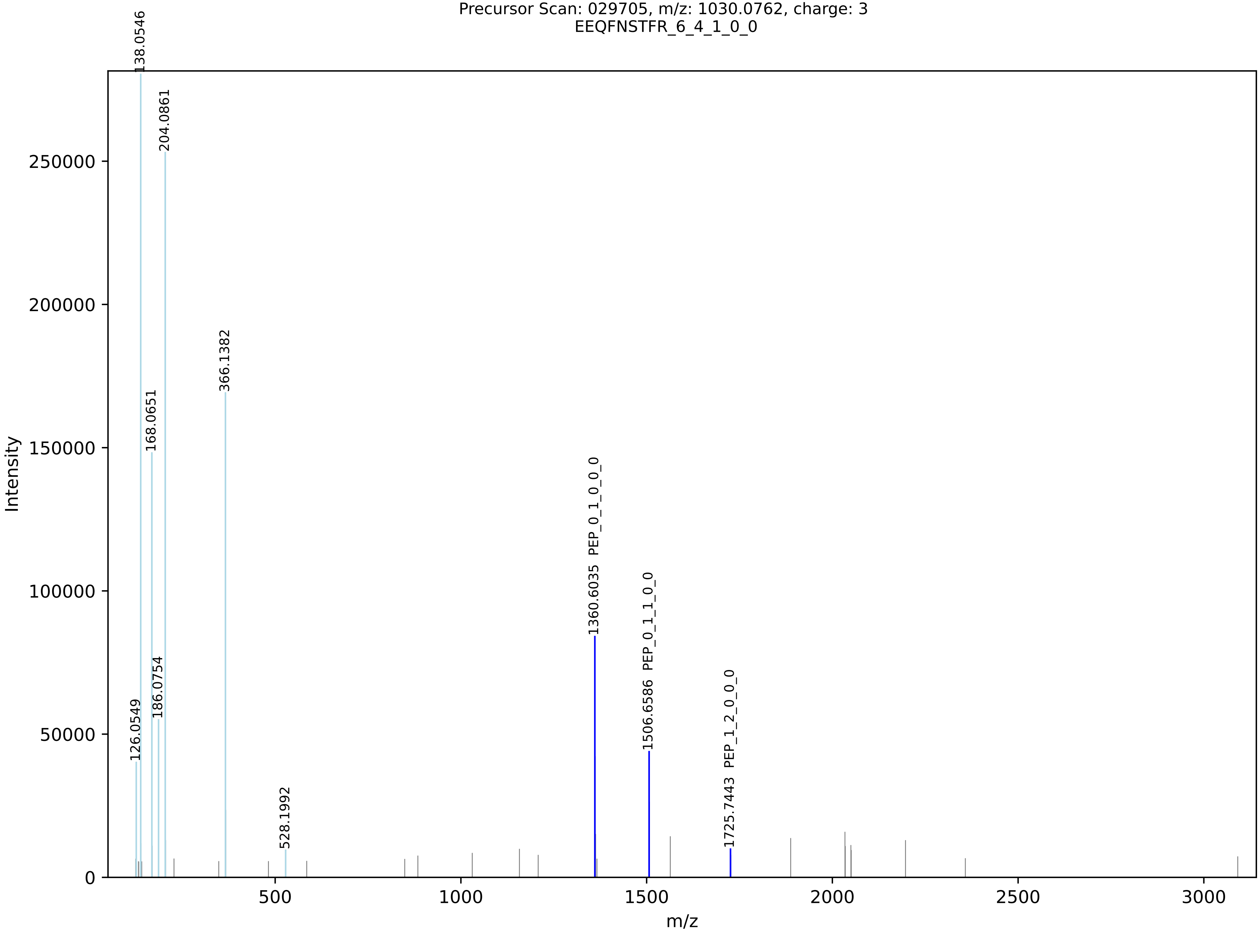

File: 231129_OE_RK_4, RT: 25.258, Scan: 029852
Type: HCD, FTMS + c NSI d Full ms2 1211.1382@hcd30.00 [120.0000-3728.2246]
Precursor Scan: 029777, m/z: 1210.4698, charge: 3
MDGASNVTCINSR_5_4_0_2_0

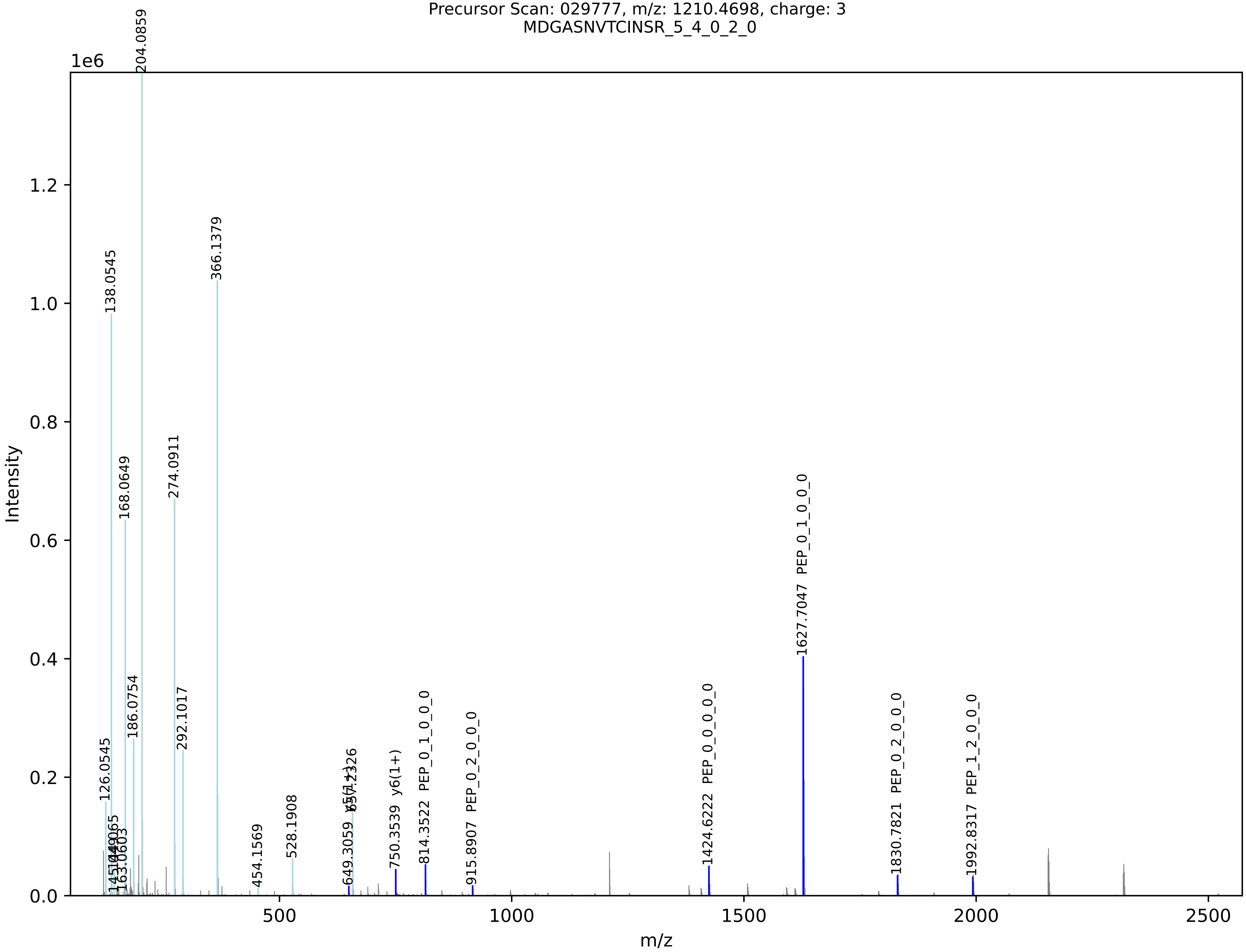

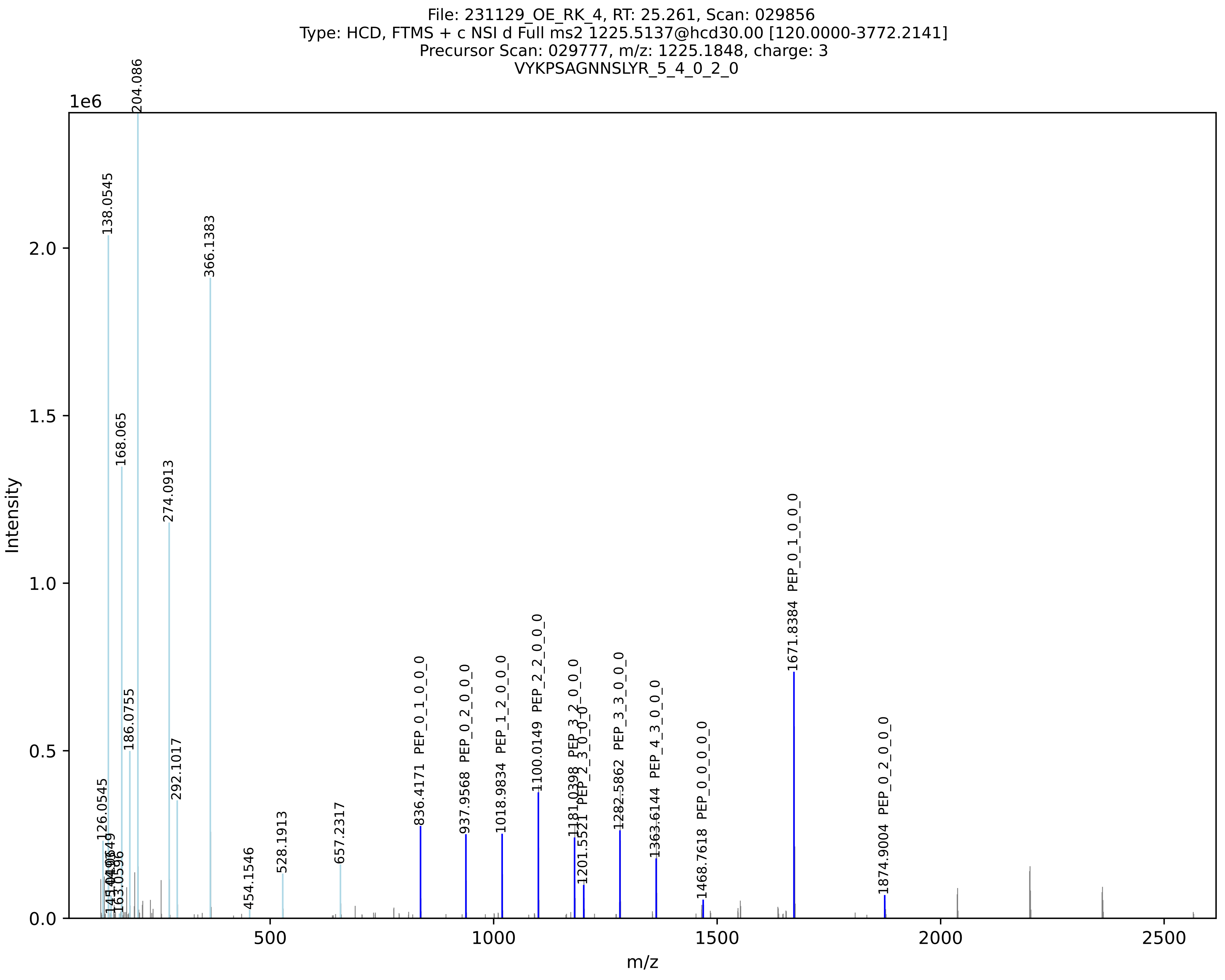

File: 231129_OE_RK_4, RT: 25.261, Scan: 029856
Type: HCD, FTMS + c NSI d Full ms2 1225.5137@hcd30.00 [120.0000-3772.2141]
Precursor Scan: 029777, m/z: 1225.1848, charge: 3
VYKPSAGNNSLYR_5_4_0_2_0
1e6
Intensity
m/z
204.086
138.0545
366.1383
168.065
274.0913
186.0755
292.1017
126.0545
454.1546
528.1913
657.2317
836.4171 PEP_0_1_0_0_0
937.9568 PEP_0_2_0_0_0
1018.9834 PEP_1_2_0_0_0
1100.0149 PEP_2_2_0_0_0
1181.0398 PEP_3_2_0_0_0
1201.5521 PEP_2_3_0_0_0
1282.5862 PEP_3_3_0_0_0
1363.6144 PEP_4_3_0_0_0
1468.7618 PEP_0_0_0_0_0
1671.8384 PEP_0_1_0_0_0
1874.9004 PEP_0_2_0_0_0

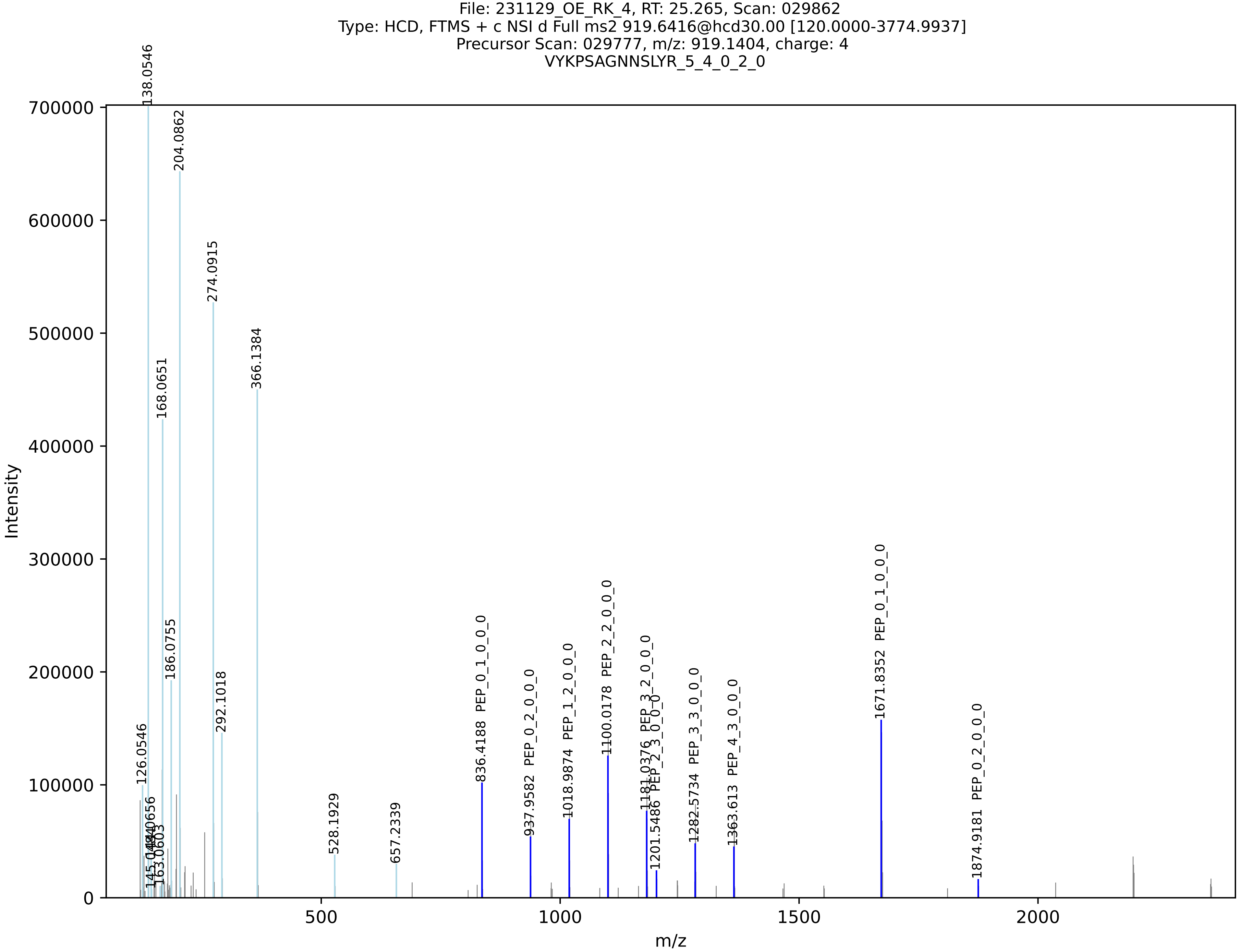
File: 231129_OE_RK_4, RT: 25.265, Scan: 029862
Type: HCD, FTMS + c NSI d Full ms2 919.6416@hcd30.00 [120.0000-3774.9937]
Precursor Scan: 029777, m/z: 919.1404, charge: 4
VYKPSAGNNSLYR_5_4_0_2_0
Intensity
m/z
138.0546
204.0862
274.0915
366.1384
168.0651
186.0755
292.1018
126.0546
163.0603
528.1929
657.2339
836.4188 PEP_0_1_0_0_0
937.9582 PEP_0_2_0_0_0
1018.9874 PEP_1_2_0_0_0
1100.0178 PEP_2_2_0_0_0
1181.0376 PEP_3_2_0_0_0
1201.5486 PEP_2_3_0_0_0
1282.5734 PEP_3_3_0_0_0
1363.613 PEP_4_3_0_0_0
1671.8352 PEP_0_1_0_0_0
1874.9181 PEP_0_2_0_0_0

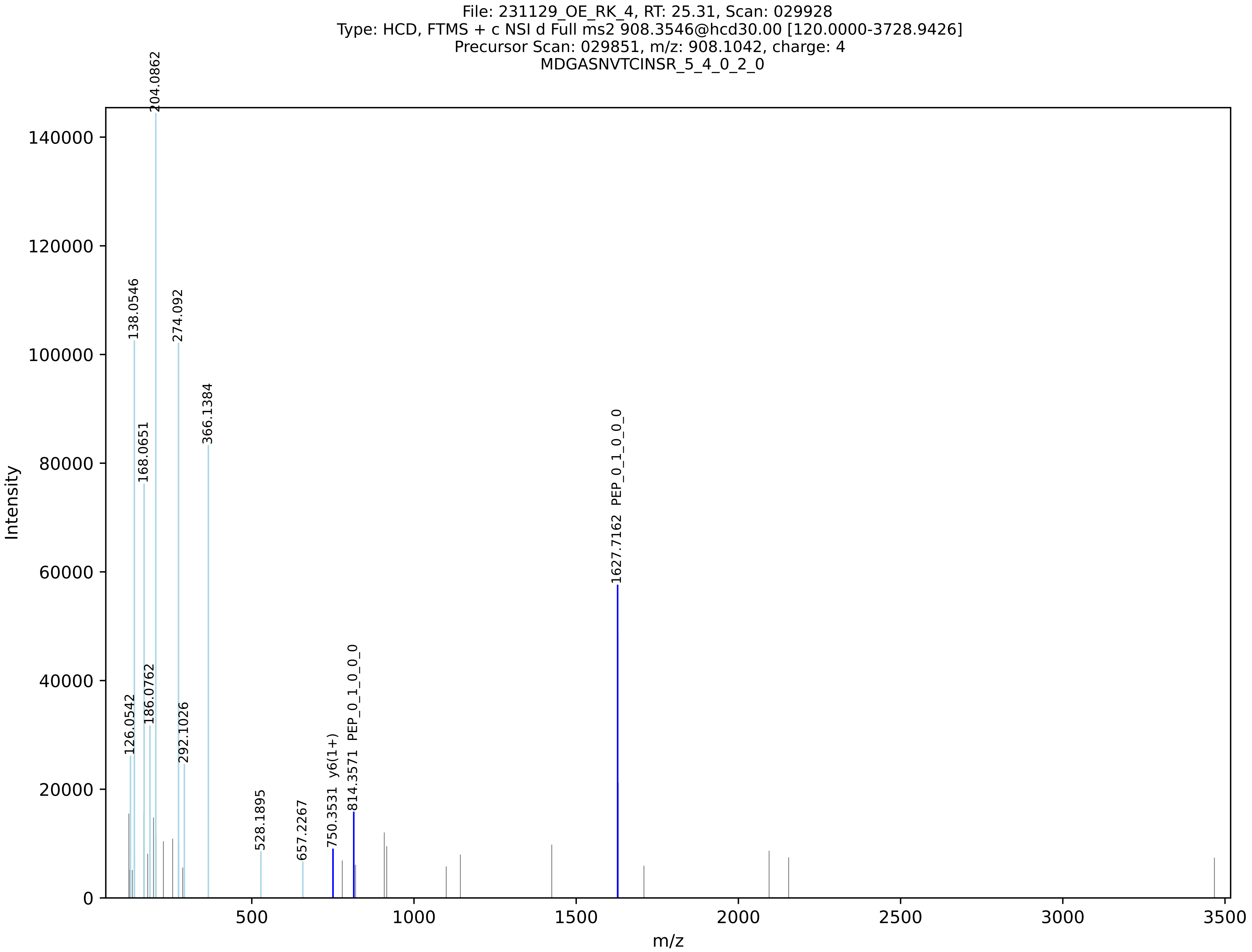
File: 231129_OE_RK_4, RT: 25.31, Scan: 029928
Type: HCD, FTMS + c NSI d Full ms2 908.3546@hcd30.00 [120.0000-3728.9426]
Precursor Scan: 029851, m/z: 908.1042, charge: 4
MDGASNVTCINSR_5_4_0_2_0
Intensity
m/z
204.0862
138.0546
274.092
366.1384
168.0651
126.0542
186.0762
292.1026
528.1895
657.2267
750.3531 y6(1+)
814.3571 PEP_0_1_0_0_0
1627.7162 PEP_0_1_0_0_0_

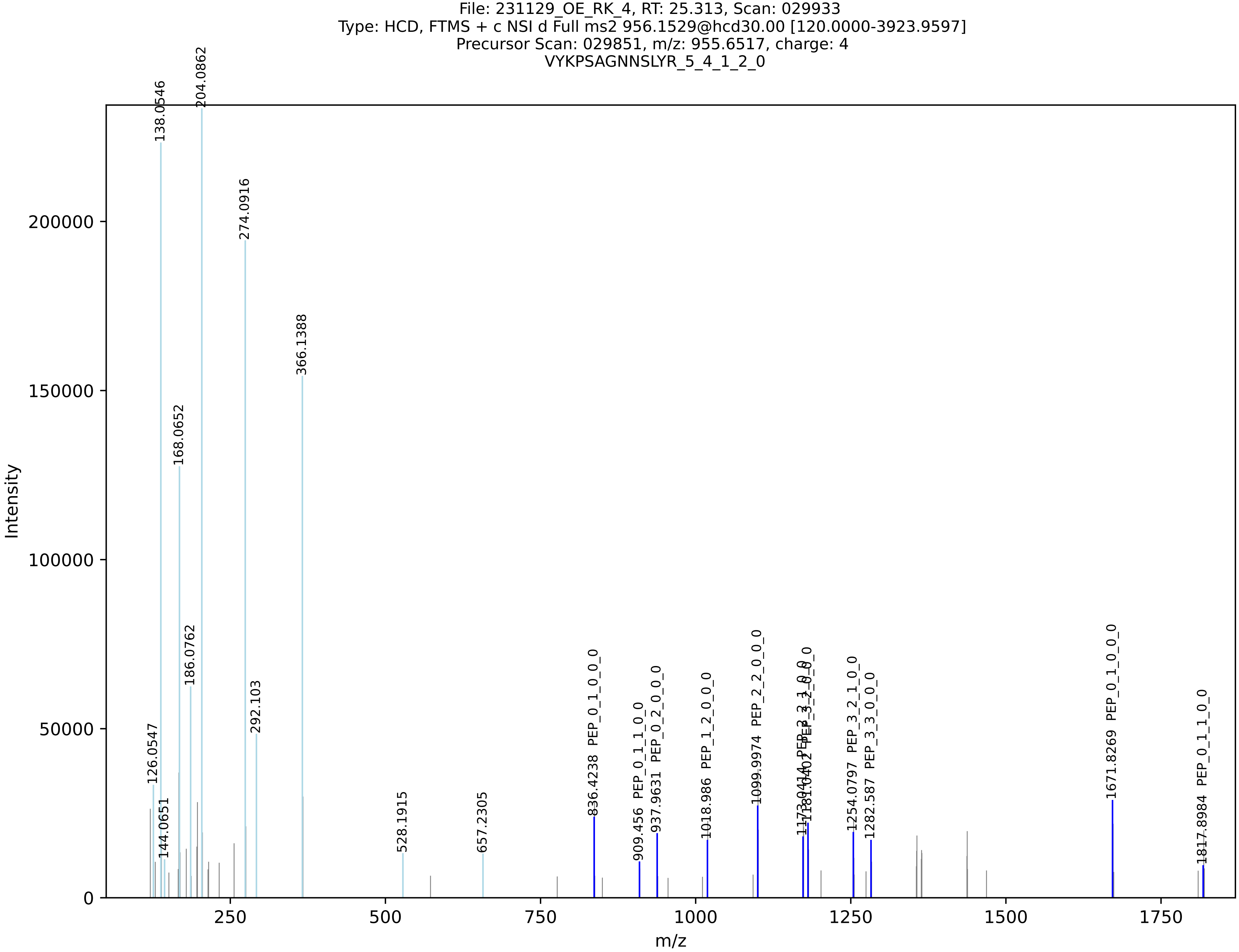

File: 231129_OE_RK_4, RT: 25.313, Scan: 029933
Type: HCD, FTMS + c NSI d Full ms2 956.1529@hcd30.00 [120.0000-3923.9597]
Precursor Scan: 029851, m/z: 955.6517, charge: 4
VYKPSAGNNSLYR_5_4_1_2_0
Intensity
m/z
0
50000
100000
150000
200000
250
500
750
1000
1250
1500
1750
126.0547
138.0546
144.0651
168.0652
186.0762
204.0862
274.0916
292.103
366.1388
528.1915
657.2305
836.4238 PEP_0_1_0_0_0
909.456 PEP_0_1_1_0_0
937.9631 PEP_0_2_0_0_0
1018.986 PEP_1_2_0_0_0
1099.9974 PEP_2_2_0_0_0
1254.0797 PEP_3_2_1_0_0
1282.587 PEP_3_3_0_0_0
1671.8269 PEP_0_1_0_0_0
1817.8984 PEP_0_1_1_0_0

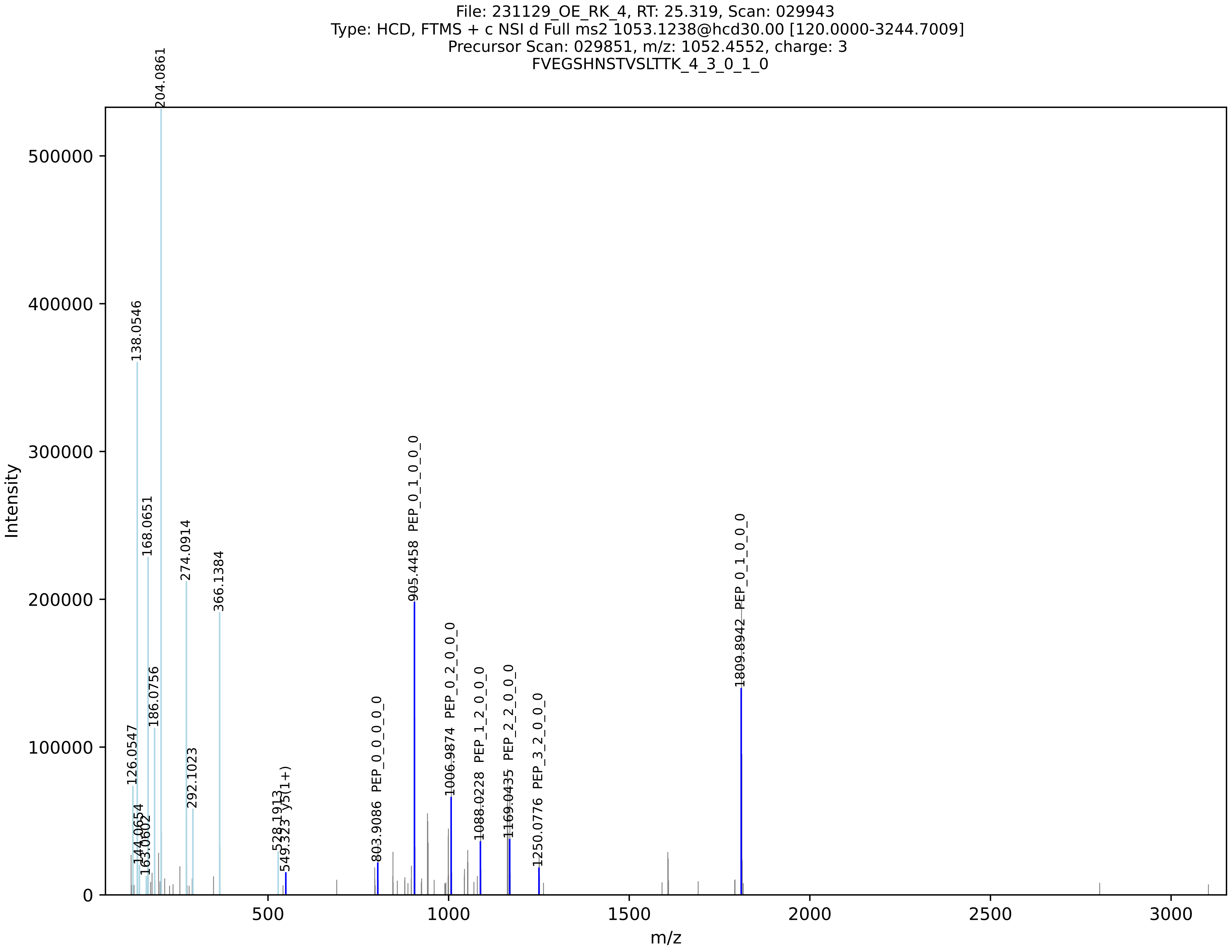
File: 231129_OE_RK_4, RT: 25.319, Scan: 029943
Type: HCD, FTMS + c NSI d Full ms2 1053.1238@hcd30.00 [120.0000-3244.7009]
Precursor Scan: 029851, m/z: 1052.4552, charge: 3
FVEGSHNSTVSLTTK_4_3_0_1_0
Intensity
m/z
0
100000
200000
300000
400000
500000
500
1000
1500
2000
2500
3000
204.0861
138.0546
168.0651
274.0914
366.1384
186.0756
126.0547
292.1023
144.0654
163.0602
528.1913
549.323 y5(1+)
803.9086 PEP_0_0_0_0_0
905.4458 PEP_0_1_0_0_0
1006.9874 PEP_0_2_0_0_0
1088.0228 PEP_1_2_0_0_0
1169.0435 PEP_2_2_0_0_0
1250.0776 PEP_3_2_0_0_0
1809.8942 PEP_0_1_0_0_0

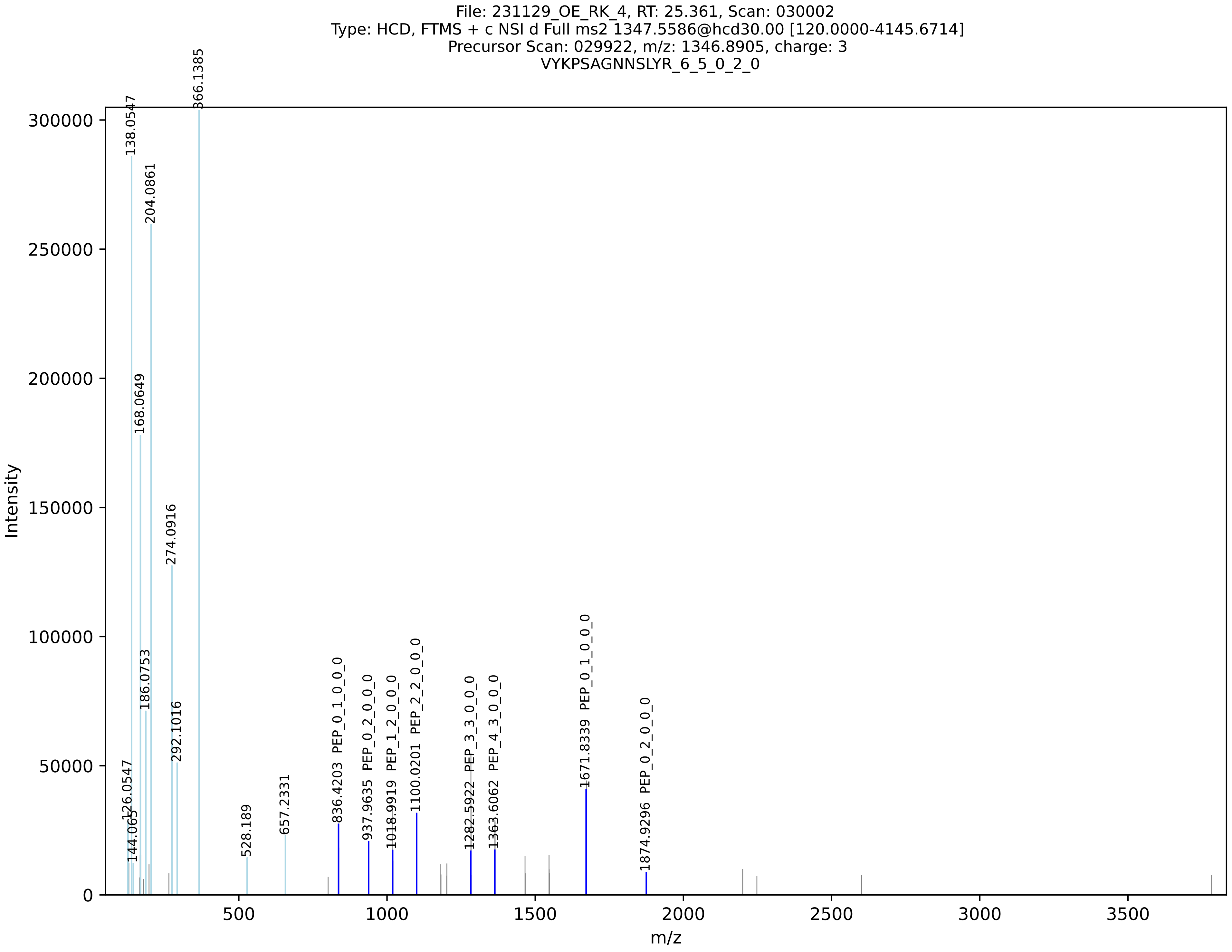

File: 231129_OE_RK_4, RT: 25.361, Scan: 030002
Type: HCD, FTMS + c NSI d Full ms2 1347.5586@hcd30.00 [120.0000-4145.6714]
Precursor Scan: 029922, m/z: 1346.8905, charge: 3
VYKPSAGNNSLYR_6_5_0_2_0
Intensity
m/z
366.1385
138.0547
204.0861
168.0649
274.0916
186.0753
292.1016
126.0547
144.065
528.189
657.2331
836.4203 PEP_0_1_0_0_0
937.9635 PEP_0_2_0_0_0
1018.9919 PEP_1_2_0_0_0
1100.0201 PEP_2_2_0_0_0
1282.5922 PEP_3_3_0_0_0
1363.6062 PEP_4_3_0_0_0
1671.8339 PEP_0_1_0_0_0
1874.9296 PEP_0_2_0_0_0

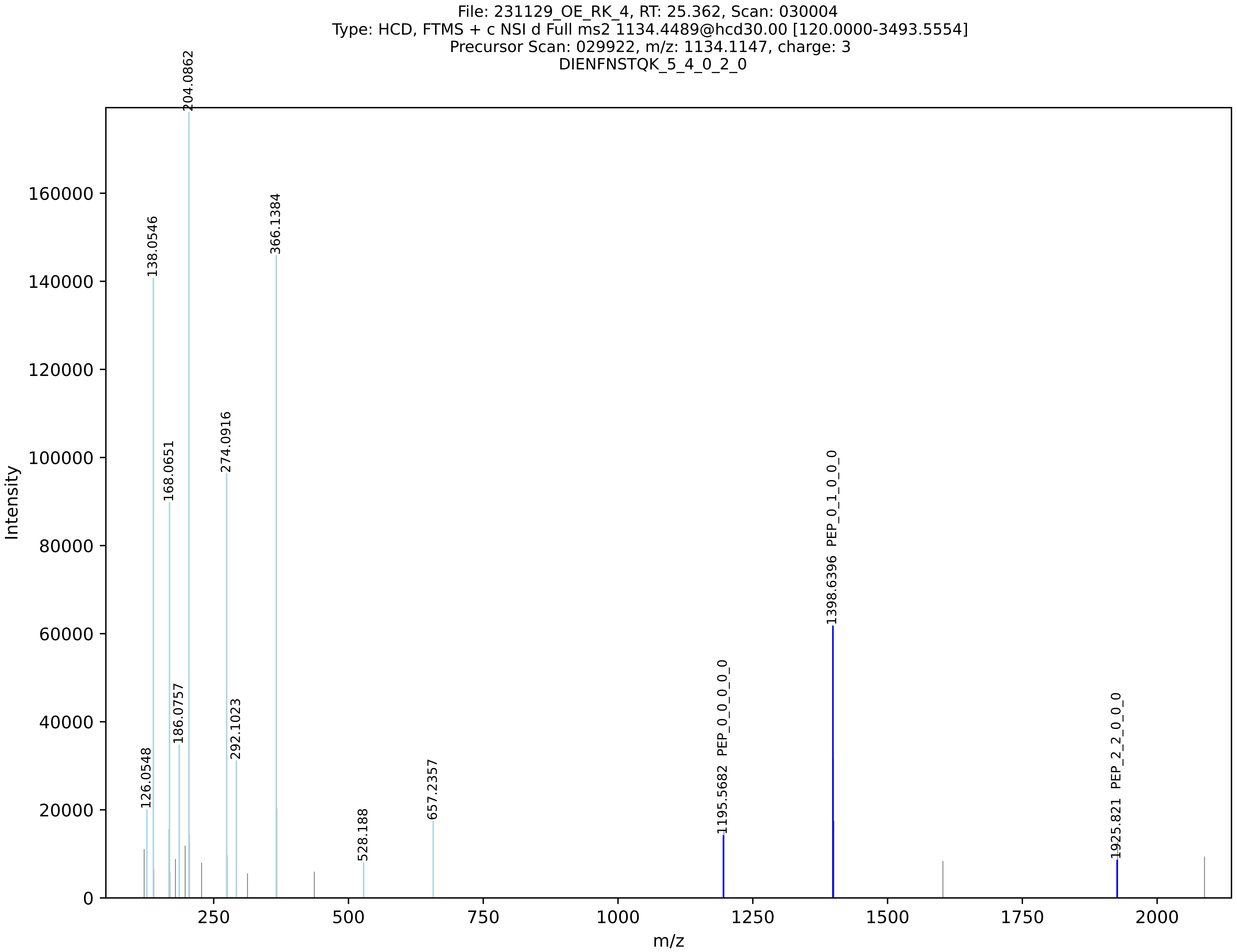
File: 231129_OE_RK_4, RT: 25.362, Scan: 030004
Type: HCD, FTMS + c NSI d Full ms2 1134.4489@hcd30.00 [120.0000-3493.5554]
Precursor Scan: 029922, m/z: 1134.1147, charge: 3
DIENFNSTQK_5_4_0_2_0
Intensity
m/z
204.0862
138.0546
366.1384
168.0651
274.0916
186.0757
292.1023
126.0548
528.188
657.2357
1195.5682 PEP_0_0_0_0_0
1398.6396 PEP_0_1_0_0_0
1925.821 PEP_2_2_0_0_0
0
20000
40000
60000
80000
100000
120000
140000
160000
250
500
750
1000
1250
1500
1750
2000

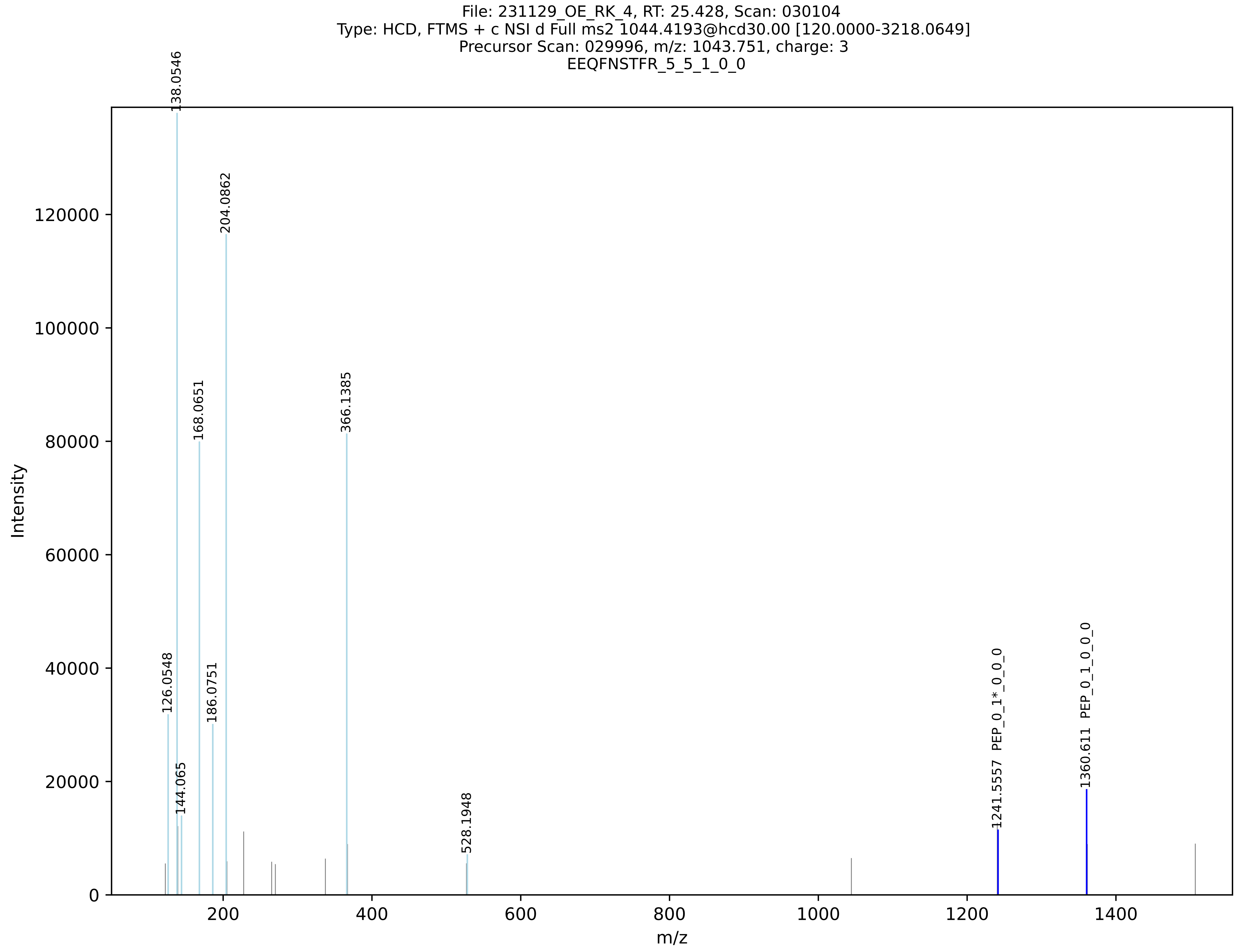
File: 231129_OE_RK_4, RT: 25.428, Scan: 030104
Type: HCD, FTMS + c NSI d Full ms2 1044.4193@hcd30.00 [120.0000-3218.0649]
Precursor Scan: 029996, m/z: 1043.751, charge: 3
EEQFNSTFR_5_5_1_0_0
138.0546
204.0862
168.0651
366.1385
126.0548
186.0751
144.065
528.1948
1241.5557 PEP_0_1*_0_0_0
1360.611 PEP_0_1_0_0_0
Intensity
m/z
0
20000
40000
60000
80000
100000
120000
200
400
600
800
1000
1200
1400

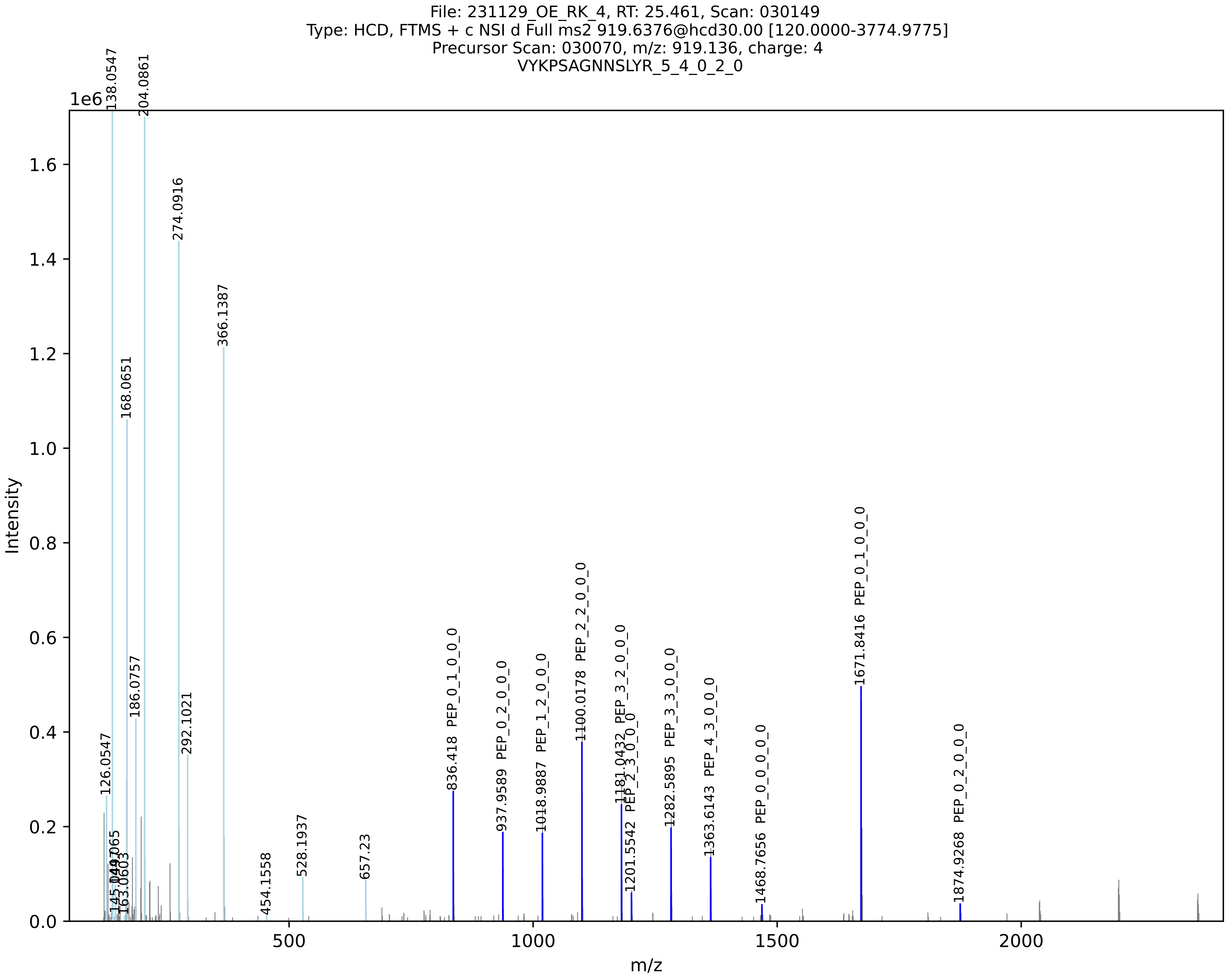
File: 231129_OE_RK_4, RT: 25.461, Scan: 030149
Type: HCD, FTMS + c NSI d Full ms2 919.6376@hcd30.00 [120.0000-3774.9775]
Precursor Scan: 030070, m/z: 919.136, charge: 4
VYKPSAGNNSLYR_5_4_0_2_0
1e6
Intensity
m/z
138.0547
204.0861
274.0916
366.1387
168.0651
186.0757
292.1021
126.0547
163.0603
454.1558
528.1937
657.23
836.418 PEP_0_1_0_0_0
937.9589 PEP_0_2_0_0_0
1018.9887 PEP_1_2_0_0_0
1100.0178 PEP_2_2_0_0_0
1181.0432 PEP_3_2_0_0_0
1201.5542 PEP_2_3_0_0_0
1282.5895 PEP_3_3_0_0_0
1363.6143 PEP_4_3_0_0_0
1468.7656 PEP_0_0_0_0_0
1671.8416 PEP_0_1_0_0_0
1874.9268 PEP_0_2_0_0_0
0.0
0.2
0.4
0.6
0.8
1.0
1.2
1.4
1.6
500
1000
1500
2000

File: 231129_OE_RK_4, RT: 25.462, Scan: 030151
Type: HCD, FTMS + c NSI d Full ms2 1210.8038@hcd30.00 [120.0000-3727.2017]
Precursor Scan: 030070, m/z: 1210.4697, charge: 3
MDGASNVTCINSR_5_4_0_2_0

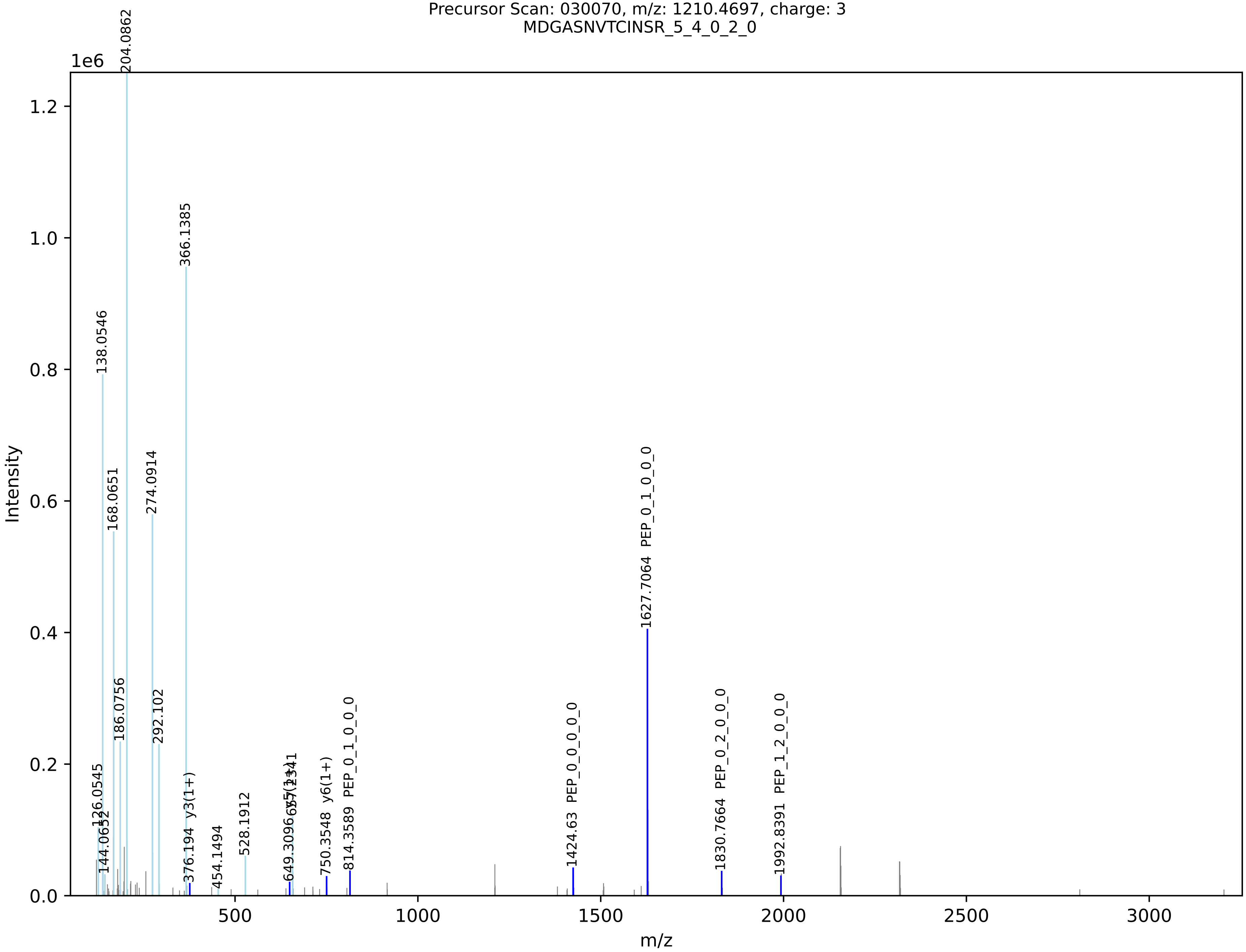

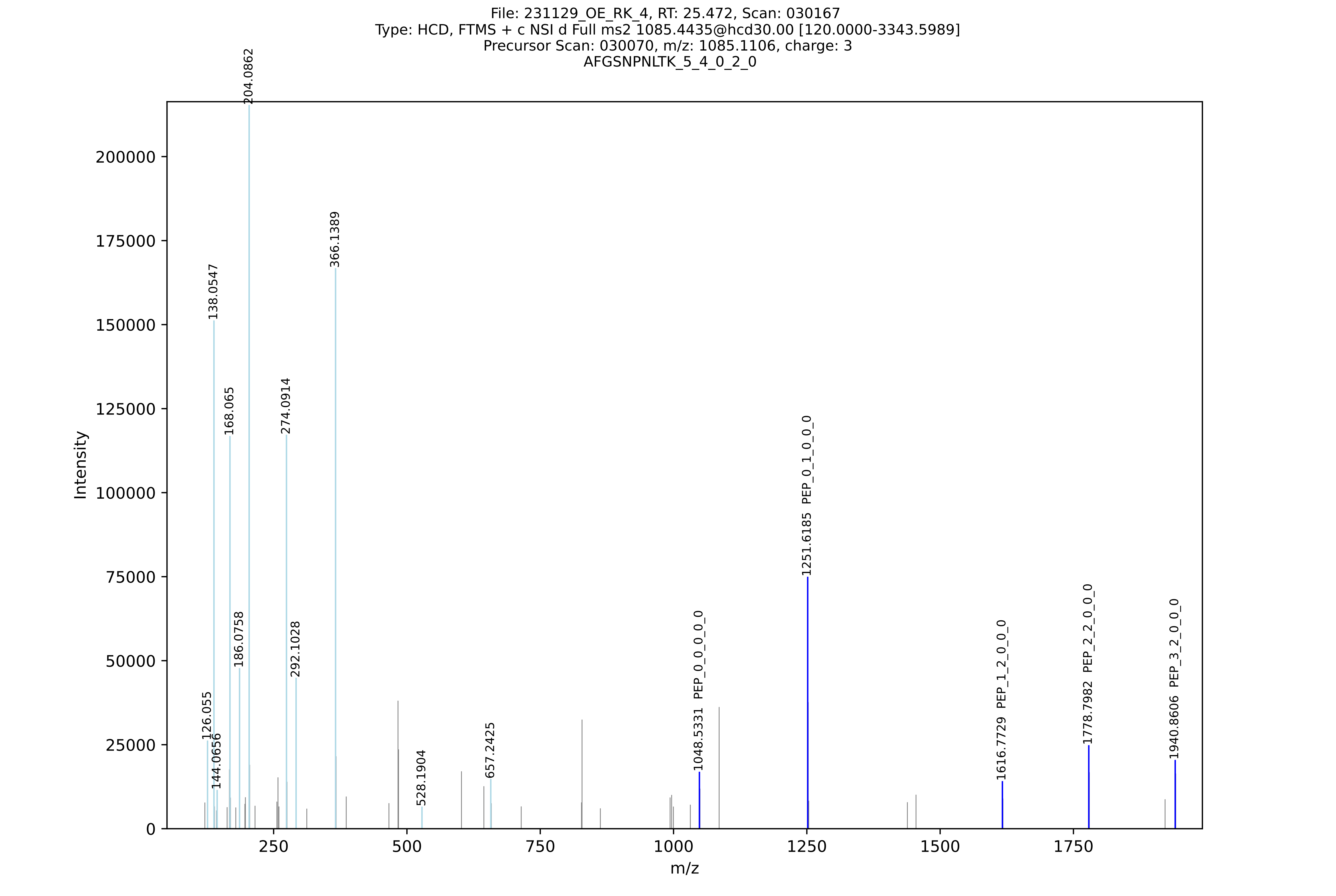

File: 231129_OE_RK_4, RT: 25.472, Scan: 030167
Type: HCD, FTMS + c NSI d Full ms2 1085.4435@hcd30.00 [120.0000-3343.5989]
Precursor Scan: 030070, m/z: 1085.1106, charge: 3
AFGSNPNLTK_5_4_0_2_0
Intensity
m/z
204.0862
366.1389
138.0547
168.065
274.0914
186.0758
292.1028
126.055
144.0656
528.1904
657.2425
1048.5331 PEP_0_0_0_0_0
1251.6185 PEP_0_1_0_0_0
1616.7729 PEP_1_2_0_0_0
1778.7982 PEP_2_2_0_0_0
1940.8606 PEP_3_2_0_0_0

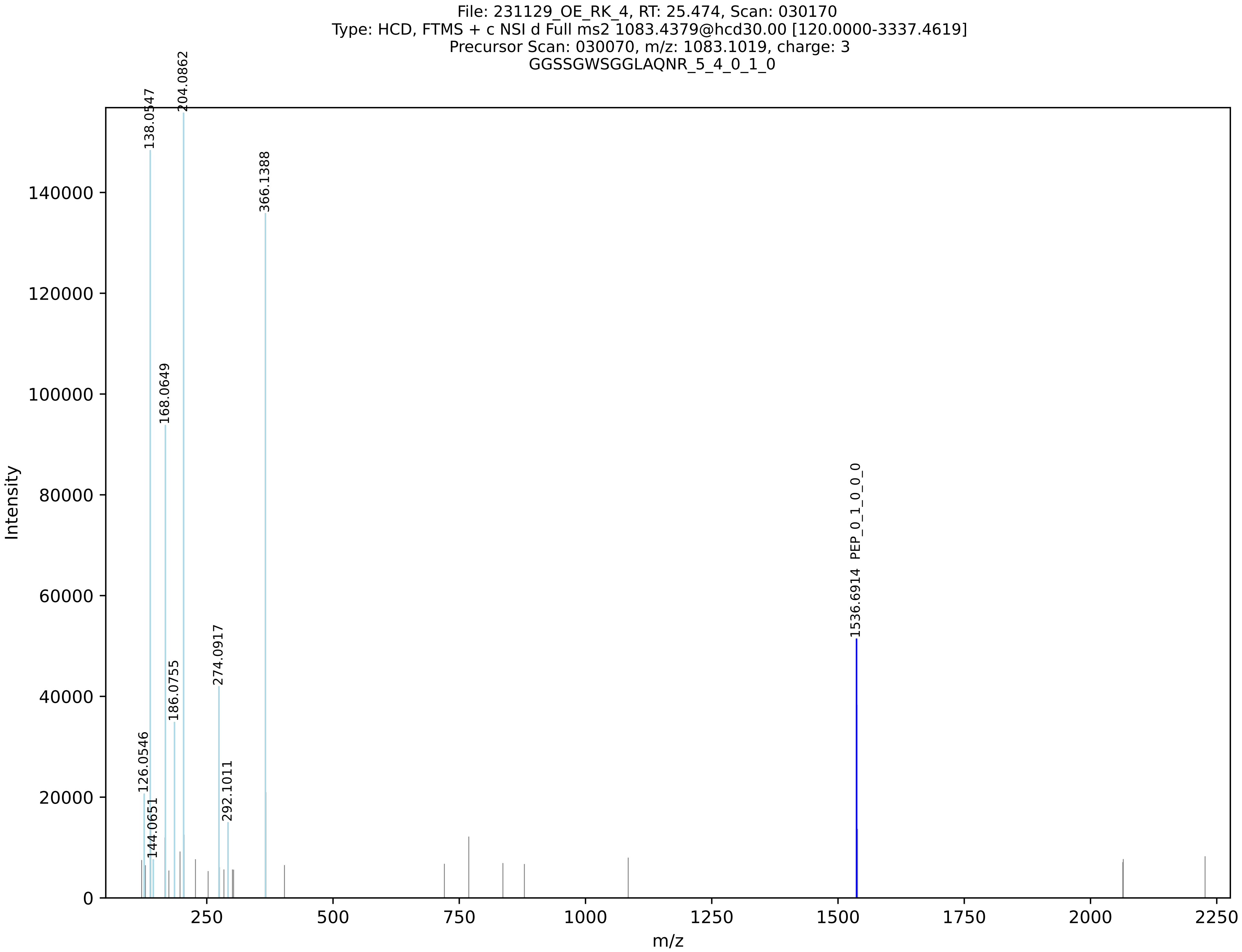

File: 231129_OE_RK_4, RT: 25.474, Scan: 030170
Type: HCD, FTMS + c NSI d Full ms2 1083.4379@hcd30.00 [120.0000-3337.4619]
Precursor Scan: 030070, m/z: 1083.1019, charge: 3
GGSSGWSGGLAQNR_5_4_0_1_0
204.0862
138.0547
366.1388
168.0649
1536.6914 PEP_0_1_0_0_0
274.0917
186.0755
126.0546
292.1011
144.0651
Intensity
m/z
0
20000
40000
60000
80000
100000
120000
140000
250
500
750
1000
1250
1500
1750
2000
2250

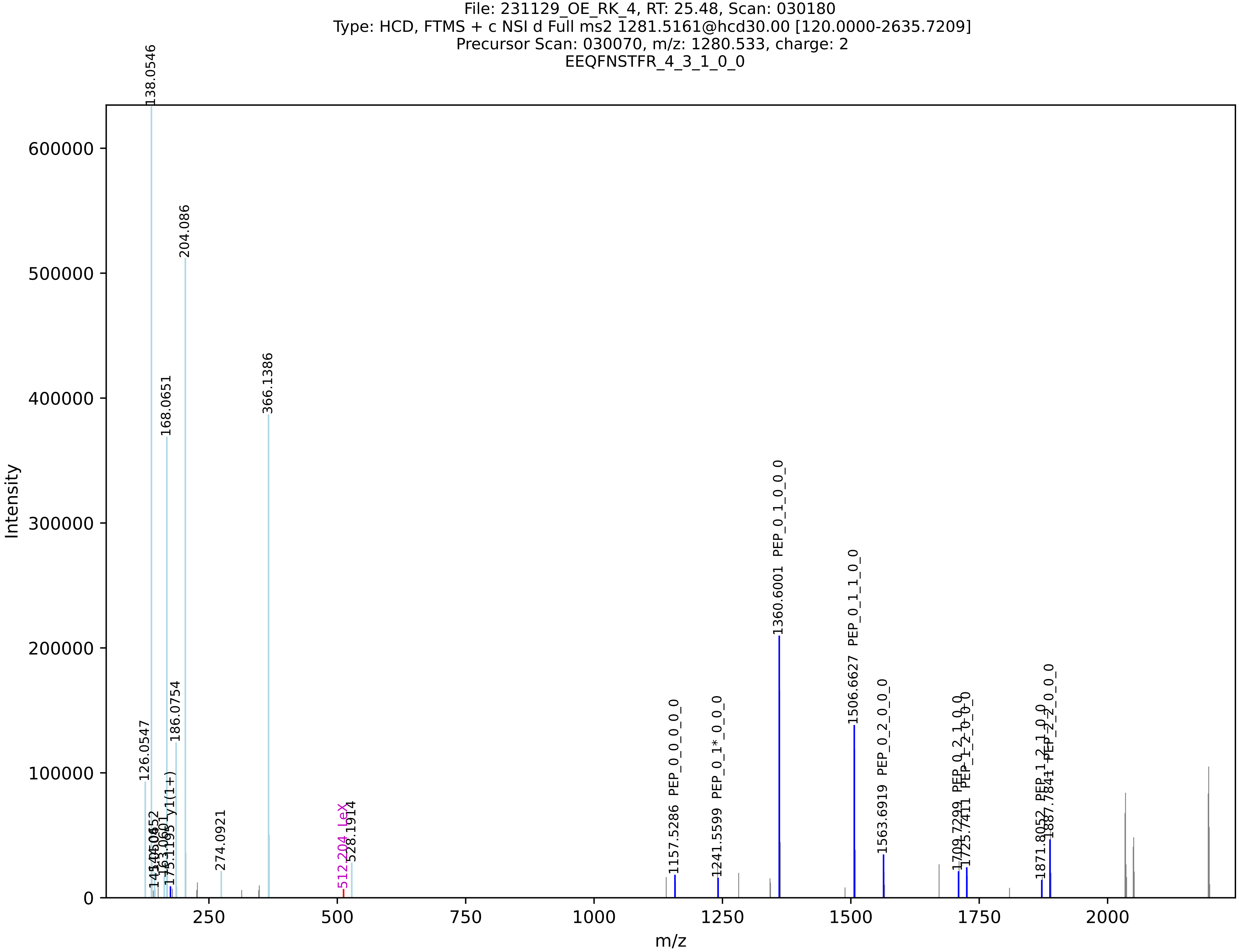
File: 231129_OE_RK_4, RT: 25.48, Scan: 030180
Type: HCD, FTMS + c NSI d Full ms2 1281.5161@hcd30.00 [120.0000-2635.7209]
Precursor Scan: 030070, m/z: 1280.533, charge: 2
EEQFNSTFR_4_3_1_0_0
138.0546
204.086
366.1386
168.0651
186.0754
126.0547
y1(1+)
274.0921
512.204 LeX
528.1914
1157.5286 PEP_0_0_0_0_0
1241.5599 PEP_0_1*_0_0_0
1360.6001 PEP_0_1_0_0_0
1506.6627 PEP_0_1_1_0_0
1563.6919 PEP_0_2_0_0_0
1871.8052 PEP_1_2_1_0_0
Intensity
m/z
0
100000
200000
300000
400000
500000
600000
250
500
750
1000
1250
1500
1750
2000

File: 231129_OE_RK_4, RT: 25.51, Scan: 030220
Type: HCD, FTMS + c NSI d Full ms2 1383.0565@hcd30.00 [120.0000-2842.8633]
Precursor Scan: 030144, m/z: 1382.5558, charge: 2
EEQFNSTFR_4_4_1_0_0

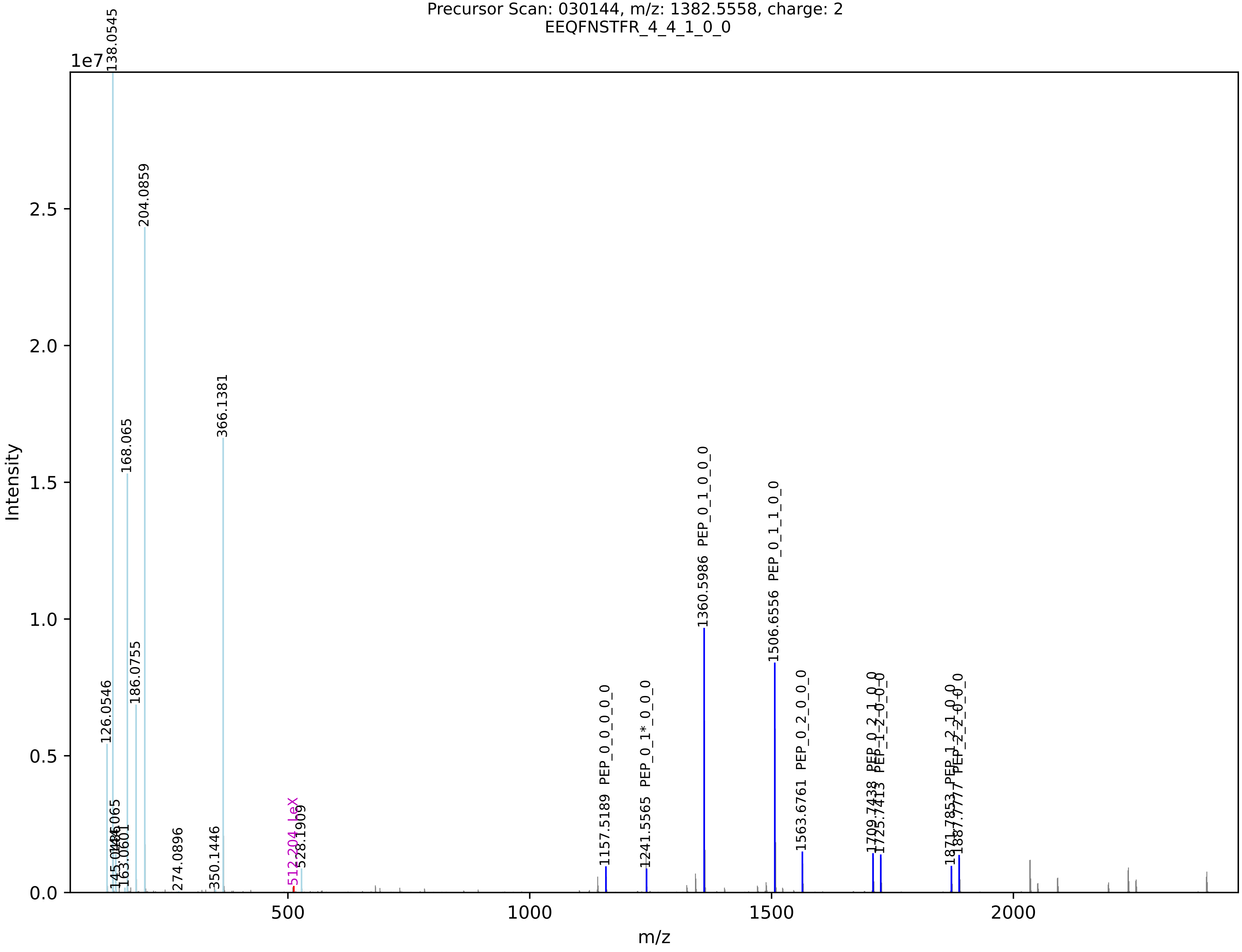

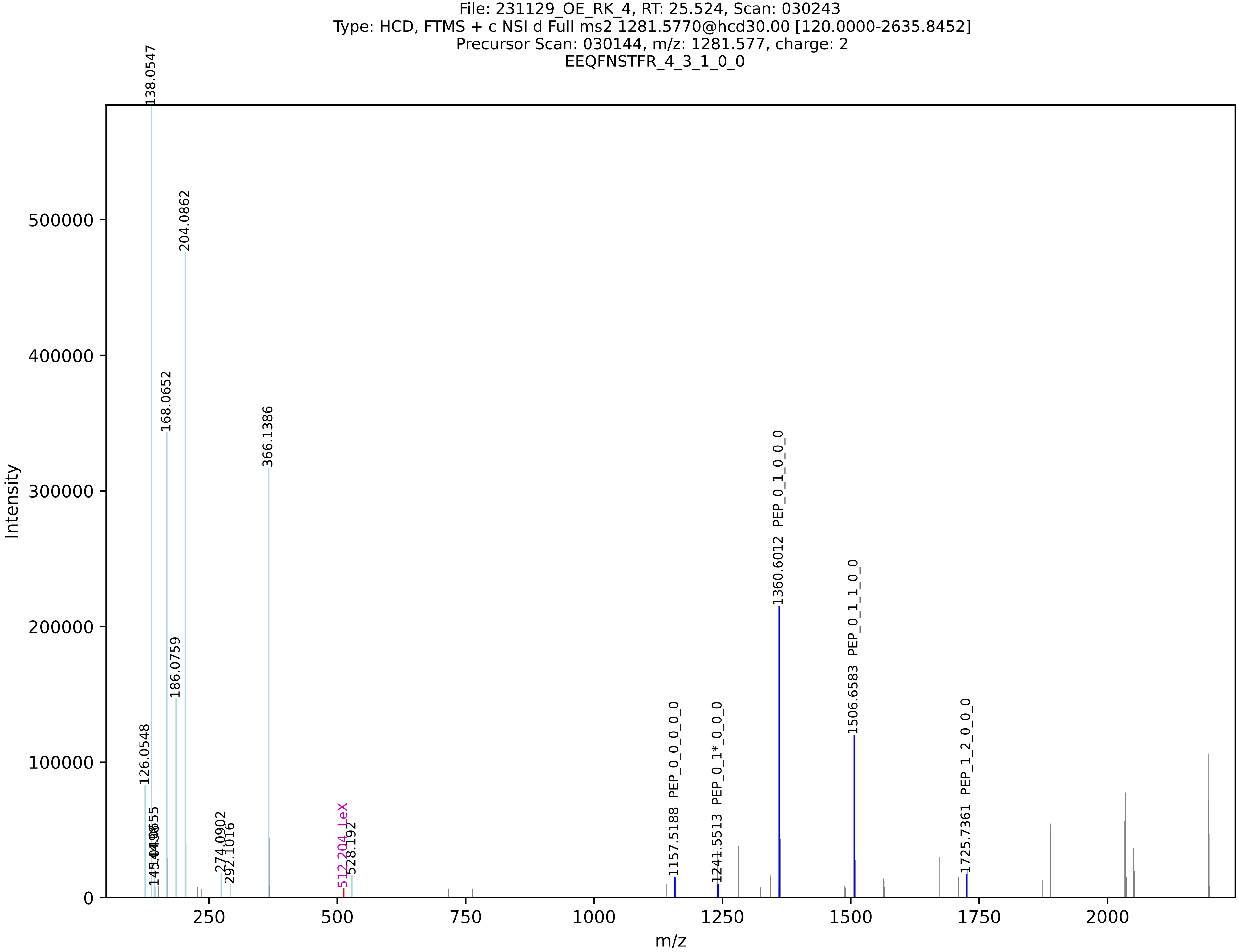
File: 231129_OE_RK_4, RT: 25.524, Scan: 030243
Type: HCD, FTMS + c NSI d Full ms2 1281.5770@hcd30.00 [120.0000-2635.8452]
Precursor Scan: 030144, m/z: 1281.577, charge: 2
EEQFNSTFR_4_3_1_0_0
138.0547
204.0862
168.0652
366.1386
186.0759
126.0548
274.0902
292.1016
512.204_LeX
528.192
1157.5188 PEP_0_0_0_0_0
1241.5513 PEP_0_1*_0_0_0
1360.6012 PEP_0_1_0_0_0
1506.6583 PEP_0_1_1_0_0
1725.7361 PEP_1_2_0_0_0
Intensity
0
100000
200000
300000
400000
500000
250
500
750
1000
1250
1500
1750
2000
m/z

File: 231129_OE_RK_4, RT: 25.569, Scan: 030308
Type: HCD, FTMS + c NSI d Full ms2 1347.5587@hcd30.00 [120.0000-4145.6719]
Precursor Scan: 030218, m/z: 1346.8915, charge: 3
VYKPSAGNNSLYR_6_5_0_2_0

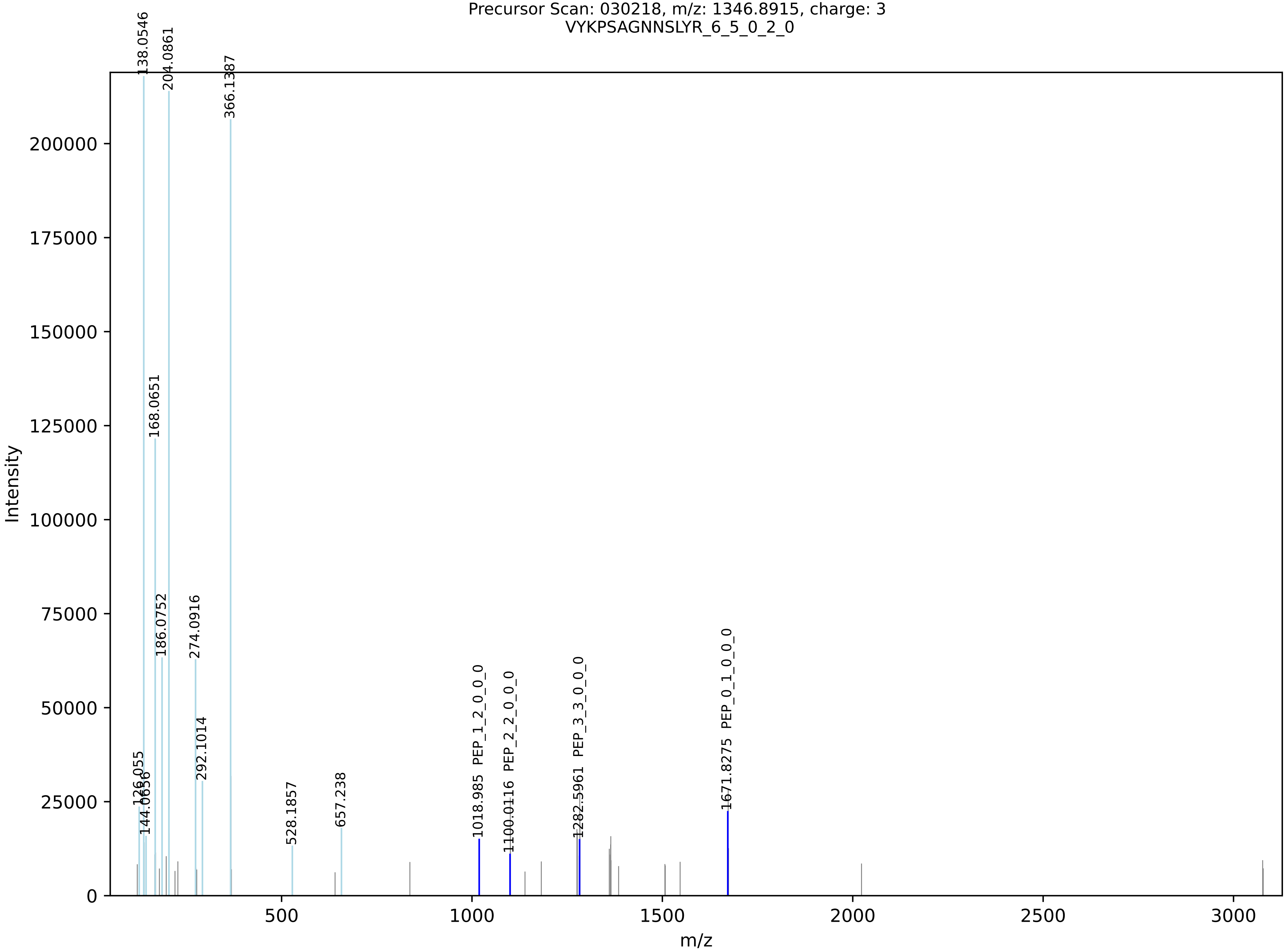

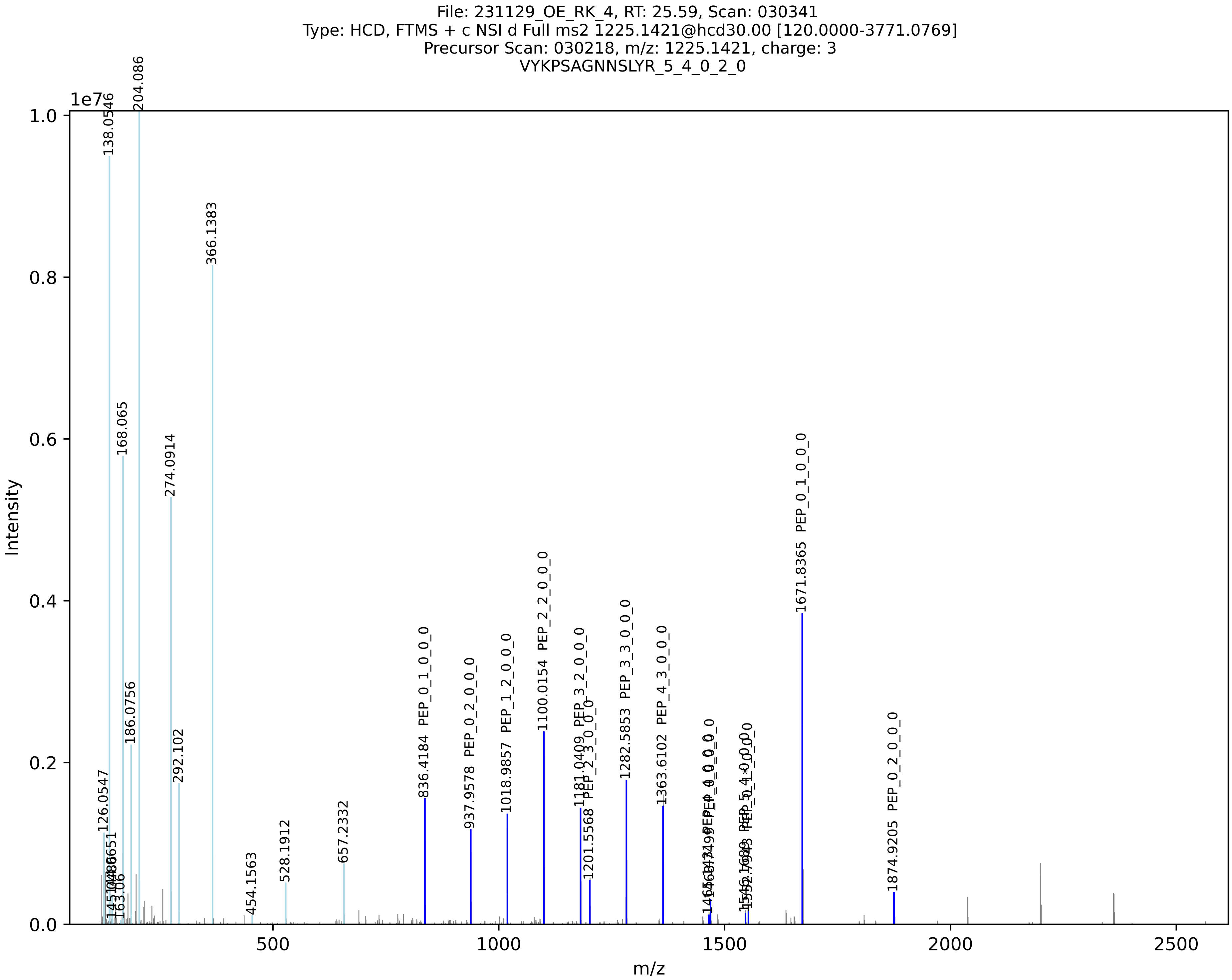

File: 231129_OE_RK_4, RT: 25.59, Scan: 030341
Type: HCD, FTMS + c NSI d Full ms2 1225.1421@hcd30.00 [120.0000-3771.0769]
Precursor Scan: 030218, m/z: 1225.1421, charge: 3
VYKPSAGNNSLYR_5_4_0_2_0
1e7
Intensity
m/z
204.086
138.0546
366.1383
168.065
274.0914
186.0756
292.102
126.0547
454.1563
528.1912
657.2332
836.4184 PEP_0_1_0_0_0
937.9578 PEP_0_2_0_0_0
1018.9857 PEP_1_2_0_0_0
1100.0154 PEP_2_2_0_0_0
1181.0409 PEP_3_2_0_0_0
1201.5568 PEP_2_3_0_0_0
1282.5853 PEP_3_3_0_0_0
1363.6102 PEP_4_3_0_0_0
1671.8365 PEP_0_1_0_0_0
1874.9205 PEP_0_2_0_0_0

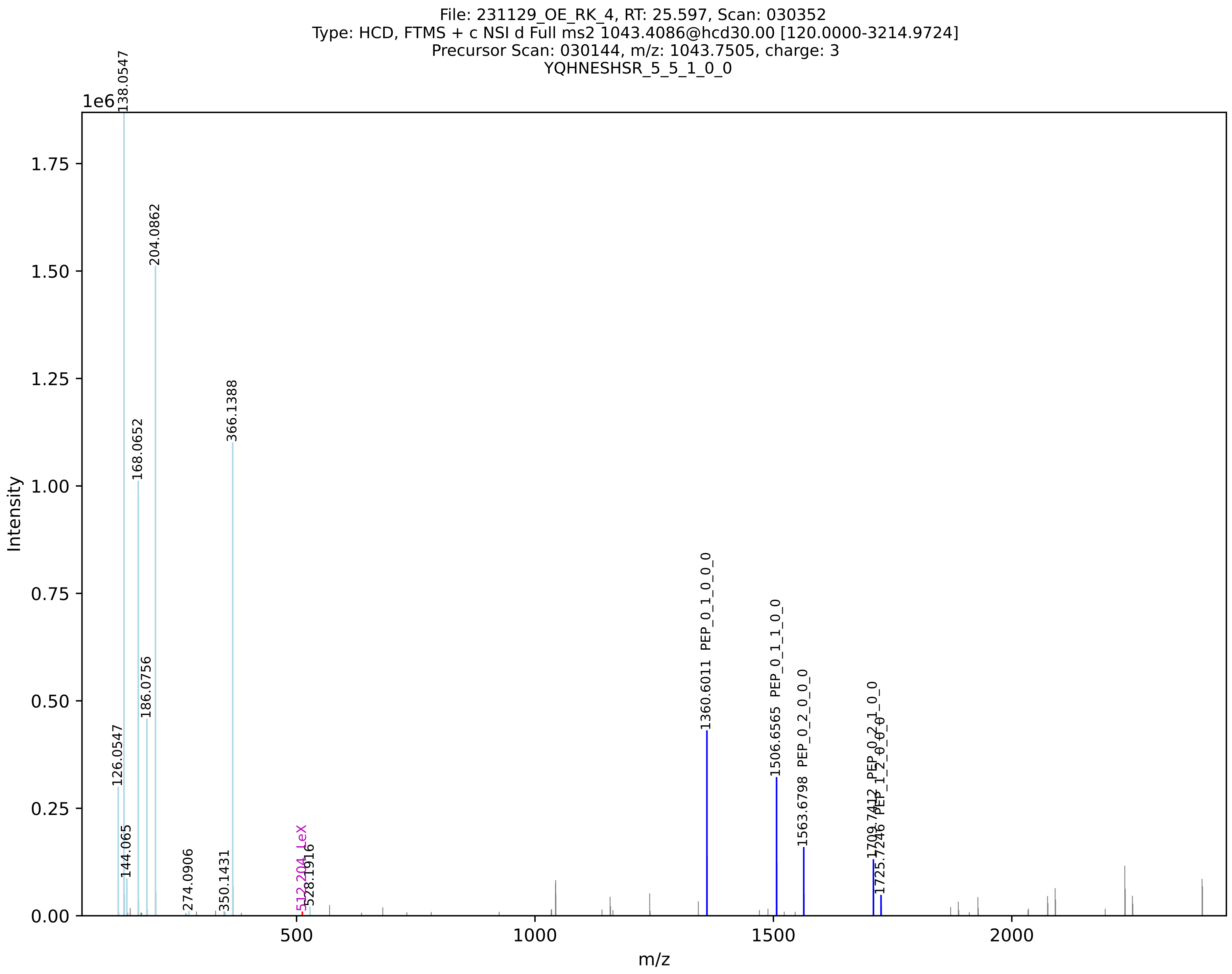
File: 231129_OE_RK_4, RT: 25.597, Scan: 030352
Type: HCD, FTMS + c NSI d Full ms2 1043.4086@hcd30.00 [120.0000-3214.9724]
Precursor Scan: 030144, m/z: 1043.7505, charge: 3
YQHNESHSR_5_5_1_0_0
1e6
Intensity
m/z
138.0547
204.0862
366.1388
168.0652
186.0756
126.0547
144.065
274.0906
350.1431
512.204_LeX
528.1916
1360.6011 PEP_0_1_0_0_0
1506.6565 PEP_0_1_1_0_0
1563.6798 PEP_0_2_0_0_0
1709.7412 PEP_0_2_1_0_0
1725.7246 PEP_1_2_0_0

File: 231129_OE_RK_4, RT: 25.61, Scan: 030366
Type: HCD, FTMS + c NSI d Full ms2 1044.4177@hcd30.00 [120.0000-3218.0603]
Precursor Scan: 030292, m/z: 1043.7496, charge: 3
EEQFNSTFR_5_5_1_0_0

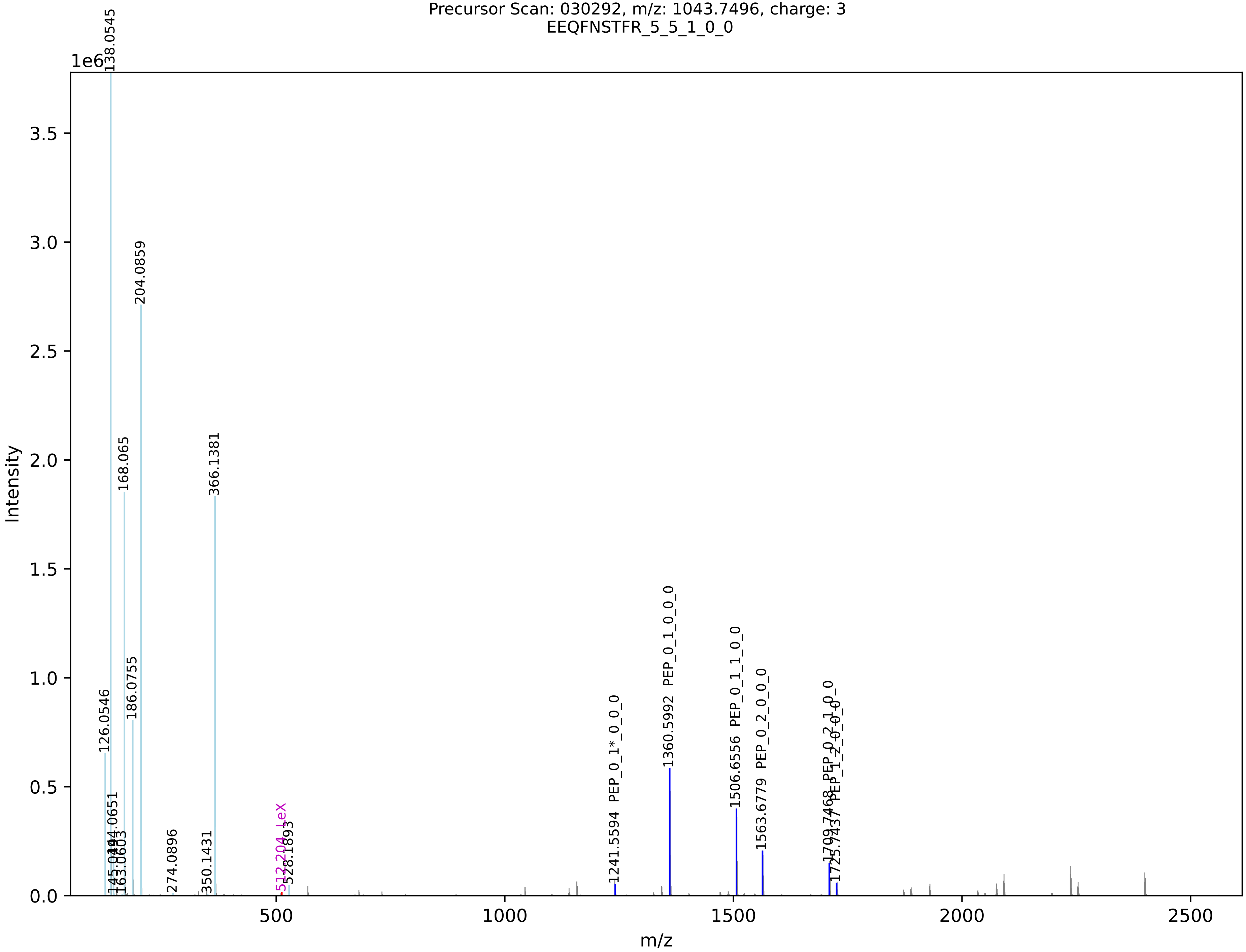

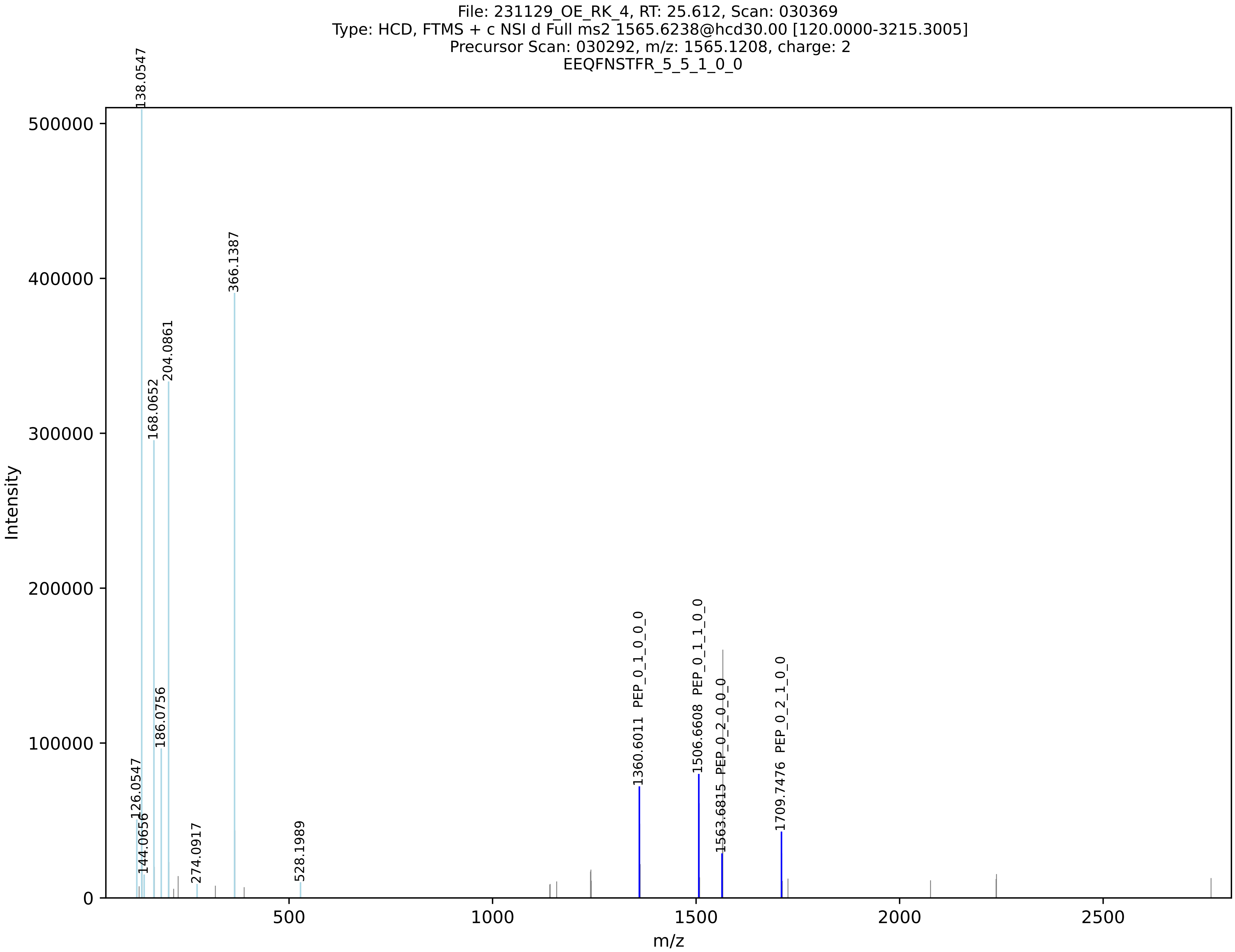
File: 231129_OE_RK_4, RT: 25.612, Scan: 030369
Type: HCD, FTMS + c NSI d Full ms2 1565.6238@hcd30.00 [120.0000-3215.3005]
Precursor Scan: 030292, m/z: 1565.1208, charge: 2
EEQFNSTFR_5_5_1_0_0
Intensity
m/z
138.0547
366.1387
204.0861
168.0652
186.0756
126.0547
144.0656
274.0917
528.1989
1360.6011 PEP_0_1_0_0_0
1506.6608 PEP_0_1_1_0_0
1563.6815 PEP_0_2_0_0_0
1709.7476 PEP_0_2_1_0_0

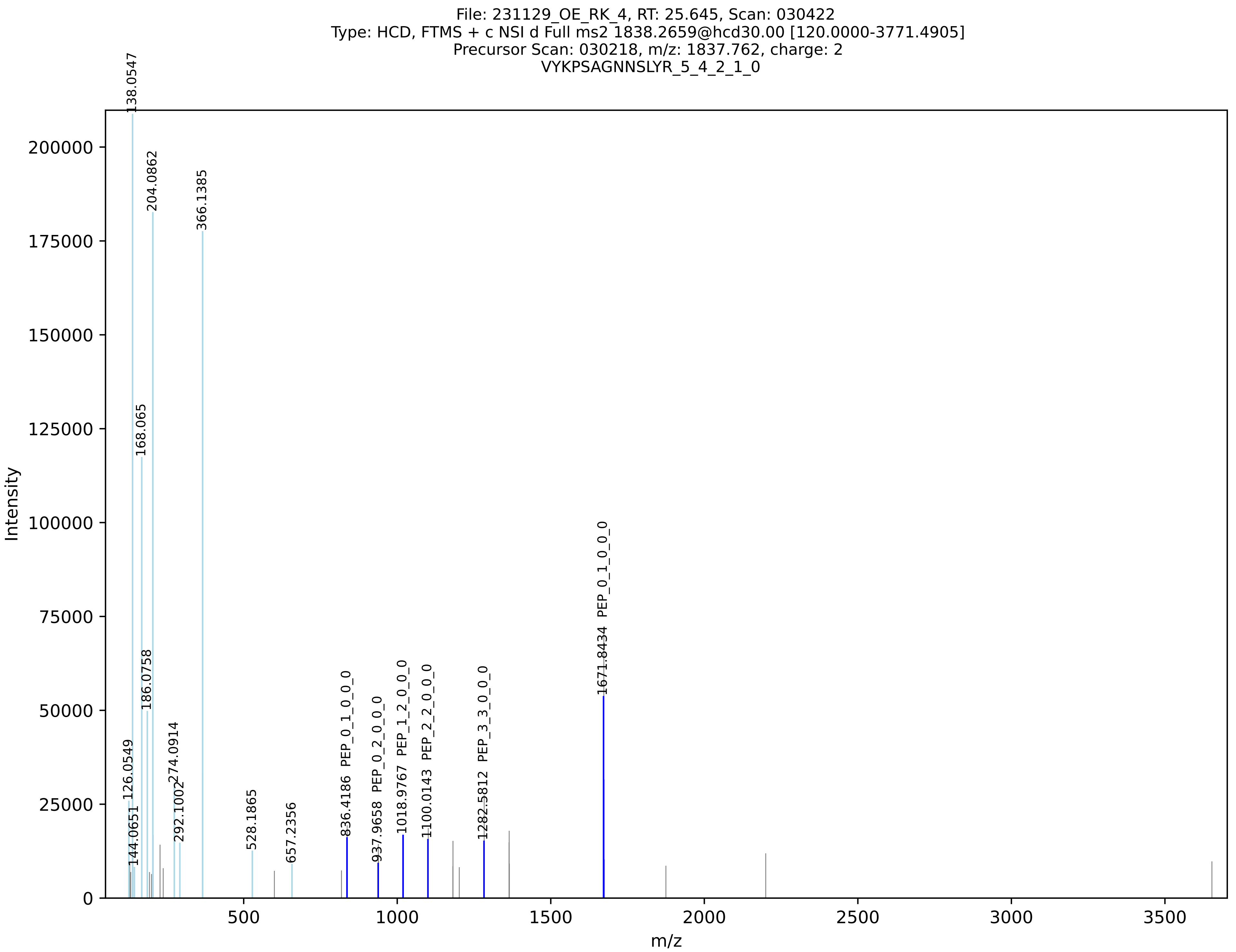
File: 231129_OE_RK_4, RT: 25.645, Scan: 030422
Type: HCD, FTMS + c NSI d Full ms2 1838.2659@hcd30.00 [120.0000-3771.4905]
Precursor Scan: 030218, m/z: 1837.762, charge: 2
VYKPSAGNNSLYR_5_4_2_1_0
Intensity
m/z
138.0547
204.0862
366.1385
168.065
186.0758
126.0549
144.0651
274.0914
292.1002
528.1865
657.2356
836.4186 PEP_0_1_0_0_0
937.9658 PEP_0_2_0_0_0
1018.9767 PEP_1_2_0_0_0
1100.0143 PEP_2_2_0_0_0
1282.5812 PEP_3_3_0_0_0
1671.8434 PEP_0_1_0_0_0

File: 231129_OE_RK_4, RT: 25.664, Scan: 030446
Type: HCD, FTMS + c NSI d Full ms2 1210.8036@hcd30.00 [120.0000-3727.2009]
Precursor Scan: 030365, m/z: 1210.4696, charge: 3
MDGASNVTCINSR_5_4_0_2_0

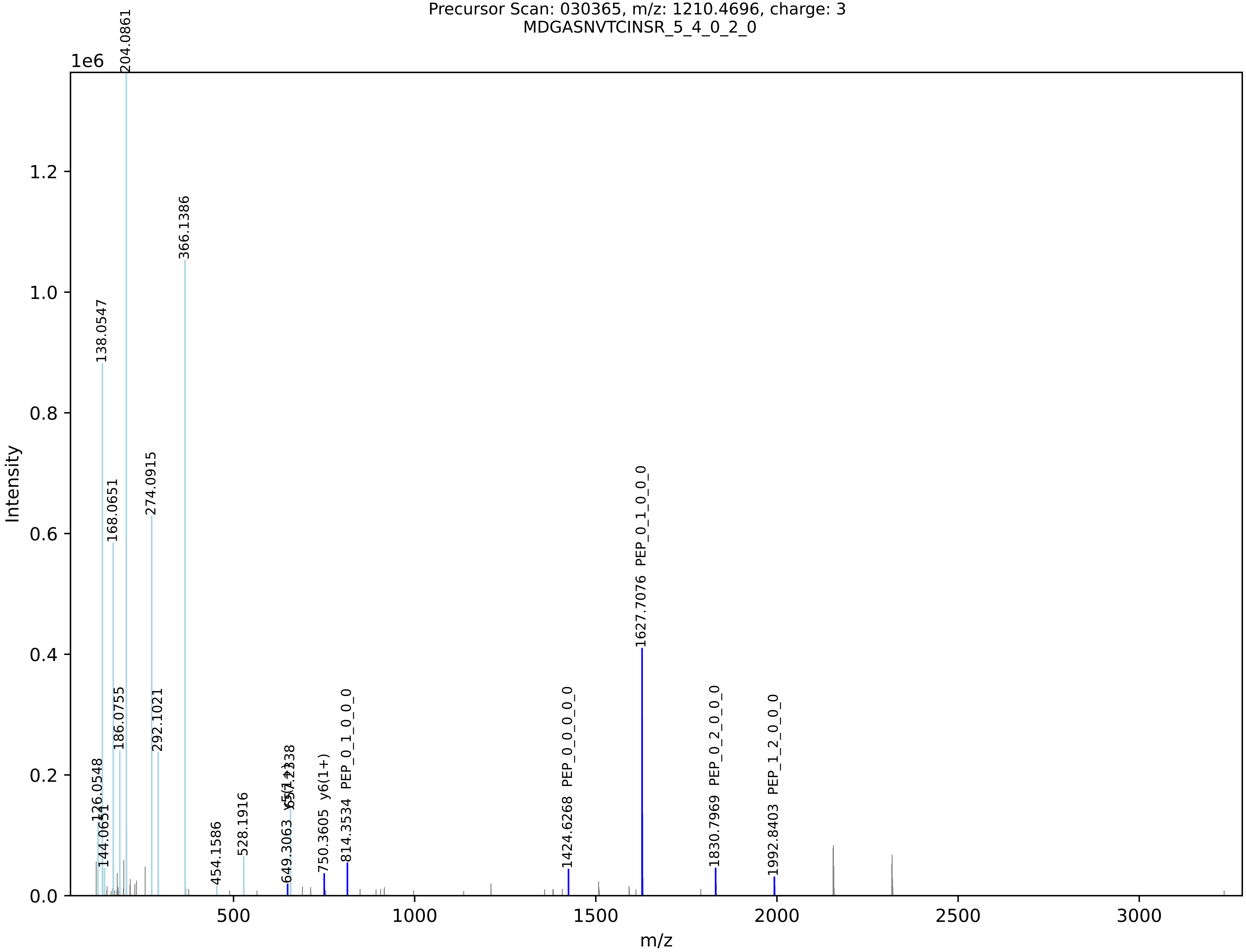

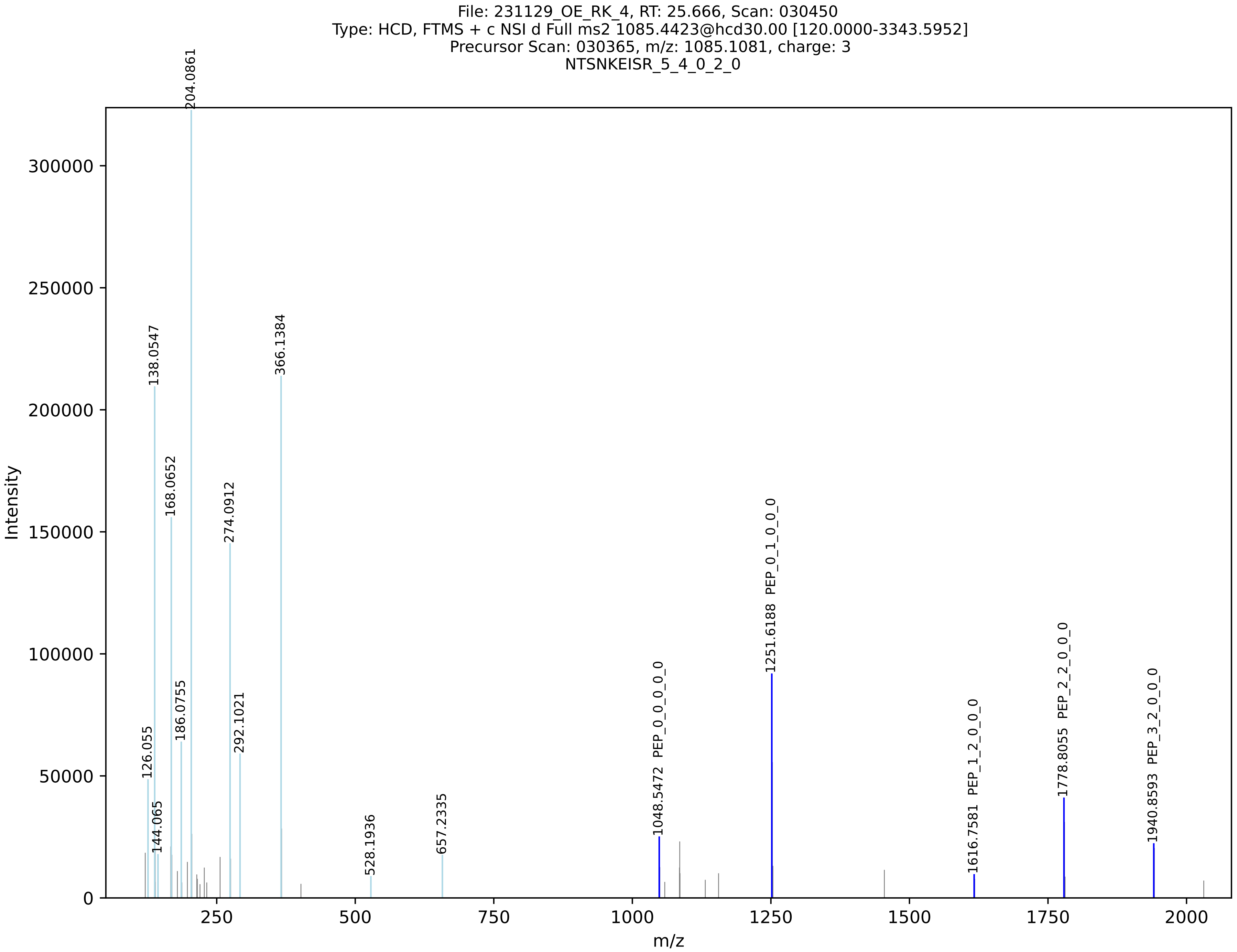
File: 231129_OE_RK_4, RT: 25.666, Scan: 030450
Type: HCD, FTMS + c NSI d Full ms2 1085.4423@hcd30.00 [120.0000-3343.5952]
Precursor Scan: 030365, m/z: 1085.1081, charge: 3
NTSNKEISR_5_4_0_2_0
Intensity
m/z
204.0861
138.0547
366.1384
168.0652
274.0912
186.0755
292.1021
126.055
144.065
528.1936
657.2335
1048.5472 PEP_0_0_0_0_0
1251.6188 PEP_0_1_0_0_0
1616.7581 PEP_1_2_0_0_0
1778.8055 PEP_2_2_0_0_0
1940.8593 PEP_3_2_0_0_0
0
50000
100000
150000
200000
250000
300000
250
500
750
1000
1250
1500
1750
2000

File: 231129_OE_RK_4, RT: 25.668, Scan: 030452
Type: HCD, FTMS + c NSI d Full ms2 1083.4384@hcd30.00 [120.0000-3337.4634]
Precursor Scan: 030365, m/z: 1083.1032, charge: 3
GGSSGWSGGLAQNR_5_4_0_1_0

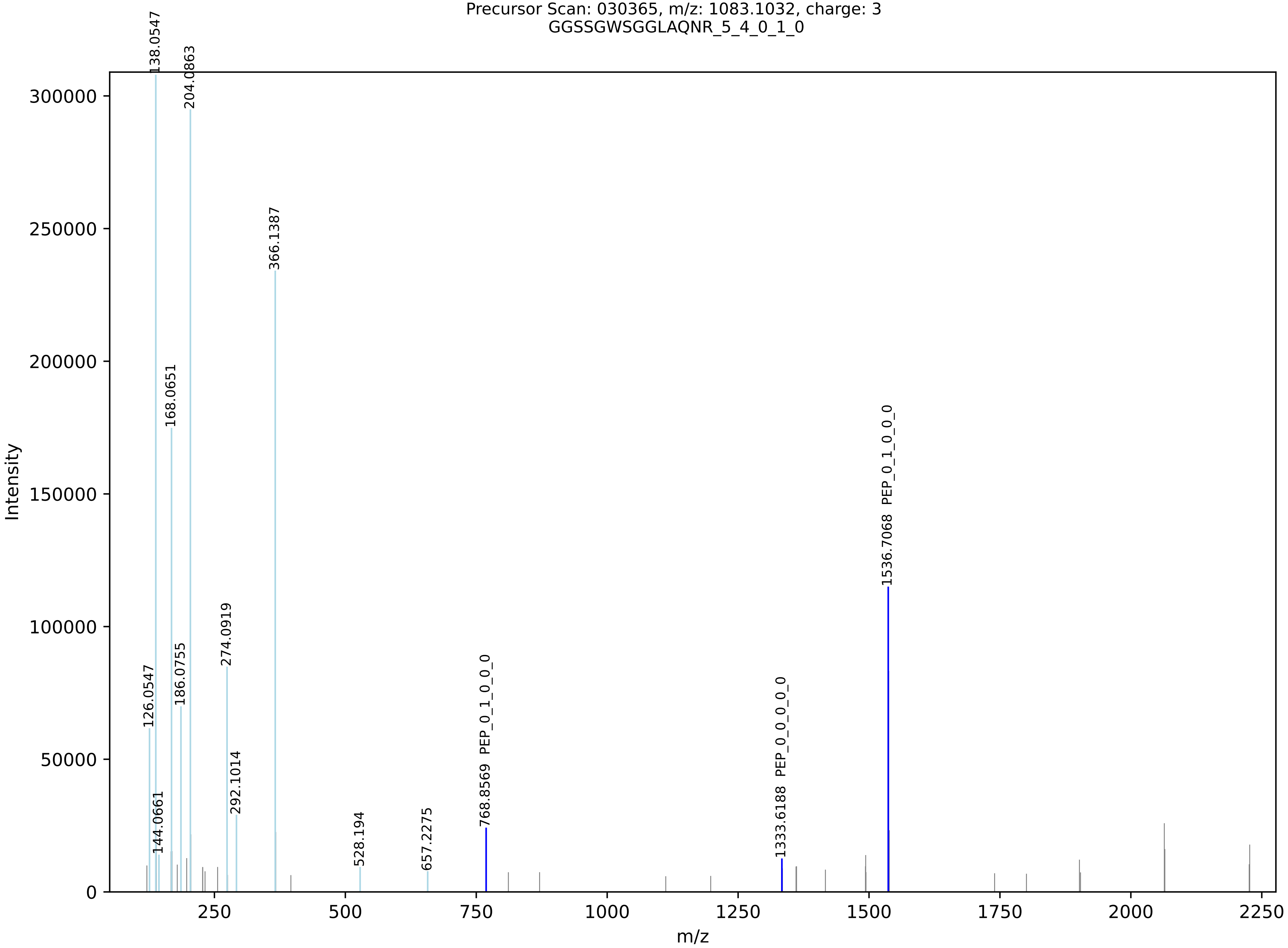

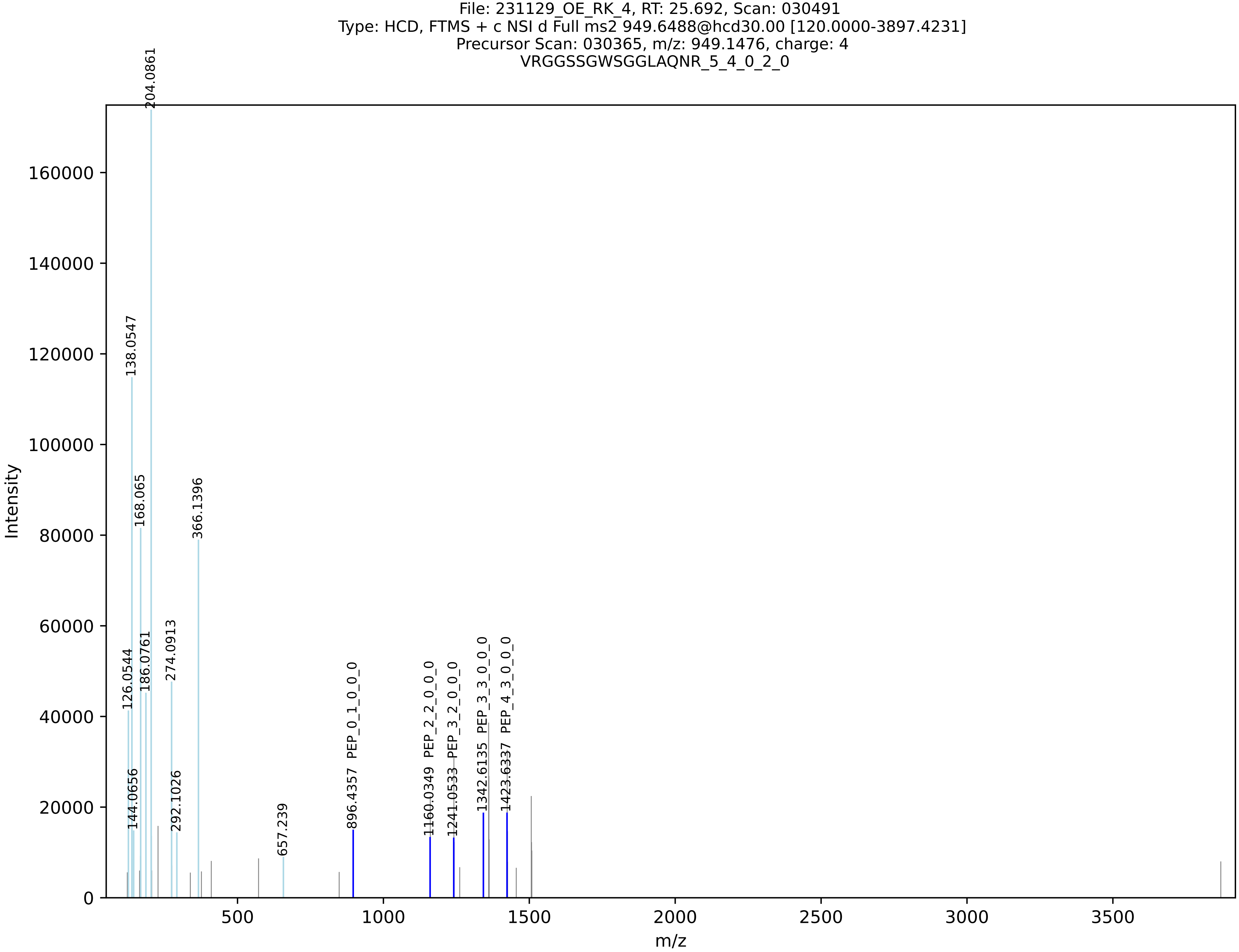

File: 231129_OE_RK_4, RT: 25.692, Scan: 030491
Type: HCD, FTMS + c NSI d Full ms2 949.6488@hcd30.00 [120.0000-3897.4231]
Precursor Scan: 030365, m/z: 949.1476, charge: 4
VRGGSSGWSGGLAQNR_5_4_0_2_0
204.0861
138.0547
168.065
366.1396
126.0544
186.0761
274.0913
144.0656
292.1026
657.239
896.4357 PEP_0_1_0_0_0
1160.0349 PEP_2_2_0_0_0
1241.0533 PEP_3_2_0_0_0
1342.6135 PEP_3_3_0_0_0
1423.6337 PEP_4_3_0_0_0
Intensity
m/z

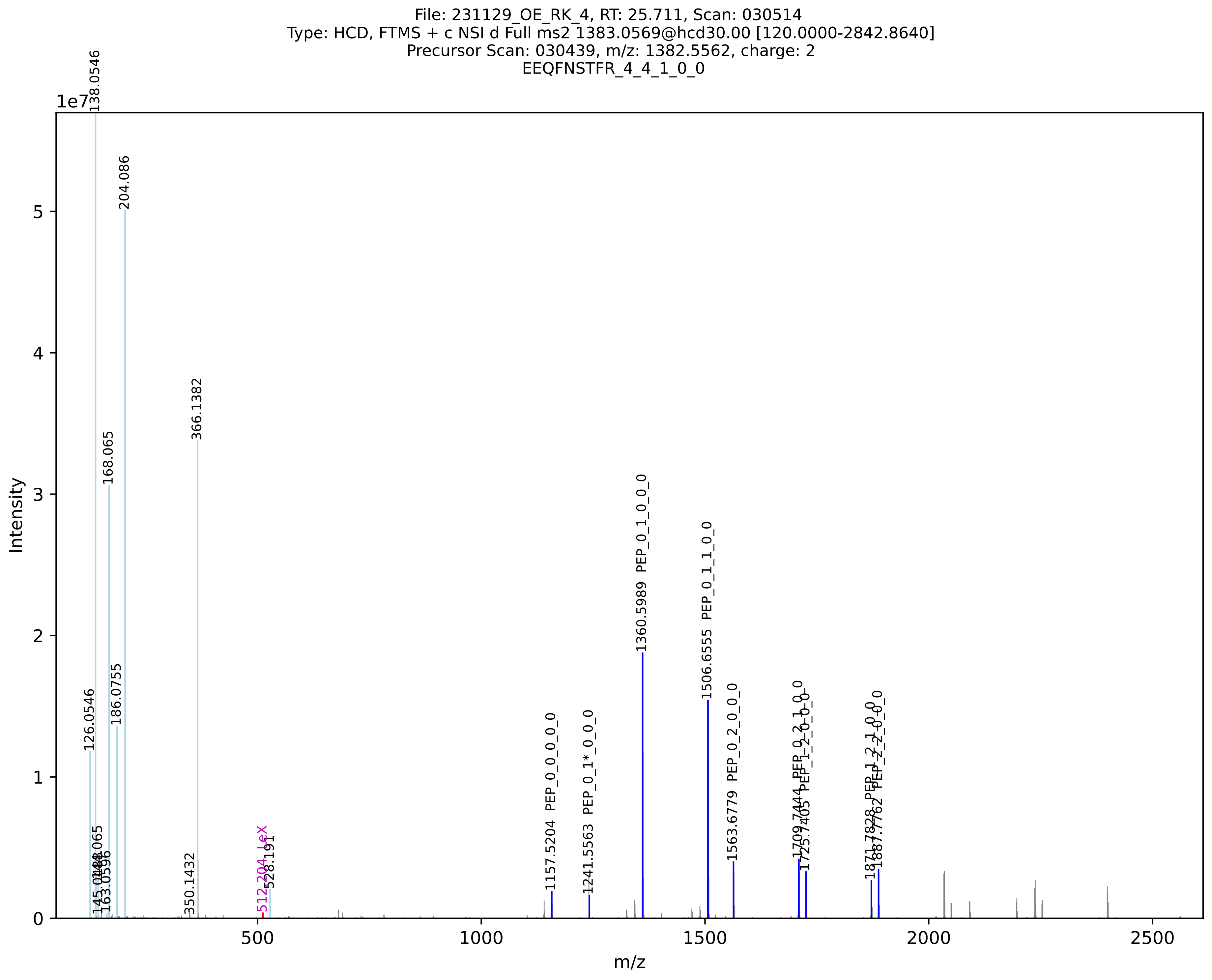

File: 231129_OE_RK_4, RT: 25.711, Scan: 030514
Type: HCD, FTMS + c NSI d Full ms2 1383.0569@hcd30.00 [120.0000-2842.8640]
Precursor Scan: 030439, m/z: 1382.5562, charge: 2
EEQFNSTFR_4_4_1_0_0
1e7
Intensity
m/z
138.0546
204.086
366.1382
168.065
186.0755
126.0546
350.1432
512.204_LeX
528.191
1157.5204 PEP_0_0_0_0_0
1241.5563 PEP_0_1*_0_0_0
1360.5989 PEP_0_1_0_0_0
1506.6555 PEP_0_1_1_0_0
1563.6779 PEP_0_2_0_0_0
1709.7444 PEP_0_2_1_0_0
1725.7405 PEP_1_2_0_0_0
1871.7828 PEP_1_2_1_0_0
1887.7762 PEP_2_2_0_0_0

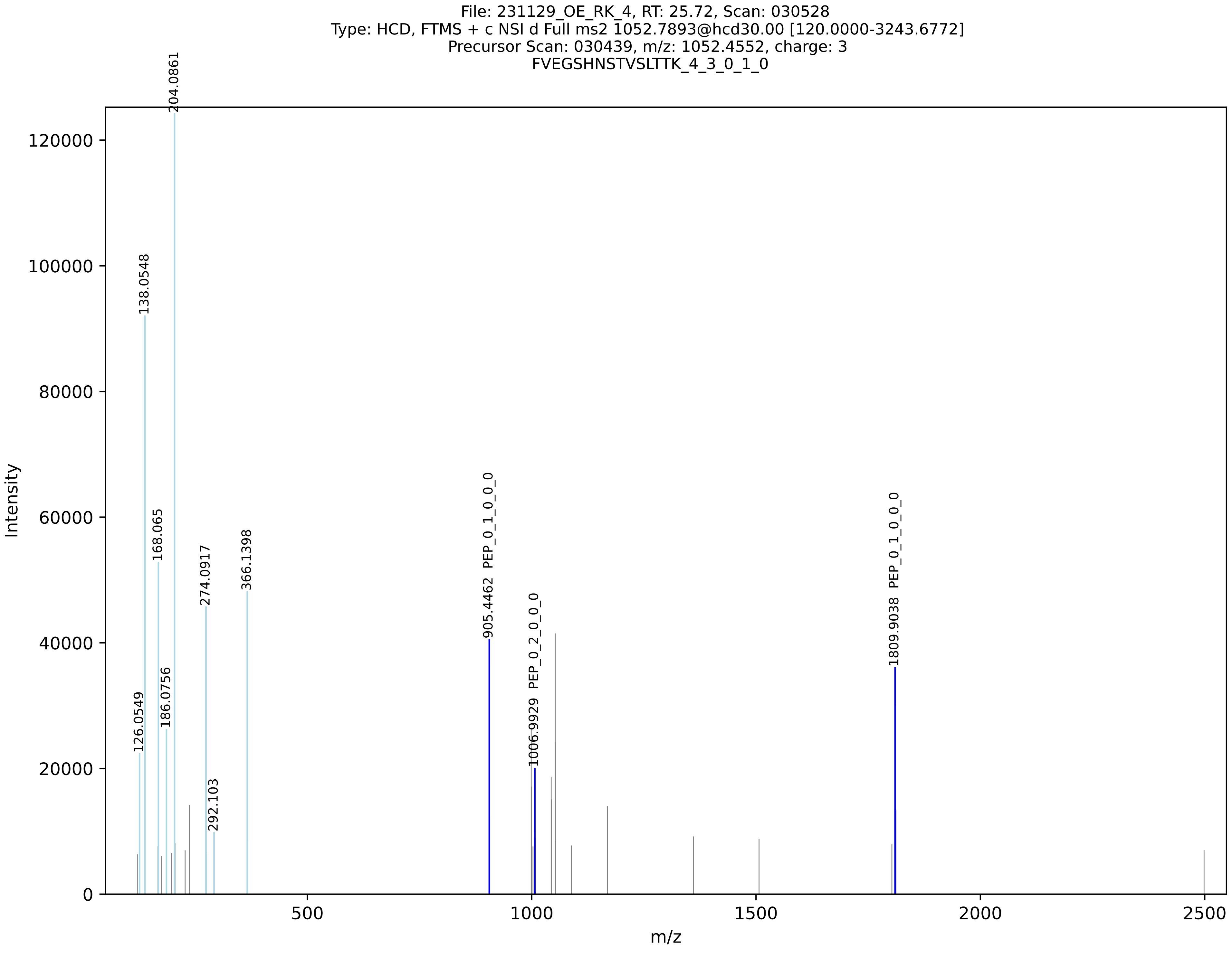
File: 231129_OE_RK_4, RT: 25.72, Scan: 030528
Type: HCD, FTMS + c NSI d Full ms2 1052.7893@hcd30.00 [120.0000-3243.6772]
Precursor Scan: 030439, m/z: 1052.4552, charge: 3
FVEGSHNSTVSLTTK_4_3_0_1_0
204.0861
138.0548
168.065
274.0917
366.1398
126.0549
186.0756
292.103
905.4462 PEP_0_1_0_0_0_
1006.9929 PEP_0_2_0_0_0_
1809.9038 PEP_0_1_0_0_0_
Intensity
m/z
0
20000
40000
60000
80000
100000
120000
500
1000
1500
2000
2500

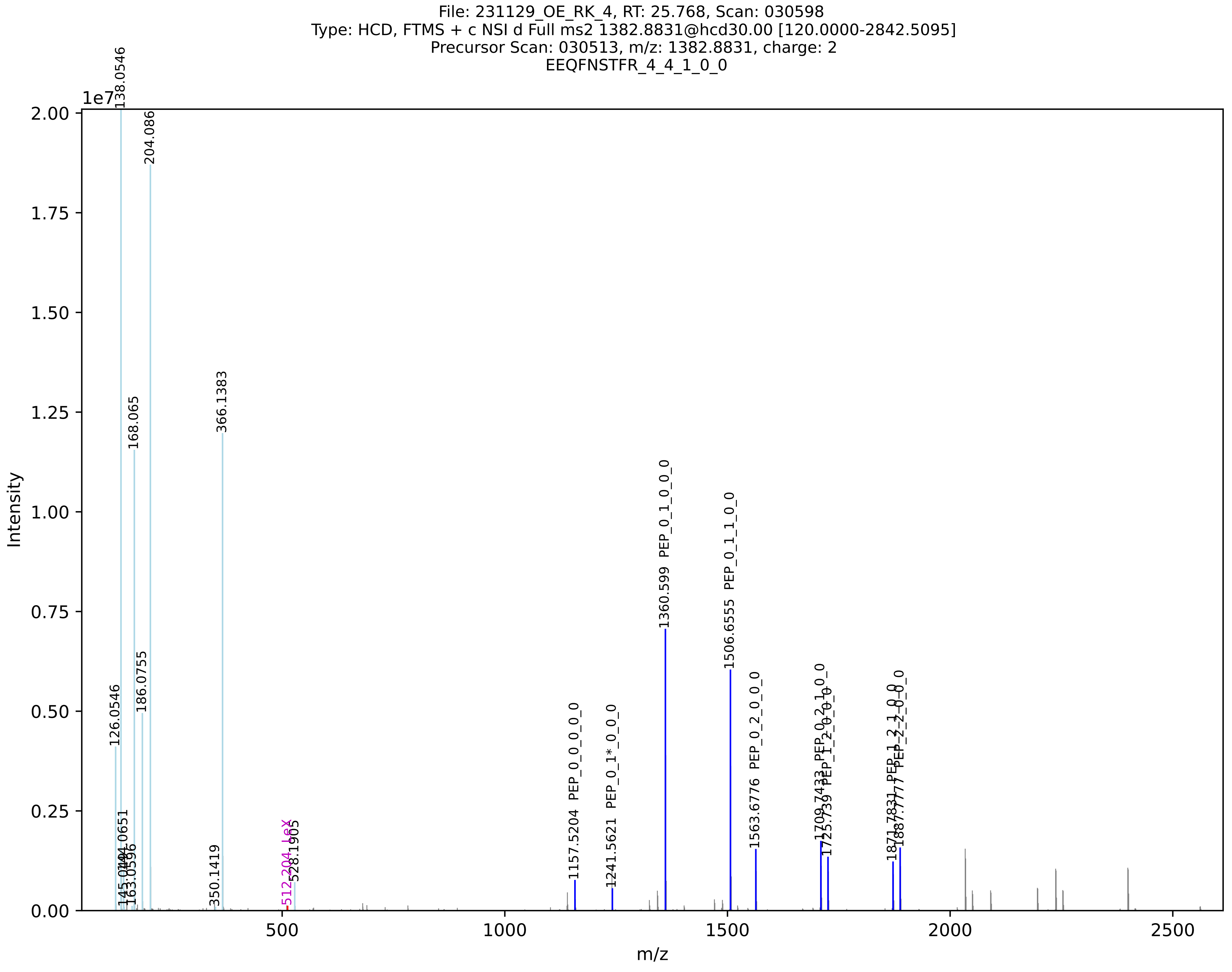
File: 231129_OE_RK_4, RT: 25.768, Scan: 030598
Type: HCD, FTMS + c NSI d Full ms2 1382.8831@hcd30.00 [120.0000-2842.5095]
Precursor Scan: 030513, m/z: 1382.8831, charge: 2
EEQFNSTFR_4_4_1_0_0
1e7
Intensity
m/z
138.0546
204.086
168.065
366.1383
126.0546
186.0755
350.1419
512.204_LeX
528.1905
163.0596
1157.5204 PEP_0_0_0_0_0
1241.5621 PEP_0_1*_0_0_0
1360.599 PEP_0_1_0_0_0
1506.6555 PEP_0_1_1_0_0
1563.6776 PEP_0_2_0_0_0
1709.7433 PEP_0_2_1_0_0
1725.739 PEP_1_2_0_0_0
1871.7831 PEP_1_2_1_0_0
1887.7777 PEP_2_2_0_0_0

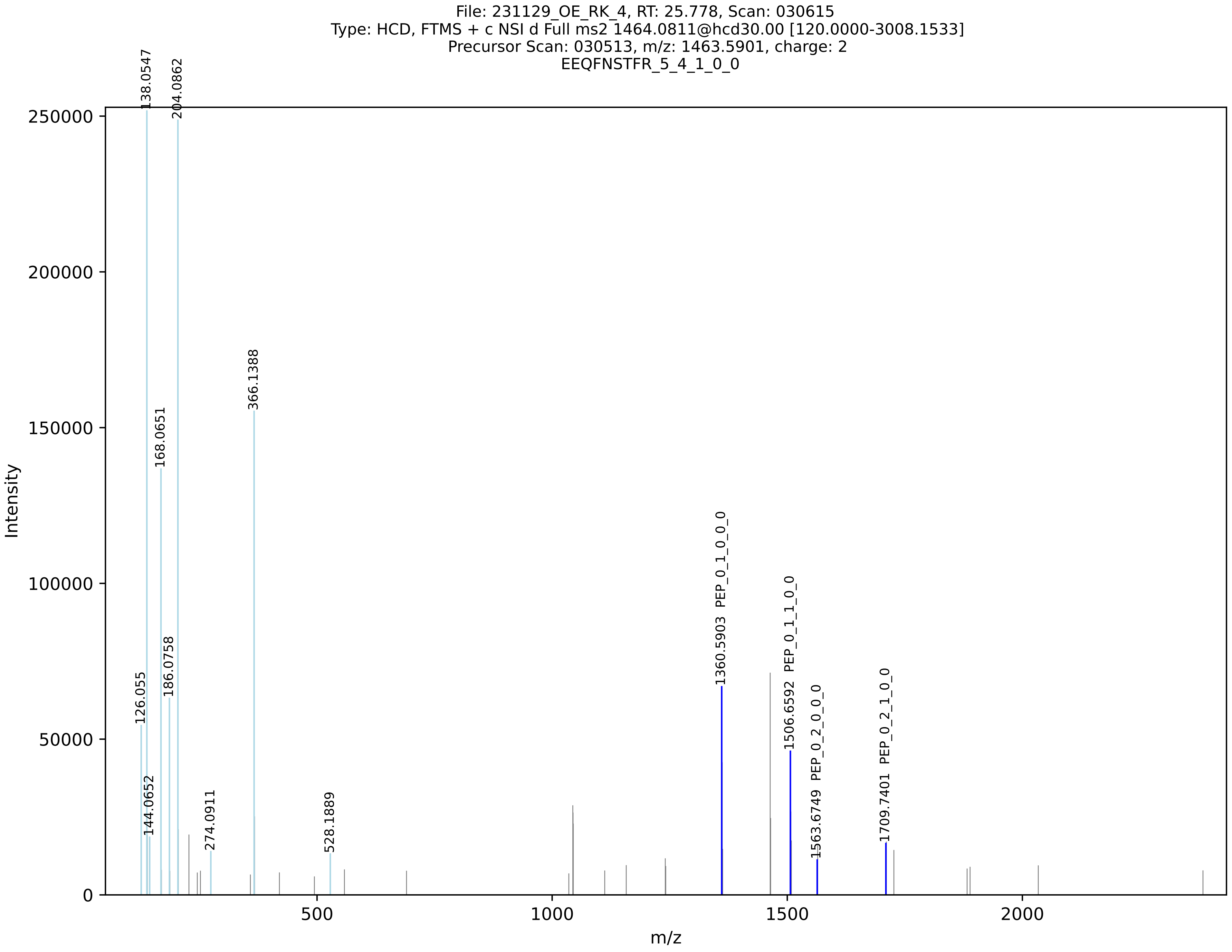
File: 231129_OE_RK_4, RT: 25.778, Scan: 030615
Type: HCD, FTMS + c NSI d Full ms2 1464.0811@hcd30.00 [120.0000-3008.1533]
Precursor Scan: 030513, m/z: 1463.5901, charge: 2
EEQFNSTFR_5_4_1_0_0
138.0547
204.0862
366.1388
168.0651
186.0758
126.055
144.0652
274.0911
528.1889
1360.5903 PEP_0_1_0_0_0
1506.6592 PEP_0_1_1_0_0
1563.6749 PEP_0_2_0_0_0
1709.7401 PEP_0_2_1_0_0
Intensity
m/z
250000
200000
150000
100000
50000
0
500
1000
1500
2000

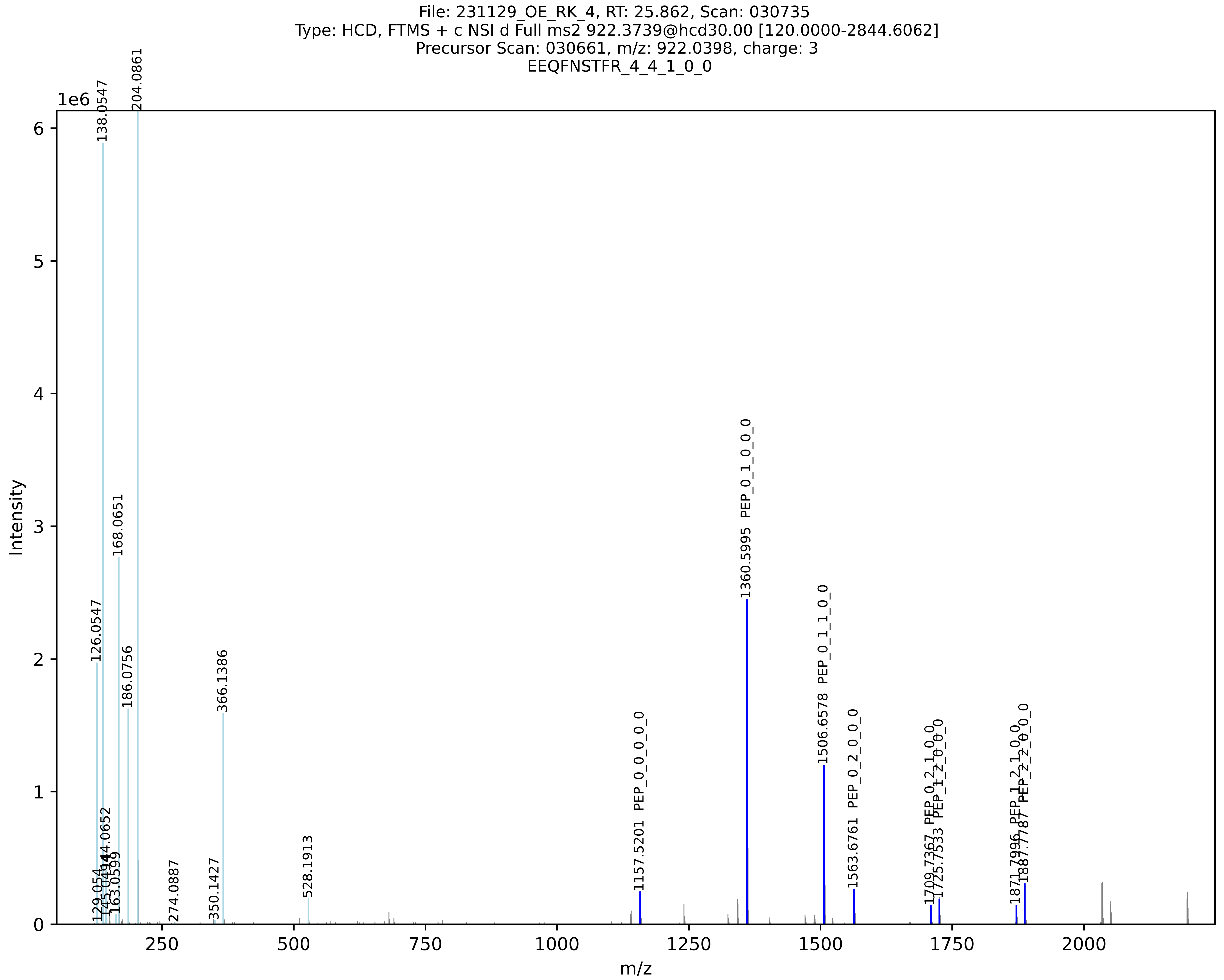
File: 231129_OE_RK_4, RT: 25.862, Scan: 030735
Type: HCD, FTMS + c NSI d Full ms2 922.3739@hcd30.00 [120.0000-2844.6062]
Precursor Scan: 030661, m/z: 922.0398, charge: 3
EEQFNSTFR_4_4_1_0_0
1e6
Intensity
m/z
204.0861
138.0547
168.0651
126.0547
186.0756
366.1386
528.1913
274.0887
350.1427
1157.5201 PEP_0_0_0_0_0
1360.5995 PEP_0_1_0_0_0
1506.6578 PEP_0_1_1_0_0
1563.6761 PEP_0_2_0_0_0
1709.7367 PEP_0_2_1_0_0
1725.7533 PEP_1_2_0_0
1871.7996 PEP_1_2_1_0_0
1887.7787 PEP_2_2_0_0_0

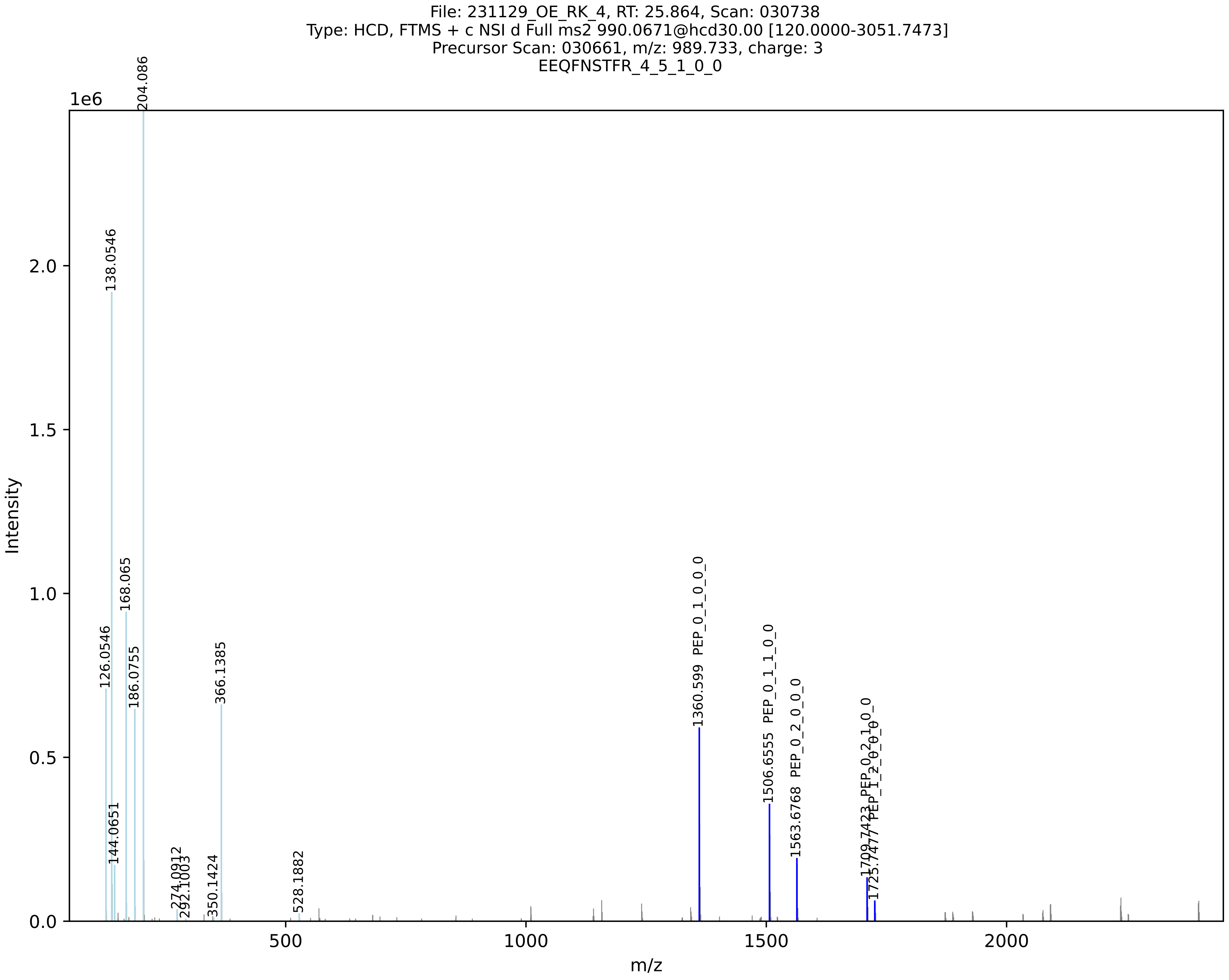
File: 231129_OE_RK_4, RT: 25.864, Scan: 030738
Type: HCD, FTMS + c NSI d Full ms2 990.0671@hcd30.00 [120.0000-3051.7473]
Precursor Scan: 030661, m/z: 989.733, charge: 3
EEQFNSTFR_4_5_1_0_0
1e6
Intensity
m/z
204.086
138.0546
168.065
126.0546
186.0755
366.1385
144.0651
274.0912
292.1003
350.1424
528.1882
1360.599 PEP_0_1_0_0_0
1506.6555 PEP_0_1_1_0_0
1563.6768 PEP_0_2_0_0_0
1709.7423 PEP_0_2_1_0_0
1725.7477 PEP_1_2_0_0_0

File: 231129_OE_RK_4, RT: 25.864, Scan: 030739
Type: HCD, FTMS + c NSI d Full ms2 919.6372@hcd30.00 [120.0000-3774.9758]
Precursor Scan: 030661, m/z: 919.1358, charge: 4
VYKPSAGNNSLYR_5_4_0_2_0

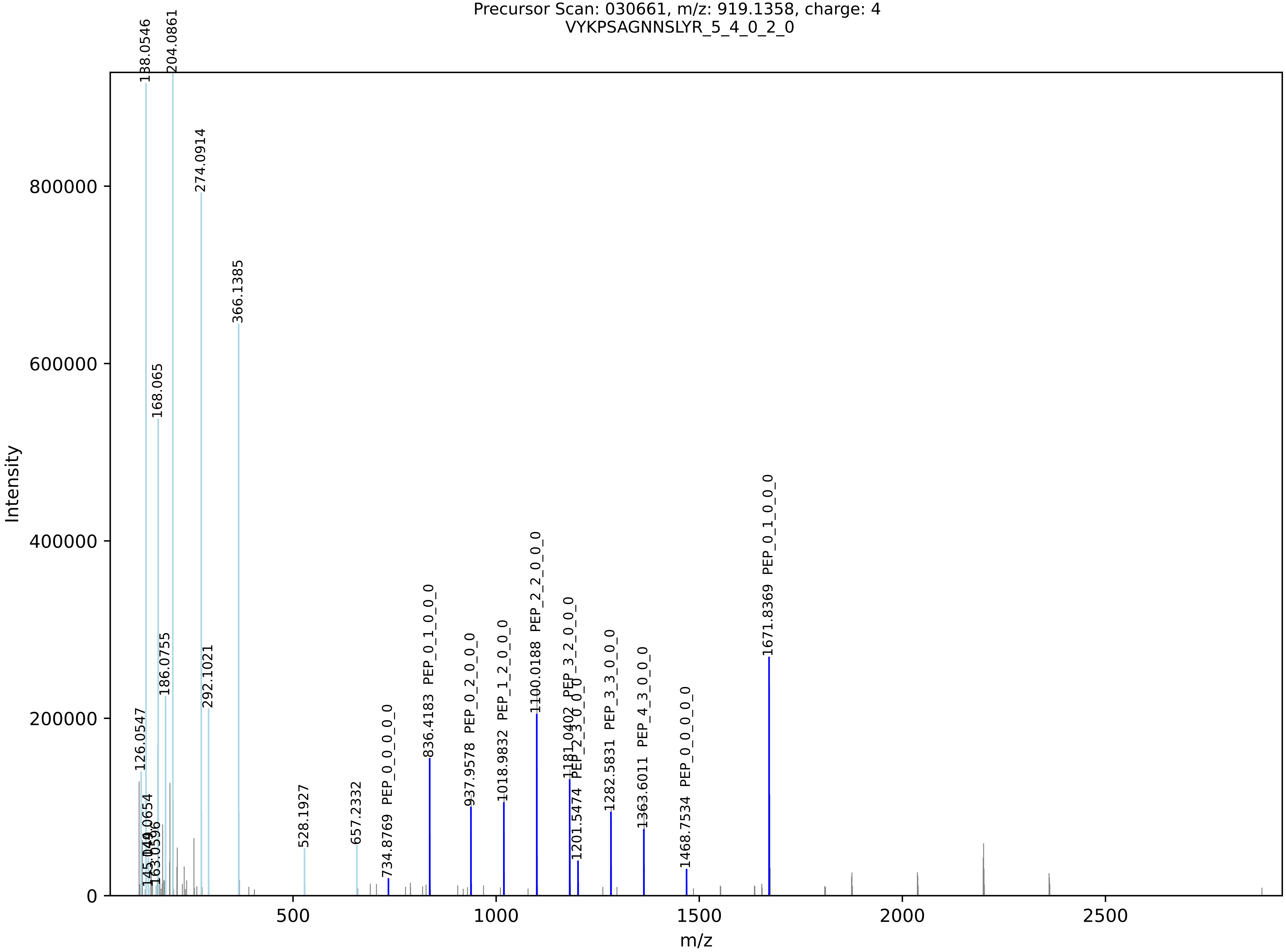

File: 231129_OE_RK_4, RT: 25.865, Scan: 030740
Type: HCD, FTMS + c NSI d Full ms2 1274.5341@hcd30.00 [120.0000-3922.2163]
Precursor Scan: 030661, m/z: 1273.8659, charge: 3
VYKPSAGNNSLYR_5_4_1_2_0

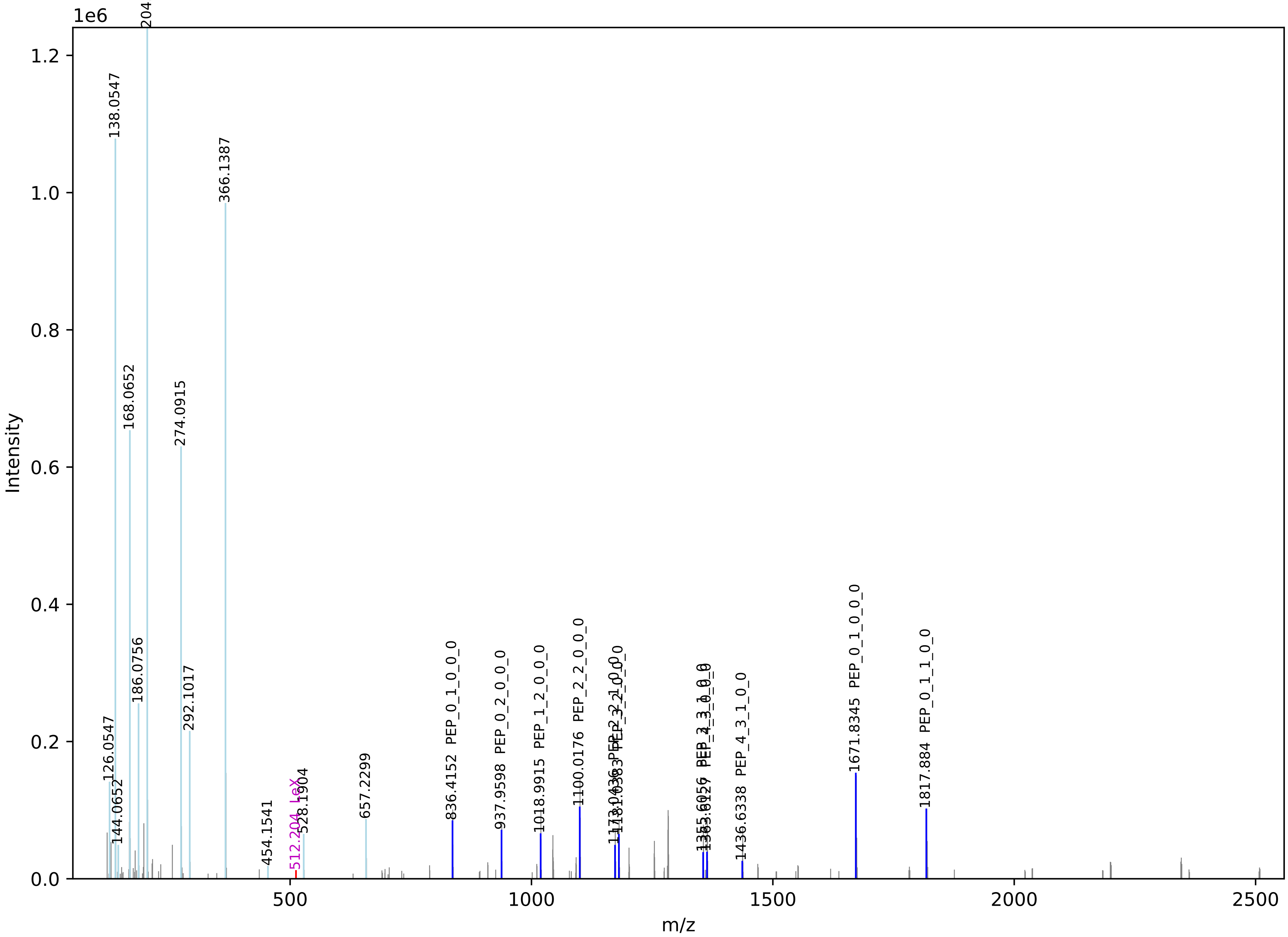

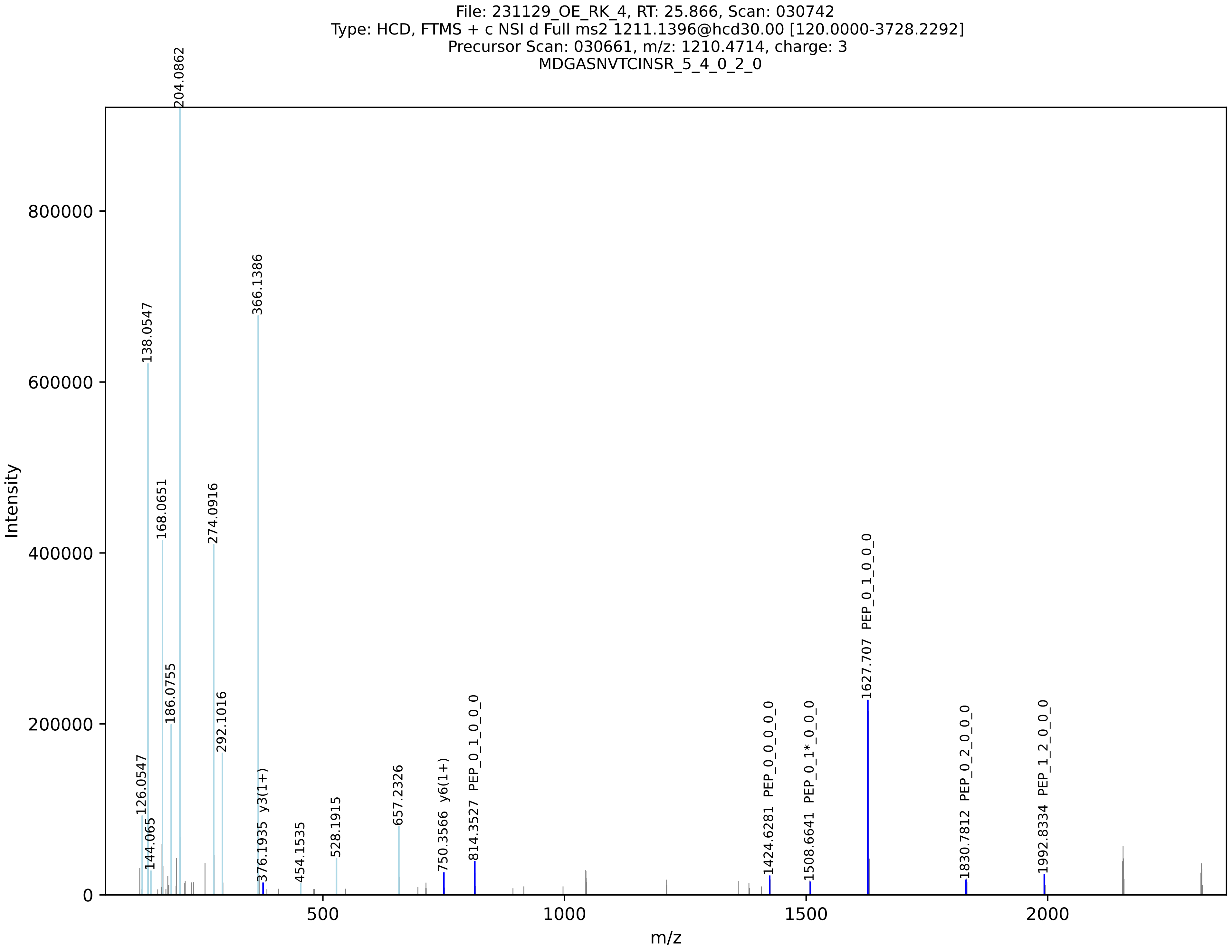
File: 231129_OE_RK_4, RT: 25.866, Scan: 030742
Type: HCD, FTMS + c NSI d Full ms2 1211.1396@hcd30.00 [120.0000-3728.2292]
Precursor Scan: 030661, m/z: 1210.4714, charge: 3
MDGASNVTCINSR_5_4_0_2_0
Intensity
m/z
204.0862
366.1386
138.0547
168.0651
274.0916
186.0755
292.1016
126.0547
144.065
376.1935 y3(1+)
454.1535
528.1915
657.2326
750.3566 y6(1+)
814.3527 PEP_0_1_0_0_0
1424.6281 PEP_0_0_0_0_0
1508.6641 PEP_0_1*_0_0_0
1627.707 PEP_0_1_0_0_0
1830.7812 PEP_0_2_0_0_0
1992.8334 PEP_1_2_0_0_0
0
200000
400000
600000
800000
500
1000
1500
2000

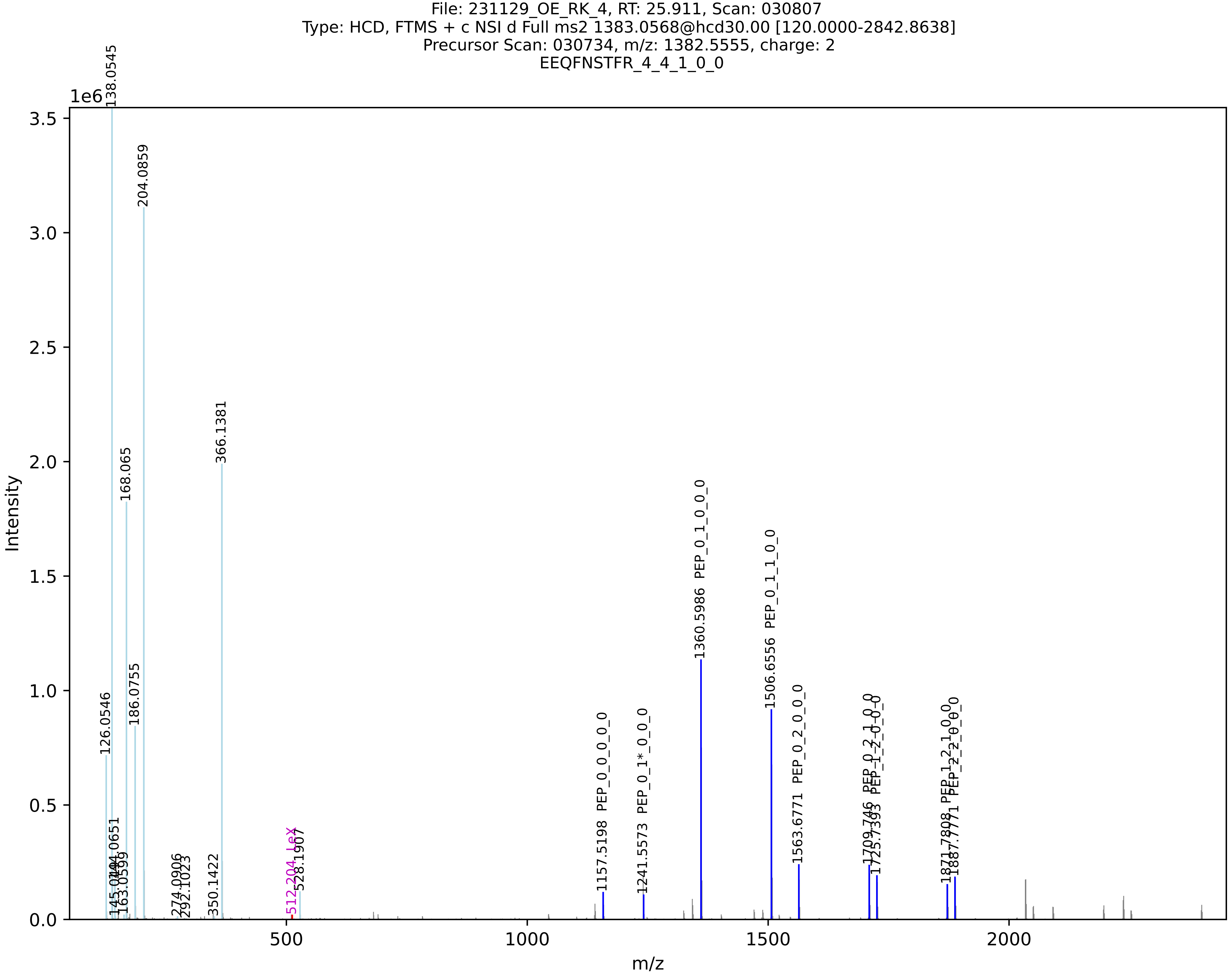
File: 231129_OE_RK_4, RT: 25.911, Scan: 030807
Type: HCD, FTMS + c NSI d Full ms2 1383.0568@hcd30.00 [120.0000-2842.8638]
Precursor Scan: 030734, m/z: 1382.5555, charge: 2
EEQFNSTFR_4_4_1_0_0
1e6
Intensity
m/z
138.0545
204.0859
366.1381
168.065
186.0755
126.0546
512.204_LeX
528.1907
1360.5986 PEP_0_1_0_0_0
1506.6556 PEP_0_1_1_0_0
1563.6771 PEP_0_2_0_0_0
1157.5198 PEP_0_0_0_0_0
1241.5573 PEP_0_1*_0_0_0
274.0906
292.1023
350.1422
163.0599

File: 231129_OE_RK_4, RT: 25.916, Scan: 030814
Type: HCD, FTMS + c NSI d Full ms2 956.1520@hcd30.00 [120.0000-3923.9563]
Precursor Scan: 030734, m/z: 955.3993, charge: 4
VYKPSAGNNSLYR_5_4_1_2_0

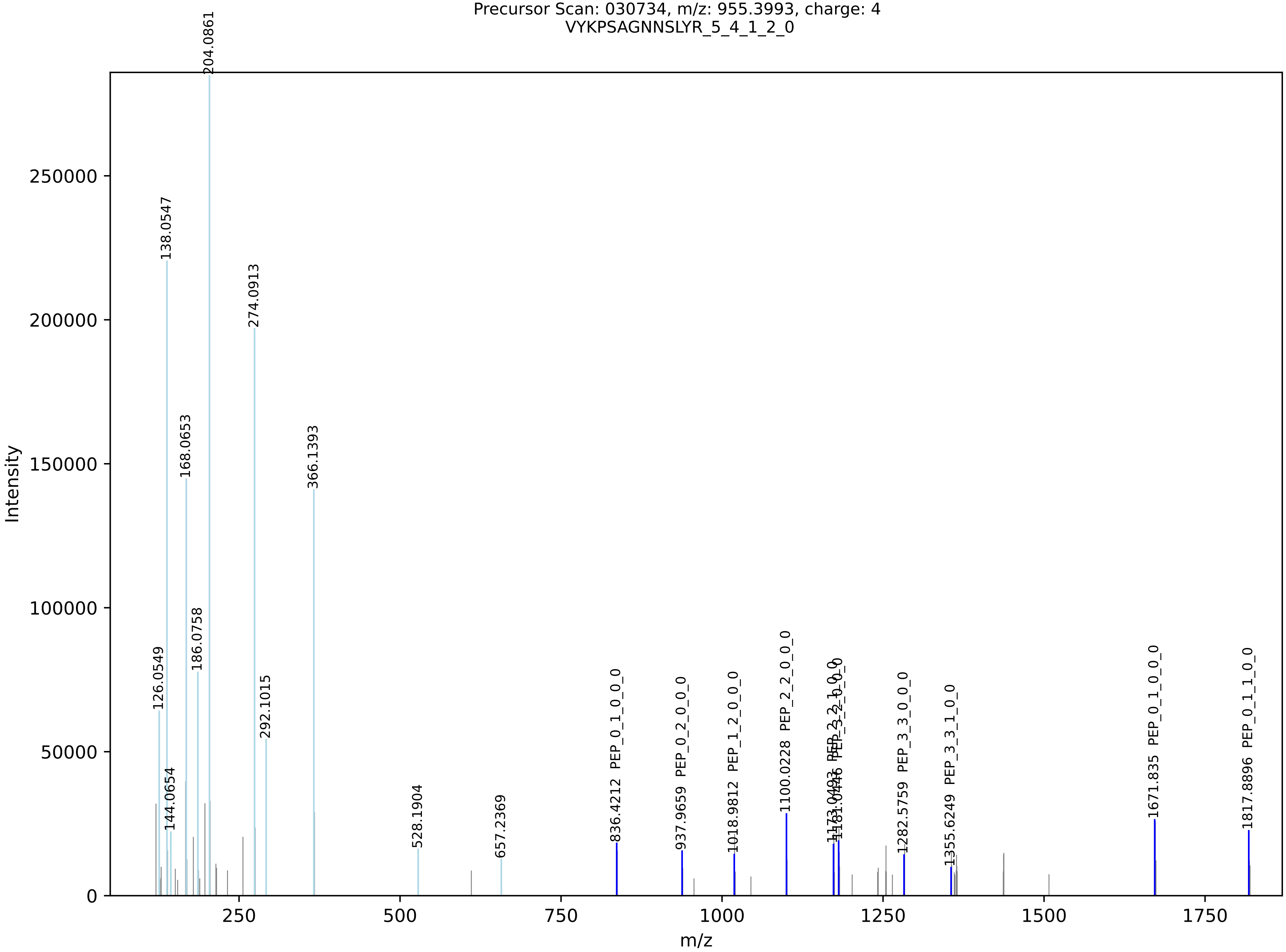

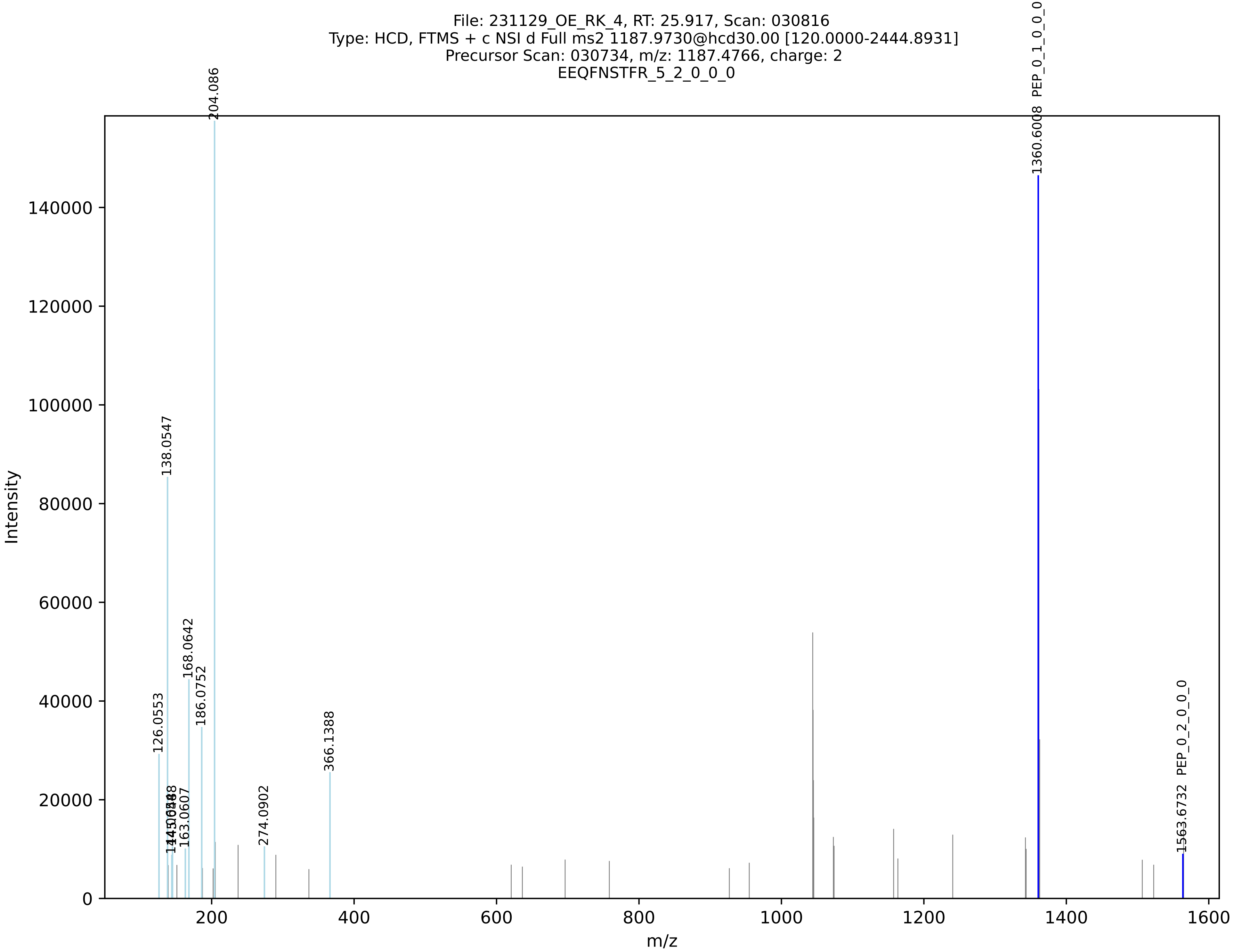

File: 231129_OE_RK_4, RT: 25.917, Scan: 030816
Type: HCD, FTMS + c NSI d Full ms2 1187.9730@hcd30.00 [120.0000-2444.8931]
Precursor Scan: 030734, m/z: 1187.4766, charge: 2
EEQFNSTFR_5_2_0_0_0
Intensity
m/z
204.086
138.0547
168.0642
186.0752
126.0553
163.0607
274.0902
366.1388
1360.6008 PEP_0_1_0_0_0
1563.6732 PEP_0_2_0_0_0

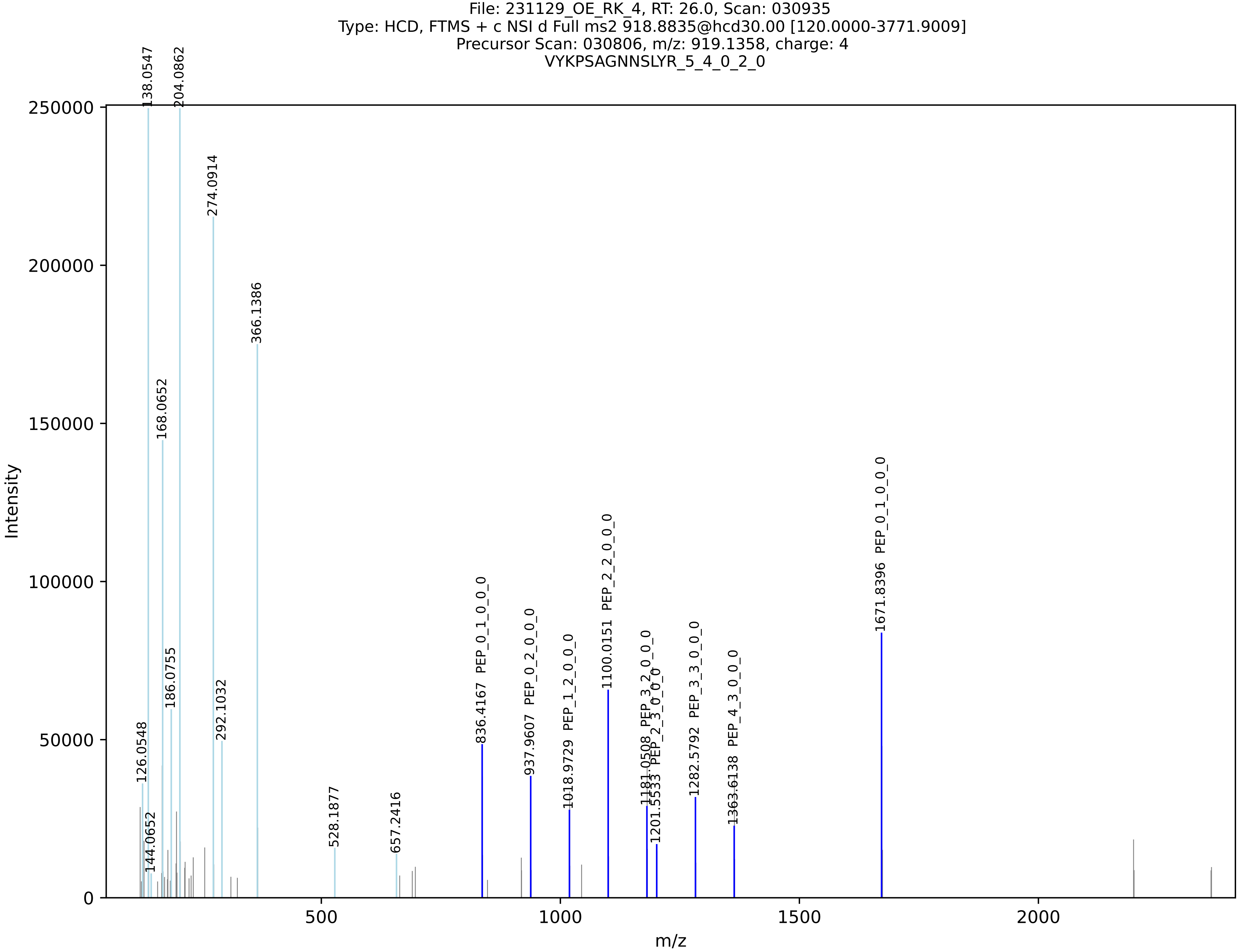
File: 231129_OE_RK_4, RT: 26.0, Scan: 030935
Type: HCD, FTMS + c NSI d Full ms2 918.8835@hcd30.00 [120.0000-3771.9009]
Precursor Scan: 030806, m/z: 919.1358, charge: 4
VYKPSAGNNSLYR_5_4_0_2_0
Intensity
m/z
138.0547
204.0862
274.0914
366.1386
168.0652
186.0755
126.0548
144.0652
292.1032
528.1877
657.2416
836.4167 PEP_0_1_0_0_0
937.9607 PEP_0_2_0_0_0
1018.9729 PEP_1_2_0_0_0
1100.0151 PEP_2_2_0_0_0
1181.0508 PEP_3_2_0_0_0
1201.5533 PEP_2_3_0_0_0
1282.5792 PEP_3_3_0_0_0
1363.6138 PEP_4_3_0_0_0
1671.8396 PEP_0_1_0_0_0

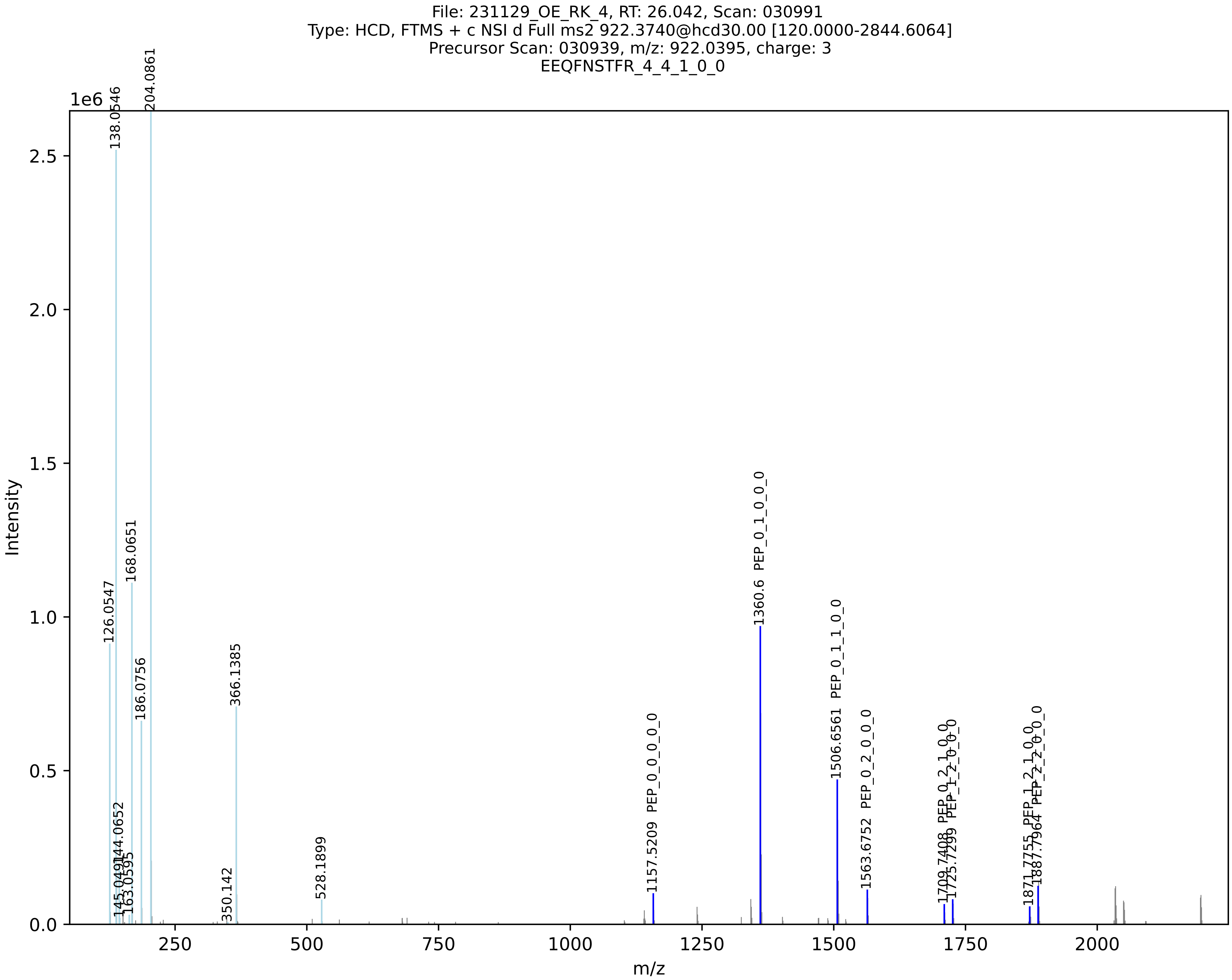
File: 231129_OE_RK_4, RT: 26.042, Scan: 030991
Type: HCD, FTMS + c NSI d Full ms2 922.3740@hcd30.00 [120.0000-2844.6064]
Precursor Scan: 030939, m/z: 922.0395, charge: 3
EEQFNSTFR_4_4_1_0_0
1e6
Intensity
m/z
204.0861
138.0546
168.0651
126.0547
186.0756
366.1385
144.0652
145.0491
163.0595
350.142
528.1899
1157.5209 PEP_0_0_0_0_0
1360.6 PEP_0_1_0_0_0
1506.6561 PEP_0_1_1_0_0
1563.6752 PEP_0_2_0_0_0
1709.7408 PEP_0_2_1_0_0
1725.7299 PEP_1_2_0_0_0
1871.7755 PEP_1_2_1_0_0
1887.7964 PEP_2_2_0_0_0

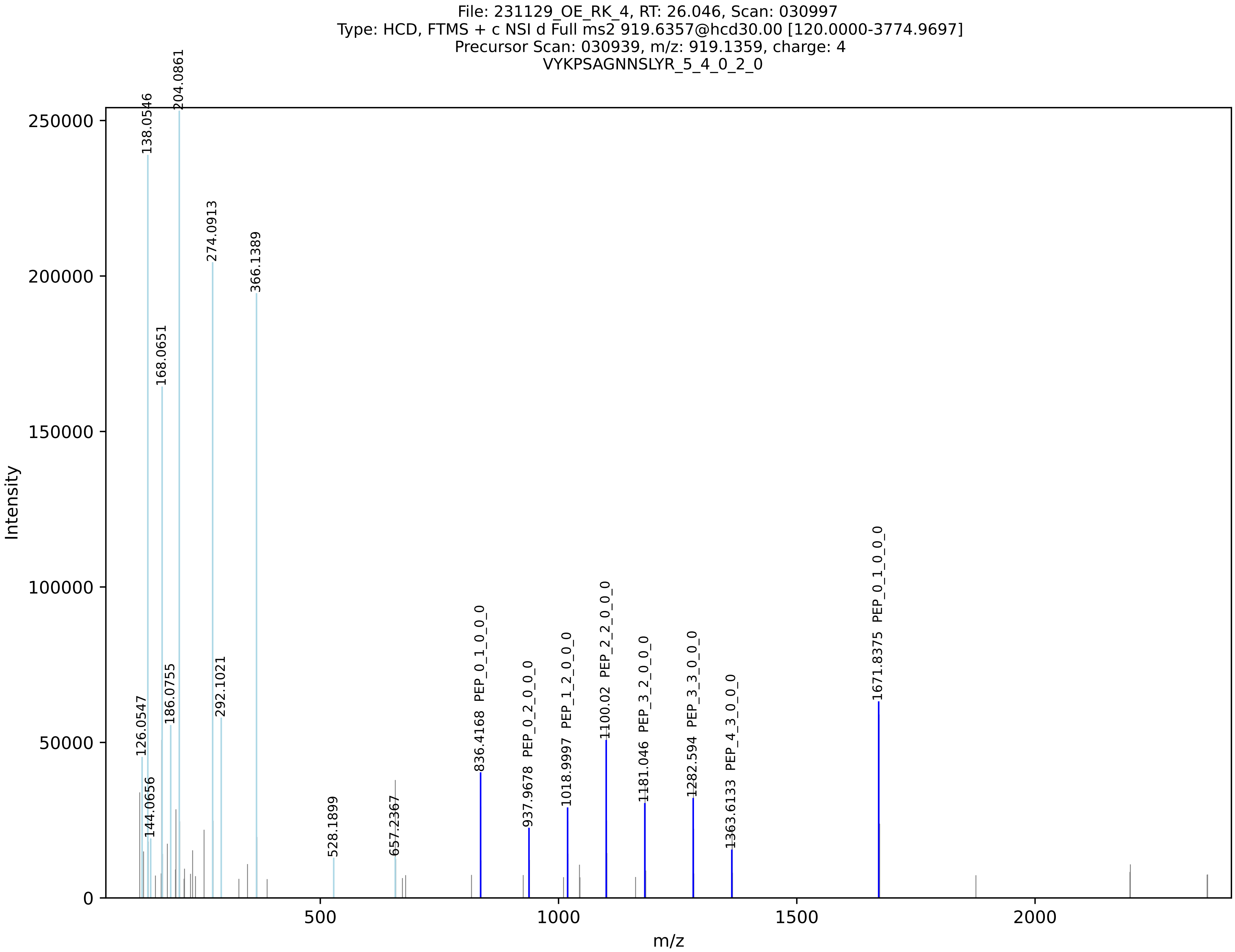
File: 231129_OE_RK_4, RT: 26.046, Scan: 030997
Type: HCD, FTMS + c NSI d Full ms2 919.6357@hcd30.00 [120.0000-3774.9697]
Precursor Scan: 030939, m/z: 919.1359, charge: 4
VYKPSAGNNSLYR_5_4_0_2_0
Intensity
m/z
0
50000
100000
150000
200000
250000
500
1000
1500
2000
126.0547
138.0546
144.0656
168.0651
186.0755
204.0861
274.0913
292.1021
366.1389
528.1899
657.2367
836.4168 PEP_0_1_0_0_0
937.9678 PEP_0_2_0_0_0
1018.9997 PEP_1_2_0_0_0
1100.02 PEP_2_2_0_0_0
1181.046 PEP_3_2_0_0_0
1282.594 PEP_3_3_0_0_0
1363.6133 PEP_4_3_0_0_0
1671.8375 PEP_0_1_0_0_0

File: 231129_OE_RK_4, RT: 26.049, Scan: 031002
Type: HCD, FTMS + c NSI d Full ms2 1484.5966@hcd30.00 [120.0000-3050.0049]
Precursor Scan: 030939, m/z: 1484.0948, charge: 2
EEQFNSTFR_4_5_1_0_0

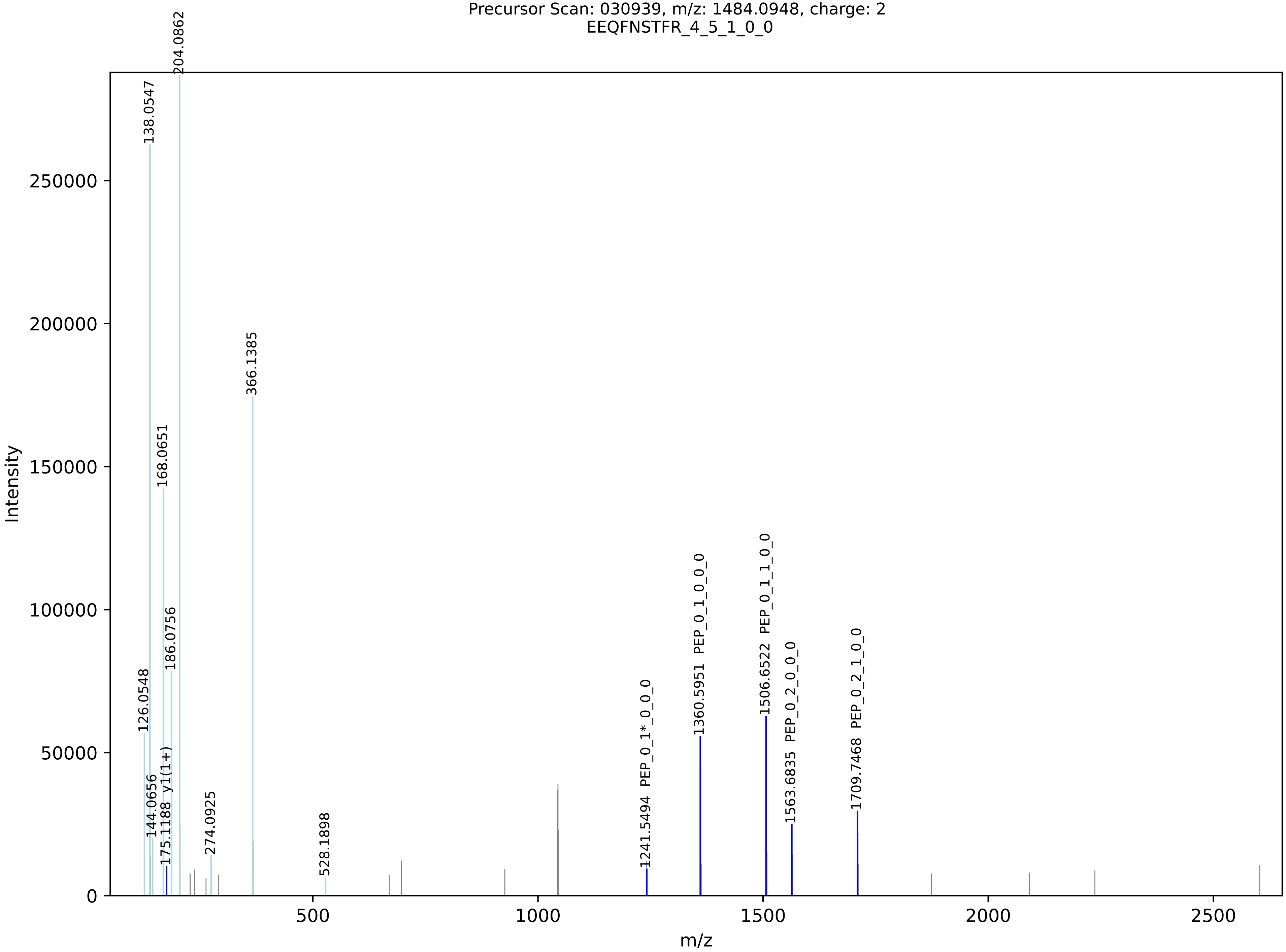

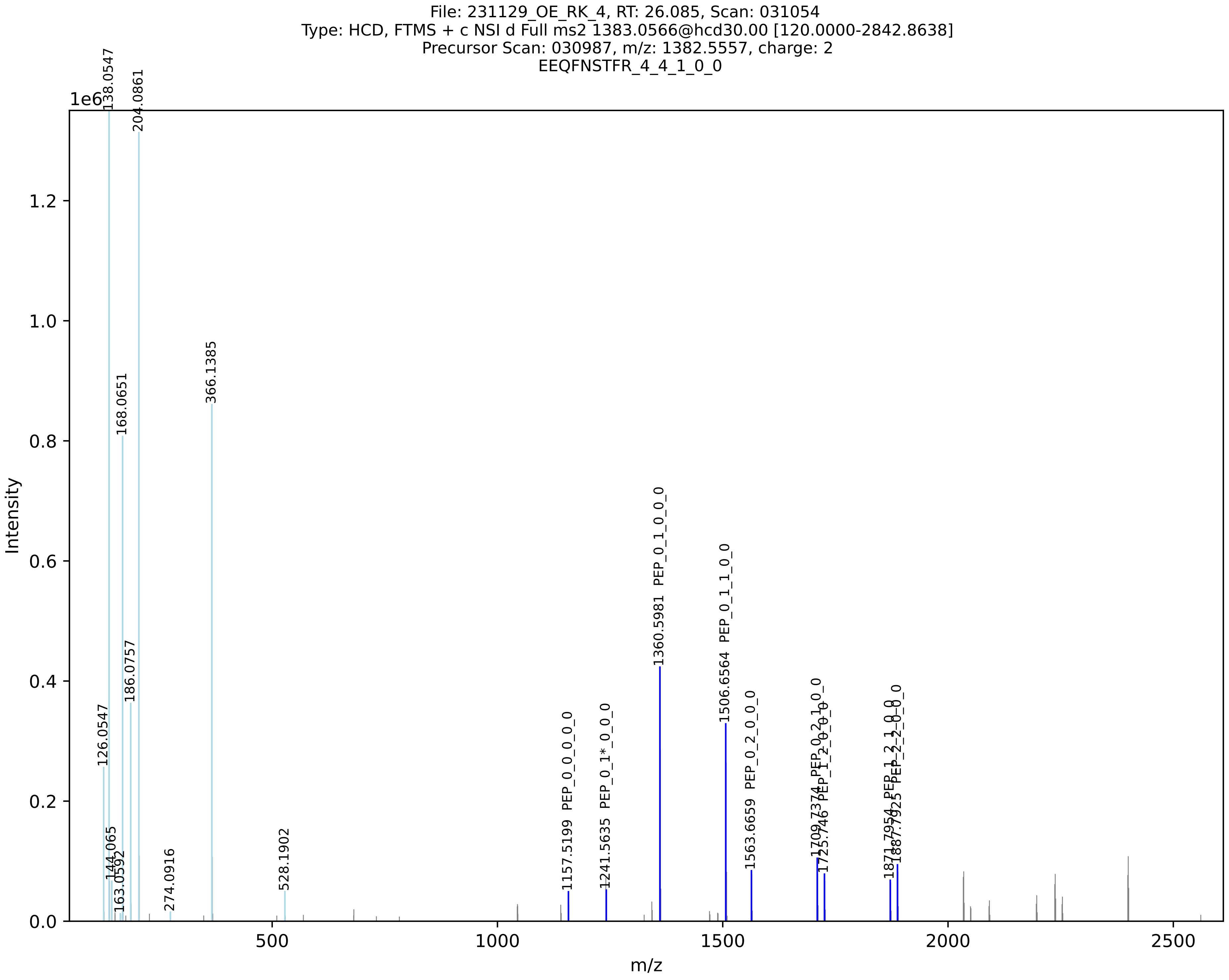

File: 231129_OE_RK_4, RT: 26.085, Scan: 031054
Type: HCD, FTMS + c NSI d Full ms2 1383.0566@hcd30.00 [120.0000-2842.8638]
Precursor Scan: 030987, m/z: 1382.5557, charge: 2
EEQFNSTFR_4_4_1_0_0
1e6
Intensity
m/z
138.0547
204.0861
366.1385
168.0651
186.0757
126.0547
144.065
163.0592
274.0916
528.1902
1157.5199 PEP_0_0_0_0_0
1241.5635 PEP_0_1*_0_0_0
1360.5981 PEP_0_1_0_0_0
1506.6564 PEP_0_1_1_0_0
1563.6659 PEP_0_2_0_0_0
1709.7374 PEP_0_2_1_0_0
1725.746 PEP_1_2_0_0_0
1871.7954 PEP_1_2_1_0_0
1887.7925 PEP_2_2_0_0_0

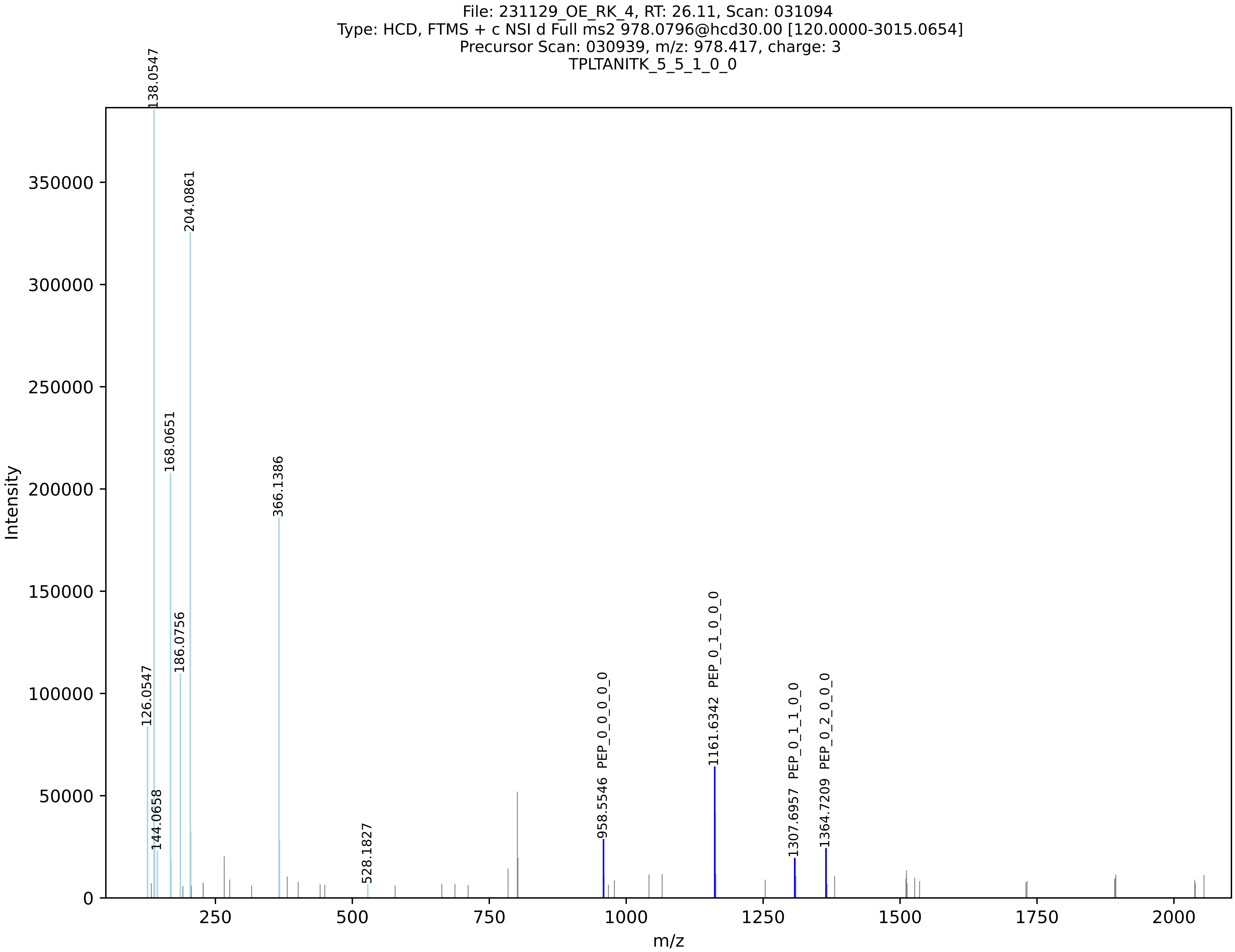
File: 231129_OE_RK_4, RT: 26.11, Scan: 031094
Type: HCD, FTMS + c NSI d Full ms2 978.0796@hcd30.00 [120.0000-3015.0654]
Precursor Scan: 030939, m/z: 978.417, charge: 3
TPLTANITK_5_5_1_0_0
Intensity
m/z
138.0547
204.0861
168.0651
366.1386
186.0756
126.0547
144.0658
528.1827
958.5546 PEP_0_0_0_0_0_
1161.6342 PEP_0_1_0_0_0
1307.6957 PEP_0_1_1_0_0
1364.7209 PEP_0_2_0_0_0
350000
300000
250000
200000
150000
100000
50000
0
250
500
750
1000
1250
1500
1750
2000

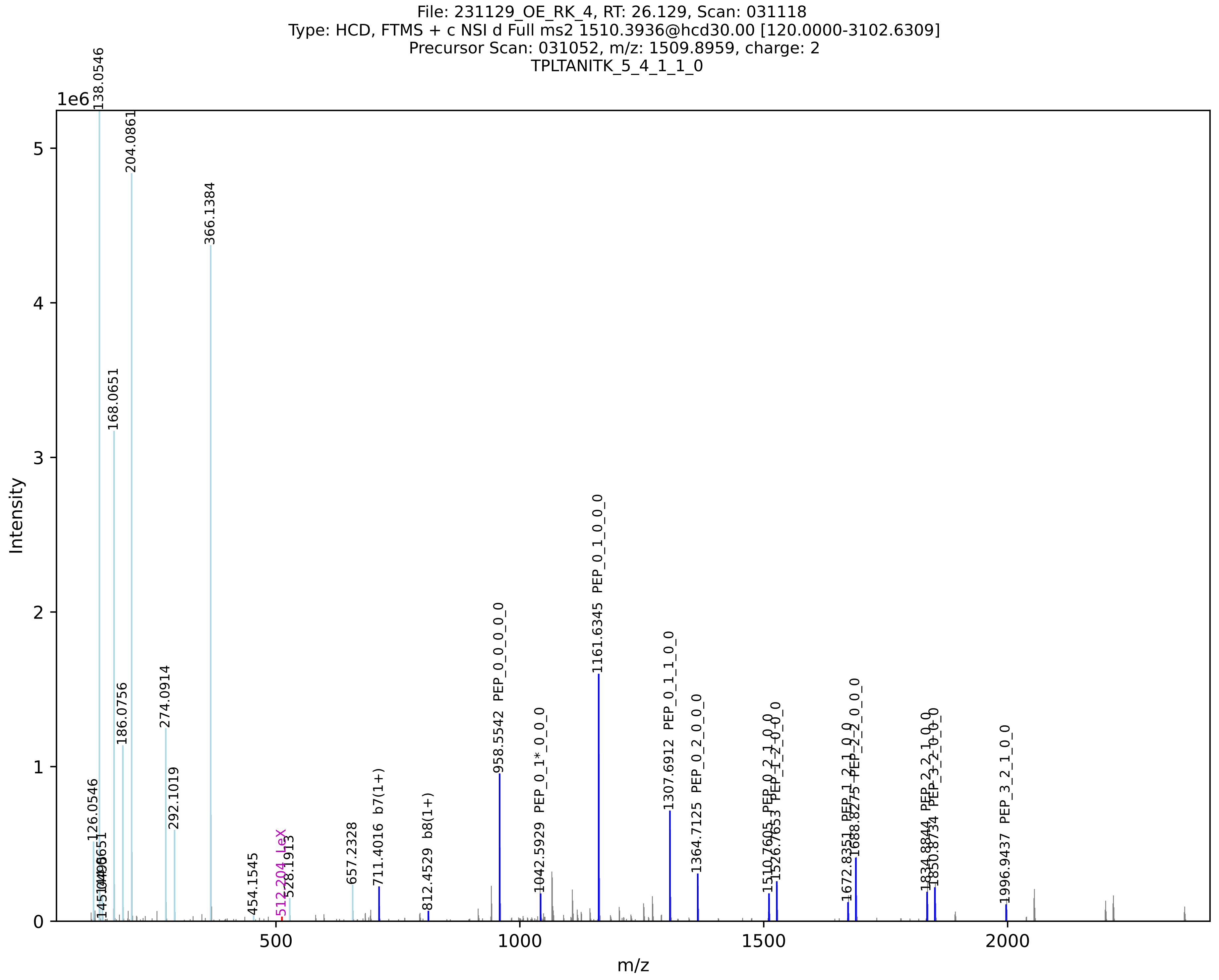

File: 231129_OE_RK_4, RT: 26.129, Scan: 031118
Type: HCD, FTMS + c NSI d Full ms2 1510.3936@hcd30.00 [120.0000-3102.6309]
Precursor Scan: 031052, m/z: 1509.8959, charge: 2
TPLTANITK_5_4_1_1_0
1e6
Intensity
m/z
138.0546
204.0861
366.1384
168.0651
186.0756
274.0914
292.1019
126.0546
454.1545
512.204_LeX
528.1913
657.2328
711.4016 b7(1+)
812.4529 b8(1+)
958.5542 PEP_0_0_0_0_0_
1042.5929 PEP_0_1*_0_0_0_
1161.6345 PEP_0_1_0_0_0_
1307.6912 PEP_0_1_1_0_0_
1364.7125 PEP_0_2_0_0_0_
1996.9437 PEP_3_2_1_0_0_

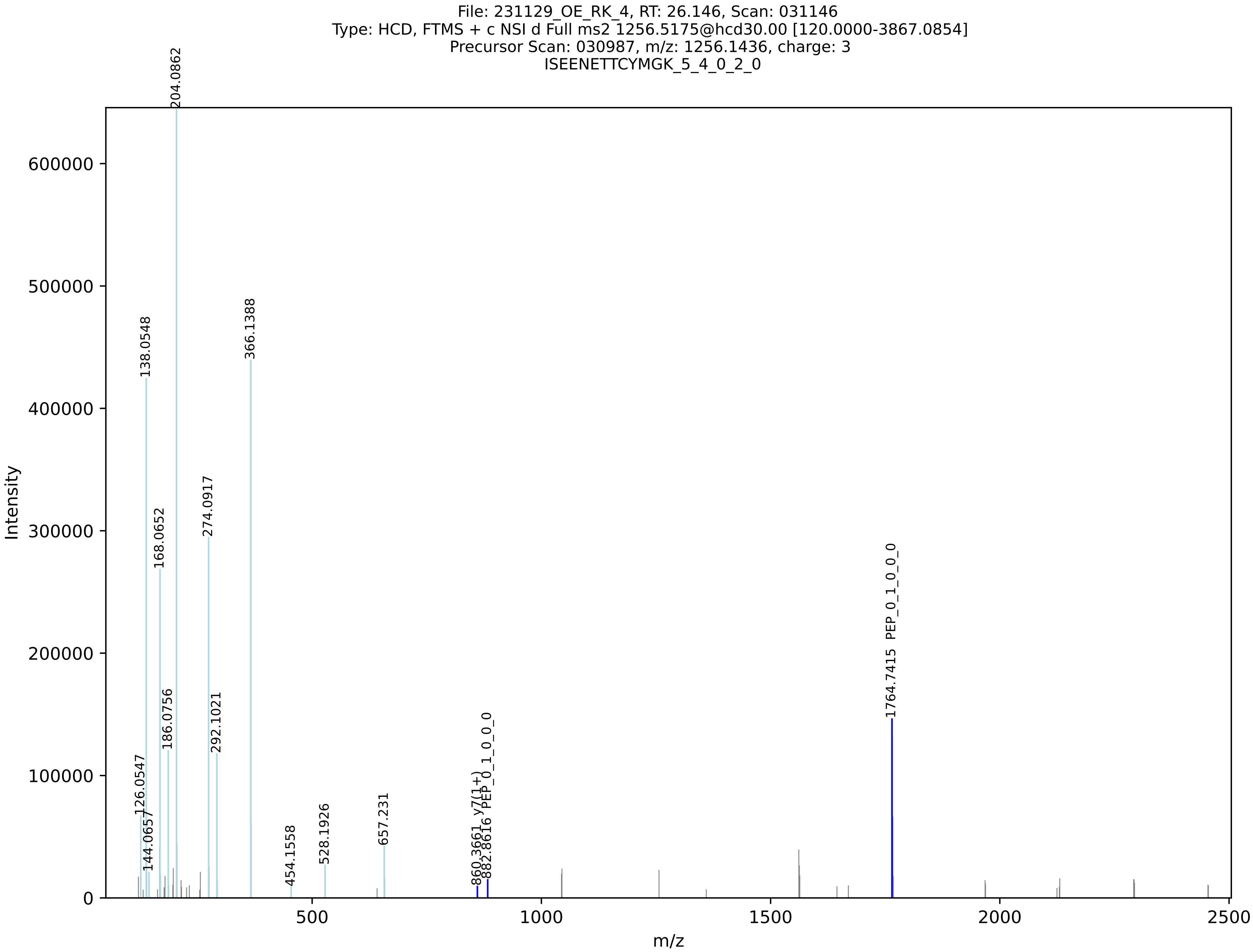

File: 231129_OE_RK_4, RT: 26.146, Scan: 031146
Type: HCD, FTMS + c NSI d Full ms2 1256.5175@hcd30.00 [120.0000-3867.0854]
Precursor Scan: 030987, m/z: 1256.1436, charge: 3
ISEENETTCYMGK_5_4_0_2_0
Intensity
m/z
204.0862
138.0548
366.1388
274.0917
168.0652
186.0756
292.1021
126.0547
144.0657
454.1558
528.1926
657.231
860.3661 y7(1+)
882.8616 PEP_0_1_0_0_0
1764.7415 PEP_0_1_0_0_0

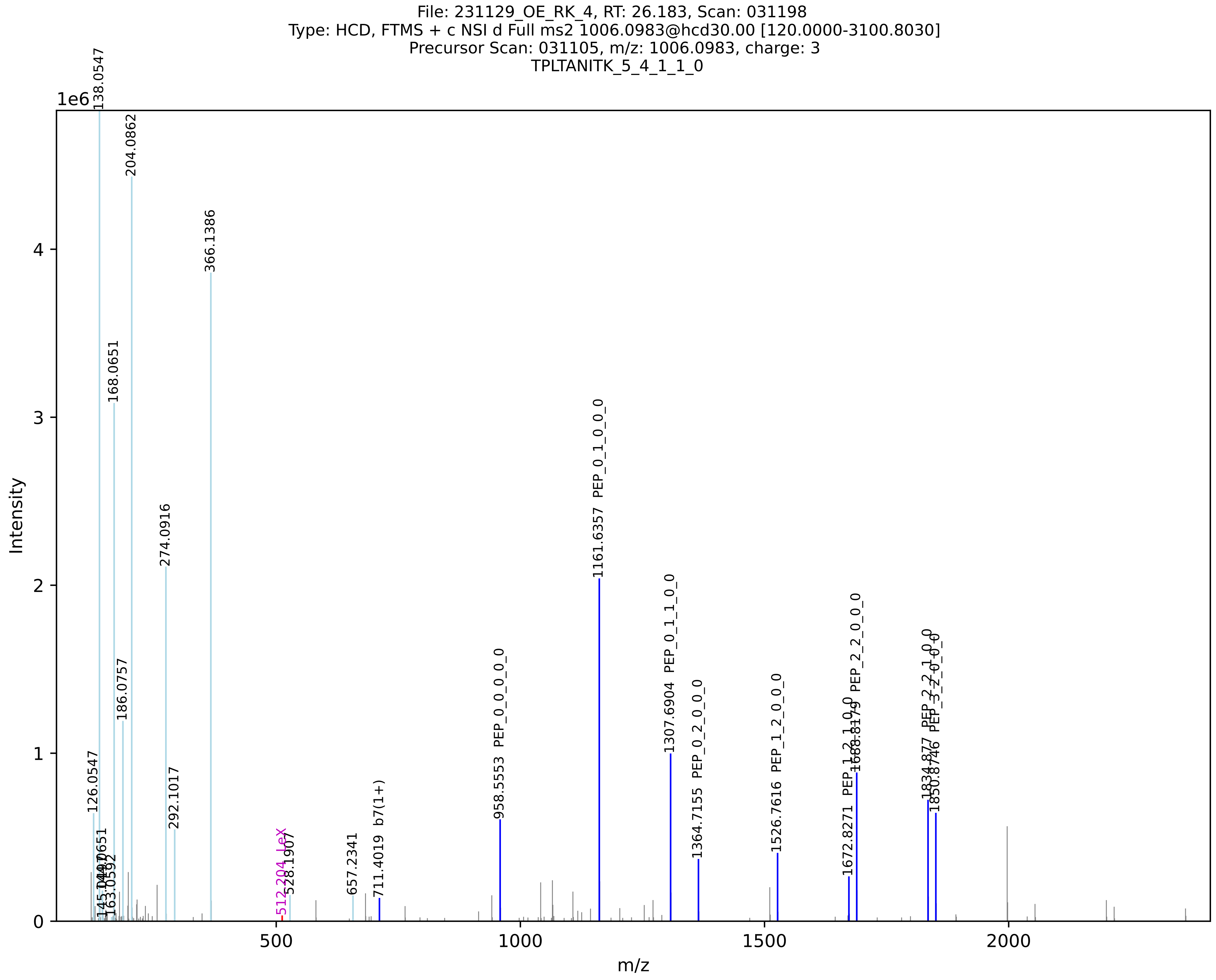

File: 231129_OE_RK_4, RT: 26.183, Scan: 031198
Type: HCD, FTMS + c NSI d Full ms2 1006.0983@hcd30.00 [120.0000-3100.8030]
Precursor Scan: 031105, m/z: 1006.0983, charge: 3
TPLTANITK_5_4_1_1_0
1e6
Intensity
m/z
138.0547
204.0862
366.1386
168.0651
274.0916
186.0757
126.0547
292.1017
163.0592
512.204_LeX
528.1907
657.2341
711.4019 b7(1+)
958.5553 PEP_0_0_0_0_0
1161.6357 PEP_0_1_0_0_0
1307.6904 PEP_0_1_1_0_0
1364.7155 PEP_0_2_0_0_0
1526.7616 PEP_1_2_0_0_0
1672.8271 PEP_1_2_1_0_0
1688.8179 PEP_2_2_0_0_0
1834.877 PEP_2_2_1_0_0
1850.8746 PEP_3_2_0_0_0

File: 231129_OE_RK_4, RT: 26.246, Scan: 031287
Type: HCD, FTMS + c NSI d Full ms2 1007.1018@hcd30.00 [120.0000-3103.8735]
Precursor Scan: 031221, m/z: 1006.7677, charge: 3
TPLTANITK_5_4_1_1_0

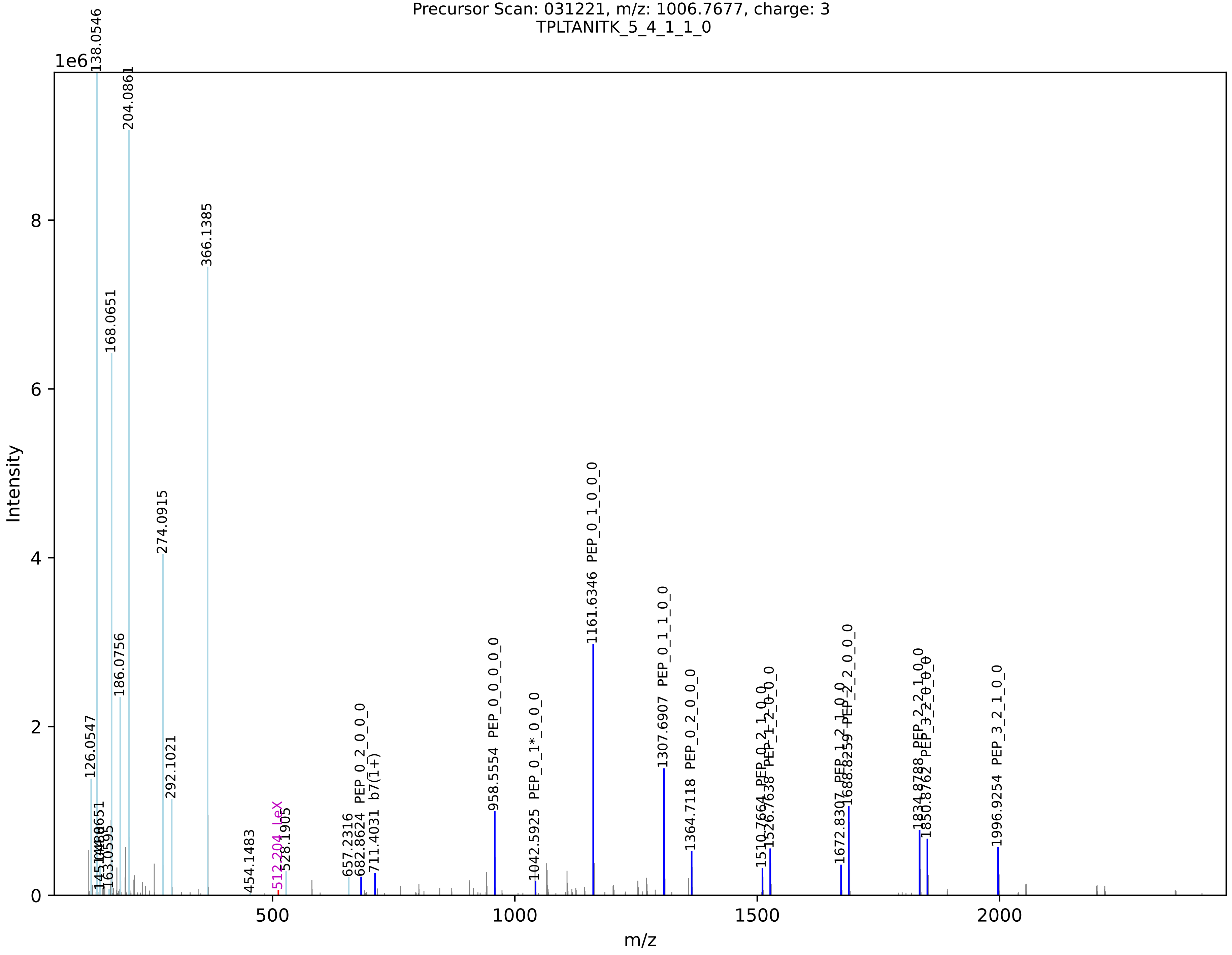

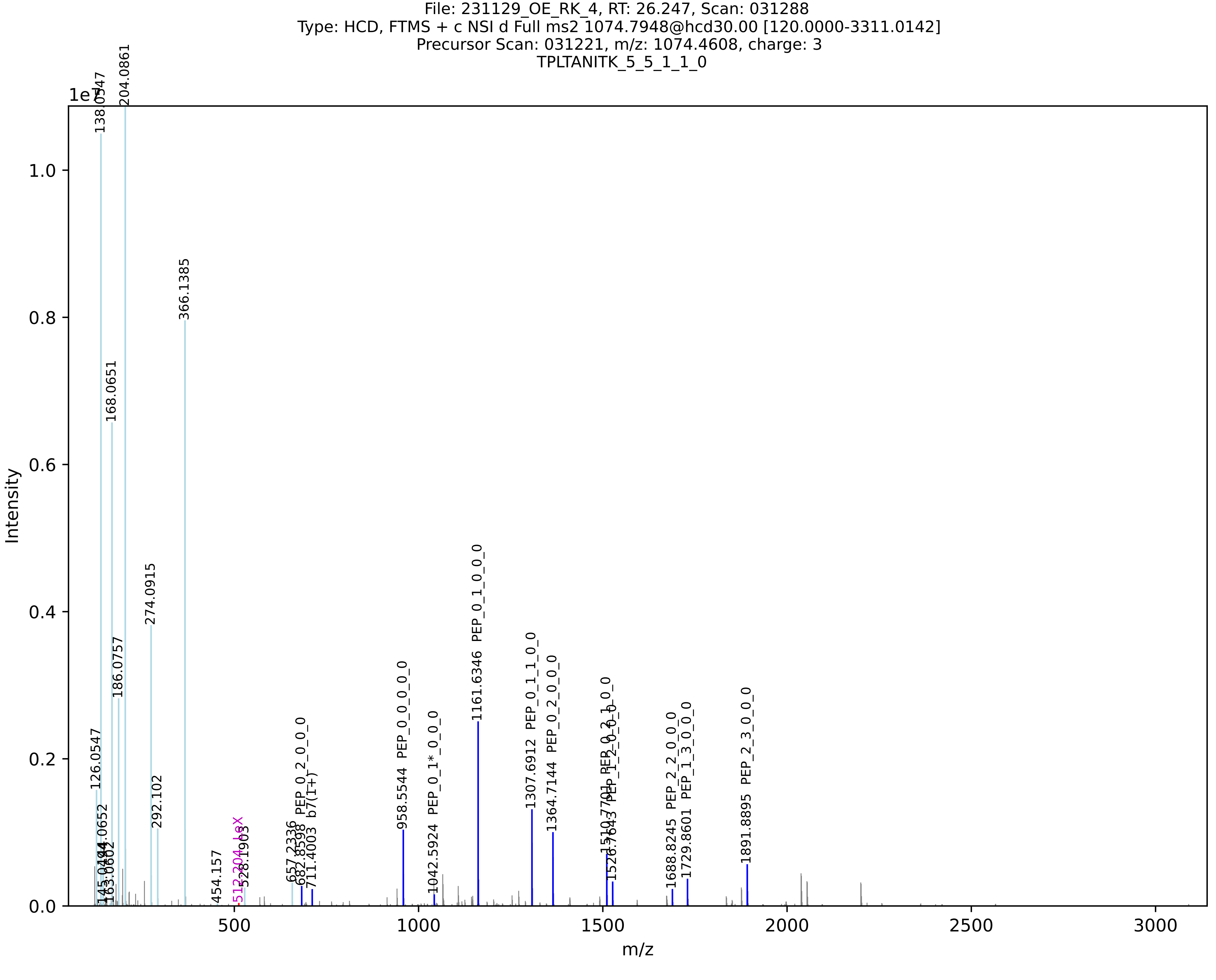

File: 231129_OE_RK_4, RT: 26.247, Scan: 031288
Type: HCD, FTMS + c NSI d Full ms2 1074.7948@hcd30.00 [120.0000-3311.0142]
Precursor Scan: 031221, m/z: 1074.4608, charge: 3
TPLTANITK_5_5_1_1_0
1e7
Intensity
m/z
204.0861
138.0547
366.1385
168.0651
274.0915
186.0757
126.0547
292.102
454.157
512.204 LeX
528.1903
657.2336
682.8598 PEP_0_2_0_0_0
711.4003 b7(1+)
958.5544 PEP_0_0_0_0_0
1042.5924 PEP_0_1*_0_0_0
1161.6346 PEP_0_1_0_0_0
1307.6912 PEP_0_1_1_0_0
1364.7144 PEP_0_2_0_0_0
1688.8245 PEP_2_2_0_0_0
1729.8601 PEP_1_3_0_0_0
1891.8895 PEP_2_3_0_0_0

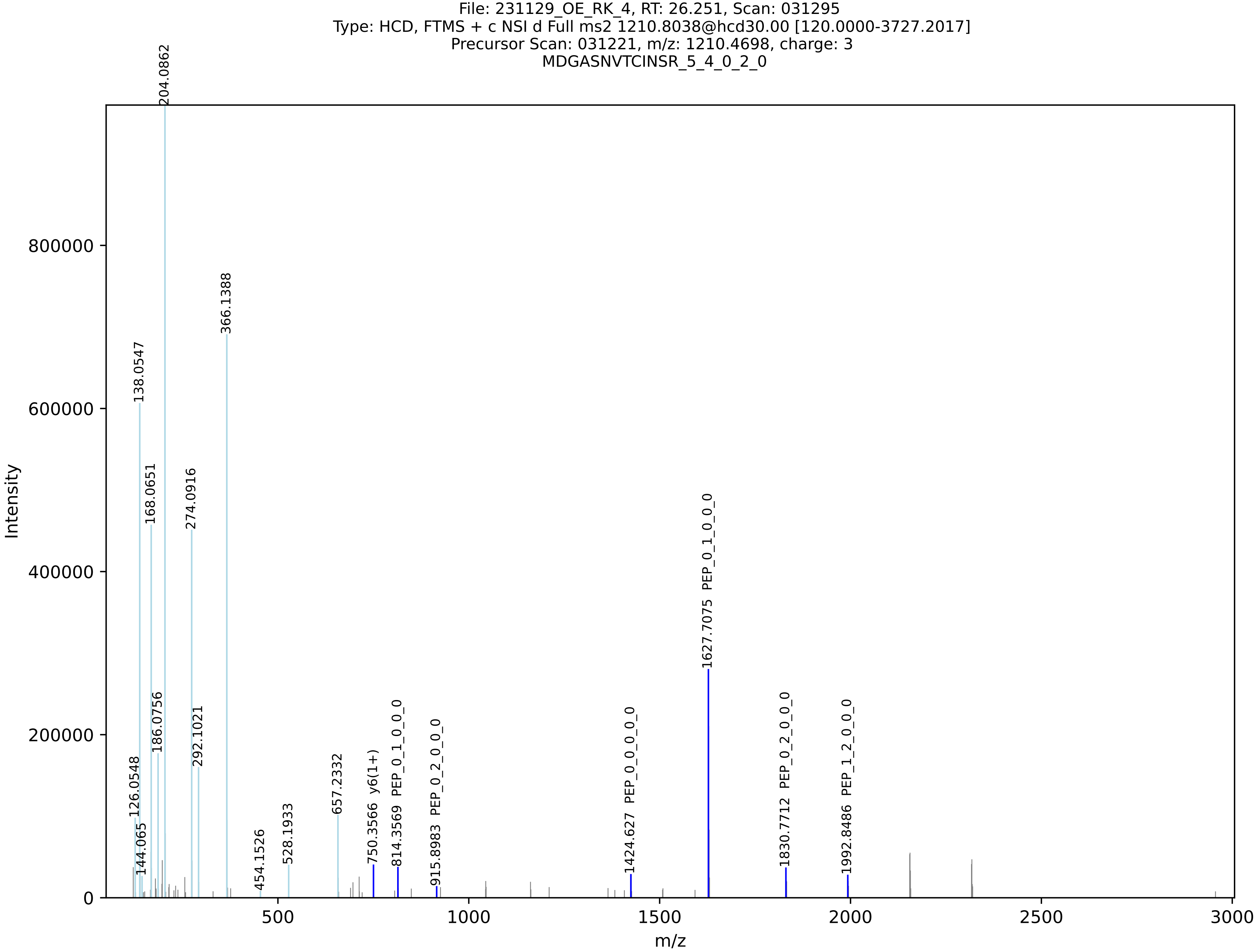
File: 231129_OE_RK_4, RT: 26.251, Scan: 031295
Type: HCD, FTMS + c NSI d Full ms2 1210.8038@hcd30.00 [120.0000-3727.2017]
Precursor Scan: 031221, m/z: 1210.4698, charge: 3
MDGASNVTCINSR_5_4_0_2_0
Intensity
m/z
204.0862
366.1388
138.0547
168.0651
274.0916
186.0756
292.1021
126.0548
144.065
454.1526
528.1933
657.2332
750.3566 y6(1+)
814.3569 PEP_0_1_0_0_0
915.8983 PEP_0_2_0_0_0
1424.627 PEP_0_0_0_0_0
1627.7075 PEP_0_1_0_0_
1830.7712 PEP_0_2_0_0_0
1992.8486 PEP_1_2_0_0_0

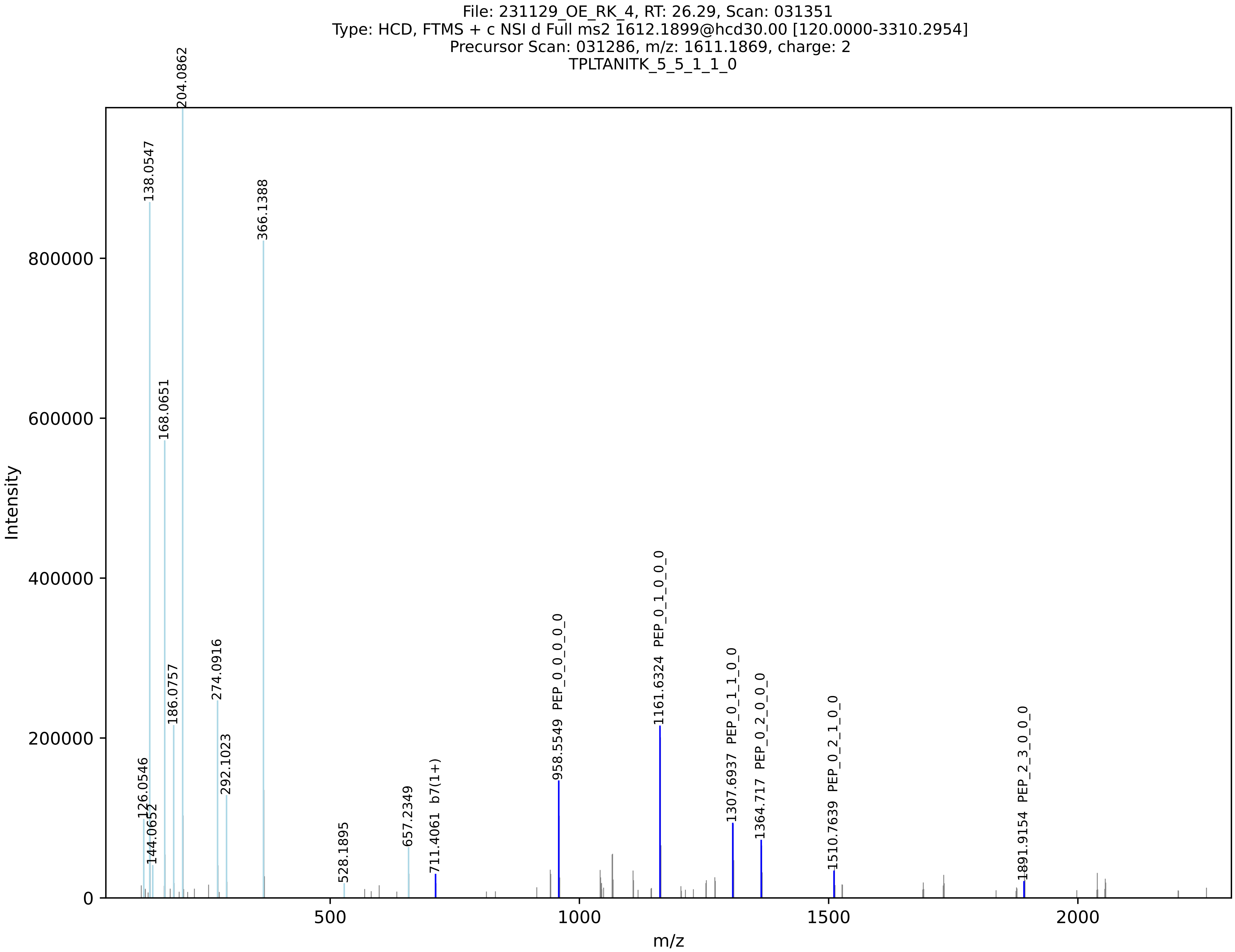
File: 231129_OE_RK_4, RT: 26.29, Scan: 031351
Type: HCD, FTMS + c NSI d Full ms2 1612.1899@hcd30.00 [120.0000-3310.2954]
Precursor Scan: 031286, m/z: 1611.1869, charge: 2
TPLTANITK_5_5_1_1_0
Intensity
m/z
204.0862
138.0547
366.1388
168.0651
186.0757
274.0916
292.1023
126.0546
144.0652
528.1895
657.2349
711.4061 b7(1+)
958.5549 PEP_0_0_0_0_0
1161.6324 PEP_0_1_0_0_0
1307.6937 PEP_0_1_1_0_0
1364.717 PEP_0_2_0_0_0
1510.7639 PEP_0_2_1_0_0
1891.9154 PEP_2_3_0_0_0
0
200000
400000
600000
800000
500
1000
1500
2000

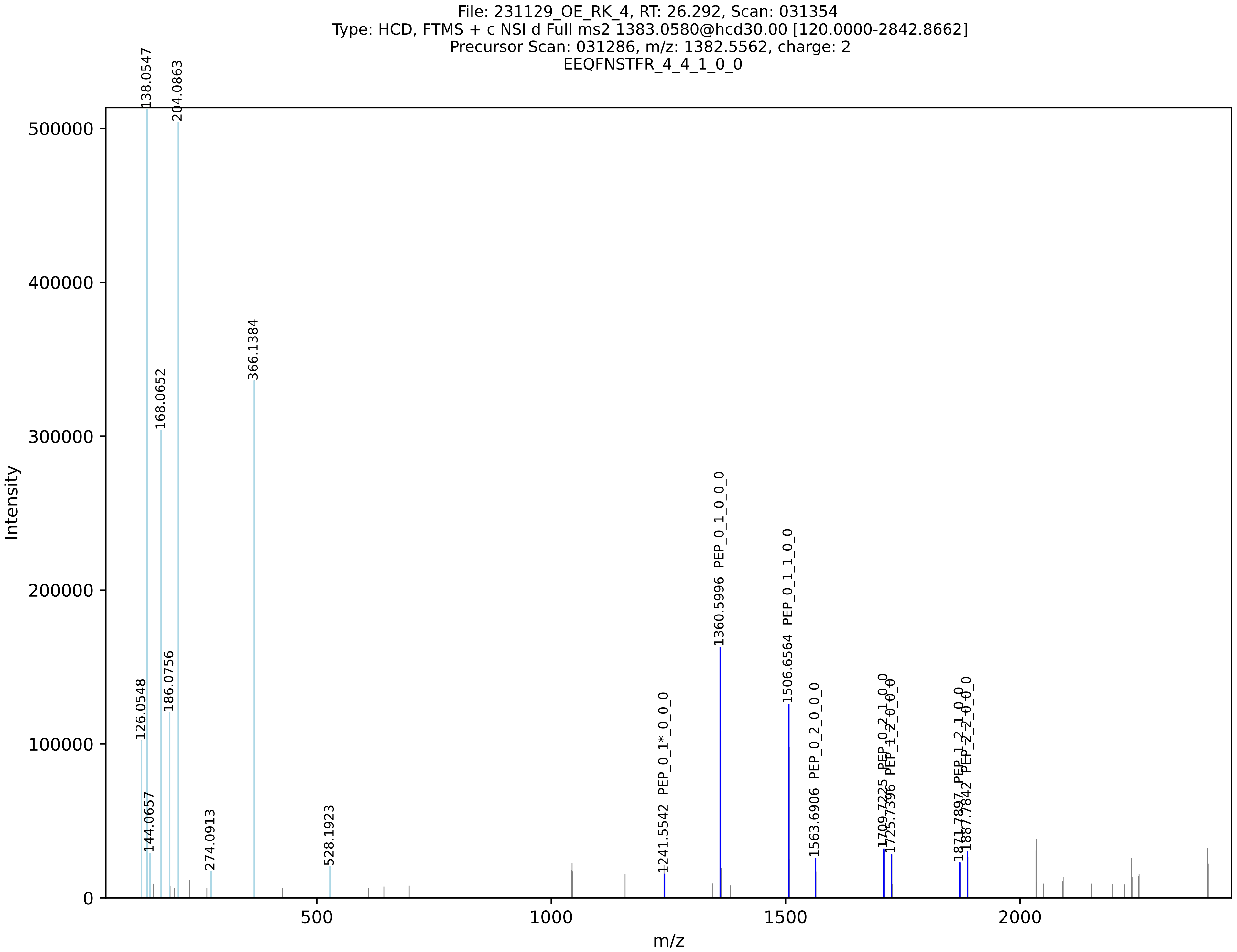
File: 231129_OE_RK_4, RT: 26.292, Scan: 031354
Type: HCD, FTMS + c NSI d Full ms2 1383.0580@hcd30.00 [120.0000-2842.8662]
Precursor Scan: 031286, m/z: 1382.5562, charge: 2
EEQFNSTFR_4_4_1_0_0
Intensity
m/z
138.0547
204.0863
366.1384
168.0652
186.0756
126.0548
144.0657
274.0913
528.1923
1241.5542 PEP_0_1*_0_0_0
1360.5996 PEP_0_1_0_0_0
1506.6564 PEP_0_1_1_0_0
1563.6906 PEP_0_2_0_0_0
1709.7225 PEP_0_2_1_0_0
1725.7396 PEP_1_2_0_0_0
1871.7897 PEP_1_2_1_0_0
1887.7842 PEP_2_2_0_0_0

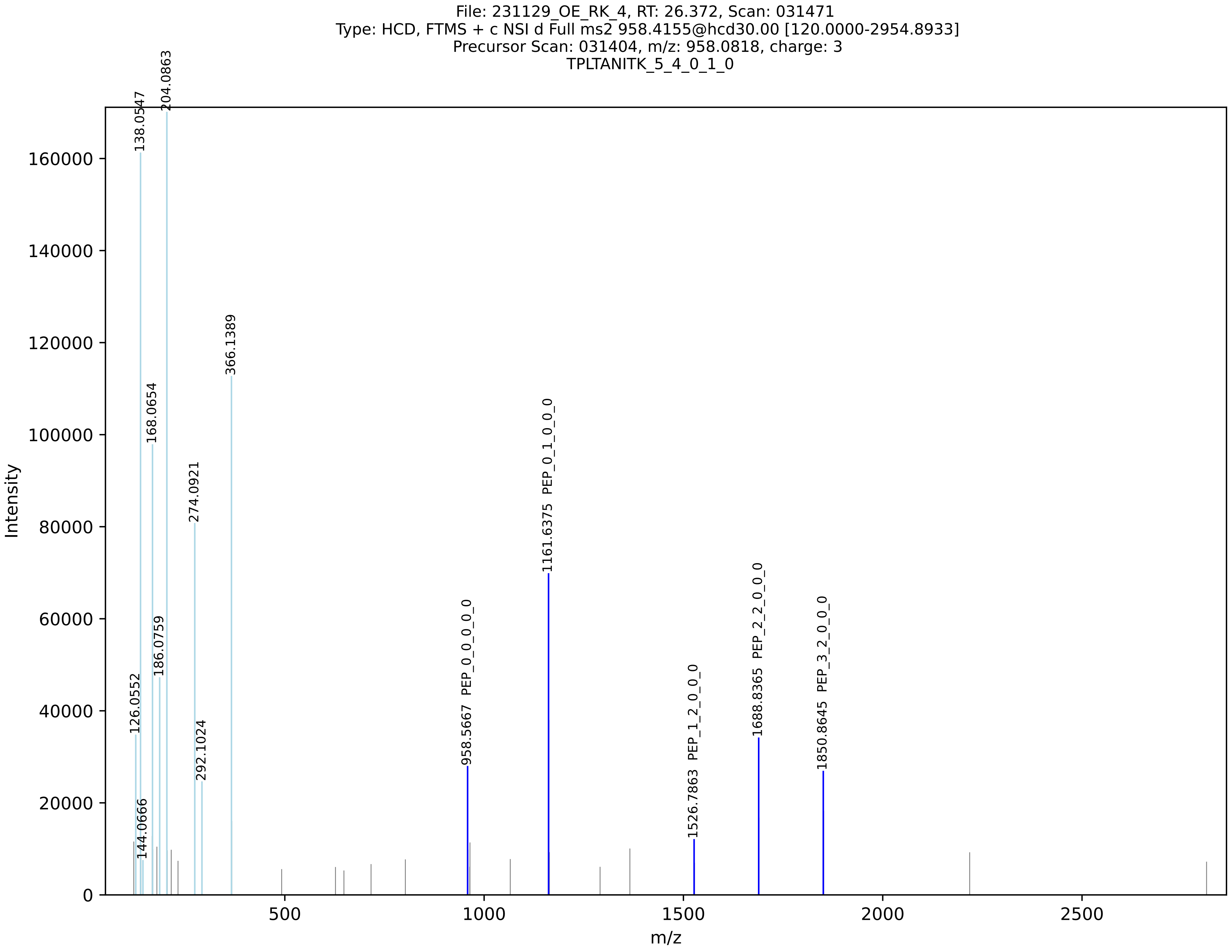
File: 231129_OE_RK_4, RT: 26.372, Scan: 031471
Type: HCD, FTMS + c NSI d Full ms2 958.4155@hcd30.00 [120.0000-2954.8933]
Precursor Scan: 031404, m/z: 958.0818, charge: 3
TPLTANITK_5_4_0_1_0
Intensity
m/z
204.0863
138.0547
168.0654
186.0759
126.0552
144.0666
274.0921
292.1024
366.1389
958.5667 PEP_0_0_0_0_0
1161.6375 PEP_0_1_0_0_0
1526.7863 PEP_1_2_0_0_0
1688.8365 PEP_2_2_0_0_0
1850.8645 PEP_3_2_0_0_0

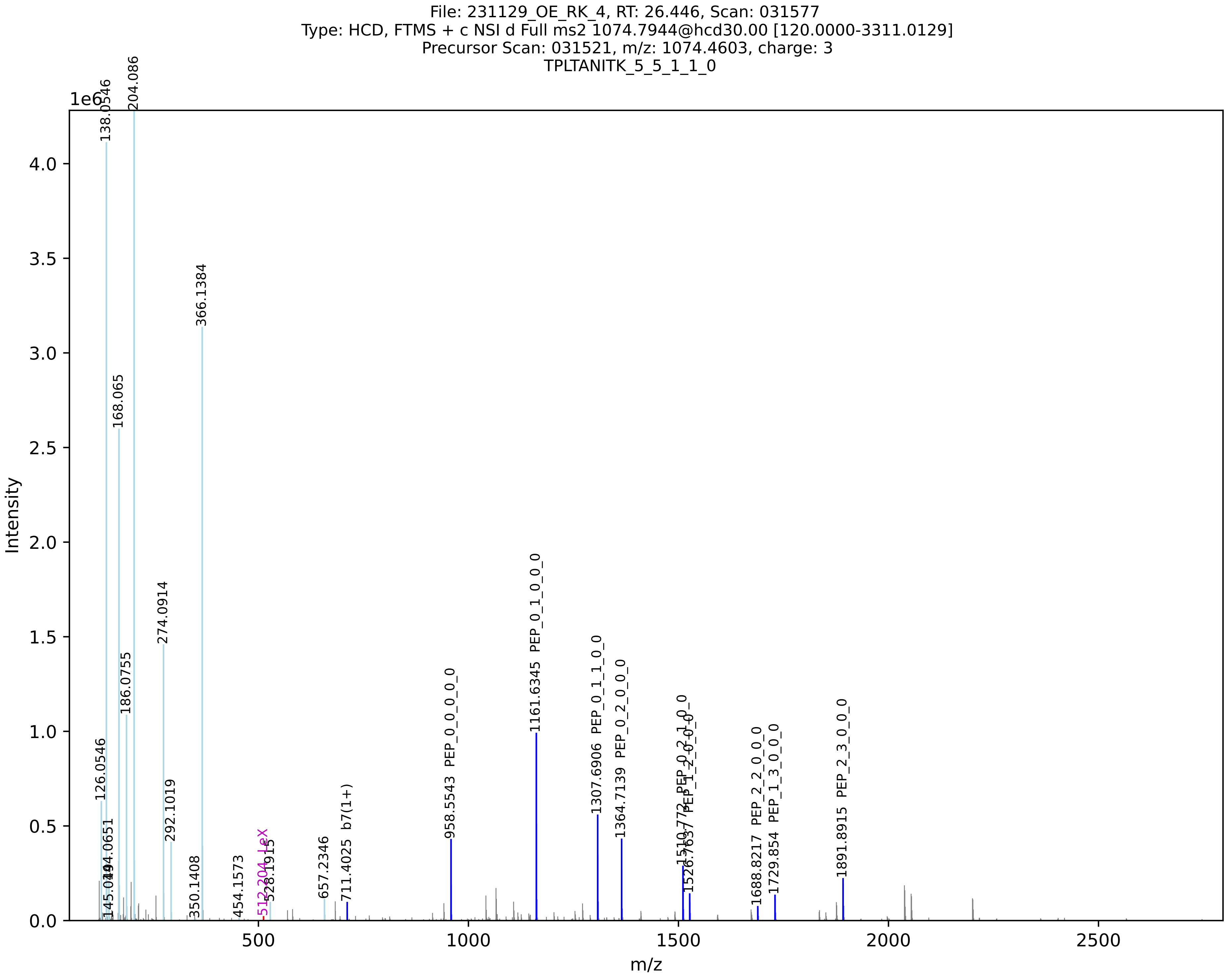

File: 231129_OE_RK_4, RT: 26.446, Scan: 031577
Type: HCD, FTMS + c NSI d Full ms2 1074.7944@hcd30.00 [120.0000-3311.0129]
Precursor Scan: 031521, m/z: 1074.4603, charge: 3
TPLTANITK_5_5_1_1_0
1e6
Intensity
m/z
204.086
138.0546
366.1384
168.065
274.0914
186.0755
126.0546
292.1019
144.0651
350.1408
454.1573
512.204_LeX
528.1915
657.2346
711.4025 b7(1+)
958.5543 PEP_0_0_0_0_0_
1161.6345 PEP_0_1_0_0_0_
1307.6906 PEP_0_1_1_0_0
1364.7139 PEP_0_2_0_0_0_
1510.772 PEP_0_2_1_0_0
1688.8217 PEP_2_2_0_0_0
1729.854 PEP_1_3_0_0_0
1891.8915 PEP_2_3_0_0_0_

File: 231129_OE_RK_4, RT: 26.446, Scan: 031578
Type: HCD, FTMS + c NSI d Full ms2 1007.1016@hcd30.00 [120.0000-3103.8730]
Precursor Scan: 031521, m/z: 1006.7675, charge: 3
TPLTANITK_5_4_1_1_0

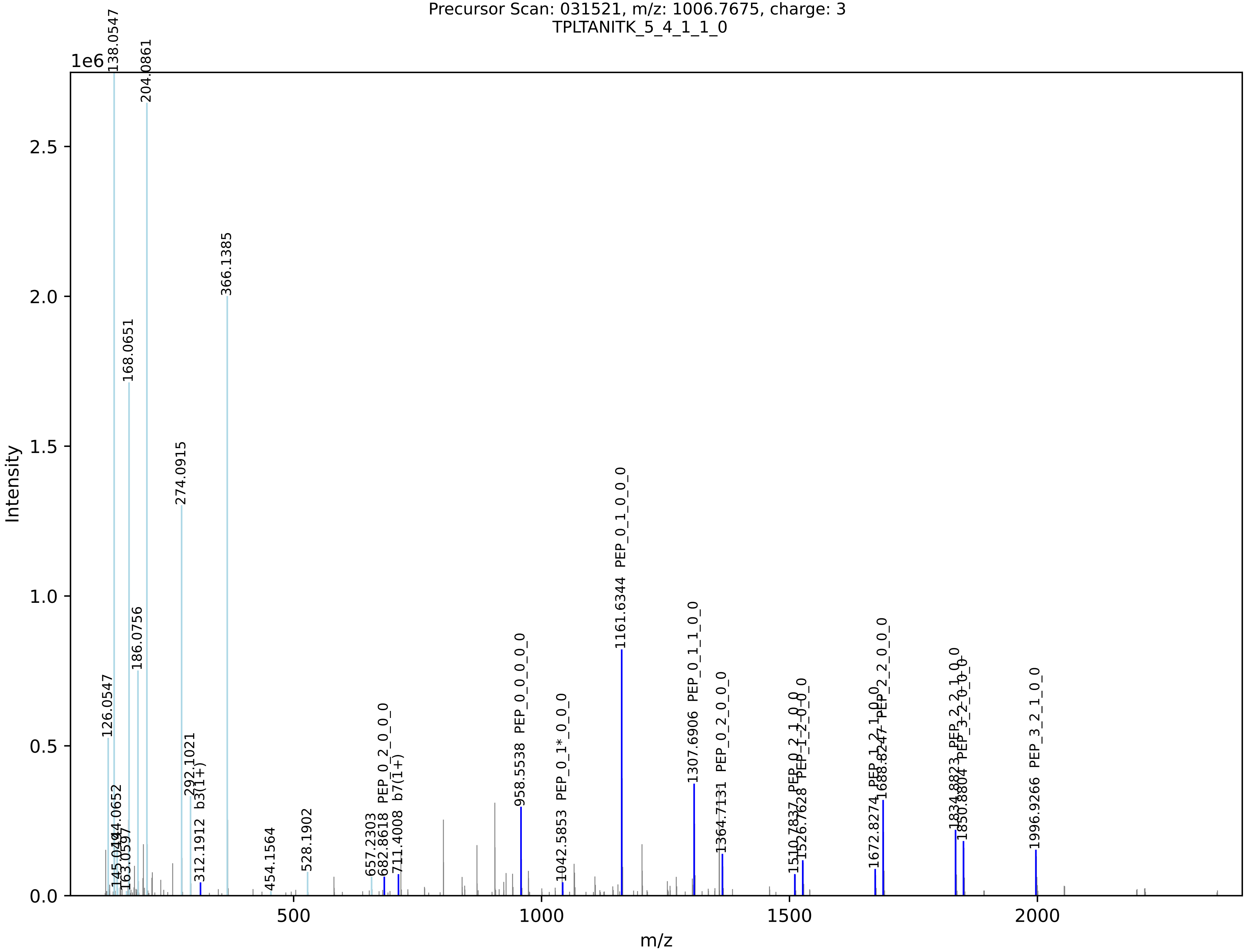

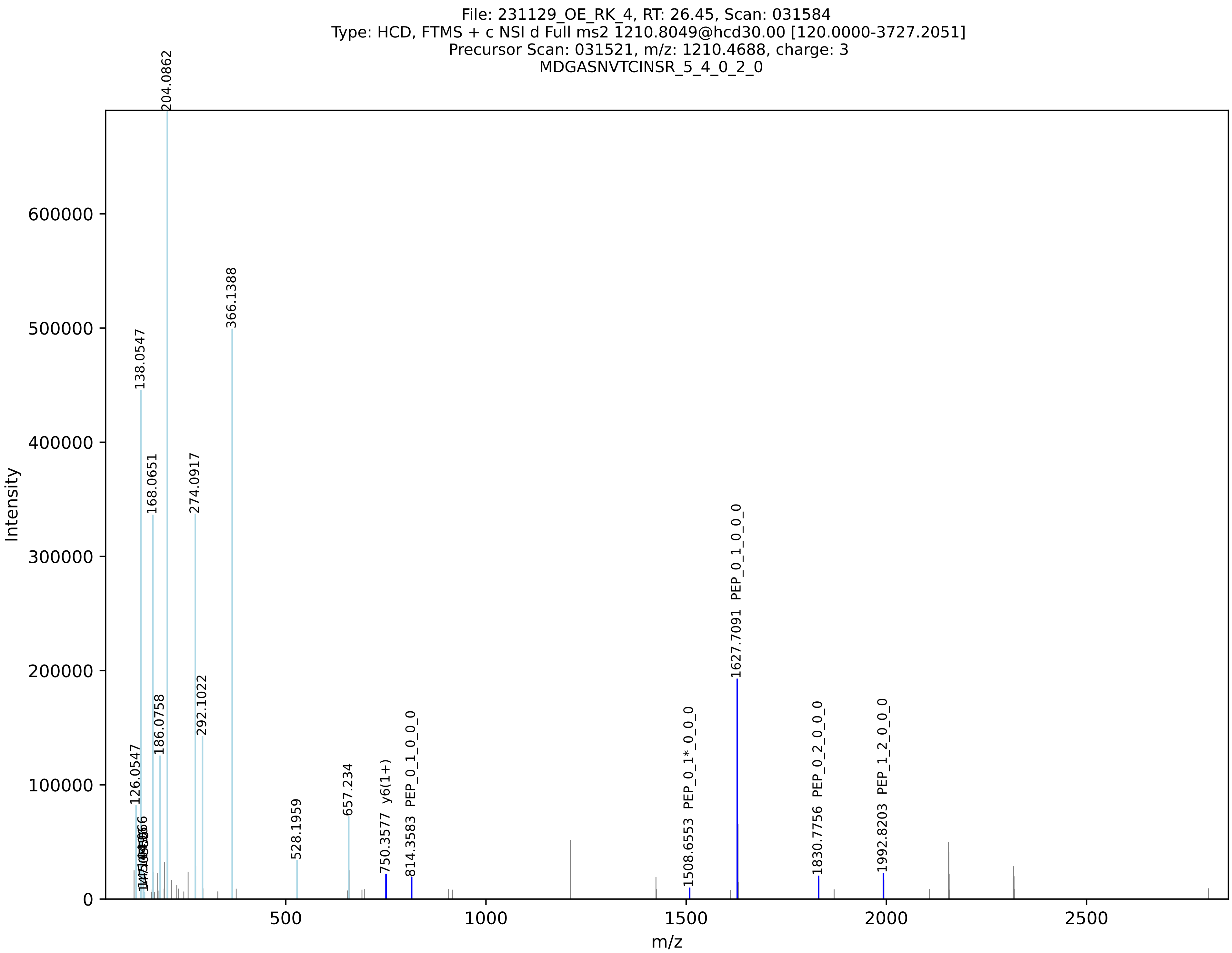

File: 231129_OE_RK_4, RT: 26.45, Scan: 031584
Type: HCD, FTMS + c NSI d Full ms2 1210.8049@hcd30.00 [120.0000-3727.2051]
Precursor Scan: 031521, m/z: 1210.4688, charge: 3
MDGASNVTCINSR_5_4_0_2_0
Intensity
m/z
204.0862
366.1388
138.0547
168.0651
274.0917
292.1022
186.0758
126.0547
528.1959
657.234
750.3577 y6(1+)
814.3583 PEP_0_1_0_0_0
1508.6553 PEP_0_1*_0_0_0
1627.7091 PEP_0_1_0_0_0
1830.7756 PEP_0_2_0_0_0
1992.8203 PEP_1_2_0_0_0

File: 231129_OE_RK_4, RT: 26.451, Scan: 031585
Type: HCD, FTMS + c NSI d Full ms2 922.3740@hcd30.00 [120.0000-2844.6064]
Precursor Scan: 031521, m/z: 922.0393, charge: 3
EEQFNSTFR_4_4_1_0_0

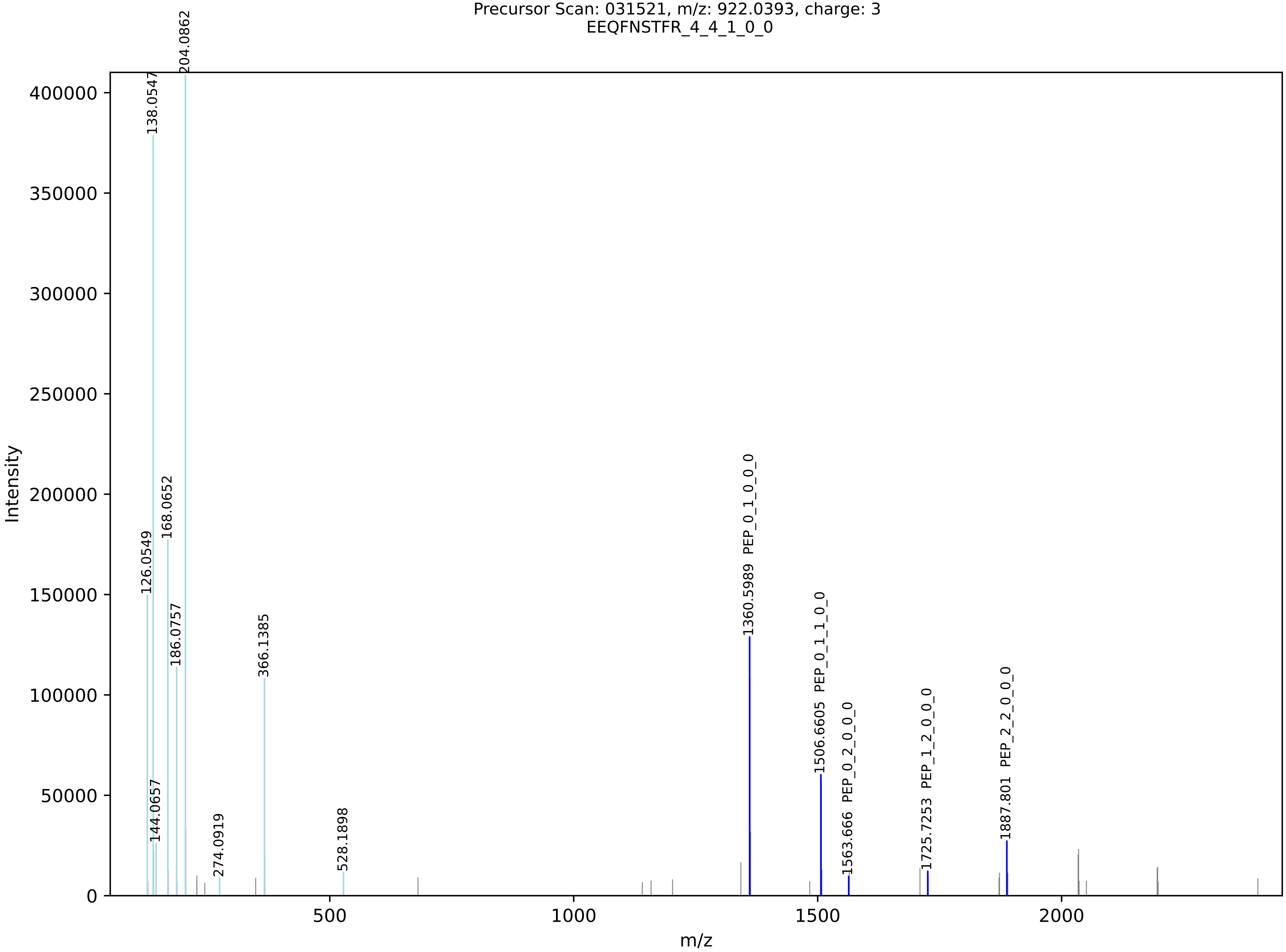

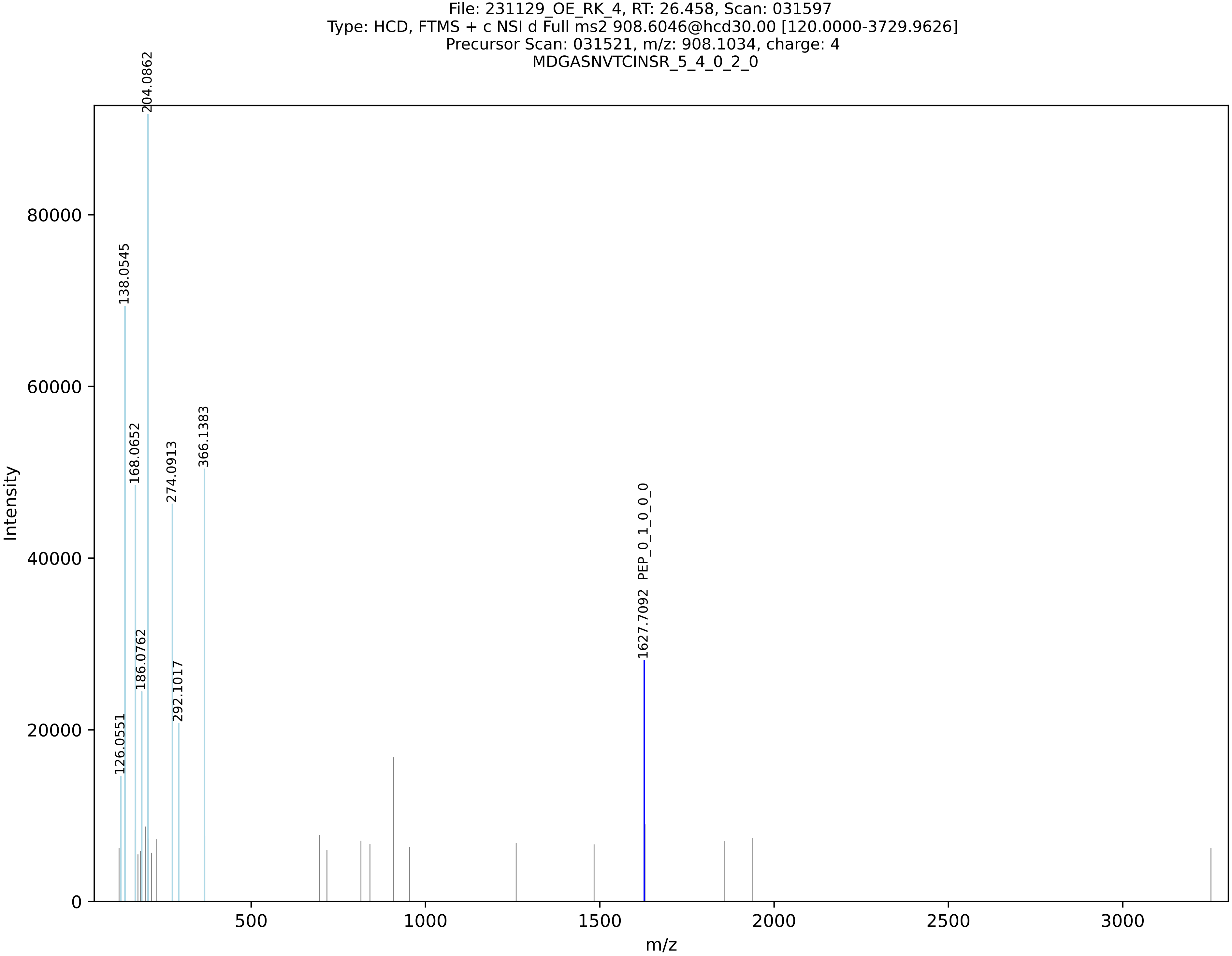
File: 231129_OE_RK_4, RT: 26.458, Scan: 031597
Type: HCD, FTMS + c NSI d Full ms2 908.6046@hcd30.00 [120.0000-3729.9626]
Precursor Scan: 031521, m/z: 908.1034, charge: 4
MDGASNVTCINSR_5_4_0_2_0
204.0862
138.0545
168.0652
274.0913
366.1383
186.0762
292.1017
126.0551
1627.7092 PEP_0_1_0_0_0
Intensity
m/z
0
20000
40000
60000
80000
500
1000
1500
2000
2500
3000

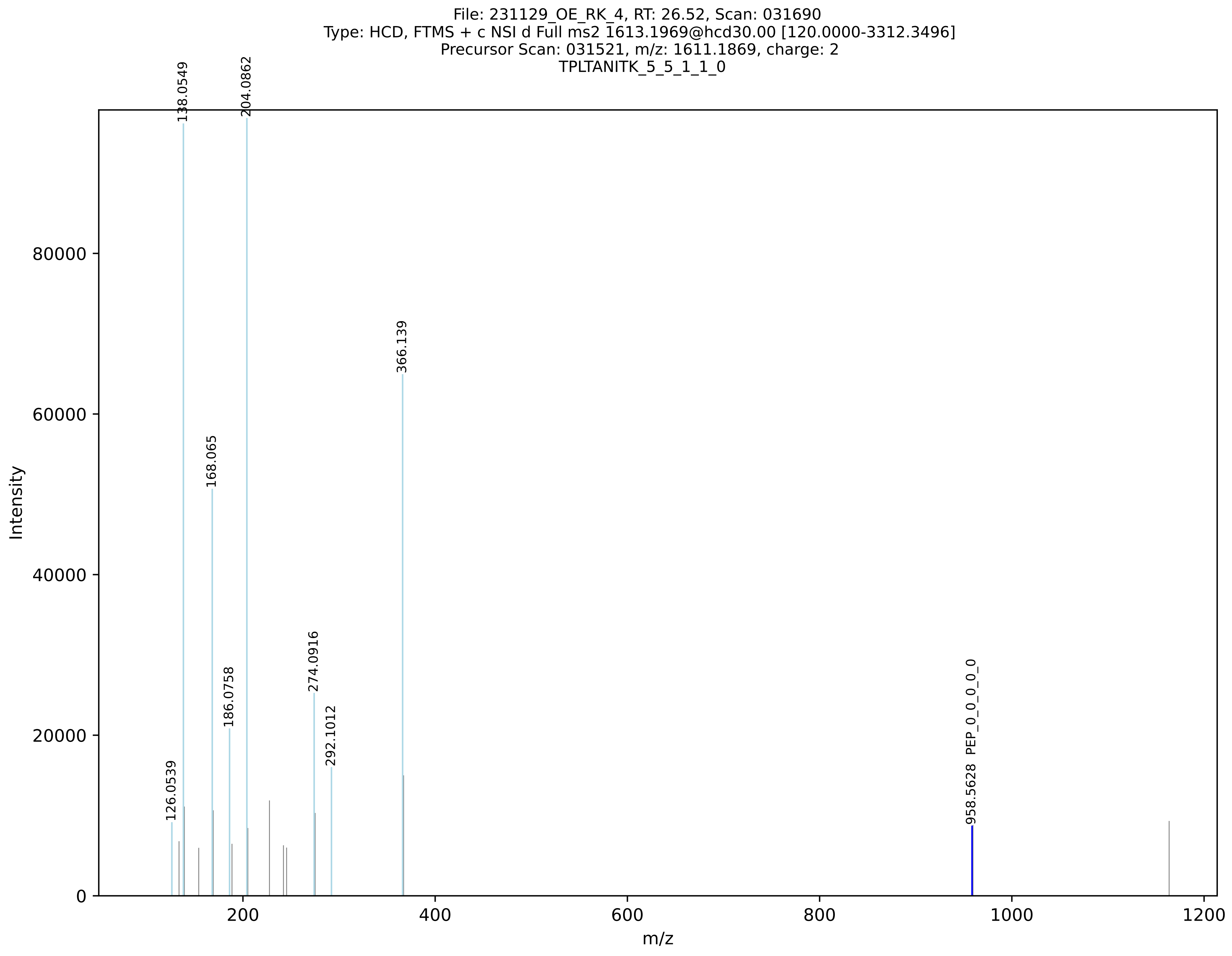

File: 231129_OE_RK_4, RT: 26.52, Scan: 031690
Type: HCD, FTMS + c NSI d Full ms2 1613.1969@hcd30.00 [120.0000-3312.3496]
Precursor Scan: 031521, m/z: 1611.1869, charge: 2
TPLTANITK_5_5_1_1_0
138.0549
204.0862
366.139
168.065
274.0916
186.0758
292.1012
126.0539
958.5628 PEP_0_0_0_0_
Intensity
m/z
0
20000
40000
60000
80000
200
400
600
800
1000
1200

File: 231129_OE_RK_4, RT: 26.544, Scan: 031723
Type: HCD, FTMS + c NSI d Full ms2 1429.1205@hcd30.00 [120.0000-2936.8337]
Precursor Scan: 031638, m/z: 1428.6212, charge: 2
TPLTANITK_4_4_1_1_0

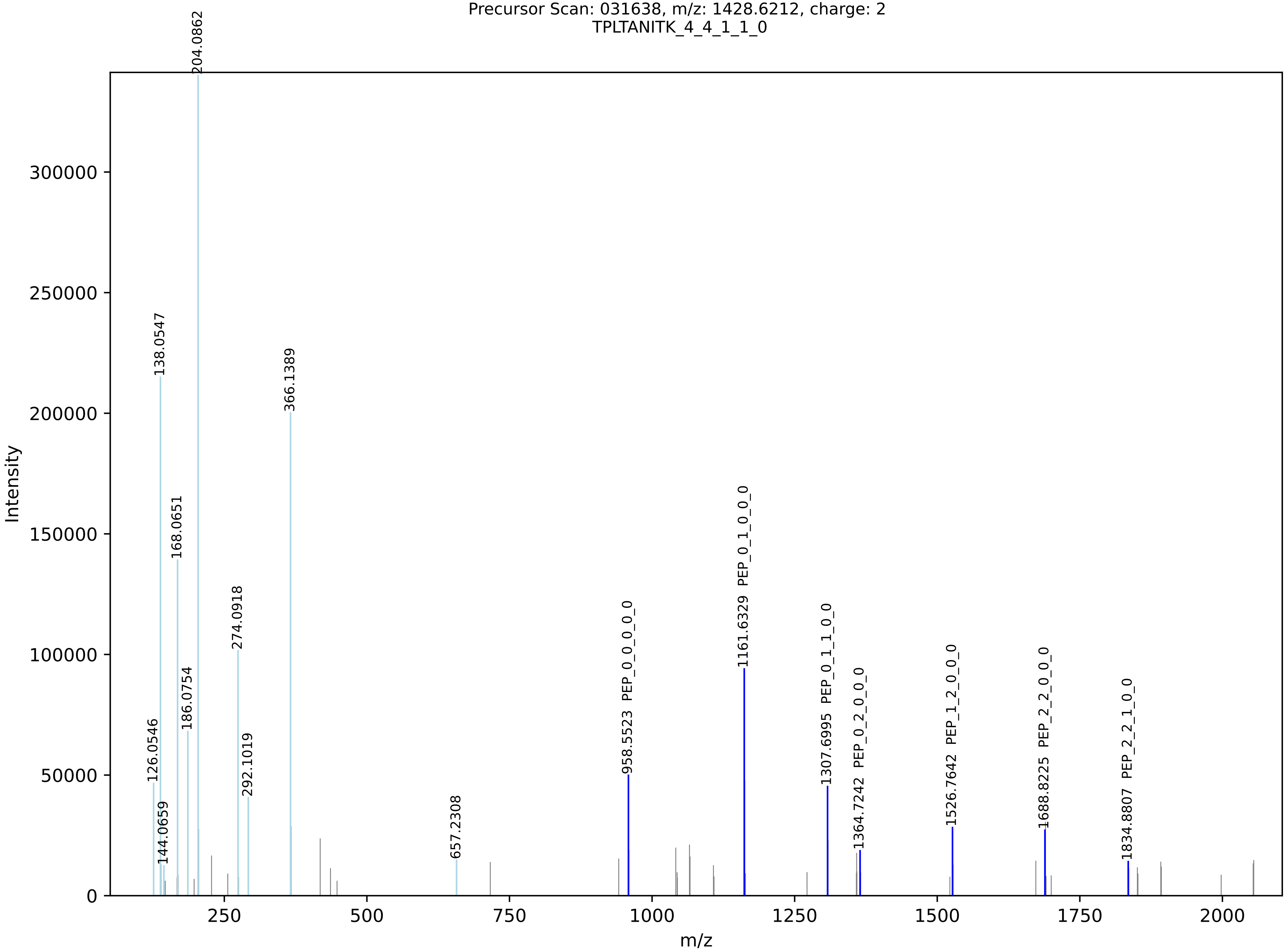

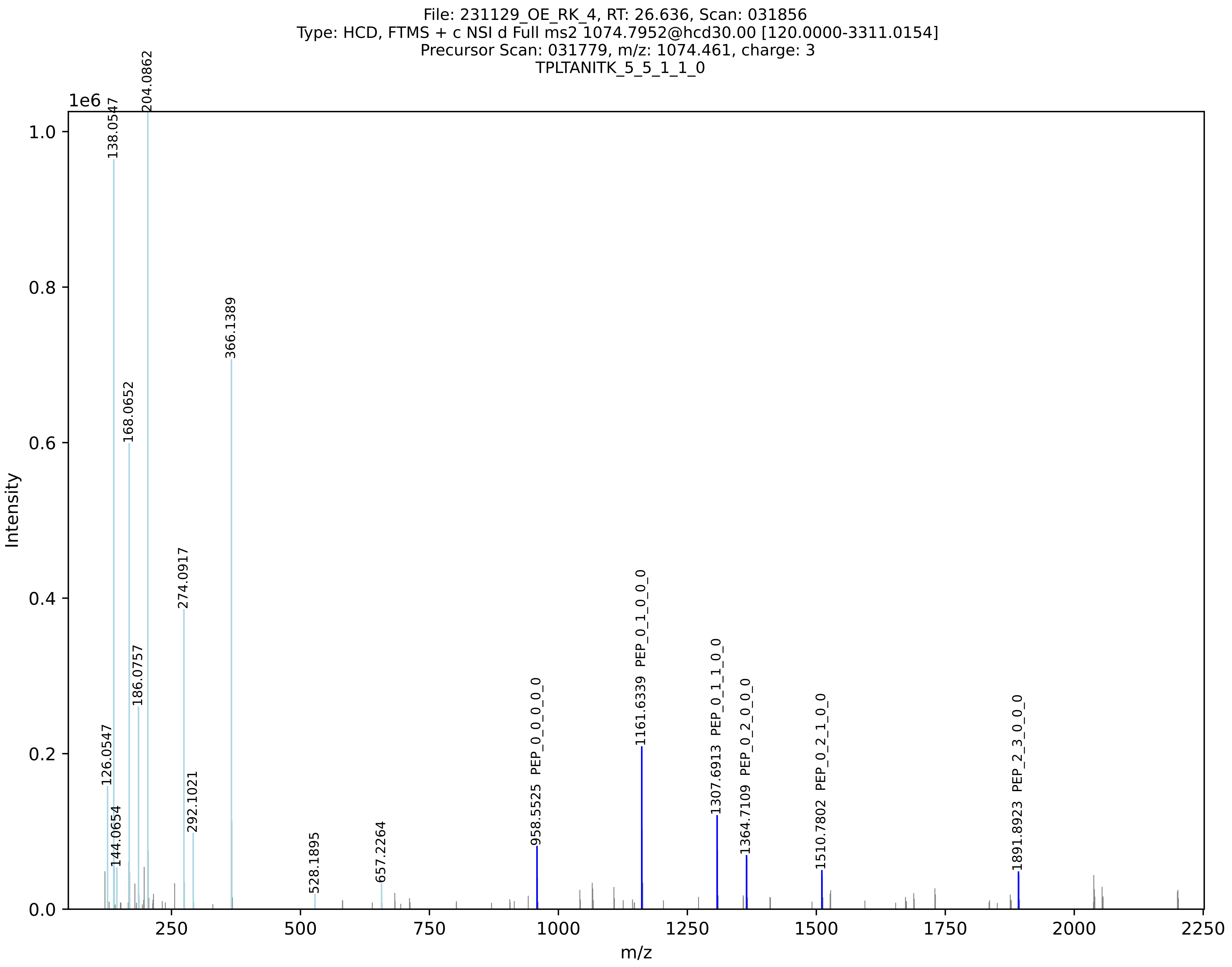
File: 231129_OE_RK_4, RT: 26.636, Scan: 031856
Type: HCD, FTMS + c NSI d Full ms2 1074.7952@hcd30.00 [120.0000-3311.0154]
Precursor Scan: 031779, m/z: 1074.461, charge: 3
TPLTANITK_5_5_1_1_0
1e6
Intensity
m/z
204.0862
138.0547
366.1389
168.0652
274.0917
186.0757
126.0547
144.0654
292.1021
528.1895
657.2264
958.5525 PEP_0_0_0_0_0
1161.6339 PEP_0_1_0_0_0
1307.6913 PEP_0_1_1_0_0
1364.7109 PEP_0_2_0_0_0
1510.7802 PEP_0_2_1_0_0
1891.8923 PEP_2_3_0_0_0

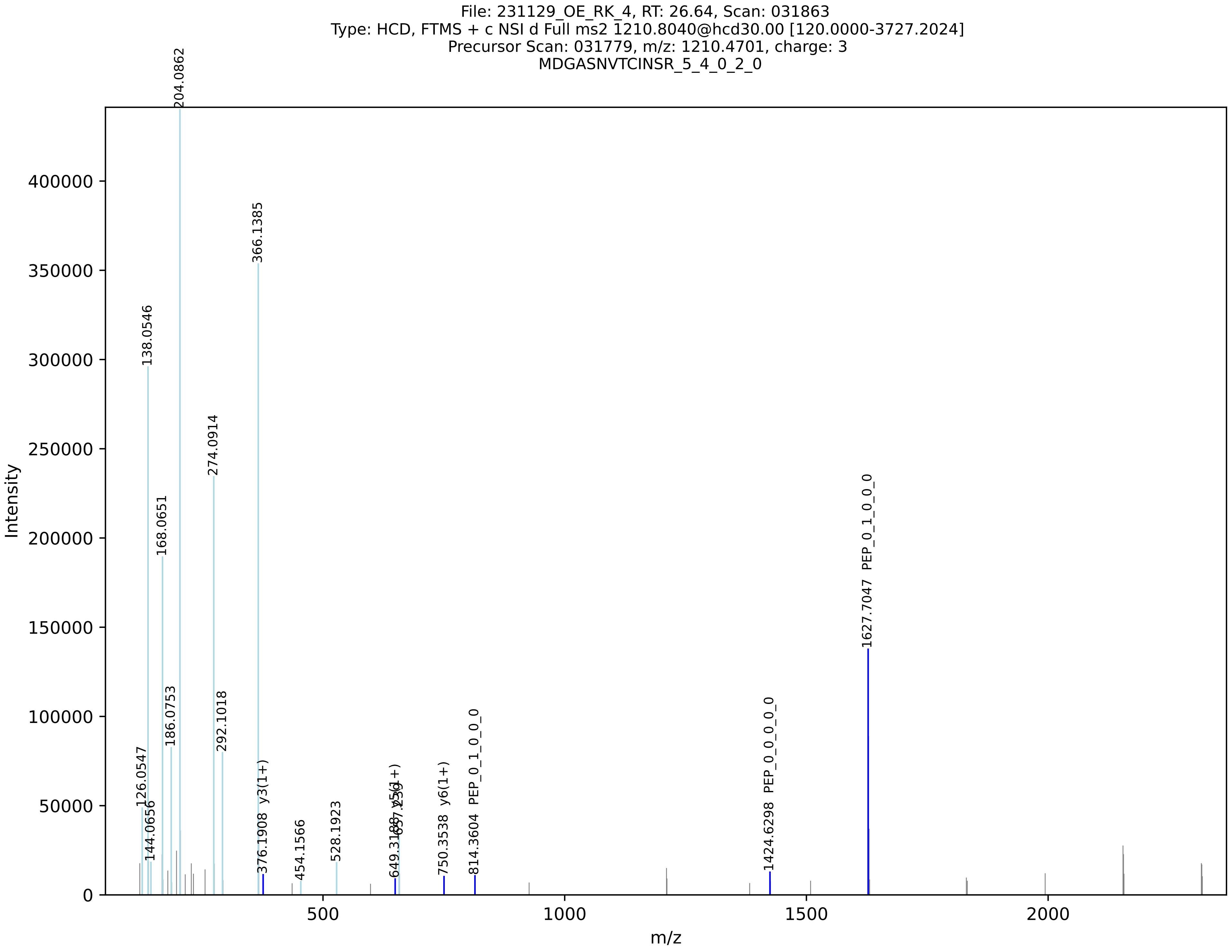
File: 231129_OE_RK_4, RT: 26.64, Scan: 031863
Type: HCD, FTMS + c NSI d Full ms2 1210.8040@hcd30.00 [120.0000-3727.2024]
Precursor Scan: 031779, m/z: 1210.4701, charge: 3
MDGASNVTCINSR_5_4_0_2_0
Intensity
m/z
0
50000
100000
150000
200000
250000
300000
350000
400000
500
1000
1500
2000
204.0862
366.1385
138.0546
274.0914
168.0651
186.0753
292.1018
126.0547
144.0656
376.1908 y3(1+)
454.1566
528.1923
649.3188 y5(1+)
657.239
750.3538 y6(1+)
814.3604 PEP_0_1_0_0_0
1424.6298 PEP_0_0_0_0_0
1627.7047 PEP_0_1_0_0_0

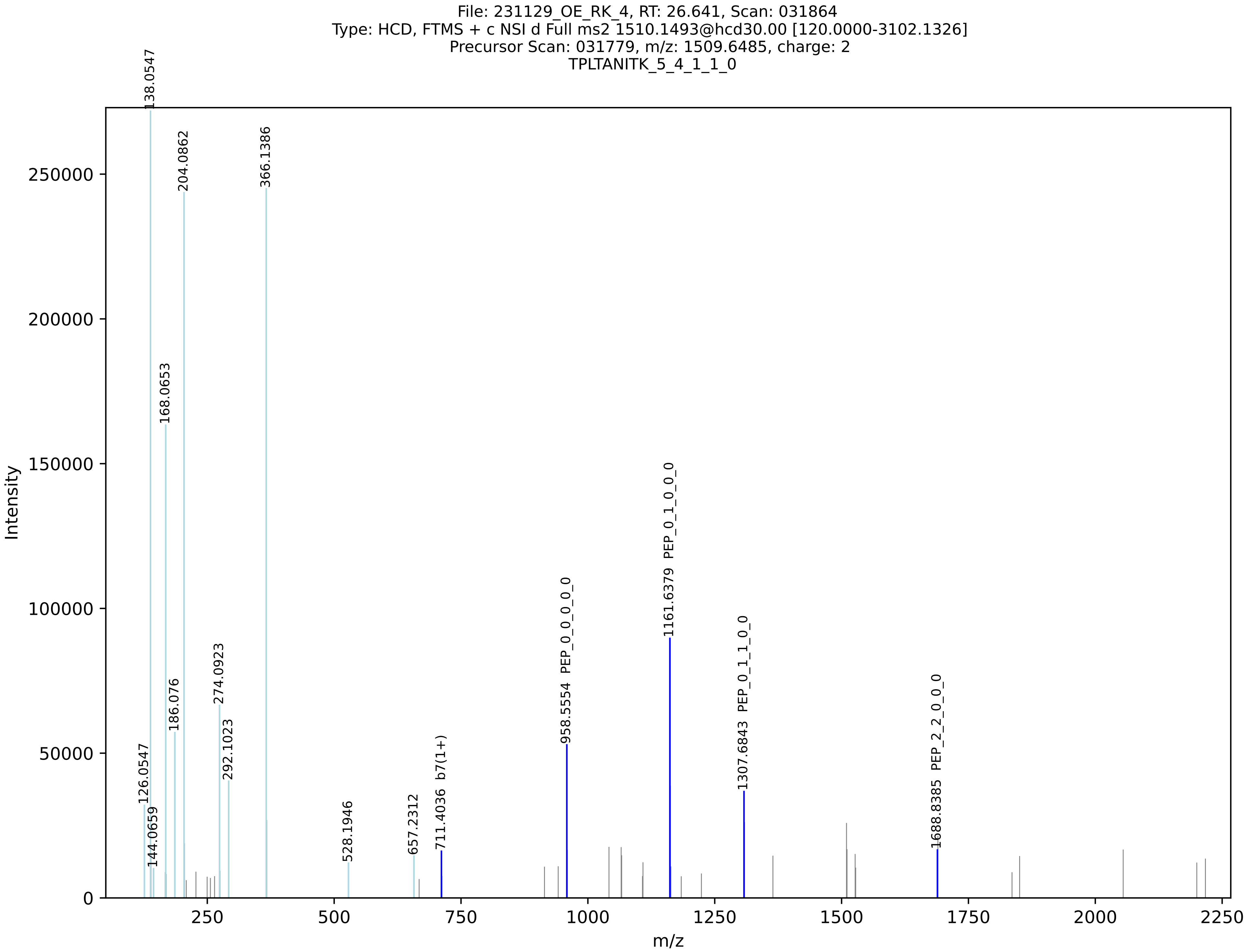

File: 231129_OE_RK_4, RT: 26.641, Scan: 031864
Type: HCD, FTMS + c NSI d Full ms2 1510.1493@hcd30.00 [120.0000-3102.1326]
Precursor Scan: 031779, m/z: 1509.6485, charge: 2
TPLTANITK_5_4_1_1_0
Intensity
m/z
138.0547
204.0862
366.1386
168.0653
186.076
274.0923
292.1023
126.0547
144.0659
528.1946
657.2312
711.4036 b7(1+)
958.5554 PEP_0_0_0_0_0
1161.6379 PEP_0_1_0_0_0
1307.6843 PEP_0_1_1_0_0
1688.8385 PEP_2_2_0_0_0

File: 231129_OE_RK_4, RT: 26.694, Scan: 031942
Type: HCD, FTMS + c NSI d Full ms2 927.0494@hcd30.00 [120.0000-2858.9131]
Precursor Scan: 031853, m/z: 926.7165, charge: 3
GVNFNVSK_5_4_0_1_0

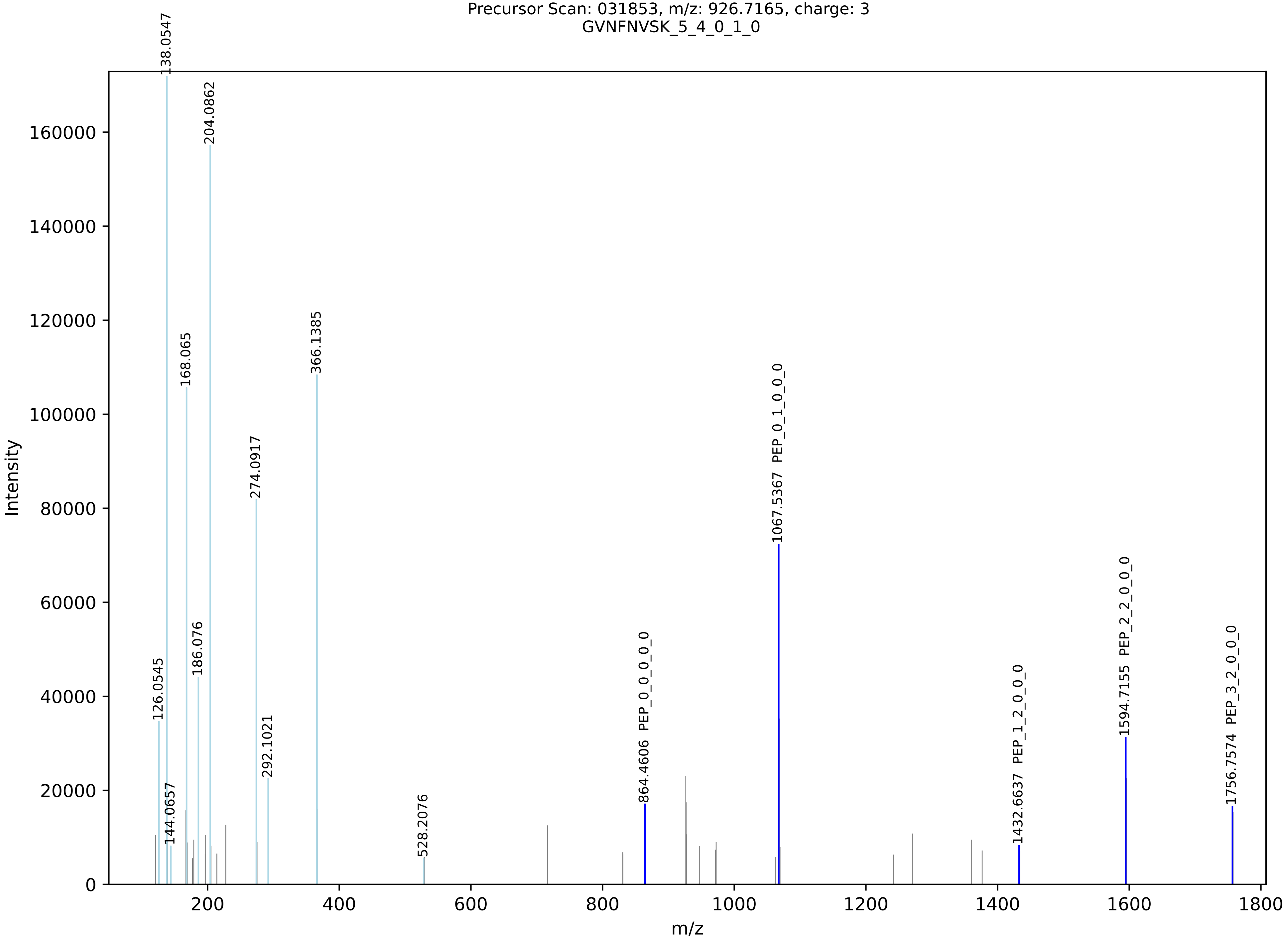

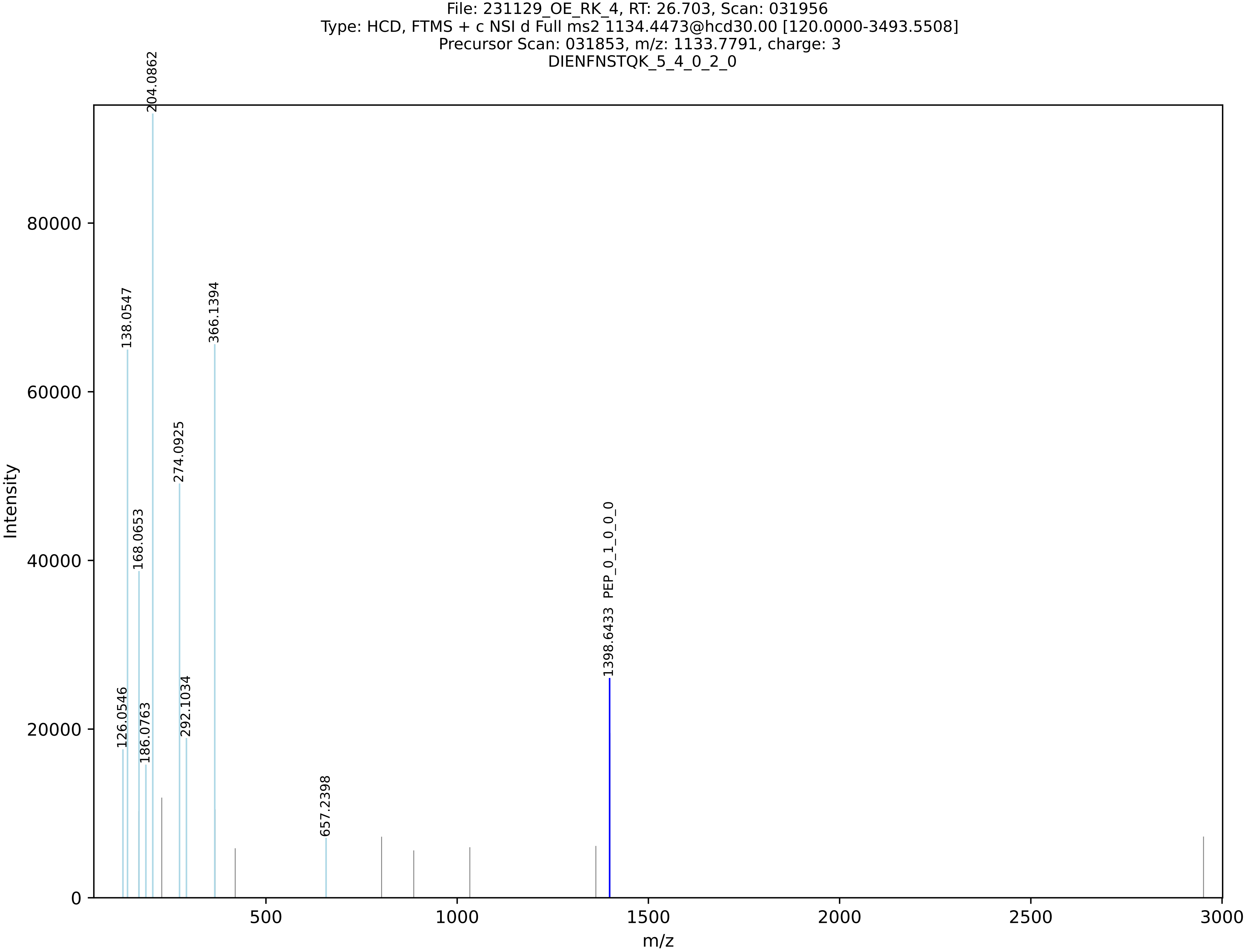
File: 231129_OE_RK_4, RT: 26.703, Scan: 031956
Type: HCD, FTMS + c NSI d Full ms2 1134.4473@hcd30.00 [120.0000-3493.5508]
Precursor Scan: 031853, m/z: 1133.7791, charge: 3
DIENFNSTQK_5_4_0_2_0
204.0862
138.0547
366.1394
274.0925
168.0653
126.0546
186.0763
292.1034
657.2398
1398.6433 PEP_0_1_0_0_0
Intensity
m/z
0
20000
40000
60000
80000
500
1000
1500
2000
2500
3000

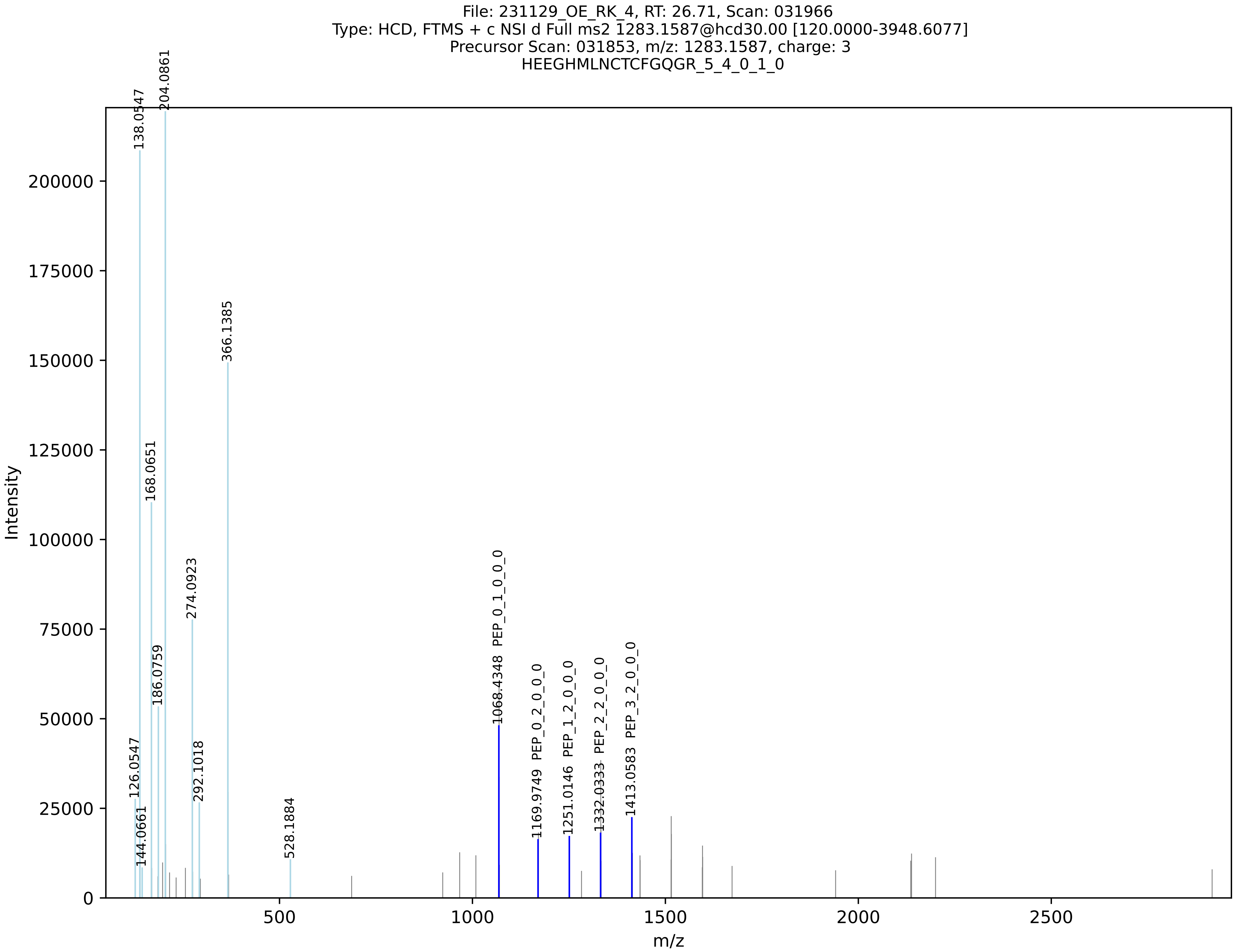
File: 231129_OE_RK_4, RT: 26.71, Scan: 031966
Type: HCD, FTMS + c NSI d Full ms2 1283.1587@hcd30.00 [120.0000-3948.6077]
Precursor Scan: 031853, m/z: 1283.1587, charge: 3
HEEGHMLNCTCFGQGR_5_4_0_1_0
Intensity
m/z
138.0547
204.0861
366.1385
168.0651
274.0923
186.0759
126.0547
144.0661
292.1018
528.1884
1068.4348 PEP_0_1_0_0_0
1169.9749 PEP_0_2_0_0_0
1251.0146 PEP_1_2_0_0_0
1332.0333 PEP_2_2_0_0_0
1413.0583 PEP_3_2_0_0_0

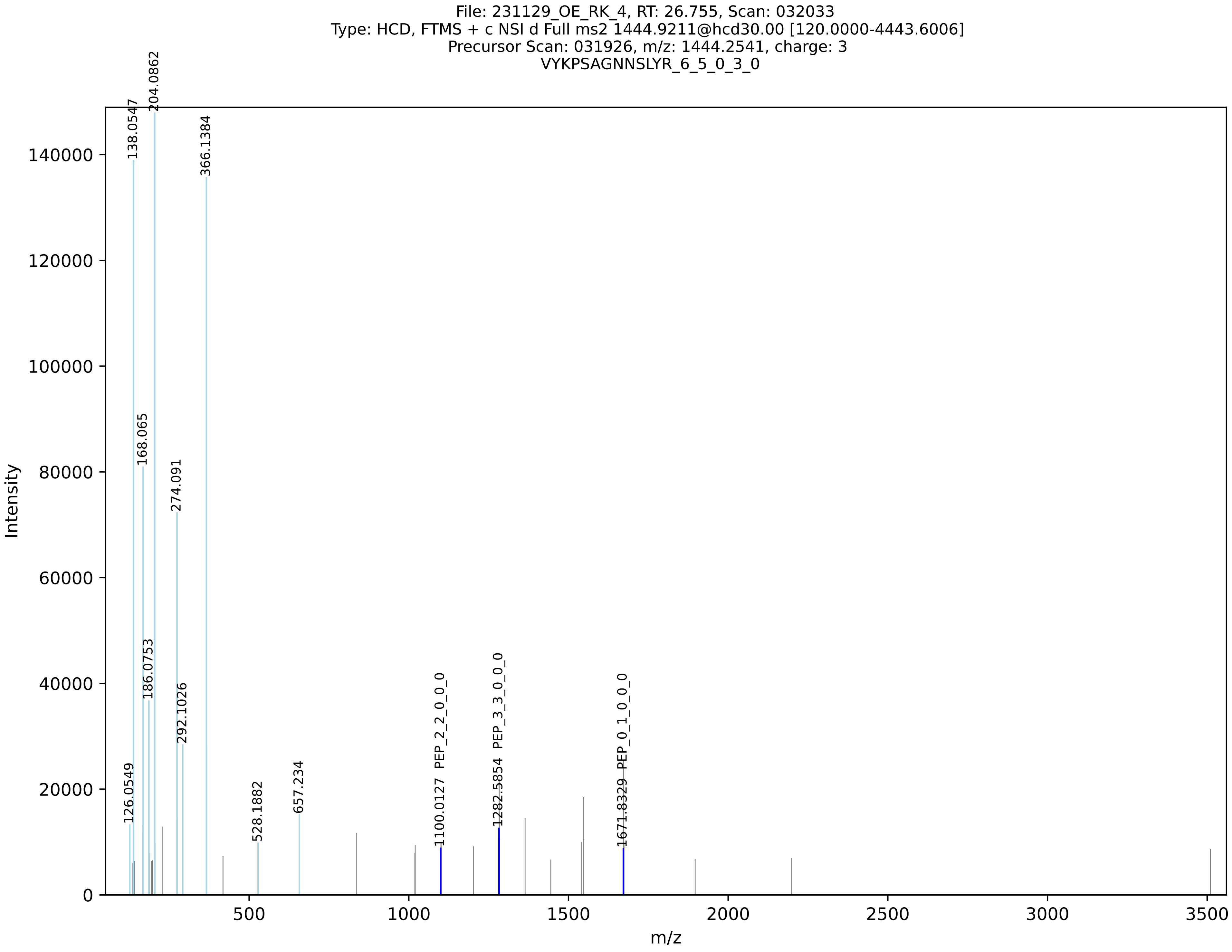
File: 231129_OE_RK_4, RT: 26.755, Scan: 032033
Type: HCD, FTMS + c NSI d Full ms2 1444.9211@hcd30.00 [120.0000-4443.6006]
Precursor Scan: 031926, m/z: 1444.2541, charge: 3
VYKPSAGNNSLYR_6_5_0_3_0
Intensity
m/z
138.0547
204.0862
366.1384
168.065
274.091
186.0753
292.1026
126.0549
528.1882
657.234
1100.0127 PEP_2_2_0_0_0
1282.5854 PEP_3_3_0_0_0
1671.8329 PEP_0_1_0_0_0

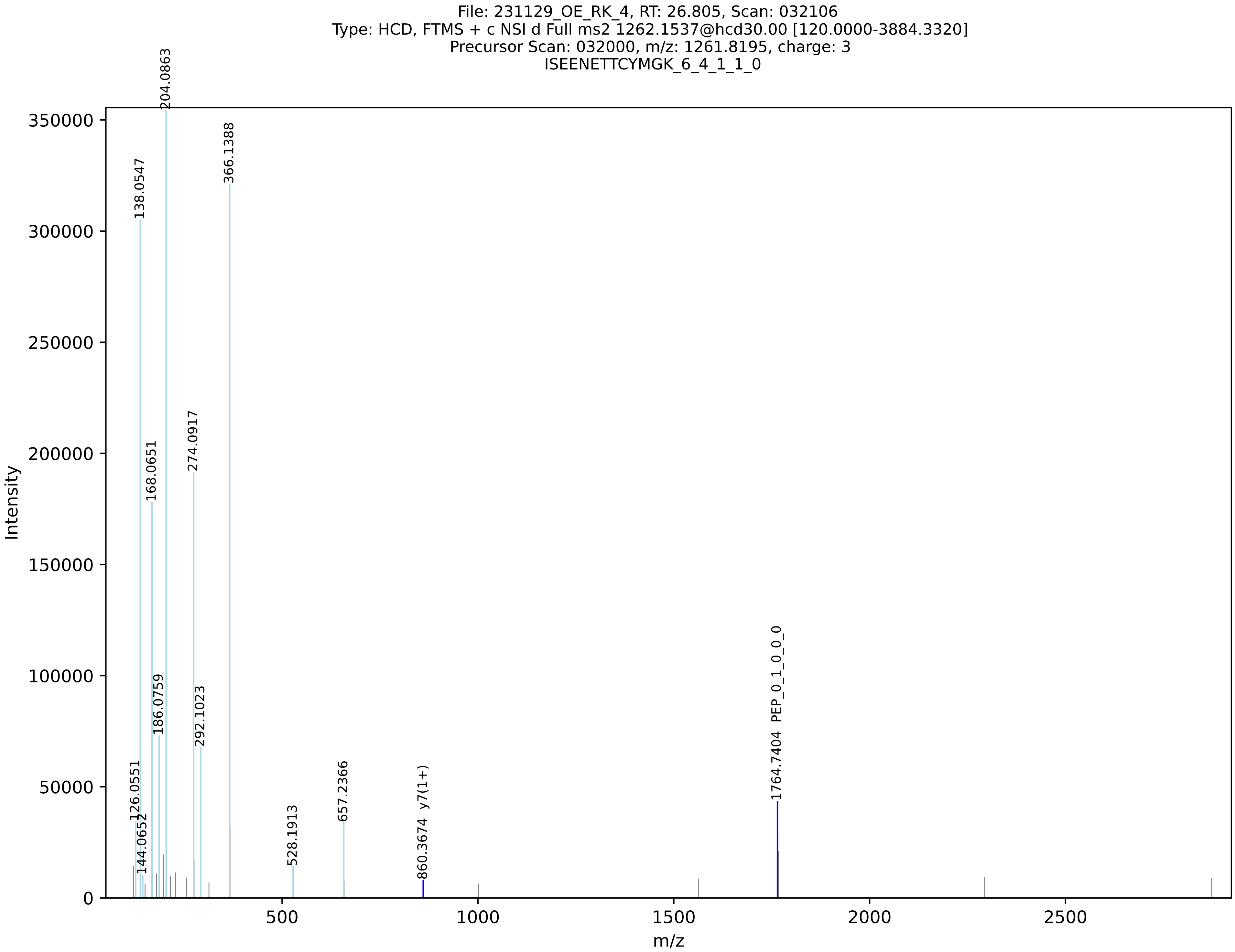
File: 231129_OE_RK_4, RT: 26.805, Scan: 032106
Type: HCD, FTMS + c NSI d Full ms2 1262.1537@hcd30.00 [120.0000-3884.3320]
Precursor Scan: 032000, m/z: 1261.8195, charge: 3
ISEENETTCYMGK_6_4_1_1_0
Intensity
m/z
204.0863
366.1388
138.0547
274.0917
168.0651
186.0759
292.1023
126.0551
144.0652
528.1913
657.2366
860.3674 y7(1+)
1764.7404 PEP_0_1_0_0_
0
50000
100000
150000
200000
250000
300000
350000
500
1000
1500
2000
2500

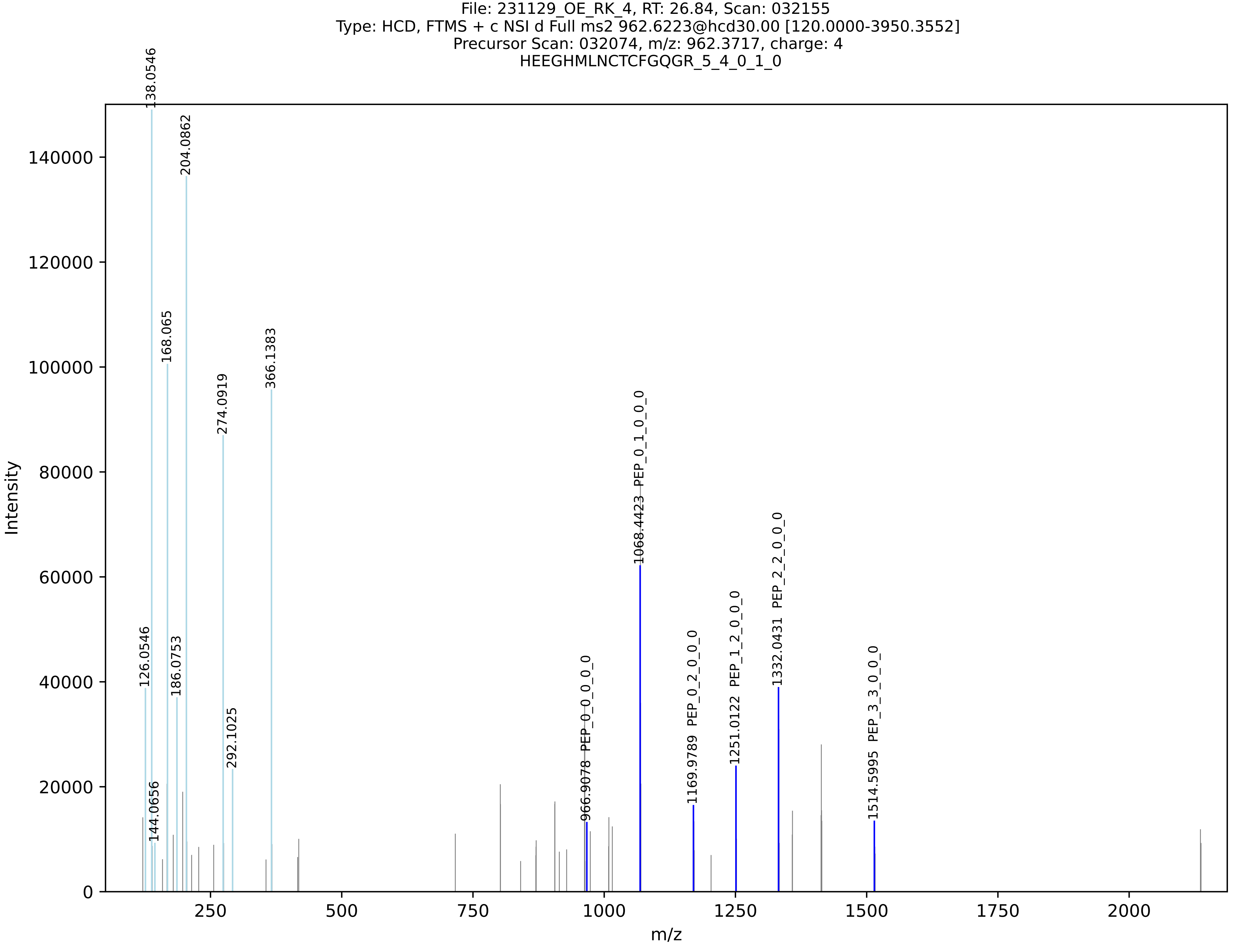
File: 231129_OE_RK_4, RT: 26.84, Scan: 032155
Type: HCD, FTMS + c NSI d Full ms2 962.6223@hcd30.00 [120.0000-3950.3552]
Precursor Scan: 032074, m/z: 962.3717, charge: 4
HEEGHMLNCTCFGQGR_5_4_0_1_0
Intensity
m/z
138.0546
204.0862
168.065
366.1383
274.0919
126.0546
186.0753
292.1025
144.0656
1068.4423 PEP_0_1_0_0_0
1332.0431 PEP_2_2_0_0_0
1251.0122 PEP_1_2_0_0_0
1169.9789 PEP_0_2_0_0_0
966.9078 PEP_0_0_0_0_0
1514.5995 PEP_3_3_0_0_0

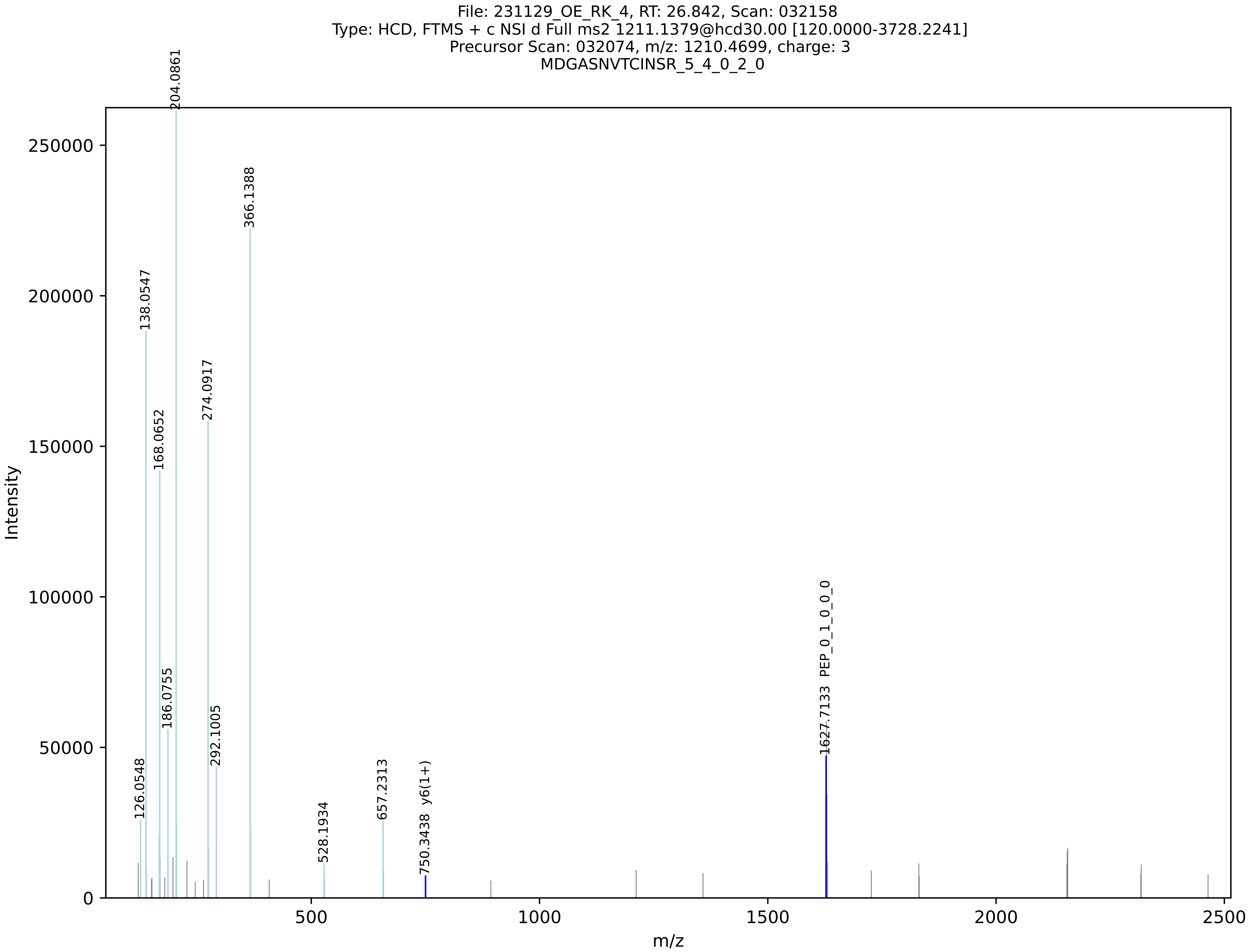

File: 231129_OE_RK_4, RT: 26.842, Scan: 032158
Type: HCD, FTMS + c NSI d Full ms2 1211.1379@hcd30.00 [120.0000-3728.2241]
Precursor Scan: 032074, m/z: 1210.4699, charge: 3
MDGASNVTCINSR_5_4_0_2_0
Intensity
m/z
204.0861
366.1388
138.0547
274.0917
168.0652
186.0755
292.1005
126.0548
528.1934
657.2313
750.3438 y6(1+)
1627.7133 PEP_0_1_0_0_0

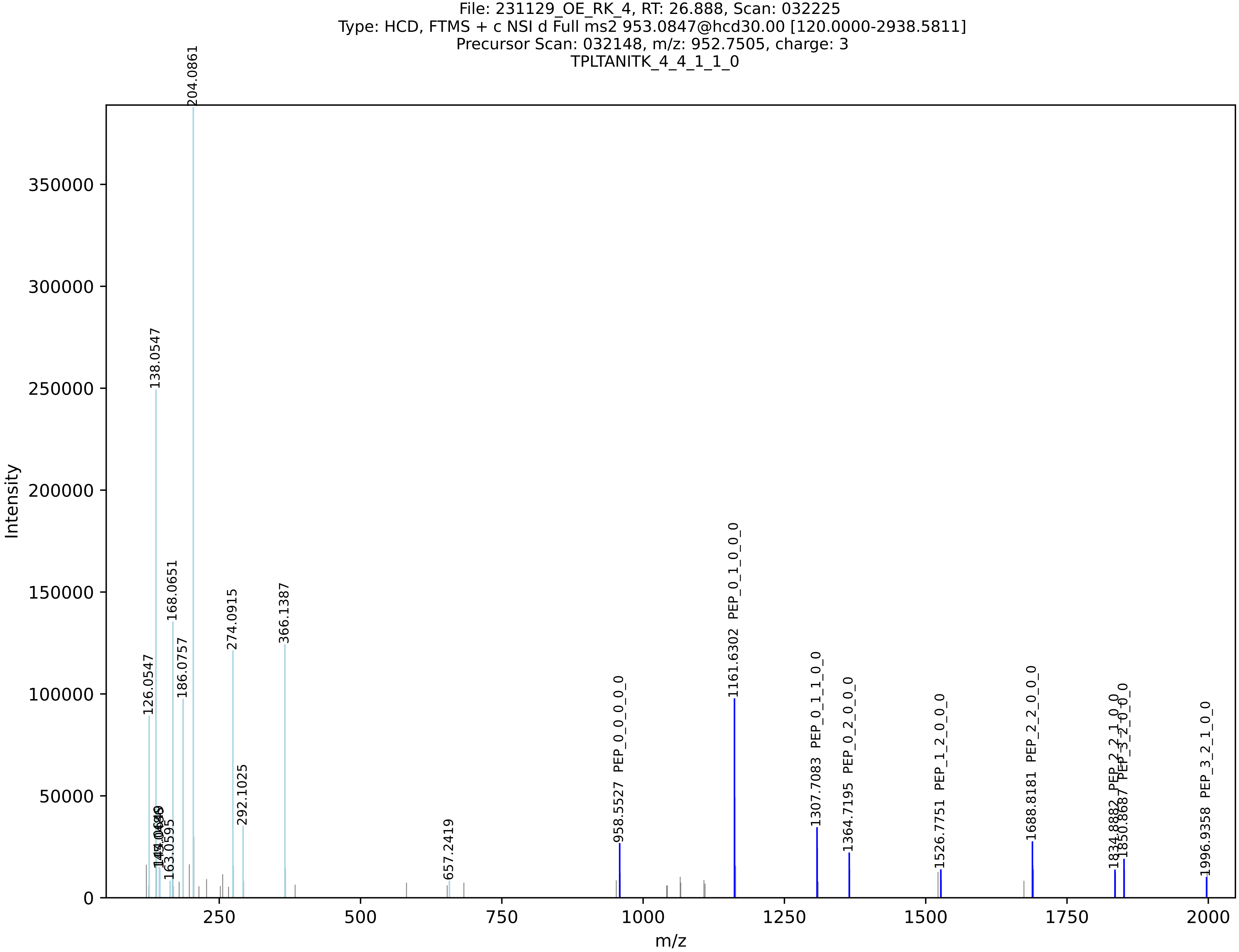

File: 231129_OE_RK_4, RT: 26.888, Scan: 032225
Type: HCD, FTMS + c NSI d Full ms2 953.0847@hcd30.00 [120.0000-2938.5811]
Precursor Scan: 032148, m/z: 952.7505, charge: 3
TPLTANITK_4_4_1_1_0
Intensity
m/z
204.0861
138.0547
168.0651
126.0547
186.0757
274.0915
366.1387
292.1025
163.0595
657.2419
958.5527 PEP_0_0_0_0_0
1161.6302 PEP_0_1_0_0_0
1307.7083 PEP_0_1_1_0_0
1364.7195 PEP_0_2_0_0_0
1526.7751 PEP_1_2_0_0_0
1688.8181 PEP_2_2_0_0_0
1834.8882 PEP_2_2_1_0_0
1850.8687 PEP_3_2_0_0_0
1996.9358 PEP_3_2_1_0_0

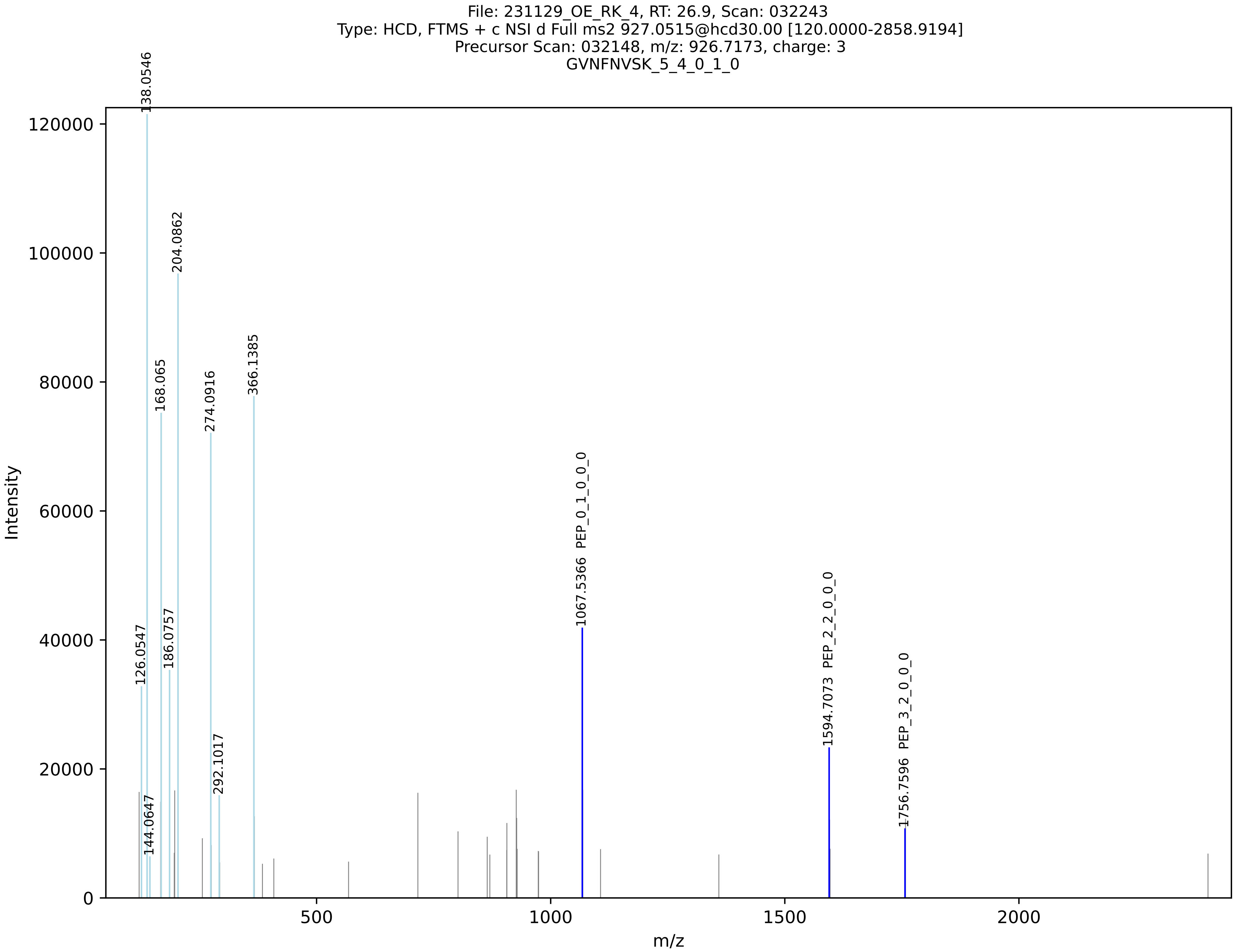
File: 231129_OE_RK_4, RT: 26.9, Scan: 032243
Type: HCD, FTMS + c NSI d Full ms2 927.0515@hcd30.00 [120.0000-2858.9194]
Precursor Scan: 032148, m/z: 926.7173, charge: 3
GVNFNVSK_5_4_0_1_0
Intensity
m/z
0
20000
40000
60000
80000
100000
120000
500
1000
1500
2000
138.0546
204.0862
168.065
274.0916
366.1385
126.0547
186.0757
292.1017
144.0647
1067.5366 PEP_0_1_0_0_0
1594.7073 PEP_2_2_0_0_0
1756.7596 PEP_3_2_0_0_0

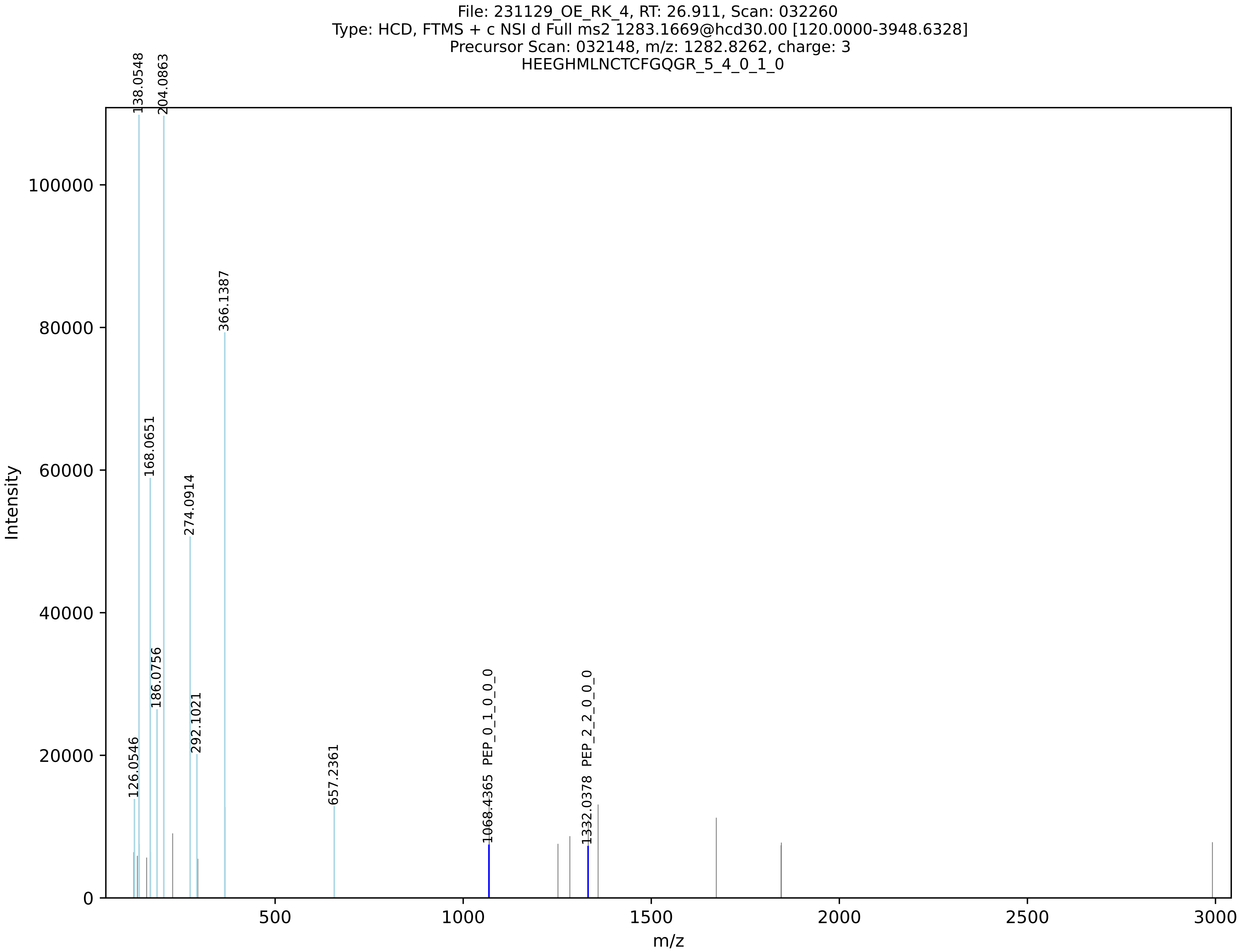

File: 231129_OE_RK_4, RT: 26.911, Scan: 032260
Type: HCD, FTMS + c NSI d Full ms2 1283.1669@hcd30.00 [120.0000-3948.6328]
Precursor Scan: 032148, m/z: 1282.8262, charge: 3
HEEGHMLNCTCFGQGR_5_4_0_1_0
138.0548
204.0863
366.1387
168.0651
274.0914
186.0756
292.1021
126.0546
657.2361
1068.4365 PEP_0_1_0_0_0
1332.0378 PEP_2_2_0_0_0
Intensity
m/z

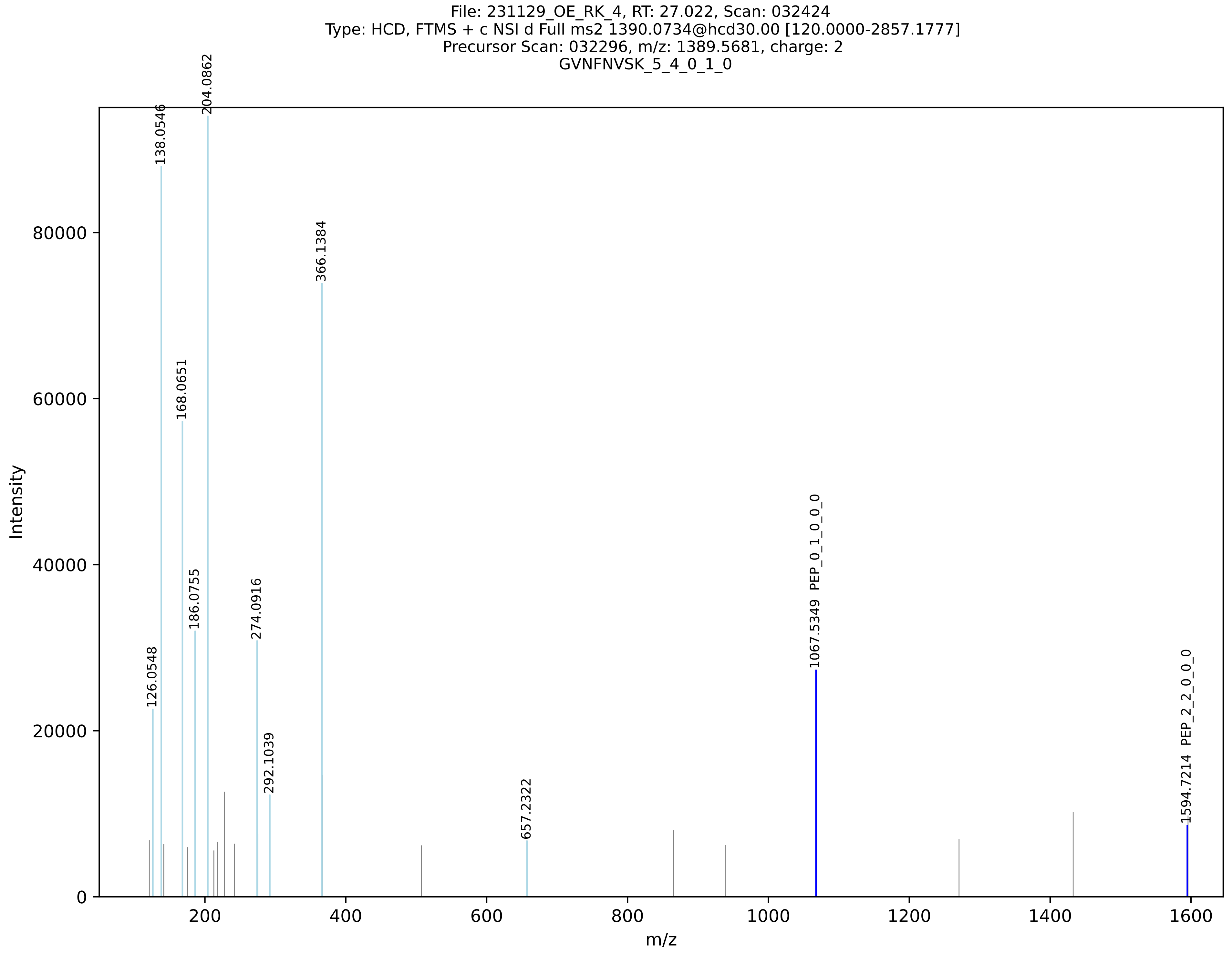
File: 231129_OE_RK_4, RT: 27.022, Scan: 032424
Type: HCD, FTMS + c NSI d Full ms2 1390.0734@hcd30.00 [120.0000-2857.1777]
Precursor Scan: 032296, m/z: 1389.5681, charge: 2
GVNFNVSK_5_4_0_1_0
Intensity
m/z
0
20000
40000
60000
80000
200
400
600
800
1000
1200
1400
1600
126.0548
138.0546
168.0651
186.0755
204.0862
274.0916
292.1039
366.1384
657.2322
1067.5349 PEP_0_1_0_0_0
1594.7214 PEP_2_2_0_0_0

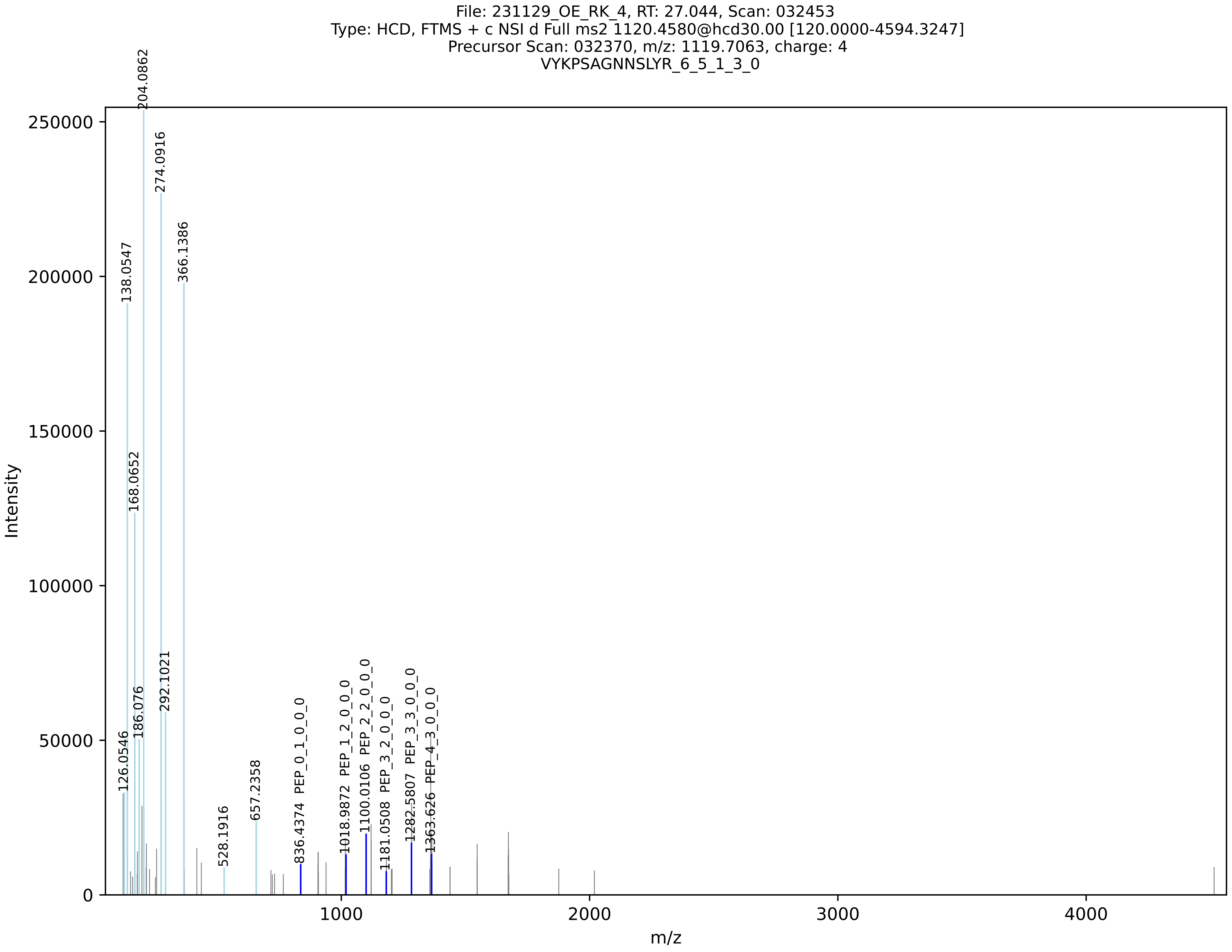
File: 231129_OE_RK_4, RT: 27.044, Scan: 032453
Type: HCD, FTMS + c NSI d Full ms2 1120.4580@hcd30.00 [120.0000-4594.3247]
Precursor Scan: 032370, m/z: 1119.7063, charge: 4
VYKPSAGNNSLYR_6_5_1_3_0
Intensity
m/z
250000
200000
150000
100000
50000
0
1000
2000
3000
4000
204.0862
274.0916
366.1386
138.0547
168.0652
292.1021
186.076
126.0546
528.1916
657.2358
836.4374 PEP_0_1_0_0_0
1018.9872 PEP_1_2_0_0_0
1100.0106 PEP_2_2_0_0_0
1181.0508 PEP_3_2_0_0_0
1282.5807 PEP_3_3_0_0_0
1363.626 PEP_4_3_0_0_0

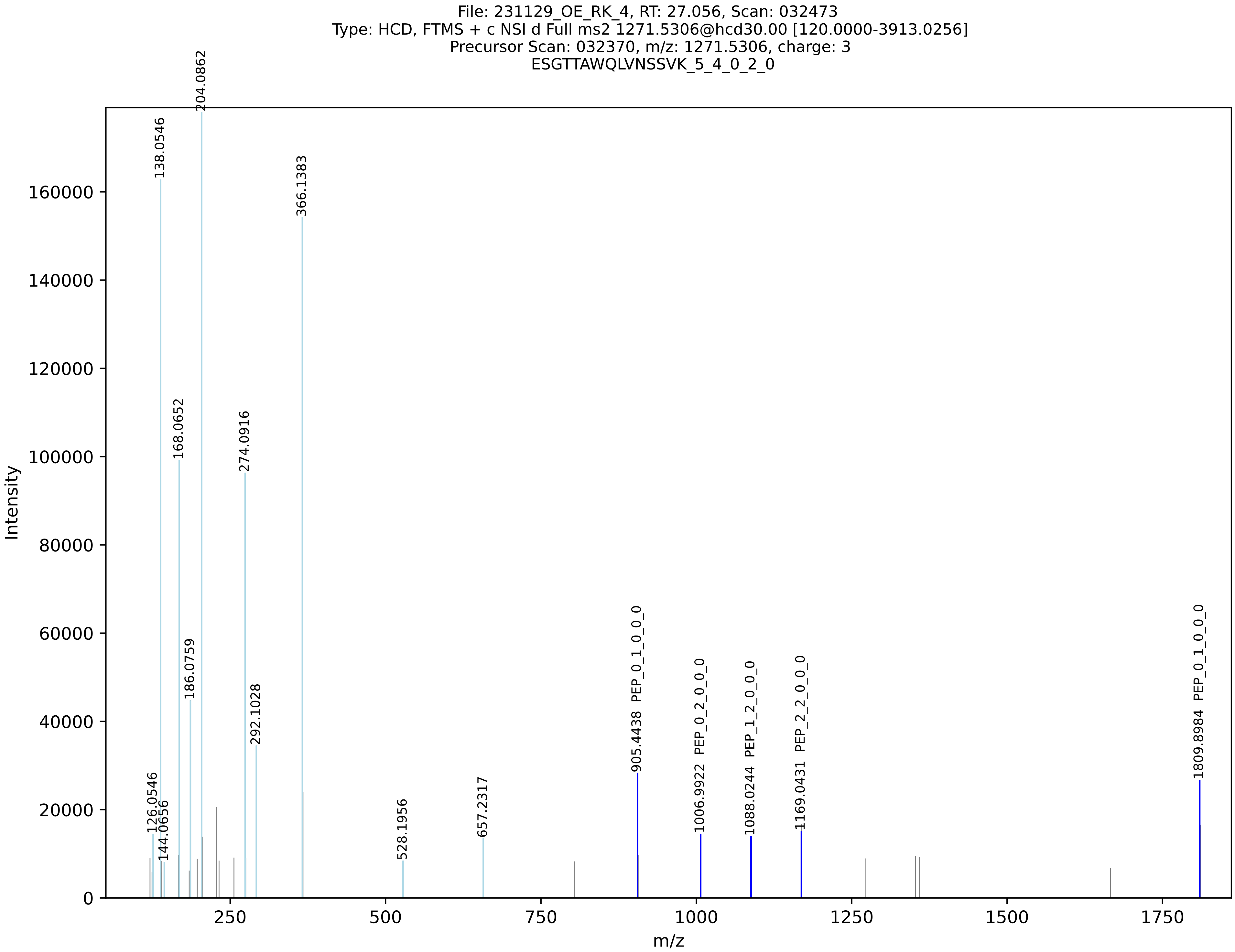
File: 231129_OE_RK_4, RT: 27.056, Scan: 032473
Type: HCD, FTMS + c NSI d Full ms2 1271.5306@hcd30.00 [120.0000-3913.0256]
Precursor Scan: 032370, m/z: 1271.5306, charge: 3
ESGTTAWQLVNSSVK_5_4_0_2_0
Intensity
m/z
204.0862
138.0546
366.1383
168.0652
274.0916
186.0759
292.1028
126.0546
144.0656
528.1956
657.2317
905.4438 PEP_0_1_0_0_0
1006.9922 PEP_0_2_0_0_0
1088.0244 PEP_1_2_0_0_0
1169.0431 PEP_2_2_0_0_0
1809.8984 PEP_0_1_0_0_0
0
20000
40000
60000
80000
100000
120000
140000
160000
250
500
750
1000
1250
1500
1750

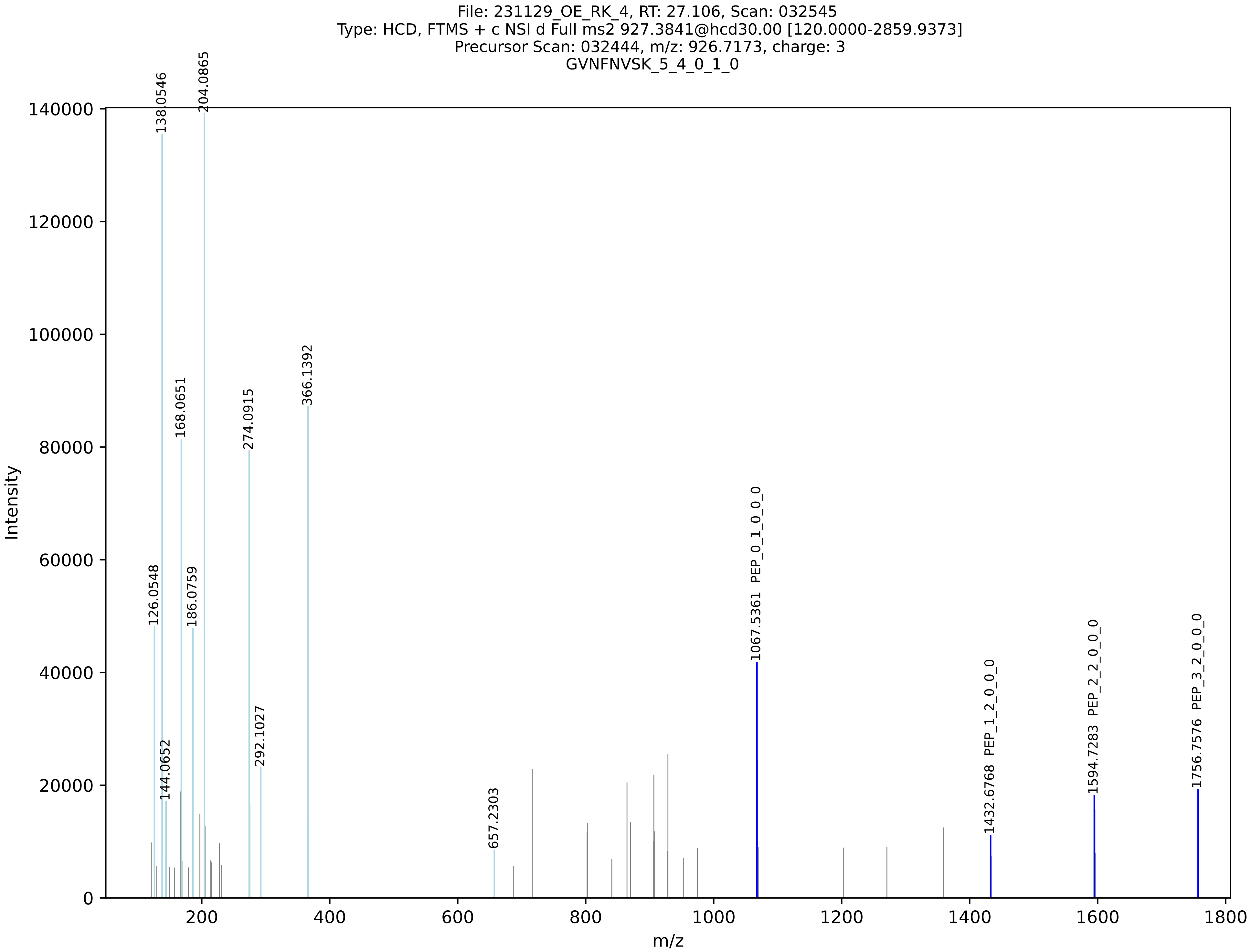
File: 231129_OE_RK_4, RT: 27.106, Scan: 032545
Type: HCD, FTMS + c NSI d Full ms2 927.3841@hcd30.00 [120.0000-2859.9373]
Precursor Scan: 032444, m/z: 926.7173, charge: 3
GVNFNVSK_5_4_0_1_0
Intensity
m/z
138.0546
204.0865
168.0651
274.0915
366.1392
126.0548
186.0759
144.0652
292.1027
657.2303
1067.5361 PEP_0_1_0_0_0
1432.6768 PEP_1_2_0_0_0
1594.7283 PEP_2_2_0_0_0
1756.7576 PEP_3_2_0_0_0
0
20000
40000
60000
80000
100000
120000
140000
200
400
600
800
1000
1200
1400
1600
1800

File: 231129_OE_RK_4, RT: 27.113, Scan: 032557
Type: HCD, FTMS + c NSI d Full ms2 1271.8596@hcd30.00 [120.0000-3914.0325]
Precursor Scan: 032444, m/z: 1271.197, charge: 3
ESGTTAWQLVNSSVK_5_4_0_2_0

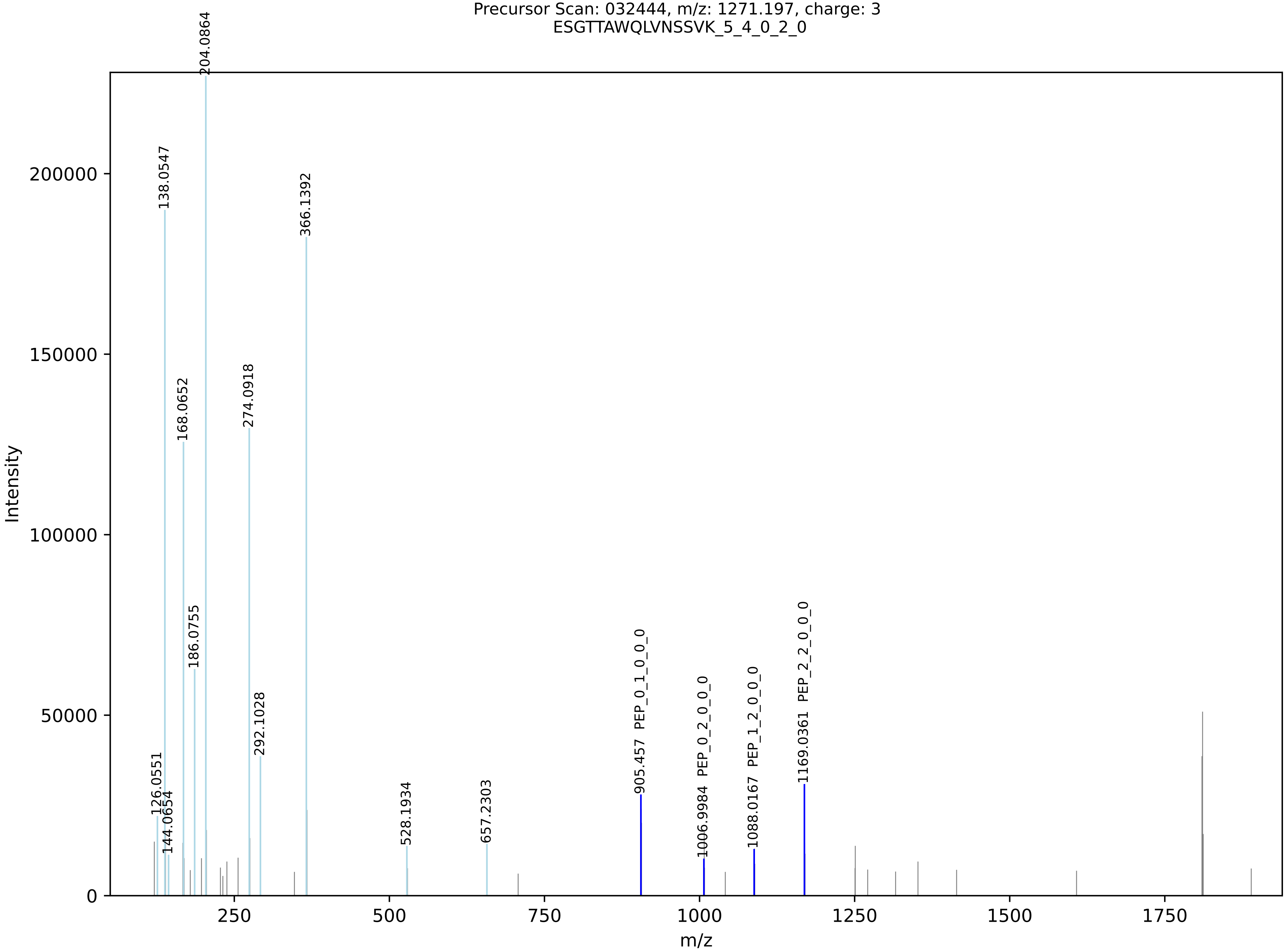

File: 231129_OE_RK_4, RT: 27.305, Scan: 032837
Type: HCD, FTMS + c NSI d Full ms2 1180.8054@hcd30.00 [120.0000-3635.4065]
Precursor Scan: 032739, m/z: 1180.1372, charge: 3
GGSSGWSGGLAQNR_5_4_0_2_0

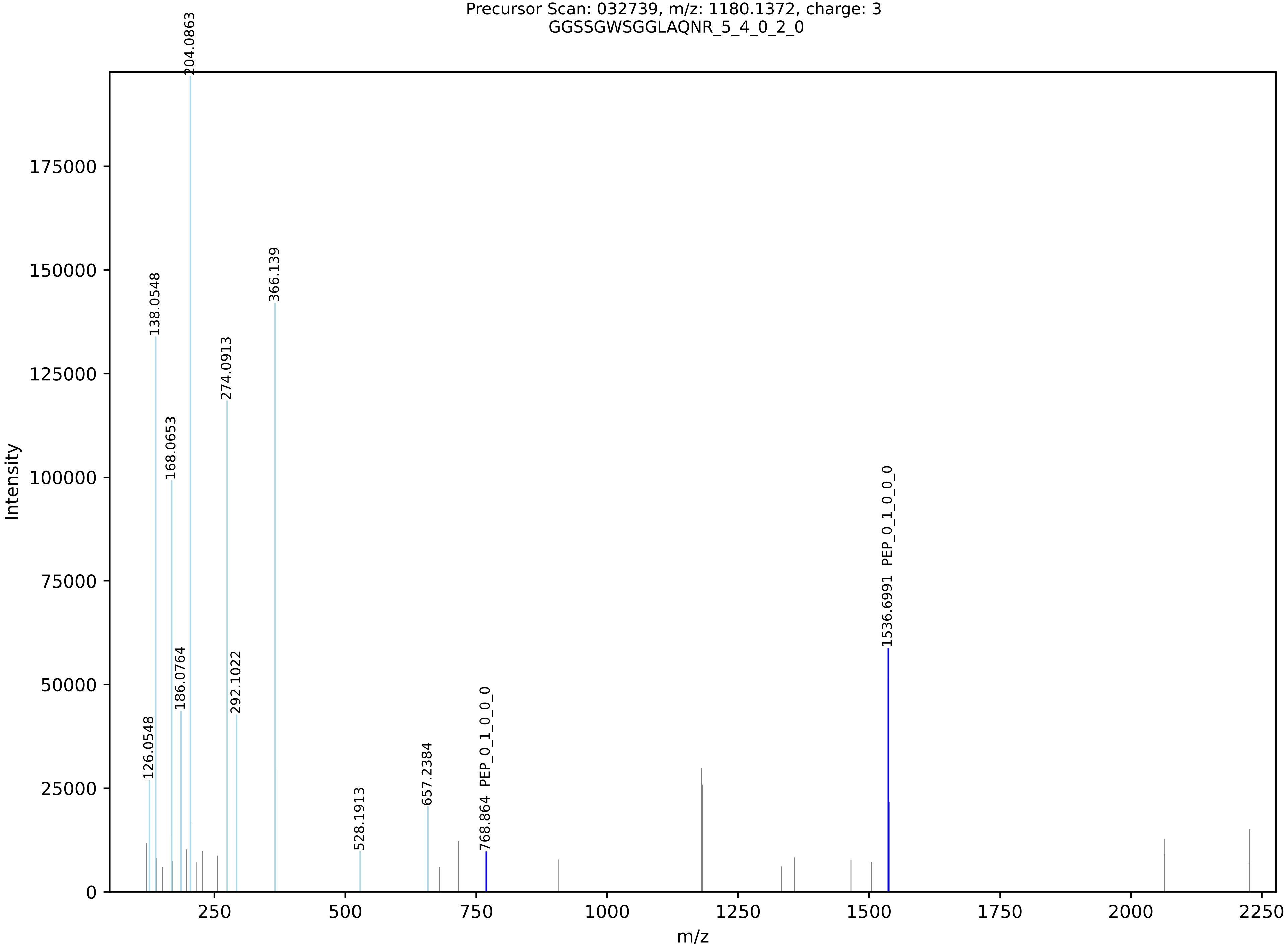

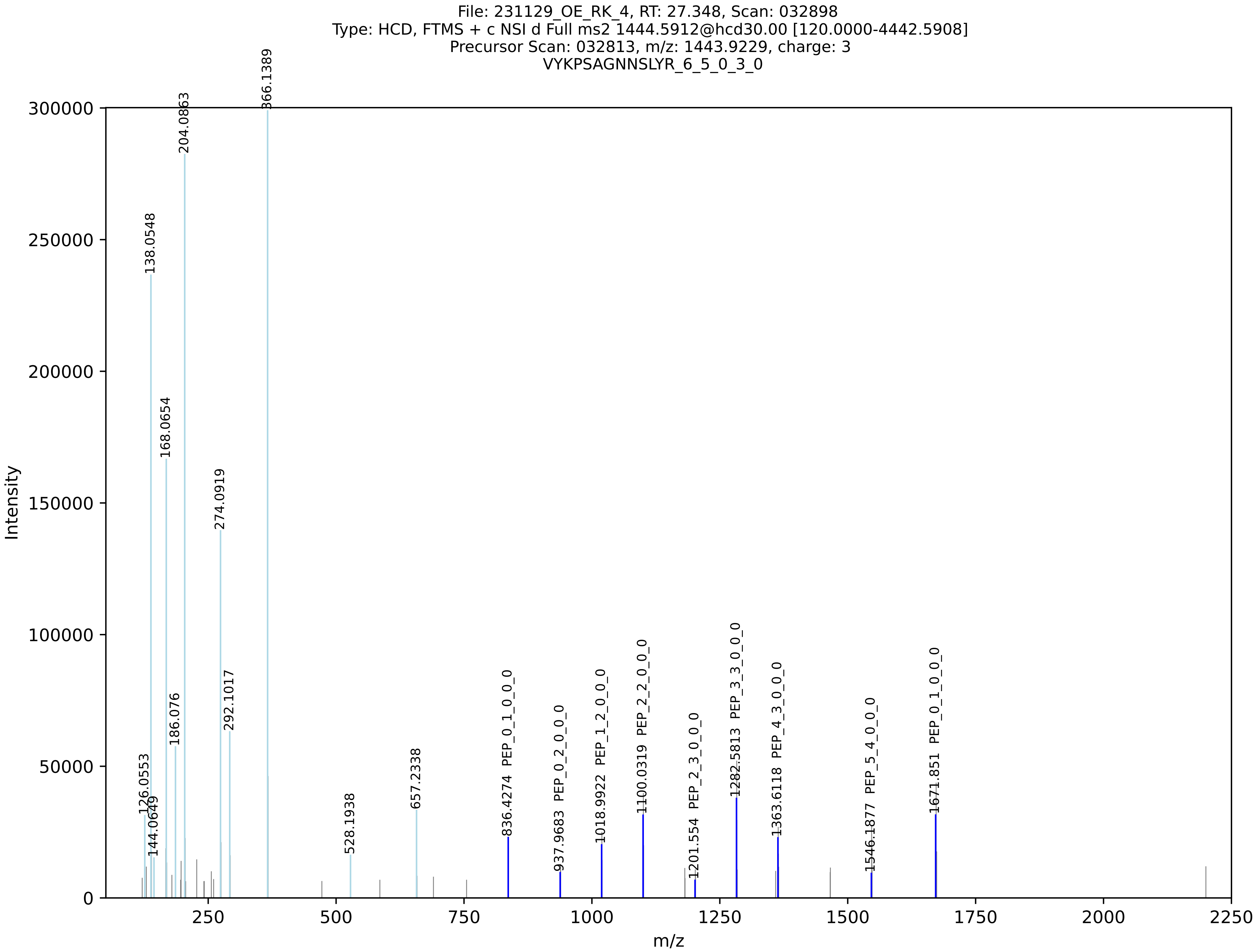

File: 231129_OE_RK_4, RT: 27.348, Scan: 032898
Type: HCD, FTMS + c NSI d Full ms2 1444.5912@hcd30.00 [120.0000-4442.5908]
Precursor Scan: 032813, m/z: 1443.9229, charge: 3
VYKPSAGNNSLYR_6_5_0_3_0
Intensity
m/z
366.1389
204.0863
138.0548
168.0654
274.0919
292.1017
186.076
126.0553
144.0649
528.1938
657.2338
836.4274 PEP_0_1_0_0_0
937.9683 PEP_0_2_0_0_0
1018.9922 PEP_1_2_0_0_0
1100.0319 PEP_2_2_0_0_0
1201.554 PEP_2_3_0_0_0
1282.5813 PEP_3_3_0_0_0
1363.6118 PEP_4_3_0_0_0
1546.1877 PEP_5_4_0_0_0
1671.851 PEP_0_1_0_0_0

File: 231129_OE_RK_4, RT: 27.401, Scan: 032977
Type: HCD, FTMS + c NSI d Full ms2 1055.6771@hcd30.00 [120.0000-4330.0186]
Precursor Scan: 032887, m/z: 1055.176, charge: 4
INNDFNYEFYNSTWSYVK_5_4_0_1_0

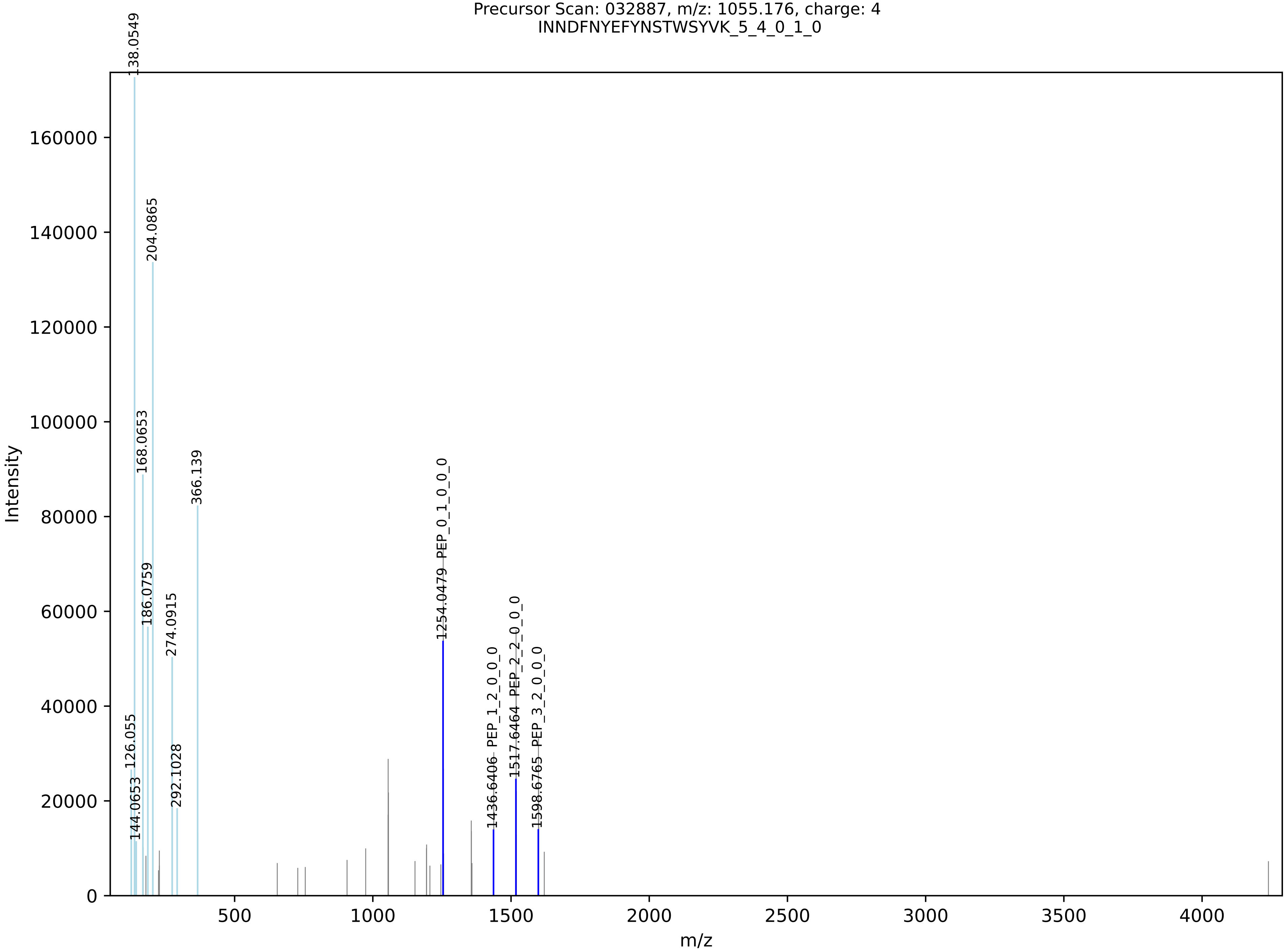

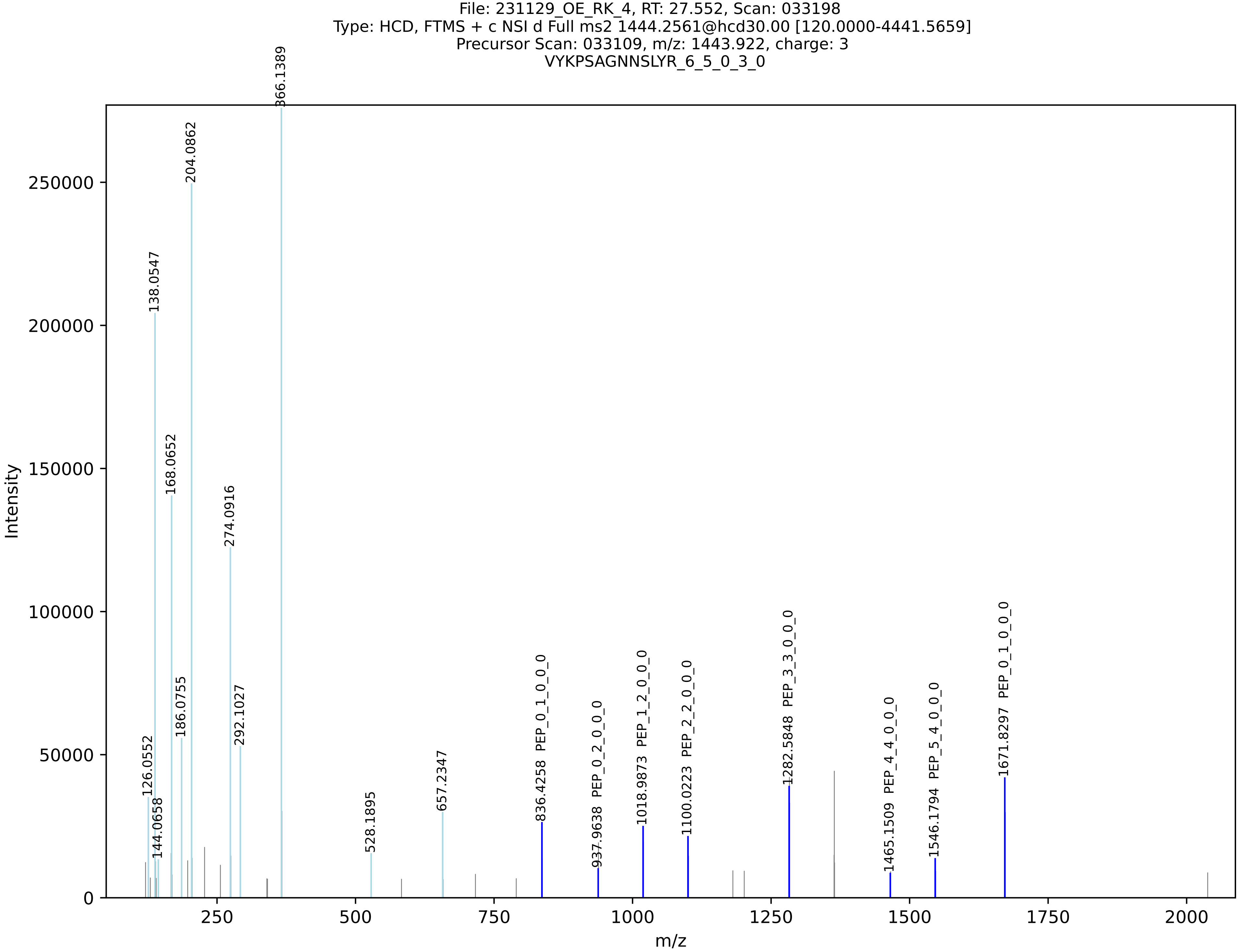

File: 231129_OE_RK_4, RT: 27.552, Scan: 033198
Type: HCD, FTMS + c NSI d Full ms2 1444.2561@hcd30.00 [120.0000-4441.5659]
Precursor Scan: 033109, m/z: 1443.922, charge: 3
VYKPSAGNNSLYR_6_5_0_3_0
Intensity
m/z
366.1389
204.0862
138.0547
168.0652
274.0916
186.0755
292.1027
126.0552
144.0658
528.1895
657.2347
836.4258 PEP_0_1_0_0_0
937.9638 PEP_0_2_0_0_0
1018.9873 PEP_1_2_0_0_0
1100.0223 PEP_2_2_0_0_0
1282.5848 PEP_3_3_0_0_0
1465.1509 PEP_4_4_0_0_0
1546.1794 PEP_5_4_0_0_0
1671.8297 PEP_0_1_0_0_0

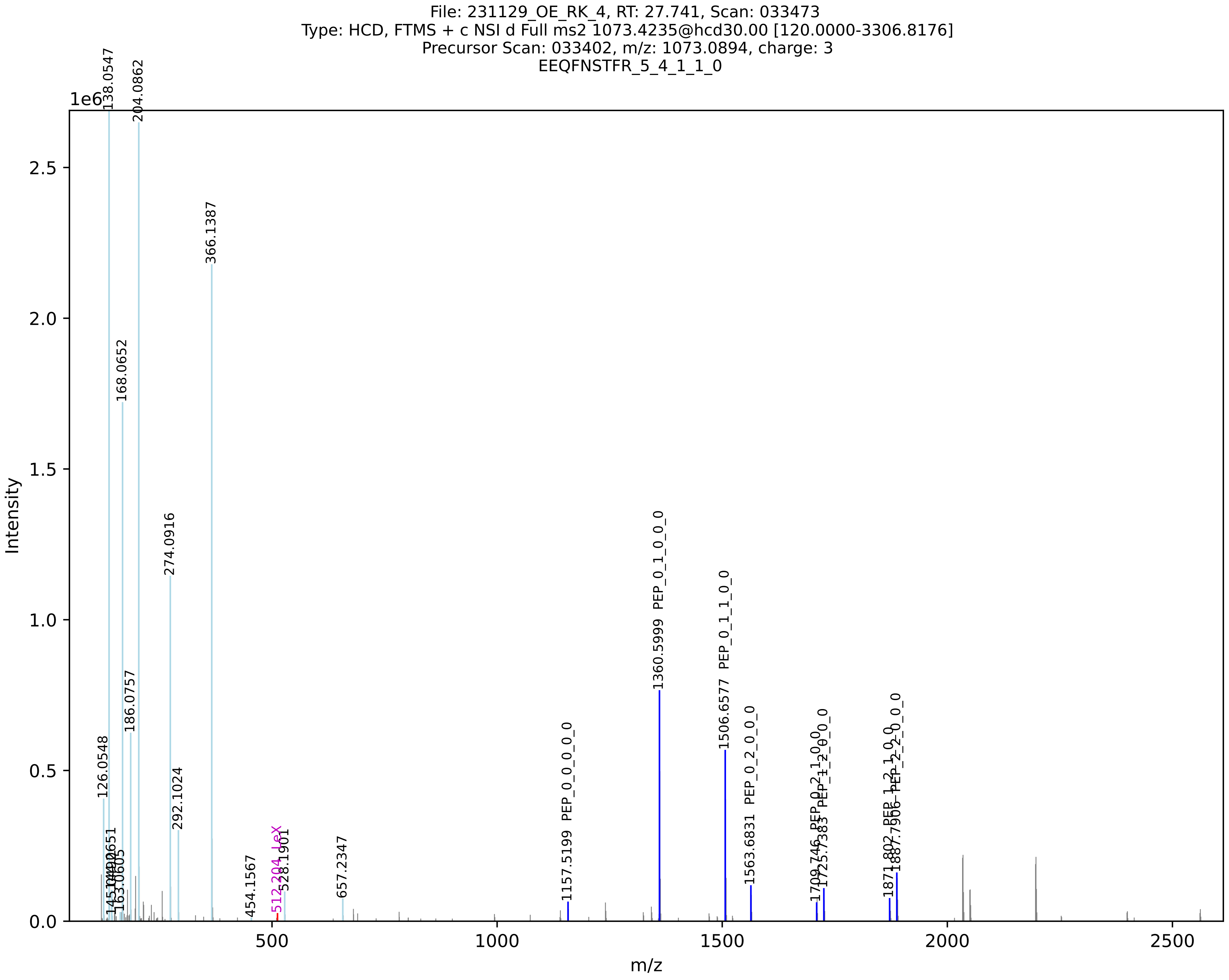
File: 231129_OE_RK_4, RT: 27.741, Scan: 033473
Type: HCD, FTMS + c NSI d Full ms2 1073.4235@hcd30.00 [120.0000-3306.8176]
Precursor Scan: 033402, m/z: 1073.0894, charge: 3
EEQFNSTFR_5_4_1_1_0
1e6
Intensity
m/z
0.0
0.5
1.0
1.5
2.0
2.5
500
1000
1500
2000
2500
138.0547
204.0862
366.1387
168.0652
274.0916
186.0757
126.0548
292.1024
163.0605
454.1567
512.204_LeX
528.1901
657.2347
1157.5199 PEP_0_0_0_0_0
1360.5999 PEP_0_1_0_0_0
1506.6577 PEP_0_1_1_0_0
1563.6831 PEP_0_2_0_0_0
1709.746 PEP_0_2_1_0_0
1725.7383 PEP_1_2_0_0_0
1871.802 PEP_1_2_1_0_0
1887.7906 PEP_2_2_0_0_0

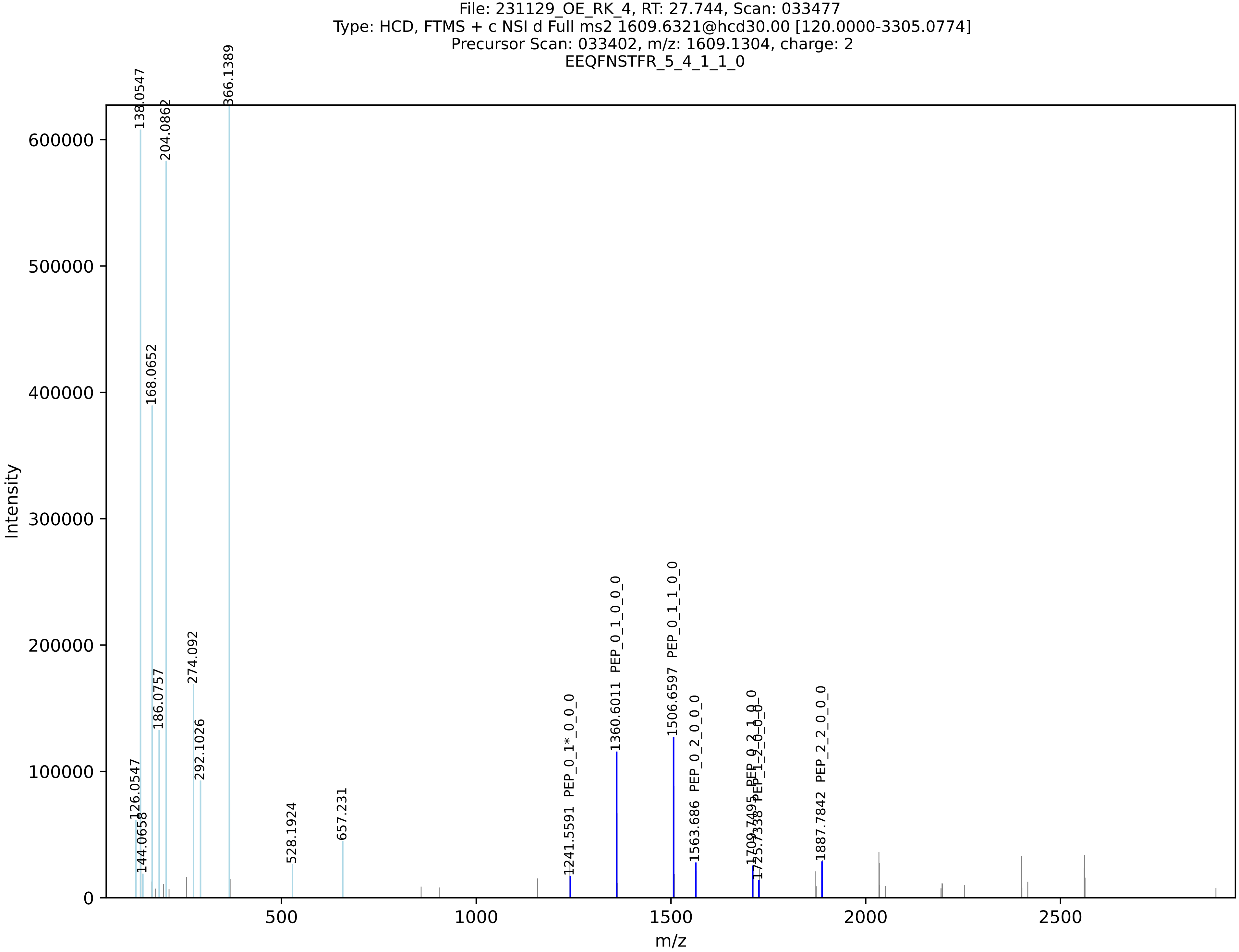

File: 231129_OE_RK_4, RT: 27.744, Scan: 033477
Type: HCD, FTMS + c NSI d Full ms2 1609.6321@hcd30.00 [120.0000-3305.0774]
Precursor Scan: 033402, m/z: 1609.1304, charge: 2
EEQFNSTFR_5_4_1_1_0
Intensity
m/z
138.0547
204.0862
366.1389
168.0652
186.0757
274.092
292.1026
126.0547
144.0658
528.1924
657.231
1241.5591 PEP_0_1*_0_0_0
1360.6011 PEP_0_1_0_0_0
1506.6597 PEP_0_1_1_0_0
1563.686 PEP_0_2_0_0_0
1709.7495 PEP_0_2_1_0_0
1725.7338 PEP_1_2_0_0_0
1887.7842 PEP_2_2_0_0_0

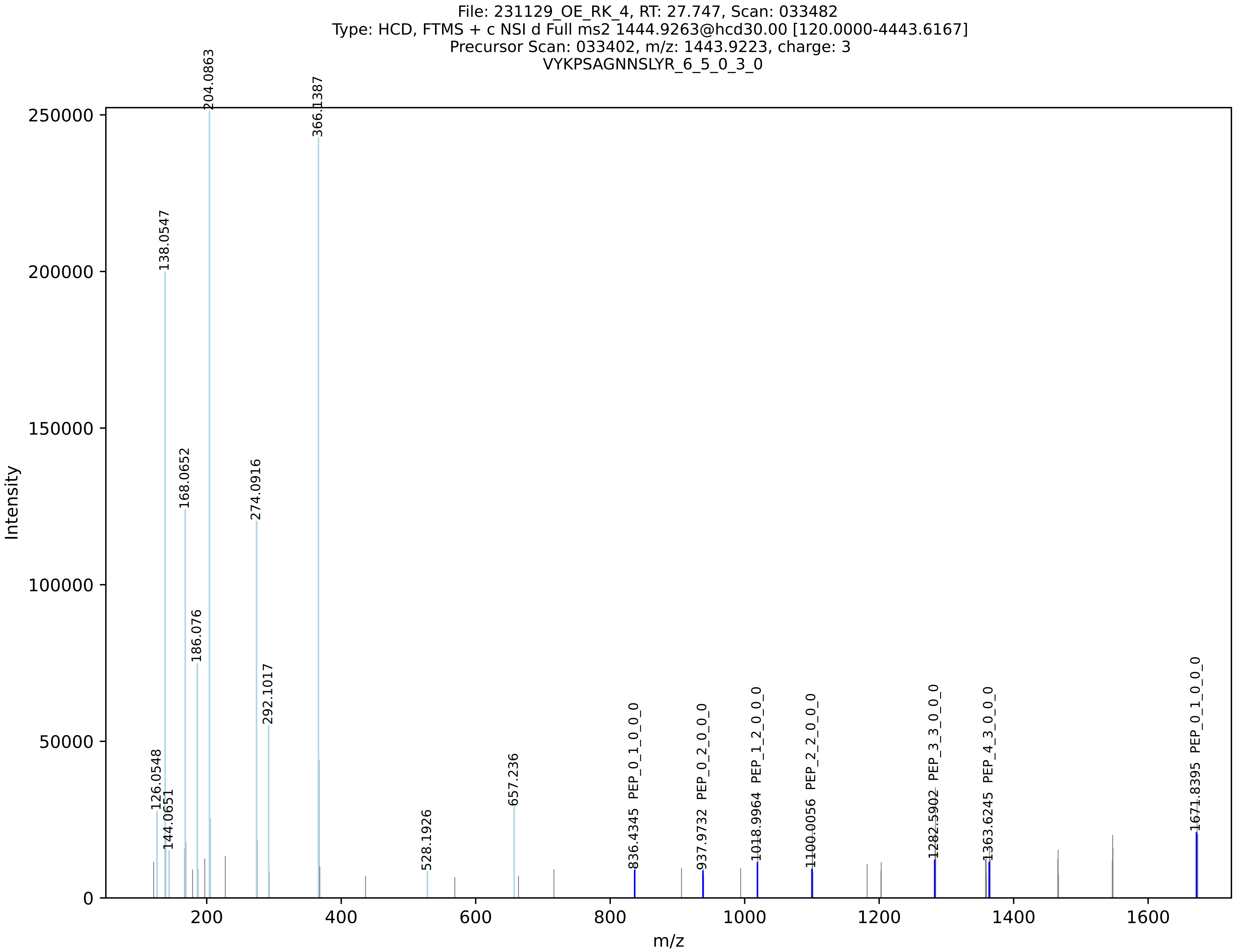
File: 231129_OE_RK_4, RT: 27.747, Scan: 033482
Type: HCD, FTMS + c NSI d Full ms2 1444.9263@hcd30.00 [120.0000-4443.6167]
Precursor Scan: 033402, m/z: 1443.9223, charge: 3
VYKPSAGNNSLYR_6_5_0_3_0
Intensity
m/z
204.0863
366.1387
138.0547
168.0652
274.0916
186.076
292.1017
126.0548
144.0651
528.1926
657.236
836.4345 PEP_0_1_0_0_0
937.9732 PEP_0_2_0_0_0
1018.9964 PEP_1_2_0_0_0
1100.0056 PEP_2_2_0_0_0
1282.5902 PEP_3_3_0_0_0
1363.6245 PEP_4_3_0_0_0
1671.8395 PEP_0_1_0_0_0

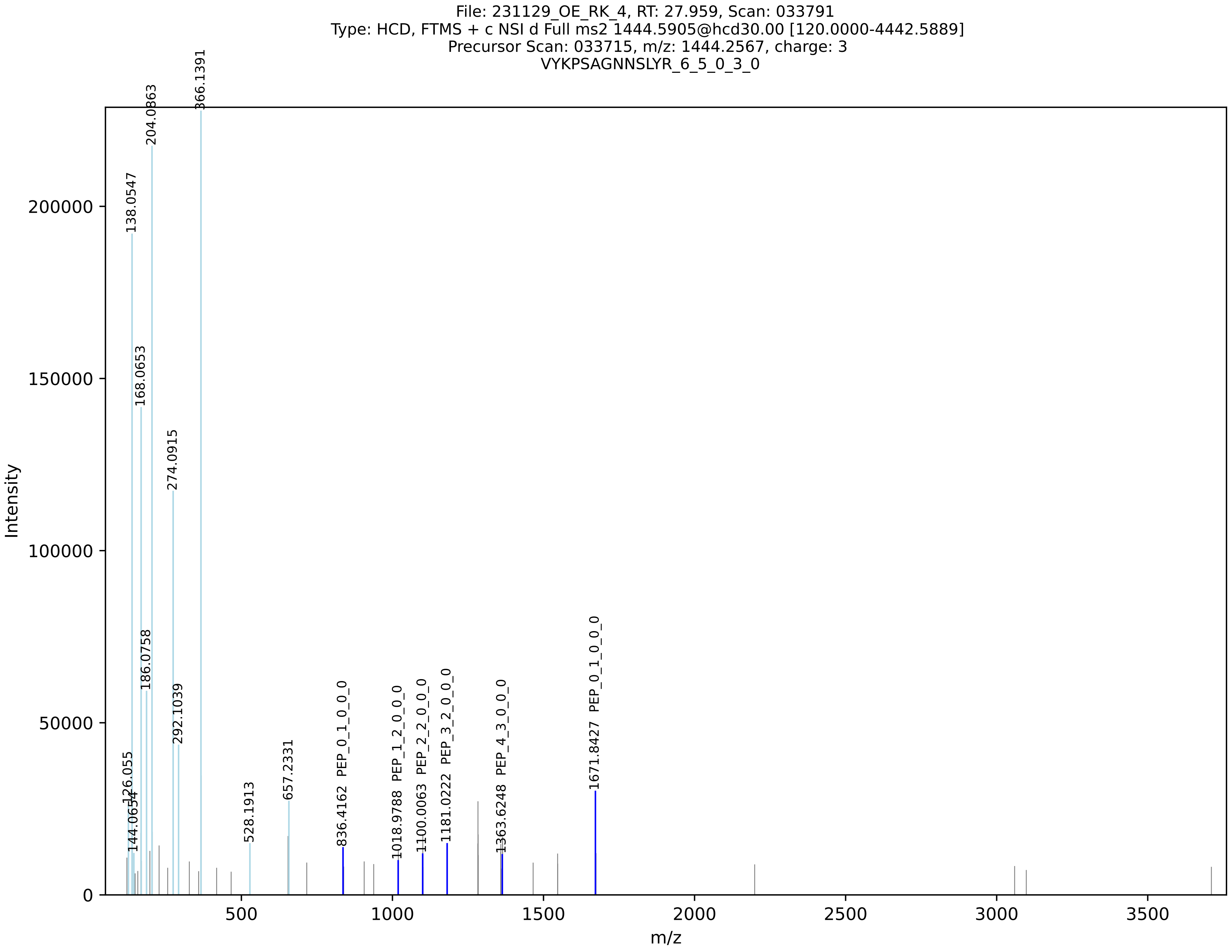

File: 231129_OE_RK_4, RT: 27.959, Scan: 033791
Type: HCD, FTMS + c NSI d Full ms2 1444.5905@hcd30.00 [120.0000-4442.5889]
Precursor Scan: 033715, m/z: 1444.2567, charge: 3
VYKPSAGNNSLYR_6_5_0_3_0
Intensity
m/z
366.1391
204.0863
138.0547
168.0653
274.0915
186.0758
292.1039
126.055
144.0654
528.1913
657.2331
836.4162 PEP_0_1_0_0_0
1018.9788 PEP_1_2_0_0_0
1100.0063 PEP_2_2_0_0_0
1181.0222 PEP_3_2_0_0_0
1363.6248 PEP_4_3_0_0_0
1671.8427 PEP_0_1_0_0_0

File: 231129_OE_RK_4, RT: 27.997, Scan: 033846
Type: HCD, FTMS + c NSI d Full ms2 1019.7391@hcd30.00 [120.0000-3142.5437]
Precursor Scan: 033776, m/z: 1019.0709, charge: 3
EEQFNSTFR_4_4_1_1_0

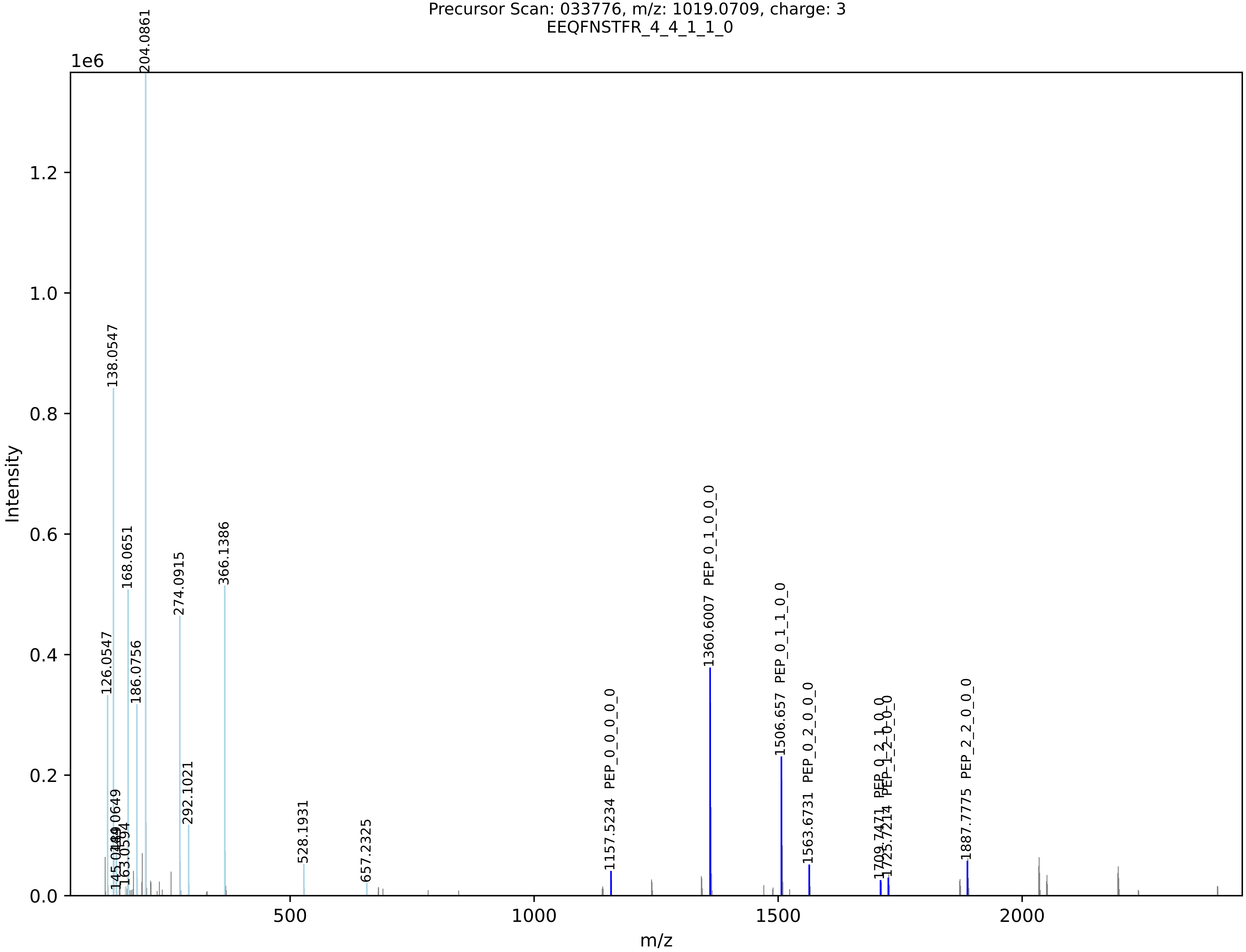

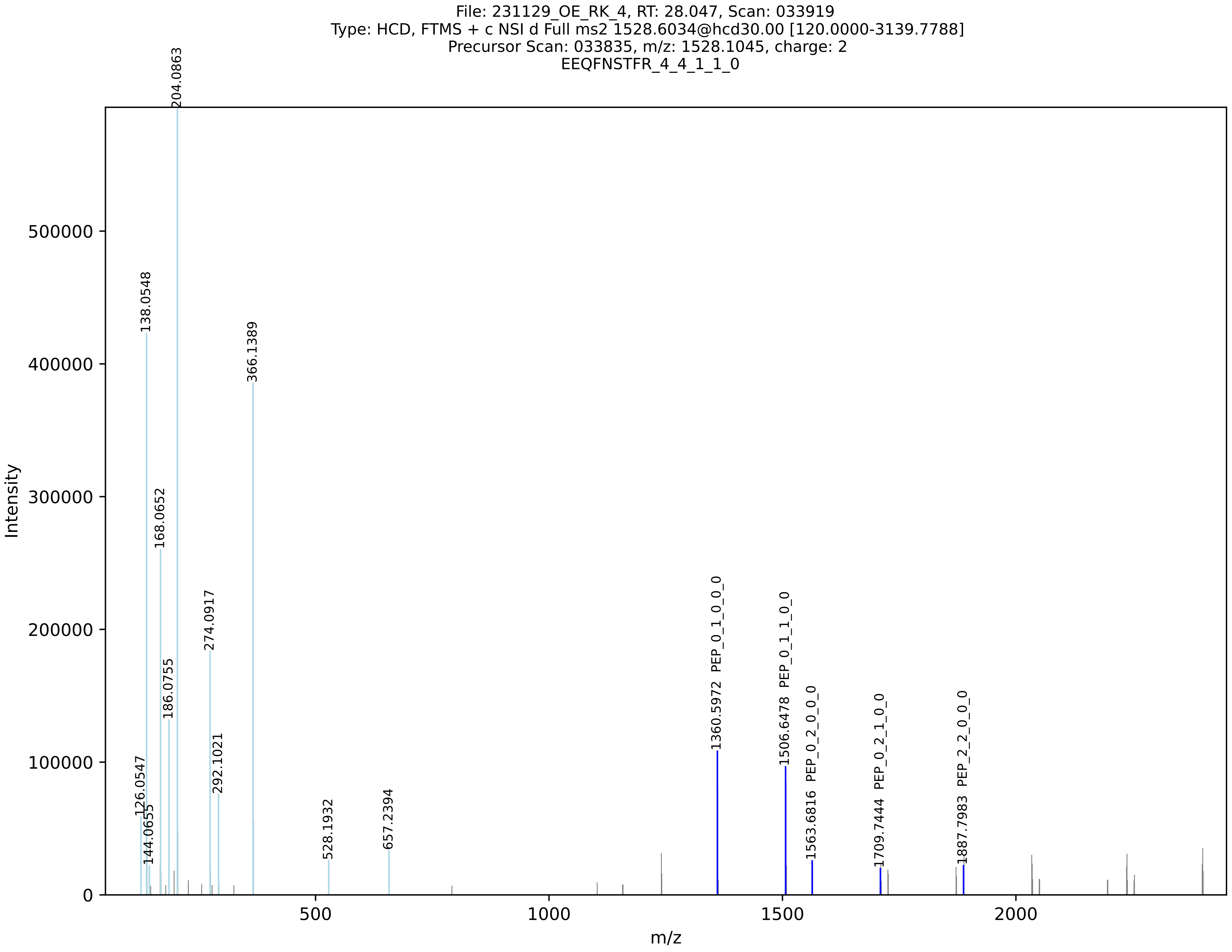
File: 231129_OE_RK_4, RT: 28.047, Scan: 033919
Type: HCD, FTMS + c NSI d Full ms2 1528.6034@hcd30.00 [120.0000-3139.7788]
Precursor Scan: 033835, m/z: 1528.1045, charge: 2
EEQFNSTFR_4_4_1_1_0
204.0863
138.0548
366.1389
168.0652
274.0917
186.0755
292.1021
126.0547
144.0655
528.1932
657.2394
1360.5972 PEP_0_1_0_0_0
1506.6478 PEP_0_1_1_0_0
1563.6816 PEP_0_2_0_0_0
1709.7444 PEP_0_2_1_0_0
1887.7983 PEP_2_2_0_0_0
Intensity
m/z
0
100000
200000
300000
400000
500000
500
1000
1500
2000

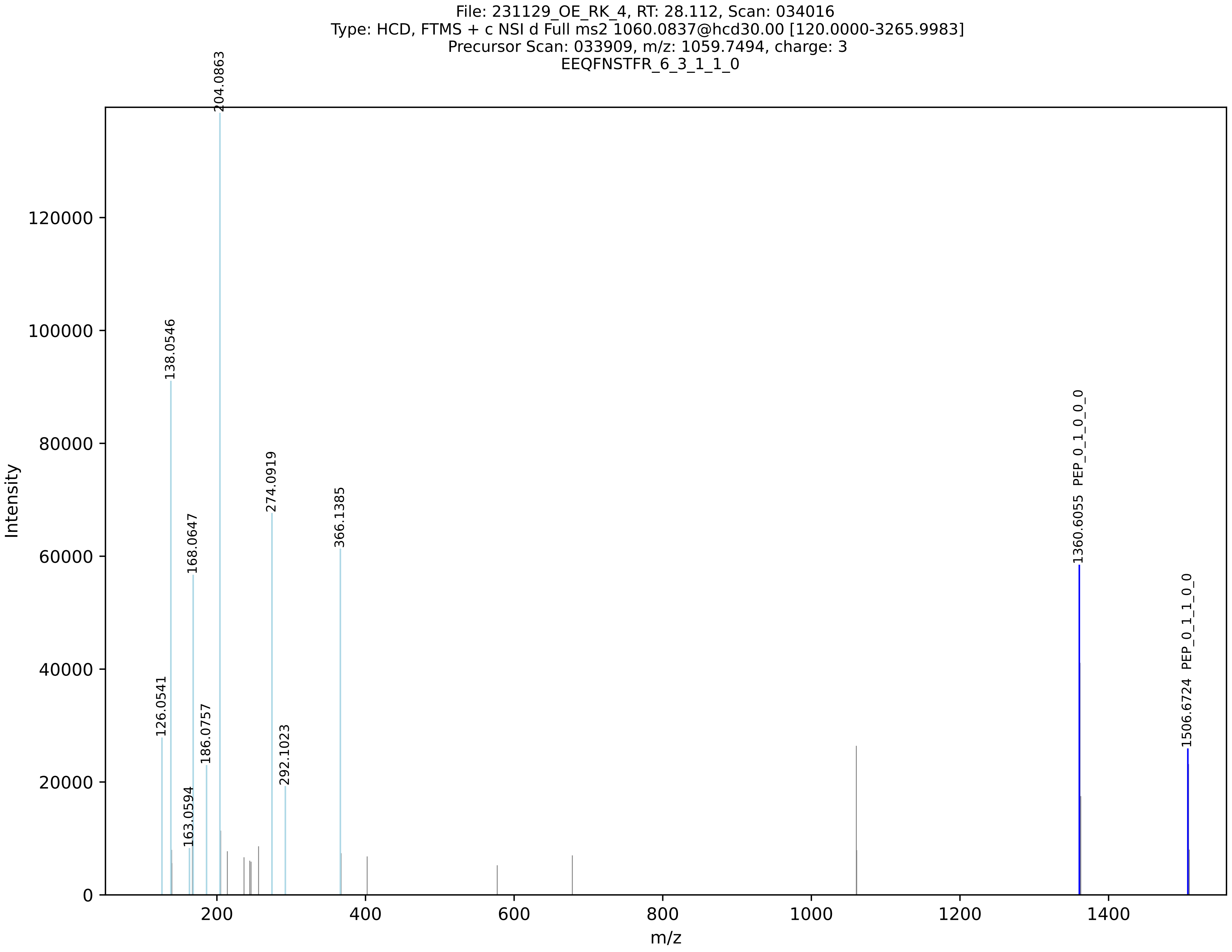
File: 231129_OE_RK_4, RT: 28.112, Scan: 034016
Type: HCD, FTMS + c NSI d Full ms2 1060.0837@hcd30.00 [120.0000-3265.9983]
Precursor Scan: 033909, m/z: 1059.7494, charge: 3
EEQFNSTFR_6_3_1_1_0
Intensity
m/z
204.0863
138.0546
168.0647
274.0919
366.1385
126.0541
163.0594
186.0757
292.1023
1360.6055 PEP_0_1_0_0_0
1506.6724 PEP_0_1_1_0_0

File: 231129_OE_RK_4, RT: 28.13, Scan: 034045
Type: HCD, FTMS + c NSI d Full ms2 1096.4209@hcd30.00 [120.0000-3377.1899]
Precursor Scan: 033835, m/z: 1096.0867, charge: 3
FNGSNSNGER_5_4_0_2_0

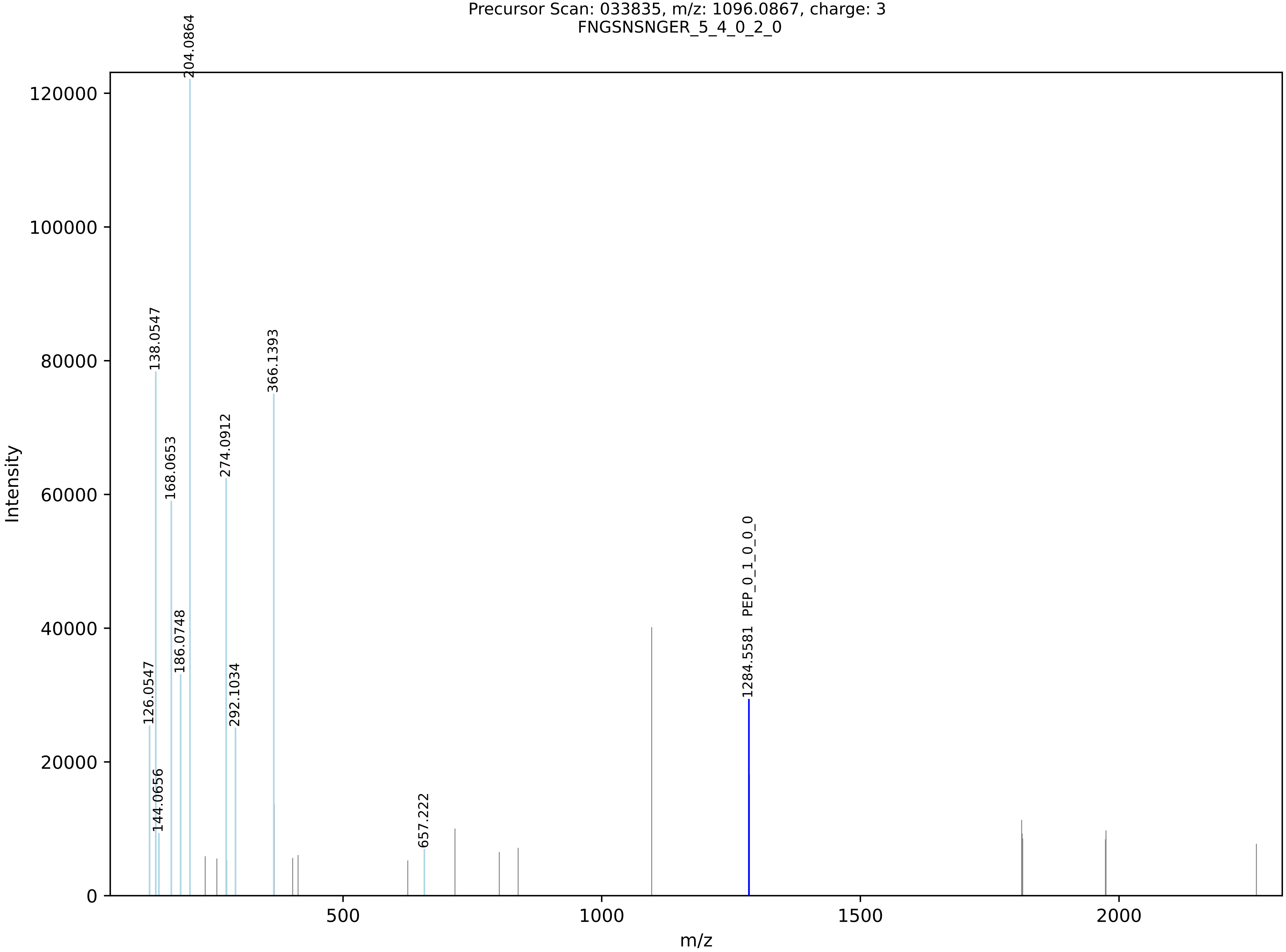

File: 231129_OE_RK_4, RT: 28.132, Scan: 034048
Type: HCD, FTMS + c NSI d Full ms2 1608.6652@hcd30.00 [120.0000-3303.1047]
Precursor Scan: 033835, m/z: 1609.1303, charge: 2
EEQFNSTFR_5_4_1_1_0

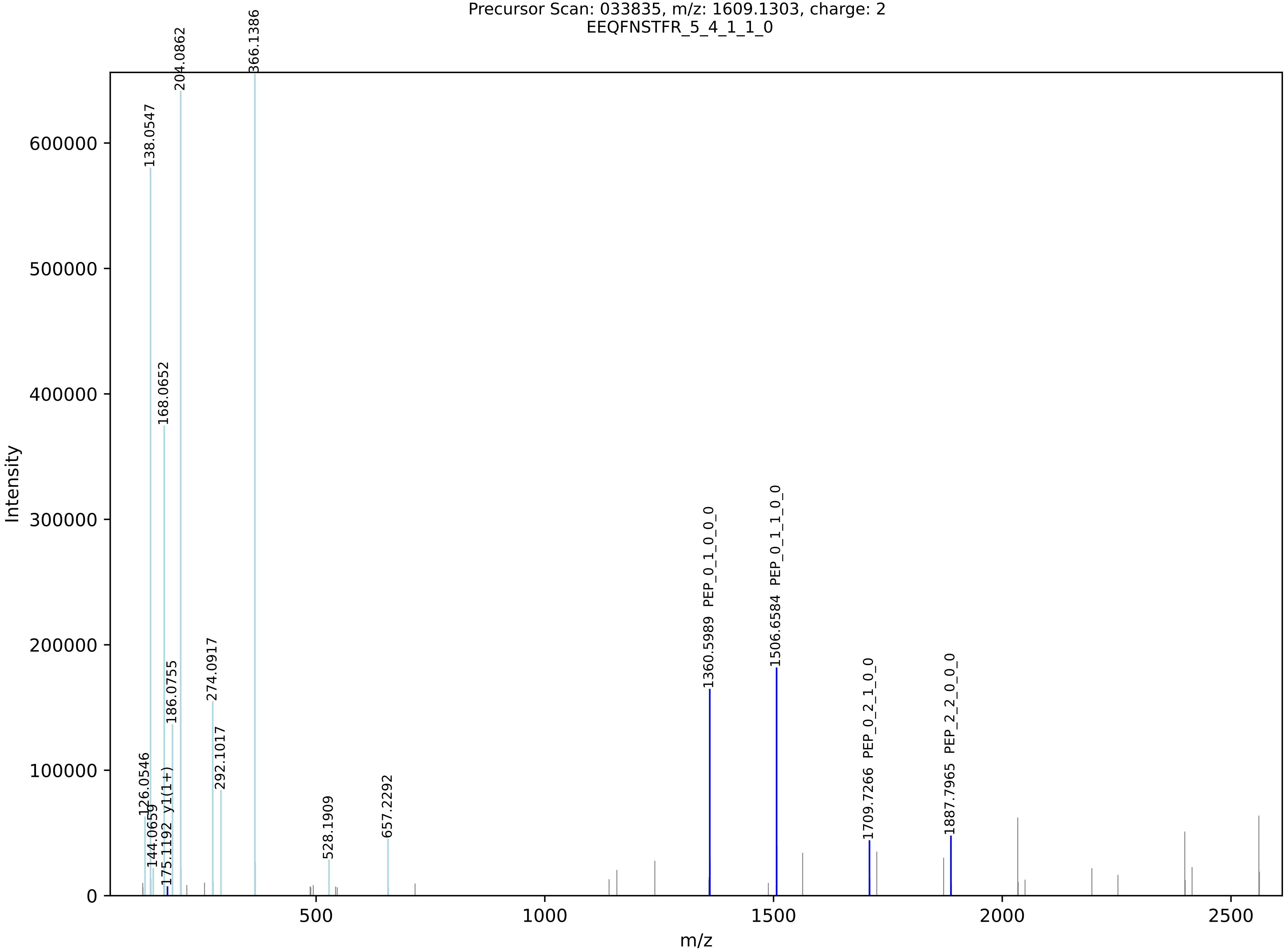

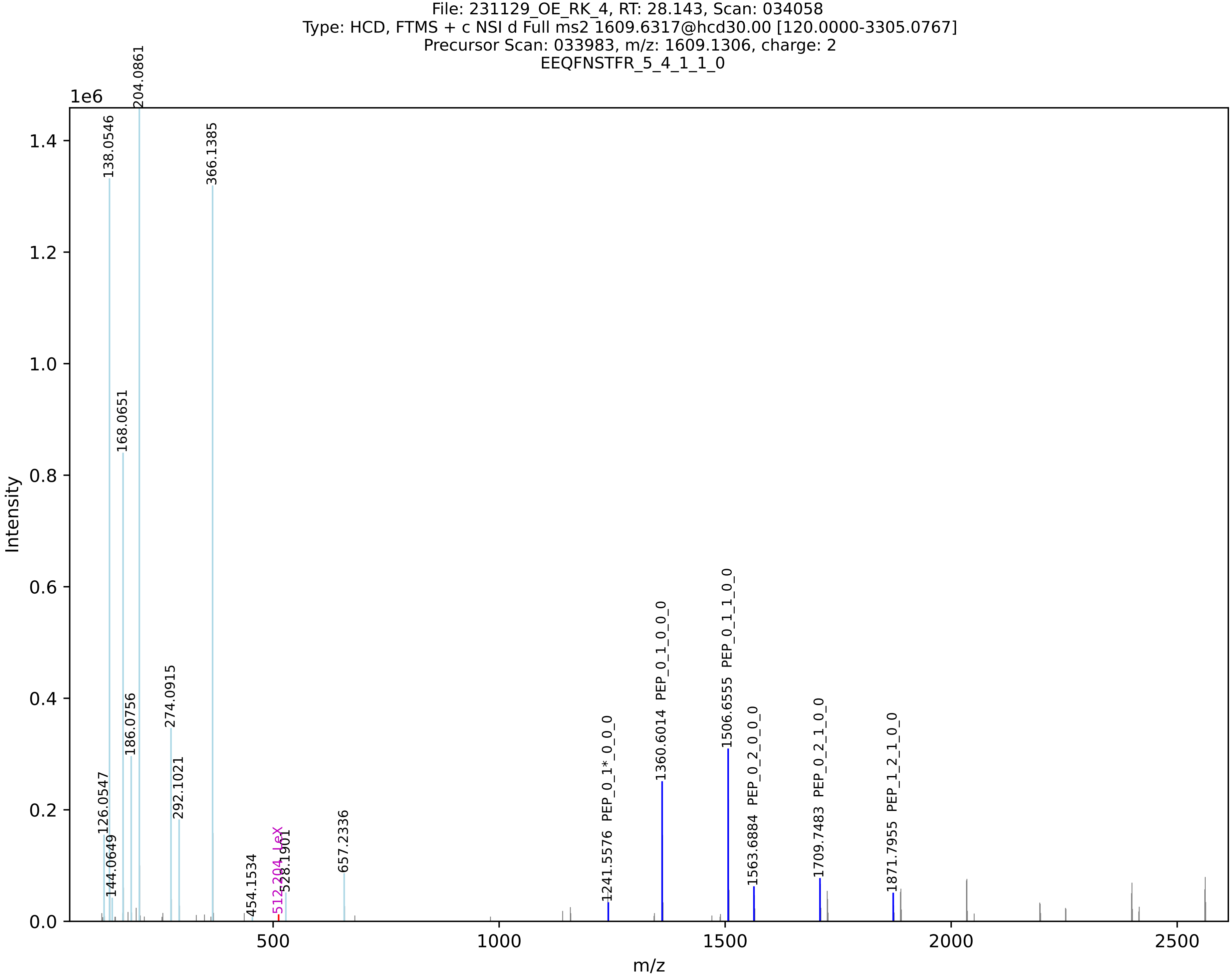

File: 231129_OE_RK_4, RT: 28.143, Scan: 034058
Type: HCD, FTMS + c NSI d Full ms2 1609.6317@hcd30.00 [120.0000-3305.0767]
Precursor Scan: 033983, m/z: 1609.1306, charge: 2
EEQFNSTFR_5_4_1_1_0
1e6
Intensity
m/z
204.0861
138.0546
366.1385
168.0651
274.0915
186.0756
292.1021
126.0547
144.0649
454.1534
512.204_LeX
528.1901
657.2336
1241.5576 PEP_0_1*_0_0_0
1360.6014 PEP_0_1_0_0_0
1506.6555 PEP_0_1_1_0_0
1563.6884 PEP_0_2_0_0_0
1709.7483 PEP_0_2_1_0_0
1871.7955 PEP_1_2_1_0_0

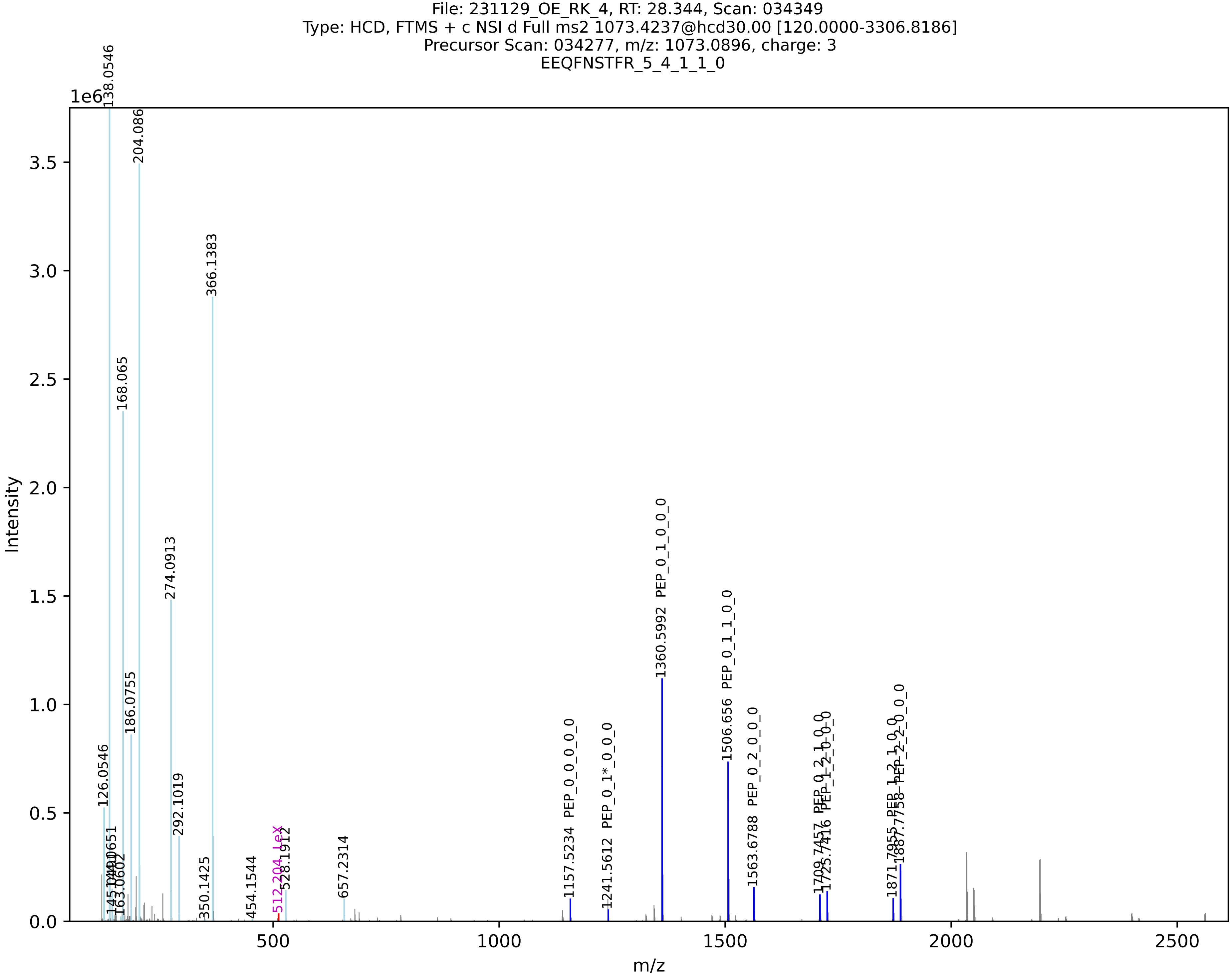
File: 231129_OE_RK_4, RT: 28.344, Scan: 034349
Type: HCD, FTMS + c NSI d Full ms2 1073.4237@hcd30.00 [120.0000-3306.8186]
Precursor Scan: 034277, m/z: 1073.0896, charge: 3
EEQFNSTFR_5_4_1_1_0
1e6
Intensity
m/z
138.0546
204.086
366.1383
168.065
274.0913
186.0755
126.0546
292.1019
350.1425
454.1544
512.204_LeX
528.1912
657.2314
1157.5234 PEP_0_0_0_0_0
1241.5612 PEP_0_1*_0_0_0
1360.5992 PEP_0_1_0_0_0
1506.656 PEP_0_1_1_0_0
1563.6788 PEP_0_2_0_0_0
1709.7457 PEP_0_2_1_0_0
1725.7416 PEP_1_2_0_0_0
1871.7955 PEP_1_2_1_0_0
1887.7758 PEP_2_2_0_0_0

File: 231129_OE_RK_4, RT: 28.452, Scan: 034508
Type: HCD, FTMS + c NSI d Full ms2 1256.8119@hcd30.00 [120.0000-3867.9861]
Precursor Scan: 034422, m/z: 1256.1436, charge: 3
ISEENETTCYMGK_5_4_0_2_0

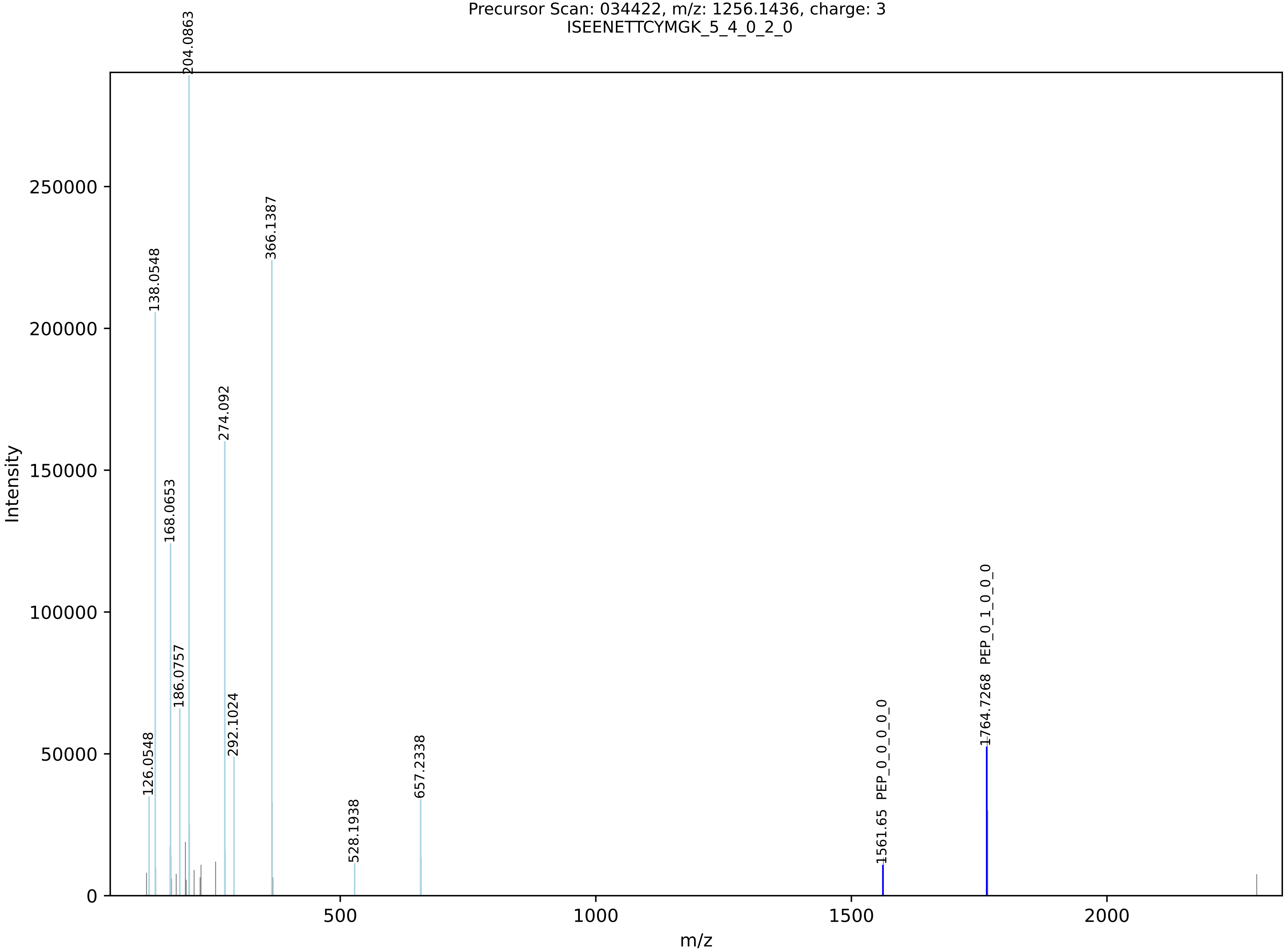

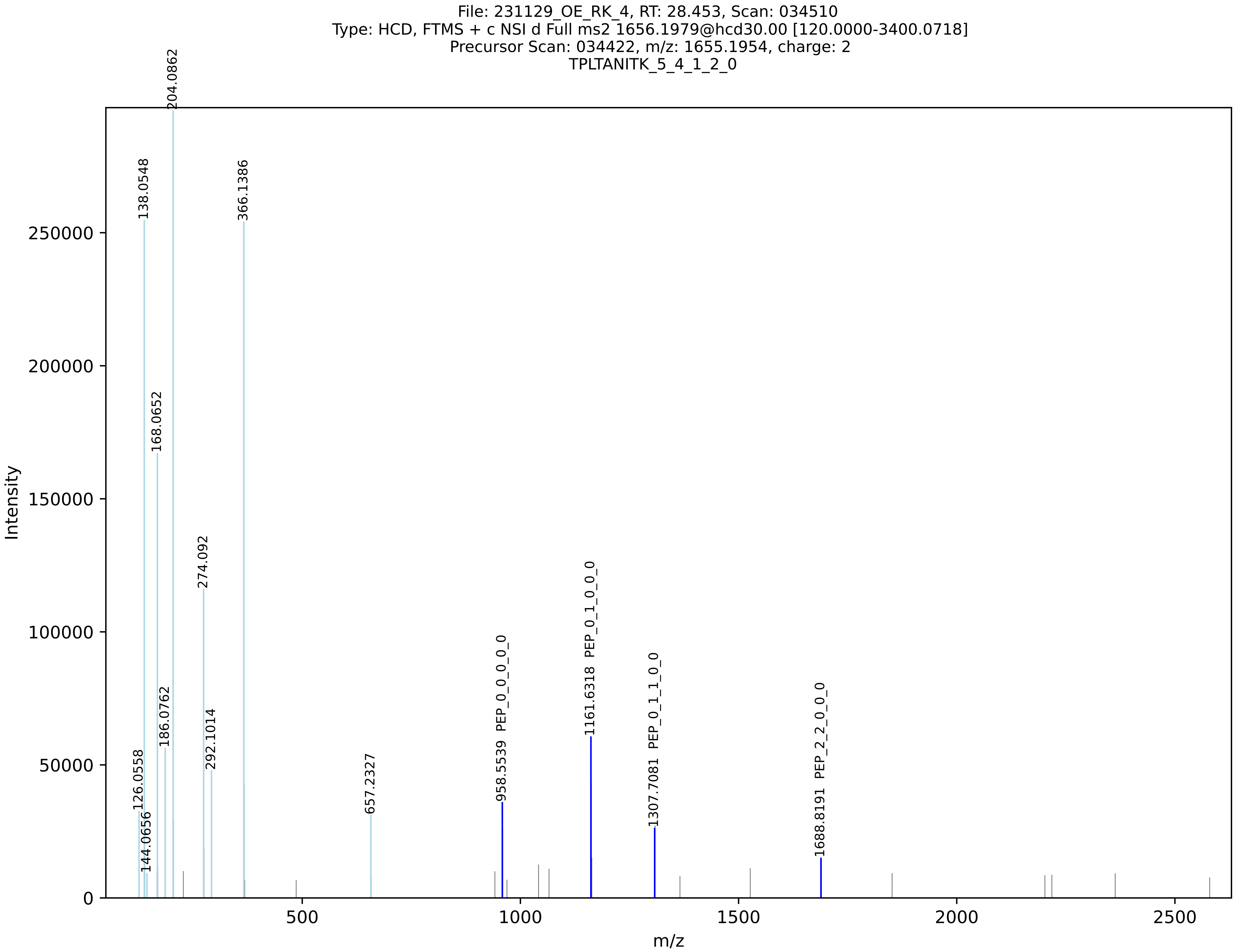
File: 231129_OE_RK_4, RT: 28.453, Scan: 034510
Type: HCD, FTMS + c NSI d Full ms2 1656.1979@hcd30.00 [120.0000-3400.0718]
Precursor Scan: 034422, m/z: 1655.1954, charge: 2
TPLTANITK_5_4_1_2_0
Intensity
m/z
204.0862
138.0548
366.1386
168.0652
274.092
186.0762
292.1014
126.0558
144.0656
657.2327
958.5539 PEP_0_0_0_0_0_
1161.6318 PEP_0_1_0_0_0_
1307.7081 PEP_0_1_1_0_0_
1688.8191 PEP_2_2_0_0_0_
0
50000
100000
150000
200000
250000
500
1000
1500
2000
2500

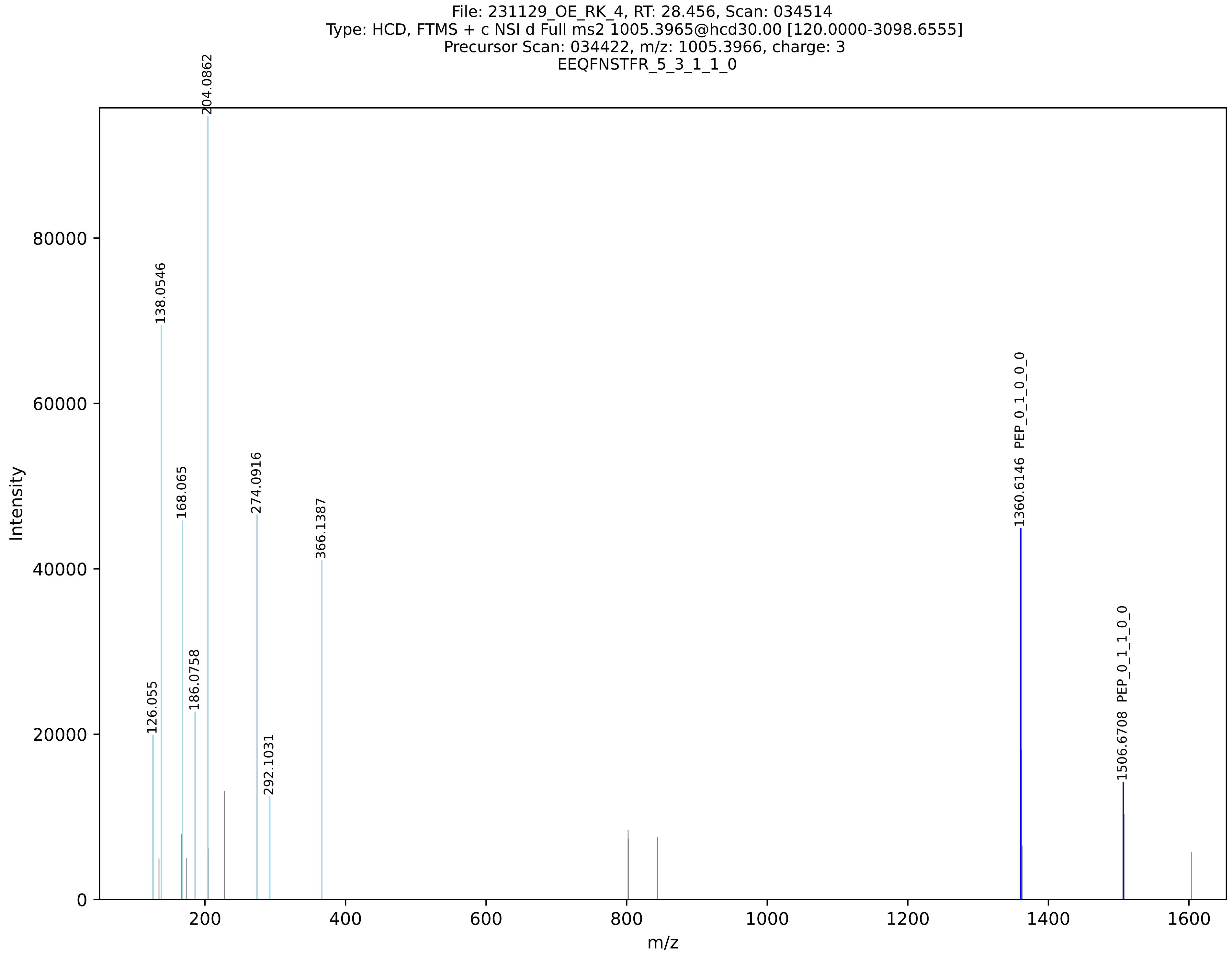

File: 231129_OE_RK_4, RT: 28.456, Scan: 034514
Type: HCD, FTMS + c NSI d Full ms2 1005.3965@hcd30.00 [120.0000-3098.6555]
Precursor Scan: 034422, m/z: 1005.3966, charge: 3
EEQFNSTFR_5_3_1_1_0
Intensity
m/z
204.0862
138.0546
168.065
274.0916
366.1387
186.0758
126.055
292.1031
1360.6146 PEP_0_1_0_0_0
1506.6708 PEP_0_1_1_0_0

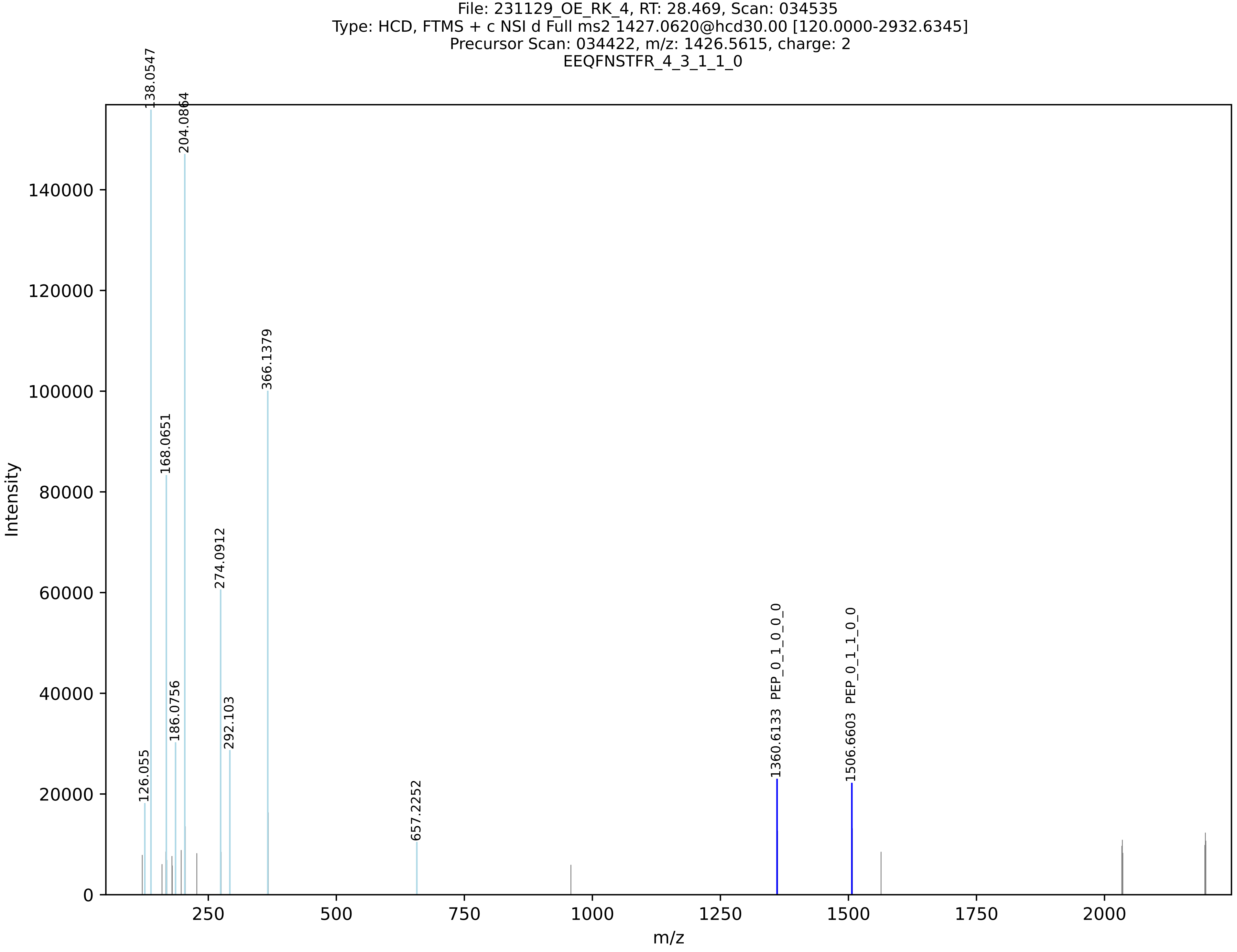
File: 231129_OE_RK_4, RT: 28.469, Scan: 034535
Type: HCD, FTMS + c NSI d Full ms2 1427.0620@hcd30.00 [120.0000-2932.6345]
Precursor Scan: 034422, m/z: 1426.5615, charge: 2
EEQFNSTFR_4_3_1_1_0
Intensity
m/z
0
20000
40000
60000
80000
100000
120000
140000
250
500
750
1000
1250
1500
1750
2000
138.0547
204.0864
366.1379
168.0651
274.0912
186.0756
292.103
126.055
657.2252
1360.6133 PEP_0_1_0_0_0
1506.6603 PEP_0_1_1_0_0

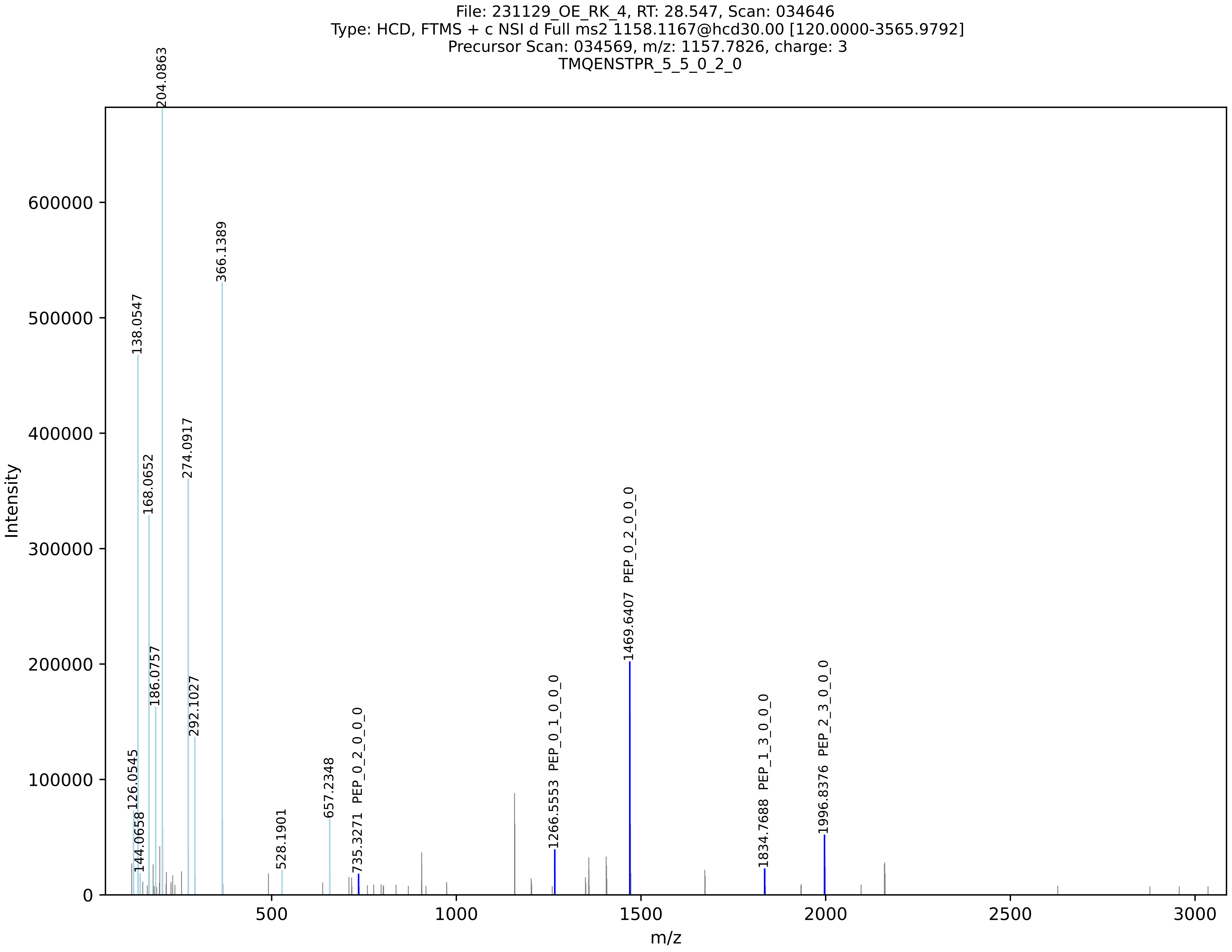
File: 231129_OE_RK_4, RT: 28.547, Scan: 034646
Type: HCD, FTMS + c NSI d Full ms2 1158.1167@hcd30.00 [120.0000-3565.9792]
Precursor Scan: 034569, m/z: 1157.7826, charge: 3
TMQENSTPR_5_5_0_2_0
Intensity
m/z
204.0863
366.1389
138.0547
274.0917
168.0652
186.0757
292.1027
126.0545
144.0658
528.1901
657.2348
735.3271 PEP_0_2_0_0_0
1266.5553 PEP_0_1_0_0_0
1469.6407 PEP_0_2_0_0_0
1834.7688 PEP_1_3_0_0_0
1996.8376 PEP_2_3_0_0_0

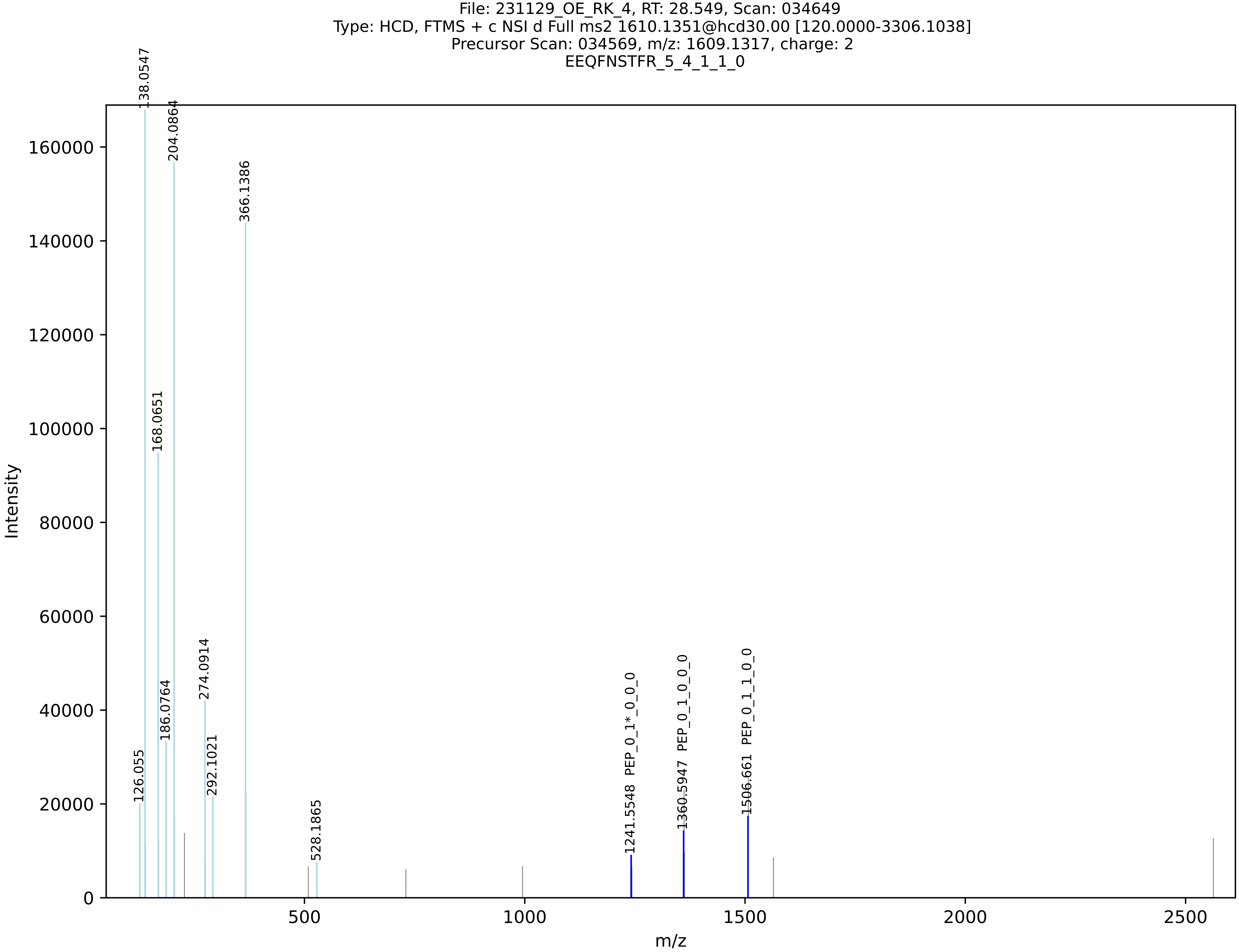
File: 231129_OE_RK_4, RT: 28.549, Scan: 034649
Type: HCD, FTMS + c NSI d Full ms2 1610.1351@hcd30.00 [120.0000-3306.1038]
Precursor Scan: 034569, m/z: 1609.1317, charge: 2
EEQFNSTFR_5_4_1_1_0
Intensity
m/z
138.0547
204.0864
366.1386
168.0651
274.0914
186.0764
292.1021
126.055
528.1865
1241.5548 PEP_0_1*_0_0_0
1360.5947 PEP_0_1_0_0_0
1506.661 PEP_0_1_1_0_0

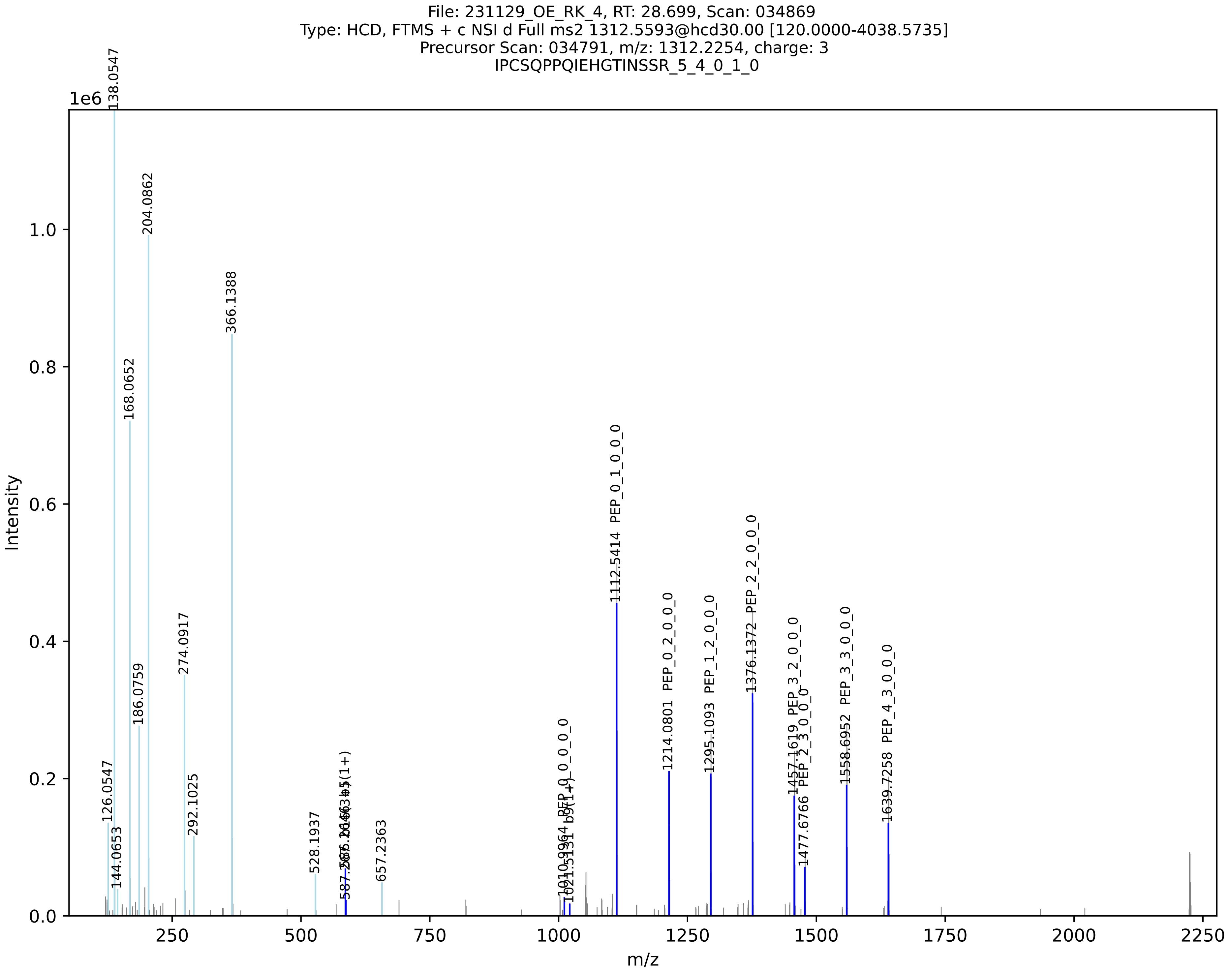
File: 231129_OE_RK_4, RT: 28.699, Scan: 034869
Type: HCD, FTMS + c NSI d Full ms2 1312.5593@hcd30.00 [120.0000-4038.5735]
Precursor Scan: 034791, m/z: 1312.2254, charge: 3
IPCSQPPQIEHGTINSSR_5_4_0_1_0
1e6
Intensity
m/z
138.0547
204.0862
366.1388
168.0652
274.0917
186.0759
126.0547
144.0653
292.1025
528.1937
657.2363
1112.5414 PEP_0_1_0_0_0
1214.0801 PEP_0_2_0_0_0
1295.1093 PEP_1_2_0_0_0
1376.1372 PEP_2_2_0_0_0
1457.1619 PEP_3_2_0_0_0
1477.6766 PEP_2_3_0_0_0
1558.6952 PEP_3_3_0_0_0
1639.7258 PEP_4_3_0_0_0

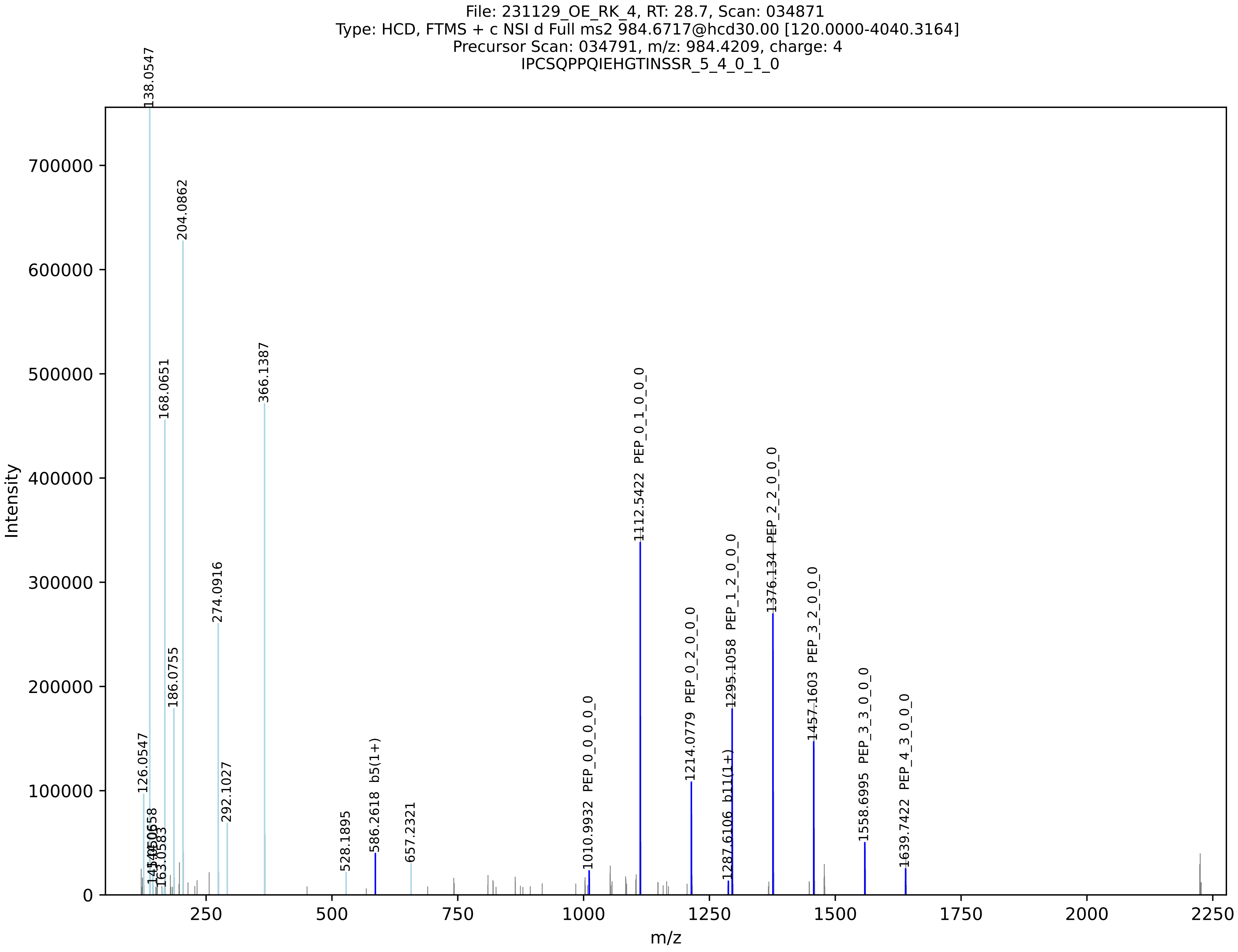
File: 231129_OE_RK_4, RT: 28.7, Scan: 034871
Type: HCD, FTMS + c NSI d Full ms2 984.6717@hcd30.00 [120.0000-4040.3164]
Precursor Scan: 034791, m/z: 984.4209, charge: 4
IPCSQPPQIEHGTINSSR_5_4_0_1_0
Intensity
m/z
138.0547
204.0862
168.0651
366.1387
274.0916
186.0755
126.0547
292.1027
163.0583
528.1895
586.2618 b5(1+)
657.2321
1010.9932 PEP_0_0_0_0_0
1112.5422 PEP_0_1_0_0_0
1214.0779 PEP_0_2_0_0_0
1287.6106 b11(1+)
1295.1058 PEP_1_2_0_0_0
1376.134 PEP_2_2_0_0_0
1457.1603 PEP_3_2_0_0_0
1558.6995 PEP_3_3_0_0_0
1639.7422 PEP_4_3_0_0_0

File: 231129_OE_RK_4, RT: 28.702, Scan: 034873
Type: HCD, FTMS + c NSI d Full ms2 1361.5790@hcd30.00 [120.0000-4188.5737]
Precursor Scan: 034791, m/z: 1360.9108, charge: 3
IPCSQPPQIEHGTINSSR_5_4_1_1_0

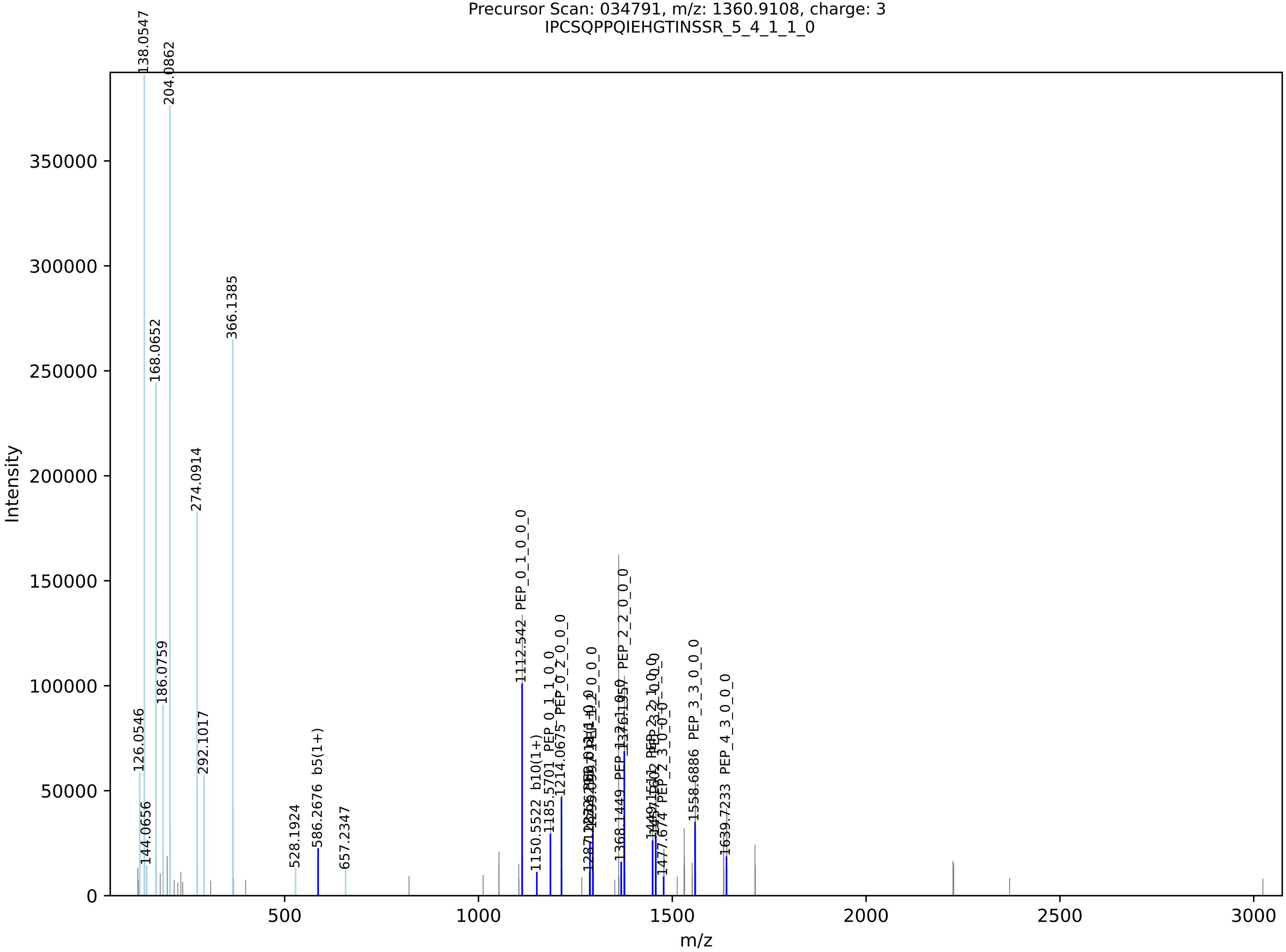

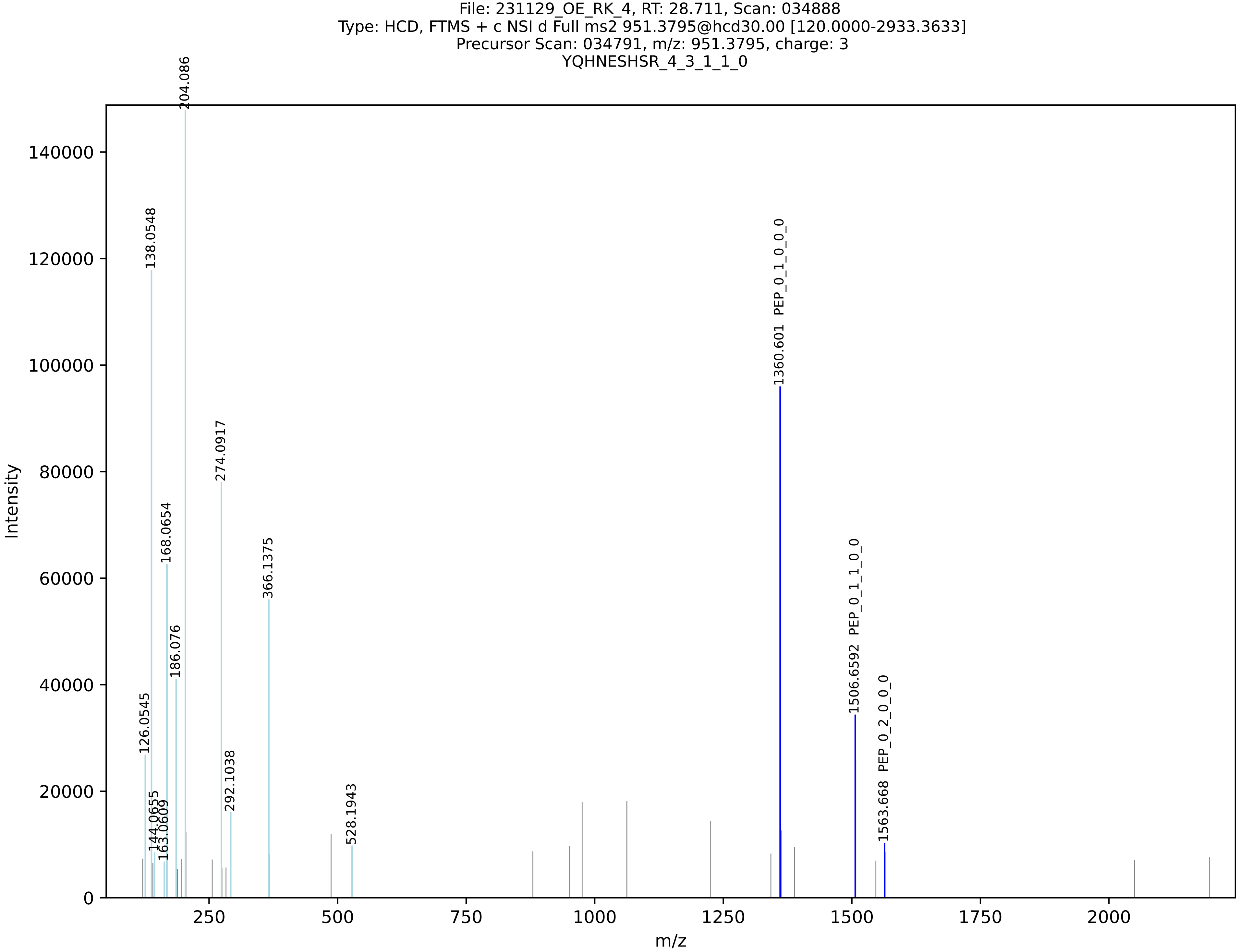
File: 231129_OE_RK_4, RT: 28.711, Scan: 034888
Type: HCD, FTMS + c NSI d Full ms2 951.3795@hcd30.00 [120.0000-2933.3633]
Precursor Scan: 034791, m/z: 951.3795, charge: 3
YQHNESHSR_4_3_1_1_0
Intensity
m/z
204.086
138.0548
274.0917
168.0654
366.1375
186.076
126.0545
292.1038
144.0655
163.0609
528.1943
1360.601 PEP_0_1_0_0_0
1506.6592 PEP_0_1_1_0_0
1563.668 PEP_0_2_0_0_0
0
20000
40000
60000
80000
100000
120000
140000
250
500
750
1000
1250
1500
1750
2000

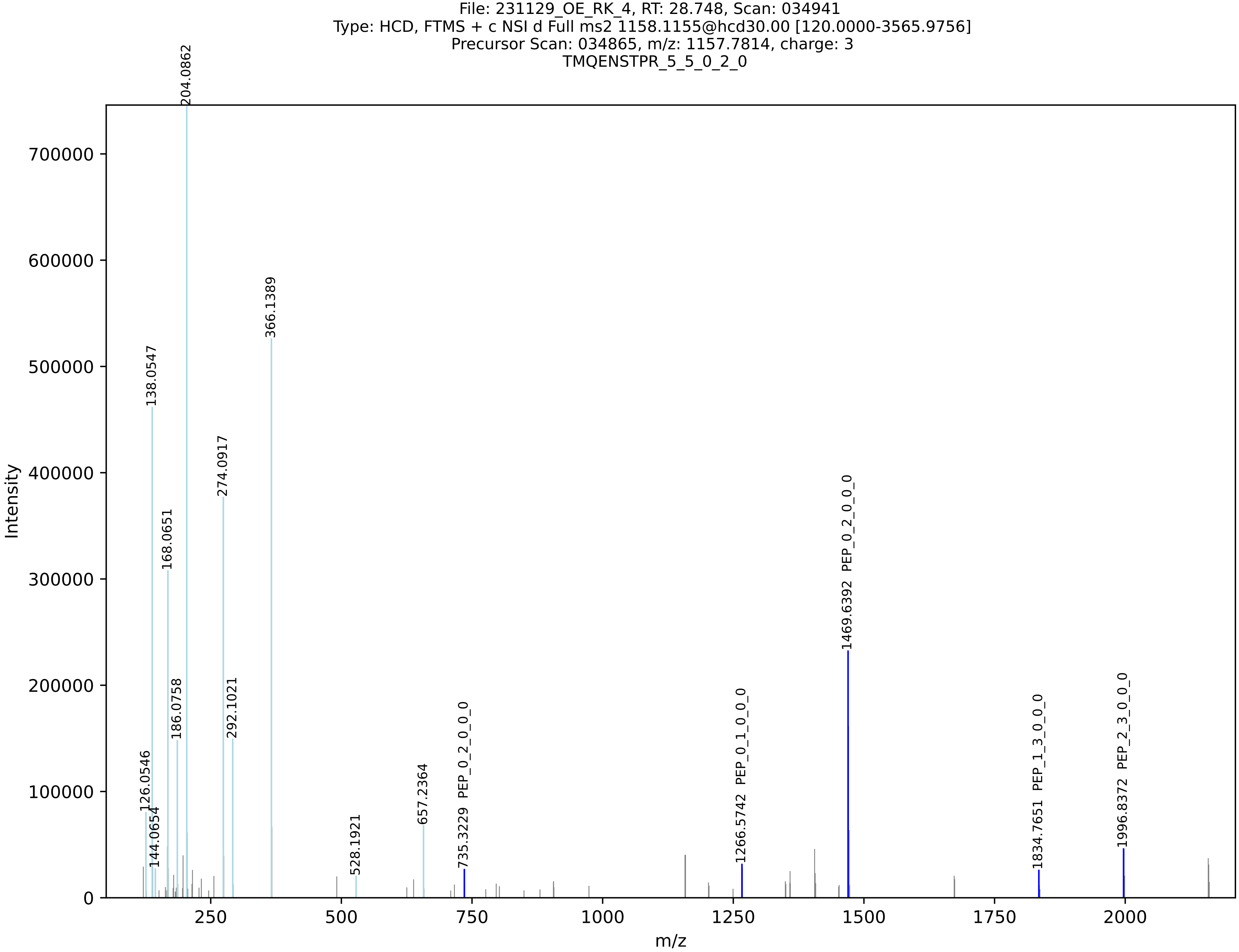
File: 231129_OE_RK_4, RT: 28.748, Scan: 034941
Type: HCD, FTMS + c NSI d Full ms2 1158.1155@hcd30.00 [120.0000-3565.9756]
Precursor Scan: 034865, m/z: 1157.7814, charge: 3
TMQENSTPR_5_5_0_2_0
Intensity
m/z
0
100000
200000
300000
400000
500000
600000
700000
250
500
750
1000
1250
1500
1750
2000
204.0862
366.1389
138.0547
274.0917
168.0651
186.0758
292.1021
126.0546
144.0654
528.1921
657.2364
735.3229 PEP_0_2_0_0_0
1266.5742 PEP_0_1_0_0_0
1469.6392 PEP_0_2_0_0_0
1834.7651 PEP_1_3_0_0_0
1996.8372 PEP_2_3_0_0_0

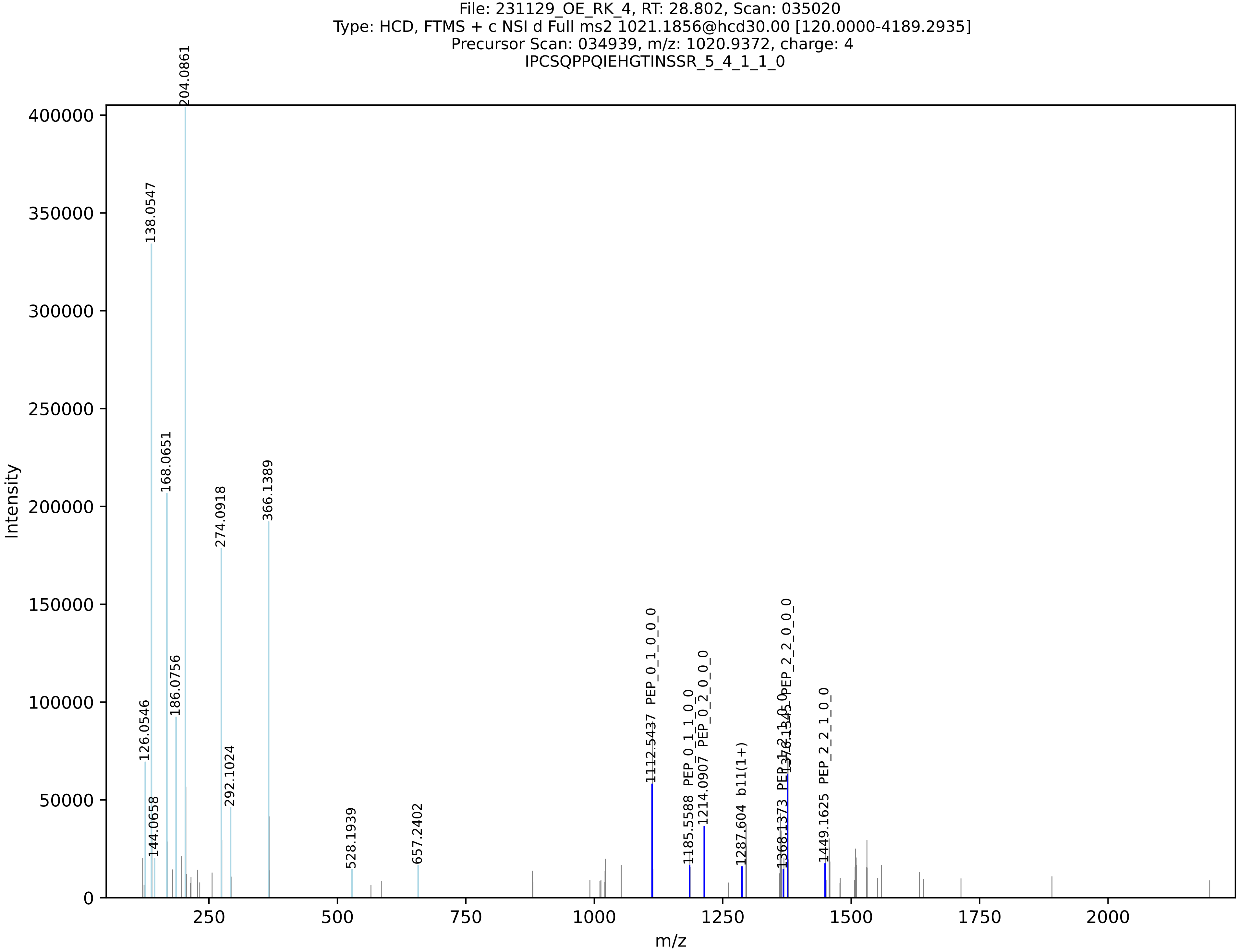

File: 231129_OE_RK_4, RT: 28.802, Scan: 035020
Type: HCD, FTMS + c NSI d Full ms2 1021.1856@hcd30.00 [120.0000-4189.2935]
Precursor Scan: 034939, m/z: 1020.9372, charge: 4
IPCSQPPQIEHGTINSSR_5_4_1_1_0
Intensity
m/z
204.0861
138.0547
168.0651
366.1389
274.0918
186.0756
126.0546
292.1024
144.0658
528.1939
657.2402
1112.5437 PEP_0_1_0_0_0
1185.5588 PEP_0_1_1_0_0
1214.0907 PEP_0_2_0_0_0
1287.604 b11(1+)
1376.1345 PEP_2_2_0_0_0
1449.1625 PEP_2_2_1_0_0

File: 231129_OE_RK_4, RT: 28.822, Scan: 035053
Type: HCD, FTMS + c NSI d Full ms2 1128.2021@hcd30.00 [120.0000-4625.9209]
Precursor Scan: 034939, m/z: 1127.9516, charge: 4
ICAMEGLPQKHNFSHCCSK_5_4_0_2_0

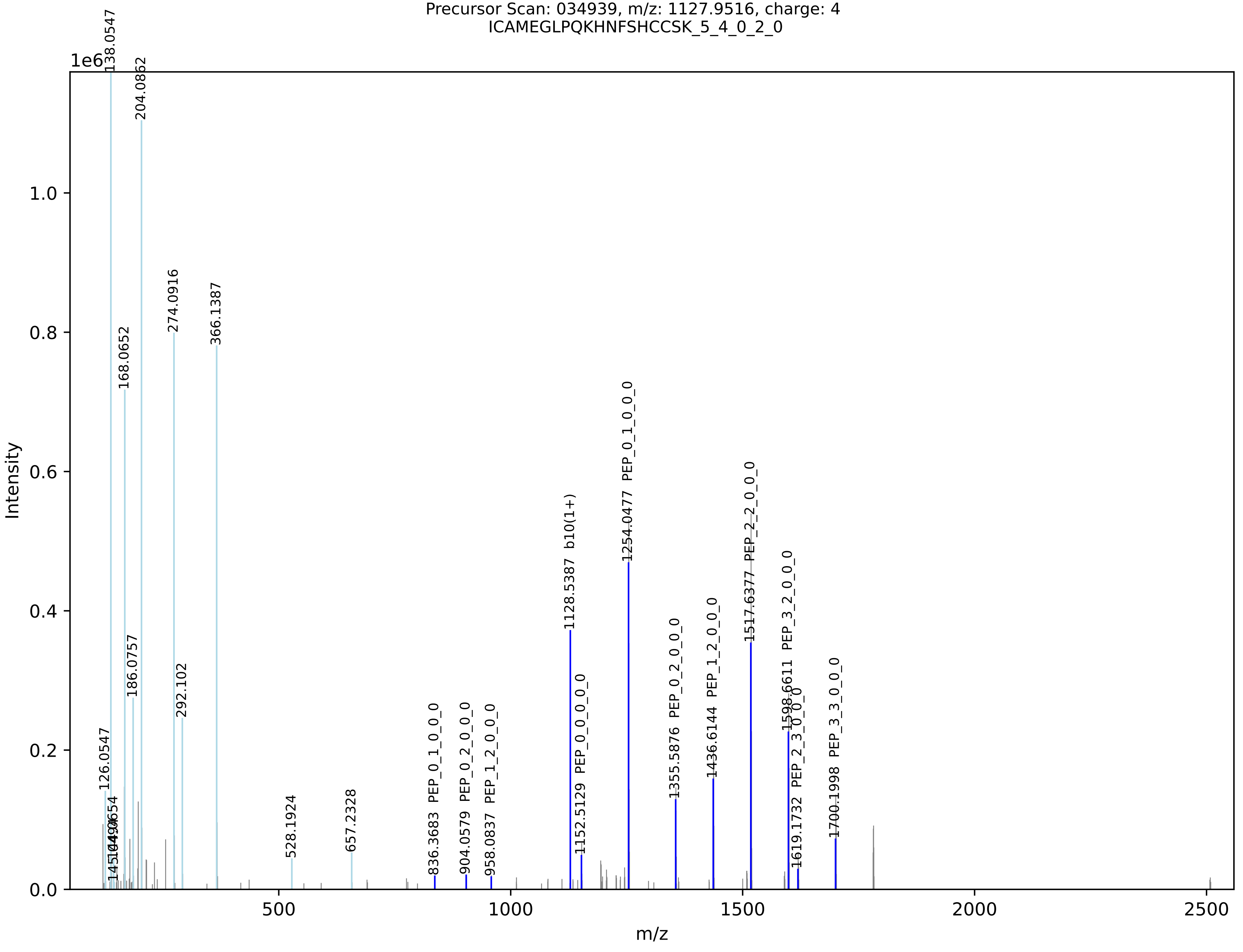

File: 231129_OE_RK_4, RT: 28.901, Scan: 035165
Type: HCD, FTMS + c NSI d Full ms2 1312.5596@hcd30.00 [120.0000-4038.5742]
Precursor Scan: 035087, m/z: 1312.2254, charge: 3
IPCSQPPQIEHGTINSSR_5_4_0_1_0

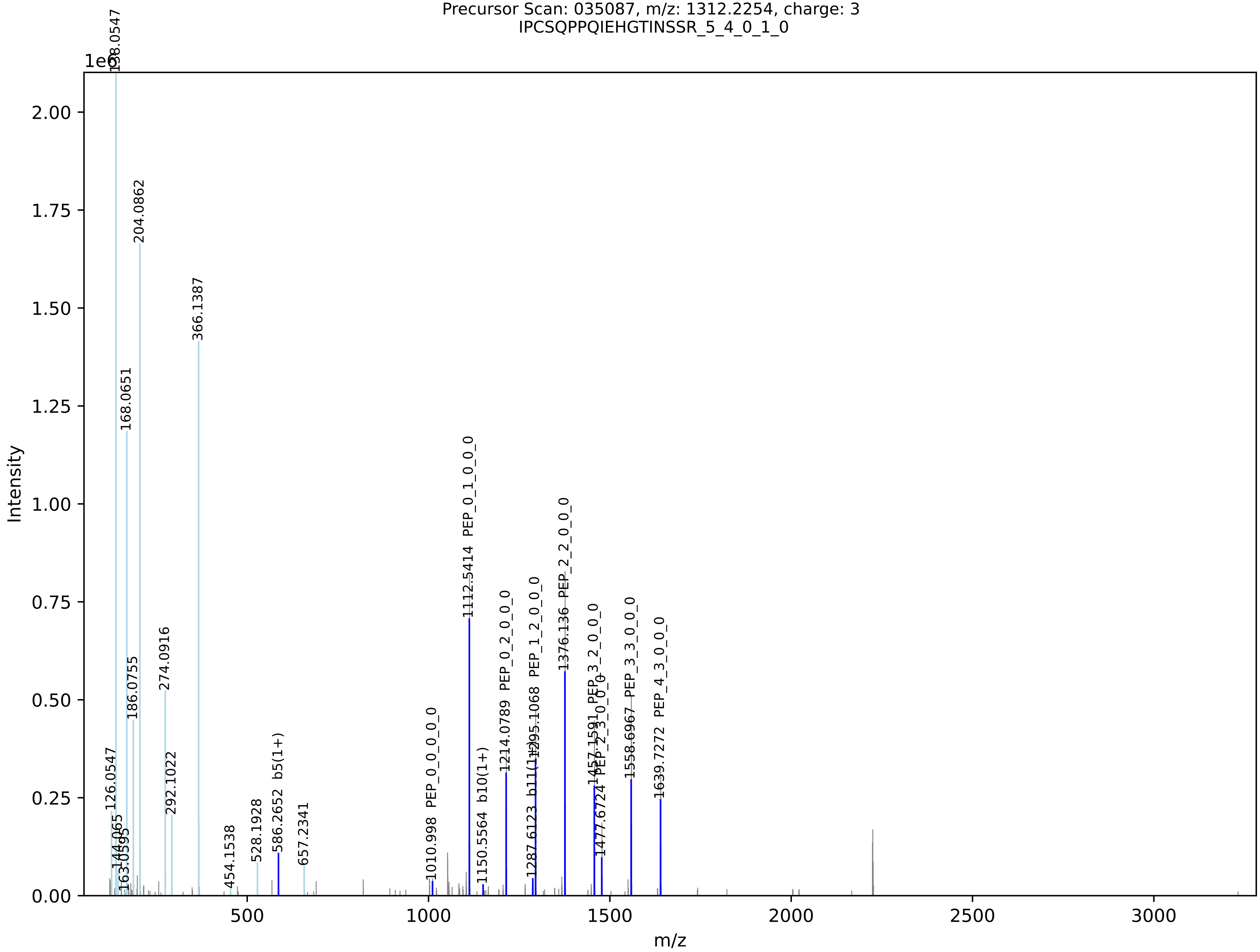

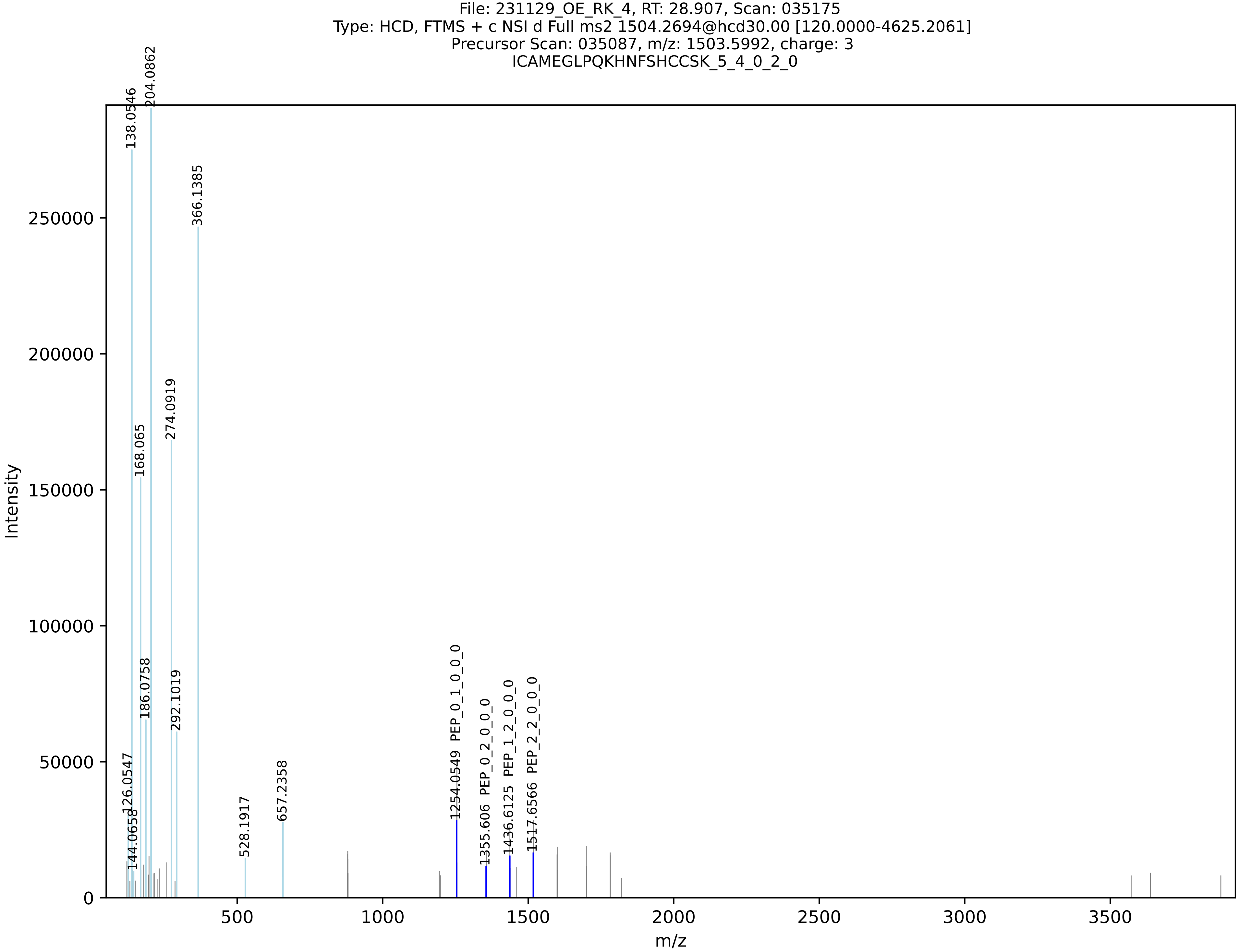

File: 231129_OE_RK_4, RT: 28.907, Scan: 035175
Type: HCD, FTMS + c NSI d Full ms2 1504.2694@hcd30.00 [120.0000-4625.2061]
Precursor Scan: 035087, m/z: 1503.5992, charge: 3
ICAMEGLPQKHNFSHCCSK_5_4_0_2_0
Intensity
m/z
138.0546
204.0862
366.1385
274.0919
168.065
186.0758
292.1019
126.0547
144.0658
528.1917
657.2358
1254.0549 PEP_0_1_0_0_0
1355.606 PEP_0_2_0_0_0
1436.6125 PEP_1_2_0_0_0
1517.6566 PEP_2_2_0_0_0

File: 231129_OE_RK_4, RT: 28.959, Scan: 035251
Type: HCD, FTMS + c NSI d Full ms2 1064.1174@hcd30.00 [120.0000-3278.3413]
Precursor Scan: 035161, m/z: 1063.4492, charge: 3
AAIPSALDTNSSK_5_4_0_1_0

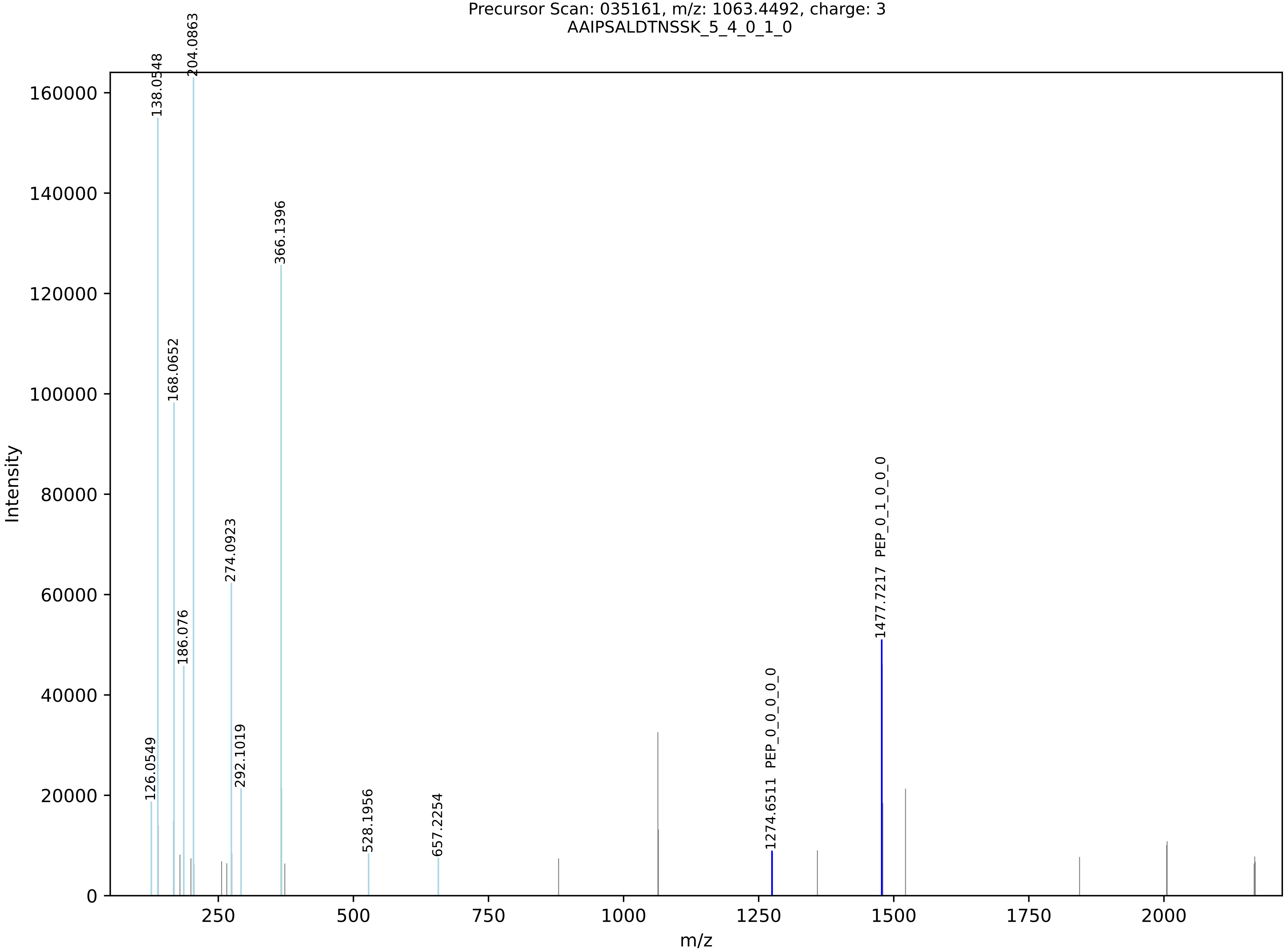

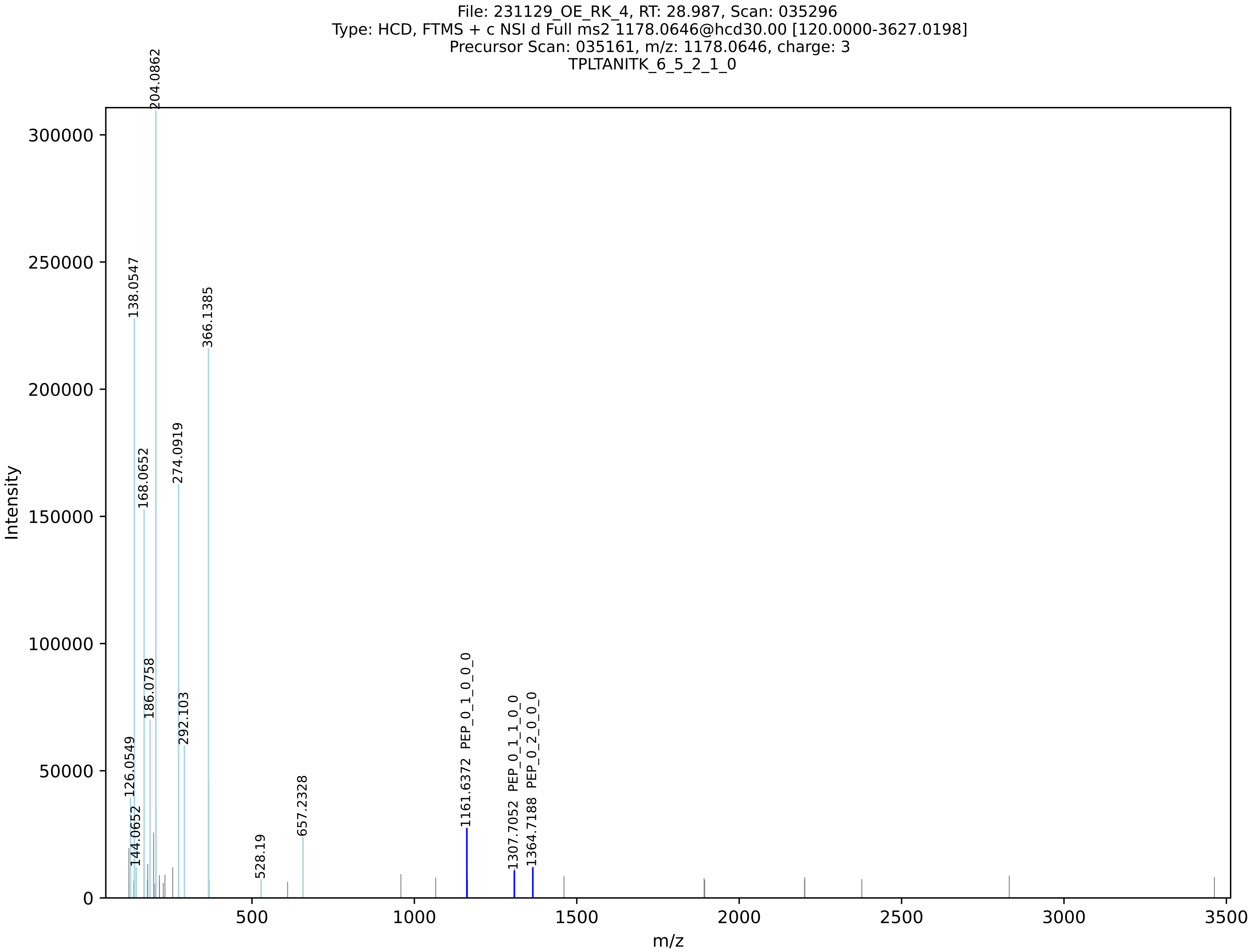
File: 231129_OE_RK_4, RT: 28.987, Scan: 035296
Type: HCD, FTMS + c NSI d Full ms2 1178.0646@hcd30.00 [120.0000-3627.0198]
Precursor Scan: 035161, m/z: 1178.0646, charge: 3
TPLTANITK_6_5_2_1_0
Intensity
m/z
204.0862
138.0547
366.1385
274.0919
168.0652
186.0758
292.103
126.0549
144.0652
528.19
657.2328
1161.6372 PEP_0_1_0_0_0
1307.7052 PEP_0_1_1_0_0
1364.7188 PEP_0_2_0_0_0
300000
250000
200000
150000
100000
50000
0
500
1000
1500
2000
2500
3000
3500

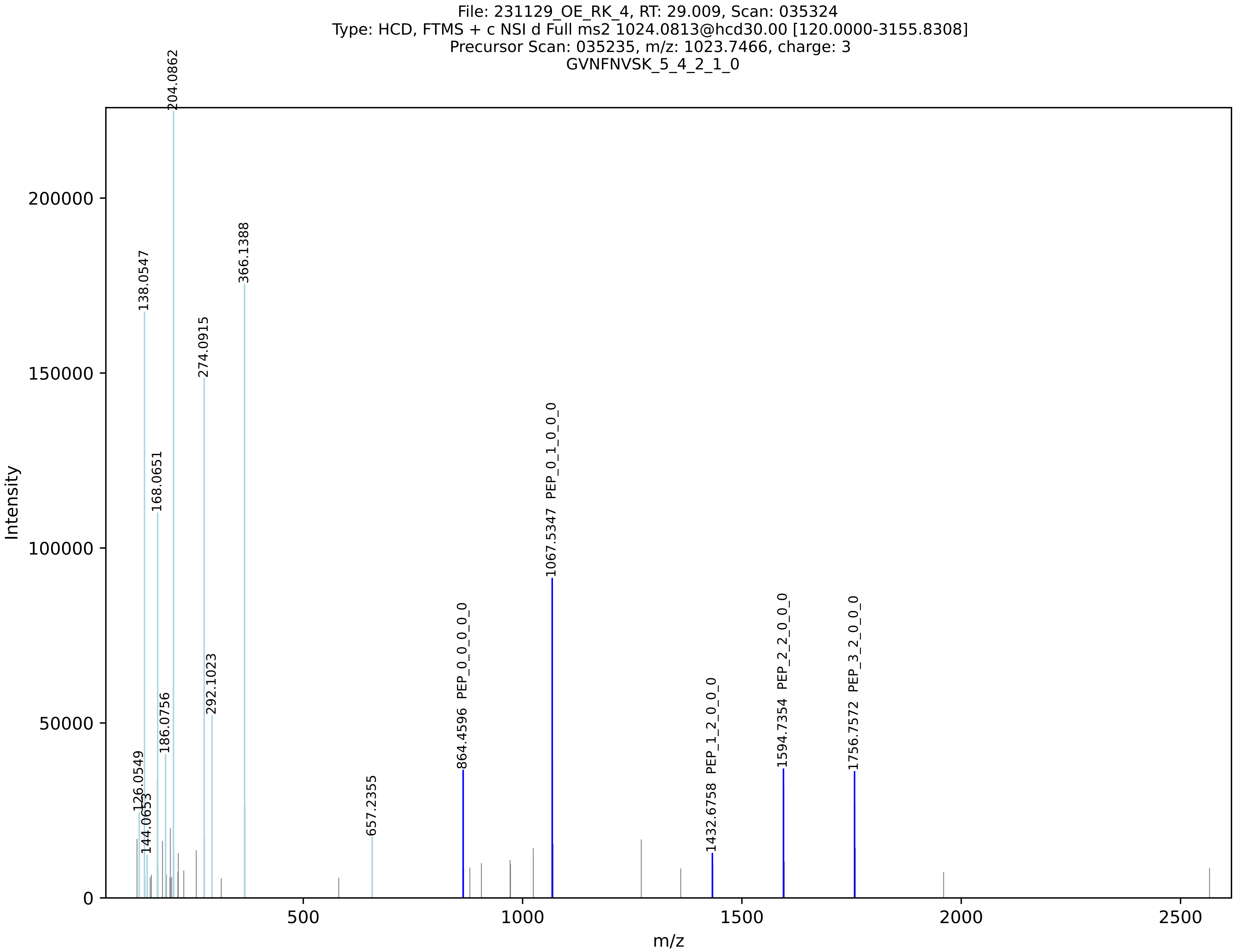
File: 231129_OE_RK_4, RT: 29.009, Scan: 035324
Type: HCD, FTMS + c NSI d Full ms2 1024.0813@hcd30.00 [120.0000-3155.8308]
Precursor Scan: 035235, m/z: 1023.7466, charge: 3
GVNFNVSK_5_4_2_1_0
204.0862
366.1388
138.0547
274.0915
168.0651
292.1023
186.0756
126.0549
144.0653
657.2355
864.4596 PEP_0_0_0_0_0
1067.5347 PEP_0_1_0_0_0
1432.6758 PEP_1_2_0_0_0
1594.7354 PEP_2_2_0_0_0
1756.7572 PEP_3_2_0_0_0
Intensity
0
50000
100000
150000
200000
500
1000
1500
2000
2500
m/z

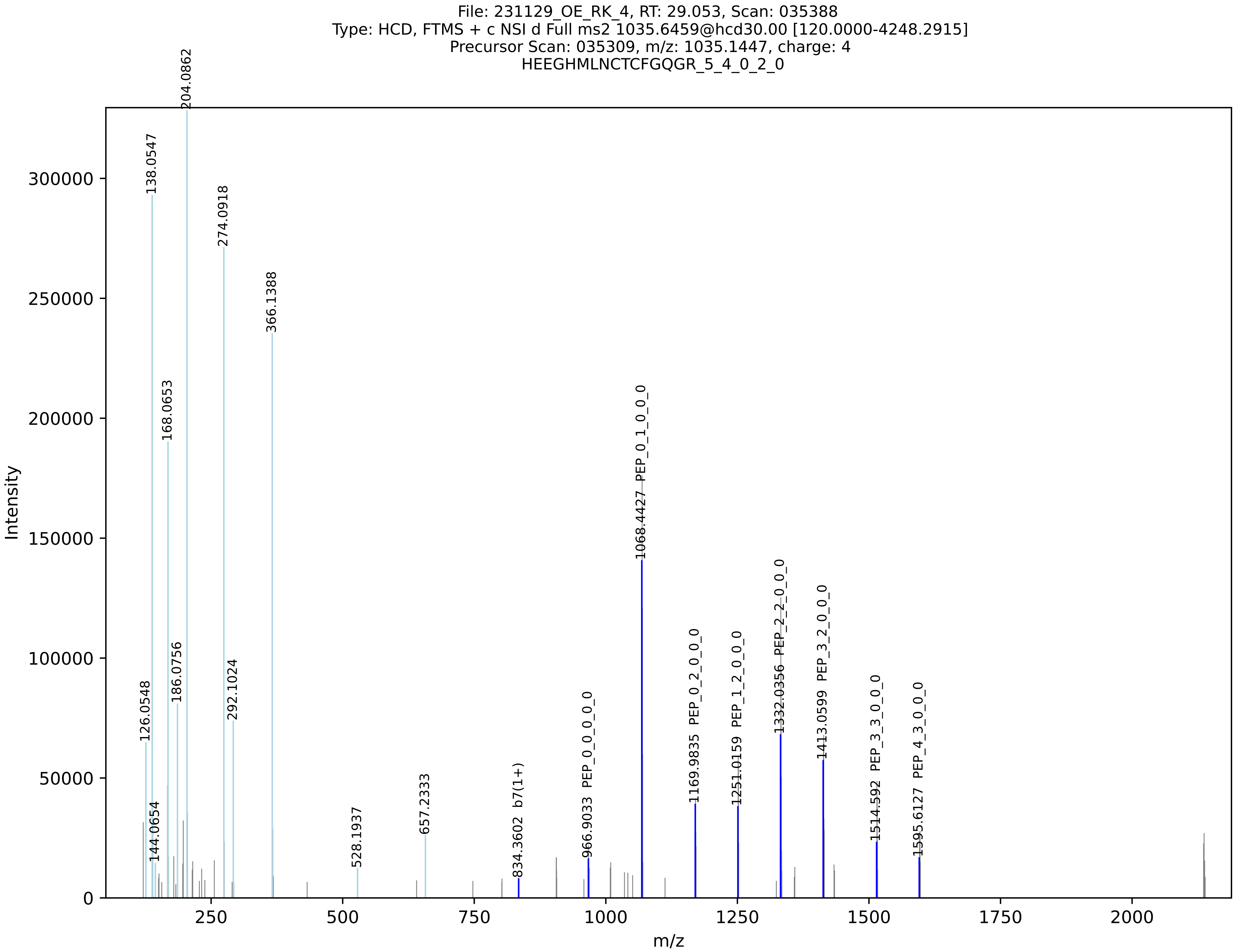
File: 231129_OE_RK_4, RT: 29.053, Scan: 035388
Type: HCD, FTMS + c NSI d Full ms2 1035.6459@hcd30.00 [120.0000-4248.2915]
Precursor Scan: 035309, m/z: 1035.1447, charge: 4
HEEGHMLNCTCFGQGR_5_4_0_2_0
Intensity
m/z
204.0862
138.0547
274.0918
366.1388
168.0653
186.0756
292.1024
126.0548
144.0654
528.1937
657.2333
834.3602 b7(1+)
966.9033 PEP_0_0_0_0_0
1068.4427 PEP_0_1_0_0_0
1169.9835 PEP_0_2_0_0_0
1251.0159 PEP_1_2_0_0_0
1332.0356 PEP_2_2_0_0_0
1413.0599 PEP_3_2_0_0_0
1514.592 PEP_3_3_0_0_0
1595.6127 PEP_4_3_0_0_0

File: 231129_OE_RK_4, RT: 29.077, Scan: 035427
Type: HCD, FTMS + c NSI d Full ms2 788.1384@hcd30.00 [120.0000-4043.0759]
Precursor Scan: 035309, m/z: 787.7377, charge: 5
IPCSQPPQIEHGTINSSR_5_4_0_1_0

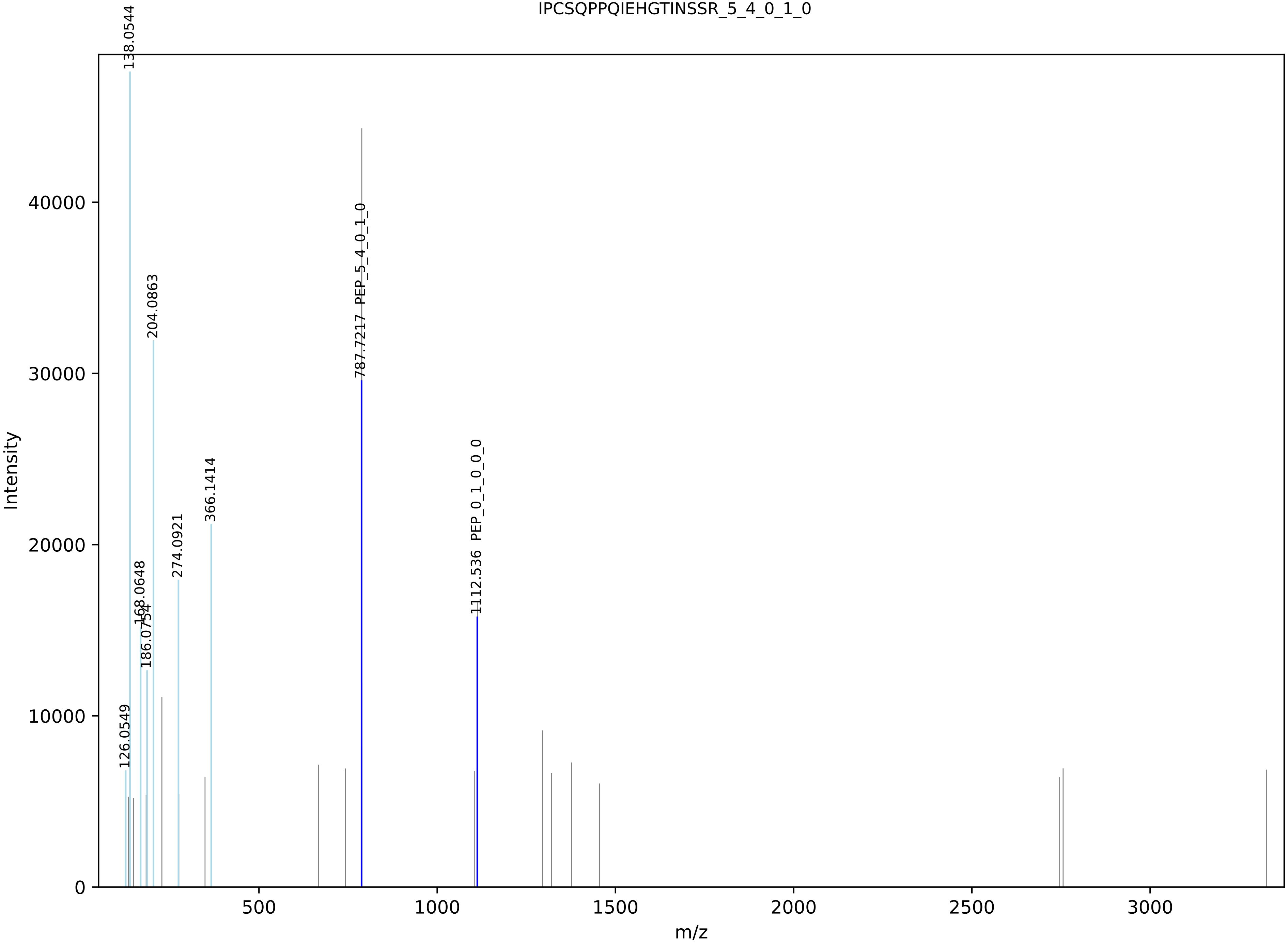

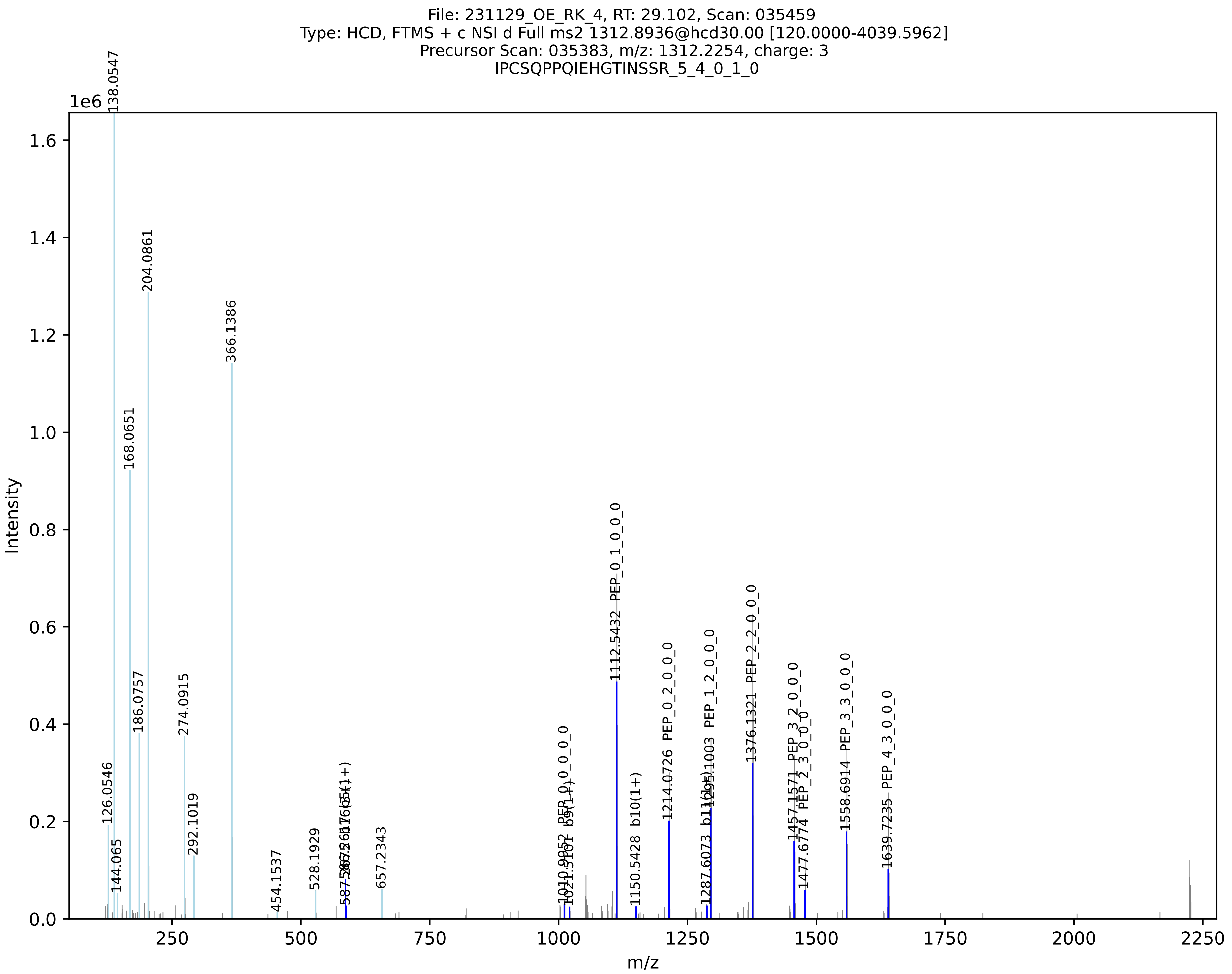
File: 231129_OE_RK_4, RT: 29.102, Scan: 035459
Type: HCD, FTMS + c NSI d Full ms2 1312.8936@hcd30.00 [120.0000-4039.5962]
Precursor Scan: 035383, m/z: 1312.2254, charge: 3
IPCSQPPQIEHGTINSSR_5_4_0_1_0
1e6
Intensity
m/z
138.0547
204.0861
366.1386
168.0651
186.0757
274.0915
126.0546
292.1019
144.065
454.1537
528.1929
657.2343
1112.5432_PEP_0_1_0_0_0
1214.0726_PEP_0_2_0_0_0
1295.1003_PEP_1_2_0_0_0
1376.1321_PEP_2_2_0_0_0
1457.1571_PEP_3_2_0_0_0
1477.6774_PEP_2_3_0_0_0
1558.6914_PEP_3_3_0_0_0
1639.7235_PEP_4_3_0_0_0
1150.5428_b10(1+)
1287.6073_b11(1+)

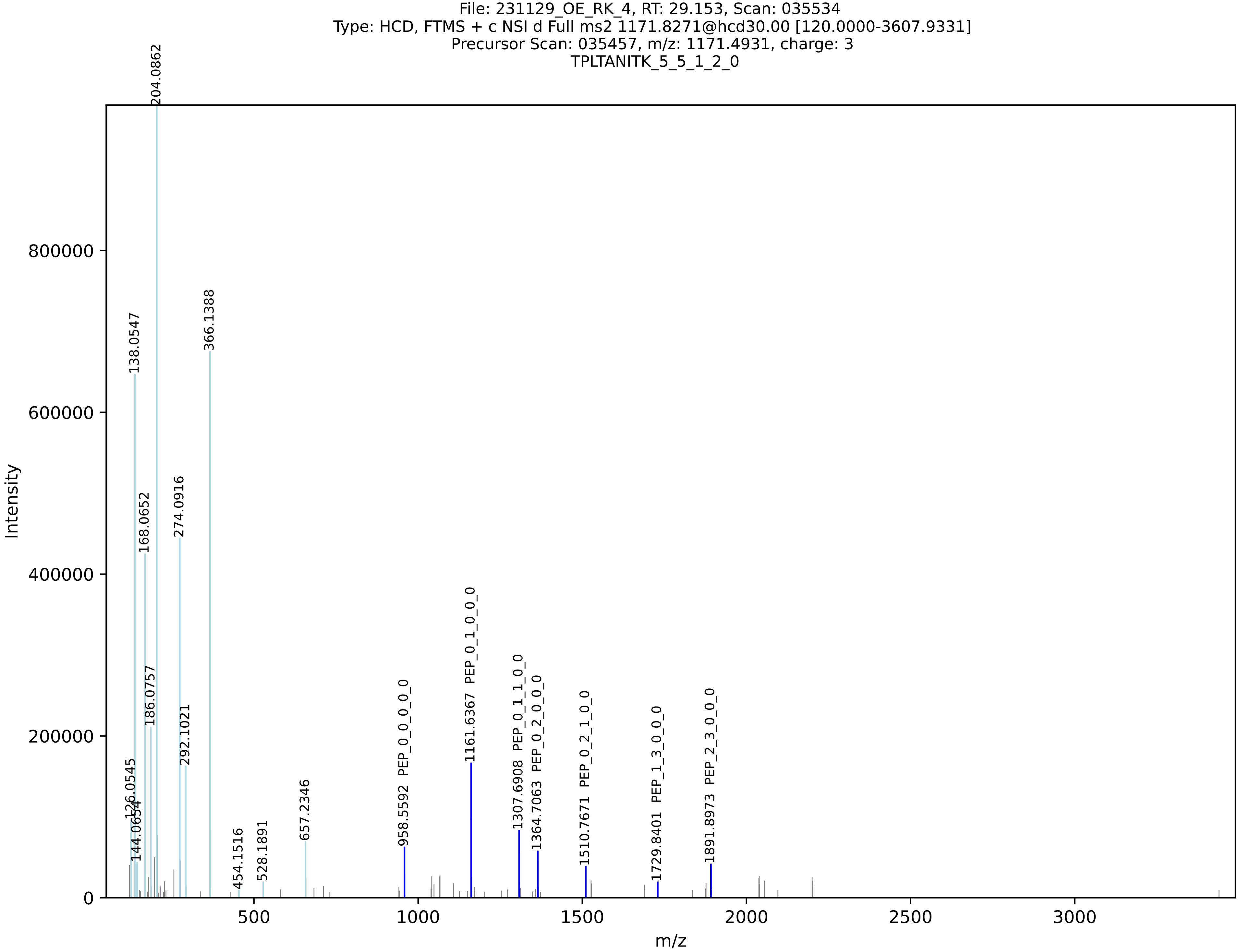

File: 231129_OE_RK_4, RT: 29.153, Scan: 035534
Type: HCD, FTMS + c NSI d Full ms2 1171.8271@hcd30.00 [120.0000-3607.9331]
Precursor Scan: 035457, m/z: 1171.4931, charge: 3
TPLTANITK_5_5_1_2_0
Intensity
m/z
204.0862
138.0547
366.1388
168.0652
274.0916
186.0757
292.1021
126.0545
144.0654
454.1516
528.1891
657.2346
958.5592 PEP_0_0_0_0_0
1161.6367 PEP_0_1_0_0_0
1307.6908 PEP_0_1_1_0_0
1364.7063 PEP_0_2_0_0_0
1510.7671 PEP_0_2_1_0_0
1729.8401 PEP_1_3_0_0_0
1891.8973 PEP_2_3_0_0_0

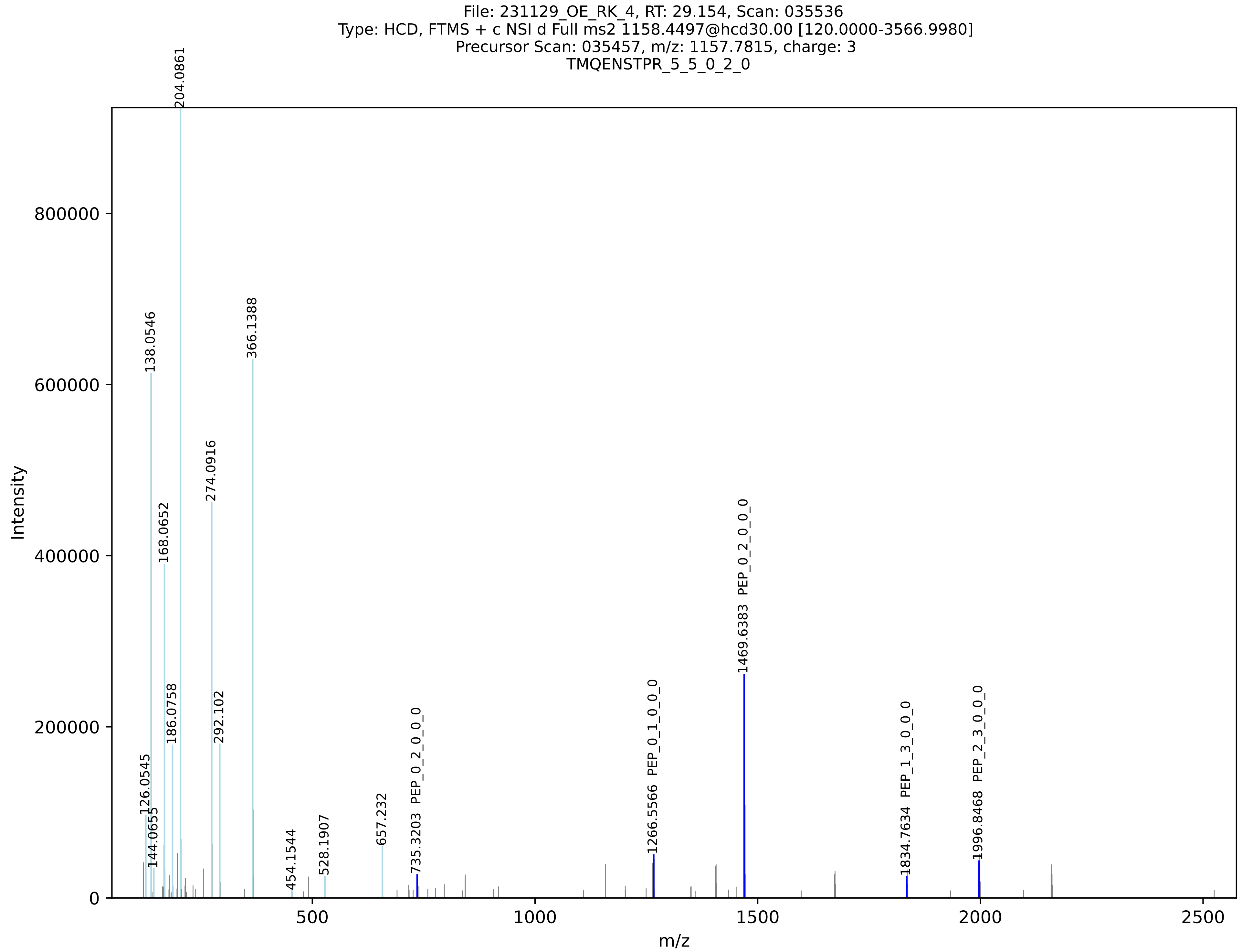

File: 231129_OE_RK_4, RT: 29.154, Scan: 035536
Type: HCD, FTMS + c NSI d Full ms2 1158.4497@hcd30.00 [120.0000-3566.9980]
Precursor Scan: 035457, m/z: 1157.7815, charge: 3
TMQENSTPR_5_5_0_2_0
204.0861
138.0546
366.1388
274.0916
168.0652
186.0758
292.102
126.0545
144.0655
454.1544
528.1907
657.232
735.3203 PEP_0_2_0_0_0
1266.5566 PEP_0_1_0_0_0
1469.6383 PEP_0_2_0_0_0
1834.7634 PEP_1_3_0_0_0
1996.8468 PEP_2_3_0_0_0
Intensity
m/z
0
200000
400000
600000
800000
500
1000
1500
2000
2500

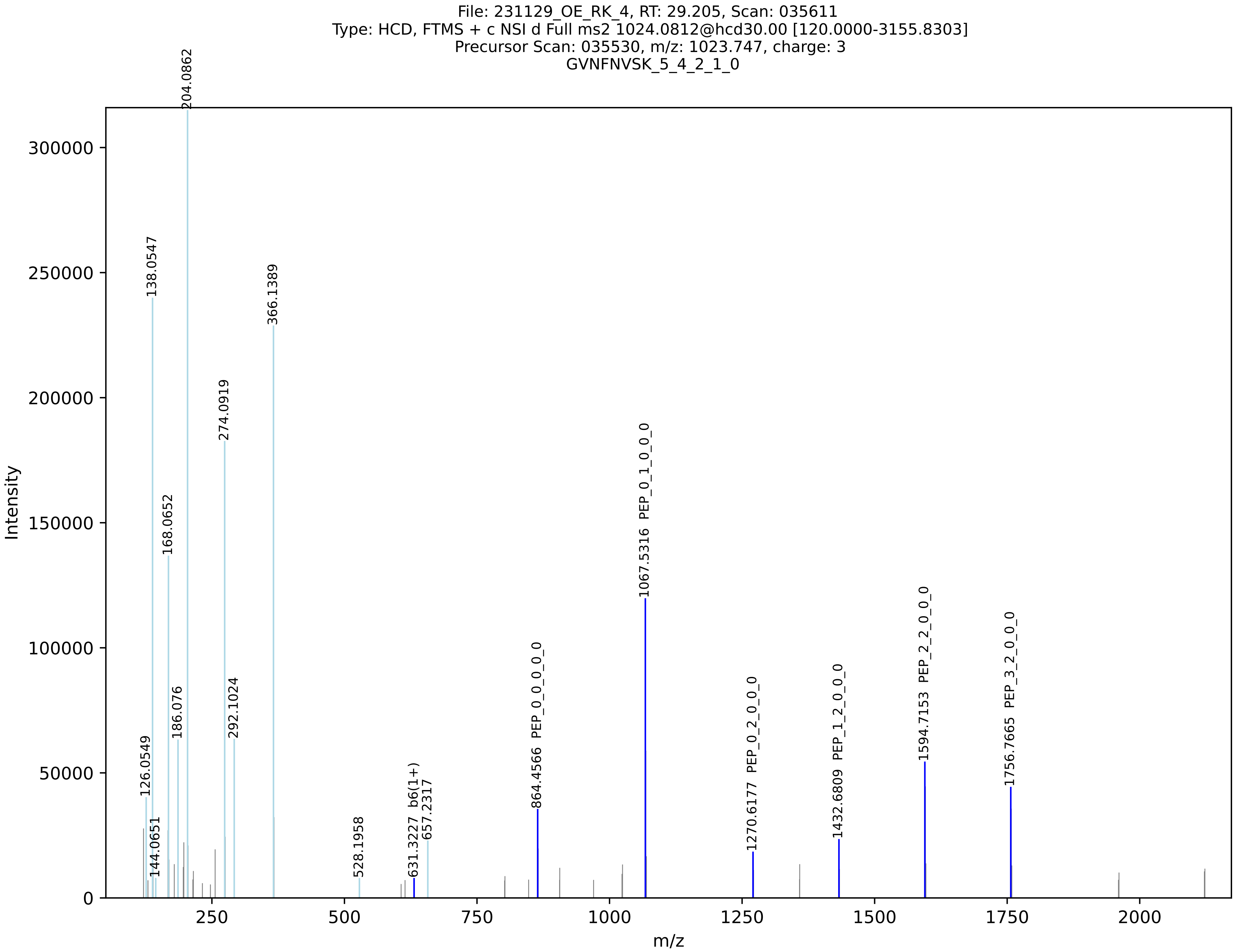

File: 231129_OE_RK_4, RT: 29.205, Scan: 035611
Type: HCD, FTMS + c NSI d Full ms2 1024.0812@hcd30.00 [120.0000-3155.8303]
Precursor Scan: 035530, m/z: 1023.747, charge: 3
GVNFNVSK_5_4_2_1_0
Intensity
m/z
204.0862
138.0547
366.1389
274.0919
168.0652
186.076
292.1024
126.0549
144.0651
528.1958
631.3227 b6(1+)
657.2317
864.4566 PEP_0_0_0_0_0
1067.5316 PEP_0_1_0_0_0
1270.6177 PEP_0_2_0_0_0
1432.6809 PEP_1_2_0_0_0
1594.7153 PEP_2_2_0_0_0
1756.7665 PEP_3_2_0_0_0

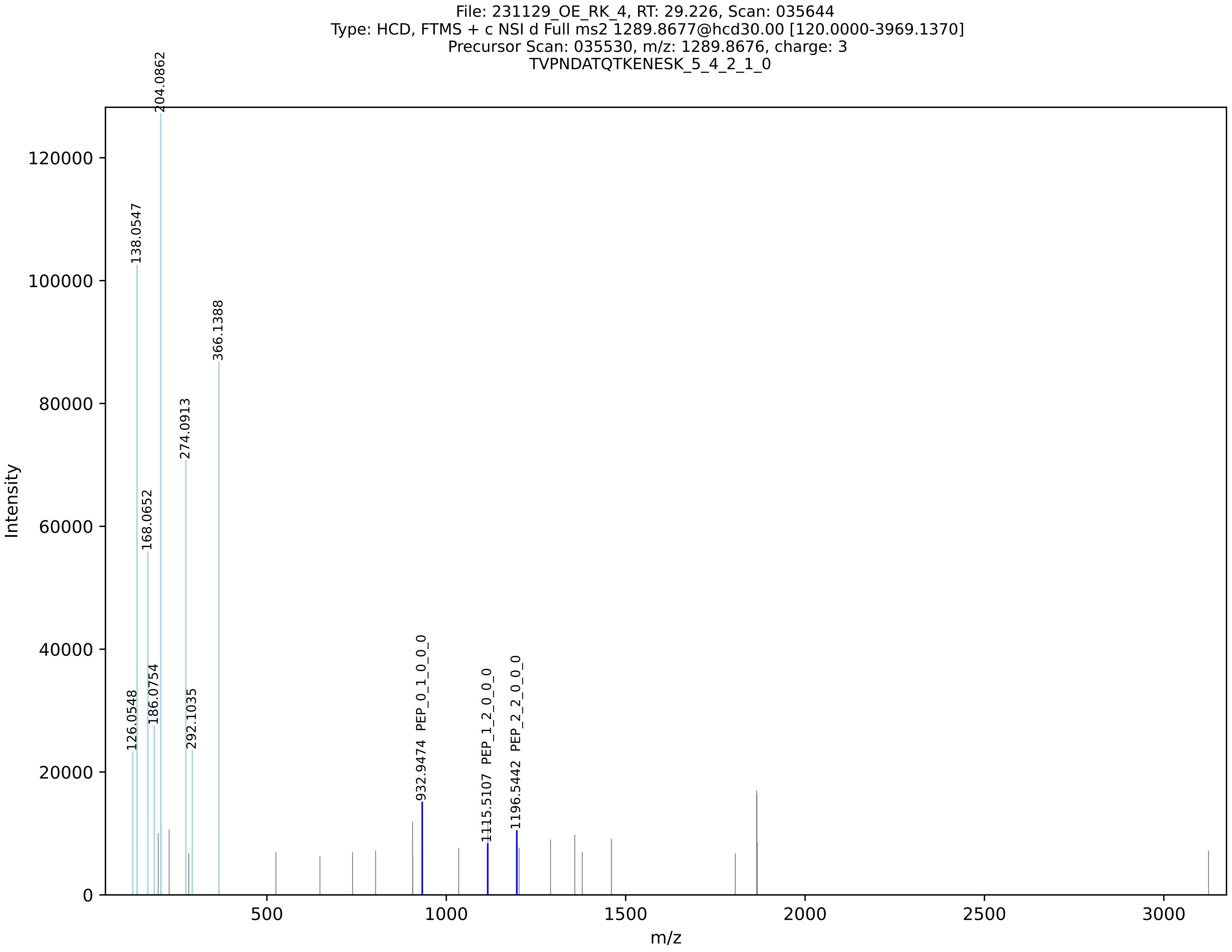
File: 231129_OE_RK_4, RT: 29.226, Scan: 035644
Type: HCD, FTMS + c NSI d Full ms2 1289.8677@hcd30.00 [120.0000-3969.1370]
Precursor Scan: 035530, m/z: 1289.8676, charge: 3
TVPNDATQTKENESK_5_4_2_1_0
204.0862
138.0547
366.1388
274.0913
168.0652
126.0548
186.0754
292.1035
932.9474 PEP_0_1_0_0_0
1115.5107 PEP_1_2_0_0_0
1196.5442 PEP_2_2_0_0_0
Intensity
m/z
0
20000
40000
60000
80000
100000
120000
500
1000
1500
2000
2500
3000

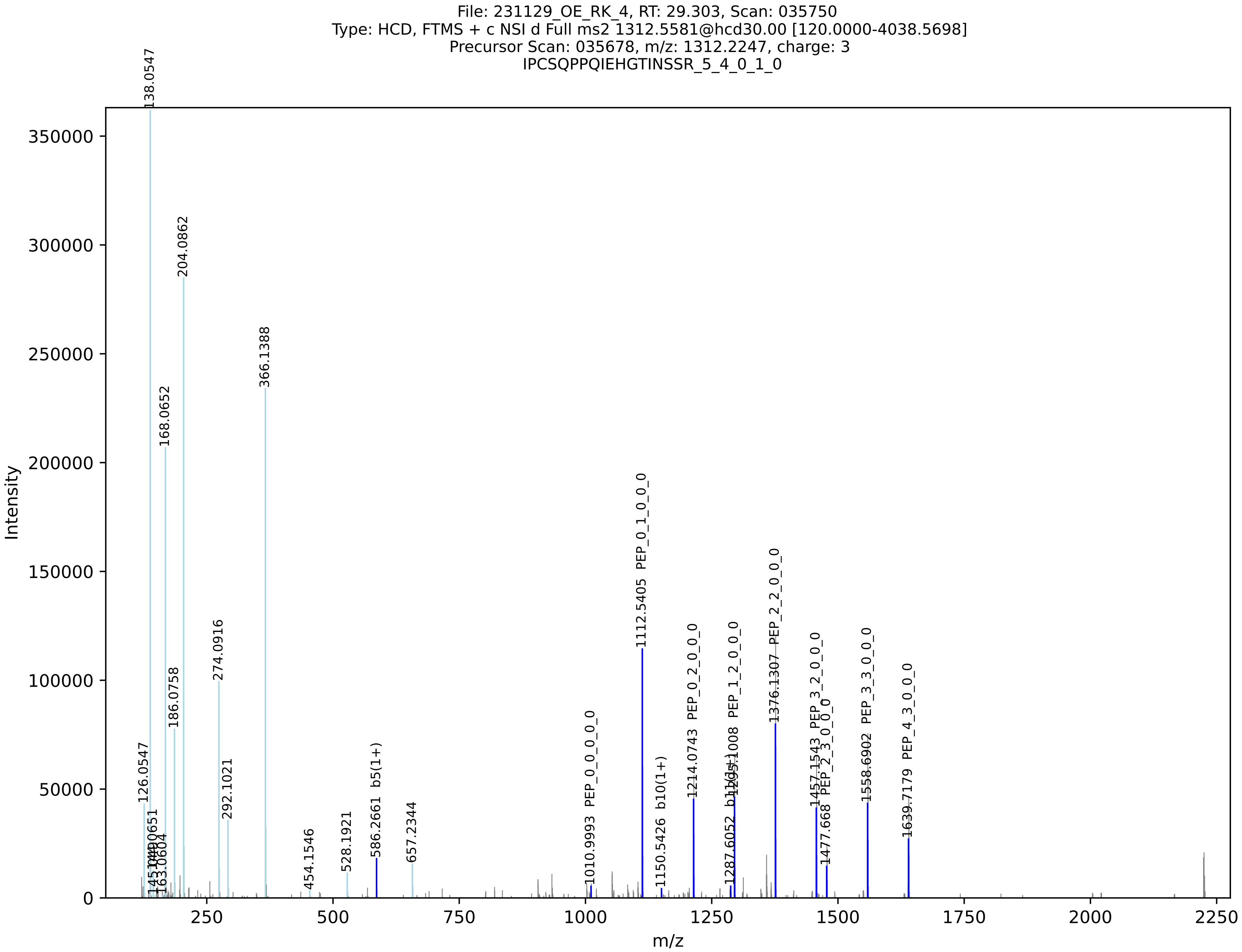

File: 231129_OE_RK_4, RT: 29.303, Scan: 035750
Type: HCD, FTMS + c NSI d Full ms2 1312.5581@hcd30.00 [120.0000-4038.5698]
Precursor Scan: 035678, m/z: 1312.2247, charge: 3
IPCSQPPQIEHGTINSSR_5_4_0_1_0
Intensity
m/z
138.0547
204.0862
366.1388
168.0652
274.0916
186.0758
126.0547
292.1021
454.1546
528.1921
586.2661 b5(1+)
657.2344
1010.9993 PEP_0_0_0_0_0
1112.5405 PEP_0_1_0_0_0
1150.5426 b10(1+)
1214.0743 PEP_0_2_0_0_0
1287.6052 b11(1+)
1295.1008 PEP_1_2_0_0_0
1376.1307 PEP_2_2_0_0_0
1457.1543 PEP_3_2_0_0_0
1477.668 PEP_2_3_0_0_0
1558.6902 PEP_3_3_0_0_0
1639.7179 PEP_4_3_0_0_0

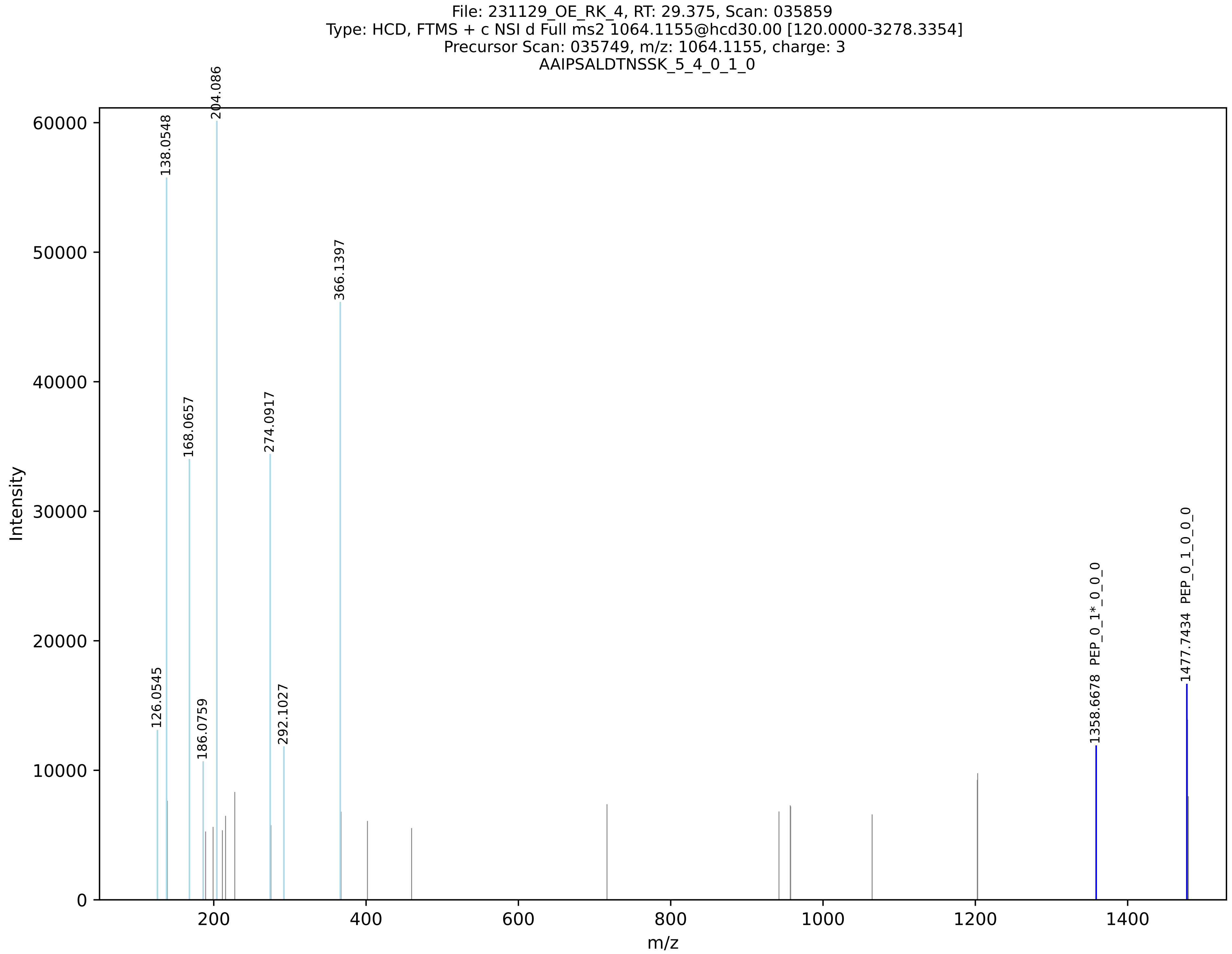

File: 231129_OE_RK_4, RT: 29.375, Scan: 035859
Type: HCD, FTMS + c NSI d Full ms2 1064.1155@hcd30.00 [120.0000-3278.3354]
Precursor Scan: 035749, m/z: 1064.1155, charge: 3
AAIPSALDTNSSK_5_4_0_1_0
Intensity
m/z
126.0545
138.0548
168.0657
186.0759
204.086
274.0917
292.1027
366.1397
1358.6678 PEP_0_1*_0_0_0
1477.7434 PEP_0_1_0_0_0

File: 231129_OE_RK_4, RT: 29.402, Scan: 035895
Type: HCD, FTMS + c NSI d Full ms2 1103.7983@hcd30.00 [120.0000-3399.7649]
Precursor Scan: 035823, m/z: 1103.4642, charge: 3
TPLTANITK_5_4_1_2_0

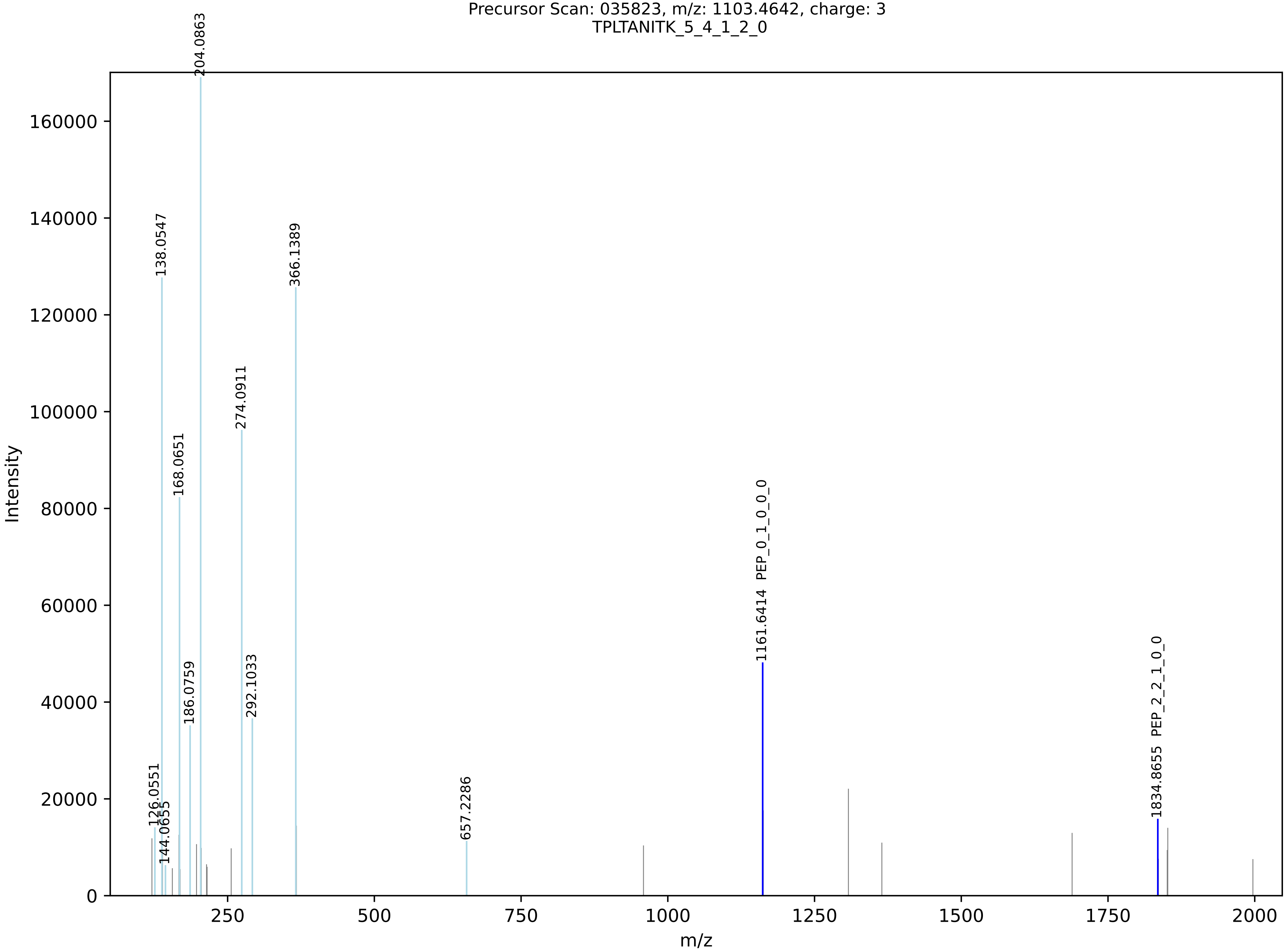

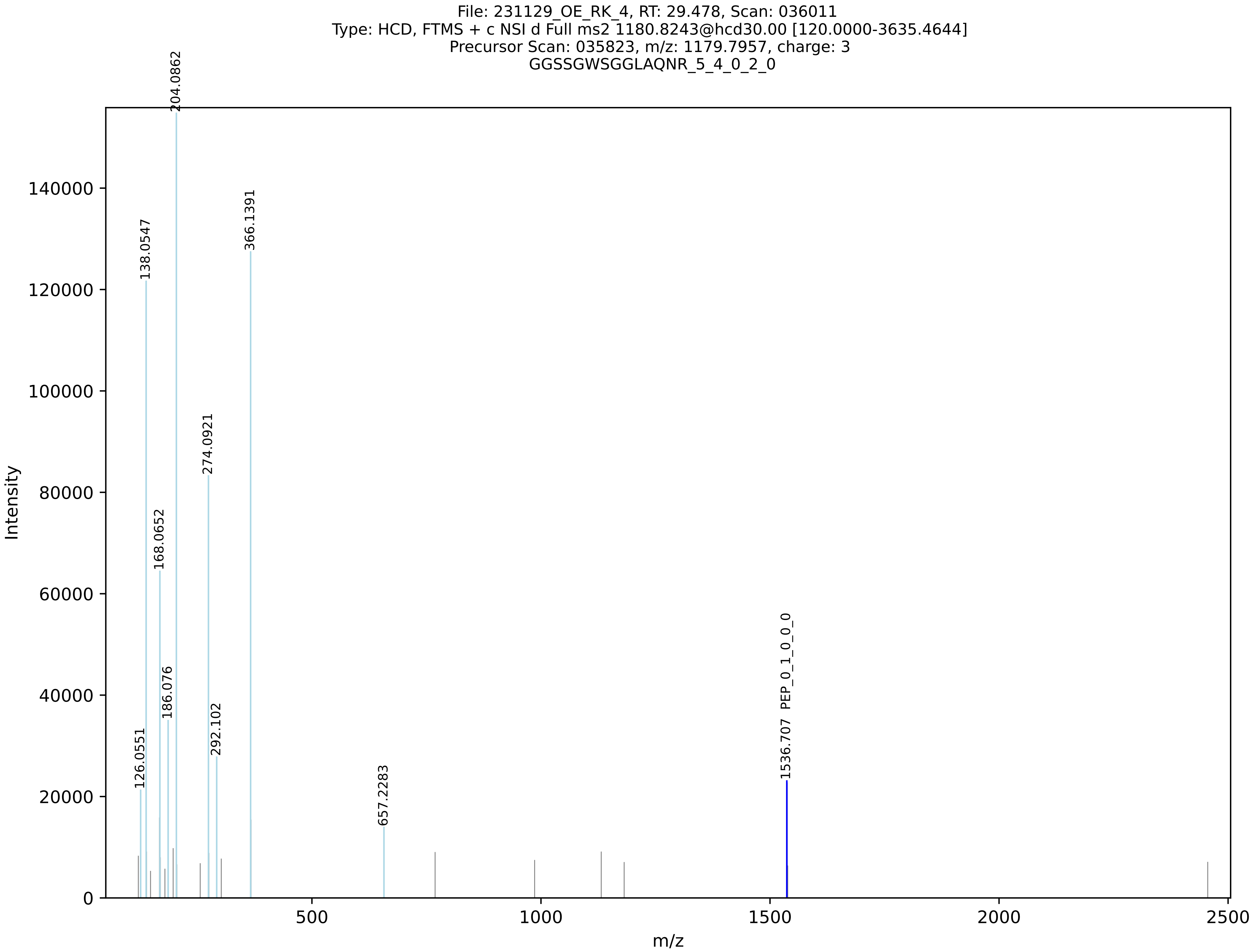

File: 231129_OE_RK_4, RT: 29.478, Scan: 036011
Type: HCD, FTMS + c NSI d Full ms2 1180.8243@hcd30.00 [120.0000-3635.4644]
Precursor Scan: 035823, m/z: 1179.7957, charge: 3
GGSSGWSGGLAQNR_5_4_0_2_0
Intensity
m/z
204.0862
138.0547
366.1391
274.0921
168.0652
186.076
292.102
126.0551
657.2283
1536.707 PEP_0_1_0_0_0
0
20000
40000
60000
80000
100000
120000
140000
500
1000
1500
2000
2500

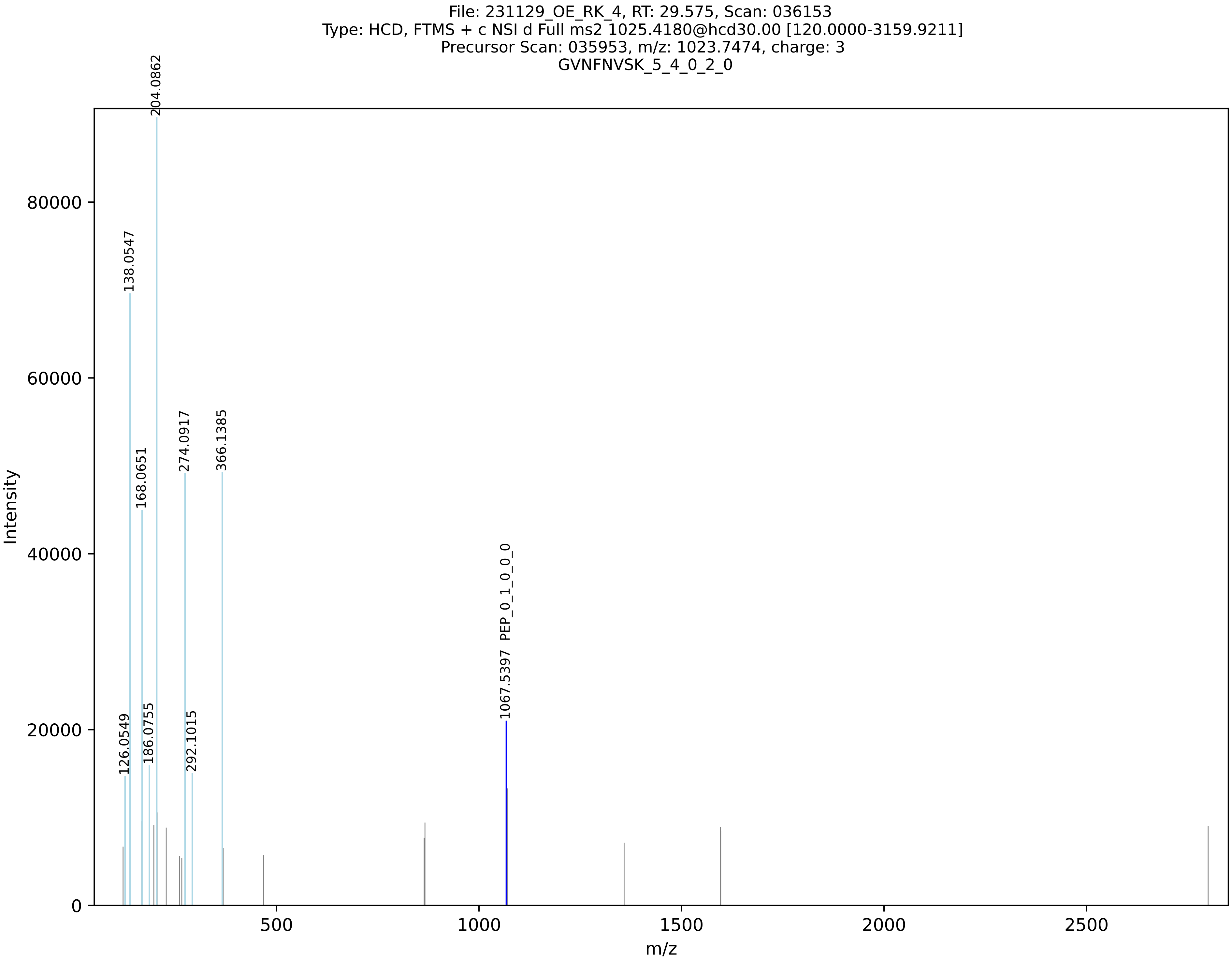

File: 231129_OE_RK_4, RT: 29.575, Scan: 036153
Type: HCD, FTMS + c NSI d Full ms2 1025.4180@hcd30.00 [120.0000-3159.9211]
Precursor Scan: 035953, m/z: 1023.7474, charge: 3
GVNFNVSK_5_4_0_2_0
204.0862
138.0547
168.0651
274.0917
366.1385
126.0549
186.0755
292.1015
1067.5397 PEP_0_1_0_0_0
Intensity
m/z
0
20000
40000
60000
80000
500
1000
1500
2000
2500

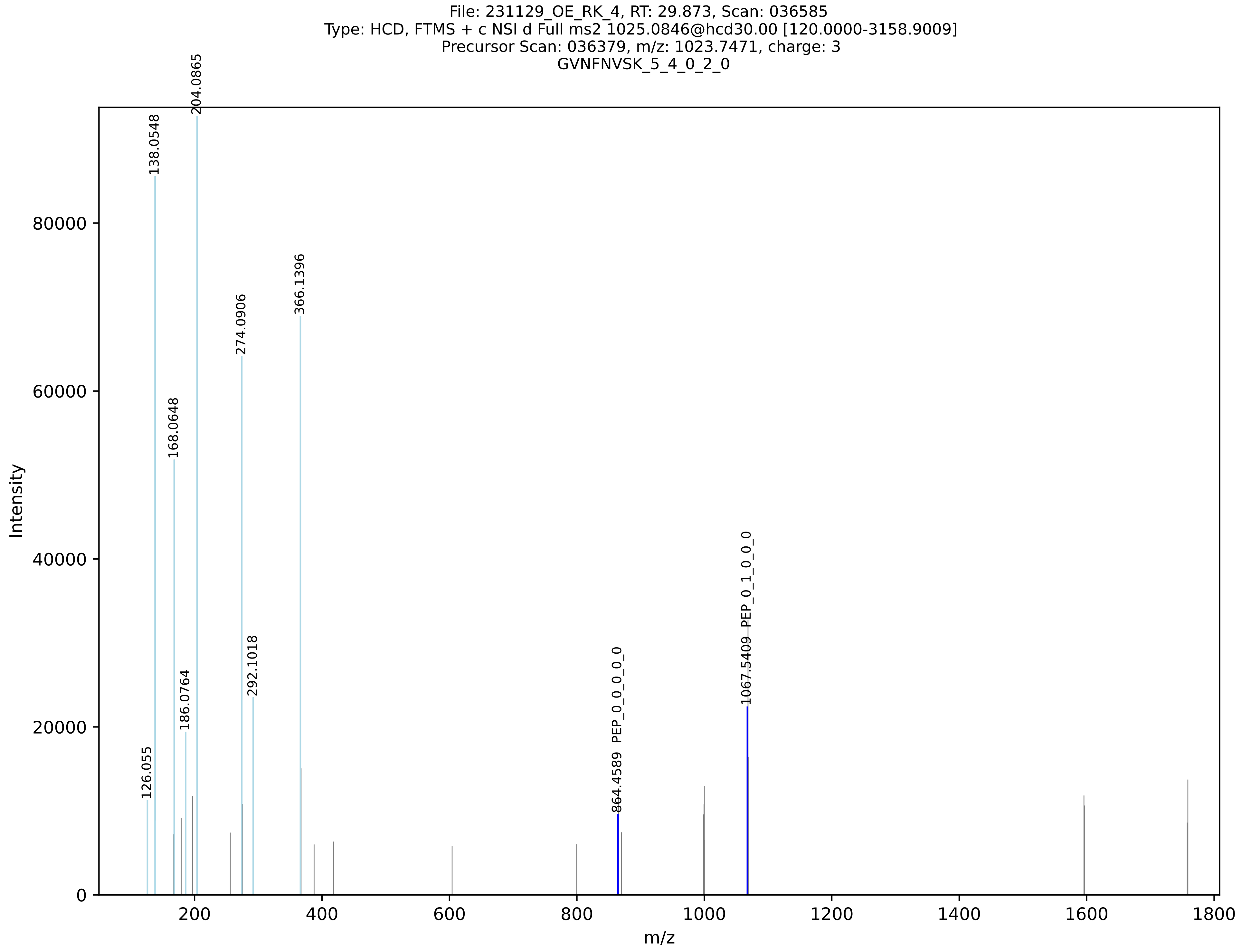
File: 231129_OE_RK_4, RT: 29.873, Scan: 036585
Type: HCD, FTMS + c NSI d Full ms2 1025.0846@hcd30.00 [120.0000-3158.9009]
Precursor Scan: 036379, m/z: 1023.7471, charge: 3
GVNFNVSK_5_4_0_2_0
Intensity
m/z
0
20000
40000
60000
80000
200
400
600
800
1000
1200
1400
1600
1800
126.055
138.0548
168.0648
186.0764
204.0865
274.0906
292.1018
366.1396
864.4589 PEP_0_0_0_0_0
1067.5409 PEP_0_1_0_0_0

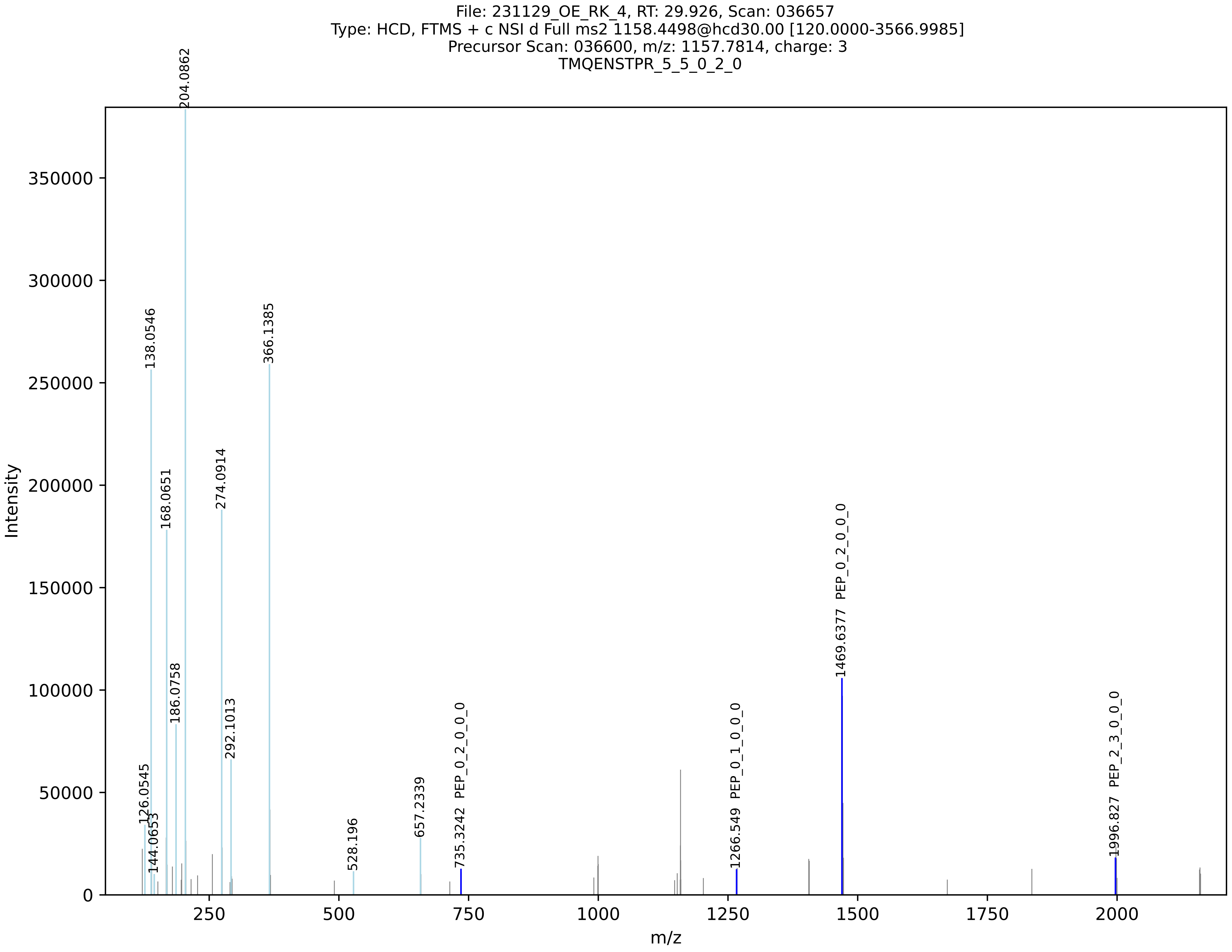

File: 231129_OE_RK_4, RT: 29.926, Scan: 036657
Type: HCD, FTMS + c NSI d Full ms2 1158.4498@hcd30.00 [120.0000-3566.9985]
Precursor Scan: 036600, m/z: 1157.7814, charge: 3
TMQENSTPR_5_5_0_2_0
Intensity
m/z
204.0862
138.0546
366.1385
274.0914
168.0651
186.0758
292.1013
126.0545
144.0653
528.196
657.2339
735.3242 PEP_0_2_0_0_
1266.549 PEP_0_1_0_0_0
1469.6377 PEP_0_2_0_0_
1996.827 PEP_2_3_0_0_

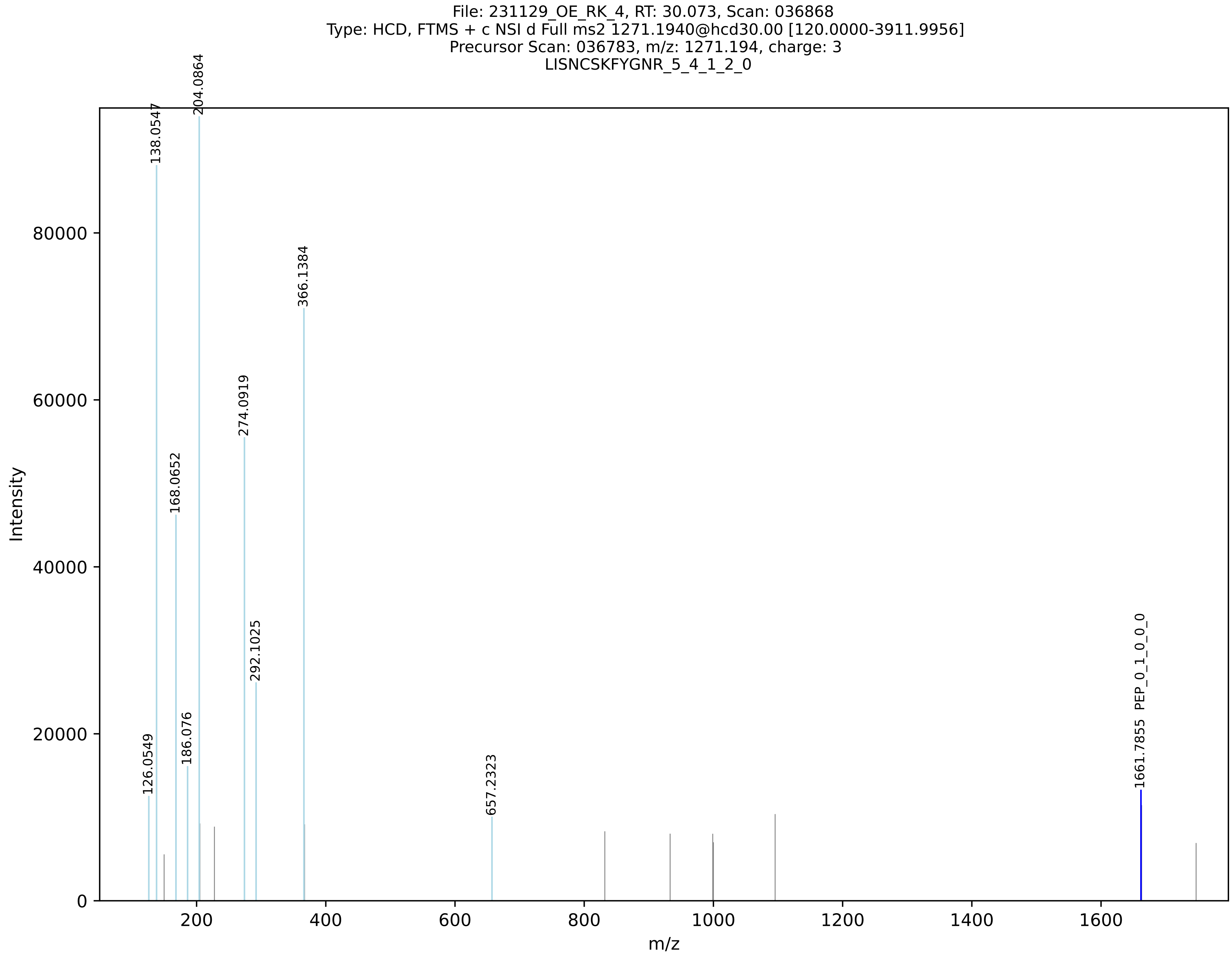
File: 231129_OE_RK_4, RT: 30.073, Scan: 036868
Type: HCD, FTMS + c NSI d Full ms2 1271.1940@hcd30.00 [120.0000-3911.9956]
Precursor Scan: 036783, m/z: 1271.194, charge: 3
LISNCSKFYGNR_5_4_1_2_0
204.0864
138.0547
366.1384
274.0919
168.0652
292.1025
186.076
126.0549
657.2323
1661.7855 PEP_0_1_0_0_0_
Intensity
m/z
0
20000
40000
60000
80000
200
400
600
800
1000
1200
1400
1600

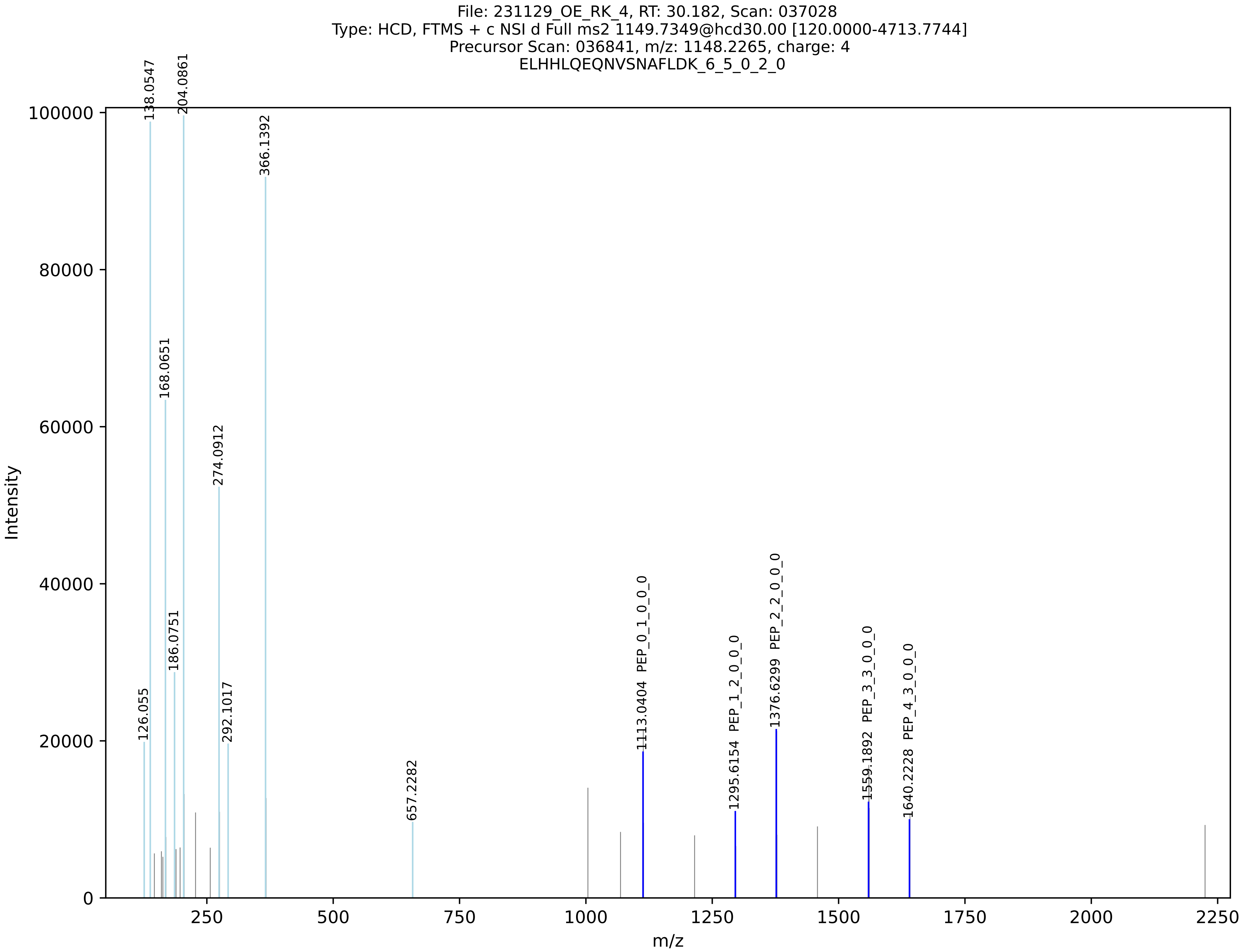

File: 231129_OE_RK_4, RT: 30.182, Scan: 037028
Type: HCD, FTMS + c NSI d Full ms2 1149.7349@hcd30.00 [120.0000-4713.7744]
Precursor Scan: 036841, m/z: 1148.2265, charge: 4
ELHHLQEQNVSNAFLDK_6_5_0_2_0
138.0547
204.0861
366.1392
168.0651
274.0912
186.0751
126.055
292.1017
657.2282
1113.0404 PEP_0_1_0_0_0
1295.6154 PEP_1_2_0_0_0
1376.6299 PEP_2_2_0_0_0
1559.1892 PEP_3_3_0_0_0
1640.2228 PEP_4_3_0_0_0
Intensity
m/z
0
20000
40000
60000
80000
100000
250
500
750
1000
1250
1500
1750
2000
2250

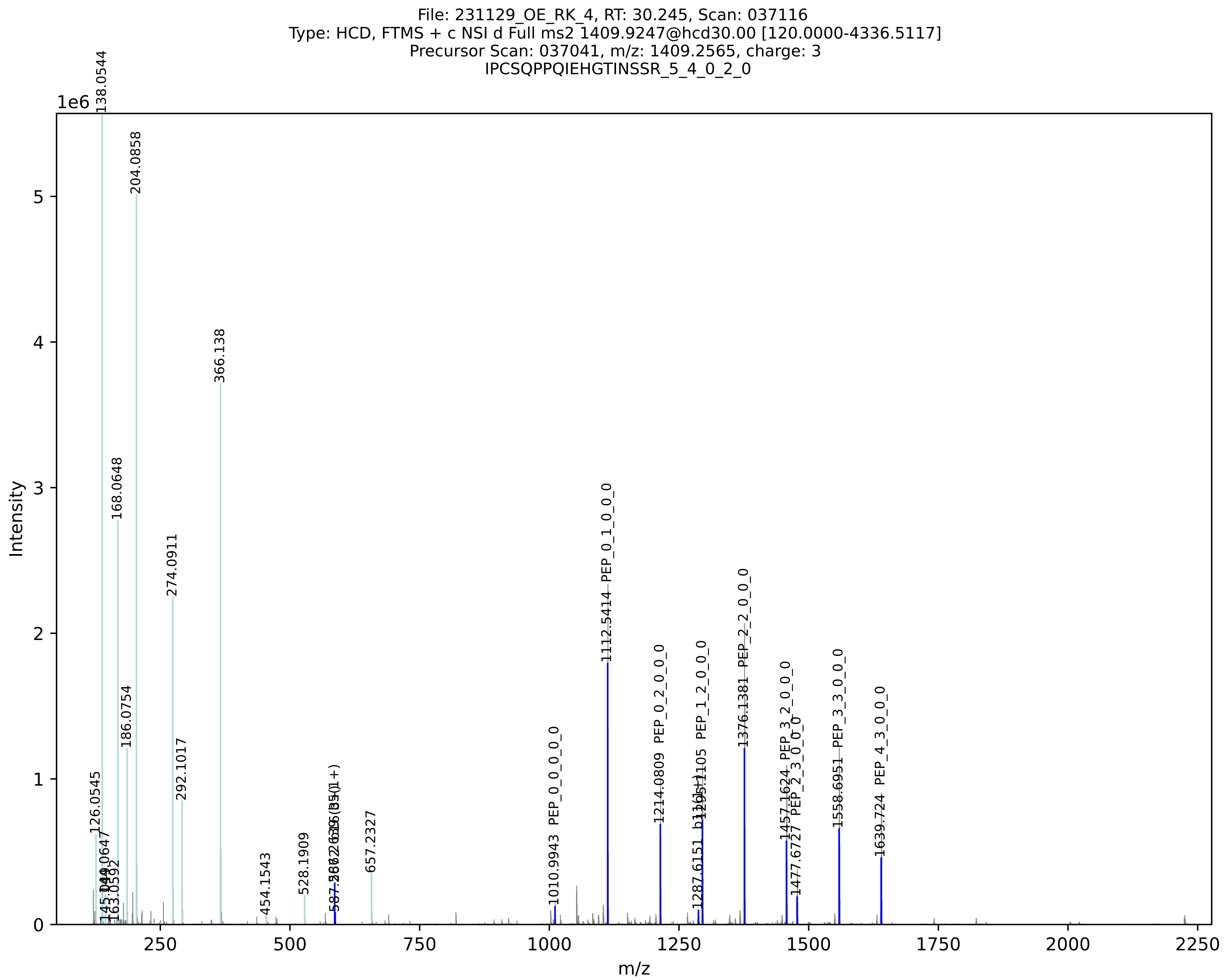
File: 231129_OE_RK_4, RT: 30.245, Scan: 037116
Type: HCD, FTMS + c NSI d Full ms2 1409.9247@hcd30.00 [120.0000-4336.5117]
Precursor Scan: 037041, m/z: 1409.2565, charge: 3
IPCSQPPQIEHGTINSSR_5_4_0_2_0
1e6
Intensity
m/z
138.0544
204.0858
366.138
168.0648
274.0911
186.0754
292.1017
126.0545
454.1543
528.1909
657.2327
1010.9943 PEP_0_0_0_0_0
1112.5414 PEP_0_1_0_0_0
1214.0809 PEP_0_2_0_0_0
1295.1105 PEP_1_2_0_0_0
1376.1381 PEP_2_2_0_0_0
1457.1624 PEP_3_2_0_0_0
1477.6727 PEP_2_3_0_0_0
1558.6951 PEP_3_3_0_0_0
1639.724 PEP_4_3_0_0_0

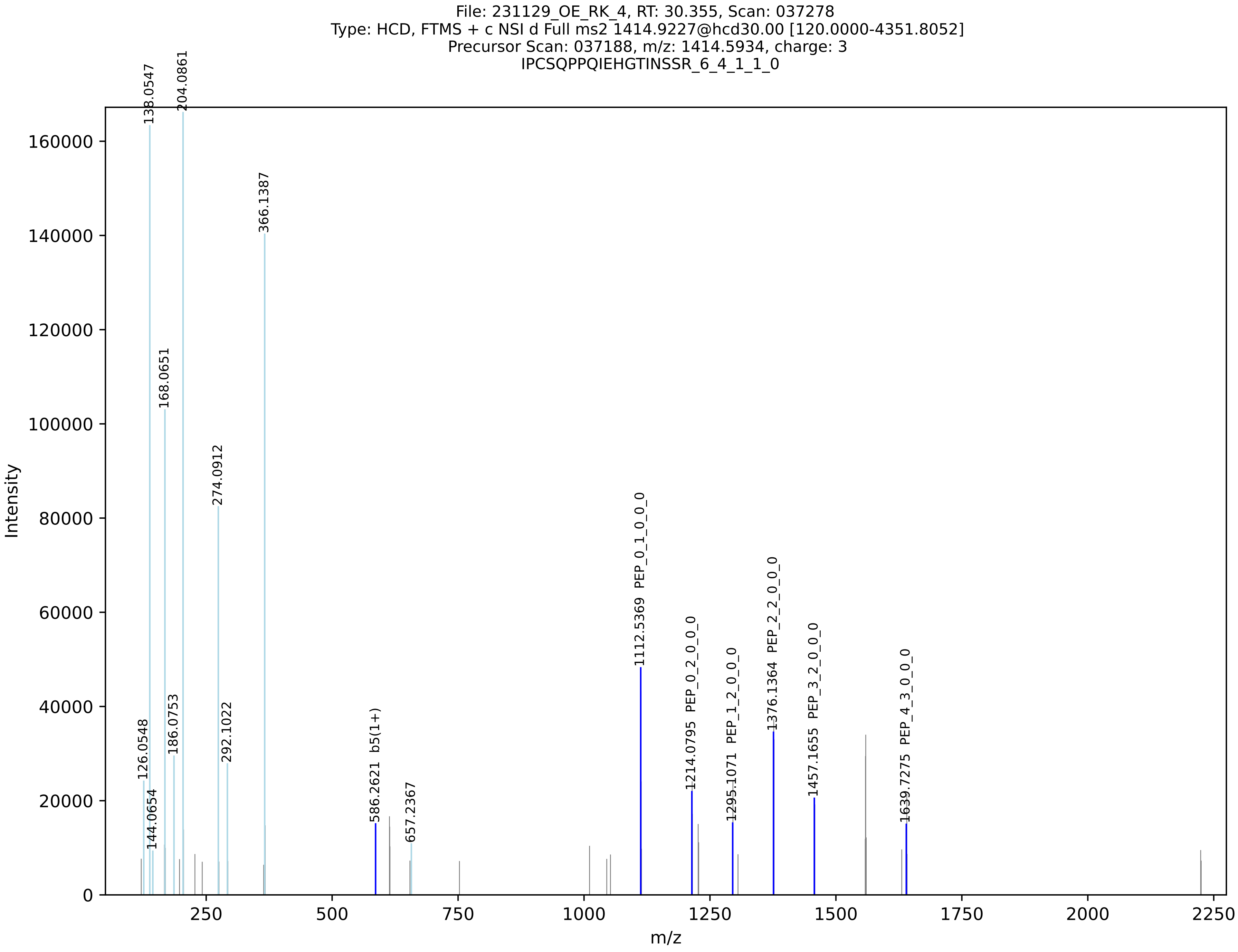

File: 231129_OE_RK_4, RT: 30.355, Scan: 037278
Type: HCD, FTMS + c NSI d Full ms2 1414.9227@hcd30.00 [120.0000-4351.8052]
Precursor Scan: 037188, m/z: 1414.5934, charge: 3
IPCSQPPQIEHGTINSSR_6_4_1_1_0
Intensity
m/z
138.0547
204.0861
366.1387
168.0651
274.0912
126.0548
144.0654
186.0753
292.1022
586.2621 b5(1+)
657.2367
1112.5369 PEP_0_1_0_0_0
1214.0795 PEP_0_2_0_0_0
1295.1071 PEP_1_2_0_0_0
1376.1364 PEP_2_2_0_0_0
1457.1655 PEP_3_2_0_0_0
1639.7275 PEP_4_3_0_0_0

File: 231129_OE_RK_4, RT: 30.435, Scan: 037393
Type: HCD, FTMS + c NSI d Full ms2 1409.9242@hcd30.00 [120.0000-4336.5103]
Precursor Scan: 037336, m/z: 1409.256, charge: 3
IPCSQPPQIEHGTINSSR_5_4_0_2_0

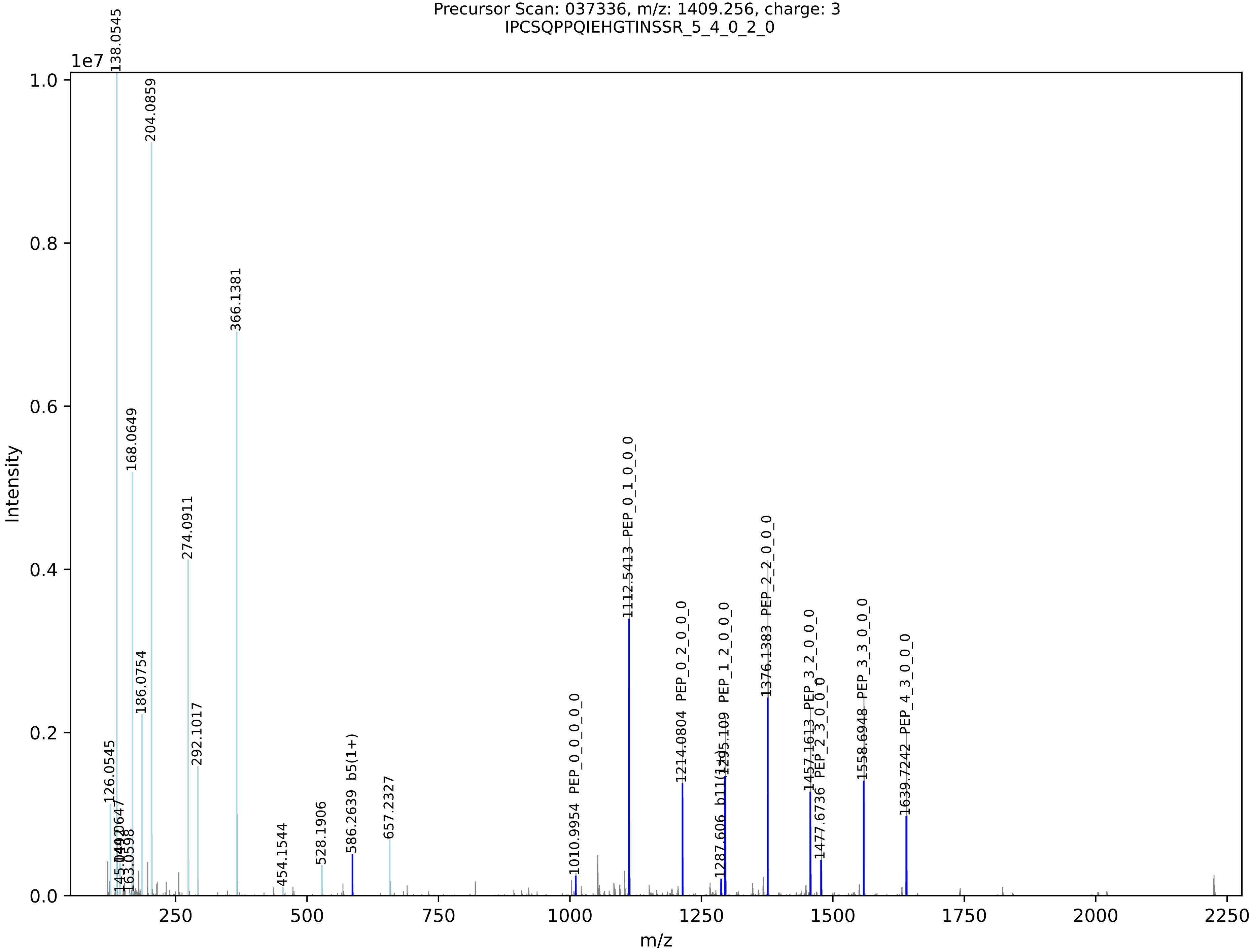

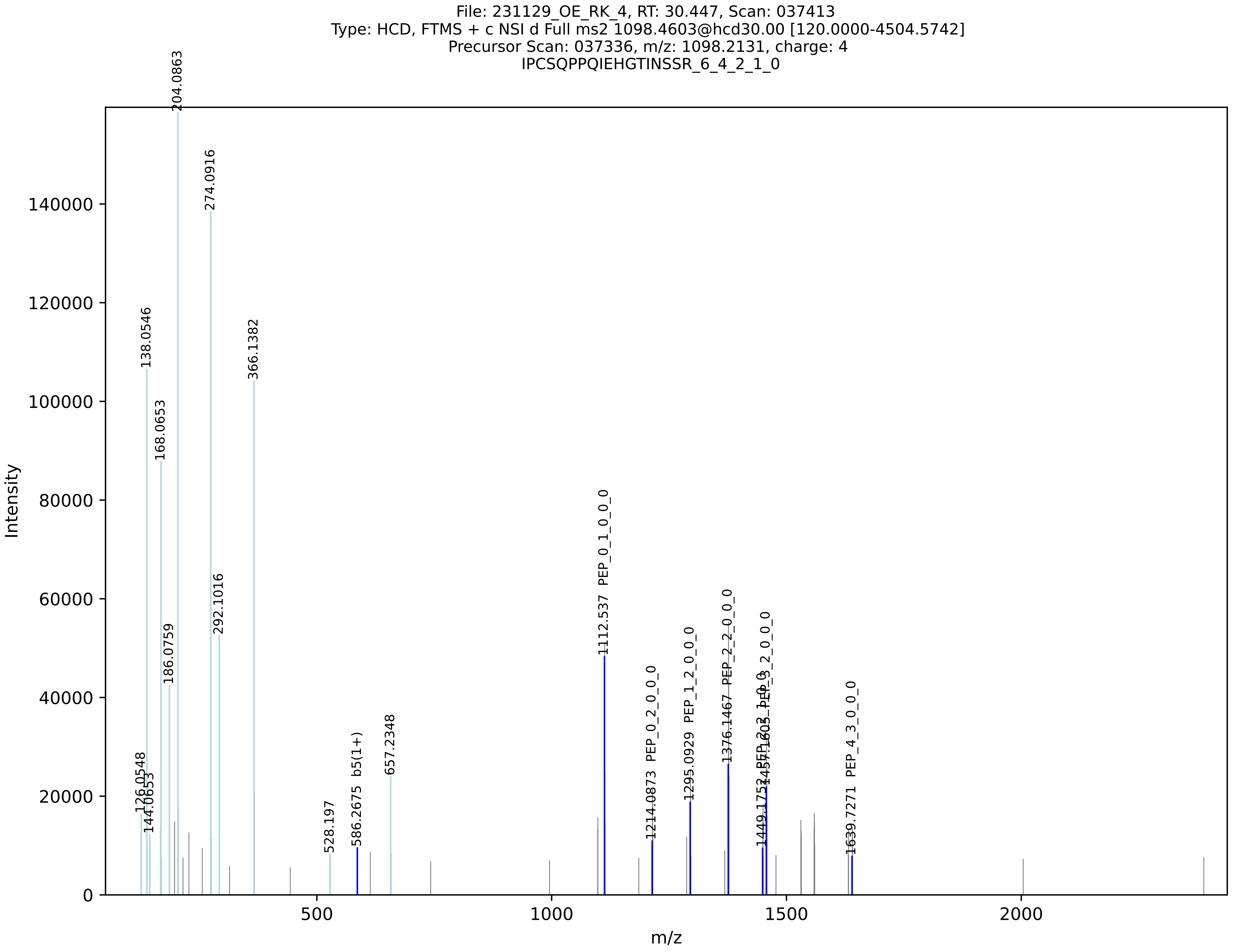
File: 231129_OE_RK_4, RT: 30.447, Scan: 037413
Type: HCD, FTMS + c NSI d Full ms2 1098.4603@hcd30.00 [120.0000-4504.5742]
Precursor Scan: 037336, m/z: 1098.2131, charge: 4
IPCSQPPQIEHGTINSSR_6_4_2_1_0
Intensity
m/z
204.0863
274.0916
138.0546
366.1382
168.0653
292.1016
186.0759
126.0548
144.0653
528.197
586.2675 b5(1+)
657.2348
1112.537 PEP_0_1_0_0_0
1214.0873 PEP_0_2_0_0_0
1295.0929 PEP_1_2_0_0_0
1376.1467 PEP_2_2_0_0_0
1639.7271 PEP_4_3_0_0_0

File: 231129_OE_RK_4, RT: 30.532, Scan: 037534
Type: HCD, FTMS + c NSI d Full ms2 1057.6959@hcd30.00 [120.0000-4338.2554]
Precursor Scan: 037463, m/z: 1057.1947, charge: 4
IPCSQPPQIEHGTINSSR_5_4_0_2_0

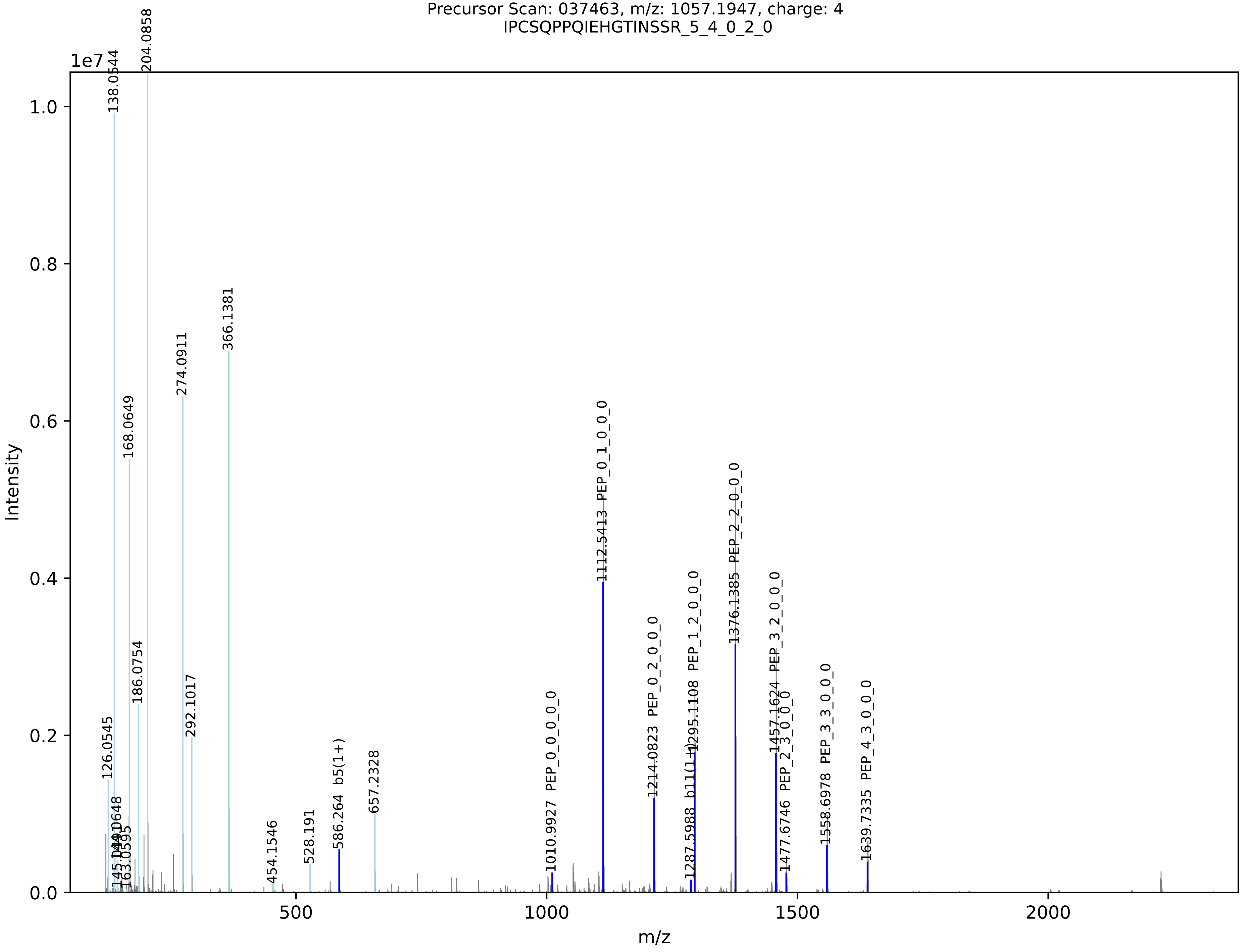

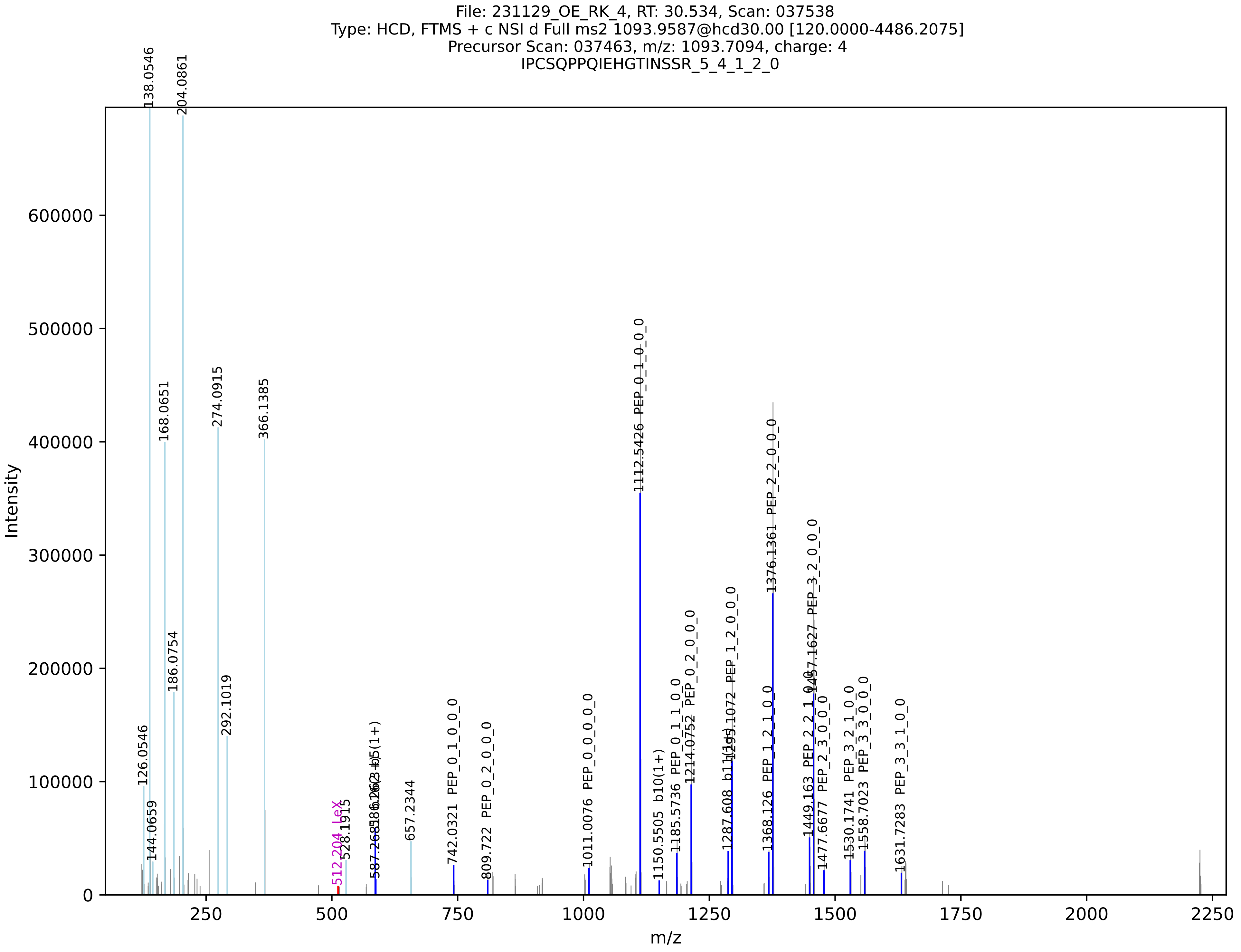

File: 231129_OE_RK_4, RT: 30.534, Scan: 037538
Type: HCD, FTMS + c NSI d Full ms2 1093.9587@hcd30.00 [120.0000-4486.2075]
Precursor Scan: 037463, m/z: 1093.7094, charge: 4
IPCSQPPQIEHGTINSSR_5_4_1_2_0
Intensity
m/z
138.0546
204.0861
168.0651
274.0915
366.1385
186.0754
292.1019
126.0546
144.0659
512.204 LeX
528.1915
657.2344
742.0321 PEP_0_1_0_0_0
809.722 PEP_0_2_0_0_0
1011.0076 PEP_0_0_0_0_0
1112.5426 PEP_0_1_0_0_0
1150.5505 b10(1+)
1185.5736 PEP_0_1_1_0_0
1214.0752 PEP_0_2_0_0_0
1287.608 b11(1+)
1297.1072 PEP_1_2_0_0_0
1368.126 PEP_1_2_1_0_0
1376.1361 PEP_2_2_0_0_0
1449.163 PEP_2_2_1_0_0
1457.1627 PEP_3_2_0_0_0
1477.6677 PEP_2_3_0_0_0
1530.1741 PEP_3_2_1_0_0
1558.7023 PEP_3_3_0_0_0
1631.7283 PEP_3_3_1_0_0

File: 231129_OE_RK_4, RT: 30.537, Scan: 037542
Type: HCD, FTMS + c NSI d Full ms2 1048.4347@hcd30.00 [120.0000-3230.3523]
Precursor Scan: 037463, m/z: 1048.1006, charge: 3
ENISDPTSPLR_5_4_0_1_0

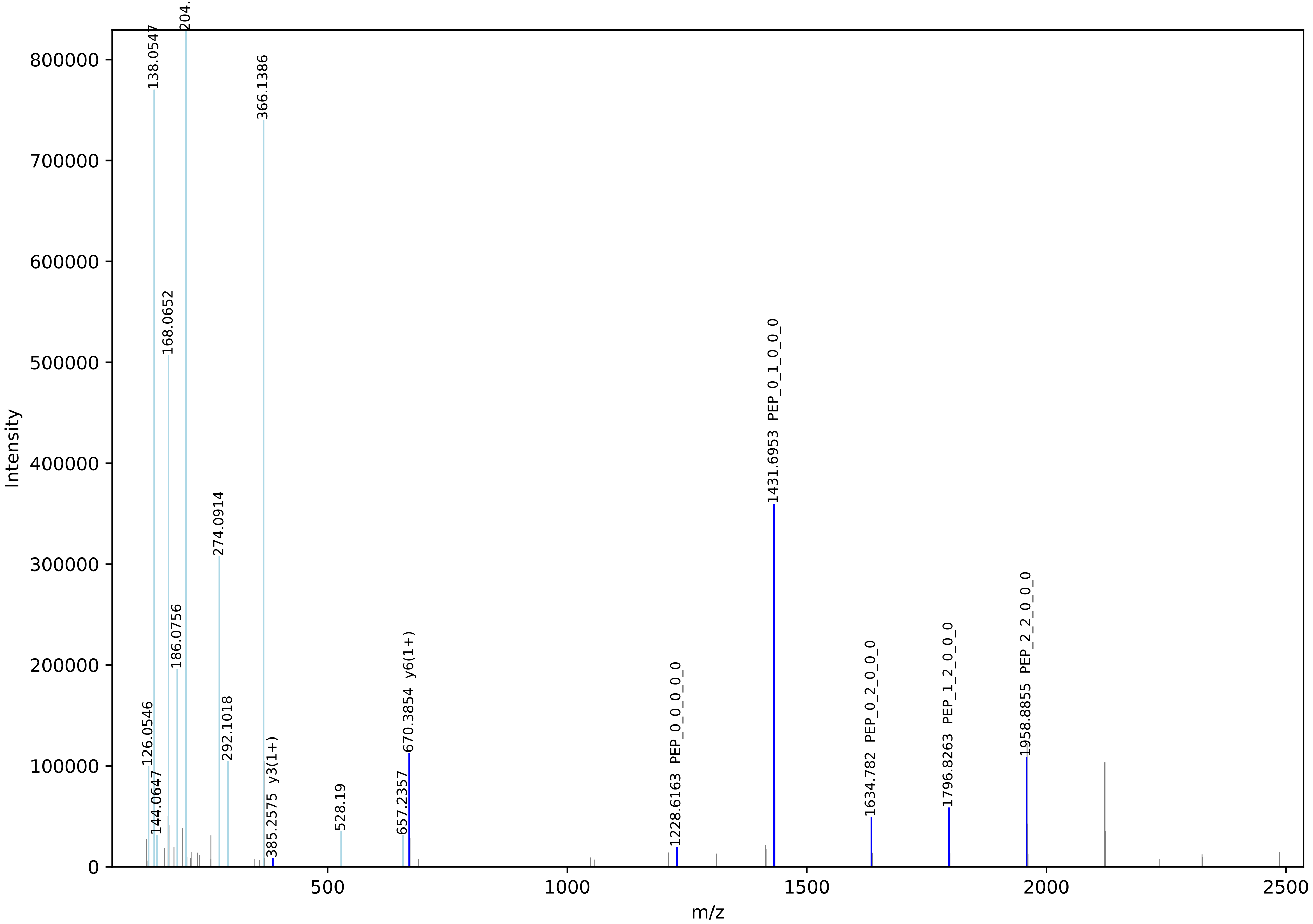

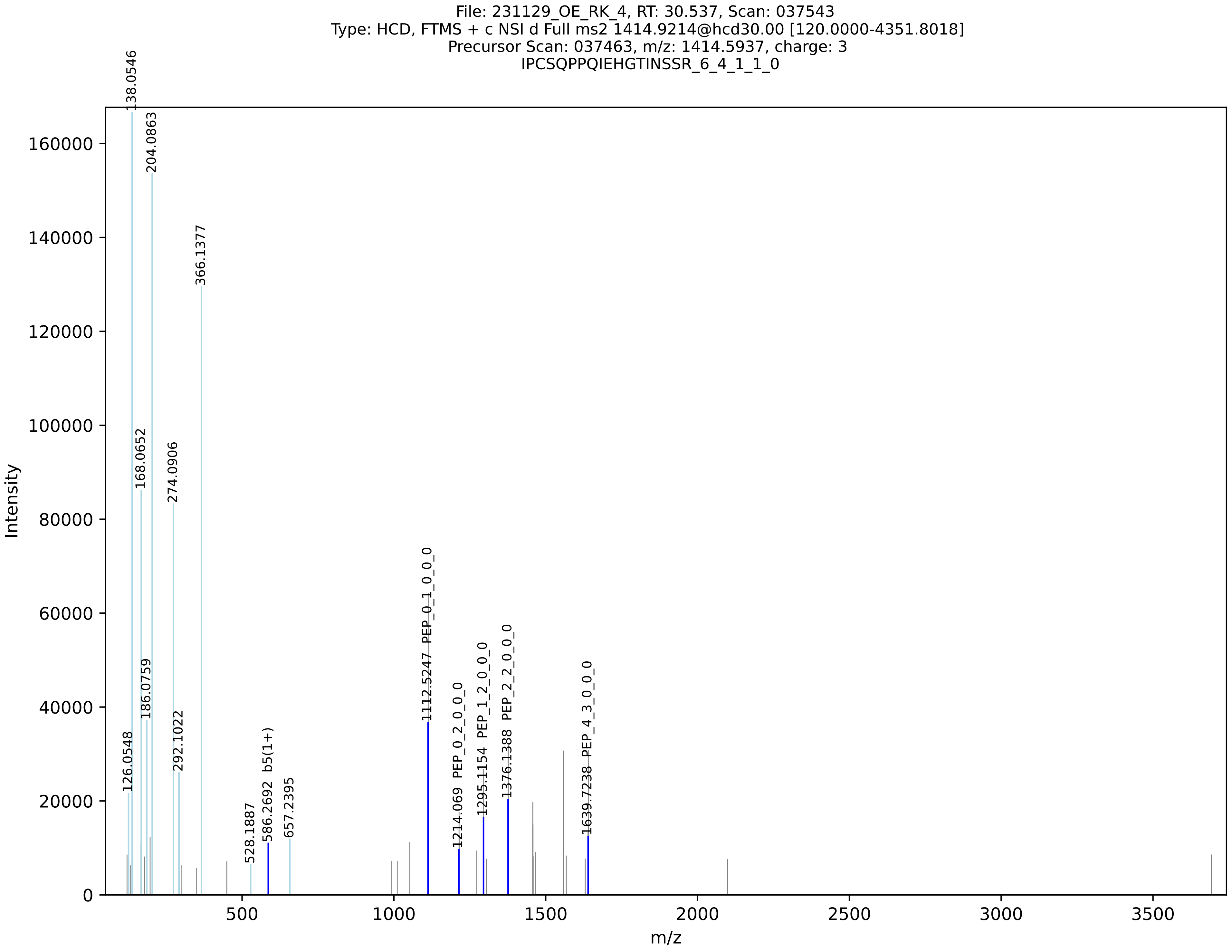
File: 231129_OE_RK_4, RT: 30.537, Scan: 037543
Type: HCD, FTMS + c NSI d Full ms2 1414.9214@hcd30.00 [120.0000-4351.8018]
Precursor Scan: 037463, m/z: 1414.5937, charge: 3
IPCSQPPQIEHGTINSSR_6_4_1_1_0
138.0546
204.0863
366.1377
168.0652
274.0906
186.0759
292.1022
126.0548
528.1887
586.2692 b5(1+)
657.2395
1112.5247 PEP_0_1_0_0_0
1214.069 PEP_0_2_0_0_0
1295.1154 PEP_1_2_0_0_0
1376.1388 PEP_2_2_0_0_0
1639.7238 PEP_4_3_0_0_0
Intensity
m/z
0
20000
40000
60000
80000
100000
120000
140000
160000
500
1000
1500
2000
2500
3000
3500

File: 231129_OE_RK_4, RT: 30.609, Scan: 037651
Type: HCD, FTMS + c NSI d Full ms2 1313.2192@hcd30.00 [120.0000-4040.5930]
Precursor Scan: 037533, m/z: 1312.8873, charge: 3
IPCSQPPQIEHGTINSSR_5_4_0_1_0

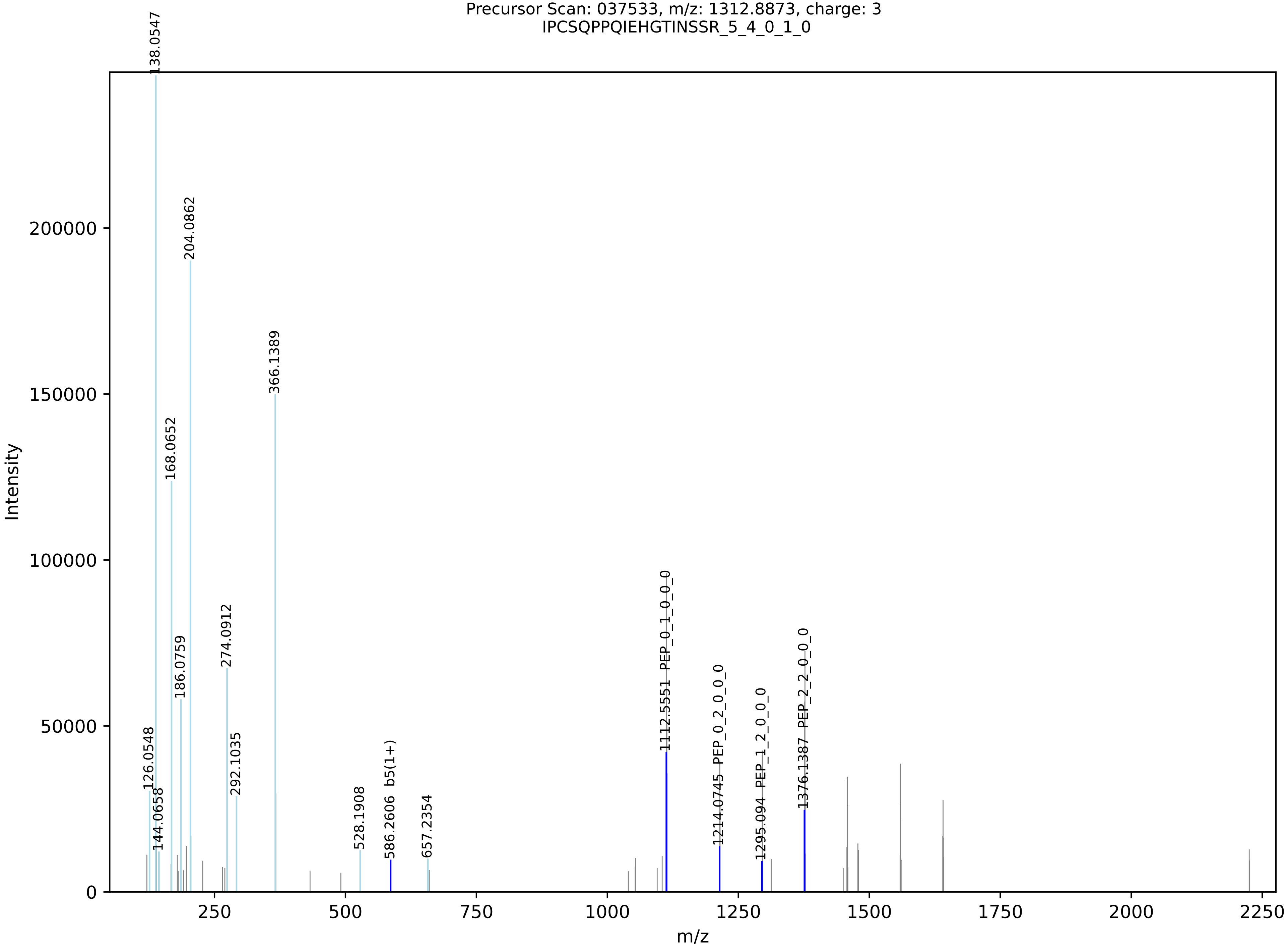

File: 231129_OE_RK_4, RT: 30.648, Scan: 037707
Type: HCD, FTMS + c NSI d Full ms2 1087.4288@hcd30.00 [120.0000-4459.5654]
Precursor Scan: 037606, m/z: 1086.6769, charge: 4
LTDTICGVGNMSANASDQER_5_4_0_2_0

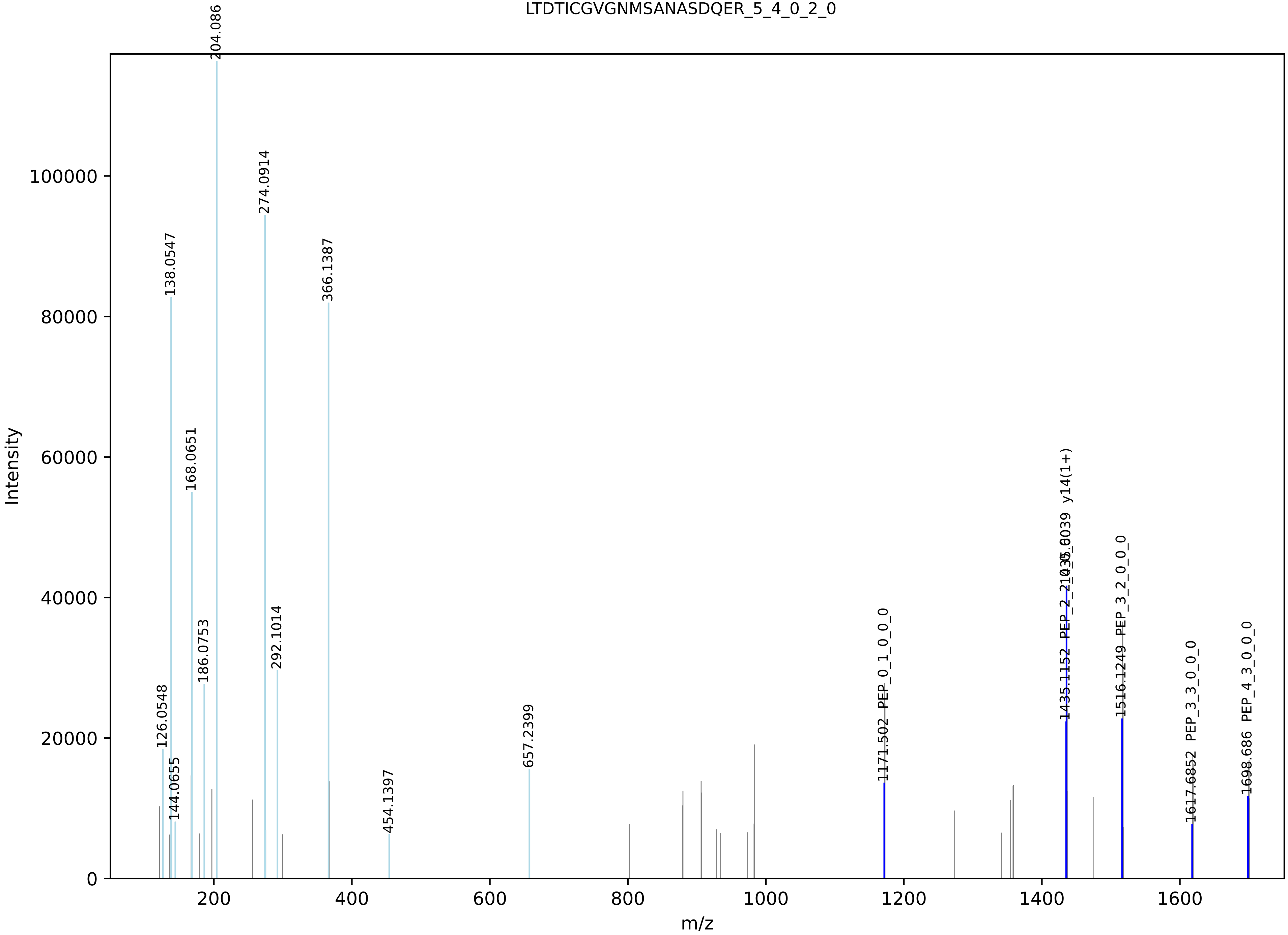

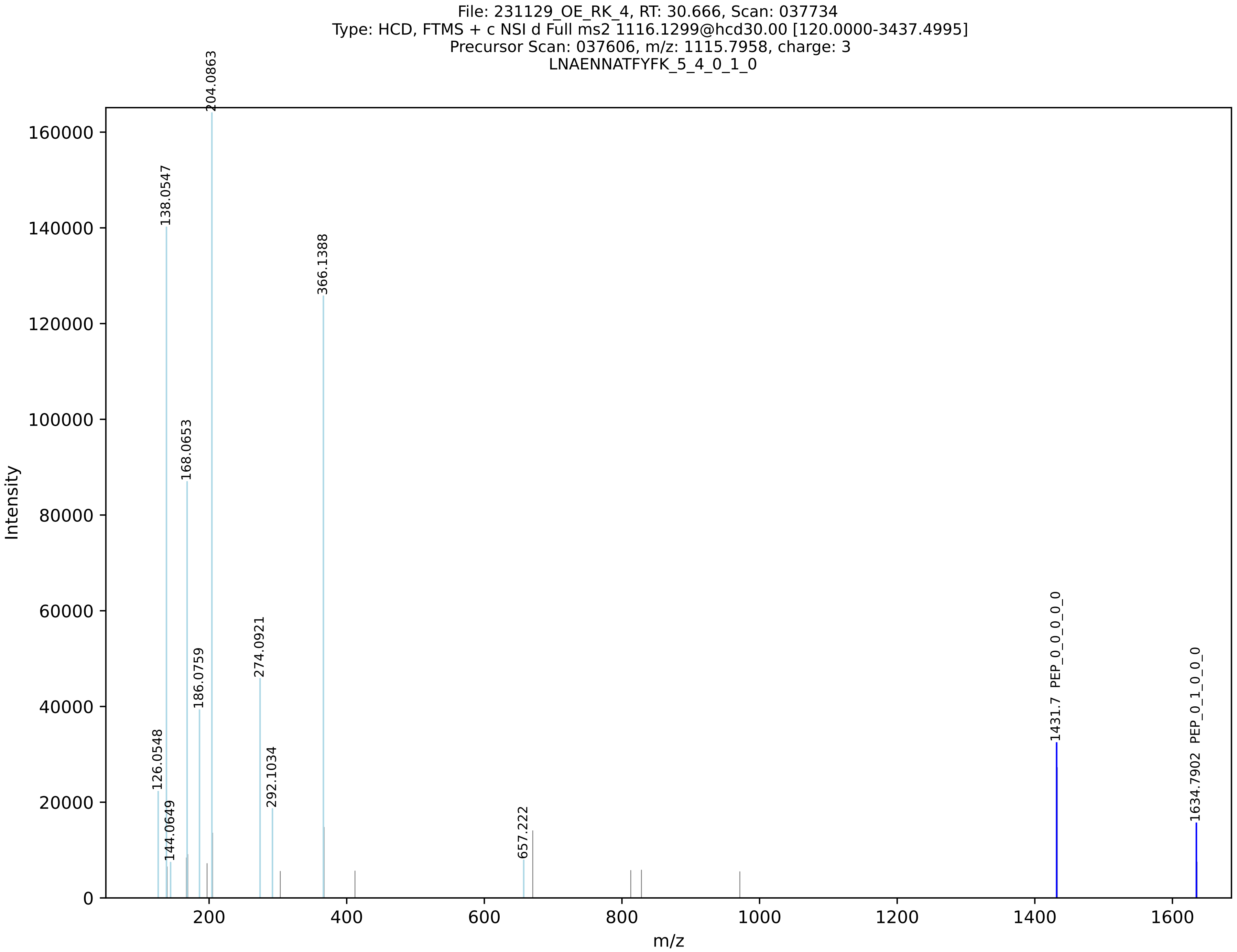
File: 231129_OE_RK_4, RT: 30.666, Scan: 037734
Type: HCD, FTMS + c NSI d Full ms2 1116.1299@hcd30.00 [120.0000-3437.4995]
Precursor Scan: 037606, m/z: 1115.7958, charge: 3
LNAENNATFYFK_5_4_0_1_0
Intensity
m/z
204.0863
138.0547
366.1388
168.0653
274.0921
186.0759
126.0548
292.1034
144.0649
657.222
1431.7 PEP_0_0_0_0_0
1634.7902 PEP_0_1_0_0_0

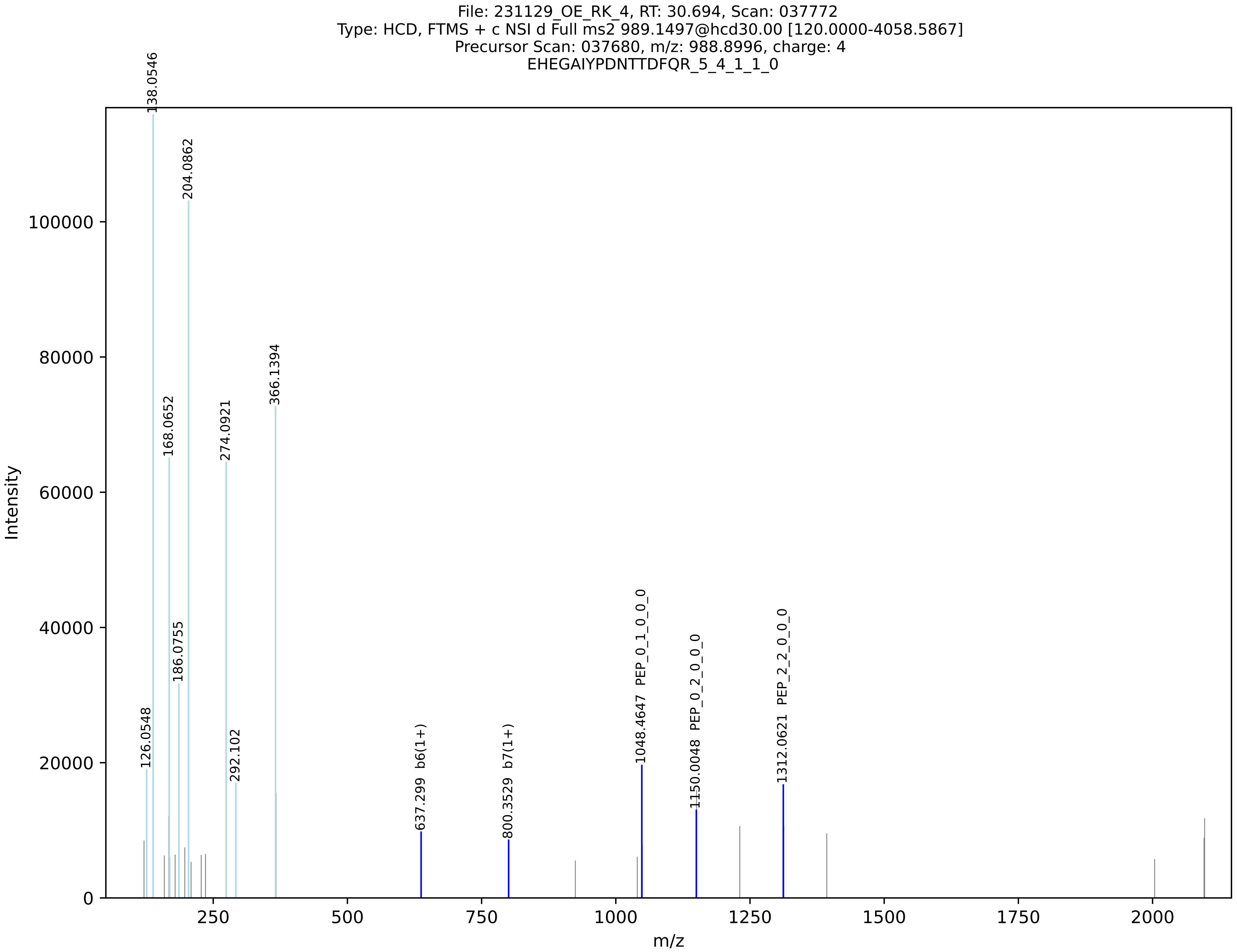
File: 231129_OE_RK_4, RT: 30.694, Scan: 037772
Type: HCD, FTMS + c NSI d Full ms2 989.1497@hcd30.00 [120.0000-4058.5867]
Precursor Scan: 037680, m/z: 988.8996, charge: 4
EHEGAIYPDNTTDFQR_5_4_1_1_0
Intensity
m/z
138.0546
204.0862
168.0652
274.0921
366.1394
186.0755
126.0548
292.102
637.299 b6(1+)
800.3529 b7(1+)
1048.4647 PEP_0_1_0_0_0
1150.0048 PEP_0_2_0_0_0
1312.0621 PEP_2_2_0_0_0

File: 231129_OE_RK_4, RT: 30.734, Scan: 037829
Type: HCD, FTMS + c NSI d Full ms2 1057.6964@hcd30.00 [120.0000-4338.2573]
Precursor Scan: 037754, m/z: 1057.1952, charge: 4
IPCSQPPQIEHGTINSSR_5_4_0_2_0

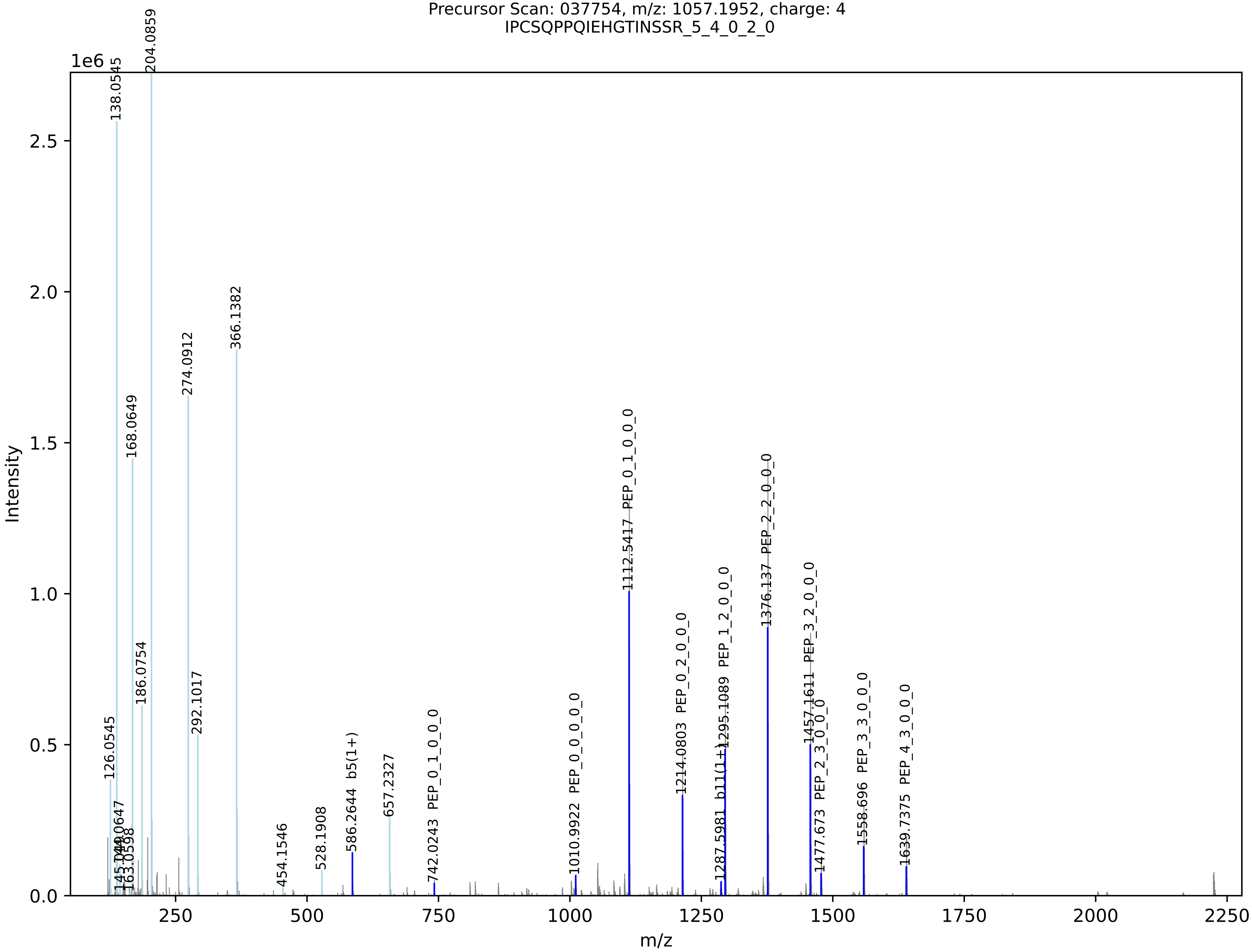

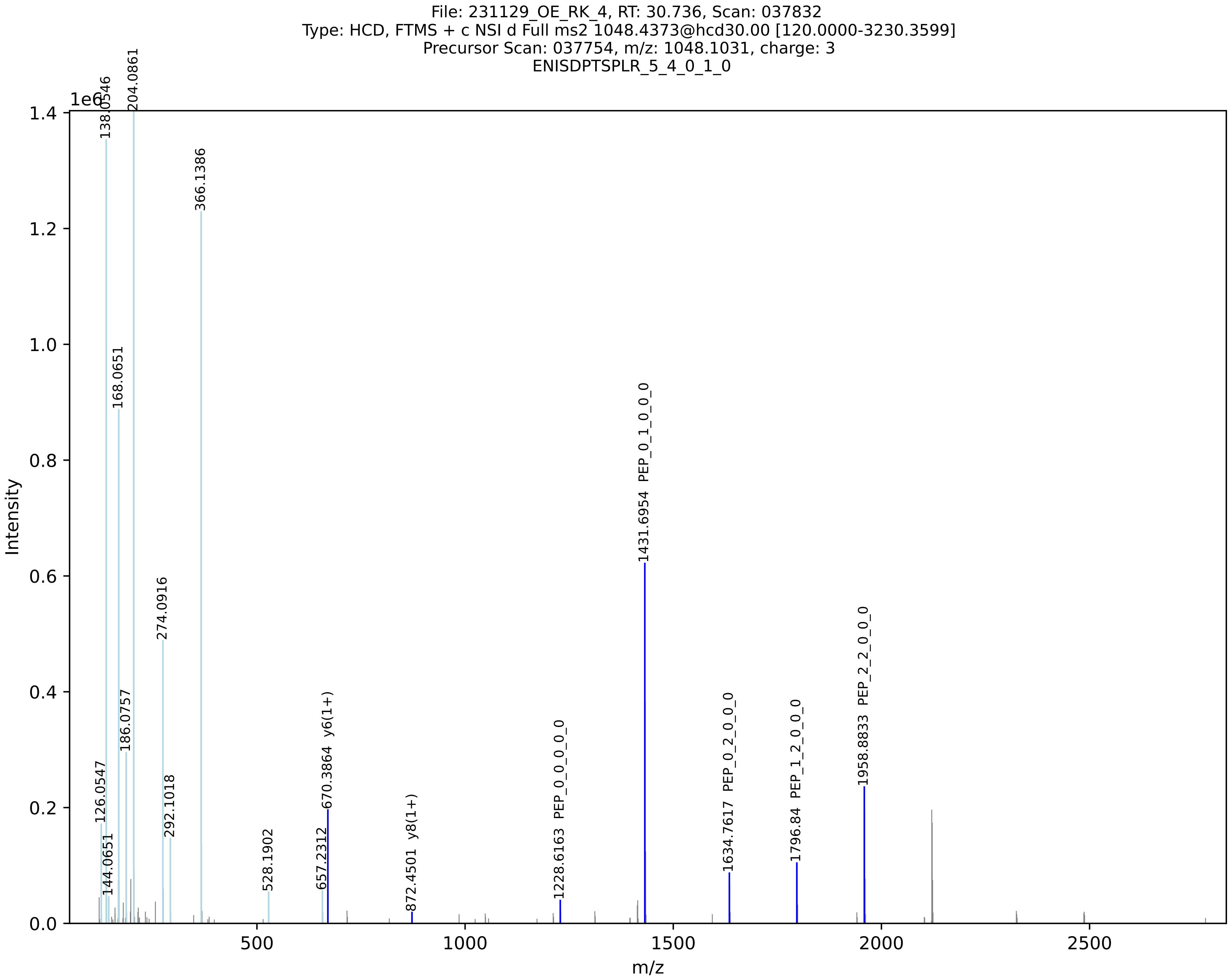
File: 231129_OE_RK_4, RT: 30.736, Scan: 037832
Type: HCD, FTMS + c NSI d Full ms2 1048.4373@hcd30.00 [120.0000-3230.3599]
Precursor Scan: 037754, m/z: 1048.1031, charge: 3
ENISDPTSPLR_5_4_0_1_0
1e6
Intensity
m/z
138.0546
204.0861
366.1386
168.0651
274.0916
186.0757
126.0547
144.0651
292.1018
528.1902
657.2312
670.3864 y6(1+)
872.4501 y8(1+)
1228.6163 PEP_0_0_0_0_0
1431.6954 PEP_0_1_0_0_0
1634.7617 PEP_0_2_0_0_0
1796.84 PEP_1_2_0_0_0
1958.8833 PEP_2_2_0_0_0

File: 231129_OE_RK_4, RT: 30.738, Scan: 037835
Type: HCD, FTMS + c NSI d Full ms2 1094.2112@hcd30.00 [120.0000-4487.2373]
Precursor Scan: 037754, m/z: 1093.709, charge: 4
IPCSQPPQIEHGTINSSR_5_4_1_2_0

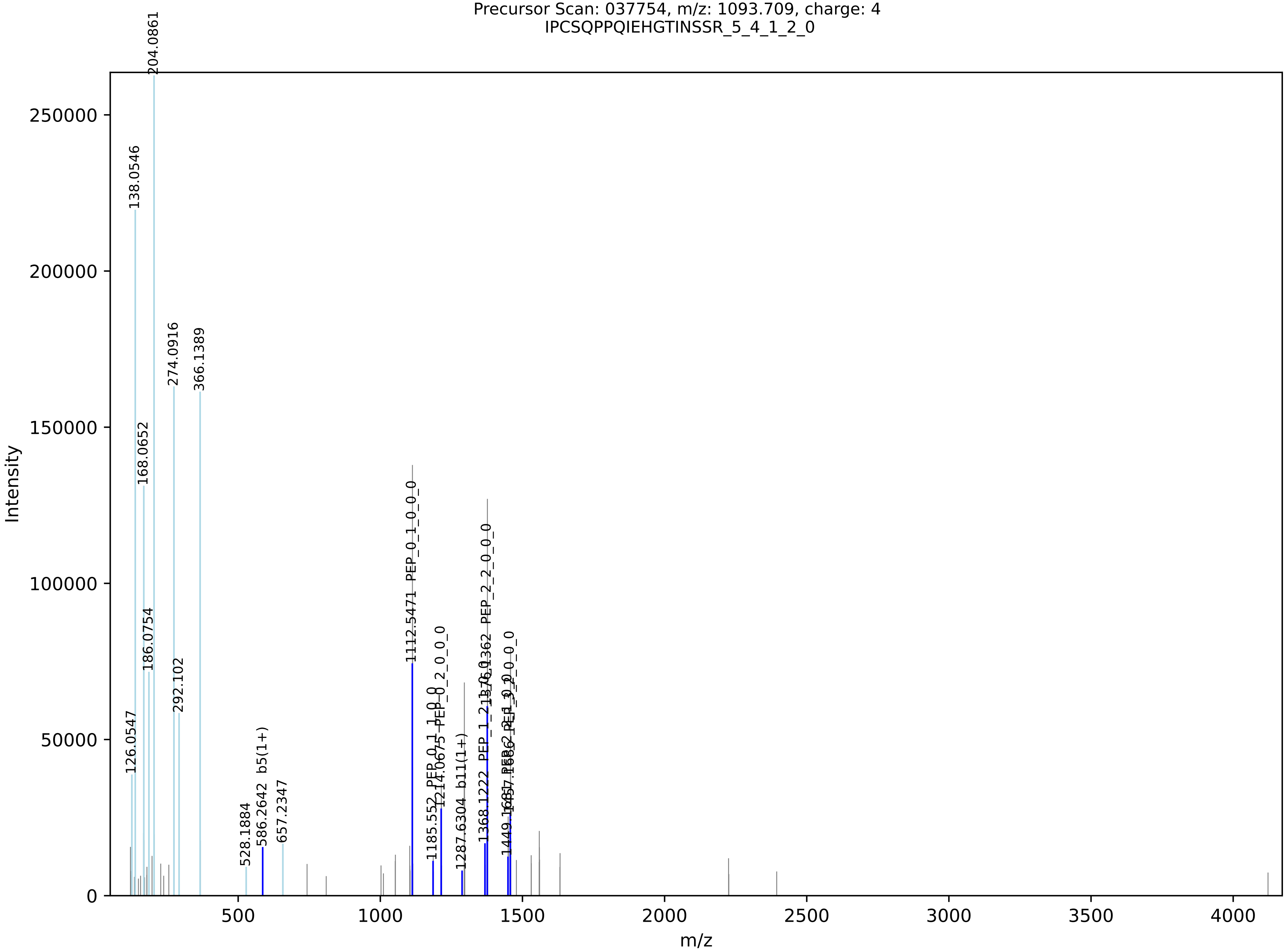

File: 231129_OE_RK_4, RT: 30.786, Scan: 037905
Type: HCD, FTMS + c NSI d Full ms2 1097.1215@hcd30.00 [120.0000-3379.3335]
Precursor Scan: 037828, m/z: 1096.7873, charge: 3
ENISDPTSPLR_5_4_1_1_0

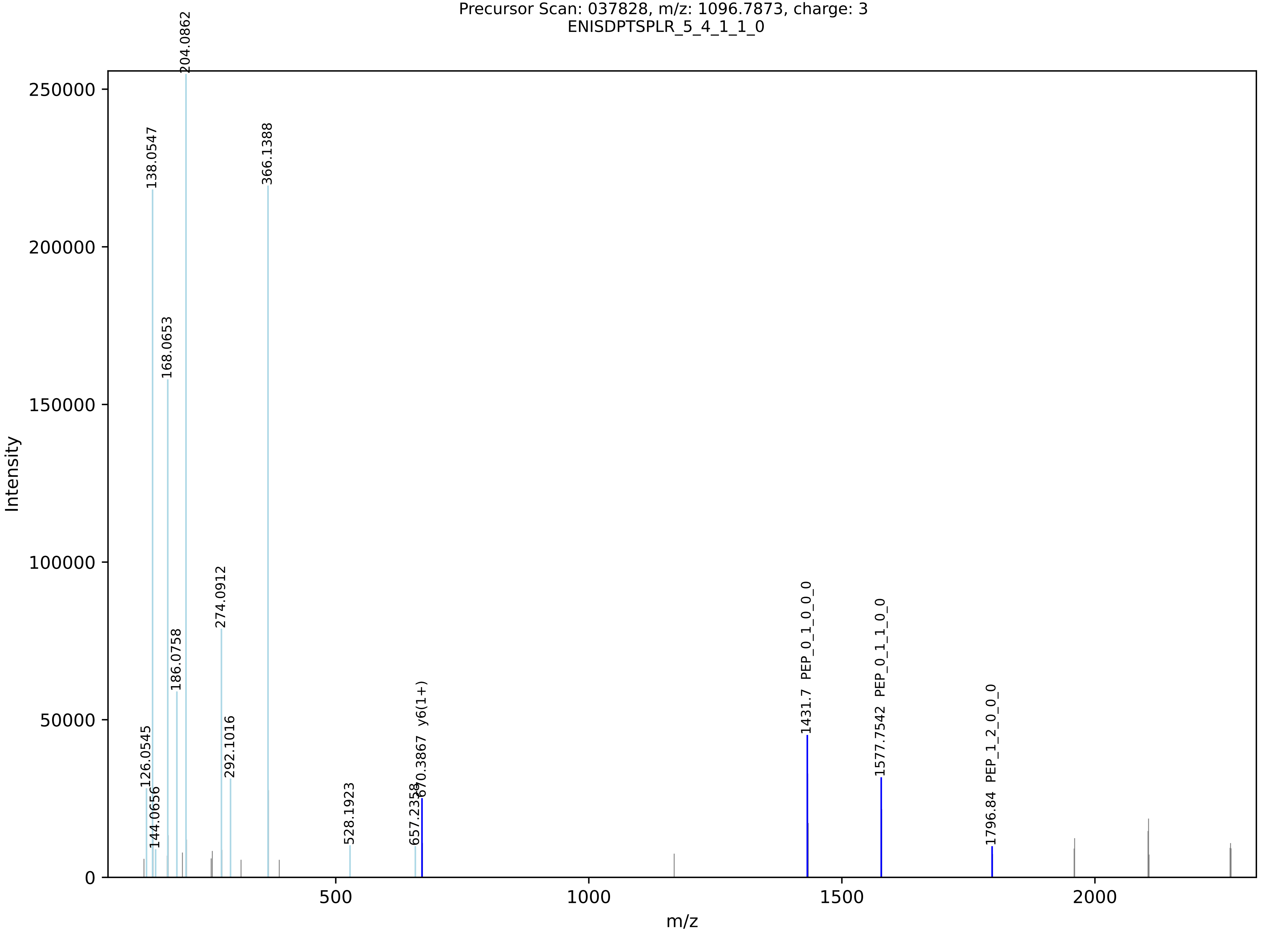

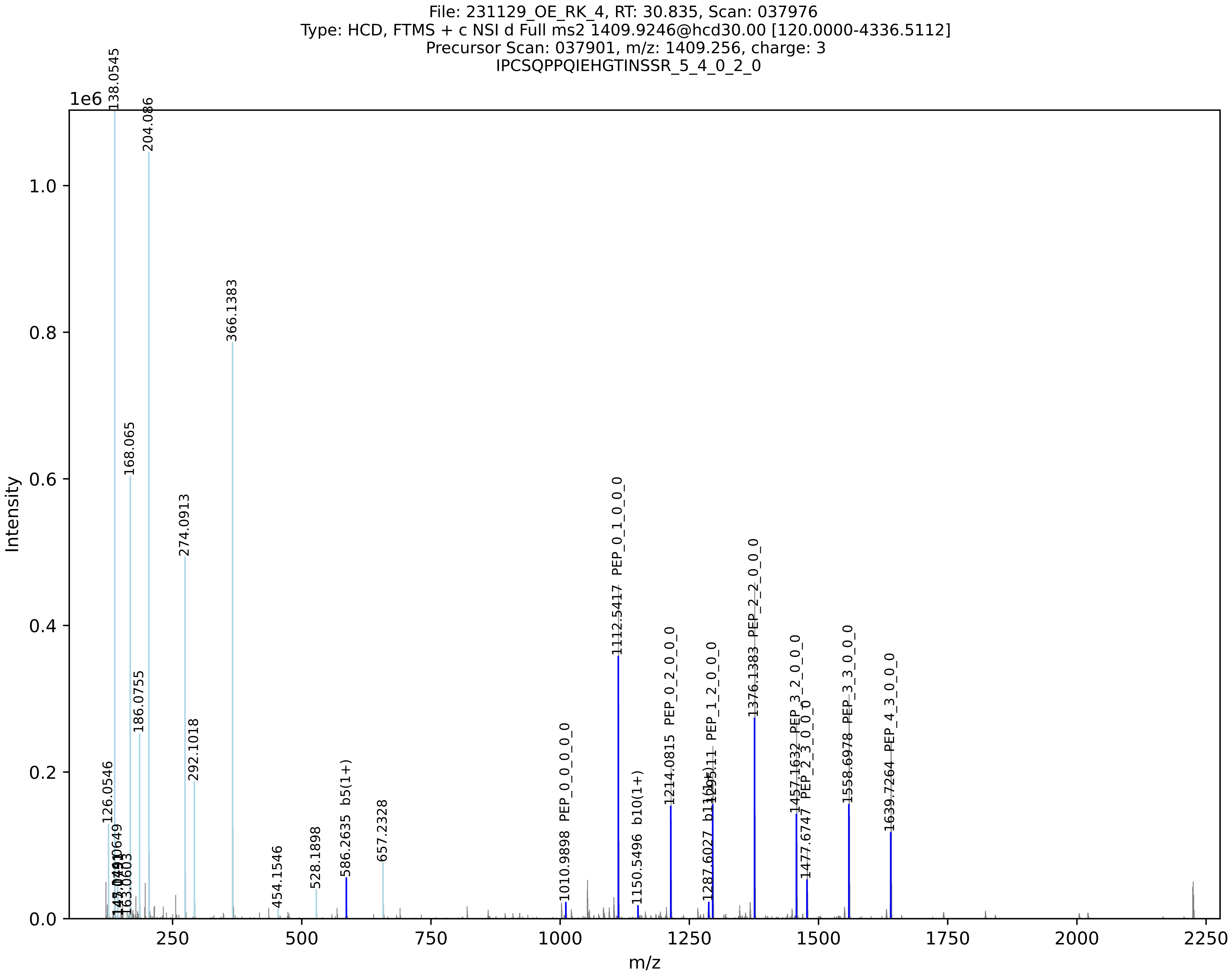

File: 231129_OE_RK_4, RT: 30.835, Scan: 037976
Type: HCD, FTMS + c NSI d Full ms2 1409.9246@hcd30.00 [120.0000-4336.5112]
Precursor Scan: 037901, m/z: 1409.256, charge: 3
IPCSQPPQIEHGTINSSR_5_4_0_2_0
1e6
Intensity
m/z
138.0545
204.086
366.1383
168.065
274.0913
186.0755
292.1018
126.0546
454.1546
528.1898
586.2635 b5(1+)
657.2328
1010.9898 PEP_0_0_0_0_0
1112.5417 PEP_0_1_0_0_0
1150.5496 b10(1+)
1214.0815 PEP_0_2_0_0_0
1287.6027 b11(1+)
1295.11 PEP_1_2_0_0_0
1376.1383 PEP_2_2_0_0_0
1457.1632 PEP_3_2_0_0_0
1477.6747 PEP_2_3_0_0_0
1558.6978 PEP_3_3_0_0_0
1639.7264 PEP_4_3_0_0_0

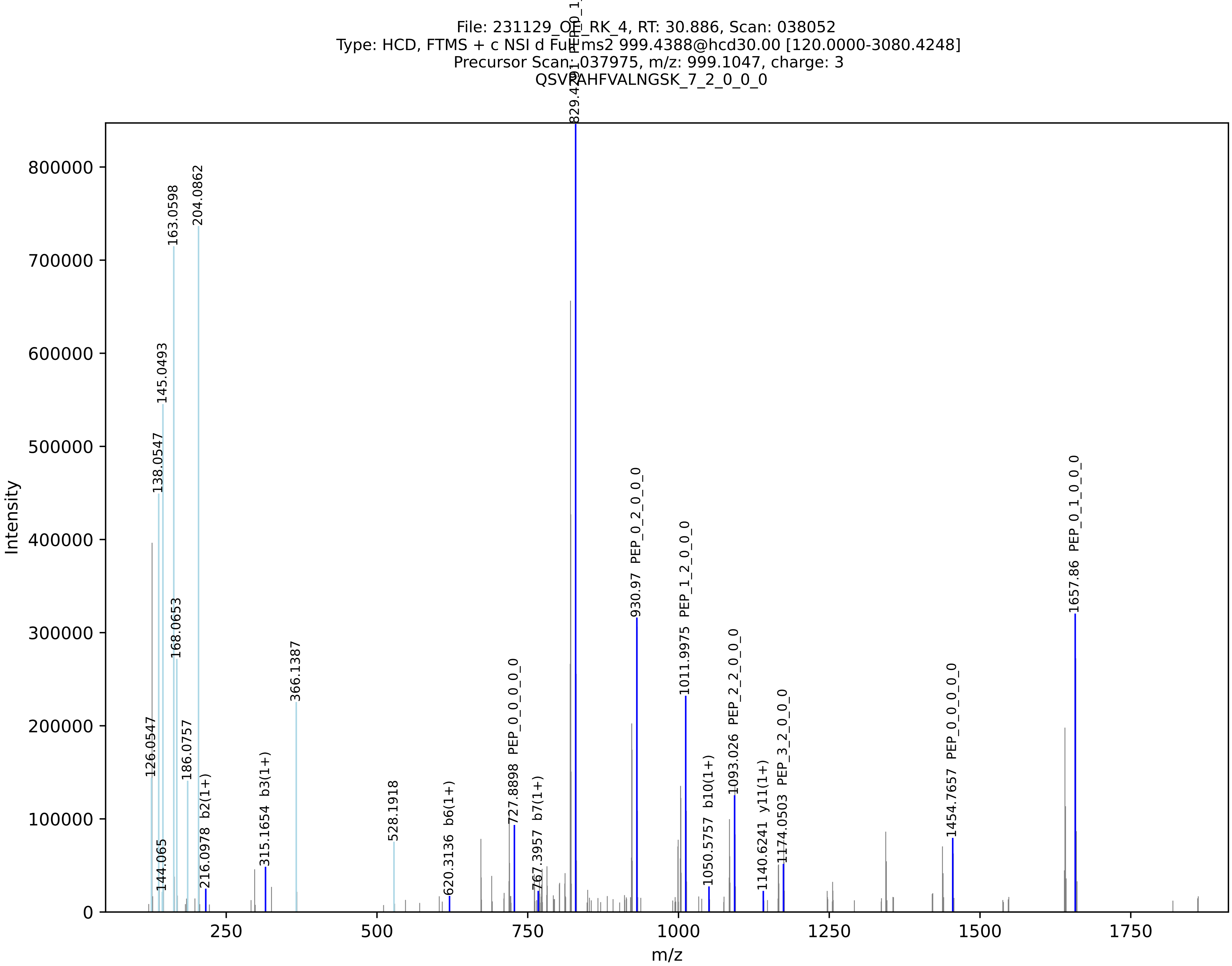
File: 231129_OE_RK_4, RT: 30.886, Scan: 038052
Type: HCD, FTMS + c NSI d Full ms2 999.4388@hcd30.00 [120.0000-3080.4248]
Precursor Scan: 037975, m/z: 999.1047, charge: 3
QSVPAHFVALNGSK_7_2_0_0_0
Intensity
m/z
126.0547
138.0547
144.065
145.0493
163.0598
168.0653
186.0757
204.0862
216.0978 b2(1+)
315.1654 b3(1+)
366.1387
528.1918
620.3136 b6(1+)
727.8898 PEP_0_0_0_0_0
767.3957 b7(1+)
930.97 PEP_0_2_0_0_0
1011.9975 PEP_1_2_0_0_0
1050.5757 b10(1+)
1093.026 PEP_2_2_0_0_0
1140.6241 y11(1+)
1174.0503 PEP_3_2_0_0_0
1454.7657 PEP_0_0_0_0_0
1657.86 PEP_0_1_0_0_0

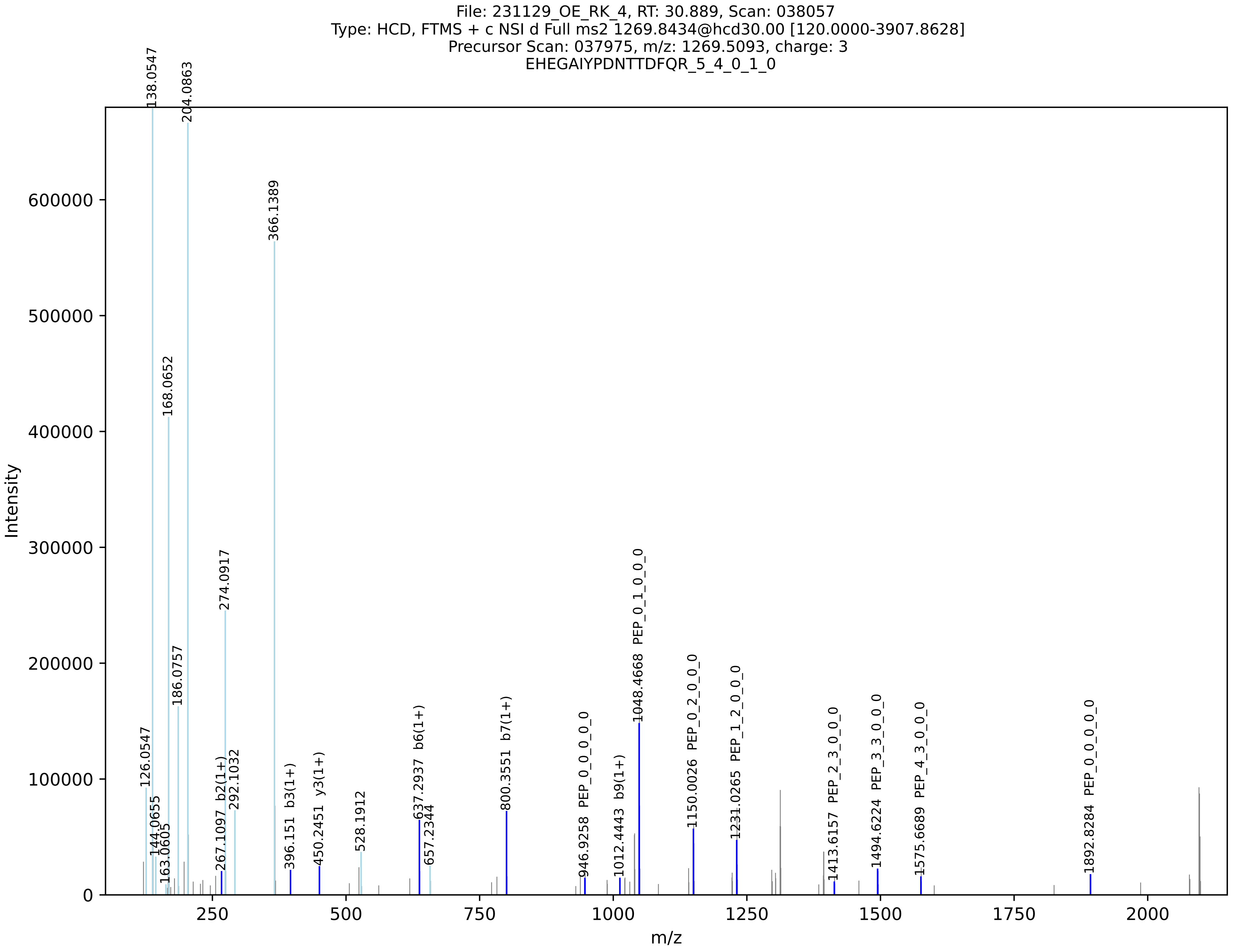

File: 231129_OE_RK_4, RT: 30.889, Scan: 038057
Type: HCD, FTMS + c NSI d Full ms2 1269.8434@hcd30.00 [120.0000-3907.8628]
Precursor Scan: 037975, m/z: 1269.5093, charge: 3
EHEGAIYPDNTTDFQR_5_4_0_1_0
Intensity
m/z
138.0547
204.0863
366.1389
168.0652
274.0917
186.0757
126.0547
144.0655
163.0605
267.1097 b2(1+)
292.1032
396.151 b3(1+)
450.2451 y3(1+)
528.1912
637.2937 b6(1+)
657.2344
800.3551 b7(1+)
946.9258 PEP_0_0_0_0_0
1012.4443 b9(1+)
1048.4668 PEP_0_1_0_0_0
1150.0026 PEP_0_2_0_0_0
1231.0265 PEP_1_2_0_0_0
1413.6157 PEP_2_3_0_0_0
1494.6224 PEP_3_3_0_0_0
1575.6689 PEP_4_3_0_0_0
1892.8284 PEP_0_0_0_0_0

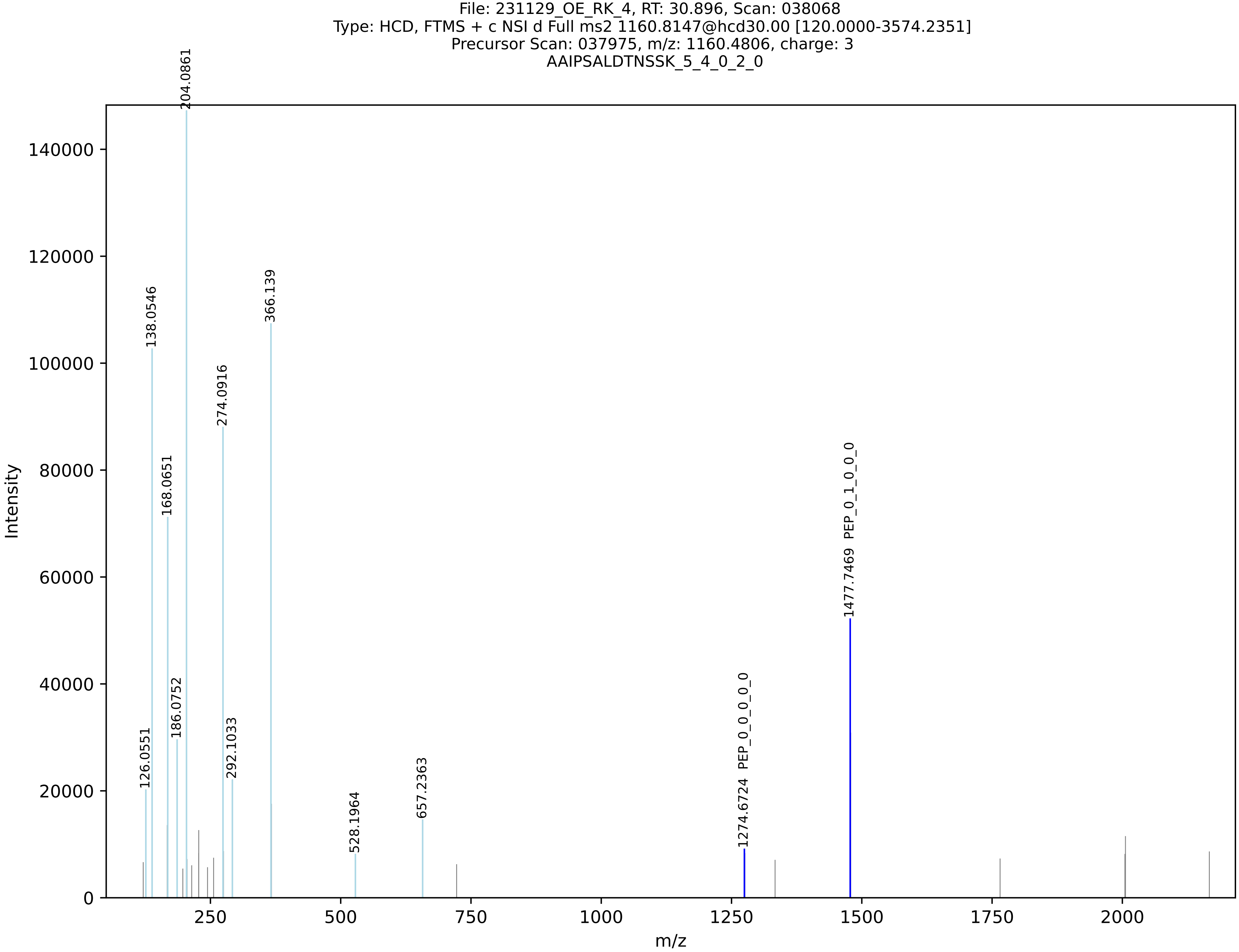

File: 231129_OE_RK_4, RT: 30.896, Scan: 038068
Type: HCD, FTMS + c NSI d Full ms2 1160.8147@hcd30.00 [120.0000-3574.2351]
Precursor Scan: 037975, m/z: 1160.4806, charge: 3
AAIPSALDTNSSK_5_4_0_2_0
204.0861
138.0546
366.139
274.0916
168.0651
186.0752
126.0551
292.1033
528.1964
657.2363
1274.6724 PEP_0_0_0_0_0
1477.7469 PEP_0_1_0_0_0
Intensity
m/z
0
20000
40000
60000
80000
100000
120000
140000
250
500
750
1000
1250
1500
1750
2000

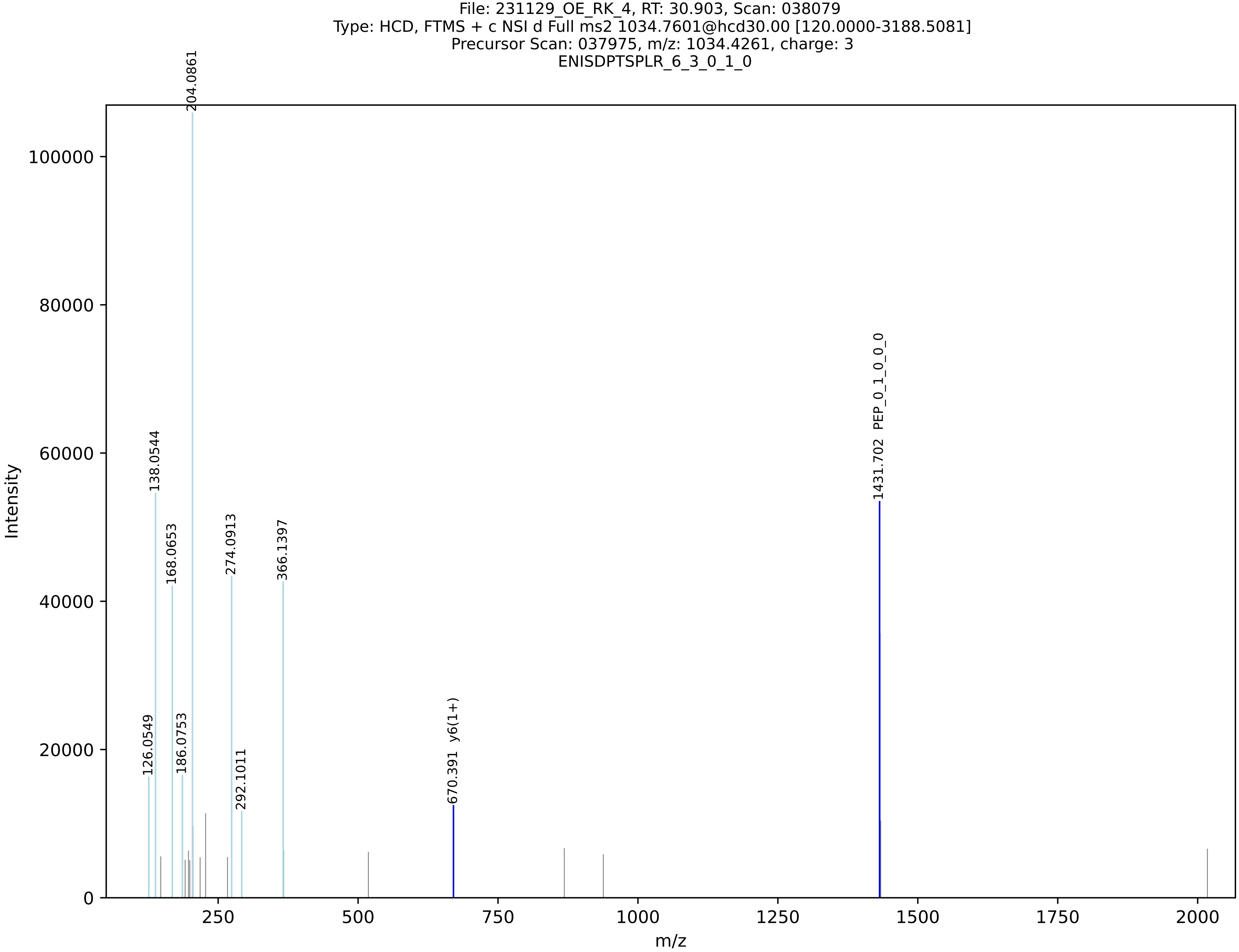
File: 231129_OE_RK_4, RT: 30.903, Scan: 038079
Type: HCD, FTMS + c NSI d Full ms2 1034.7601@hcd30.00 [120.0000-3188.5081]
Precursor Scan: 037975, m/z: 1034.4261, charge: 3
ENISDPTSPLR_6_3_0_1_0
Intensity
m/z
204.0861
138.0544
168.0653
274.0913
366.1397
126.0549
186.0753
292.1011
670.391 y6(1+)
1431.702 PEP_0_1_0_0_0
0
20000
40000
60000
80000
100000
250
500
750
1000
1250
1500
1750
2000

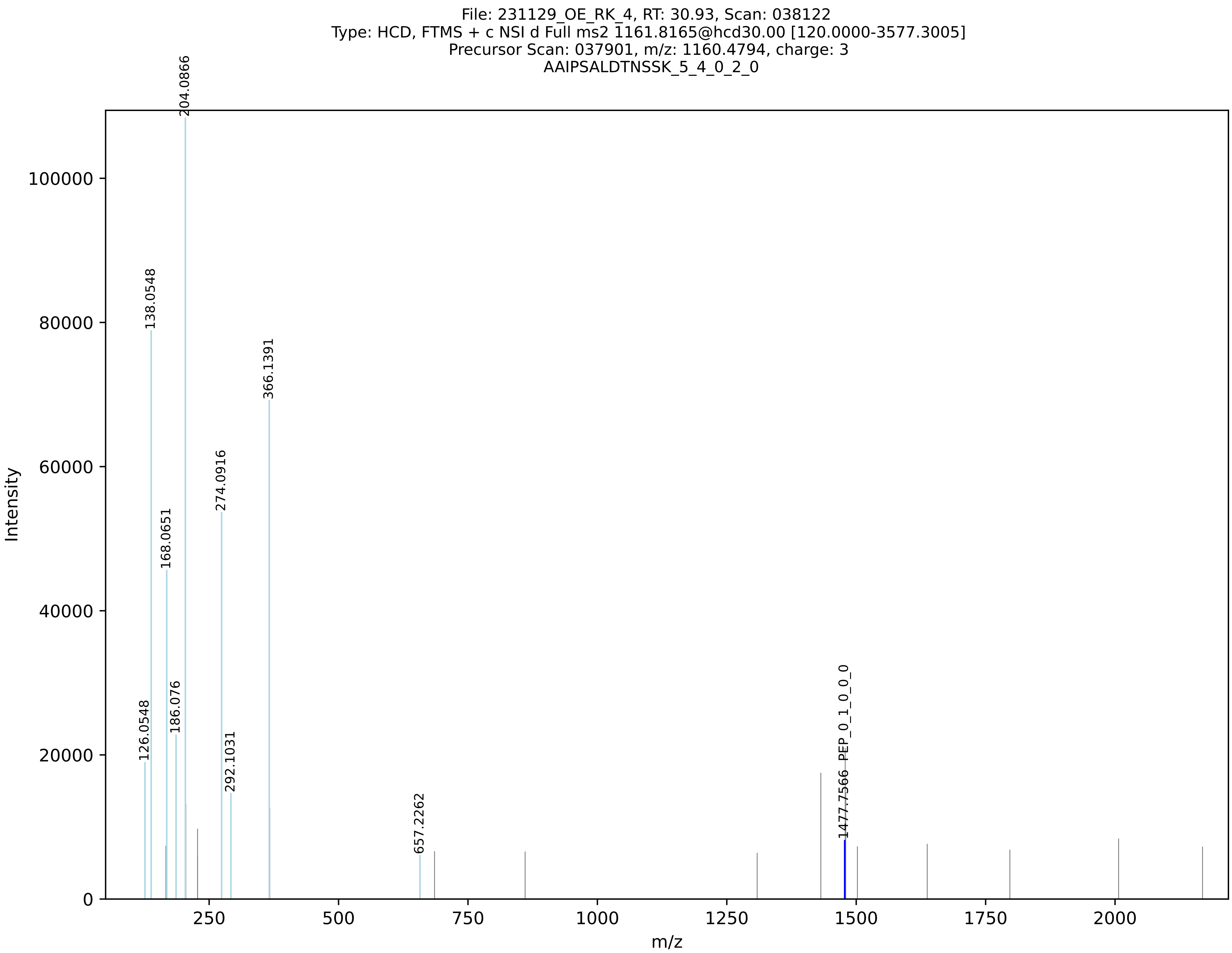
File: 231129_OE_RK_4, RT: 30.93, Scan: 038122
Type: HCD, FTMS + c NSI d Full ms2 1161.8165@hcd30.00 [120.0000-3577.3005]
Precursor Scan: 037901, m/z: 1160.4794, charge: 3
AAIPSALDTNSSK_5_4_0_2_0
Intensity
m/z
204.0866
138.0548
366.1391
274.0916
168.0651
186.076
126.0548
292.1031
657.2262
1477.7566_PEP_0_1_0_0_0
0
20000
40000
60000
80000
100000
250
500
750
1000
1250
1500
1750
2000

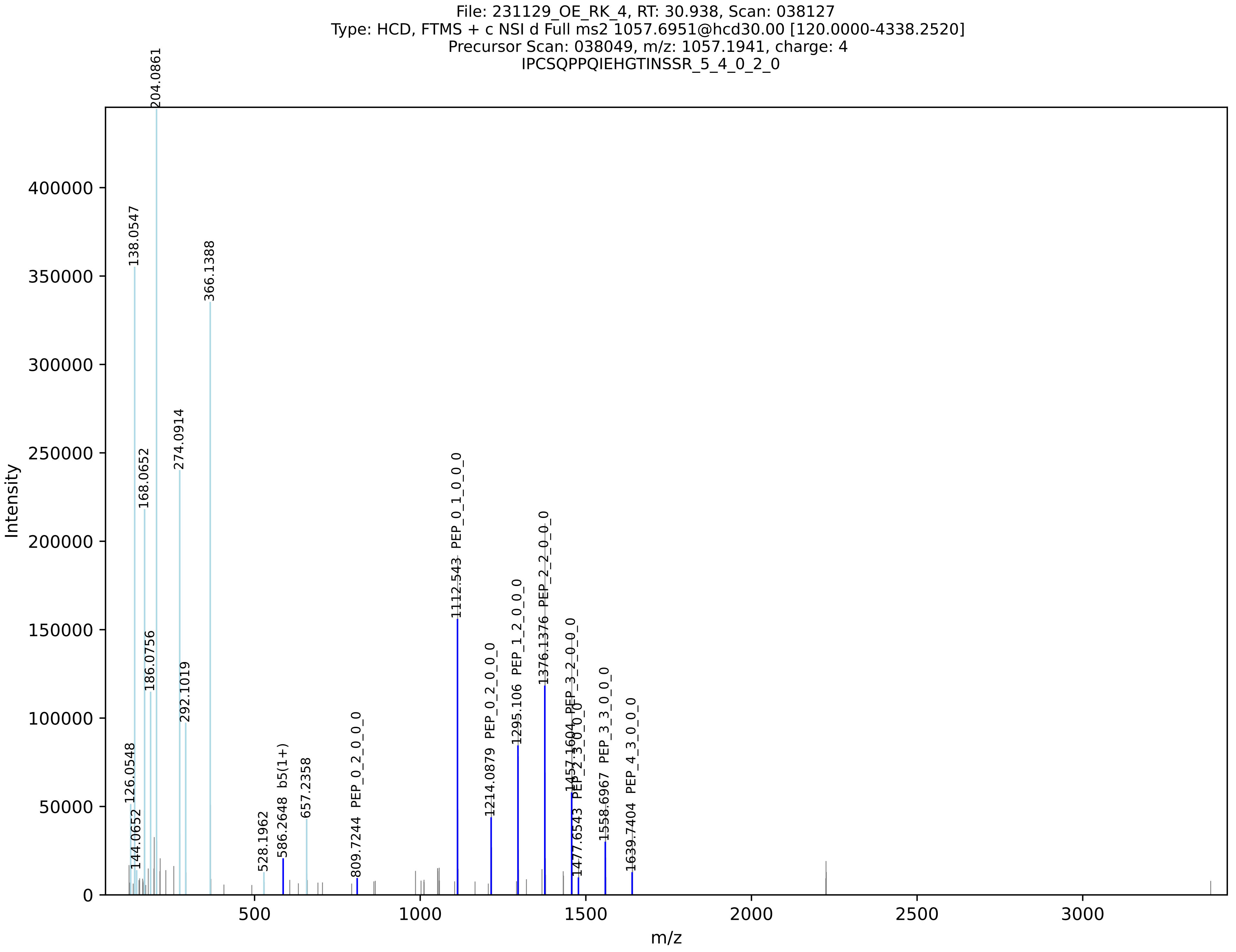
File: 231129_OE_RK_4, RT: 30.938, Scan: 038127
Type: HCD, FTMS + c NSI d Full ms2 1057.6951@hcd30.00 [120.0000-4338.2520]
Precursor Scan: 038049, m/z: 1057.1941, charge: 4
IPCSQPPQIEHGTINSSR_5_4_0_2_0
Intensity
m/z
204.0861
138.0547
366.1388
274.0914
168.0652
186.0756
292.1019
126.0548
144.0652
528.1962
586.2648 b5(1+)
657.2358
809.7244 PEP_0_2_0_0_0
1112.543 PEP_0_1_0_0_0
1214.0879 PEP_0_2_0_0_0
1295.106 PEP_1_2_0_0_0
1376.1376 PEP_2_2_0_0_0
1457.1604 PEP_3_2_0_0_0
1477.6543 PEP_2_3_0_0_0
1558.6967 PEP_3_3_0_0_0
1639.7404 PEP_4_3_0_0_0

File: 231129_OE_RK_4, RT: 31.02, Scan: 038252
Type: HCD, FTMS + c NSI d Full ms2 1035.4270@hcd30.00 [120.0000-3190.5488]
Precursor Scan: 038049, m/z: 1035.0959, charge: 3
ENISDPTSPLR_6_3_0_1_0

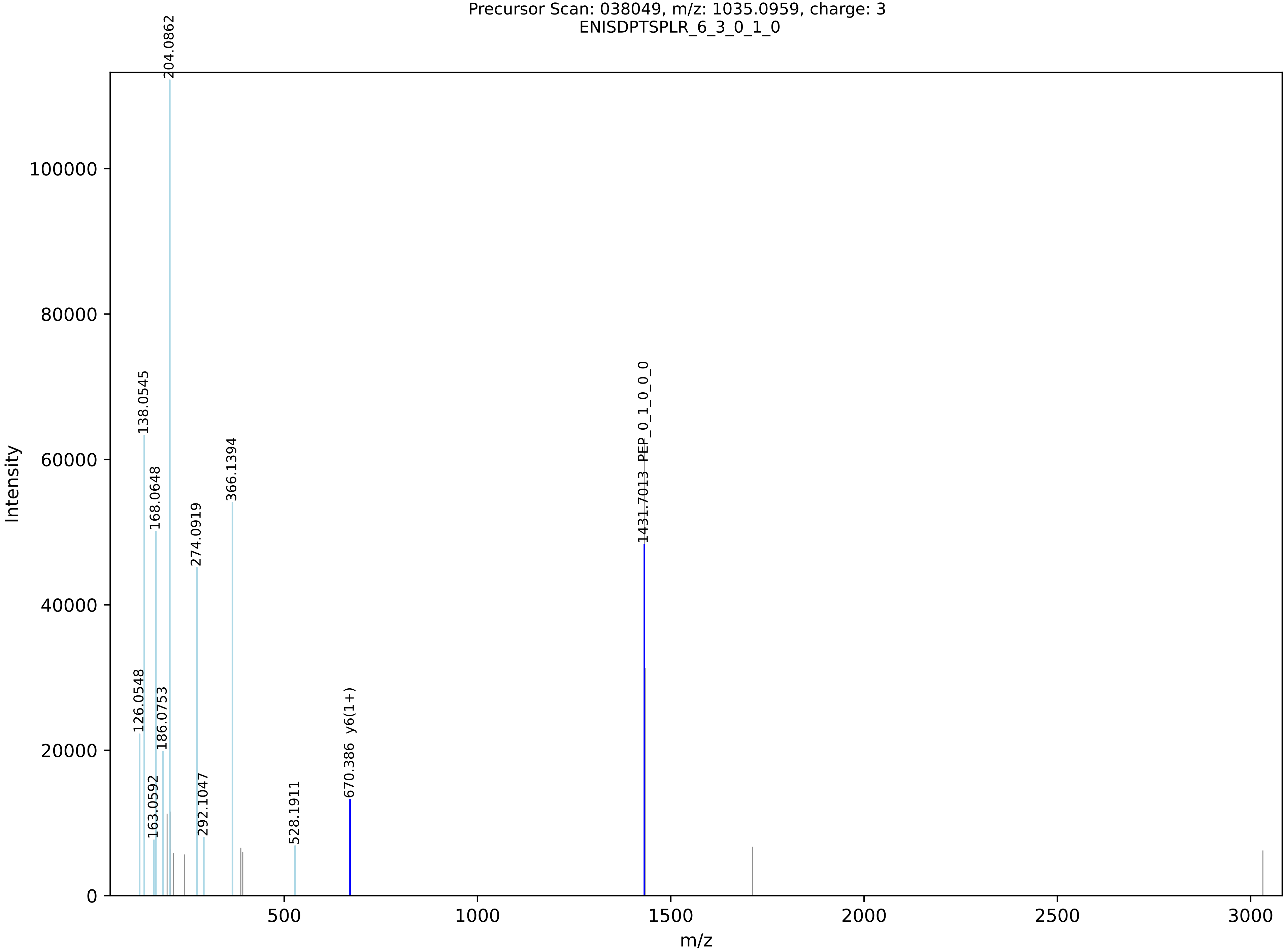

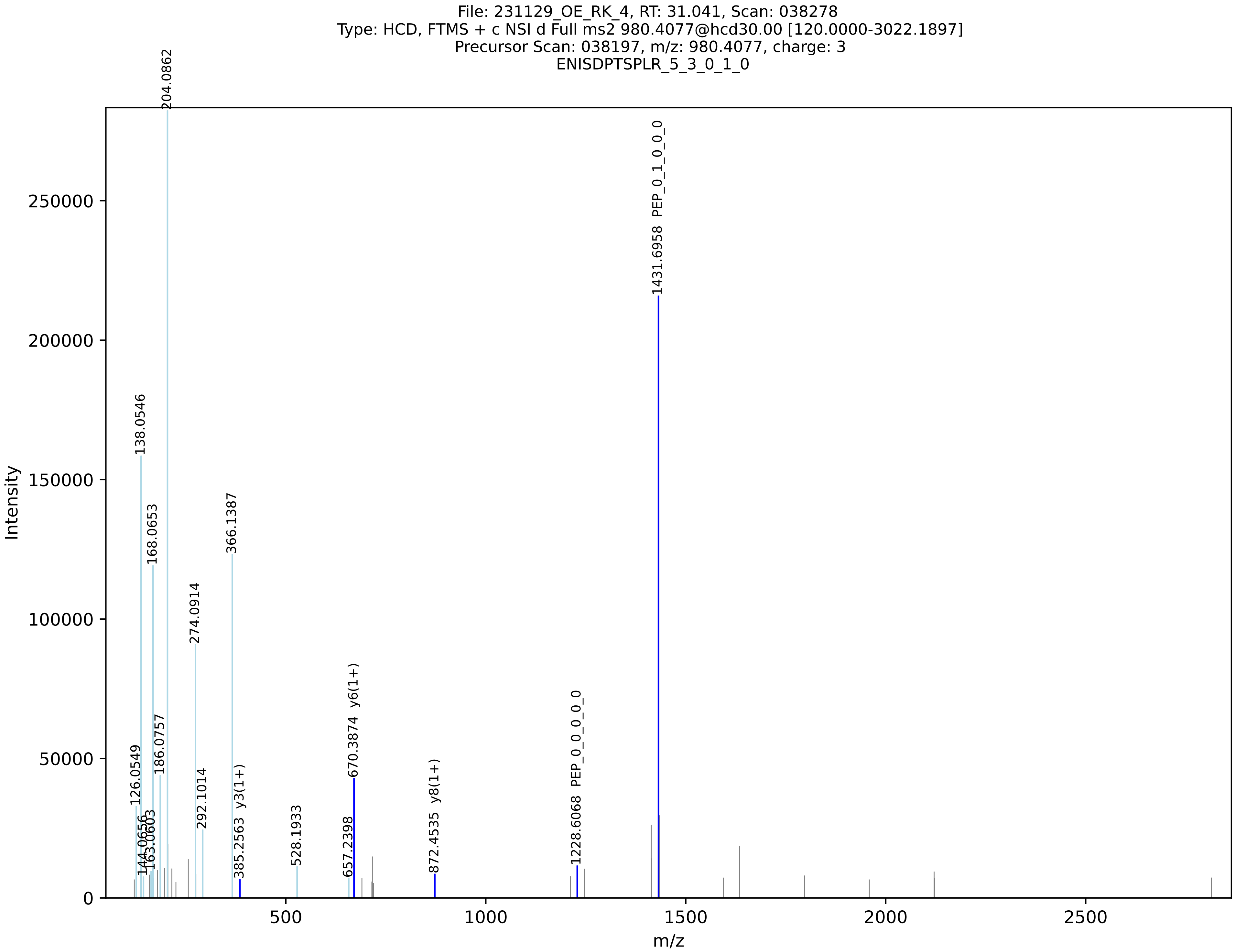
File: 231129_OE_RK_4, RT: 31.041, Scan: 038278
Type: HCD, FTMS + c NSI d Full ms2 980.4077@hcd30.00 [120.0000-3022.1897]
Precursor Scan: 038197, m/z: 980.4077, charge: 3
ENISDPTSPLR_5_3_0_1_0
204.0862
1431.6958 PEP_0_1_0_0_0
138.0546
168.0653
366.1387
274.0914
670.3874 y6(1+)
1228.6068 PEP_0_0_0_0_0
872.4535 y8(1+)
126.0549
186.0757
292.1014
385.2563 y3(1+)
528.1933
657.2398
144.0656
163.0603
Intensity
m/z
250000
200000
150000
100000
50000
0
500
1000
1500
2000
2500

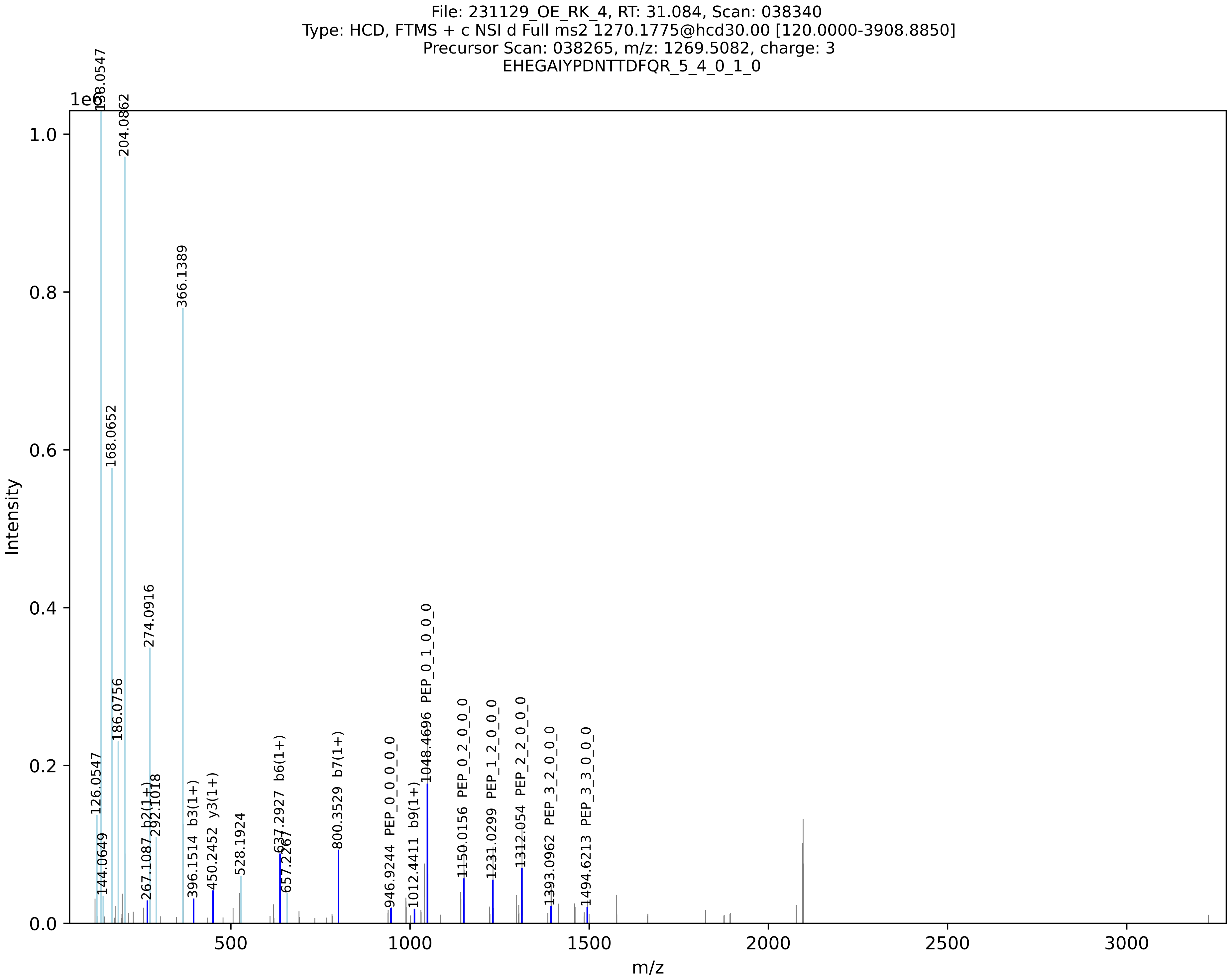
File: 231129_OE_RK_4, RT: 31.084, Scan: 038340
Type: HCD, FTMS + c NSI d Full ms2 1270.1775@hcd30.00 [120.0000-3908.8850]
Precursor Scan: 038265, m/z: 1269.5082, charge: 3
EHEGAIYPDNTTDFQR_5_4_0_1_0
1e6
Intensity
m/z
204.0862
366.1389
168.0652
274.0916
186.0756
126.0547
144.0649
267.1087 b2(1+)
292.1018
396.1514 b3(1+)
450.2452 y3(1+)
528.1924
637.2927 b6(1+)
657.2267
800.3529 b7(1+)
946.9244 PEP_0_0_0_0_0
1012.4411 b9(1+)
1048.4696 PEP_0_1_0_0_0
1150.0156 PEP_0_2_0_0_0
1231.0299 PEP_1_2_0_0_0
1312.054 PEP_2_2_0_0_0
1393.0962 PEP_3_2_0_0_0
1494.6213 PEP_3_3_0_0_0

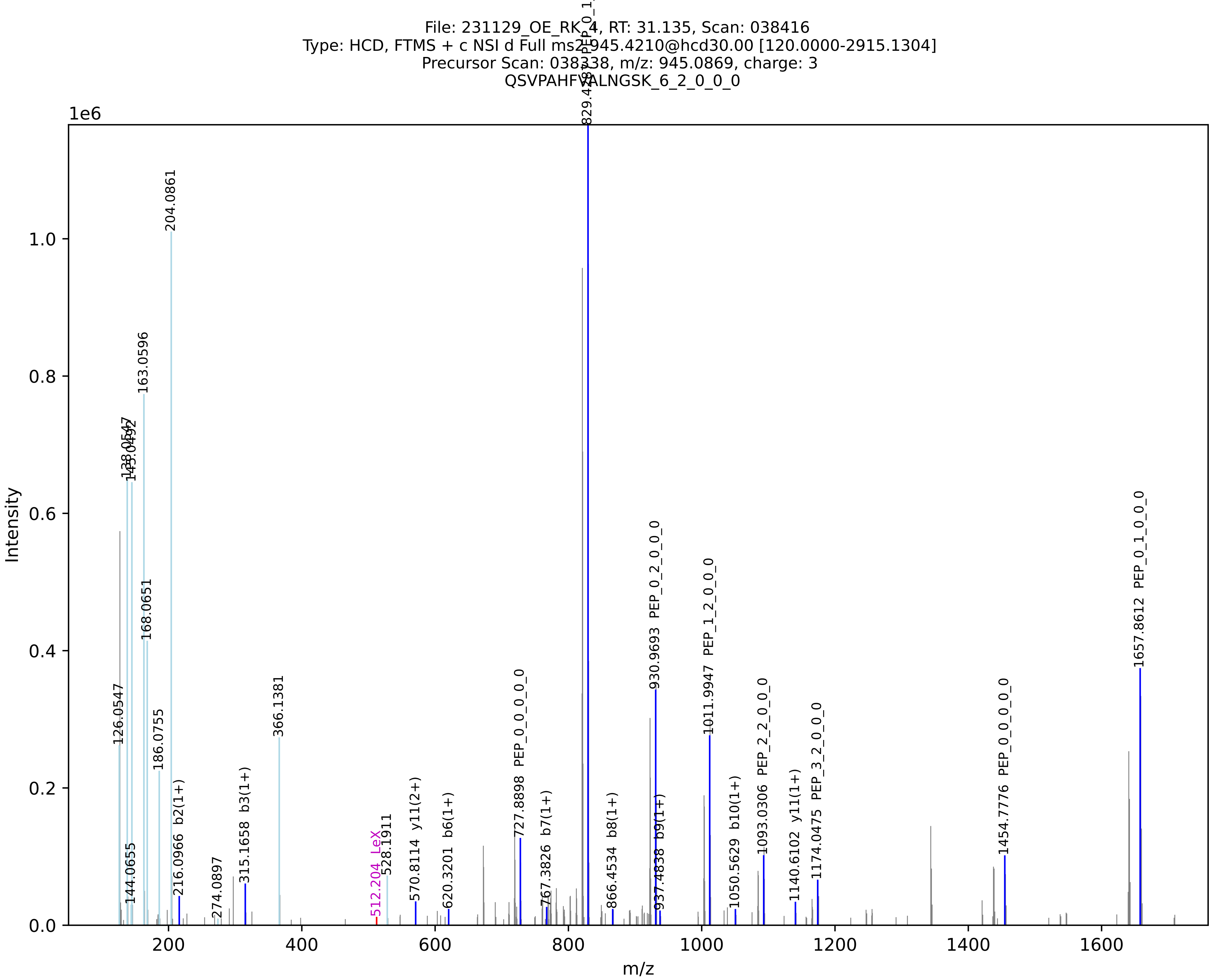
File: 231129_OE_RK_4, RT: 31.135, Scan: 038416
Type: HCD, FTMS + c NSI d Full ms2 945.4210@hcd30.00 [120.0000-2915.1304]
Precursor Scan: 038338, m/z: 945.0869, charge: 3
QSVPAHFVALNGSK_6_2_0_0_0
1e6
Intensity
m/z
204.0861
163.0596
138.0547
145.0492
168.0651
126.0547
186.0755
144.0655
216.0966 b2(1+)
274.0897
315.1658 b3(1+)
366.1381
512.204_LeX
528.1911
570.8114 y11(2+)
620.3201 b6(1+)
727.8898 PEP_0_0_0_0_0
767.3826 b7(1+)
866.4534 b8(1+)
930.9693 PEP_0_2_0_0_0
937.4838 b9(1+)
1011.9947 PEP_1_2_0_0_0
1050.5629 b10(1+)
1093.0306 PEP_2_2_0_0_0
1140.6102 y11(1+)
1174.0475 PEP_3_2_0_0_0
1454.7776 PEP_0_0_0_0_0
1657.8612 PEP_0_1_0_0_0

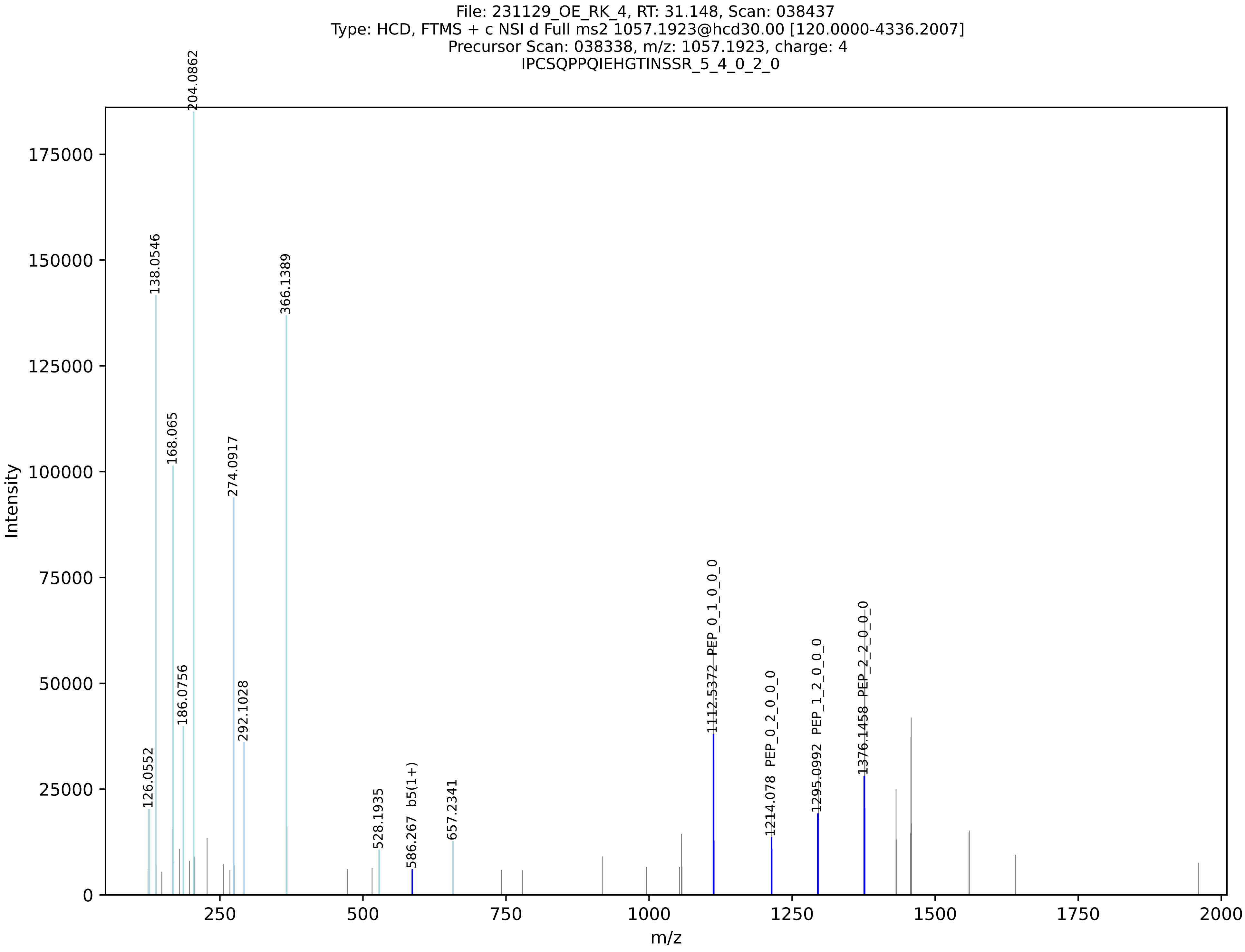
File: 231129_OE_RK_4, RT: 31.148, Scan: 038437
Type: HCD, FTMS + c NSI d Full ms2 1057.1923@hcd30.00 [120.0000-4336.2007]
Precursor Scan: 038338, m/z: 1057.1923, charge: 4
IPCSQPPQIEHGTINSSR_5_4_0_2_0
Intensity
m/z
204.0862
138.0546
366.1389
168.065
274.0917
186.0756
292.1028
126.0552
528.1935
586.267 b5(1+)
657.2341
1112.5372 PEP_0_1_0_0_0
1214.078 PEP_0_2_0_0_0
1295.0992 PEP_1_2_0_0_0
1376.1458 PEP_2_2_0_0_0

File: 231129_OE_RK_4, RT: 31.216, Scan: 038537
Type: HCD, FTMS + c NSI d Full ms2 982.0805@hcd30.00 [120.0000-3027.3083]
Precursor Scan: 038338, m/z: 980.4083, charge: 3
ENISDPTSPLR_5_3_0_1_0

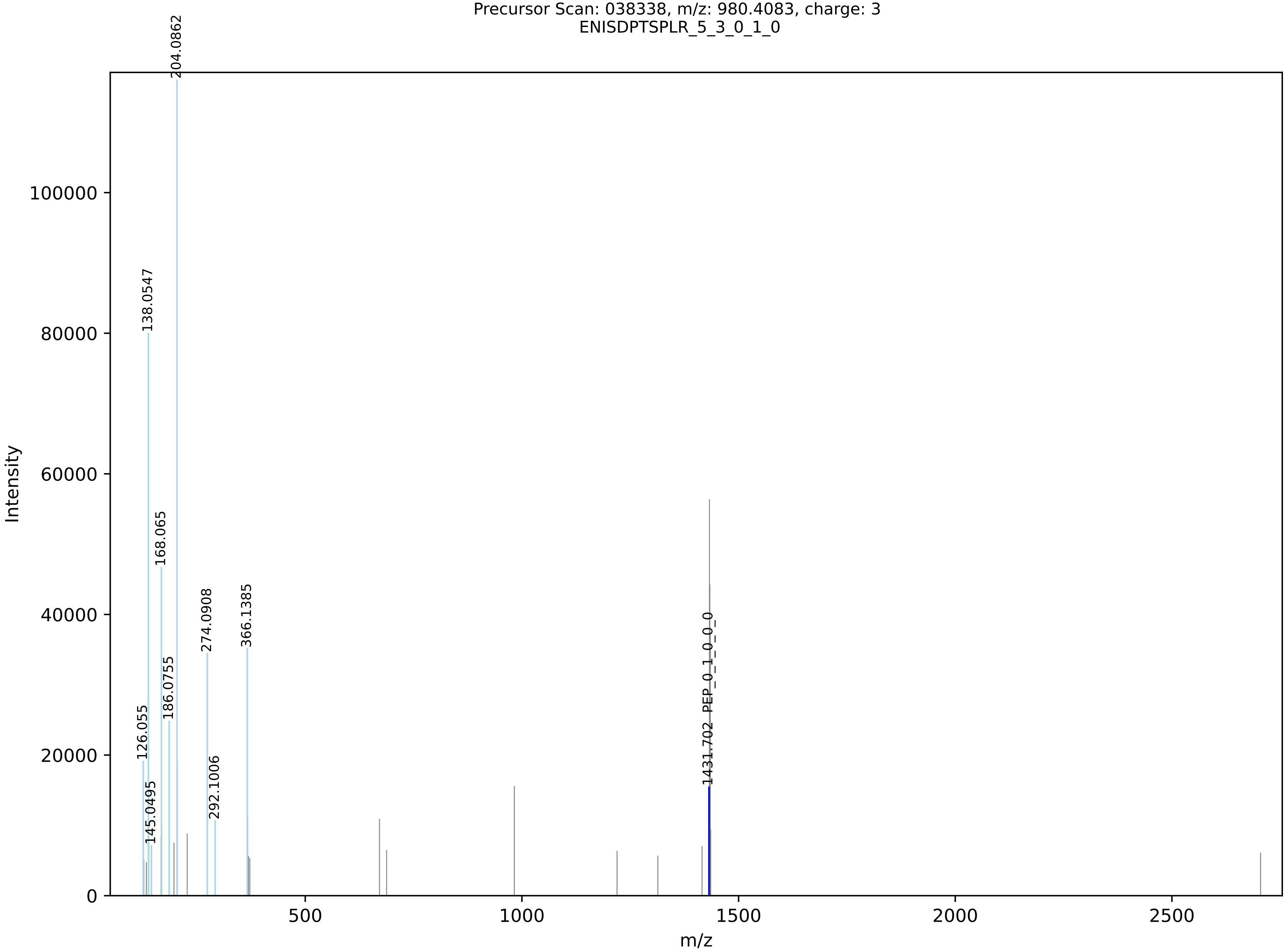

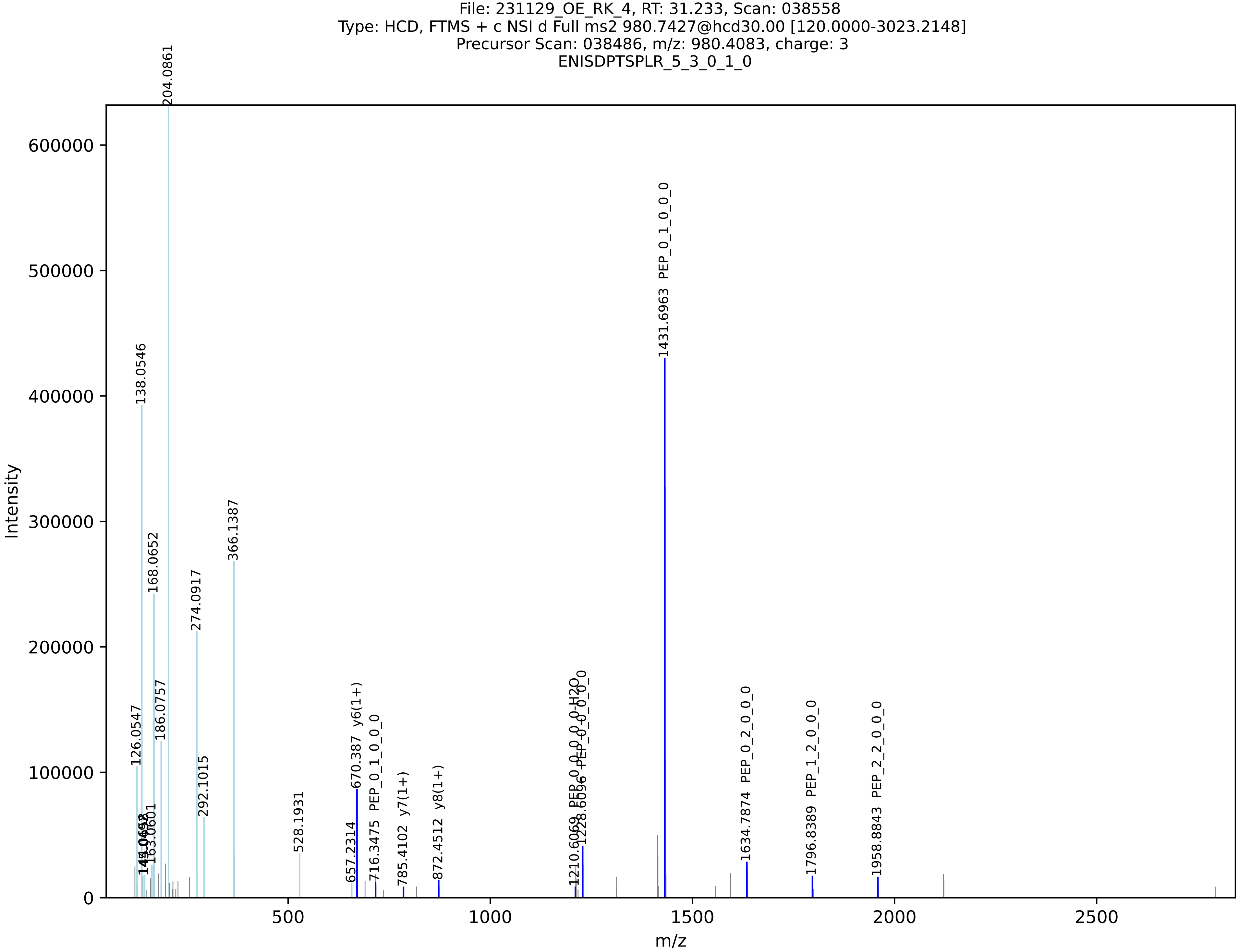
File: 231129_OE_RK_4, RT: 31.233, Scan: 038558
Type: HCD, FTMS + c NSI d Full ms2 980.7427@hcd30.00 [120.0000-3023.2148]
Precursor Scan: 038486, m/z: 980.4083, charge: 3
ENISDPTSPLR_5_3_0_1_0
Intensity
m/z
204.0861
138.0546
168.0652
274.0917
366.1387
186.0757
126.0547
292.1015
163.0601
528.1931
657.2314
670.387 y6(1+)
716.3475 PEP_0_1_0_0_0
785.4102 y7(1+)
872.4512 y8(1+)
1210.6069 PEP_0_0_0_0-H2O
1228.6096 PEP_0_0_0_0
1431.6963 PEP_0_1_0_0_0
1634.7874 PEP_0_2_0_0_0
1796.8389 PEP_1_2_0_0_0
1958.8843 PEP_2_2_0_0_0

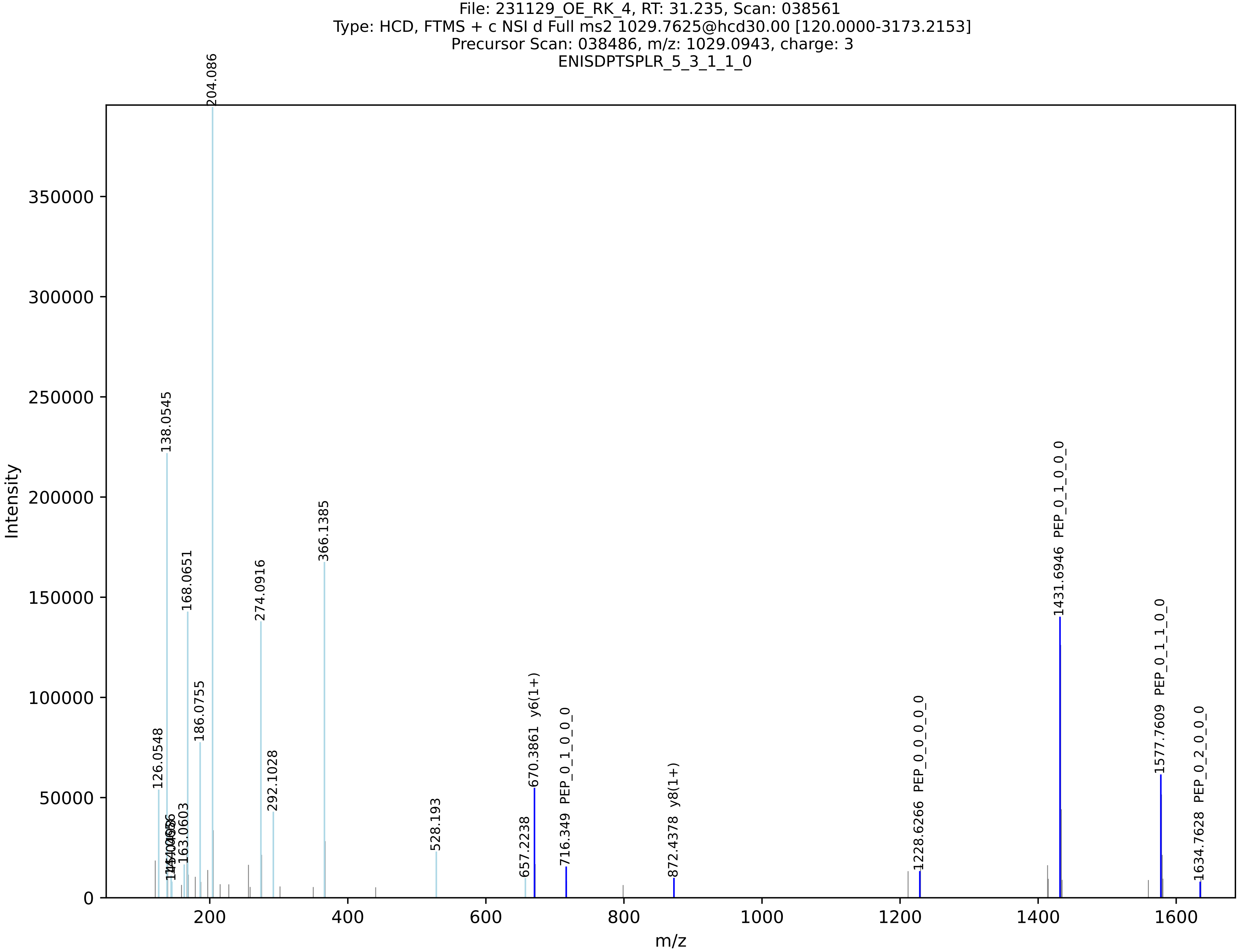
File: 231129_OE_RK_4, RT: 31.235, Scan: 038561
Type: HCD, FTMS + c NSI d Full ms2 1029.7625@hcd30.00 [120.0000-3173.2153]
Precursor Scan: 038486, m/z: 1029.0943, charge: 3
ENISDPTSPLR_5_3_1_1_0
Intensity
m/z
204.086
138.0545
168.0651
186.0755
126.0548
163.0603
274.0916
292.1028
366.1385
528.193
657.2238
670.3861 y6(1+)
716.349 PEP_0_1_0_0_0
872.4378 y8(1+)
1228.6266 PEP_0_0_0_0_0
1431.6946 PEP_0_1_0_0_0
1577.7609 PEP_0_1_1_0_0
1634.7628 PEP_0_2_0_0_0

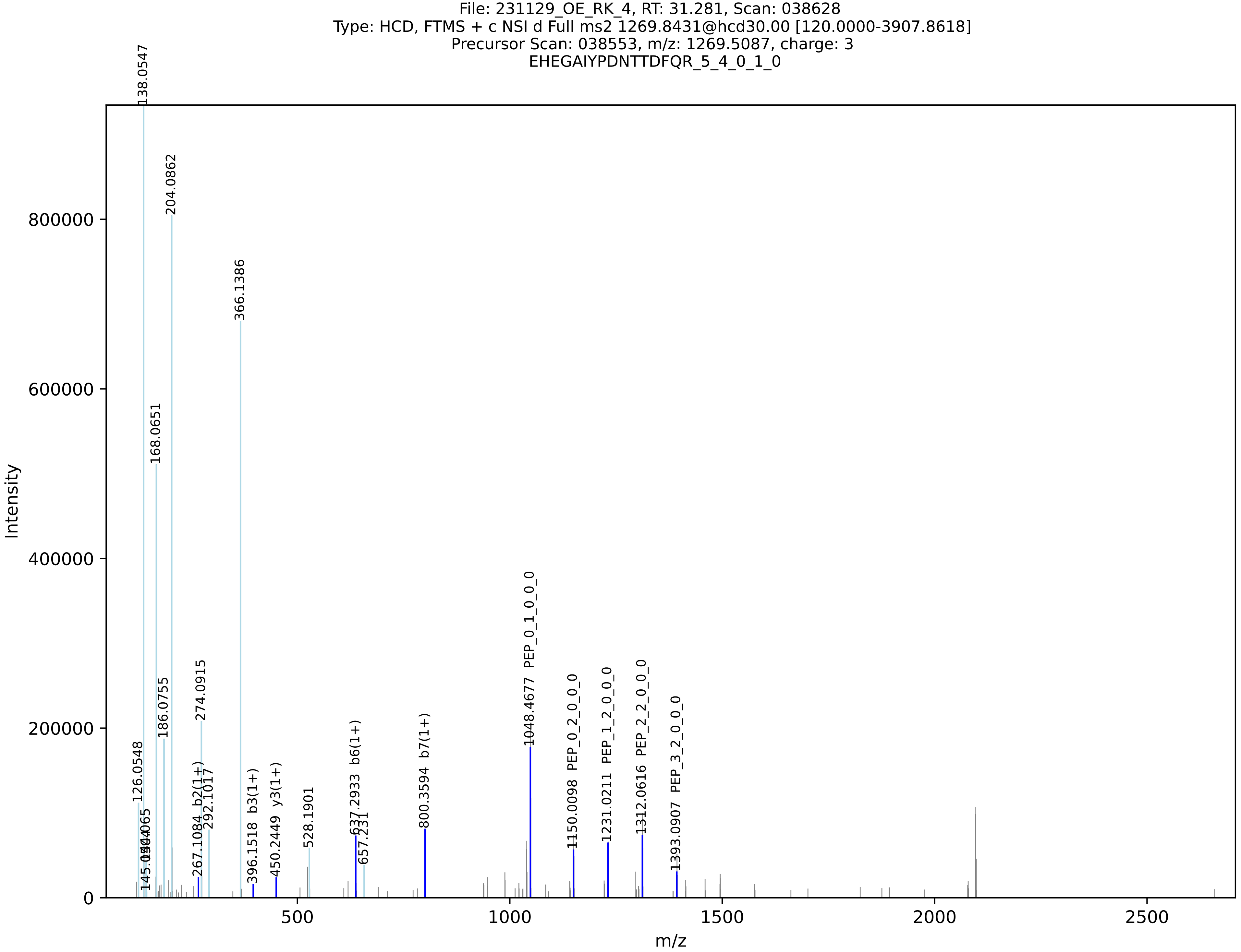
File: 231129_OE_RK_4, RT: 31.281, Scan: 038628
Type: HCD, FTMS + c NSI d Full ms2 1269.8431@hcd30.00 [120.0000-3907.8618]
Precursor Scan: 038553, m/z: 1269.5087, charge: 3
EHEGAIYPDNTTDFQR_5_4_0_1_0
Intensity
m/z
138.0547
204.0862
366.1386
168.0651
186.0755
274.0915
126.0548
267.1084 b2(1+)
292.1017
396.1518 b3(1+)
450.2449 y3(1+)
528.1901
637.2933 b6(1+)
657.231
800.3594 b7(1+)
1048.4677 PEP_0_1_0_0_0
1150.0098 PEP_0_2_0_0_0
1231.0211 PEP_1_2_0_0_0
1312.0616 PEP_2_2_0_0_0
1393.0907 PEP_3_2_0_0_0
0
200000
400000
600000
800000
500
1000
1500
2000
2500

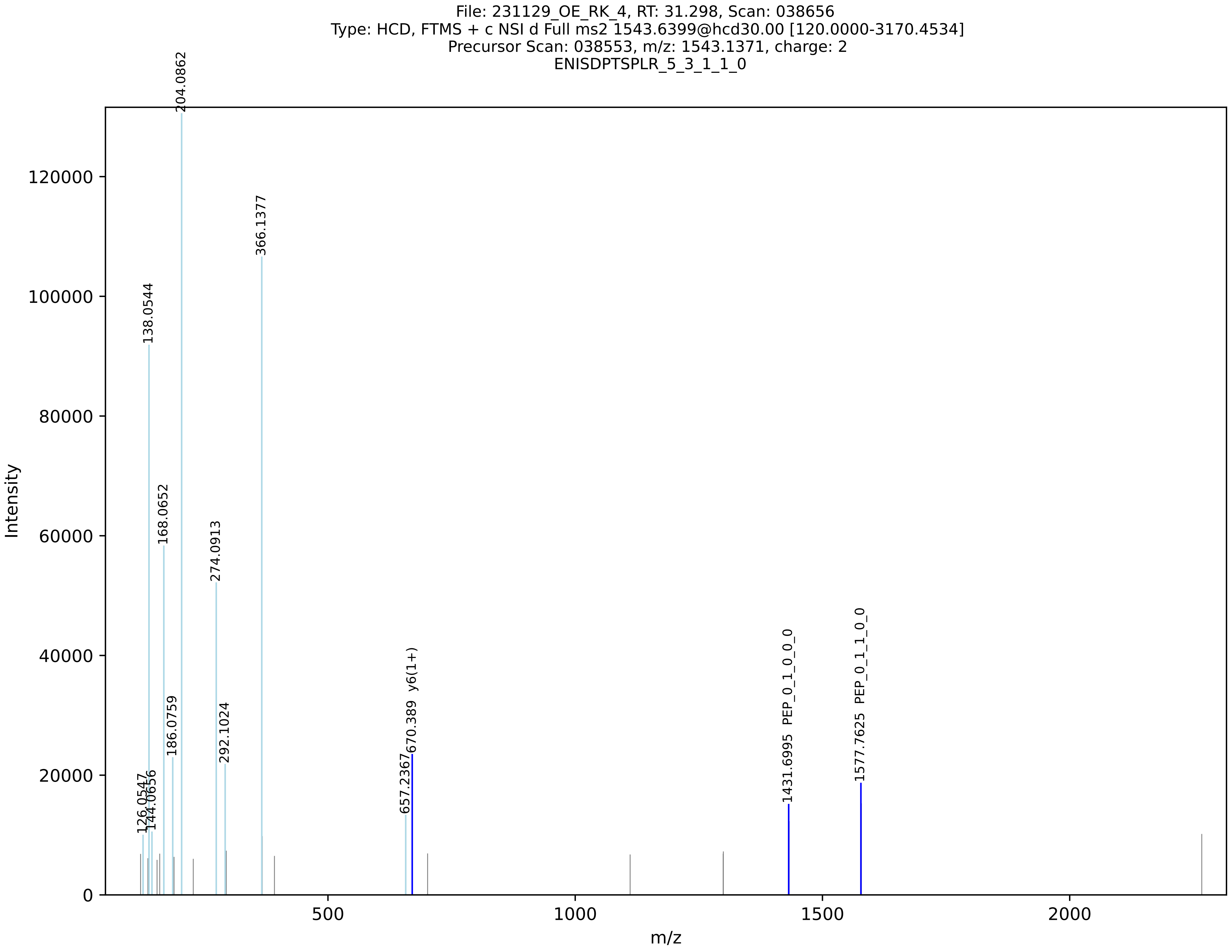

File: 231129_OE_RK_4, RT: 31.298, Scan: 038656
Type: HCD, FTMS + c NSI d Full ms2 1543.6399@hcd30.00 [120.0000-3170.4534]
Precursor Scan: 038553, m/z: 1543.1371, charge: 2
ENISDPTSPLR_5_3_1_1_0
Intensity
m/z
204.0862
366.1377
138.0544
168.0652
274.0913
186.0759
292.1024
126.0547
144.0656
657.2367
670.389 y6(1+)
1431.6995 PEP_0_1_0_0_0
1577.7625 PEP_0_1_1_0_0

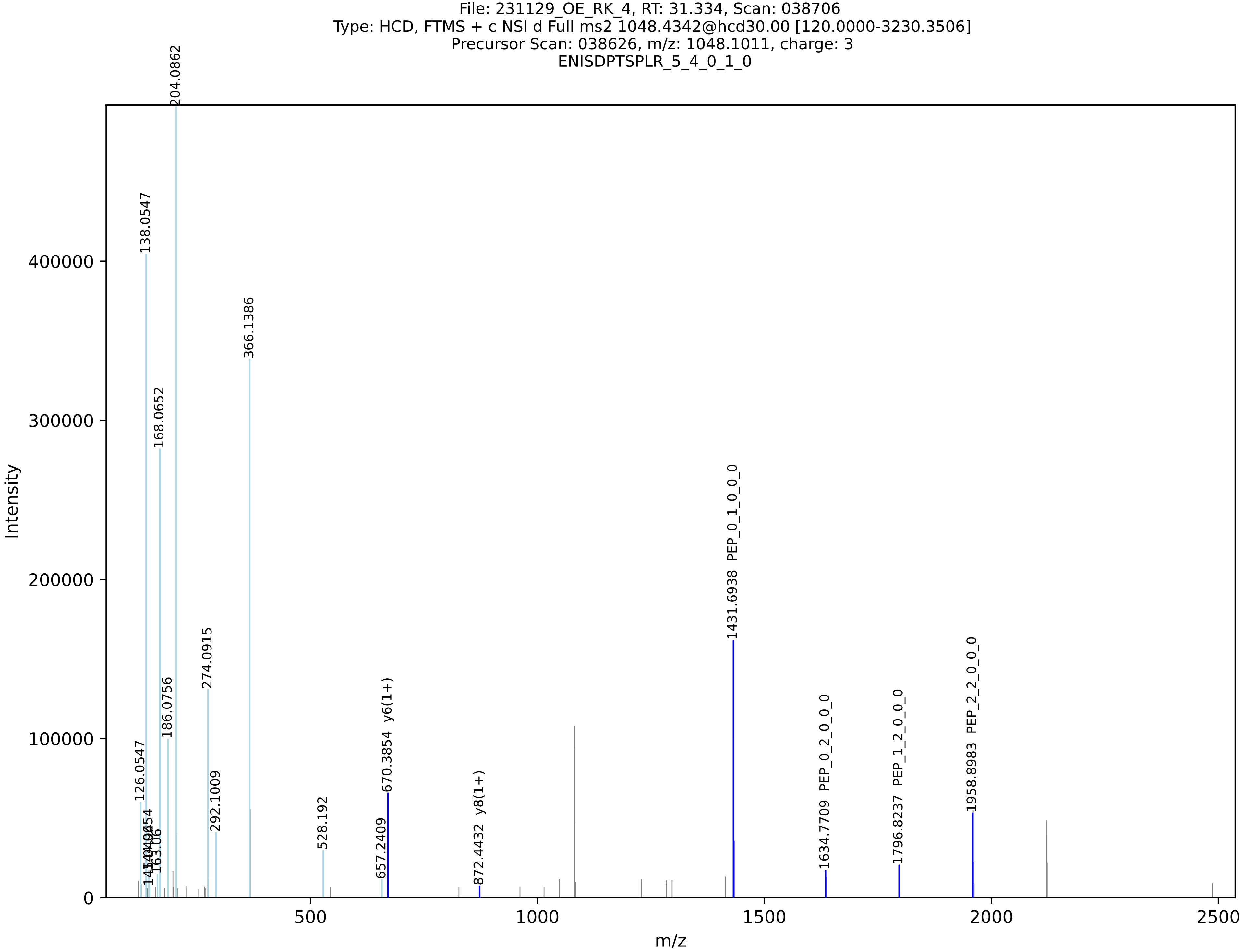
File: 231129_OE_RK_4, RT: 31.334, Scan: 038706
Type: HCD, FTMS + c NSI d Full ms2 1048.4342@hcd30.00 [120.0000-3230.3506]
Precursor Scan: 038626, m/z: 1048.1011, charge: 3
ENISDPTSPLR_5_4_0_1_0
204.0862
138.0547
366.1386
168.0652
1431.6938 PEP_0_1_0_0_0
274.0915
186.0756
670.3854 y6(1+)
1958.8983 PEP_2_2_0_0_0
126.0547
292.1009
528.192
872.4432 y8(1+)
1634.7709 PEP_0_2_0_0_0
1796.8237 PEP_1_2_0_0_0
657.2409
163.06
Intensity
m/z
0
100000
200000
300000
400000
500
1000
1500
2000
2500

File: 231129_OE_RK_4, RT: 31.344, Scan: 038722
Type: HCD, FTMS + c NSI d Full ms2 1161.1484@hcd30.00 [120.0000-3575.2563]
Precursor Scan: 038626, m/z: 1160.4803, charge: 3
AAIPSALDTNSSK_5_4_0_2_0

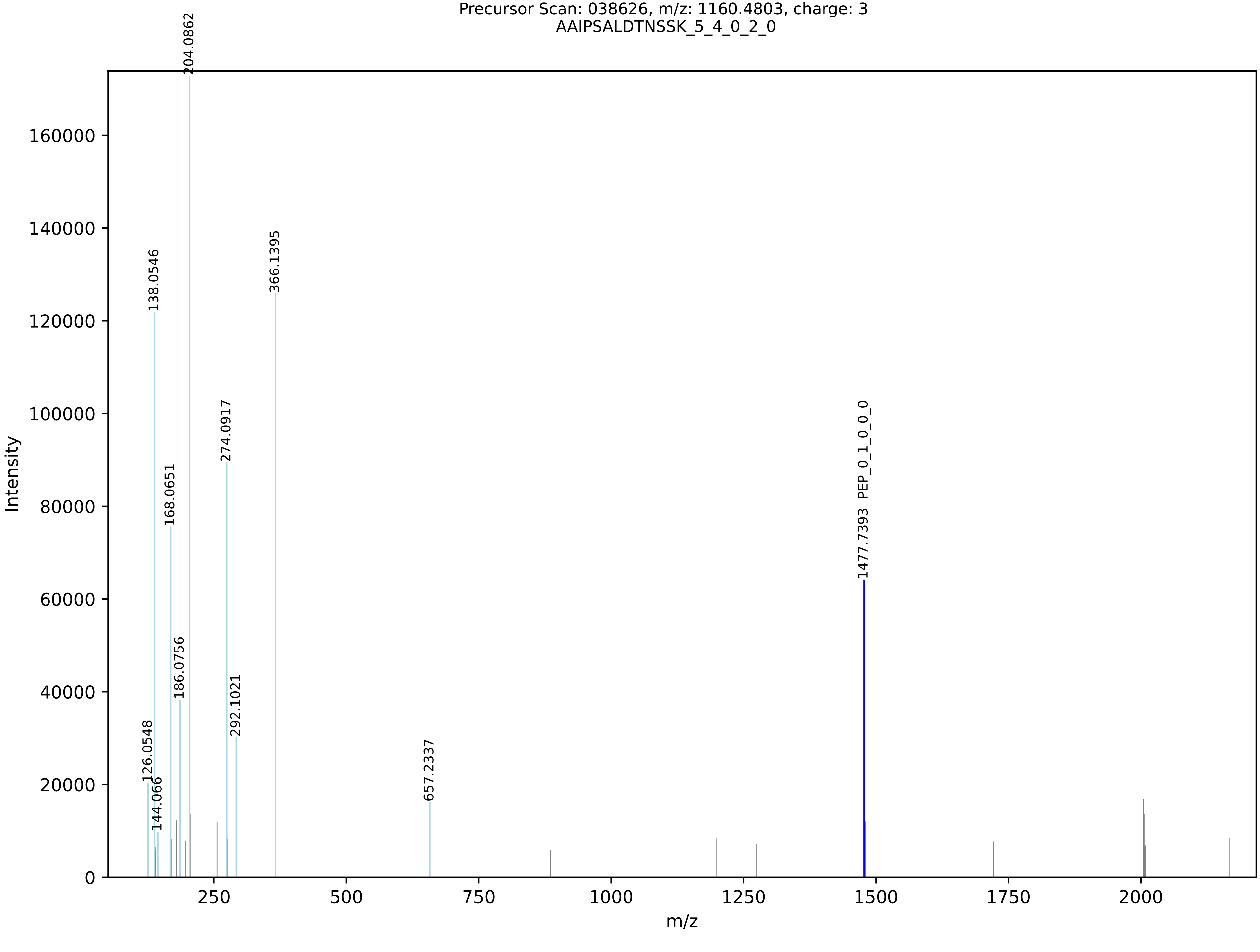

File: 231129_OE_RK_4, RT: 31.398, Scan: 038801
Type: HCD, FTMS + c NSI d Full ms2 1116.1289@hcd30.00 [120.0000-3437.4966]
Precursor Scan: 038700, m/z: 1115.7948, charge: 3
LNAENNATFYFK_5_4_0_1_0

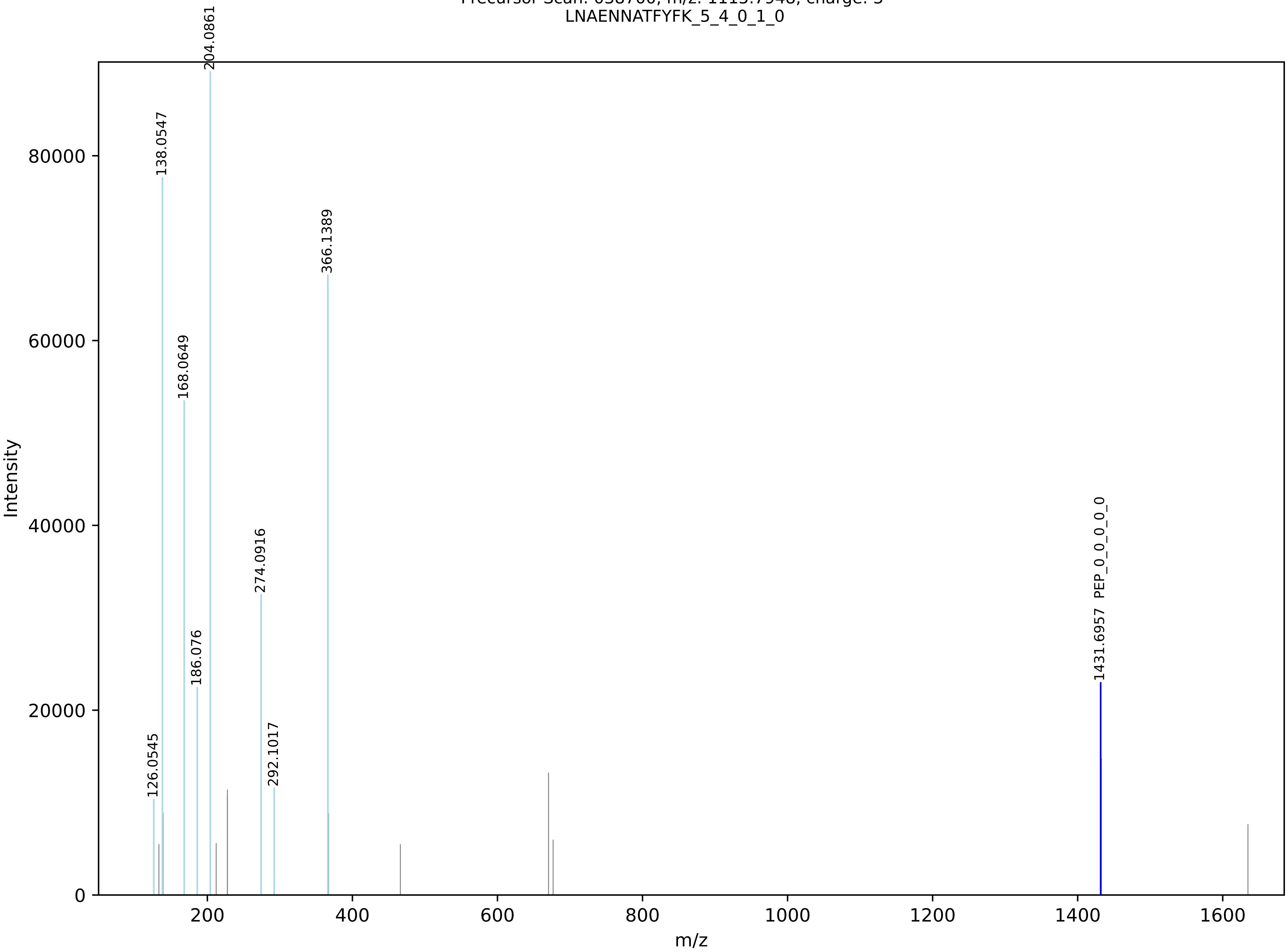

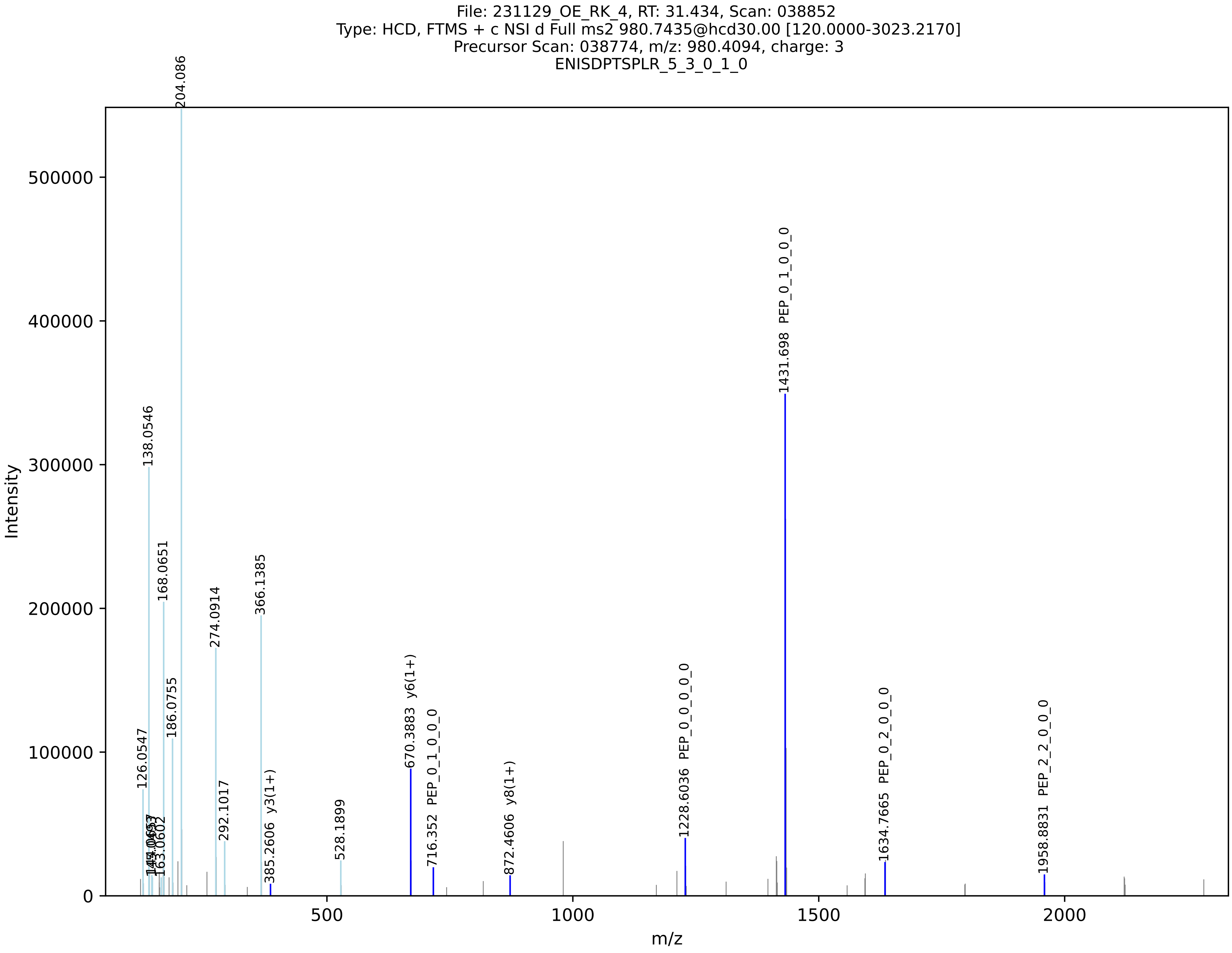
File: 231129_OE_RK_4, RT: 31.434, Scan: 038852
Type: HCD, FTMS + c NSI d Full ms2 980.7435@hcd30.00 [120.0000-3023.2170]
Precursor Scan: 038774, m/z: 980.4094, charge: 3
ENISDPTSPLR_5_3_0_1_0
204.086
138.0546
168.0651
186.0755
126.0547
163.0602
274.0914
292.1017
366.1385
385.2606 y3(1+)
528.1899
670.3883 y6(1+)
716.352 PEP_0_1_0_0_0
872.4606 y8(1+)
1228.6036 PEP_0_0_0_0_0
1431.698 PEP_0_1_0_0_0
1634.7665 PEP_0_2_0_0_0
1958.8831 PEP_2_2_0_0_0
Intensity
0
100000
200000
300000
400000
500000
500
1000
1500
2000
m/z

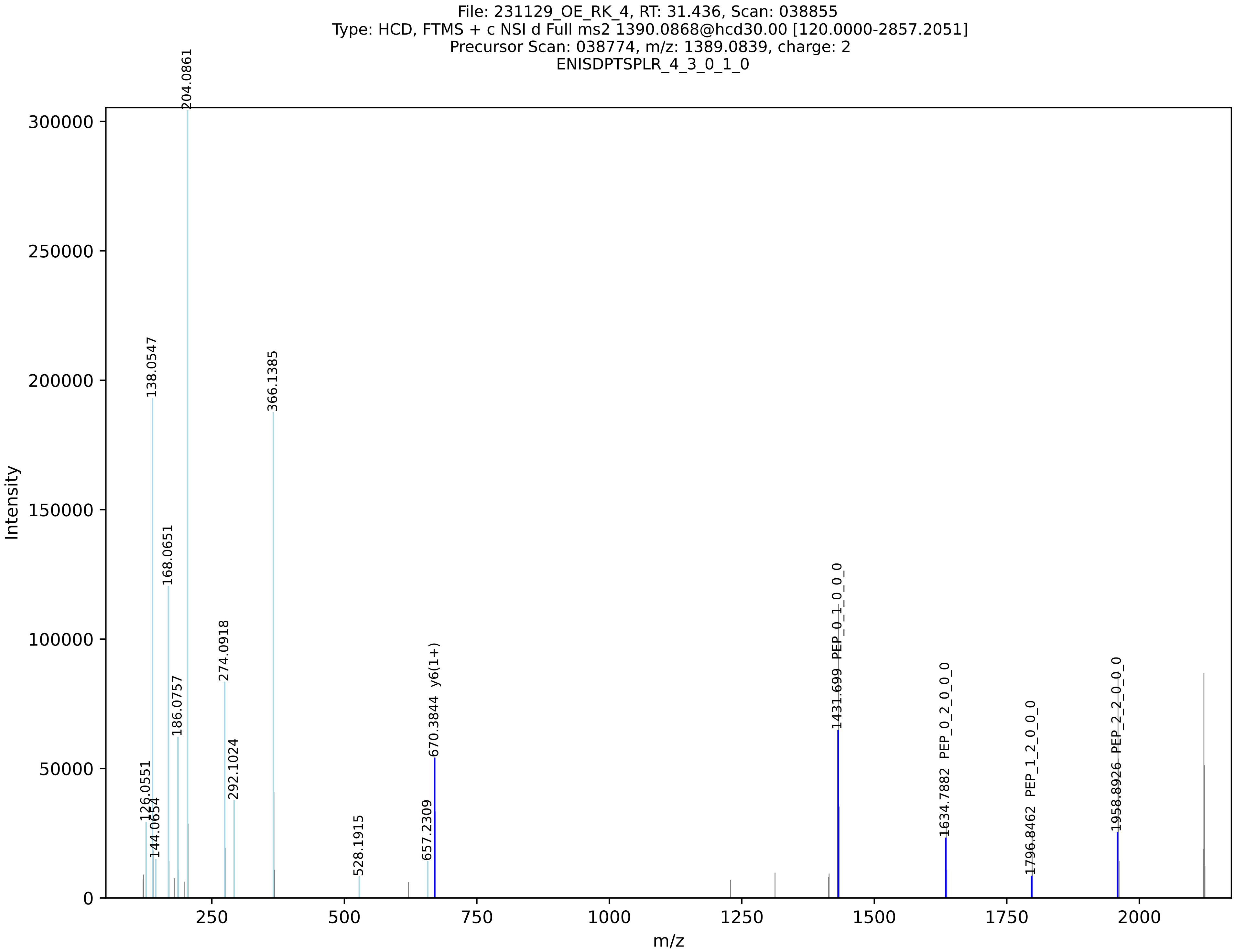

File: 231129_OE_RK_4, RT: 31.436, Scan: 038855
Type: HCD, FTMS + c NSI d Full ms2 1390.0868@hcd30.00 [120.0000-2857.2051]
Precursor Scan: 038774, m/z: 1389.0839, charge: 2
ENISDPTSPLR_4_3_0_1_0
Intensity
m/z
0
50000
100000
150000
200000
250000
300000
250
500
750
1000
1250
1500
1750
2000
204.0861
138.0547
366.1385
168.0651
274.0918
186.0757
292.1024
126.0551
144.0654
528.1915
657.2309
670.3844 y6(1+)
1431.699 PEP_0_1_0_0_0_
1634.7882 PEP_0_2_0_0_0_
1796.8462 PEP_1_2_0_0_0_
1958.8926 PEP_2_2_0_0_0_

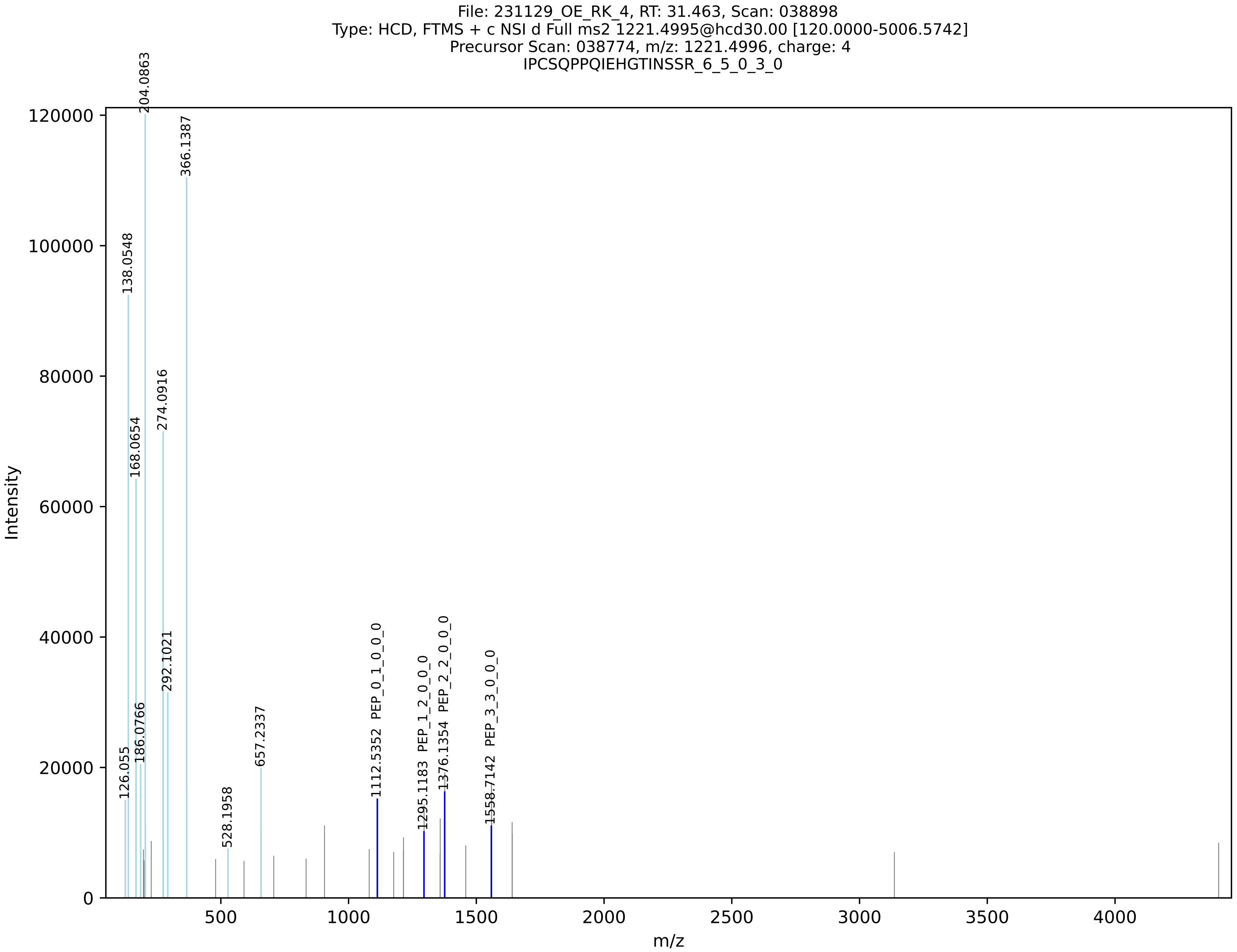
File: 231129_OE_RK_4, RT: 31.463, Scan: 038898
Type: HCD, FTMS + c NSI d Full ms2 1221.4995@hcd30.00 [120.0000-5006.5742]
Precursor Scan: 038774, m/z: 1221.4996, charge: 4
IPCSQPPQIEHGTINSSR_6_5_0_3_0
Intensity
m/z
204.0863
366.1387
138.0548
274.0916
168.0654
292.1021
186.0766
126.055
528.1958
657.2337
1112.5352 PEP_0_1_0_0_0
1295.1183 PEP_1_2_0_0_0
1376.1354 PEP_2_2_0_0_0
1558.7142 PEP_3_3_0_0_0
0
20000
40000
60000
80000
100000
120000
500
1000
1500
2000
2500
3000
3500
4000

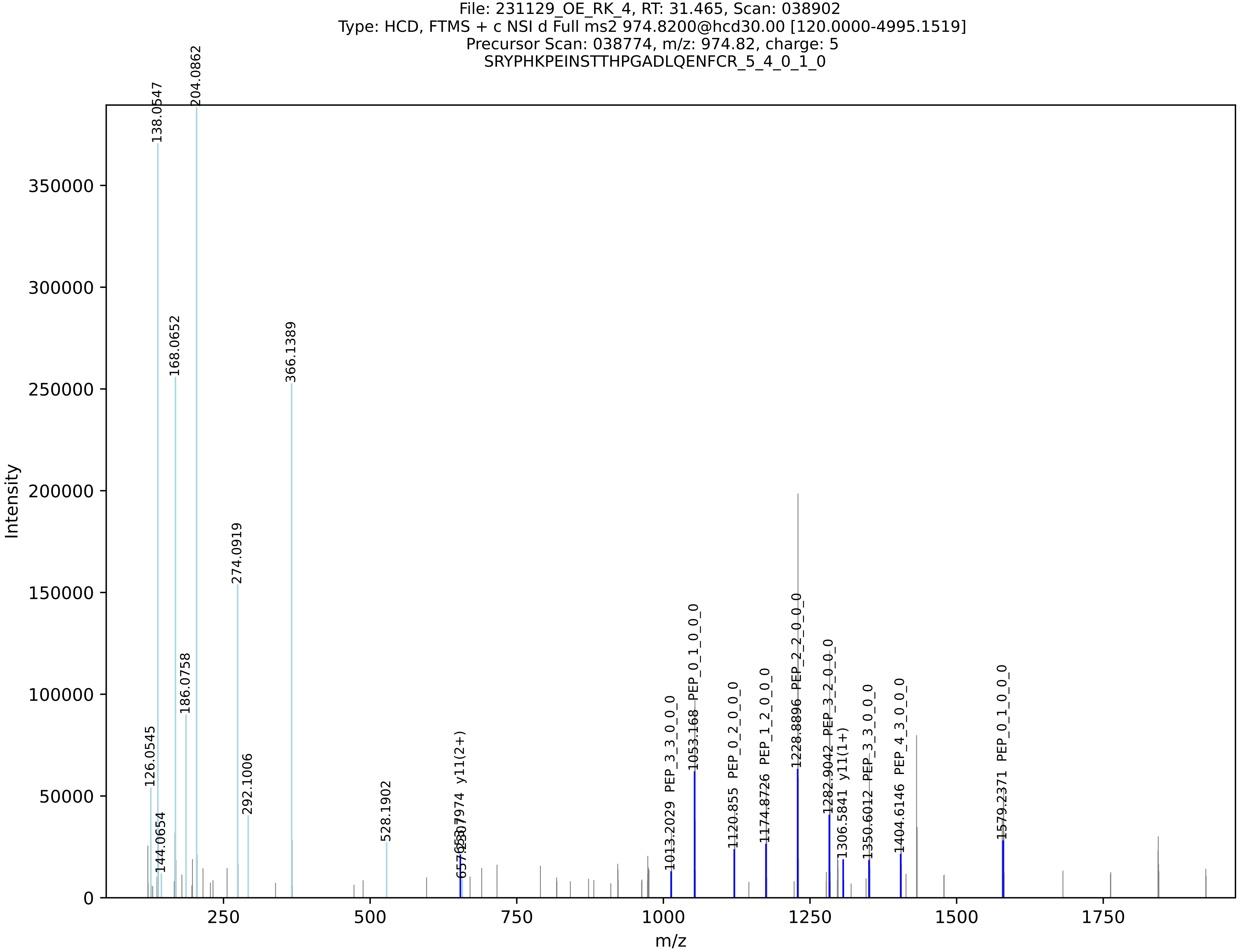
File: 231129_OE_RK_4, RT: 31.465, Scan: 038902
Type: HCD, FTMS + c NSI d Full ms2 974.8200@hcd30.00 [120.0000-4995.1519]
Precursor Scan: 038774, m/z: 974.82, charge: 5
SRYPHKPEINSTTHPGADLQENFCR_5_4_0_1_0
Intensity
m/z
0
50000
100000
150000
200000
250000
300000
350000
250
500
750
1000
1250
1500
1750
126.0545
138.0547
144.0654
168.0652
186.0758
204.0862
274.0919
292.1006
366.1389
528.1902
y11(2+)
1013.2029 PEP_3_3_0_0_0
1053.168 PEP_0_1_0_0_0
1120.855 PEP_0_2_0_0_0
1174.8726 PEP_1_2_0_0_0
1228.8896 PEP_2_2_0_0_0
1282.9042 PEP_3_2_0_0_0
1306.5841 y11(1+)
1350.6012 PEP_3_3_0_0_0
1404.6146 PEP_4_3_0_0_0
1579.2371 PEP_0_1_0_0_0

File: 231129_OE_RK_4, RT: 31.534, Scan: 038999
Type: HCD, FTMS + c NSI d Full ms2 1311.2036@hcd30.00 [120.0000-4034.4250]
Precursor Scan: 038921, m/z: 1310.5354, charge: 3
FSDGLESNSSTQFEVKK_10_2_0_0_0

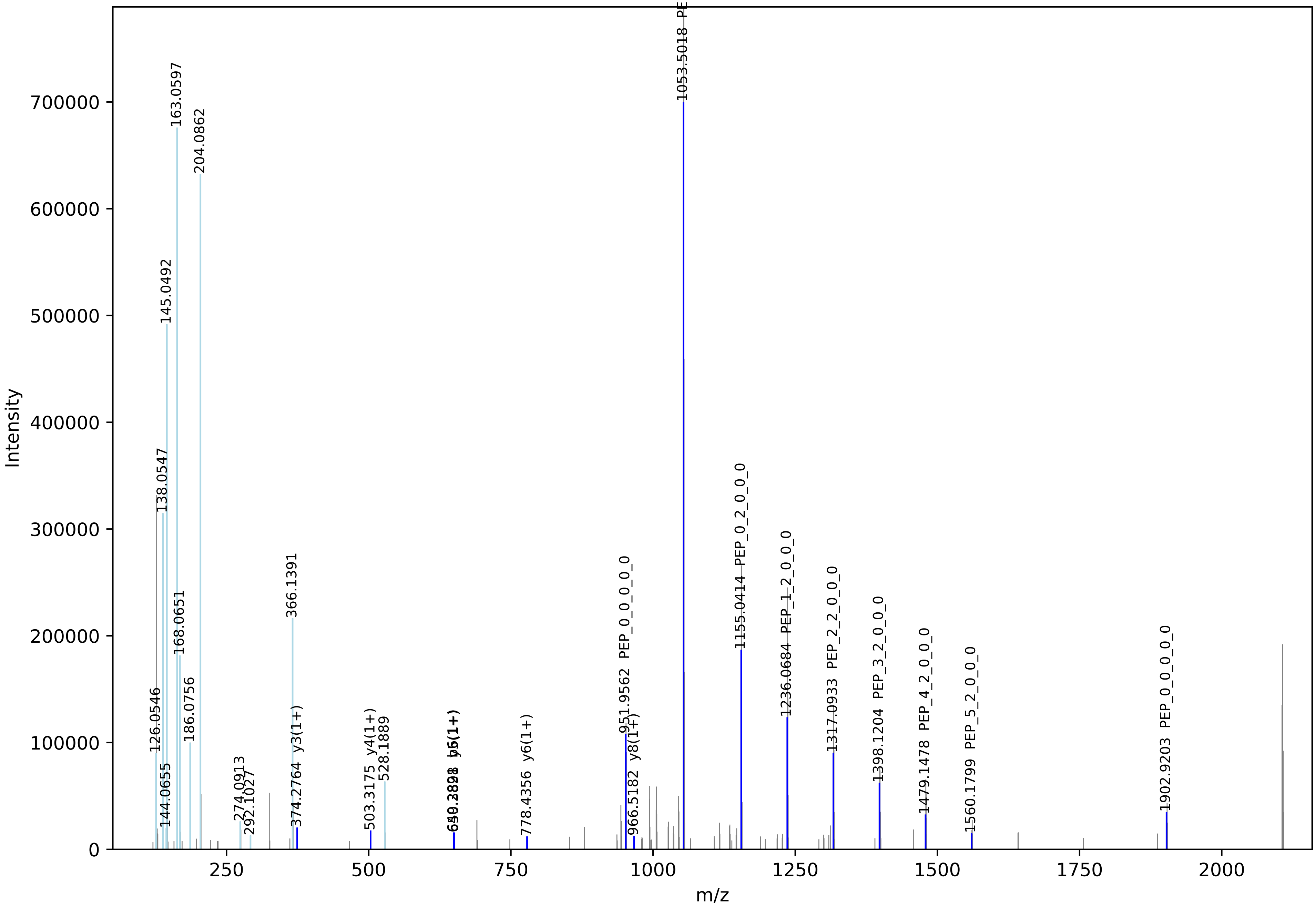

File: 231129_OE_RK_4, RT: 31.545, Scan: 039016
Type: HCD, FTMS + c NSI d Full ms2 983.1530@hcd30.00 [120.0000-4034.1204]
Precursor Scan: 038921, m/z: 983.1534, charge: 4
FSDGLESNSSTQFEVKK_10_2_0_0_0

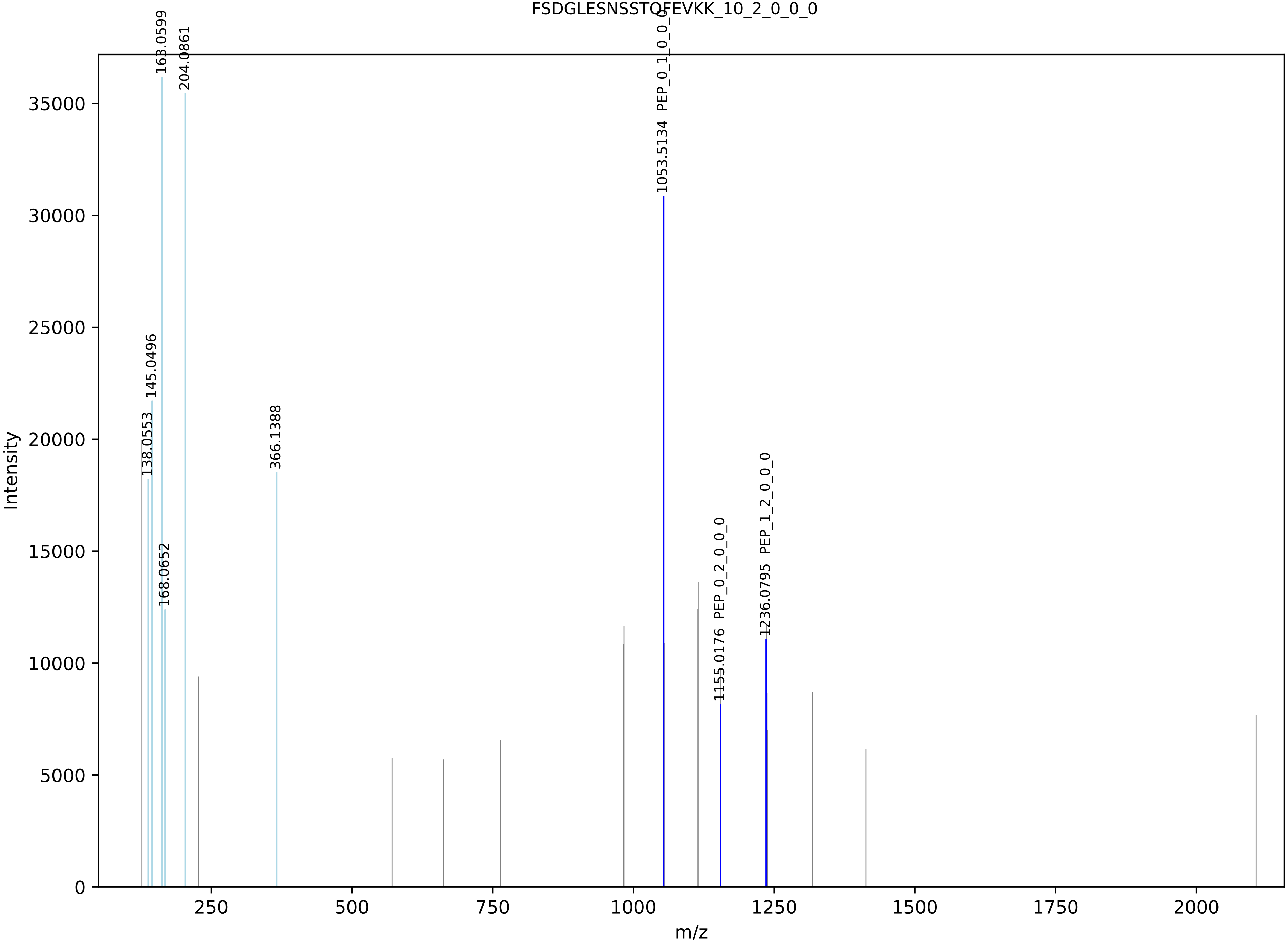

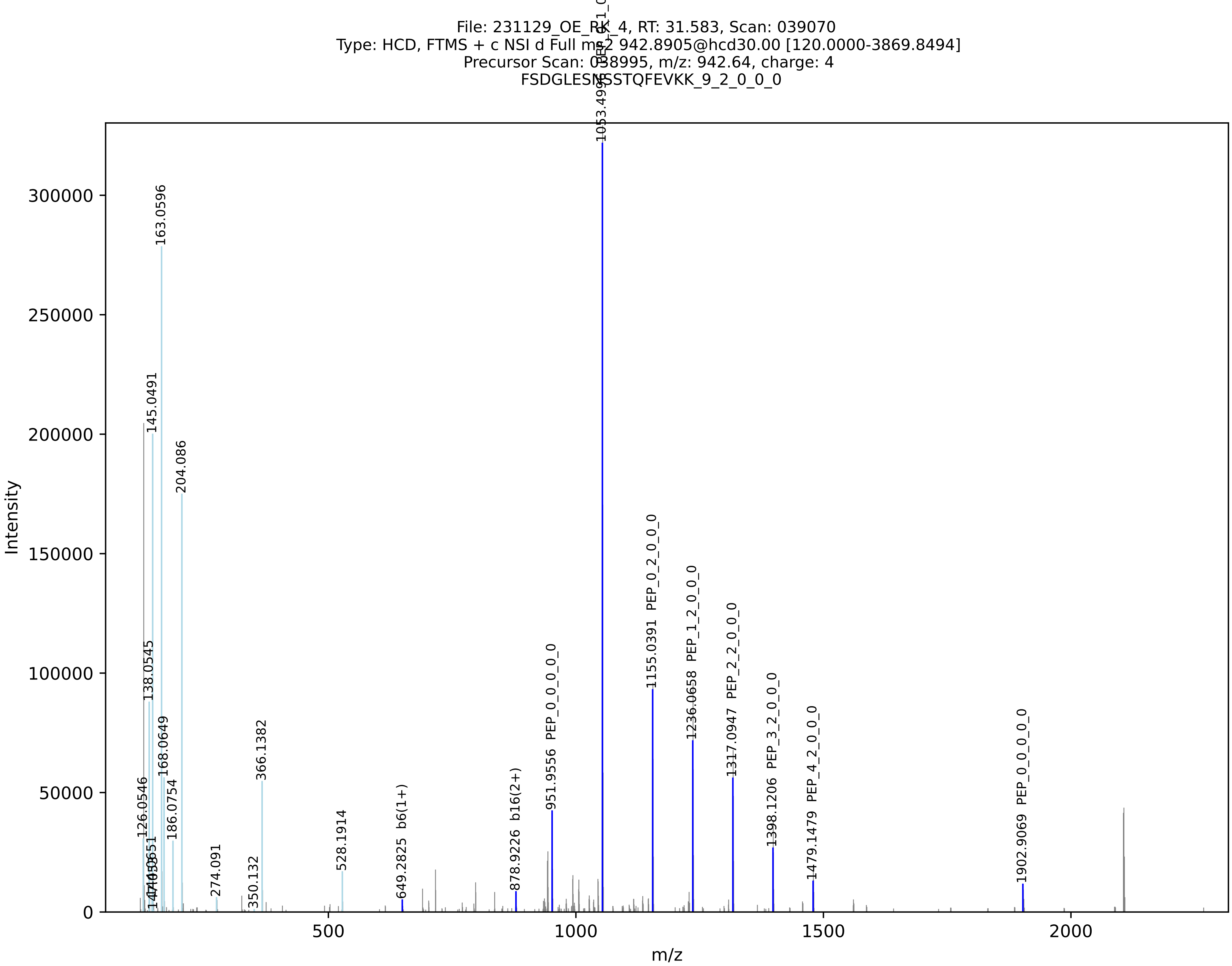
File: 231129_OE_R_K_4, RT: 31.583, Scan: 039070
Type: HCD, FTMS + c NSI d Full ms2 942.8905@hcd30.00 [120.0000-3869.8494]
Precursor Scan: 038995, m/z: 942.64, charge: 4
FSDGLESNSSTQFEVKK_9_2_0_0_0
Intensity
m/z
0
50000
100000
150000
200000
250000
300000
500
1000
1500
2000
163.0596
145.0491
204.086
138.0545
168.0649
126.0546
186.0754
366.1382
274.091
350.132
528.1914
649.2825 b6(1+)
878.9226 b16(2+)
951.9556 PEP_0_0_0_0_0
1155.0391 PEP_0_2_0_0_0
1236.0658 PEP_1_2_0_0_0
1317.0947 PEP_2_2_0_0_0
1398.1206 PEP_3_2_0_0_0
1479.1479 PEP_4_2_0_0_0
1902.9069 PEP_0_0_0_0_0

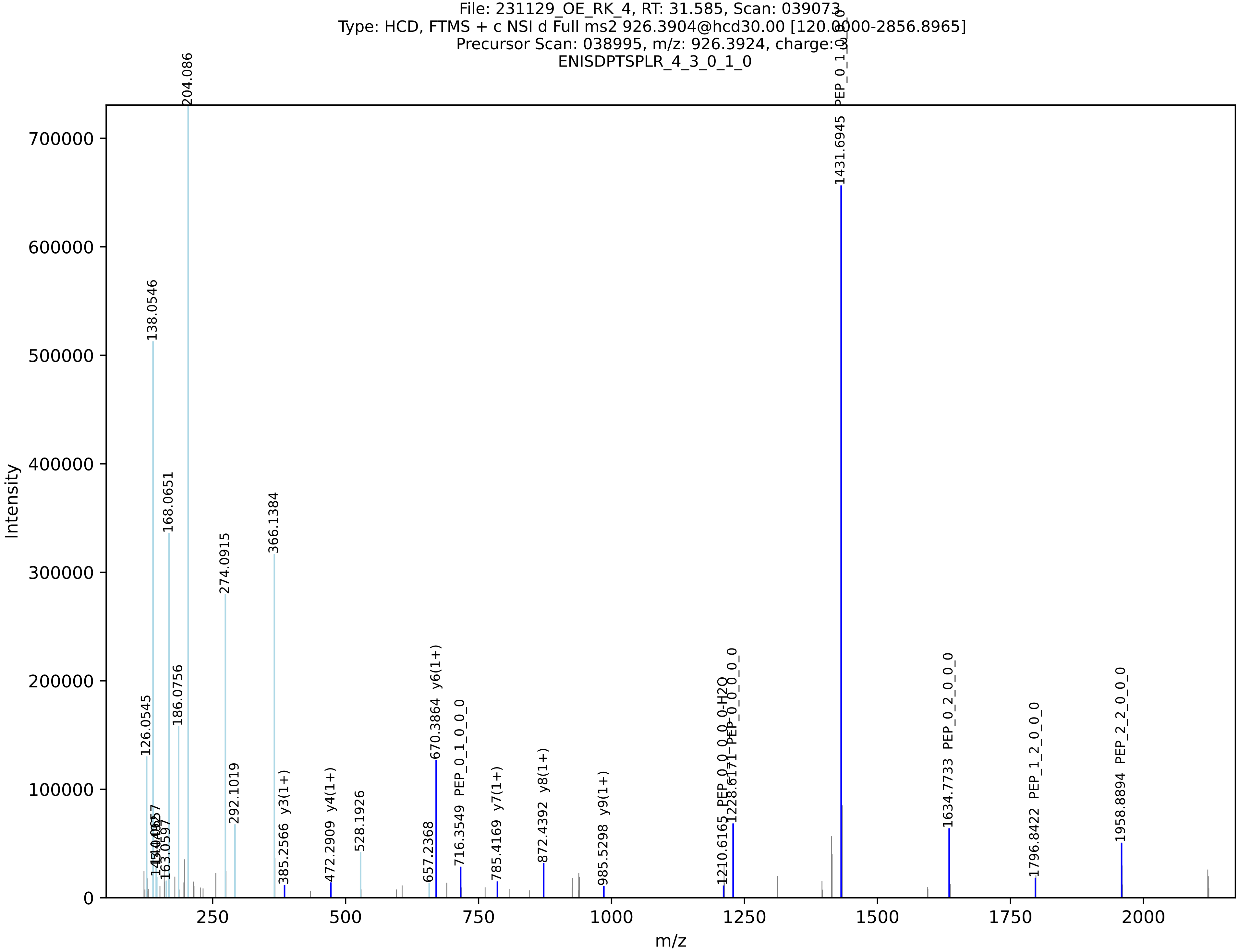
File: 231129_OE_RK_4, RT: 31.585, Scan: 039073
Type: HCD, FTMS + c NSI d Full ms2 926.3904@hcd30.00 [120.0000-2856.8965]
Precursor Scan: 038995, m/z: 926.3924, charge: 3
ENISDPTSPLR_4_3_0_1_0
Intensity
m/z
204.086
138.0546
168.0651
186.0756
126.0545
274.0915
292.1019
366.1384
385.2566 y3(1+)
472.2909 y4(1+)
528.1926
657.2368
670.3864 y6(1+)
716.3549 PEP_0_1_0_0_0
785.4169 y7(1+)
872.4392 y8(1+)
985.5298 y9(1+)
1210.6165 PEP_0_0_0_0_0-H2O
1228.6171 PEP_0_0_0_0_0
1431.6945 PEP_0_1_0_0_0
1634.7733 PEP_0_2_0_0_0
1796.8422 PEP_1_2_0_0_0
1958.8894 PEP_2_2_0_0_0
163.0597

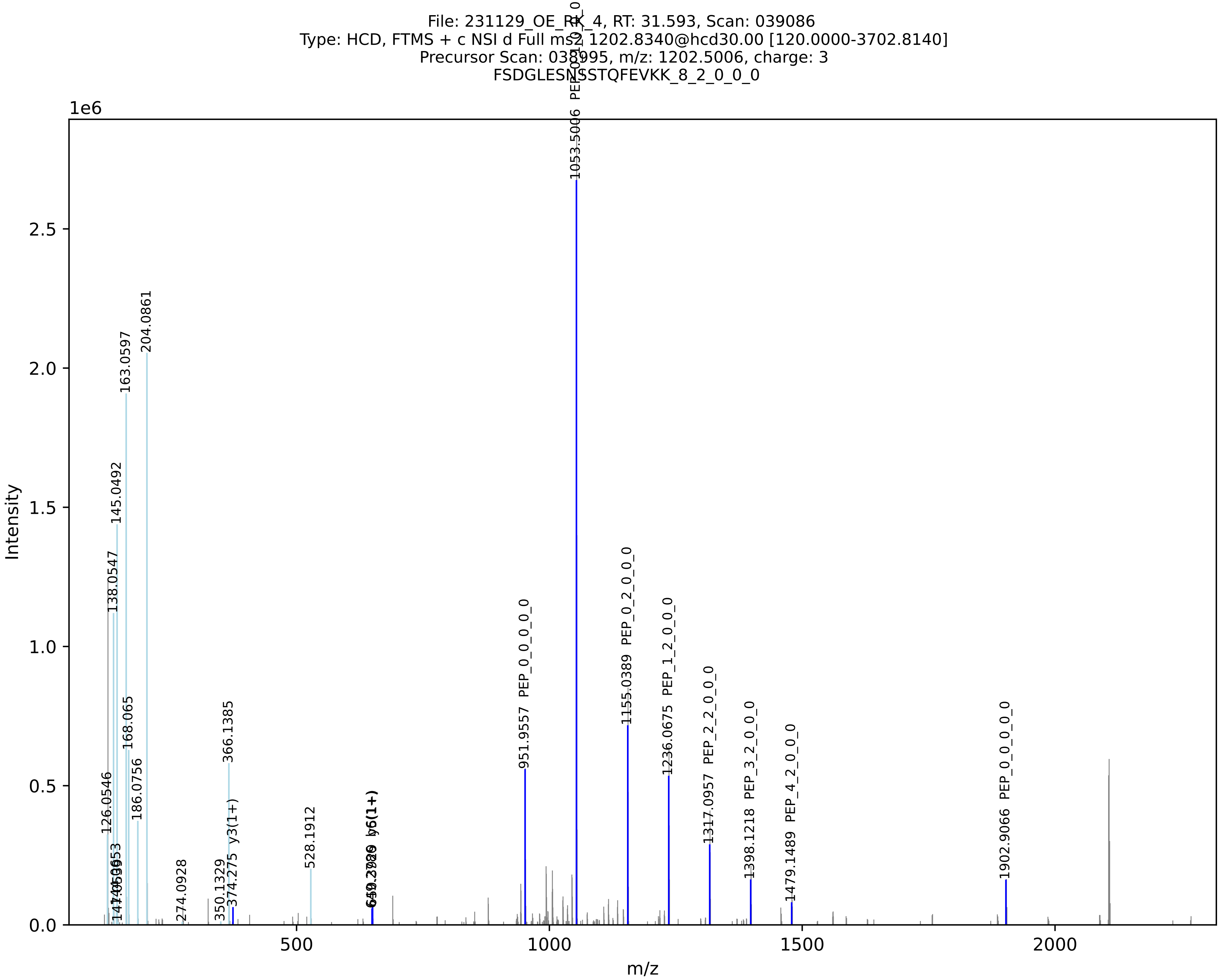

File: 231129_OE_RK_4, RT: 31.593, Scan: 039086
Type: HCD, FTMS + c NSI d Full ms2 1202.8340@hcd30.00 [120.0000-3702.8140]
Precursor Scan: 038995, m/z: 1202.5006, charge: 3
FSDGLESNSSTQFEVKK_8_2_0_0_0
1e6
Intensity
m/z
0.0
0.5
1.0
1.5
2.0
2.5
500
1000
1500
2000
1053.5006
126.0546
138.0547
145.0492
163.0597
168.065
186.0756
204.0861
274.0928
350.1329
374.275 y3(1+)
366.1385
528.1912
951.9557 PEP_0_0_0_0_0
1155.0389 PEP_0_2_0_0_0
1236.0675 PEP_1_2_0_0_0
1317.0957 PEP_2_2_0_0_0
1398.1218 PEP_3_2_0_0_0
1479.1489 PEP_4_2_0_0_0
1902.9066 PEP_0_0_0_0_0

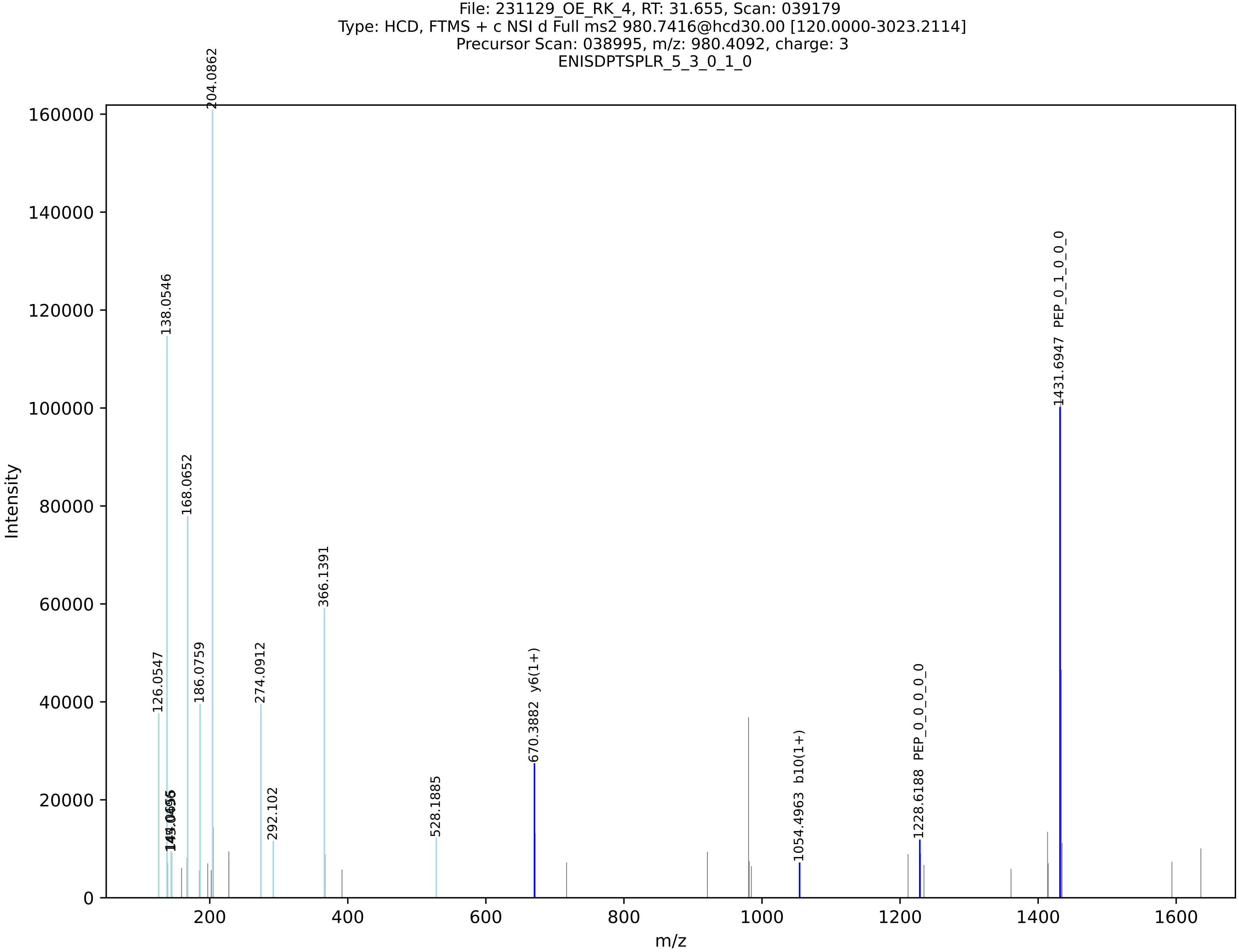
File: 231129_OE_RK_4, RT: 31.655, Scan: 039179
Type: HCD, FTMS + c NSI d Full ms2 980.7416@hcd30.00 [120.0000-3023.2114]
Precursor Scan: 038995, m/z: 980.4092, charge: 3
ENISDPTSPLR_5_3_0_1_0
Intensity
m/z
204.0862
138.0546
168.0652
126.0547
186.0759
274.0912
292.102
366.1391
528.1885
670.3882 y6(1+)
1054.4963 b10(1+)
1228.6188 PEP_0_0_0_0_0_
1431.6947 PEP_0_1_0_0_0_
0
20000
40000
60000
80000
100000
120000
140000
160000
200
400
600
800
1000
1200
1400
1600

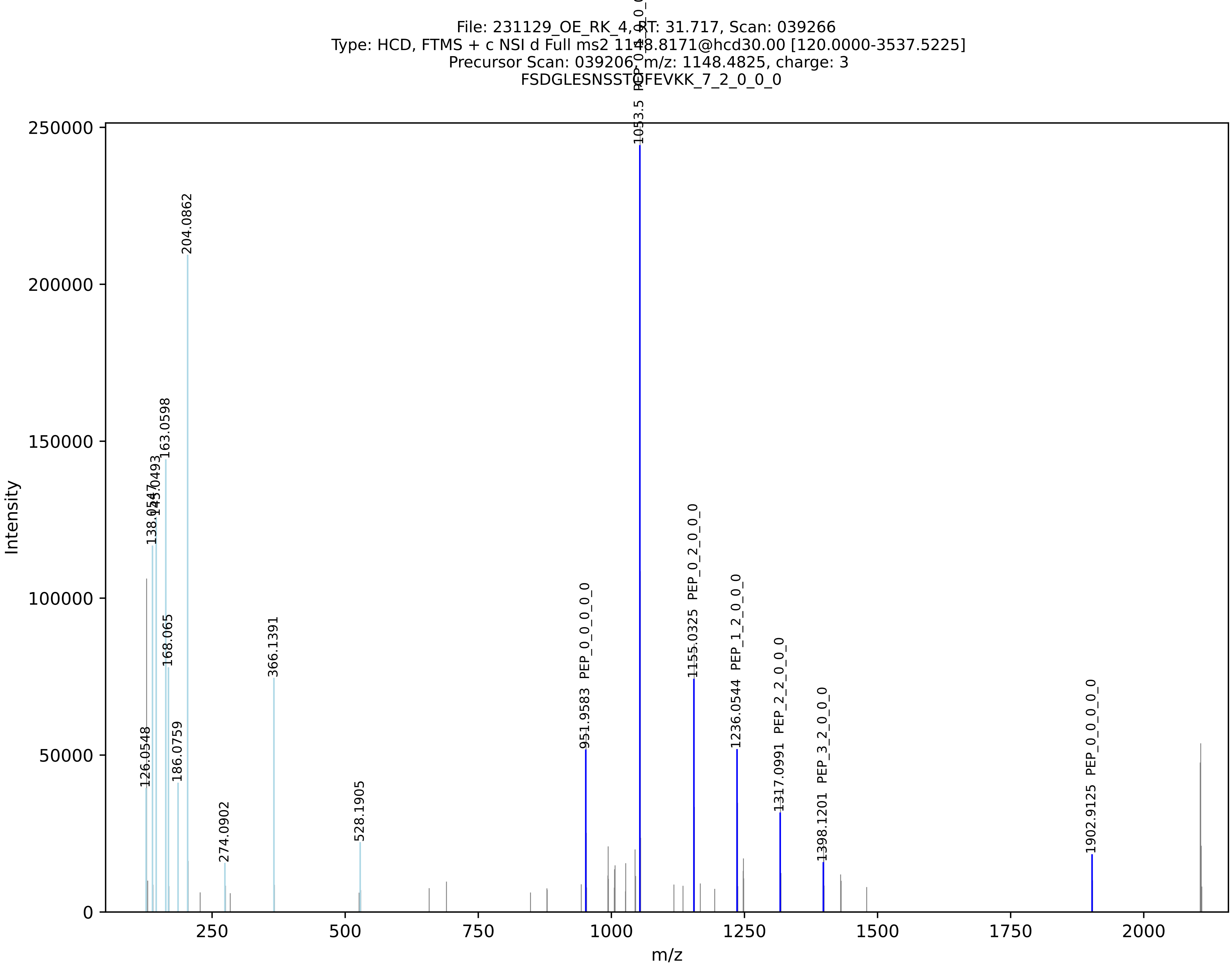
File: 231129_OE_RK_4, RT: 31.717, Scan: 039266
Type: HCD, FTMS + c NSI d Full ms2 1148.8171@hcd30.00 [120.0000-3537.5225]
Precursor Scan: 039206, m/z: 1148.4825, charge: 3
FSDGLESNSSTQFEVKK_7_2_0_0_0
1053.5
204.0862
163.0598
145.0493
138.0547
168.065
186.0759
126.0548
274.0902
366.1391
528.1905
951.9583 PEP_0_0_0_0_0
1155.0325 PEP_0_2_0_0_0
1236.0544 PEP_1_2_0_0_0
1317.0991 PEP_2_2_0_0_0
1398.1201 PEP_3_2_0_0_0
1902.9125 PEP_0_0_0_0_0
Intensity
m/z
0
50000
100000
150000
200000
250000
250
500
750
1000
1250
1500
1750
2000

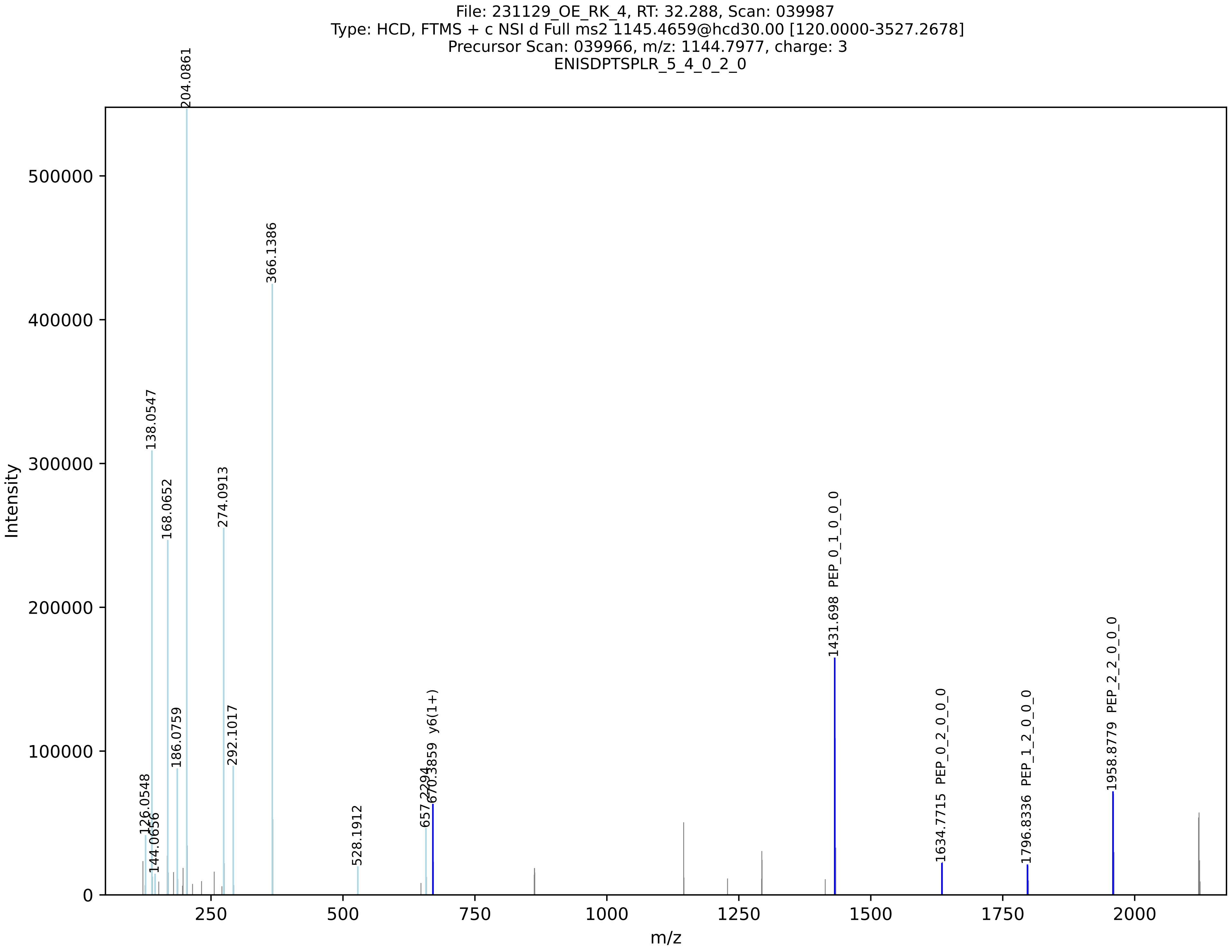
File: 231129_OE_RK_4, RT: 32.288, Scan: 039987
Type: HCD, FTMS + c NSI d Full ms2 1145.4659@hcd30.00 [120.0000-3527.2678]
Precursor Scan: 039966, m/z: 1144.7977, charge: 3
ENISDPTSPLR_5_4_0_2_0
Intensity
m/z
204.0861
366.1386
138.0547
168.0652
274.0913
186.0759
292.1017
126.0548
144.0656
528.1912
657.2294
670.3859 y6(1+)
1431.698 PEP_0_1_0_0_0
1634.7715 PEP_0_2_0_0_0
1796.8336 PEP_1_2_0_0_0
1958.8779 PEP_2_2_0_0_0

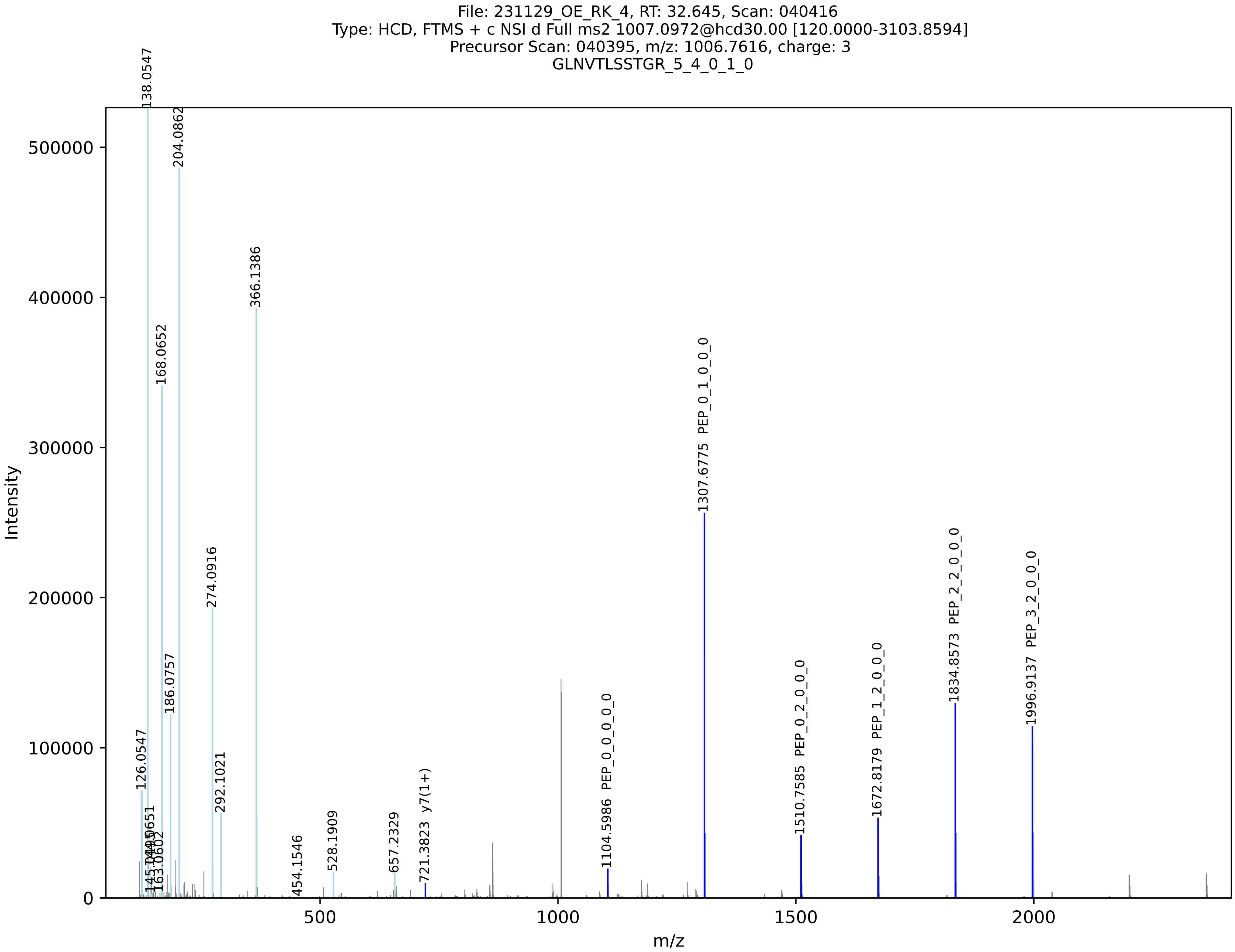
File: 231129_OE_RK_4, RT: 32.645, Scan: 040416
Type: HCD, FTMS + c NSI d Full ms2 1007.0972@hcd30.00 [120.0000-3103.8594]
Precursor Scan: 040395, m/z: 1006.7616, charge: 3
GLNVTLSSTGR_5_4_0_1_0
Intensity
m/z
138.0547
204.0862
366.1386
168.0652
274.0916
186.0757
126.0547
292.1021
163.0602
454.1546
528.1909
657.2329
721.3823 y7(1+)
1104.5986 PEP_0_0_0_0_0
1307.6775 PEP_0_1_0_0_0
1510.7585 PEP_0_2_0_0_0
1672.8179 PEP_1_2_0_0_0
1834.8573 PEP_2_2_0_0_0
1996.9137 PEP_3_2_0_0_0

File: 231129_OE_RK_4, RT: 32.71, Scan: 040499
Type: HCD, FTMS + c NSI d Full ms2 1510.6410@hcd30.00 [120.0000-3103.1357]
Precursor Scan: 040466, m/z: 1509.6415, charge: 2
GLNVTLSSTGR_5_4_0_1_0

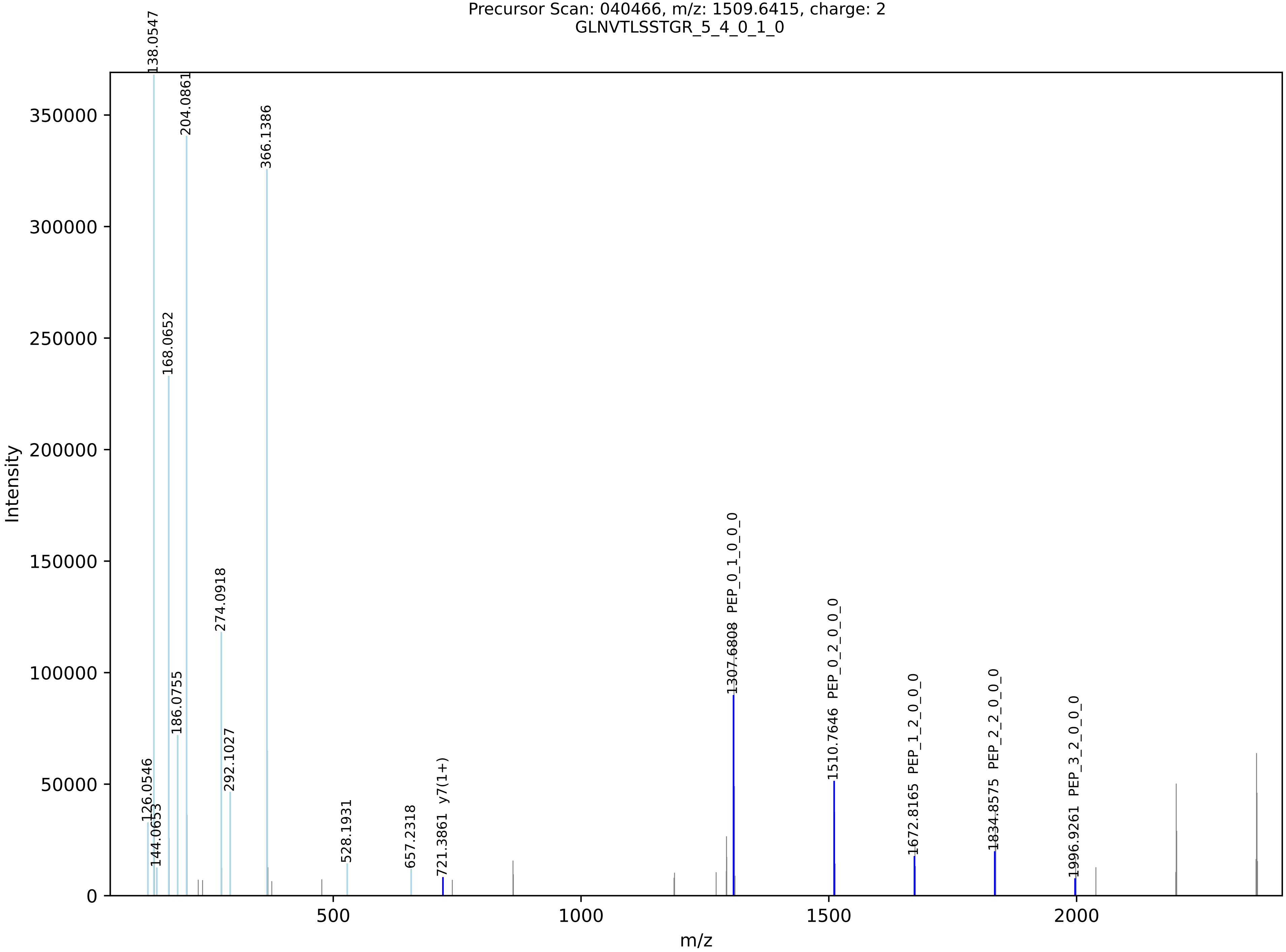

File: 231129_OE_RK_4, RT: 32.814, Scan: 040632
Type: HCD, FTMS + c NSI d Full ms2 1007.0963@hcd30.00 [120.0000-3103.8564]
Precursor Scan: 040602, m/z: 1006.7629, charge: 3
GLNVTLSSTGR_5_4_0_1_0

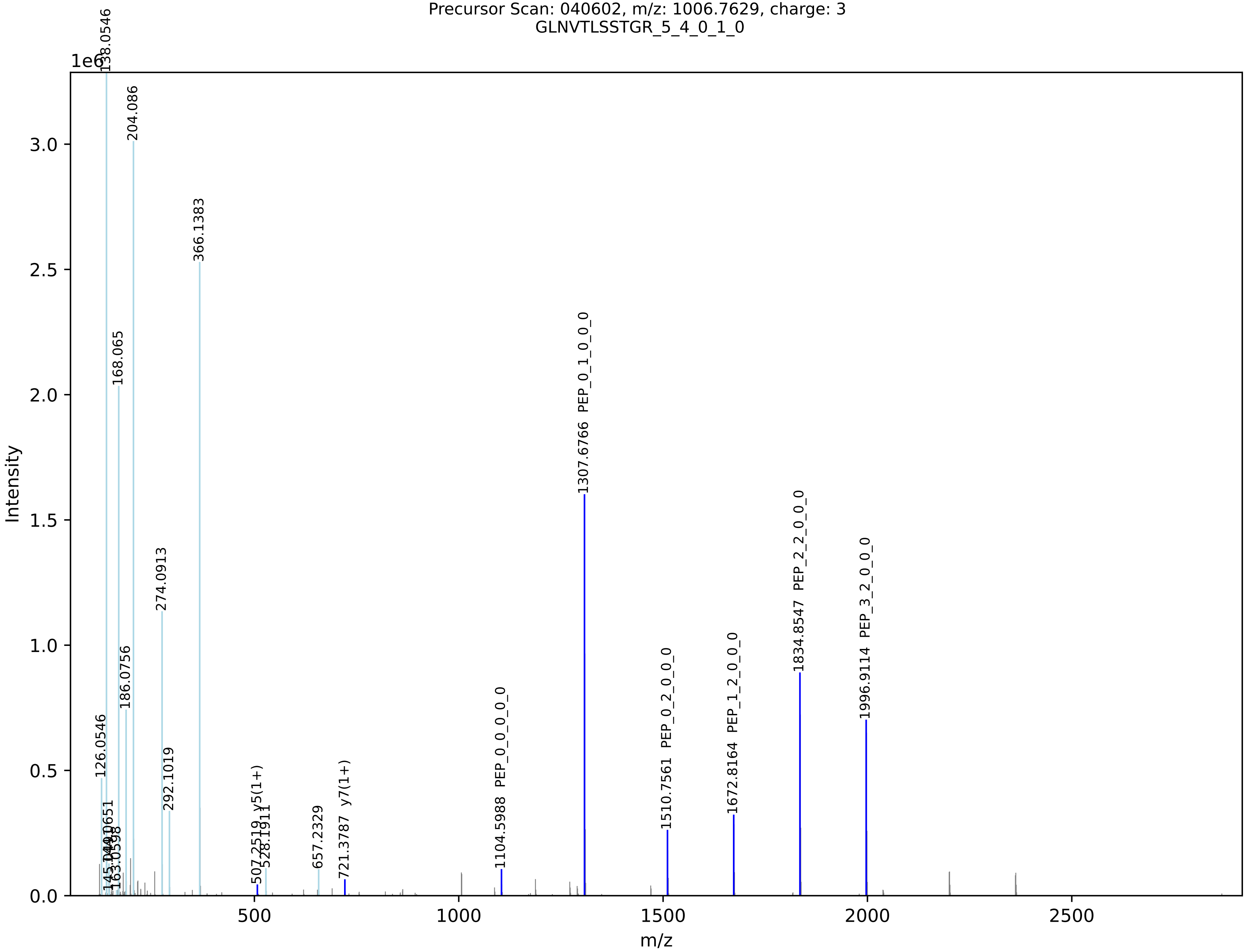

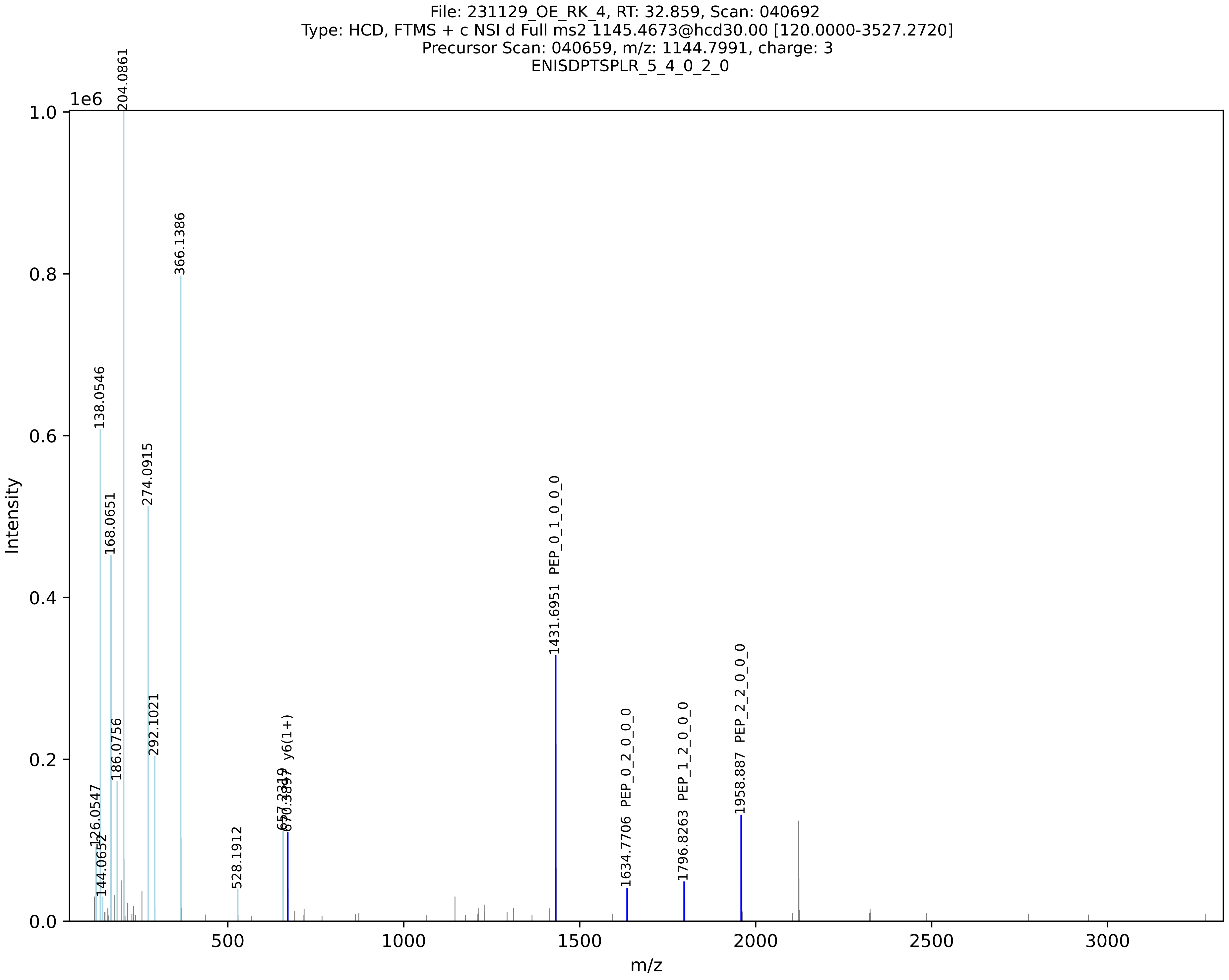

File: 231129_OE_RK_4, RT: 32.859, Scan: 040692
Type: HCD, FTMS + c NSI d Full ms2 1145.4673@hcd30.00 [120.0000-3527.2720]
Precursor Scan: 040659, m/z: 1144.7991, charge: 3
ENISDPTSPLR_5_4_0_2_0
1e6
Intensity
m/z
204.0861
366.1386
138.0546
274.0915
168.0651
186.0756
292.1021
126.0547
144.0652
528.1912
657.2319
670.3897 y6(1+)
1431.6951 PEP_0_1_0_0_0
1634.7706 PEP_0_2_0_0_0
1796.8263 PEP_1_2_0_0_0
1958.887 PEP_2_2_0_0_0

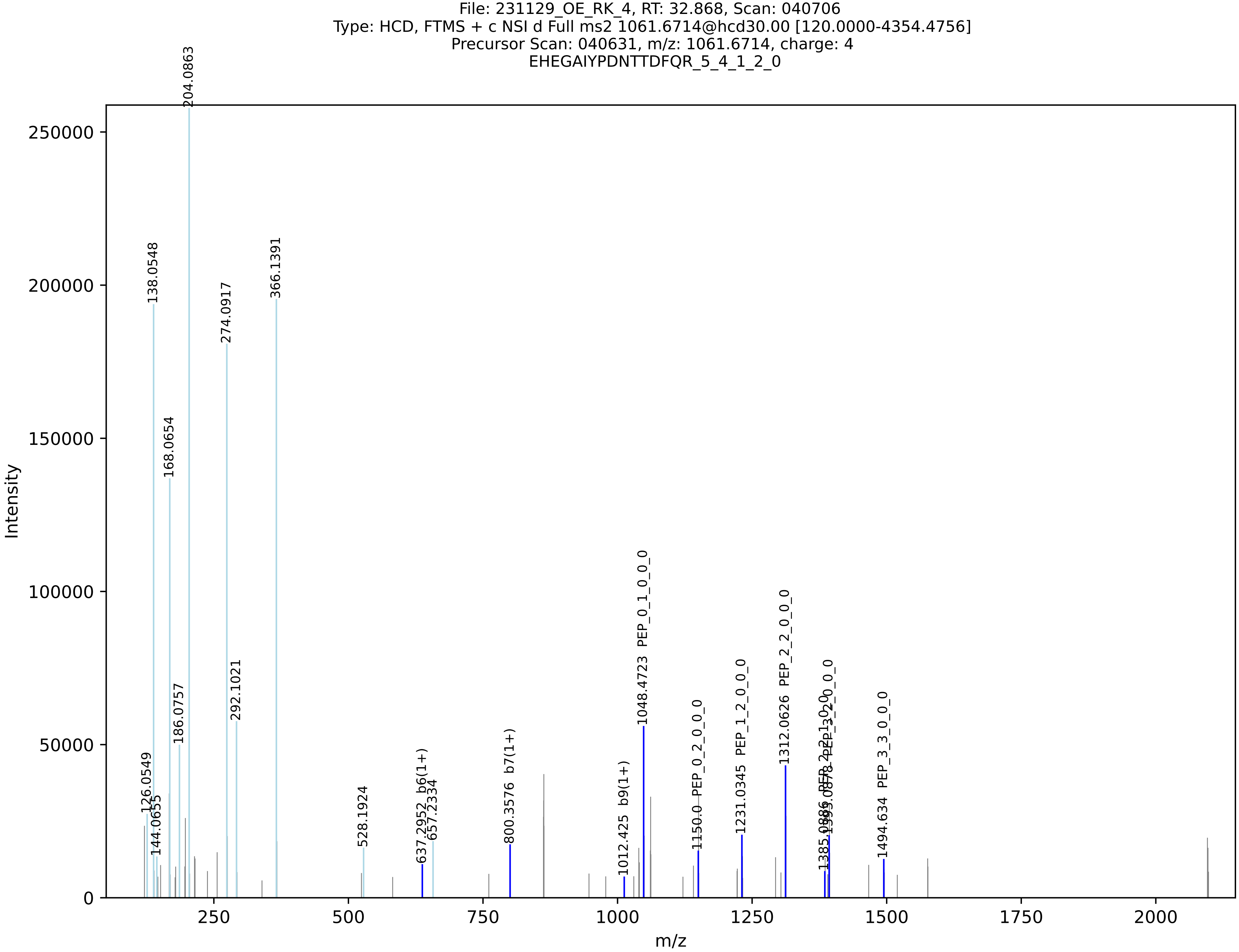
File: 231129_OE_RK_4, RT: 32.868, Scan: 040706
Type: HCD, FTMS + c NSI d Full ms2 1061.6714@hcd30.00 [120.0000-4354.4756]
Precursor Scan: 040631, m/z: 1061.6714, charge: 4
EHEGAIYPDNTTDFQR_5_4_1_2_0
204.0863
138.0548
366.1391
274.0917
168.0654
292.1021
186.0757
126.0549
144.0655
528.1924
637.2952 b6(1+)
657.2334
800.3576 b7(1+)
1012.425 b9(1+)
1048.4723 PEP_0_1_0_0_0
1150.0 PEP_0_2_0_0_0
1231.0345 PEP_1_2_0_0_0
1312.0626 PEP_2_2_0_0_0
1494.634 PEP_3_3_0_0_0
Intensity
m/z
0
50000
100000
150000
200000
250000
250
500
750
1000
1250
1500
1750
2000

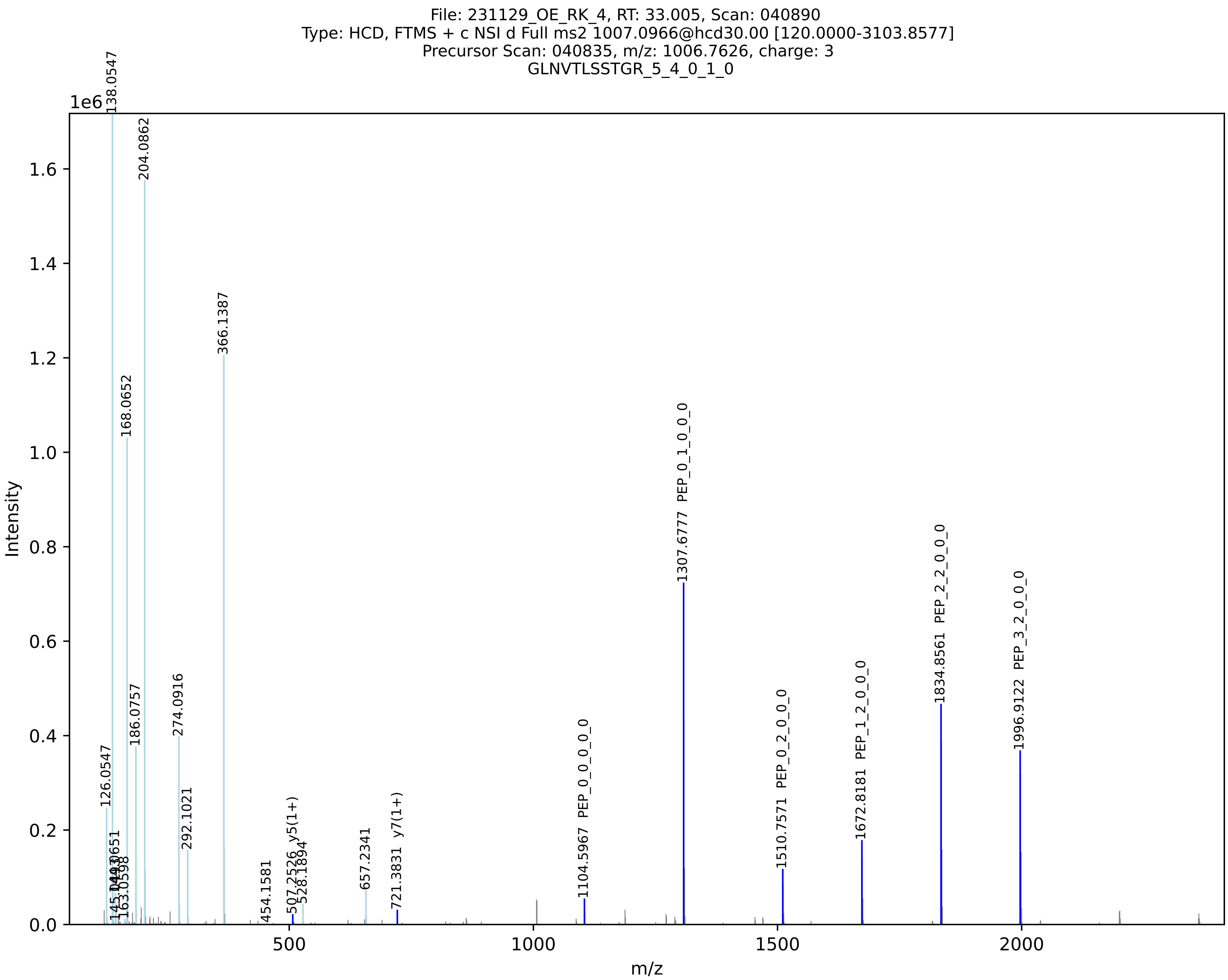

File: 231129_OE_RK_4, RT: 33.005, Scan: 040890
Type: HCD, FTMS + c NSI d Full ms2 1007.0966@hcd30.00 [120.0000-3103.8577]
Precursor Scan: 040835, m/z: 1006.7626, charge: 3
GLNVTLSSTGR_5_4_0_1_0
1e6
Intensity
m/z
138.0547
204.0862
366.1387
168.0652
186.0757
274.0916
126.0547
292.1021
163.0598
454.1581
507.2526 y5(1+)
528.1894
657.2341
721.3831 y7(1+)
1104.5967 PEP_0_0_0_0_0
1307.6777 PEP_0_1_0_0_0
1510.7571 PEP_0_2_0_0_0
1672.8181 PEP_1_2_0_0_0
1834.8561 PEP_2_2_0_0_0
1996.9122 PEP_3_2_0_0_0

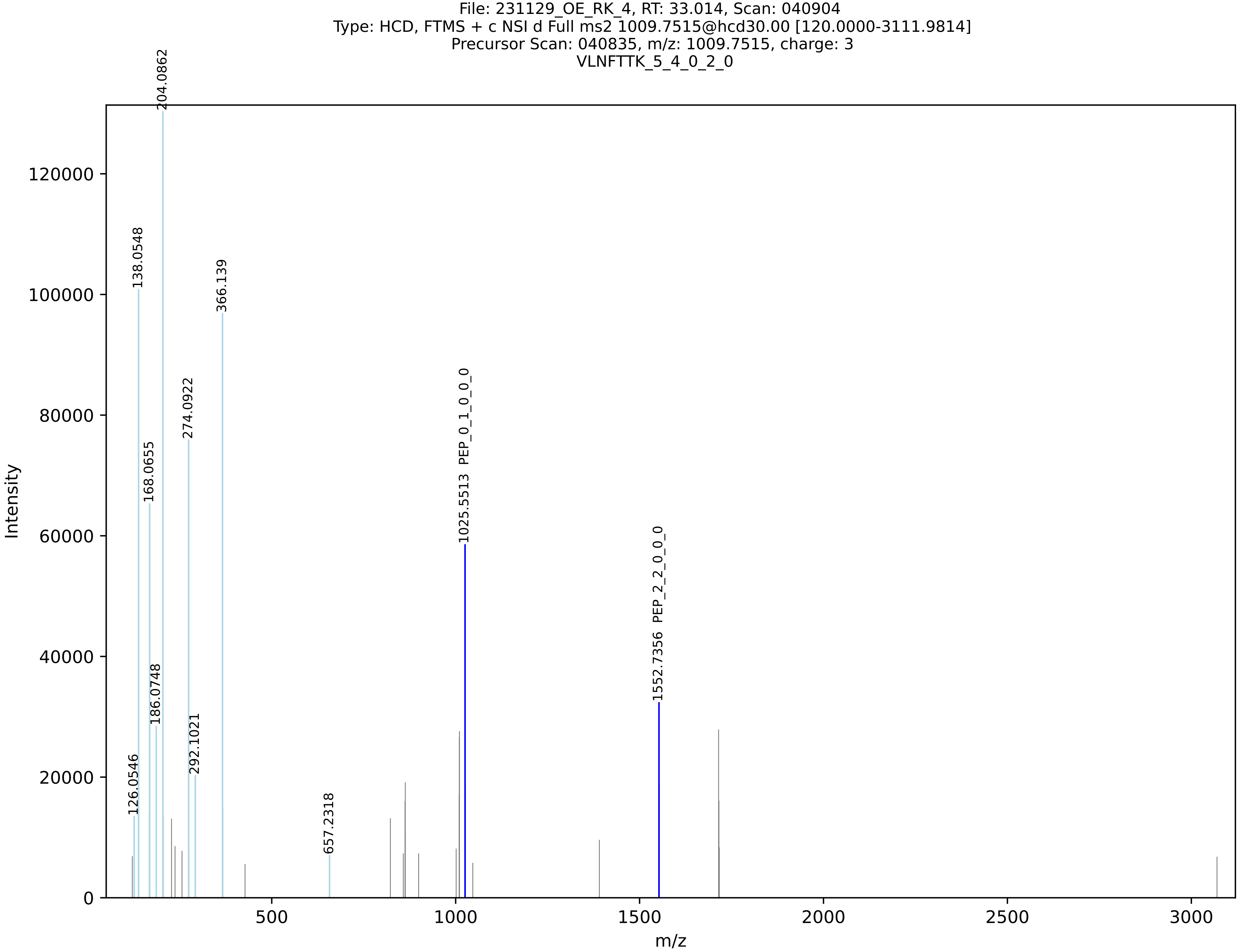
File: 231129_OE_RK_4, RT: 33.014, Scan: 040904
Type: HCD, FTMS + c NSI d Full ms2 1009.7515@hcd30.00 [120.0000-3111.9814]
Precursor Scan: 040835, m/z: 1009.7515, charge: 3
VLNFTTK_5_4_0_2_0
204.0862
138.0548
366.139
274.0922
168.0655
186.0748
292.1021
126.0546
657.2318
1025.5513 PEP_0_1_0_0_0
1552.7356 PEP_2_2_0_0_0
Intensity
m/z
0
20000
40000
60000
80000
100000
120000
500
1000
1500
2000
2500
3000

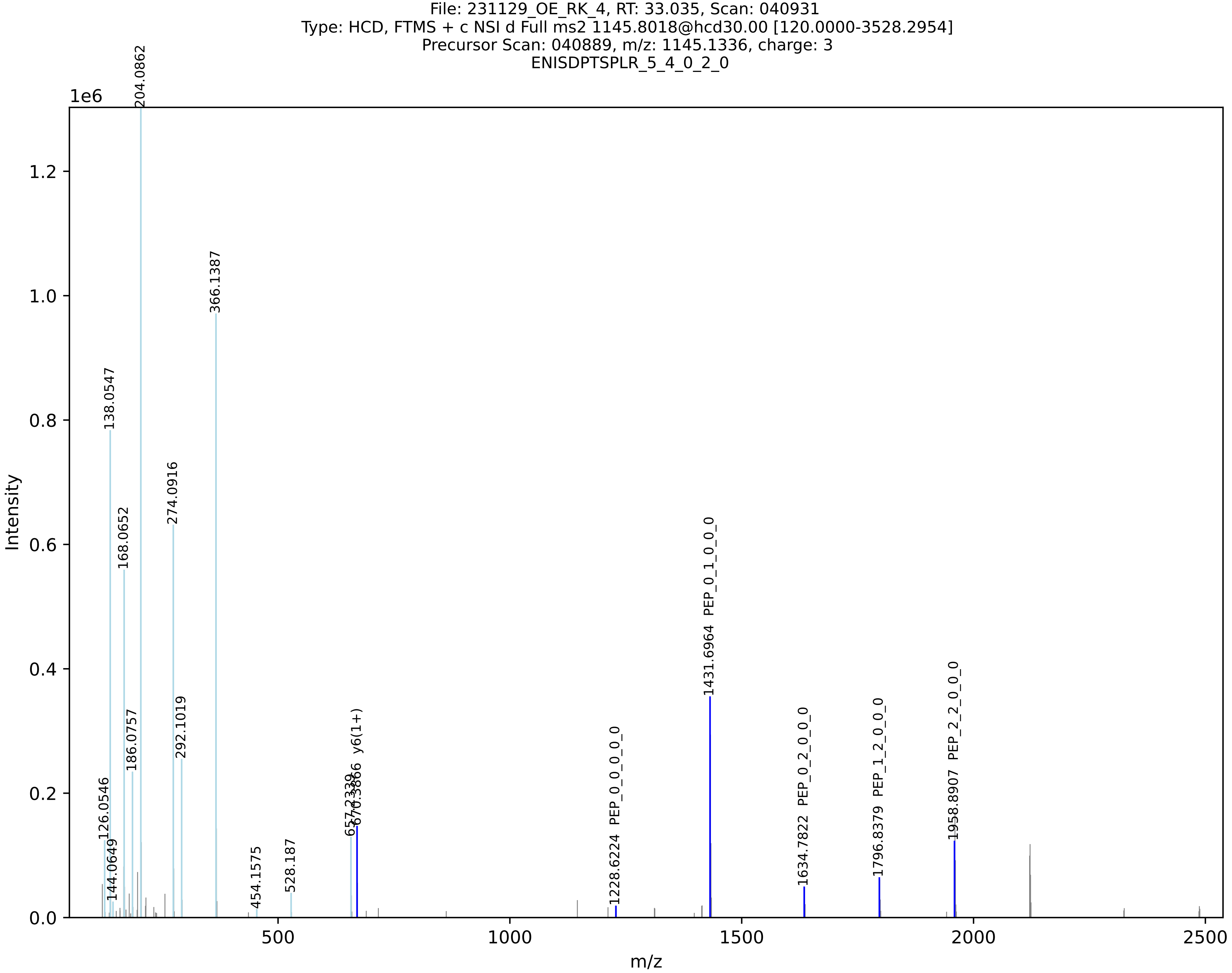

File: 231129_OE_RK_4, RT: 33.035, Scan: 040931
Type: HCD, FTMS + c NSI d Full ms2 1145.8018@hcd30.00 [120.0000-3528.2954]
Precursor Scan: 040889, m/z: 1145.1336, charge: 3
ENISDPTSPLR_5_4_0_2_0
1e6
Intensity
m/z
0.0
0.2
0.4
0.6
0.8
1.0
1.2
500
1000
1500
2000
2500
204.0862
366.1387
138.0547
274.0916
168.0652
186.0757
292.1019
126.0546
144.0649
454.1575
528.187
657.2339
670.3866 y6(1+)
1228.6224 PEP_0_0_0_0_0
1431.6964 PEP_0_1_0_0_0
1634.7822 PEP_0_2_0_0_0
1796.8379 PEP_1_2_0_0_0
1958.8907 PEP_2_2_0_0_0

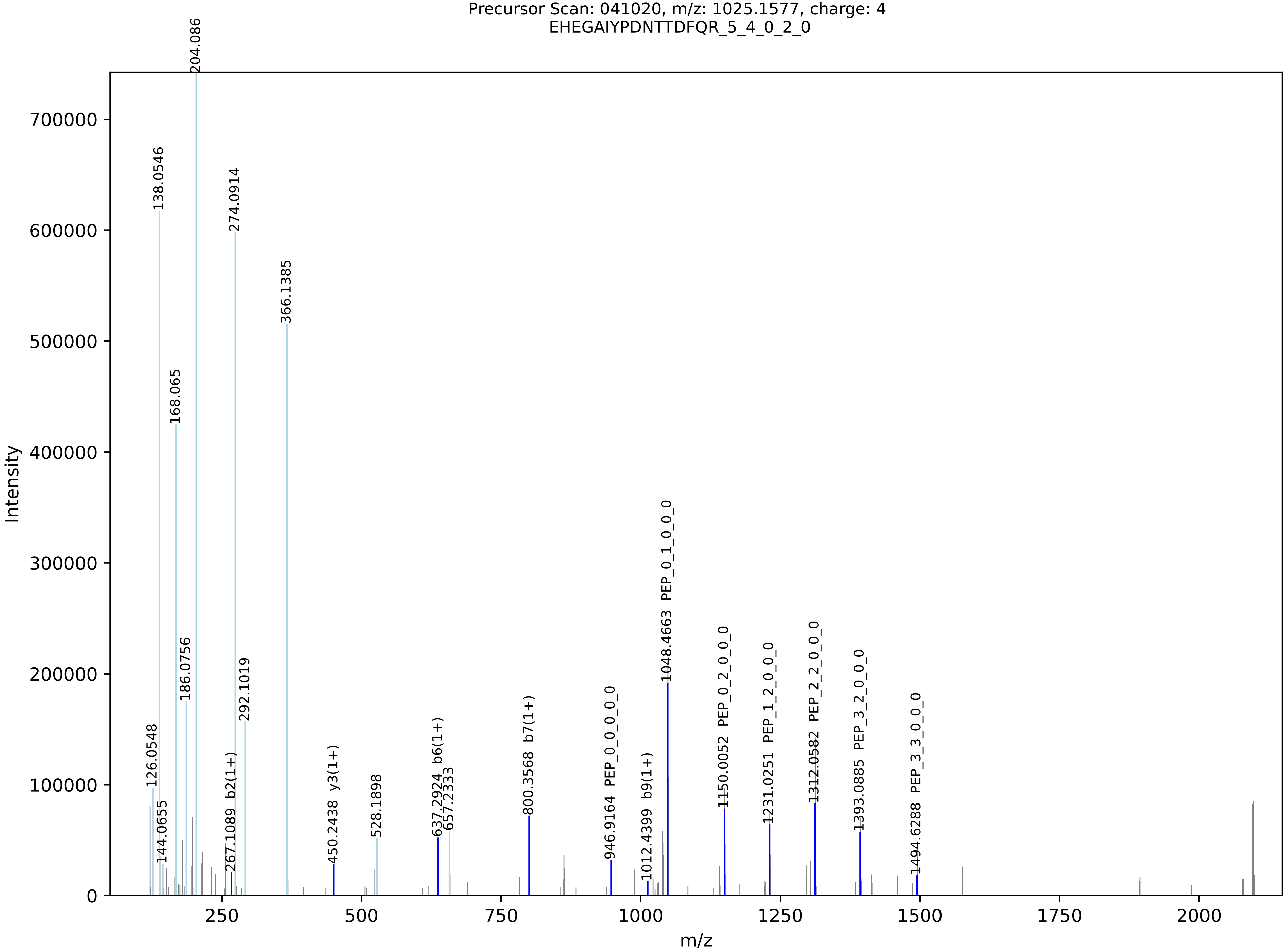
File: 231129_OE_RK_4, RT: 33.14, Scan: 041082
Type: HCD, FTMS + c NSI d Full ms2 1025.6589@hcd30.00 [120.0000-4207.5444]
Precursor Scan: 041020, m/z: 1025.1577, charge: 4
EHEGAIYPDNTTDFQR_5_4_0_2_0
Intensity
m/z
204.086
138.0546
274.0914
366.1385
168.065
186.0756
292.1019
126.0548
144.0655
267.1089 b2(1+)
450.2438 y3(1+)
528.1898
637.2924 b6(1+)
657.2333
800.3568 b7(1+)
946.9164 PEP_0_0_0_0_0
1012.4399 b9(1+)
1048.4663 PEP_0_1_0_0_0
1150.0052 PEP_0_2_0_0_0
1231.0251 PEP_1_2_0_0_0
1312.0582 PEP_2_2_0_0_0
1393.0885 PEP_3_2_0_0_0
1494.6288 PEP_3_3_0_0_0

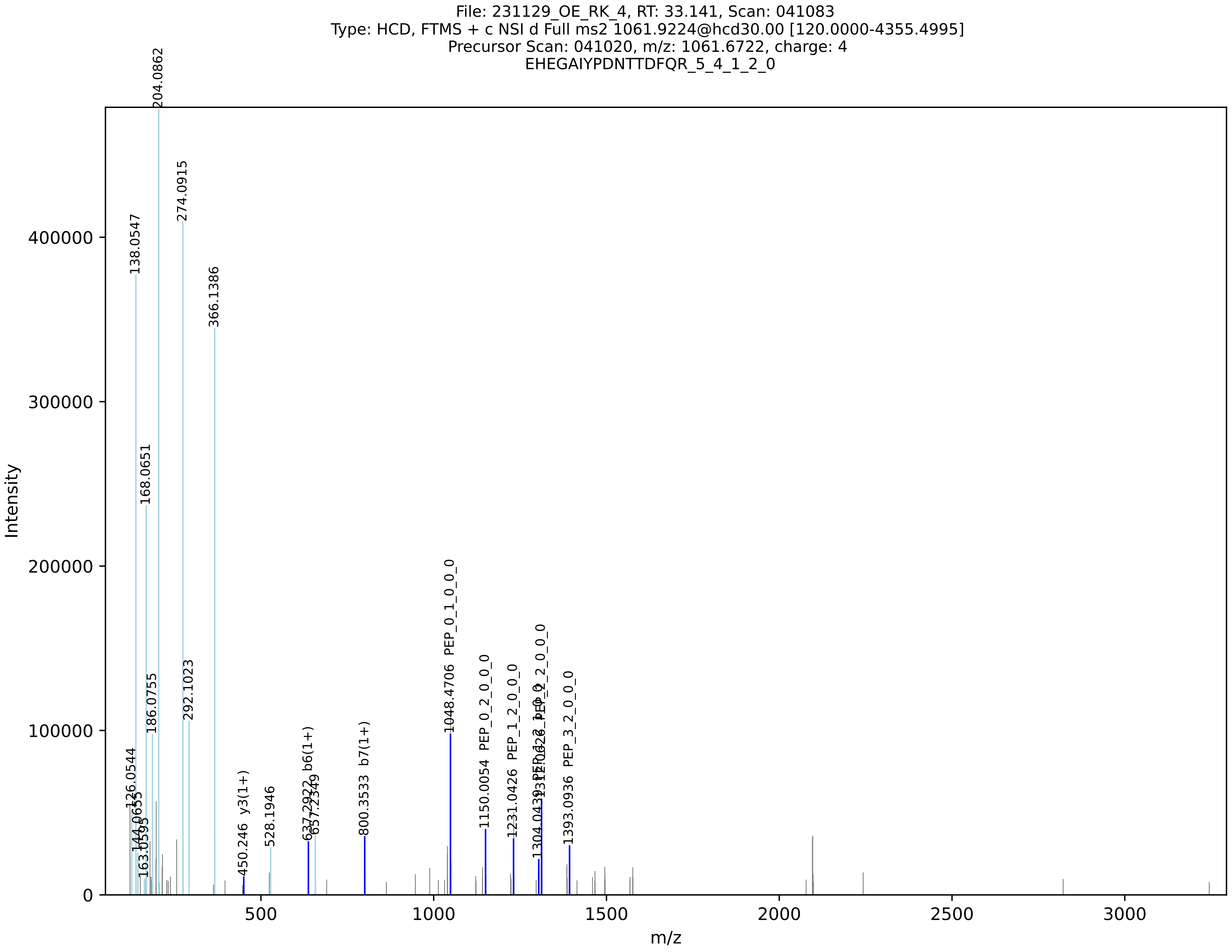
File: 231129_OE_RK_4, RT: 33.141, Scan: 041083
Type: HCD, FTMS + c NSI d Full ms2 1061.9224@hcd30.00 [120.0000-4355.4995]
Precursor Scan: 041020, m/z: 1061.6722, charge: 4
EHEGAIYPDNTTDFQR_5_4_1_2_0
Intensity
m/z
0
100000
200000
300000
400000
500
1000
1500
2000
2500
3000
204.0862
274.0915
138.0547
366.1386
168.0651
186.0755
292.1023
126.0544
144.0655
163.0595
450.246 y3(1+)
528.1946
637.2922 b6(1+)
657.2349
800.3533 b7(1+)
1048.4706 PEP_0_1_0_0_0
1150.0054 PEP_0_2_0_0_0
1231.0426 PEP_1_2_0_0_0
1304.0439
1312.0426
1393.0936 PEP_3_2_0_0_0

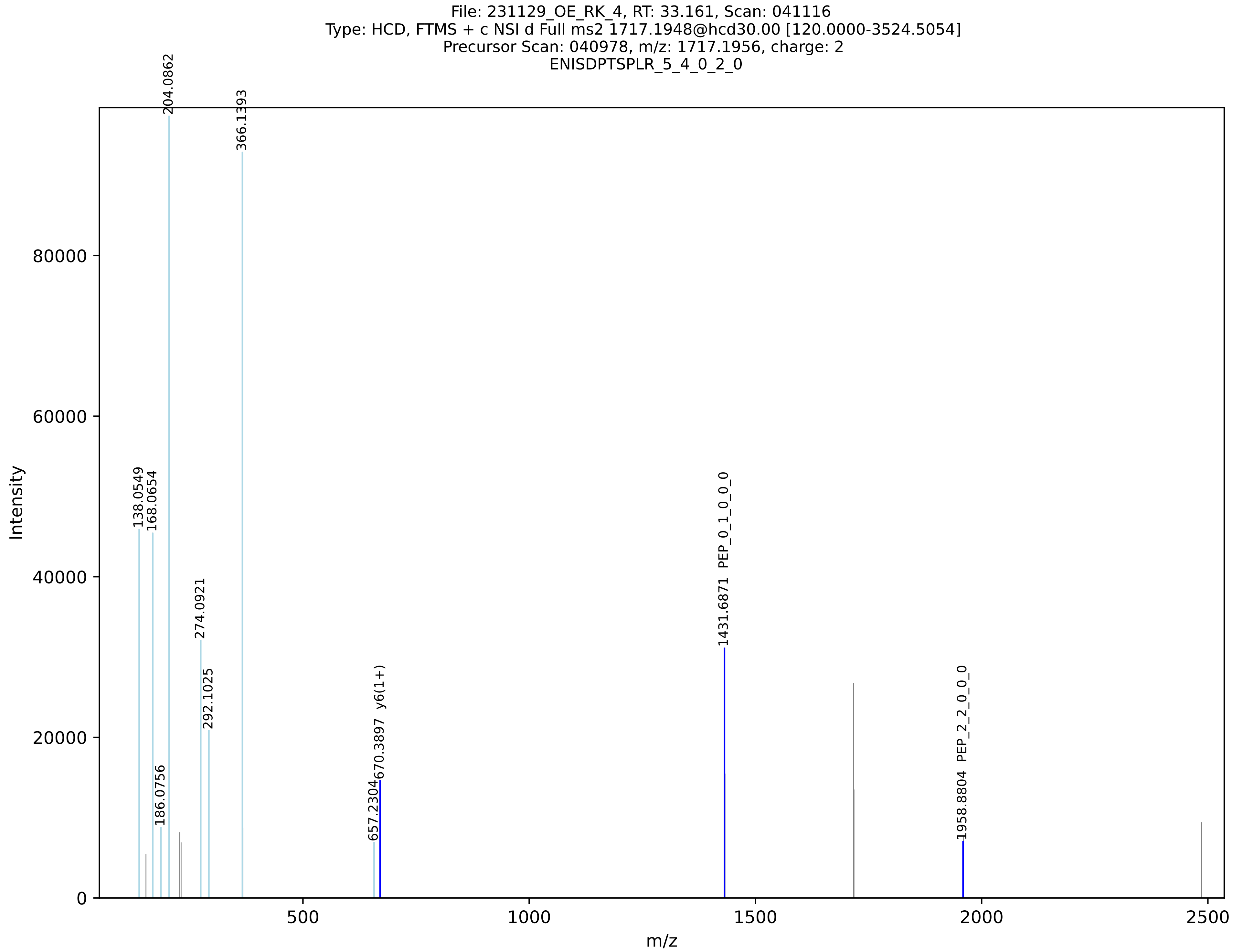
File: 231129_OE_RK_4, RT: 33.161, Scan: 041116
Type: HCD, FTMS + c NSI d Full ms2 1717.1948@hcd30.00 [120.0000-3524.5054]
Precursor Scan: 040978, m/z: 1717.1956, charge: 2
ENISDPTSPLR_5_4_0_2_0
204.0862
366.1393
138.0549
168.0654
274.0921
292.1025
186.0756
657.2304
670.3897 y6(1+)
1431.6871 PEP_0_1_0_0_0
1958.8804 PEP_2_2_0_0_0
Intensity
m/z
0
20000
40000
60000
80000
500
1000
1500
2000
2500

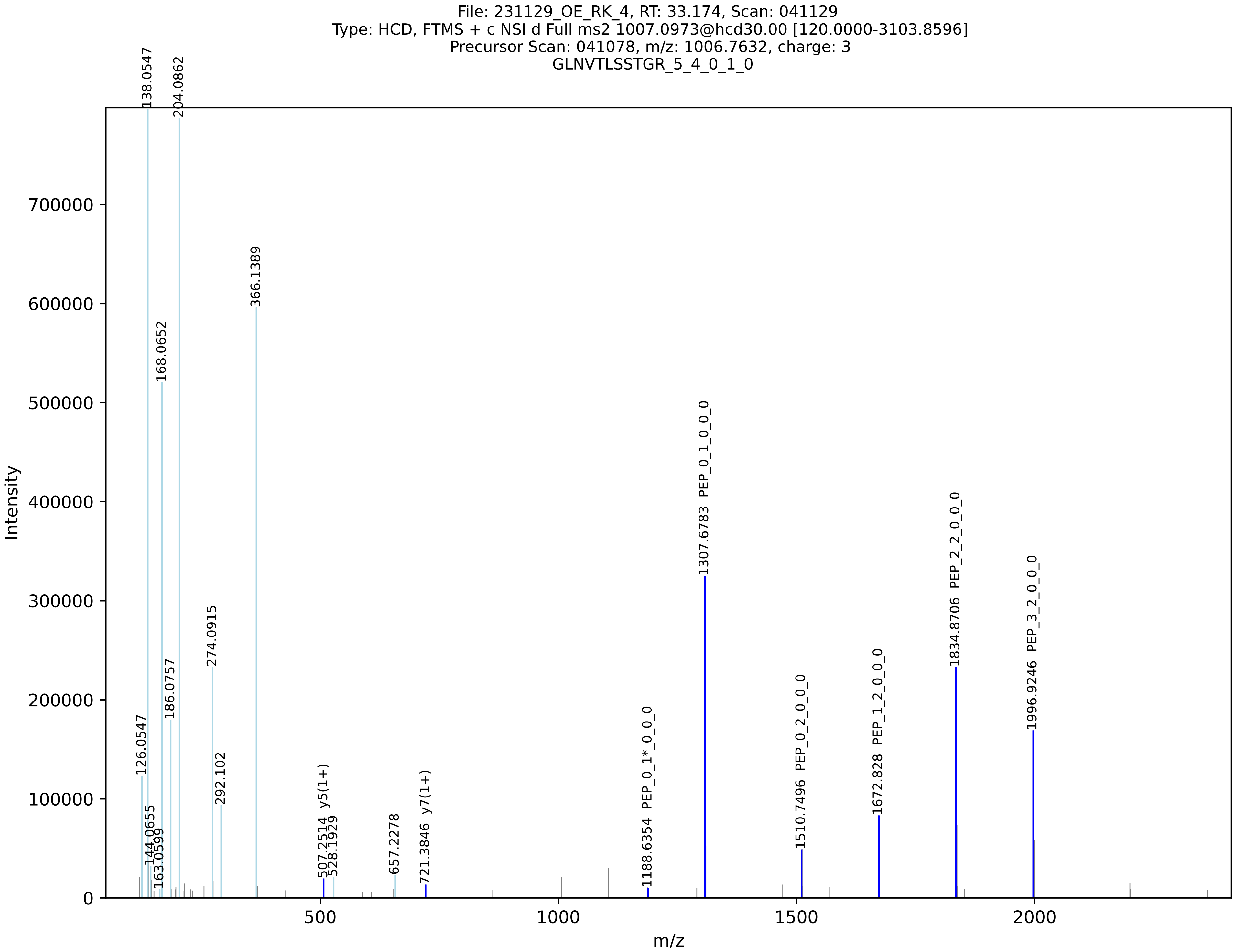
File: 231129_OE_RK_4, RT: 33.174, Scan: 041129
Type: HCD, FTMS + c NSI d Full ms2 1007.0973@hcd30.00 [120.0000-3103.8596]
Precursor Scan: 041078, m/z: 1006.7632, charge: 3
GLNVTLSSTGR_5_4_0_1_0
138.0547
204.0862
366.1389
168.0652
274.0915
186.0757
126.0547
292.102
144.0655
163.0599
507.2514 y5(1+)
528.1929
657.2278
721.3846 y7(1+)
1188.6354 PEP_0_1*_0_0_0
1307.6783 PEP_0_1_0_0_0
1510.7496 PEP_0_2_0_0_0
1672.828 PEP_1_2_0_0_0
1834.8706 PEP_2_2_0_0_0
1996.9246 PEP_3_2_0_0_0
Intensity
m/z
0
100000
200000
300000
400000
500000
600000
700000
500
1000
1500
2000

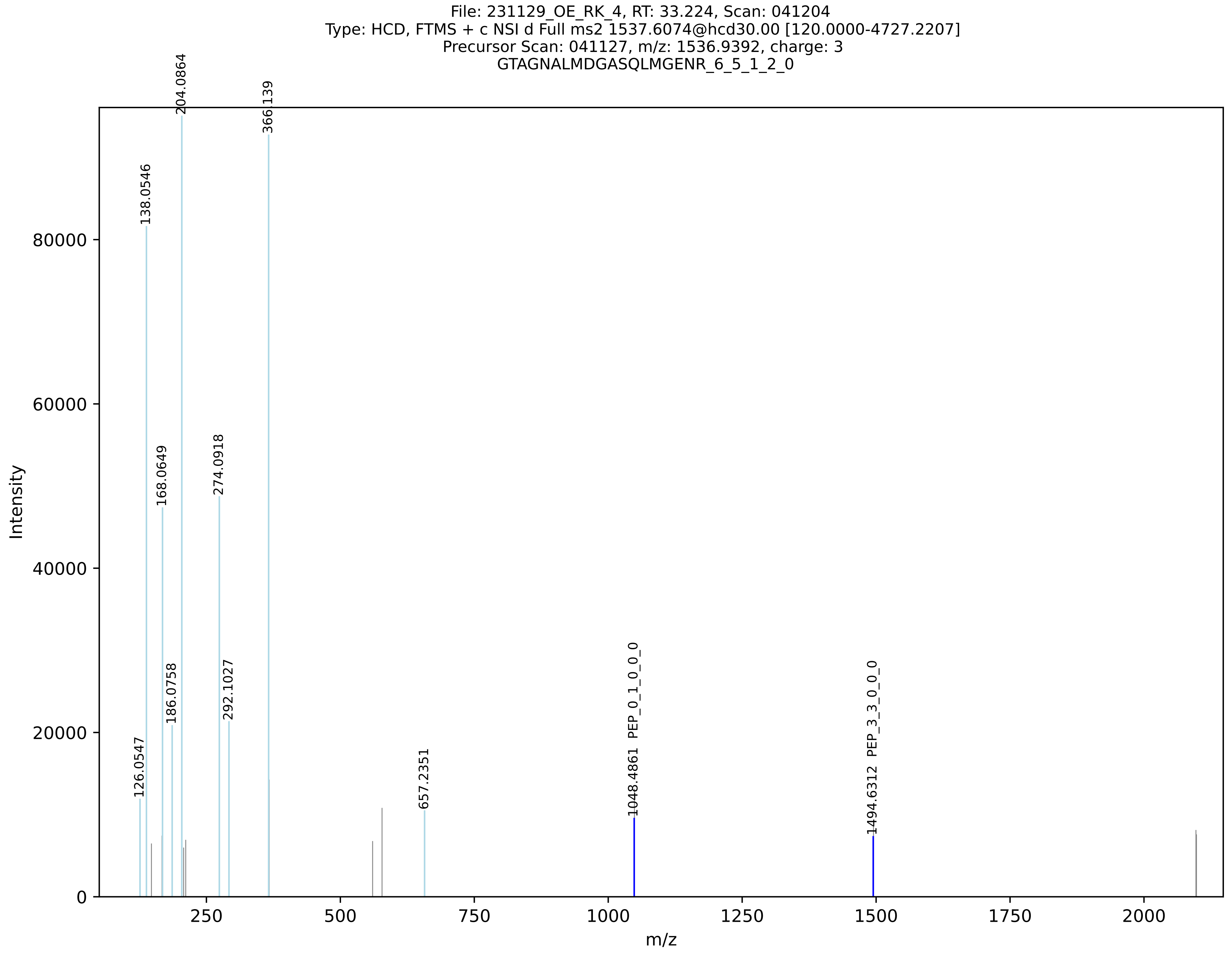

File: 231129_OE_RK_4, RT: 33.224, Scan: 041204
Type: HCD, FTMS + c NSI d Full ms2 1537.6074@hcd30.00 [120.0000-4727.2207]
Precursor Scan: 041127, m/z: 1536.9392, charge: 3
GTAGNALMDGASQLMGENR_6_5_1_2_0
204.0864
366.139
138.0546
168.0649
274.0918
186.0758
292.1027
126.0547
657.2351
1048.4861 PEP_0_1_0_0_0
1494.6312 PEP_3_3_0_0_0
Intensity
m/z
0
20000
40000
60000
80000
250
500
750
1000
1250
1500
1750
2000

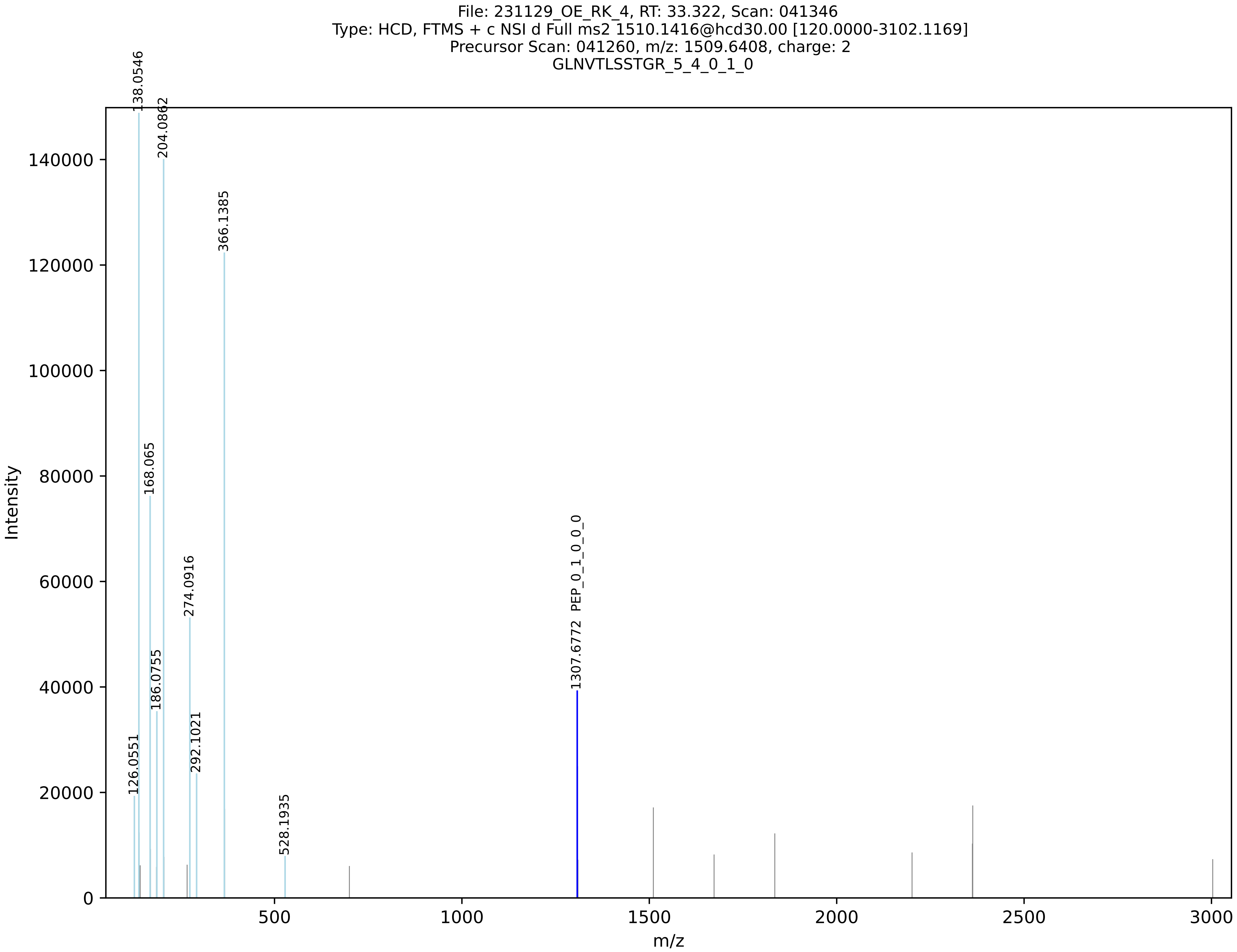

File: 231129_OE_RK_4, RT: 33.322, Scan: 041346
Type: HCD, FTMS + c NSI d Full ms2 1510.1416@hcd30.00 [120.0000-3102.1169]
Precursor Scan: 041260, m/z: 1509.6408, charge: 2
GLNVTLSSTGR_5_4_0_1_0
138.0546
204.0862
366.1385
168.065
274.0916
186.0755
126.0551
292.1021
528.1935
1307.6772 PEP_0_1_0_0_0
Intensity
m/z
0
20000
40000
60000
80000
100000
120000
140000
500
1000
1500
2000
2500
3000

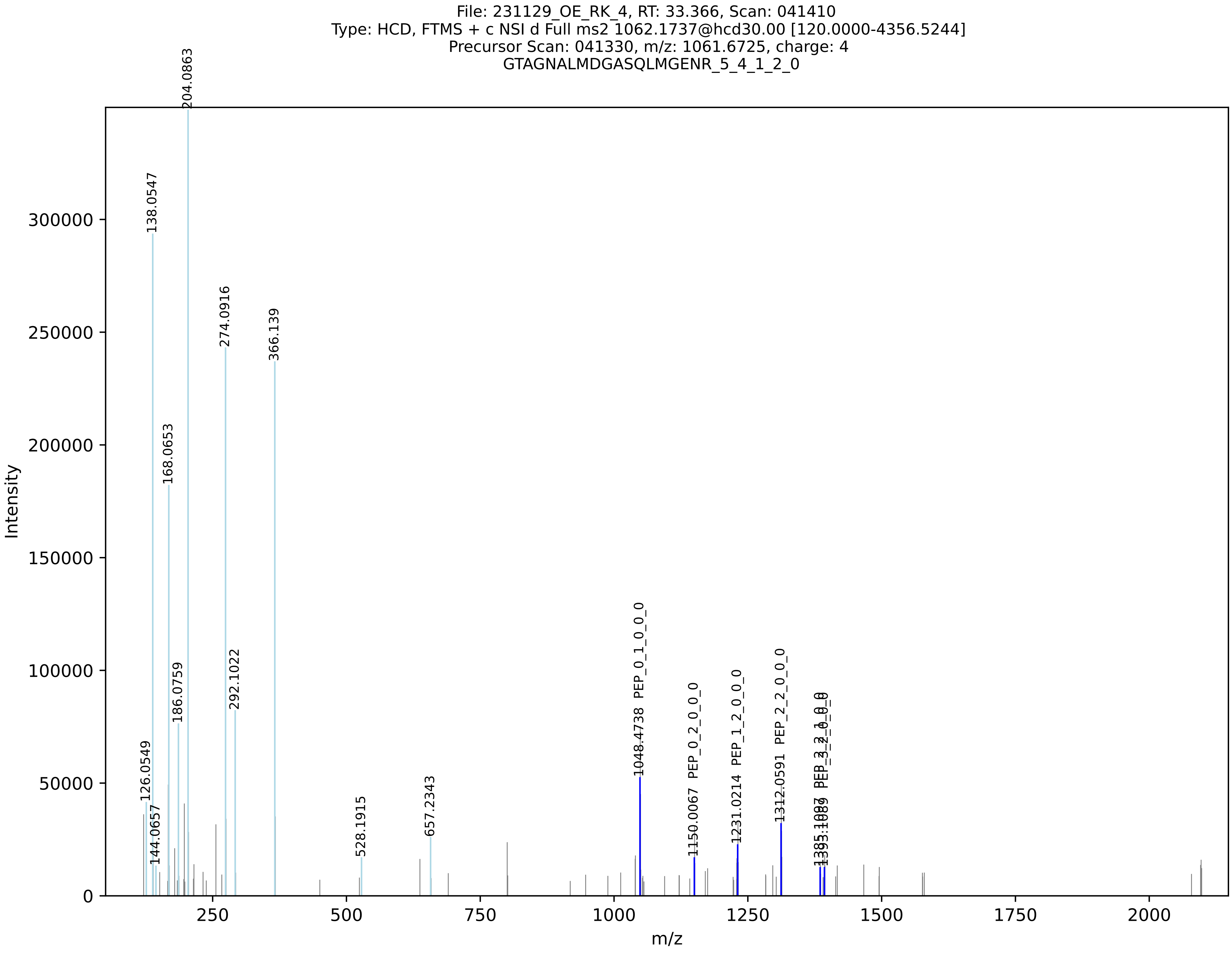
File: 231129_OE_RK_4, RT: 33.366, Scan: 041410
Type: HCD, FTMS + c NSI d Full ms2 1062.1737@hcd30.00 [120.0000-4356.5244]
Precursor Scan: 041330, m/z: 1061.6725, charge: 4
GTAGNALMDGASQLMGENR_5_4_1_2_0
Intensity
m/z
0
50000
100000
150000
200000
250000
300000
250
500
750
1000
1250
1500
1750
2000
126.0549
138.0547
144.0657
168.0653
186.0759
204.0863
274.0916
292.1022
366.139
528.1915
657.2343
1048.4738 PEP_0_1_0_0_0
1150.0067 PEP_0_2_0_0_0
1231.0214 PEP_1_2_0_0_0
1312.0591 PEP_2_2_0_0_0
1385.1087 PEP_3_2_1_0_0
1393.1089 PEP_3_2_0_0_0

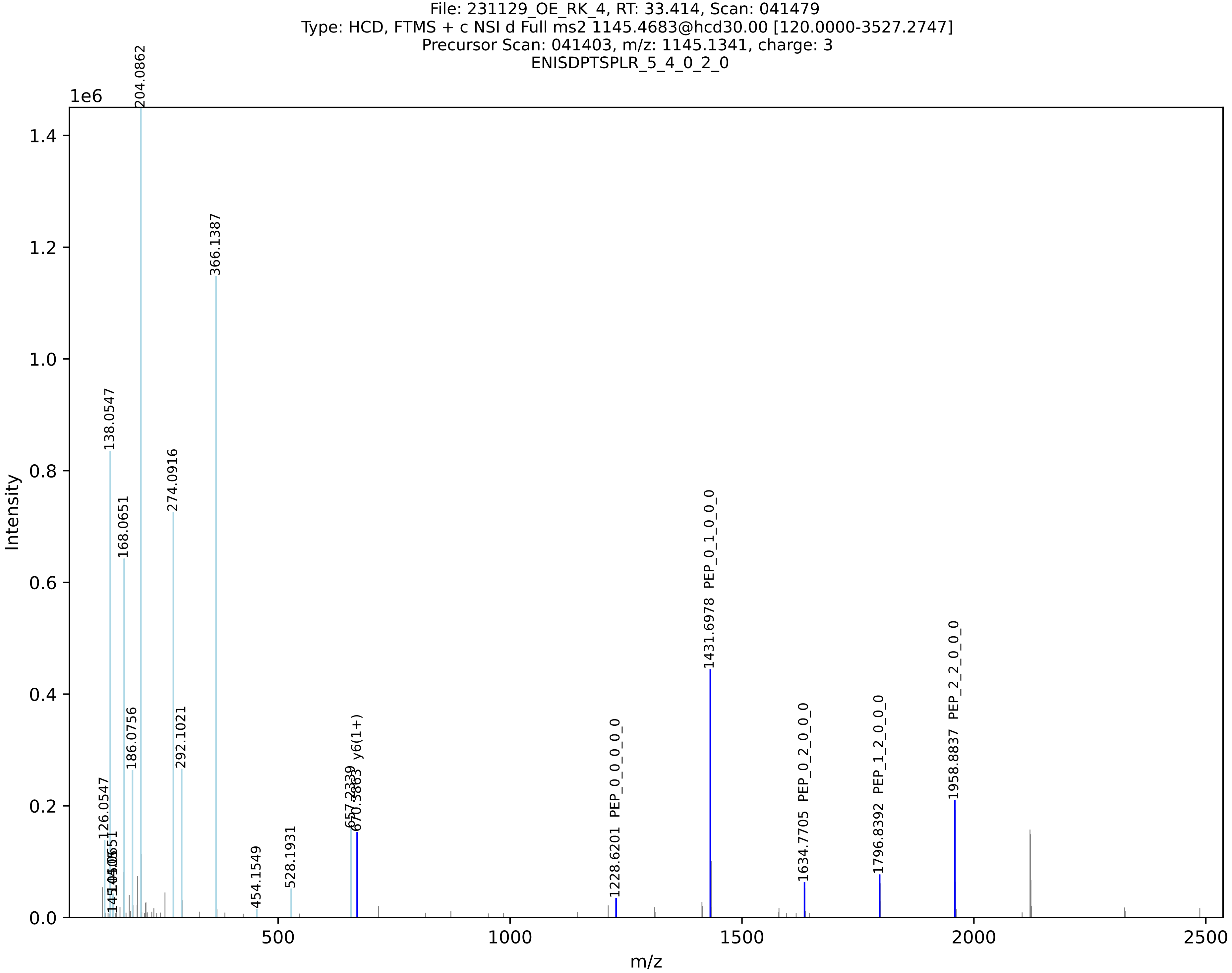
File: 231129_OE_RK_4, RT: 33.414, Scan: 041479
Type: HCD, FTMS + c NSI d Full ms2 1145.4683@hcd30.00 [120.0000-3527.2747]
Precursor Scan: 041403, m/z: 1145.1341, charge: 3
ENISDPTSPLR_5_4_0_2_0
1e6
Intensity
m/z
204.0862
366.1387
138.0547
274.0916
168.0651
186.0756
292.1021
126.0547
454.1549
528.1931
657.2339
670.3863 y6(1+)
1228.6201 PEP_0_0_0_0_0
1431.6978 PEP_0_1_0_0_0
1634.7705 PEP_0_2_0_0_0
1796.8392 PEP_1_2_0_0_0
1958.8837 PEP_2_2_0_0_0

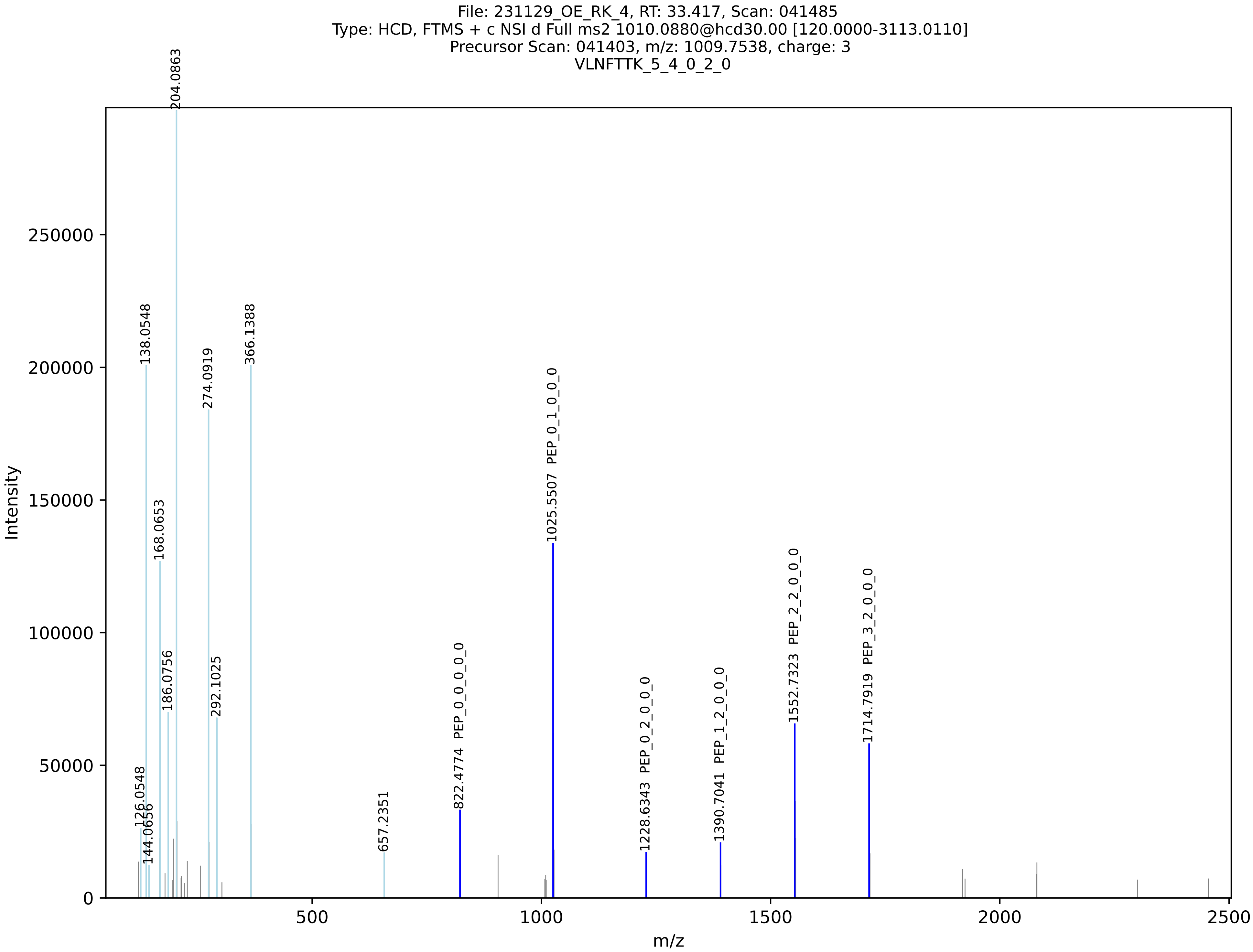
File: 231129_OE_RK_4, RT: 33.417, Scan: 041485
Type: HCD, FTMS + c NSI d Full ms2 1010.0880@hcd30.00 [120.0000-3113.0110]
Precursor Scan: 041403, m/z: 1009.7538, charge: 3
VLNFTTK_5_4_0_2_0
Intensity
m/z
204.0863
138.0548
366.1388
274.0919
168.0653
186.0756
292.1025
126.0548
144.0656
657.2351
822.4774 PEP_0_0_0_0_0
1025.5507 PEP_0_1_0_0_0
1228.6343 PEP_0_2_0_0_0
1390.7041 PEP_1_2_0_0_0
1552.7323 PEP_2_2_0_0_0
1714.7919 PEP_3_2_0_0_0
0
50000
100000
150000
200000
250000
500
1000
1500
2000
2500

File: 231129_OE_RK_4, RT: 33.439, Scan: 041520
Type: HCD, FTMS + c NSI d Full ms2 1207.1655@hcd30.00 [120.0000-3716.0686]
Precursor Scan: 041403, m/z: 1207.1656, charge: 3
ITYSIVQTNCSK_5_4_0_2_0

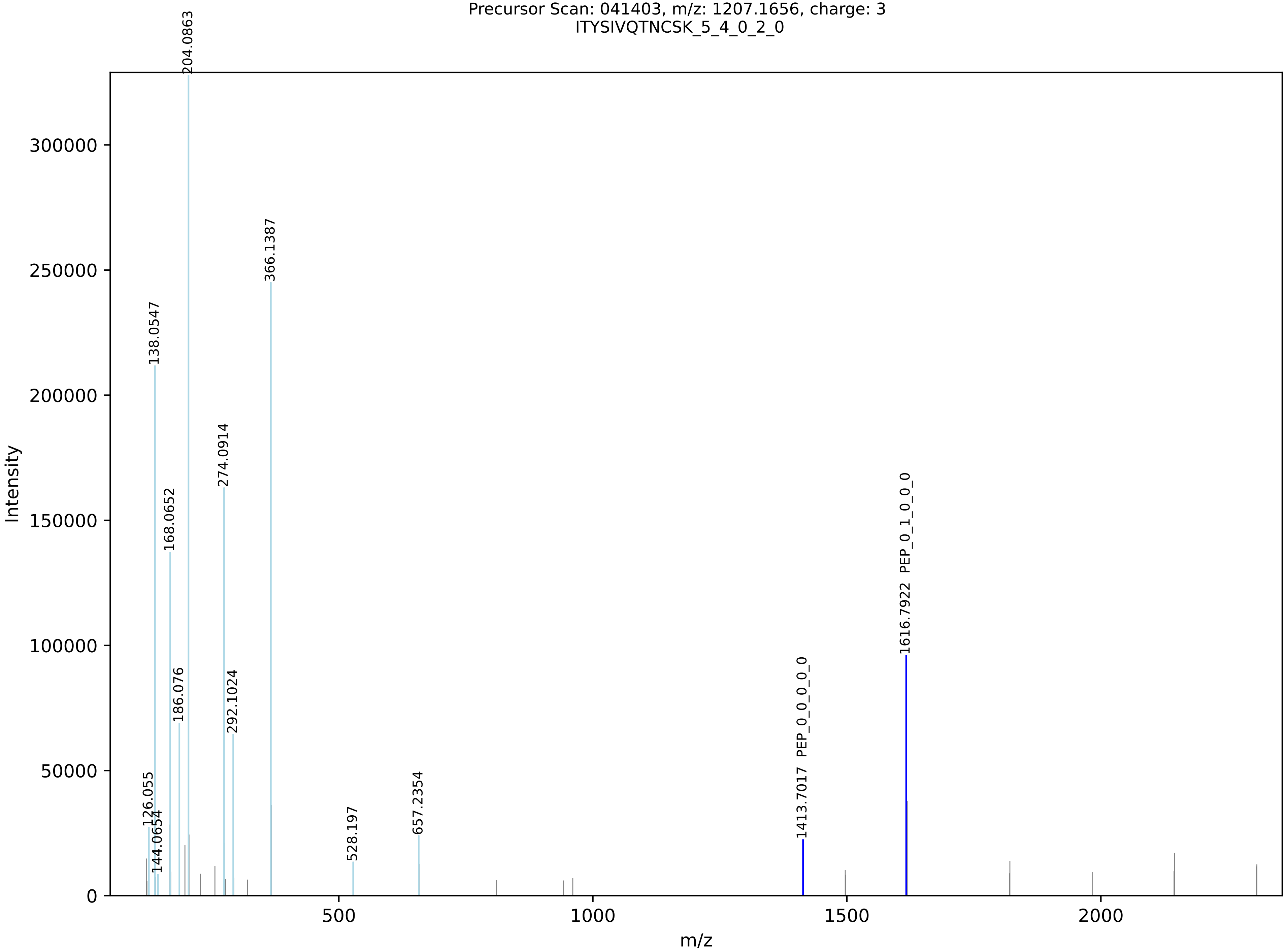

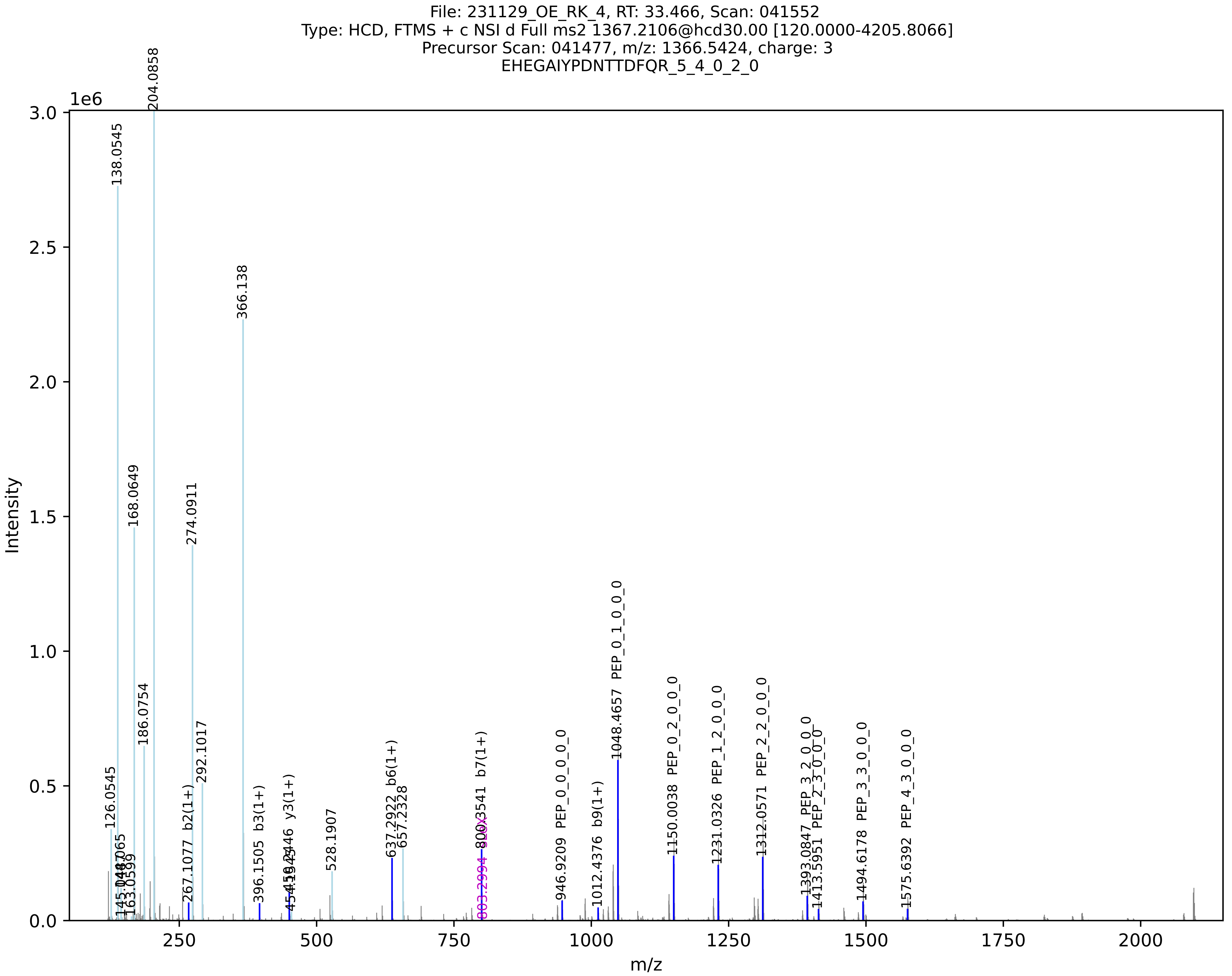
File: 231129_OE_RK_4, RT: 33.466, Scan: 041552
Type: HCD, FTMS + c NSI d Full ms2 1367.2106@hcd30.00 [120.0000-4205.8066]
Precursor Scan: 041477, m/z: 1366.5424, charge: 3
EHEGAIYPDNTTDFQR_5_4_0_2_0
1e6
Intensity
m/z
204.0858
138.0545
366.138
168.0649
274.0911
186.0754
292.1017
126.0545
267.1077 b2(1+)
396.1505 b3(1+)
y3(1+)
528.1907
637.2922 b6(1+)
657.2328
b7(1+)
946.9209 PEP_0_0_0_0_0
1012.4376 b9(1+)
1048.4657 PEP_0_1_0_0_0
1150.0038 PEP_0_2_0_0_0
1231.0326 PEP_1_2_0_0_0
1312.0571 PEP_2_2_0_0_0
1393.0847 PEP_3_2_0_0_0
1413.5951 PEP_2_3_0_0_0
1494.6178 PEP_3_3_0_0_0
1575.6392 PEP_4_3_0_0_0

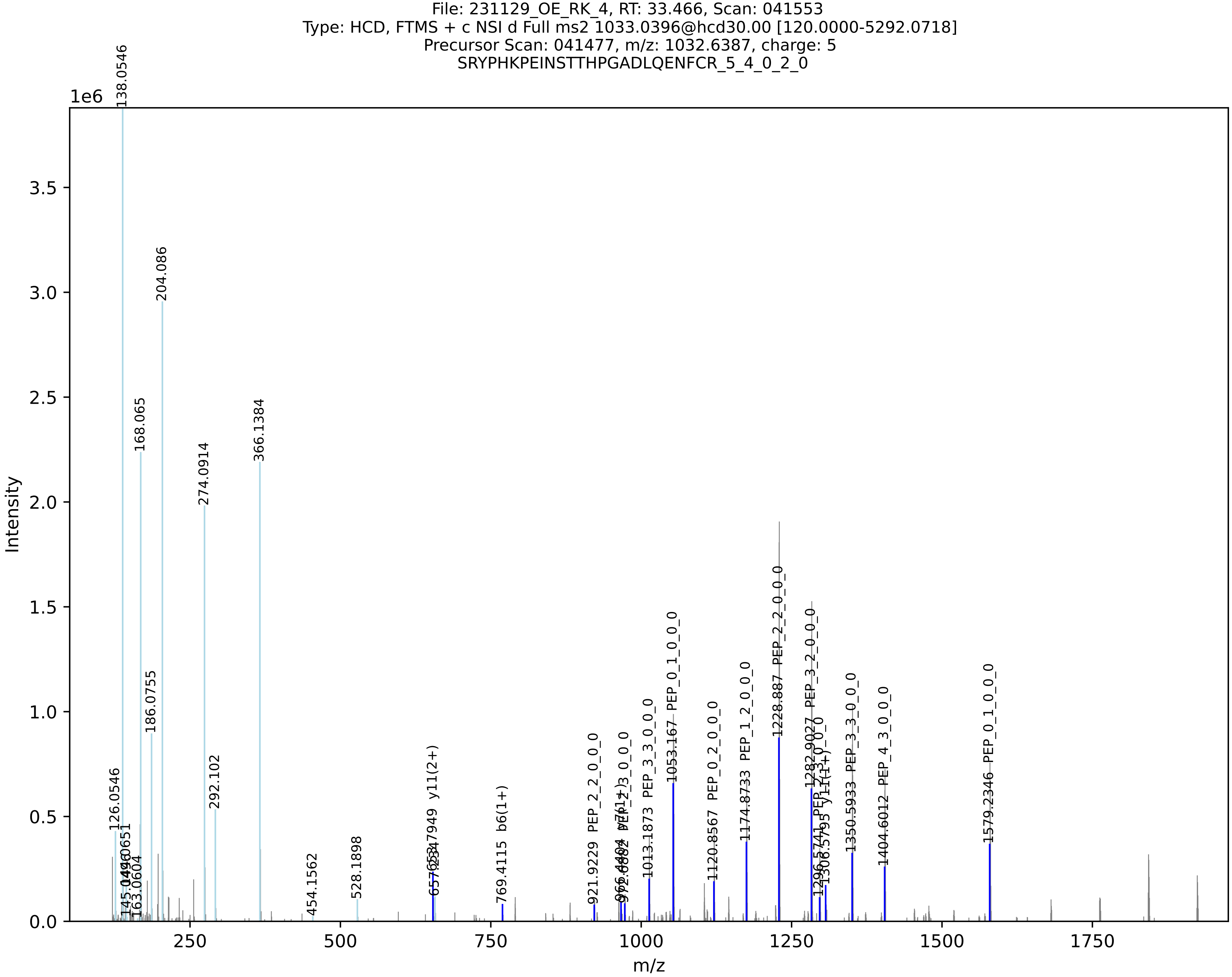

File: 231129_OE_RK_4, RT: 33.466, Scan: 041553
Type: HCD, FTMS + c NSI d Full ms2 1033.0396@hcd30.00 [120.0000-5292.0718]
Precursor Scan: 041477, m/z: 1032.6387, charge: 5
SRYPHKPEINSTTHPGADLQENFCR_5_4_0_2_0
1e6
Intensity
m/z
138.0546
204.086
168.065
366.1384
274.0914
186.0755
292.102
126.0546
454.1562
528.1898
y11(2+)
769.4115 b6(1+)
921.9229 PEP_2_2_0_0_0
1013.1873 PEP_3_3_0_0_0
1053.167 PEP_0_1_0_0_0
1120.8567 PEP_0_2_0_0_0
1174.8733 PEP_1_2_0_0_0
1228.887 PEP_2_2_0_0_0
1282.9027 PEP_3_2_0_0_0
1350.5933 PEP_3_3_0_0_0
1404.6012 PEP_4_3_0_0_0
1579.2346 PEP_0_1_0_0_0
y11(1+)

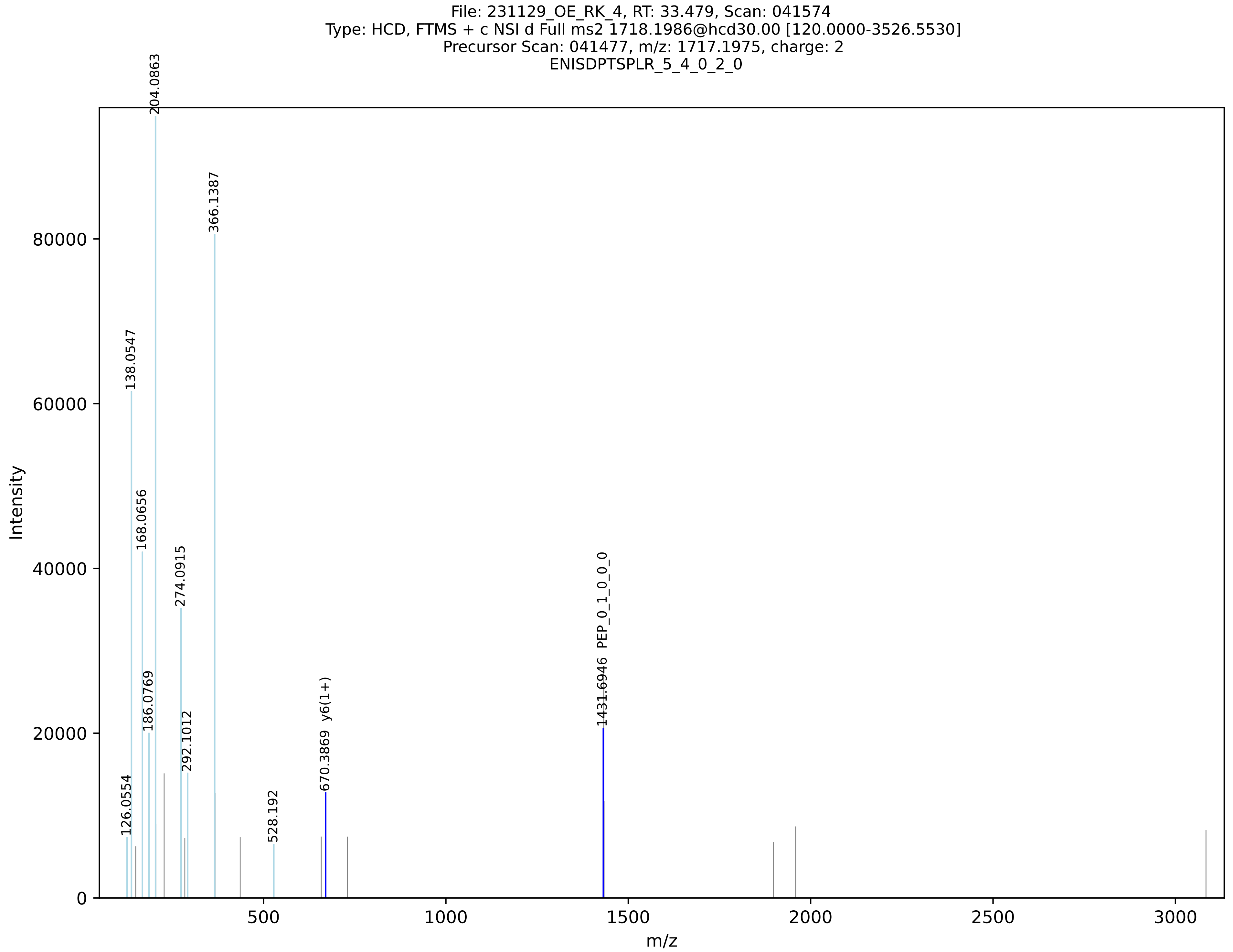
File: 231129_OE_RK_4, RT: 33.479, Scan: 041574
Type: HCD, FTMS + c NSI d Full ms2 1718.1986@hcd30.00 [120.0000-3526.5530]
Precursor Scan: 041477, m/z: 1717.1975, charge: 2
ENISDPTSPLR_5_4_0_2_0
204.0863
366.1387
138.0547
168.0656
274.0915
186.0769
292.1012
126.0554
528.192
670.3869 y6(1+)
1431.6946 PEP_0_1_0_0_0
Intensity
80000
60000
40000
20000
0
500
1000
1500
2000
2500
3000
m/z

File: 231129_OE_RK_4, RT: 33.566, Scan: 041696
Type: HCD, FTMS + c NSI d Full ms2 1025.4093@hcd30.00 [120.0000-4206.5259]
Precursor Scan: 041622, m/z: 1025.1586, charge: 4
EHEGAIYPDNTTDFQR_5_4_0_2_0

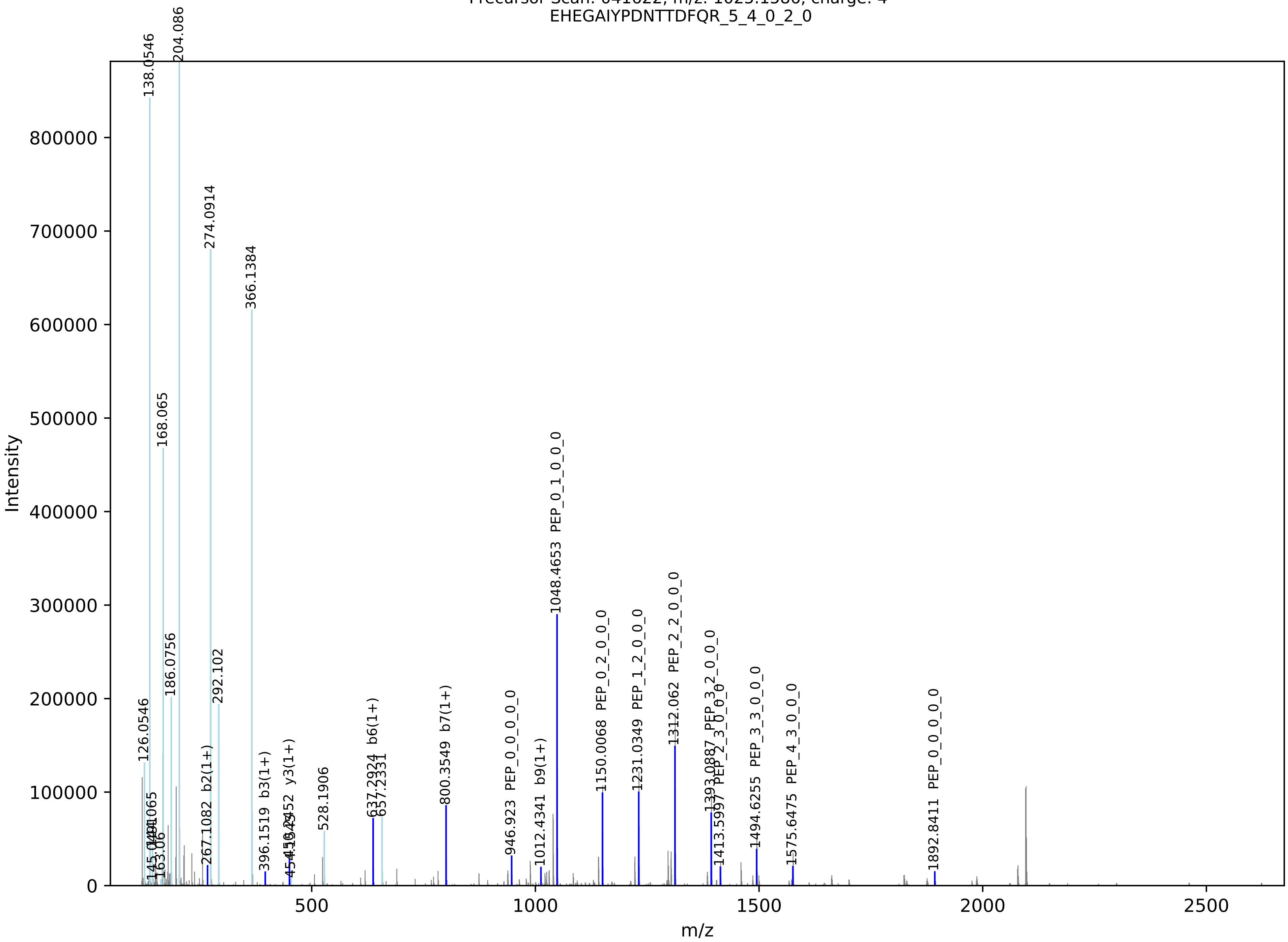

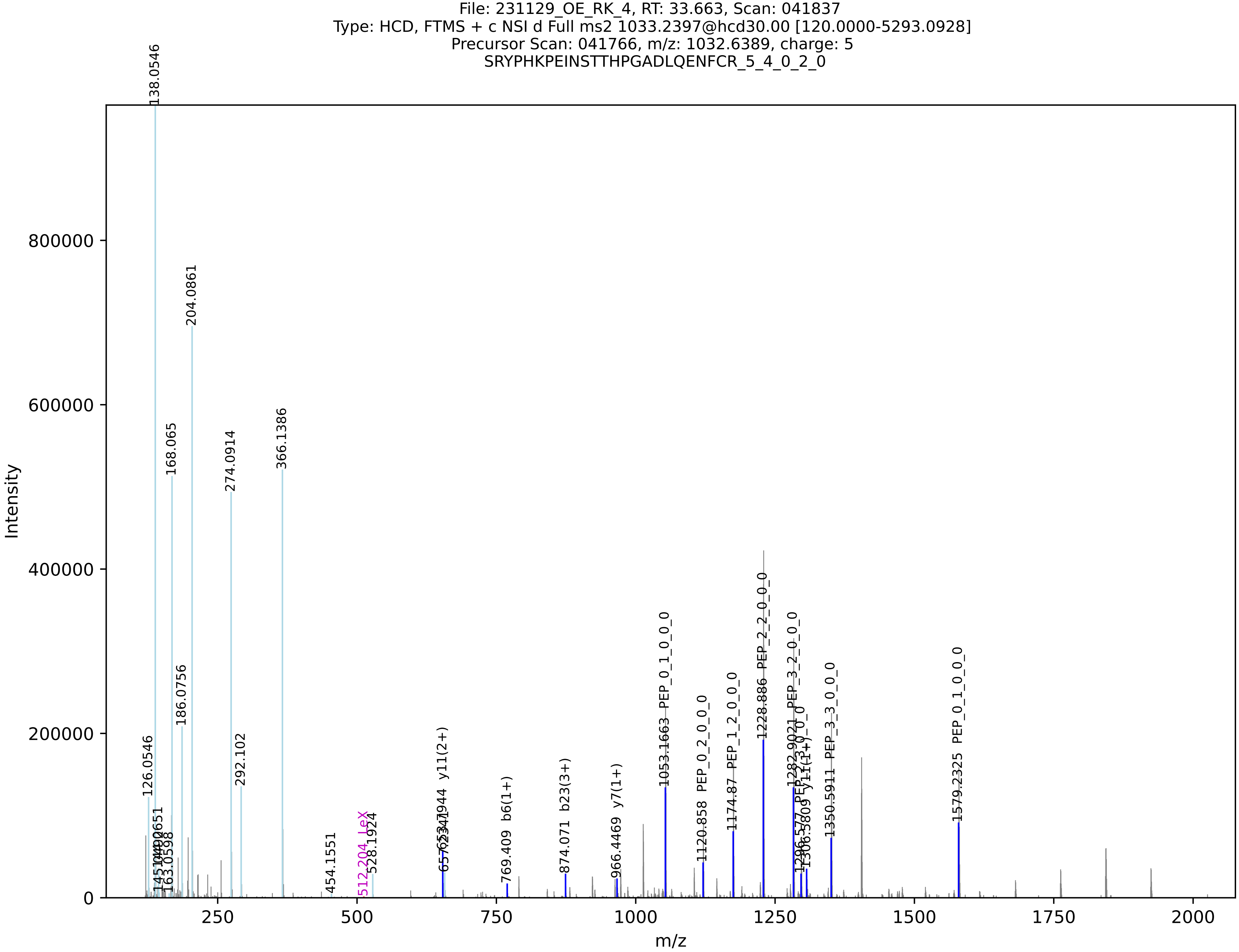

File: 231129_OE_RK_4, RT: 33.663, Scan: 041837
Type: HCD, FTMS + c NSI d Full ms2 1033.2397@hcd30.00 [120.0000-5293.0928]
Precursor Scan: 041766, m/z: 1032.6389, charge: 5
SRYPHKPEINSTTHPGADLQENFCR_5_4_0_2_0
Intensity
m/z
138.0546
204.0861
168.065
274.0914
366.1386
186.0756
292.102
126.0546
163.0598
454.1551
512.204_LeX
528.1924
y11(2+)
769.409 b6(1+)
874.071 b23(3+)
966.4469 y7(1+)
1053.1663 PEP_0_1_0_0_0
1120.858 PEP_0_2_0_0_0
1174.87 PEP_1_2_0_0_0
1228.886 PEP_2_2_0_0_0
1282.9021 PEP_3_2_0_0_0
1306.5809 y11(1+)
1350.5911 PEP_3_3_0_0_0
1579.2325 PEP_0_1_0_0_0

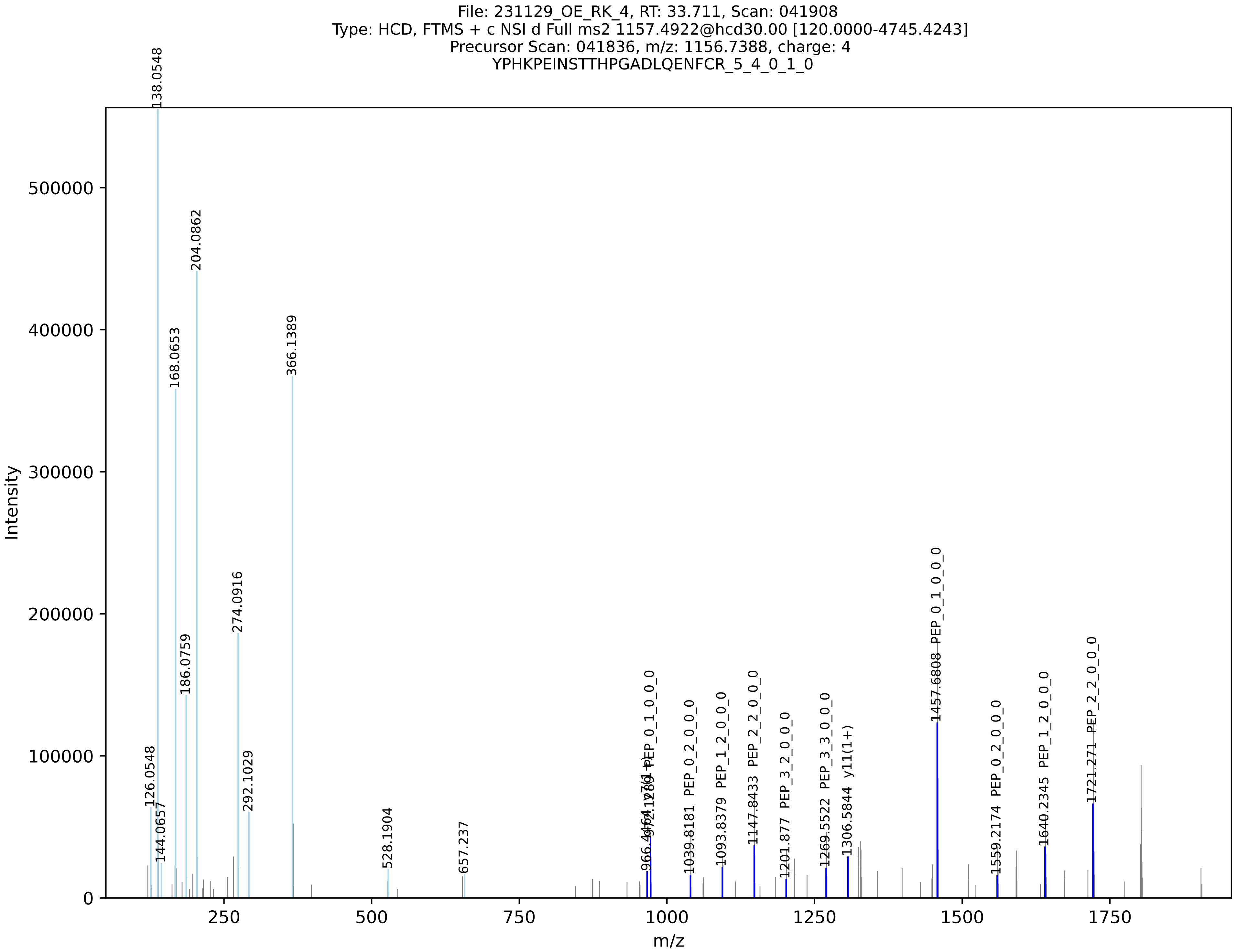

File: 231129_OE_RK_4, RT: 33.711, Scan: 041908
Type: HCD, FTMS + c NSI d Full ms2 1157.4922@hcd30.00 [120.0000-4745.4243]
Precursor Scan: 041836, m/z: 1156.7388, charge: 4
YPHKPEINSTTHPGADLQENFCR_5_4_0_1_0
Intensity
m/z
138.0548
204.0862
168.0653
366.1389
274.0916
186.0759
126.0548
144.0657
292.1029
528.1904
657.237
1039.8181 PEP_0_2_0_0_0
1093.8379 PEP_1_2_0_0_0
1147.8433 PEP_2_2_0_0_0
1201.877 PEP_3_2_0_0_0
1269.5522 PEP_3_3_0_0_0
1306.5844 y11(1+)
1457.6808 PEP_0_1_0_0_0
1559.2174 PEP_0_2_0_0_0
1640.2345 PEP_1_2_0_0_0
1721.271 PEP_2_2_0_0_0

File: 231129_OE_RK_4, RT: 33.743, Scan: 041953
Type: HCD, FTMS + c NSI d Full ms2 1025.4082@hcd30.00 [120.0000-4206.5215]
Precursor Scan: 041897, m/z: 1025.1578, charge: 4
EHEGAIYPDNTTDFQR_5_4_0_2_0

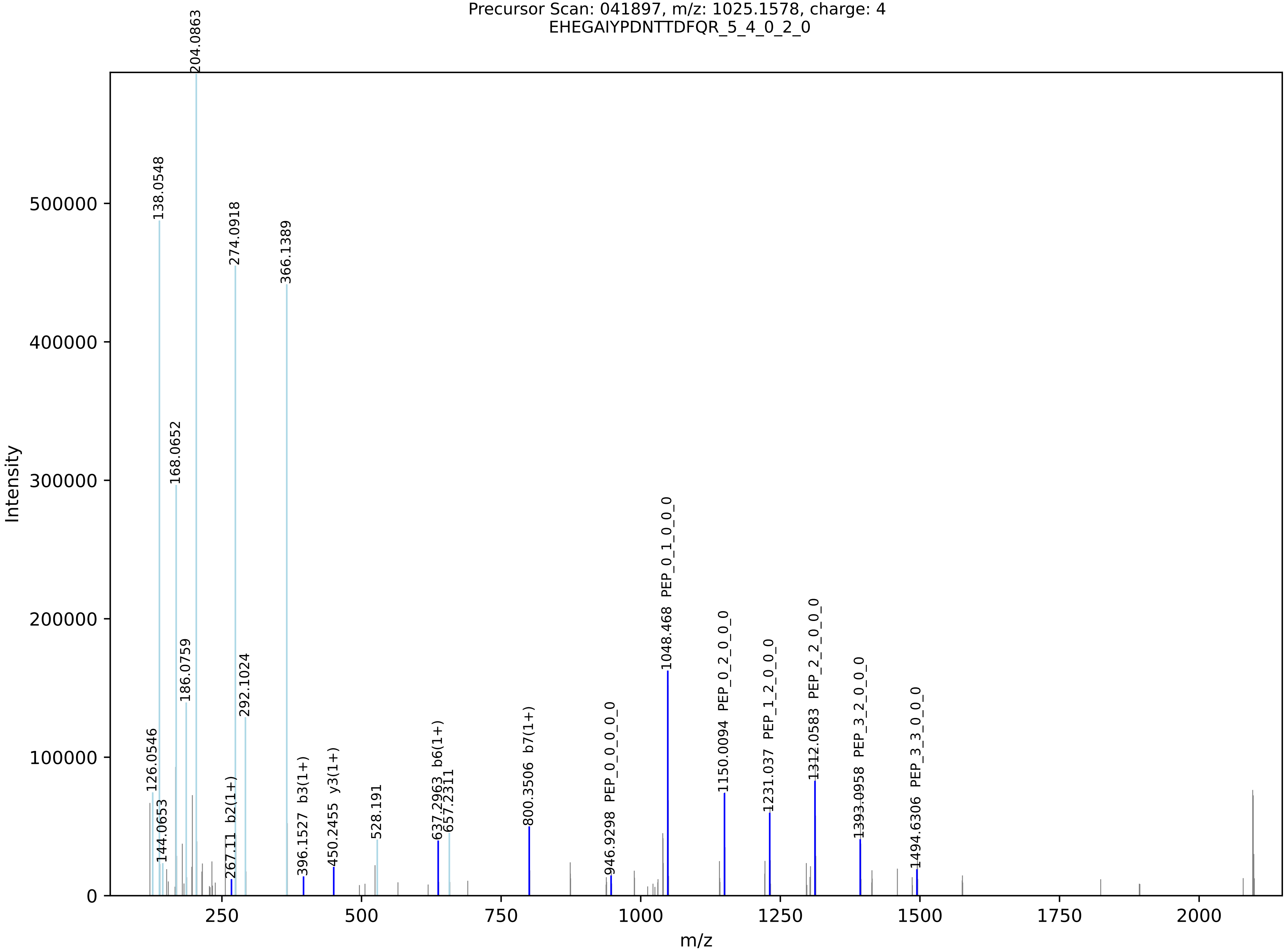

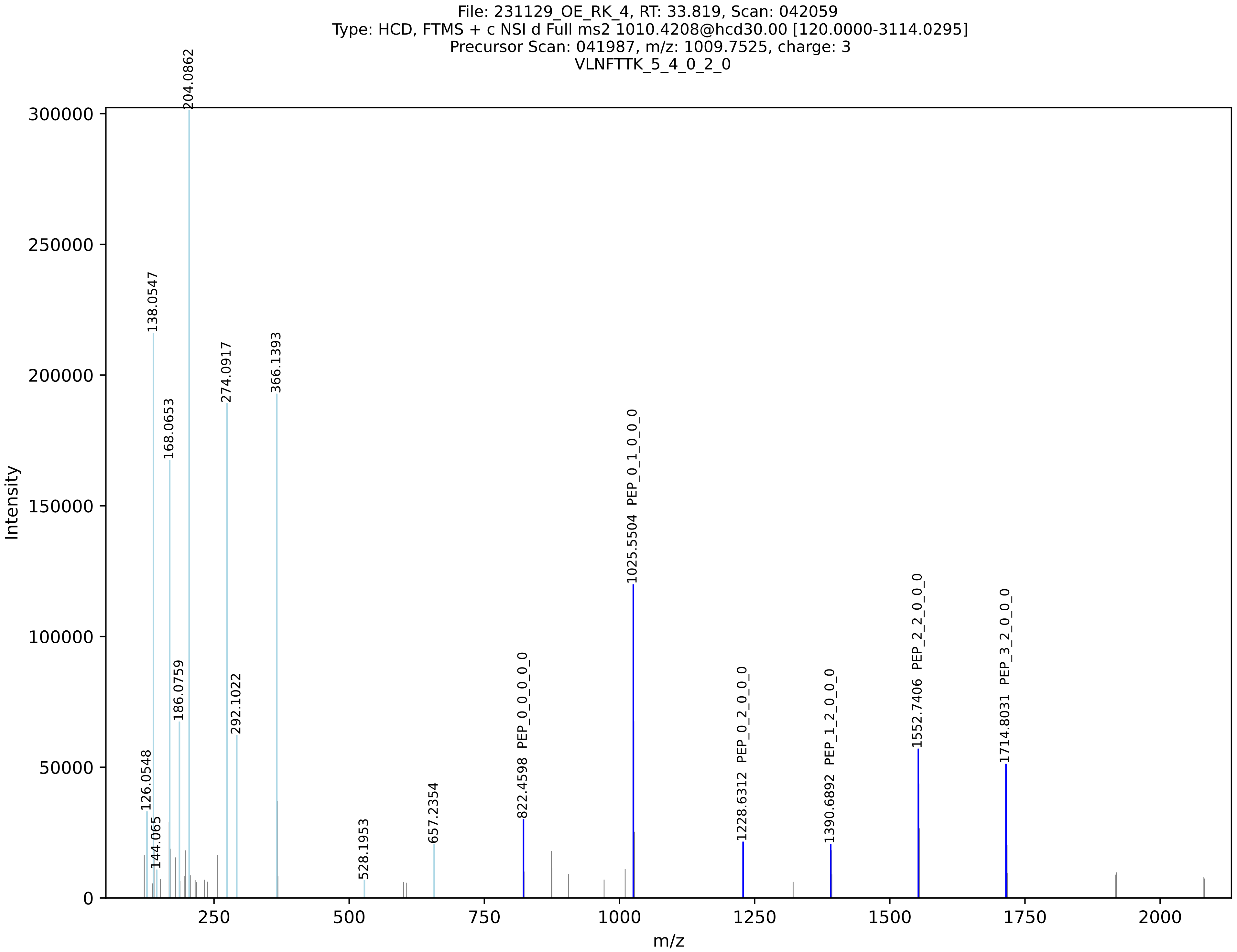
File: 231129_OE_RK_4, RT: 33.819, Scan: 042059
Type: HCD, FTMS + c NSI d Full ms2 1010.4208@hcd30.00 [120.0000-3114.0295]
Precursor Scan: 041987, m/z: 1009.7525, charge: 3
VLNFTTK_5_4_0_2_0
Intensity
m/z
204.0862
138.0547
168.0653
274.0917
366.1393
186.0759
292.1022
126.0548
144.065
528.1953
657.2354
822.4598 PEP_0_0_0_0_0_
1025.5504 PEP_0_1_0_0_0_
1228.6312 PEP_0_2_0_0_0_
1390.6892 PEP_1_2_0_0_0_
1552.7406 PEP_2_2_0_0_0_
1714.8031 PEP_3_2_0_0_0_
0
50000
100000
150000
200000
250000
300000
250
500
750
1000
1250
1500
1750
2000

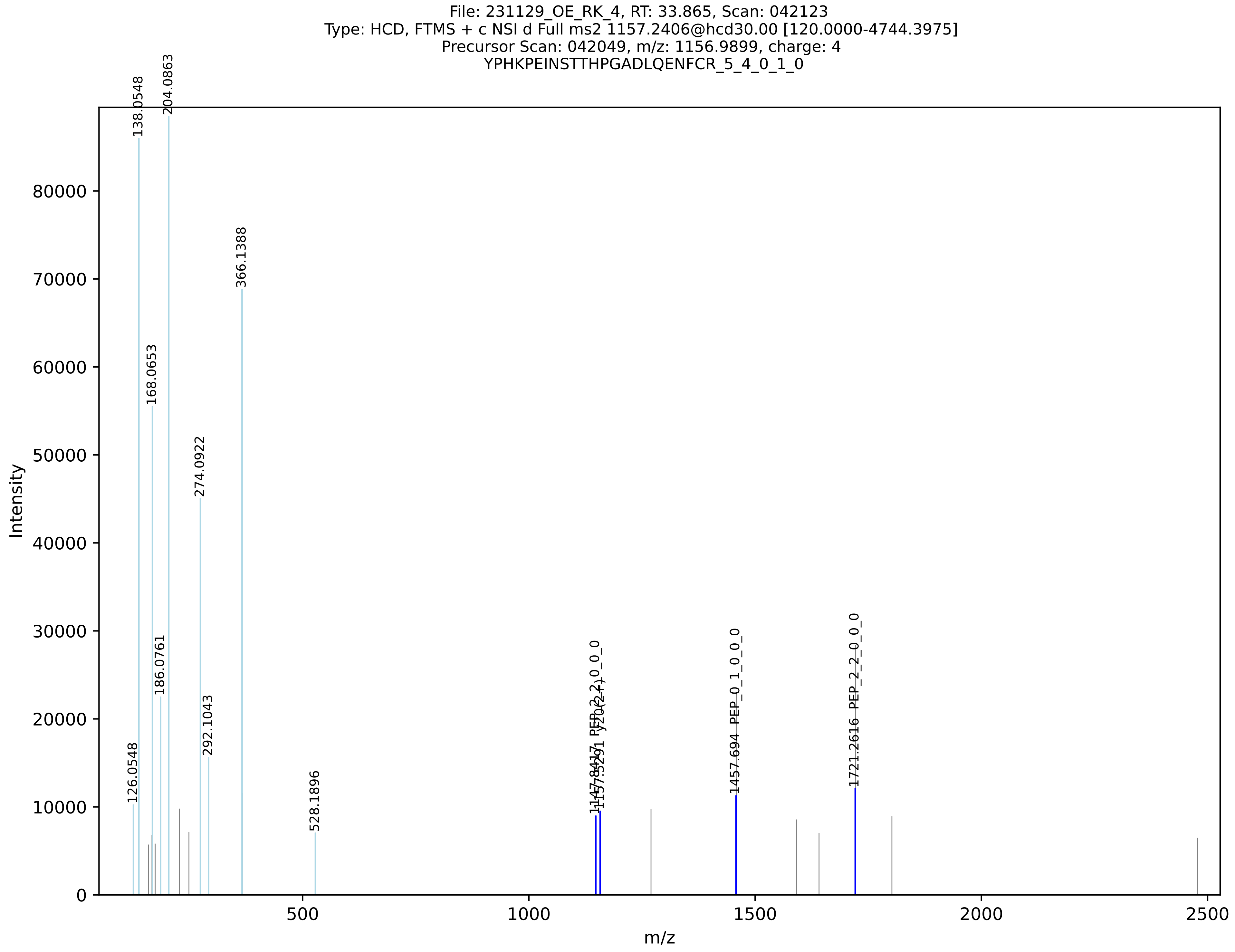

File: 231129_OE_RK_4, RT: 33.865, Scan: 042123
Type: HCD, FTMS + c NSI d Full ms2 1157.2406@hcd30.00 [120.0000-4744.3975]
Precursor Scan: 042049, m/z: 1156.9899, charge: 4
YPHKPEINSTTHPGADLQENFCR_5_4_0_1_0
Intensity
m/z
0
10000
20000
30000
40000
50000
60000
70000
80000
500
1000
1500
2000
2500
126.0548
138.0548
168.0653
186.0761
204.0863
274.0922
292.1043
366.1388
528.1896
1147.8417_PEP_2_2_0_0_0
1157.5291_y20(2+)
1457.694_PEP_0_1_0_0_0_
1721.2616_PEP_2_2_0_0_0_

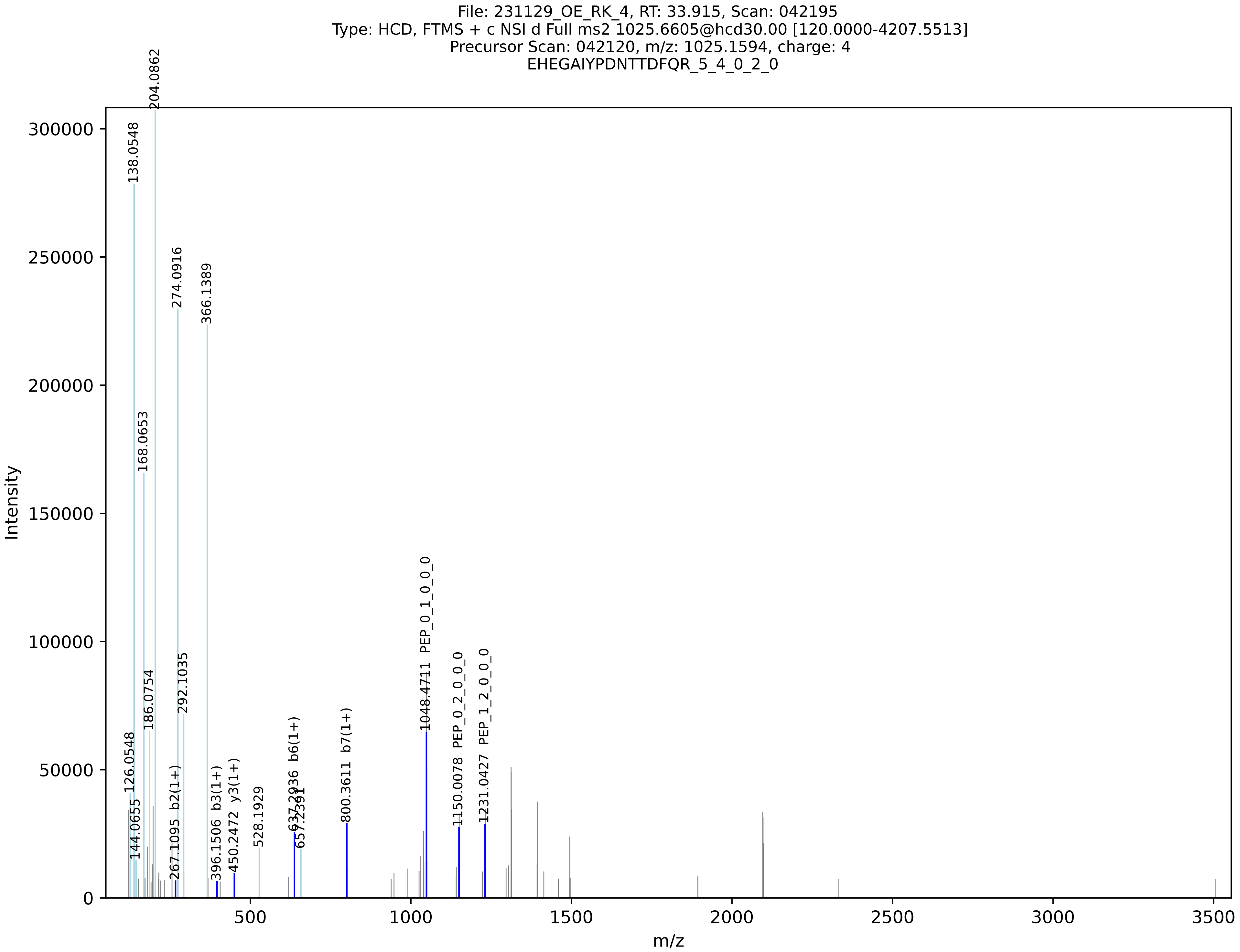
File: 231129_OE_RK_4, RT: 33.915, Scan: 042195
Type: HCD, FTMS + c NSI d Full ms2 1025.6605@hcd30.00 [120.0000-4207.5513]
Precursor Scan: 042120, m/z: 1025.1594, charge: 4
EHEGAIYPDNTTDFQR_5_4_0_2_0
Intensity
m/z
204.0862
138.0548
274.0916
366.1389
168.0653
186.0754
292.1035
126.0548
144.0655
267.1095 b2(1+)
396.1506 b3(1+)
450.2472 y3(1+)
528.1929
637.2936 b6(1+)
657.2391
800.3611 b7(1+)
1048.4711 PEP_0_1_0_0_0
1150.0078 PEP_0_2_0_0_0
1231.0427 PEP_1_2_0_0_0

File: 231129_OE_RK_4, RT: 34.027, Scan: 042360
Type: HCD, FTMS + c NSI d Full ms2 1194.4868@hcd30.00 [120.0000-3677.2717]
Precursor Scan: 042265, m/z: 1193.8219, charge: 3
ENISDPTSPLR_5_4_1_2_0

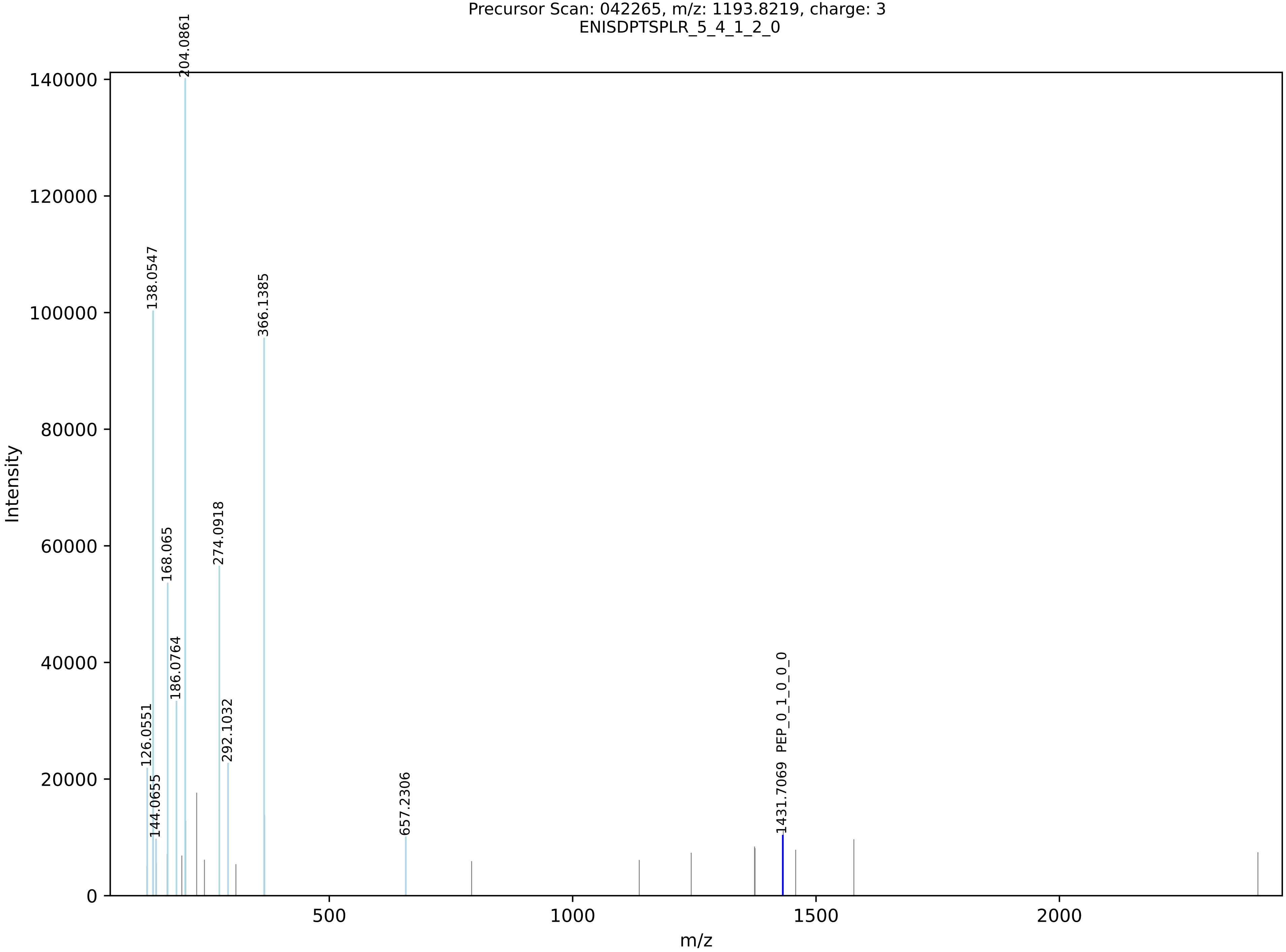

File: 231129_OE_RK_4, RT: 34.066, Scan: 042414
Type: HCD, FTMS + c NSI d Full ms2 1367.2104@hcd30.00 [120.0000-4205.8057]
Precursor Scan: 042339, m/z: 1366.5425, charge: 3
EHEGAIYPDNTTDFQR_5_4_0_2_0

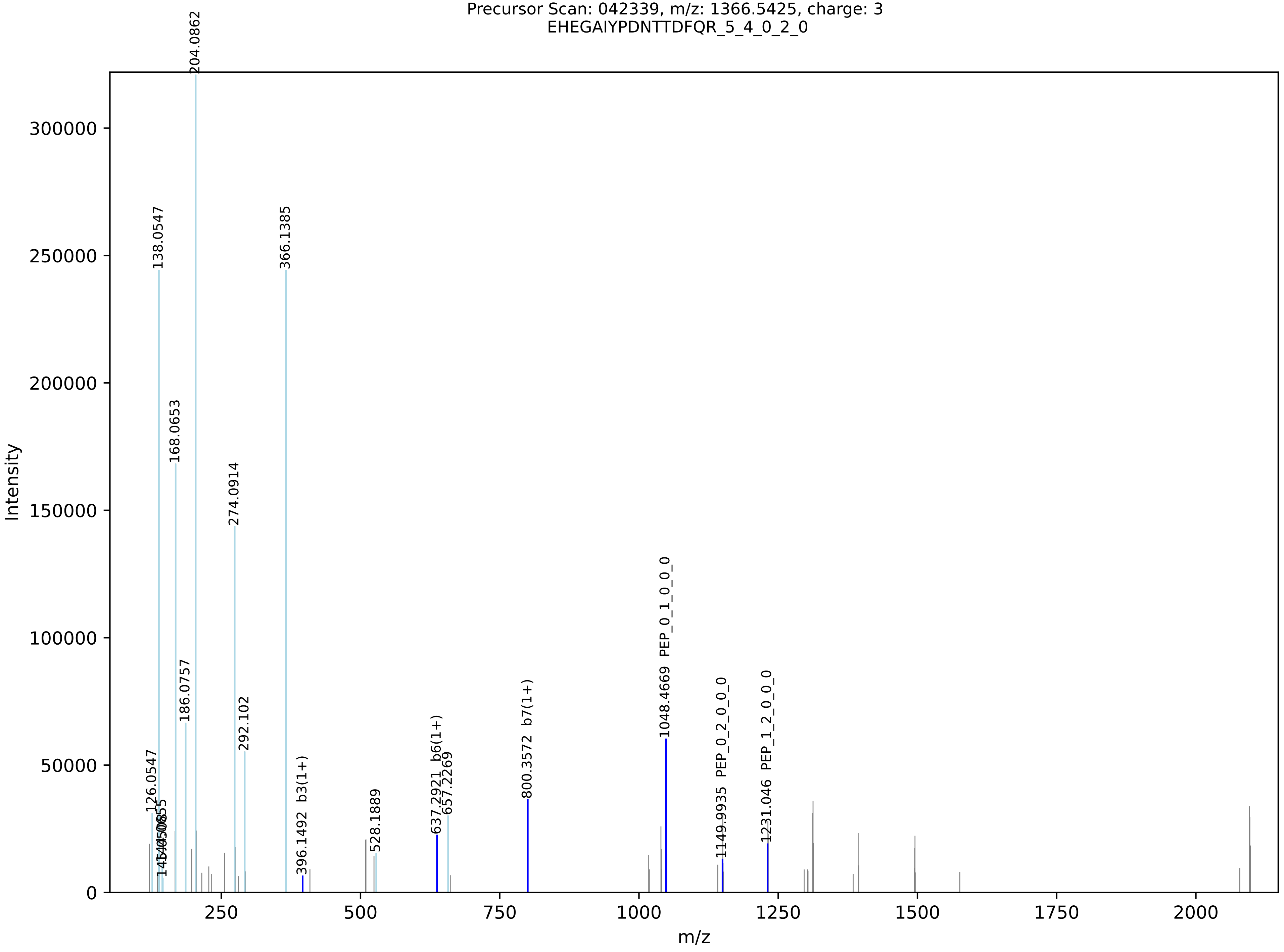

File: 231129_OE_RK_4, RT: 34.086, Scan: 042447
Type: HCD, FTMS + c NSI d Full ms2 1718.1976@hcd30.00 [120.0000-3526.5510]
Precursor Scan: 042339, m/z: 1717.1952, charge: 2
ENISDPTSPLR_5_4_0_2_0

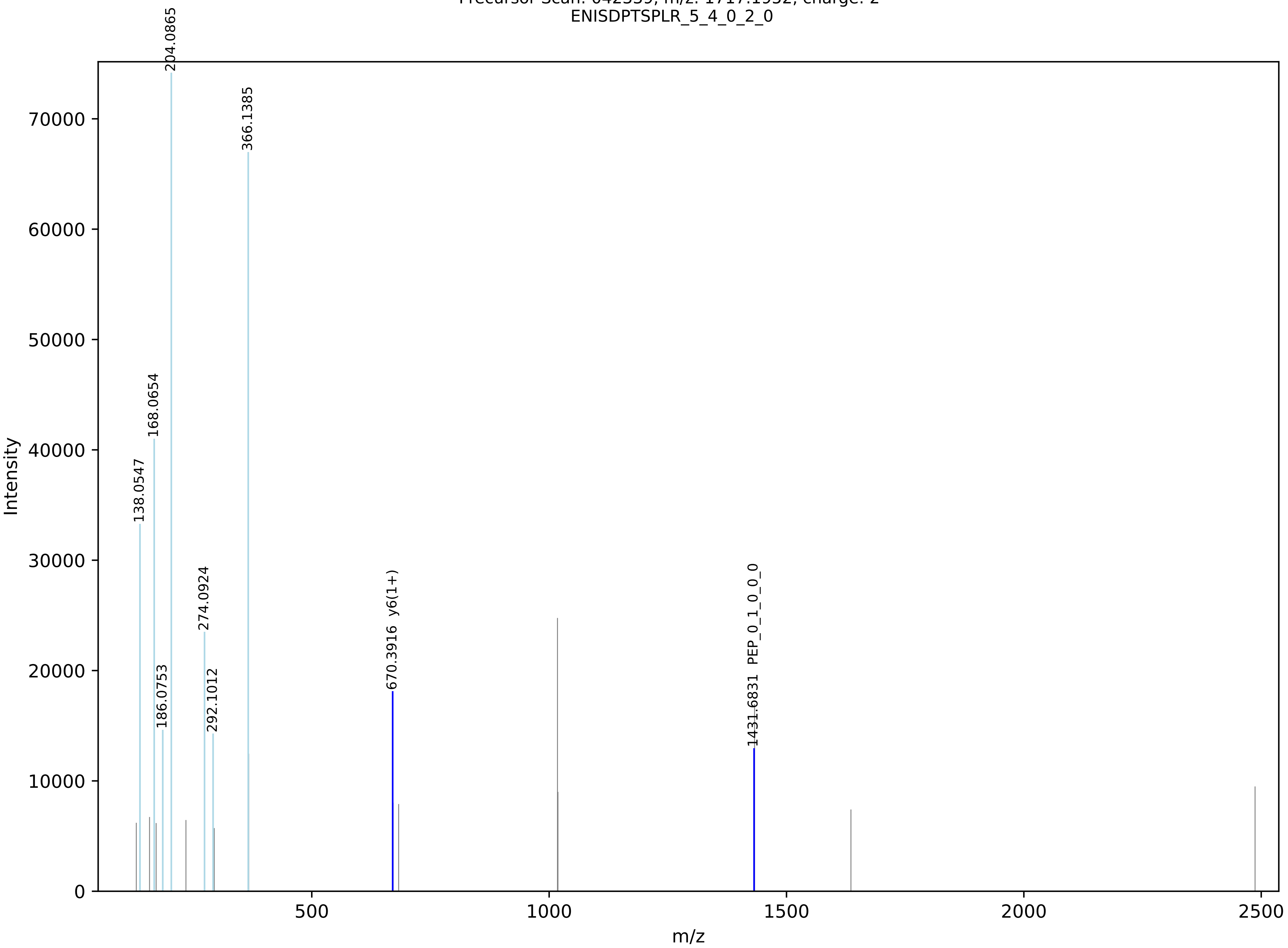

File: 231129_OE_RK_4, RT: 34.115, Scan: 042486
Type: HCD, FTMS + c NSI d Full ms2 926.1940@hcd30.00 [120.0000-4747.1597]
Precursor Scan: 042412, m/z: 925.7945, charge: 5
YPHKPEINSTTHPGADLQENFCR_5_4_0_1_0

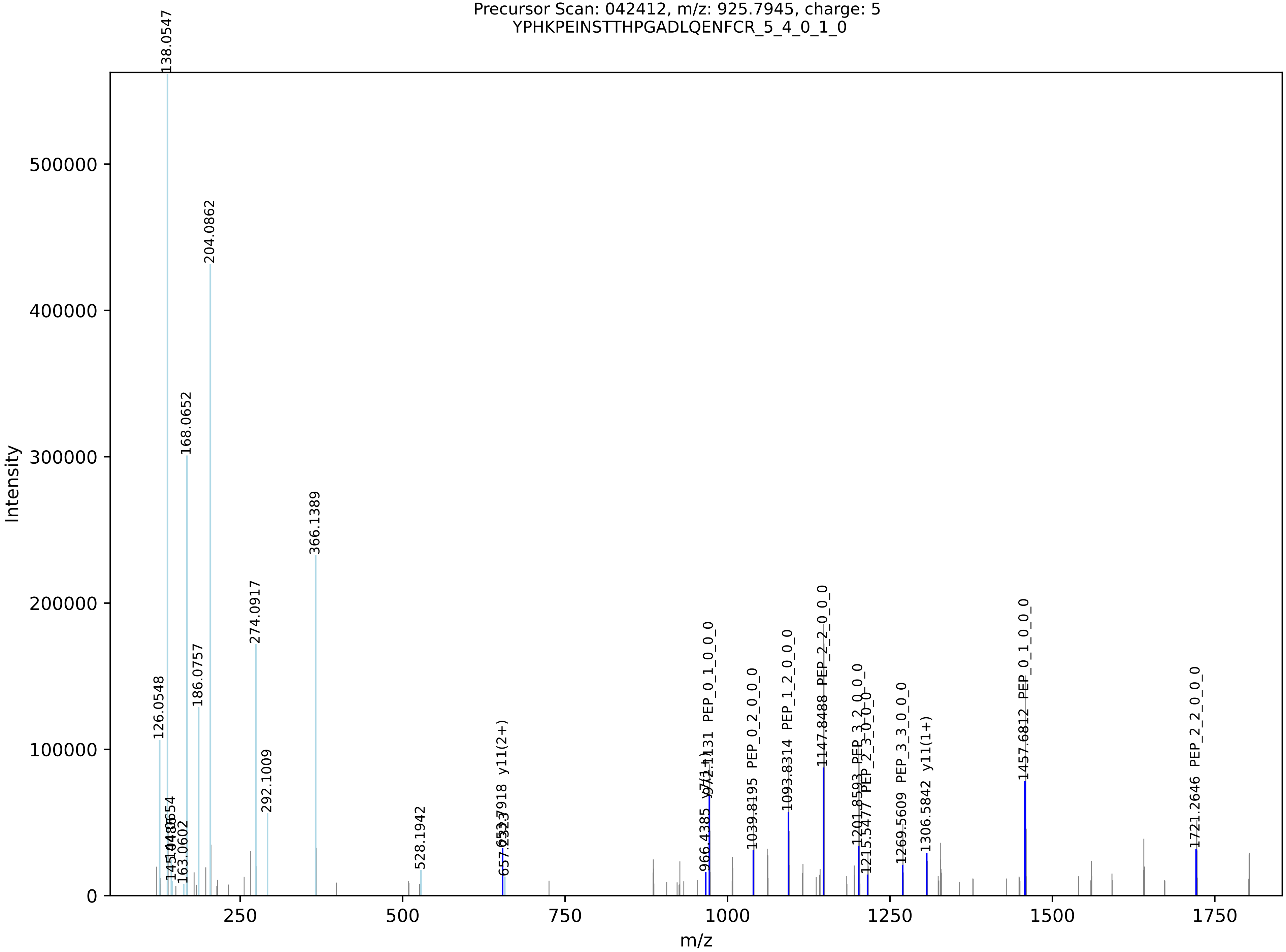

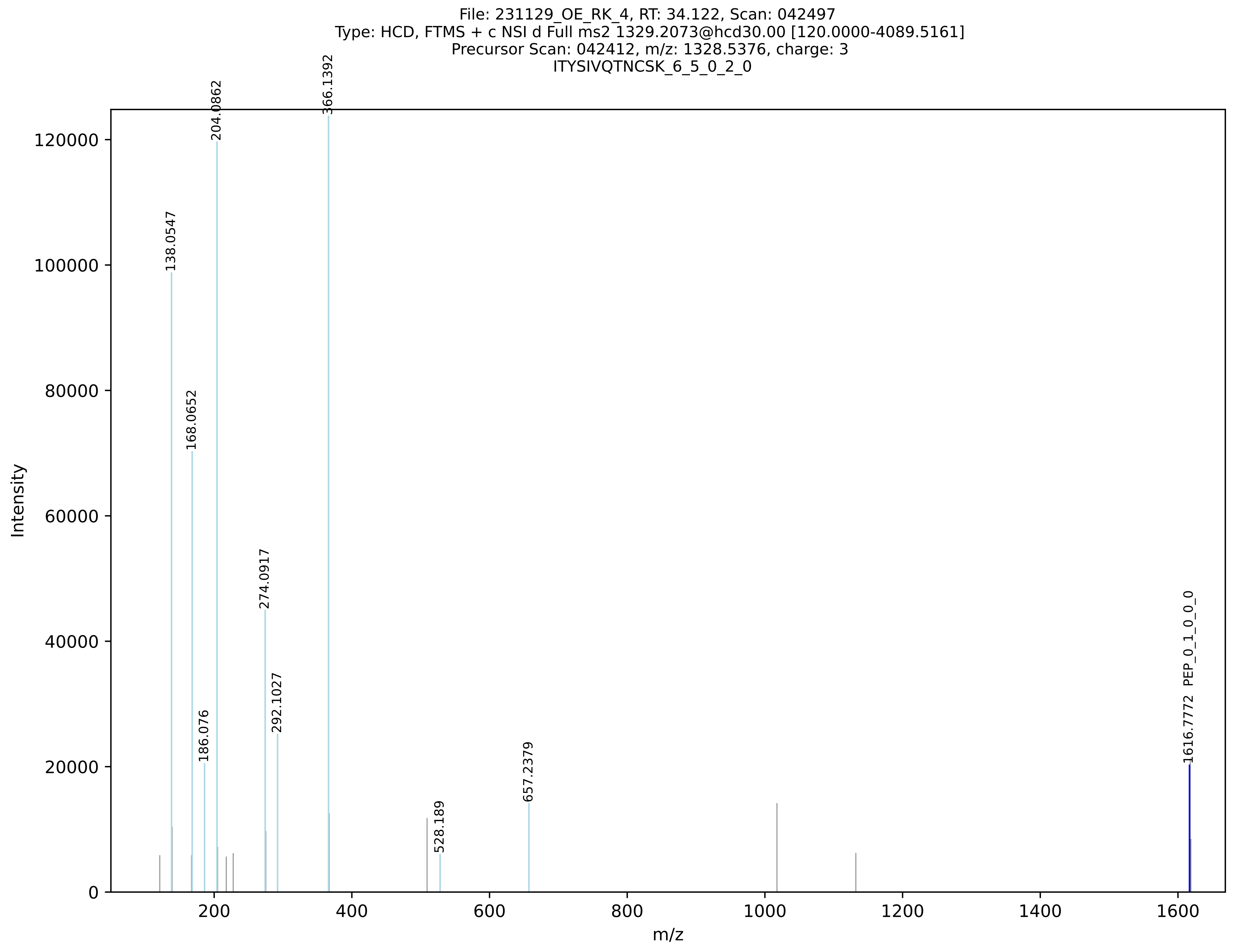
File: 231129_OE_RK_4, RT: 34.122, Scan: 042497
Type: HCD, FTMS + c NSI d Full ms2 1329.2073@hcd30.00 [120.0000-4089.5161]
Precursor Scan: 042412, m/z: 1328.5376, charge: 3
ITYSIVQTNCSK_6_5_0_2_0
Intensity
m/z
138.0547
168.0652
186.076
204.0862
274.0917
292.1027
366.1392
528.189
657.2379
1616.7772 PEP_0_1_0_0_0
0
20000
40000
60000
80000
100000
120000
200
400
600
800
1000
1200
1400
1600

File: 231129_OE_RK_4, RT: 34.207, Scan: 042618
Type: HCD, FTMS + c NSI d Full ms2 1207.4979@hcd30.00 [120.0000-3717.0857]
Precursor Scan: 042546, m/z: 1206.8298, charge: 3
ITYSIVQTNCSK_5_4_0_2_0

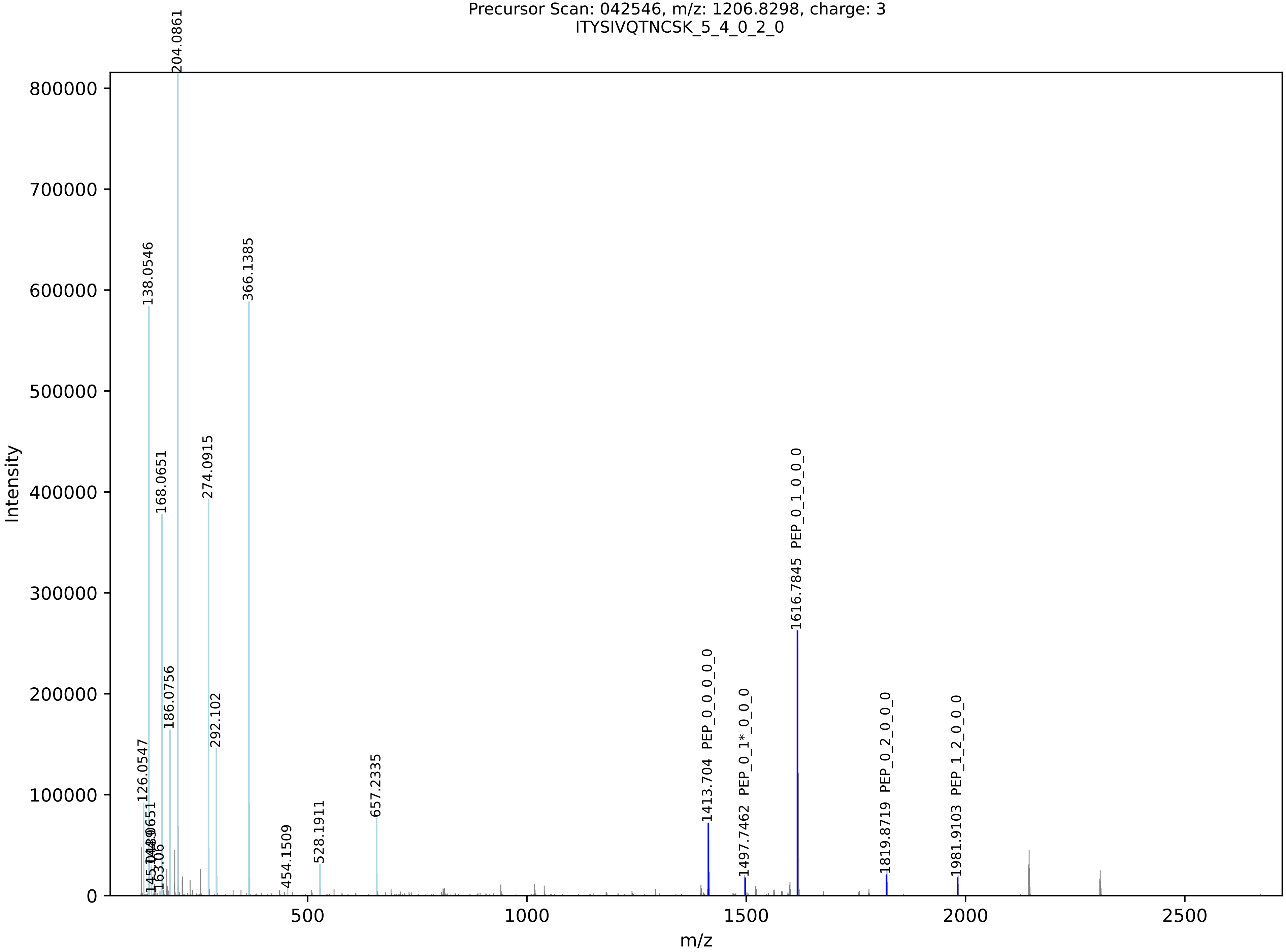

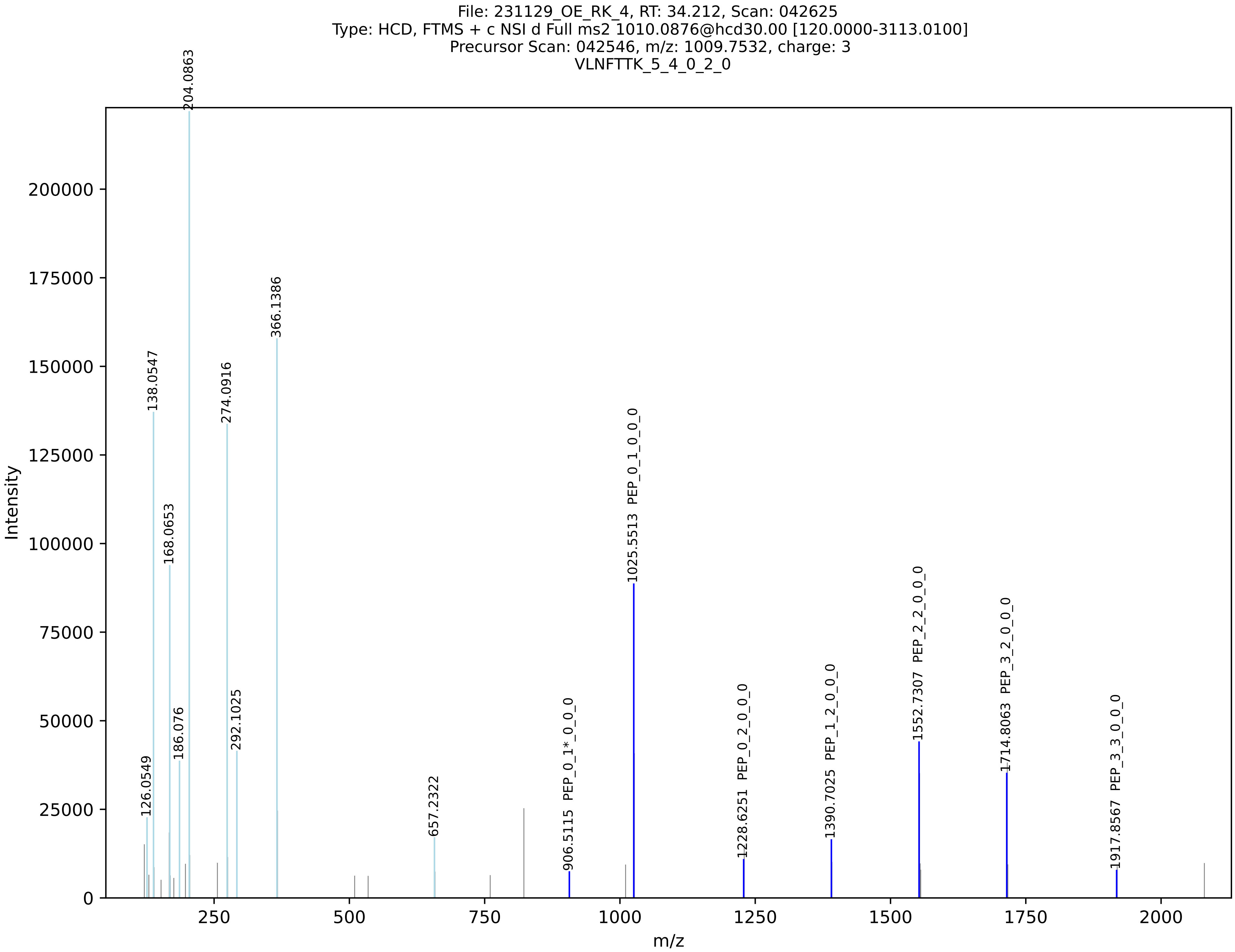

File: 231129_OE_RK_4, RT: 34.212, Scan: 042625
Type: HCD, FTMS + c NSI d Full ms2 1010.0876@hcd30.00 [120.0000-3113.0100]
Precursor Scan: 042546, m/z: 1009.7532, charge: 3
VLNFTTK_5_4_0_2_0
Intensity
m/z
0
25000
50000
75000
100000
125000
150000
175000
200000
250
500
750
1000
1250
1500
1750
2000
204.0863
366.1386
138.0547
274.0916
168.0653
186.076
292.1025
126.0549
657.2322
906.5115 PEP_0_1*_0_0_0
1025.5513 PEP_0_1_0_0_0
1228.6251 PEP_0_2_0_0_0
1390.7025 PEP_1_2_0_0_0
1552.7307 PEP_2_2_0_0_0
1714.8063 PEP_3_2_0_0_0
1917.8567 PEP_3_3_0_0_0

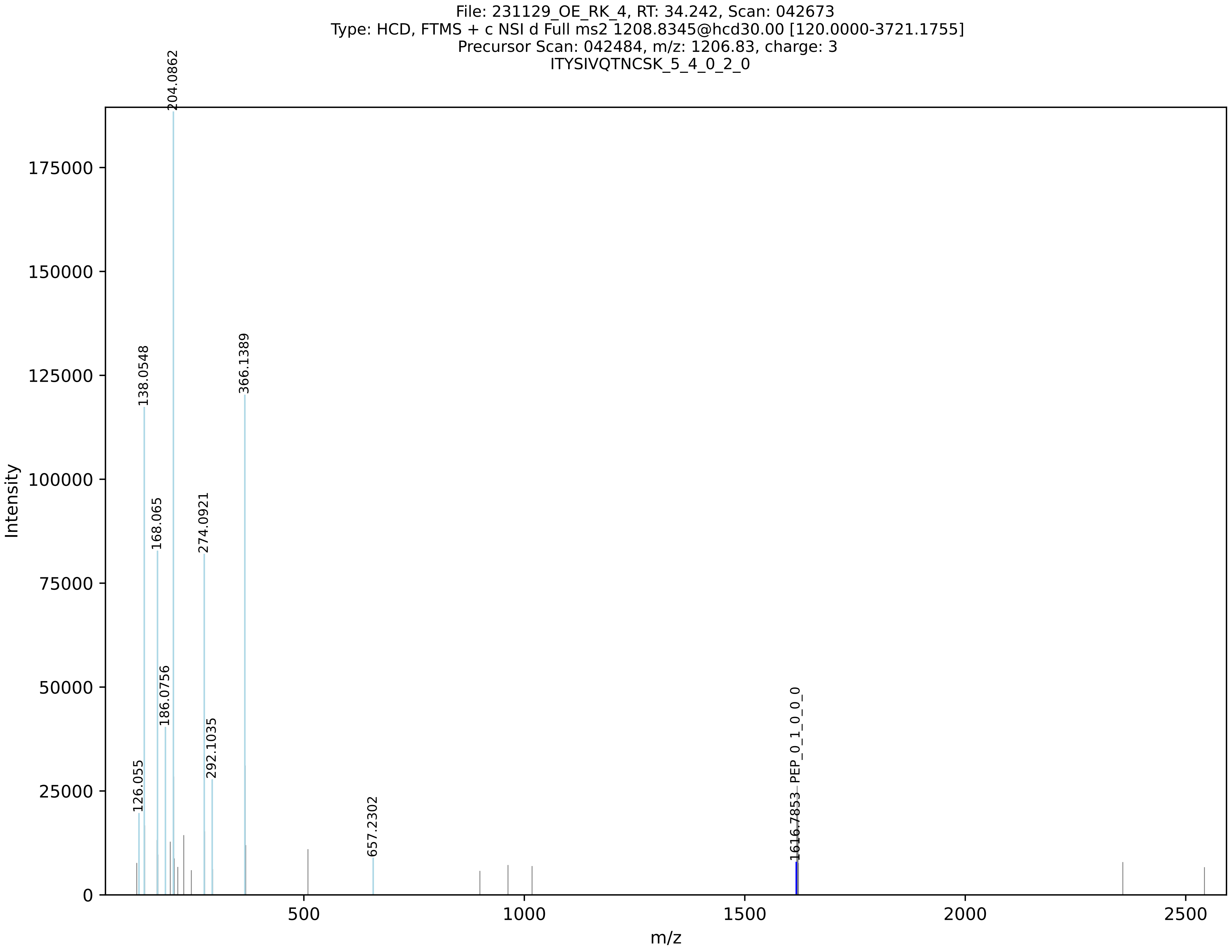
File: 231129_OE_RK_4, RT: 34.242, Scan: 042673
Type: HCD, FTMS + c NSI d Full ms2 1208.8345@hcd30.00 [120.0000-3721.1755]
Precursor Scan: 042484, m/z: 1206.83, charge: 3
ITYSIVQTNCSK_5_4_0_2_0
Intensity
m/z
204.0862
138.0548
366.1389
168.065
274.0921
186.0756
292.1035
126.055
657.2302
1616.7853_PEP_0_1_0_0_0_
175000
150000
125000
100000
75000
50000
25000
0
500
1000
1500
2000
2500

File: 231129_OE_RK_4, RT: 34.407, Scan: 042907
Type: HCD, FTMS + c NSI d Full ms2 1207.4985@hcd30.00 [120.0000-3717.0876]
Precursor Scan: 042833, m/z: 1206.8305, charge: 3
ITYSIVQTNCSK_5_4_0_2_0

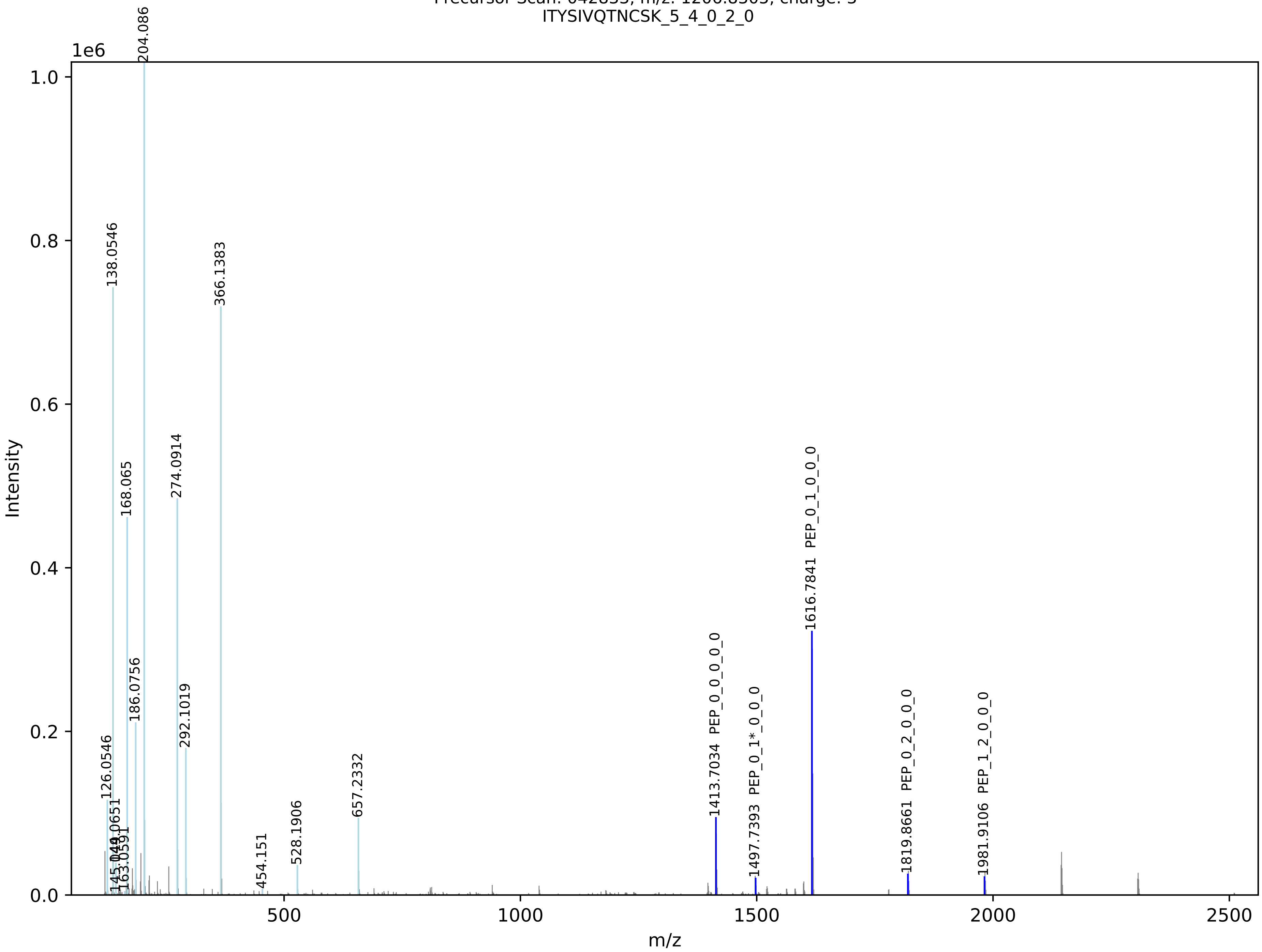

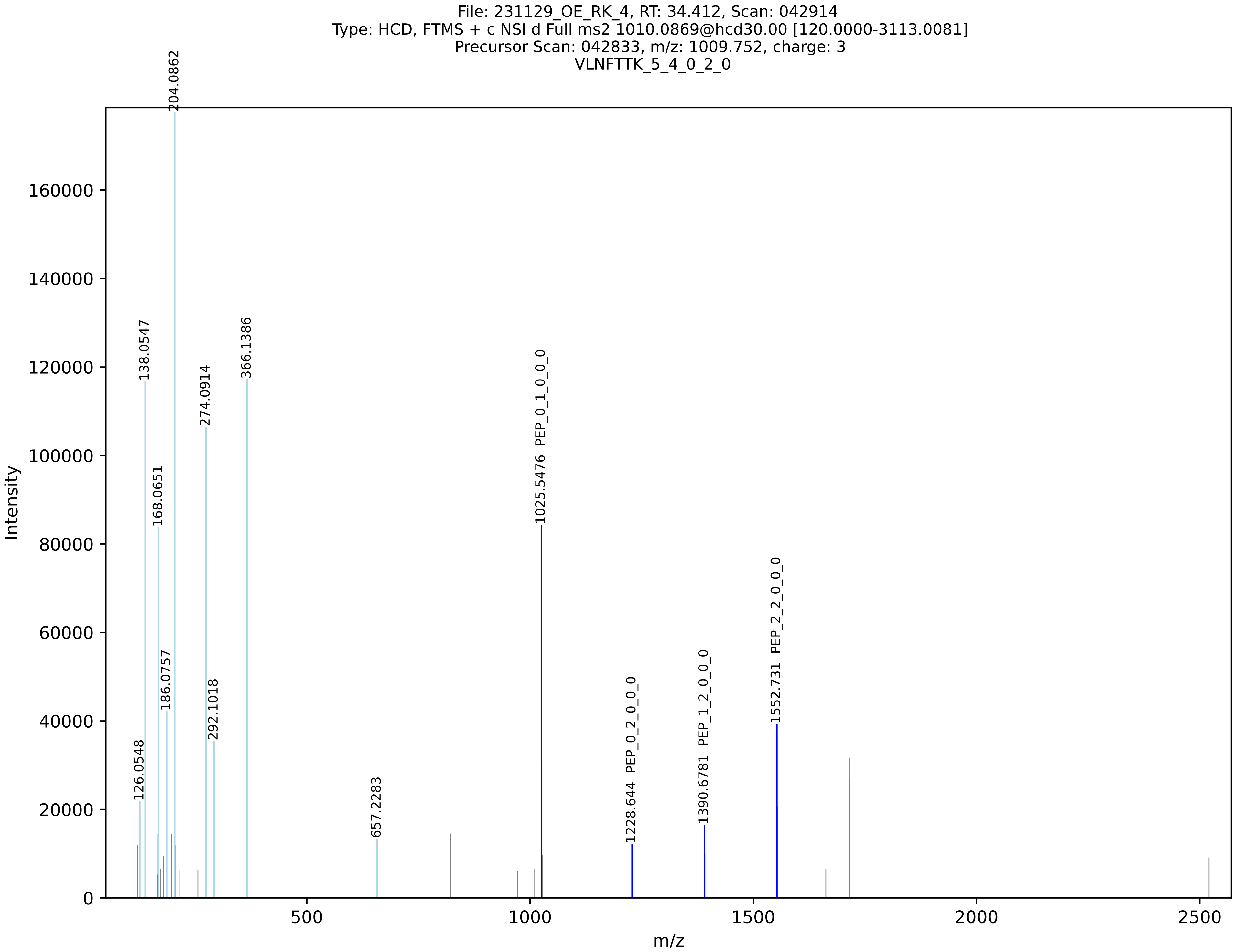
File: 231129_OE_RK_4, RT: 34.412, Scan: 042914
Type: HCD, FTMS + c NSI d Full ms2 1010.0869@hcd30.00 [120.0000-3113.0081]
Precursor Scan: 042833, m/z: 1009.752, charge: 3
VLNFTTK_5_4_0_2_0
Intensity
m/z
204.0862
138.0547
366.1386
274.0914
168.0651
186.0757
292.1018
126.0548
657.2283
1025.5476 PEP_0_1_0_0_0
1228.644 PEP_0_2_0_0_0
1390.6781 PEP_1_2_0_0_0
1552.731 PEP_2_2_0_0_0
0
20000
40000
60000
80000
100000
120000
140000
160000
500
1000
1500
2000
2500

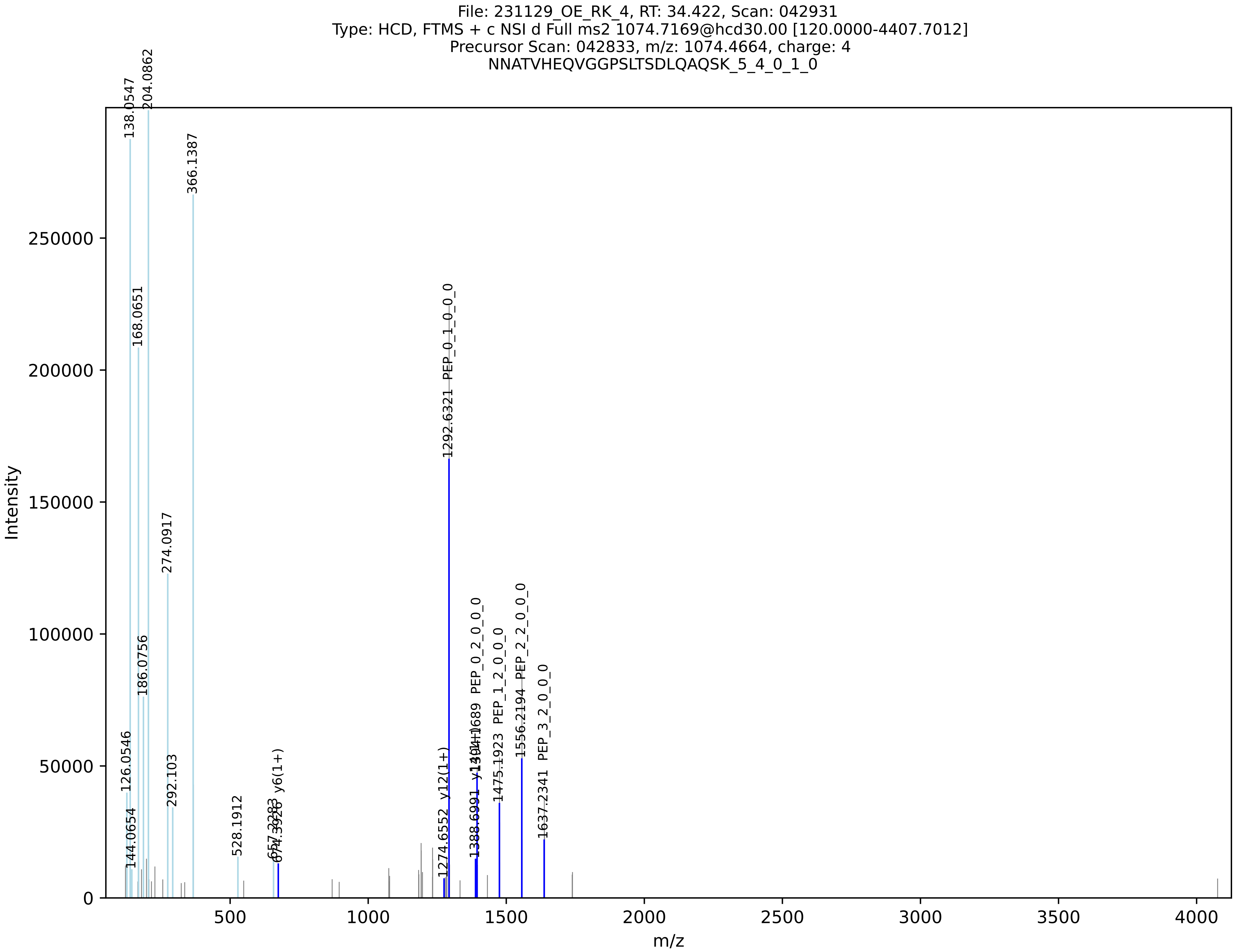

File: 231129_OE_RK_4, RT: 34.422, Scan: 042931
Type: HCD, FTMS + c NSI d Full ms2 1074.7169@hcd30.00 [120.0000-4407.7012]
Precursor Scan: 042833, m/z: 1074.4664, charge: 4
NNATVHEQVGGPSLTSDLQAQSK_5_4_0_1_0
Intensity
m/z
0
50000
100000
150000
200000
250000
500
1000
1500
2000
2500
3000
3500
4000
138.0547
204.0862
366.1387
168.0651
274.0917
186.0756
126.0546
292.103
144.0654
528.1912
y6(1+)
1274.6552 y12(1+)
1292.6321 PEP_0_1_0_0_0
1388.6991
PEP_0_2_0_0_0
1475.1923 PEP_1_2_0_0_0
1556.2194 PEP_2_2_0_0_0
1637.2341 PEP_3_2_0_0_0

File: 231129_OE_RK_4, RT: 34.61, Scan: 043200
Type: HCD, FTMS + c NSI d Full ms2 1074.9675@hcd30.00 [120.0000-4408.7236]
Precursor Scan: 043125, m/z: 1074.4665, charge: 4
NNATVHEQVGGPSLTSDLQAQSK_5_4_0_1_0

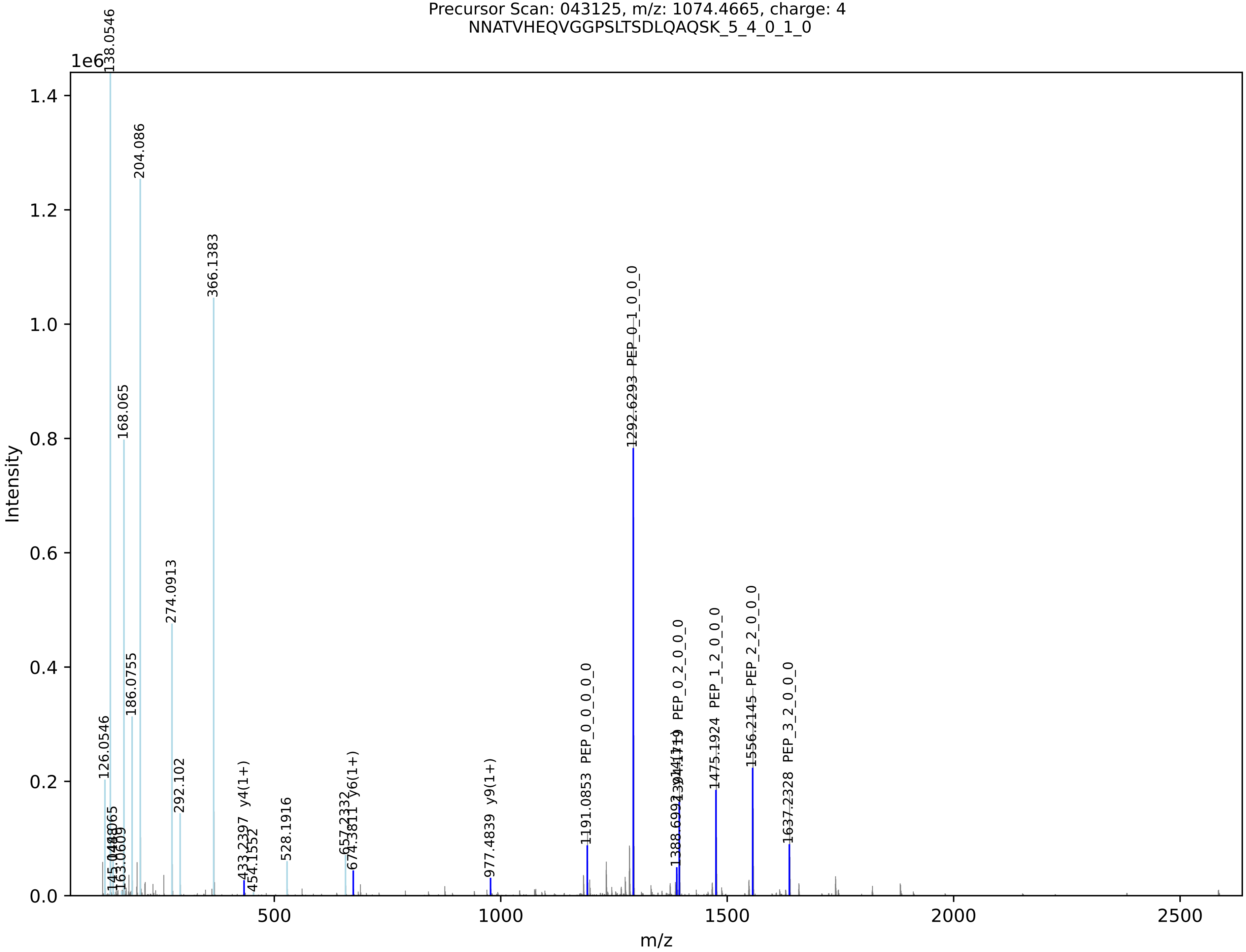

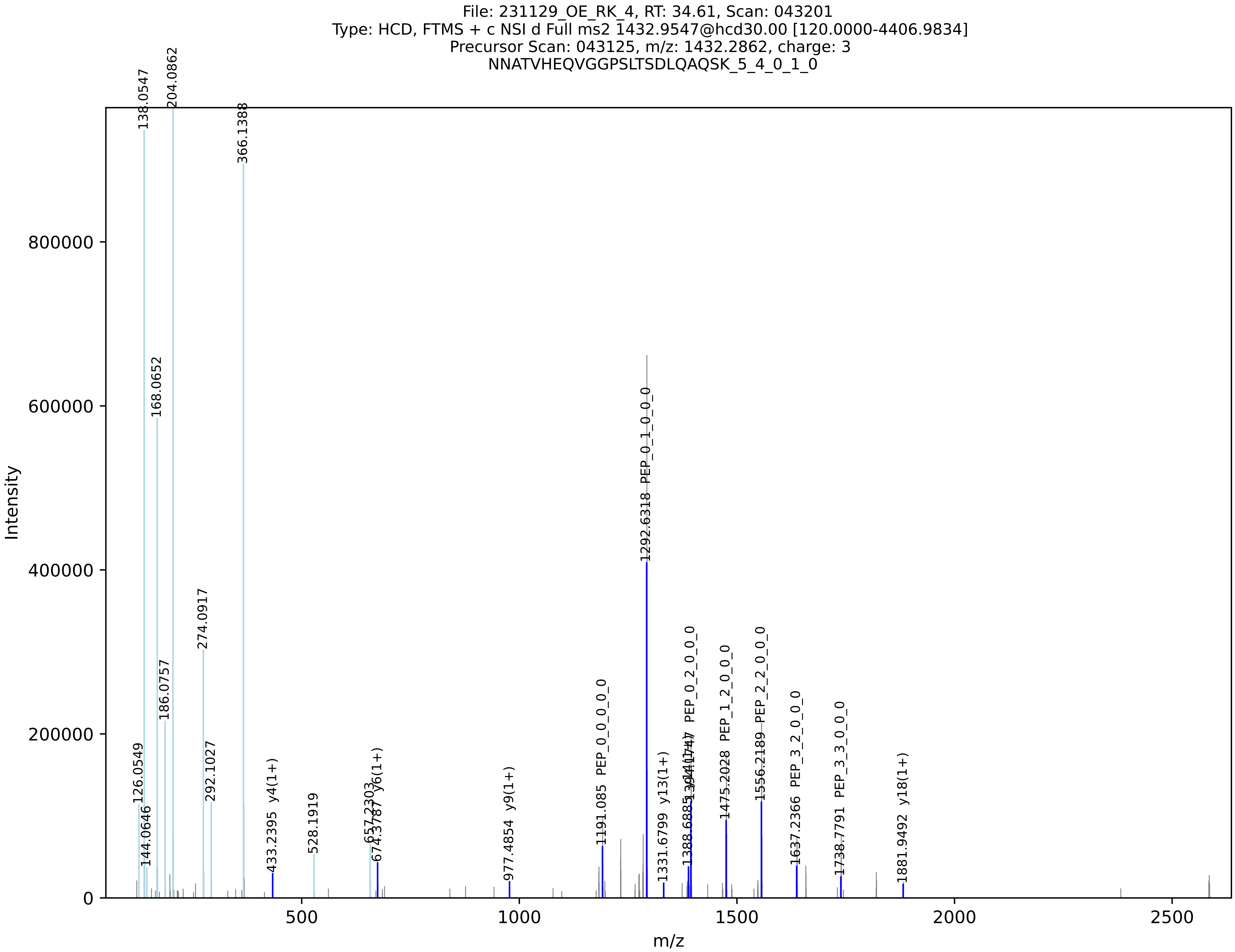
File: 231129_OE_RK_4, RT: 34.61, Scan: 043201
Type: HCD, FTMS + c NSI d Full ms2 1432.9547@hcd30.00 [120.0000-4406.9834]
Precursor Scan: 043125, m/z: 1432.2862, charge: 3
NNATVHEQVGGPSLTSDLQAQSK_5_4_0_1_0
Intensity
m/z
138.0547
204.0862
366.1388
168.0652
274.0917
186.0757
126.0549
144.0646
292.1027
433.2395 y4(1+)
528.1919
657.2303
674.3787 y6(1+)
977.4854 y9(1+)
1191.085 PEP_0_0_0_0_0
1292.6318 PEP_0_1_0_0_0
1331.6799 y13(1+)
1388.6885 y14(1+)
PEP_0_2_0_0_0
1475.2028 PEP_1_2_0_0_0
1556.2189 PEP_2_2_0_0_0
1637.2366 PEP_3_2_0_0_0
1738.7791 PEP_3_3_0_0_0
1881.9492 y18(1+)

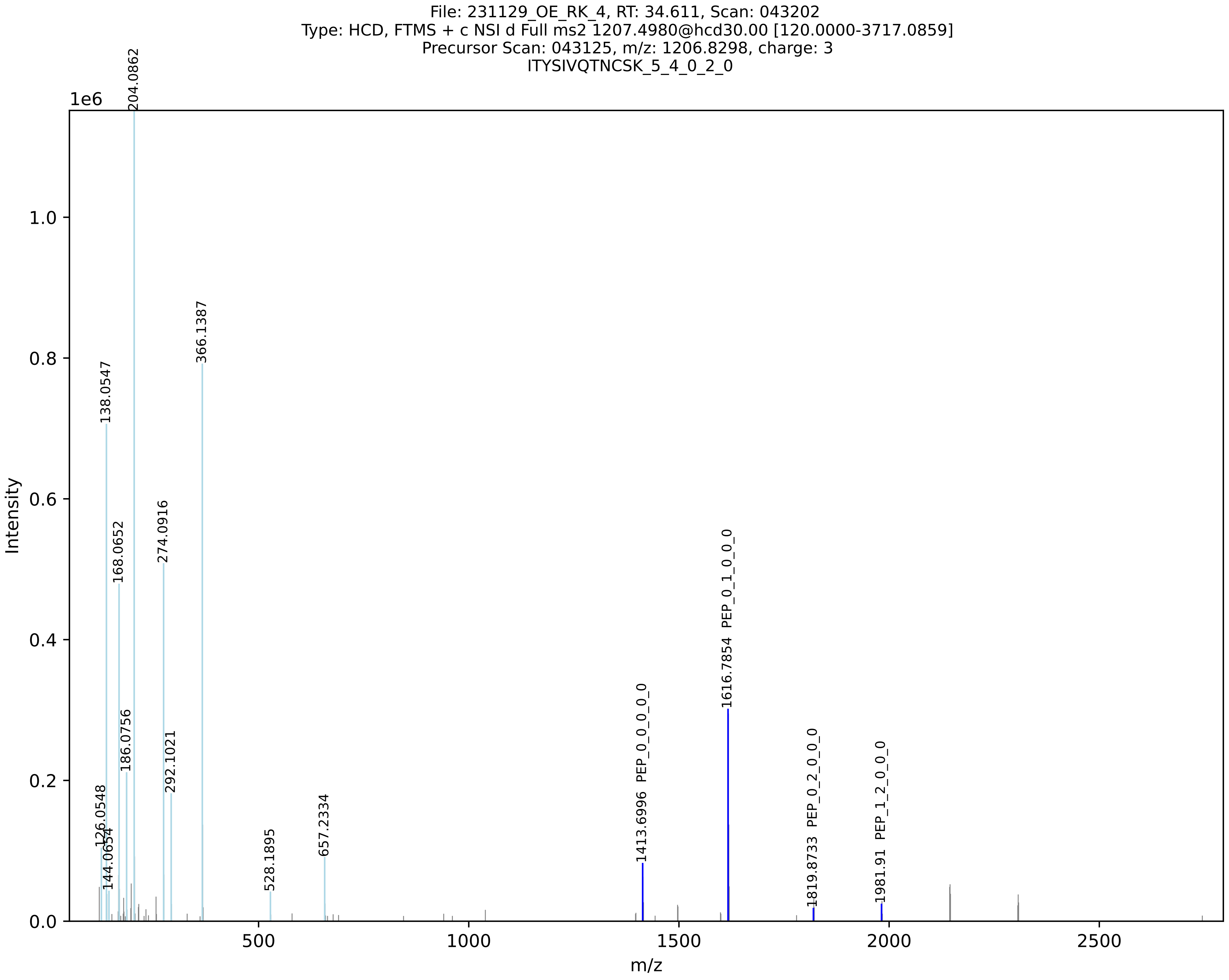
File: 231129_OE_RK_4, RT: 34.611, Scan: 043202
Type: HCD, FTMS + c NSI d Full ms2 1207.4980@hcd30.00 [120.0000-3717.0859]
Precursor Scan: 043125, m/z: 1206.8298, charge: 3
ITYSIVQTNCSK_5_4_0_2_0
1e6
Intensity
m/z
0.0
0.2
0.4
0.6
0.8
1.0
500
1000
1500
2000
2500
204.0862
366.1387
138.0547
274.0916
168.0652
186.0756
292.1021
126.0548
144.0654
528.1895
657.2334
1413.6996 PEP_0_0_0_0_0
1616.7854 PEP_0_1_0_0_0
1819.8733 PEP_0_2_0_0_0
1981.91 PEP_1_2_0_0_0

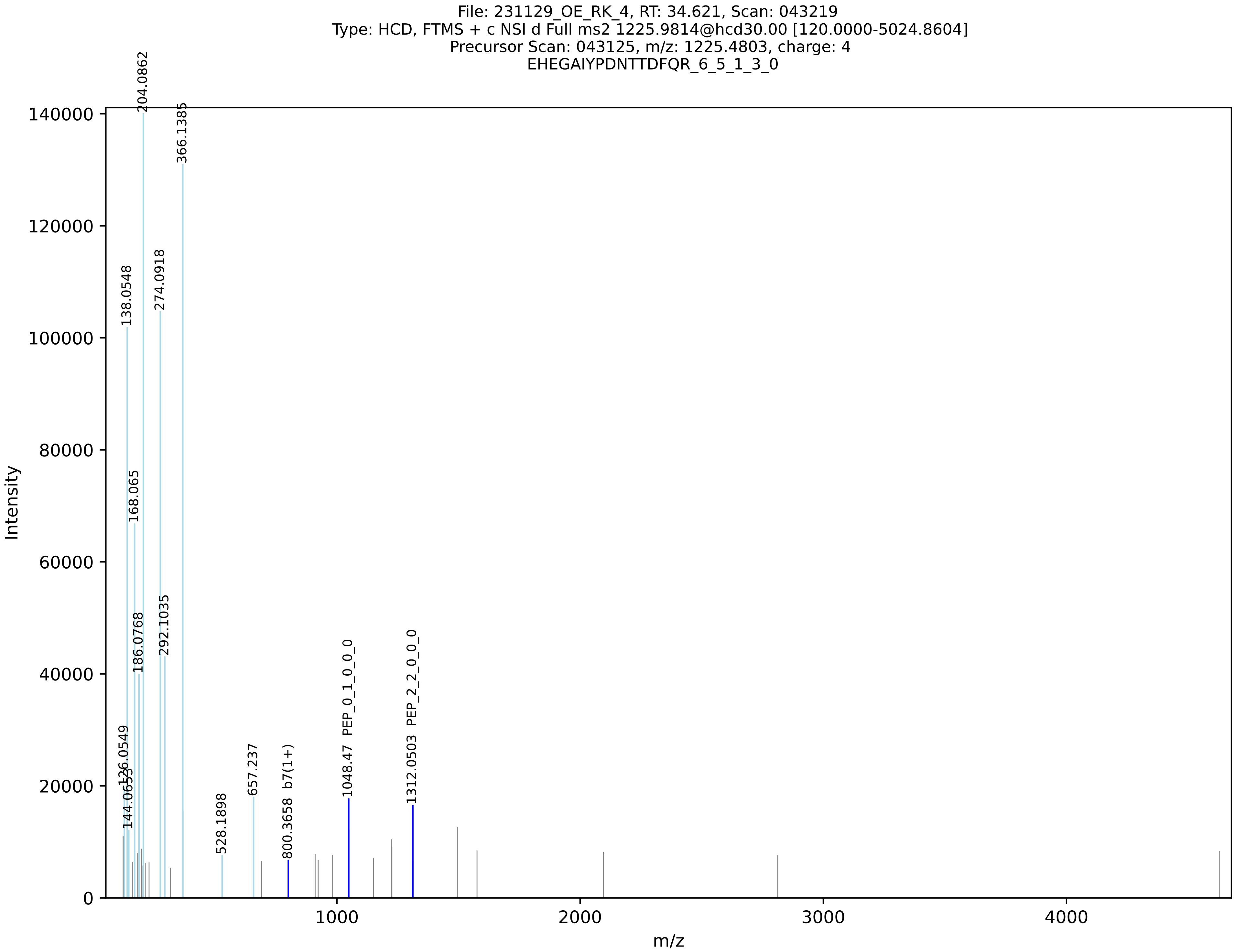
File: 231129_OE_RK_4, RT: 34.621, Scan: 043219
Type: HCD, FTMS + c NSI d Full ms2 1225.9814@hcd30.00 [120.0000-5024.8604]
Precursor Scan: 043125, m/z: 1225.4803, charge: 4
EHEGAIYPDNTTDFQR_6_5_1_3_0
204.0862
366.1385
138.0548
274.0918
168.065
186.0768
292.1035
126.0549
144.0653
528.1898
657.237
800.3658 b7(1+)
1048.47 PEP_0_1_0_0_0
1312.0503 PEP_2_2_0_0_0
Intensity
m/z
0
20000
40000
60000
80000
100000
120000
140000
1000
2000
3000
4000

File: 231129_OE_RK_4, RT: 34.668, Scan: 043287
Type: HCD, FTMS + c NSI d Full ms2 1112.4515@hcd30.00 [120.0000-3426.2439]
Precursor Scan: 043199, m/z: 1112.1202, charge: 3
HSHNNNSSDLHPHK_5_3_0_1_0

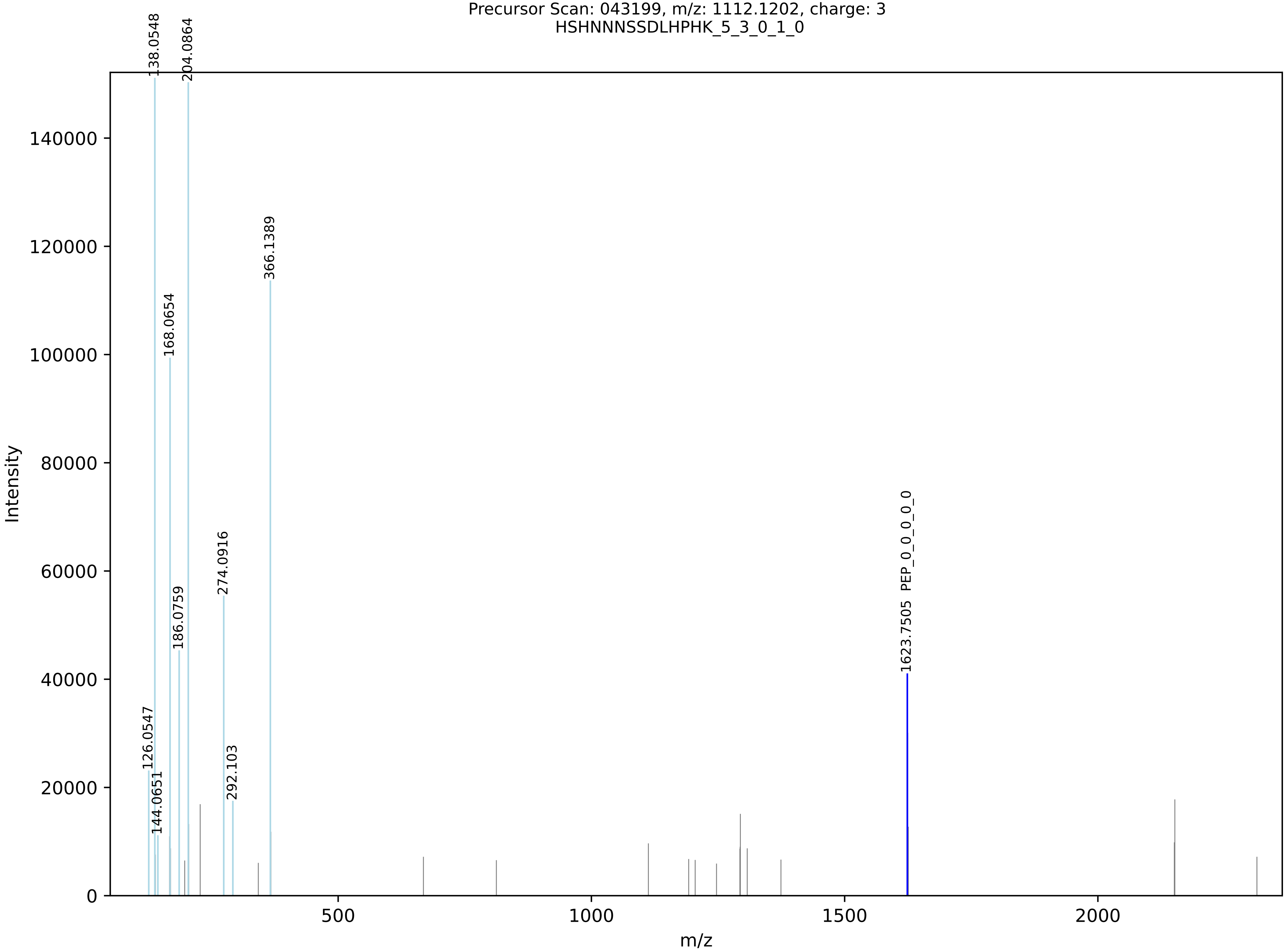

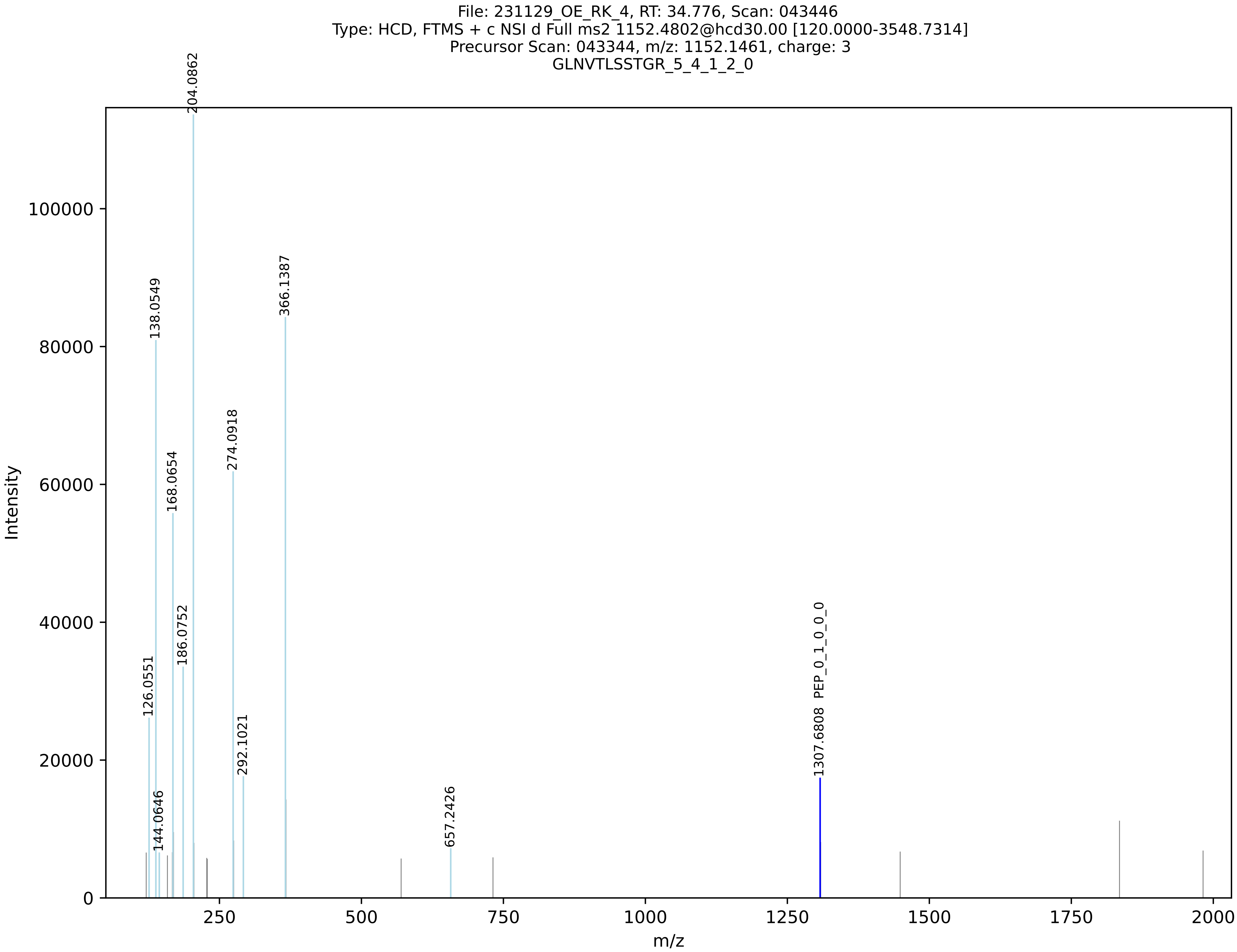
File: 231129_OE_RK_4, RT: 34.776, Scan: 043446
Type: HCD, FTMS + c NSI d Full ms2 1152.4802@hcd30.00 [120.0000-3548.7314]
Precursor Scan: 043344, m/z: 1152.1461, charge: 3
GLNVTLSSTGR_5_4_1_2_0
204.0862
138.0549
366.1387
274.0918
168.0654
186.0752
126.0551
292.1021
144.0646
657.2426
1307.6808 PEP_0_1_0_0_0
Intensity
m/z
0
20000
40000
60000
80000
100000
250
500
750
1000
1250
1500
1750
2000

File: 231129_OE_RK_4, RT: 34.81, Scan: 043493
Type: HCD, FTMS + c NSI d Full ms2 1074.9679@hcd30.00 [120.0000-4408.7251]
Precursor Scan: 043418, m/z: 1074.4668, charge: 4
NNNATVHEQVGGPSLTSDLQAQSK_5_4_0_1_0

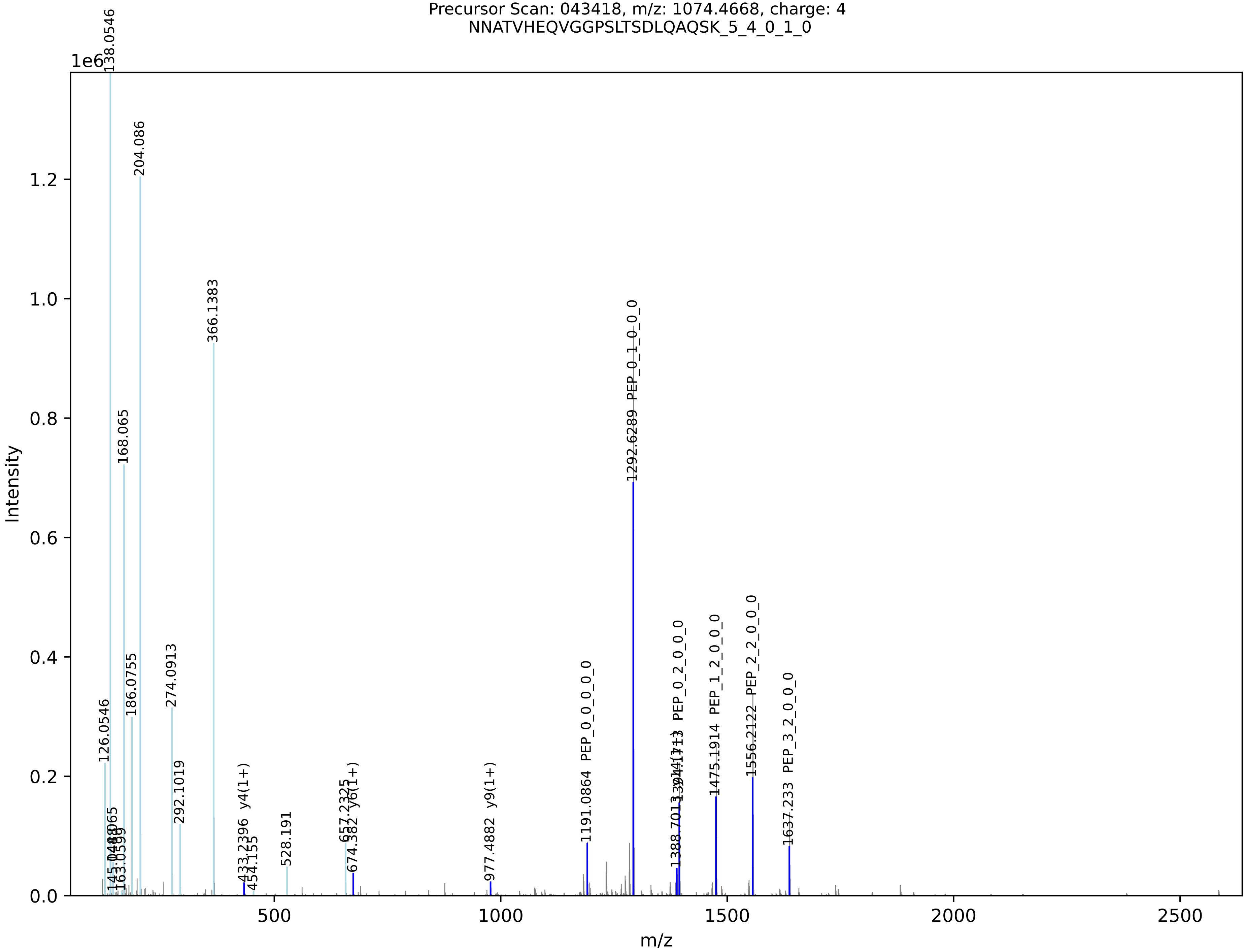

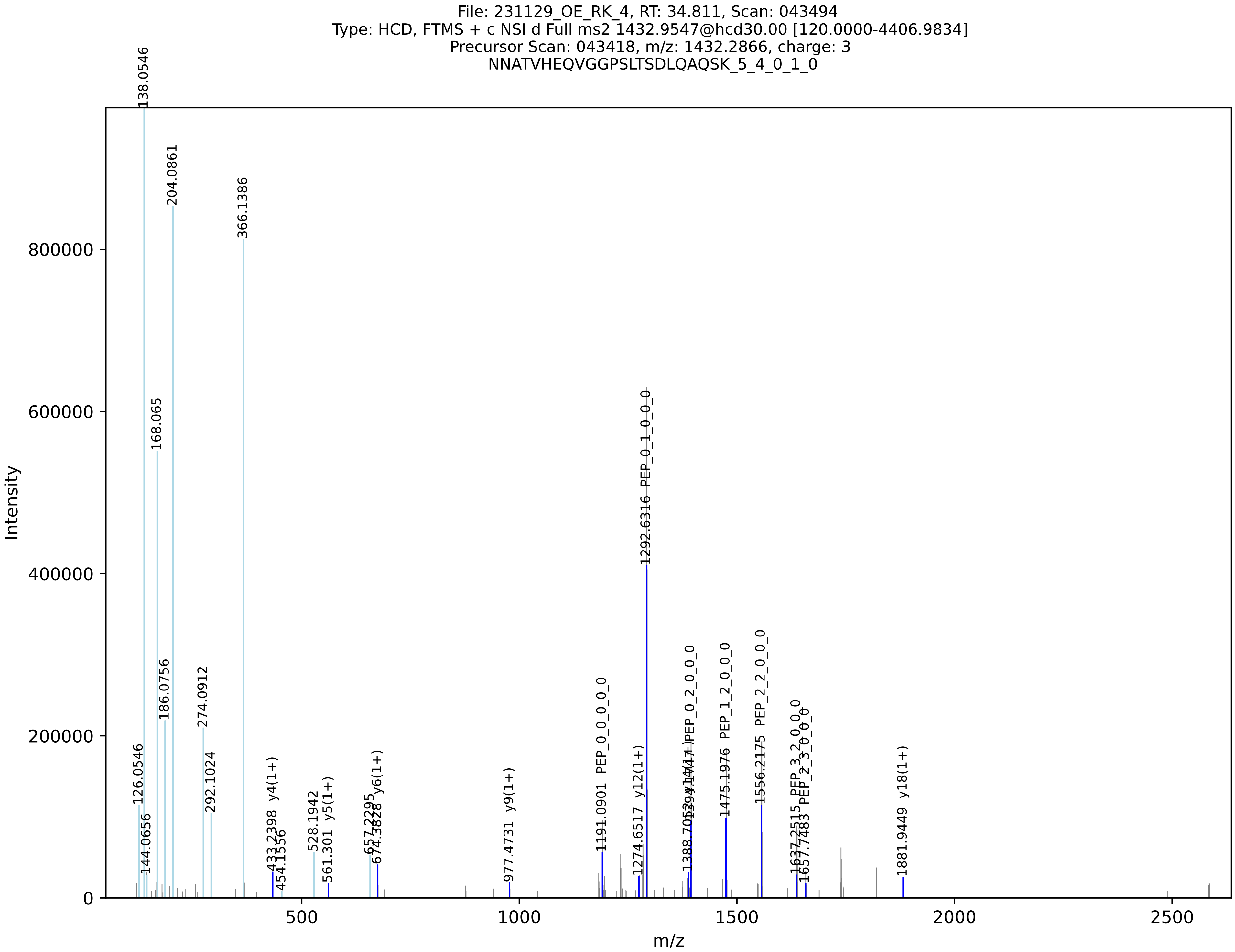

File: 231129_OE_RK_4, RT: 34.811, Scan: 043494
Type: HCD, FTMS + c NSI d Full ms2 1432.9547@hcd30.00 [120.0000-4406.9834]
Precursor Scan: 043418, m/z: 1432.2866, charge: 3
NNATVHEQVGGPSLTSDLQAQSK_5_4_0_1_0
Intensity
m/z
138.0546
204.0861
366.1386
168.065
186.0756
274.0912
292.1024
126.0546
144.0656
433.2398 y4(1+)
454.1556
528.1942
561.301 y5(1+)
657.2295
674.3828 y6(1+)
977.4731 y9(1+)
1191.0901 PEP_0_0_0_0_0
1274.6517 y12(1+)
1292.6316 PEP_0_1_0_0_0
1475.1976 PEP_1_2_0_0_0
1556.2175 PEP_2_2_0_0_0
1637.2515 PEP_3_2_0_0_0
1657.7483 PEP_2_3_0_0_0
1881.9449 y18(1+)

File: 231129_OE_RK_4, RT: 34.812, Scan: 043496
Type: HCD, FTMS + c NSI d Full ms2 1207.1648@hcd30.00 [120.0000-3716.0662]
Precursor Scan: 043418, m/z: 1206.8308, charge: 3
ITYSIVQTNCSK_5_4_0_2_0

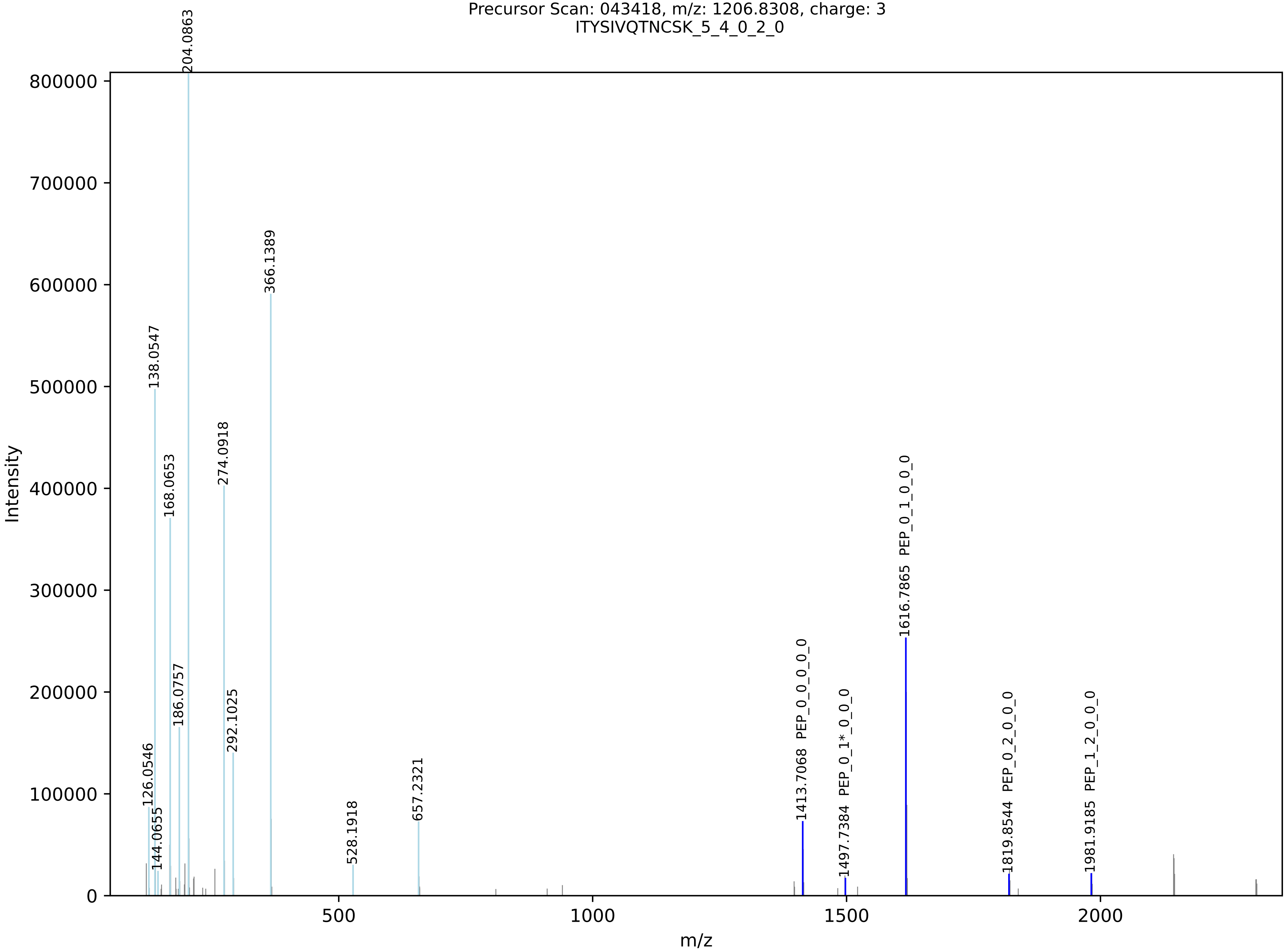

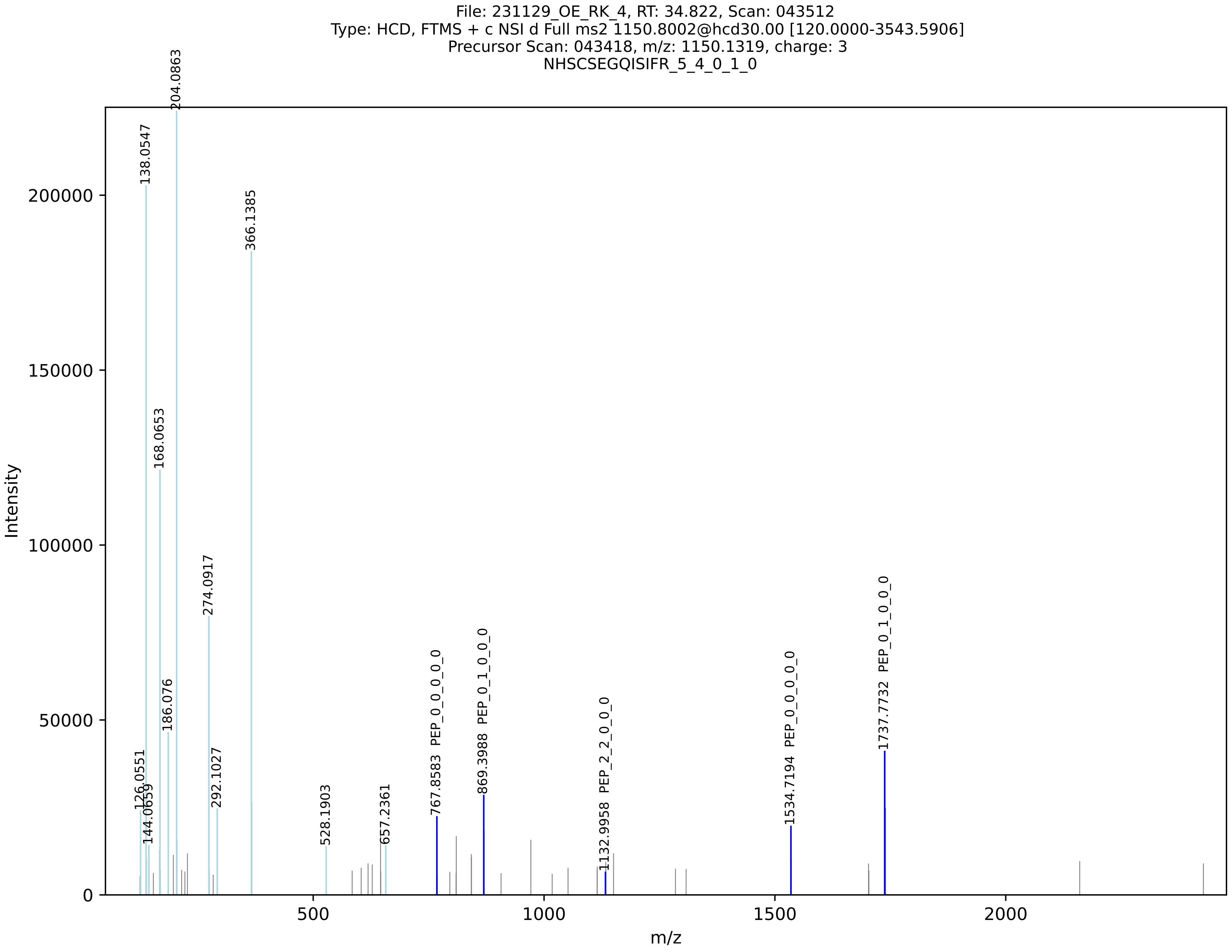

File: 231129_OE_RK_4, RT: 34.822, Scan: 043512
Type: HCD, FTMS + c NSI d Full ms2 1150.8002@hcd30.00 [120.0000-3543.5906]
Precursor Scan: 043418, m/z: 1150.1319, charge: 3
NHSCSEGQISIFR_5_4_0_1_0
Intensity
m/z
204.0863
138.0547
366.1385
168.0653
274.0917
186.076
126.0551
144.0659
292.1027
528.1903
657.2361
767.8583 PEP_0_0_0_0_0
869.3988 PEP_0_1_0_0_0
1132.9958 PEP_2_2_0_0_0
1534.7194 PEP_0_0_0_0_0
1737.7732 PEP_0_1_0_0_0

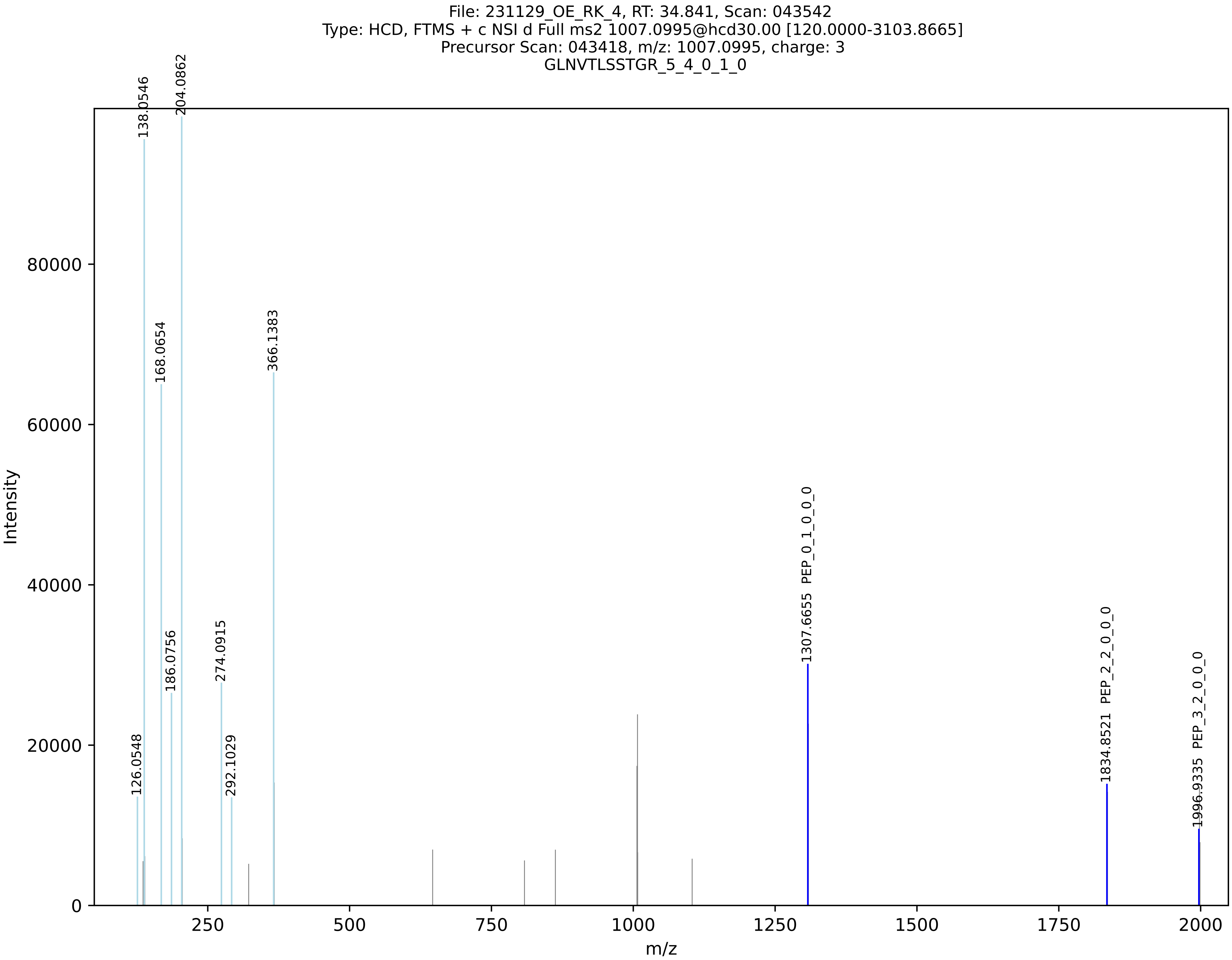
File: 231129_OE_RK_4, RT: 34.841, Scan: 043542
Type: HCD, FTMS + c NSI d Full ms2 1007.0995@hcd30.00 [120.0000-3103.8665]
Precursor Scan: 043418, m/z: 1007.0995, charge: 3
GLNVTLSSTGR_5_4_0_1_0
Intensity
m/z
0
20000
40000
60000
80000
250
500
750
1000
1250
1500
1750
2000
126.0548
138.0546
168.0654
186.0756
204.0862
274.0915
292.1029
366.1383
1307.6655 PEP_0_1_0_0_
1834.8521 PEP_2_2_0_0_0_
1996.9335 PEP_3_2_0_0_0

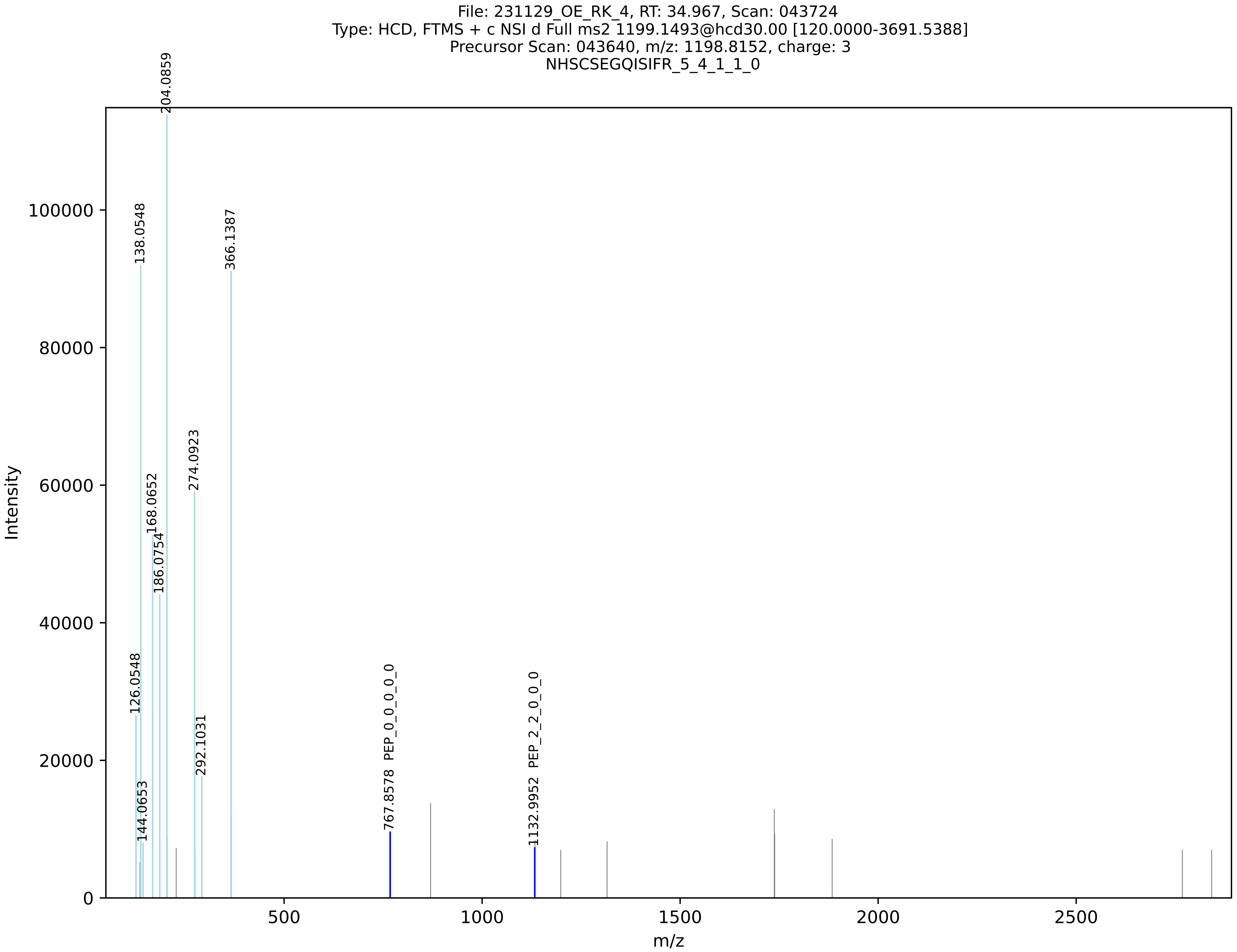

File: 231129_OE_RK_4, RT: 34.967, Scan: 043724
Type: HCD, FTMS + c NSI d Full ms2 1199.1493@hcd30.00 [120.0000-3691.5388]
Precursor Scan: 043640, m/z: 1198.8152, charge: 3
NHSCSEGQISIFR_5_4_1_1_0
Intensity
m/z
204.0859
138.0548
366.1387
274.0923
168.0652
186.0754
126.0548
292.1031
144.0653
767.8578 PEP_0_0_0_0_0
1132.9952 PEP_2_2_0_0_0
0
20000
40000
60000
80000
100000
500
1000
1500
2000
2500

File: 231129_OE_RK_4, RT: 34.986, Scan: 043754
Type: HCD, FTMS + c NSI d Full ms2 1206.4987@hcd30.00 [120.0000-3714.0278]
Precursor Scan: 043566, m/z: 1206.8294, charge: 3
ITYSIVQTNCSK_5_4_0_2_0

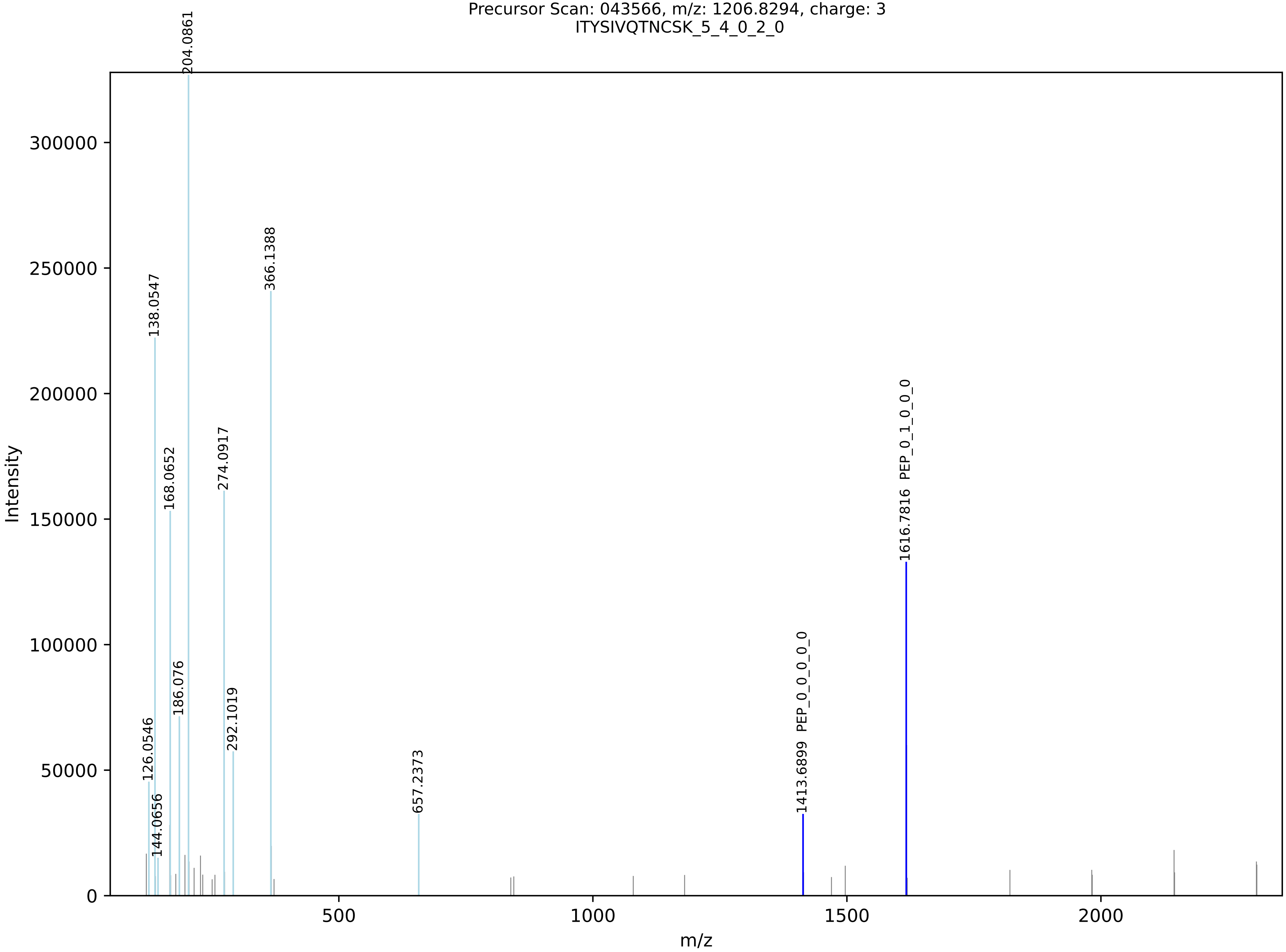

File: 231129_OE_RK_4, RT: 35.004, Scan: 043777
Type: HCD, FTMS + c NSI d Full ms2 1074.7152@hcd30.00 [120.0000-4407.6943]
Precursor Scan: 043698, m/z: 1074.4657, charge: 4
NNATVHEQVGGPSLTSDLQAQSK_5_4_0_1_0

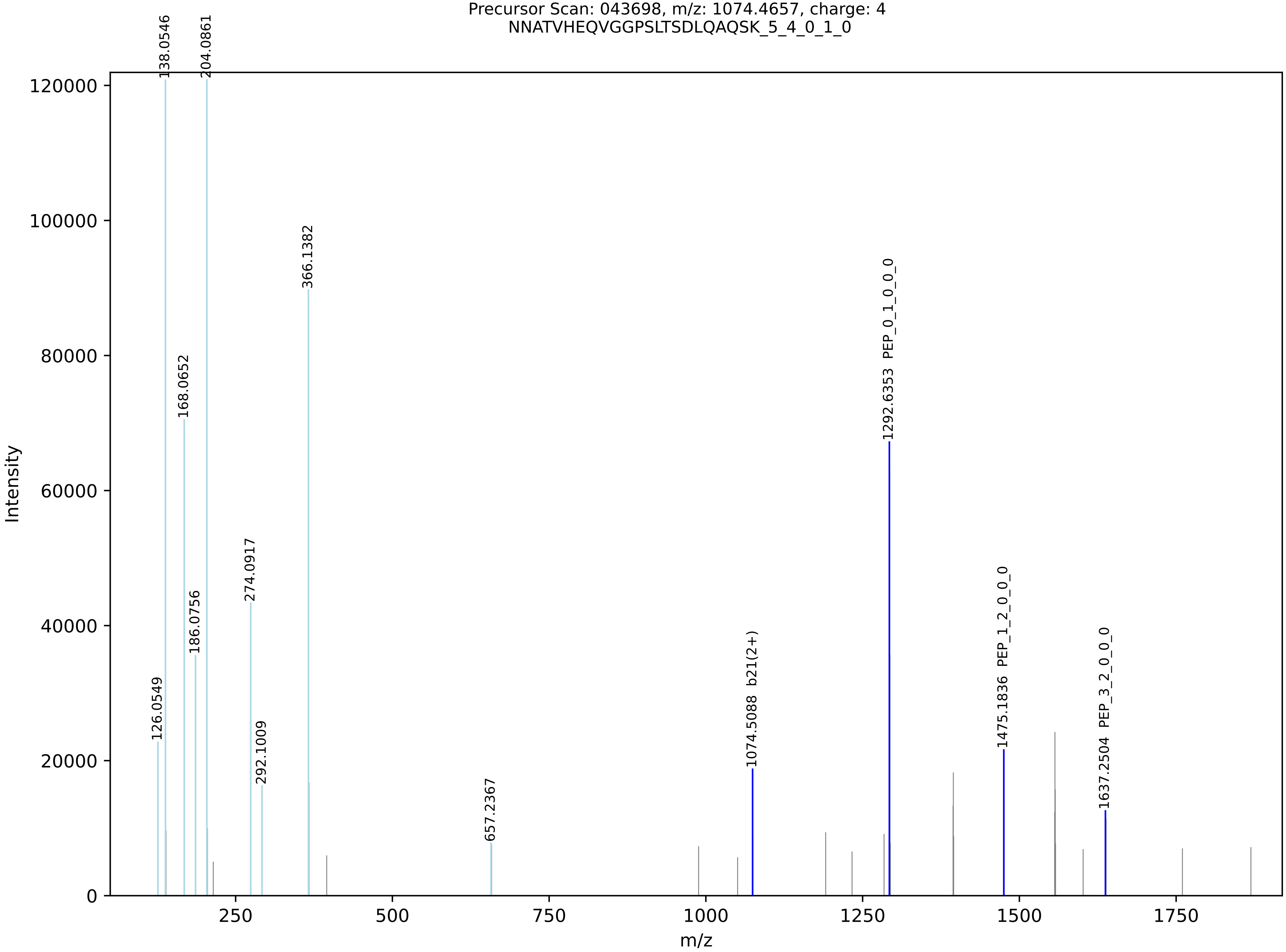

File: 231129_OE_RK_4, RT: 35.007, Scan: 043781
Type: HCD, FTMS + c NSI d Full ms2 1433.2900@hcd30.00 [120.0000-4408.0093]
Precursor Scan: 043698, m/z: 1432.2852, charge: 3
NNATVHEQVGGPSLTSDLQAQSK_5_4_0_1_0

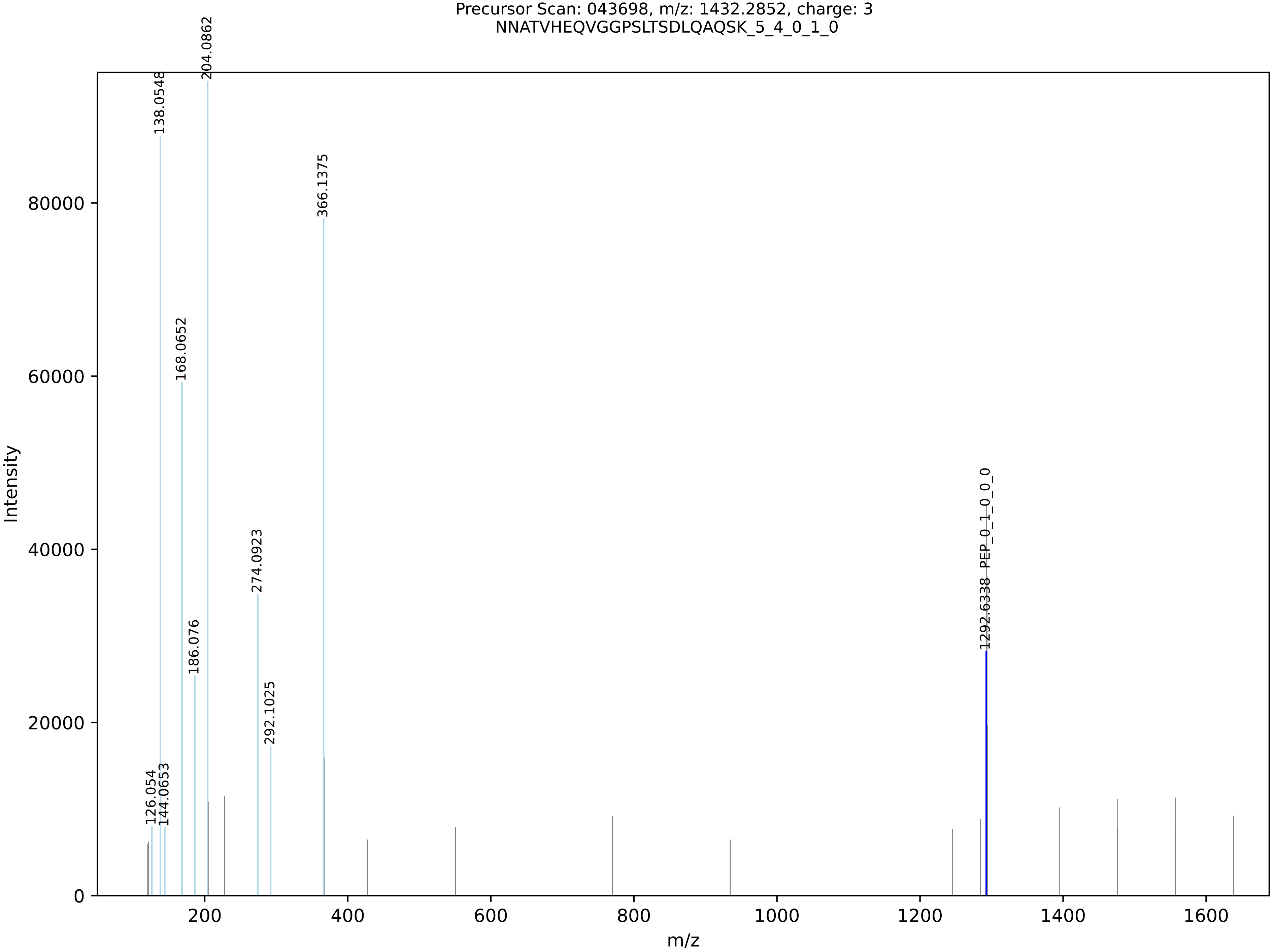

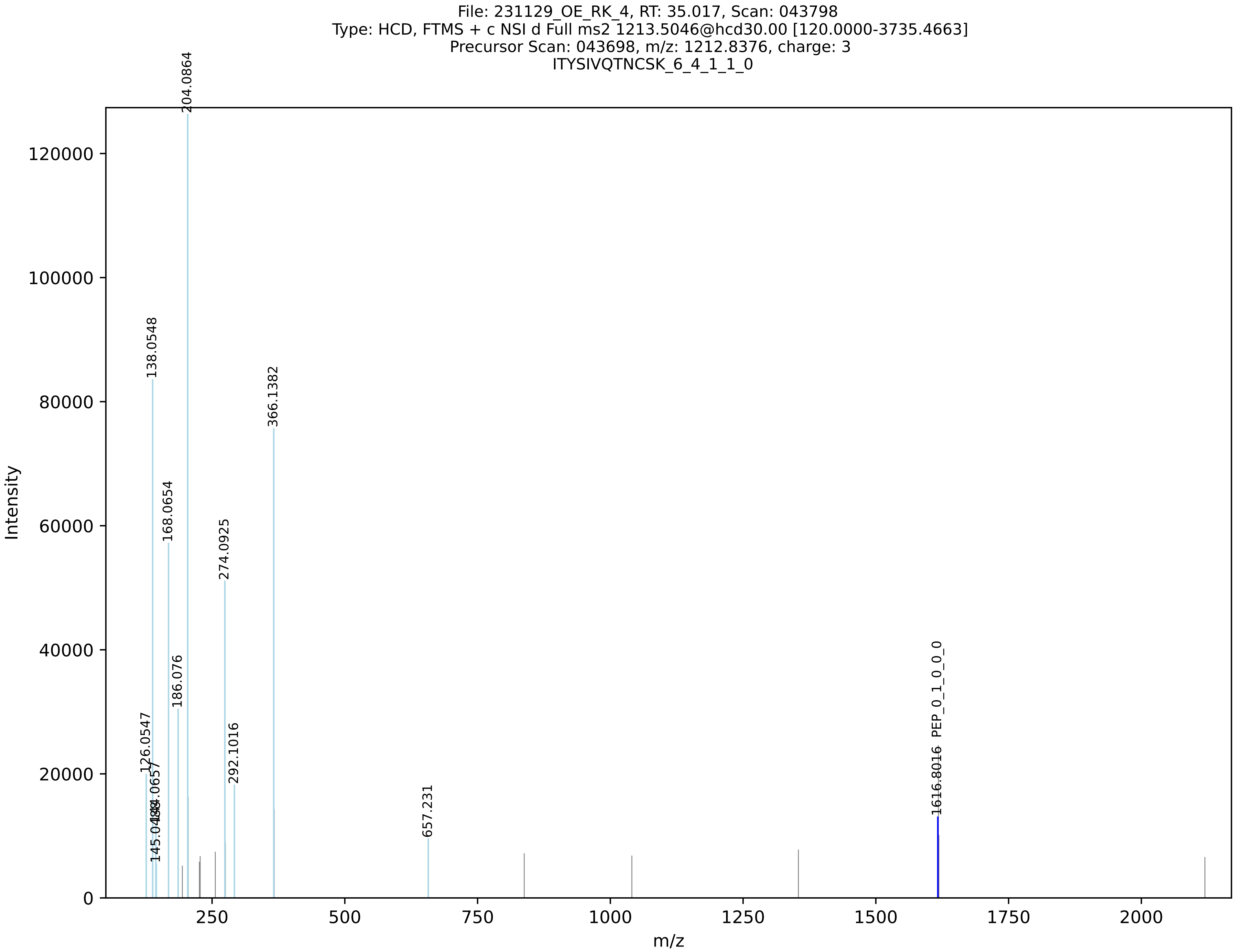
File: 231129_OE_RK_4, RT: 35.017, Scan: 043798
Type: HCD, FTMS + c NSI d Full ms2 1213.5046@hcd30.00 [120.0000-3735.4663]
Precursor Scan: 043698, m/z: 1212.8376, charge: 3
ITYSIVQTNCSK_6_4_1_1_0
Intensity
m/z
204.0864
138.0548
366.1382
168.0654
274.0925
186.076
126.0547
292.1016
657.231
1616.8016
PEP_0_1_0_0_0
0
20000
40000
60000
80000
100000
120000
250
500
750
1000
1250
1500
1750
2000

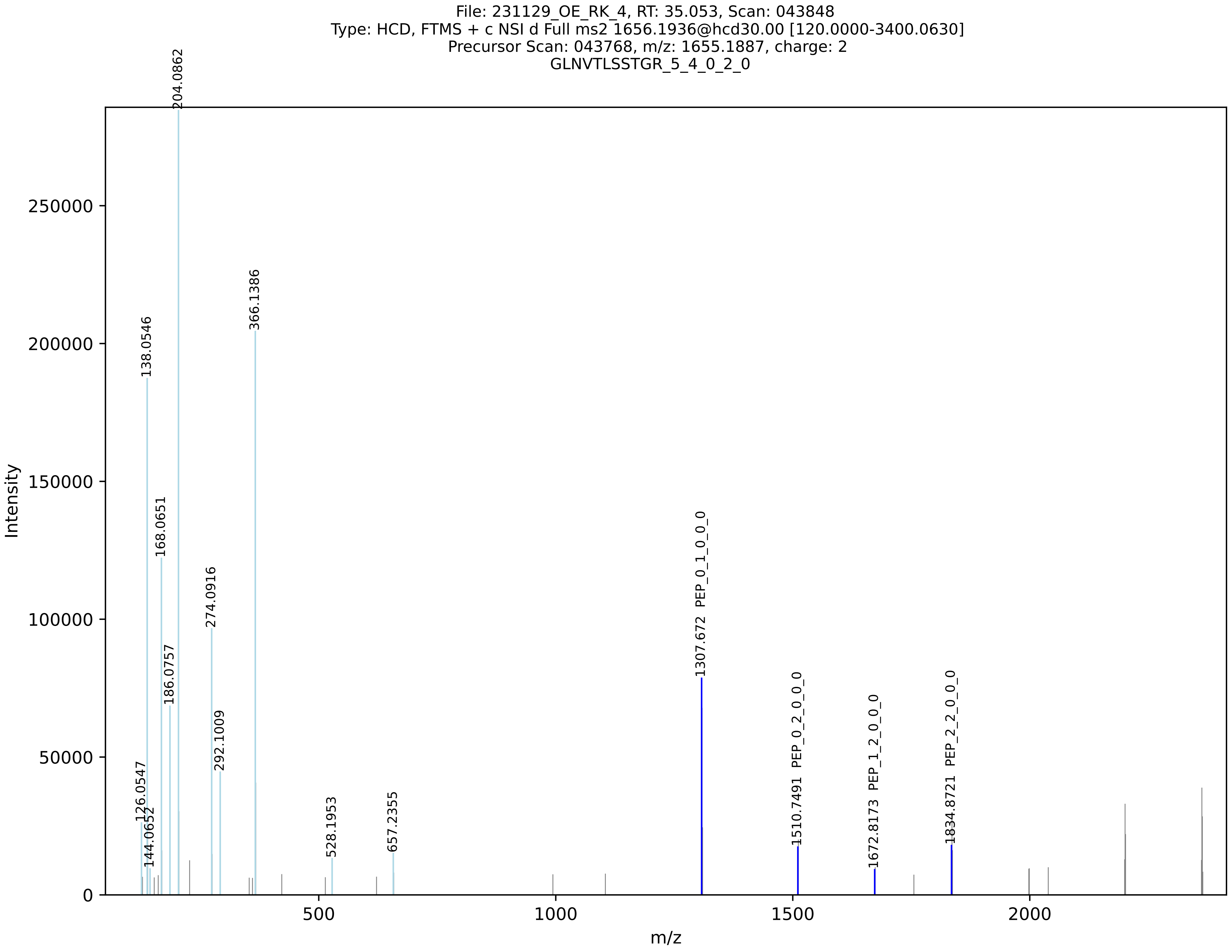

File: 231129_OE_RK_4, RT: 35.053, Scan: 043848
Type: HCD, FTMS + c NSI d Full ms2 1656.1936@hcd30.00 [120.0000-3400.0630]
Precursor Scan: 043768, m/z: 1655.1887, charge: 2
GLNVTLSSTGR_5_4_0_2_0
Intensity
m/z
204.0862
366.1386
138.0546
168.0651
274.0916
186.0757
292.1009
126.0547
144.0652
528.1953
657.2355
1307.672 PEP_0_1_0_0_0
1510.7491 PEP_0_2_0_0_0
1672.8173 PEP_1_2_0_0_0
1834.8721 PEP_2_2_0_0_0

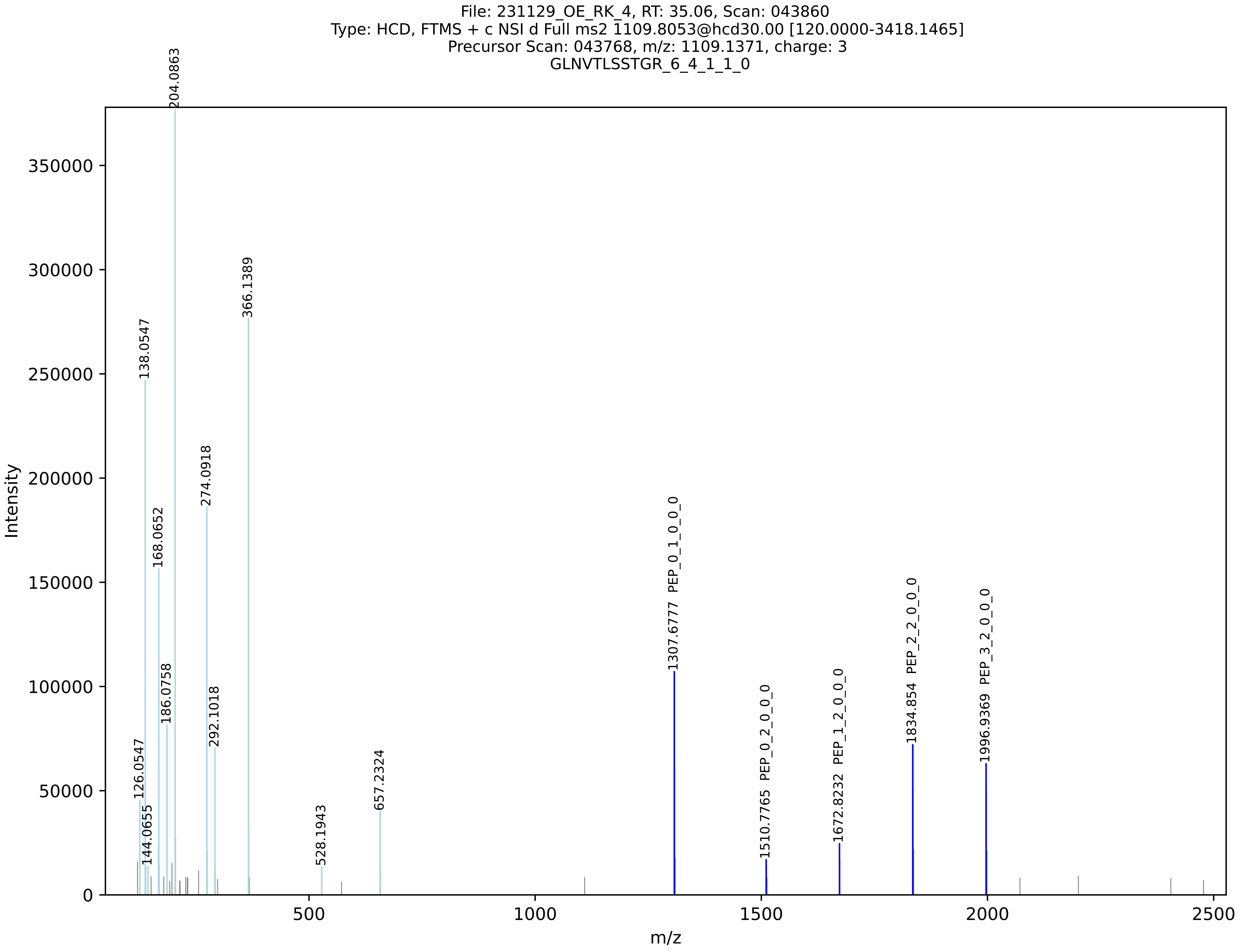

File: 231129_OE_RK_4, RT: 35.06, Scan: 043860
Type: HCD, FTMS + c NSI d Full ms2 1109.8053@hcd30.00 [120.0000-3418.1465]
Precursor Scan: 043768, m/z: 1109.1371, charge: 3
GLNVTLSSTGR_6_4_1_1_0
Intensity
m/z
204.0863
366.1389
138.0547
274.0918
168.0652
186.0758
292.1018
126.0547
144.0655
528.1943
657.2324
1307.6777 PEP_0_1_0_0_0
1510.7765 PEP_0_2_0_0_0
1672.8232 PEP_1_2_0_0_0
1834.854 PEP_2_2_0_0_0
1996.9369 PEP_3_2_0_0_0

File: 231129_OE_RK_4, RT: 35.103, Scan: 043921
Type: HCD, FTMS + c NSI d Full ms2 828.3498@hcd30.00 [120.0000-3402.5232]
Precursor Scan: 043841, m/z: 828.0985, charge: 4
GLNVTLSSTGR_5_4_0_2_0

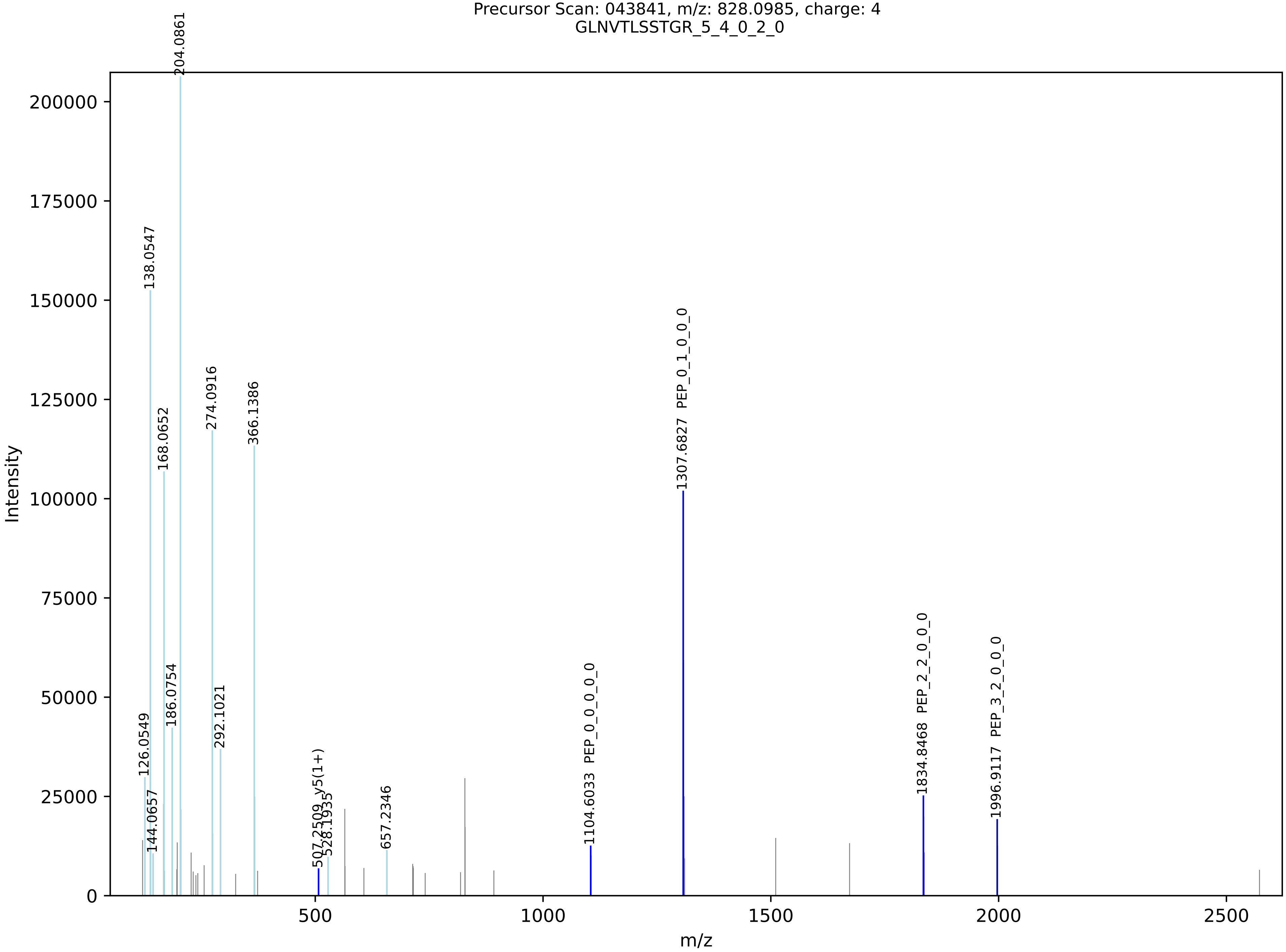

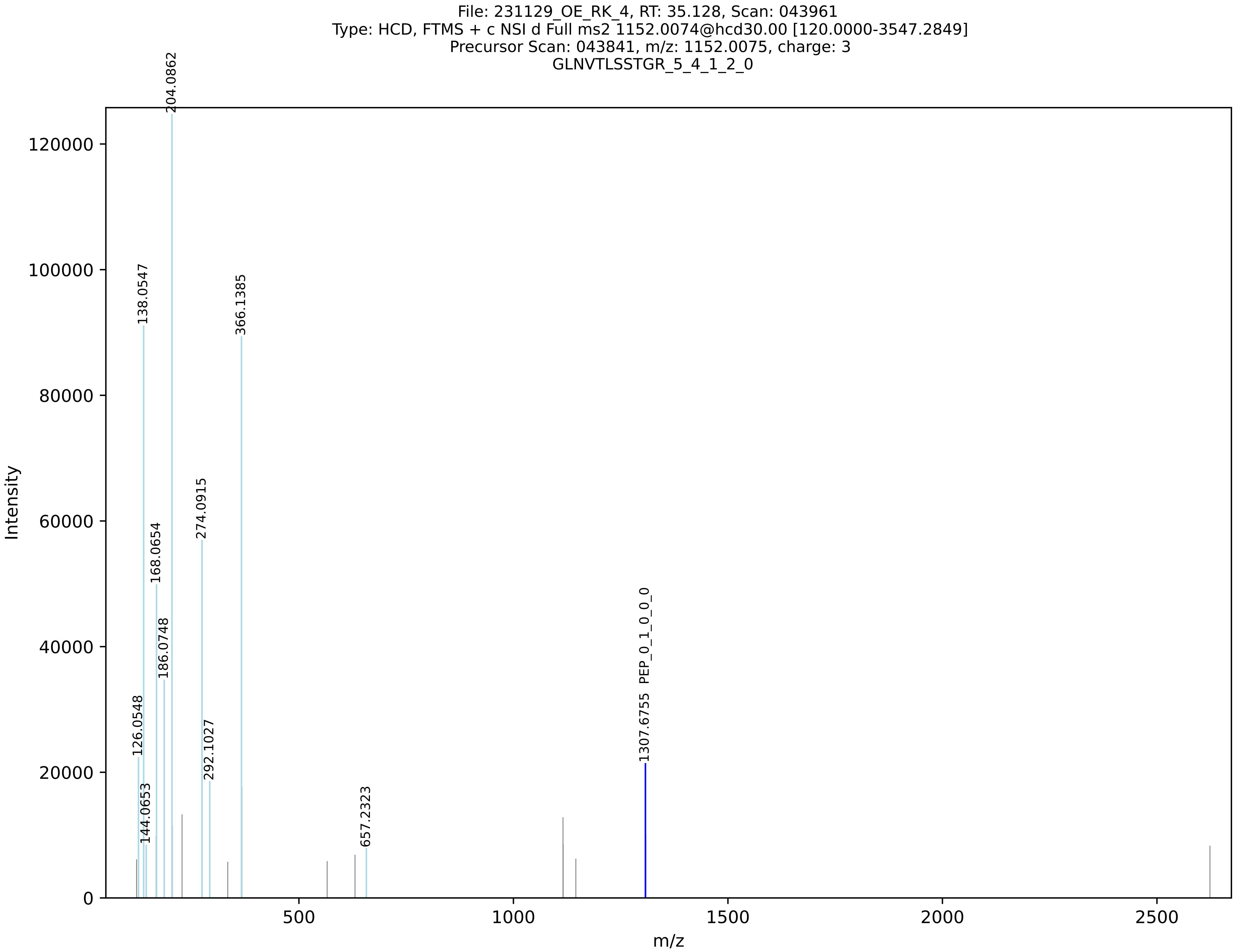
File: 231129_OE_RK_4, RT: 35.128, Scan: 043961
Type: HCD, FTMS + c NSI d Full ms2 1152.0074@hcd30.00 [120.0000-3547.2849]
Precursor Scan: 043841, m/z: 1152.0075, charge: 3
GLNVTLSSTGR_5_4_1_2_0
Intensity
m/z
204.0862
138.0547
366.1385
274.0915
168.0654
186.0748
126.0548
292.1027
144.0653
657.2323
PEP_0_1_0_0_0
1307.6755

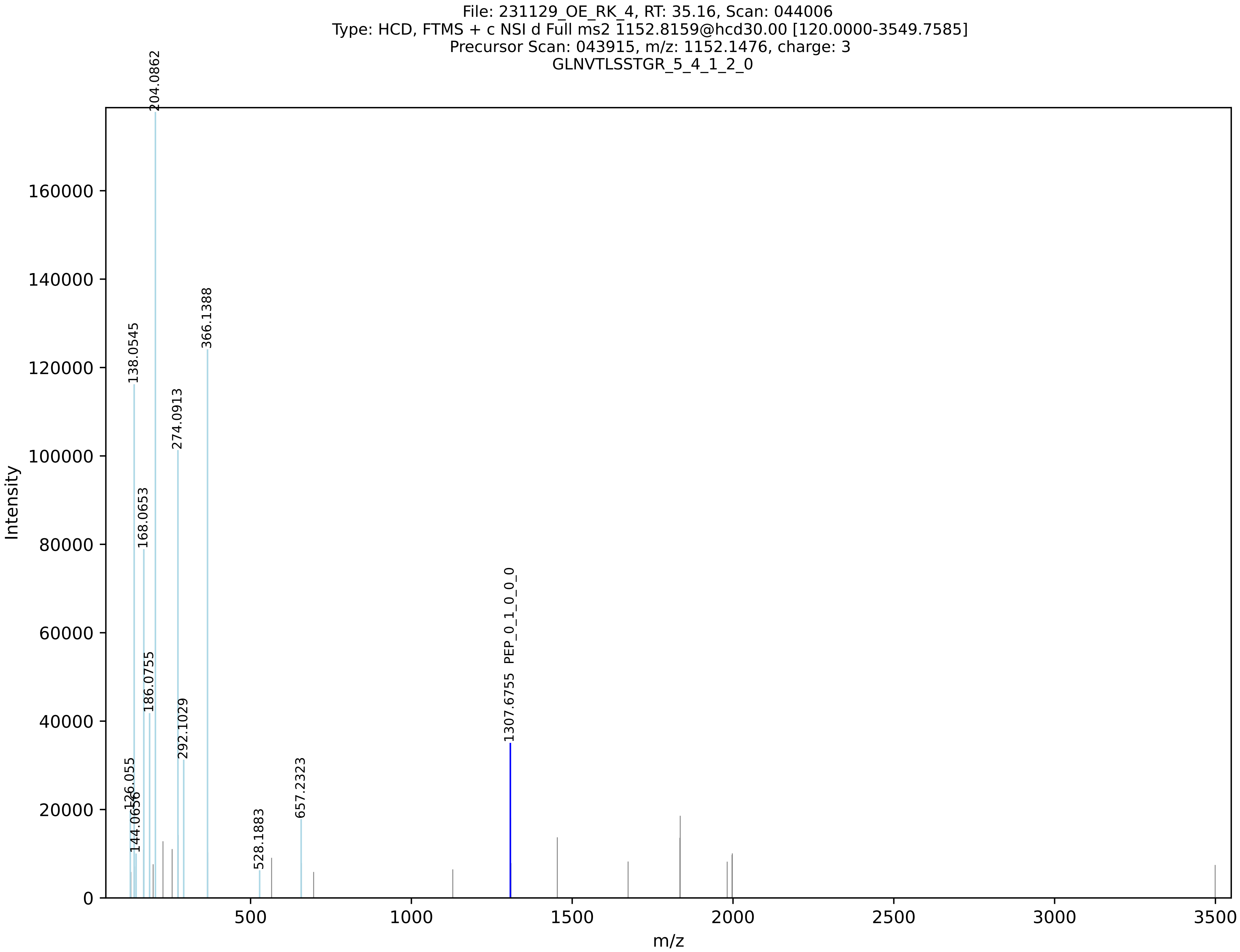
File: 231129_OE_RK_4, RT: 35.16, Scan: 044006
Type: HCD, FTMS + c NSI d Full ms2 1152.8159@hcd30.00 [120.0000-3549.7585]
Precursor Scan: 043915, m/z: 1152.1476, charge: 3
GLNVTLSSTGR_5_4_1_2_0
Intensity
m/z
204.0862
366.1388
138.0545
274.0913
168.0653
186.0755
292.1029
126.055
144.0656
528.1883
657.2323
1307.6755 PEP_0_1_0_0_0
0
20000
40000
60000
80000
100000
120000
140000
160000
500
1000
1500
2000
2500
3000
3500

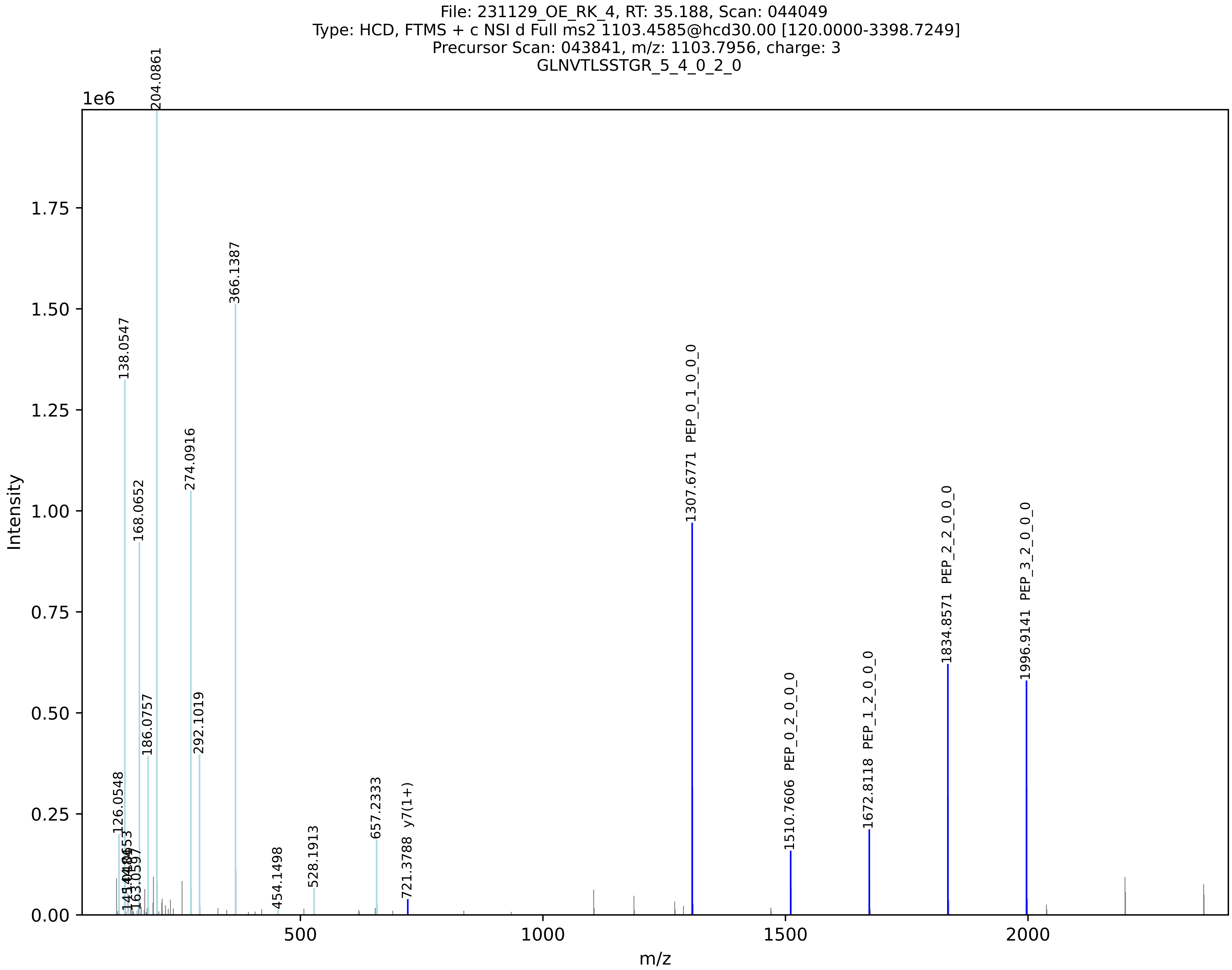
File: 231129_OE_RK_4, RT: 35.188, Scan: 044049
Type: HCD, FTMS + c NSI d Full ms2 1103.4585@hcd30.00 [120.0000-3398.7249]
Precursor Scan: 043841, m/z: 1103.7956, charge: 3
GLNVTLSSTGR_5_4_0_2_0
1e6
Intensity
m/z
204.0861
366.1387
138.0547
274.0916
168.0652
186.0757
292.1019
126.0548
163.0597
454.1498
528.1913
657.2333
721.3788 y7(1+)
1307.6771 PEP_0_1_0_0_0
1510.7606 PEP_0_2_0_0_0
1672.8118 PEP_1_2_0_0_0
1834.8571 PEP_2_2_0_0_0
1996.9141 PEP_3_2_0_0_0

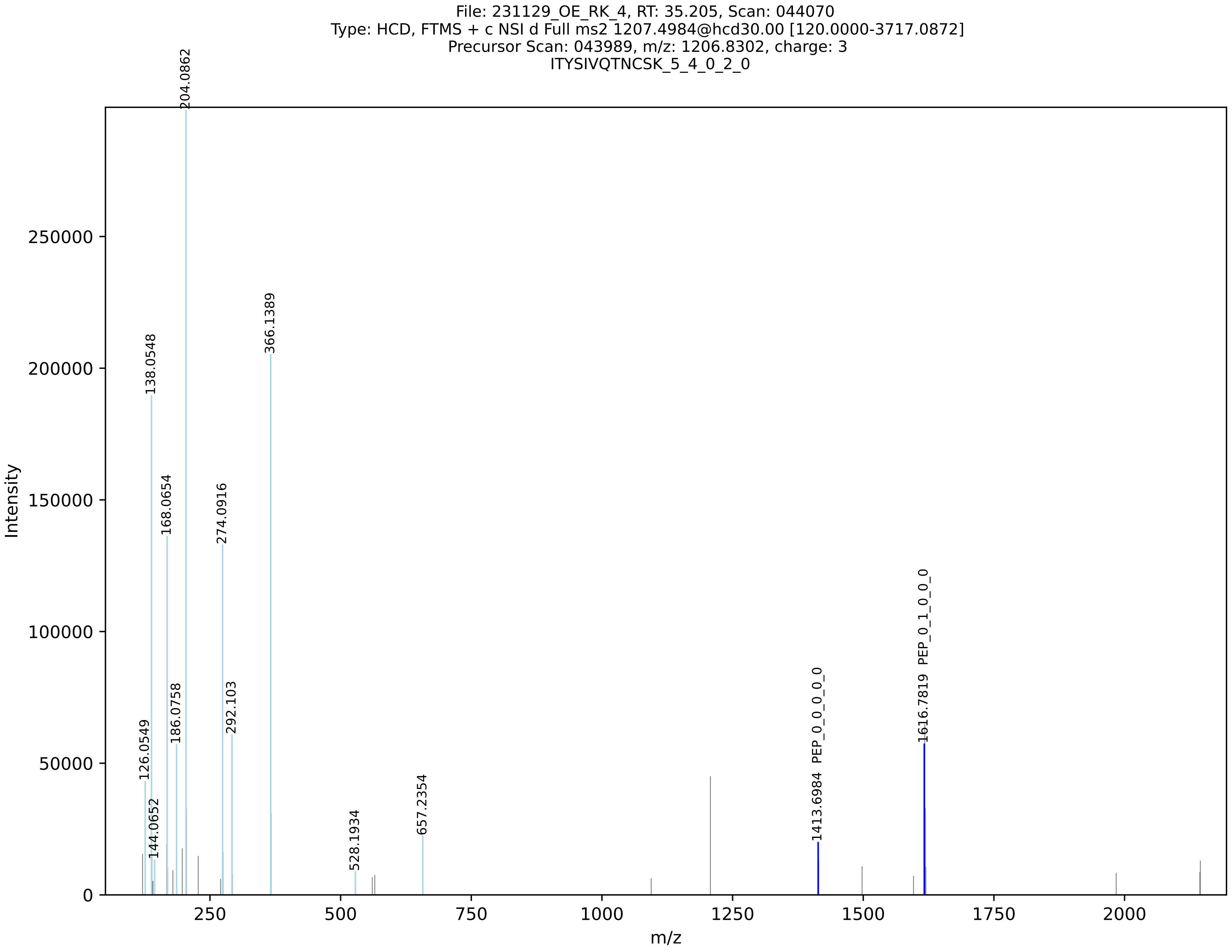
File: 231129_OE_RK_4, RT: 35.205, Scan: 044070
Type: HCD, FTMS + c NSI d Full ms2 1207.4984@hcd30.00 [120.0000-3717.0872]
Precursor Scan: 043989, m/z: 1206.8302, charge: 3
ITYSIVQTNCSK_5_4_0_2_0
204.0862
366.1389
138.0548
168.0654
274.0916
186.0758
292.103
126.0549
144.0652
528.1934
657.2354
1413.6984 PEP_0_0_0_0_0
1616.7819 PEP_0_1_0_0_
Intensity
m/z
0
50000
100000
150000
200000
250000
250
500
750
1000
1250
1500
1750
2000

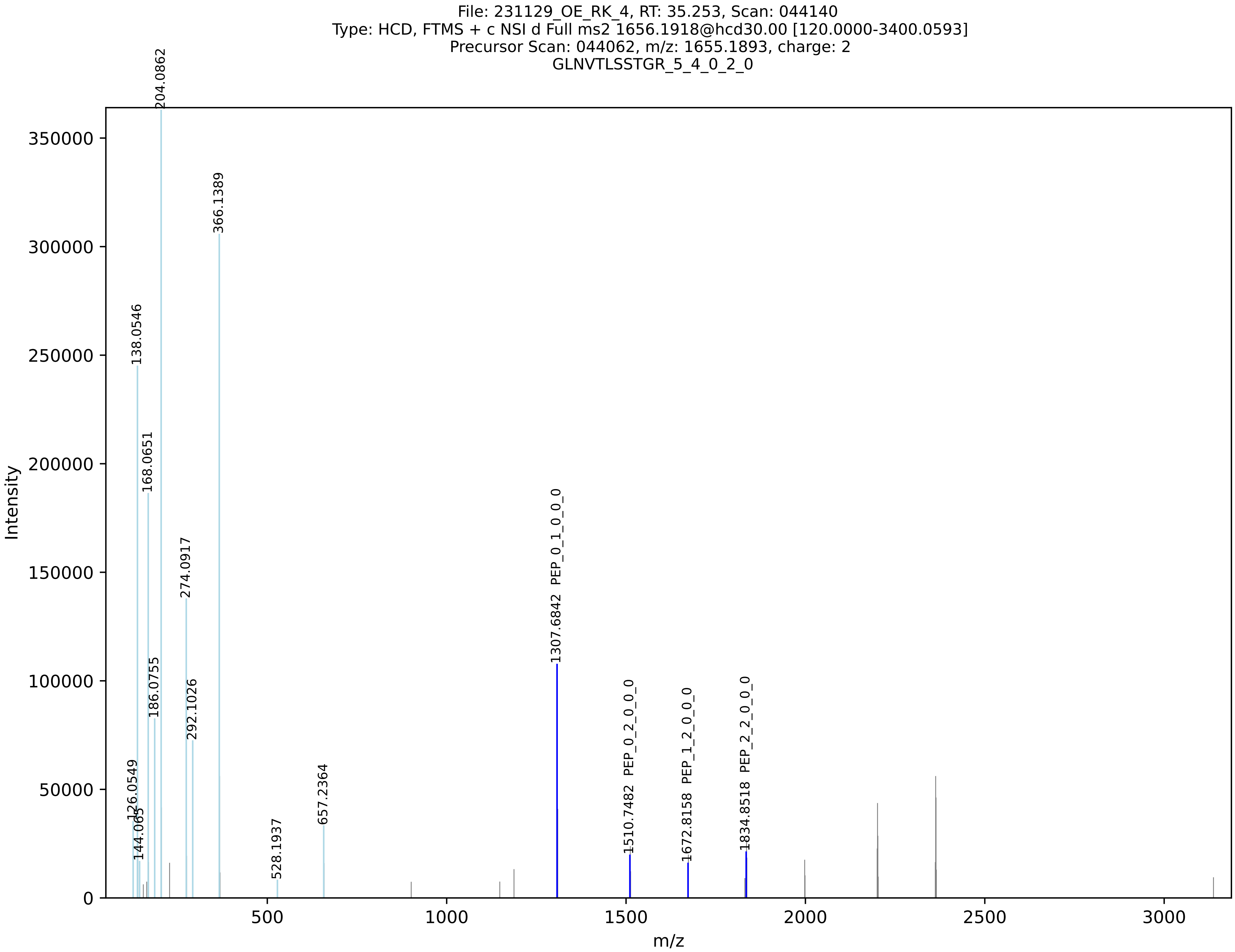

File: 231129_OE_RK_4, RT: 35.253, Scan: 044140
Type: HCD, FTMS + c NSI d Full ms2 1656.1918@hcd30.00 [120.0000-3400.0593]
Precursor Scan: 044062, m/z: 1655.1893, charge: 2
GLNVTLSSTGR_5_4_0_2_0
Intensity
m/z
204.0862
366.1389
138.0546
168.0651
274.0917
186.0755
292.1026
126.0549
144.065
528.1937
657.2364
1307.6842 PEP_0_1_0_0_0
1510.7482 PEP_0_2_0_0_0
1672.8158 PEP_1_2_0_0_0
1834.8518 PEP_2_2_0_0_0

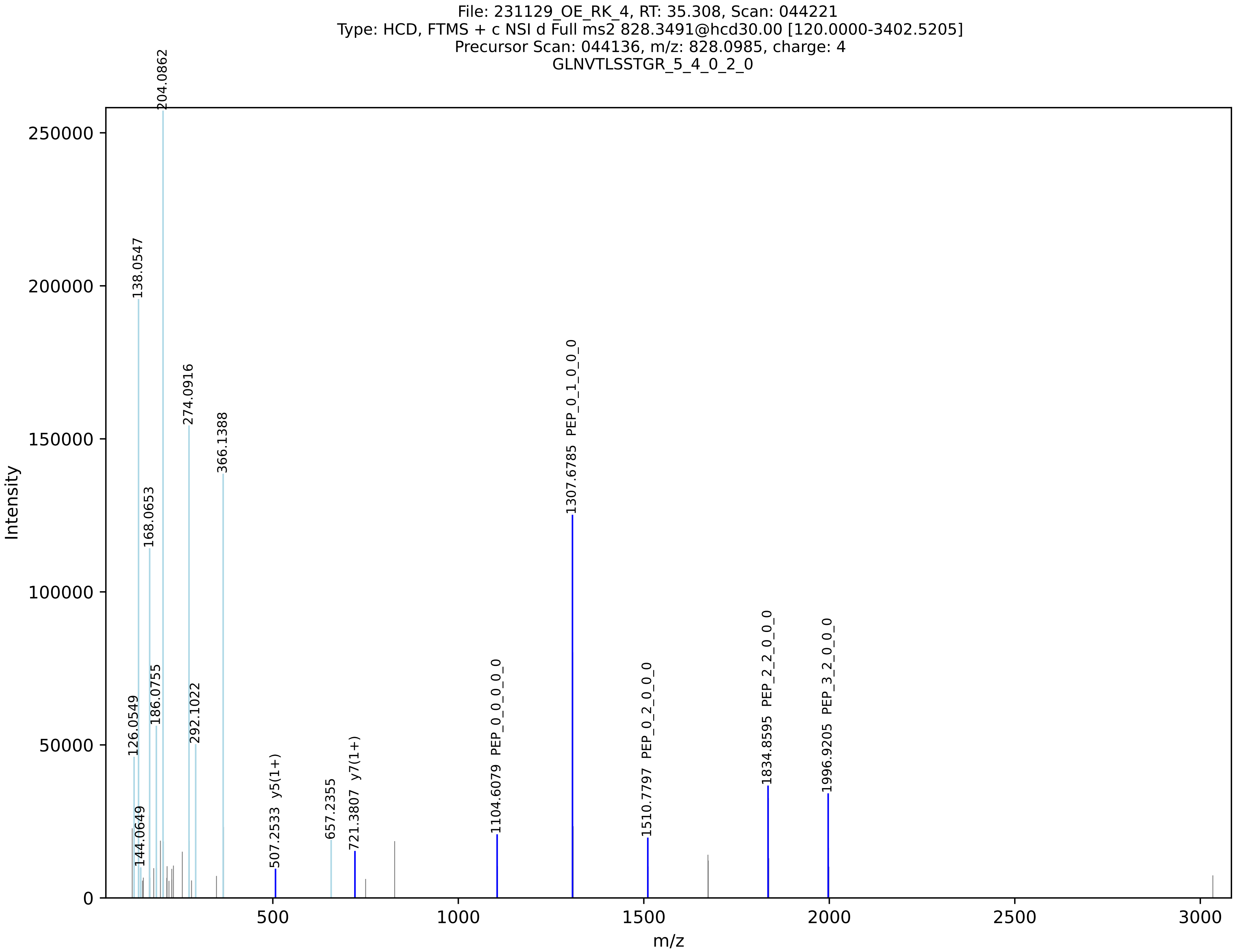
File: 231129_OE_RK_4, RT: 35.308, Scan: 044221
Type: HCD, FTMS + c NSI d Full ms2 828.3491@hcd30.00 [120.0000-3402.5205]
Precursor Scan: 044136, m/z: 828.0985, charge: 4
GLNVTLSSTGR_5_4_0_2_0
204.0862
138.0547
274.0916
366.1388
168.0653
186.0755
126.0549
292.1022
144.0649
507.2533 y5(1+)
657.2355
721.3807 y7(1+)
1104.6079 PEP_0_0_0_0_0
1307.6785 PEP_0_1_0_0_0
1510.7797 PEP_0_2_0_0_0
1834.8595 PEP_2_2_0_0_0
1996.9205 PEP_3_2_0_0_0
Intensity
m/z
0
50000
100000
150000
200000
250000
500
1000
1500
2000
2500
3000

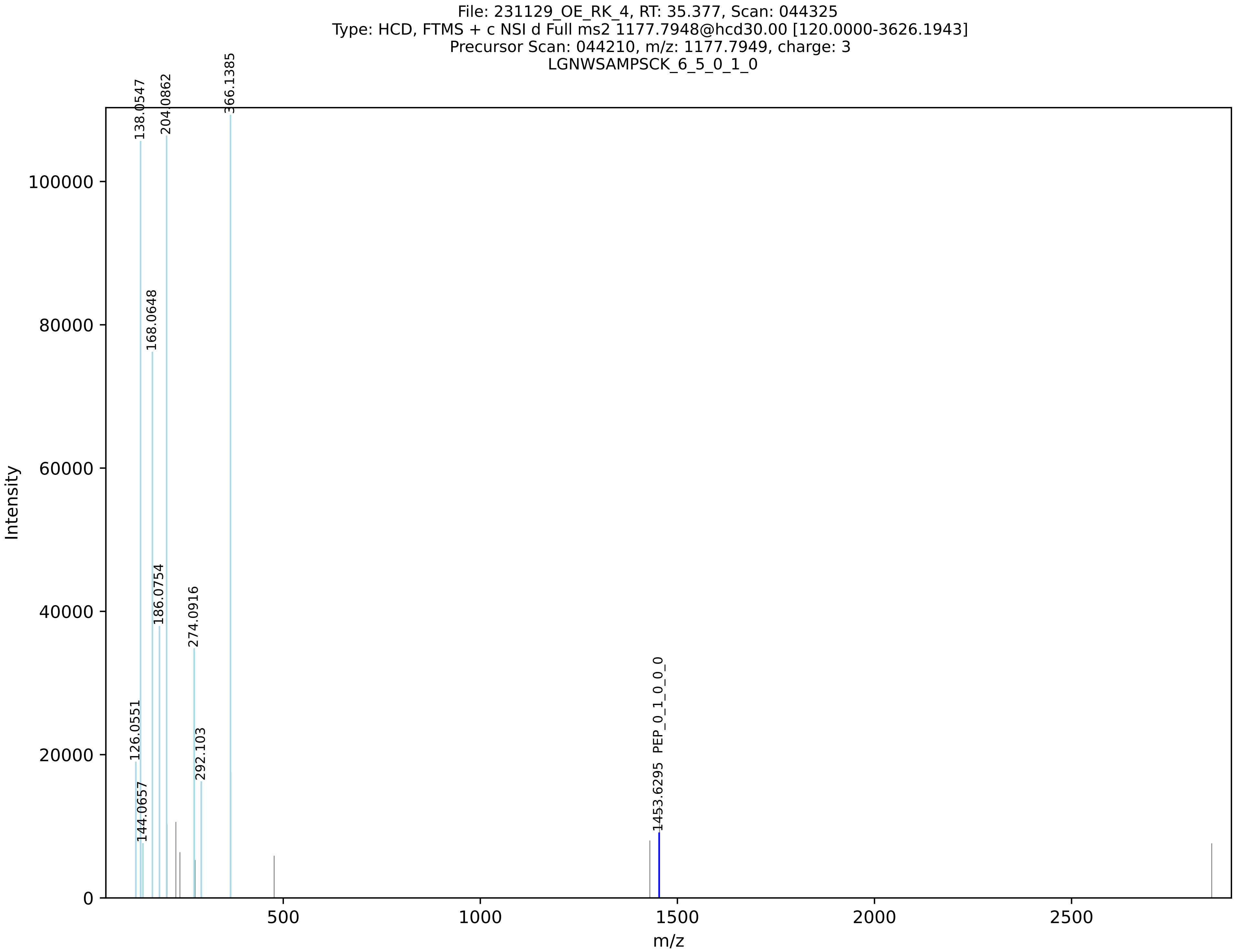

File: 231129_OE_RK_4, RT: 35.377, Scan: 044325
Type: HCD, FTMS + c NSI d Full ms2 1177.7948@hcd30.00 [120.0000-3626.1943]
Precursor Scan: 044210, m/z: 1177.7949, charge: 3
LGNWSAMPSCK_6_5_0_1_0
138.0547
204.0862
366.1385
168.0648
186.0754
274.0916
126.0551
292.103
144.0657
1453.6295 PEP_0_1_0_0_0
Intensity
m/z
0
20000
40000
60000
80000
100000
500
1000
1500
2000
2500

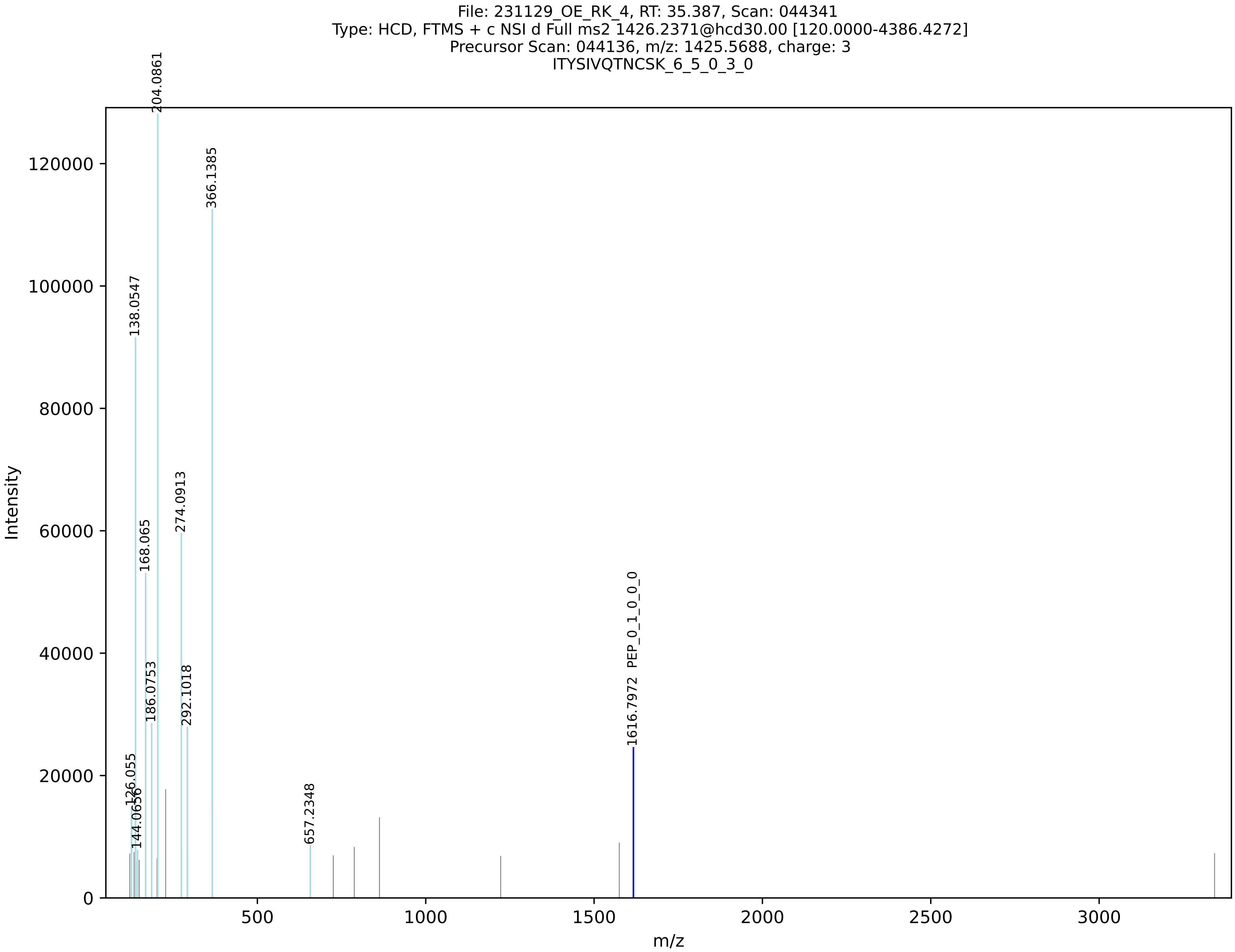
File: 231129_OE_RK_4, RT: 35.387, Scan: 044341
Type: HCD, FTMS + c NSI d Full ms2 1426.2371@hcd30.00 [120.0000-4386.4272]
Precursor Scan: 044136, m/z: 1425.5688, charge: 3
ITYSIVQTNCSK_6_5_0_3_0
Intensity
m/z
204.0861
366.1385
138.0547
274.0913
168.065
186.0753
292.1018
126.055
144.0656
657.2348
1616.7972 PEP_0_1_0_0_0

File: 231129_OE_RK_4, RT: 35.509, Scan: 044515
Type: HCD, FTMS + c NSI d Full ms2 1109.7996@hcd30.00 [120.0000-3418.1289]
Precursor Scan: 044430, m/z: 1109.4702, charge: 3
GLNVTLSSTGR_6_4_1_1_0

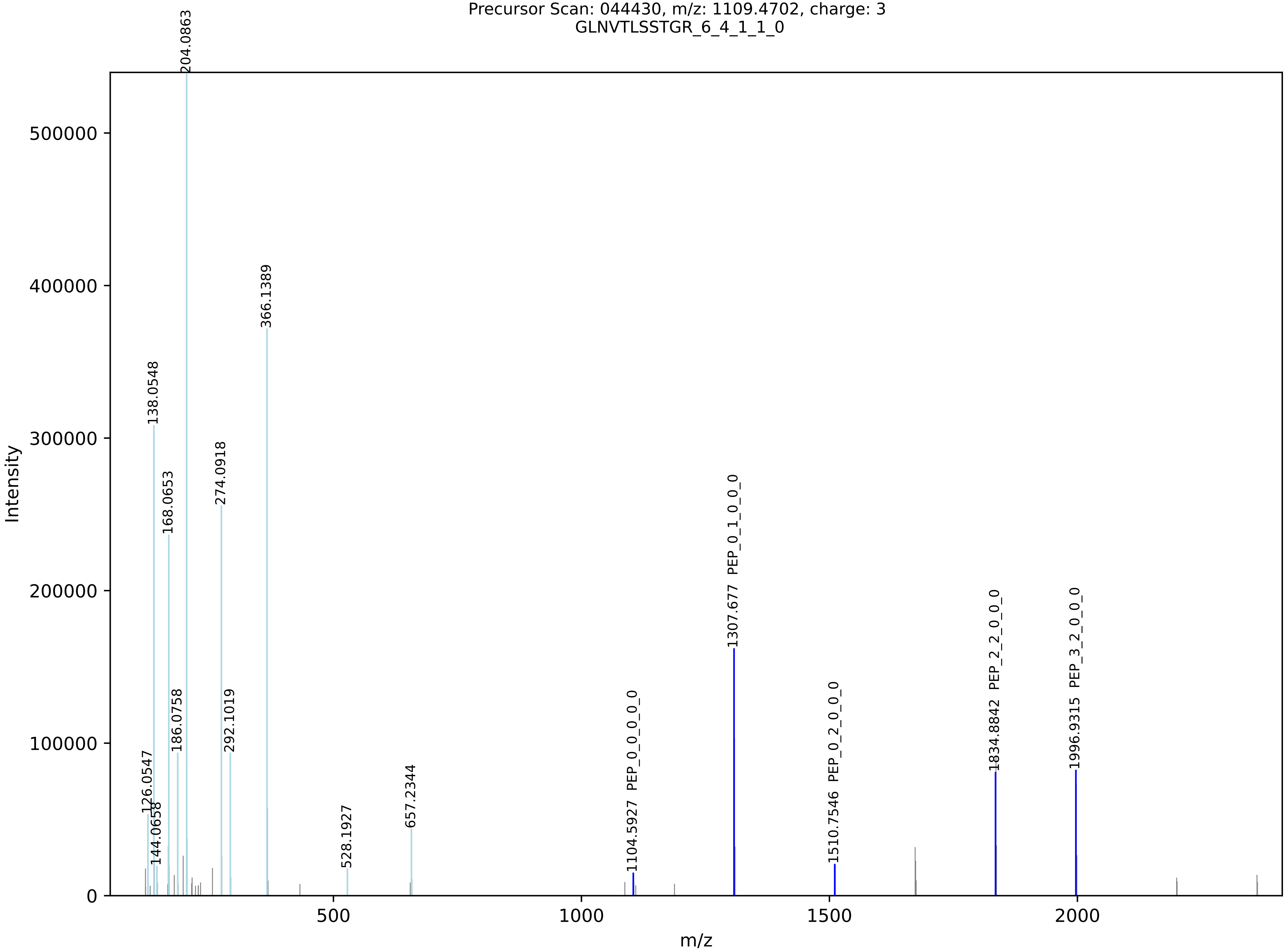

File: 231129_OE_RK_4, RT: 35.558, Scan: 044586
Type: HCD, FTMS + c NSI d Full ms2 1266.7787@hcd30.00 [120.0000-5191.3130]
Precursor Scan: 044503, m/z: 1266.2773, charge: 4
YPHKPEINSTTHPGADLQENFCR_5_4_1_2_0

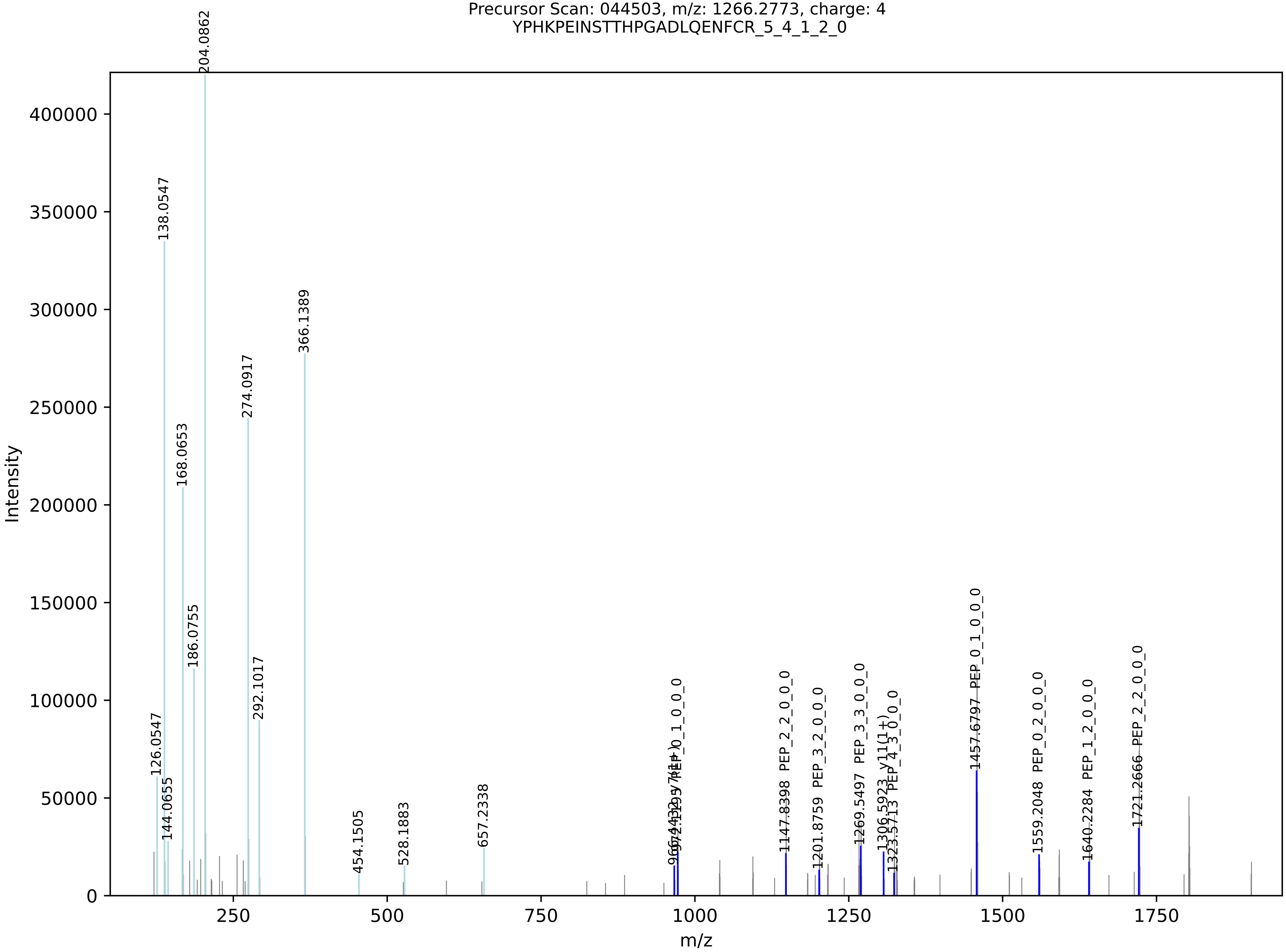

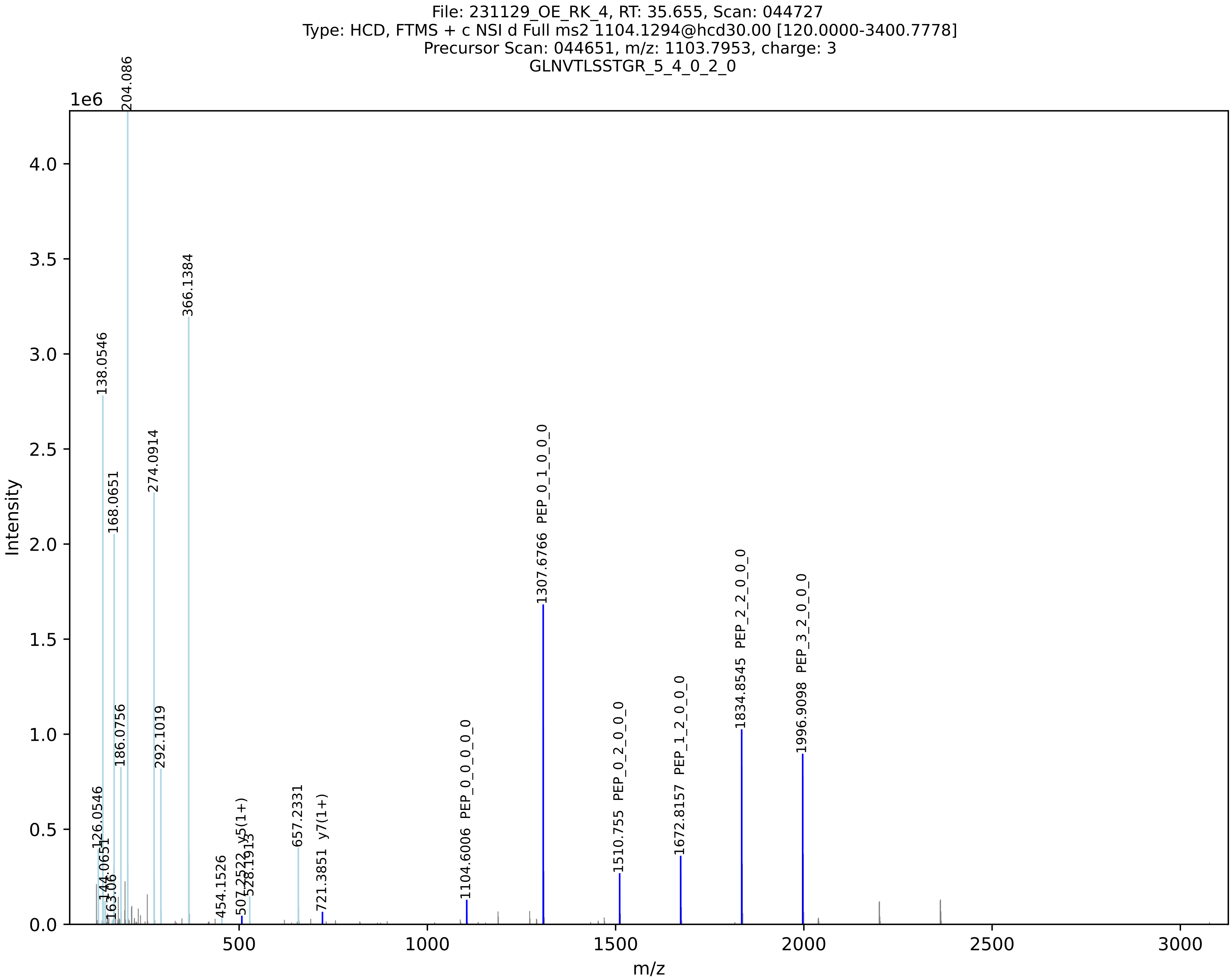
File: 231129_OE_RK_4, RT: 35.655, Scan: 044727
Type: HCD, FTMS + c NSI d Full ms2 1104.1294@hcd30.00 [120.0000-3400.7778]
Precursor Scan: 044651, m/z: 1103.7953, charge: 3
GLNVTLSSTGR_5_4_0_2_0
1e6
Intensity
m/z
204.086
366.1384
138.0546
274.0914
168.0651
186.0756
292.1019
126.0546
144.0651
163.06
454.1526
507.2522 y5(1+)
528.1913
657.2331
721.3851 y7(1+)
1104.6006 PEP_0_0_0_0_0
1307.6766 PEP_0_1_0_0_0
1510.755 PEP_0_2_0_0_0
1672.8157 PEP_1_2_0_0_0
1834.8545 PEP_2_2_0_0_0
1996.9098 PEP_3_2_0_0_0

File: 231129_OE_RK_4, RT: 35.657, Scan: 044730
Type: HCD, FTMS + c NSI d Full ms2 1655.6925@hcd30.00 [120.0000-3399.0408]
Precursor Scan: 044651, m/z: 1655.1894, charge: 2
GLNVTLSSTGR_5_4_0_2_0

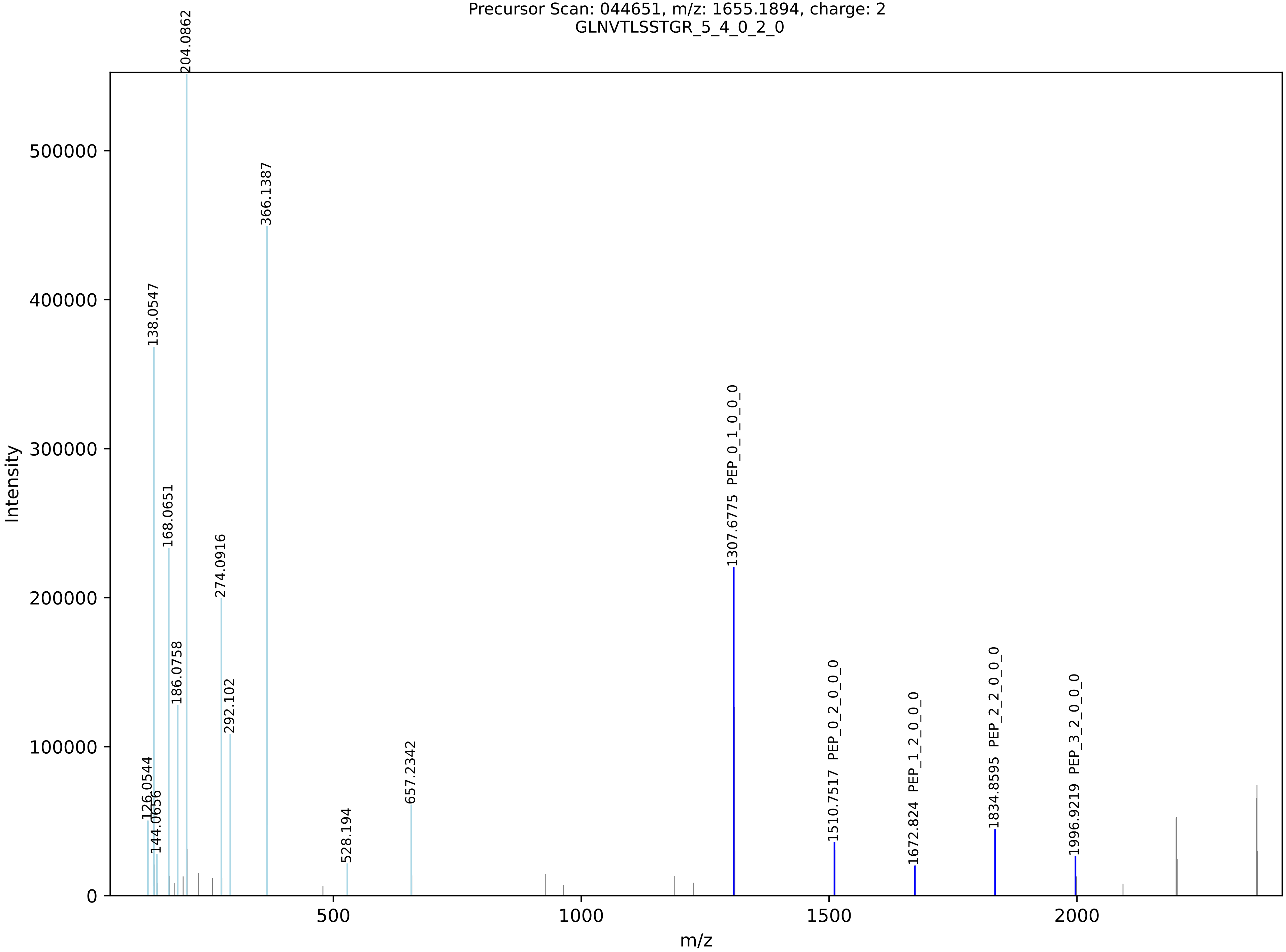

File: 231129_OE_RK_4, RT: 35.716, Scan: 044818
Type: HCD, FTMS + c NSI d Full ms2 1109.8007@hcd30.00 [120.0000-3418.1321]
Precursor Scan: 044725, m/z: 1109.1324, charge: 3
GLNVTLSSTGR_6_4_1_1_0

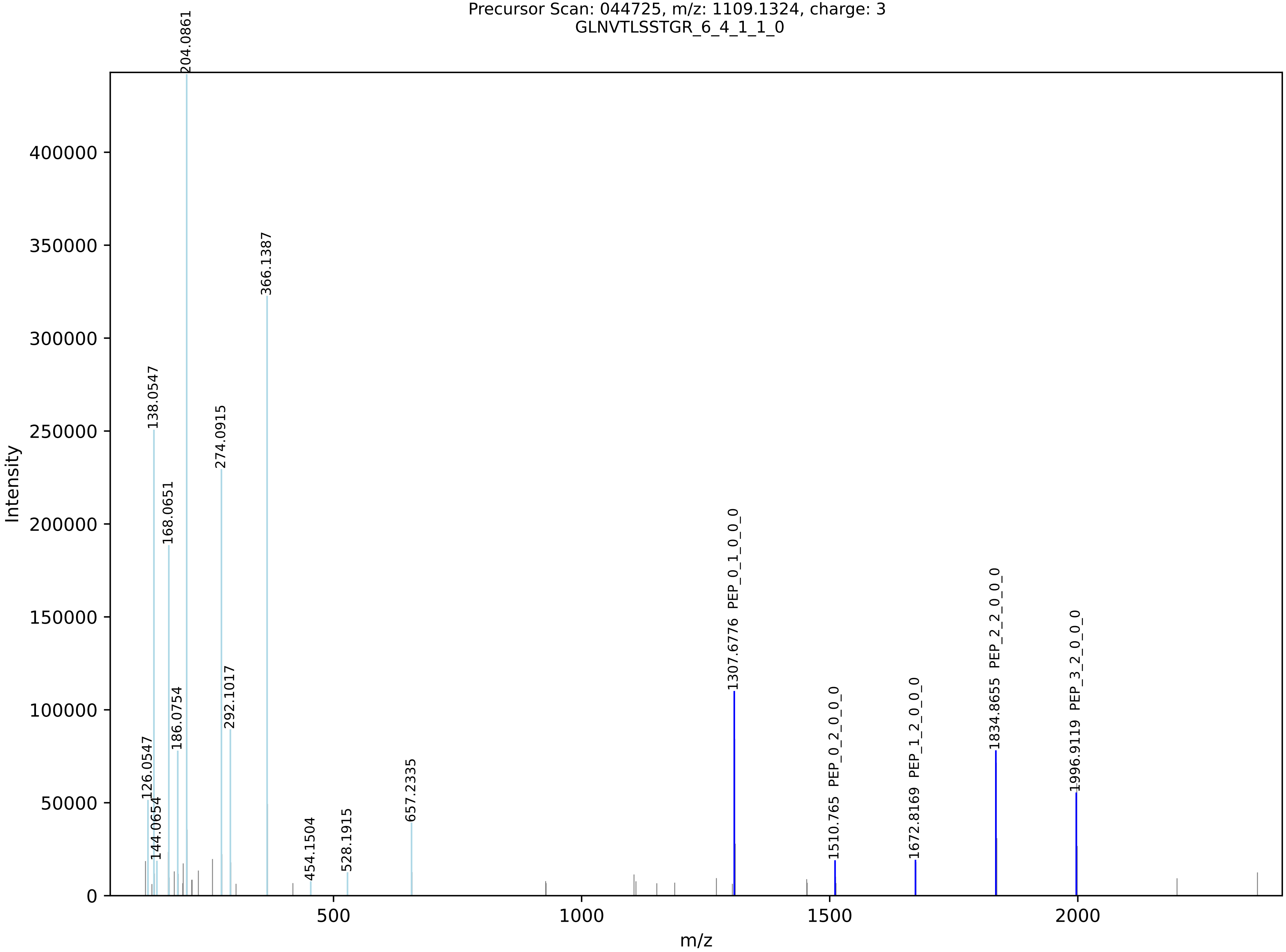

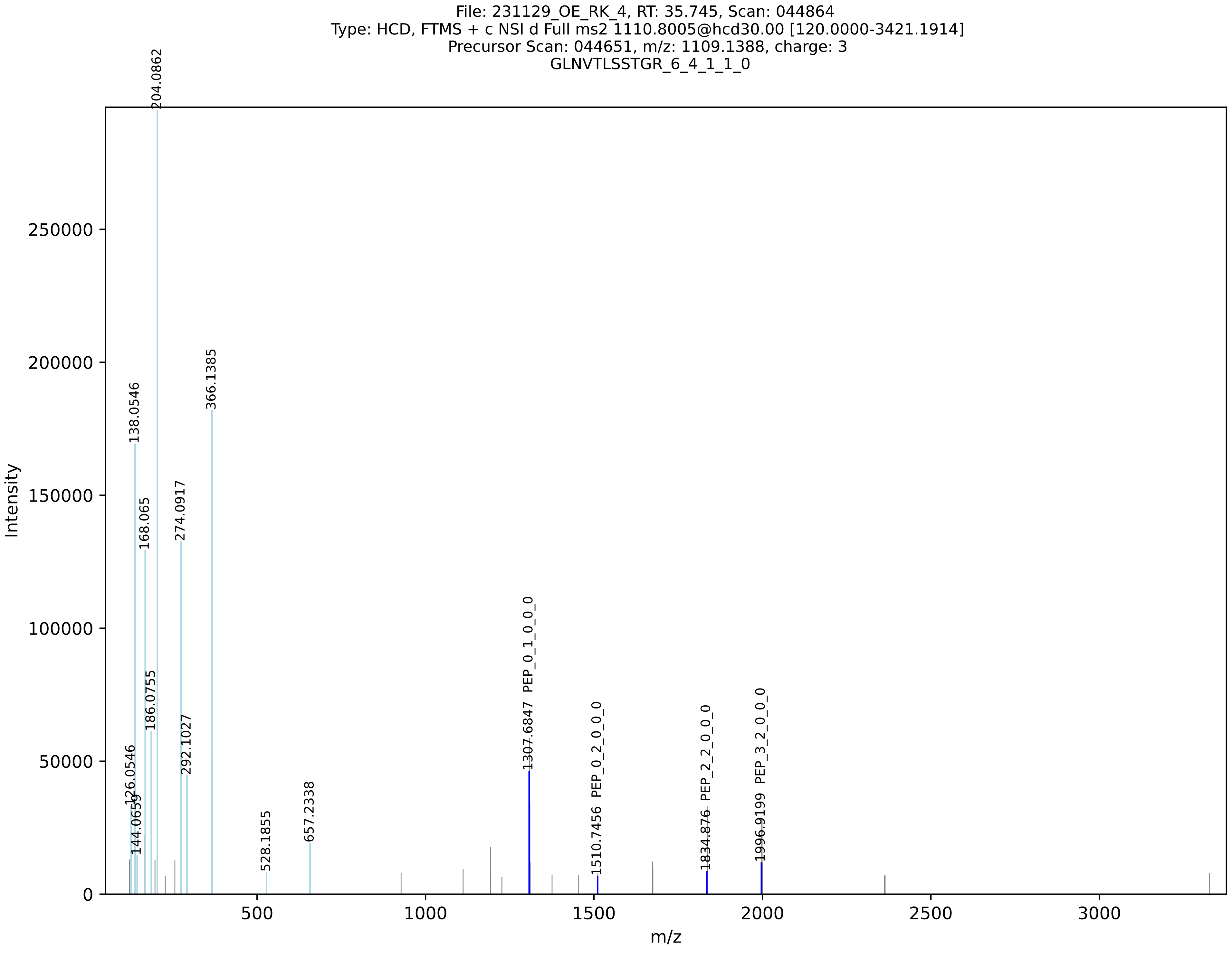

File: 231129_OE_RK_4, RT: 35.745, Scan: 044864
Type: HCD, FTMS + c NSI d Full ms2 1110.8005@hcd30.00 [120.0000-3421.1914]
Precursor Scan: 044651, m/z: 1109.1388, charge: 3
GLNVTLSSTGR_6_4_1_1_0
204.0862
138.0546
366.1385
168.065
274.0917
186.0755
292.1027
126.0546
144.0659
528.1855
657.2338
1307.6847 PEP_0_1_0_0_
1510.7456 PEP_0_2_0_0_
1834.876 PEP_2_2_0_0_
1996.9199 PEP_3_2_0_0_
Intensity
m/z
0
50000
100000
150000
200000
250000
500
1000
1500
2000
2500
3000

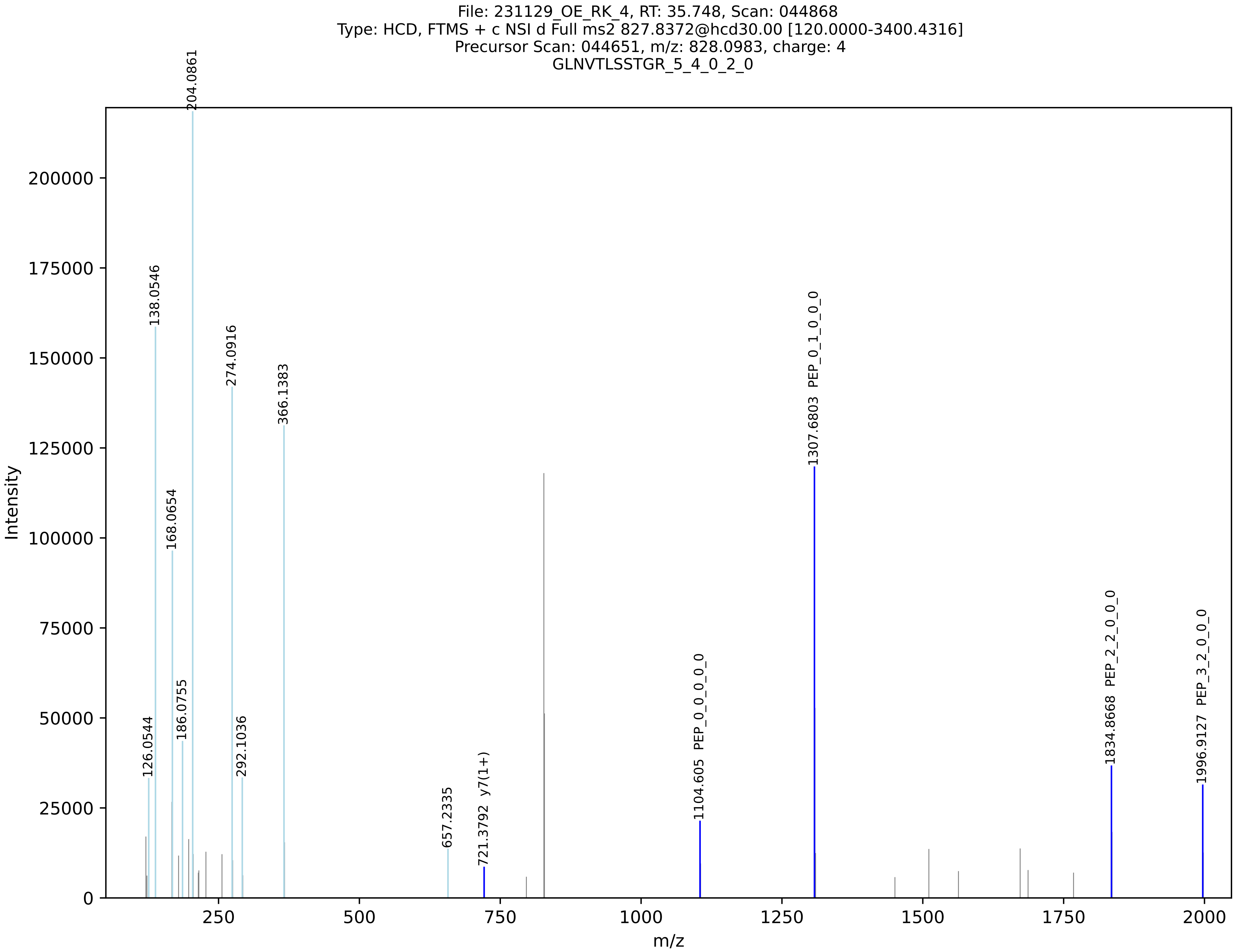
File: 231129_OE_RK_4, RT: 35.748, Scan: 044868
Type: HCD, FTMS + c NSI d Full ms2 827.8372@hcd30.00 [120.0000-3400.4316]
Precursor Scan: 044651, m/z: 828.0983, charge: 4
GLNVTLSSTGR_5_4_0_2_0
204.0861
138.0546
274.0916
366.1383
168.0654
186.0755
126.0544
292.1036
657.2335
721.3792 y7(1+)
1104.605 PEP_0_0_0_0_0
1307.6803 PEP_0_1_0_0_0
1834.8668 PEP_2_2_0_0_0
1996.9127 PEP_3_2_0_0_0
Intensity
m/z
0
25000
50000
75000
100000
125000
150000
175000
200000
250
500
750
1000
1250
1500
1750
2000

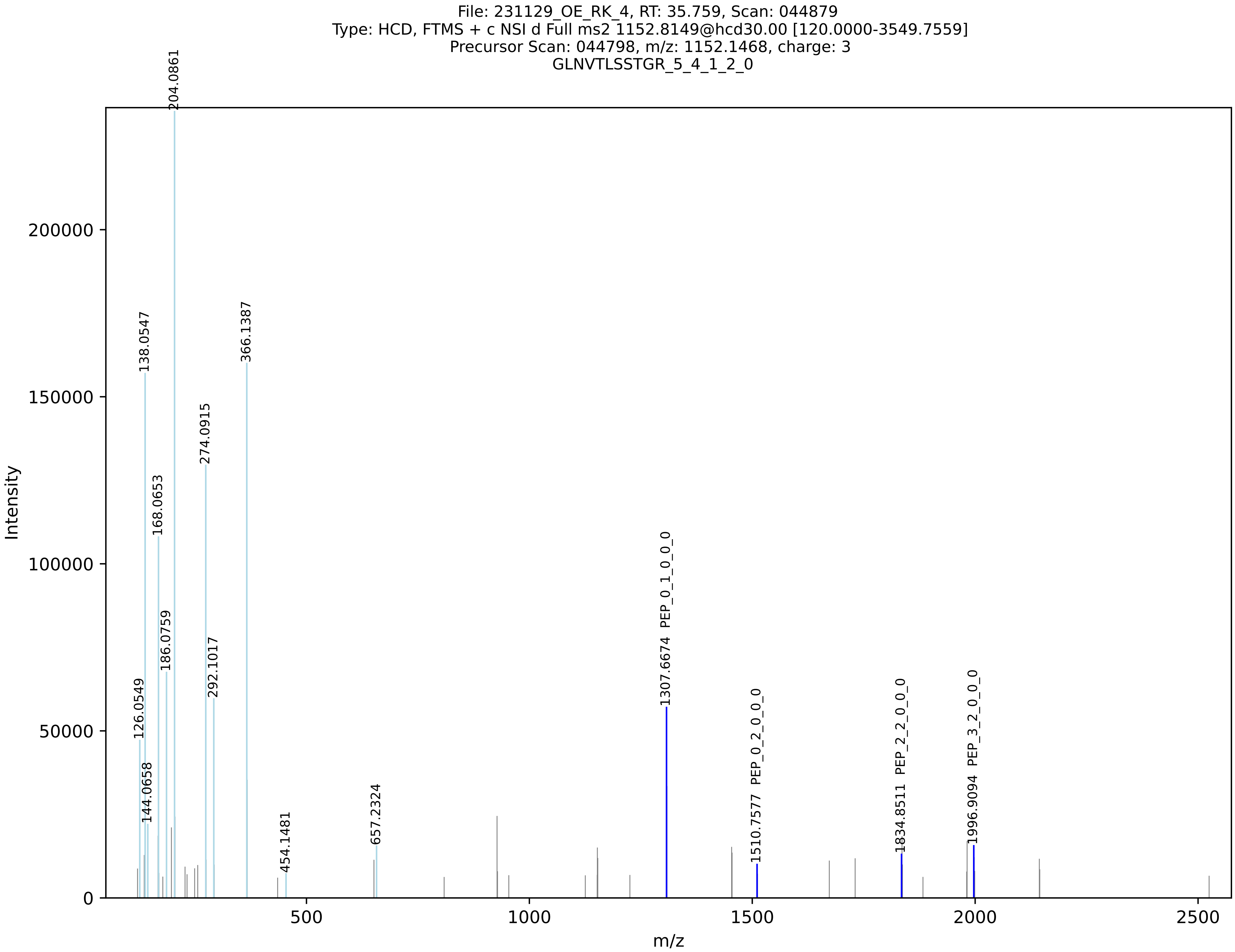
File: 231129_OE_RK_4, RT: 35.759, Scan: 044879
Type: HCD, FTMS + c NSI d Full ms2 1152.8149@hcd30.00 [120.0000-3549.7559]
Precursor Scan: 044798, m/z: 1152.1468, charge: 3
GLNVTLSSTGR_5_4_1_2_0
204.0861
138.0547
366.1387
274.0915
168.0653
186.0759
292.1017
126.0549
144.0658
454.1481
657.2324
1307.6674 PEP_0_1_0_0_0
1510.7577 PEP_0_2_0_0_0
1834.8511 PEP_2_2_0_0_0
1996.9094 PEP_3_2_0_0_0
Intensity
m/z
0
50000
100000
150000
200000
500
1000
1500
2000
2500

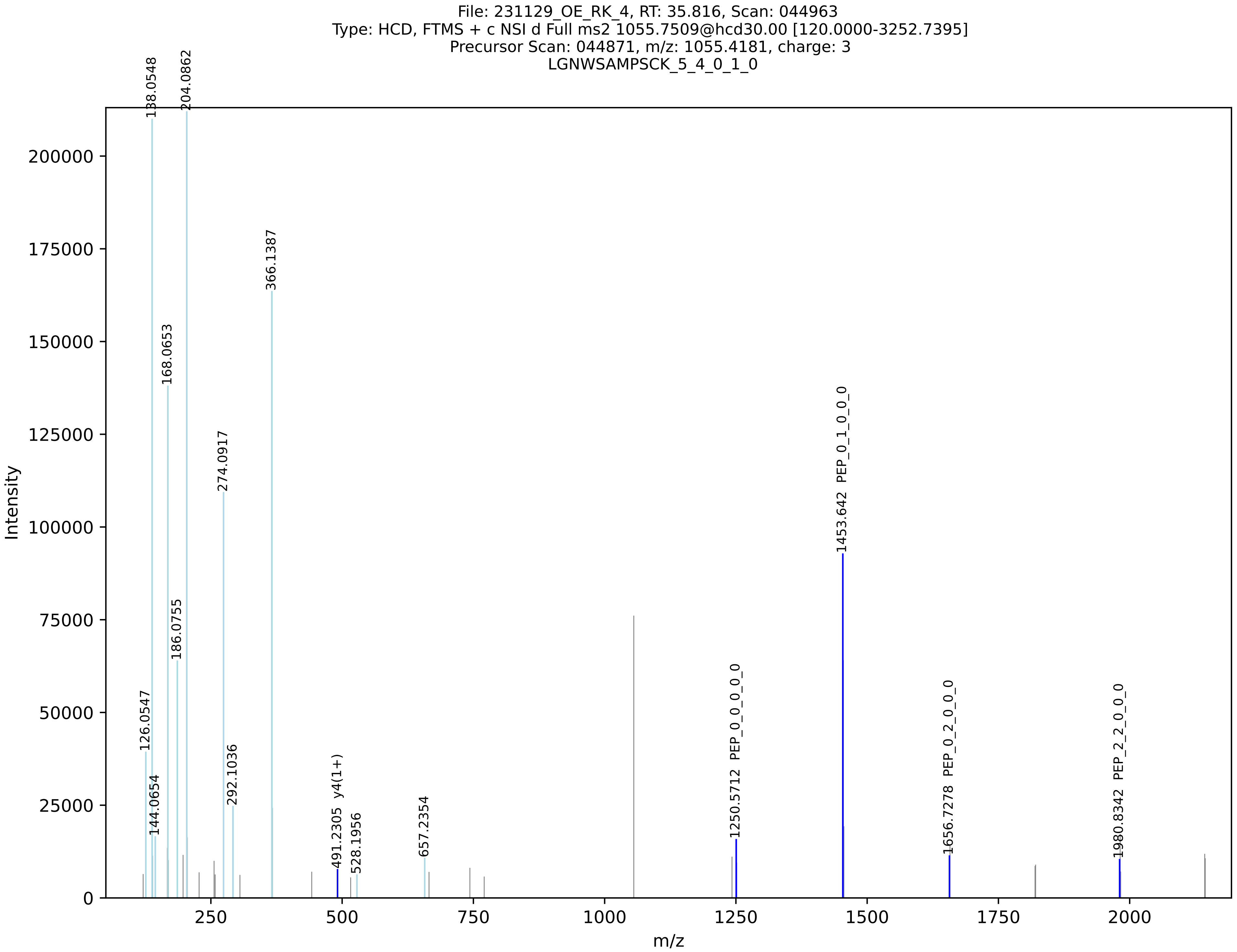
File: 231129_OE_RK_4, RT: 35.816, Scan: 044963
Type: HCD, FTMS + c NSI d Full ms2 1055.7509@hcd30.00 [120.0000-3252.7395]
Precursor Scan: 044871, m/z: 1055.4181, charge: 3
LGNWSAMPSCK_5_4_0_1_0
Intensity
m/z
138.0548
204.0862
366.1387
168.0653
274.0917
186.0755
126.0547
144.0654
292.1036
491.2305 y4(1+)
528.1956
657.2354
1250.5712 PEP_0_0_0_0_0
1453.642 PEP_0_1_0_0_0
1656.7278 PEP_0_2_0_0_0
1980.8342 PEP_2_2_0_0_0
0
25000
50000
75000
100000
125000
150000
175000
200000
250
500
750
1000
1250
1500
1750
2000

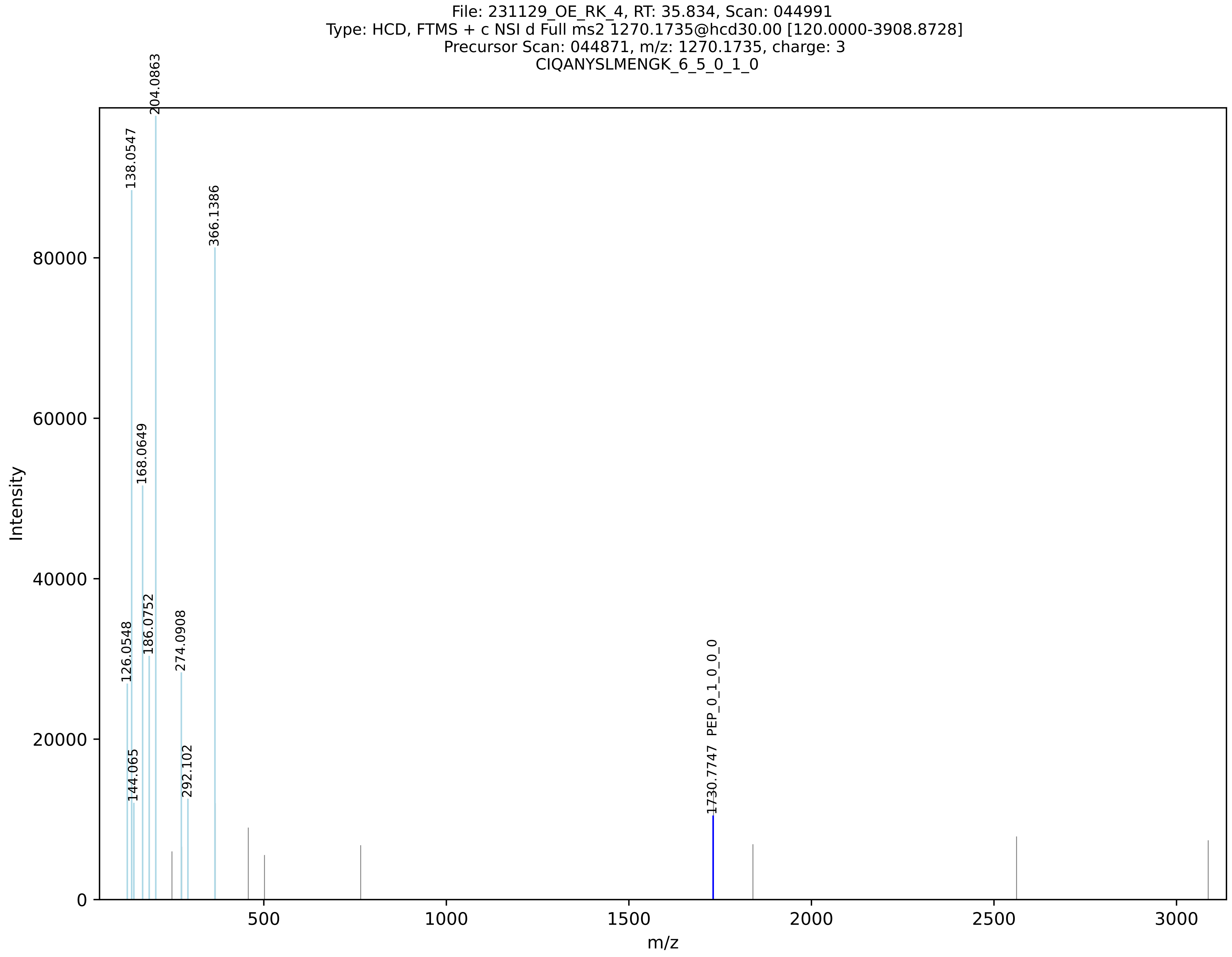
File: 231129_OE_RK_4, RT: 35.834, Scan: 044991
Type: HCD, FTMS + c NSI d Full ms2 1270.1735@hcd30.00 [120.0000-3908.8728]
Precursor Scan: 044871, m/z: 1270.1735, charge: 3
CIQANYSLMENGK_6_5_0_1_0
204.0863
138.0547
366.1386
168.0649
126.0548
186.0752
274.0908
144.065
292.102
1730.7747 PEP_0_1_0_0_0
Intensity
m/z
0
20000
40000
60000
80000
500
1000
1500
2000
2500
3000

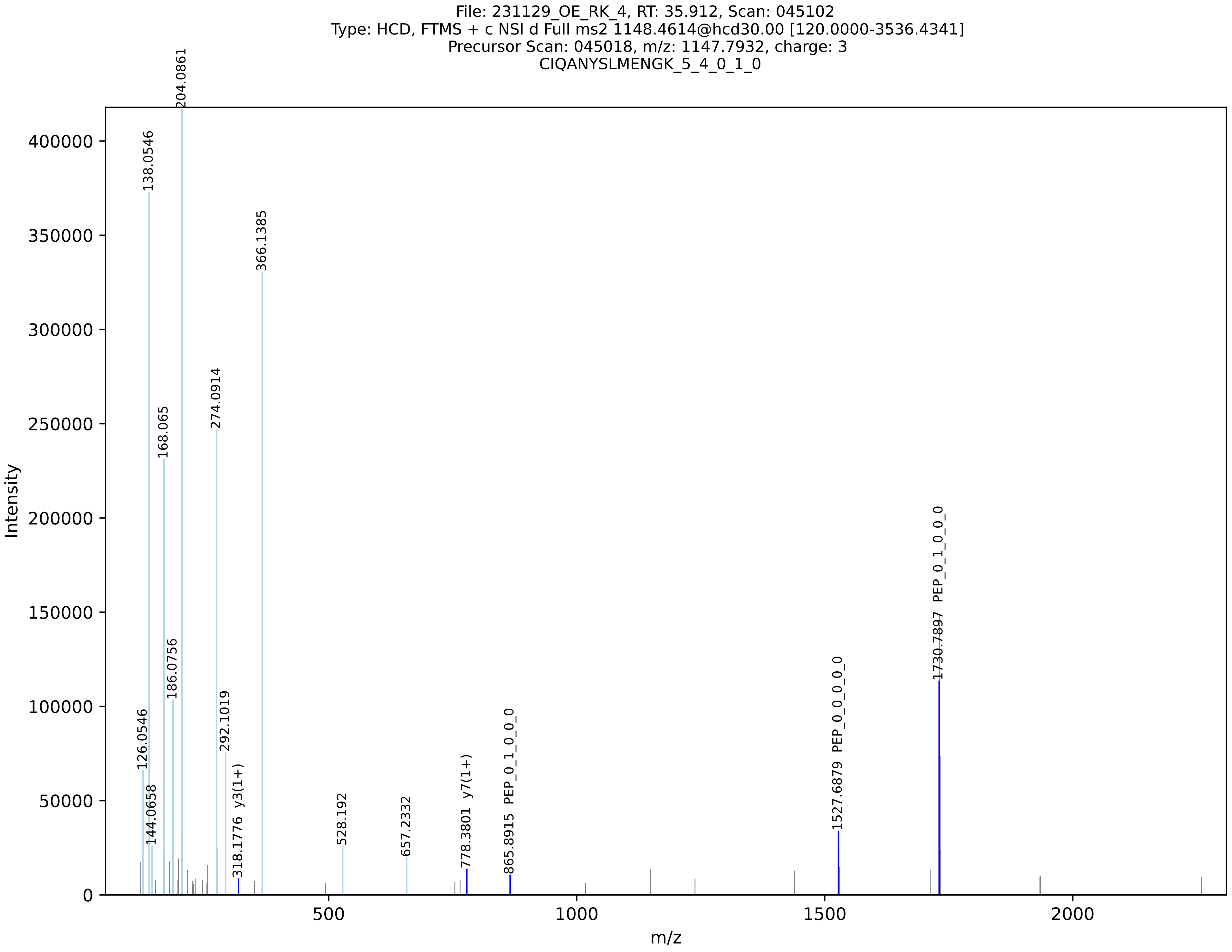
File: 231129_OE_RK_4, RT: 35.912, Scan: 045102
Type: HCD, FTMS + c NSI d Full ms2 1148.4614@hcd30.00 [120.0000-3536.4341]
Precursor Scan: 045018, m/z: 1147.7932, charge: 3
CIQANYSLMENGK_5_4_0_1_0
Intensity
m/z
204.0861
138.0546
366.1385
274.0914
168.065
186.0756
126.0546
292.1019
144.0658
318.1776 y3(1+)
528.192
657.2332
778.3801 y7(1+)
865.8915 PEP_0_1_0_0_0
1527.6879 PEP_0_0_0_0_0
1730.7897 PEP_0_1_0_0_0
0
50000
100000
150000
200000
250000
300000
350000
400000
500
1000
1500
2000

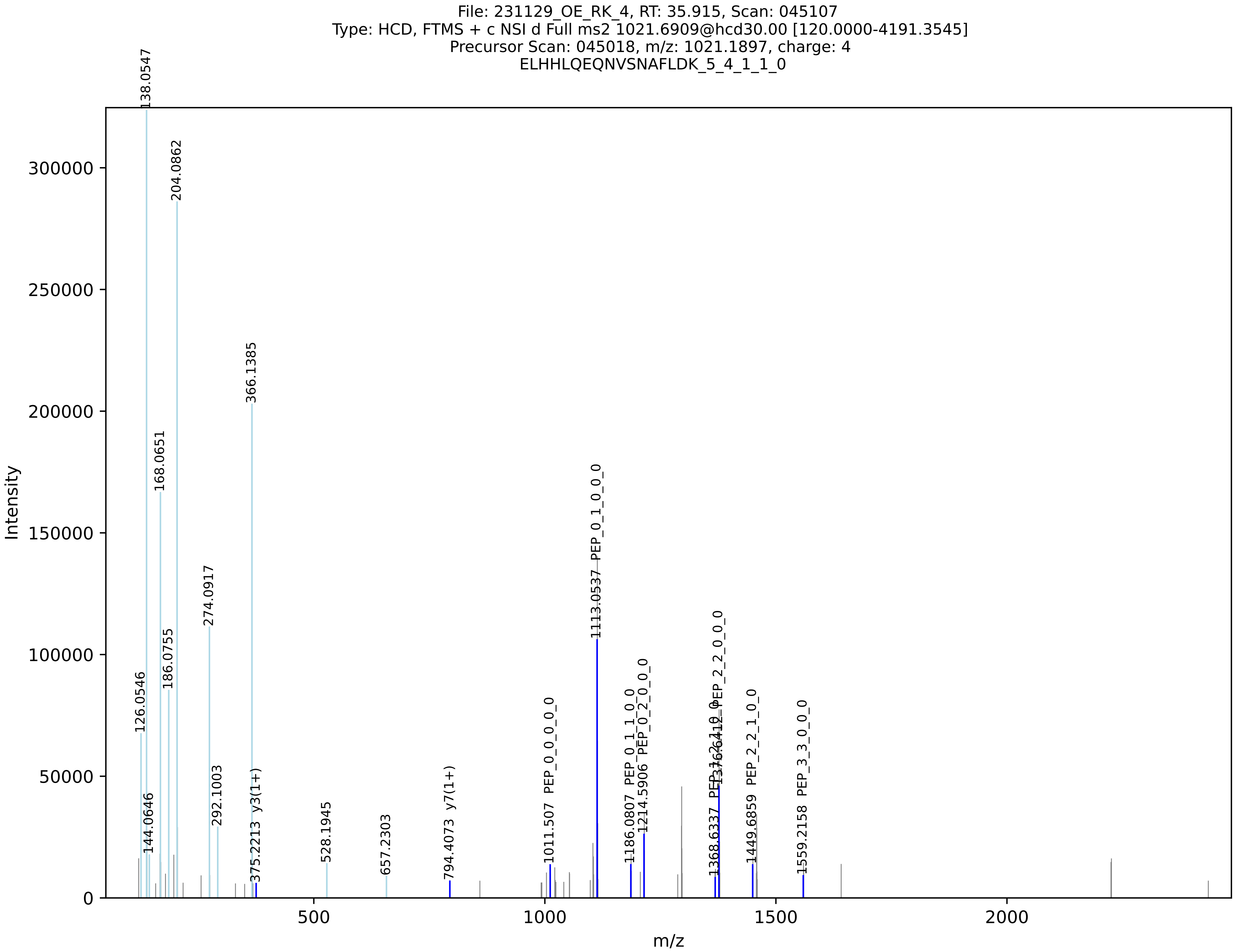

File: 231129_OE_RK_4, RT: 35.915, Scan: 045107
Type: HCD, FTMS + c NSI d Full ms2 1021.6909@hcd30.00 [120.0000-4191.3545]
Precursor Scan: 045018, m/z: 1021.1897, charge: 4
ELHHLQEQNVSNAFLDK_5_4_1_1_0
Intensity
m/z
138.0547
204.0862
366.1385
168.0651
274.0917
186.0755
126.0546
144.0646
292.1003
375.2213 y3(1+)
528.1945
657.2303
794.4073 y7(1+)
1011.507 PEP_0_0_0_0_0
1113.0537 PEP_0_1_0_0_0
1186.0807 PEP_0_1_1_0_0
1214.5906 PEP_0_2_0_0_0
1368.6337 PEP_1_2_1_0_0
1376.6412 PEP_2_2_0_0_0
1449.6859 PEP_2_2_1_0_0
1559.2158 PEP_3_3_0_0_0

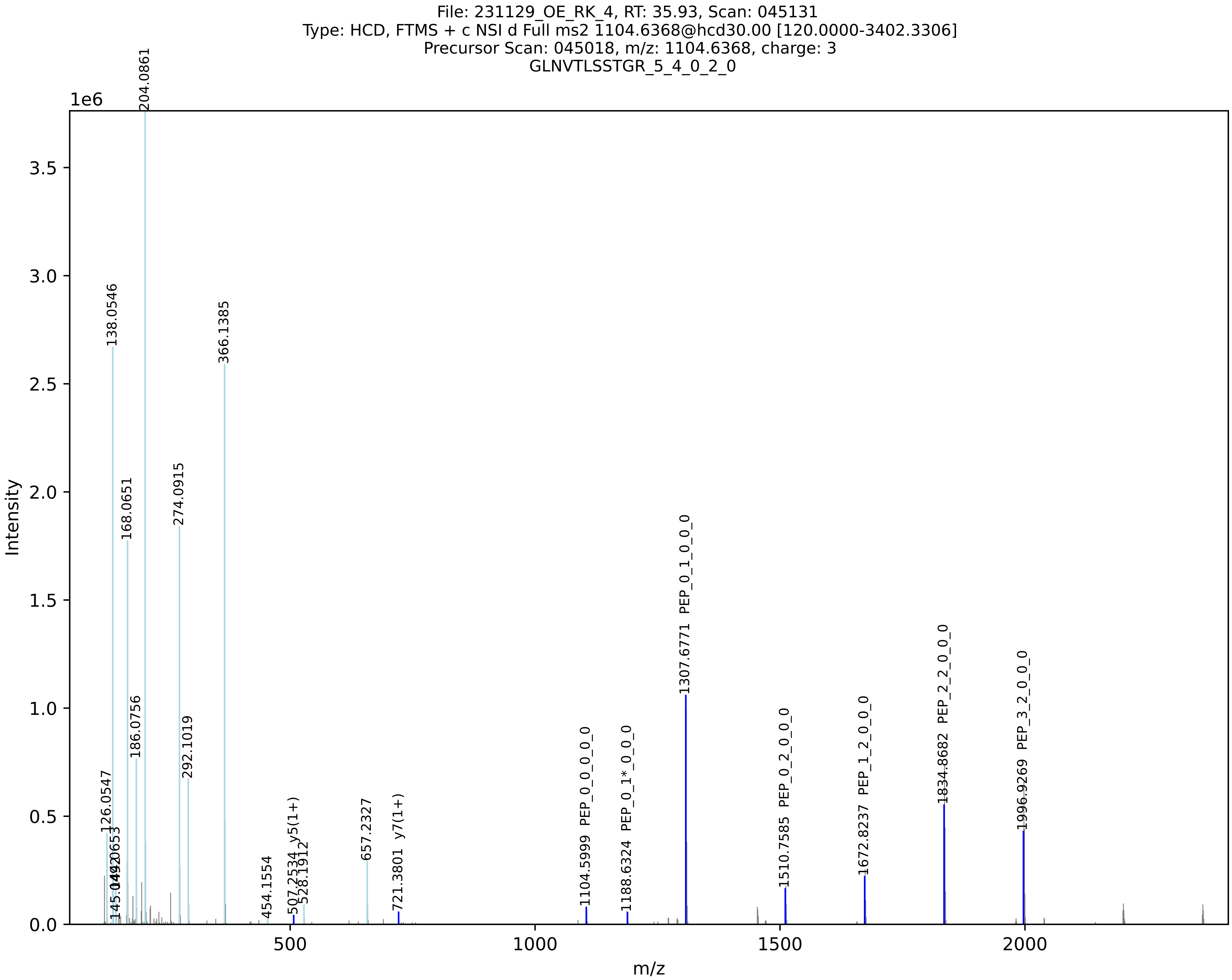
File: 231129_OE_RK_4, RT: 35.93, Scan: 045131
Type: HCD, FTMS + c NSI d Full ms2 1104.6368@hcd30.00 [120.0000-3402.3306]
Precursor Scan: 045018, m/z: 1104.6368, charge: 3
GLNVTLSSTGR_5_4_0_2_0
1e6
Intensity
m/z
204.0861
138.0546
366.1385
274.0915
168.0651
186.0756
292.1019
126.0547
454.1554
507.2534 y5(1+)
528.1912
657.2327
721.3801 y7(1+)
1104.5999 PEP_0_0_0_0_0
1188.6324 PEP_0_1*_0_0_0
1307.6771 PEP_0_1_0_0_0
1510.7585 PEP_0_2_0_0_0
1672.8237 PEP_1_2_0_0_0
1834.8682 PEP_2_2_0_0_0
1996.9269 PEP_3_2_0_0_0

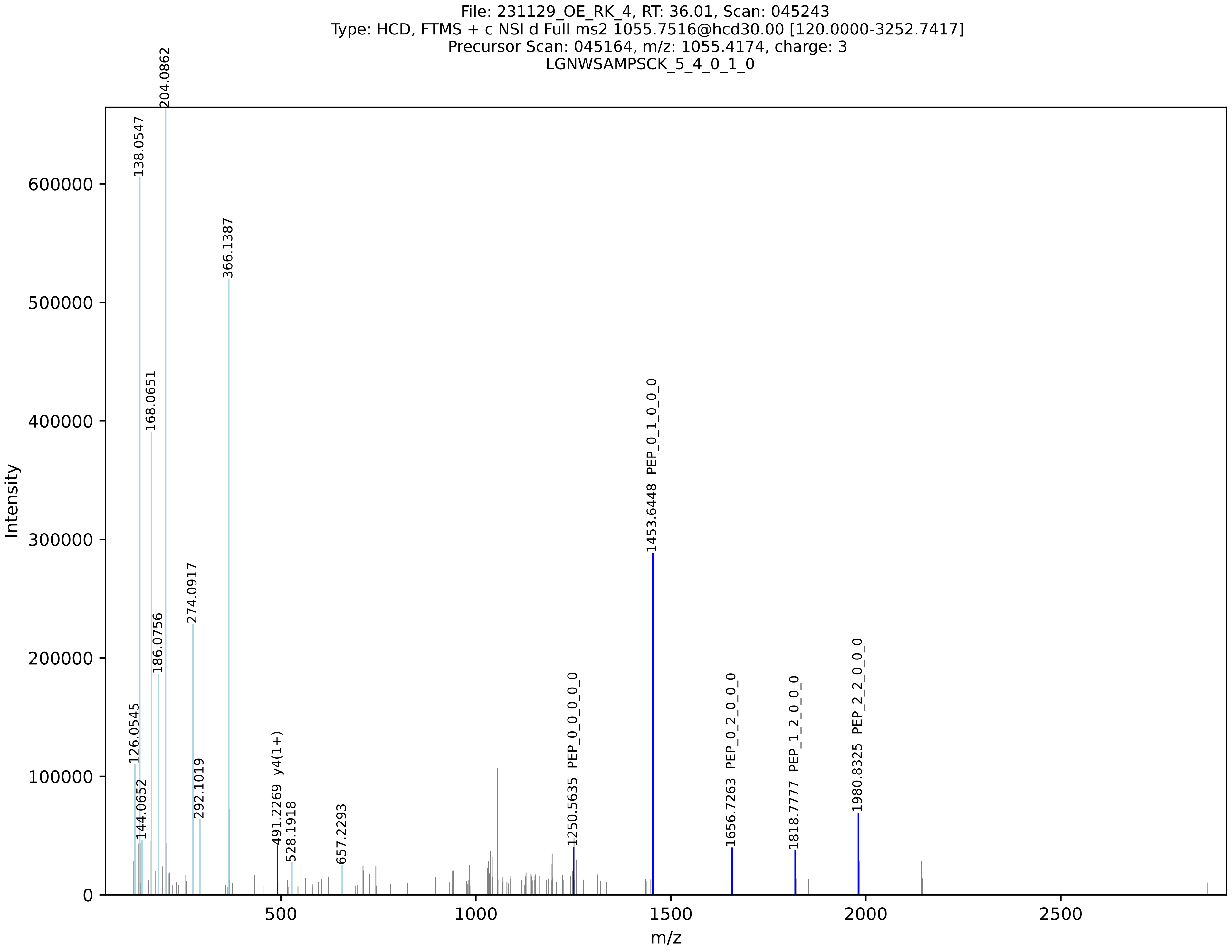

File: 231129_OE_RK_4, RT: 36.01, Scan: 045243
Type: HCD, FTMS + c NSI d Full ms2 1055.7516@hcd30.00 [120.0000-3252.7417]
Precursor Scan: 045164, m/z: 1055.4174, charge: 3
LGNWSAMPSCK_5_4_0_1_0
Intensity
m/z
204.0862
138.0547
366.1387
168.0651
186.0756
274.0917
126.0545
144.0652
292.1019
491.2269 y4(1+)
528.1918
657.2293
1250.5635 PEP_0_0_0_0_0
1453.6448 PEP_0_1_0_0_0
1656.7263 PEP_0_2_0_0_0
1818.7777 PEP_1_2_0_0_0
1980.8325 PEP_2_2_0_0_0

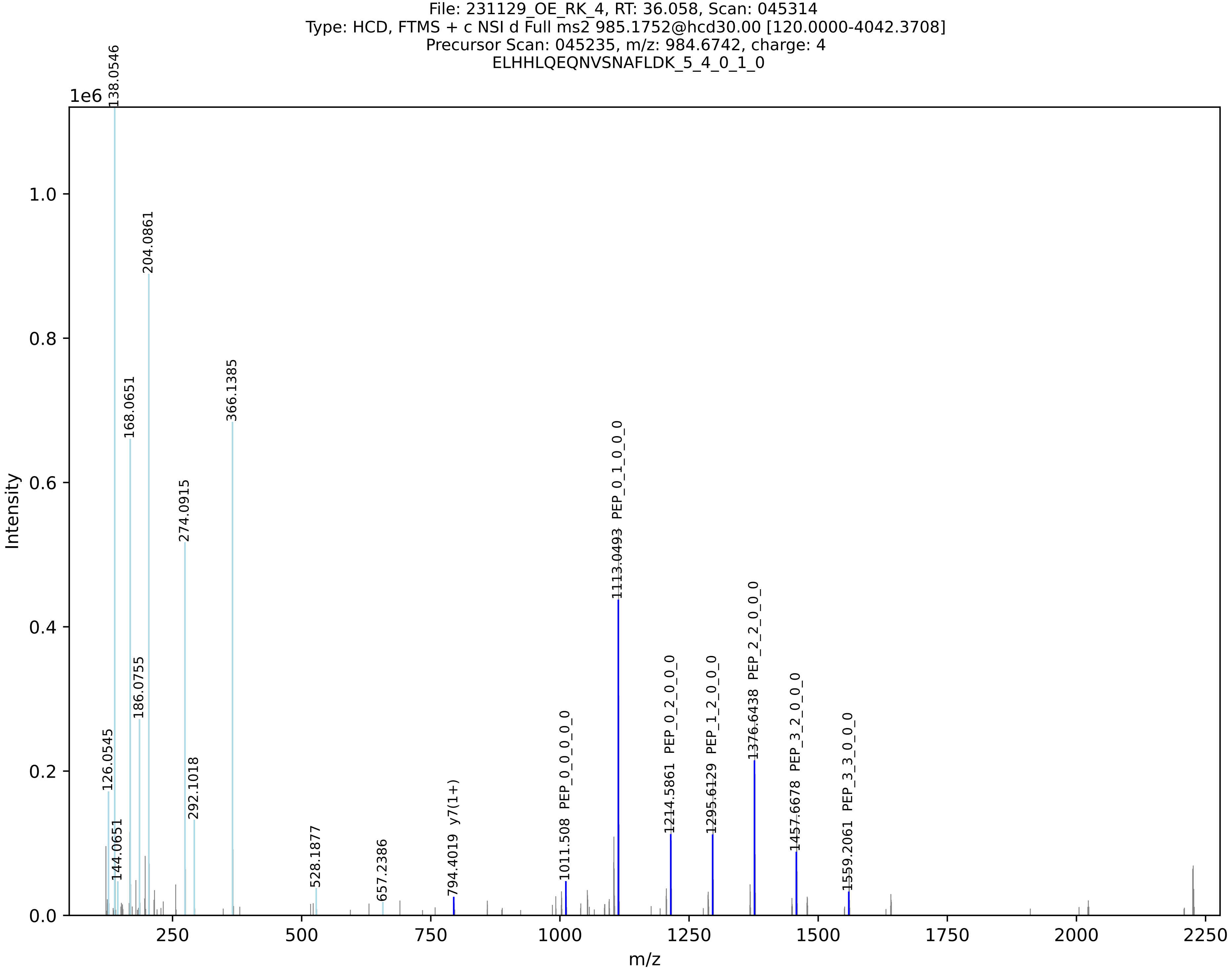
File: 231129_OE_RK_4, RT: 36.058, Scan: 045314
Type: HCD, FTMS + c NSI d Full ms2 985.1752@hcd30.00 [120.0000-4042.3708]
Precursor Scan: 045235, m/z: 984.6742, charge: 4
ELHHLQEQNVSNAFLDK_5_4_0_1_0
1e6
Intensity
m/z
138.0546
204.0861
168.0651
366.1385
274.0915
186.0755
126.0545
144.0651
292.1018
528.1877
657.2386
794.4019 y7(1+)
1011.508 PEP_0_0_0_0_0
1113.0493 PEP_0_1_0_0_0
1214.5861 PEP_0_2_0_0_0
1295.6129 PEP_1_2_0_0_0
1376.6438 PEP_2_2_0_0_0
1457.6678 PEP_3_2_0_0_0
1559.2061 PEP_3_3_0_0_0

File: 231129_OE_RK_4, RT: 36.117, Scan: 045401
Type: HCD, FTMS + c NSI d Full ms2 1313.2301@hcd30.00 [120.0000-4040.6260]
Precursor Scan: 045309, m/z: 1312.5624, charge: 3
ELHHLQEQNVSNAFLDK_5_4_0_1_0

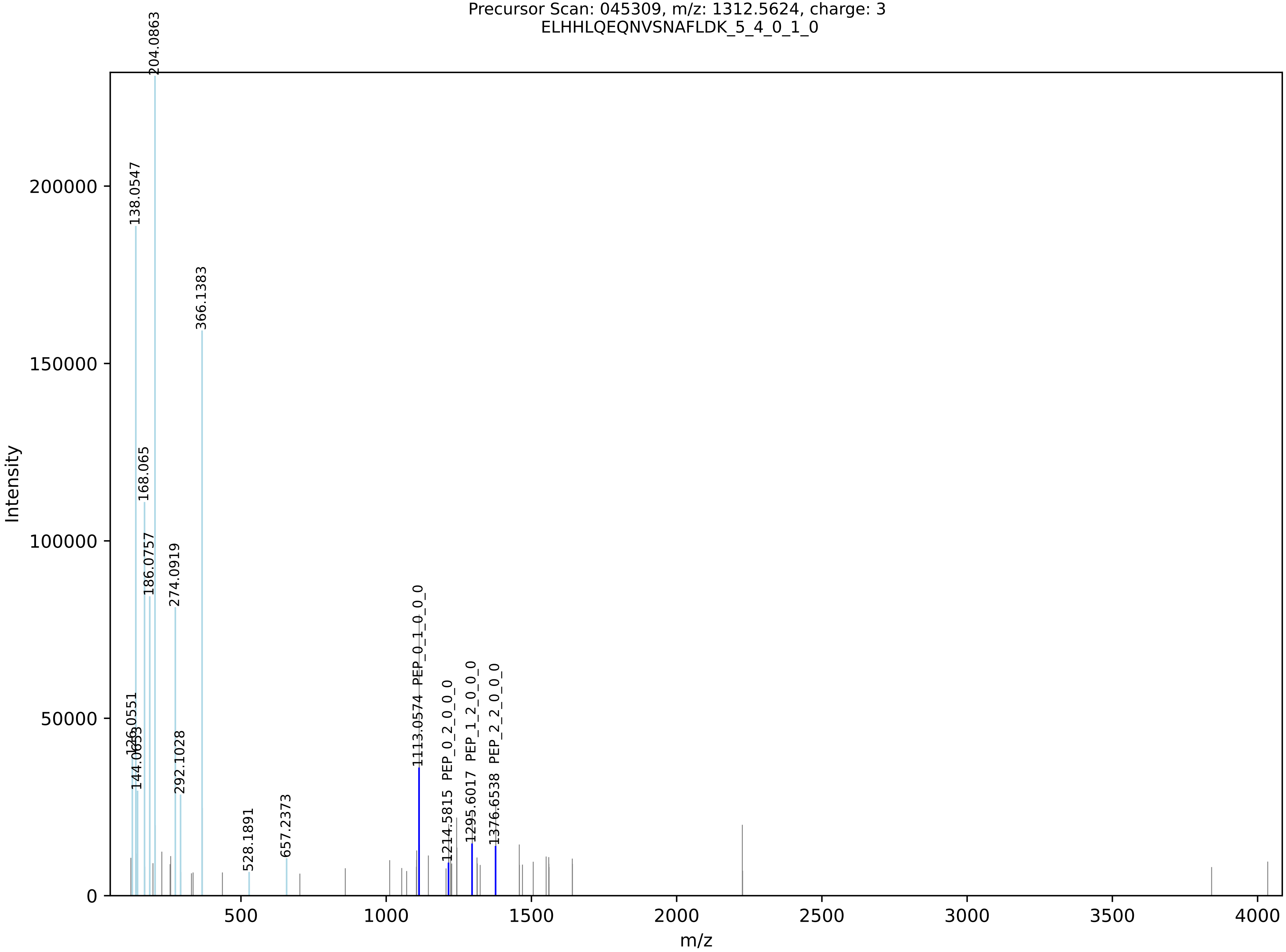

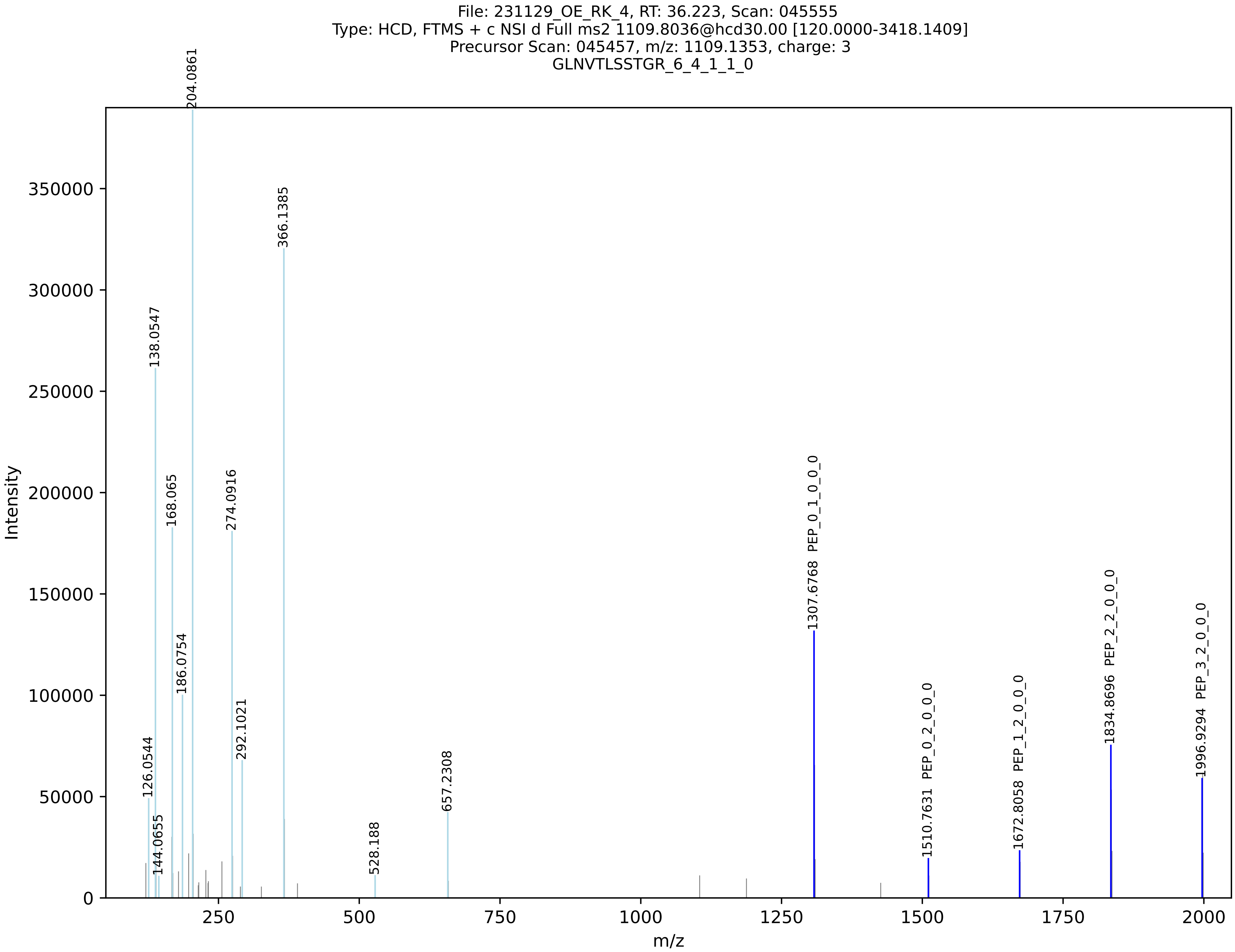
File: 231129_OE_RK_4, RT: 36.223, Scan: 045555
Type: HCD, FTMS + c NSI d Full ms2 1109.8036@hcd30.00 [120.0000-3418.1409]
Precursor Scan: 045457, m/z: 1109.1353, charge: 3
GLNVTLSSTGR_6_4_1_1_0
Intensity
m/z
204.0861
366.1385
138.0547
168.065
274.0916
186.0754
292.1021
126.0544
144.0655
528.188
657.2308
1307.6768 PEP_0_1_0_0_0
1510.7631 PEP_0_2_0_0_0
1672.8058 PEP_1_2_0_0_0
1834.8696 PEP_2_2_0_0_0
1996.9294 PEP_3_2_0_0_0

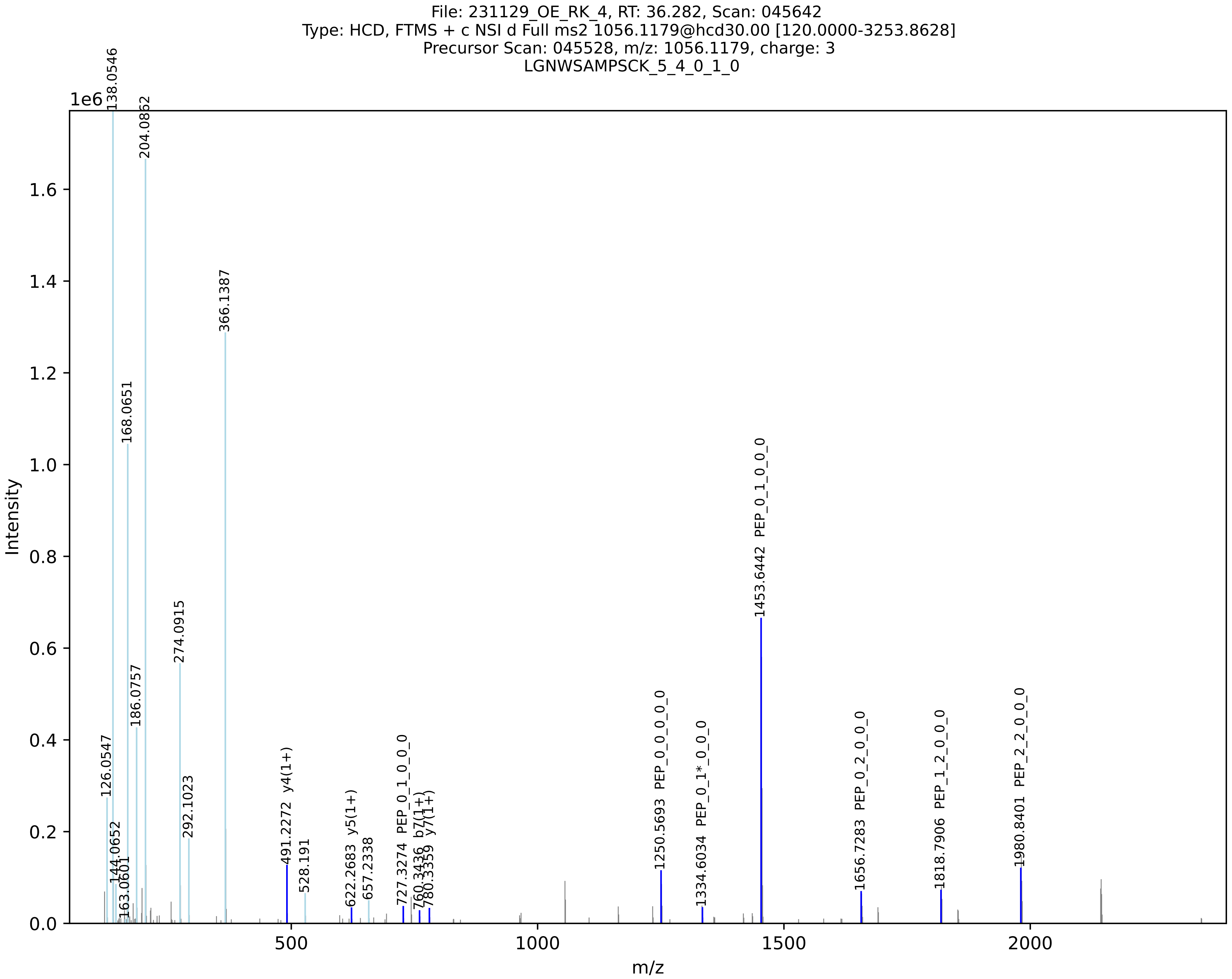
File: 231129_OE_RK_4, RT: 36.282, Scan: 045642
Type: HCD, FTMS + c NSI d Full ms2 1056.1179@hcd30.00 [120.0000-3253.8628]
Precursor Scan: 045528, m/z: 1056.1179, charge: 3
LGNWSAMPSCK_5_4_0_1_0
1e6
Intensity
m/z
138.0546
204.0862
366.1387
168.0651
274.0915
186.0757
126.0547
292.1023
144.0652
163.0601
491.2272 y4(1+)
528.191
622.2683 y5(1+)
657.2338
727.3274 PEP_0_1_0_0_0
760.3436 b7(1+)
780.3359 y7(1+)
1250.5693 PEP_0_0_0_0_0
1334.6034 PEP_0_1*_0_0_0
1453.6442 PEP_0_1_0_0_0
1656.7283 PEP_0_2_0_0_0
1818.7906 PEP_1_2_0_0_0
1980.8401 PEP_2_2_0_0_0

File: 231129_OE_RK_4, RT: 36.306, Scan: 045673
Type: HCD, FTMS + c NSI d Full ms2 1147.7413@hcd30.00 [120.0000-4705.6406]
Precursor Scan: 045598, m/z: 1147.24, charge: 4
NNATVHEQVGGPSLTSDLQAQSK_5_4_0_2_0

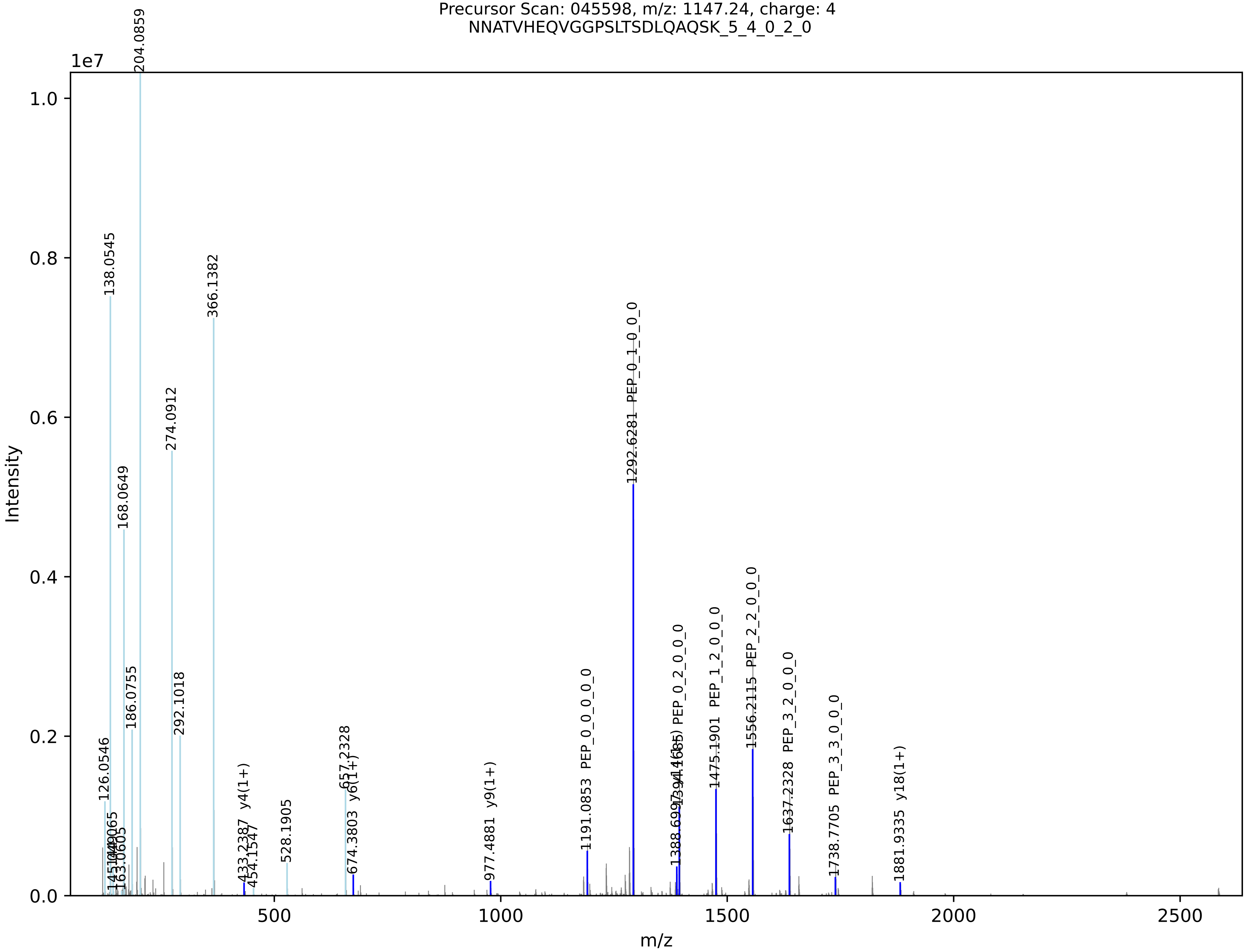

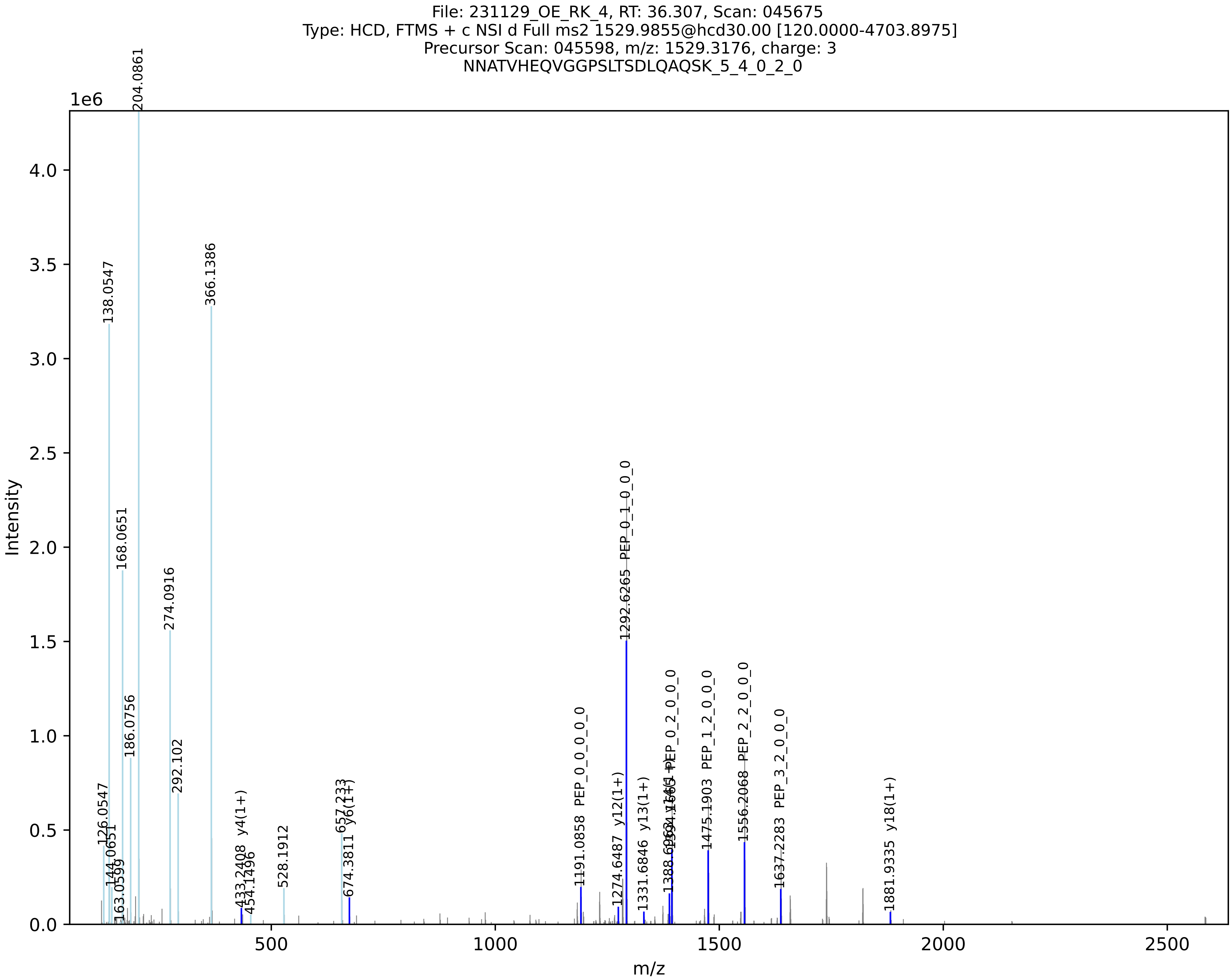
File: 231129_OE_RK_4, RT: 36.307, Scan: 045675
Type: HCD, FTMS + c NSI d Full ms2 1529.9855@hcd30.00 [120.0000-4703.8975]
Precursor Scan: 045598, m/z: 1529.3176, charge: 3
NNATVHEQVGGPSLTSDLQAQSK_5_4_0_2_0
1e6
Intensity
m/z
204.0861
138.0547
366.1386
168.0651
274.0916
186.0756
292.102
126.0547
144.0651
163.0599
433.2408 y4(1+)
454.1496
528.1912
657.233
674.3811 y6(1+)
1191.0858 PEP_0_0_0_0_0
1274.6487 y12(1+)
1292.6265 PEP_0_1_0_0_0
1331.6846 y13(1+)
PEP_0_2_0_0_0
1475.1903 PEP_1_2_0_0_0
1556.2068 PEP_2_2_0_0_0
1637.2283 PEP_3_2_0_0_0
1881.9335 y18(1+)

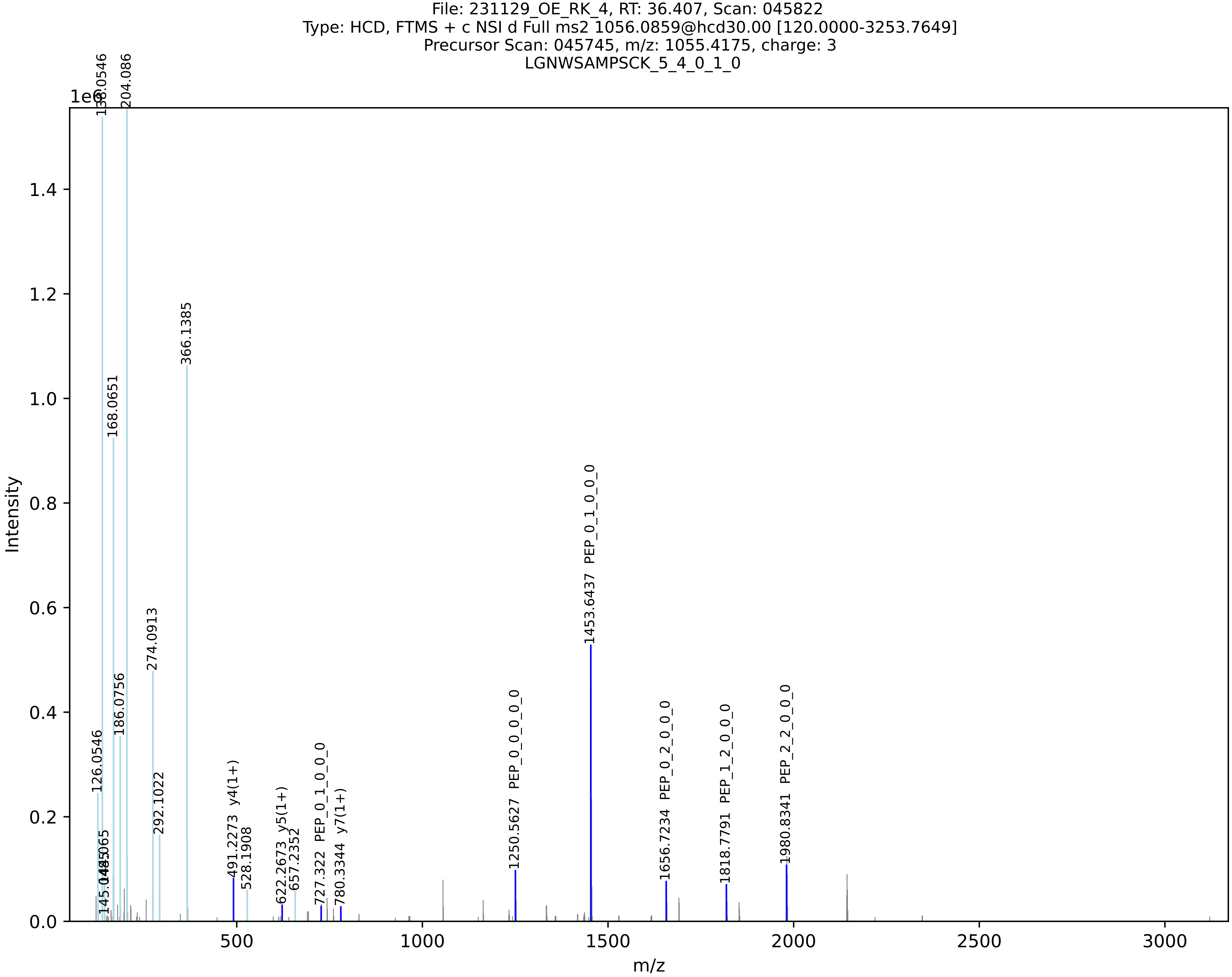
File: 231129_OE_RK_4, RT: 36.407, Scan: 045822
Type: HCD, FTMS + c NSI d Full ms2 1056.0859@hcd30.00 [120.0000-3253.7649]
Precursor Scan: 045745, m/z: 1055.4175, charge: 3
LGNWSAMPSCK_5_4_0_1_0
1e6
Intensity
m/z
204.086
168.0651
366.1385
274.0913
186.0756
126.0546
292.1022
491.2273 y4(1+)
528.1908
622.2673 y5(1+)
657.2352
727.322 PEP_0_1_0_0_0
780.3344 y7(1+)
1250.5627 PEP_0_0_0_0_0
1453.6437 PEP_0_1_0_0_0
1656.7234 PEP_0_2_0_0_0
1818.7791 PEP_1_2_0_0_0
1980.8341 PEP_2_2_0_0_0

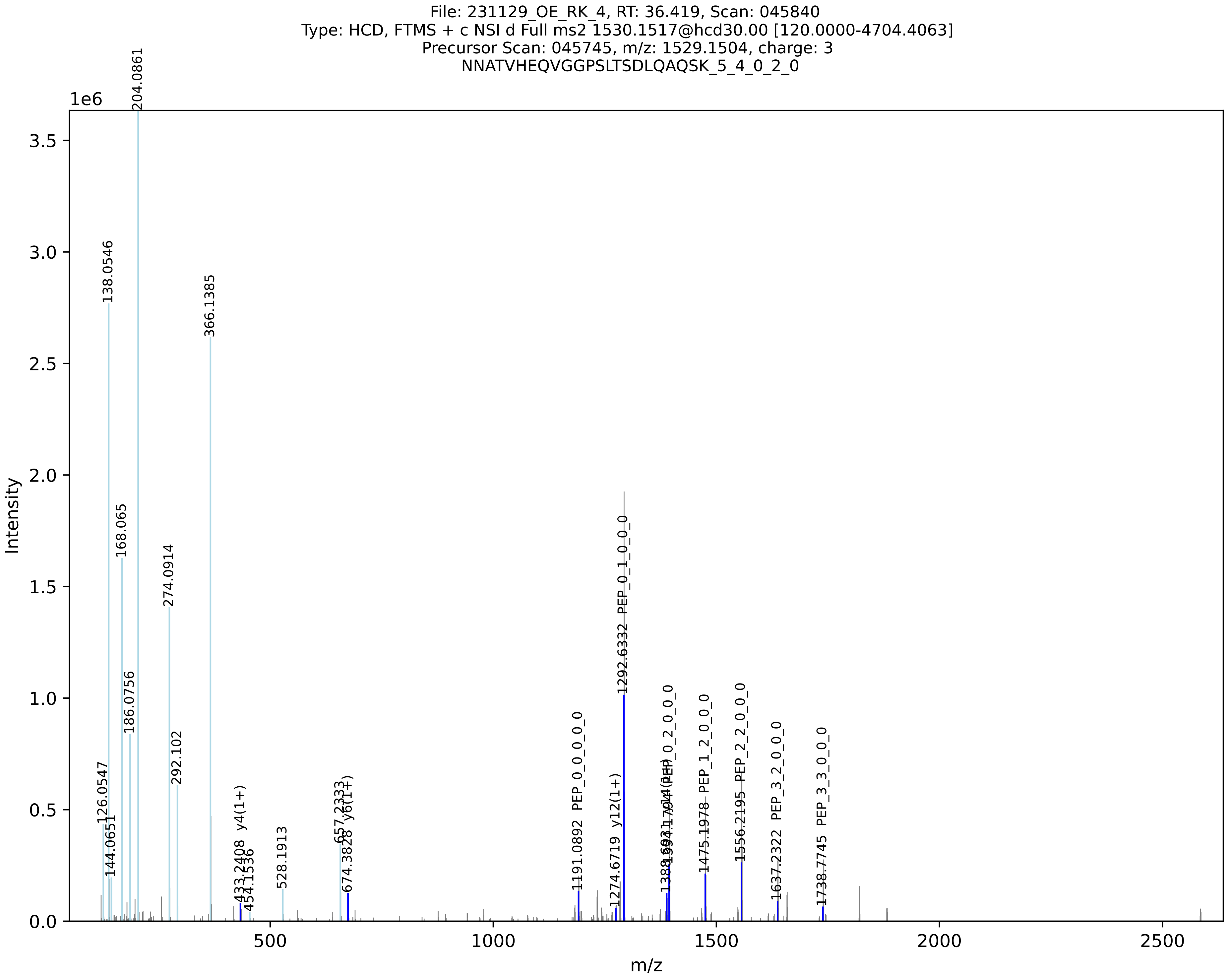

File: 231129_OE_RK_4, RT: 36.419, Scan: 045840
Type: HCD, FTMS + c NSI d Full ms2 1530.1517@hcd30.00 [120.0000-4704.4063]
Precursor Scan: 045745, m/z: 1529.1504, charge: 3
NNATVHEQVGGPSLTSDLQAQSK_5_4_0_2_0
1e6
Intensity
m/z
204.0861
138.0546
366.1385
168.065
274.0914
186.0756
292.102
126.0547
144.0651
433.2408 y4(1+)
454.1536
528.1913
657.2333
674.3828 y6(1+)
1191.0892 PEP_0_0_0_0_0
1274.6719 y12(1+)
1292.6332 PEP_0_1_0_0_0
1475.1978 PEP_1_2_0_0_0
1556.2195 PEP_2_2_0_0_0
1637.2322 PEP_3_2_0_0_0
1738.7745 PEP_3_3_0_0_0

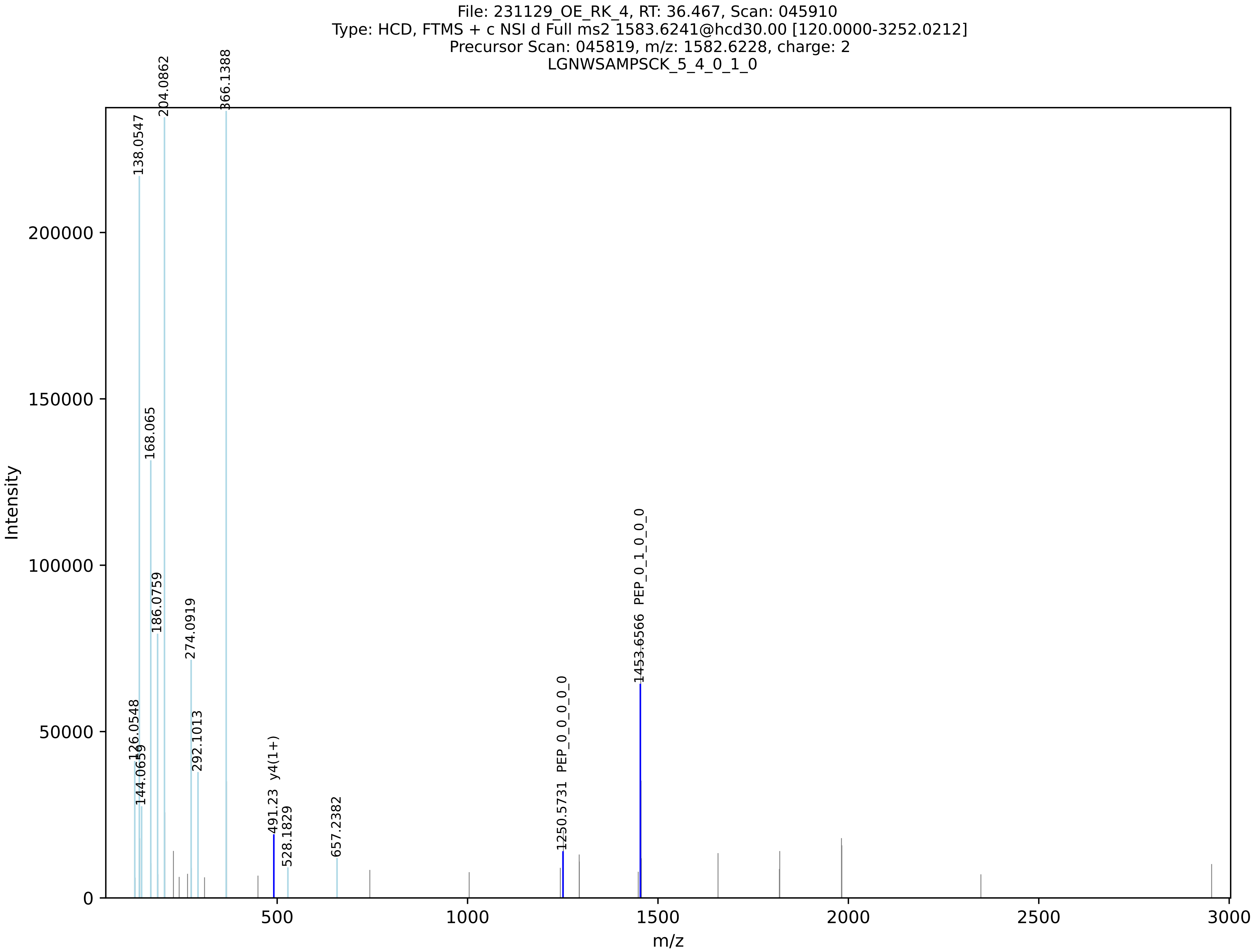
File: 231129_OE_RK_4, RT: 36.467, Scan: 045910
Type: HCD, FTMS + c NSI d Full ms2 1583.6241@hcd30.00 [120.0000-3252.0212]
Precursor Scan: 045819, m/z: 1582.6228, charge: 2
LGNWSAMPSCK_5_4_0_1_0
204.0862
366.1388
138.0547
168.065
186.0759
274.0919
126.0548
144.0659
292.1013
491.23 y4(1+)
528.1829
657.2382
1250.5731 PEP_0_0_0_0_0_
1453.6566 PEP_0_1_0_0_0_
Intensity
m/z
0
50000
100000
150000
200000
500
1000
1500
2000
2500
3000

File: 231129_OE_RK_4, RT: 36.484, Scan: 045937
Type: HCD, FTMS + c NSI d Full ms2 1056.1155@hcd30.00 [120.0000-3253.8552]
Precursor Scan: 045819, m/z: 1056.1154, charge: 3
LGNWSAMPSCK_5_4_0_1_0

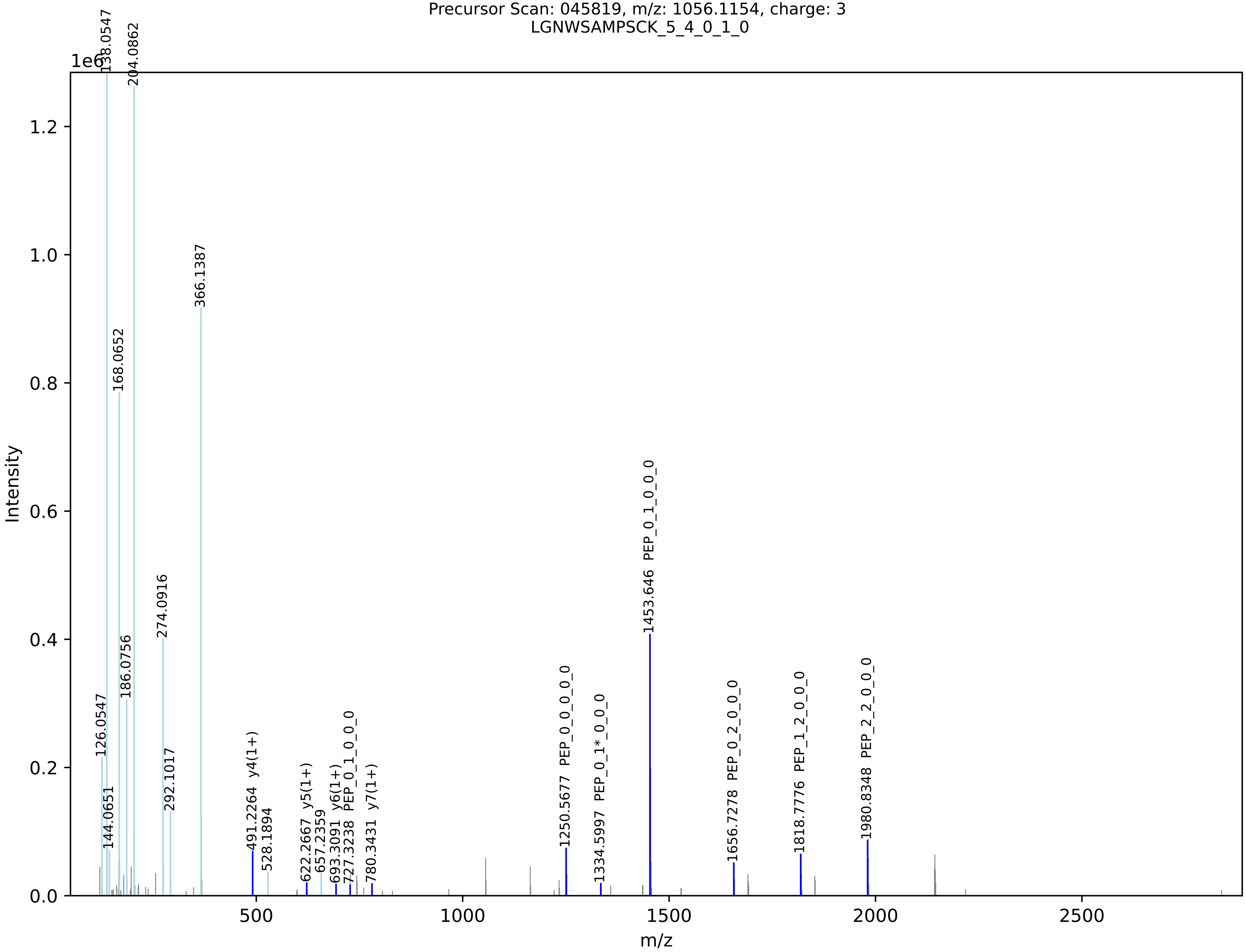

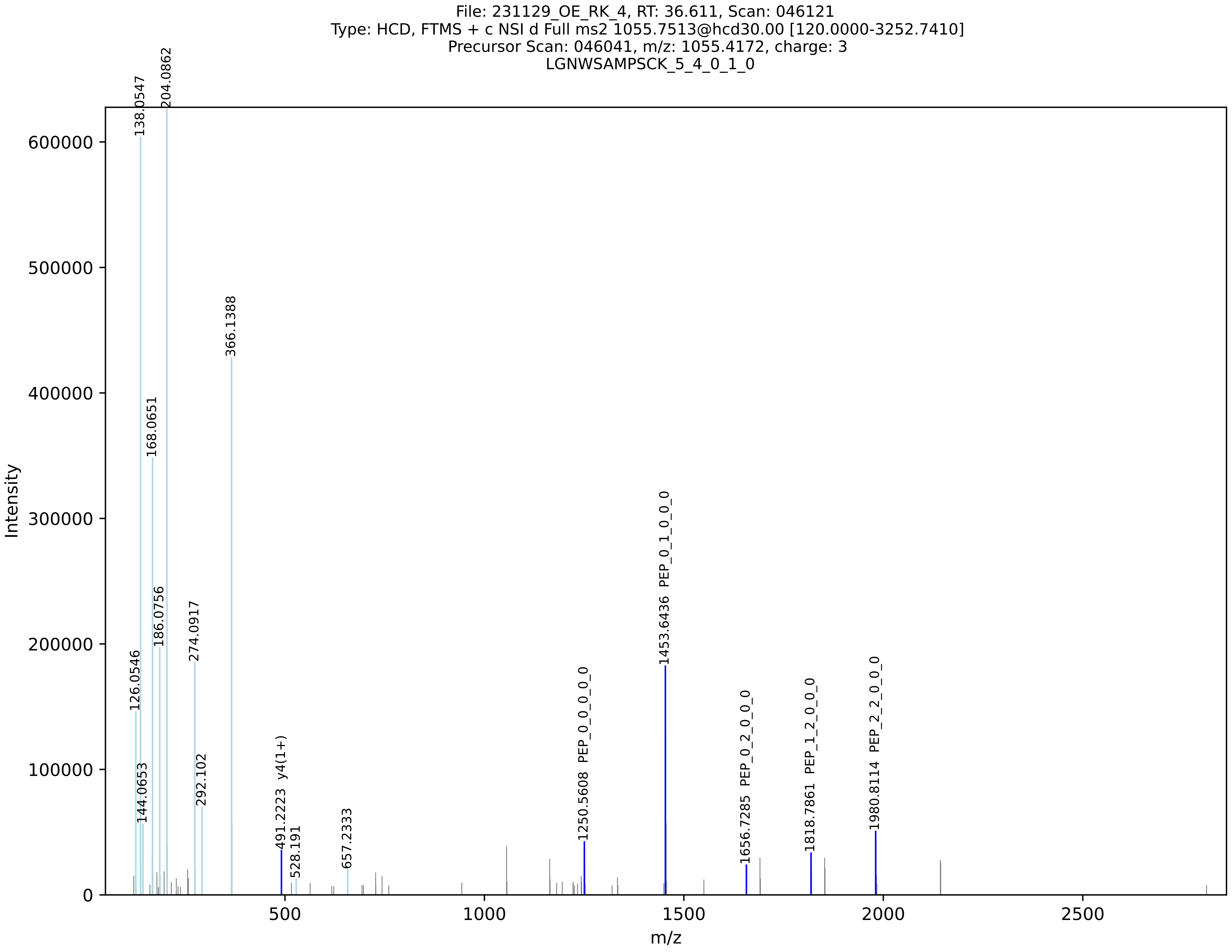

File: 231129_OE_RK_4, RT: 36.611, Scan: 046121
Type: HCD, FTMS + c NSI d Full ms2 1055.7513@hcd30.00 [120.0000-3252.7410]
Precursor Scan: 046041, m/z: 1055.4172, charge: 3
LGNWSAMPSCK_5_4_0_1_0
Intensity
m/z
138.0547
204.0862
366.1388
168.0651
186.0756
274.0917
126.0546
292.102
144.0653
491.2223 y4(1+)
528.191
657.2333
1250.5608 PEP_0_0_0_0_0
1453.6436 PEP_0_1_0_0_0
1656.7285 PEP_0_2_0_0_0
1818.7861 PEP_1_2_0_0_0
1980.8114 PEP_2_2_0_0_0

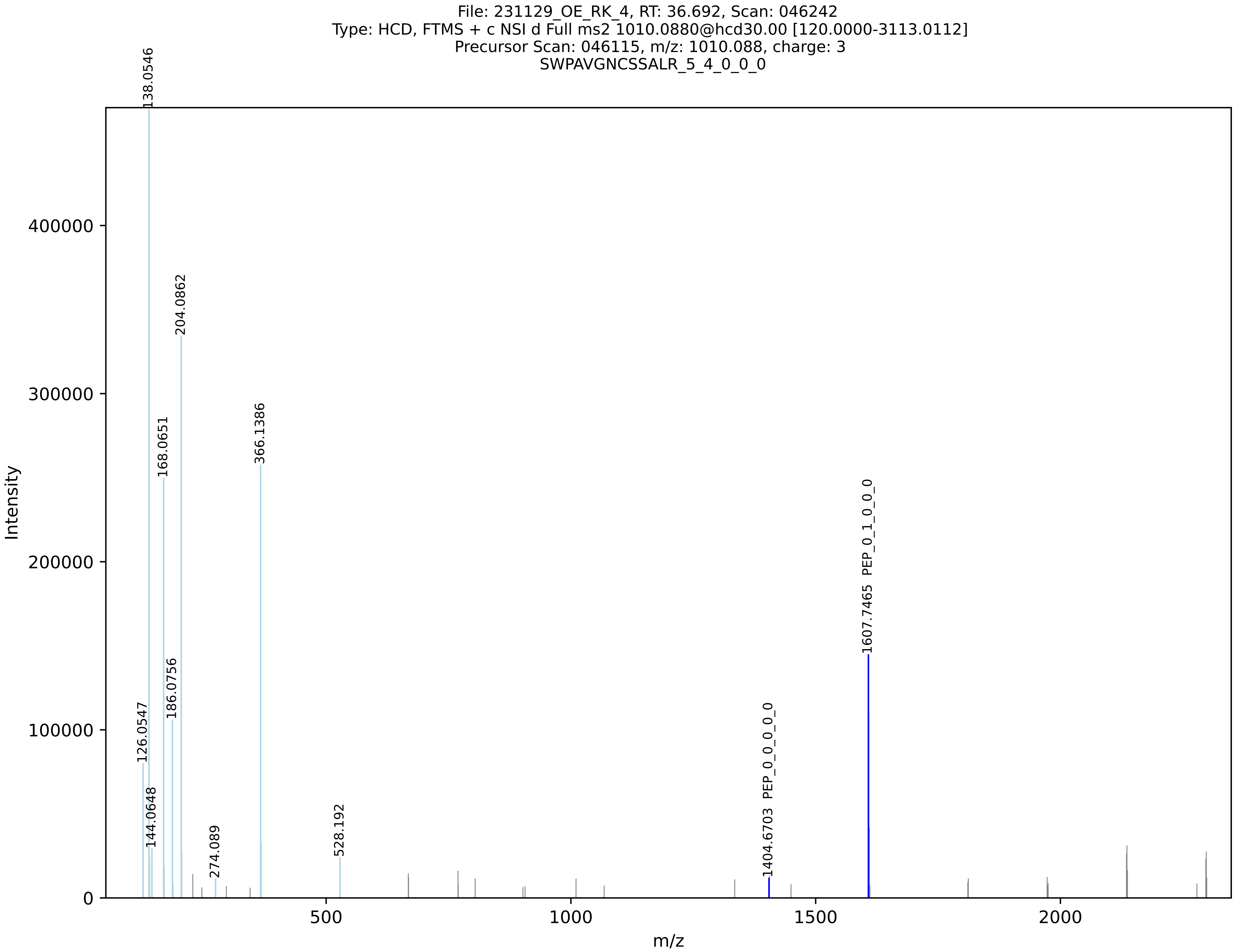
File: 231129_OE_RK_4, RT: 36.692, Scan: 046242
Type: HCD, FTMS + c NSI d Full ms2 1010.0880@hcd30.00 [120.0000-3113.0112]
Precursor Scan: 046115, m/z: 1010.088, charge: 3
SWPAVGNCSSALR_5_4_0_0_0
Intensity
m/z
138.0546
204.0862
168.0651
366.1386
186.0756
126.0547
144.0648
274.089
528.192
1404.6703 PEP_0_0_0_0_0
1607.7465 PEP_0_1_0_0_0
0
100000
200000
300000
400000
500
1000
1500
2000

File: 231129_OE_RK_4, RT: 36.711, Scan: 046266
Type: HCD, FTMS + c NSI d Full ms2 1147.7412@hcd30.00 [120.0000-4705.6401]
Precursor Scan: 046189, m/z: 1147.2416, charge: 4
NNATVHEQVGGPSLTSDLQAQSK_5_4_0_2_0

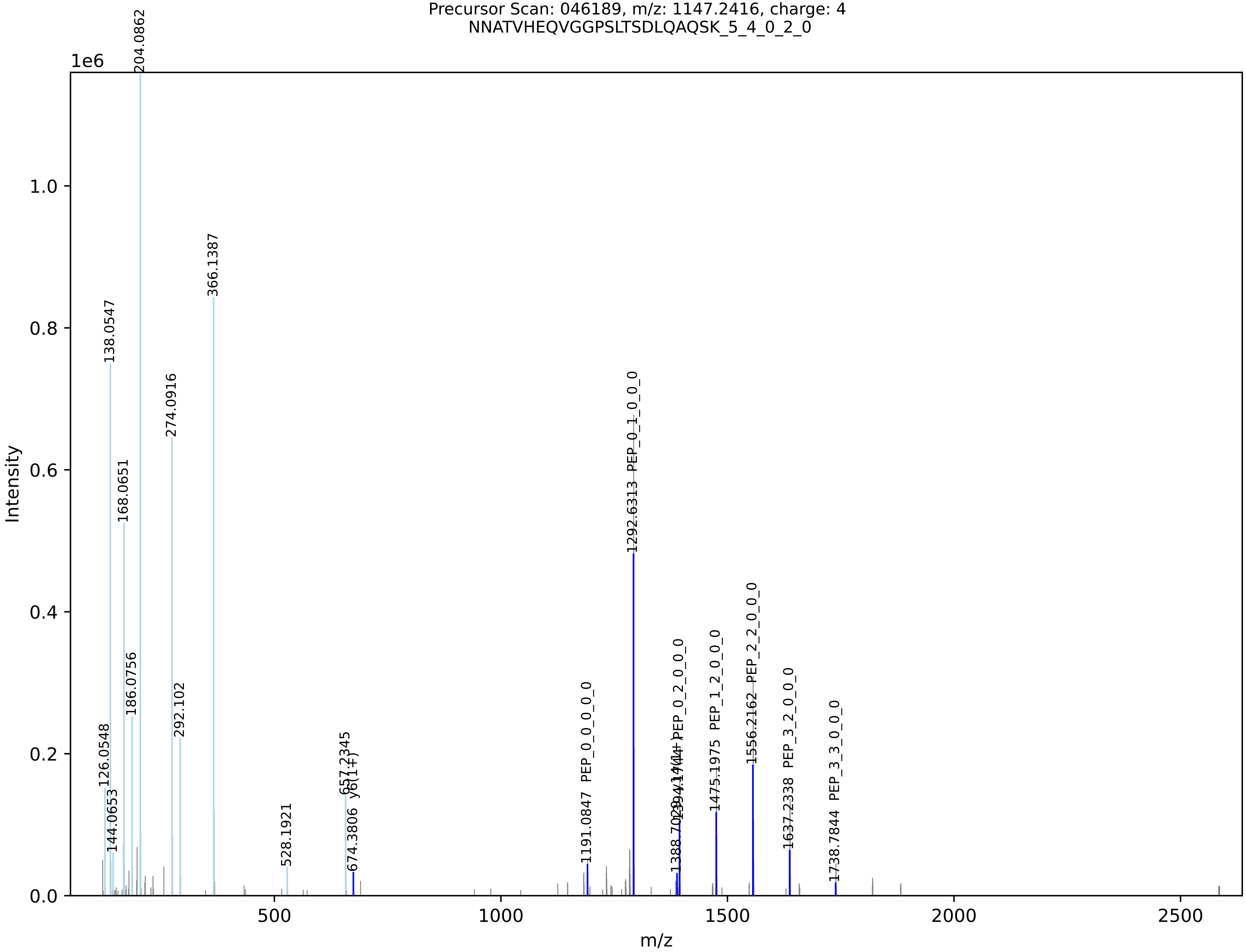

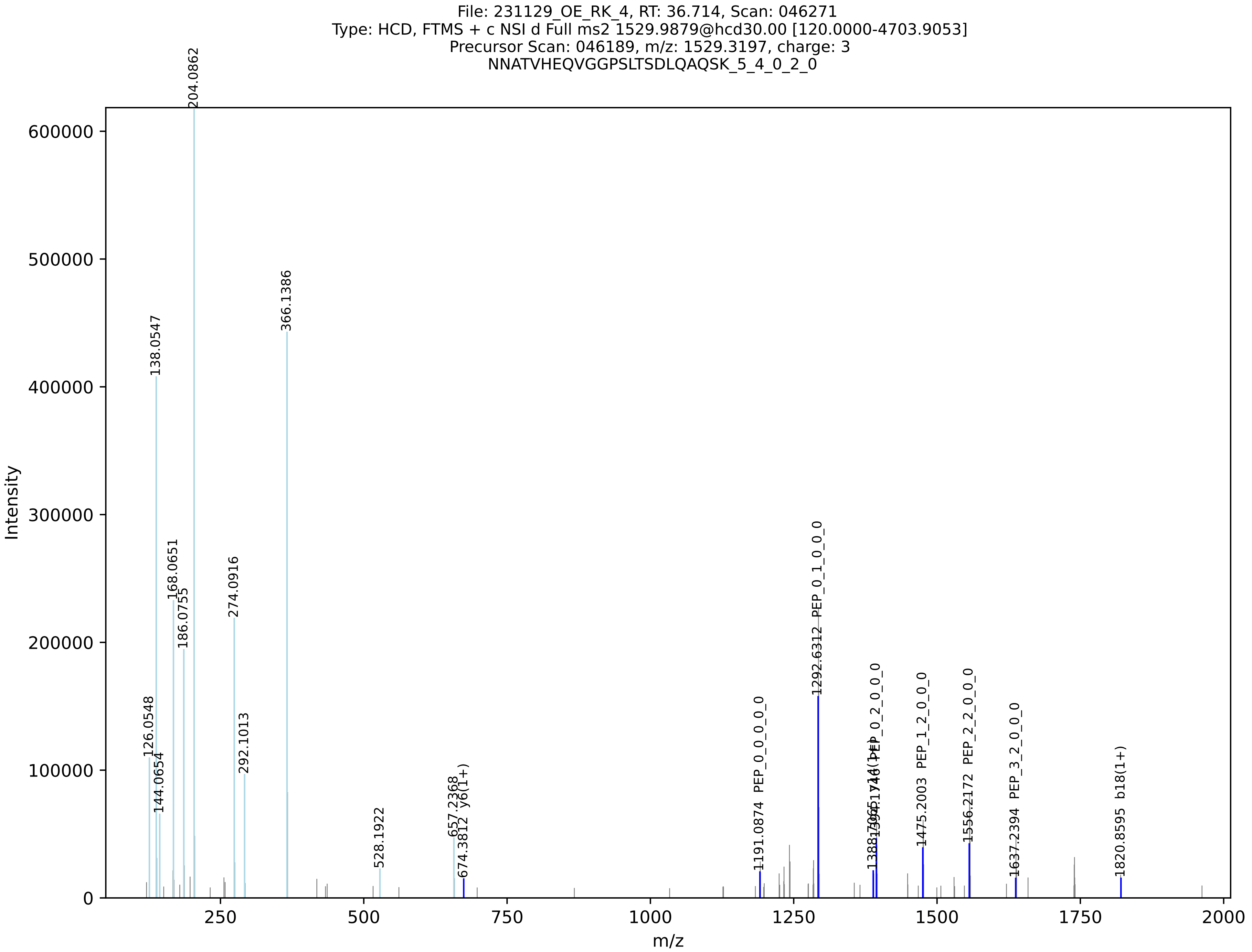
File: 231129_OE_RK_4, RT: 36.714, Scan: 046271
Type: HCD, FTMS + c NSI d Full ms2 1529.9879@hcd30.00 [120.0000-4703.9053]
Precursor Scan: 046189, m/z: 1529.3197, charge: 3
NNATVHEQVGGPSLTSDLQAQSK_5_4_0_2_0
Intensity
m/z
204.0862
138.0547
366.1386
168.0651
186.0755
274.0916
126.0548
144.0654
292.1013
528.1922
657.2368
674.3812 y6(1+)
1191.0874 PEP_0_0_0_0_0
1292.6312 PEP_0_1_0_0_0
PEP_0_2_0_0_0
1475.2003 PEP_1_2_0_0_0
1556.2172 PEP_2_2_0_0_0
1637.2394 PEP_3_2_0_0_0
1820.8595 b18(1+)

File: 231129_OE_RK_4, RT: 36.863, Scan: 046489
Type: HCD, FTMS + c NSI d Full ms2 1104.4634@hcd30.00 [120.0000-3401.7998]
Precursor Scan: 046410, m/z: 1103.7951, charge: 3
GLNVTLSSTGR_5_4_0_2_0

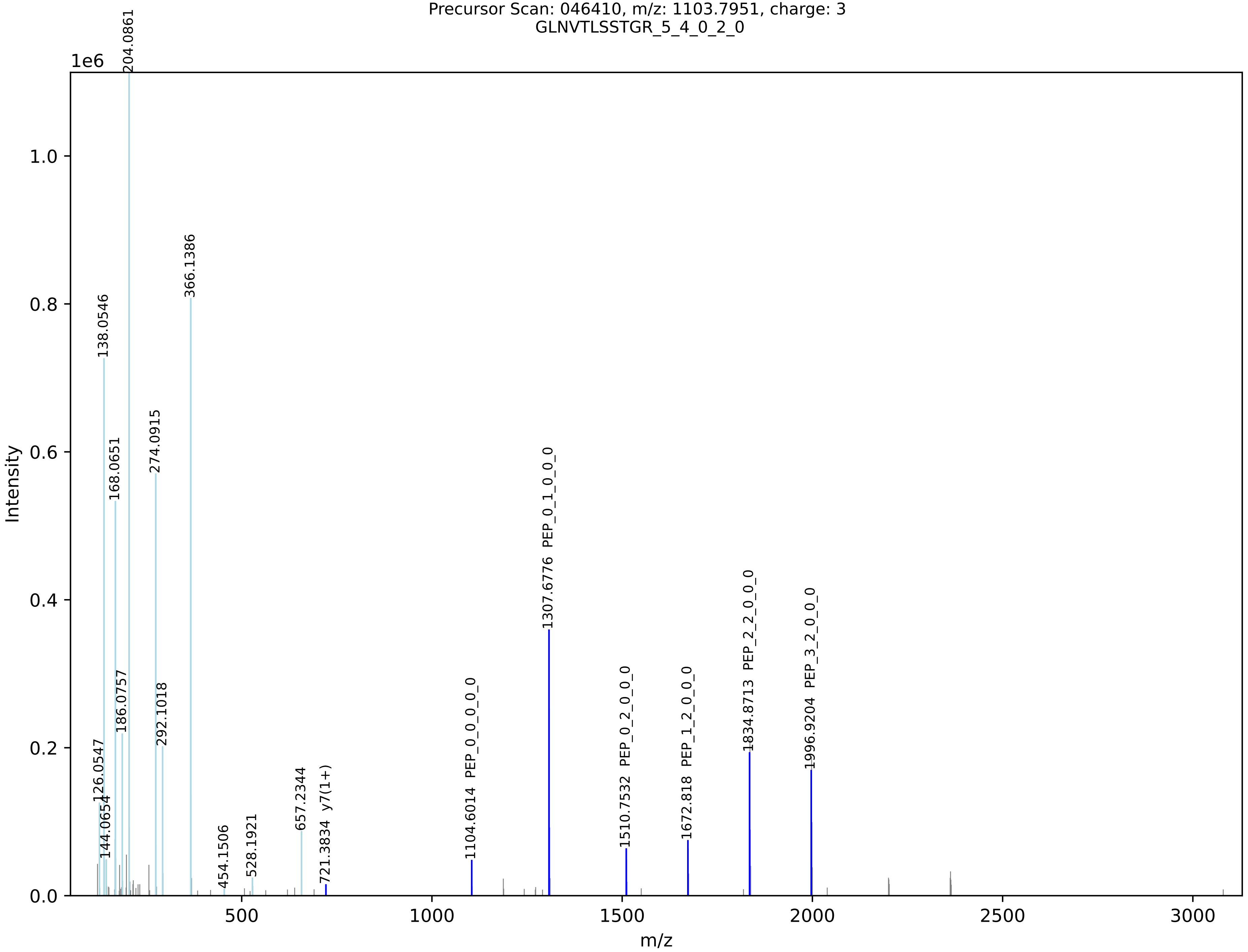

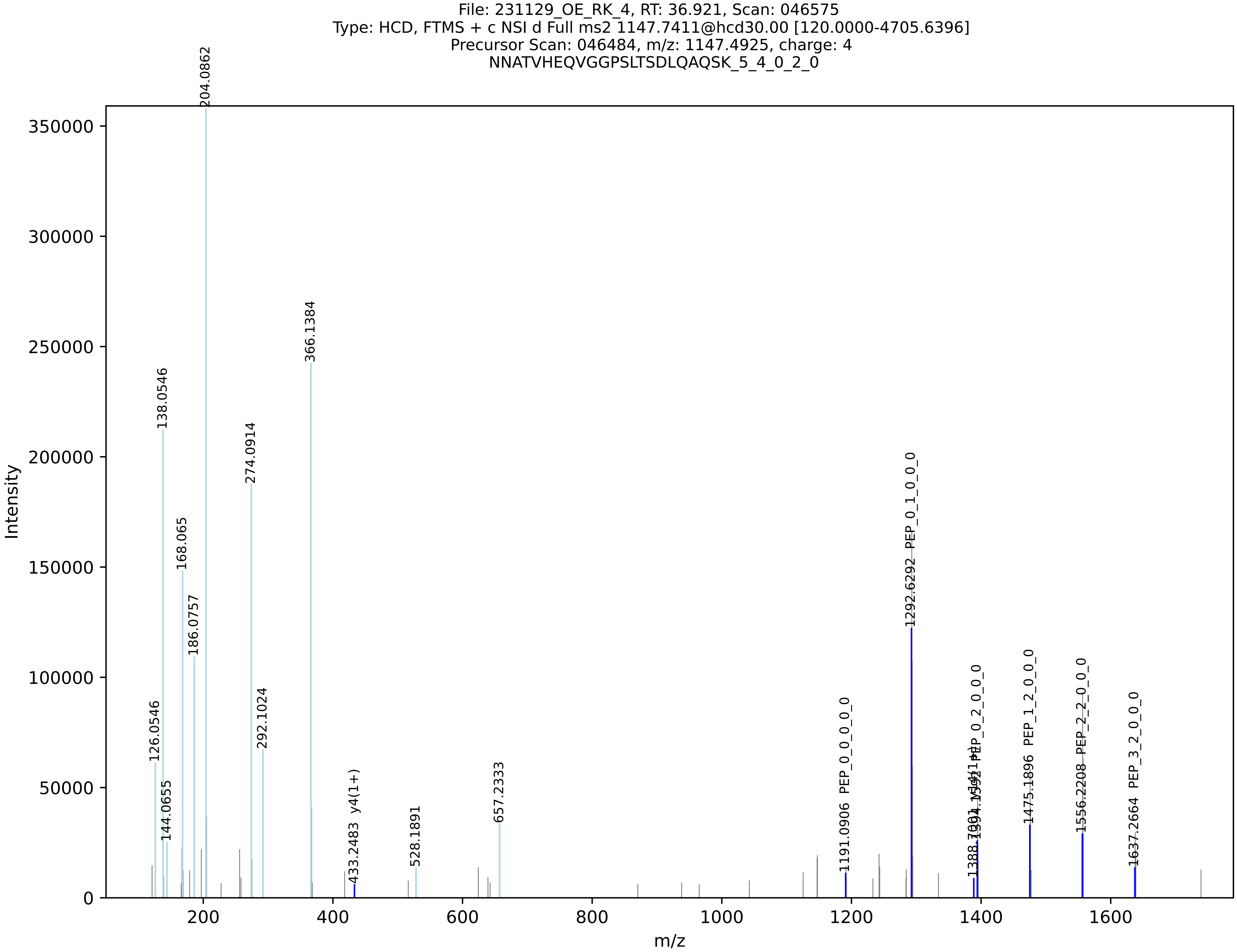
File: 231129_OE_RK_4, RT: 36.921, Scan: 046575
Type: HCD, FTMS + c NSI d Full ms2 1147.7411@hcd30.00 [120.0000-4705.6396]
Precursor Scan: 046484, m/z: 1147.4925, charge: 4
NNATVHEQVGGPSLTSDLQAQSK_5_4_0_2_0
Intensity
m/z
0
50000
100000
150000
200000
250000
300000
350000
200
400
600
800
1000
1200
1400
1600
126.0546
138.0546
144.0655
168.065
186.0757
204.0862
274.0914
292.1024
366.1384
433.2483 y4(1+)
528.1891
657.2333
1191.0906 PEP_0_0_0_0_0
1292.6292 PEP_0_1_0_0_0
1388.7001 y14(1+)
1394.1592 PEP_0_2_0_0_0
1475.1896 PEP_1_2_0_0_0
1556.2208 PEP_2_2_0_0_0
1637.2664 PEP_3_2_0_0_0

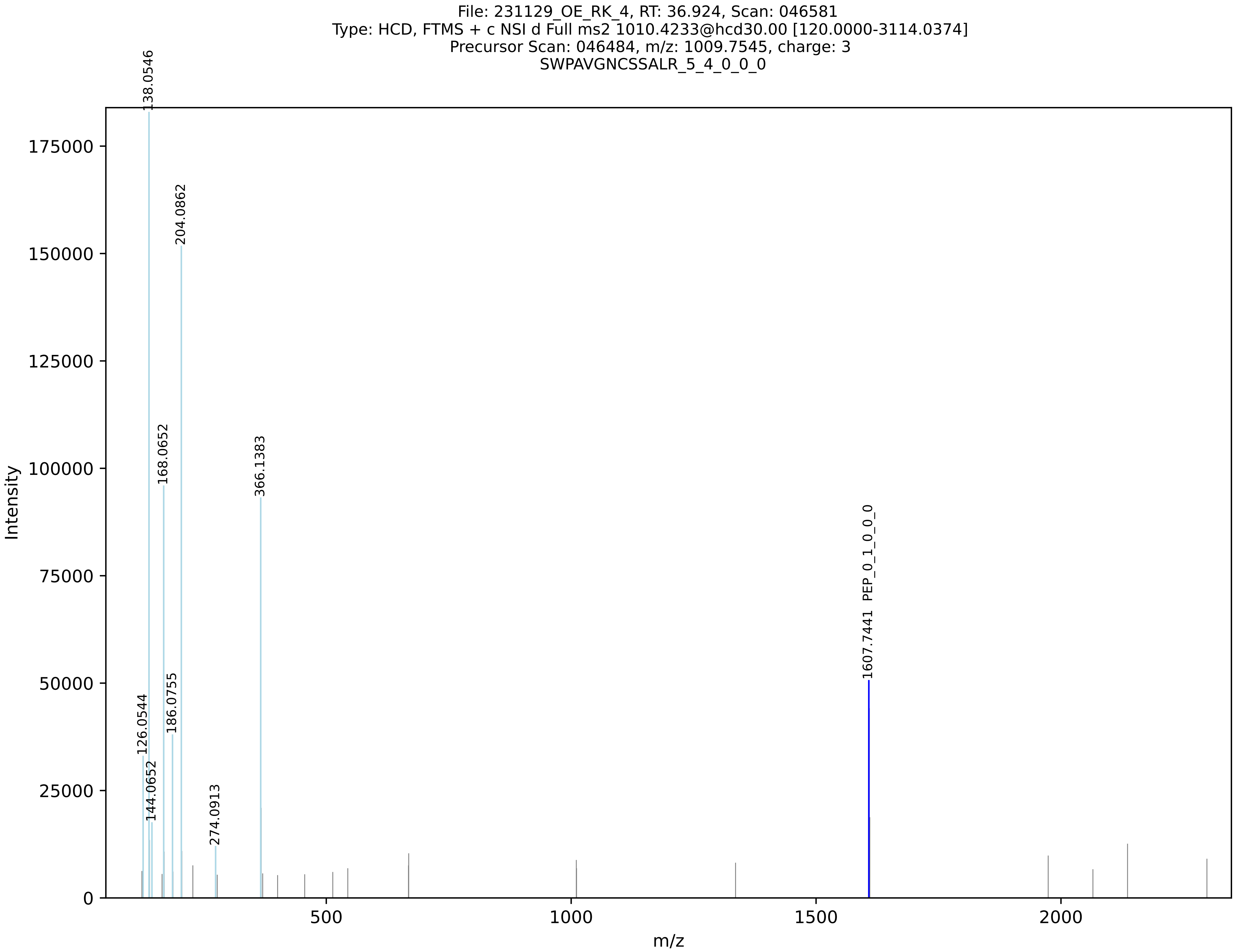
File: 231129_OE_RK_4, RT: 36.924, Scan: 046581
Type: HCD, FTMS + c NSI d Full ms2 1010.4233@hcd30.00 [120.0000-3114.0374]
Precursor Scan: 046484, m/z: 1009.7545, charge: 3
SWPAVGNCSSALR_5_4_0_0_0
138.0546
204.0862
168.0652
366.1383
PEP_0_1_0_0_0
1607.7441
186.0755
126.0544
144.0652
274.0913
Intensity
m/z
175000
150000
125000
100000
75000
50000
25000
0
500
1000
1500
2000

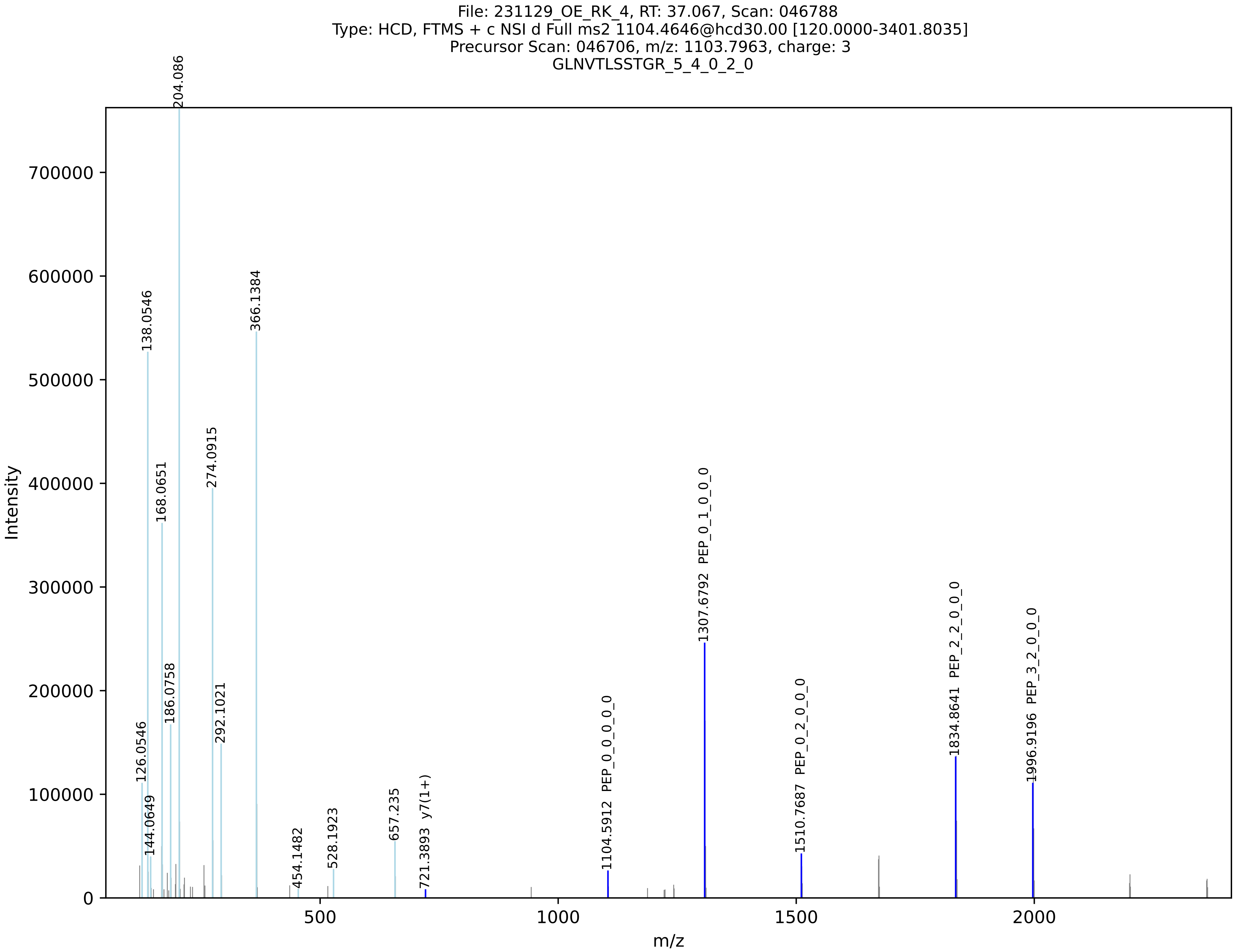
File: 231129_OE_RK_4, RT: 37.067, Scan: 046788
Type: HCD, FTMS + c NSI d Full ms2 1104.4646@hcd30.00 [120.0000-3401.8035]
Precursor Scan: 046706, m/z: 1103.7963, charge: 3
GLNVTLSSTGR_5_4_0_2_0
Intensity
m/z
204.086
138.0546
366.1384
168.0651
274.0915
186.0758
292.1021
126.0546
144.0649
454.1482
528.1923
657.235
721.3893 y7(1+)
1104.5912 PEP_0_0_0_0_0
1307.6792 PEP_0_1_0_0_0
1510.7687 PEP_0_2_0_0_0
1834.8641 PEP_2_2_0_0_0
1996.9196 PEP_3_2_0_0_0

File: 231129_OE_RK_4, RT: 37.671, Scan: 047669
Type: HCD, FTMS + c NSI d Full ms2 1094.4646@hcd30.00 [120.0000-4488.2715]
Precursor Scan: 047588, m/z: 1093.9634, charge: 4
ELHHLQEQNVSNAFLDK_5_4_1_2_0

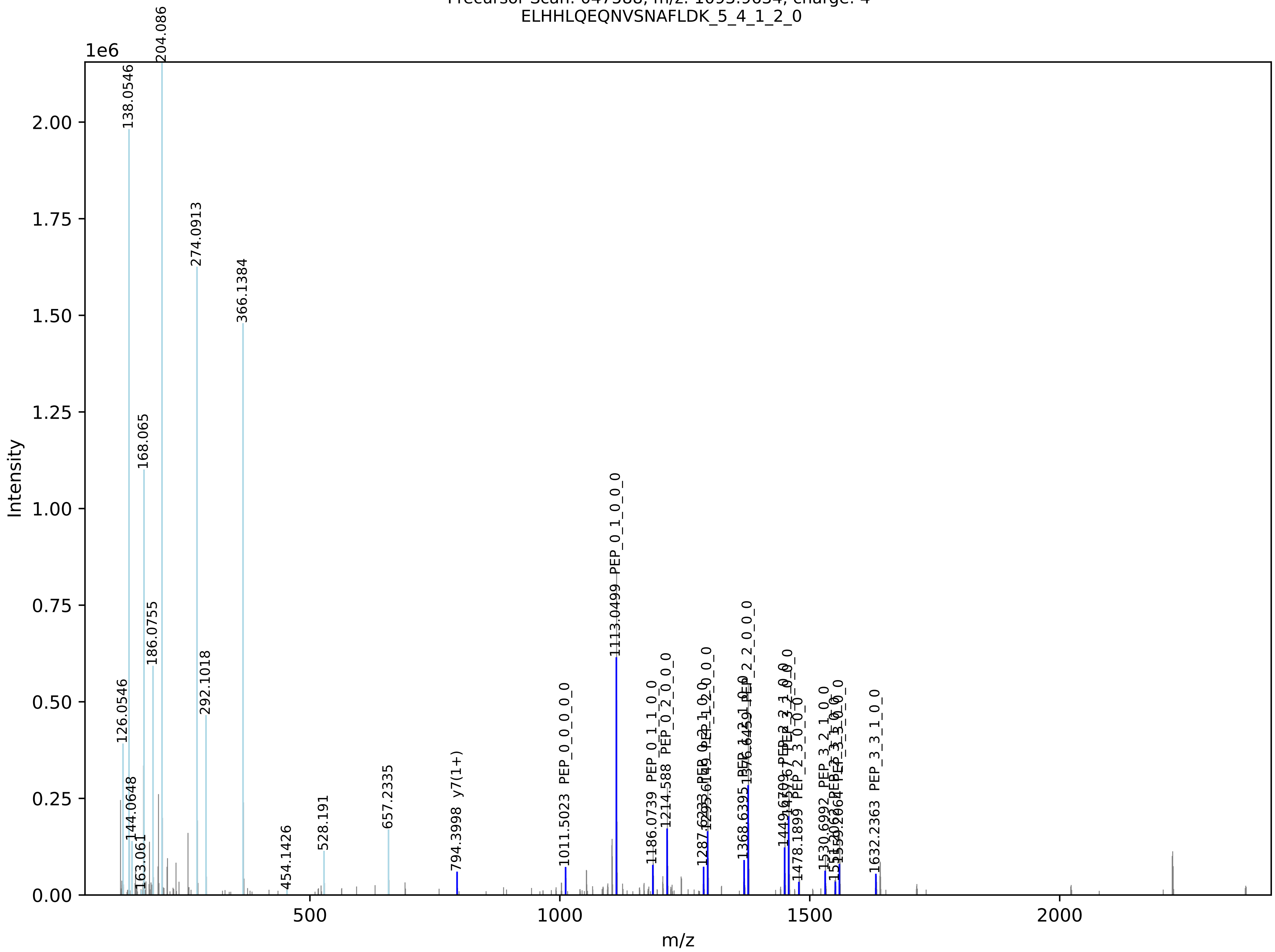

File: 231129_OE_RK_4, RT: 37.878, Scan: 047974
Type: HCD, FTMS + c NSI d Full ms2 1094.4633@hcd30.00 [120.0000-4488.2661]
Precursor Scan: 047882, m/z: 1093.9624, charge: 4
ELHHLQEQNVSNAFLDK_5_4_1_2_0

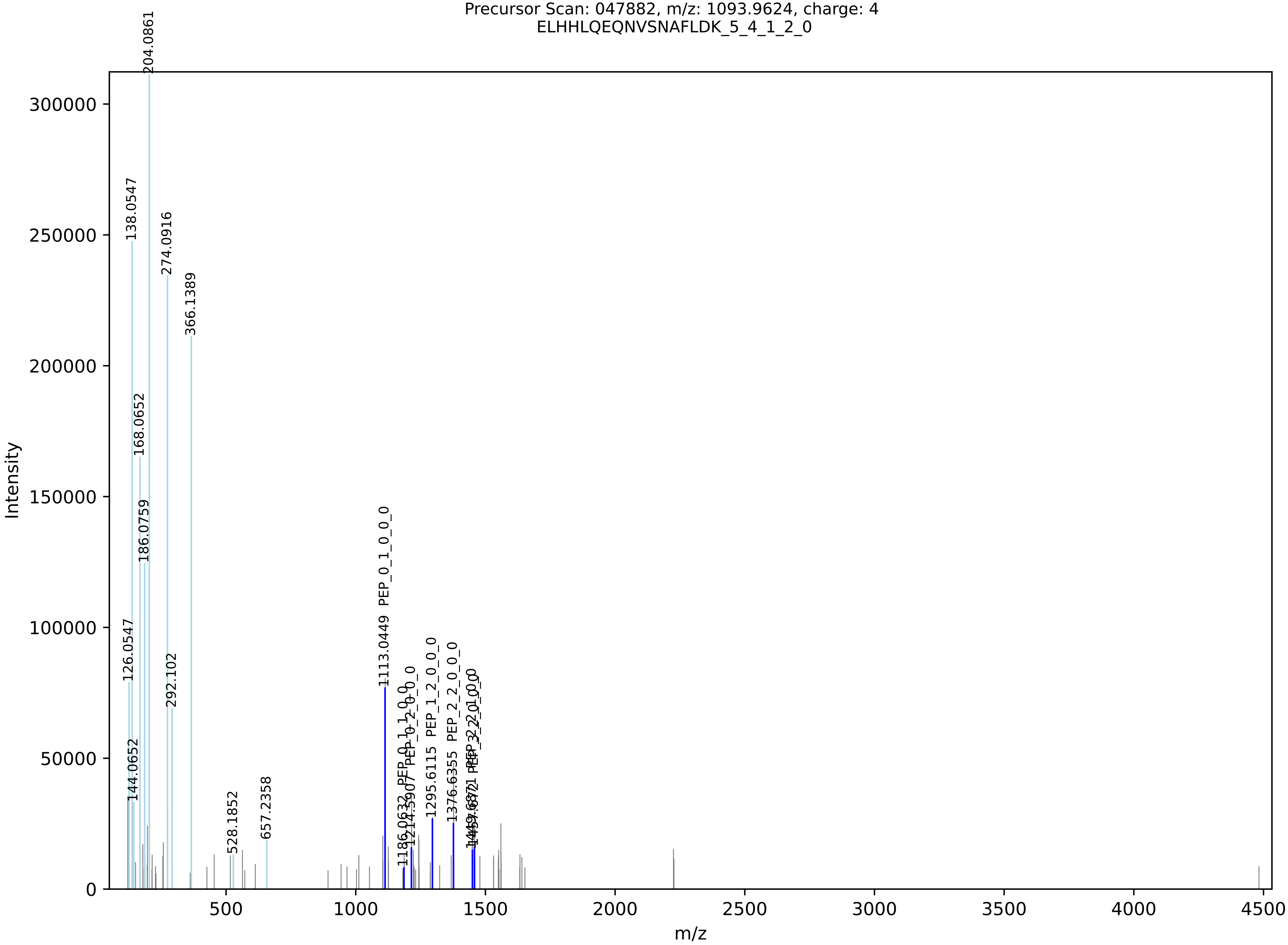

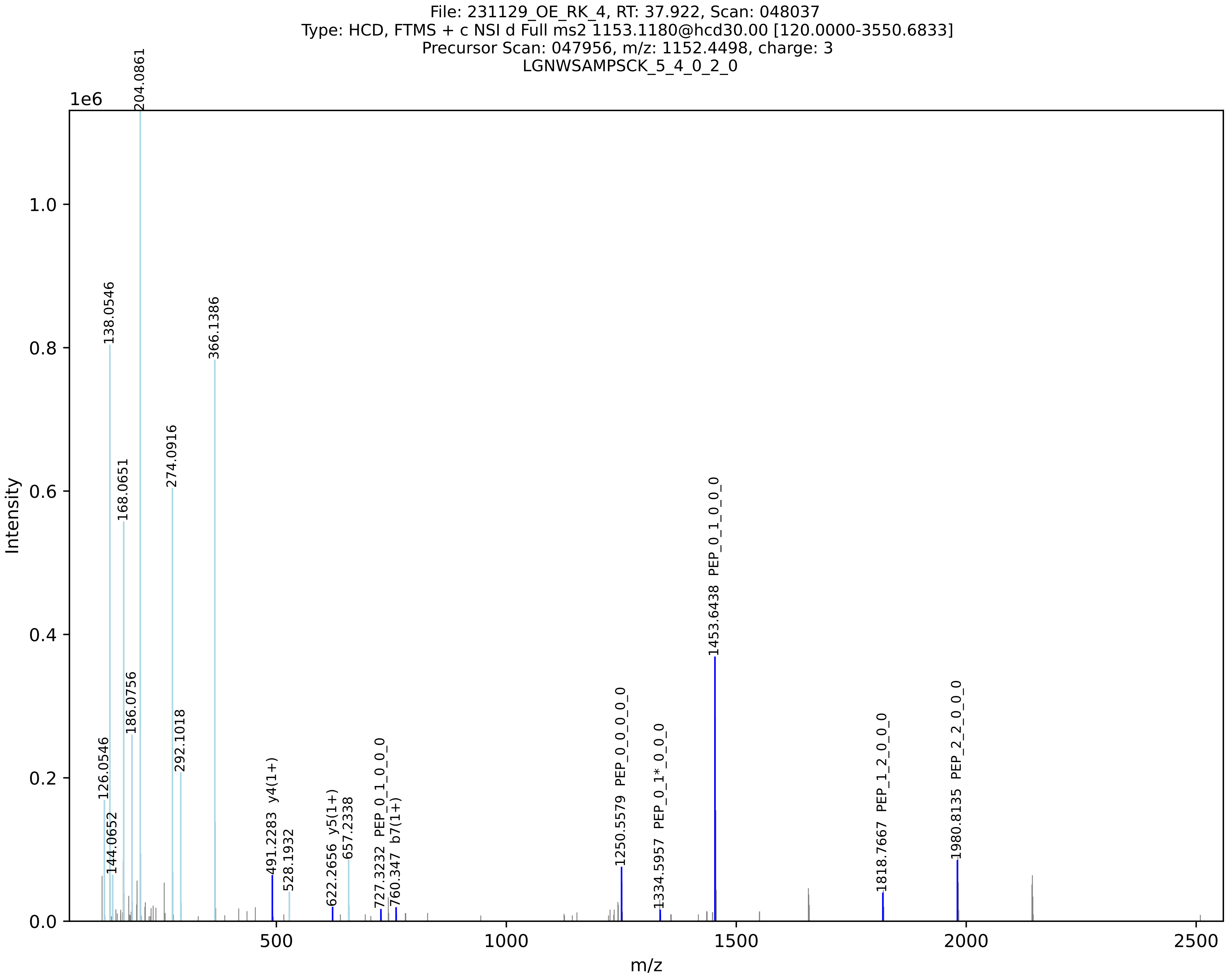
File: 231129_OE_RK_4, RT: 37.922, Scan: 048037
Type: HCD, FTMS + c NSI d Full ms2 1153.1180@hcd30.00 [120.0000-3550.6833]
Precursor Scan: 047956, m/z: 1152.4498, charge: 3
LGNWSAMPSCK_5_4_0_2_0
1e6
Intensity
m/z
204.0861
138.0546
366.1386
274.0916
168.0651
186.0756
292.1018
126.0546
144.0652
491.2283 y4(1+)
528.1932
622.2656 y5(1+)
657.2338
727.3232 PEP_0_1_0_0_0
760.347 b7(1+)
1250.5579 PEP_0_0_0_0_0
1334.5957 PEP_0_1*_0_0_0
1453.6438 PEP_0_1_0_0_0
1818.7667 PEP_1_2_0_0_0
1980.8135 PEP_2_2_0_0_0

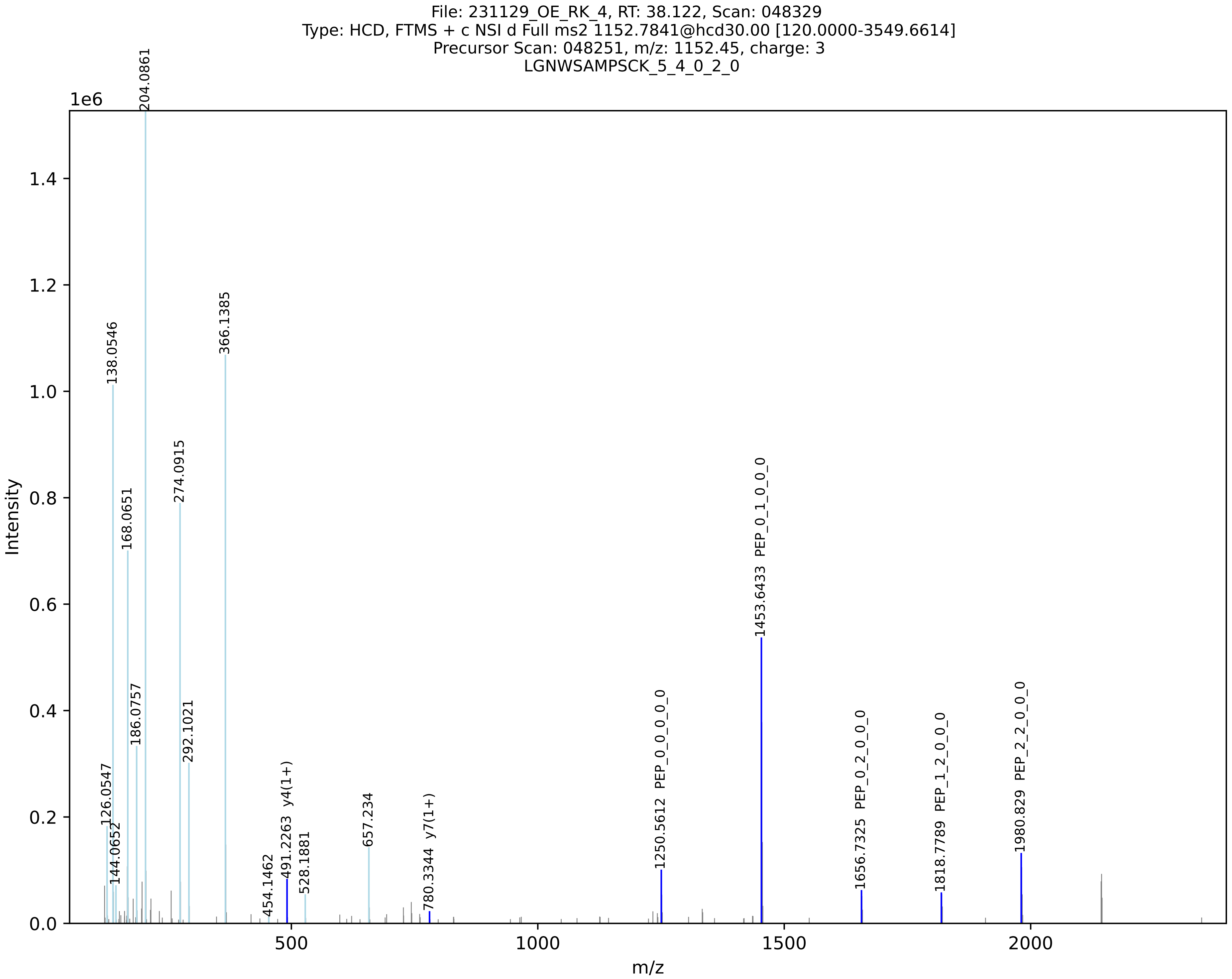
File: 231129_OE_RK_4, RT: 38.122, Scan: 048329
Type: HCD, FTMS + c NSI d Full ms2 1152.7841@hcd30.00 [120.0000-3549.6614]
Precursor Scan: 048251, m/z: 1152.45, charge: 3
LGNWSAMPSCK_5_4_0_2_0
1e6
Intensity
m/z
204.0861
366.1385
138.0546
274.0915
168.0651
186.0757
292.1021
126.0547
144.0652
454.1462
491.2263 y4(1+)
528.1881
657.234
780.3344 y7(1+)
1250.5612 PEP_0_0_0_0_0
1453.6433 PEP_0_1_0_0_0
1656.7325 PEP_0_2_0_0_0
1818.7789 PEP_1_2_0_0_0
1980.829 PEP_2_2_0_0_0

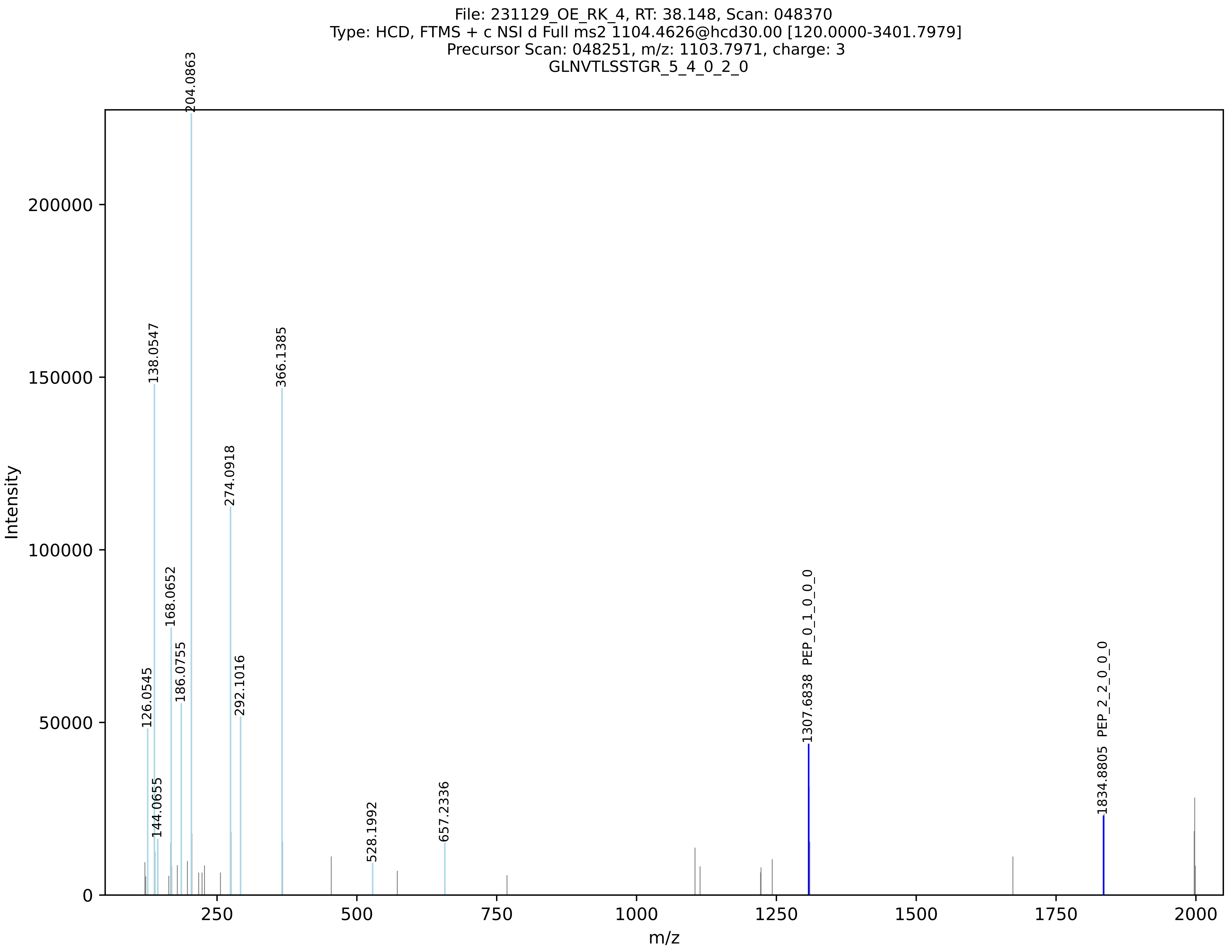
File: 231129_OE_RK_4, RT: 38.148, Scan: 048370
Type: HCD, FTMS + c NSI d Full ms2 1104.4626@hcd30.00 [120.0000-3401.7979]
Precursor Scan: 048251, m/z: 1103.7971, charge: 3
GLNVTLSSTGR_5_4_0_2_0
204.0863
138.0547
366.1385
274.0918
168.0652
186.0755
292.1016
126.0545
144.0655
528.1992
657.2336
1307.6838 PEP_0_1_0_0_0
1834.8805 PEP_2_2_0_0_0
Intensity
m/z
0
50000
100000
150000
200000
250
500
750
1000
1250
1500
1750
2000

File: 231129_OE_RK_4, RT: 38.225, Scan: 048478
Type: HCD, FTMS + c NSI d Full ms2 1409.9307@hcd30.00 [120.0000-4336.5298]
Precursor Scan: 048398, m/z: 1409.5958, charge: 3
ELHHLQEQNVSNAFLDK_5_4_0_2_0

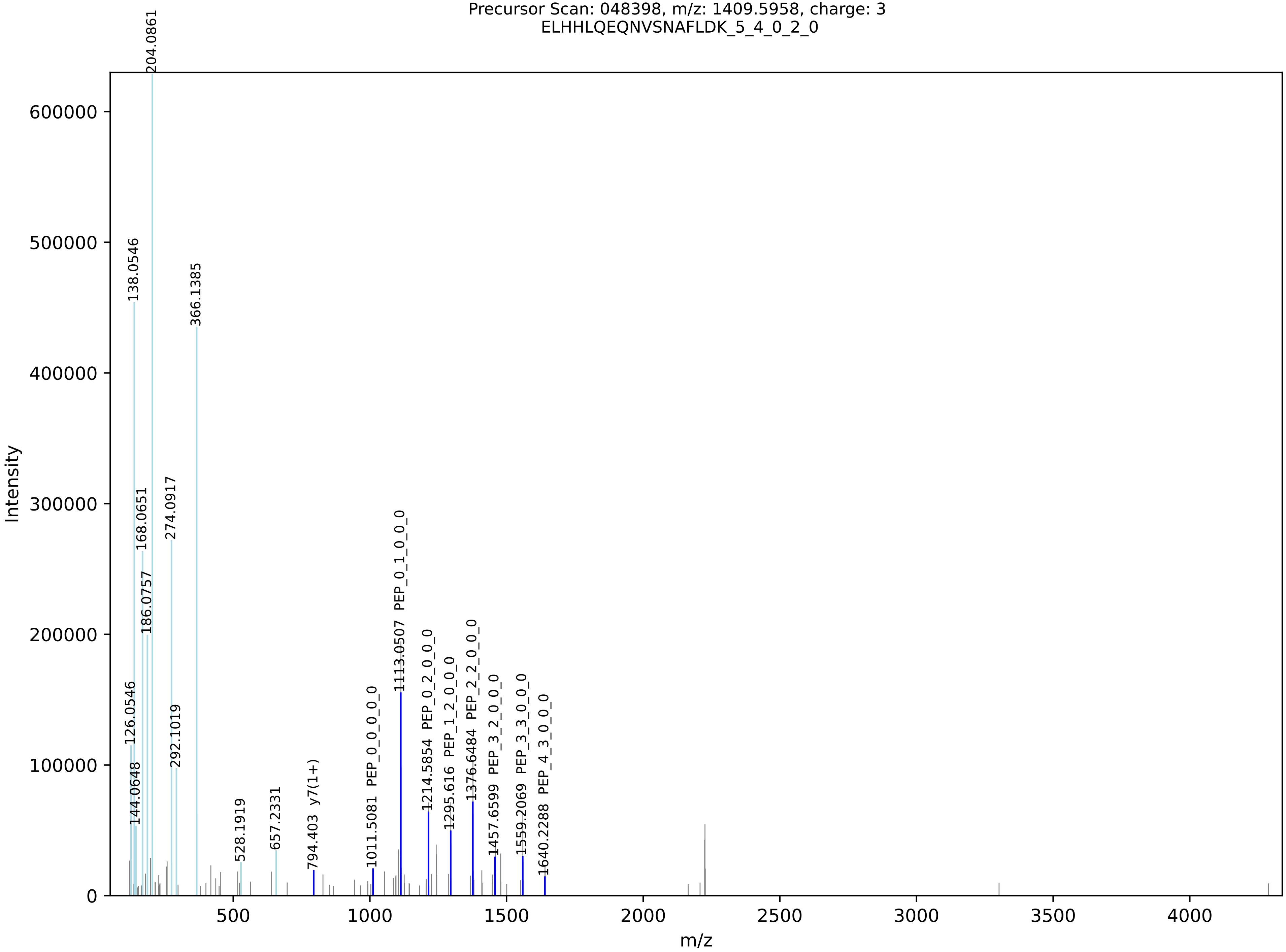

File: 231129_OE_RK_4, RT: 38.233, Scan: 048491
Type: HCD, FTMS + c NSI d Full ms2 1311.7950@hcd30.00 [120.0000-5374.9800]
Precursor Scan: 048398, m/z: 1311.5485, charge: 4
NNATVHEQVGGPSLTSDLQAQSK_6_5_2_2_0

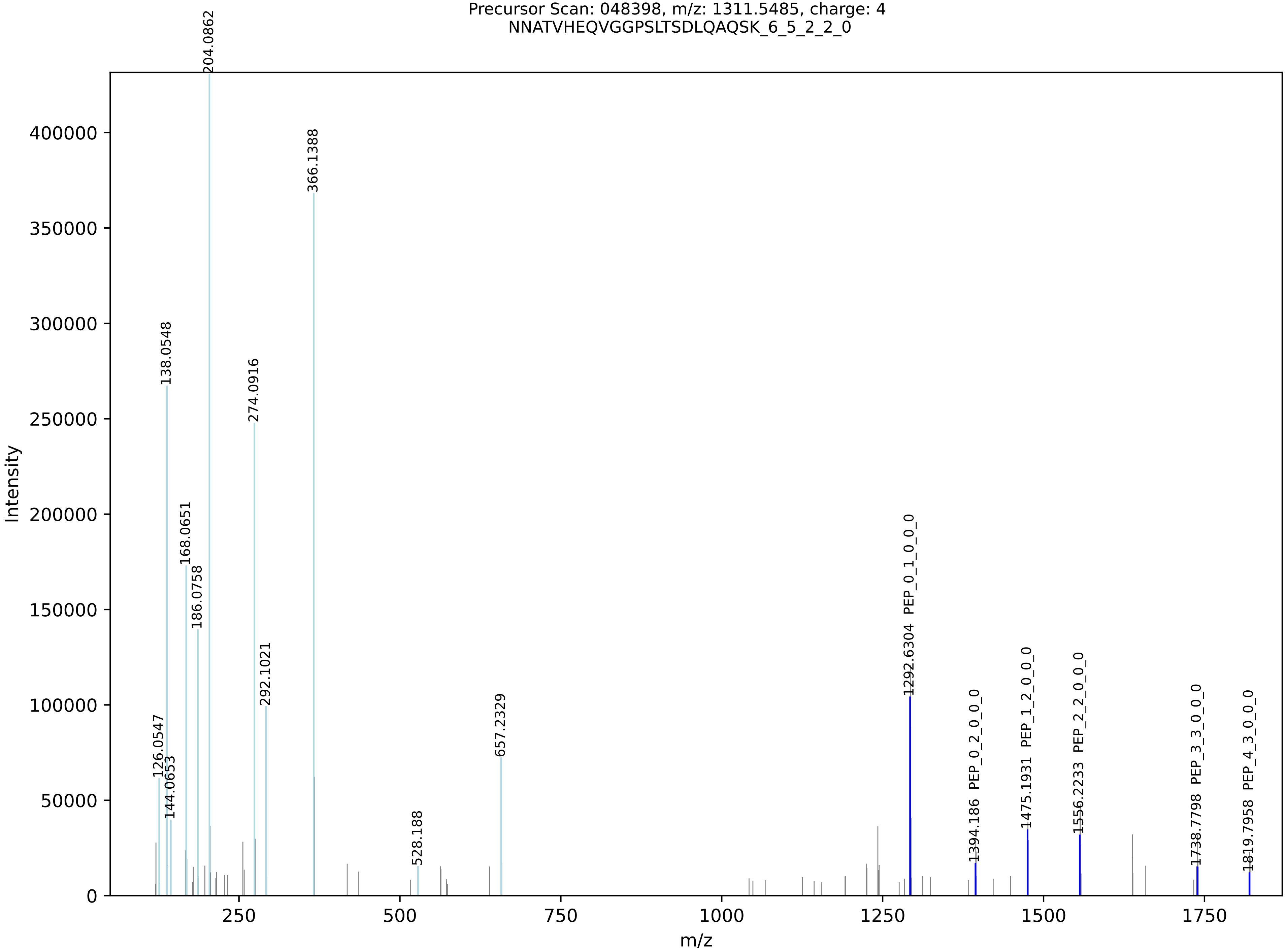

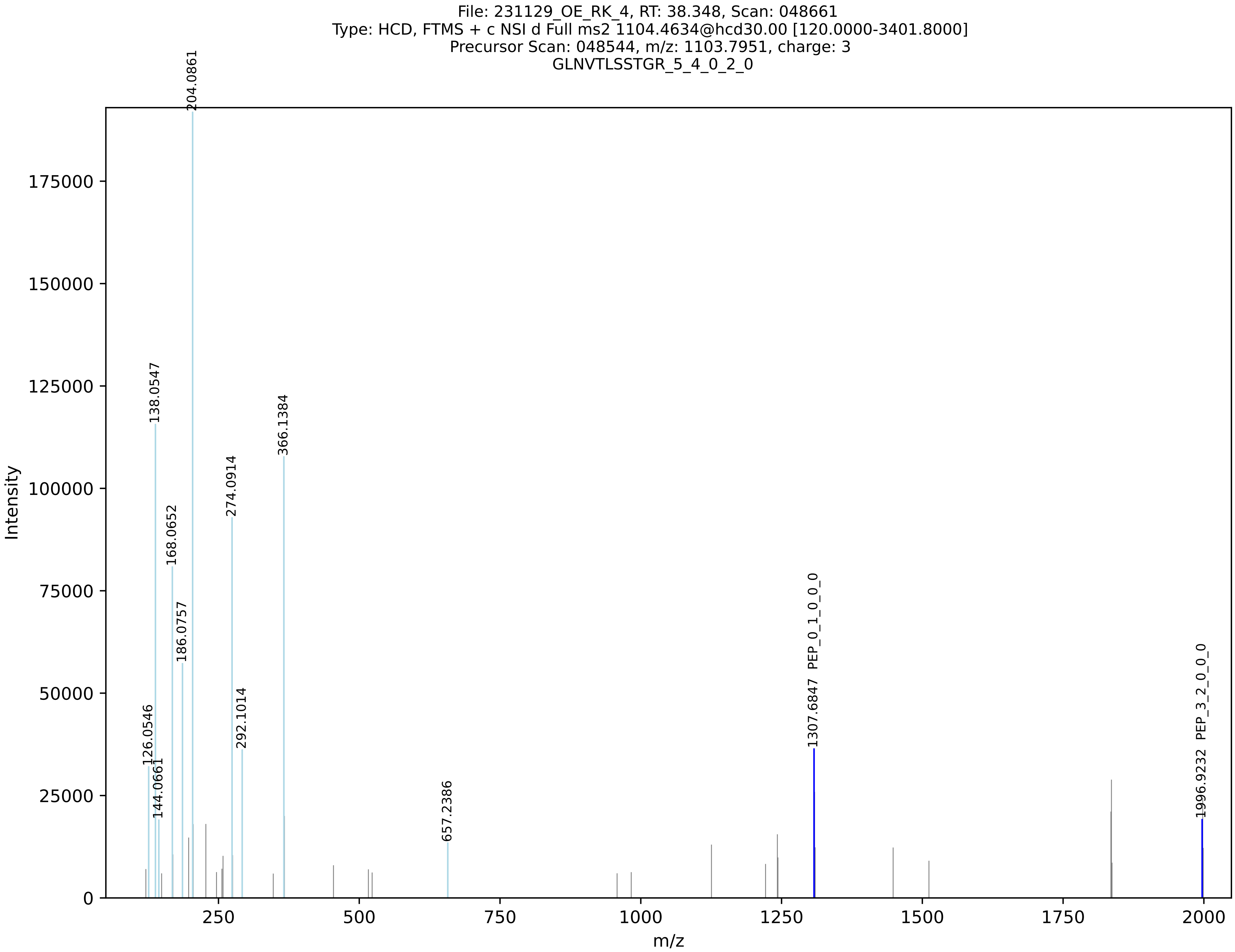

File: 231129_OE_RK_4, RT: 38.348, Scan: 048661
Type: HCD, FTMS + c NSI d Full ms2 1104.4634@hcd30.00 [120.0000-3401.8000]
Precursor Scan: 048544, m/z: 1103.7951, charge: 3
GLNVTLSSTGR_5_4_0_2_0
Intensity
m/z
204.0861
138.0547
366.1384
274.0914
168.0652
186.0757
126.0546
144.0661
292.1014
657.2386
1307.6847 PEP_0_1_0_0_
1996.9232 PEP_3_2_0_0_
0
25000
50000
75000
100000
125000
150000
175000
250
500
750
1000
1250
1500
1750
2000

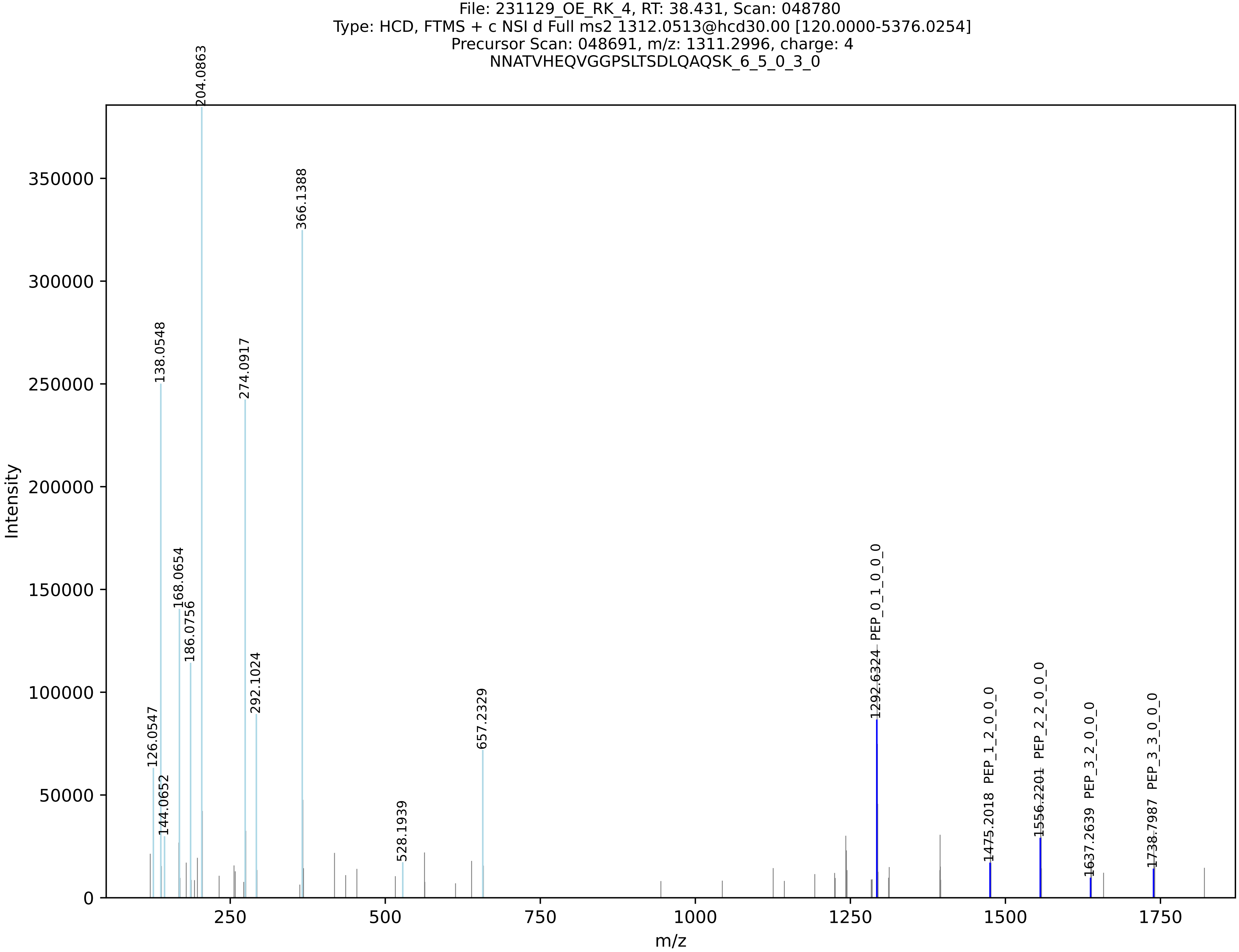

File: 231129_OE_RK_4, RT: 38.431, Scan: 048780
Type: HCD, FTMS + c NSI d Full ms2 1312.0513@hcd30.00 [120.0000-5376.0254]
Precursor Scan: 048691, m/z: 1311.2996, charge: 4
NNATVHEQVGGPSLTSDLQAQSK_6_5_0_3_0
Intensity
m/z
0
50000
100000
150000
200000
250000
300000
350000
250
500
750
1000
1250
1500
1750
204.0863
366.1388
138.0548
274.0917
168.0654
186.0756
292.1024
126.0547
144.0652
528.1939
657.2329
1292.6324 PEP_0_1_0_0_0
1475.2018 PEP_1_2_0_0_0
1556.2201 PEP_2_2_0_0_0
1637.2639 PEP_3_2_0_0_0
1738.7987 PEP_3_3_0_0_0

File: 231129_OE_RK_4, RT: 38.525, Scan: 048917
Type: HCD, FTMS + c NSI d Full ms2 1152.7847@hcd30.00 [120.0000-3549.6628]
Precursor Scan: 048838, m/z: 1152.4504, charge: 3
LGNWSAMPSCK_5_4_0_2_0

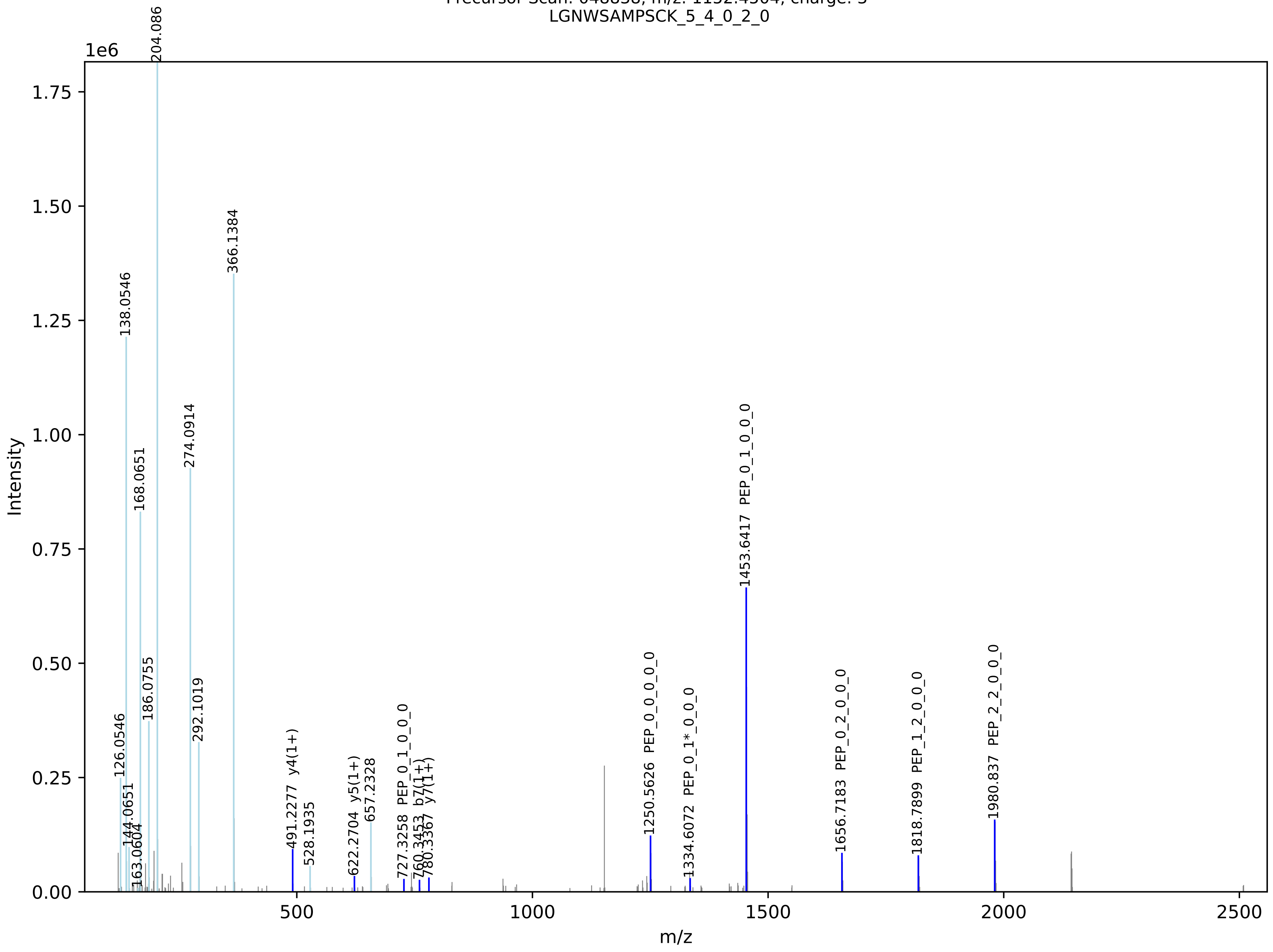

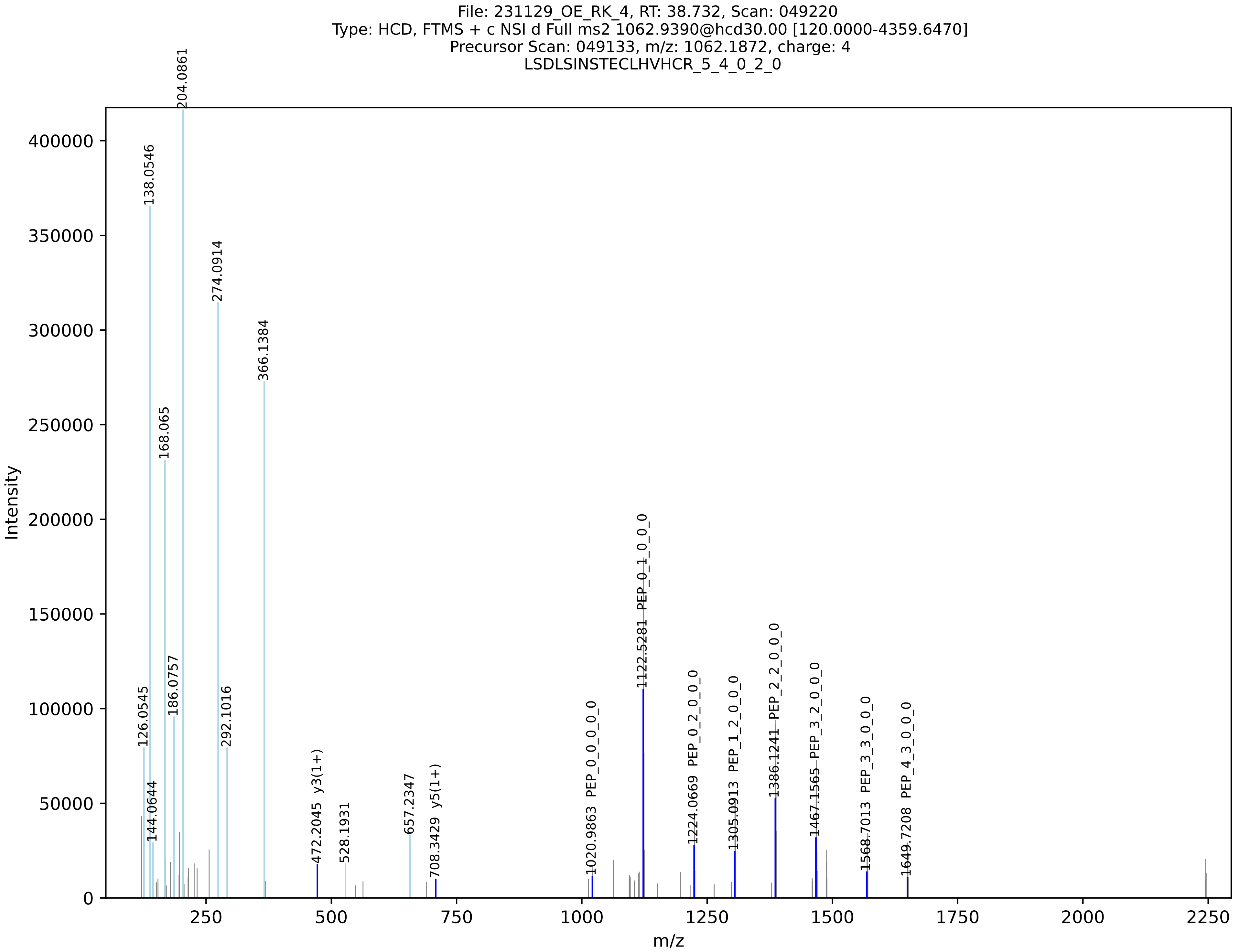

File: 231129_OE_RK_4, RT: 38.732, Scan: 049220
Type: HCD, FTMS + c NSI d Full ms2 1062.9390@hcd30.00 [120.0000-4359.6470]
Precursor Scan: 049133, m/z: 1062.1872, charge: 4
LSDLSINSTECLHVHCR_5_4_0_2_0
Intensity
m/z
204.0861
138.0546
274.0914
366.1384
168.065
186.0757
126.0545
292.1016
144.0644
472.2045 y3(1+)
528.1931
657.2347
708.3429 y5(1+)
1020.9863 PEP_0_0_0_0_0
1122.5281 PEP_0_1_0_0_0
1224.0669 PEP_0_2_0_0_0
1305.0913 PEP_1_2_0_0_0
1386.1241 PEP_2_2_0_0_0
1467.1565 PEP_3_2_0_0_0
1568.7013 PEP_3_3_0_0_0
1649.7208 PEP_4_3_0_0_0

File: 231129_OE_RK_4, RT: 38.787, Scan: 049301
Type: HCD, FTMS + c NSI d Full ms2 1107.1204@hcd30.00 [120.0000-3409.9302]
Precursor Scan: 049207, m/z: 1106.4521, charge: 3
SWPAVGNCSSALR_5_4_0_1_0

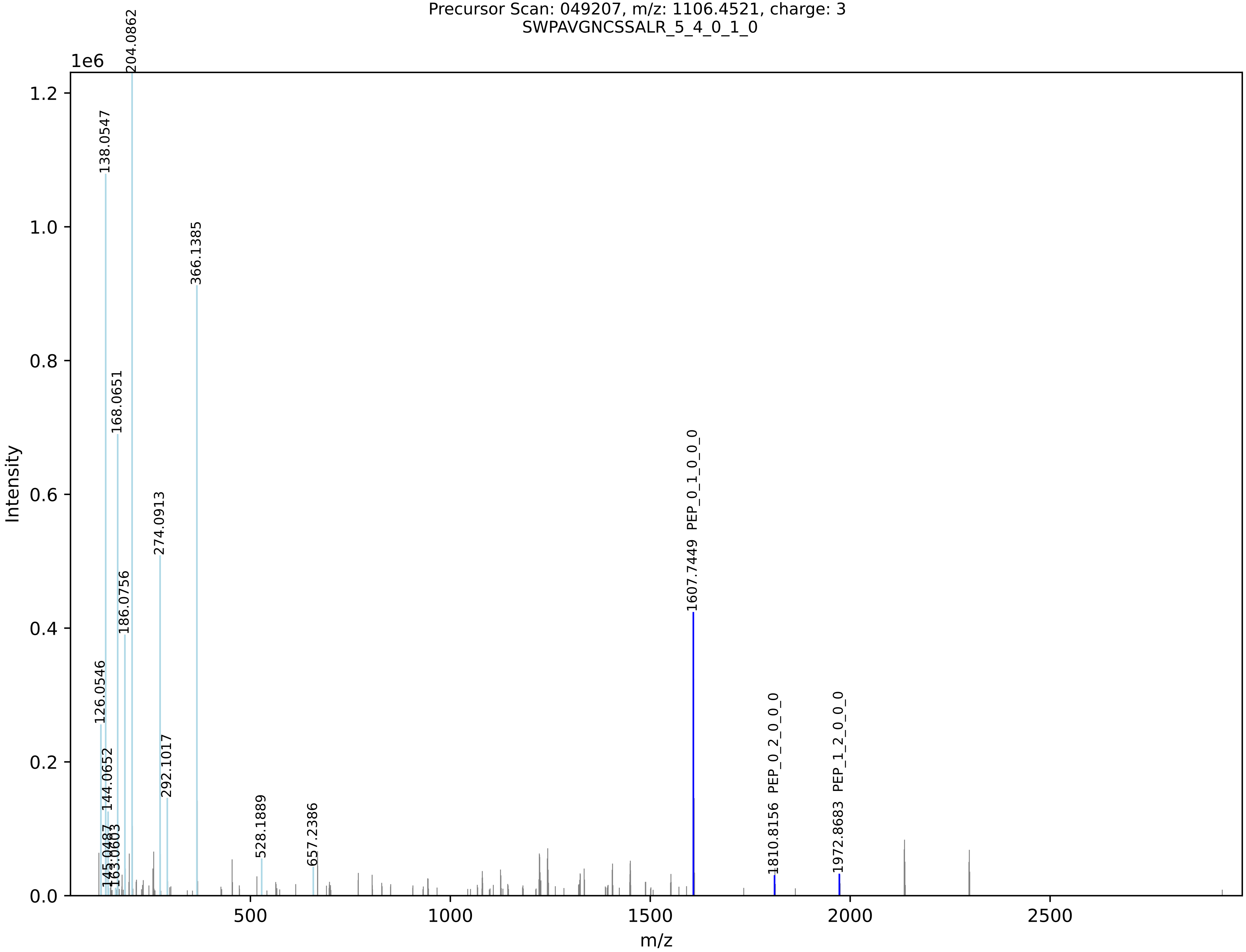

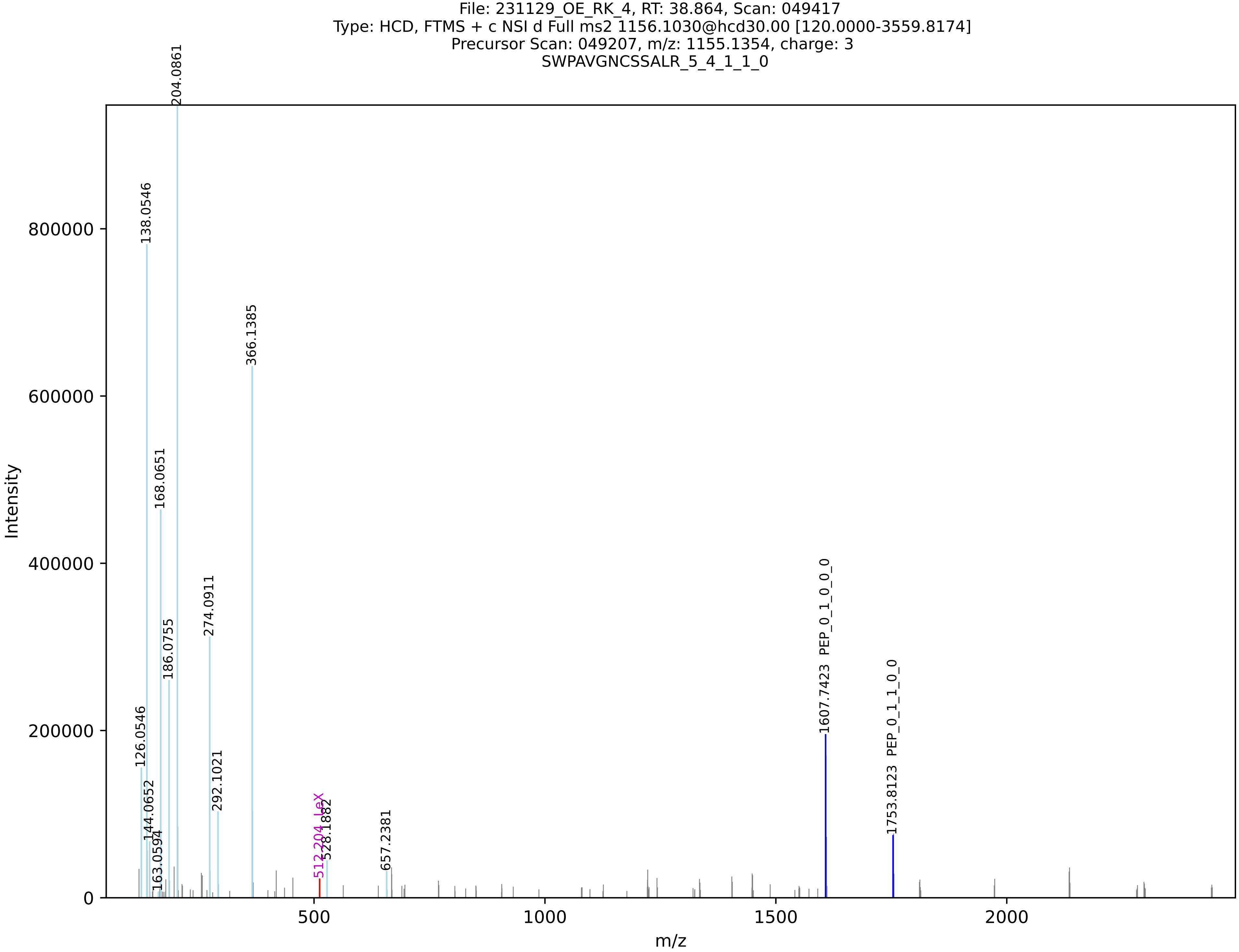

File: 231129_OE_RK_4, RT: 38.864, Scan: 049417
Type: HCD, FTMS + c NSI d Full ms2 1156.1030@hcd30.00 [120.0000-3559.8174]
Precursor Scan: 049207, m/z: 1155.1354, charge: 3
SWPAVGNCSSALR_5_4_1_1_0
Intensity
m/z
0
200000
400000
600000
800000
500
1000
1500
2000
204.0861
138.0546
366.1385
168.0651
274.0911
186.0755
126.0546
292.1021
144.0652
163.0594
512.204_LeX
528.1882
657.2381
1607.7423 PEP_0_1_0_0_0_
1753.8123 PEP_0_1_1_0_0

File: 231129_OE_RK_4, RT: 38.977, Scan: 049577
Type: HCD, FTMS + c NSI d Full ms2 1107.1194@hcd30.00 [120.0000-3409.9275]
Precursor Scan: 049502, m/z: 1106.7856, charge: 3
SWPAVGNCSSALR_5_4_0_1_0

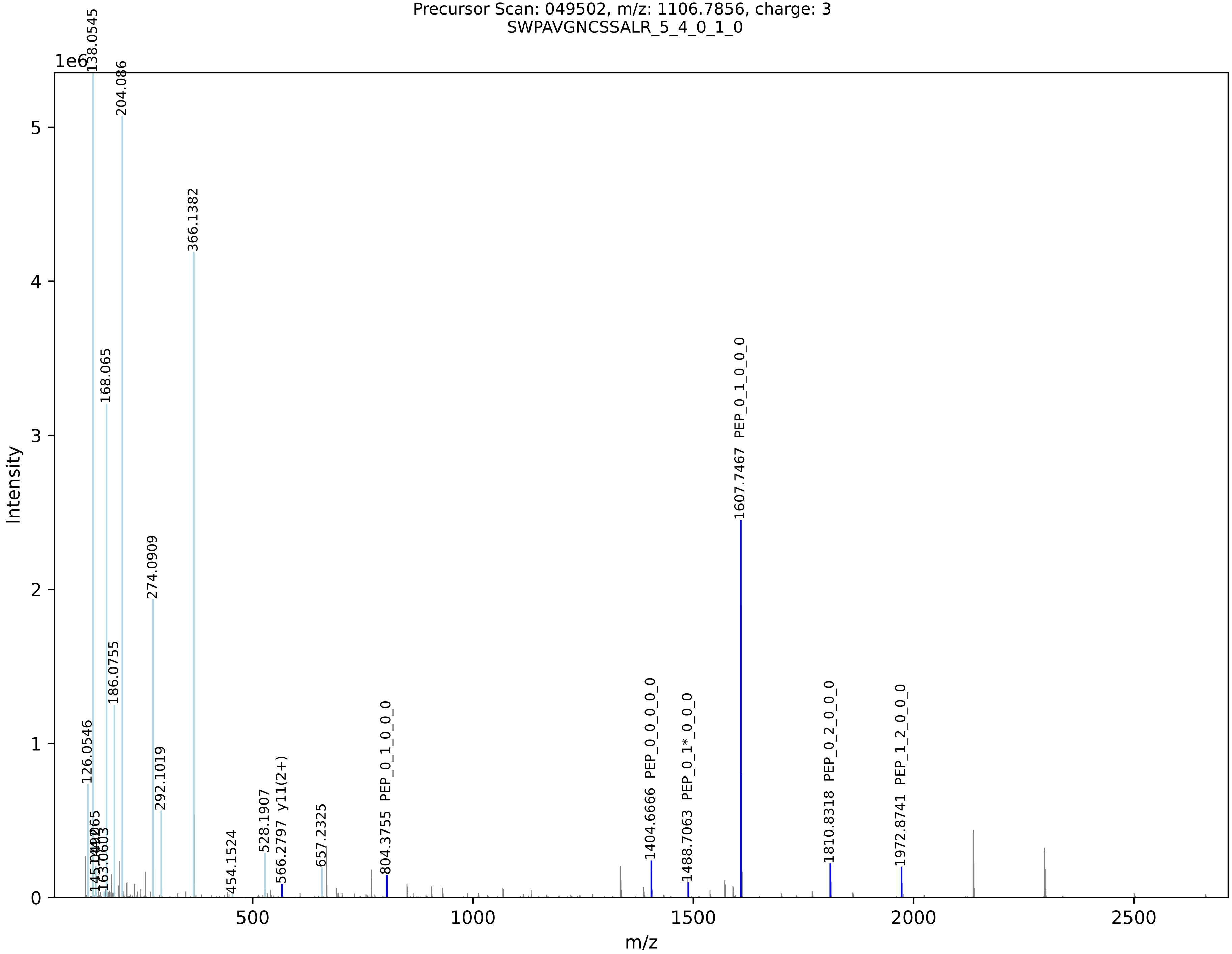

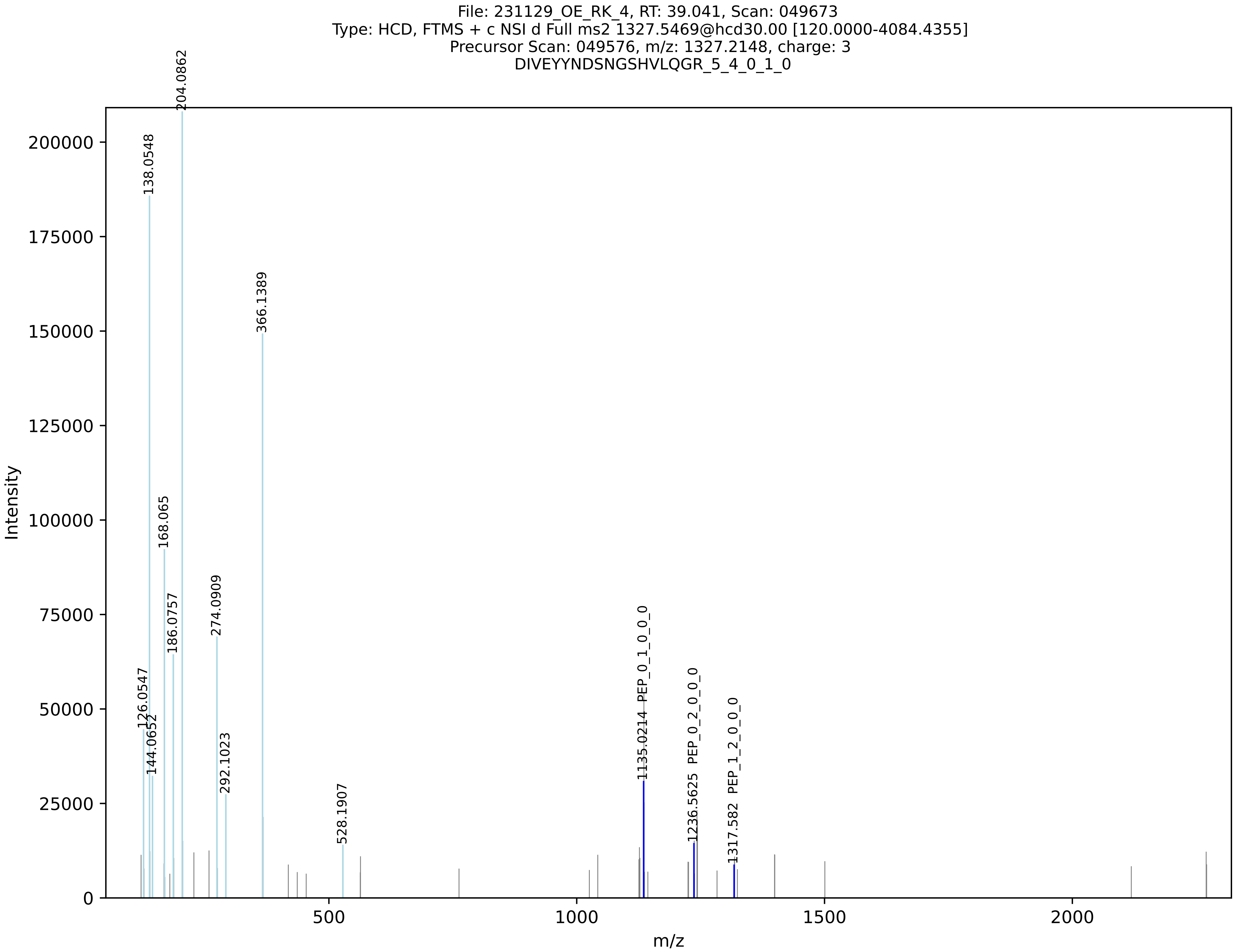

File: 231129_OE_RK_4, RT: 39.041, Scan: 049673
Type: HCD, FTMS + c NSI d Full ms2 1327.5469@hcd30.00 [120.0000-4084.4355]
Precursor Scan: 049576, m/z: 1327.2148, charge: 3
DIVEYYNDSNGSHVLQGR_5_4_0_1_0
Intensity
m/z
204.0862
138.0548
366.1389
168.065
186.0757
274.0909
126.0547
144.0652
292.1023
528.1907
1135.0214 PEP_0_1_0_0_0
1236.5625 PEP_0_2_0_0_0
1317.582 PEP_1_2_0_0_0

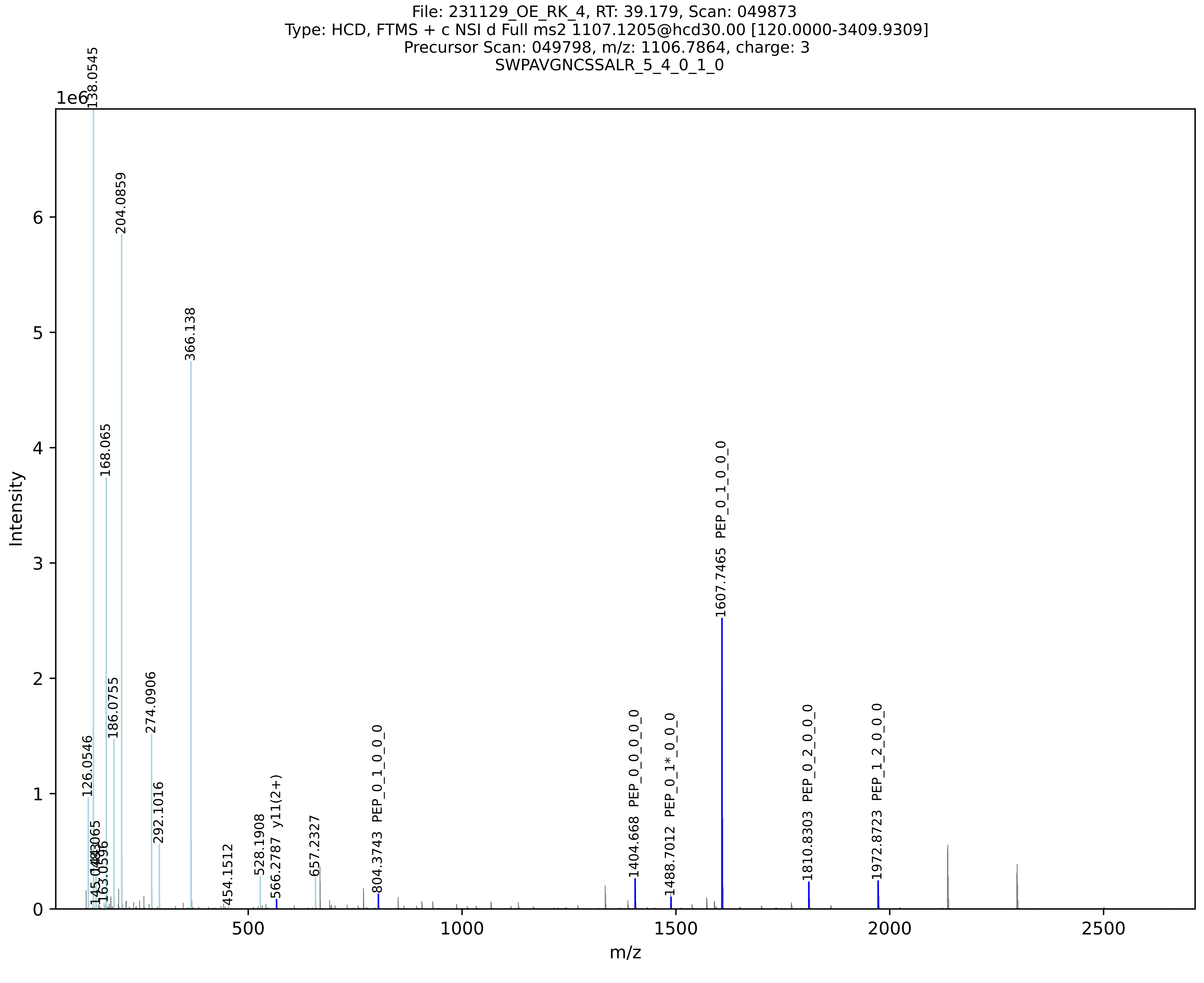

File: 231129_OE_RK_4, RT: 39.179, Scan: 049873
Type: HCD, FTMS + c NSI d Full ms2 1107.1205@hcd30.00 [120.0000-3409.9309]
Precursor Scan: 049798, m/z: 1106.7864, charge: 3
SWPAVGNCSSALR_5_4_0_1_0
1e6
Intensity
m/z
138.0545
204.0859
366.138
168.065
186.0755
274.0906
126.0546
292.1016
163.0596
454.1512
528.1908
566.2787 y11(2+)
657.2327
804.3743 PEP_0_1_0_0_0
1404.668 PEP_0_0_0_0_0
1488.7012 PEP_0_1*_0_0_0
1607.7465 PEP_0_1_0_0_0
1810.8303 PEP_0_2_0_0_0
1972.8723 PEP_1_2_0_0_0

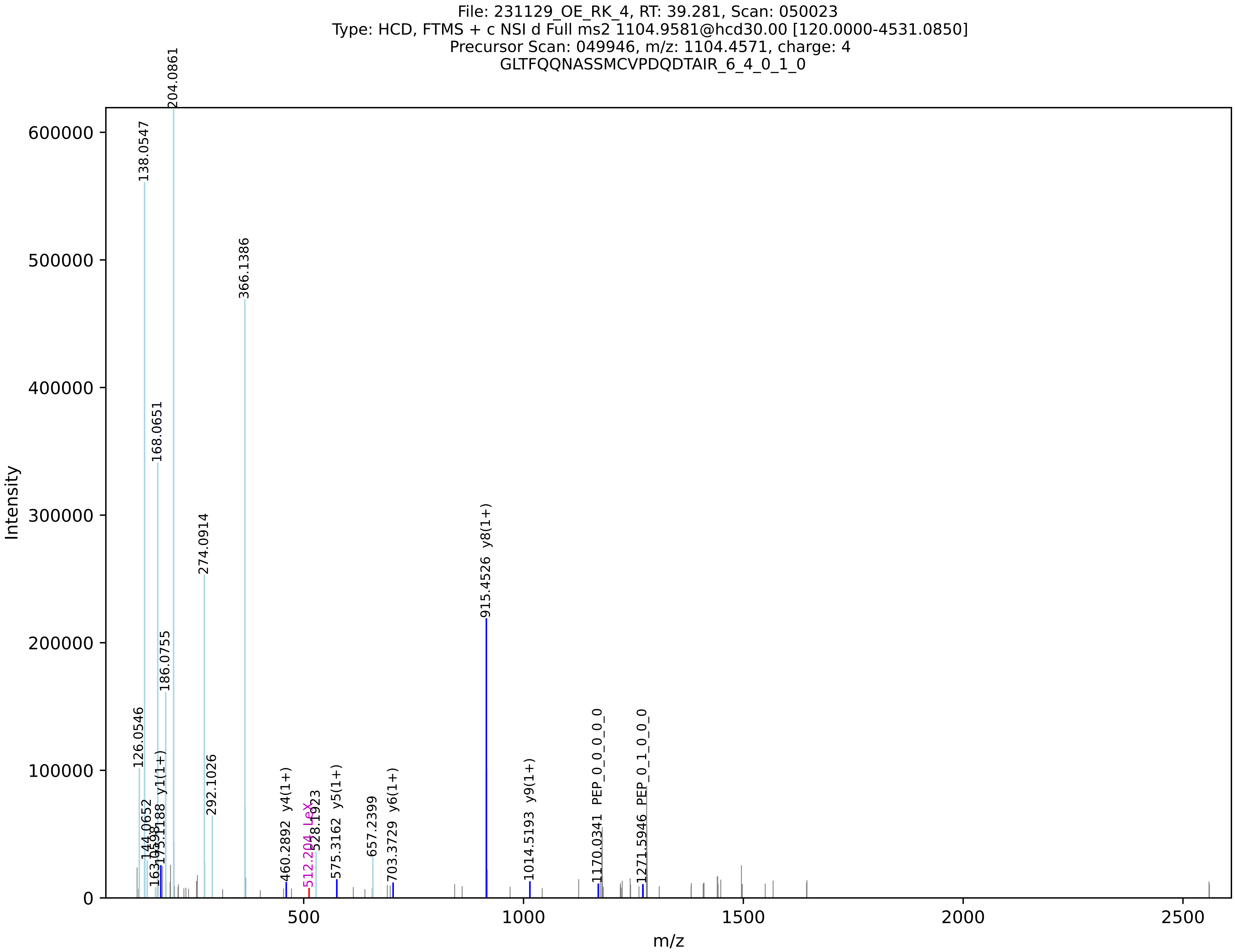

File: 231129_OE_RK_4, RT: 39.281, Scan: 050023
Type: HCD, FTMS + c NSI d Full ms2 1104.9581@hcd30.00 [120.0000-4531.0850]
Precursor Scan: 049946, m/z: 1104.4571, charge: 4
GLTFQQNASSMCVPDQDTAIR_6_4_0_1_0
Intensity
m/z
204.0861
138.0547
366.1386
168.0651
274.0914
186.0755
126.0546
292.1026
144.0652
163.0598
175.1188 y1(1+)
460.2892 y4(1+)
512.204 LeX
528.1923
575.3162 y5(1+)
657.2399
703.3729 y6(1+)
915.4526 y8(1+)
1014.5193 y9(1+)
1170.0341 PEP_0_0_0_0_0
1271.5946 PEP_0_1_0_0_0

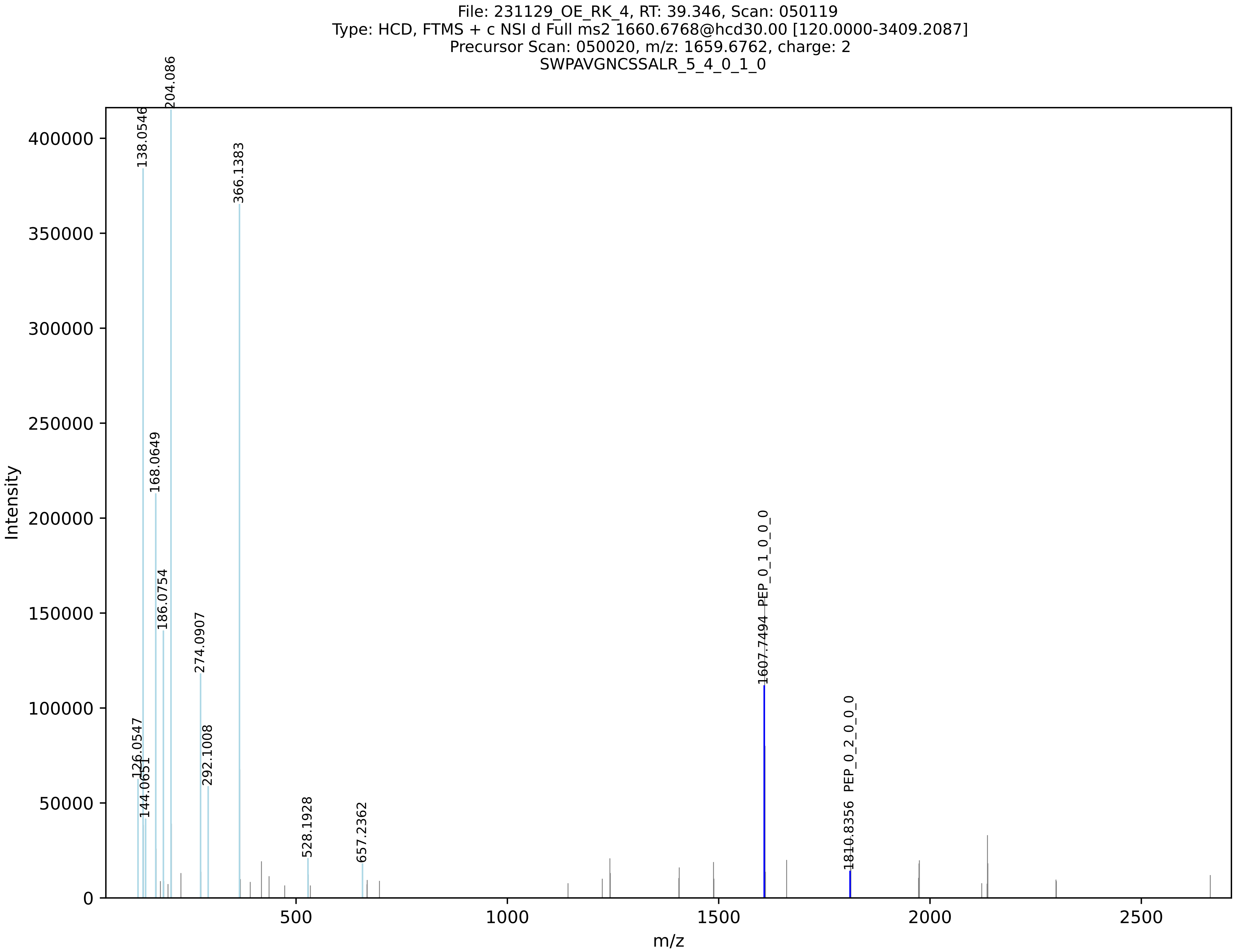
File: 231129_OE_RK_4, RT: 39.346, Scan: 050119
Type: HCD, FTMS + c NSI d Full ms2 1660.6768@hcd30.00 [120.0000-3409.2087]
Precursor Scan: 050020, m/z: 1659.6762, charge: 2
SWPAVGNCSSALR_5_4_0_1_0
Intensity
m/z
204.086
138.0546
366.1383
168.0649
186.0754
274.0907
292.1008
126.0547
144.0651
528.1928
657.2362
1607.7494 PEP_0_1_0_0_0
1810.8356 PEP_0_2_0_0_0
0
50000
100000
150000
200000
250000
300000
350000
400000
500
1000
1500
2000
2500

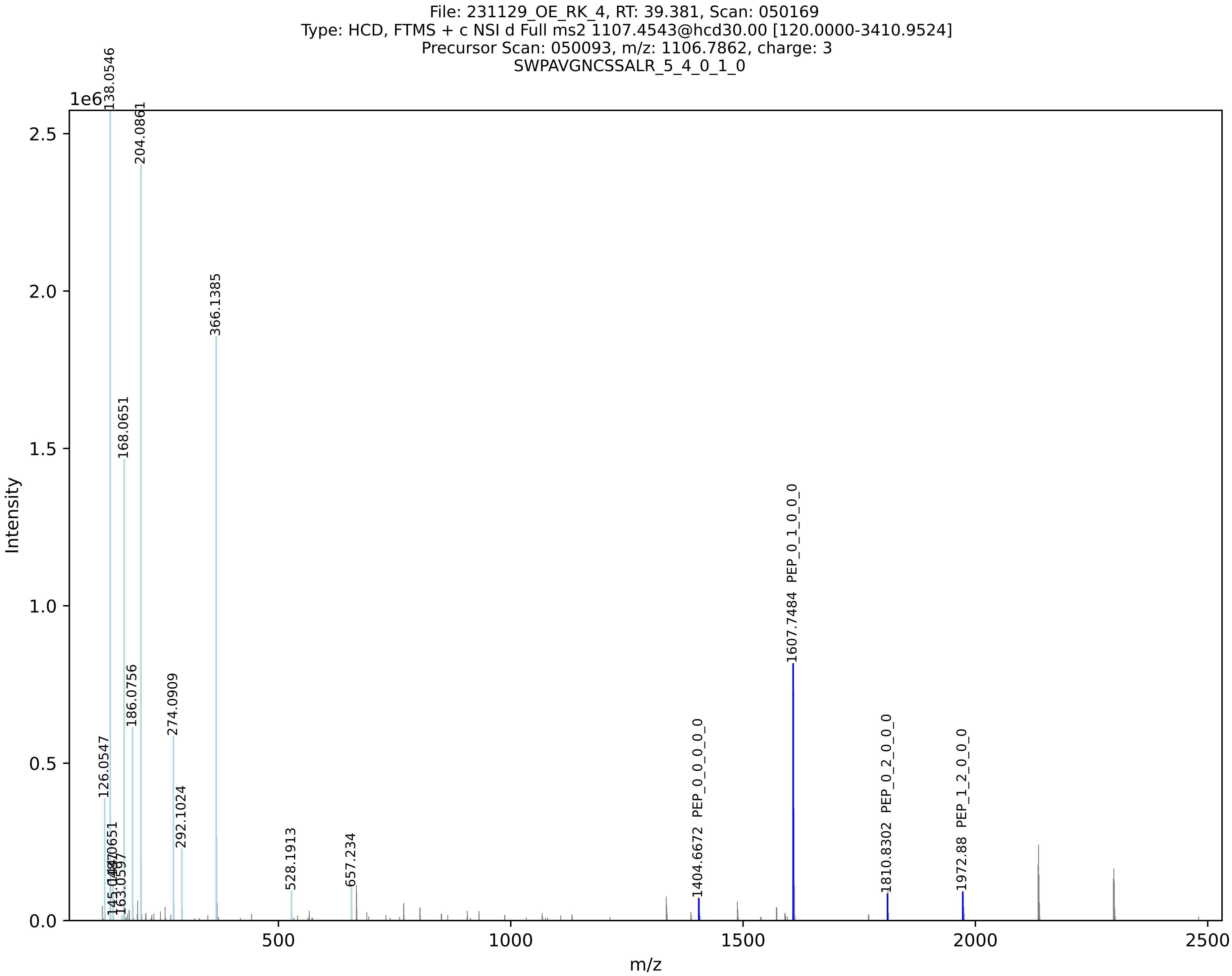
File: 231129_OE_RK_4, RT: 39.381, Scan: 050169
Type: HCD, FTMS + c NSI d Full ms2 1107.4543@hcd30.00 [120.0000-3410.9524]
Precursor Scan: 050093, m/z: 1106.7862, charge: 3
SWPAVGNCSSALR_5_4_0_1_0
1e6
Intensity
m/z
138.0546
204.0861
366.1385
168.0651
186.0756
274.0909
126.0547
292.1024
528.1913
657.234
163.0597
1404.6672 PEP_0_0_0_0_0
1607.7484 PEP_0_1_0_0_0
1810.8302 PEP_0_2_0_0_0
1972.88 PEP_1_2_0_0_0

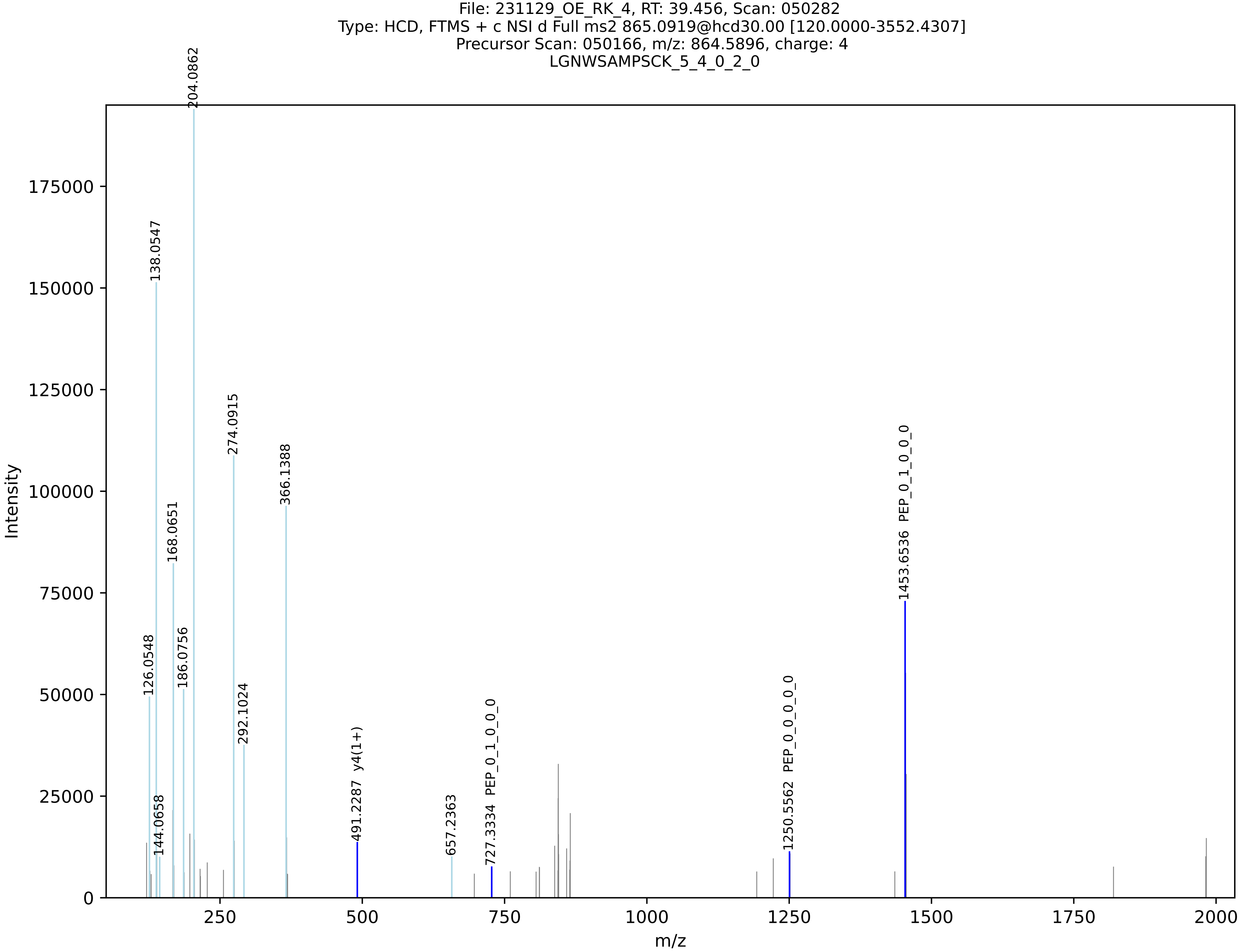
File: 231129_OE_RK_4, RT: 39.456, Scan: 050282
Type: HCD, FTMS + c NSI d Full ms2 865.0919@hcd30.00 [120.0000-3552.4307]
Precursor Scan: 050166, m/z: 864.5896, charge: 4
LGNWSAMPSCK_5_4_0_2_0
Intensity
m/z
0
25000
50000
75000
100000
125000
150000
175000
250
500
750
1000
1250
1500
1750
2000
204.0862
138.0547
274.0915
366.1388
168.0651
126.0548
186.0756
292.1024
144.0658
491.2287 y4(1+)
657.2363
727.3334 PEP_0_1_0_0_0_
1250.5562 PEP_0_0_0_0_0_
1453.6536 PEP_0_1_0_0_0_

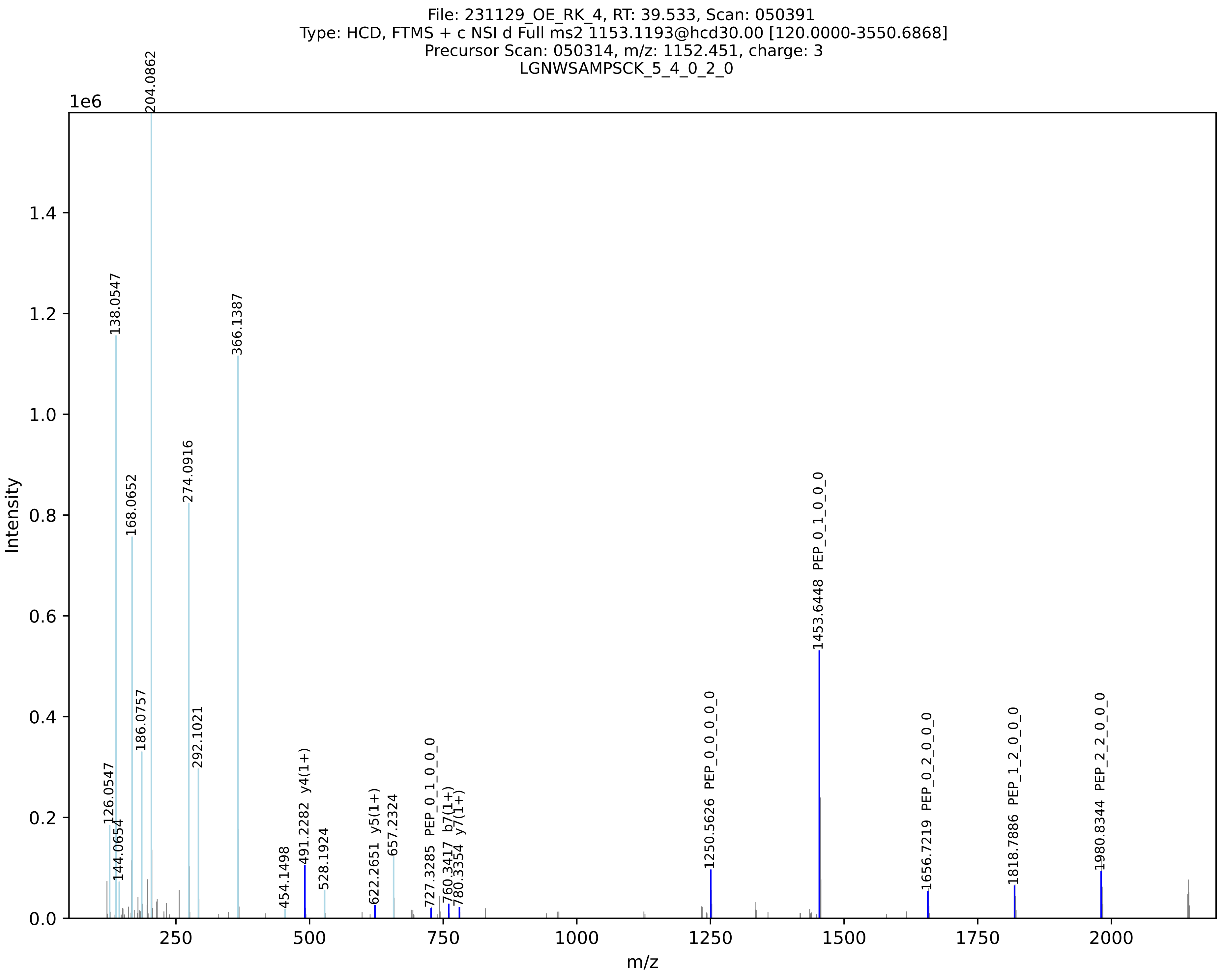

File: 231129_OE_RK_4, RT: 39.533, Scan: 050391
Type: HCD, FTMS + c NSI d Full ms2 1153.1193@hcd30.00 [120.0000-3550.6868]
Precursor Scan: 050314, m/z: 1152.451, charge: 3
LGNWSAMPSCK_5_4_0_2_0
1e6
Intensity
m/z
204.0862
138.0547
366.1387
274.0916
168.0652
186.0757
292.1021
126.0547
144.0654
454.1498
491.2282 y4(1+)
528.1924
622.2651 y5(1+)
657.2324
727.3285 PEP_0_1_0_0_0
760.3417 b7(1+)
780.3354 y7(1+)
1250.5626 PEP_0_0_0_0_0
1453.6448 PEP_0_1_0_0_0
1656.7219 PEP_0_2_0_0_0
1818.7886 PEP_1_2_0_0_0
1980.8344 PEP_2_2_0_0_0

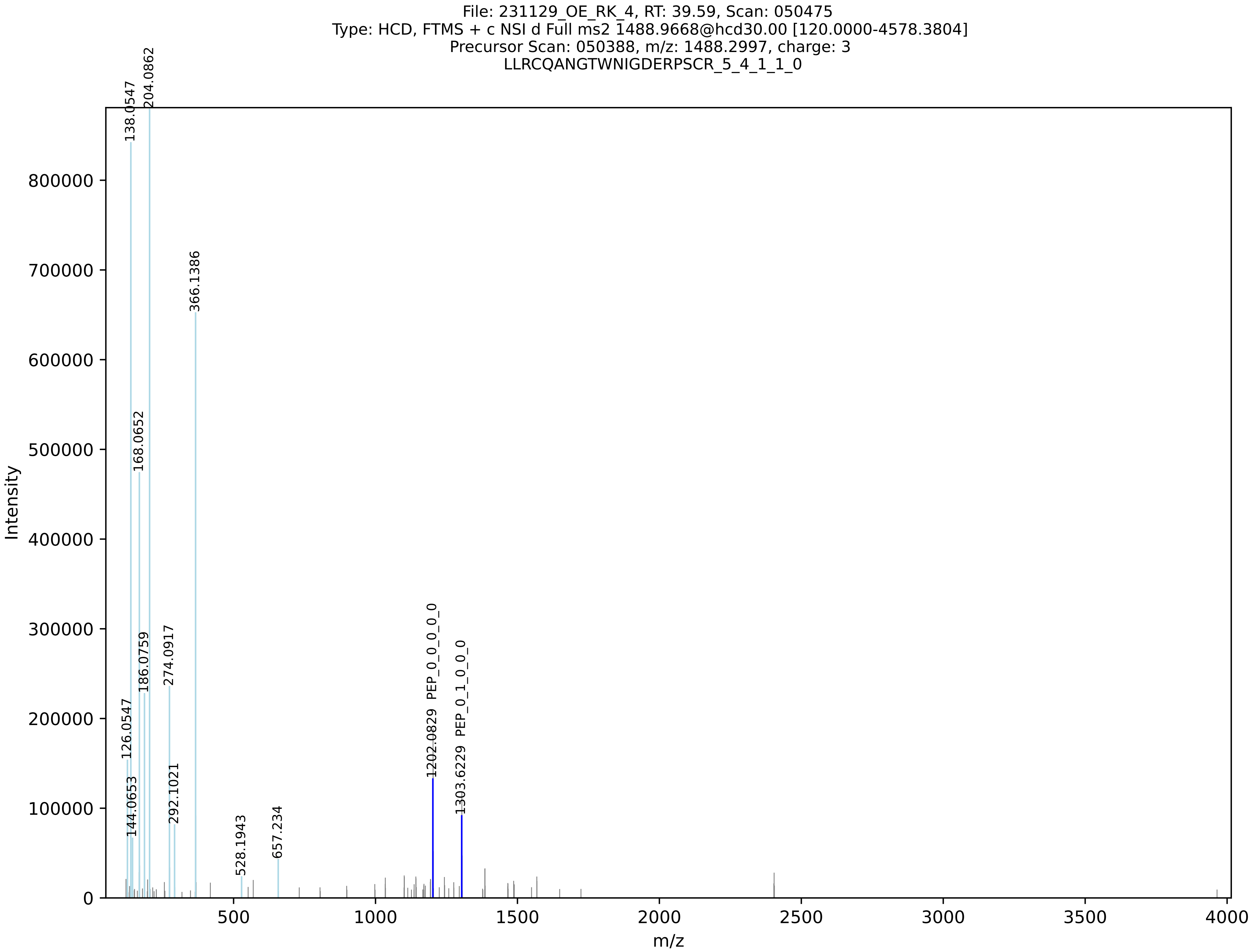
File: 231129_OE_RK_4, RT: 39.59, Scan: 050475
Type: HCD, FTMS + c NSI d Full ms2 1488.9668@hcd30.00 [120.0000-4578.3804]
Precursor Scan: 050388, m/z: 1488.2997, charge: 3
LLRCQANGTWNIGDERPSCR_5_4_1_1_0
Intensity
m/z
204.0862
138.0547
366.1386
168.0652
186.0759
274.0917
126.0547
144.0653
292.1021
528.1943
657.234
1202.0829 PEP_0_0_0_0_0
1303.6229 PEP_0_1_0_0_0
0
100000
200000
300000
400000
500000
600000
700000
800000
500
1000
1500
2000
2500
3000
3500
4000

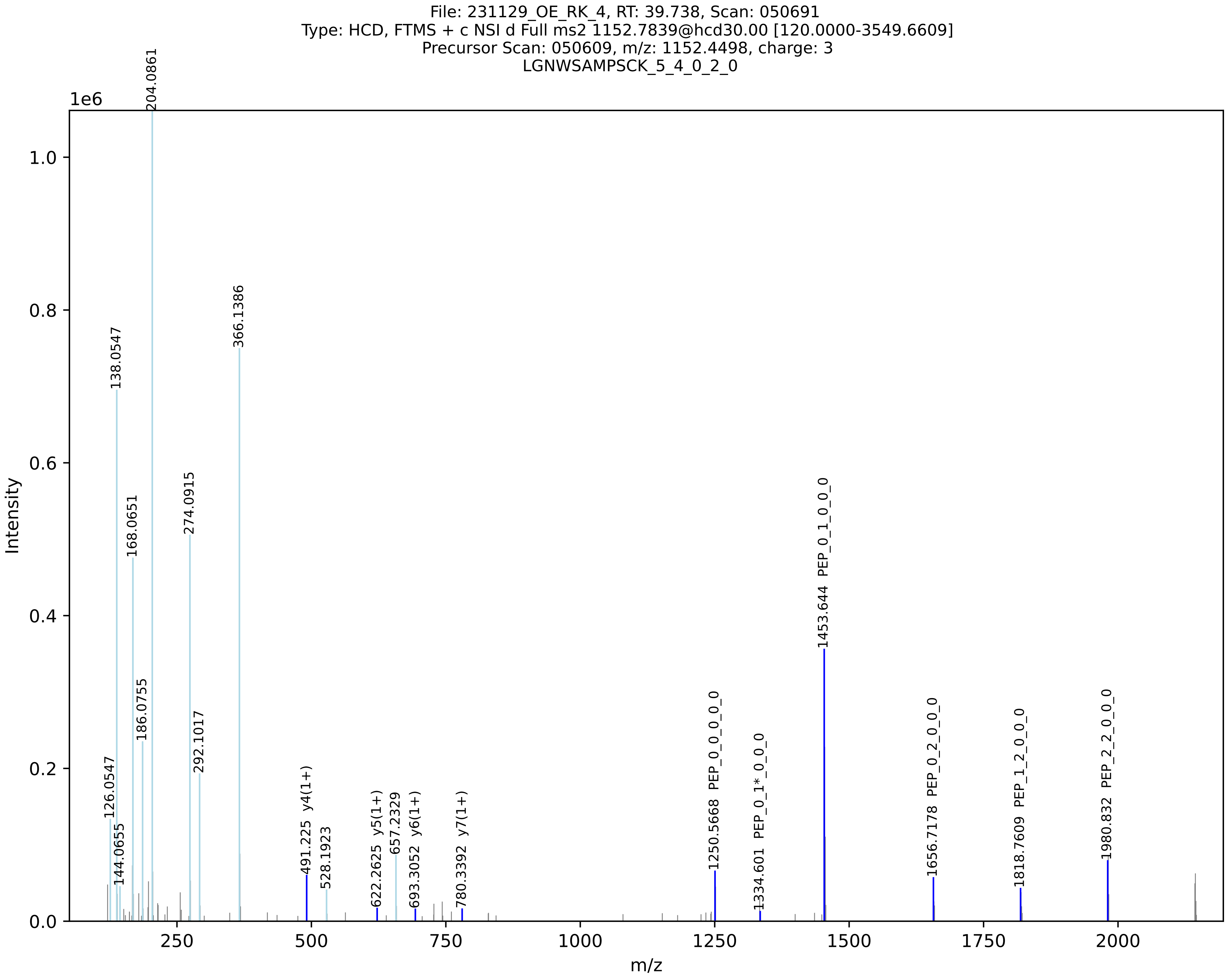
File: 231129_OE_RK_4, RT: 39.738, Scan: 050691
Type: HCD, FTMS + c NSI d Full ms2 1152.7839@hcd30.00 [120.0000-3549.6609]
Precursor Scan: 050609, m/z: 1152.4498, charge: 3
LGNWSAMPSCK_5_4_0_2_0
1e6
Intensity
m/z
204.0861
138.0547
366.1386
274.0915
168.0651
186.0755
292.1017
126.0547
144.0655
491.225 y4(1+)
528.1923
622.2625 y5(1+)
657.2329
693.3052 y6(1+)
780.3392 y7(1+)
1250.5668 PEP_0_0_0_0_0
1334.601 PEP_0_1*_0_0_0
1453.644 PEP_0_1_0_0_0
1656.7178 PEP_0_2_0_0_0
1818.7609 PEP_1_2_0_0_0
1980.832 PEP_2_2_0_0_0

File: 231129_OE_RK_4, RT: 39.94, Scan: 050988
Type: HCD, FTMS + c NSI d Full ms2 1153.1200@hcd30.00 [120.0000-3550.6890]
Precursor Scan: 050905, m/z: 1152.4517, charge: 3
LGNWSAMPSCK_5_4_0_2_0

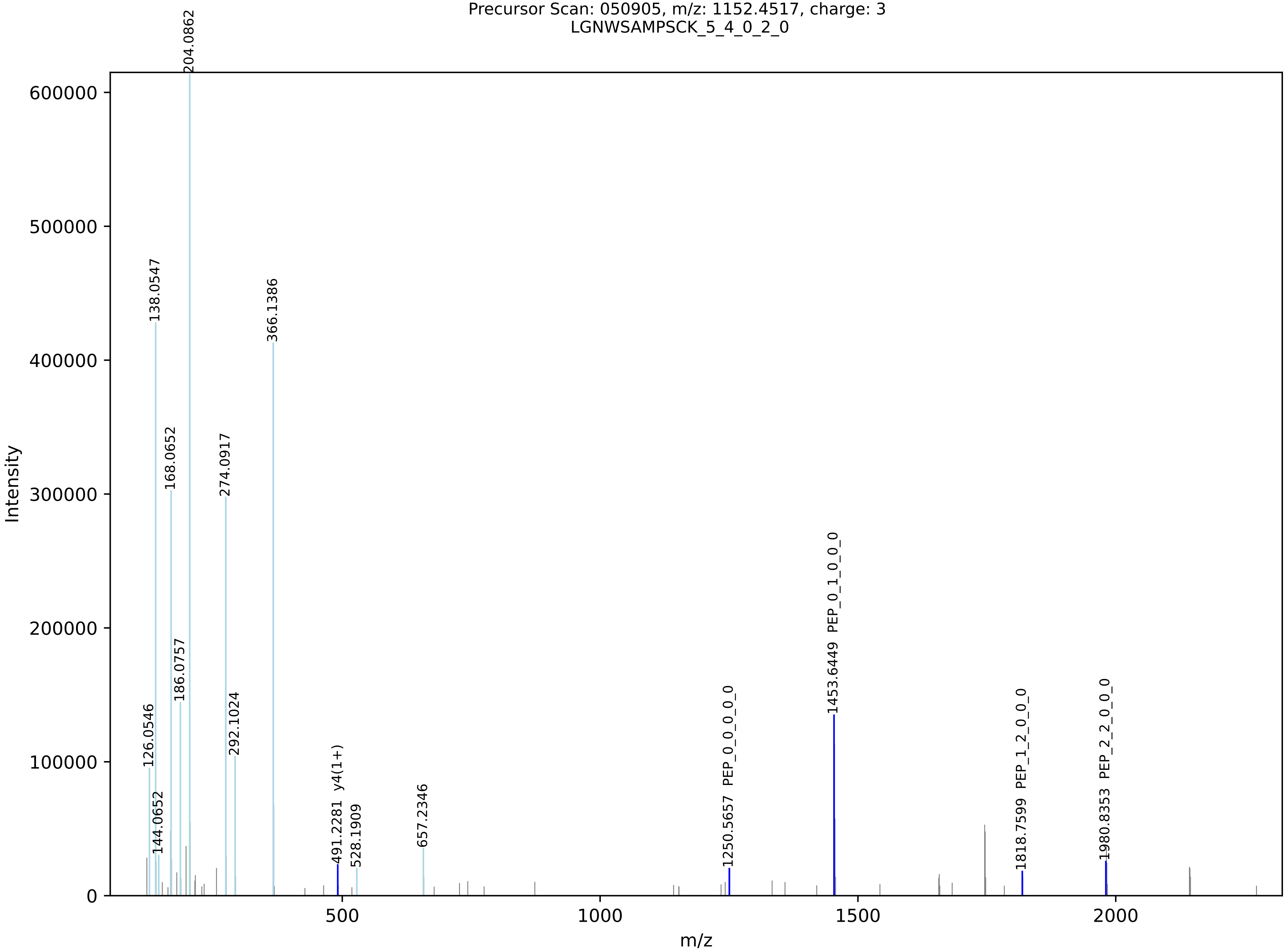

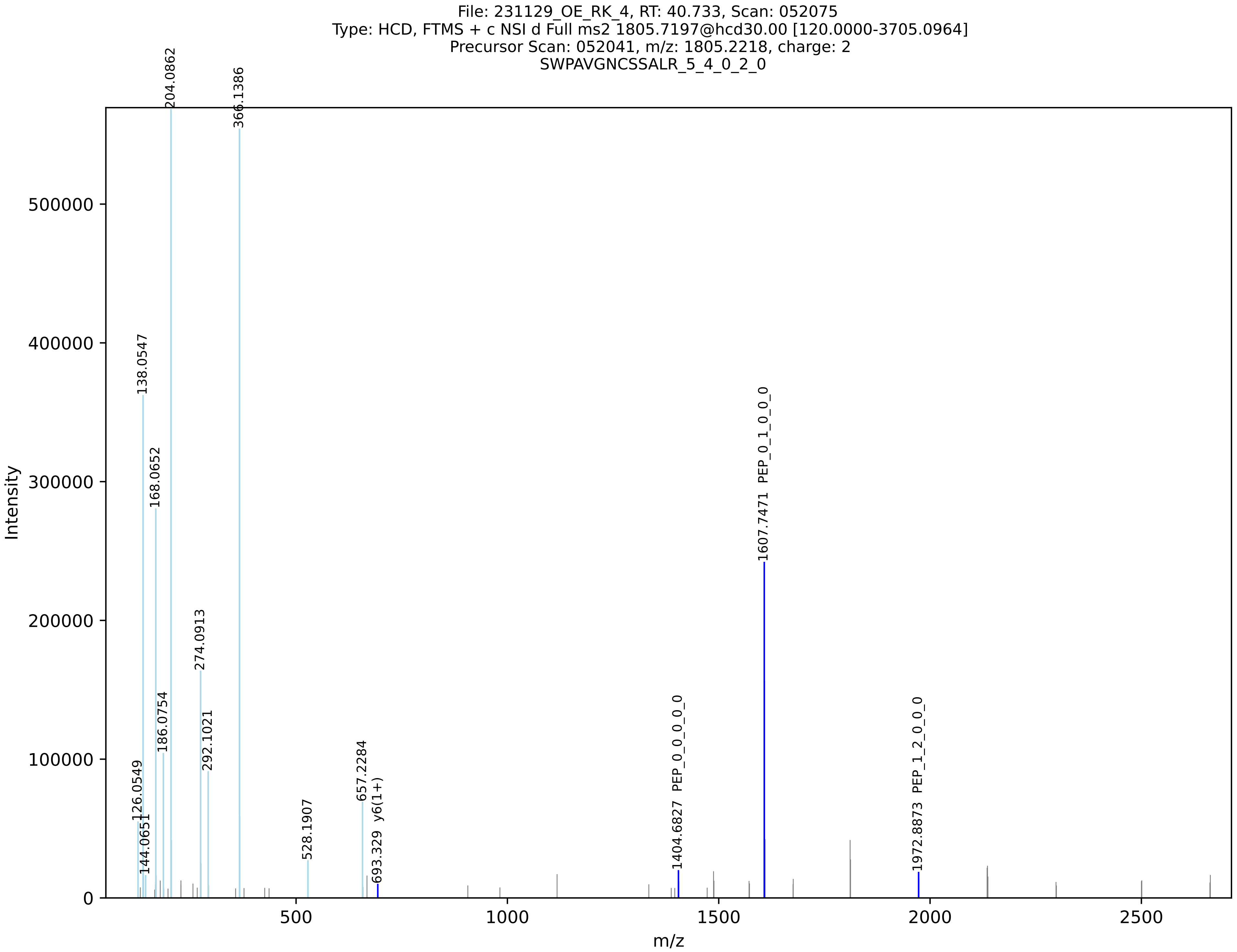

File: 231129_OE_RK_4, RT: 40.733, Scan: 052075
Type: HCD, FTMS + c NSI d Full ms2 1805.7197@hcd30.00 [120.0000-3705.0964]
Precursor Scan: 052041, m/z: 1805.2218, charge: 2
SWPAVGNCSSALR_5_4_0_2_0
Intensity
m/z
204.0862
366.1386
138.0547
168.0652
274.0913
186.0754
292.1021
126.0549
144.0651
528.1907
657.2284
693.329 y6(1+)
1404.6827 PEP_0_0_0_0_0
1607.7471 PEP_0_1_0_0_0
1972.8873 PEP_1_2_0_0_0

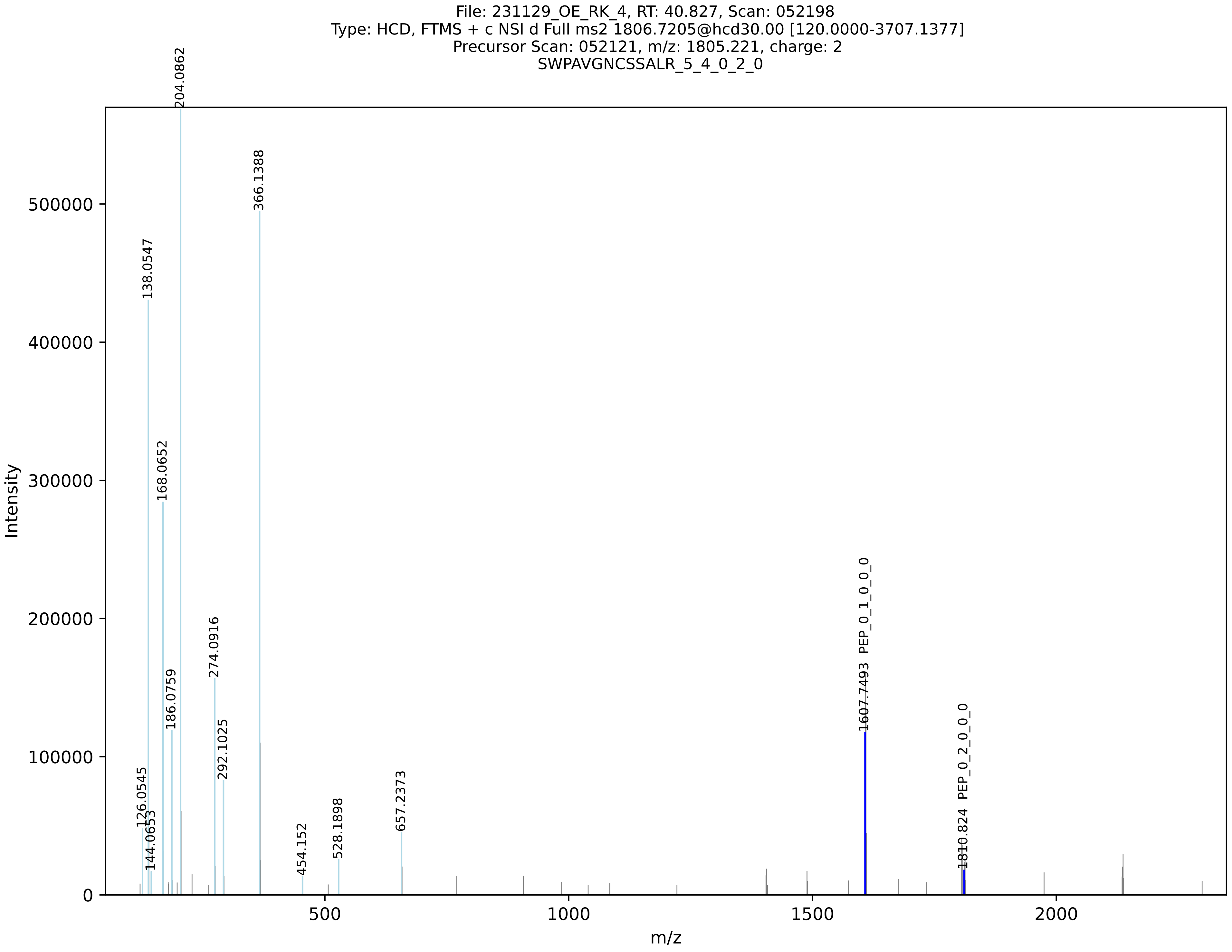
File: 231129_OE_RK_4, RT: 40.827, Scan: 052198
Type: HCD, FTMS + c NSI d Full ms2 1806.7205@hcd30.00 [120.0000-3707.1377]
Precursor Scan: 052121, m/z: 1805.221, charge: 2
SWPAVGNCSSALR_5_4_0_2_0
Intensity
m/z
204.0862
366.1388
138.0547
168.0652
274.0916
186.0759
292.1025
126.0545
144.0653
454.152
528.1898
657.2373
1607.7493 PEP_0_1_0_0_0
1810.824 PEP_0_2_0_0_0
0
100000
200000
300000
400000
500000
500
1000
1500
2000

File: 231129_OE_RK_4, RT: 40.887, Scan: 052274
Type: HCD, FTMS + c NSI d Full ms2 1807.7236@hcd30.00 [120.0000-3709.1843]
Precursor Scan: 052202, m/z: 1805.2222, charge: 2
SWPAVGNCSSALR_5_4_0_2_0

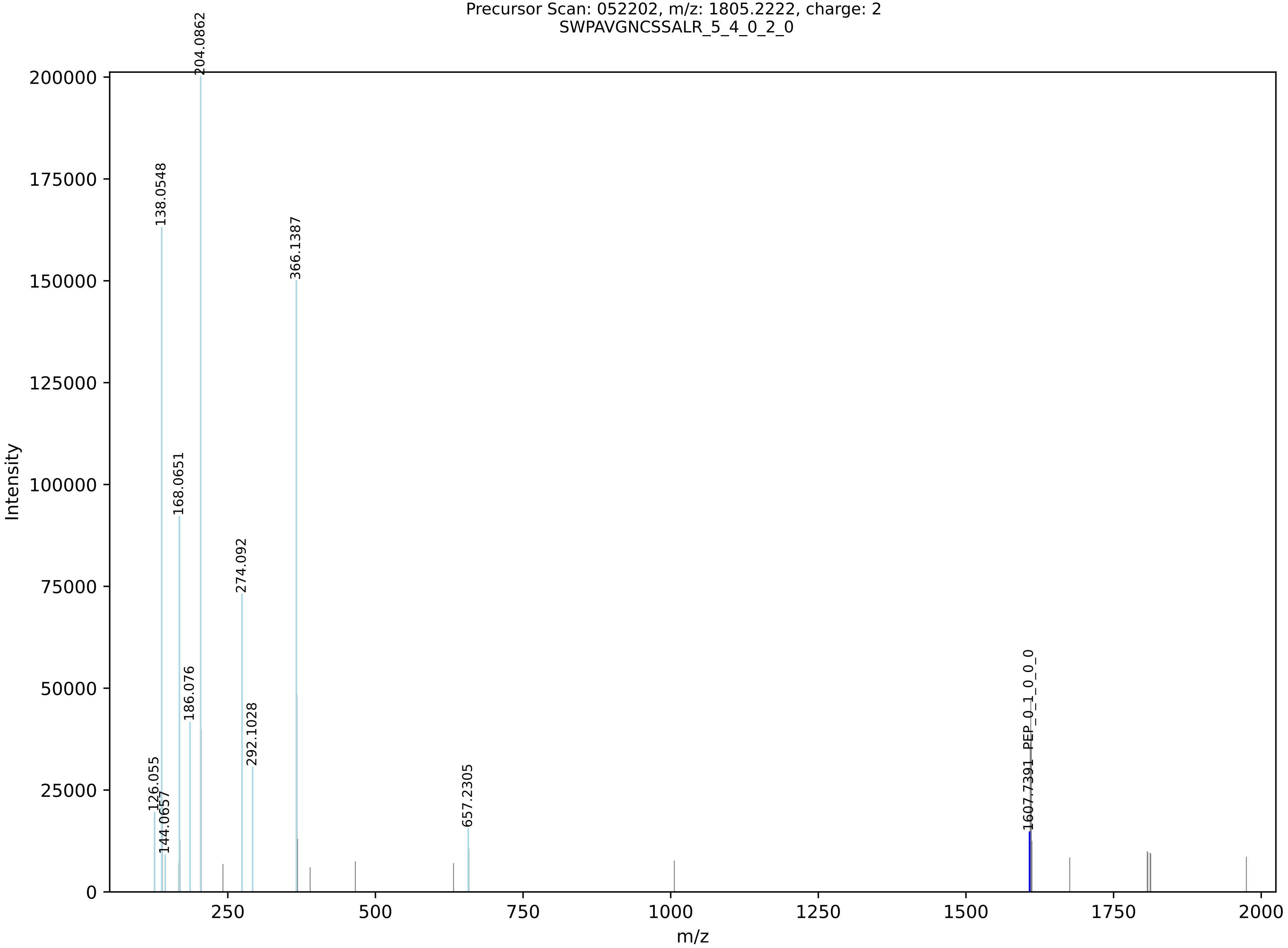

File: 231129_OE_RK_4, RT: 40.923, Scan: 052318
Type: HCD, FTMS + c NSI d Full ms2 1424.9144@hcd30.00 [120.0000-4382.3799]
Precursor Scan: 052285, m/z: 1424.2462, charge: 3
DIVEYYNDSNGSHVLQGR_5_4_0_2_0

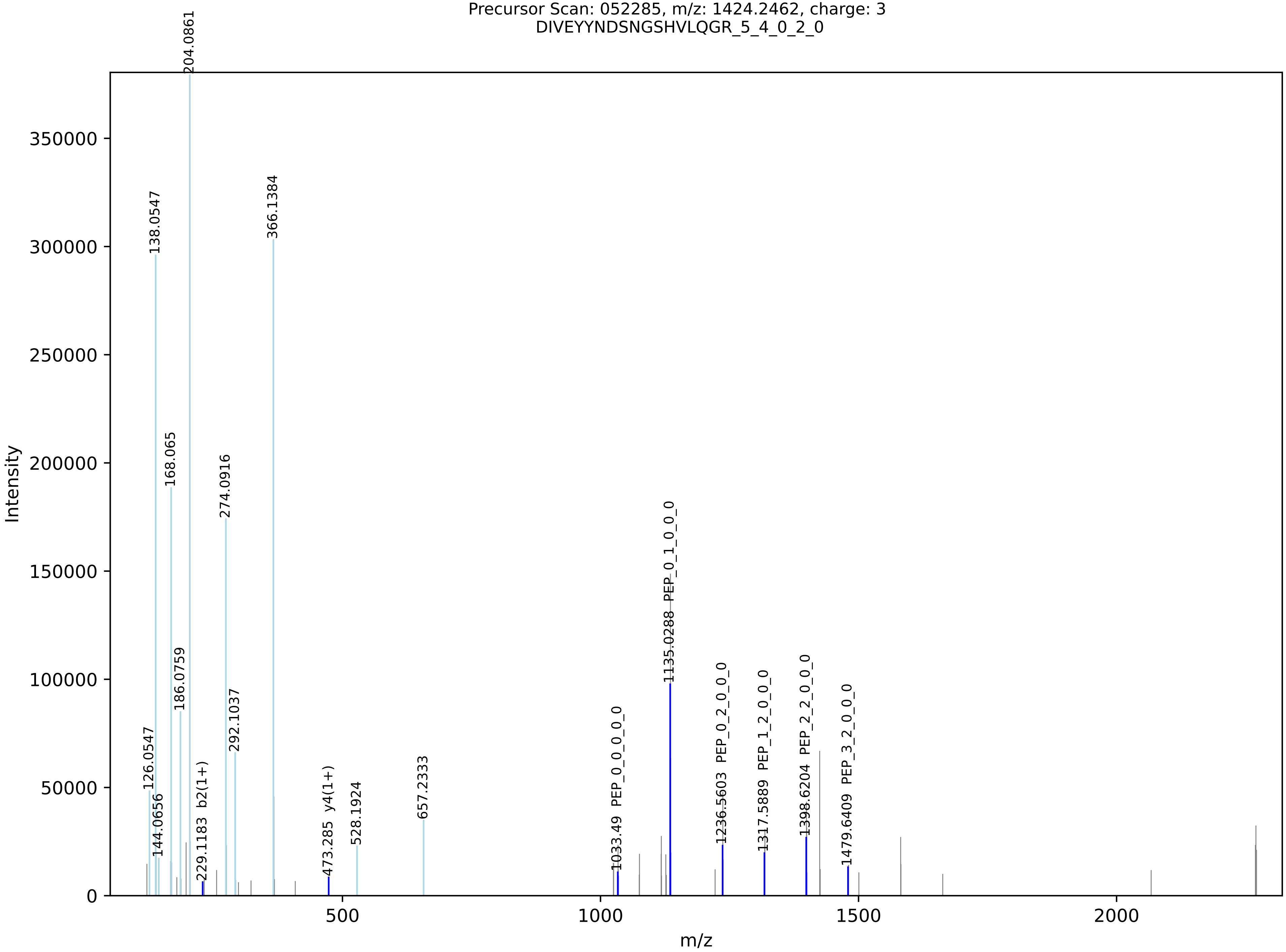

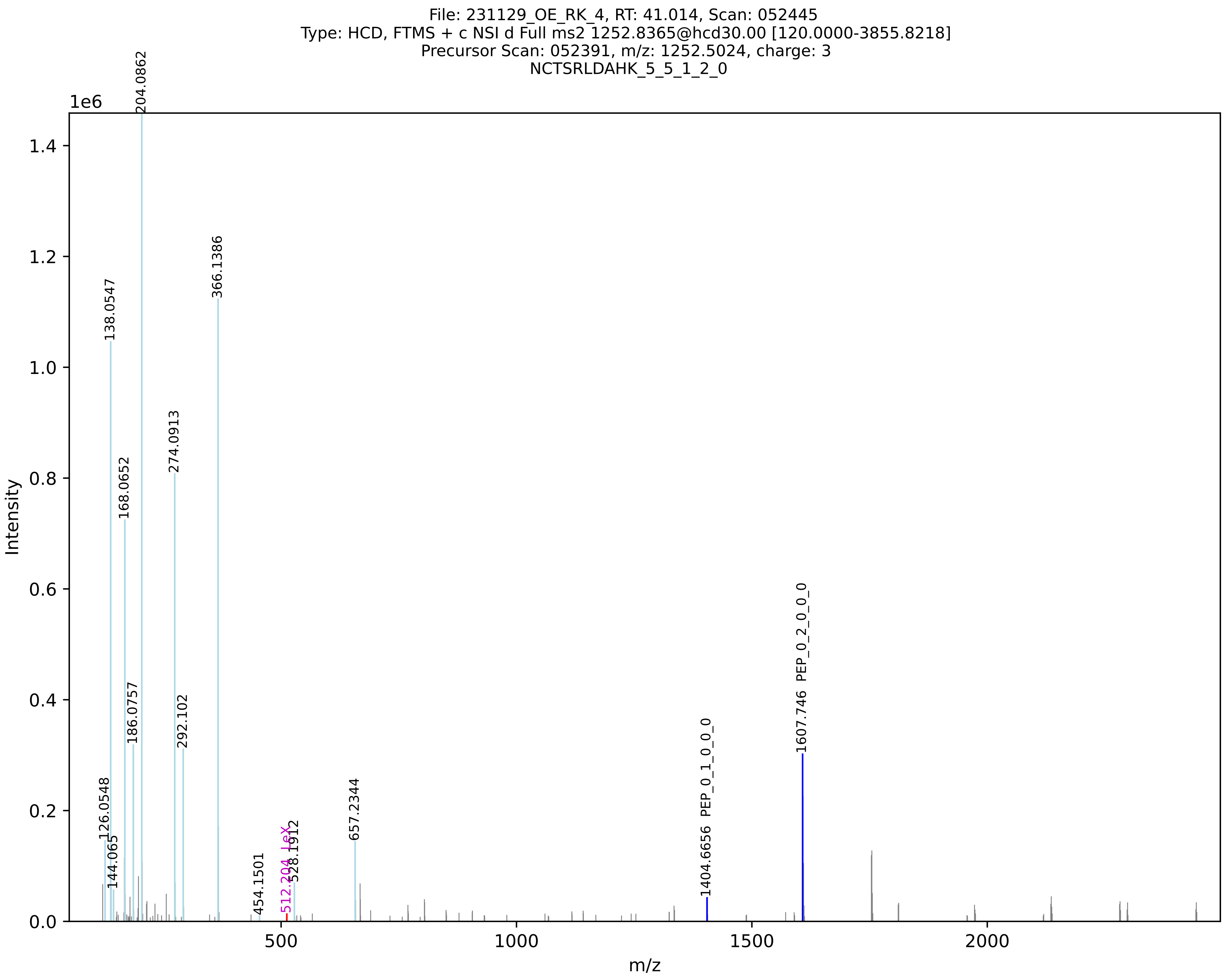

File: 231129_OE_RK_4, RT: 41.014, Scan: 052445
Type: HCD, FTMS + c NSI d Full ms2 1252.8365@hcd30.00 [120.0000-3855.8218]
Precursor Scan: 052391, m/z: 1252.5024, charge: 3
NCTSRLDAHK_5_5_1_2_0
1e6
Intensity
m/z
204.0862
366.1386
138.0547
274.0913
168.0652
186.0757
292.102
126.0548
144.065
454.1501
512.204_LeX
528.1912
657.2344
1404.6656 PEP_0_1_0_0_0
1607.746 PEP_0_2_0_0_0

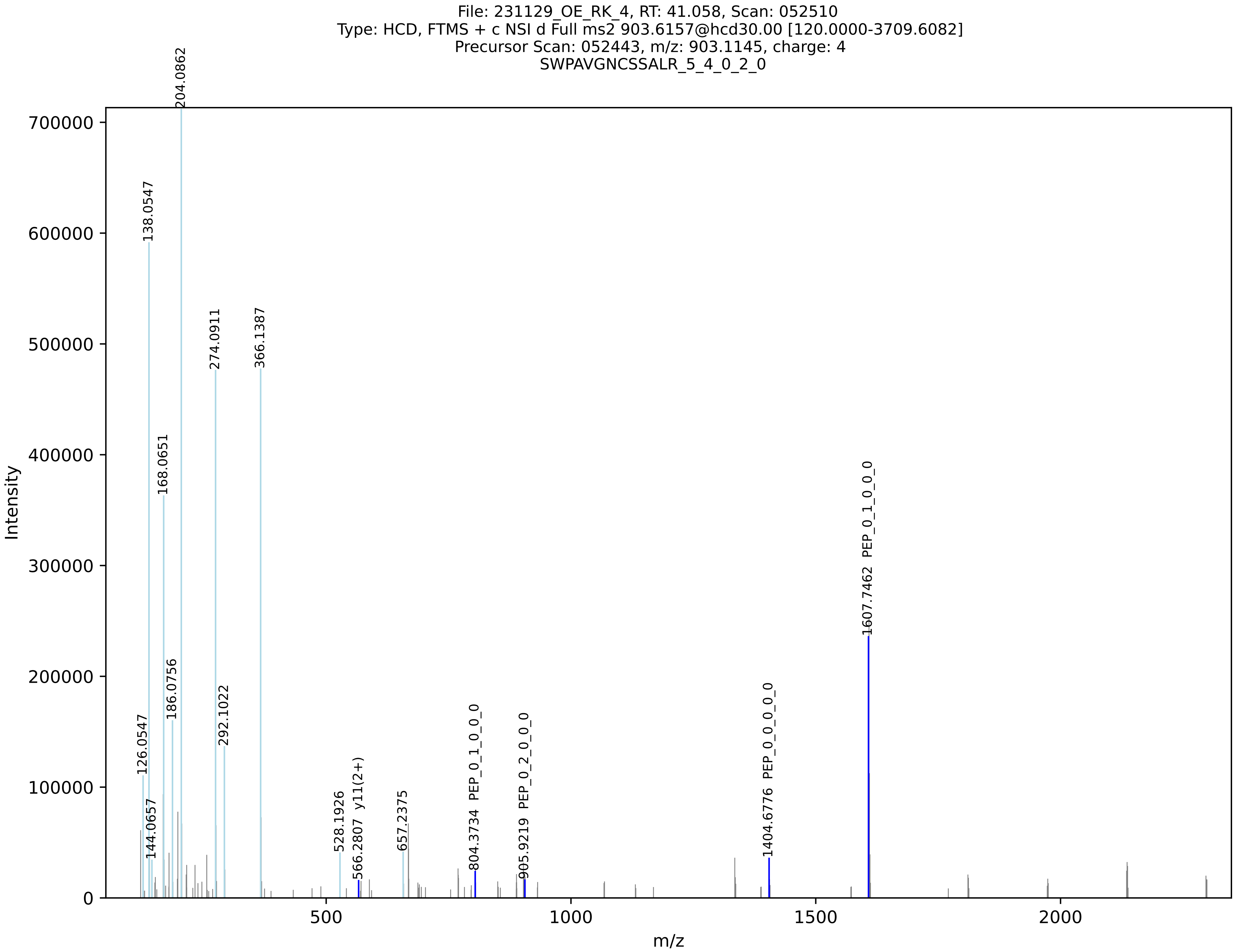
File: 231129_OE_RK_4, RT: 41.058, Scan: 052510
Type: HCD, FTMS + c NSI d Full ms2 903.6157@hcd30.00 [120.0000-3709.6082]
Precursor Scan: 052443, m/z: 903.1145, charge: 4
SWPAVGNCSSALR_5_4_0_2_0
Intensity
m/z
204.0862
138.0547
274.0911
366.1387
168.0651
186.0756
292.1022
126.0547
144.0657
528.1926
566.2807 y11(2+)
657.2375
804.3734 PEP_0_1_0_0_0
905.9219 PEP_0_2_0_0_0
1404.6776 PEP_0_0_0_0_0
1607.7462 PEP_0_1_0_0_0

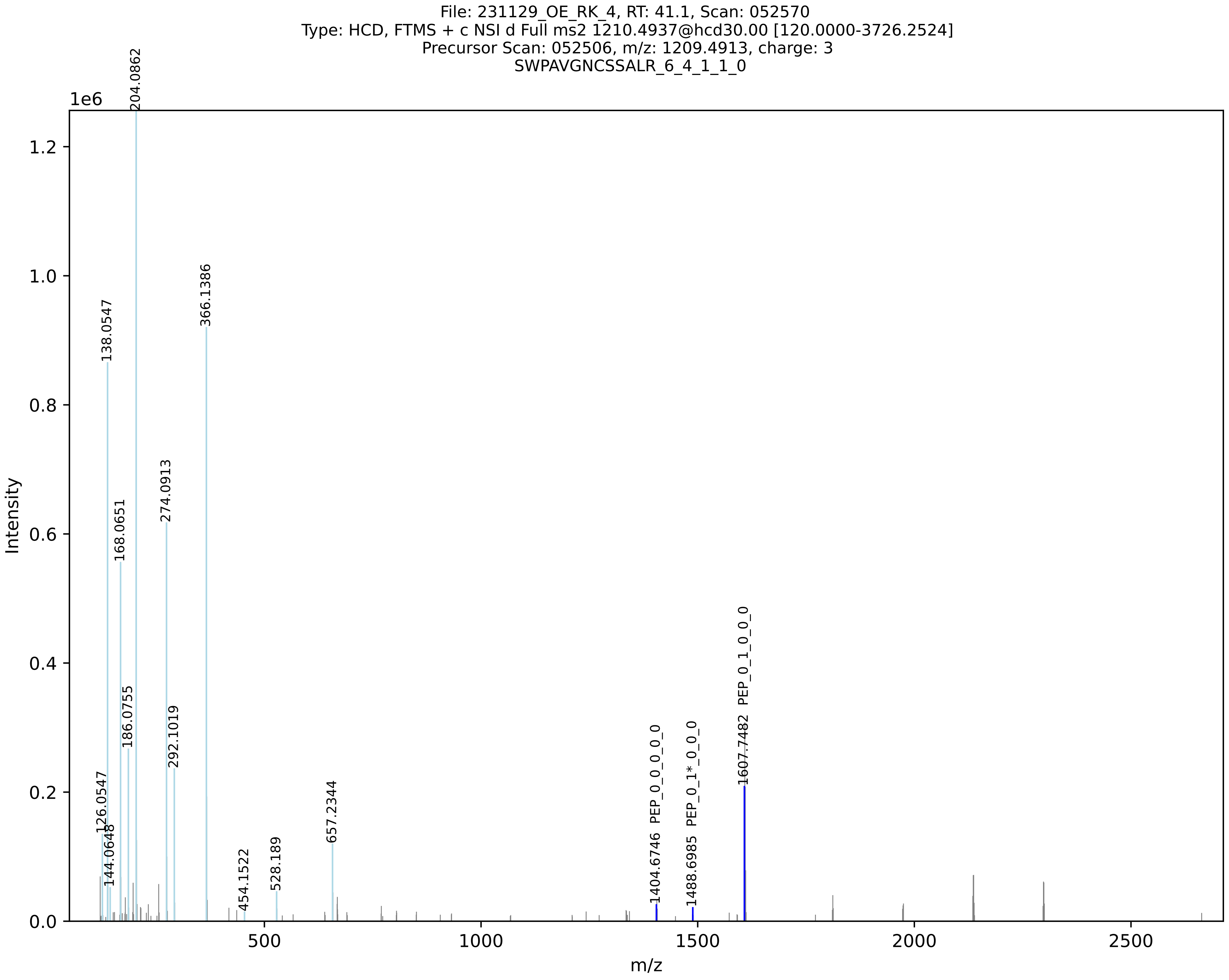
File: 231129_OE_RK_4, RT: 41.1, Scan: 052570
Type: HCD, FTMS + c NSI d Full ms2 1210.4937@hcd30.00 [120.0000-3726.2524]
Precursor Scan: 052506, m/z: 1209.4913, charge: 3
SWPAVGNCSSALR_6_4_1_1_0
1e6
Intensity
m/z
204.0862
138.0547
366.1386
168.0651
274.0913
186.0755
292.1019
126.0547
144.0648
454.1522
528.189
657.2344
1404.6746 PEP_0_0_0_0_0
1488.6985 PEP_0_1*_0_0_0
1607.7482 PEP_0_1_0_0_0

File: 231129_OE_RK_4, RT: 41.191, Scan: 052704
Type: HCD, FTMS + c NSI d Full ms2 1253.1708@hcd30.00 [120.0000-3856.8445]
Precursor Scan: 052627, m/z: 1252.5025, charge: 3
SWPAVGNCSSALR_5_4_1_2_0

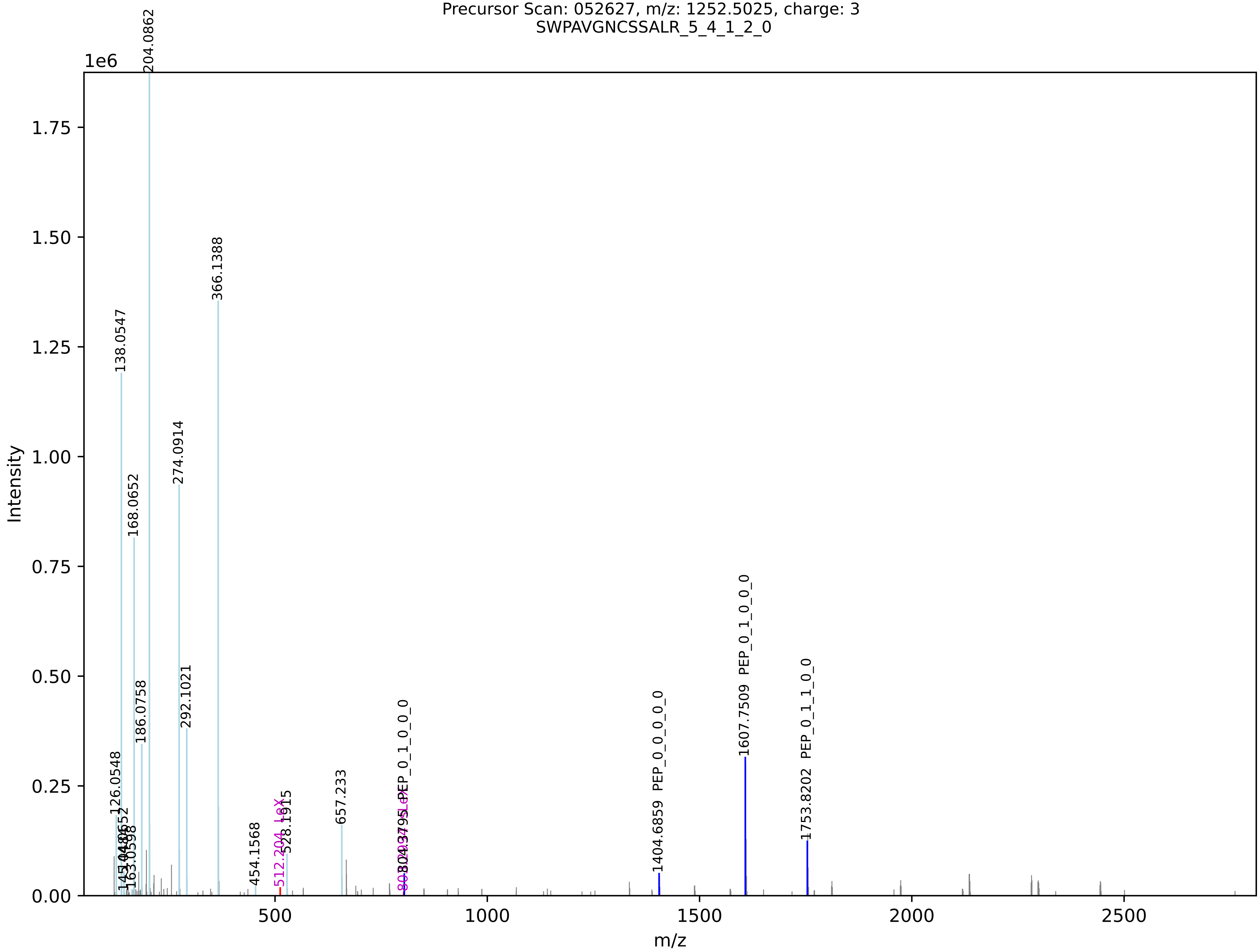

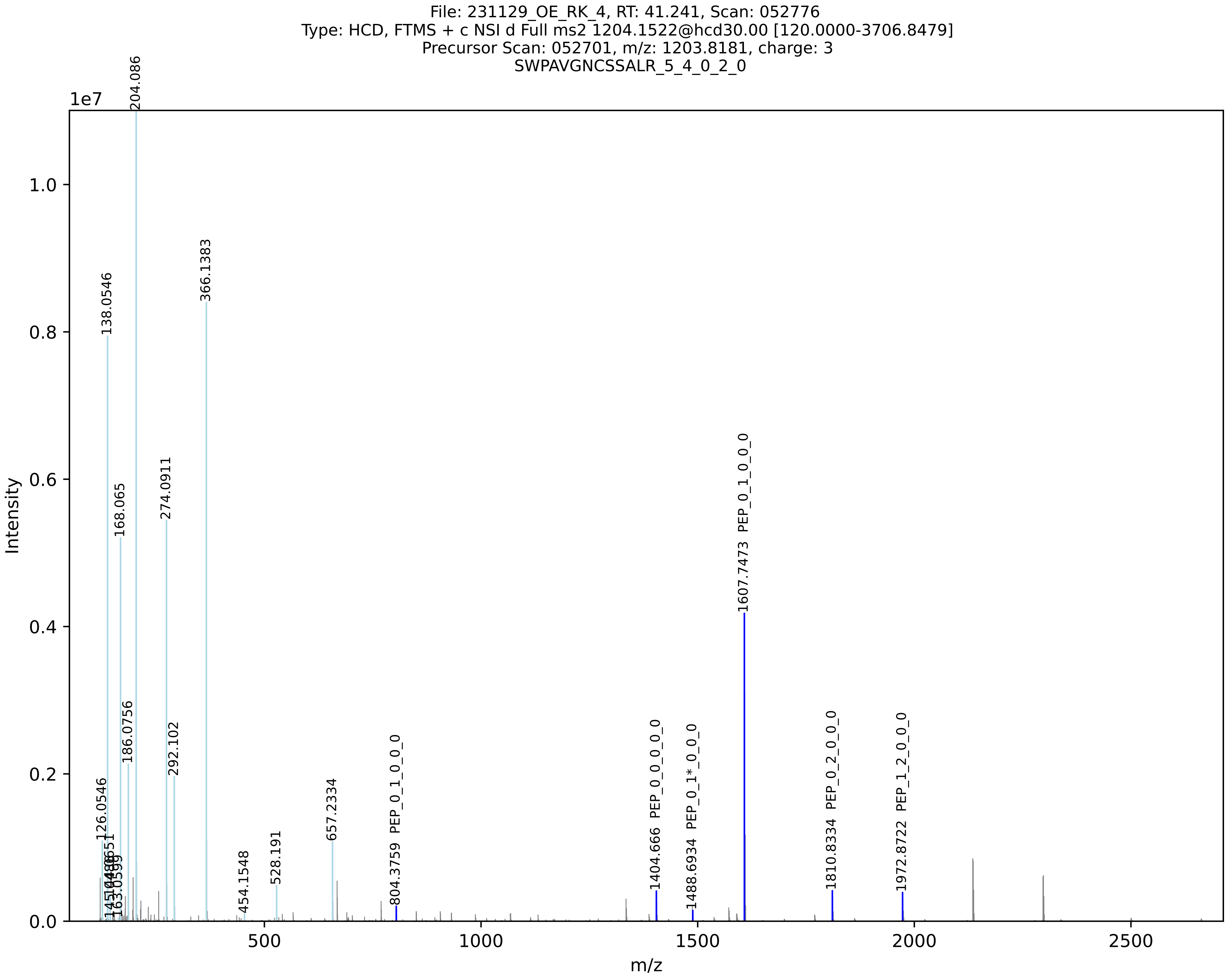
File: 231129_OE_RK_4, RT: 41.241, Scan: 052776
Type: HCD, FTMS + c NSI d Full ms2 1204.1522@hcd30.00 [120.0000-3706.8479]
Precursor Scan: 052701, m/z: 1203.8181, charge: 3
SWPAVGNCSSALR_5_4_0_2_0
1e7
Intensity
m/z
0.0
0.2
0.4
0.6
0.8
1.0
500
1000
1500
2000
2500
204.086
138.0546
366.1383
168.065
274.0911
186.0756
292.102
126.0546
454.1548
528.191
657.2334
804.3759 PEP_0_1_0_0_0
1404.666 PEP_0_0_0_0_0
1488.6934 PEP_0_1*_0_0_0
1607.7473 PEP_0_1_0_0_
1810.8334 PEP_0_2_0_0_0
1972.8722 PEP_1_2_0_0_0

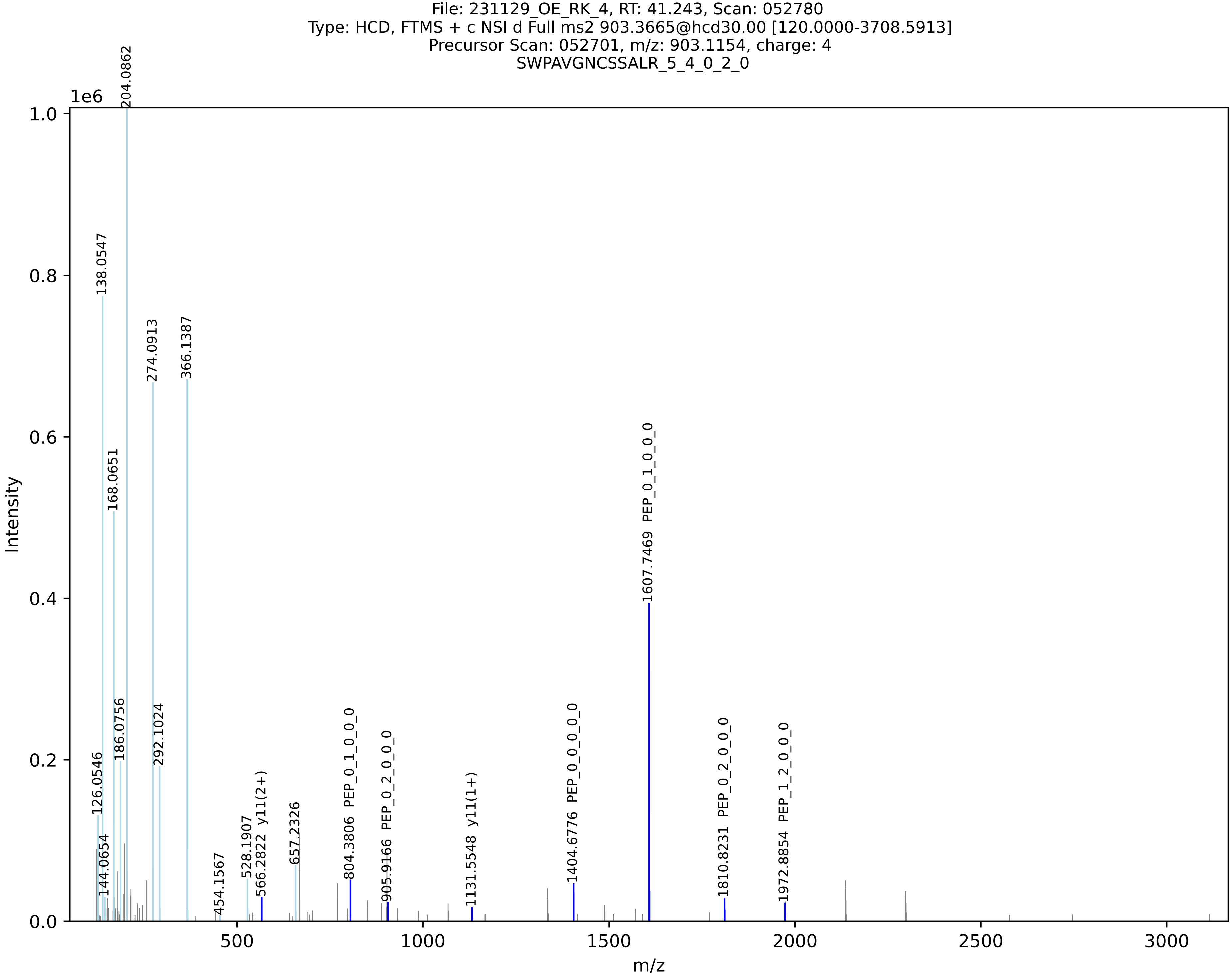

File: 231129_OE_RK_4, RT: 41.243, Scan: 052780
Type: HCD, FTMS + c NSI d Full ms2 903.3665@hcd30.00 [120.0000-3708.5913]
Precursor Scan: 052701, m/z: 903.1154, charge: 4
SWPAVGNCSSALR_5_4_0_2_0
1e6
Intensity
m/z
204.0862
138.0547
274.0913
366.1387
168.0651
186.0756
292.1024
126.0546
144.0654
454.1567
528.1907
566.2822 y11(2+)
657.2326
804.3806 PEP_0_1_0_0_0_
905.9166 PEP_0_2_0_0_0_
1131.5548 y11(1+)
1404.6776 PEP_0_0_0_0_0_
1607.7469 PEP_0_1_0_0_0_
1810.8231 PEP_0_2_0_0_0_
1972.8854 PEP_1_2_0_0_0_

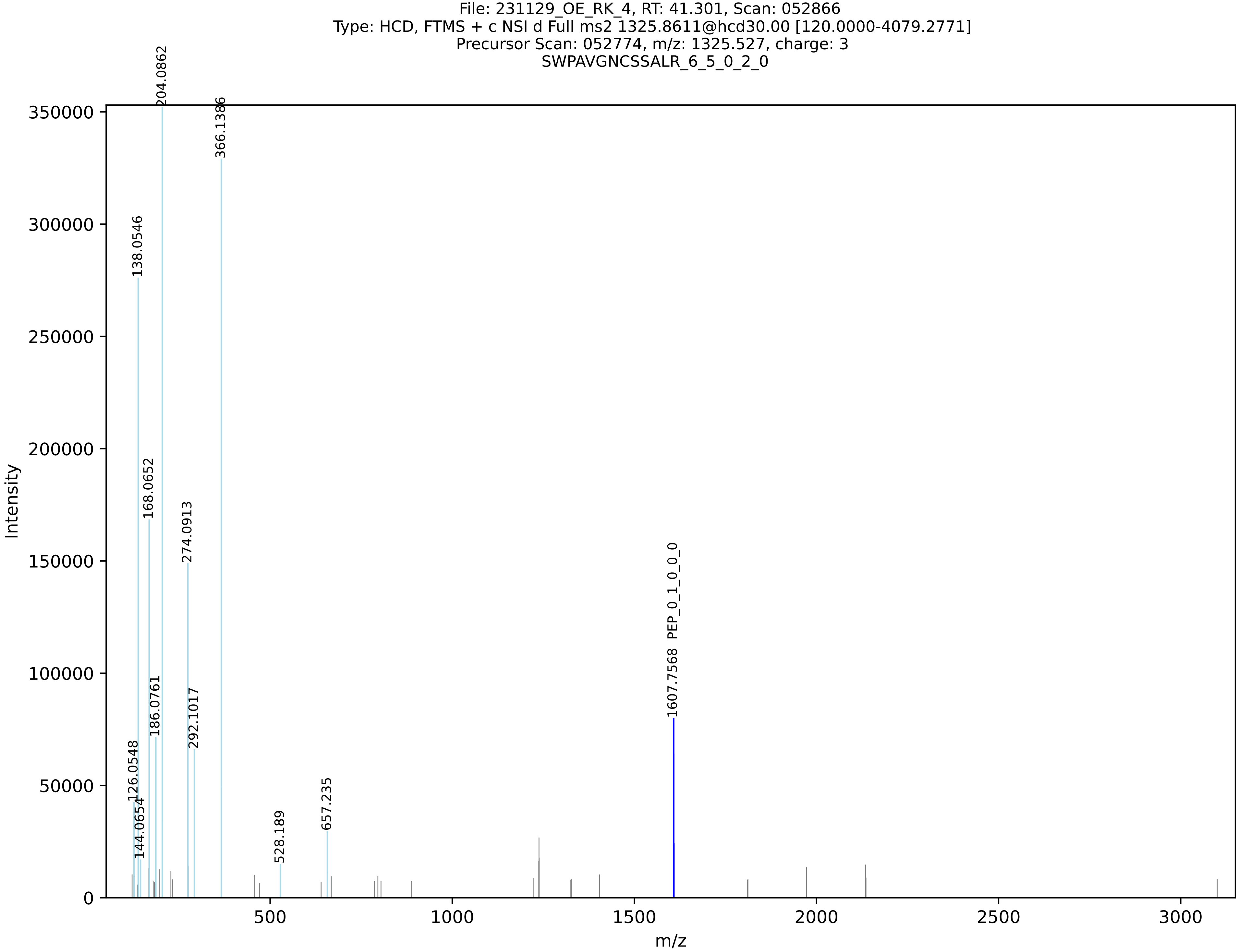
File: 231129_OE_RK_4, RT: 41.301, Scan: 052866
Type: HCD, FTMS + c NSI d Full ms2 1325.8611@hcd30.00 [120.0000-4079.2771]
Precursor Scan: 052774, m/z: 1325.527, charge: 3
SWPAVGNCSSALR_6_5_0_2_0
Intensity
m/z
204.0862
366.1386
138.0546
168.0652
274.0913
186.0761
292.1017
126.0548
144.0654
528.189
657.235
1607.7568 PEP_0_1_0_0_0
350000
300000
250000
200000
150000
100000
50000
0
500
1000
1500
2000
2500
3000

File: 231129_OE_RK_4, RT: 41.326, Scan: 052906
Type: HCD, FTMS + c NSI d Full ms2 1807.7288@hcd30.00 [120.0000-3709.1948]
Precursor Scan: 052701, m/z: 1805.2235, charge: 2
SWPAVGNCSSALR_5_4_0_2_0

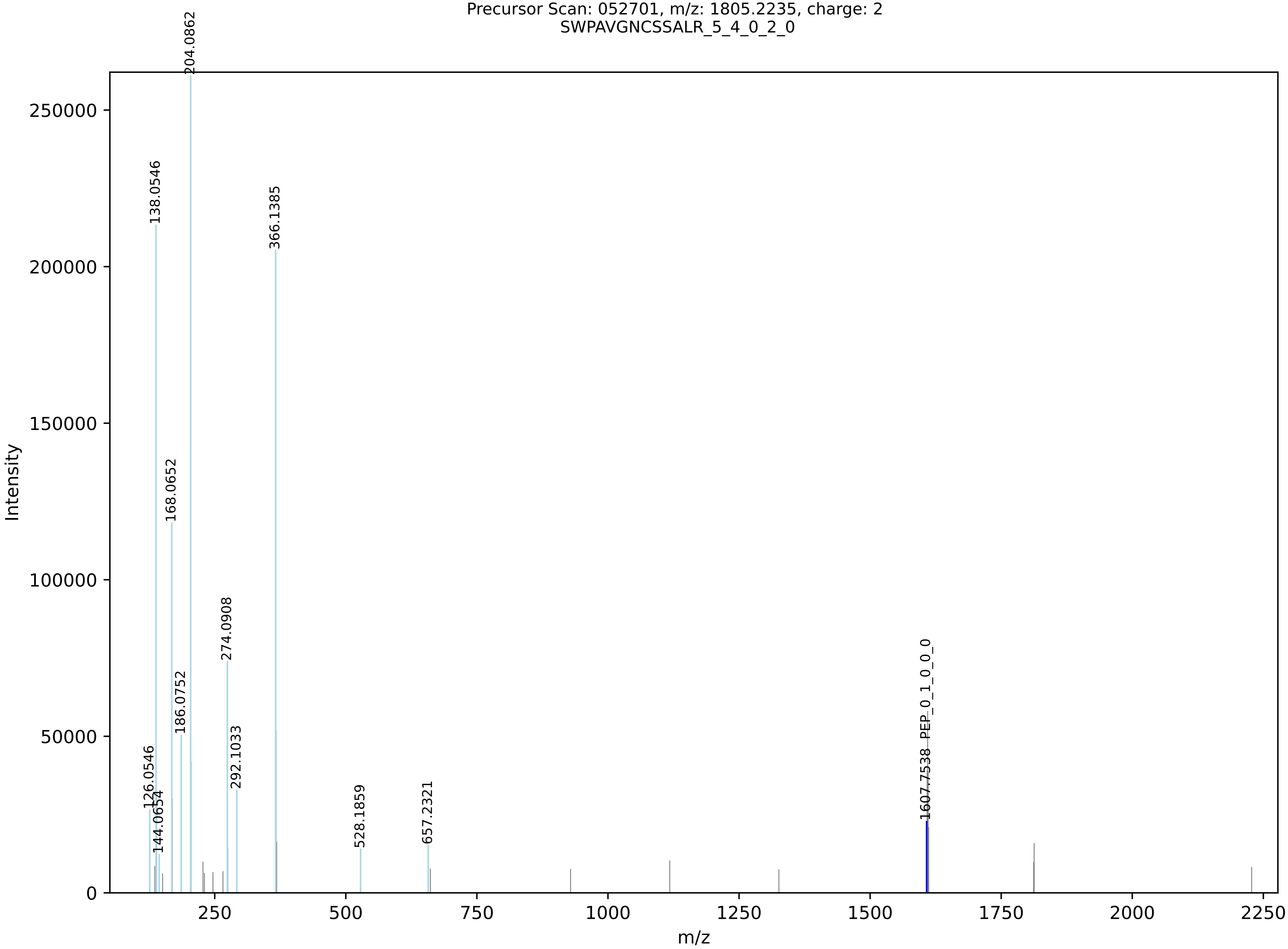

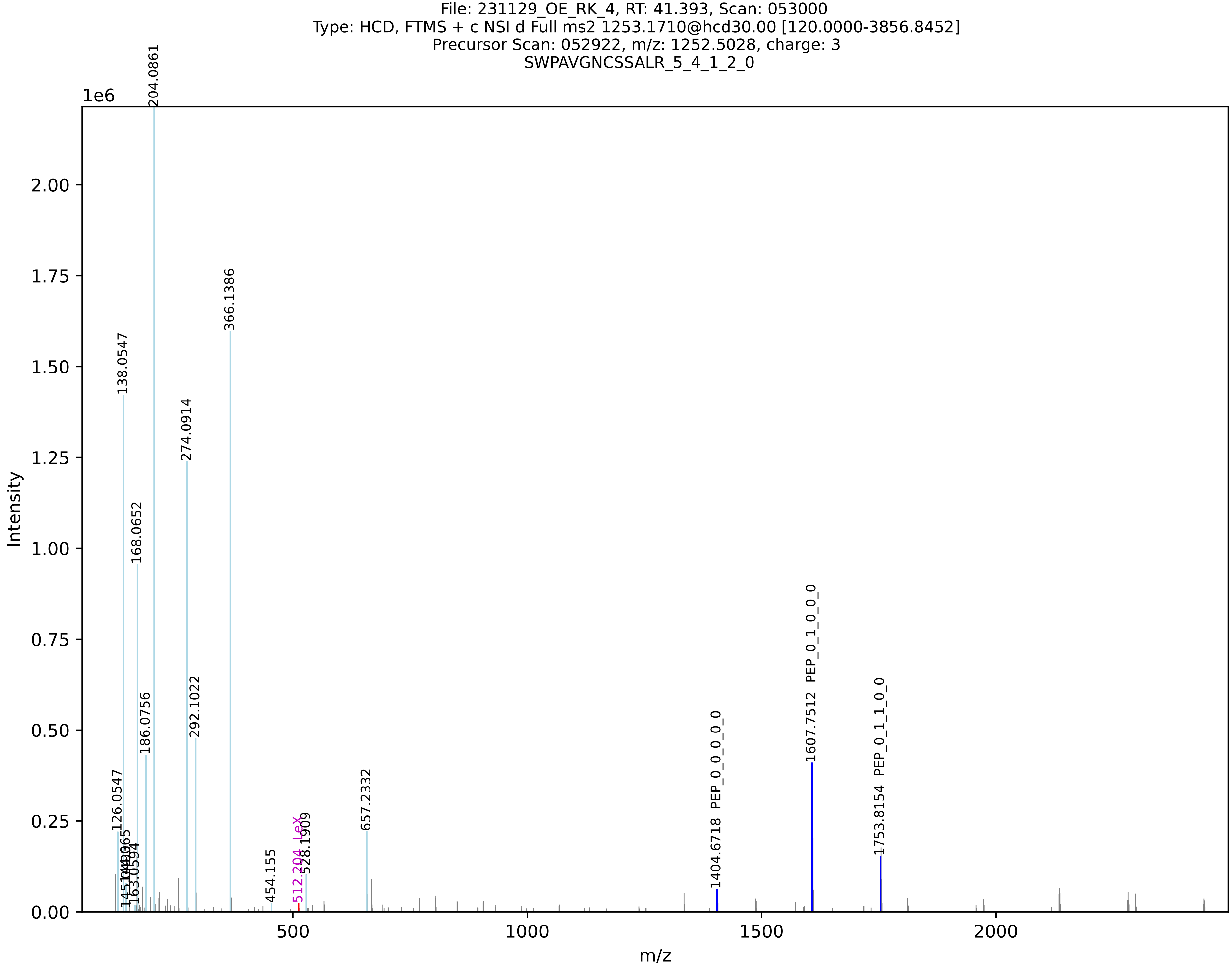
File: 231129_OE_RK_4, RT: 41.393, Scan: 053000
Type: HCD, FTMS + c NSI d Full ms2 1253.1710@hcd30.00 [120.0000-3856.8452]
Precursor Scan: 052922, m/z: 1252.5028, charge: 3
SWPAVGNCSSALR_5_4_1_2_0
1e6
Intensity
m/z
204.0861
366.1386
138.0547
274.0914
168.0652
186.0756
292.1022
126.0547
163.0594
454.155
512.204_LeX
528.1909
657.2332
1404.6718 PEP_0_0_0_0_0
1607.7512 PEP_0_1_0_0_0
1753.8154 PEP_0_1_1_0_0

File: 231129_OE_RK_4, RT: 41.404, Scan: 053018
Type: HCD, FTMS + c NSI d Full ms2 1325.5260@hcd30.00 [120.0000-4078.2515]
Precursor Scan: 052922, m/z: 1325.526, charge: 3
SWPAVGNCSSALR_6_5_0_2_0

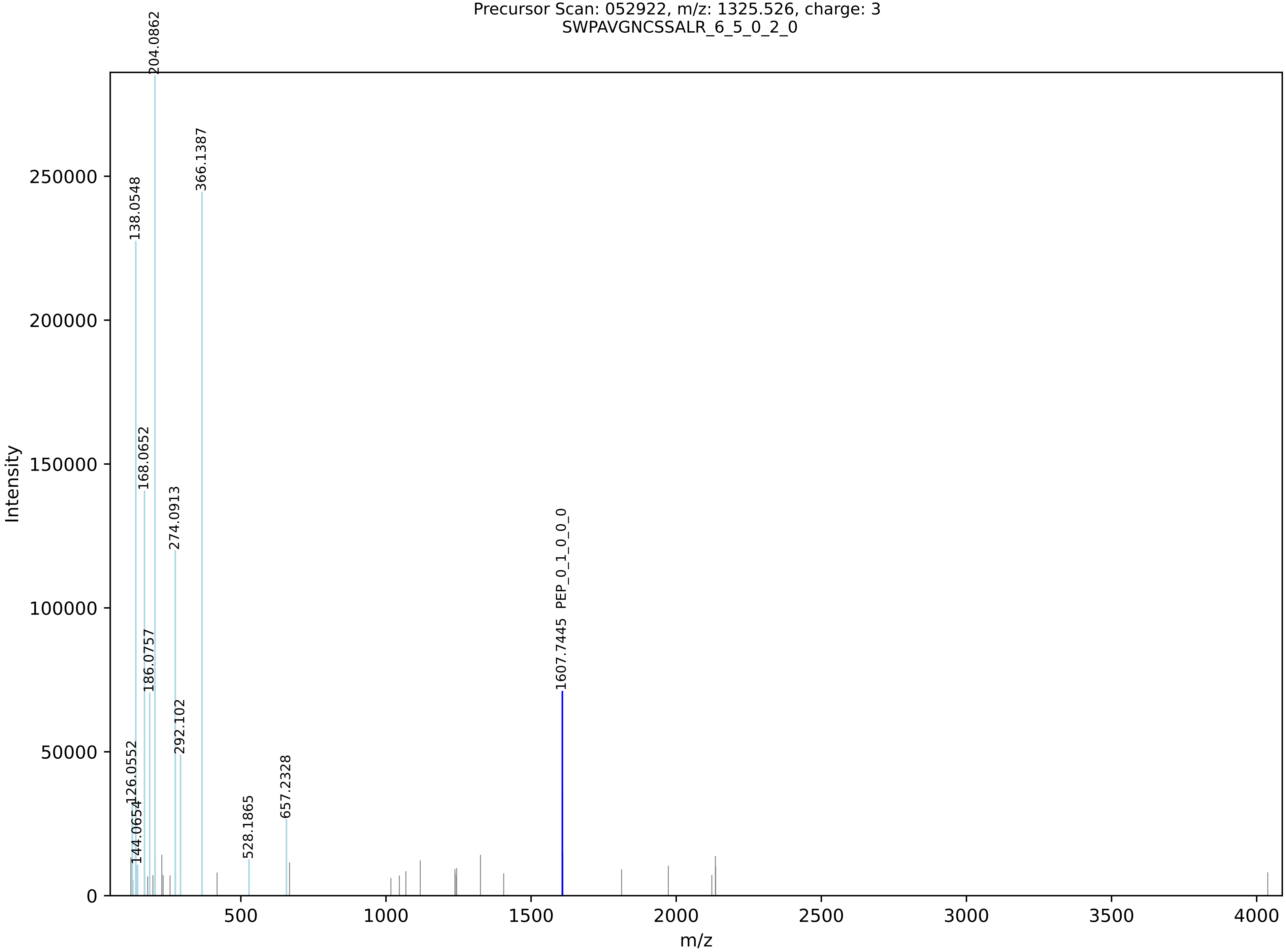

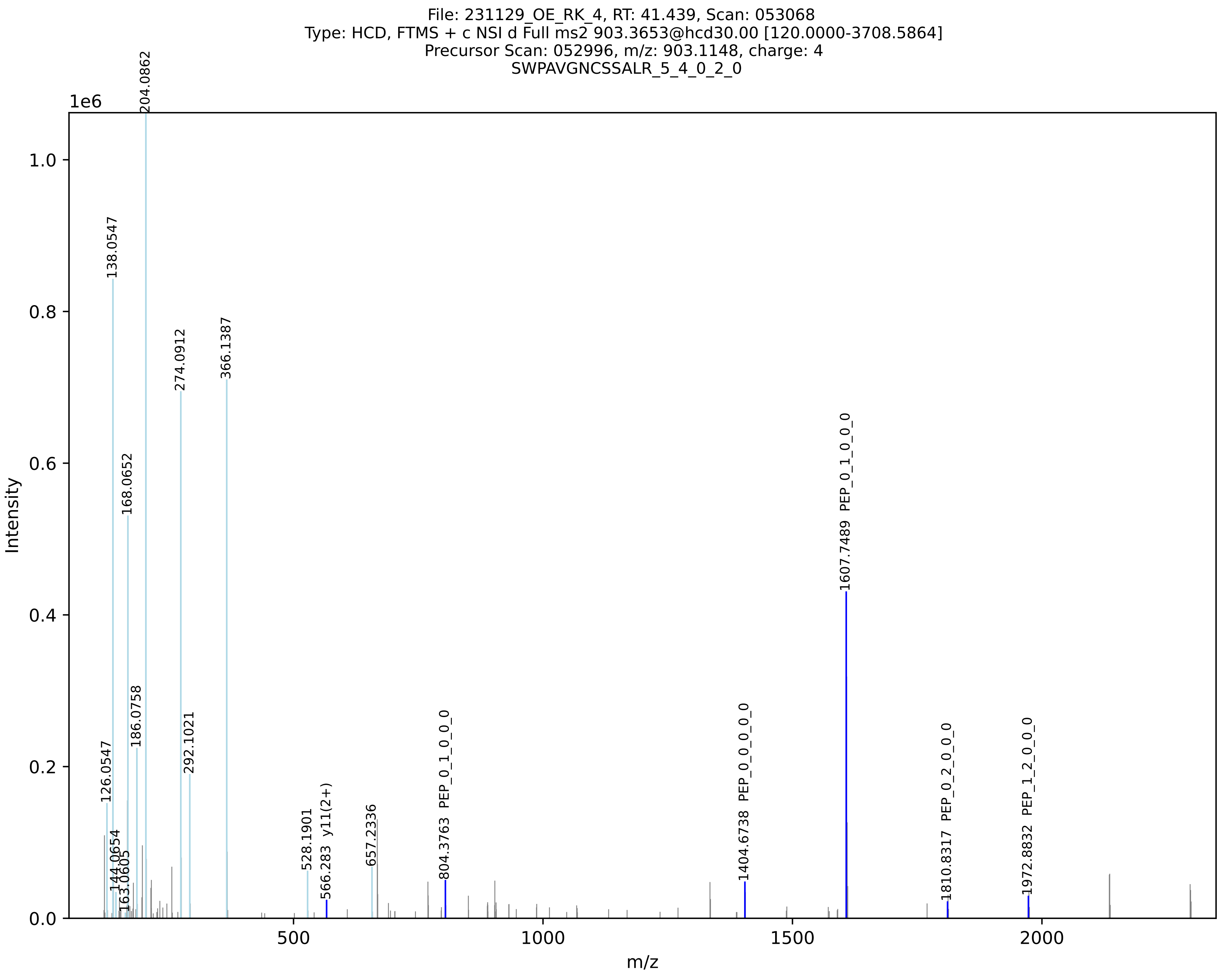

File: 231129_OE_RK_4, RT: 41.439, Scan: 053068
Type: HCD, FTMS + c NSI d Full ms2 903.3653@hcd30.00 [120.0000-3708.5864]
Precursor Scan: 052996, m/z: 903.1148, charge: 4
SWPAVGNCSSALR_5_4_0_2_0
1e6
Intensity
m/z
204.0862
138.0547
168.0652
274.0912
366.1387
186.0758
292.1021
126.0547
144.0654
163.0605
528.1901
566.283 y11(2+)
657.2336
804.3763 PEP_0_1_0_0_0
1404.6738 PEP_0_0_0_0_0
1607.7489 PEP_0_1_0_0_0
1810.8317 PEP_0_2_0_0_0
1972.8832 PEP_1_2_0_0_0

File: 231129_OE_RK_4, RT: 41.489, Scan: 053138
Type: HCD, FTMS + c NSI d Full ms2 1190.0018@hcd30.00 [120.0000-4878.0635]
Precursor Scan: 053063, m/z: 1189.2498, charge: 4
LLRCQANGTWNIGDERPSCR_5_4_1_2_0

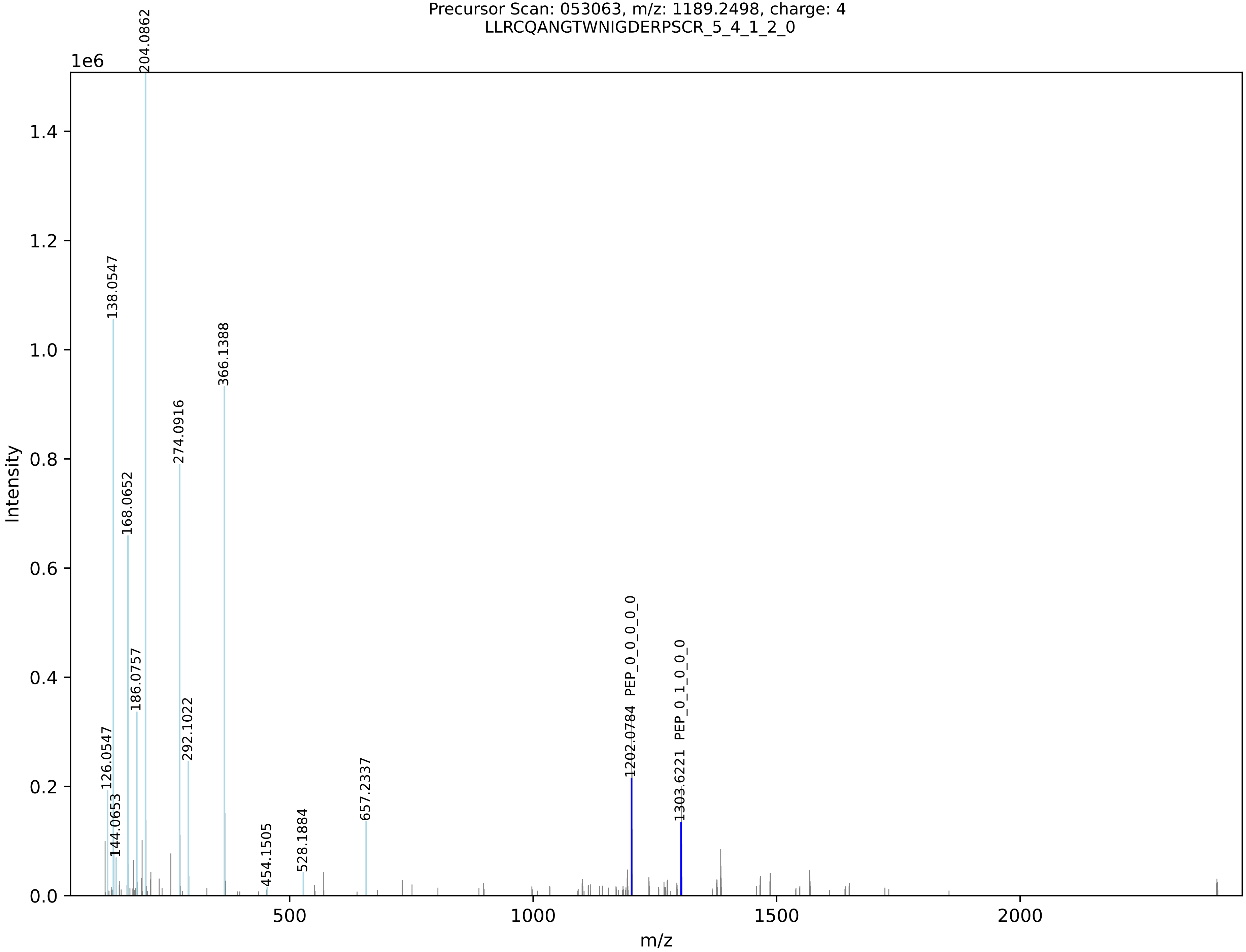

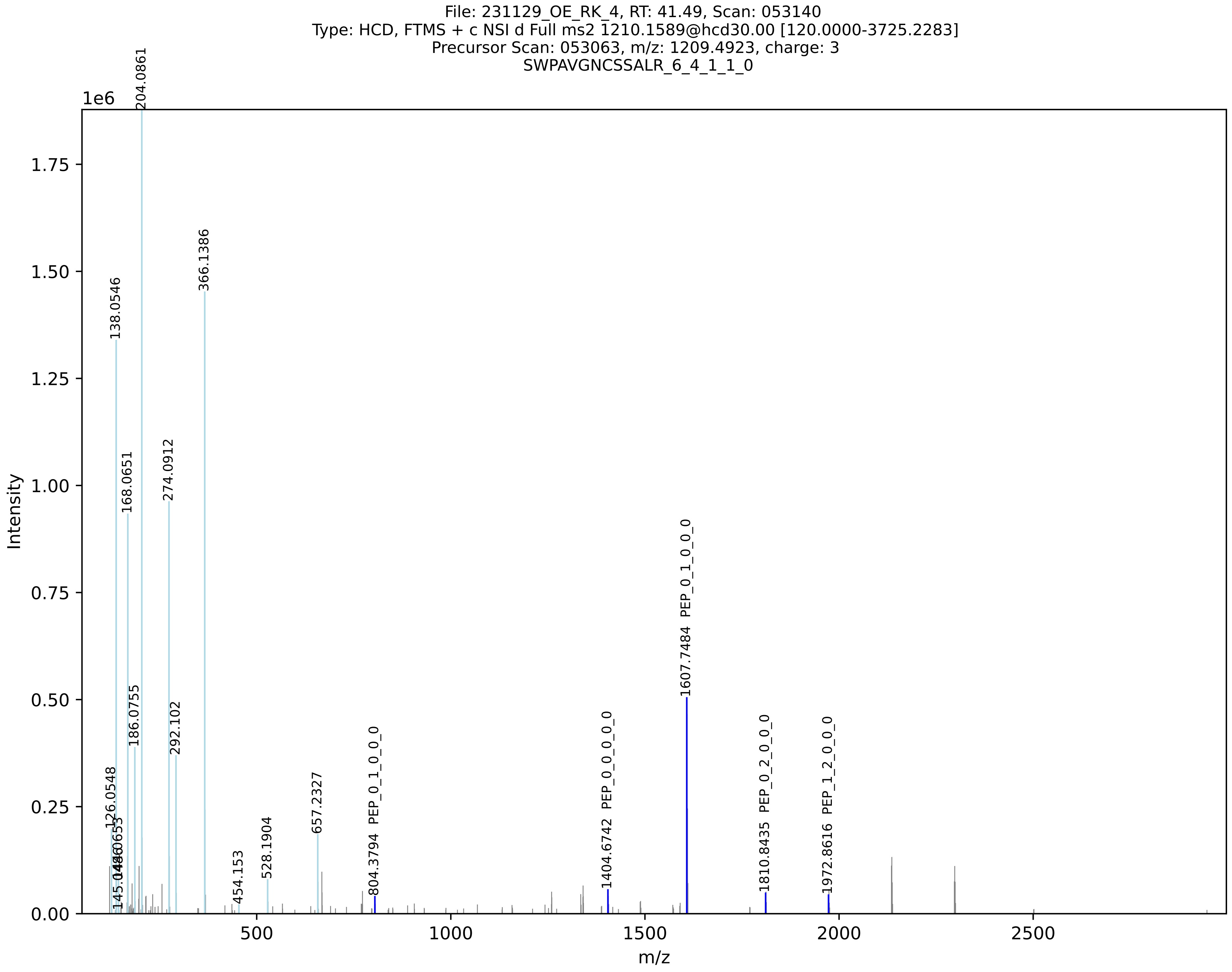

File: 231129_OE_RK_4, RT: 41.49, Scan: 053140
Type: HCD, FTMS + c NSI d Full ms2 1210.1589@hcd30.00 [120.0000-3725.2283]
Precursor Scan: 053063, m/z: 1209.4923, charge: 3
SWPAVGNCSSALR_6_4_1_1_0
1e6
Intensity
m/z
204.0861
366.1386
138.0546
168.0651
274.0912
186.0755
292.102
126.0548
657.2327
528.1904
454.153
804.3794 PEP_0_1_0_0_0
1404.6742 PEP_0_0_0_0_0
1607.7484 PEP_0_1_0_0_0
1810.8435 PEP_0_2_0_0_0
1972.8616 PEP_1_2_0_0_0

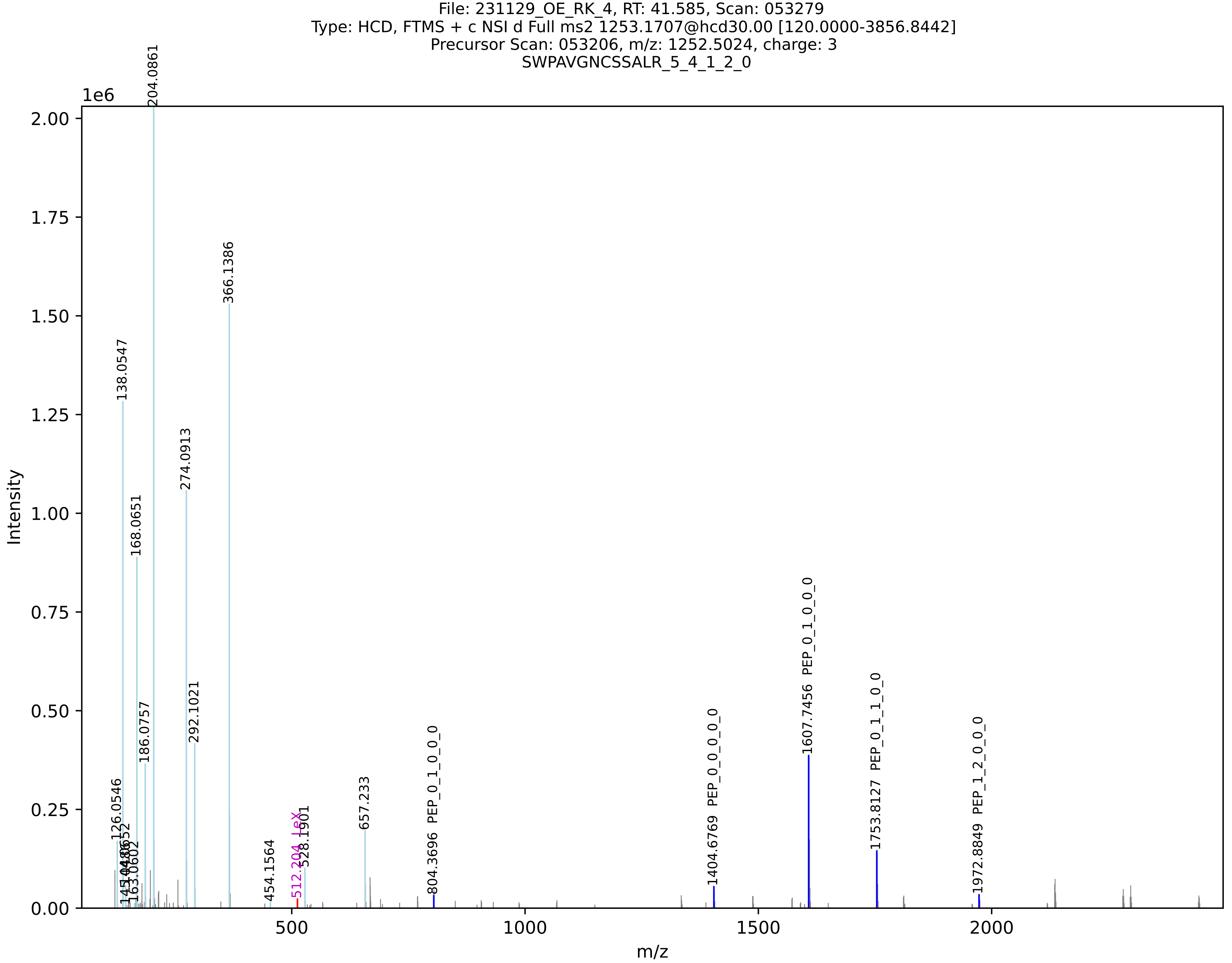
File: 231129_OE_RK_4, RT: 41.585, Scan: 053279
Type: HCD, FTMS + c NSI d Full ms2 1253.1707@hcd30.00 [120.0000-3856.8442]
Precursor Scan: 053206, m/z: 1252.5024, charge: 3
SWPAVGNCSSALR_5_4_1_2_0
1e6
Intensity
m/z
204.0861
366.1386
138.0547
274.0913
168.0651
292.1021
186.0757
126.0546
163.0602
454.1564
512.204_LeX
528.1901
657.233
804.3696 PEP_0_1_0_0_0
1404.6769 PEP_0_0_0_0_0
1607.7456 PEP_0_1_0_0_0
1753.8127 PEP_0_1_1_0_0
1972.8849 PEP_1_2_0_0_0

File: 231129_OE_RK_4, RT: 41.634, Scan: 053350
Type: HCD, FTMS + c NSI d Full ms2 1204.1521@hcd30.00 [120.0000-3706.8477]
Precursor Scan: 053275, m/z: 1203.8179, charge: 3
SWPAVGNCSSALR_5_4_0_2_0

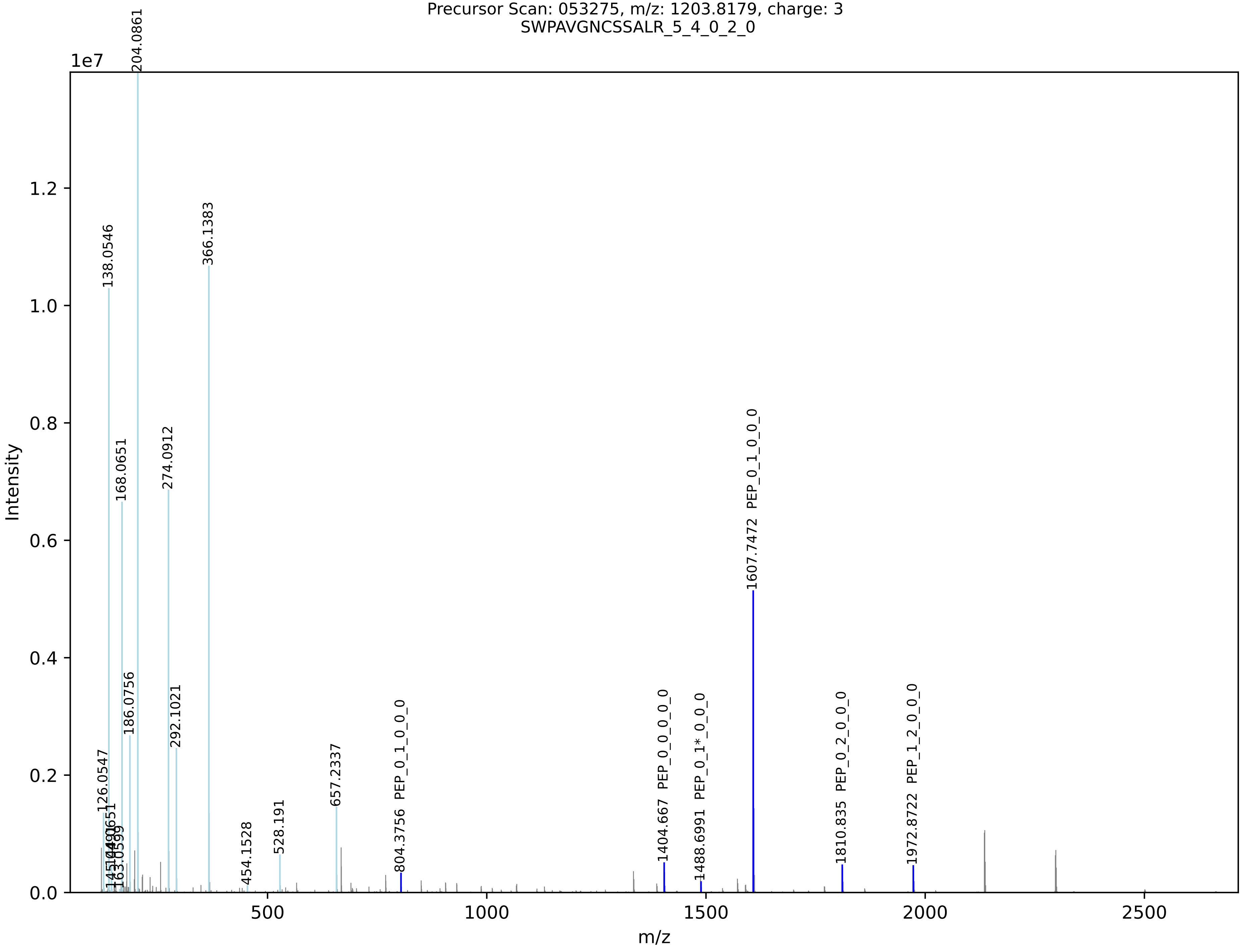

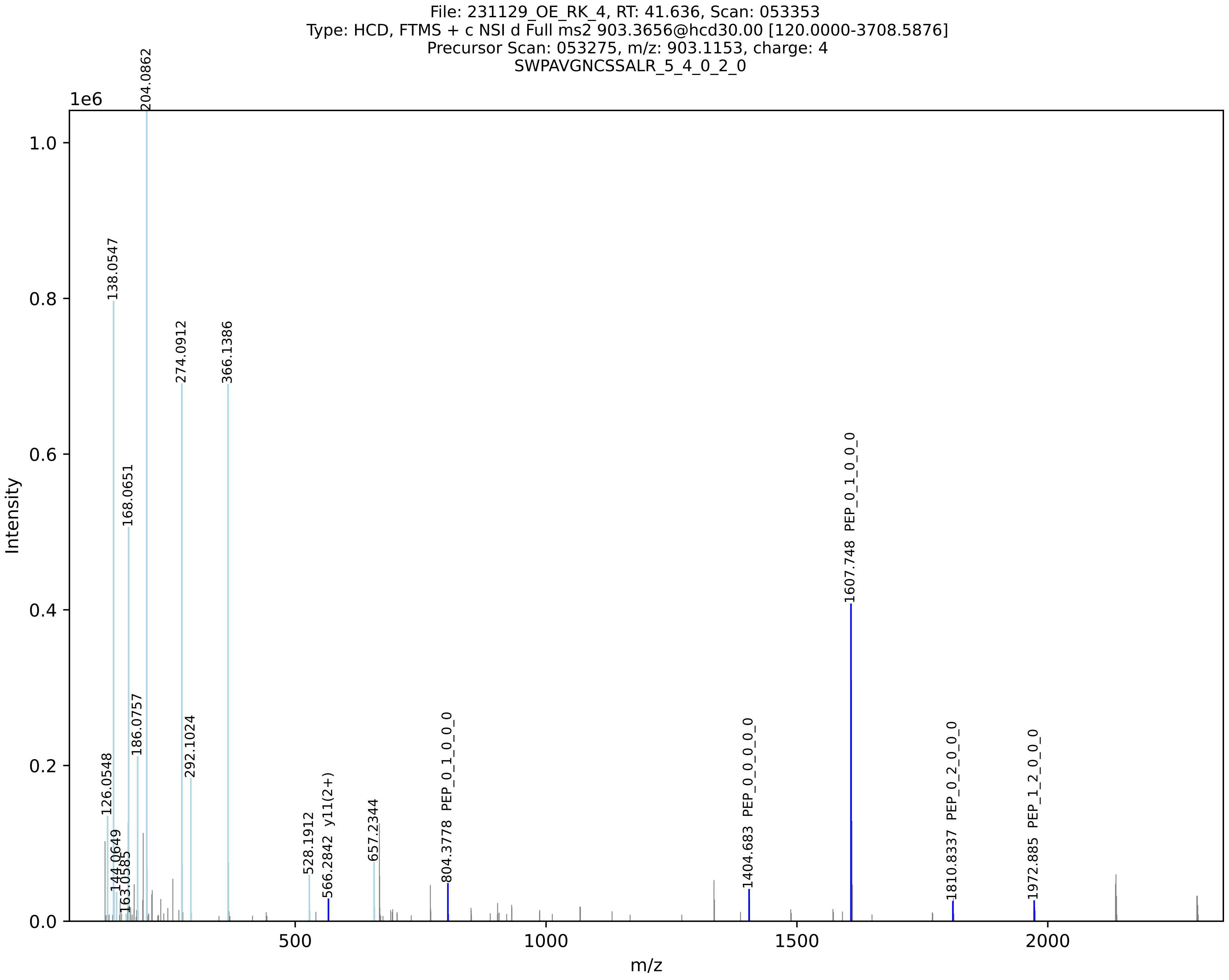

File: 231129_OE_RK_4, RT: 41.636, Scan: 053353
Type: HCD, FTMS + c NSI d Full ms2 903.3656@hcd30.00 [120.0000-3708.5876]
Precursor Scan: 053275, m/z: 903.1153, charge: 4
SWPAVGNCSSALR_5_4_0_2_0
1e6
Intensity
m/z
204.0862
138.0547
168.0651
274.0912
366.1386
186.0757
292.1024
126.0548
144.0649
163.0585
528.1912
566.2842 y11(2+)
657.2344
804.3778 PEP_0_1_0_0_0
1404.683 PEP_0_0_0_0_0
1607.748 PEP_0_1_0_0_0
1810.8337 PEP_0_2_0_0_0
1972.885 PEP_1_2_0_0_0

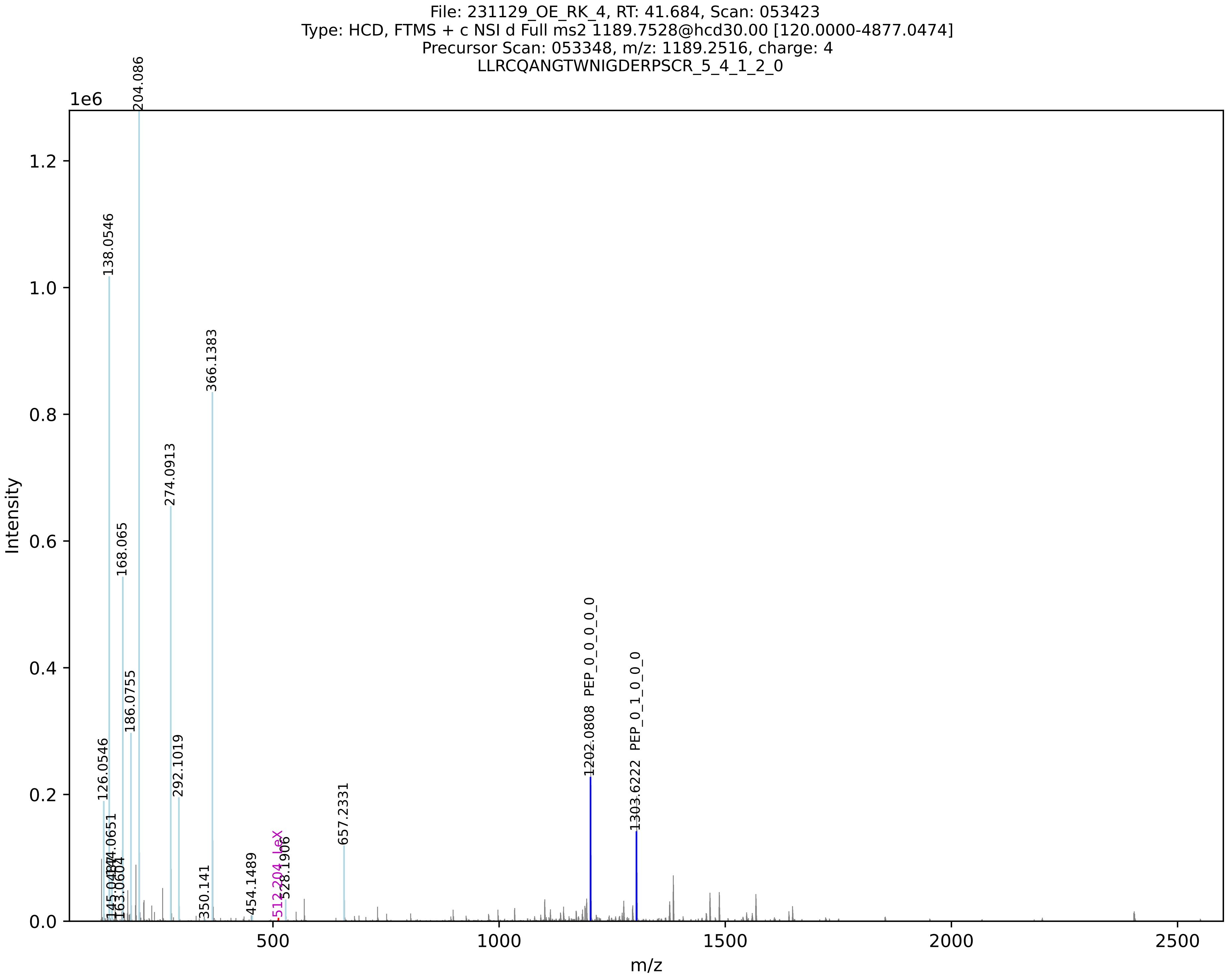
File: 231129_OE_RK_4, RT: 41.684, Scan: 053423
Type: HCD, FTMS + c NSI d Full ms2 1189.7528@hcd30.00 [120.0000-4877.0474]
Precursor Scan: 053348, m/z: 1189.2516, charge: 4
LLRCQANGTWNIGDERPSCR_5_4_1_2_0
1e6
Intensity
m/z
204.086
138.0546
366.1383
274.0913
168.065
186.0755
292.1019
126.0546
657.2331
1202.0808 PEP_0_0_0_0_0
1303.6222 PEP_0_1_0_0_0
350.141
454.1489
528.1906
512.204 LeX
163.0604
0.0
0.2
0.4
0.6
0.8
1.0
1.2
500
1000
1500
2000
2500

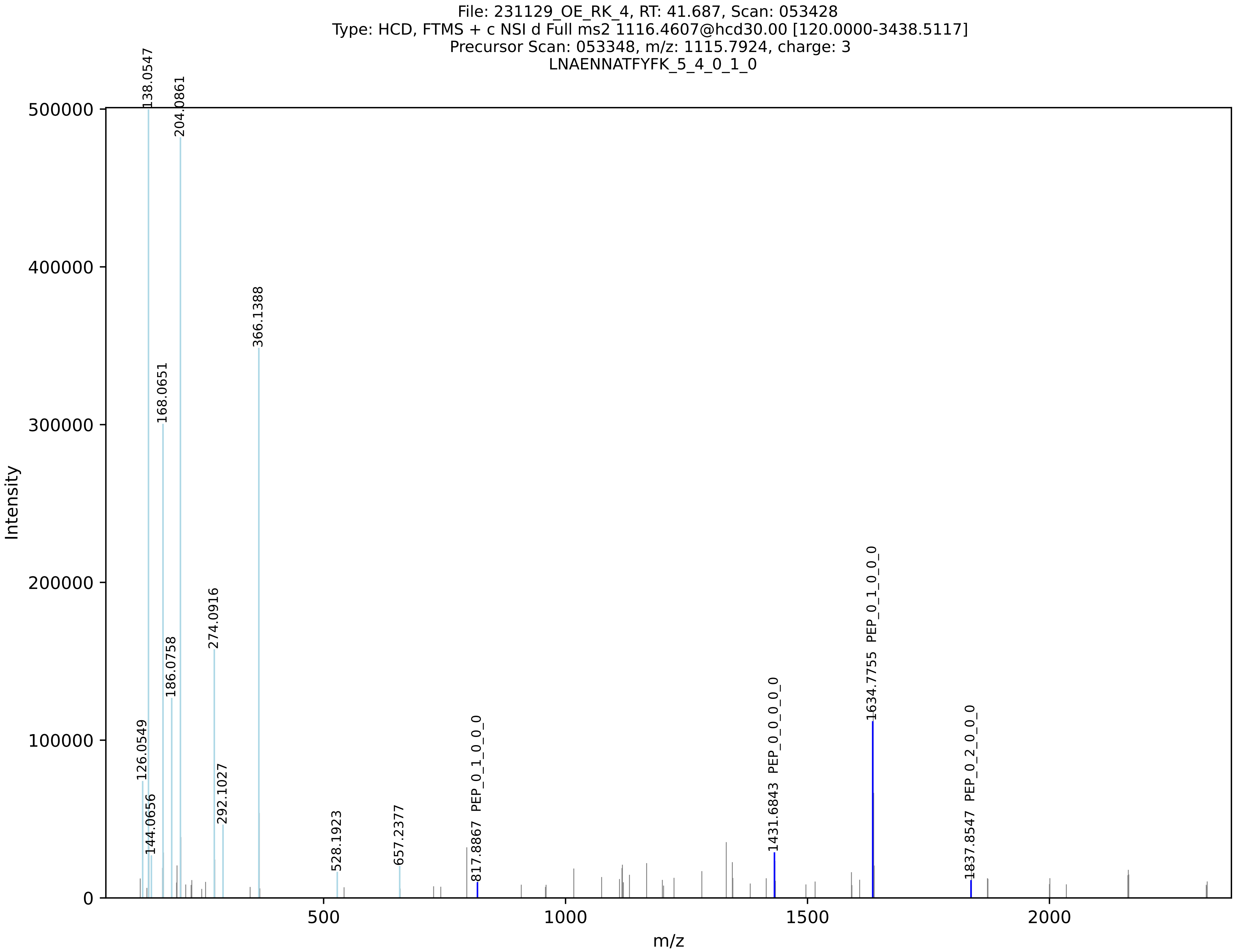
File: 231129_OE_RK_4, RT: 41.687, Scan: 053428
Type: HCD, FTMS + c NSI d Full ms2 1116.4607@hcd30.00 [120.0000-3438.5117]
Precursor Scan: 053348, m/z: 1115.7924, charge: 3
LNAENNATFYFK_5_4_0_1_0
Intensity
m/z
0
100000
200000
300000
400000
500000
500
1000
1500
2000
138.0547
204.0861
366.1388
168.0651
274.0916
186.0758
126.0549
292.1027
144.0656
528.1923
657.2377
817.8867 PEP_0_1_0_0_0
1431.6843 PEP_0_0_0_0_0
1634.7755 PEP_0_1_0_0_0
1837.8547 PEP_0_2_0_0_0

File: 231129_OE_RK_4, RT: 41.695, Scan: 053441
Type: HCD, FTMS + c NSI d Full ms2 1203.4817@hcd30.00 [120.0000-3704.7959]
Precursor Scan: 053348, m/z: 1203.4816, charge: 3
SWPAVGNCSSALR_5_4_0_2_0

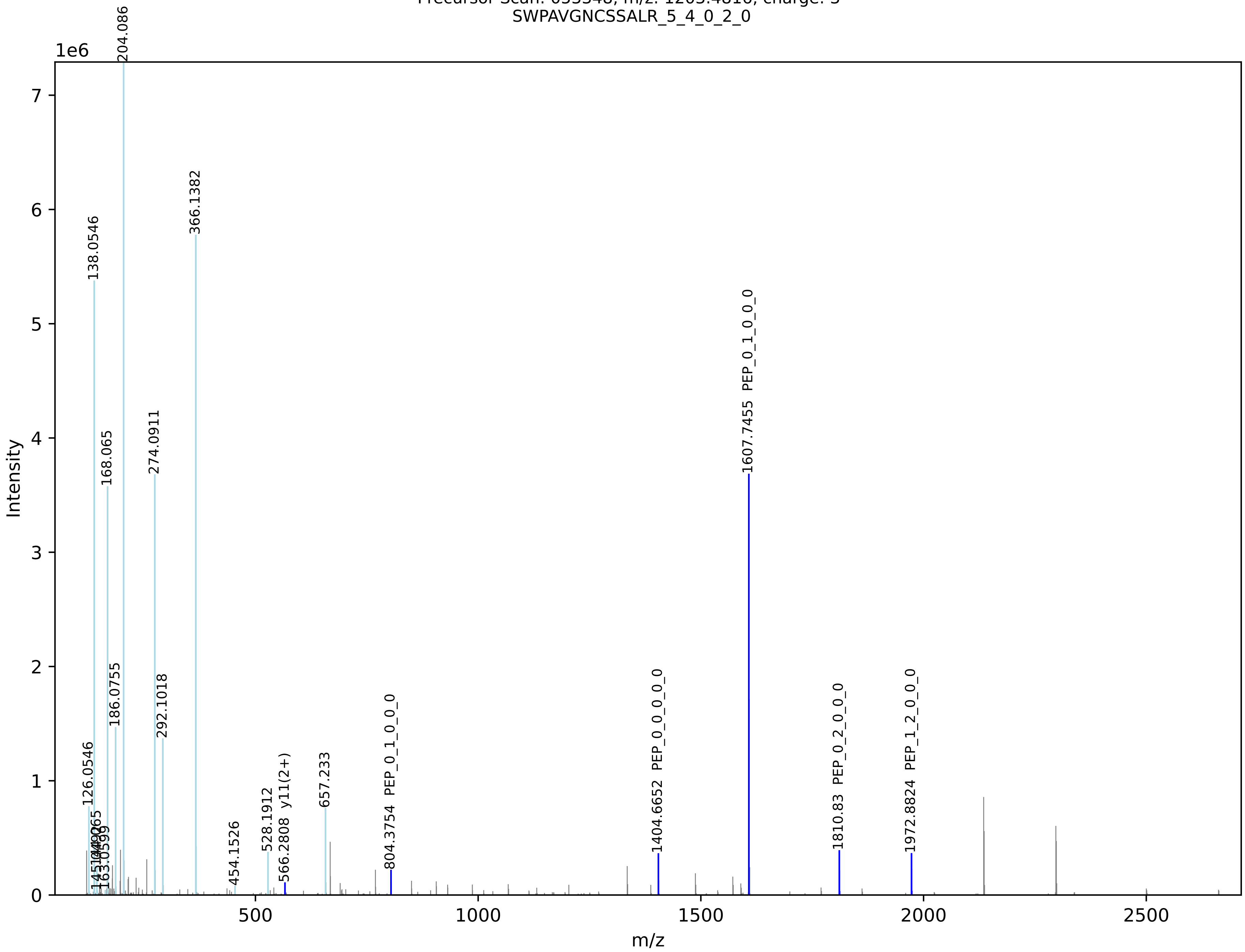

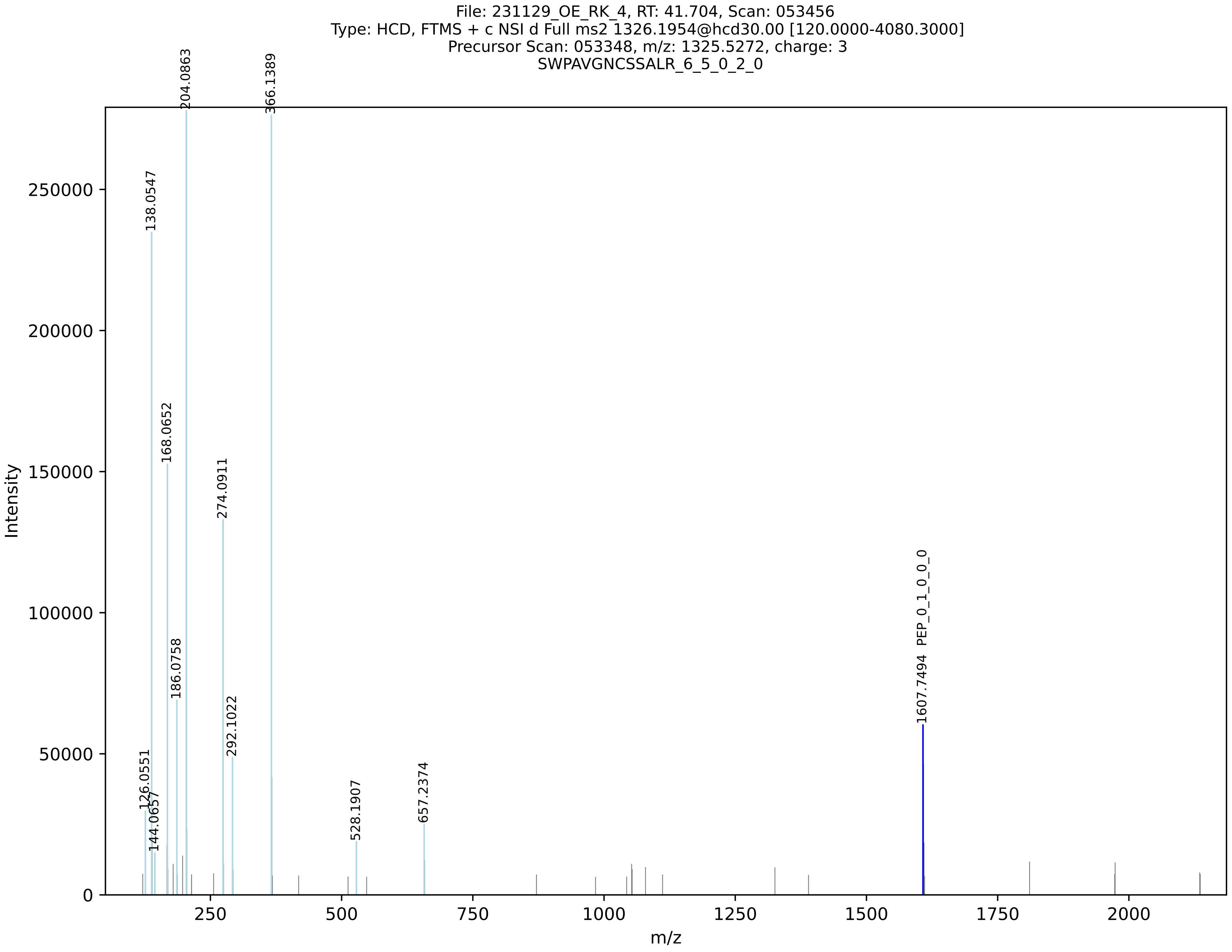

File: 231129_OE_RK_4, RT: 41.704, Scan: 053456
Type: HCD, FTMS + c NSI d Full ms2 1326.1954@hcd30.00 [120.0000-4080.3000]
Precursor Scan: 053348, m/z: 1325.5272, charge: 3
SWPAVGNCSSALR_6_5_0_2_0
Intensity
m/z
204.0863
366.1389
138.0547
168.0652
274.0911
186.0758
292.1022
126.0551
144.0657
528.1907
657.2374
1607.7494 PEP_0_1_0_0_0
250000
200000
150000
100000
50000
0
250
500
750
1000
1250
1500
1750
2000

File: 231129_OE_RK_4, RT: 41.758, Scan: 053536
Type: HCD, FTMS + c NSI d Full ms2 1209.8854@hcd30.00 [120.0000-3724.3914]
Precursor Scan: 053422, m/z: 1209.5533, charge: 3
SWPAVGNCSSALR_6_4_1_1_0

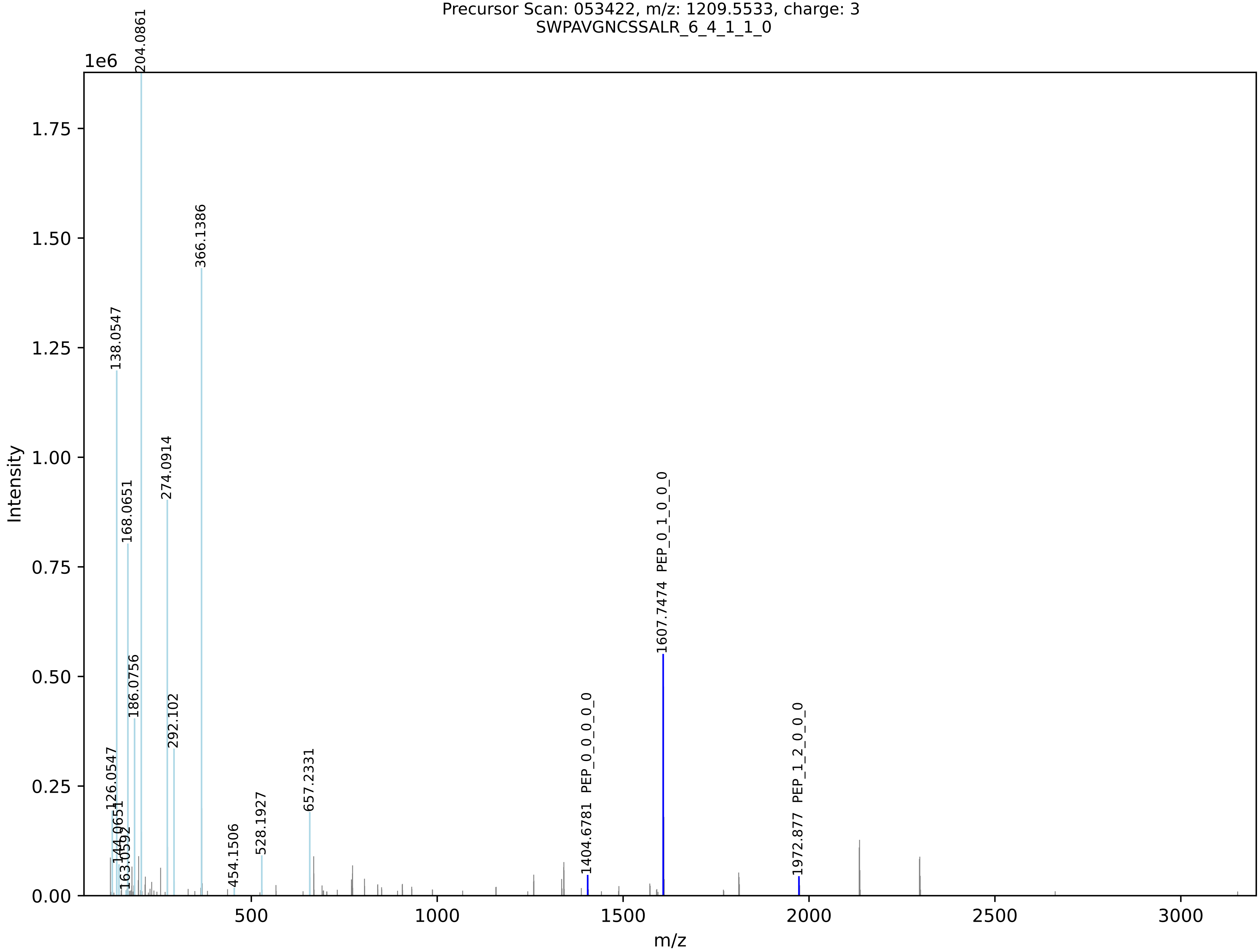

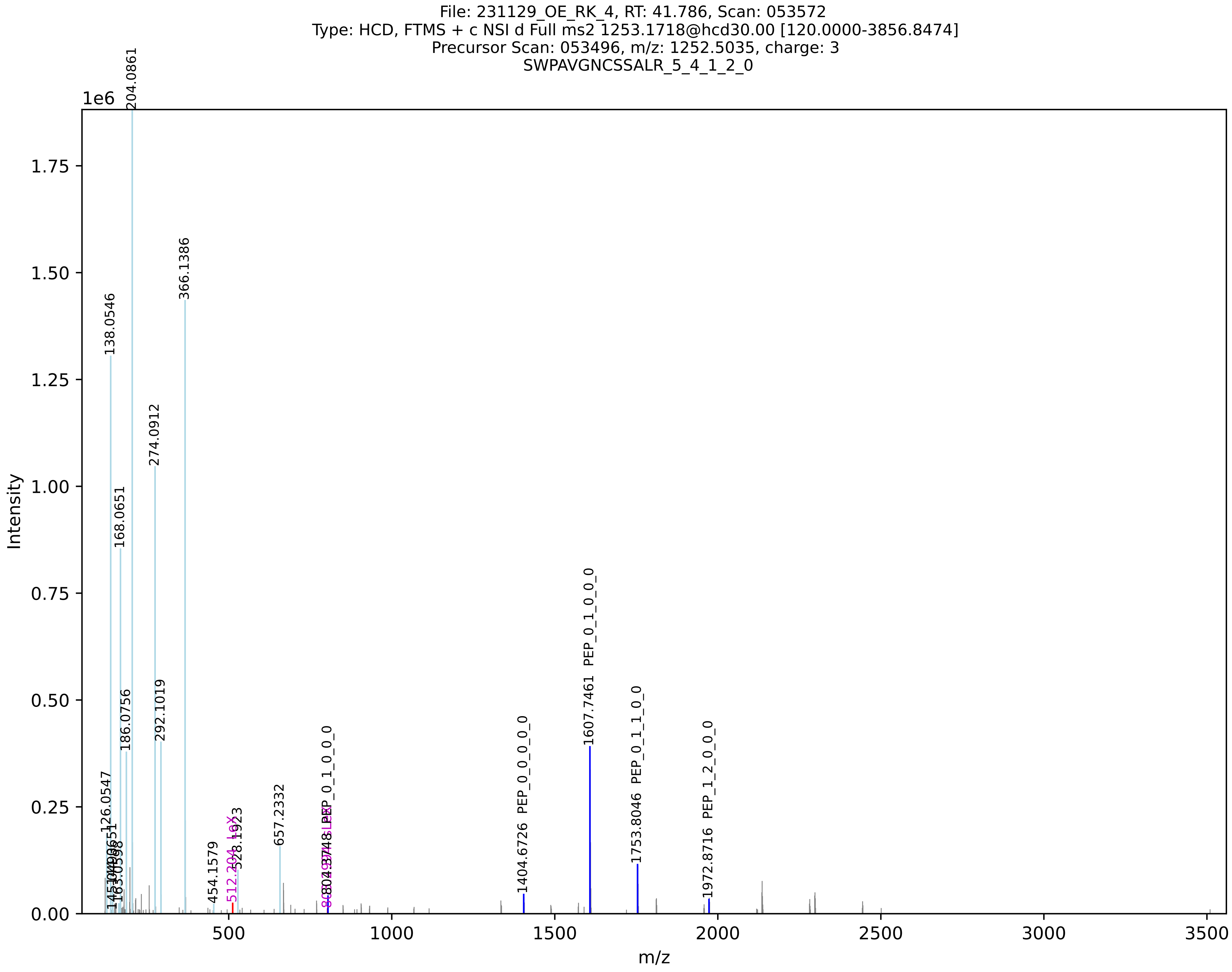
File: 231129_OE_RK_4, RT: 41.786, Scan: 053572
Type: HCD, FTMS + c NSI d Full ms2 1253.1718@hcd30.00 [120.0000-3856.8474]
Precursor Scan: 053496, m/z: 1252.5035, charge: 3
SWPAVGNCSSALR_5_4_1_2_0
1e6
Intensity
m/z
204.0861
366.1386
138.0546
274.0912
168.0651
186.0756
292.1019
126.0547
454.1579
512.204_LeX
528.1923
657.2332
PEP_0_1_0_0_0
1404.6726 PEP_0_0_0_0_0
1607.7461 PEP_0_1_0_0_0
1753.8046 PEP_0_1_1_0_0
1972.8716 PEP_1_2_0_0_0
0.00
0.25
0.50
0.75
1.00
1.25
1.50
1.75
500
1000
1500
2000
2500
3000
3500

File: 231129_OE_RK_4, RT: 41.816, Scan: 053621
Type: HCD, FTMS + c NSI d Full ms2 1210.2255@hcd30.00 [120.0000-3725.4319]
Precursor Scan: 053496, m/z: 1209.5572, charge: 3
SWPAVGNCSSALR_6_4_1_1_0

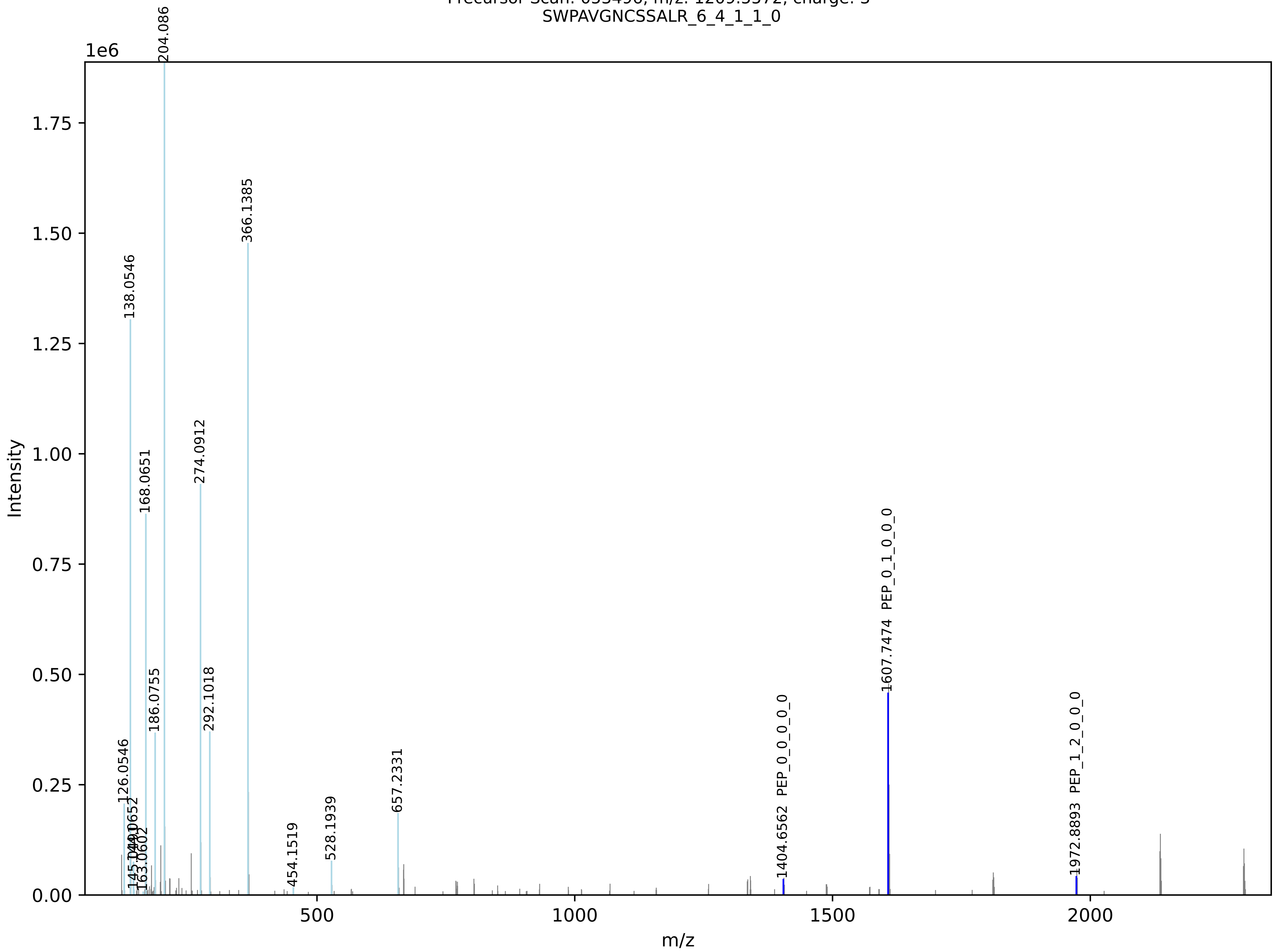

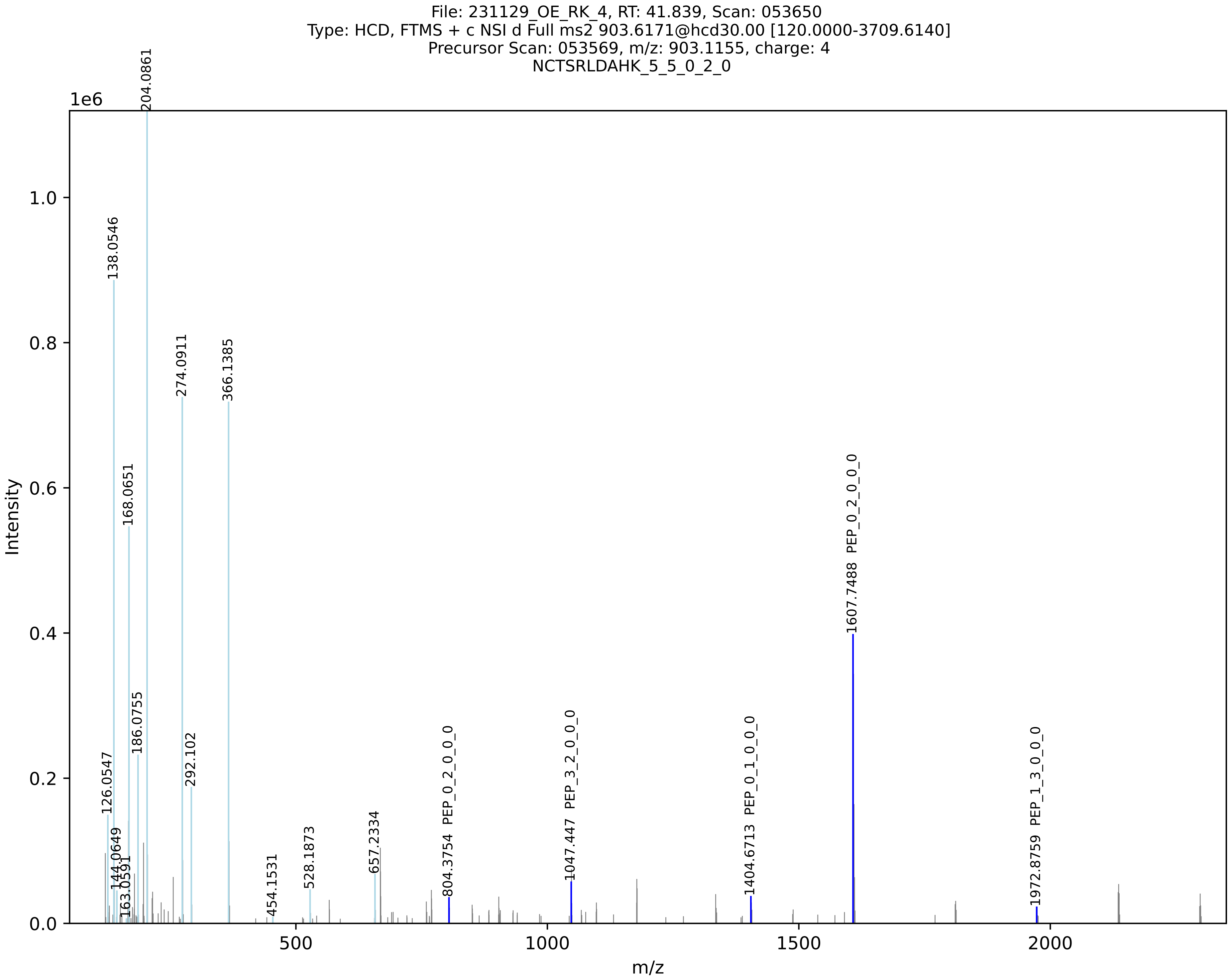
File: 231129_OE_RK_4, RT: 41.839, Scan: 053650
Type: HCD, FTMS + c NSI d Full ms2 903.6171@hcd30.00 [120.0000-3709.6140]
Precursor Scan: 053569, m/z: 903.1155, charge: 4
NCTSRLDAHK_5_5_0_2_0
1e6
Intensity
m/z
204.0861
138.0546
274.0911
366.1385
168.0651
186.0755
292.102
126.0547
144.0649
163.0591
454.1531
528.1873
657.2334
804.3754 PEP_0_2_0_0_0
1047.447 PEP_3_2_0_0_0
1404.6713 PEP_0_1_0_0_0
1607.7488 PEP_0_2_0_0_0
1972.8759 PEP_1_3_0_0_0

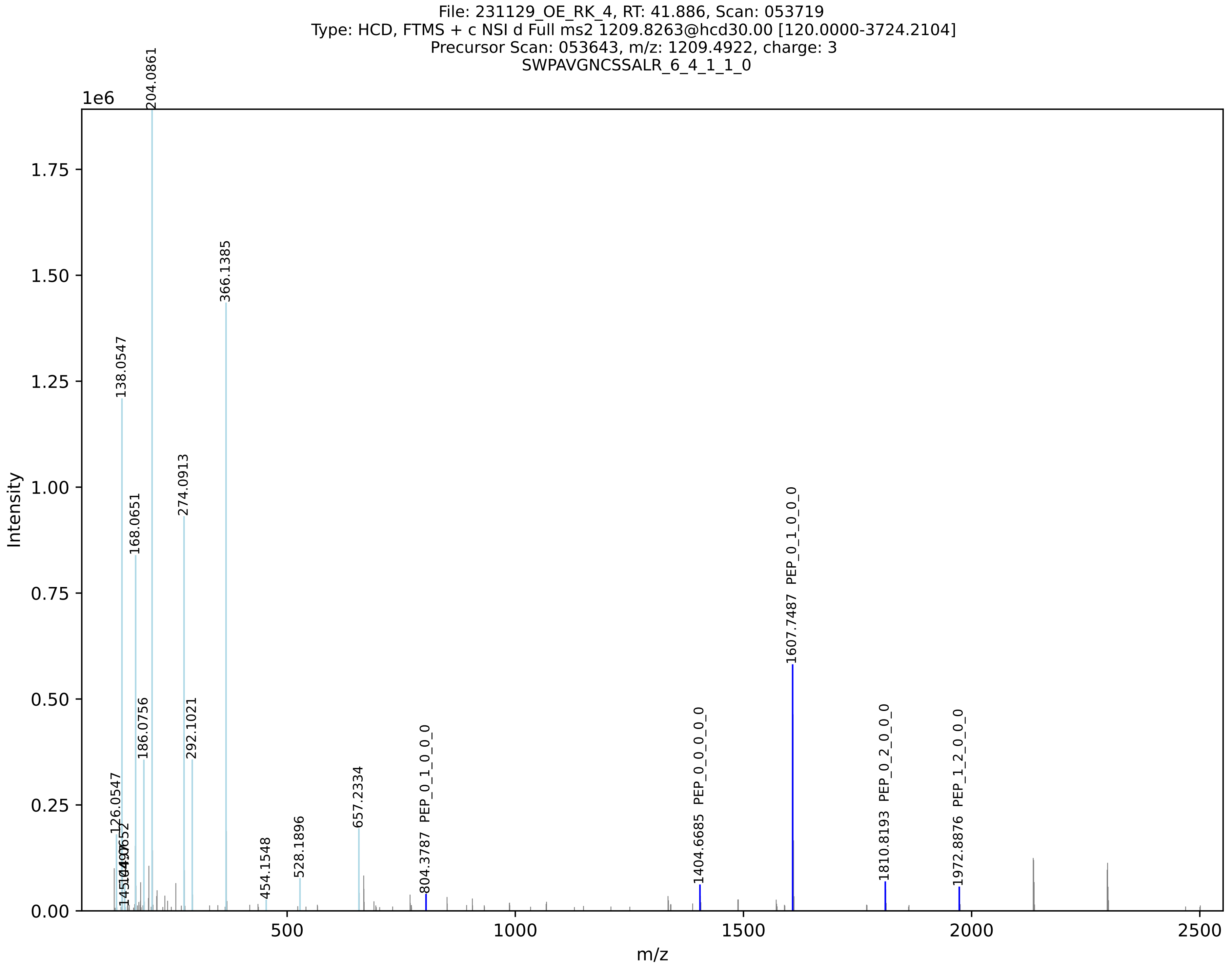

File: 231129_OE_RK_4, RT: 41.886, Scan: 053719
Type: HCD, FTMS + c NSI d Full ms2 1209.8263@hcd30.00 [120.0000-3724.2104]
Precursor Scan: 053643, m/z: 1209.4922, charge: 3
SWPAVGNCSSALR_6_4_1_1_0
1e6
Intensity
m/z
0.00
0.25
0.50
0.75
1.00
1.25
1.50
1.75
500
1000
1500
2000
2500
204.0861
366.1385
138.0547
274.0913
168.0651
186.0756
292.1021
126.0547
454.1548
528.1896
657.2334
804.3787 PEP_0_1_0_0_0
1404.6685 PEP_0_0_0_0_0
1607.7487 PEP_0_1_0_0_0
1810.8193 PEP_0_2_0_0_0
1972.8876 PEP_1_2_0_0_0

File: 231129_OE_RK_4, RT: 41.887, Scan: 053721
Type: HCD, FTMS + c NSI d Full ms2 1806.7284@hcd30.00 [120.0000-3707.1538]
Precursor Scan: 053643, m/z: 1805.2233, charge: 2
SWPAVGNCSSALR_5_4_0_2_0

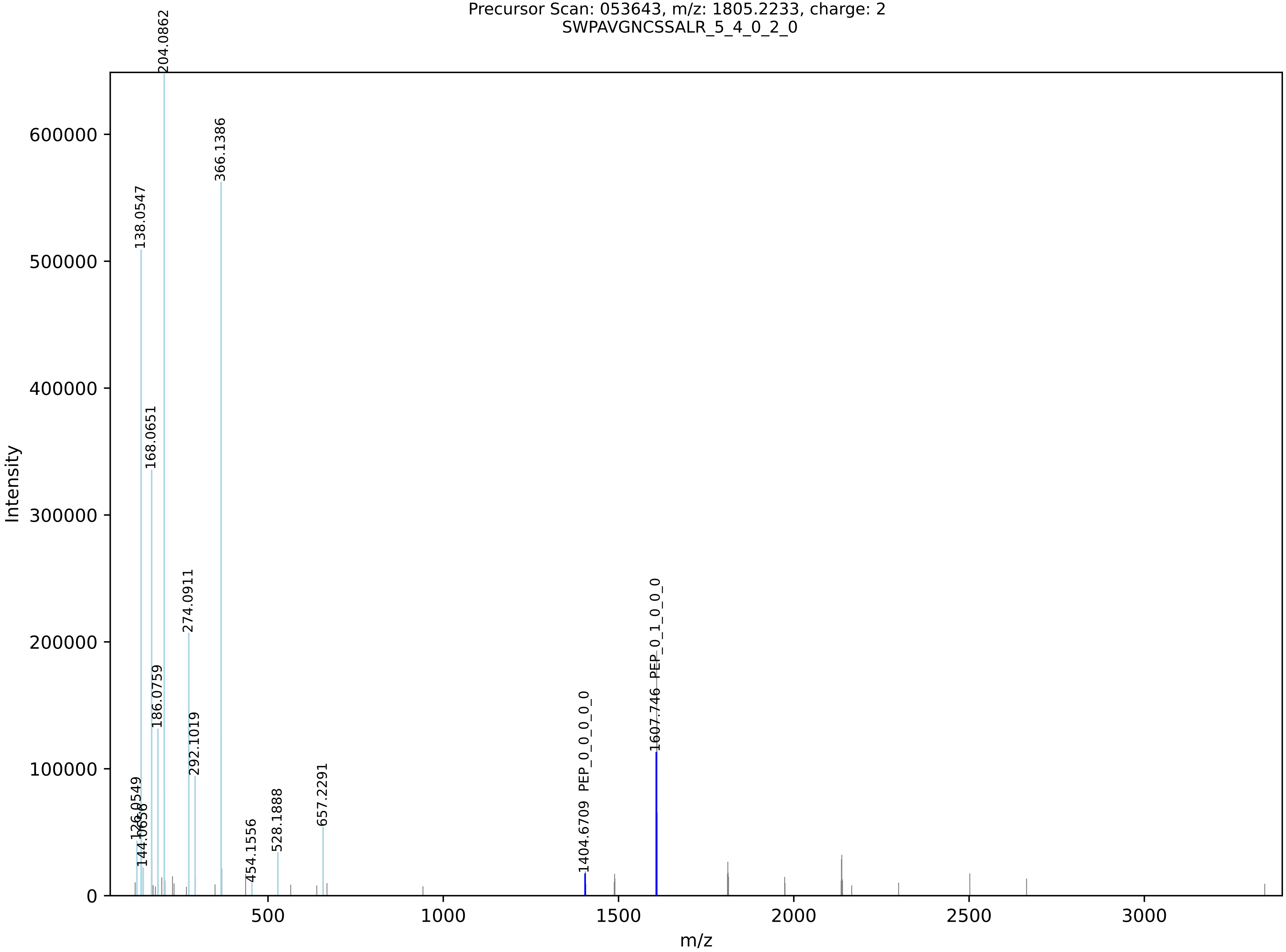

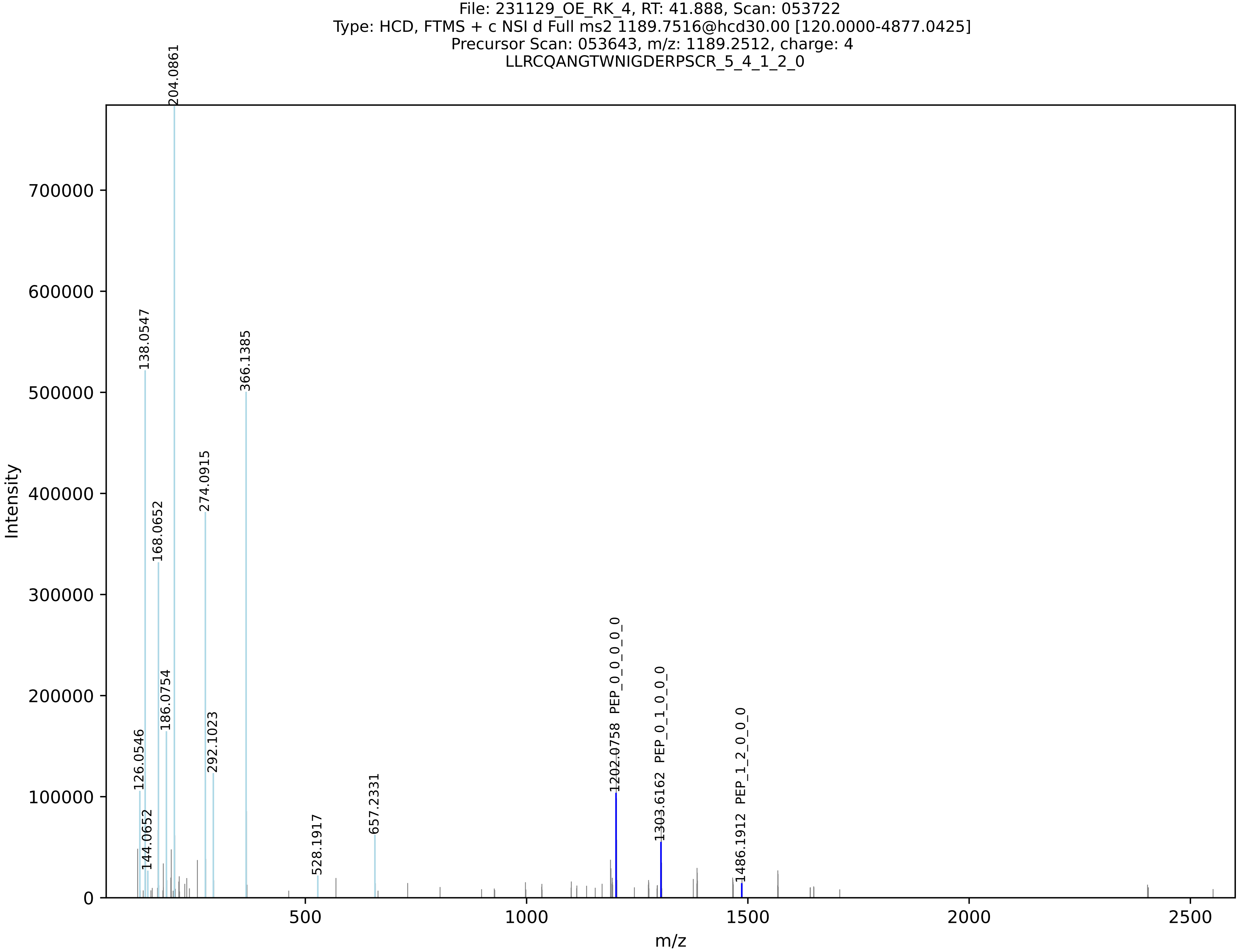
File: 231129_OE_RK_4, RT: 41.888, Scan: 053722
Type: HCD, FTMS + c NSI d Full ms2 1189.7516@hcd30.00 [120.0000-4877.0425]
Precursor Scan: 053643, m/z: 1189.2512, charge: 4
LLRCQANGTWNIGDERPSCR_5_4_1_2_0
Intensity
m/z
204.0861
138.0547
366.1385
274.0915
168.0652
186.0754
126.0546
292.1023
144.0652
528.1917
657.2331
1202.0758 PEP_0_0_0_0_0_
1303.6162 PEP_0_1_0_0_0
1486.1912 PEP_1_2_0_0_0_

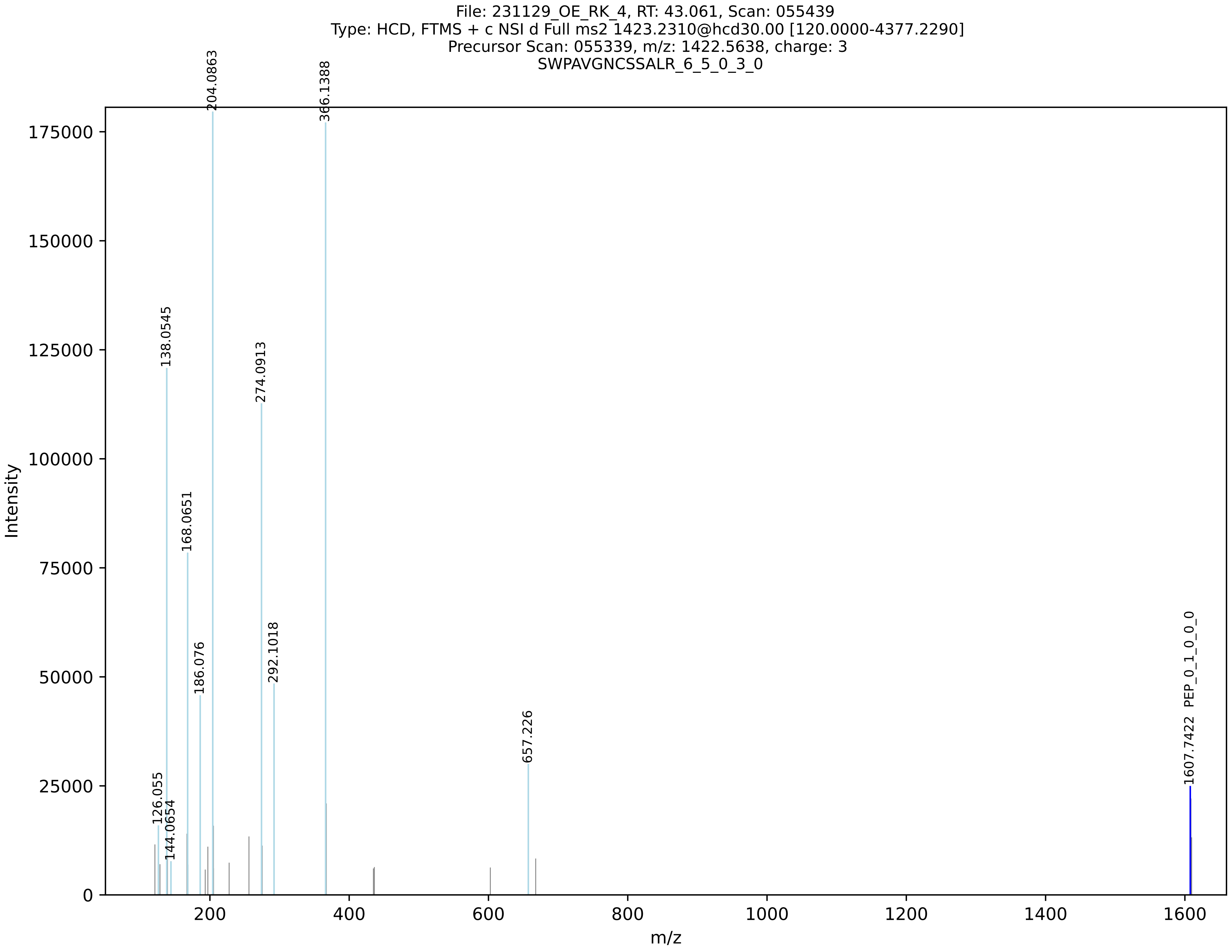
File: 231129_OE_RK_4, RT: 43.061, Scan: 055439
Type: HCD, FTMS + c NSI d Full ms2 1423.2310@hcd30.00 [120.0000-4377.2290]
Precursor Scan: 055339, m/z: 1422.5638, charge: 3
SWPAVGNCSSALR_6_5_0_3_0
Intensity
m/z
204.0863
366.1388
138.0545
274.0913
168.0651
186.076
292.1018
657.226
126.055
144.0654
1607.7422 PEP_0_1_0_0_0
0
25000
50000
75000
100000
125000
150000
175000
200
400
600
800
1000
1200
1400
1600

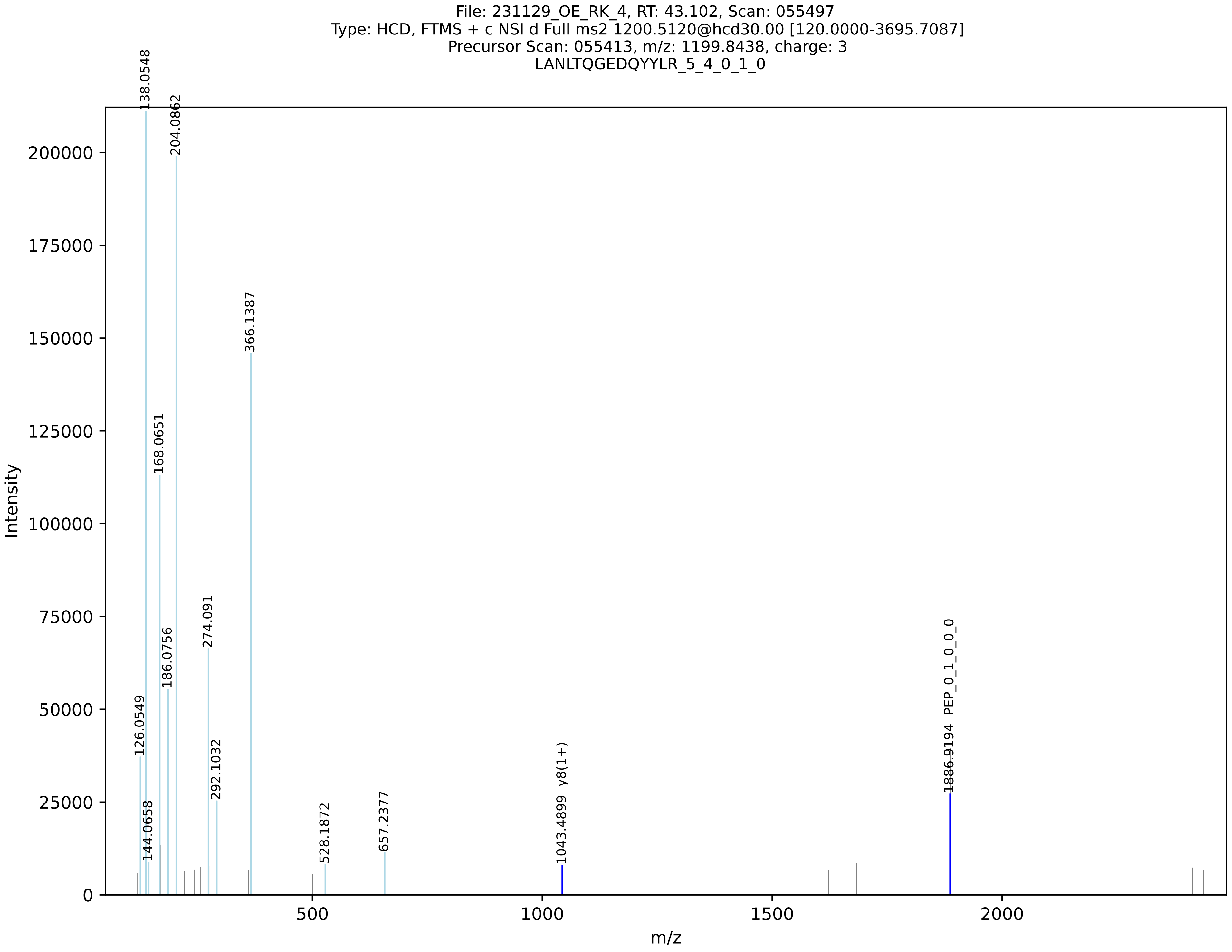

File: 231129_OE_RK_4, RT: 43.102, Scan: 055497
Type: HCD, FTMS + c NSI d Full ms2 1200.5120@hcd30.00 [120.0000-3695.7087]
Precursor Scan: 055413, m/z: 1199.8438, charge: 3
LANLTQGEDQYYLR_5_4_0_1_0
138.0548
204.0862
366.1387
168.0651
274.091
186.0756
126.0549
292.1032
144.0658
528.1872
657.2377
1043.4899 y8(1+)
1886.9194 PEP_0_1_0_0_0
Intensity
m/z
0
25000
50000
75000
100000
125000
150000
175000
200000
500
1000
1500
2000

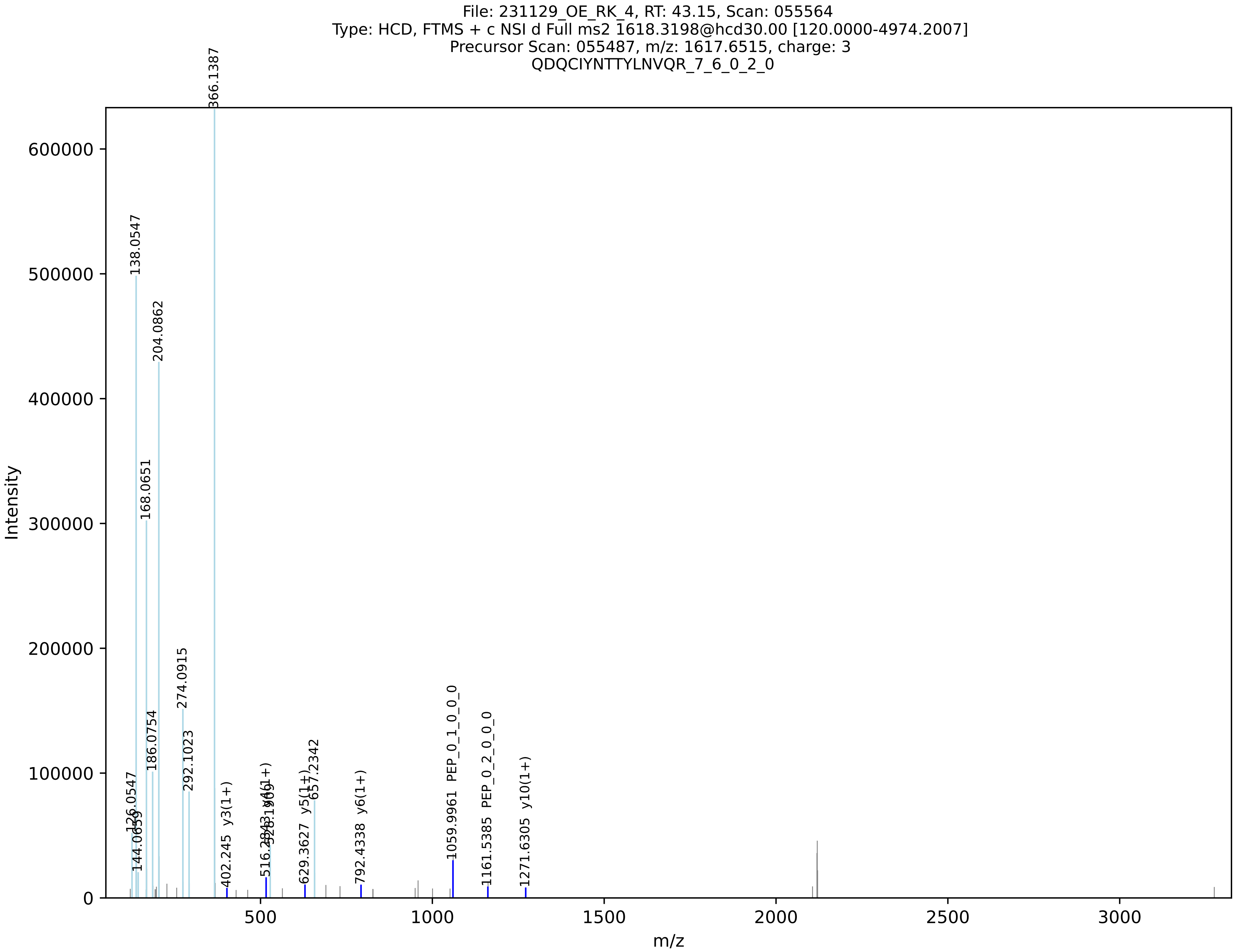

File: 231129_OE_RK_4, RT: 43.15, Scan: 055564
Type: HCD, FTMS + c NSI d Full ms2 1618.3198@hcd30.00 [120.0000-4974.2007]
Precursor Scan: 055487, m/z: 1617.6515, charge: 3
QDQCIYNTTYLNVQR_7_6_0_2_0
366.1387
138.0547
204.0862
168.0651
274.0915
186.0754
292.1023
126.0547
144.0659
402.245 y3(1+)
516.2843 y4(1+)
528.1909
629.3627 y5(1+)
657.2342
792.4338 y6(1+)
1059.9961 PEP_0_1_0_0_0
1161.5385 PEP_0_2_0_0_0
1271.6305 y10(1+)
Intensity
m/z
0
100000
200000
300000
400000
500000
600000
500
1000
1500
2000
2500
3000

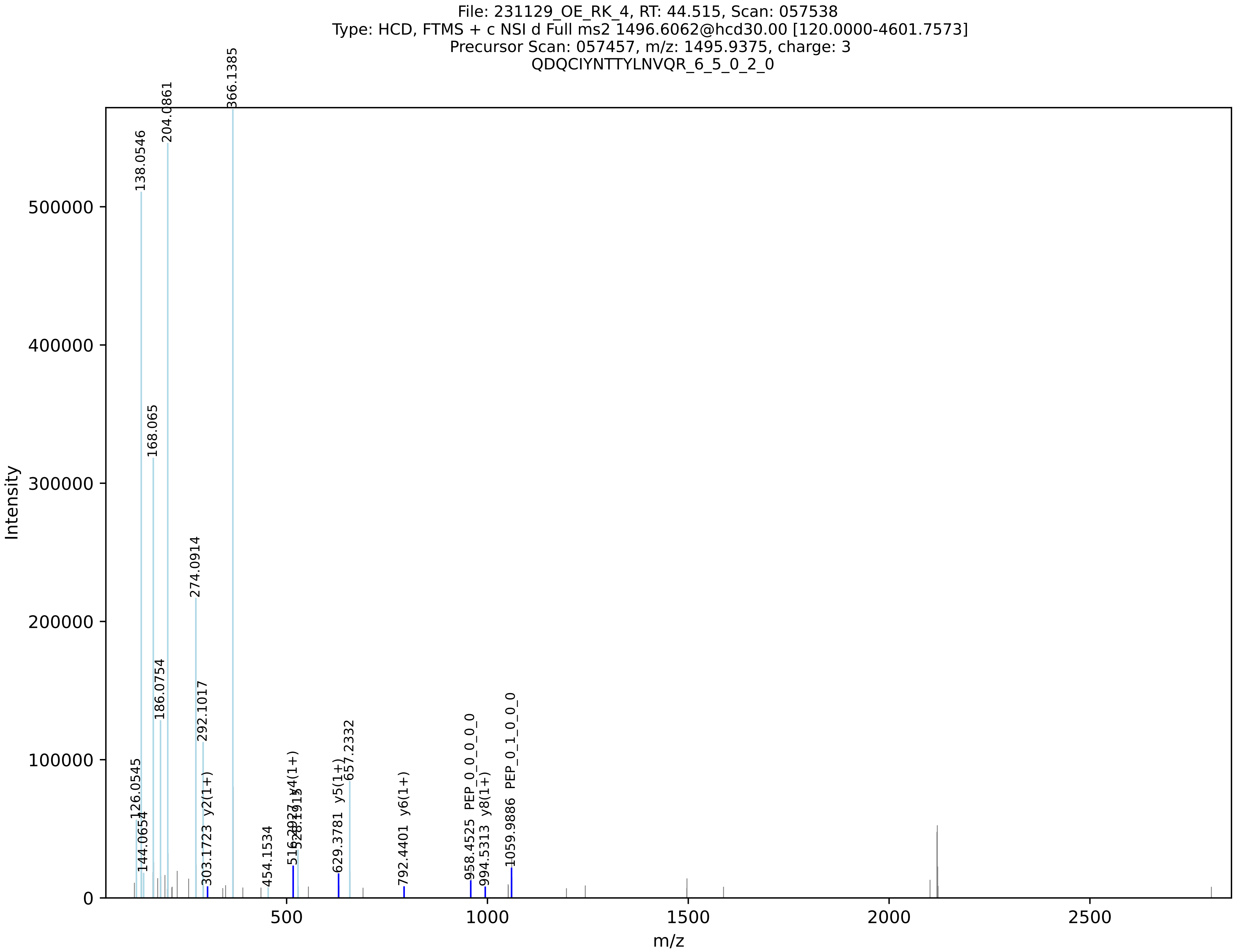
File: 231129_OE_RK_4, RT: 44.515, Scan: 057538
Type: HCD, FTMS + c NSI d Full ms2 1496.6062@hcd30.00 [120.0000-4601.7573]
Precursor Scan: 057457, m/z: 1495.9375, charge: 3
QDQCIYNTTYLNVQR_6_5_0_2_0
Intensity
m/z
366.1385
204.0861
138.0546
168.065
274.0914
186.0754
292.1017
126.0545
144.0654
303.1723 y2(1+)
454.1534
516.2927 y4(1+)
528.1915
629.3781 y5(1+)
657.2332
792.4401 y6(1+)
958.4525 PEP_0_0_0_0_0
994.5313 y8(1+)
1059.9886 PEP_0_1_0_0_0

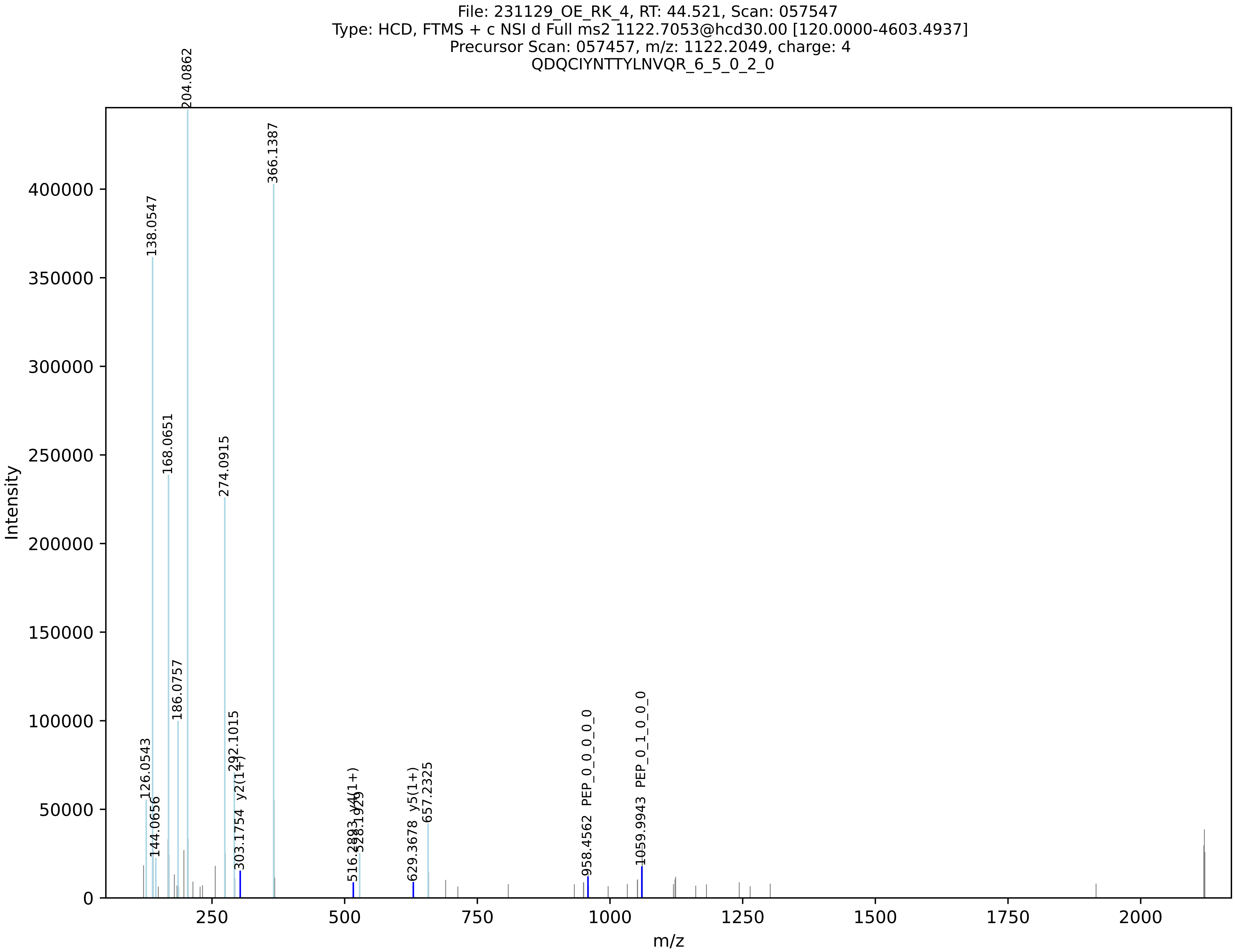

File: 231129_OE_RK_4, RT: 44.521, Scan: 057547
Type: HCD, FTMS + c NSI d Full ms2 1122.7053@hcd30.00 [120.0000-4603.4937]
Precursor Scan: 057457, m/z: 1122.2049, charge: 4
QDQCIYNTTYLNVQR_6_5_0_2_0
204.0862
366.1387
138.0547
168.0651
274.0915
186.0757
126.0543
144.0656
292.1015
303.1754 y2(1+)
516.2883 y4(1+)
528.1929
629.3678 y5(1+)
657.2325
958.4562 PEP_0_0_0_0_0_
1059.9943 PEP_0_1_0_0_0_
Intensity
m/z
0
50000
100000
150000
200000
250000
300000
350000
400000
250
500
750
1000
1250
1500
1750
2000

File: 231129_OE_RK_4, RT: 44.612, Scan: 057679
Type: HCD, FTMS + c NSI d Full ms2 1213.4928@hcd30.00 [120.0000-3735.4299]
Precursor Scan: 057601, m/z: 1212.8237, charge: 3
LNAENNATFYFK_5_4_0_2_0

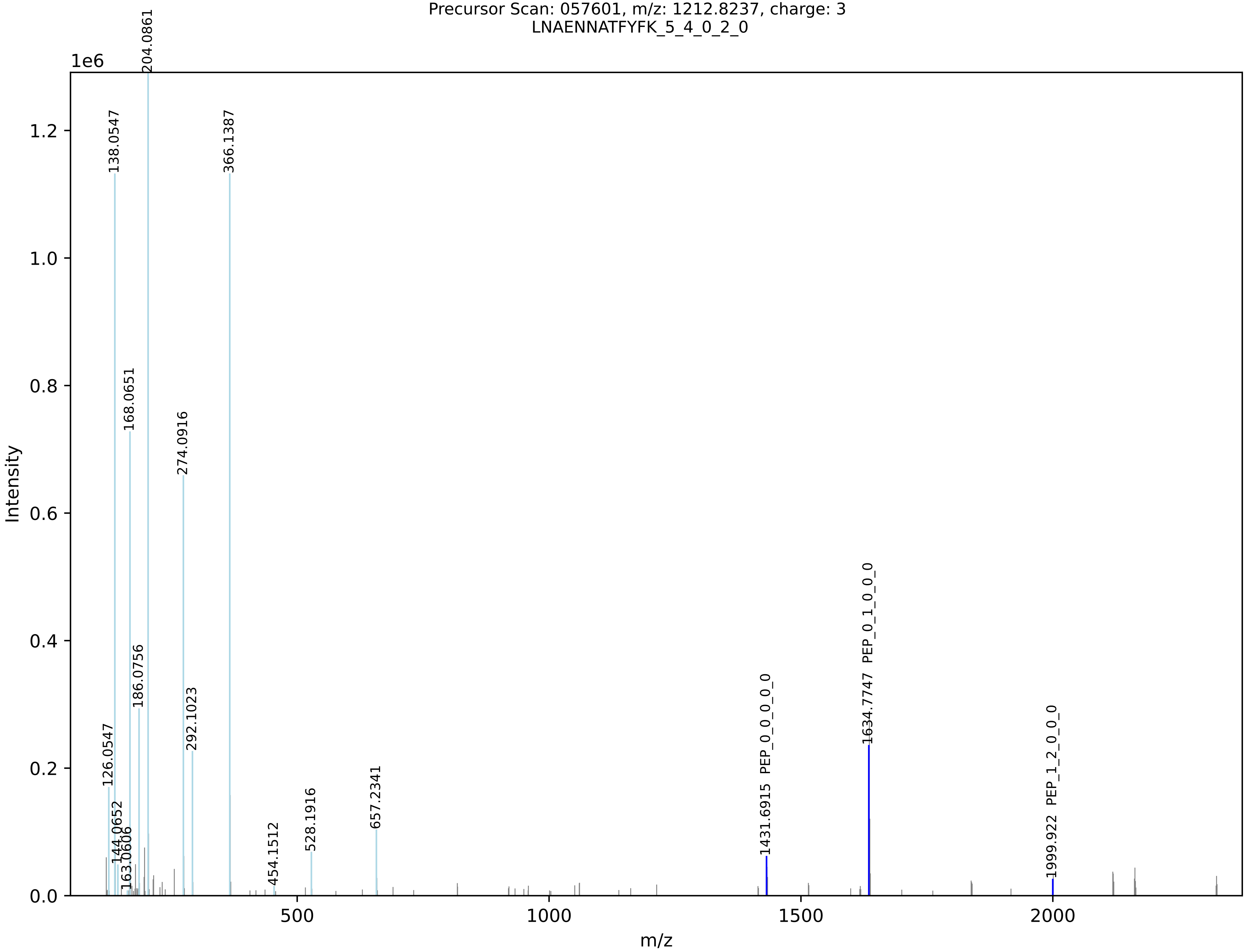

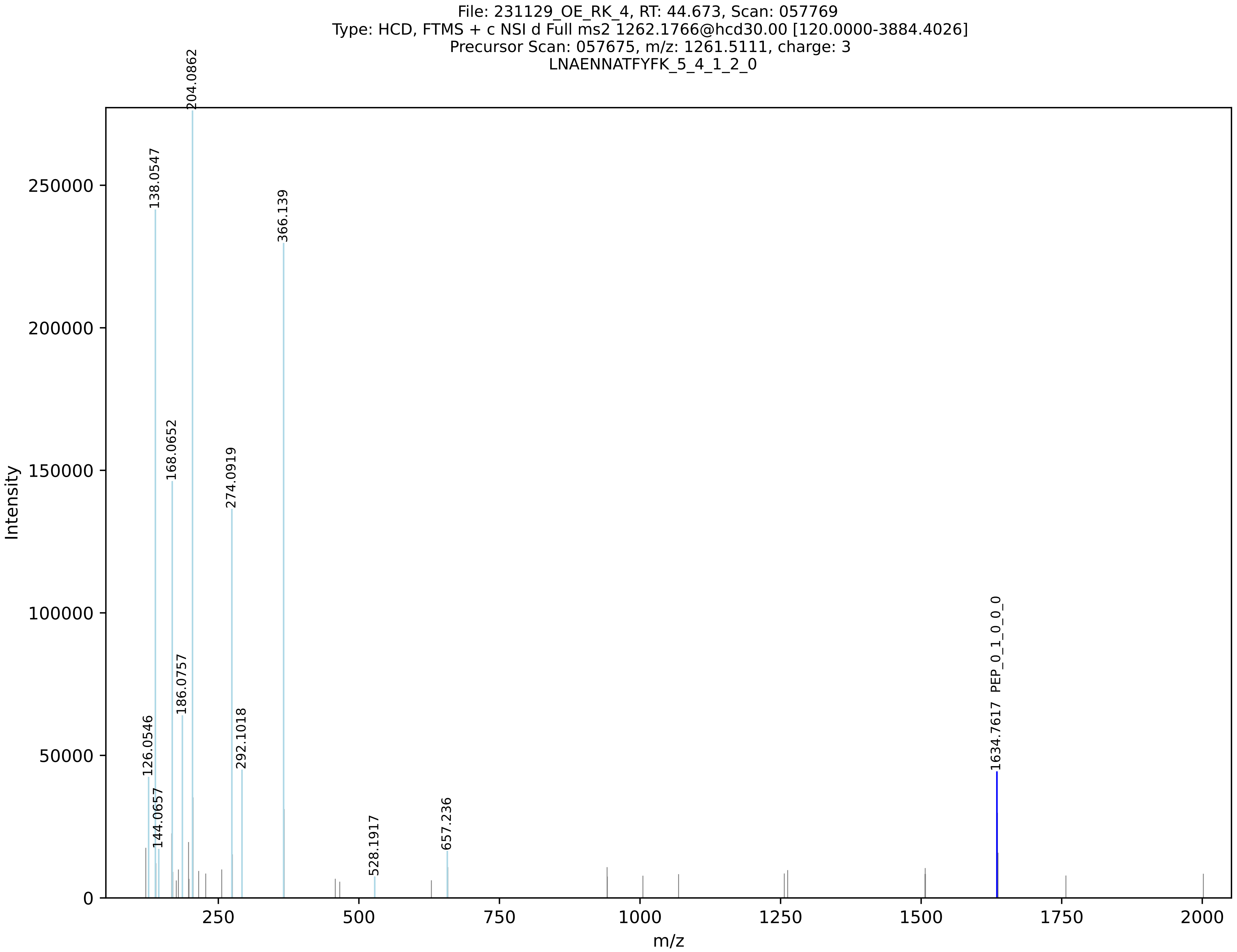
File: 231129_OE_RK_4, RT: 44.673, Scan: 057769
Type: HCD, FTMS + c NSI d Full ms2 1262.1766@hcd30.00 [120.0000-3884.4026]
Precursor Scan: 057675, m/z: 1261.5111, charge: 3
LNAENNATFYFK_5_4_1_2_0
204.0862
138.0547
366.139
168.0652
274.0919
186.0757
126.0546
292.1018
144.0657
528.1917
657.236
1634.7617 PEP_0_1_0_0_0
Intensity
m/z
0
50000
100000
150000
200000
250000
250
500
750
1000
1250
1500
1750
2000

File: 231129_OE_RK_4, RT: 44.715, Scan: 057827
Type: HCD, FTMS + c NSI d Full ms2 1287.0126@hcd30.00 [120.0000-5273.8677]
Precursor Scan: 057748, m/z: 1286.2609, charge: 4
QDQCIYNTTYLNVQR_7_6_0_3_0

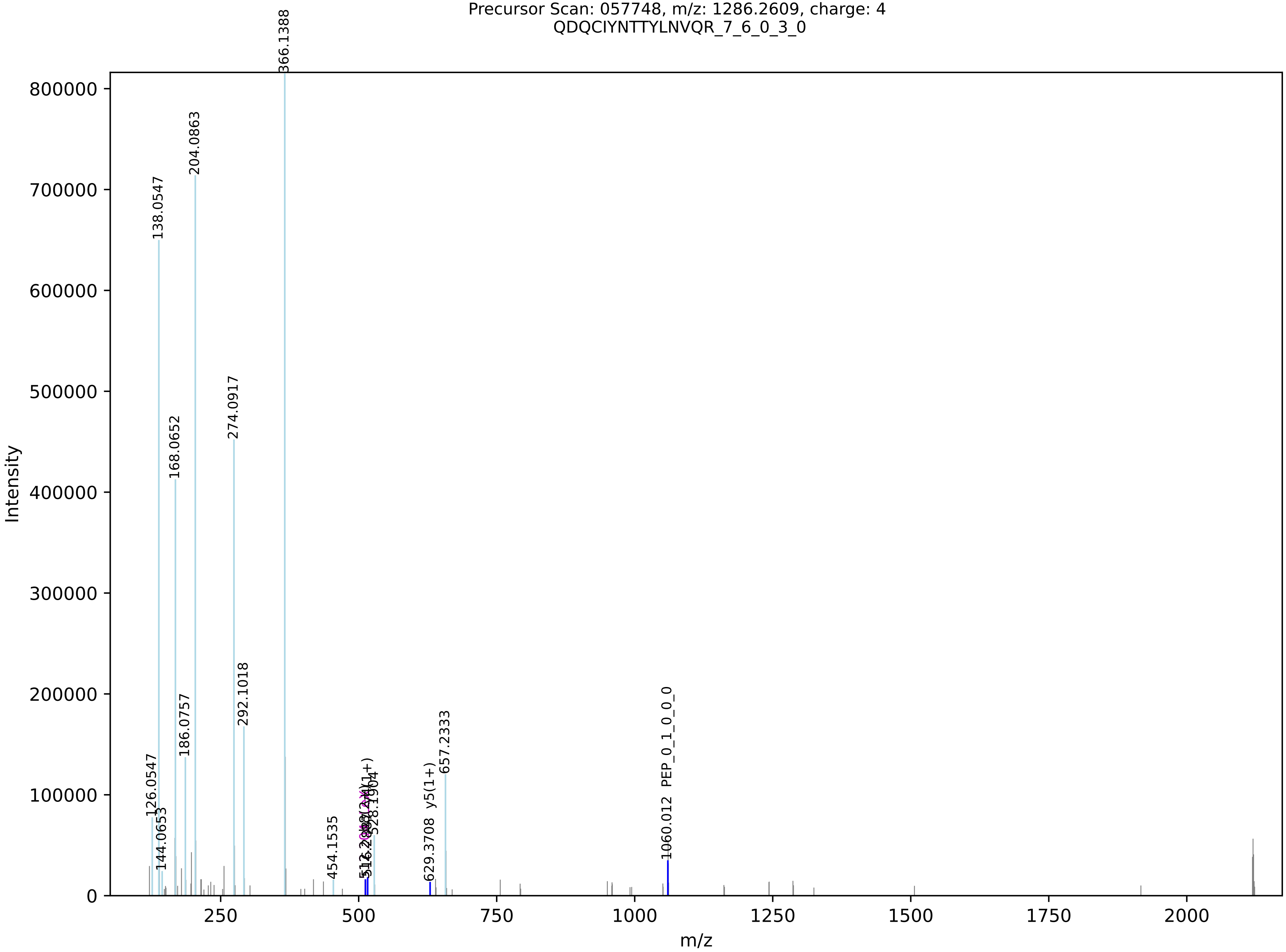

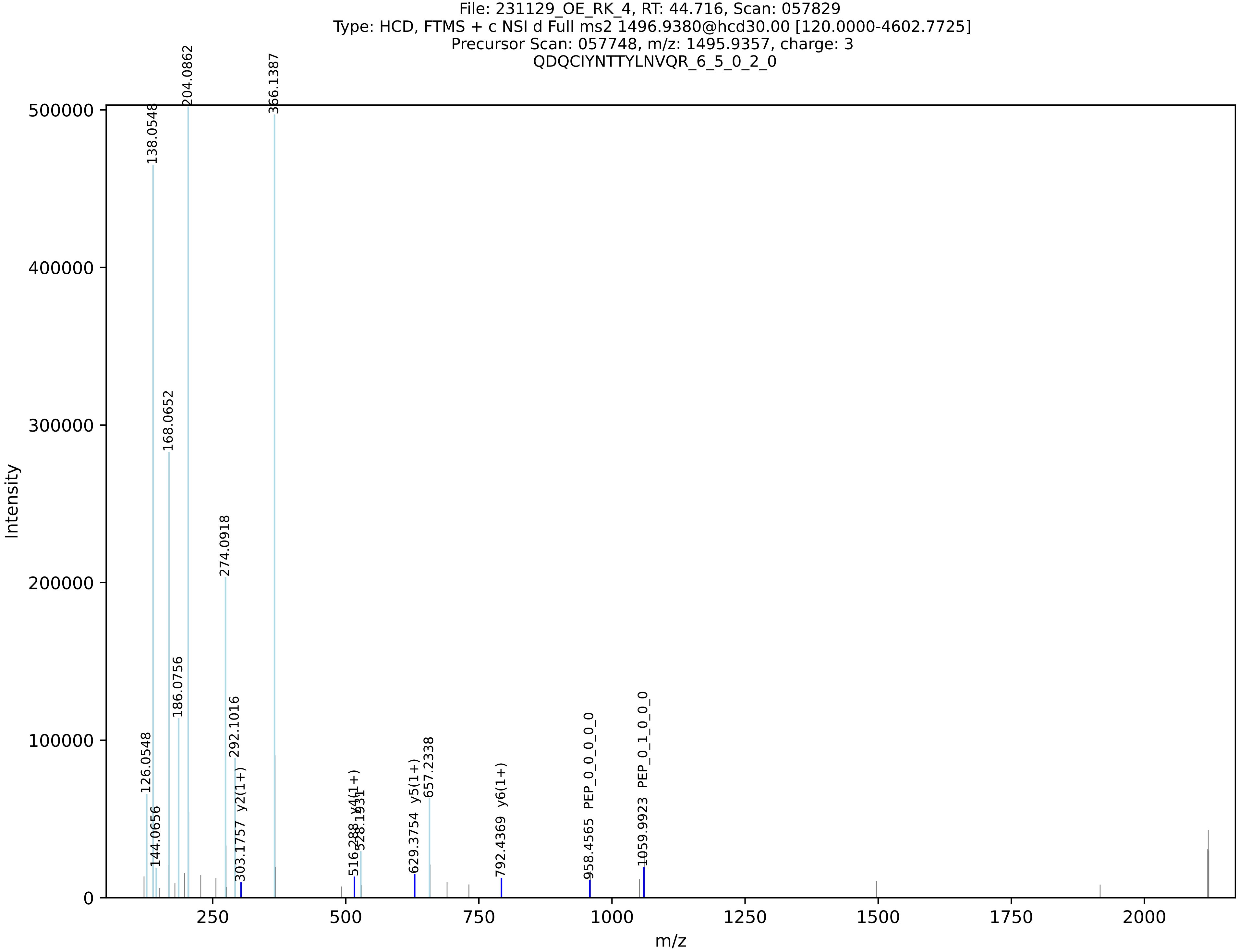
File: 231129_OE_RK_4, RT: 44.716, Scan: 057829
Type: HCD, FTMS + c NSI d Full ms2 1496.9380@hcd30.00 [120.0000-4602.7725]
Precursor Scan: 057748, m/z: 1495.9357, charge: 3
QDQCIYNTTYLNVQR_6_5_0_2_0
Intensity
m/z
204.0862
366.1387
138.0548
168.0652
274.0918
186.0756
292.1016
126.0548
144.0656
303.1757 y2(1+)
516.288 y4(1+)
528.1931
629.3754 y5(1+)
657.2338
792.4369 y6(1+)
958.4565 PEP_0_0_0_0_0_
1059.9923 PEP_0_1_0_0_0_

File: 231129_OE_RK_4, RT: 44.718, Scan: 057831
Type: HCD, FTMS + c NSI d Full ms2 1667.3373@hcd30.00 [120.0000-5124.1938]
Precursor Scan: 057748, m/z: 1666.3343, charge: 3
QDQCIYNTTYLNVQR_7_6_1_2_0

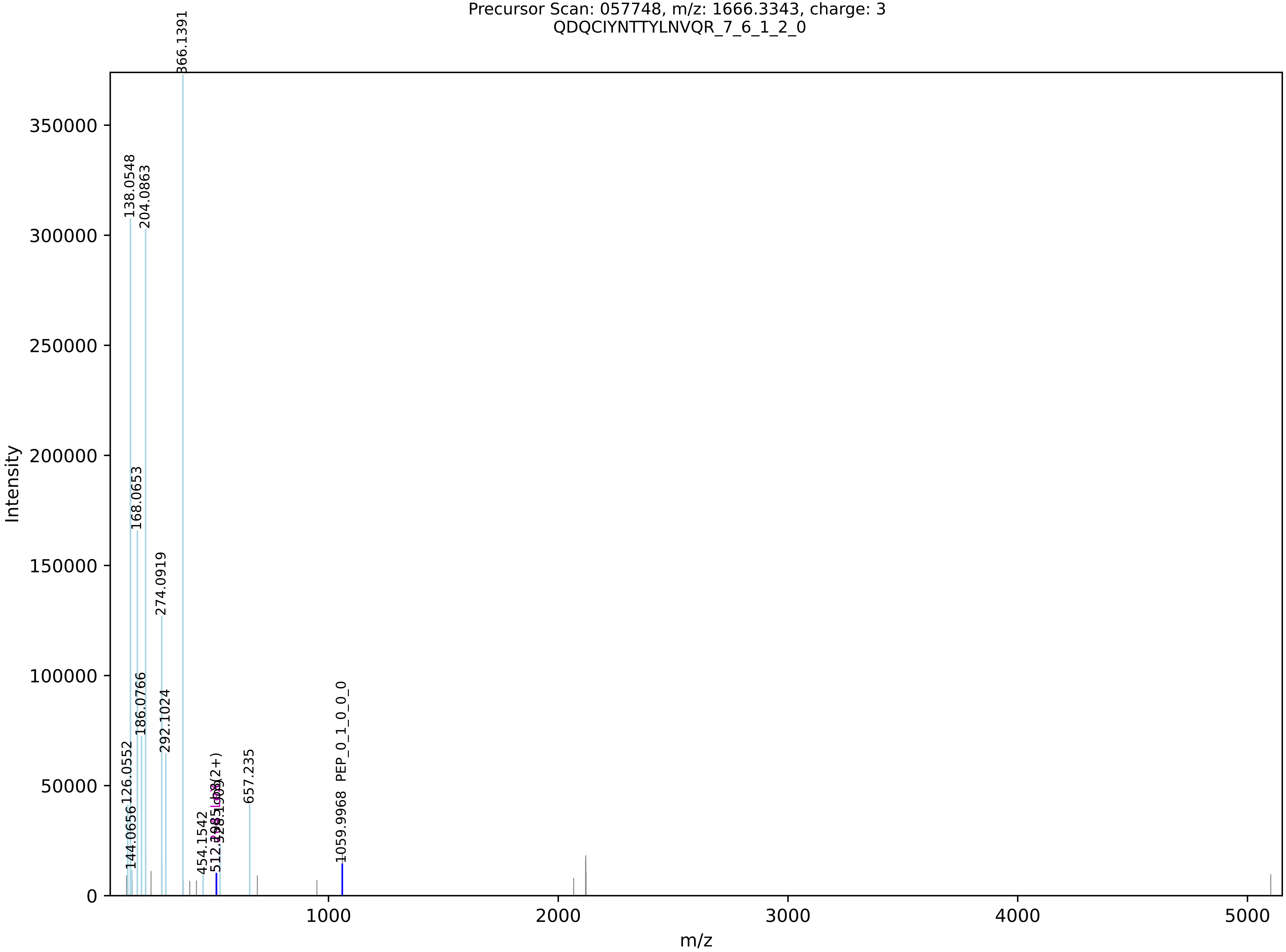

File: 231129_OE_RK_4, RT: 44.815, Scan: 057973
Type: HCD, FTMS + c NSI d Full ms2 1618.3167@hcd30.00 [120.0000-4974.1909]
Precursor Scan: 057893, m/z: 1617.6484, charge: 3
QDQCIYNTTYLNVQR_7_6_0_2_0

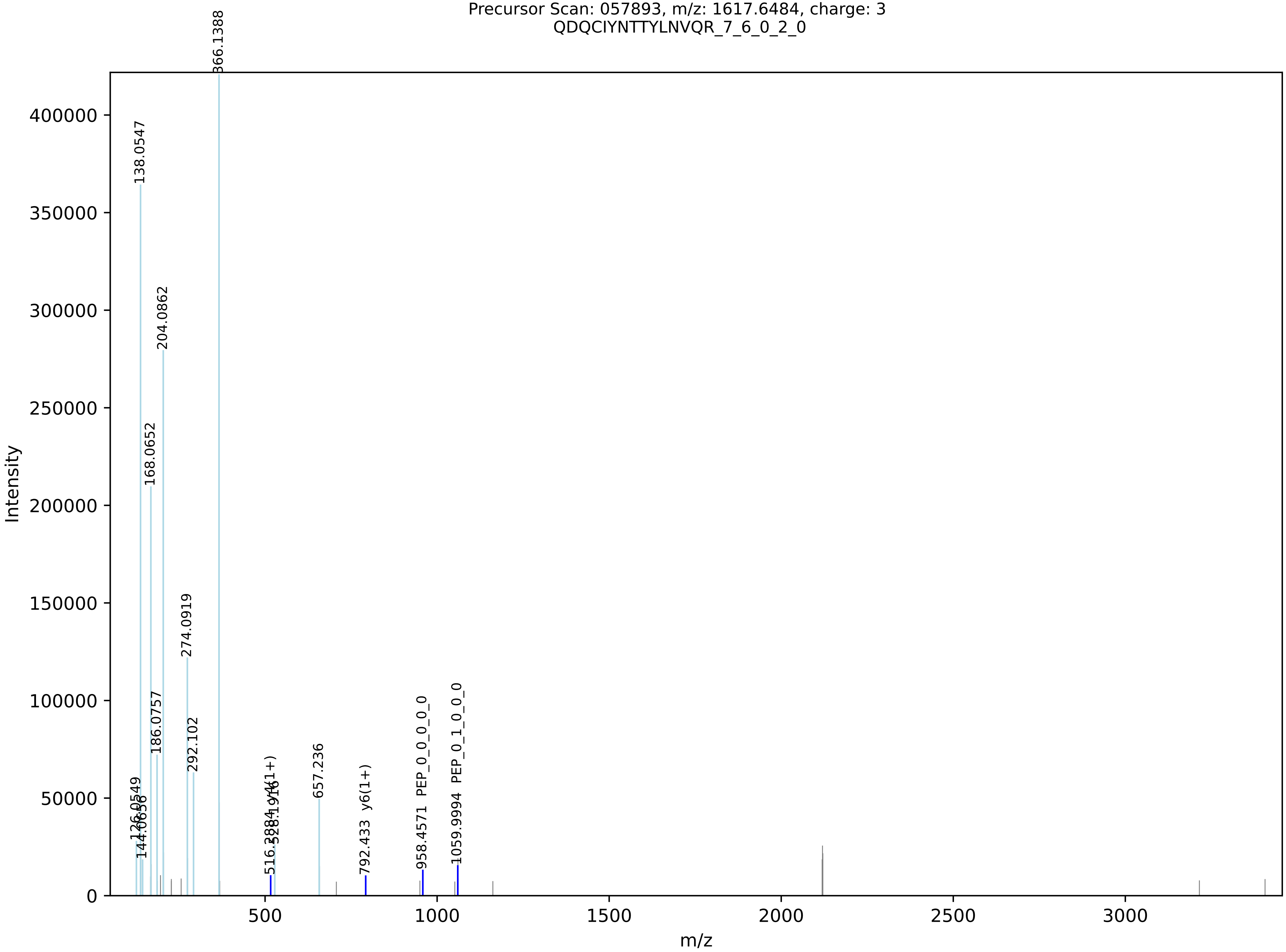

File: 231129_OE_RK_4, RT: 44.867, Scan: 058050
Type: HCD, FTMS + c NSI d Full ms2 1031.4241@hcd30.00 [120.0000-4231.0664]
Precursor Scan: 057967, m/z: 1030.923, charge: 4
QDQCIYNTTYLNVQR_5_4_0_2_0

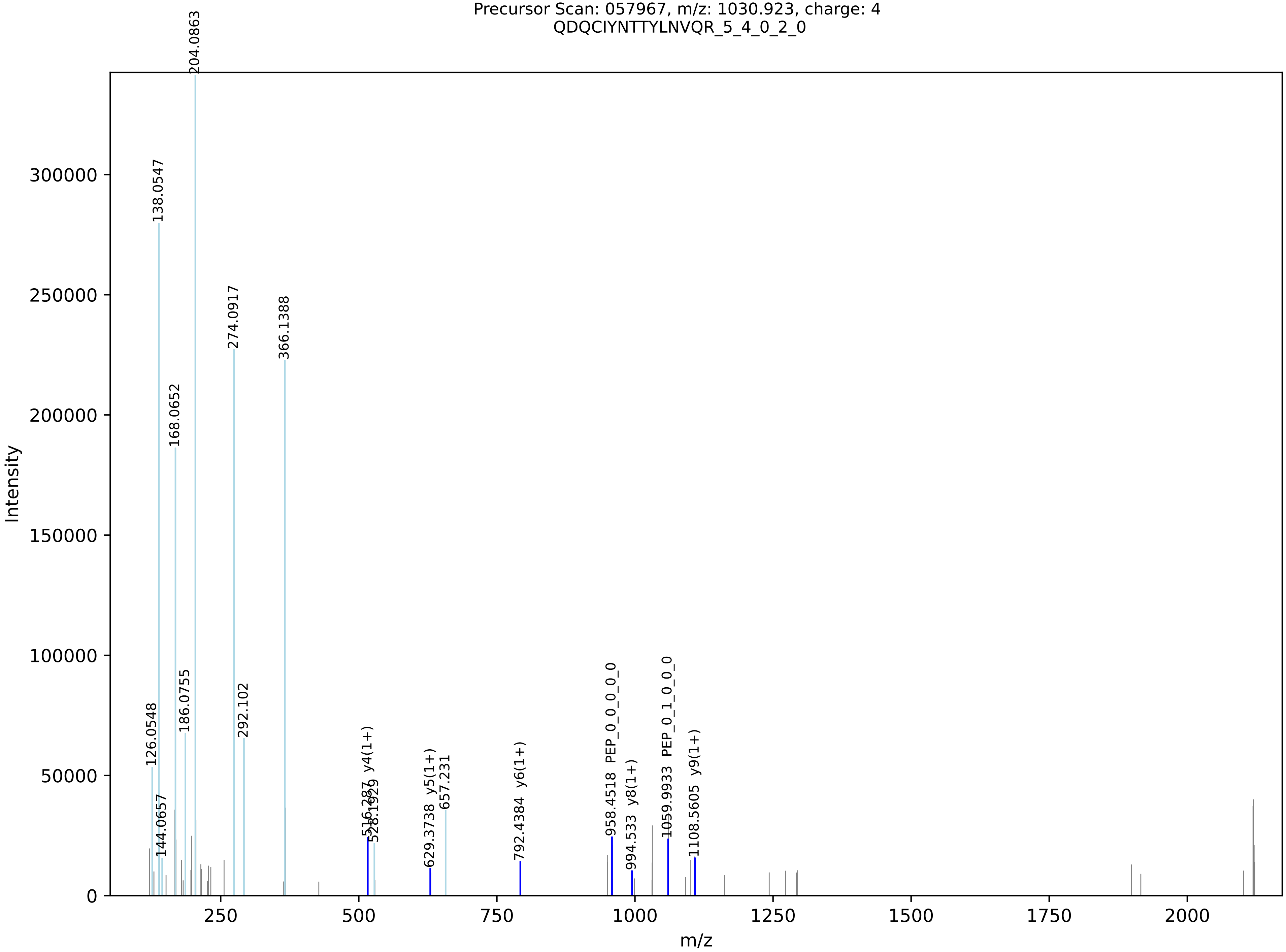

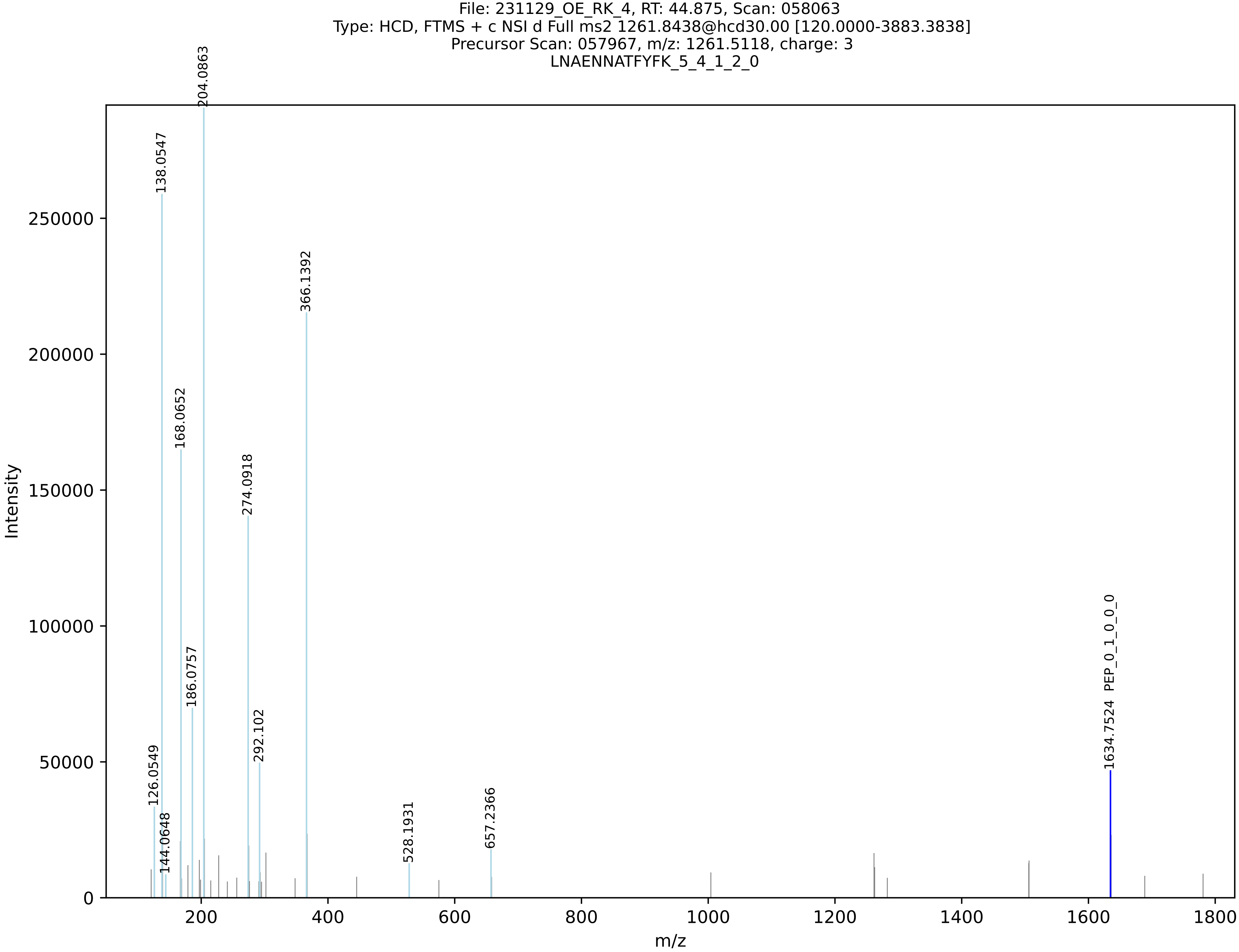
File: 231129_OE_RK_4, RT: 44.875, Scan: 058063
Type: HCD, FTMS + c NSI d Full ms2 1261.8438@hcd30.00 [120.0000-3883.3838]
Precursor Scan: 057967, m/z: 1261.5118, charge: 3
LNAENNATFYFK_5_4_1_2_0
Intensity
m/z
0
50000
100000
150000
200000
250000
200
400
600
800
1000
1200
1400
1600
1800
204.0863
138.0547
366.1392
168.0652
274.0918
186.0757
292.102
126.0549
144.0648
528.1931
657.2366
1634.7524 PEP_0_1_0_0_0

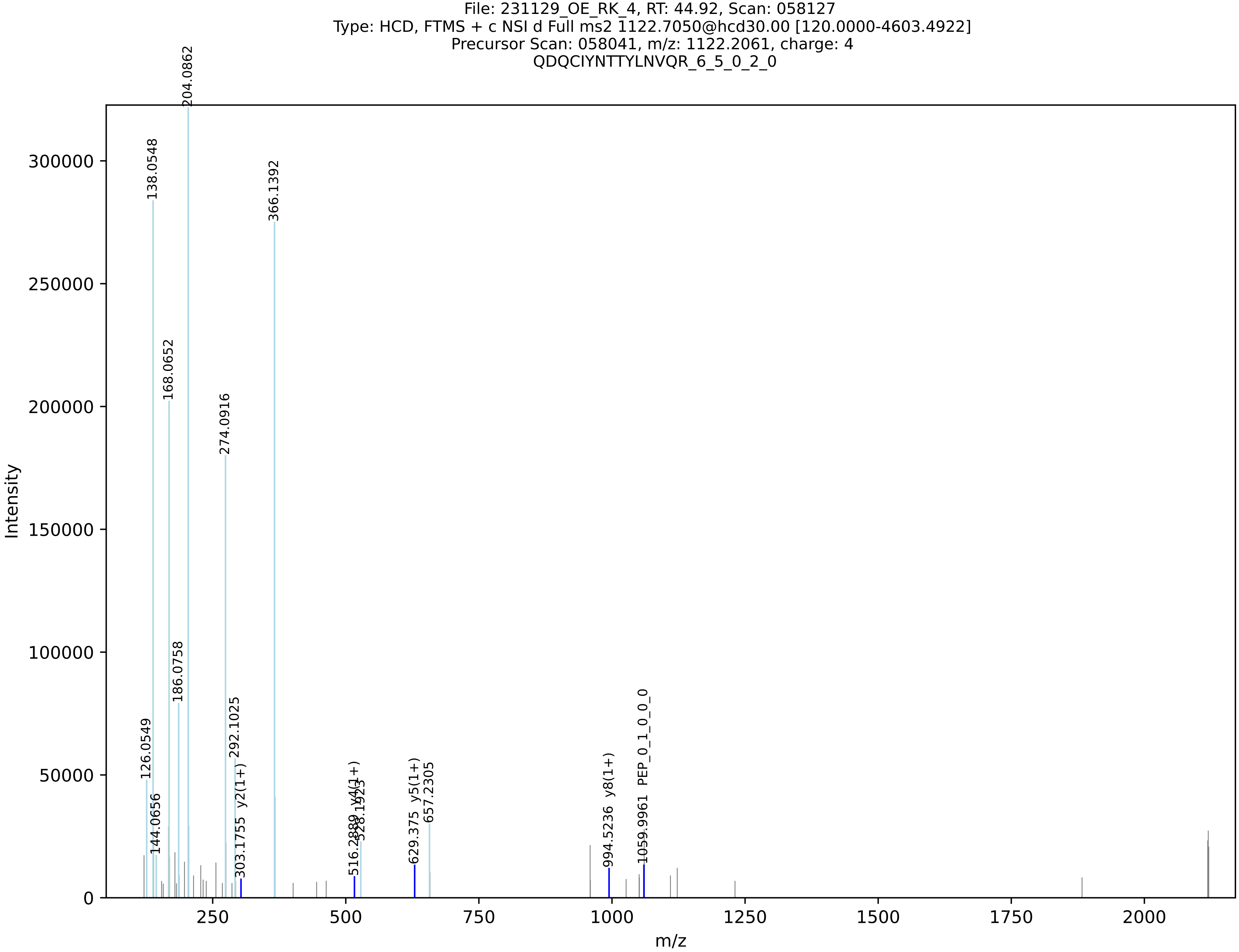
File: 231129_OE_RK_4, RT: 44.92, Scan: 058127
Type: HCD, FTMS + c NSI d Full ms2 1122.7050@hcd30.00 [120.0000-4603.4922]
Precursor Scan: 058041, m/z: 1122.2061, charge: 4
QDQCIYNTTYLNVQR_6_5_0_2_0
Intensity
m/z
204.0862
138.0548
366.1392
168.0652
274.0916
186.0758
126.0549
292.1025
144.0656
303.1755 y2(1+)
516.2889 y4(1+)
528.1923
629.375 y5(1+)
657.2305
994.5236 y8(1+)
1059.9961 PEP_0_1_0_0_0_
0
50000
100000
150000
200000
250000
300000
250
500
750
1000
1250
1500
1750
2000

File: 231129_OE_RK_4, RT: 45.067, Scan: 058342
Type: HCD, FTMS + c NSI d Full ms2 1297.2080@hcd30.00 [120.0000-3991.5986]
Precursor Scan: 058262, m/z: 1296.8743, charge: 3
LANLTQGEDQYYLR_5_4_0_2_0

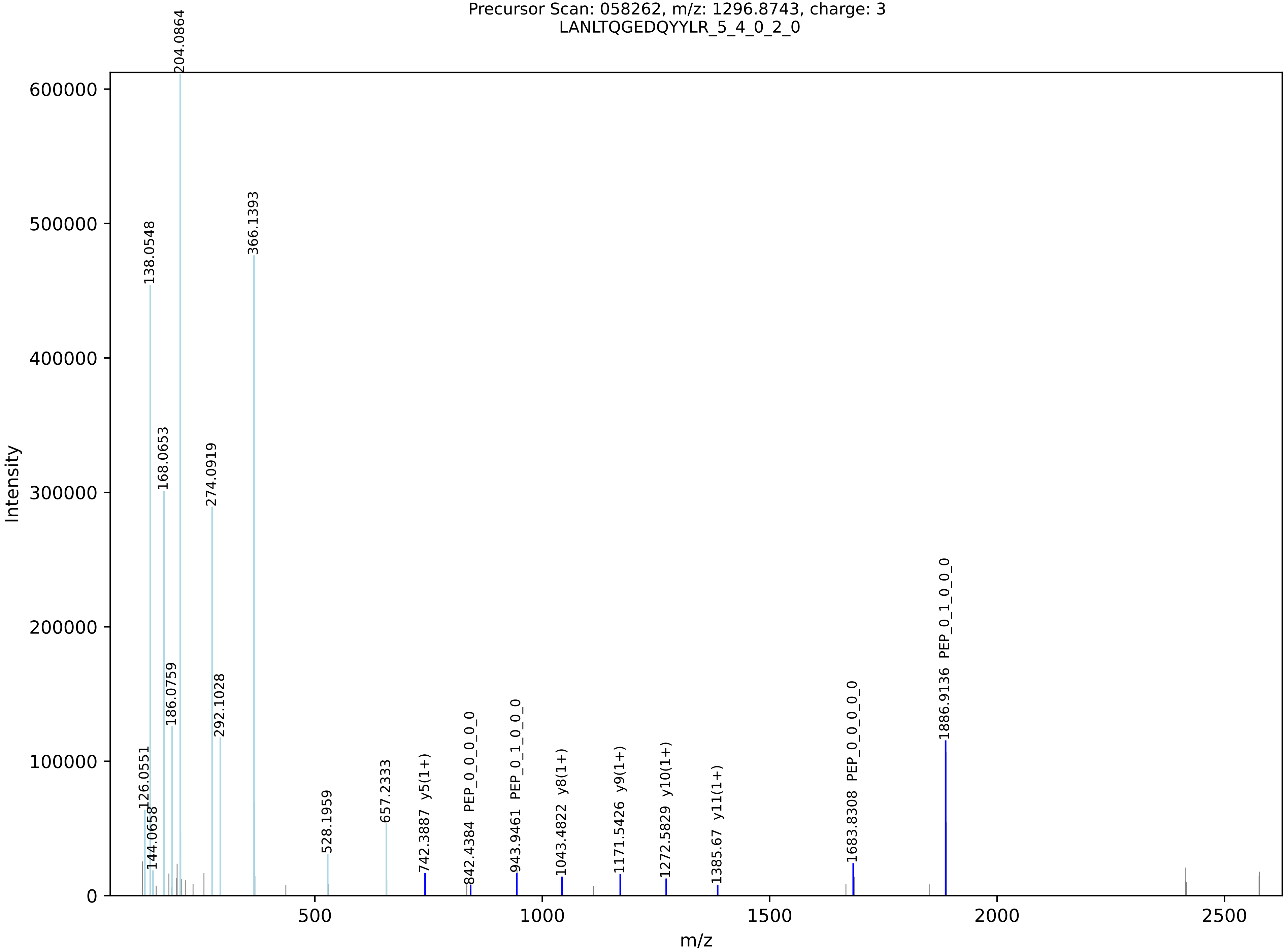

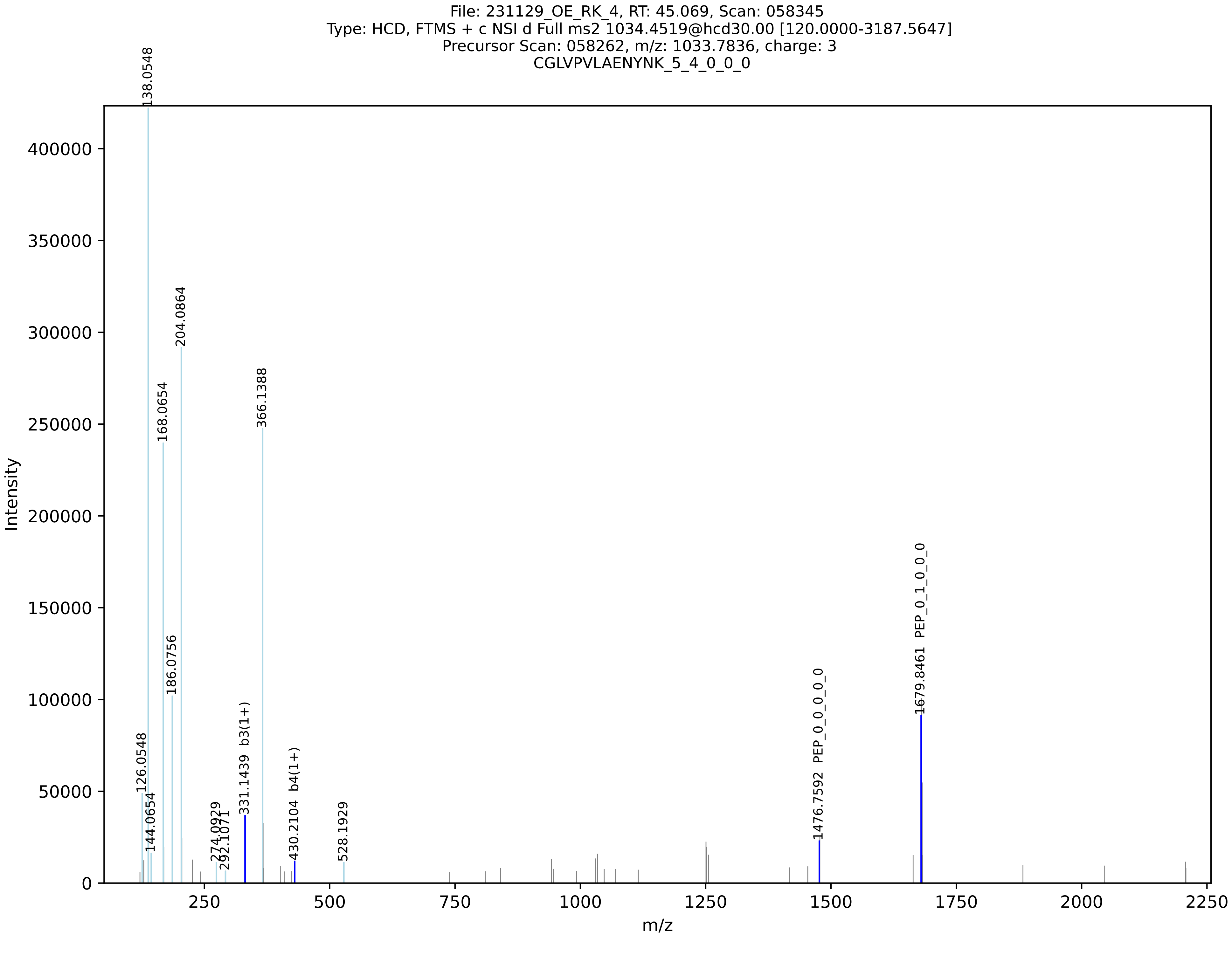
File: 231129_OE_RK_4, RT: 45.069, Scan: 058345
Type: HCD, FTMS + c NSI d Full ms2 1034.4519@hcd30.00 [120.0000-3187.5647]
Precursor Scan: 058262, m/z: 1033.7836, charge: 3
CGLVPVLAENYNK_5_4_0_0_0
Intensity
m/z
138.0548
204.0864
366.1388
168.0654
186.0756
126.0548
144.0654
274.0929
292.1071
331.1439 b3(1+)
430.2104 b4(1+)
528.1929
1476.7592 PEP_0_0_0_0_0
1679.8461 PEP_0_1_0_0_0
0
50000
100000
150000
200000
250000
300000
350000
400000
250
500
750
1000
1250
1500
1750
2000
2250

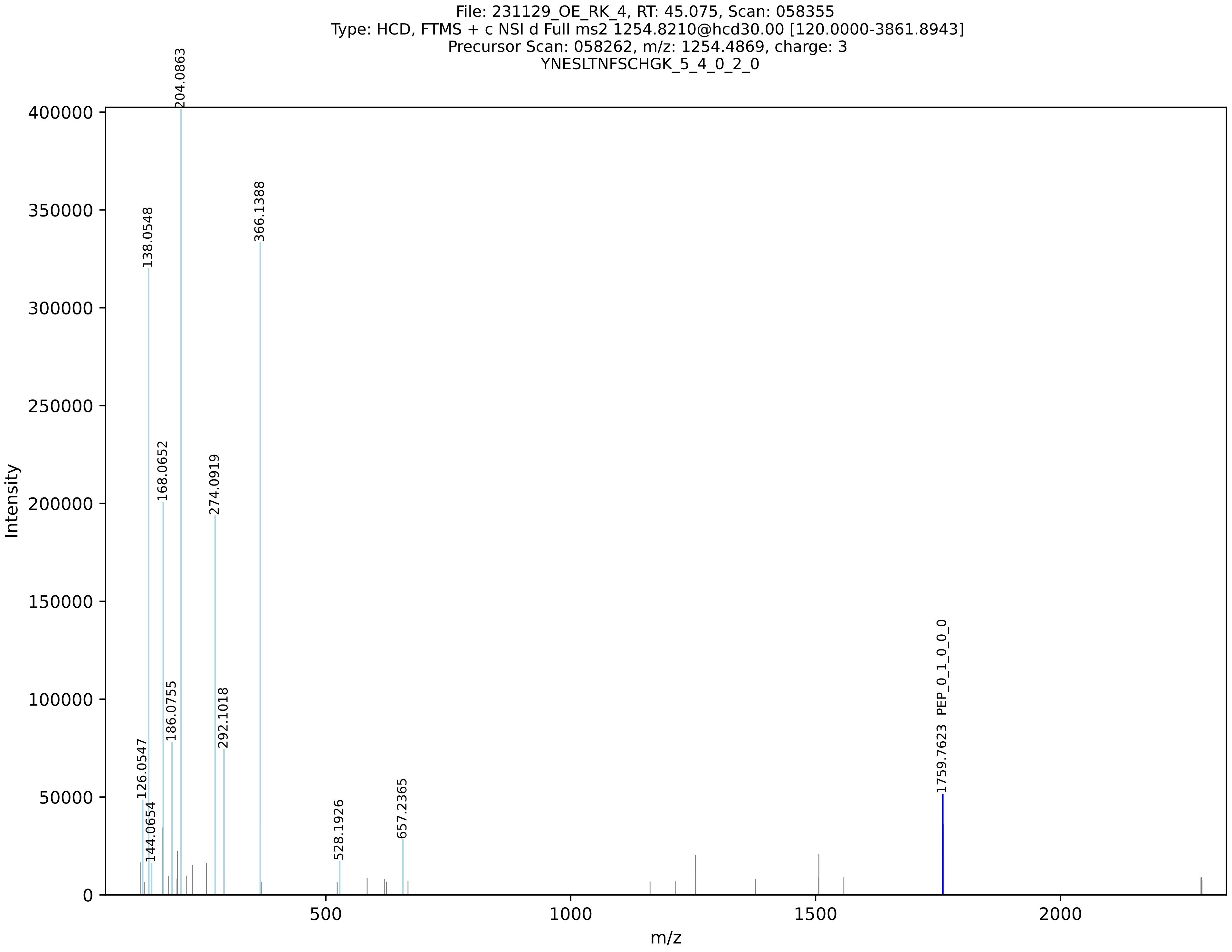
File: 231129_OE_RK_4, RT: 45.075, Scan: 058355
Type: HCD, FTMS + c NSI d Full ms2 1254.8210@hcd30.00 [120.0000-3861.8943]
Precursor Scan: 058262, m/z: 1254.4869, charge: 3
YNESLTNFSCHGK_5_4_0_2_0
Intensity
m/z
204.0863
138.0548
366.1388
168.0652
274.0919
186.0755
292.1018
126.0547
144.0654
528.1926
657.2365
1759.7623 PEP_0_1_0_0_0
0
50000
100000
150000
200000
250000
300000
350000
400000
500
1000
1500
2000

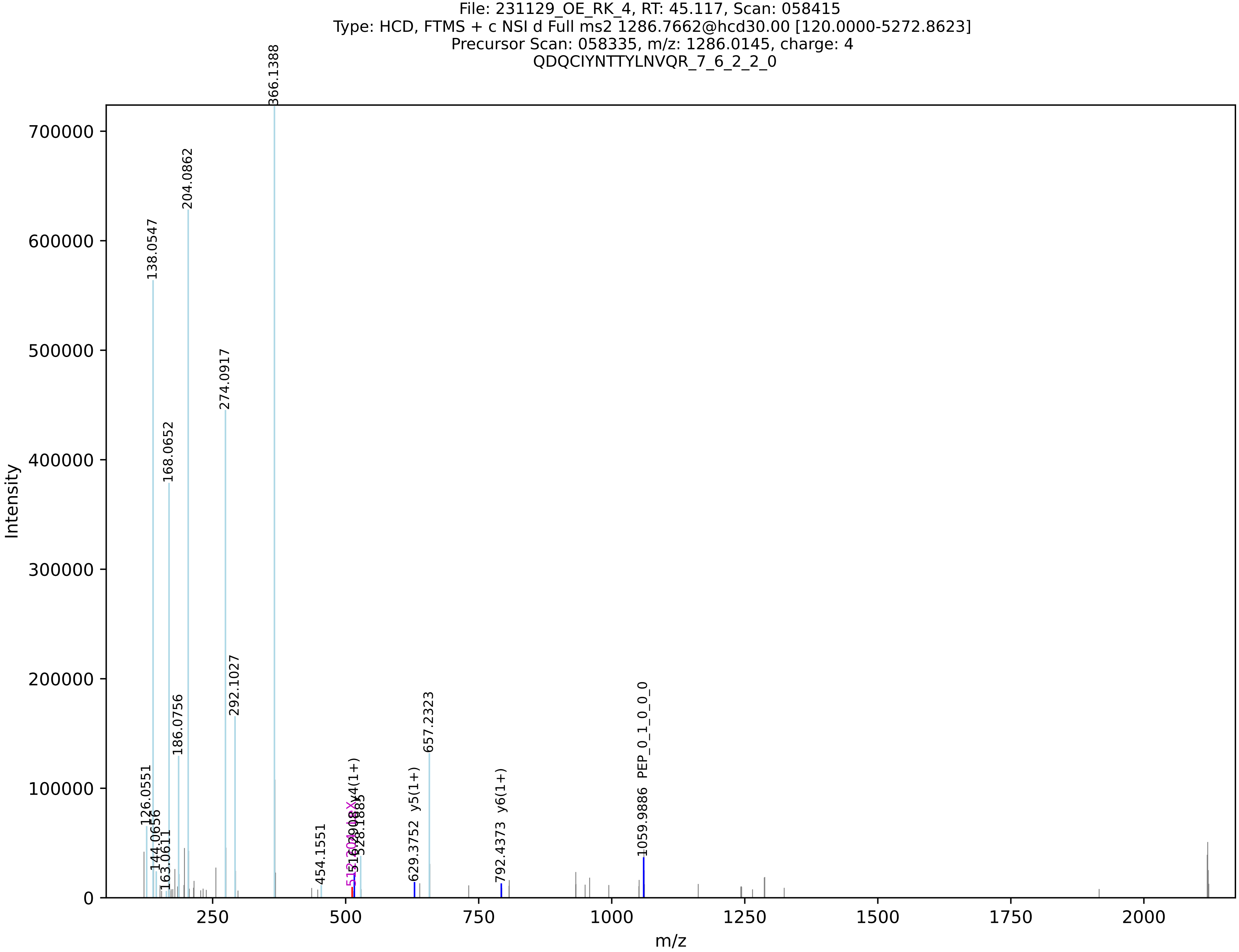

File: 231129_OE_RK_4, RT: 45.117, Scan: 058415
Type: HCD, FTMS + c NSI d Full ms2 1286.7662@hcd30.00 [120.0000-5272.8623]
Precursor Scan: 058335, m/z: 1286.0145, charge: 4
QDQCIYNTTYLNVQR_7_6_2_2_0
Intensity
m/z
366.1388
204.0862
138.0547
274.0917
168.0652
292.1027
186.0756
126.0551
144.0656
163.0611
454.1551
516.2908 y4(1+)
528.1885
629.3752 y5(1+)
657.2323
792.4373 y6(1+)
1059.9886 PEP_0_1_0_0_0_
0
100000
200000
300000
400000
500000
600000
700000
250
500
750
1000
1250
1500
1750
2000

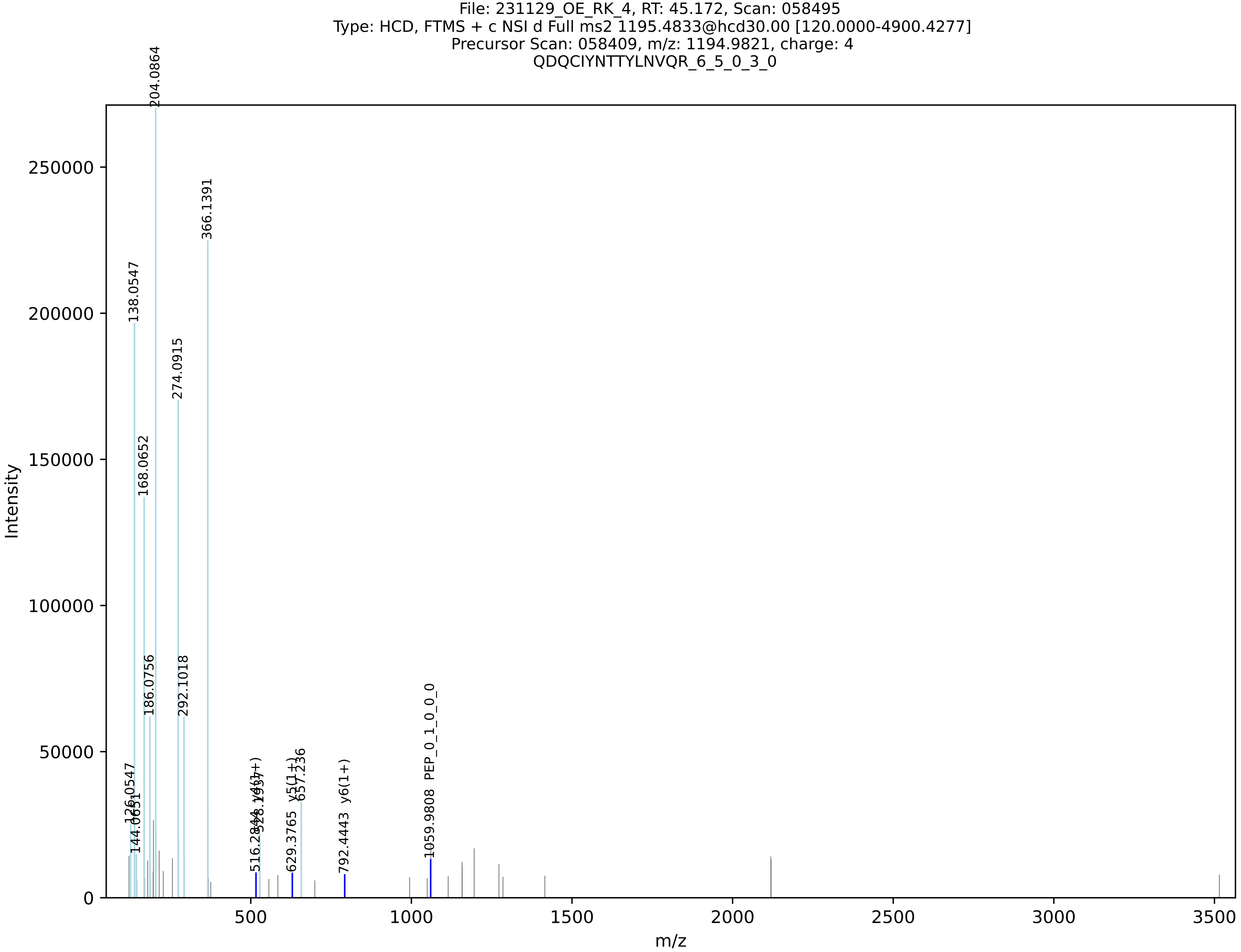

File: 231129_OE_RK_4, RT: 45.172, Scan: 058495
Type: HCD, FTMS + c NSI d Full ms2 1195.4833@hcd30.00 [120.0000-4900.4277]
Precursor Scan: 058409, m/z: 1194.9821, charge: 4
QDQCIYNTTYLNVQR_6_5_0_3_0
204.0864
366.1391
138.0547
274.0915
168.0652
186.0756
292.1018
126.0547
144.0651
516.2844 y4(1+)
528.1937
629.3765 y5(1+)
657.236
792.4443 y6(1+)
1059.9808 PEP_0_1_0_0_
Intensity
0
50000
100000
150000
200000
250000
500
1000
1500
2000
2500
3000
3500
m/z

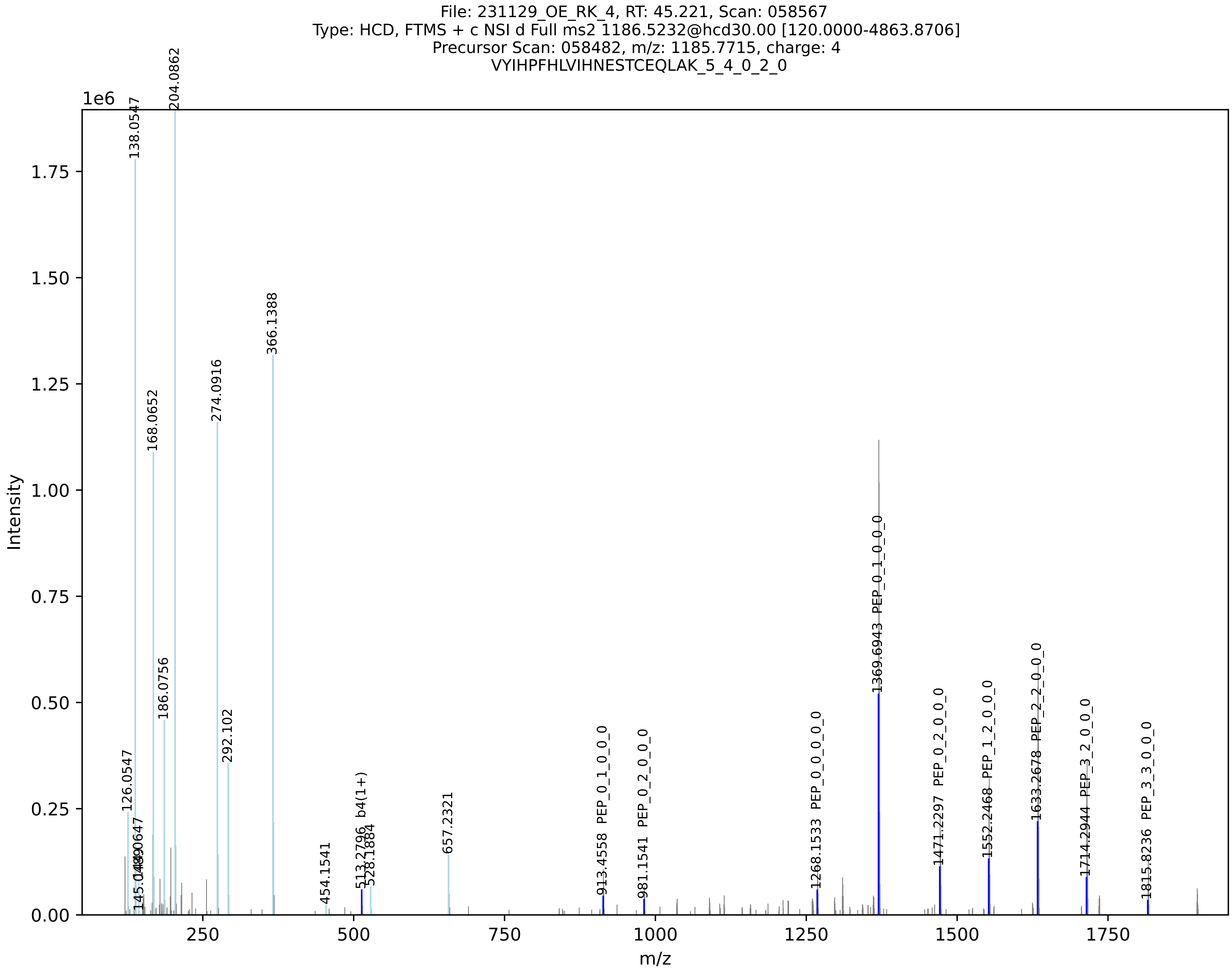

File: 231129_OE_RK_4, RT: 45.221, Scan: 058567
Type: HCD, FTMS + c NSI d Full ms2 1186.5232@hcd30.00 [120.0000-4863.8706]
Precursor Scan: 058482, m/z: 1185.7715, charge: 4
VYIHPFHLVIHNESTCEQLAK_5_4_0_2_0
1e6
Intensity
m/z
204.0862
138.0547
168.0652
186.0756
126.0547
274.0916
292.102
366.1388
454.1541
513.2796 b4(1+)
528.1884
657.2321
913.4558 PEP_0_1_0_0_0
981.1541 PEP_0_2_0_0_0
1268.1533 PEP_0_0_0_0_0
1369.6943 PEP_0_1_0_0_0
1471.2297 PEP_0_2_0_0_0
1552.2468 PEP_1_2_0_0_0
1633.2678 PEP_2_2_0_0_0
1714.2944 PEP_3_2_0_0_0
1815.8236 PEP_3_3_0_0_0

File: 231129_OE_RK_4, RT: 45.247, Scan: 058608
Type: HCD, FTMS + c NSI d Full ms2 1312.0396@hcd30.00 [120.0000-2697.9885]
Precursor Scan: 058482, m/z: 1312.0395, charge: 2
NGSLFAFR_5_3_0_1_0

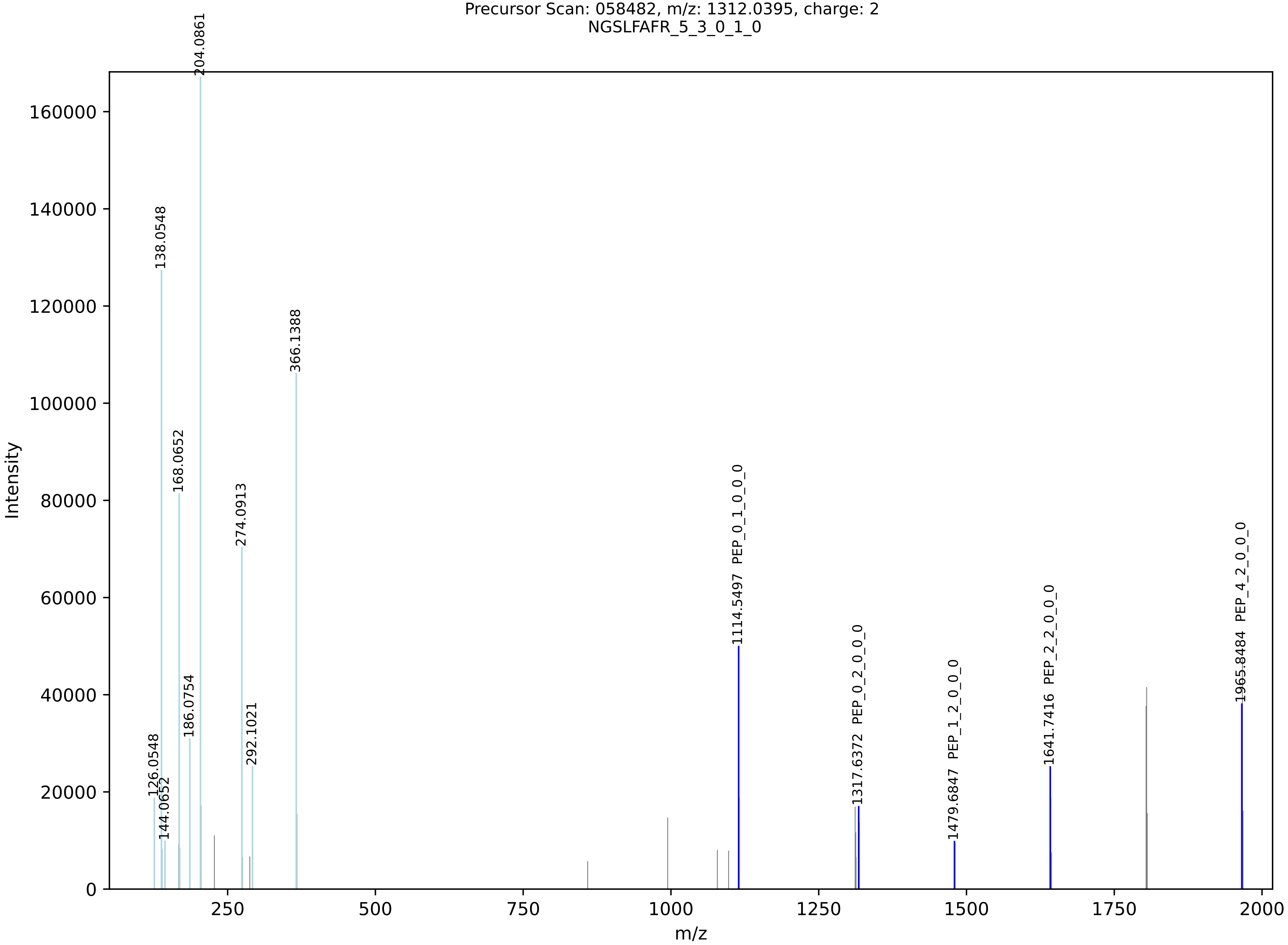

File: 231129_OE_RK_4, RT: 45.269, Scan: 058638
Type: HCD, FTMS + c NSI d Full ms2 1359.7925@hcd30.00 [120.0000-5570.8096]
Precursor Scan: 058556, m/z: 1359.2913, charge: 4
QDQCIYNTTYLNVQR_7_6_2_3_0

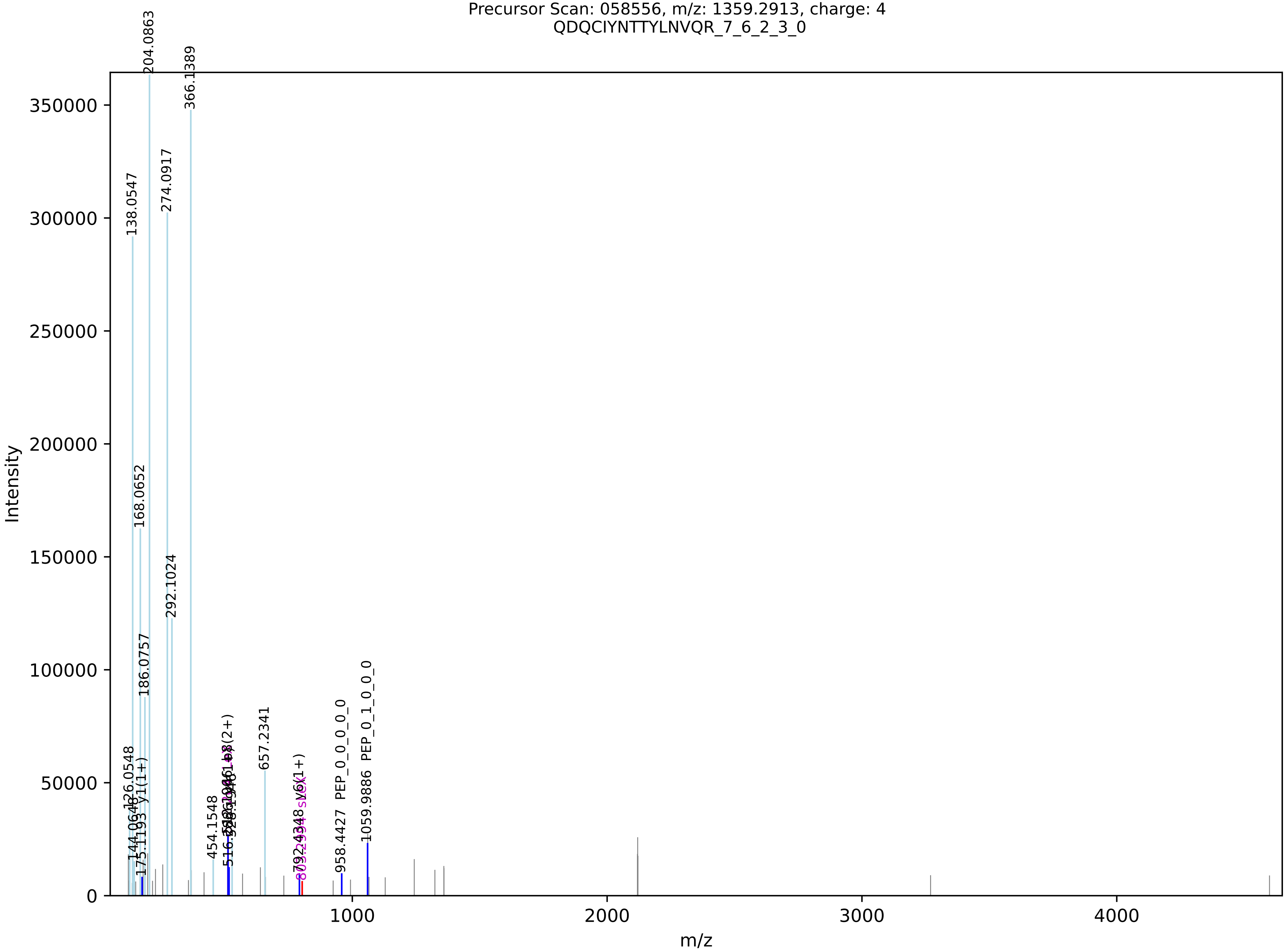

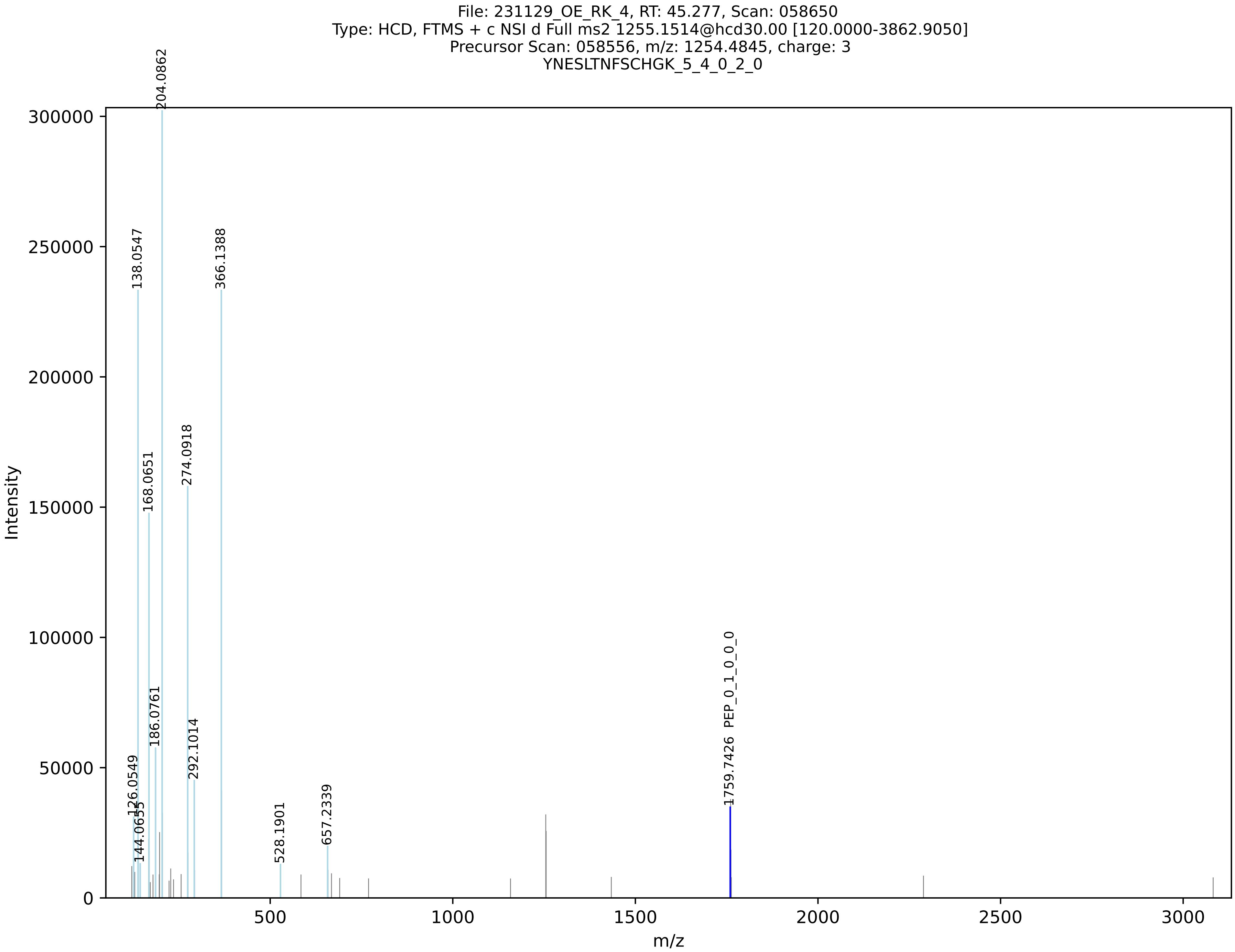
File: 231129_OE_RK_4, RT: 45.277, Scan: 058650
Type: HCD, FTMS + c NSI d Full ms2 1255.1514@hcd30.00 [120.0000-3862.9050]
Precursor Scan: 058556, m/z: 1254.4845, charge: 3
YNESLTNFSCHGK_5_4_0_2_0
204.0862
138.0547
366.1388
274.0918
168.0651
186.0761
292.1014
126.0549
144.0655
528.1901
657.2339
1759.7426 PEP_0_1_0_0_0
Intensity
m/z
0
50000
100000
150000
200000
250000
300000
500
1000
1500
2000
2500
3000

File: 231129_OE_RK_4, RT: 45.322, Scan: 058716
Type: HCD, FTMS + c NSI d Full ms2 942.3885@hcd30.00 [120.0000-2905.8506]
Precursor Scan: 058630, m/z: 942.3885, charge: 3
NGSLFAFR_5_4_0_1_0

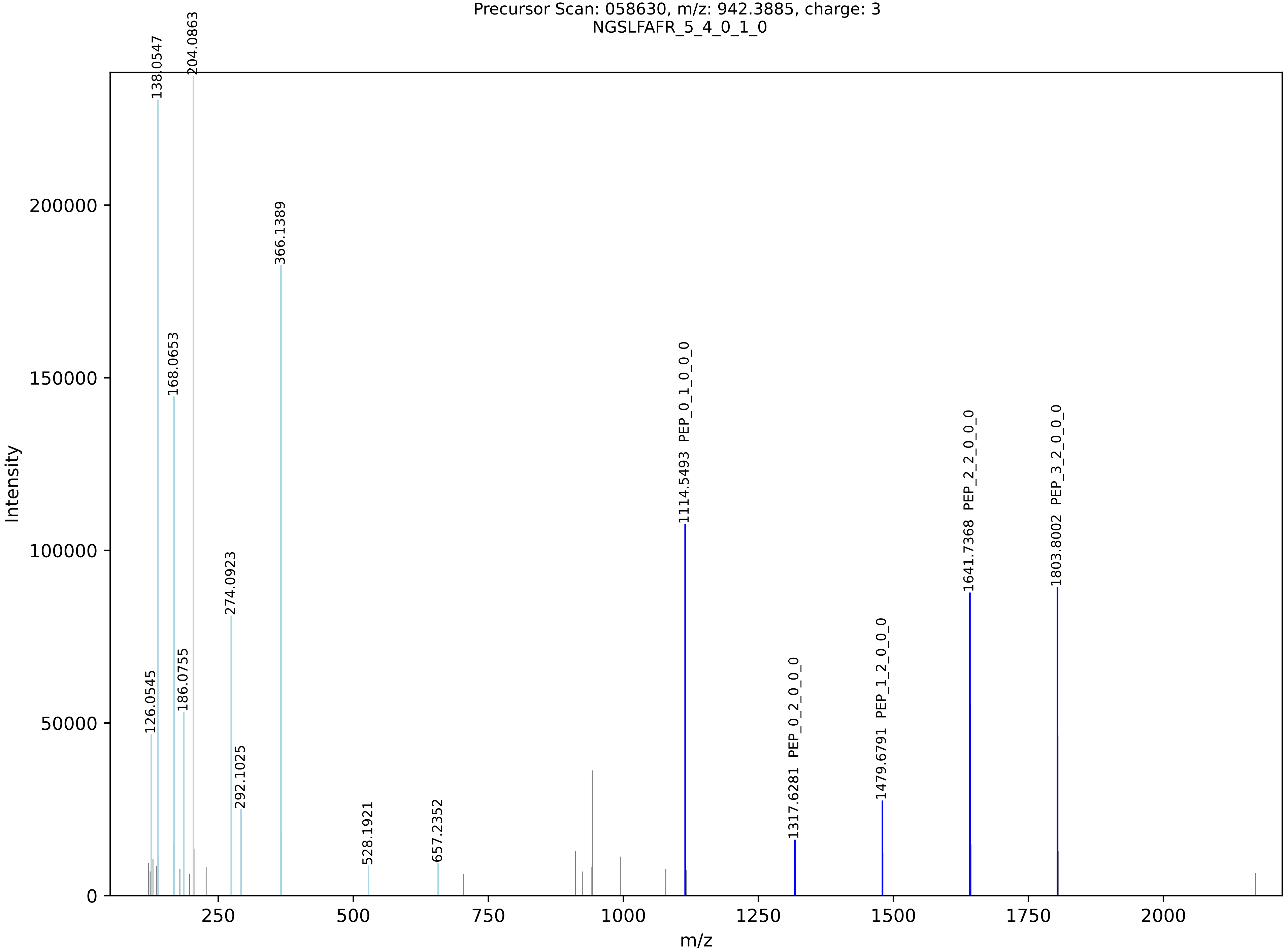

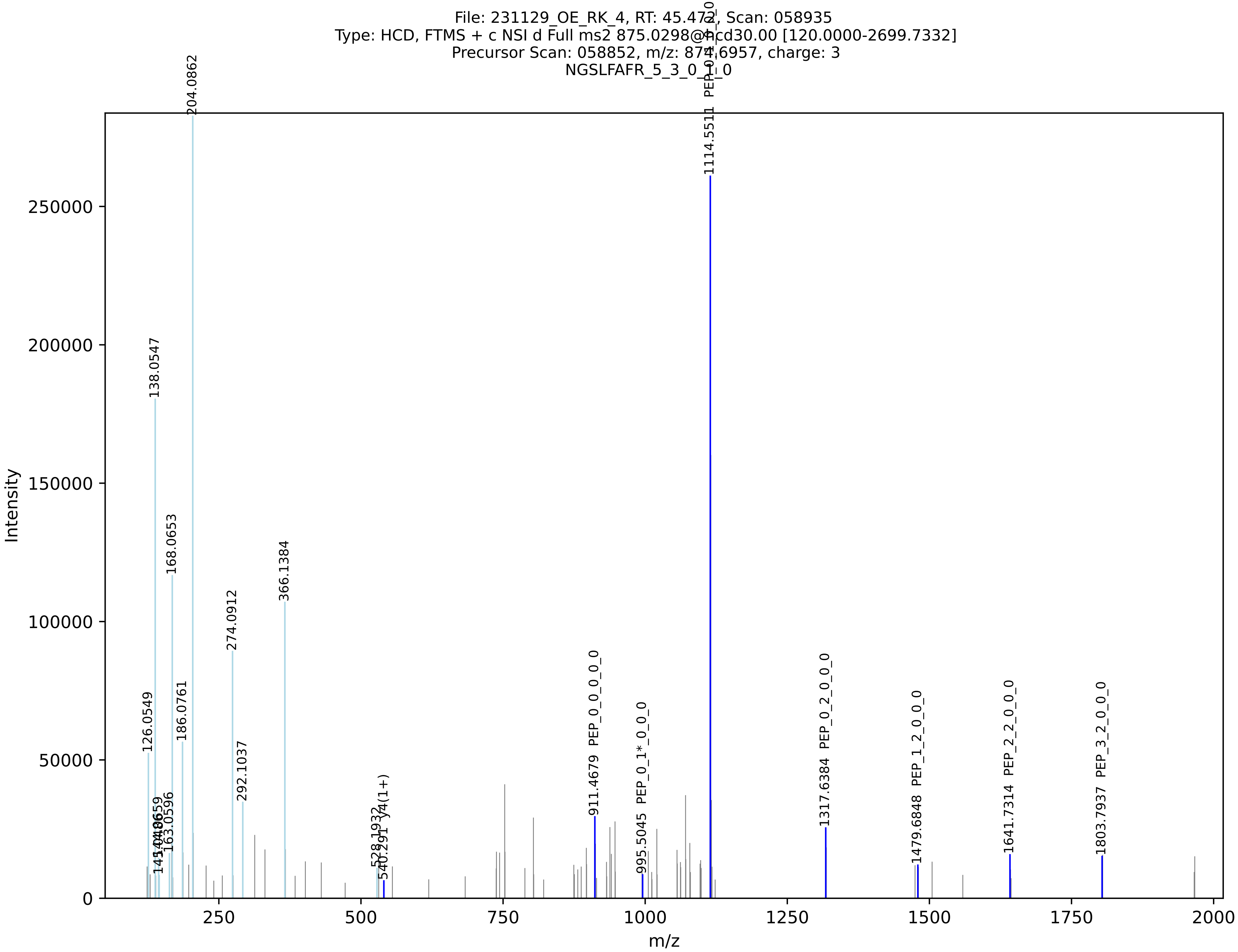
File: 231129_OE_RK_4, RT: 45.472, Scan: 058935
Type: HCD, FTMS + c NSI d Full ms2 875.0298@hcd30.00 [120.0000-2699.7332]
Precursor Scan: 058852, m/z: 874.6957, charge: 3
NGSLFAFR_5_3_0_1_0
Intensity
m/z
204.0862
138.0547
168.0653
366.1384
274.0912
126.0549
186.0761
292.1037
163.0596
528.1932
540.291 y4(1+)
911.4679 PEP_0_0_0_0_0
995.5045 PEP_0_1*_0_0_0
1114.5511 PEP_0_1_1_0
1317.6384 PEP_0_2_0_0_0
1479.6848 PEP_1_2_0_0_0
1641.7314 PEP_2_2_0_0_0
1803.7937 PEP_3_2_0_0_0

File: 231129_OE_RK_4, RT: 45.519, Scan: 059003
Type: HCD, FTMS + c NSI d Full ms2 1287.0151@hcd30.00 [120.0000-5273.8779]
Precursor Scan: 058926, m/z: 1286.2614, charge: 4
QDQCIYNTTYLNVQR_7_6_0_3_0

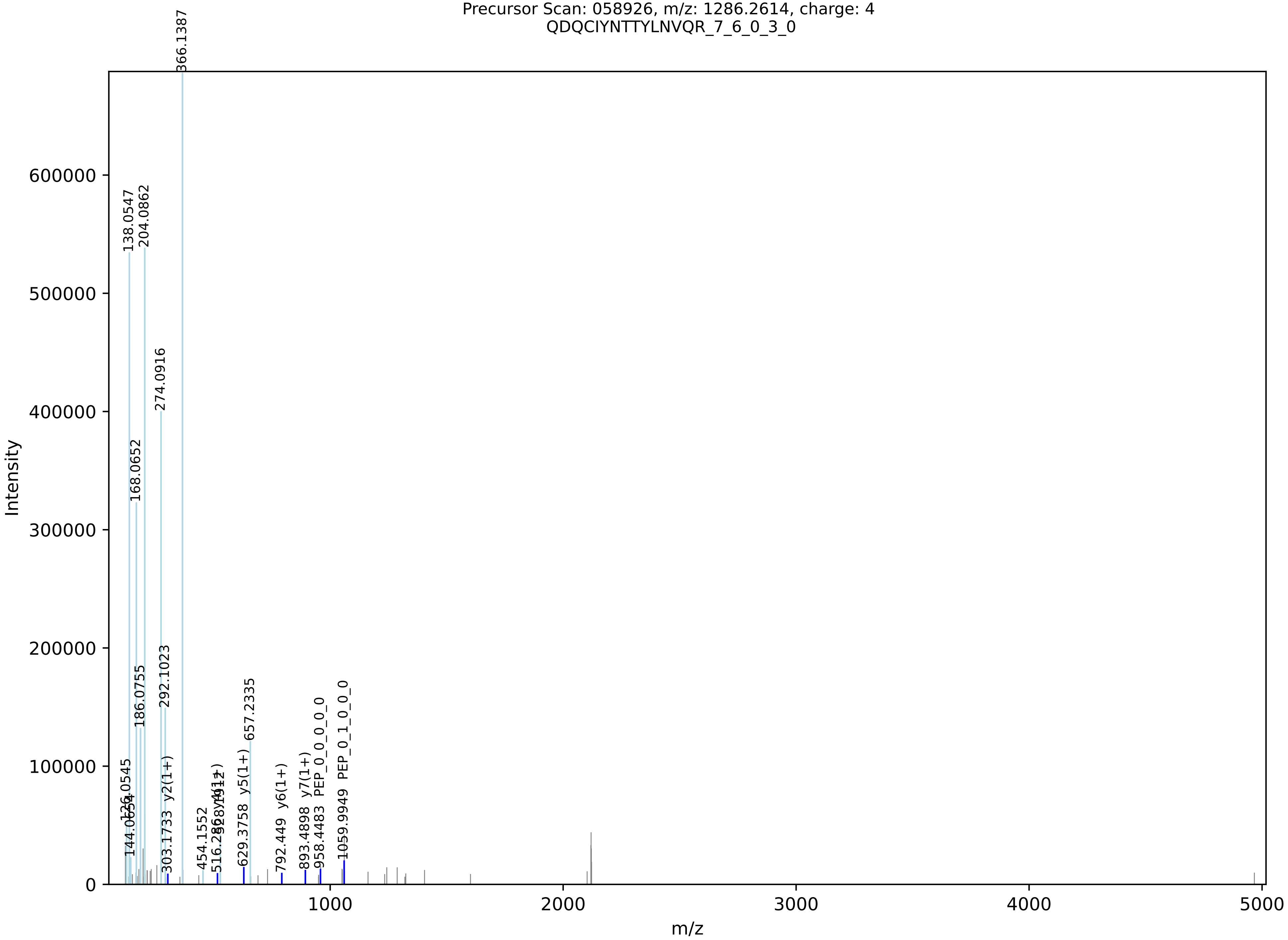

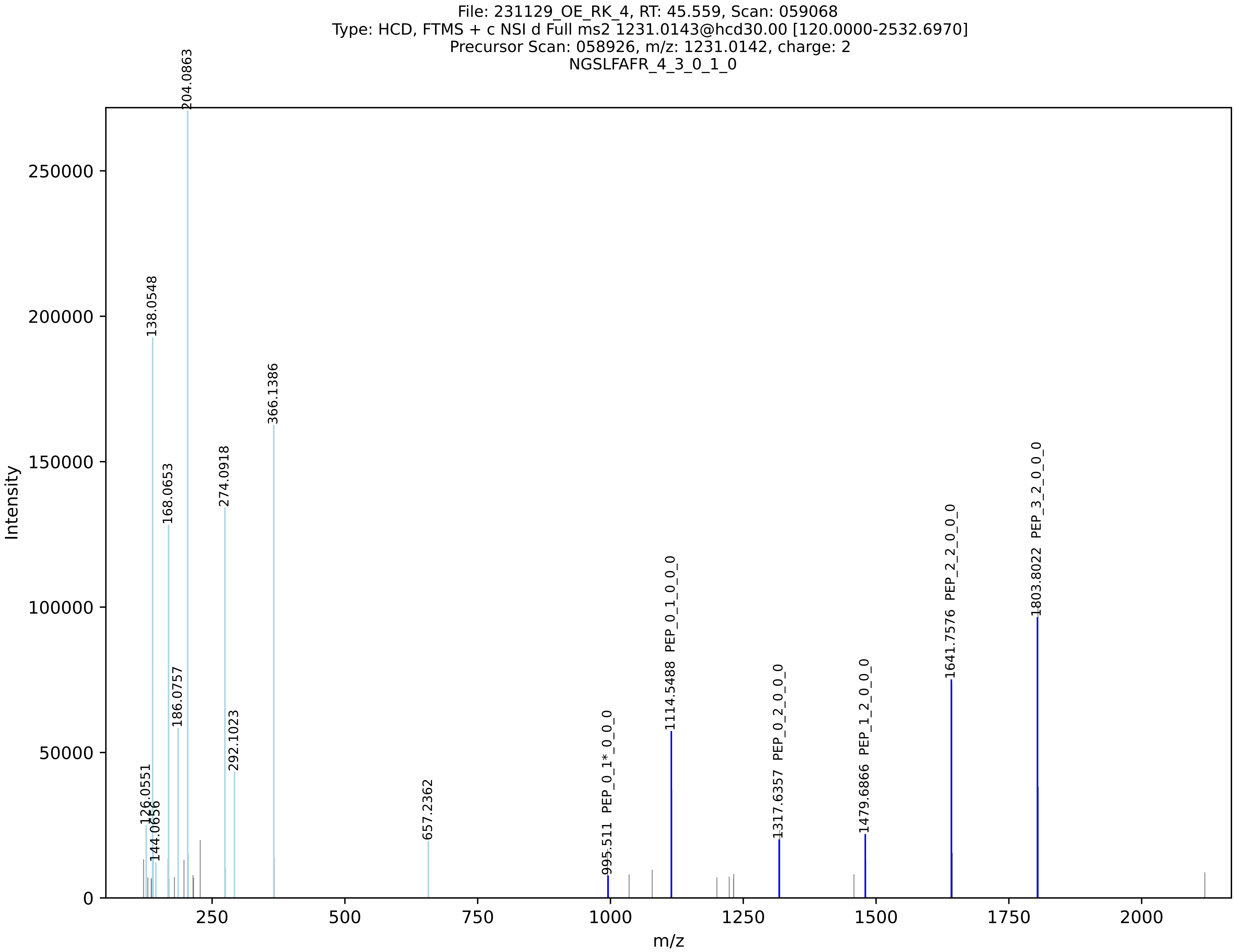
File: 231129_OE_RK_4, RT: 45.559, Scan: 059068
Type: HCD, FTMS + c NSI d Full ms2 1231.0143@hcd30.00 [120.0000-2532.6970]
Precursor Scan: 058926, m/z: 1231.0142, charge: 2
NGSLFAFR_4_3_0_1_0
Intensity
m/z
204.0863
138.0548
366.1386
274.0918
168.0653
186.0757
292.1023
126.0551
144.0656
657.2362
995.511 PEP_0_1*_0_0_0
1114.5488 PEP_0_1_0_0_0
1317.6357 PEP_0_2_0_0_0
1479.6866 PEP_1_2_0_0_0
1641.7576 PEP_2_2_0_0_0
1803.8022 PEP_3_2_0_0_0

File: 231129_OE_RK_4, RT: 45.628, Scan: 059163
Type: HCD, FTMS + c NSI d Full ms2 1423.2284@hcd30.00 [120.0000-4377.2207]
Precursor Scan: 059074, m/z: 1422.8986, charge: 3
SWPAVGNCSSALR_6_5_2_2_0

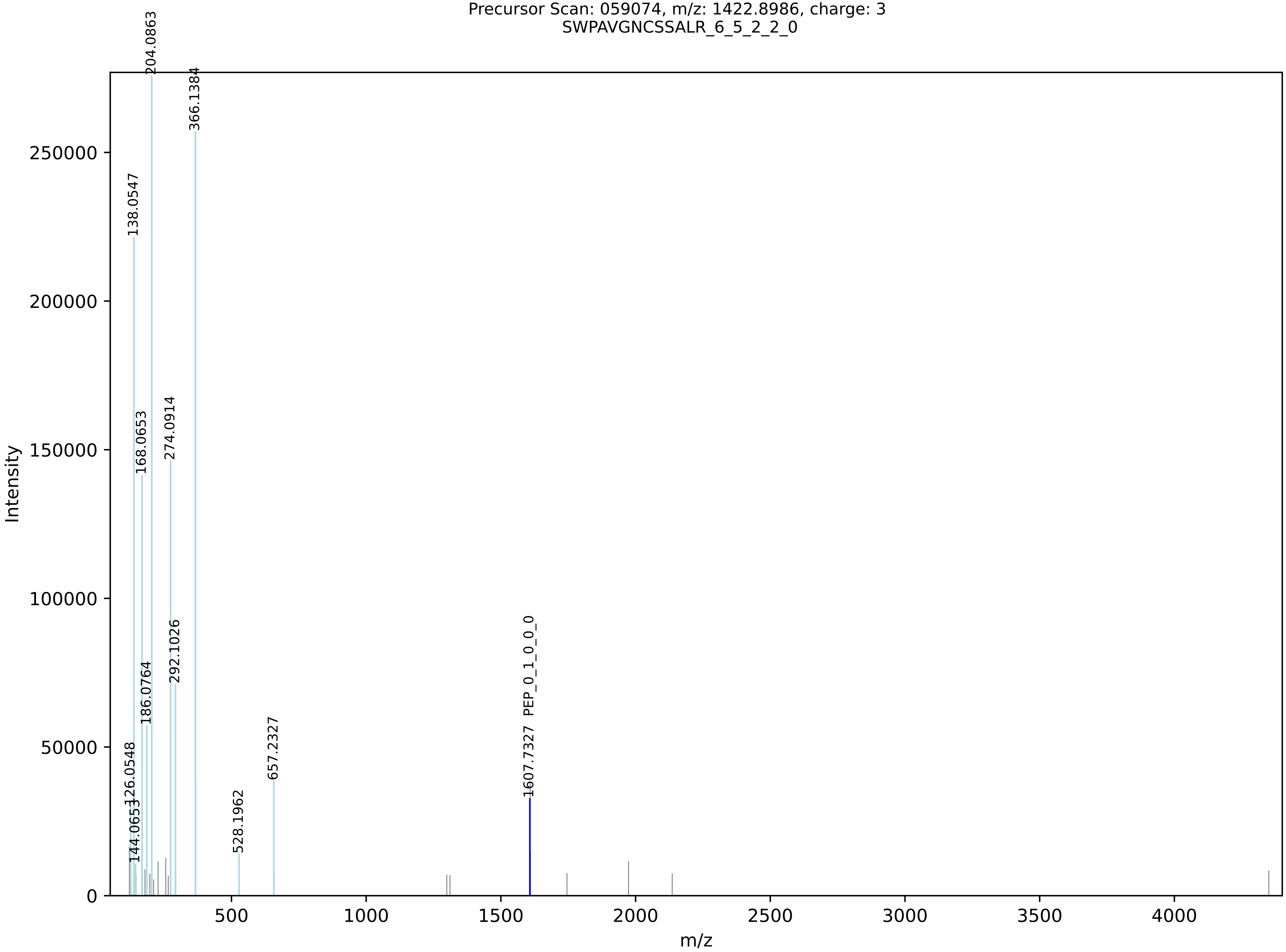

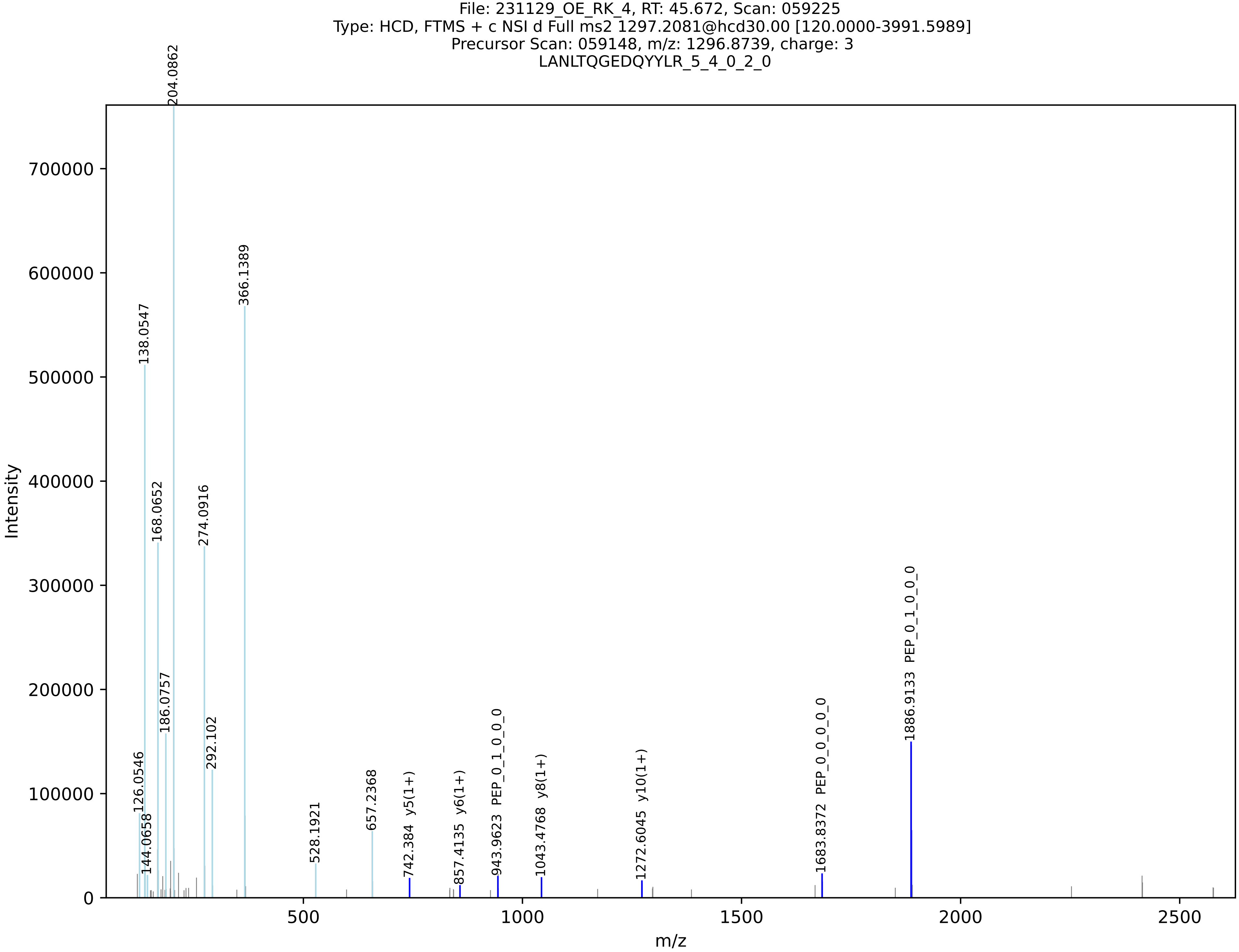

File: 231129_OE_RK_4, RT: 45.672, Scan: 059225
Type: HCD, FTMS + c NSI d Full ms2 1297.2081@hcd30.00 [120.0000-3991.5989]
Precursor Scan: 059148, m/z: 1296.8739, charge: 3
LANLTQGEDQYYLR_5_4_0_2_0
Intensity
m/z
204.0862
366.1389
138.0547
168.0652
274.0916
186.0757
292.102
126.0546
144.0658
528.1921
657.2368
742.384 y5(1+)
857.4135 y6(1+)
943.9623 PEP_0_1_0_0_0
1043.4768 y8(1+)
1272.6045 y10(1+)
1683.8372 PEP_0_0_0_0_0
1886.9133 PEP_0_1_0_0_0

File: 231129_OE_RK_4, RT: 45.672, Scan: 059226
Type: HCD, FTMS + c NSI d Full ms2 875.0306@hcd30.00 [120.0000-2699.7358]
Precursor Scan: 059148, m/z: 874.6962, charge: 3
NGSLFAFR_5_3_0_1_0

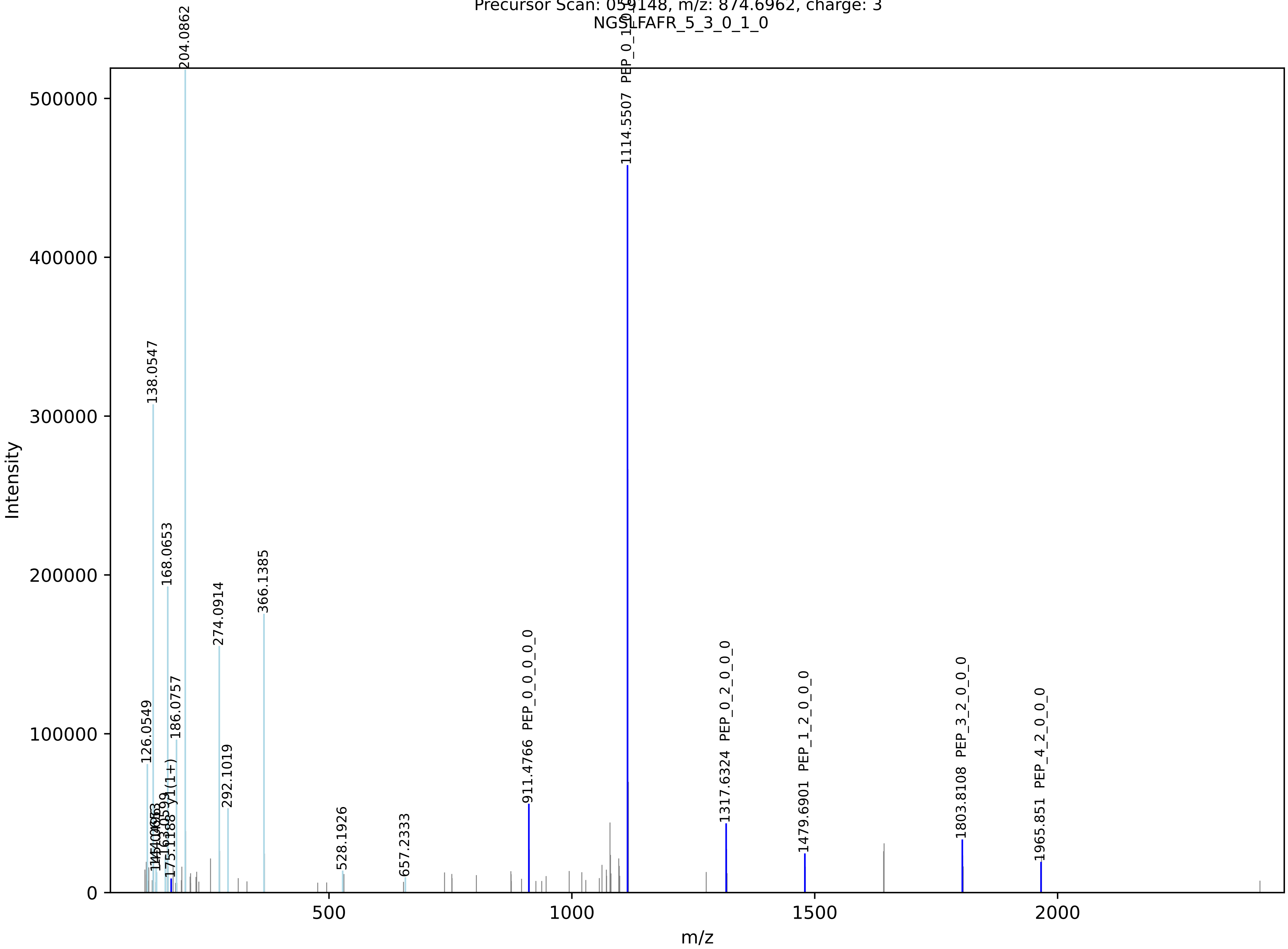

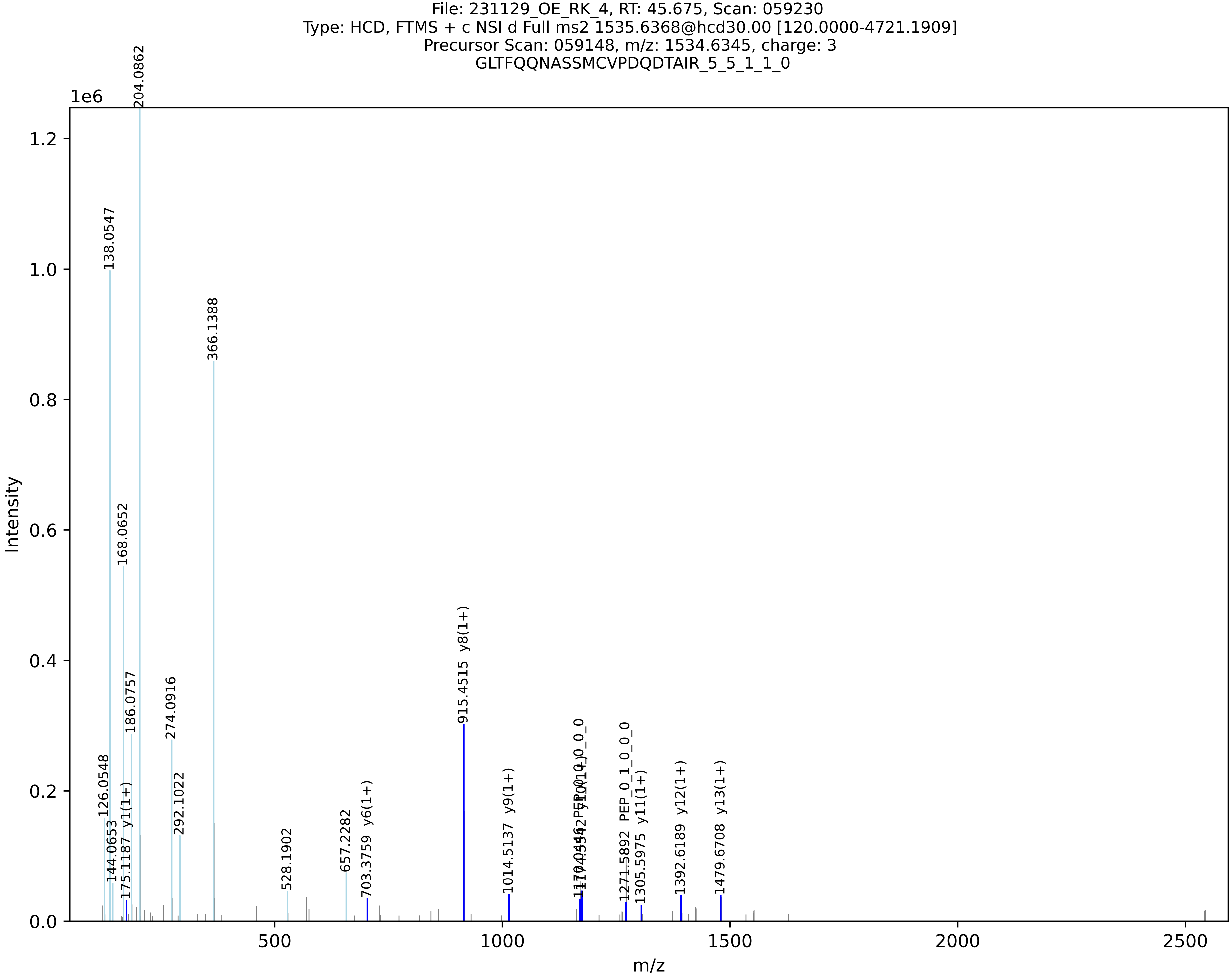
File: 231129_OE_RK_4, RT: 45.675, Scan: 059230
Type: HCD, FTMS + c NSI d Full ms2 1535.6368@hcd30.00 [120.0000-4721.1909]
Precursor Scan: 059148, m/z: 1534.6345, charge: 3
GLTFQQNASSMCVPDQDTAIR_5_5_1_1_0
1e6
Intensity
m/z
204.0862
138.0547
366.1388
168.0652
186.0757
274.0916
126.0548
292.1022
144.0653
175.1187 y1(1+)
528.1902
657.2282
703.3759 y6(1+)
915.4515 y8(1+)
1014.5137 y9(1+)
1305.5975 y11(1+)
1392.6189 y12(1+)
1479.6708 y13(1+)

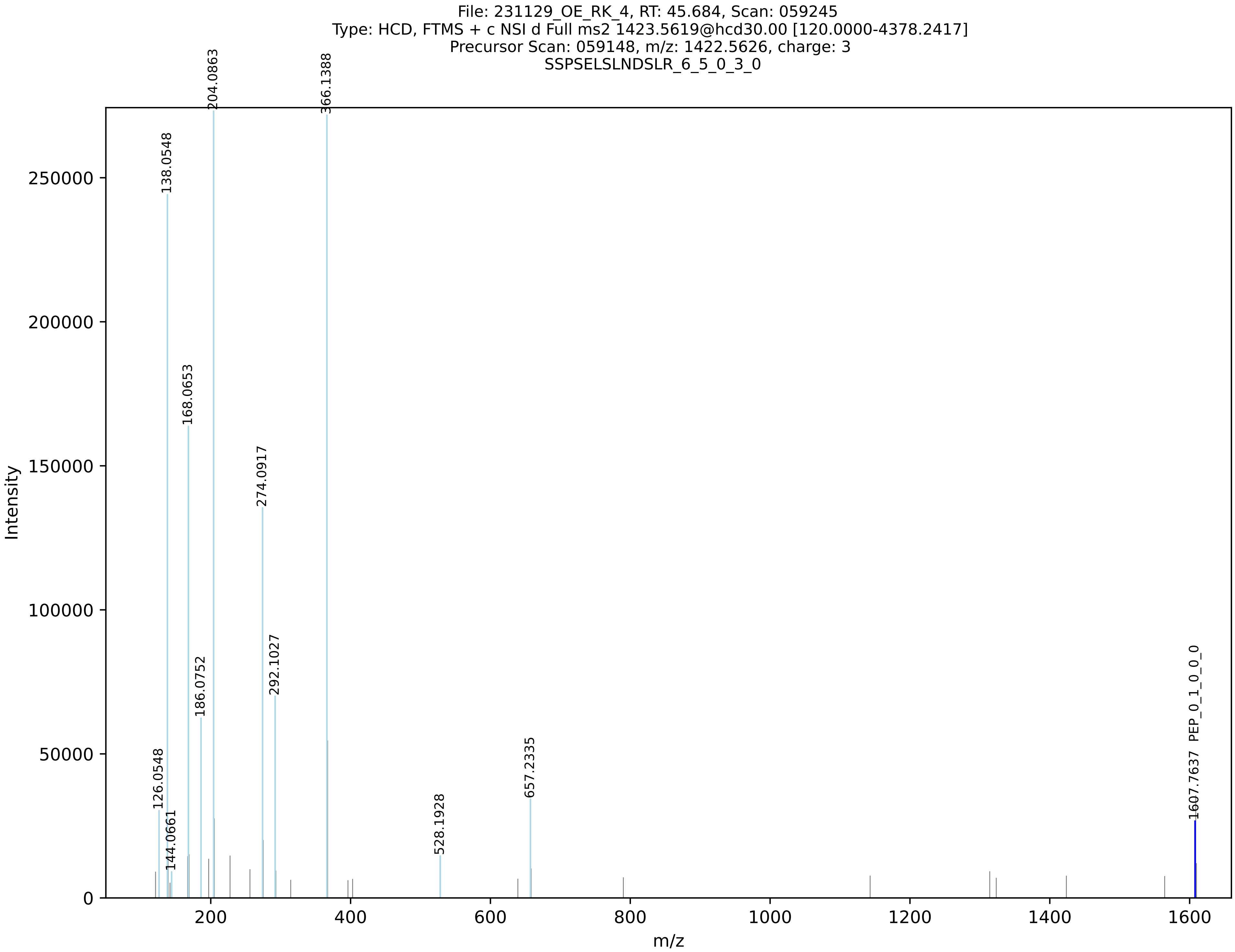

File: 231129_OE_RK_4, RT: 45.684, Scan: 059245
Type: HCD, FTMS + c NSI d Full ms2 1423.5619@hcd30.00 [120.0000-4378.2417]
Precursor Scan: 059148, m/z: 1422.5626, charge: 3
SSPSELSLNDSLR_6_5_0_3_0
Intensity
m/z
204.0863
366.1388
138.0548
168.0653
274.0917
186.0752
292.1027
126.0548
144.0661
528.1928
657.2335
1607.7637 PEP_0_1_0_0_0
0
50000
100000
150000
200000
250000
200
400
600
800
1000
1200
1400
1600

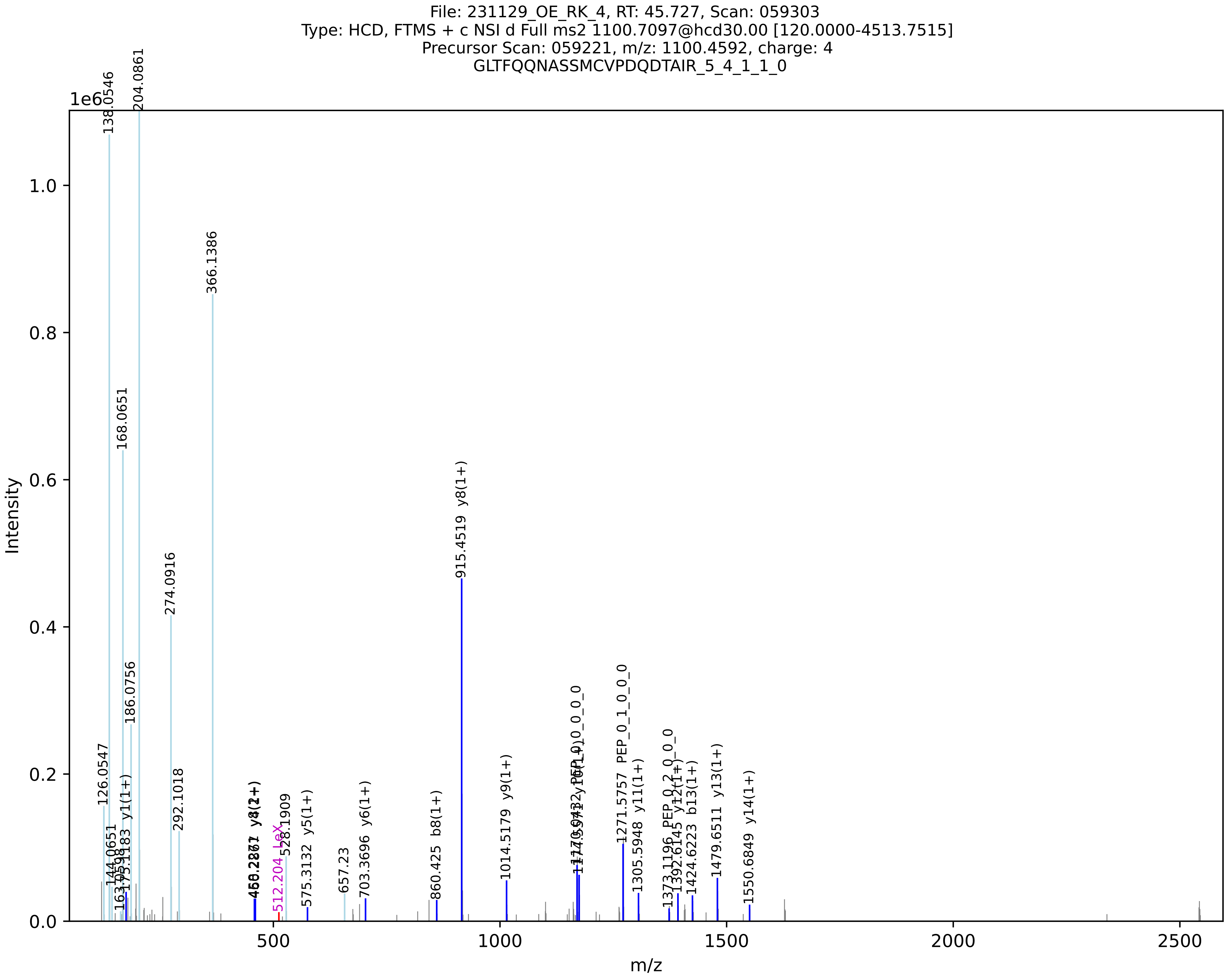

File: 231129_OE_RK_4, RT: 45.727, Scan: 059303
Type: HCD, FTMS + c NSI d Full ms2 1100.7097@hcd30.00 [120.0000-4513.7515]
Precursor Scan: 059221, m/z: 1100.4592, charge: 4
GLTFQQNASSMCVPDQDTAIR_5_4_1_1_0
1e6
Intensity
m/z
138.0546
204.0861
366.1386
168.0651
274.0916
186.0756
126.0547
292.1018
915.4519 y8(1+)
1014.5179 y9(1+)
1271.5757 PEP_0_1_0_0_0
1305.5948 y11(1+)
1424.6223 b13(1+)
1479.6511 y13(1+)
1550.6849 y14(1+)
860.425 b8(1+)
703.3696 y6(1+)
575.3132 y5(1+)
657.23
528.1909
512.204 LeX

File: 231129_OE_RK_4, RT: 45.738, Scan: 059322
Type: HCD, FTMS + c NSI d Full ms2 875.4221@hcd30.00 [120.0000-2700.9336]
Precursor Scan: 059221, m/z: 875.4221, charge: 3
NGSLFAFR_5_3_0_1_0

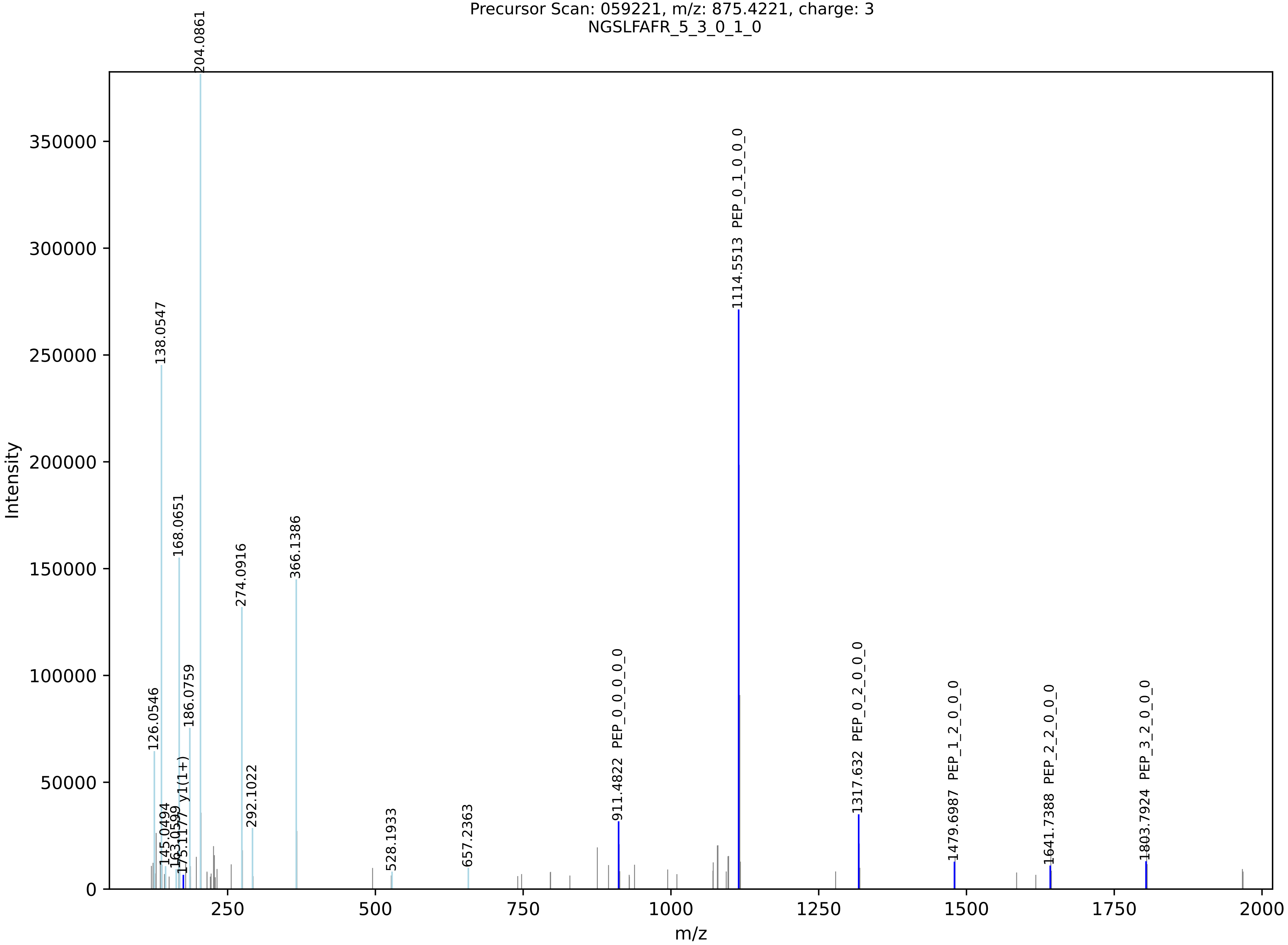

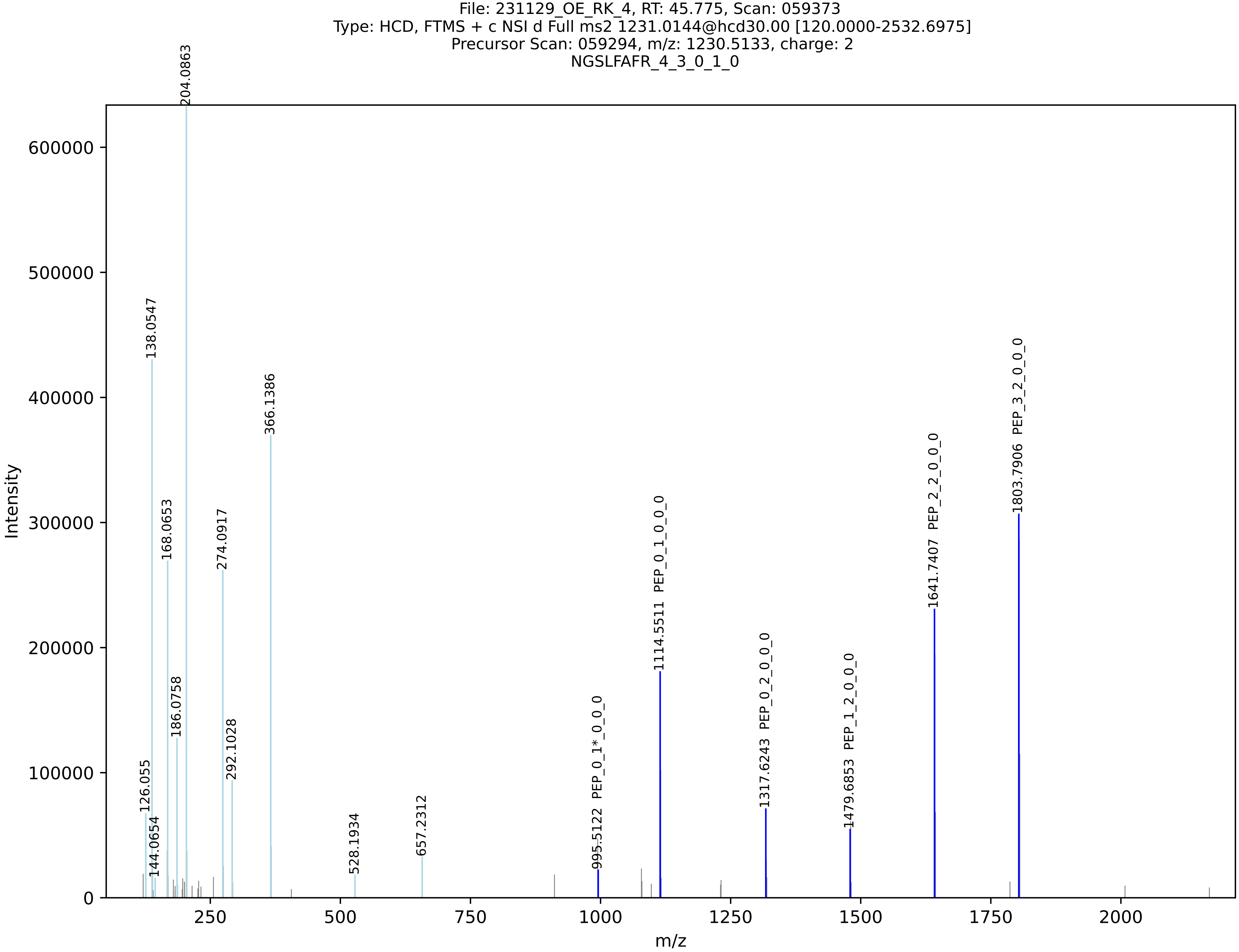
File: 231129_OE_RK_4, RT: 45.775, Scan: 059373
Type: HCD, FTMS + c NSI d Full ms2 1231.0144@hcd30.00 [120.0000-2532.6975]
Precursor Scan: 059294, m/z: 1230.5133, charge: 2
NGSLFAFR_4_3_0_1_0
Intensity
m/z
204.0863
138.0547
366.1386
168.0653
274.0917
186.0758
292.1028
126.055
144.0654
528.1934
657.2312
995.5122 PEP_0_1*_0_0_0
1114.5511 PEP_0_1_0_0_0
1317.6243 PEP_0_2_0_0_0
1479.6853 PEP_1_2_0_0_0
1641.7407 PEP_2_2_0_0_0
1803.7906 PEP_3_2_0_0_0

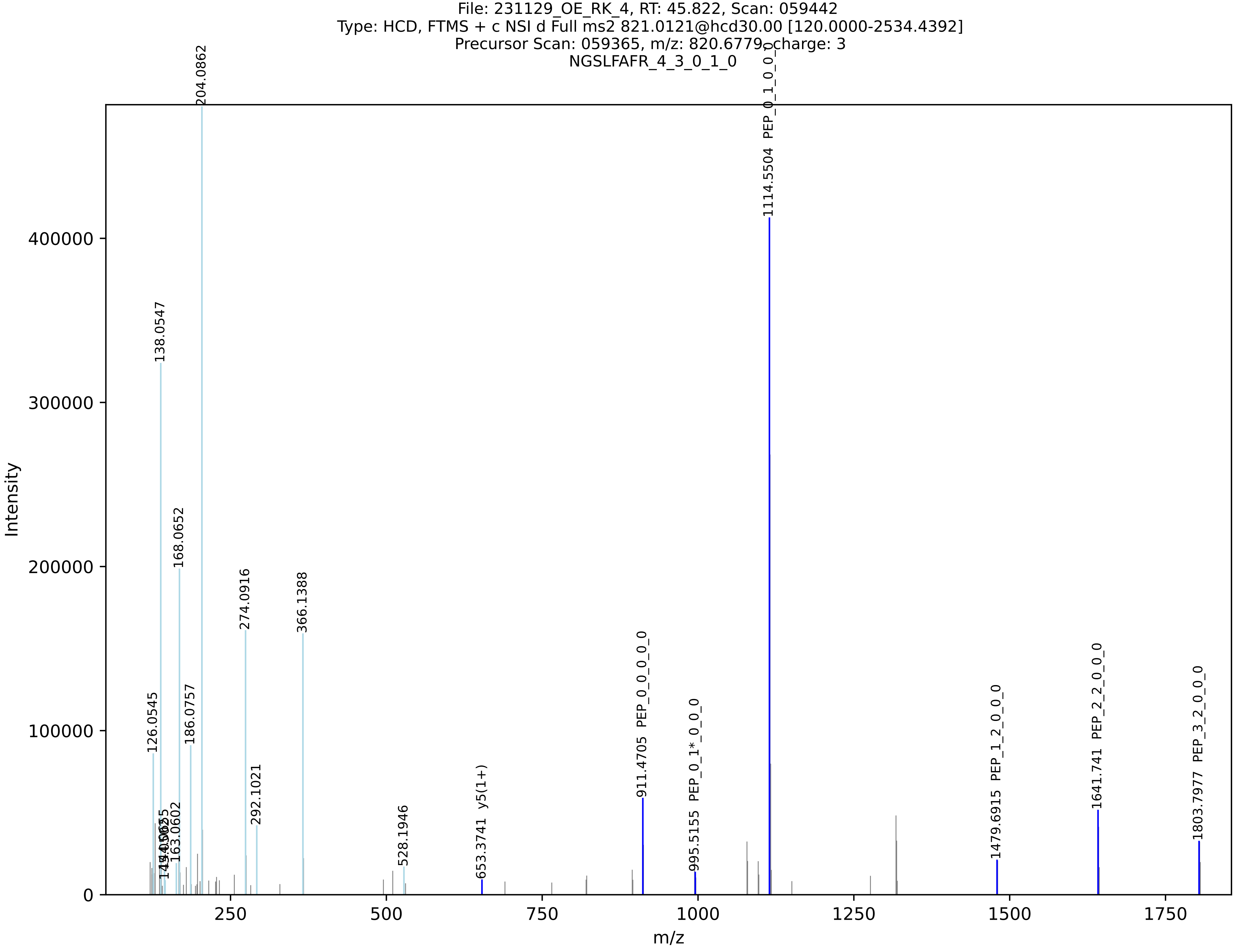
File: 231129_OE_RK_4, RT: 45.822, Scan: 059442
Type: HCD, FTMS + c NSI d Full ms2 821.0121@hcd30.00 [120.0000-2534.4392]
Precursor Scan: 059365, m/z: 820.6779, charge: 3
NGSLFAFR_4_3_0_1_0
204.0862
1114.5504 PEP_0_1_0_0_0
138.0547
168.0652
274.0916
366.1388
126.0545
186.0757
292.1021
163.0602
528.1946
653.3741 y5(1+)
911.4705 PEP_0_0_0_0_0
995.5155 PEP_0_1*_0_0_0
1479.6915 PEP_1_2_0_0_0
1641.741 PEP_2_2_0_0_0
1803.7977 PEP_3_2_0_0_0
Intensity
m/z
0
100000
200000
300000
400000
250
500
750
1000
1250
1500
1750

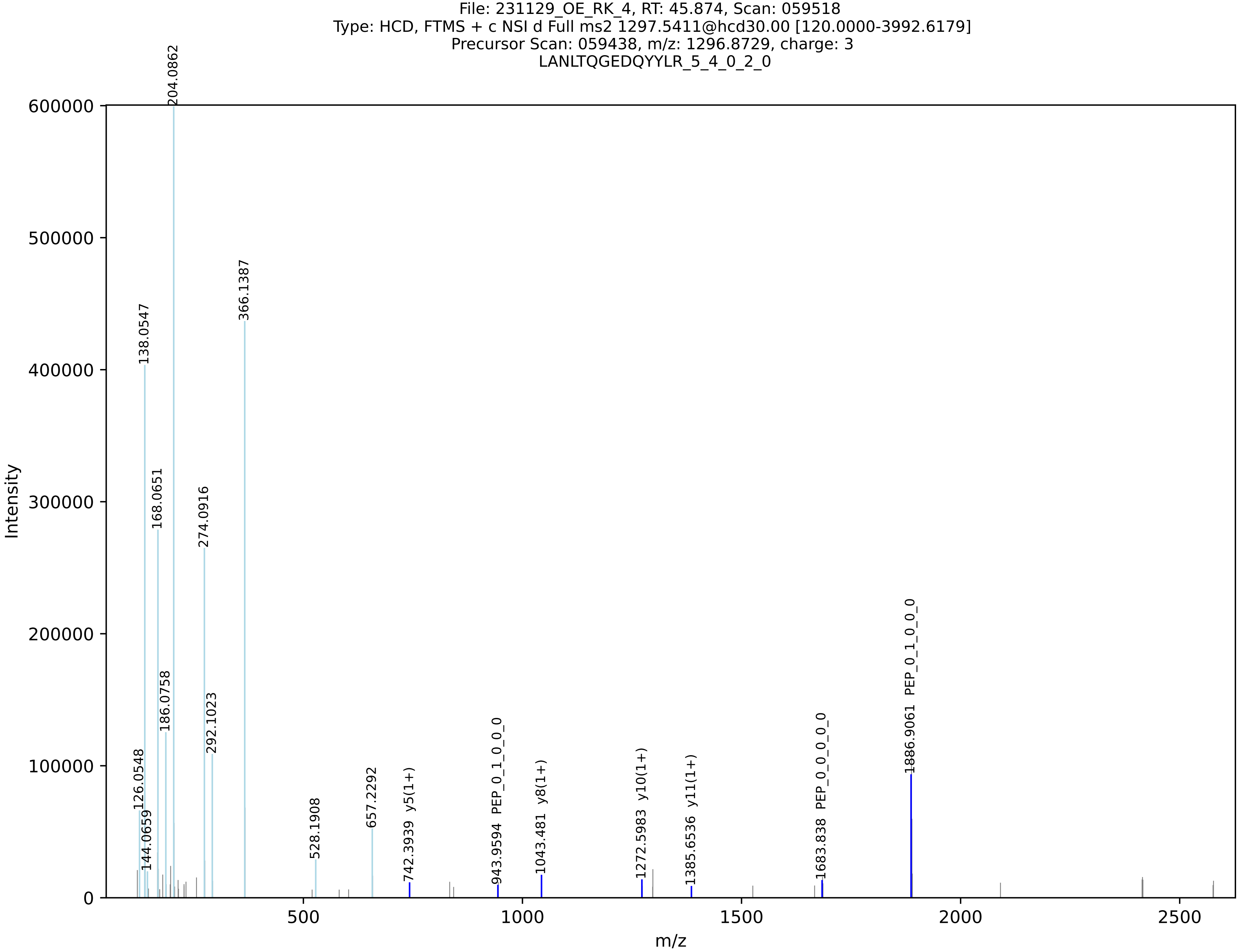
File: 231129_OE_RK_4, RT: 45.874, Scan: 059518
Type: HCD, FTMS + c NSI d Full ms2 1297.5411@hcd30.00 [120.0000-3992.6179]
Precursor Scan: 059438, m/z: 1296.8729, charge: 3
LANLTQGEDQYYLR_5_4_0_2_0
Intensity
m/z
204.0862
366.1387
138.0547
168.0651
274.0916
186.0758
292.1023
126.0548
144.0659
528.1908
657.2292
742.3939 y5(1+)
943.9594 PEP_0_1_0_0_0
1043.481 y8(1+)
1272.5983 y10(1+)
1385.6536 y11(1+)
1683.838 PEP_0_0_0_0_0
1886.9061 PEP_0_1_0_0_0
0
100000
200000
300000
400000
500000
600000
500
1000
1500
2000
2500

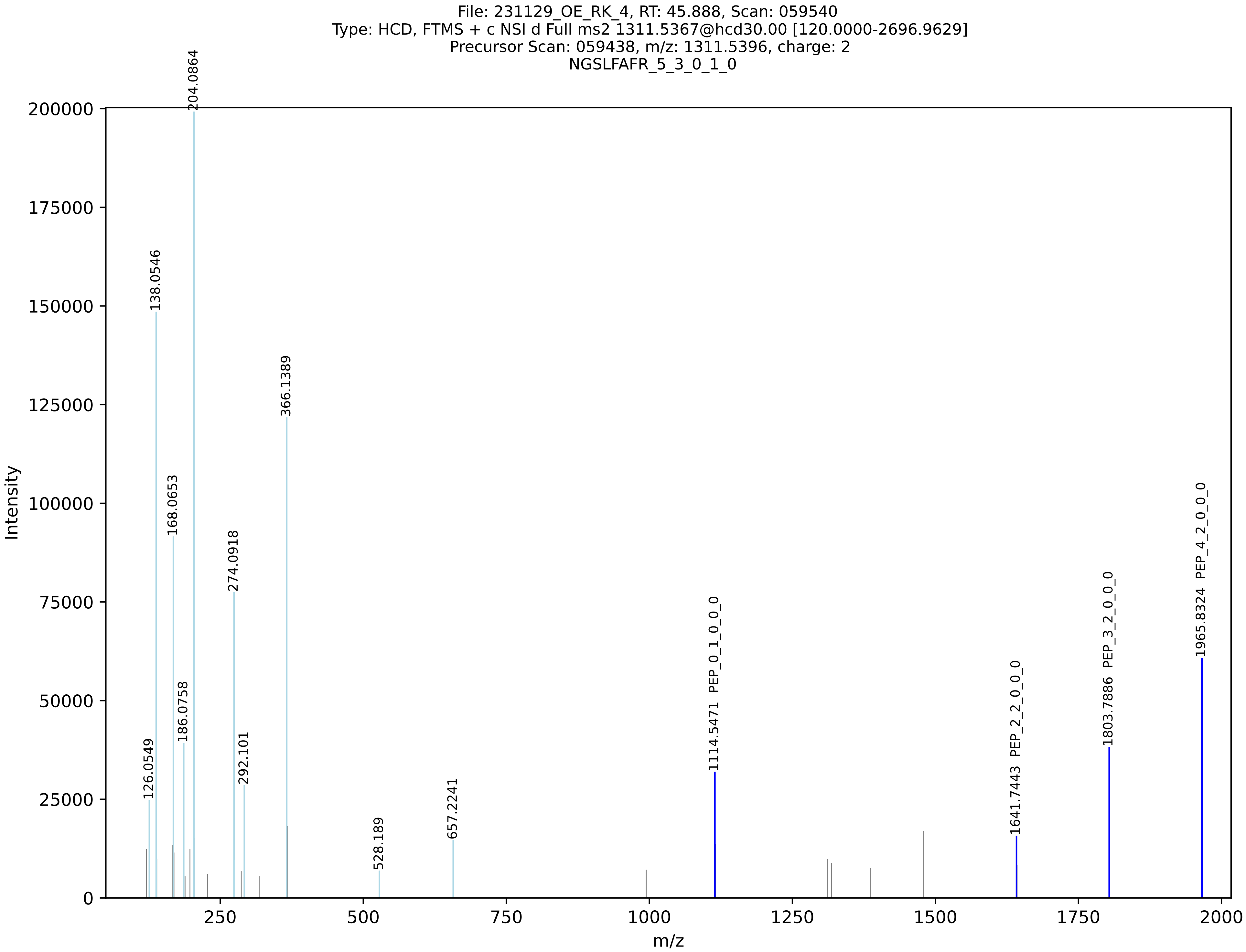

File: 231129_OE_RK_4, RT: 45.888, Scan: 059540
Type: HCD, FTMS + c NSI d Full ms2 1311.5367@hcd30.00 [120.0000-2696.9629]
Precursor Scan: 059438, m/z: 1311.5396, charge: 2
NGSLFAFR_5_3_0_1_0
Intensity
m/z
204.0864
138.0546
366.1389
168.0653
274.0918
186.0758
292.101
126.0549
528.189
657.2241
1114.5471 PEP_0_1_0_0_0
1641.7443 PEP_2_2_0_0_0
1803.7886 PEP_3_2_0_0_0
1965.8324 PEP_4_2_0_0_0

File: 231129_OE_RK_4, RT: 45.922, Scan: 059588
Type: HCD, FTMS + c NSI d Full ms2 1467.6091@hcd30.00 [120.0000-4513.0259]
Precursor Scan: 059512, m/z: 1466.941, charge: 3
GLTFQQNASSMCVPDQDTAIR_5_4_1_1_0

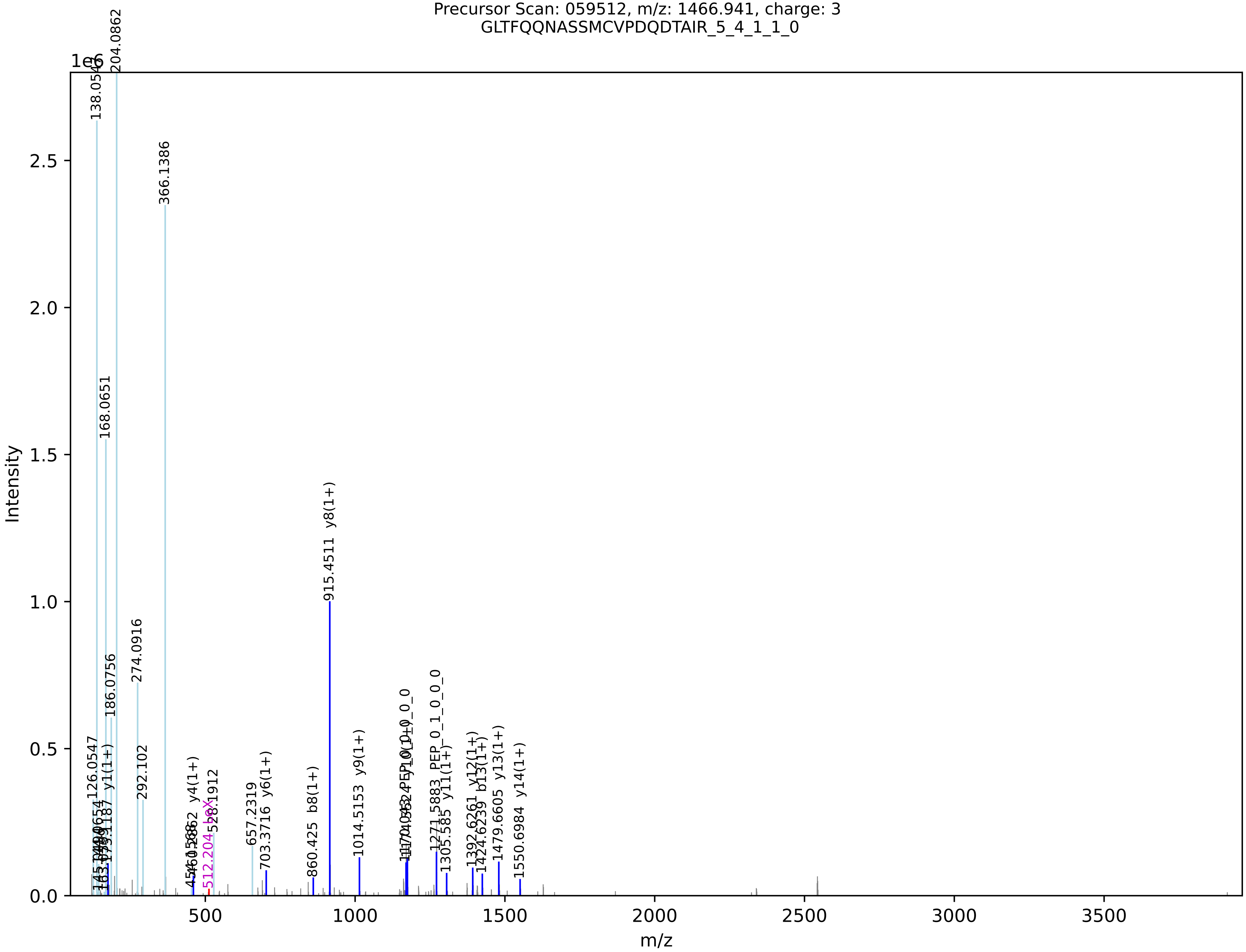

File: 231129_OE_RK_4, RT: 45.923, Scan: 059589
Type: HCD, FTMS + c NSI d Full ms2 1100.9589@hcd30.00 [120.0000-4514.7681]
Precursor Scan: 059512, m/z: 1100.4576, charge: 4
GLTFQQNASSMCVPDQDTAIR_5_4_1_1_0

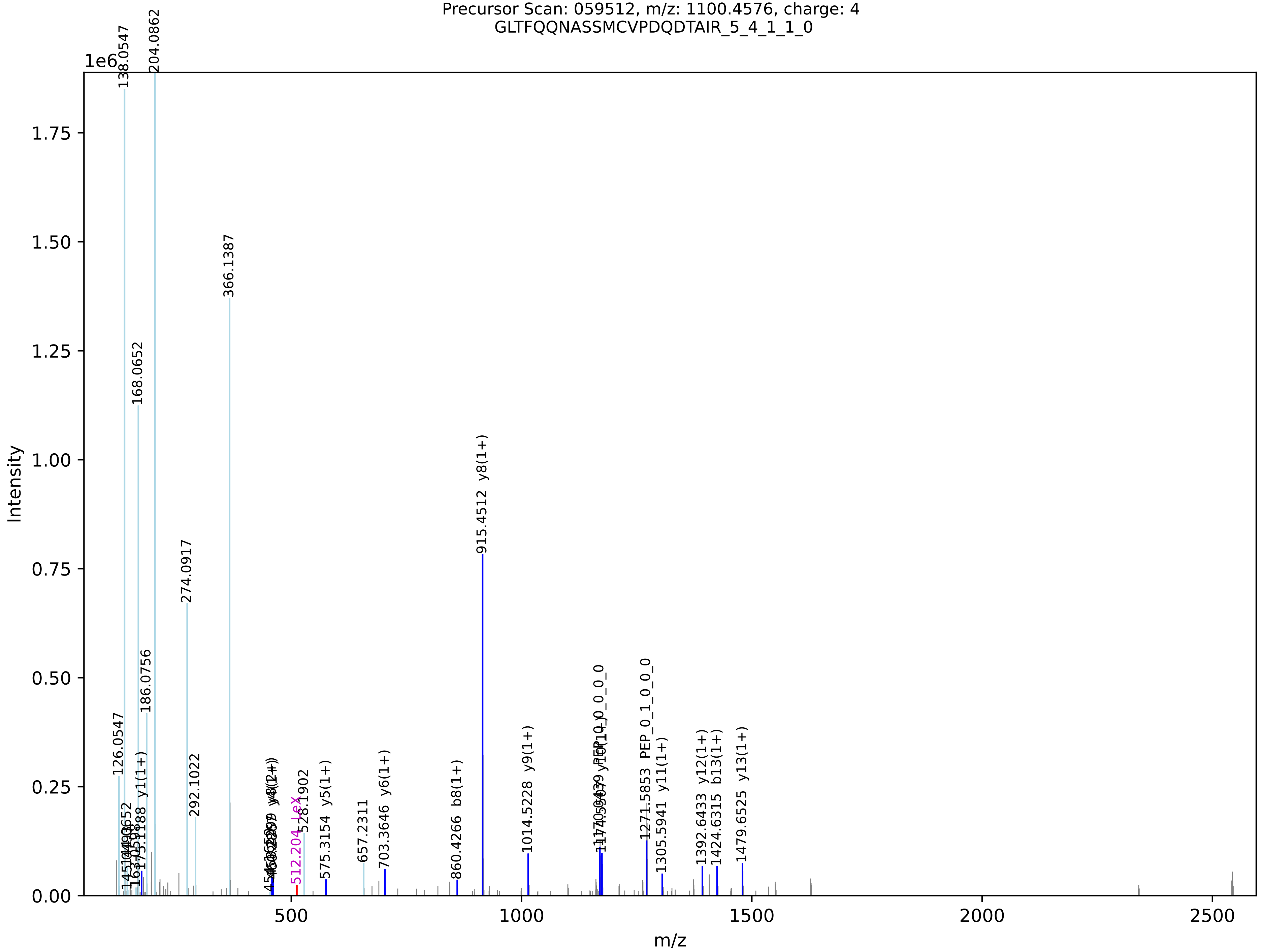

File: 231129_OE_RK_4, RT: 45.933, Scan: 059605
Type: HCD, FTMS + c NSI d Full ms2 972.9055@hcd30.00 [120.0000-3992.3105]
Precursor Scan: 059512, m/z: 972.9056, charge: 4
LANLTQGEDQYYLR_5_4_0_2_0

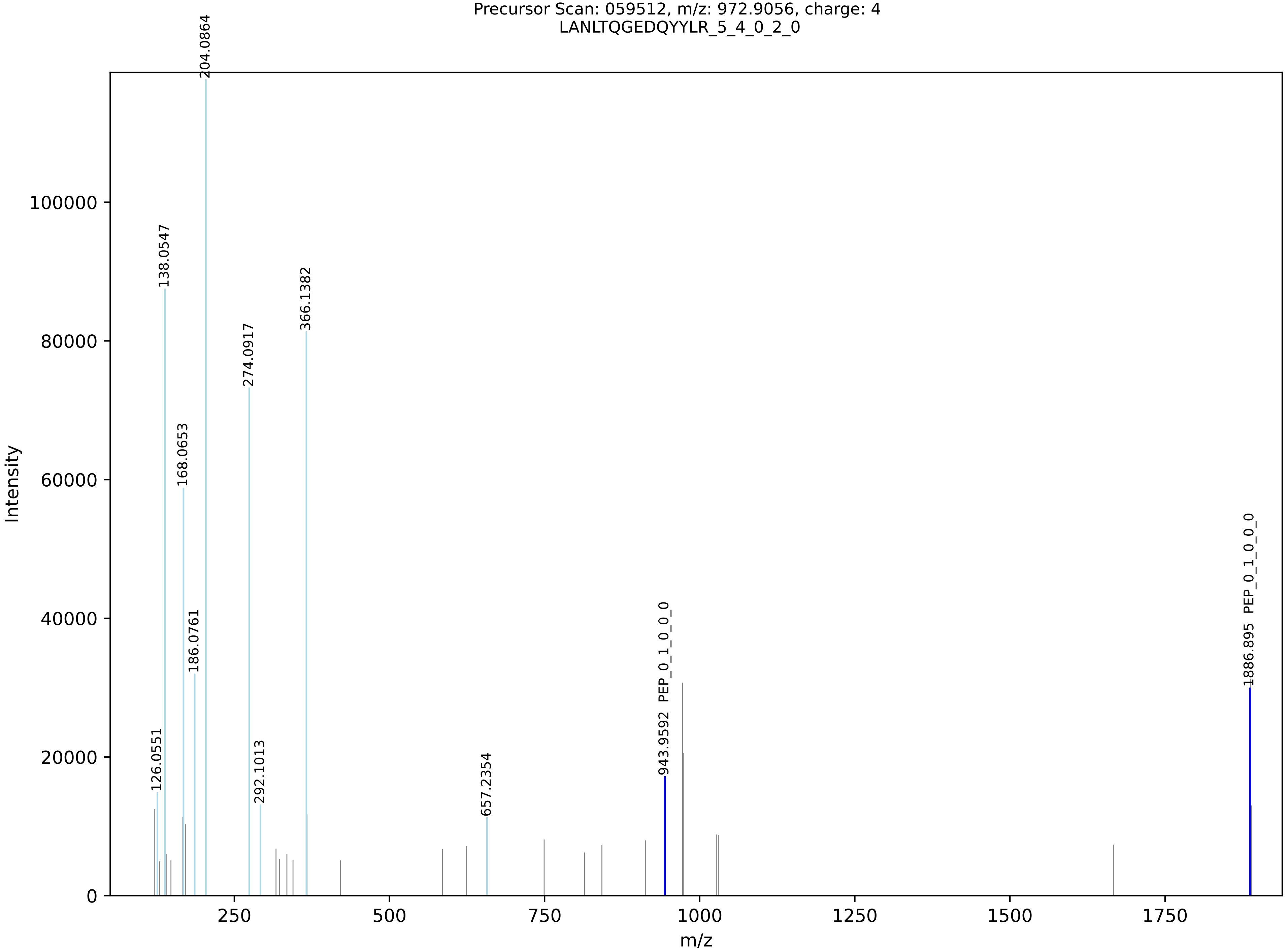

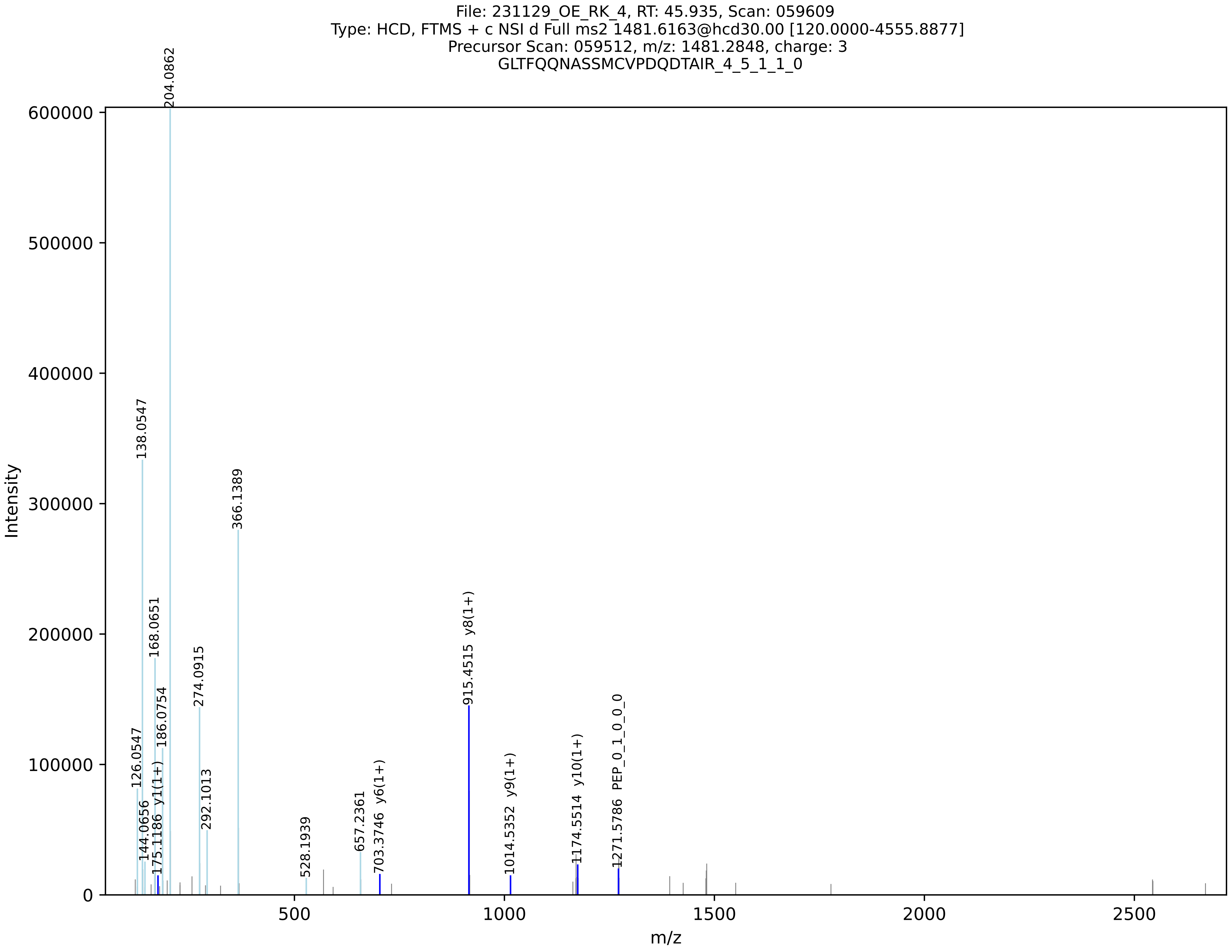

File: 231129_OE_RK_4, RT: 45.935, Scan: 059609
Type: HCD, FTMS + c NSI d Full ms2 1481.6163@hcd30.00 [120.0000-4555.8877]
Precursor Scan: 059512, m/z: 1481.2848, charge: 3
GLTFQQNASSMCVPDQDTAIR_4_5_1_1_0
Intensity
m/z
204.0862
138.0547
366.1389
168.0651
274.0915
186.0754
126.0547
y1(1+)
144.0656
175.1186
292.1013
528.1939
657.2361
703.3746 y6(1+)
915.4515 y8(1+)
1014.5352 y9(1+)
1174.5514 y10(1+)
1271.5786 PEP_0_1_0_0_0

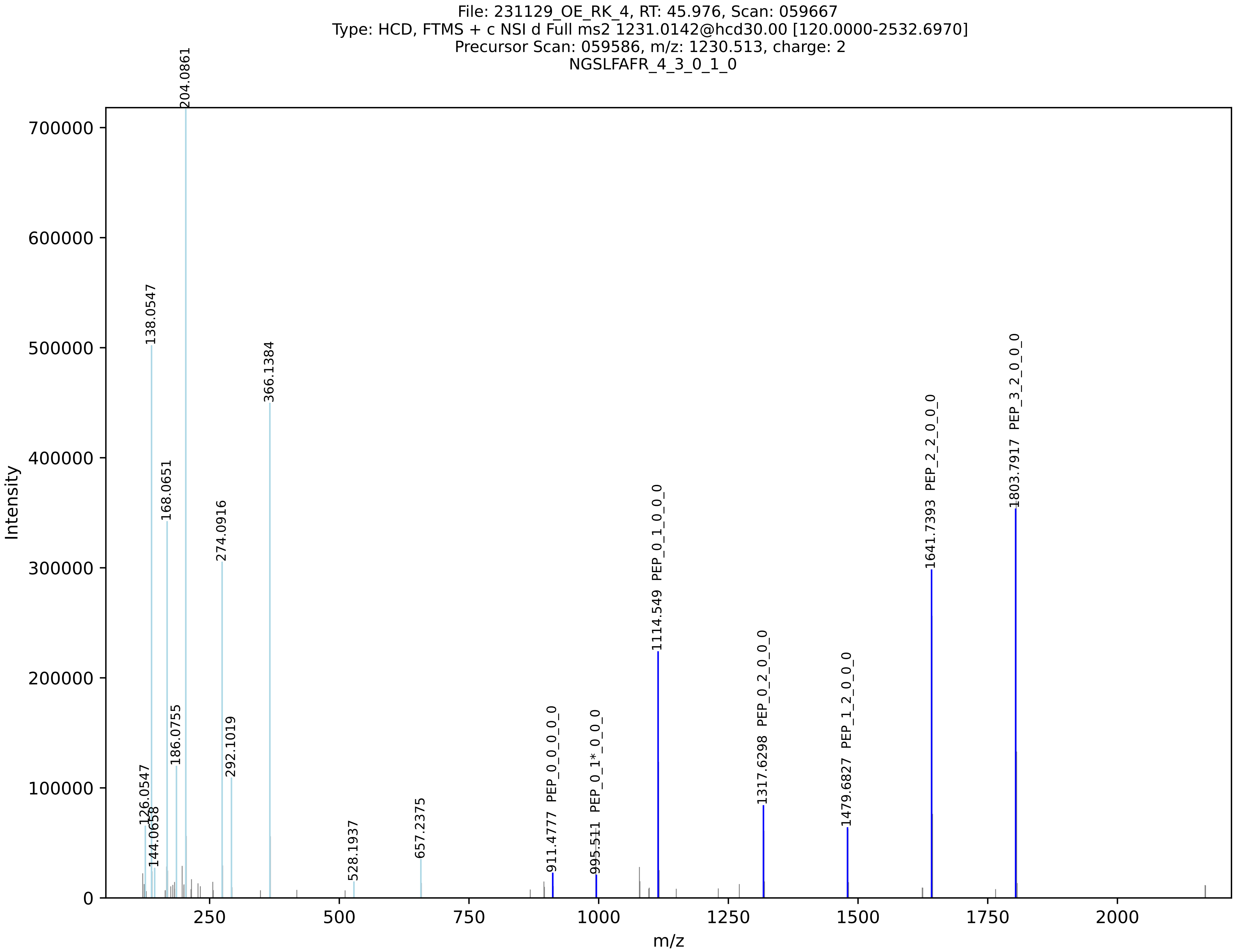

File: 231129_OE_RK_4, RT: 45.976, Scan: 059667
Type: HCD, FTMS + c NSI d Full ms2 1231.0142@hcd30.00 [120.0000-2532.6970]
Precursor Scan: 059586, m/z: 1230.513, charge: 2
NGSLFAFR_4_3_0_1_0
Intensity
m/z
204.0861
138.0547
366.1384
168.0651
274.0916
186.0755
292.1019
126.0547
144.0658
528.1937
657.2375
911.4777 PEP_0_0_0_0_0
995.511 PEP_0_1*_0_0_0
1114.549 PEP_0_1_0_0_0
1317.6298 PEP_0_2_0_0_0
1479.6827 PEP_1_2_0_0_0
1641.7393 PEP_2_2_0_0_0
1803.7917 PEP_3_2_0_0_0

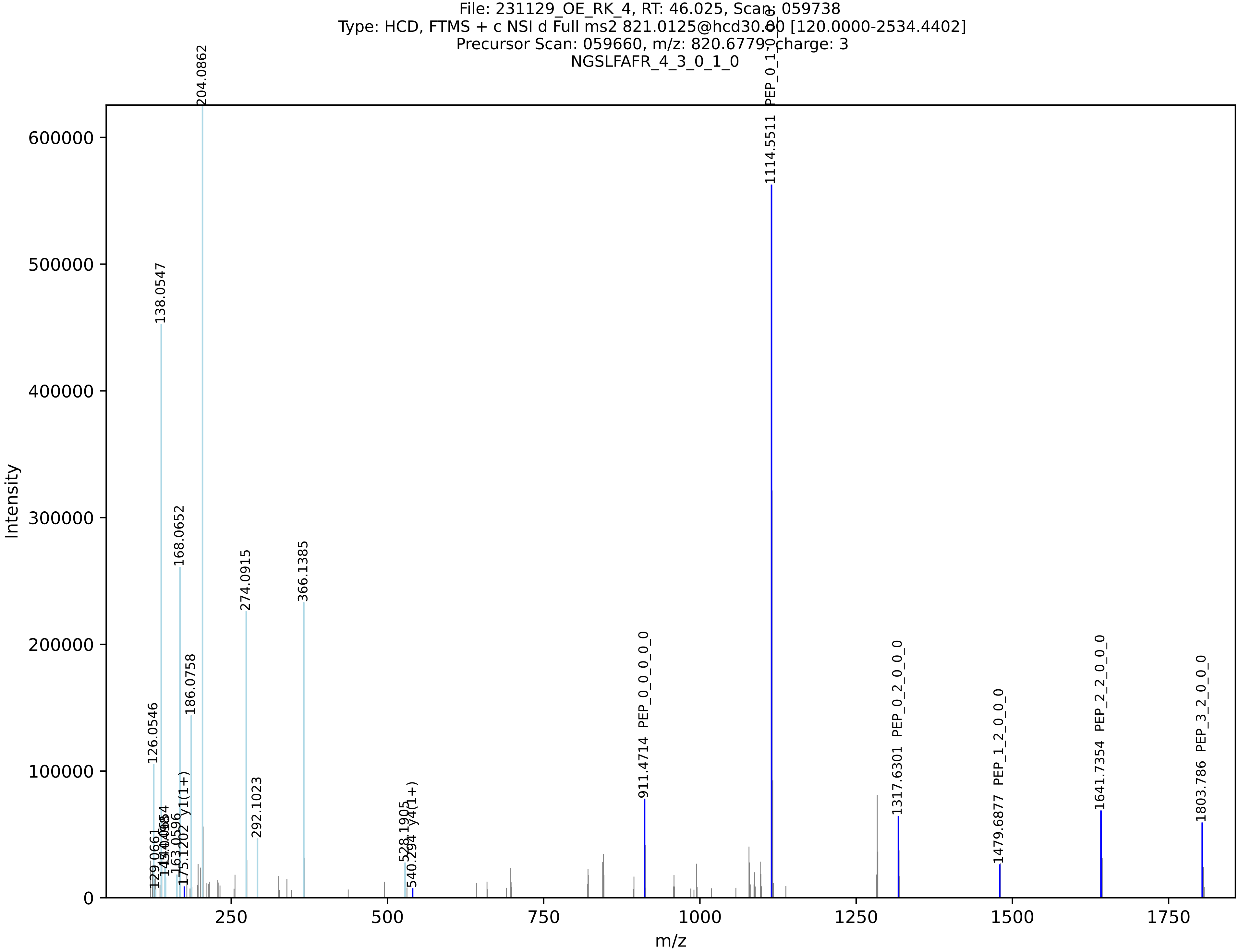
File: 231129_OE_RK_4, RT: 46.025, Scan: 059738
Type: HCD, FTMS + c NSI d Full ms2 821.0125@hcd30.00 [120.0000-2534.4402]
Precursor Scan: 059660, m/z: 820.6779, charge: 3
NGSLFAFR_4_3_0_1_0
Intensity
m/z
0
100000
200000
300000
400000
500000
600000
250
500
750
1000
1250
1500
1750
204.0862
138.0547
168.0652
186.0758
126.0546
274.0915
366.1385
292.1023
175.1202 y1(1+)
528.1905
540.294 y4(1+)
911.4714 PEP_0_0_0_0_0
1114.5511
1317.6301 PEP_0_2_0_0_0
1479.6877 PEP_1_2_0_0_0
1641.7354 PEP_2_2_0_0_0
1803.786 PEP_3_2_0_0_0

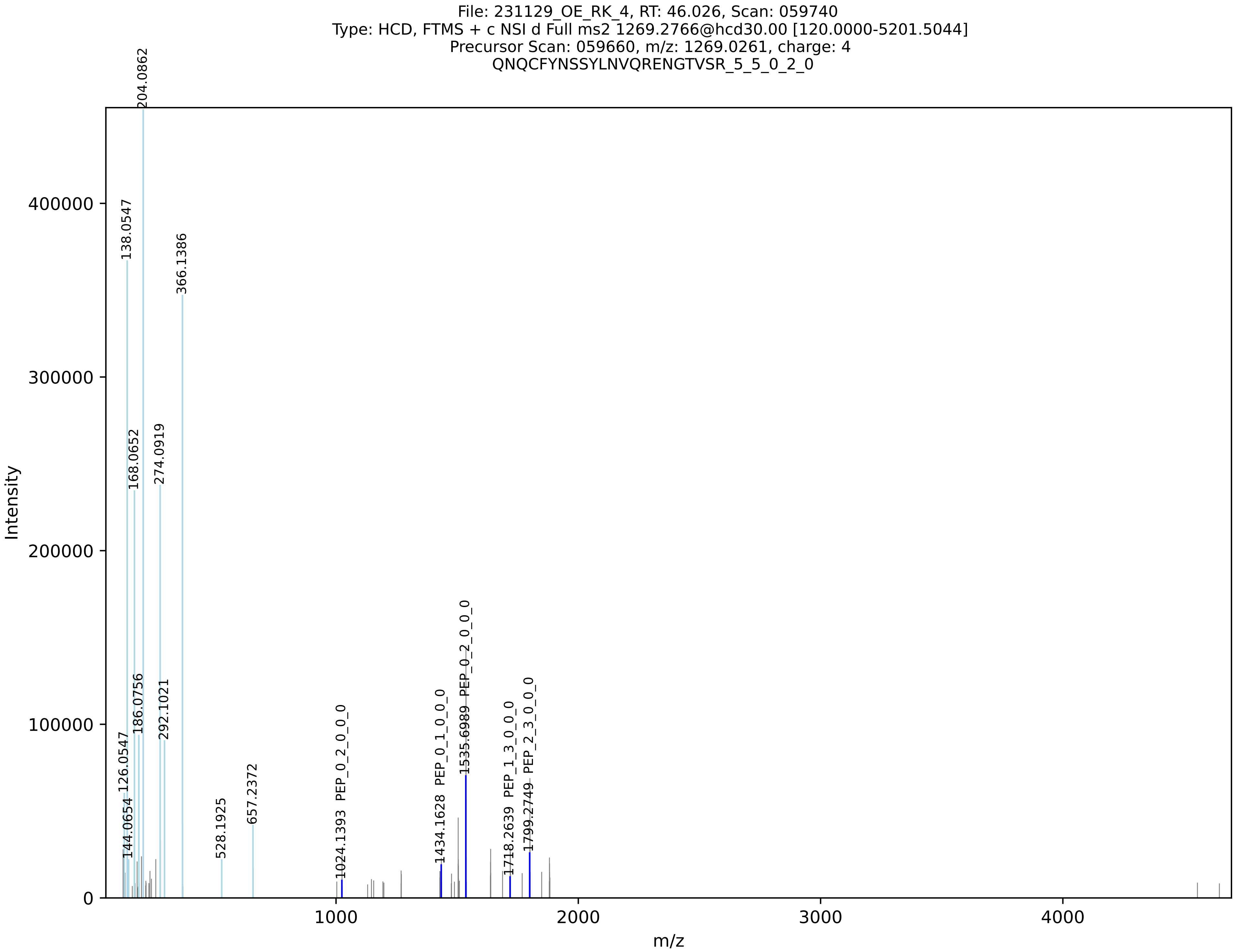
File: 231129_OE_RK_4, RT: 46.026, Scan: 059740
Type: HCD, FTMS + c NSI d Full ms2 1269.2766@hcd30.00 [120.0000-5201.5044]
Precursor Scan: 059660, m/z: 1269.0261, charge: 4
QNQCFYNSSYLNVQRENGTVSR_5_5_0_2_0
Intensity
m/z
204.0862
138.0547
366.1386
168.0652
274.0919
186.0756
292.1021
126.0547
144.0654
528.1925
657.2372
1024.1393 PEP_0_2_0_0_0
1434.1628 PEP_0_1_0_0_0
1535.6989 PEP_0_2_0_0_0
1718.2639 PEP_1_3_0_0_0
1799.2749 PEP_2_3_0_0_0

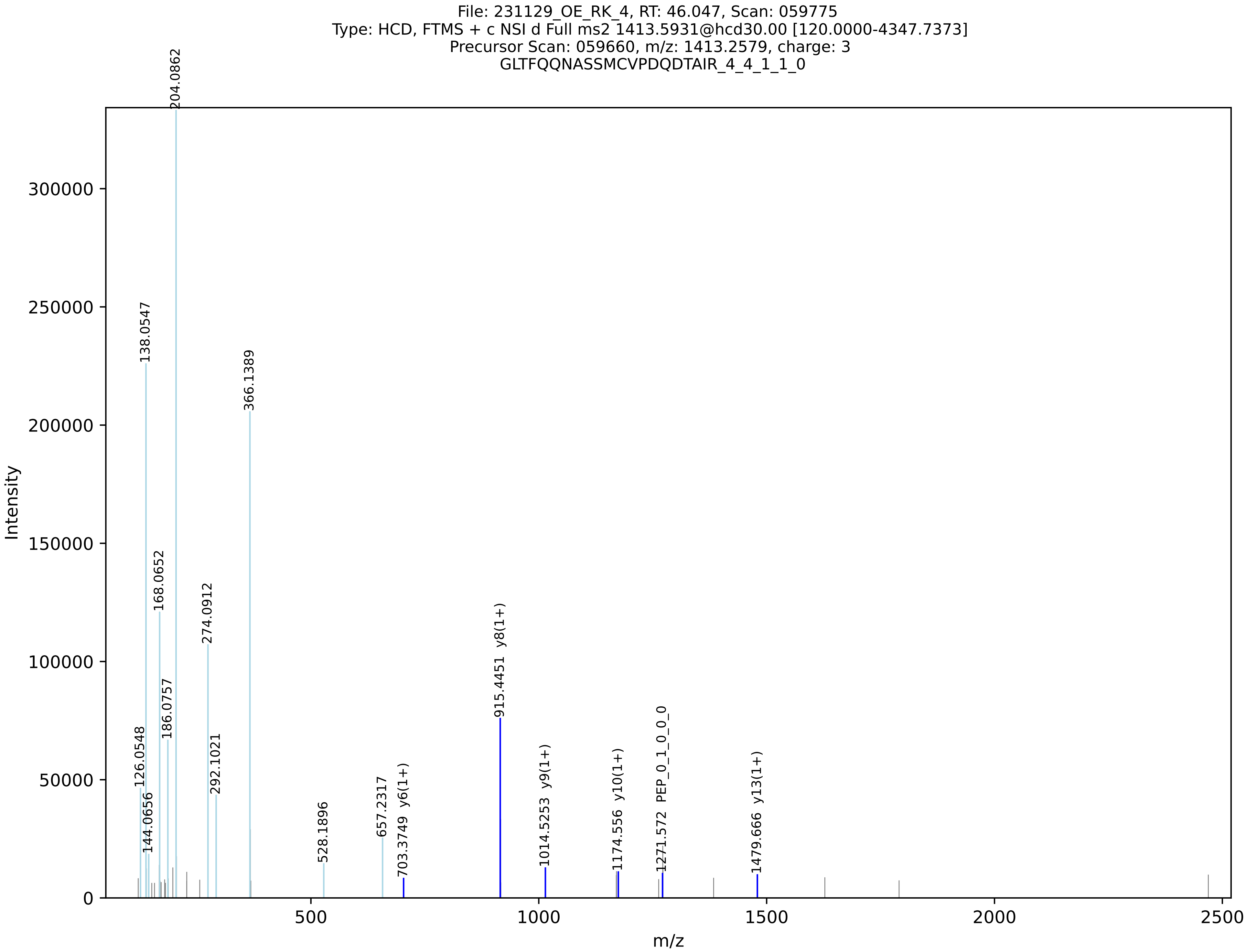
File: 231129_OE_RK_4, RT: 46.047, Scan: 059775
Type: HCD, FTMS + c NSI d Full ms2 1413.5931@hcd30.00 [120.0000-4347.7373]
Precursor Scan: 059660, m/z: 1413.2579, charge: 3
GLTFQQNASSMCVPDQDTAIR_4_4_1_1_0
Intensity
m/z
0
50000
100000
150000
200000
250000
300000
500
1000
1500
2000
2500
204.0862
138.0547
366.1389
168.0652
274.0912
186.0757
126.0548
292.1021
144.0656
528.1896
657.2317
703.3749 y6(1+)
915.4451 y8(1+)
1014.5253 y9(1+)
1174.556 y10(1+)
1271.572 PEP_0_1_0_0_0
1479.666 y13(1+)

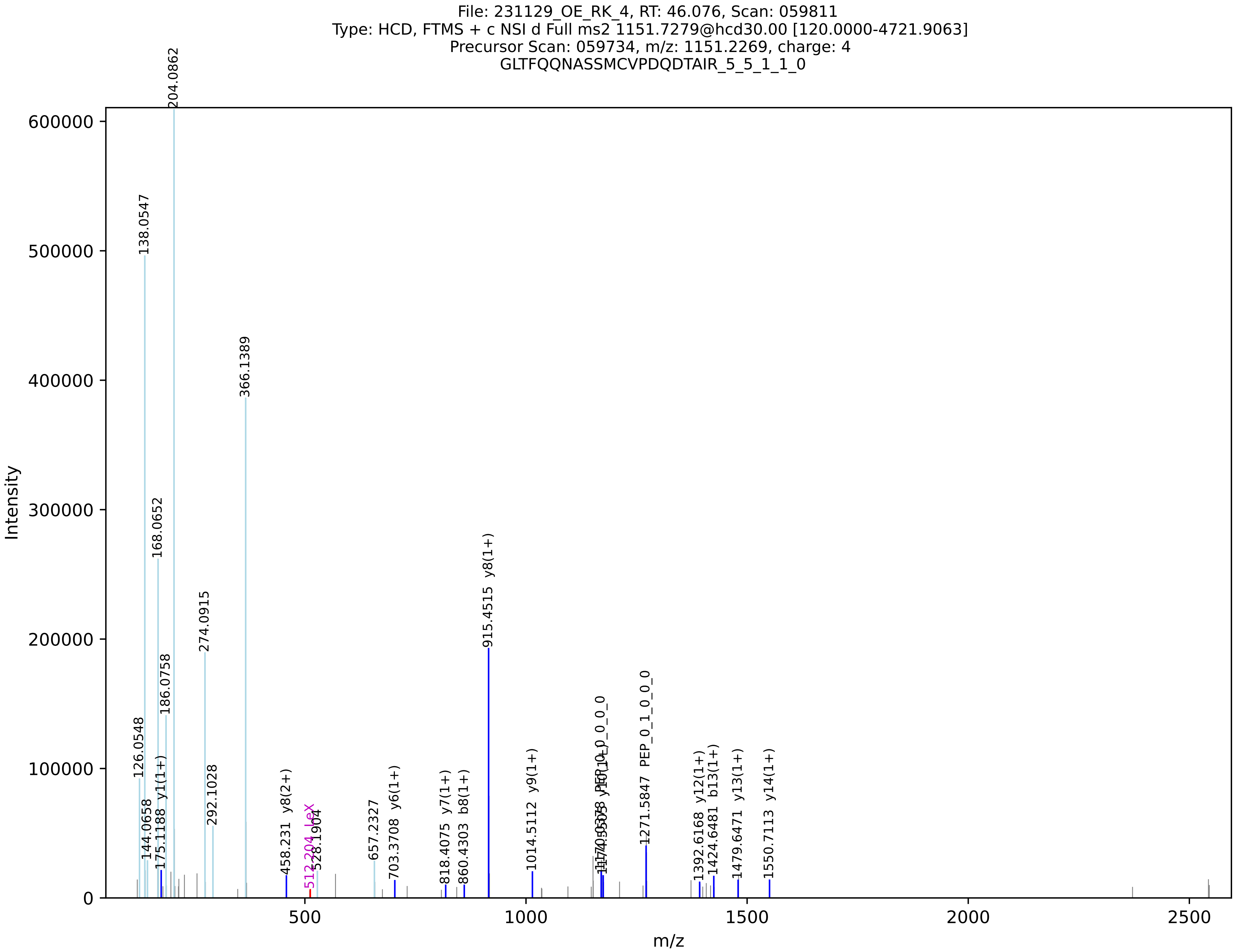
File: 231129_OE_RK_4, RT: 46.076, Scan: 059811
Type: HCD, FTMS + c NSI d Full ms2 1151.7279@hcd30.00 [120.0000-4721.9063]
Precursor Scan: 059734, m/z: 1151.2269, charge: 4
GLTFQQNASSMCVPDQDTAIR_5_5_1_1_0
Intensity
m/z
204.0862
138.0547
366.1389
168.0652
274.0915
186.0758
126.0548
144.0658
175.1188 y1(1+)
292.1028
458.231 y8(2+)
512.204 LeX
528.1904
657.2327
703.3708 y6(1+)
818.4075 y7(1+)
860.4303 b8(1+)
915.4515 y8(1+)
1014.5112 y9(1+)
PEP_0_0_0_0_0
y10(1+)
1271.5847 PEP_0_1_0_0_0
1392.6168 y12(1+)
1424.6481 b13(1+)
1479.6471 y13(1+)
1550.7113 y14(1+)

File: 231129_OE_RK_4, RT: 46.082, Scan: 059820
Type: HCD, FTMS + c NSI d Full ms2 1419.2588@hcd30.00 [120.0000-4365.0737]
Precursor Scan: 059734, m/z: 1418.2564, charge: 3
GLTFQQNASSMCVPDQDTAIR_5_4_0_1_0

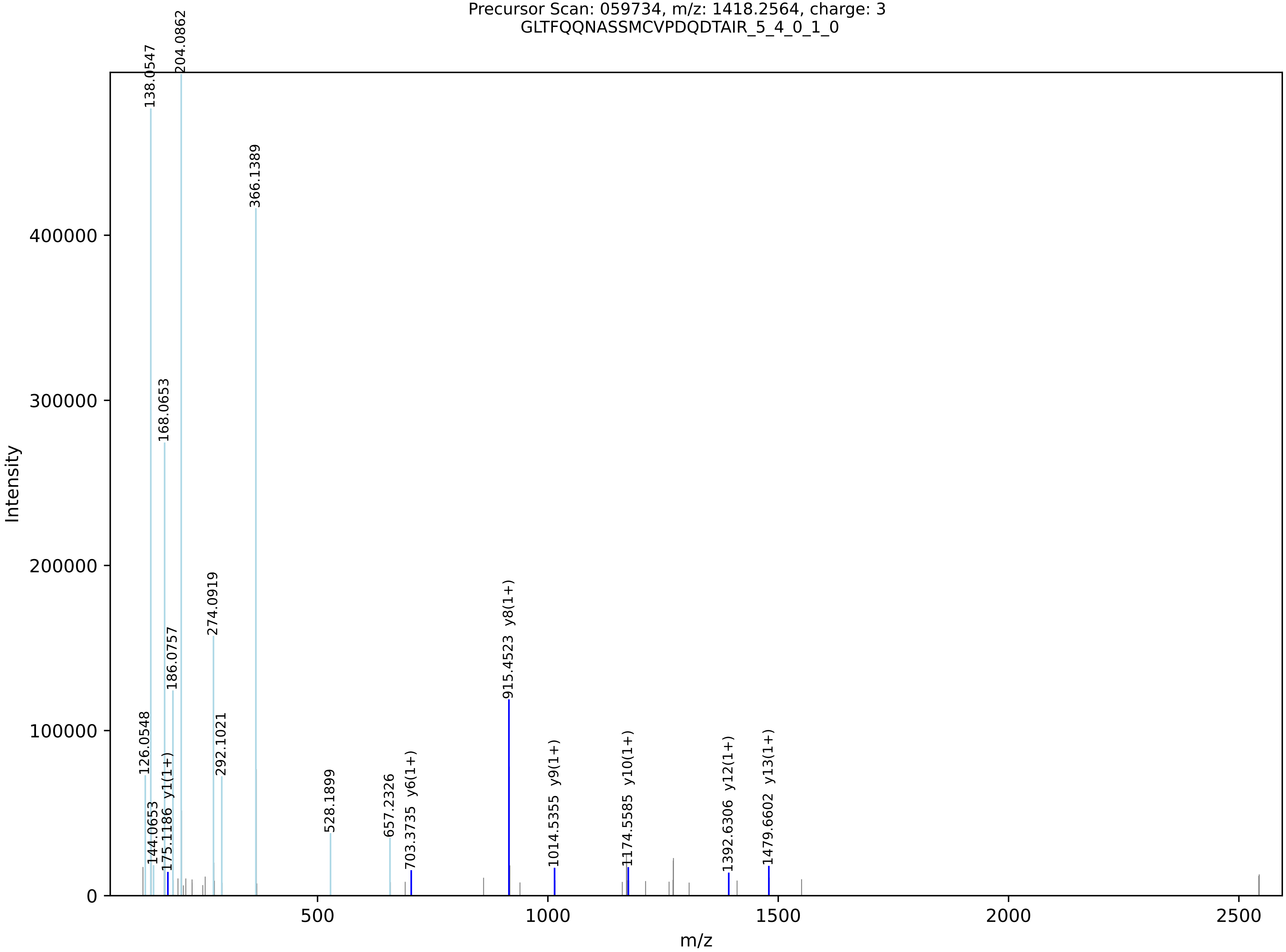

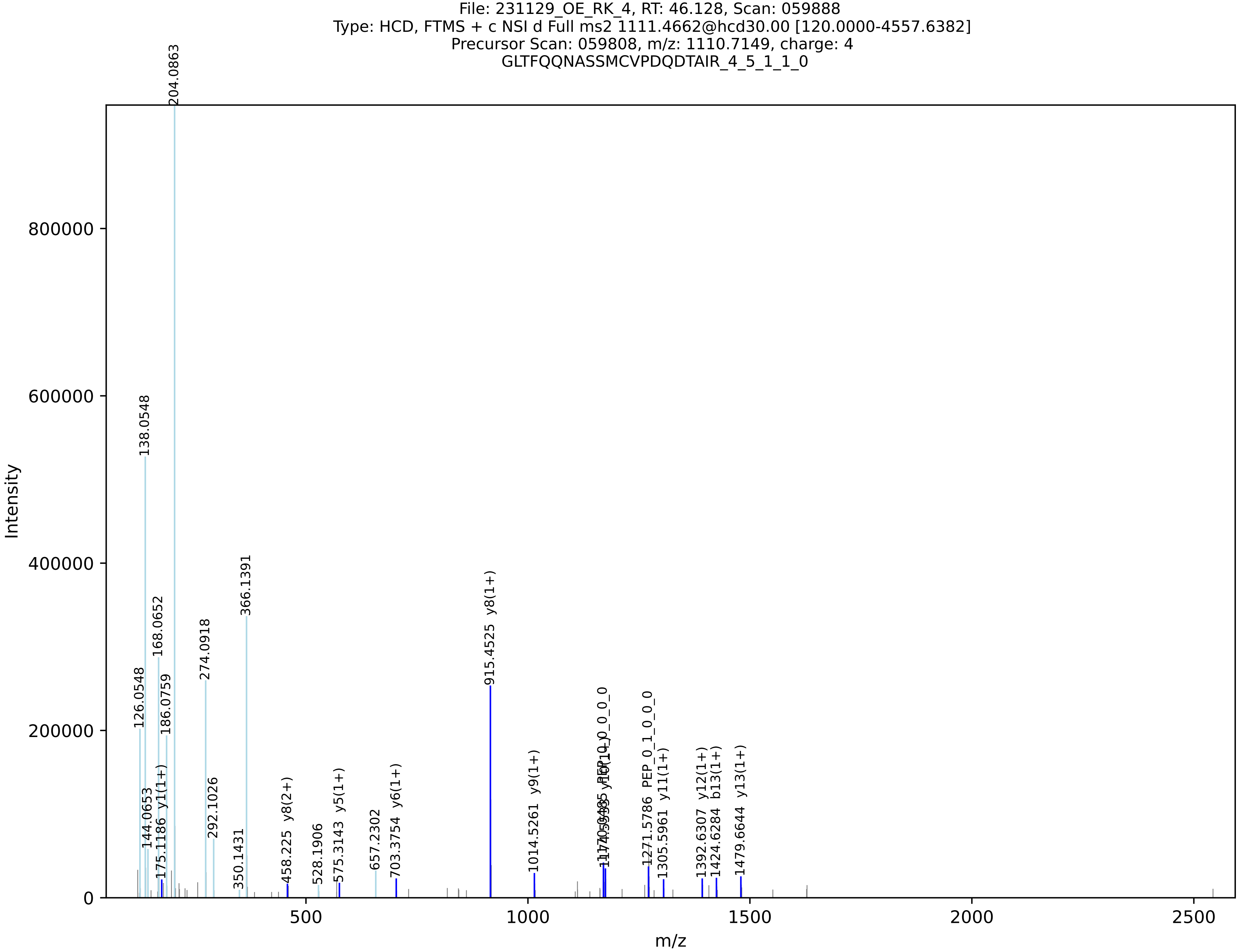
File: 231129_OE_RK_4, RT: 46.128, Scan: 059888
Type: HCD, FTMS + c NSI d Full ms2 1111.4662@hcd30.00 [120.0000-4557.6382]
Precursor Scan: 059808, m/z: 1110.7149, charge: 4
GLTFQQNASSMCVPDQDTAIR_4_5_1_1_0
204.0863
138.0548
366.1391
168.0652
274.0918
126.0548
186.0759
915.4525 y8(1+)
1014.5261 y9(1+)
1271.5786 PEP_0_1_0_0_0
1305.5961 y11(1+)
1392.6307 y12(1+)
1424.6284 b13(1+)
1479.6644 y13(1+)
144.0653
175.1186 y1(1+)
292.1026
350.1431
458.225 y8(2+)
528.1906
575.3143 y5(1+)
657.2302
703.3754 y6(1+)
Intensity
m/z
0
200000
400000
600000
800000
500
1000
1500
2000
2500

File: 231129_OE_RK_4, RT: 46.132, Scan: 059894
Type: HCD, FTMS + c NSI d Full ms2 1369.9124@hcd30.00 [120.0000-4214.0737]
Precursor Scan: 059808, m/z: 1368.9101, charge: 3
KVCQDCPLLAPLNDTR_5_4_0_2_0

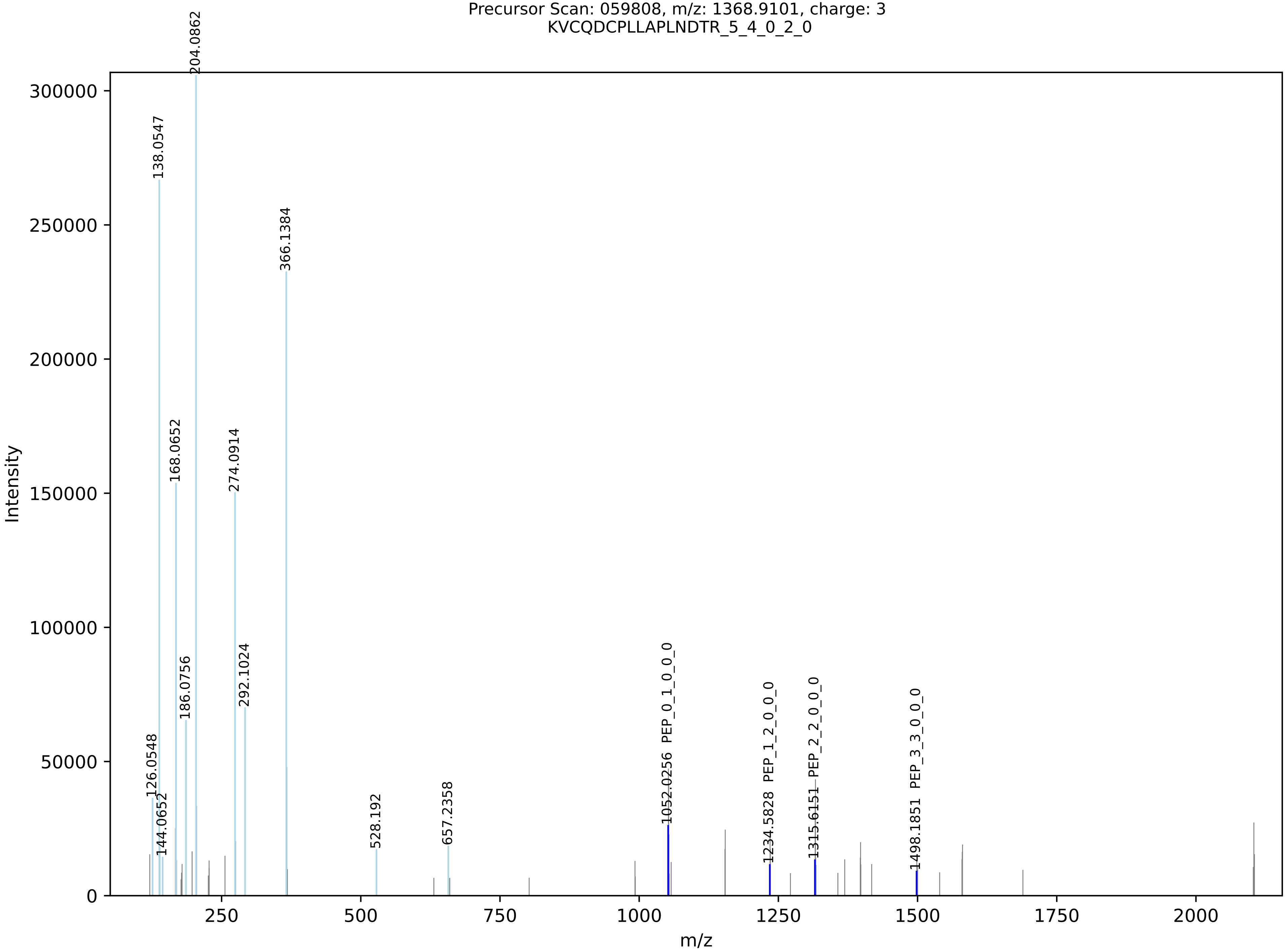

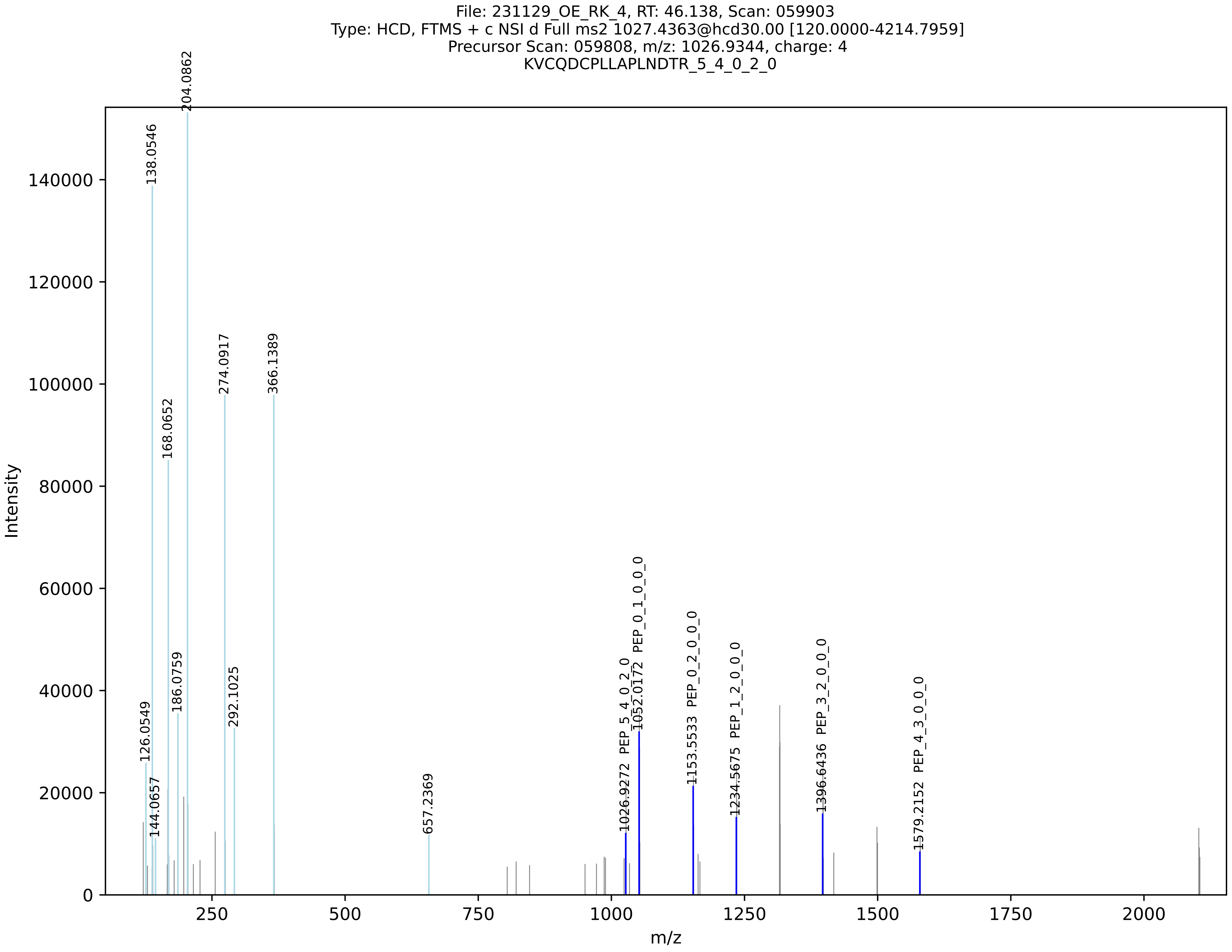
File: 231129_OE_RK_4, RT: 46.138, Scan: 059903
Type: HCD, FTMS + c NSI d Full ms2 1027.4363@hcd30.00 [120.0000-4214.7959]
Precursor Scan: 059808, m/z: 1026.9344, charge: 4
KVCQDCPLLAPLNDTR_5_4_0_2_0
Intensity
m/z
204.0862
138.0546
168.0652
274.0917
366.1389
186.0759
292.1025
126.0549
144.0657
657.2369
1026.9272 PEP_5_4_0_2_0
1052.0172 PEP_0_1_0_0_0
1153.5533 PEP_0_2_0_0_0
1234.5675 PEP_1_2_0_0_0
1396.6436 PEP_3_2_0_0_0
1579.2152 PEP_4_3_0_0_0
0
20000
40000
60000
80000
100000
120000
140000
250
500
750
1000
1250
1500
1750
2000

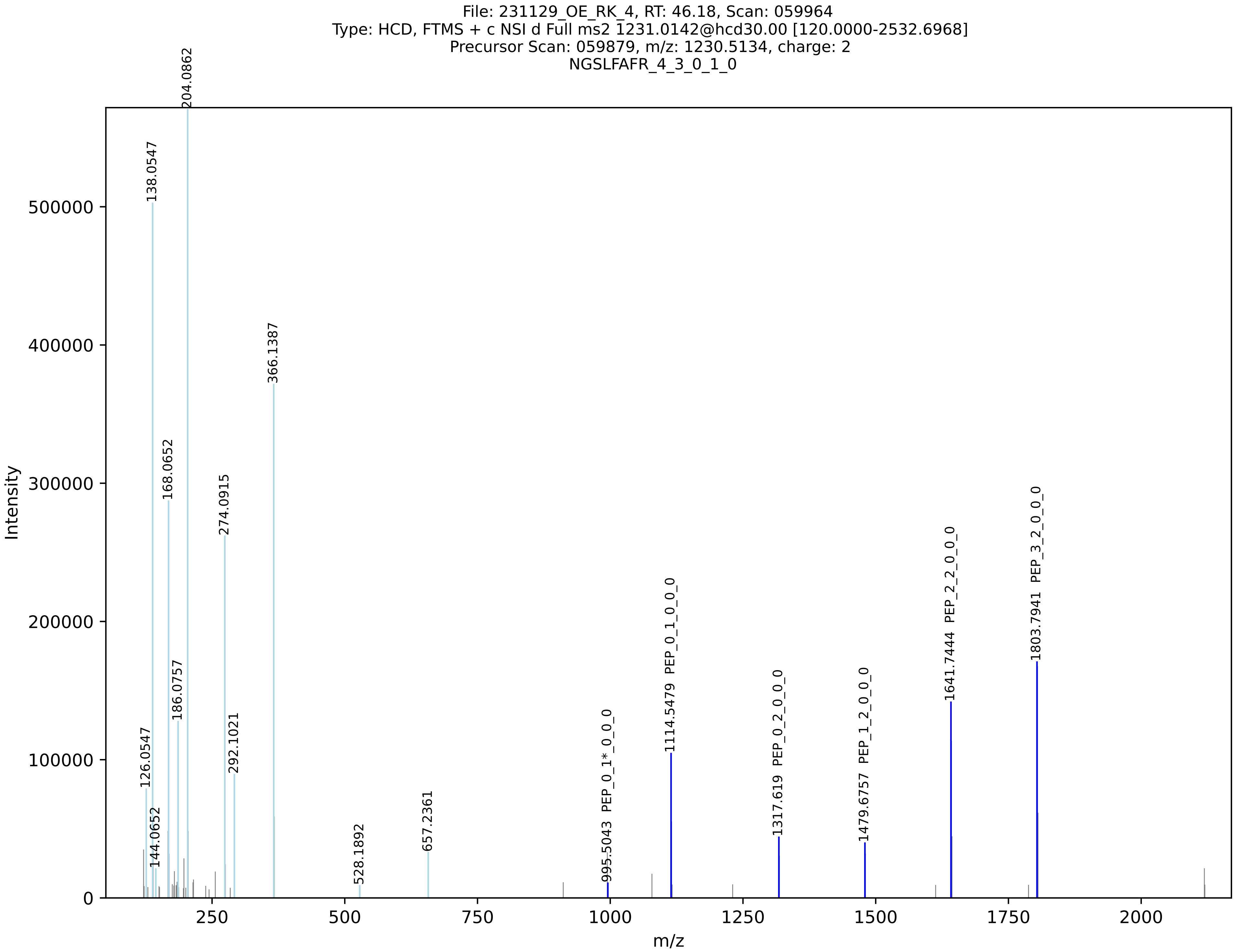
File: 231129_OE_RK_4, RT: 46.18, Scan: 059964
Type: HCD, FTMS + c NSI d Full ms2 1231.0142@hcd30.00 [120.0000-2532.6968]
Precursor Scan: 059879, m/z: 1230.5134, charge: 2
NGSLFAFR_4_3_0_1_0
Intensity
m/z
204.0862
138.0547
366.1387
168.0652
274.0915
186.0757
292.1021
126.0547
144.0652
528.1892
657.2361
995.5043 PEP_0_1*_0_0_0
1114.5479 PEP_0_1_0_0_0
1317.619 PEP_0_2_0_0_0
1479.6757 PEP_1_2_0_0_0
1641.7444 PEP_2_2_0_0_0
1803.7941 PEP_3_2_0_0_0

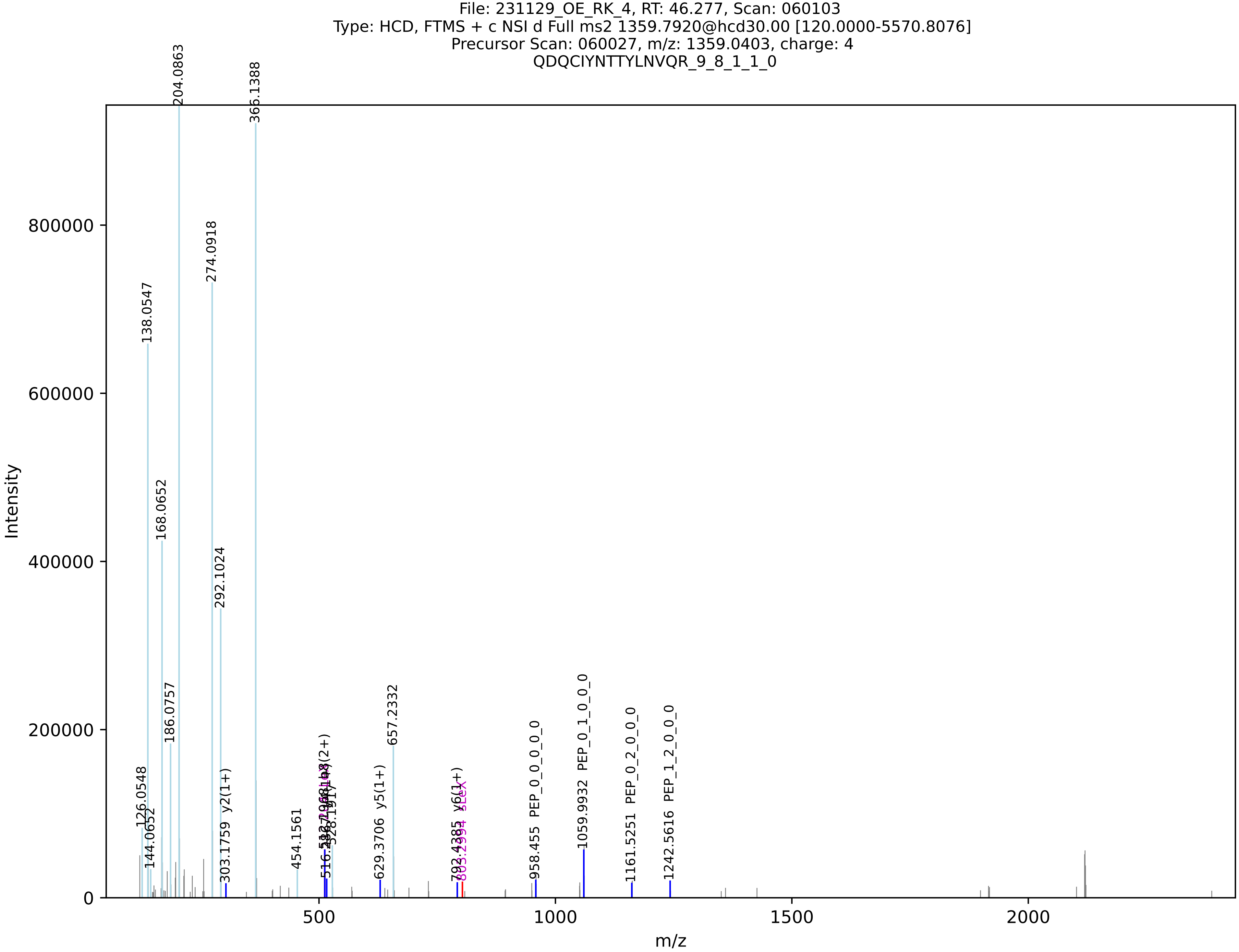
File: 231129_OE_RK_4, RT: 46.277, Scan: 060103
Type: HCD, FTMS + c NSI d Full ms2 1359.7920@hcd30.00 [120.0000-5570.8076]
Precursor Scan: 060027, m/z: 1359.0403, charge: 4
QDQCIYNTTYLNVQR_9_8_1_1_0
Intensity
m/z
204.0863
366.1388
274.0918
138.0547
168.0652
292.1024
186.0757
657.2332
126.0548
144.0652
303.1759 y2(1+)
454.1561
629.3706 y5(1+)
793.4385 y6(1+)
958.455 PEP_0_0_0_0_0
1059.9932 PEP_0_1_0_0_0
1161.5251 PEP_0_2_0_0_0
1242.5616 PEP_1_2_0_0_0
528.1917
sLeX

File: 231129_OE_RK_4, RT: 46.582, Scan: 060543
Type: HCD, FTMS + c NSI d Full ms2 1195.4814@hcd30.00 [120.0000-4900.4204]
Precursor Scan: 060461, m/z: 1194.9795, charge: 4
QDQCIYNTTYLNVQR_6_5_0_3_0

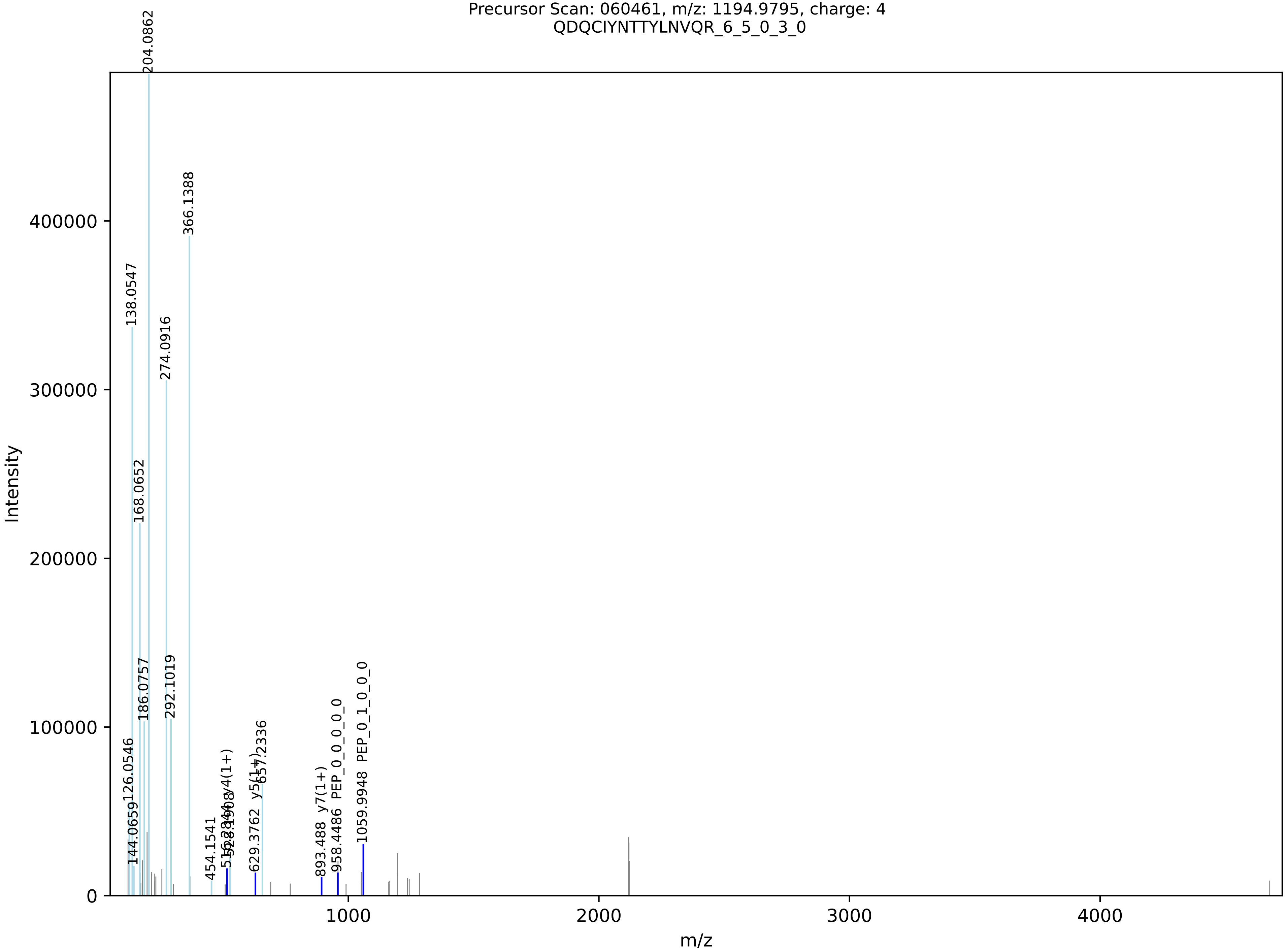

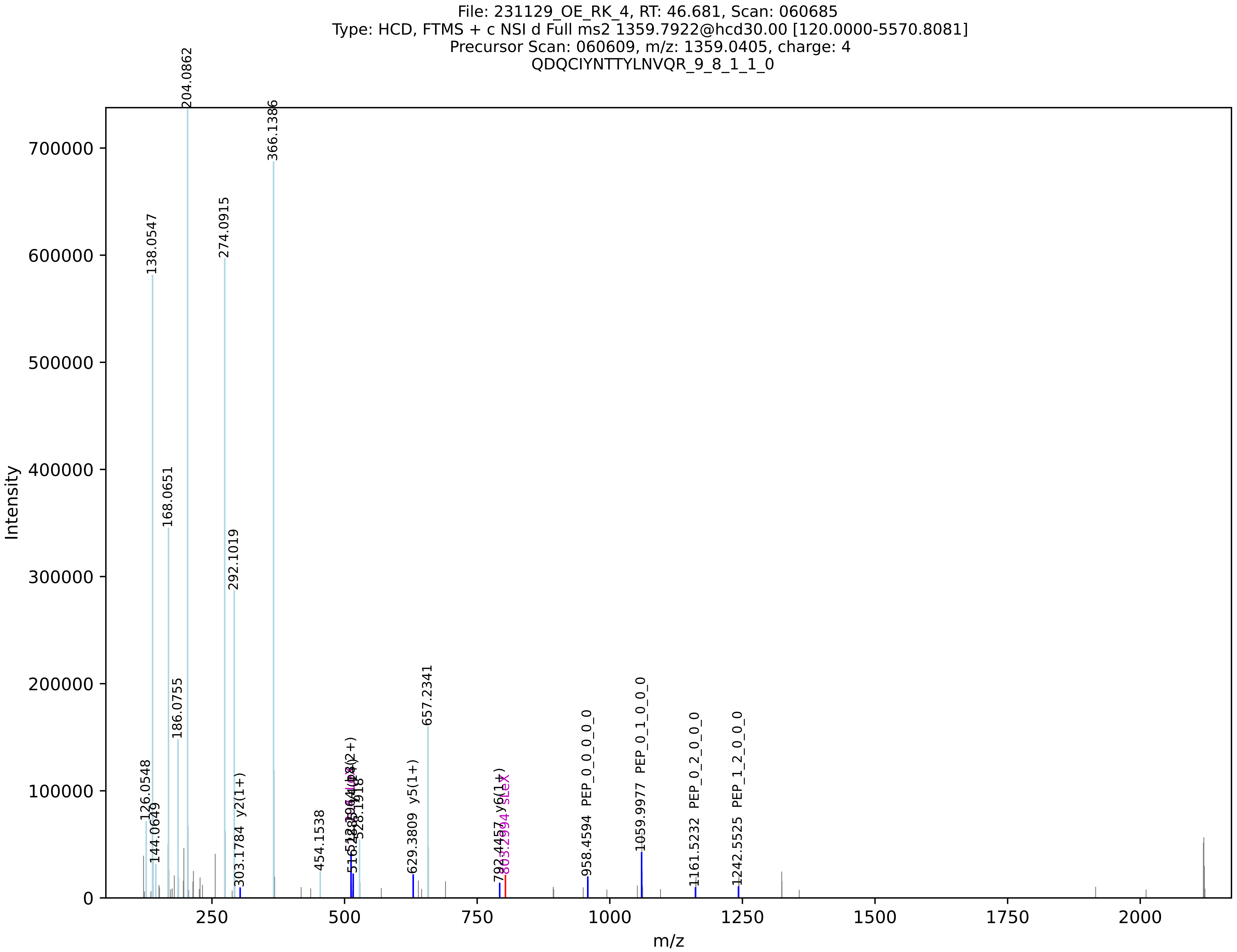

File: 231129_OE_RK_4, RT: 46.681, Scan: 060685
Type: HCD, FTMS + c NSI d Full ms2 1359.7922@hcd30.00 [120.0000-5570.8081]
Precursor Scan: 060609, m/z: 1359.0405, charge: 4
QDQCIYNTTYLNVQR_9_8_1_1_0
Intensity
m/z
204.0862
366.1386
138.0547
274.0915
168.0651
292.1019
186.0755
126.0548
144.0649
303.1784 y2(1+)
454.1538
657.2341
629.3809 y5(1+)
792.4457 y6(1+)
803.2994 SLeX
958.4594 PEP_0_0_0_0_0
1059.9977 PEP_0_1_0_0_0
1161.5232 PEP_0_2_0_0_0
1242.5525 PEP_1_2_0_0_0
528.1918

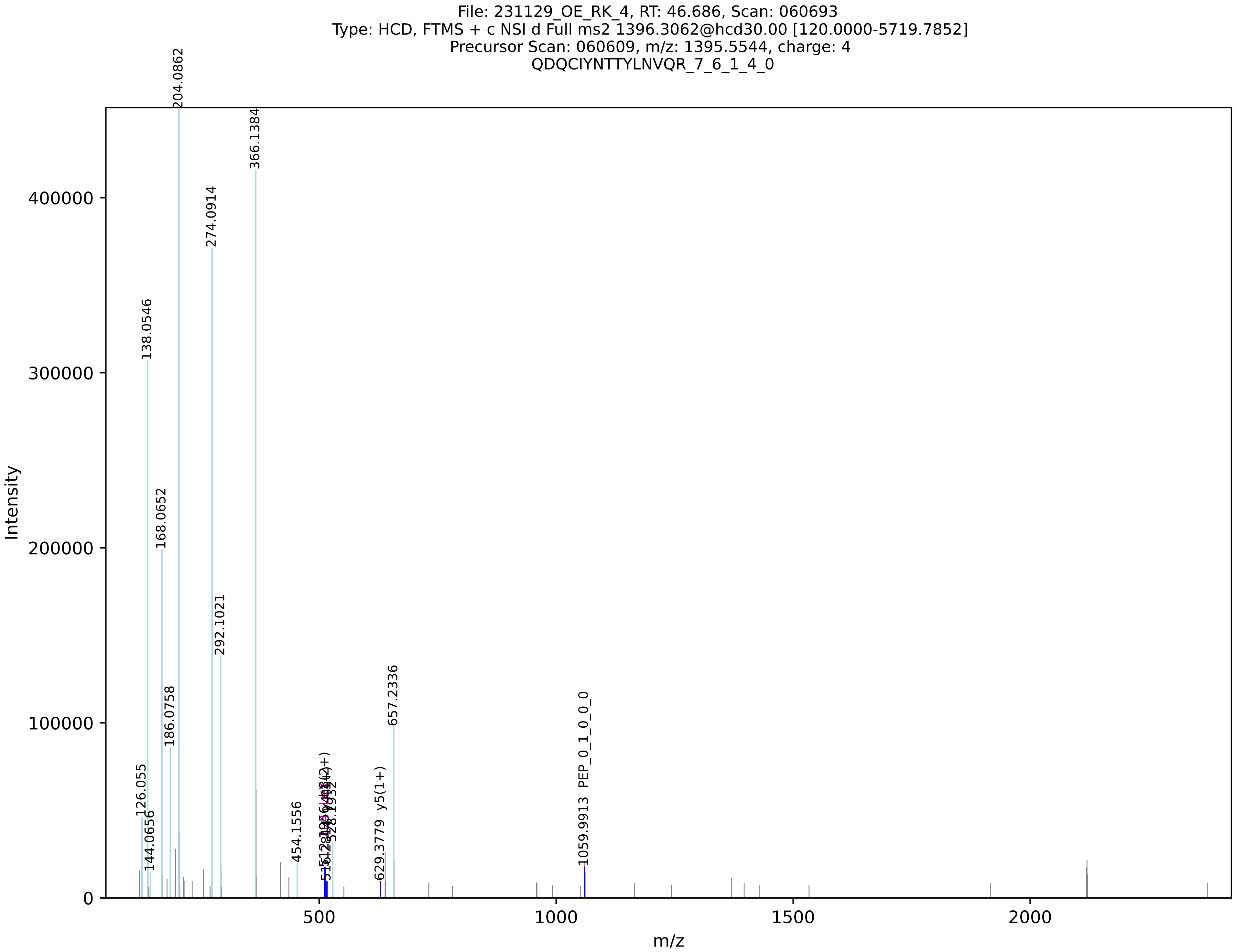
File: 231129_OE_RK_4, RT: 46.686, Scan: 060693
Type: HCD, FTMS + c NSI d Full ms2 1396.3062@hcd30.00 [120.0000-5719.7852]
Precursor Scan: 060609, m/z: 1395.5544, charge: 4
QDQCIYNTTYLNVQR_7_6_1_4_0
Intensity
m/z
204.0862
366.1384
274.0914
138.0546
168.0652
292.1021
186.0758
126.055
144.0656
454.1556
528.1932
629.3779 y5(1+)
657.2336
1059.9913 PEP_0_1_0_0_0
0
100000
200000
300000
400000
500
1000
1500
2000

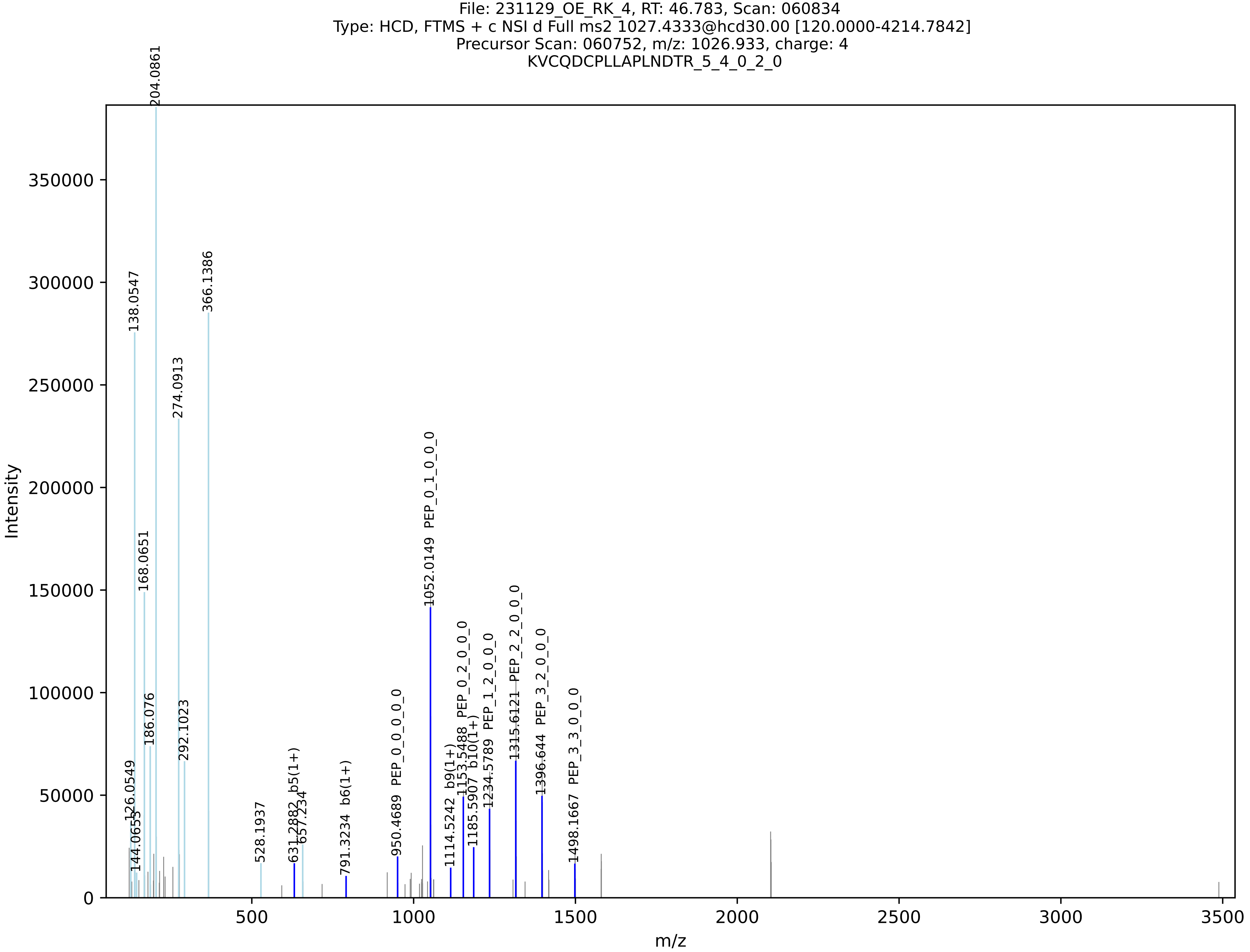
File: 231129_OE_RK_4, RT: 46.783, Scan: 060834
Type: HCD, FTMS + c NSI d Full ms2 1027.4333@hcd30.00 [120.0000-4214.7842]
Precursor Scan: 060752, m/z: 1026.933, charge: 4
KVCQDCPLLAPLNDTR_5_4_0_2_0
Intensity
m/z
204.0861
138.0547
366.1386
274.0913
168.0651
186.076
292.1023
126.0549
144.0653
528.1937
631.2882 b5(1+)
657.234
791.3234 b6(1+)
950.4689 PEP_0_0_0_0_0
1052.0149 PEP_0_1_0_0_0
1114.5242 b9(1+)
1153.5488 PEP_0_2_0_0_0
1185.5907 b10(1+)
1234.5789 PEP_1_2_0_0_0
1315.6121 PEP_2_2_0_0_0
1396.644 PEP_3_2_0_0_0
1498.1667 PEP_3_3_0_0_0

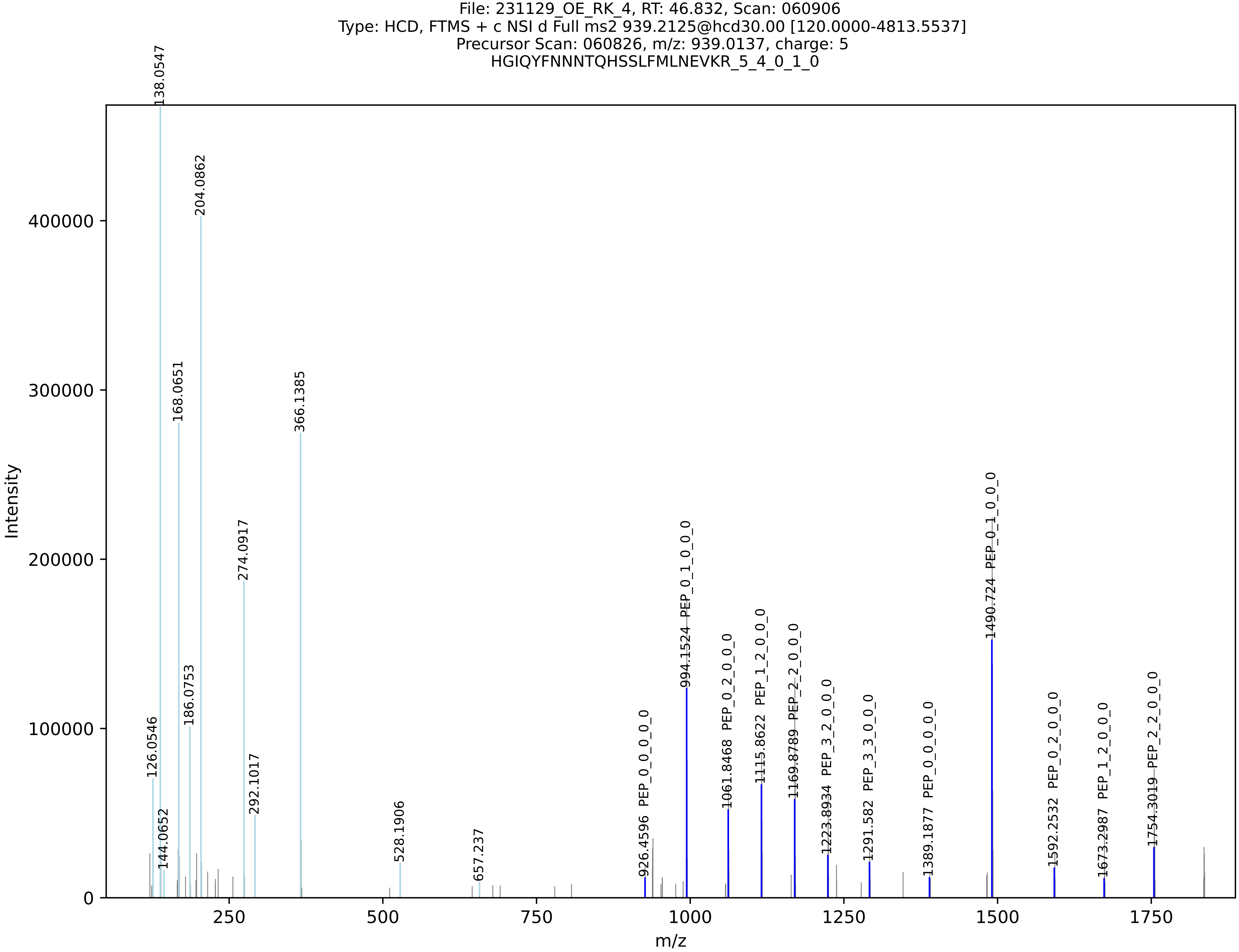
File: 231129_OE_RK_4, RT: 46.832, Scan: 060906
Type: HCD, FTMS + c NSI d Full ms2 939.2125@hcd30.00 [120.0000-4813.5537]
Precursor Scan: 060826, m/z: 939.0137, charge: 5
HGIQYFNNNTQHSSLFMLNEVKR_5_4_0_1_0
Intensity
m/z
138.0547
204.0862
168.0651
366.1385
274.0917
186.0753
126.0546
292.1017
144.0652
528.1906
657.237
926.4596 PEP_0_0_0_0_0
994.1524 PEP_0_1_0_0_0
1061.8468 PEP_0_2_0_0_0
1115.8622 PEP_1_2_0_0_0
1169.8789 PEP_2_2_0_0_0
1223.8934 PEP_3_2_0_0_0
1291.582 PEP_3_3_0_0_0
1389.1877 PEP_0_0_0_0_0
1490.724 PEP_0_1_0_0_0
1592.2532 PEP_0_2_0_0_0
1673.2987 PEP_1_2_0_0_0
1754.3019 PEP_2_2_0_0_0
0
100000
200000
300000
400000
250
500
750
1000
1250
1500
1750

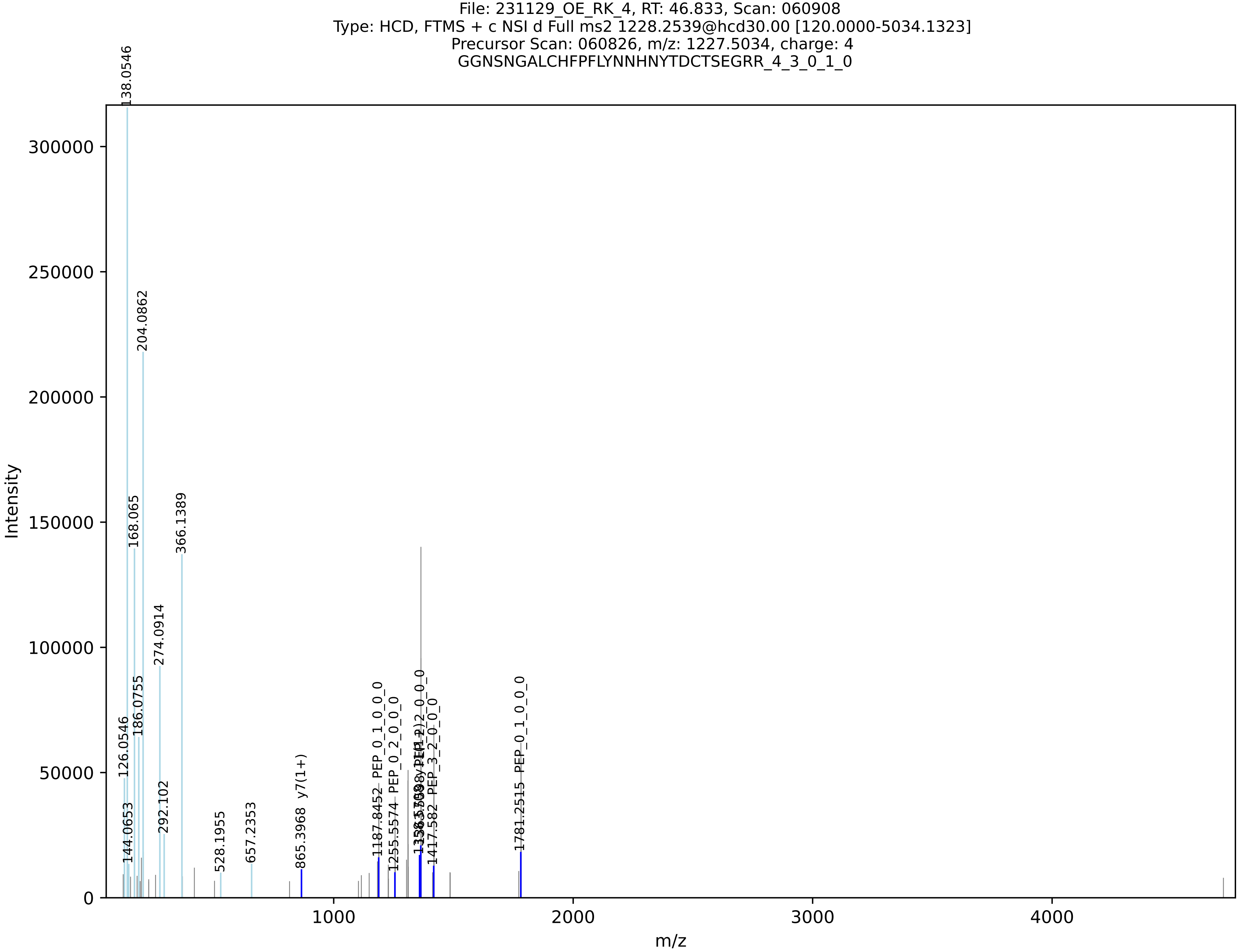
File: 231129_OE_RK_4, RT: 46.833, Scan: 060908
Type: HCD, FTMS + c NSI d Full ms2 1228.2539@hcd30.00 [120.0000-5034.1323]
Precursor Scan: 060826, m/z: 1227.5034, charge: 4
GGNSNGALCHFPFLYNNHNYTDCTSEGRR_4_3_0_1_0
Intensity
m/z
0
50000
100000
150000
200000
250000
300000
1000
2000
3000
4000
138.0546
204.0862
168.065
366.1389
274.0914
186.0755
126.0546
292.102
144.0653
528.1955
657.2353
865.3968 y7(1+)
1187.8452 PEP_0_1_0_0_0
1255.5574 PEP_0_2_0_0_0
1417.582 PEP_3_2_0_0_0
1781.2515 PEP_0_1_0_0_0

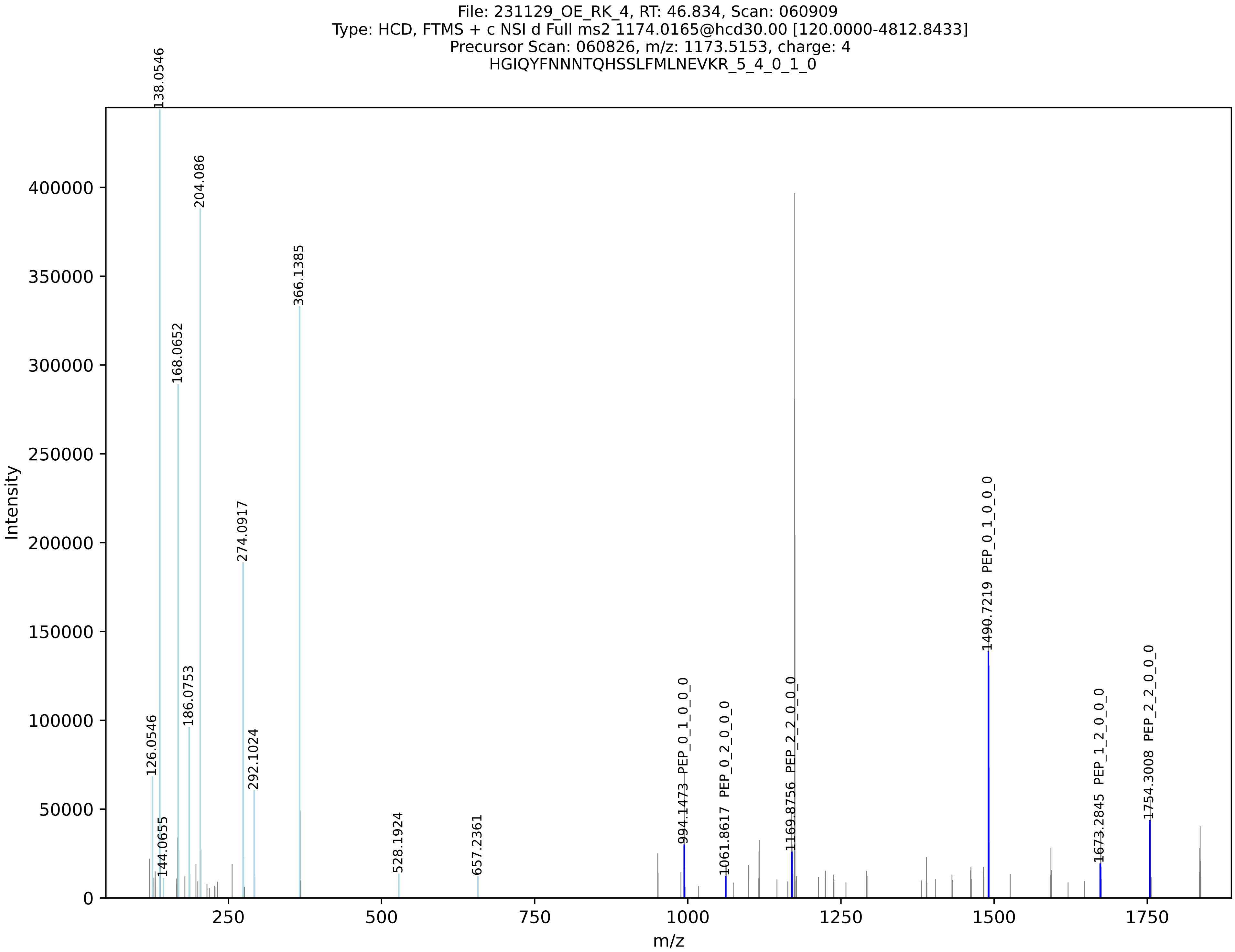
File: 231129_OE_RK_4, RT: 46.834, Scan: 060909
Type: HCD, FTMS + c NSI d Full ms2 1174.0165@hcd30.00 [120.0000-4812.8433]
Precursor Scan: 060826, m/z: 1173.5153, charge: 4
HGIQYFNNNTQHSSLFMLNEVKR_5_4_0_1_0
Intensity
m/z
0
50000
100000
150000
200000
250000
300000
350000
400000
250
500
750
1000
1250
1500
1750
138.0546
204.086
366.1385
168.0652
274.0917
186.0753
126.0546
292.1024
144.0655
528.1924
657.2361
994.1473 PEP_0_1_0_0_0
1061.8617 PEP_0_2_0_0_0
1169.8756 PEP_2_2_0_0_0
1490.7219 PEP_0_1_0_0_0
1673.2845 PEP_1_2_0_0_0
1754.3008 PEP_2_2_0_0_0

File: 231129_OE_RK_4, RT: 46.891, Scan: 060990
Type: HCD, FTMS + c NSI d Full ms2 1404.3110@hcd30.00 [120.0000-4319.3340]
Precursor Scan: 060900, m/z: 1403.3087, charge: 3
IIVPLNNRENISDPTSPLR_5_4_1_1_0

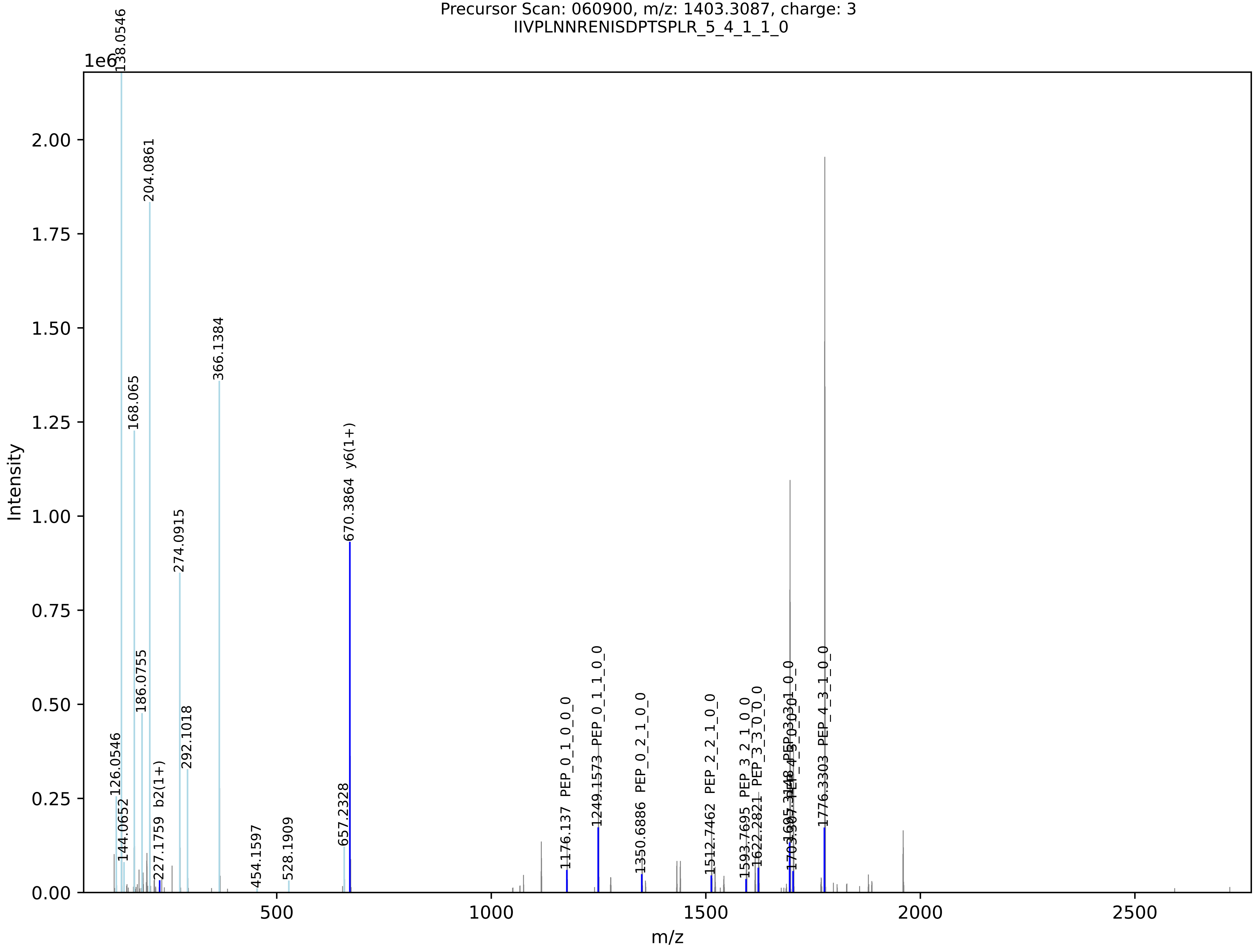

File: 231129_OE_RK_4, RT: 46.932, Scan: 061049
Type: HCD, FTMS + c NSI d Full ms2 1053.2335@hcd30.00 [120.0000-4320.0483]
Precursor Scan: 060974, m/z: 1052.732, charge: 4
IIVPLNNRENISDPTSPLR_5_4_1_1_0

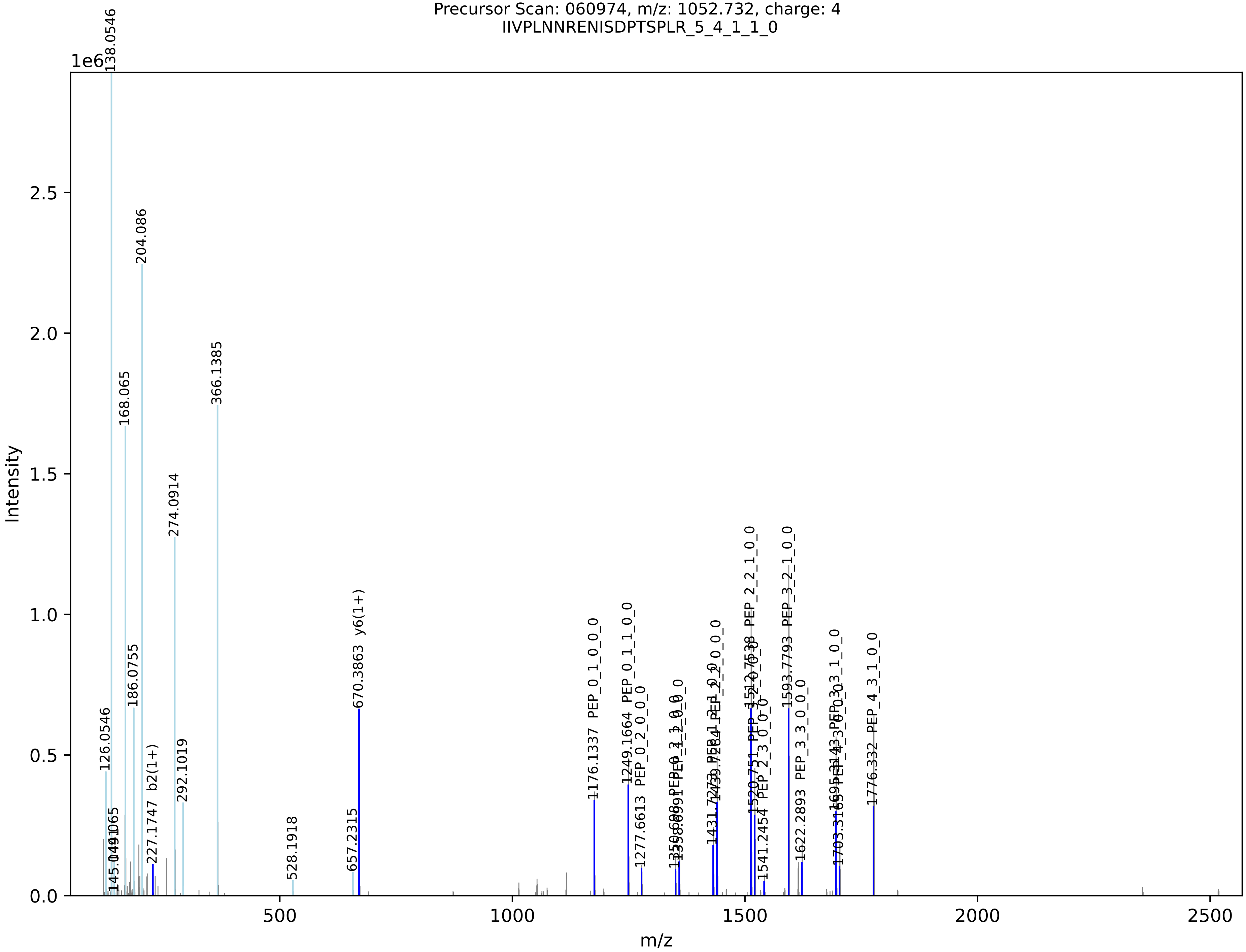

File: 231129_OE_RK_4, RT: 46.935, Scan: 061054
Type: HCD, FTMS + c NSI d Full ms2 1396.3033@hcd30.00 [120.0000-5719.7734]
Precursor Scan: 060974, m/z: 1395.5515, charge: 4
QDQCIYNTTYLNVQR_7_6_1_4_0

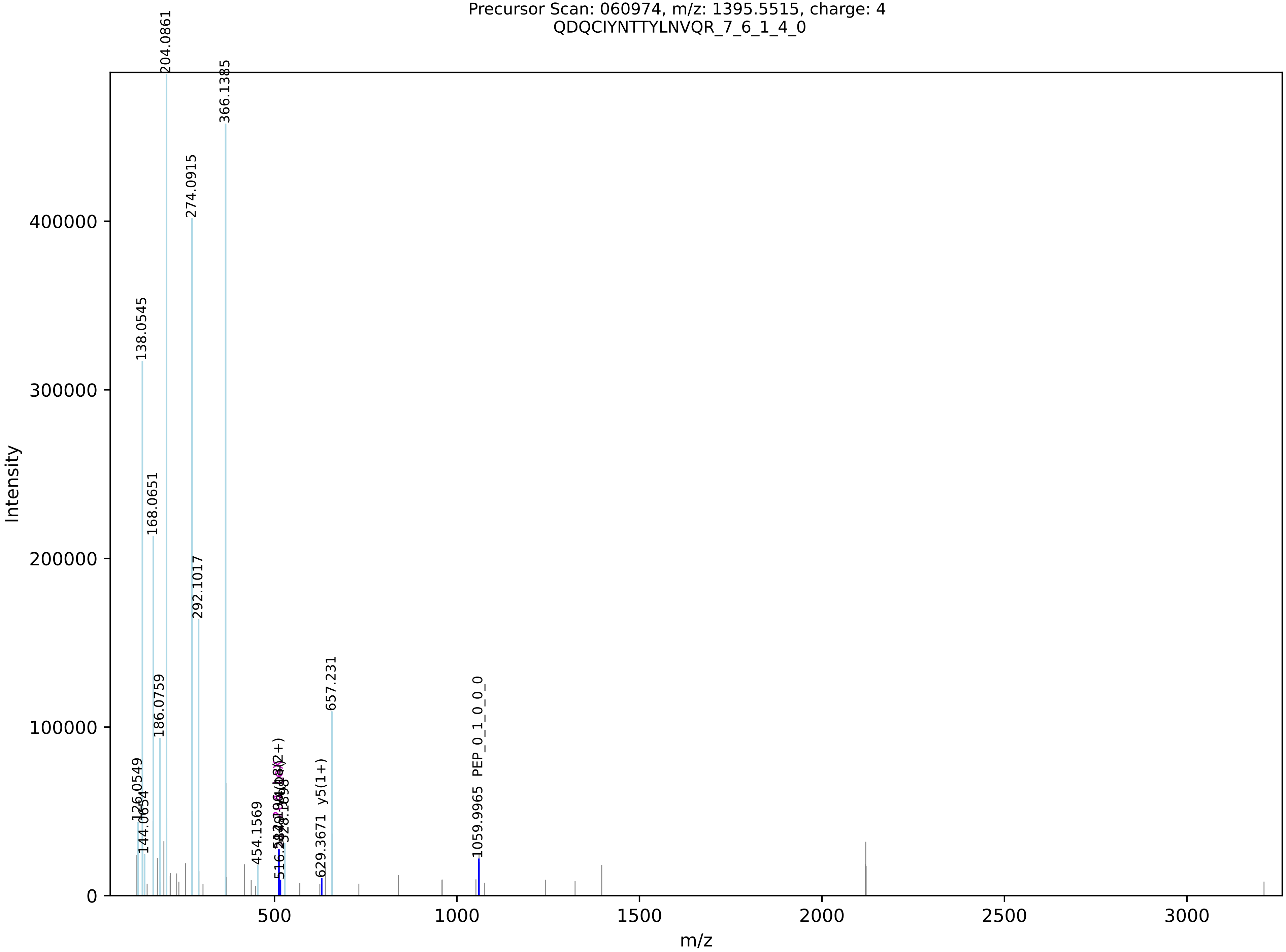

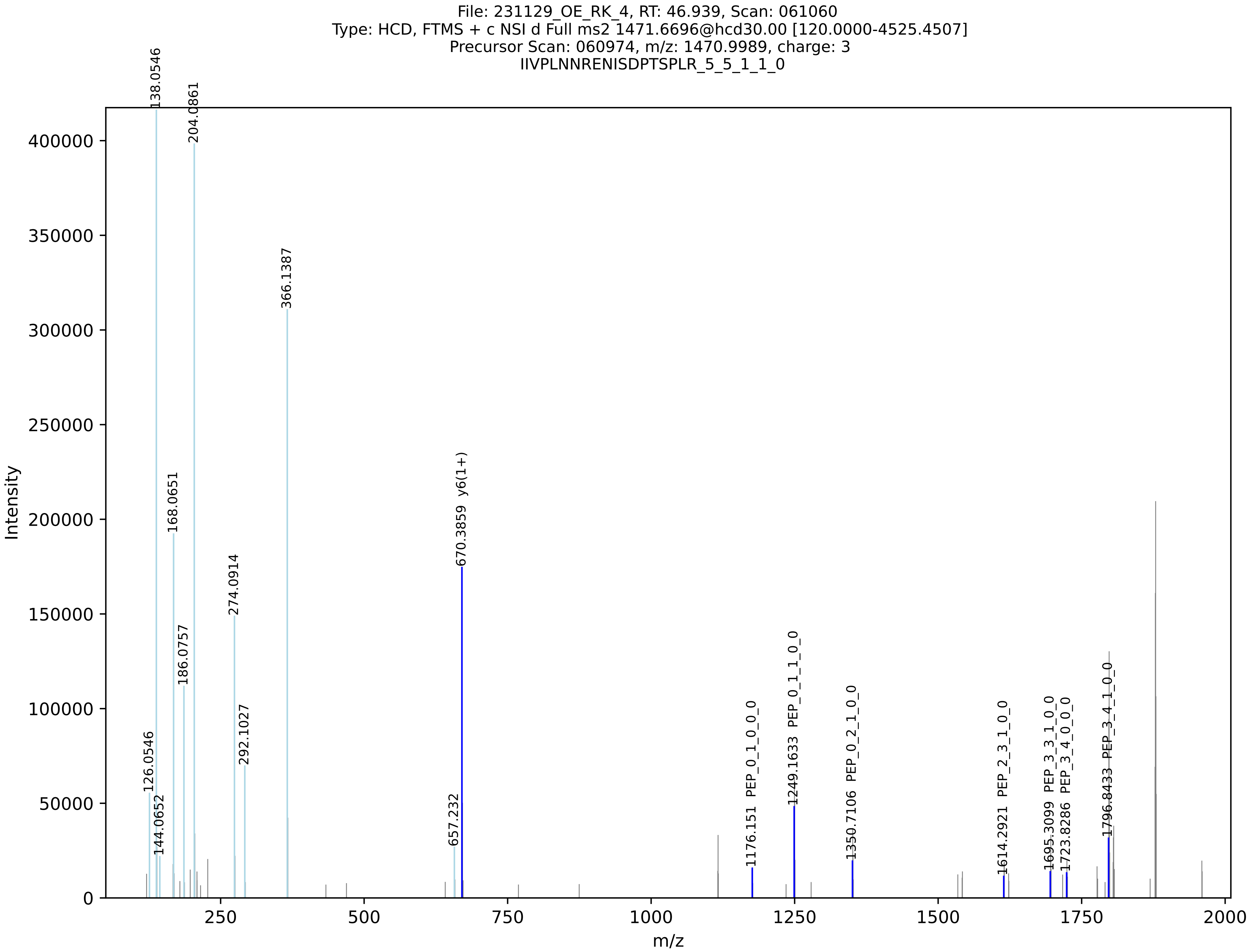

File: 231129_OE_RK_4, RT: 46.939, Scan: 061060
Type: HCD, FTMS + c NSI d Full ms2 1471.6696@hcd30.00 [120.0000-4525.4507]
Precursor Scan: 060974, m/z: 1470.9989, charge: 3
IIVPLNNRENISDPTSPLR_5_5_1_1_0
Intensity
m/z
138.0546
204.0861
366.1387
670.3859 y6(1+)
168.0651
274.0914
186.0757
292.1027
126.0546
144.0652
657.232
1176.151 PEP_0_1_0_0_0_
1249.1633 PEP_0_1_1_0_0_
1350.7106 PEP_0_2_1_0_0_
1614.2921 PEP_2_3_1_0_0_
1695.3099 PEP_3_3_1_0_0_
1723.8286 PEP_3_4_0_0_0_
1796.8433 PEP_3_4_1_0_0_

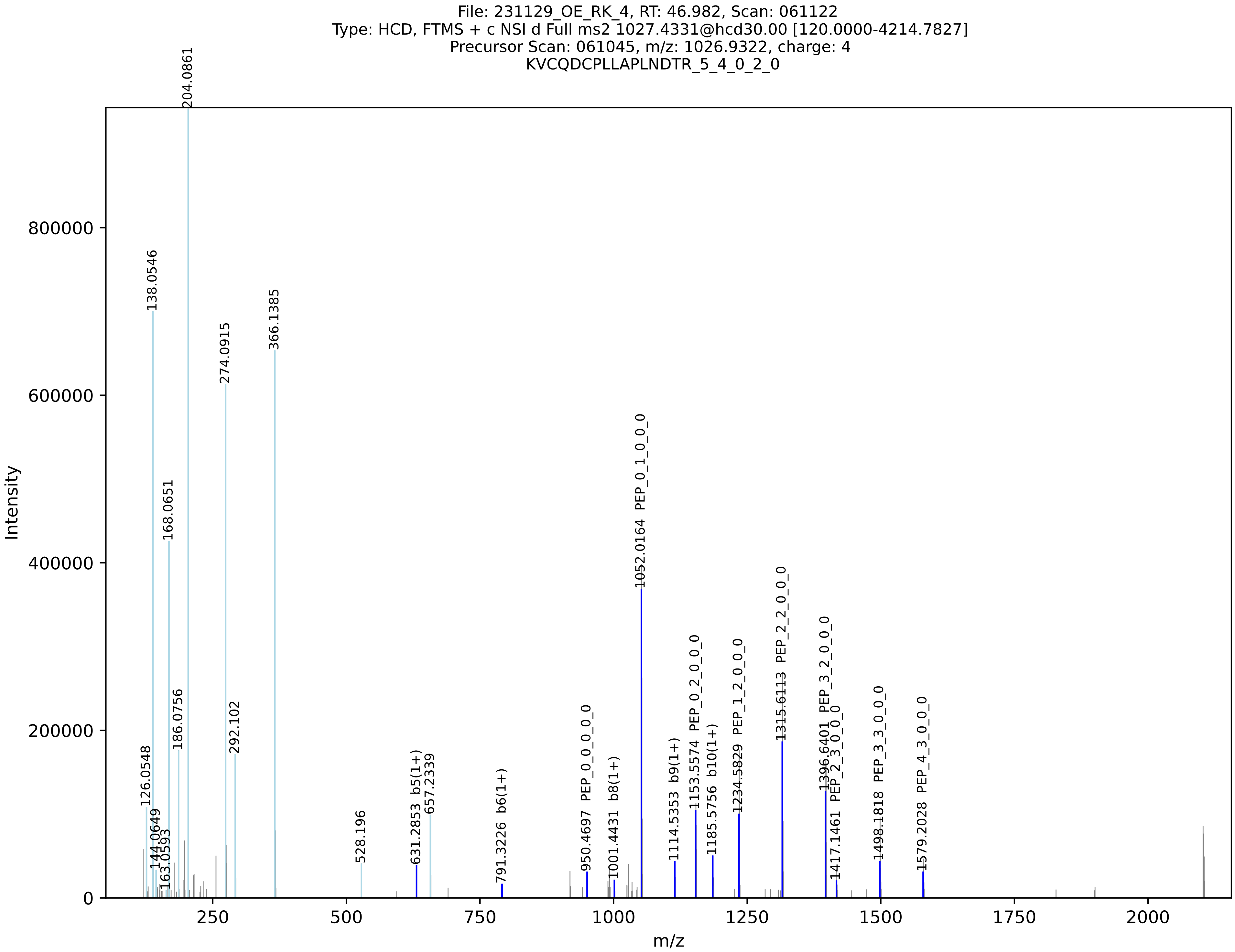
File: 231129_OE_RK_4, RT: 46.982, Scan: 061122
Type: HCD, FTMS + c NSI d Full ms2 1027.4331@hcd30.00 [120.0000-4214.7827]
Precursor Scan: 061045, m/z: 1026.9322, charge: 4
KVCQDCPLLAPLNDTR_5_4_0_2_0
Intensity
m/z
204.0861
138.0546
168.0651
186.0756
126.0548
144.0649
163.0593
274.0915
292.102
366.1385
528.196
631.2853 b5(1+)
657.2339
791.3226 b6(1+)
950.4697 PEP_0_0_0_0_0
1001.4431 b8(1+)
1052.0164 PEP_0_1_0_0_0
1114.5353 b9(1+)
1153.5574 PEP_0_2_0_0_0
1185.5756 b10(1+)
1234.5829 PEP_1_2_0_0_0
1315.6113 PEP_2_2_0_0_0
1396.6401 PEP_3_2_0_0_0
1417.1461 PEP_2_3_0_0_0
1498.1818 PEP_3_3_0_0_0
1579.2028 PEP_4_3_0_0_0

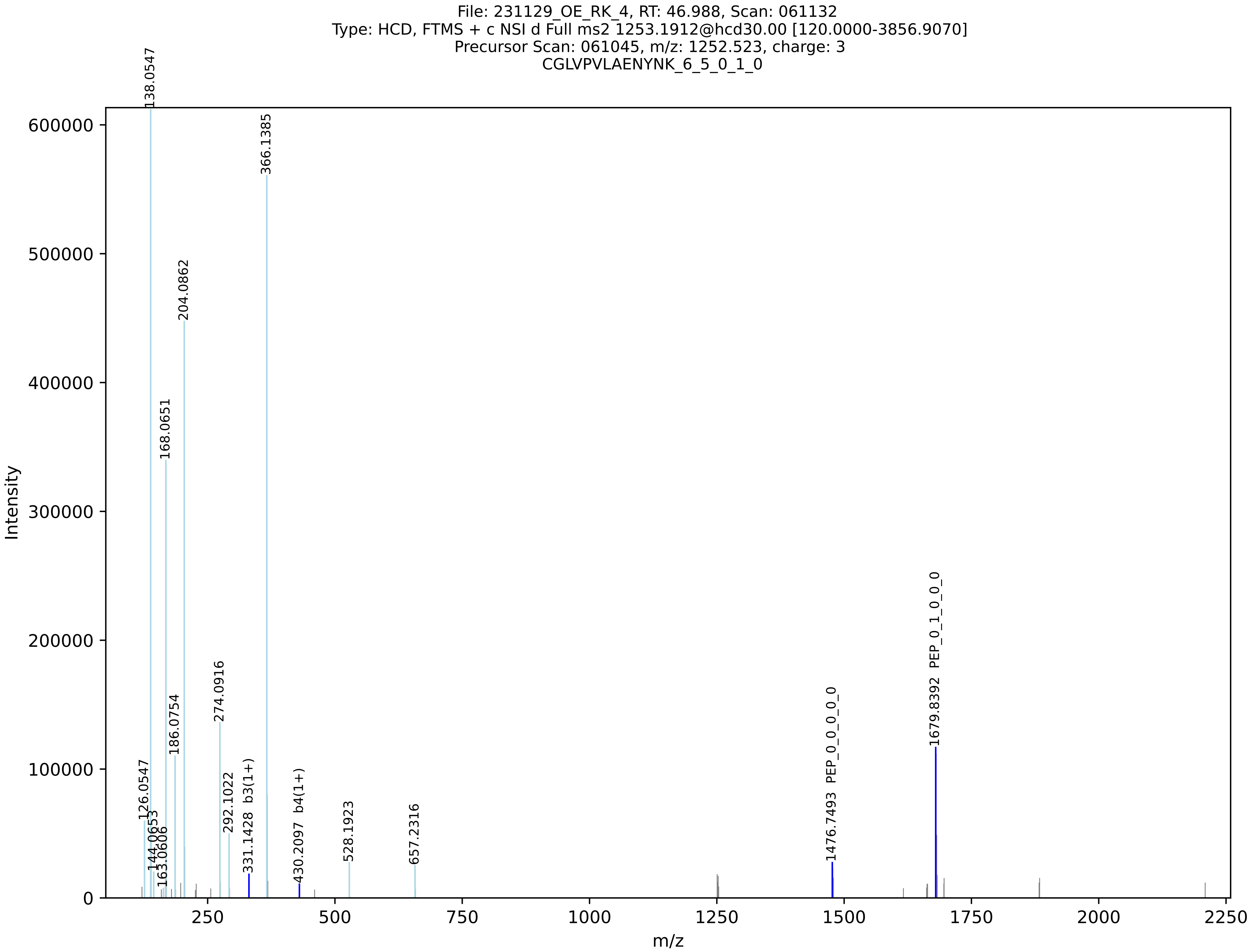
File: 231129_OE_RK_4, RT: 46.988, Scan: 061132
Type: HCD, FTMS + c NSI d Full ms2 1253.1912@hcd30.00 [120.0000-3856.9070]
Precursor Scan: 061045, m/z: 1252.523, charge: 3
CGLVPVLAENYNK_6_5_0_1_0
Intensity
m/z
138.0547
366.1385
204.0862
168.0651
186.0754
274.0916
126.0547
144.0653
163.0606
292.1022
331.1428 b3(1+)
430.2097 b4(1+)
528.1923
657.2316
1476.7493 PEP_0_0_0_0_0_
1679.8392 PEP_0_1_0_0_0_

File: 231129_OE_RK_4, RT: 47.032, Scan: 061195
Type: HCD, FTMS + c NSI d Full ms2 1369.5756@hcd30.00 [120.0000-4213.0430]
Precursor Scan: 061119, m/z: 1368.9077, charge: 3
KVCQDCPLLAPLNDTR_5_4_0_2_0

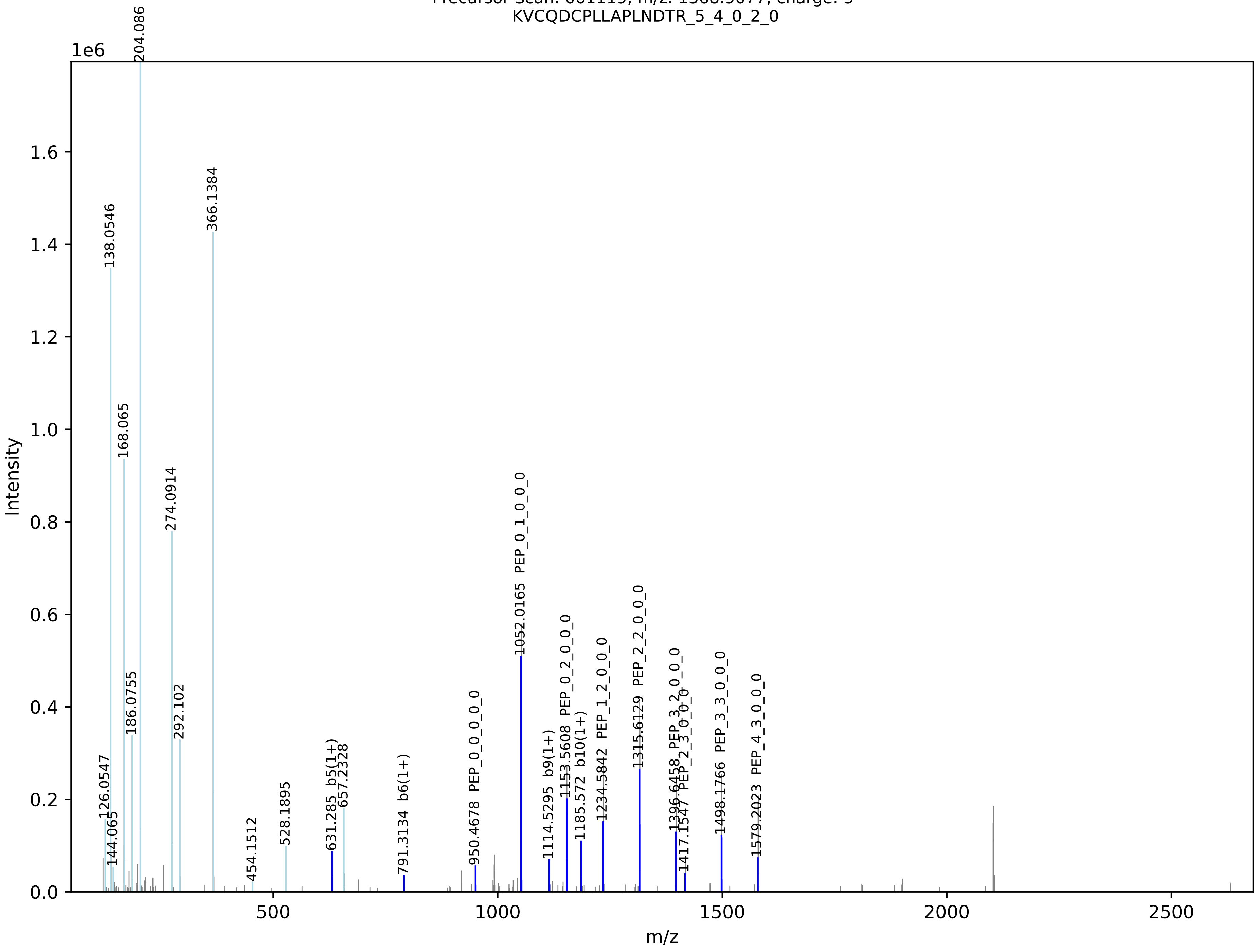

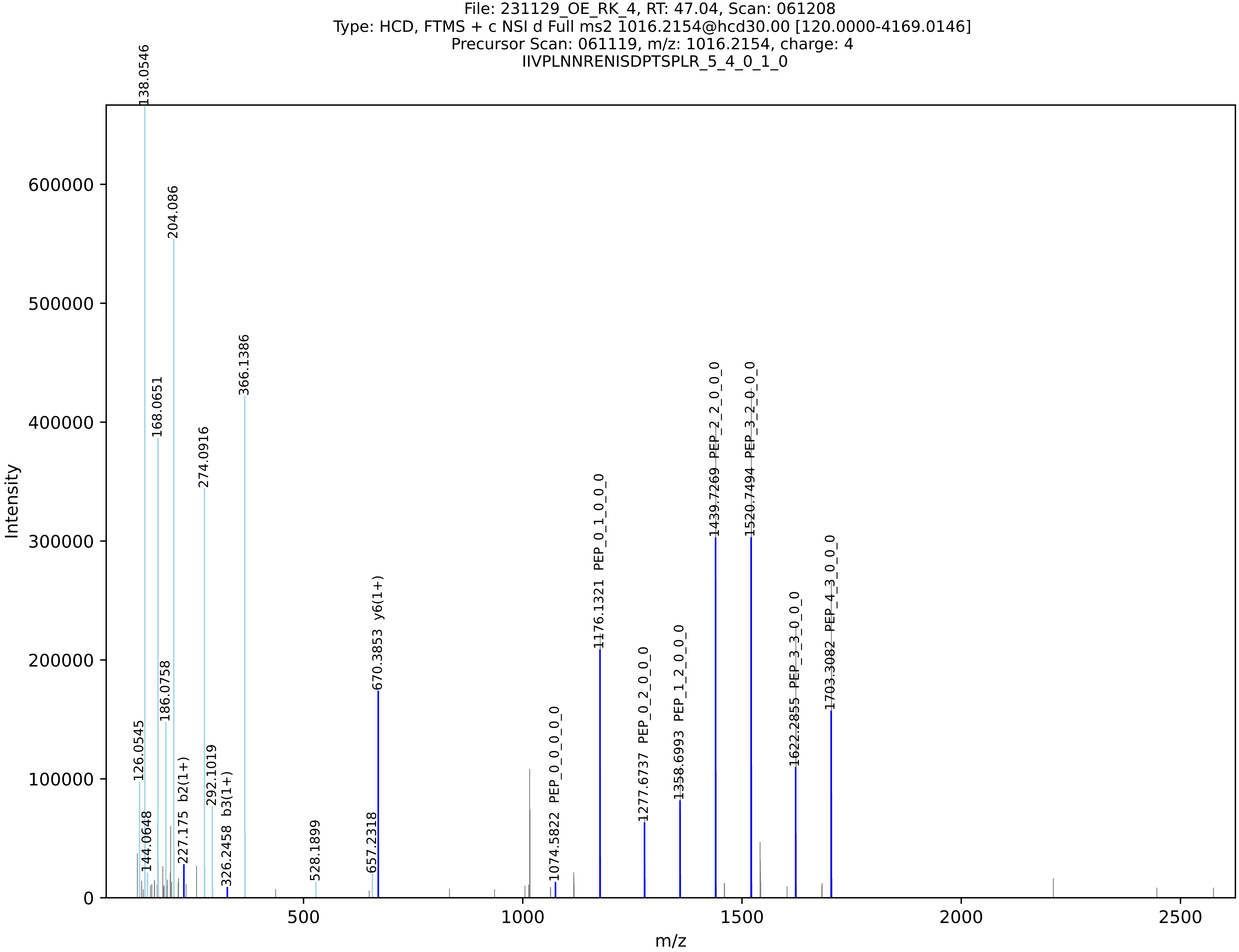

File: 231129_OE_RK_4, RT: 47.04, Scan: 061208
Type: HCD, FTMS + c NSI d Full ms2 1016.2154@hcd30.00 [120.0000-4169.0146]
Precursor Scan: 061119, m/z: 1016.2154, charge: 4
IIVPLNNRENISDPTSPLR_5_4_0_1_0
Intensity
m/z
138.0546
204.086
366.1386
168.0651
274.0916
186.0758
126.0545
144.0648
227.175 b2(1+)
292.1019
326.2458 b3(1+)
528.1899
657.2318
670.3853 y6(1+)
1074.5822 PEP_0_0_0_0_0
1176.1321 PEP_0_1_0_0_0
1277.6737 PEP_0_2_0_0_0
1358.6993 PEP_1_2_0_0_0
1439.7269 PEP_2_2_0_0_0
1520.7494 PEP_3_2_0_0_0
1622.2855 PEP_3_3_0_0_0
1703.3082 PEP_4_3_0_0_0

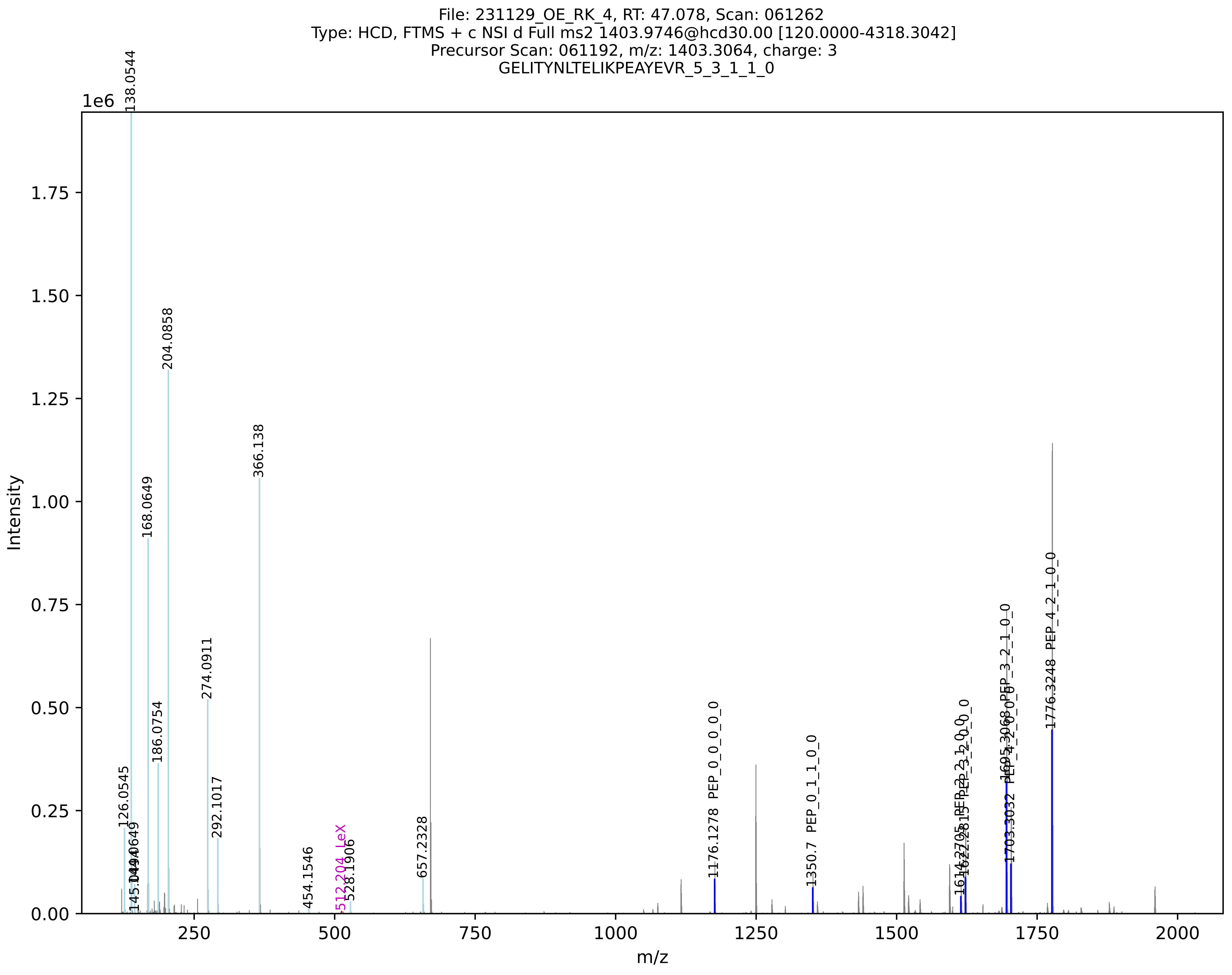
File: 231129_OE_RK_4, RT: 47.078, Scan: 061262
Type: HCD, FTMS + c NSI d Full ms2 1403.9746@hcd30.00 [120.0000-4318.3042]
Precursor Scan: 061192, m/z: 1403.3064, charge: 3
GELITYNLTELIKPEAYEVR_5_3_1_1_0
1e6
Intensity
m/z
138.0544
204.0858
366.138
168.0649
274.0911
186.0754
126.0545
292.1017
454.1546
512.204_LeX
528.1906
657.2328
1176.1278 PEP_0_0_0_0_0
1350.7 PEP_0_1_1_0_0
1776.3248 PEP_4_2_1_0_0
1703.3032 PEP_4_2_0_0_0
PEP_3_2_1_0_0
1614.2705 PEP_2_2_1_0_0
PEP_3_2_0_0_0

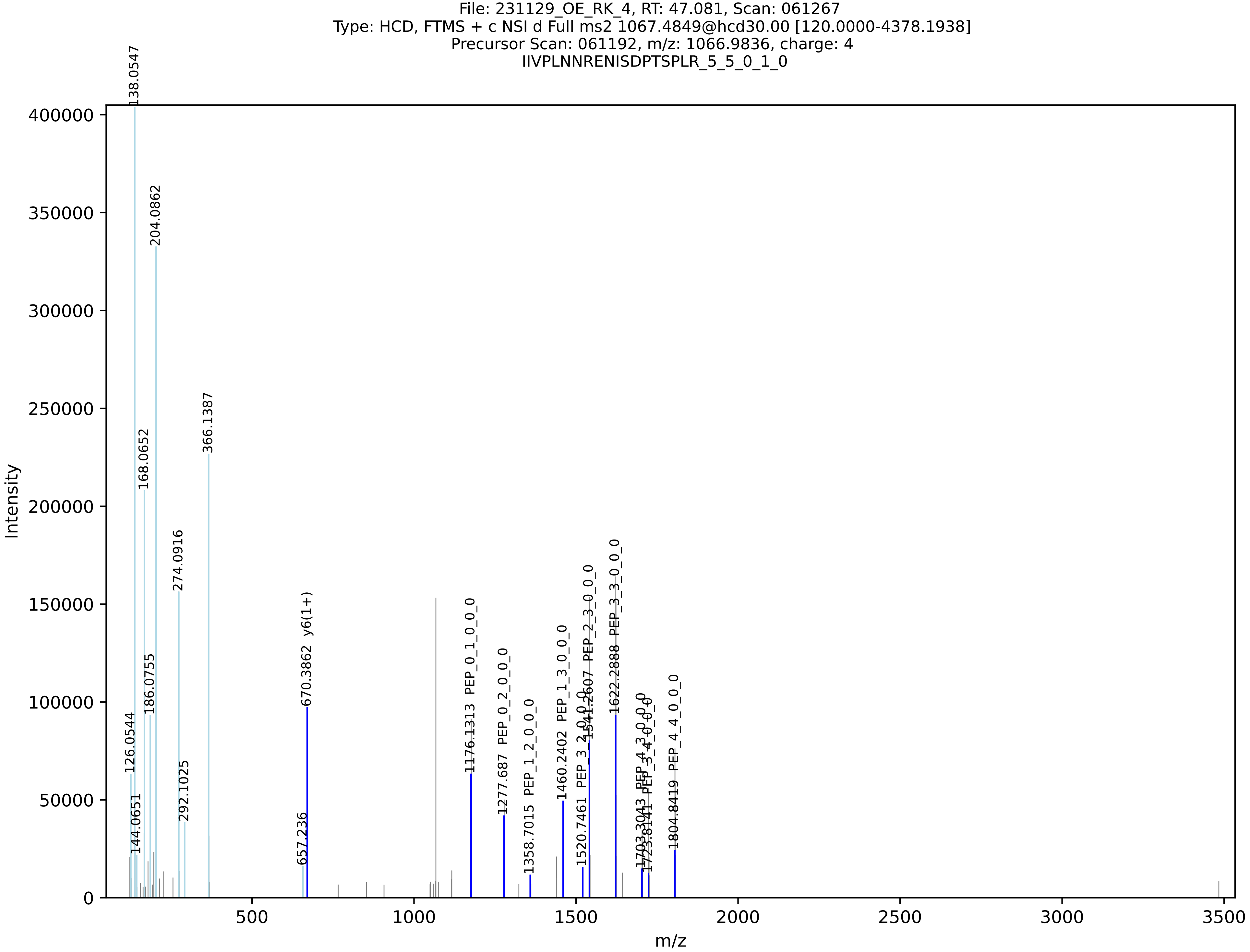
File: 231129_OE_RK_4, RT: 47.081, Scan: 061267
Type: HCD, FTMS + c NSI d Full ms2 1067.4849@hcd30.00 [120.0000-4378.1938]
Precursor Scan: 061192, m/z: 1066.9836, charge: 4
IIVPLNNRENISDPTSPLR_5_5_0_1_0
Intensity
m/z
138.0547
204.0862
366.1387
168.0652
274.0916
186.0755
126.0544
292.1025
144.0651
657.236
670.3862 y6(1+)
1176.1313 PEP_0_1_0_0_0_
1277.687 PEP_0_2_0_0_0_
1358.7015 PEP_1_2_0_0_0_
1460.2402 PEP_1_3_0_0_0_
1520.7461 PEP_3_2_0_0_0_
1541.2607 PEP_2_3_0_0_0_
1622.2888 PEP_3_3_0_0_0_
1703.3043 PEP_4_3_0_0_0_
1723.8141 PEP_3_4_0_0_0_
1804.8419 PEP_4_4_0_0_0_

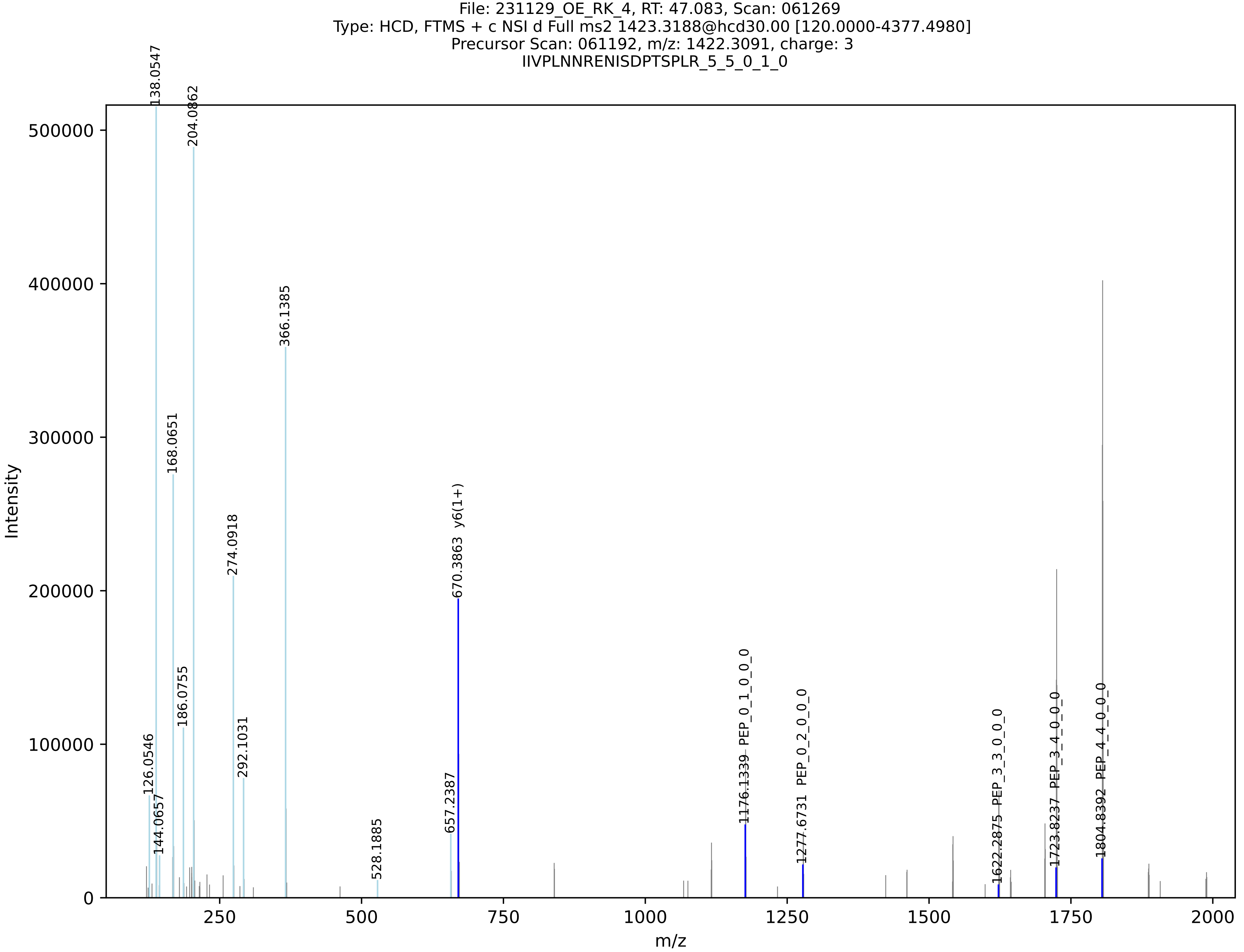

File: 231129_OE_RK_4, RT: 47.083, Scan: 061269
Type: HCD, FTMS + c NSI d Full ms2 1423.3188@hcd30.00 [120.0000-4377.4980]
Precursor Scan: 061192, m/z: 1422.3091, charge: 3
IIVPLNNRENISDPTSPLR_5_5_0_1_0
Intensity
m/z
138.0547
204.0862
366.1385
168.0651
274.0918
186.0755
292.1031
126.0546
144.0657
528.1885
657.2387
670.3863 y6(1+)
1176.1339 PEP_0_1_0_0_0
1277.6731 PEP_0_2_0_0_0
1622.2875 PEP_3_3_0_0_0
1723.8237 PEP_3_4_0_0_0
1804.8392 PEP_4_4_0_0_0

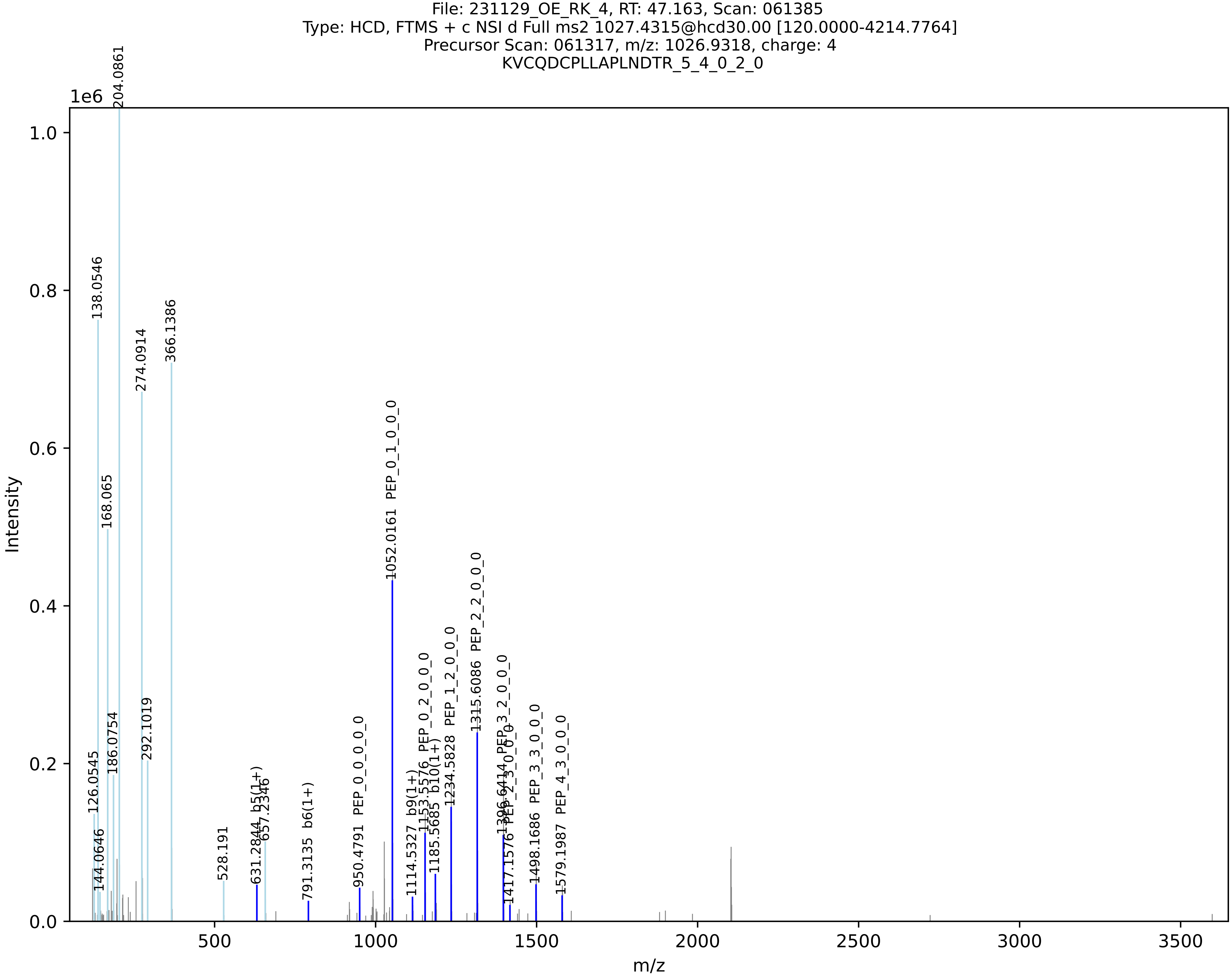
File: 231129_OE_RK_4, RT: 47.163, Scan: 061385
Type: HCD, FTMS + c NSI d Full ms2 1027.4315@hcd30.00 [120.0000-4214.7764]
Precursor Scan: 061317, m/z: 1026.9318, charge: 4
KVCQDCPLLAPLNDTR_5_4_0_2_0
1e6
Intensity
m/z
204.0861
138.0546
366.1386
274.0914
168.065
292.1019
186.0754
126.0545
144.0646
528.191
631.2844 b5(1+)
657.2346
791.3135 b6(1+)
950.4791 PEP_0_0_0_0_0
1052.0161 PEP_0_1_0_0_0
1114.5327 b9(1+)
1153.5576 PEP_0_2_0_0_0
1185.5685 b10(1+)
1234.5828 PEP_1_2_0_0_0
1315.6086 PEP_2_2_0_0_0
1396.6414 PEP_3_2_0_0_0
1417.1576 PEP_2_3_0_0_0
1498.1686 PEP_3_3_0_0_0
1579.1987 PEP_4_3_0_0_0

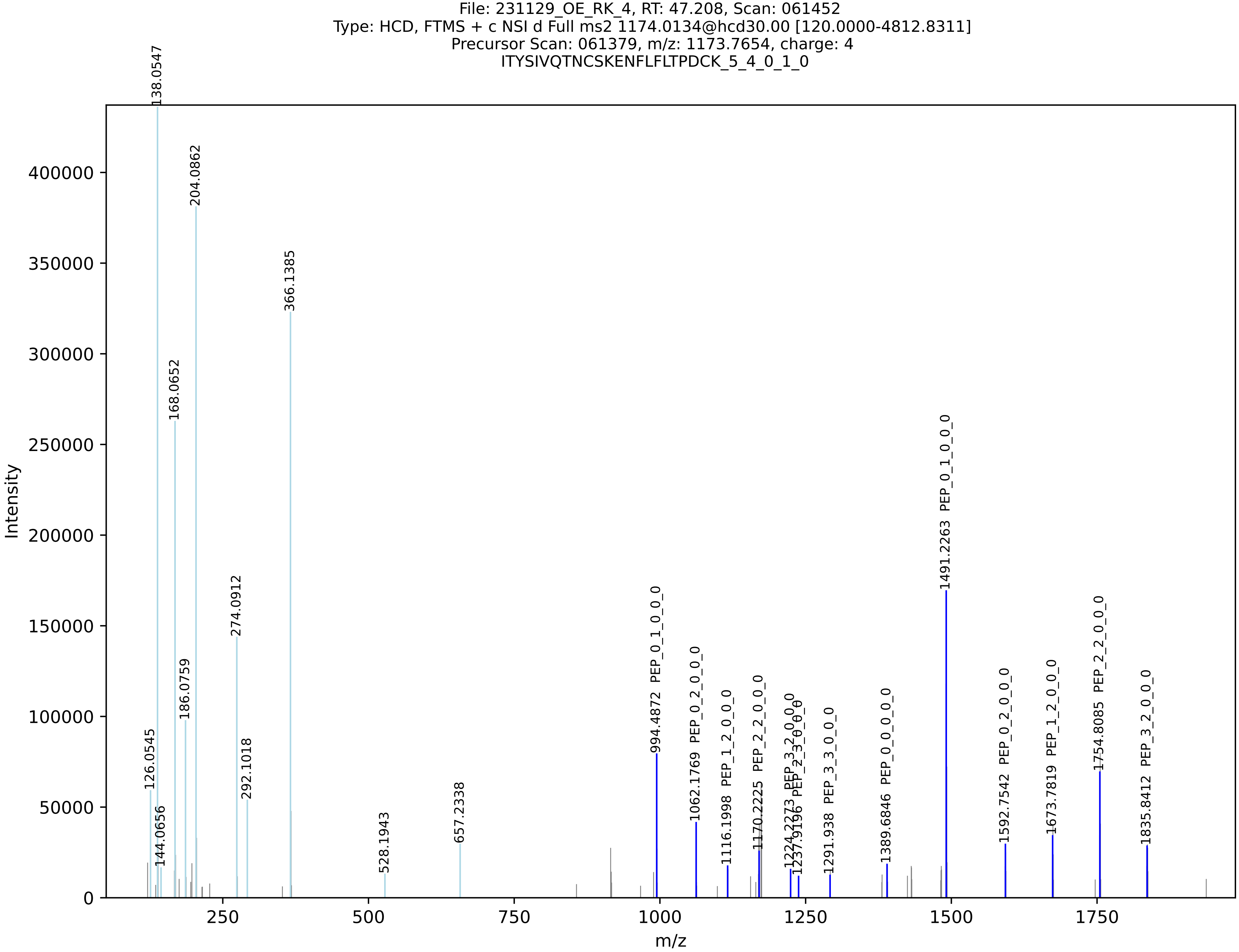
File: 231129_OE_RK_4, RT: 47.208, Scan: 061452
Type: HCD, FTMS + c NSI d Full ms2 1174.0134@hcd30.00 [120.0000-4812.8311]
Precursor Scan: 061379, m/z: 1173.7654, charge: 4
ITYSIVQTNCSKENFLFLTPDCK_5_4_0_1_0
Intensity
m/z
0
50000
100000
150000
200000
250000
300000
350000
400000
250
500
750
1000
1250
1500
1750
138.0547
204.0862
366.1385
168.0652
274.0912
186.0759
126.0545
292.1018
144.0656
528.1943
657.2338
994.4872 PEP_0_1_0_0_0
1062.1769 PEP_0_2_0_0_0
1116.1998 PEP_1_2_0_0_0
1170.2225 PEP_2_2_0_0_0
1224.2273 PEP_3_2_0_0_0
1237.9196 PEP_2_3_0_0_0
1291.938 PEP_3_3_0_0_0
1389.6846 PEP_0_0_0_0_0
1491.2263 PEP_0_1_0_0_0
1592.7542 PEP_0_2_0_0_0
1673.7819 PEP_1_2_0_0_0
1754.8085 PEP_2_2_0_0_0
1835.8412 PEP_3_2_0_0_0

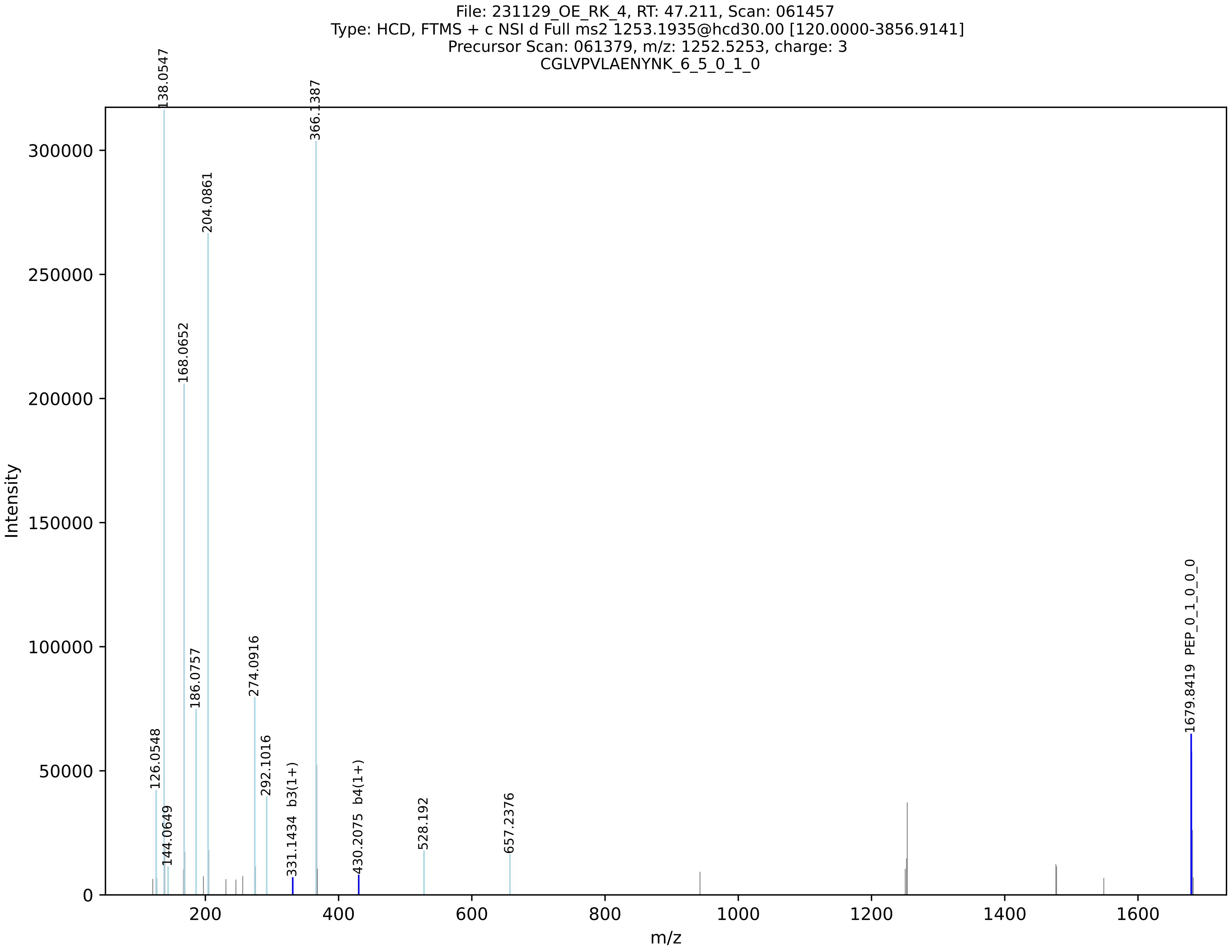
File: 231129_OE_RK_4, RT: 47.211, Scan: 061457
Type: HCD, FTMS + c NSI d Full ms2 1253.1935@hcd30.00 [120.0000-3856.9141]
Precursor Scan: 061379, m/z: 1252.5253, charge: 3
CGLVPVLAENYNK_6_5_0_1_0
138.0547
366.1387
204.0861
168.0652
186.0757
274.0916
126.0548
292.1016
144.0649
331.1434 b3(1+)
430.2075 b4(1+)
528.192
657.2376
1679.8419 PEP_0_1_0_0_0
Intensity
m/z
0
50000
100000
150000
200000
250000
300000
200
400
600
800
1000
1200
1400
1600

File: 231129_OE_RK_4, RT: 47.253, Scan: 061517
Type: HCD, FTMS + c NSI d Full ms2 1369.2405@hcd30.00 [120.0000-4212.0181]
Precursor Scan: 061441, m/z: 1368.9066, charge: 3
KVCQDCPLLAPLNDTR_5_4_0_2_0

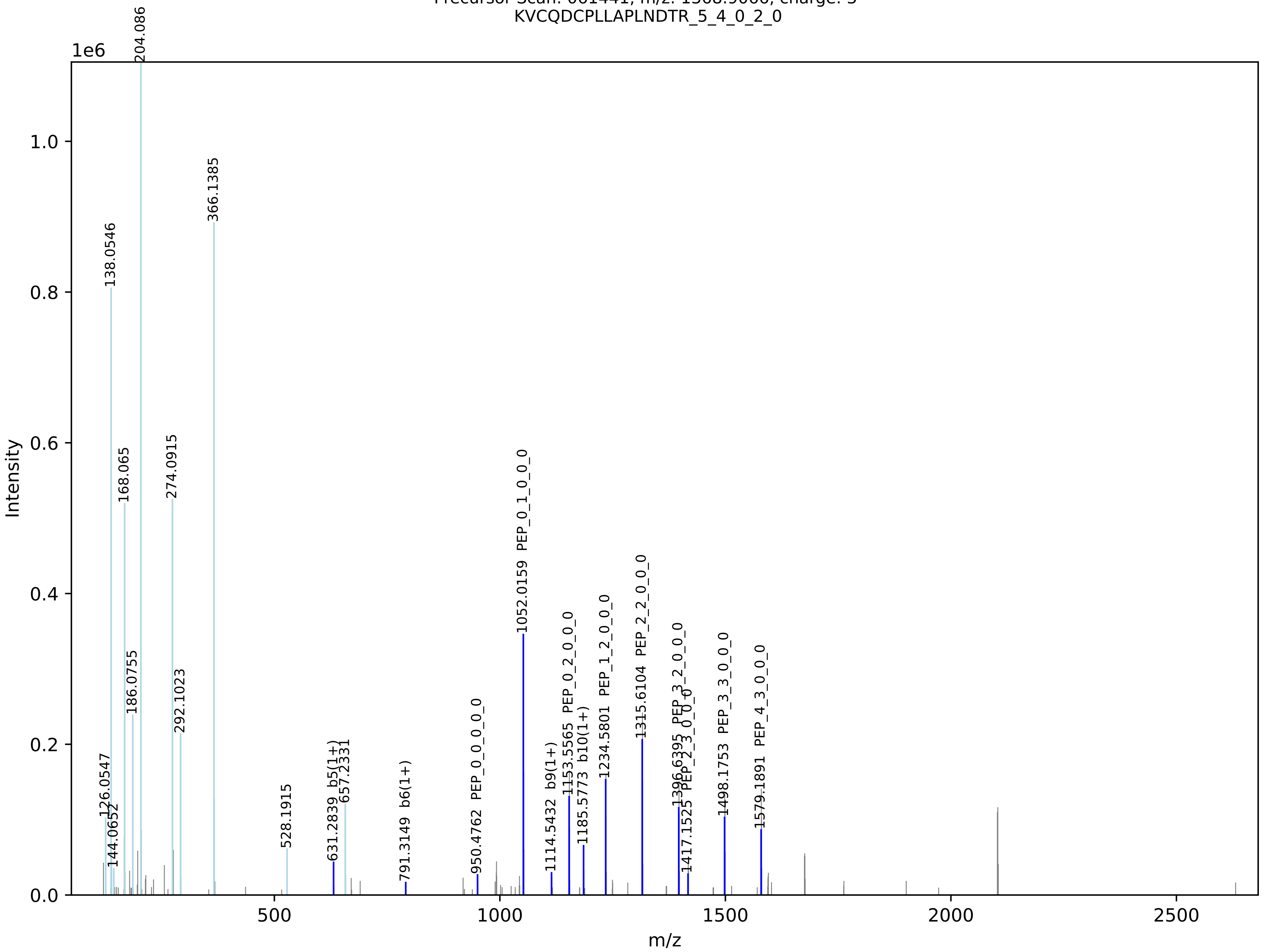

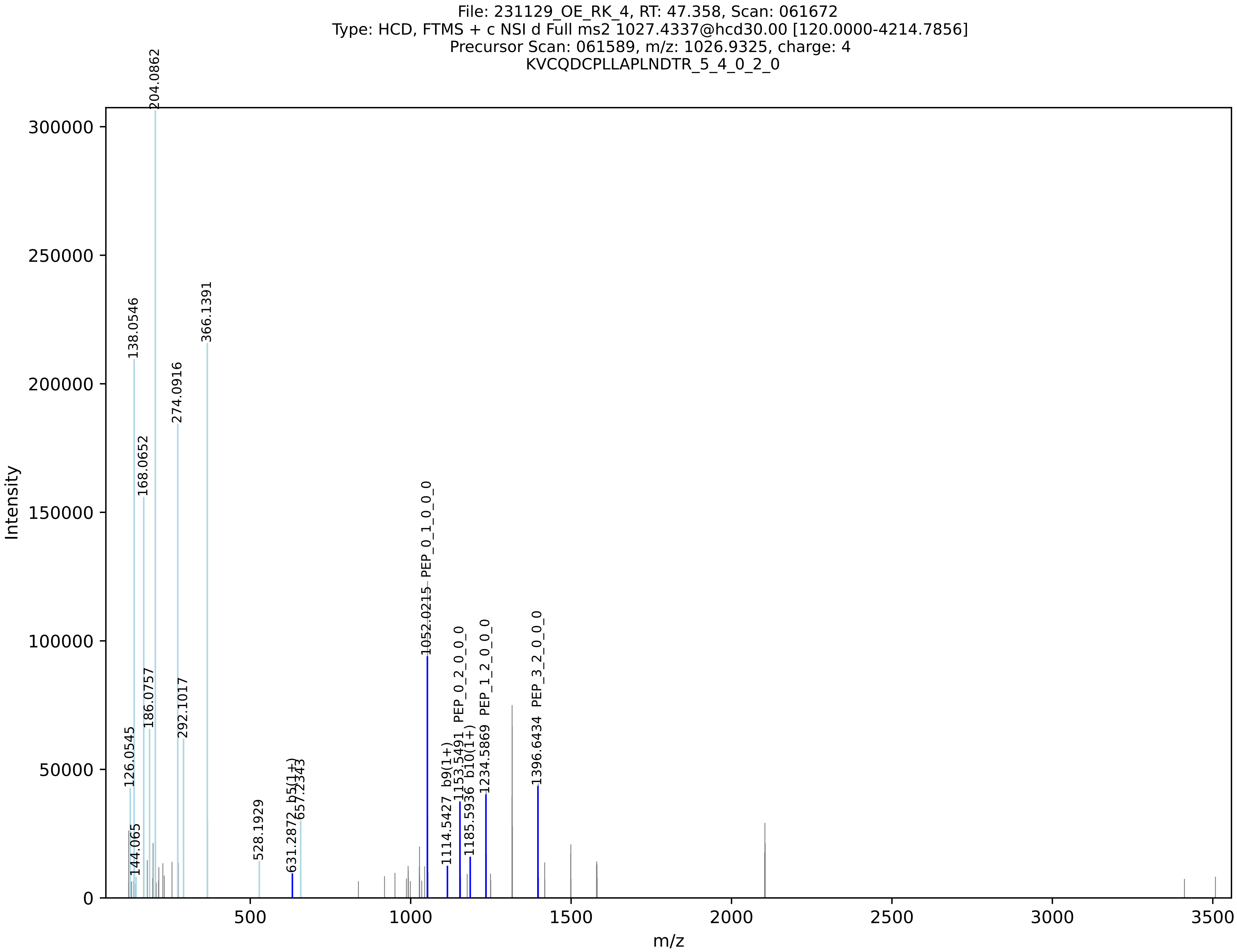

File: 231129_OE_RK_4, RT: 47.358, Scan: 061672
Type: HCD, FTMS + c NSI d Full ms2 1027.4337@hcd30.00 [120.0000-4214.7856]
Precursor Scan: 061589, m/z: 1026.9325, charge: 4
KVCQDCPLLAPLNDTR_5_4_0_2_0
Intensity
m/z
204.0862
138.0546
366.1391
274.0916
168.0652
186.0757
292.1017
126.0545
144.065
528.1929
631.2872 b5(1+)
657.2343
1052.0215 PEP_0_1_0_0_0
1114.5427 b9(1+)
1153.5491 PEP_0_2_0_0_0
1185.5936 b10(1+)
1234.5869 PEP_1_2_0_0_0
1396.6434 PEP_3_2_0_0_0

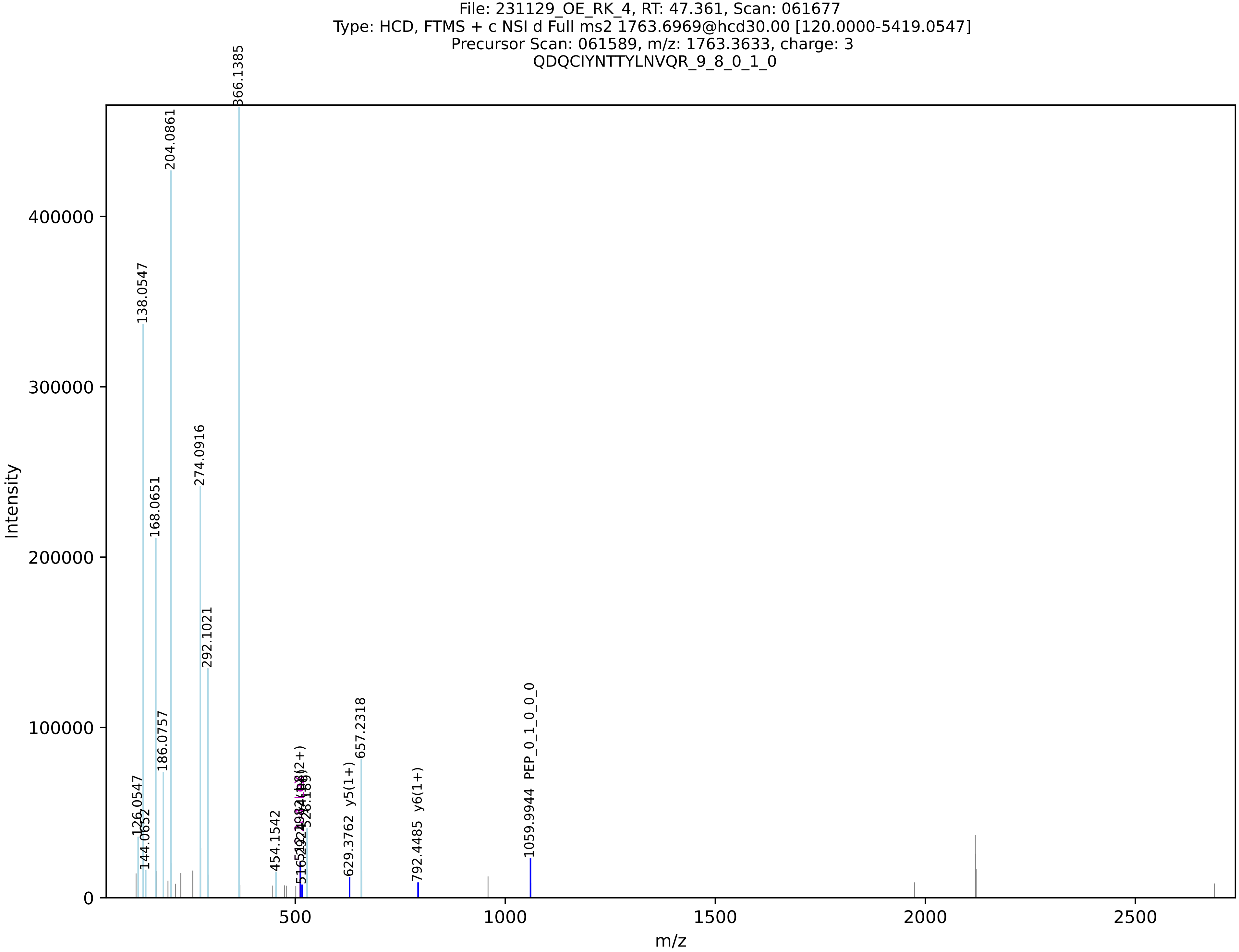
File: 231129_OE_RK_4, RT: 47.361, Scan: 061677
Type: HCD, FTMS + c NSI d Full ms2 1763.6969@hcd30.00 [120.0000-5419.0547]
Precursor Scan: 061589, m/z: 1763.3633, charge: 3
QDQCIYNTTYLNVQR_9_8_0_1_0
Intensity
m/z
0
100000
200000
300000
400000
500
1000
1500
2000
2500
366.1385
204.0861
138.0547
168.0651
274.0916
292.1021
186.0757
126.0547
144.0652
454.1542
629.3762 y5(1+)
657.2318
792.4485 y6(1+)
1059.9944 PEP_0_1_0_0_0

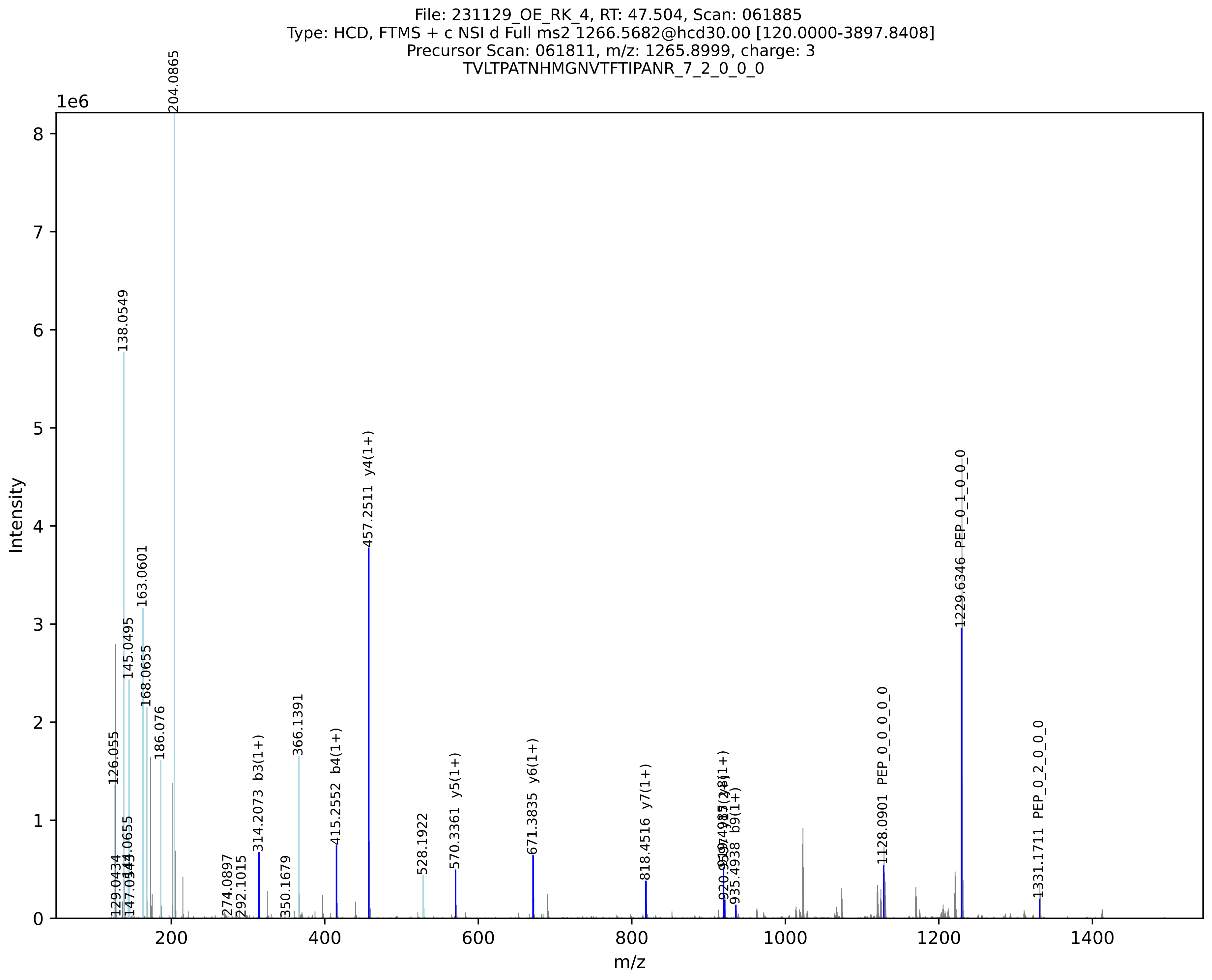

File: 231129_OE_RK_4, RT: 47.504, Scan: 061885
Type: HCD, FTMS + c NSI d Full ms2 1266.5682@hcd30.00 [120.0000-3897.8408]
Precursor Scan: 061811, m/z: 1265.8999, charge: 3
TVLTPATNHMGNVTFTIPANR_7_2_0_0_0
1e6
Intensity
m/z
204.0865
138.0549
163.0601
145.0495
168.0655
186.076
126.055
129.0434
147.0544
143.0655
274.0897
292.1015
314.2073 b3(1+)
350.1679
366.1391
415.2552 b4(1+)
457.2511 y4(1+)
528.1922
570.3361 y5(1+)
671.3835 y6(1+)
818.4516 y7(1+)
935.4938 b9(1+)
1128.0901 PEP_0_0_0_0_0
1229.6346 PEP_0_1_0_0_0
1331.1711 PEP_0_2_0_0_0

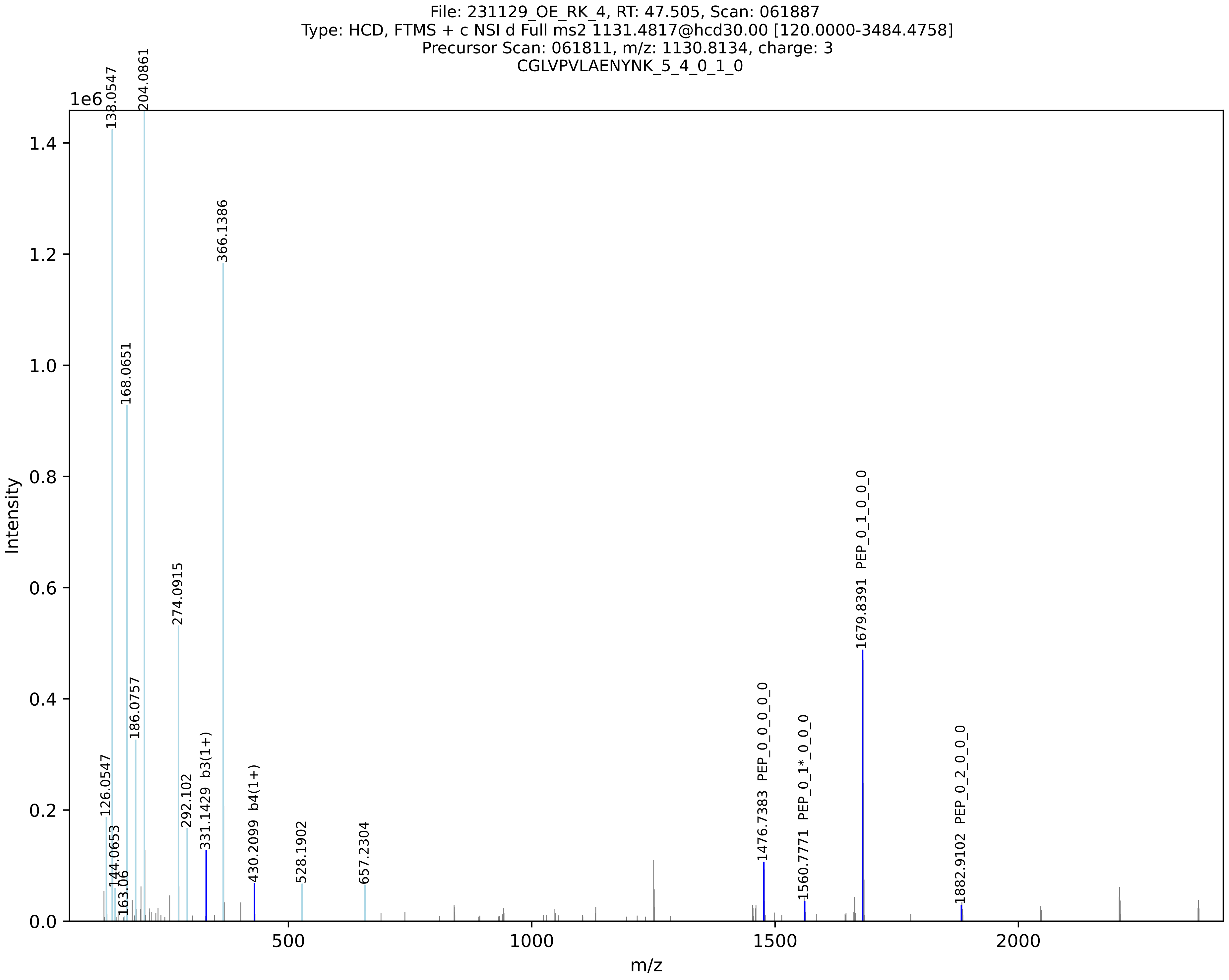
File: 231129_OE_RK_4, RT: 47.505, Scan: 061887
Type: HCD, FTMS + c NSI d Full ms2 1131.4817@hcd30.00 [120.0000-3484.4758]
Precursor Scan: 061811, m/z: 1130.8134, charge: 3
CGLVPVLAENYNK_5_4_0_1_0
1e6
Intensity
m/z
138.0547
204.0861
366.1386
168.0651
274.0915
186.0757
126.0547
144.0653
163.06
292.102
331.1429 b3(1+)
430.2099 b4(1+)
528.1902
657.2304
1476.7383 PEP_0_0_0_0_0
1560.7771 PEP_0_1*_0_0_0
1679.8391 PEP_0_1_0_0_0
1882.9102 PEP_0_2_0_0_0

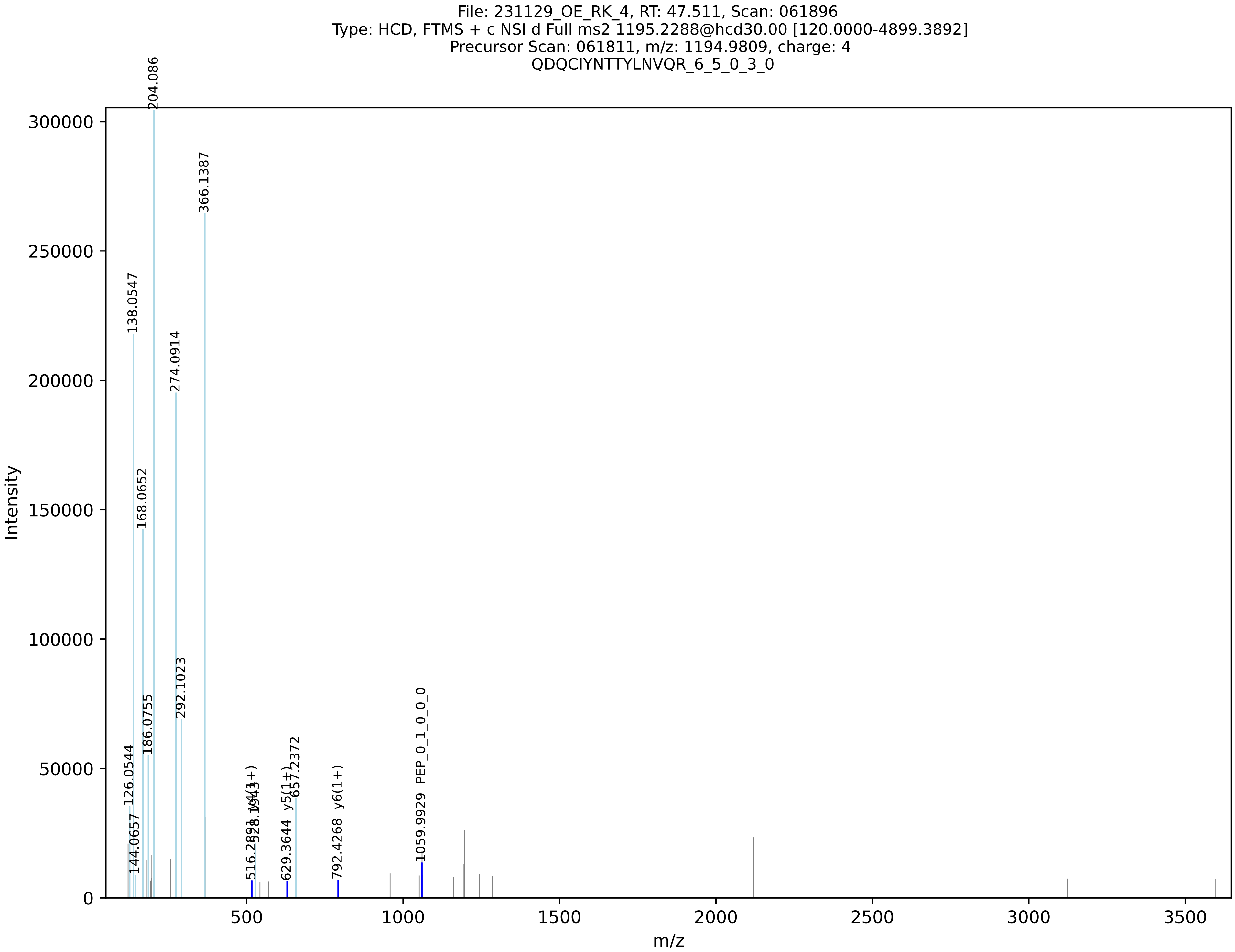

File: 231129_OE_RK_4, RT: 47.511, Scan: 061896
Type: HCD, FTMS + c NSI d Full ms2 1195.2288@hcd30.00 [120.0000-4899.3892]
Precursor Scan: 061811, m/z: 1194.9809, charge: 4
QDQCIYNTTYLNVQR_6_5_0_3_0
Intensity
m/z
204.086
366.1387
138.0547
274.0914
168.0652
292.1023
186.0755
126.0544
144.0657
657.2372
629.3644 y5(1+)
792.4268 y6(1+)
1059.9929 PEP_0_1_0_0_0
300000
250000
200000
150000
100000
50000
0
500
1000
1500
2000
2500
3000
3500

File: 231129_OE_RK_4, RT: 47.517, Scan: 061906
Type: HCD, FTMS + c NSI d Full ms2 1233.2405@hcd30.00 [120.0000-3795.8579]
Precursor Scan: 061811, m/z: 1232.91, charge: 3
IIVPLNNRENISDPTSPLR_4_3_0_1_0

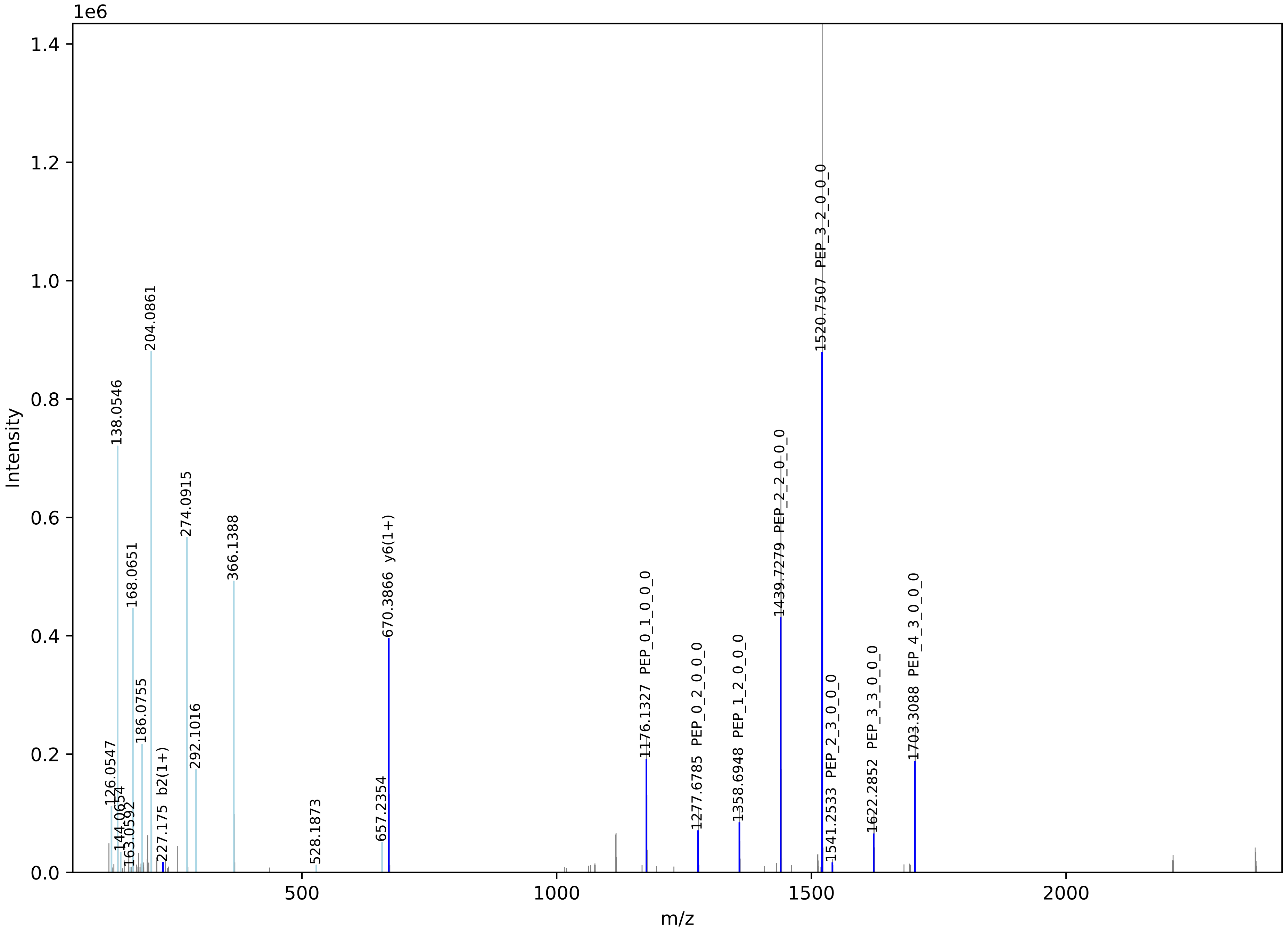

File: 231129_OE_RK_4, RT: 47.566, Scan: 061978
Type: HCD, FTMS + c NSI d Full ms2 1212.2147@hcd30.00 [120.0000-3731.5190]
Precursor Scan: 061884, m/z: 1211.5474, charge: 3
DFVNASSKYEITTIHNLFR_6_2_0_0_0

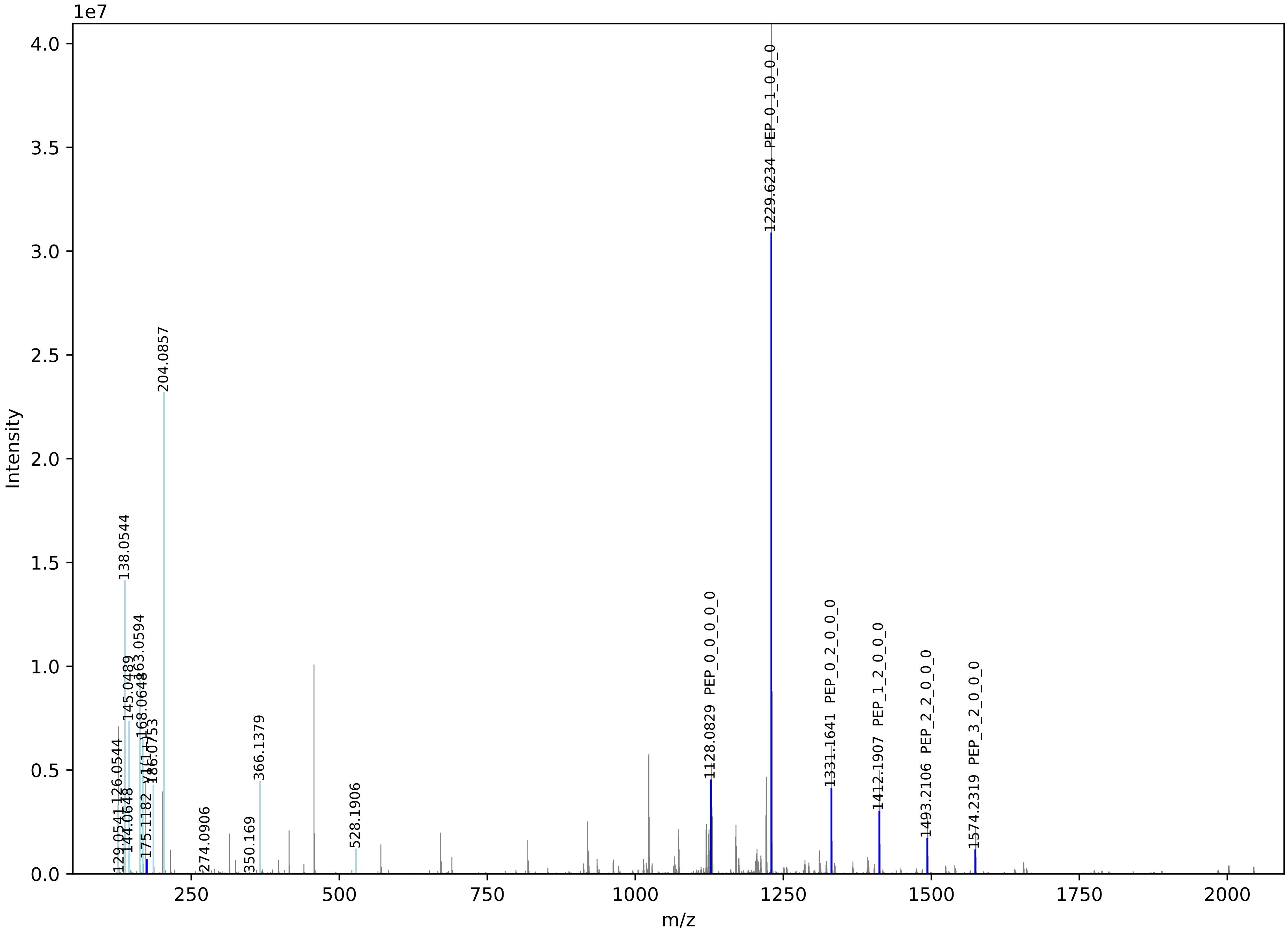

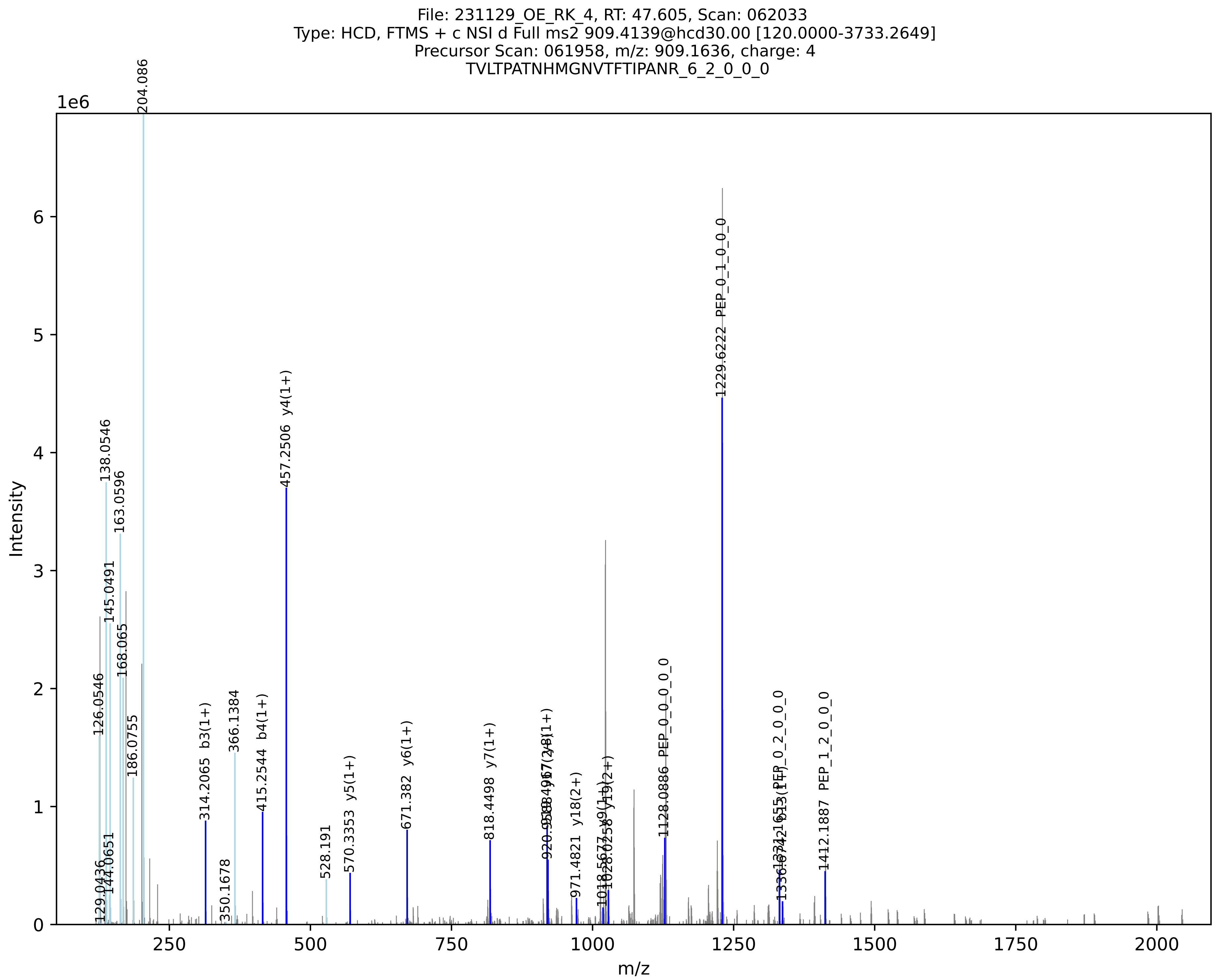

File: 231129_OE_RK_4, RT: 47.605, Scan: 062033
Type: HCD, FTMS + c NSI d Full ms2 909.4139@hcd30.00 [120.0000-3733.2649]
Precursor Scan: 061958, m/z: 909.1636, charge: 4
TVLTPATNHMGNVTFTIPANR_6_2_0_0_0
1e6
Intensity
m/z
204.086
138.0546
163.0596
145.0491
168.065
126.0546
186.0755
129.0436
144.0651
314.2065 b3(1+)
350.1678
366.1384
415.2544 b4(1+)
457.2506 y4(1+)
528.191
570.3353 y5(1+)
671.382 y6(1+)
818.4498 y7(1+)
971.4821 y18(2+)
1028.0258 y19(2+)
1128.0886 PEP_0_0_0_0_0
1229.6222 PEP_0_1_0_0_0
1412.1887 PEP_1_2_0_0_0

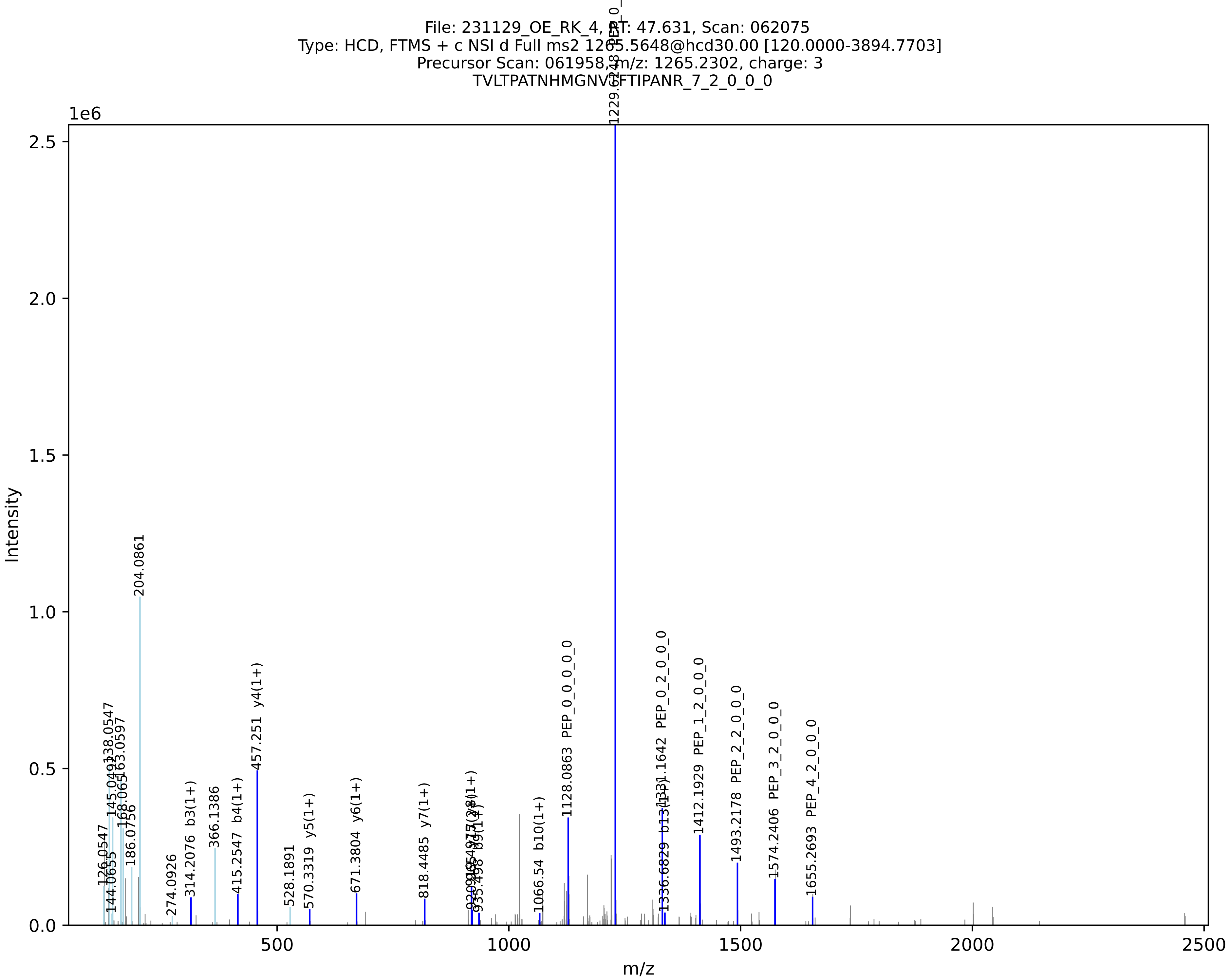

File: 231129_OE_RK_4, RT: 47.631, Scan: 062075
Type: HCD, FTMS + c NSI d Full ms2 1265.5648@hcd30.00 [120.0000-3894.7703]
Precursor Scan: 061958, m/z: 1265.2302, charge: 3
TVLTPATNHMGNVTFTIPANR_7_2_0_0_0
1e6
Intensity
m/z
204.0861
457.251 y4(1+)
314.2076 b3(1+)
366.1386
415.2547 b4(1+)
528.1891
570.3319 y5(1+)
671.3804 y6(1+)
818.4485 y7(1+)
1066.54 b10(1+)
1128.0863 PEP_0_0_0_0_0
1331.1642 PEP_0_2_0_0_0
1412.1929 PEP_1_2_0_0_0
1493.2178 PEP_2_2_0_0_0
1574.2406 PEP_3_2_0_0_0
1655.2693 PEP_4_2_0_0_0

File: 231129_OE_RK_4, RT: 47.673, Scan: 062135
Type: HCD, FTMS + c NSI d Full ms2 1013.4727@hcd30.00 [120.0000-3123.3684]
Precursor Scan: 062032, m/z: 1012.8064, charge: 3
VFHIHNESWVLLTPK_5_2_0_0_0

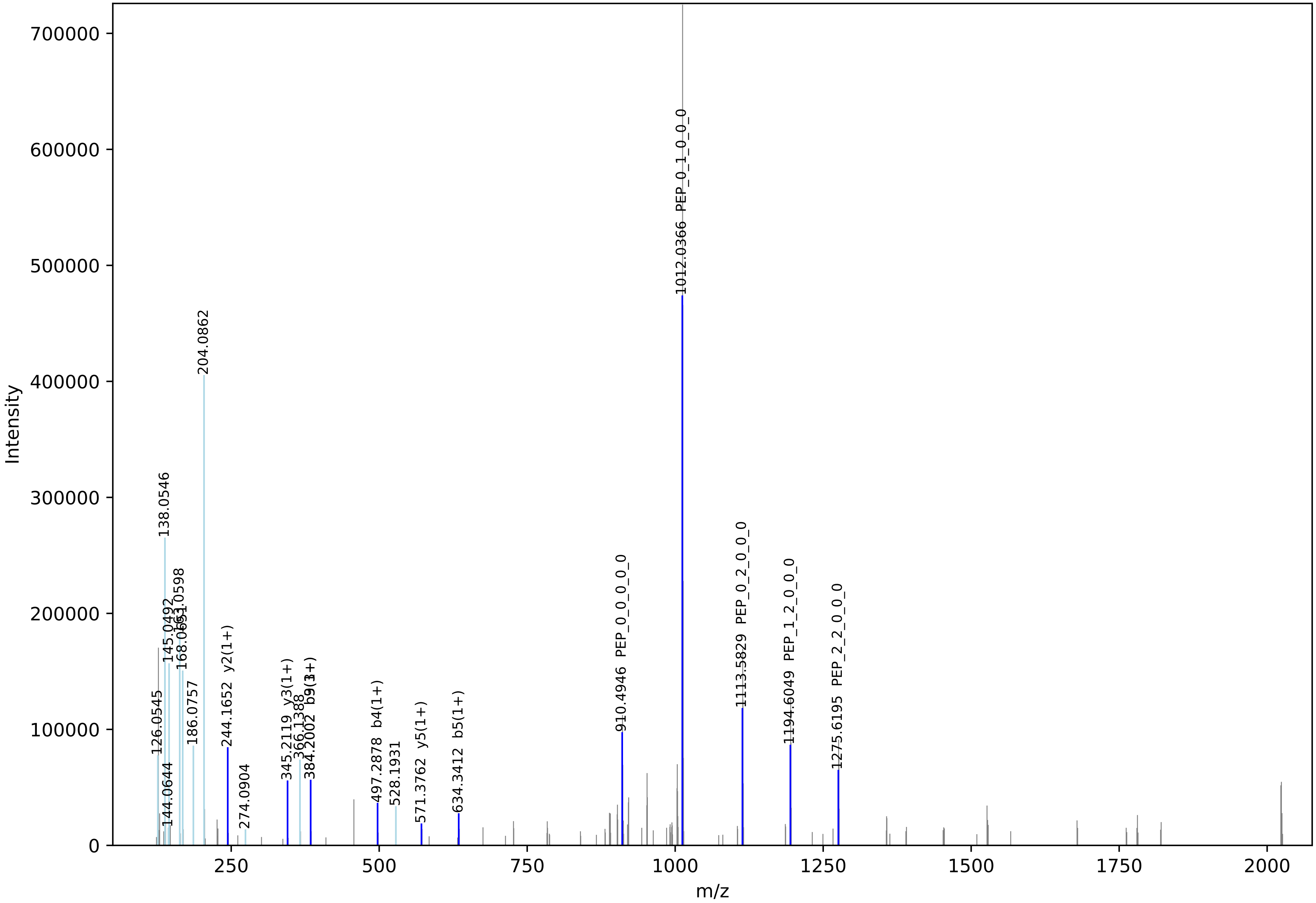

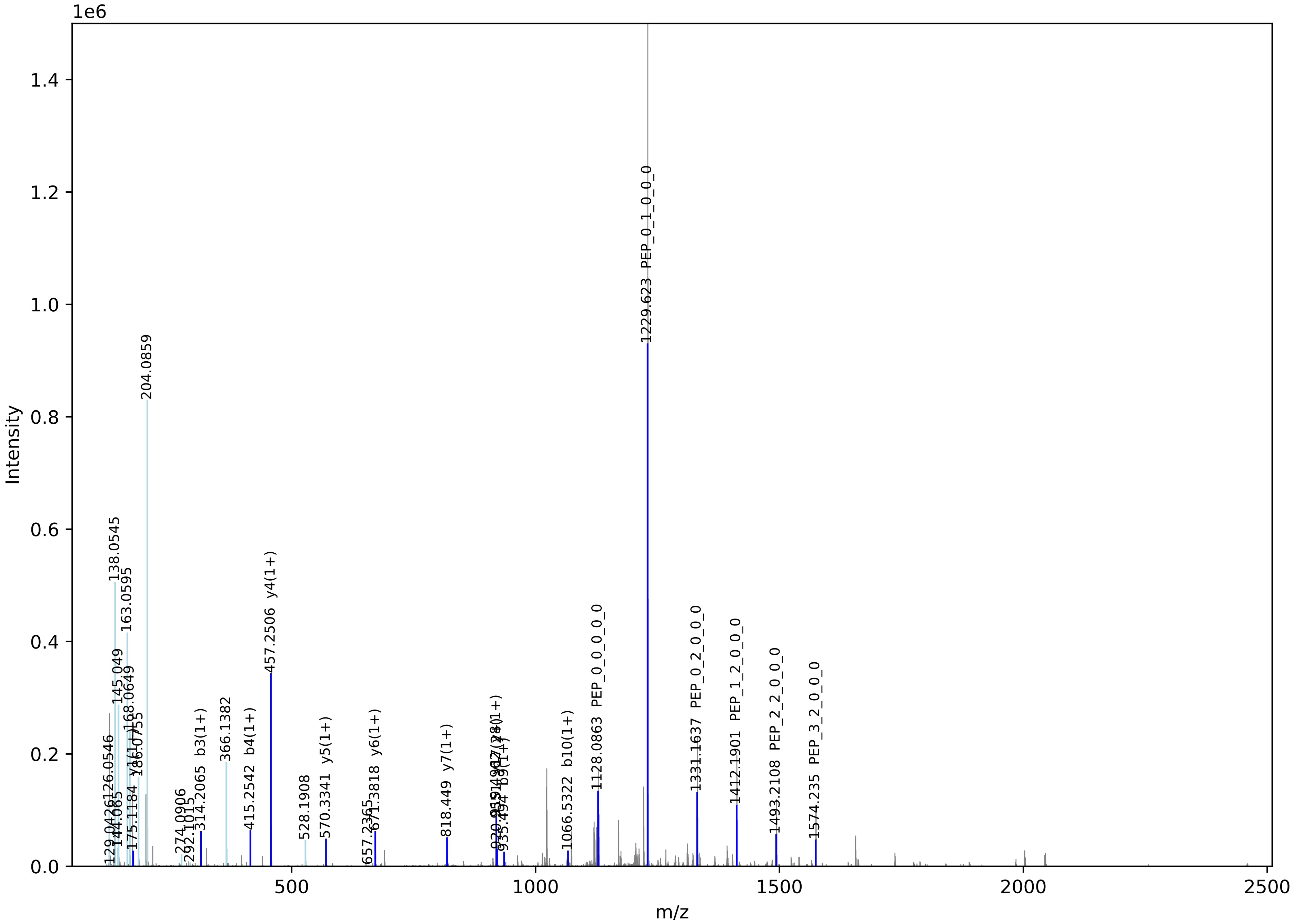
File: 231129_OE_RK_4, RT: 47.708, Scan: 062181
Type: HCD, FTMS + c NSI d Full ms2 1266.5669@hcd30.00 [120.0000-3897.8367]
Precursor Scan: 062106, m/z: 1265.8989, charge: 3
TVLTPATNHMGNVTFTIPANR_7_2_0_0_0
1e6
Intensity
m/z
204.0859
138.0545
163.0595
145.049
168.0649
186.0755
126.0546
144.065
129.0426
175.1184 y1(1+)
274.0906
292.1015
314.2065 b3(1+)
366.1382
415.2542 b4(1+)
457.2506 y4(1+)
528.1908
570.3341 y5(1+)
657.2365
671.3818 y6(1+)
818.449 y7(1+)
935.494 b9(1+)
1066.5322 b10(1+)
1128.0863 PEP_0_0_0_0_0
1229.623 PEP_0_1_0_0_0
1331.1637 PEP_0_2_0_0_0
1412.1901 PEP_1_2_0_0_0
1493.2108 PEP_2_2_0_0_0
1574.235 PEP_3_2_0_0_0

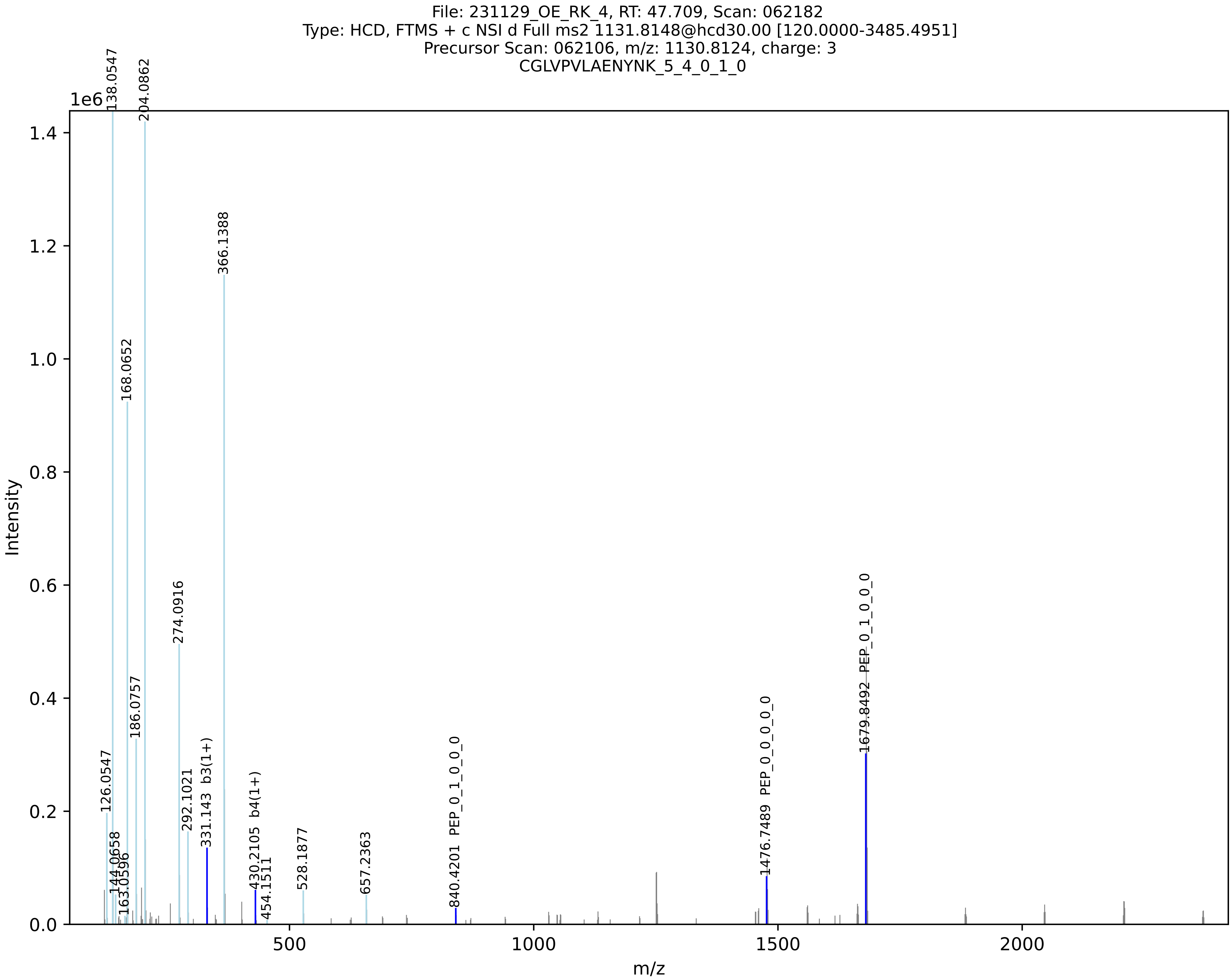
File: 231129_OE_RK_4, RT: 47.709, Scan: 062182
Type: HCD, FTMS + c NSI d Full ms2 1131.8148@hcd30.00 [120.0000-3485.4951]
Precursor Scan: 062106, m/z: 1130.8124, charge: 3
CGLVPVLAENYNK_5_4_0_1_0
1e6
Intensity
m/z
138.0547
204.0862
366.1388
168.0652
274.0916
186.0757
126.0547
144.0658
163.0596
292.1021
331.143 b3(1+)
430.2105 b4(1+)
454.1511
528.1877
657.2363
840.4201 PEP_0_1_0_0_0
1476.7489 PEP_0_0_0_0_0
1679.8492 PEP_0_1_0_0_0

File: 231129_OE_RK_4, RT: 47.716, Scan: 062193
Type: HCD, FTMS + c NSI d Full ms2 949.9277@hcd30.00 [120.0000-3898.5610]
Precursor Scan: 062106, m/z: 949.676, charge: 4
TVLTPATNHMGNVTFTIPANR_7_2_0_0_0

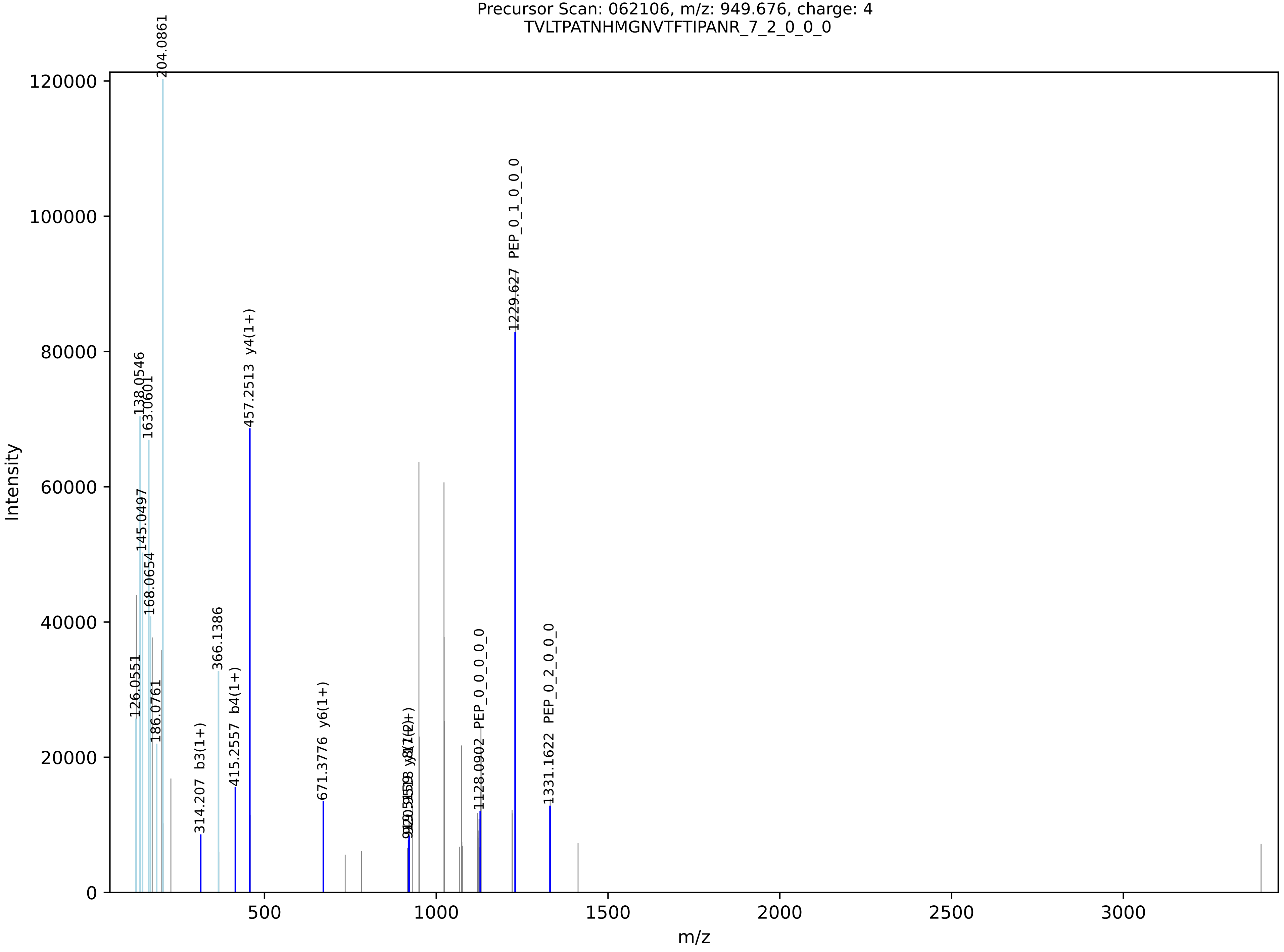

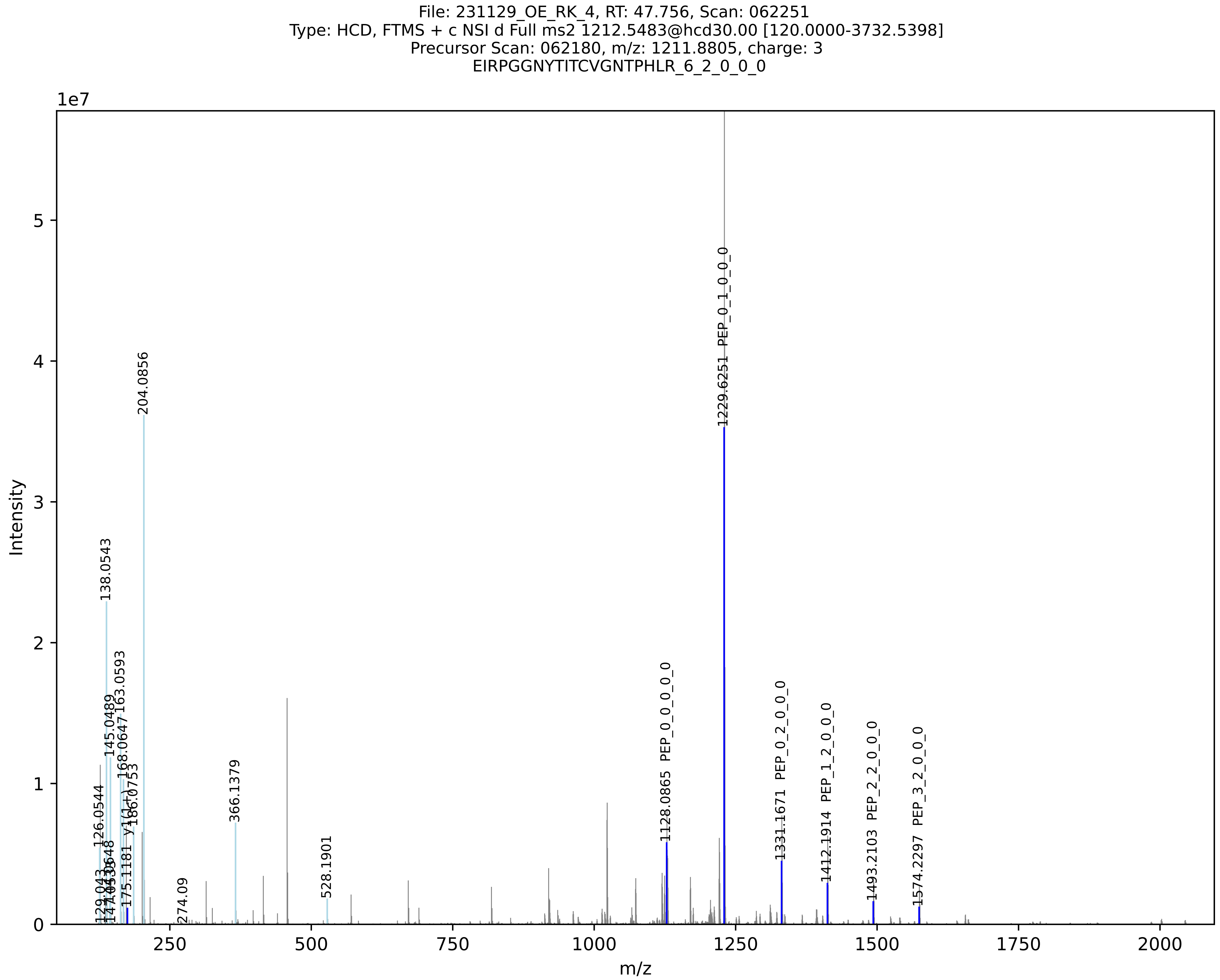
File: 231129_OE_RK_4, RT: 47.756, Scan: 062251
Type: HCD, FTMS + c NSI d Full ms2 1212.5483@hcd30.00 [120.0000-3732.5398]
Precursor Scan: 062180, m/z: 1211.8805, charge: 3
EIRPGGNYTITCVGNTPHLR_6_2_0_0_0
1e7
Intensity
m/z
0
1
2
3
4
5
250
500
750
1000
1250
1500
1750
2000
204.0856
138.0543
163.0593
145.0489
168.0647
186.0753
126.0544
175.1181 y1(1+)
129.043
366.1379
274.09
528.1901
1229.6251 PEP_0_1_0_0_0
1128.0865 PEP_0_0_0_0_0
1331.1671 PEP_0_2_0_0_0
1412.1914 PEP_1_2_0_0_0
1493.2103 PEP_2_2_0_0_0
1574.2297 PEP_3_2_0_0_0

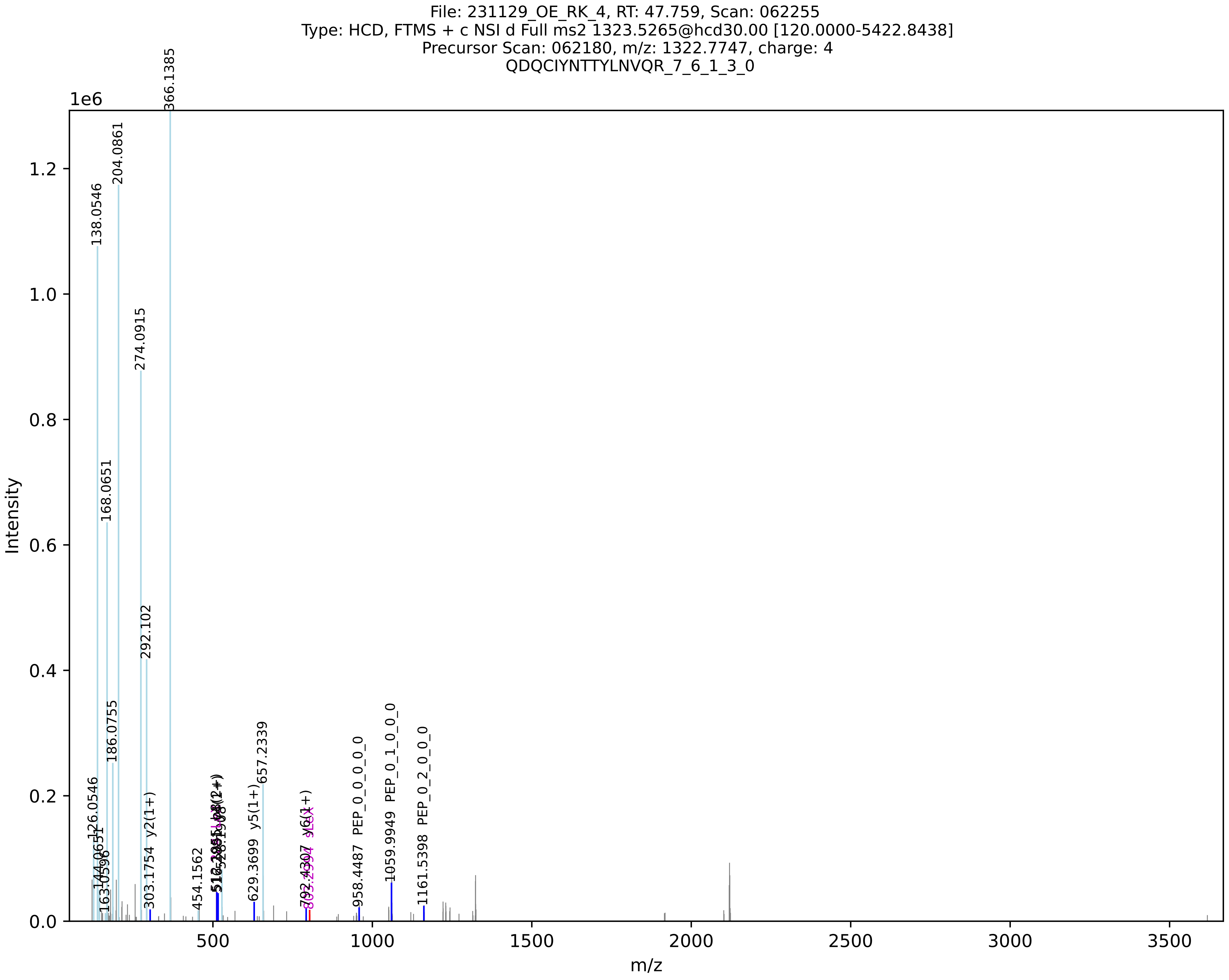
File: 231129_OE_RK_4, RT: 47.759, Scan: 062255
Type: HCD, FTMS + c NSI d Full ms2 1323.5265@hcd30.00 [120.0000-5422.8438]
Precursor Scan: 062180, m/z: 1322.7747, charge: 4
QDQCIYNTTYLNVQR_7_6_1_3_0
1e6
Intensity
m/z
366.1385
204.0861
138.0546
274.0915
168.0651
292.102
186.0755
126.0546
144.0651
163.0596
303.1754
y2(1+)
454.1562
629.3699
y5(1+)
657.2339
792.4307
y6(1+)
958.4487
PEP_0_0_0_0_0
1059.9949
PEP_0_1_0_0_0
1161.5398
PEP_0_2_0_0_0
0.0
0.2
0.4
0.6
0.8
1.0
1.2
500
1000
1500
2000
2500
3000
3500

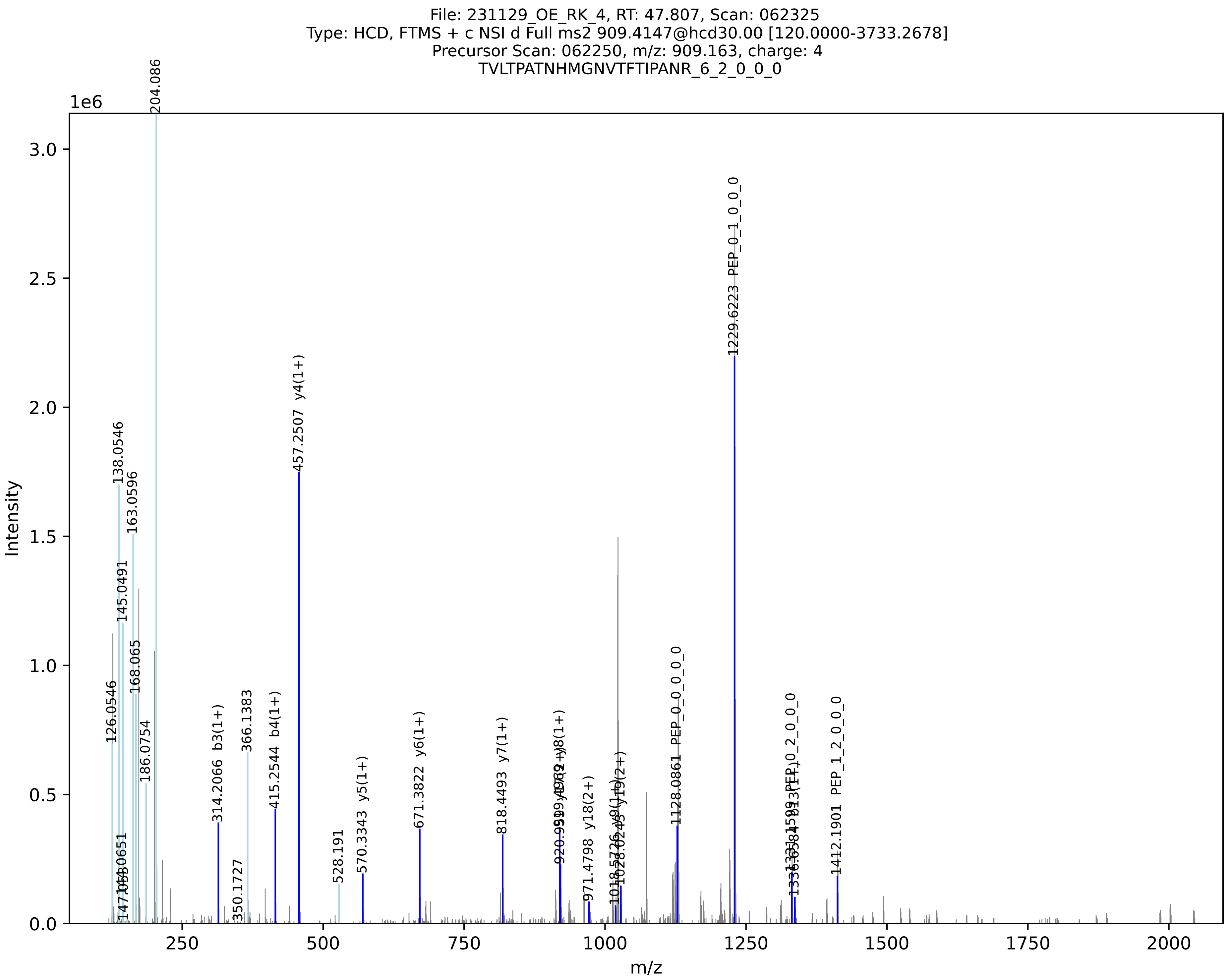

File: 231129_OE_RK_4, RT: 47.807, Scan: 062325
Type: HCD, FTMS + c NSI d Full ms2 909.4147@hcd30.00 [120.0000-3733.2678]
Precursor Scan: 062250, m/z: 909.163, charge: 4
TVLTPATNHMGNVTFTIPANR_6_2_0_0_0
1e6
Intensity
m/z
204.086
138.0546
163.0596
145.0491
168.065
126.0546
186.0754
314.2066 b3(1+)
366.1383
415.2544 b4(1+)
457.2507 y4(1+)
350.1727
528.191
570.3343 y5(1+)
671.3822 y6(1+)
818.4493 y7(1+)
971.4798 y18(2+)
1128.0861 PEP_0_0_0_0_0
1229.6223 PEP_0_1_0_0_0
PEP_0_2_0_0_0
1412.1901 PEP_1_2_0_0_0

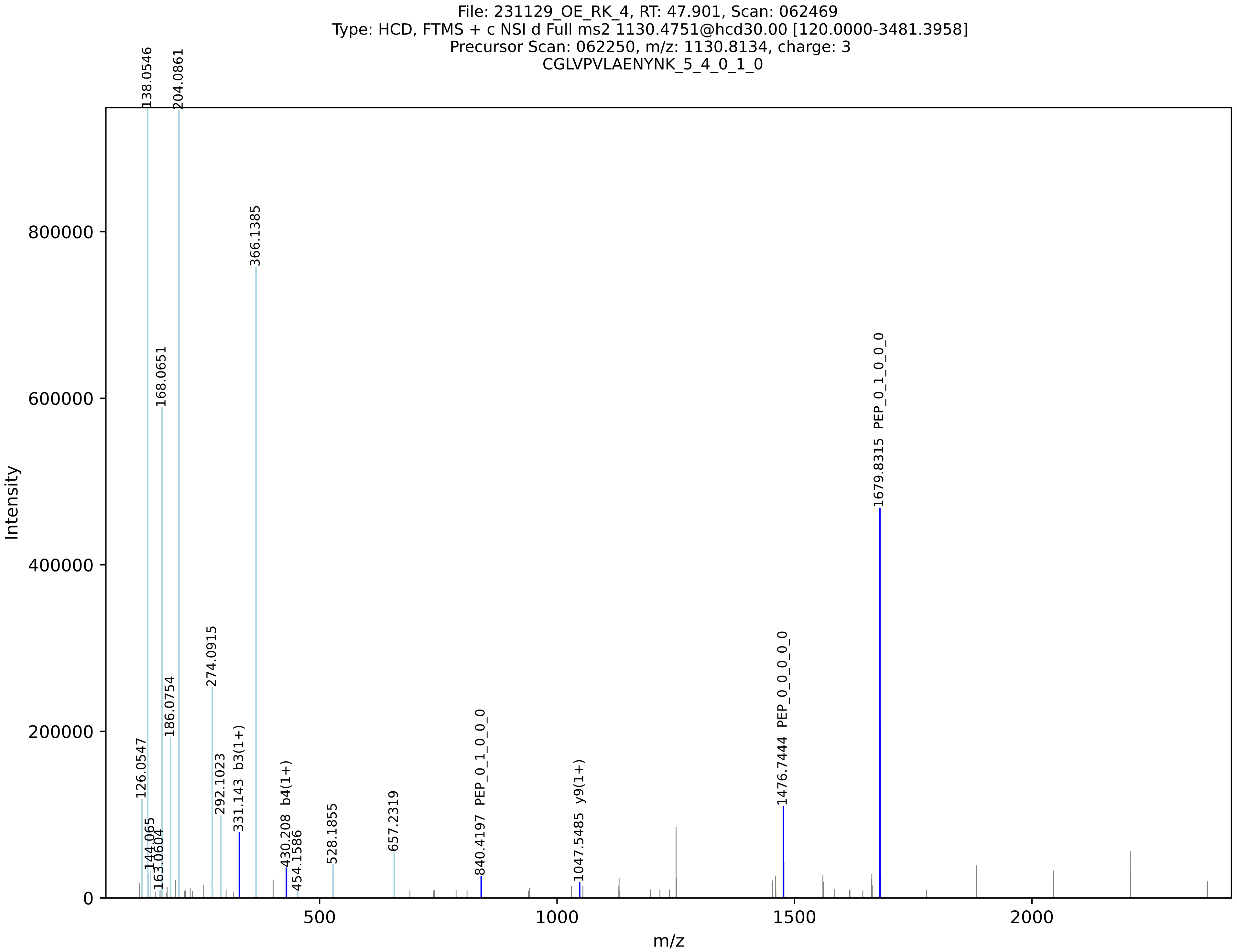
File: 231129_OE_RK_4, RT: 47.901, Scan: 062469
Type: HCD, FTMS + c NSI d Full ms2 1130.4751@hcd30.00 [120.0000-3481.3958]
Precursor Scan: 062250, m/z: 1130.8134, charge: 3
CGLVPVLAENYNK_5_4_0_1_0
138.0546
204.0861
366.1385
168.0651
1679.8315 PEP_0_1_0_0_0
274.0915
186.0754
1476.7444 PEP_0_0_0_0_0
126.0547
292.1023
331.143 b3(1+)
430.208 b4(1+)
454.1586
144.065
163.0604
528.1855
657.2319
840.4197 PEP_0_1_0_0_0
1047.5485 y9(1+)
Intensity
800000
600000
400000
200000
0
500
1000
1500
2000
m/z

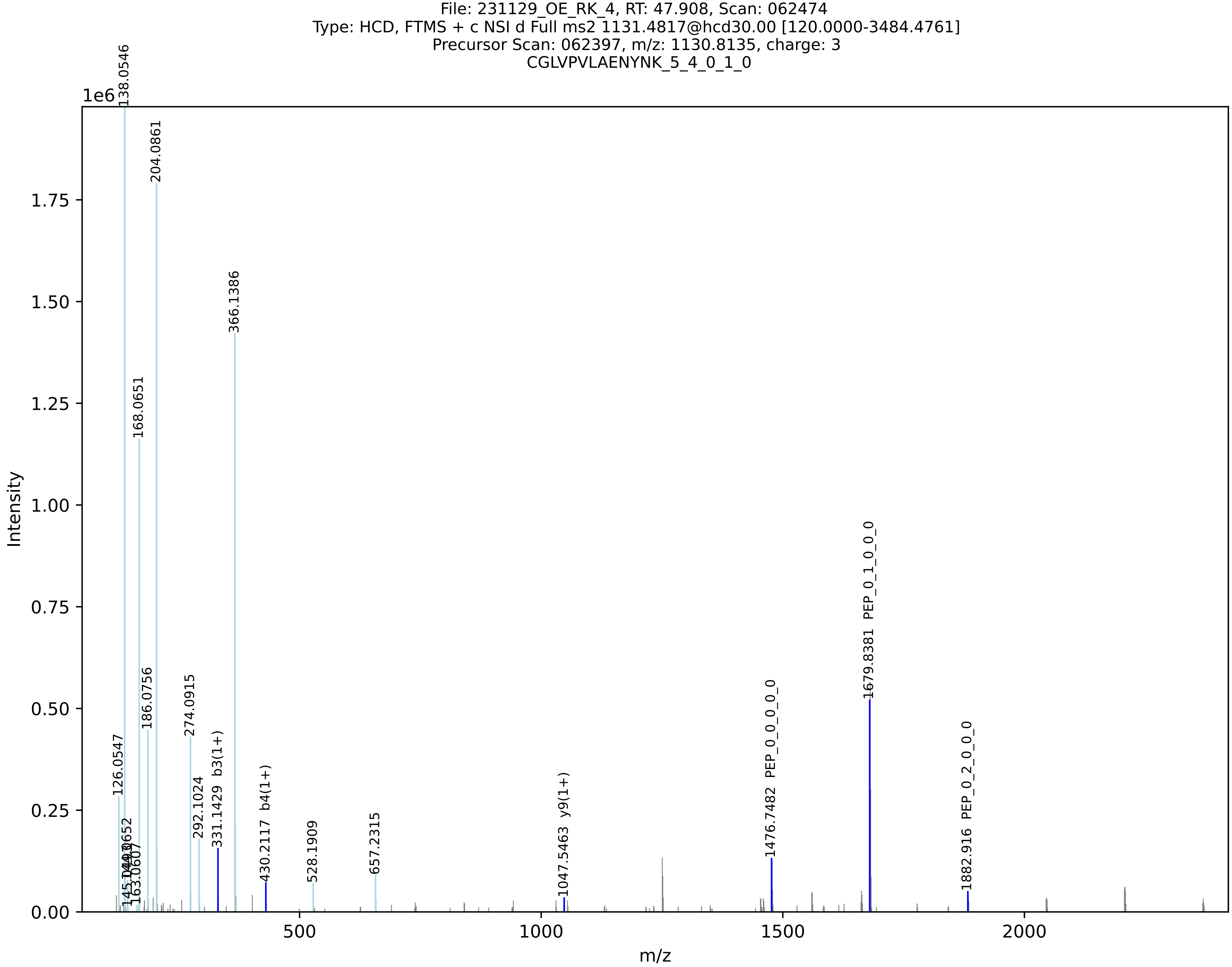
File: 231129_OE_RK_4, RT: 47.908, Scan: 062474
Type: HCD, FTMS + c NSI d Full ms2 1131.4817@hcd30.00 [120.0000-3484.4761]
Precursor Scan: 062397, m/z: 1130.8135, charge: 3
CGLVPVLAENYNK_5_4_0_1_0
1e6
Intensity
m/z
0.00
0.25
0.50
0.75
1.00
1.25
1.50
1.75
500
1000
1500
2000
138.0546
204.0861
366.1386
168.0651
186.0756
274.0915
126.0547
163.0607
292.1024
331.1429 b3(1+)
430.2117 b4(1+)
528.1909
657.2315
1047.5463 y9(1+)
1476.7482 PEP_0_0_0_0_0
1679.8381 PEP_0_1_0_0_0
1882.916 PEP_0_2_0_0_0

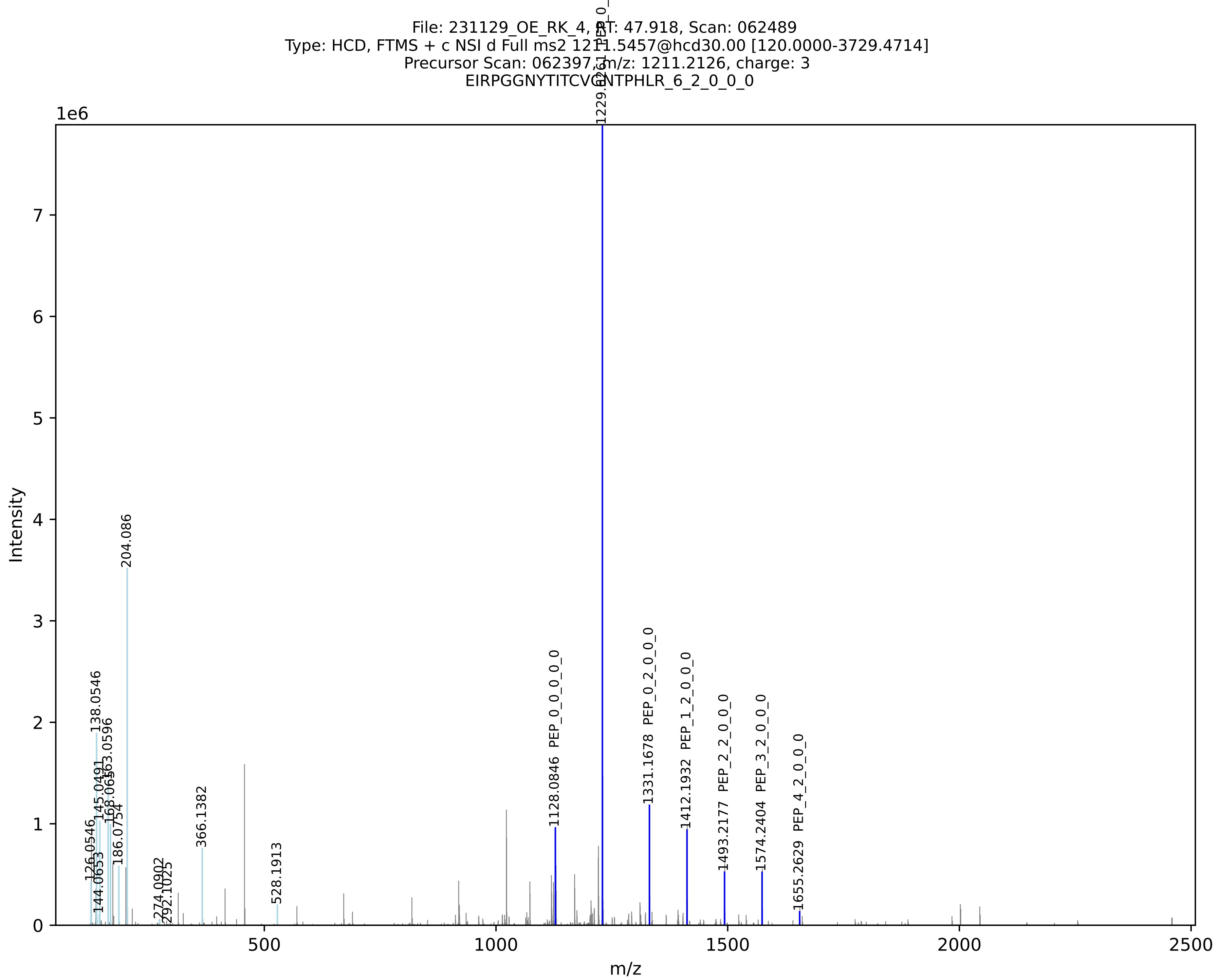

File: 231129_OE_RK_4, RT: 47.918, Scan: 062489
Type: HCD, FTMS + c NSI d Full ms2 1211.5457@hcd30.00 [120.0000-3729.4714]
Precursor Scan: 062397, m/z: 1211.2126, charge: 3
EIRPGGNYTITCVGNTPHLR_6_2_0_0_0
1e6
Intensity
m/z
204.086
138.0546
163.0596
145.0491
168.065
186.0754
126.0546
144.0653
274.0902
292.1025
366.1382
528.1913
1128.0846 PEP_0_0_0_0_0
1331.1678 PEP_0_2_0_0_0
1412.1932 PEP_1_2_0_0_0
1493.2177 PEP_2_2_0_0_0
1574.2404 PEP_3_2_0_0_0
1655.2629 PEP_4_2_0_0_0

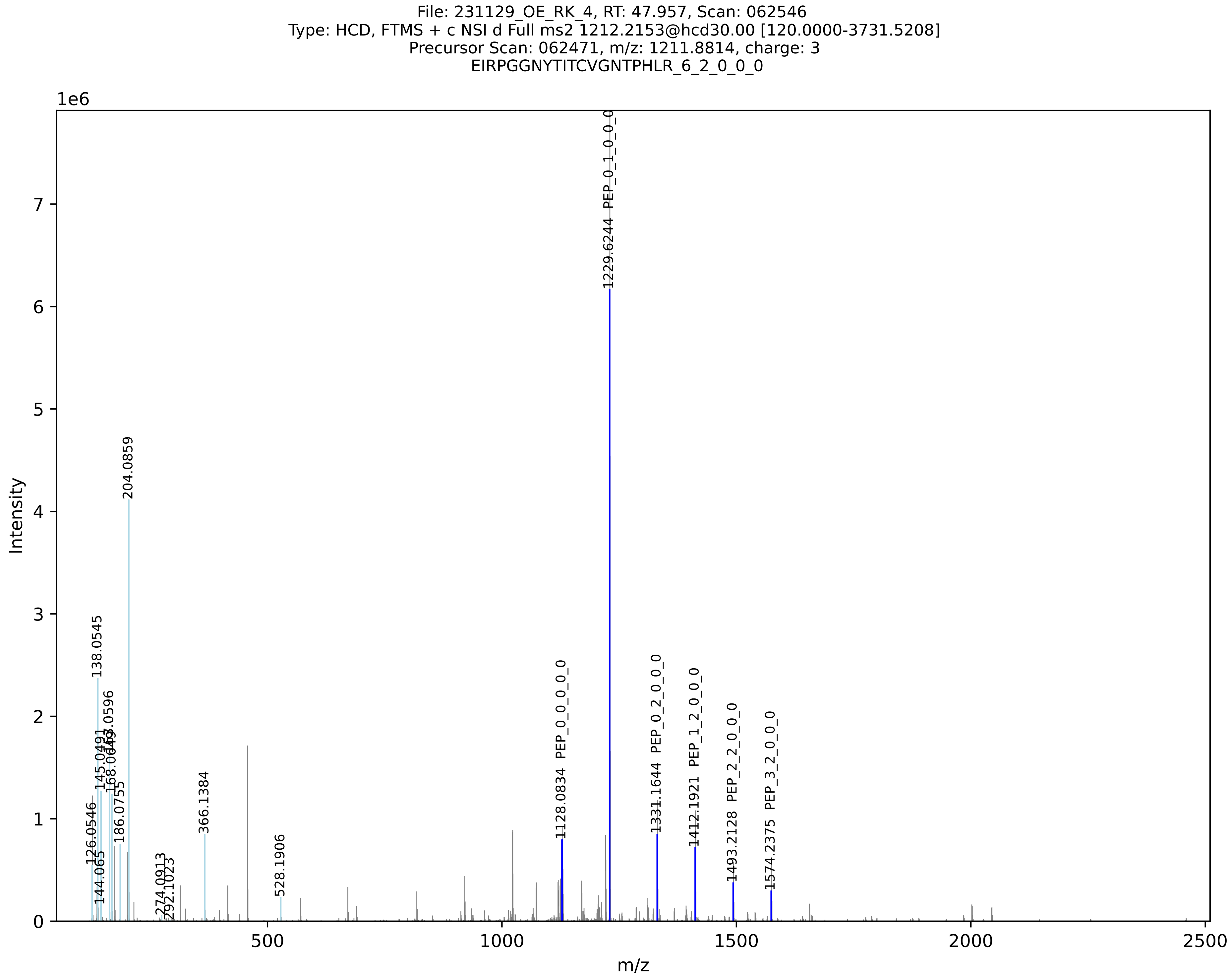
File: 231129_OE_RK_4, RT: 47.957, Scan: 062546
Type: HCD, FTMS + c NSI d Full ms2 1212.2153@hcd30.00 [120.0000-3731.5208]
Precursor Scan: 062471, m/z: 1211.8814, charge: 3
EIRPGGNYTITCVGNTPHLR_6_2_0_0_0
1e6
Intensity
m/z
0
1
2
3
4
5
6
7
500
1000
1500
2000
2500
204.0859
138.0545
145.0491
168.0649
186.0755
126.0546
144.065
366.1384
274.0913
292.1023
528.1906
1229.6244 PEP_0_1_0_0_0
1128.0834 PEP_0_0_0_0_0
1331.1644 PEP_0_2_0_0_0
1412.1921 PEP_1_2_0_0_0
1493.2128 PEP_2_2_0_0_0
1574.2375 PEP_3_2_0_0_0

File: 231129_OE_RK_4, RT: 47.96, Scan: 062550
Type: HCD, FTMS + c NSI d Full ms2 1221.5477@hcd30.00 [120.0000-3760.0781]
Precursor Scan: 062471, m/z: 1221.2138, charge: 3
QDNTTQLVQDDQVKGPLR_4_4_1_0_0

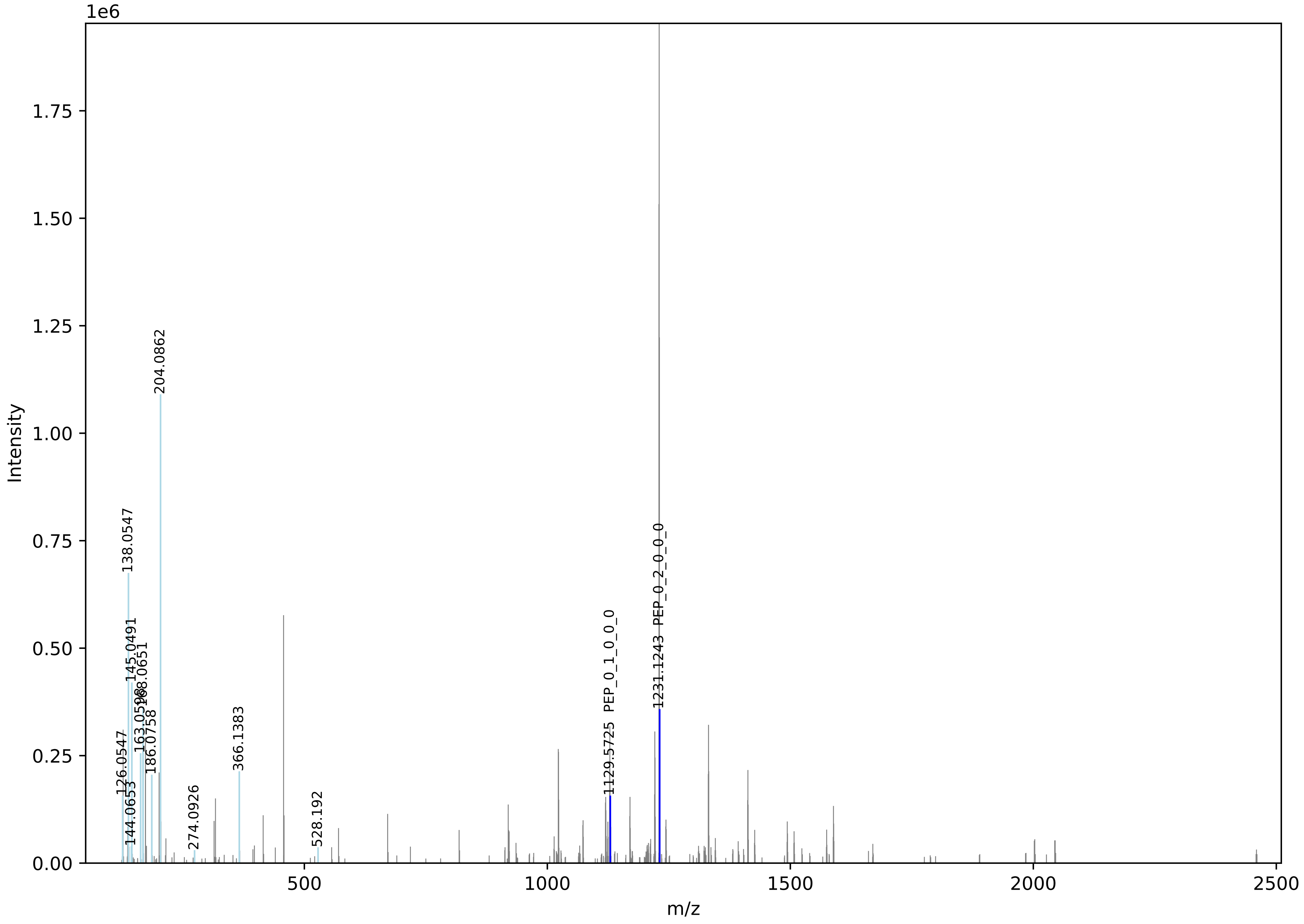

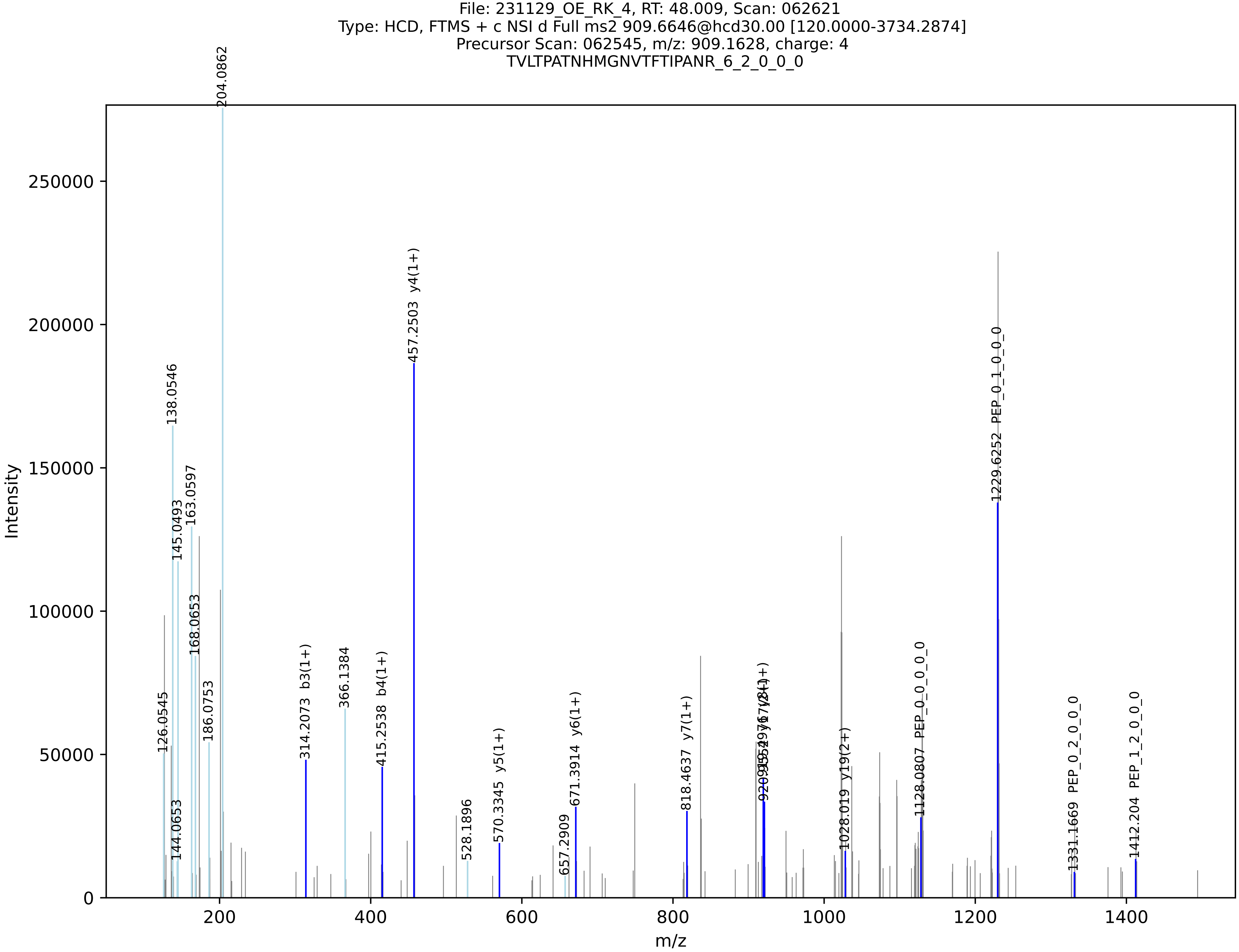
File: 231129_OE_RK_4, RT: 48.009, Scan: 062621
Type: HCD, FTMS + c NSI d Full ms2 909.6646@hcd30.00 [120.0000-3734.2874]
Precursor Scan: 062545, m/z: 909.1628, charge: 4
TVLTPATNHMGNVTFTIPANR_6_2_0_0_0
Intensity
m/z
0
50000
100000
150000
200000
250000
200
400
600
800
1000
1200
1400
204.0862
138.0546
145.0493
163.0597
168.0653
126.0545
186.0753
144.0653
314.2073 b3(1+)
366.1384
415.2538 b4(1+)
457.2503 y4(1+)
528.1896
570.3345 y5(1+)
657.2909
671.3914 y6(1+)
818.4637 y7(1+)
1028.019 y19(2+)
1128.0807 PEP_0_0_0_0_0
1229.6252 PEP_0_1_0_0_0
1331.1669 PEP_0_2_0_0_0
1412.204 PEP_1_2_0_0_0

File: 231129_OE_RK_4, RT: 48.08, Scan: 062722
Type: HCD, FTMS + c NSI d Full ms2 1158.5322@hcd30.00 [120.0000-3567.2505]
Precursor Scan: 062680, m/z: 1157.864, charge: 3
TVLTPATNHMGNVTFTIPANR_5_2_0_0_0

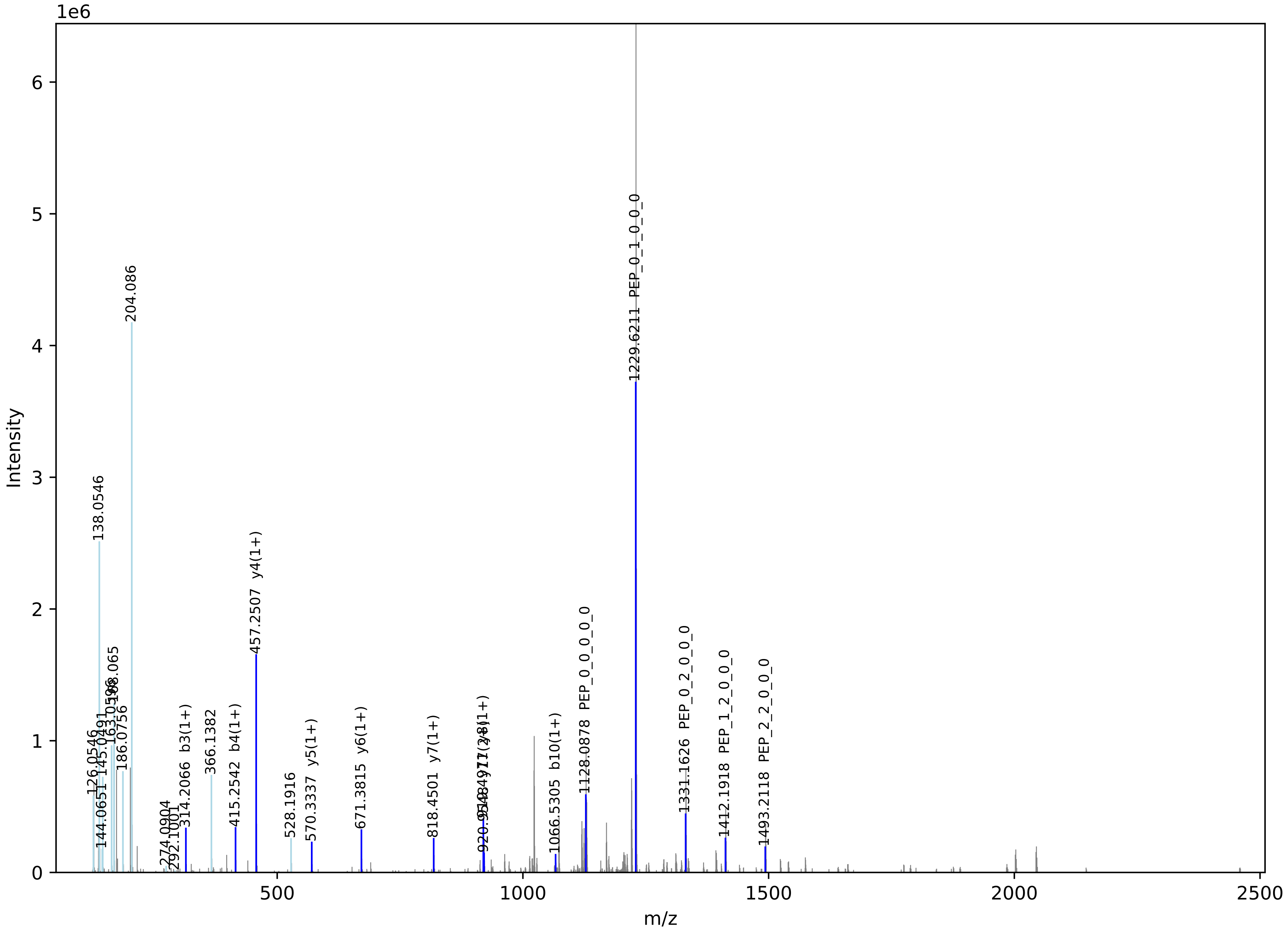

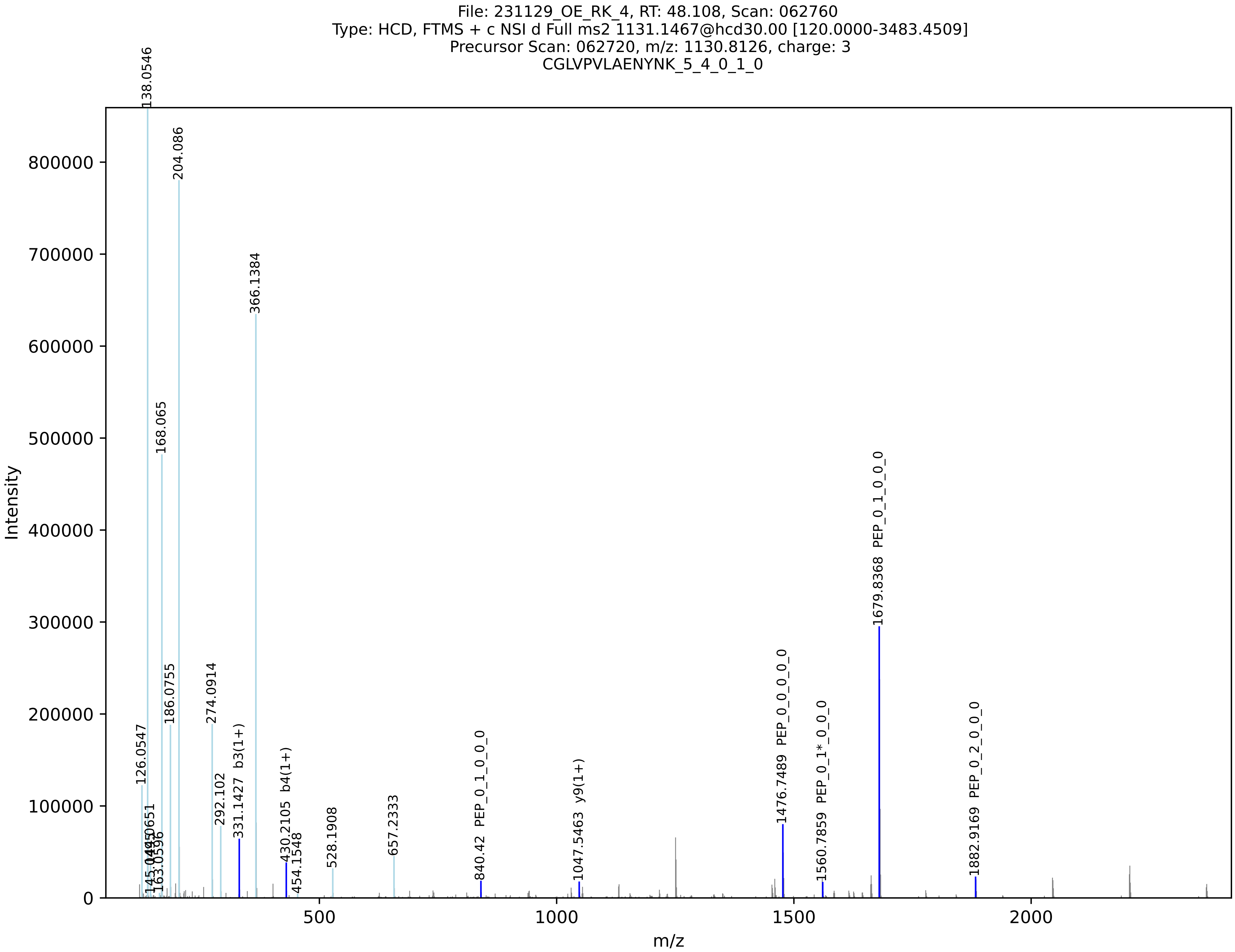

File: 231129_OE_RK_4, RT: 48.108, Scan: 062760
Type: HCD, FTMS + c NSI d Full ms2 1131.1467@hcd30.00 [120.0000-3483.4509]
Precursor Scan: 062720, m/z: 1130.8126, charge: 3
CGLVPVLAENYNK_5_4_0_1_0
Intensity
m/z
138.0546
204.086
366.1384
168.065
186.0755
274.0914
126.0547
292.102
331.1427 b3(1+)
430.2105 b4(1+)
454.1548
528.1908
657.2333
840.42 PEP_0_1_0_0_0
1047.5463 y9(1+)
1476.7489 PEP_0_0_0_0_0
1560.7859 PEP_0_1*_0_0_0
1679.8368 PEP_0_1_0_0_0
1882.9169 PEP_0_2_0_0_0
163.0596

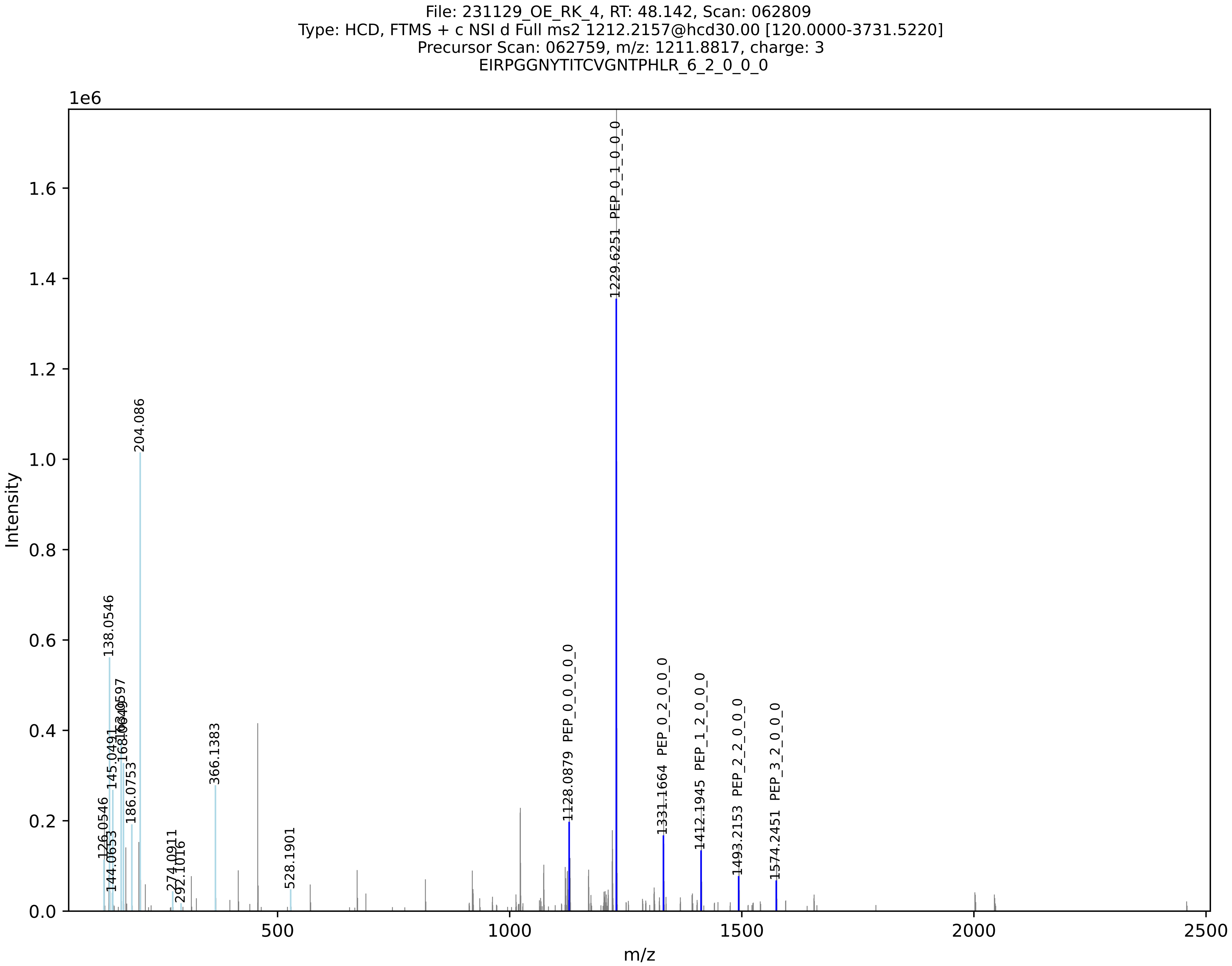
File: 231129_OE_RK_4, RT: 48.142, Scan: 062809
Type: HCD, FTMS + c NSI d Full ms2 1212.2157@hcd30.00 [120.0000-3731.5220]
Precursor Scan: 062759, m/z: 1211.8817, charge: 3
EIRPGGNYTITCVGNTPHLR_6_2_0_0_0
1e6
Intensity
m/z
1229.6251 PEP_0_1_0_0_0
204.086
138.0546
1128.0879 PEP_0_0_0_0_0
1331.1664 PEP_0_2_0_0_0
1412.1945 PEP_1_2_0_0_0
1493.2153 PEP_2_2_0_0_0
1574.2451 PEP_3_2_0_0_0
366.1383
186.0753
145.0491
126.0546
144.0653
274.0911
292.1016
528.1901

File: 231129_OE_RK_4, RT: 48.147, Scan: 062817
Type: HCD, FTMS + c NSI d Full ms2 1764.3629@hcd30.00 [120.0000-5421.0928]
Precursor Scan: 062759, m/z: 1763.3606, charge: 3
QDQCIYNTTYLNVQR_7_6_1_3_0

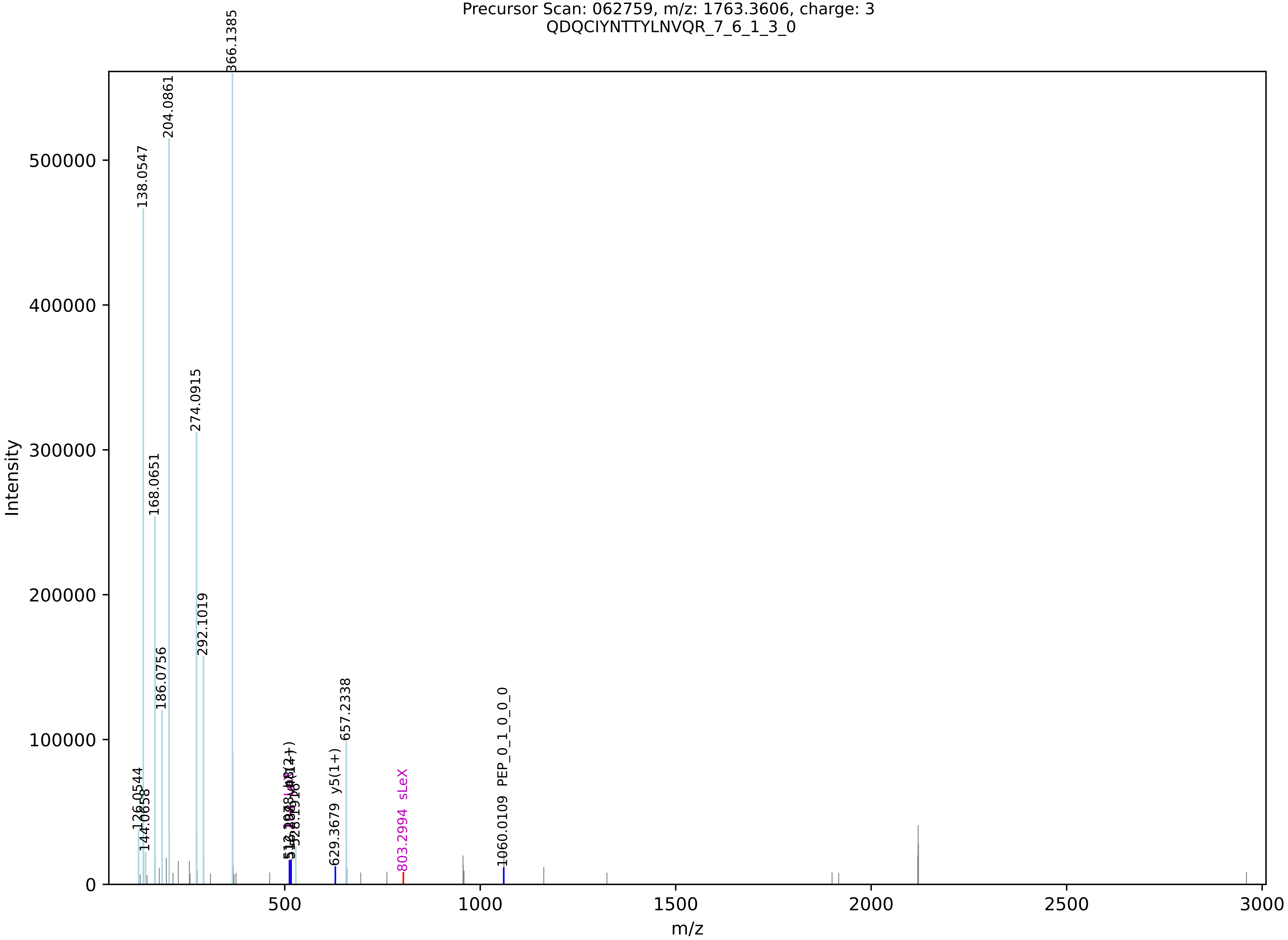

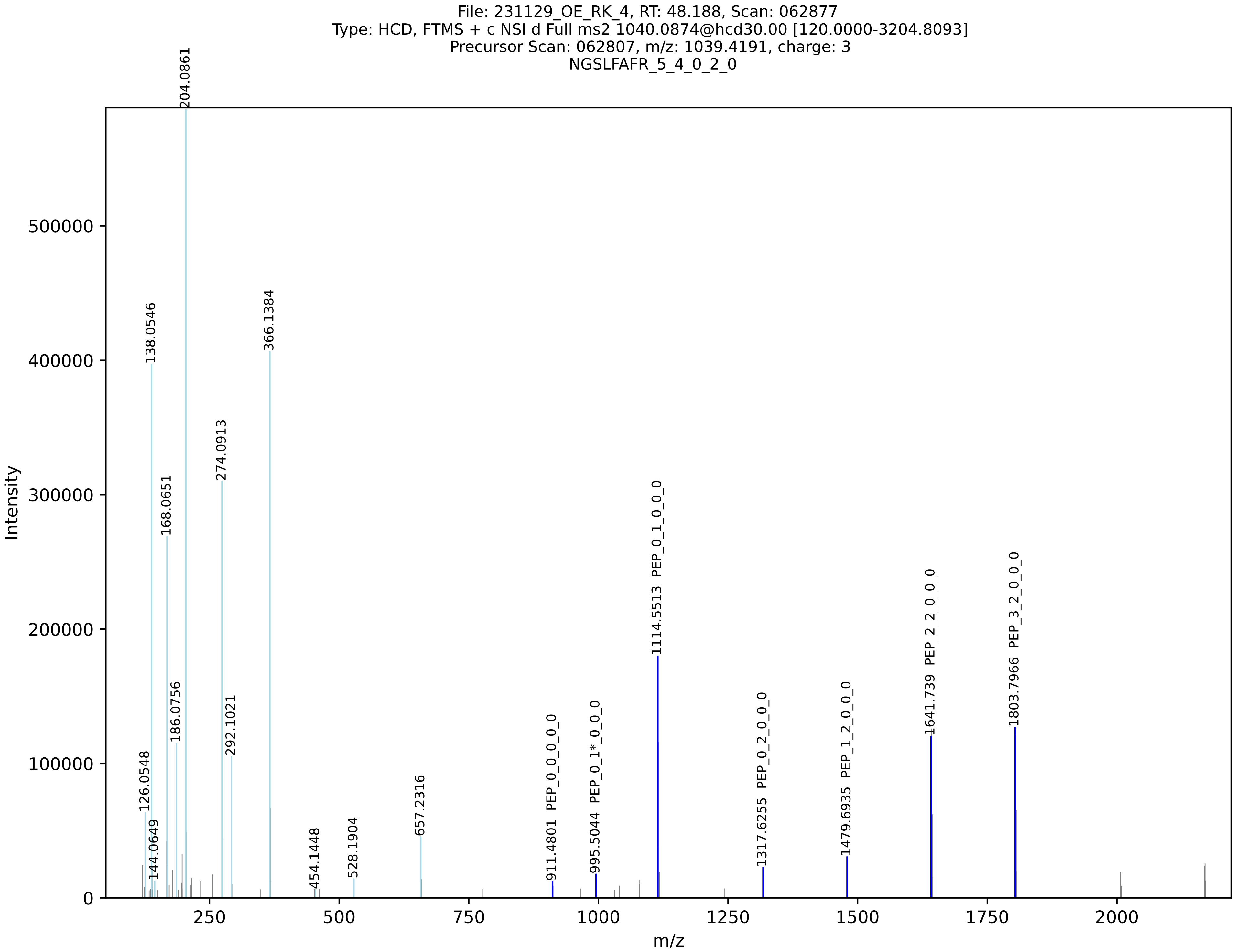

File: 231129_OE_RK_4, RT: 48.188, Scan: 062877
Type: HCD, FTMS + c NSI d Full ms2 1040.0874@hcd30.00 [120.0000-3204.8093]
Precursor Scan: 062807, m/z: 1039.4191, charge: 3
NGSLFAFR_5_4_0_2_0
Intensity
m/z
204.0861
138.0546
366.1384
274.0913
168.0651
186.0756
292.1021
126.0548
144.0649
454.1448
528.1904
657.2316
911.4801 PEP_0_0_0_0_0
995.5044 PEP_0_1*_0_0_0
1114.5513 PEP_0_1_0_0_0
1317.6255 PEP_0_2_0_0_0
1479.6935 PEP_1_2_0_0_0
1641.739 PEP_2_2_0_0_0
1803.7966 PEP_3_2_0_0_0

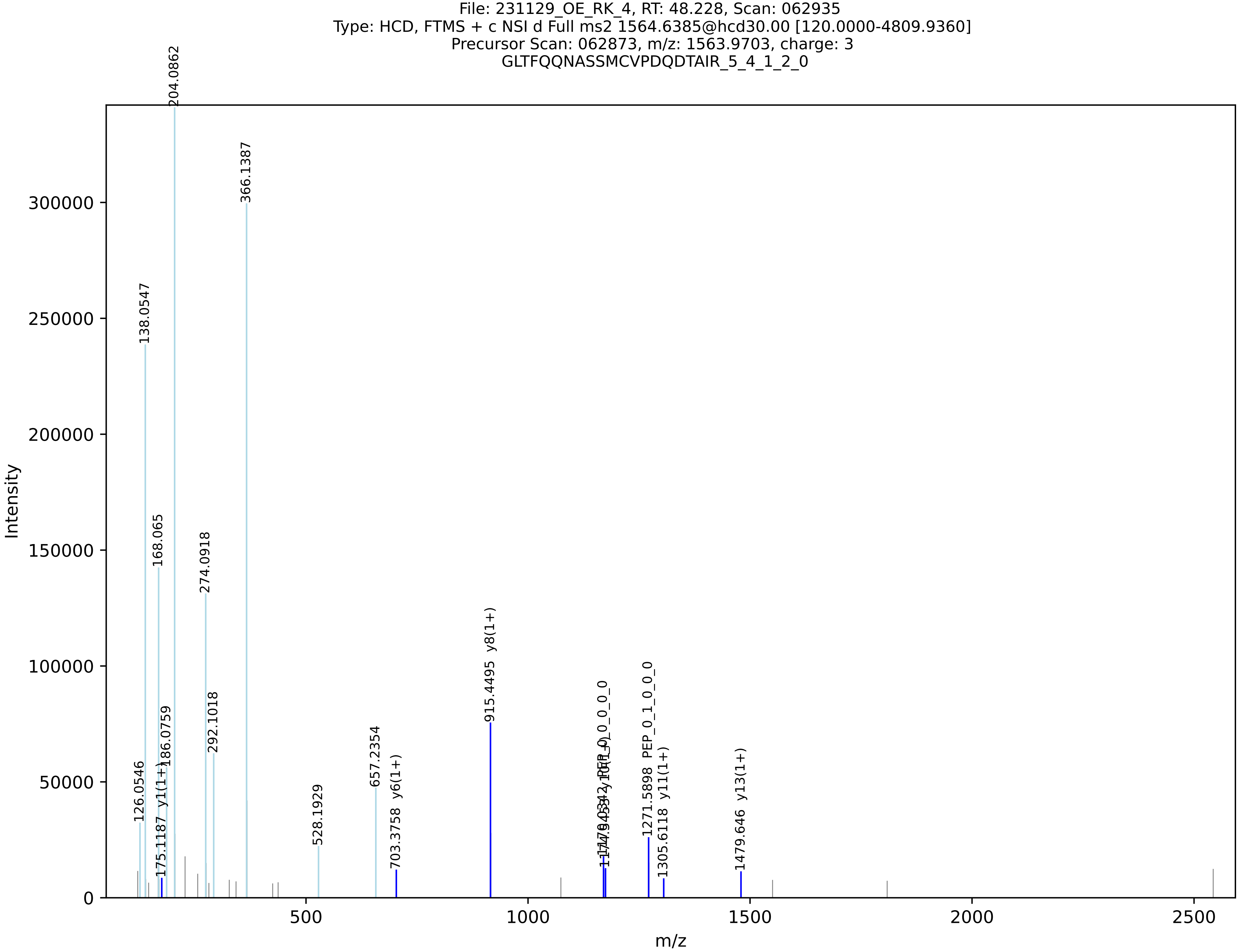
File: 231129_OE_RK_4, RT: 48.228, Scan: 062935
Type: HCD, FTMS + c NSI d Full ms2 1564.6385@hcd30.00 [120.0000-4809.9360]
Precursor Scan: 062873, m/z: 1563.9703, charge: 3
GLTFQQNASSMCVPDQDTAIR_5_4_1_2_0
Intensity
m/z
0
50000
100000
150000
200000
250000
300000
500
1000
1500
2000
2500
204.0862
366.1387
138.0547
168.065
274.0918
292.1018
186.0759
126.0546
175.1187 y1(1+)
528.1929
657.2354
703.3758 y6(1+)
915.4495 y8(1+)
1271.5898 PEP_0_1_0_0_0
1305.6118 y11(1+)
1479.646 y13(1+)

File: 231129_OE_RK_4, RT: 48.23, Scan: 062938
Type: HCD, FTMS + c NSI d Full ms2 1173.9800@hcd30.00 [120.0000-4812.6943]
Precursor Scan: 062873, m/z: 1173.2295, charge: 4
GLTFQQNASSMCVPDQDTAIR_5_4_1_2_0

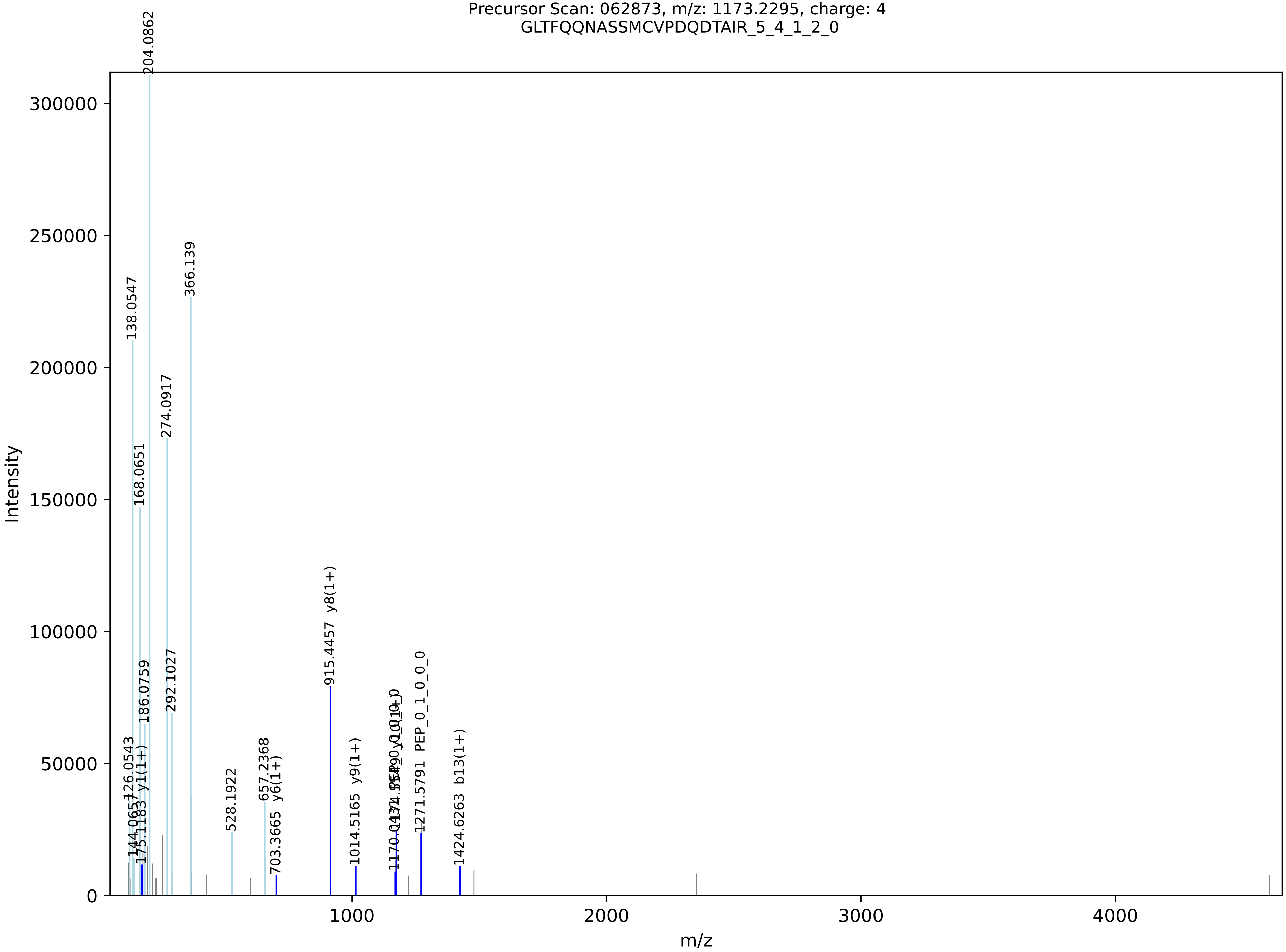

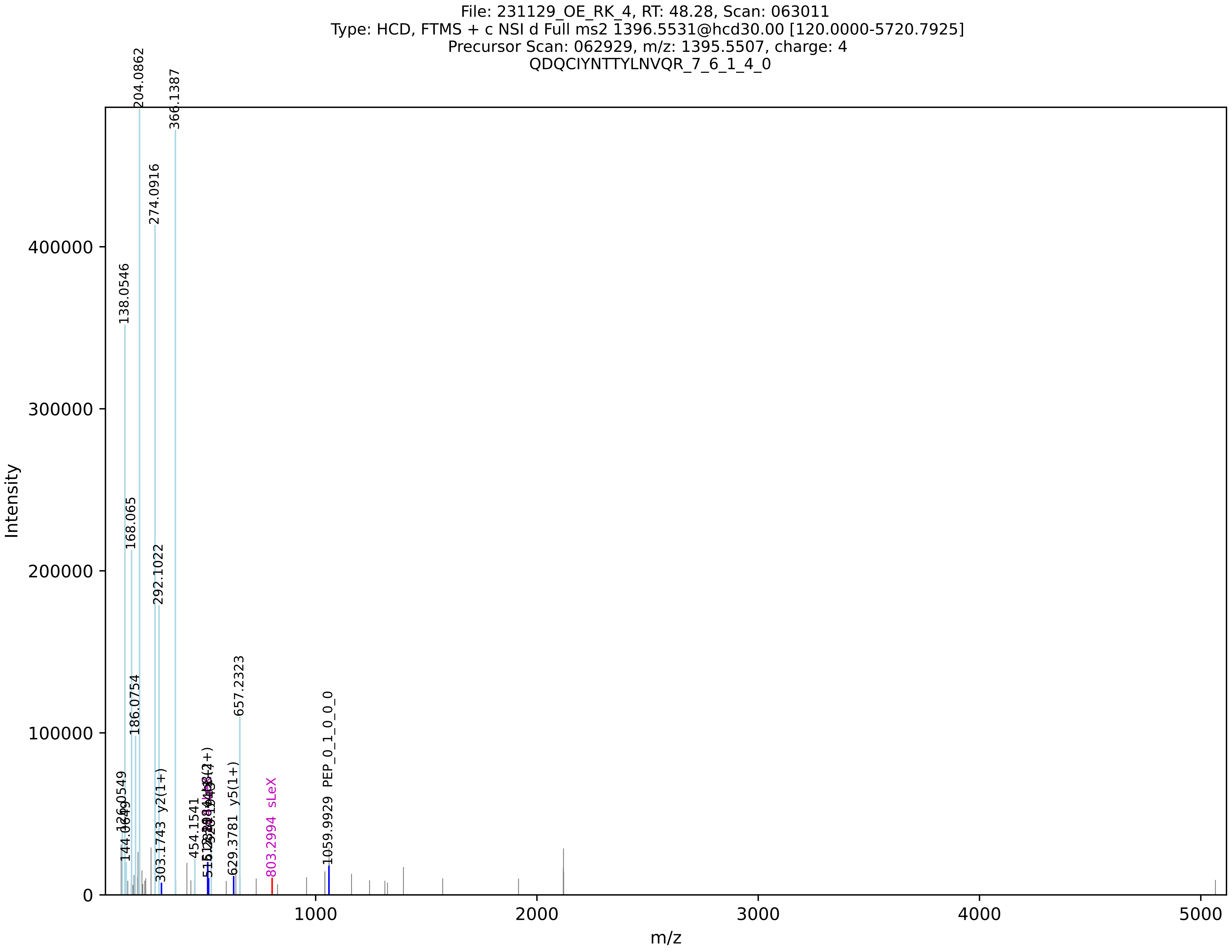
File: 231129_OE_RK_4, RT: 48.28, Scan: 063011
Type: HCD, FTMS + c NSI d Full ms2 1396.5531@hcd30.00 [120.0000-5720.7925]
Precursor Scan: 062929, m/z: 1395.5507, charge: 4
QDQCIYNTTYLNVQR_7_6_1_4_0
204.0862
366.1387
274.0916
138.0546
168.065
292.1022
186.0754
657.2323
126.0549
144.0649
303.1743 y2(1+)
454.1541
629.3781 y5(1+)
803.2994 sLeX
1059.9929 PEP_0_1_0_0_0
Intensity
m/z
0
100000
200000
300000
400000
1000
2000
3000
4000
5000

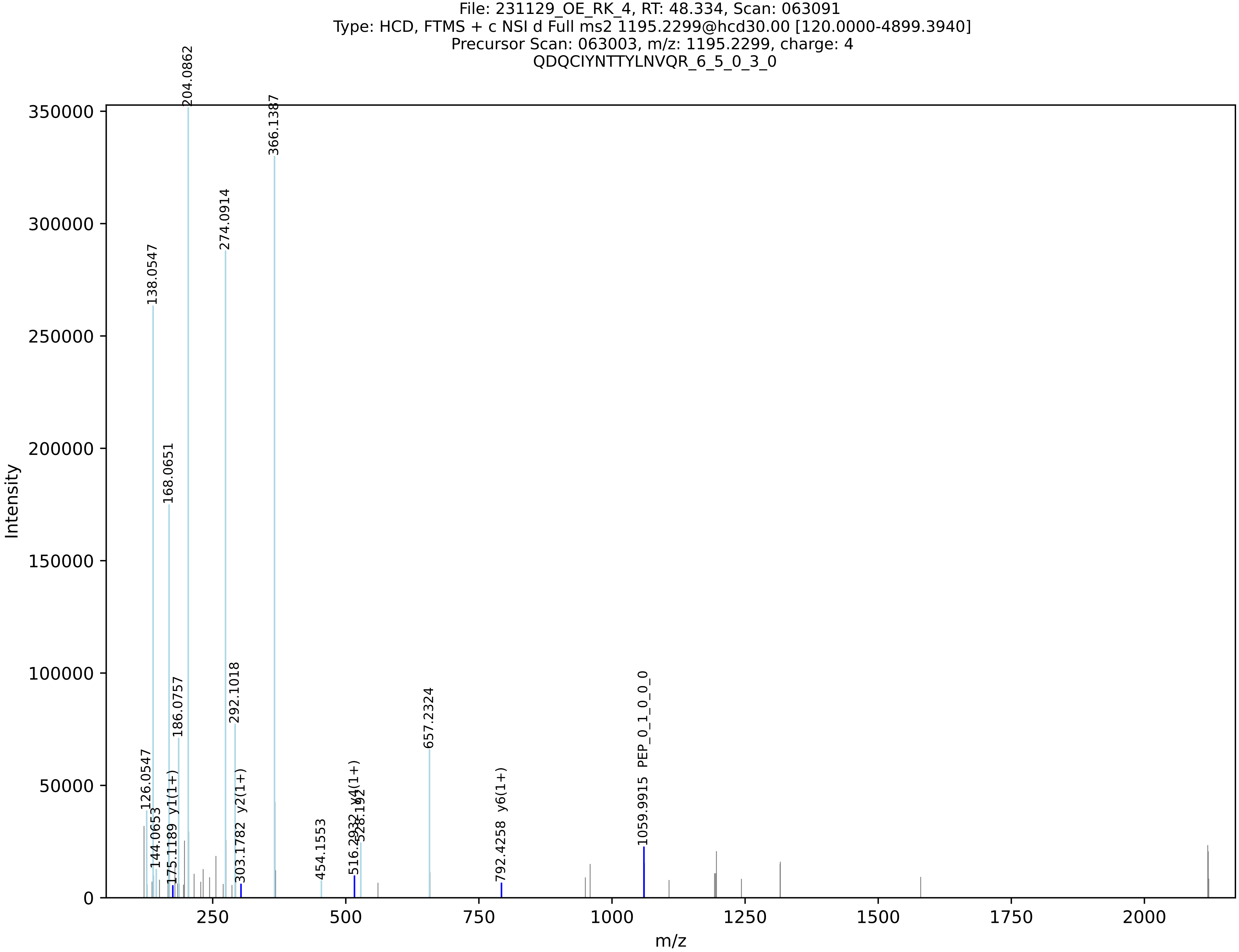
File: 231129_OE_RK_4, RT: 48.334, Scan: 063091
Type: HCD, FTMS + c NSI d Full ms2 1195.2299@hcd30.00 [120.0000-4899.3940]
Precursor Scan: 063003, m/z: 1195.2299, charge: 4
QDQCIYNTTYLNVQR_6_5_0_3_0
Intensity
m/z
204.0862
366.1387
274.0914
138.0547
168.0651
186.0757
292.1018
657.2324
1059.9915 PEP_0_1_0_0_0
126.0547
144.0653
175.1189 y1(1+)
303.1782 y2(1+)
454.1553
516.2932 y4(1+)
792.4258 y6(1+)

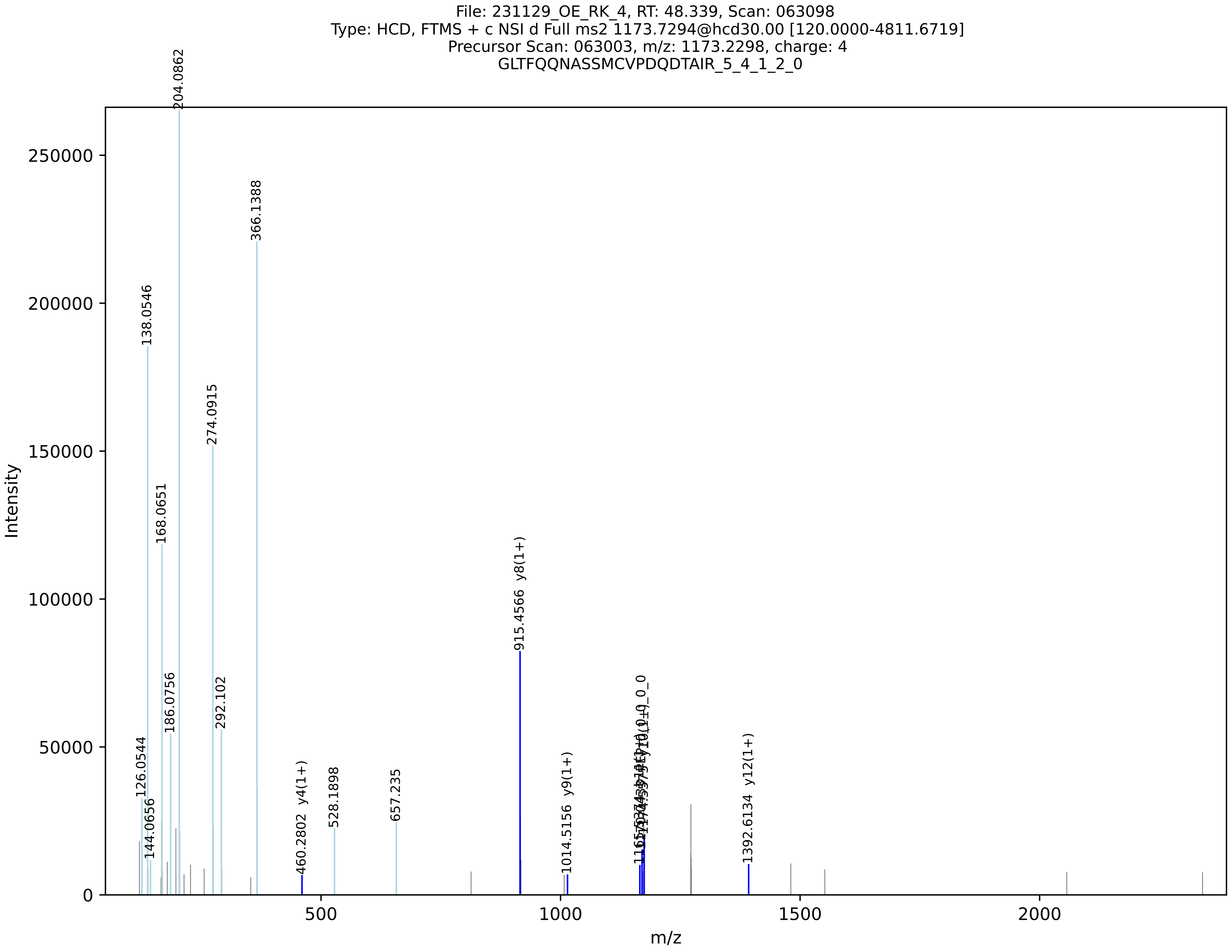

File: 231129_OE_RK_4, RT: 48.339, Scan: 063098
Type: HCD, FTMS + c NSI d Full ms2 1173.7294@hcd30.00 [120.0000-4811.6719]
Precursor Scan: 063003, m/z: 1173.2298, charge: 4
GLTFQQNASSMCVPDQDTAIR_5_4_1_2_0
Intensity
m/z
0
50000
100000
150000
200000
250000
500
1000
1500
2000
204.0862
366.1388
138.0546
274.0915
168.0651
186.0756
292.102
126.0544
144.0656
460.2802 y4(1+)
528.1898
657.235
915.4566 y8(1+)
1014.5156 y9(1+)
1392.6134 y12(1+)

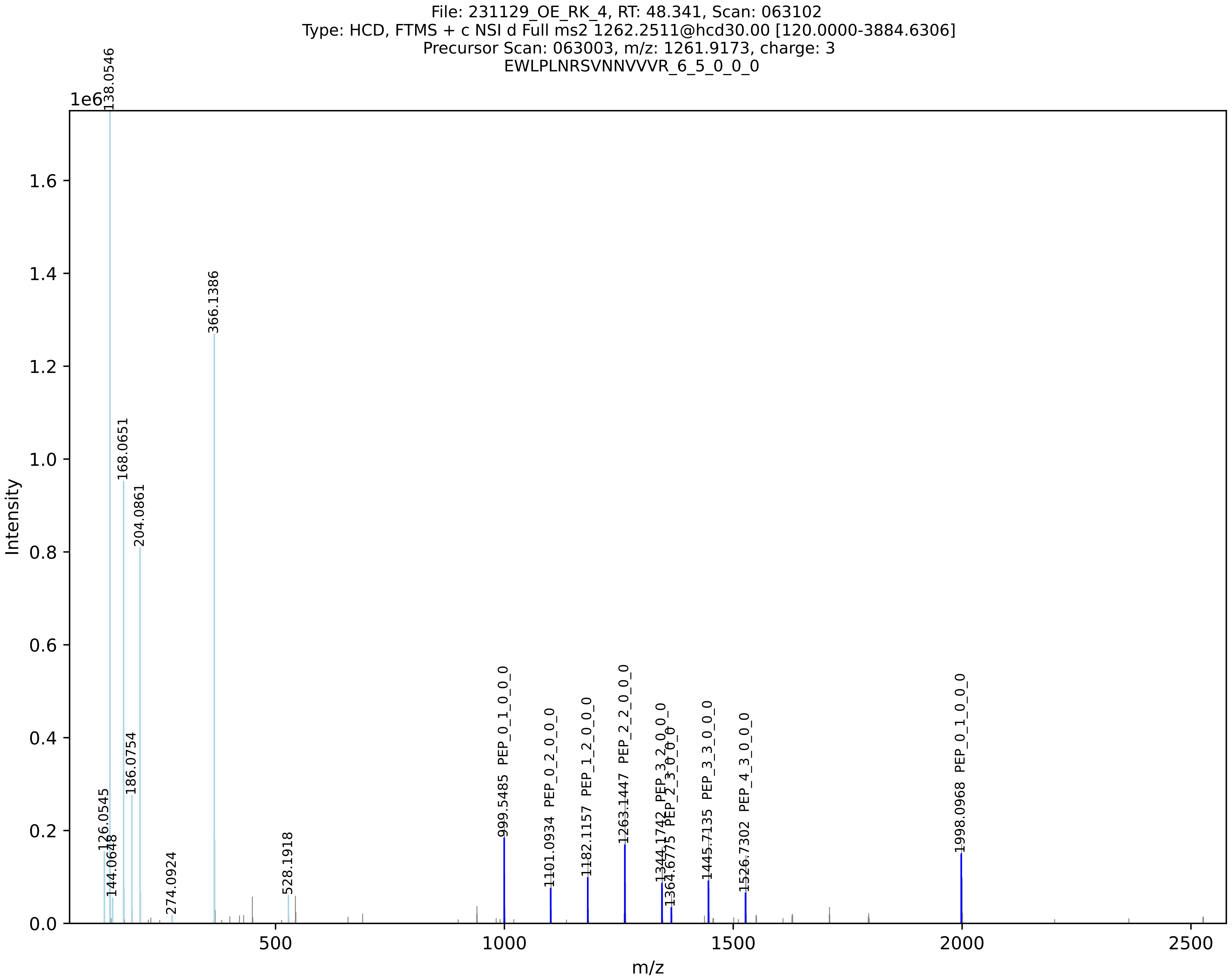

File: 231129_OE_RK_4, RT: 48.341, Scan: 063102
Type: HCD, FTMS + c NSI d Full ms2 1262.2511@hcd30.00 [120.0000-3884.6306]
Precursor Scan: 063003, m/z: 1261.9173, charge: 3
EWLPLNRSVNNVVVR_6_5_0_0_0
1e6
Intensity
m/z
138.0546
366.1386
168.0651
204.0861
186.0754
126.0545
144.0648
274.0924
528.1918
999.5485 PEP_0_1_0_0_0
1101.0934 PEP_0_2_0_0_0
1182.1157 PEP_1_2_0_0_0
1263.1447 PEP_2_2_0_0_0
1344.1742 PEP_3_2_0_0_0
1364.6775 PEP_2_3_0_0_0
1445.7135 PEP_3_3_0_0_0
1526.7302 PEP_4_3_0_0_0
1998.0968 PEP_0_1_0_0_0

File: 231129_OE_RK_4, RT: 48.48, Scan: 063304
Type: HCD, FTMS + c NSI d Full ms2 1396.3037@hcd30.00 [120.0000-5719.7749]
Precursor Scan: 063224, m/z: 1395.5519, charge: 4
QDQCIYNTTYLNVQR_7_6_1_4_0

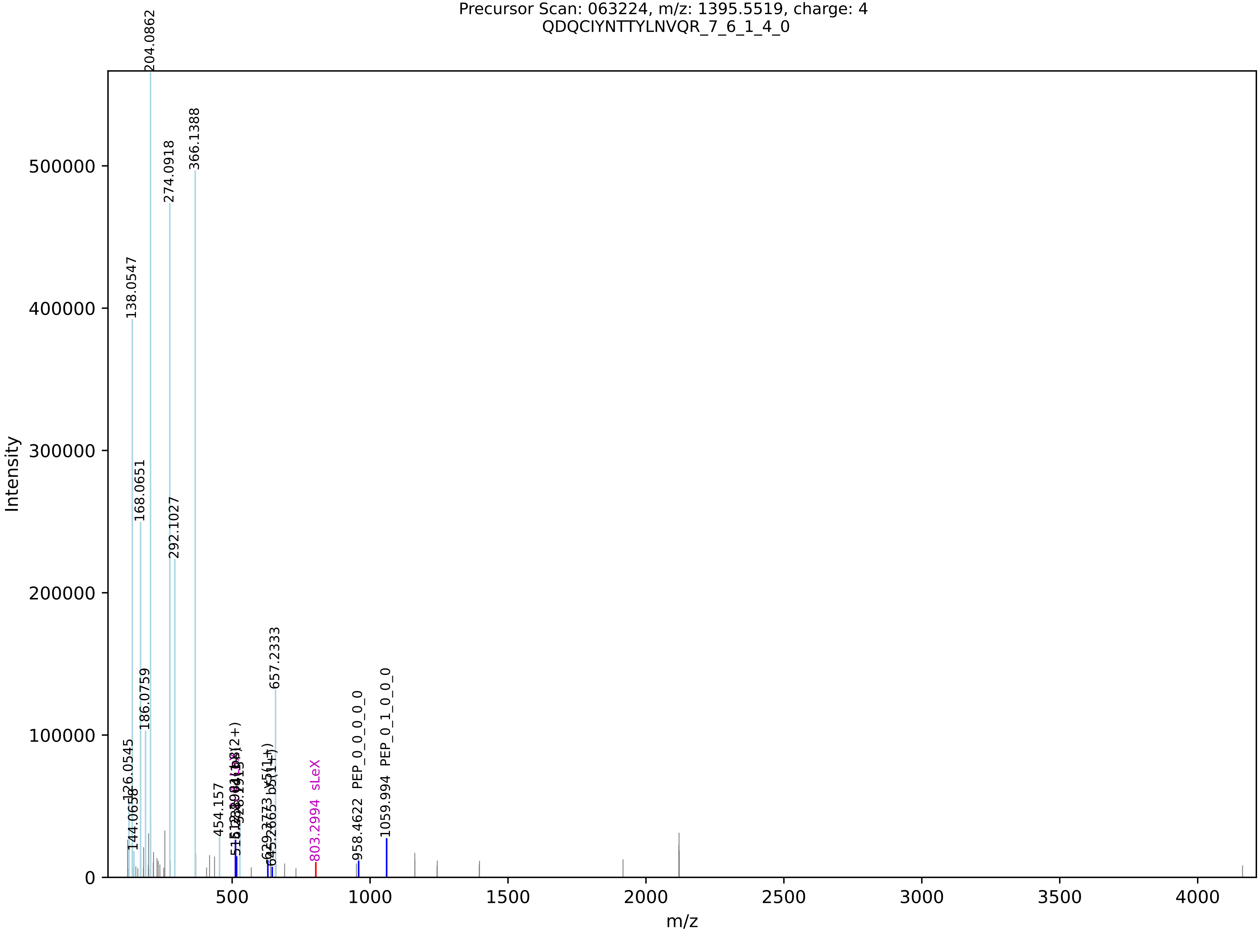

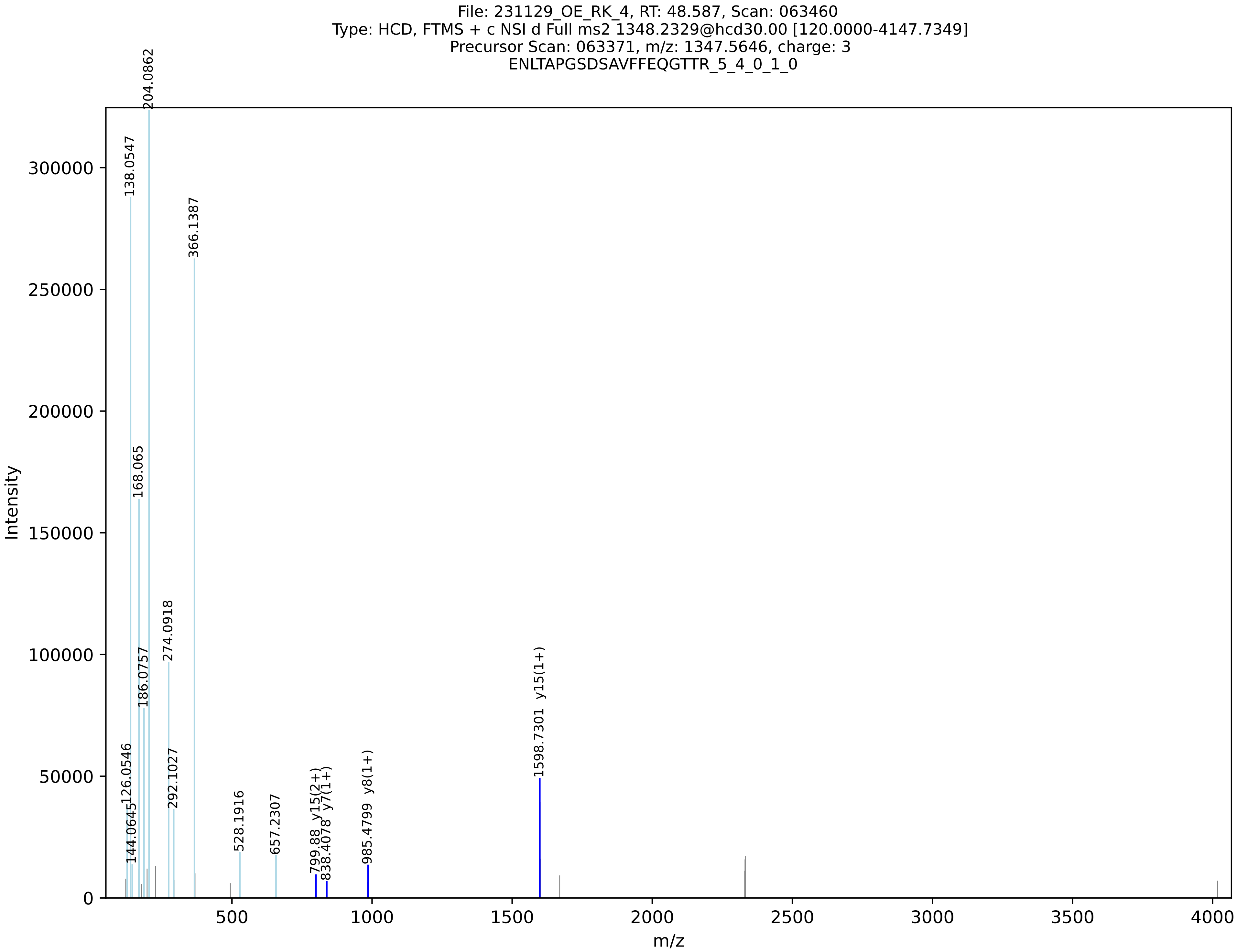
File: 231129_OE_RK_4, RT: 48.587, Scan: 063460
Type: HCD, FTMS + c NSI d Full ms2 1348.2329@hcd30.00 [120.0000-4147.7349]
Precursor Scan: 063371, m/z: 1347.5646, charge: 3
ENLTAPGSDSAVFFEQGTTR_5_4_0_1_0
204.0862
138.0547
366.1387
168.065
274.0918
186.0757
126.0546
292.1027
144.0645
528.1916
657.2307
799.88 y15(2+)
838.4078 y7(1+)
985.4799 y8(1+)
1598.7301 y15(1+)
Intensity
m/z
0
50000
100000
150000
200000
250000
300000
500
1000
1500
2000
2500
3000
3500
4000

File: 231129_OE_RK_4, RT: 48.685, Scan: 063600
Type: HCD, FTMS + c NSI d Full ms2 1080.5013@hcd30.00 [120.0000-4431.3018]
Precursor Scan: 063519, m/z: 1079.7496, charge: 4
LATALSLSNKFVEGSHNSTVSLTTK_5_3_0_1_0

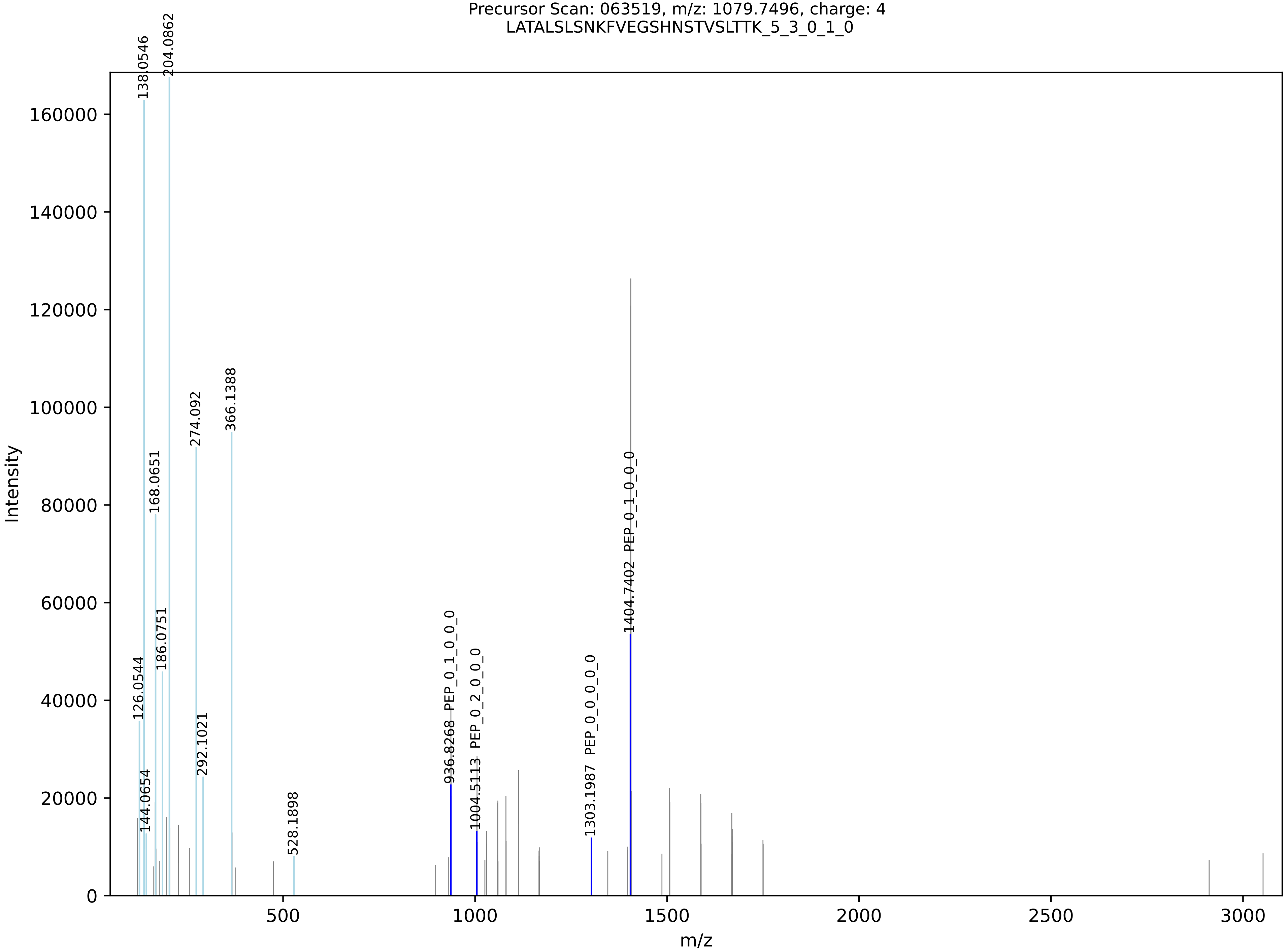

File: 231129_OE_RK_4, RT: 48.833, Scan: 063817
Type: HCD, FTMS + c NSI d Full ms2 964.8278@hcd30.00 [120.0000-4944.1919]
Precursor Scan: 063738, m/z: 964.2264, charge: 5
ELHHLQEQNVSNAFLDKGEFYIGSK_5_4_0_1_0

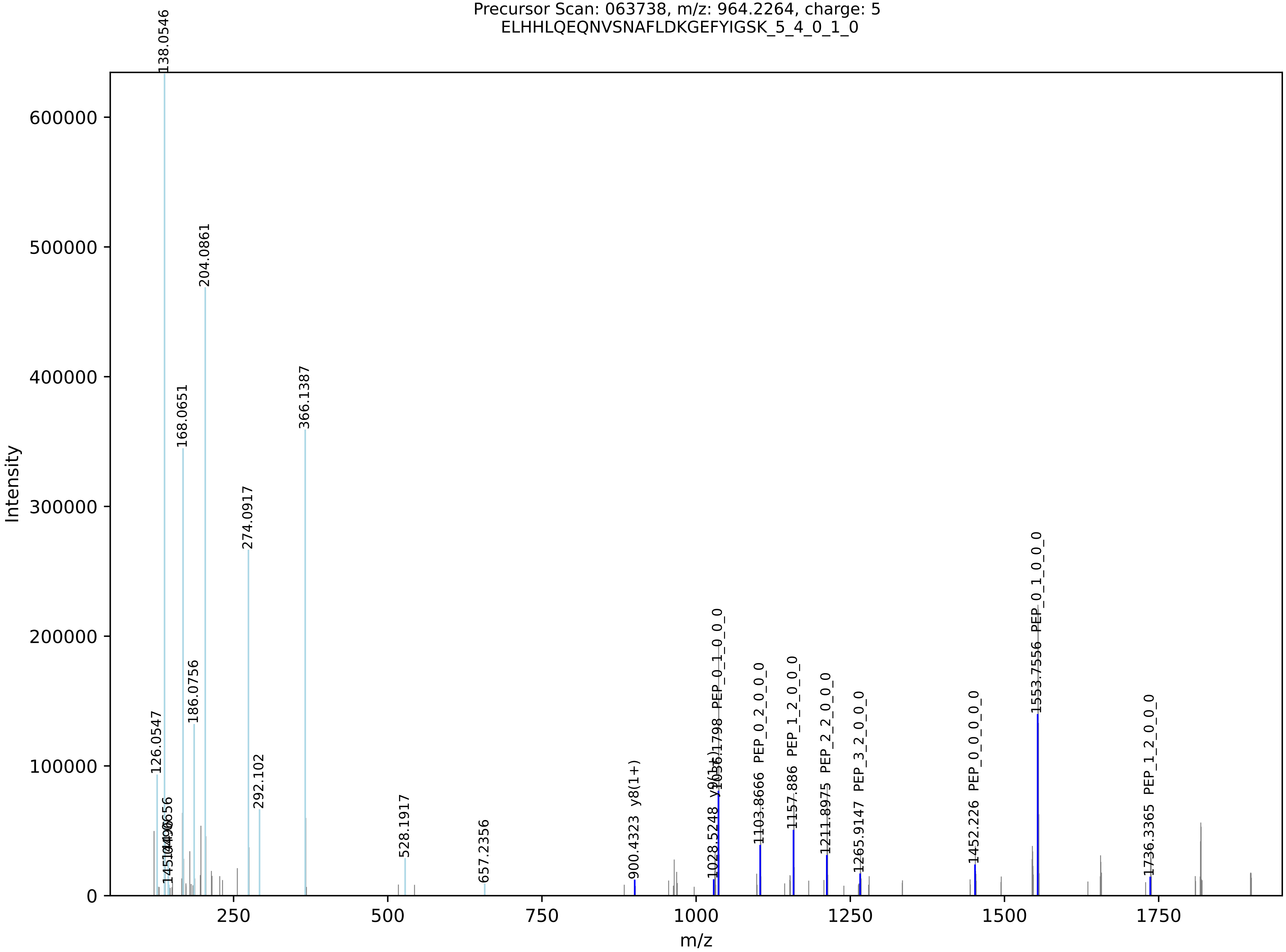

File: 231129_OE_RK_4, RT: 48.882, Scan: 063888
Type: HCD, FTMS + c NSI d Full ms2 1126.0071@hcd30.00 [120.0000-4616.9648]
Precursor Scan: 063812, m/z: 1125.5059, charge: 4
IIVPLNNRENISDPTSPLR_5_4_1_2_0

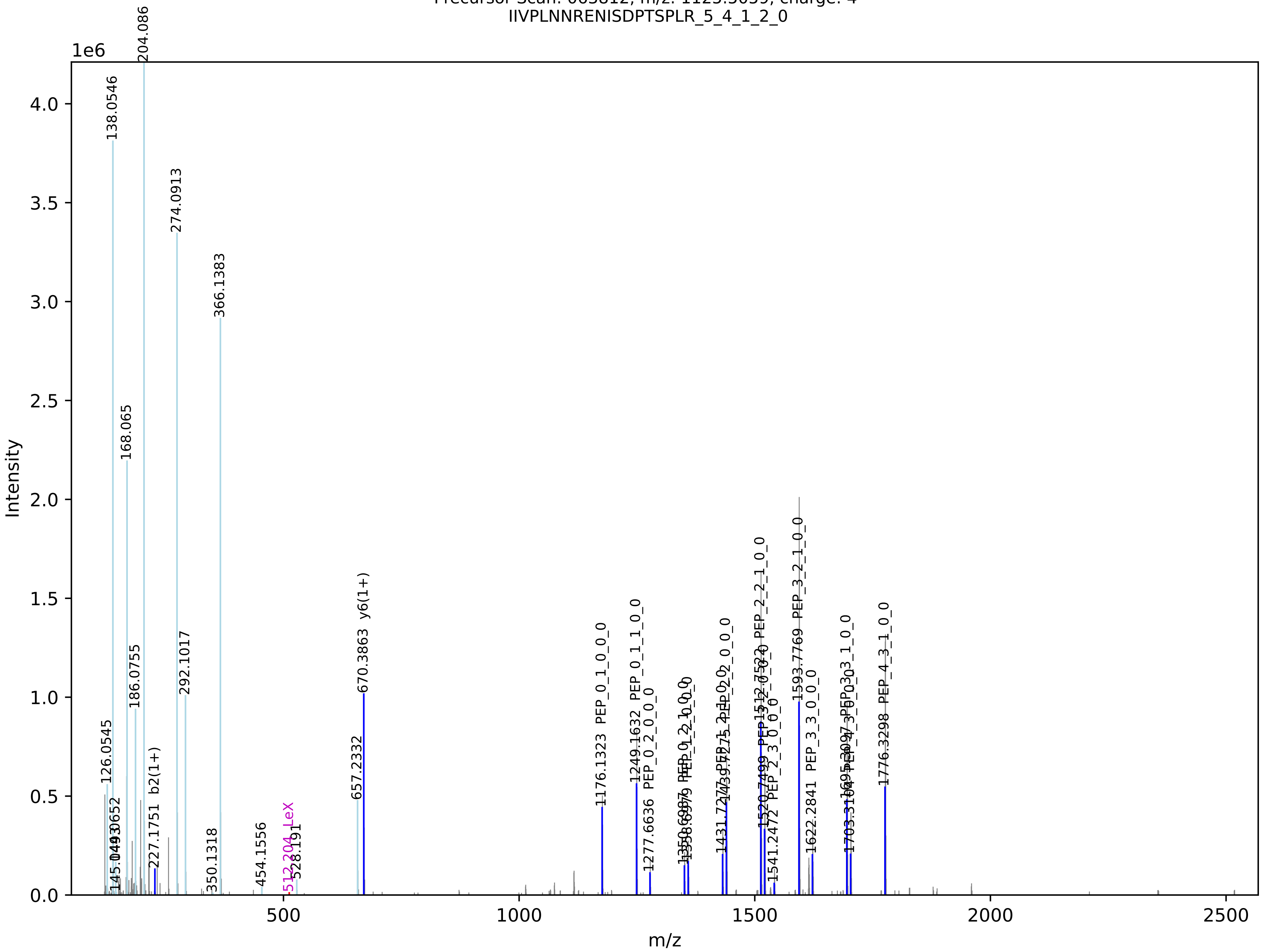

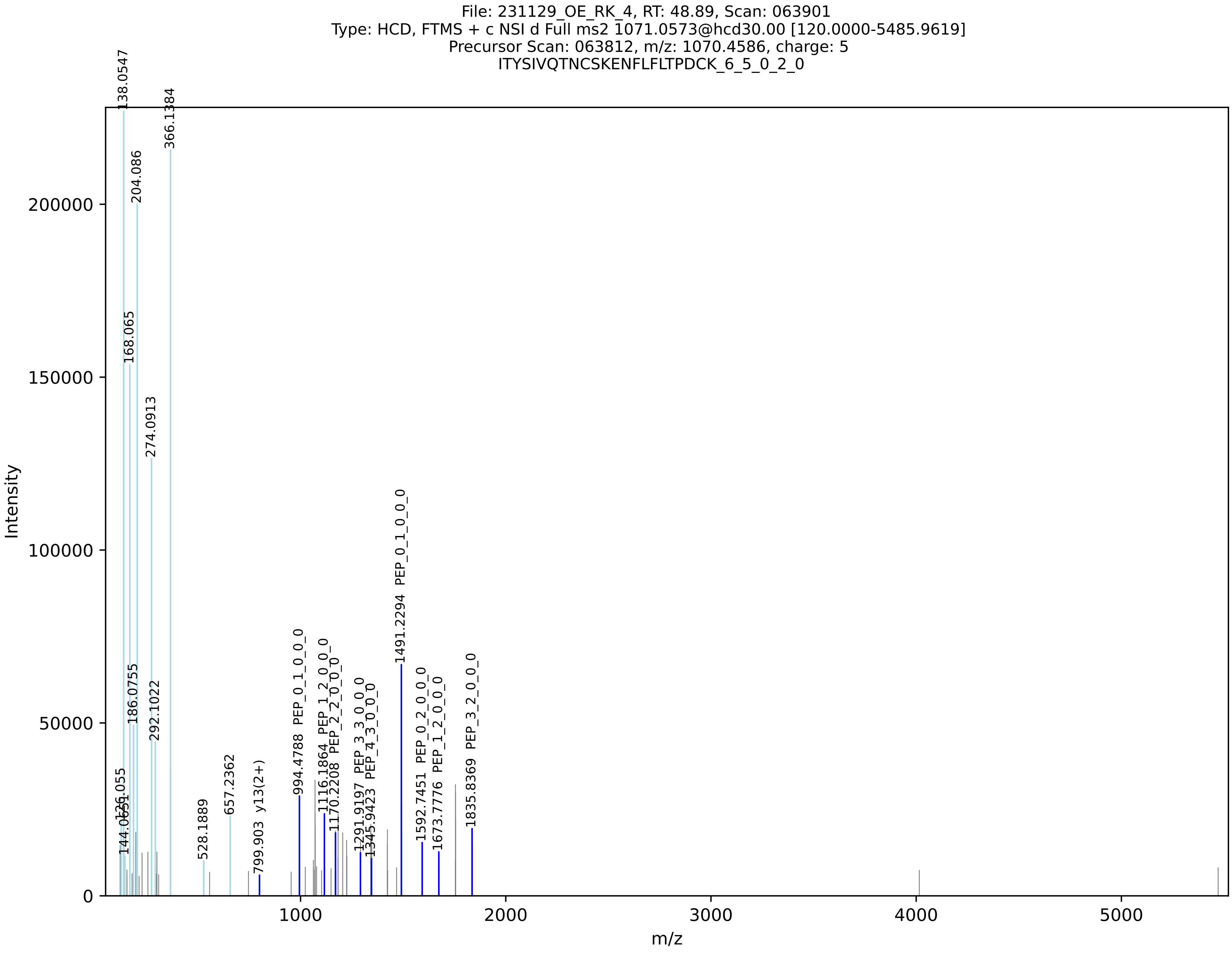
File: 231129_OE_RK_4, RT: 48.89, Scan: 063901
Type: HCD, FTMS + c NSI d Full ms2 1071.0573@hcd30.00 [120.0000-5485.9619]
Precursor Scan: 063812, m/z: 1070.4586, charge: 5
ITYSIVQTNCSKENFLFLTPDCK_6_5_0_2_0
Intensity
m/z
138.0547
366.1384
204.086
168.065
274.0913
186.0755
292.1022
126.055
144.0651
528.1889
657.2362
799.903 y13(2+)
994.4788 PEP_0_1_0_0_0
1116.1864 PEP_1_2_0_0_0
1170.2208 PEP_2_2_0_0_0
1291.9197 PEP_3_3_0_0_0
1345.9423 PEP_4_3_0_0_0
1491.2294 PEP_0_1_0_0_0
1592.7451 PEP_0_2_0_0_0
1673.7776 PEP_1_2_0_0_0
1835.8369 PEP_3_2_0_0_0

File: 231129_OE_RK_4, RT: 48.983, Scan: 064034
Type: HCD, FTMS + c NSI d Full ms2 1286.7628@hcd30.00 [120.0000-5272.8486]
Precursor Scan: 063959, m/z: 1286.2617, charge: 4
QDQCIYNTTYLNVQR_7_6_0_3_0

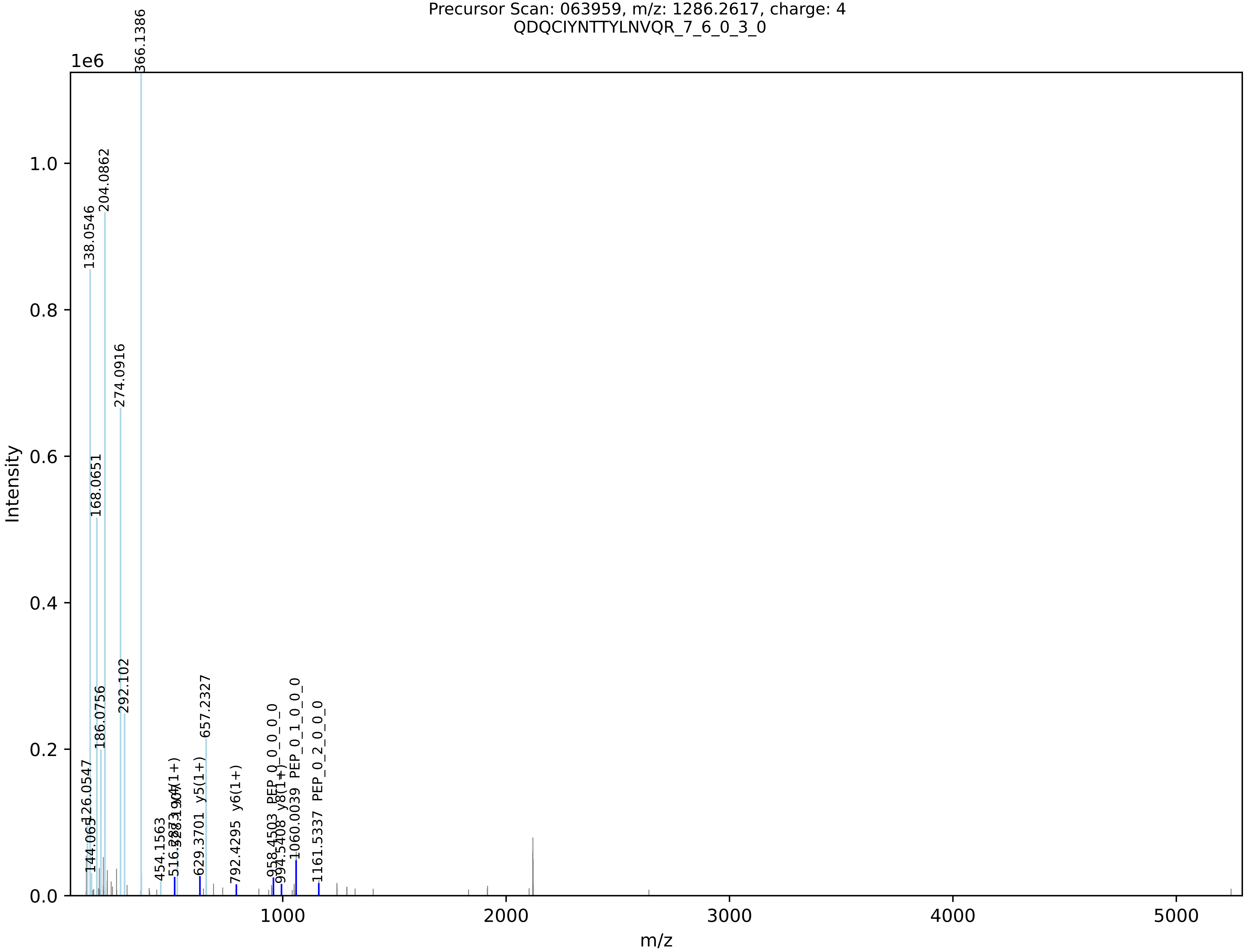

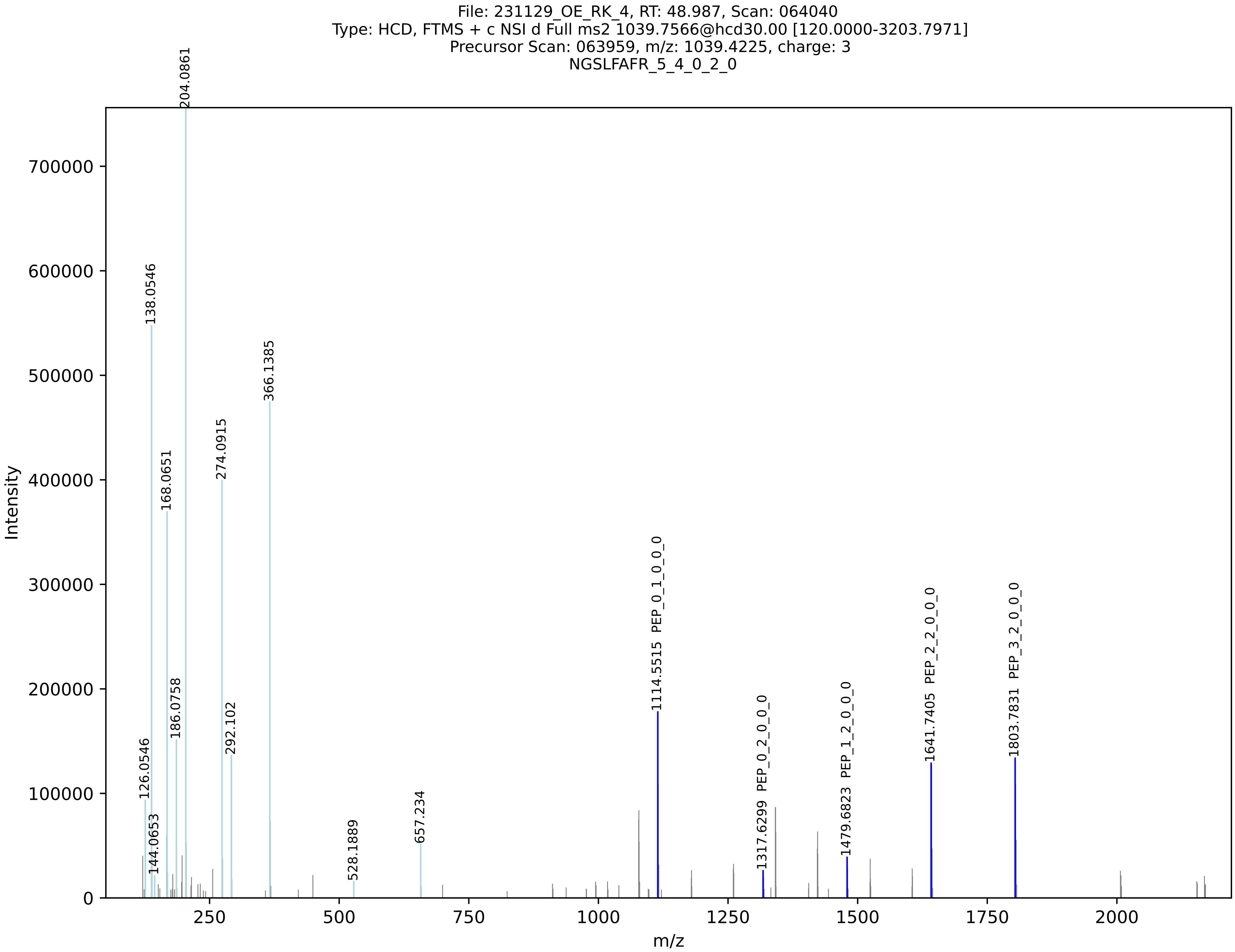
File: 231129_OE_RK_4, RT: 48.987, Scan: 064040
Type: HCD, FTMS + c NSI d Full ms2 1039.7566@hcd30.00 [120.0000-3203.7971]
Precursor Scan: 063959, m/z: 1039.4225, charge: 3
NGSLFAFR_5_4_0_2_0
Intensity
m/z
204.0861
138.0546
366.1385
274.0915
168.0651
186.0758
292.102
126.0546
144.0653
528.1889
657.234
1114.5515 PEP_0_1_0_0_0
1317.6299 PEP_0_2_0_0_0
1479.6823 PEP_1_2_0_0_0
1641.7405 PEP_2_2_0_0_0
1803.7831 PEP_3_2_0_0_0

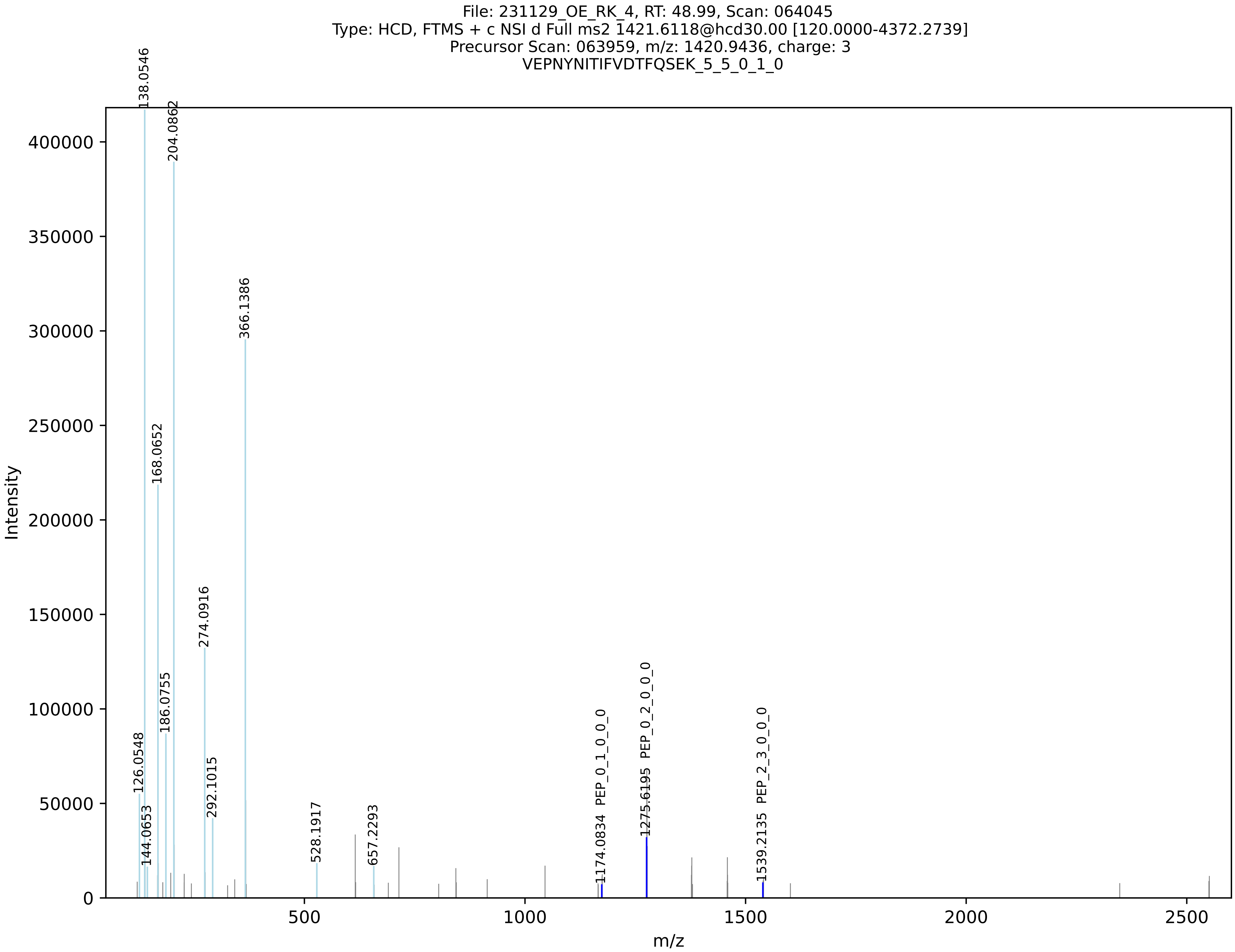
File: 231129_OE_RK_4, RT: 48.99, Scan: 064045
Type: HCD, FTMS + c NSI d Full ms2 1421.6118@hcd30.00 [120.0000-4372.2739]
Precursor Scan: 063959, m/z: 1420.9436, charge: 3
VEPNYNITIFVDTFQSEK_5_5_0_1_0
Intensity
m/z
0
50000
100000
150000
200000
250000
300000
350000
400000
500
1000
1500
2000
2500
138.0546
204.0862
366.1386
168.0652
274.0916
186.0755
126.0548
292.1015
144.0653
528.1917
657.2293
1174.0834 PEP_0_1_0_0_0
1275.6195 PEP_0_2_0_0_0
1539.2135 PEP_2_3_0_0_0

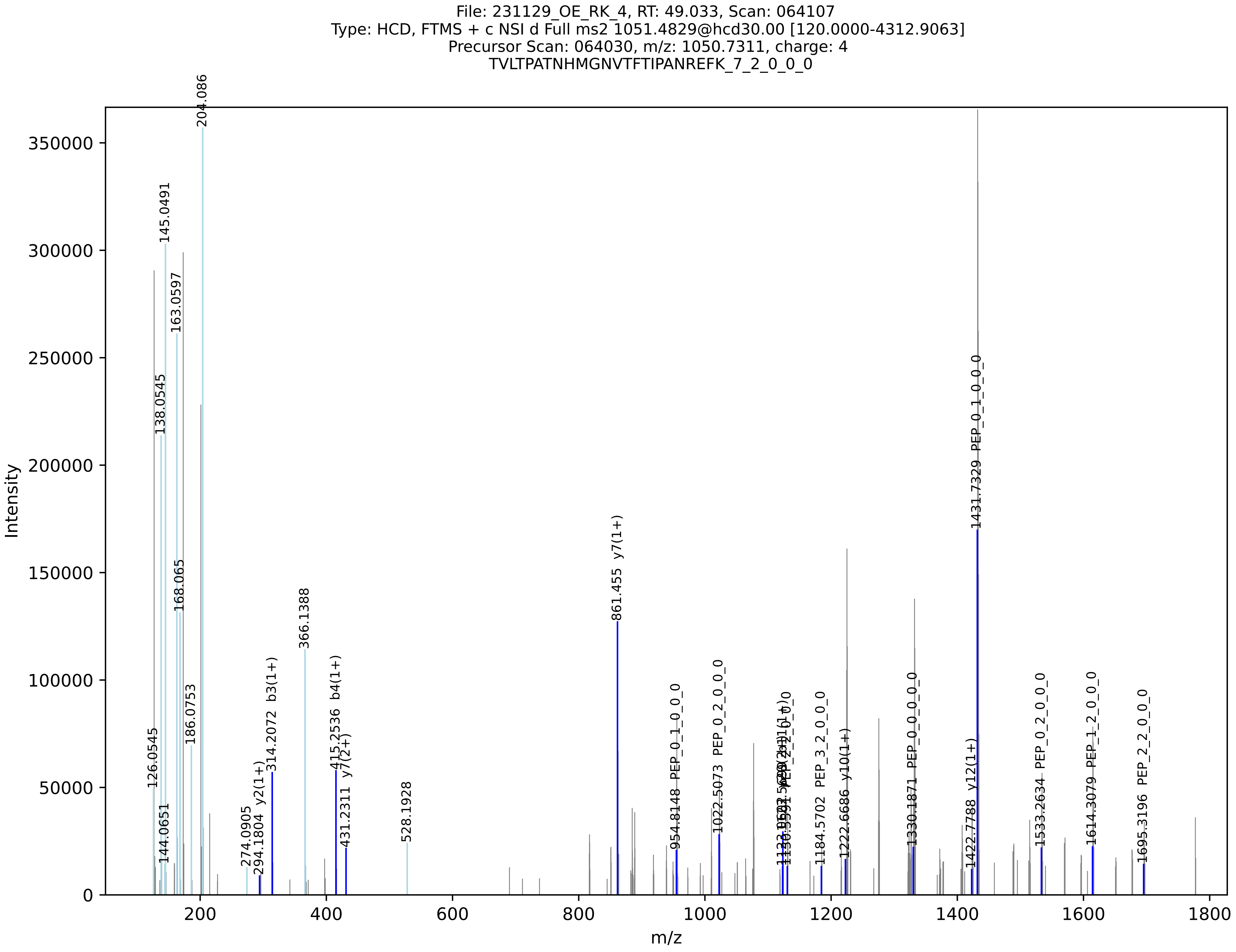
File: 231129_OE_RK_4, RT: 49.033, Scan: 064107
Type: HCD, FTMS + c NSI d Full ms2 1051.4829@hcd30.00 [120.0000-4312.9063]
Precursor Scan: 064030, m/z: 1050.7311, charge: 4
TVLTPATNHMGNVTFTIPANREFK_7_2_0_0_0
Intensity
m/z
126.0545
138.0545
144.0651
145.0491
163.0597
168.065
186.0753
204.086
274.0905
294.1804 y2(1+)
314.2072 b3(1+)
366.1388
415.2536 b4(1+)
431.2311 y7(2+)
528.1928
861.455 y7(1+)
954.8148 PEP_0_1_0_0_0
1022.5073 PEP_0_2_0_0_0
1184.5702 PEP_3_2_0_0_0
1222.6686 y10(1+)
1330.1871 PEP_0_0_0_0_0
1422.7788 y12(1+)
1431.7329 PEP_0_1_0_0_0
1533.2634 PEP_0_2_0_0_0
1614.3079 PEP_1_2_0_0_0
1695.3196 PEP_2_2_0_0_0

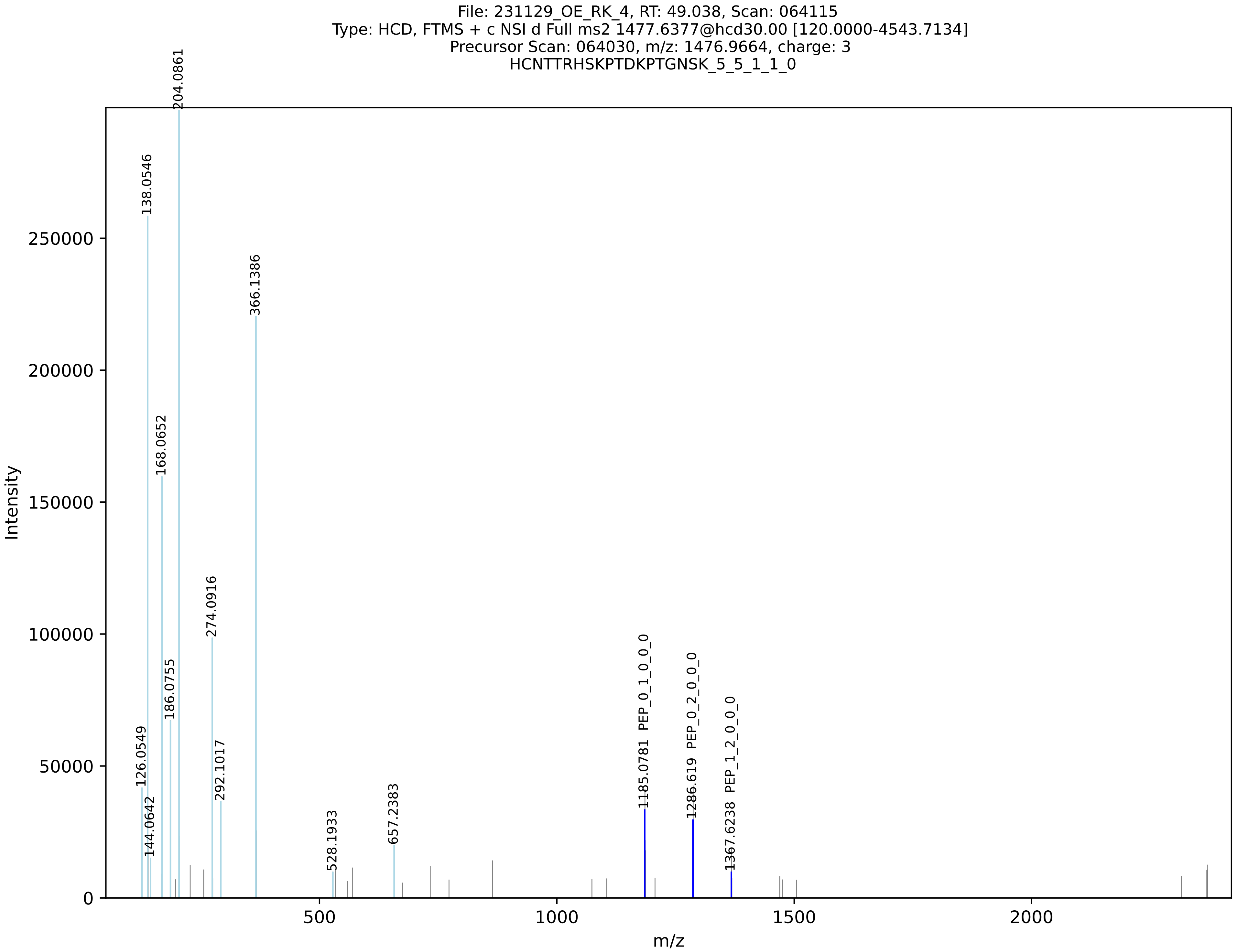
File: 231129_OE_RK_4, RT: 49.038, Scan: 064115
Type: HCD, FTMS + c NSI d Full ms2 1477.6377@hcd30.00 [120.0000-4543.7134]
Precursor Scan: 064030, m/z: 1476.9664, charge: 3
HCNTTRHSKPTDKPTGNSK_5_5_1_1_0
Intensity
m/z
204.0861
138.0546
366.1386
168.0652
274.0916
186.0755
126.0549
292.1017
144.0642
528.1933
657.2383
1185.0781 PEP_0_1_0_0_
1286.619 PEP_0_2_0_0_0
1367.6238 PEP_1_2_0_0_0
0
50000
100000
150000
200000
250000
500
1000
1500
2000

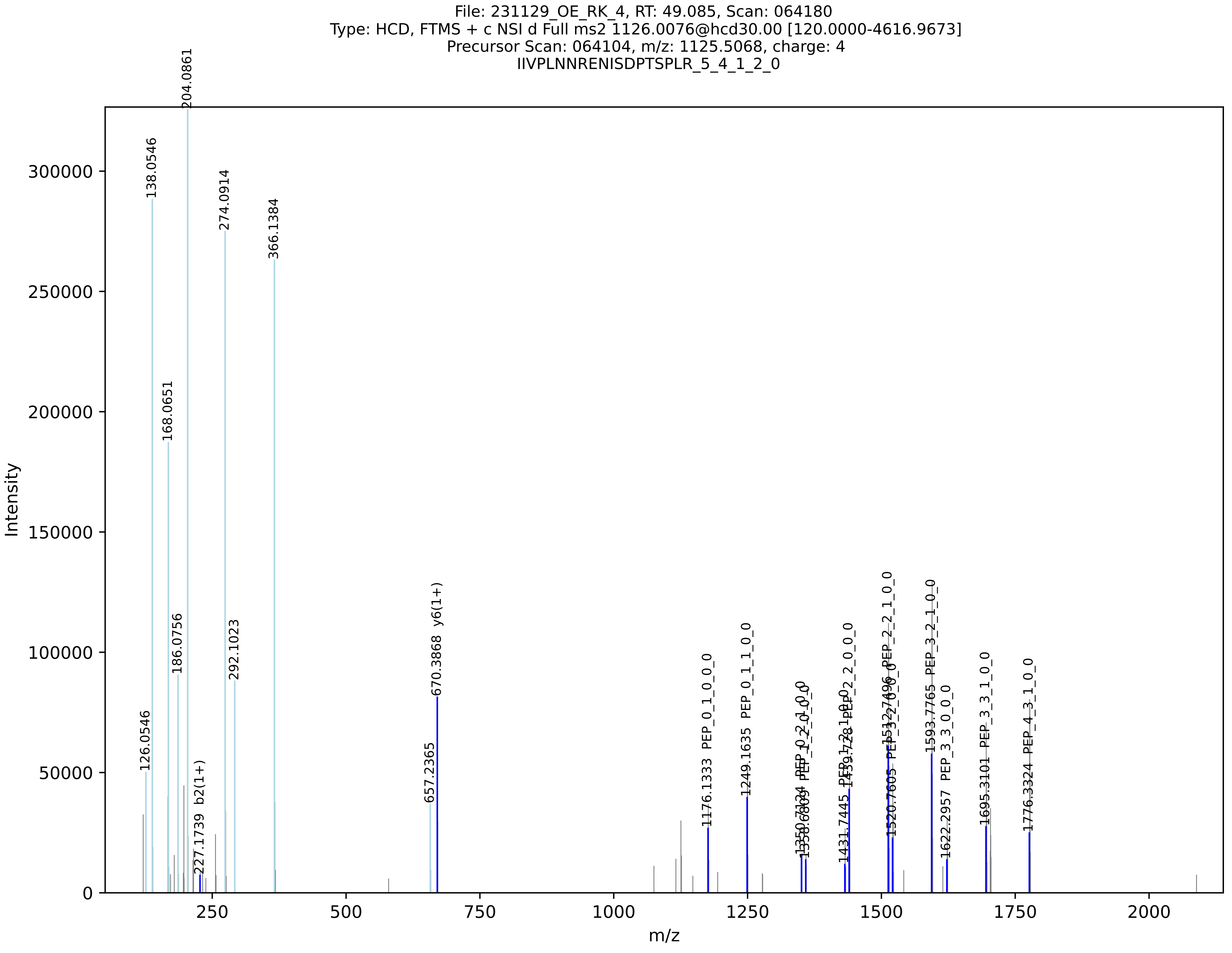
File: 231129_OE_RK_4, RT: 49.085, Scan: 064180
Type: HCD, FTMS + c NSI d Full ms2 1126.0076@hcd30.00 [120.0000-4616.9673]
Precursor Scan: 064104, m/z: 1125.5068, charge: 4
IIVPLNNRENISDPTSPLR_5_4_1_2_0
Intensity
m/z
204.0861
138.0546
274.0914
366.1384
168.0651
186.0756
292.1023
126.0546
227.1739 b2(1+)
657.2365
670.3868 y6(1+)
1176.1333 PEP_0_1_0_0_0
1249.1635 PEP_0_1_1_0_0
1431.7445
1512.7496 PEP_2_2_1_0_0
1520.7605
1593.7765 PEP_3_2_1_0_0
1622.2957 PEP_3_3_0_0_0
1695.3101 PEP_3_3_1_0_0
1776.3324 PEP_4_3_1_0_0

File: 231129_OE_RK_4, RT: 49.087, Scan: 064183
Type: HCD, FTMS + c NSI d Full ms2 964.6277@hcd30.00 [120.0000-4943.1714]
Precursor Scan: 064104, m/z: 964.2268, charge: 5
ELHHLQEQNVSNAFLDKGEFYIGSK_5_4_0_1_0

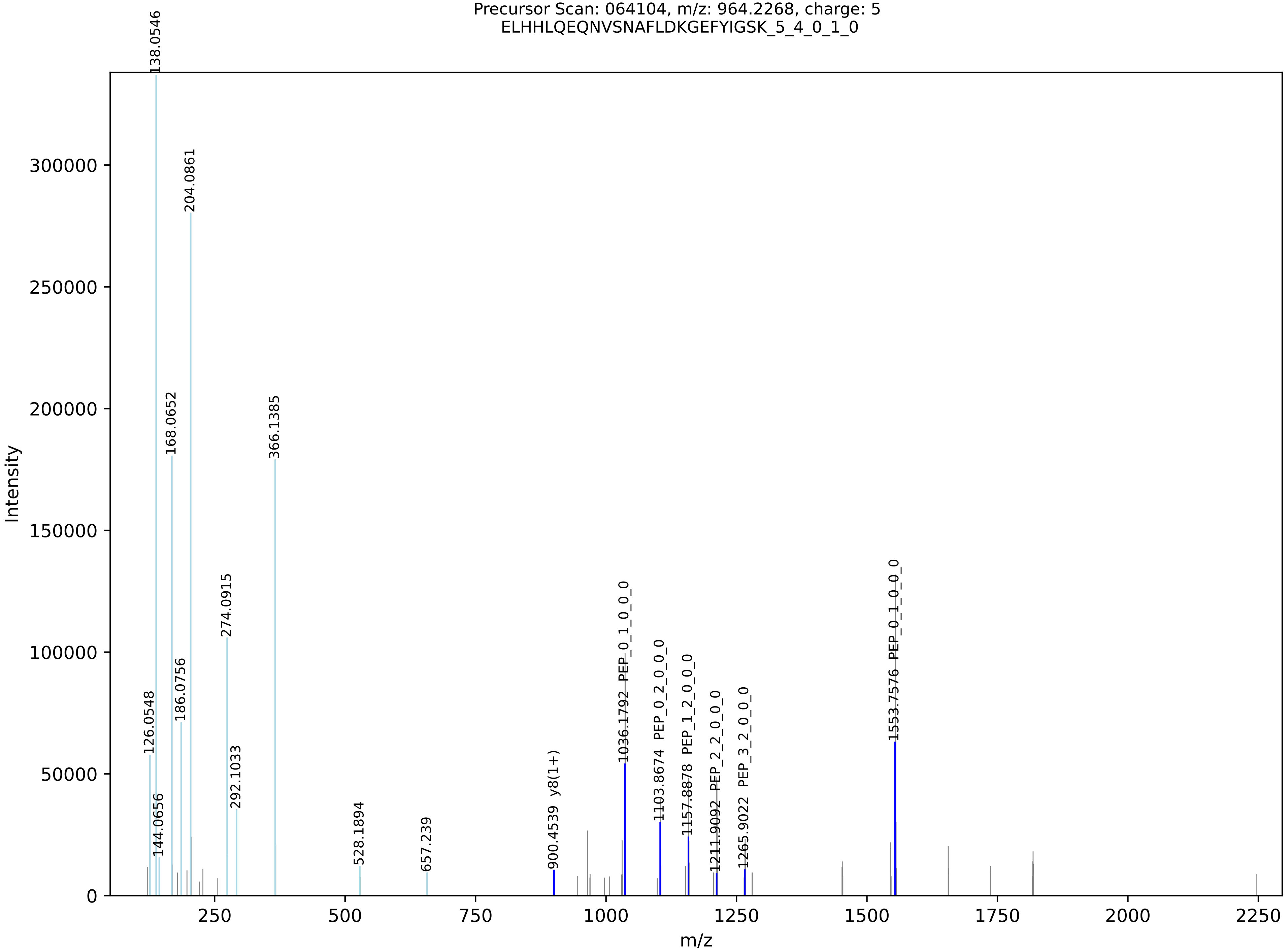

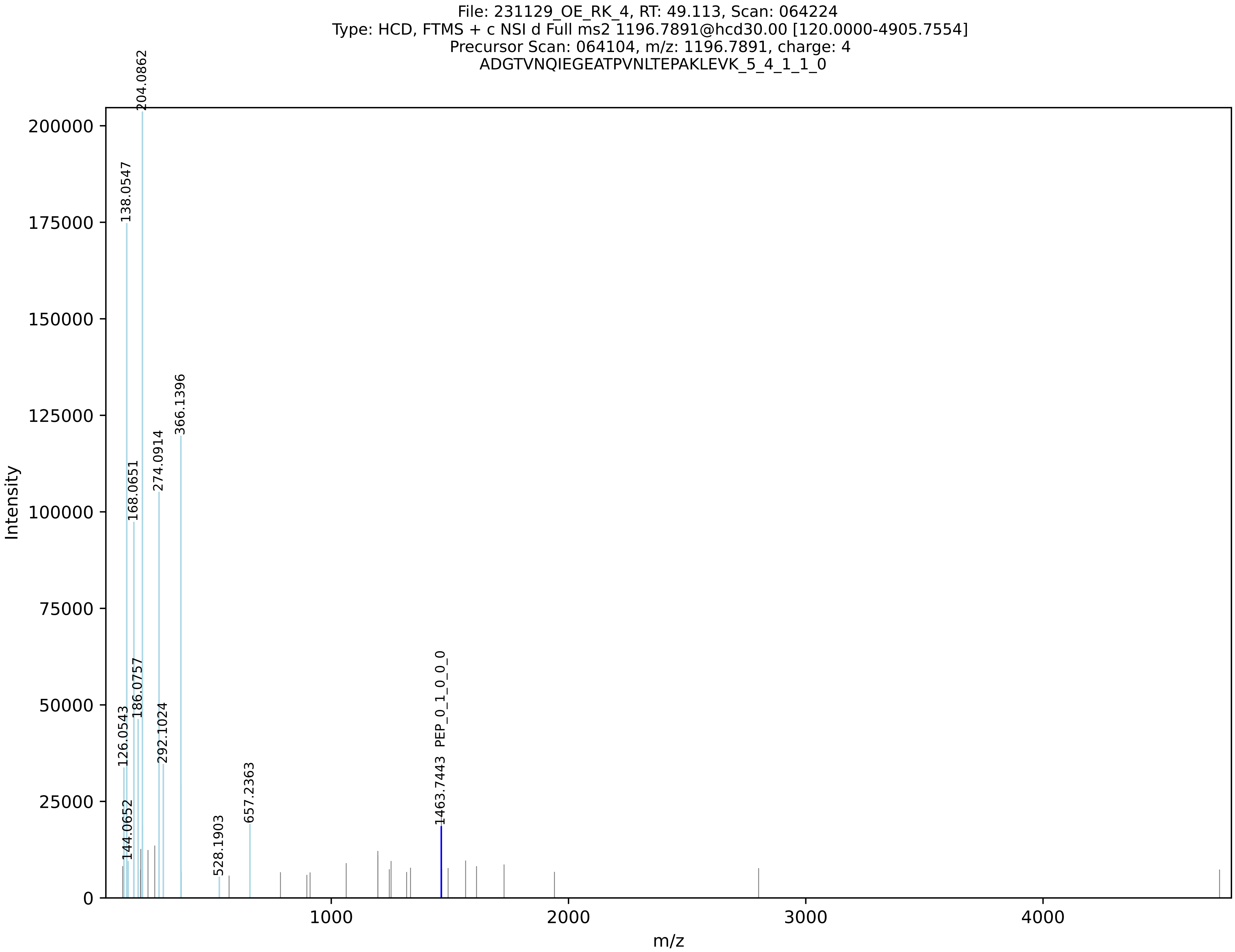
File: 231129_OE_RK_4, RT: 49.113, Scan: 064224
Type: HCD, FTMS + c NSI d Full ms2 1196.7891@hcd30.00 [120.0000-4905.7554]
Precursor Scan: 064104, m/z: 1196.7891, charge: 4
ADGTVNQIEGEATPVNLTEPAKLEVK_5_4_1_1_0
Intensity
m/z
0
25000
50000
75000
100000
125000
150000
175000
200000
1000
2000
3000
4000
204.0862
138.0547
366.1396
274.0914
168.0651
186.0757
126.0543
292.1024
144.0652
528.1903
657.2363
1463.7443 PEP_0_1_0_0_0_

File: 231129_OE_RK_4, RT: 49.134, Scan: 064251
Type: HCD, FTMS + c NSI d Full ms2 1089.4907@hcd30.00 [120.0000-4467.9780]
Precursor Scan: 064178, m/z: 1088.9895, charge: 4
IIVPLNNRENISDPTSPLR_5_4_0_2_0

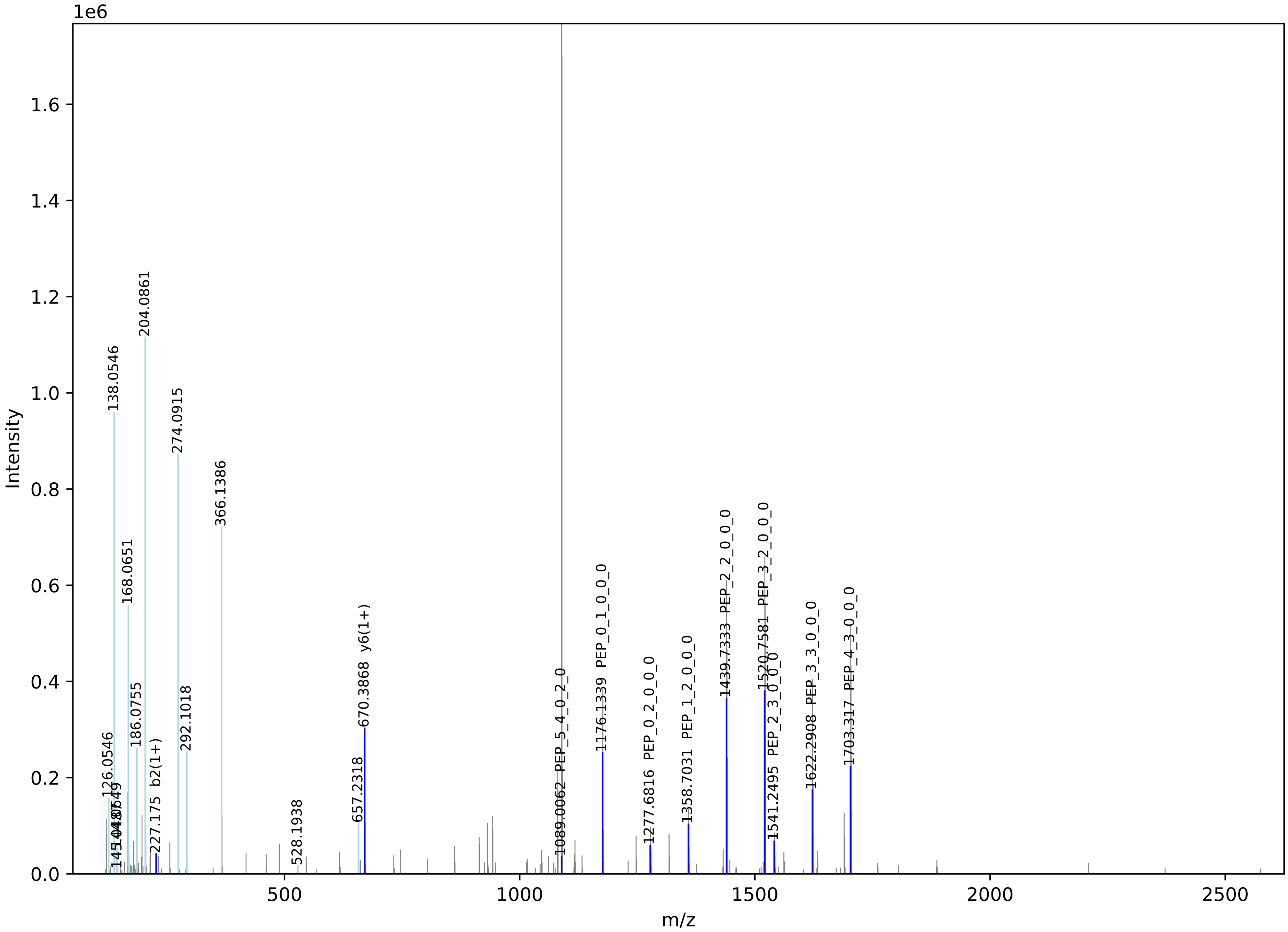

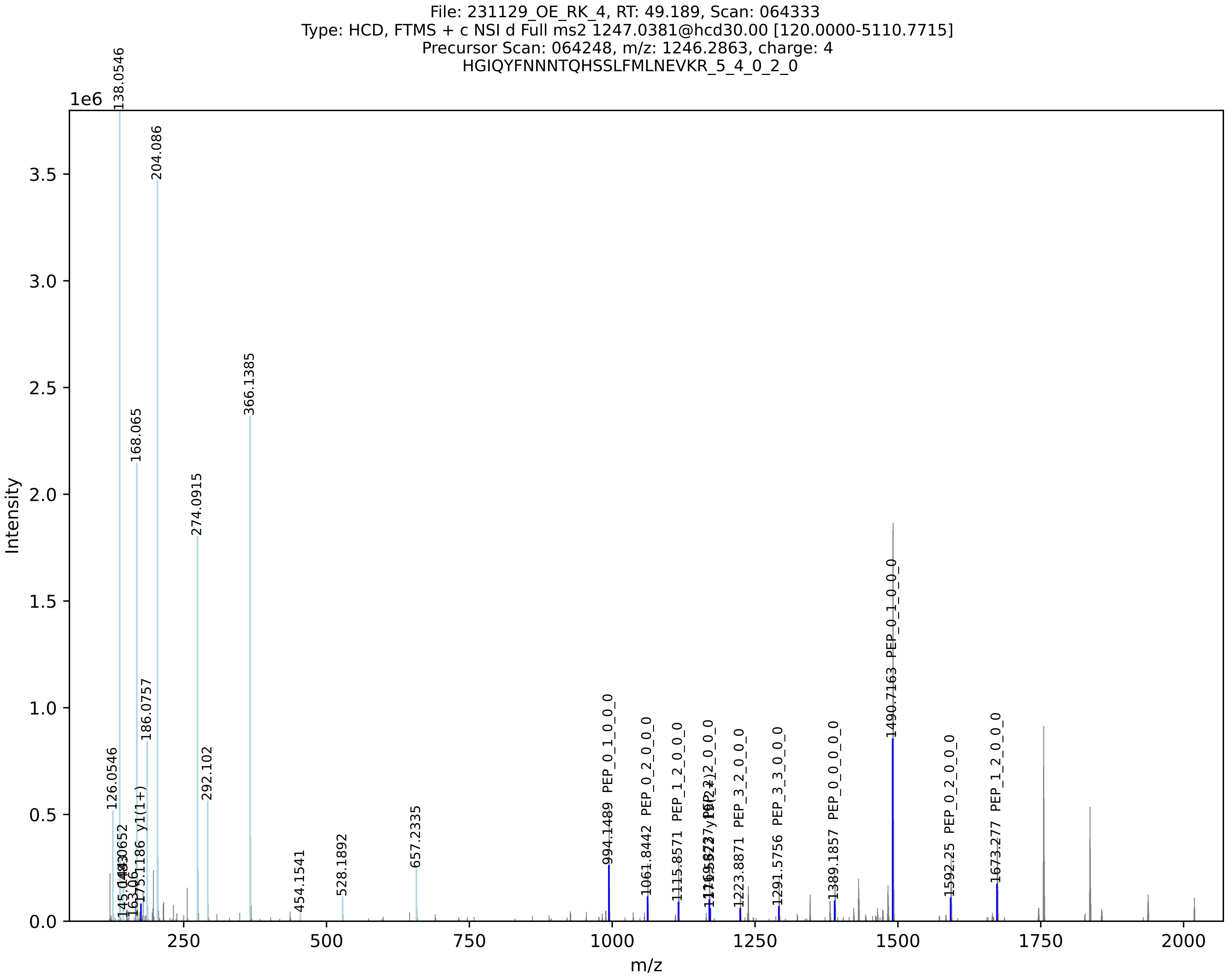
File: 231129_OE_RK_4, RT: 49.189, Scan: 064333
Type: HCD, FTMS + c NSI d Full ms2 1247.0381@hcd30.00 [120.0000-5110.7715]
Precursor Scan: 064248, m/z: 1246.2863, charge: 4
HGIQYFNNNTQHSSLFMLNEVKR_5_4_0_2_0
1e6
Intensity
m/z
138.0546
204.086
366.1385
168.065
274.0915
186.0757
126.0546
292.102
y1(1+)
175.1186
657.2335
528.1892
454.1541
994.1489 PEP_0_1_0_0_0
1061.8442 PEP_0_2_0_0_0
1115.8571 PEP_1_2_0_0_0
1223.8871 PEP_3_2_0_0_0
1291.5756 PEP_3_3_0_0_0
1389.1857 PEP_0_0_0_0_0
1490.7163 PEP_0_1_0_0_0
1592.25 PEP_0_2_0_0_0
1673.277 PEP_1_2_0_0_0

File: 231129_OE_RK_4, RT: 49.285, Scan: 064472
Type: HCD, FTMS + c NSI d Full ms2 1359.7903@hcd30.00 [120.0000-5570.8003]
Precursor Scan: 064395, m/z: 1359.039, charge: 4
QDQCIYNTTYLNVQR_7_6_0_4_0

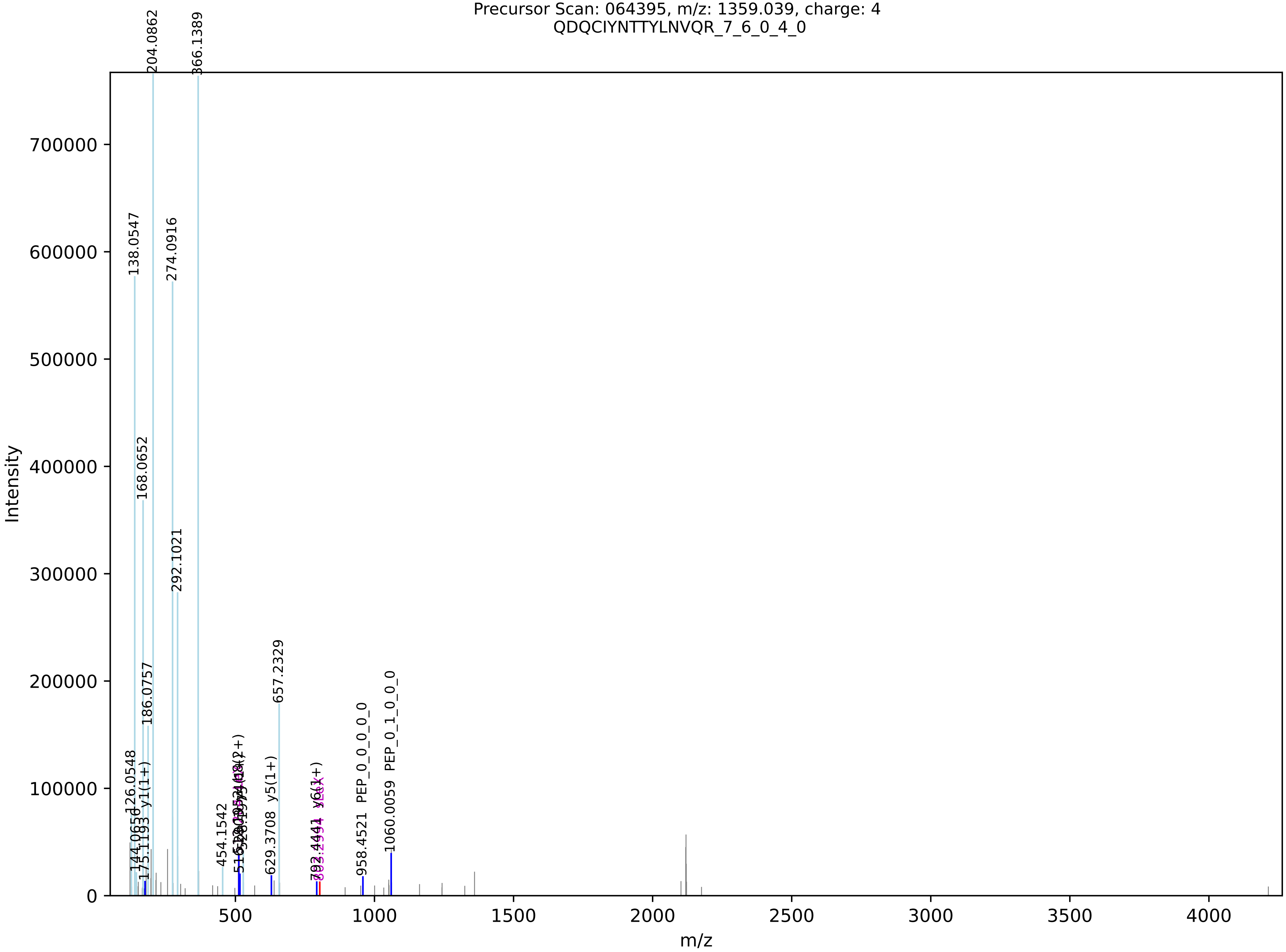

File: 231129_OE_RK_4, RT: 49.286, Scan: 064474
Type: HCD, FTMS + c NSI d Full ms2 970.2031@hcd30.00 [120.0000-3981.2844]
Precursor Scan: 064395, m/z: 969.7027, charge: 4
TVLTPATNHMGNVTFTIPANREFK_5_2_0_0_0

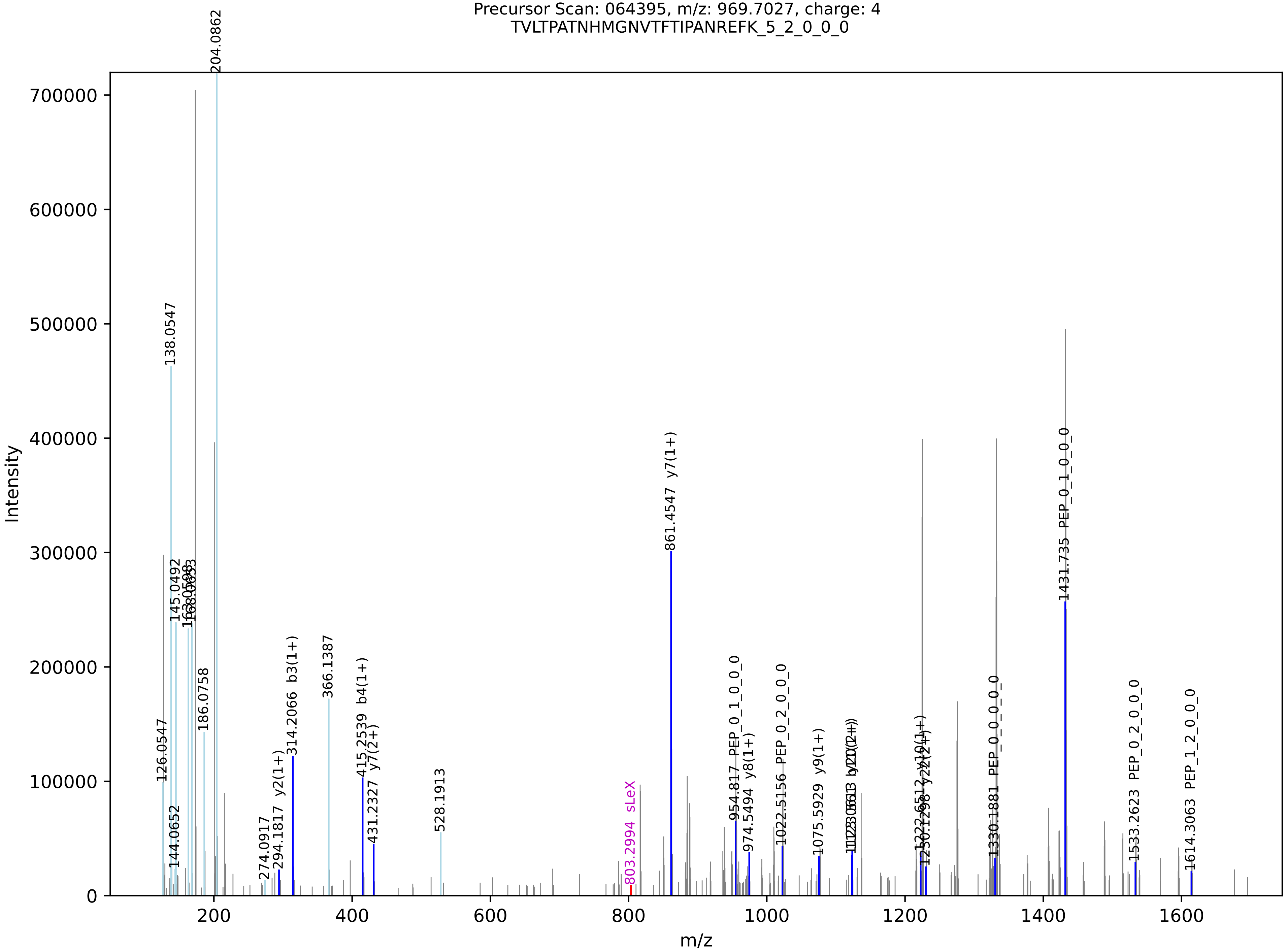

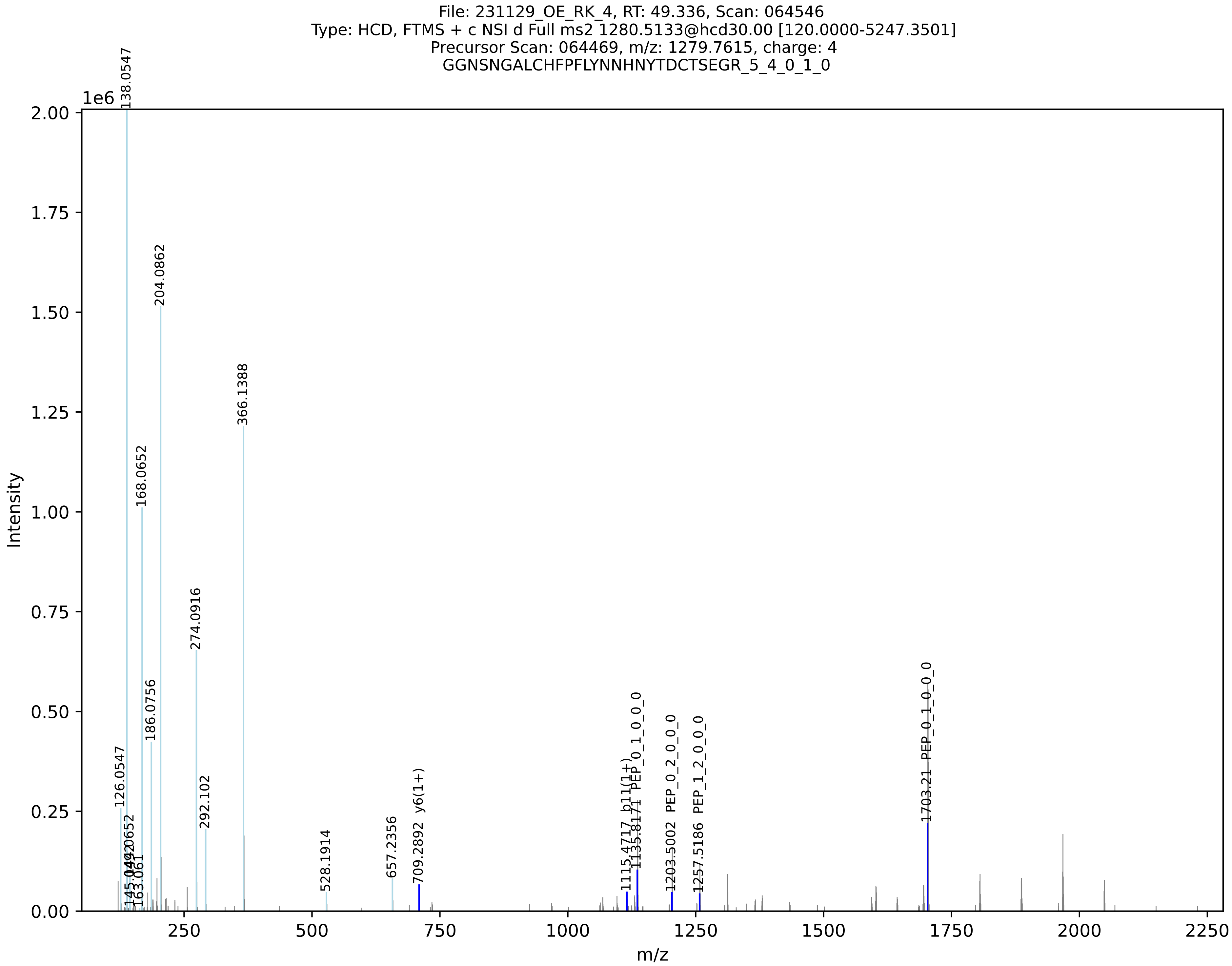
File: 231129_OE_RK_4, RT: 49.336, Scan: 064546
Type: HCD, FTMS + c NSI d Full ms2 1280.5133@hcd30.00 [120.0000-5247.3501]
Precursor Scan: 064469, m/z: 1279.7615, charge: 4
GGNSNGALCHFPFLYNNHNYTDCTSEGR_5_4_0_1_0
1e6
Intensity
m/z
138.0547
204.0862
366.1388
168.0652
274.0916
186.0756
126.0547
292.102
528.1914
657.2356
709.2892 y6(1+)
1115.4717 b11(1+)
1135.8171 PEP_0_1_0_0_0
1203.5002 PEP_0_2_0_0_0
1257.5186 PEP_1_2_0_0_0
1703.21 PEP_0_1_0_0_0
163.061
145.049

File: 231129_OE_RK_4, RT: 49.339, Scan: 064551
Type: HCD, FTMS + c NSI d Full ms2 1396.5536@hcd30.00 [120.0000-5720.7944]
Precursor Scan: 064469, m/z: 1395.5512, charge: 4
QDQCIYNTTYLNVQR_7_6_1_4_0

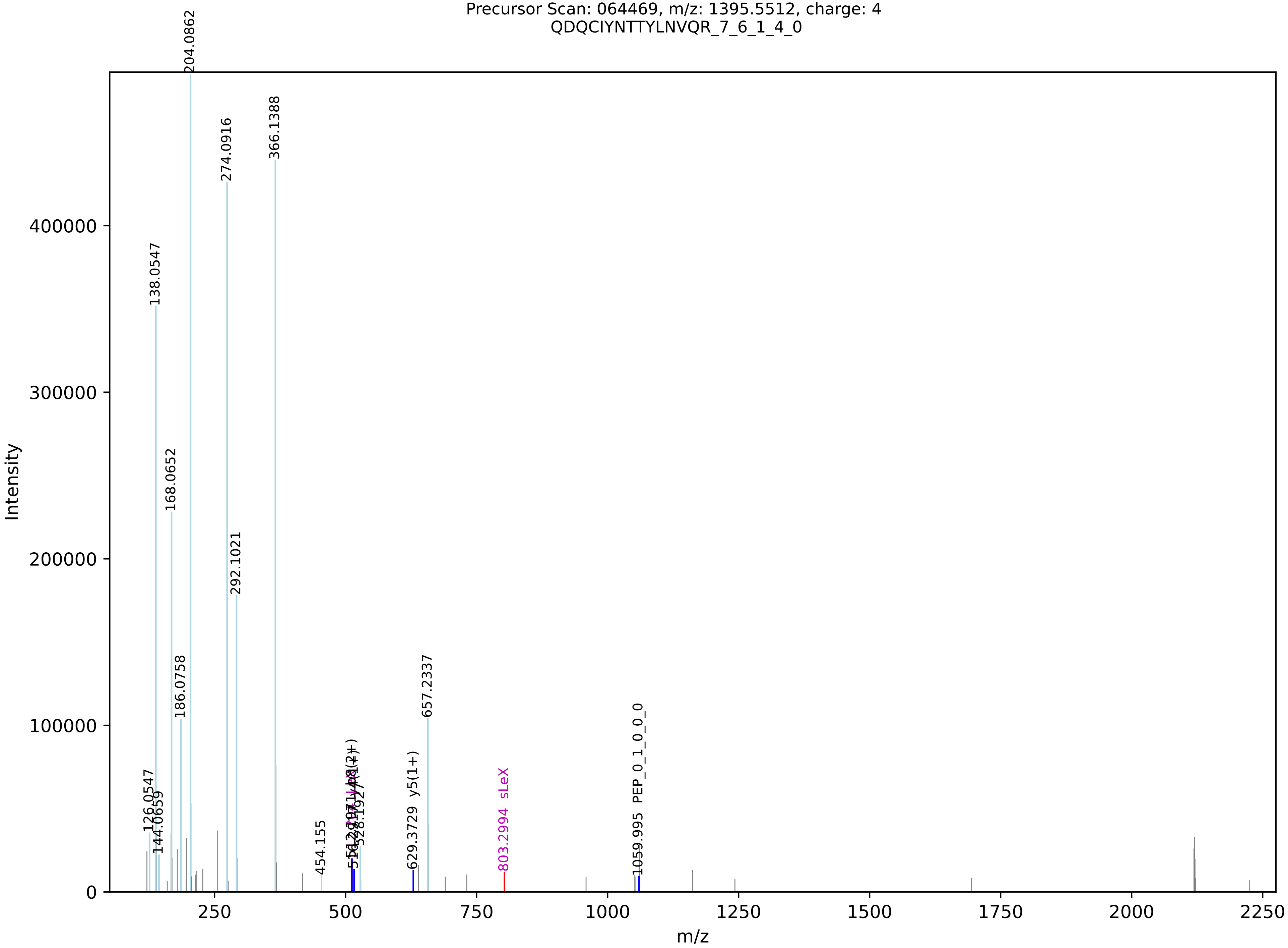

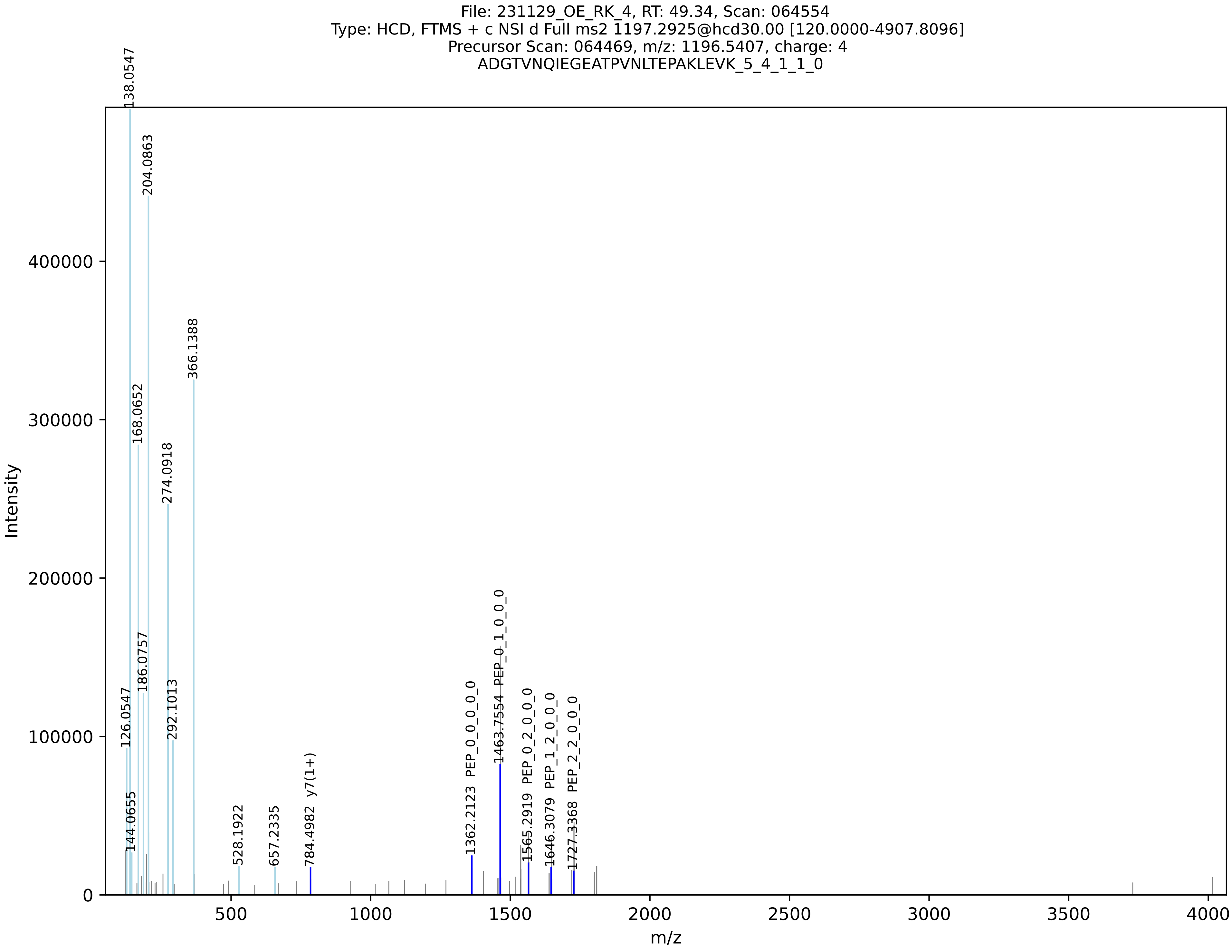
File: 231129_OE_RK_4, RT: 49.34, Scan: 064554
Type: HCD, FTMS + c NSI d Full ms2 1197.2925@hcd30.00 [120.0000-4907.8096]
Precursor Scan: 064469, m/z: 1196.5407, charge: 4
ADGTVNQIEGEATPVNLTEPAKLEVK_5_4_1_1_0
138.0547
204.0863
366.1388
168.0652
274.0918
186.0757
126.0547
292.1013
144.0655
528.1922
657.2335
784.4982 y7(1+)
1362.2123 PEP_0_0_0_0_0
1463.7554 PEP_0_1_0_0_0
1565.2919 PEP_0_2_0_0_0
1646.3079 PEP_1_2_0_0_0
1727.3368 PEP_2_2_0_0_0
Intensity
m/z
0
100000
200000
300000
400000
500
1000
1500
2000
2500
3000
3500
4000

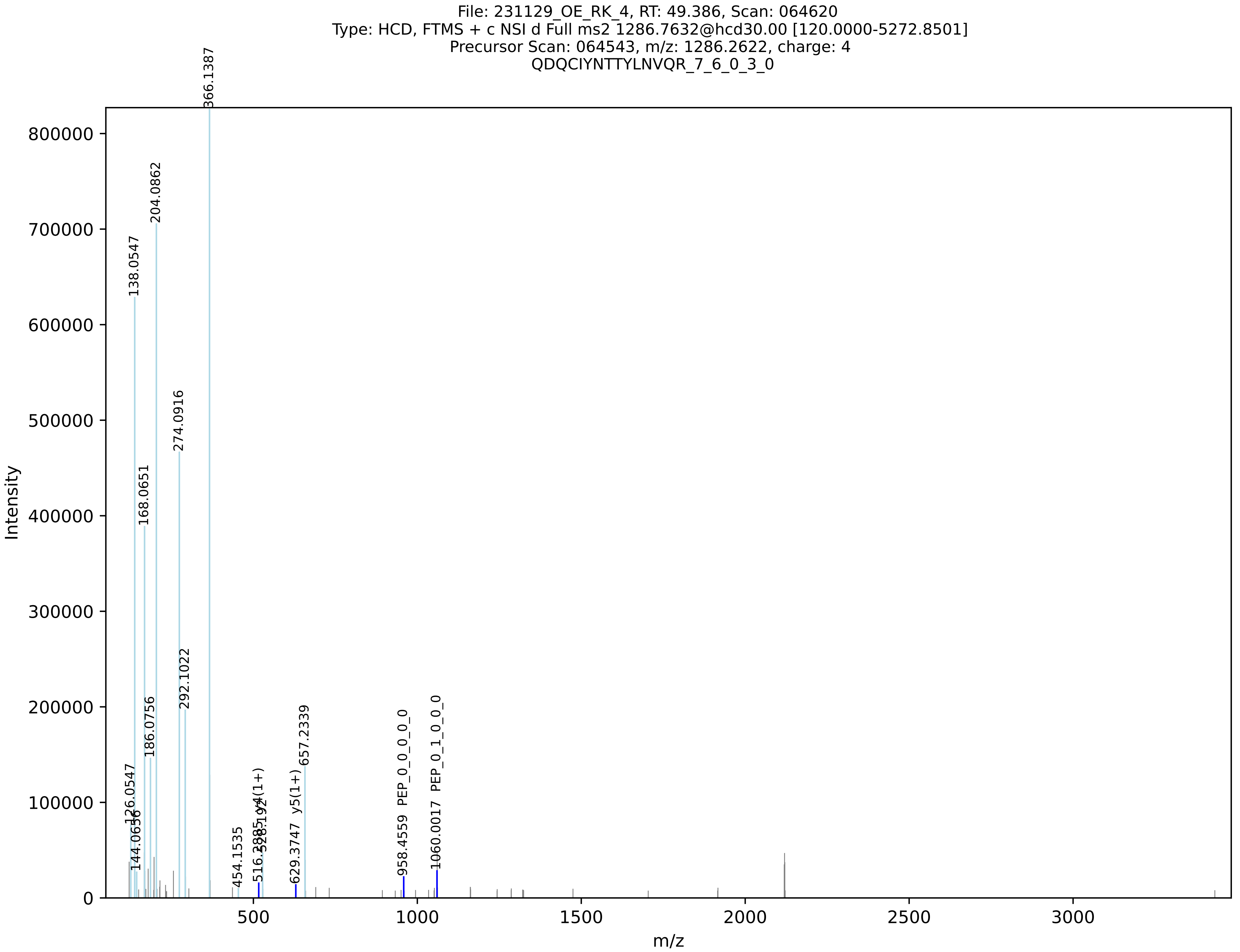

File: 231129_OE_RK_4, RT: 49.386, Scan: 064620
Type: HCD, FTMS + c NSI d Full ms2 1286.7632@hcd30.00 [120.0000-5272.8501]
Precursor Scan: 064543, m/z: 1286.2622, charge: 4
QDQCIYNTTYLNVQR_7_6_0_3_0
Intensity
m/z
366.1387
204.0862
138.0547
274.0916
168.0651
292.1022
186.0756
126.0547
144.0656
454.1535
516.2885 y4(1+)
528.192
629.3747 y5(1+)
657.2339
958.4559 PEP_0_0_0_0_0
1060.0017 PEP_0_1_0_0_0

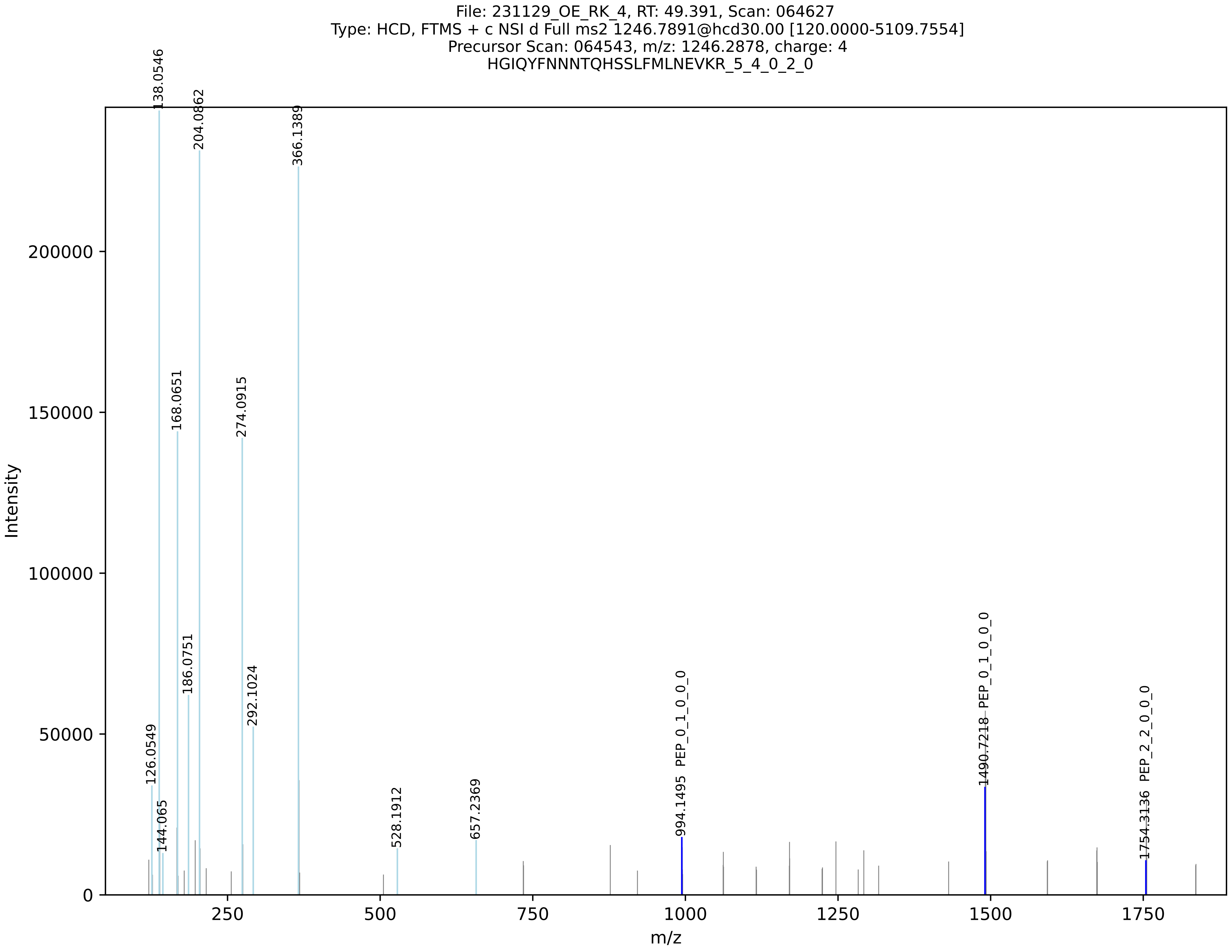
File: 231129_OE_RK_4, RT: 49.391, Scan: 064627
Type: HCD, FTMS + c NSI d Full ms2 1246.7891@hcd30.00 [120.0000-5109.7554]
Precursor Scan: 064543, m/z: 1246.2878, charge: 4
HGIQYFNNNTQHSSLFMLNEVKR_5_4_0_2_0
Intensity
m/z
0
50000
100000
150000
200000
250
500
750
1000
1250
1500
1750
138.0546
204.0862
366.1389
168.0651
274.0915
186.0751
292.1024
126.0549
144.065
528.1912
657.2369
994.1495 PEP_0_1_0_0_0
1490.7218 PEP_0_1_0_0_
1754.3136 PEP_2_2_0_0_0

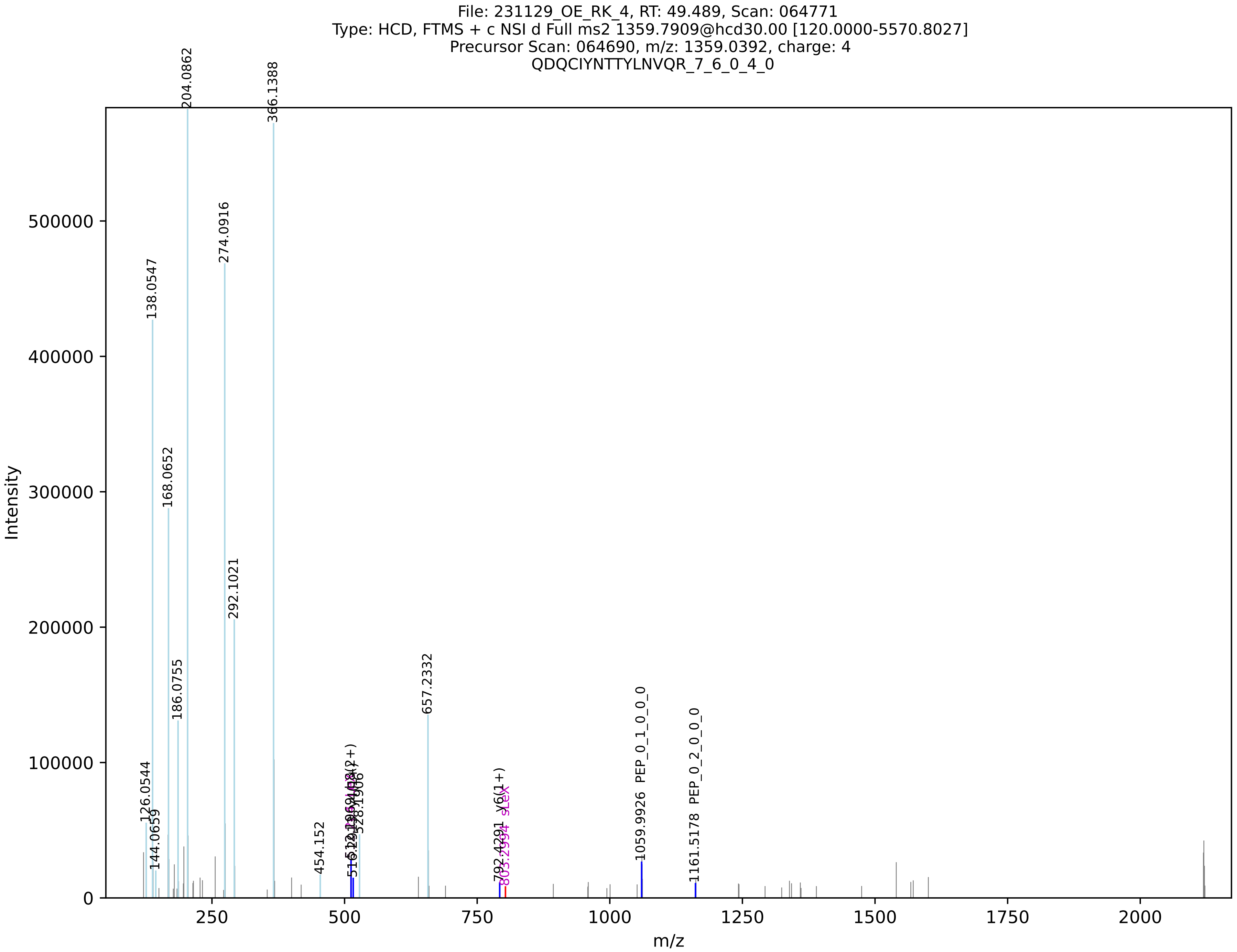
File: 231129_OE_RK_4, RT: 49.489, Scan: 064771
Type: HCD, FTMS + c NSI d Full ms2 1359.7909@hcd30.00 [120.0000-5570.8027]
Precursor Scan: 064690, m/z: 1359.0392, charge: 4
QDQCIYNTTYLNVQR_7_6_0_4_0
Intensity
m/z
204.0862
366.1388
274.0916
138.0547
168.0652
292.1021
186.0755
657.2332
126.0544
144.0659
454.152
528.1906
792.4291 y6(1+)
803.2994 sLeX
1059.9926 PEP_0_1_0_0_0_
1161.5178 PEP_0_2_0_0_0_

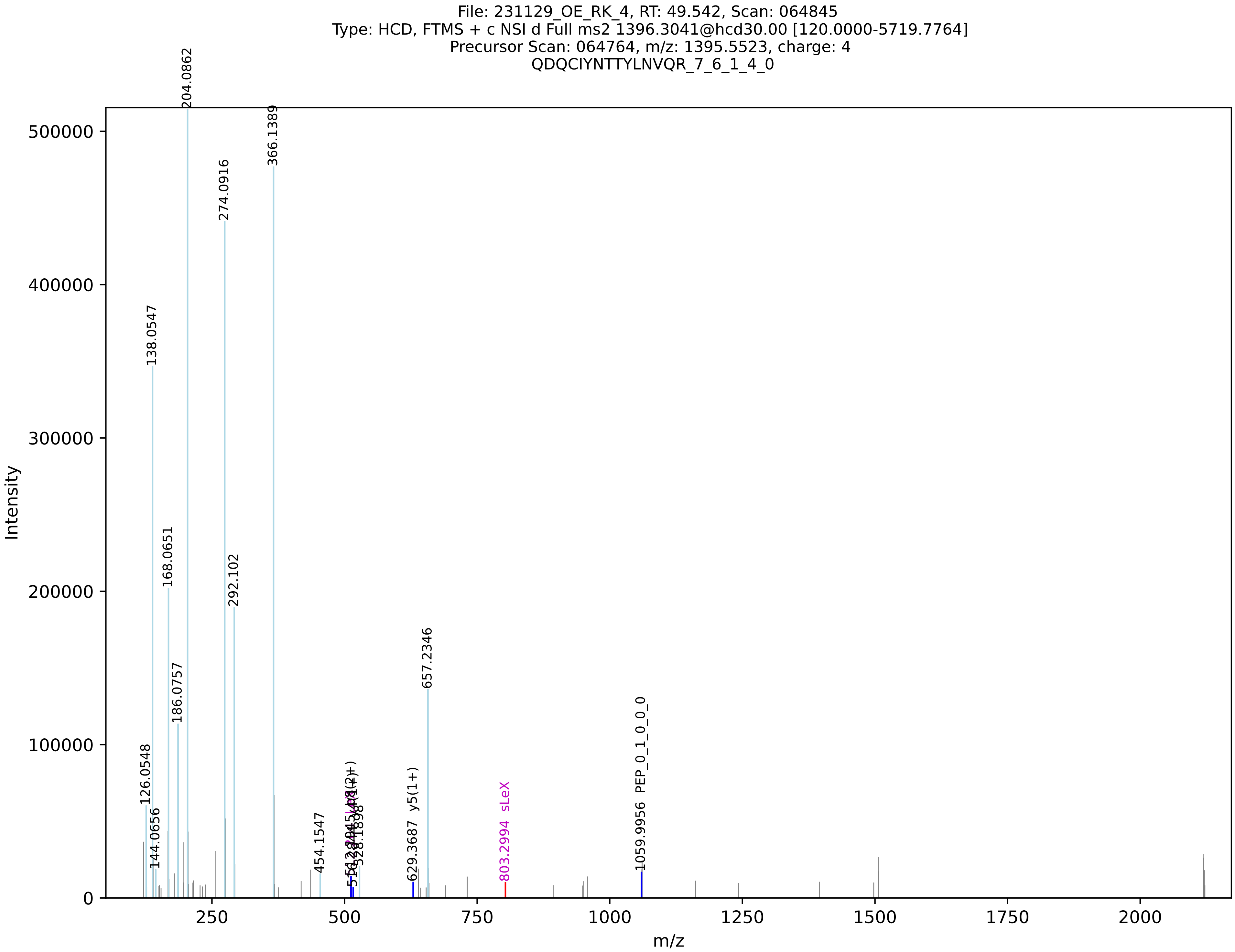
File: 231129_OE_RK_4, RT: 49.542, Scan: 064845
Type: HCD, FTMS + c NSI d Full ms2 1396.3041@hcd30.00 [120.0000-5719.7764]
Precursor Scan: 064764, m/z: 1395.5523, charge: 4
QDQCIYNTTYLNVQR_7_6_1_4_0
Intensity
m/z
204.0862
366.1389
274.0916
138.0547
168.0651
292.102
186.0757
657.2346
126.0548
144.0656
454.1547
528.1898
629.3687 y5(1+)
803.2994 sLeX
1059.9956 PEP_0_1_0_0_0
0
100000
200000
300000
400000
500000
250
500
750
1000
1250
1500
1750
2000

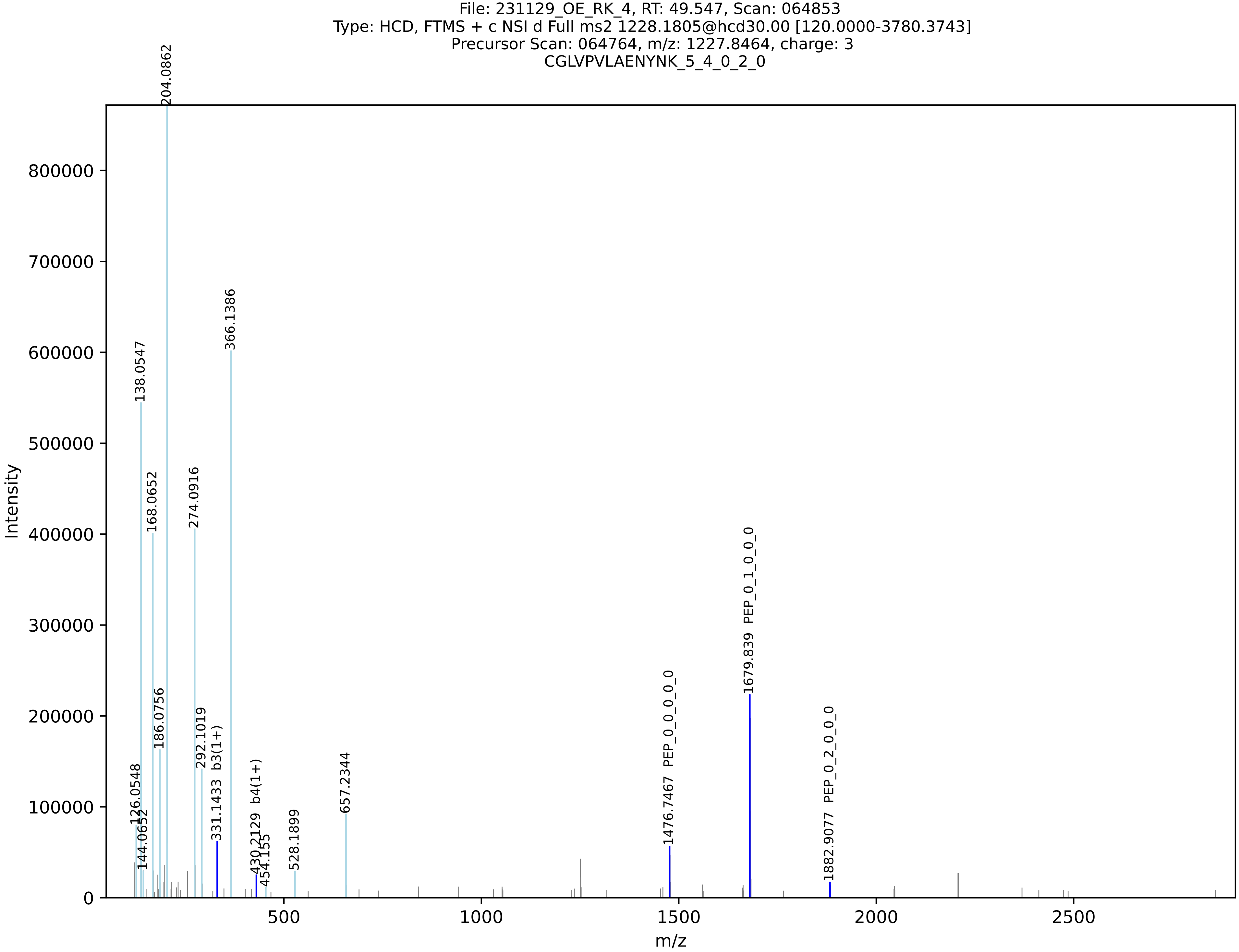
File: 231129_OE_RK_4, RT: 49.547, Scan: 064853
Type: HCD, FTMS + c NSI d Full ms2 1228.1805@hcd30.00 [120.0000-3780.3743]
Precursor Scan: 064764, m/z: 1227.8464, charge: 3
CGLVPVLAENYNK_5_4_0_2_0
Intensity
m/z
204.0862
366.1386
138.0547
168.0652
274.0916
186.0756
292.1019
331.1433 b3(1+)
126.0548
144.0652
430.2129 b4(1+)
454.155
528.1899
657.2344
1476.7467 PEP_0_0_0_0_0
1679.839 PEP_0_1_0_0_0
1882.9077 PEP_0_2_0_0_0

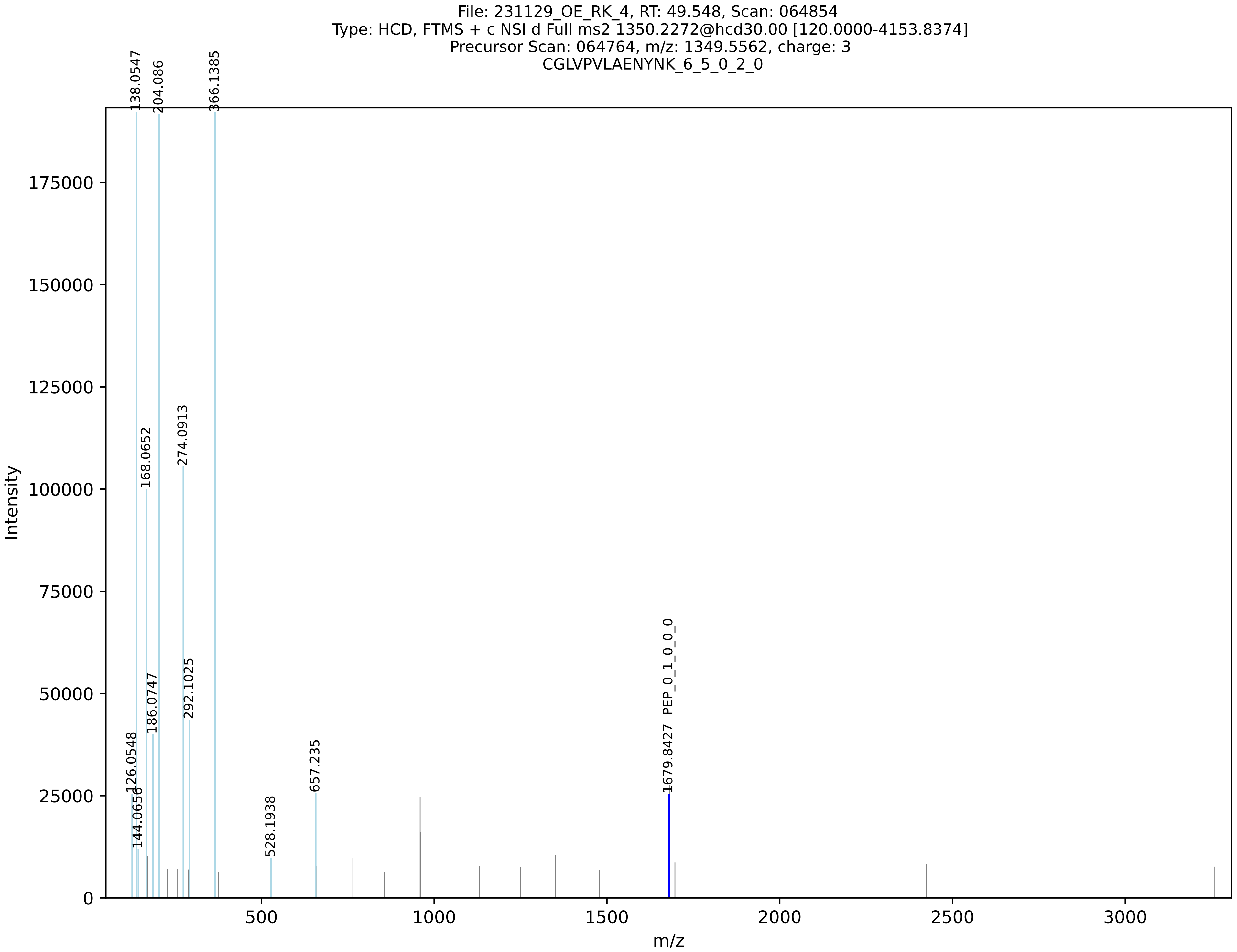
File: 231129_OE_RK_4, RT: 49.548, Scan: 064854
Type: HCD, FTMS + c NSI d Full ms2 1350.2272@hcd30.00 [120.0000-4153.8374]
Precursor Scan: 064764, m/z: 1349.5562, charge: 3
CGLVPVLAENYNK_6_5_0_2_0
138.0547
204.086
366.1385
168.0652
274.0913
186.0747
292.1025
126.0548
144.0656
528.1938
657.235
PEP_0_1_0_0_0
1679.8427
Intensity
m/z
0
25000
50000
75000
100000
125000
150000
175000
500
1000
1500
2000
2500
3000

File: 231129_OE_RK_4, RT: 49.587, Scan: 064910
Type: HCD, FTMS + c NSI d Full ms2 1286.7651@hcd30.00 [120.0000-5272.8574]
Precursor Scan: 064837, m/z: 1286.2626, charge: 4
QDQCIYNTTYLNVQR_7_6_0_3_0

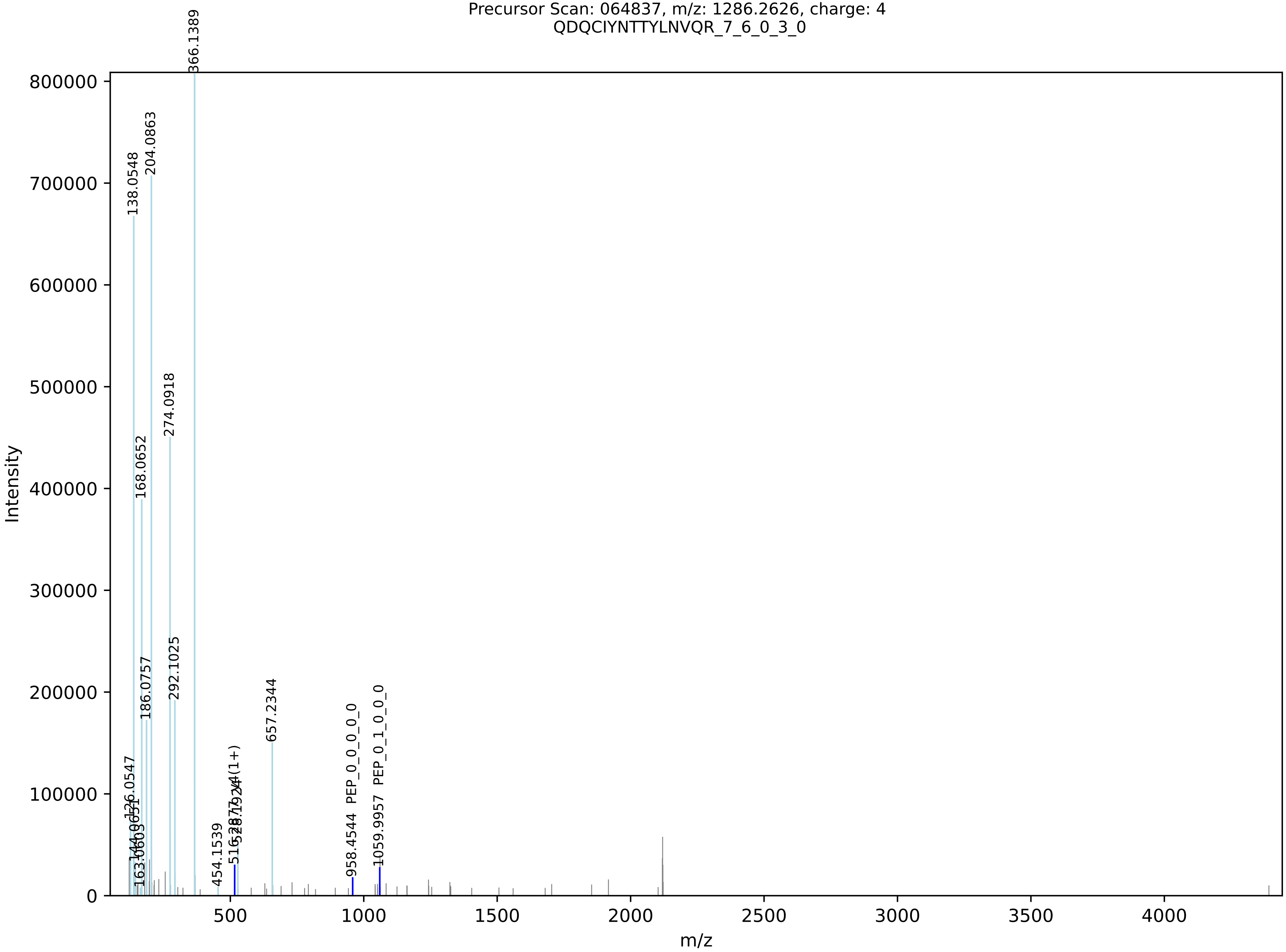

File: 231129_OE_RK_4, RT: 49.62, Scan: 064963
Type: HCD, FTMS + c NSI d Full ms2 997.6328@hcd30.00 [120.0000-5111.4976]
Precursor Scan: 064837, m/z: 997.2321, charge: 5
HGIQYFNNNTQHSSLFMLNEVKR_5_4_0_2_0

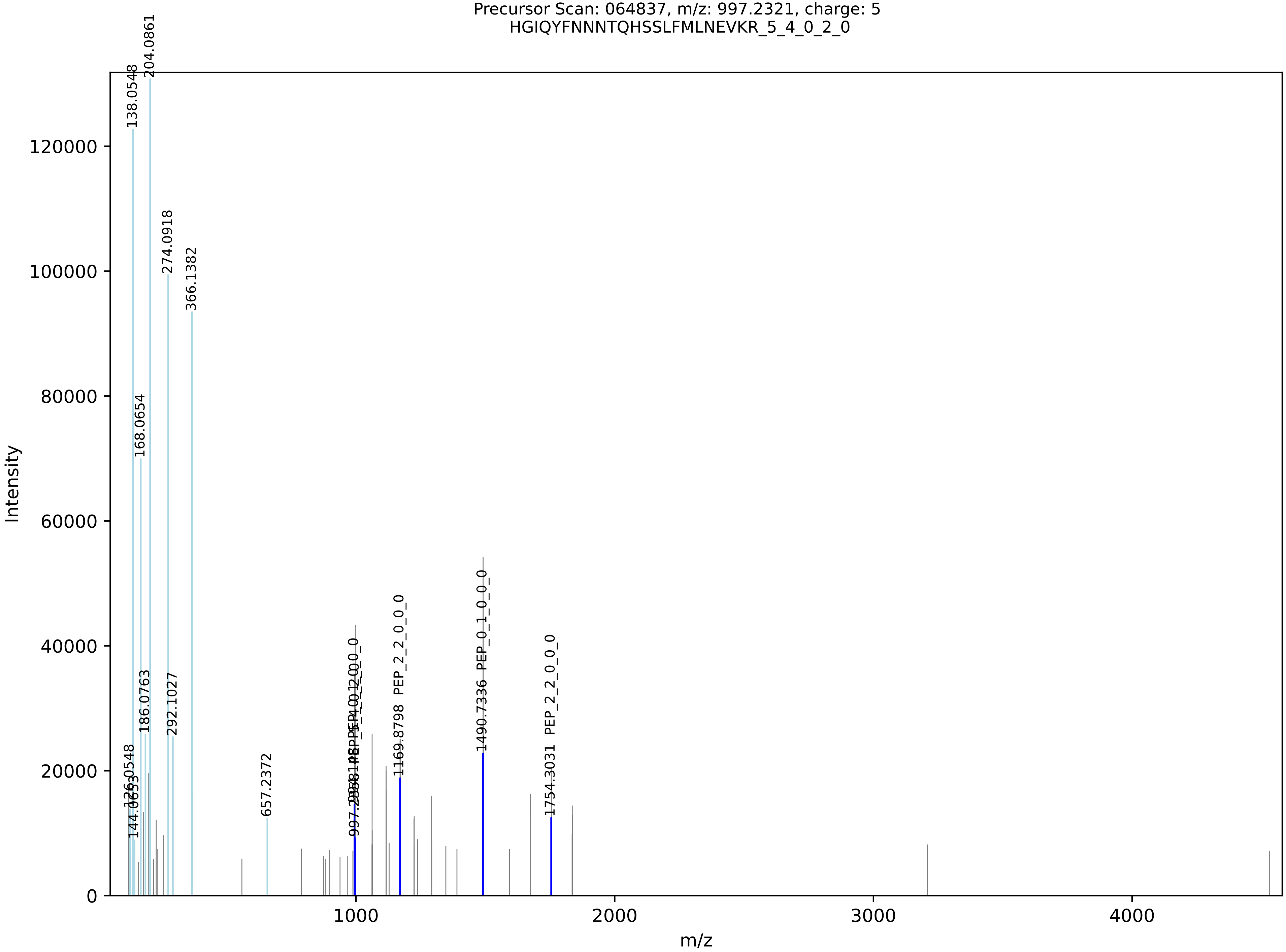

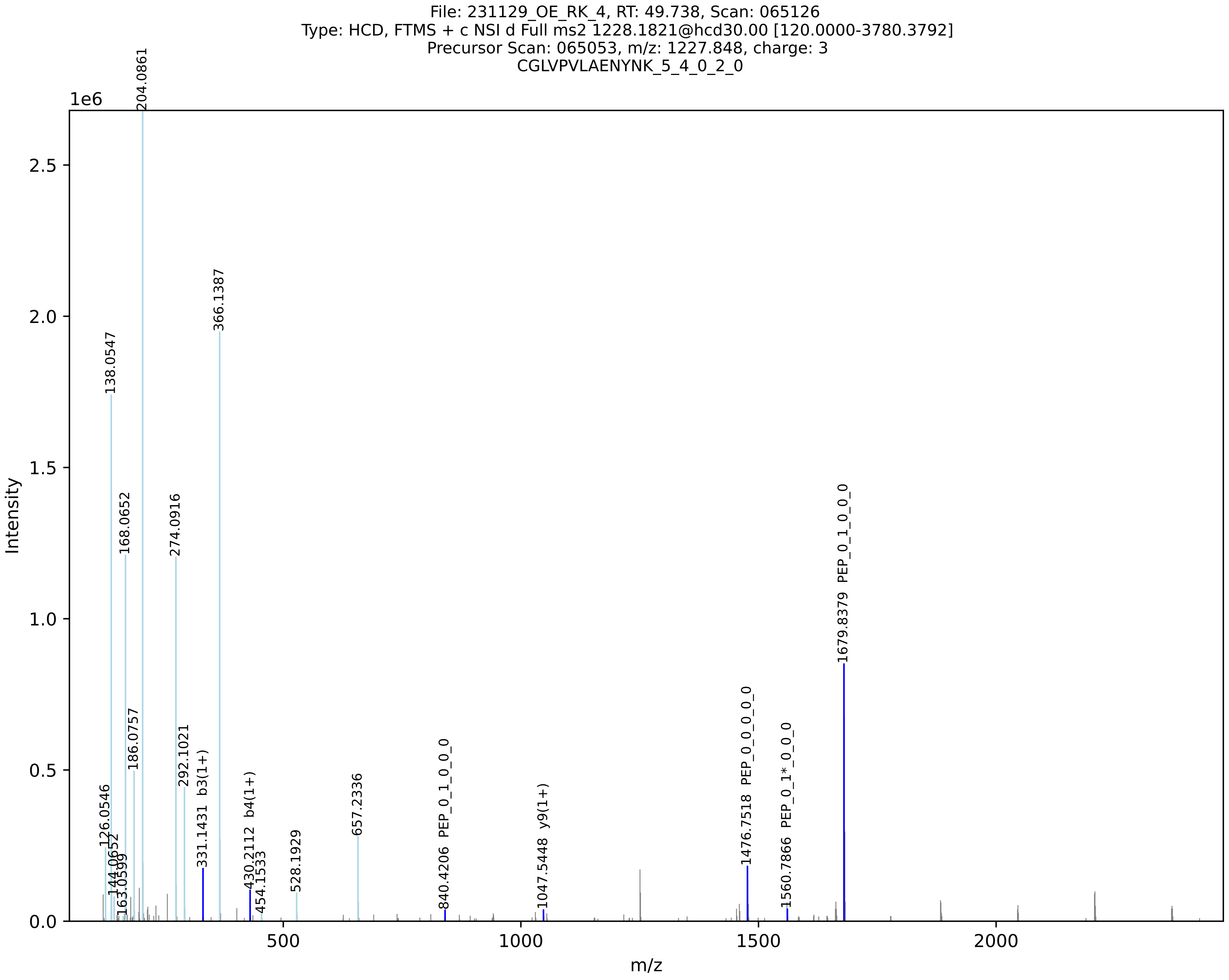
File: 231129_OE_RK_4, RT: 49.738, Scan: 065126
Type: HCD, FTMS + c NSI d Full ms2 1228.1821@hcd30.00 [120.0000-3780.3792]
Precursor Scan: 065053, m/z: 1227.848, charge: 3
CGLVPVLAENYNK_5_4_0_2_0
1e6
Intensity
m/z
204.0861
366.1387
138.0547
168.0652
274.0916
186.0757
292.1021
126.0546
144.0652
163.0599
331.1431 b3(1+)
430.2112 b4(1+)
454.1533
528.1929
657.2336
840.4206 PEP_0_1_0_0_0
1047.5448 y9(1+)
1476.7518 PEP_0_0_0_0_0
1560.7866 PEP_0_1*_0_0_0
1679.8379 PEP_0_1_0_0_0

File: 231129_OE_RK_4, RT: 49.743, Scan: 065134
Type: HCD, FTMS + c NSI d Full ms2 1396.3052@hcd30.00 [120.0000-5719.7813]
Precursor Scan: 065053, m/z: 1395.5535, charge: 4
QDQCIYNTTYLNVQR_7_6_1_4_0

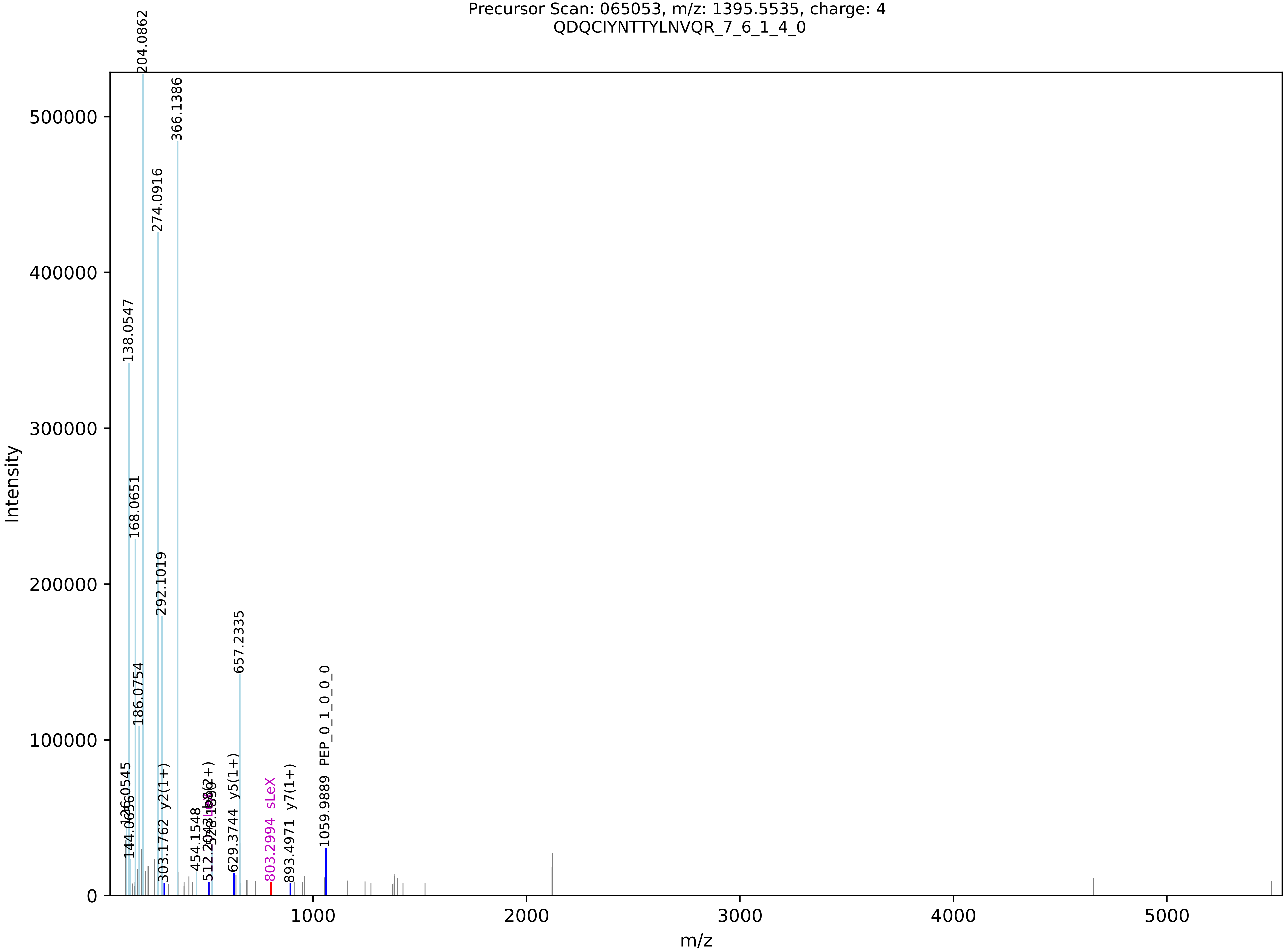

File: 231129_OE_RK_4, RT: 49.799, Scan: 065216
Type: HCD, FTMS + c NSI d Full ms2 921.6396@hcd30.00 [120.0000-3783.1458]
Precursor Scan: 065124, m/z: 921.1373, charge: 4
CGLVPVLAENYNK_5_4_0_2_0

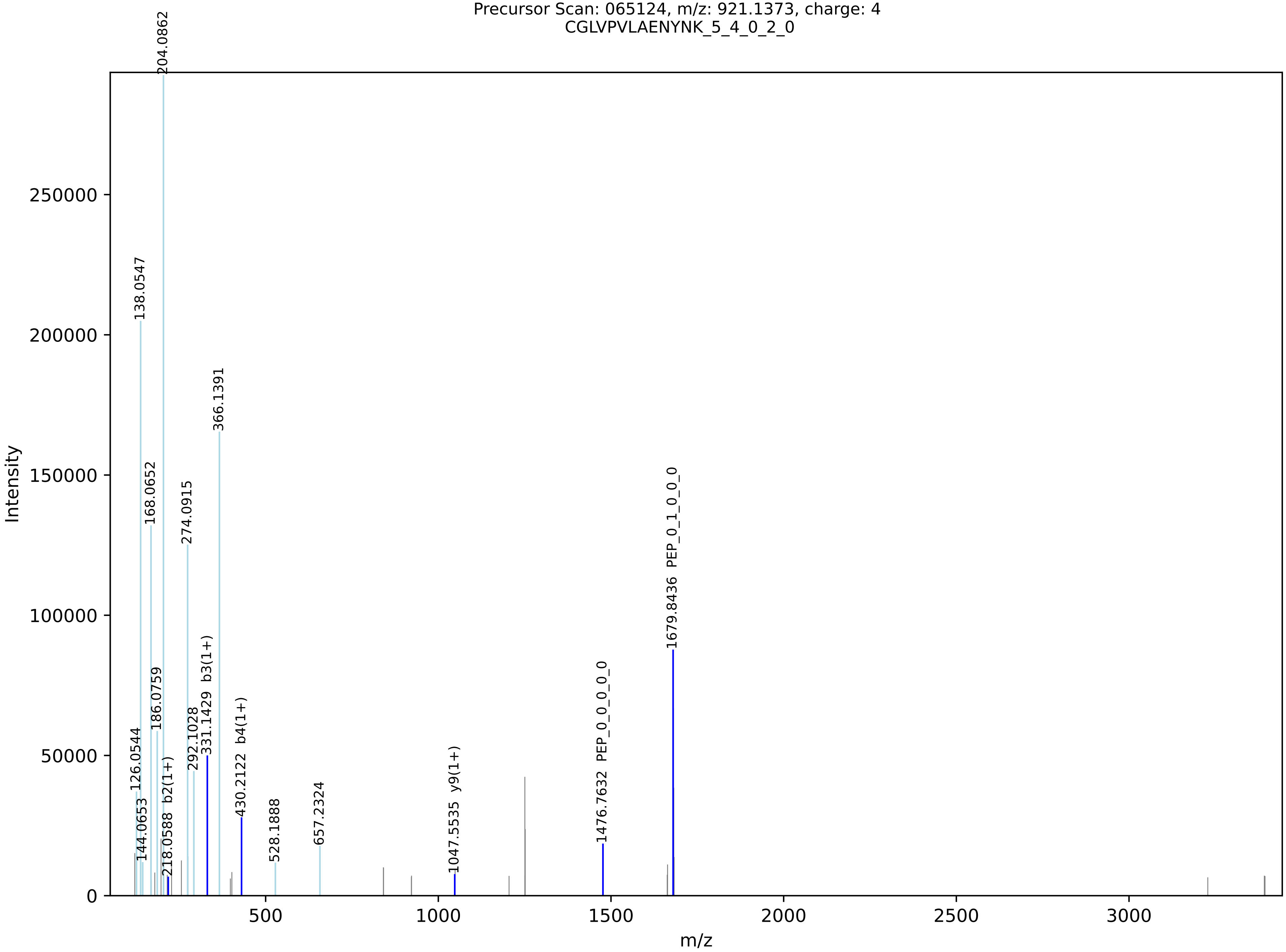

File: 231129_OE_RK_4, RT: 49.877, Scan: 065334
Type: HCD, FTMS + c NSI d Full ms2 1842.2620@hcd30.00 [120.0000-3779.6426]
Precursor Scan: 065198, m/z: 1841.2672, charge: 2
CGLVPVLAENYNK_5_4_2_1_0

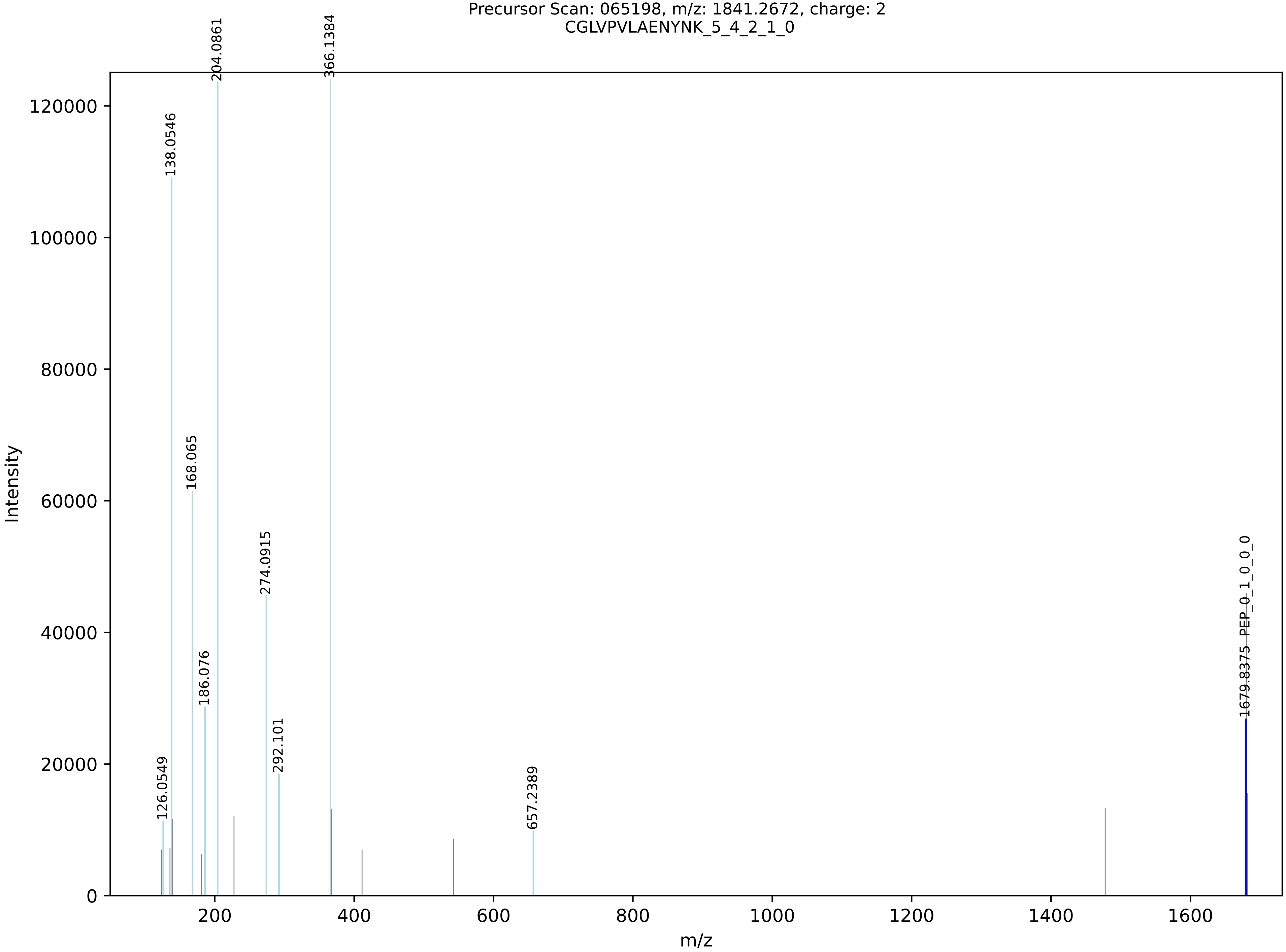

File: 231129_OE_RK_4, RT: 49.991, Scan: 065494
Type: HCD, FTMS + c NSI d Full ms2 1310.8892@hcd30.00 [120.0000-4033.4629]
Precursor Scan: 065416, m/z: 1310.2206, charge: 3
VIDFNCTTSSVSSALANTK_5_4_0_1_0

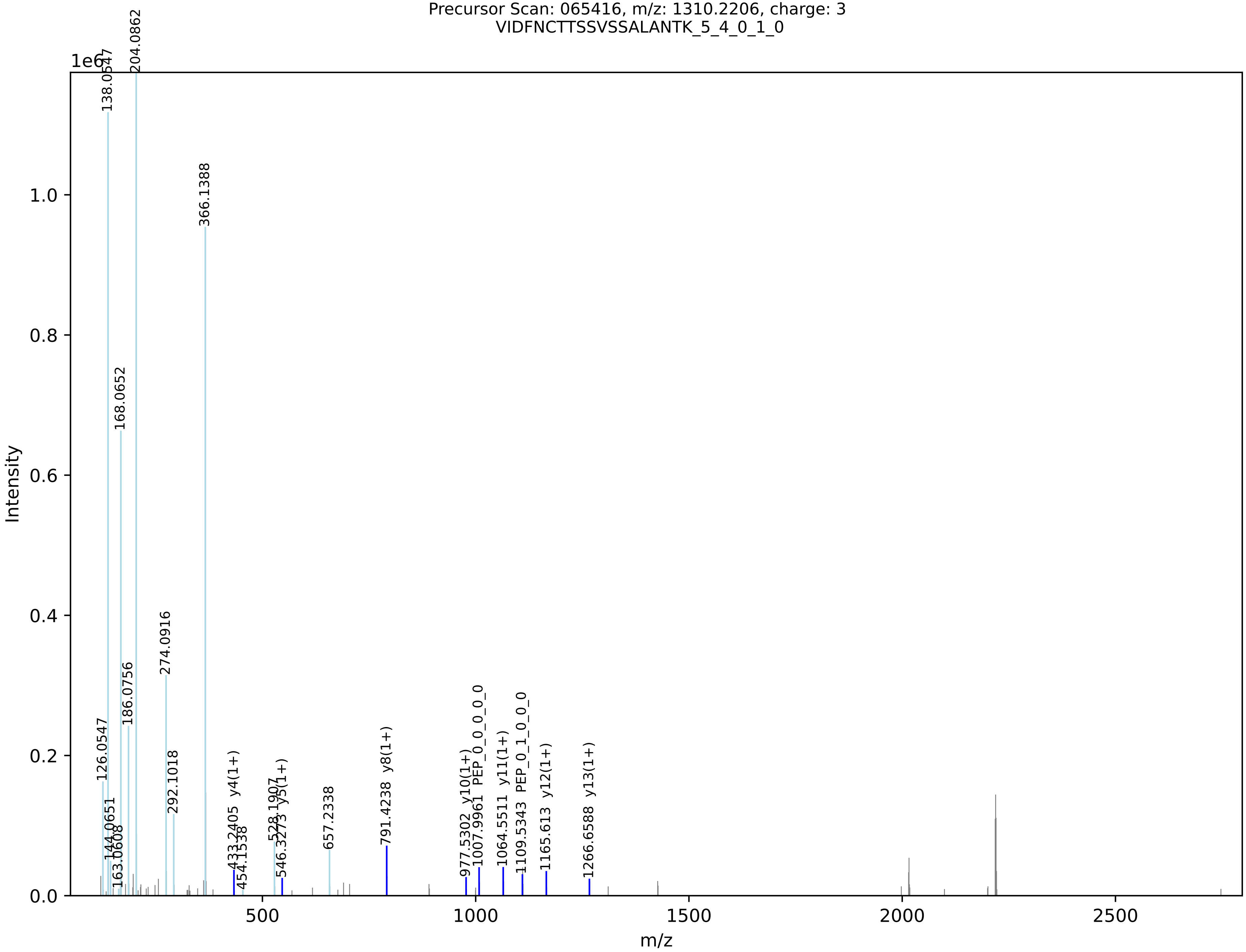

File: 231129_OE_RK_4, RT: 49.993, Scan: 065496
Type: HCD, FTMS + c NSI d Full ms2 1377.2745@hcd30.00 [120.0000-4236.6021]
Precursor Scan: 065416, m/z: 1376.6063, charge: 3
TVLTPATNHMGNVTFTIPANR_6_3_0_1_0

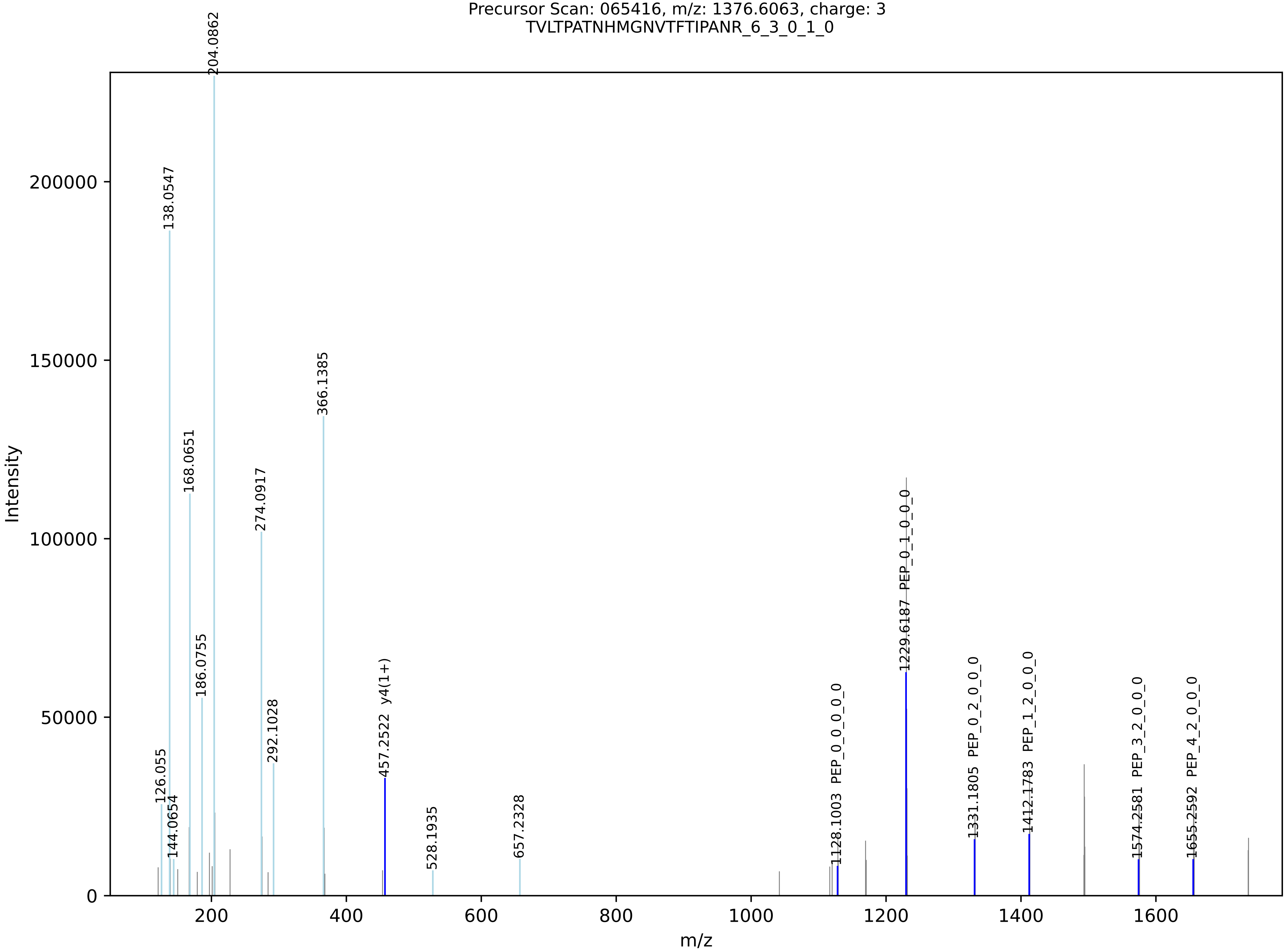

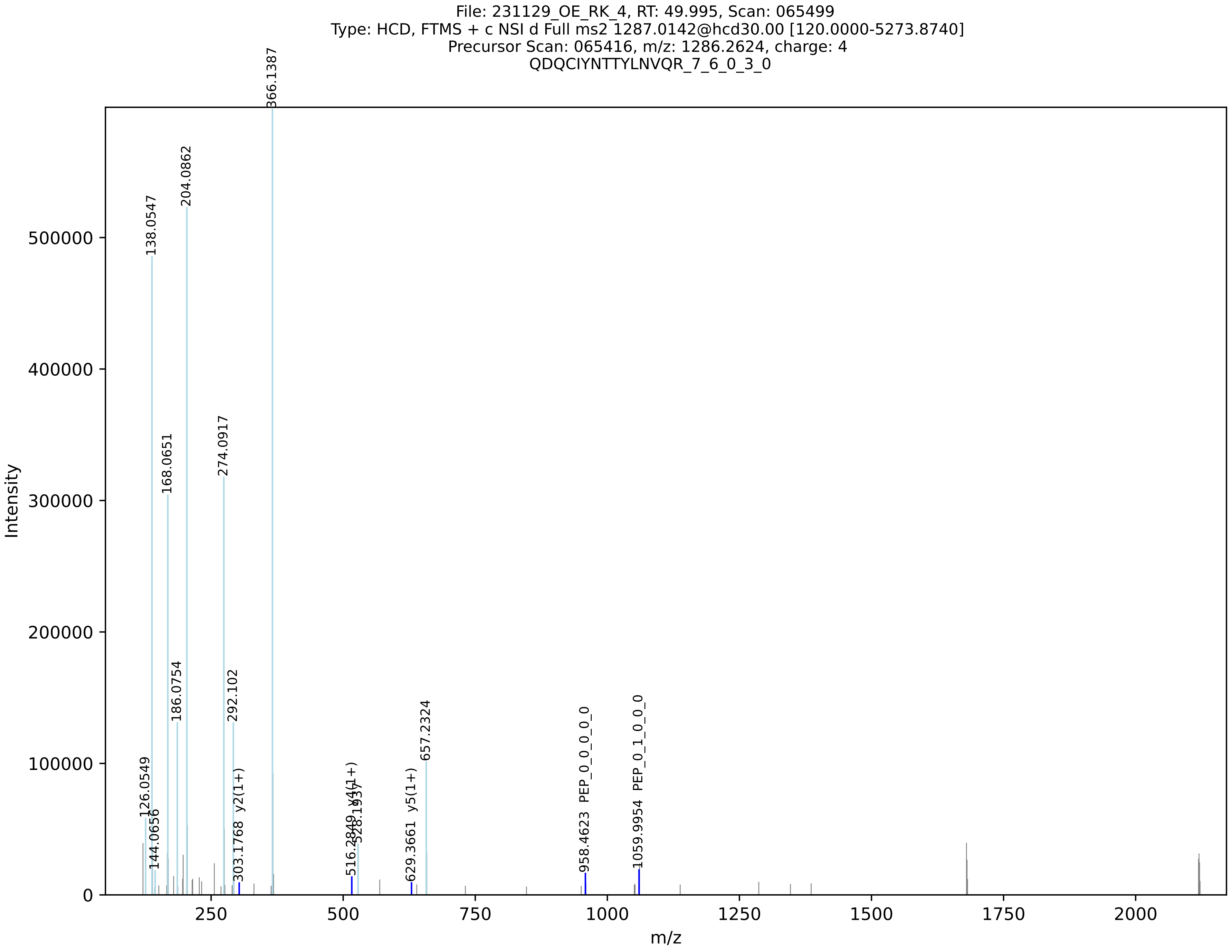

File: 231129_OE_RK_4, RT: 49.995, Scan: 065499
Type: HCD, FTMS + c NSI d Full ms2 1287.0142@hcd30.00 [120.0000-5273.8740]
Precursor Scan: 065416, m/z: 1286.2624, charge: 4
QDQCIYNTTYLNVQR_7_6_0_3_0
Intensity
m/z
366.1387
204.0862
138.0547
168.0651
274.0917
186.0754
292.102
126.0549
144.0656
303.1768 y2(1+)
516.2849 y4(1+)
528.1937
629.3661 y5(1+)
657.2324
958.4623 PEP_0_0_0_0_0_
1059.9954 PEP_0_1_0_0_0

File: 231129_OE_RK_4, RT: 49.995, Scan: 065500
Type: HCD, FTMS + c NSI d Full ms2 921.3860@hcd30.00 [120.0000-3782.1111]
Precursor Scan: 065416, m/z: 921.136, charge: 4
CGLVPVLAENYNK_5_4_0_2_0

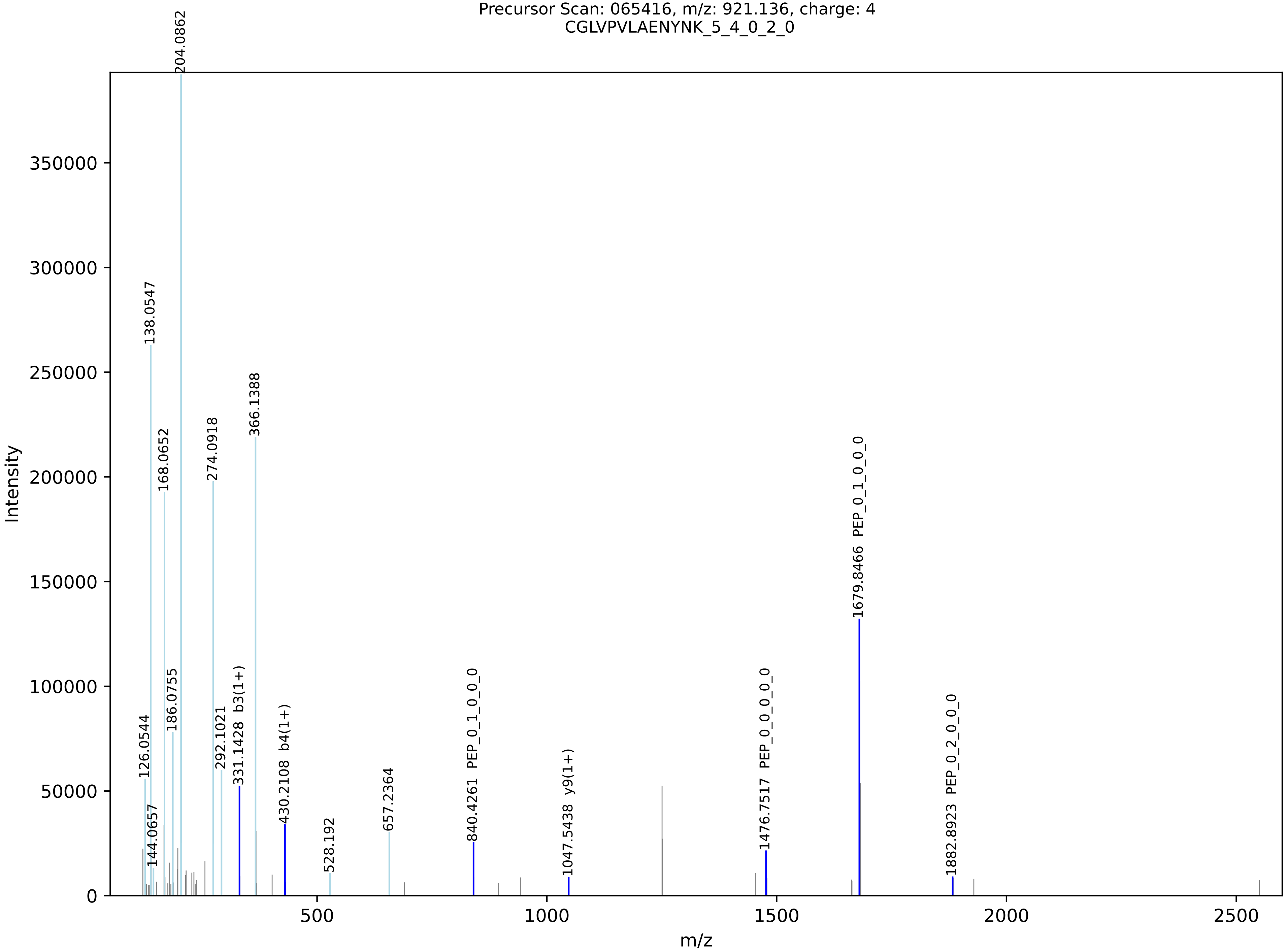

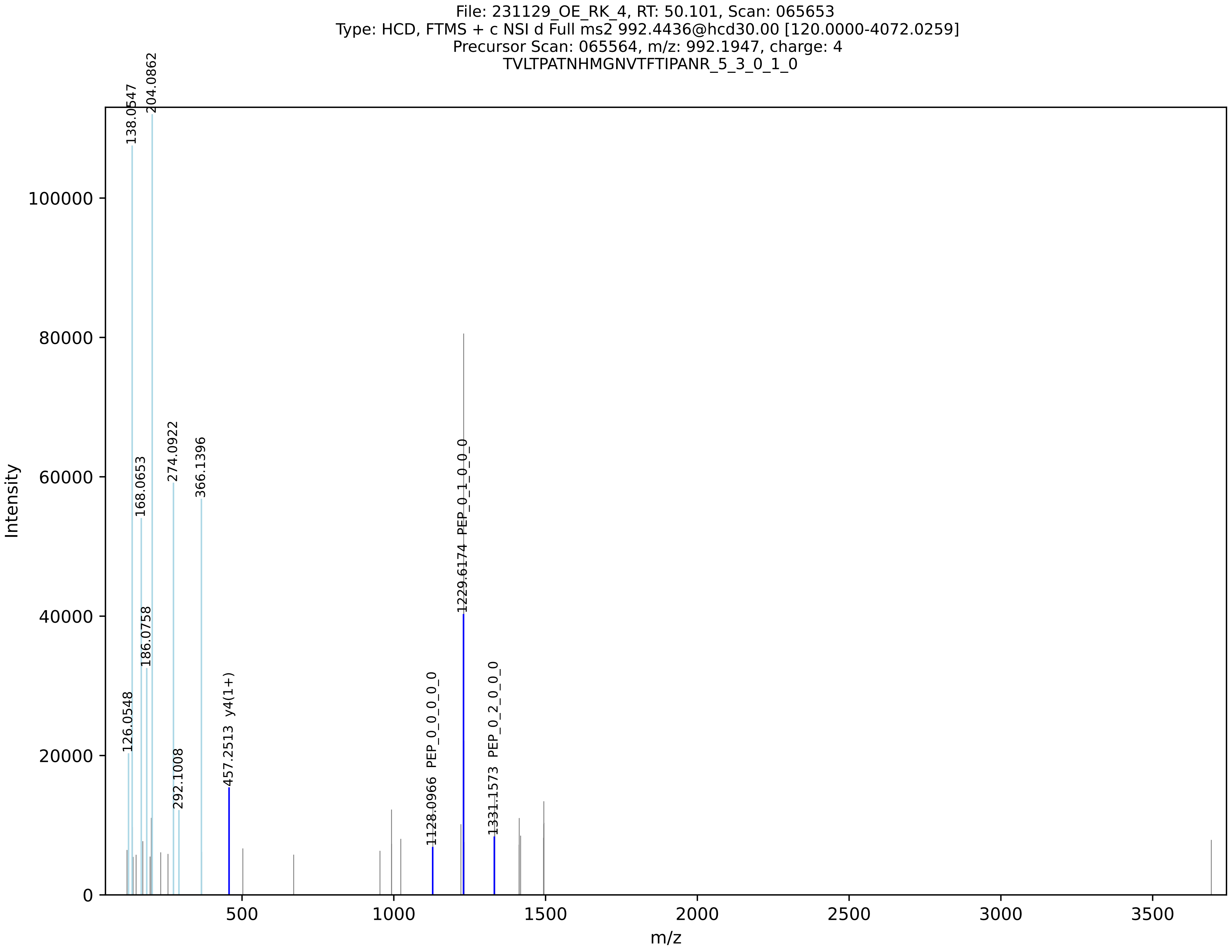
File: 231129_OE_RK_4, RT: 50.101, Scan: 065653
Type: HCD, FTMS + c NSI d Full ms2 992.4436@hcd30.00 [120.0000-4072.0259]
Precursor Scan: 065564, m/z: 992.1947, charge: 4
TVLTPATNHMGNVTFTIPANR_5_3_0_1_0
Intensity
m/z
138.0547
204.0862
168.0653
274.0922
366.1396
186.0758
126.0548
292.1008
457.2513 y4(1+)
1128.0966 PEP_0_0_0_0_0
1229.6174 PEP_0_1_0_0_0
1331.1573 PEP_0_2_0_0_0
0
20000
40000
60000
80000
100000
500
1000
1500
2000
2500
3000
3500

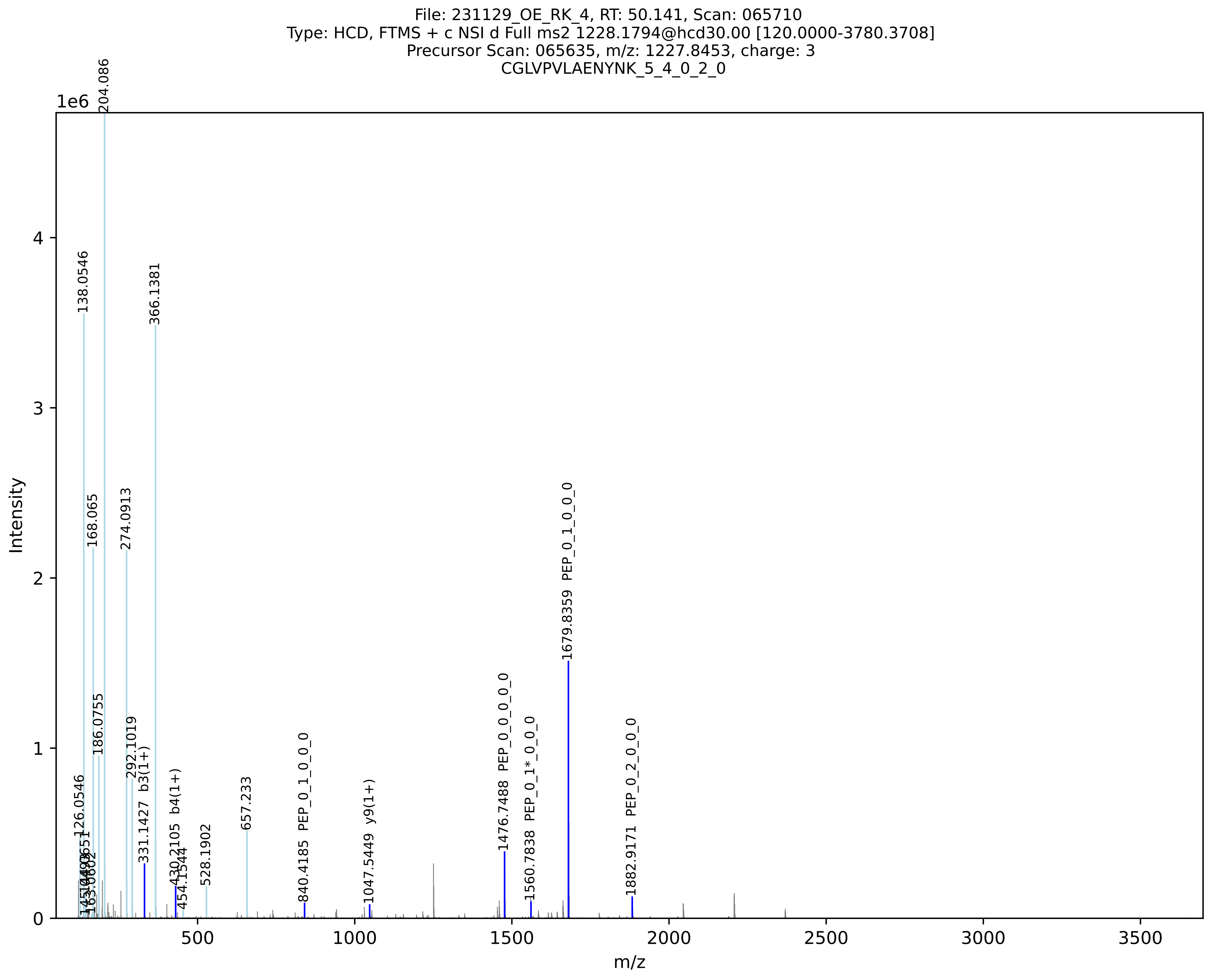
File: 231129_OE_RK_4, RT: 50.141, Scan: 065710
Type: HCD, FTMS + c NSI d Full ms2 1228.1794@hcd30.00 [120.0000-3780.3708]
Precursor Scan: 065635, m/z: 1227.8453, charge: 3
CGLVPVLAENYNK_5_4_0_2_0
1e6
Intensity
m/z
204.086
138.0546
366.1381
168.065
274.0913
186.0755
292.1019
331.1427 b3(1+)
430.2105 b4(1+)
454.1544
528.1902
657.233
126.0546
840.4185 PEP_0_1_0_0_0
1047.5449 y9(1+)
1476.7488 PEP_0_0_0_0_0
1560.7838 PEP_0_1*_0_0_0
1679.8359 PEP_0_1_0_0_0_
1882.9171 PEP_0_2_0_0_0

File: 231129_OE_RK_4, RT: 50.156, Scan: 065735
Type: HCD, FTMS + c NSI d Full ms2 1841.7646@hcd30.00 [120.0000-3778.6277]
Precursor Scan: 065635, m/z: 1841.2643, charge: 2
CGLVPVLAENYNK_5_4_2_1_0

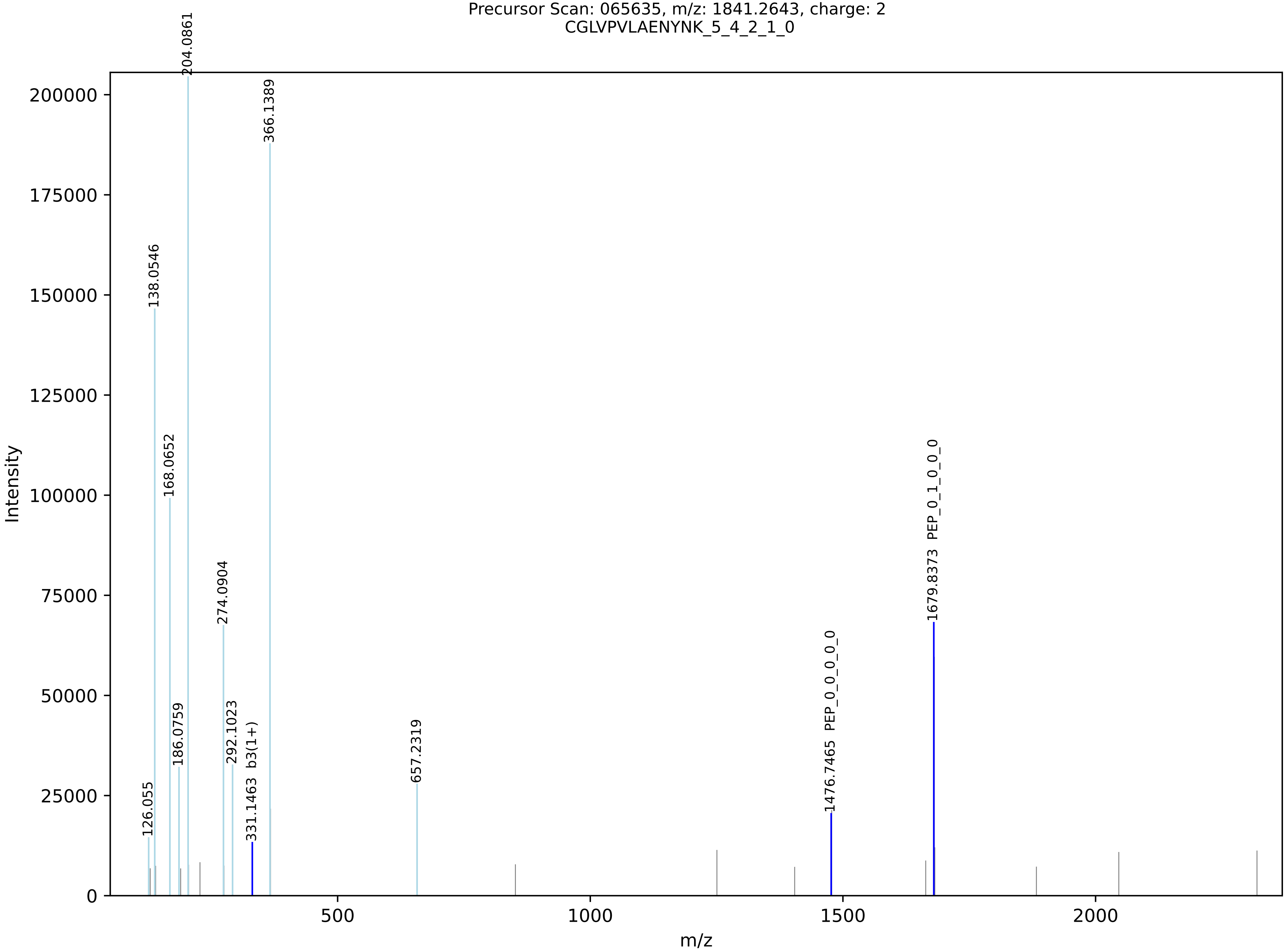

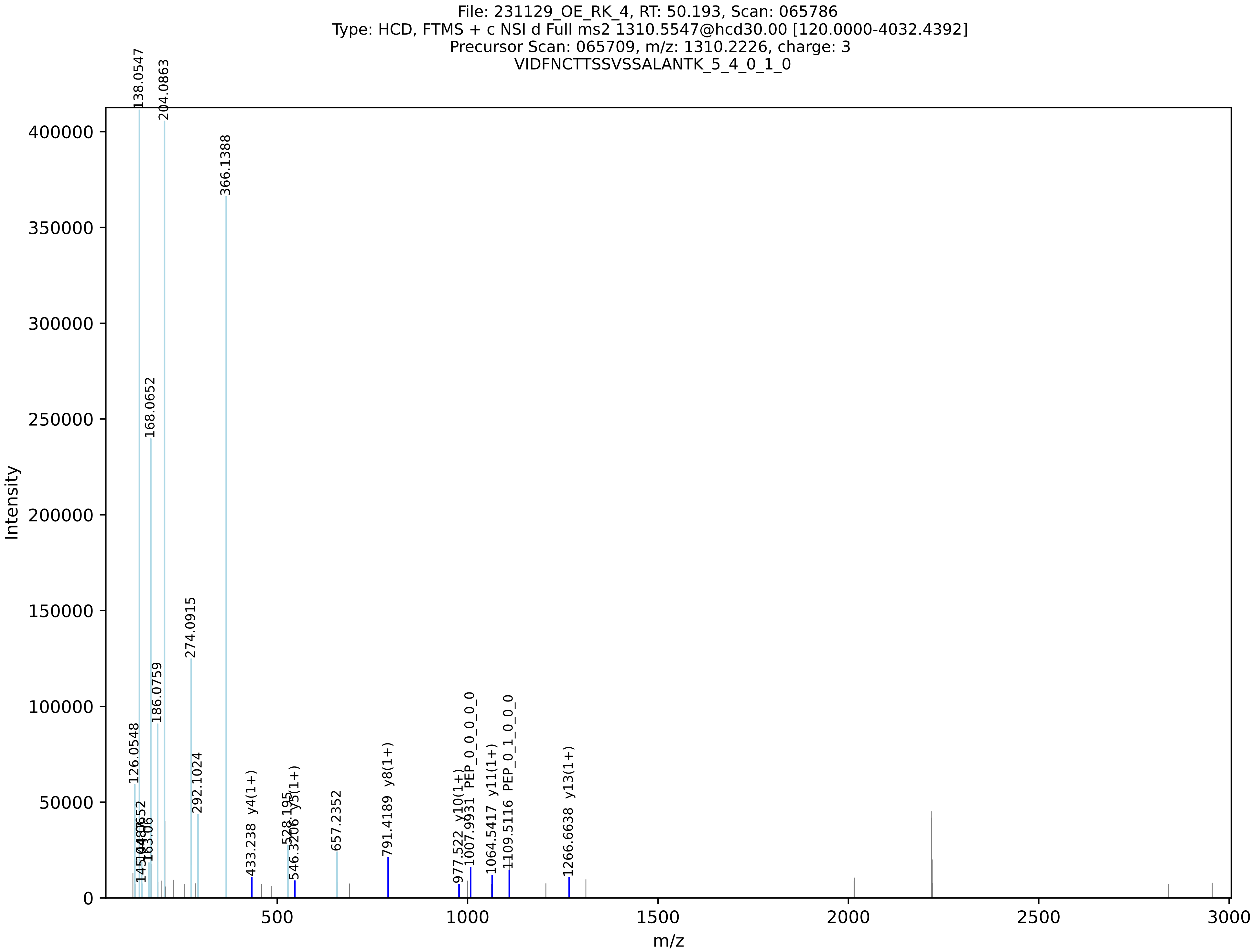
File: 231129_OE_RK_4, RT: 50.193, Scan: 065786
Type: HCD, FTMS + c NSI d Full ms2 1310.5547@hcd30.00 [120.0000-4032.4392]
Precursor Scan: 065709, m/z: 1310.2226, charge: 3
VIDFNCTTSSVSSALANTK_5_4_0_1_0
Intensity
m/z
138.0547
204.0863
366.1388
168.0652
274.0915
186.0759
126.0548
292.1024
163.06
433.238 y4(1+)
528.195
546.3206 y5(1+)
657.2352
791.4189 y8(1+)
977.522 y10(1+)
1007.9931 PEP_0_0_0_0_0
1064.5417 y11(1+)
1109.5116 PEP_0_1_0_0_0
1266.6638 y13(1+)

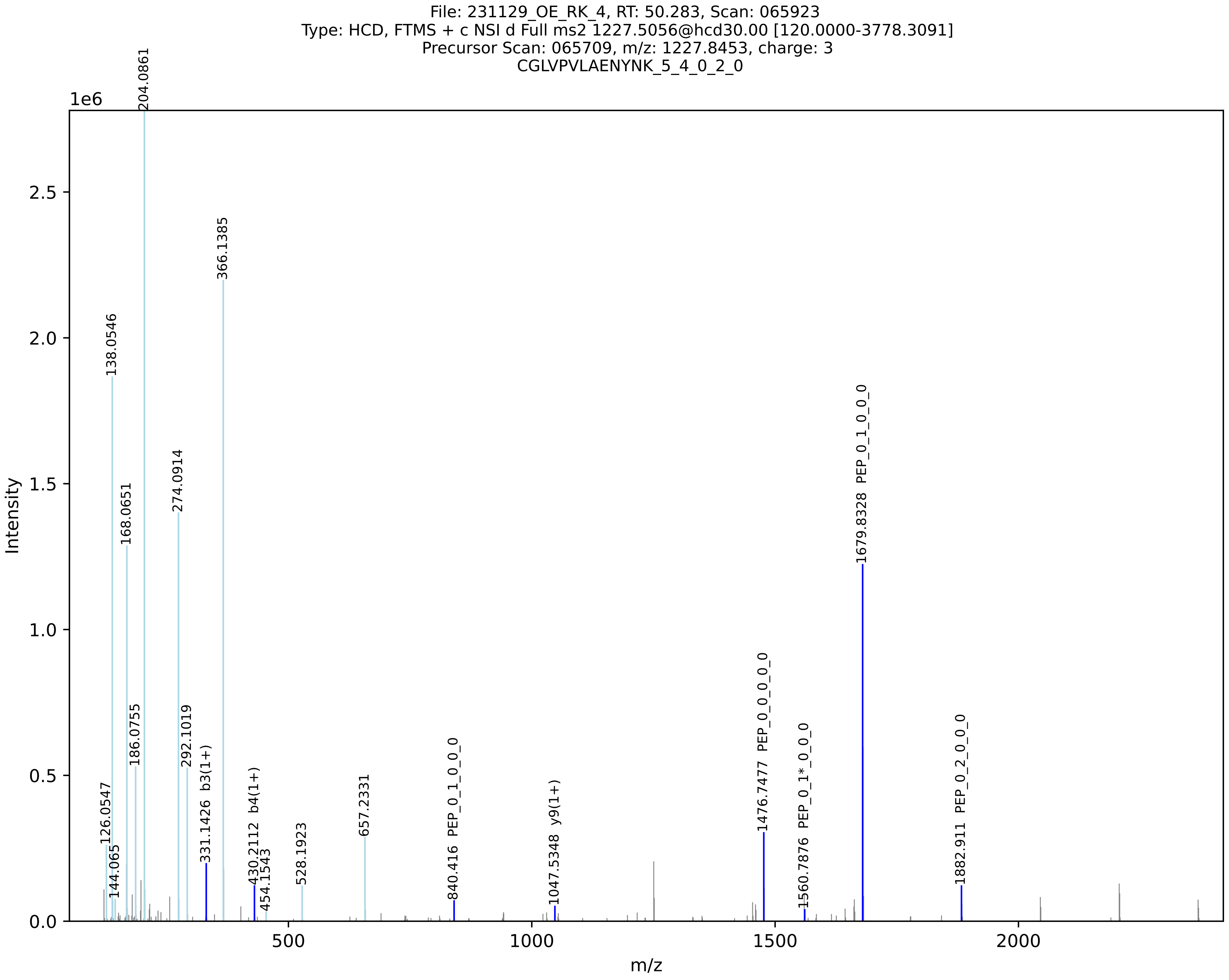

File: 231129_OE_RK_4, RT: 50.283, Scan: 065923
Type: HCD, FTMS + c NSI d Full ms2 1227.5056@hcd30.00 [120.0000-3778.3091]
Precursor Scan: 065709, m/z: 1227.8453, charge: 3
CGLVPVLAENYNK_5_4_0_2_0
1e6
Intensity
m/z
204.0861
366.1385
138.0546
274.0914
168.0651
186.0755
292.1019
126.0547
144.065
331.1426 b3(1+)
430.2112 b4(1+)
454.1543
528.1923
657.2331
840.416 PEP_0_1_0_0_0
1047.5348 y9(1+)
1476.7477 PEP_0_0_0_0_0
1560.7876 PEP_0_1*_0_0_0
1679.8328 PEP_0_1_0_0_0
1882.911 PEP_0_2_0_0_0

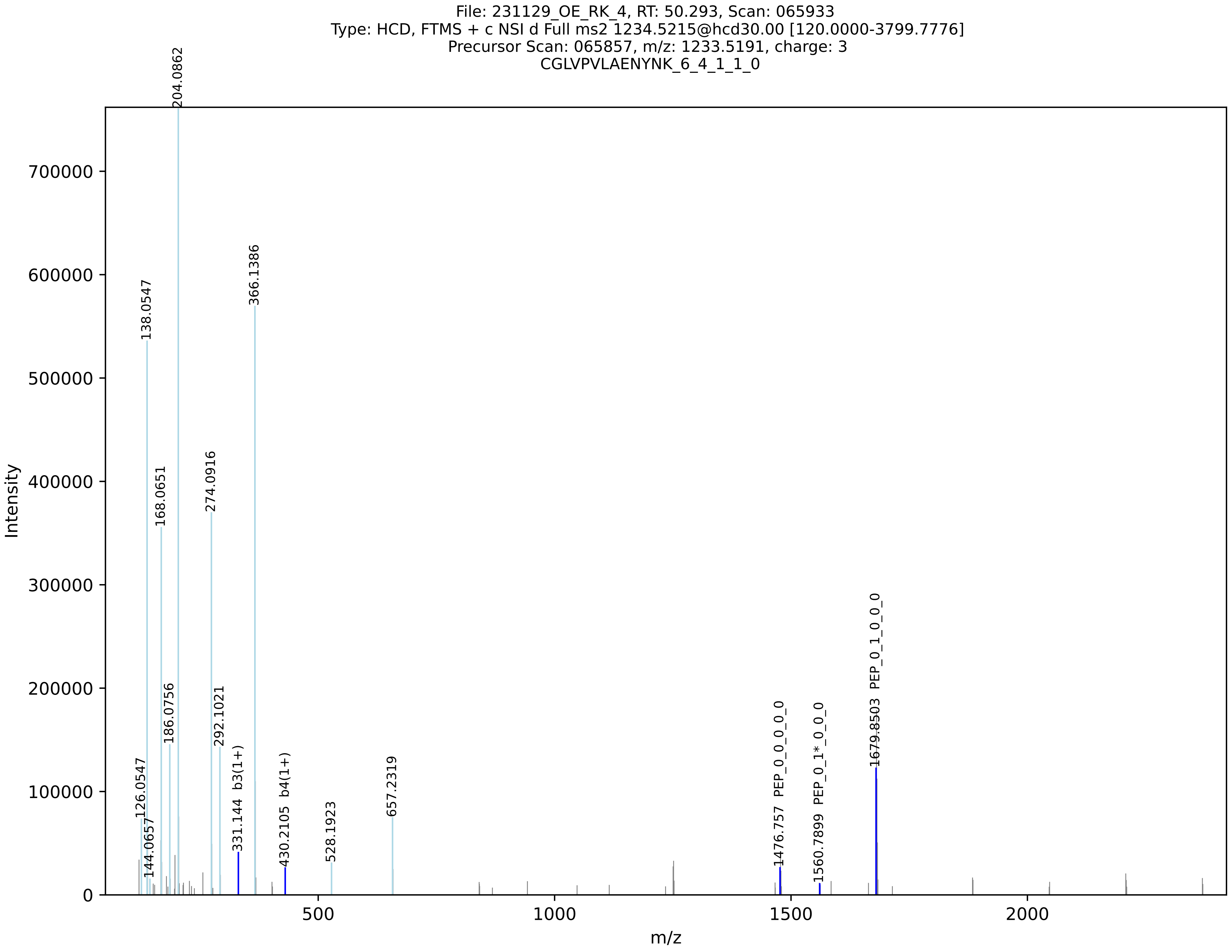
File: 231129_OE_RK_4, RT: 50.293, Scan: 065933
Type: HCD, FTMS + c NSI d Full ms2 1234.5215@hcd30.00 [120.0000-3799.7776]
Precursor Scan: 065857, m/z: 1233.5191, charge: 3
CGLVPVLAENYNK_6_4_1_1_0
204.0862
138.0547
366.1386
168.0651
274.0916
186.0756
292.1021
126.0547
144.0657
331.144 b3(1+)
430.2105 b4(1+)
528.1923
657.2319
1476.757 PEP_0_0_0_0_0_
1560.7899 PEP_0_1*_0_0_0
1679.8503 PEP_0_1_0_0_0_
Intensity
m/z
0
100000
200000
300000
400000
500000
600000
700000
500
1000
1500
2000

File: 231129_OE_RK_4, RT: 50.343, Scan: 066005
Type: HCD, FTMS + c NSI d Full ms2 1228.5135@hcd30.00 [120.0000-3781.3933]
Precursor Scan: 065930, m/z: 1227.8452, charge: 3
CGLVPVLAENYNK_5_4_0_2_0

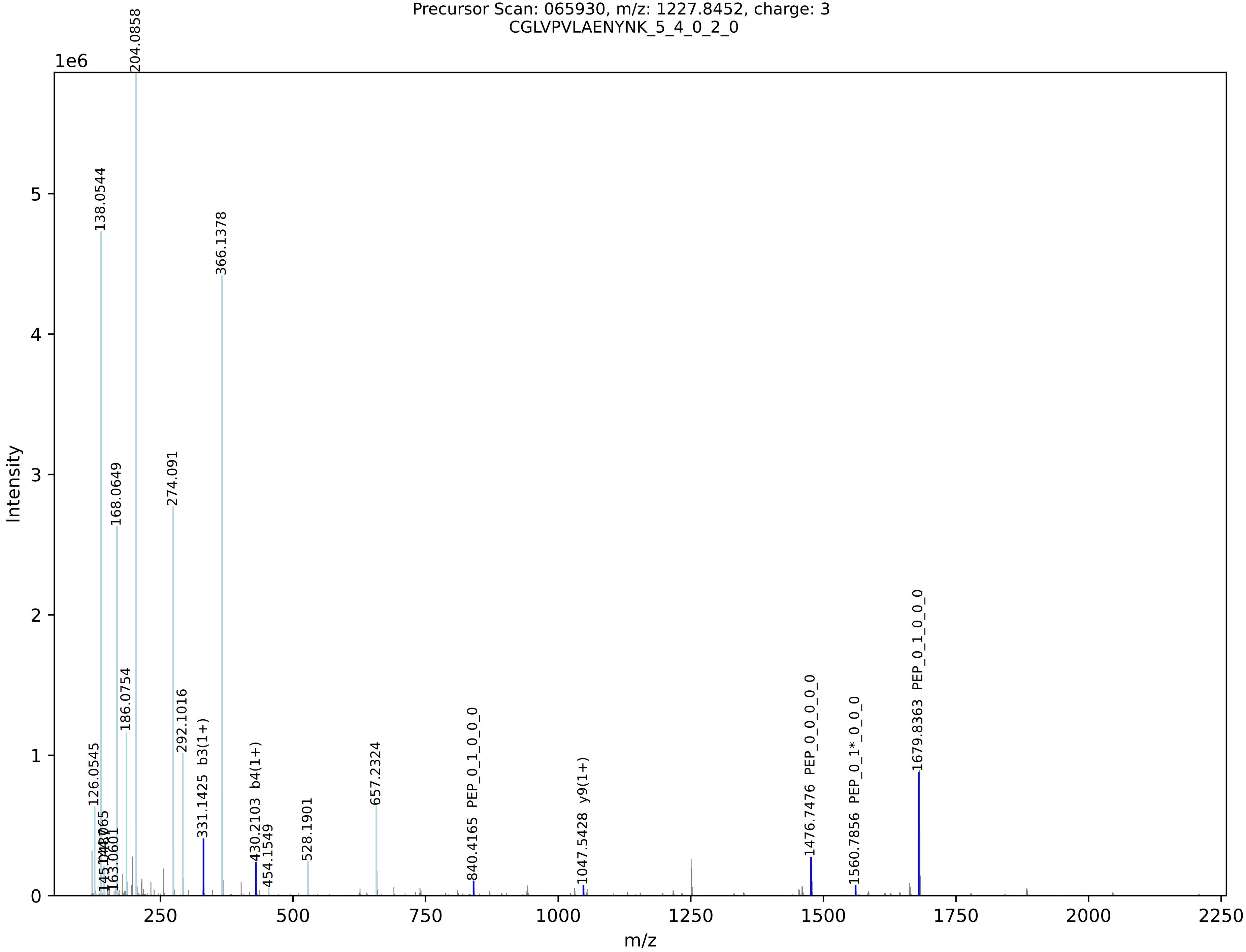

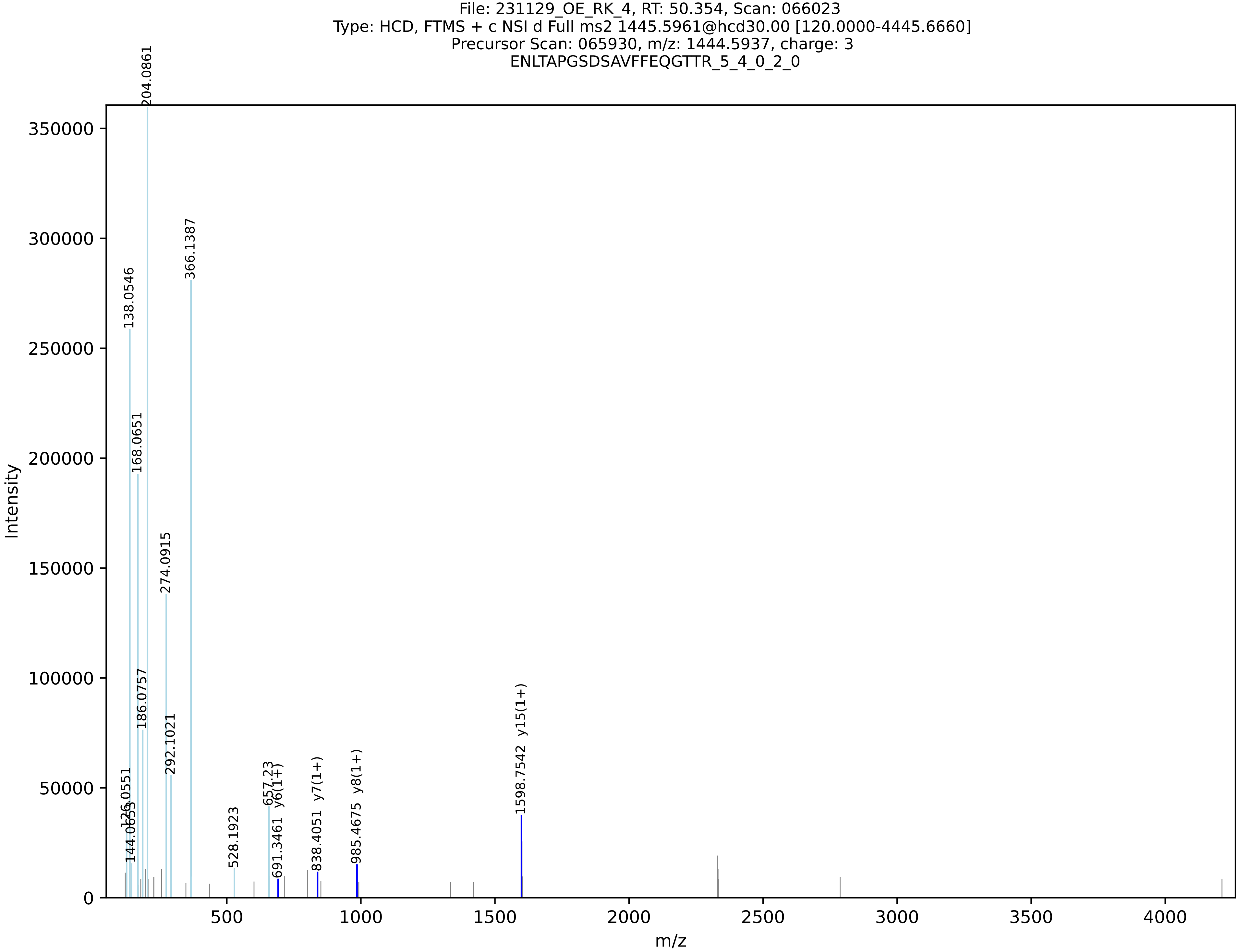
File: 231129_OE_RK_4, RT: 50.354, Scan: 066023
Type: HCD, FTMS + c NSI d Full ms2 1445.5961@hcd30.00 [120.0000-4445.6660]
Precursor Scan: 065930, m/z: 1444.5937, charge: 3
ENLTAPGSDSAVFFEQGTTR_5_4_0_2_0
Intensity
m/z
204.0861
366.1387
138.0546
168.0651
274.0915
186.0757
292.1021
126.0551
528.1923
657.23
y6(1+)
691.3461
838.4051 y7(1+)
985.4675 y8(1+)
1598.7542 y15(1+)
0
50000
100000
150000
200000
250000
300000
350000
500
1000
1500
2000
2500
3000
3500
4000

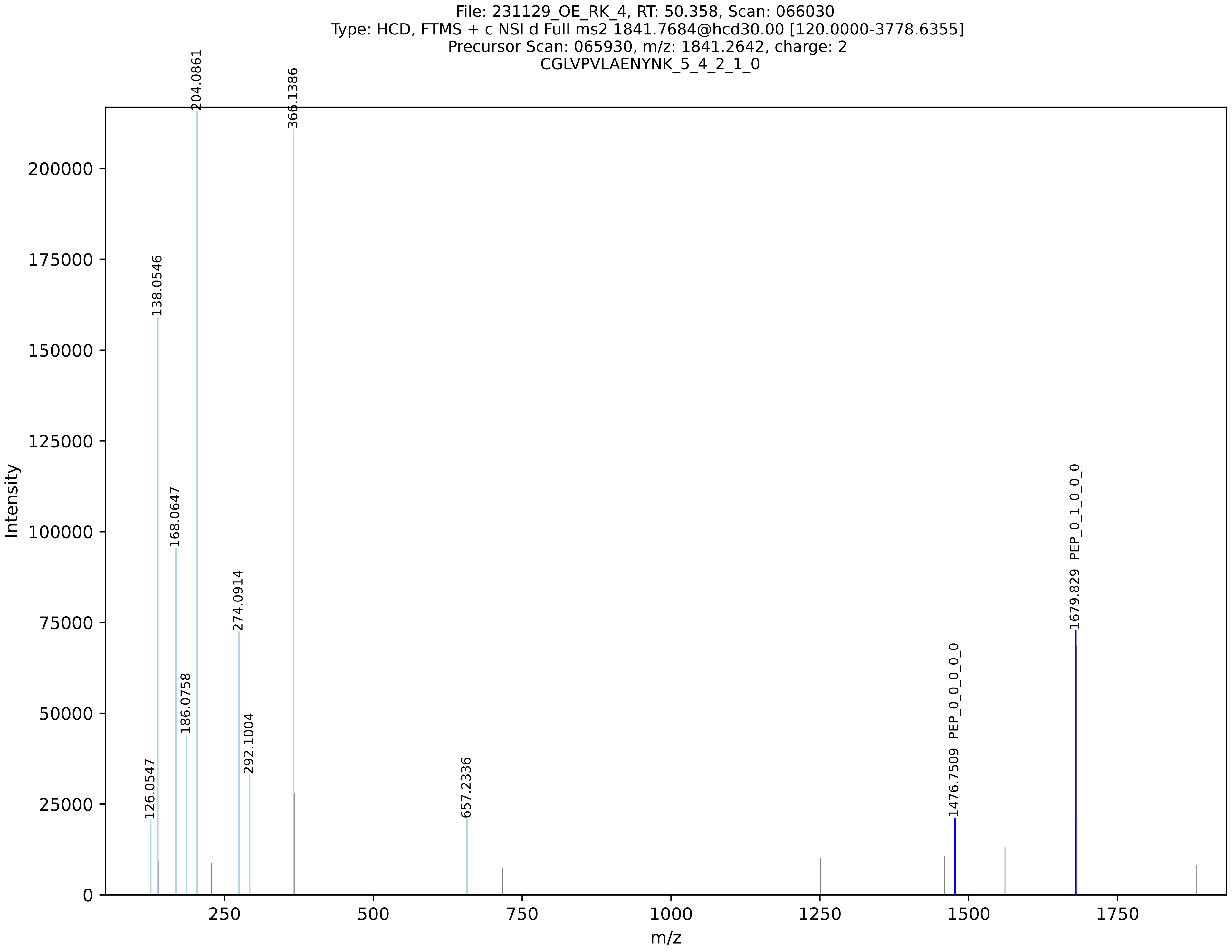

File: 231129_OE_RK_4, RT: 50.358, Scan: 066030
Type: HCD, FTMS + c NSI d Full ms2 1841.7684@hcd30.00 [120.0000-3778.6355]
Precursor Scan: 065930, m/z: 1841.2642, charge: 2
CGLVPVLAENYNK_5_4_2_1_0
Intensity
m/z
204.0861
366.1386
138.0546
168.0647
274.0914
186.0758
292.1004
126.0547
657.2336
1476.7509 PEP_0_0_0_0_0
1679.829 PEP_0_1_0_0_0

File: 231129_OE_RK_4, RT: 50.397, Scan: 066082
Type: HCD, FTMS + c NSI d Full ms2 921.6375@hcd30.00 [120.0000-3783.1370]
Precursor Scan: 066004, m/z: 921.1364, charge: 4
CGLVPVLAENYNK_5_4_0_2_0

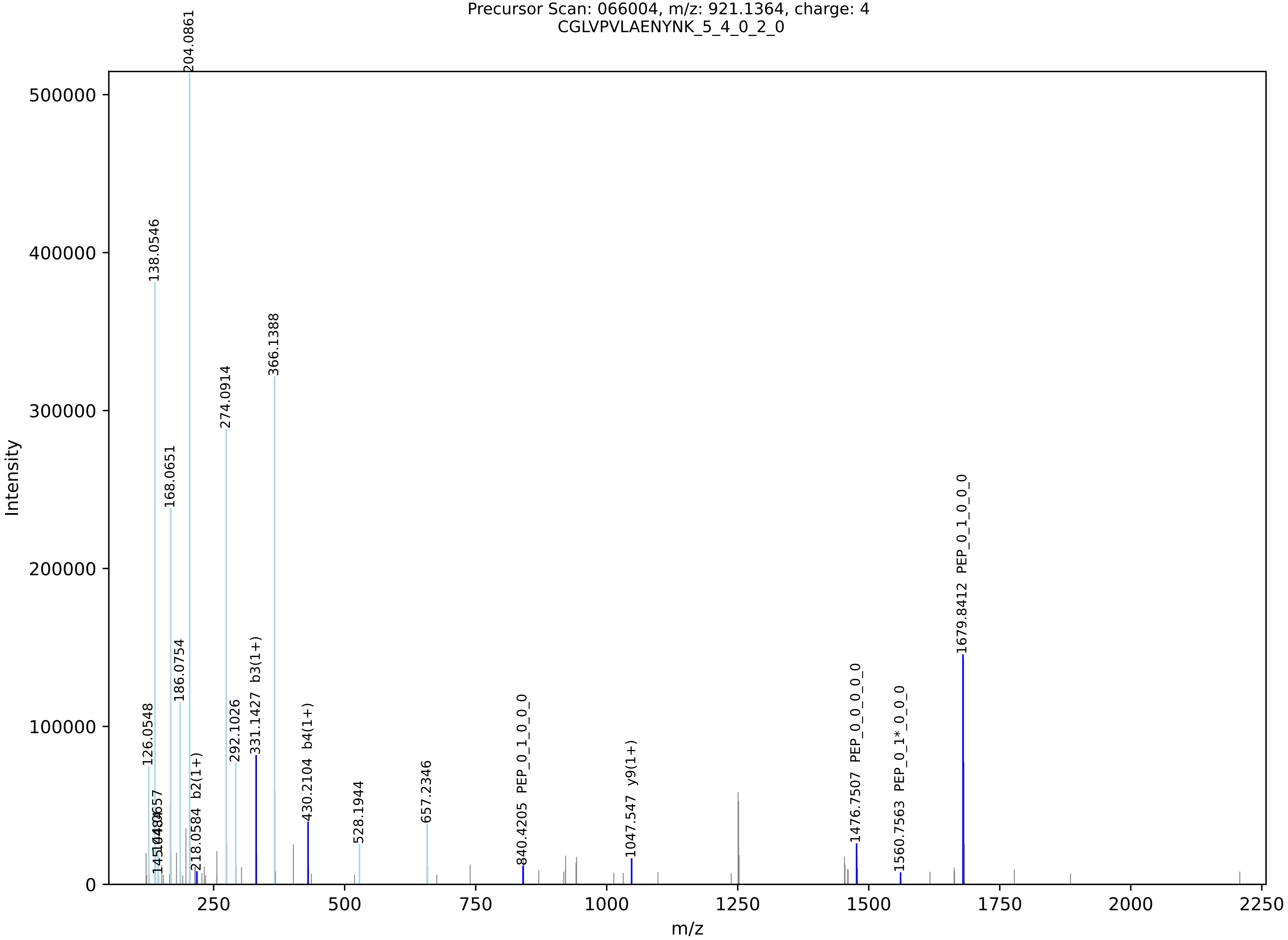

File: 231129_OE_RK_4, RT: 50.407, Scan: 066099
Type: HCD, FTMS + c NSI d Full ms2 1349.8903@hcd30.00 [120.0000-4152.8062]
Precursor Scan: 066004, m/z: 1349.5562, charge: 3
CGLVPVLAENYNK_6_5_0_2_0

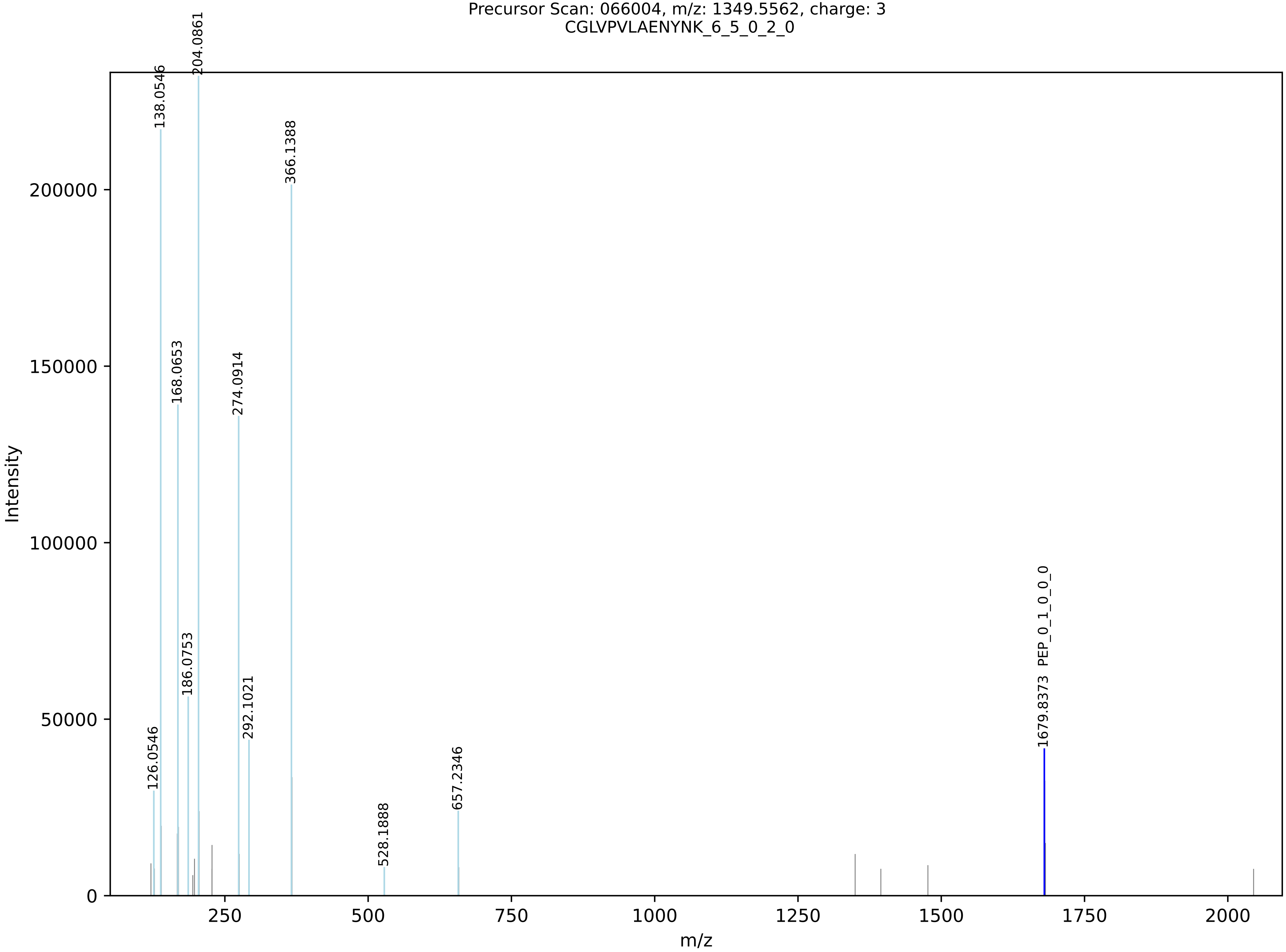

File: 231129_OE_RK_4, RT: 50.503, Scan: 066238
Type: HCD, FTMS + c NSI d Full ms2 1553.6895@hcd30.00 [120.0000-4776.4316]
Precursor Scan: 066149, m/z: 1553.3572, charge: 3
EWLPLNRSVNNVVVR_6_5_2_2_0

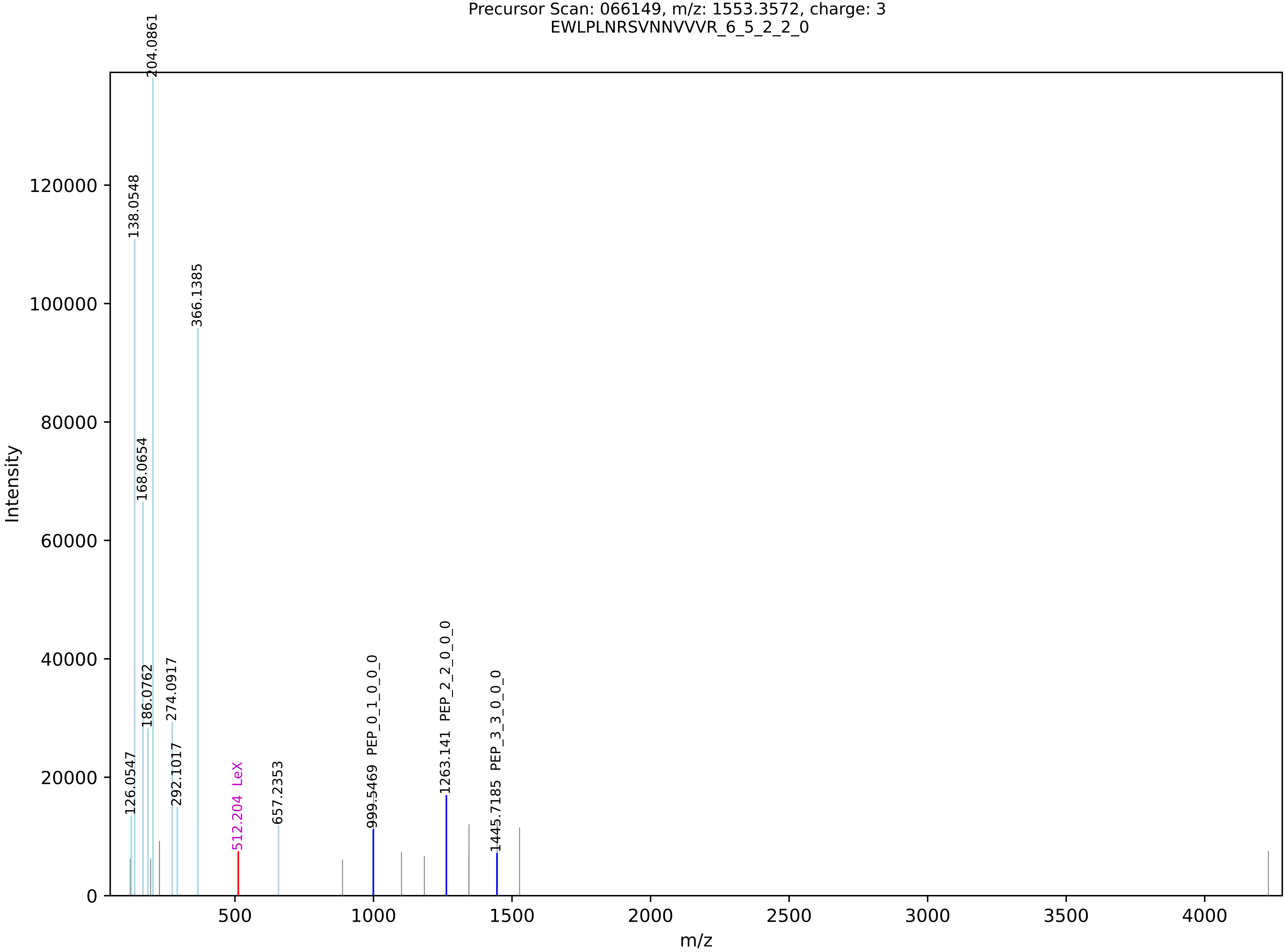

File: 231129_OE_RK_4, RT: 50.545, Scan: 066298
Type: HCD, FTMS + c NSI d Full ms2 1228.5143@hcd30.00 [120.0000-3781.3960]
Precursor Scan: 066223, m/z: 1227.8461, charge: 3
CGLVPVLAENYNK_5_4_0_2_0

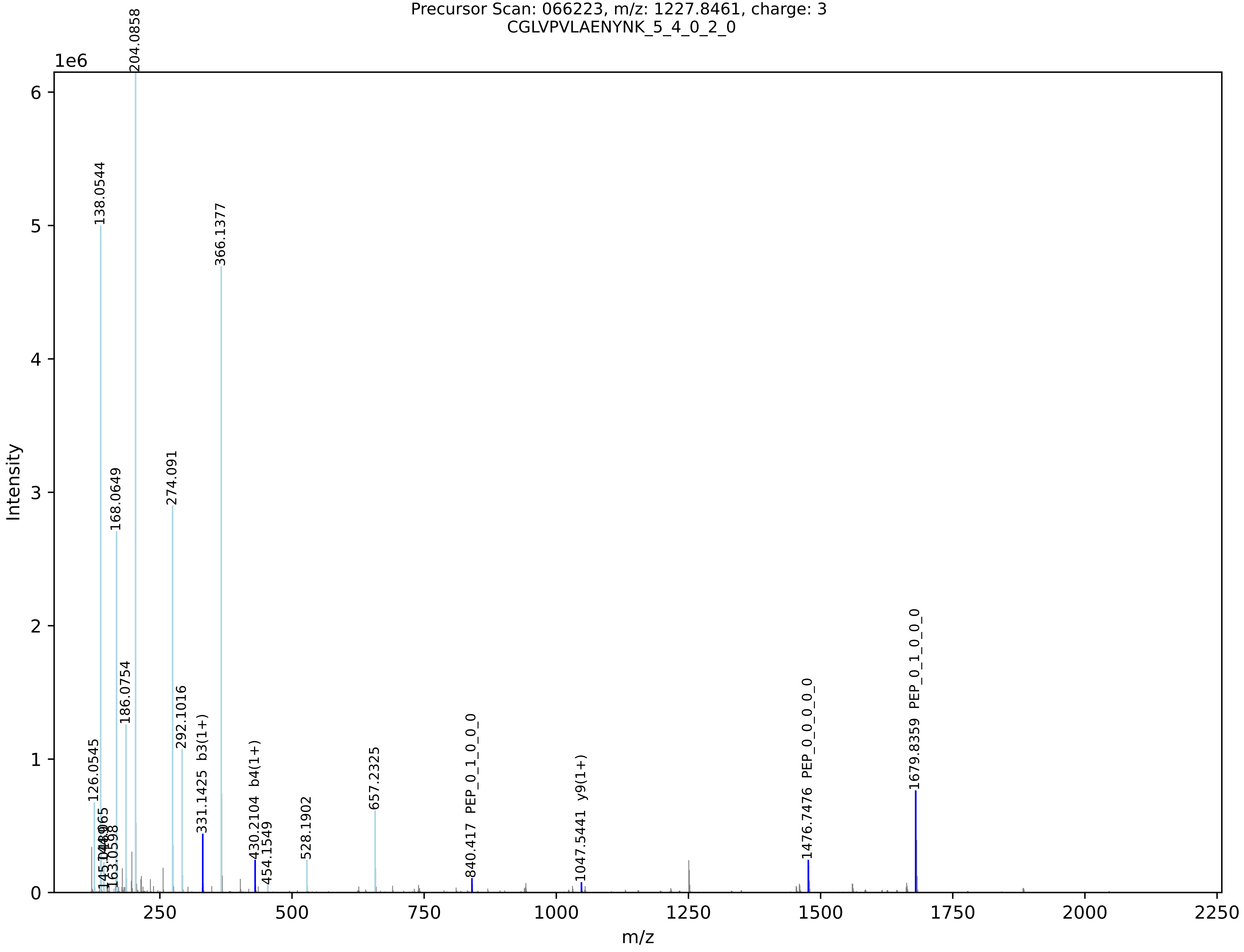

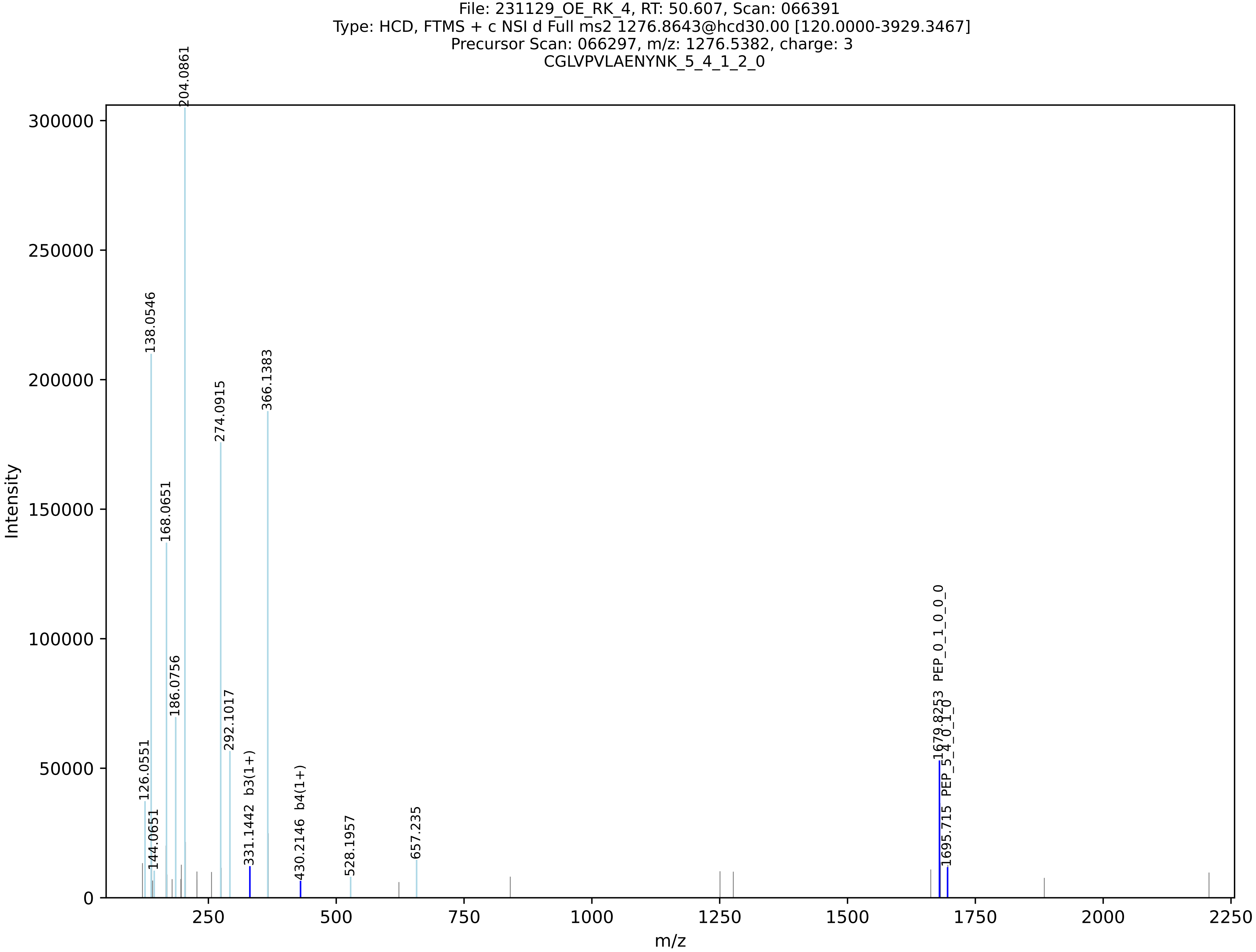
File: 231129_OE_RK_4, RT: 50.607, Scan: 066391
Type: HCD, FTMS + c NSI d Full ms2 1276.8643@hcd30.00 [120.0000-3929.3467]
Precursor Scan: 066297, m/z: 1276.5382, charge: 3
CGLVPVLAENYNK_5_4_1_2_0
Intensity
m/z
204.0861
138.0546
366.1383
274.0915
168.0651
186.0756
292.1017
126.0551
144.0651
331.1442 b3(1+)
430.2146 b4(1+)
528.1957
657.235
1679.8253 PEP_0_1_0_0_0
1695.715 PEP_5_4_0_1_0

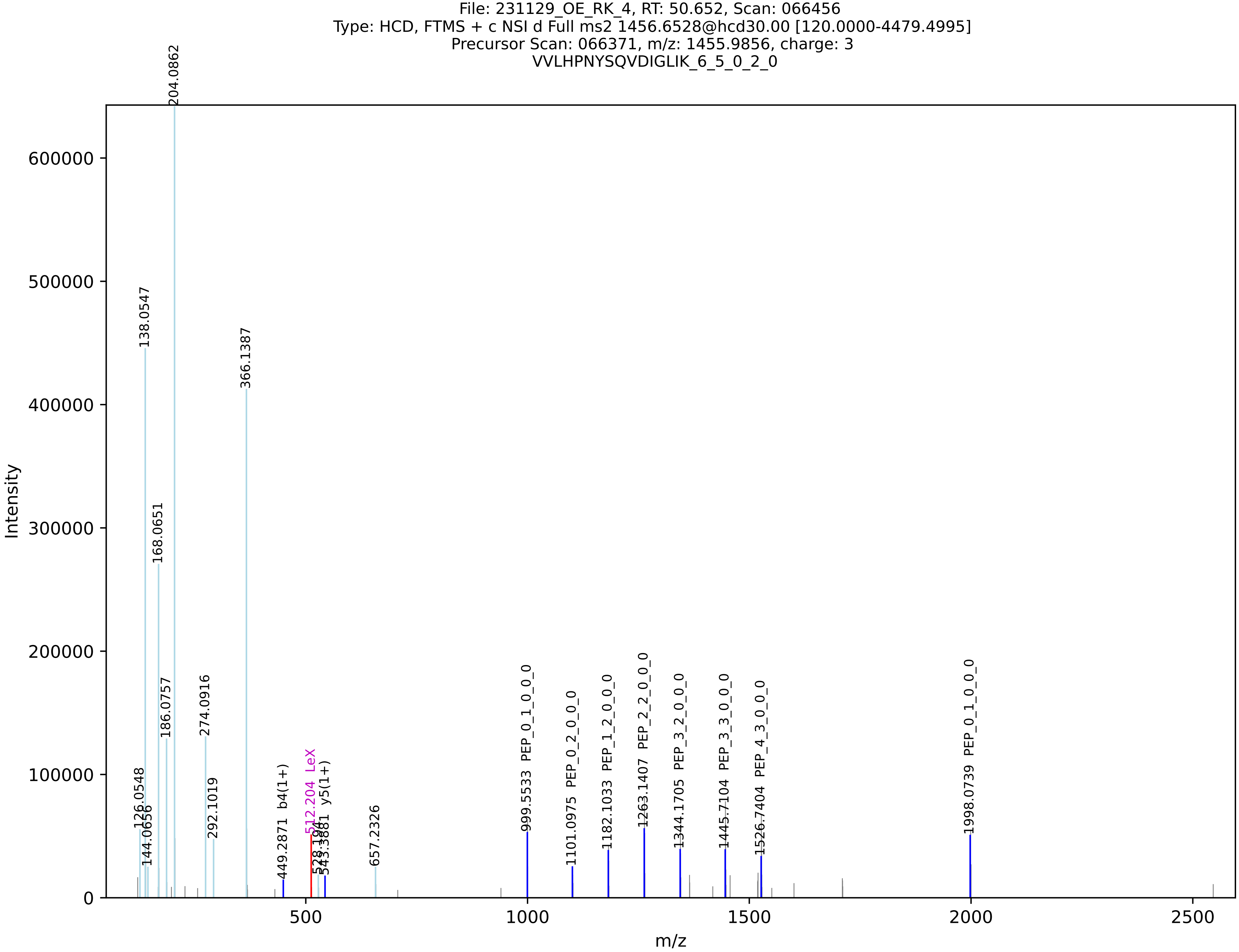

File: 231129_OE_RK_4, RT: 50.652, Scan: 066456
Type: HCD, FTMS + c NSI d Full ms2 1456.6528@hcd30.00 [120.0000-4479.4995]
Precursor Scan: 066371, m/z: 1455.9856, charge: 3
VVLHPNYSQVDIGLIK_6_5_0_2_0
Intensity
m/z
204.0862
138.0547
366.1387
168.0651
186.0757
274.0916
292.1019
126.0548
144.0656
449.2871 b4(1+)
512.204 LeX
543.3881 y5(1+)
657.2326
999.5533 PEP_0_1_0_0_0_
1101.0975 PEP_0_2_0_0_0_
1182.1033 PEP_1_2_0_0_0_
1263.1407 PEP_2_2_0_0_0_
1344.1705 PEP_3_2_0_0_0_
1445.7104 PEP_3_3_0_0_0_
1526.7404 PEP_4_3_0_0_0_
1998.0739 PEP_0_1_0_0_0_

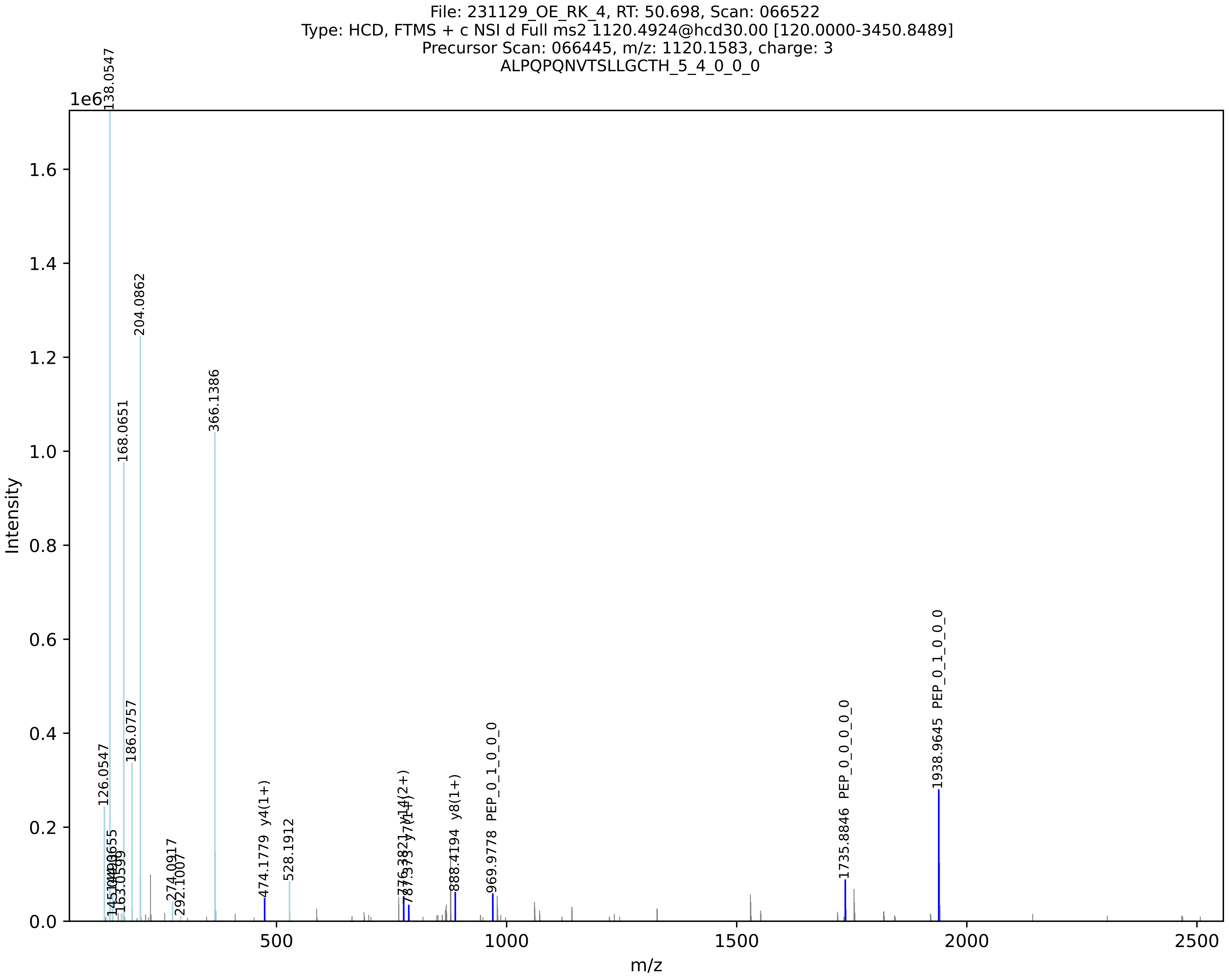
File: 231129_OE_RK_4, RT: 50.698, Scan: 066522
Type: HCD, FTMS + c NSI d Full ms2 1120.4924@hcd30.00 [120.0000-3450.8489]
Precursor Scan: 066445, m/z: 1120.1583, charge: 3
ALPQPQNVTSLLGCTH_5_4_0_0_0
1e6
Intensity
m/z
138.0547
204.0862
366.1386
168.0651
186.0757
126.0547
274.0917
292.1007
163.0599
474.1779 y4(1+)
528.1912
787.373 y7(1+)
888.4194 y8(1+)
969.9778 PEP_0_1_0_0_0
1735.8846 PEP_0_0_0_0_0
1938.9645 PEP_0_1_0_0_0

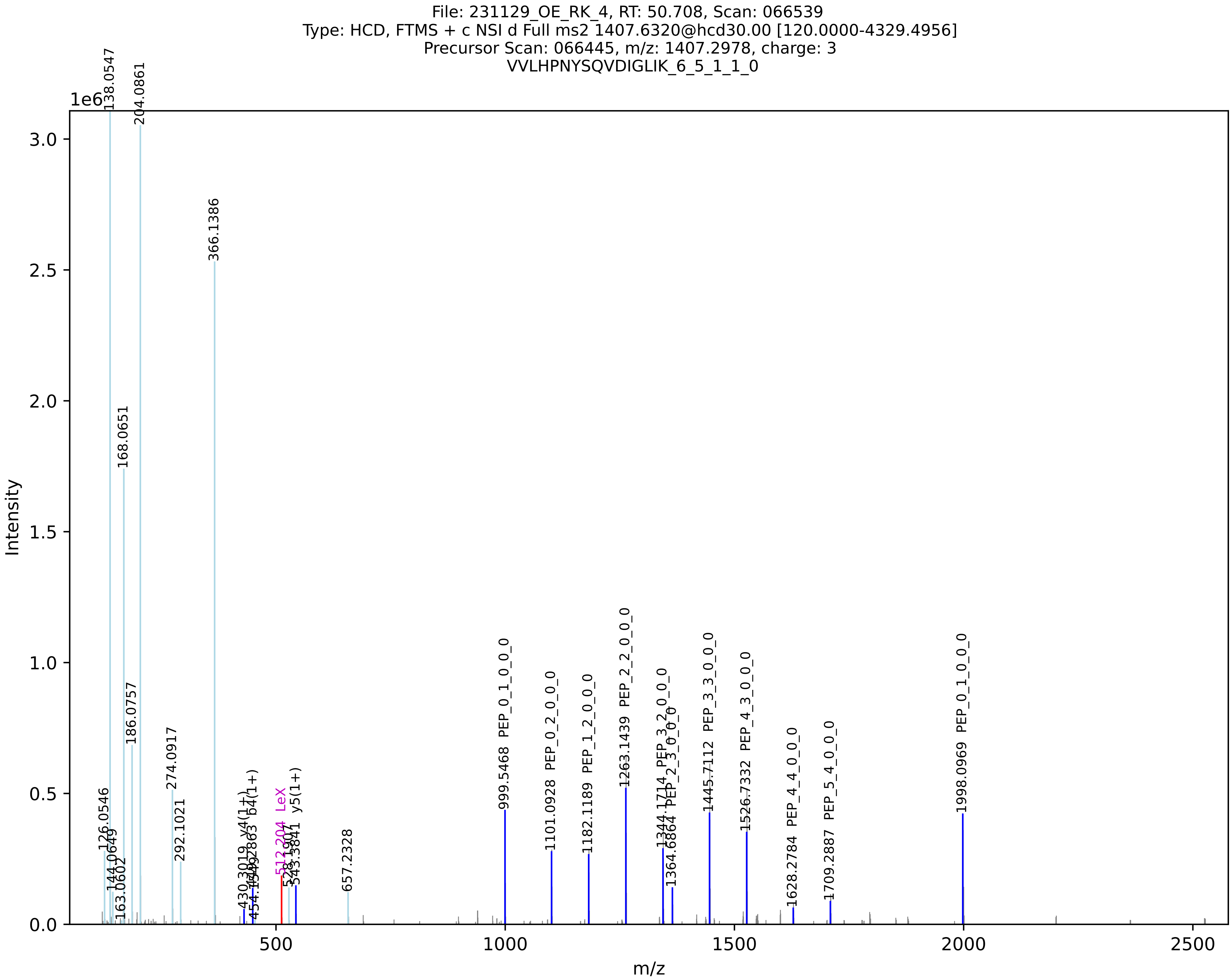
File: 231129_OE_RK_4, RT: 50.708, Scan: 066539
Type: HCD, FTMS + c NSI d Full ms2 1407.6320@hcd30.00 [120.0000-4329.4956]
Precursor Scan: 066445, m/z: 1407.2978, charge: 3
VVLHPNYSQVDIGLIK_6_5_1_1_0
1e6
Intensity
m/z
138.0547
204.0861
366.1386
168.0651
186.0757
274.0917
292.1021
126.0546
144.0649
163.0602
430.3019 y4(1+)
454.1549
447.2863 b4(1+)
512.204 LeX
528.1907
543.3841 y5(1+)
657.2328
999.5468 PEP_0_1_0_0_0
1101.0928 PEP_0_2_0_0_0
1182.1189 PEP_1_2_0_0_0
1263.1439 PEP_2_2_0_0_0
1344.1714 PEP_3_2_0_0_0
1364.6864 PEP_2_3_0_0_0
1445.7112 PEP_3_3_0_0_0
1526.7332 PEP_4_3_0_0_0
1628.2784 PEP_4_4_0_0_0
1709.2887 PEP_5_4_0_0_0
1998.0969 PEP_0_1_0_0_0

File: 231129_OE_RK_4, RT: 50.716, Scan: 066552
Type: HCD, FTMS + c NSI d Full ms2 1056.7275@hcd30.00 [120.0000-4334.3042]
Precursor Scan: 066445, m/z: 1055.7252, charge: 4
VVLHPNYSQVDIGLIK_6_5_1_1_0

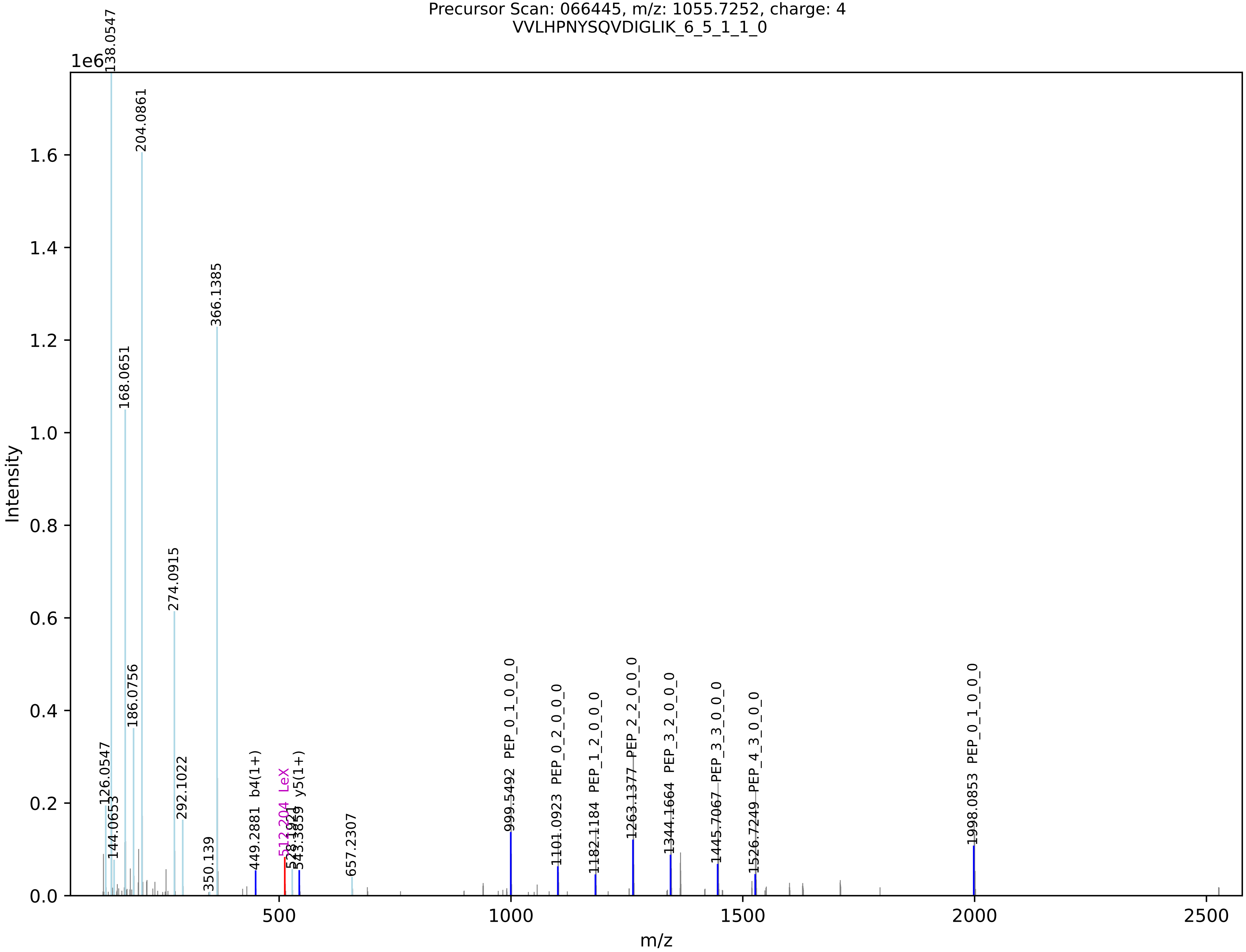

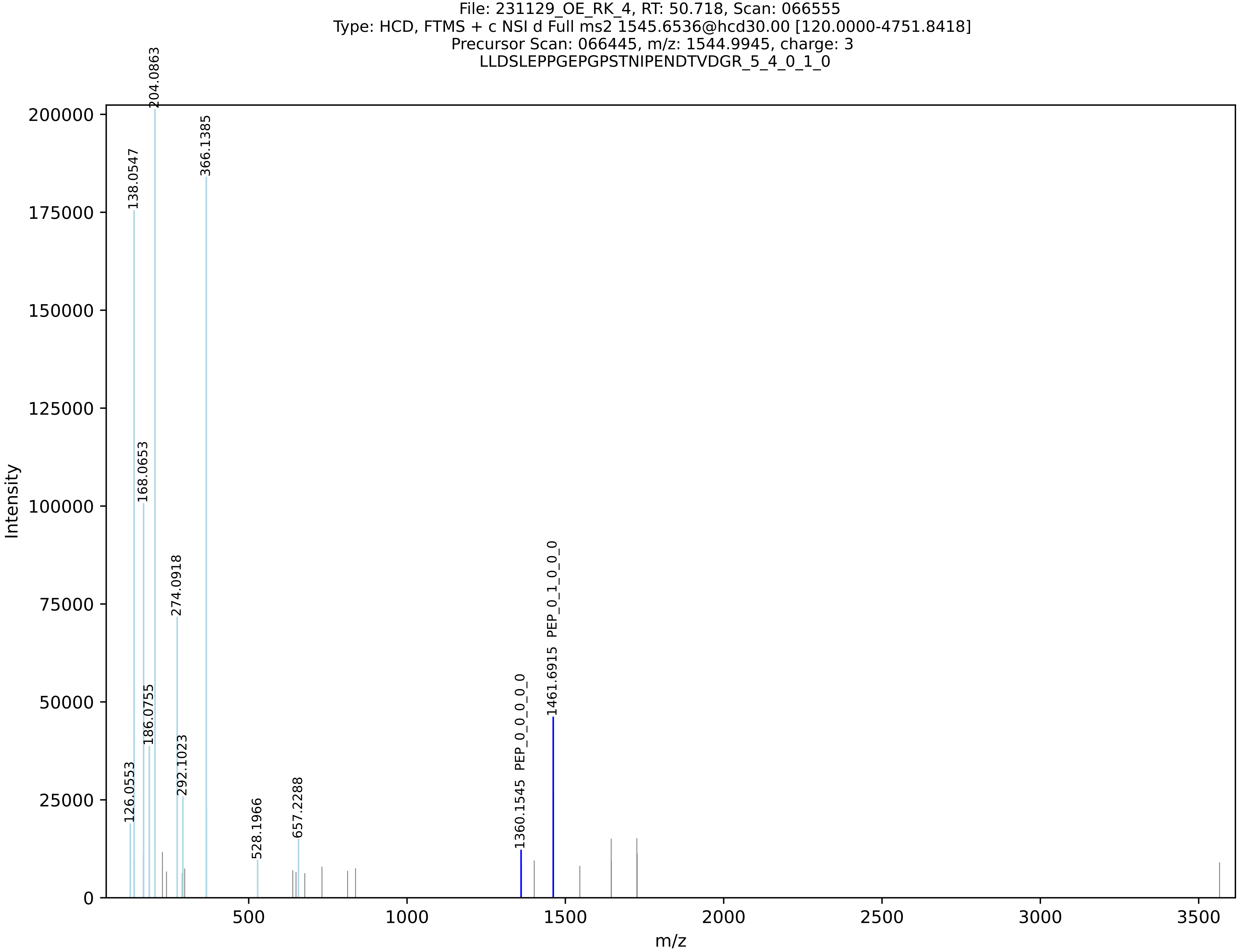

File: 231129_OE_RK_4, RT: 50.718, Scan: 066555
Type: HCD, FTMS + c NSI d Full ms2 1545.6536@hcd30.00 [120.0000-4751.8418]
Precursor Scan: 066445, m/z: 1544.9945, charge: 3
LLDSLEPPGEPGPSTNIPENDTVDGR_5_4_0_1_0
Intensity
m/z
204.0863
366.1385
138.0547
168.0653
274.0918
186.0755
292.1023
126.0553
528.1966
657.2288
1360.1545 PEP_0_0_0_0_0
1461.6915 PEP_0_1_0_0_0
0
25000
50000
75000
100000
125000
150000
175000
200000
500
1000
1500
2000
2500
3000
3500

File: 231129_OE_RK_4, RT: 50.747, Scan: 066594
Type: HCD, FTMS + c NSI d Full ms2 1228.5138@hcd30.00 [120.0000-3781.3943]
Precursor Scan: 066519, m/z: 1227.8456, charge: 3
CGLVPVLAENYNK_5_4_0_2_0

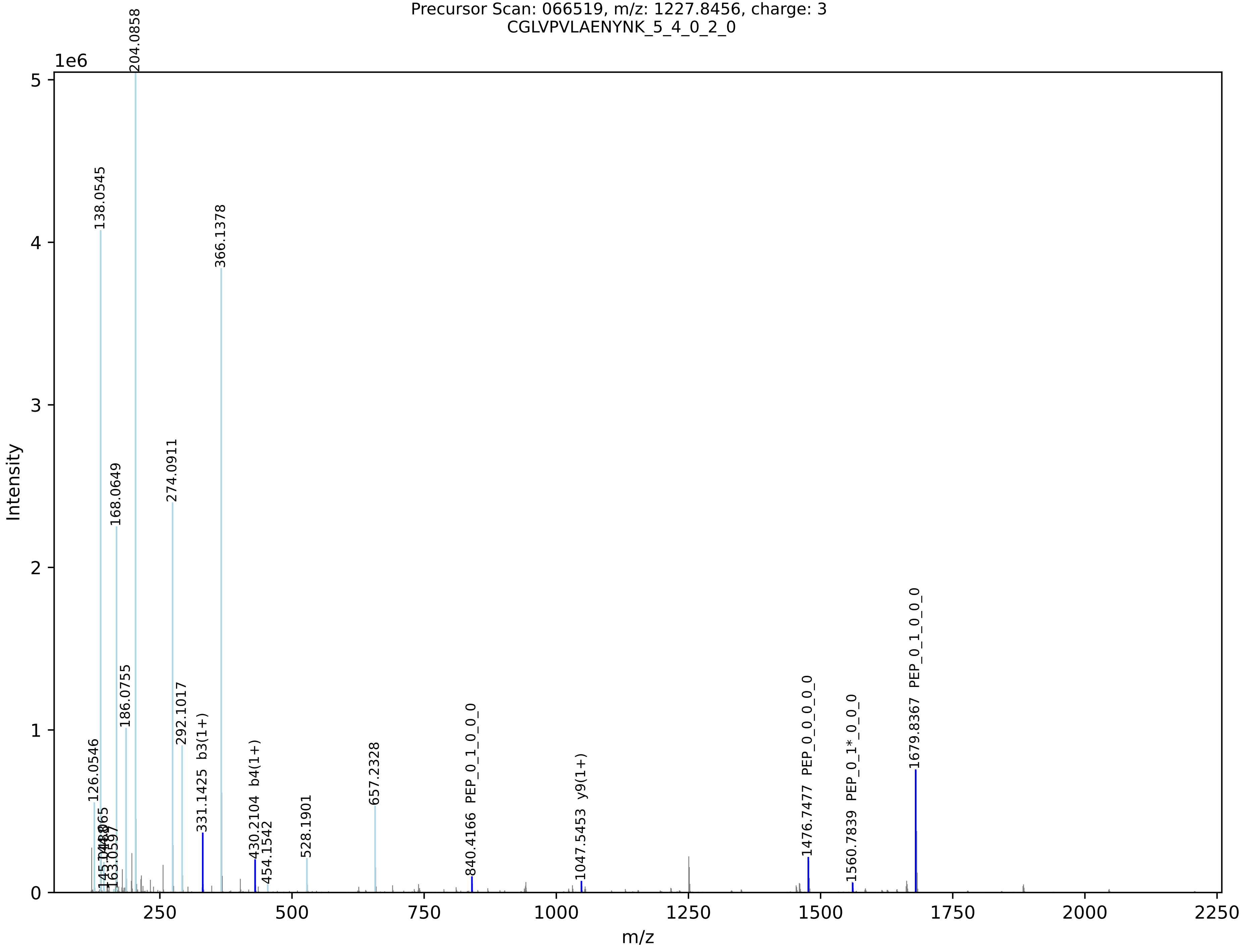

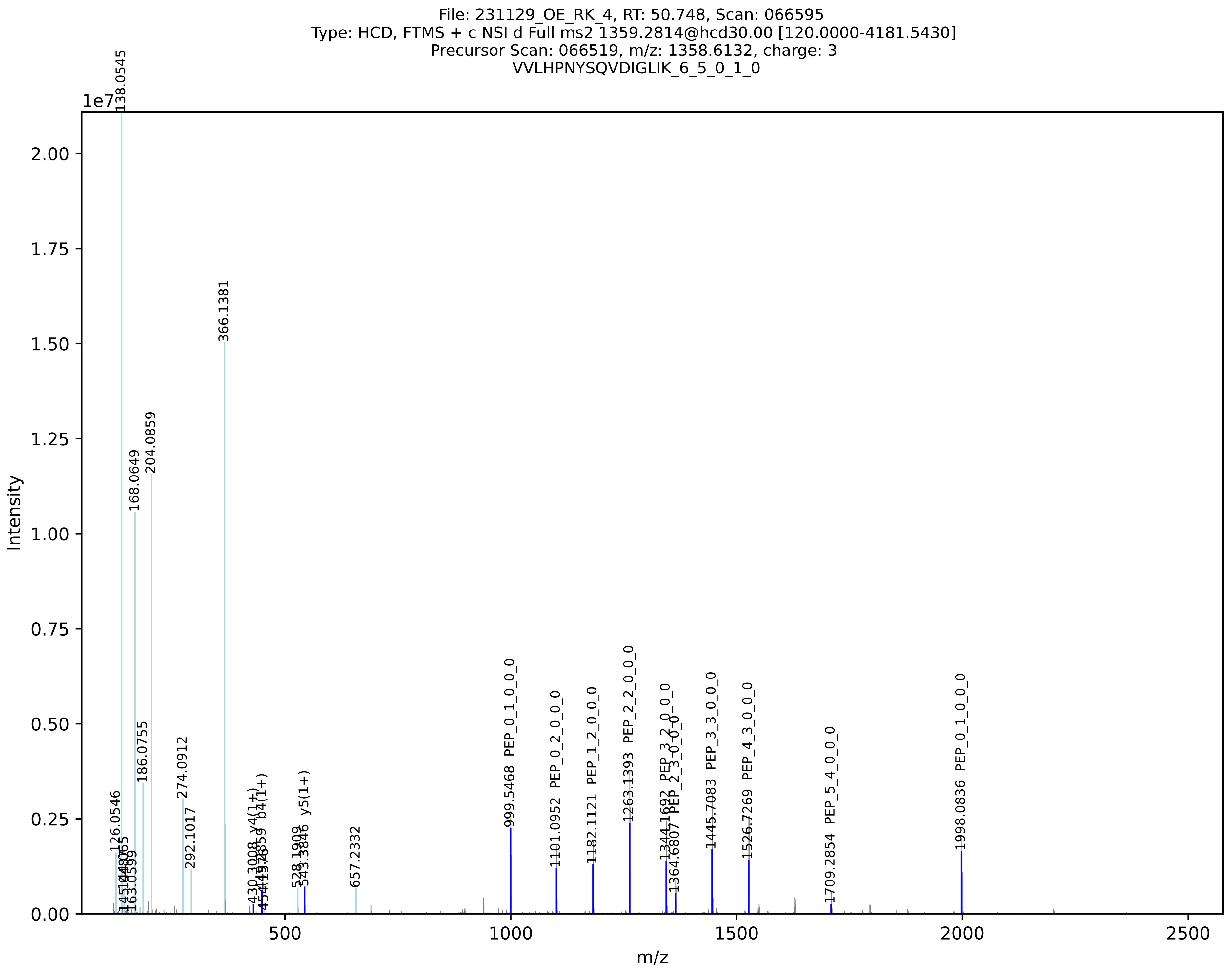

File: 231129_OE_RK_4, RT: 50.748, Scan: 066595
Type: HCD, FTMS + c NSI d Full ms2 1359.2814@hcd30.00 [120.0000-4181.5430]
Precursor Scan: 066519, m/z: 1358.6132, charge: 3
VVLHPNYSQVDIGLIK_6_5_0_1_0
1e7
Intensity
m/z
138.0545
366.1381
204.0859
168.0649
186.0755
274.0912
292.1017
126.0546
163.0599
430.3008 y4(1+)
b4(1+)
y5(1+)
543.3846
657.2332
999.5468 PEP_0_1_0_0_0
1101.0952 PEP_0_2_0_0_0
1182.1121 PEP_1_2_0_0_0
1263.1393 PEP_2_2_0_0_0
1344.1692 PEP_3_2_0_0_0
1364.6807 PEP_2_3_0_0_0
1445.7083 PEP_3_3_0_0_0
1526.7269 PEP_4_3_0_0_0
1709.2854 PEP_5_4_0_0_0
1998.0836 PEP_0_1_0_0_0

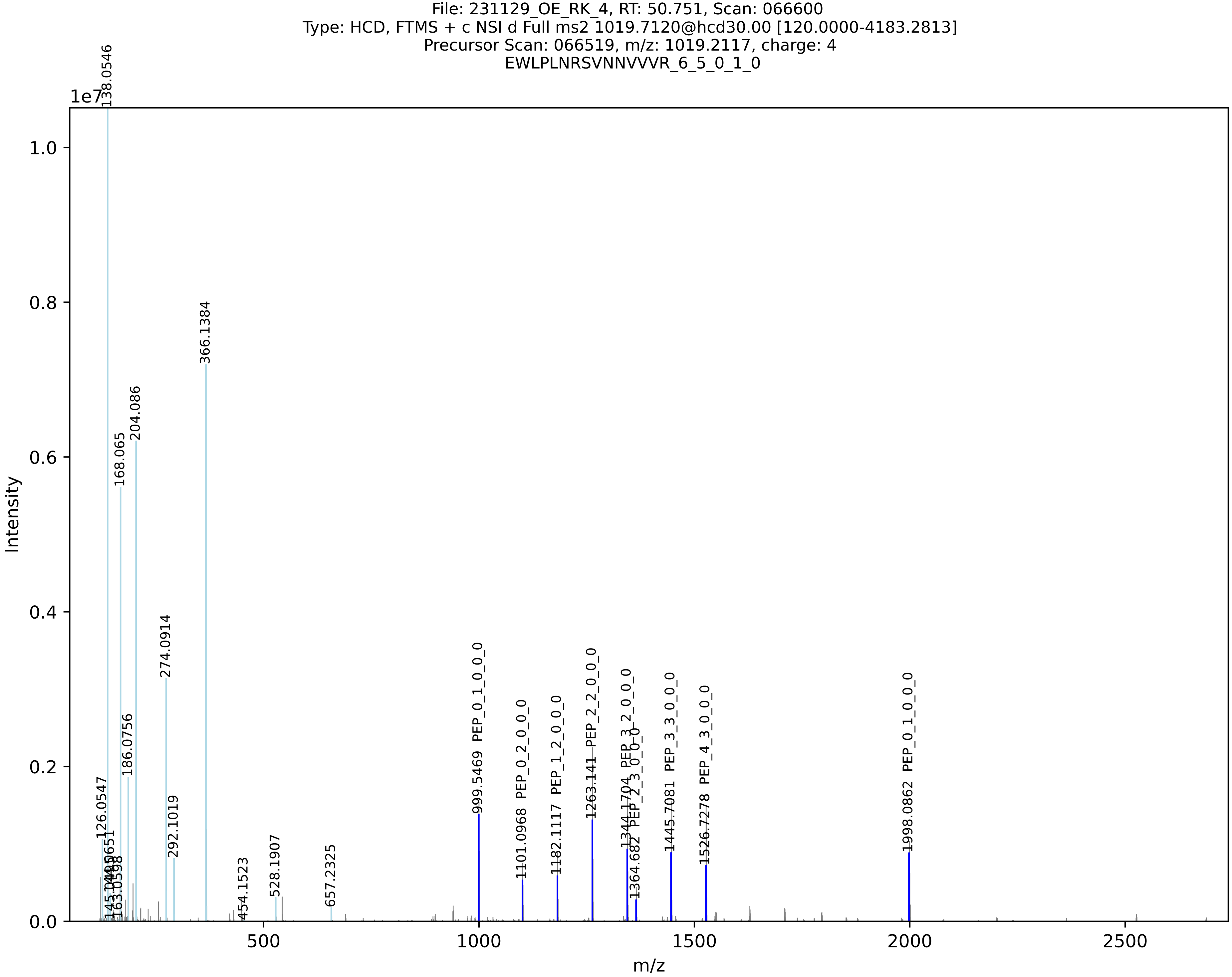

File: 231129_OE_RK_4, RT: 50.751, Scan: 066600
Type: HCD, FTMS + c NSI d Full ms2 1019.7120@hcd30.00 [120.0000-4183.2813]
Precursor Scan: 066519, m/z: 1019.2117, charge: 4
EWLPLNRSVNNVVVR_6_5_0_1_0
1e7
Intensity
m/z
138.0546
366.1384
204.086
168.065
274.0914
186.0756
126.0547
292.1019
163.0598
454.1523
528.1907
657.2325
999.5469 PEP_0_1_0_0_0
1101.0968 PEP_0_2_0_0_0
1182.1117 PEP_1_2_0_0_0
1263.141 PEP_2_2_0_0_0
1344.1704 PEP_3_2_0_0_0
1364.682 PEP_2_3_0_0_0
1445.7081 PEP_3_3_0_0_0
1526.7278 PEP_4_3_0_0_0
1998.0862 PEP_0_1_0_0_0

File: 231129_OE_RK_4, RT: 50.799, Scan: 066671
Type: HCD, FTMS + c NSI d Full ms2 921.3868@hcd30.00 [120.0000-3782.1143]
Precursor Scan: 066593, m/z: 921.1362, charge: 4
CGLVPVLAENYNK_5_4_0_2_0

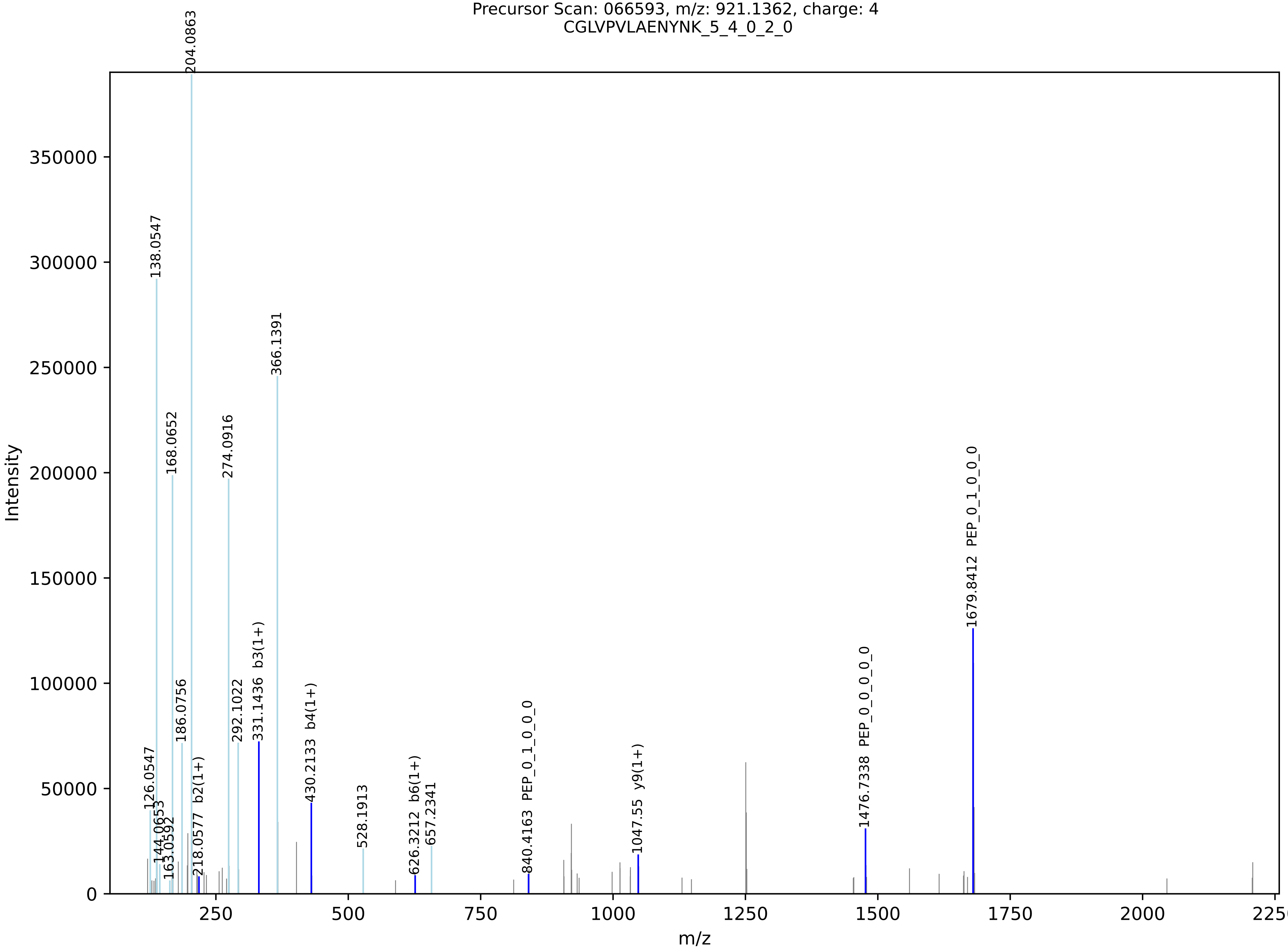

File: 231129_OE_RK_4, RT: 50.802, Scan: 066675
Type: HCD, FTMS + c NSI d Full ms2 995.4116@hcd30.00 [120.0000-4084.1355]
Precursor Scan: 066593, m/z: 994.9095, charge: 4
VCQDCPLLAPLNDTR_5_4_0_2_0

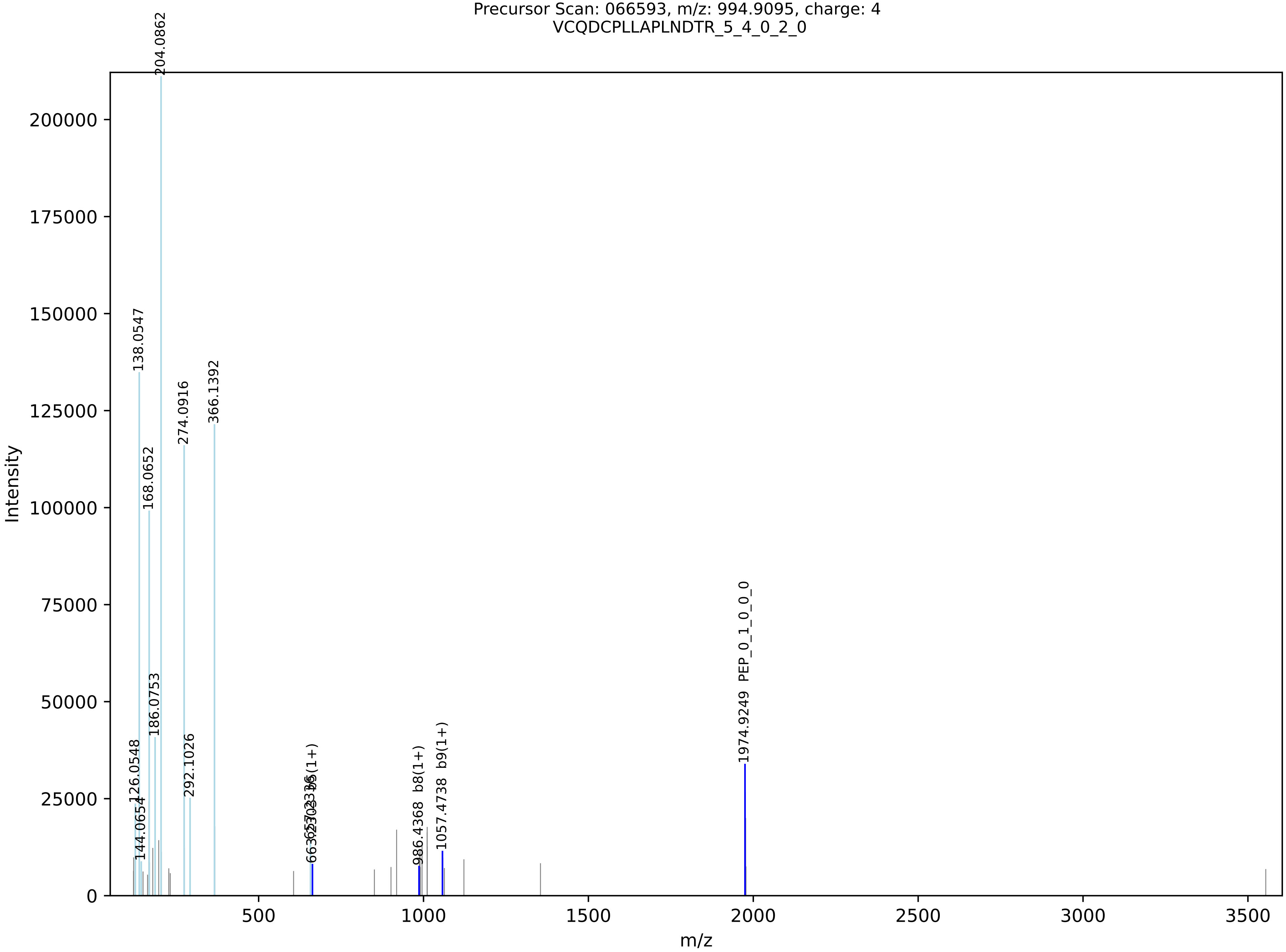

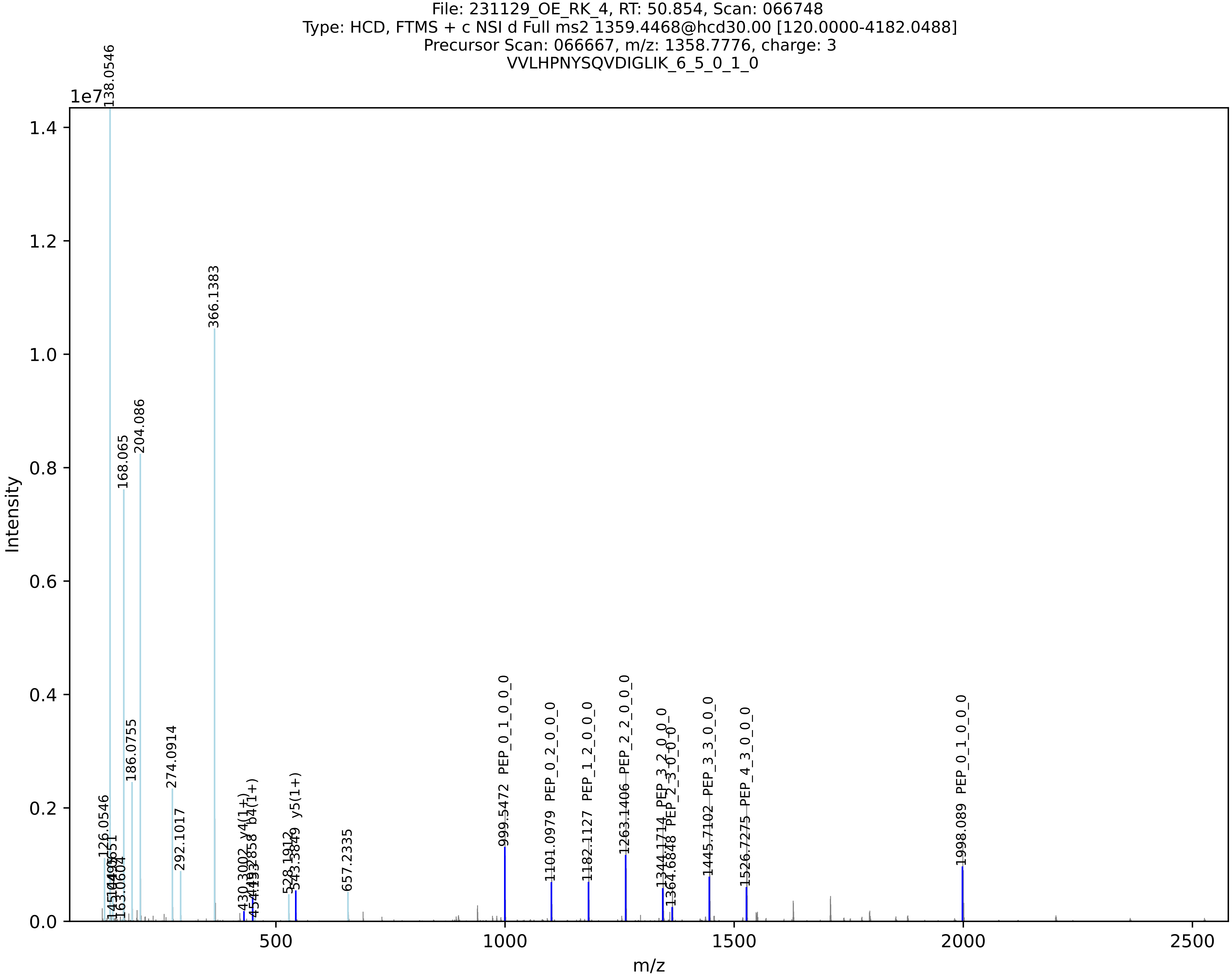

File: 231129_OE_RK_4, RT: 50.854, Scan: 066748
Type: HCD, FTMS + c NSI d Full ms2 1359.4468@hcd30.00 [120.0000-4182.0488]
Precursor Scan: 066667, m/z: 1358.7776, charge: 3
VVLHPNYSQVDIGLIK_6_5_0_1_0
1e7
Intensity
m/z
138.0546
366.1383
204.086
168.065
186.0755
274.0914
292.1017
126.0546
163.0604
430.3002 y4(1+)
454.2858 b4(1+)
528.1912
543.3849 y5(1+)
657.2335
999.5472 PEP_0_1_0_0_0
1101.0979 PEP_0_2_0_0_0
1182.1127 PEP_1_2_0_0_0
1263.1406 PEP_2_2_0_0_0
1344.1714 PEP_3_2_0_0_0
1364.6848 PEP_2_3_0_0_0
1445.7102 PEP_3_3_0_0_0
1526.7275 PEP_4_3_0_0_0
1998.089 PEP_0_1_0_0_0

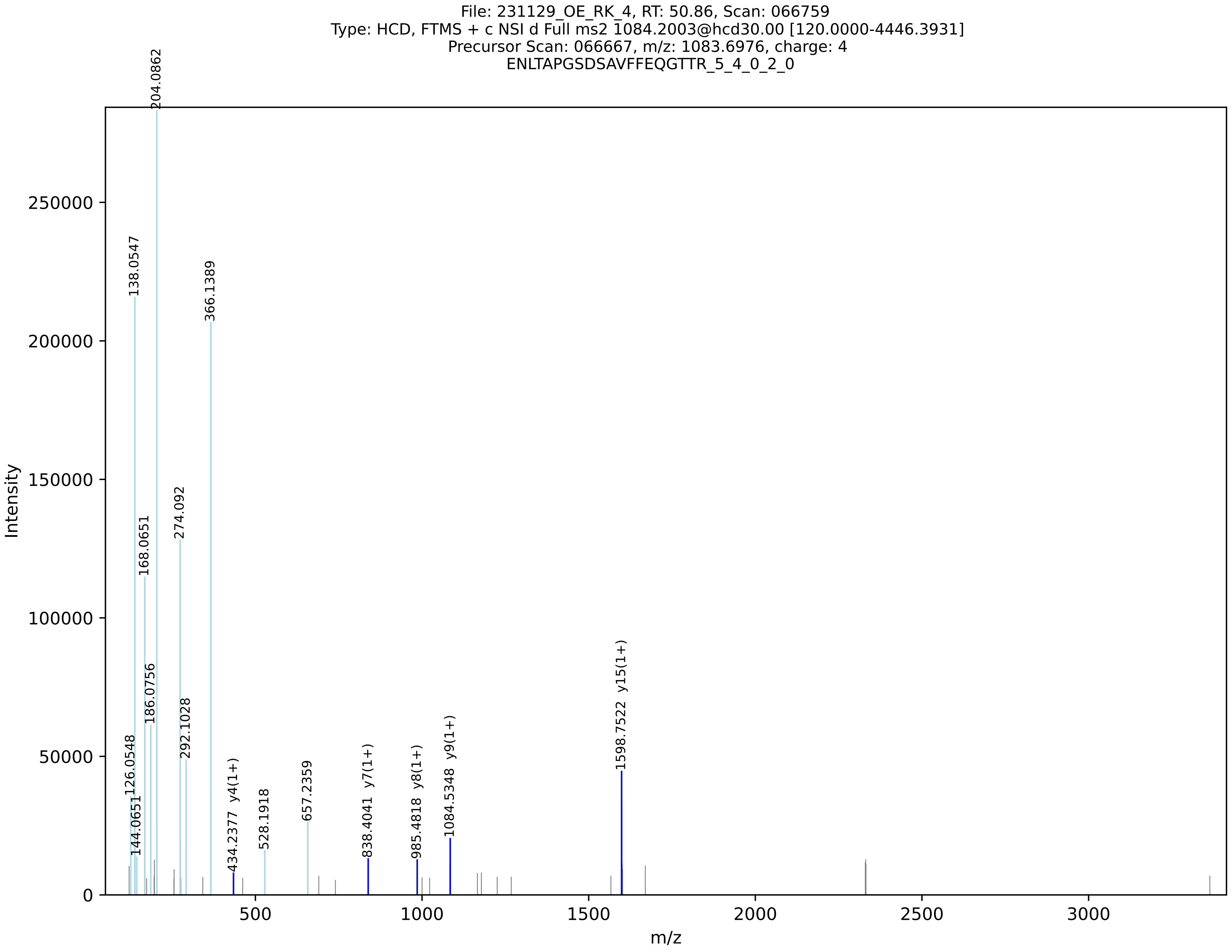
File: 231129_OE_RK_4, RT: 50.86, Scan: 066759
Type: HCD, FTMS + c NSI d Full ms2 1084.2003@hcd30.00 [120.0000-4446.3931]
Precursor Scan: 066667, m/z: 1083.6976, charge: 4
ENLTAPGSDSAVFFEQGTTR_5_4_0_2_0
Intensity
m/z
204.0862
138.0547
366.1389
274.092
168.0651
186.0756
292.1028
126.0548
144.0651
434.2377 y4(1+)
528.1918
657.2359
838.4041 y7(1+)
985.4818 y8(1+)
1084.5348 y9(1+)
1598.7522 y15(1+)
0
50000
100000
150000
200000
250000
500
1000
1500
2000
2500
3000

File: 231129_OE_RK_4, RT: 50.903, Scan: 066820
Type: HCD, FTMS + c NSI d Full ms2 1158.4874@hcd30.00 [120.0000-5931.8560]
Precursor Scan: 066741, m/z: 1157.8865, charge: 5
ITYSIVQTNCSKENFLFLTPDCK_6_5_1_3_0

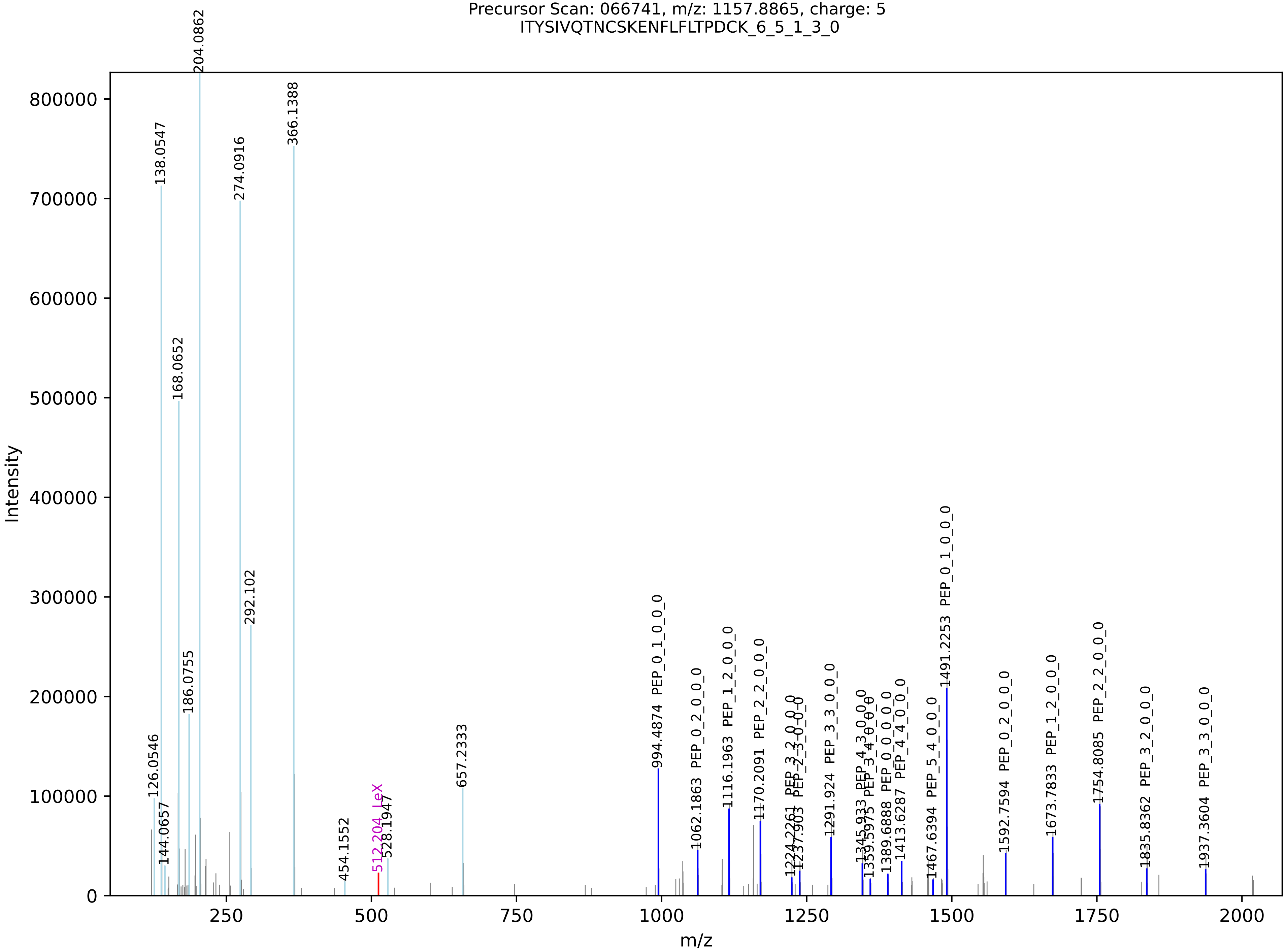

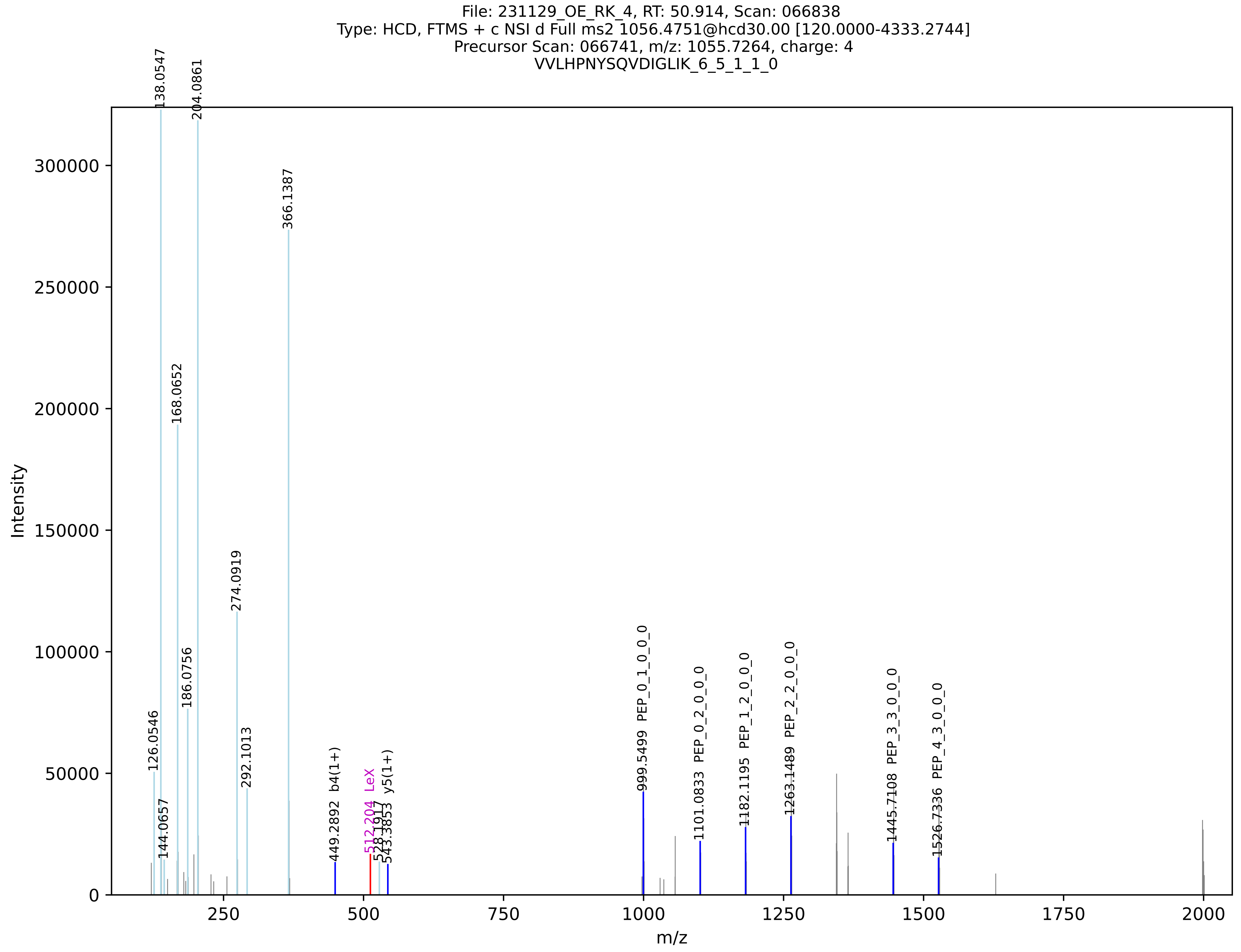

File: 231129_OE_RK_4, RT: 50.914, Scan: 066838
Type: HCD, FTMS + c NSI d Full ms2 1056.4751@hcd30.00 [120.0000-4333.2744]
Precursor Scan: 066741, m/z: 1055.7264, charge: 4
VVLHPNYSQVDIGLIK_6_5_1_1_0
Intensity
m/z
138.0547
204.0861
366.1387
168.0652
274.0919
186.0756
126.0546
292.1013
144.0657
449.2892 b4(1+)
512.204 LeX
528.1917
543.3853 y5(1+)
999.5499 PEP_0_1_0_0_0
1101.0833 PEP_0_2_0_0_0
1182.1195 PEP_1_2_0_0_0
1263.1489 PEP_2_2_0_0_0
1445.7108 PEP_3_3_0_0_0
1526.7336 PEP_4_3_0_0_0

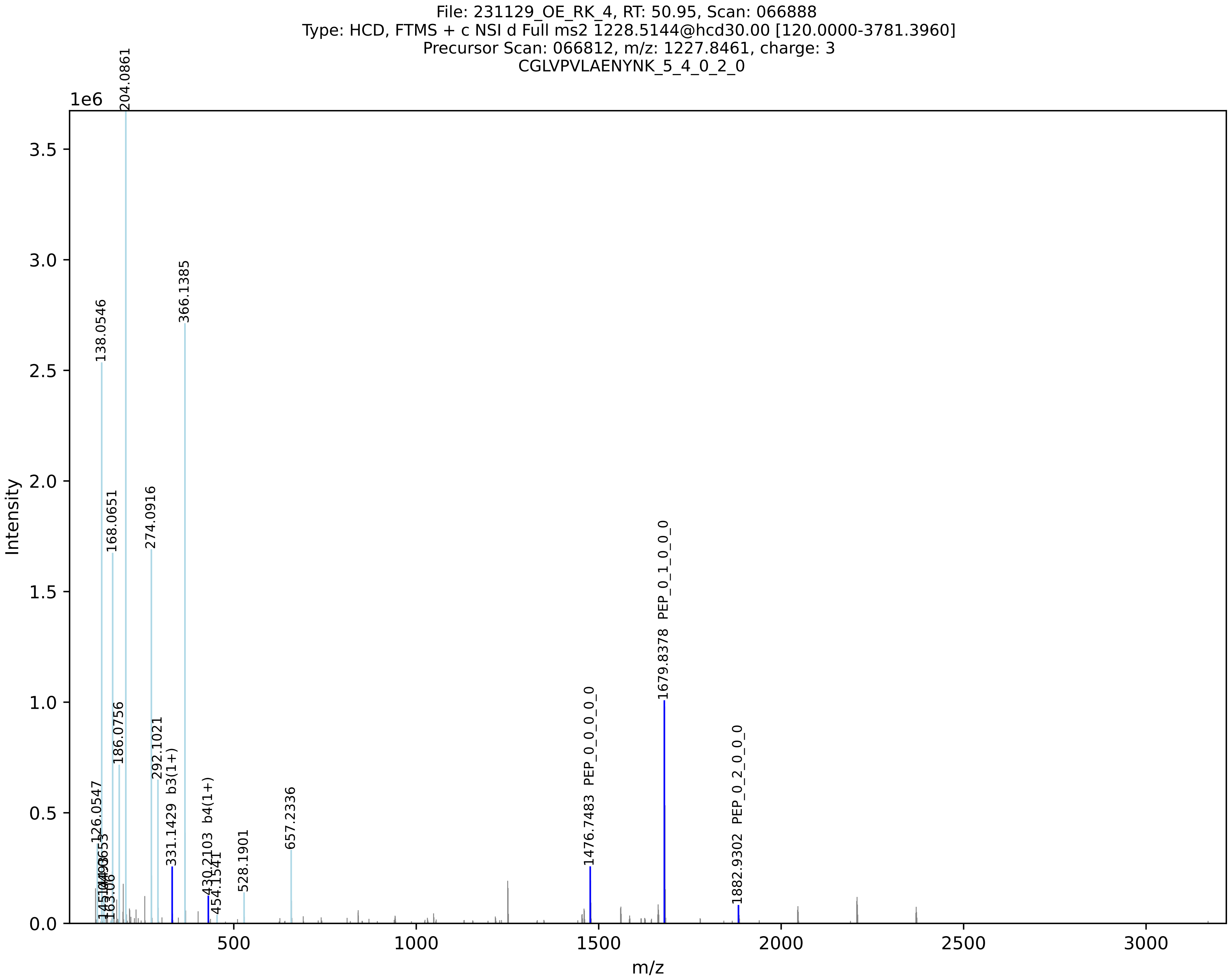

File: 231129_OE_RK_4, RT: 50.95, Scan: 066888
Type: HCD, FTMS + c NSI d Full ms2 1228.5144@hcd30.00 [120.0000-3781.3960]
Precursor Scan: 066812, m/z: 1227.8461, charge: 3
CGLVPVLAENYNK_5_4_0_2_0
1e6
Intensity
m/z
204.0861
366.1385
138.0546
168.0651
274.0916
186.0756
292.1021
331.1429 b3(1+)
126.0547
430.2103 b4(1+)
454.1541
528.1901
657.2336
1476.7483 PEP_0_0_0_0_0
1679.8378 PEP_0_1_0_0_0
1882.9302 PEP_0_2_0_0_0

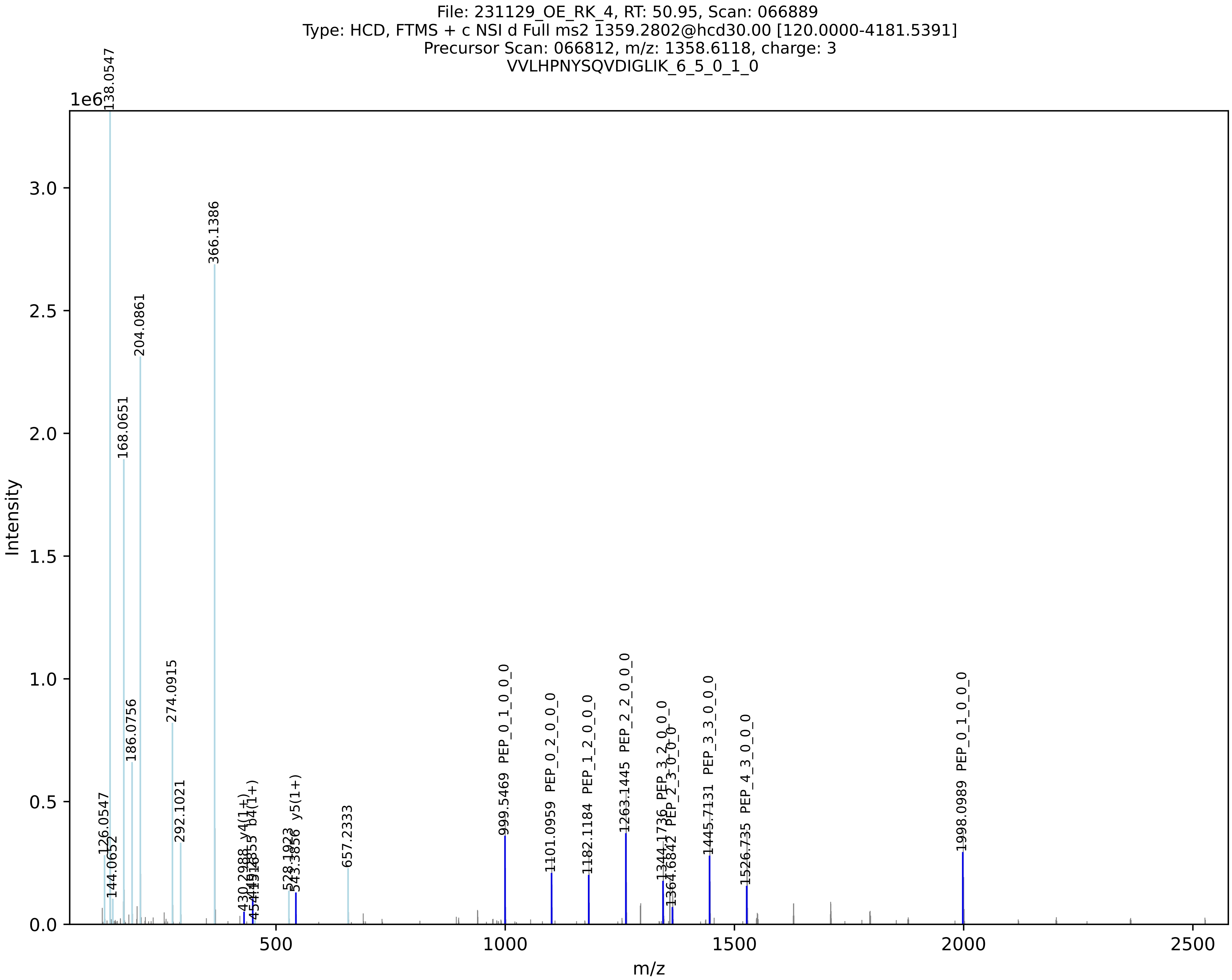
File: 231129_OE_RK_4, RT: 50.95, Scan: 066889
Type: HCD, FTMS + c NSI d Full ms2 1359.2802@hcd30.00 [120.0000-4181.5391]
Precursor Scan: 066812, m/z: 1358.6118, charge: 3
VVLHPNYSQVDIGLIK_6_5_0_1_0
1e6
Intensity
m/z
138.0547
366.1386
204.0861
168.0651
274.0915
186.0756
292.1021
126.0547
144.0652
430.2988 y4(1+)
b4(1+)
543.3856 y5(1+)
657.2333
999.5469 PEP_0_1_0_0_0
1101.0959 PEP_0_2_0_0_0
1182.1184 PEP_1_2_0_0_0
1263.1445 PEP_2_2_0_0_0
1344.1736 PEP_3_2_0_0_0
1364.6842 PEP_2_3_0_0_0
1445.7131 PEP_3_3_0_0_0
1526.735 PEP_4_3_0_0_0
1998.0989 PEP_0_1_0_0_0

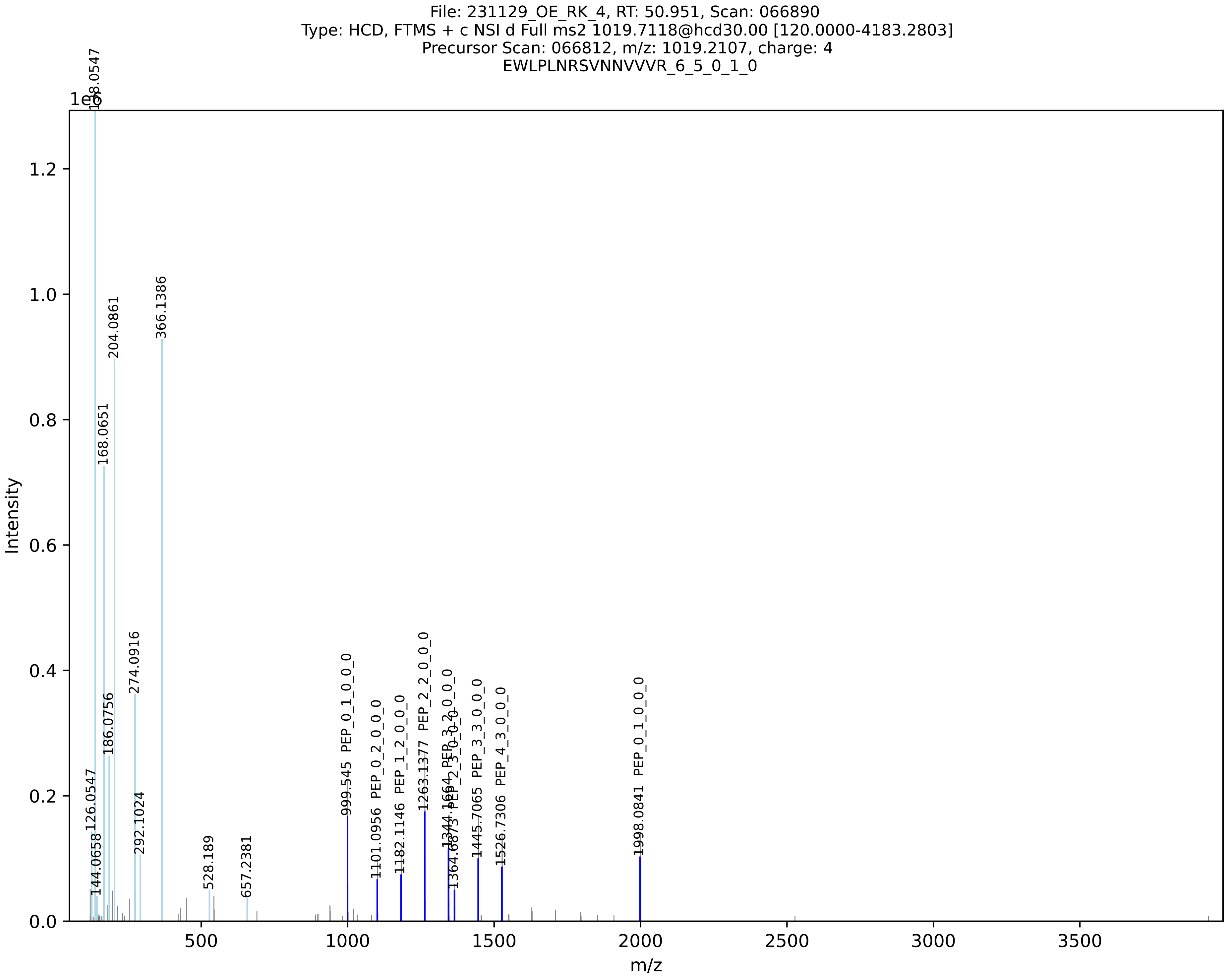
File: 231129_OE_RK_4, RT: 50.951, Scan: 066890
Type: HCD, FTMS + c NSI d Full ms2 1019.7118@hcd30.00 [120.0000-4183.2803]
Precursor Scan: 066812, m/z: 1019.2107, charge: 4
EWLPLNRSVNNVVVR_6_5_0_1_0
1e6
138.0547
366.1386
204.0861
168.0651
274.0916
186.0756
126.0547
292.1024
144.0658
528.189
657.2381
999.545 PEP_0_1_0_0_0
1101.0956 PEP_0_2_0_0_0
1182.1146 PEP_1_2_0_0_0
1263.1377 PEP_2_2_0_0_0
1445.7065 PEP_3_3_0_0_0
1526.7306 PEP_4_3_0_0_0
1998.0841 PEP_0_1_0_0_0
Intensity
m/z

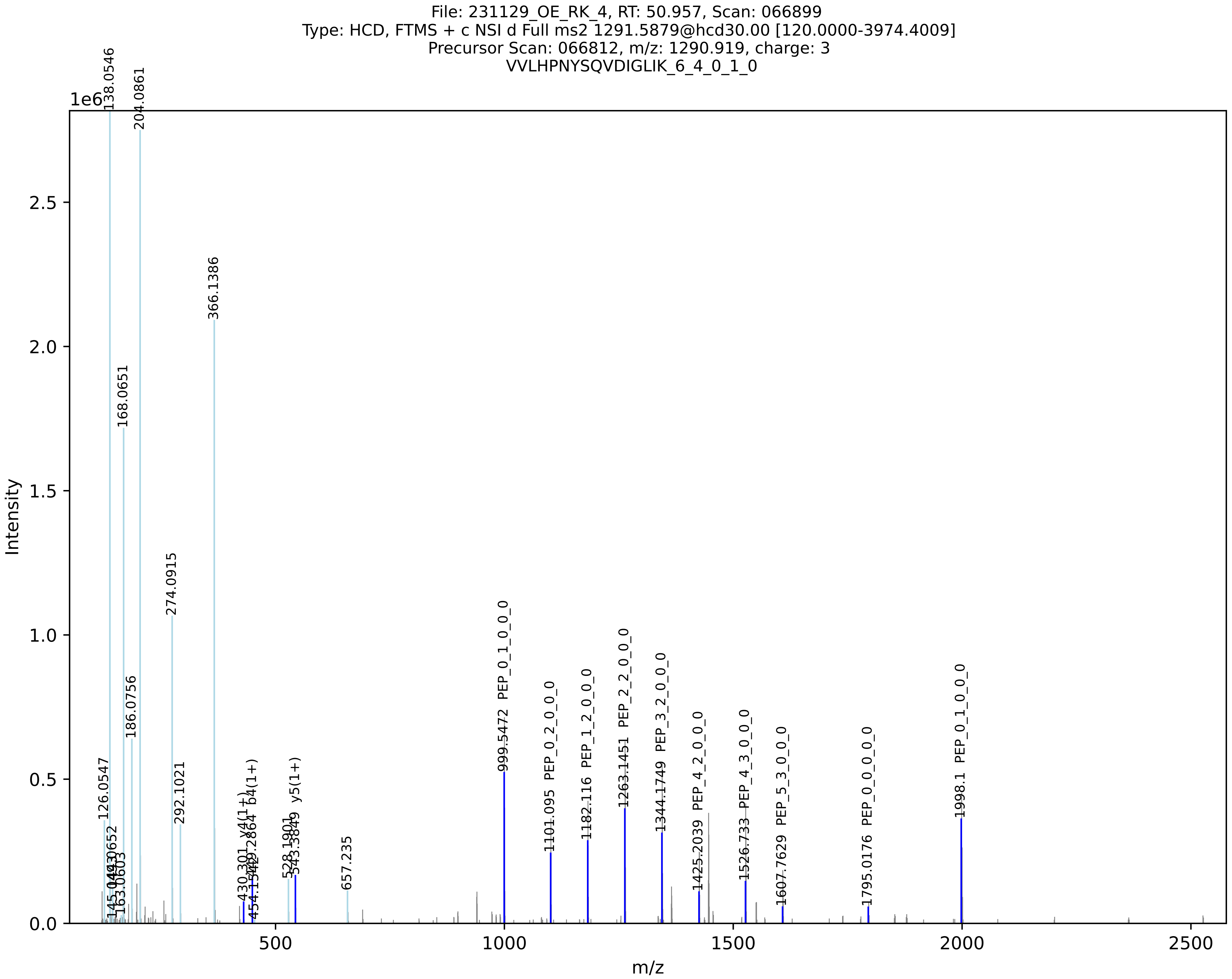
File: 231129_OE_RK_4, RT: 50.957, Scan: 066899
Type: HCD, FTMS + c NSI d Full ms2 1291.5879@hcd30.00 [120.0000-3974.4009]
Precursor Scan: 066812, m/z: 1290.919, charge: 3
VVLHPNYSQVDIGLIK_6_4_0_1_0
1e6
Intensity
m/z
138.0546
204.0861
366.1386
168.0651
274.0915
186.0756
126.0547
292.1021
163.0603
657.235
999.5472 PEP_0_1_0_0_0
1101.095 PEP_0_2_0_0_0
1182.116 PEP_1_2_0_0_0
1263.1451 PEP_2_2_0_0_0
1344.1749 PEP_3_2_0_0_0
1425.2039 PEP_4_2_0_0_0
1526.733 PEP_4_3_0_0_0
1607.7629 PEP_5_3_0_0_0
1795.0176 PEP_0_0_0_0_0
1998.1 PEP_0_1_0_0_0
0.0
0.5
1.0
1.5
2.0
2.5
500
1000
1500
2000
2500

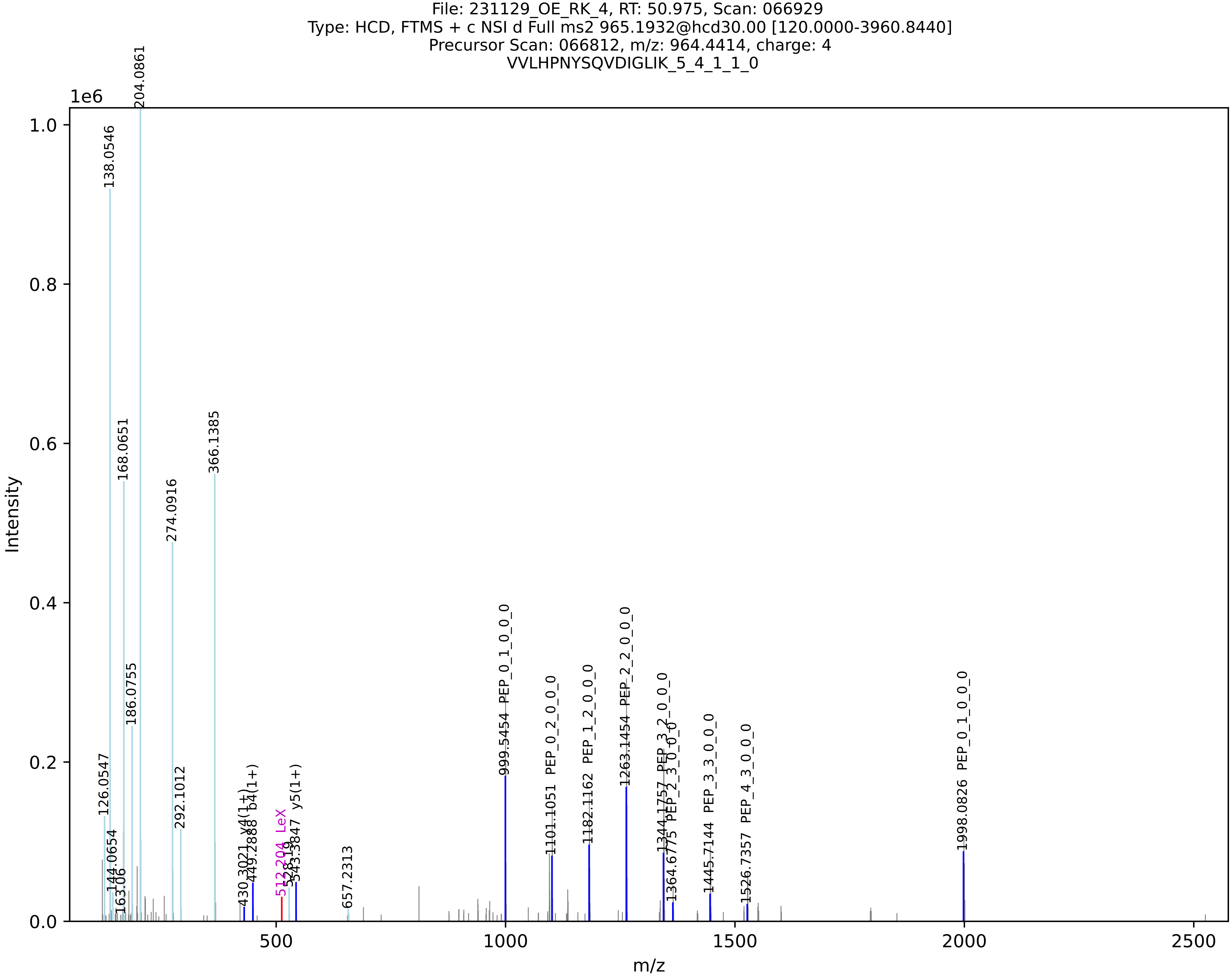
File: 231129_OE_RK_4, RT: 50.975, Scan: 066929
Type: HCD, FTMS + c NSI d Full ms2 965.1932@hcd30.00 [120.0000-3960.8440]
Precursor Scan: 066812, m/z: 964.4414, charge: 4
VVLHPNYSQVDIGLIK_5_4_1_1_0
1e6
Intensity
m/z
204.0861
138.0546
168.0651
366.1385
274.0916
186.0755
126.0547
292.1012
144.0654
163.06
430.3021 y4(1+)
449.2888 b4(1+)
512.204 LeX
528.19
543.3847 y5(1+)
657.2313
999.5454 PEP_0_1_0_0_0
1101.1051 PEP_0_2_0_0_0
1182.1162 PEP_1_2_0_0_0
1263.1454 PEP_2_2_0_0_0
1344.1757 PEP_3_2_0_0_0
1364.6775 PEP_2_3_0_0_0
1445.7144 PEP_3_3_0_0_0
1526.7357 PEP_4_3_0_0_0
1998.0826 PEP_0_1_0_0_0

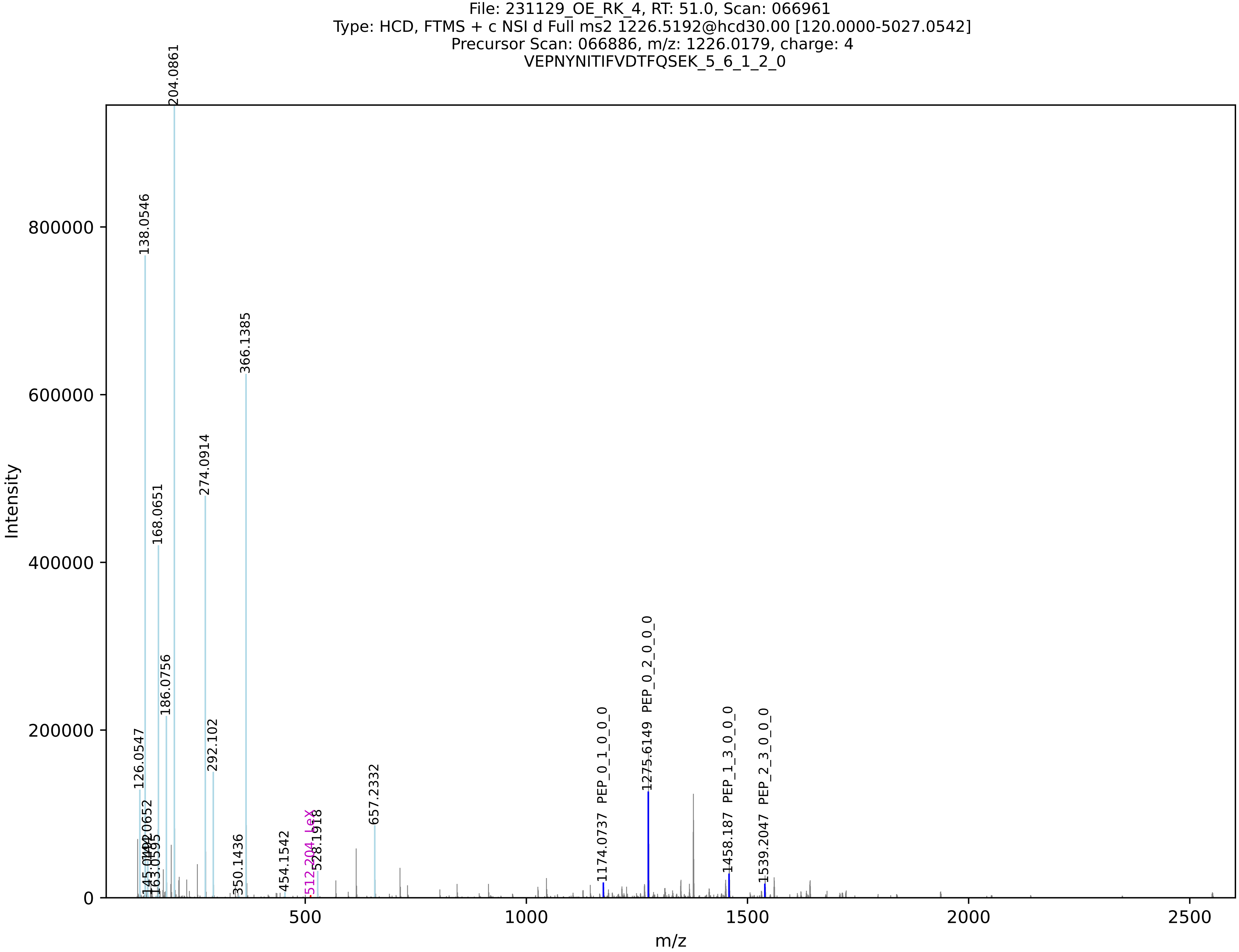
File: 231129_OE_RK_4, RT: 51.0, Scan: 066961
Type: HCD, FTMS + c NSI d Full ms2 1226.5192@hcd30.00 [120.0000-5027.0542]
Precursor Scan: 066886, m/z: 1226.0179, charge: 4
VEPNYNITIFVDTFQSEK_5_6_1_2_0
Intensity
m/z
204.0861
138.0546
366.1385
274.0914
168.0651
186.0756
292.102
126.0547
657.2332
528.1918
512.204_LeX
454.1542
350.1436
163.0595
1174.0737 PEP_0_1_0_0_0
1275.6149 PEP_0_2_0_0_0
1458.187 PEP_1_3_0_0_0
1539.2047 PEP_2_3_0_0_0
0
200000
400000
600000
800000
500
1000
1500
2000
2500

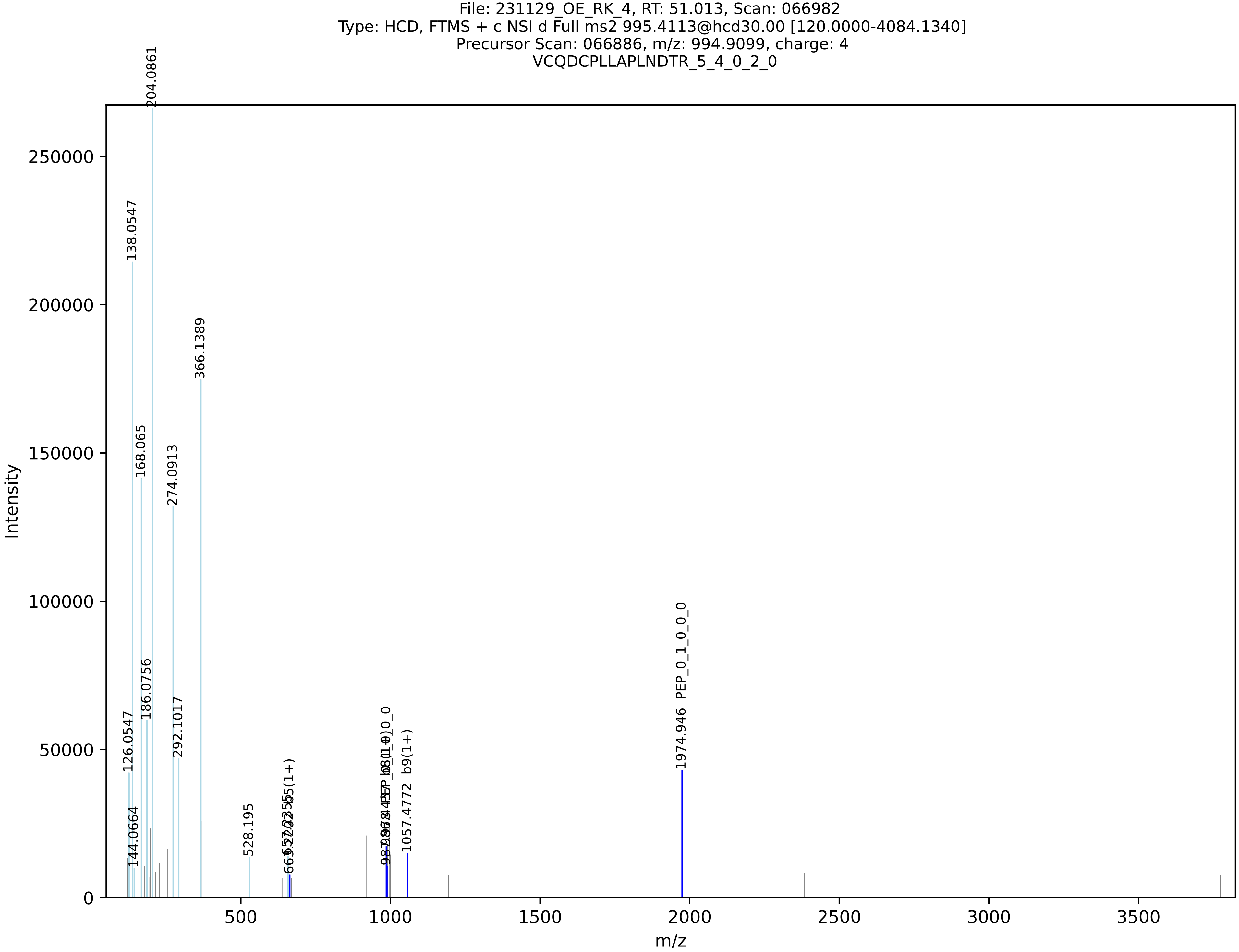
File: 231129_OE_RK_4, RT: 51.013, Scan: 066982
Type: HCD, FTMS + c NSI d Full ms2 995.4113@hcd30.00 [120.0000-4084.1340]
Precursor Scan: 066886, m/z: 994.9099, charge: 4
VCQDCPLLAPLNDTR_5_4_0_2_0
204.0861
138.0547
366.1389
168.065
274.0913
186.0756
292.1017
126.0547
144.0664
528.195
1974.946 PEP_0_1_0_0_0
1057.4772 b9(1+)
Intensity
m/z
0
50000
100000
150000
200000
250000
500
1000
1500
2000
2500
3000
3500

File: 231129_OE_RK_4, RT: 51.019, Scan: 066993
Type: HCD, FTMS + c NSI d Full ms2 1068.1989@hcd30.00 [120.0000-4381.1074]
Precursor Scan: 066886, m/z: 1067.4471, charge: 4
DQCIVDDITYNVNDTFHKR_5_4_0_1_0

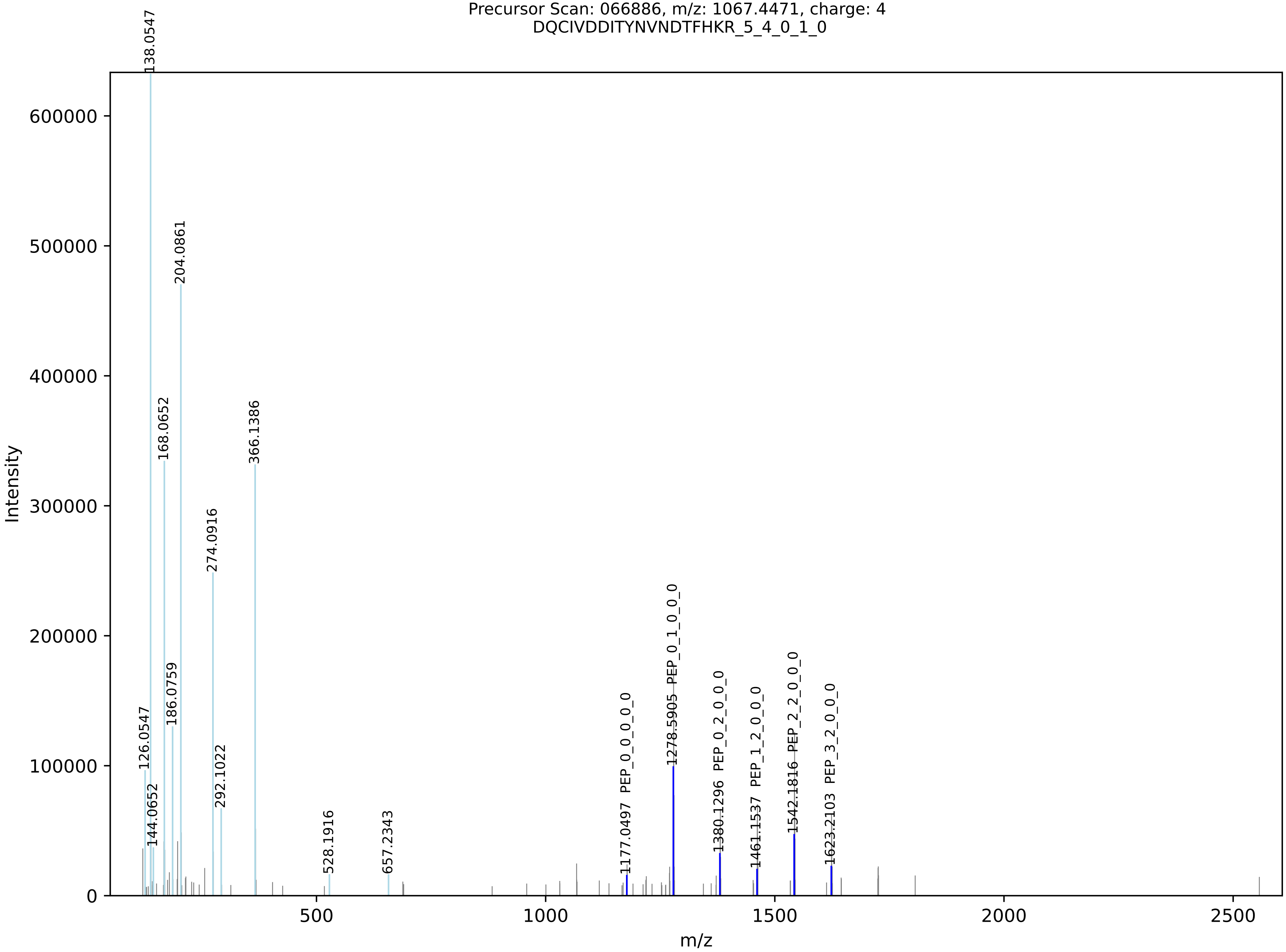

File: 231129_OE_RK_4, RT: 51.05, Scan: 067035
Type: HCD, FTMS + c NSI d Full ms2 1237.5690@hcd30.00 [120.0000-3809.1030]
Precursor Scan: 066960, m/z: 1236.9007, charge: 3
VVLHPNYSQVDIGLIK_5_4_0_1_0

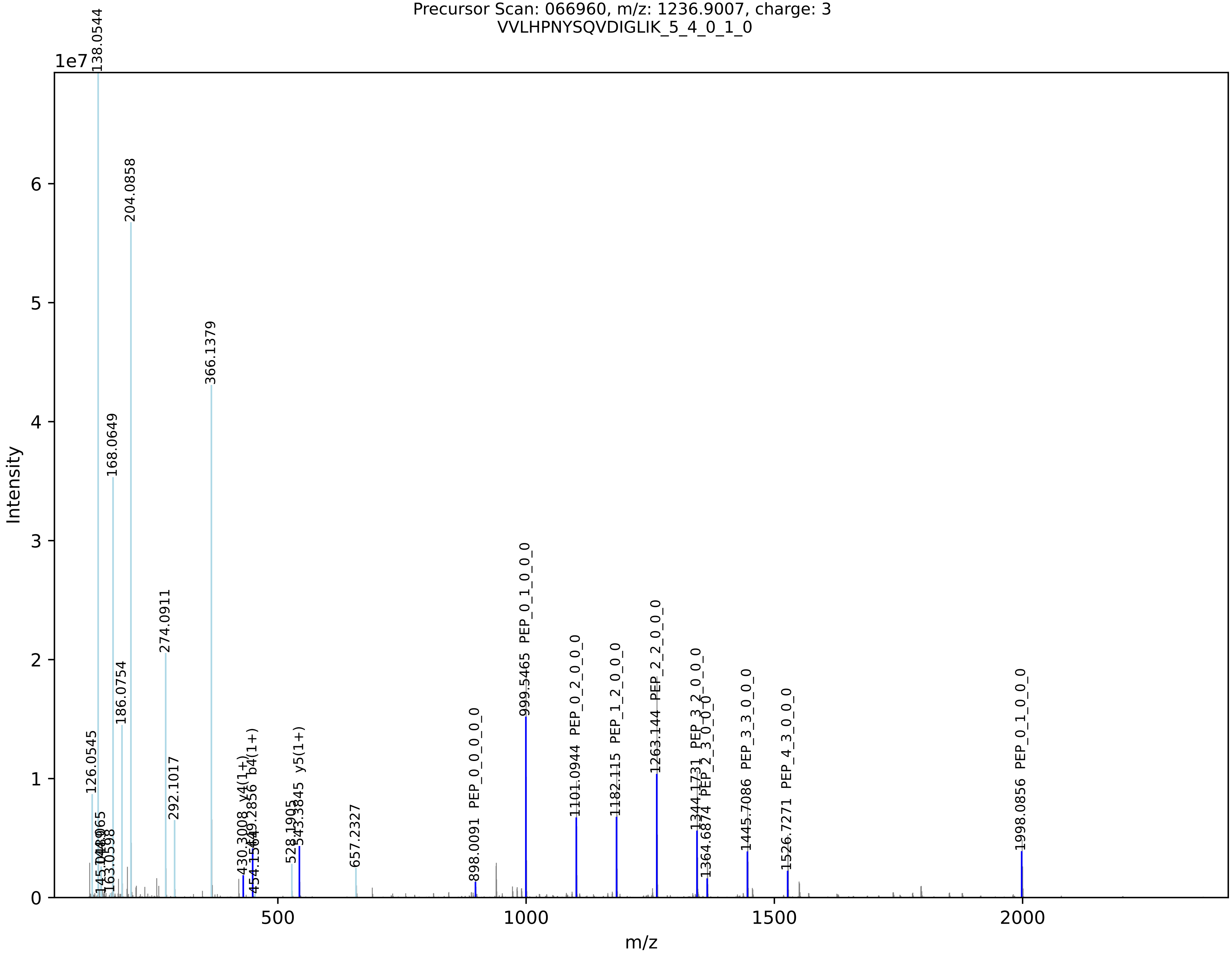

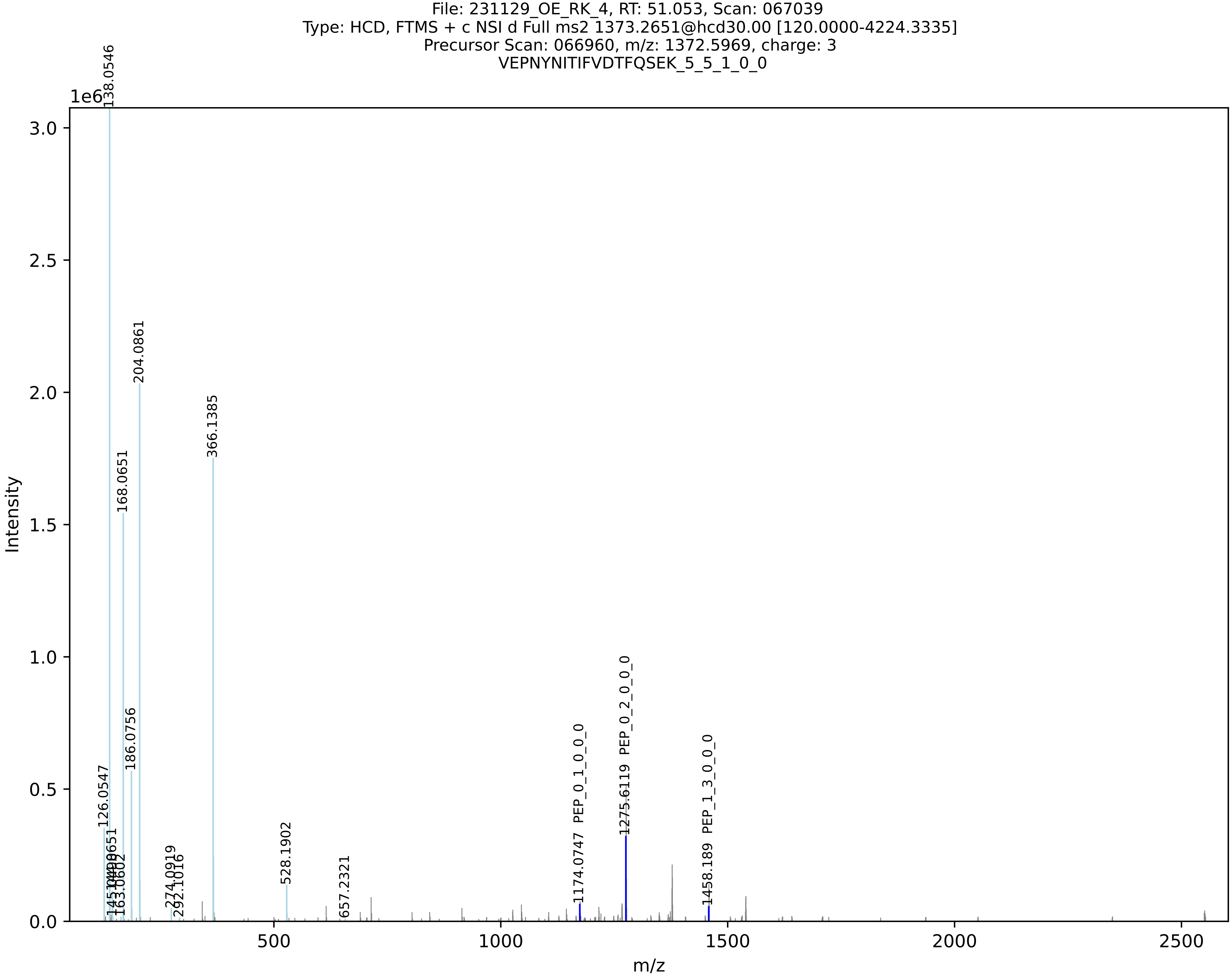

File: 231129_OE_RK_4, RT: 51.053, Scan: 067039
Type: HCD, FTMS + c NSI d Full ms2 1373.2651@hcd30.00 [120.0000-4224.3335]
Precursor Scan: 066960, m/z: 1372.5969, charge: 3
VEPNYNITIFVDTFQSEK_5_5_1_0_0
1e6
Intensity
m/z
138.0546
204.0861
366.1385
168.0651
186.0756
126.0547
528.1902
657.2321
274.0919
292.1016
163.0602
1174.0747 PEP_0_1_0_0_0
1275.6119 PEP_0_2_0_0_0
1458.189 PEP_1_3_0_0_0

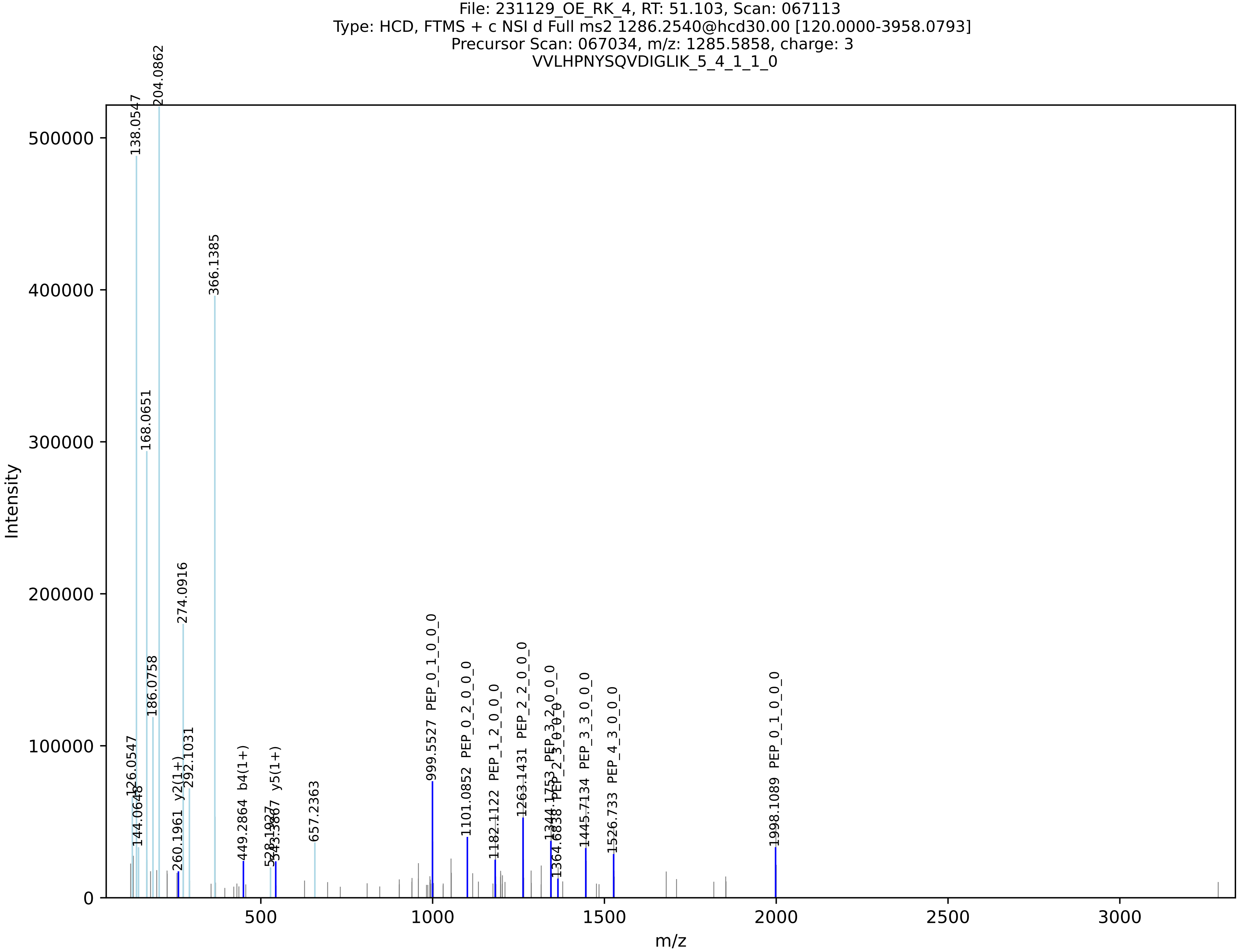

File: 231129_OE_RK_4, RT: 51.103, Scan: 067113
Type: HCD, FTMS + c NSI d Full ms2 1286.2540@hcd30.00 [120.0000-3958.0793]
Precursor Scan: 067034, m/z: 1285.5858, charge: 3
VVLHPNYSQVDIGLIK_5_4_1_1_0
Intensity
m/z
204.0862
138.0547
366.1385
168.0651
274.0916
186.0758
126.0547
144.0648
292.1031
260.1961 y2(1+)
449.2864 b4(1+)
543.3367 y5(1+)
657.2363
999.5527 PEP_0_1_0_0_0
1101.0852 PEP_0_2_0_0_0
1182.1122 PEP_1_2_0_0_0
1263.1431 PEP_2_2_0_0_0
1344.1753 PEP_3_2_0_0_0
1364.6838 PEP_2_3_0_0_0
1445.7134 PEP_3_3_0_0_0
1526.733 PEP_4_3_0_0_0
1998.1089 PEP_0_1_0_0_0

File: 231129_OE_RK_4, RT: 51.105, Scan: 067116
Type: HCD, FTMS + c NSI d Full ms2 1855.3481@hcd30.00 [120.0000-3806.3381]
Precursor Scan: 067034, m/z: 1854.8452, charge: 2
VVLHPNYSQVDIGLIK_5_4_0_1_0

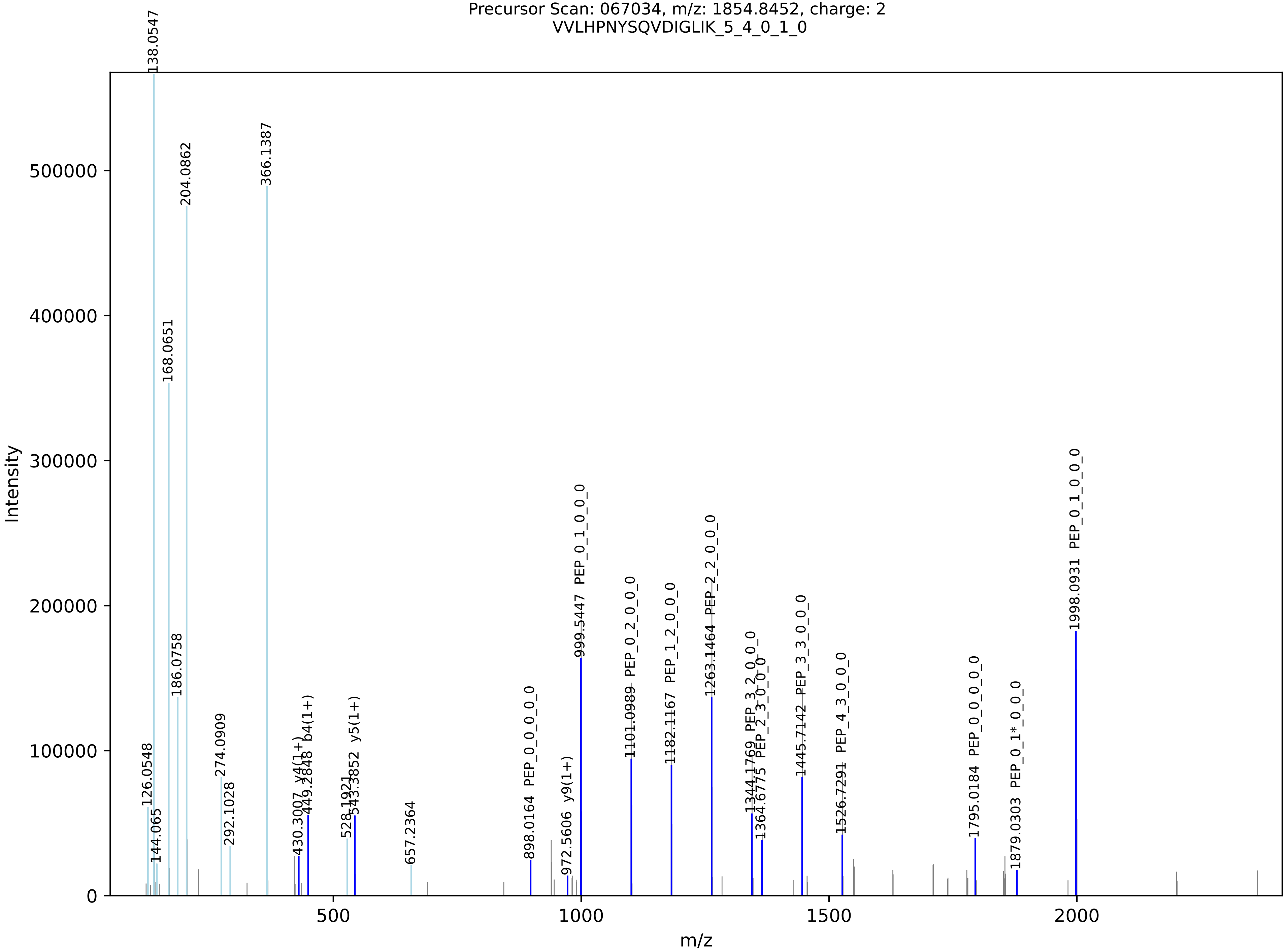

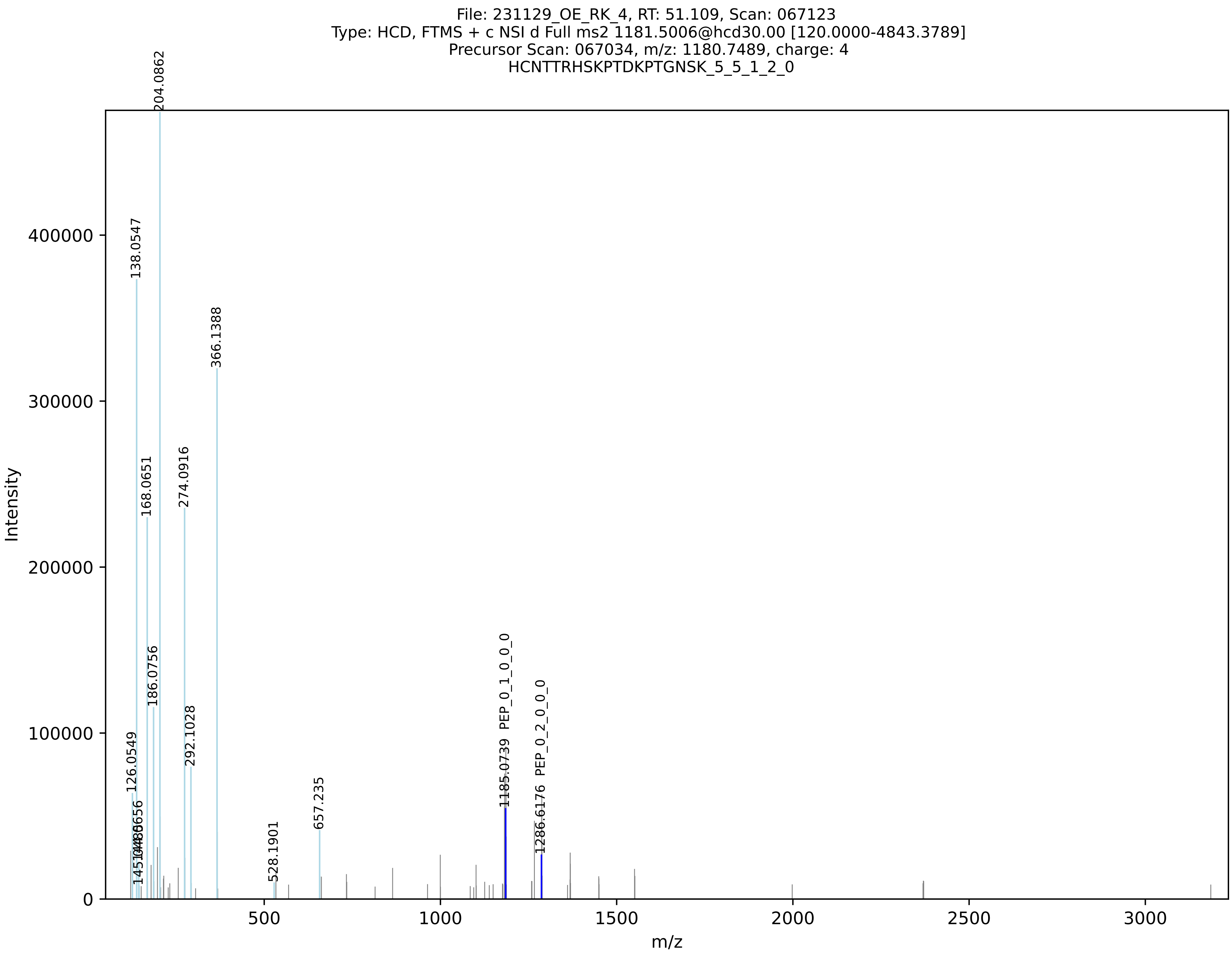
File: 231129_OE_RK_4, RT: 51.109, Scan: 067123
Type: HCD, FTMS + c NSI d Full ms2 1181.5006@hcd30.00 [120.0000-4843.3789]
Precursor Scan: 067034, m/z: 1180.7489, charge: 4
HCNTTRHSKPTDKPTGNSK_5_5_1_2_0
204.0862
138.0547
366.1388
274.0916
168.0651
186.0756
292.1028
126.0549
657.235
528.1901
1185.0739 PEP_0_1_0_0_0
1286.6176 PEP_0_2_0_0_0
Intensity
m/z
0
100000
200000
300000
400000
500
1000
1500
2000
2500
3000

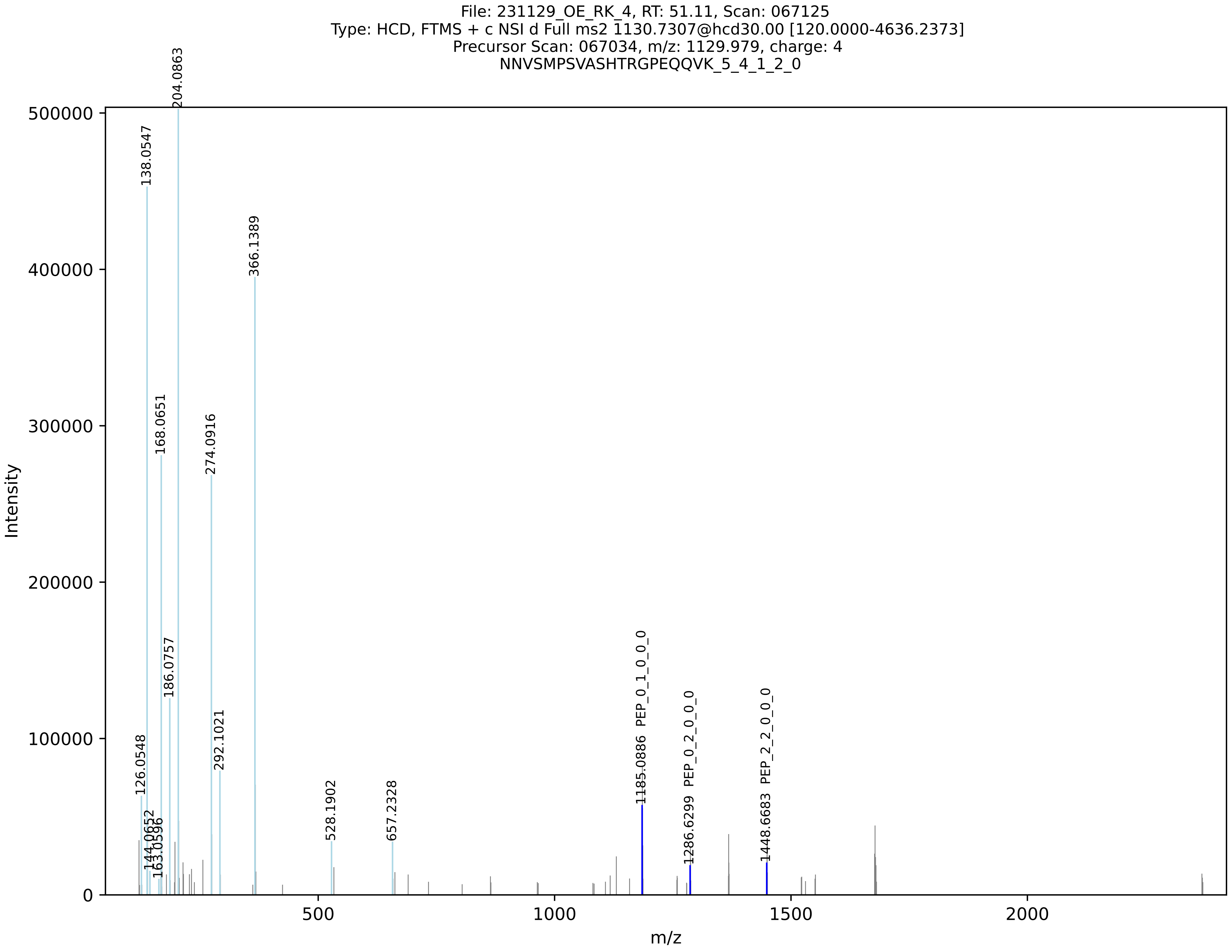
File: 231129_OE_RK_4, RT: 51.11, Scan: 067125
Type: HCD, FTMS + c NSI d Full ms2 1130.7307@hcd30.00 [120.0000-4636.2373]
Precursor Scan: 067034, m/z: 1129.979, charge: 4
NNVSMPSVASHTRGPEQQVK_5_4_1_2_0
204.0863
138.0547
366.1389
168.0651
274.0916
186.0757
292.1021
126.0548
144.0652
163.0596
528.1902
657.2328
1185.0886 PEP_0_1_0_0_0_
1286.6299 PEP_0_2_0_0_0_
1448.6683 PEP_2_2_0_0_0_
Intensity
m/z
500000
400000
300000
200000
100000
0
500
1000
1500
2000

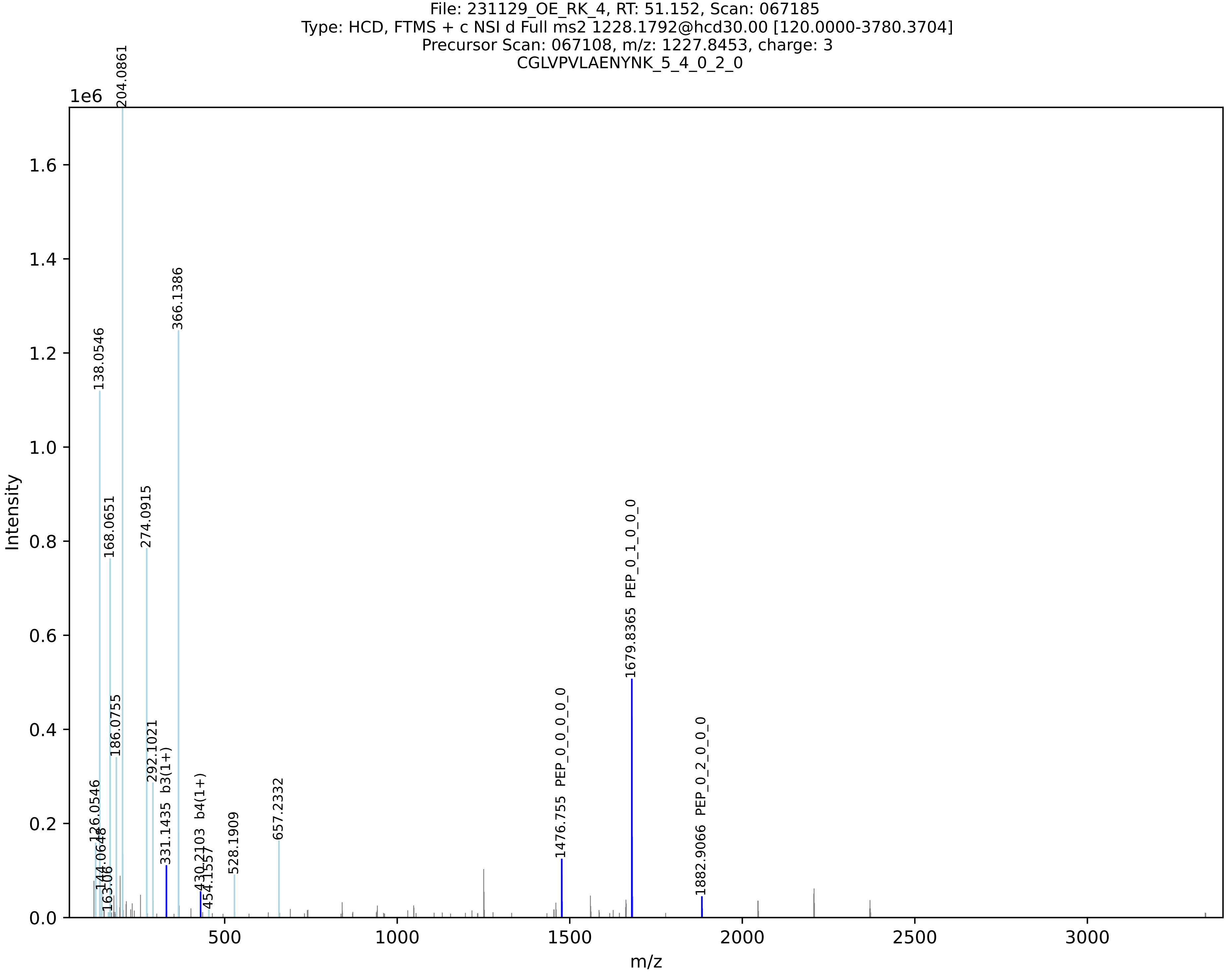
File: 231129_OE_RK_4, RT: 51.152, Scan: 067185
Type: HCD, FTMS + c NSI d Full ms2 1228.1792@hcd30.00 [120.0000-3780.3704]
Precursor Scan: 067108, m/z: 1227.8453, charge: 3
CGLVPVLAENYNK_5_4_0_2_0
1e6
Intensity
m/z
204.0861
366.1386
138.0546
168.0651
274.0915
186.0755
292.1021
331.1435 b3(1+)
430.2103 b4(1+)
454.1557
528.1909
657.2332
126.0546
144.0648
163.06
1476.755 PEP_0_0_0_0_0
1679.8365 PEP_0_1_0_0_0
1882.9066 PEP_0_2_0_0_0

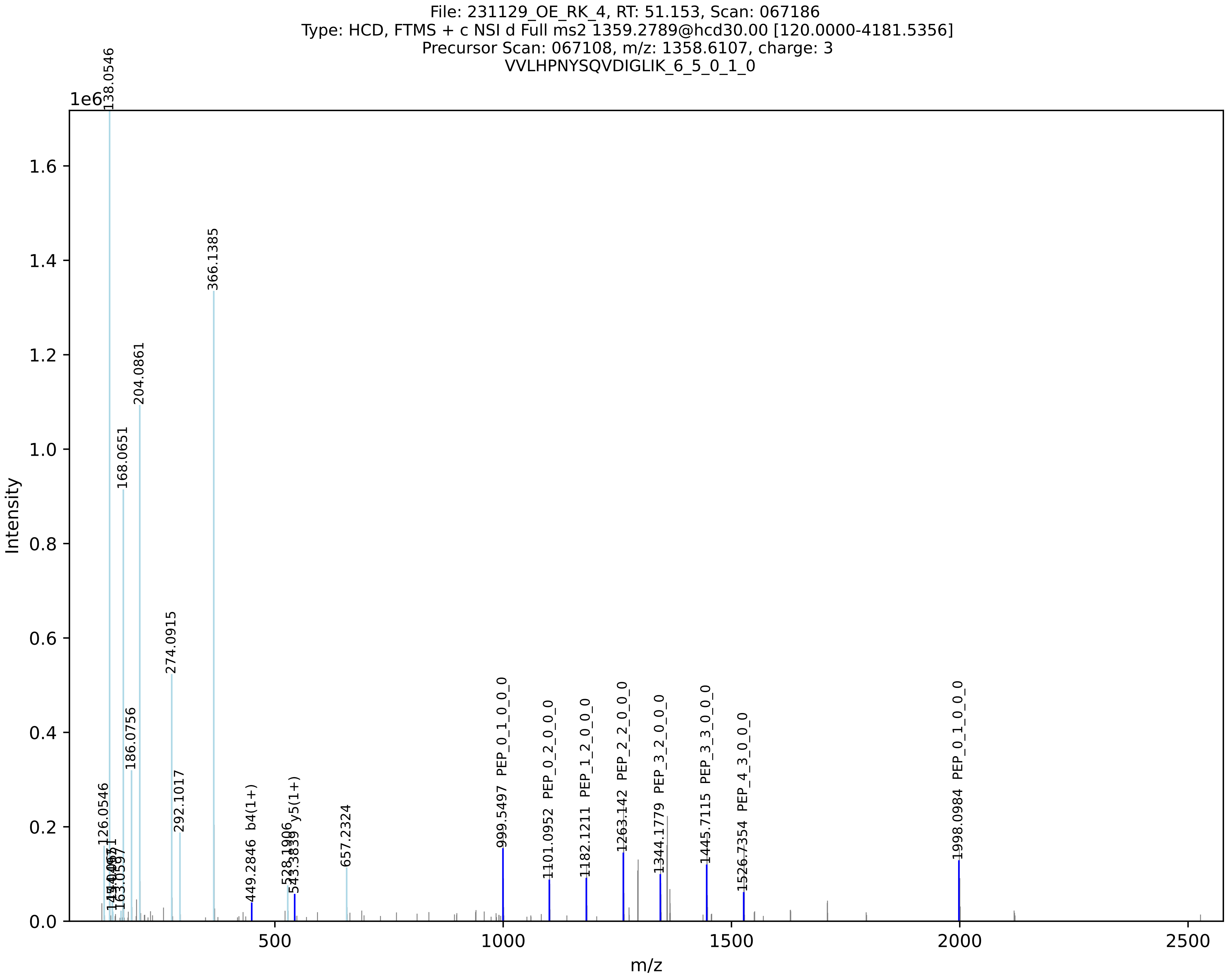

File: 231129_OE_RK_4, RT: 51.153, Scan: 067186
Type: HCD, FTMS + c NSI d Full ms2 1359.2789@hcd30.00 [120.0000-4181.5356]
Precursor Scan: 067108, m/z: 1358.6107, charge: 3
VVLHPNYSQVDIGLIK_6_5_0_1_0
1e6
Intensity
m/z
0.0
0.2
0.4
0.6
0.8
1.0
1.2
1.4
1.6
500
1000
1500
2000
2500
138.0546
366.1385
204.0861
168.0651
274.0915
186.0756
292.1017
126.0546
163.0597
449.2846 b4(1+)
528.1906
543.3839 y5(1+)
657.2324
999.5497 PEP_0_1_0_0_0
1101.0952 PEP_0_2_0_0_0
1182.1211 PEP_1_2_0_0_0
1263.142 PEP_2_2_0_0_0
1344.1779 PEP_3_2_0_0_0
1445.7115 PEP_3_3_0_0_0
1526.7354 PEP_4_3_0_0_0
1998.0984 PEP_0_1_0_0_0

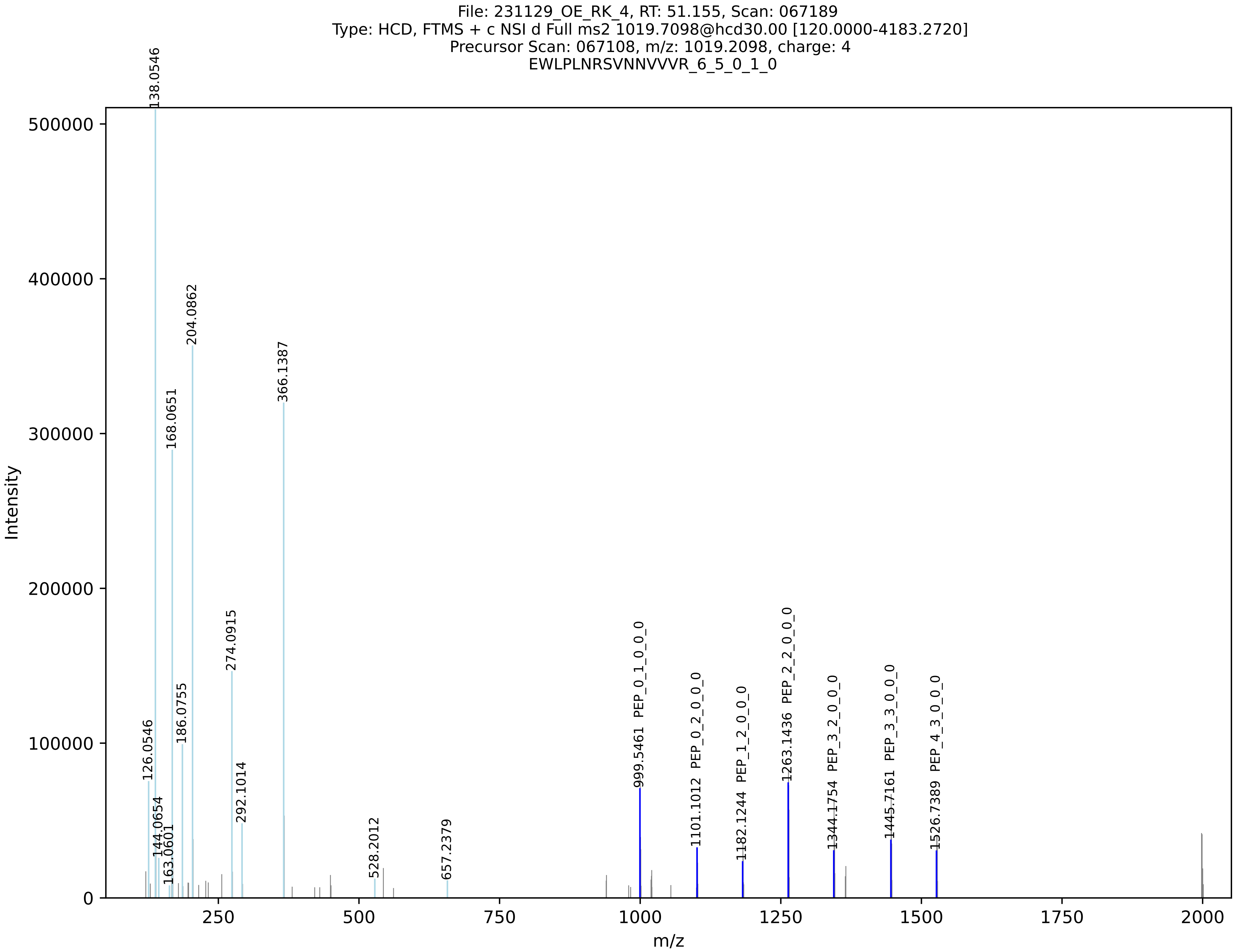
File: 231129_OE_RK_4, RT: 51.155, Scan: 067189
Type: HCD, FTMS + c NSI d Full ms2 1019.7098@hcd30.00 [120.0000-4183.2720]
Precursor Scan: 067108, m/z: 1019.2098, charge: 4
EWLPLNRSVNNVVVR_6_5_0_1_0
Intensity
m/z
138.0546
204.0862
366.1387
168.0651
274.0915
186.0755
126.0546
292.1014
144.0654
163.0601
528.2012
657.2379
999.5461 PEP_0_1_0_0_0
1101.1012 PEP_0_2_0_0_0
1182.1244 PEP_1_2_0_0_0
1263.1436 PEP_2_2_0_0_0
1344.1754 PEP_3_2_0_0_0
1445.7161 PEP_3_3_0_0_0
1526.7389 PEP_4_3_0_0_0

File: 231129_OE_RK_4, RT: 51.167, Scan: 067209
Type: HCD, FTMS + c NSI d Full ms2 1291.5858@hcd30.00 [120.0000-3974.3945]
Precursor Scan: 067108, m/z: 1291.5858, charge: 3
EWLPLNRSVNNVVVR_6_4_0_1_0

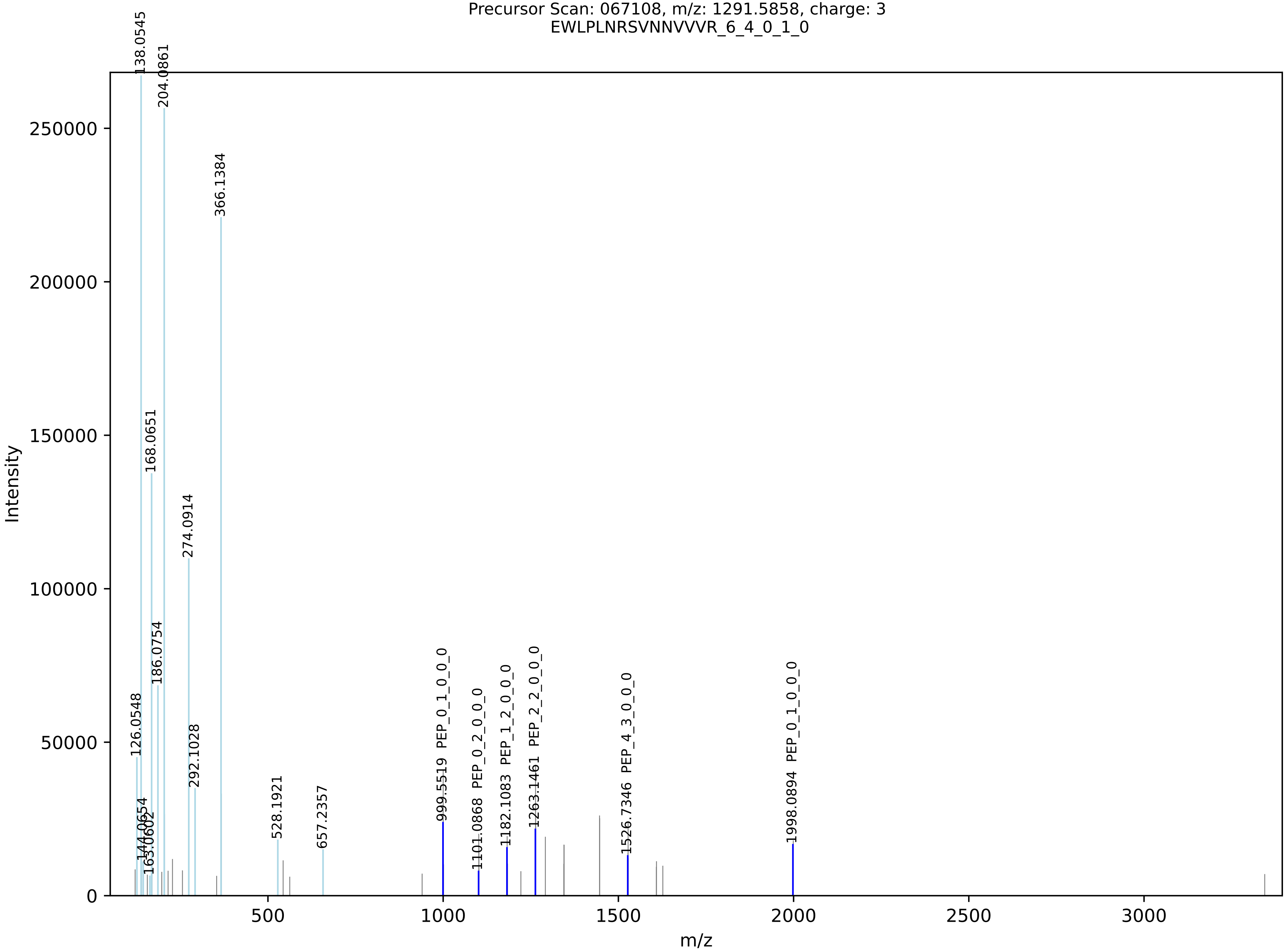

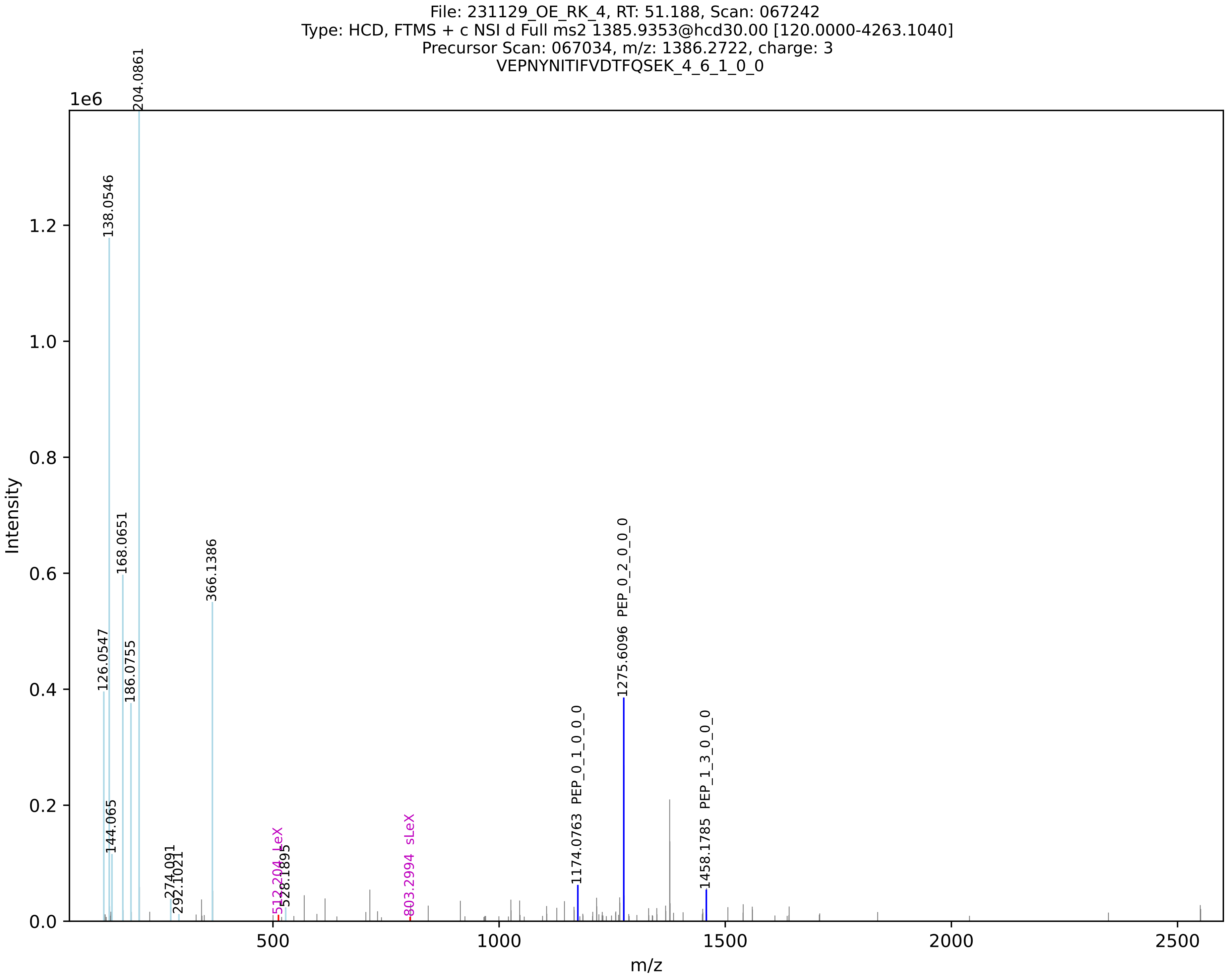

File: 231129_OE_RK_4, RT: 51.188, Scan: 067242
Type: HCD, FTMS + c NSI d Full ms2 1385.9353@hcd30.00 [120.0000-4263.1040]
Precursor Scan: 067034, m/z: 1386.2722, charge: 3
VEPNYNITIFVDTFQSEK_4_6_1_0_0
1e6
Intensity
m/z
204.0861
138.0546
168.0651
366.1386
126.0547
186.0755
144.065
274.091
292.1021
512.204 LeX
528.1895
803.2994 sLeX
1174.0763 PEP_0_1_0_0_0
1275.6096 PEP_0_2_0_0_0
1458.1785 PEP_1_3_0_0_0
0.0
0.2
0.4
0.6
0.8
1.0
1.2
500
1000
1500
2000
2500

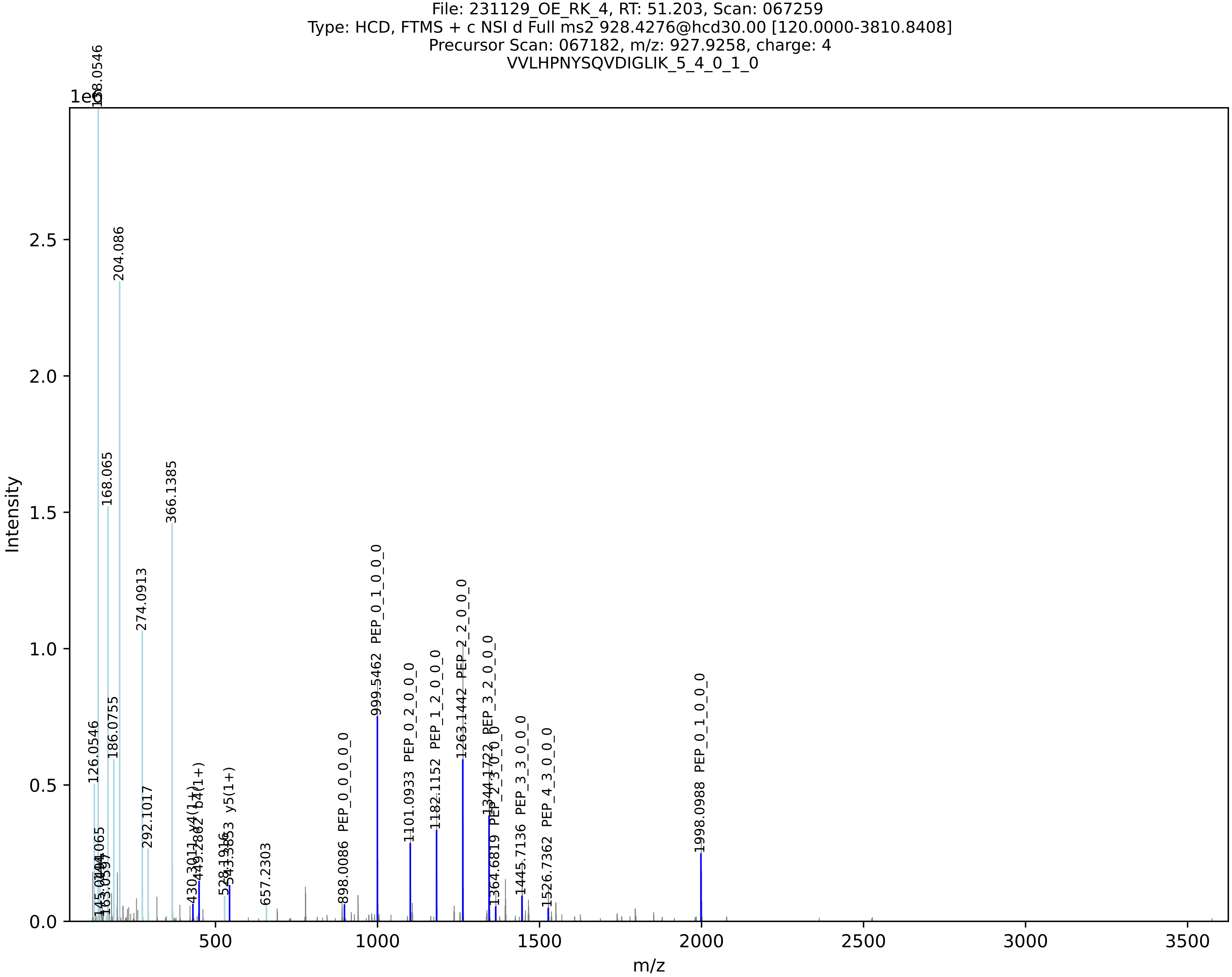

File: 231129_OE_RK_4, RT: 51.203, Scan: 067259
Type: HCD, FTMS + c NSI d Full ms2 928.4276@hcd30.00 [120.0000-3810.8408]
Precursor Scan: 067182, m/z: 927.9258, charge: 4
VVLHPNYSQVDIGLIK_5_4_0_1_0
1e6
Intensity
m/z
138.0546
204.086
168.065
366.1385
274.0913
186.0755
126.0546
292.1017
430.3011 y4(1+)
449.2862 b4(1+)
543.3653 y5(1+)
657.2303
898.0086 PEP_0_0_0_0_0
999.5462 PEP_0_1_0_0_0
1101.0933 PEP_0_2_0_0_0
1182.1152 PEP_1_2_0_0_0
1263.1442 PEP_2_2_0_0_0
1364.6819 PEP_2_3_0_0_0
1445.7136 PEP_3_3_0_0_0
1526.7362 PEP_4_3_0_0_0
1998.0988 PEP_0_1_0_0_0

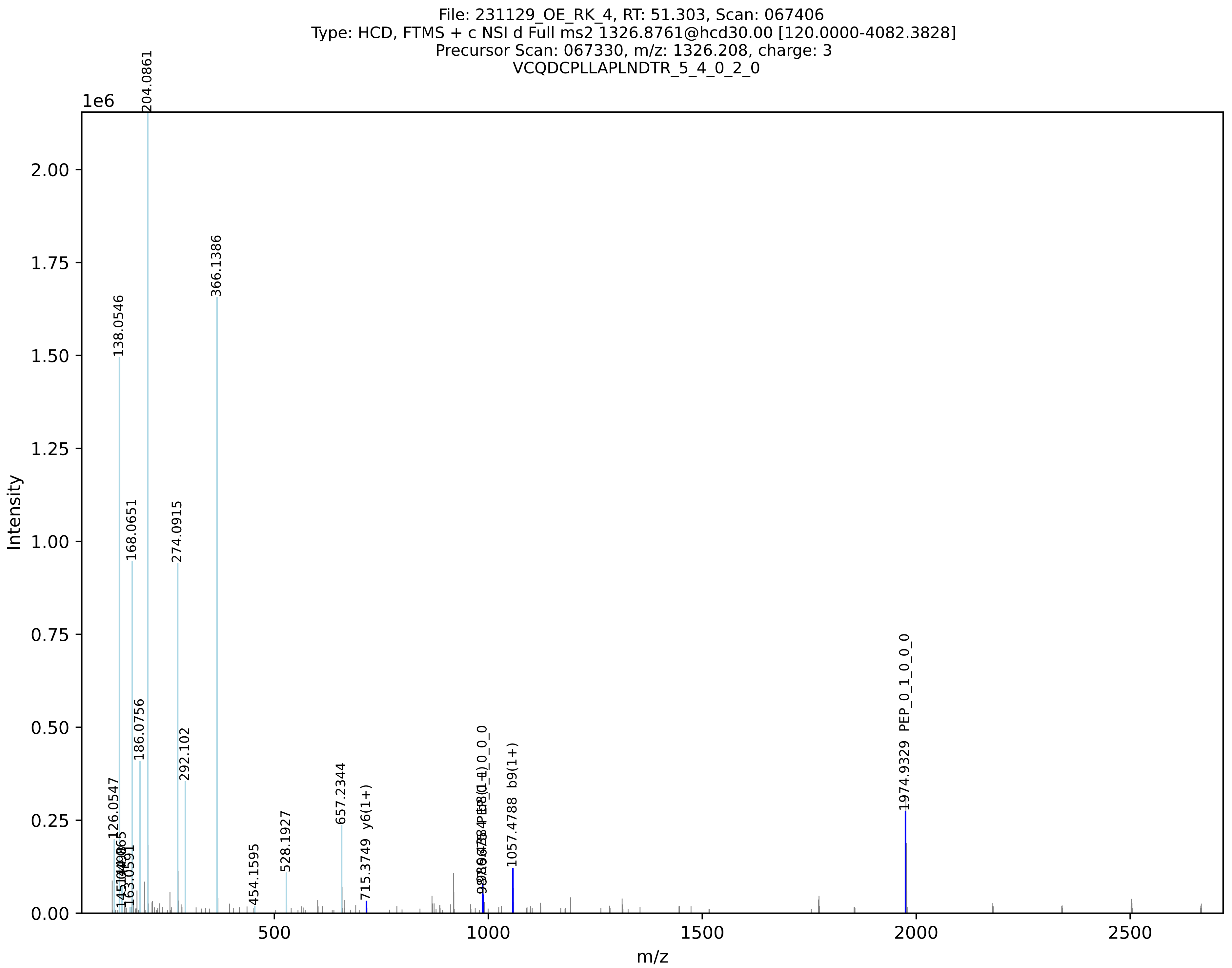
File: 231129_OE_RK_4, RT: 51.303, Scan: 067406
Type: HCD, FTMS + c NSI d Full ms2 1326.8761@hcd30.00 [120.0000-4082.3828]
Precursor Scan: 067330, m/z: 1326.208, charge: 3
VCQDCPLLAPLNDTR_5_4_0_2_0
1e6
Intensity
m/z
204.0861
366.1386
138.0546
168.0651
274.0915
186.0756
292.102
126.0547
163.0591
454.1595
528.1927
657.2344
715.3749 y6(1+)
1057.4788 b9(1+)
1974.9329 PEP_0_1_0_0_0

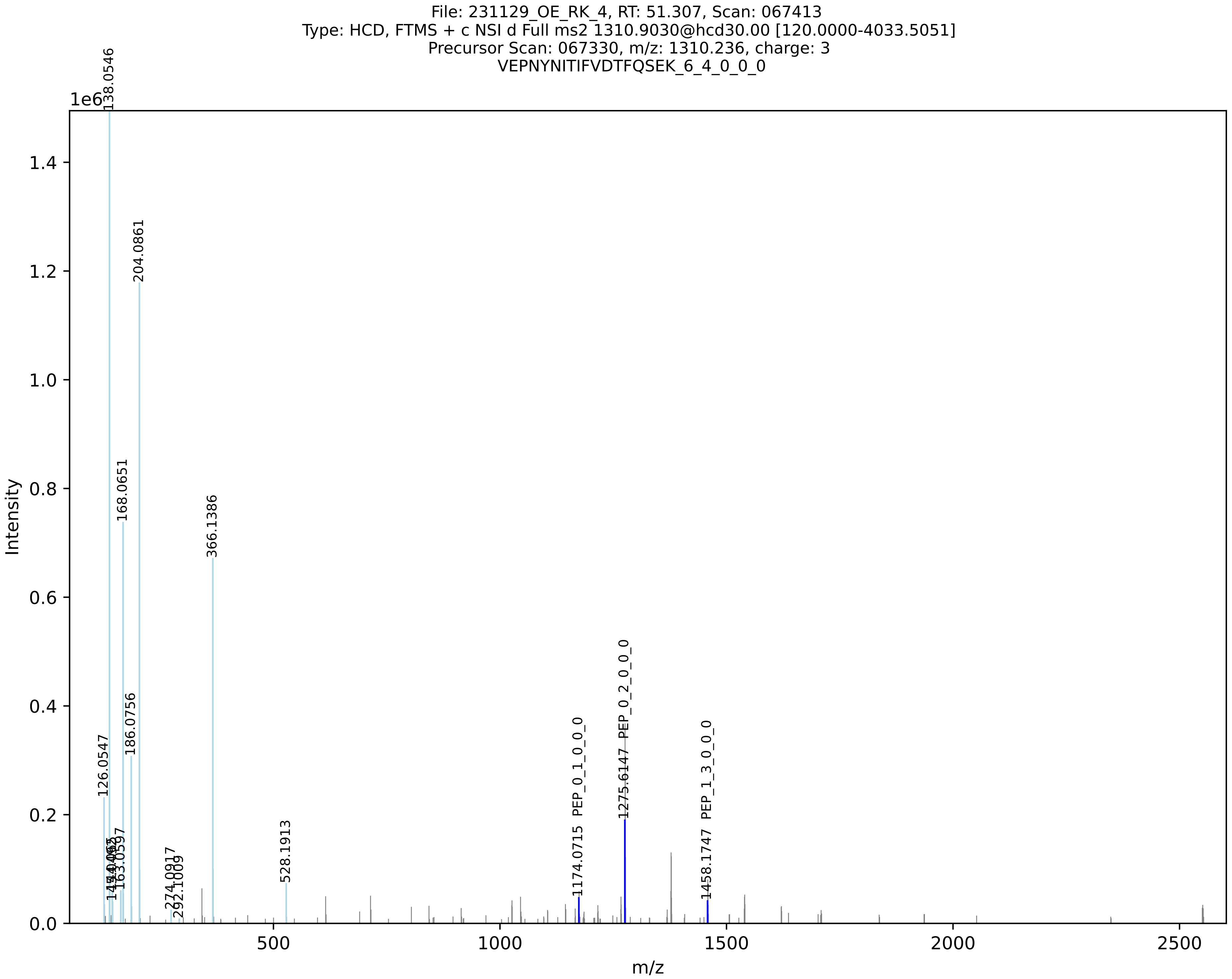

File: 231129_OE_RK_4, RT: 51.307, Scan: 067413
Type: HCD, FTMS + c NSI d Full ms2 1310.9030@hcd30.00 [120.0000-4033.5051]
Precursor Scan: 067330, m/z: 1310.236, charge: 3
VEPNYNITIFVDTFQSEK_6_4_0_0_0
1e6
Intensity
m/z
138.0546
204.0861
168.0651
366.1386
186.0756
126.0547
163.0597
274.0917
292.1009
528.1913
1174.0715 PEP_0_1_0_0_0
1275.6147 PEP_0_2_0_0_0
1458.1747 PEP_1_3_0_0_0

File: 231129_OE_RK_4, RT: 51.313, Scan: 067422
Type: HCD, FTMS + c NSI d Full ms2 1855.8435@hcd30.00 [120.0000-3807.3486]
Precursor Scan: 067330, m/z: 1854.8451, charge: 2
VVLHPNYSQVDIGLIK_5_4_0_1_0

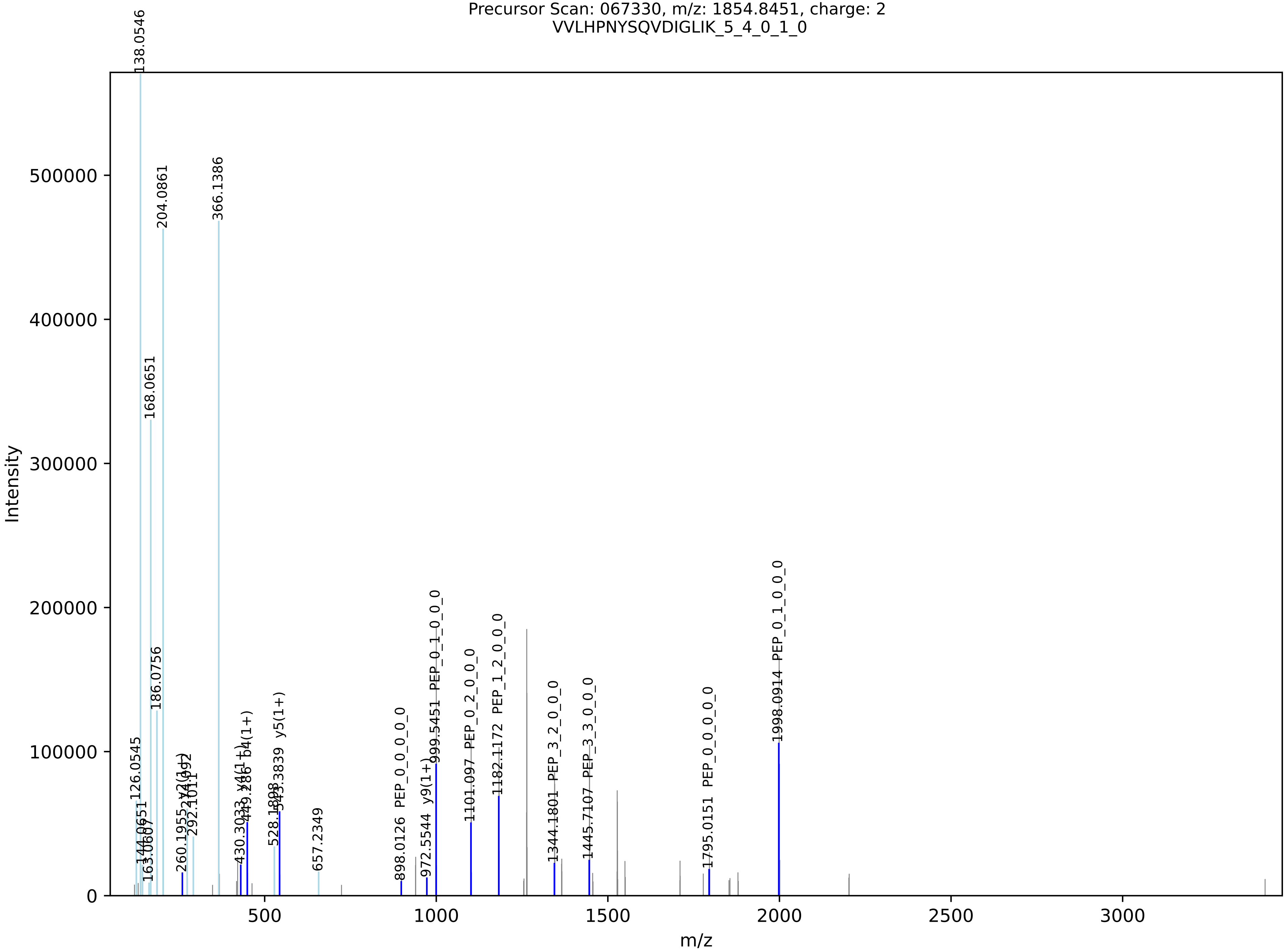

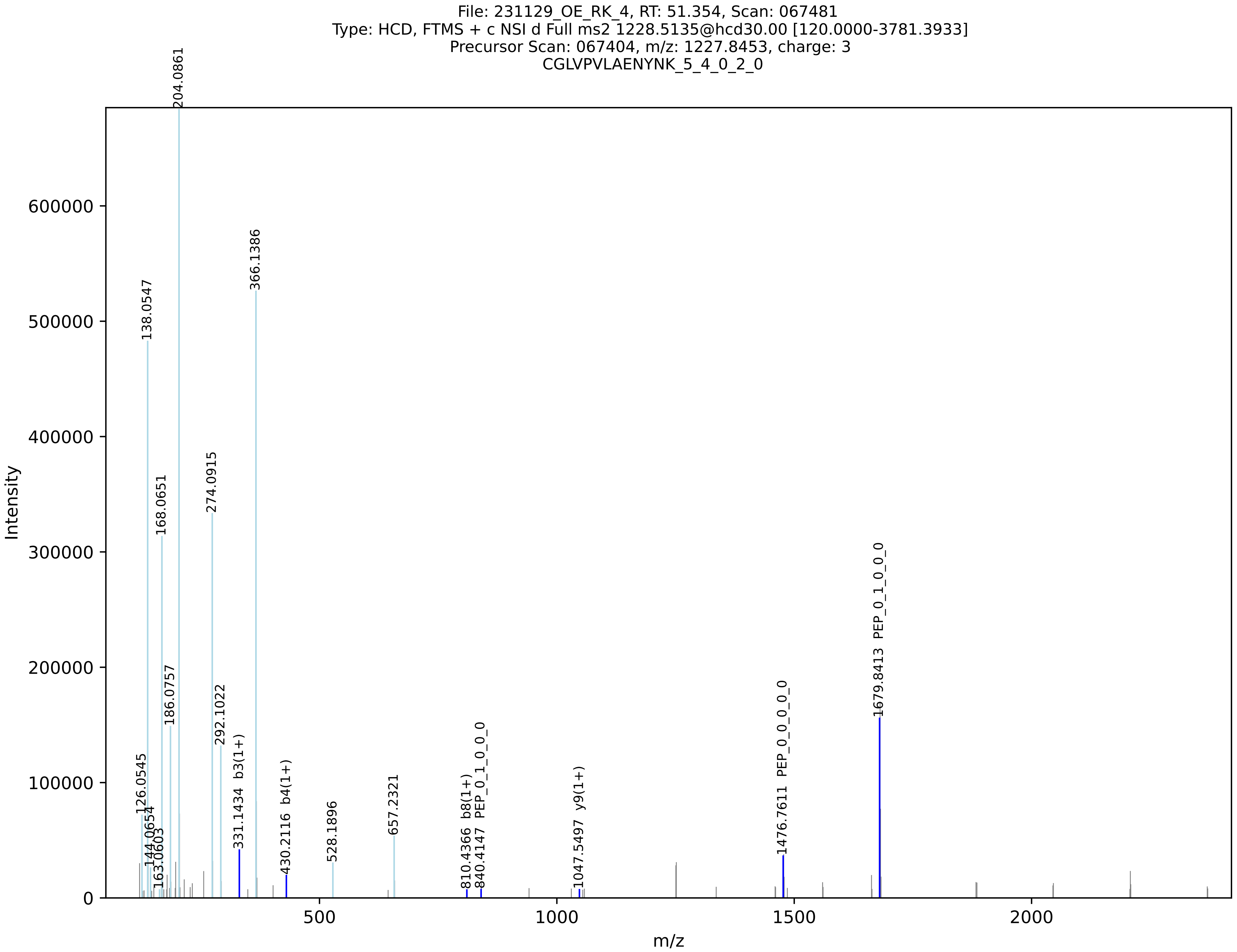
File: 231129_OE_RK_4, RT: 51.354, Scan: 067481
Type: HCD, FTMS + c NSI d Full ms2 1228.5135@hcd30.00 [120.0000-3781.3933]
Precursor Scan: 067404, m/z: 1227.8453, charge: 3
CGLVPVLAENYNK_5_4_0_2_0
Intensity
m/z
0
100000
200000
300000
400000
500000
600000
500
1000
1500
2000
204.0861
366.1386
138.0547
274.0915
168.0651
186.0757
292.1022
126.0545
144.0654
163.0603
331.1434 b3(1+)
430.2116 b4(1+)
528.1896
657.2321
810.4366 b8(1+)
840.4147 PEP_0_1_0_0_0
1047.5497 y9(1+)
1476.7611 PEP_0_0_0_0_0
1679.8413 PEP_0_1_0_0_0

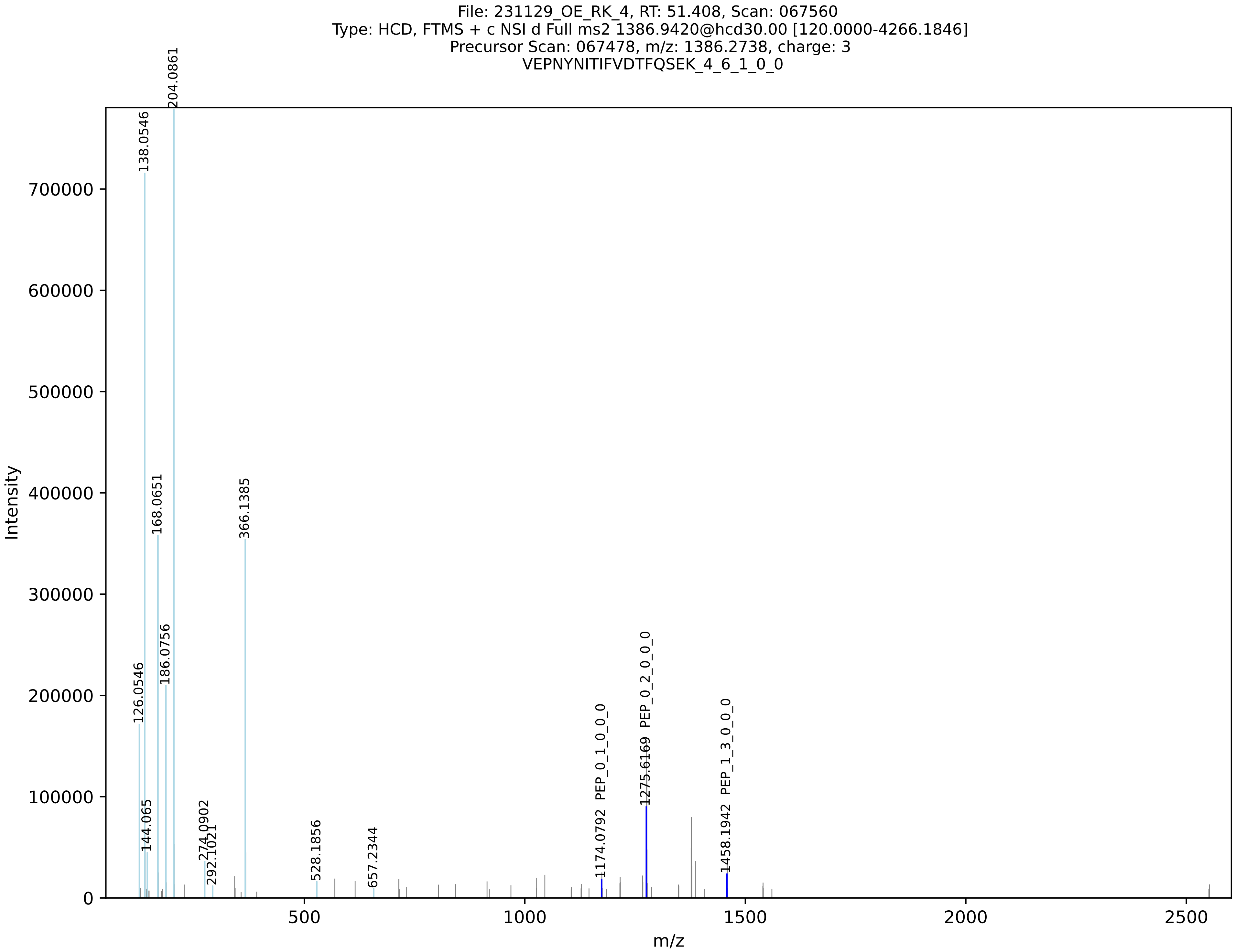
File: 231129_OE_RK_4, RT: 51.408, Scan: 067560
Type: HCD, FTMS + c NSI d Full ms2 1386.9420@hcd30.00 [120.0000-4266.1846]
Precursor Scan: 067478, m/z: 1386.2738, charge: 3
VEPNYNITIFVDTFQSEK_4_6_1_0_0
204.0861
138.0546
168.0651
366.1385
186.0756
126.0546
144.065
274.0902
292.1021
528.1856
657.2344
1174.0792 PEP_0_1_0_0_0
1275.6169 PEP_0_2_0_0_0
1458.1942 PEP_1_3_0_0_0
Intensity
m/z
0
100000
200000
300000
400000
500000
600000
700000
500
1000
1500
2000
2500

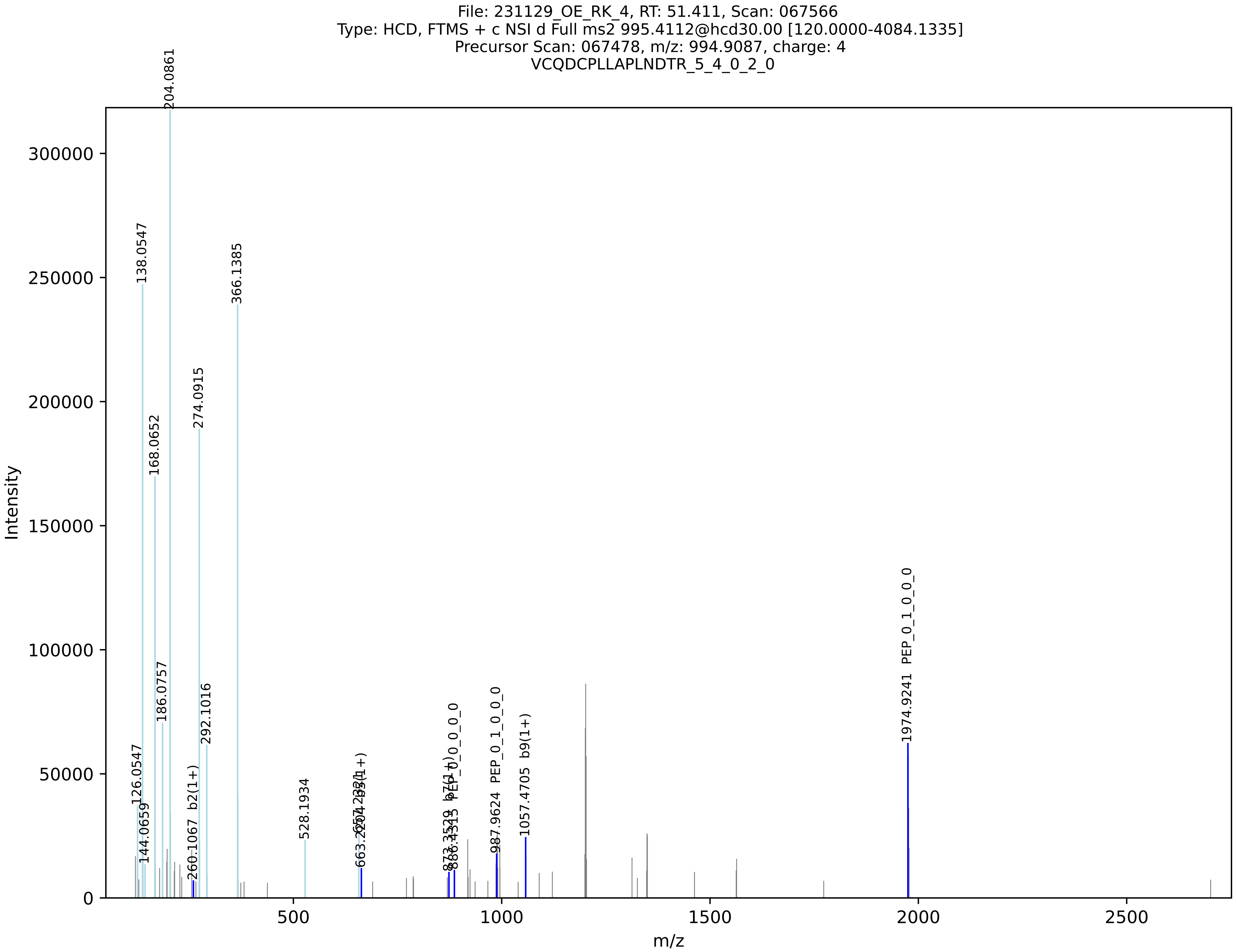
File: 231129_OE_RK_4, RT: 51.411, Scan: 067566
Type: HCD, FTMS + c NSI d Full ms2 995.4112@hcd30.00 [120.0000-4084.1335]
Precursor Scan: 067478, m/z: 994.9087, charge: 4
VCQDCPLLAPLNDTR_5_4_0_2_0
Intensity
m/z
204.0861
138.0547
366.1385
274.0915
168.0652
186.0757
292.1016
126.0547
144.0659
260.1067 b2(1+)
528.1934
663.2604
873.3529 b7(1+)
886.4515 PEP_0_0_0_0_0
987.9624 PEP_0_1_0_0_0
1057.4705 b9(1+)
1974.9241 PEP_0_1_0_0_0
0
50000
100000
150000
200000
250000
300000
500
1000
1500
2000
2500

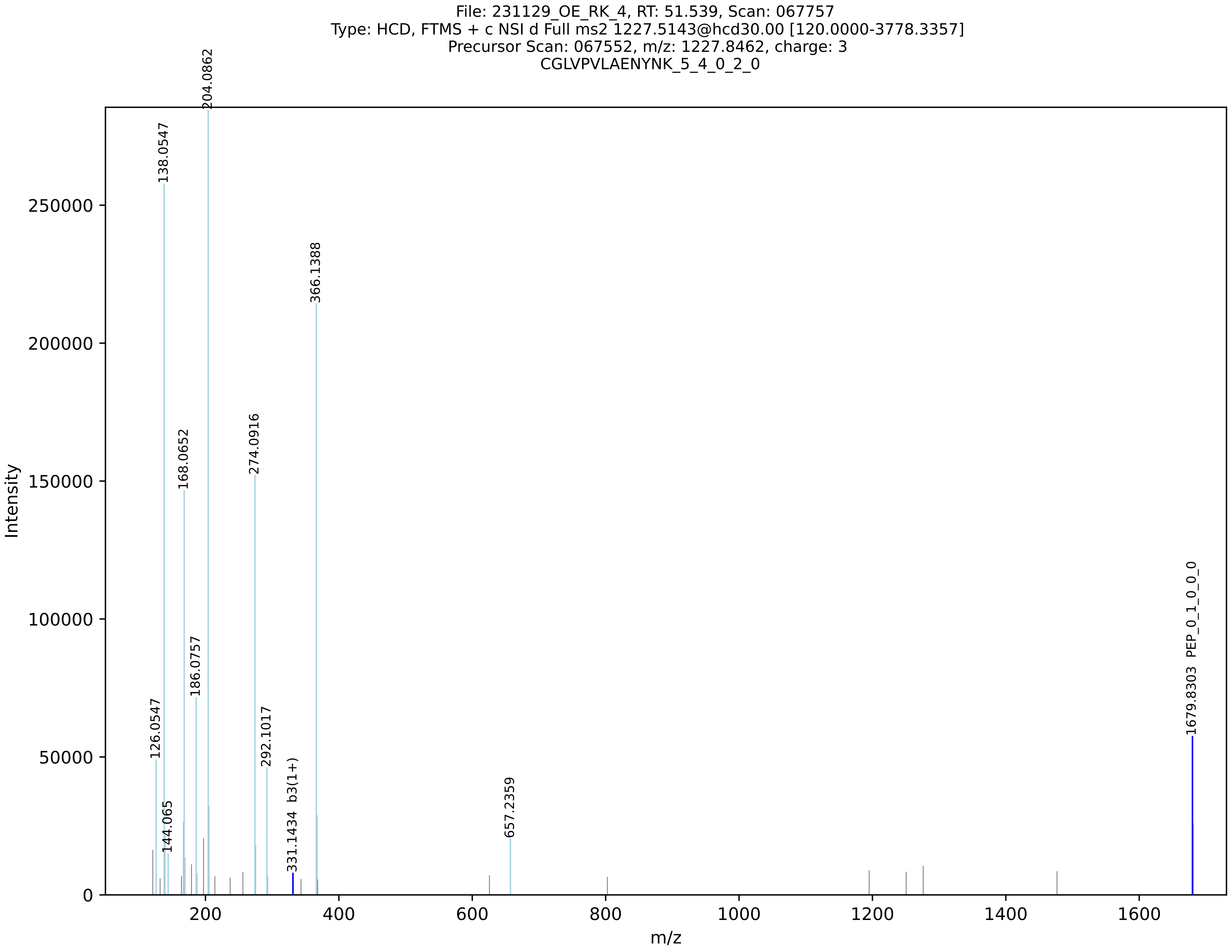

File: 231129_OE_RK_4, RT: 51.539, Scan: 067757
Type: HCD, FTMS + c NSI d Full ms2 1227.5143@hcd30.00 [120.0000-3778.3357]
Precursor Scan: 067552, m/z: 1227.8462, charge: 3
CGLVPVLAENYNK_5_4_0_2_0
Intensity
m/z
204.0862
138.0547
366.1388
168.0652
274.0916
186.0757
126.0547
292.1017
144.065
331.1434 b3(1+)
657.2359
1679.8303 PEP_0_1_0_0_0

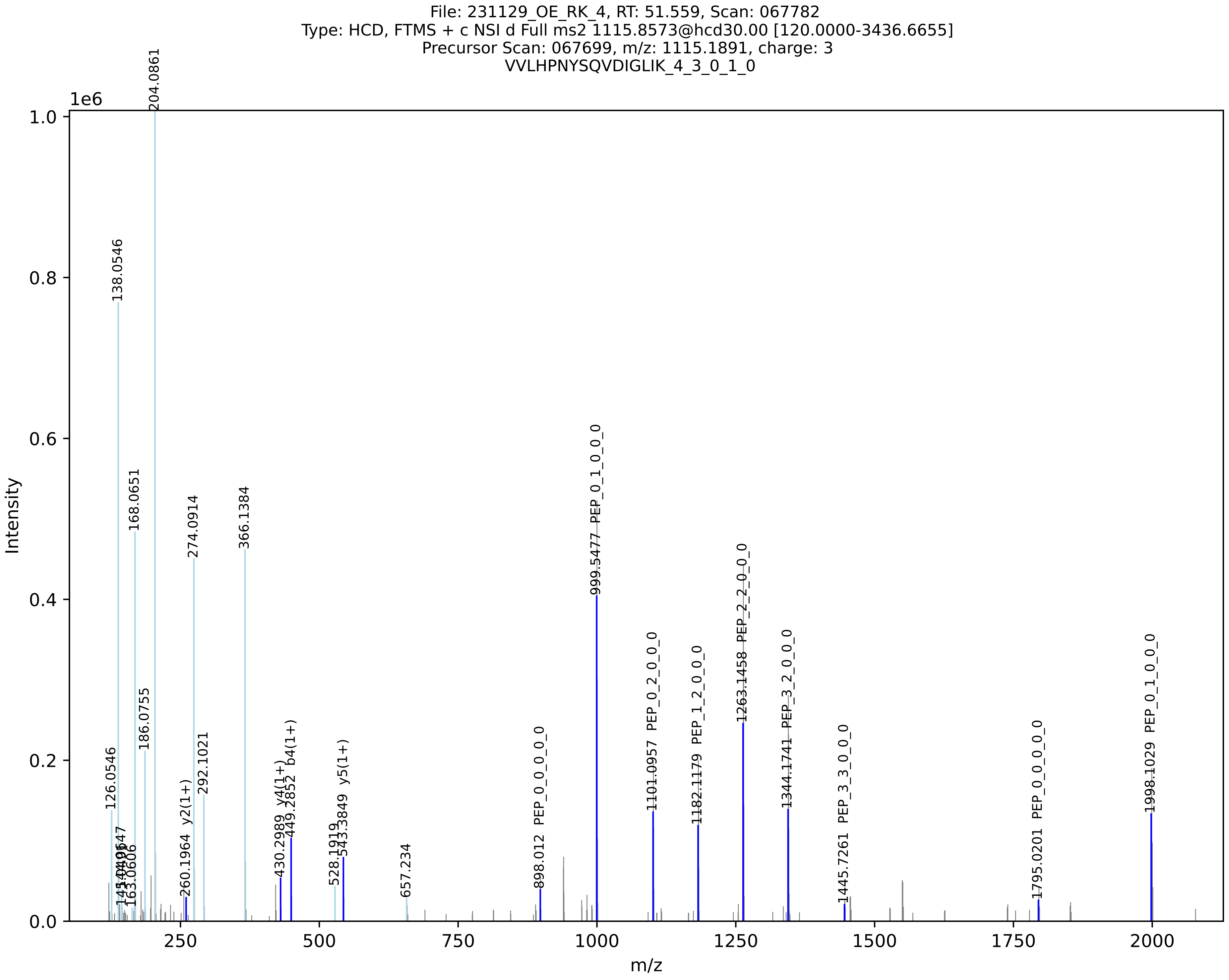

File: 231129_OE_RK_4, RT: 51.559, Scan: 067782
Type: HCD, FTMS + c NSI d Full ms2 1115.8573@hcd30.00 [120.0000-3436.6655]
Precursor Scan: 067699, m/z: 1115.1891, charge: 3
VVLHPNYSQVDIGLIK_4_3_0_1_0
1e6
Intensity
m/z
204.0861
138.0546
168.0651
274.0914
366.1384
186.0755
126.0546
292.1021
260.1964 y2(1+)
163.0606
430.2989 y4(1+)
449.2852 b4(1+)
528.1919
543.3849 y5(1+)
657.234
898.012 PEP_0_0_0_0_0
999.5477 PEP_0_1_0_0_0
1101.0957 PEP_0_2_0_0_0
1182.1179 PEP_1_2_0_0_0
1263.1458 PEP_2_2_0_0_0
1344.1741 PEP_3_2_0_0_0
1445.7261 PEP_3_3_0_0_0
1795.0201 PEP_0_0_0_0_0
1998.1029 PEP_0_1_0_0_0

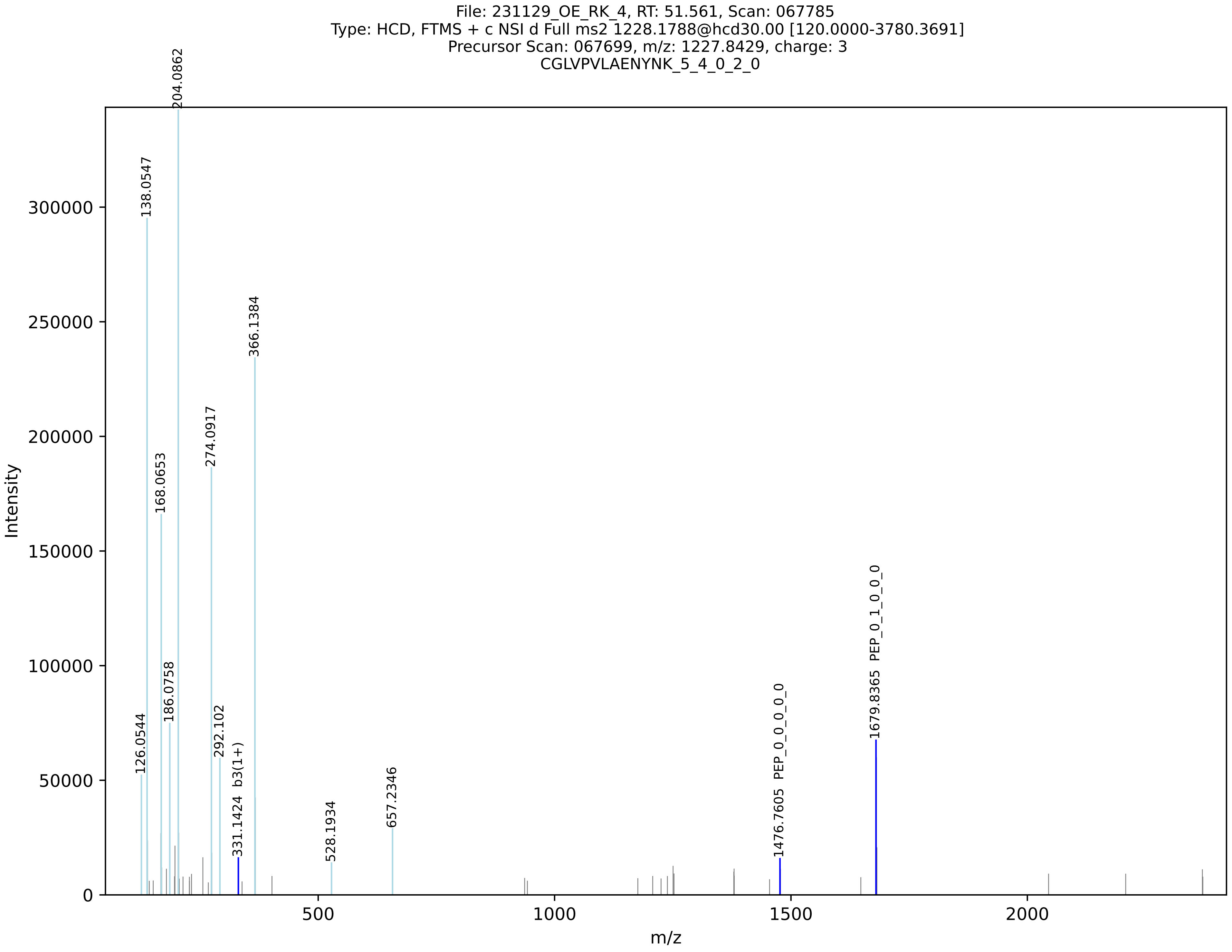
File: 231129_OE_RK_4, RT: 51.561, Scan: 067785
Type: HCD, FTMS + c NSI d Full ms2 1228.1788@hcd30.00 [120.0000-3780.3691]
Precursor Scan: 067699, m/z: 1227.8429, charge: 3
CGLVPVLAENYNK_5_4_0_2_0
Intensity
m/z
204.0862
138.0547
366.1384
274.0917
168.0653
186.0758
126.0544
292.102
331.1424 b3(1+)
528.1934
657.2346
1476.7605 PEP_0_0_0_0_0
1679.8365 PEP_0_1_0_0_0
0
50000
100000
150000
200000
250000
300000
500
1000
1500
2000

File: 231129_OE_RK_4, RT: 51.762, Scan: 068074
Type: HCD, FTMS + c NSI d Full ms2 1084.1996@hcd30.00 [120.0000-4446.3901]
Precursor Scan: 067990, m/z: 1083.6983, charge: 4
ENLTAPGSDSAVFFEQGTTR_5_4_0_2_0

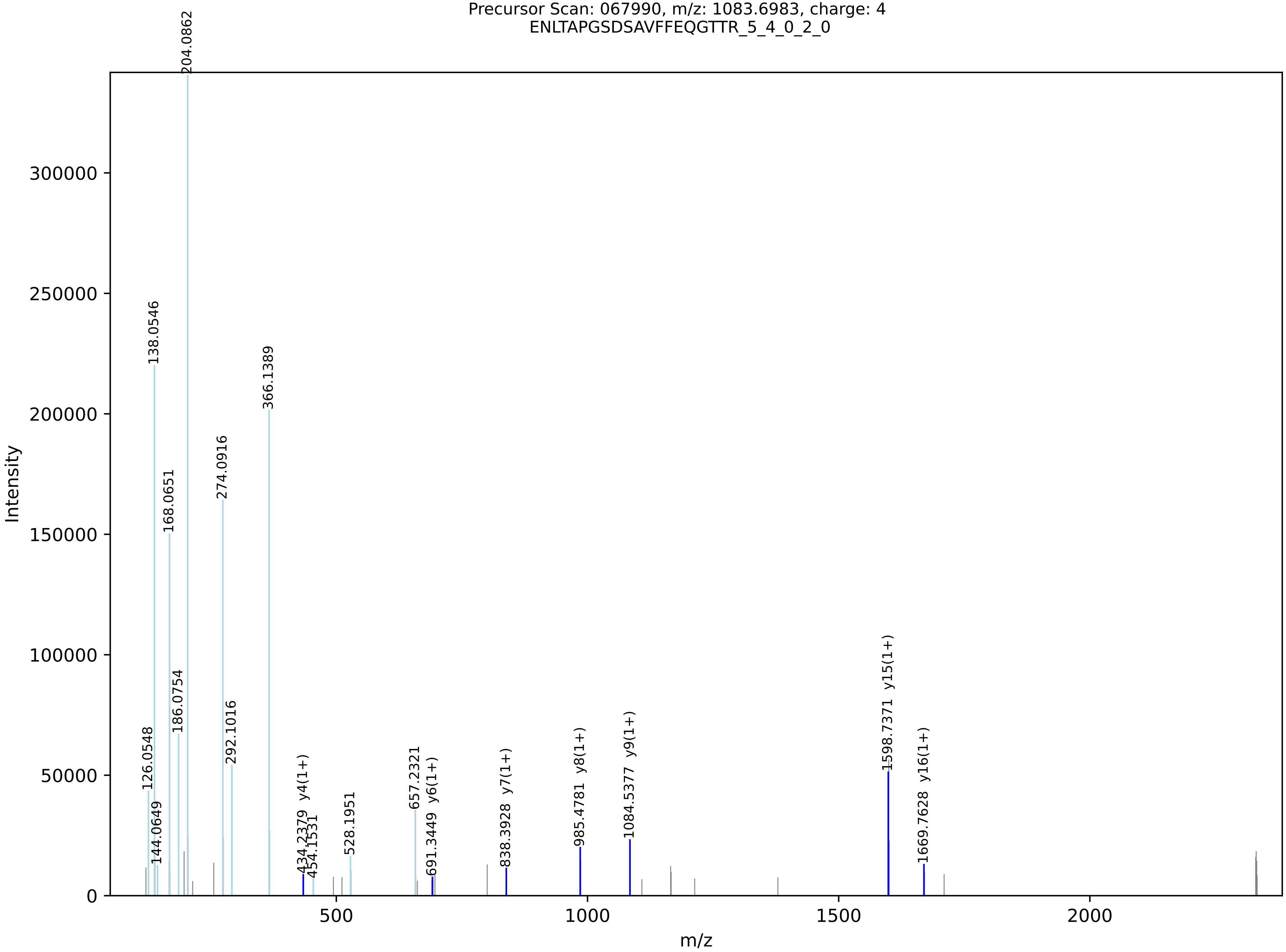

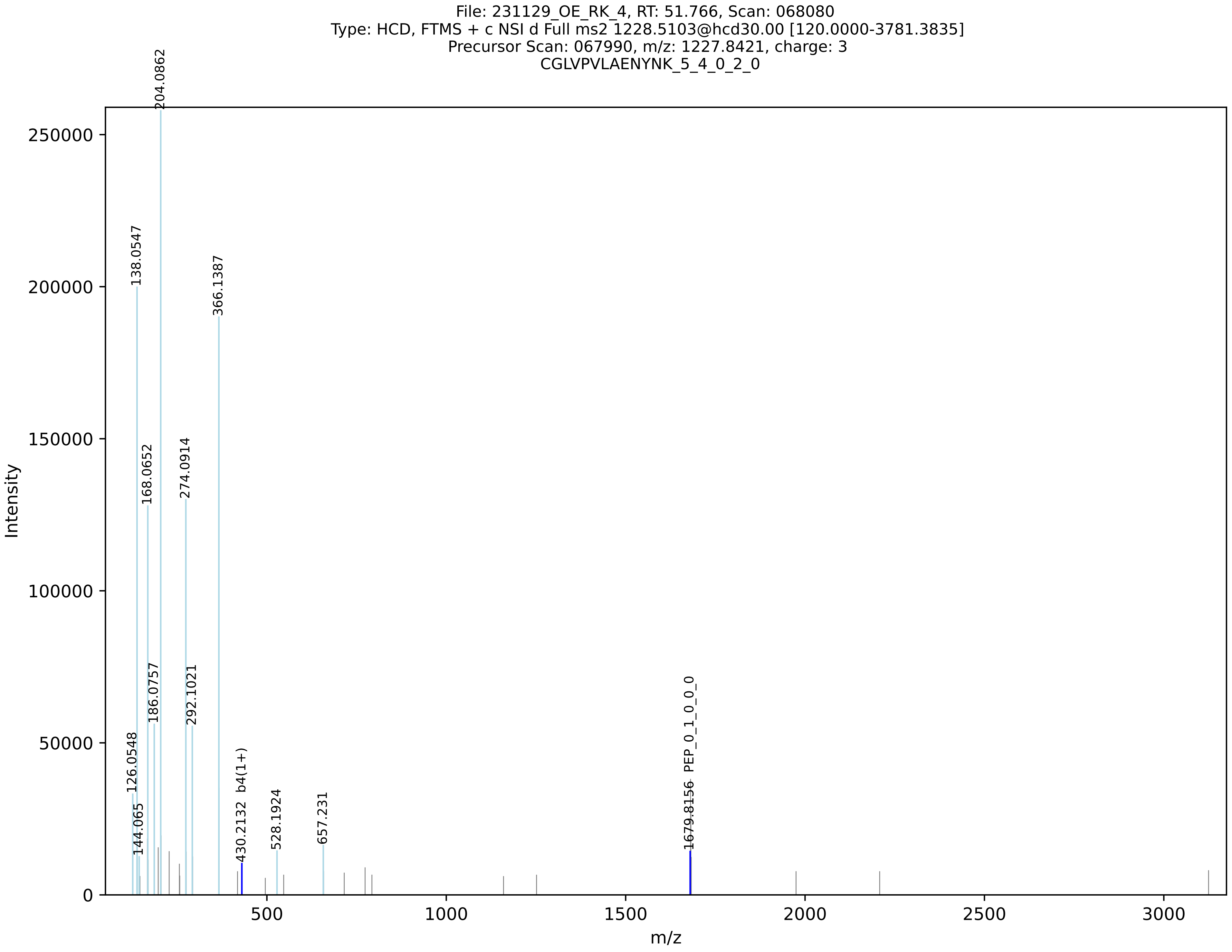
File: 231129_OE_RK_4, RT: 51.766, Scan: 068080
Type: HCD, FTMS + c NSI d Full ms2 1228.5103@hcd30.00 [120.0000-3781.3835]
Precursor Scan: 067990, m/z: 1227.8421, charge: 3
CGLVPVLAENYNK_5_4_0_2_0
204.0862
138.0547
366.1387
168.0652
274.0914
186.0757
292.1021
126.0548
144.065
430.2132 b4(1+)
528.1924
657.231
1679.8156 PEP_0_1_0_0_0
Intensity
m/z
0
50000
100000
150000
200000
250000
500
1000
1500
2000
2500
3000

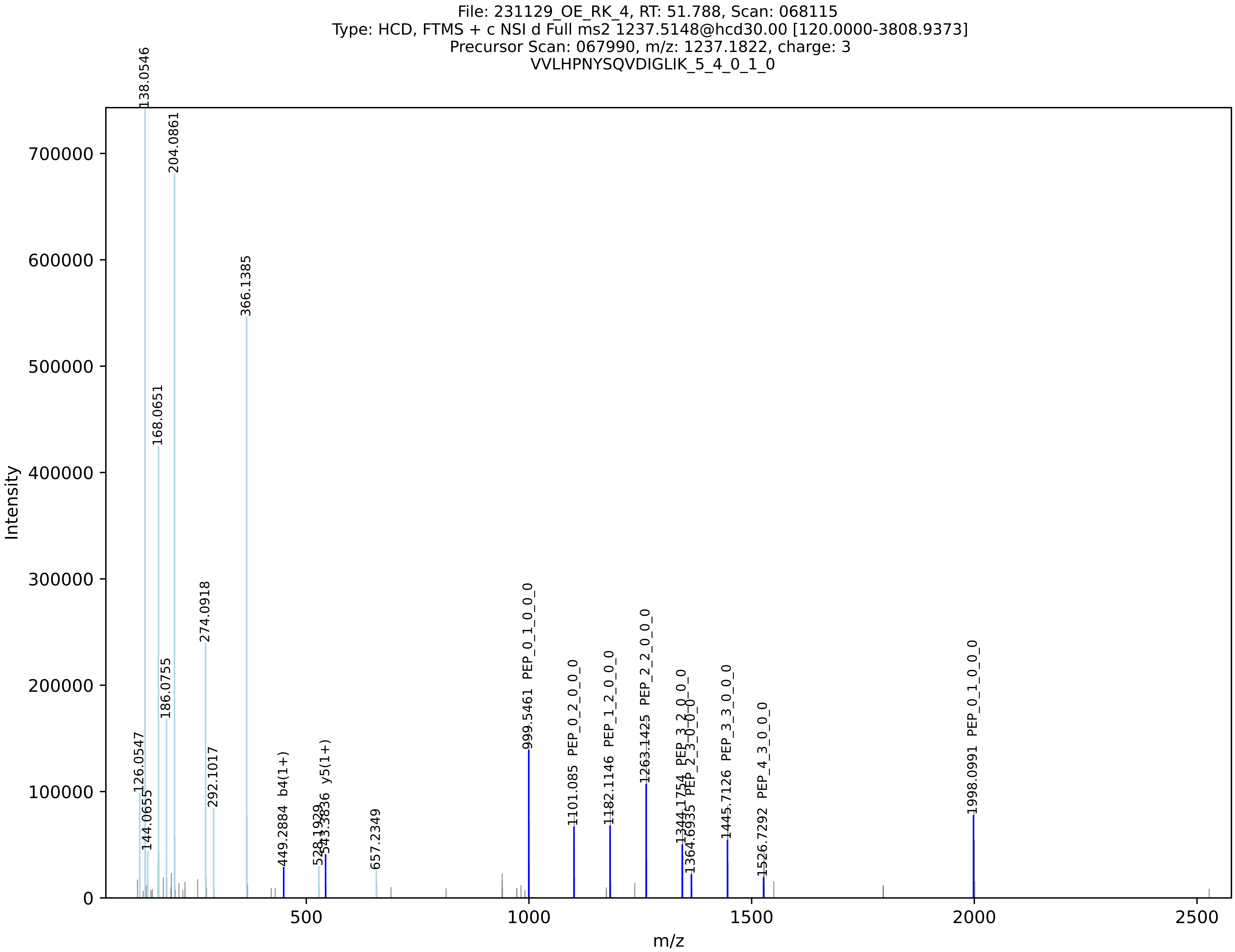

File: 231129_OE_RK_4, RT: 51.788, Scan: 068115
Type: HCD, FTMS + c NSI d Full ms2 1237.5148@hcd30.00 [120.0000-3808.9373]
Precursor Scan: 067990, m/z: 1237.1822, charge: 3
VVLHPNYSQVDIGLIK_5_4_0_1_0
Intensity
m/z
138.0546
204.0861
168.0651
366.1385
274.0918
186.0755
126.0547
144.0655
292.1017
449.2884 b4(1+)
528.1929
543.3836 y5(1+)
657.2349
999.5461 PEP_0_1_0_0_0
1101.085 PEP_0_2_0_0_0
1182.1146 PEP_1_2_0_0_0
1263.1425 PEP_2_2_0_0_0
1344.1754 PEP_3_2_0_0_0
1364.6935 PEP_2_3_0_0_0
1445.7126 PEP_3_3_0_0_0
1526.7292 PEP_4_3_0_0_0
1998.0991 PEP_0_1_0_0_0

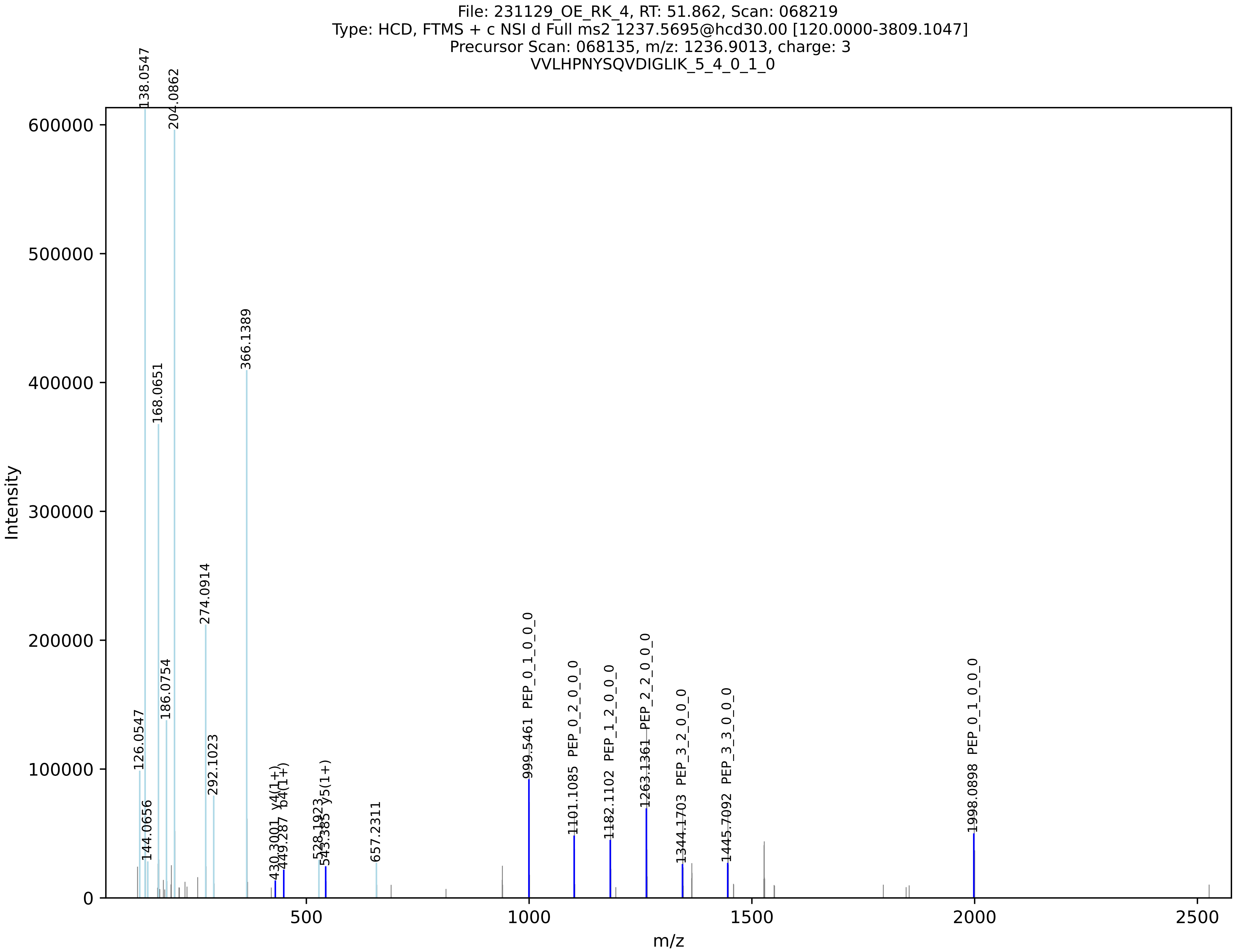

File: 231129_OE_RK_4, RT: 51.862, Scan: 068219
Type: HCD, FTMS + c NSI d Full ms2 1237.5695@hcd30.00 [120.0000-3809.1047]
Precursor Scan: 068135, m/z: 1236.9013, charge: 3
VVLHPNYSQVDIGLIK_5_4_0_1_0
Intensity
m/z
138.0547
204.0862
168.0651
366.1389
274.0914
186.0754
126.0547
292.1023
144.0656
430.3001 y4(1+)
449.287 b4(1+)
528.1923
543.385 y5(1+)
657.2311
999.5461 PEP_0_1_0_0_0
1101.1085 PEP_0_2_0_0_0
1182.1102 PEP_1_2_0_0_0
1263.1361 PEP_2_2_0_0_0
1344.1703 PEP_3_2_0_0_0
1445.7092 PEP_3_3_0_0_0
1998.0898 PEP_0_1_0_0_0

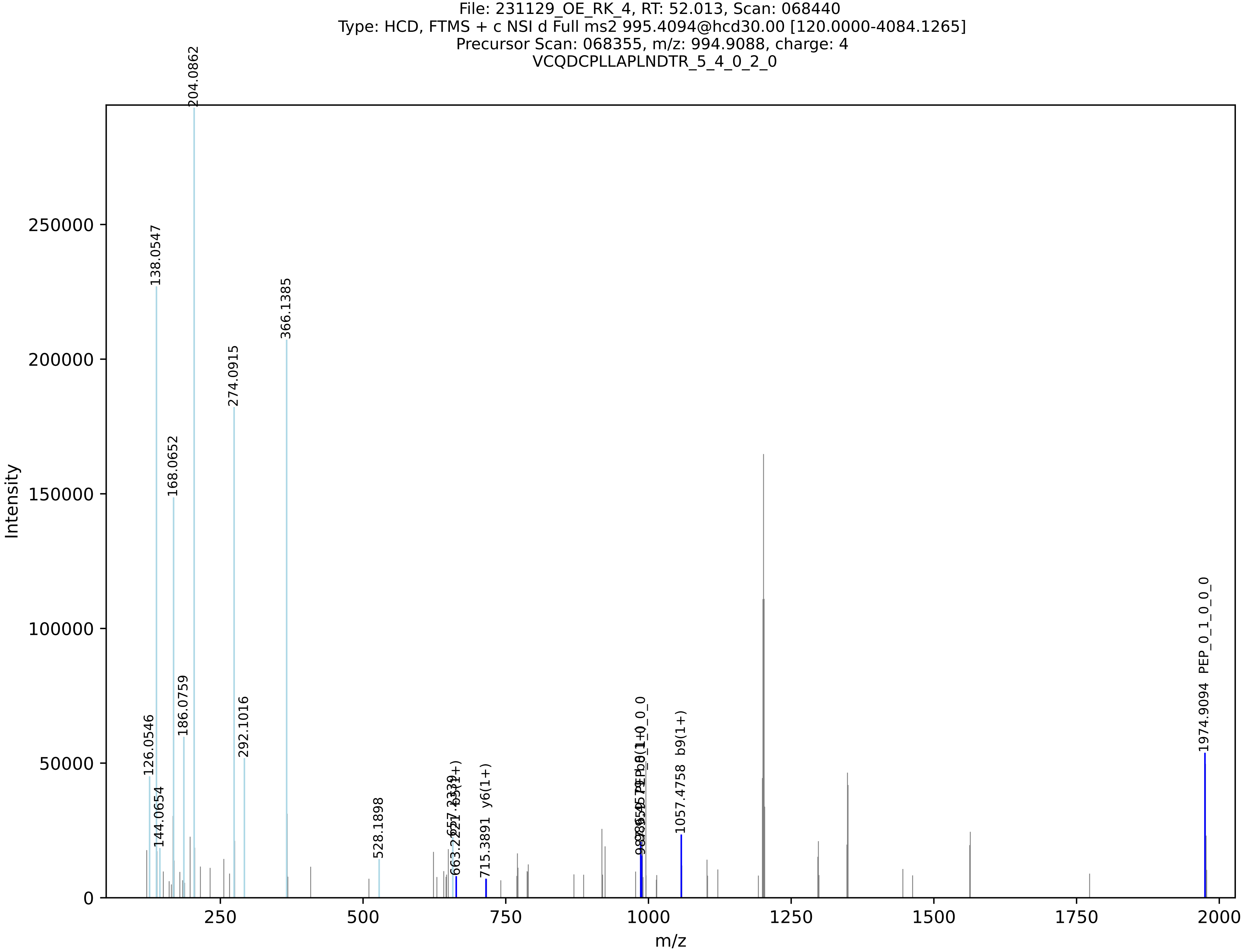

File: 231129_OE_RK_4, RT: 52.013, Scan: 068440
Type: HCD, FTMS + c NSI d Full ms2 995.4094@hcd30.00 [120.0000-4084.1265]
Precursor Scan: 068355, m/z: 994.9088, charge: 4
VCQDCPLLAPLNDTR_5_4_0_2_0
Intensity
m/z
204.0862
138.0547
366.1385
274.0915
168.0652
186.0759
292.1016
126.0546
144.0654
528.1898
663.2221 b3(1+)
715.3891 y6(1+)
1057.4758 b9(1+)
1974.9094 PEP_0_1_0_0_0
0
50000
100000
150000
200000
250000
250
500
750
1000
1250
1500
1750
2000

File: 231129_OE_RK_4, RT: 52.167, Scan: 068666
Type: HCD, FTMS + c NSI d Full ms2 1083.9458@hcd30.00 [120.0000-4445.3550]
Precursor Scan: 068574, m/z: 1083.6965, charge: 4
ENLTAPGSDSAVFFEQGTTR_5_4_0_2_0

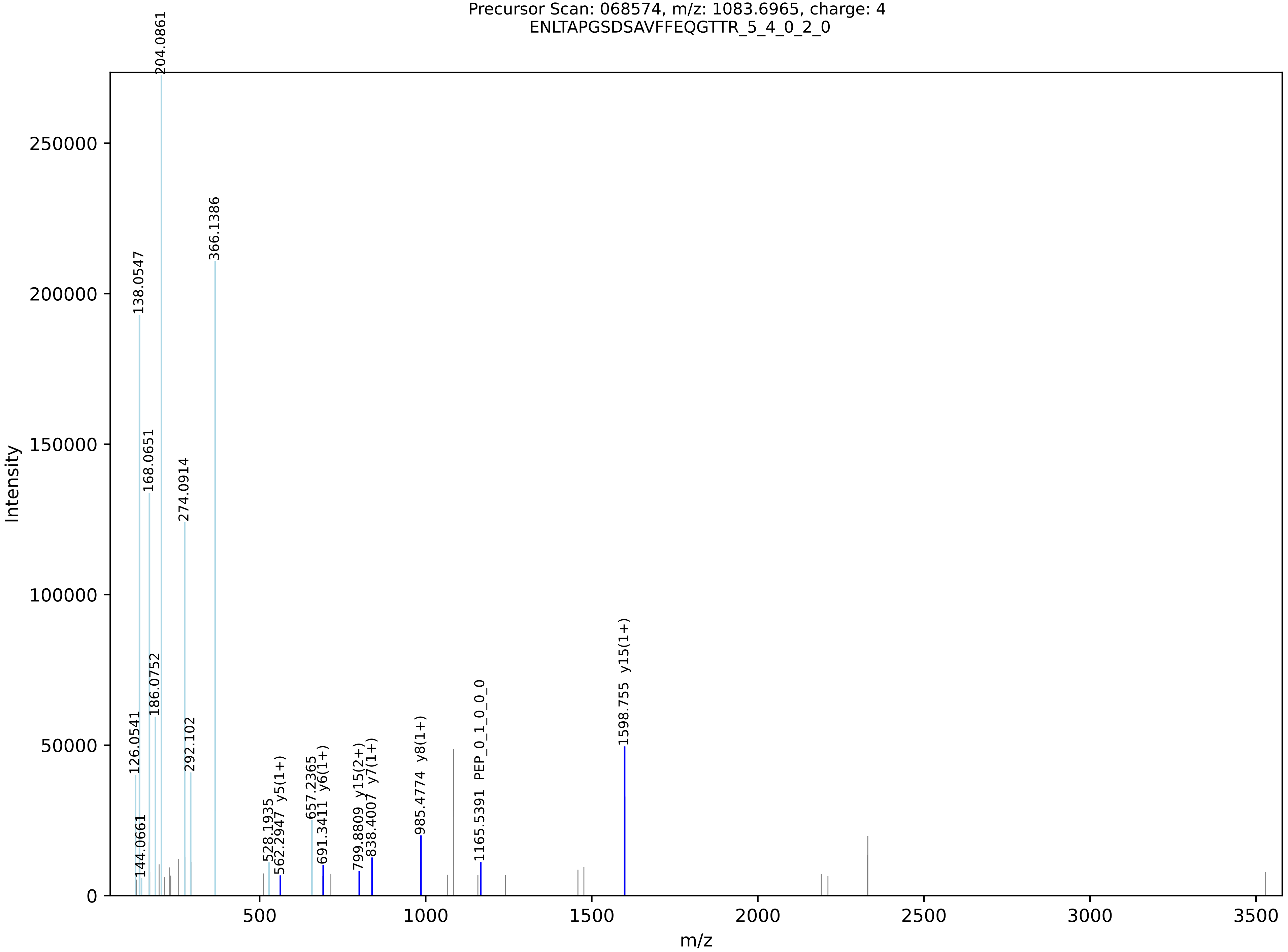

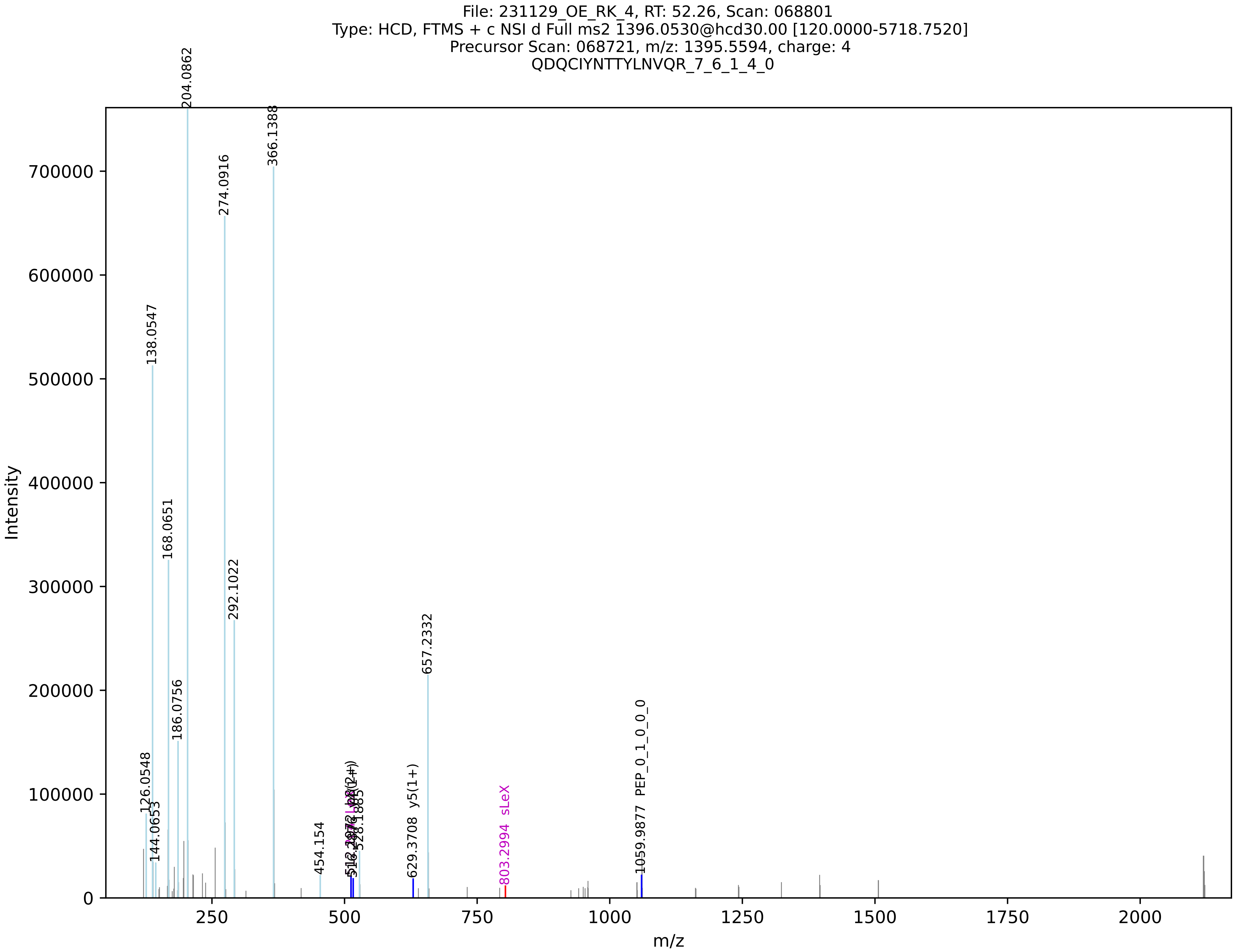
File: 231129_OE_RK_4, RT: 52.26, Scan: 068801
Type: HCD, FTMS + c NSI d Full ms2 1396.0530@hcd30.00 [120.0000-5718.7520]
Precursor Scan: 068721, m/z: 1395.5594, charge: 4
QDQCIYNTTYLNVQR_7_6_1_4_0
Intensity
m/z
204.0862
366.1388
274.0916
138.0547
168.0651
292.1022
657.2332
186.0756
126.0548
144.0653
454.154
528.1885
629.3708 y5(1+)
803.2994 sLeX
1059.9877 PEP_0_1_0_0_0
0
100000
200000
300000
400000
500000
600000
700000
250
500
750
1000
1250
1500
1750
2000

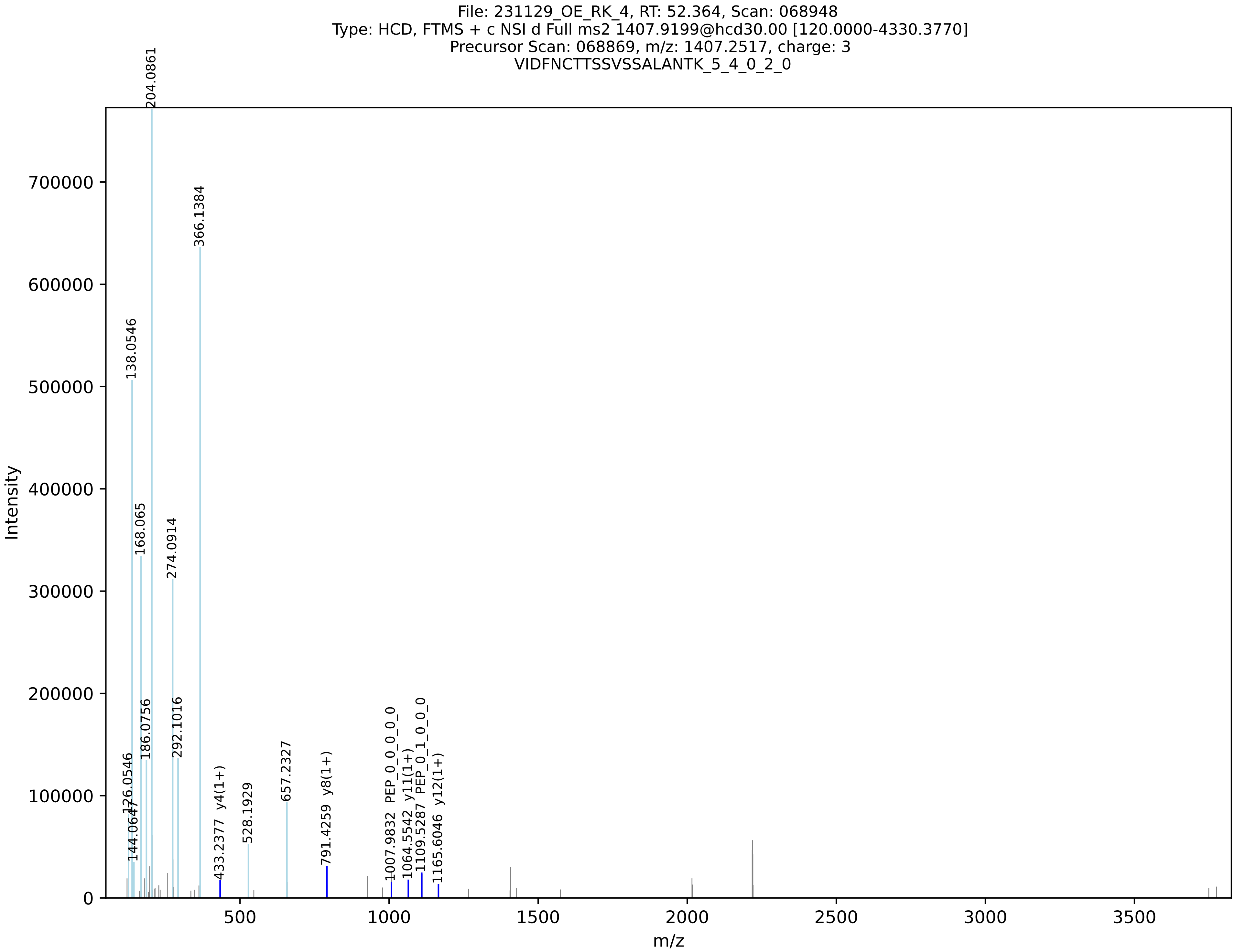

File: 231129_OE_RK_4, RT: 52.364, Scan: 068948
Type: HCD, FTMS + c NSI d Full ms2 1407.9199@hcd30.00 [120.0000-4330.3770]
Precursor Scan: 068869, m/z: 1407.2517, charge: 3
VIDFNCTTSSVSSALANTK_5_4_0_2_0
Intensity
m/z
204.0861
366.1384
138.0546
168.065
274.0914
186.0756
292.1016
126.0546
144.0647
433.2377 y4(1+)
528.1929
657.2327
791.4259 y8(1+)
1007.9832 PEP_0_0_0_0_0
1064.5542 y11(1+)
1109.5287 PEP_0_1_0_0_0
1165.6046 y12(1+)
0
100000
200000
300000
400000
500000
600000
700000
500
1000
1500
2000
2500
3000
3500

File: 231129_OE_RK_4, RT: 52.416, Scan: 069024
Type: HCD, FTMS + c NSI d Full ms2 960.4613@hcd30.00 [120.0000-4921.9224]
Precursor Scan: 068940, m/z: 959.6594, charge: 5
EVSFDVELPKTAFITNFTLTIDGVTYPGNVK_6_2_0_0_0

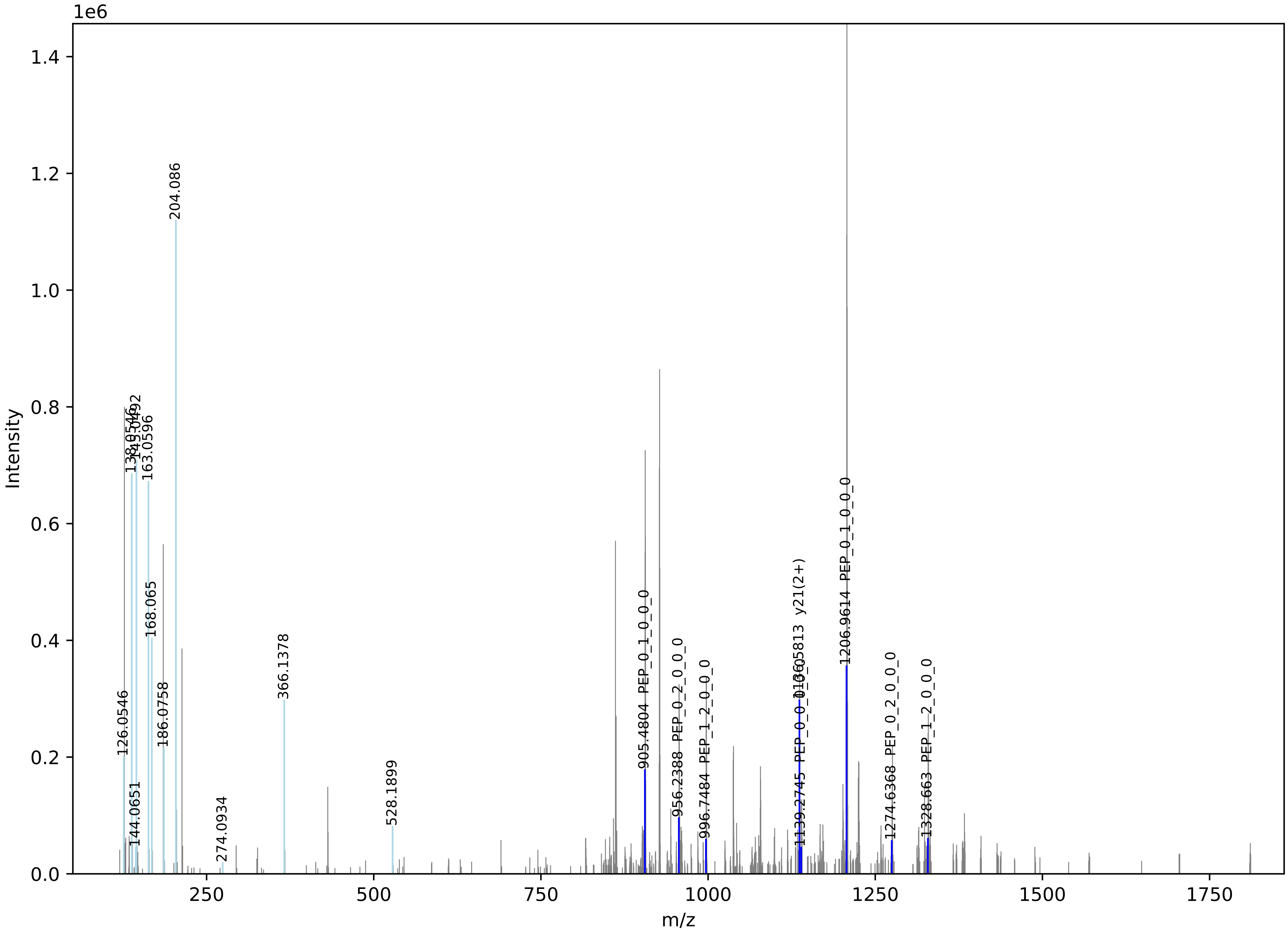

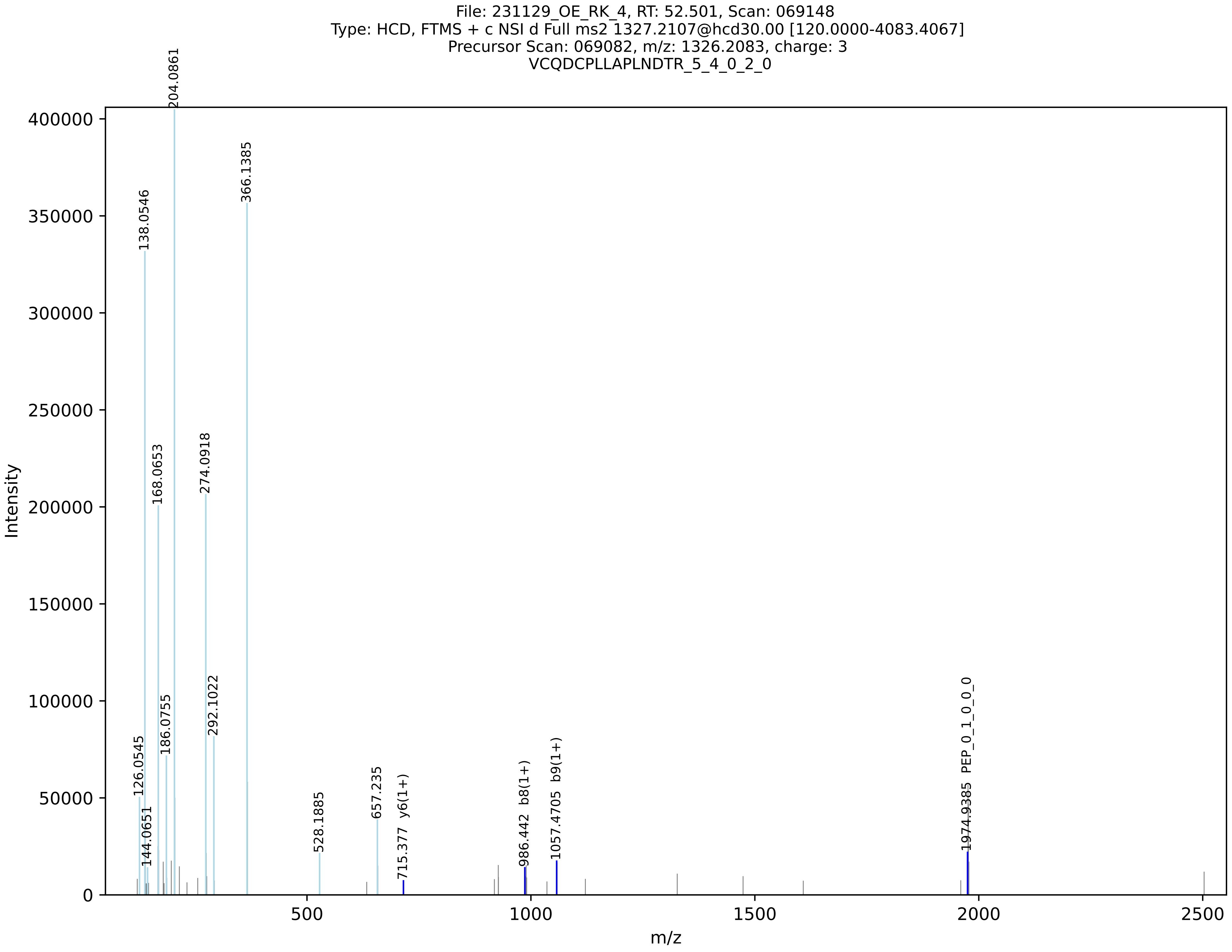
File: 231129_OE_RK_4, RT: 52.501, Scan: 069148
Type: HCD, FTMS + c NSI d Full ms2 1327.2107@hcd30.00 [120.0000-4083.4067]
Precursor Scan: 069082, m/z: 1326.2083, charge: 3
VCQDCPLLAPLNDTR_5_4_0_2_0
Intensity
m/z
204.0861
366.1385
138.0546
168.0653
274.0918
292.1022
186.0755
126.0545
144.0651
528.1885
657.235
715.377 y6(1+)
986.442 b8(1+)
1057.4705 b9(1+)
1974.9385 PEP_0_1_0_0_0
0
50000
100000
150000
200000
250000
300000
350000
400000
500
1000
1500
2000
2500

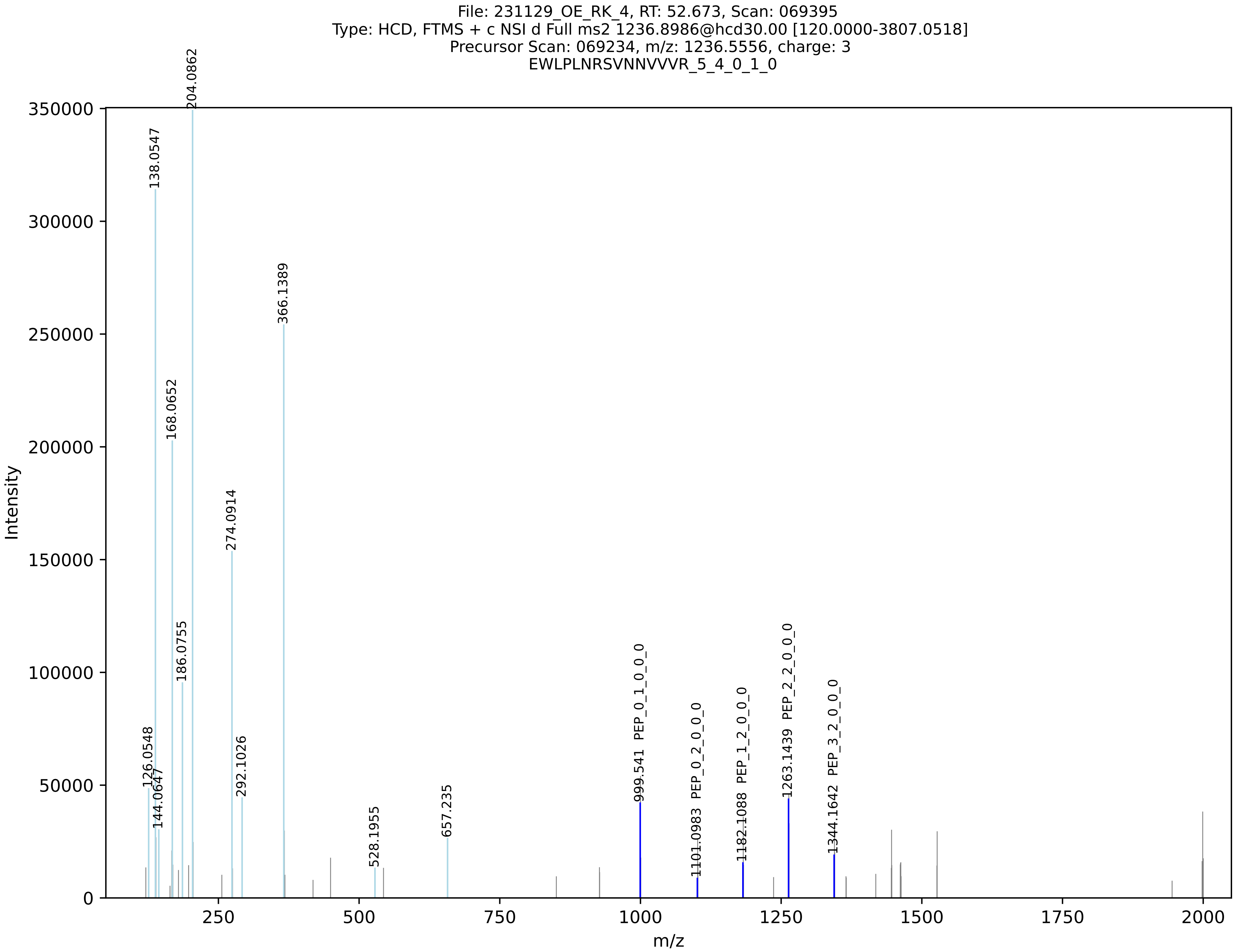
File: 231129_OE_RK_4, RT: 52.673, Scan: 069395
Type: HCD, FTMS + c NSI d Full ms2 1236.8986@hcd30.00 [120.0000-3807.0518]
Precursor Scan: 069234, m/z: 1236.5556, charge: 3
EWLPLNRSVNNVVVR_5_4_0_1_0
Intensity
m/z
204.0862
138.0547
366.1389
168.0652
274.0914
186.0755
126.0548
144.0647
292.1026
528.1955
657.235
999.541 PEP_0_1_0_0_0
1101.0983 PEP_0_2_0_0_0
1182.1088 PEP_1_2_0_0_0
1263.1439 PEP_2_2_0_0_0
1344.1642 PEP_3_2_0_0_0

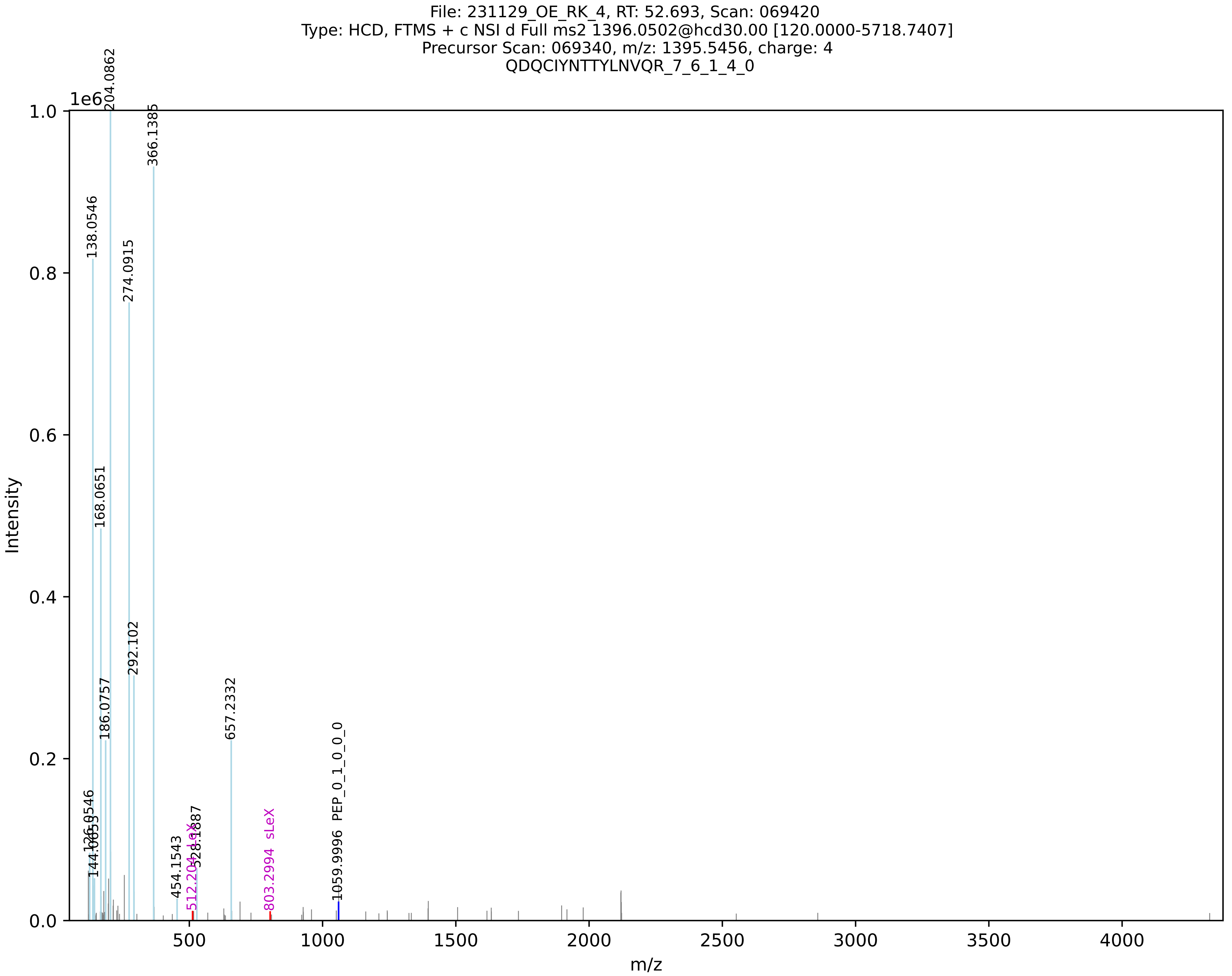

File: 231129_OE_RK_4, RT: 52.693, Scan: 069420
Type: HCD, FTMS + c NSI d Full ms2 1396.0502@hcd30.00 [120.0000-5718.7407]
Precursor Scan: 069340, m/z: 1395.5456, charge: 4
QDQCIYNTTYLNVQR_7_6_1_4_0
1e6
Intensity
m/z
204.0862
366.1385
138.0546
274.0915
168.0651
292.102
186.0757
657.2332
126.0546
144.0653
454.1543
512.204 LeX
528.1887
803.2994 sLeX
1059.9996 PEP_0_1_0_0_0_

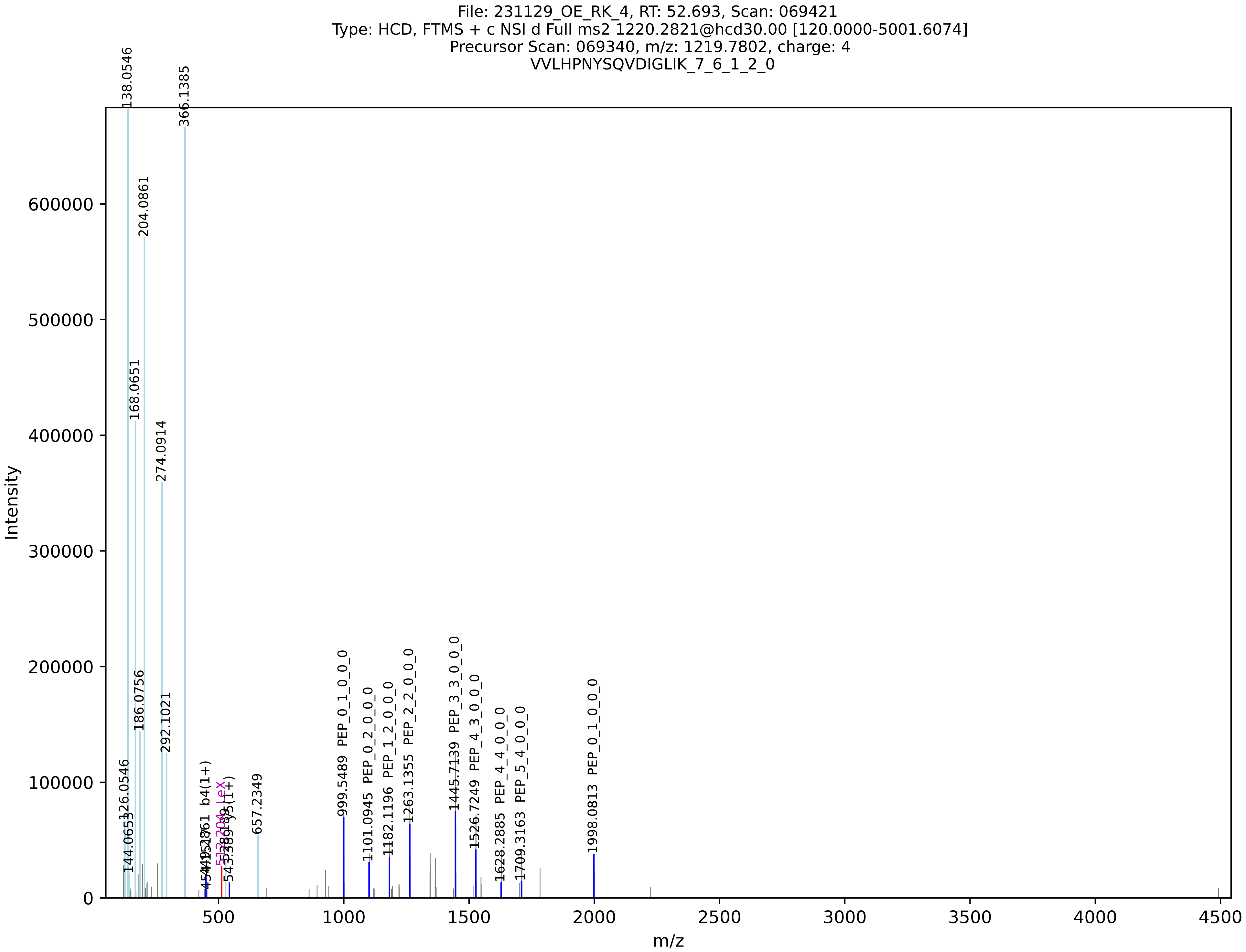

File: 231129_OE_RK_4, RT: 52.693, Scan: 069421
Type: HCD, FTMS + c NSI d Full ms2 1220.2821@hcd30.00 [120.0000-5001.6074]
Precursor Scan: 069340, m/z: 1219.7802, charge: 4
VVLHPNYSQVDIGLIK_7_6_1_2_0
Intensity
m/z
138.0546
366.1385
204.0861
168.0651
274.0914
186.0756
292.1021
126.0546
144.0653
657.2349
999.5489 PEP_0_1_0_0_0_
1101.0945 PEP_0_2_0_0_0_
1182.1196 PEP_1_2_0_0_0_
1263.1355 PEP_2_2_0_0_0_
1445.7139 PEP_3_3_0_0_0_
1526.7249 PEP_4_3_0_0_0_
1628.2885 PEP_4_4_0_0_0_
1709.3163 PEP_5_4_0_0_0_
1998.0813 PEP_0_1_0_0_0_
b4(1+)
y5(1+)
LeX

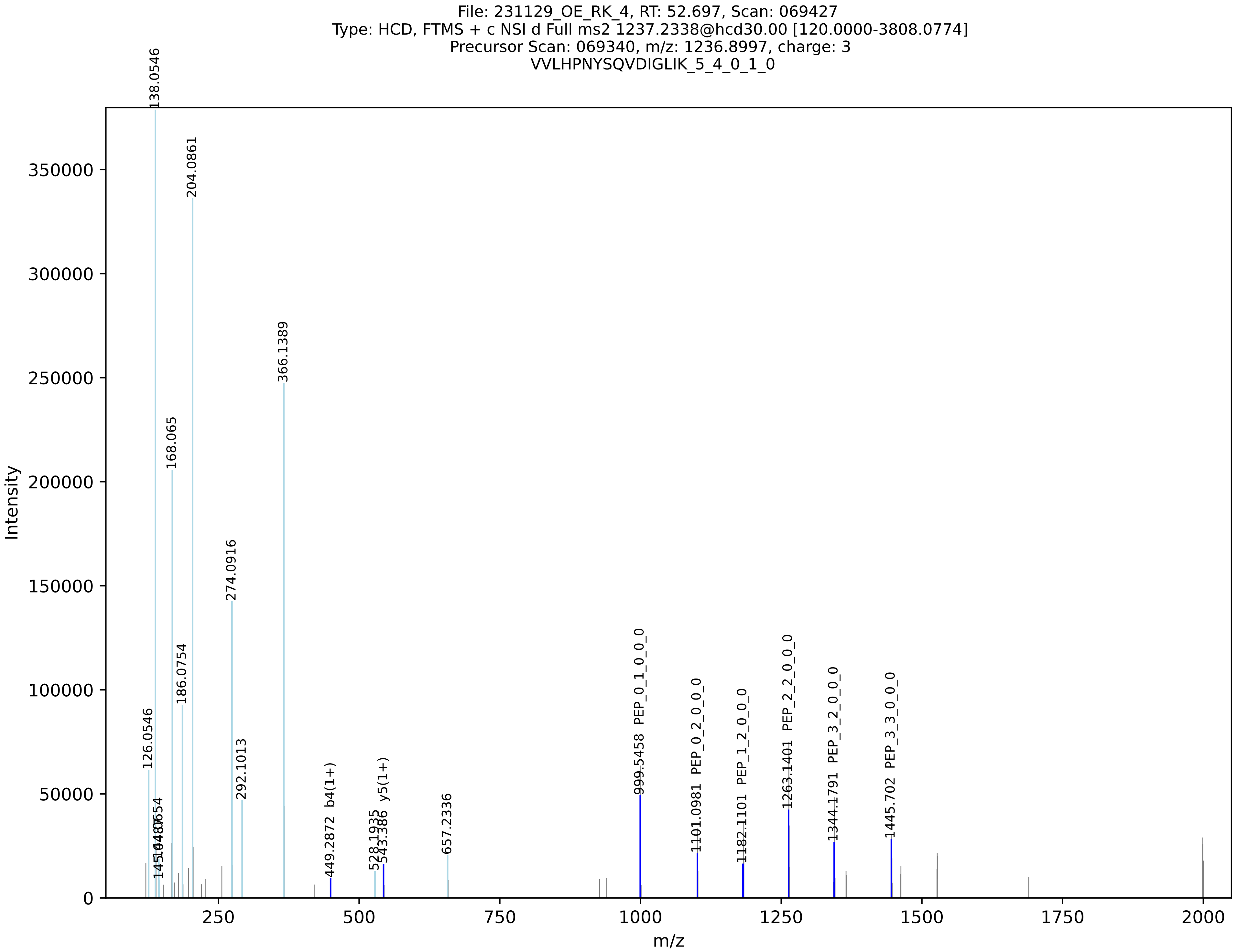

File: 231129_OE_RK_4, RT: 52.697, Scan: 069427
Type: HCD, FTMS + c NSI d Full ms2 1237.2338@hcd30.00 [120.0000-3808.0774]
Precursor Scan: 069340, m/z: 1236.8997, charge: 3
VVLHPNYSQVDIGLIK_5_4_0_1_0
Intensity
m/z
138.0546
204.0861
366.1389
168.065
274.0916
186.0754
126.0546
292.1013
449.2872 b4(1+)
528.1935
543.386 y5(1+)
657.2336
999.5458 PEP_0_1_0_0_0
1101.0981 PEP_0_2_0_0_0
1182.1101 PEP_1_2_0_0_0
1263.1401 PEP_2_2_0_0_0
1344.1791 PEP_3_2_0_0_0
1445.702 PEP_3_3_0_0_0

File: 231129_OE_RK_4, RT: 52.698, Scan: 069428
Type: HCD, FTMS + c NSI d Full ms2 1327.2101@hcd30.00 [120.0000-4083.4050]
Precursor Scan: 069340, m/z: 1326.2101, charge: 3
VCQDCPLLAPLNDTR_5_4_0_2_0

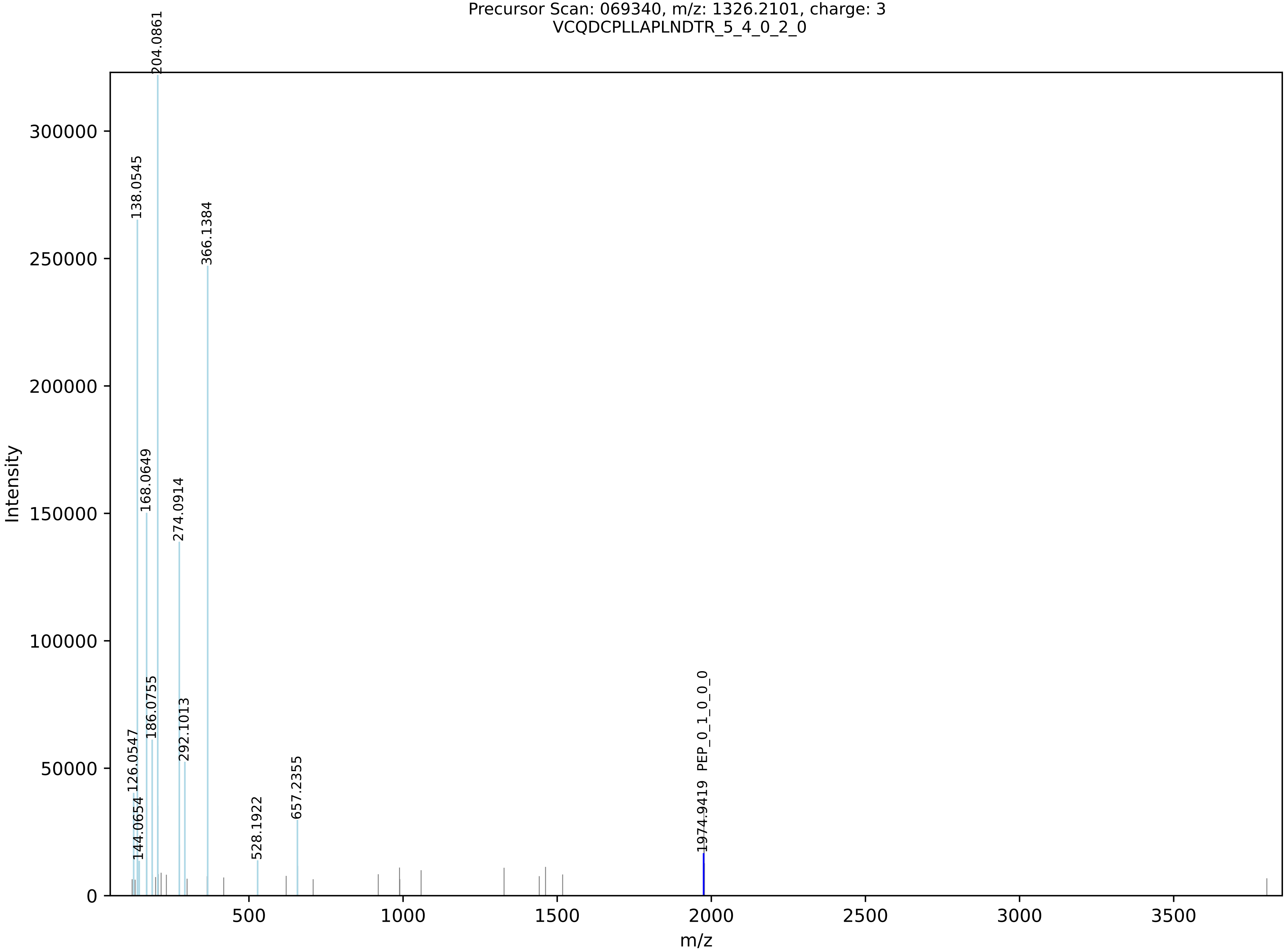

File: 231129_OE_RK_4, RT: 52.742, Scan: 069492
Type: HCD, FTMS + c NSI d Full ms2 1217.8593@hcd30.00 [120.0000-3748.7913]
Precursor Scan: 069414, m/z: 1217.191, charge: 3
ALPQPQNVTSLLGCTH_5_4_0_1_0

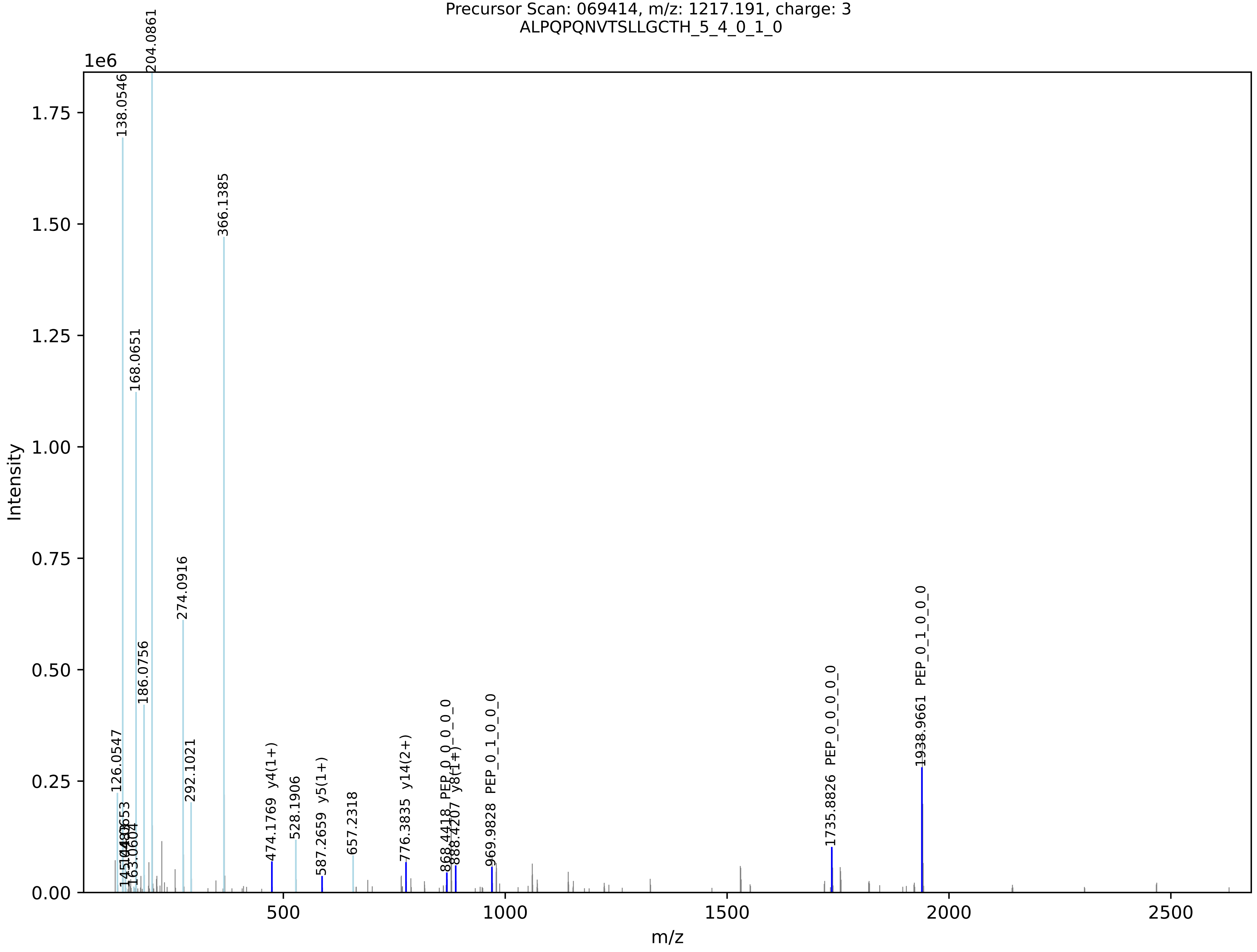

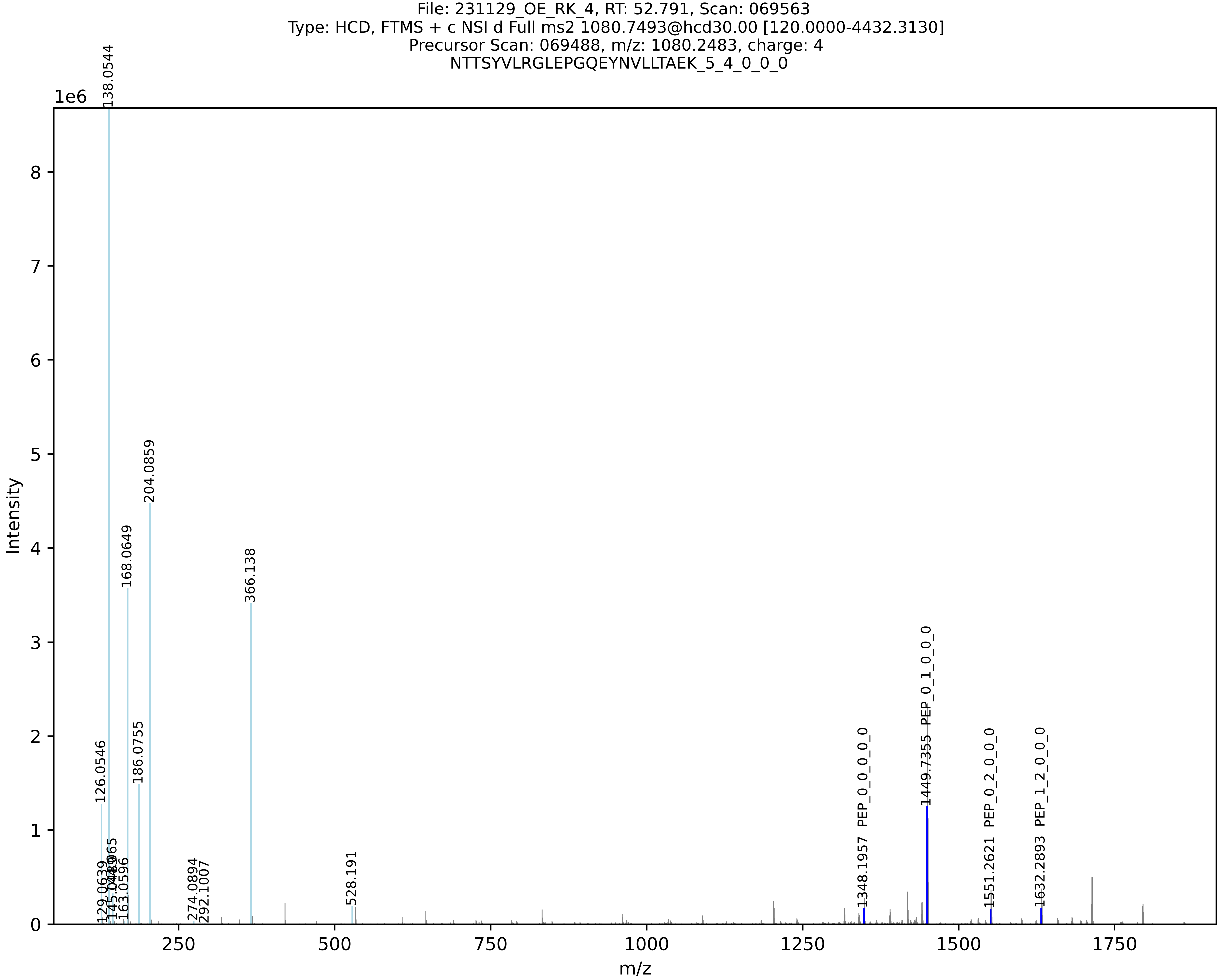

File: 231129_OE_RK_4, RT: 52.791, Scan: 069563
Type: HCD, FTMS + c NSI d Full ms2 1080.7493@hcd30.00 [120.0000-4432.3130]
Precursor Scan: 069488, m/z: 1080.2483, charge: 4
NTTSYVLRGLEPGQEYNVLLTAEK_5_4_0_0_0
1e6
Intensity
m/z
138.0544
204.0859
168.0649
366.138
186.0755
126.0546
129.0639
145.0495
163.0596
274.0894
292.1007
528.191
1348.1957 PEP_0_0_0_0_0
1449.7355 PEP_0_1_0_0_0
1551.2621 PEP_0_2_0_0_0
1632.2893 PEP_1_2_0_0_0

File: 231129_OE_RK_4, RT: 52.794, Scan: 069568
Type: HCD, FTMS + c NSI d Full ms2 1427.2811@hcd30.00 [120.0000-4389.6221]
Precursor Scan: 069488, m/z: 1426.949, charge: 3
AALAAFNAQNNGSNFQLEEISR_5_4_0_1_0

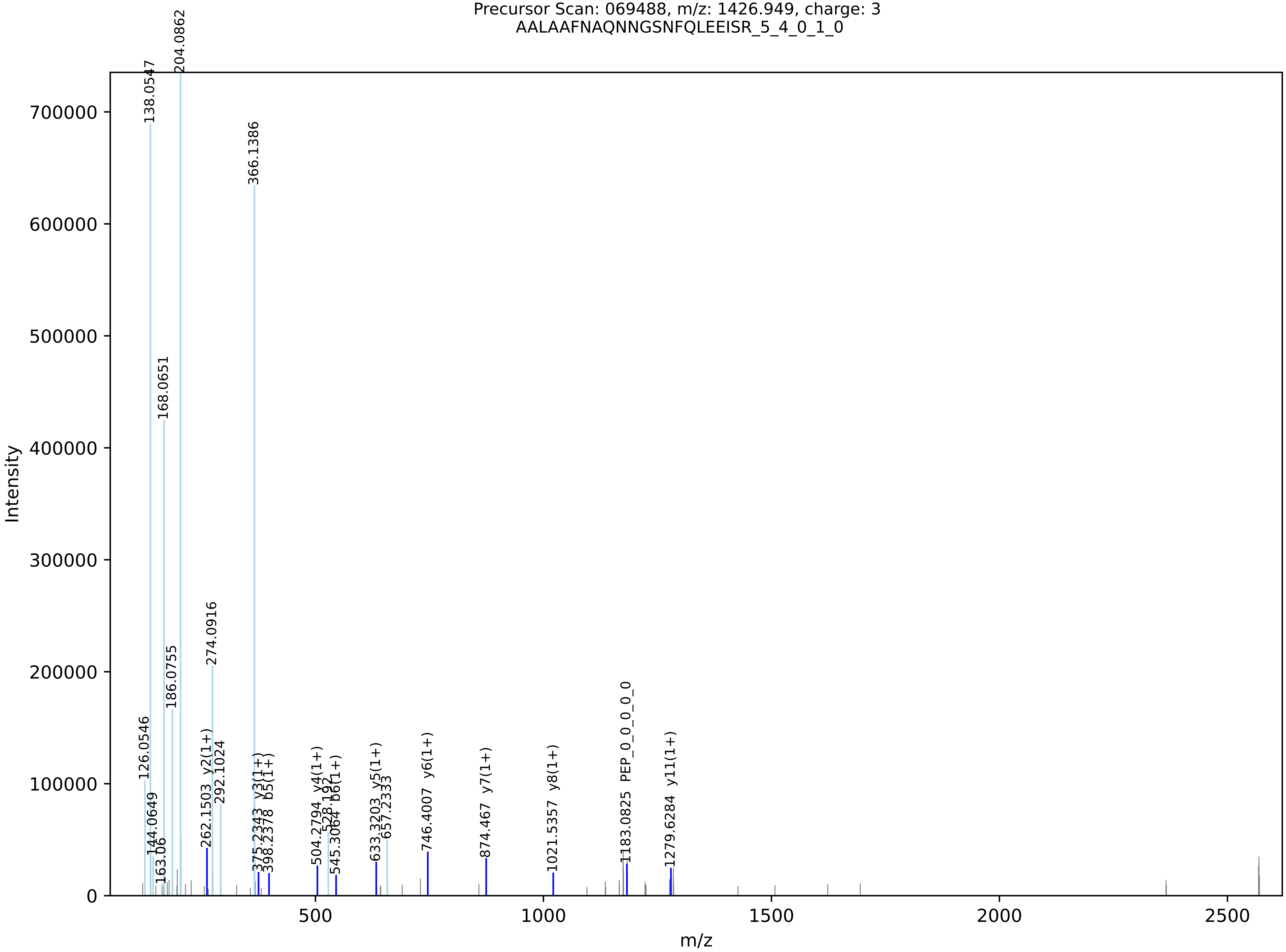

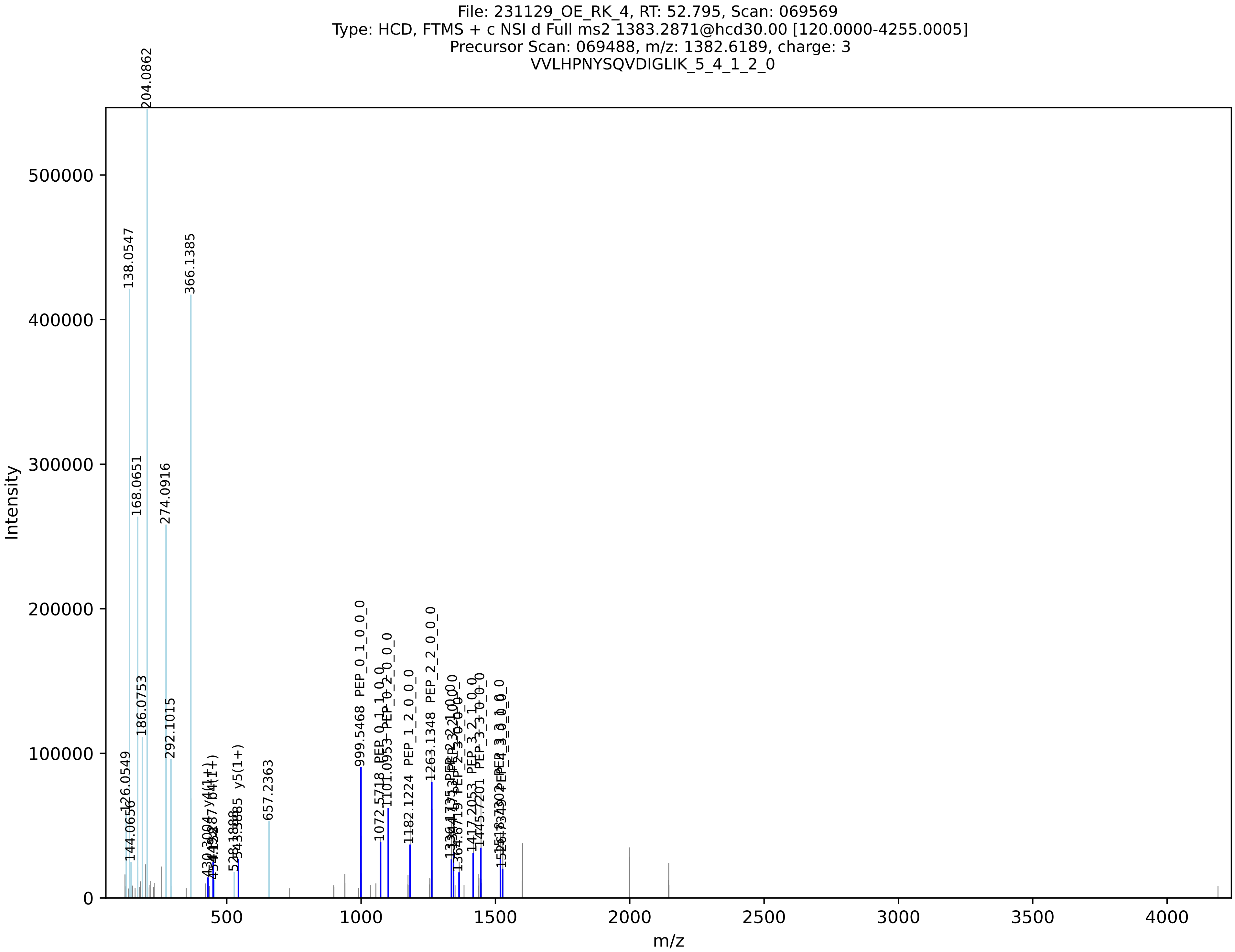

File: 231129_OE_RK_4, RT: 52.795, Scan: 069569
Type: HCD, FTMS + c NSI d Full ms2 1383.2871@hcd30.00 [120.0000-4255.0005]
Precursor Scan: 069488, m/z: 1382.6189, charge: 3
VVLHPNYSQVDIGLIK_5_4_1_2_0
Intensity
m/z
204.0862
138.0547
366.1385
168.0651
274.0916
186.0753
292.1015
126.0549
144.0656
657.2363
999.5468 PEP_0_1_0_0_0
1263.1348 PEP_2_2_0_0_0
1182.1224 PEP_1_2_0_0_0
1417.2053 PEP_3_2_1_0_0

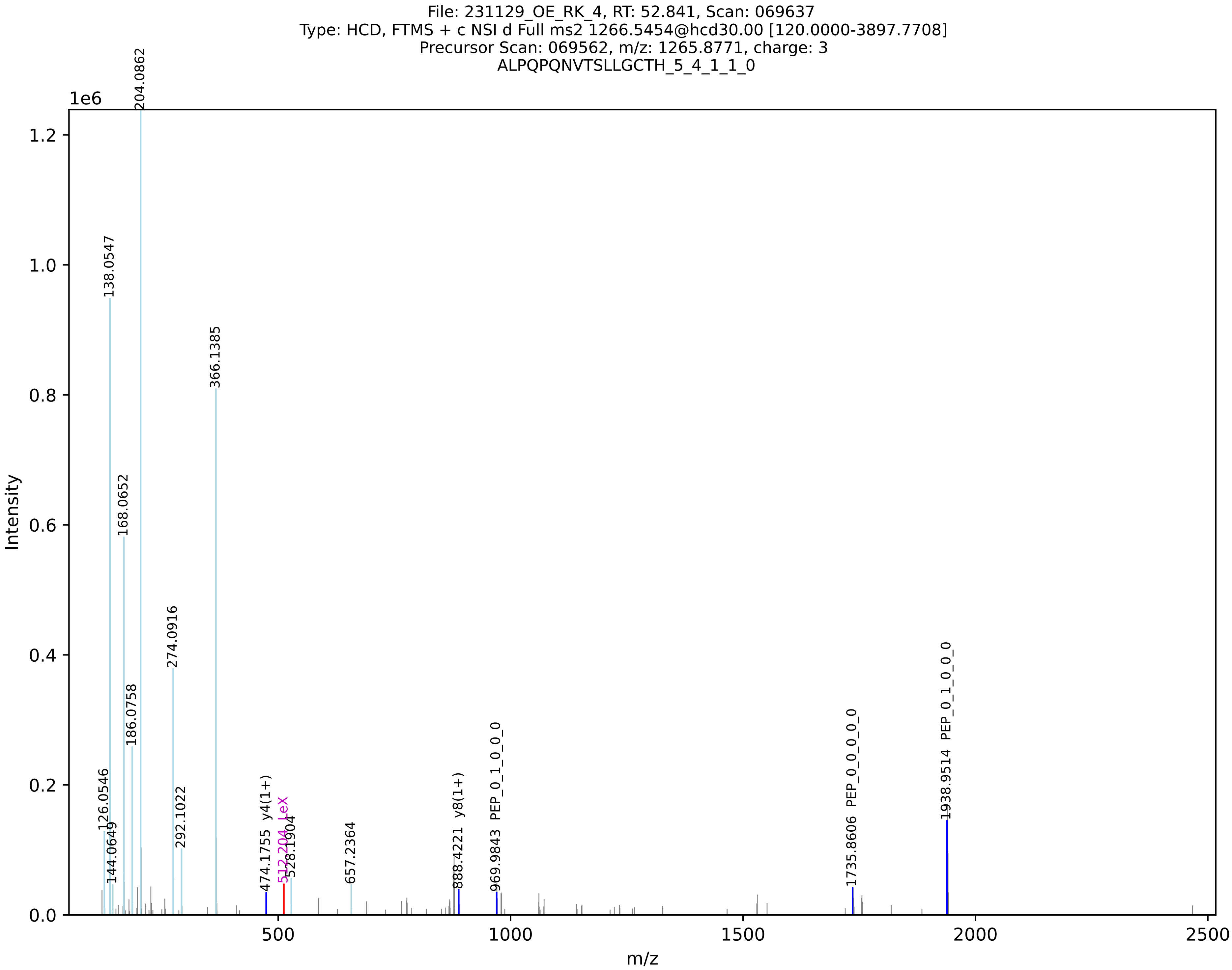
File: 231129_OE_RK_4, RT: 52.841, Scan: 069637
Type: HCD, FTMS + c NSI d Full ms2 1266.5454@hcd30.00 [120.0000-3897.7708]
Precursor Scan: 069562, m/z: 1265.8771, charge: 3
ALPQPQNVTSLLGCTH_5_4_1_1_0
1e6
Intensity
m/z
204.0862
138.0547
366.1385
168.0652
274.0916
186.0758
126.0546
144.0649
292.1022
474.1755 y4(1+)
512.204 LeX
528.1904
657.2364
888.4221 y8(1+)
969.9843 PEP_0_1_0_0_0_0
1735.8606 PEP_0_0_0_0_0
1938.9514 PEP_0_1_0_0_0

File: 231129_OE_RK_4, RT: 52.847, Scan: 069646
Type: HCD, FTMS + c NSI d Full ms2 1371.8976@hcd30.00 [120.0000-4220.1489]
Precursor Scan: 069562, m/z: 1370.8953, charge: 3
DQCIVDDITYNVNDTFHK_5_4_0_1_0

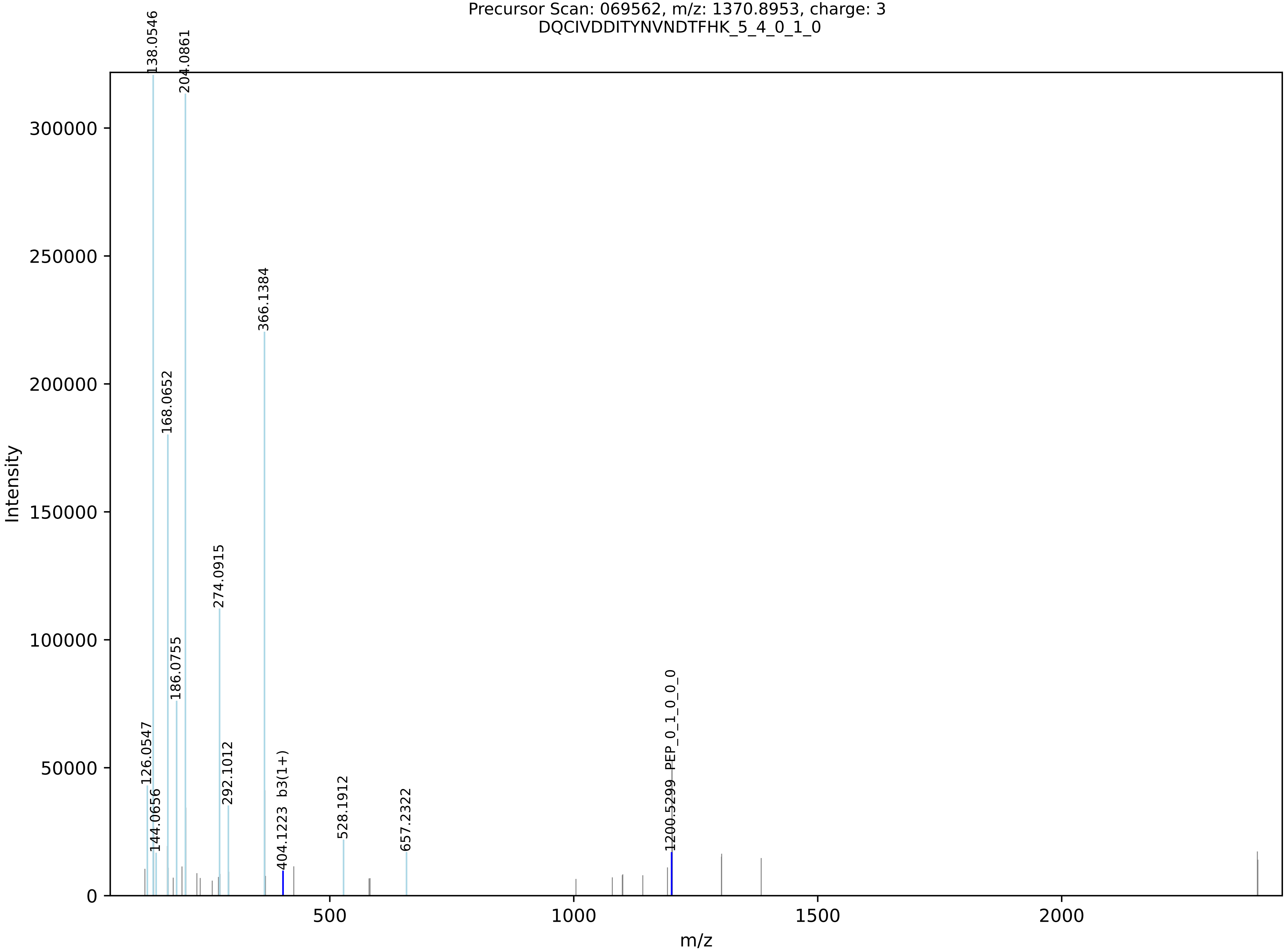

File: 231129_OE_RK_4, RT: 52.899, Scan: 069719
Type: HCD, FTMS + c NSI d Full ms2 1220.0356@hcd30.00 [120.0000-5000.6011]
Precursor Scan: 069635, m/z: 1219.783, charge: 4
VVLHPNYSQVDIGLIK_7_6_1_2_0

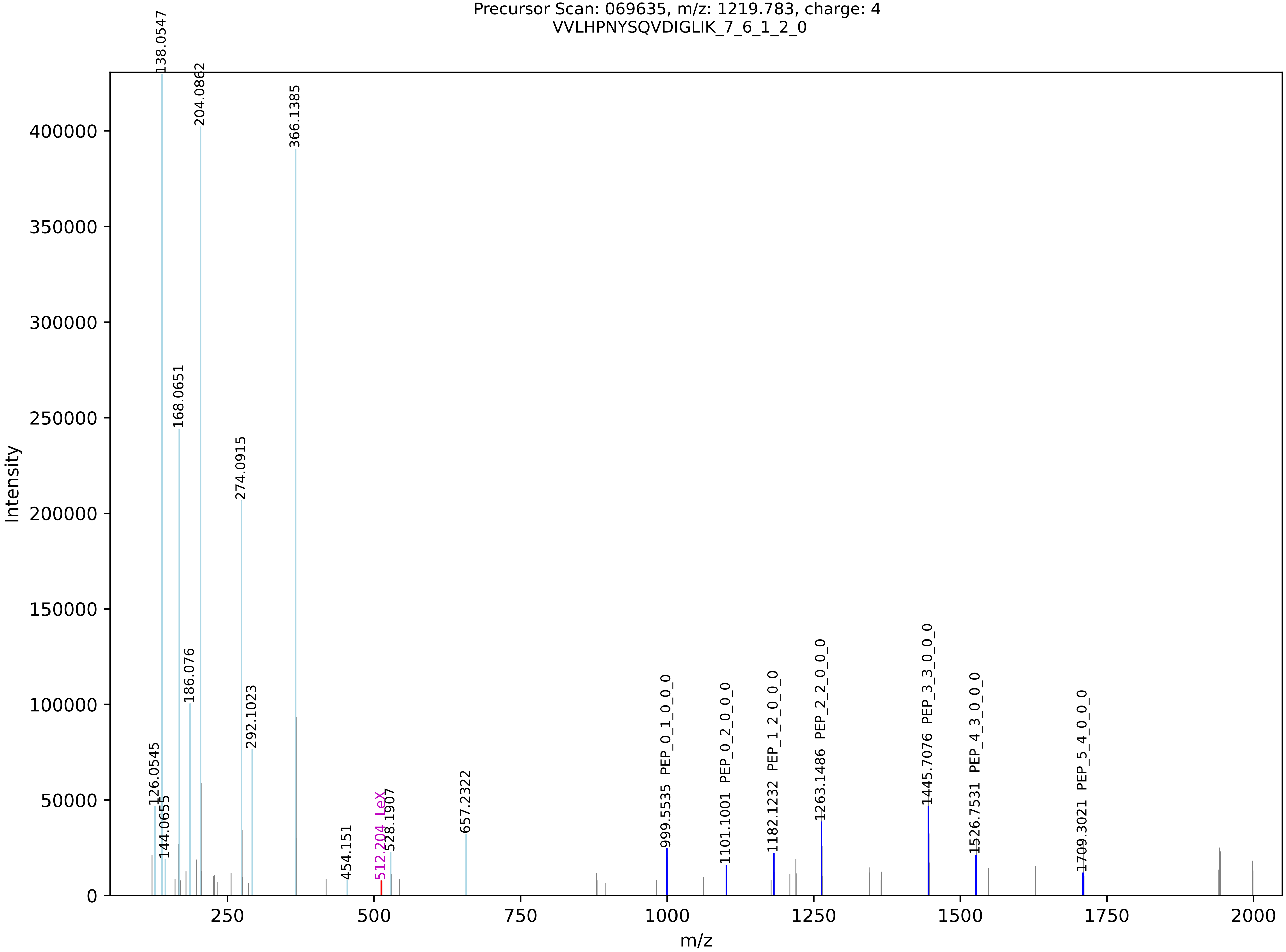

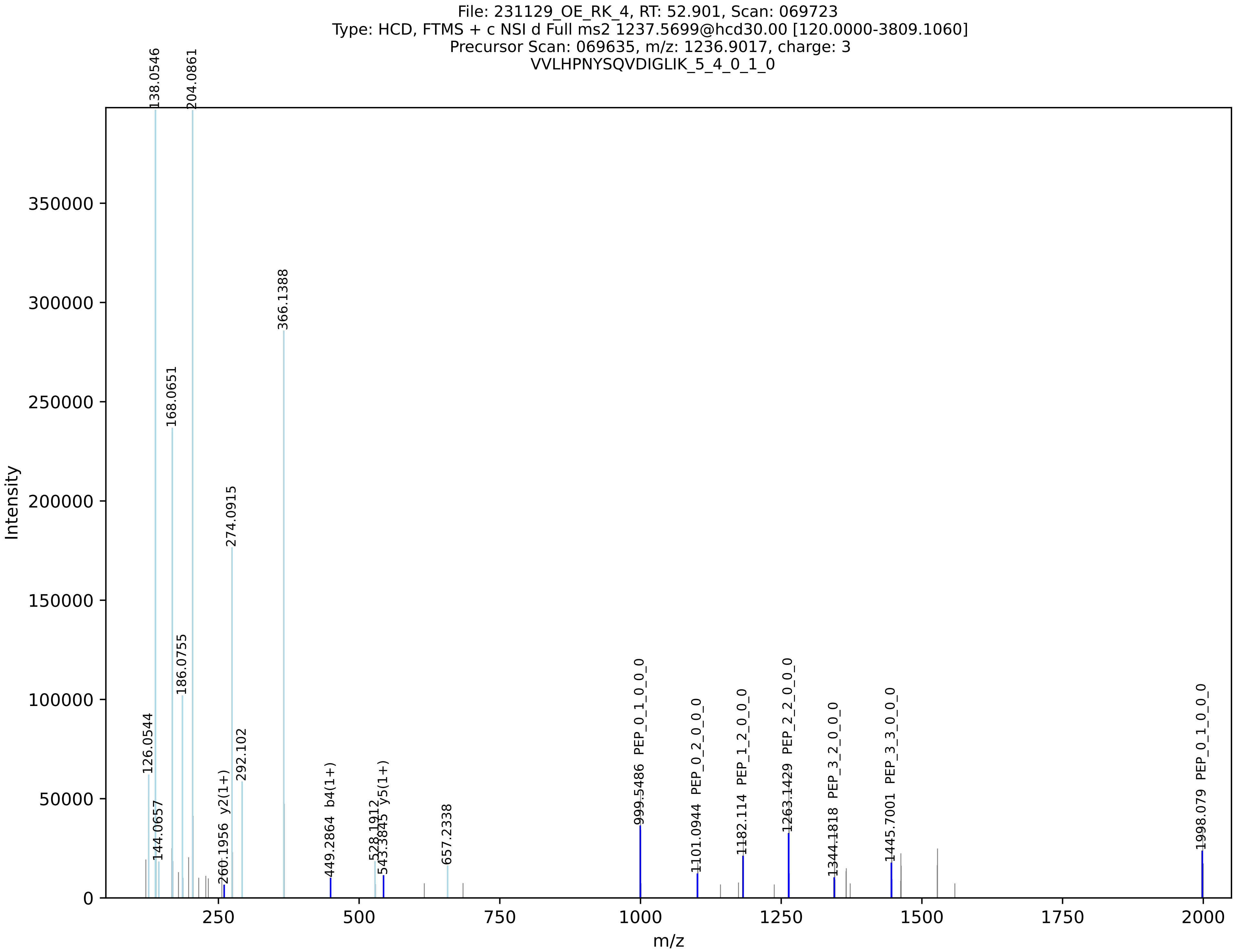
File: 231129_OE_RK_4, RT: 52.901, Scan: 069723
Type: HCD, FTMS + c NSI d Full ms2 1237.5699@hcd30.00 [120.0000-3809.1060]
Precursor Scan: 069635, m/z: 1236.9017, charge: 3
VVLHPNYSQVDIGLIK_5_4_0_1_0
138.0546
204.0861
366.1388
168.0651
274.0915
186.0755
126.0544
292.102
144.0657
260.1956 y2(1+)
449.2864 b4(1+)
528.1912
543.3845 y5(1+)
657.2338
999.5486 PEP_0_1_0_0_0
1101.0944 PEP_0_2_0_0_0
1182.114 PEP_1_2_0_0_0
1263.1429 PEP_2_2_0_0_0
1344.1818 PEP_3_2_0_0_0
1445.7001 PEP_3_3_0_0_0
1998.079 PEP_0_1_0_0_0
Intensity
m/z
0
50000
100000
150000
200000
250000
300000
350000
250
500
750
1000
1250
1500
1750
2000

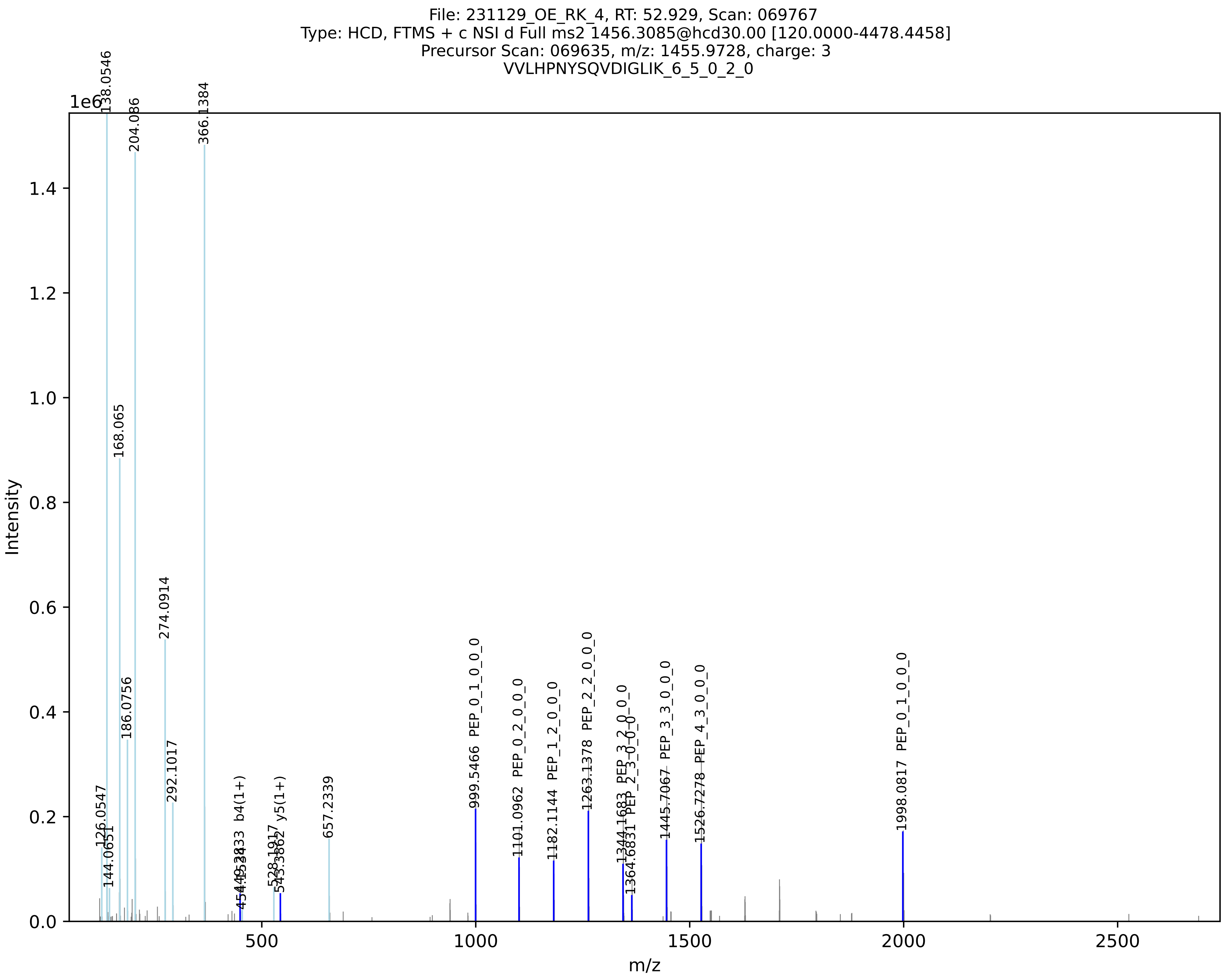
File: 231129_OE_RK_4, RT: 52.929, Scan: 069767
Type: HCD, FTMS + c NSI d Full ms2 1456.3085@hcd30.00 [120.0000-4478.4458]
Precursor Scan: 069635, m/z: 1455.9728, charge: 3
VVLHPNYSQVDIGLIK_6_5_0_2_0
1e6
Intensity
m/z
138.0546
204.086
366.1384
168.065
274.0914
186.0756
292.1017
126.0547
144.0651
b4(1+)
y5(1+)
528.1917
543.3862
657.2339
999.5466 PEP_0_1_0_0_0
1101.0962 PEP_0_2_0_0_0
1182.1144 PEP_1_2_0_0_0
1263.1378 PEP_2_2_0_0_0
1344.1683 PEP_3_2_0_0_0
1364.6831 PEP_2_3_0_0_0
1445.7067 PEP_3_3_0_0_0
1526.7278 PEP_4_3_0_0_0
1998.0817 PEP_0_1_0_0_0

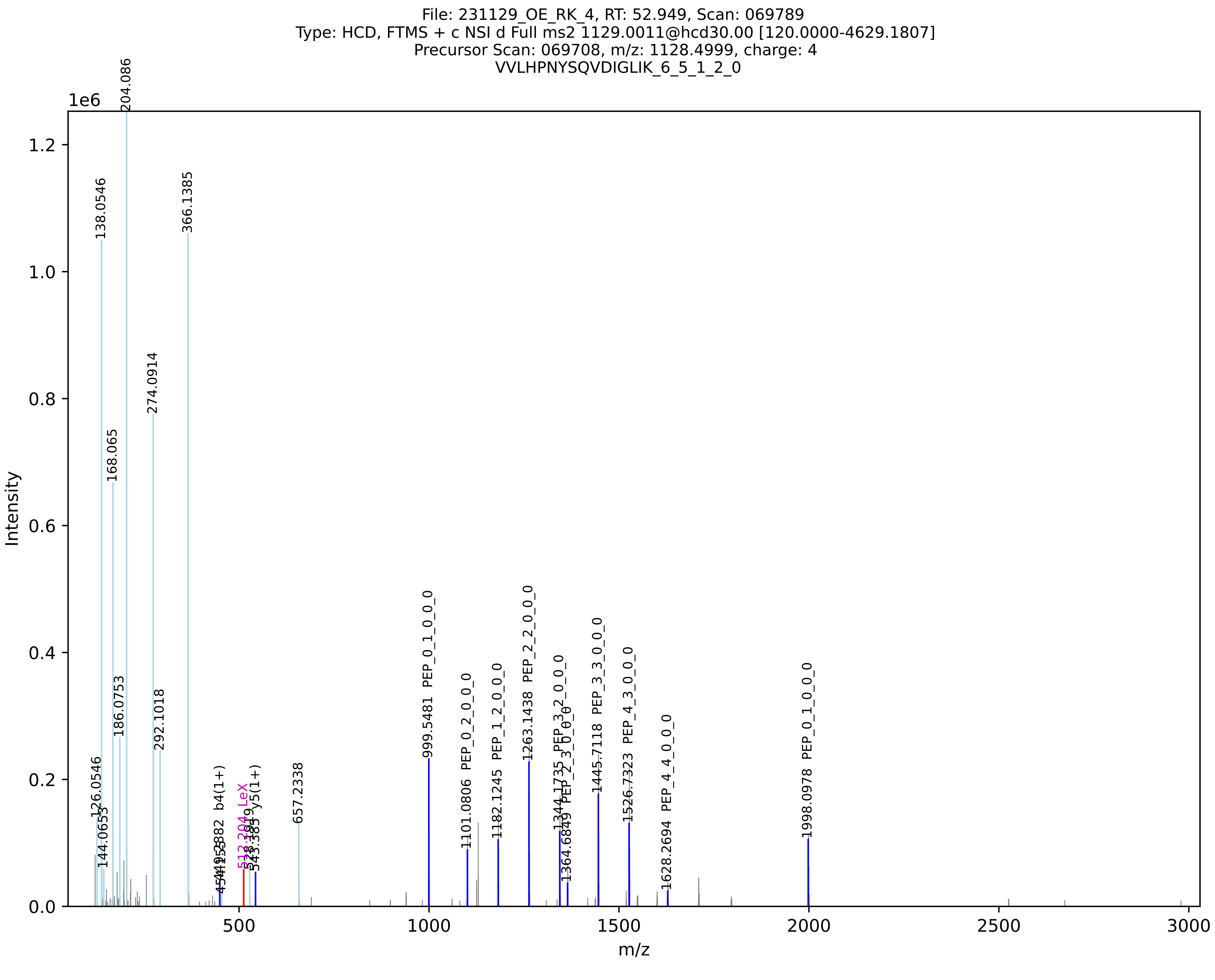
File: 231129_OE_RK_4, RT: 52.949, Scan: 069789
Type: HCD, FTMS + c NSI d Full ms2 1129.0011@hcd30.00 [120.0000-4629.1807]
Precursor Scan: 069708, m/z: 1128.4999, charge: 4
VVLHPNYSQVDIGLIK_6_5_1_2_0
1e6
Intensity
m/z
204.086
138.0546
366.1385
274.0914
168.065
186.0753
292.1018
126.0546
144.0653
b4(1+)
512.204_LeX
y5(1+)
543.385
657.2338
999.5481 PEP_0_1_0_0_0_
1101.0806 PEP_0_2_0_0_0_
1182.1245 PEP_1_2_0_0_0_
1263.1438 PEP_2_2_0_0_0_
1344.1735 PEP_3_2_0_0_0_
1364.6849 PEP_2_3_0_0_0_
1445.7118 PEP_3_3_0_0_0_
1526.7323 PEP_4_3_0_0_0_
1628.2694 PEP_4_4_0_0_0_
1998.0978 PEP_0_1_0_0_0_

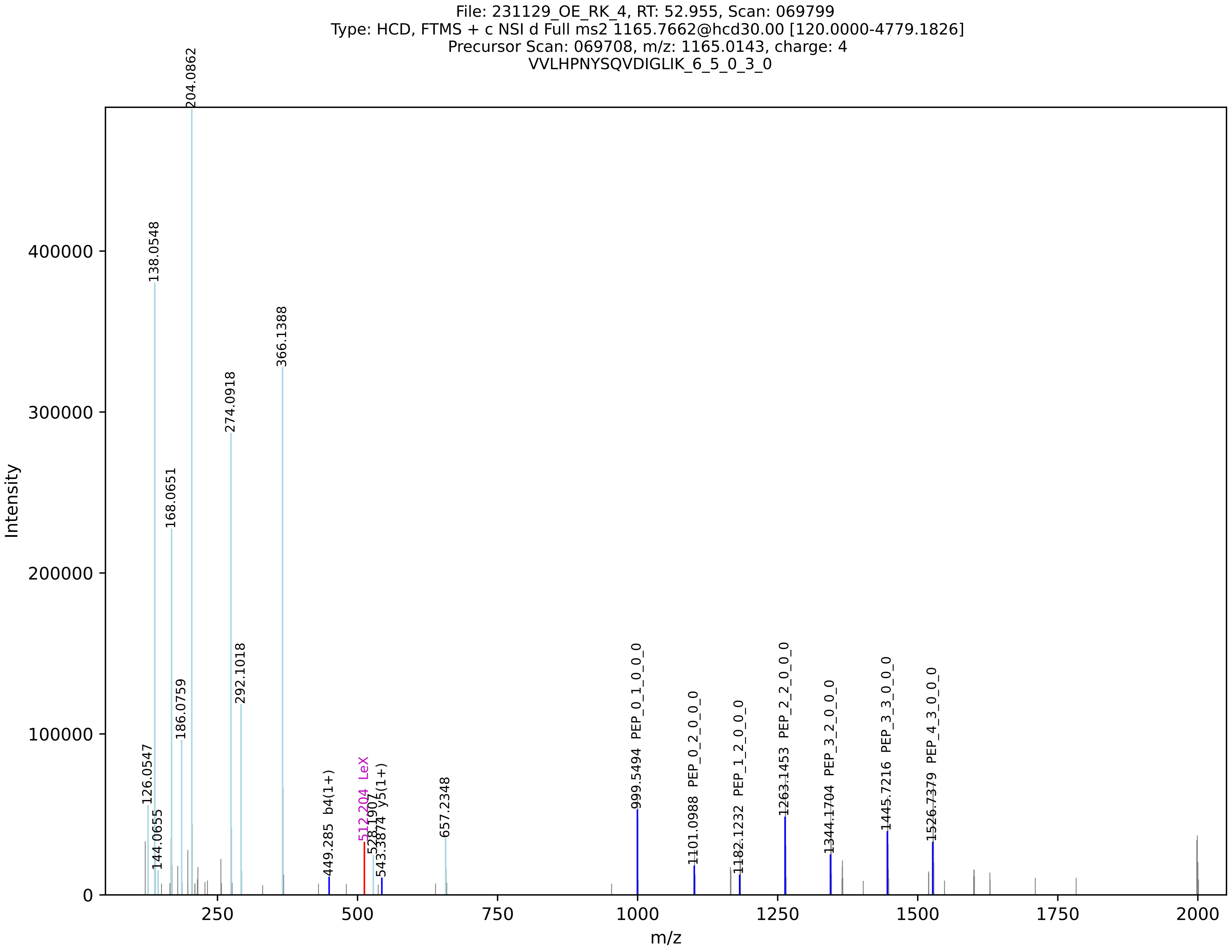

File: 231129_OE_RK_4, RT: 52.955, Scan: 069799
Type: HCD, FTMS + c NSI d Full ms2 1165.7662@hcd30.00 [120.0000-4779.1826]
Precursor Scan: 069708, m/z: 1165.0143, charge: 4
VVLHPNYSQVDIGLIK_6_5_0_3_0
Intensity
m/z
204.0862
138.0548
366.1388
274.0918
168.0651
292.1018
186.0759
126.0547
144.0655
449.285 b4(1+)
512.204 LeX
528.1907
543.3874 y5(1+)
657.2348
999.5494 PEP_0_1_0_0_0
1101.0988 PEP_0_2_0_0_0
1182.1232 PEP_1_2_0_0_0
1263.1453 PEP_2_2_0_0_0
1344.1704 PEP_3_2_0_0_0
1445.7216 PEP_3_3_0_0_0
1526.7379 PEP_4_3_0_0_0

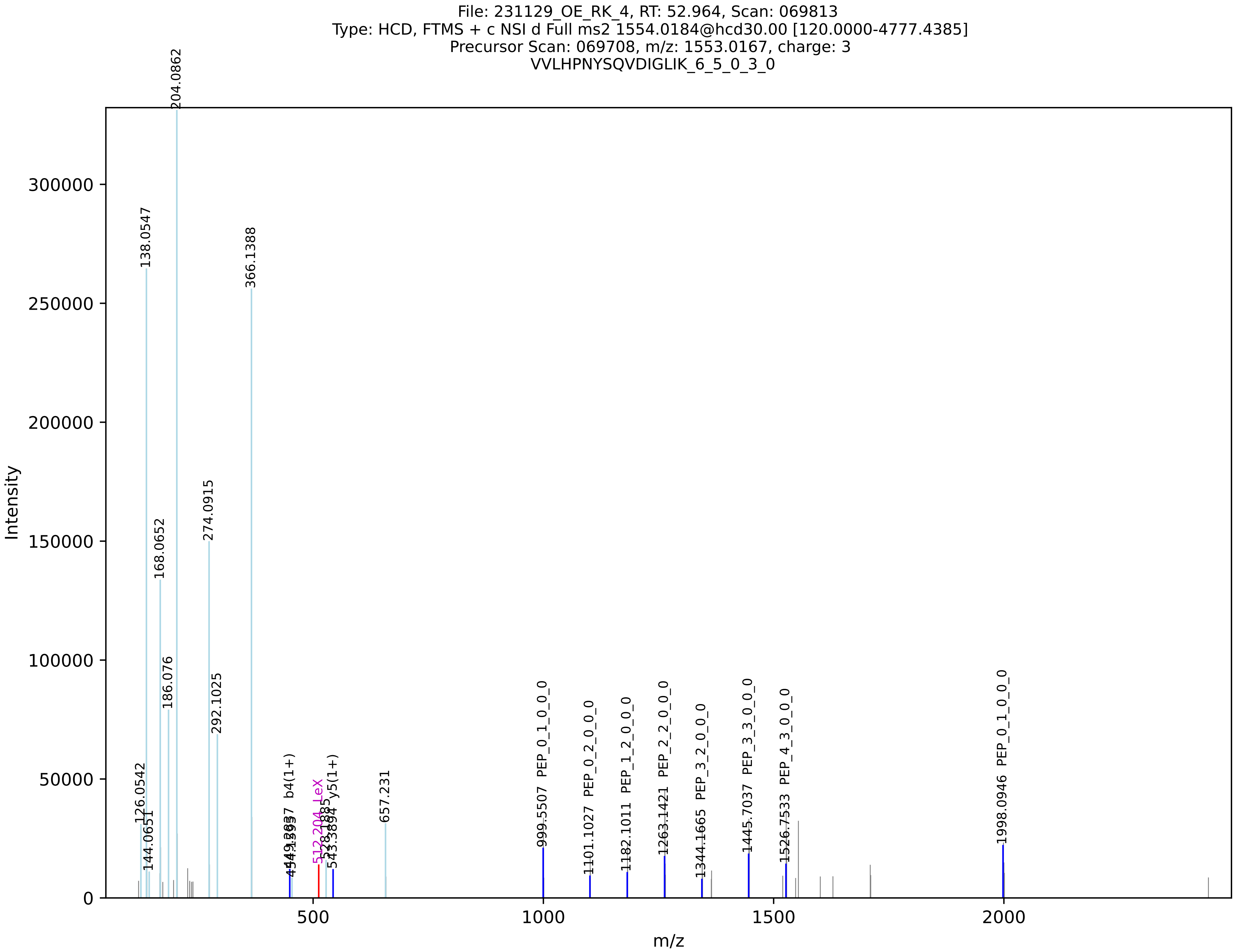
File: 231129_OE_RK_4, RT: 52.964, Scan: 069813
Type: HCD, FTMS + c NSI d Full ms2 1554.0184@hcd30.00 [120.0000-4777.4385]
Precursor Scan: 069708, m/z: 1553.0167, charge: 3
VVLHPNYSQVDIGLIK_6_5_0_3_0
Intensity
m/z
204.0862
138.0547
366.1388
274.0915
168.0652
186.076
292.1025
126.0542
144.0651
b4(1+)
512.204_LeX
y5(1+)
543.3894
657.231
999.5507 PEP_0_1_0_0_0
1101.1027 PEP_0_2_0_0_0
1182.1011 PEP_1_2_0_0_0
1263.1421 PEP_2_2_0_0_0
1344.1665 PEP_3_2_0_0_0
1445.7037 PEP_3_3_0_0_0
1526.7533 PEP_4_3_0_0_0
1998.0946 PEP_0_1_0_0_0

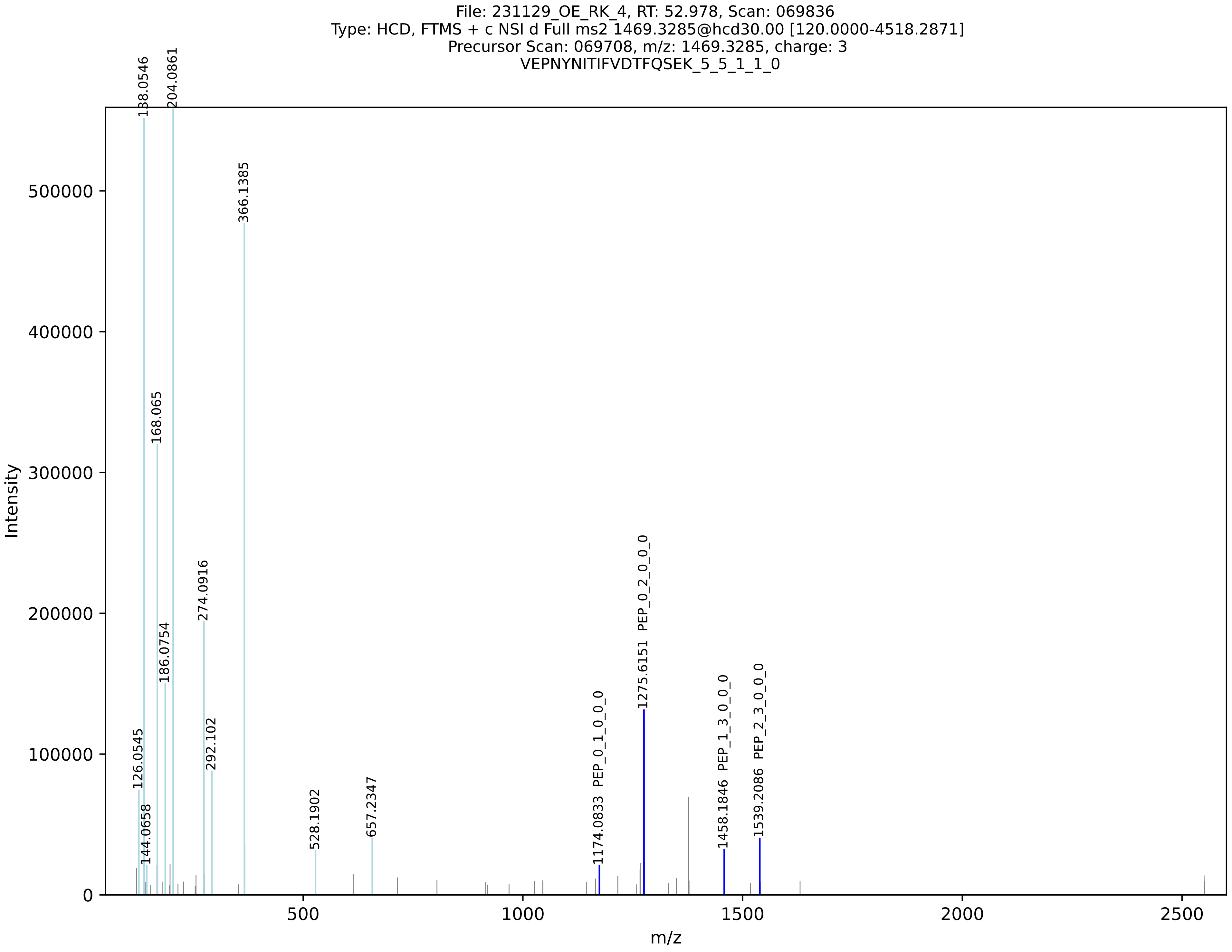

File: 231129_OE_RK_4, RT: 52.978, Scan: 069836
Type: HCD, FTMS + c NSI d Full ms2 1469.3285@hcd30.00 [120.0000-4518.2871]
Precursor Scan: 069708, m/z: 1469.3285, charge: 3
VEPNYNITIFVDTFQSEK_5_5_1_1_0
138.0546
204.0861
366.1385
168.065
274.0916
186.0754
126.0545
144.0658
292.102
528.1902
657.2347
1174.0833 PEP_0_1_0_0_0
1275.6151 PEP_0_2_0_0_0
1458.1846 PEP_1_3_0_0_0
1539.2086 PEP_2_3_0_0_0
Intensity
m/z
0
100000
200000
300000
400000
500000
500
1000
1500
2000
2500

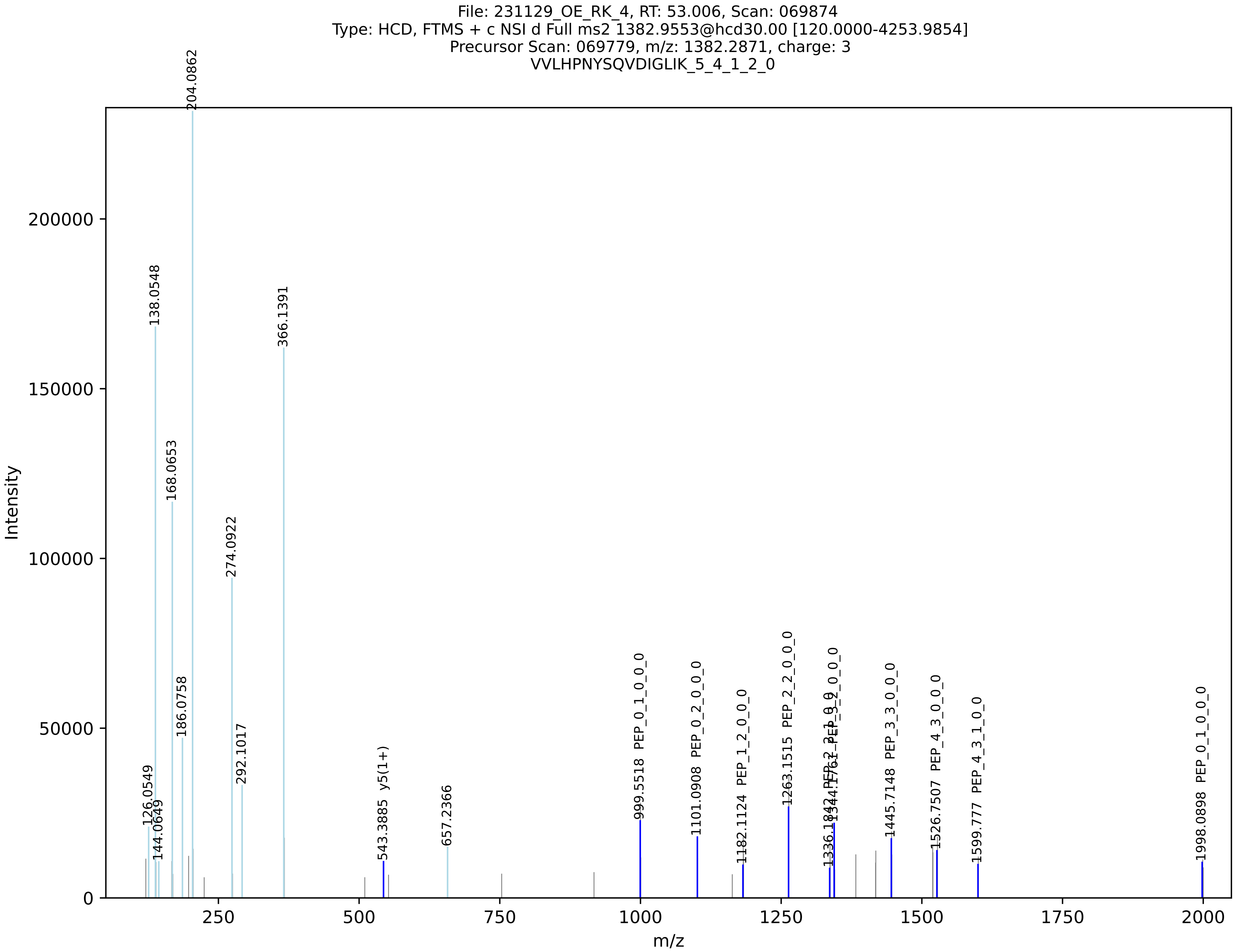
File: 231129_OE_RK_4, RT: 53.006, Scan: 069874
Type: HCD, FTMS + c NSI d Full ms2 1382.9553@hcd30.00 [120.0000-4253.9854]
Precursor Scan: 069779, m/z: 1382.2871, charge: 3
VVLHPNYSQVDIGLIK_5_4_1_2_0
Intensity
m/z
0
50000
100000
150000
200000
250
500
750
1000
1250
1500
1750
2000
204.0862
138.0548
366.1391
168.0653
274.0922
186.0758
292.1017
126.0549
144.0649
543.3885 y5(1+)
657.2366
999.5518 PEP_0_1_0_0_0
1101.0908 PEP_0_2_0_0_0
1182.1124 PEP_1_2_0_0_0
1263.1515 PEP_2_2_0_0_0
1336.1842 PEP_2_3_1_0_0
1344.1761 PEP_3_2_0_0_0
1445.7148 PEP_3_3_0_0_0
1526.7507 PEP_4_3_0_0_0
1599.777 PEP_4_3_1_0_0
1998.0898 PEP_0_1_0_0_0

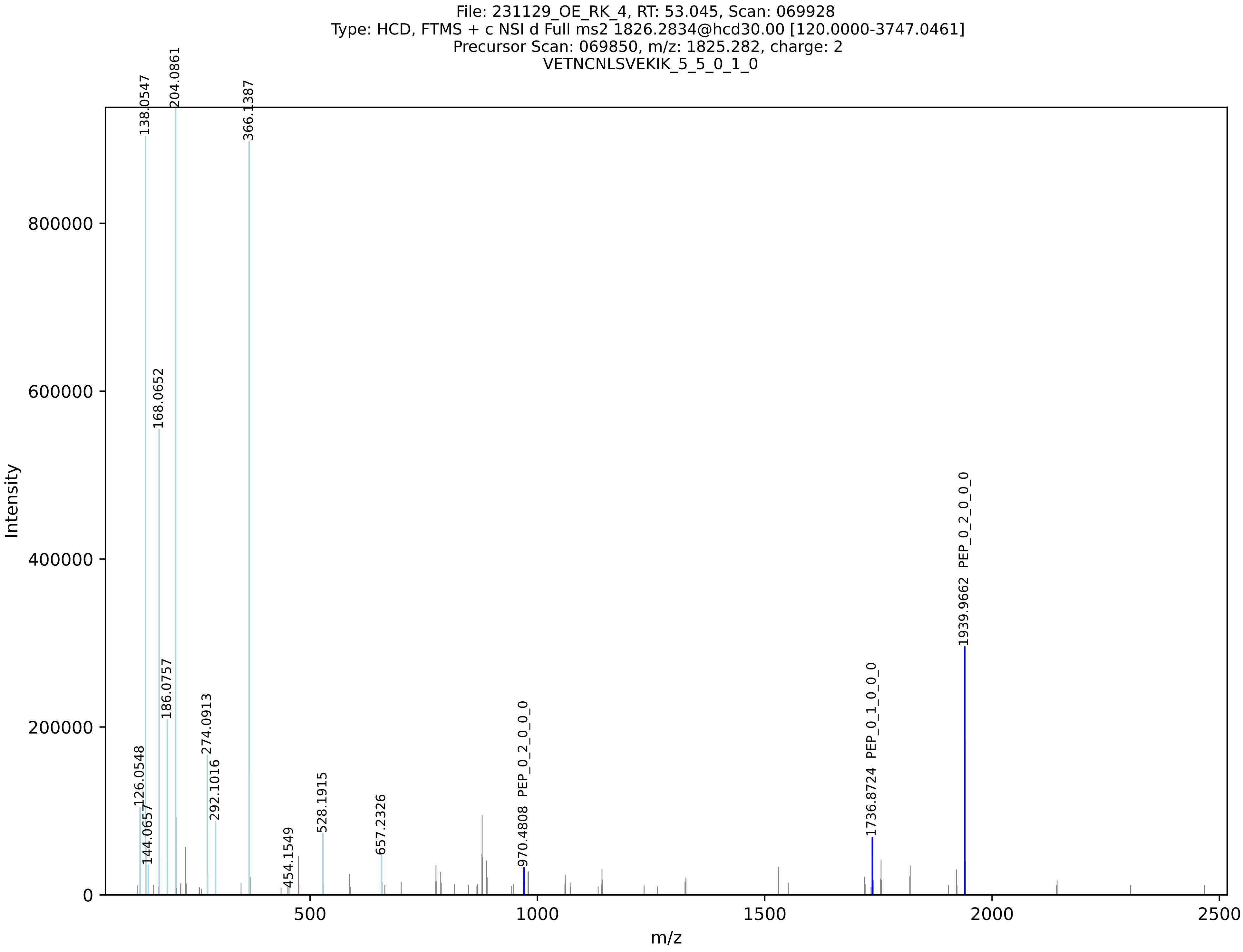

File: 231129_OE_RK_4, RT: 53.045, Scan: 069928
Type: HCD, FTMS + c NSI d Full ms2 1826.2834@hcd30.00 [120.0000-3747.0461]
Precursor Scan: 069850, m/z: 1825.282, charge: 2
VETNCNLSVEKIK_5_5_0_1_0
138.0547
204.0861
366.1387
168.0652
186.0757
274.0913
292.1016
126.0548
144.0657
454.1549
528.1915
657.2326
970.4808 PEP_0_2_0_0_0
1736.8724 PEP_0_1_0_0_0
1939.9662 PEP_0_2_0_0_0
Intensity
800000
600000
400000
200000
0
500
1000
1500
2000
2500
m/z

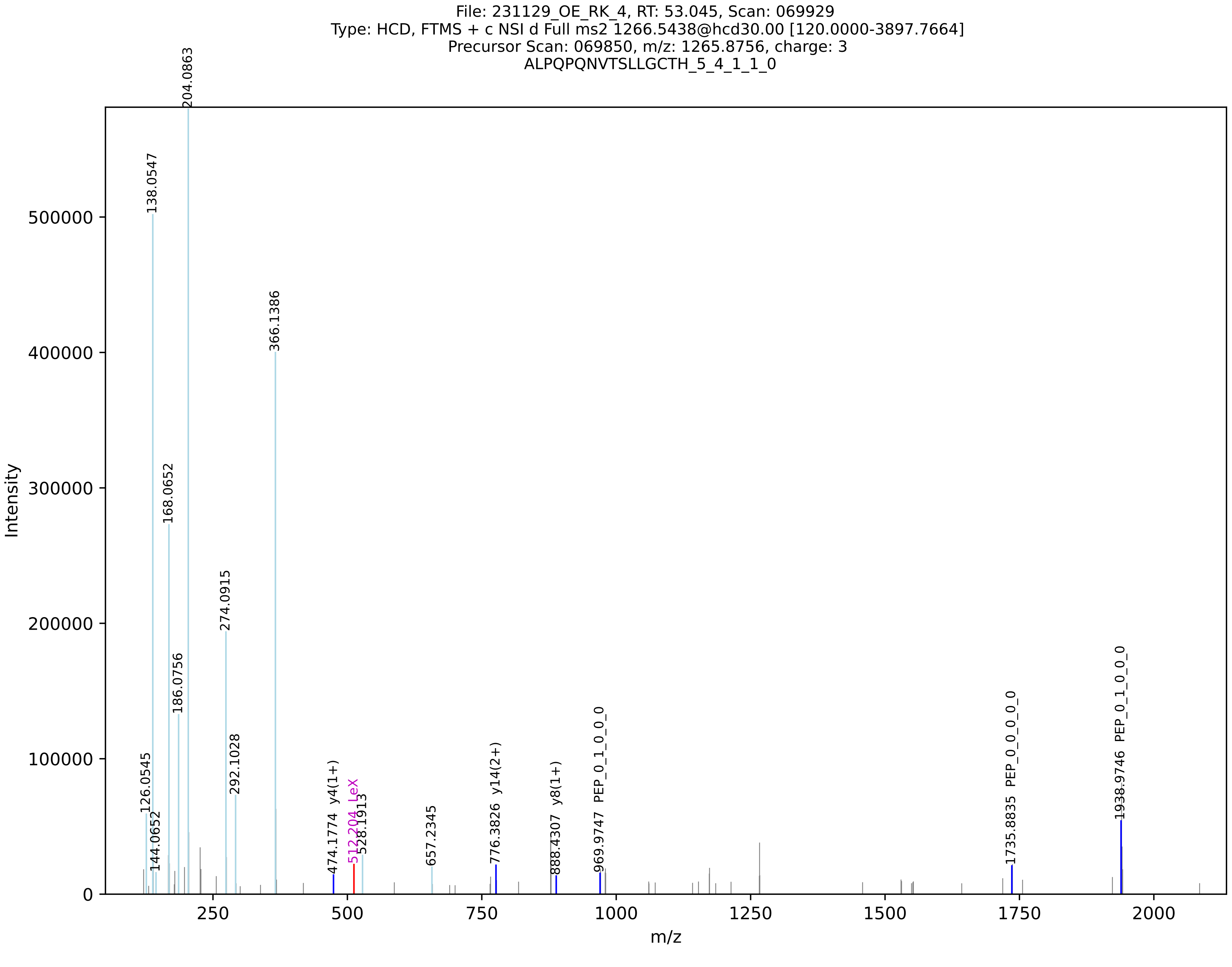
File: 231129_OE_RK_4, RT: 53.045, Scan: 069929
Type: HCD, FTMS + c NSI d Full ms2 1266.5438@hcd30.00 [120.0000-3897.7664]
Precursor Scan: 069850, m/z: 1265.8756, charge: 3
ALPQPQNVTSLLGCTH_5_4_1_1_0
Intensity
m/z
204.0863
138.0547
366.1386
168.0652
274.0915
186.0756
292.1028
126.0545
144.0652
474.1774 y4(1+)
512.204 LeX
528.1913
657.2345
776.3826 y14(2+)
888.4307 y8(1+)
969.9747 PEP_0_1_0_0_0
1735.8835 PEP_0_0_0_0_0
1938.9746 PEP_0_1_0_0_0

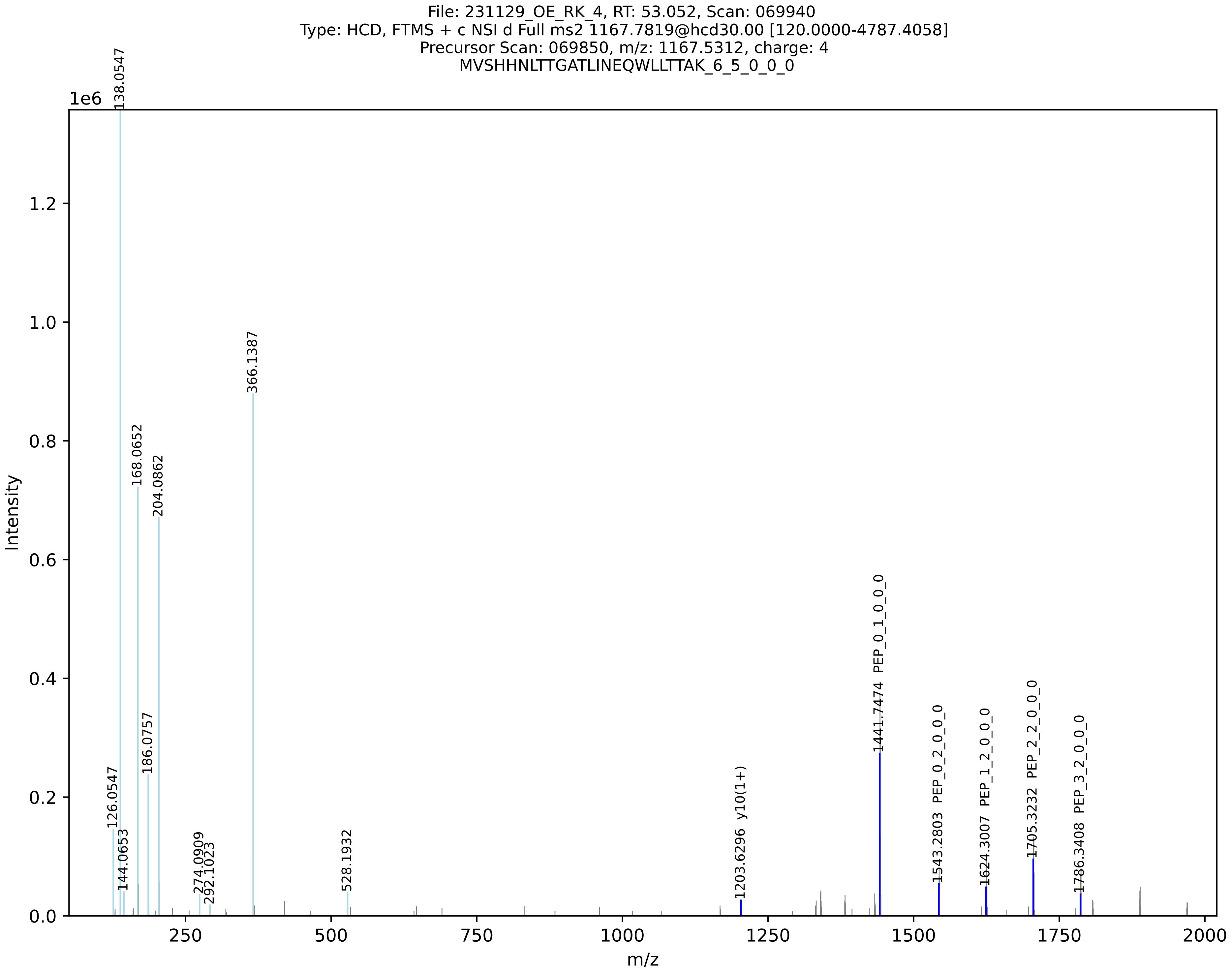
File: 231129_OE_RK_4, RT: 53.052, Scan: 069940
Type: HCD, FTMS + c NSI d Full ms2 1167.7819@hcd30.00 [120.0000-4787.4058]
Precursor Scan: 069850, m/z: 1167.5312, charge: 4
MVSHHNLTTGATLINEQWLLTTAK_6_5_0_0_0
1e6
Intensity
m/z
138.0547
366.1387
168.0652
204.0862
186.0757
126.0547
144.0653
274.0909
292.1023
528.1932
1203.6296 y10(1+)
1441.7474 PEP_0_1_0_0_0
1543.2803 PEP_0_2_0_0_0
1624.3007 PEP_1_2_0_0_0
1705.3232 PEP_2_2_0_0_0
1786.3408 PEP_3_2_0_0_0

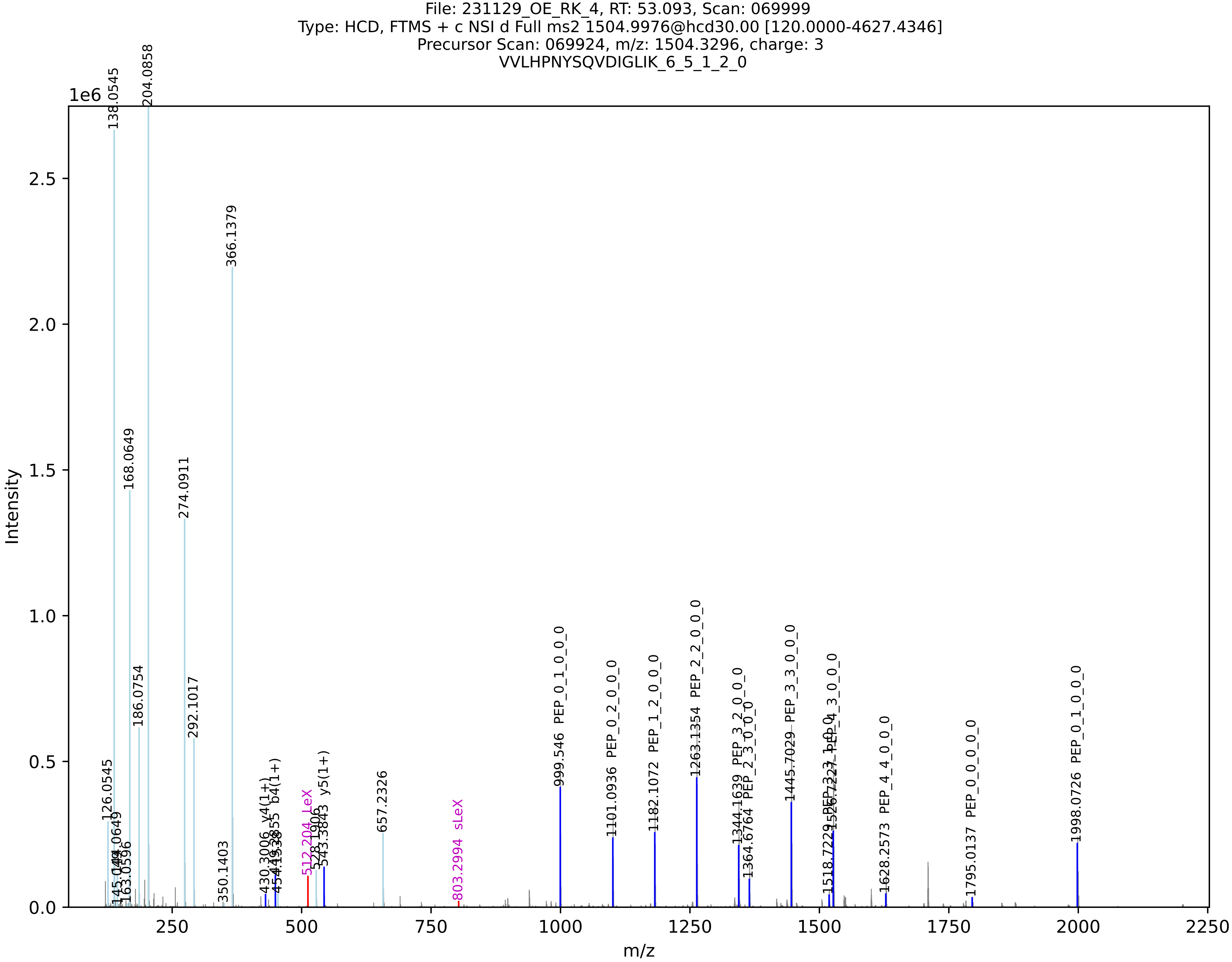
File: 231129_OE_RK_4, RT: 53.093, Scan: 069999
Type: HCD, FTMS + c NSI d Full ms2 1504.9976@hcd30.00 [120.0000-4627.4346]
Precursor Scan: 069924, m/z: 1504.3296, charge: 3
VVLHPNYSQVDIGLIK_6_5_1_2_0
1e6
Intensity
m/z
138.0545
204.0858
366.1379
168.0649
274.0911
186.0754
292.1017
126.0545
350.1403
430.3006 y4(1+)
449.2855 b4(1+)
512.204 LeX
543.3843 y5(1+)
657.2326
803.2994 sLeX
999.546 PEP_0_1_0_0_0
1101.0936 PEP_0_2_0_0_0
1182.1072 PEP_1_2_0_0_0
1263.1354 PEP_2_2_0_0_0
1344.1639 PEP_3_2_0_0_0
1364.6764 PEP_2_3_0_0_0
1445.7029 PEP_3_3_0_0_0
1628.2573 PEP_4_4_0_0_0
1795.0137 PEP_0_0_0_0_0
1998.0726 PEP_0_1_0_0_0

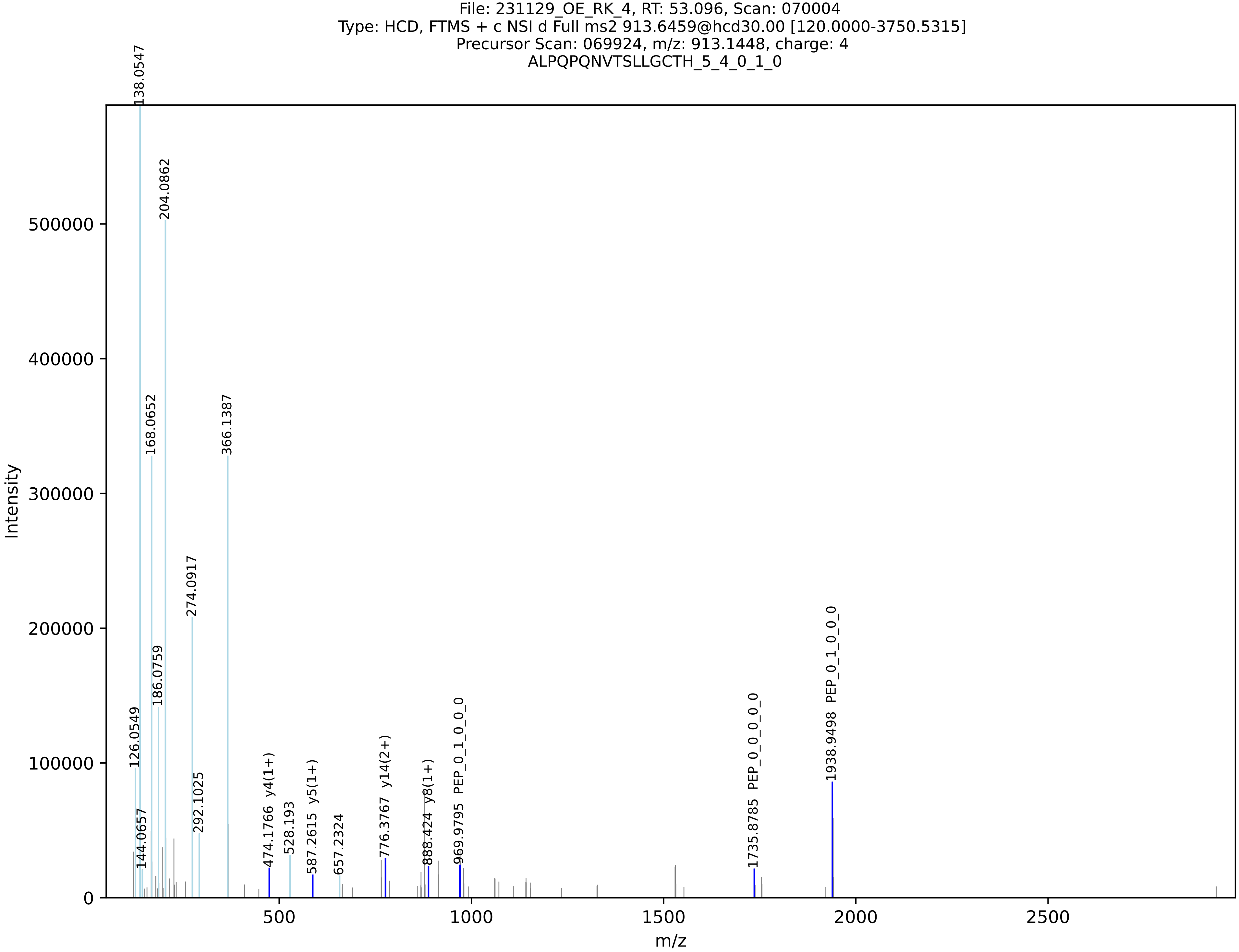
File: 231129_OE_RK_4, RT: 53.096, Scan: 070004
Type: HCD, FTMS + c NSI d Full ms2 913.6459@hcd30.00 [120.0000-3750.5315]
Precursor Scan: 069924, m/z: 913.1448, charge: 4
ALPQPQNVTSLLGCTH_5_4_0_1_0
Intensity
m/z
138.0547
204.0862
168.0652
366.1387
274.0917
186.0759
126.0549
144.0657
292.1025
474.1766 y4(1+)
528.193
587.2615 y5(1+)
657.2324
776.3767 y14(2+)
888.424 y8(1+)
969.9795 PEP_0_1_0_0_0
1735.8785 PEP_0_0_0_0_0
1938.9498 PEP_0_1_0_0_0
0
100000
200000
300000
400000
500000
500
1000
1500
2000
2500

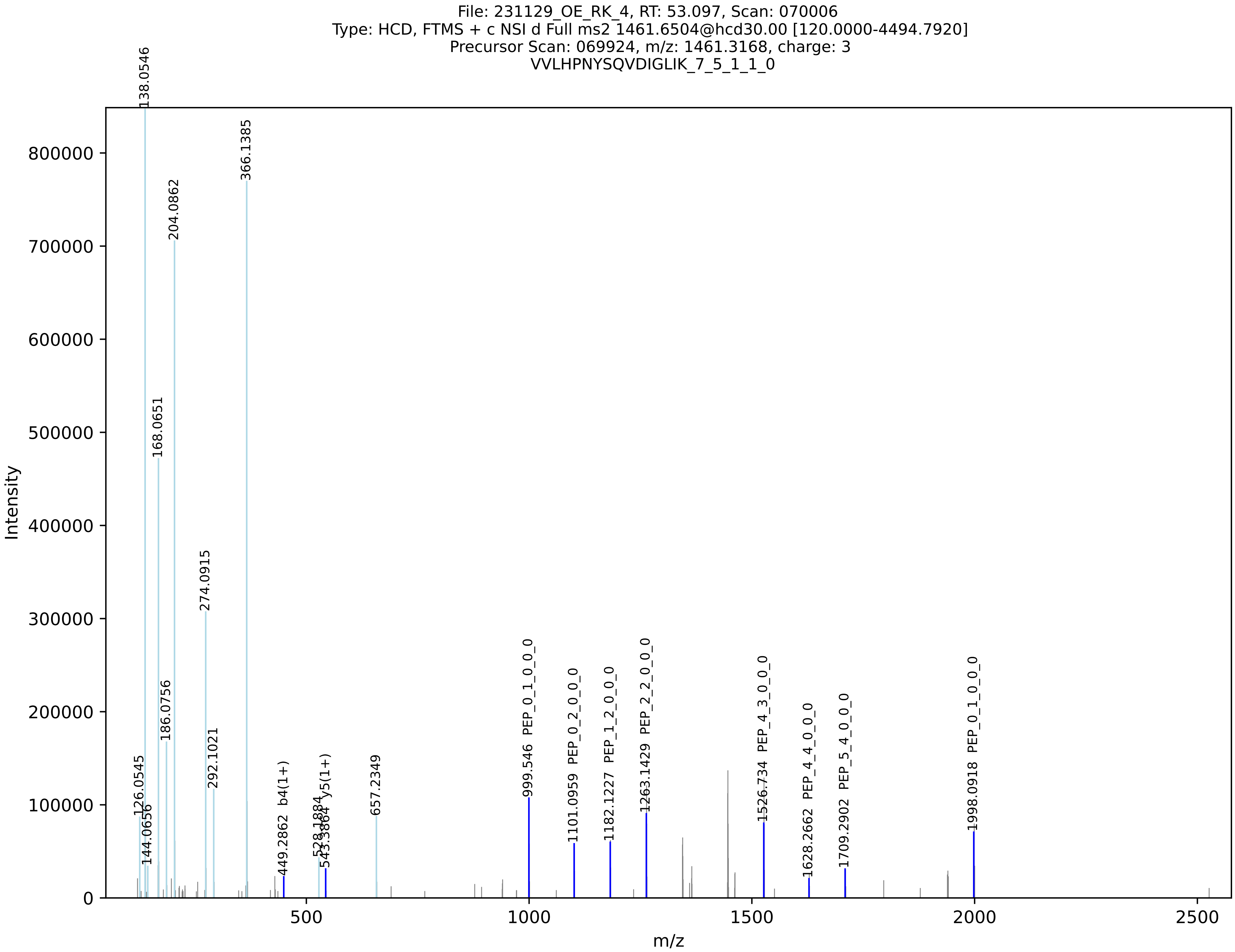
File: 231129_OE_RK_4, RT: 53.097, Scan: 070006
Type: HCD, FTMS + c NSI d Full ms2 1461.6504@hcd30.00 [120.0000-4494.7920]
Precursor Scan: 069924, m/z: 1461.3168, charge: 3
VVLHPNYSQVDIGLIK_7_5_1_1_0
Intensity
m/z
138.0546
366.1385
204.0862
168.0651
274.0915
186.0756
292.1021
126.0545
144.0656
449.2862 b4(1+)
529.1884
543.3864 y5(1+)
657.2349
999.546 PEP_0_1_0_0_0
1101.0959 PEP_0_2_0_0_0
1182.1227 PEP_1_2_0_0_0
1263.1429 PEP_2_2_0_0_0
1526.734 PEP_4_3_0_0_0
1628.2662 PEP_4_4_0_0_0
1709.2902 PEP_5_4_0_0_0
1998.0918 PEP_0_1_0_0_0

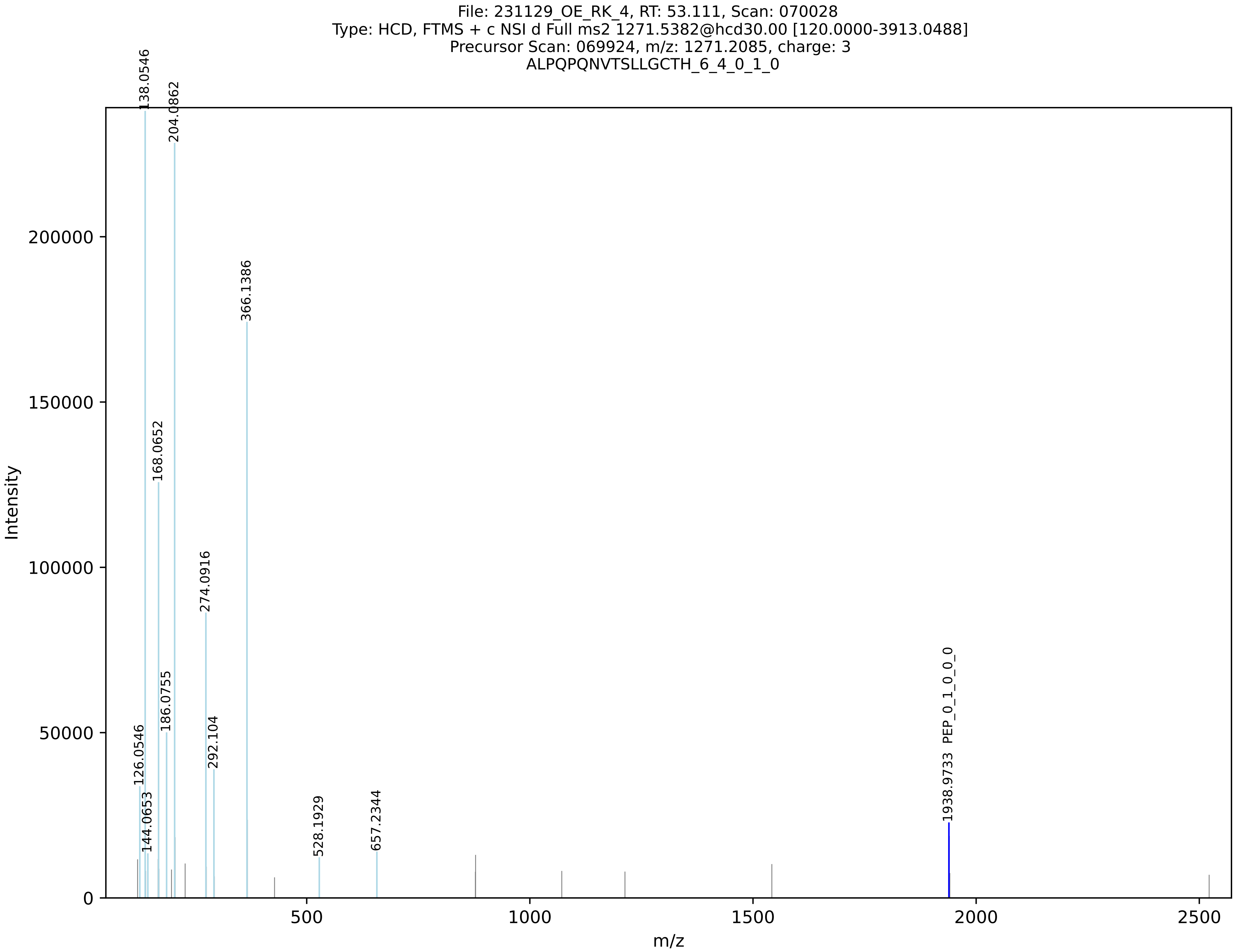

File: 231129_OE_RK_4, RT: 53.111, Scan: 070028
Type: HCD, FTMS + c NSI d Full ms2 1271.5382@hcd30.00 [120.0000-3913.0488]
Precursor Scan: 069924, m/z: 1271.2085, charge: 3
ALPQPQNVTSLLGCTH_6_4_0_1_0
Intensity
m/z
138.0546
204.0862
366.1386
168.0652
274.0916
186.0755
292.104
126.0546
144.0653
528.1929
657.2344
1938.9733 PEP_0_1_0_0_0
0
50000
100000
150000
200000
500
1000
1500
2000
2500

File: 231129_OE_RK_4, RT: 53.144, Scan: 070073
Type: HCD, FTMS + c NSI d Full ms2 1217.5245@hcd30.00 [120.0000-3747.7671]
Precursor Scan: 069998, m/z: 1217.1904, charge: 3
ALPQPQNVTSLLGCTH_5_4_0_1_0

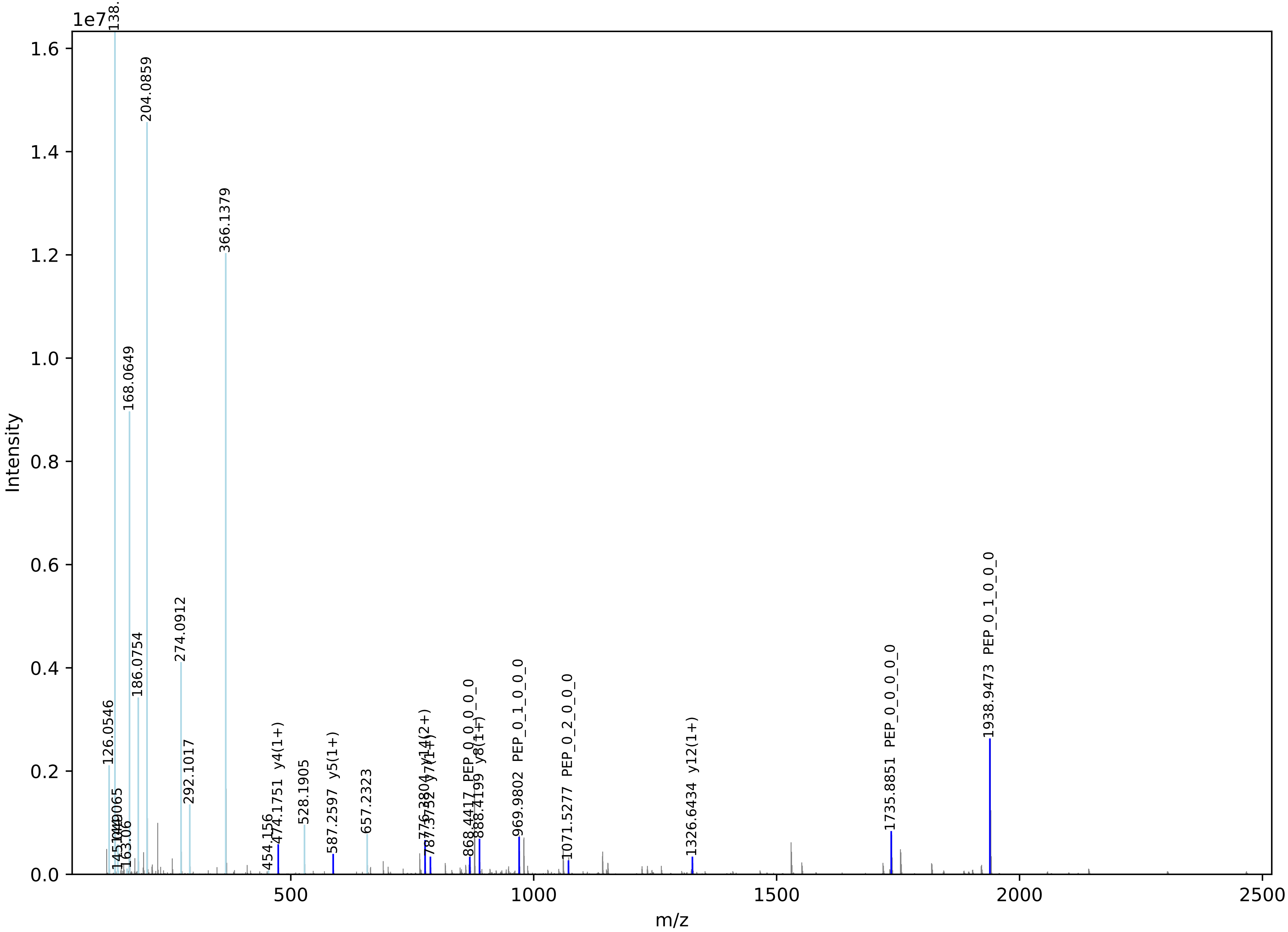

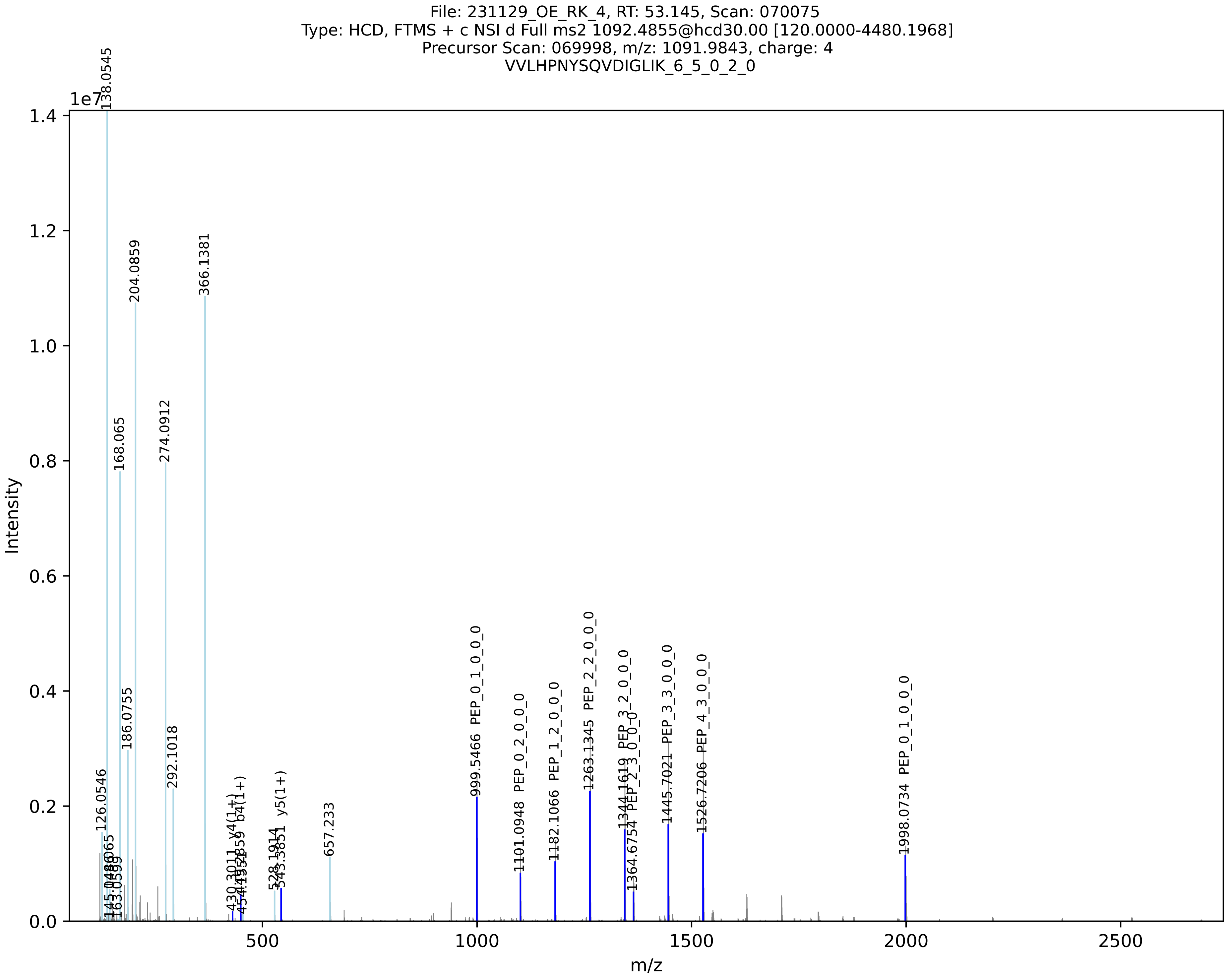
File: 231129_OE_RK_4, RT: 53.145, Scan: 070075
Type: HCD, FTMS + c NSI d Full ms2 1092.4855@hcd30.00 [120.0000-4480.1968]
Precursor Scan: 069998, m/z: 1091.9843, charge: 4
VVLHPNYSQVDIGLIK_6_5_0_2_0
1e7
Intensity
m/z
138.0545
204.0859
366.1381
168.065
274.0912
186.0755
292.1018
126.0546
528.1914
543.3851 y5(1+)
657.233
999.5466 PEP_0_1_0_0_
1101.0948 PEP_0_2_0_0_0
1182.1066 PEP_1_2_0_0_0
1263.1345 PEP_2_2_0_0_0
1344.1619 PEP_3_2_0_0_0
1364.6754 PEP_2_3_0_0_0
1445.7021 PEP_3_3_0_0_0
1526.7206 PEP_4_3_0_0_0
1998.0734 PEP_0_1_0_0_0

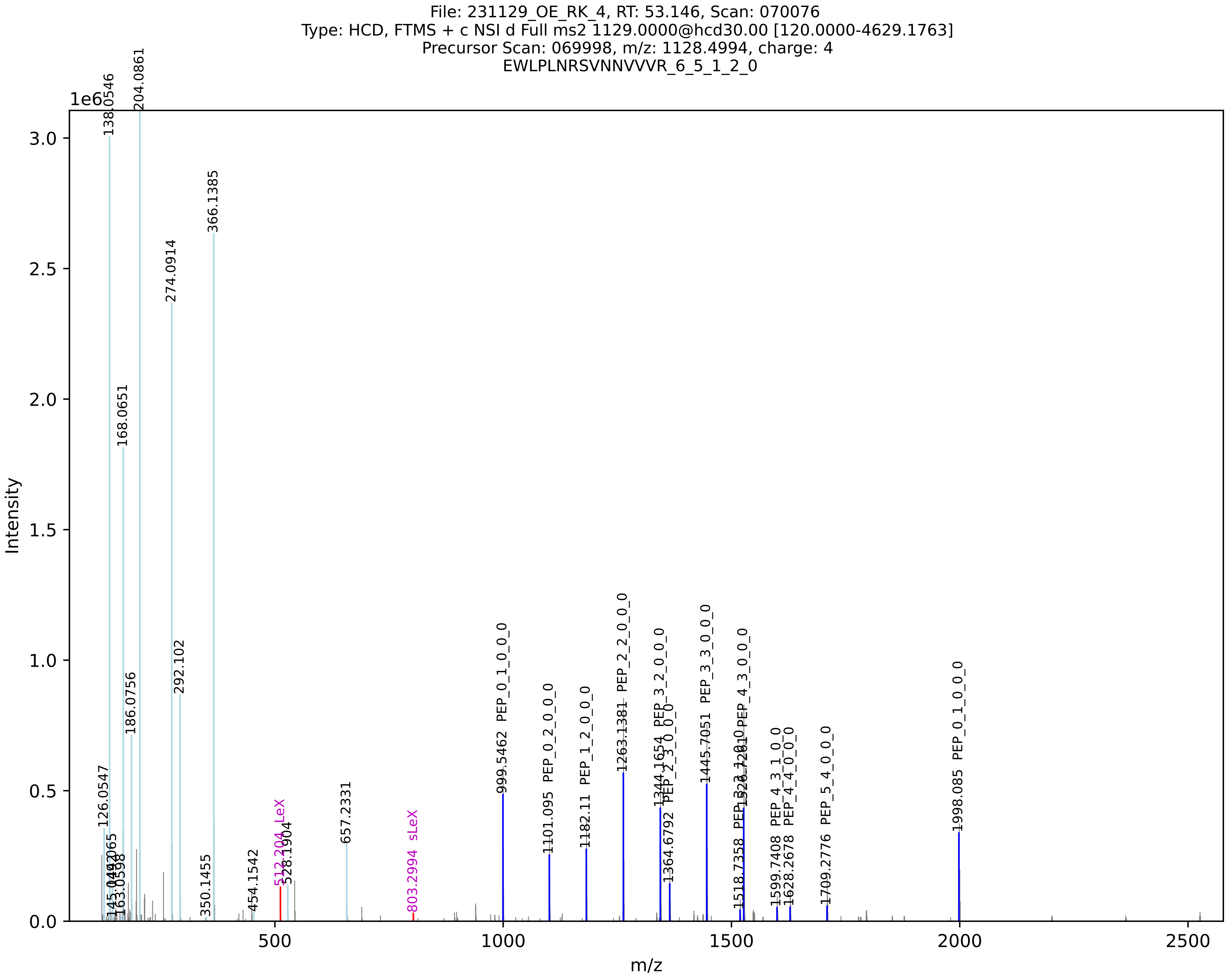
File: 231129_OE_RK_4, RT: 53.146, Scan: 070076
Type: HCD, FTMS + c NSI d Full ms2 1129.0000@hcd30.00 [120.0000-4629.1763]
Precursor Scan: 069998, m/z: 1128.4994, charge: 4
EWLPLNRSVNNVVVR_6_5_1_2_0
1e6
Intensity
m/z
138.0546
204.0861
366.1385
274.0914
168.0651
292.102
186.0756
126.0547
163.0598
350.1455
454.1542
512.204 LeX
528.1904
657.2331
803.2994 sLeX
999.5462 PEP_0_1_0_0_
1101.095 PEP_0_2_0_0_0
1182.11 PEP_1_2_0_0_0
1263.1381 PEP_2_2_0_0_0
1344.1654 PEP_3_2_0_0_0
1364.6792 PEP_2_3_0_0_0
1445.7051 PEP_3_3_0_0_0
1518.7358
1526.7201 PEP_4_3_0_0_0
1599.7408 PEP_4_3_1_0_0
1628.2678 PEP_4_4_0_0_0
1709.2776 PEP_5_4_0_0_0
1998.085 PEP_0_1_0_0_0

File: 231129_OE_RK_4, RT: 53.149, Scan: 070081
Type: HCD, FTMS + c NSI d Full ms2 1165.7642@hcd30.00 [120.0000-4779.1738]
Precursor Scan: 069998, m/z: 1165.0111, charge: 4
VVLHPNYSQVDIGLIK_6_5_0_3_0

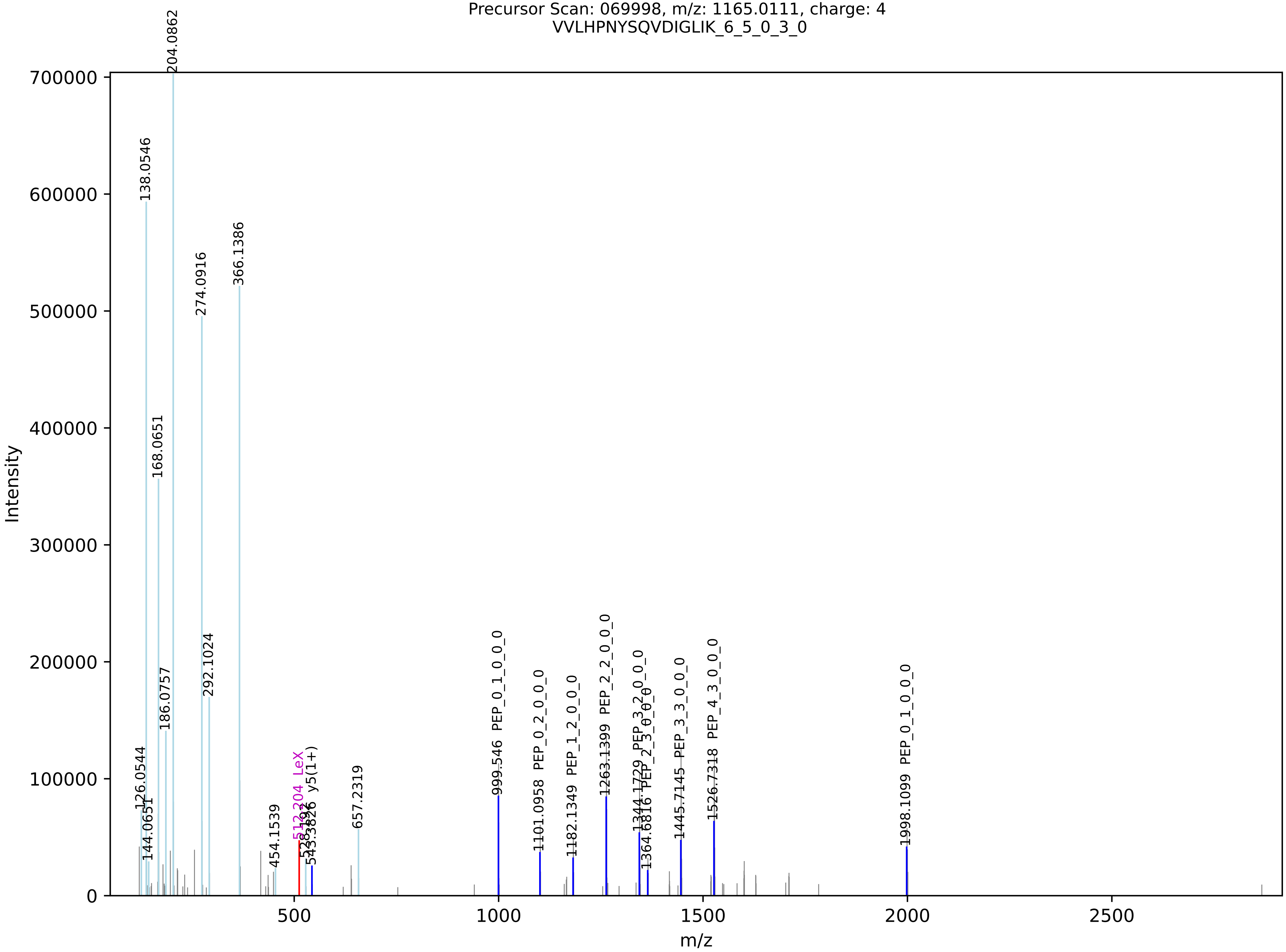

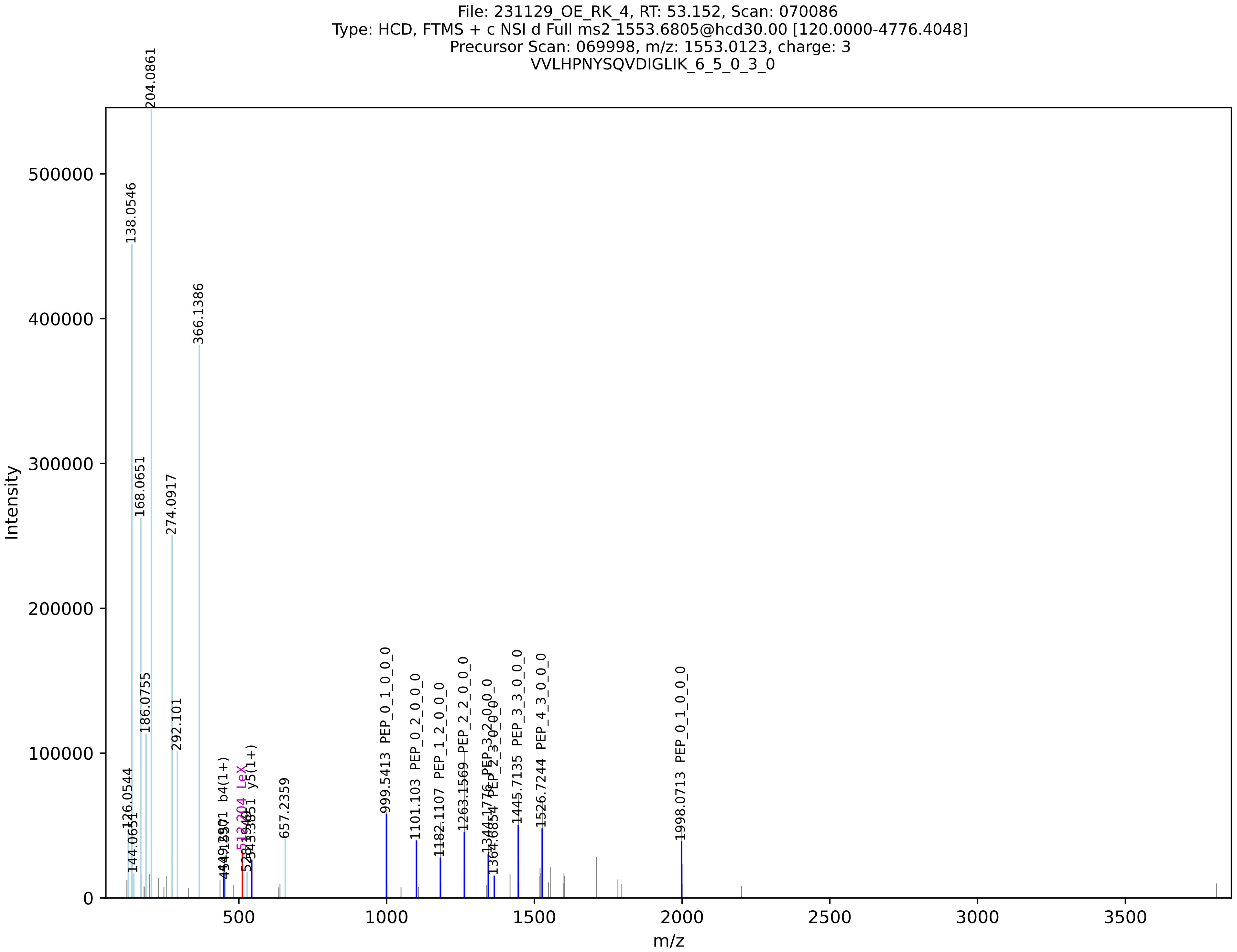
File: 231129_OE_RK_4, RT: 53.152, Scan: 070086
Type: HCD, FTMS + c NSI d Full ms2 1553.6805@hcd30.00 [120.0000-4776.4048]
Precursor Scan: 069998, m/z: 1553.0123, charge: 3
VVLHPNYSQVDIGLIK_6_5_0_3_0
Intensity
m/z
204.0861
138.0546
366.1386
168.0651
274.0917
186.0755
292.101
126.0544
144.0651
b4(1+)
LeX
y5(1+)
543.3651
657.2359
999.5413 PEP_0_1_0_0_0
1101.103 PEP_0_2_0_0_0
1182.1107 PEP_1_2_0_0_0
1263.1569 PEP_2_2_0_0_0
1364.6854
1445.7135 PEP_3_3_0_0_0
1526.7244 PEP_4_3_0_0_0
1998.0713 PEP_0_1_0_0_0
0
100000
200000
300000
400000
500000
500
1000
1500
2000
2500
3000
3500

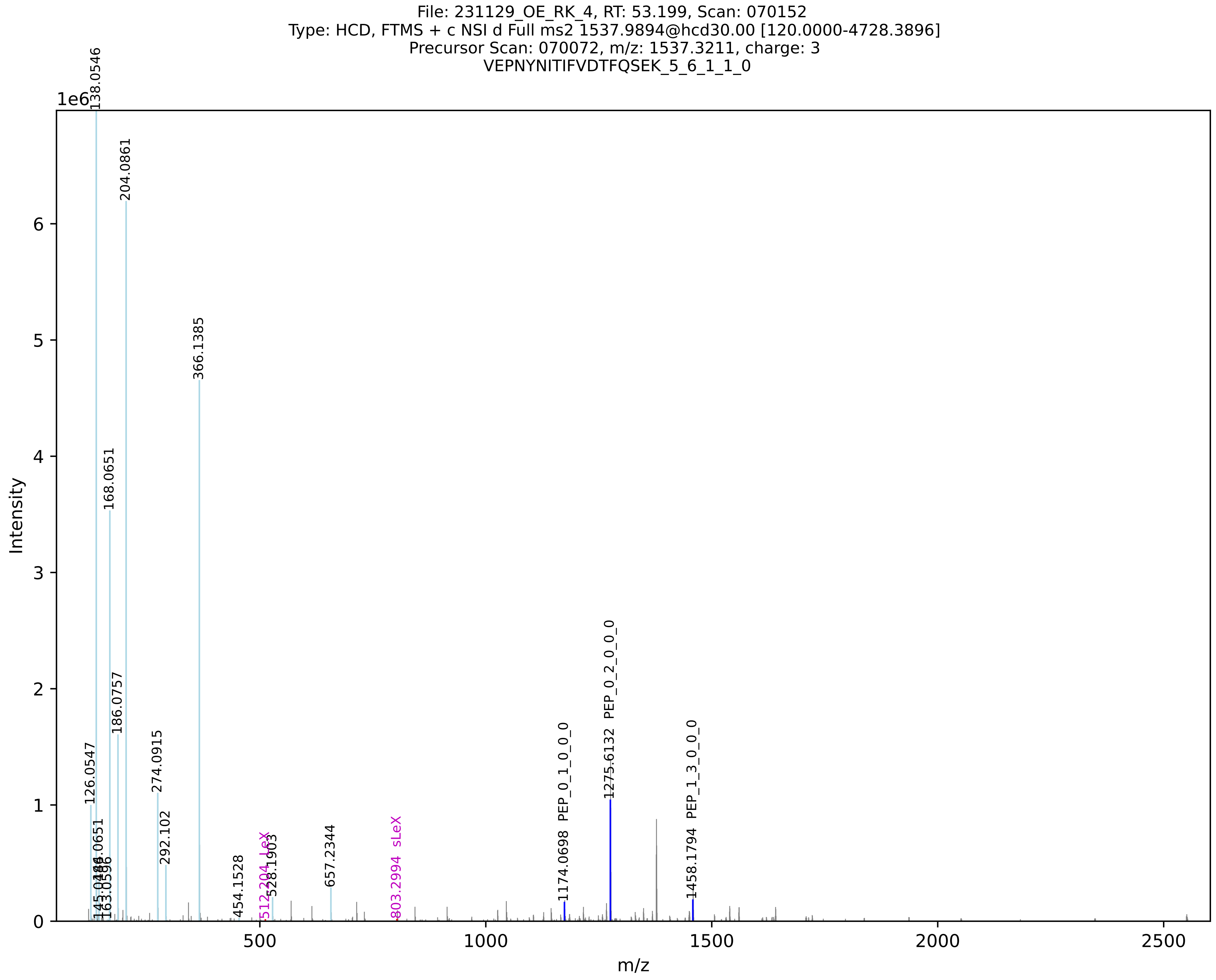
File: 231129_OE_RK_4, RT: 53.199, Scan: 070152
Type: HCD, FTMS + c NSI d Full ms2 1537.9894@hcd30.00 [120.0000-4728.3896]
Precursor Scan: 070072, m/z: 1537.3211, charge: 3
VEPNYNITIFVDTFQSEK_5_6_1_1_0
1e6
Intensity
m/z
138.0546
204.0861
366.1385
168.0651
186.0757
274.0915
126.0547
292.102
454.1528
512.204 LeX
528.1903
657.2344
803.2994 sLeX
1174.0698 PEP_0_1_0_0_0
1275.6132 PEP_0_2_0_0_0
1458.1794 PEP_1_3_0_0_0
163.0596
0
1
2
3
4
5
6
500
1000
1500
2000
2500

File: 231129_OE_RK_4, RT: 53.203, Scan: 070159
Type: HCD, FTMS + c NSI d Full ms2 1396.0516@hcd30.00 [120.0000-5718.7471]
Precursor Scan: 070072, m/z: 1395.5485, charge: 4
QDQCIYNTTYLNVQR_7_6_1_4_0

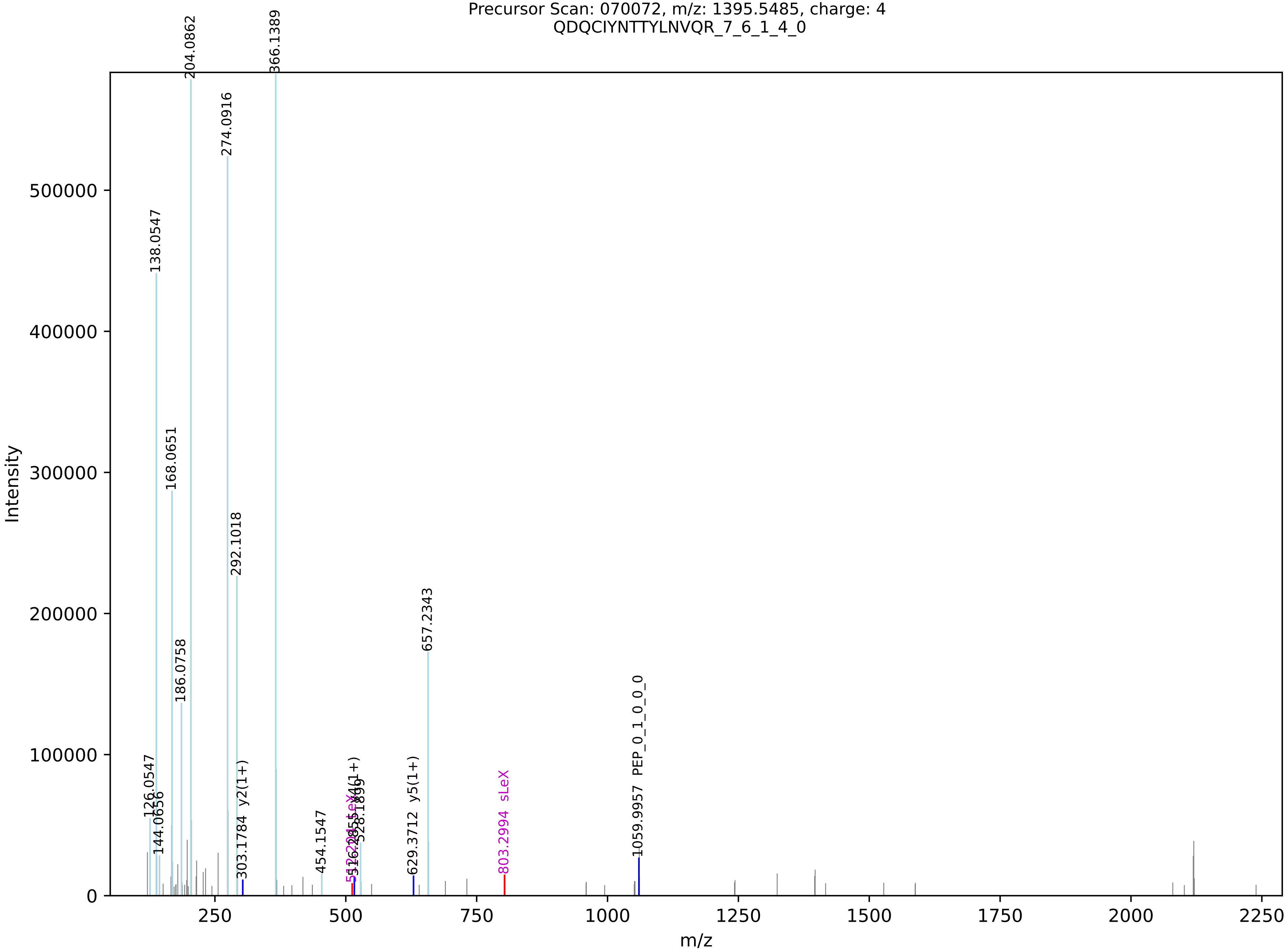

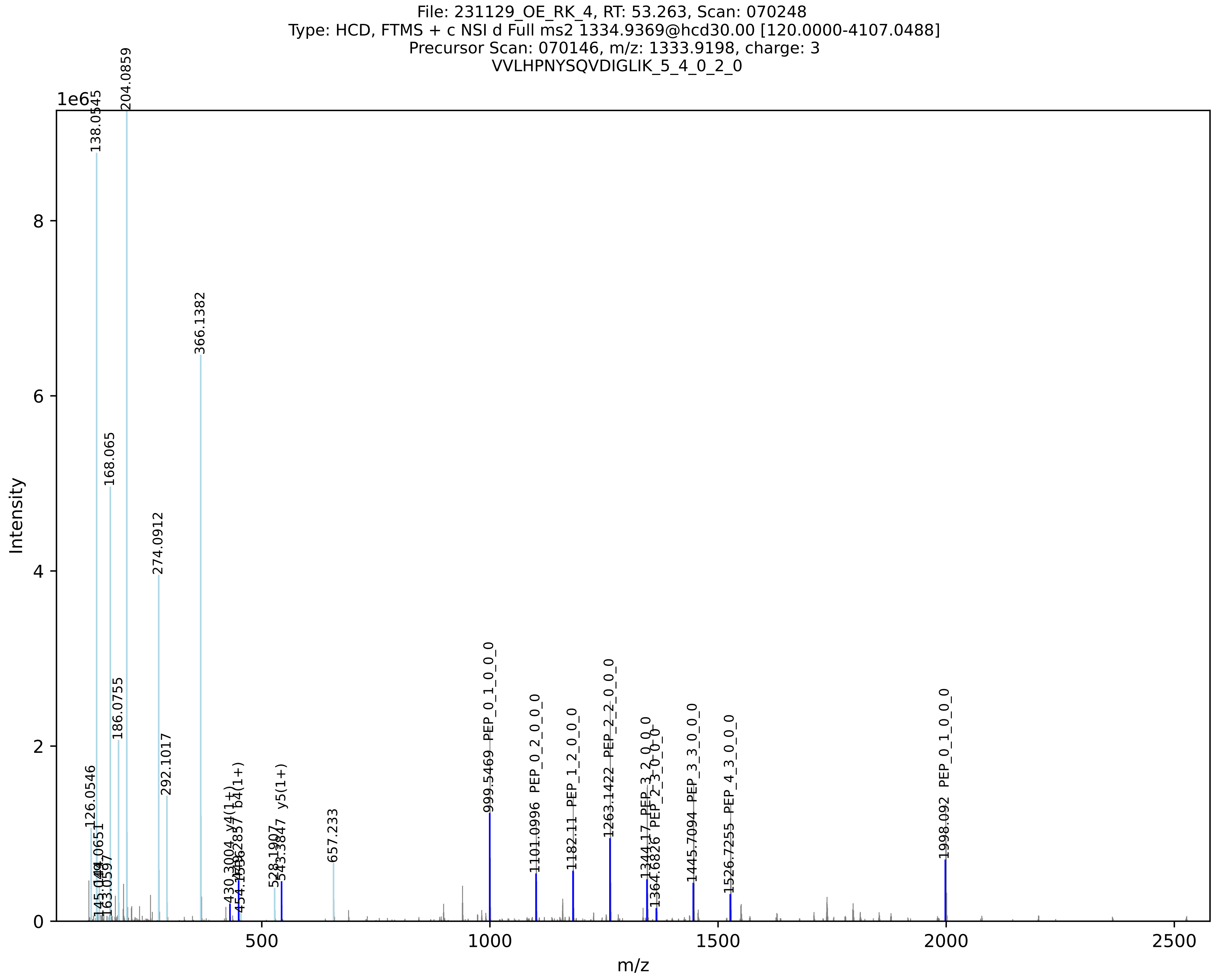

File: 231129_OE_RK_4, RT: 53.263, Scan: 070248
Type: HCD, FTMS + c NSI d Full ms2 1334.9369@hcd30.00 [120.0000-4107.0488]
Precursor Scan: 070146, m/z: 1333.9198, charge: 3
VVLHPNYSQVDIGLIK_5_4_0_2_0
1e6
Intensity
m/z
204.0859
138.0545
366.1382
168.065
274.0912
186.0755
292.1017
126.0546
999.5469 PEP_0_1_0_0_
1263.1422 PEP_2_2_0_0_
1101.0996 PEP_0_2_0_0_
1182.11 PEP_1_2_0_0_
1445.7094 PEP_3_3_0_0_
1526.7255 PEP_4_3_0_0_
1998.092 PEP_0_1_0_0_
657.233
543.3847 y5(1+)
430.3004 y4(1+)
454.1558
528.1907

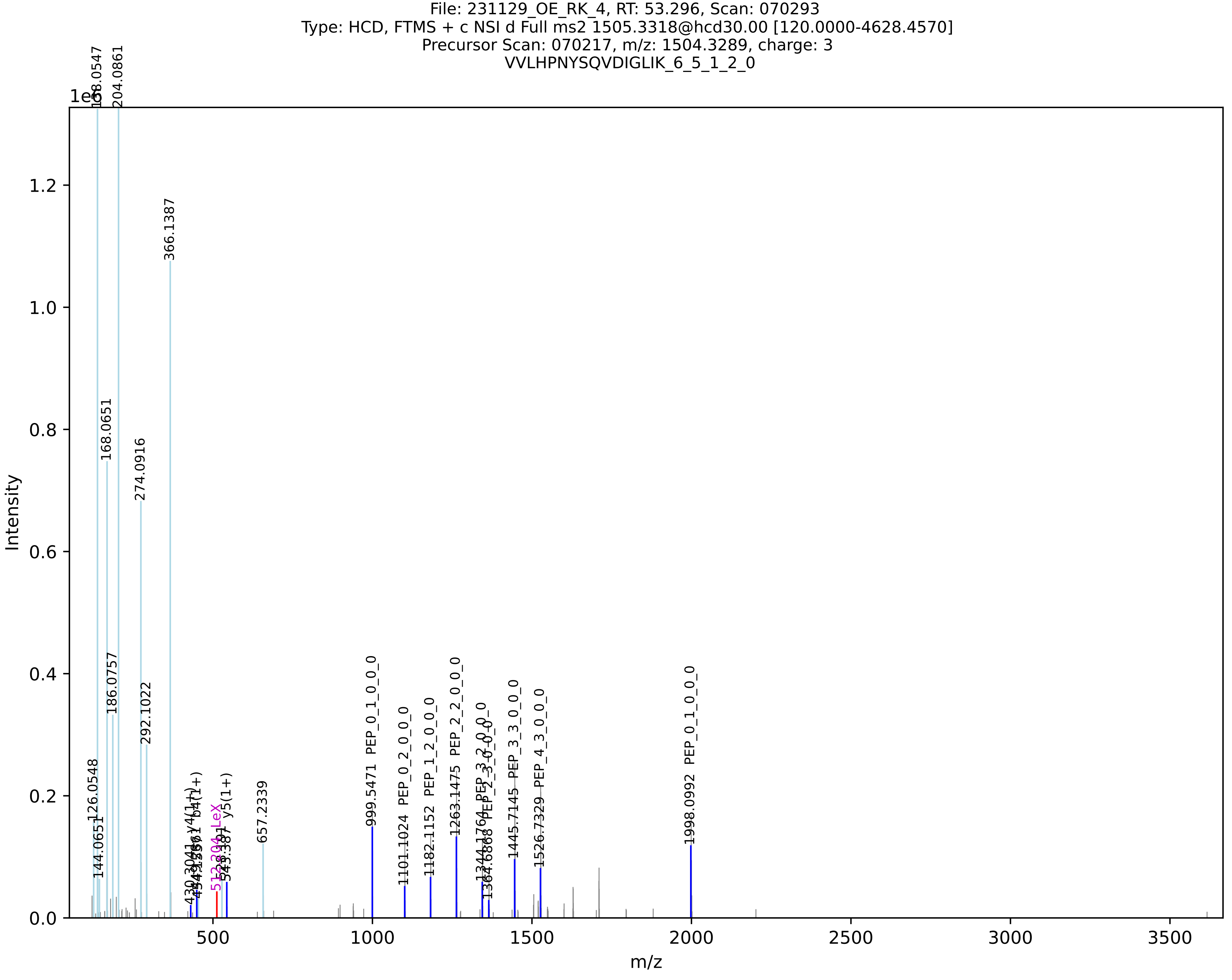
File: 231129_OE_RK_4, RT: 53.296, Scan: 070293
Type: HCD, FTMS + c NSI d Full ms2 1505.3318@hcd30.00 [120.0000-4628.4570]
Precursor Scan: 070217, m/z: 1504.3289, charge: 3
VVLHPNYSQVDIGLIK_6_5_1_2_0
1e6
Intensity
m/z
138.0547
204.0861
366.1387
168.0651
274.0916
186.0757
292.1022
126.0548
144.0651
657.2339
512.204 LeX
543.387 y5(1+)
999.5471 PEP_0_1_0_0_0
1101.1024 PEP_0_2_0_0_0
1182.1152 PEP_1_2_0_0_0
1263.1475 PEP_2_2_0_0_0
1344.1764 PEP_3_2_0_0_0
1445.7145 PEP_3_3_0_0_0
1526.7329 PEP_4_3_0_0_0
1998.0992 PEP_0_1_0_0_0

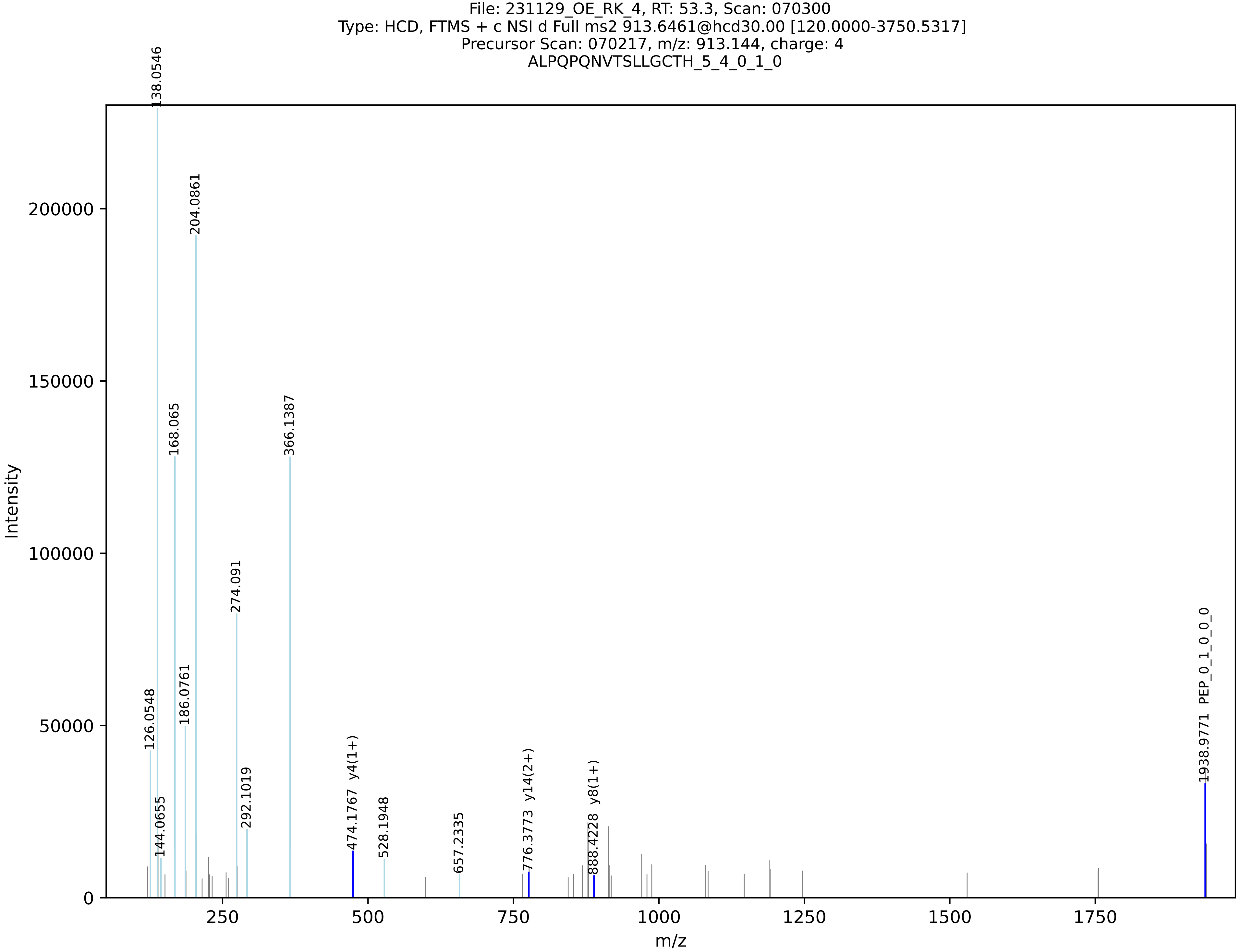
File: 231129_OE_RK_4, RT: 53.3, Scan: 070300
Type: HCD, FTMS + c NSI d Full ms2 913.6461@hcd30.00 [120.0000-3750.5317]
Precursor Scan: 070217, m/z: 913.144, charge: 4
ALPQPQNVTSLLGCTH_5_4_0_1_0
Intensity
m/z
138.0546
204.0861
168.065
366.1387
274.091
186.0761
126.0548
144.0655
292.1019
474.1767 y4(1+)
528.1948
657.2335
776.3773 y14(2+)
888.4228 y8(1+)
1938.9771 PEP_0_1_0_0_0
0
50000
100000
150000
200000
250
500
750
1000
1250
1500
1750

File: 231129_OE_RK_4, RT: 53.303, Scan: 070304
Type: HCD, FTMS + c NSI d Full ms2 1456.4769@hcd30.00 [120.0000-4478.9614]
Precursor Scan: 070217, m/z: 1455.4746, charge: 3
VVLHPNYSQVDIGLIK_6_5_0_2_0

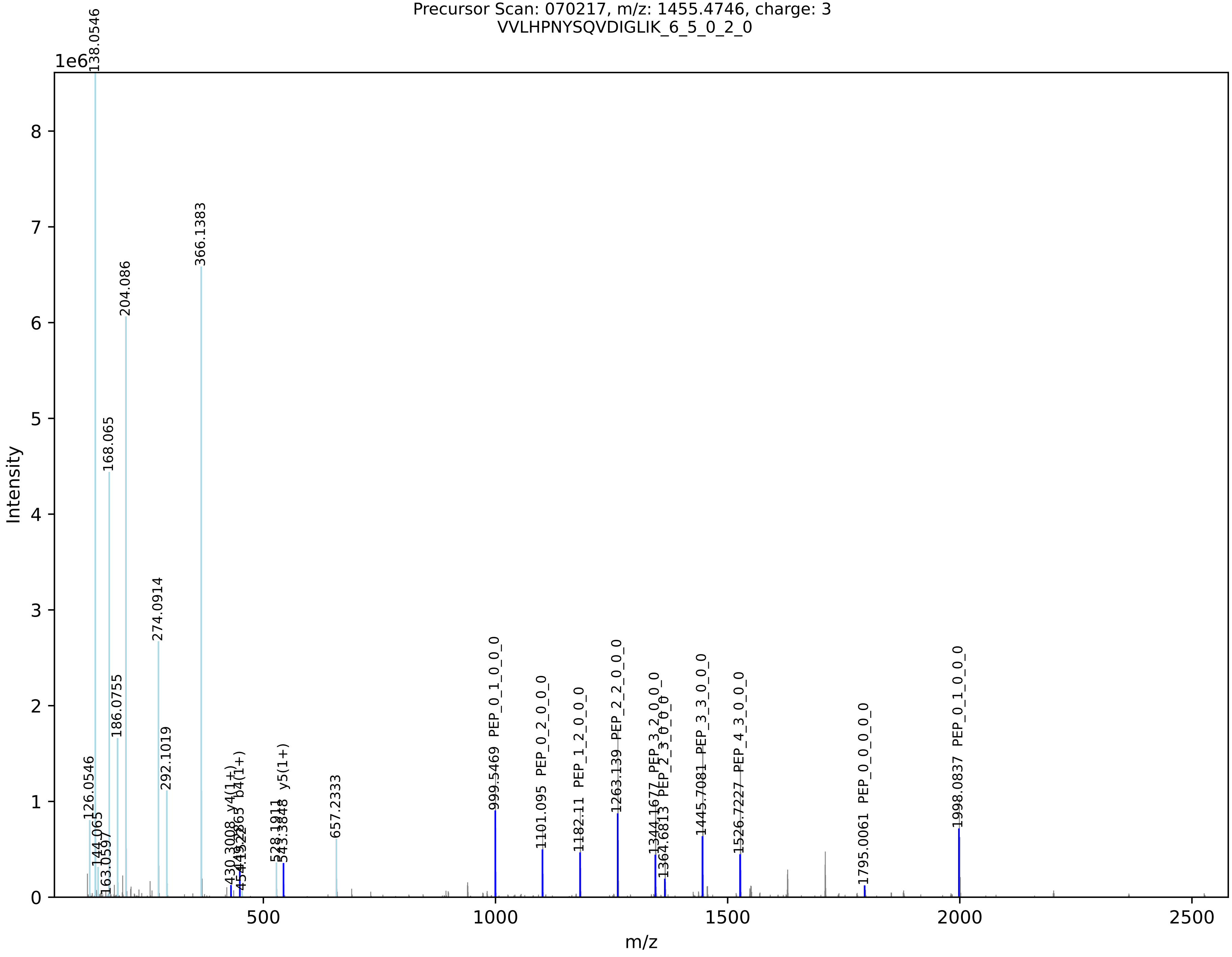

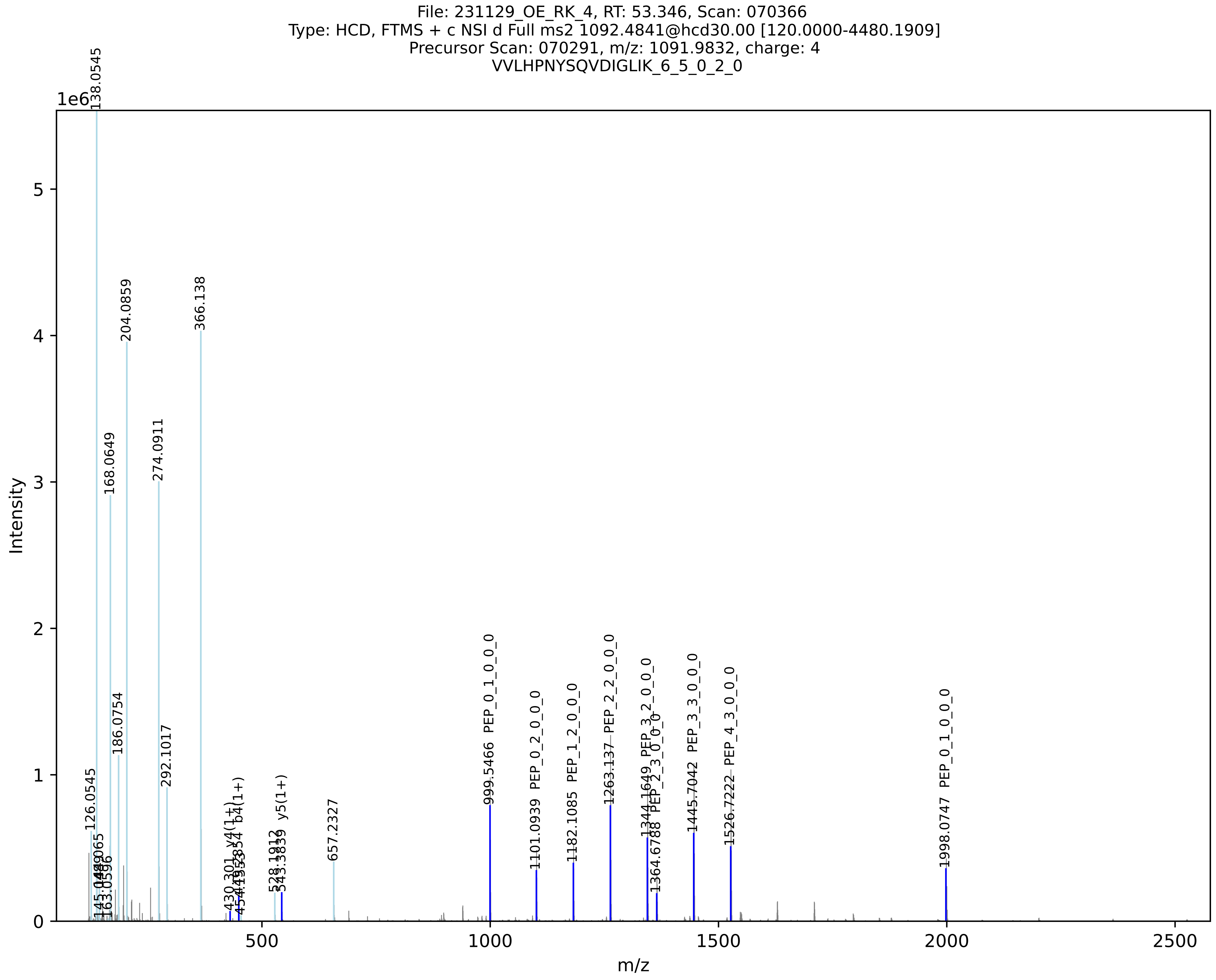
File: 231129_OE_RK_4, RT: 53.346, Scan: 070366
Type: HCD, FTMS + c NSI d Full ms2 1092.4841@hcd30.00 [120.0000-4480.1909]
Precursor Scan: 070291, m/z: 1091.9832, charge: 4
VVLHPNYSQVDIGLIK_6_5_0_2_0
1e6
Intensity
m/z
138.0545
204.0859
366.138
168.0649
274.0911
186.0754
292.1017
126.0545
657.2327
999.5466 PEP_0_1_0_0_0
1101.0939 PEP_0_2_0_0_0
1182.1085 PEP_1_2_0_0_0
1263.137 PEP_2_2_0_0_0
1344.1649 PEP_3_2_0_0_0
1364.6788 PEP_2_3_0_0_0
1445.7042 PEP_3_3_0_0_0
1526.7222 PEP_4_3_0_0_0
1998.0747 PEP_0_1_0_0_0
430.301 y4(1+)
b4(1+)
543.3839 y5(1+)
528.1912
163.0596

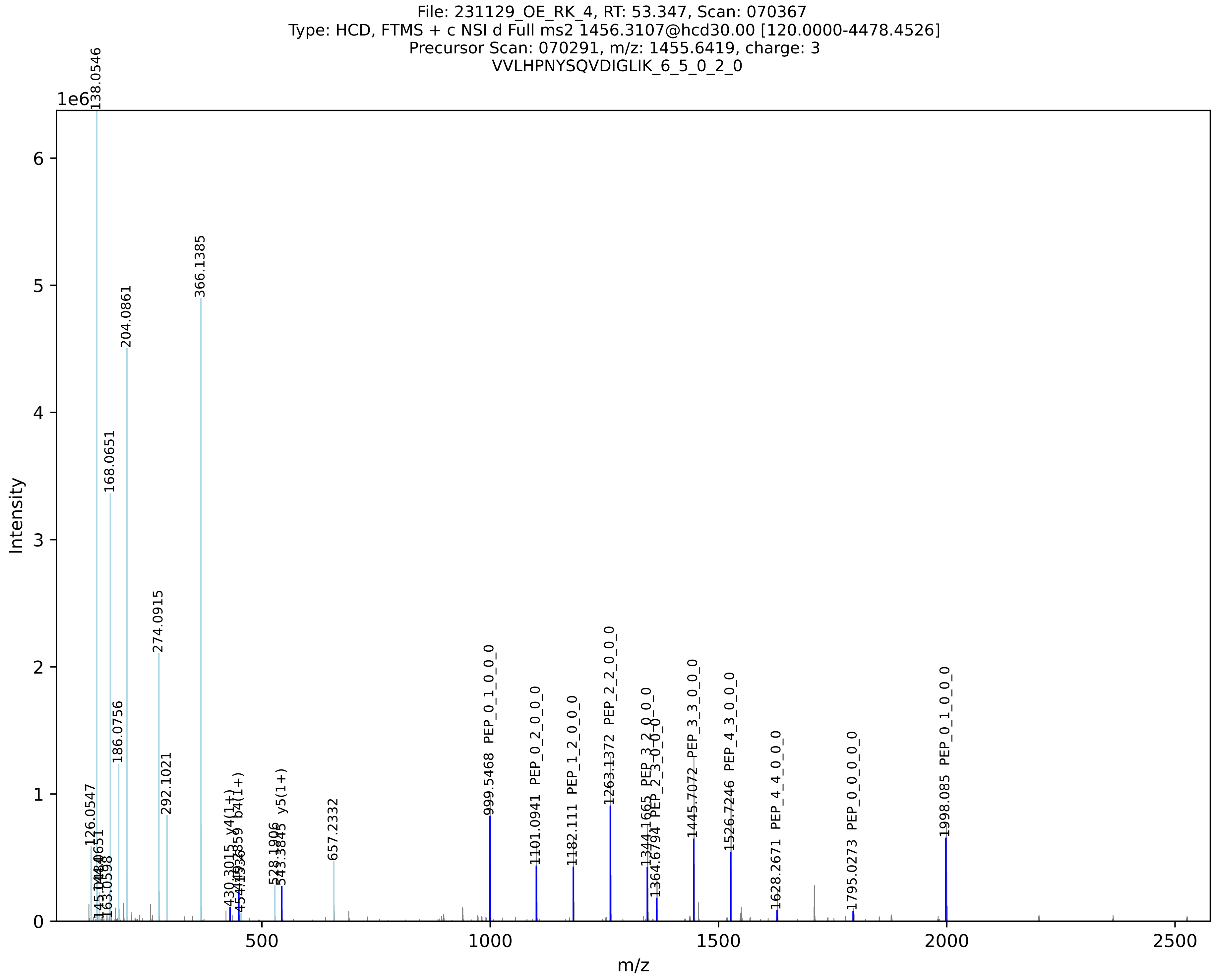

File: 231129_OE_RK_4, RT: 53.347, Scan: 070367
Type: HCD, FTMS + c NSI d Full ms2 1456.3107@hcd30.00 [120.0000-4478.4526]
Precursor Scan: 070291, m/z: 1455.6419, charge: 3
VVLHPNYSQVDIGLIK_6_5_0_2_0
1e6
Intensity
m/z
138.0546
366.1385
204.0861
168.0651
274.0915
186.0756
292.1021
126.0547
163.0598
430.3015 y4(1+)
b4(1+)
y5(1+)
543.3845
528.1906
657.2332
999.5468 PEP_0_1_0_0_0
1101.0941 PEP_0_2_0_0_0
1182.111 PEP_1_2_0_0_0
1263.1372 PEP_2_2_0_0_0
1344.1665 PEP_3_2_0_0_0
1364.6794 PEP_2_3_0_0_0
1445.7072 PEP_3_3_0_0_0
1526.7246 PEP_4_3_0_0_0
1628.2671 PEP_4_4_0_0_0
1795.0273 PEP_0_0_0_0_0
1998.085 PEP_0_1_0_0_0

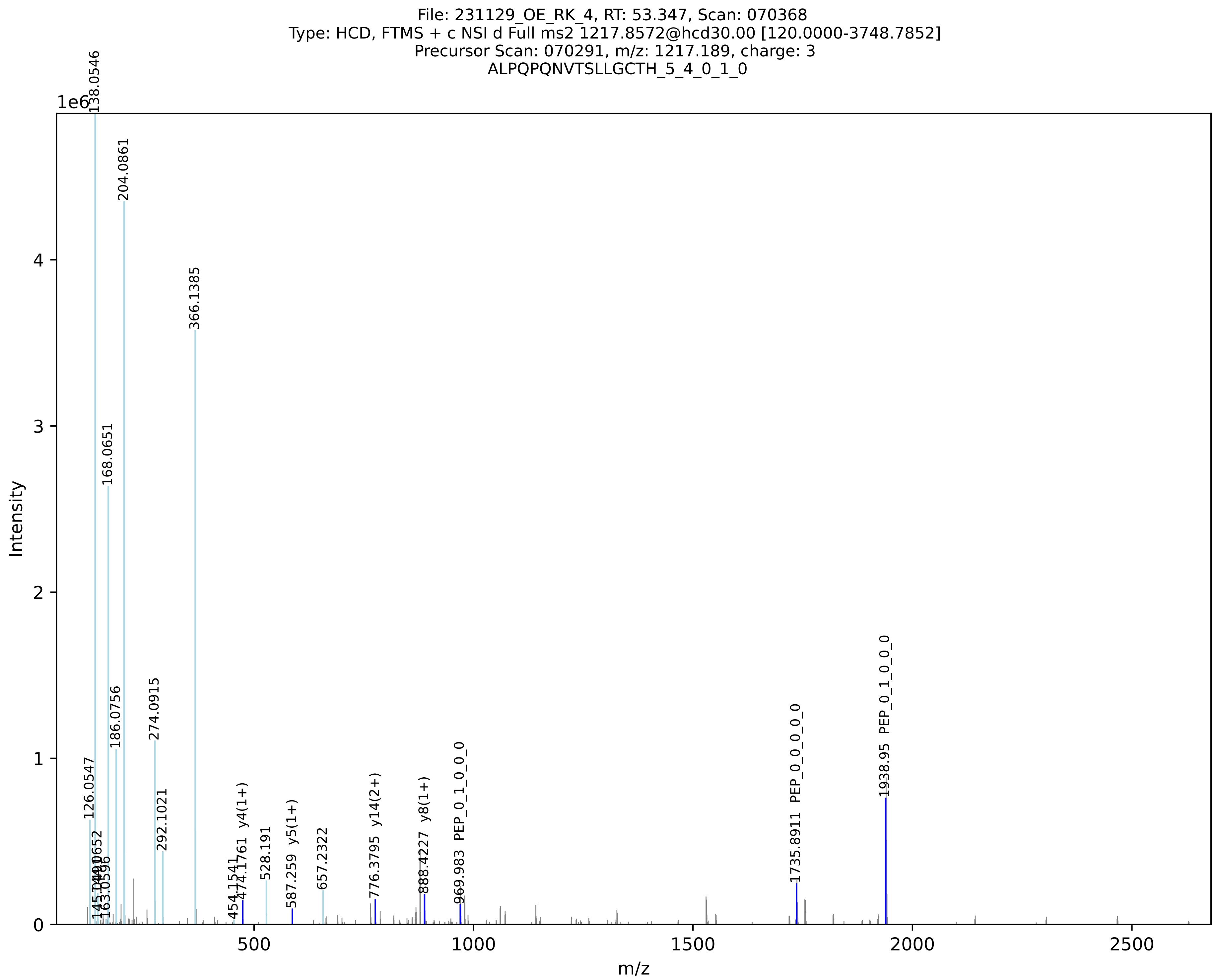
File: 231129_OE_RK_4, RT: 53.347, Scan: 070368
Type: HCD, FTMS + c NSI d Full ms2 1217.8572@hcd30.00 [120.0000-3748.7852]
Precursor Scan: 070291, m/z: 1217.189, charge: 3
ALPQPQNVTSLLGCTH_5_4_0_1_0
1e6
Intensity
m/z
138.0546
204.0861
366.1385
168.0651
186.0756
274.0915
126.0547
292.1021
454.1541
474.1761 y4(1+)
528.191
587.259 y5(1+)
657.2322
776.3795 y14(2+)
888.4227 y8(1+)
969.983 PEP_0_1_0_0_0
1735.8911 PEP_0_0_0_0_0
1938.95 PEP_0_1_0_0_0

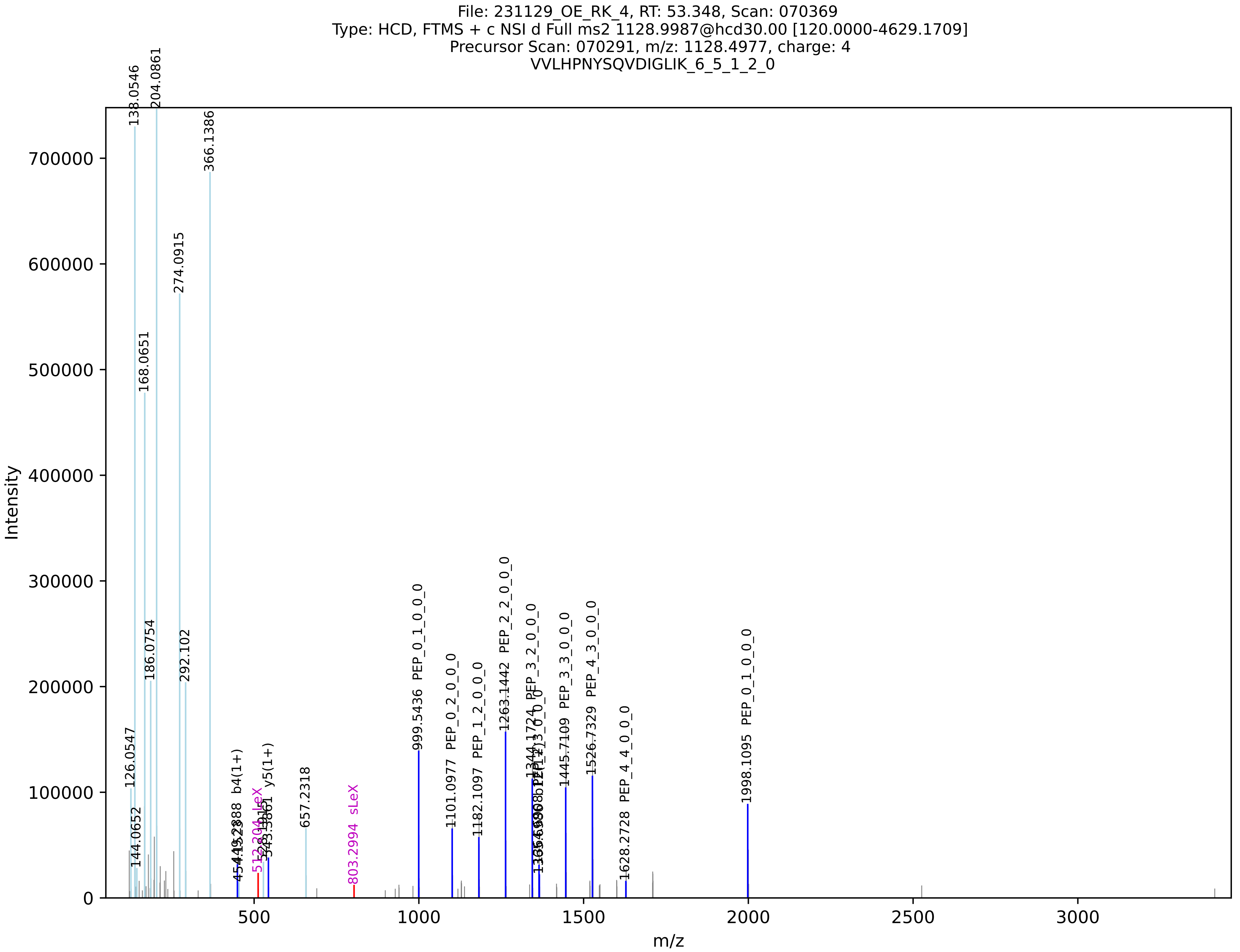
File: 231129_OE_RK_4, RT: 53.348, Scan: 070369
Type: HCD, FTMS + c NSI d Full ms2 1128.9987@hcd30.00 [120.0000-4629.1709]
Precursor Scan: 070291, m/z: 1128.4977, charge: 4
VVLHPNYSQVDIGLIK_6_5_1_2_0
Intensity
m/z
138.0546
204.0861
366.1386
274.0915
168.0651
186.0754
292.102
126.0547
144.0652
b4(1+)
y5(1+)
657.2318
803.2994 sLeX
999.5436 PEP_0_1_0_0_0
1101.0977 PEP_0_2_0_0_0
1182.1097 PEP_1_2_0_0_0
1263.1442 PEP_2_2_0_0_0
PEP_3_2_0_0_0
1445.7109 PEP_3_3_0_0_0
1526.7329 PEP_4_3_0_0_0
1628.2728 PEP_4_4_0_0_0
1998.1095 PEP_0_1_0_0_0

File: 231129_OE_RK_4, RT: 53.353, Scan: 070377
Type: HCD, FTMS + c NSI d Full ms2 1383.2850@hcd30.00 [120.0000-4254.9941]
Precursor Scan: 070291, m/z: 1382.6168, charge: 3
VVLHPNYSQVDIGLIK_5_4_1_2_0

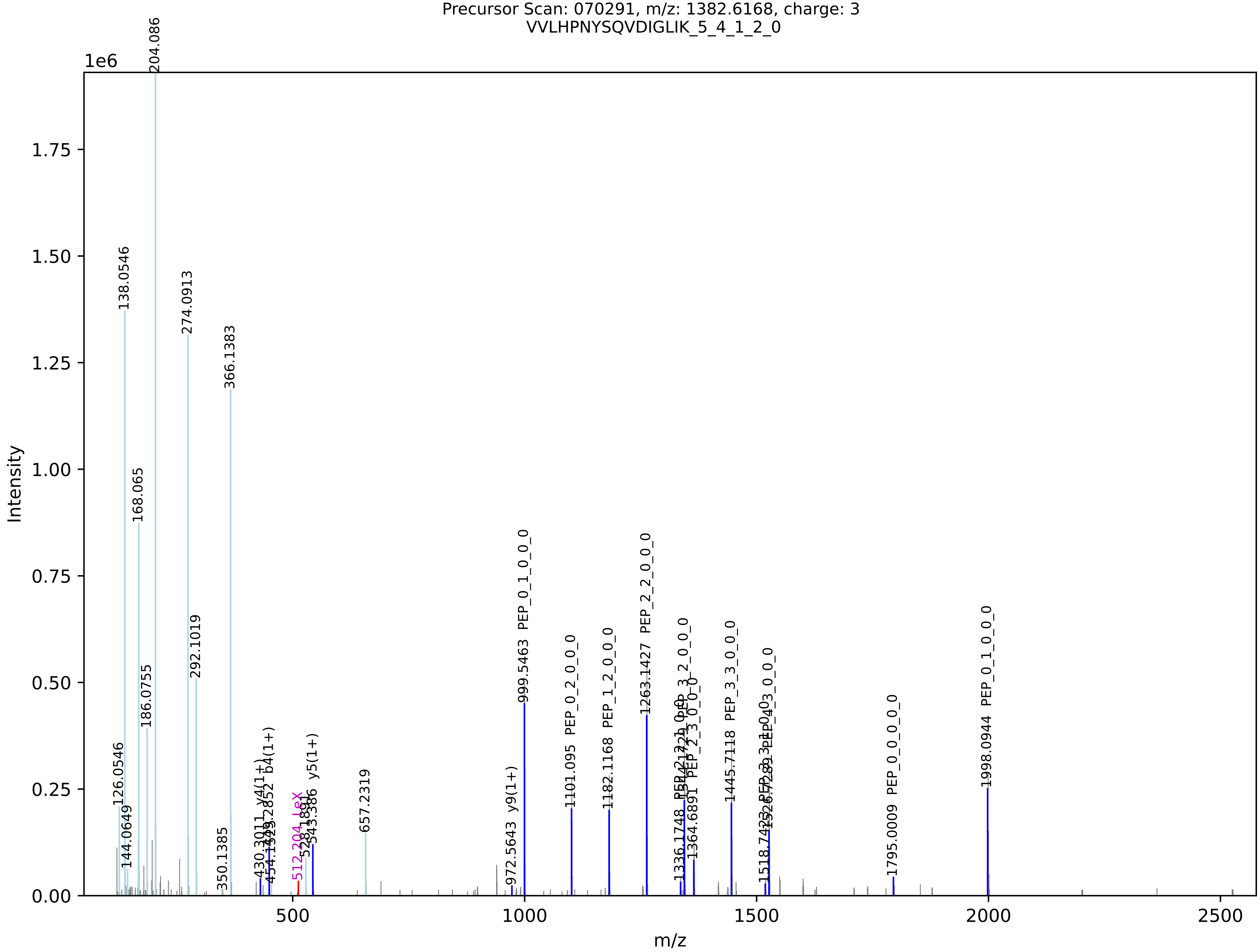

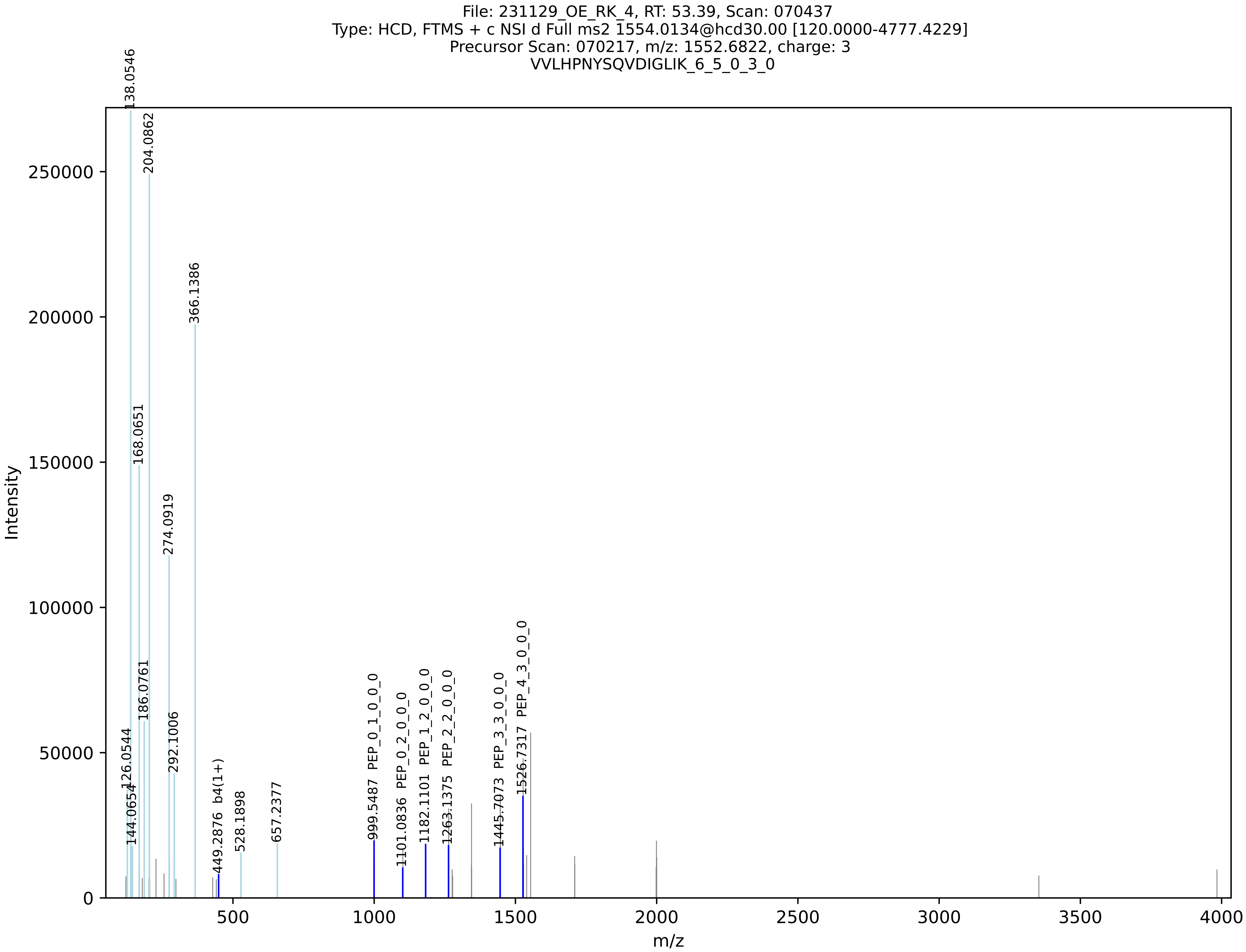
File: 231129_OE_RK_4, RT: 53.39, Scan: 070437
Type: HCD, FTMS + c NSI d Full ms2 1554.0134@hcd30.00 [120.0000-4777.4229]
Precursor Scan: 070217, m/z: 1552.6822, charge: 3
VVLHPNYSQVDIGLIK_6_5_0_3_0
Intensity
m/z
138.0546
204.0862
366.1386
168.0651
274.0919
186.0761
292.1006
126.0544
144.0654
449.2876 b4(1+)
528.1898
657.2377
999.5487 PEP_0_1_0_0_0
1101.0836 PEP_0_2_0_0_0
1182.1101 PEP_1_2_0_0_0
1263.1375 PEP_2_2_0_0_0
1445.7073 PEP_3_3_0_0_0
1526.7317 PEP_4_3_0_0_0

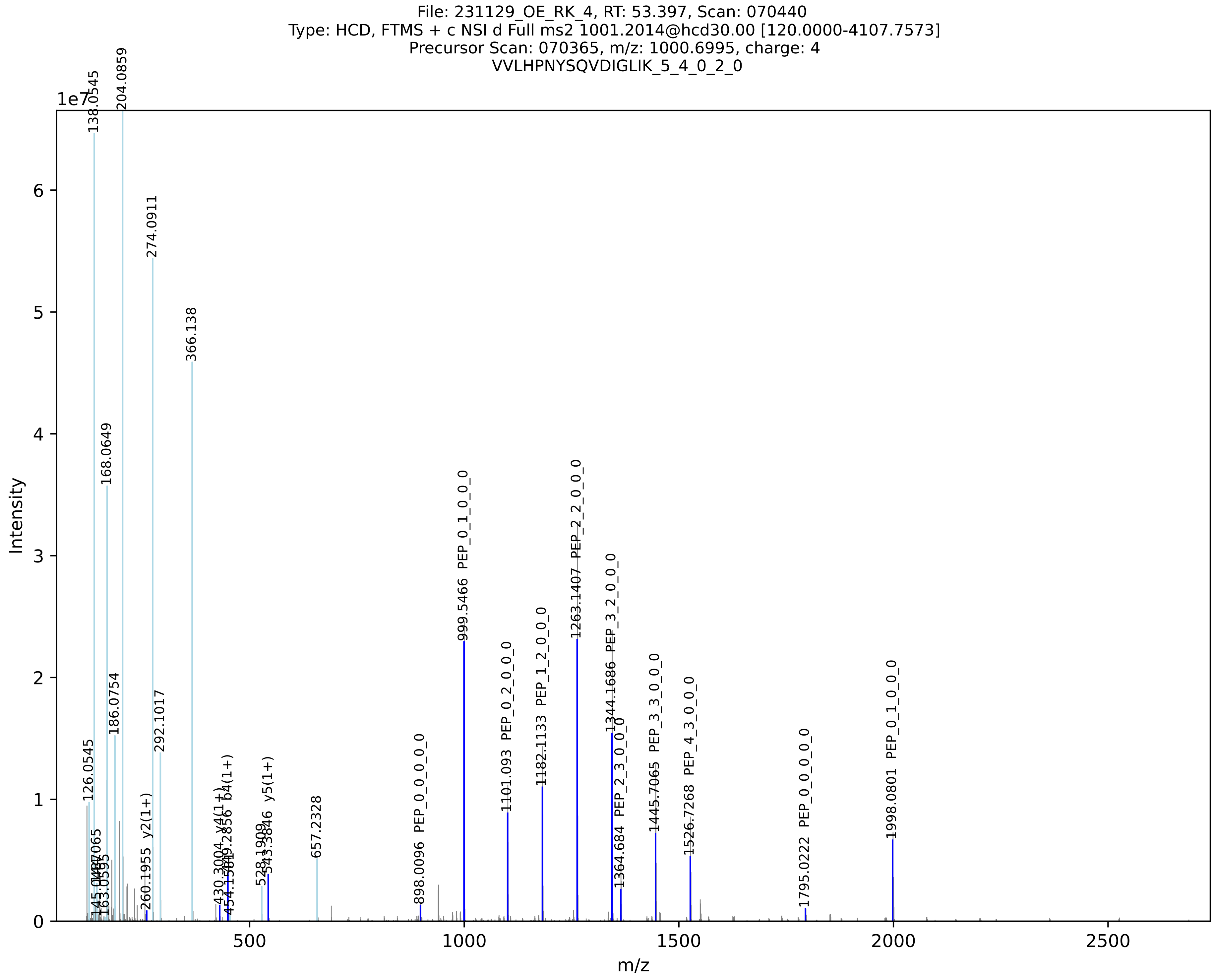
File: 231129_OE_RK_4, RT: 53.397, Scan: 070440
Type: HCD, FTMS + c NSI d Full ms2 1001.2014@hcd30.00 [120.0000-4107.7573]
Precursor Scan: 070365, m/z: 1000.6995, charge: 4
VVLHPNYSQVDIGLIK_5_4_0_2_0
1e7
Intensity
m/z
204.0859
138.0545
274.0911
366.138
168.0649
186.0754
292.1017
126.0545
260.1955 y2(1+)
449.2856 b4(1+)
543.3846 y5(1+)
528.1909
657.2328
898.0096 PEP_0_0_0_0_0
999.5466 PEP_0_1_0_0_0
1101.093 PEP_0_2_0_0_0
1182.1133 PEP_1_2_0_0_0
1263.1407 PEP_2_2_0_0_0
1344.1686 PEP_3_2_0_0_0
1364.684 PEP_2_3_0_0_0
1445.7065 PEP_3_3_0_0_0
1526.7268 PEP_4_3_0_0_0
1795.0222 PEP_0_0_0_0_0
1998.0801 PEP_0_1_0_0_0

File: 231129_OE_RK_4, RT: 53.399, Scan: 070444
Type: HCD, FTMS + c NSI d Full ms2 1537.9897@hcd30.00 [120.0000-4728.3906]
Precursor Scan: 070365, m/z: 1537.3216, charge: 3
VEPNYNITIFVDTFQSEK_5_6_1_1_0

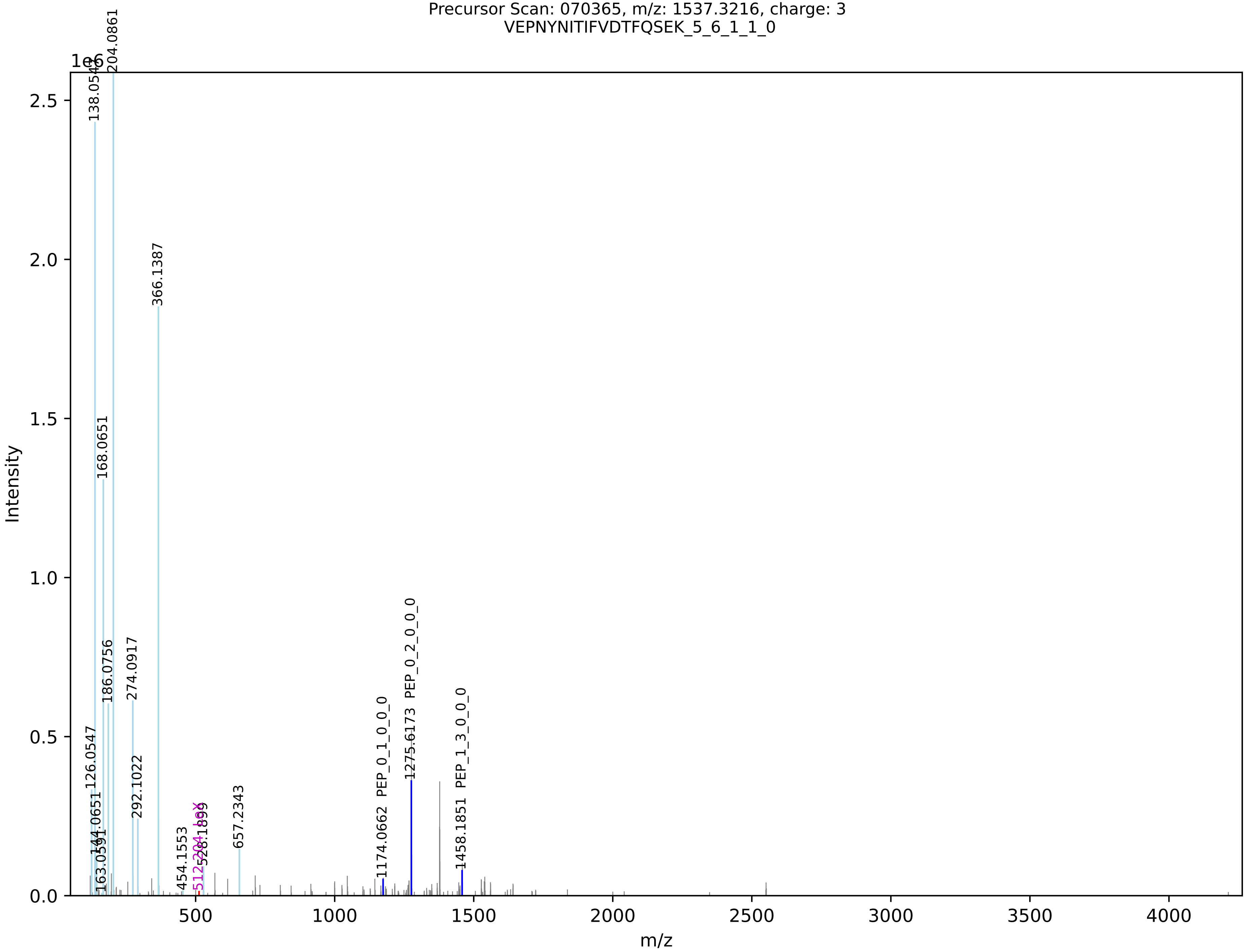

File: 231129_OE_RK_4, RT: 53.403, Scan: 070450
Type: HCD, FTMS + c NSI d Full ms2 1066.4598@hcd30.00 [120.0000-4374.0122]
Precursor Scan: 070365, m/z: 1065.9585, charge: 4
VEPNYNITIFVDTFQSEK_5_5_0_1_0

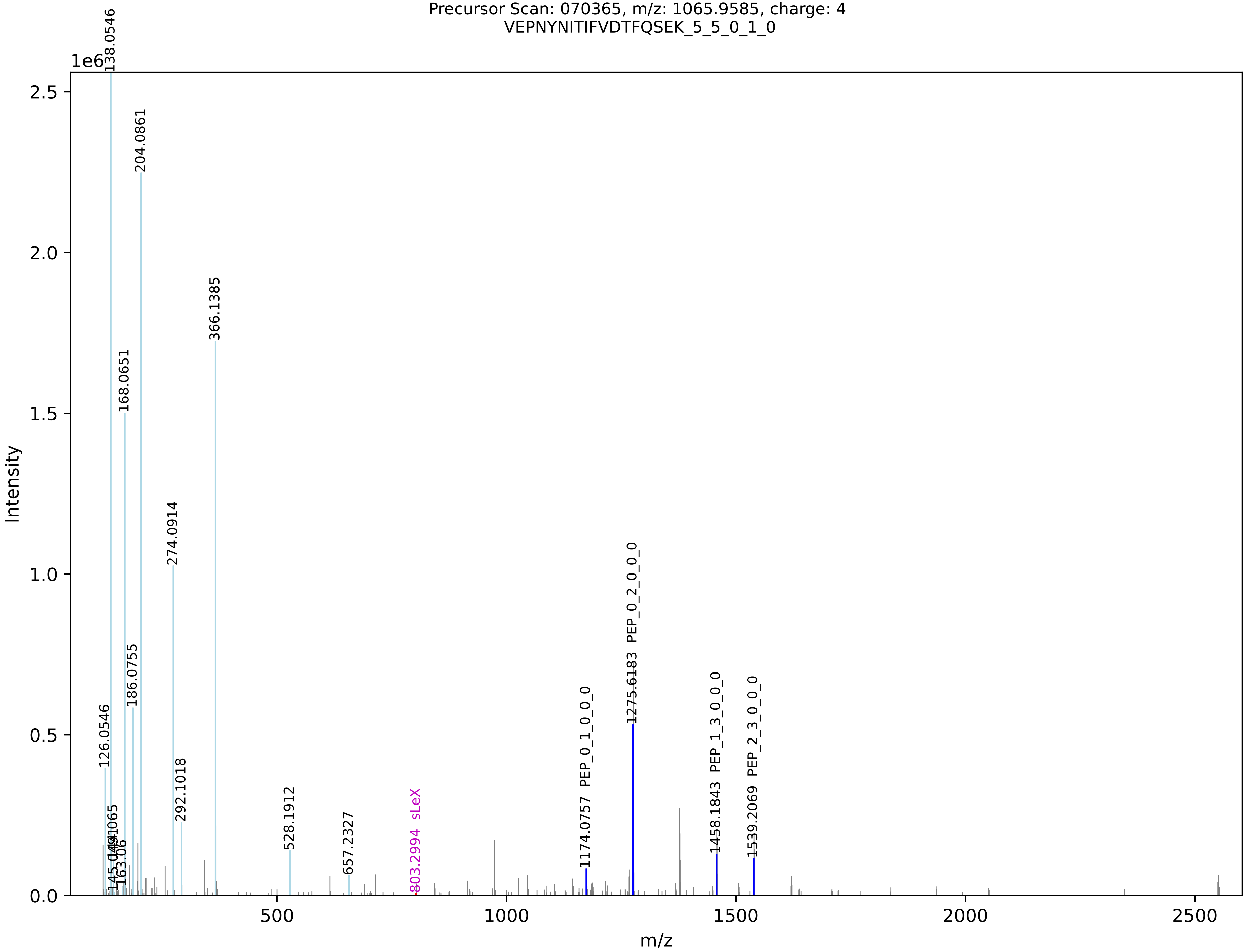

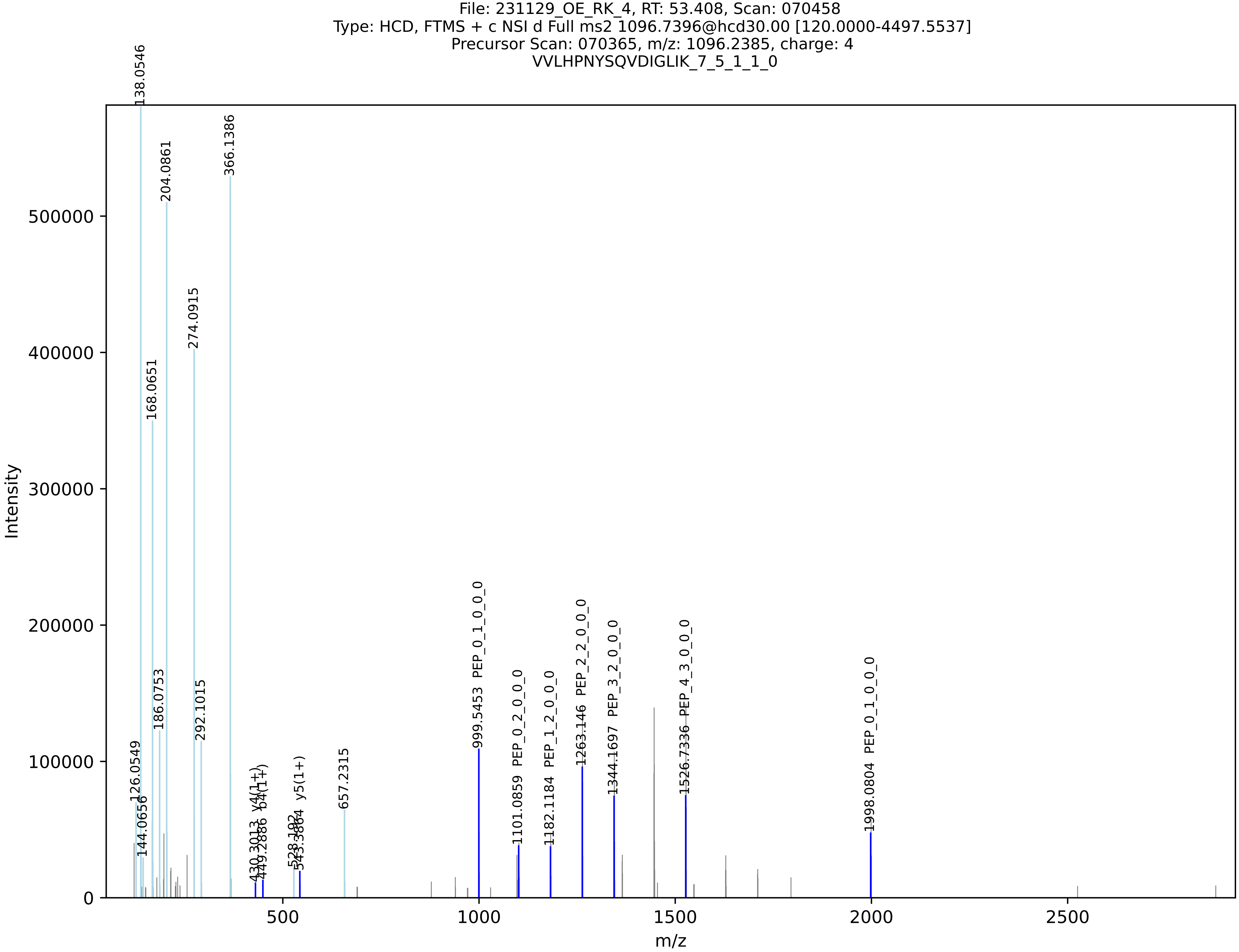
File: 231129_OE_RK_4, RT: 53.408, Scan: 070458
Type: HCD, FTMS + c NSI d Full ms2 1096.7396@hcd30.00 [120.0000-4497.5537]
Precursor Scan: 070365, m/z: 1096.2385, charge: 4
VVLHPNYSQVDIGLIK_7_5_1_1_0
Intensity
m/z
138.0546
366.1386
204.0861
274.0915
168.0651
186.0753
292.1015
126.0549
144.0656
430.3013 y4(1+)
449.2886 b4(1+)
528.192
543.3864 y5(1+)
657.2315
999.5453 PEP_0_1_0_0_0
1101.0859 PEP_0_2_0_0_0
1182.1184 PEP_1_2_0_0_0
1263.146 PEP_2_2_0_0_0
1344.1697 PEP_3_2_0_0_0
1526.7336 PEP_4_3_0_0_0
1998.0804 PEP_0_1_0_0_0

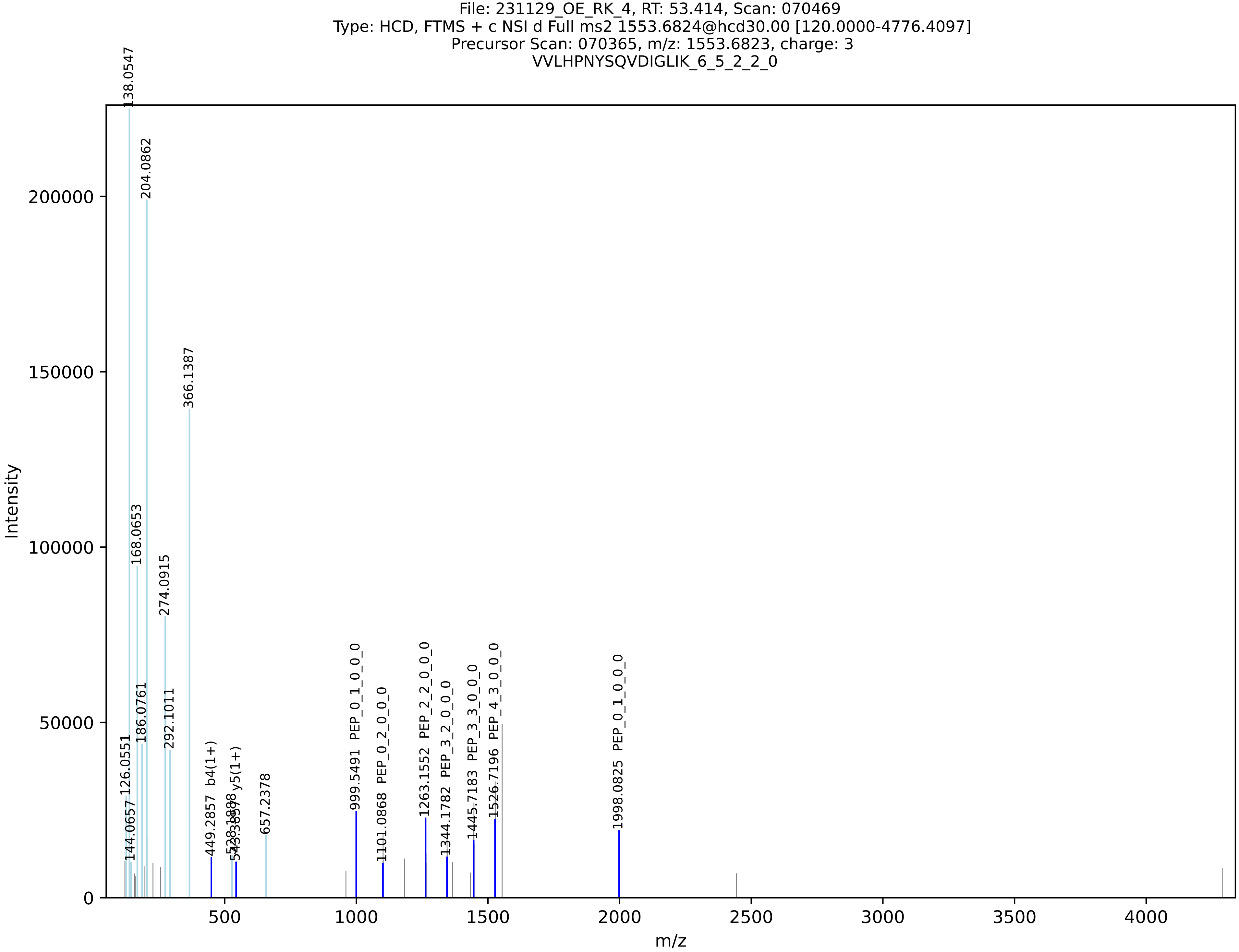
File: 231129_OE_RK_4, RT: 53.414, Scan: 070469
Type: HCD, FTMS + c NSI d Full ms2 1553.6824@hcd30.00 [120.0000-4776.4097]
Precursor Scan: 070365, m/z: 1553.6823, charge: 3
VVLHPNYSQVDIGLIK_6_5_2_2_0
Intensity
m/z
138.0547
204.0862
366.1387
168.0653
274.0915
186.0761
292.1011
126.0551
144.0657
449.2857 b4(1+)
543.3857 y5(1+)
657.2378
999.5491 PEP_0_1_0_0_
1101.0868 PEP_0_2_0_0_0
1263.1552 PEP_2_2_0_0_
1344.1782 PEP_3_2_0_0_
1445.7183 PEP_3_3_0_0_
1526.7196 PEP_4_3_0_0_
1998.0825 PEP_0_1_0_0_

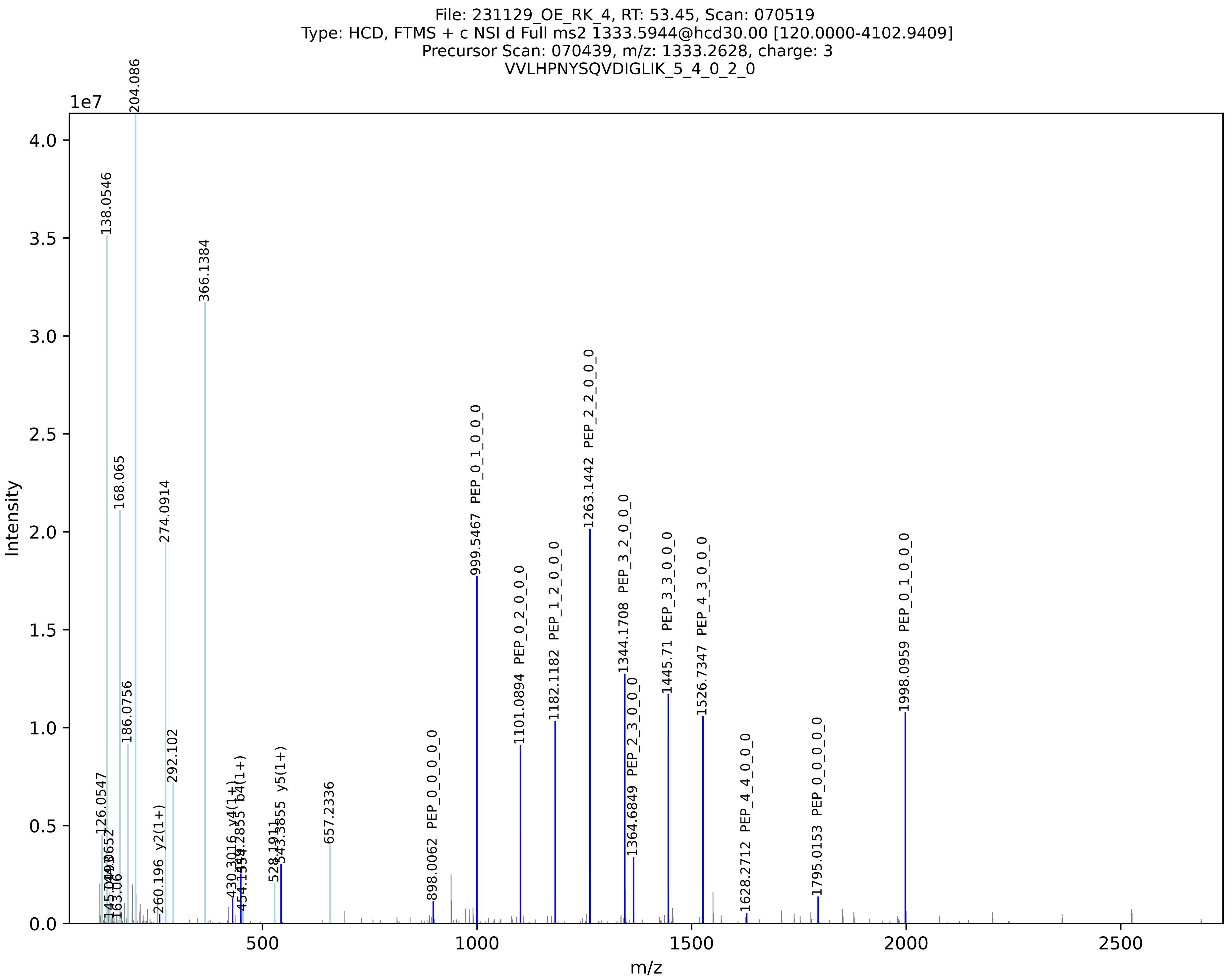
File: 231129_OE_RK_4, RT: 53.45, Scan: 070519
Type: HCD, FTMS + c NSI d Full ms2 1333.5944@hcd30.00 [120.0000-4102.9409]
Precursor Scan: 070439, m/z: 1333.2628, charge: 3
VVLHPNYSQVDIGLIK_5_4_0_2_0
1e7
Intensity
m/z
204.086
138.0546
366.1384
168.065
274.0914
186.0756
292.102
126.0547
260.196 y2(1+)
430.3016 y4(1+)
442.2855 b4(1+)
543.3855 y5(1+)
528.1911
657.2336
898.0062 PEP_0_0_0_0_0
999.5467 PEP_0_1_0_0_0
1101.0894 PEP_0_2_0_0_0
1182.1182 PEP_1_2_0_0_0
1263.1442 PEP_2_2_0_0_0
1344.1708 PEP_3_2_0_0_0
1364.6849 PEP_2_3_0_0_0
1445.71 PEP_3_3_0_0_0
1526.7347 PEP_4_3_0_0_0
1628.2712 PEP_4_4_0_0_0
1795.0153 PEP_0_0_0_0_0
1998.0959 PEP_0_1_0_0_0

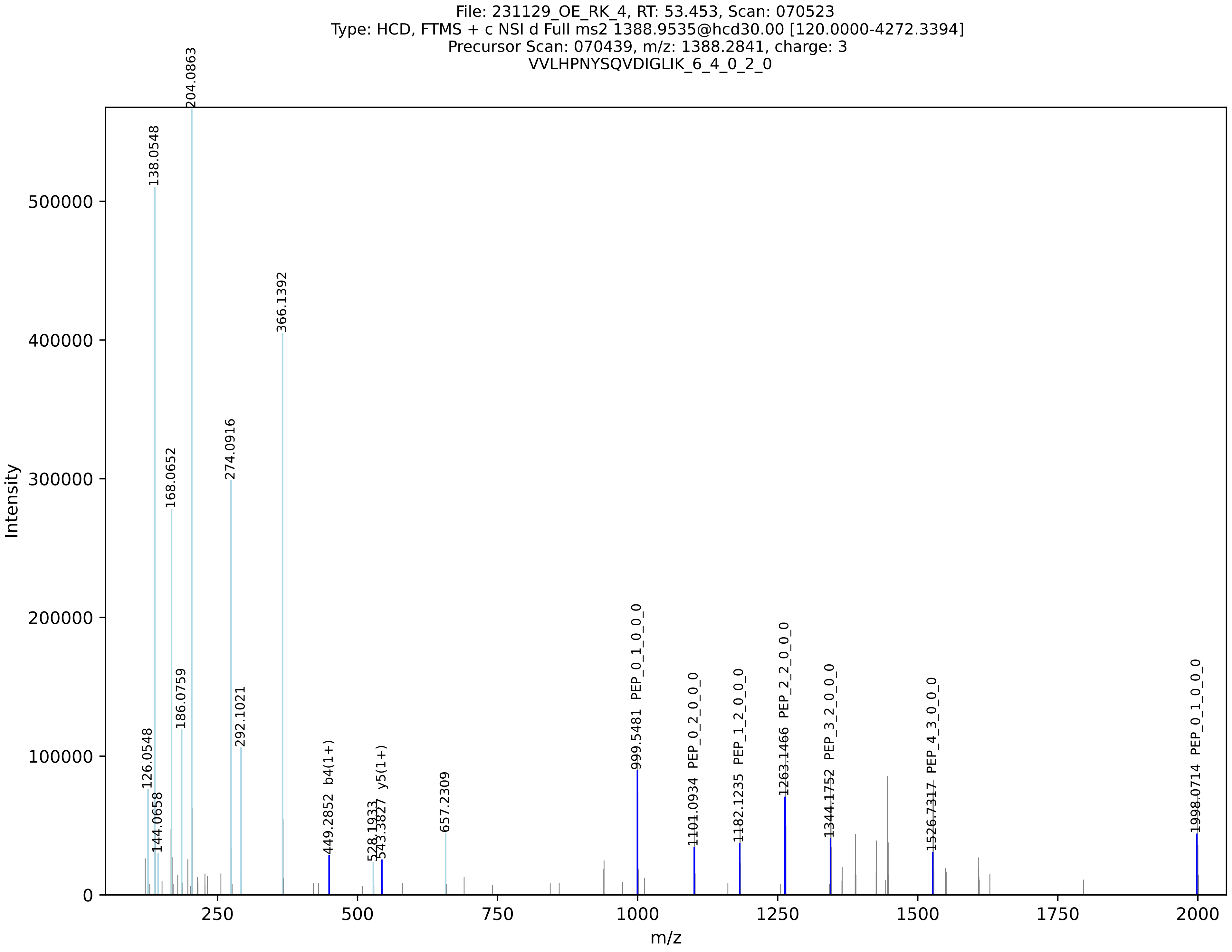

File: 231129_OE_RK_4, RT: 53.453, Scan: 070523
Type: HCD, FTMS + c NSI d Full ms2 1388.9535@hcd30.00 [120.0000-4272.3394]
Precursor Scan: 070439, m/z: 1388.2841, charge: 3
VVLHPNYSQVDIGLIK_6_4_0_2_0
Intensity
m/z
204.0863
138.0548
366.1392
274.0916
168.0652
186.0759
292.1021
126.0548
144.0658
449.2852 b4(1+)
528.1933
543.3827 y5(1+)
657.2309
999.5481 PEP_0_1_0_0_0
1101.0934 PEP_0_2_0_0_0
1182.1235 PEP_1_2_0_0_0
1263.1466 PEP_2_2_0_0_0
1344.1752 PEP_3_2_0_0_0
1526.7317 PEP_4_3_0_0_0
1998.0714 PEP_0_1_0_0_

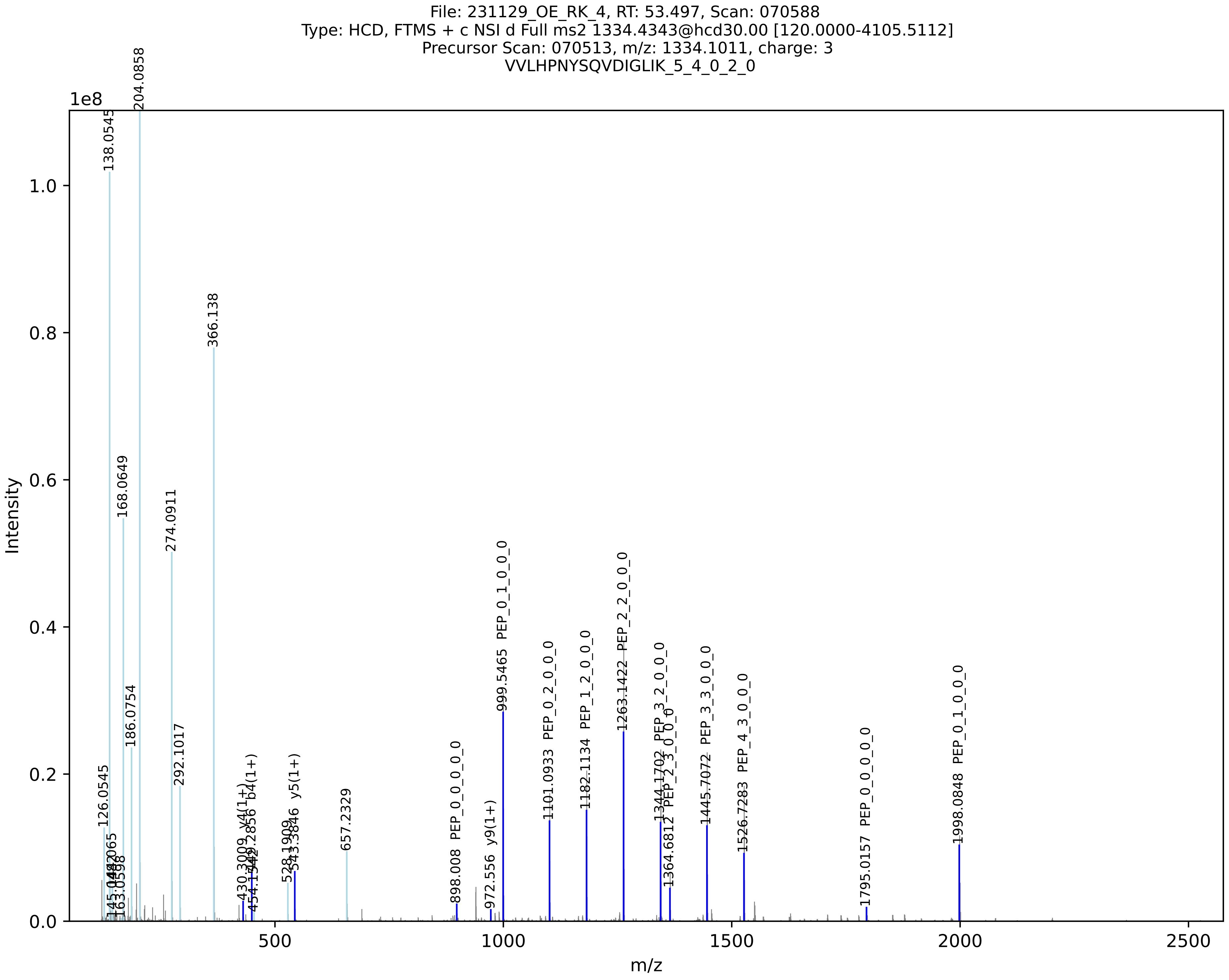

File: 231129_OE_RK_4, RT: 53.497, Scan: 070588
Type: HCD, FTMS + c NSI d Full ms2 1334.4343@hcd30.00 [120.0000-4105.5112]
Precursor Scan: 070513, m/z: 1334.1011, charge: 3
VVLHPNYSQVDIGLIK_5_4_0_2_0
1e8
Intensity
m/z
0.0
0.2
0.4
0.6
0.8
1.0
500
1000
1500
2000
2500
204.0858
138.0545
366.138
168.0649
274.0911
186.0754
292.1017
126.0545
163.0598
430.3009 y4(1+)
442.2856 b4(1+)
454.1542
528.1909
543.3846 y5(1+)
657.2329
898.008 PEP_0_0_0_0_0
972.556 y9(1+)
999.5465 PEP_0_1_0_0_0
1101.0933 PEP_0_2_0_0_0
1182.1134 PEP_1_2_0_0_0
1263.1422 PEP_2_2_0_0_0
1344.1702 PEP_3_2_0_0_0
1364.6812 PEP_2_3_0_0_0
1445.7072 PEP_3_3_0_0_0
1526.7283 PEP_4_3_0_0_0
1795.0157 PEP_0_0_0_0_0
1998.0848 PEP_0_1_0_0_0

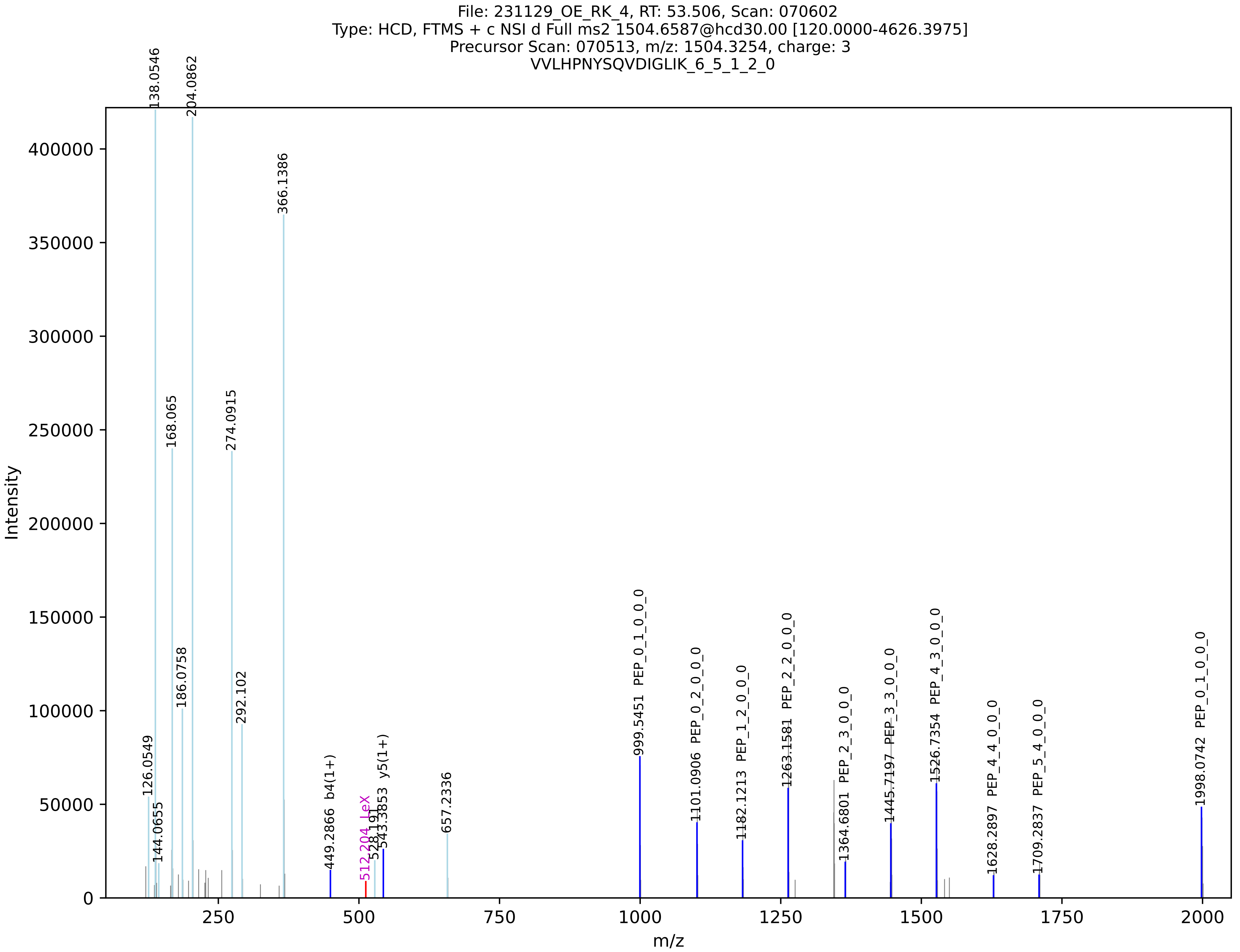
File: 231129_OE_RK_4, RT: 53.506, Scan: 070602
Type: HCD, FTMS + c NSI d Full ms2 1504.6587@hcd30.00 [120.0000-4626.3975]
Precursor Scan: 070513, m/z: 1504.3254, charge: 3
VVLHPNYSQVDIGLIK_6_5_1_2_0
Intensity
m/z
138.0546
204.0862
366.1386
168.065
274.0915
186.0758
292.102
126.0549
144.0655
449.2866 b4(1+)
512.204 LeX
528.191
543.3853 y5(1+)
657.2336
999.5451 PEP_0_1_0_0_0
1101.0906 PEP_0_2_0_0_0
1182.1213 PEP_1_2_0_0_0
1263.1581 PEP_2_2_0_0_0
1364.6801 PEP_2_3_0_0_0
1445.7197 PEP_3_3_0_0_0
1526.7354 PEP_4_3_0_0_0
1628.2897 PEP_4_4_0_0_0
1709.2837 PEP_5_4_0_0_0
1998.0742 PEP_0_1_0_0_0

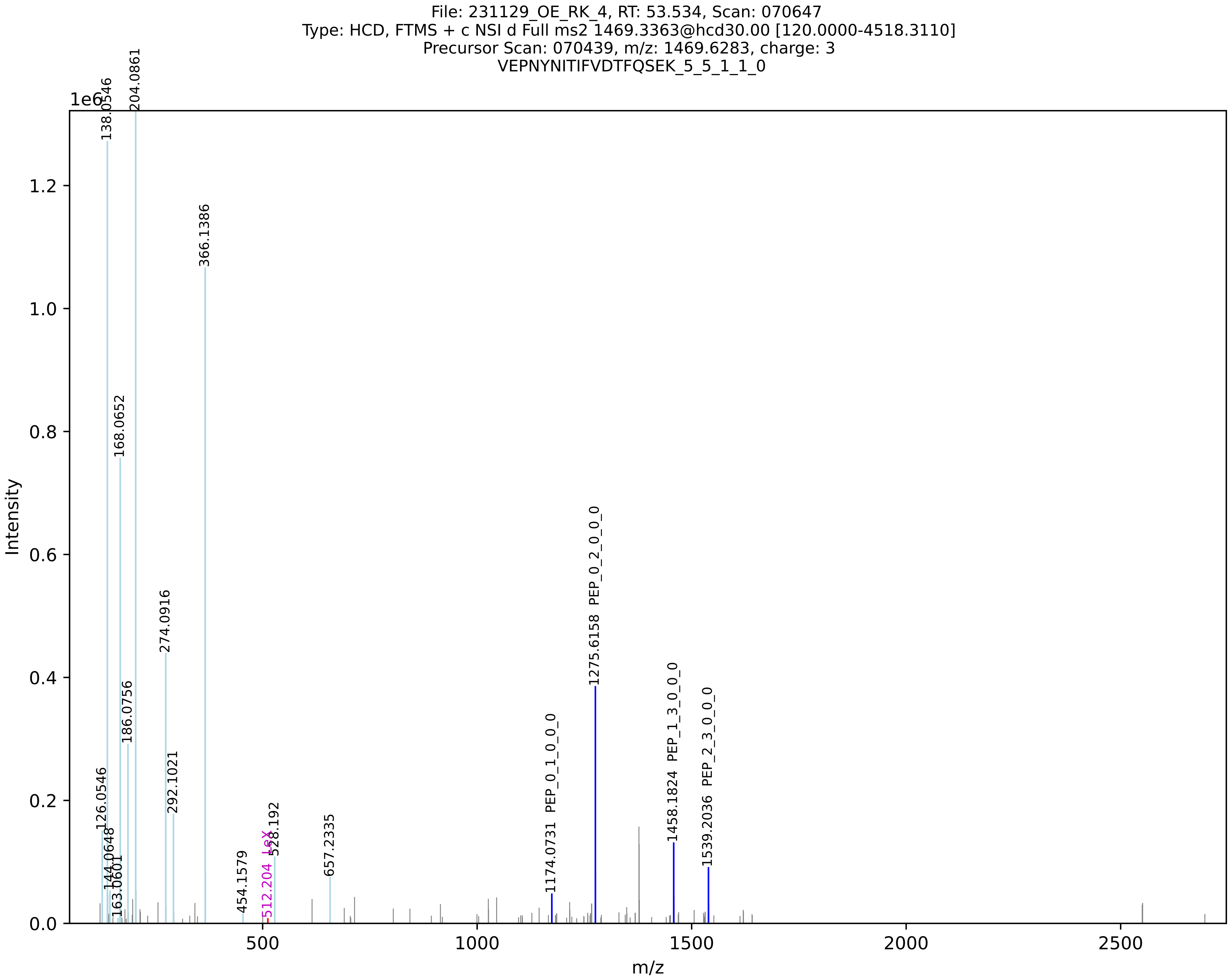
File: 231129_OE_RK_4, RT: 53.534, Scan: 070647
Type: HCD, FTMS + c NSI d Full ms2 1469.3363@hcd30.00 [120.0000-4518.3110]
Precursor Scan: 070439, m/z: 1469.6283, charge: 3
VEPNYNITIFVDTFQSEK_5_5_1_1_0
1e6
Intensity
m/z
138.0546
204.0861
366.1386
168.0652
274.0916
186.0756
292.1021
126.0546
144.0648
163.0601
454.1579
512.204 LeX
528.192
657.2335
1174.0731 PEP_0_1_0_0_0
1275.6158 PEP_0_2_0_0_0
1458.1824 PEP_1_3_0_0_0
1539.2036 PEP_2_3_0_0_0

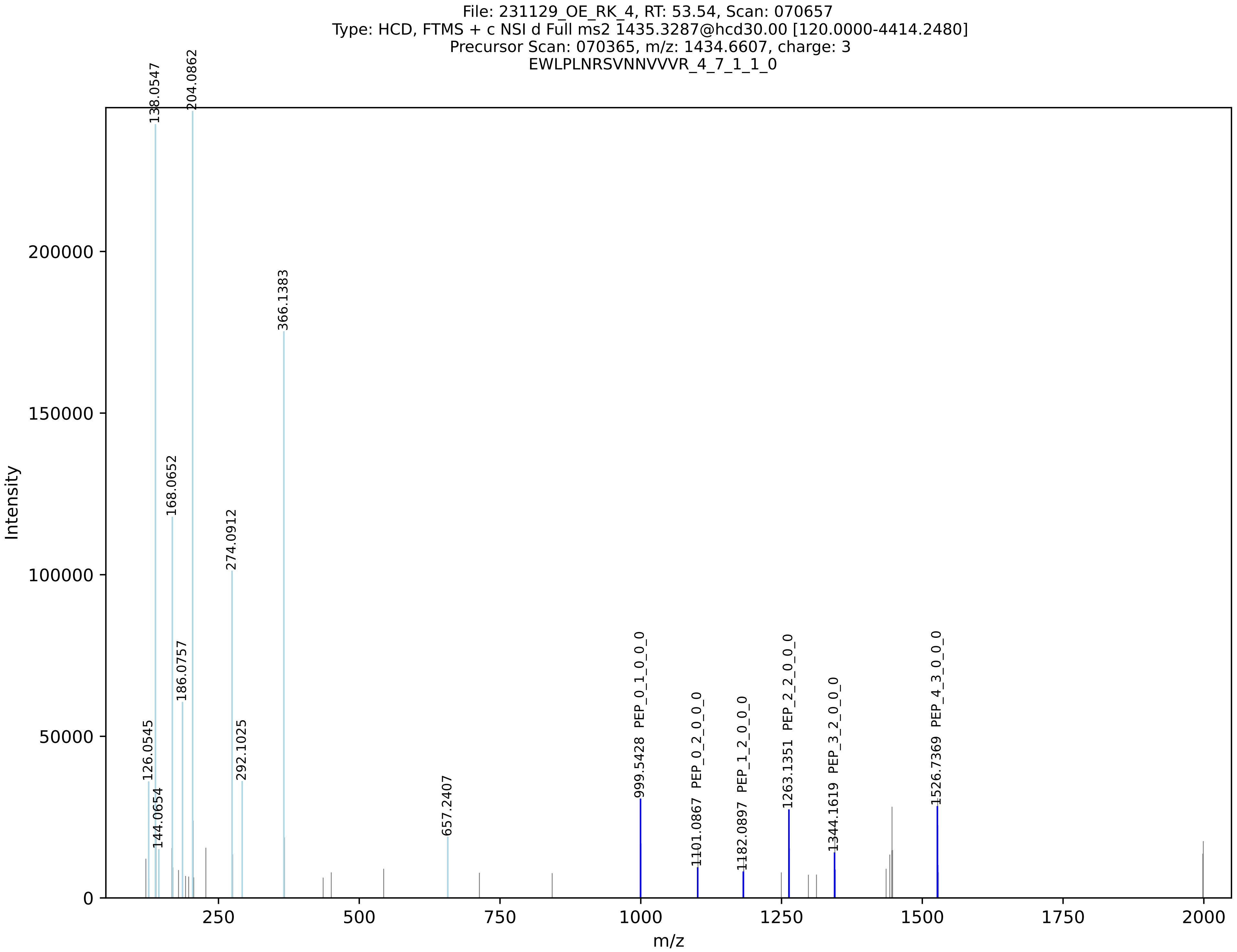

File: 231129_OE_RK_4, RT: 53.54, Scan: 070657
Type: HCD, FTMS + c NSI d Full ms2 1435.3287@hcd30.00 [120.0000-4414.2480]
Precursor Scan: 070365, m/z: 1434.6607, charge: 3
EWLPLNRSVNNVVVR_4_7_1_1_0
138.0547
204.0862
366.1383
168.0652
274.0912
186.0757
126.0545
292.1025
144.0654
657.2407
999.5428 PEP_0_1_0_0_0
1101.0867 PEP_0_2_0_0_0
1182.0897 PEP_1_2_0_0_0
1263.1351 PEP_2_2_0_0_0
1344.1619 PEP_3_2_0_0_0
1526.7369 PEP_4_3_0_0_0
Intensity
m/z

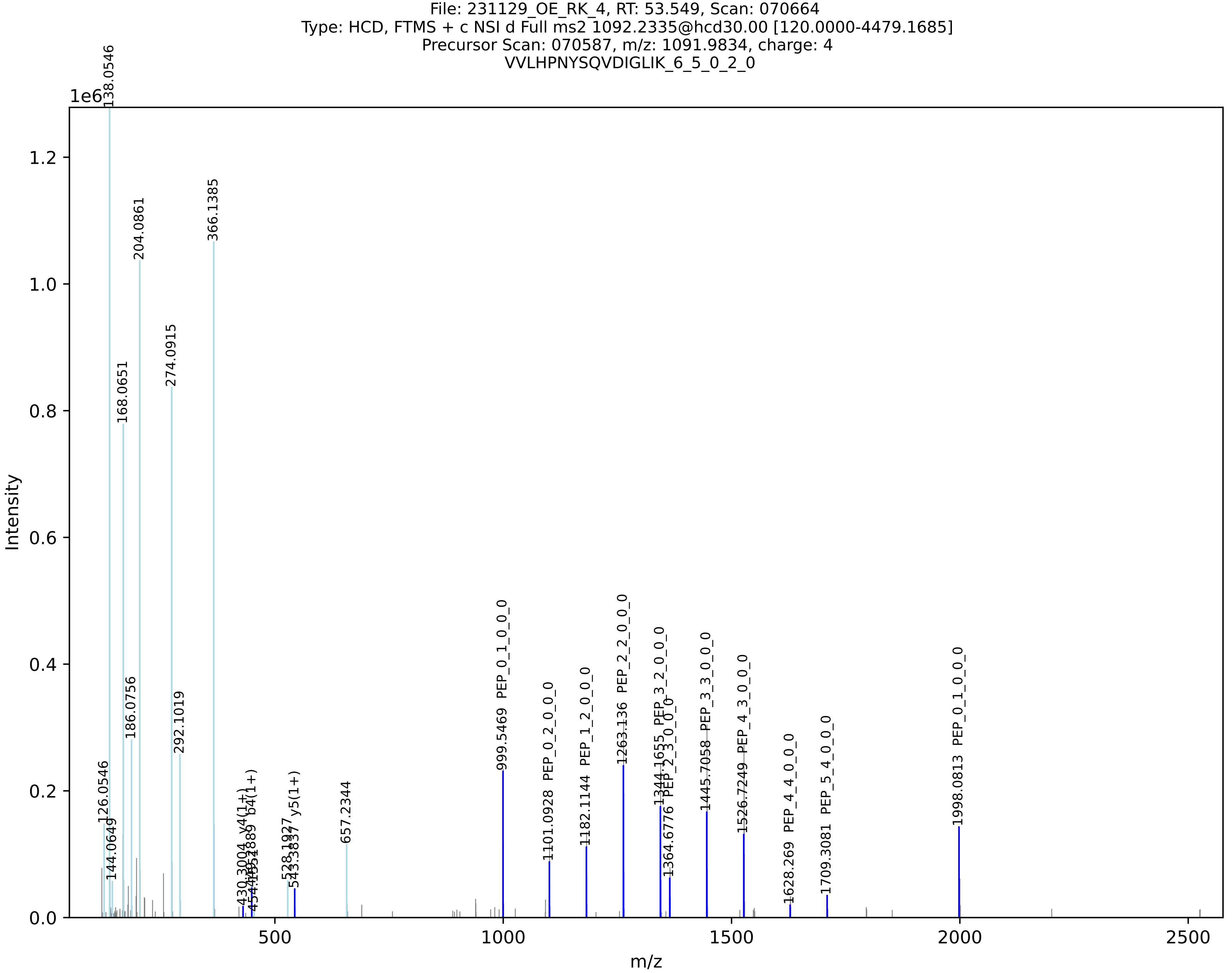
File: 231129_OE_RK_4, RT: 53.549, Scan: 070664
Type: HCD, FTMS + c NSI d Full ms2 1092.2335@hcd30.00 [120.0000-4479.1685]
Precursor Scan: 070587, m/z: 1091.9834, charge: 4
VVLHPNYSQVDIGLIK_6_5_0_2_0
1e6
Intensity
m/z
138.0546
366.1385
204.0861
274.0915
168.0651
186.0756
292.1019
126.0546
144.0649
657.2344
999.5469 PEP_0_1_0_0_0
1101.0928 PEP_0_2_0_0_0
1182.1144 PEP_1_2_0_0_0
1263.136 PEP_2_2_0_0_0
1344.1655 PEP_3_2_0_0_0
1364.6776 PEP_2_3_0_0_0
1445.7058 PEP_3_3_0_0_0
1526.7249 PEP_4_3_0_0_0
1628.269 PEP_4_4_0_0_0
1709.3081 PEP_5_4_0_0_0
1998.0813 PEP_0_1_0_0_0
y4(1+)
b4(1+)
y5(1+)
543.3837

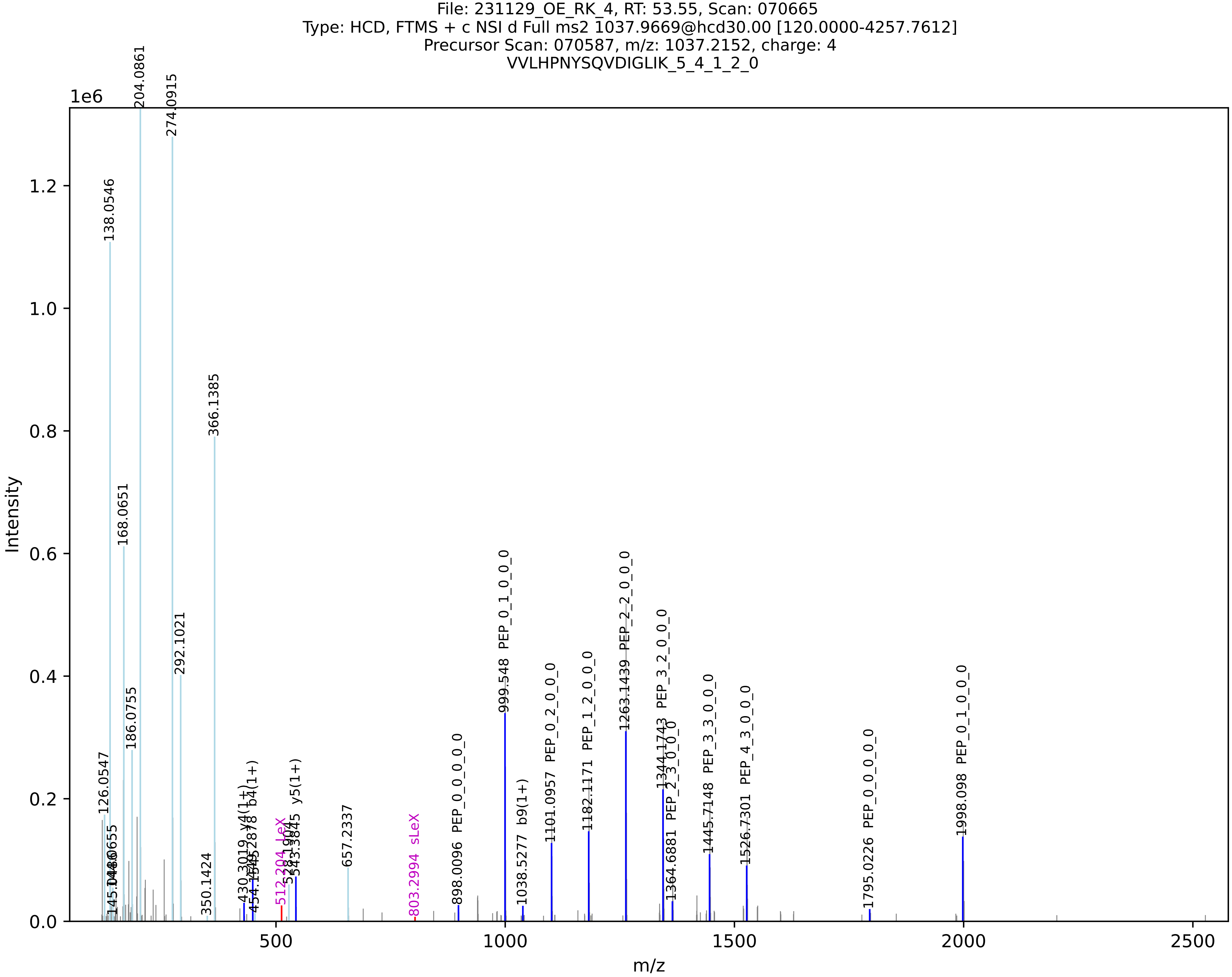

File: 231129_OE_RK_4, RT: 53.55, Scan: 070665
Type: HCD, FTMS + c NSI d Full ms2 1037.9669@hcd30.00 [120.0000-4257.7612]
Precursor Scan: 070587, m/z: 1037.2152, charge: 4
VVLHPNYSQVDIGLIK_5_4_1_2_0
1e6
Intensity
m/z
204.0861
274.0915
138.0546
366.1385
168.0651
292.1021
186.0755
126.0547
350.1424
y4(1+)
b4(1+)
y5(1+)
543.3845
512.204 LeX
657.2337
803.2994 sLeX
898.0096 PEP_0_0_0_0_0
999.548 PEP_0_1_0_0_0
1038.5277 b9(1+)
1101.0957 PEP_0_2_0_0_0
1182.1171 PEP_1_2_0_0_0
1263.1439 PEP_2_2_0_0_0
1344.1743 PEP_3_2_0_0_0
1364.6881 PEP_2_3_0_0_0
1445.7148 PEP_3_3_0_0_0
1526.7301 PEP_4_3_0_0_0
1795.0226 PEP_0_0_0_0_0
1998.098 PEP_0_1_0_0_0

File: 231129_OE_RK_4, RT: 53.585, Scan: 070721
Type: HCD, FTMS + c NSI d Full ms2 1038.9717@hcd30.00 [120.0000-4261.8604]
Precursor Scan: 070513, m/z: 1037.2142, charge: 4
VVLHPNYSQVDIGLIK_5_4_1_2_0

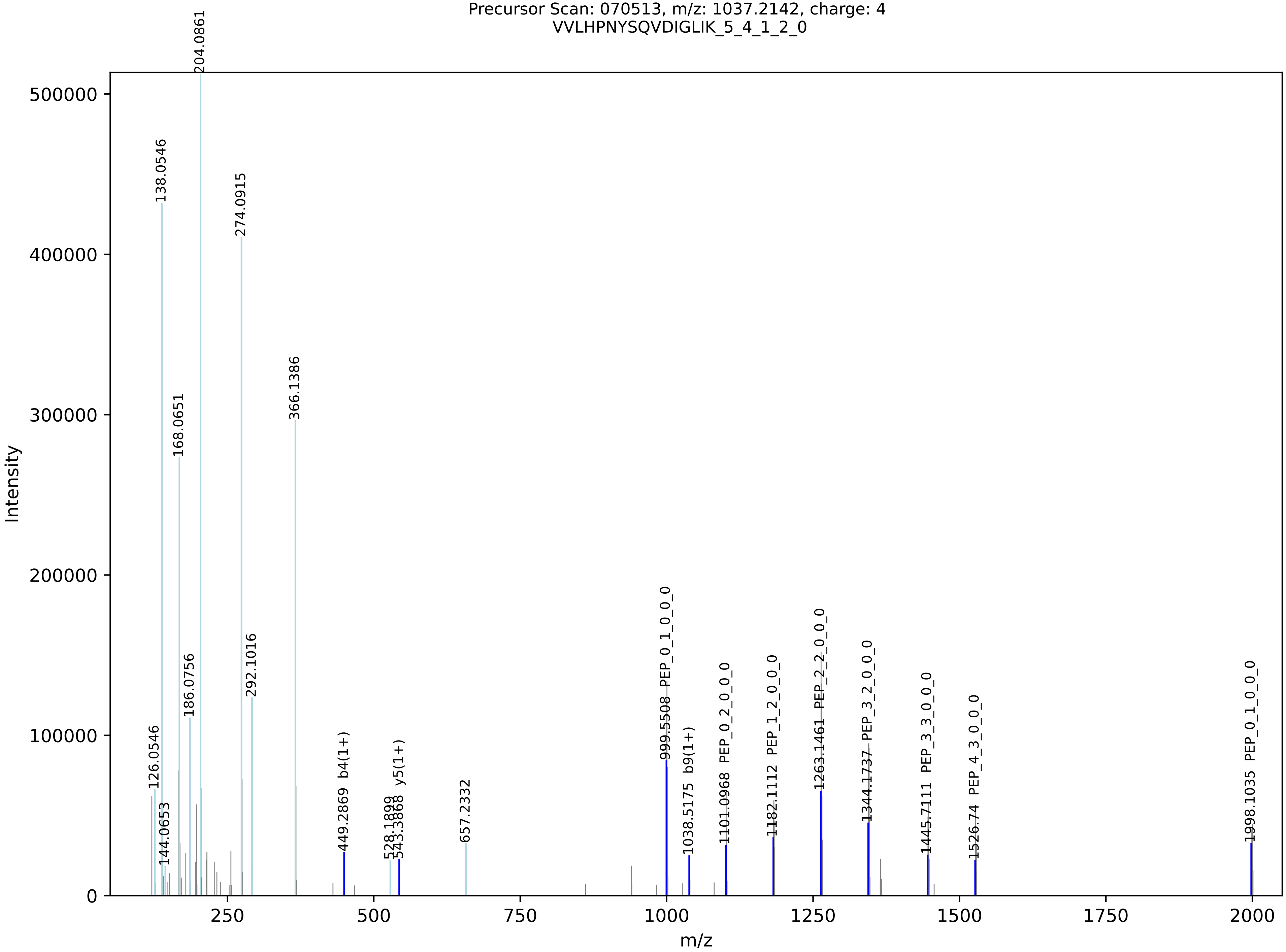

File: 231129_OE_RK_4, RT: 53.585, Scan: 070722
Type: HCD, FTMS + c NSI d Full ms2 1382.3032@hcd30.00 [120.0000-4251.9897]
Precursor Scan: 070513, m/z: 1382.6166, charge: 3
VVLHPNYSQVDIGLIK_5_4_1_2_0

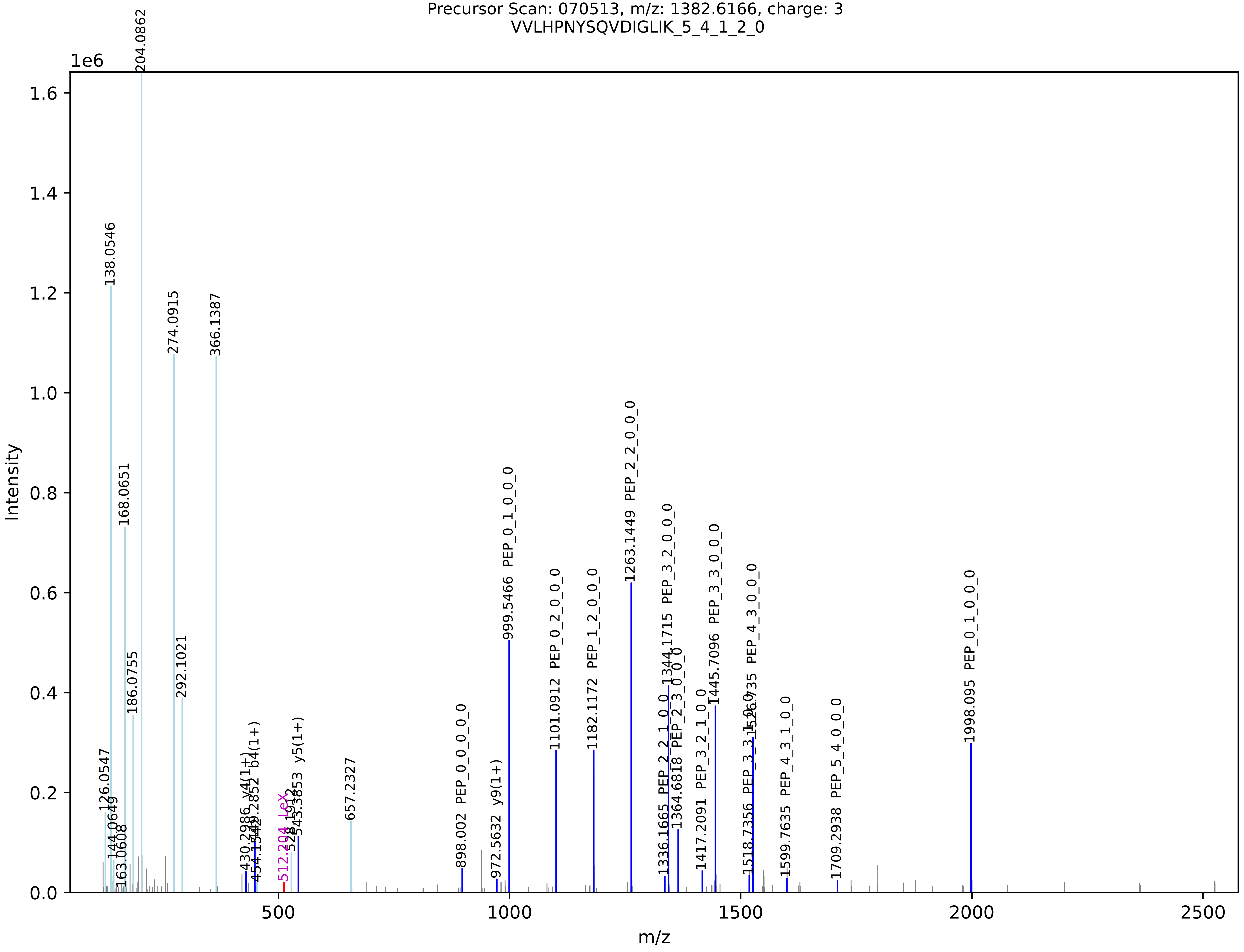

File: 231129_OE_RK_4, RT: 53.598, Scan: 070736
Type: HCD, FTMS + c NSI d Full ms2 1001.2011@hcd30.00 [120.0000-4107.7568]
Precursor Scan: 070661, m/z: 1000.6996, charge: 4
VVLHPNYSQVDIGLIK_5_4_0_2_0

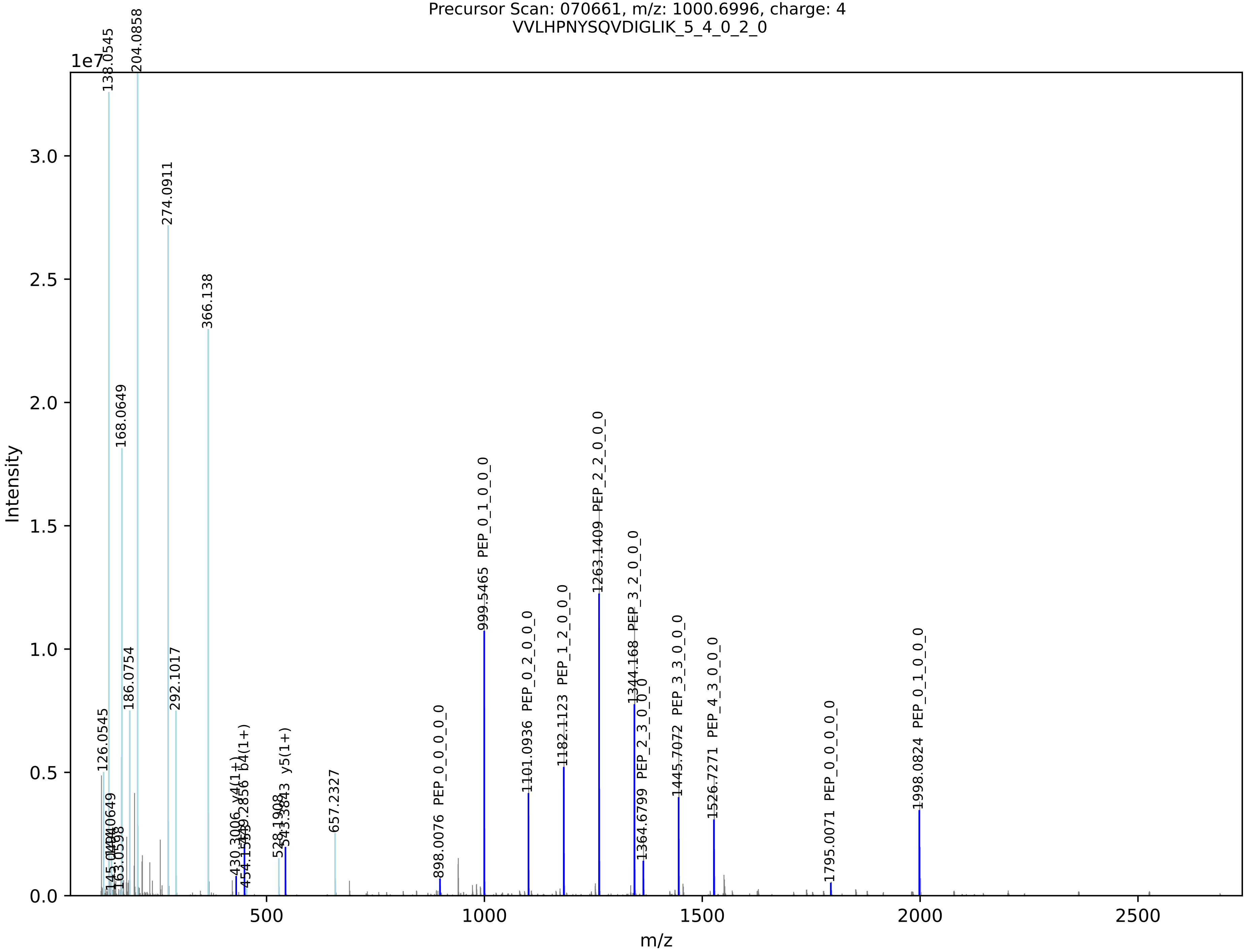

File: 231129_OE_RK_4, RT: 53.599, Scan: 070737
Type: HCD, FTMS + c NSI d Full ms2 1470.2966@hcd30.00 [120.0000-4521.2495]
Precursor Scan: 070661, m/z: 1469.6278, charge: 3
VEPNYNITIFVDTFQSEK_5_5_1_1_0

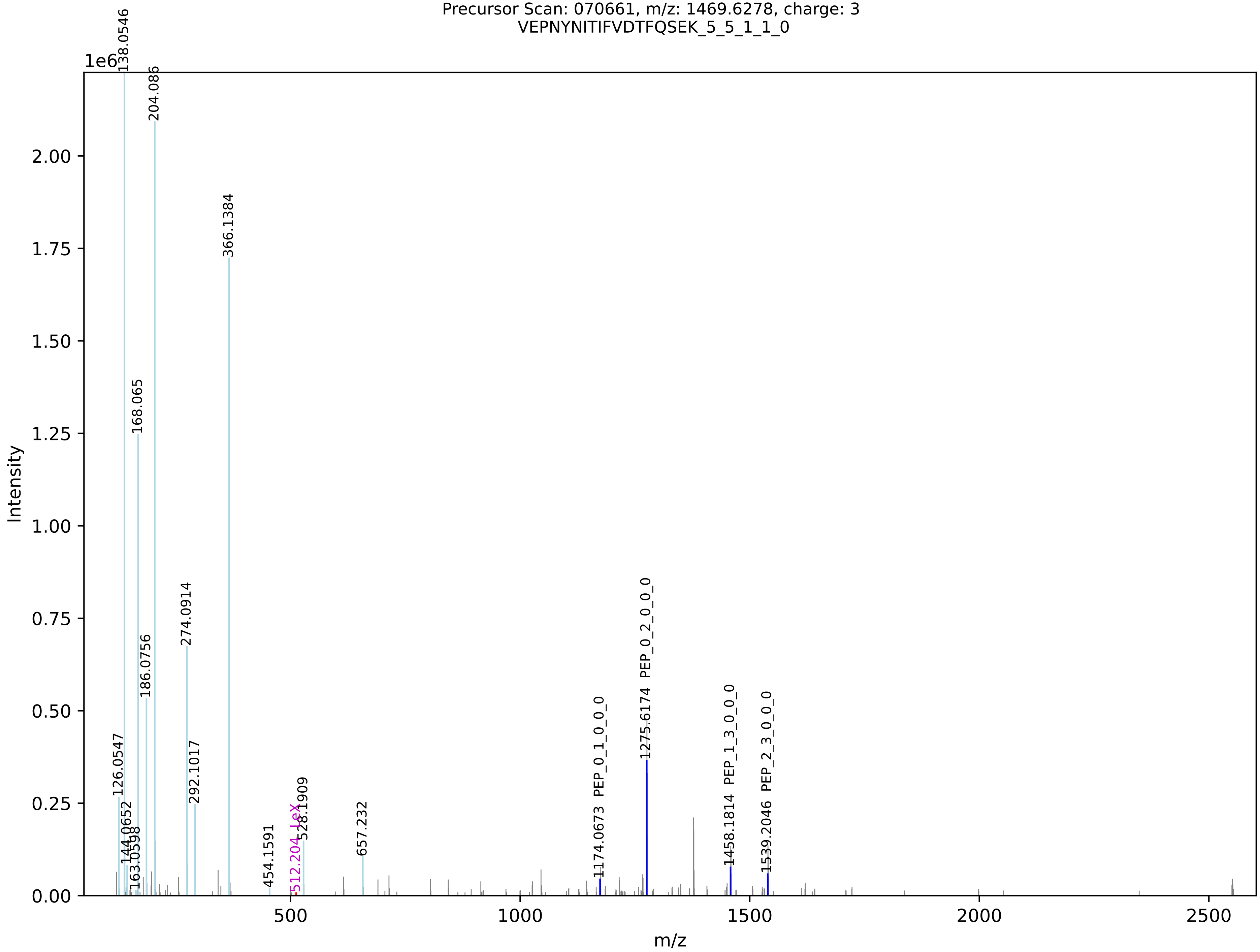

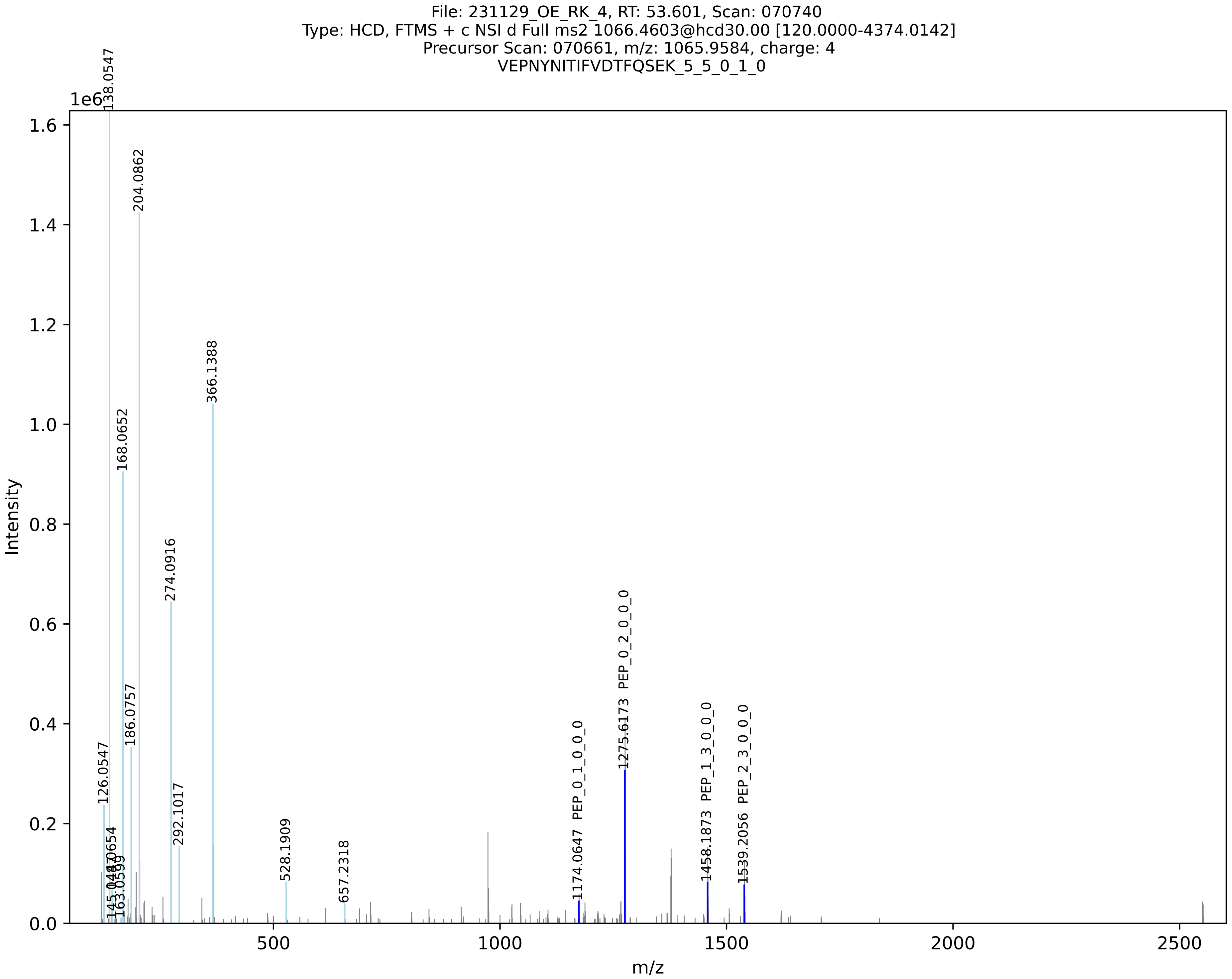

File: 231129_OE_RK_4, RT: 53.601, Scan: 070740
Type: HCD, FTMS + c NSI d Full ms2 1066.4603@hcd30.00 [120.0000-4374.0142]
Precursor Scan: 070661, m/z: 1065.9584, charge: 4
VEPNYNITIFVDTFQSEK_5_5_0_1_0
1e6
138.0547
204.0862
366.1388
168.0652
274.0916
186.0757
126.0547
292.1017
528.1909
657.2318
1174.0647 PEP_0_1_0_0_0
1275.6173 PEP_0_2_0_0_0
1458.1873 PEP_1_3_0_0_0
1539.2056 PEP_2_3_0_0_0
Intensity
m/z

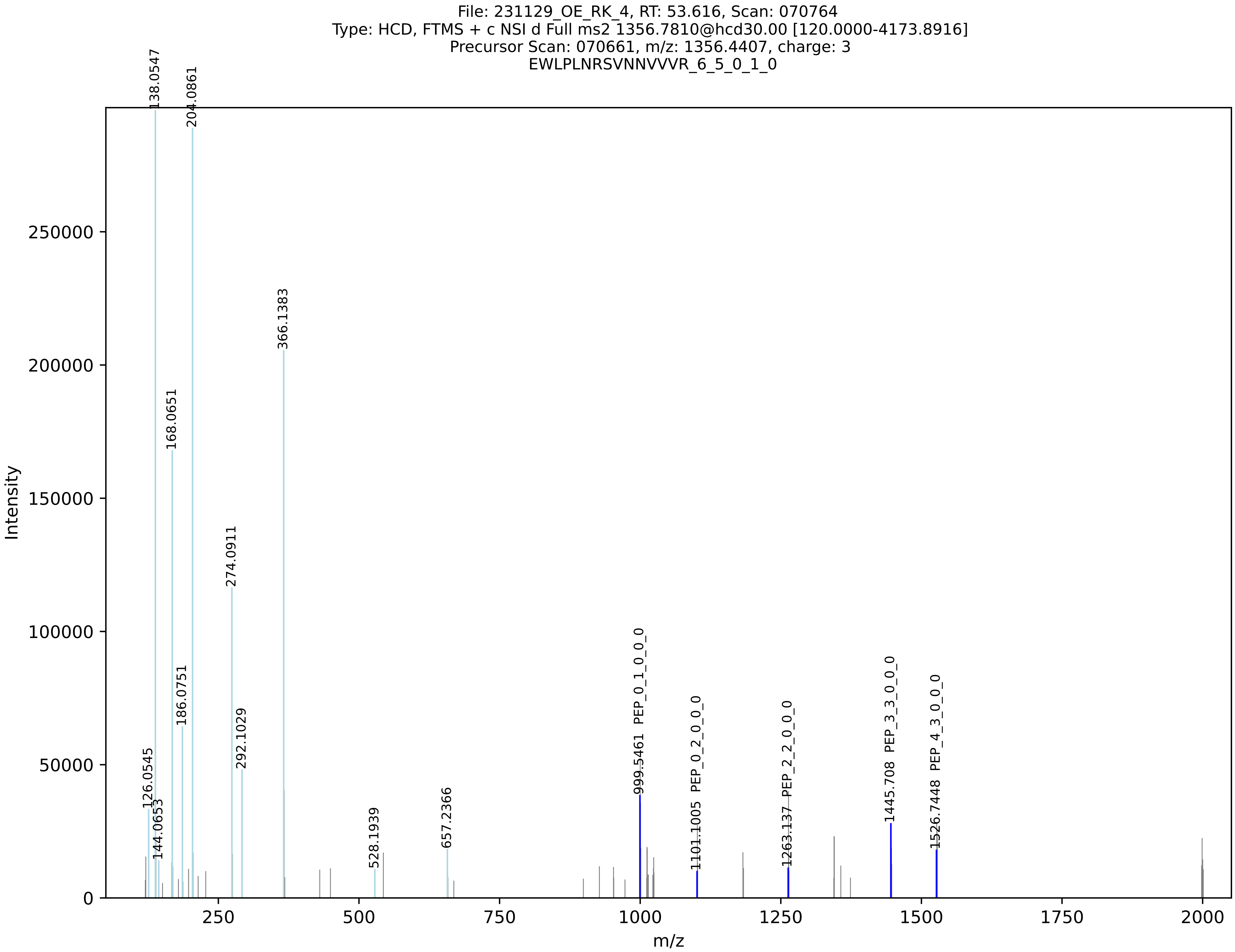
File: 231129_OE_RK_4, RT: 53.616, Scan: 070764
Type: HCD, FTMS + c NSI d Full ms2 1356.7810@hcd30.00 [120.0000-4173.8916]
Precursor Scan: 070661, m/z: 1356.4407, charge: 3
EWLPLNRSVNNVVVR_6_5_0_1_0
138.0547
204.0861
366.1383
168.0651
274.0911
186.0751
292.1029
126.0545
144.0653
528.1939
657.2366
999.5461 PEP_0_1_0_0_0
1101.1005 PEP_0_2_0_0_0
1263.137 PEP_2_2_0_0_0
1445.708 PEP_3_3_0_0_0
1526.7448 PEP_4_3_0_0_0
Intensity
0
50000
100000
150000
200000
250000
250
500
750
1000
1250
1500
1750
2000
m/z

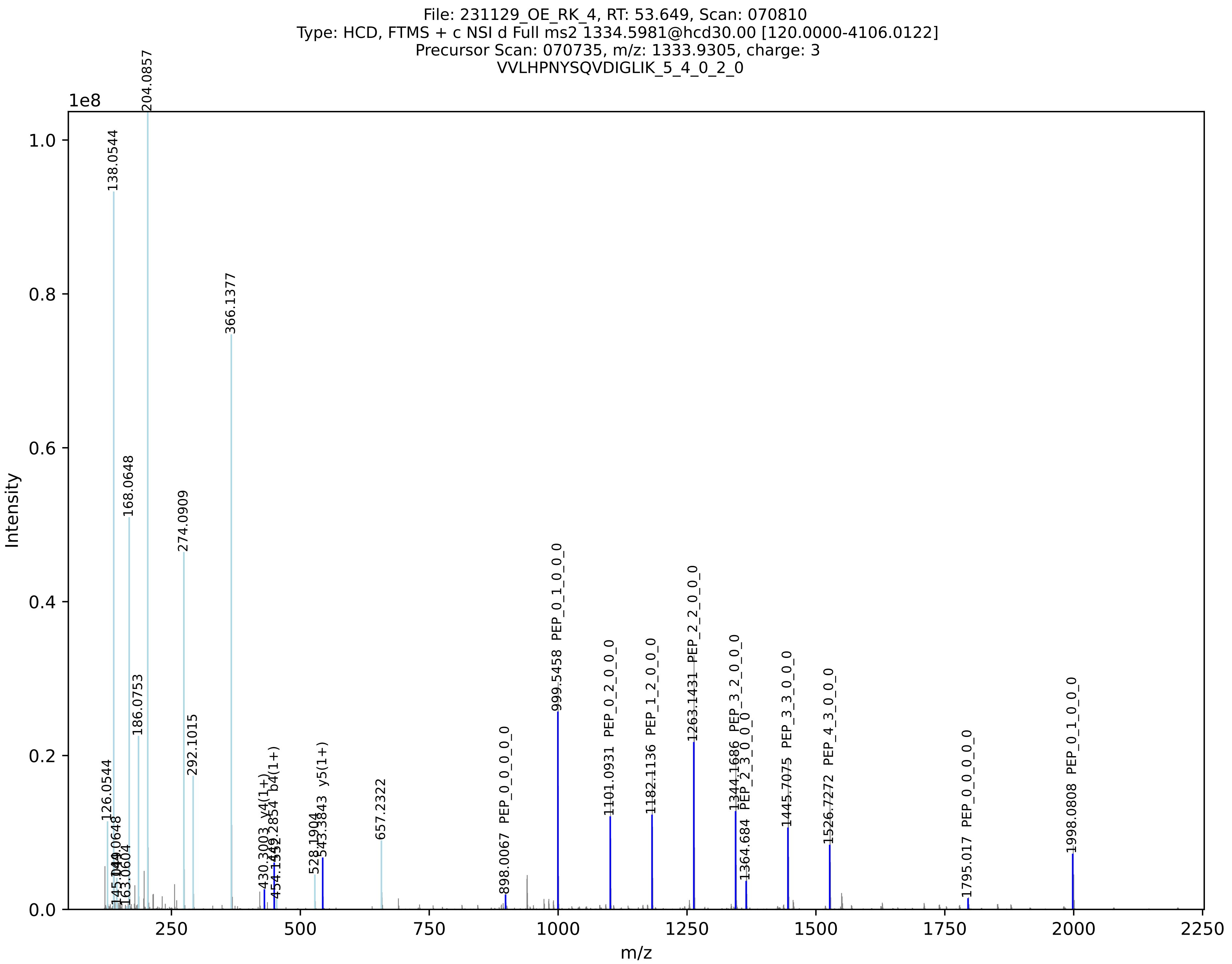
File: 231129_OE_RK_4, RT: 53.649, Scan: 070810
Type: HCD, FTMS + c NSI d Full ms2 1334.5981@hcd30.00 [120.0000-4106.0122]
Precursor Scan: 070735, m/z: 1333.9305, charge: 3
VVLHPNYSQVDIGLIK_5_4_0_2_0
1e8
Intensity
m/z
204.0857
138.0544
366.1377
168.0648
274.0909
186.0753
292.1015
126.0544
430.3003 y4(1+)
449.2854 b4(1+)
454.1592
528.1904
543.3843 y5(1+)
657.2322
898.0067 PEP_0_0_0_0_0
999.5458 PEP_0_1_0_0_0
1101.0931 PEP_0_2_0_0_0
1182.1136 PEP_1_2_0_0_0
1263.1431 PEP_2_2_0_0_0
1344.1686 PEP_3_2_0_0_0
1364.684 PEP_2_3_0_0_0
1445.7075 PEP_3_3_0_0_0
1526.7272 PEP_4_3_0_0_0
1795.017 PEP_0_0_0_0_0
1998.0808 PEP_0_1_0_0_0

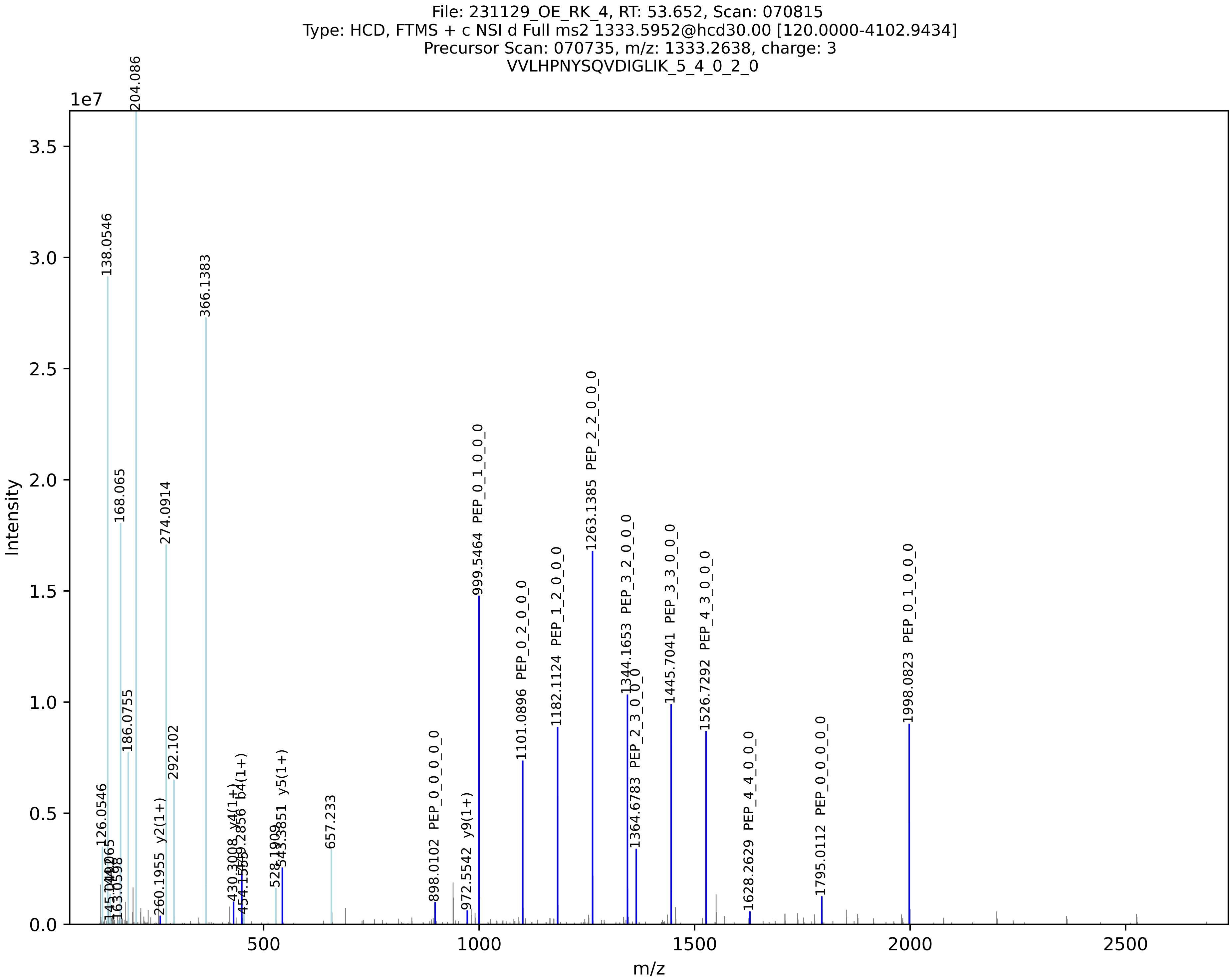
File: 231129_OE_RK_4, RT: 53.652, Scan: 070815
Type: HCD, FTMS + c NSI d Full ms2 1333.5952@hcd30.00 [120.0000-4102.9434]
Precursor Scan: 070735, m/z: 1333.2638, charge: 3
VVLHPNYSQVDIGLIK_5_4_0_2_0
1e7
Intensity
m/z
204.086
138.0546
366.1383
168.065
274.0914
186.0755
292.102
126.0546
260.1955 y2(1+)
430.3008 y4(1+)
454.1559
449.2856 b4(1+)
543.3851 y5(1+)
528.1909
657.233
898.0102 PEP_0_0_0_0_0_
972.5542 y9(1+)
999.5464 PEP_0_1_0_0_0_
1101.0896 PEP_0_2_0_0_0_
1182.1124 PEP_1_2_0_0_0_
1263.1385 PEP_2_2_0_0_0_
1344.1653 PEP_3_2_0_0_0_
1364.6783 PEP_2_3_0_0_0_
1445.7041 PEP_3_3_0_0_0_
1526.7292 PEP_4_3_0_0_0_
1628.2629 PEP_4_4_0_0_0_
1795.0112 PEP_0_0_0_0_0_
1998.0823 PEP_0_1_0_0_0_

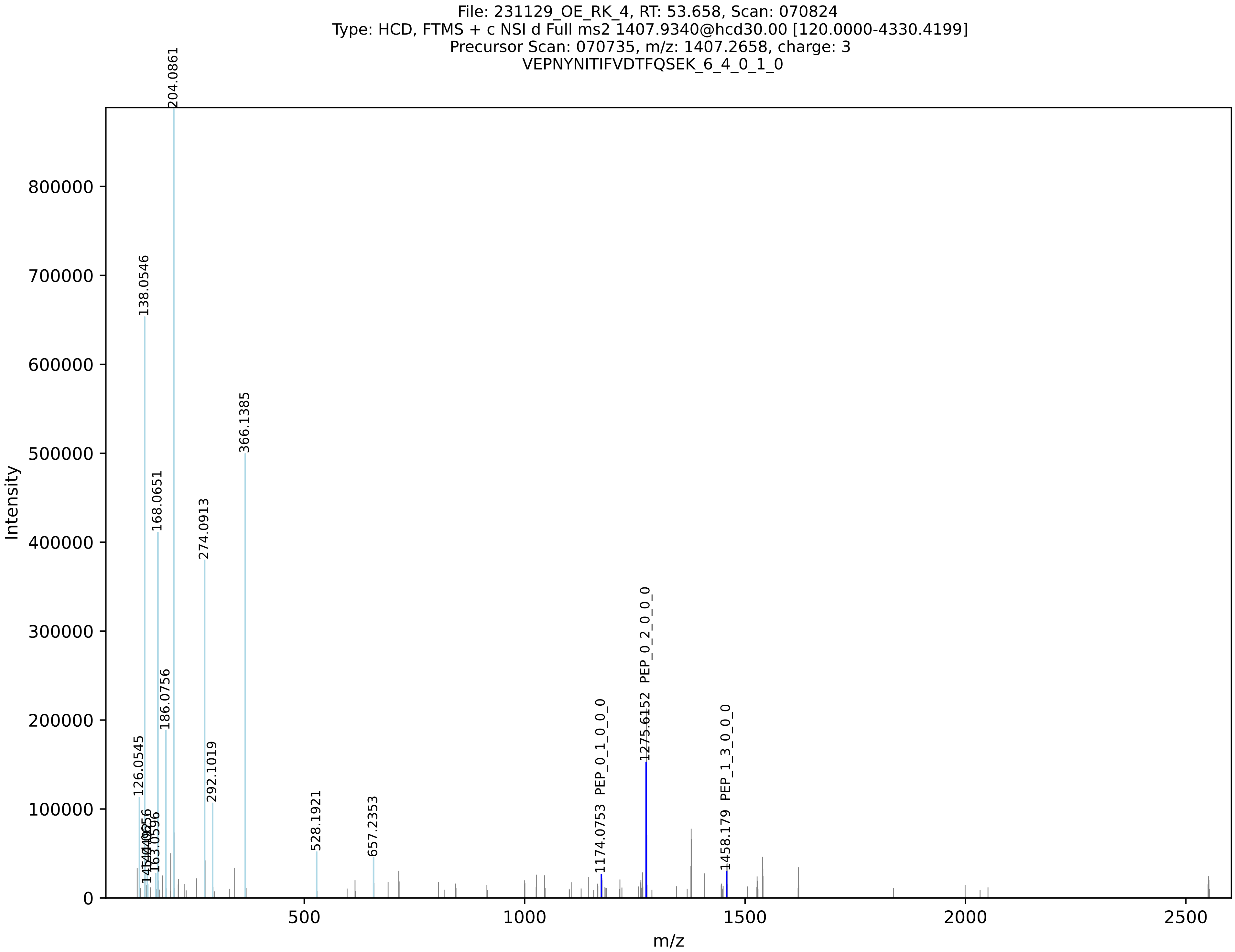
File: 231129_OE_RK_4, RT: 53.658, Scan: 070824
Type: HCD, FTMS + c NSI d Full ms2 1407.9340@hcd30.00 [120.0000-4330.4199]
Precursor Scan: 070735, m/z: 1407.2658, charge: 3
VEPNYNITIFVDTFQSEK_6_4_0_1_0
Intensity
m/z
204.0861
138.0546
366.1385
168.0651
274.0913
186.0756
126.0545
292.1019
163.0596
528.1921
657.2353
1174.0753
PEP_0_1_0_0_0
1275.6152
PEP_0_2_0_0_0
1458.179
PEP_1_3_0_0_0

File: 231129_OE_RK_4, RT: 53.672, Scan: 070848
Type: HCD, FTMS + c NSI d Full ms2 1115.8743@hcd30.00 [120.0000-3436.7173]
Precursor Scan: 070735, m/z: 1115.187, charge: 3
VVLHPNYSQVDIGLIK_4_3_0_1_0

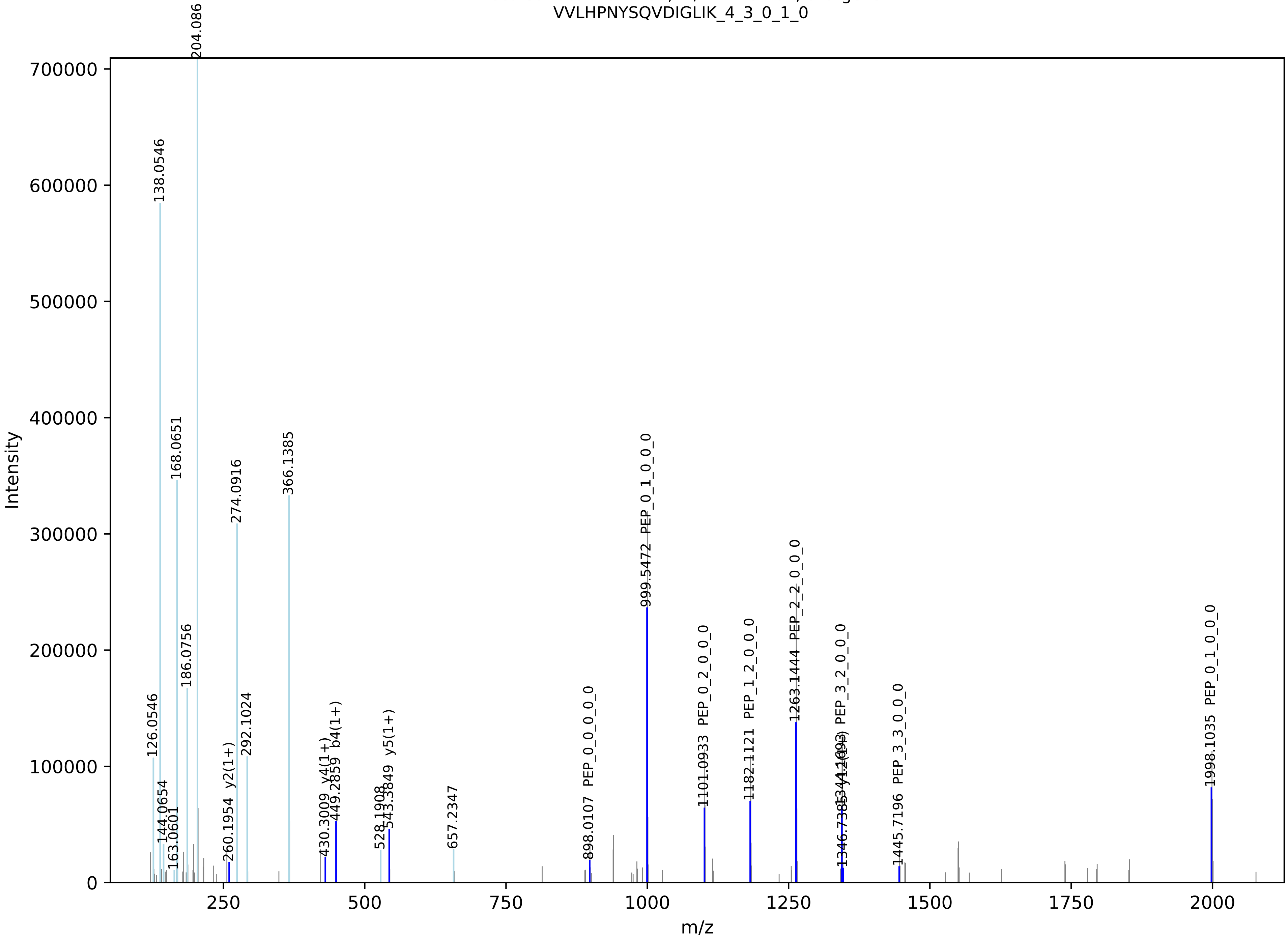

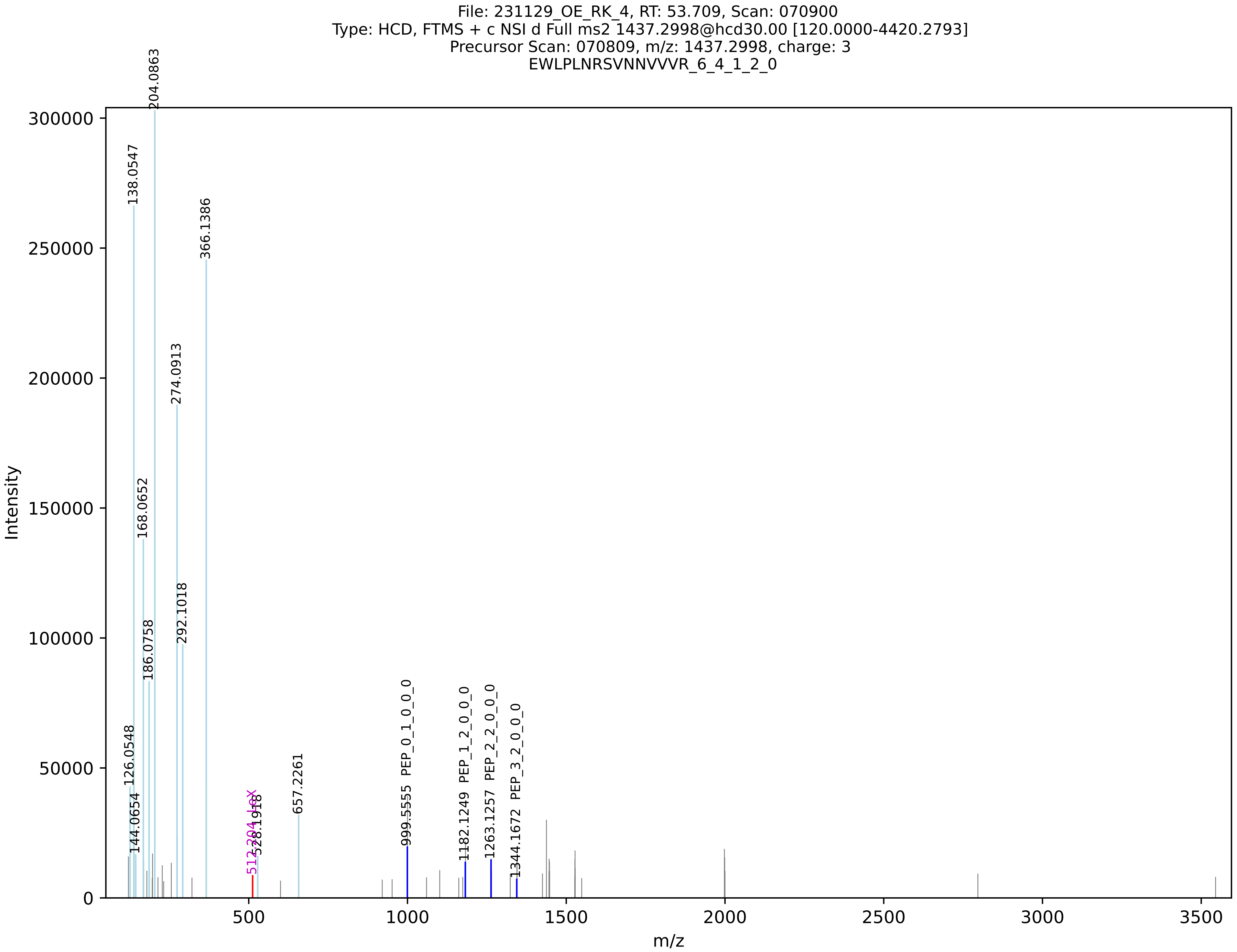

File: 231129_OE_RK_4, RT: 53.709, Scan: 070900
Type: HCD, FTMS + c NSI d Full ms2 1437.2998@hcd30.00 [120.0000-4420.2793]
Precursor Scan: 070809, m/z: 1437.2998, charge: 3
EWLPLNRSVNNVVVR_6_4_1_2_0
Intensity
m/z
204.0863
138.0547
366.1386
274.0913
168.0652
292.1018
186.0758
126.0548
144.0654
512.204_LeX
528.1918
657.2261
999.5555 PEP_0_1_0_0_0_
1182.1249 PEP_1_2_0_0_0_
1263.1257 PEP_2_2_0_0_0_
1344.1672 PEP_3_2_0_0_0_

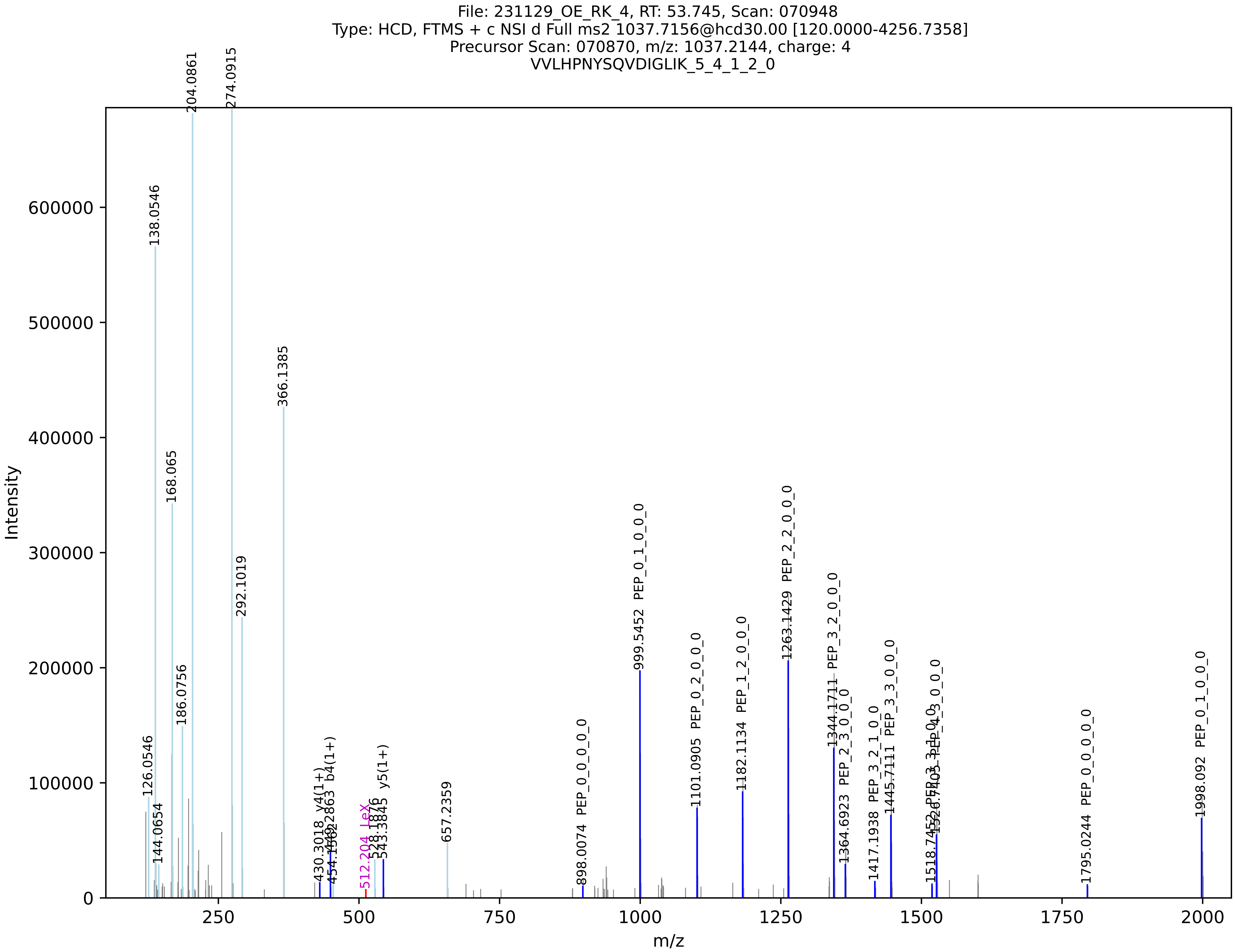

File: 231129_OE_RK_4, RT: 53.745, Scan: 070948
Type: HCD, FTMS + c NSI d Full ms2 1037.7156@hcd30.00 [120.0000-4256.7358]
Precursor Scan: 070870, m/z: 1037.2144, charge: 4
VVLHPNYSQVDIGLIK_5_4_1_2_0
Intensity
m/z
204.0861
274.0915
138.0546
366.1385
168.065
292.1019
186.0756
126.0546
144.0654
430.3018 y4(1+)
449.2863 b4(1+)
454.1562
512.204 LeX
528.1876
543.3845 y5(1+)
657.2359
898.0074 PEP_0_0_0_0_0
999.5452 PEP_0_1_0_0_0
1101.0905 PEP_0_2_0_0_0
1182.1134 PEP_1_2_0_0_0
1263.1429 PEP_2_2_0_0_0
1344.1711 PEP_3_2_0_0_0
1364.6923 PEP_2_3_0_0_0
1417.1938 PEP_3_2_1_0_0
1445.7111 PEP_3_3_0_0_0
1795.0244 PEP_0_0_0_0_0
1998.092 PEP_0_1_0_0_0

File: 231129_OE_RK_4, RT: 53.792, Scan: 071016
Type: HCD, FTMS + c NSI d Full ms2 1000.9512@hcd30.00 [120.0000-4106.7368]
Precursor Scan: 070944, m/z: 1000.7, charge: 4
VVLHPNYSQVDIGLIK_5_4_0_2_0

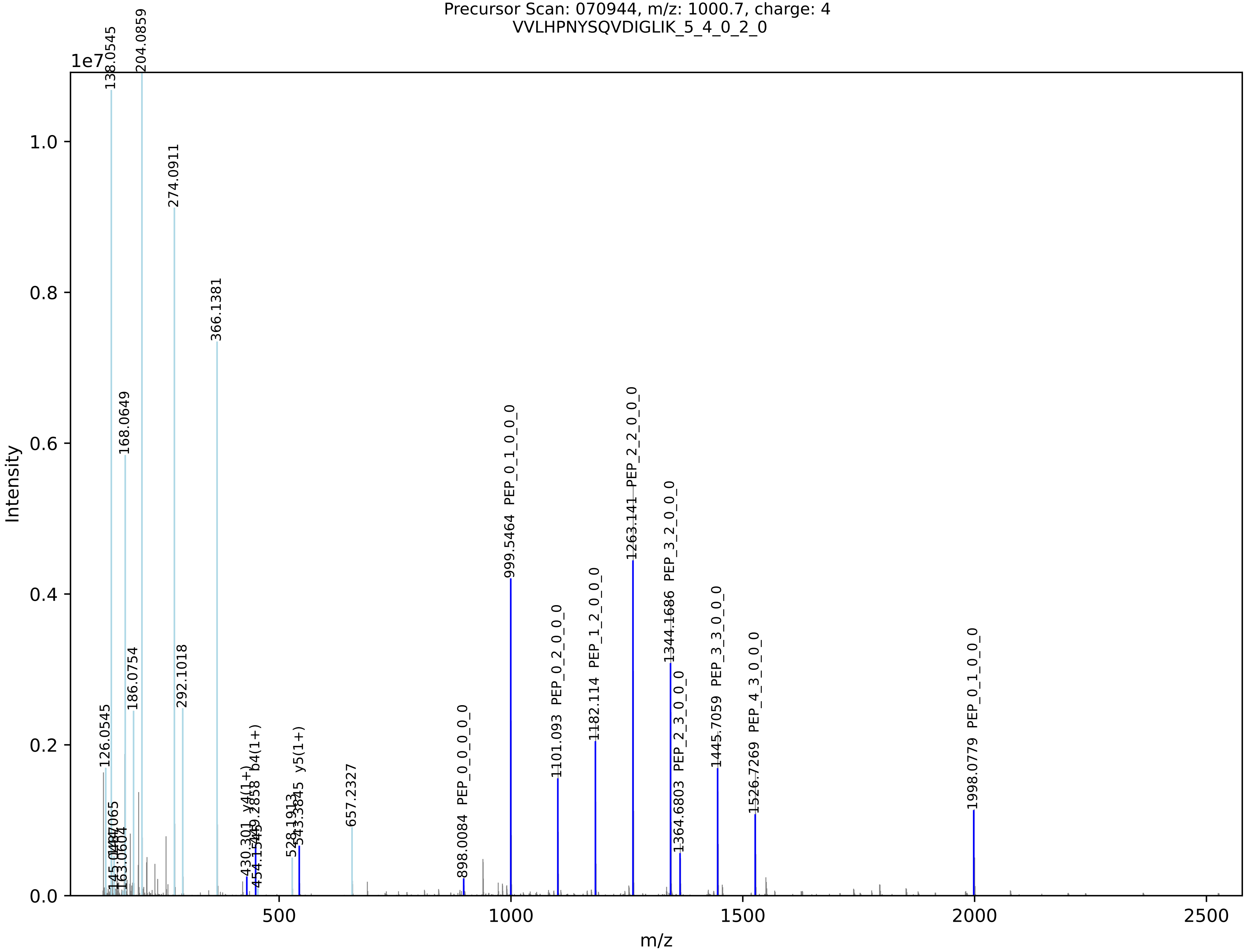

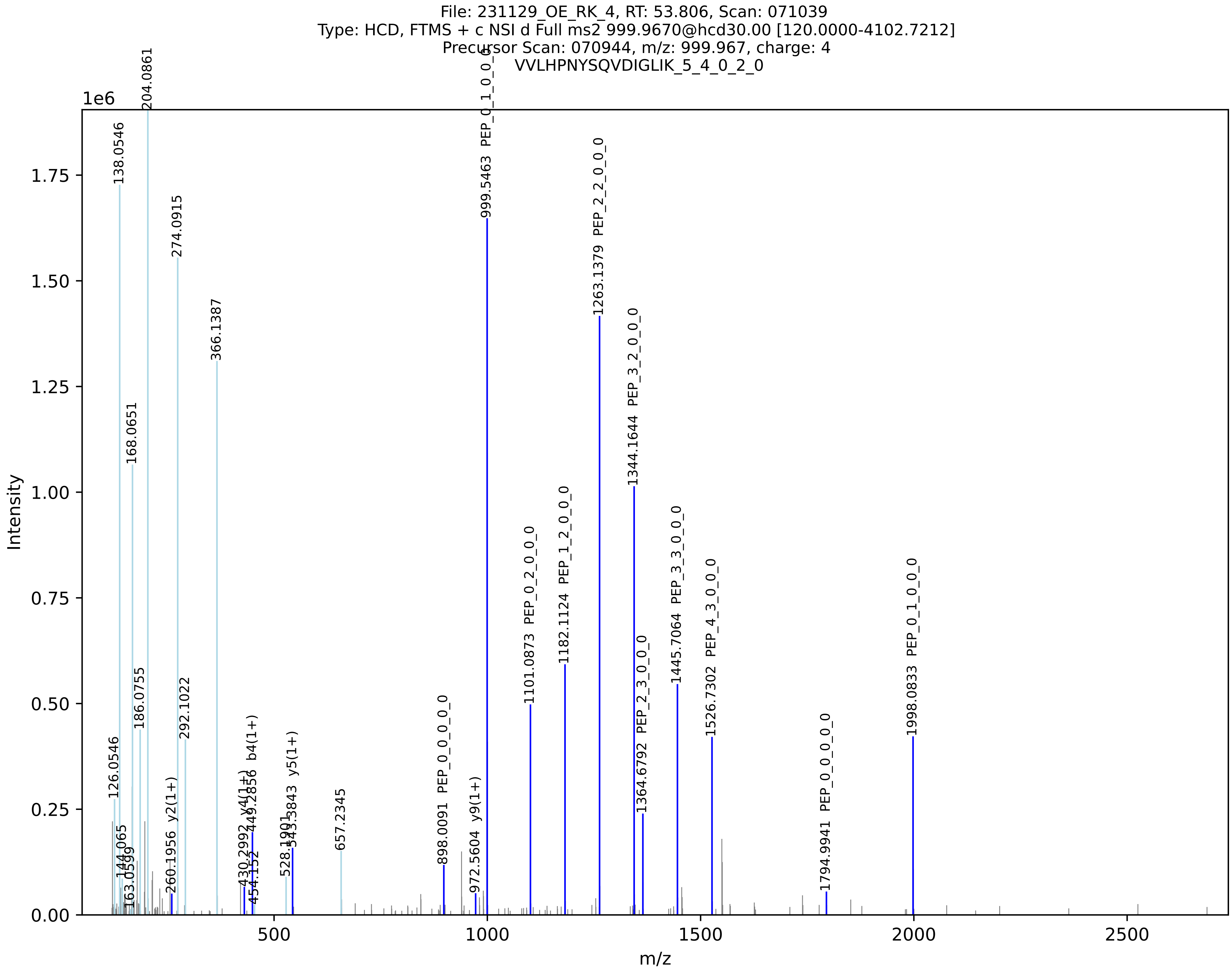
File: 231129_OE_RK_4, RT: 53.806, Scan: 071039
Type: HCD, FTMS + c NSI d Full ms2 999.9670@hcd30.00 [120.0000-4102.7212]
Precursor Scan: 070944, m/z: 999.967, charge: 4
VVLHPNYSQVDIGLIK_5_4_0_2_0
1e6
Intensity
m/z
204.0861
138.0546
168.0651
274.0915
366.1387
186.0755
292.1022
126.0546
144.065
163.0599
260.1956 y2(1+)
430.2992 y4(1+)
449.2856 b4(1+)
454.152
528.1901
543.3843 y5(1+)
657.2345
898.0091 PEP_0_0_0_0_0
972.5604 y9(1+)
999.5463 PEP_0_1_0_0_0
1101.0873 PEP_0_2_0_0_0
1182.1124 PEP_1_2_0_0_0
1263.1379 PEP_2_2_0_0_0
1344.1644 PEP_3_2_0_0_0
1364.6792 PEP_2_3_0_0_0
1445.7064 PEP_3_3_0_0_0
1526.7302 PEP_4_3_0_0_0
1794.9941 PEP_0_0_0_0_0
1998.0833 PEP_0_1_0_0_0

File: 231129_OE_RK_4, RT: 53.832, Scan: 071080
Type: HCD, FTMS + c NSI d Full ms2 1610.0164@hcd30.00 [120.0000-4948.7920]
Precursor Scan: 070870, m/z: 1610.0164, charge: 3
LMLQNISPSDAGEYTAVVGQLECK_5_4_0_2_0

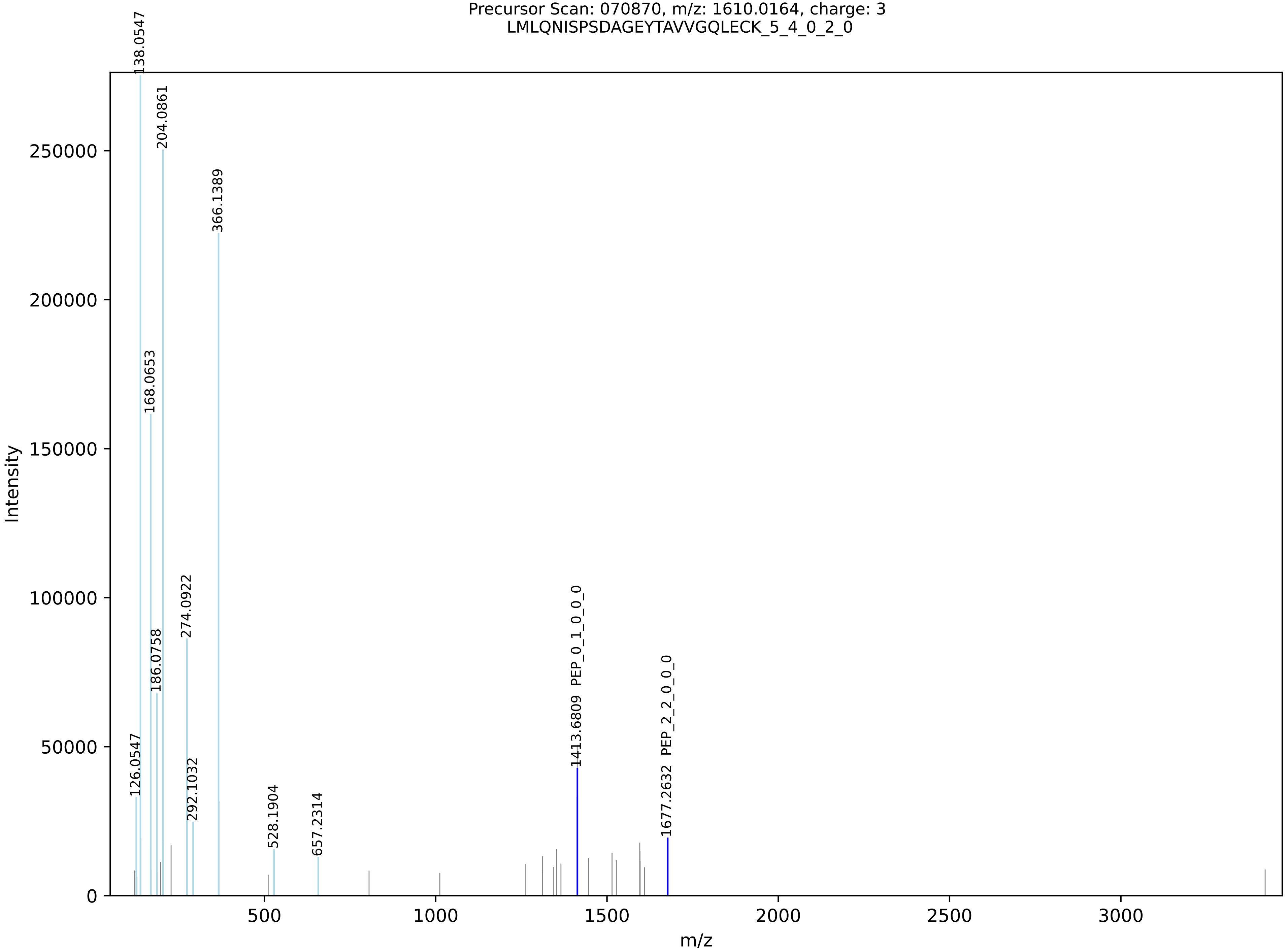

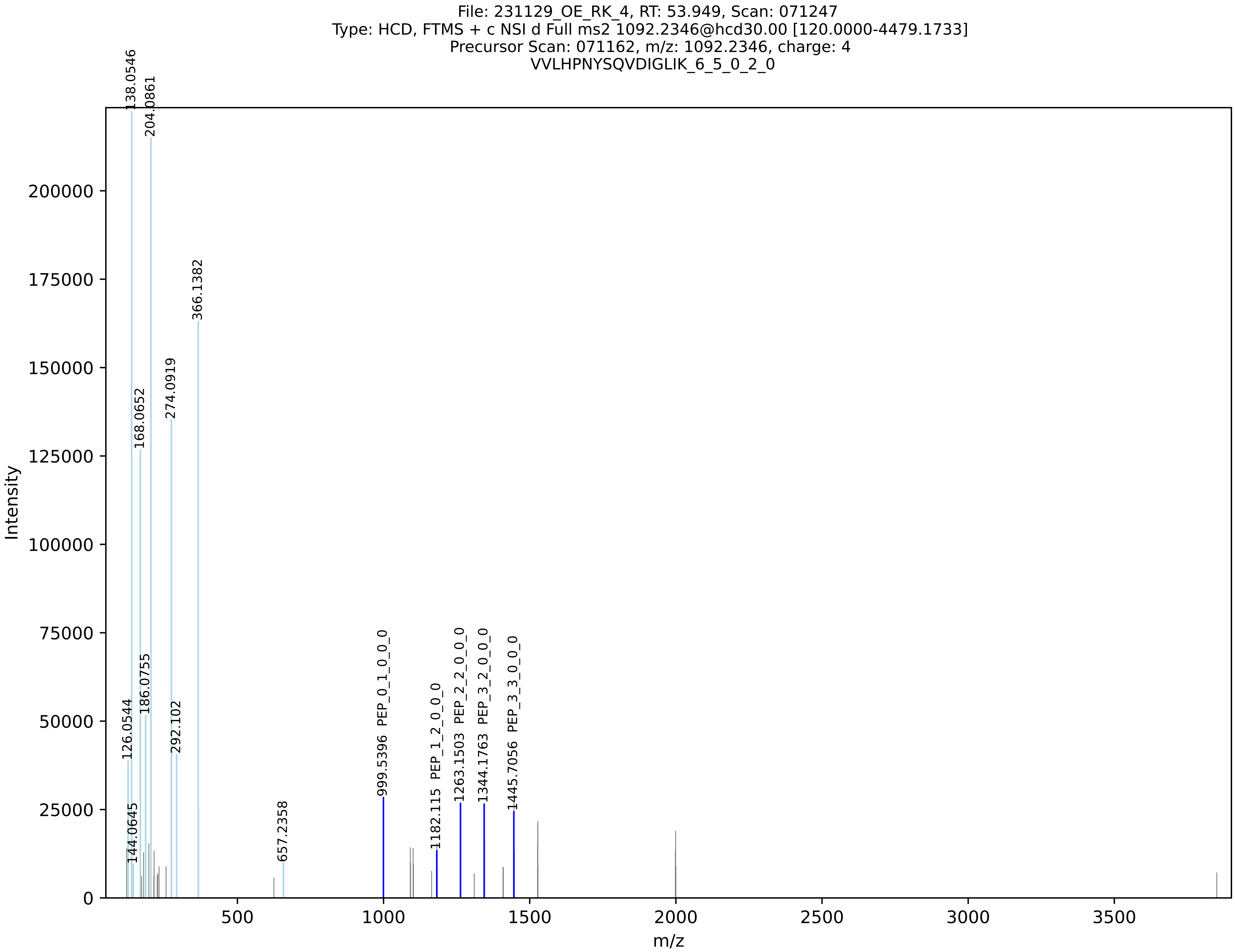
File: 231129_OE_RK_4, RT: 53.949, Scan: 071247
Type: HCD, FTMS + c NSI d Full ms2 1092.2346@hcd30.00 [120.0000-4479.1733]
Precursor Scan: 071162, m/z: 1092.2346, charge: 4
VVLHPNYSQVDIGLIK_6_5_0_2_0
138.0546
204.0861
366.1382
274.0919
168.0652
186.0755
126.0544
292.102
144.0645
657.2358
999.5396 PEP_0_1_0_0_0
1182.115 PEP_1_2_0_0_0
1263.1503 PEP_2_2_0_0_0
1344.1763 PEP_3_2_0_0_0
1445.7056 PEP_3_3_0_0_0
Intensity
m/z
0
25000
50000
75000
100000
125000
150000
175000
200000
500
1000
1500
2000
2500
3000
3500

File: 231129_OE_RK_4, RT: 54.1, Scan: 071463
Type: HCD, FTMS + c NSI d Full ms2 1359.7865@hcd30.00 [120.0000-5570.7847]
Precursor Scan: 071382, m/z: 1359.0347, charge: 4
QDQCIYNTTYLNVQR_7_6_0_4_0

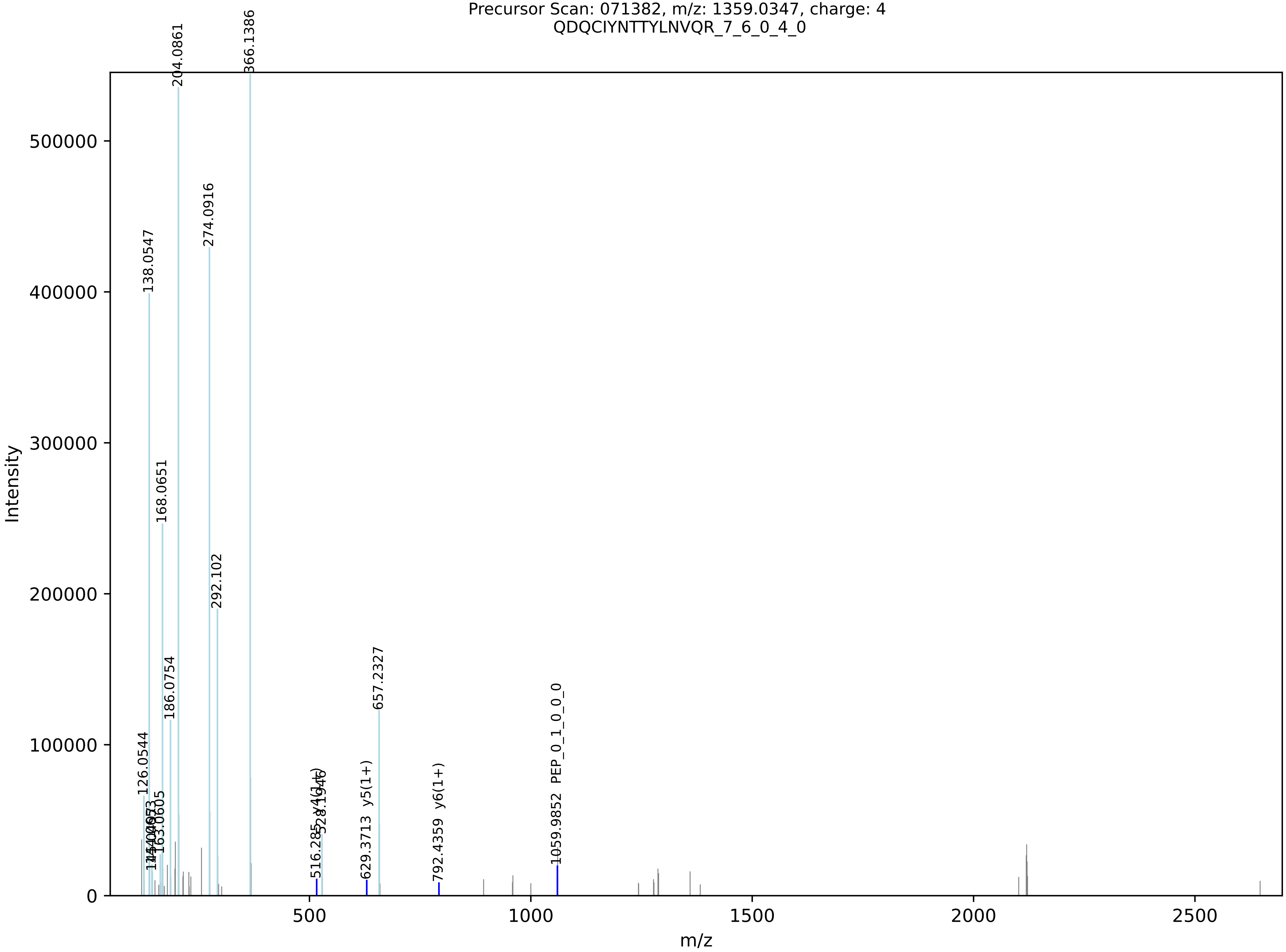

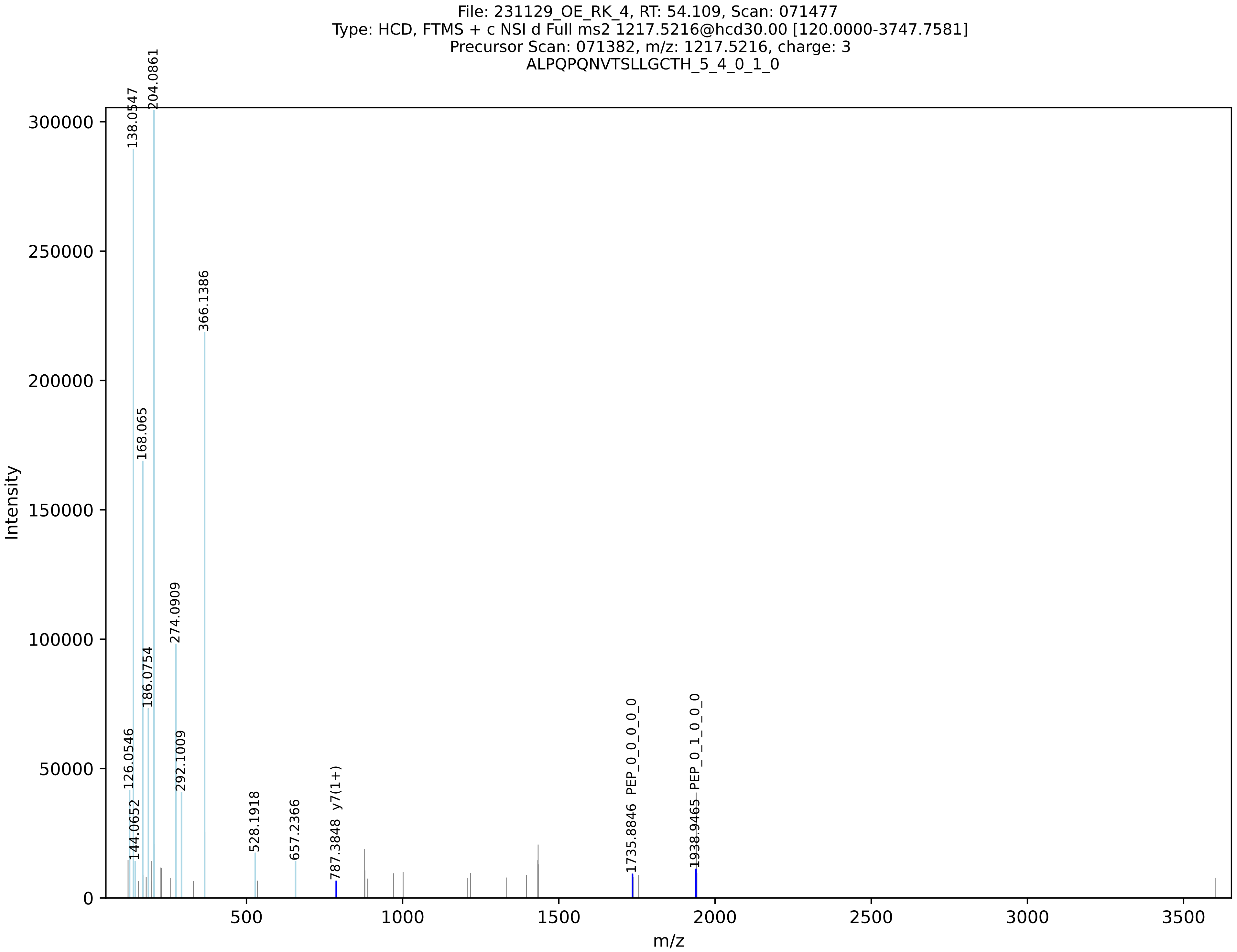

File: 231129_OE_RK_4, RT: 54.109, Scan: 071477
Type: HCD, FTMS + c NSI d Full ms2 1217.5216@hcd30.00 [120.0000-3747.7581]
Precursor Scan: 071382, m/z: 1217.5216, charge: 3
ALPQPQNVTSLLGCTH_5_4_0_1_0
Intensity
m/z
138.0547
204.0861
366.1386
168.065
274.0909
186.0754
126.0546
292.1009
144.0652
528.1918
657.2366
787.3848 y7(1+)
1735.8846 PEP_0_0_0_0_0
1938.9465 PEP_0_1_0_0_0

File: 231129_OE_RK_4, RT: 54.402, Scan: 071901
Type: HCD, FTMS + c NSI d Full ms2 1001.2017@hcd30.00 [120.0000-4107.7593]
Precursor Scan: 071820, m/z: 1000.701, charge: 4
VVLHPNYSQVDIGLIK_5_4_0_2_0

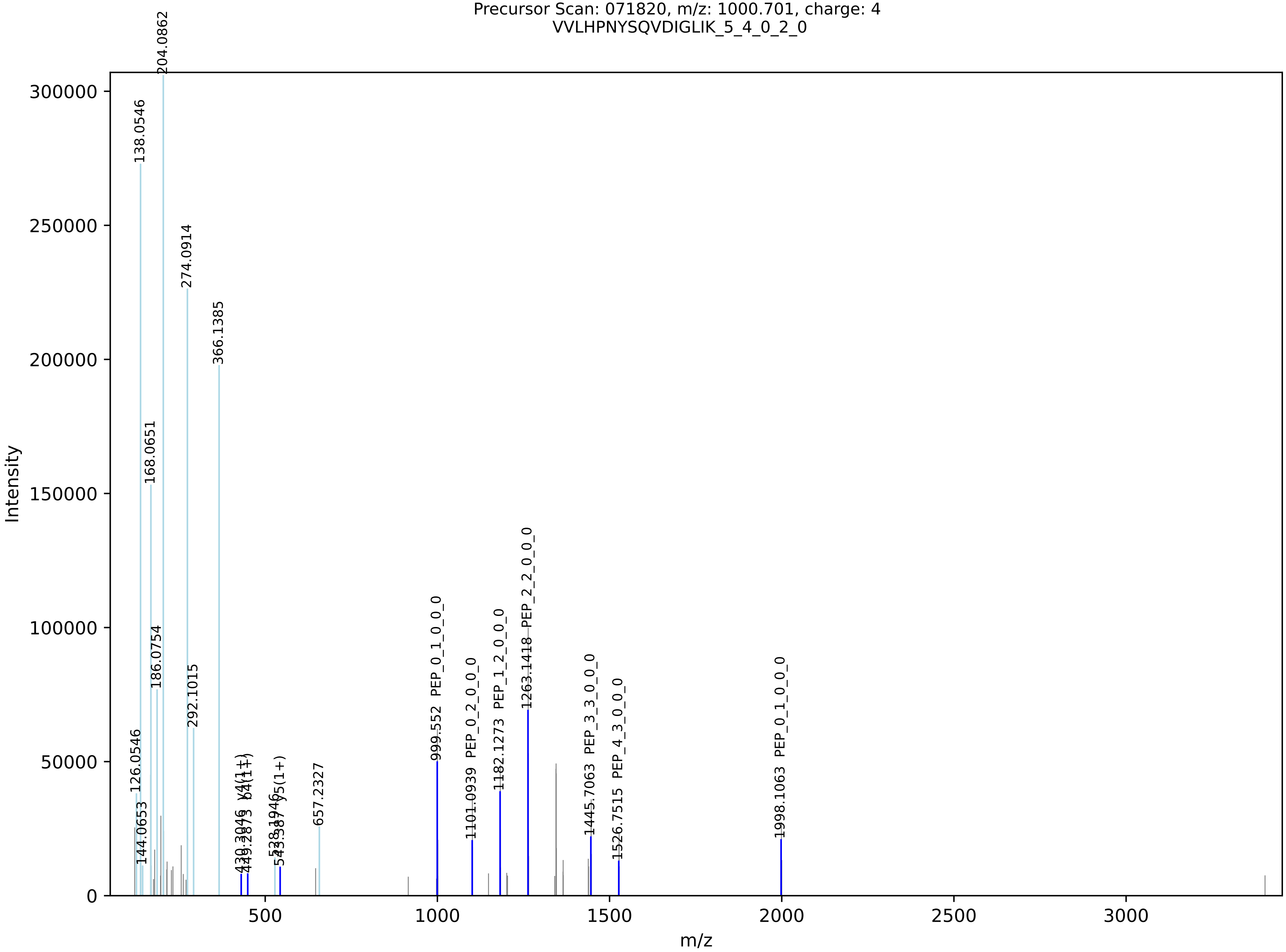

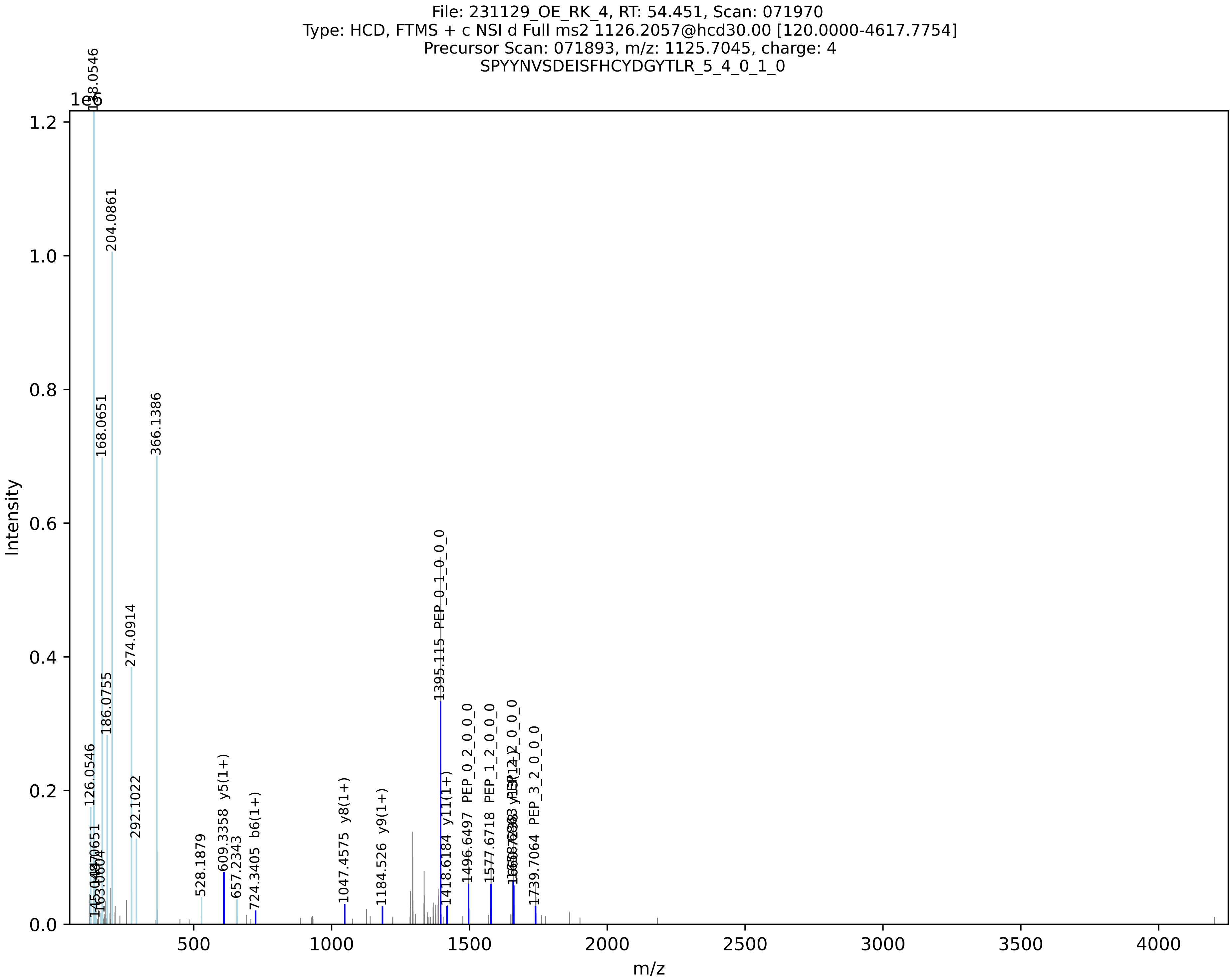

File: 231129_OE_RK_4, RT: 54.451, Scan: 071970
Type: HCD, FTMS + c NSI d Full ms2 1126.2057@hcd30.00 [120.0000-4617.7754]
Precursor Scan: 071893, m/z: 1125.7045, charge: 4
SPYYNVSDEISFHCYDGYTLR_5_4_0_1_0
1e6
Intensity
m/z
204.0861
168.0651
366.1386
274.0914
186.0755
126.0546
292.1022
528.1879
609.3358 y5(1+)
657.2343
724.3405 b6(1+)
1047.4575 y8(1+)
1184.526 y9(1+)
1395.115 PEP_0_1_0_0_0
1418.6184 y11(1+)
1496.6497 PEP_0_2_0_0_0
1577.6718 PEP_1_2_0_0_0
1739.7064 PEP_3_2_0_0_0

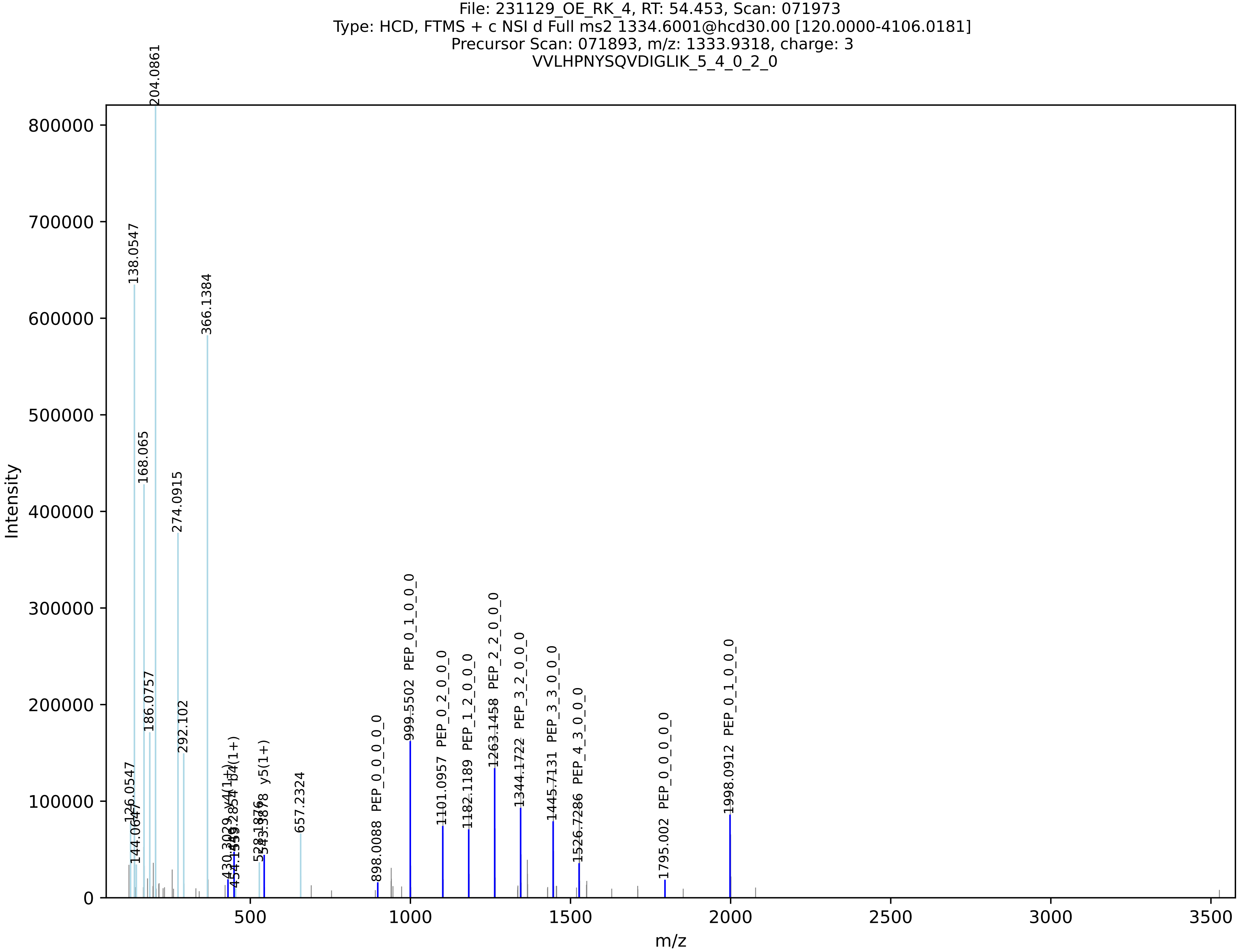

File: 231129_OE_RK_4, RT: 54.453, Scan: 071973
Type: HCD, FTMS + c NSI d Full ms2 1334.6001@hcd30.00 [120.0000-4106.0181]
Precursor Scan: 071893, m/z: 1333.9318, charge: 3
VVLHPNYSQVDIGLIK_5_4_0_2_0
Intensity
m/z
204.0861
138.0547
366.1384
168.065
274.0915
186.0757
292.102
126.0547
144.0647
657.2324
y5(1+)
y4(1+)
b4(1+)
999.5502 PEP_0_1_0_0_0
1101.0957 PEP_0_2_0_0_0
1182.1189 PEP_1_2_0_0_0
1263.1458 PEP_2_2_0_0_0
1344.1722 PEP_3_2_0_0_0
1445.7131 PEP_3_3_0_0_0
1526.7286 PEP_4_3_0_0_0
1795.002 PEP_0_0_0_0_0
1998.0912 PEP_0_1_0_0_0
898.0088 PEP_0_0_0_0_0

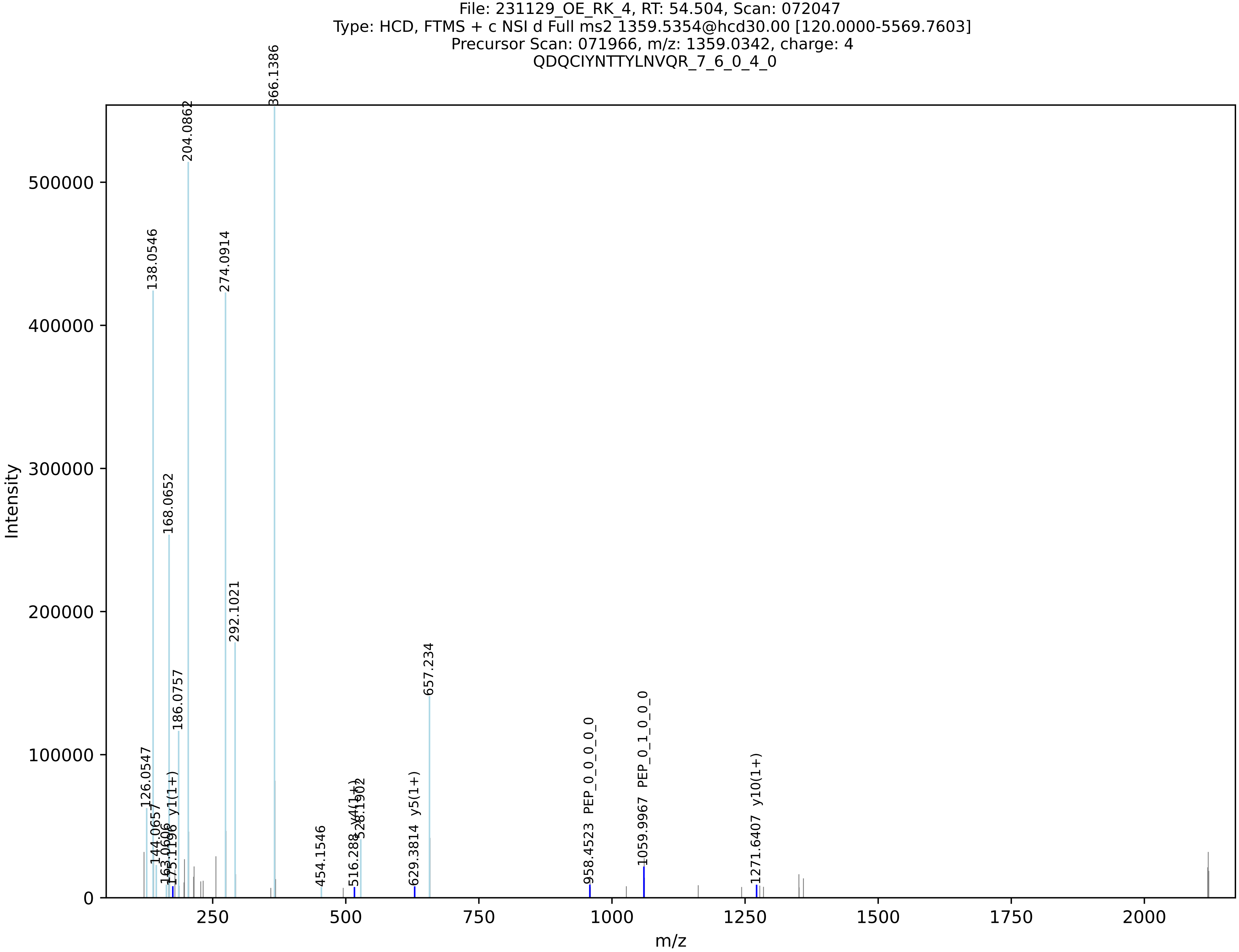

File: 231129_OE_RK_4, RT: 54.504, Scan: 072047
Type: HCD, FTMS + c NSI d Full ms2 1359.5354@hcd30.00 [120.0000-5569.7603]
Precursor Scan: 071966, m/z: 1359.0342, charge: 4
QDQCIYNTTYLNVQR_7_6_0_4_0
366.1386
204.0862
138.0546
274.0914
168.0652
292.1021
186.0757
657.234
126.0547
144.0657
163.0606
175.1196 y1(1+)
454.1546
516.288 y4(1+)
528.1902
629.3814 y5(1+)
958.4523 PEP_0_0_0_0_0
1059.9967 PEP_0_1_0_0_0
1271.6407 y10(1+)
Intensity
0
100000
200000
300000
400000
500000
250
500
750
1000
1250
1500
1750
2000
m/z

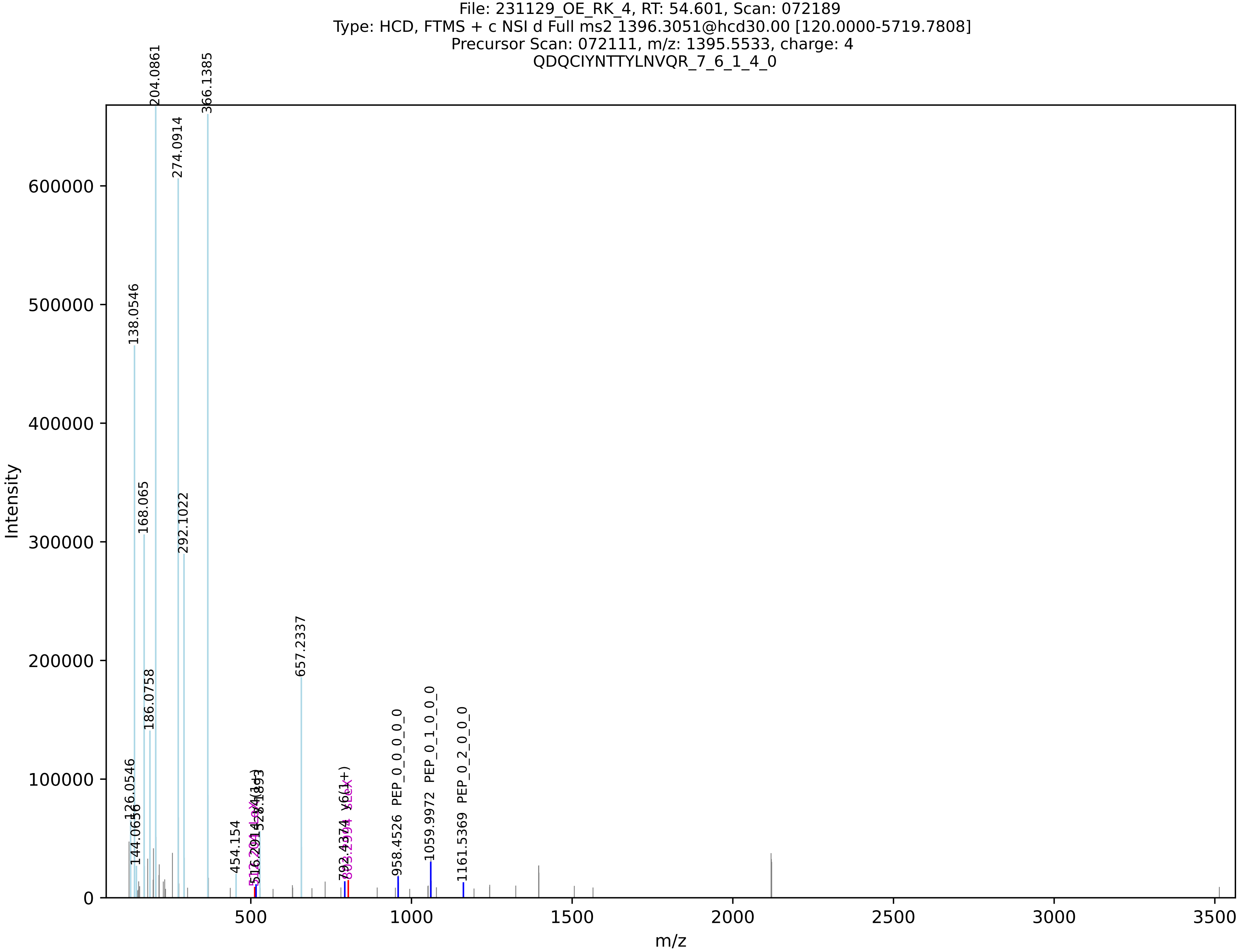

File: 231129_OE_RK_4, RT: 54.601, Scan: 072189
Type: HCD, FTMS + c NSI d Full ms2 1396.3051@hcd30.00 [120.0000-5719.7808]
Precursor Scan: 072111, m/z: 1395.5533, charge: 4
QDQCIYNTTYLNVQR_7_6_1_4_0
Intensity
m/z
204.0861
366.1385
274.0914
138.0546
168.065
292.1022
186.0758
657.2337
126.0546
144.0656
454.154
958.4526 PEP_0_0_0_0_
1059.9972 PEP_0_1_0_0_0
1161.5369 PEP_0_2_0_0_0

File: 231129_OE_RK_4, RT: 54.651, Scan: 072261
Type: HCD, FTMS + c NSI d Full ms2 1501.6055@hcd30.00 [120.0000-4617.0547]
Precursor Scan: 072185, m/z: 1500.6025, charge: 3
SPYYNVSDEISFHCYDGYTLR_5_4_0_1_0

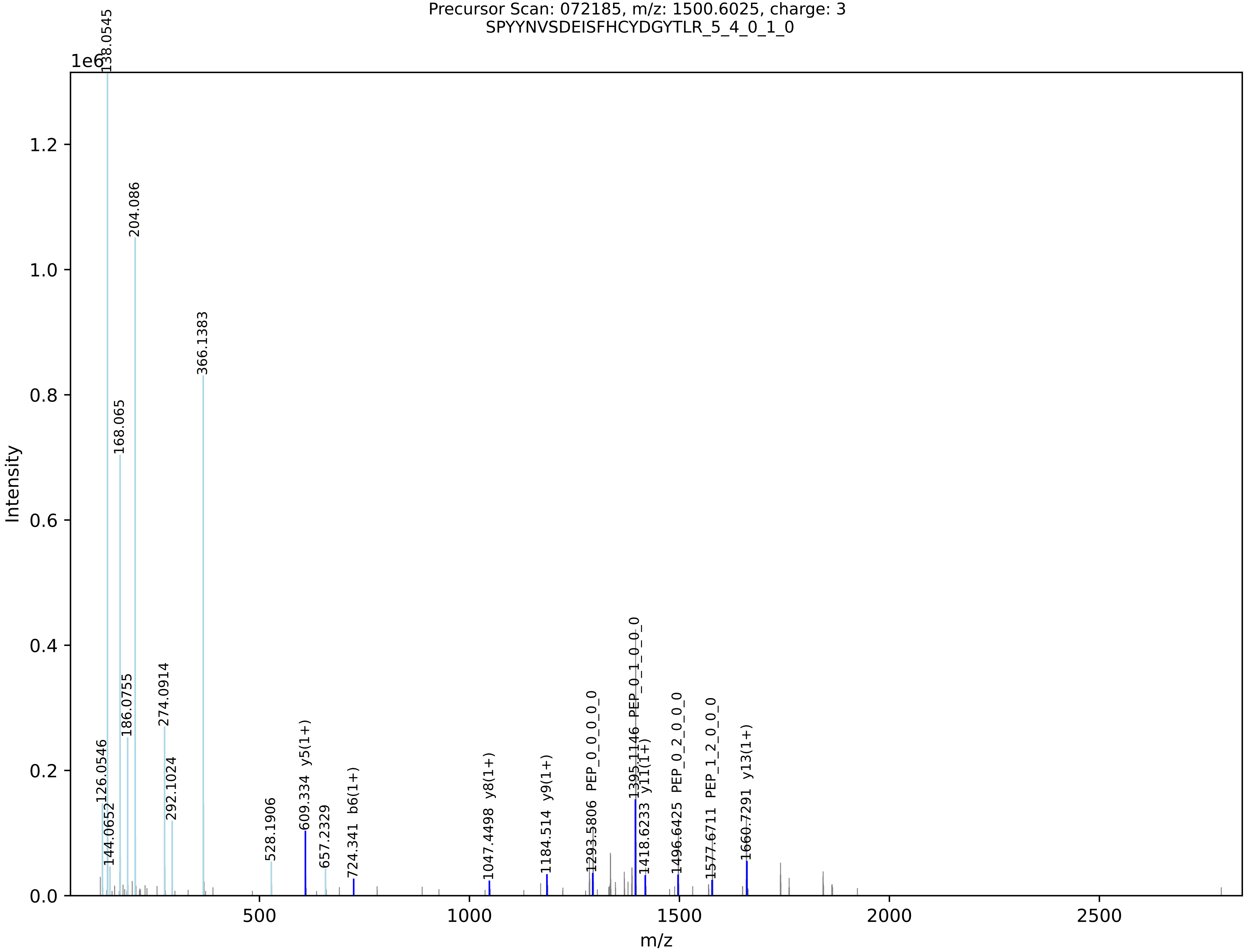

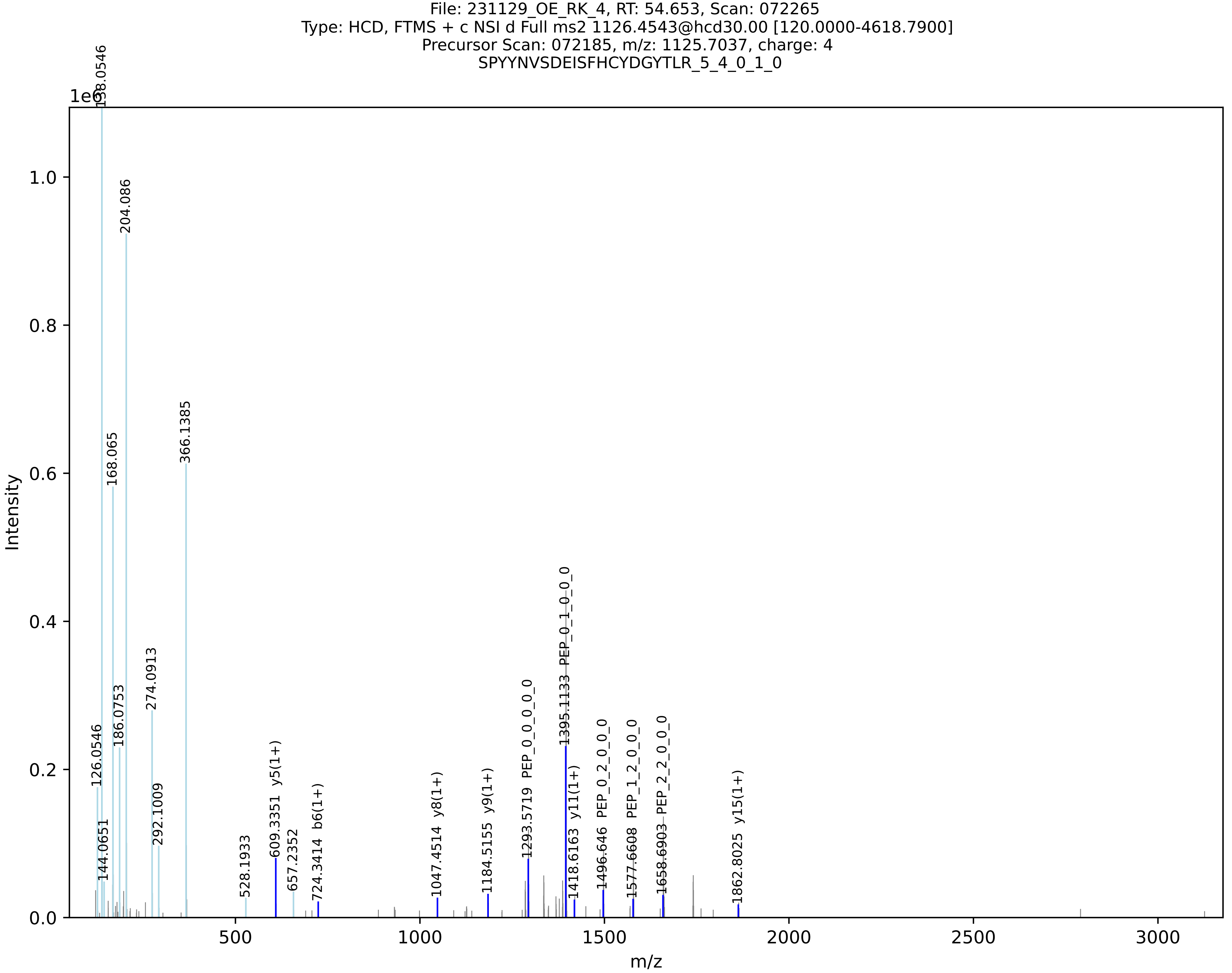
File: 231129_OE_RK_4, RT: 54.653, Scan: 072265
Type: HCD, FTMS + c NSI d Full ms2 1126.4543@hcd30.00 [120.0000-4618.7900]
Precursor Scan: 072185, m/z: 1125.7037, charge: 4
SPYYNVSDEISFHCYDGYTLR_5_4_0_1_0
1e6
Intensity
m/z
138.0546
204.086
168.065
366.1385
274.0913
186.0753
126.0546
292.1009
144.0651
528.1933
609.3351 y5(1+)
657.2352
724.3414 b6(1+)
1047.4514 y8(1+)
1184.5155 y9(1+)
1293.5719 PEP_0_0_0_0_0
1395.1133 PEP_0_1_0_0_0_
1418.6163 y11(1+)
1496.646 PEP_0_2_0_0_0_
1577.6608 PEP_1_2_0_0_0_
1658.6903 PEP_2_2_0_0_0_
1862.8025 y15(1+)

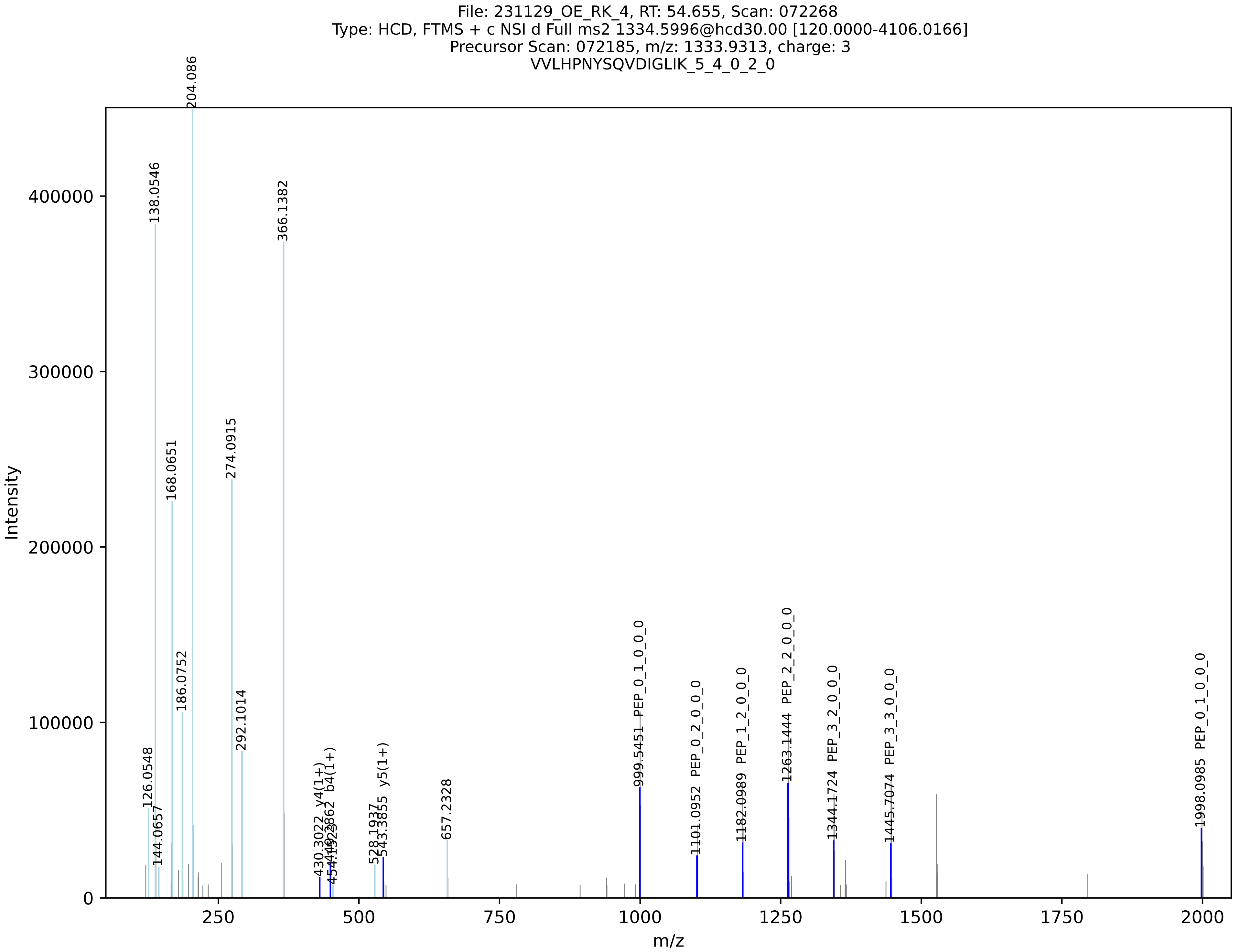

File: 231129_OE_RK_4, RT: 54.655, Scan: 072268
Type: HCD, FTMS + c NSI d Full ms2 1334.5996@hcd30.00 [120.0000-4106.0166]
Precursor Scan: 072185, m/z: 1333.9313, charge: 3
VVLHPNYSQVDIGLIK_5_4_0_2_0
Intensity
m/z
204.086
138.0546
366.1382
168.0651
274.0915
186.0752
292.1014
126.0548
144.0657
430.3022 y4(1+)
454.2862 b4(1+)
528.1937
543.3855 y5(1+)
657.2328
999.5451 PEP_0_1_0_0_0
1101.0952 PEP_0_2_0_0_0
1182.0989 PEP_1_2_0_0_0
1263.1444 PEP_2_2_0_0_0
1344.1724 PEP_3_2_0_0_0
1445.7074 PEP_3_3_0_0_0
1998.0985 PEP_0_1_0_0_0

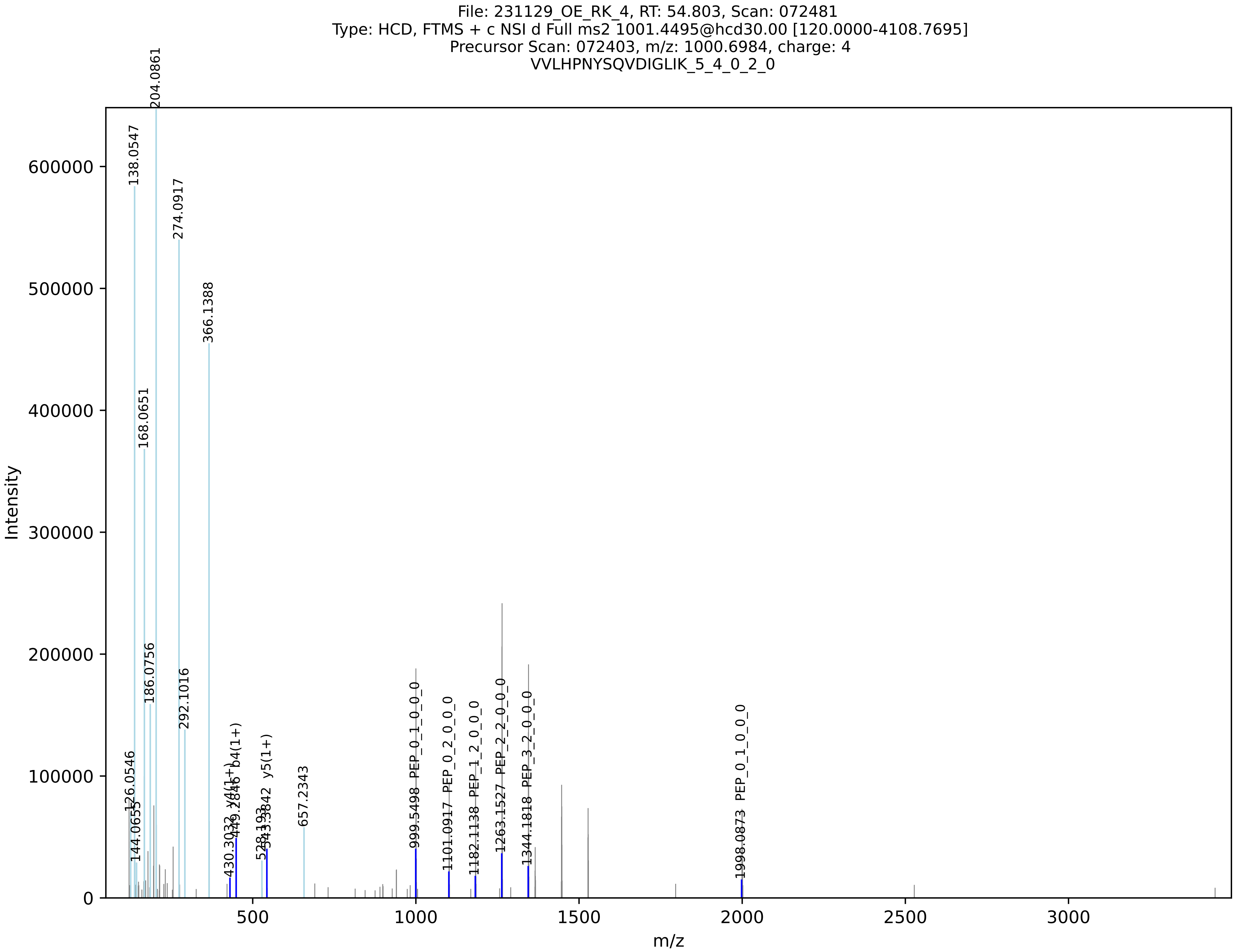
File: 231129_OE_RK_4, RT: 54.803, Scan: 072481
Type: HCD, FTMS + c NSI d Full ms2 1001.4495@hcd30.00 [120.0000-4108.7695]
Precursor Scan: 072403, m/z: 1000.6984, charge: 4
VVLHPNYSQVDIGLIK_5_4_0_2_0
Intensity
m/z
204.0861
138.0547
274.0917
366.1388
168.0651
186.0756
292.1016
126.0546
144.0655
430.3032_y4(1+)
449.2846_b4(1+)
528.193
543.3842_y5(1+)
657.2343
999.5498_PEP_0_1_0_0_0
1101.0917_PEP_0_2_0_0_0
1182.1138_PEP_1_2_0_0_0
1263.1527_PEP_2_2_0_0_0
1344.1818_PEP_3_2_0_0_0
1998.0873_PEP_0_1_0_0_0

File: 231129_OE_RK_4, RT: 54.804, Scan: 072482
Type: HCD, FTMS + c NSI d Full ms2 1143.7384@hcd30.00 [120.0000-4689.3086]
Precursor Scan: 072403, m/z: 1143.2372, charge: 4
AALAAFNAQNNGSNFQLEEISR_5_4_0_2_0

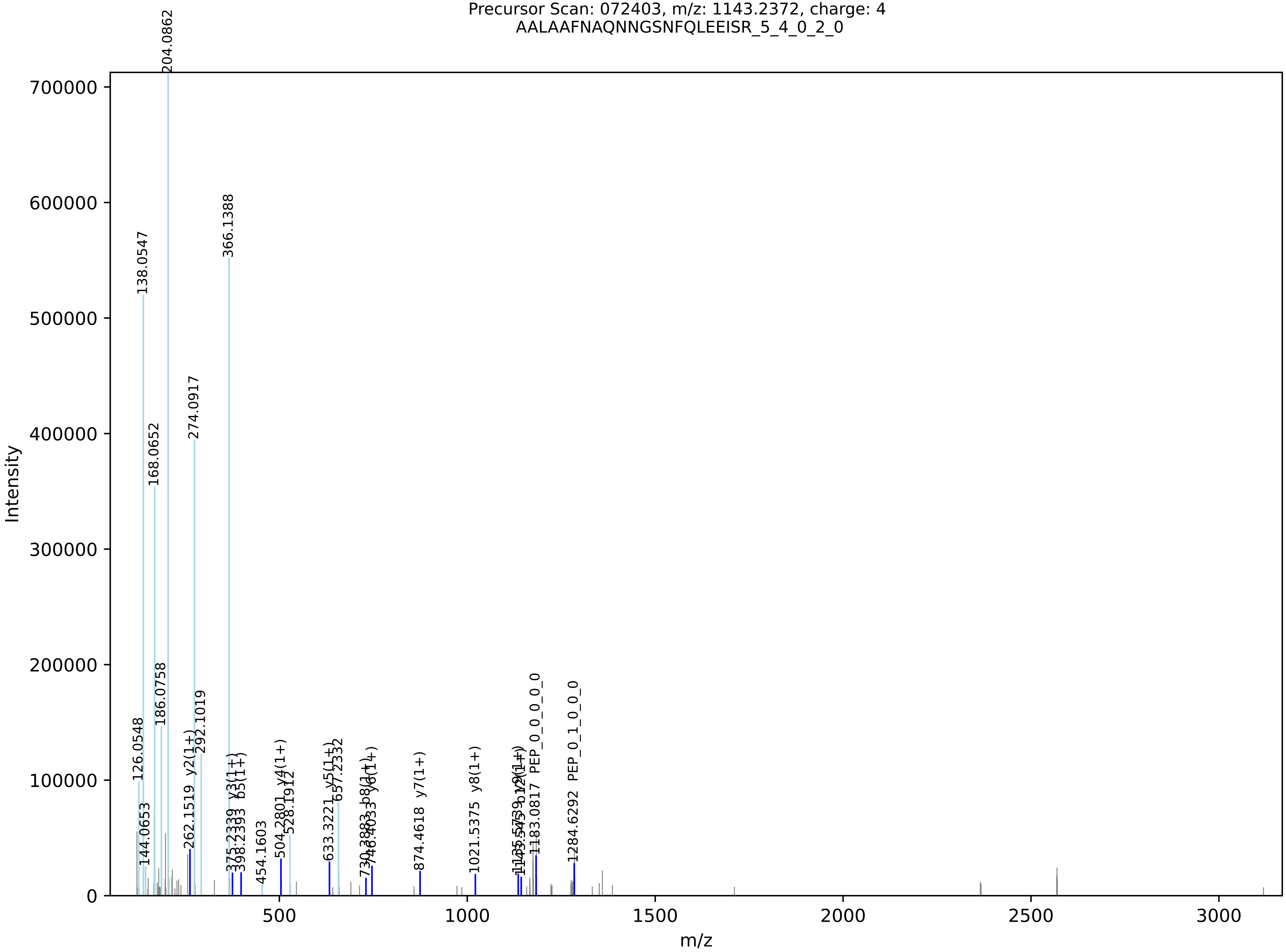

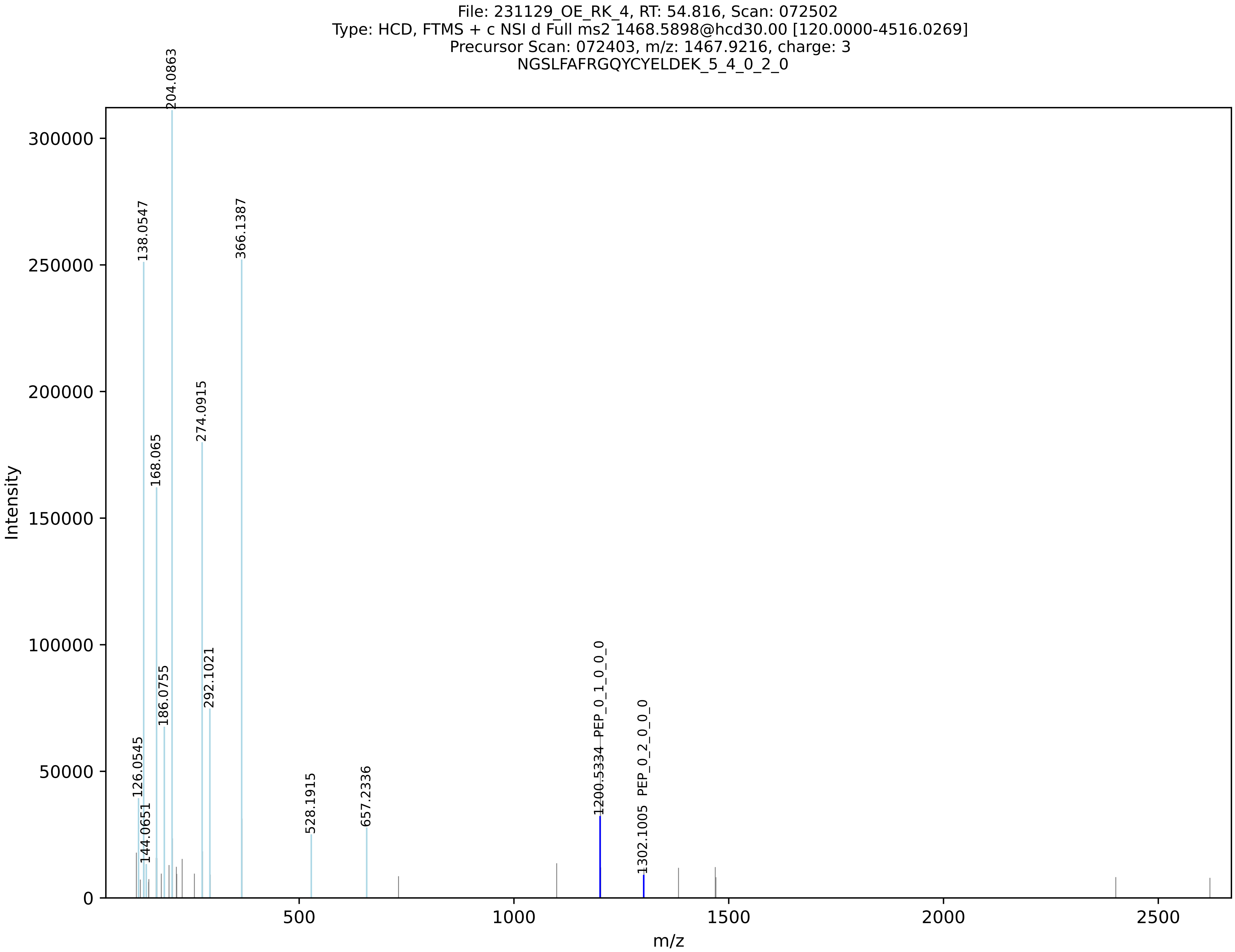

File: 231129_OE_RK_4, RT: 54.816, Scan: 072502
Type: HCD, FTMS + c NSI d Full ms2 1468.5898@hcd30.00 [120.0000-4516.0269]
Precursor Scan: 072403, m/z: 1467.9216, charge: 3
NGSLFAFRGQYCYELDEK_5_4_0_2_0
Intensity
m/z
204.0863
138.0547
366.1387
274.0915
168.065
292.1021
186.0755
126.0545
144.0651
528.1915
657.2336
1200.5334_PEP_0_1_0_0_0
1302.1005_PEP_0_2_0_0_0
0
50000
100000
150000
200000
250000
300000
500
1000
1500
2000
2500

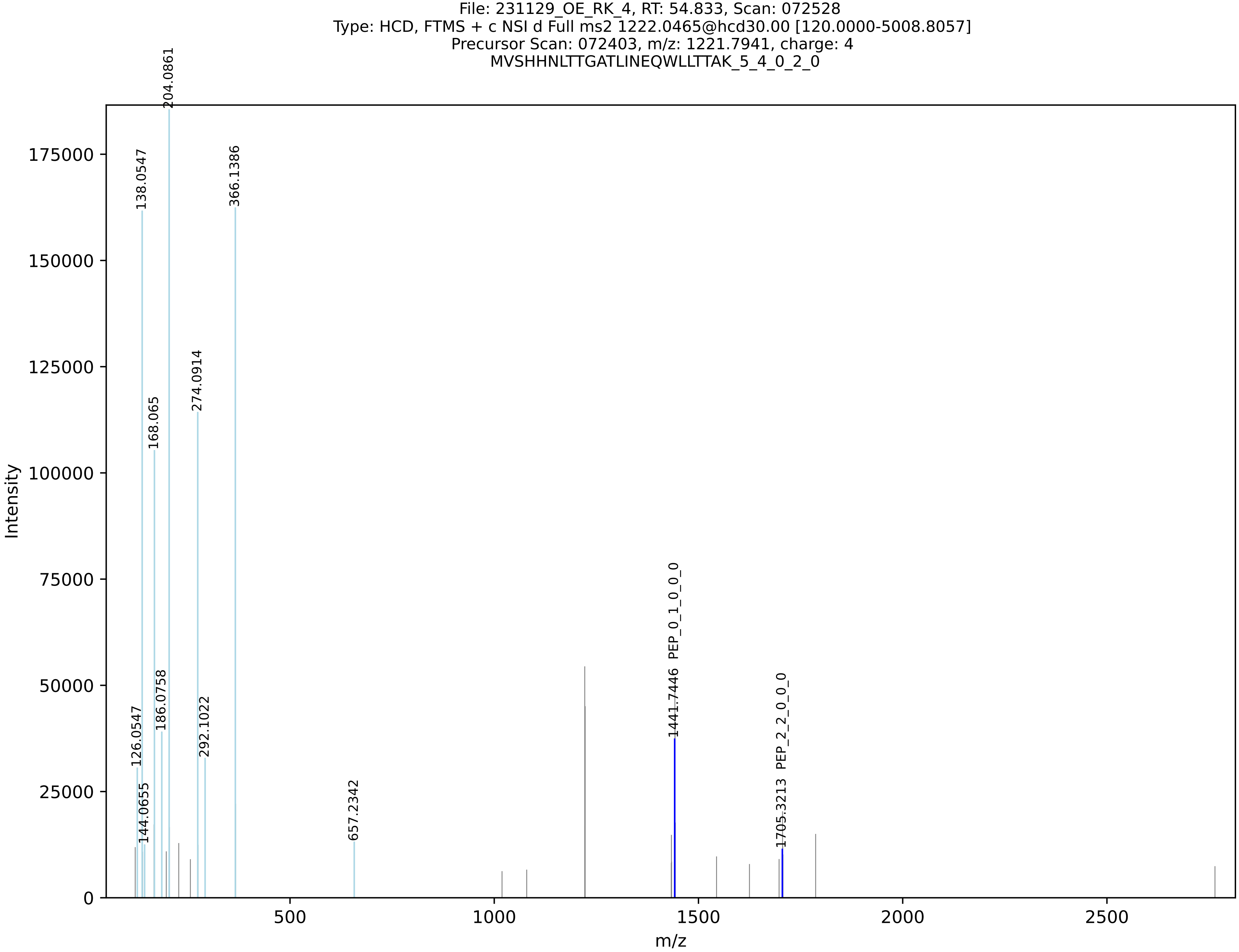

File: 231129_OE_RK_4, RT: 54.833, Scan: 072528
Type: HCD, FTMS + c NSI d Full ms2 1222.0465@hcd30.00 [120.0000-5008.8057]
Precursor Scan: 072403, m/z: 1221.7941, charge: 4
MVSHHNLTTGATLINEQWLLTTAK_5_4_0_2_0
Intensity
m/z
204.0861
138.0547
366.1386
274.0914
168.065
186.0758
126.0547
292.1022
144.0655
657.2342
1441.7446 PEP_0_1_0_0_0
1705.3213 PEP_2_2_0_0_0

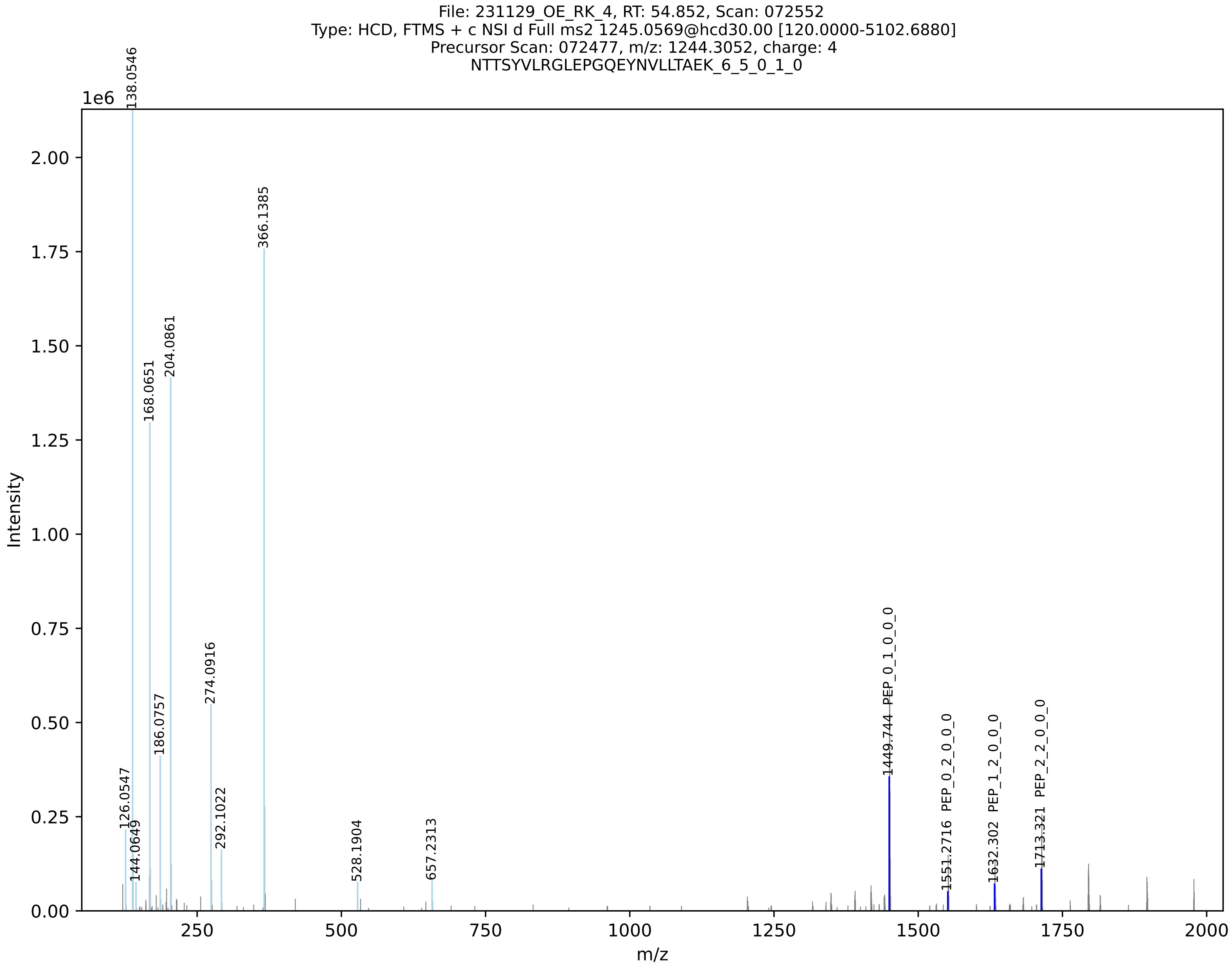
File: 231129_OE_RK_4, RT: 54.852, Scan: 072552
Type: HCD, FTMS + c NSI d Full ms2 1245.0569@hcd30.00 [120.0000-5102.6880]
Precursor Scan: 072477, m/z: 1244.3052, charge: 4
NTTSYVLRGLEPGQEYNVLLTAEK_6_5_0_1_0
1e6
Intensity
m/z
138.0546
366.1385
204.0861
168.0651
274.0916
186.0757
126.0547
144.0649
292.1022
528.1904
657.2313
1449.744 PEP_0_1_0_0_0
1551.2716 PEP_0_2_0_0_0
1632.302 PEP_1_2_0_0_0
1713.321 PEP_2_2_0_0_0

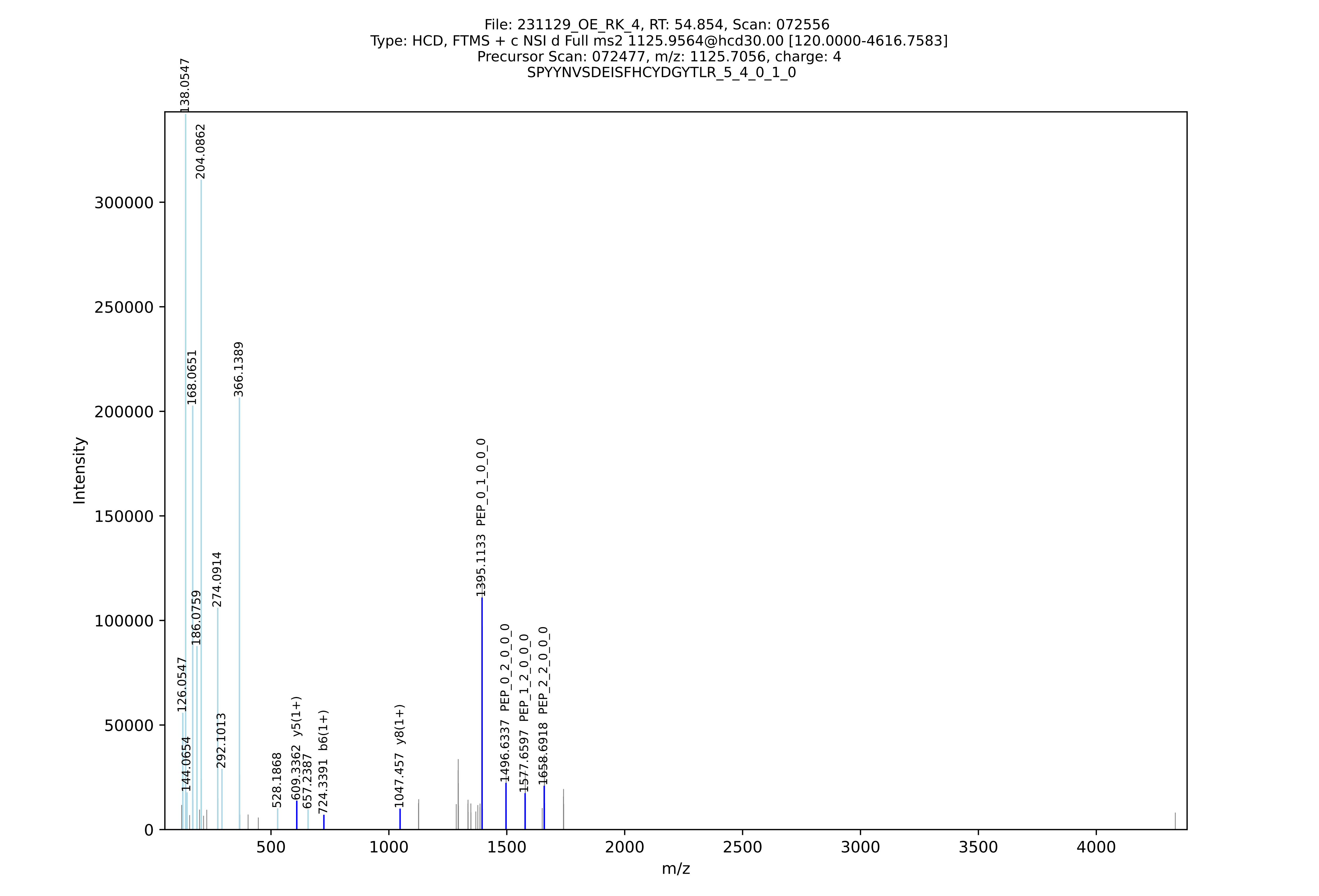
File: 231129_OE_RK_4, RT: 54.854, Scan: 072556
Type: HCD, FTMS + c NSI d Full ms2 1125.9564@hcd30.00 [120.0000-4616.7583]
Precursor Scan: 072477, m/z: 1125.7056, charge: 4
SPYYNVSDEISFHCYDGYTLR_5_4_0_1_0
138.0547
204.0862
168.0651
366.1389
274.0914
186.0759
126.0547
144.0654
292.1013
528.1868
609.3362 y5(1+)
657.2387
724.3391 b6(1+)
1047.457 y8(1+)
1395.1133 PEP_0_1_0_0_0
1496.6337 PEP_0_2_0_0_0
1577.6597 PEP_1_2_0_0_0
1658.6918 PEP_2_2_0_0_0
Intensity
m/z
0
50000
100000
150000
200000
250000
300000
500
1000
1500
2000
2500
3000
3500
4000

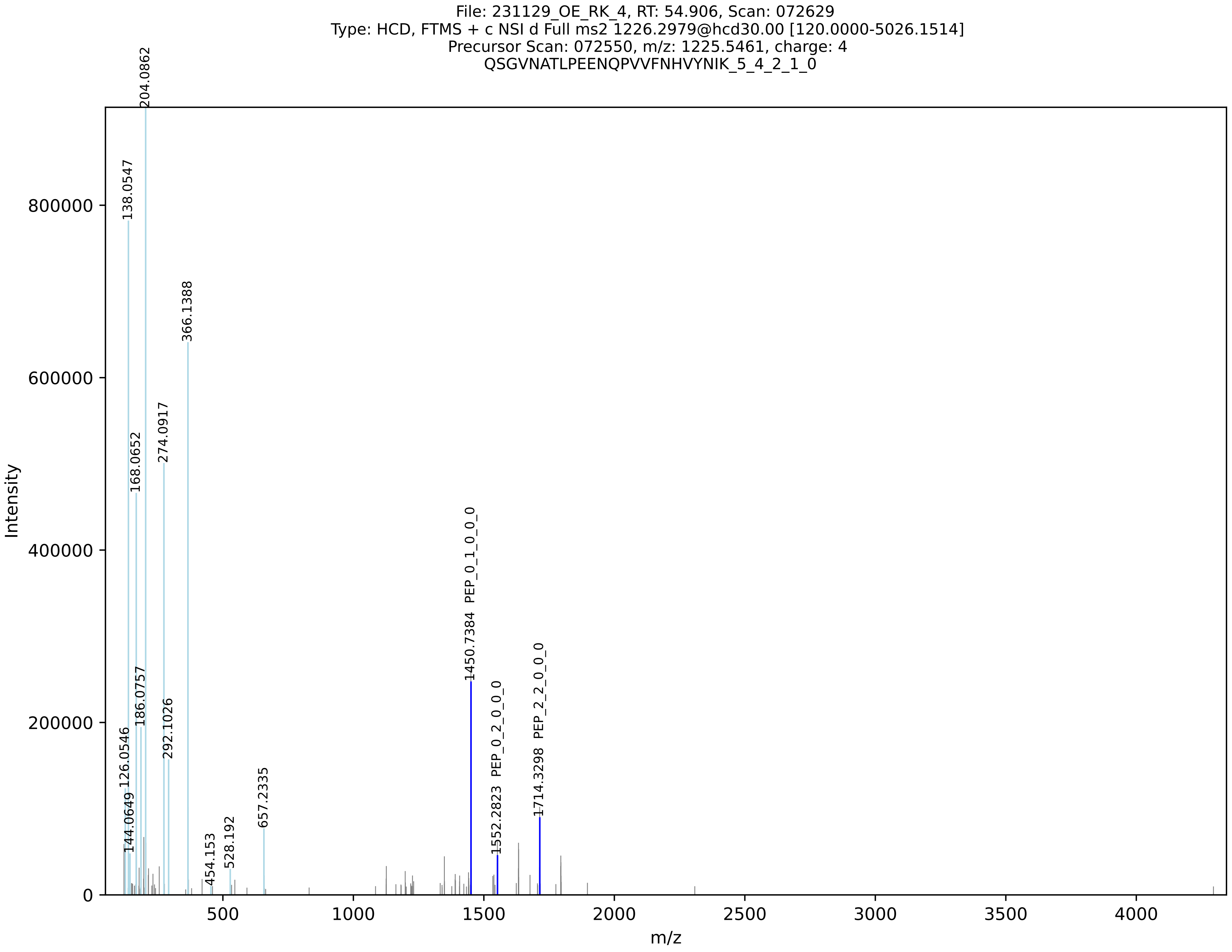

File: 231129_OE_RK_4, RT: 54.906, Scan: 072629
Type: HCD, FTMS + c NSI d Full ms2 1226.2979@hcd30.00 [120.0000-5026.1514]
Precursor Scan: 072550, m/z: 1225.5461, charge: 4
QSGVNATLPEENQPVVFNHVYNIK_5_4_2_1_0
204.0862
138.0547
366.1388
274.0917
168.0652
186.0757
292.1026
126.0546
144.0649
454.153
528.192
657.2335
1450.7384 PEP_0_1_0_0_0
1552.2823 PEP_0_2_0_0_0
1714.3298 PEP_2_2_0_0_0
Intensity
m/z
0
200000
400000
600000
800000
500
1000
1500
2000
2500
3000
3500
4000

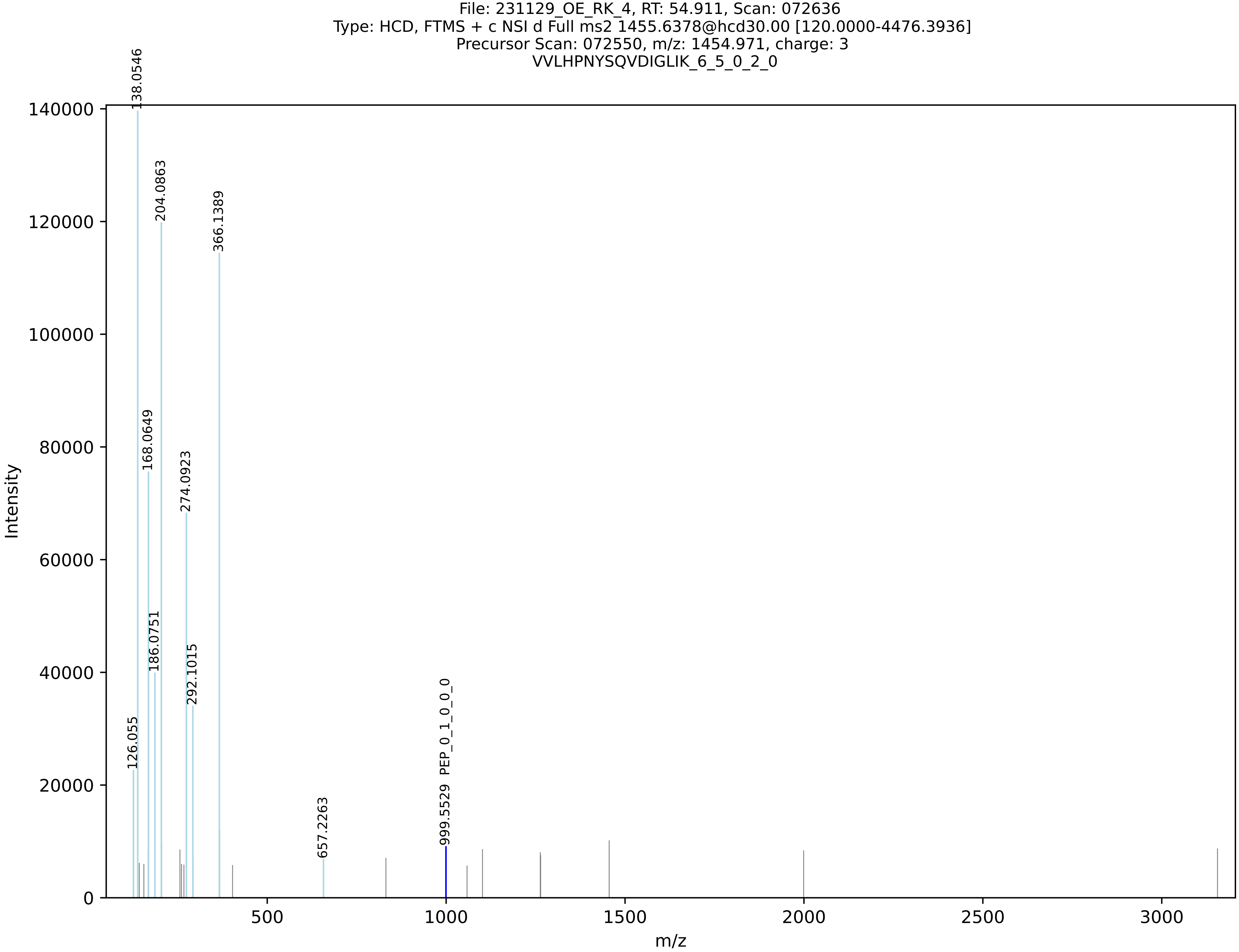
File: 231129_OE_RK_4, RT: 54.911, Scan: 072636
Type: HCD, FTMS + c NSI d Full ms2 1455.6378@hcd30.00 [120.0000-4476.3936]
Precursor Scan: 072550, m/z: 1454.971, charge: 3
VVLHPNYSQVDIGLIK_6_5_0_2_0
138.0546
204.0863
366.1389
168.0649
274.0923
186.0751
292.1015
126.055
657.2263
999.5529 PEP_0_1_0_0_0
Intensity
m/z
140000
120000
100000
80000
60000
40000
20000
0
500
1000
1500
2000
2500
3000

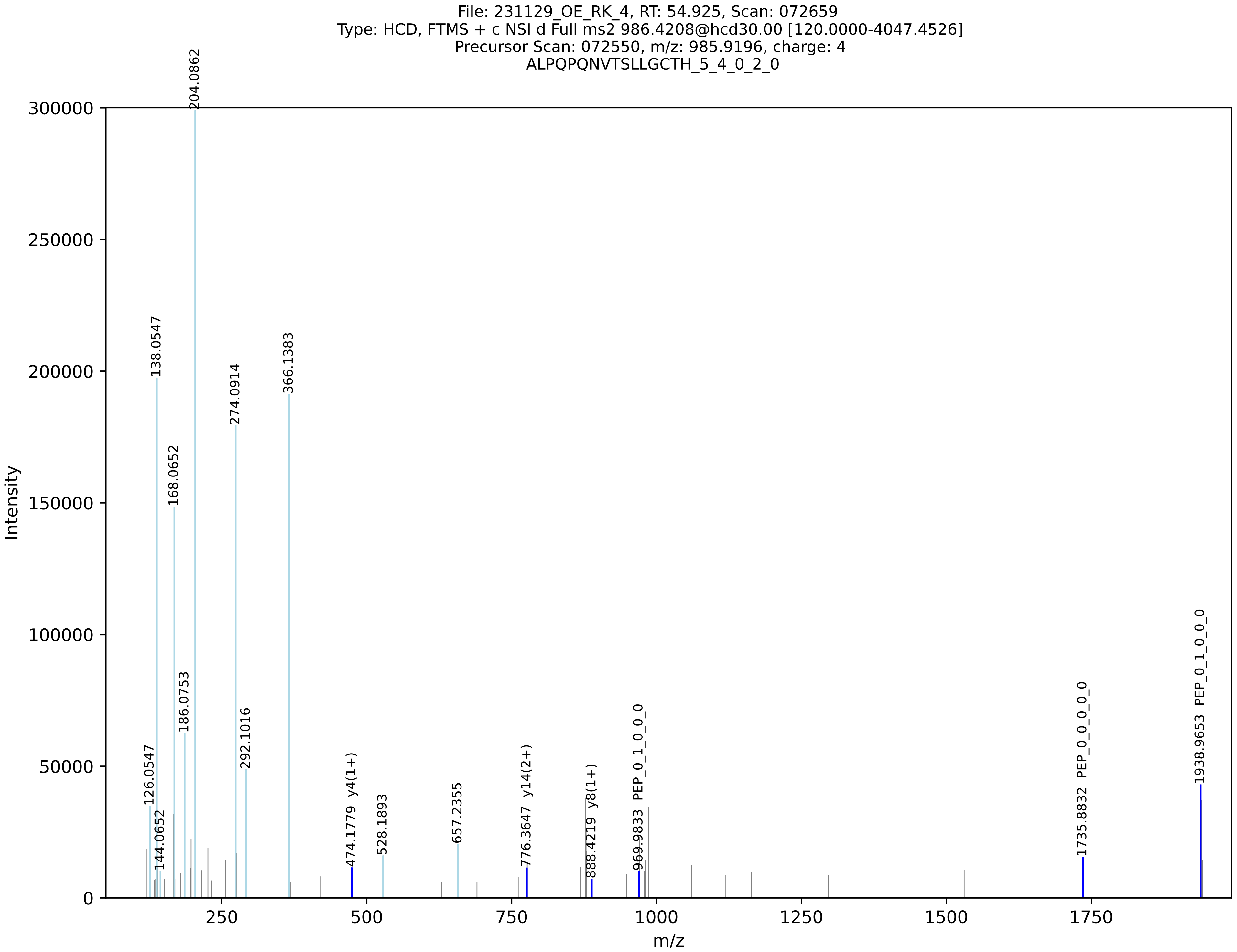

File: 231129_OE_RK_4, RT: 54.925, Scan: 072659
Type: HCD, FTMS + c NSI d Full ms2 986.4208@hcd30.00 [120.0000-4047.4526]
Precursor Scan: 072550, m/z: 985.9196, charge: 4
ALPQPQNVTSLLGCTH_5_4_0_2_0
Intensity
m/z
204.0862
138.0547
168.0652
186.0753
126.0547
144.0652
274.0914
292.1016
366.1383
474.1779 y4(1+)
528.1893
657.2355
776.3647 y14(2+)
888.4219 y8(1+)
969.9833 PEP_0_1_0_0_0
1735.8832 PEP_0_0_0_0_0
1938.9653 PEP_0_1_0_0_0

File: 231129_OE_RK_4, RT: 54.955, Scan: 072697
Type: HCD, FTMS + c NSI d Full ms2 1255.0361@hcd30.00 [120.0000-5143.4033]
Precursor Scan: 072623, m/z: 1254.2844, charge: 4
LPTQNITFQTESSVAEQEAEFQSPK_5_4_0_2_0

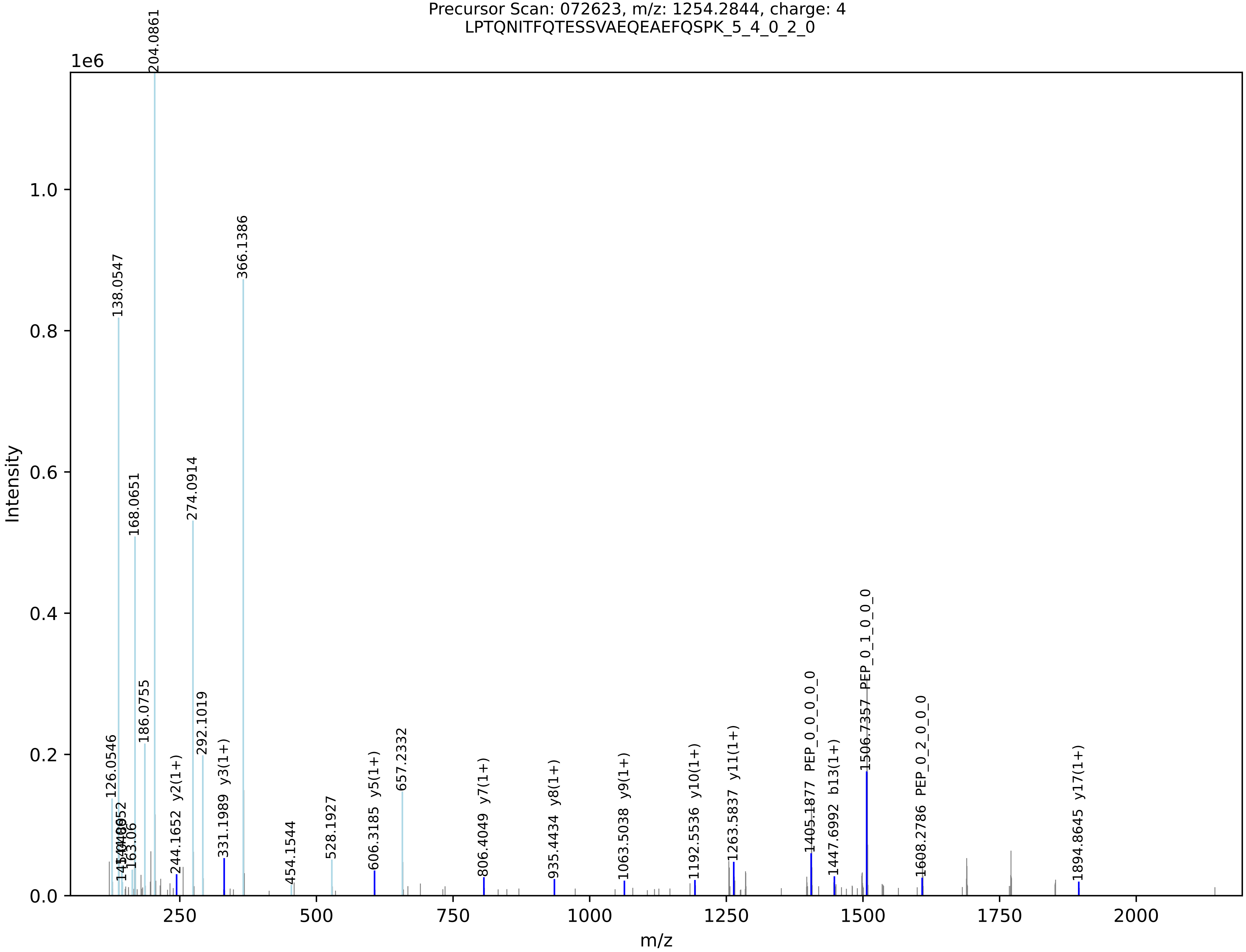

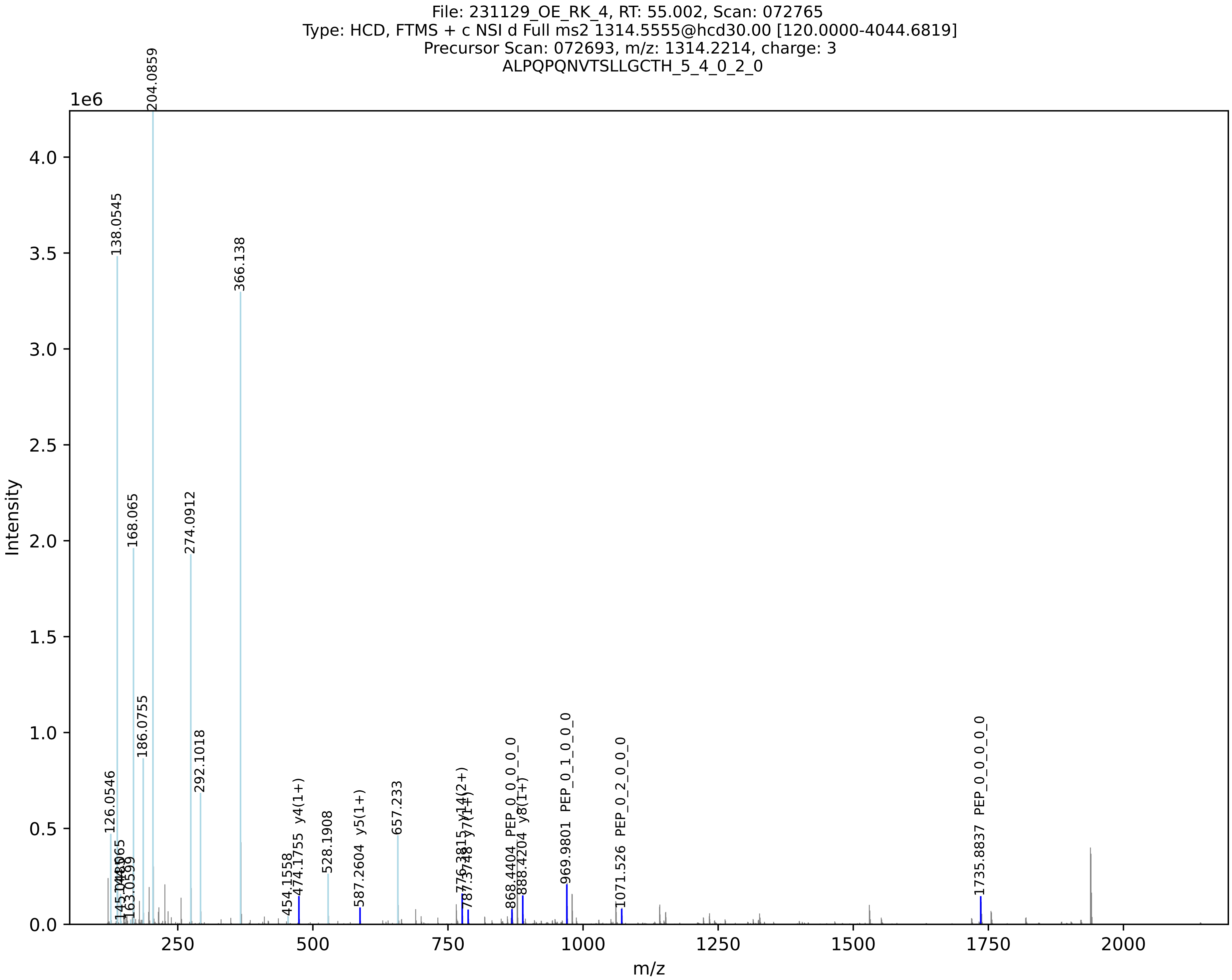
File: 231129_OE_RK_4, RT: 55.002, Scan: 072765
Type: HCD, FTMS + c NSI d Full ms2 1314.5555@hcd30.00 [120.0000-4044.6819]
Precursor Scan: 072693, m/z: 1314.2214, charge: 3
ALPQPQNVTSLLGCTH_5_4_0_2_0
1e6
Intensity
m/z
204.0859
138.0545
366.138
168.065
274.0912
186.0755
292.1018
126.0546
454.1558
474.1755 y4(1+)
528.1908
587.2604 y5(1+)
657.233
787.3748 y7(1+)
868.4404 PEP_0_0_0_0_0
888.4204 y8(1+)
969.9801 PEP_0_1_0_0_0
1071.526 PEP_0_2_0_0_0
1735.8837 PEP_0_0_0_0_0

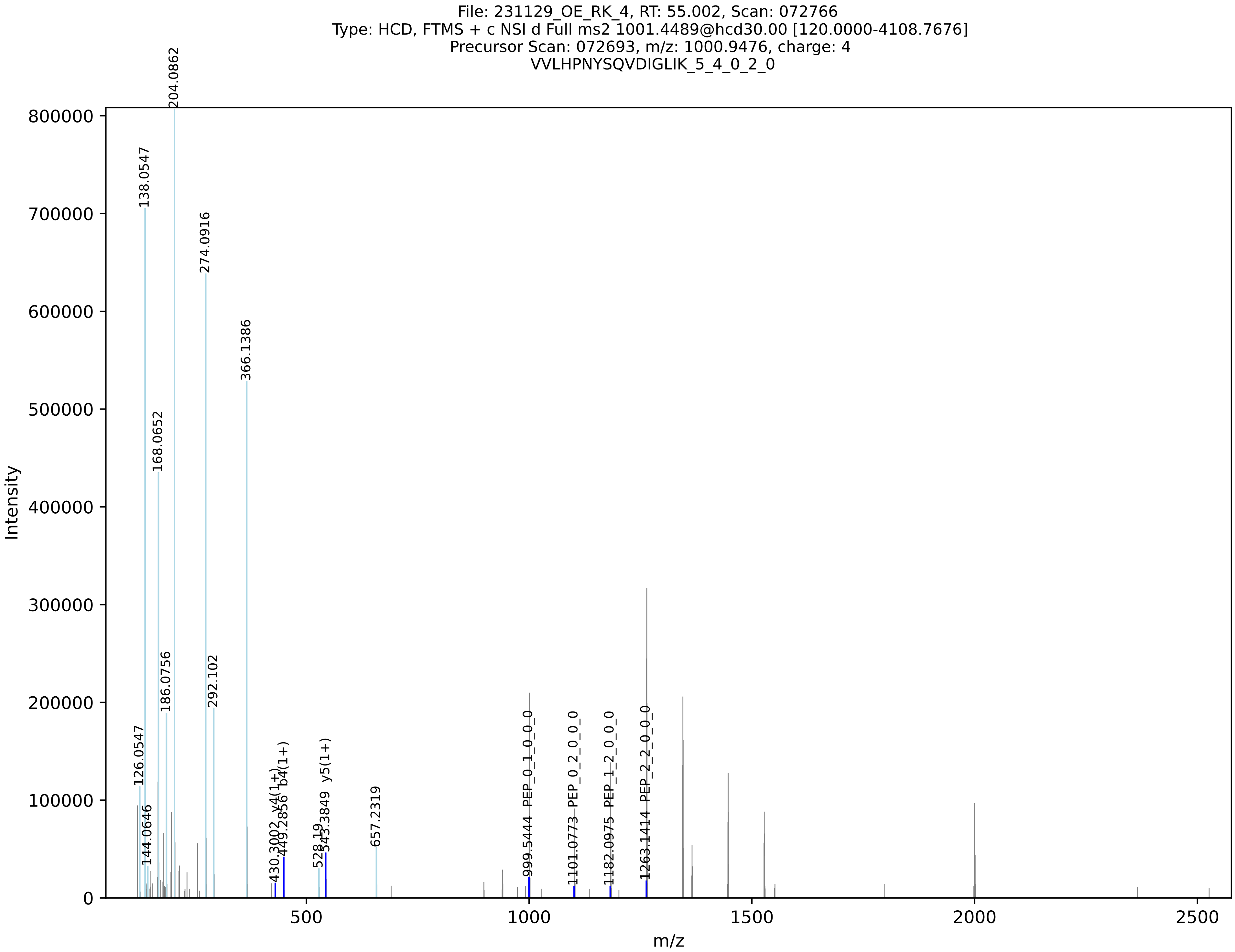
File: 231129_OE_RK_4, RT: 55.002, Scan: 072766
Type: HCD, FTMS + c NSI d Full ms2 1001.4489@hcd30.00 [120.0000-4108.7676]
Precursor Scan: 072693, m/z: 1000.9476, charge: 4
VVLHPNYSQVDIGLIK_5_4_0_2_0
Intensity
m/z
204.0862
138.0547
274.0916
366.1386
168.0652
186.0756
292.102
126.0547
144.0646
430.3002 y4(1+)
449.2856 b4(1+)
528.19
543.3849 y5(1+)
657.2319
999.5444 PEP_0_1_0_0_0_
1101.0773 PEP_0_2_0_0_0_
1182.0975 PEP_1_2_0_0_0_
1263.1414 PEP_2_2_0_0_0_
0
100000
200000
300000
400000
500000
600000
700000
800000
500
1000
1500
2000
2500

File: 231129_OE_RK_4, RT: 55.006, Scan: 072772
Type: HCD, FTMS + c NSI d Full ms2 1477.6072@hcd30.00 [120.0000-4543.6201]
Precursor Scan: 072693, m/z: 1476.9379, charge: 3
QQQHLFGSNVTDCSGNFCLFR_5_4_0_1_0

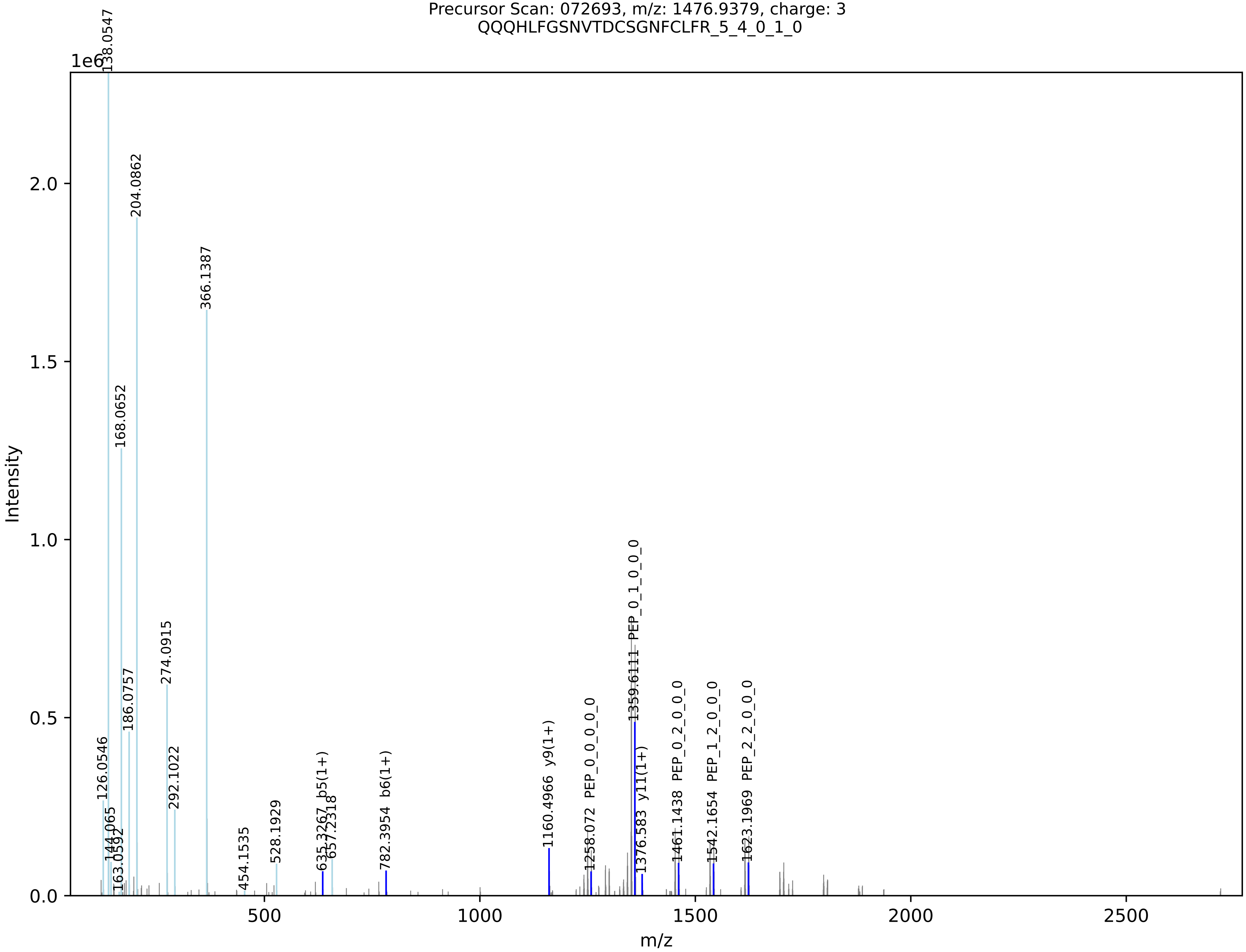

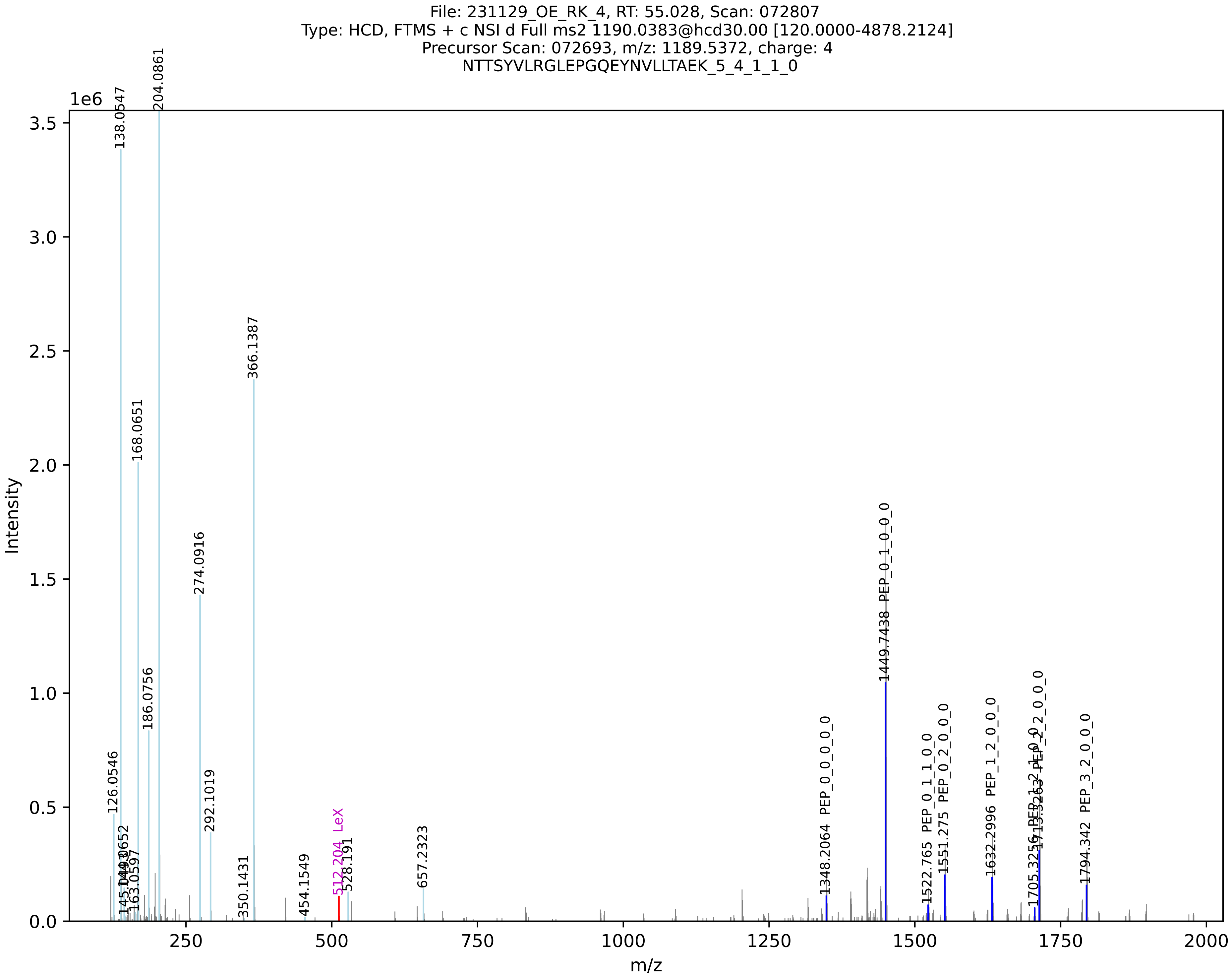
File: 231129_OE_RK_4, RT: 55.028, Scan: 072807
Type: HCD, FTMS + c NSI d Full ms2 1190.0383@hcd30.00 [120.0000-4878.2124]
Precursor Scan: 072693, m/z: 1189.5372, charge: 4
NTTSYVLRGLEPGQEYNVLLTAEK_5_4_1_1_0
1e6
Intensity
m/z
204.0861
138.0547
366.1387
168.0651
274.0916
186.0756
126.0546
292.1019
163.0597
350.1431
454.1549
512.204 LeX
528.191
657.2323
1348.2064 PEP_0_0_0_0_0
1449.7438 PEP_0_1_0_0_0
1522.765 PEP_0_1_1_0_0
1551.275 PEP_0_2_0_0_0
1632.2996 PEP_1_2_0_0_0
1794.342 PEP_3_2_0_0_0

File: 231129_OE_RK_4, RT: 55.069, Scan: 072865
Type: HCD, FTMS + c NSI d Full ms2 1586.7158@hcd30.00 [120.0000-4877.4922]
Precursor Scan: 072764, m/z: 1585.7118, charge: 3
NTTTSYVLRGLEPGQEYNVLLTAEK_5_4_1_1_0

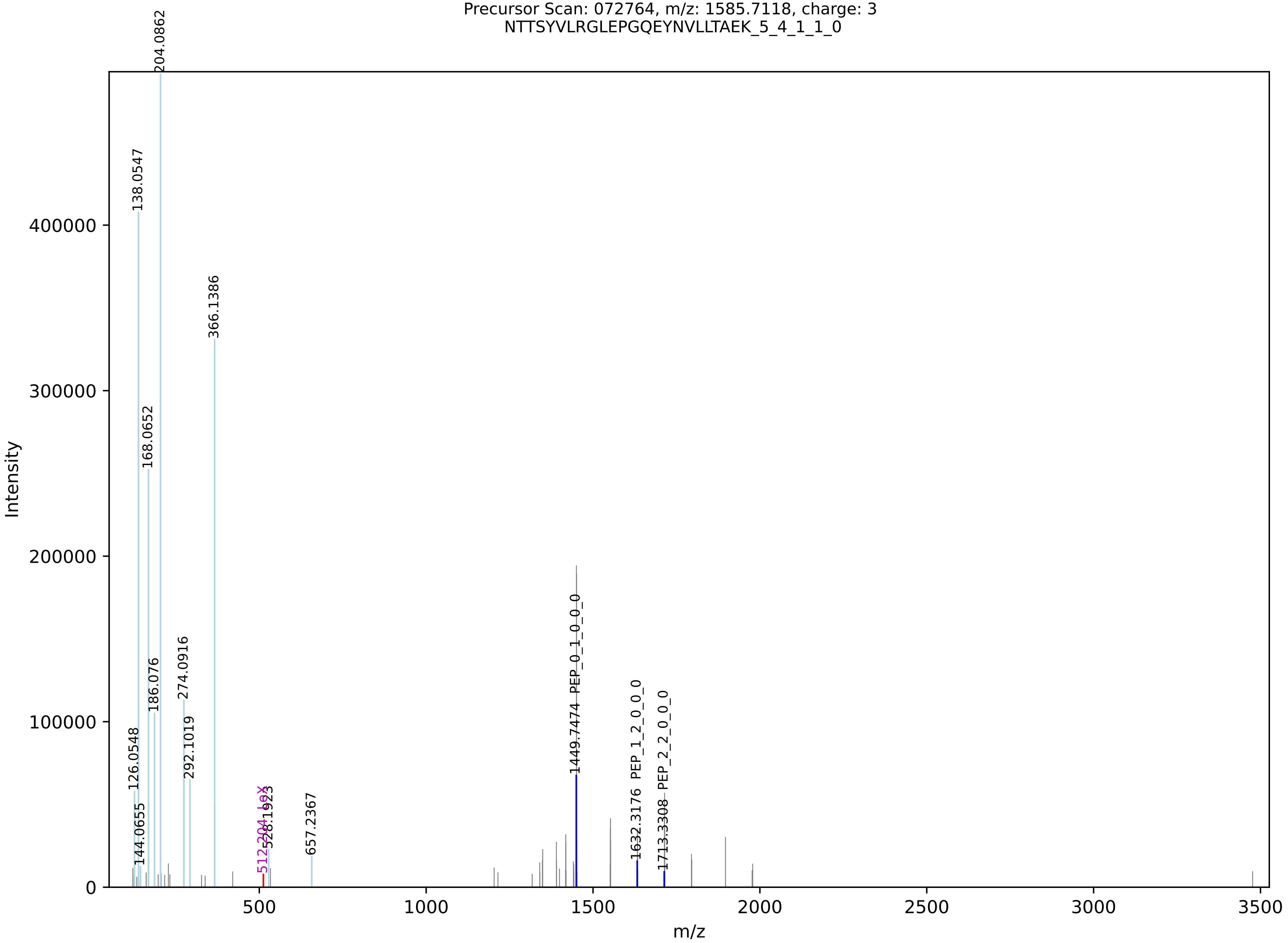

File: 231129_OE_RK_4, RT: 55.105, Scan: 072916
Type: HCD, FTMS + c NSI d Full ms2 1334.5956@hcd30.00 [120.0000-4106.0044]
Precursor Scan: 072838, m/z: 1333.9274, charge: 3
VVLHPNYSQVDIGLIK_5_4_0_2_0

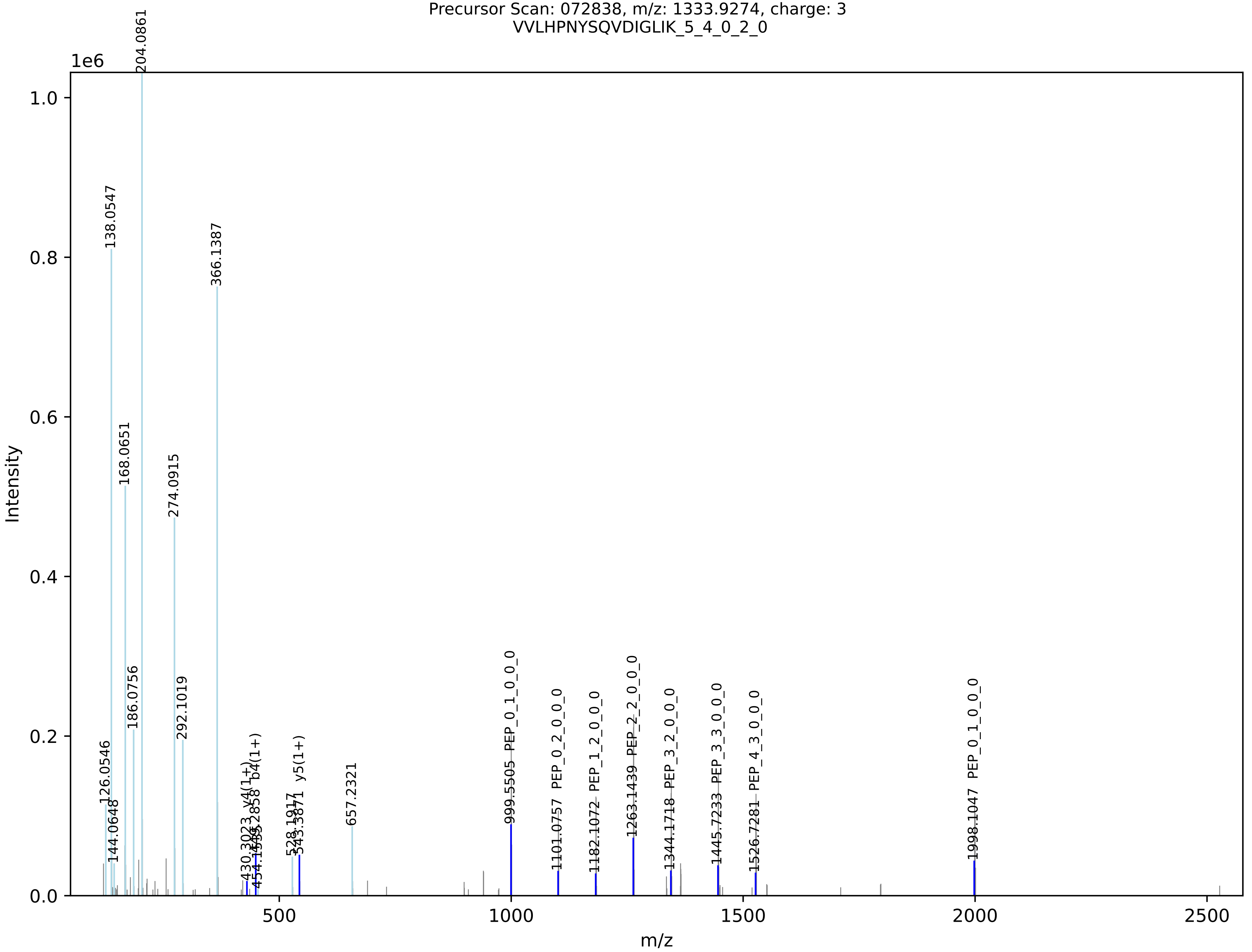

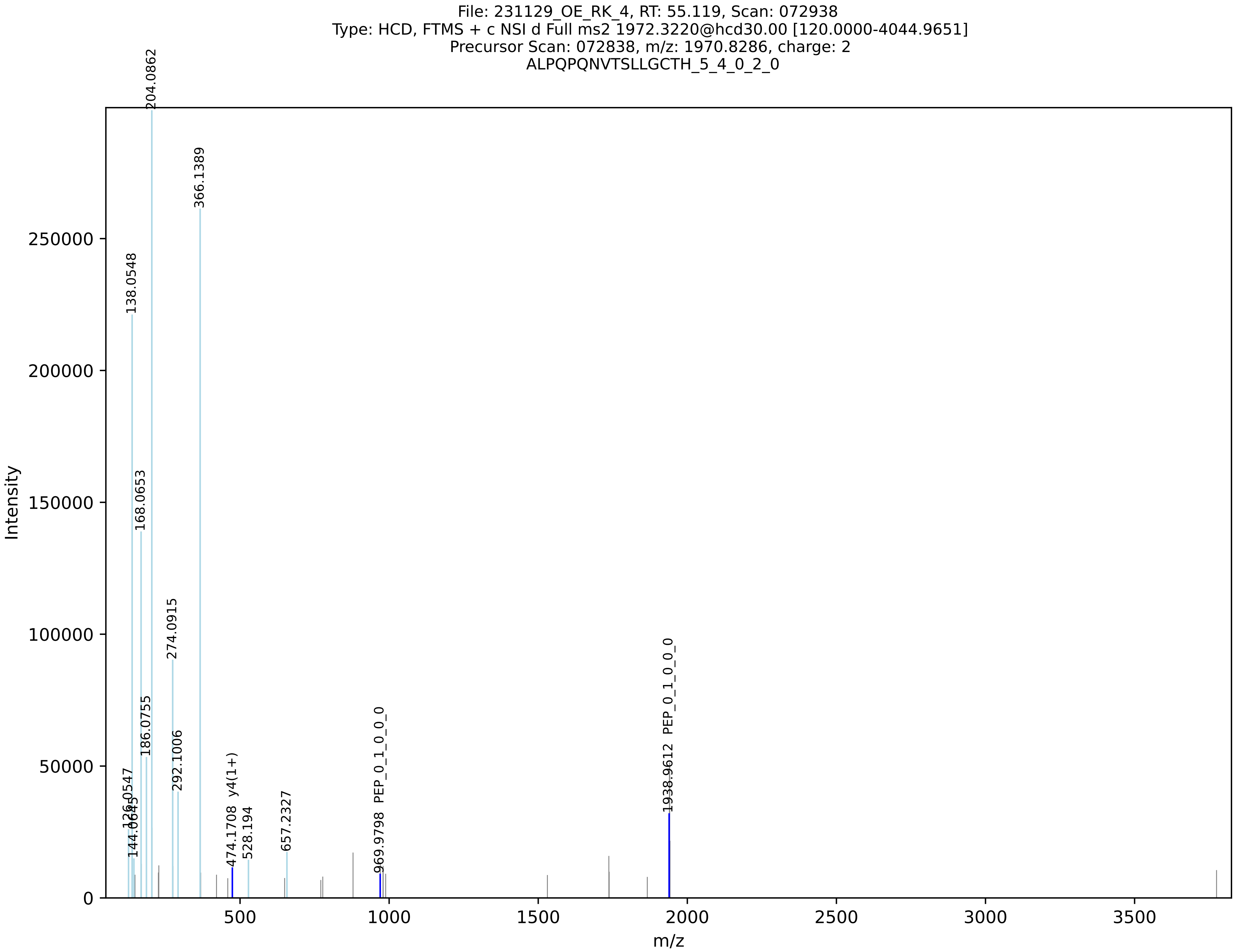
File: 231129_OE_RK_4, RT: 55.119, Scan: 072938
Type: HCD, FTMS + c NSI d Full ms2 1972.3220@hcd30.00 [120.0000-4044.9651]
Precursor Scan: 072838, m/z: 1970.8286, charge: 2
ALPQPQNVTSLLGCTH_5_4_0_2_0
Intensity
m/z
204.0862
366.1389
138.0548
168.0653
274.0915
186.0755
292.1006
126.0547
144.0645
474.1708 y4(1+)
528.194
657.2327
969.9798 PEP_0_1_0_0_0
1938.9612 PEP_0_1_0_0_0
0
50000
100000
150000
200000
250000
500
1000
1500
2000
2500
3000
3500

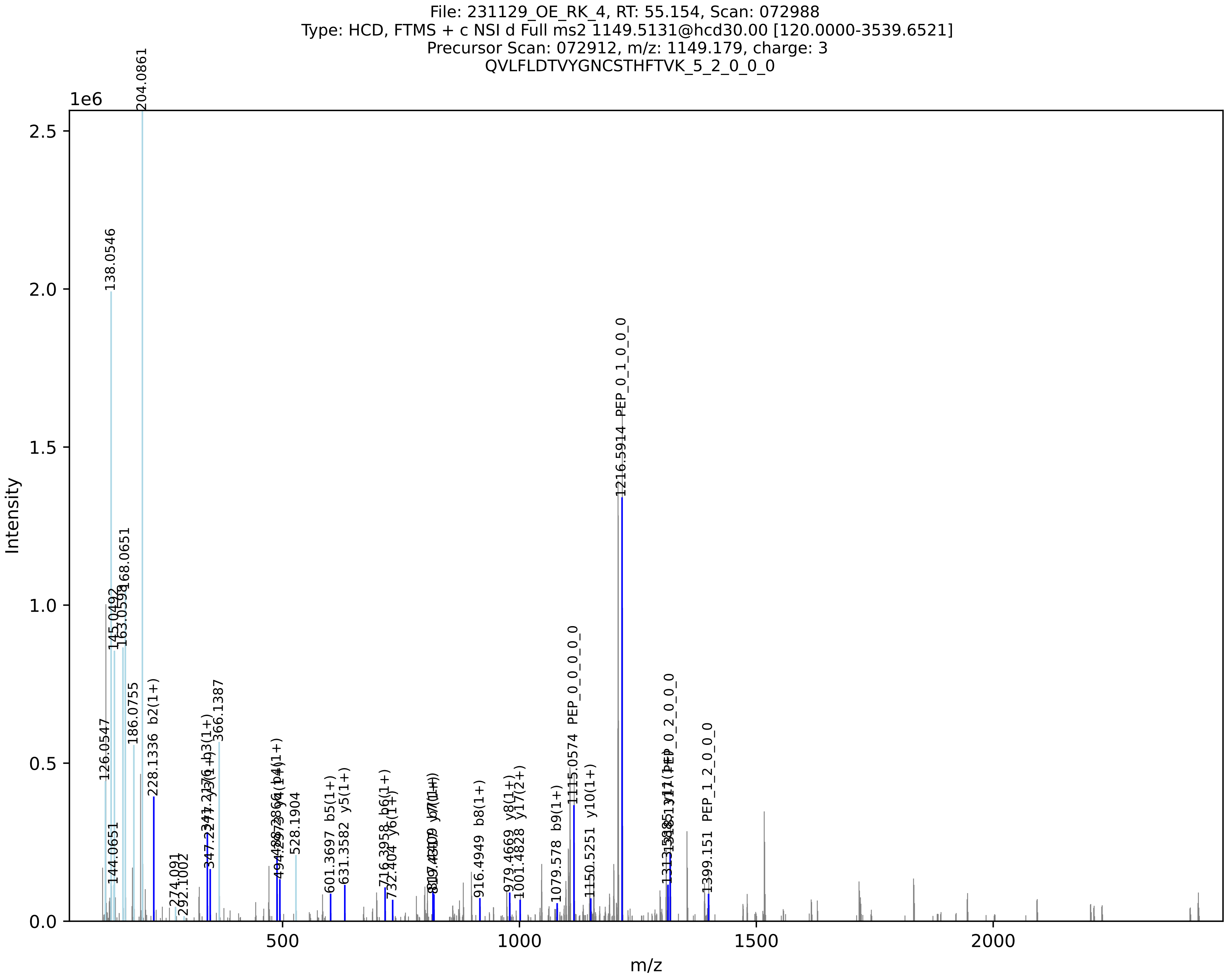

File: 231129_OE_RK_4, RT: 55.154, Scan: 072988
Type: HCD, FTMS + c NSI d Full ms2 1149.5131@hcd30.00 [120.0000-3539.6521]
Precursor Scan: 072912, m/z: 1149.179, charge: 3
QVLFLDTVYGNCSTHFTVK_5_2_0_0_0
1e6
Intensity
m/z
204.0861
138.0546
168.0651
145.0492
163.0592
126.0547
186.0755
228.1336 b2(1+)
144.0651
274.091
292.1002
366.1387
347.2277 y3(1+)
528.1904
601.3697 b5(1+)
631.3582 y5(1+)
716.3958 b6(1+)
732.404 y6(1+)
916.4949 b8(1+)
979.4669 y8(1+)
1001.4828 y17(2+)
1079.578 b9(1+)
1115.0574 PEP_0_0_0_0_0
1150.5251 y10(1+)
1216.5914 PEP_0_1_0_0_0
PEP_0_2_0_0_0
1399.151 PEP_1_2_0_0_0

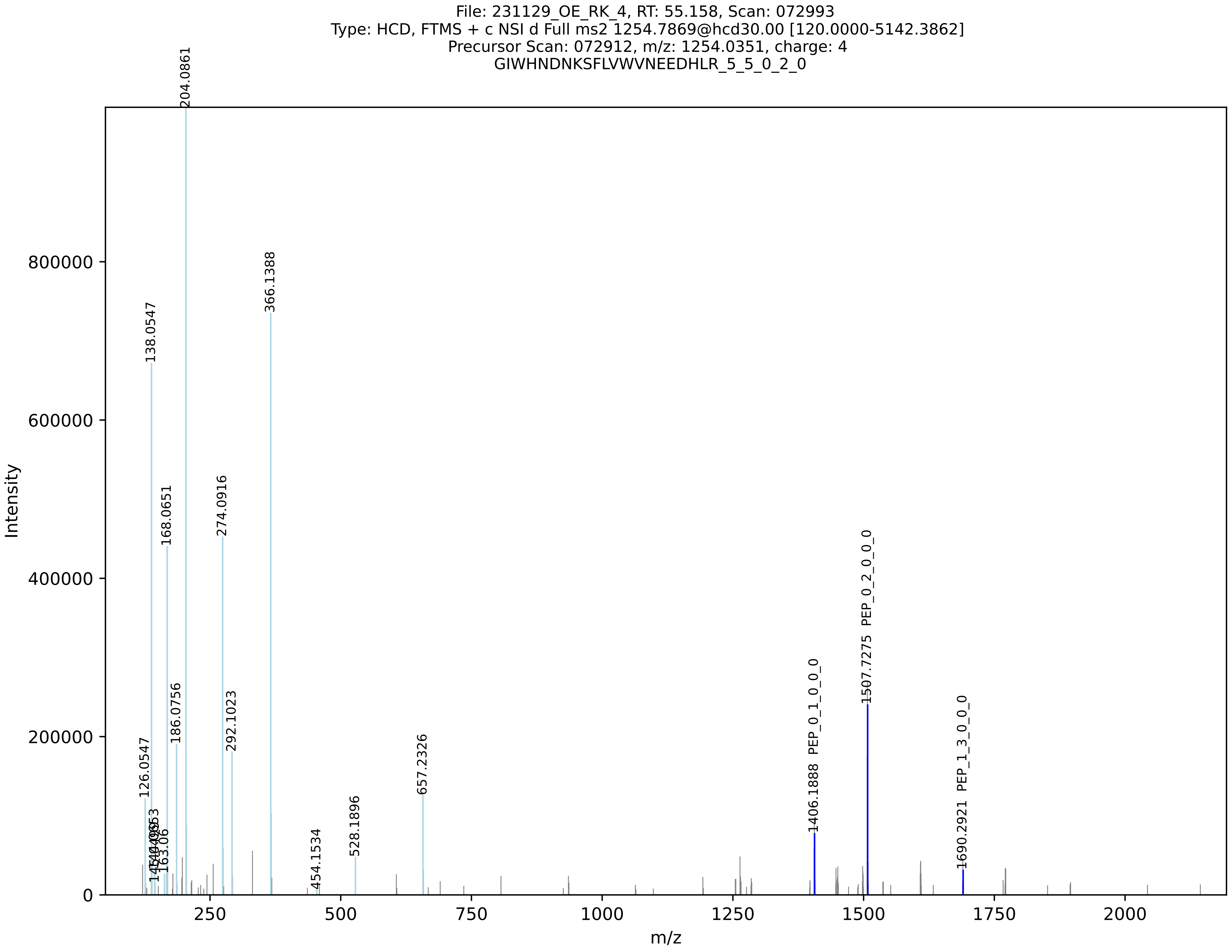
File: 231129_OE_RK_4, RT: 55.158, Scan: 072993
Type: HCD, FTMS + c NSI d Full ms2 1254.7869@hcd30.00 [120.0000-5142.3862]
Precursor Scan: 072912, m/z: 1254.0351, charge: 4
GIWHNDNKSFLVWVNEEDHLR_5_5_0_2_0
204.0861
138.0547
366.1388
168.0651
274.0916
186.0756
292.1023
126.0547
163.06
454.1534
528.1896
657.2326
1406.1888 PEP_0_1_0_0_0
1507.7275 PEP_0_2_0_0_0
1690.2921 PEP_1_3_0_0_0
Intensity
800000
600000
400000
200000
0
250
500
750
1000
1250
1500
1750
2000
m/z

File: 231129_OE_RK_4, RT: 55.183, Scan: 073034
Type: HCD, FTMS + c NSI d Full ms2 1164.2571@hcd30.00 [120.0000-4773.0249]
Precursor Scan: 072912, m/z: 1164.0112, charge: 4
EWLPLNRSVNNVVVR_6_5_0_3_0

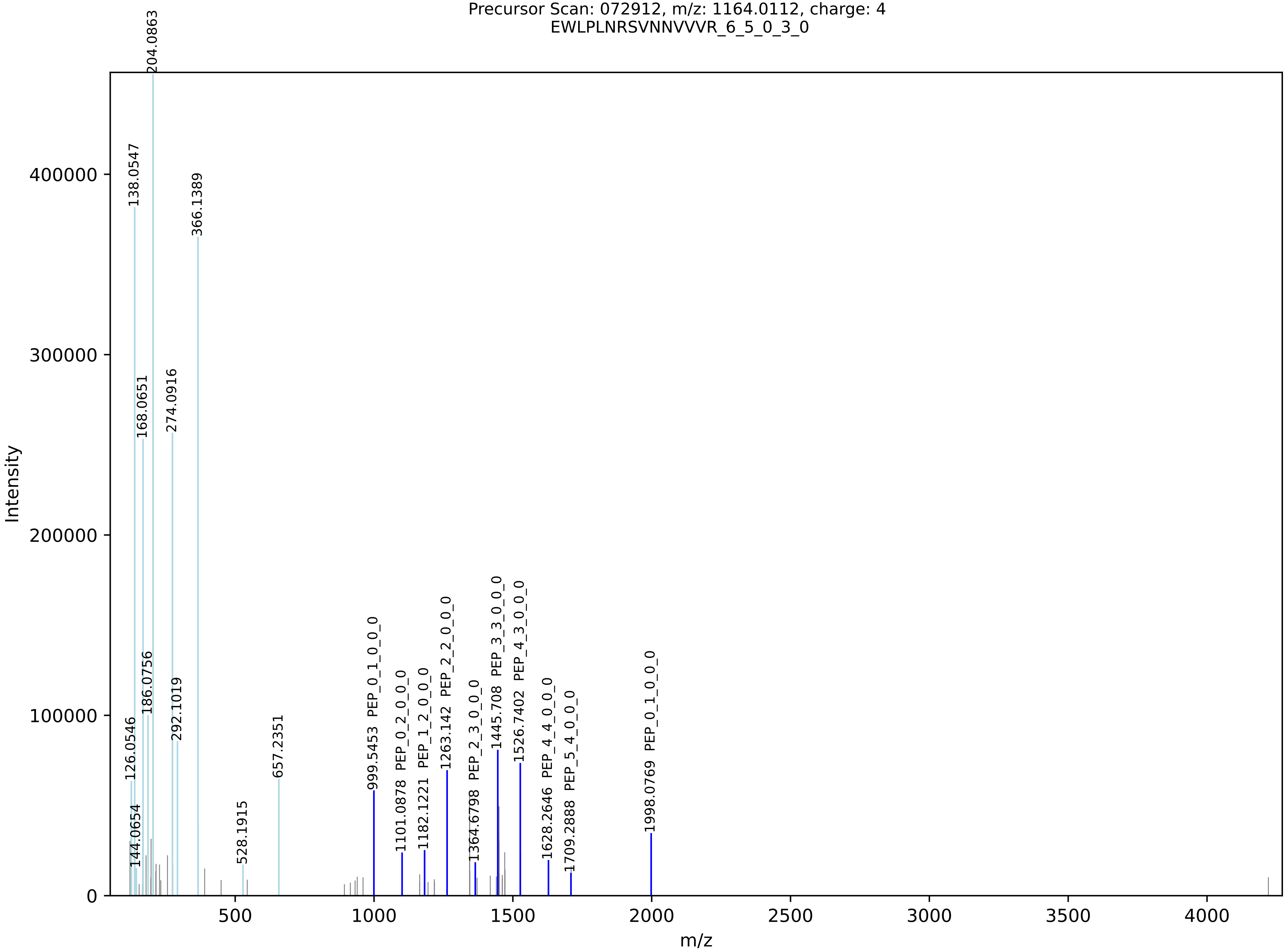

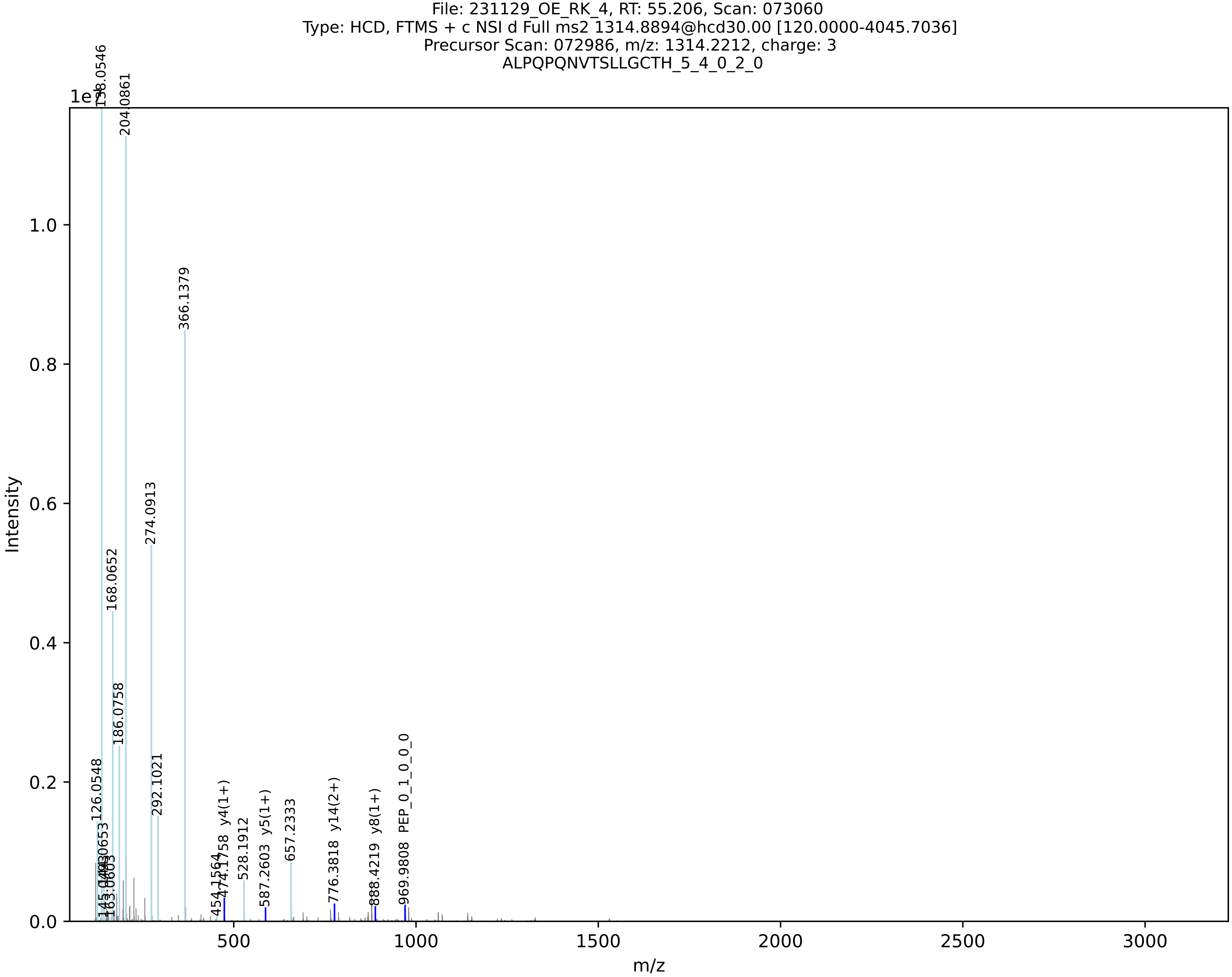
File: 231129_OE_RK_4, RT: 55.206, Scan: 073060
Type: HCD, FTMS + c NSI d Full ms2 1314.8894@hcd30.00 [120.0000-4045.7036]
Precursor Scan: 072986, m/z: 1314.2212, charge: 3
ALPQPQNVTSLLGCTH_5_4_0_2_0
1e7
138.0546
204.0861
366.1379
274.0913
168.0652
186.0758
126.0548
292.1021
474.1758 y4(1+)
528.1912
587.2603 y5(1+)
657.2333
776.3818 y14(2+)
888.4219 y8(1+)
969.9808 PEP_0_1_0_0_0
454.1564
Intensity
m/z
0.0
0.2
0.4
0.6
0.8
1.0
500
1000
1500
2000
2500
3000

File: 231129_OE_RK_4, RT: 55.206, Scan: 073061
Type: HCD, FTMS + c NSI d Full ms2 1477.9399@hcd30.00 [120.0000-4544.6382]
Precursor Scan: 072986, m/z: 1476.9374, charge: 3
QQQHLFGSNVTDCSGNFCLFR_5_4_0_1_0

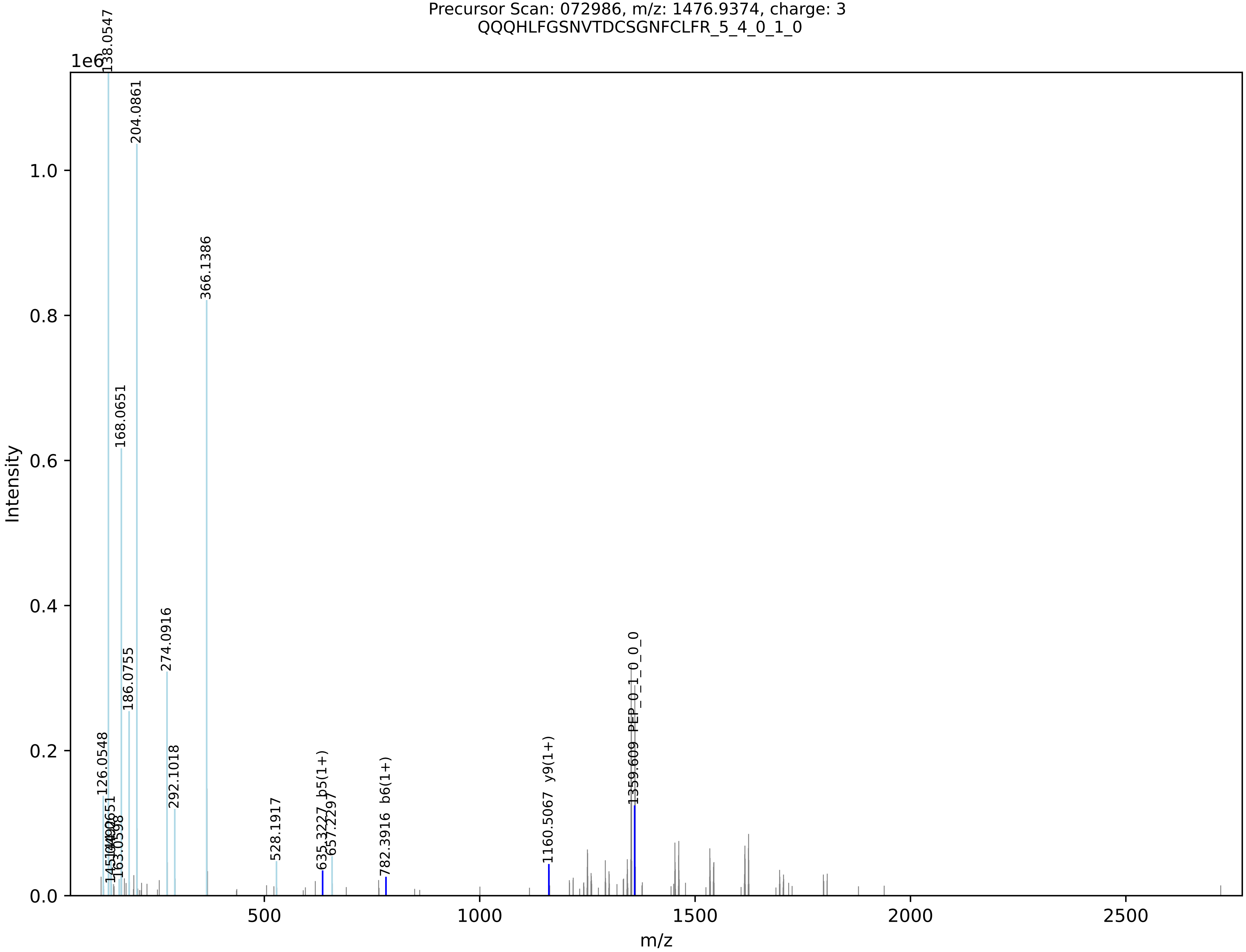

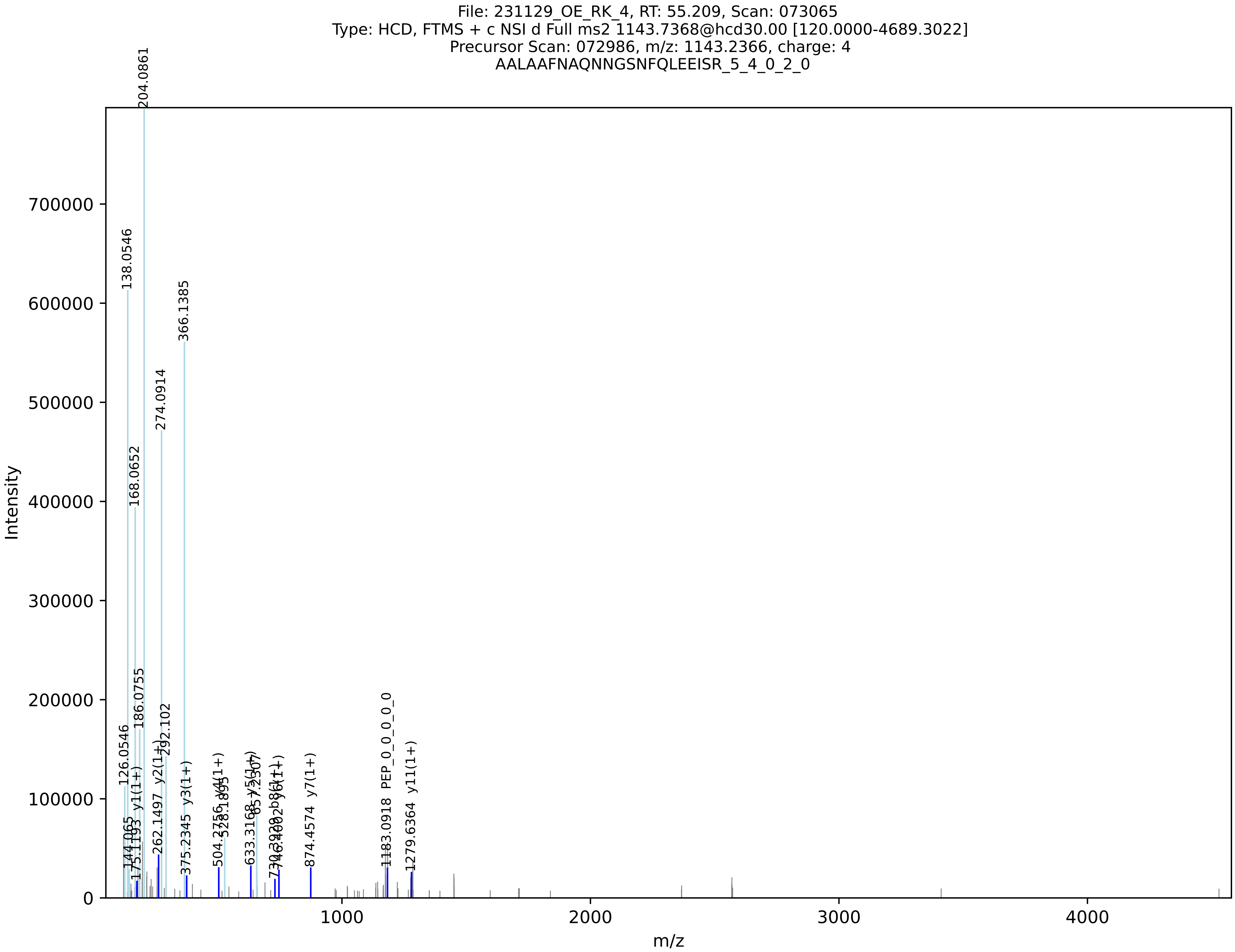
File: 231129_OE_RK_4, RT: 55.209, Scan: 073065
Type: HCD, FTMS + c NSI d Full ms2 1143.7368@hcd30.00 [120.0000-4689.3022]
Precursor Scan: 072986, m/z: 1143.2366, charge: 4
AALAAFNAQNNGSNFQLEEISR_5_4_0_2_0
Intensity
m/z
204.0861
138.0546
366.1385
274.0914
168.0652
186.0755
292.102
126.0546
144.065
175.1193 y1(1+)
262.1497 y2(1+)
375.2345 y3(1+)
504.2756 y4(1+)
528.1895
633.3168 y5(1+)
657.2507
746.4002 y6(1+)
874.4574 y7(1+)
1183.0918 PEP_0_0_0_0_0_
1279.6364 y11(1+)

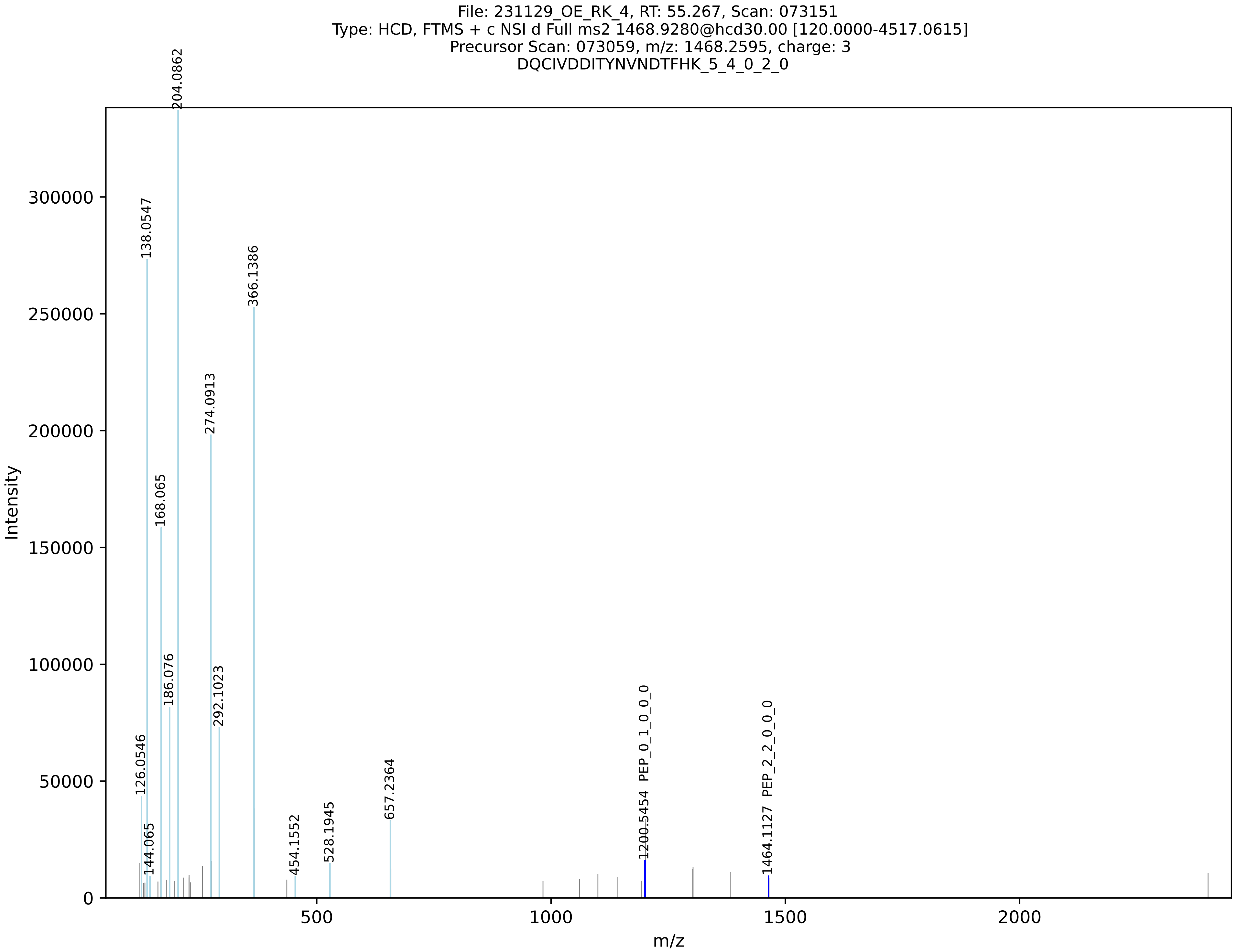
File: 231129_OE_RK_4, RT: 55.267, Scan: 073151
Type: HCD, FTMS + c NSI d Full ms2 1468.9280@hcd30.00 [120.0000-4517.0615]
Precursor Scan: 073059, m/z: 1468.2595, charge: 3
DQCIVDDITYNVNDTFHK_5_4_0_2_0
Intensity
m/z
204.0862
138.0547
366.1386
274.0913
168.065
186.076
292.1023
126.0546
144.065
454.1552
528.1945
657.2364
1200.5454 PEP_0_1_0_0_0
1464.1127 PEP_2_2_0_0_0
0
50000
100000
150000
200000
250000
300000
500
1000
1500
2000

File: 231129_OE_RK_4, RT: 55.31, Scan: 073210
Type: HCD, FTMS + c NSI d Full ms2 1202.0243@hcd30.00 [120.0000-4927.1152]
Precursor Scan: 073130, m/z: 1201.2726, charge: 4
VVLHPNYSQVDIGLIK_6_5_1_3_0

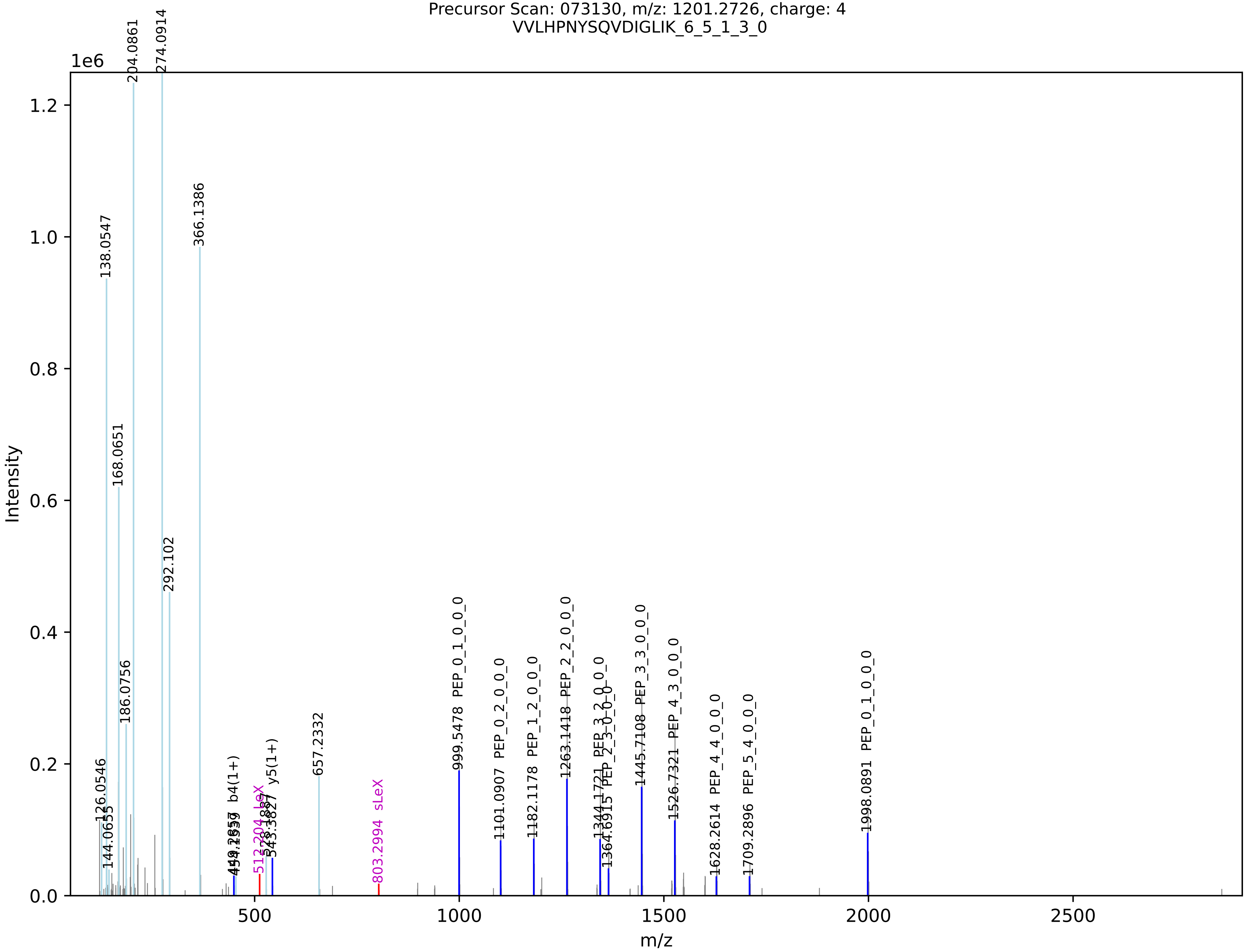

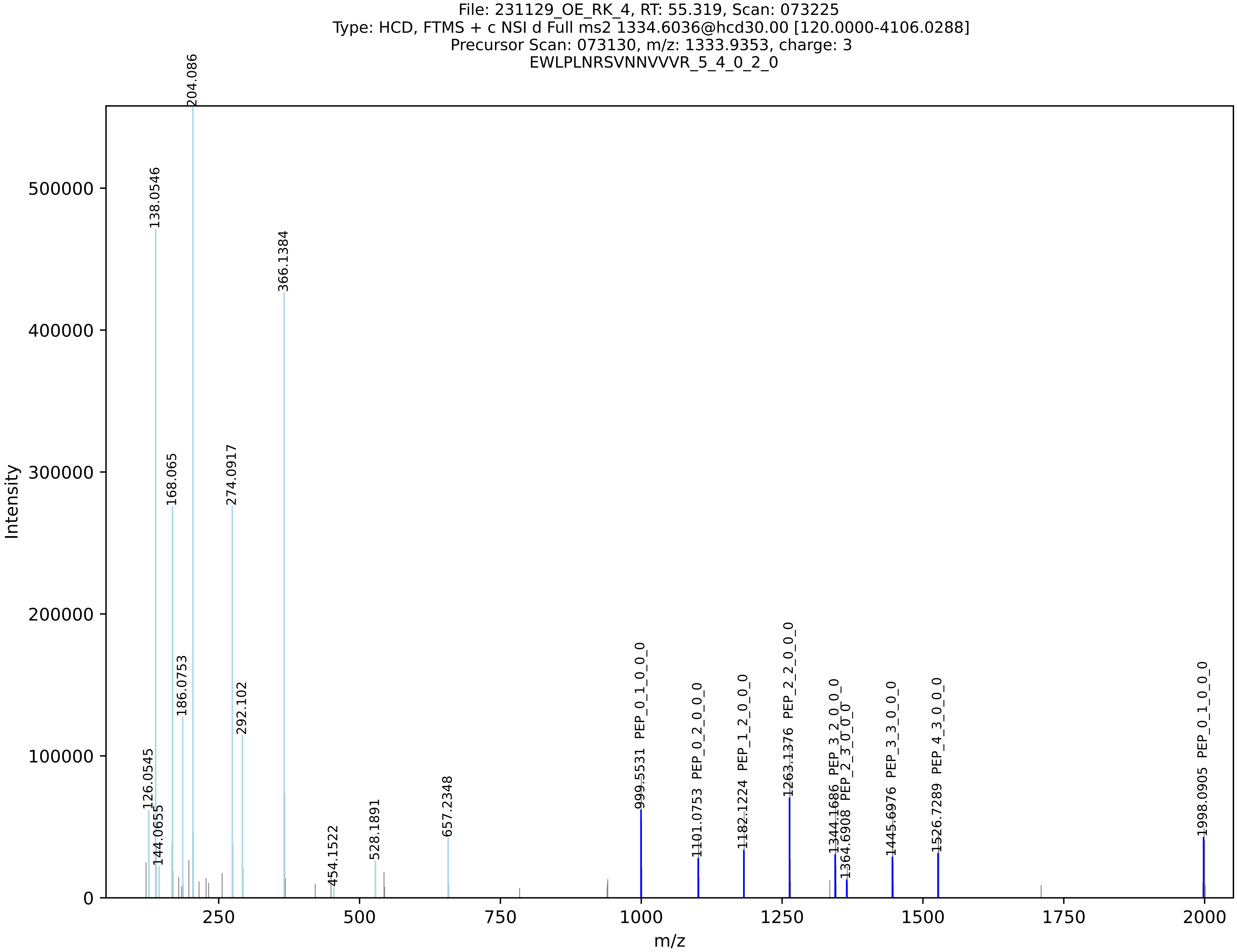

File: 231129_OE_RK_4, RT: 55.319, Scan: 073225
Type: HCD, FTMS + c NSI d Full ms2 1334.6036@hcd30.00 [120.0000-4106.0288]
Precursor Scan: 073130, m/z: 1333.9353, charge: 3
EWLPLNRSVNNVVVR_5_4_0_2_0
Intensity
m/z
204.086
138.0546
366.1384
168.065
274.0917
186.0753
292.102
126.0545
144.0655
454.1522
528.1891
657.2348
999.5531 PEP_0_1_0_0_0
1101.0753 PEP_0_2_0_0_0
1182.1224 PEP_1_2_0_0_0
1263.1376 PEP_2_2_0_0_0
1344.1686 PEP_3_2_0_0_0
1364.6908 PEP_2_3_0_0_0
1445.6976 PEP_3_3_0_0_0
1526.7289 PEP_4_3_0_0_0
1998.0905 PEP_0_1_0_0_

File: 231129_OE_RK_4, RT: 55.358, Scan: 073280
Type: HCD, FTMS + c NSI d Full ms2 1567.3282@hcd30.00 [120.0000-4818.1665]
Precursor Scan: 073204, m/z: 1566.6602, charge: 3
VEPNYNITIFVDTFQSEK_5_5_1_2_0

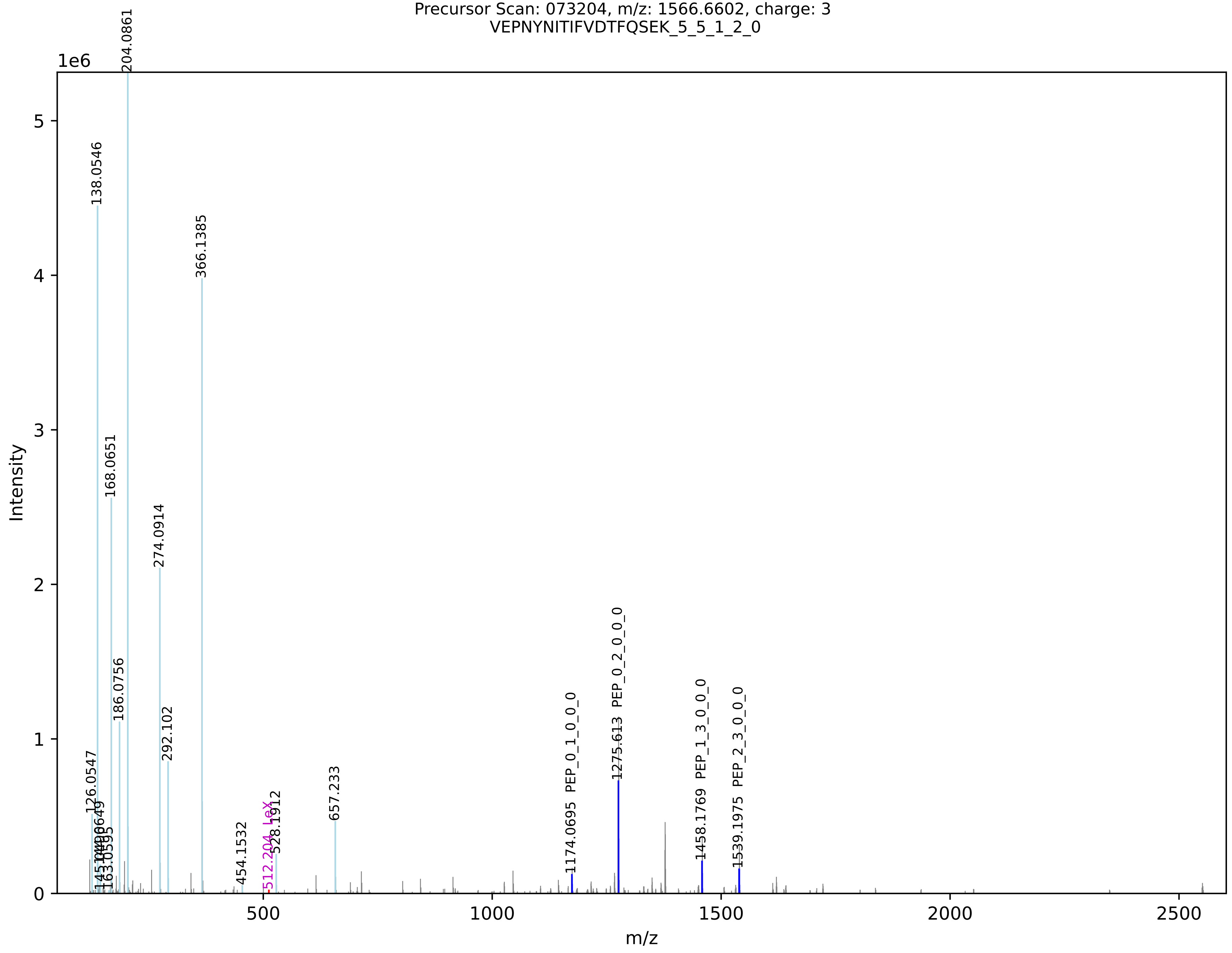

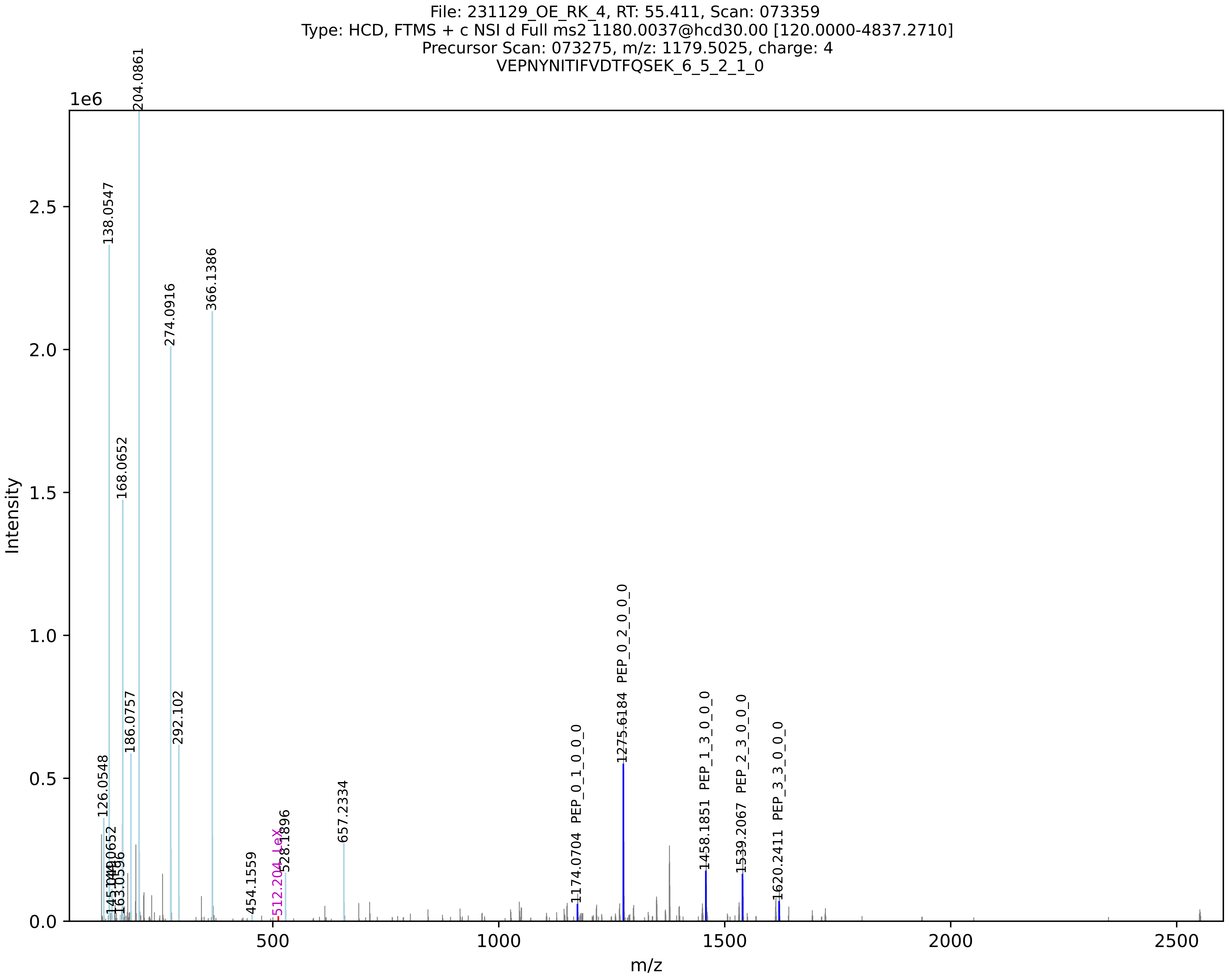

File: 231129_OE_RK_4, RT: 55.411, Scan: 073359
Type: HCD, FTMS + c NSI d Full ms2 1180.0037@hcd30.00 [120.0000-4837.2710]
Precursor Scan: 073275, m/z: 1179.5025, charge: 4
VEPNYNITIFVDTFQSEK_6_5_2_1_0
1e6
Intensity
m/z
204.0861
138.0547
366.1386
274.0916
168.0652
186.0757
292.102
126.0548
163.0596
454.1559
512.204 LeX
528.1896
657.2334
1174.0704 PEP_0_1_0_0_0
1275.6184 PEP_0_2_0_0_0
1458.1851 PEP_1_3_0_0_0
1539.2067 PEP_2_3_0_0_0
1620.2411 PEP_3_3_0_0_0

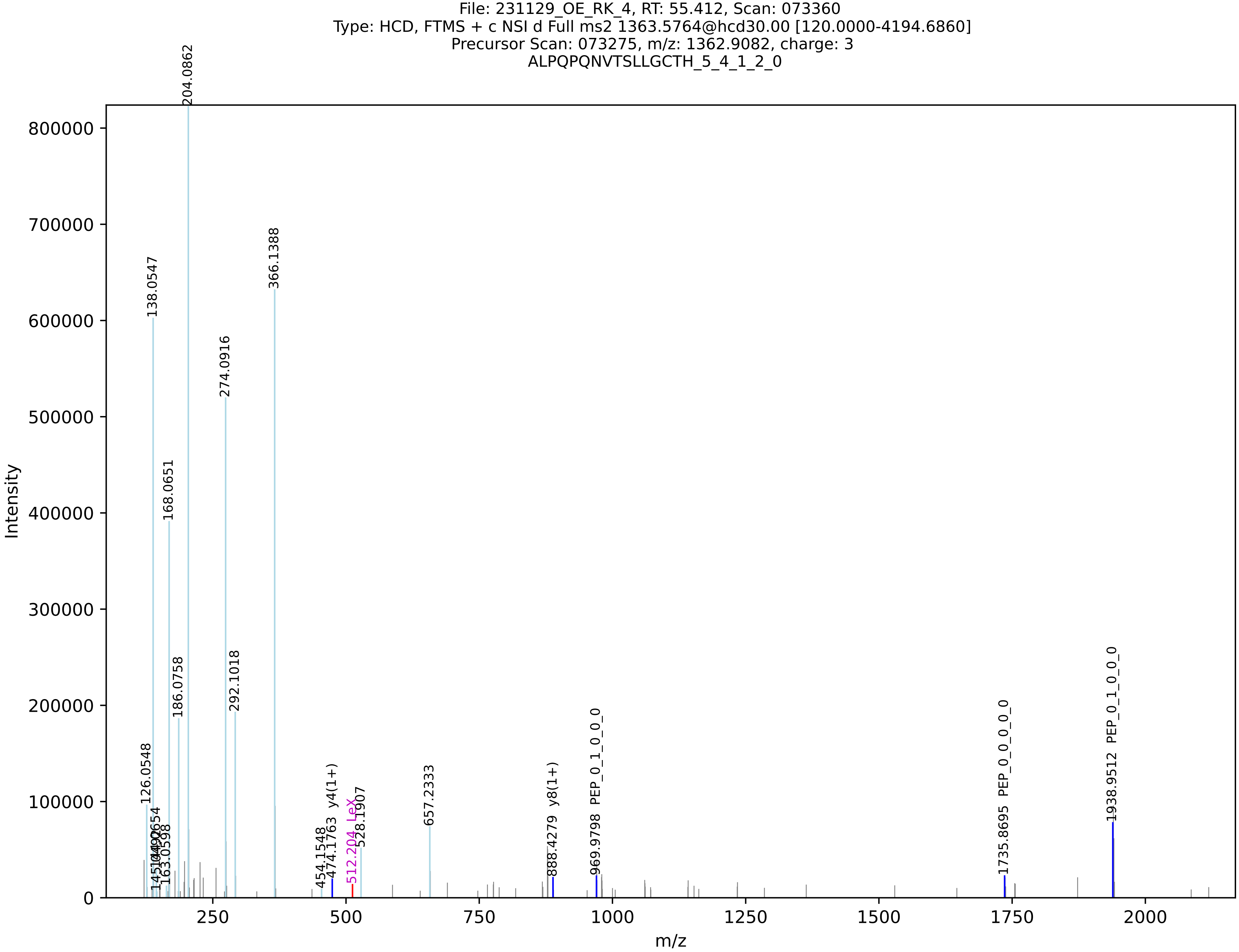

File: 231129_OE_RK_4, RT: 55.412, Scan: 073360
Type: HCD, FTMS + c NSI d Full ms2 1363.5764@hcd30.00 [120.0000-4194.6860]
Precursor Scan: 073275, m/z: 1362.9082, charge: 3
ALPQPQNVTSLLGCTH_5_4_1_2_0
Intensity
m/z
204.0862
138.0547
366.1388
274.0916
168.0651
186.0758
292.1018
126.0548
163.0598
454.1548
474.1763 y4(1+)
512.204 LeX
528.1907
657.2333
888.4279 y8(1+)
969.9798 PEP_0_1_0_0_0
1735.8695 PEP_0_0_0_0_0
1938.9512 PEP_0_1_0_0_0

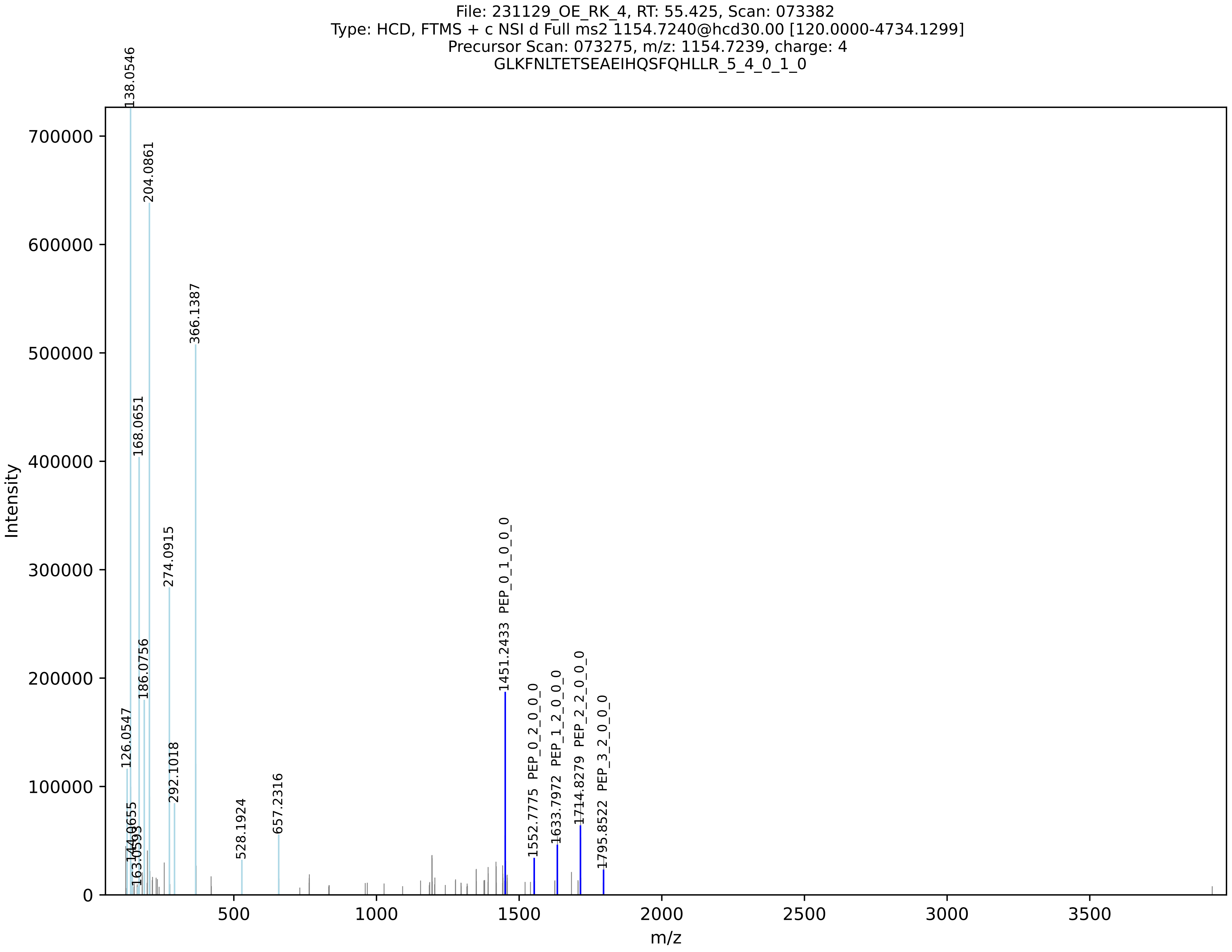

File: 231129_OE_RK_4, RT: 55.425, Scan: 073382
Type: HCD, FTMS + c NSI d Full ms2 1154.7240@hcd30.00 [120.0000-4734.1299]
Precursor Scan: 073275, m/z: 1154.7239, charge: 4
GLKFNLTETSEAEIHQSFQHLLR_5_4_0_1_0
Intensity
m/z
138.0546
204.0861
366.1387
168.0651
274.0915
186.0756
126.0547
292.1018
528.1924
657.2316
1451.2433 PEP_0_1_0_0_0
1552.7775 PEP_0_2_0_0_0
1633.7972 PEP_1_2_0_0_0
1714.8279 PEP_2_2_0_0_0
1795.8522 PEP_3_2_0_0_0

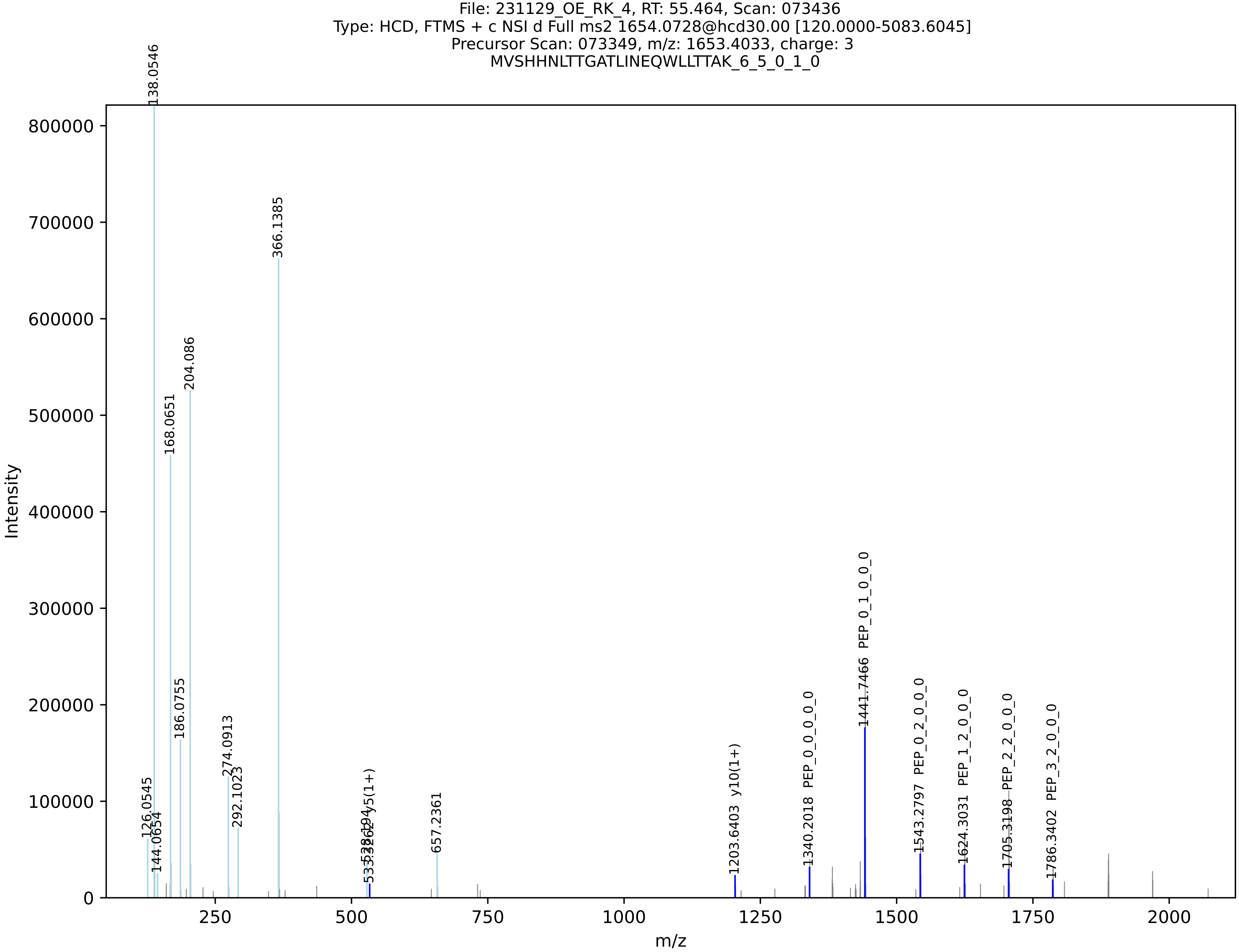
File: 231129_OE_RK_4, RT: 55.464, Scan: 073436
Type: HCD, FTMS + c NSI d Full ms2 1654.0728@hcd30.00 [120.0000-5083.6045]
Precursor Scan: 073349, m/z: 1653.4033, charge: 3
MVSHHNLTTGATLINEQWLLTTAK_6_5_0_1_0
Intensity
m/z
0
100000
200000
300000
400000
500000
600000
700000
800000
250
500
750
1000
1250
1500
1750
2000
138.0546
366.1385
204.086
168.0651
186.0755
274.0913
292.1023
126.0545
144.0654
657.2361
1203.6403 y10(1+)
1340.2018 PEP_0_0_0_0_0
1441.7466 PEP_0_1_0_0_0
1543.2797 PEP_0_2_0_0_0
1624.3031 PEP_1_2_0_0_0
1705.3198 PEP_2_2_0_0_0
1786.3402 PEP_3_2_0_0_0

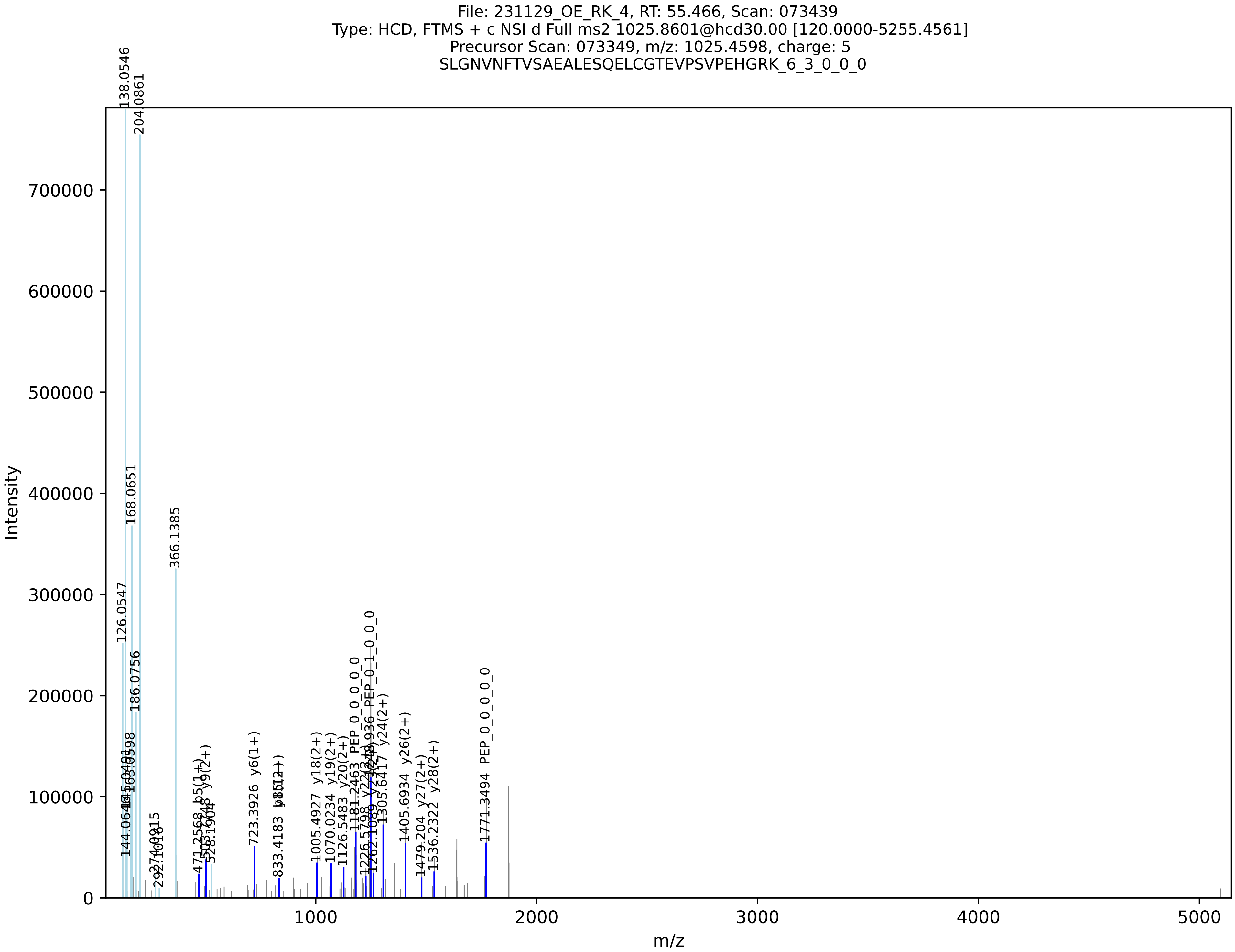
File: 231129_OE_RK_4, RT: 55.466, Scan: 073439
Type: HCD, FTMS + c NSI d Full ms2 1025.8601@hcd30.00 [120.0000-5255.4561]
Precursor Scan: 073349, m/z: 1025.4598, charge: 5
SLGNVNFTVSAEALESQELCGTEVPSVPEHGRK_6_3_0_0_0
138.0546
204.0861
168.0651
366.1385
126.0547
186.0756
723.3926 y6(1+)
1005.4927 y18(2+)
1070.0234 y19(2+)
1126.5483 y20(2+)
1181.2463 PEP_0_0_0_0_0
1305.6417 y24(2+)
1405.6934 y26(2+)
1479.204 y27(2+)
1536.2322 y28(2+)
1771.3494 PEP_0_0_0_0_0
833.4183
471.2568 b5(1+)
528.1904
Intensity
m/z
0
100000
200000
300000
400000
500000
600000
700000
1000
2000
3000
4000
5000

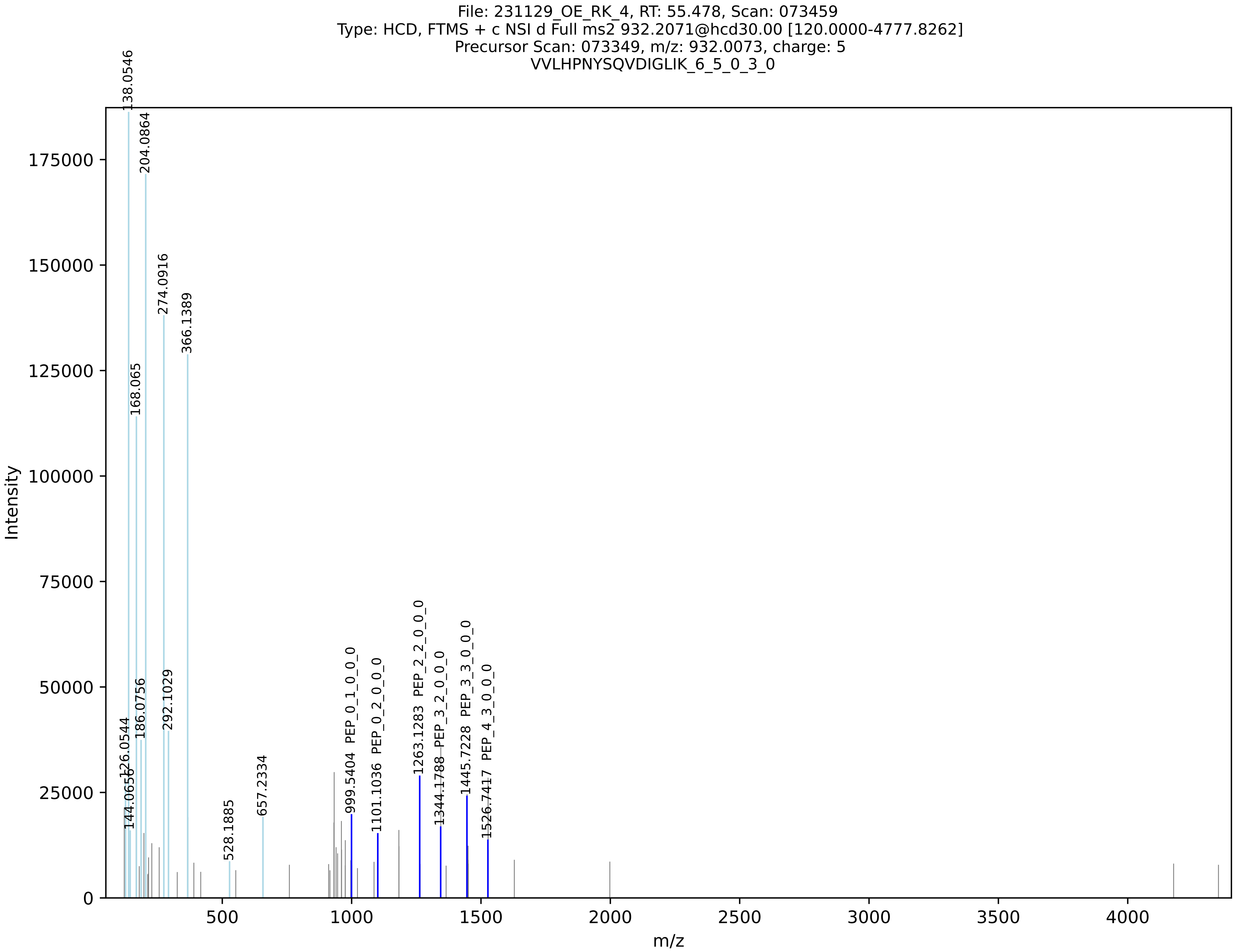
File: 231129_OE_RK_4, RT: 55.478, Scan: 073459
Type: HCD, FTMS + c NSI d Full ms2 932.2071@hcd30.00 [120.0000-4777.8262]
Precursor Scan: 073349, m/z: 932.0073, charge: 5
VVLHPNYSQVDIGLIK_6_5_0_3_0
138.0546
204.0864
274.0916
366.1389
168.065
186.0756
292.1029
126.0544
144.0656
528.1885
657.2334
999.5404 PEP_0_1_0_0_0
1101.1036 PEP_0_2_0_0_0
1263.1283 PEP_2_2_0_0_0
1344.1788 PEP_3_2_0_0_0
1445.7228 PEP_3_3_0_0_0
1526.7417 PEP_4_3_0_0_0
Intensity
m/z
0
25000
50000
75000
100000
125000
150000
175000
500
1000
1500
2000
2500
3000
3500
4000

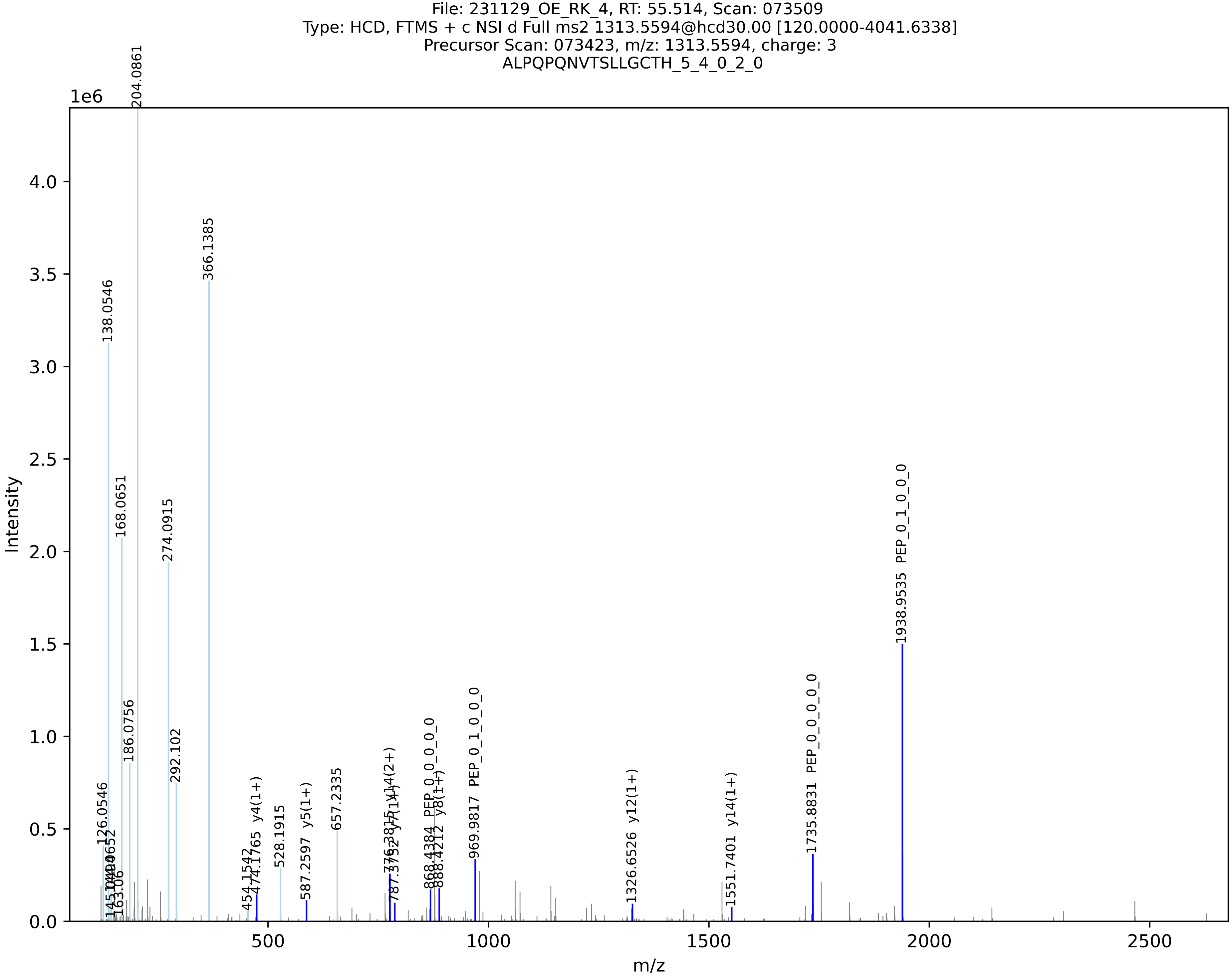
File: 231129_OE_RK_4, RT: 55.514, Scan: 073509
Type: HCD, FTMS + c NSI d Full ms2 1313.5594@hcd30.00 [120.0000-4041.6338]
Precursor Scan: 073423, m/z: 1313.5594, charge: 3
ALPQPQNVTSLLGCTH_5_4_0_2_0
1e6
Intensity
m/z
204.0861
366.1385
138.0546
168.0651
274.0915
186.0756
292.102
126.0546
528.1915
657.2335
454.1542
474.1765 y4(1+)
587.2597 y5(1+)
776.3815 y14(2+)
787.3752 y7(1+)
868.4384 PEP_0_0_0_0_0_
888.4212 y8(1+)
969.9817 PEP_0_1_0_0_0_
1326.6526 y12(1+)
1551.7401 y14(1+)
1735.8831 PEP_0_0_0_0_0_
1938.9535 PEP_0_1_0_0_0_

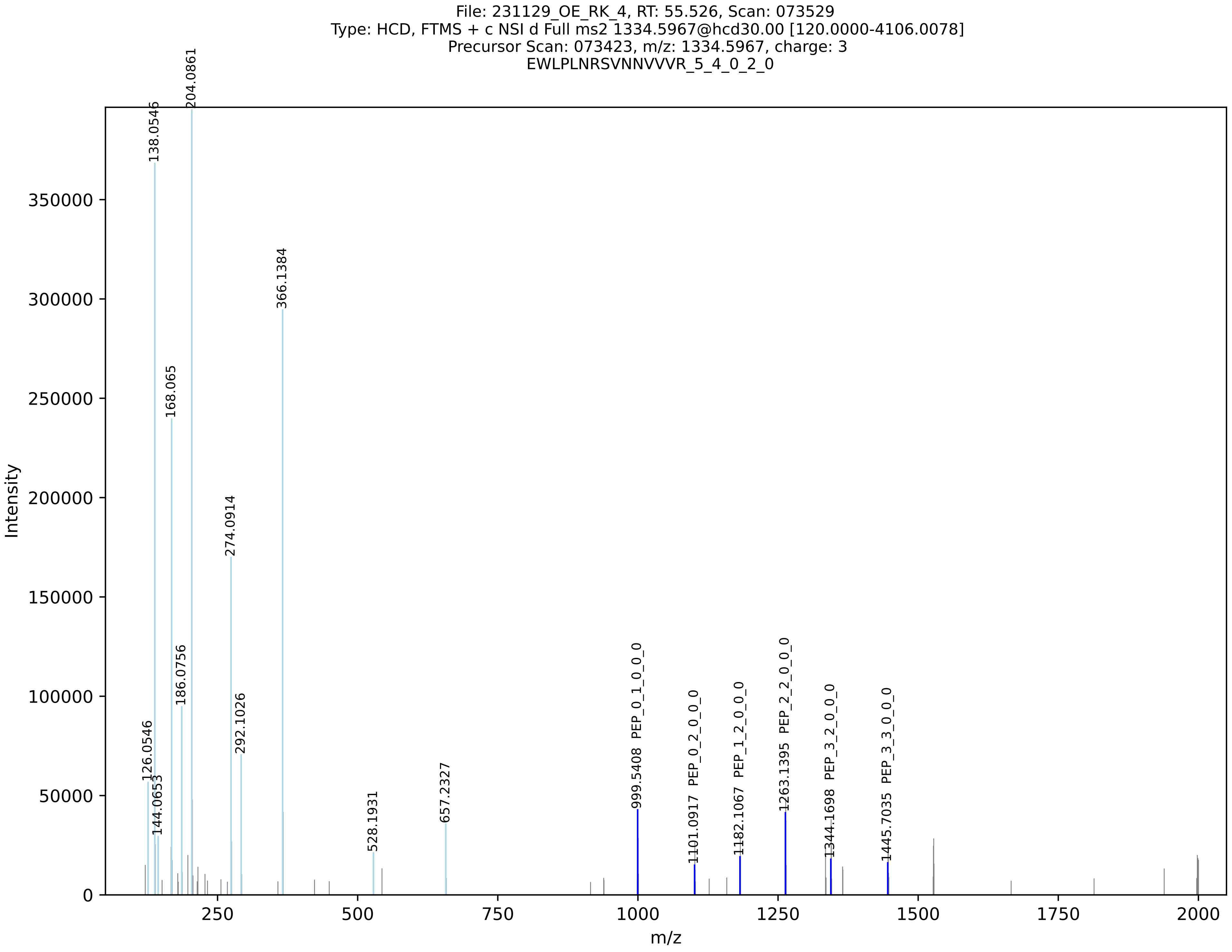

File: 231129_OE_RK_4, RT: 55.526, Scan: 073529
Type: HCD, FTMS + c NSI d Full ms2 1334.5967@hcd30.00 [120.0000-4106.0078]
Precursor Scan: 073423, m/z: 1334.5967, charge: 3
EWLPLNRSVNNVVVR_5_4_0_2_0
Intensity
m/z
204.0861
138.0546
366.1384
168.065
274.0914
186.0756
292.1026
126.0546
144.0653
528.1931
657.2327
999.5408 PEP_0_1_0_0_0
1101.0917 PEP_0_2_0_0_0
1182.1067 PEP_1_2_0_0_0
1263.1395 PEP_2_2_0_0_0
1344.1698 PEP_3_2_0_0_0
1445.7035 PEP_3_3_0_0_0

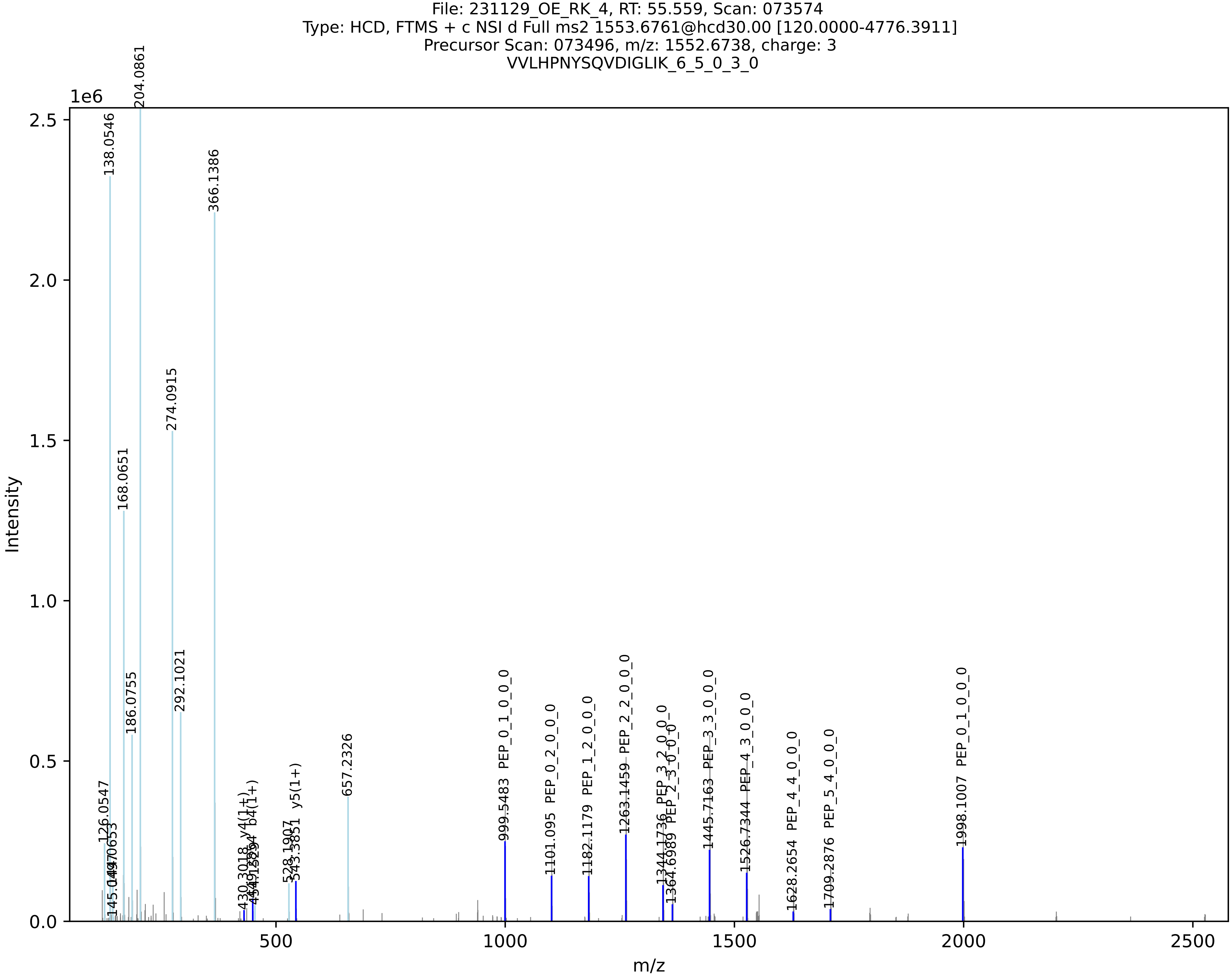
File: 231129_OE_RK_4, RT: 55.559, Scan: 073574
Type: HCD, FTMS + c NSI d Full ms2 1553.6761@hcd30.00 [120.0000-4776.3911]
Precursor Scan: 073496, m/z: 1552.6738, charge: 3
VVLHPNYSQVDIGLIK_6_5_0_3_0
1e6
Intensity
m/z
204.0861
138.0546
366.1386
274.0915
168.0651
292.1021
186.0755
126.0547
657.2326
430.3018 y4(1+)
543.3851 y5(1+)
528.1907
999.5483 PEP_0_1_0_0_
1101.095 PEP_0_2_0_0_0_
1182.1179 PEP_1_2_0_0_0_
1263.1459 PEP_2_2_0_0_0_
1344.1736 PEP_3_2_0_0_0_
1364.6989 PEP_2_3_0_0_0_
1445.7163 PEP_3_3_0_0_0_
1526.7344 PEP_4_3_0_0_0_
1628.2654 PEP_4_4_0_0_0_
1709.2876 PEP_5_4_0_0_0_
1998.1007 PEP_0_1_0_0_0_

File: 231129_OE_RK_4, RT: 55.56, Scan: 073576
Type: HCD, FTMS + c NSI d Full ms2 1201.7722@hcd30.00 [120.0000-4926.0864]
Precursor Scan: 073496, m/z: 1201.0171, charge: 4
VVLHPNYSQVDIGLIK_6_5_1_3_0

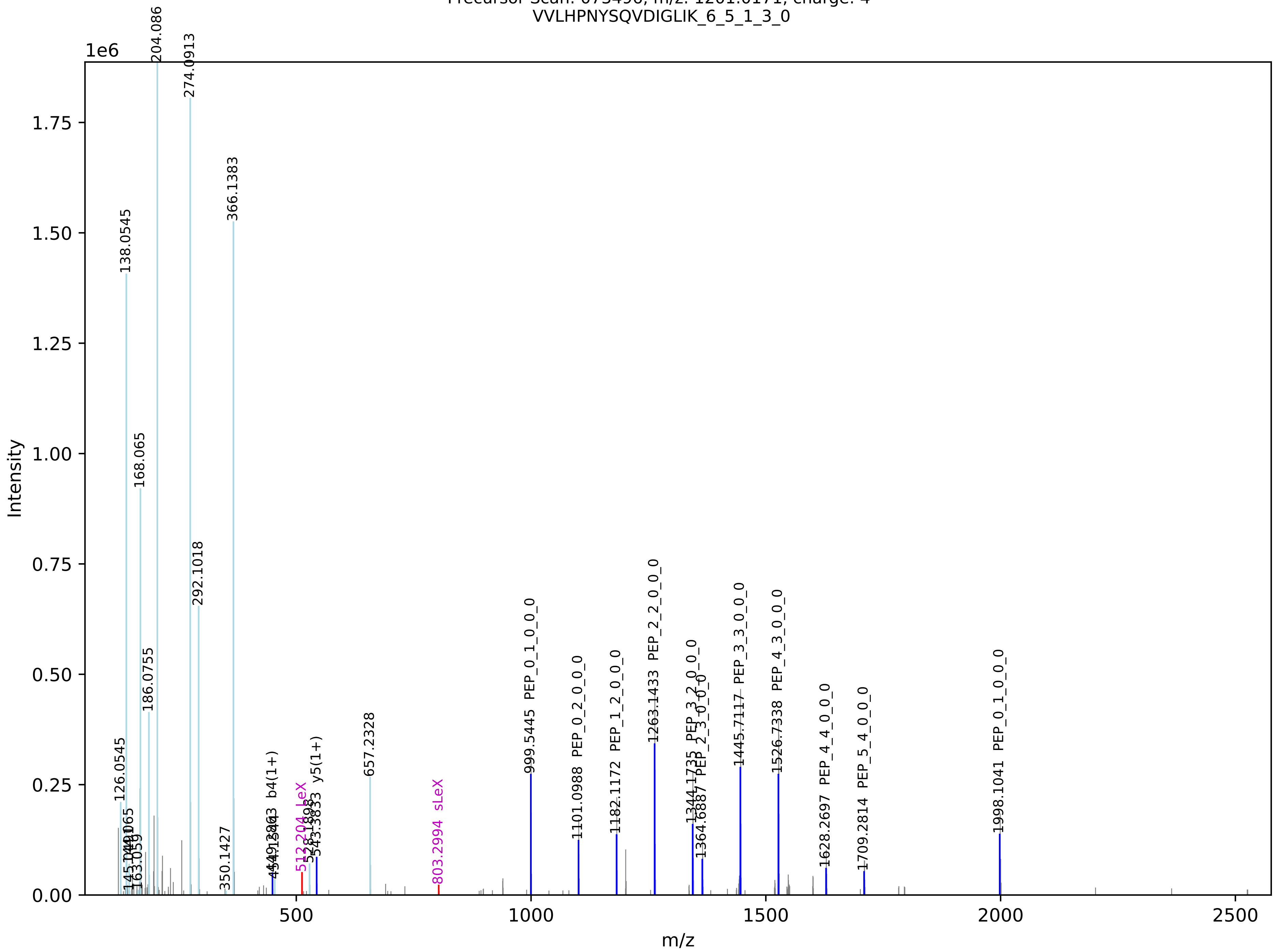

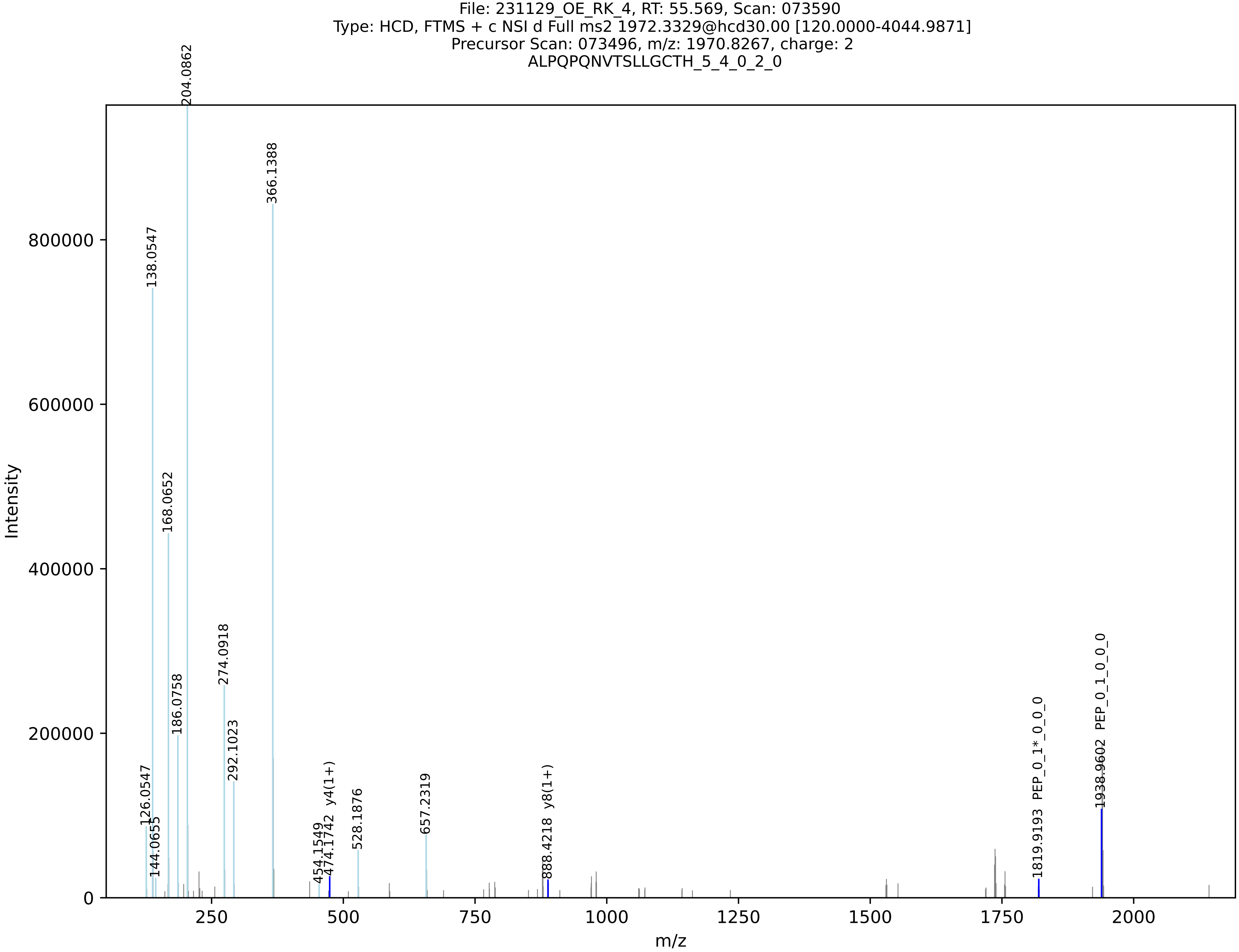
File: 231129_OE_RK_4, RT: 55.569, Scan: 073590
Type: HCD, FTMS + c NSI d Full ms2 1972.3329@hcd30.00 [120.0000-4044.9871]
Precursor Scan: 073496, m/z: 1970.8267, charge: 2
ALPQPQNVTSLLGCTH_5_4_0_2_0
Intensity
m/z
204.0862
366.1388
138.0547
168.0652
274.0918
186.0758
292.1023
126.0547
144.0655
454.1549
474.1742 y4(1+)
528.1876
657.2319
888.4218 y8(1+)
1819.9193 PEP_0_1*_0_0_0
1938.9602 PEP_0_1_0_0_0

File: 231129_OE_RK_4, RT: 55.607, Scan: 073644
Type: HCD, FTMS + c NSI d Full ms2 1226.5181@hcd30.00 [120.0000-5027.0498]
Precursor Scan: 073569, m/z: 1226.017, charge: 4
VEPNYNITIFVDTFQSEK_5_6_1_2_0

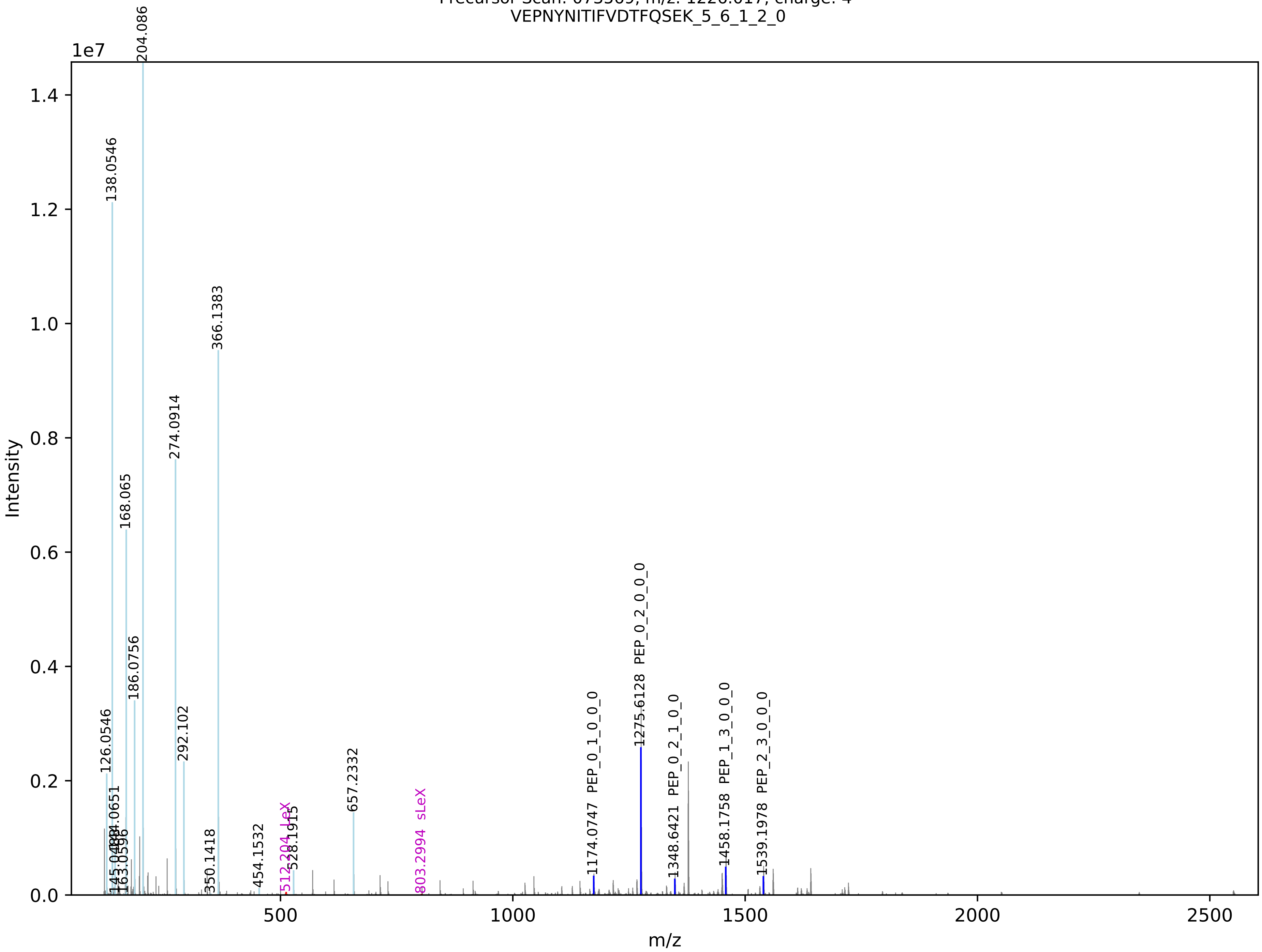

File: 231129_OE_RK_4, RT: 55.608, Scan: 073645
Type: HCD, FTMS + c NSI d Full ms2 1165.2590@hcd30.00 [120.0000-4777.1128]
Precursor Scan: 073569, m/z: 1164.7579, charge: 4
VVLHPNYSQVDIGLIK_6_5_0_3_0

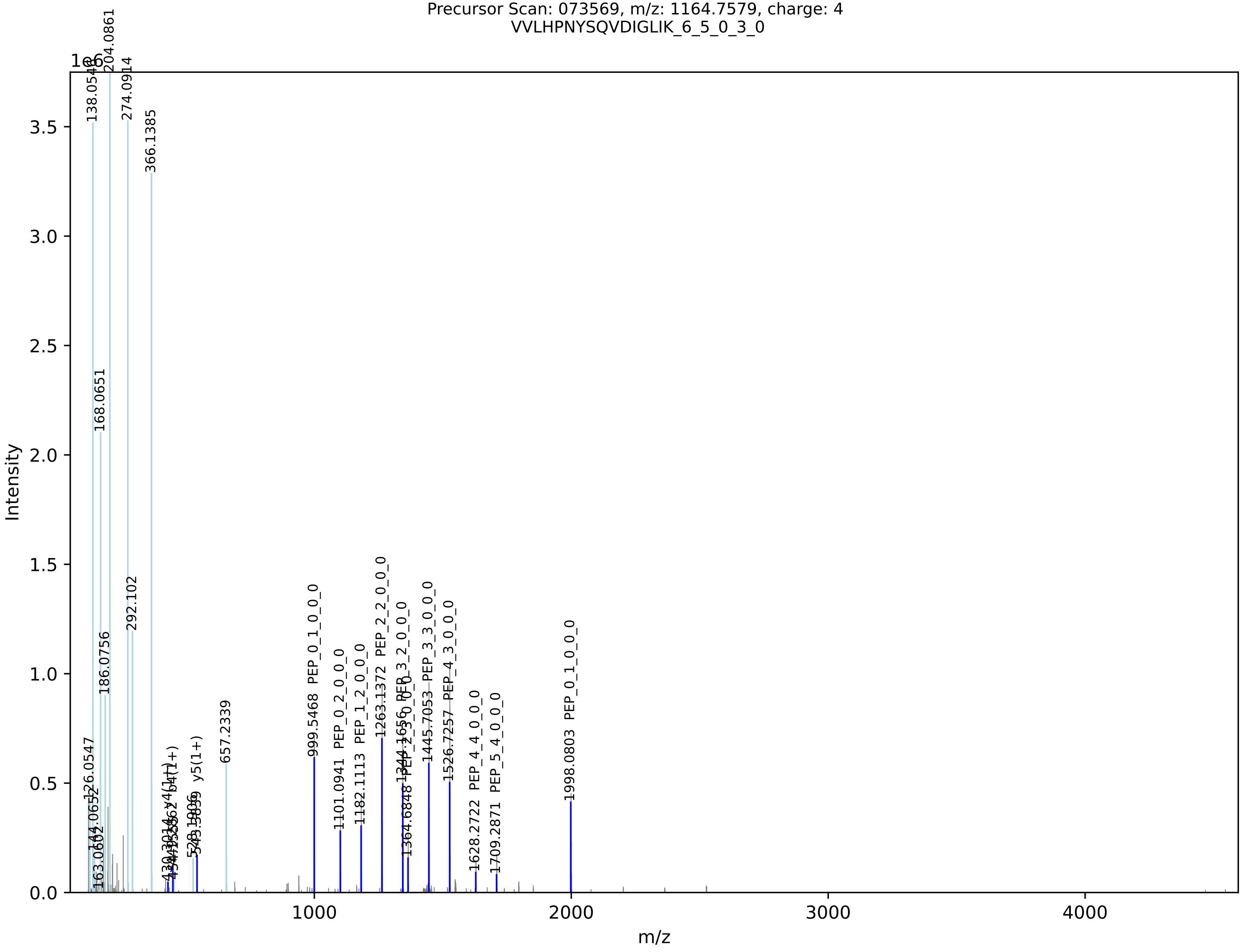

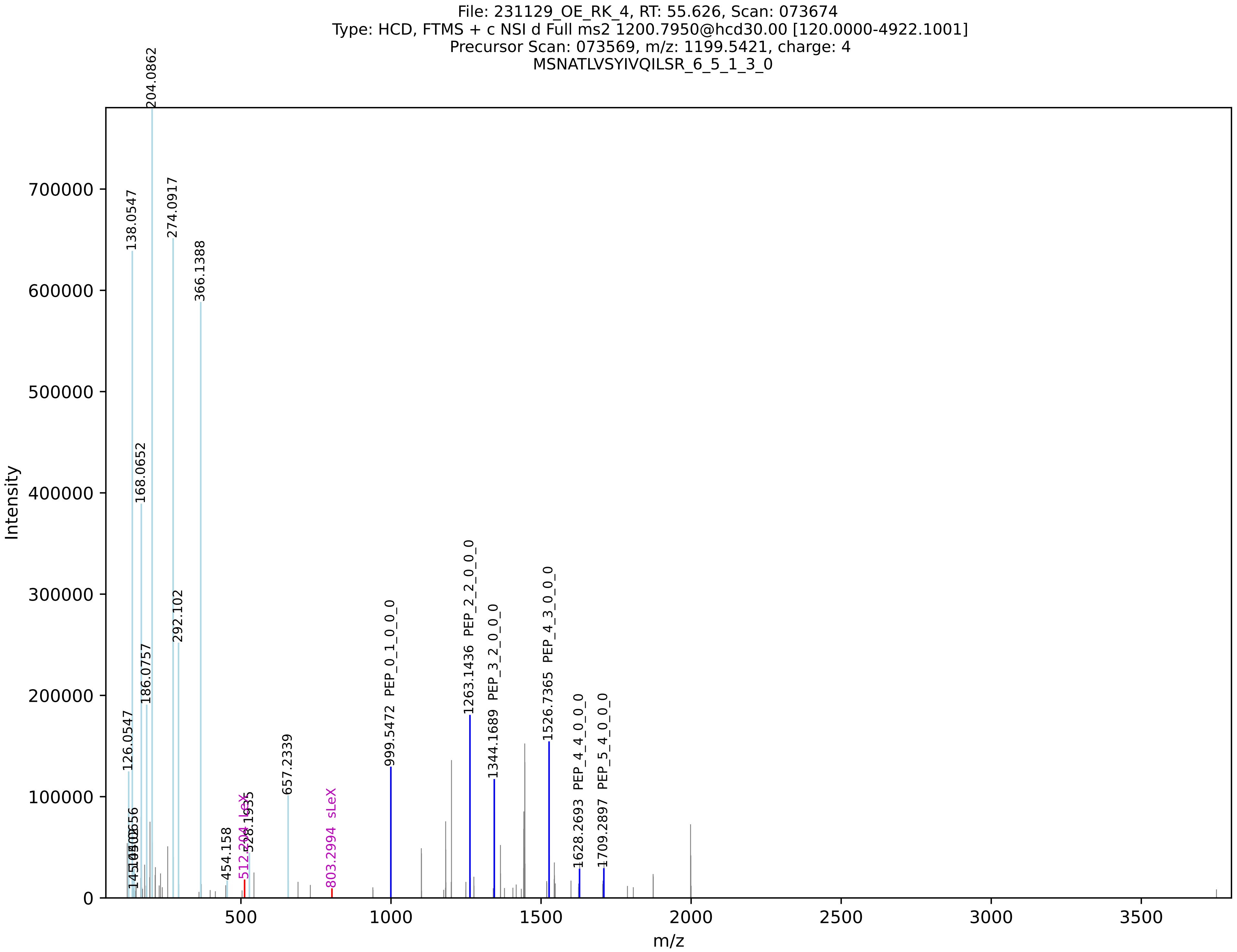
File: 231129_OE_RK_4, RT: 55.626, Scan: 073674
Type: HCD, FTMS + c NSI d Full ms2 1200.7950@hcd30.00 [120.0000-4922.1001]
Precursor Scan: 073569, m/z: 1199.5421, charge: 4
MSNATLVSYIVQILSR_6_5_1_3_0
Intensity
m/z
204.0862
138.0547
274.0917
366.1388
168.0652
292.102
186.0757
126.0547
454.158
512.204 LeX
528.1935
657.2339
803.2994 sLeX
999.5472 PEP_0_1_0_0_0
1263.1436 PEP_2_2_0_0_0
1344.1689 PEP_3_2_0_0_0
1526.7365 PEP_4_3_0_0_0
1628.2693 PEP_4_4_0_0_0
1709.2897 PEP_5_4_0_0_0

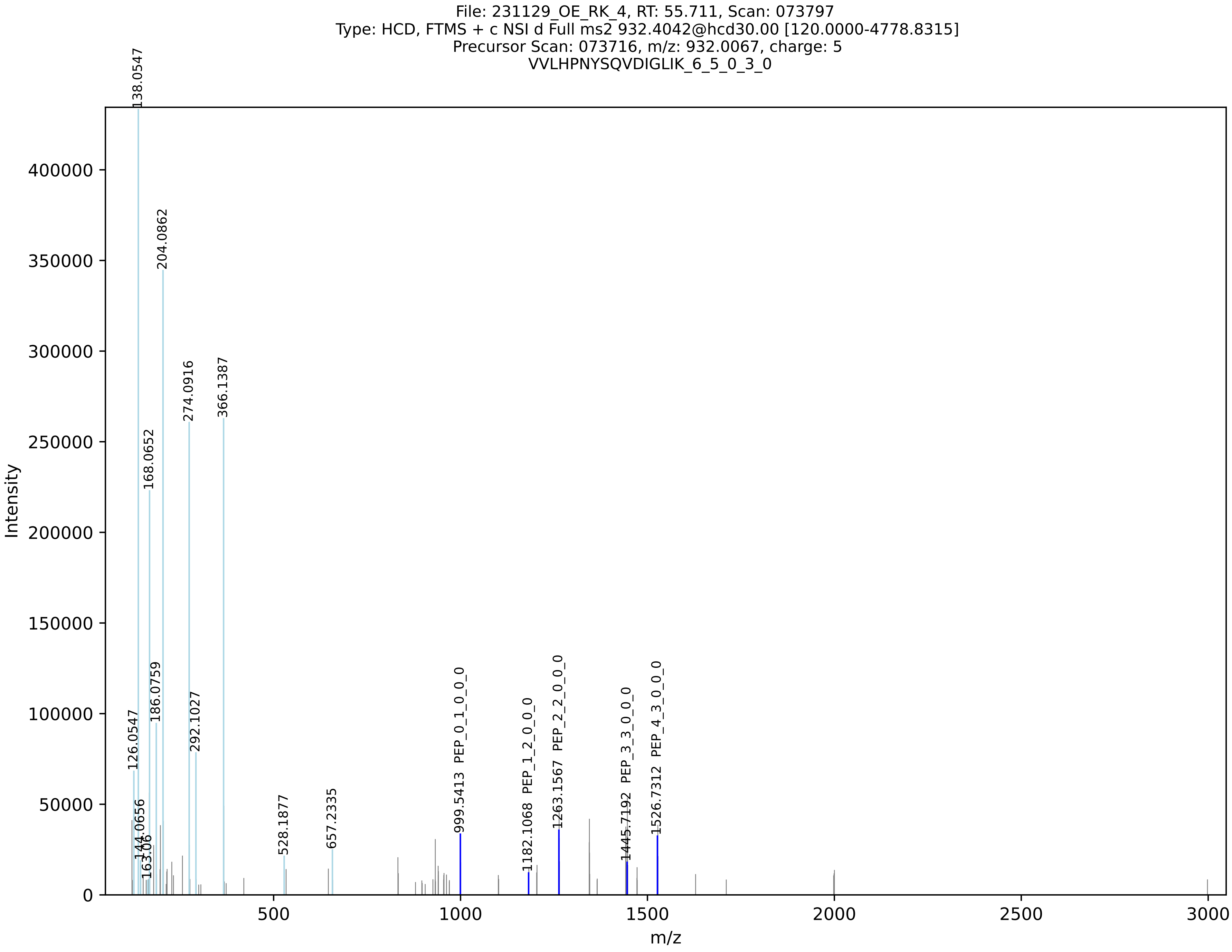
File: 231129_OE_RK_4, RT: 55.711, Scan: 073797
Type: HCD, FTMS + c NSI d Full ms2 932.4042@hcd30.00 [120.0000-4778.8315]
Precursor Scan: 073716, m/z: 932.0067, charge: 5
VVLHPNYSQVDIGLIK_6_5_0_3_0
Intensity
m/z
138.0547
204.0862
274.0916
366.1387
168.0652
186.0759
292.1027
126.0547
144.0656
163.06
528.1877
657.2335
999.5413 PEP_0_1_0_0_0
1182.1068 PEP_1_2_0_0_0
1263.1567 PEP_2_2_0_0_0
1445.7192 PEP_3_3_0_0_0
1526.7312 PEP_4_3_0_0_0

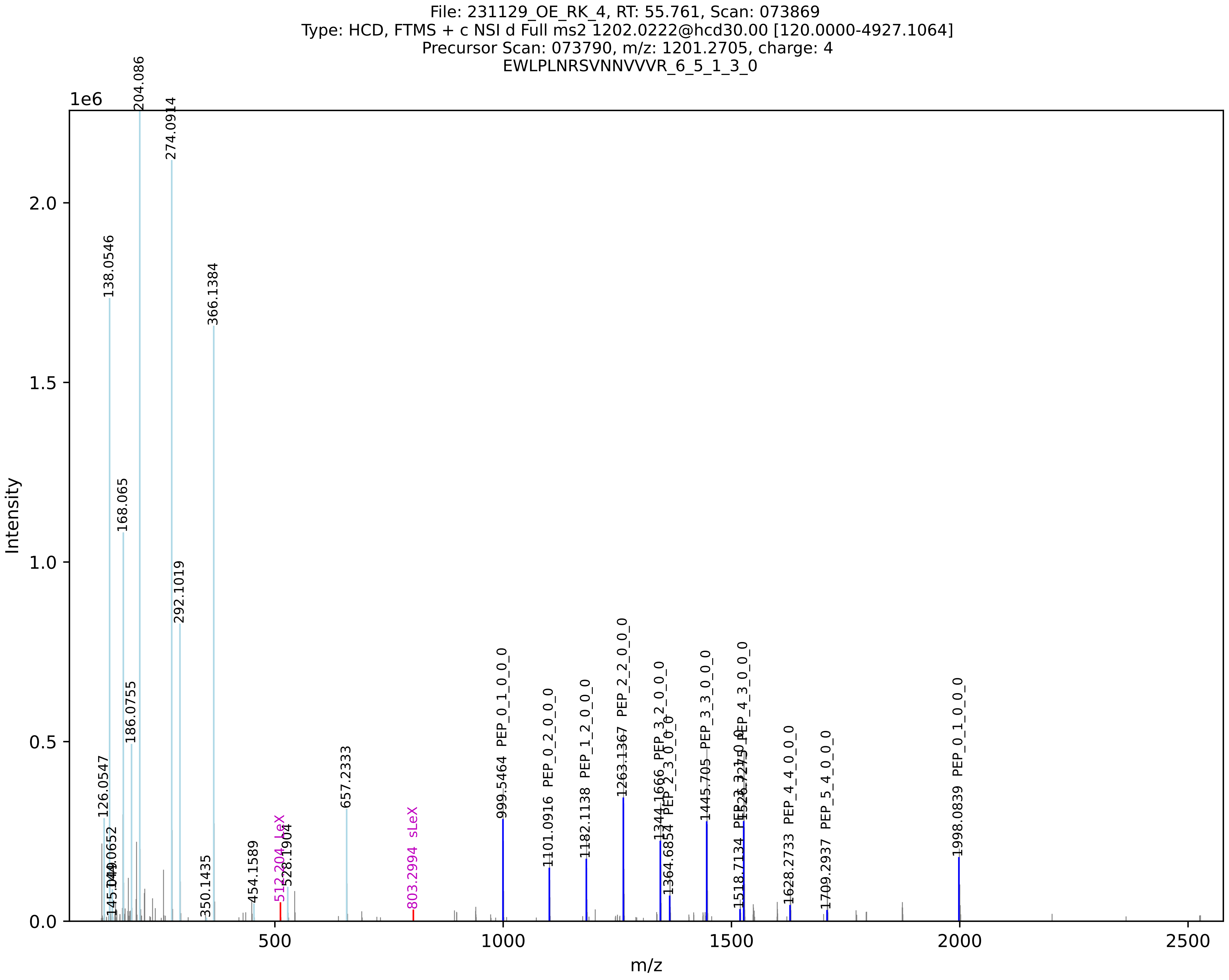

File: 231129_OE_RK_4, RT: 55.761, Scan: 073869
Type: HCD, FTMS + c NSI d Full ms2 1202.0222@hcd30.00 [120.0000-4927.1064]
Precursor Scan: 073790, m/z: 1201.2705, charge: 4
EWLPLNRSVNNVVVR_6_5_1_3_0
1e6
Intensity
m/z
0.0
0.5
1.0
1.5
2.0
500
1000
1500
2000
2500
204.086
274.0914
138.0546
366.1384
168.065
292.1019
186.0755
126.0547
657.2333
350.1435
454.1589
528.1904
512.204 LeX
803.2994 sLeX
999.5464 PEP_0_1_0_0_0
1101.0916 PEP_0_2_0_0_0
1182.1138 PEP_1_2_0_0_0
1263.1367 PEP_2_2_0_0_0
1344.1666 PEP_3_2_0_0_0
1364.6854 PEP_2_3_0_0_0
1445.705 PEP_3_3_0_0_0
1518.7134
1526.7275 PEP_4_3_0_0_0
1628.2733 PEP_4_4_0_0_0
1709.2937 PEP_5_4_0_0_0
1998.0839 PEP_0_1_0_0_0

File: 231129_OE_RK_4, RT: 55.768, Scan: 073881
Type: HCD, FTMS + c NSI d Full ms2 1971.8282@hcd30.00 [120.0000-4043.9578]
Precursor Scan: 073790, m/z: 1970.8257, charge: 2
ALPQPQNVTSLLGCTH_5_4_0_2_0

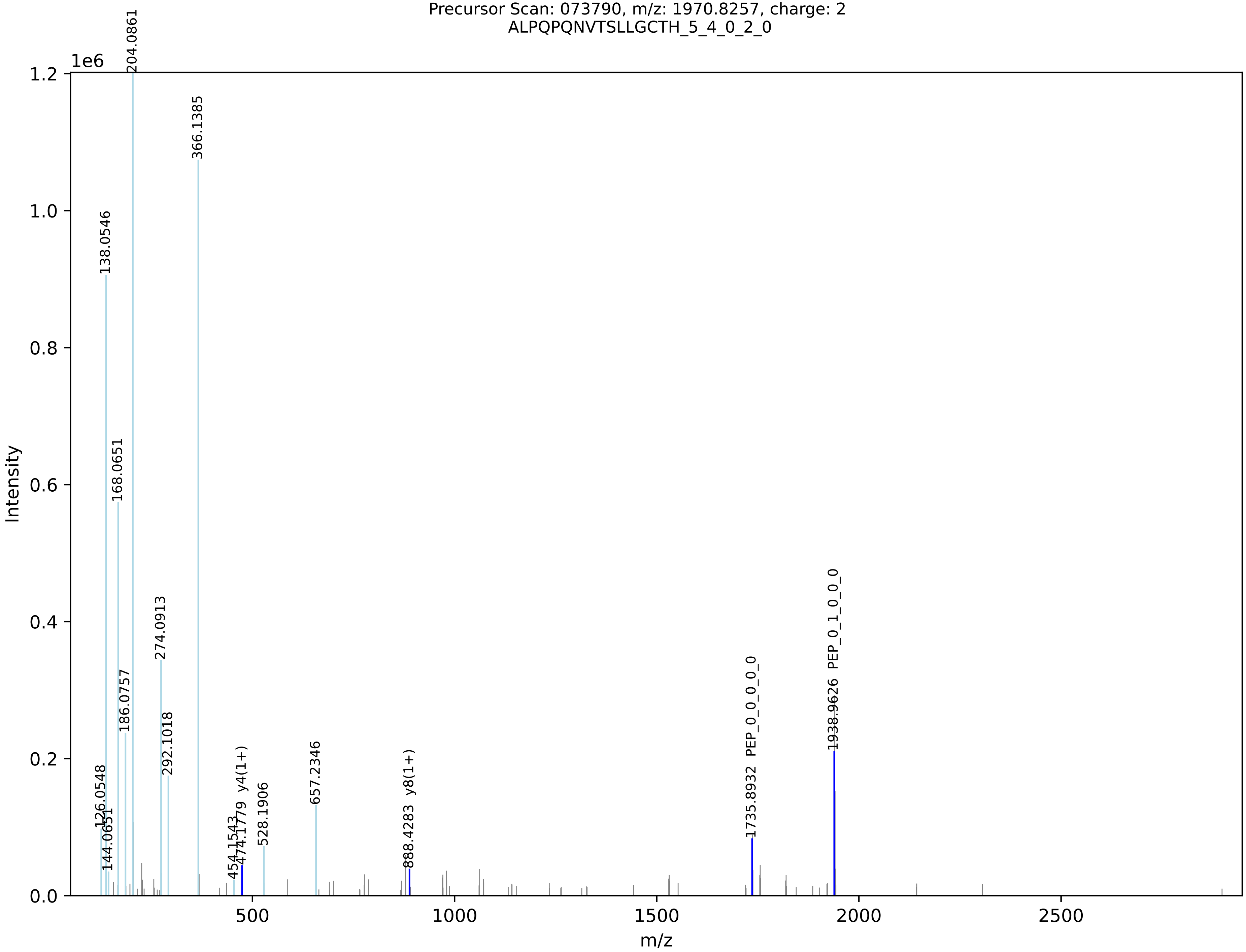

File: 231129_OE_RK_4, RT: 55.809, Scan: 073939
Type: HCD, FTMS + c NSI d Full ms2 1314.8881@hcd30.00 [120.0000-4045.6997]
Precursor Scan: 073864, m/z: 1314.2198, charge: 3
ALPQPQNVTSLLGCTH_5_4_0_2_0

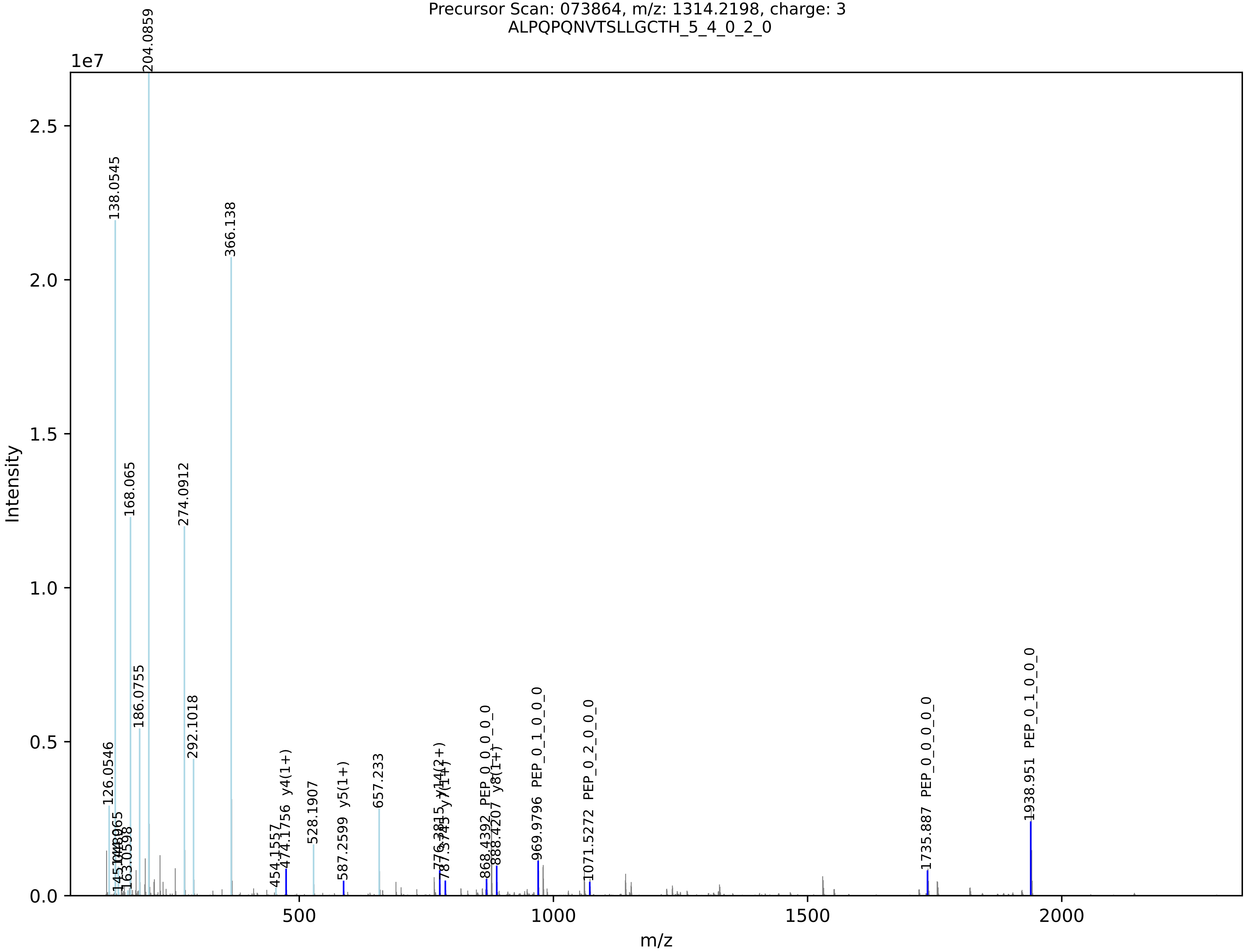

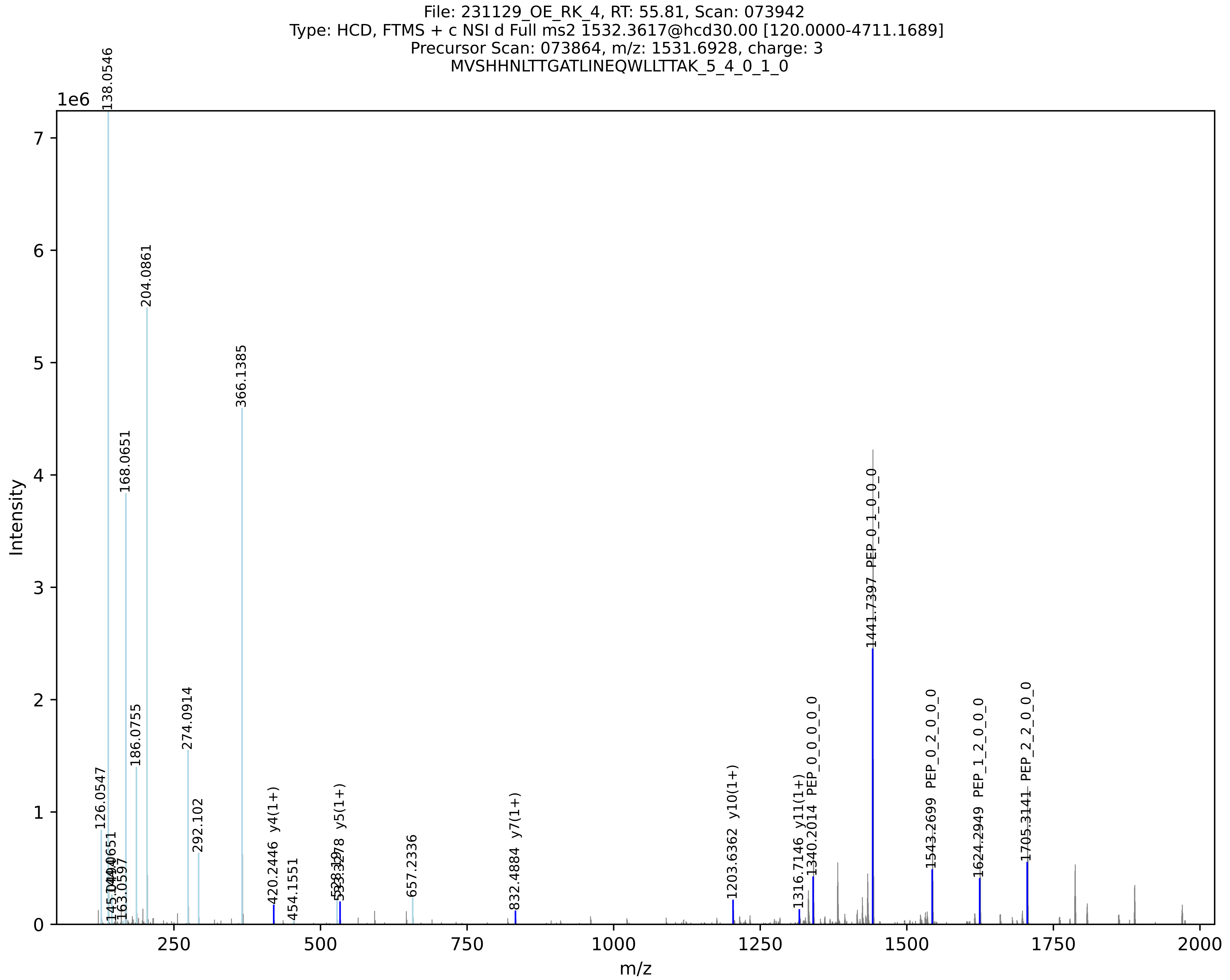
File: 231129_OE_RK_4, RT: 55.81, Scan: 073942
Type: HCD, FTMS + c NSI d Full ms2 1532.3617@hcd30.00 [120.0000-4711.1689]
Precursor Scan: 073864, m/z: 1531.6928, charge: 3
MVSHHNLTTGATLINEQWLLTTAK_5_4_0_1_0
1e6
Intensity
m/z
138.0546
204.0861
366.1385
168.0651
186.0755
274.0914
126.0547
292.102
163.0597
420.2446 y4(1+)
454.1551
533.3278 y5(1+)
657.2336
832.4884 y7(1+)
1203.6362 y10(1+)
1316.7146 y11(1+)
1340.2014 PEP_0_0_0_0_0
1441.7397 PEP_0_1_0_0_0
1543.2699 PEP_0_2_0_0_0
1624.2949 PEP_1_2_0_0_0
1705.3141 PEP_2_2_0_0_0

File: 231129_OE_RK_4, RT: 55.818, Scan: 073954
Type: HCD, FTMS + c NSI d Full ms2 1363.5726@hcd30.00 [120.0000-4194.6743]
Precursor Scan: 073864, m/z: 1362.9045, charge: 3
ALPQPQNVTSLLGCTH_5_4_1_2_0

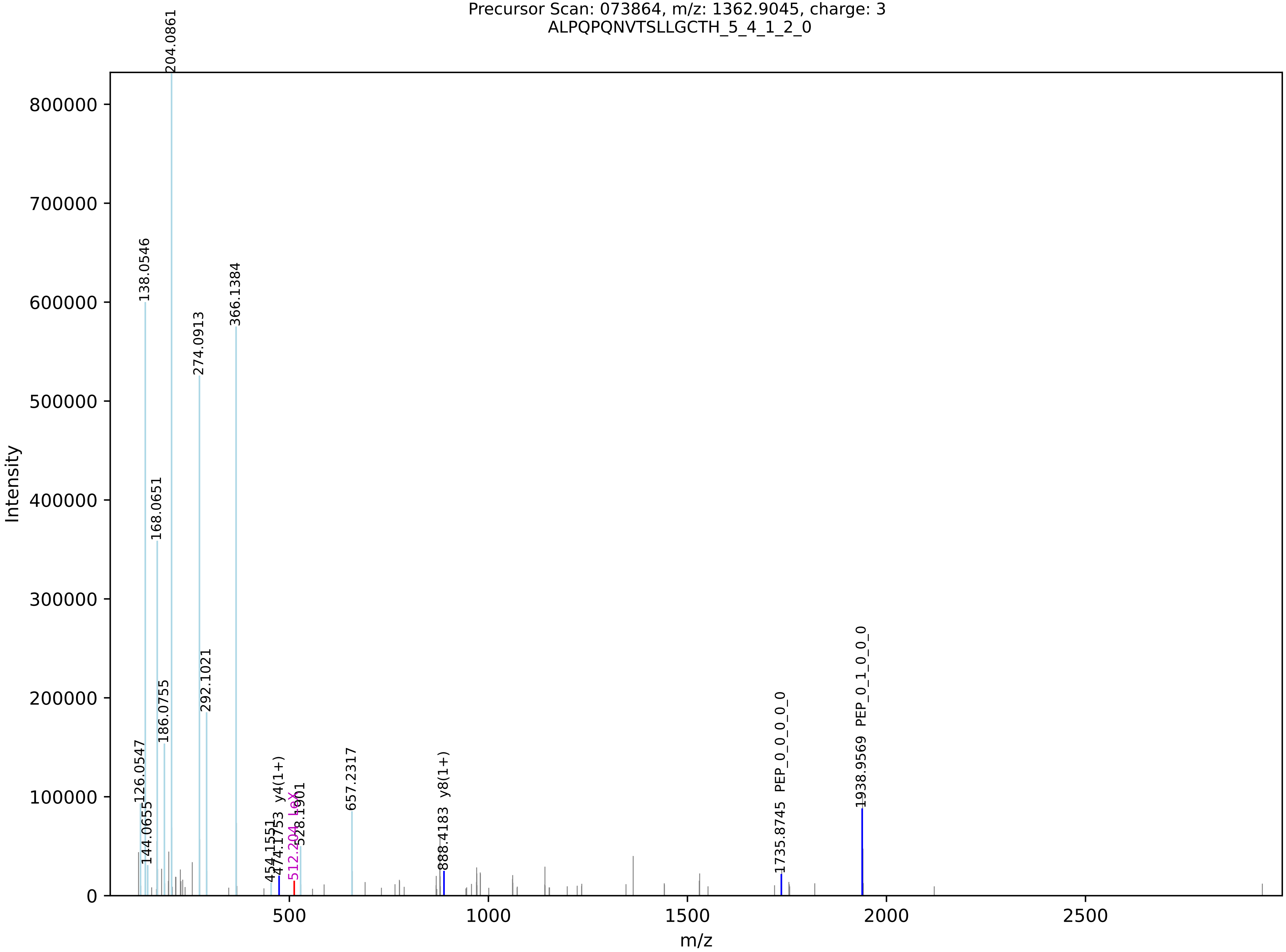

File: 231129_OE_RK_4, RT: 55.82, Scan: 073957
Type: HCD, FTMS + c NSI d Full ms2 1282.5245@hcd30.00 [120.0000-3946.6670]
Precursor Scan: 073864, m/z: 1282.1904, charge: 3
AGLQAFFQVQECNK_5_4_0_2_0

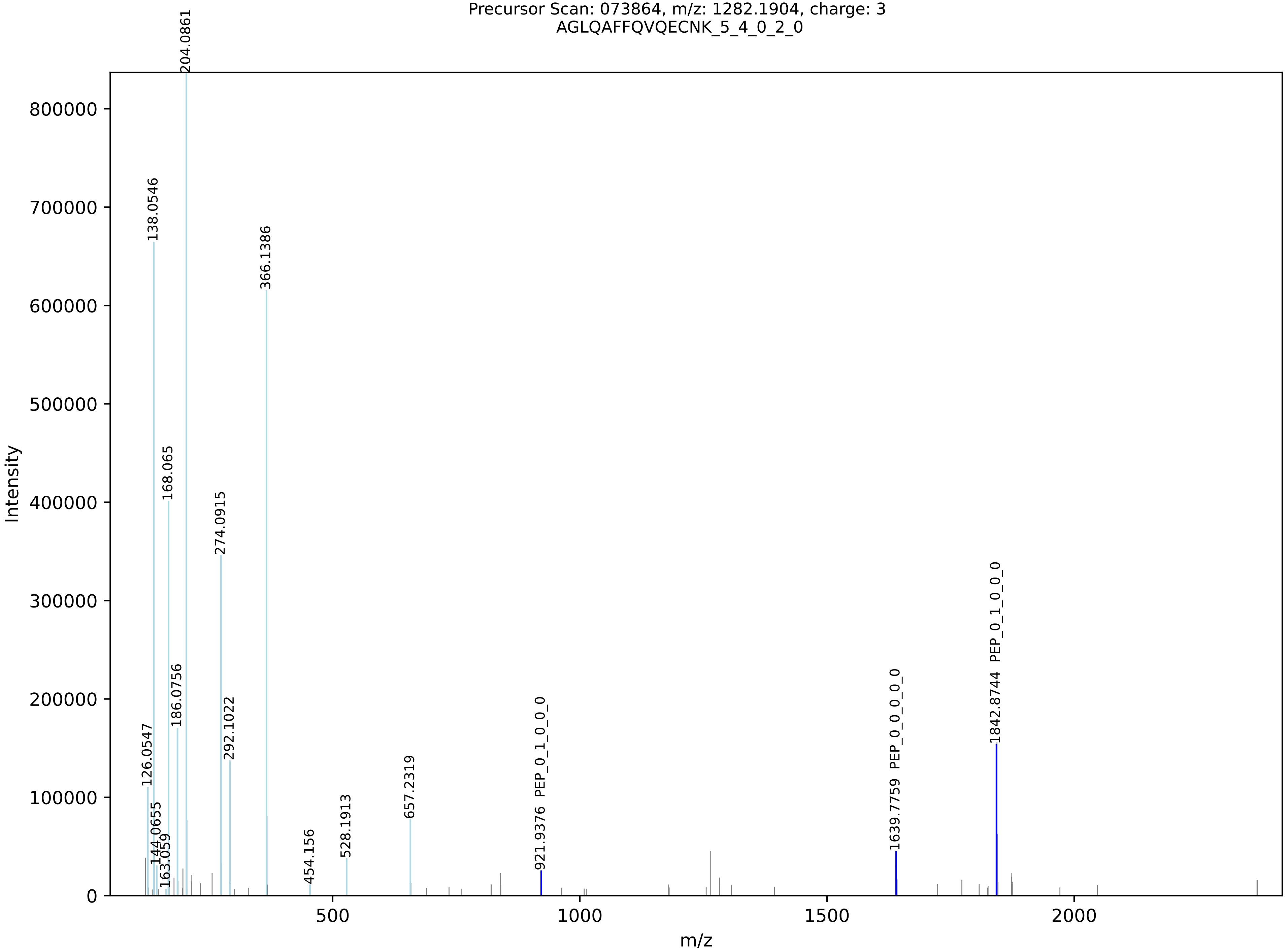

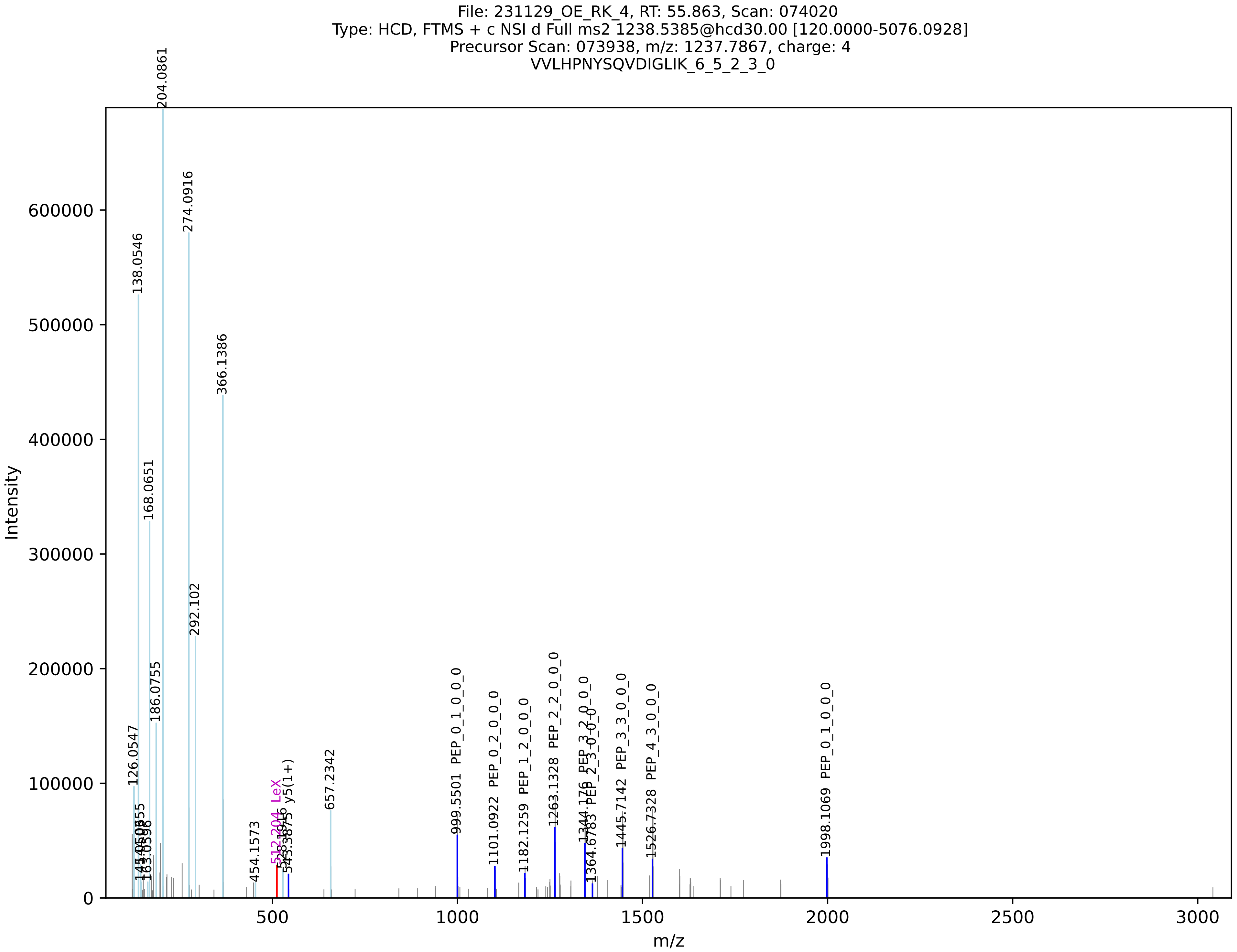
File: 231129_OE_RK_4, RT: 55.863, Scan: 074020
Type: HCD, FTMS + c NSI d Full ms2 1238.5385@hcd30.00 [120.0000-5076.0928]
Precursor Scan: 073938, m/z: 1237.7867, charge: 4
VVLHPNYSQVDIGLIK_6_5_2_3_0
Intensity
m/z
204.0861
274.0916
138.0546
366.1386
168.0651
292.102
186.0755
126.0547
454.1573
512.204 LeX
545.3875 y5(1+)
657.2342
999.5501 PEP_0_1_0_0_0_
1101.0922 PEP_0_2_0_0_0_
1182.1259 PEP_1_2_0_0_0_
1263.1328 PEP_2_2_0_0_0_
1364.6783 PEP_2_3_0_0_0_
1445.7142 PEP_3_3_0_0_0_
1526.7328 PEP_4_3_0_0_0_
1998.1069 PEP_0_1_0_0_0_

File: 231129_OE_RK_4, RT: 55.91, Scan: 074088
Type: HCD, FTMS + c NSI d Full ms2 986.1674@hcd30.00 [120.0000-4046.4187]
Precursor Scan: 074012, m/z: 985.9169, charge: 4
ALPQPQNVTSLLGCTH_5_4_0_2_0

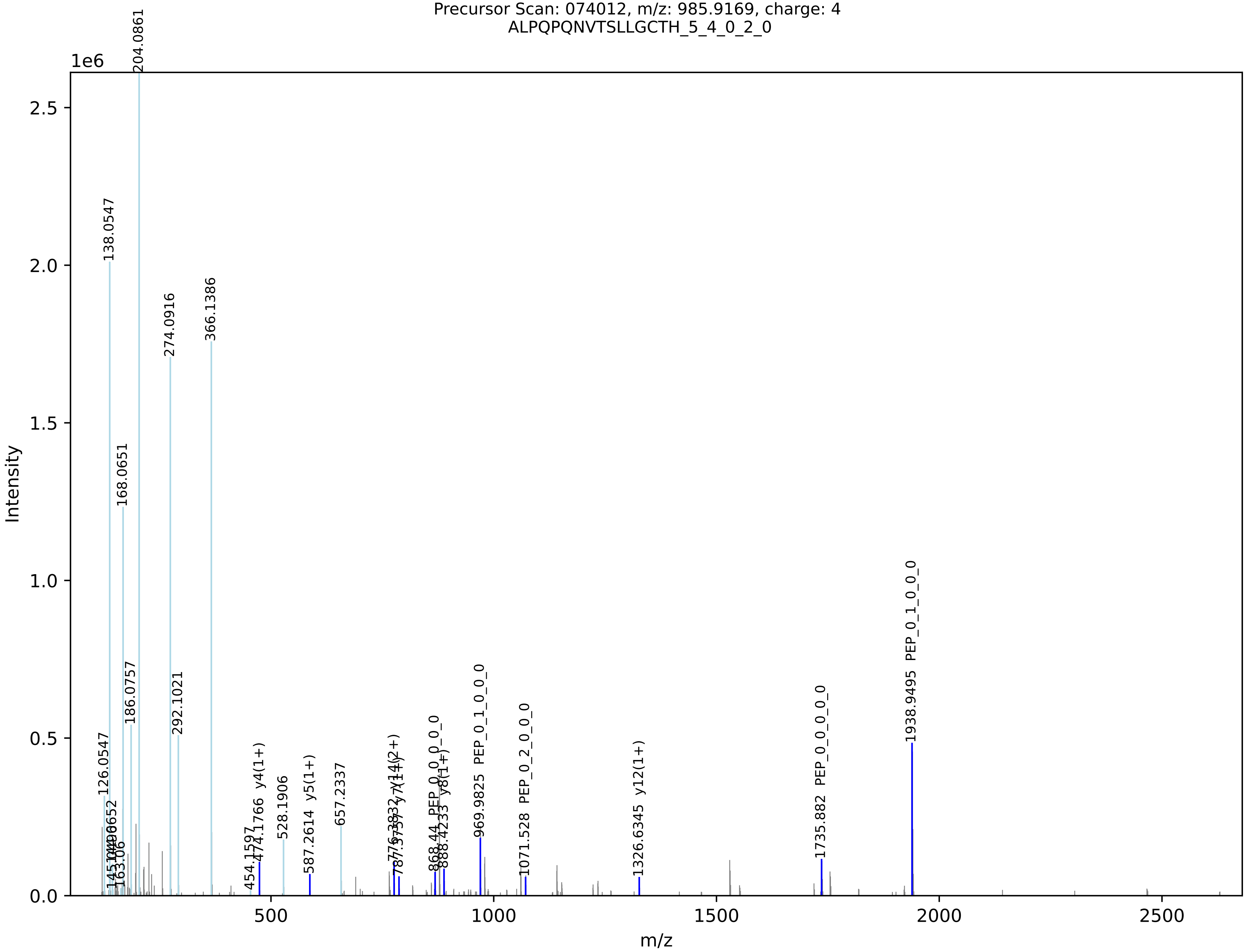

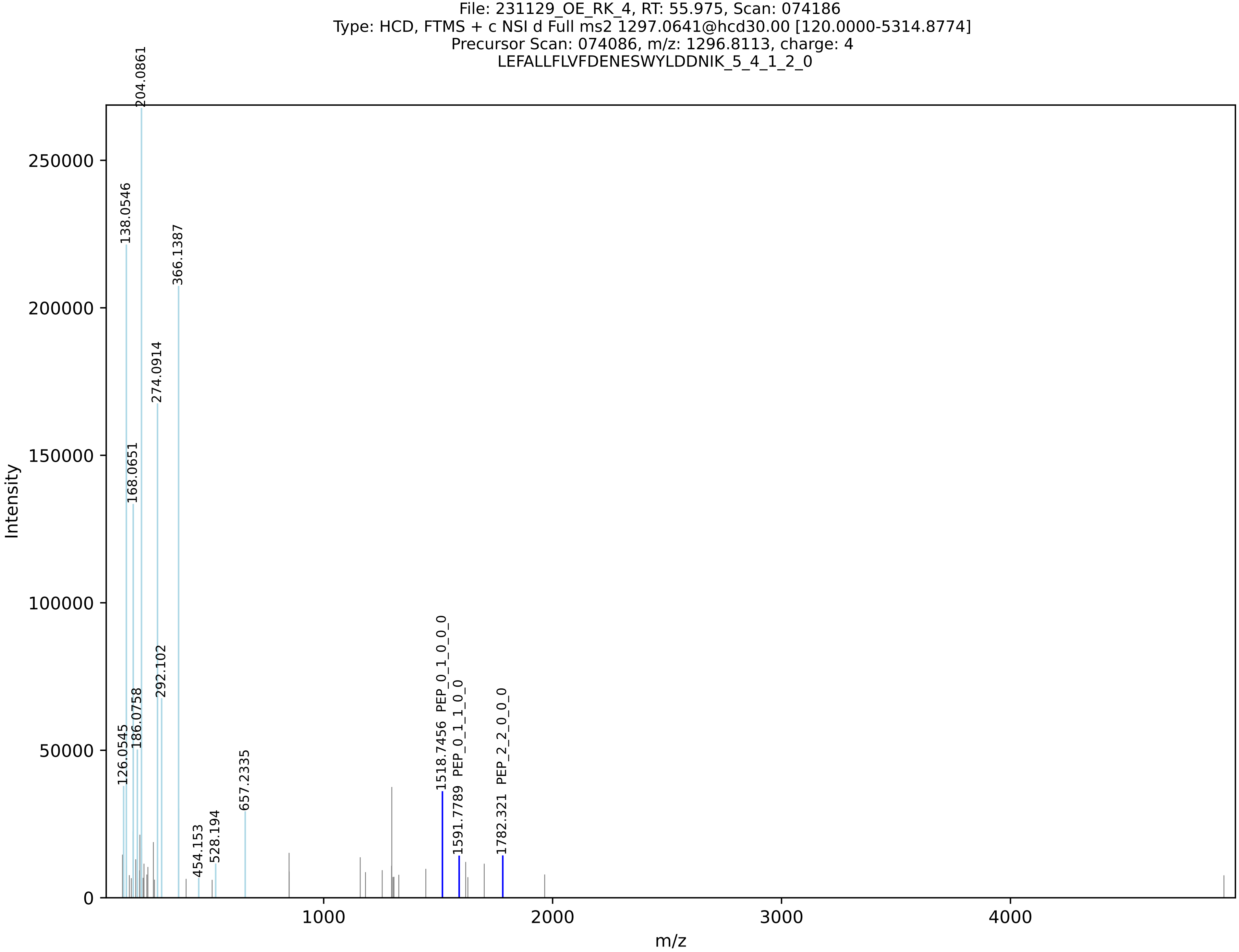
File: 231129_OE_RK_4, RT: 55.975, Scan: 074186
Type: HCD, FTMS + c NSI d Full ms2 1297.0641@hcd30.00 [120.0000-5314.8774]
Precursor Scan: 074086, m/z: 1296.8113, charge: 4
LEFALLFLVFDENESWYLDDNIK_5_4_1_2_0
204.0861
138.0546
366.1387
274.0914
168.0651
292.102
186.0758
126.0545
657.2335
454.153
528.194
1518.7456 PEP_0_1_0_0_
1591.7789 PEP_0_1_1_0_0
1782.321 PEP_2_2_0_0_0
Intensity
250000
200000
150000
100000
50000
0
1000
2000
3000
4000
m/z

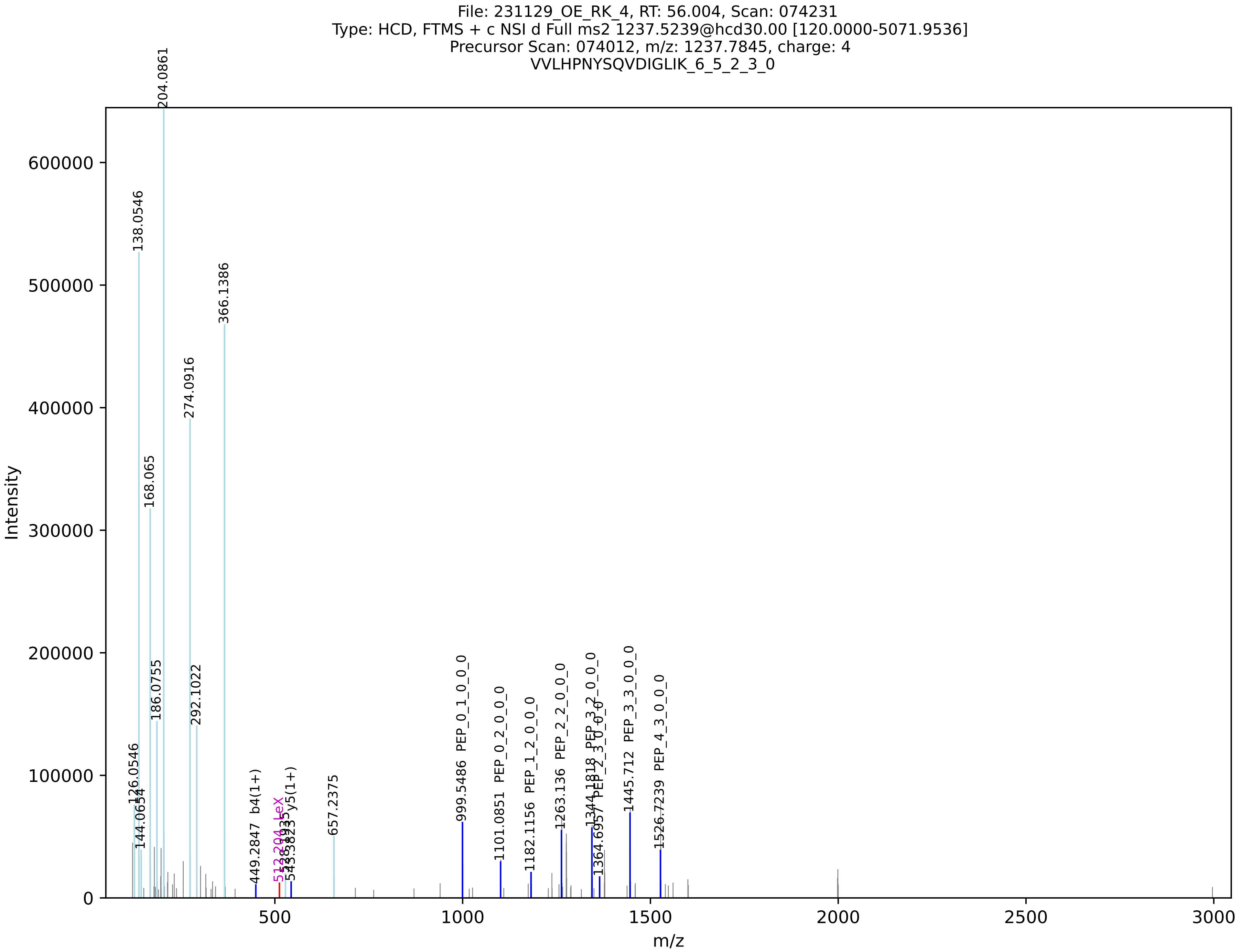
File: 231129_OE_RK_4, RT: 56.004, Scan: 074231
Type: HCD, FTMS + c NSI d Full ms2 1237.5239@hcd30.00 [120.0000-5071.9536]
Precursor Scan: 074012, m/z: 1237.7845, charge: 4
VVLHPNYSQVDIGLIK_6_5_2_3_0
204.0861
138.0546
366.1386
274.0916
168.065
186.0755
292.1022
126.0546
144.0654
449.2847 b4(1+)
543.3823 y5(1+)
657.2375
999.5486 PEP_0_1_0_0_0
1101.0851 PEP_0_2_0_0_0
1182.1156 PEP_1_2_0_0_0
1263.136 PEP_2_2_0_0_0
1344.1818 PEP_3_2_0_0_0
1364.6957 PEP_2_3_0_0_0
1445.712 PEP_3_3_0_0_0
1526.7239 PEP_4_3_0_0_0
Intensity
m/z

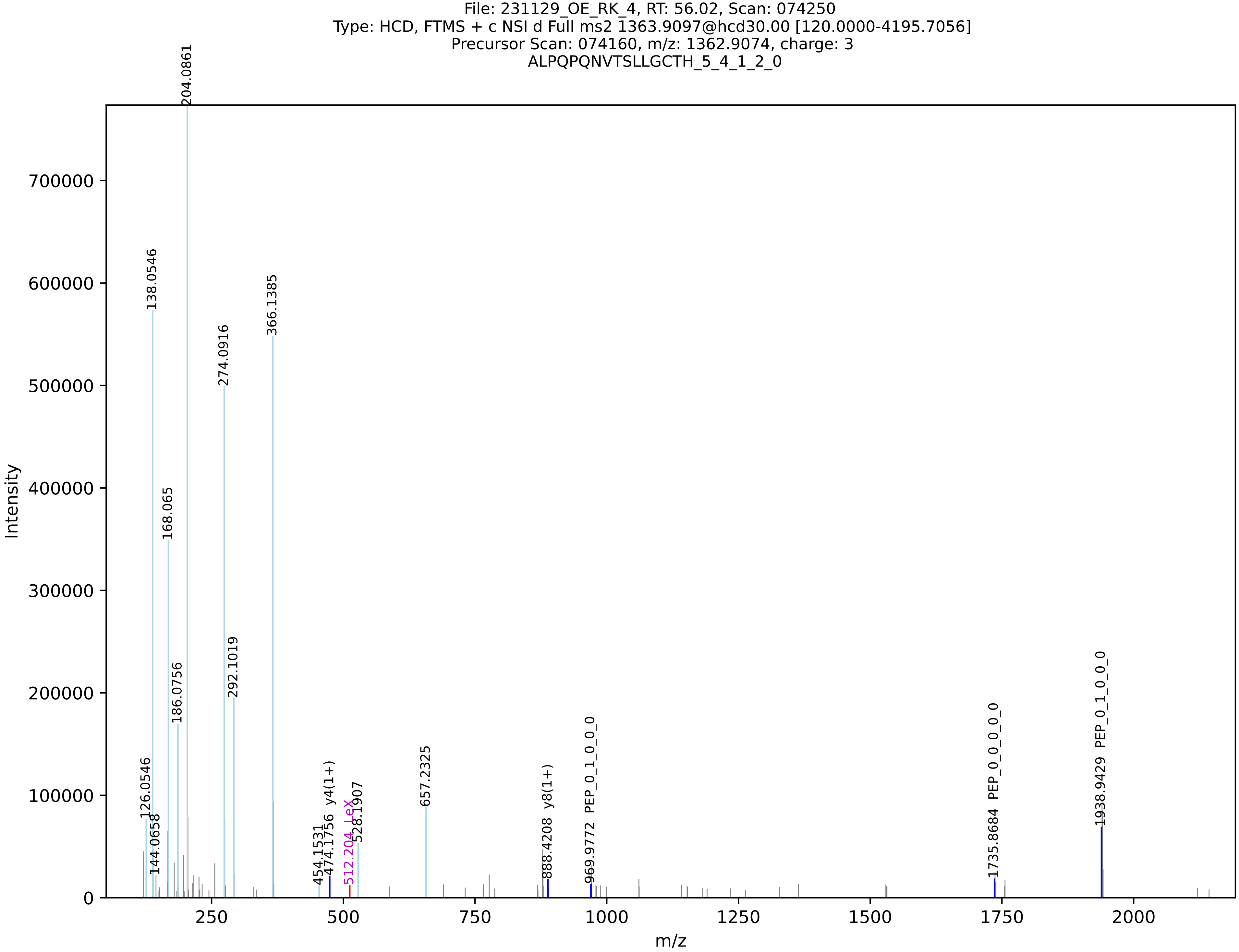

File: 231129_OE_RK_4, RT: 56.02, Scan: 074250
Type: HCD, FTMS + c NSI d Full ms2 1363.9097@hcd30.00 [120.0000-4195.7056]
Precursor Scan: 074160, m/z: 1362.9074, charge: 3
ALPQPQNVTSLLGCTH_5_4_1_2_0
Intensity
m/z
204.0861
138.0546
366.1385
274.0916
168.065
292.1019
186.0756
126.0546
144.0658
454.1531
474.1756 y4(1+)
512.204_LeX
528.1907
657.2325
888.4208 y8(1+)
969.9772 PEP_0_1_0_0_0
1735.8684 PEP_0_0_0_0_0
1938.9429 PEP_0_1_0_0_0

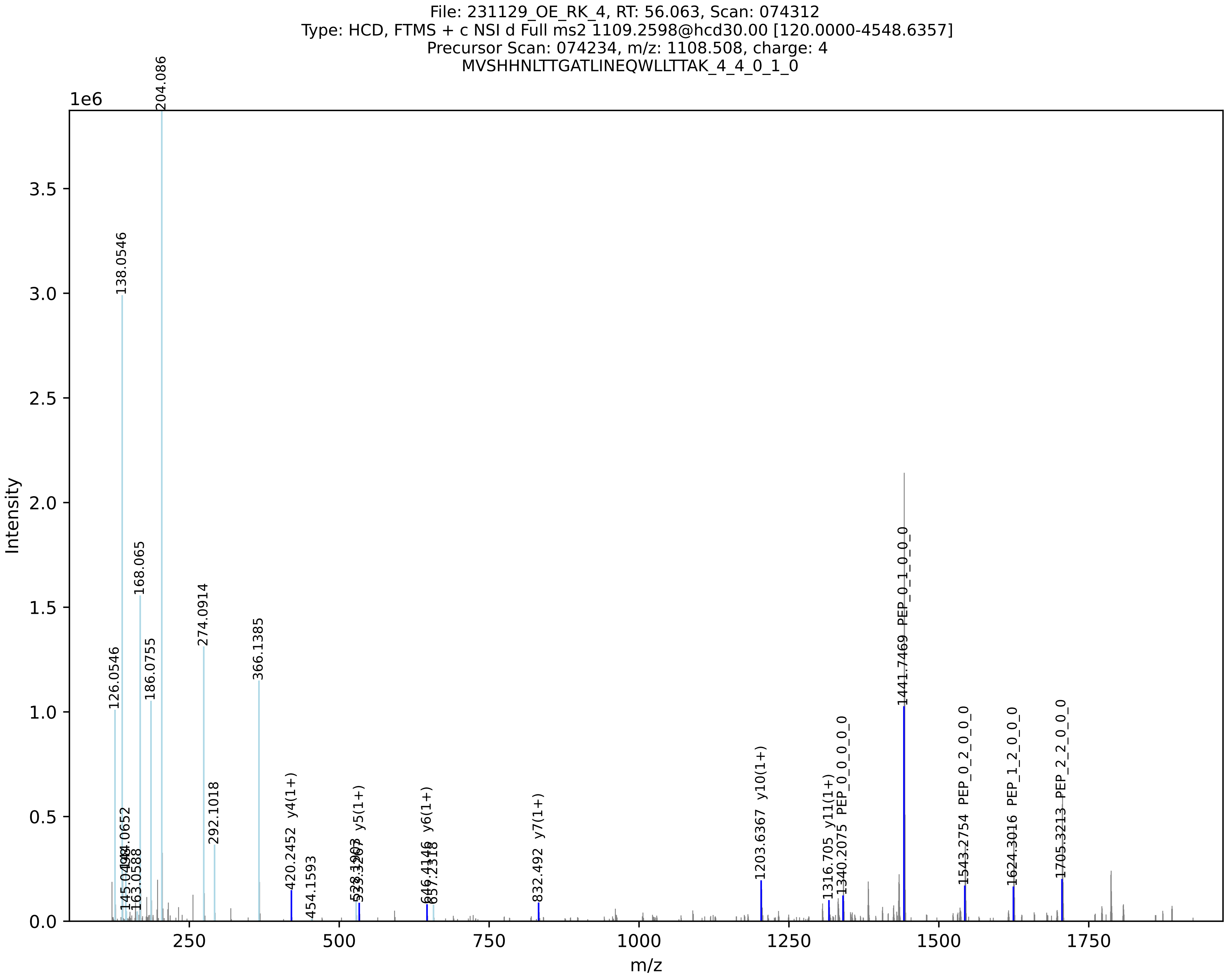
File: 231129_OE_RK_4, RT: 56.063, Scan: 074312
Type: HCD, FTMS + c NSI d Full ms2 1109.2598@hcd30.00 [120.0000-4548.6357]
Precursor Scan: 074234, m/z: 1108.508, charge: 4
MVSHHNLTTGATLINEQWLLTTAK_4_4_0_1_0
1e6
Intensity
m/z
204.086
138.0546
168.065
274.0914
366.1385
126.0546
186.0755
292.1018
163.0588
420.2452 y4(1+)
454.1593
y5(1+)
646.4146 y6(1+)
657.2318
832.492 y7(1+)
1203.6367 y10(1+)
1316.705 y11(1+)
1340.2075 PEP_0_0_0_0_0
1441.7469 PEP_0_1_0_0_0
1543.2754 PEP_0_2_0_0_0
1624.3016 PEP_1_2_0_0_0
1705.3213 PEP_2_2_0_0_0

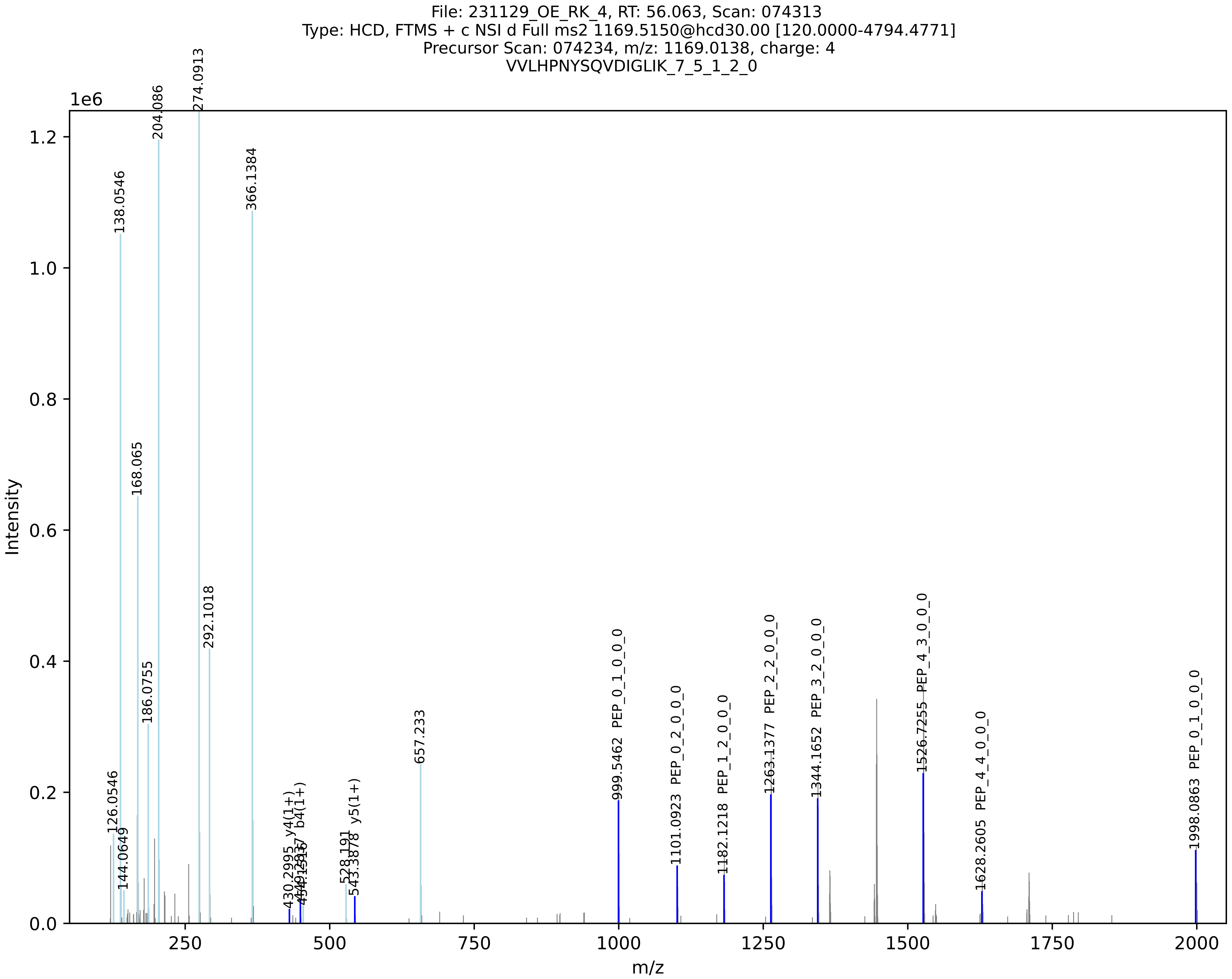
File: 231129_OE_RK_4, RT: 56.063, Scan: 074313
Type: HCD, FTMS + c NSI d Full ms2 1169.5150@hcd30.00 [120.0000-4794.4771]
Precursor Scan: 074234, m/z: 1169.0138, charge: 4
VVLHPNYSQVDIGLIK_7_5_1_2_0
1e6
Intensity
m/z
274.0913
204.086
366.1384
138.0546
168.065
292.1018
186.0755
126.0546
144.0649
430.2995_y4(1+)
b4(1+)
528.191
543.3878_y5(1+)
657.233
999.5462_PEP_0_1_0_0_0
1101.0923_PEP_0_2_0_0_0
1182.1218_PEP_1_2_0_0_0
1263.1377_PEP_2_2_0_0_0
1344.1652_PEP_3_2_0_0_0
1526.7255_PEP_4_3_0_0_0
1628.2605_PEP_4_4_0_0_0
1998.0863_PEP_0_1_0_0_0

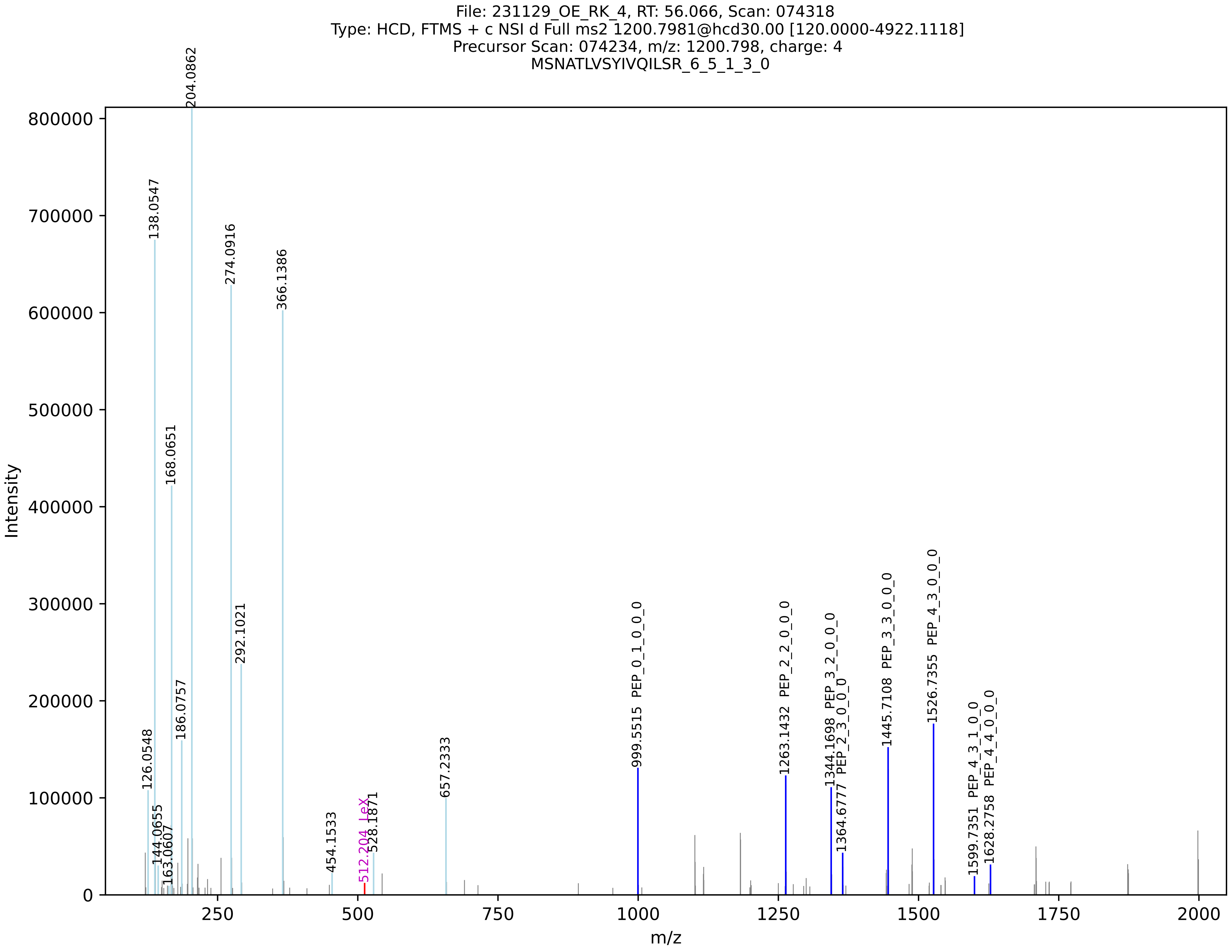

File: 231129_OE_RK_4, RT: 56.066, Scan: 074318
Type: HCD, FTMS + c NSI d Full ms2 1200.7981@hcd30.00 [120.0000-4922.1118]
Precursor Scan: 074234, m/z: 1200.798, charge: 4
MSNATLVSYIVQILSR_6_5_1_3_0
Intensity
m/z
204.0862
138.0547
274.0916
366.1386
168.0651
292.1021
186.0757
126.0548
144.0655
163.0607
454.1533
512.204_LeX
528.1871
657.2333
999.5515 PEP_0_1_0_0_0
1263.1432 PEP_2_2_0_0_0
1344.1698 PEP_3_2_0_0_0
1364.6777 PEP_2_3_0_0_0
1445.7108 PEP_3_3_0_0_0
1526.7355 PEP_4_3_0_0_0
1599.7351 PEP_4_3_1_0_0
1628.2758 PEP_4_4_0_0_0

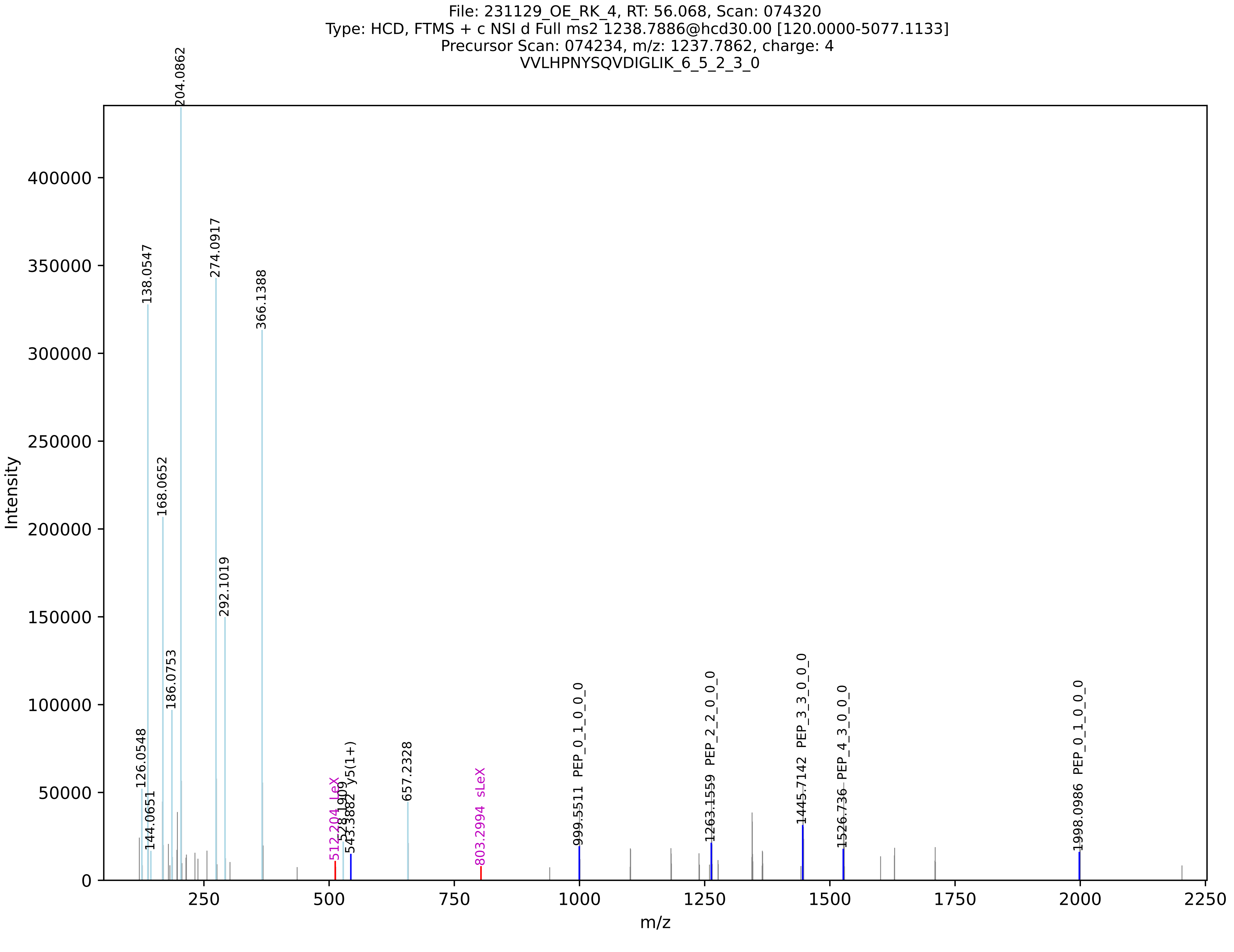
File: 231129_OE_RK_4, RT: 56.068, Scan: 074320
Type: HCD, FTMS + c NSI d Full ms2 1238.7886@hcd30.00 [120.0000-5077.1133]
Precursor Scan: 074234, m/z: 1237.7862, charge: 4
VVLHPNYSQVDIGLIK_6_5_2_3_0
Intensity
m/z
204.0862
138.0547
274.0917
366.1388
168.0652
292.1019
186.0753
126.0548
144.0651
512.204 LeX
543.3882 y5(1+)
657.2328
803.2994 sLeX
999.5511 PEP_0_1_0_0_0
1263.1559 PEP_2_2_0_0_0
1445.7142 PEP_3_3_0_0_0
1526.736 PEP_4_3_0_0_0
1998.0986 PEP_0_1_0_0_0

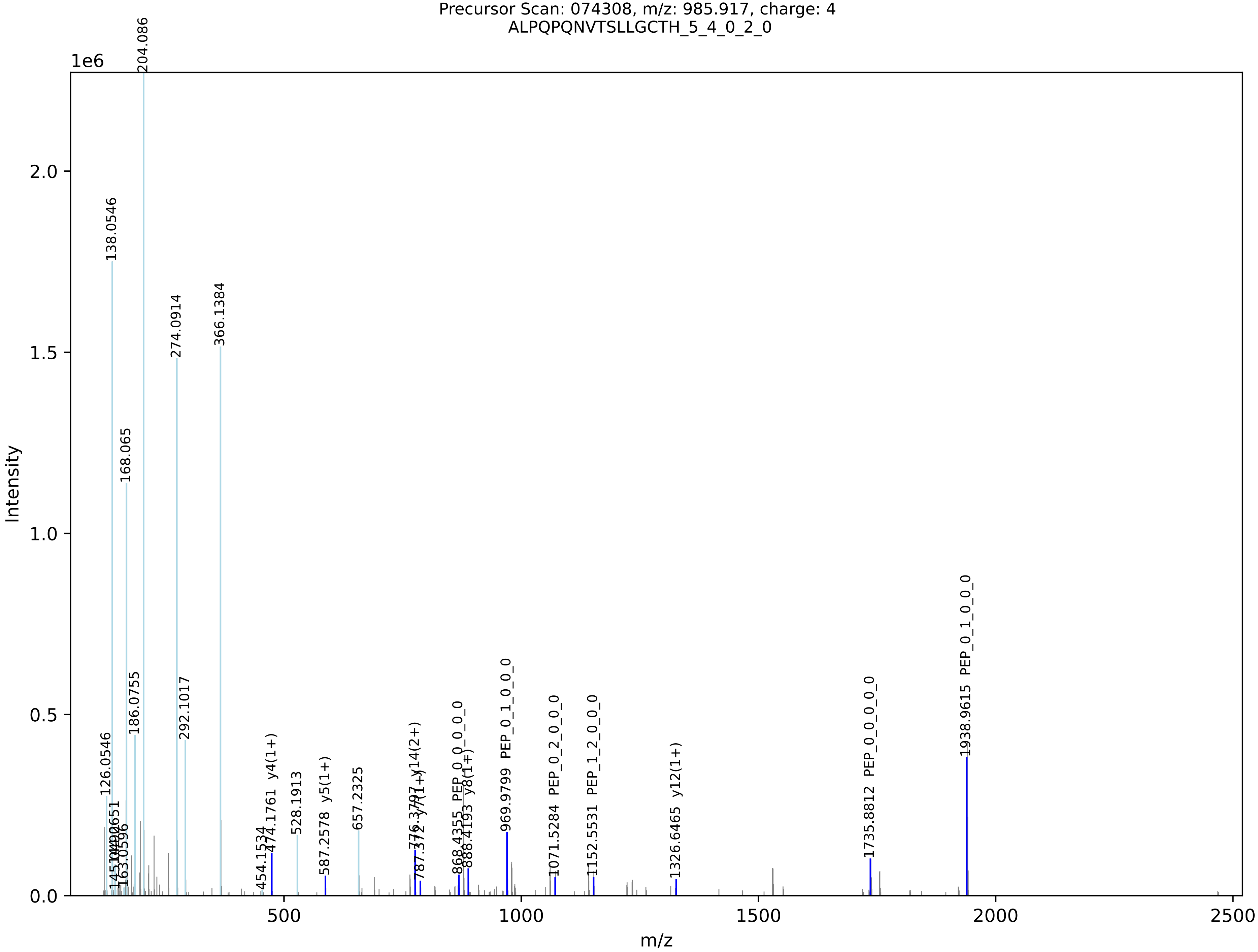
File: 231129_OE_RK_4, RT: 56.112, Scan: 074384
Type: HCD, FTMS + c NSI d Full ms2 986.4180@hcd30.00 [120.0000-4047.4417]
Precursor Scan: 074308, m/z: 985.917, charge: 4
ALPQPQNVTSLLGCTH_5_4_0_2_0
1e6
Intensity
m/z
204.086
138.0546
168.065
274.0914
366.1384
186.0755
292.1017
126.0546
163.0596
454.1534
474.1761 y4(1+)
528.1913
587.2578 y5(1+)
657.2325
776.3797 y14(2+)
787.372 y7(1+)
868.4355 PEP_0_0_0_0_0
888.4193 y8(1+)
969.9799 PEP_0_1_0_0_0
1071.5284 PEP_0_2_0_0_0
1152.5531 PEP_1_2_0_0_0
1326.6465 y12(1+)
1735.8812 PEP_0_0_0_0_0
1938.9615 PEP_0_1_0_0_0

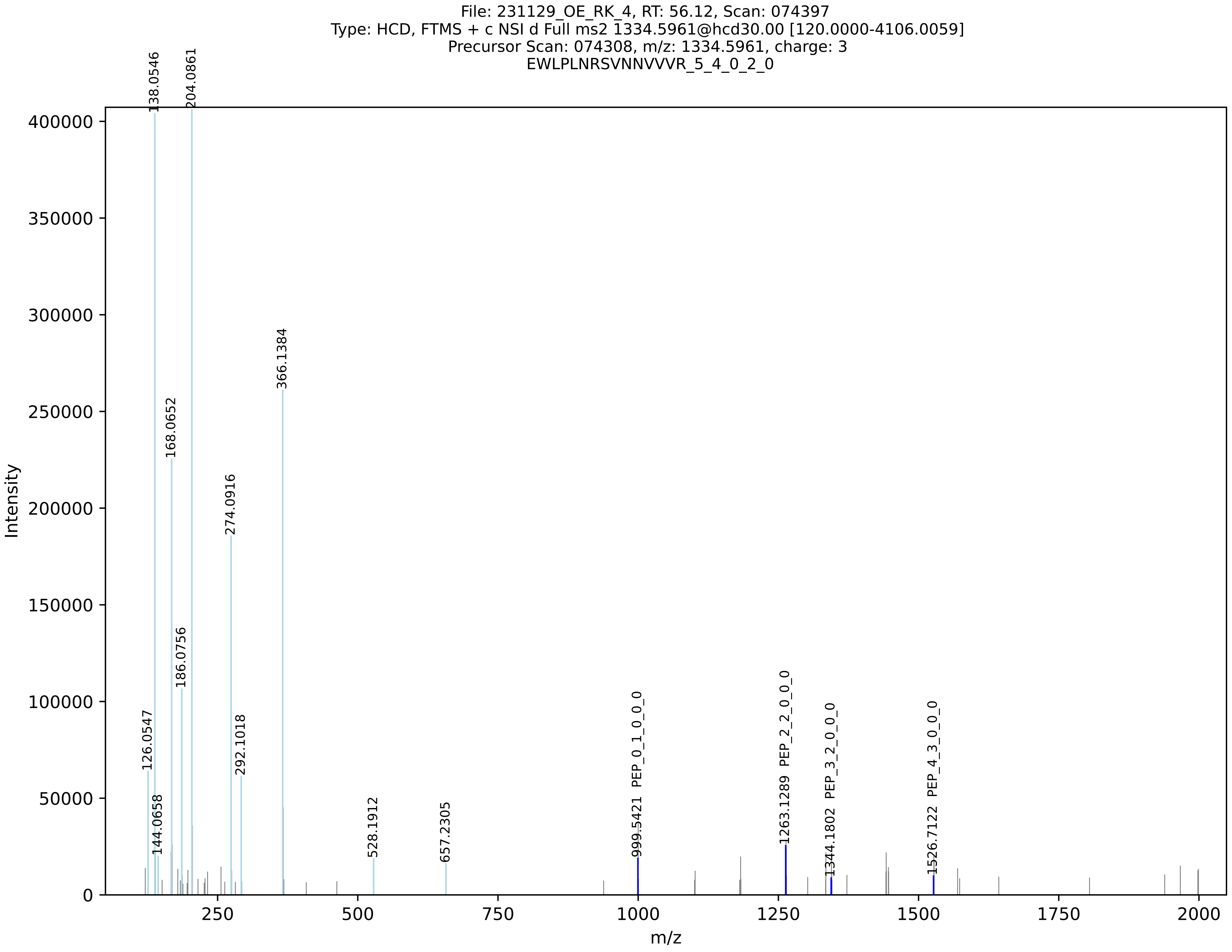
File: 231129_OE_RK_4, RT: 56.12, Scan: 074397
Type: HCD, FTMS + c NSI d Full ms2 1334.5961@hcd30.00 [120.0000-4106.0059]
Precursor Scan: 074308, m/z: 1334.5961, charge: 3
EWLPLNRSVNNVVVR_5_4_0_2_0
Intensity
m/z
138.0546
204.0861
366.1384
168.0652
274.0916
186.0756
126.0547
292.1018
144.0658
528.1912
657.2305
999.5421 PEP_0_1_0_0_0
1263.1289 PEP_2_2_0_0_0
1344.1802 PEP_3_2_0_0_0
1526.7122 PEP_4_3_0_0_0

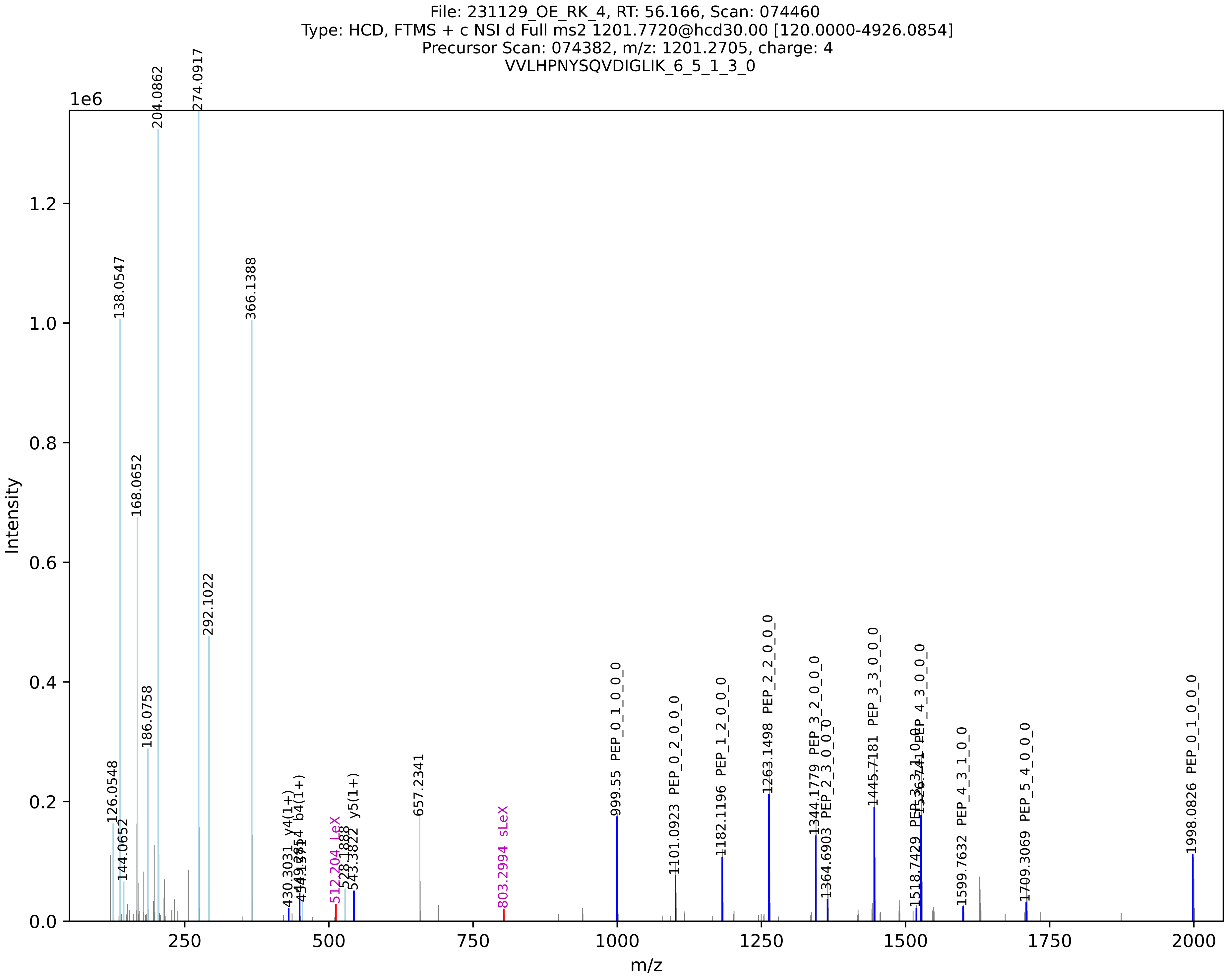

File: 231129_OE_RK_4, RT: 56.166, Scan: 074460
Type: HCD, FTMS + c NSI d Full ms2 1201.7720@hcd30.00 [120.0000-4926.0854]
Precursor Scan: 074382, m/z: 1201.2705, charge: 4
VVLHPNYSQVDIGLIK_6_5_1_3_0
1e6
Intensity
m/z
0.0
0.2
0.4
0.6
0.8
1.0
1.2
250
500
750
1000
1250
1500
1750
2000
274.0917
204.0862
138.0547
366.1388
168.0652
292.1022
186.0758
126.0548
144.0652
657.2341
430.3031 y4(1+)
543.3822 y5(1+)
528.1888
512.204 LeX
803.2994 sLeX
999.55 PEP_0_1_0_0_0
1101.0923 PEP_0_2_0_0_0
1182.1196 PEP_1_2_0_0_0
1263.1498 PEP_2_2_0_0_0
1344.1779 PEP_3_2_0_0_0
1364.6903 PEP_2_3_0_0_0
1445.7181 PEP_3_3_0_0_0
PEP_4_3_0_0_0
1599.7632 PEP_4_3_1_0_0
1709.3069 PEP_5_4_0_0_0
1998.0826 PEP_0_1_0_0_0

File: 231129_OE_RK_4, RT: 56.195, Scan: 074506
Type: HCD, FTMS + c NSI d Full ms2 1319.2675@hcd30.00 [120.0000-4059.1003]
Precursor Scan: 074382, m/z: 1319.2674, charge: 3
ALPQPQNVTSLLGCTH_6_4_1_1_0

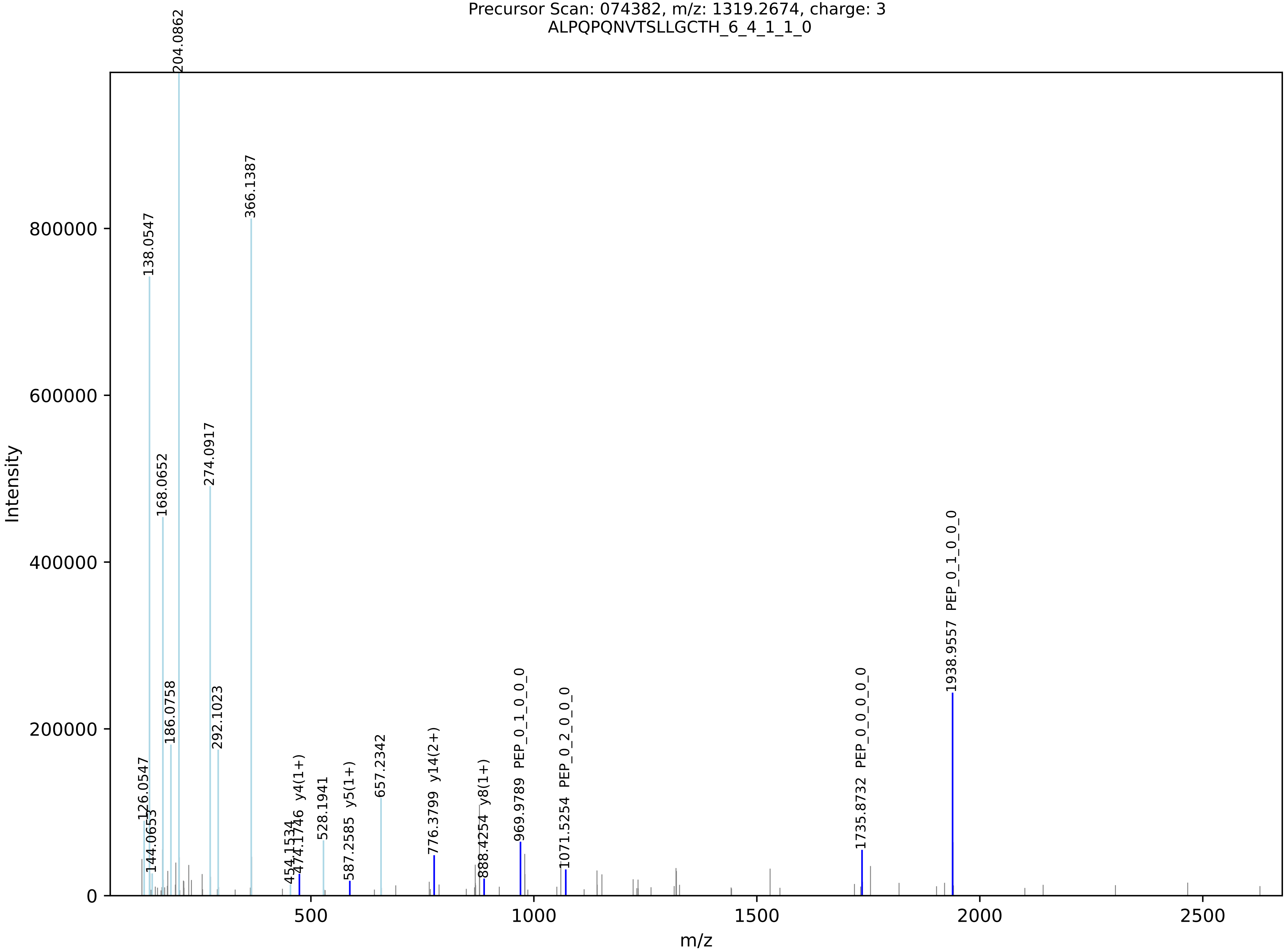

File: 231129_OE_RK_4, RT: 56.212, Scan: 074527
Type: HCD, FTMS + c NSI d Full ms2 1314.8889@hcd30.00 [120.0000-4045.7021]
Precursor Scan: 074456, m/z: 1314.2207, charge: 3
ALPQPQNVTSLLGCTH_5_4_0_2_0

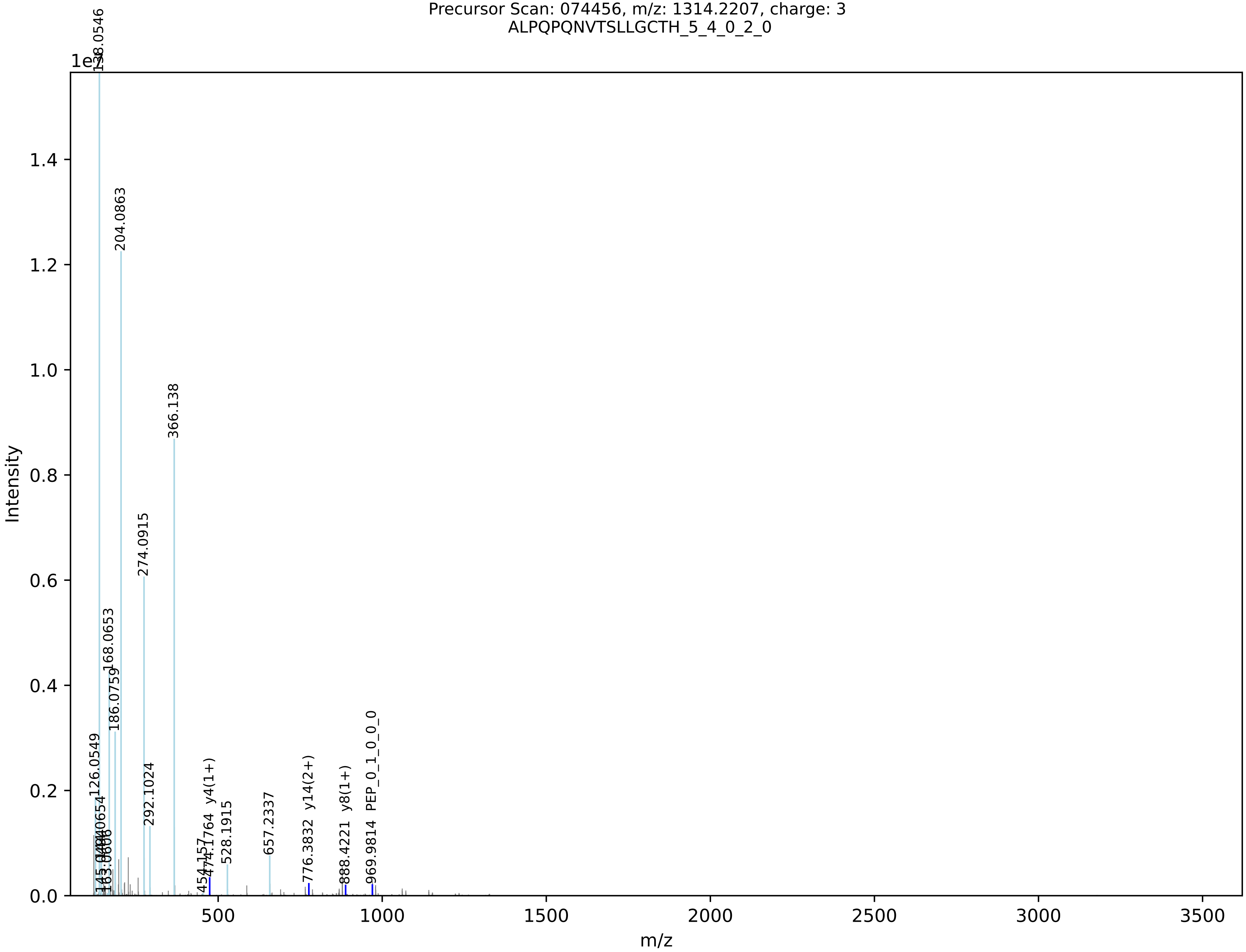

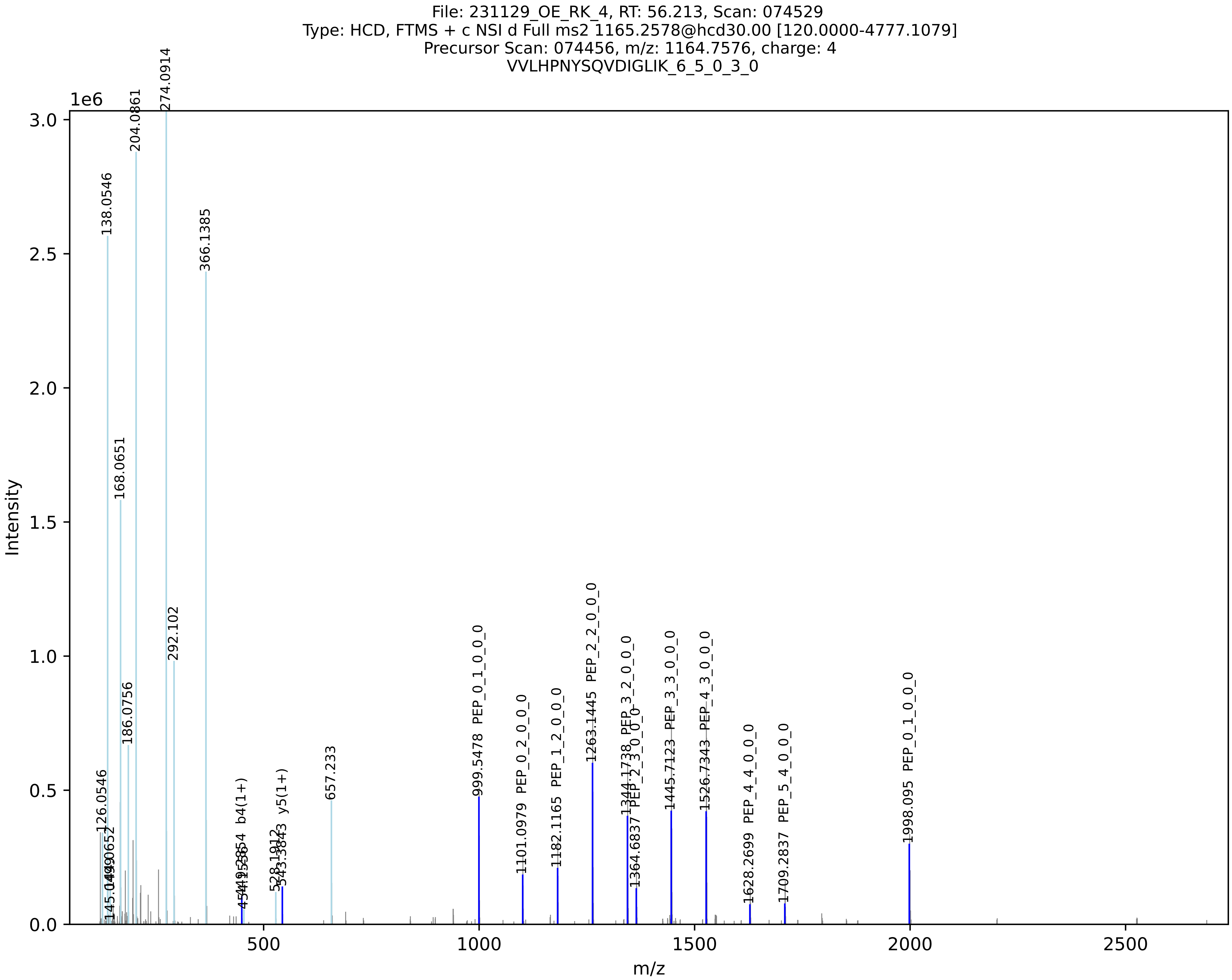
File: 231129_OE_RK_4, RT: 56.213, Scan: 074529
Type: HCD, FTMS + c NSI d Full ms2 1165.2578@hcd30.00 [120.0000-4777.1079]
Precursor Scan: 074456, m/z: 1164.7576, charge: 4
VVLHPNYSQVDIGLIK_6_5_0_3_0
1e6
Intensity
m/z
274.0914
204.0861
138.0546
366.1385
168.0651
292.102
186.0756
126.0556
657.233
999.5478 PEP_0_1_0_0_0
1101.0979 PEP_0_2_0_0_0
1182.1165 PEP_1_2_0_0_0
1263.1445 PEP_2_2_0_0_0
1344.1738 PEP_3_2_0_0_0
1364.6837 PEP_2_3_0_0_0
1445.7123 PEP_3_3_0_0_0
1526.7343 PEP_4_3_0_0_0
1628.2699 PEP_4_4_0_0_0
1709.2837 PEP_5_4_0_0_0
1998.095 PEP_0_1_0_0_0
b4(1+)
y5(1+)
528.1912
543.3843

File: 231129_OE_RK_4, RT: 56.214, Scan: 074531
Type: HCD, FTMS + c NSI d Full ms2 1320.2295@hcd30.00 [120.0000-4062.0442]
Precursor Scan: 074456, m/z: 1319.8954, charge: 3
ALPQPQNVTSLLGCTH_6_4_1_1_0

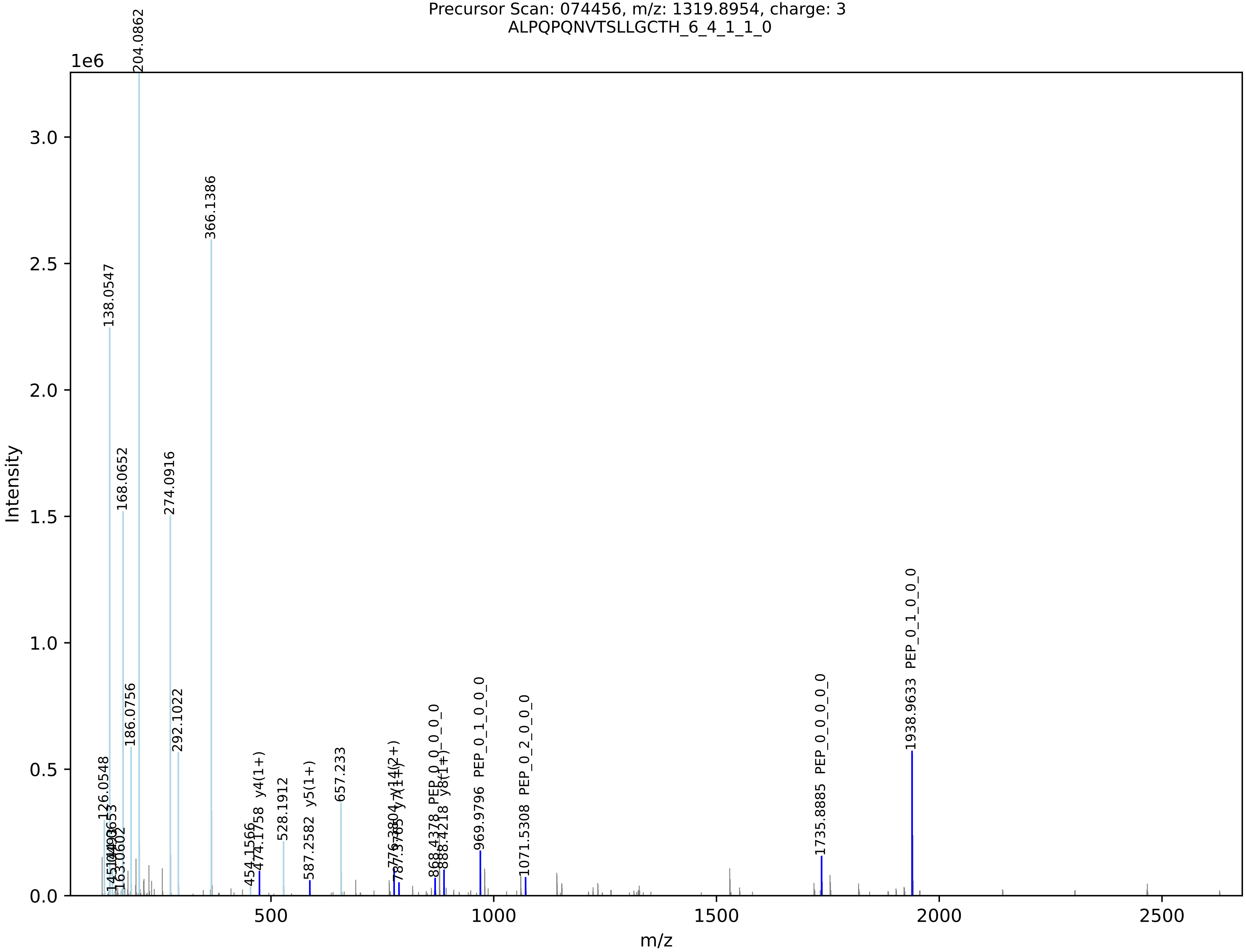

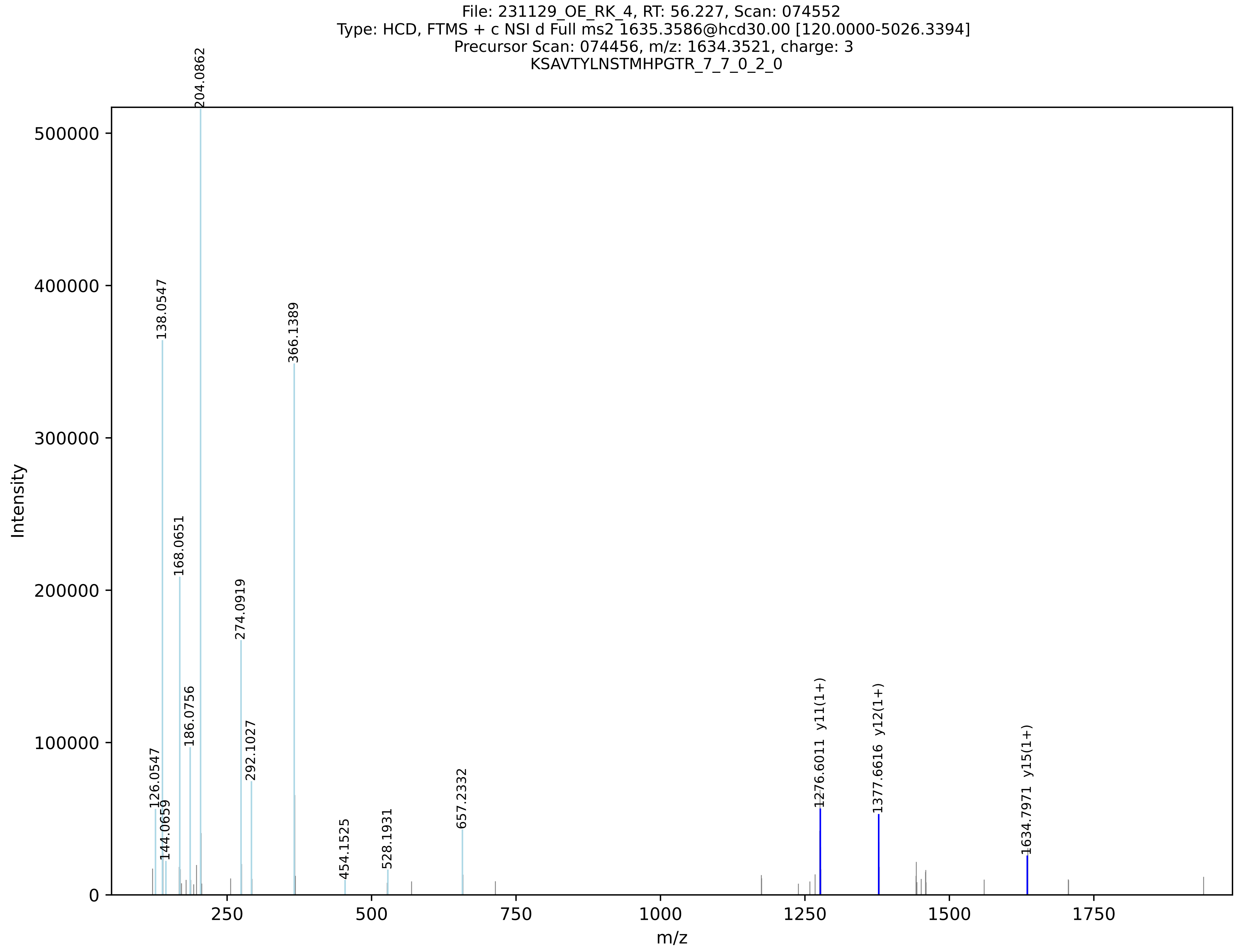

File: 231129_OE_RK_4, RT: 56.227, Scan: 074552
Type: HCD, FTMS + c NSI d Full ms2 1635.3586@hcd30.00 [120.0000-5026.3394]
Precursor Scan: 074456, m/z: 1634.3521, charge: 3
KSAVTYLNSTMHPGTR_7_7_0_2_0
Intensity
m/z
204.0862
138.0547
366.1389
168.0651
274.0919
186.0756
292.1027
126.0547
144.0659
454.1525
528.1931
657.2332
1276.6011 y11(1+)
1377.6616 y12(1+)
1634.7971 y15(1+)
0
100000
200000
300000
400000
500000
250
500
750
1000
1250
1500
1750

File: 231129_OE_RK_4, RT: 56.264, Scan: 074603
Type: HCD, FTMS + c NSI d Full ms2 1058.2399@hcd30.00 [120.0000-4340.4746]
Precursor Scan: 074526, m/z: 1057.7387, charge: 4
MVSHHNLTTGATLINEQWLLTTAK_4_3_0_1_0

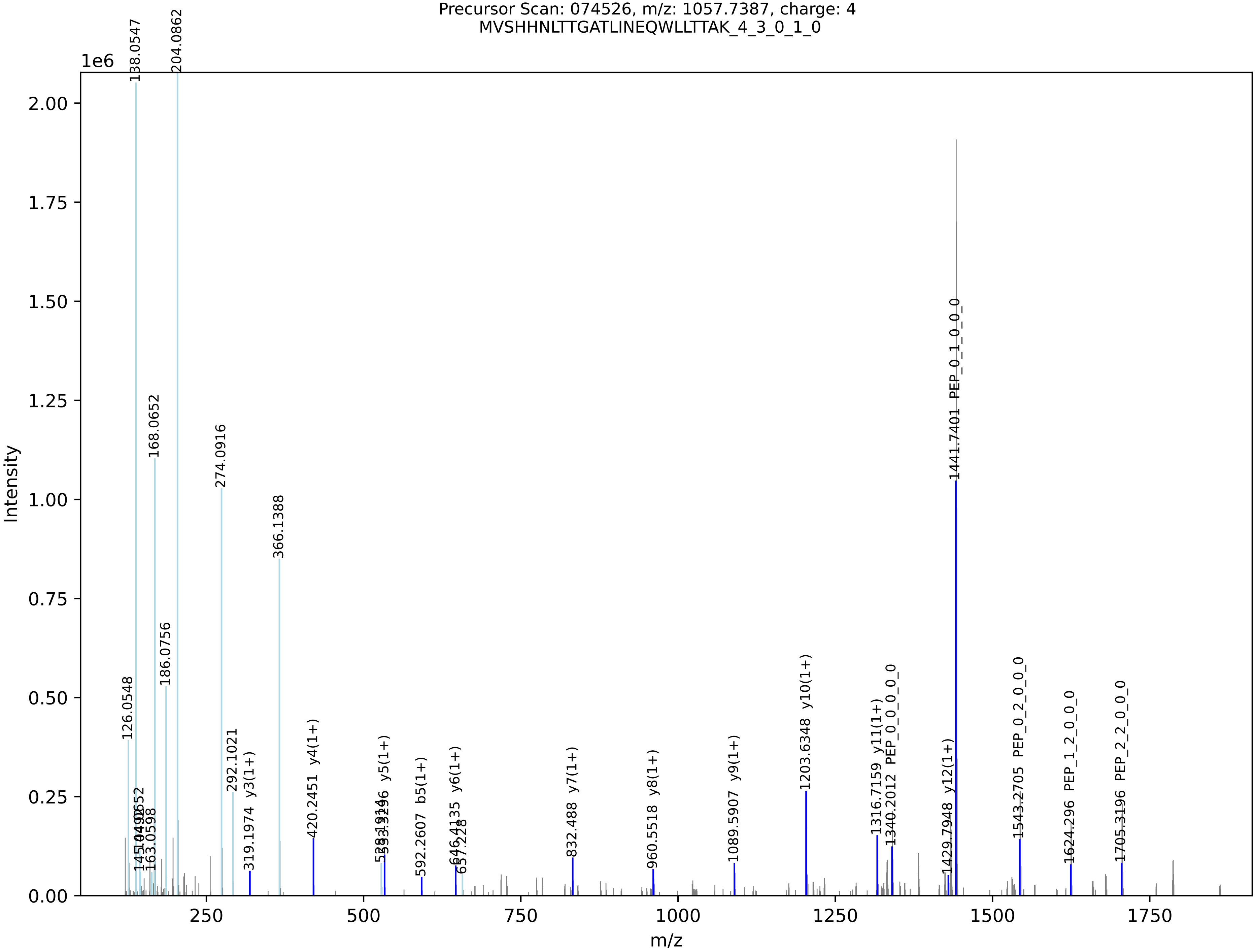

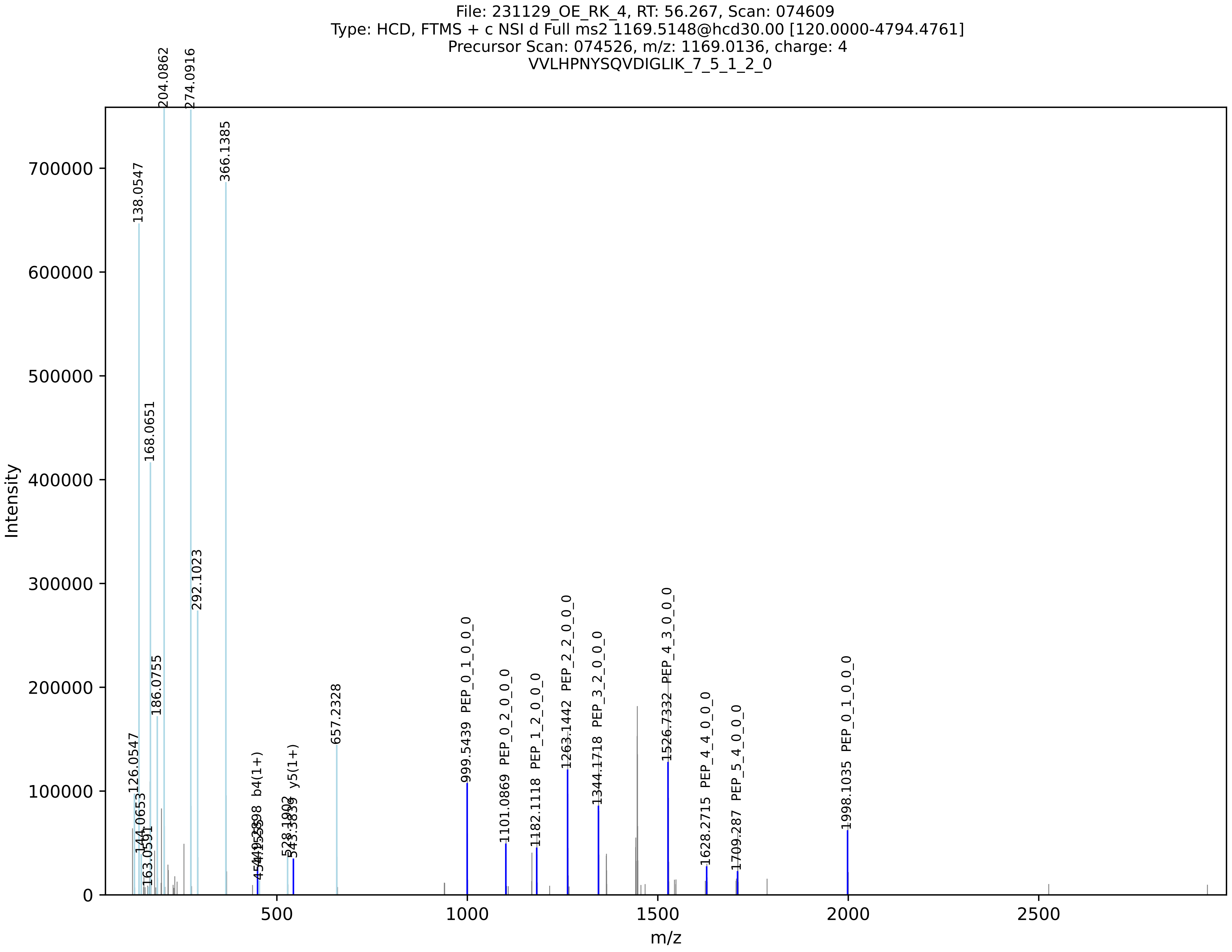

File: 231129_OE_RK_4, RT: 56.267, Scan: 074609
Type: HCD, FTMS + c NSI d Full ms2 1169.5148@hcd30.00 [120.0000-4794.4761]
Precursor Scan: 074526, m/z: 1169.0136, charge: 4
VVLHPNYSQVDIGLIK_7_5_1_2_0
Intensity
m/z
204.0862
274.0916
366.1385
138.0547
168.0651
292.1023
186.0755
126.0547
144.0653
163.0591
657.2328
b4(1+)
y5(1+)
528.1902
543.3839
999.5439 PEP_0_1_0_0_0
1101.0869 PEP_0_2_0_0_0
1182.1118 PEP_1_2_0_0_0
1263.1442 PEP_2_2_0_0_0
1344.1718 PEP_3_2_0_0_0
1526.7332 PEP_4_3_0_0_0
1628.2715 PEP_4_4_0_0_0
1709.287 PEP_5_4_0_0_0
1998.1035 PEP_0_1_0_0_0

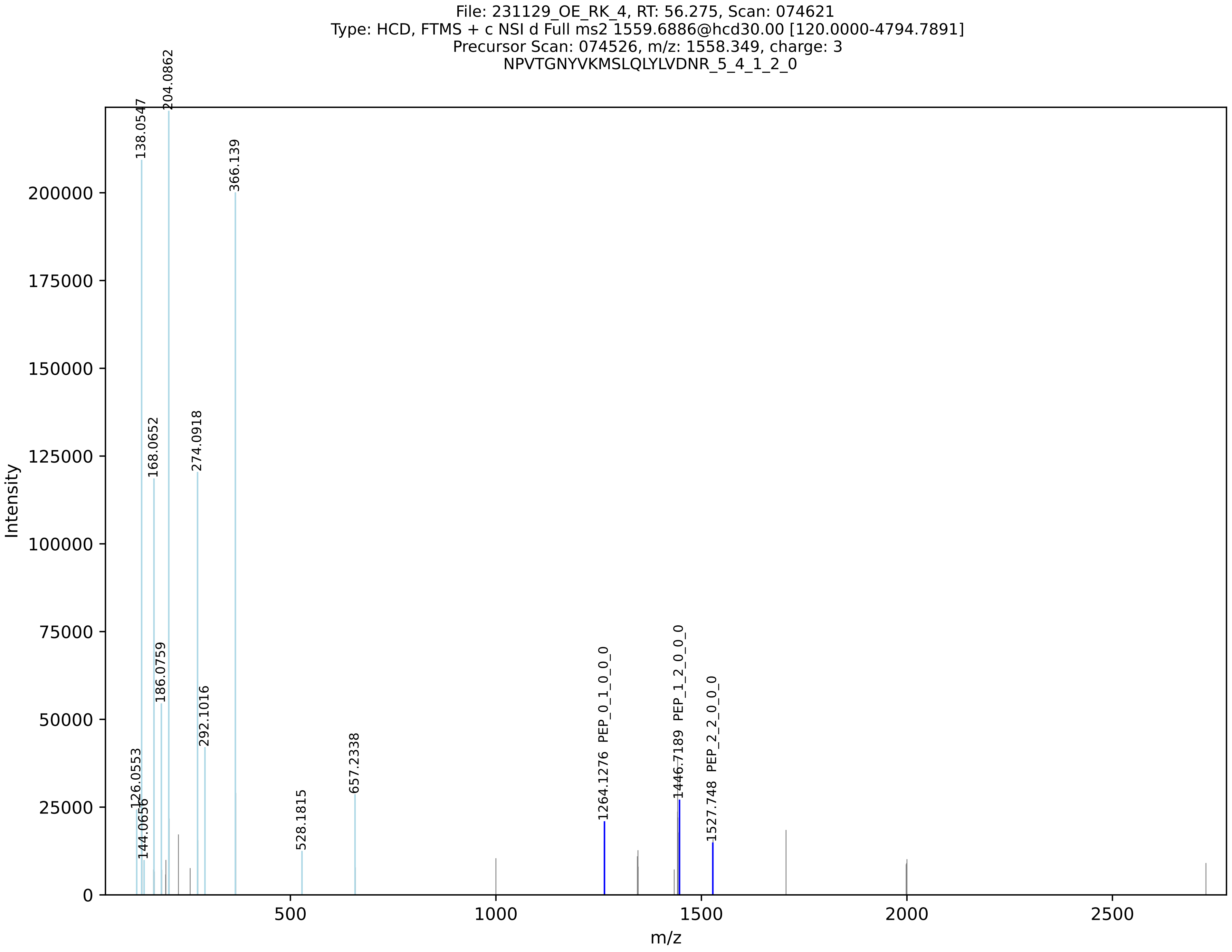
File: 231129_OE_RK_4, RT: 56.275, Scan: 074621
Type: HCD, FTMS + c NSI d Full ms2 1559.6886@hcd30.00 [120.0000-4794.7891]
Precursor Scan: 074526, m/z: 1558.349, charge: 3
NPVTGNYVKMSLQLYLVDNR_5_4_1_2_0
Intensity
m/z
204.0862
138.0547
366.139
168.0652
274.0918
186.0759
292.1016
126.0553
144.0656
528.1815
657.2338
1264.1276 PEP_0_1_0_0_0
1446.7189 PEP_1_2_0_0_0
1527.748 PEP_2_2_0_0_0
0
25000
50000
75000
100000
125000
150000
175000
200000
500
1000
1500
2000
2500

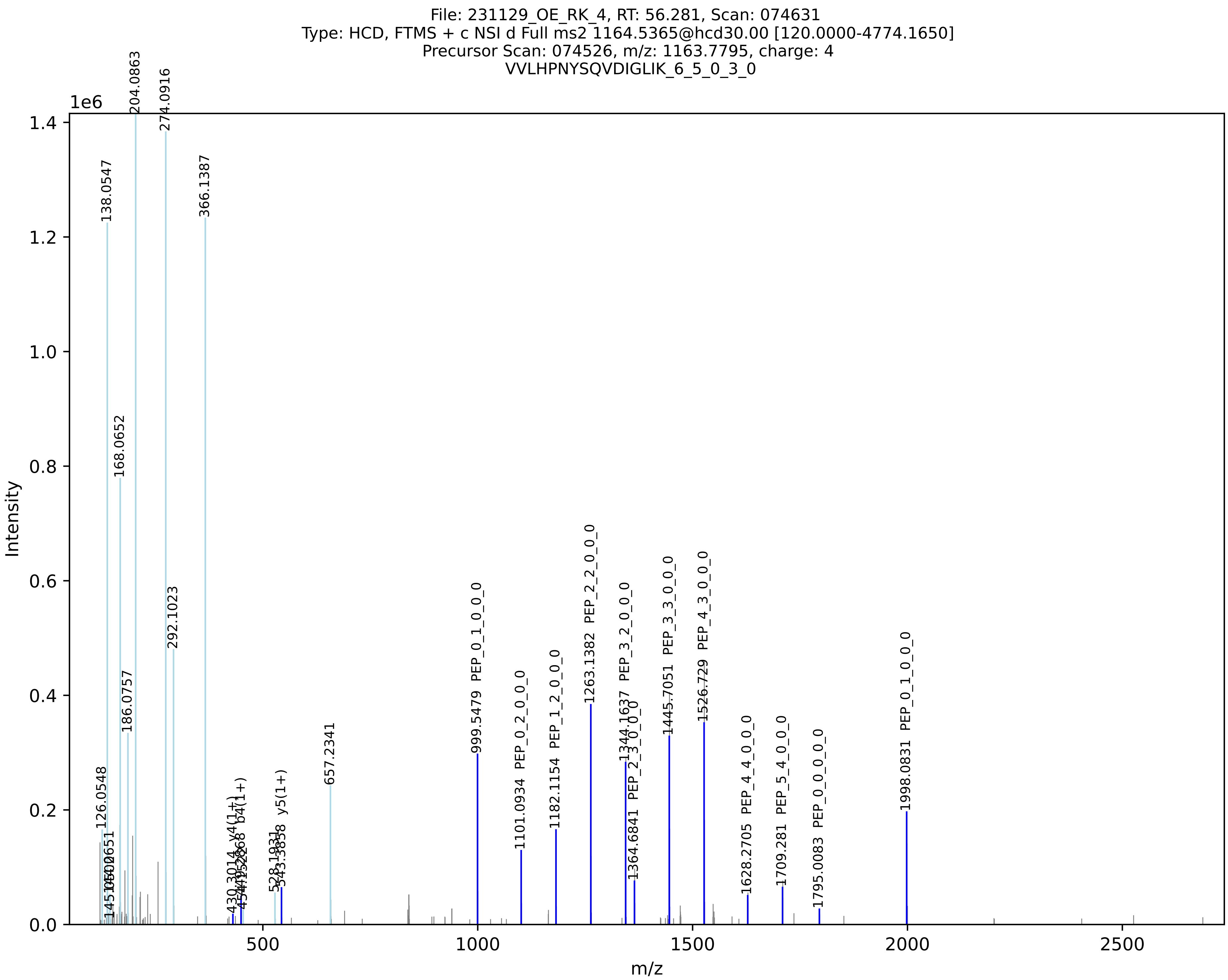
File: 231129_OE_RK_4, RT: 56.281, Scan: 074631
Type: HCD, FTMS + c NSI d Full ms2 1164.5365@hcd30.00 [120.0000-4774.1650]
Precursor Scan: 074526, m/z: 1163.7795, charge: 4
VVLHPNYSQVDIGLIK_6_5_0_3_0
1e6
Intensity
m/z
204.0863
274.0916
138.0547
366.1387
168.0652
292.1023
186.0757
126.0548
657.2341
999.5479 PEP_0_1_0_0_0
1101.0934 PEP_0_2_0_0_0
1182.1154 PEP_1_2_0_0_0
1263.1382 PEP_2_2_0_0_0
1344.1637 PEP_3_2_0_0_0
1364.6841 PEP_2_3_0_0_0
1445.7051 PEP_3_3_0_0_0
1526.729 PEP_4_3_0_0_0
1628.2705 PEP_4_4_0_0_0
1709.281 PEP_5_4_0_0_0
1795.0083 PEP_0_0_0_0_0
1998.0831 PEP_0_1_0_0_0
y4(1+)
b4(1+)
528.1931
543.3858 y5(1+)
0.0
0.2
0.4
0.6
0.8
1.0
1.2
1.4
500
1000
1500
2000
2500

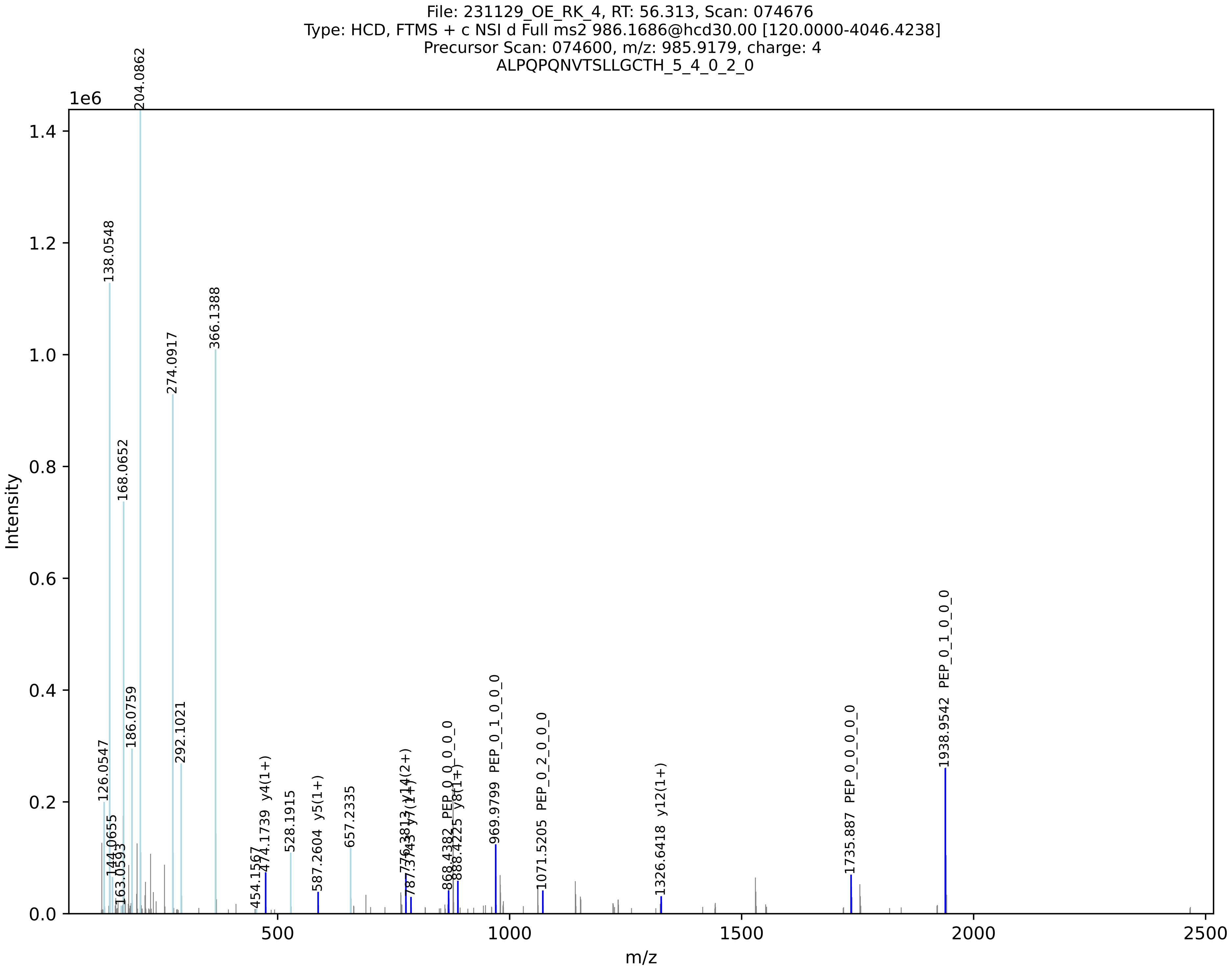
File: 231129_OE_RK_4, RT: 56.313, Scan: 074676
Type: HCD, FTMS + c NSI d Full ms2 986.1686@hcd30.00 [120.0000-4046.4238]
Precursor Scan: 074600, m/z: 985.9179, charge: 4
ALPQPQNVTSLLGCTH_5_4_0_2_0
1e6
Intensity
m/z
204.0862
138.0548
366.1388
274.0917
168.0652
186.0759
292.1021
126.0547
144.0655
163.0593
454.1567
474.1739 y4(1+)
528.1915
587.2604 y5(1+)
657.2335
776.3813 y14(2+)
787.3743 y7(1+)
868.4382 PEP_0_0_0_0_0
888.4225 y8(1+)
969.9799 PEP_0_1_0_0_0
1071.5205 PEP_0_2_0_0_0
1326.6418 y12(1+)
1735.887 PEP_0_0_0_0_0
1938.9542 PEP_0_1_0_0_0

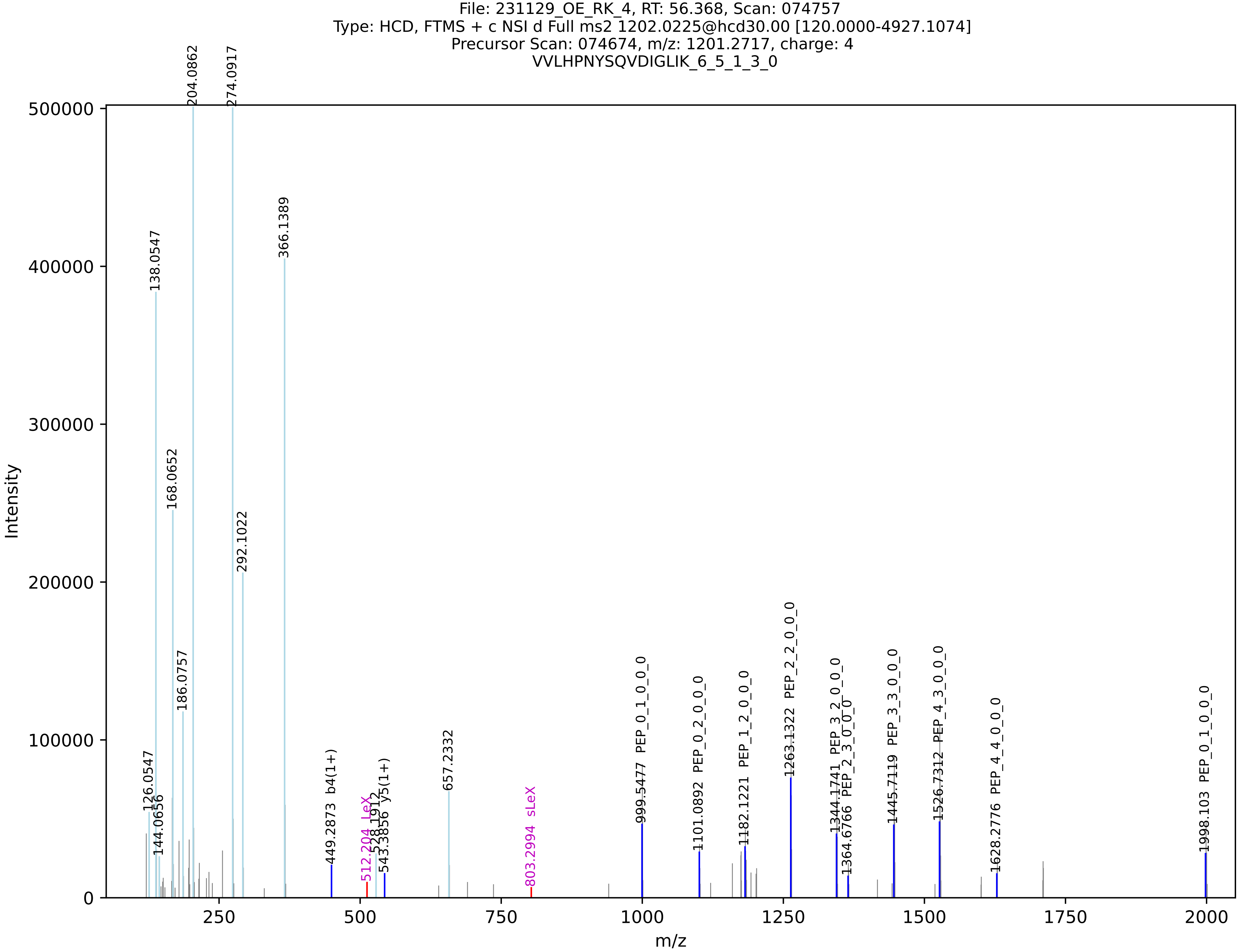
File: 231129_OE_RK_4, RT: 56.368, Scan: 074757
Type: HCD, FTMS + c NSI d Full ms2 1202.0225@hcd30.00 [120.0000-4927.1074]
Precursor Scan: 074674, m/z: 1201.2717, charge: 4
VVLHPNYSQVDIGLIK_6_5_1_3_0
Intensity
m/z
204.0862
274.0917
366.1389
138.0547
168.0652
292.1022
186.0757
126.0547
144.0656
449.2873 b4(1+)
512.204 LeX
528.1912
543.3856 y5(1+)
657.2332
803.2994 sLeX
999.5477 PEP_0_1_0_0_0
1101.0892 PEP_0_2_0_0_0
1182.1221 PEP_1_2_0_0_0
1263.1322 PEP_2_2_0_0_0
1344.1741 PEP_3_2_0_0_0
1364.6766 PEP_2_3_0_0_0
1445.7119 PEP_3_3_0_0_0
1526.7312 PEP_4_3_0_0_0
1628.2776 PEP_4_4_0_0_0
1998.103 PEP_0_1_0_0_0

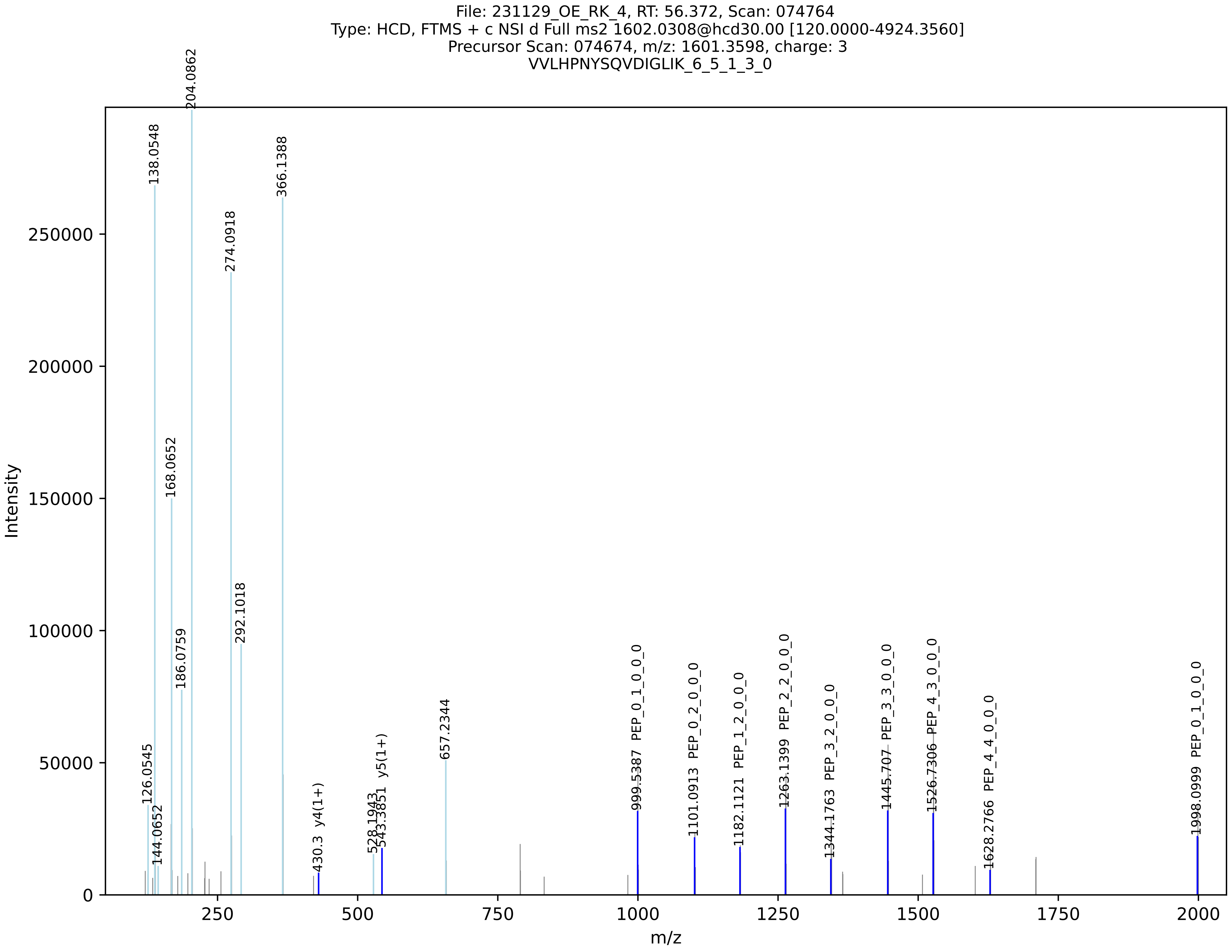
File: 231129_OE_RK_4, RT: 56.372, Scan: 074764
Type: HCD, FTMS + c NSI d Full ms2 1602.0308@hcd30.00 [120.0000-4924.3560]
Precursor Scan: 074674, m/z: 1601.3598, charge: 3
VVLHPNYSQVDIGLIK_6_5_1_3_0
Intensity
m/z
204.0862
138.0548
366.1388
274.0918
168.0652
292.1018
186.0759
126.0545
144.0652
430.3 y4(1+)
528.1943
543.3851 y5(1+)
657.2344
999.5387 PEP_0_1_0_0_0
1101.0913 PEP_0_2_0_0_0
1182.1121 PEP_1_2_0_0_0
1263.1399 PEP_2_2_0_0_0
1344.1763 PEP_3_2_0_0_0
1445.707 PEP_3_3_0_0_0
1526.7306 PEP_4_3_0_0_0
1628.2766 PEP_4_4_0_0_0
1998.0999 PEP_0_1_0_0_0

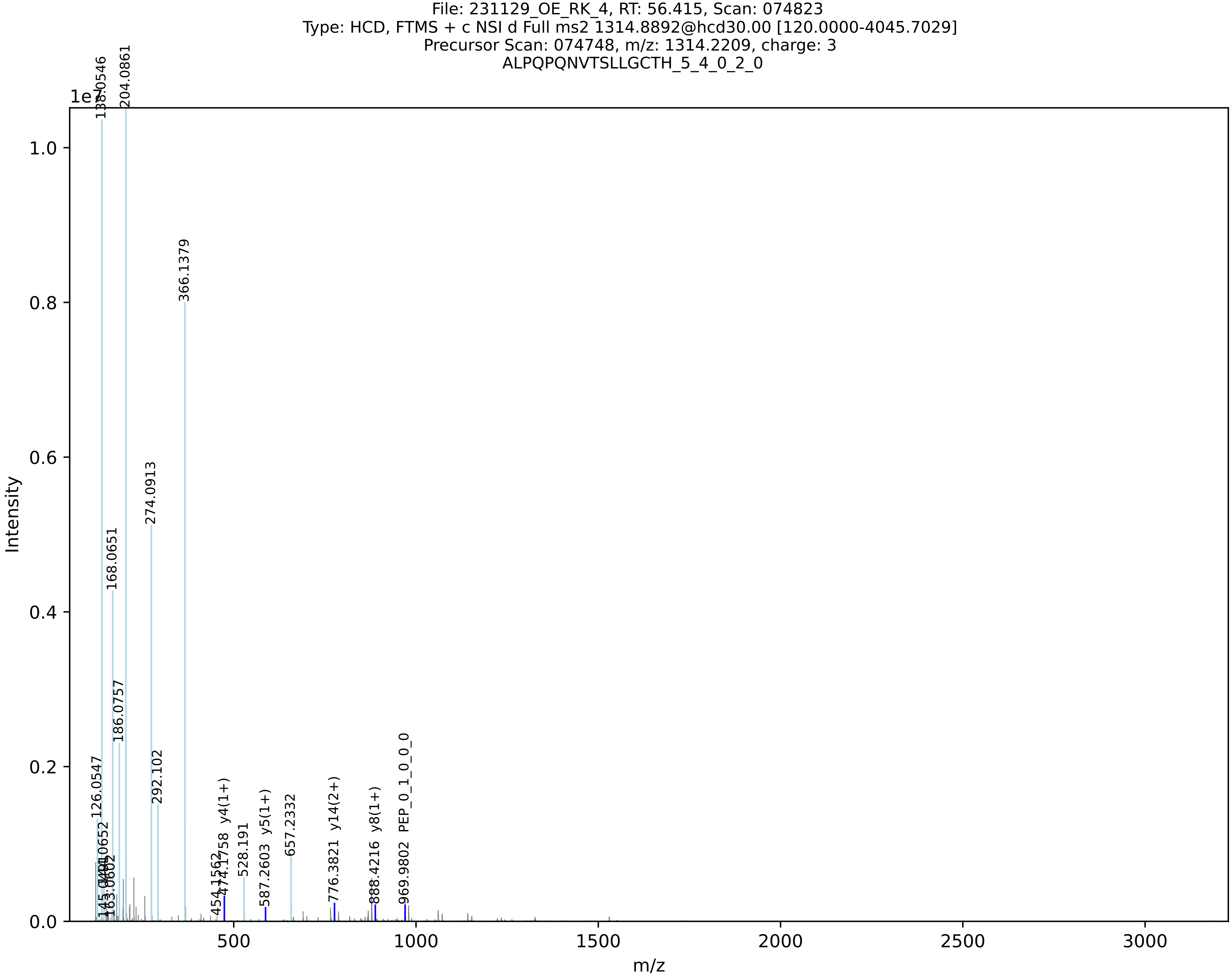
File: 231129_OE_RK_4, RT: 56.415, Scan: 074823
Type: HCD, FTMS + c NSI d Full ms2 1314.8892@hcd30.00 [120.0000-4045.7029]
Precursor Scan: 074748, m/z: 1314.2209, charge: 3
ALPQPQNVTSLLGCTH_5_4_0_2_0
1e7
138.0546
204.0861
366.1379
274.0913
168.0651
186.0757
126.0547
292.102
454.1562
474.1758 y4(1+)
528.191
587.2603 y5(1+)
657.2332
776.3821 y14(2+)
888.4216 y8(1+)
969.9802 PEP_0_1_0_0_0_
Intensity
m/z

File: 231129_OE_RK_4, RT: 56.419, Scan: 074828
Type: HCD, FTMS + c NSI d Full ms2 1282.8577@hcd30.00 [120.0000-3947.6865]
Precursor Scan: 074748, m/z: 1282.1895, charge: 3
AGLQAFFQVQECNK_5_4_0_2_0

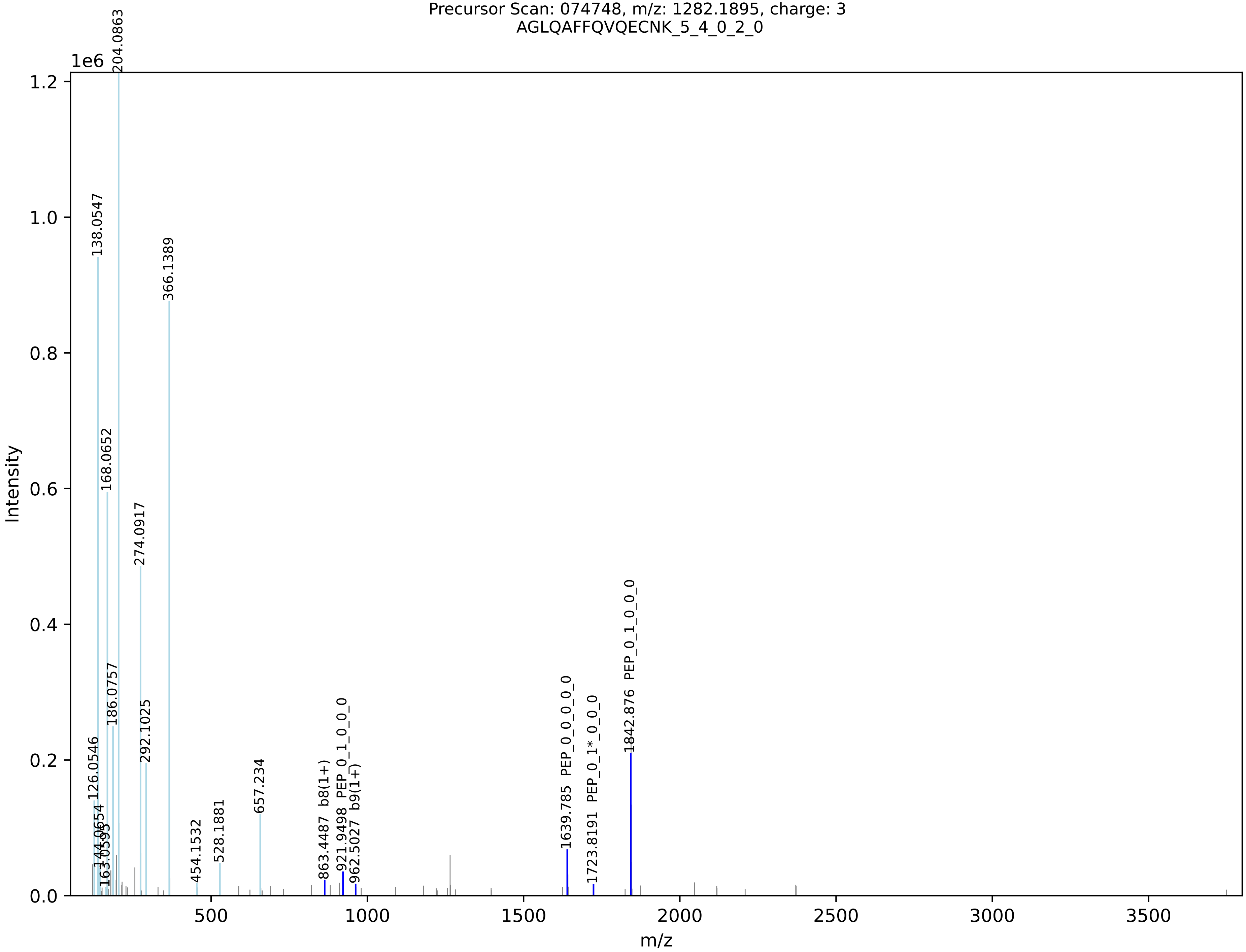

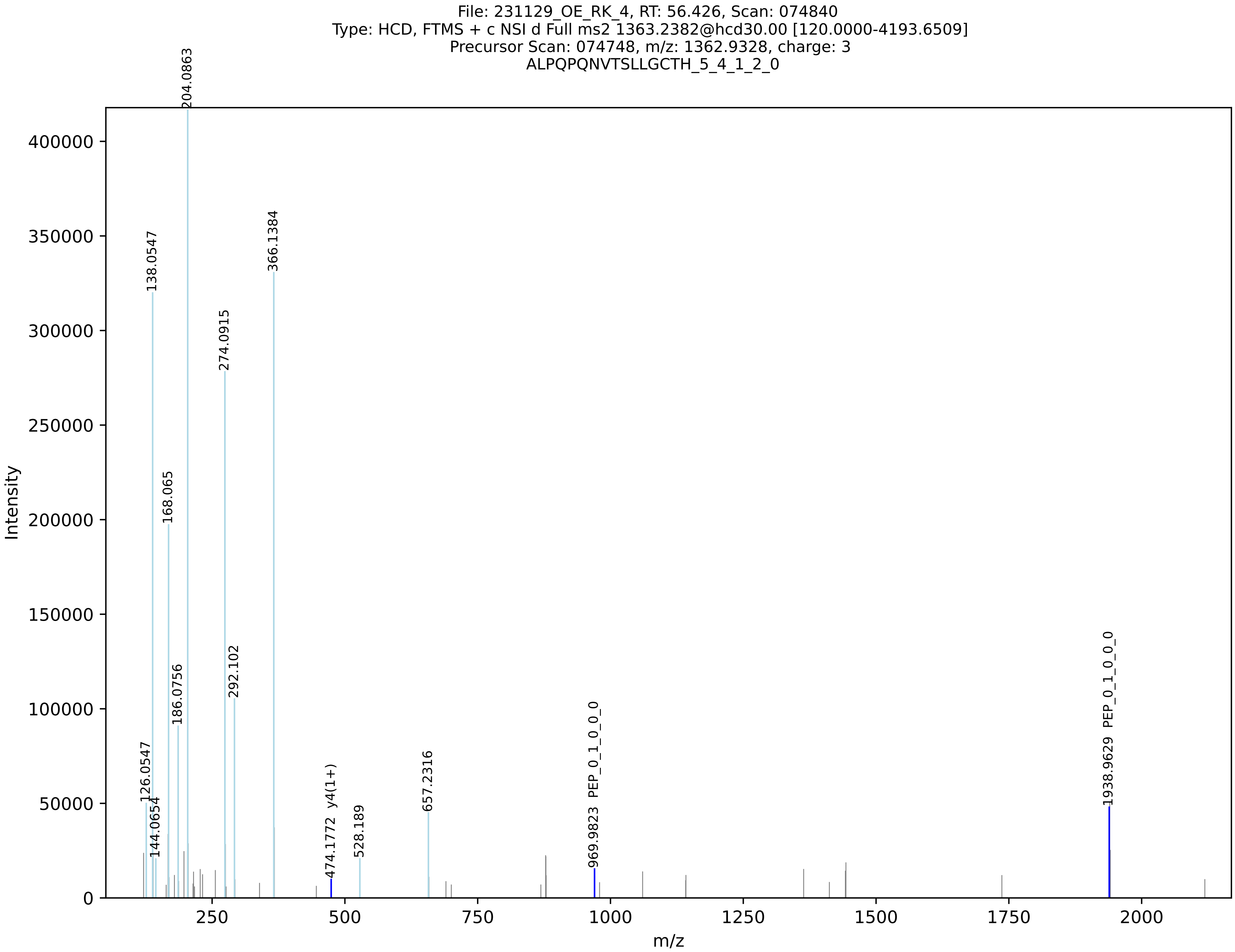
File: 231129_OE_RK_4, RT: 56.426, Scan: 074840
Type: HCD, FTMS + c NSI d Full ms2 1363.2382@hcd30.00 [120.0000-4193.6509]
Precursor Scan: 074748, m/z: 1362.9328, charge: 3
ALPQPQNVTSLLGCTH_5_4_1_2_0
Intensity
m/z
204.0863
138.0547
366.1384
274.0915
168.065
292.102
186.0756
126.0547
144.0654
474.1772 y4(1+)
528.189
657.2316
969.9823 PEP_0_1_0_0_0
1938.9629 PEP_0_1_0_0_0

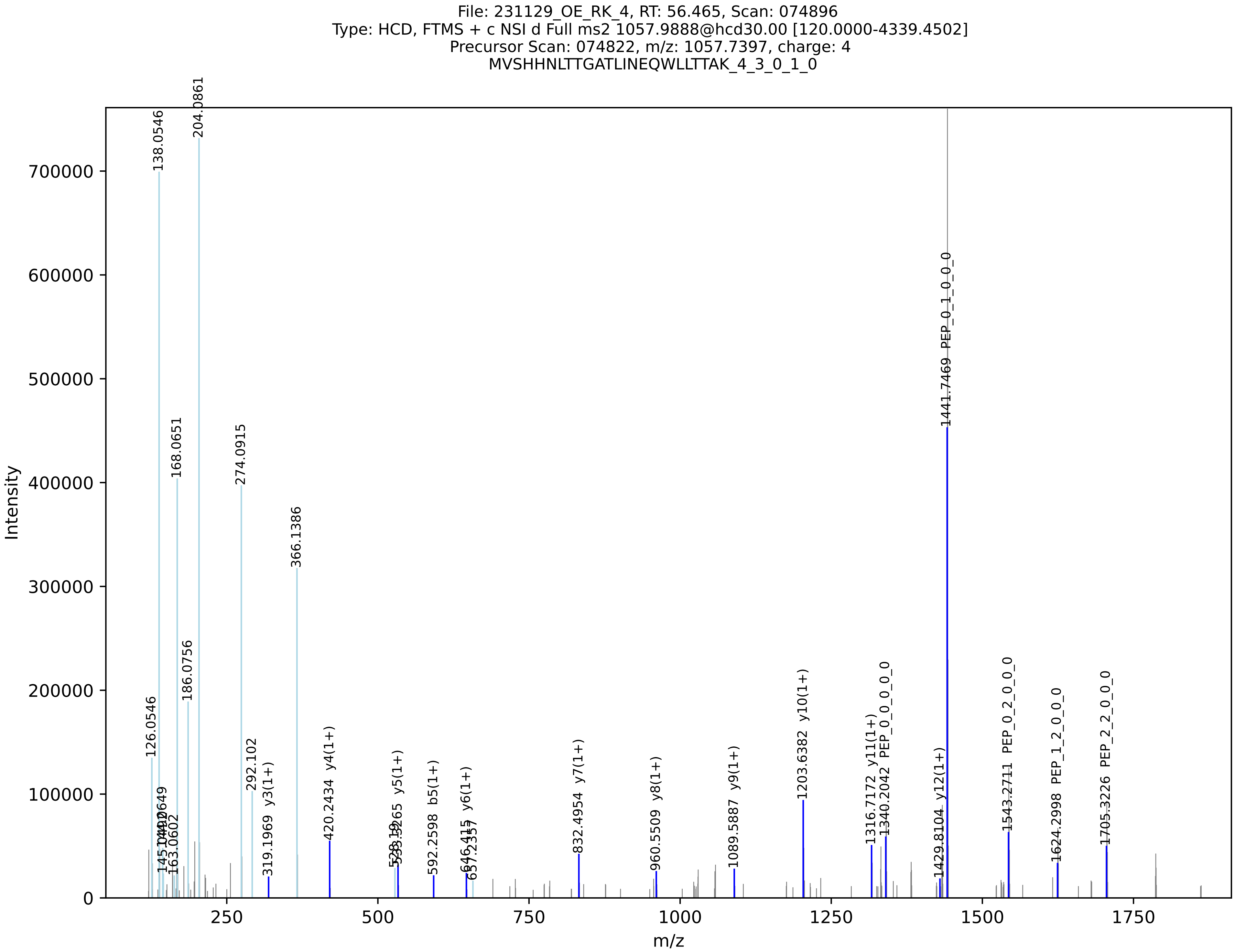
File: 231129_OE_RK_4, RT: 56.465, Scan: 074896
Type: HCD, FTMS + c NSI d Full ms2 1057.9888@hcd30.00 [120.0000-4339.4502]
Precursor Scan: 074822, m/z: 1057.7397, charge: 4
MVSHHNLTTGATLINEQWLLTTAK_4_3_0_1_0
Intensity
m/z
138.0546
204.0861
168.0651
274.0915
366.1386
186.0756
126.0546
292.102
319.1969 y3(1+)
420.2434 y4(1+)
592.2598 b5(1+)
832.4954 y7(1+)
960.5509 y8(1+)
1089.5887 y9(1+)
1203.6382 y10(1+)
1316.7172 y11(1+)
1340.2042 PEP_0_0_0_0_0
1429.8104 y12(1+)
1441.7469 PEP_0_1_0_0_0
1543.2711 PEP_0_2_0_0_0
1624.2998 PEP_1_2_0_0_0
1705.3226 PEP_2_2_0_0_0

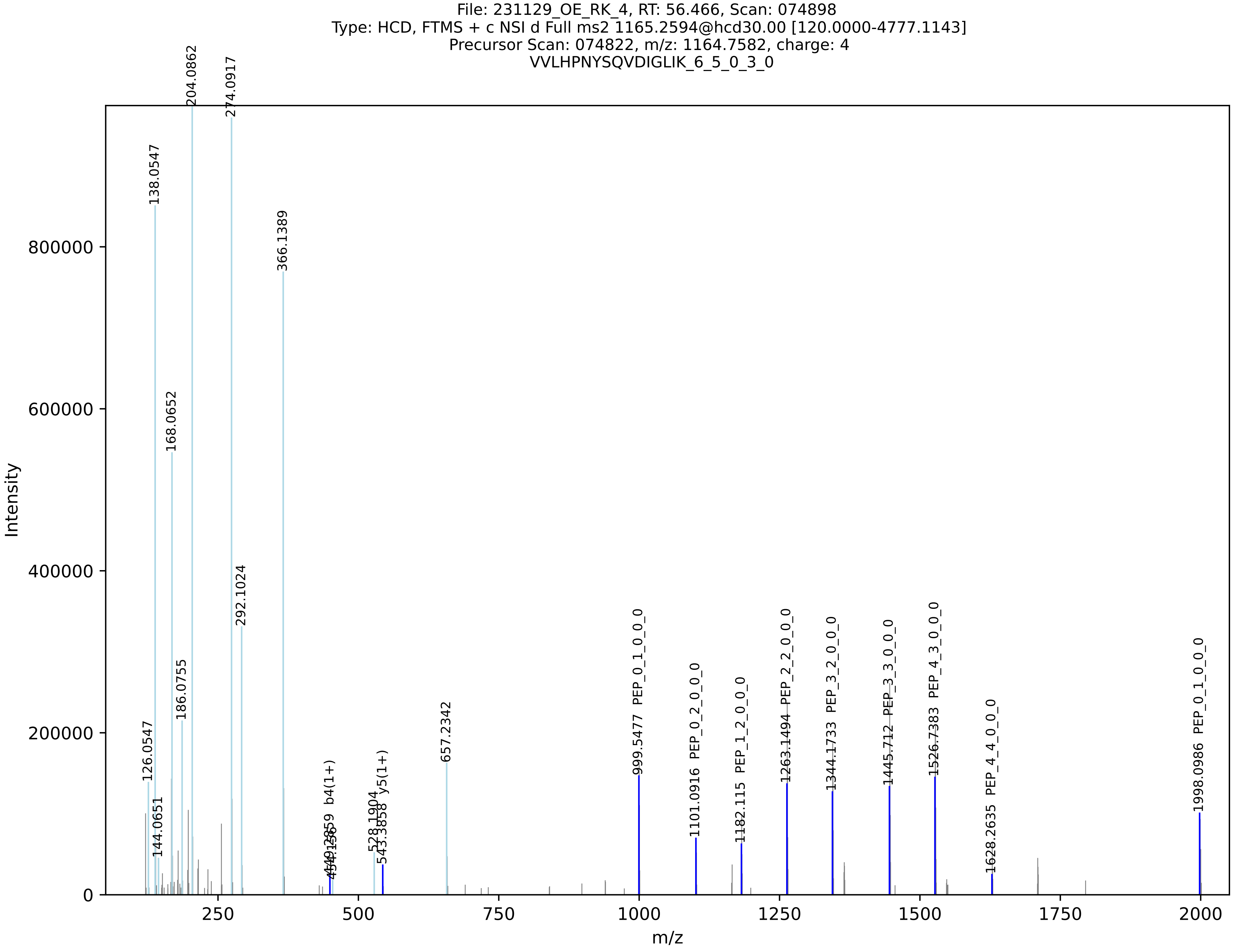
File: 231129_OE_RK_4, RT: 56.466, Scan: 074898
Type: HCD, FTMS + c NSI d Full ms2 1165.2594@hcd30.00 [120.0000-4777.1143]
Precursor Scan: 074822, m/z: 1164.7582, charge: 4
VVLHPNYSQVDIGLIK_6_5_0_3_0
204.0862
274.0917
138.0547
366.1389
168.0652
292.1024
186.0755
126.0547
144.0651
b4(1+)
528.1904
543.3858 y5(1+)
657.2342
999.5477 PEP_0_1_0_0_0
1101.0916 PEP_0_2_0_0_0
1182.115 PEP_1_2_0_0_0
1263.1494 PEP_2_2_0_0_0
1344.1733 PEP_3_2_0_0_0
1445.712 PEP_3_3_0_0_0
1526.7383 PEP_4_3_0_0_0
1628.2635 PEP_4_4_0_0_0
1998.0986 PEP_0_1_0_0_0
Intensity
800000
600000
400000
200000
0
250
500
750
1000
1250
1500
1750
2000
m/z

File: 231129_OE_RK_4, RT: 56.517, Scan: 074972
Type: HCD, FTMS + c NSI d Full ms2 1199.2311@hcd30.00 [120.0000-4915.7188]
Precursor Scan: 074893, m/z: 1198.4792, charge: 4
SPYYNVSDEISFHCYDGYTLR_5_4_0_2_0

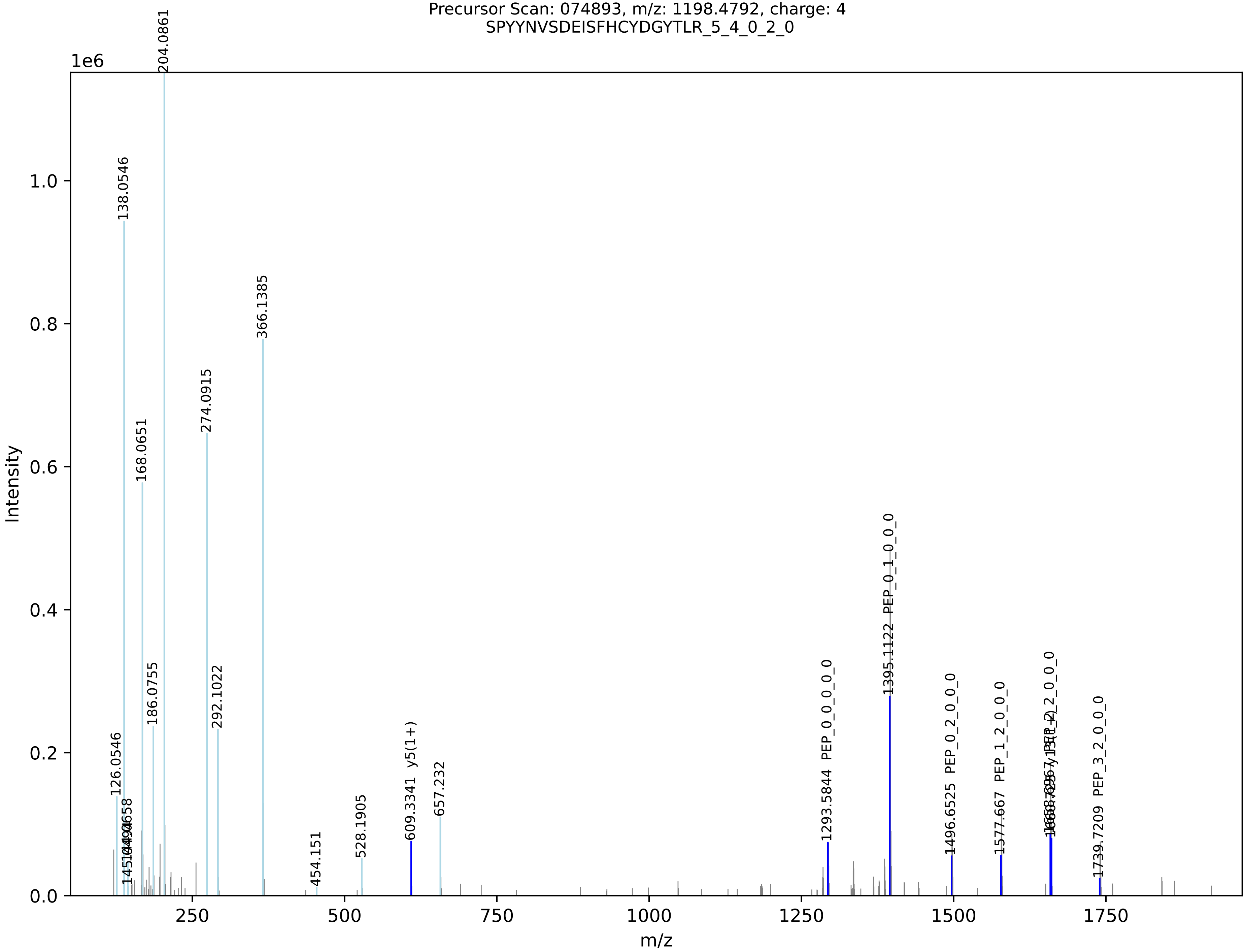

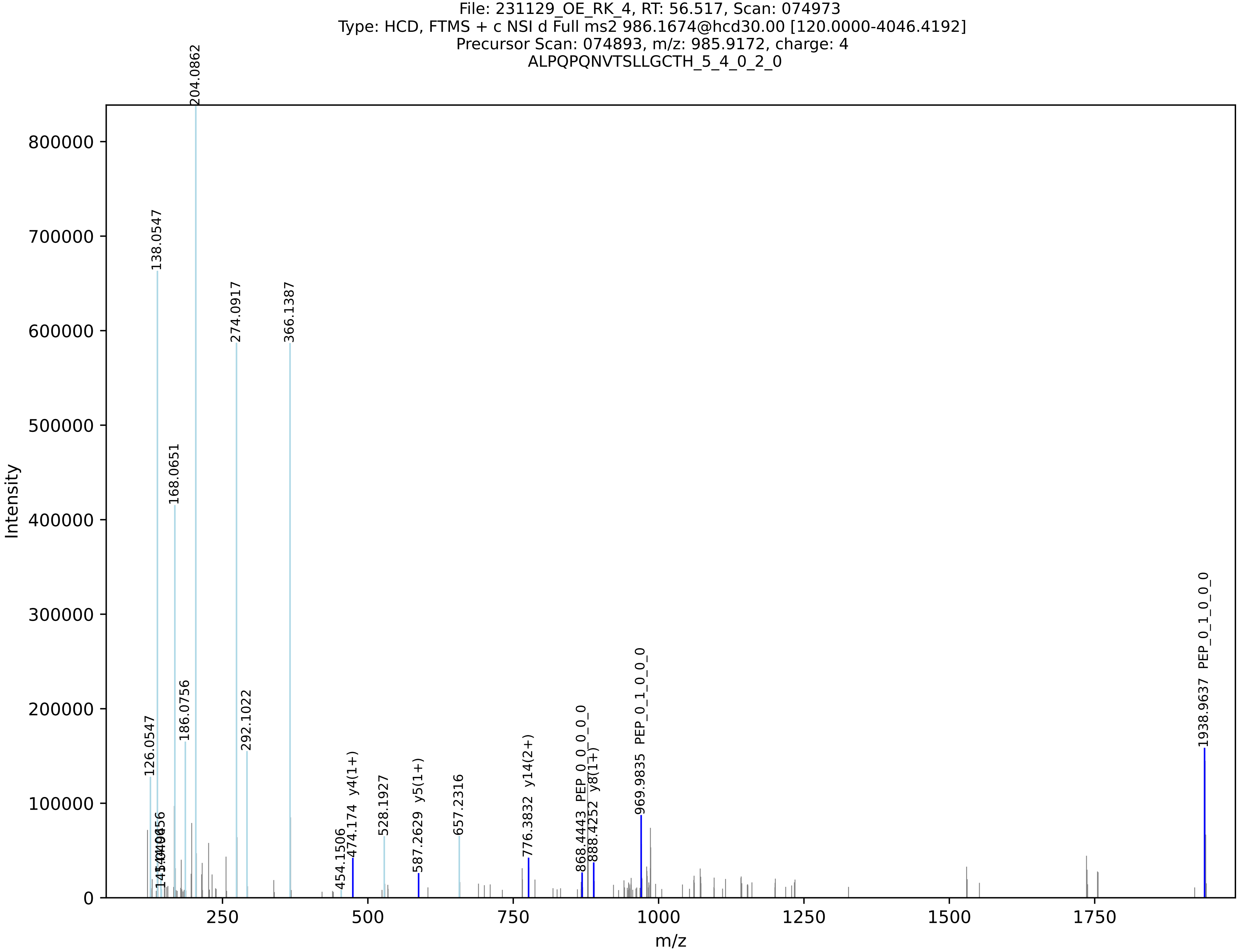
File: 231129_OE_RK_4, RT: 56.517, Scan: 074973
Type: HCD, FTMS + c NSI d Full ms2 986.1674@hcd30.00 [120.0000-4046.4192]
Precursor Scan: 074893, m/z: 985.9172, charge: 4
ALPQPQNVTSLLGCTH_5_4_0_2_0
Intensity
m/z
204.0862
138.0547
274.0917
366.1387
168.0651
186.0756
292.1022
126.0547
454.1506
474.174 y4(1+)
528.1927
587.2629 y5(1+)
657.2316
776.3832 y14(2+)
868.4443 PEP_0_0_0_0_0
888.4252 y8(1+)
969.9835 PEP_0_1_0_0_0
1938.9637 PEP_0_1_0_0_0

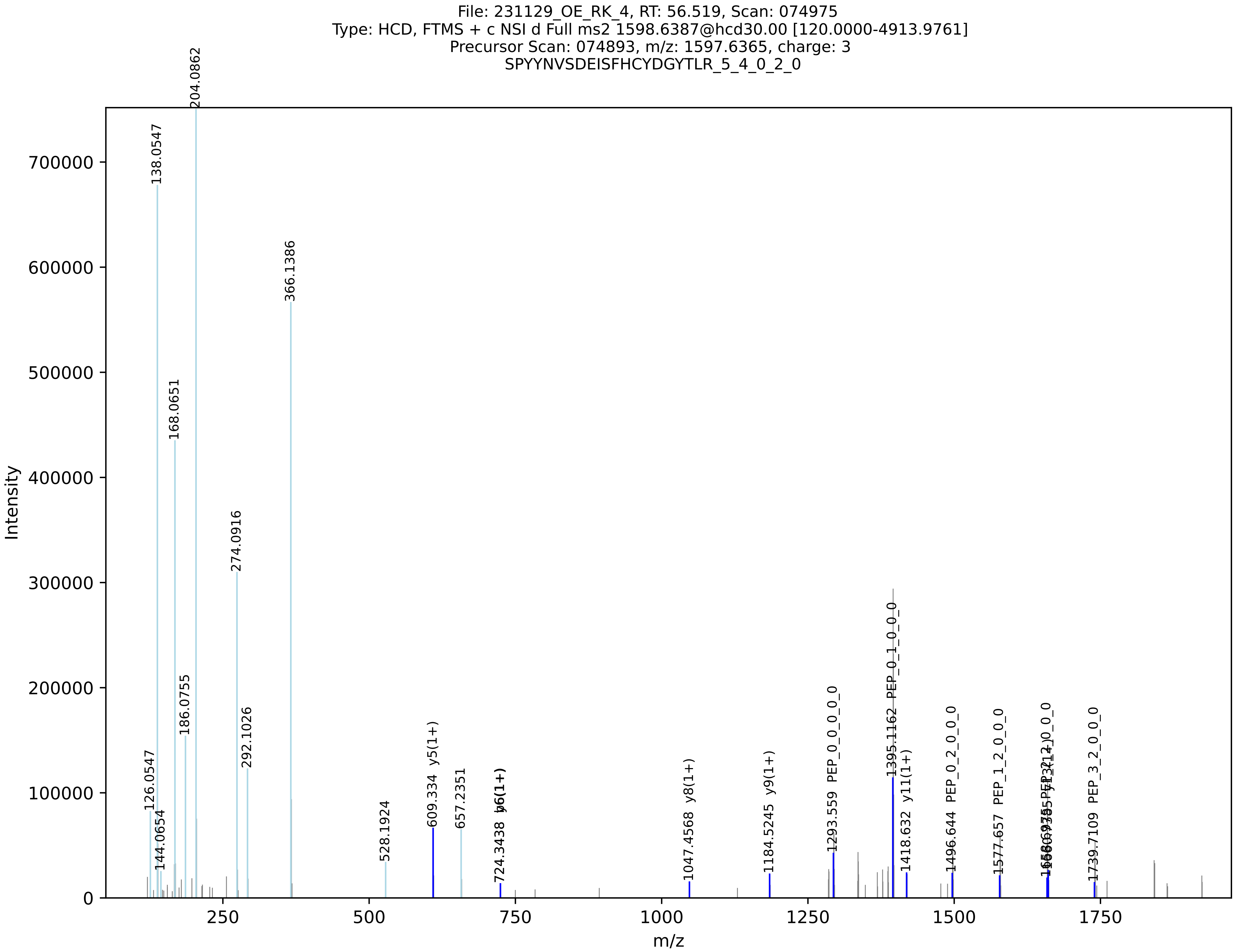
File: 231129_OE_RK_4, RT: 56.519, Scan: 074975
Type: HCD, FTMS + c NSI d Full ms2 1598.6387@hcd30.00 [120.0000-4913.9761]
Precursor Scan: 074893, m/z: 1597.6365, charge: 3
SPYYNVSDEISFHCYDGYTLR_5_4_0_2_0
Intensity
m/z
204.0862
138.0547
366.1386
168.0651
274.0916
186.0755
292.1026
126.0547
144.0654
528.1924
609.334 y5(1+)
657.2351
724.3438 y6(1+)
1047.4568 y8(1+)
1184.5245 y9(1+)
1293.559 PEP_0_0_0_0_0
1395.1162 PEP_0_1_0_0_0
1418.632 y11(1+)
1496.644 PEP_0_2_0_0_0
1577.657 PEP_1_2_0_0_0
1739.7109 PEP_3_2_0_0_0

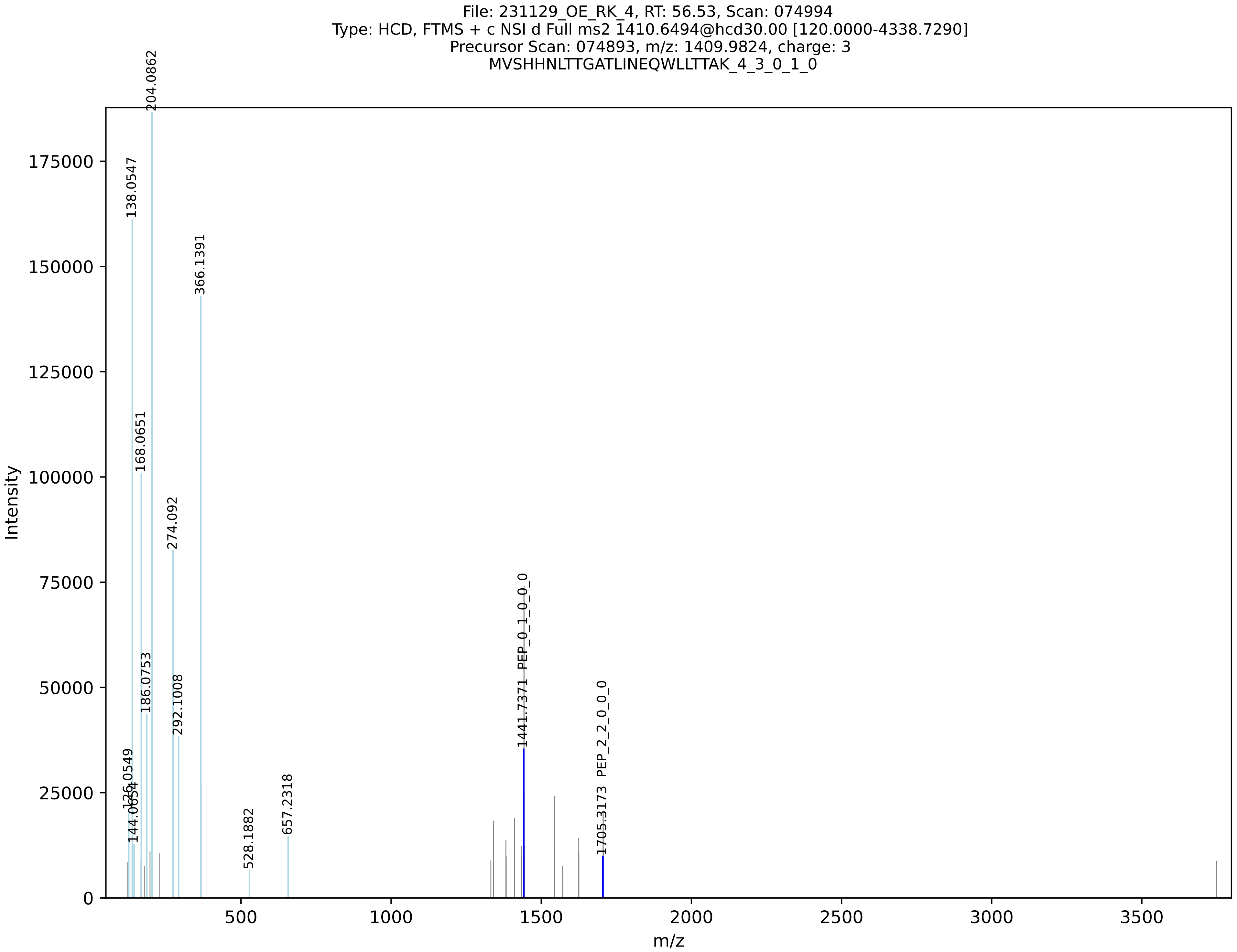
File: 231129_OE_RK_4, RT: 56.53, Scan: 074994
Type: HCD, FTMS + c NSI d Full ms2 1410.6494@hcd30.00 [120.0000-4338.7290]
Precursor Scan: 074893, m/z: 1409.9824, charge: 3
MVSHHNLTTGATLINEQWLLTTAK_4_3_0_1_0
Intensity
m/z
204.0862
138.0547
366.1391
168.0651
274.092
186.0753
292.1008
126.0549
144.0654
528.1882
657.2318
1441.7371 PEP_0_1_0_0_0_
1705.3173 PEP_2_2_0_0_0_

File: 231129_OE_RK_4, RT: 56.533, Scan: 074998
Type: HCD, FTMS + c NSI d Full ms2 1143.9867@hcd30.00 [120.0000-4690.3218]
Precursor Scan: 074893, m/z: 1143.235, charge: 4
AALAAFNAQNNGSNFQLEEISR_5_4_0_2_0

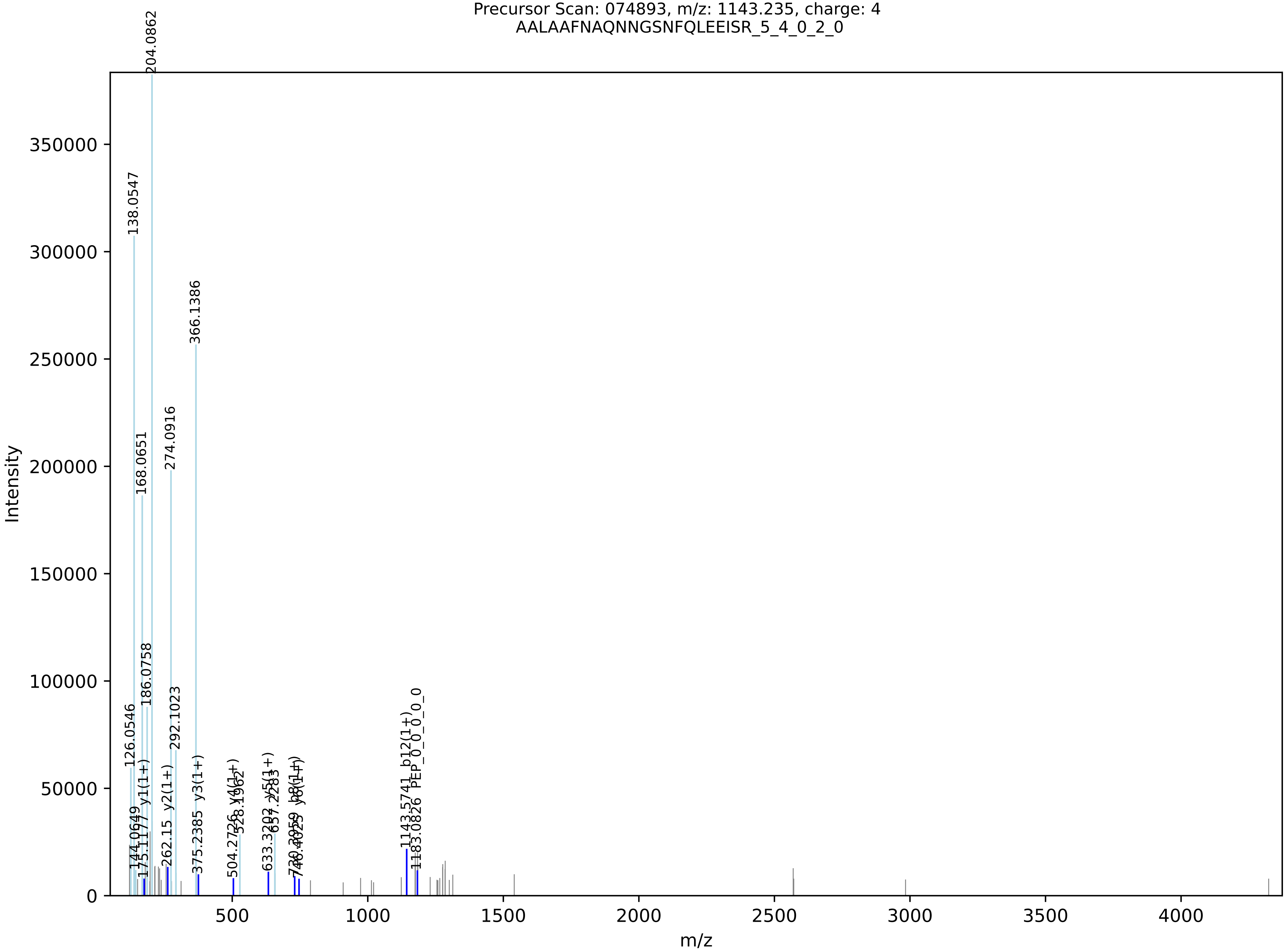

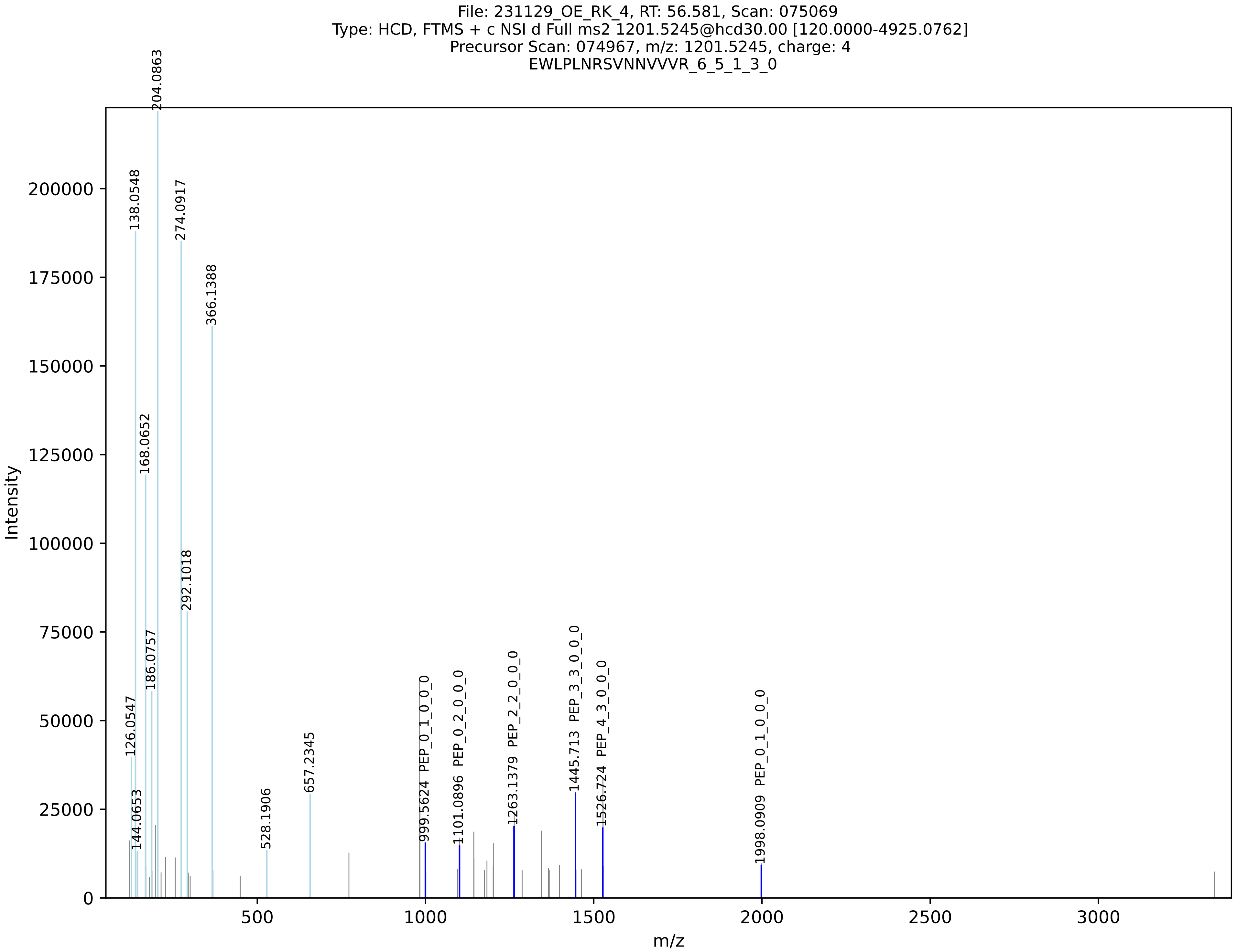

File: 231129_OE_RK_4, RT: 56.581, Scan: 075069
Type: HCD, FTMS + c NSI d Full ms2 1201.5245@hcd30.00 [120.0000-4925.0762]
Precursor Scan: 074967, m/z: 1201.5245, charge: 4
EWLPLNRSVNNVVVR_6_5_1_3_0
Intensity
m/z
204.0863
138.0548
274.0917
366.1388
168.0652
292.1018
186.0757
126.0547
144.0653
528.1906
657.2345
999.5624 PEP_0_1_0_0_0
1101.0896 PEP_0_2_0_0_0
1263.1379 PEP_2_2_0_0_0
1445.713 PEP_3_3_0_0_0
1526.724 PEP_4_3_0_0_0
1998.0909 PEP_0_1_0_0_0

File: 231129_OE_RK_4, RT: 56.615, Scan: 075116
Type: HCD, FTMS + c NSI d Full ms2 1314.5563@hcd30.00 [120.0000-4044.6843]
Precursor Scan: 075041, m/z: 1314.2222, charge: 3
ALPQPQNVTSLLGCTH_5_4_0_2_0

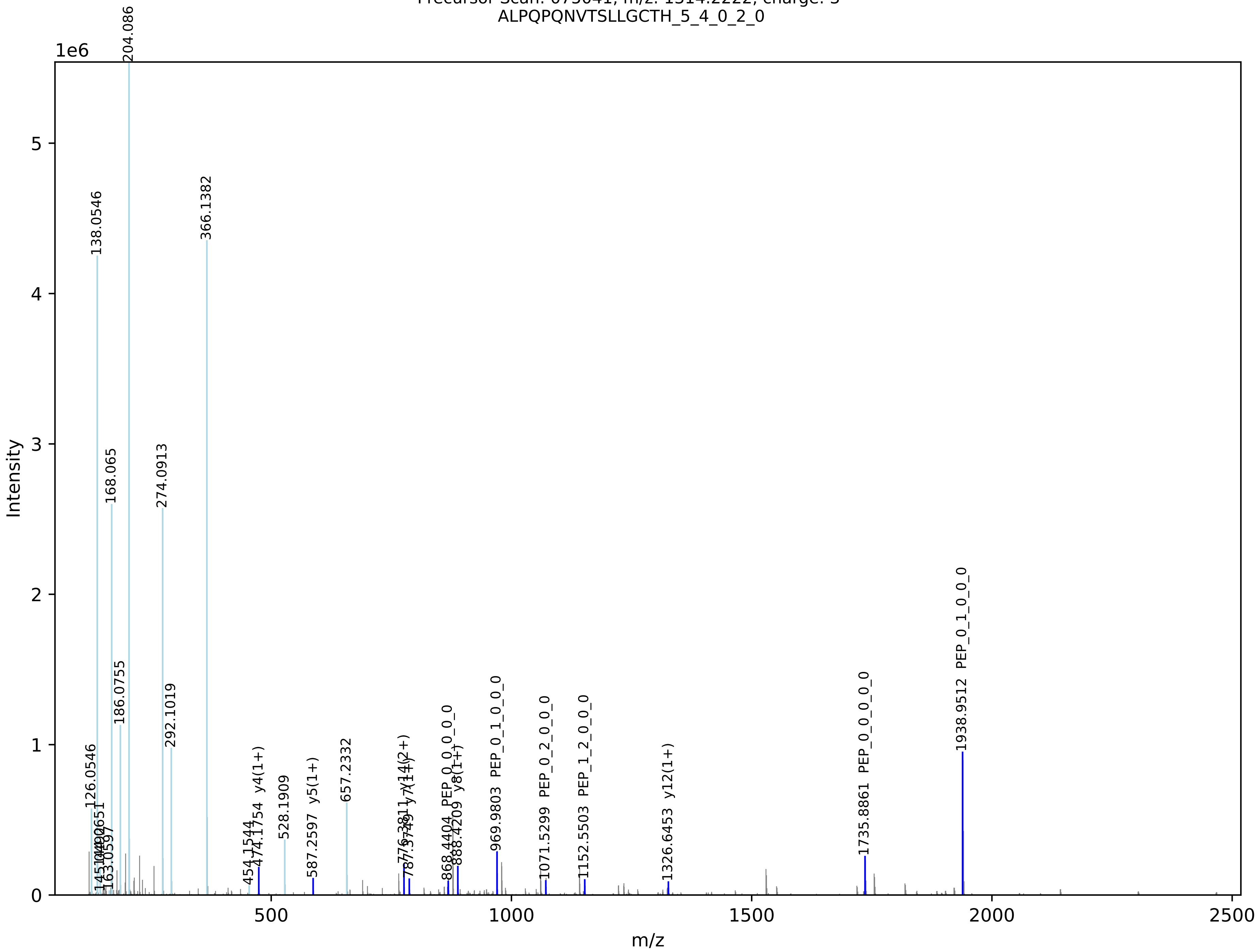

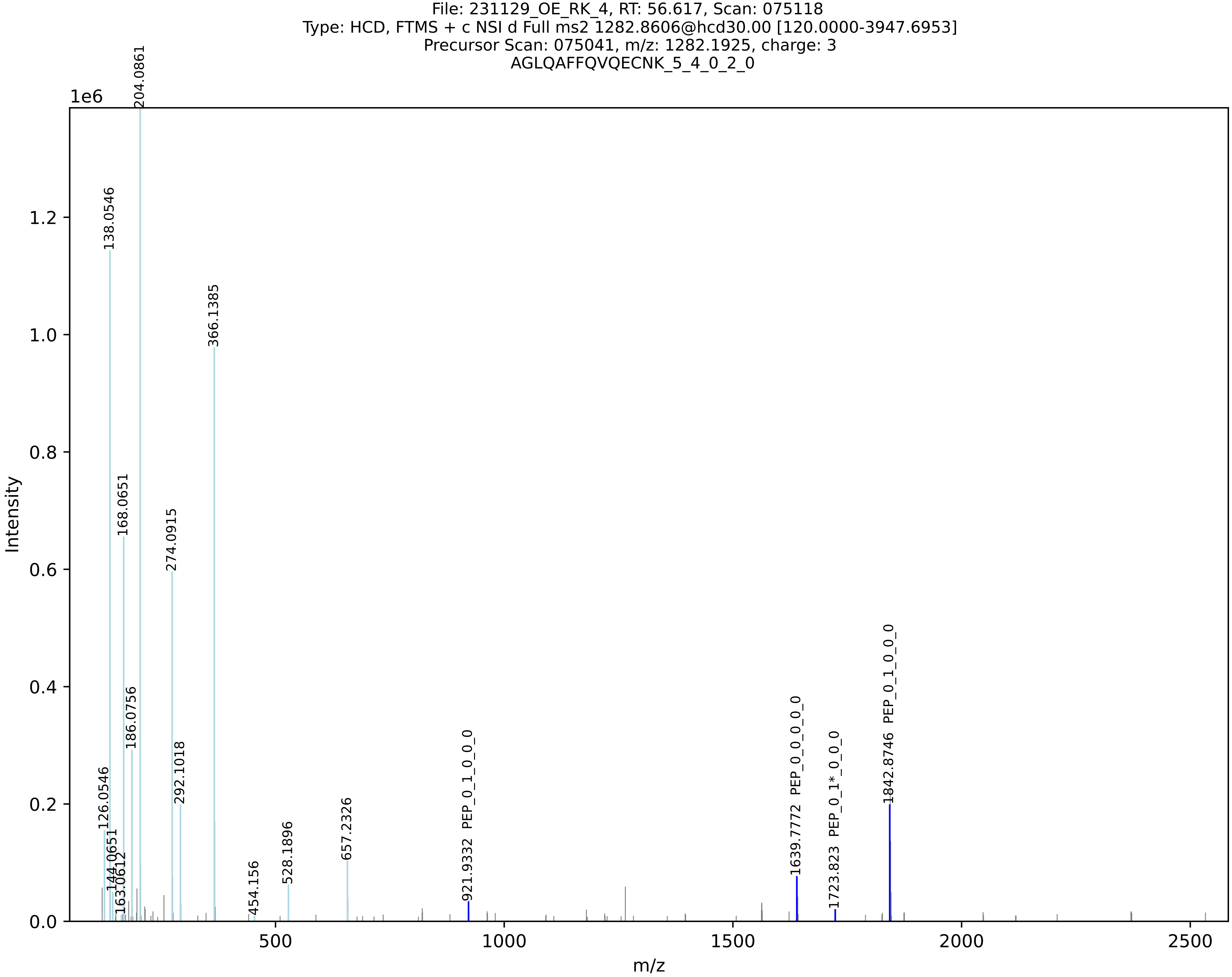
File: 231129_OE_RK_4, RT: 56.617, Scan: 075118
Type: HCD, FTMS + c NSI d Full ms2 1282.8606@hcd30.00 [120.0000-3947.6953]
Precursor Scan: 075041, m/z: 1282.1925, charge: 3
AGLQAFFQVQECNK_5_4_0_2_0
1e6
Intensity
m/z
204.0861
138.0546
366.1385
168.0651
274.0915
186.0756
292.1018
126.0546
144.0651
163.0612
454.156
528.1896
657.2326
921.9332 PEP_0_1_0_0_
1639.7772 PEP_0_0_0_0_0
1723.823 PEP_0_1*_0_0_
1842.8746 PEP_0_1_0_0_

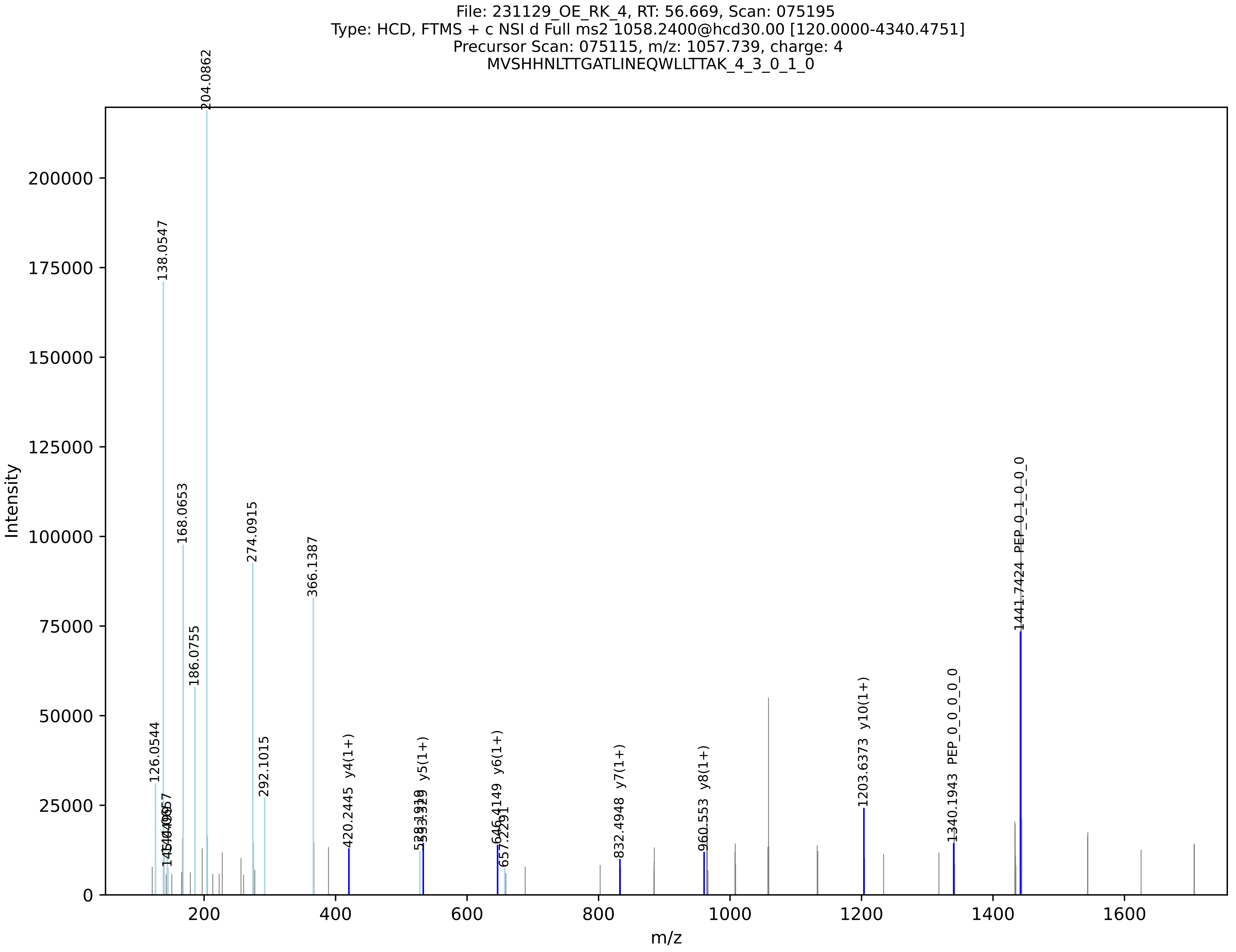

File: 231129_OE_RK_4, RT: 56.669, Scan: 075195
Type: HCD, FTMS + c NSI d Full ms2 1058.2400@hcd30.00 [120.0000-4340.4751]
Precursor Scan: 075115, m/z: 1057.739, charge: 4
MVSHHNLTTGATLINEQWLLTTAK_4_3_0_1_0
Intensity
m/z
204.0862
138.0547
168.0653
186.0755
274.0915
366.1387
126.0544
292.1015
420.2445 y4(1+)
533.329 y5(1+)
646.4149 y6(1+)
657.2291
832.4948 y7(1+)
960.553 y8(1+)
1203.6373 y10(1+)
1340.1943 PEP_0_0_0_0_0
1441.7424 PEP_0_1_0_0_0

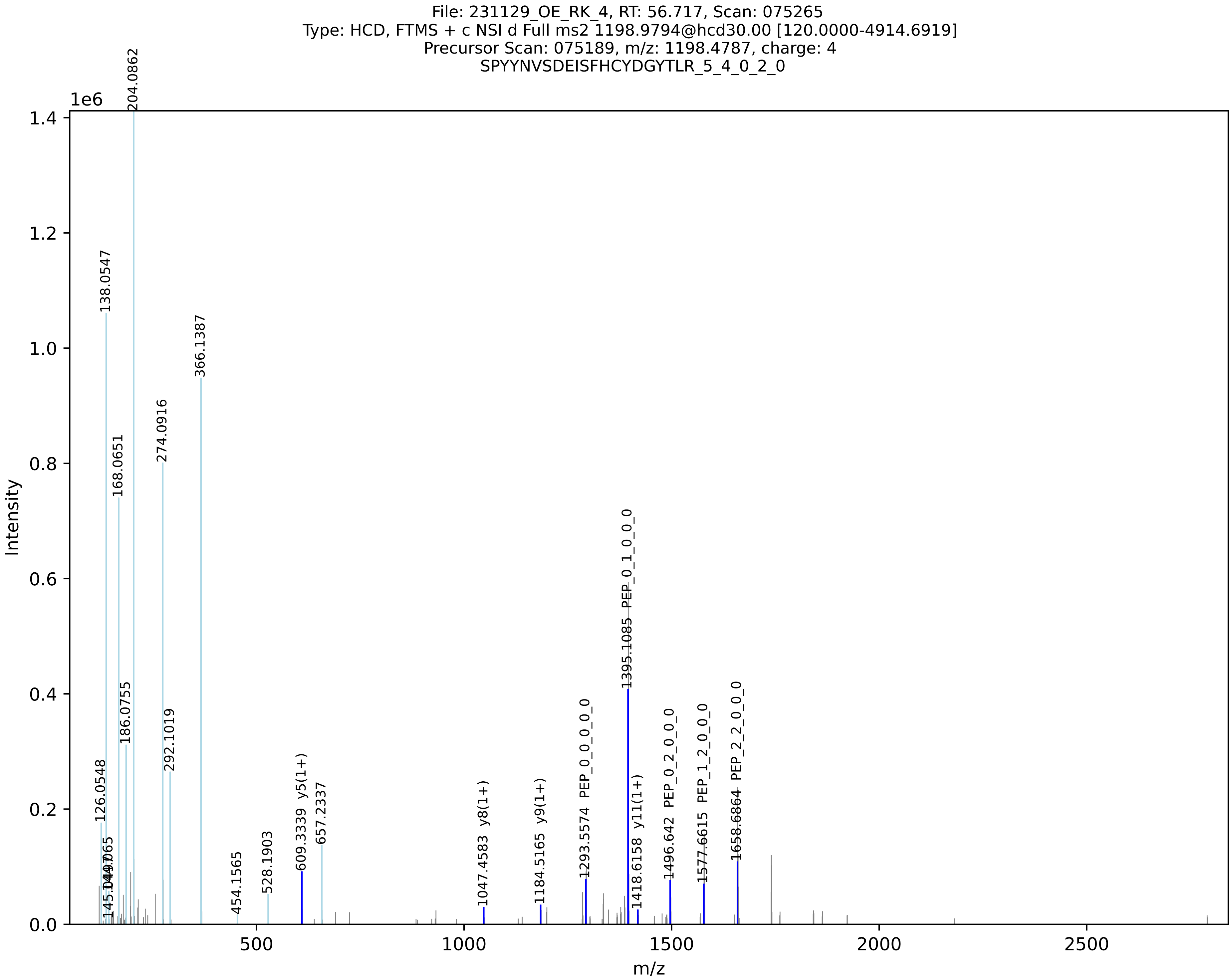

File: 231129_OE_RK_4, RT: 56.717, Scan: 075265
Type: HCD, FTMS + c NSI d Full ms2 1198.9794@hcd30.00 [120.0000-4914.6919]
Precursor Scan: 075189, m/z: 1198.4787, charge: 4
SPYYNVSDEISFHCYDGYTLR_5_4_0_2_0
1e6
Intensity
m/z
204.0862
138.0547
366.1387
274.0916
168.0651
186.0755
292.1019
126.0548
145.0
149.1065
454.1565
528.1903
609.3339 y5(1+)
657.2337
1047.4583 y8(1+)
1184.5165 y9(1+)
1293.5574 PEP_0_0_0_0_0
1395.1085 PEP_0_1_0_0_0
1418.6158 y11(1+)
1496.642 PEP_0_2_0_0_0
1577.6615 PEP_1_2_0_0_0
1658.6864 PEP_2_2_0_0_0

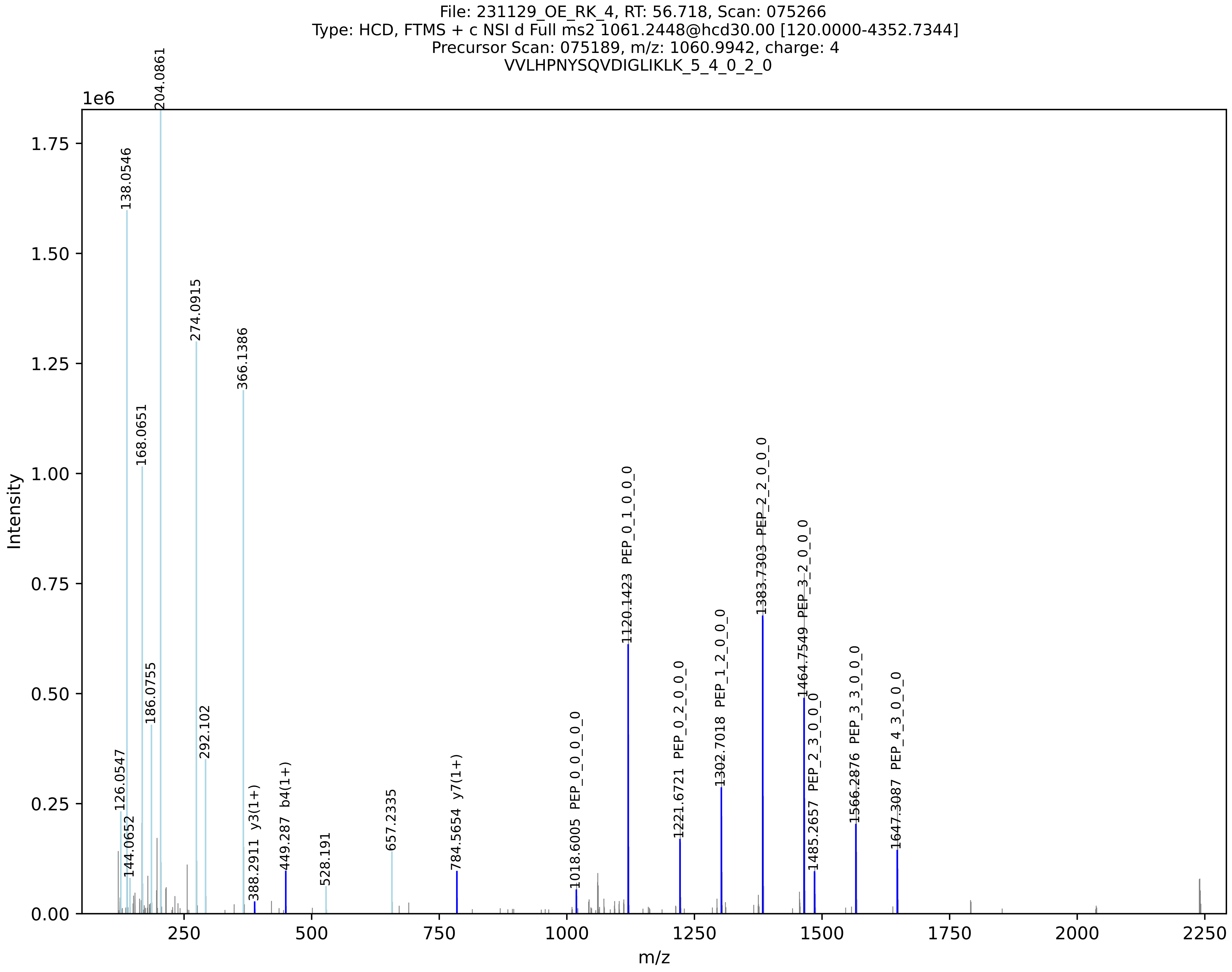
File: 231129_OE_RK_4, RT: 56.718, Scan: 075266
Type: HCD, FTMS + c NSI d Full ms2 1061.2448@hcd30.00 [120.0000-4352.7344]
Precursor Scan: 075189, m/z: 1060.9942, charge: 4
VVLHPNYSQVDIGLIKLK_5_4_0_2_0
1e6
Intensity
m/z
204.0861
138.0546
274.0915
366.1386
168.0651
186.0755
292.102
126.0547
144.0652
388.2911 y3(1+)
449.287 b4(1+)
528.191
657.2335
784.5654 y7(1+)
1018.6005 PEP_0_0_0_0_0
1120.1423 PEP_0_1_0_0_0
1221.6721 PEP_0_2_0_0_0
1302.7018 PEP_1_2_0_0_0
1383.7303 PEP_2_2_0_0_0
1464.7549 PEP_3_2_0_0_0
1485.2657 PEP_2_3_0_0_0
1566.2876 PEP_3_3_0_0_0
1647.3087 PEP_4_3_0_0_0

File: 231129_OE_RK_4, RT: 56.719, Scan: 075267
Type: HCD, FTMS + c NSI d Full ms2 1598.3049@hcd30.00 [120.0000-4912.9551]
Precursor Scan: 075189, m/z: 1597.6358, charge: 3
SPYYNVSDEISFHCYDGYTLR_5_4_0_2_0

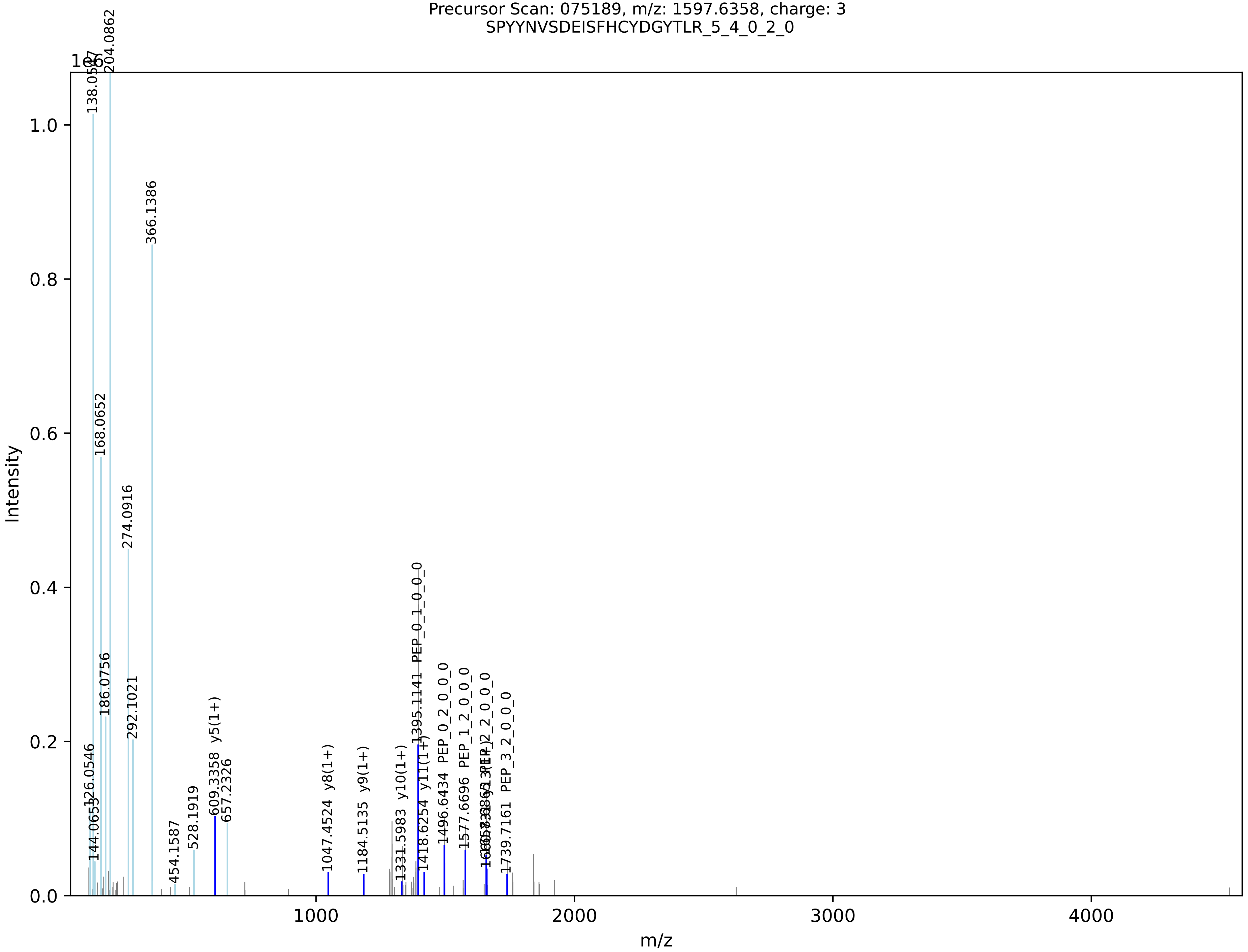

File: 231129_OE_RK_4, RT: 56.723, Scan: 075275
Type: HCD, FTMS + c NSI d Full ms2 986.1631@hcd30.00 [120.0000-4046.4019]
Precursor Scan: 075189, m/z: 985.9169, charge: 4
ALPQPQNVTSLLGCTH_5_4_0_2_0

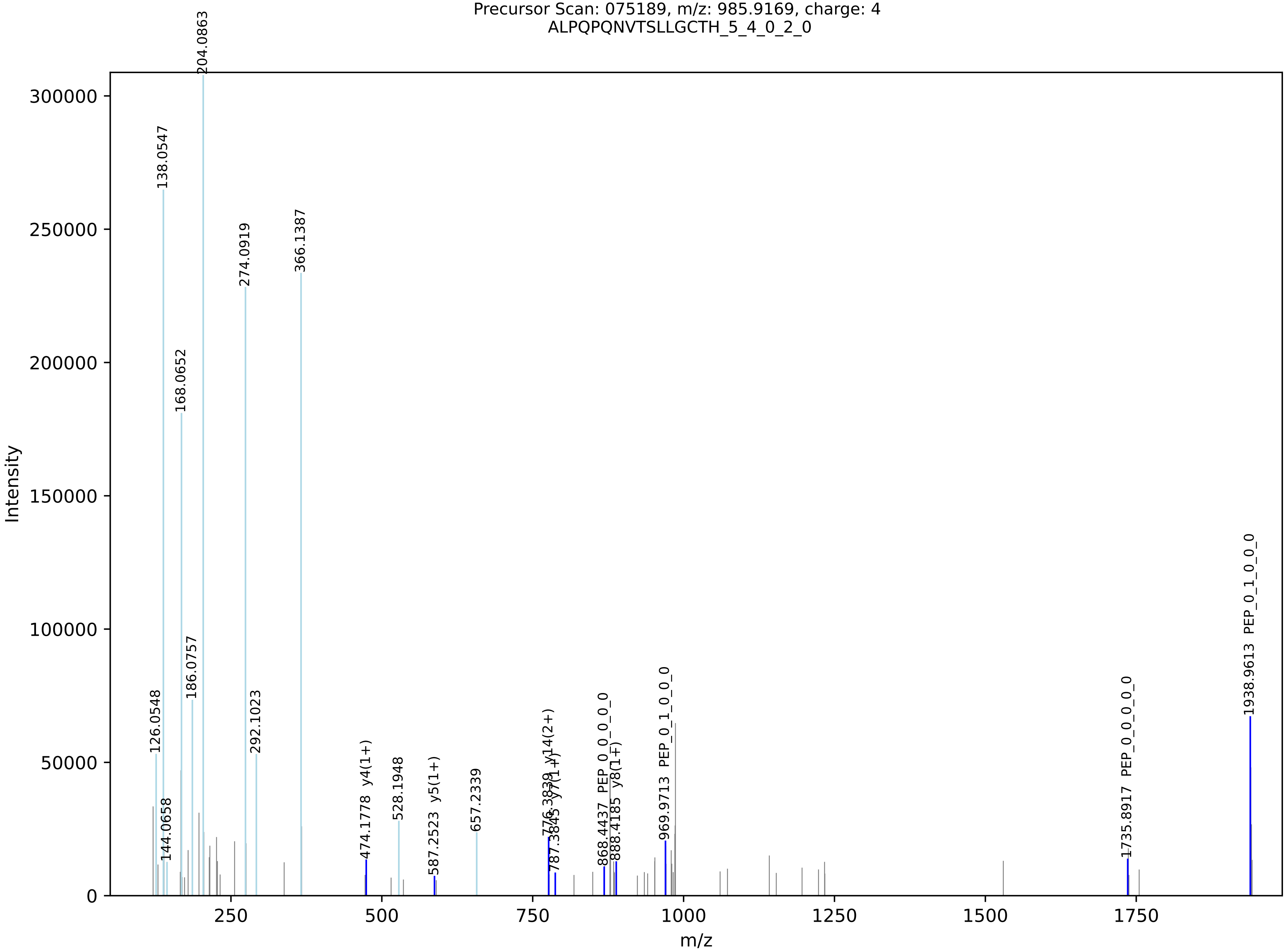

File: 231129_OE_RK_4, RT: 56.757, Scan: 075328
Type: HCD, FTMS + c NSI d Full ms2 1143.9897@hcd30.00 [120.0000-4690.3340]
Precursor Scan: 075115, m/z: 1143.2394, charge: 4
AALAAFNAQNNGSNFQLEEISR_5_4_0_2_0

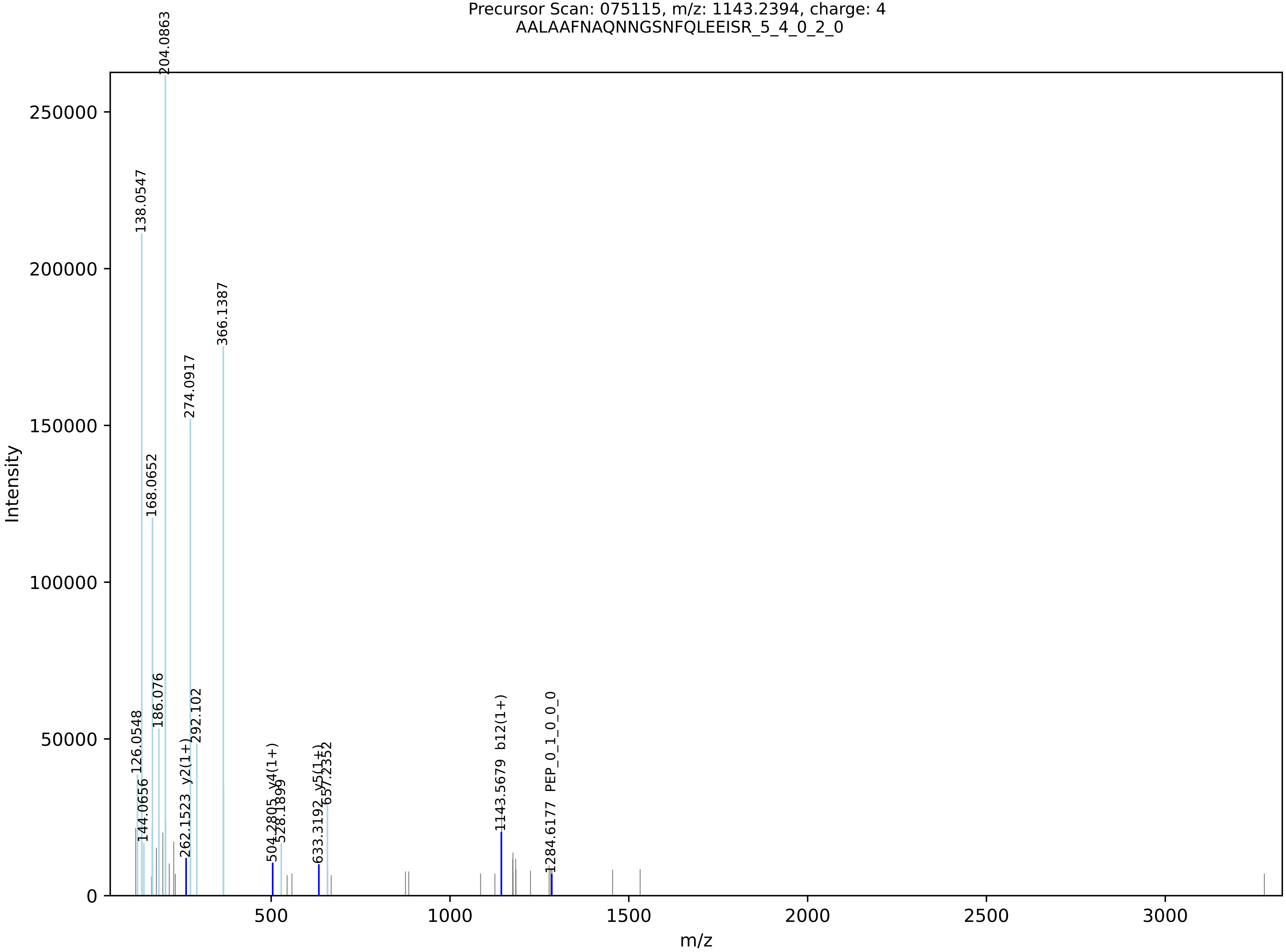

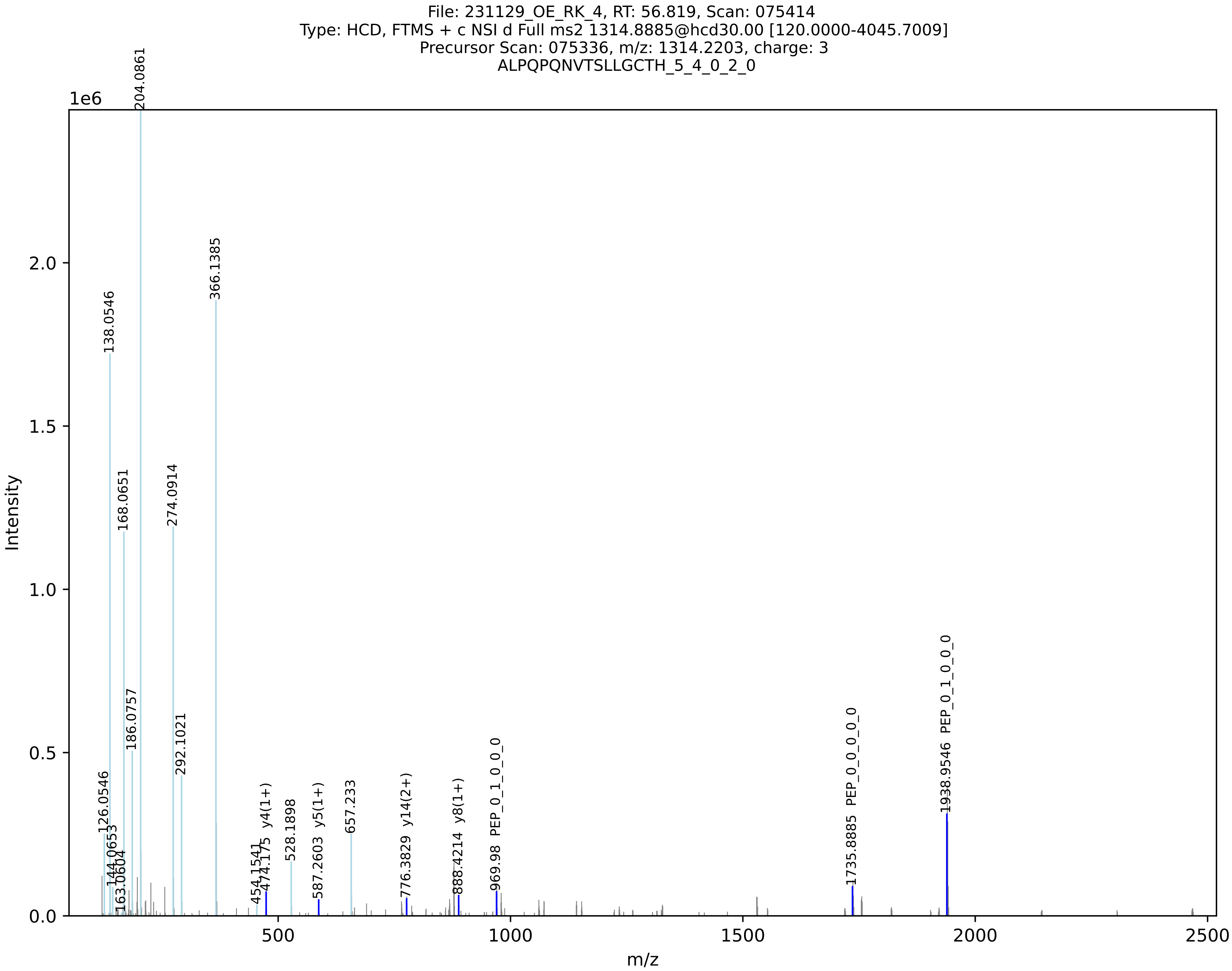
File: 231129_OE_RK_4, RT: 56.819, Scan: 075414
Type: HCD, FTMS + c NSI d Full ms2 1314.8885@hcd30.00 [120.0000-4045.7009]
Precursor Scan: 075336, m/z: 1314.2203, charge: 3
ALPQPQNVTSLLGCTH_5_4_0_2_0
1e6
Intensity
m/z
204.0861
366.1385
138.0546
168.0651
274.0914
186.0757
292.1021
126.0546
144.0653
163.0604
454.1541
474.175 y4(1+)
528.1898
587.2603 y5(1+)
657.233
776.3829 y14(2+)
888.4214 y8(1+)
969.98 PEP_0_1_0_0_0
1735.8885 PEP_0_0_0_0_0
1938.9546 PEP_0_1_0_0_0
0.0
0.5
1.0
1.5
2.0
500
1000
1500
2000
2500

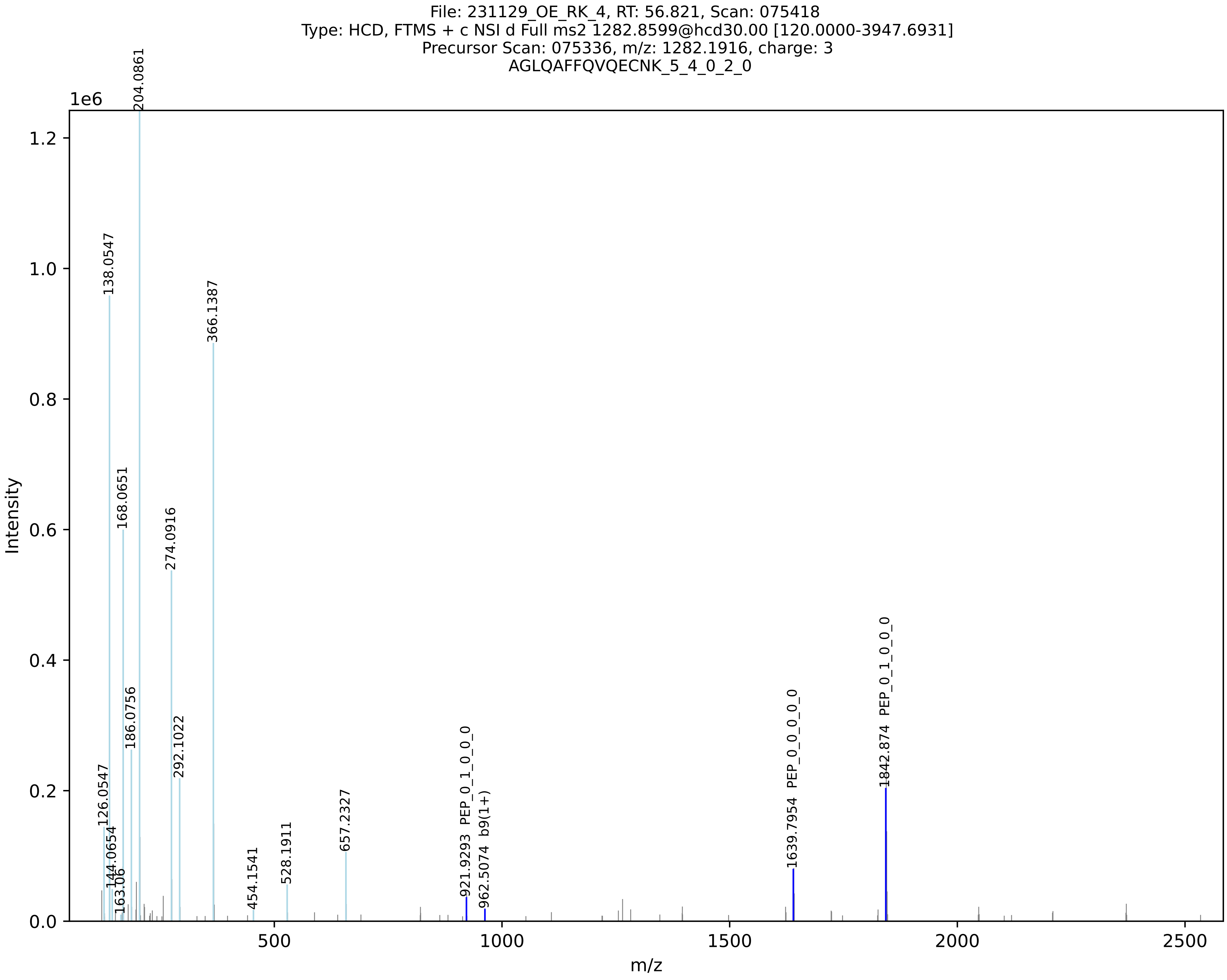

File: 231129_OE_RK_4, RT: 56.821, Scan: 075418
Type: HCD, FTMS + c NSI d Full ms2 1282.8599@hcd30.00 [120.0000-3947.6931]
Precursor Scan: 075336, m/z: 1282.1916, charge: 3
AGLQAFFQVQECNK_5_4_0_2_0
1e6
Intensity
m/z
204.0861
138.0547
366.1387
168.0651
274.0916
186.0756
292.1022
126.0547
144.0654
163.06
454.1541
528.1911
657.2327
921.9293 PEP_0_1_0_0_0
962.5074 b9(1+)
1639.7954 PEP_0_0_0_0_0
1842.874 PEP_0_1_0_0_0

File: 231129_OE_RK_4, RT: 56.83, Scan: 075432
Type: HCD, FTMS + c NSI d Full ms2 1491.2885@hcd30.00 [120.0000-4585.4844]
Precursor Scan: 075336, m/z: 1490.2837, charge: 3
YTGNASALFILPDQDK_6_5_1_2_0

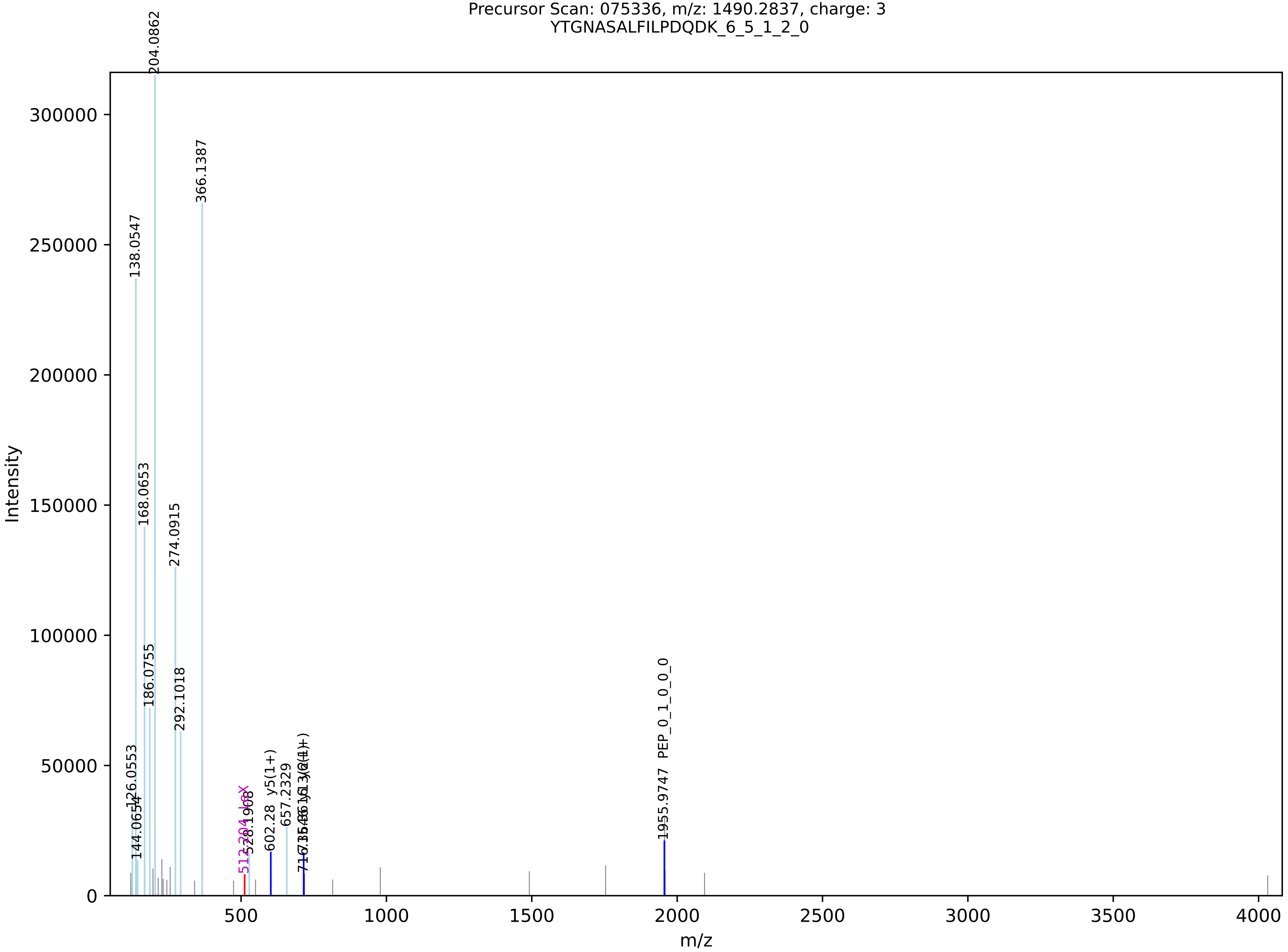

File: 231129_OE_RK_4, RT: 56.871, Scan: 075490
Type: HCD, FTMS + c NSI d Full ms2 1149.5223@hcd30.00 [120.0000-4712.9072]
Precursor Scan: 075410, m/z: 1148.7706, charge: 4
MVSHHNLTTGATLINEQWLLTTAK_5_4_0_1_0

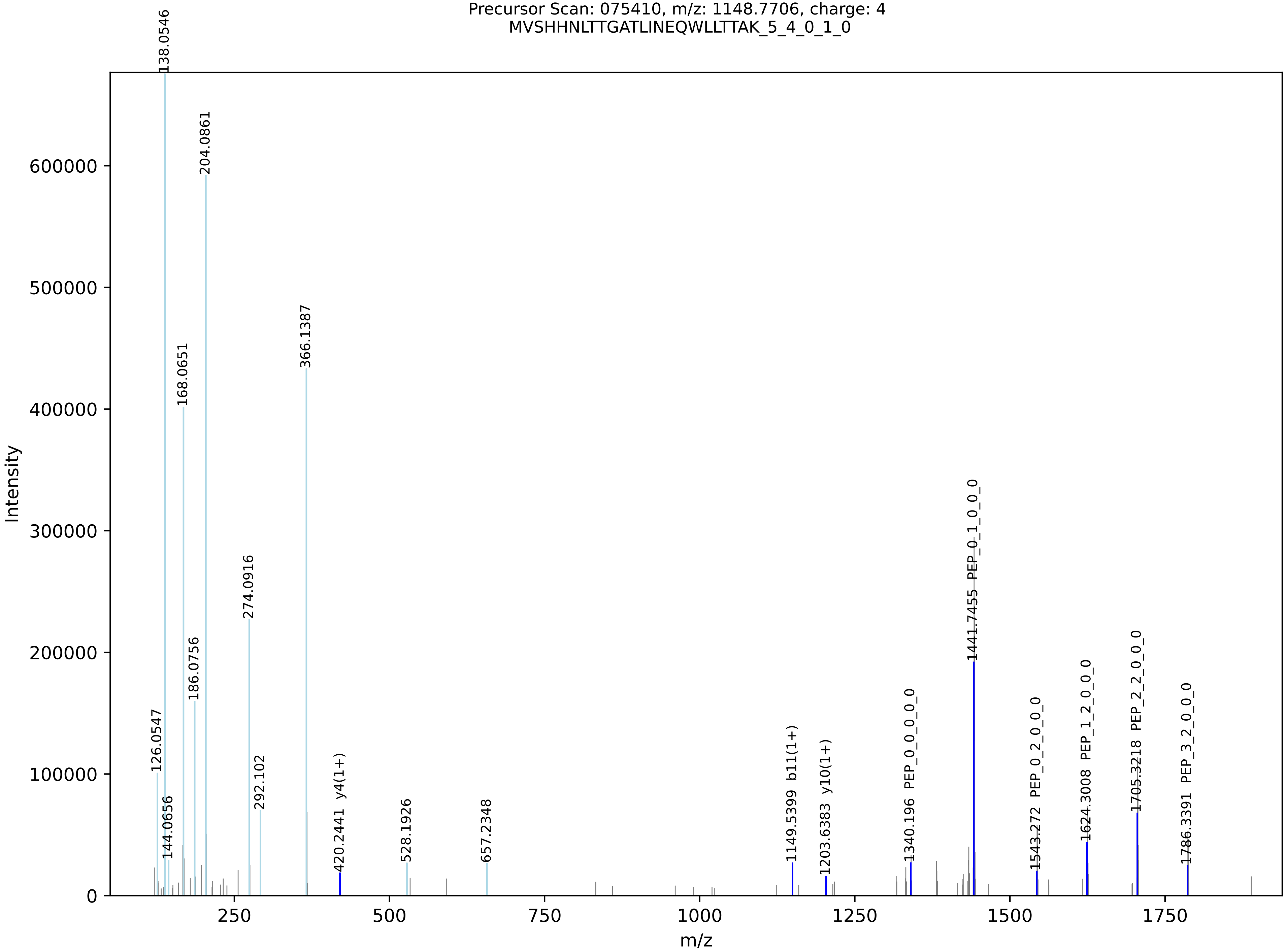

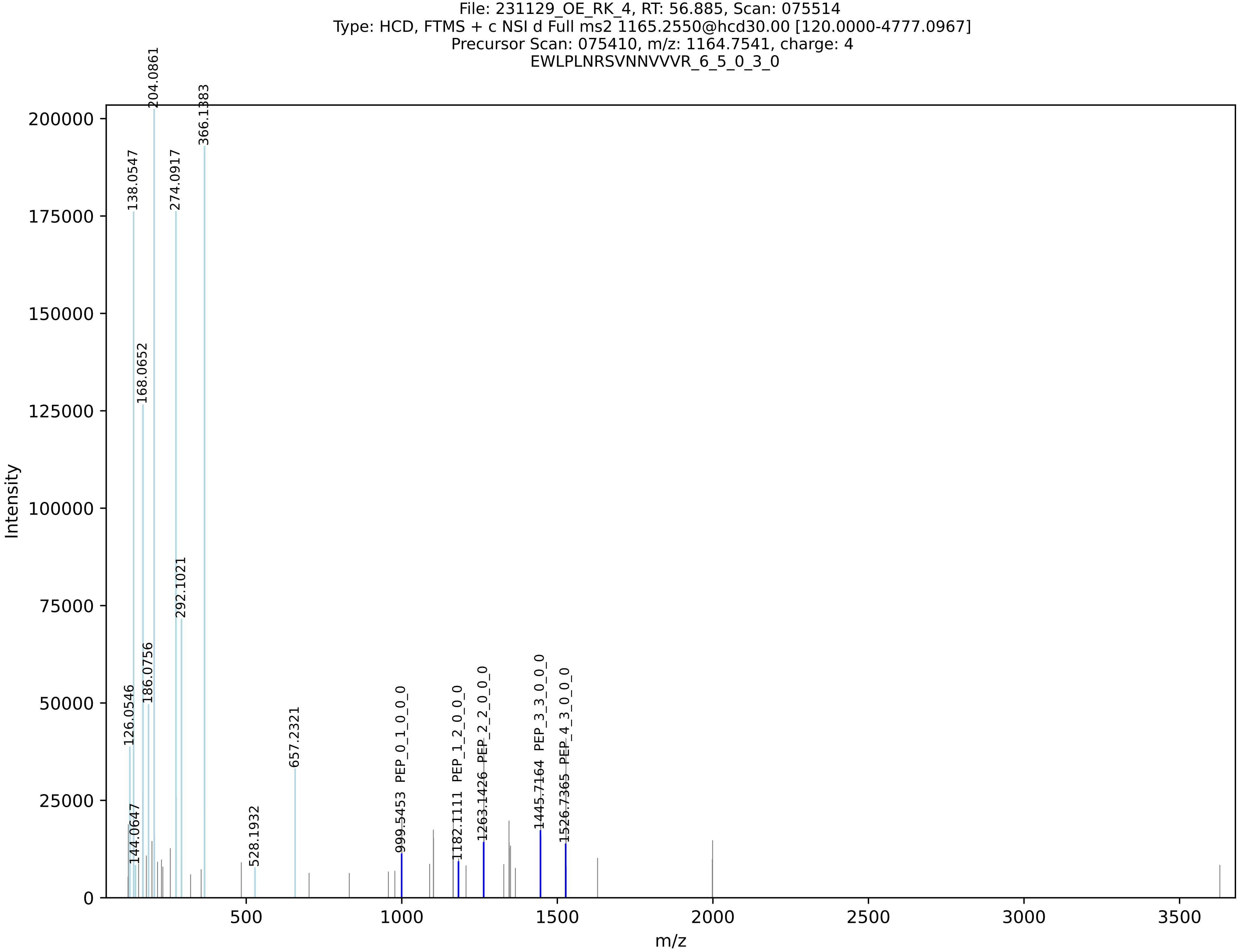
File: 231129_OE_RK_4, RT: 56.885, Scan: 075514
Type: HCD, FTMS + c NSI d Full ms2 1165.2550@hcd30.00 [120.0000-4777.0967]
Precursor Scan: 075410, m/z: 1164.7541, charge: 4
EWLPLNRSVNNVVVR_6_5_0_3_0
204.0861
366.1383
138.0547
274.0917
168.0652
292.1021
186.0756
126.0546
144.0647
528.1932
657.2321
999.5453 PEP_0_1_0_0_0_
1182.1111 PEP_1_2_0_0_0_
1263.1426 PEP_2_2_0_0_0_
1445.7164 PEP_3_3_0_0_0_
1526.7365 PEP_4_3_0_0_0_
Intensity
200000
175000
150000
125000
100000
75000
50000
25000
0
500
1000
1500
2000
2500
3000
3500
m/z

File: 231129_OE_RK_4, RT: 57.019, Scan: 075703
Type: HCD, FTMS + c NSI d Full ms2 1315.2214@hcd30.00 [120.0000-4046.7195]
Precursor Scan: 075631, m/z: 1314.2191, charge: 3
ALPQPQNVTSLLGCTH_5_4_0_2_0

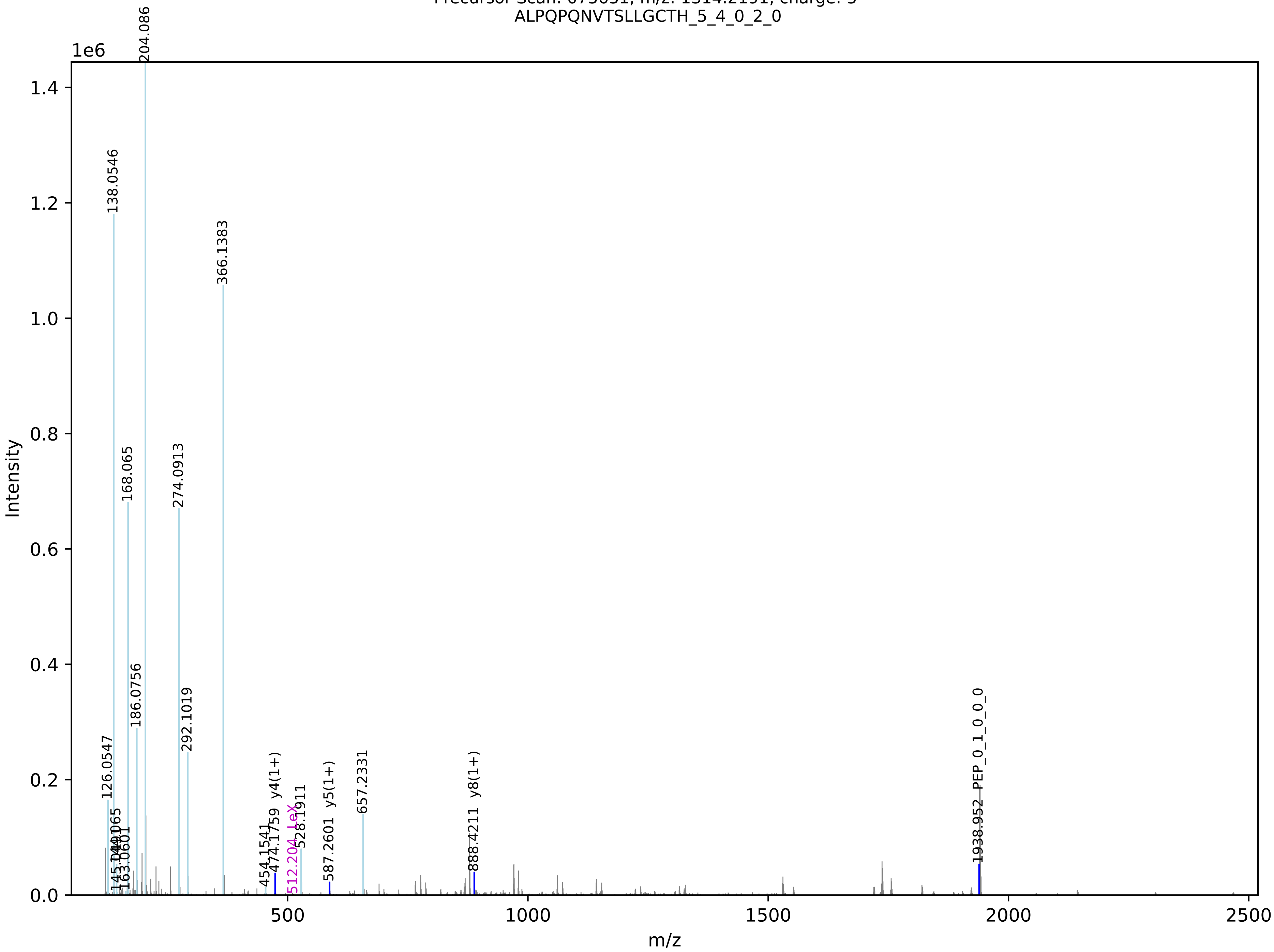

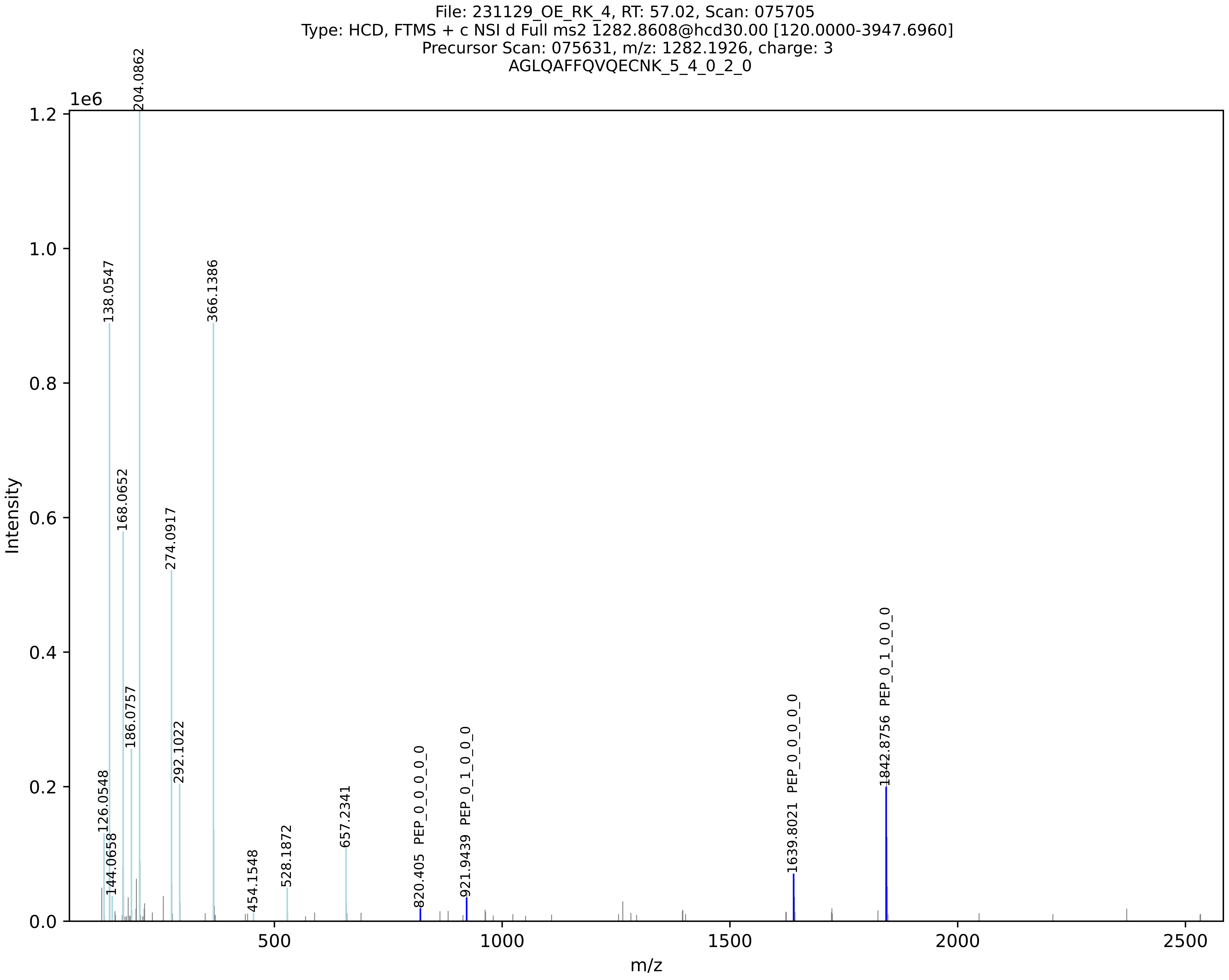

File: 231129_OE_RK_4, RT: 57.02, Scan: 075705
Type: HCD, FTMS + c NSI d Full ms2 1282.8608@hcd30.00 [120.0000-3947.6960]
Precursor Scan: 075631, m/z: 1282.1926, charge: 3
AGLQAFFQVQECNK_5_4_0_2_0
1e6
Intensity
m/z
204.0862
138.0547
366.1386
168.0652
274.0917
186.0757
292.1022
126.0548
144.0658
454.1548
528.1872
657.2341
820.405 PEP_0_0_0_0_0
921.9439 PEP_0_1_0_0_0
1639.8021 PEP_0_0_0_0_0
1842.8756 PEP_0_1_0_0_0

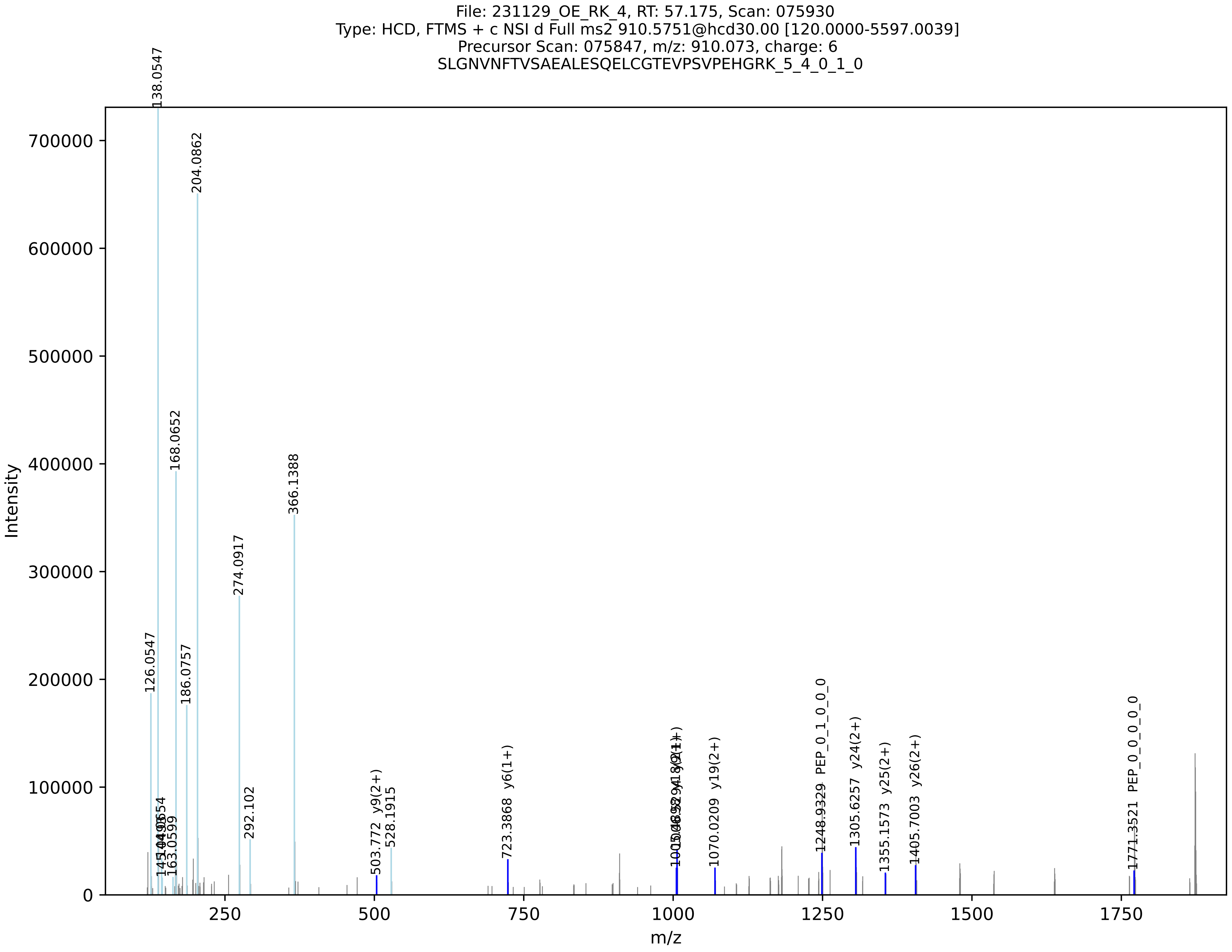
File: 231129_OE_RK_4, RT: 57.175, Scan: 075930
Type: HCD, FTMS + c NSI d Full ms2 910.5751@hcd30.00 [120.0000-5597.0039]
Precursor Scan: 075847, m/z: 910.073, charge: 6
SLGNVNFTVSAEALESQELCGTEVPSVPEHGRK_5_4_0_1_0
Intensity
m/z
0
100000
200000
300000
400000
500000
600000
700000
250
500
750
1000
1250
1500
1750
138.0547
204.0862
168.0652
366.1388
274.0917
126.0547
186.0757
292.102
163.0599
503.772 y9(2+)
528.1915
723.3868 y6(1+)
1070.0209 y19(2+)
1248.9329 PEP_0_1_0_0_0
1305.6257 y24(2+)
1355.1573 y25(2+)
1405.7003 y26(2+)
1771.3521 PEP_0_0_0_0_0

File: 231129_OE_RK_4, RT: 57.224, Scan: 076001
Type: HCD, FTMS + c NSI d Full ms2 1282.8605@hcd30.00 [120.0000-3947.6951]
Precursor Scan: 075921, m/z: 1282.1923, charge: 3
AGLQAFFQVQECNK_5_4_0_2_0

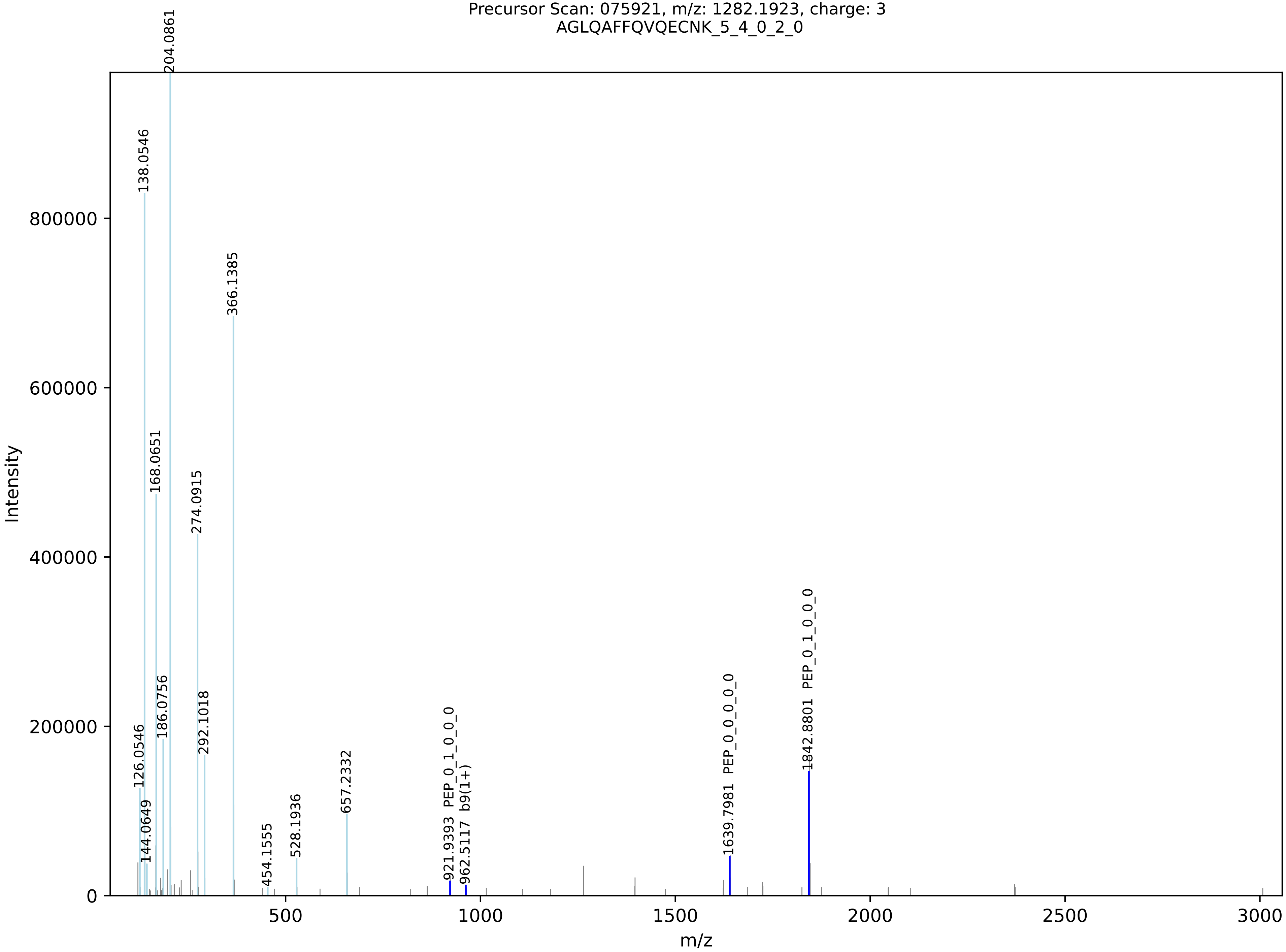

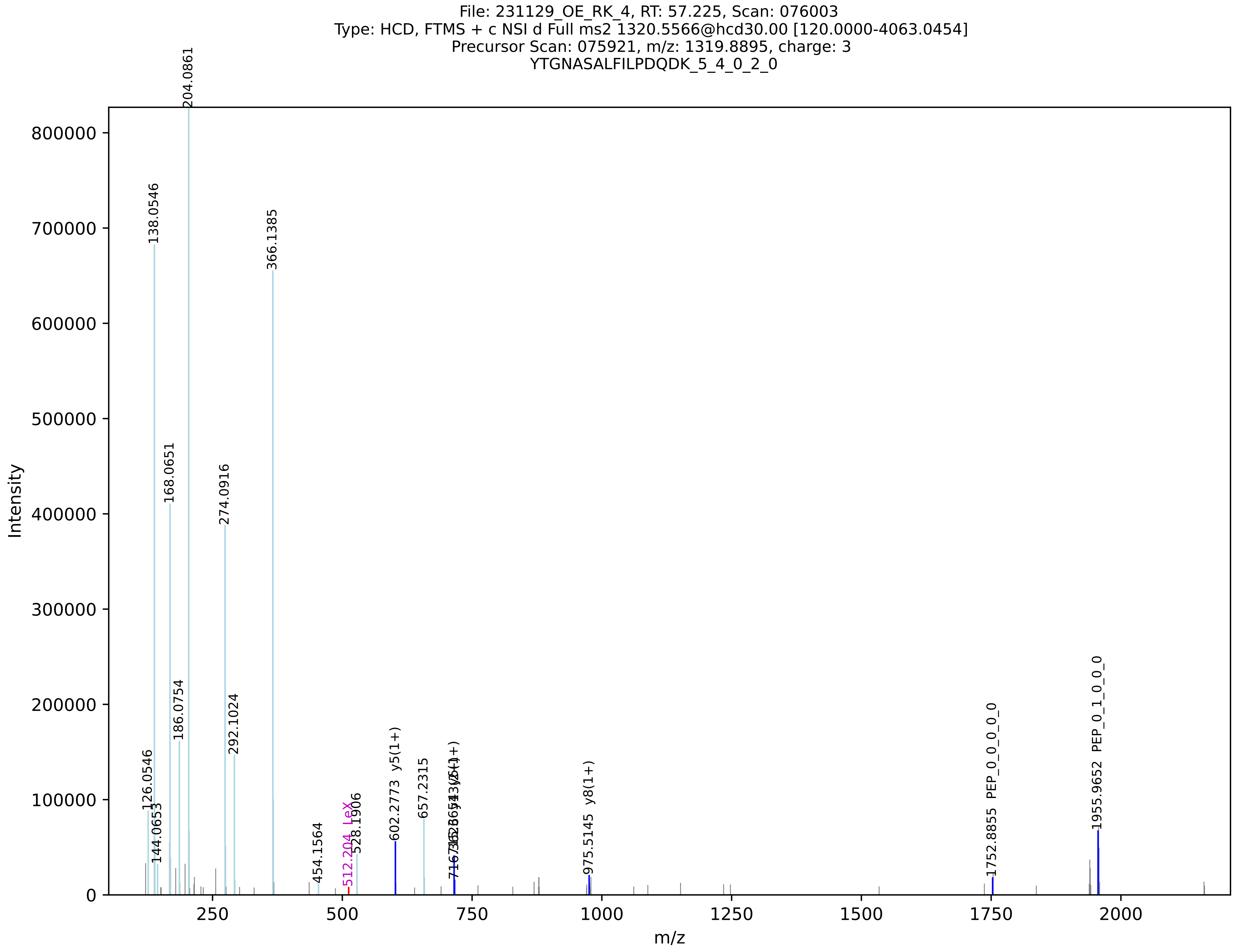
File: 231129_OE_RK_4, RT: 57.225, Scan: 076003
Type: HCD, FTMS + c NSI d Full ms2 1320.5566@hcd30.00 [120.0000-4063.0454]
Precursor Scan: 075921, m/z: 1319.8895, charge: 3
YTGNASALFILPDQDK_5_4_0_2_0
204.0861
138.0546
366.1385
168.0651
274.0916
186.0754
292.1024
126.0546
144.0653
454.1564
512.204 LeX
528.1906
602.2773 y5(1+)
657.2315
975.5145 y8(1+)
1752.8855 PEP_0_0_0_0_0
1955.9652 PEP_0_1_0_0_0
Intensity
m/z
0
100000
200000
300000
400000
500000
600000
700000
800000
250
500
750
1000
1250
1500
1750
2000

File: 231129_OE_RK_4, RT: 57.232, Scan: 076014
Type: HCD, FTMS + c NSI d Full ms2 1114.2471@hcd30.00 [120.0000-4568.9839]
Precursor Scan: 075921, m/z: 1113.7459, charge: 4
LQAPLNYTEFQKPICLPSK_5_4_0_2_0

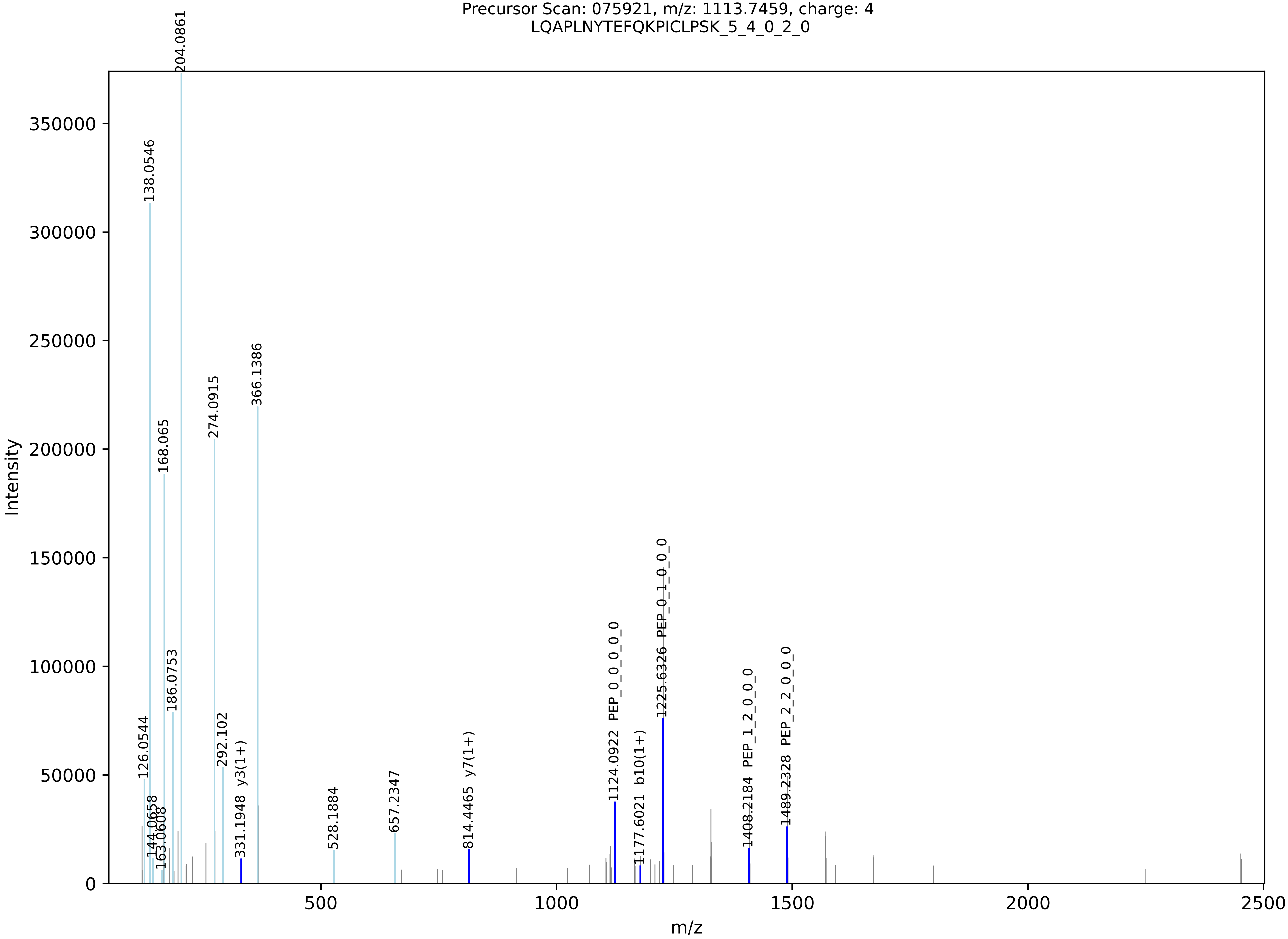

File: 231129_OE_RK_4, RT: 57.322, Scan: 076144
Type: HCD, FTMS + c NSI d Full ms2 1198.9807@hcd30.00 [120.0000-4914.6973]
Precursor Scan: 076068, m/z: 1198.4795, charge: 4
SPYYNVSDEISFHCYDGYTLR_5_4_0_2_0

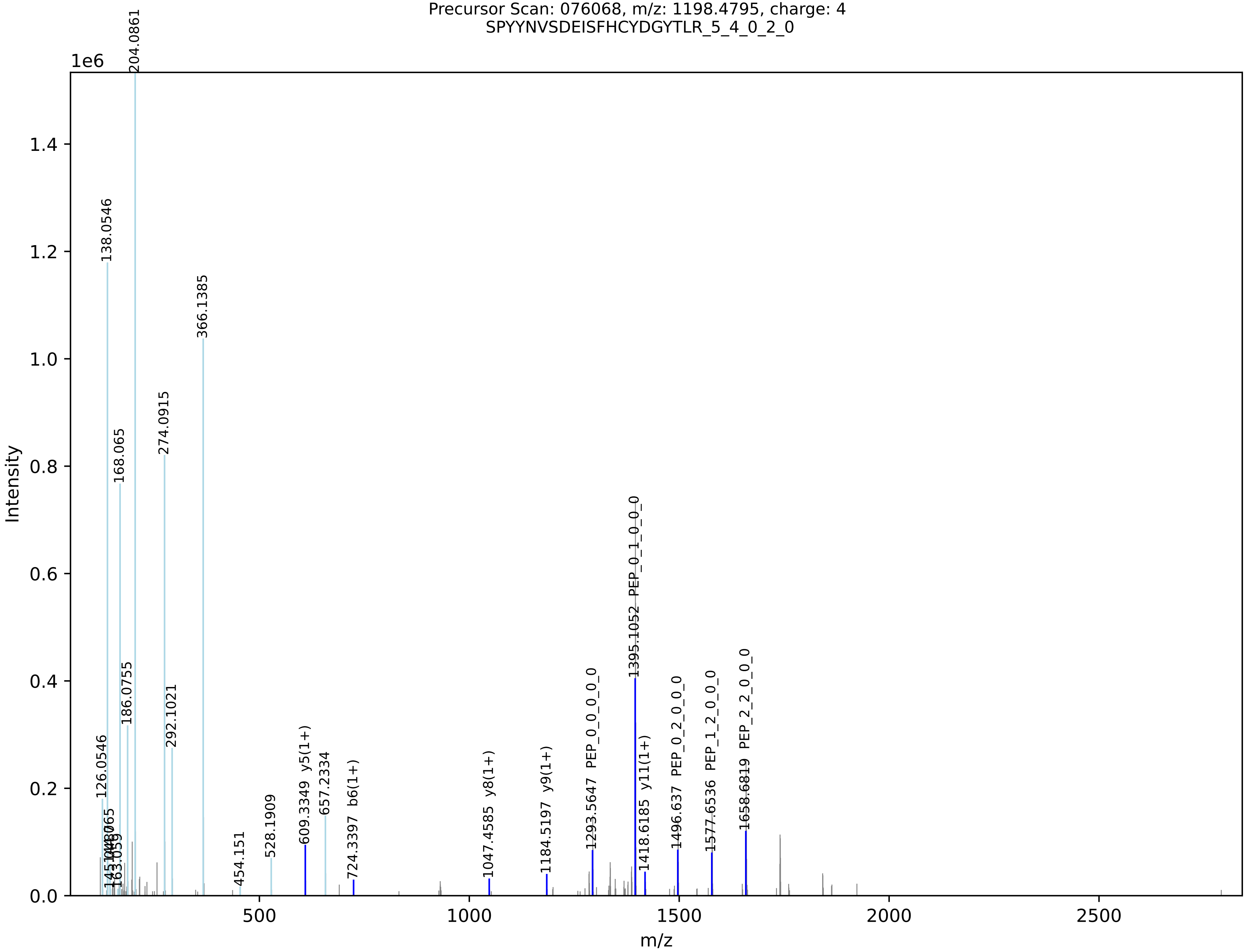

File: 231129_OE_RK_4, RT: 57.372, Scan: 076218
Type: HCD, FTMS + c NSI d Full ms2 1217.9950@hcd30.00 [120.0000-4992.2759]
Precursor Scan: 076141, m/z: 1217.2434, charge: 4
HMNHSEPRPGLGADGDAADPVDTR_5_4_1_2_0

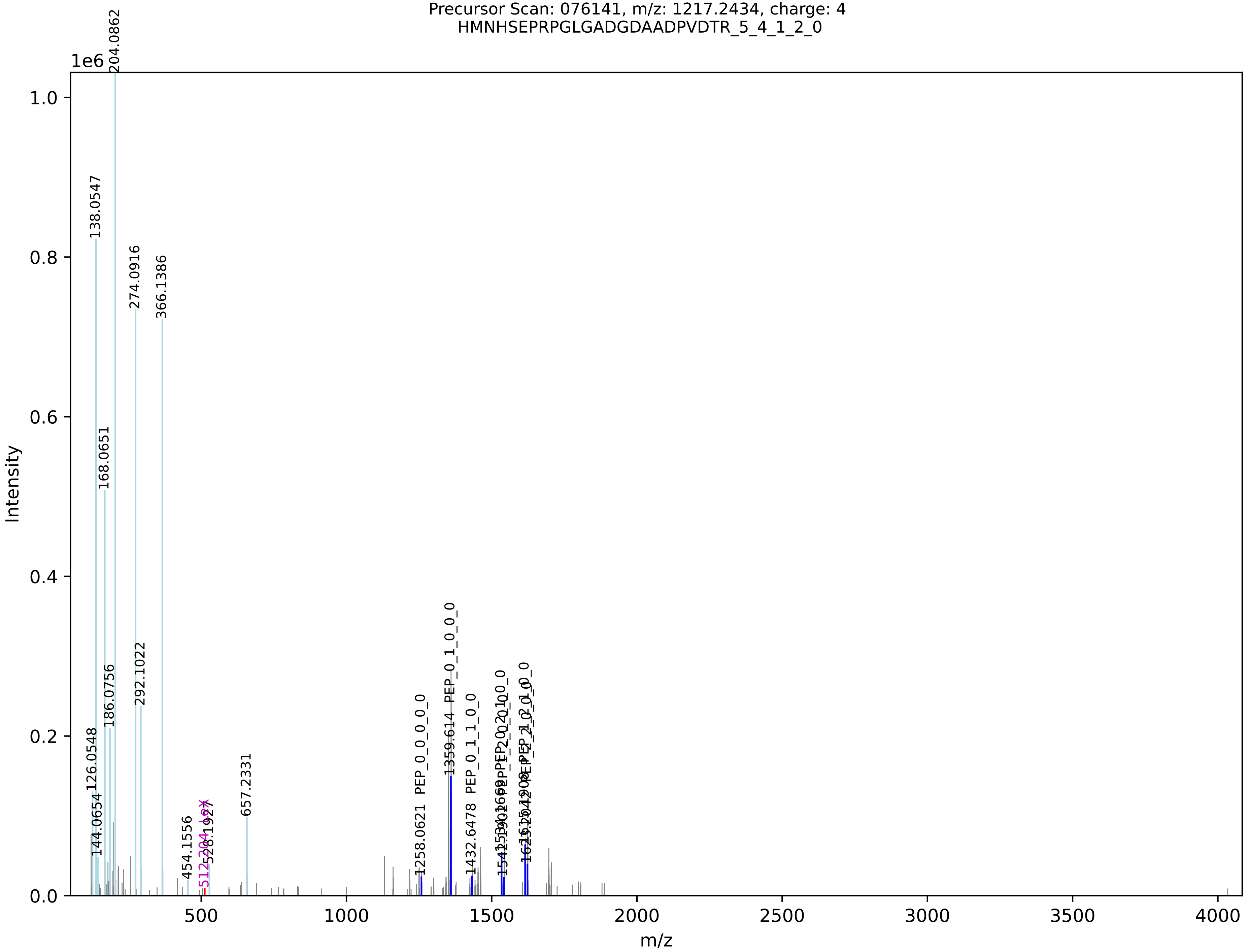

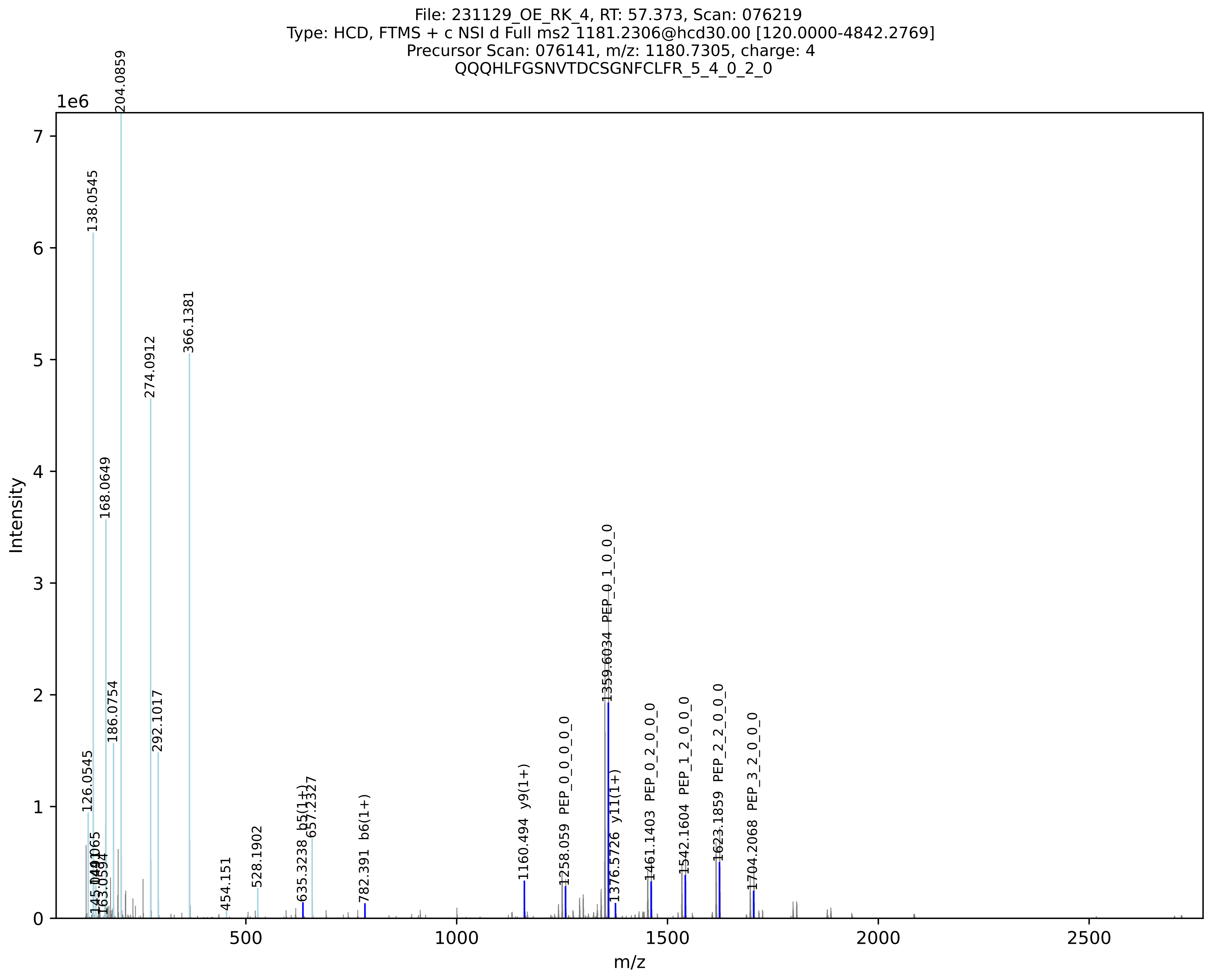

File: 231129_OE_RK_4, RT: 57.373, Scan: 076219
Type: HCD, FTMS + c NSI d Full ms2 1181.2306@hcd30.00 [120.0000-4842.2769]
Precursor Scan: 076141, m/z: 1180.7305, charge: 4
QQQHLFGSNVTDCSGNFCLFR_5_4_0_2_0
1e6
Intensity
m/z
204.0859
138.0545
366.1381
274.0912
168.0649
186.0754
292.1017
126.0545
454.151
528.1902
635.3238 b5(1+)
657.2327
782.391 b6(1+)
1160.494 y9(1+)
1258.059 PEP_0_0_0_0_0
1359.6034 PEP_0_1_0_0_0
1376.5726 y11(1+)
1461.1403 PEP_0_2_0_0_0
1542.1604 PEP_1_2_0_0_0
1623.1859 PEP_2_2_0_0_0
1704.2068 PEP_3_2_0_0_0

File: 231129_OE_RK_4, RT: 57.378, Scan: 076227
Type: HCD, FTMS + c NSI d Full ms2 1262.8107@hcd30.00 [120.0000-5175.1235]
Precursor Scan: 076141, m/z: 1262.3117, charge: 4
NTTSYVLRGLEPGQEYNVLLTAEK_5_4_1_2_0

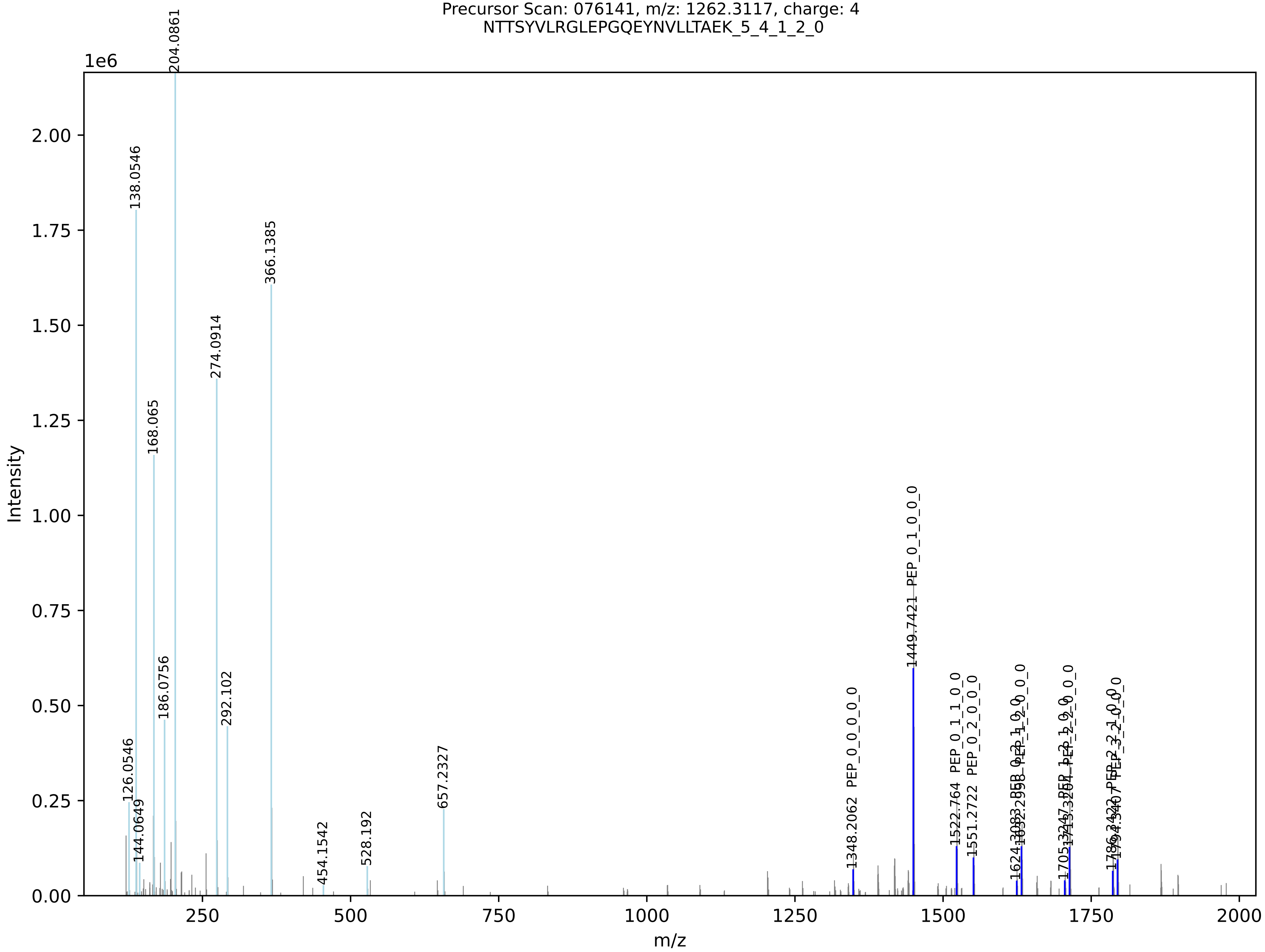

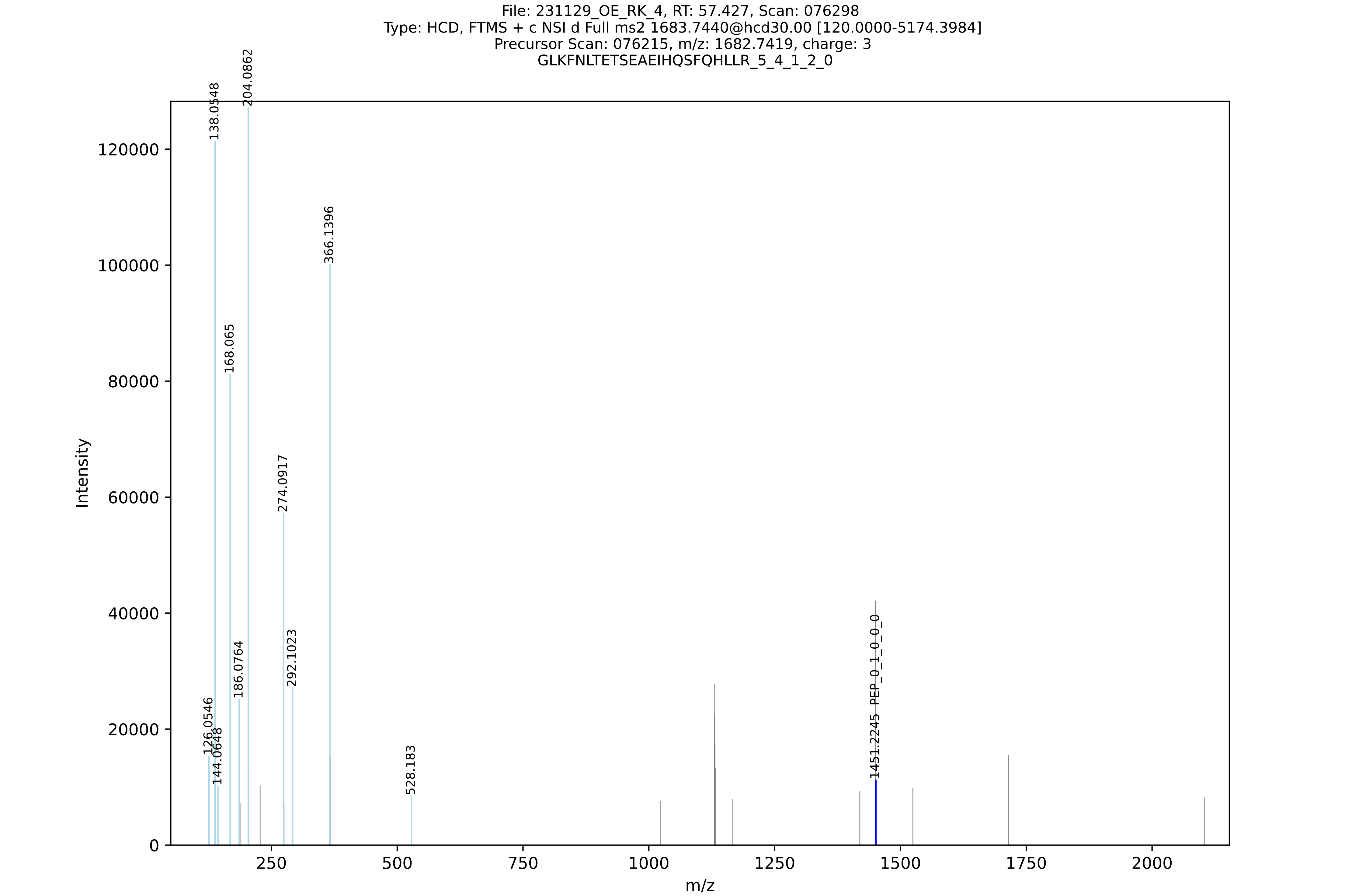
File: 231129_OE_RK_4, RT: 57.427, Scan: 076298
Type: HCD, FTMS + c NSI d Full ms2 1683.7440@hcd30.00 [120.0000-5174.3984]
Precursor Scan: 076215, m/z: 1682.7419, charge: 3
GLKFNLTETSEAEIHQSFQHLLR_5_4_1_2_0
Intensity
m/z
204.0862
138.0548
366.1396
168.065
274.0917
292.1023
186.0764
126.0546
144.0648
528.183
1451.2245_PEP_0_1_0_0_0
0
20000
40000
60000
80000
100000
120000
250
500
750
1000
1250
1500
1750
2000

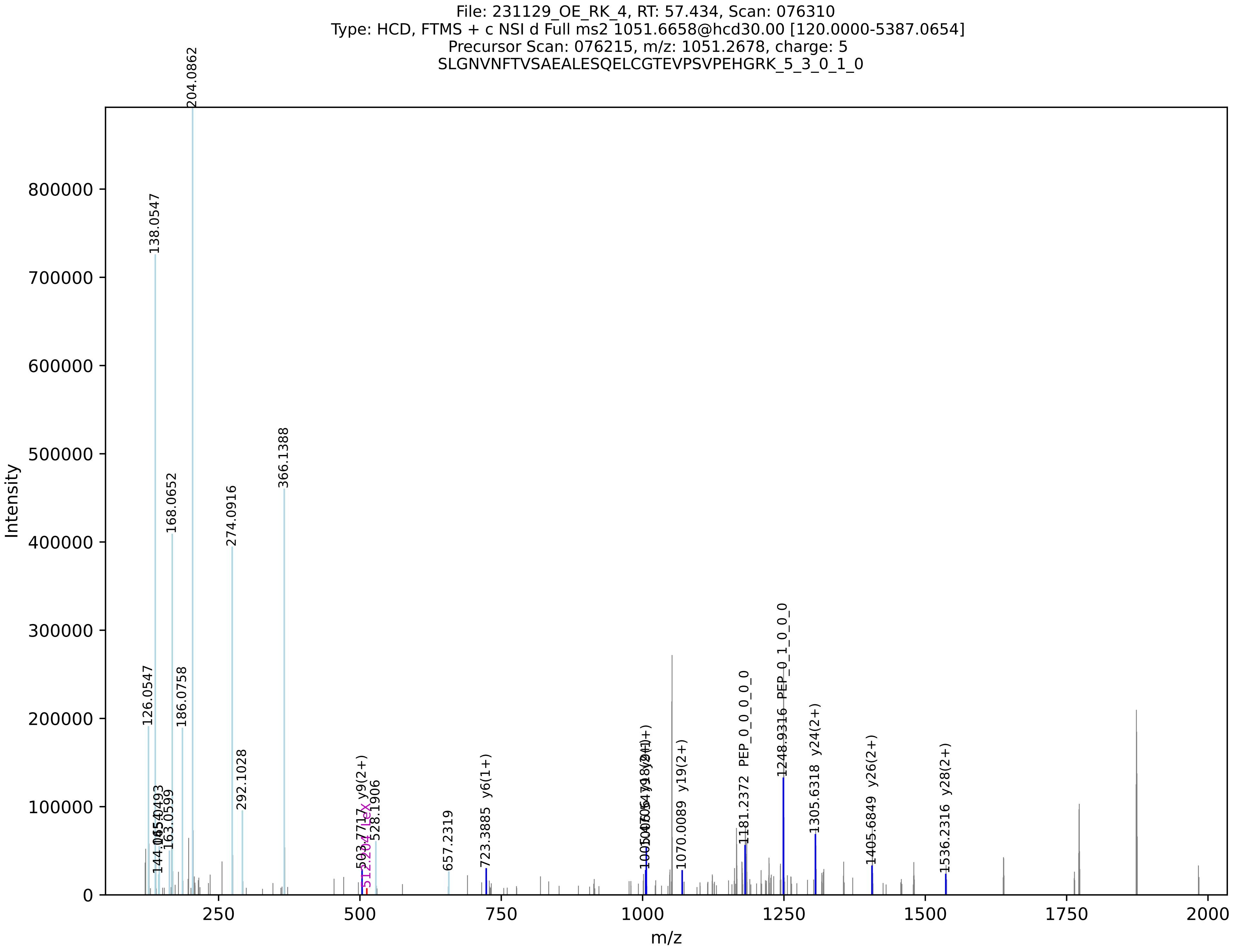
File: 231129_OE_RK_4, RT: 57.434, Scan: 076310
Type: HCD, FTMS + c NSI d Full ms2 1051.6658@hcd30.00 [120.0000-5387.0654]
Precursor Scan: 076215, m/z: 1051.2678, charge: 5
SLGNVNFTVSAEALESQELCGTEVPSVPEHGRK_5_3_0_1_0
Intensity
m/z
204.0862
138.0547
168.0652
274.0916
366.1388
126.0547
186.0758
292.1028
163.0599
528.1906
657.2319
723.3885 y6(1+)
1070.0089 y19(2+)
1181.2372 PEP_0_0_0_0_0
1248.9316 PEP_0_1_0_0_0
1305.6318 y24(2+)
1405.6849 y26(2+)
1536.2316 y28(2+)
y9(2+)

File: 231129_OE_RK_4, RT: 57.474, Scan: 076366
Type: HCD, FTMS + c NSI d Full ms2 981.4365@hcd30.00 [120.0000-5028.8960]
Precursor Scan: 076289, m/z: 980.8359, charge: 5
NTTSYVLRGLEPGQEYNVLLTAEK_5_4_0_2_0

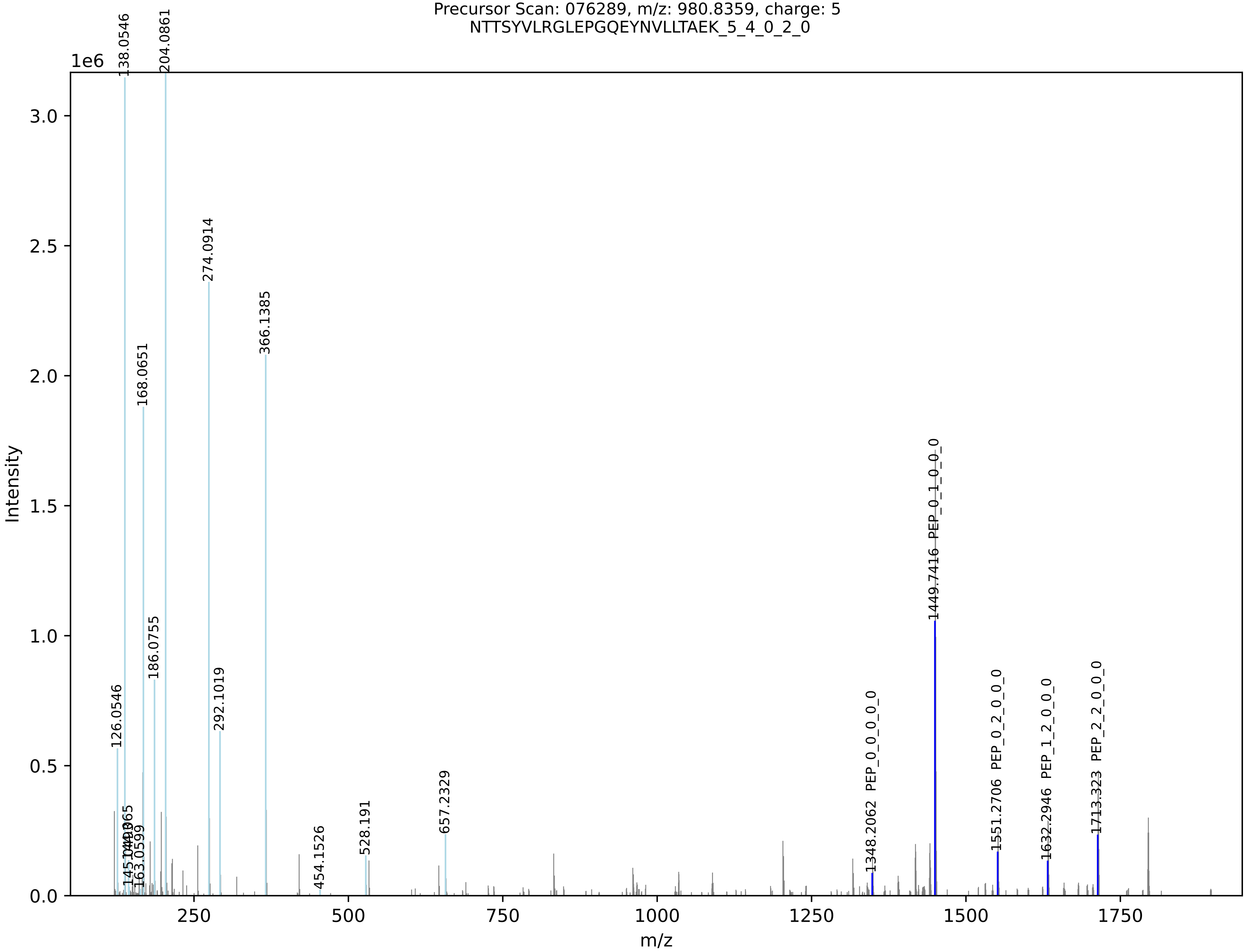

File: 231129_OE_RK_4, RT: 57.527, Scan: 076444
Type: HCD, FTMS + c NSI d Full ms2 1198.9783@hcd30.00 [120.0000-4914.6875]
Precursor Scan: 076363, m/z: 1198.4778, charge: 4
SPYYNVSDEISFHCYDGYTLR_5_4_0_2_0

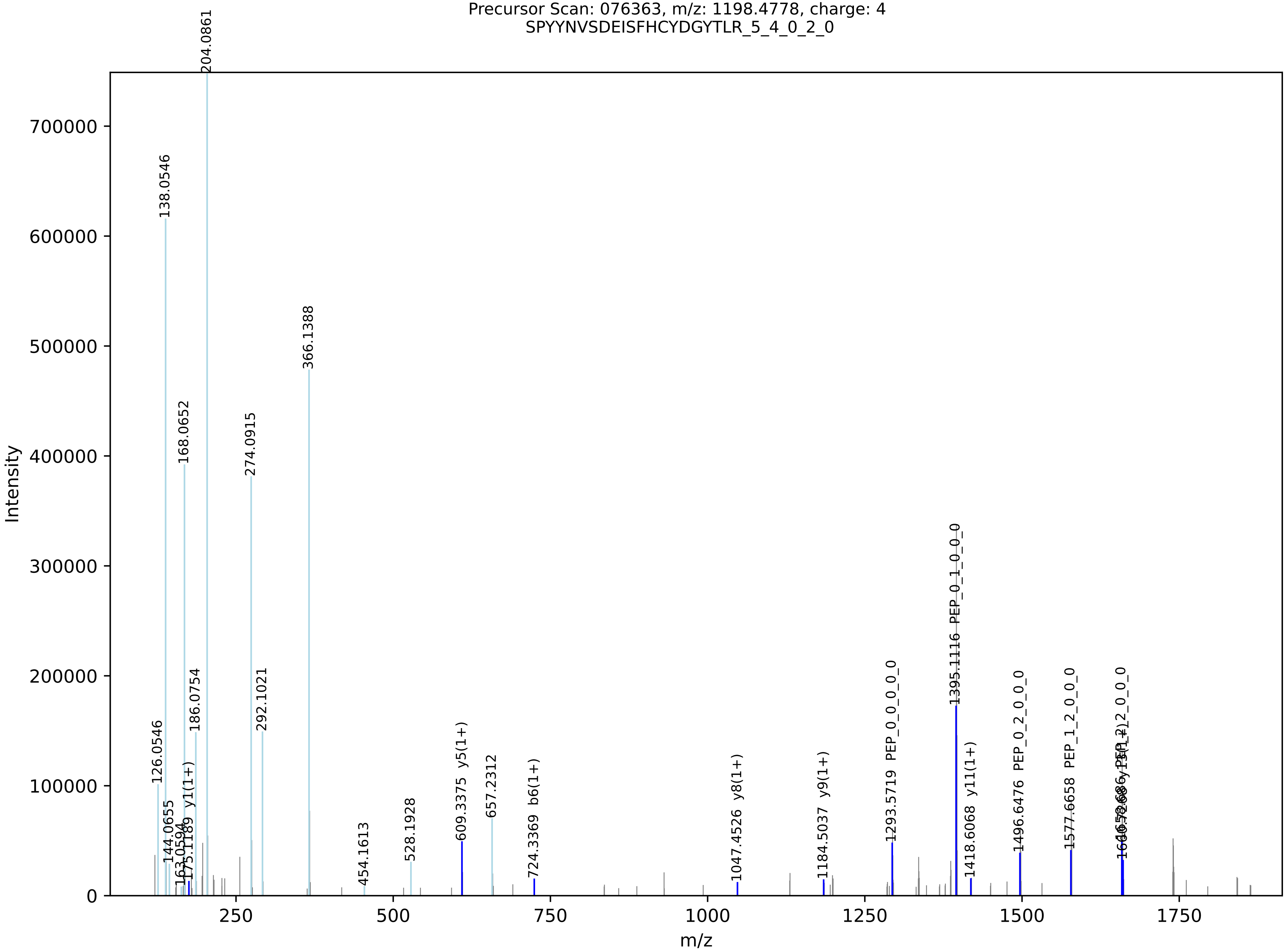

File: 231129_OE_RK_4, RT: 57.57, Scan: 076507
Type: HCD, FTMS + c NSI d Full ms2 1060.0752@hcd30.00 [120.0000-5429.9531]
Precursor Scan: 076437, m/z: 1059.4737, charge: 5
SLGNVNFTVSAEALESQELCGTEVPSVPEHGRK_4_4_0_1_0

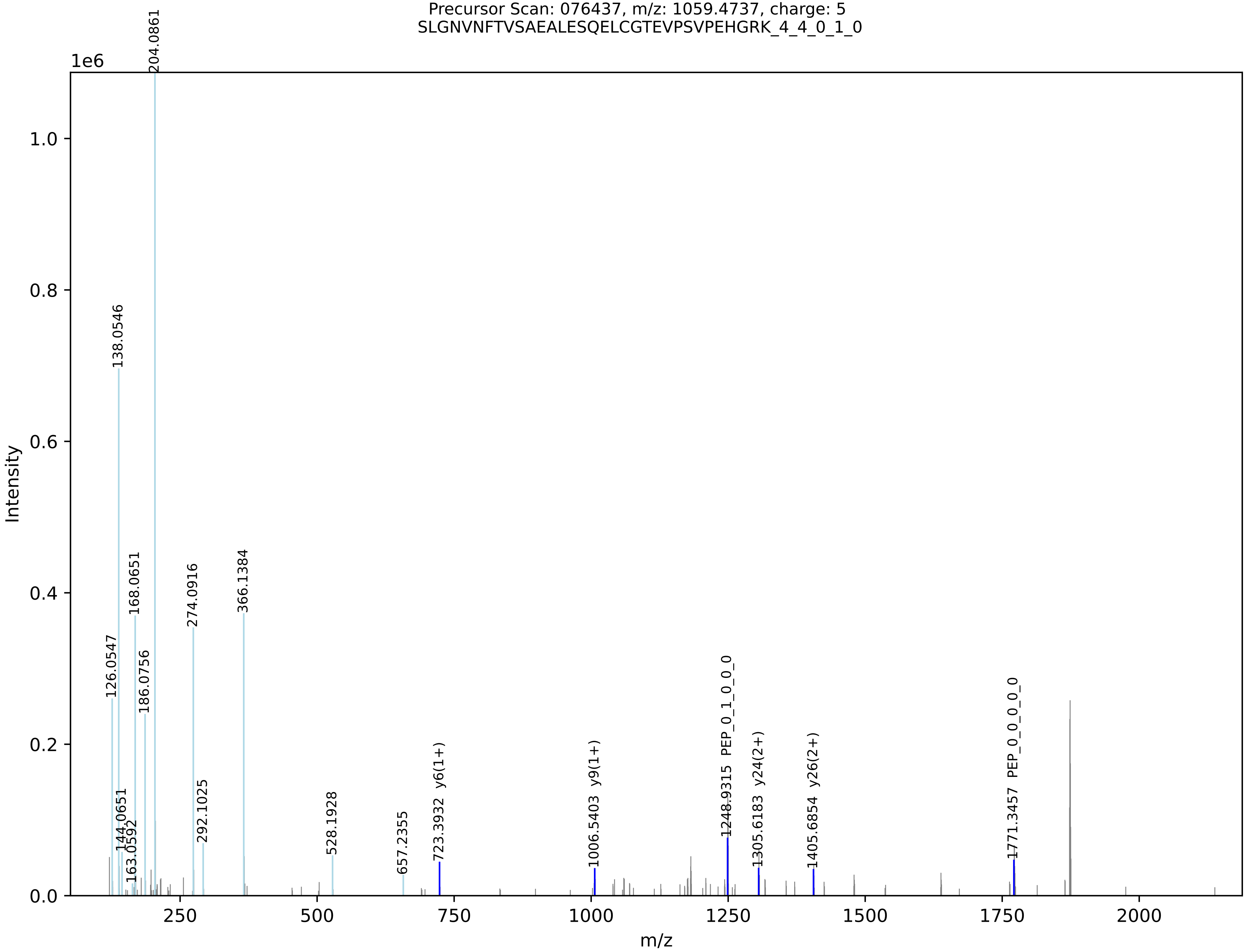

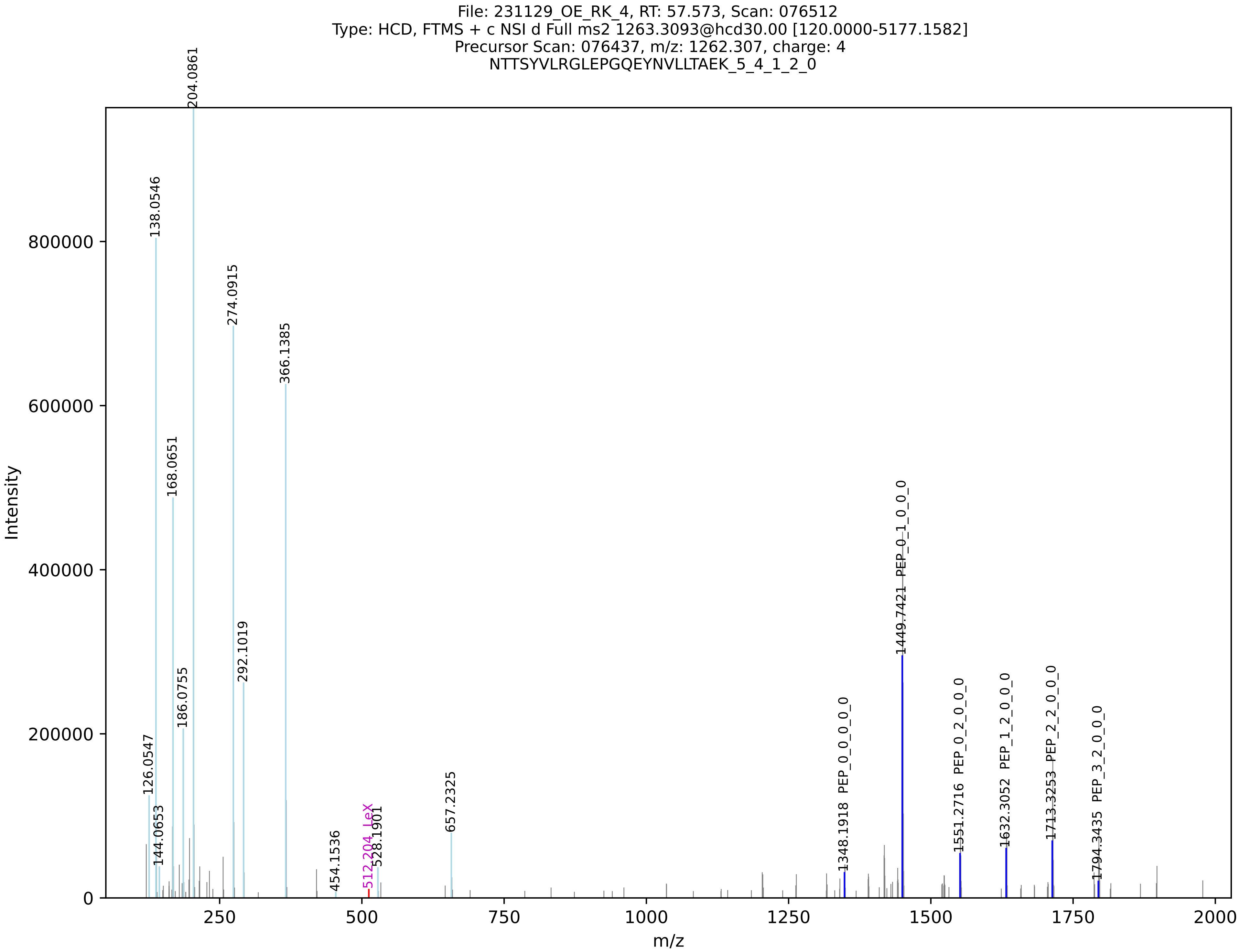
File: 231129_OE_RK_4, RT: 57.573, Scan: 076512
Type: HCD, FTMS + c NSI d Full ms2 1263.3093@hcd30.00 [120.0000-5177.1582]
Precursor Scan: 076437, m/z: 1262.307, charge: 4
NTTSYVLRGLEPGQEYNVLLTAEK_5_4_1_2_0
204.0861
138.0546
274.0915
366.1385
168.0651
292.1019
186.0755
126.0547
144.0653
454.1536
512.204_LeX
528.1901
657.2325
1348.1918 PEP_0_0_0_0_0
1449.7421 PEP_0_1_0_0_0
1551.2716 PEP_0_2_0_0_0
1632.3052 PEP_1_2_0_0_0
1713.3253 PEP_2_2_0_0_0
1794.3435 PEP_3_2_0_0_0
Intensity
m/z
0
200000
400000
600000
800000
250
500
750
1000
1250
1500
1750
2000

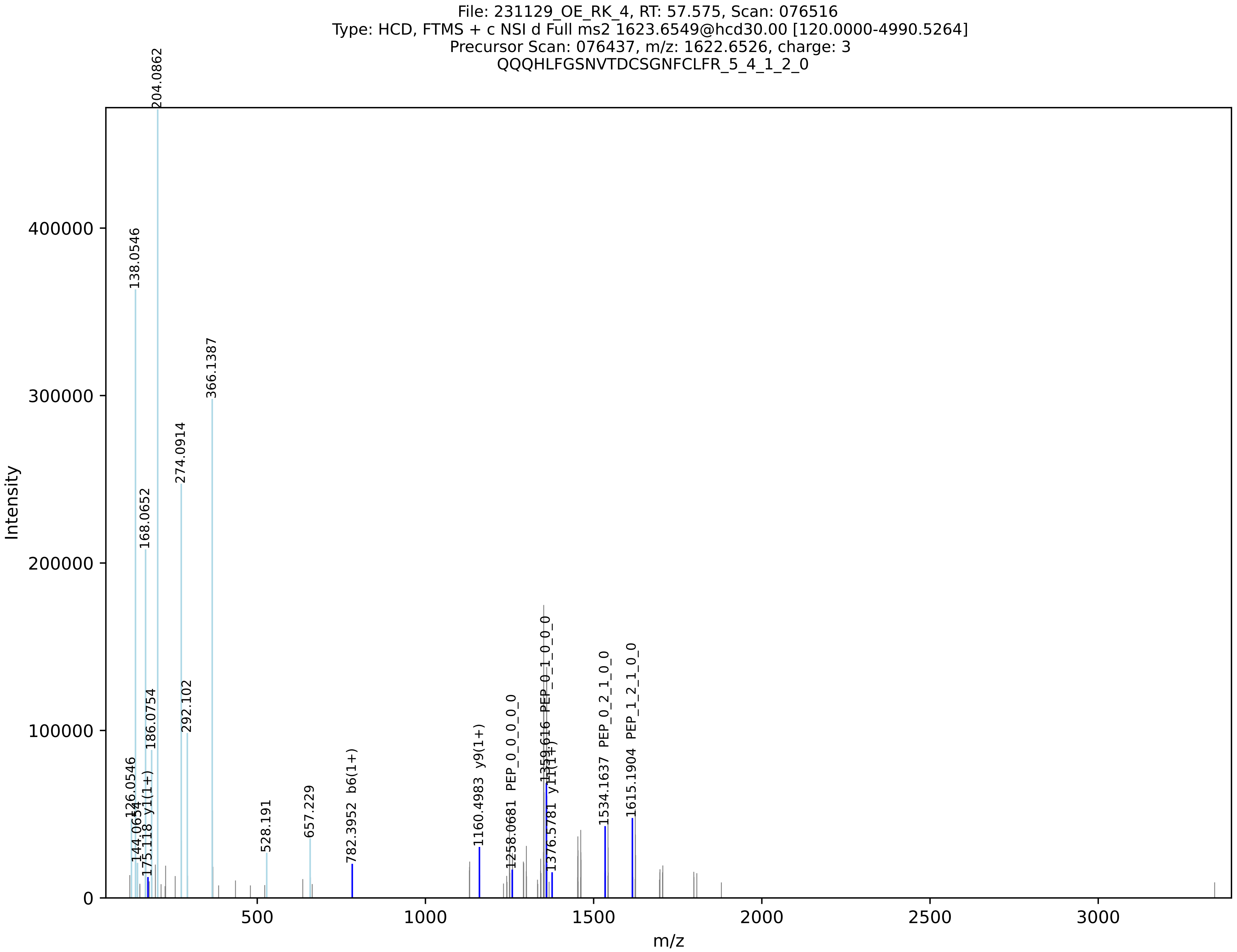

File: 231129_OE_RK_4, RT: 57.575, Scan: 076516
Type: HCD, FTMS + c NSI d Full ms2 1623.6549@hcd30.00 [120.0000-4990.5264]
Precursor Scan: 076437, m/z: 1622.6526, charge: 3
QQQHLFGSNVTDCSGNFCLFR_5_4_1_2_0
Intensity
m/z
204.0862
138.0546
366.1387
274.0914
168.0652
186.0754
292.102
126.0546
144.0654
175.118 y1(1+)
528.191
657.229
782.3952 b6(1+)
1160.4983 y9(1+)
1258.0681 PEP_0_0_0_0_0_
1359.616 PEP_0_1_0_0_0_
1376.5781 y11(1+)
1534.1637 PEP_0_2_1_0_0_
1615.1904 PEP_1_2_1_0_0_

File: 231129_OE_RK_4, RT: 57.669, Scan: 076653
Type: HCD, FTMS + c NSI d Full ms2 981.2365@hcd30.00 [120.0000-5027.8760]
Precursor Scan: 076576, m/z: 980.8361, charge: 5
NTTSYVLRGLEPGQEYNVLLTAEK_5_4_0_2_0

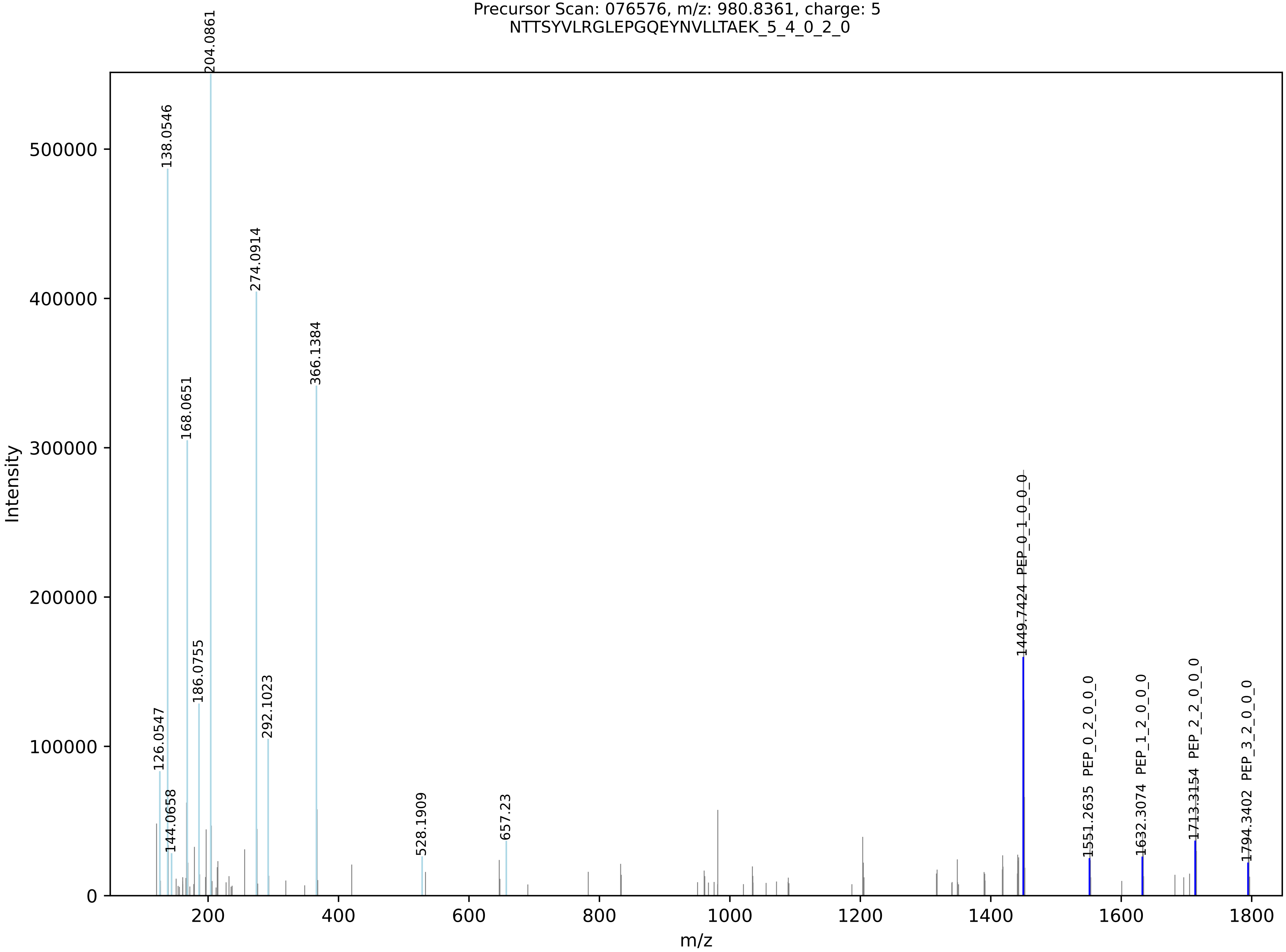

File: 231129_OE_RK_4, RT: 57.76, Scan: 076786
Type: HCD, FTMS + c NSI d Full ms2 1623.6571@hcd30.00 [120.0000-4990.5327]
Precursor Scan: 076704, m/z: 1622.6548, charge: 3
QQQHLFGSNVTDCSGNFCLFR_5_4_1_2_0

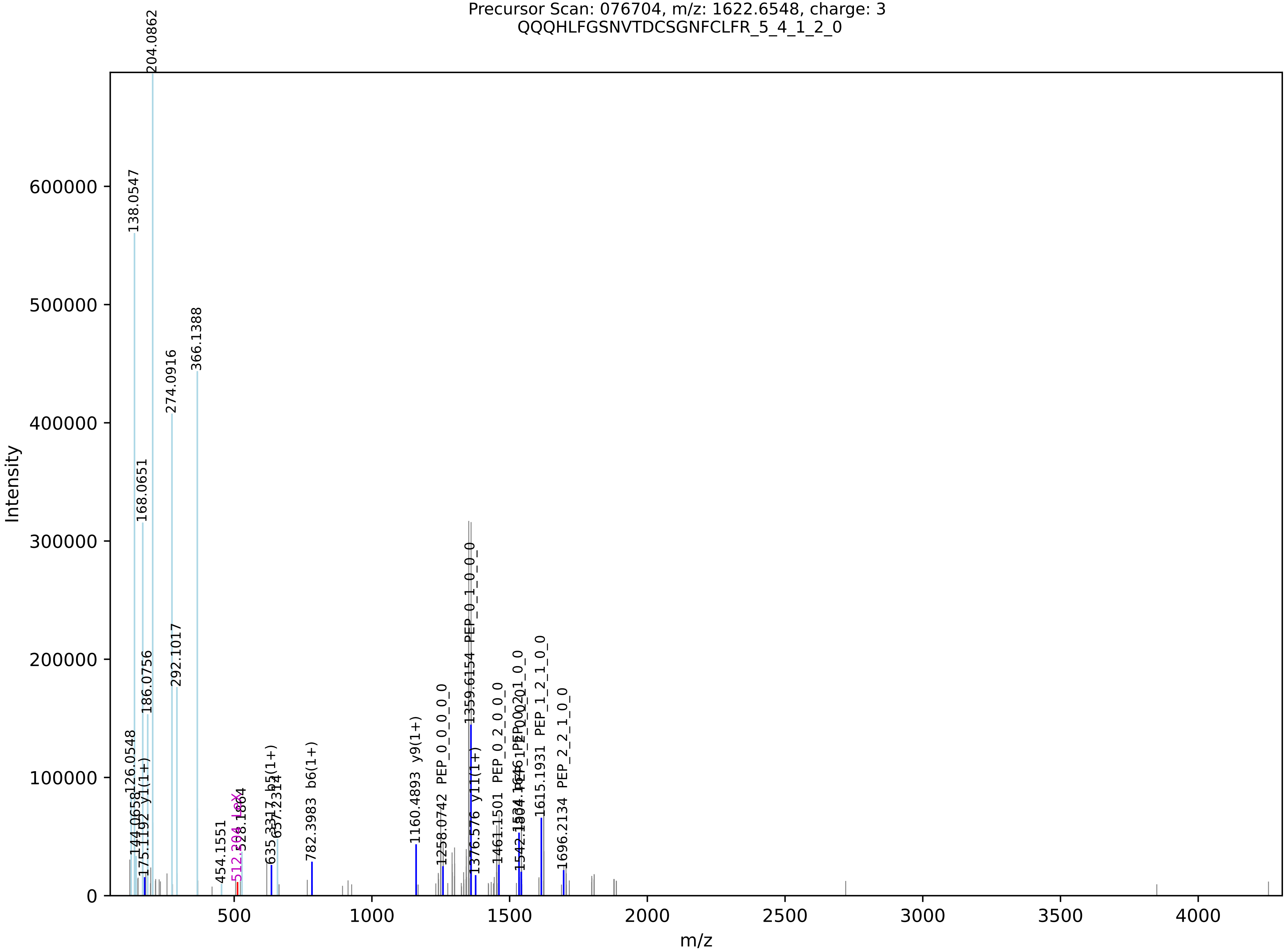

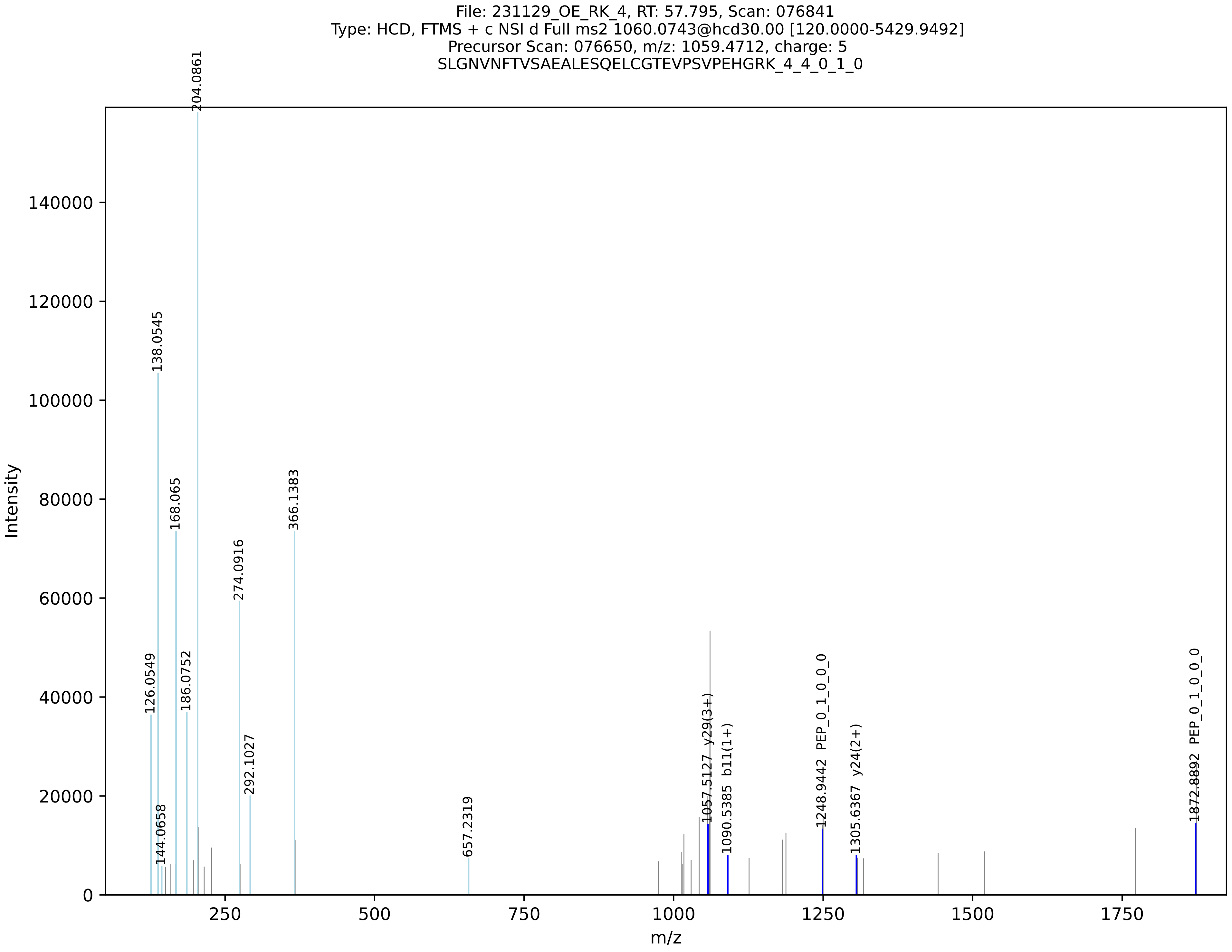

File: 231129_OE_RK_4, RT: 57.795, Scan: 076841
Type: HCD, FTMS + c NSI d Full ms2 1060.0743@hcd30.00 [120.0000-5429.9492]
Precursor Scan: 076650, m/z: 1059.4712, charge: 5
SLGNVNFTVSAEALESQELCGTEVPSVPEHGRK_4_4_0_1_0
Intensity
m/z
204.0861
138.0545
168.065
366.1383
274.0916
126.0549
186.0752
292.1027
144.0658
657.2319
1057.5127 y29(3+)
1090.5385 b11(1+)
1248.9442 PEP_0_1_0_0_0
1305.6367 y24(2+)
1872.8892 PEP_0_1_0_0_0

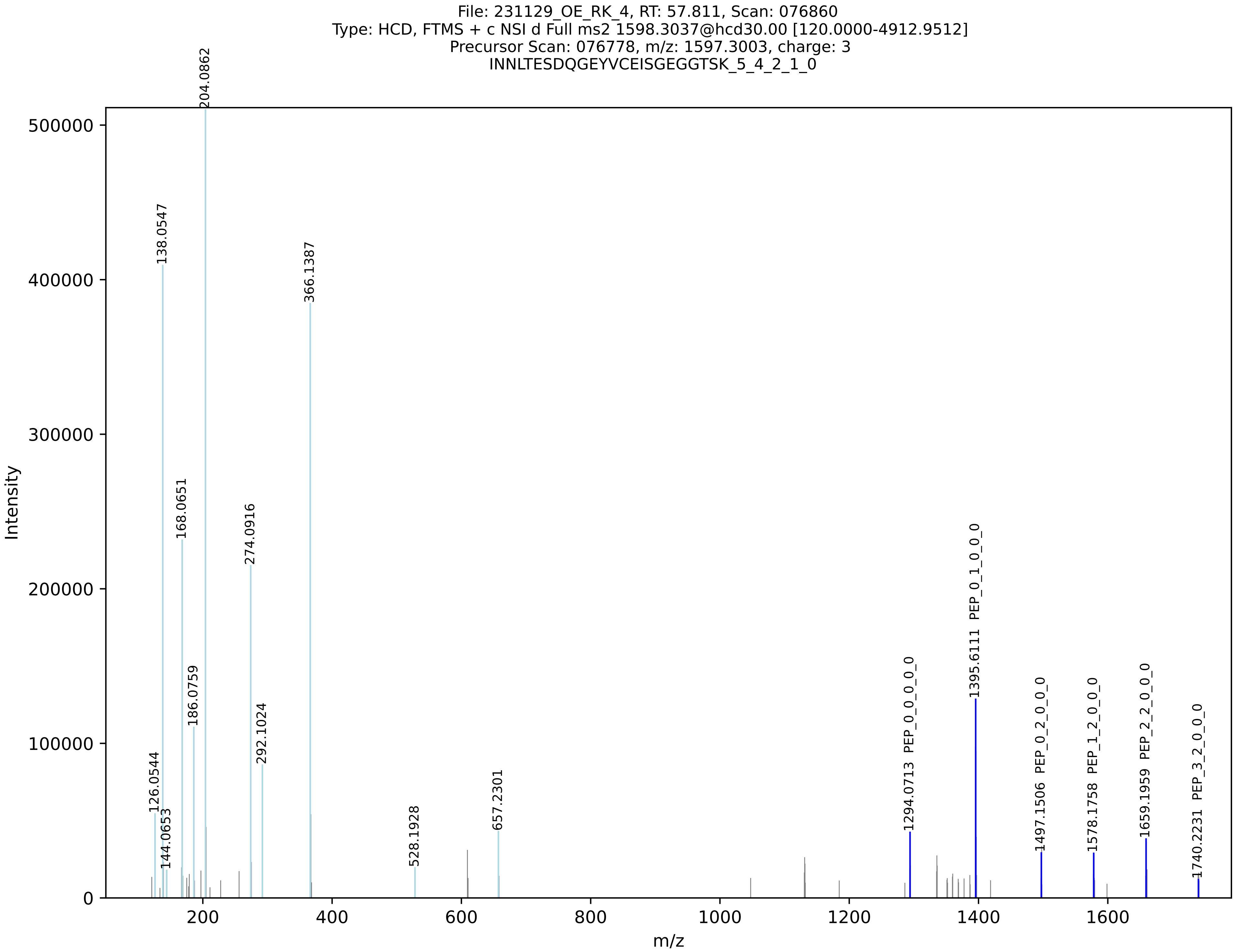
File: 231129_OE_RK_4, RT: 57.811, Scan: 076860
Type: HCD, FTMS + c NSI d Full ms2 1598.3037@hcd30.00 [120.0000-4912.9512]
Precursor Scan: 076778, m/z: 1597.3003, charge: 3
INNLTESDQGEYVCEISGEGGTSK_5_4_2_1_0
Intensity
m/z
204.0862
138.0547
366.1387
168.0651
274.0916
186.0759
292.1024
126.0544
144.0653
528.1928
657.2301
1294.0713 PEP_0_0_0_0_0
1395.6111 PEP_0_1_0_0_0
1497.1506 PEP_0_2_0_0_0
1578.1758 PEP_1_2_0_0_0
1659.1959 PEP_2_2_0_0_0
1740.2231 PEP_3_2_0_0_0

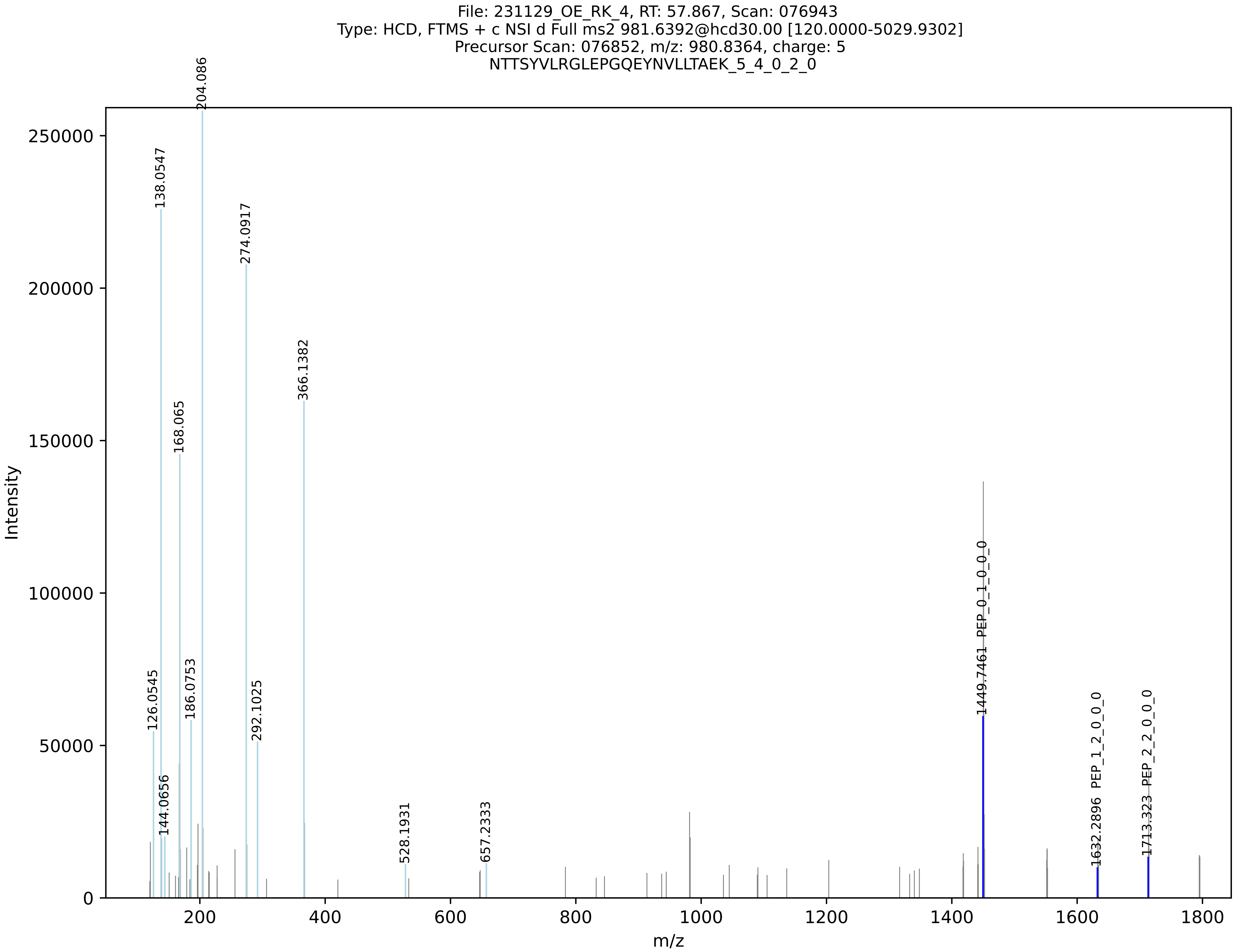
File: 231129_OE_RK_4, RT: 57.867, Scan: 076943
Type: HCD, FTMS + c NSI d Full ms2 981.6392@hcd30.00 [120.0000-5029.9302]
Precursor Scan: 076852, m/z: 980.8364, charge: 5
NTTSYVLRGLEPGQEYNVLLTAEK_5_4_0_2_0
Intensity
m/z
204.086
138.0547
274.0917
366.1382
168.065
126.0545
186.0753
292.1025
144.0656
528.1931
657.2333
1449.7461_PEP_0_1_0_0_0_
1632.2896_PEP_1_2_0_0_0_
1713.323_PEP_2_2_0_0_0_

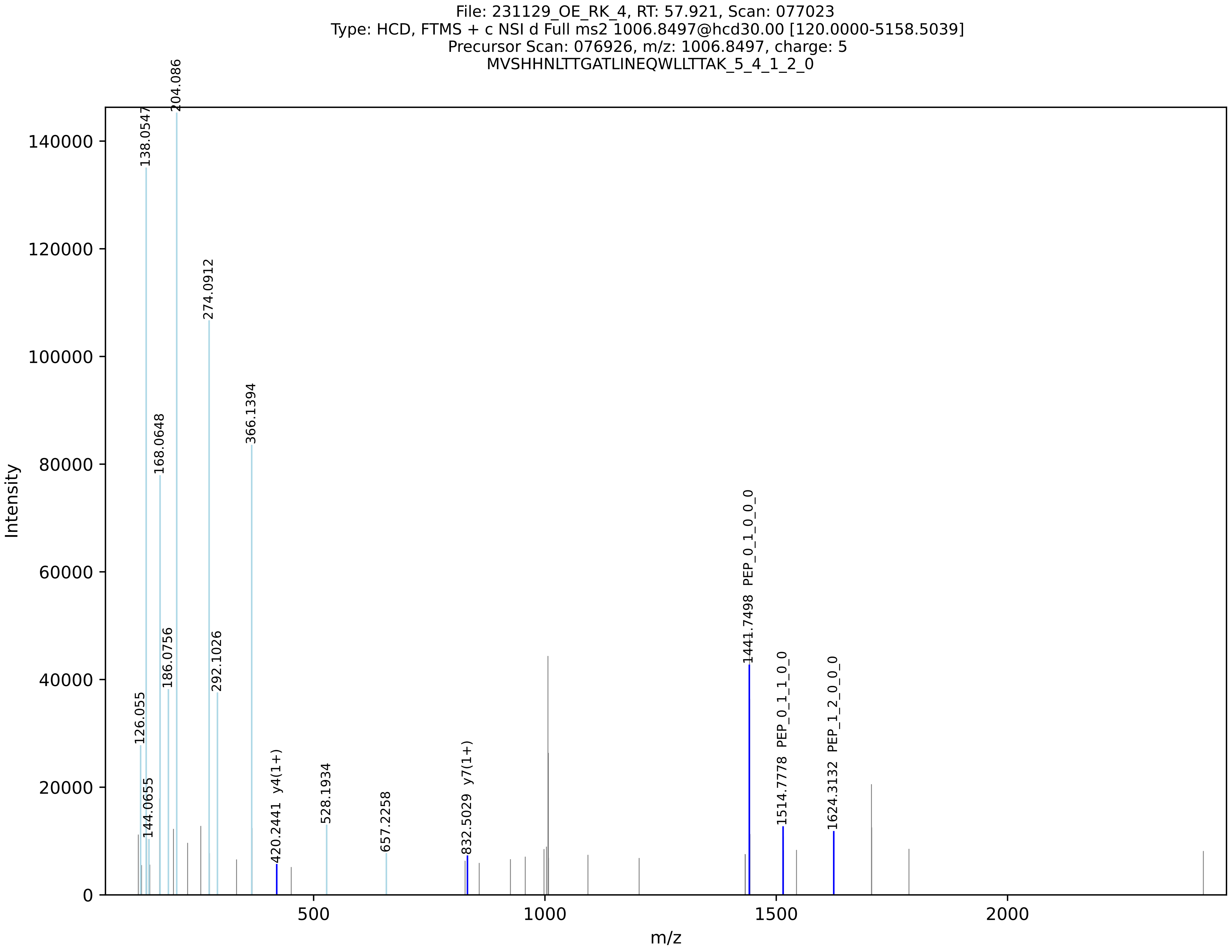

File: 231129_OE_RK_4, RT: 57.921, Scan: 077023
Type: HCD, FTMS + c NSI d Full ms2 1006.8497@hcd30.00 [120.0000-5158.5039]
Precursor Scan: 076926, m/z: 1006.8497, charge: 5
MVSHHNLTTGATLINEQWLLTTAK_5_4_1_2_0
Intensity
m/z
204.086
138.0547
274.0912
366.1394
168.0648
186.0756
292.1026
126.055
144.0655
420.2441 y4(1+)
528.1934
657.2258
832.5029 y7(1+)
1441.7498 PEP_0_1_0_0_0
1514.7778 PEP_0_1_1_0_0
1624.3132 PEP_1_2_0_0_0

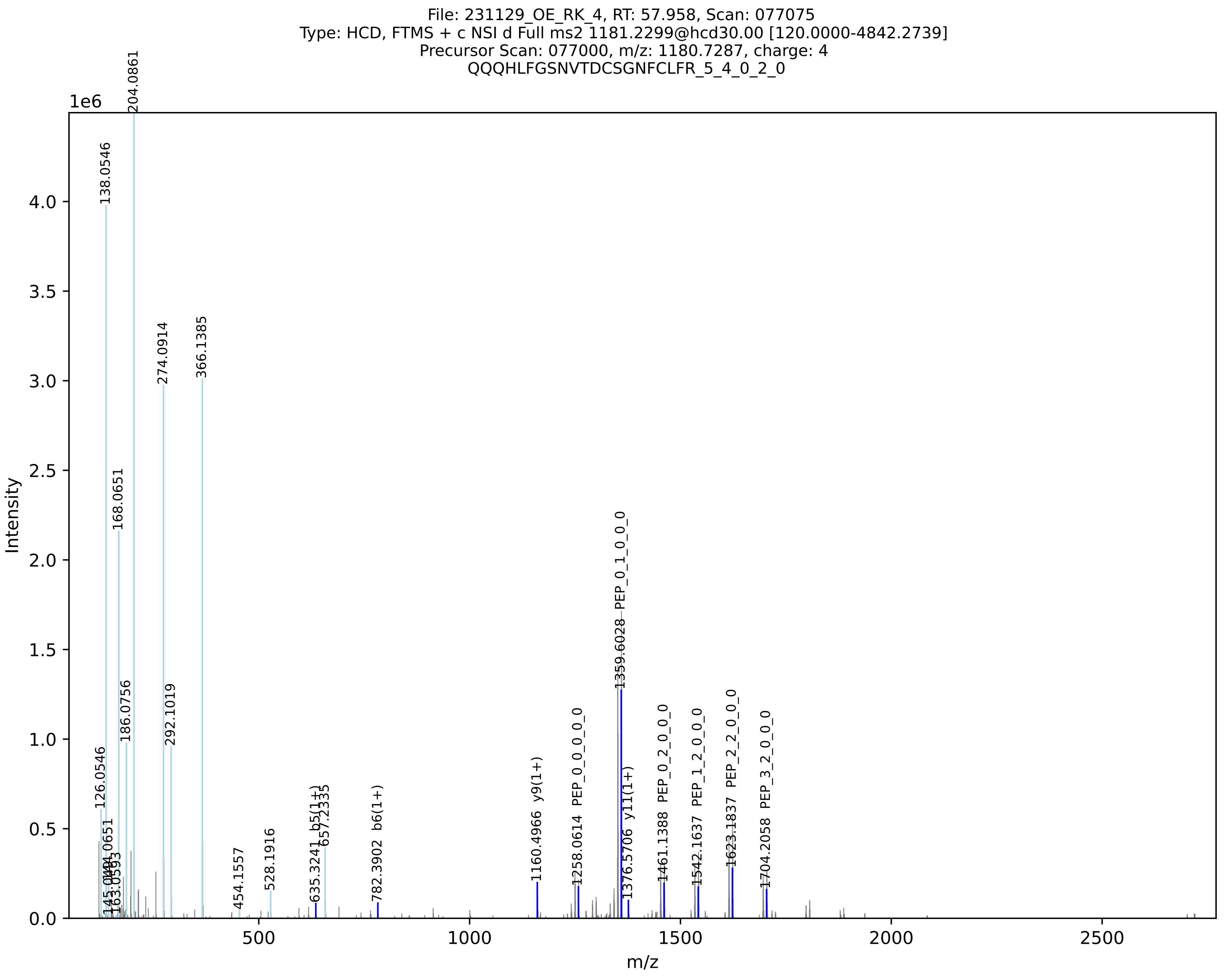
File: 231129_OE_RK_4, RT: 57.958, Scan: 077075
Type: HCD, FTMS + c NSI d Full ms2 1181.2299@hcd30.00 [120.0000-4842.2739]
Precursor Scan: 077000, m/z: 1180.7287, charge: 4
QQQHLFGSNVTDCSGNFCLFR_5_4_0_2_0
1e6
Intensity
m/z
204.0861
138.0546
168.0651
274.0914
366.1385
186.0756
292.1019
126.0546
454.1557
528.1916
635.3241 b5(1+)
657.2335
782.3902 b6(1+)
1160.4966 y9(1+)
1258.0614 PEP_0_0_0_0_0_
1359.6028 PEP_0_1_0_0_0_
1376.5706 y11(1+)
1461.1388 PEP_0_2_0_0_0_
1542.1637 PEP_1_2_0_0_0_
1623.1837 PEP_2_2_0_0_0_
1704.2058 PEP_3_2_0_0_0_

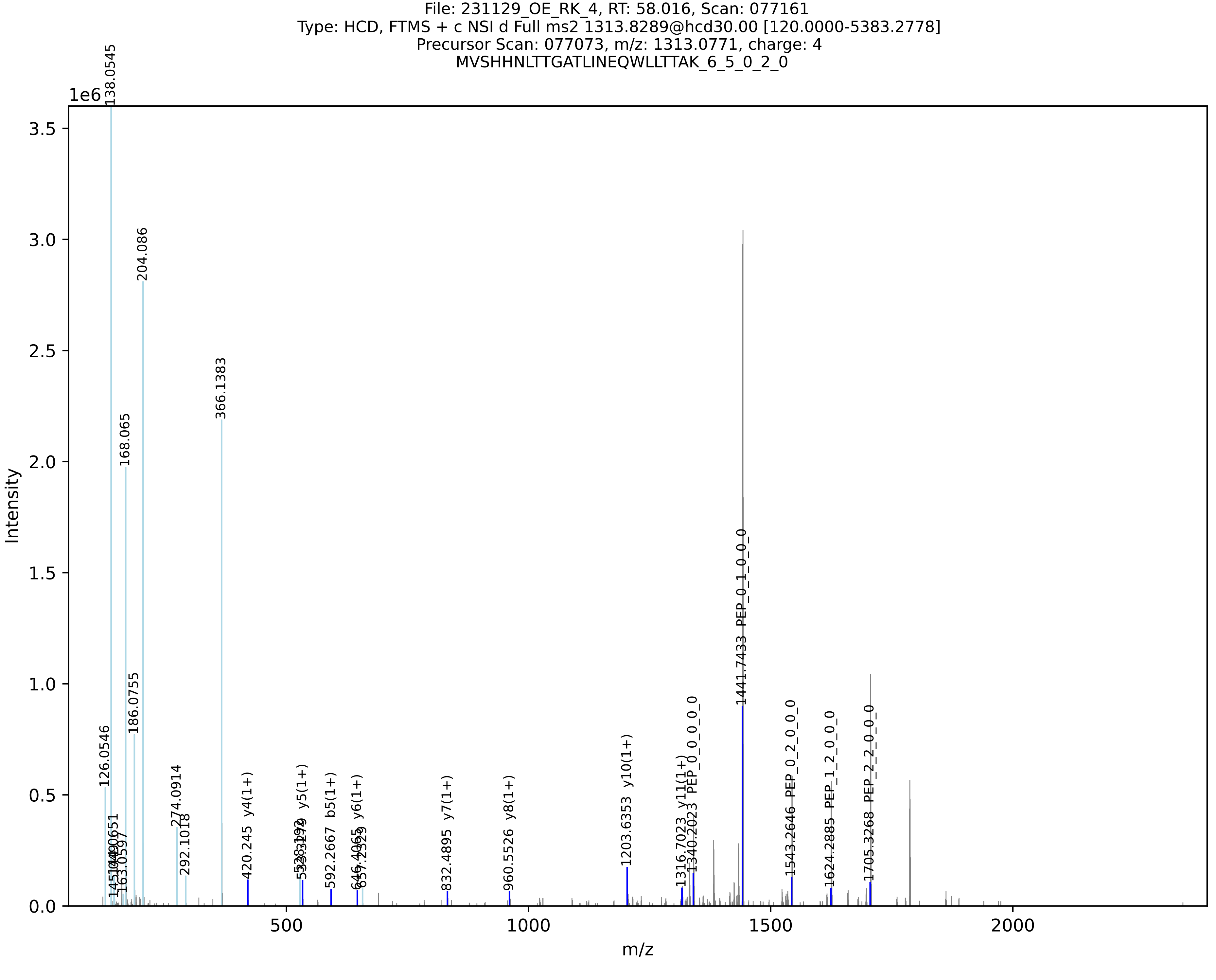
File: 231129_OE_RK_4, RT: 58.016, Scan: 077161
Type: HCD, FTMS + c NSI d Full ms2 1313.8289@hcd30.00 [120.0000-5383.2778]
Precursor Scan: 077073, m/z: 1313.0771, charge: 4
MVSHHNLTTGATLINEQWLLTTAK_6_5_0_2_0
1e6
Intensity
m/z
138.0545
204.086
366.1383
168.065
186.0755
126.0546
274.0914
292.1018
163.0597
420.245 y4(1+)
592.2667 b5(1+)
832.4895 y7(1+)
960.5526 y8(1+)
1203.6353 y10(1+)
1316.7023 y11(1+)
1340.2023 PEP_0_0_0_0_0
1441.7433 PEP_0_1_0_0_0
1543.2646 PEP_0_2_0_0_0
1624.2885 PEP_1_2_0_0_0
1705.3268 PEP_2_2_0_0_0

File: 231129_OE_RK_4, RT: 58.096, Scan: 077282
Type: HCD, FTMS + c NSI d Full ms2 1272.0406@hcd30.00 [120.0000-5212.7822]
Precursor Scan: 077073, m/z: 1272.5738, charge: 4
MVSHHNLTTGATLINEQWLLTTAK_5_5_0_2_0

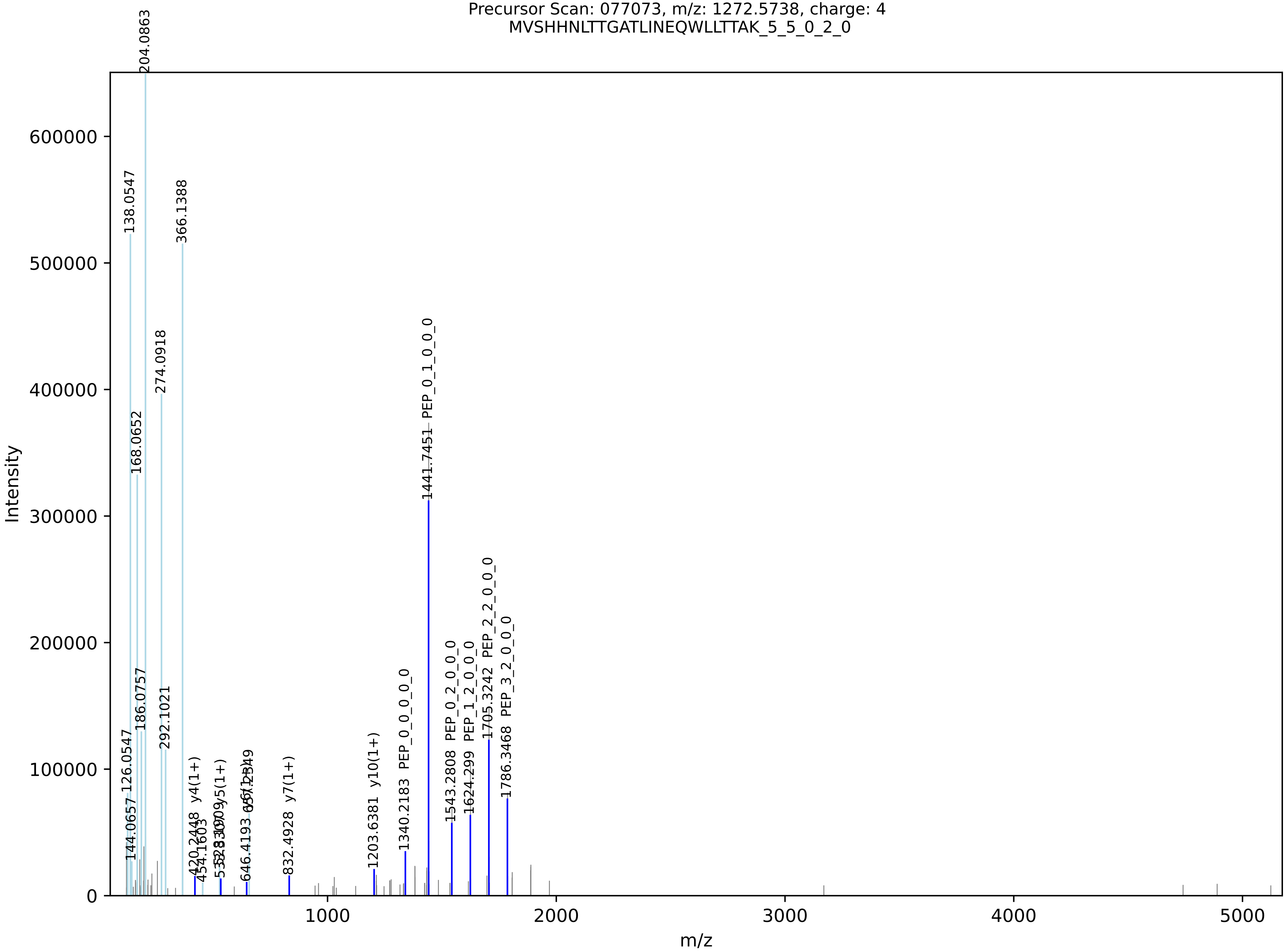

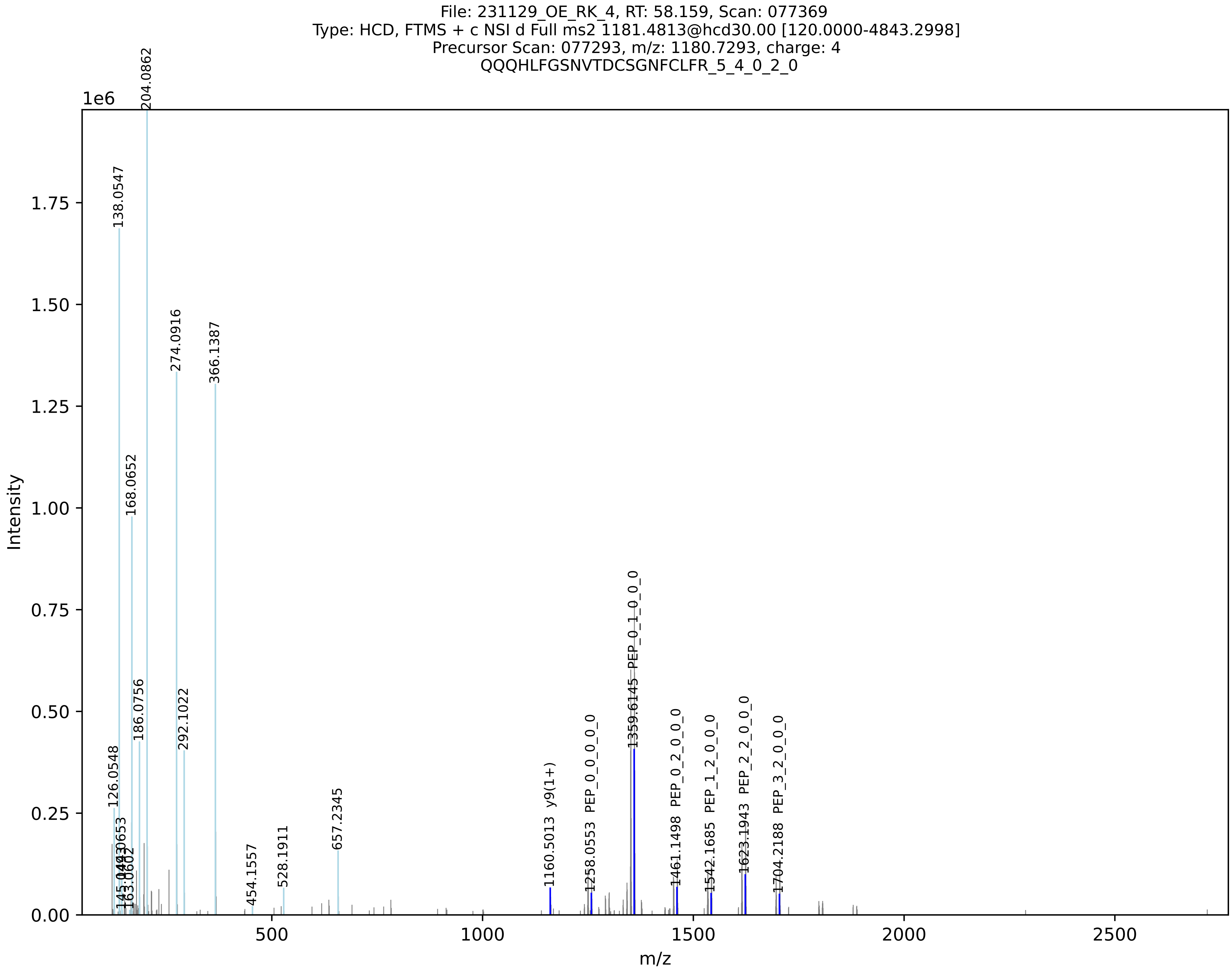
File: 231129_OE_RK_4, RT: 58.159, Scan: 077369
Type: HCD, FTMS + c NSI d Full ms2 1181.4813@hcd30.00 [120.0000-4843.2998]
Precursor Scan: 077293, m/z: 1180.7293, charge: 4
QQQHLFGSNVTDCSGNFCLFR_5_4_0_2_0
1e6
Intensity
m/z
204.0862
138.0547
274.0916
366.1387
168.0652
186.0756
292.1022
126.0548
657.2345
528.1911
454.1557
1160.5013 y9(1+)
1258.0553 PEP_0_0_0_0_0
1359.6145 PEP_0_1_0_0_0
1461.1498 PEP_0_2_0_0_0
1542.1685 PEP_1_2_0_0_0
1623.1943 PEP_2_2_0_0_0
1704.2188 PEP_3_2_0_0_0
0.00
0.25
0.50
0.75
1.00
1.25
1.50
1.75
500
1000
1500
2000
2500

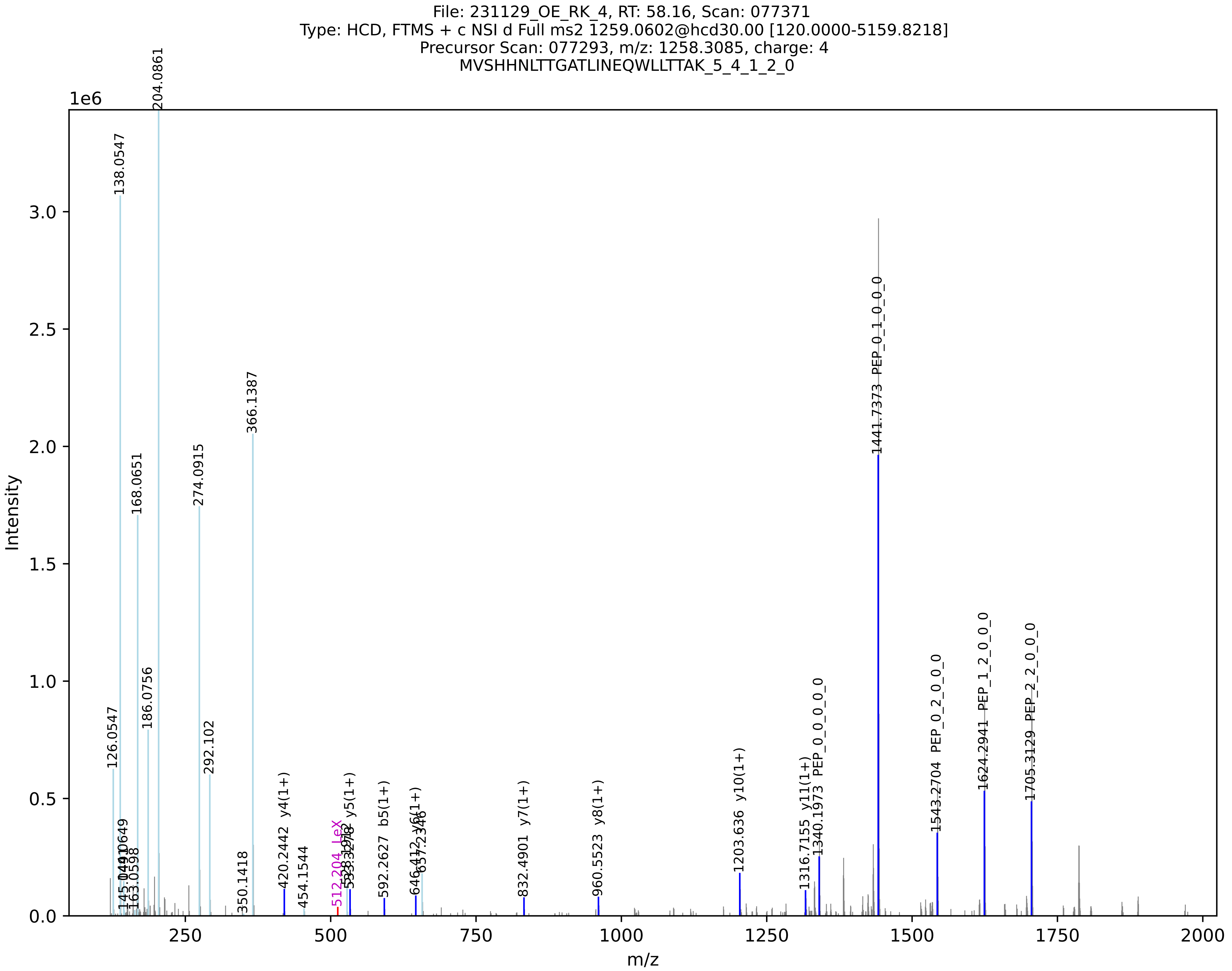

File: 231129_OE_RK_4, RT: 58.16, Scan: 077371
Type: HCD, FTMS + c NSI d Full ms2 1259.0602@hcd30.00 [120.0000-5159.8218]
Precursor Scan: 077293, m/z: 1258.3085, charge: 4
MVSHHNLTTGATLINEQWLLTTAK_5_4_1_2_0
1e6
Intensity
m/z
204.0861
138.0547
168.0651
274.0915
366.1387
186.0756
126.0547
292.102
350.1418
163.0598
420.2442 y4(1+)
454.1544
512.204 LeX
592.2627 b5(1+)
832.4901 y7(1+)
960.5523 y8(1+)
1203.636 y10(1+)
1316.7155 y11(1+)
1340.1973 PEP_0_0_0_0_0
1441.7373 PEP_0_1_0_0_0
1543.2704 PEP_0_2_0_0_0
1624.2941 PEP_1_2_0_0_0
1705.3129 PEP_2_2_0_0_0

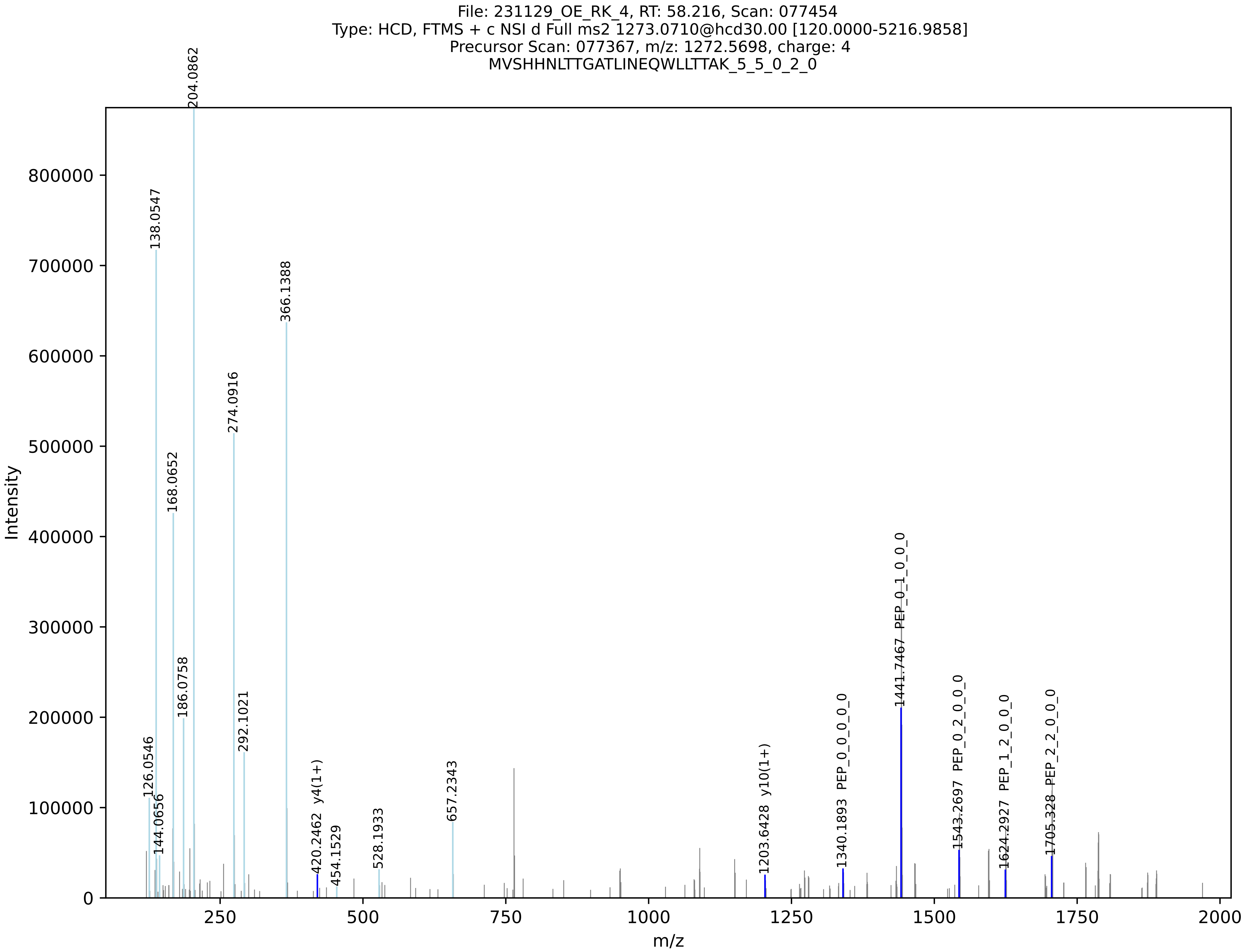
File: 231129_OE_RK_4, RT: 58.216, Scan: 077454
Type: HCD, FTMS + c NSI d Full ms2 1273.0710@hcd30.00 [120.0000-5216.9858]
Precursor Scan: 077367, m/z: 1272.5698, charge: 4
MVSHHNLTTGATLINEQWLLTTAK_5_5_0_2_0
Intensity
m/z
204.0862
138.0547
366.1388
274.0916
168.0652
186.0758
292.1021
126.0546
144.0656
420.2462 y4(1+)
454.1529
528.1933
657.2343
1203.6428 y10(1+)
1340.1893 PEP_0_0_0_0_0_
1441.7467 PEP_0_1_0_0_0_
1543.2697 PEP_0_2_0_0_0_
1624.2927 PEP_1_2_0_0_0_
1705.328 PEP_2_2_0_0_0_

File: 231129_OE_RK_4, RT: 58.229, Scan: 077474
Type: HCD, FTMS + c NSI d Full ms2 1198.7333@hcd30.00 [120.0000-4913.6880]
Precursor Scan: 077367, m/z: 1198.2321, charge: 4
INNLTESDQGEYVCEISGEGGTSK_5_4_0_2_0

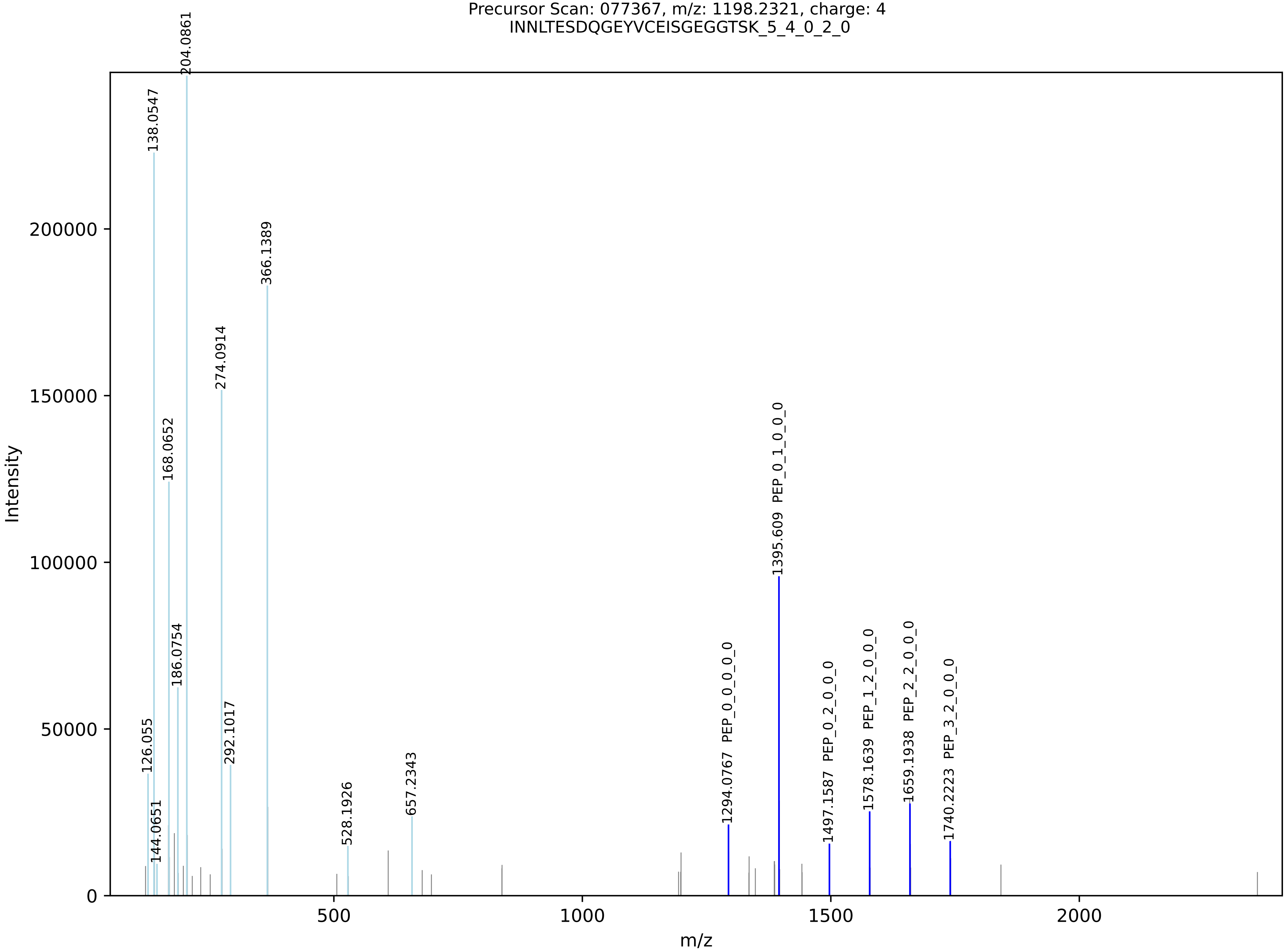

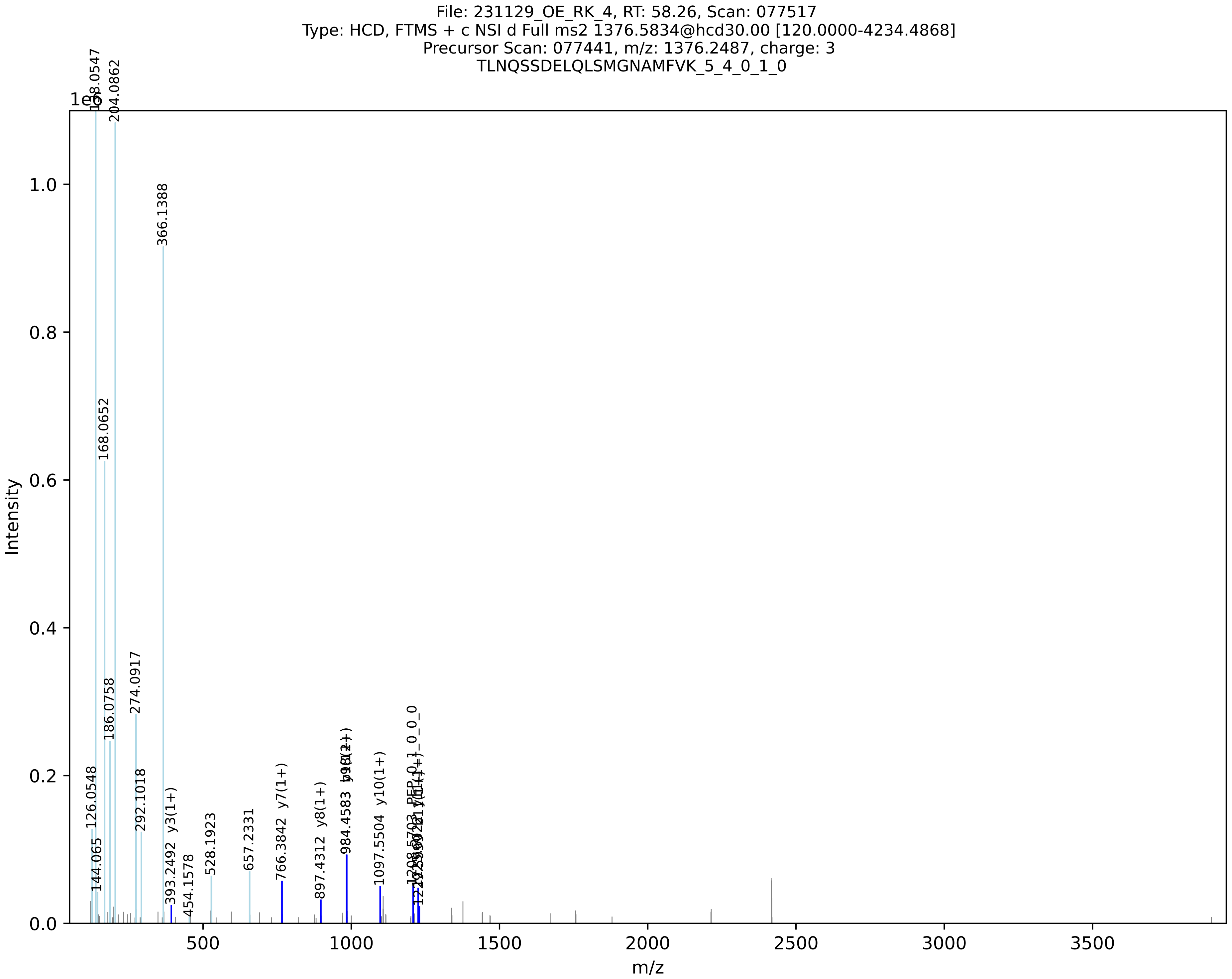

File: 231129_OE_RK_4, RT: 58.26, Scan: 077517
Type: HCD, FTMS + c NSI d Full ms2 1376.5834@hcd30.00 [120.0000-4234.4868]
Precursor Scan: 077441, m/z: 1376.2487, charge: 3
TLNQSSDELQLSMGNAMFVK_5_4_0_1_0
1e6
Intensity
m/z
204.0862
366.1388
168.0652
274.0917
186.0758
126.0548
292.1018
144.065
393.2492 y3(1+)
454.1578
528.1923
657.2331
766.3842 y7(1+)
897.4312 y8(1+)
984.4583
1097.5504 y10(1+)

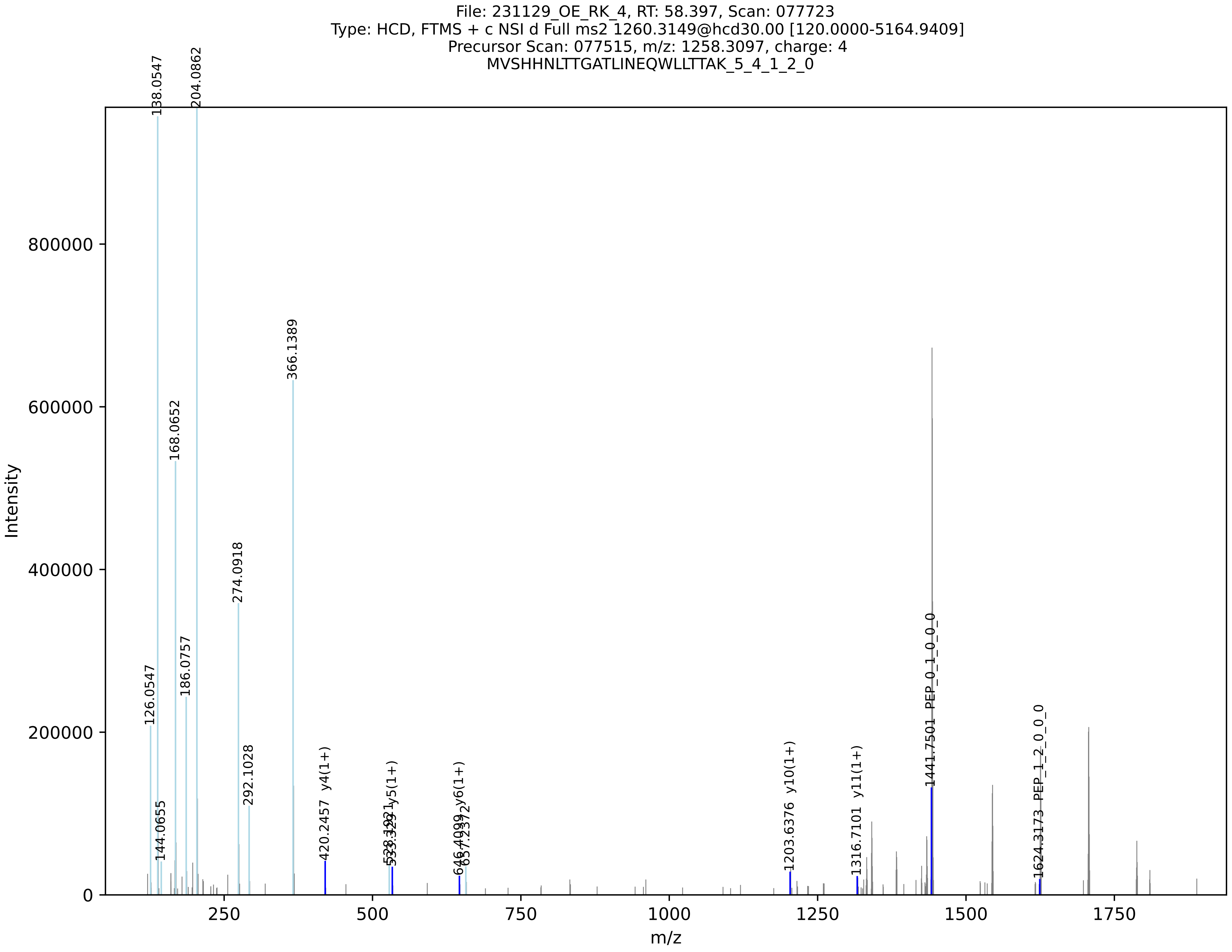

File: 231129_OE_RK_4, RT: 58.397, Scan: 077723
Type: HCD, FTMS + c NSI d Full ms2 1260.3149@hcd30.00 [120.0000-5164.9409]
Precursor Scan: 077515, m/z: 1258.3097, charge: 4
MVSHHNLTTGATLINEQWLLTTAK_5_4_1_2_0
138.0547
204.0862
366.1389
168.0652
274.0918
186.0757
126.0547
292.1028
144.0655
420.2457 y4(1+)
y5(1+)
y6(1+)
1203.6376 y10(1+)
1316.7101 y11(1+)
1441.7501 PEP_0_1_0_0_0
1624.3173 PEP_1_2_0_0_0
Intensity
m/z
0
200000
400000
600000
800000
250
500
750
1000
1250
1500
1750

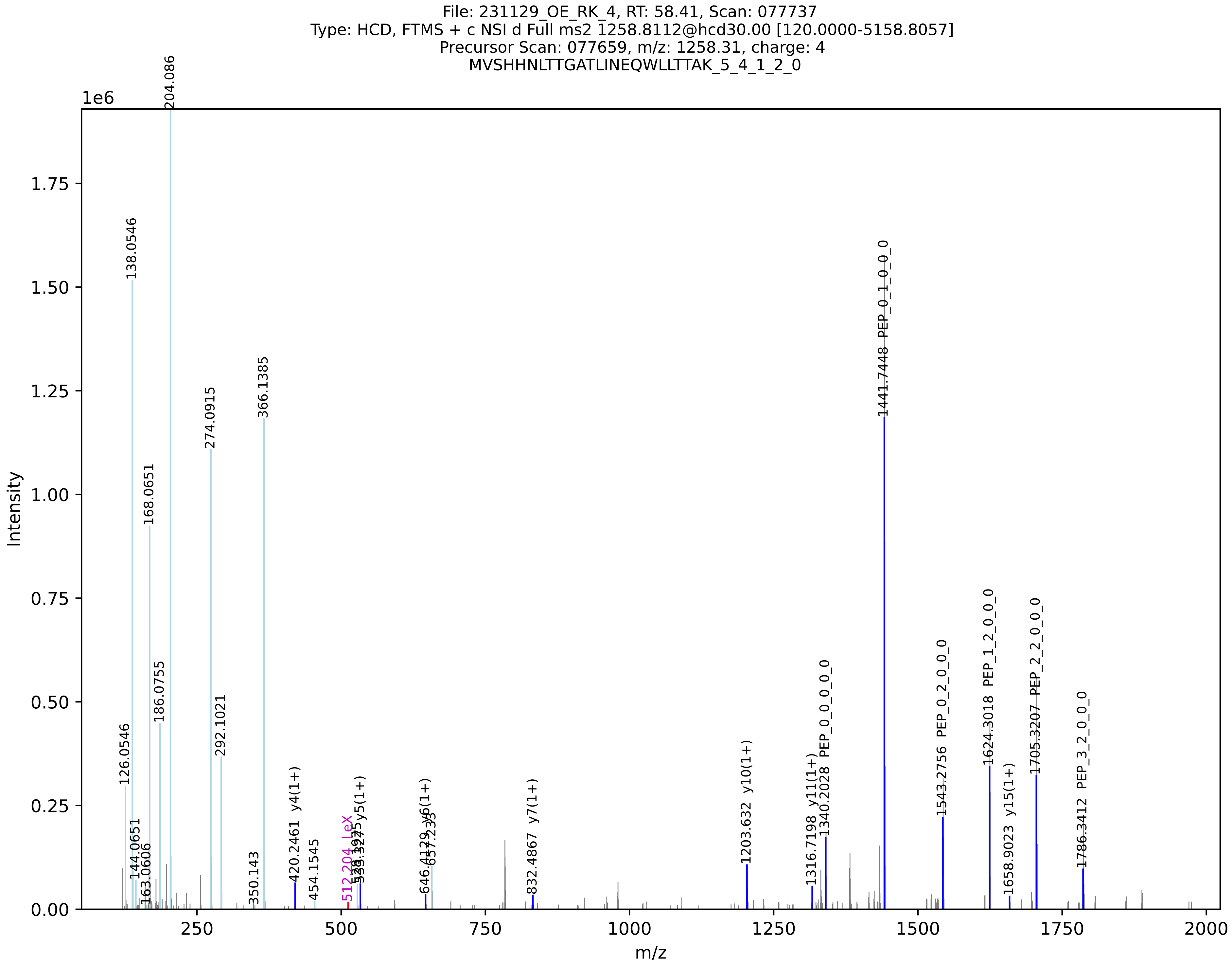
File: 231129_OE_RK_4, RT: 58.41, Scan: 077737
Type: HCD, FTMS + c NSI d Full ms2 1258.8112@hcd30.00 [120.0000-5158.8057]
Precursor Scan: 077659, m/z: 1258.31, charge: 4
MVSHHNLTTGATLINEQWLLTTAK_5_4_1_2_0
1e6
Intensity
m/z
0.00
0.25
0.50
0.75
1.00
1.25
1.50
1.75
250
500
750
1000
1250
1500
1750
2000
126.0546
138.0546
144.0651
163.0606
168.0651
186.0755
204.086
274.0915
292.1021
350.143
366.1385
420.2461 y4(1+)
454.1545
512.204 LeX
y5(1+)
646.4129 y6(1+)
832.4867 y7(1+)
1203.632 y10(1+)
1316.7198 y11(1+)
1340.2028 PEP_0_0_0_0_0
1441.7448 PEP_0_1_0_0_0
1543.2756 PEP_0_2_0_0_0
1624.3018 PEP_1_2_0_0_0
1658.9023 y15(1+)
1705.3207 PEP_2_2_0_0_0
1786.3412 PEP_3_2_0_0_0

File: 231129_OE_RK_4, RT: 58.462, Scan: 077813
Type: HCD, FTMS + c NSI d Full ms2 1376.9155@hcd30.00 [120.0000-4235.5034]
Precursor Scan: 077731, m/z: 1376.2493, charge: 3
TLNQSSDELQLSMGNAMFVK_5_4_0_1_0

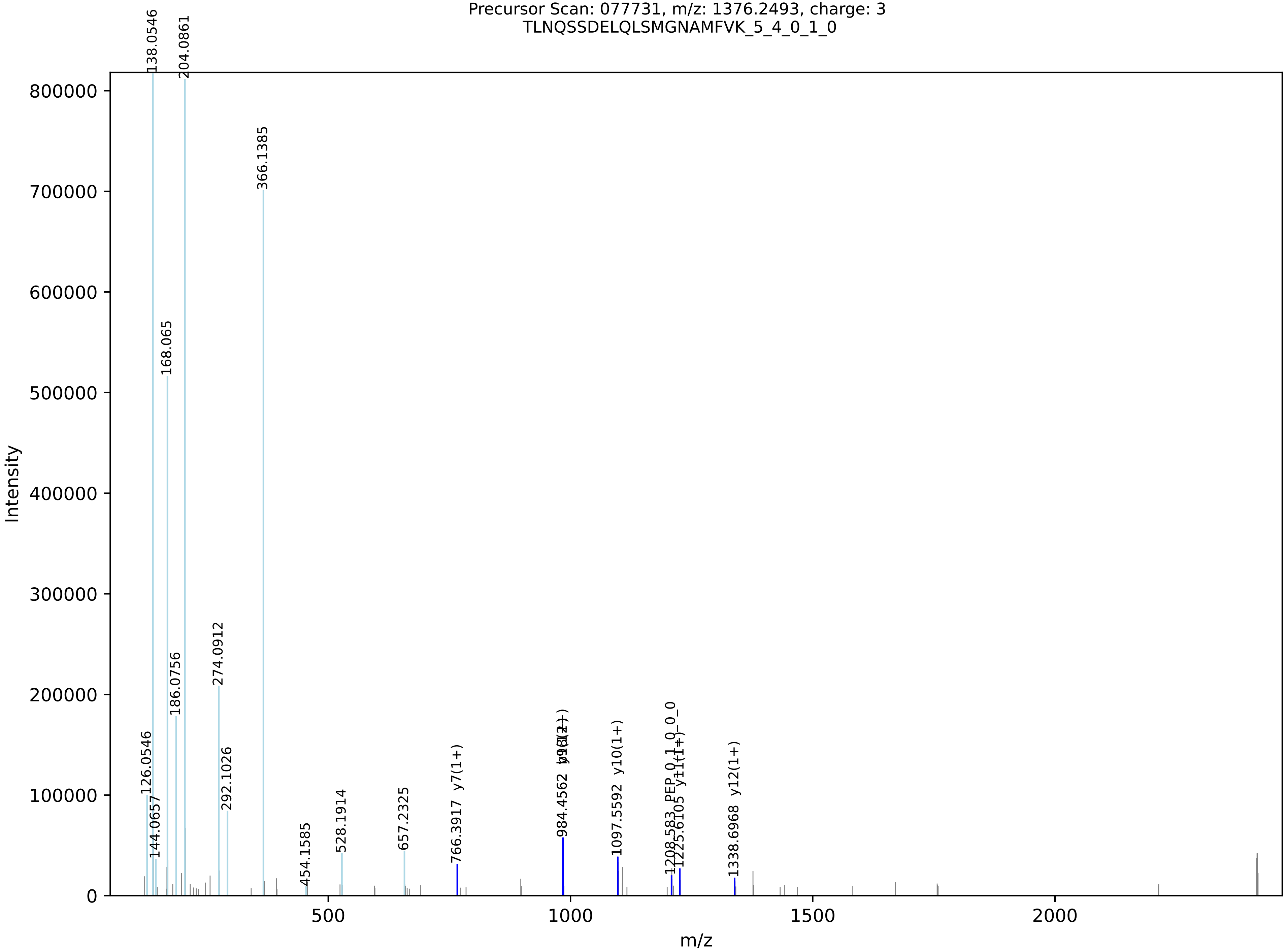

File: 231129_OE_RK_4, RT: 58.611, Scan: 078031
Type: HCD, FTMS + c NSI d Full ms2 1259.0640@hcd30.00 [120.0000-5159.8369]
Precursor Scan: 077952, m/z: 1258.3122, charge: 4
MVSHHNLTTGATLINEQWLLTTAK_5_4_1_2_0

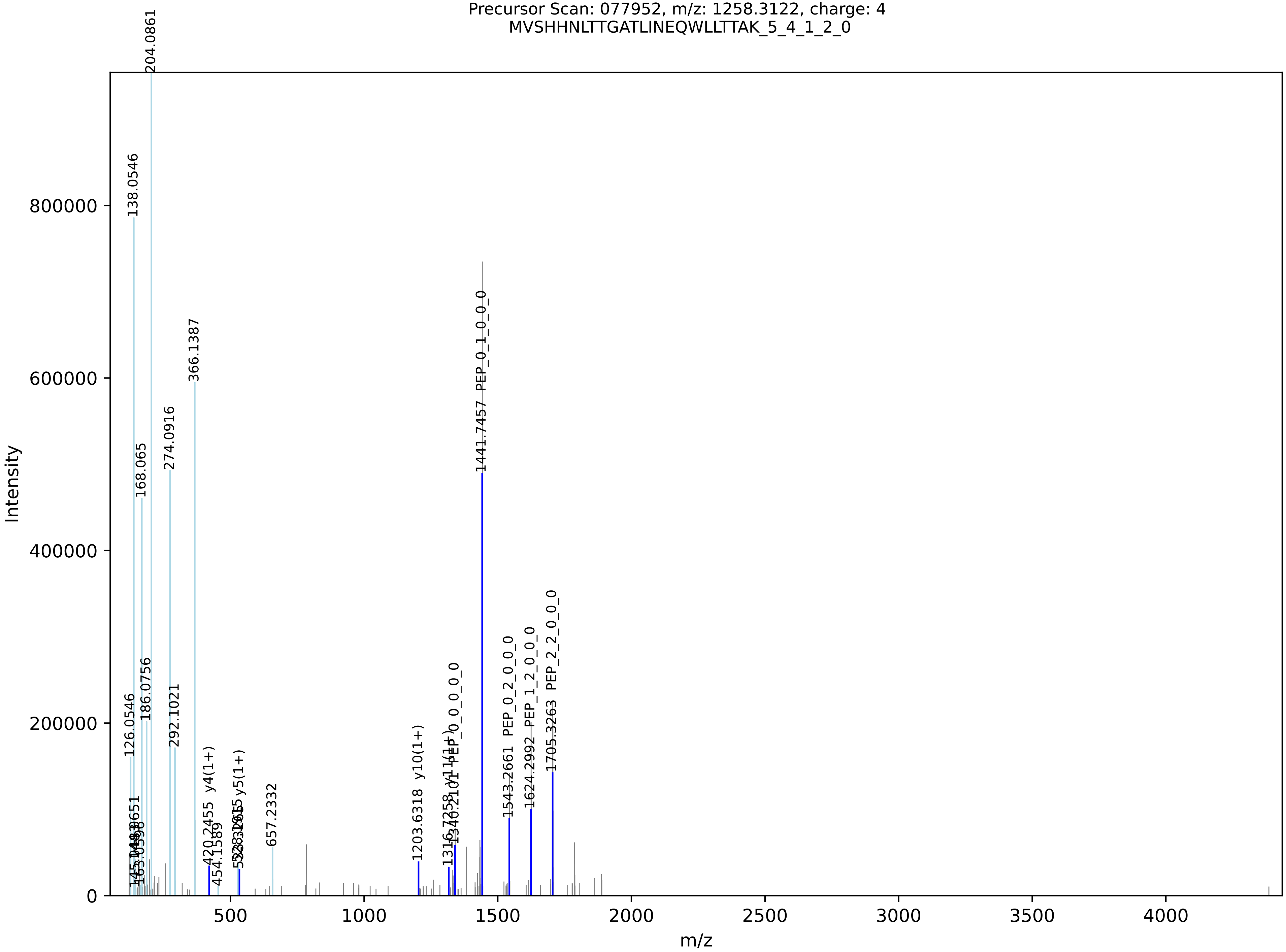

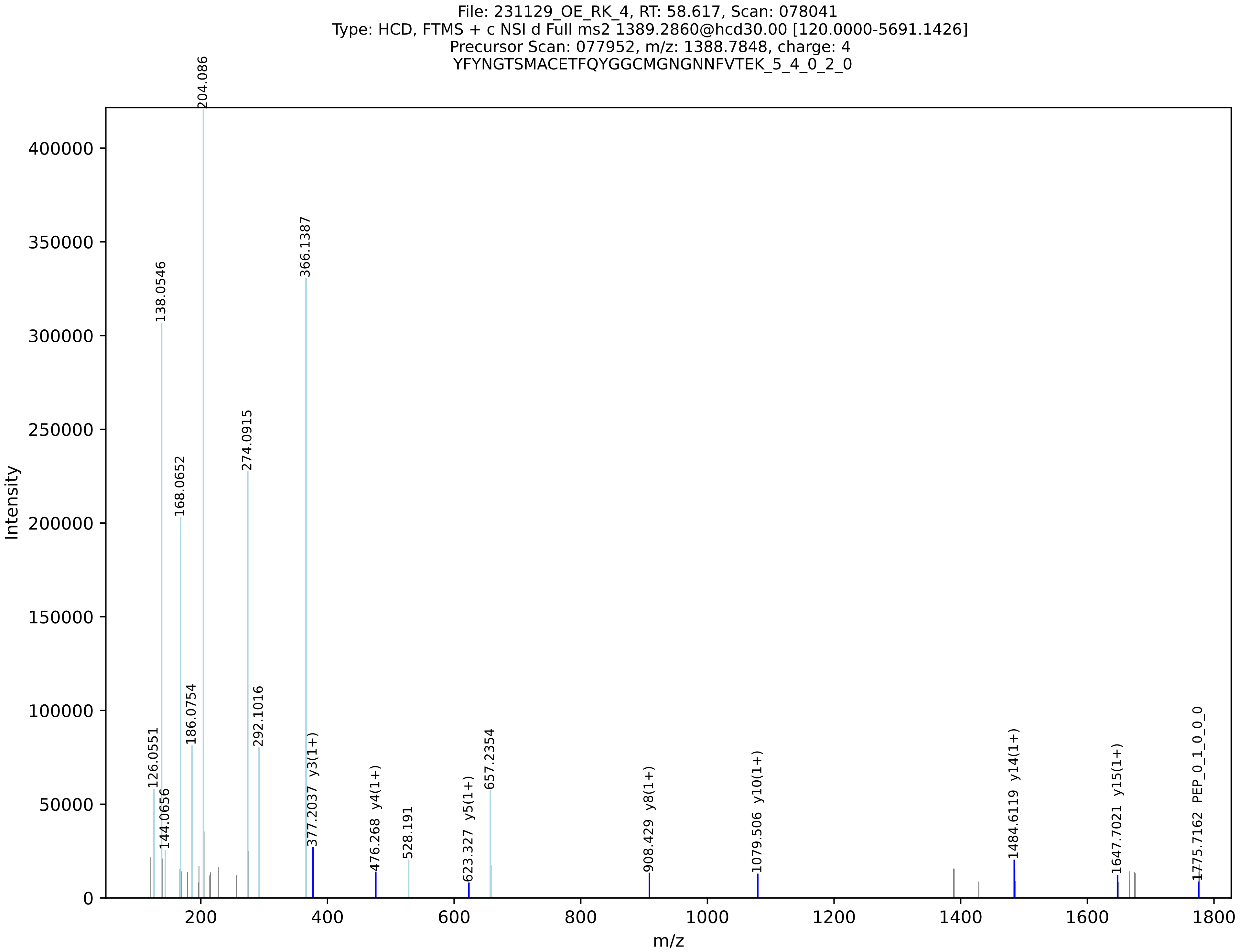
File: 231129_OE_RK_4, RT: 58.617, Scan: 078041
Type: HCD, FTMS + c NSI d Full ms2 1389.2860@hcd30.00 [120.0000-5691.1426]
Precursor Scan: 077952, m/z: 1388.7848, charge: 4
YFYNGTSMACETFQYGGCMGNGNNFVTEK_5_4_0_2_0
Intensity
m/z
204.086
366.1387
138.0546
274.0915
168.0652
186.0754
292.1016
126.0551
144.0656
377.2037 y3(1+)
476.268 y4(1+)
528.191
623.327 y5(1+)
657.2354
908.429 y8(1+)
1079.506 y10(1+)
1484.6119 y14(1+)
1647.7021 y15(1+)
1775.7162 PEP_0_1_0_0_0

File: 231129_OE_RK_4, RT: 58.652, Scan: 078097
Type: HCD, FTMS + c NSI d Full ms2 1260.0614@hcd30.00 [120.0000-5163.9063]
Precursor Scan: 077879, m/z: 1258.3086, charge: 4
MVSHHNLTTGATLINEQWLLTTAK_5_4_1_2_0

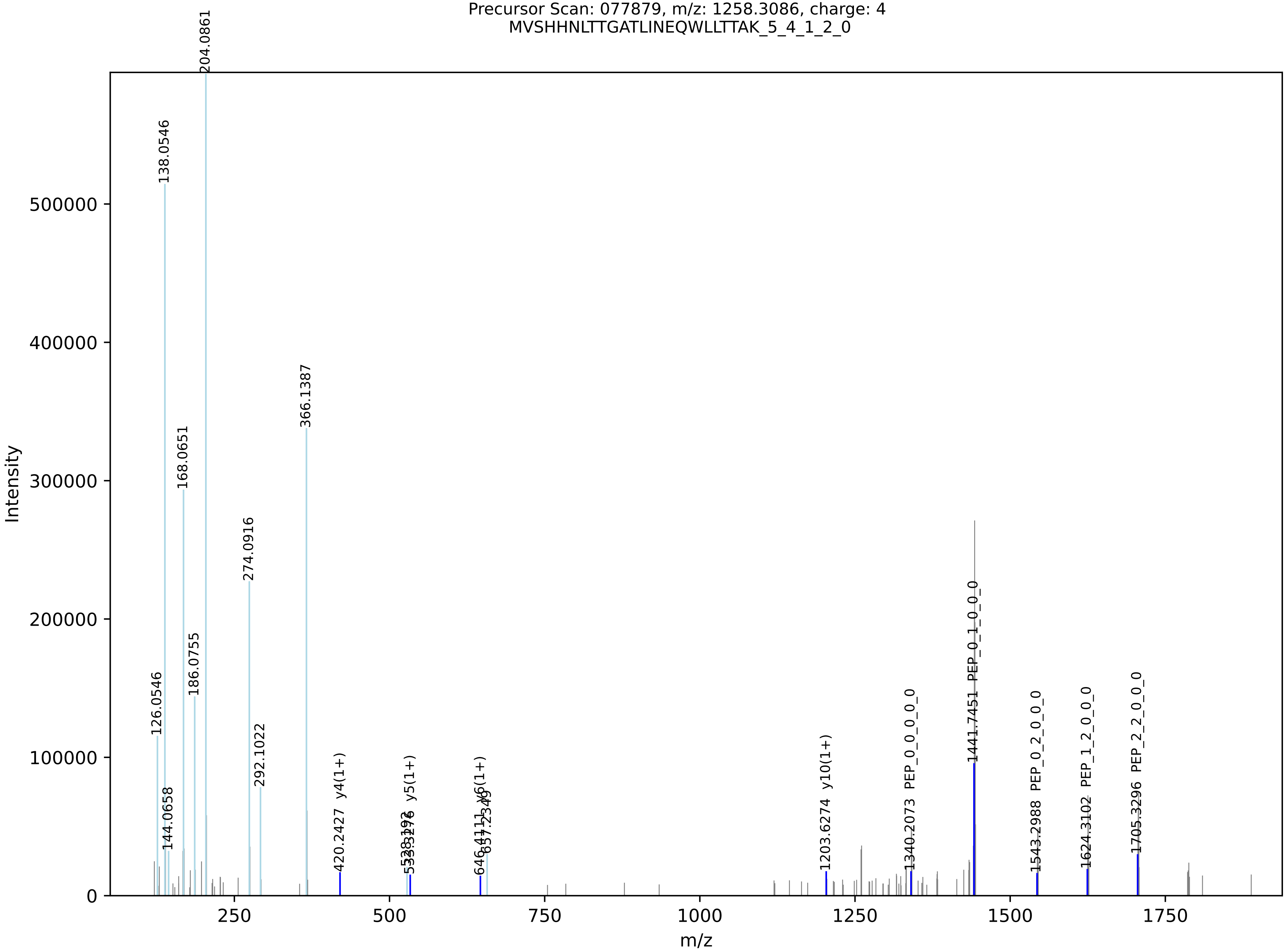

File: 231129_OE_RK_4, RT: 58.76, Scan: 078246
Type: HCD, FTMS + c NSI d Full ms2 1629.3940@hcd30.00 [120.0000-5008.0879]
Precursor Scan: 078174, m/z: 1628.7265, charge: 3
MLFVEPILEVSSLPTTNSTTNSATK_5_4_0_2_0

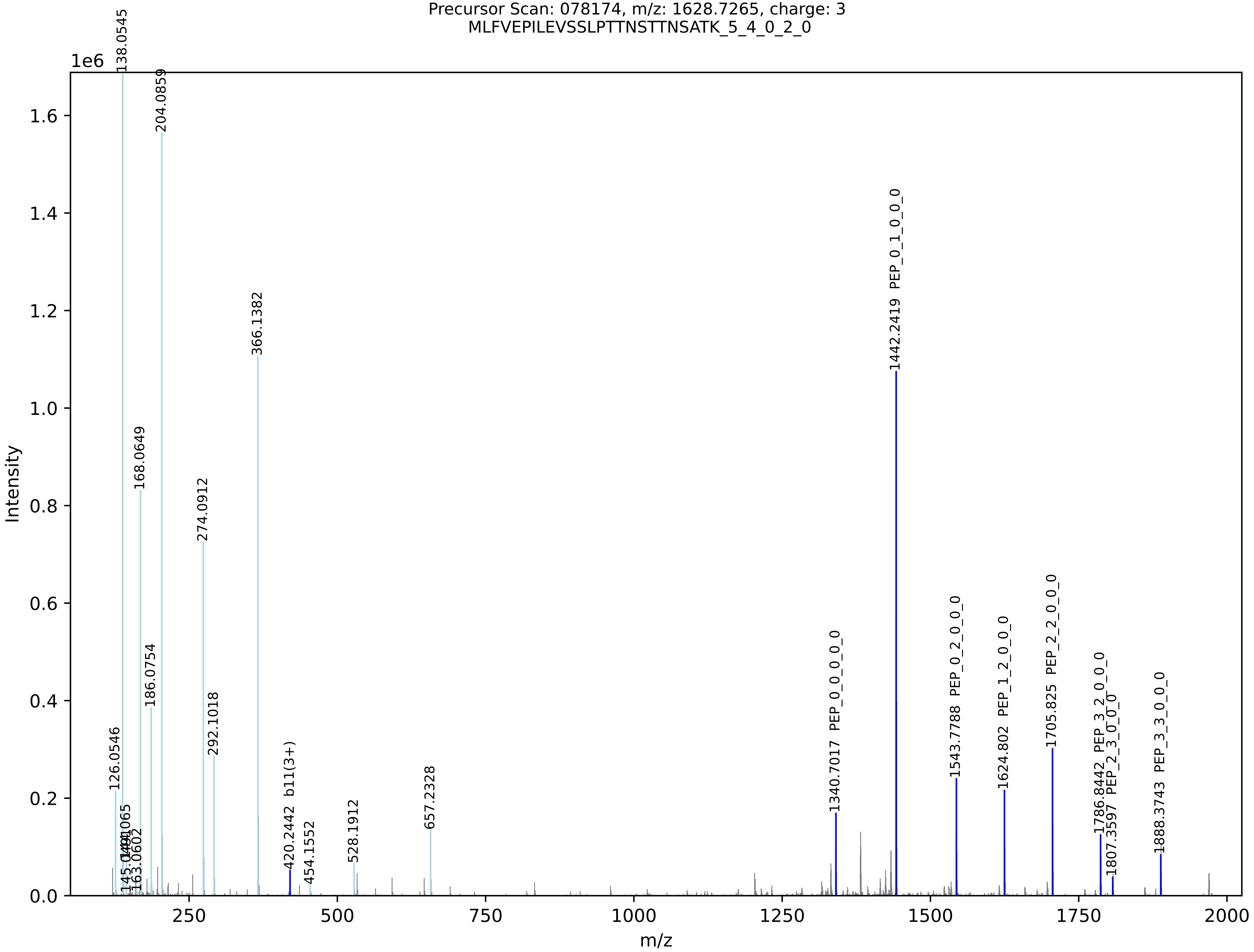

File: 231129_OE_RK_4, RT: 58.811, Scan: 078320
Type: HCD, FTMS + c NSI d Full ms2 1222.2986@hcd30.00 [120.0000-5009.8340]
Precursor Scan: 078245, m/z: 1221.7973, charge: 4
MVSHHNLTTGATLINEQWLLTTAK_5_4_0_2_0

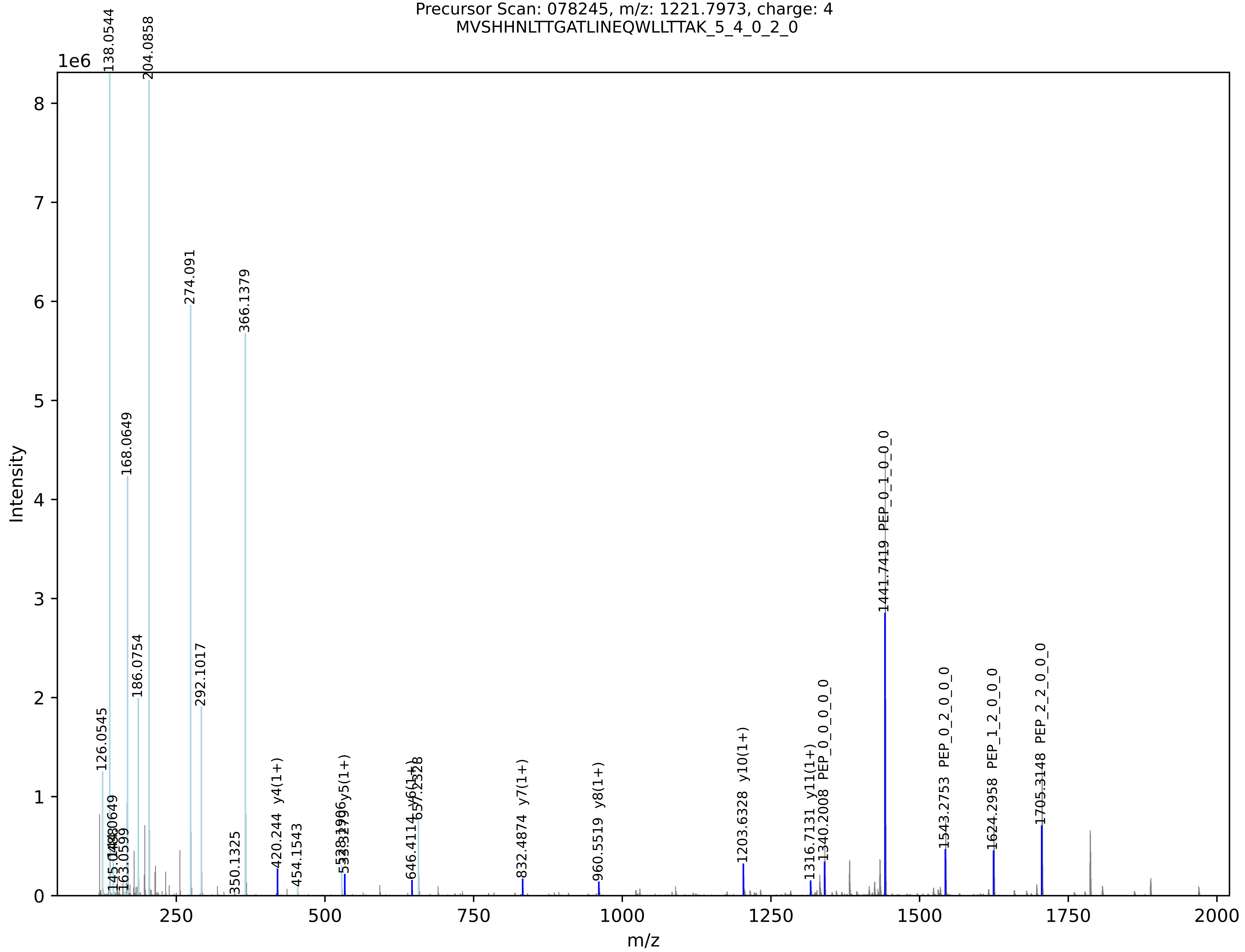

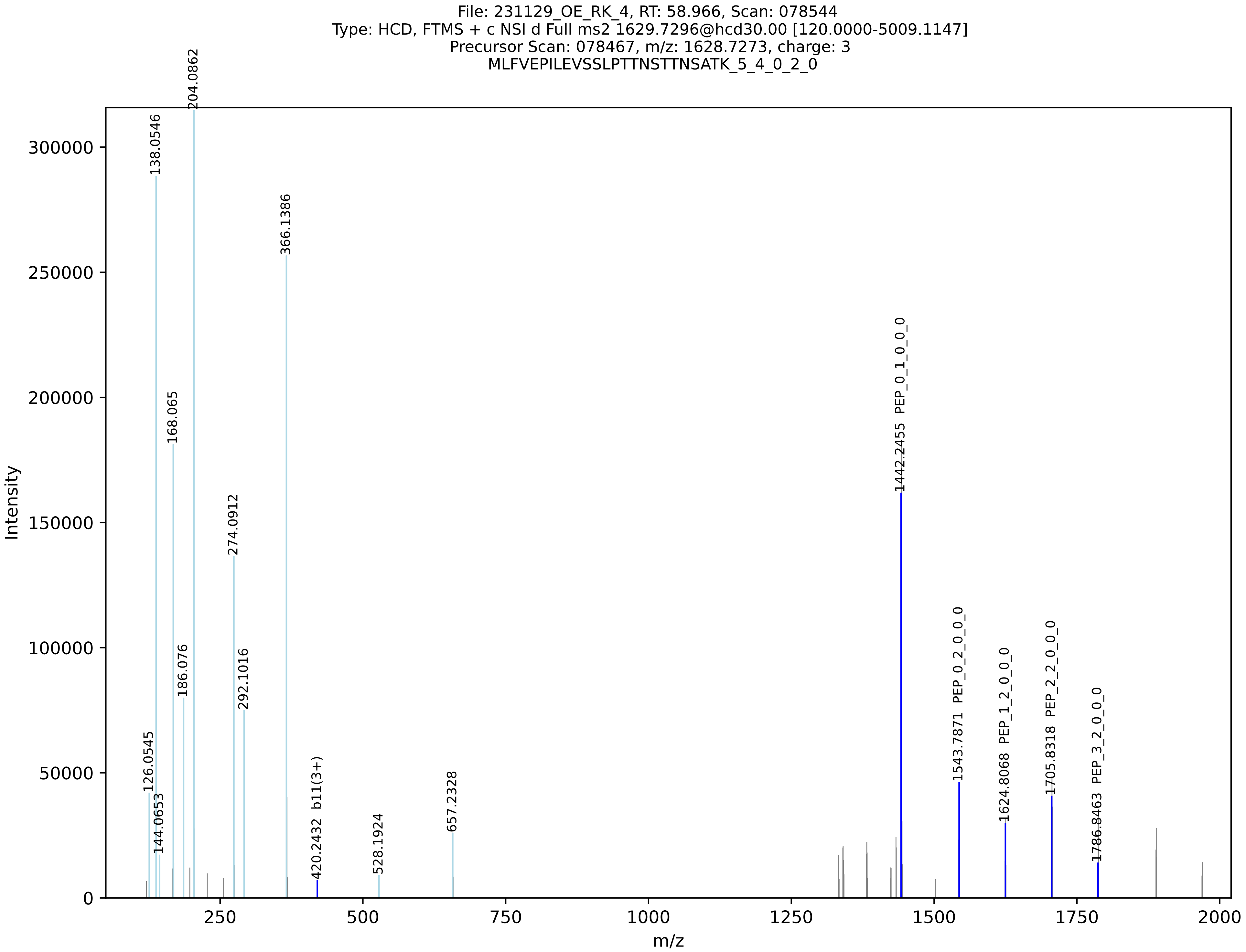

File: 231129_OE_RK_4, RT: 58.966, Scan: 078544
Type: HCD, FTMS + c NSI d Full ms2 1629.7296@hcd30.00 [120.0000-5009.1147]
Precursor Scan: 078467, m/z: 1628.7273, charge: 3
MLFVEPILEVSSLPTTNSTTNSATK_5_4_0_2_0
Intensity
m/z
204.0862
138.0546
366.1386
168.065
274.0912
292.1016
186.076
126.0545
144.0653
420.2432 b11(3+)
528.1924
657.2328
1442.2455 PEP_0_1_0_0_0_
1543.7871 PEP_0_2_0_0_0_
1624.8068 PEP_1_2_0_0_0_
1705.8318 PEP_2_2_0_0_0_
1786.8463 PEP_3_2_0_0_0_

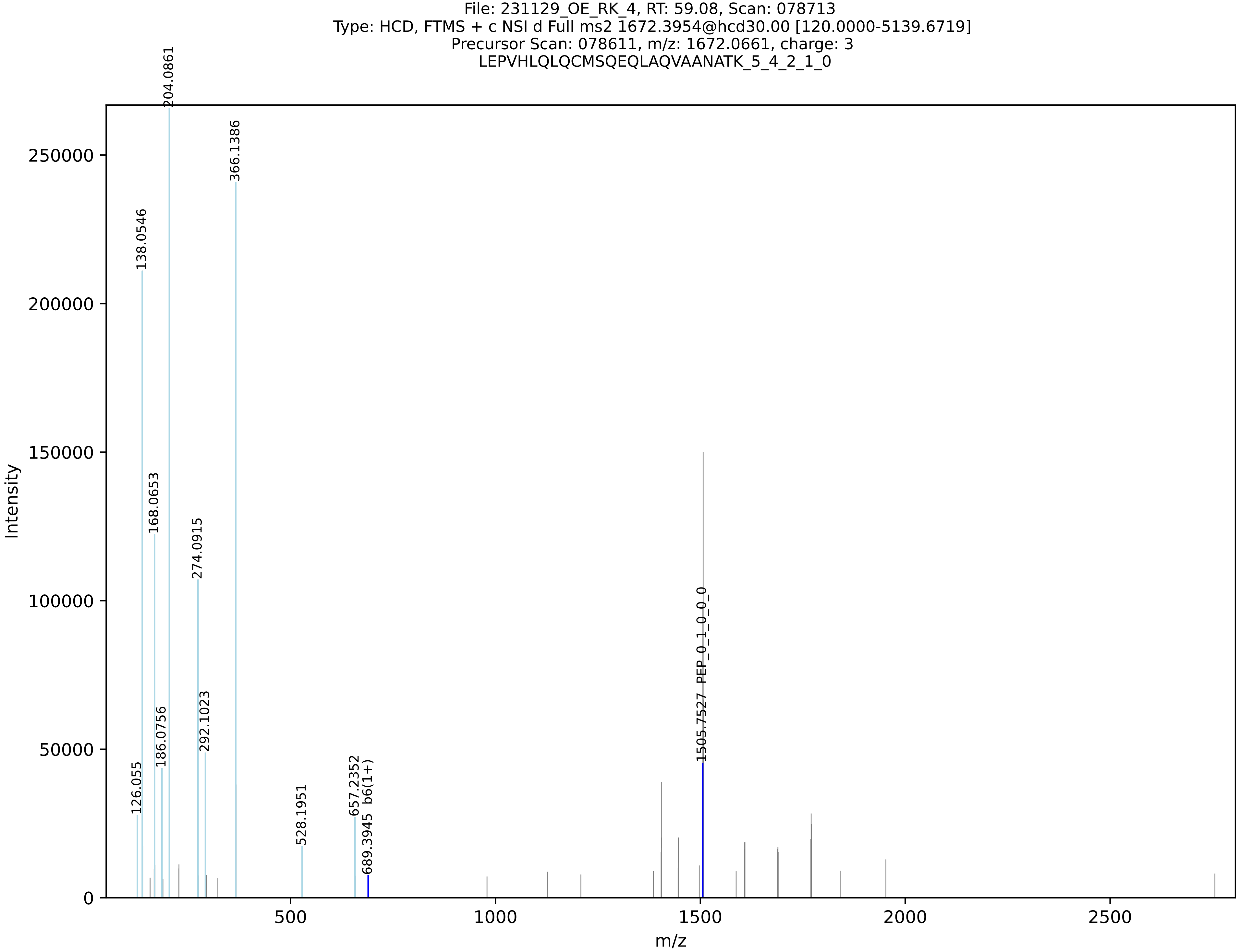

File: 231129_OE_RK_4, RT: 59.08, Scan: 078713
Type: HCD, FTMS + c NSI d Full ms2 1672.3954@hcd30.00 [120.0000-5139.6719]
Precursor Scan: 078611, m/z: 1672.0661, charge: 3
LEPVHLQLQCMSQEQLAQVAANATK_5_4_2_1_0
Intensity
m/z
204.0861
366.1386
138.0546
168.0653
274.0915
292.1023
186.0756
126.055
528.1951
657.2352
689.3945 b6(1+)
1505.7527 PEP_0_1_0_0_0
250000
200000
150000
100000
50000
0
500
1000
1500
2000
2500

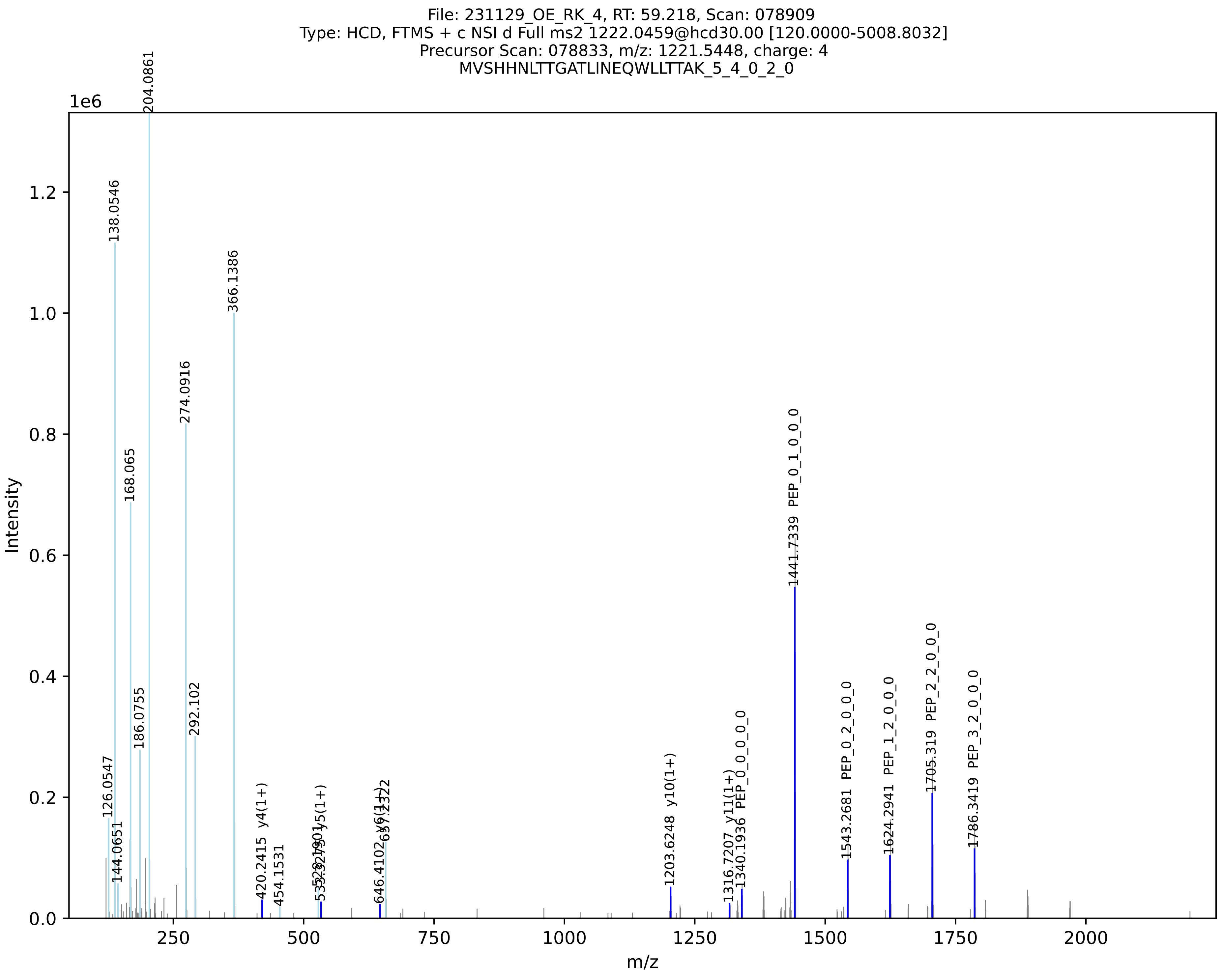

File: 231129_OE_RK_4, RT: 59.218, Scan: 078909
Type: HCD, FTMS + c NSI d Full ms2 1222.0459@hcd30.00 [120.0000-5008.8032]
Precursor Scan: 078833, m/z: 1221.5448, charge: 4
MVSHHNLTTGATLINEQWLLTTAK_5_4_0_2_0
1e6
Intensity
m/z
204.0861
138.0546
366.1386
274.0916
168.065
186.0755
292.102
126.0547
144.0651
420.2415 y4(1+)
454.1531
533.3275 y5(1+)
646.4102 y6(1+)
657.2322
1203.6248 y10(1+)
1316.7207 y11(1+)
1340.1936 PEP_0_0_0_0_0
1441.7339 PEP_0_1_0_0_0
1543.2681 PEP_0_2_0_0_0
1624.2941 PEP_1_2_0_0_0
1705.319 PEP_2_2_0_0_0
1786.3419 PEP_3_2_0_0_0

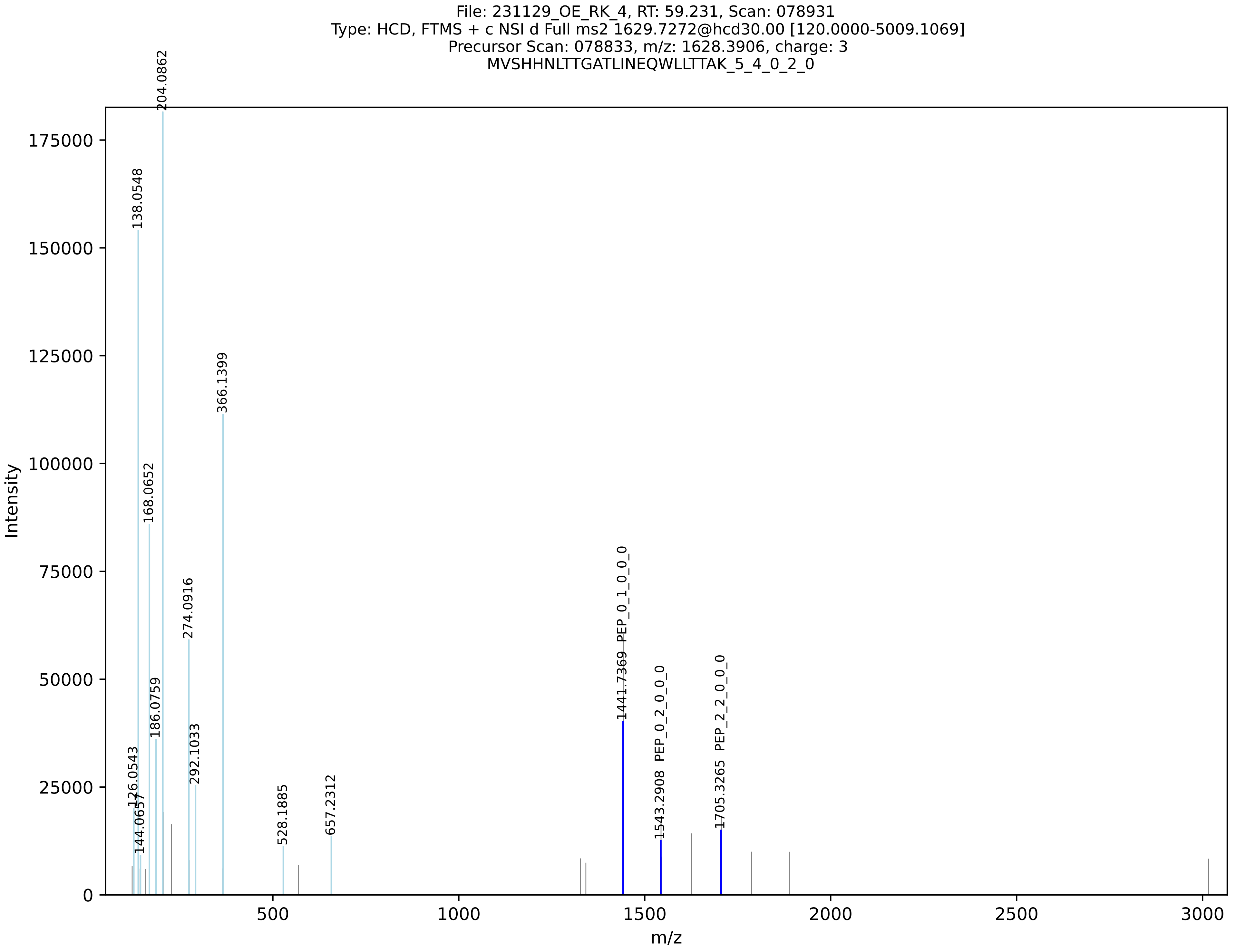
File: 231129_OE_RK_4, RT: 59.231, Scan: 078931
Type: HCD, FTMS + c NSI d Full ms2 1629.7272@hcd30.00 [120.0000-5009.1069]
Precursor Scan: 078833, m/z: 1628.3906, charge: 3
MVSHHNLTTGATLINEQWLLTTAK_5_4_0_2_0
Intensity
m/z
204.0862
138.0548
366.1399
168.0652
274.0916
186.0759
292.1033
126.0543
144.0657
528.1885
657.2312
1441.7369 PEP_0_1_0_0_0
1543.2908 PEP_0_2_0_0_0
1705.3265 PEP_2_2_0_0_0

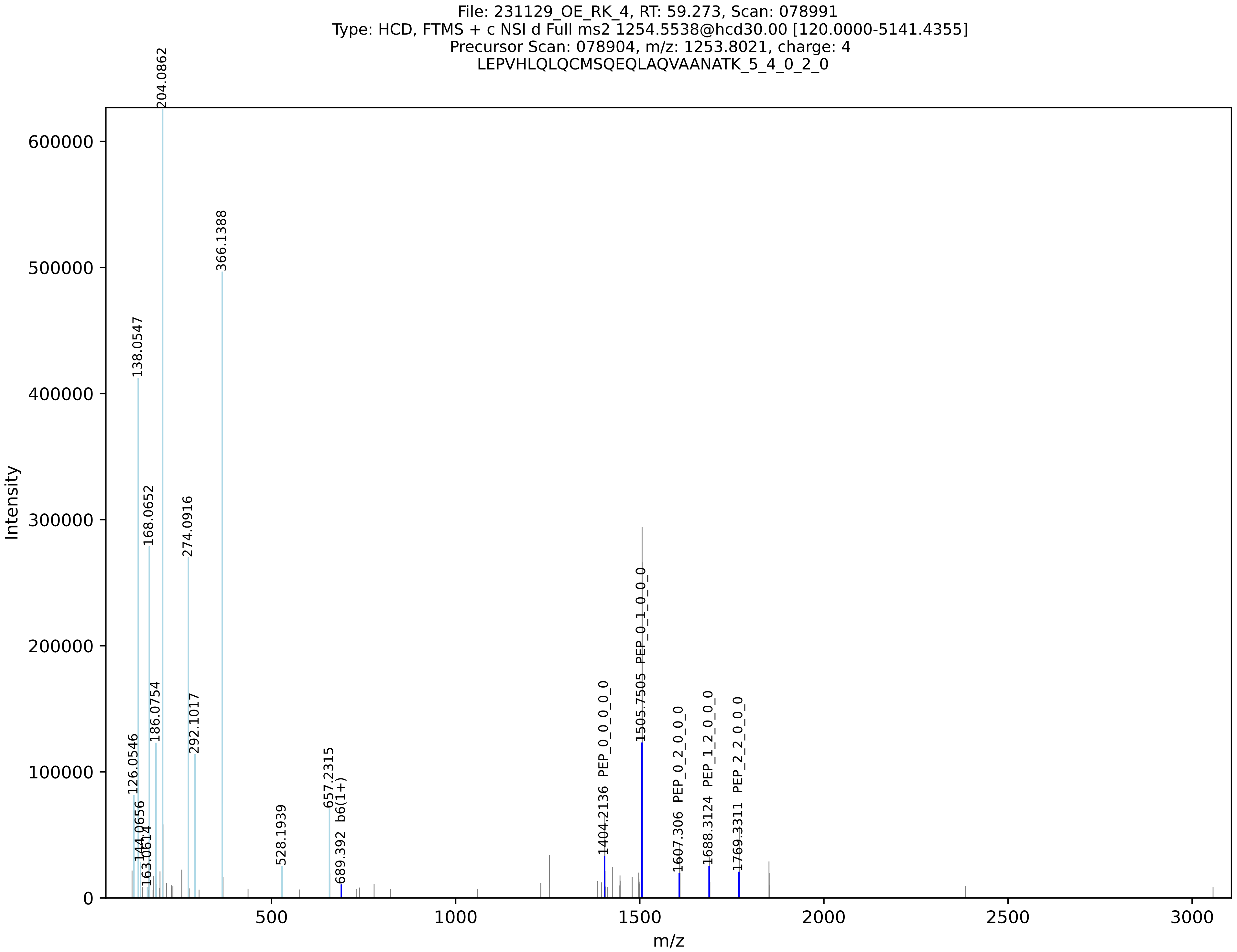
File: 231129_OE_RK_4, RT: 59.273, Scan: 078991
Type: HCD, FTMS + c NSI d Full ms2 1254.5538@hcd30.00 [120.0000-5141.4355]
Precursor Scan: 078904, m/z: 1253.8021, charge: 4
LEPVHLQLQCMSQEQLAQVAANATK_5_4_0_2_0
Intensity
m/z
204.0862
366.1388
138.0547
168.0652
274.0916
186.0754
292.1017
126.0546
144.0656
163.0614
528.1939
657.2315
689.392 b6(1+)
1404.2136 PEP_0_0_0_0_0
1505.7505 PEP_0_1_0_0_0
1607.306 PEP_0_2_0_0_0
1688.3124 PEP_1_2_0_0_0
1769.3311 PEP_2_2_0_0_0

File: 231129_OE_RK_4, RT: 59.275, Scan: 078995
Type: HCD, FTMS + c NSI d Full ms2 1389.2852@hcd30.00 [120.0000-5691.1392]
Precursor Scan: 078904, m/z: 1388.7839, charge: 4
YFYNGTSMACETFQYGGCMGNGNNFVTEK_5_4_0_2_0

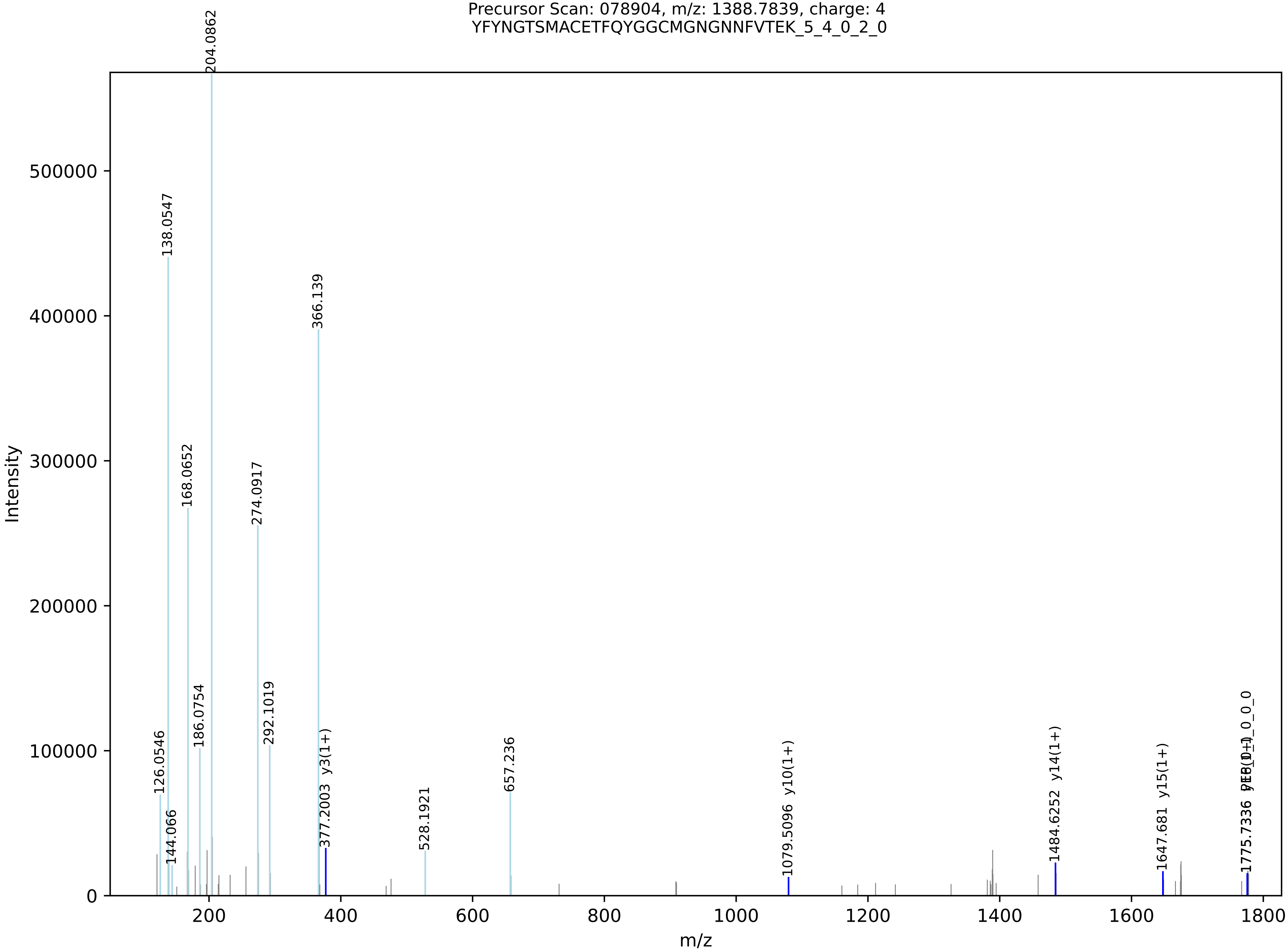

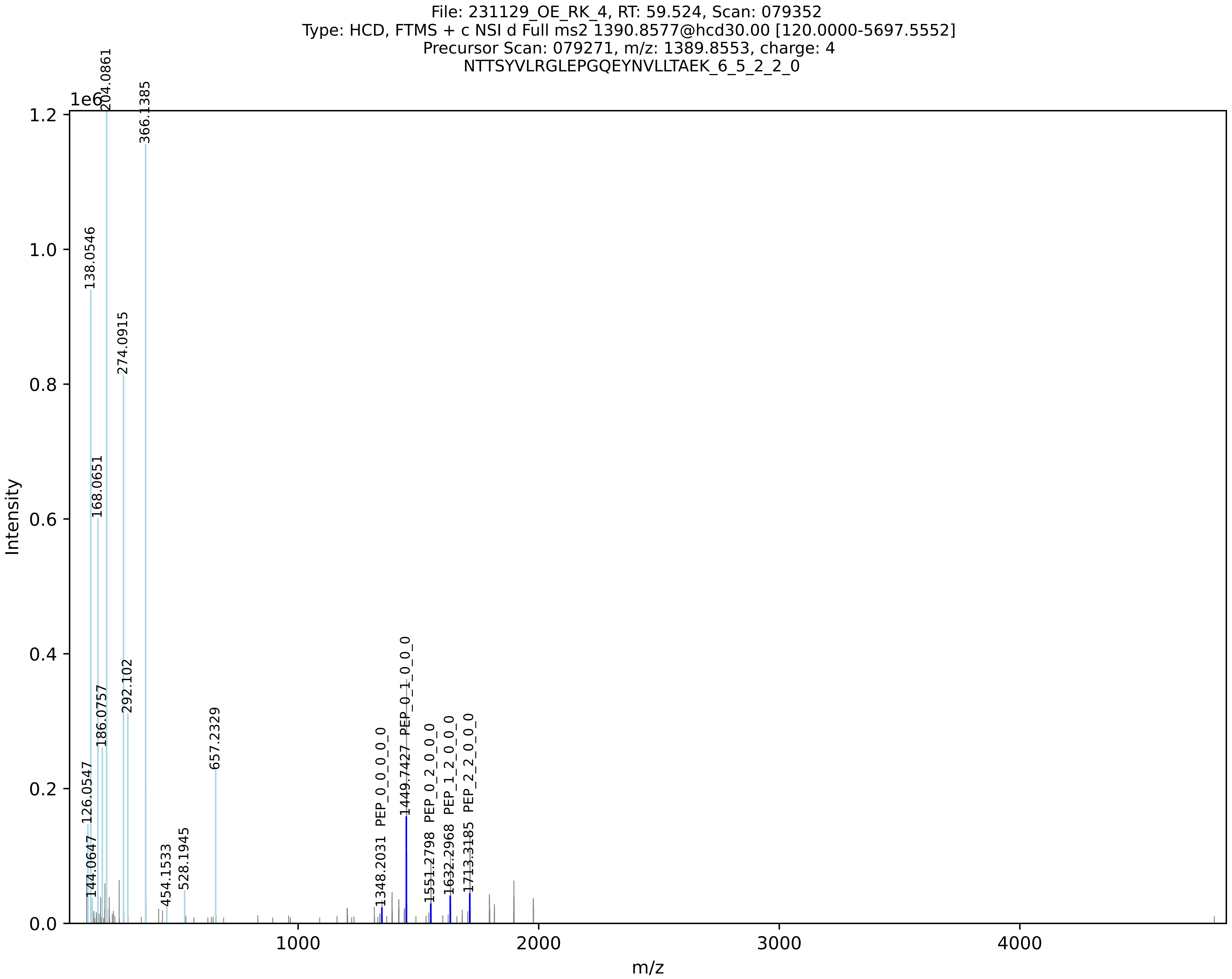
File: 231129_OE_RK_4, RT: 59.524, Scan: 079352
Type: HCD, FTMS + c NSI d Full ms2 1390.8577@hcd30.00 [120.0000-5697.5552]
Precursor Scan: 079271, m/z: 1389.8553, charge: 4
NTTSYVLRGLEPGQEYNVLLTAEK_6_5_2_2_0
1e6
Intensity
m/z
204.0861
366.1385
138.0546
274.0915
168.0651
292.102
186.0757
126.0547
144.0647
454.1533
528.1945
657.2329
1348.2031 PEP_0_0_0_0_0
1449.7427 PEP_0_1_0_0_0
1551.2798 PEP_0_2_0_0_0
1632.2968 PEP_1_2_0_0_0
1713.3185 PEP_2_2_0_0_0

File: 231129_OE_RK_4, RT: 59.672, Scan: 079565
Type: HCD, FTMS + c NSI d Full ms2 1222.5443@hcd30.00 [120.0000-5010.8364]
Precursor Scan: 079490, m/z: 1221.7916, charge: 4
MVSHHNLTTGATLINEQWLLTTAK_5_4_0_2_0

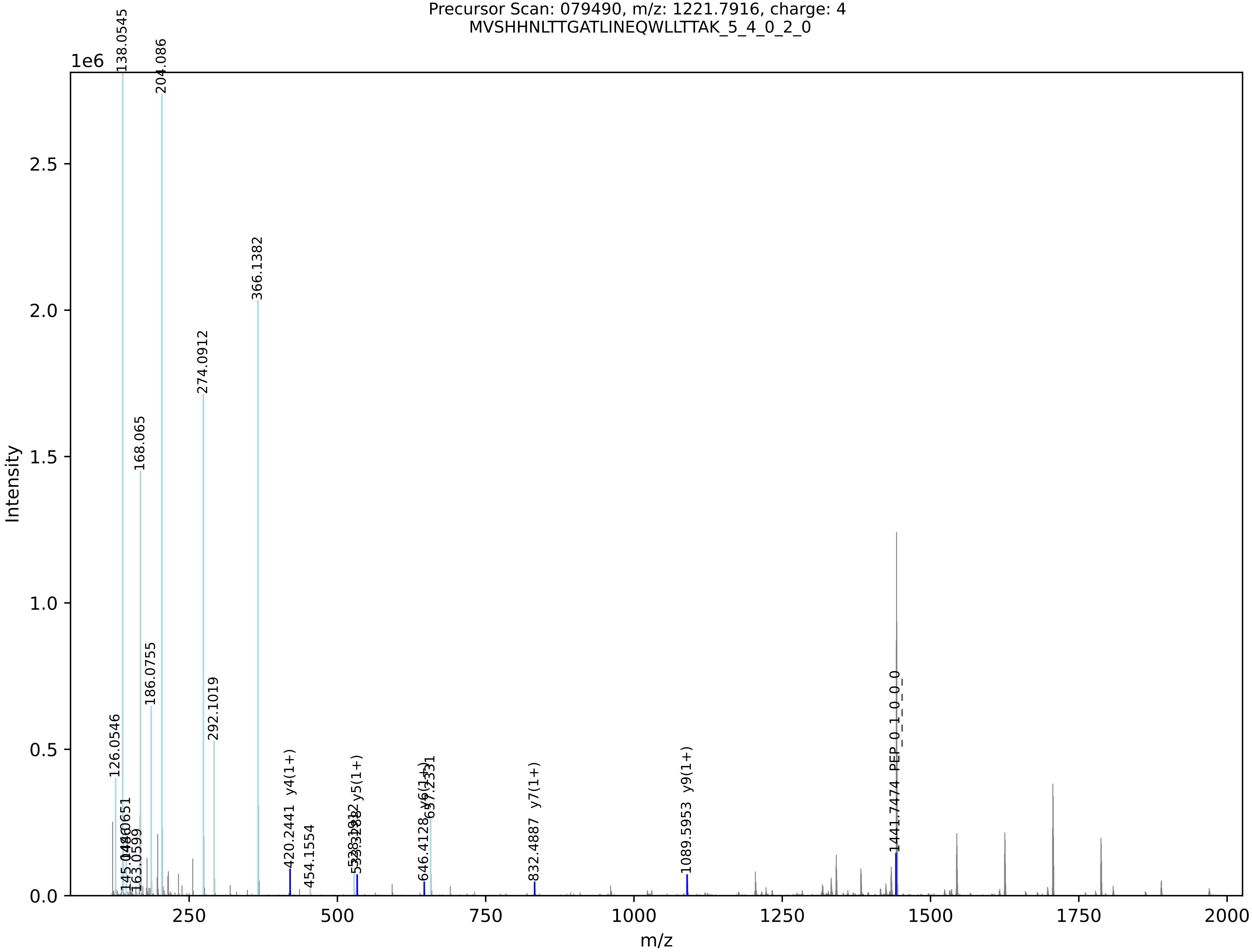

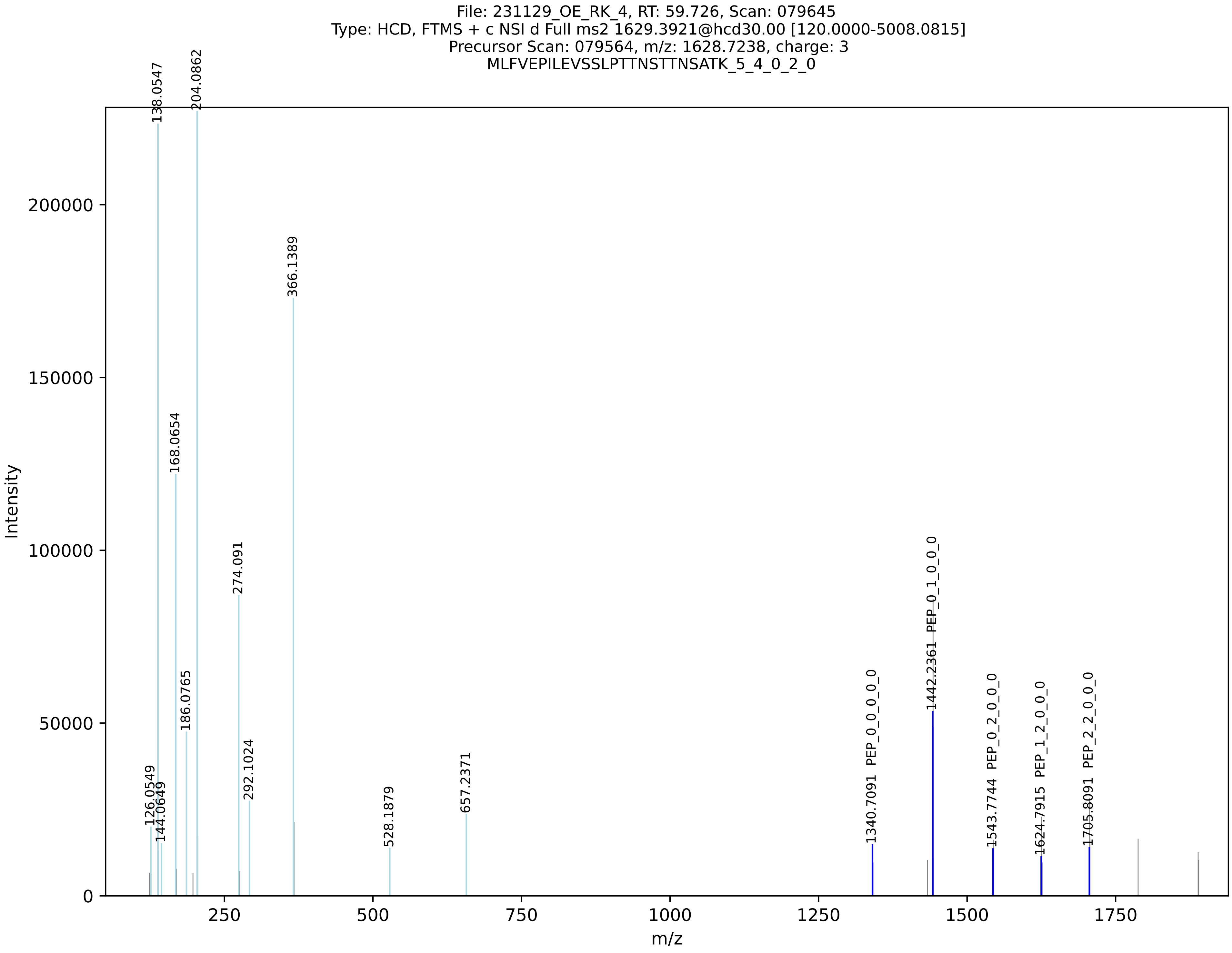
File: 231129_OE_RK_4, RT: 59.726, Scan: 079645
Type: HCD, FTMS + c NSI d Full ms2 1629.3921@hcd30.00 [120.0000-5008.0815]
Precursor Scan: 079564, m/z: 1628.7238, charge: 3
MLFVEPILEVSSLPTTNSTTNSATK_5_4_0_2_0
Intensity
m/z
0
50000
100000
150000
200000
250
500
750
1000
1250
1500
1750
126.0549
138.0547
144.0649
168.0654
186.0765
204.0862
274.091
292.1024
366.1389
528.1879
657.2371
1340.7091 PEP_0_0_0_0_0
1442.2361 PEP_0_1_0_0_0
1543.7744 PEP_0_2_0_0_0
1624.7915 PEP_1_2_0_0_0
1705.8091 PEP_2_2_0_0_0

File: 231129_OE_RK_4, RT: 59.874, Scan: 079861
Type: HCD, FTMS + c NSI d Full ms2 1222.2938@hcd30.00 [120.0000-5009.8145]
Precursor Scan: 079781, m/z: 1221.7926, charge: 4
MVSHHNLTTGATLINEQWLLTTAK_5_4_0_2_0

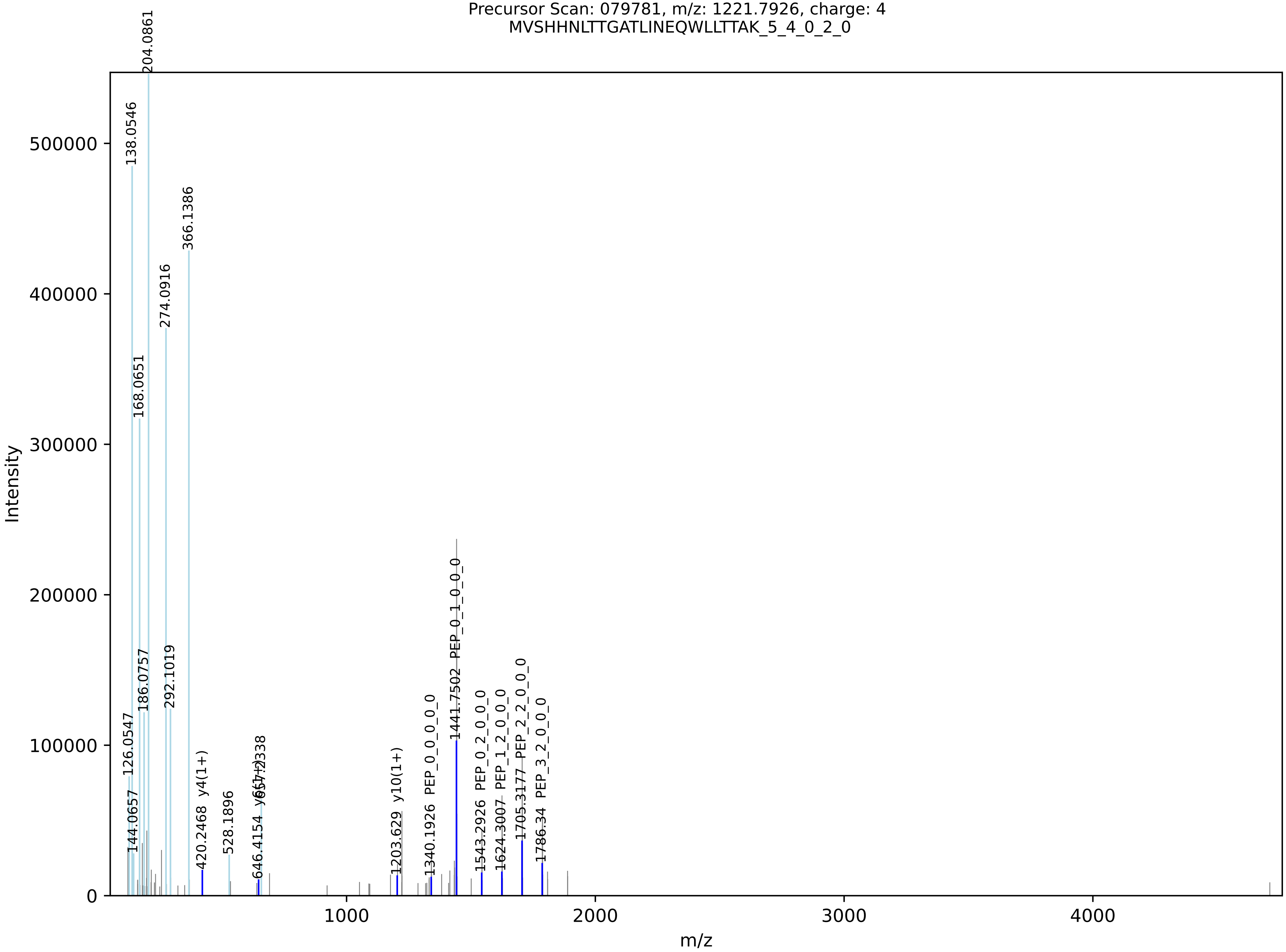

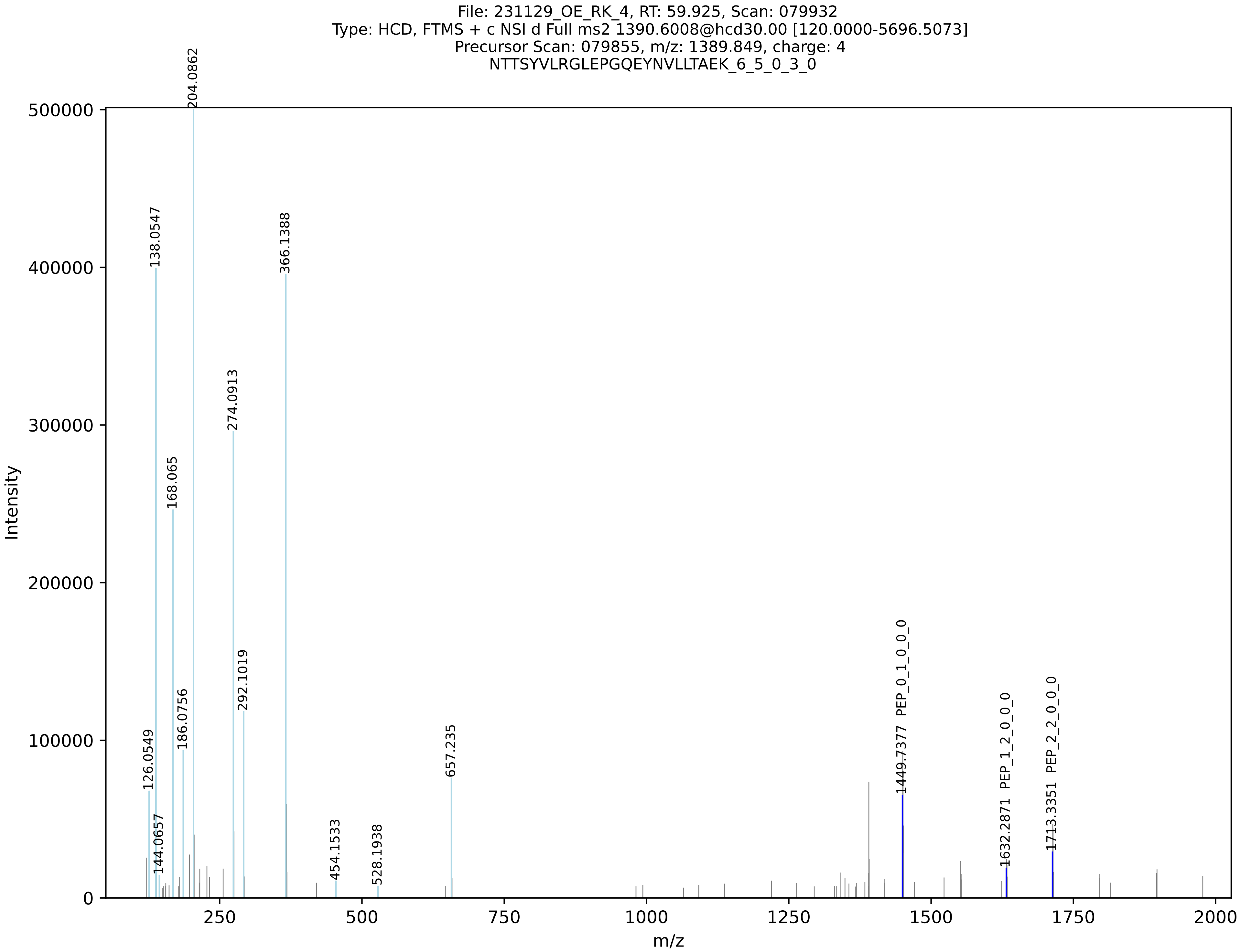

File: 231129_OE_RK_4, RT: 59.925, Scan: 079932
Type: HCD, FTMS + c NSI d Full ms2 1390.6008@hcd30.00 [120.0000-5696.5073]
Precursor Scan: 079855, m/z: 1389.849, charge: 4
NTTSYVLRGLEPGQEYNVLLTAEK_6_5_0_3_0
Intensity
m/z
204.0862
138.0547
366.1388
274.0913
168.065
292.1019
186.0756
126.0549
144.0657
454.1533
528.1938
657.235
1449.7377 PEP_0_1_0_0_0
1632.2871 PEP_1_2_0_0_0
1713.3351 PEP_2_2_0_0_0

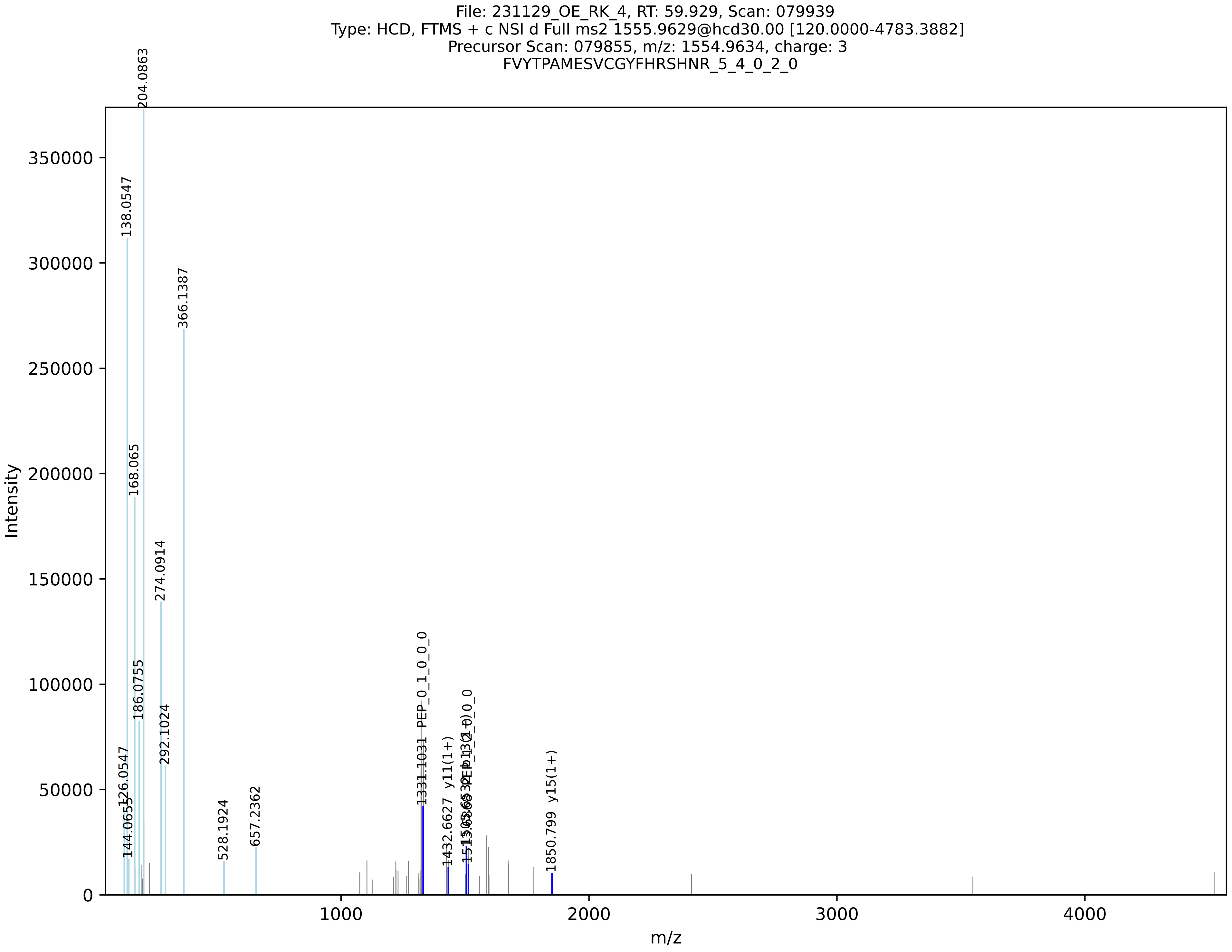
File: 231129_OE_RK_4, RT: 59.929, Scan: 079939
Type: HCD, FTMS + c NSI d Full ms2 1555.9629@hcd30.00 [120.0000-4783.3882]
Precursor Scan: 079855, m/z: 1554.9634, charge: 3
FVYTPAMESVCGYFHRSHNR_5_4_0_2_0
204.0863
138.0547
366.1387
168.065
274.0914
186.0755
292.1024
126.0547
144.0655
528.1924
657.2362
1331.1031 PEP_0_1_0_0_0_
1432.6627 y11(1+)
1850.799 y15(1+)
Intensity
m/z
0
50000
100000
150000
200000
250000
300000
350000
1000
2000
3000
4000

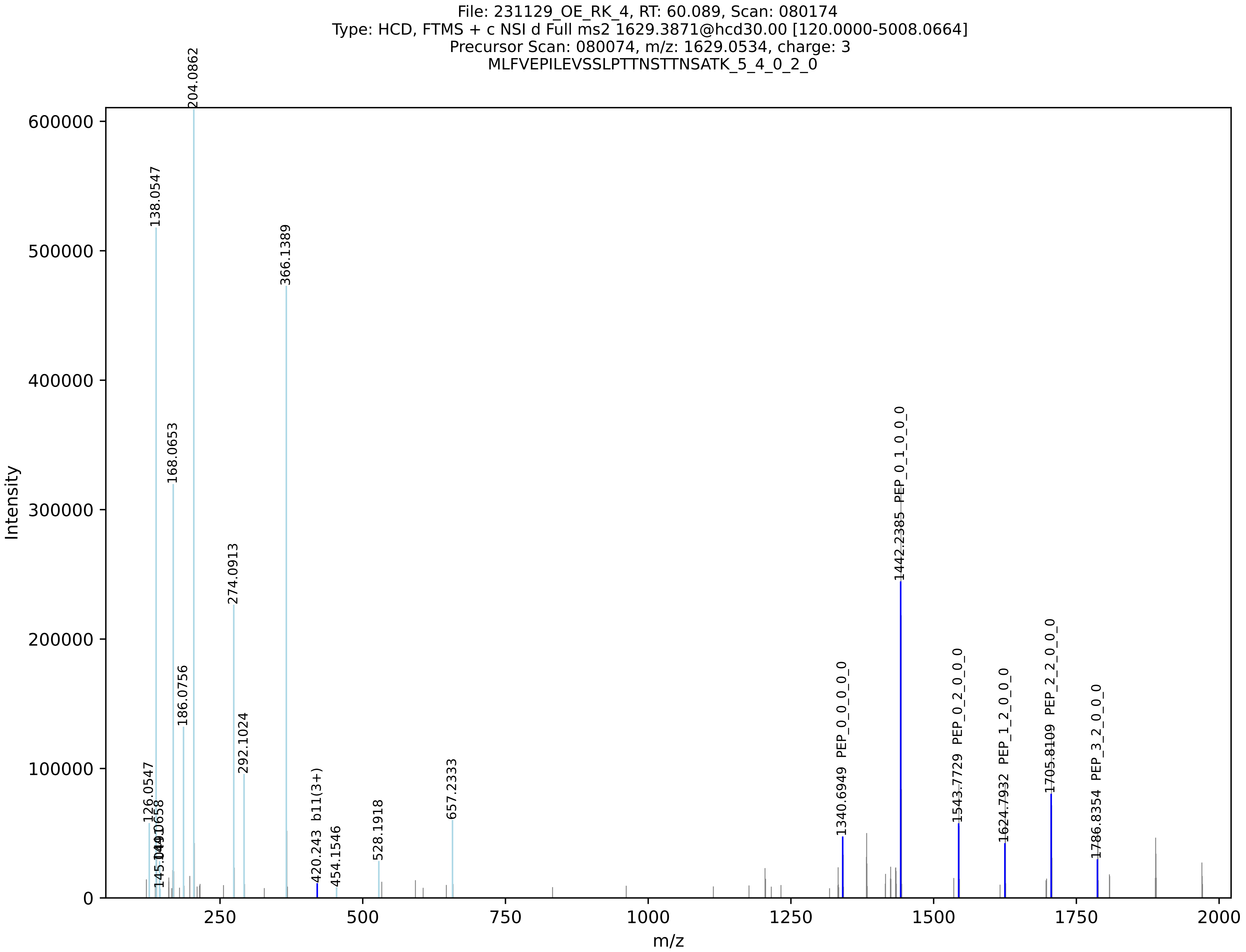

File: 231129_OE_RK_4, RT: 60.089, Scan: 080174
Type: HCD, FTMS + c NSI d Full ms2 1629.3871@hcd30.00 [120.0000-5008.0664]
Precursor Scan: 080074, m/z: 1629.0534, charge: 3
MLFVEPILEVSSLPTTNSTTNSATK_5_4_0_2_0
Intensity
m/z
204.0862
138.0547
366.1389
168.0653
274.0913
186.0756
292.1024
126.0547
420.243 b11(3+)
454.1546
528.1918
657.2333
1340.6949 PEP_0_0_0_0_0
1442.2385 PEP_0_1_0_0_0
1543.7729 PEP_0_2_0_0_0
1624.7932 PEP_1_2_0_0_0
1705.8109 PEP_2_2_0_0_0
1786.8354 PEP_3_2_0_0_0

File: 231129_OE_RK_4, RT: 60.176, Scan: 080297
Type: HCD, FTMS + c NSI d Full ms2 1323.0774@hcd30.00 [120.0000-5421.0117]
Precursor Scan: 080222, m/z: 1322.3257, charge: 4
SLGNVNFTVSAEALESQELCGTEVPSVPEHGR_6_3_0_1_0

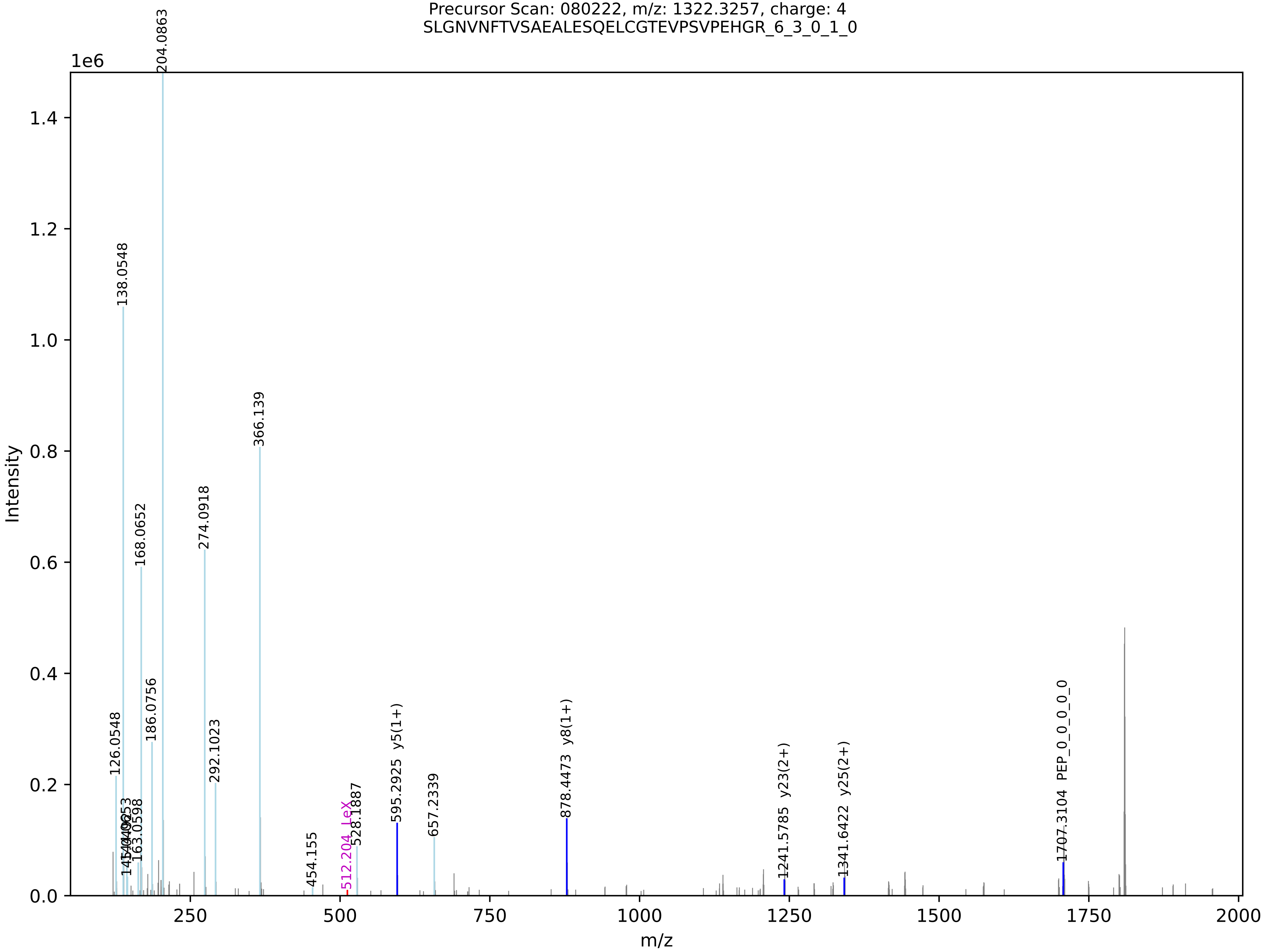

File: 231129_OE_RK_4, RT: 60.228, Scan: 080374
Type: HCD, FTMS + c NSI d Full ms2 1109.2833@hcd30.00 [120.0000-5680.9150]
Precursor Scan: 080293, m/z: 1108.8843, charge: 5
MVSHHNLTTGATLINEQWLLTTAK_6_5_0_3_0

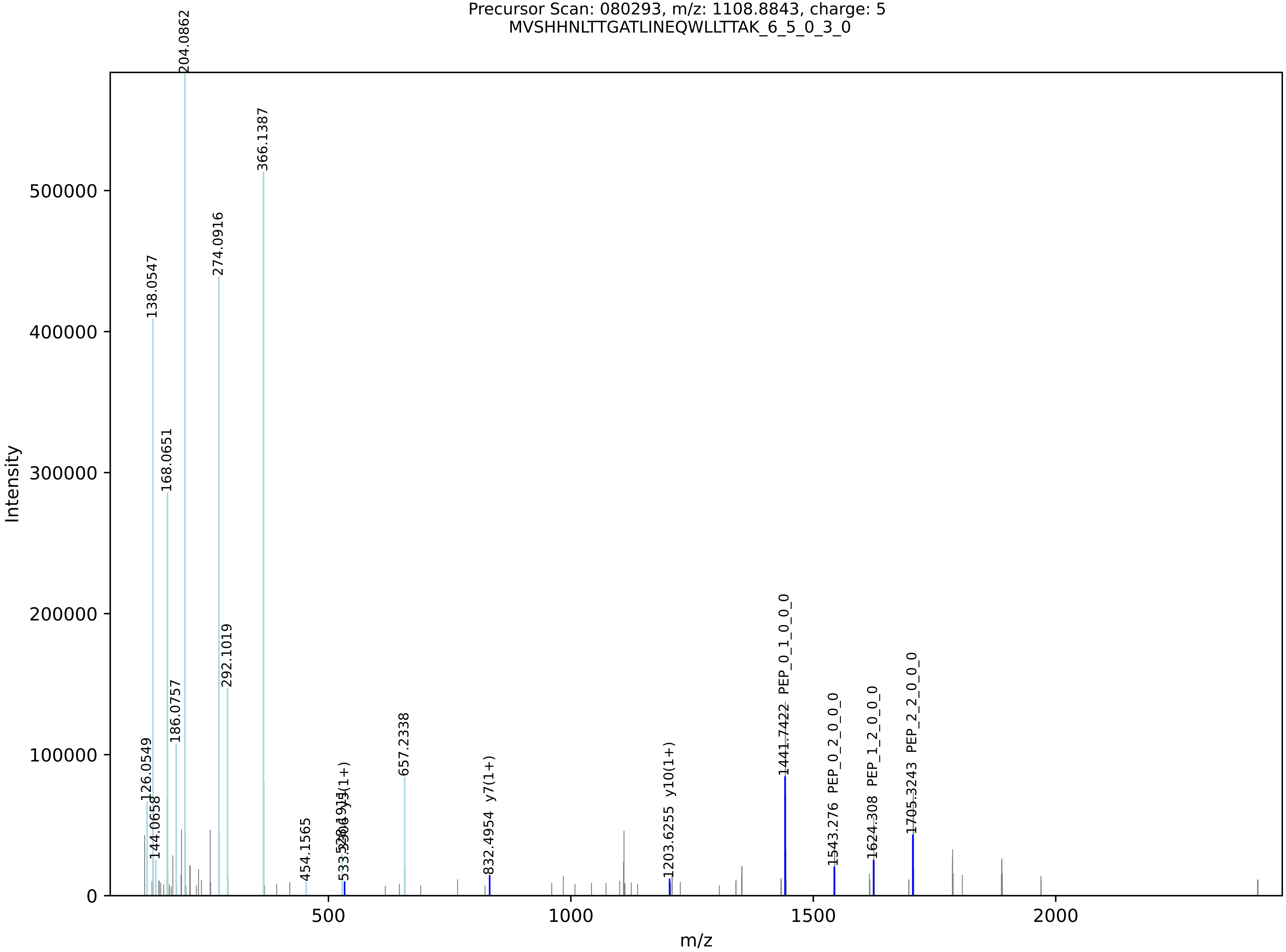

File: 231129_OE_RK_4, RT: 60.278, Scan: 080446
Type: HCD, FTMS + c NSI d Full ms2 1105.4629@hcd30.00 [120.0000-4533.1445]
Precursor Scan: 080366, m/z: 1105.2125, charge: 4
TLNQSSDELQLSMGNAMFVK_5_4_0_2_0

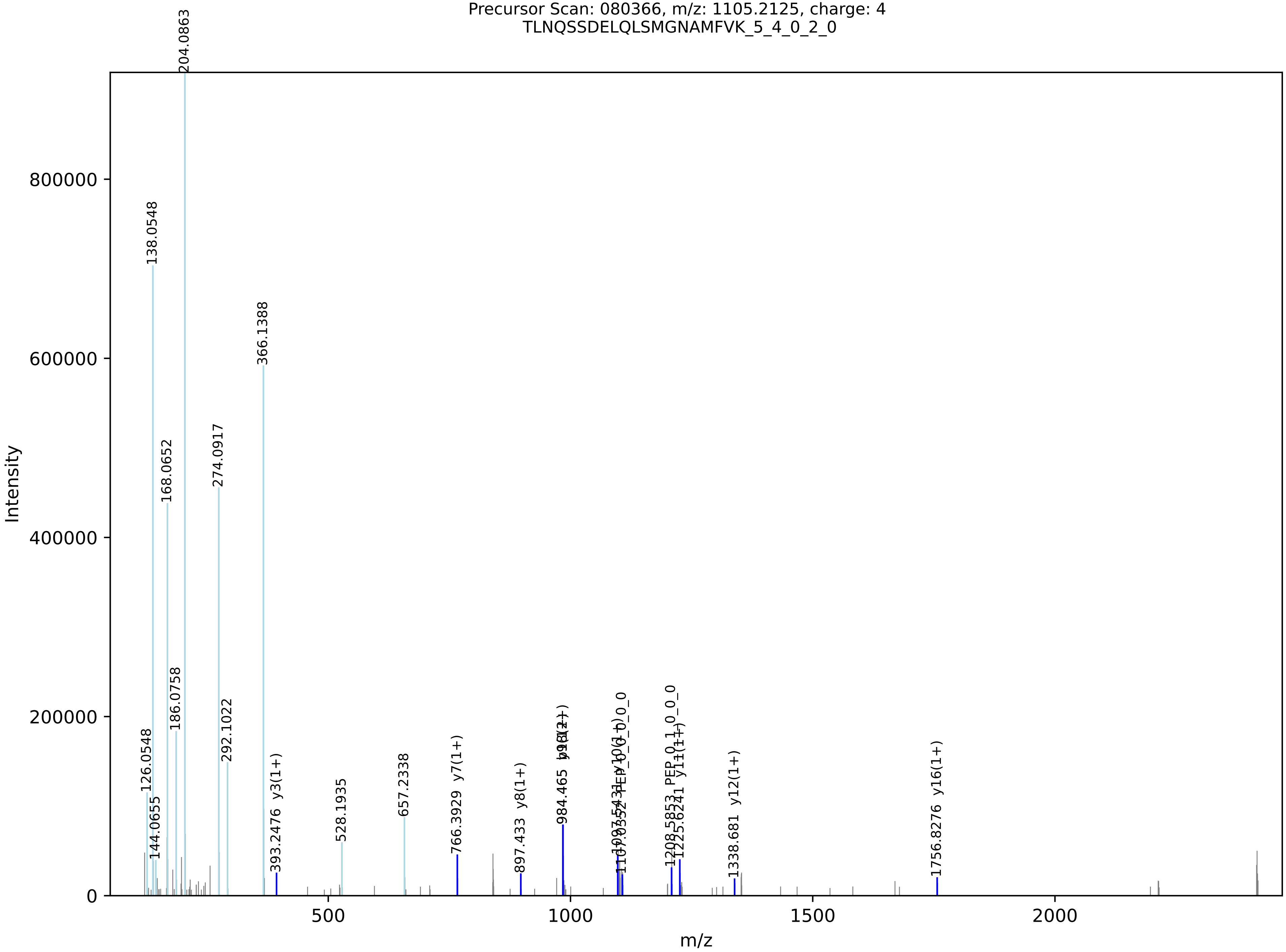

File: 231129_OE_RK_4, RT: 60.278, Scan: 080447
Type: HCD, FTMS + c NSI d Full ms2 1138.6967@hcd30.00 [120.0000-5830.9229]
Precursor Scan: 080366, m/z: 1138.0957, charge: 5
MVSHHNLTTGATLINEQWLLTTAK_6_5_1_3_0

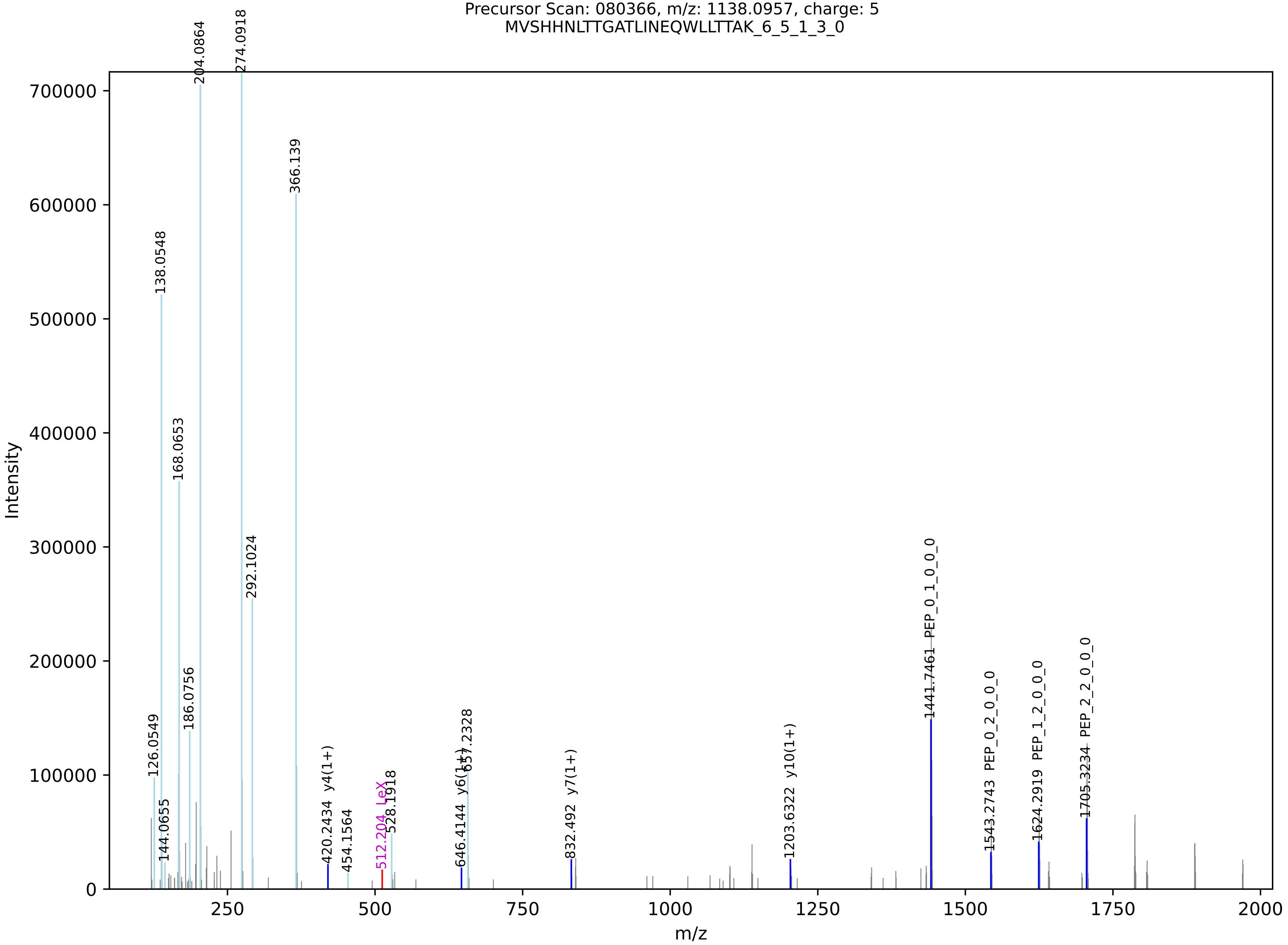

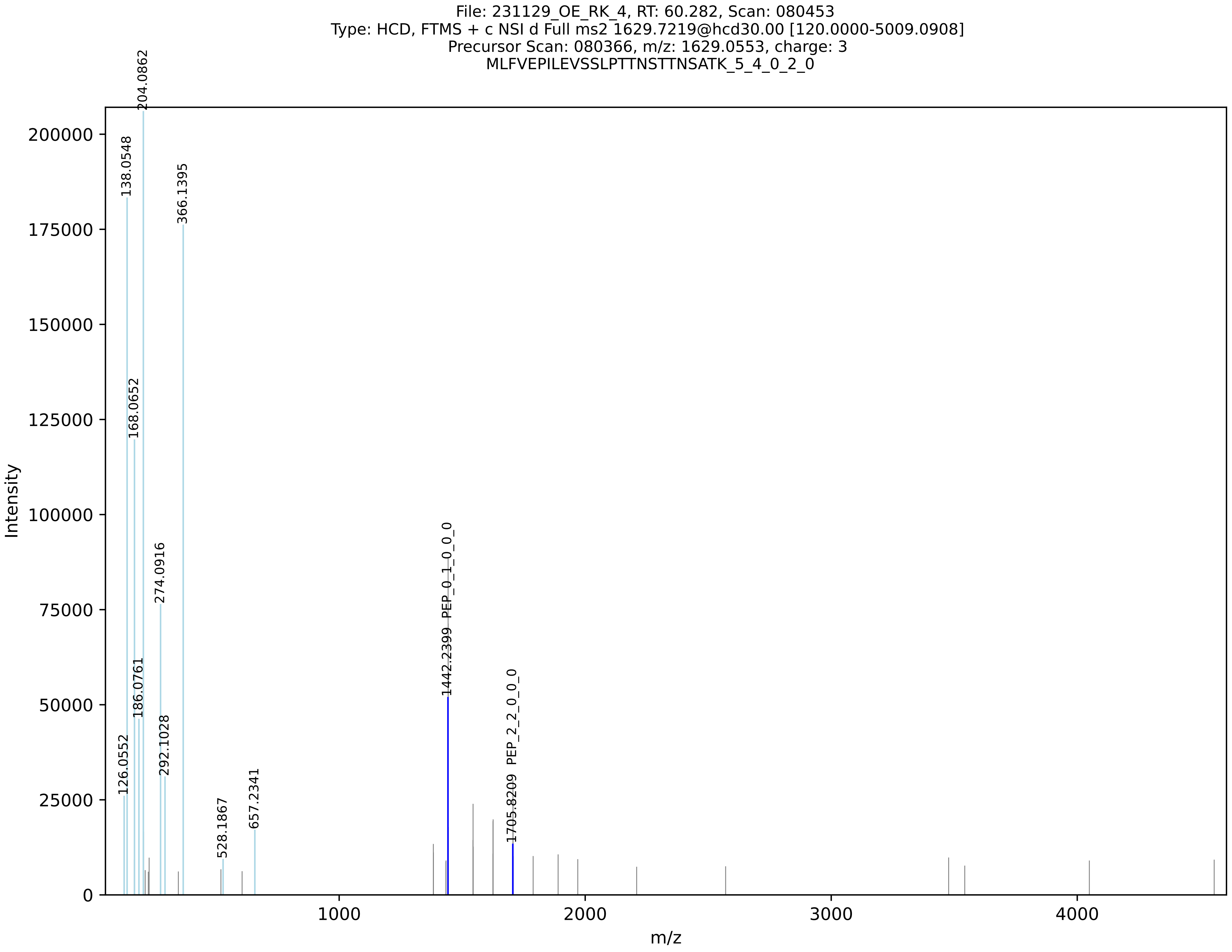
File: 231129_OE_RK_4, RT: 60.282, Scan: 080453
Type: HCD, FTMS + c NSI d Full ms2 1629.7219@hcd30.00 [120.0000-5009.0908]
Precursor Scan: 080366, m/z: 1629.0553, charge: 3
MLFVEPILEVSSLPTTNSTTNSATK_5_4_0_2_0
204.0862
138.0548
366.1395
168.0652
274.0916
186.0761
292.1028
126.0552
528.1867
657.2341
1442.2399 PEP_0_1_0_0_0
1705.8209 PEP_2_2_0_0_0
Intensity
m/z
0
25000
50000
75000
100000
125000
150000
175000
200000
1000
2000
3000
4000

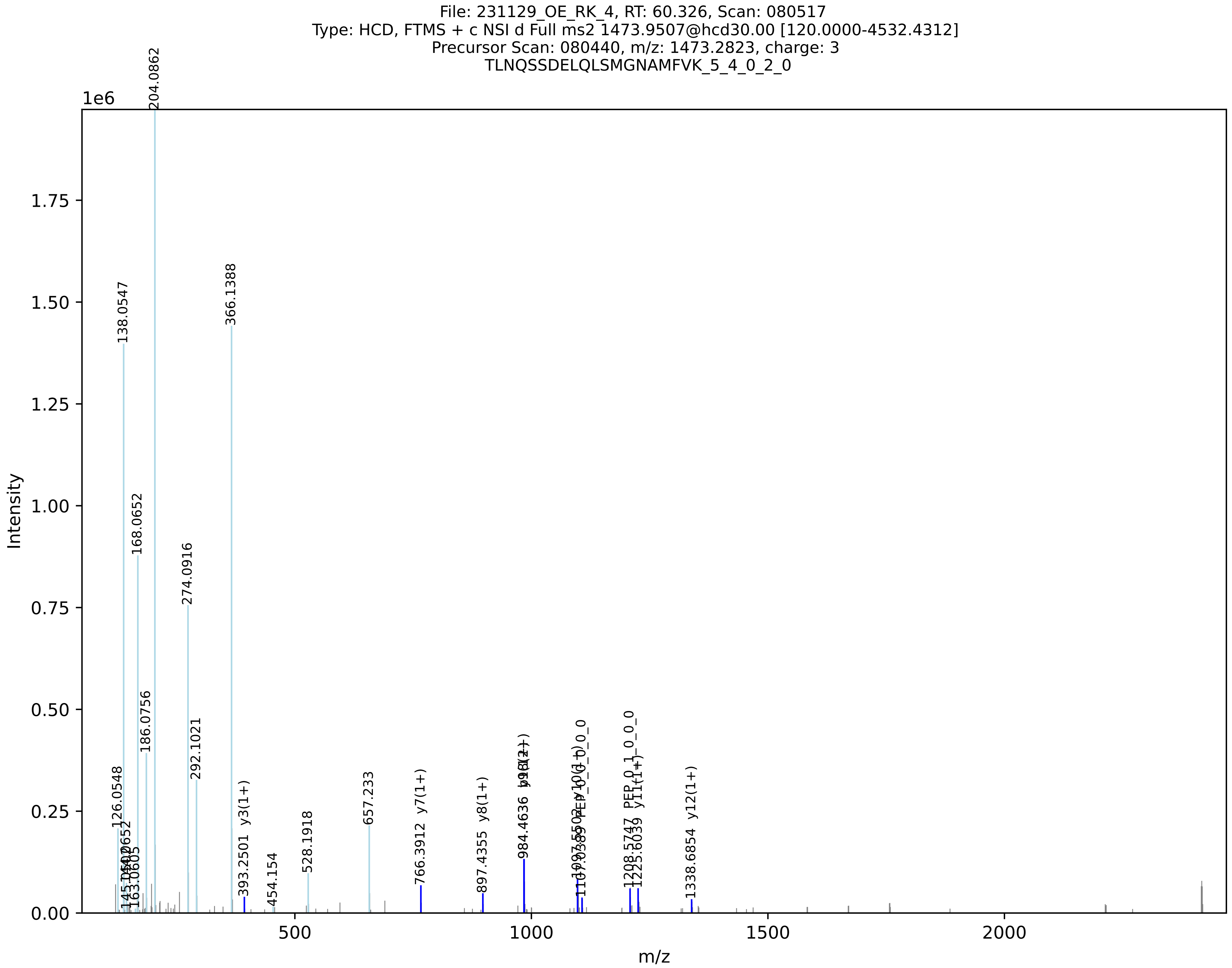

File: 231129_OE_RK_4, RT: 60.326, Scan: 080517
Type: HCD, FTMS + c NSI d Full ms2 1473.9507@hcd30.00 [120.0000-4532.4312]
Precursor Scan: 080440, m/z: 1473.2823, charge: 3
TLNQSSDELQLSMGNAMFVK_5_4_0_2_0
1e6
204.0862
138.0547
366.1388
168.0652
274.0916
186.0756
292.1021
126.0548
657.233
528.1918
393.2501 y3(1+)
454.154
766.3912 y7(1+)
897.4355 y8(1+)
984.4636 y9(1+)
1097.5502 y10(1+)
1208.5747 PEP_0_1_0_0_0
1225.6039 y11(1+)
1338.6854 y12(1+)
Intensity
m/z

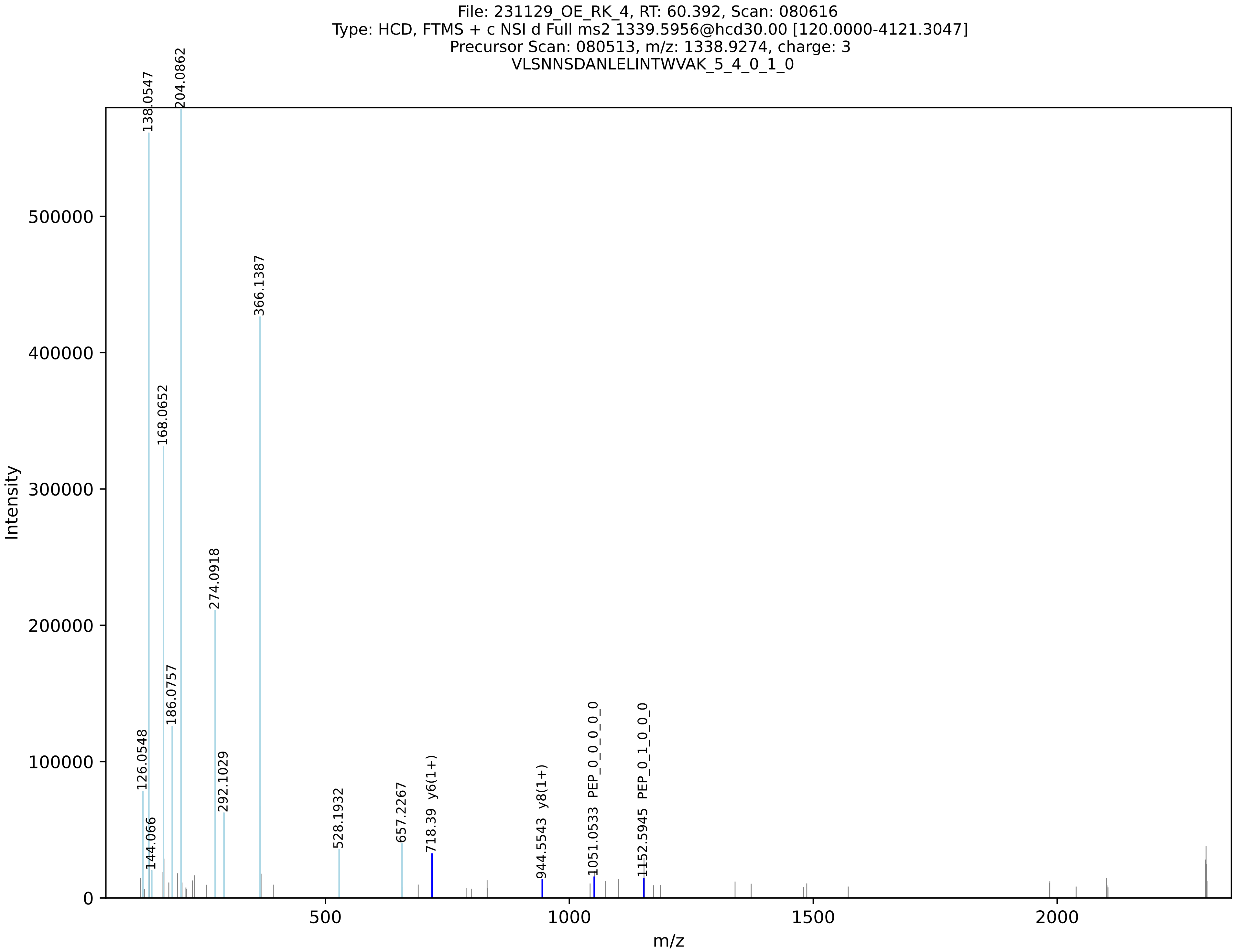
File: 231129_OE_RK_4, RT: 60.392, Scan: 080616
Type: HCD, FTMS + c NSI d Full ms2 1339.5956@hcd30.00 [120.0000-4121.3047]
Precursor Scan: 080513, m/z: 1338.9274, charge: 3
VLSNNSDANLELINTWVAK_5_4_0_1_0
Intensity
m/z
138.0547
204.0862
366.1387
168.0652
274.0918
186.0757
126.0548
292.1029
144.066
528.1932
657.2267
718.39 y6(1+)
944.5543 y8(1+)
1051.0533 PEP_0_0_0_0_0_
1152.5945 PEP_0_1_0_0_0_
0
100000
200000
300000
400000
500000
500
1000
1500
2000

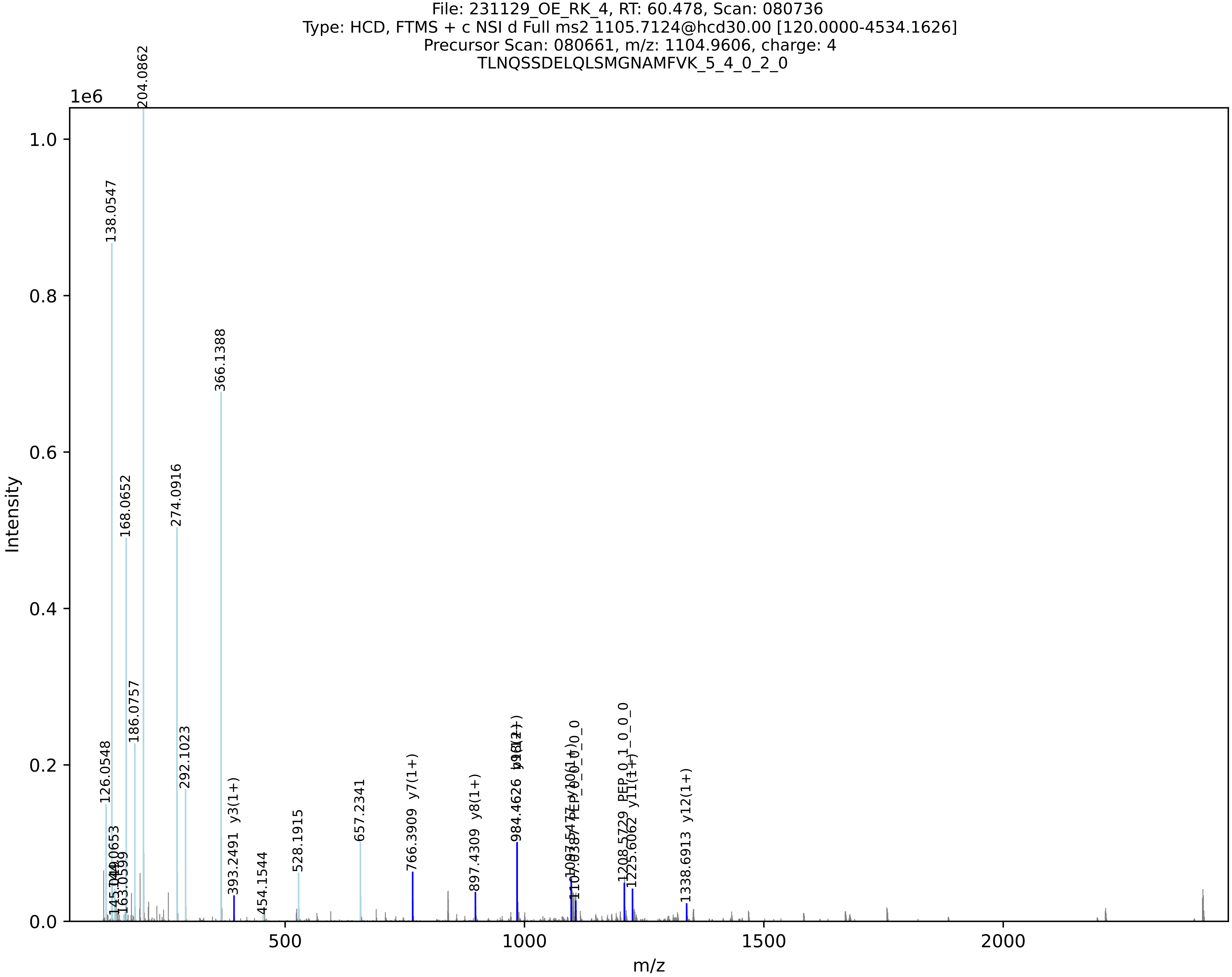

File: 231129_OE_RK_4, RT: 60.478, Scan: 080736
Type: HCD, FTMS + c NSI d Full ms2 1105.7124@hcd30.00 [120.0000-4534.1626]
Precursor Scan: 080661, m/z: 1104.9606, charge: 4
TLNQSSDELQLSMGNAMFVK_5_4_0_2_0
1e6
Intensity
m/z
204.0862
138.0547
366.1388
274.0916
168.0652
186.0757
292.1023
126.0548
657.2341
528.1915
393.2491 y3(1+)
454.1544
766.3909 y7(1+)
897.4309 y8(1+)
984.4626 y9(1+)
1097.5477 y10(1+)
1107.0387 PEP_0_0_0_0
1208.5729 PEP_0_1_0_0_0
1225.6062 y11(1+)
1338.6913 y12(1+)

File: 231129_OE_RK_4, RT: 60.578, Scan: 080884
Type: HCD, FTMS + c NSI d Full ms2 1339.5953@hcd30.00 [120.0000-4121.3037]
Precursor Scan: 080806, m/z: 1338.9271, charge: 3
VLSNNSDANLELINTWVAK_5_4_0_1_0

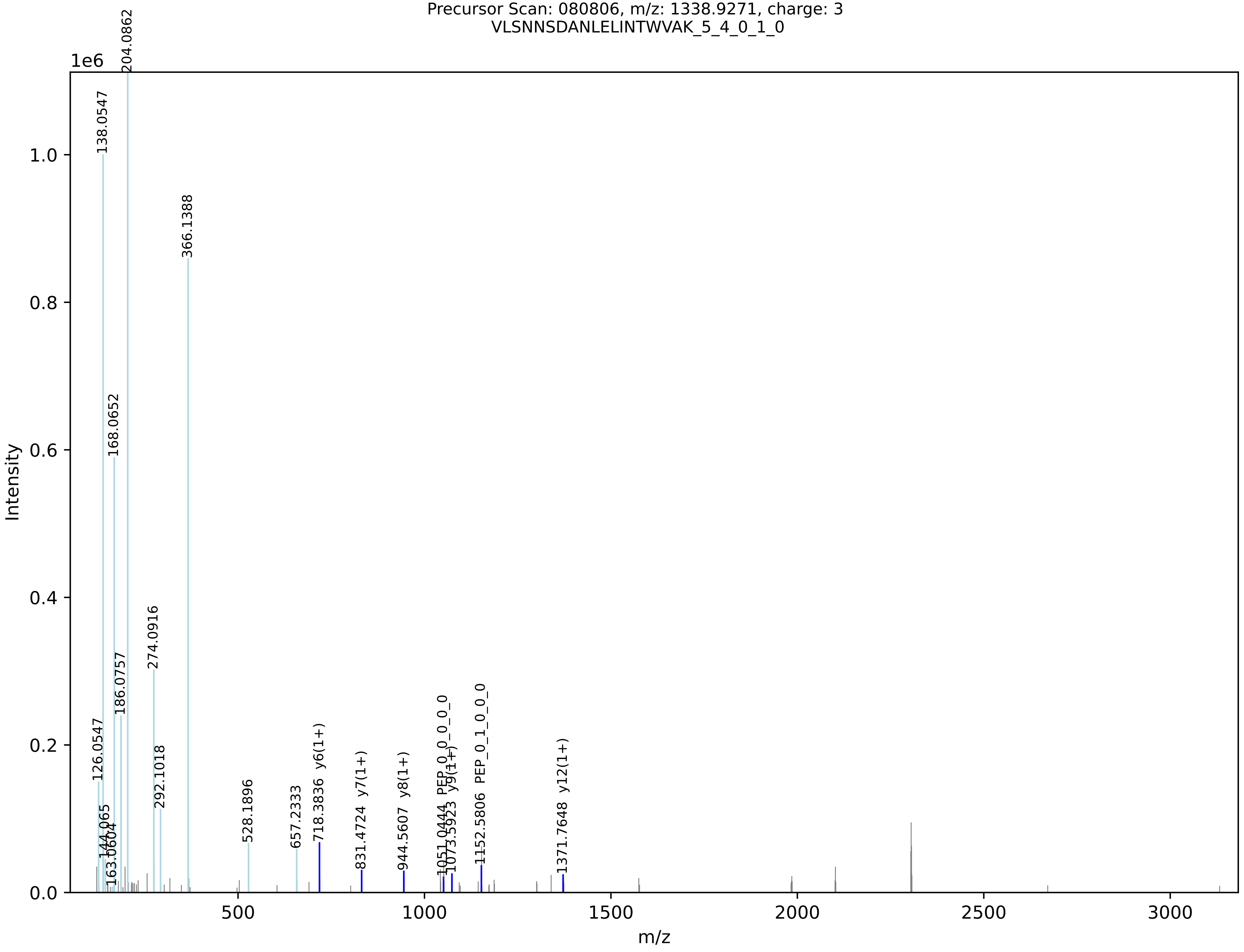

File: 231129_OE_RK_4, RT: 60.797, Scan: 081204
Type: HCD, FTMS + c NSI d Full ms2 1106.1678@hcd30.00 [120.0000-4536.0210]
Precursor Scan: 081005, m/z: 1105.2114, charge: 4
TLNQSSDELQLSMGNAMFVK_5_4_0_2_0

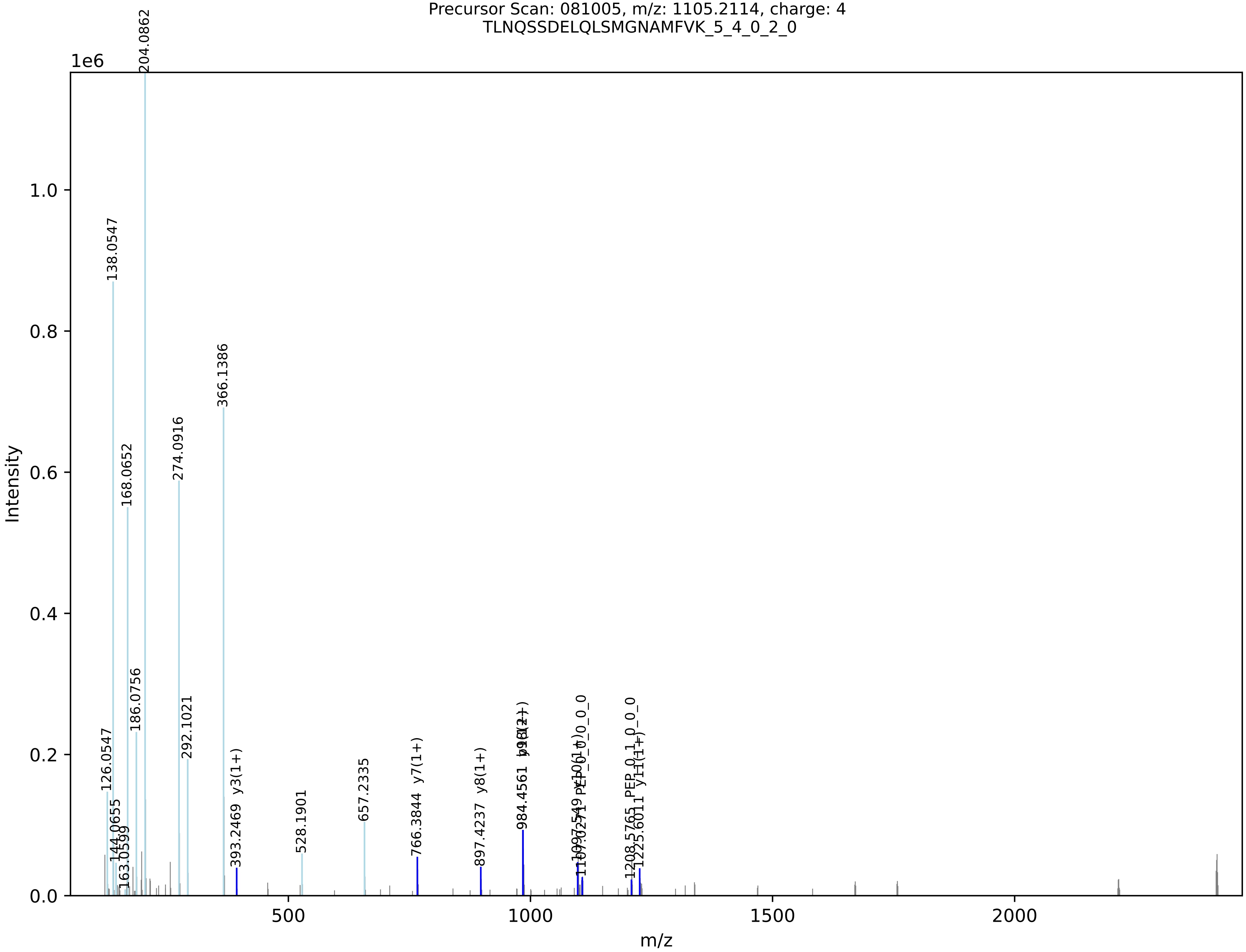

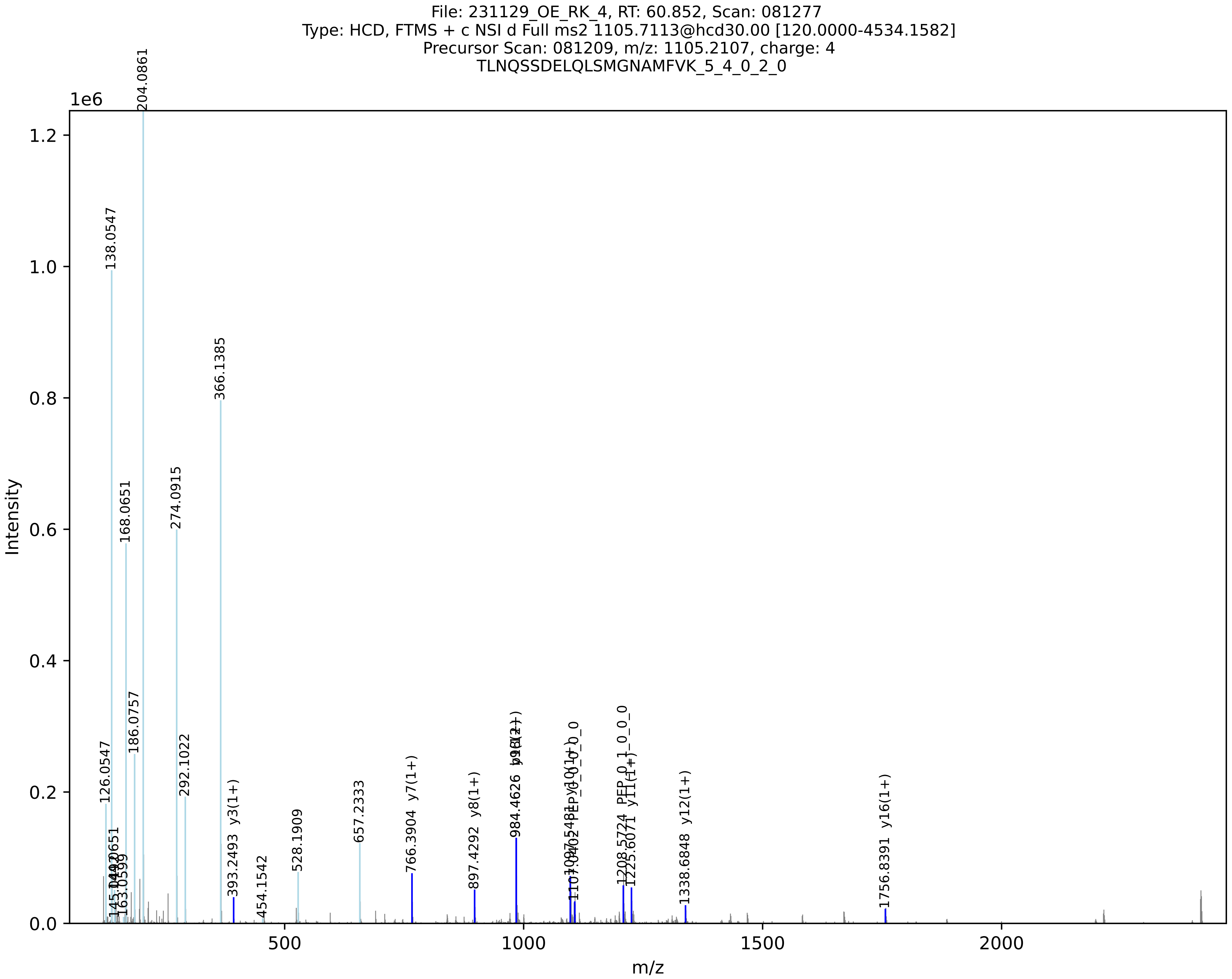
File: 231129_OE_RK_4, RT: 60.852, Scan: 081277
Type: HCD, FTMS + c NSI d Full ms2 1105.7113@hcd30.00 [120.0000-4534.1582]
Precursor Scan: 081209, m/z: 1105.2107, charge: 4
TLNQSSDELQLSMGNAMFVK_5_4_0_2_0
1e6
Intensity
m/z
204.0861
138.0547
366.1385
274.0915
168.0651
186.0757
292.1022
126.0547
393.2493 y3(1+)
454.1542
528.1909
657.2333
766.3904 y7(1+)
897.4292 y8(1+)
984.4626 y9(1+)
1208.5724 PEP_0_1_0_0_0
1225.6071 y11(1+)
1338.6848 y12(1+)
1756.8391 y16(1+)

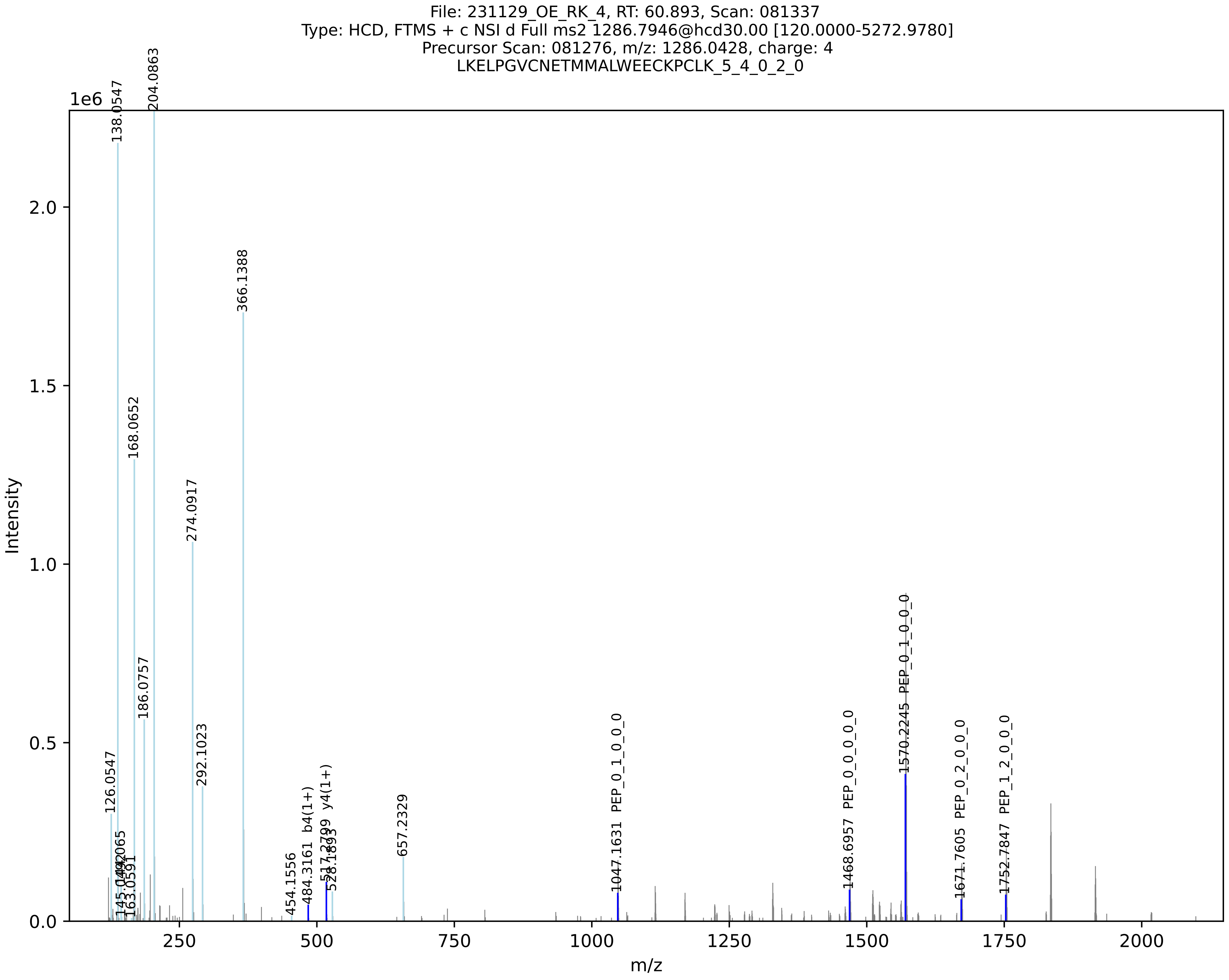
File: 231129_OE_RK_4, RT: 60.893, Scan: 081337
Type: HCD, FTMS + c NSI d Full ms2 1286.7946@hcd30.00 [120.0000-5272.9780]
Precursor Scan: 081276, m/z: 1286.0428, charge: 4
LKELPGVCNETMMALWEECKPCLK_5_4_0_2_0
1e6
Intensity
m/z
138.0547
204.0863
366.1388
168.0652
274.0917
186.0757
292.1023
126.0547
163.0591
454.1556
484.3161 b4(1+)
517.2799 y4(1+)
528.1893
657.2329
1047.1631 PEP_0_1_0_0_0
1468.6957 PEP_0_0_0_0_0
1570.2245 PEP_0_1_0_0_
1671.7605 PEP_0_2_0_0_0
1752.7847 PEP_1_2_0_0_0

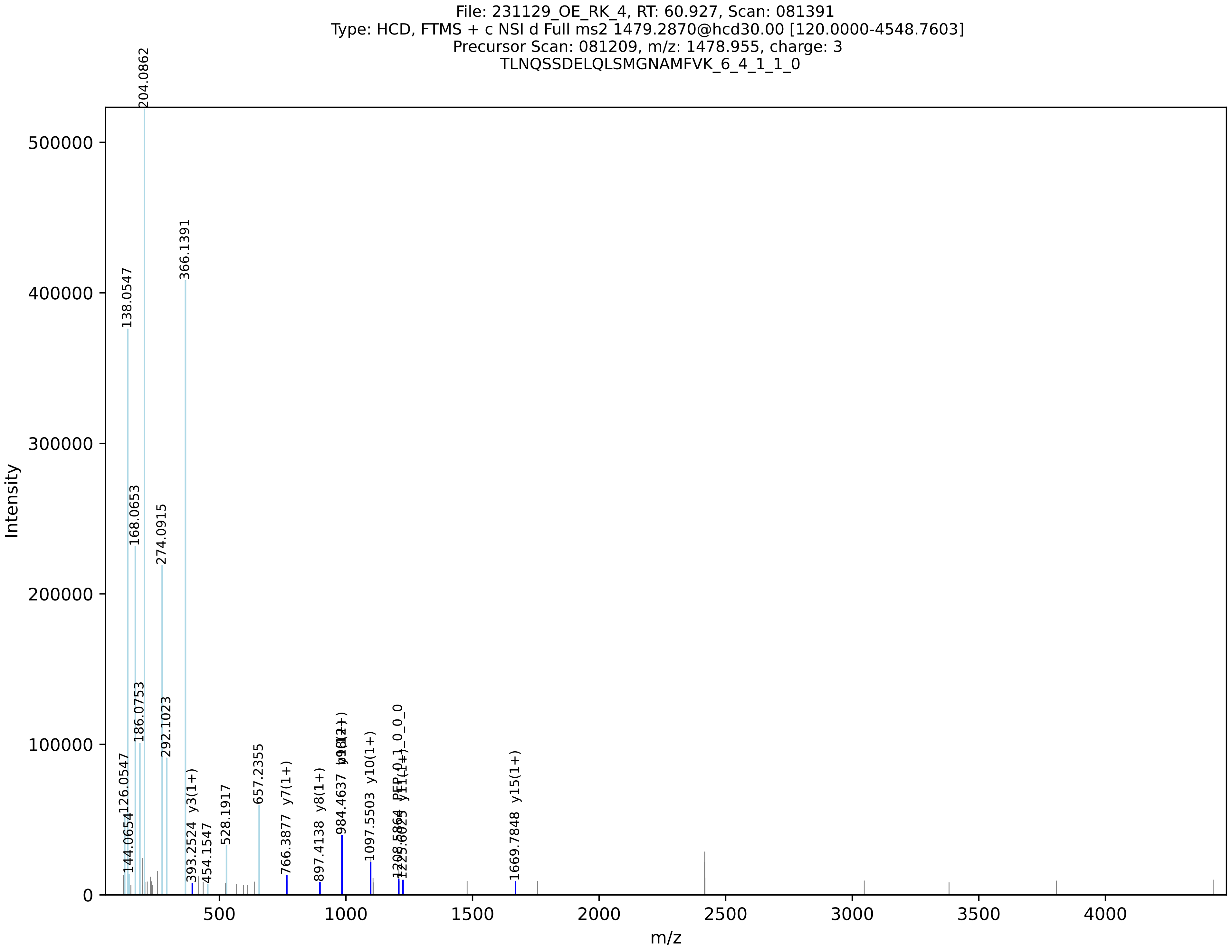
File: 231129_OE_RK_4, RT: 60.927, Scan: 081391
Type: HCD, FTMS + c NSI d Full ms2 1479.2870@hcd30.00 [120.0000-4548.7603]
Precursor Scan: 081209, m/z: 1478.955, charge: 3
TLNQSSDELQLSMGNAMFVK_6_4_1_1_0
204.0862
366.1391
138.0547
168.0653
274.0915
186.0753
292.1023
126.0547
144.0654
393.2524 y3(1+)
454.1547
528.1917
657.2355
766.3877 y7(1+)
897.4138 y8(1+)
984.4637
1097.5503 y10(1+)
1669.7848 y15(1+)
Intensity
m/z

File: 231129_OE_RK_4, RT: 61.044, Scan: 081557
Type: HCD, FTMS + c NSI d Full ms2 1105.7128@hcd30.00 [120.0000-4534.1641]
Precursor Scan: 081481, m/z: 1105.2116, charge: 4
TLNQSSDELQLSMGNAMFVK_5_4_0_2_0

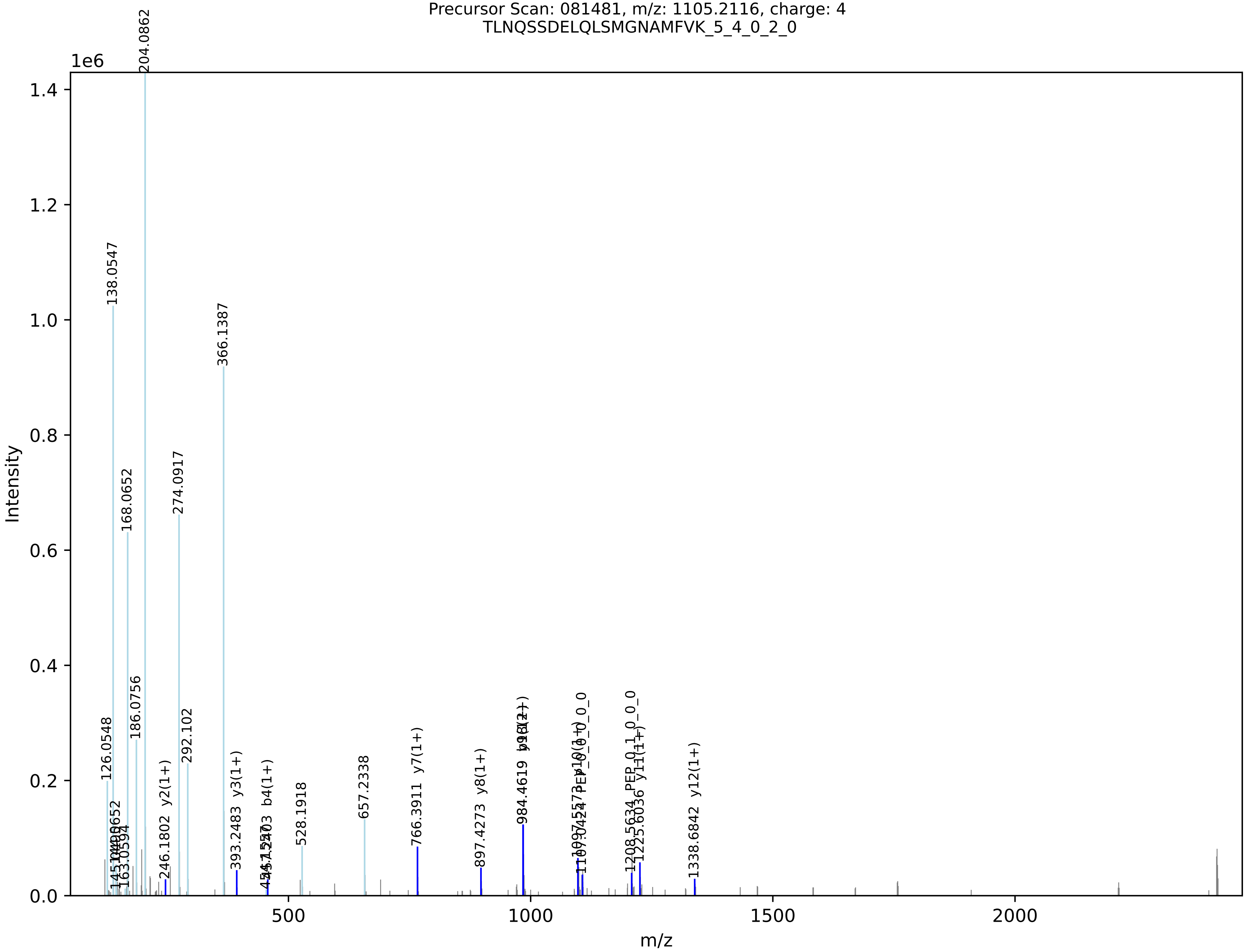

File: 231129_OE_RK_4, RT: 61.346, Scan: 082000
Type: HCD, FTMS + c NSI d Full ms2 1474.2828@hcd30.00 [120.0000-4533.4473]
Precursor Scan: 081925, m/z: 1473.2805, charge: 3
TLNQSSDELQLSMGNAMFVK_5_4_0_2_0

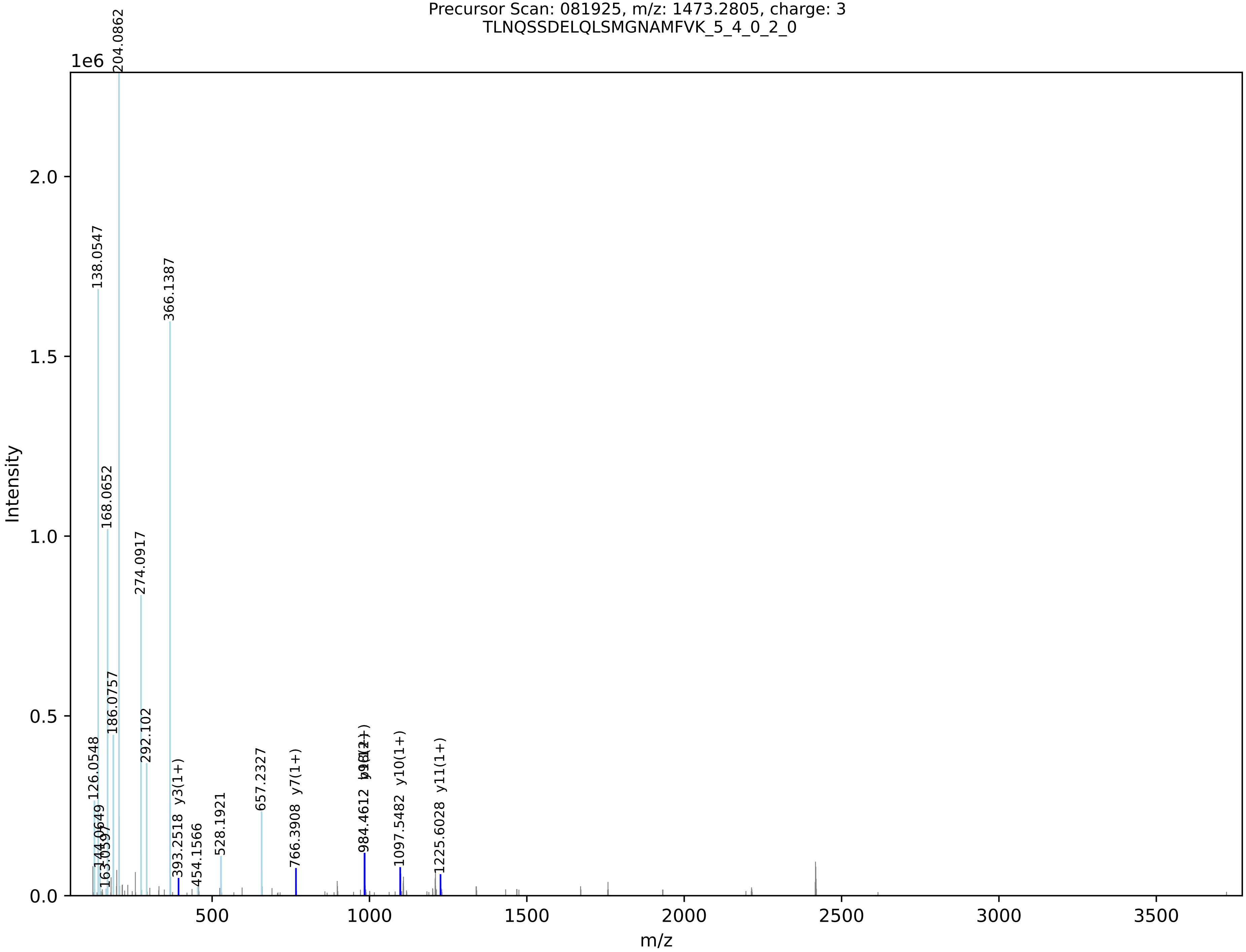

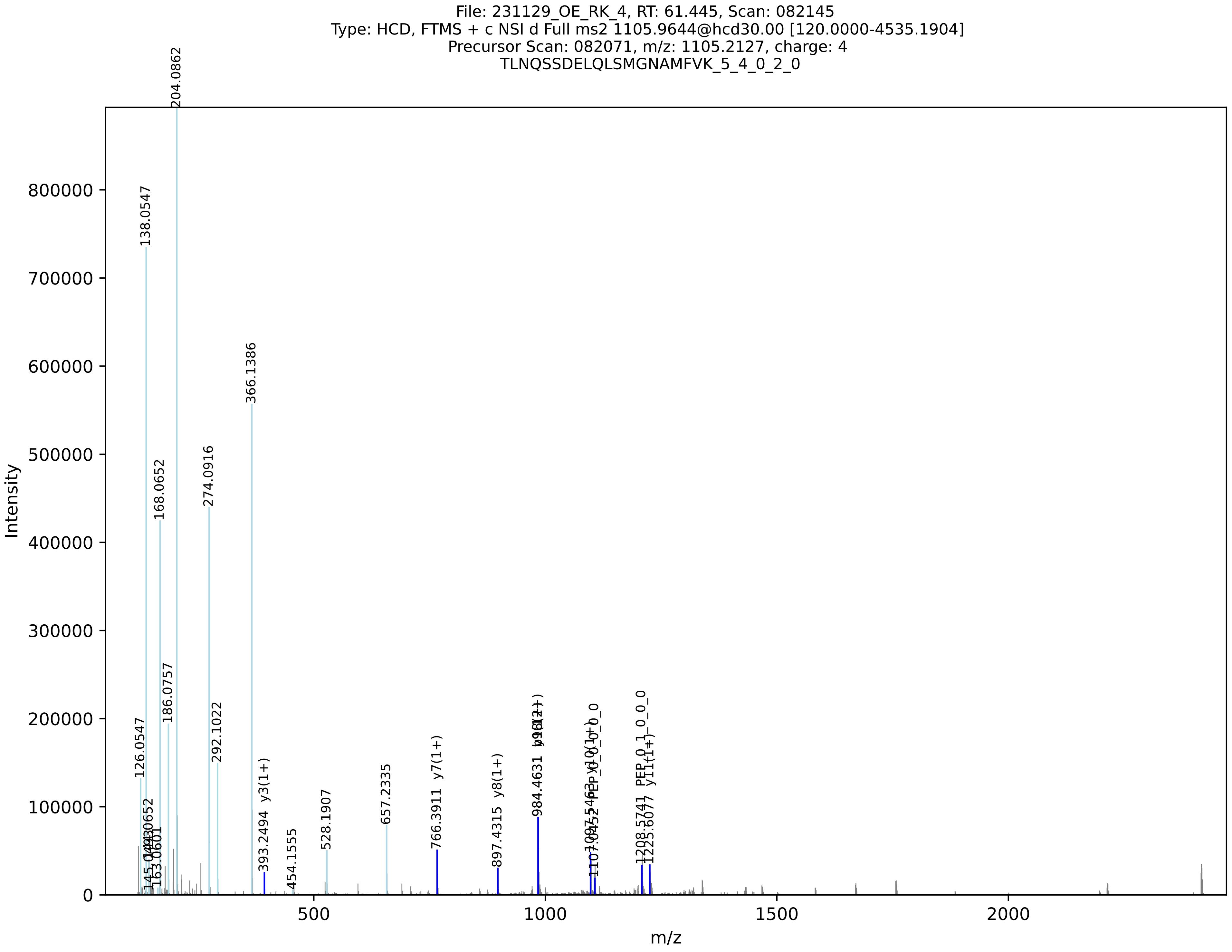
File: 231129_OE_RK_4, RT: 61.445, Scan: 082145
Type: HCD, FTMS + c NSI d Full ms2 1105.9644@hcd30.00 [120.0000-4535.1904]
Precursor Scan: 082071, m/z: 1105.2127, charge: 4
TLNQSSDELQLSMGNAMFVK_5_4_0_2_0
Intensity
m/z
0
100000
200000
300000
400000
500000
600000
700000
800000
500
1000
1500
2000
204.0862
138.0547
366.1386
274.0916
168.0652
186.0757
292.1022
126.0547
163.0601
393.2494 y3(1+)
454.1555
528.1907
657.2335
766.3911 y7(1+)
897.4315 y8(1+)
984.4631
1208.5741 PEP_0_1_0_0_0
1225.6077 y11(1+)

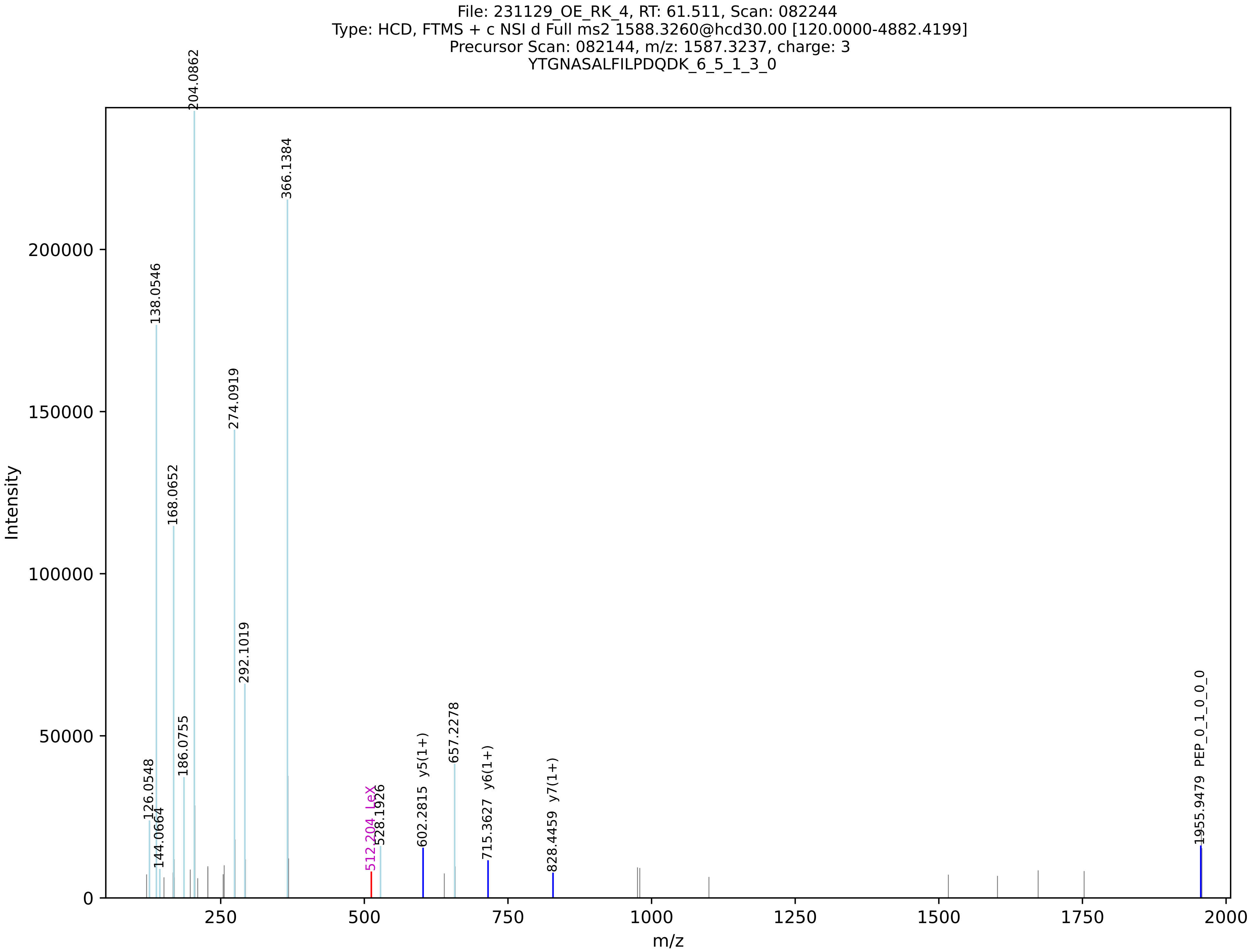

File: 231129_OE_RK_4, RT: 61.511, Scan: 082244
Type: HCD, FTMS + c NSI d Full ms2 1588.3260@hcd30.00 [120.0000-4882.4199]
Precursor Scan: 082144, m/z: 1587.3237, charge: 3
YTGNASALFILPDQDK_6_5_1_3_0
Intensity
m/z
204.0862
366.1384
138.0546
274.0919
168.0652
292.1019
186.0755
126.0548
144.0664
512.204 LeX
528.1926
602.2815 y5(1+)
657.2278
715.3627 y6(1+)
828.4459 y7(1+)
1955.9479 PEP_0_1_0_0_0
0
50000
100000
150000
200000
250
500
750
1000
1250
1500
1750
2000

File: 231129_OE_RK_4, RT: 61.548, Scan: 082293
Type: HCD, FTMS + c NSI d Full ms2 1473.6183@hcd30.00 [120.0000-4531.4141]
Precursor Scan: 082218, m/z: 1473.2841, charge: 3
TLNQSSDELQLSMGNAMFVK_5_4_0_2_0

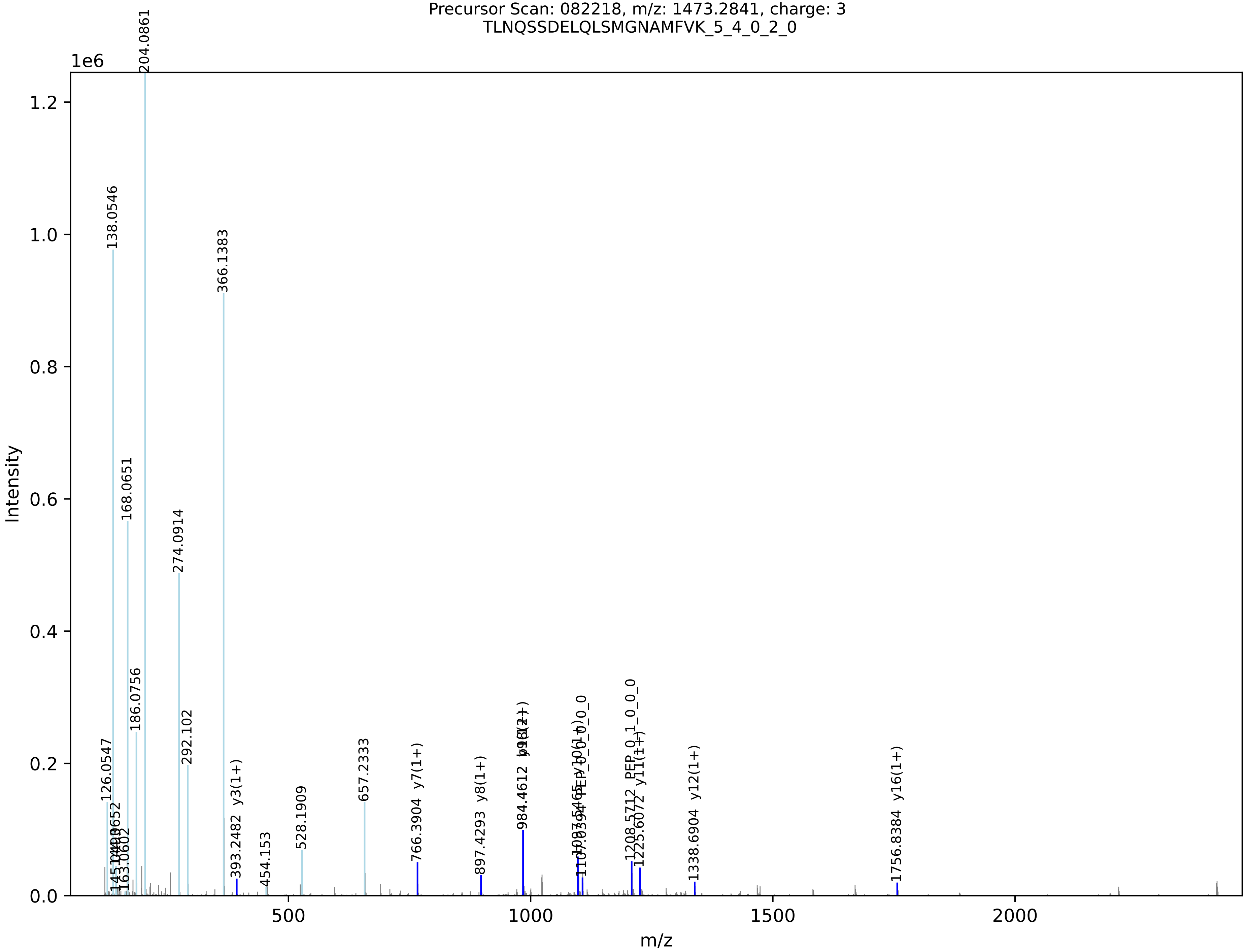

File: 231129_OE_RK_4, RT: 61.732, Scan: 082558
Type: HCD, FTMS + c NSI d Full ms2 1474.2811@hcd30.00 [120.0000-4533.4424]
Precursor Scan: 082495, m/z: 1473.2791, charge: 3
TLNQSSDELQLSMGNAMFVK_5_4_0_2_0

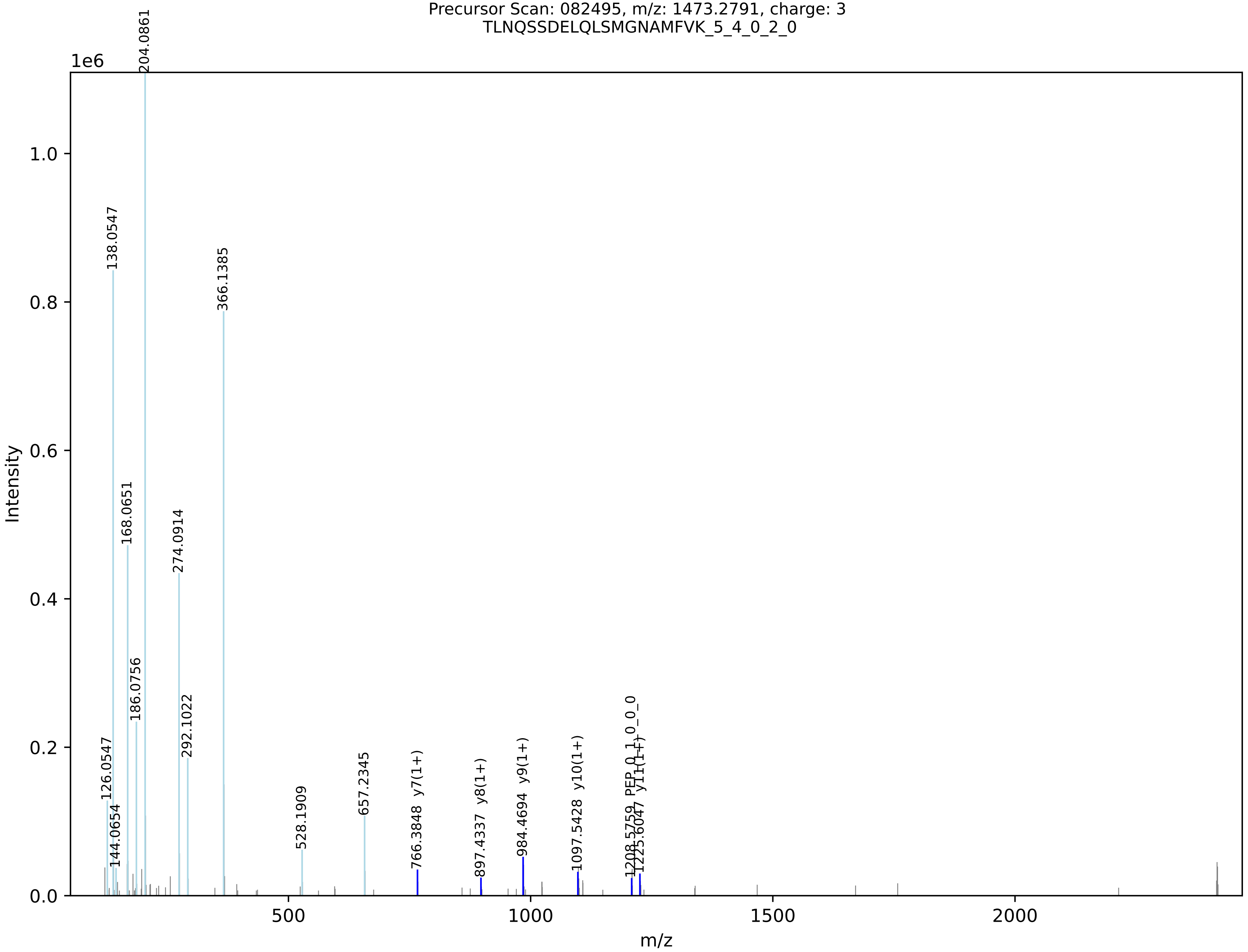

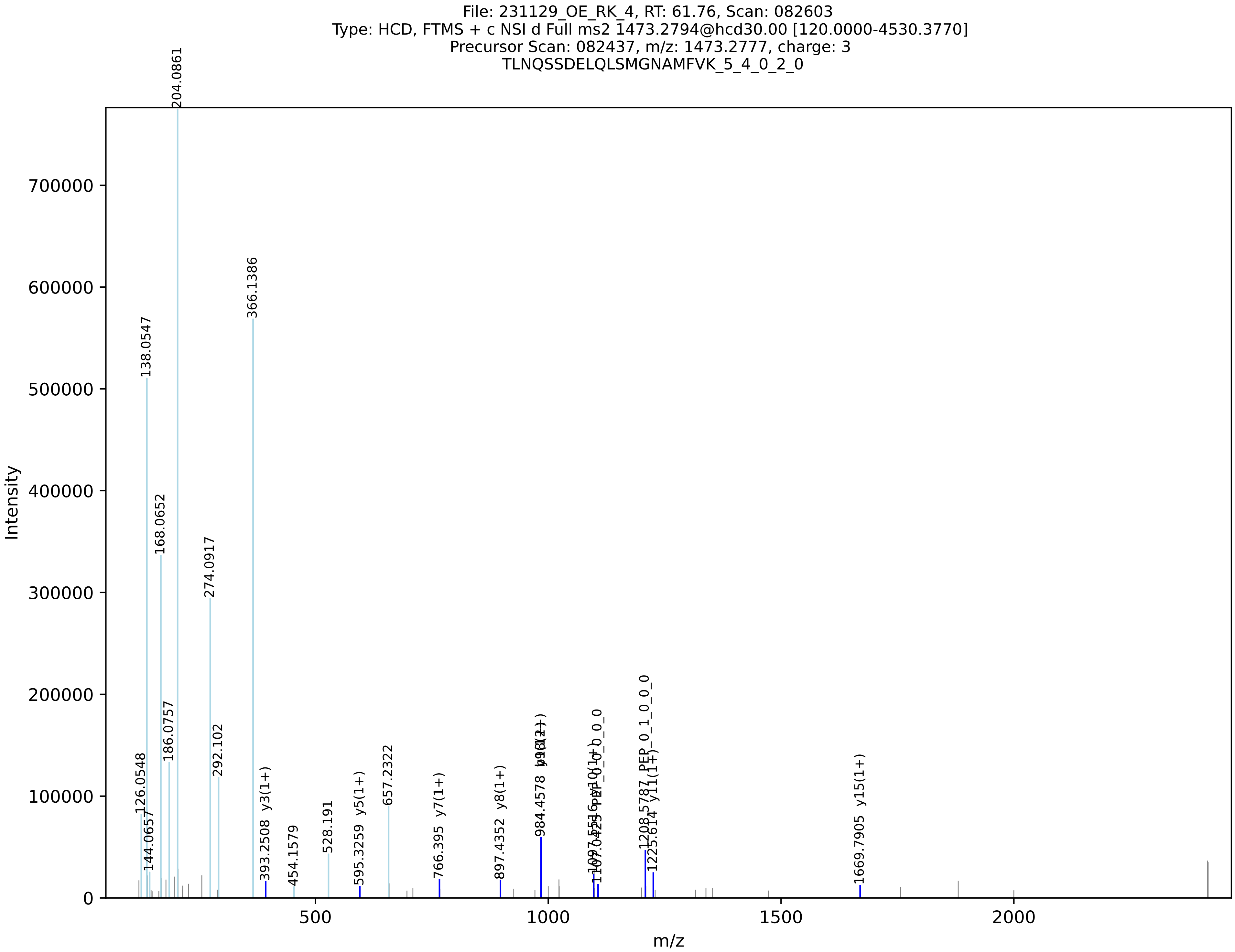
File: 231129_OE_RK_4, RT: 61.76, Scan: 082603
Type: HCD, FTMS + c NSI d Full ms2 1473.2794@hcd30.00 [120.0000-4530.3770]
Precursor Scan: 082437, m/z: 1473.2777, charge: 3
TLNQSSDELQLSMGNAMFVK_5_4_0_2_0
Intensity
m/z
0
100000
200000
300000
400000
500000
600000
700000
500
1000
1500
2000
204.0861
366.1386
138.0547
168.0652
274.0917
186.0757
292.102
126.0548
144.0657
393.2508 y3(1+)
454.1579
528.191
595.3259 y5(1+)
657.2322
766.395 y7(1+)
897.4352 y8(1+)
984.4578
1097.5516 y10(1+)
1107.0425 PEP_0_0_0_0
1208.5787 PEP_0_1_0_0_0
1225.614 y11(1+)
1669.7905 y15(1+)

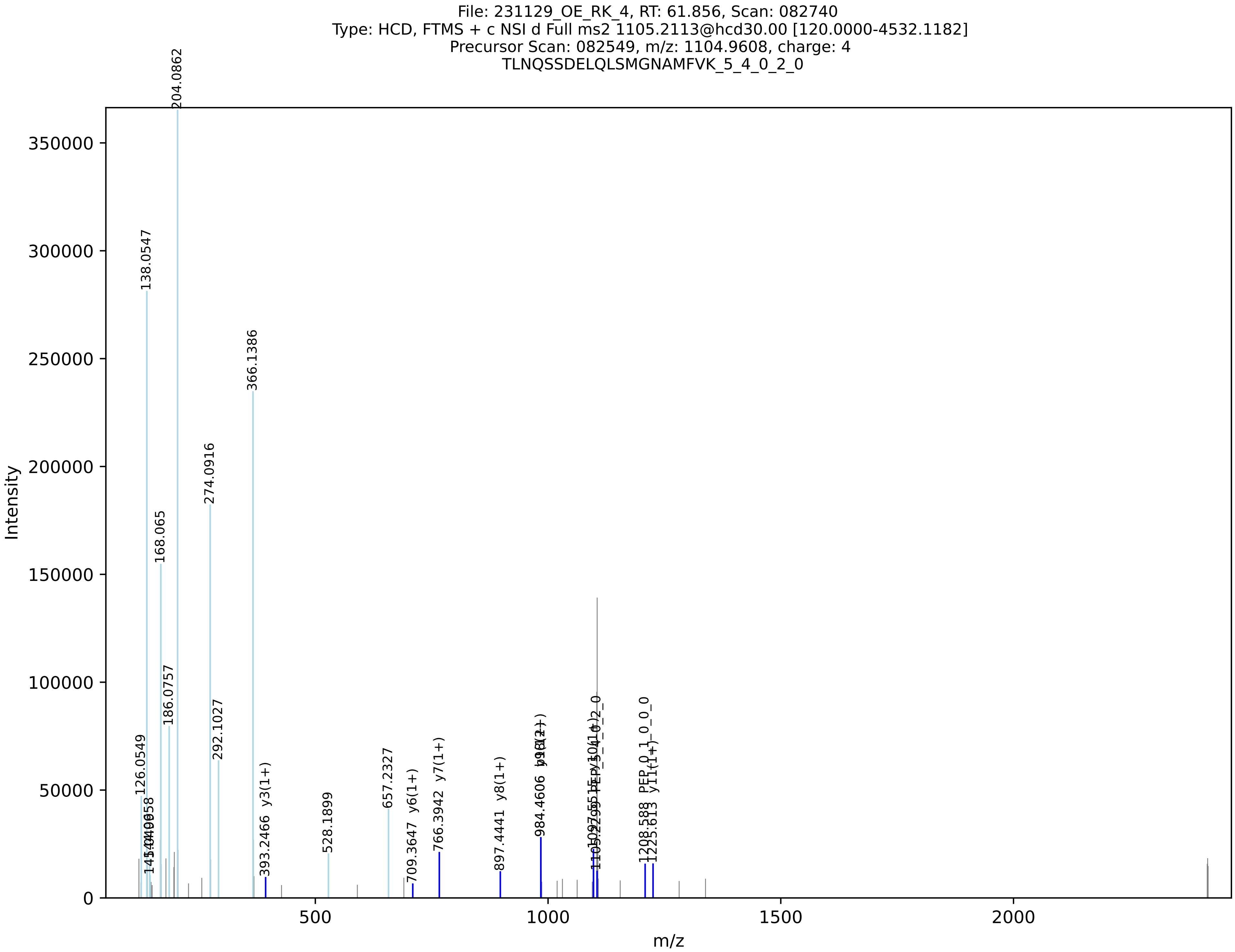
File: 231129_OE_RK_4, RT: 61.856, Scan: 082740
Type: HCD, FTMS + c NSI d Full ms2 1105.2113@hcd30.00 [120.0000-4532.1182]
Precursor Scan: 082549, m/z: 1104.9608, charge: 4
TLNQSSDELQLSMGNAMFVK_5_4_0_2_0
204.0862
138.0547
366.1386
274.0916
168.065
186.0757
292.1027
126.0549
393.2466 y3(1+)
528.1899
657.2327
709.3647 y6(1+)
766.3942 y7(1+)
897.4441 y8(1+)
984.4606
1208.588 PEP_0_1_0_0_0
1225.613 y11(1+)
Intensity
m/z
0
50000
100000
150000
200000
250000
300000
350000
500
1000
1500
2000

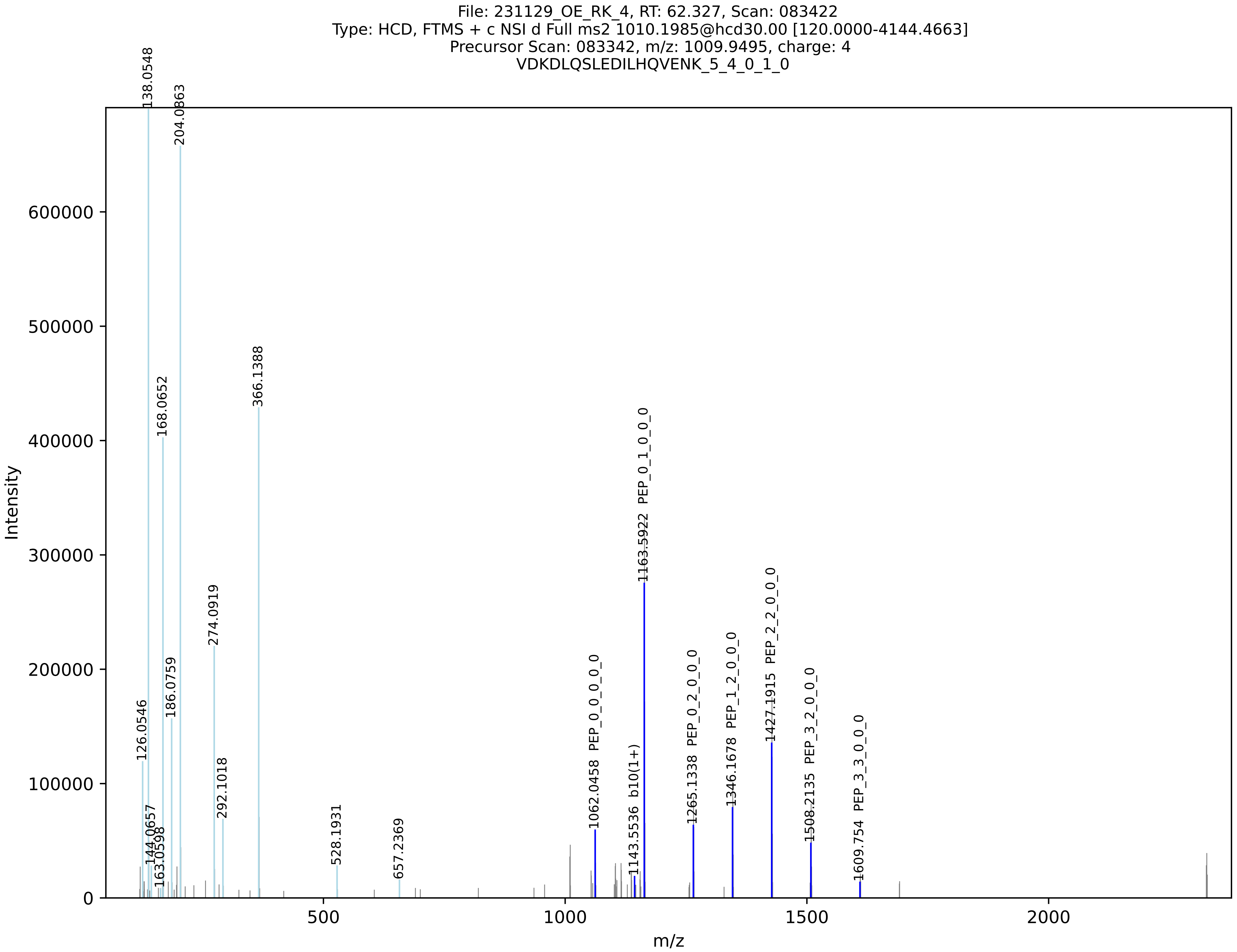
File: 231129_OE_RK_4, RT: 62.327, Scan: 083422
Type: HCD, FTMS + c NSI d Full ms2 1010.1985@hcd30.00 [120.0000-4144.4663]
Precursor Scan: 083342, m/z: 1009.9495, charge: 4
VDKDLQSLEDILHQVENK_5_4_0_1_0
Intensity
m/z
138.0548
204.0863
168.0652
366.1388
274.0919
186.0759
126.0546
292.1018
144.0657
163.0598
528.1931
657.2369
1062.0458 PEP_0_0_0_0_0
1143.5536 b10(1+)
1163.5922 PEP_0_1_0_0_0
1265.1338 PEP_0_2_0_0_0
1346.1678 PEP_1_2_0_0_0
1427.1915 PEP_2_2_0_0_0
1508.2135 PEP_3_2_0_0_0
1609.754 PEP_3_3_0_0_0

File: 231129_OE_RK_4, RT: 62.436, Scan: 083582
Type: HCD, FTMS + c NSI d Full ms2 1247.0389@hcd30.00 [120.0000-5110.7749]
Precursor Scan: 083490, m/z: 1246.7912, charge: 4
ITYSIVQTNCSKENFLFLTPDCK_5_4_0_2_0

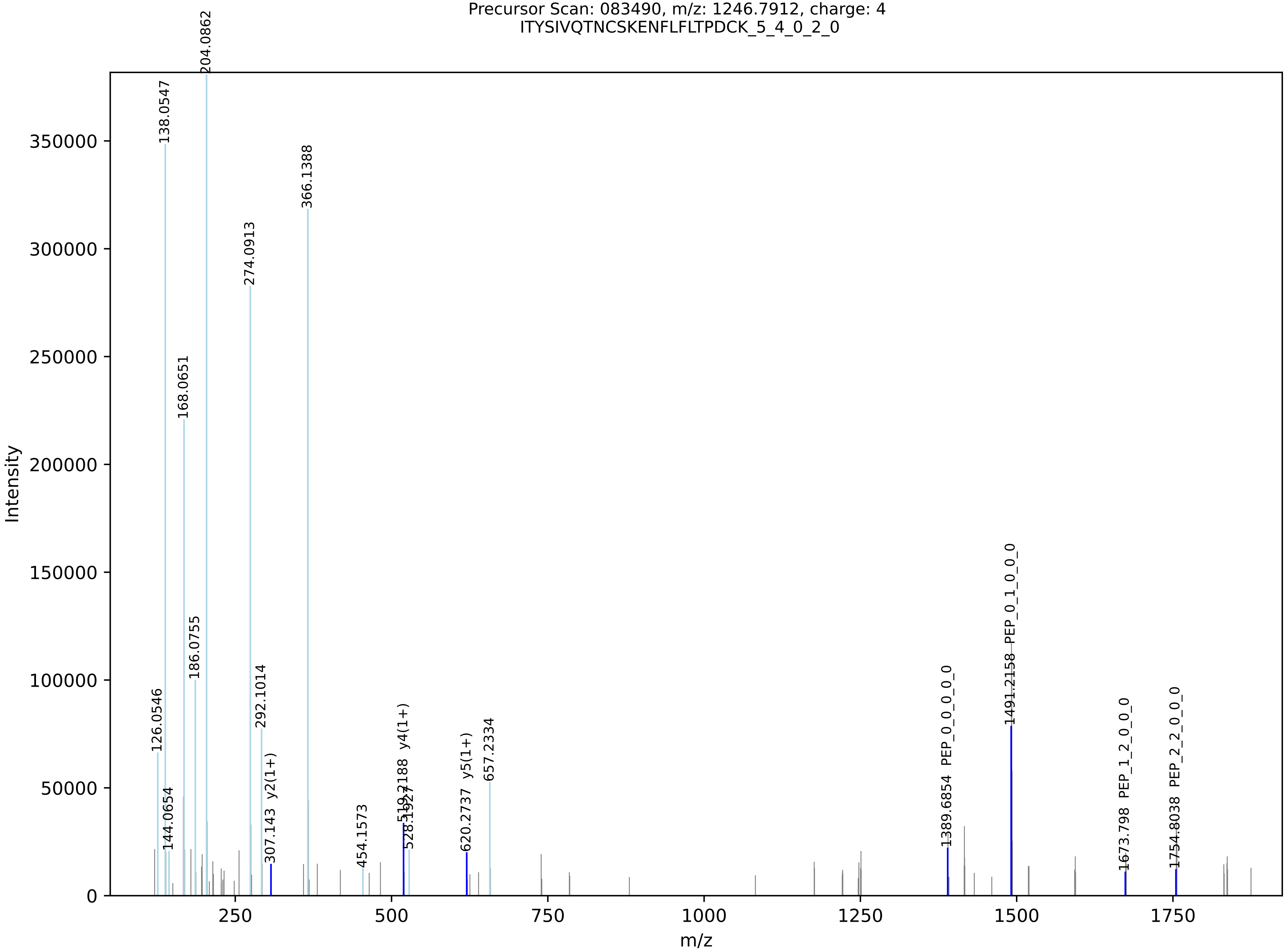

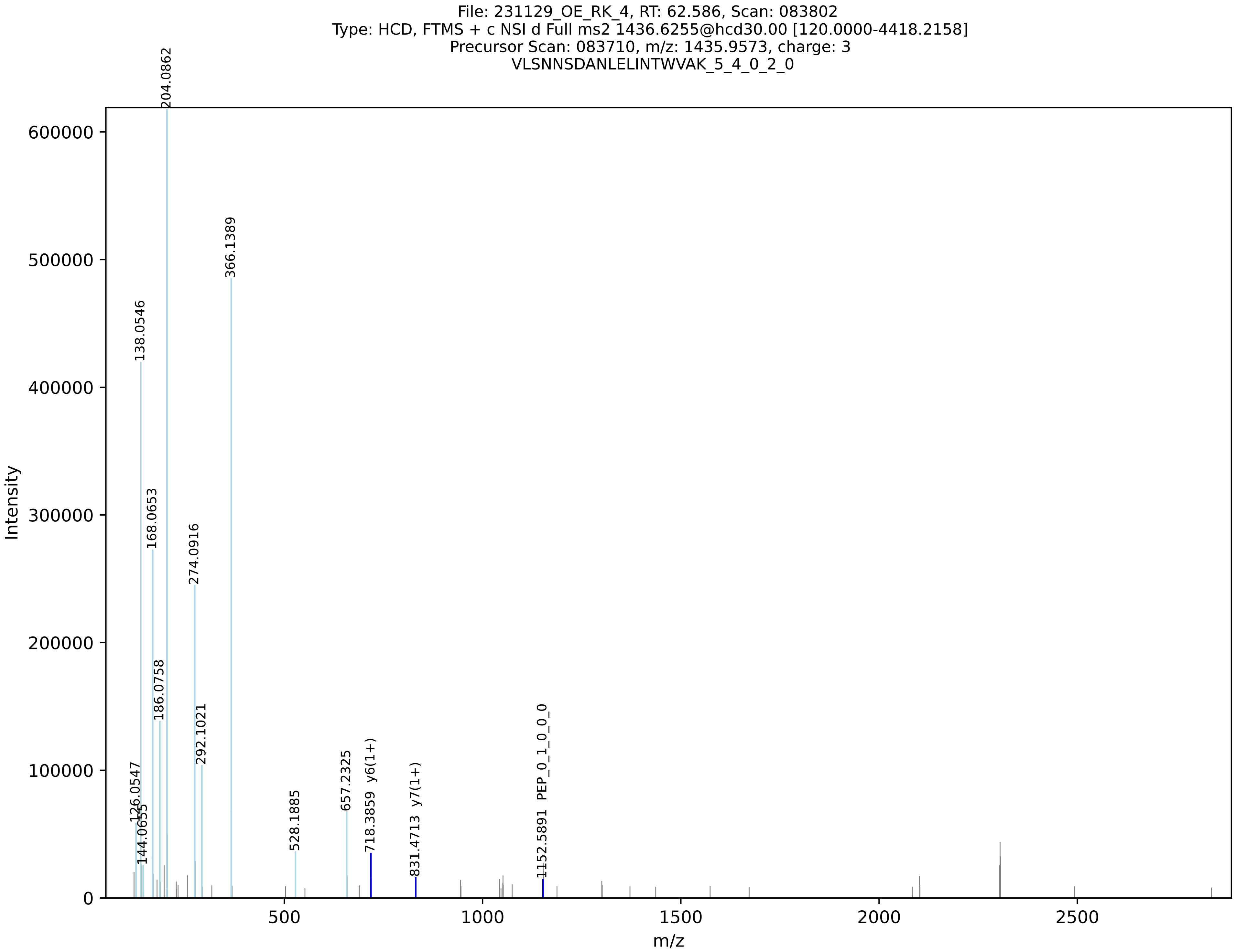
File: 231129_OE_RK_4, RT: 62.586, Scan: 083802
Type: HCD, FTMS + c NSI d Full ms2 1436.6255@hcd30.00 [120.0000-4418.2158]
Precursor Scan: 083710, m/z: 1435.9573, charge: 3
VLSNNSDANLELINTWVAK_5_4_0_2_0
204.0862
366.1389
138.0546
168.0653
274.0916
186.0758
292.1021
126.0547
144.0655
528.1885
657.2325
718.3859 y6(1+)
831.4713 y7(1+)
1152.5891 PEP_0_1_0_0_0
Intensity
m/z
0
100000
200000
300000
400000
500000
600000
500
1000
1500
2000
2500

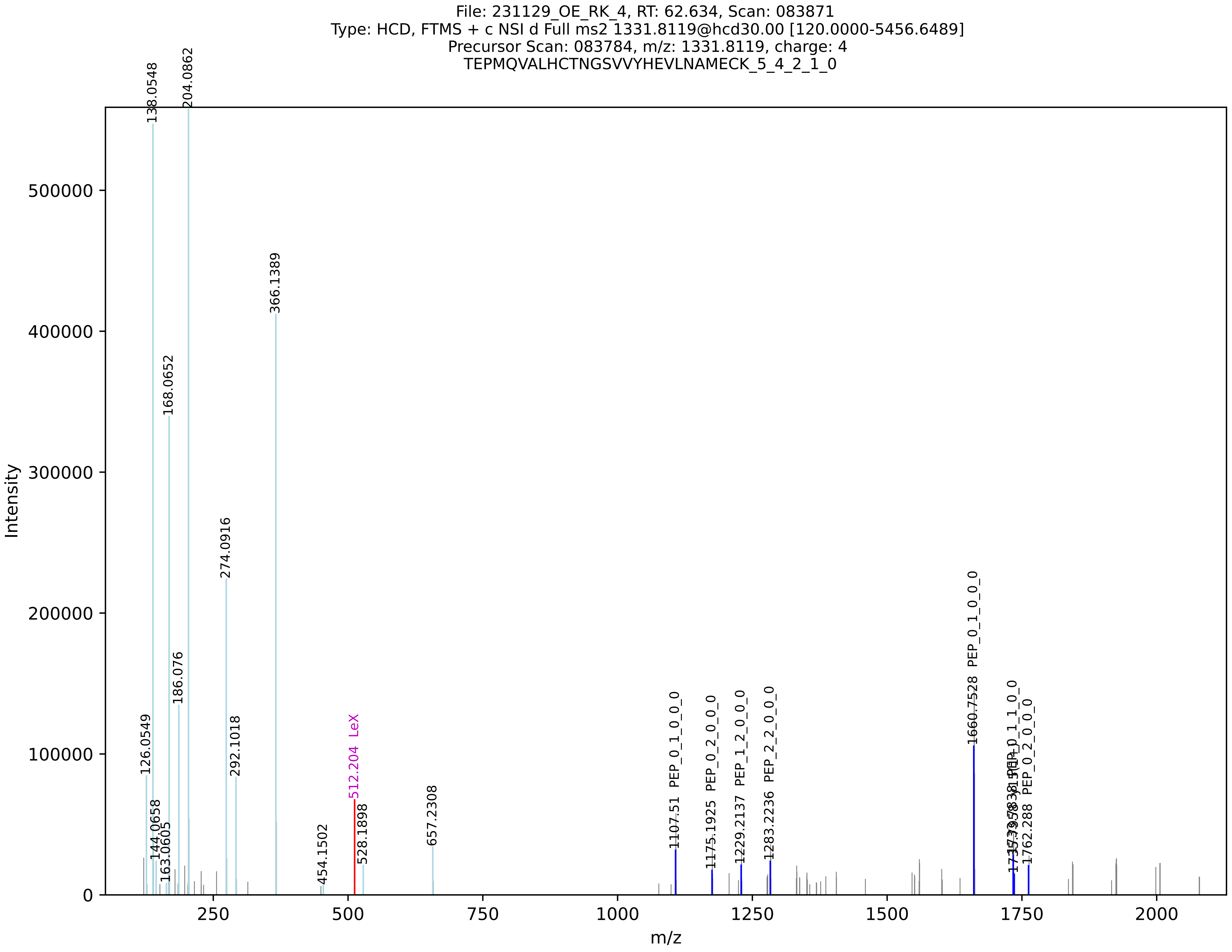
File: 231129_OE_RK_4, RT: 62.634, Scan: 083871
Type: HCD, FTMS + c NSI d Full ms2 1331.8119@hcd30.00 [120.0000-5456.6489]
Precursor Scan: 083784, m/z: 1331.8119, charge: 4
TEPMQVALHCTNGSVVYHEVLNAMECK_5_4_2_1_0
Intensity
m/z
0
100000
200000
300000
400000
500000
250
500
750
1000
1250
1500
1750
2000
138.0548
204.0862
366.1389
168.0652
274.0916
186.076
126.0549
292.1018
144.0658
163.0605
512.204 LeX
528.1898
454.1502
657.2308
1107.51 PEP_0_1_0_0_0
1175.1925 PEP_0_2_0_0_0
1229.2137 PEP_1_2_0_0_0
1283.2236 PEP_2_2_0_0_0
1660.7528 PEP_0_1_0_0_0
PEP_0_1_1_0_0
1762.288 PEP_0_2_0_0_0

File: 231129_OE_RK_4, RT: 62.685, Scan: 083944
Type: HCD, FTMS + c NSI d Full ms2 1395.6115@hcd30.00 [120.0000-5716.9507]
Precursor Scan: 083858, m/z: 1395.3603, charge: 4
FSLLGHASISCTVENETIGVWRPSPPTCEK_5_4_0_2_0

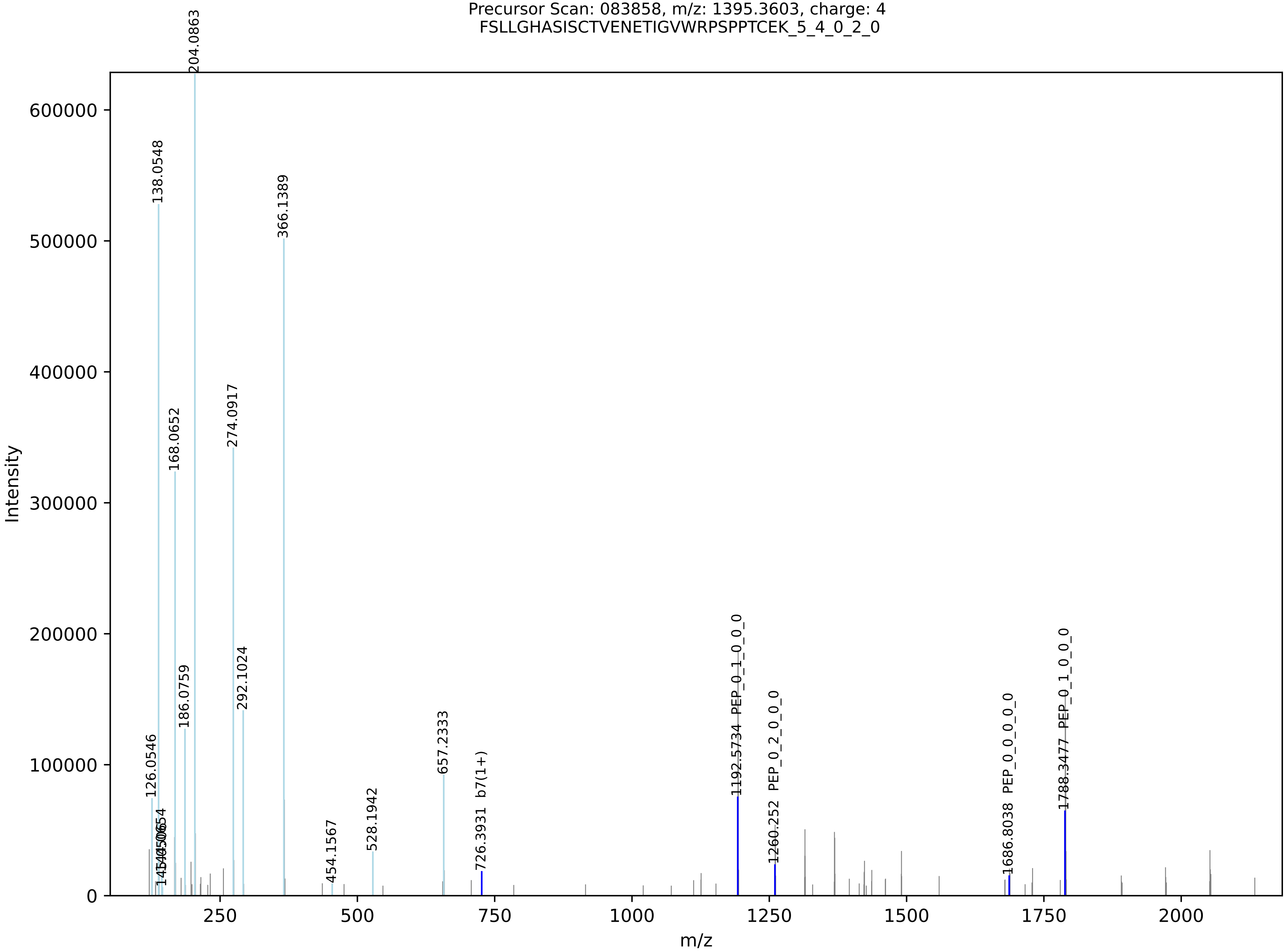

File: 231129_OE_RK_4, RT: 62.779, Scan: 084080
Type: HCD, FTMS + c NSI d Full ms2 1436.6295@hcd30.00 [120.0000-4418.2285]
Precursor Scan: 084003, m/z: 1435.9609, charge: 3
VLSNNSDANLELINTWVAK_5_4_0_2_0

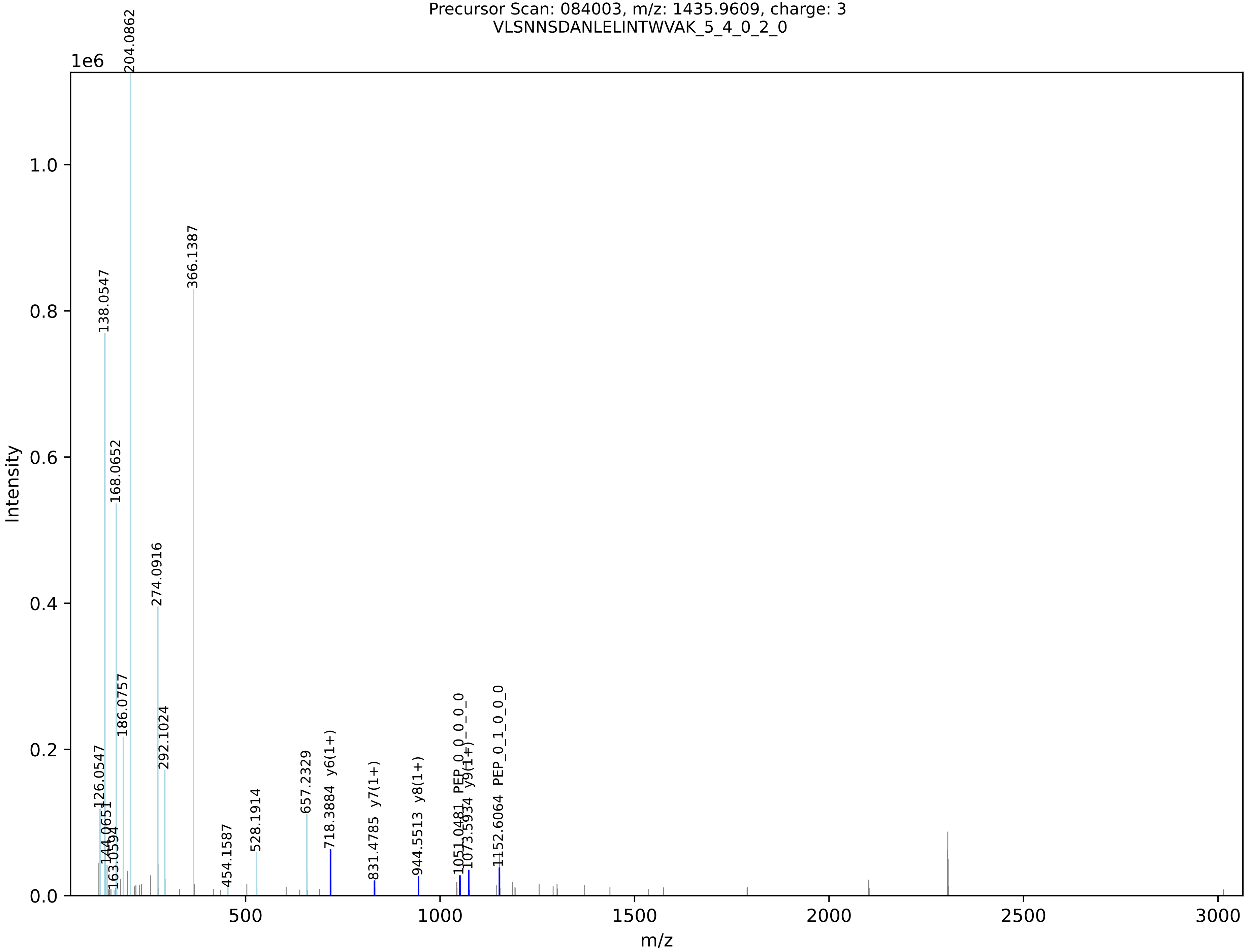

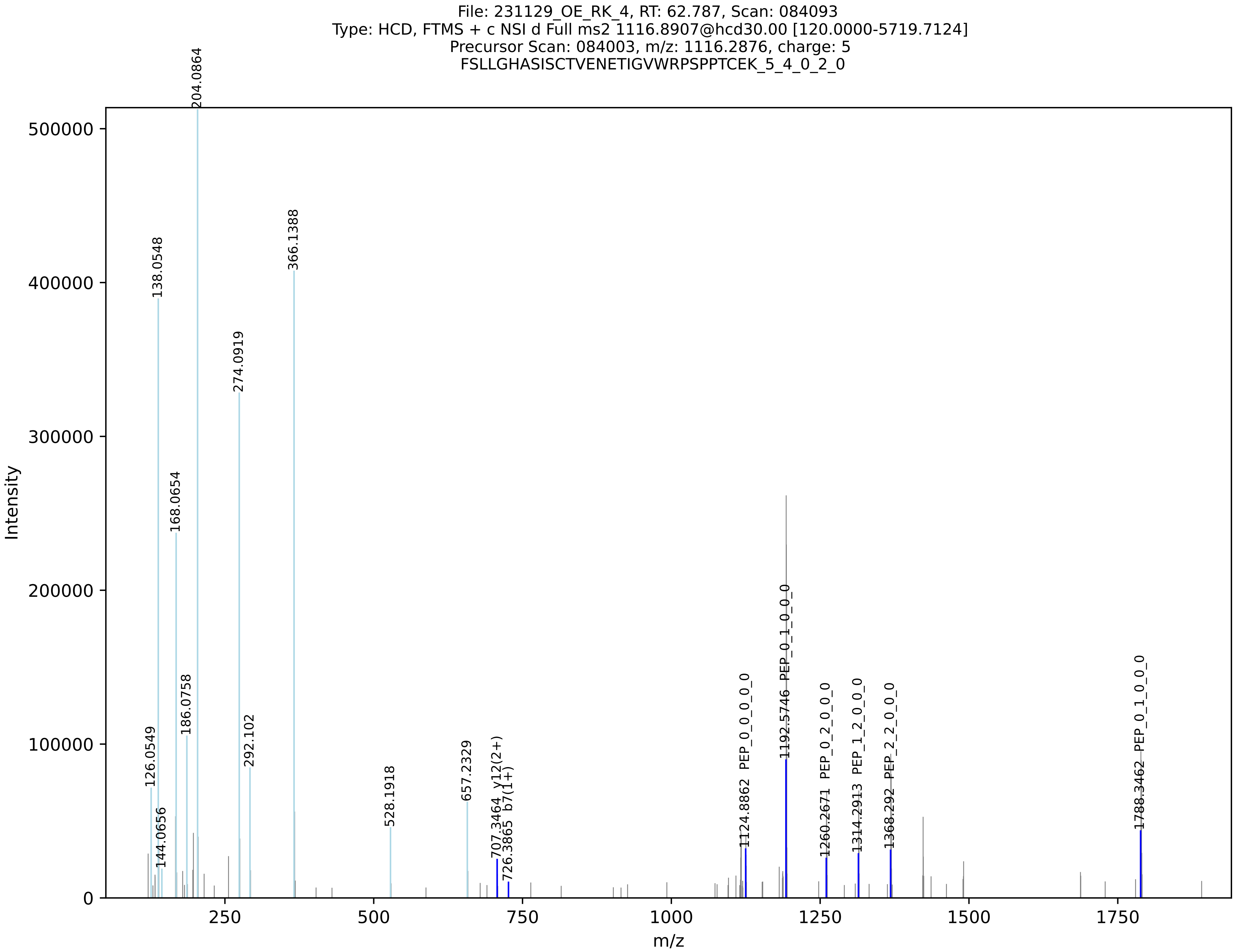
File: 231129_OE_RK_4, RT: 62.787, Scan: 084093
Type: HCD, FTMS + c NSI d Full ms2 1116.8907@hcd30.00 [120.0000-5719.7124]
Precursor Scan: 084003, m/z: 1116.2876, charge: 5
FSLLGHASISCTVENETIGVWRPSPPTCEK_5_4_0_2_0
Intensity
m/z
204.0864
138.0548
366.1388
274.0919
168.0654
186.0758
126.0549
144.0656
292.102
528.1918
657.2329
707.3464 y12(2+)
726.3865 b7(1+)
1124.8862 PEP_0_0_0_0_0
1192.5746 PEP_0_1_0_0_0
1260.2671 PEP_0_2_0_0_0
1314.2913 PEP_1_2_0_0_0
1368.292 PEP_2_2_0_0_0
1788.3462 PEP_0_1_0_0_0
0
100000
200000
300000
400000
500000
250
500
750
1000
1250
1500
1750

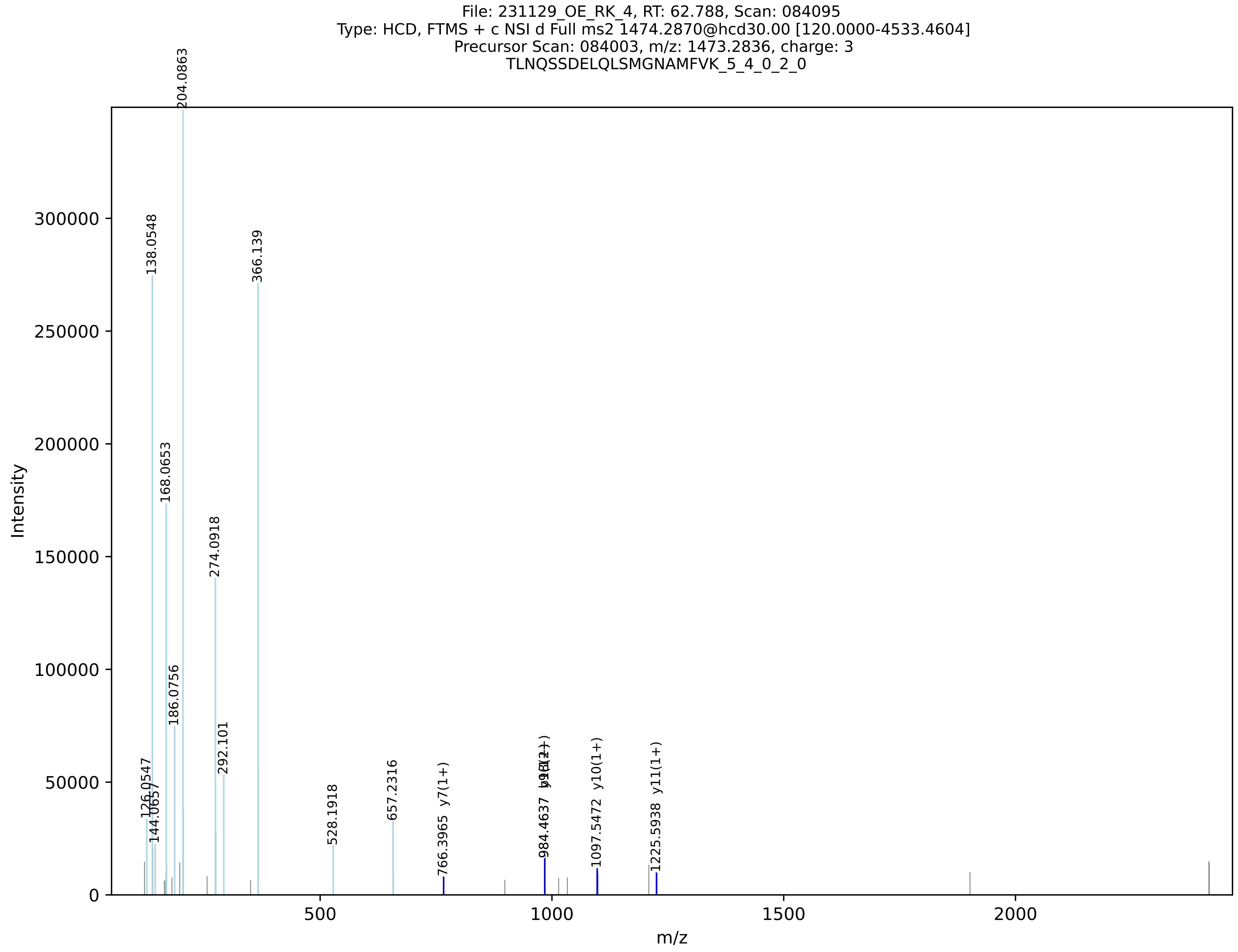

File: 231129_OE_RK_4, RT: 62.788, Scan: 084095
Type: HCD, FTMS + c NSI d Full ms2 1474.2870@hcd30.00 [120.0000-4533.4604]
Precursor Scan: 084003, m/z: 1473.2836, charge: 3
TLNQSSDELQLSMGNAMFVK_5_4_0_2_0
Intensity
m/z
204.0863
138.0548
366.139
168.0653
274.0918
186.0756
292.101
126.0547
144.0657
528.1918
657.2316
766.3965 y7(1+)
984.4637
1097.5472 y10(1+)
1225.5938 y11(1+)
0
50000
100000
150000
200000
250000
300000
500
1000
1500
2000

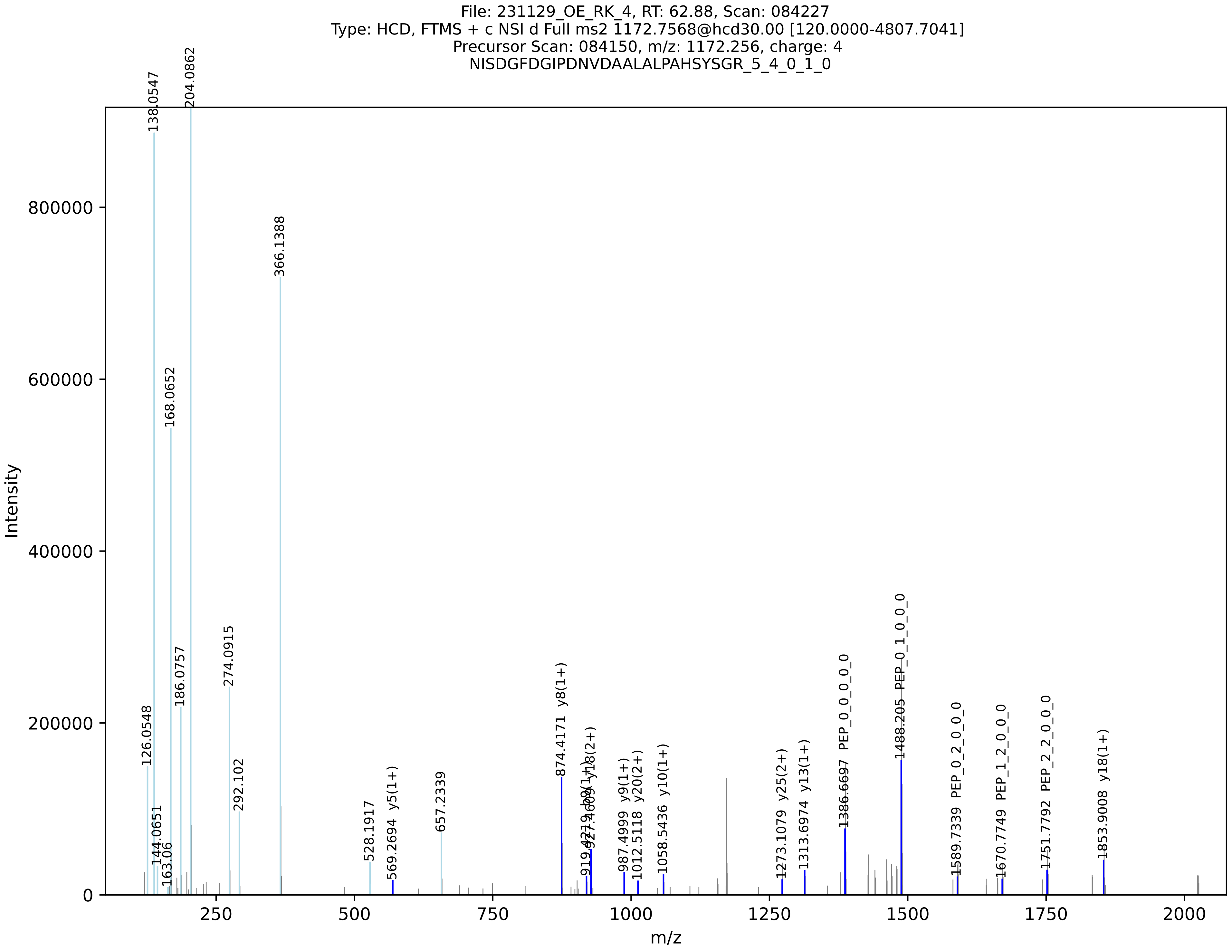

File: 231129_OE_RK_4, RT: 62.88, Scan: 084227
Type: HCD, FTMS + c NSI d Full ms2 1172.7568@hcd30.00 [120.0000-4807.7041]
Precursor Scan: 084150, m/z: 1172.256, charge: 4
NISDGFDGIPDNVDAALALPAHSYSGR_5_4_0_1_0
Intensity
m/z
138.0547
204.0862
366.1388
168.0652
186.0757
274.0915
126.0548
292.102
144.0651
163.06
528.1917
569.2694 y5(1+)
657.2339
874.4171 y8(1+)
919.4219 b9(1+)
927.4609 y18(2+)
987.4999 y9(1+)
1012.5118 y20(2+)
1058.5436 y10(1+)
1273.1079 y25(2+)
1313.6974 y13(1+)
1386.6697 PEP_0_0_0_0_0
1488.205 PEP_0_1_0_0_0
1589.7339 PEP_0_2_0_0_0
1670.7749 PEP_1_2_0_0_0
1751.7792 PEP_2_2_0_0_0
1853.9008 y18(1+)

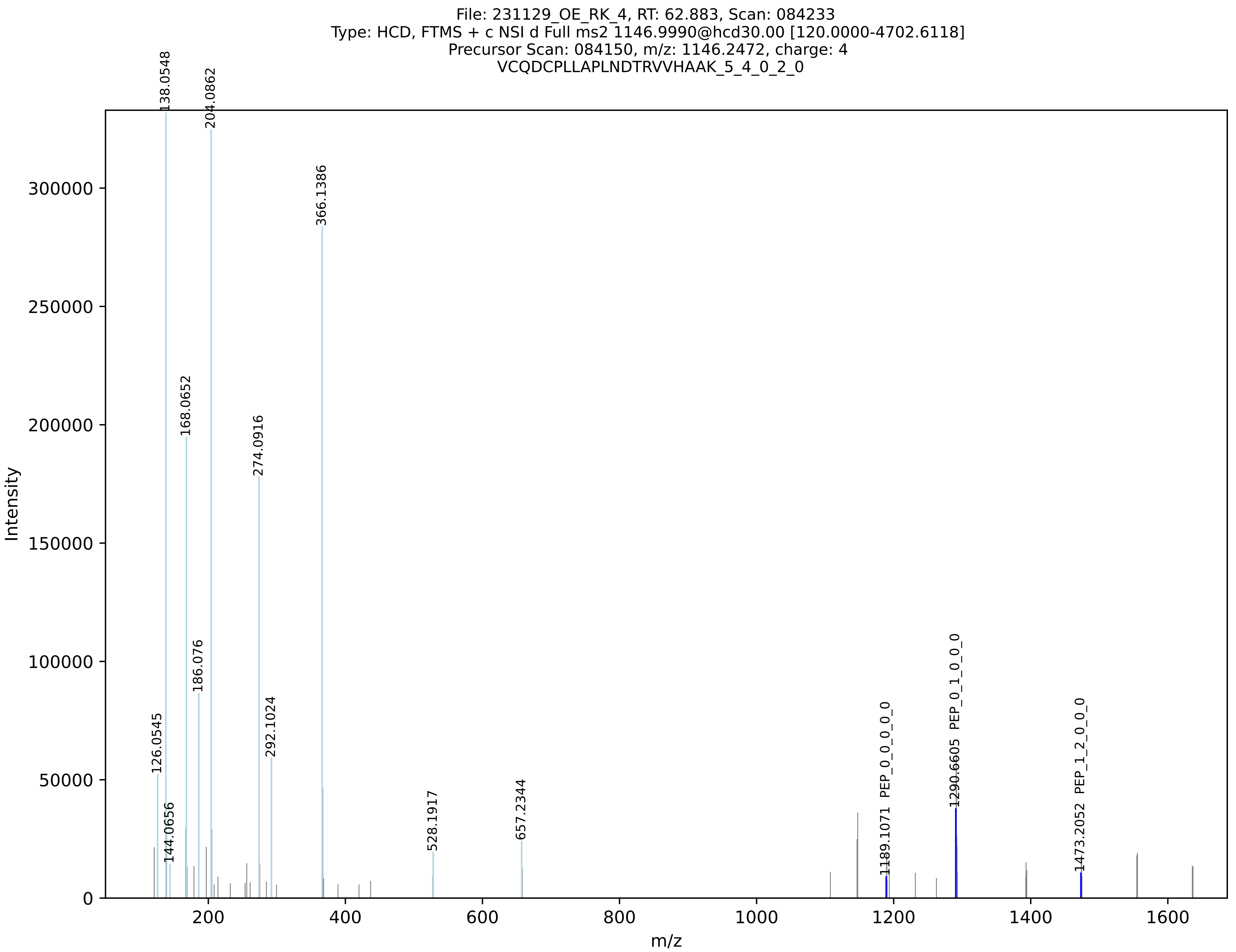
File: 231129_OE_RK_4, RT: 62.883, Scan: 084233
Type: HCD, FTMS + c NSI d Full ms2 1146.9990@hcd30.00 [120.0000-4702.6118]
Precursor Scan: 084150, m/z: 1146.2472, charge: 4
VCQDCPLLAPLNDTRVVHAAK_5_4_0_2_0
138.0548
204.0862
366.1386
168.0652
274.0916
186.076
126.0545
292.1024
144.0656
528.1917
657.2344
1189.1071 PEP_0_0_0_0_0
1290.6605 PEP_0_1_0_0_0
1473.2052 PEP_1_2_0_0_0
Intensity
m/z
0
50000
100000
150000
200000
250000
300000
200
400
600
800
1000
1200
1400
1600

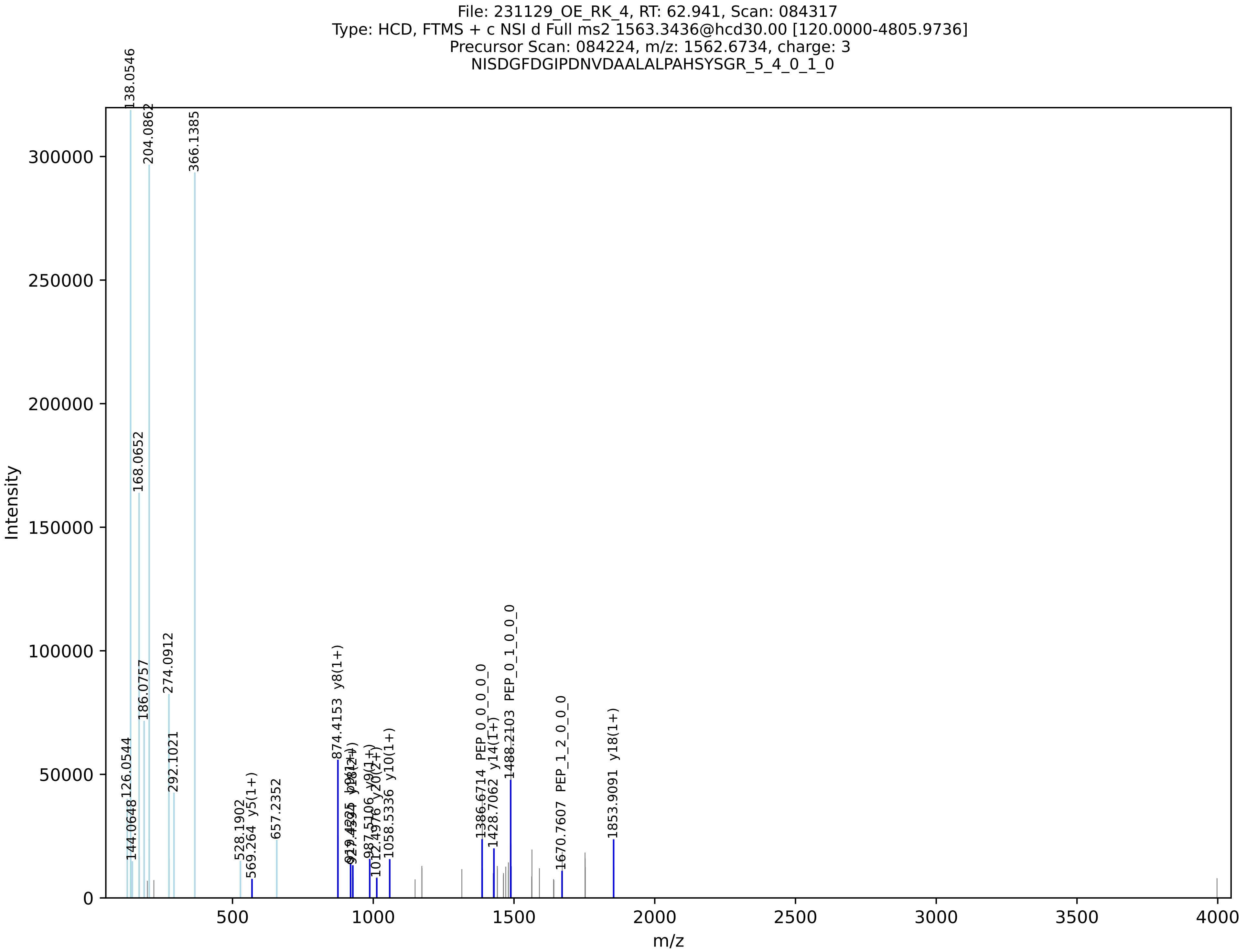

File: 231129_OE_RK_4, RT: 62.941, Scan: 084317
Type: HCD, FTMS + c NSI d Full ms2 1563.3436@hcd30.00 [120.0000-4805.9736]
Precursor Scan: 084224, m/z: 1562.6734, charge: 3
NISDGFDGIPDNVDAALALPAHSYSGR_5_4_0_1_0
Intensity
m/z
138.0546
204.0862
366.1385
168.0652
186.0757
274.0912
292.1021
126.0544
144.0648
528.1902
569.264 y5(1+)
657.2352
874.4153 y8(1+)
987.5106 y9(1+)
1012.4976 y20(2+)
1058.5336 y10(1+)
1386.6714 PEP_0_0_0_0_0
1428.7062 y14(1+)
1488.2103 PEP_0_1_0_0_0
1670.7607 PEP_1_2_0_0_0
1853.9091 y18(1+)

File: 231129_OE_RK_4, RT: 62.981, Scan: 084374
Type: HCD, FTMS + c NSI d Full ms2 1436.6302@hcd30.00 [120.0000-4418.2305]
Precursor Scan: 084298, m/z: 1435.962, charge: 3
VLSNNSDANLELINTWVAK_5_4_0_2_0

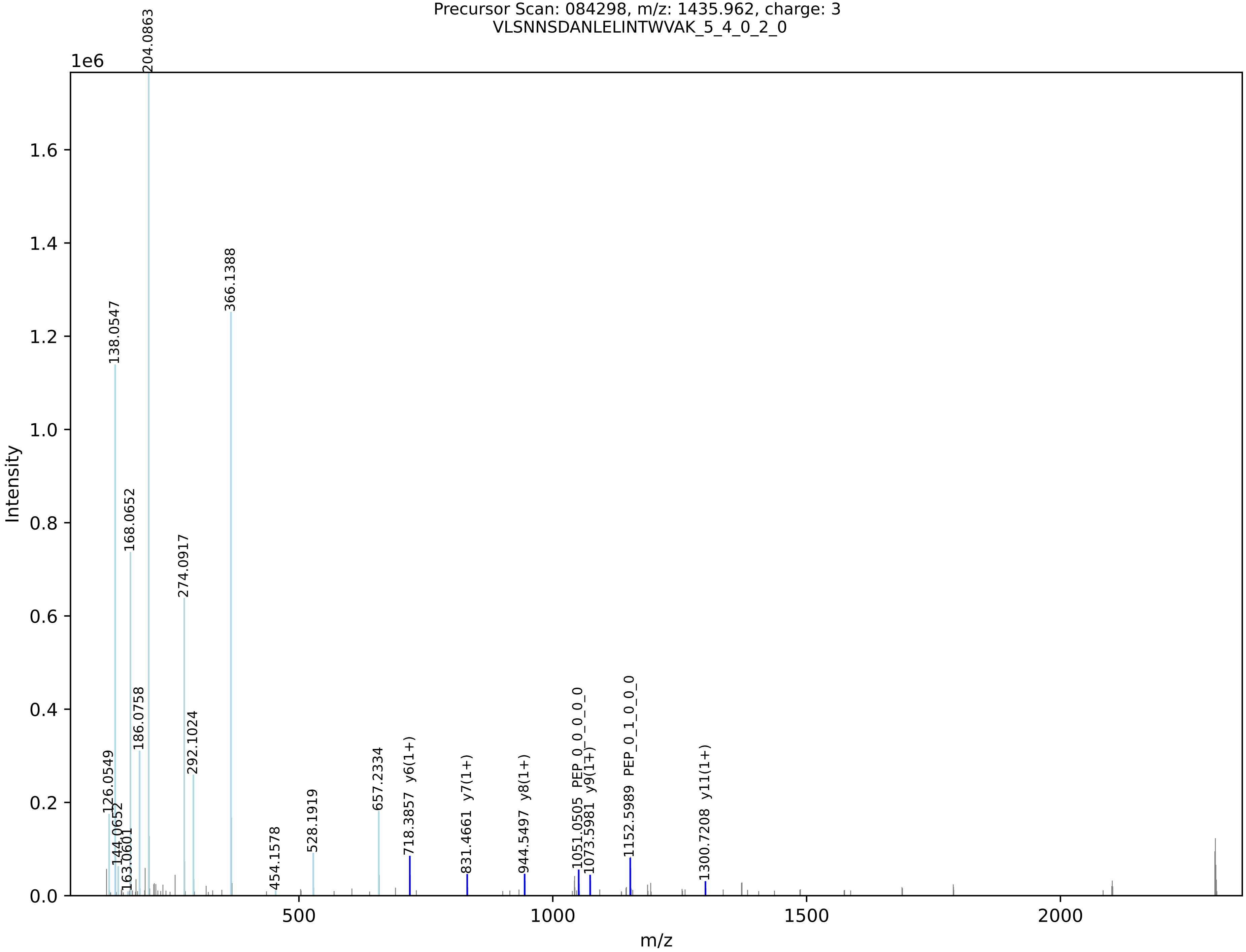

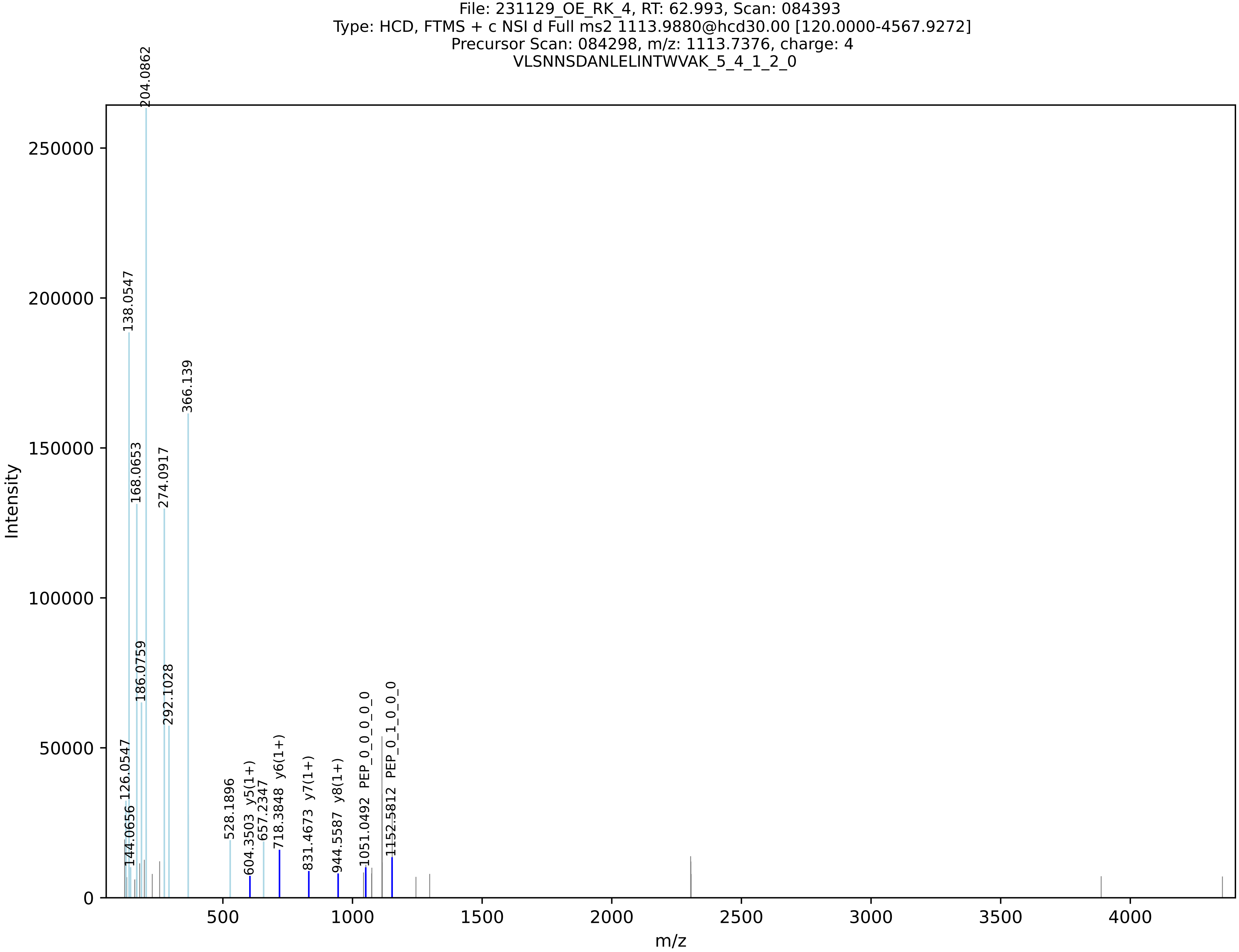
File: 231129_OE_RK_4, RT: 62.993, Scan: 084393
Type: HCD, FTMS + c NSI d Full ms2 1113.9880@hcd30.00 [120.0000-4567.9272]
Precursor Scan: 084298, m/z: 1113.7376, charge: 4
VLSNNSDANLELINTWVAK_5_4_1_2_0
Intensity
m/z
204.0862
138.0547
366.139
168.0653
274.0917
186.0759
292.1028
126.0547
144.0656
528.1896
604.3503 y5(1+)
657.2347
718.3848 y6(1+)
831.4673 y7(1+)
944.5587 y8(1+)
1051.0492 PEP_0_0_0_0_0
1152.5812 PEP_0_1_0_0_0
0
50000
100000
150000
200000
250000
500
1000
1500
2000
2500
3000
3500
4000

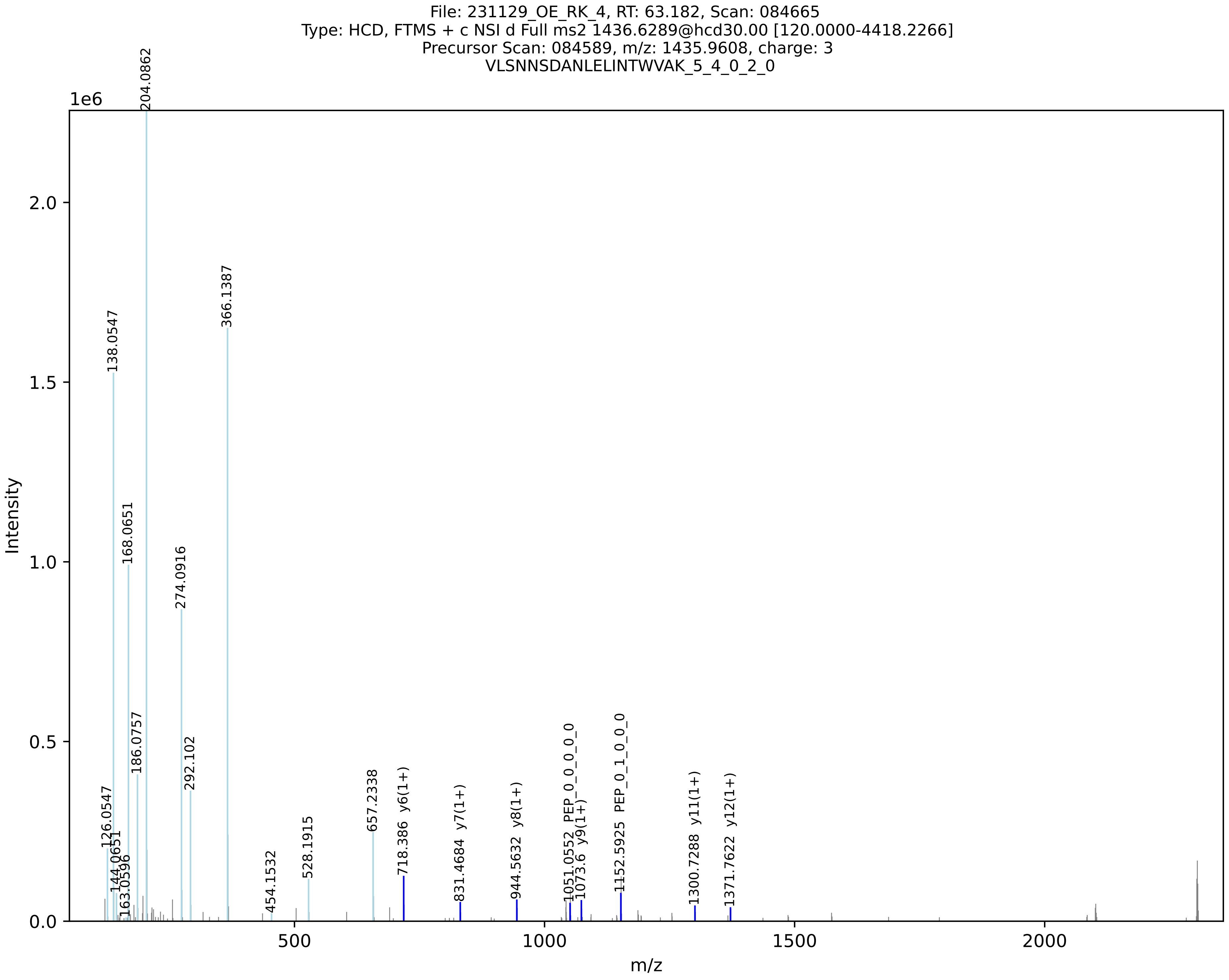
File: 231129_OE_RK_4, RT: 63.182, Scan: 084665
Type: HCD, FTMS + c NSI d Full ms2 1436.6289@hcd30.00 [120.0000-4418.2266]
Precursor Scan: 084589, m/z: 1435.9608, charge: 3
VLSNNSDANLELINTWVAK_5_4_0_2_0
1e6
Intensity
m/z
204.0862
366.1387
138.0547
168.0651
274.0916
186.0757
292.102
126.0547
144.0651
163.0596
454.1532
528.1915
657.2338
718.386 y6(1+)
831.4684 y7(1+)
944.5632 y8(1+)
1051.0552 PEP_0_0_0_0_0
1073.6 y9(1+)
1152.5925 PEP_0_1_0_0_0
1300.7288 y11(1+)
1371.7622 y12(1+)

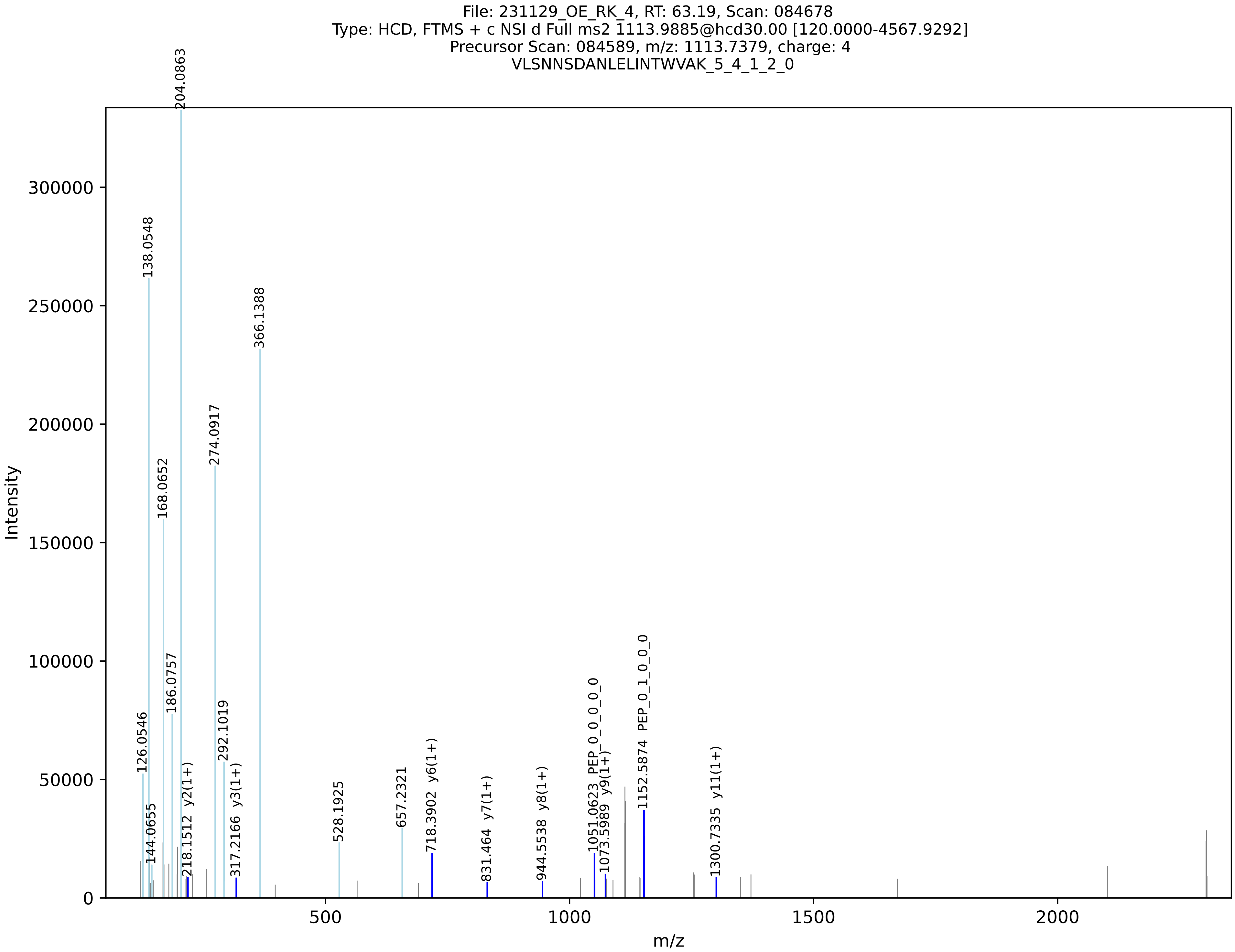
File: 231129_OE_RK_4, RT: 63.19, Scan: 084678
Type: HCD, FTMS + c NSI d Full ms2 1113.9885@hcd30.00 [120.0000-4567.9292]
Precursor Scan: 084589, m/z: 1113.7379, charge: 4
VLSNNSDANLELINTWVAK_5_4_1_2_0
204.0863
138.0548
366.1388
274.0917
168.0652
186.0757
126.0546
292.1019
144.0655
218.1512 y2(1+)
317.2166 y3(1+)
528.1925
657.2321
718.3902 y6(1+)
831.464 y7(1+)
944.5538 y8(1+)
1051.0623 PEP_0_0_0_0_0
1073.5989 y9(1+)
1152.5874 PEP_0_1_0_0_0
1300.7335 y11(1+)
Intensity
m/z
0
50000
100000
150000
200000
250000
300000
500
1000
1500
2000

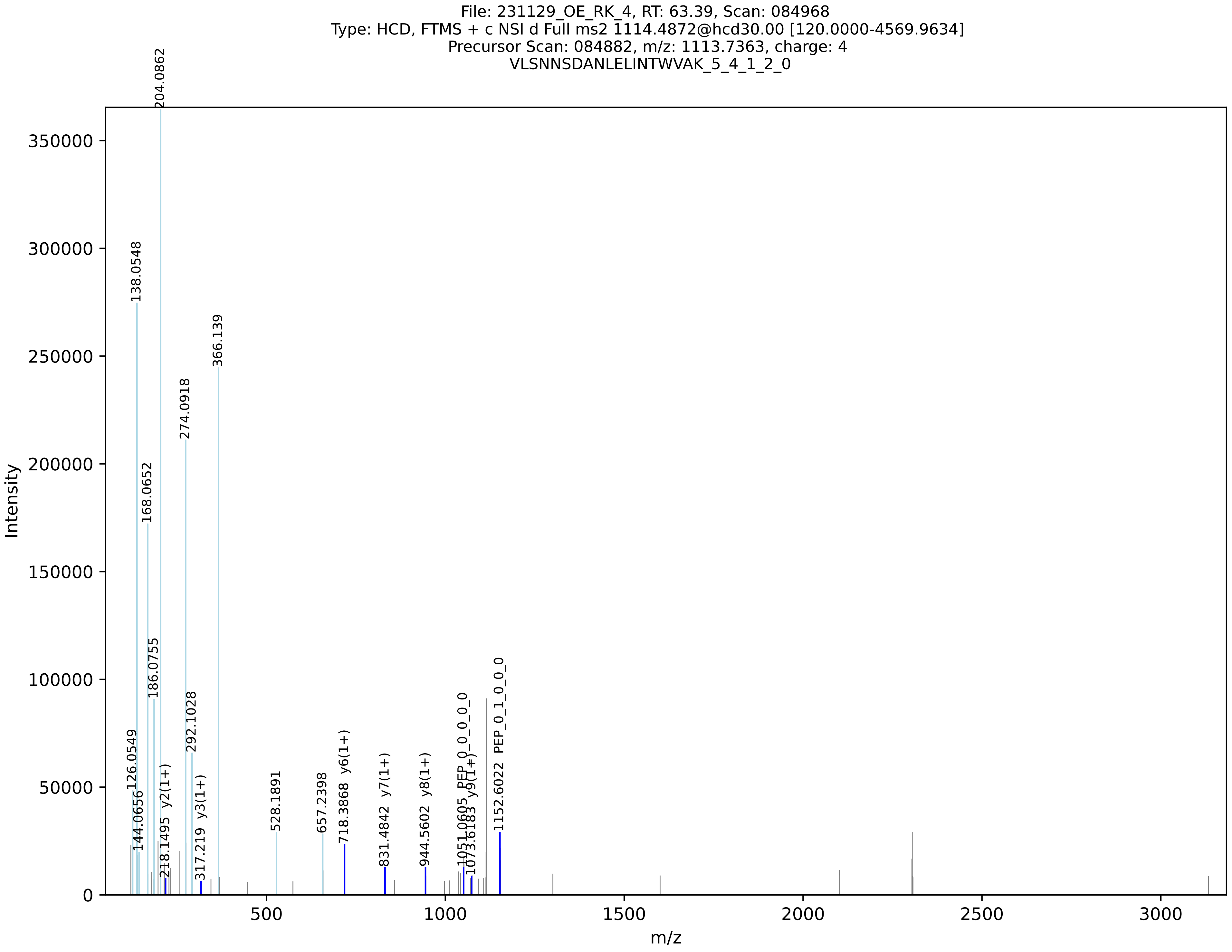

File: 231129_OE_RK_4, RT: 63.39, Scan: 084968
Type: HCD, FTMS + c NSI d Full ms2 1114.4872@hcd30.00 [120.0000-4569.9634]
Precursor Scan: 084882, m/z: 1113.7363, charge: 4
VLSNNSDANLELINTWVAK_5_4_1_2_0
Intensity
m/z
204.0862
138.0548
366.139
274.0918
168.0652
186.0755
292.1028
126.0549
144.0656
218.1495 y2(1+)
317.219 y3(1+)
528.1891
657.2398
718.3868 y6(1+)
831.4842 y7(1+)
944.5602 y8(1+)
1051.0605 PEP_0_0_0_0_0
1073.6183 y9(1+)
1152.6022 PEP_0_1_0_0_0

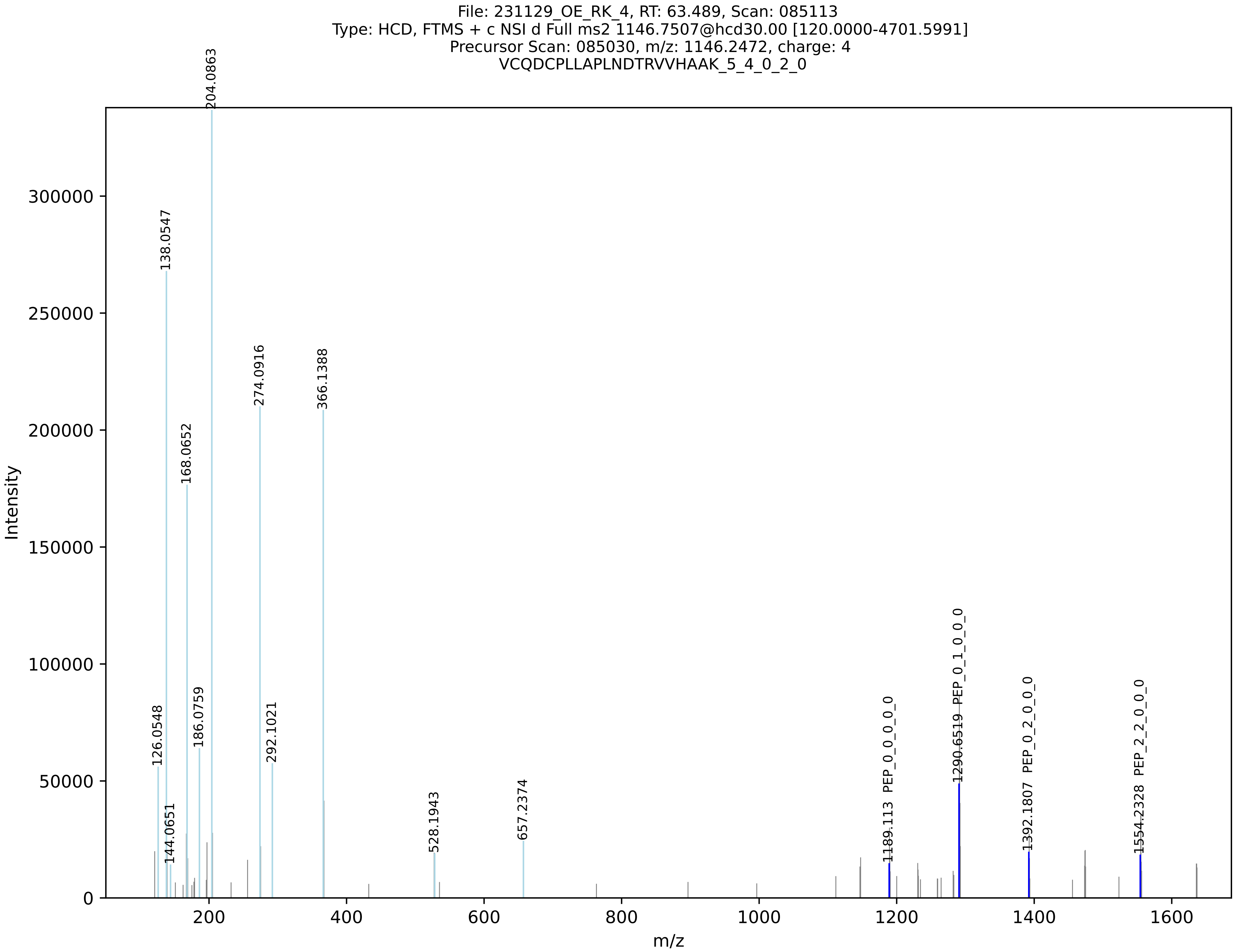
File: 231129_OE_RK_4, RT: 63.489, Scan: 085113
Type: HCD, FTMS + c NSI d Full ms2 1146.7507@hcd30.00 [120.0000-4701.5991]
Precursor Scan: 085030, m/z: 1146.2472, charge: 4
VCQDCPLLAPLNDTRVVHAAK_5_4_0_2_0
Intensity
m/z
204.0863
138.0547
274.0916
366.1388
168.0652
126.0548
186.0759
292.1021
144.0651
528.1943
657.2374
1189.113 PEP_0_0_0_0_0
1290.6519 PEP_0_1_0_0_0
1392.1807 PEP_0_2_0_0_0
1554.2328 PEP_2_2_0_0_0
0
50000
100000
150000
200000
250000
300000
200
400
600
800
1000
1200
1400
1600

File: 231129_OE_RK_4, RT: 63.586, Scan: 085254
Type: HCD, FTMS + c NSI d Full ms2 1436.6282@hcd30.00 [120.0000-4418.2241]
Precursor Scan: 085177, m/z: 1435.96, charge: 3
VLSNNSDANLELINTWVAK_5_4_0_2_0

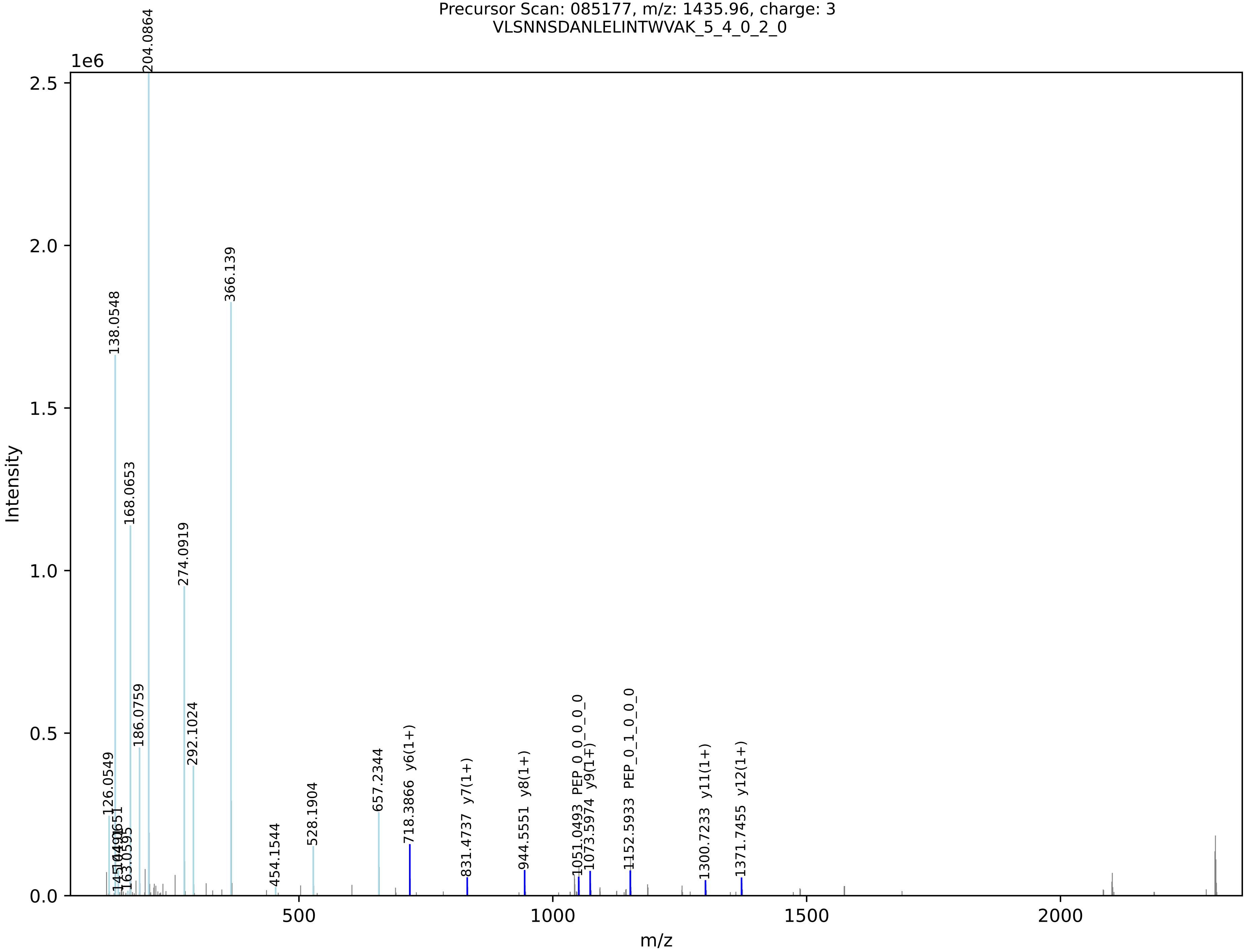

File: 231129_OE_RK_4, RT: 63.588, Scan: 085257
Type: HCD, FTMS + c NSI d Full ms2 1484.9790@hcd30.00 [120.0000-4566.1777]
Precursor Scan: 085177, m/z: 1484.6477, charge: 3
VLSNNSDANLELINTWVAK_5_4_1_2_0

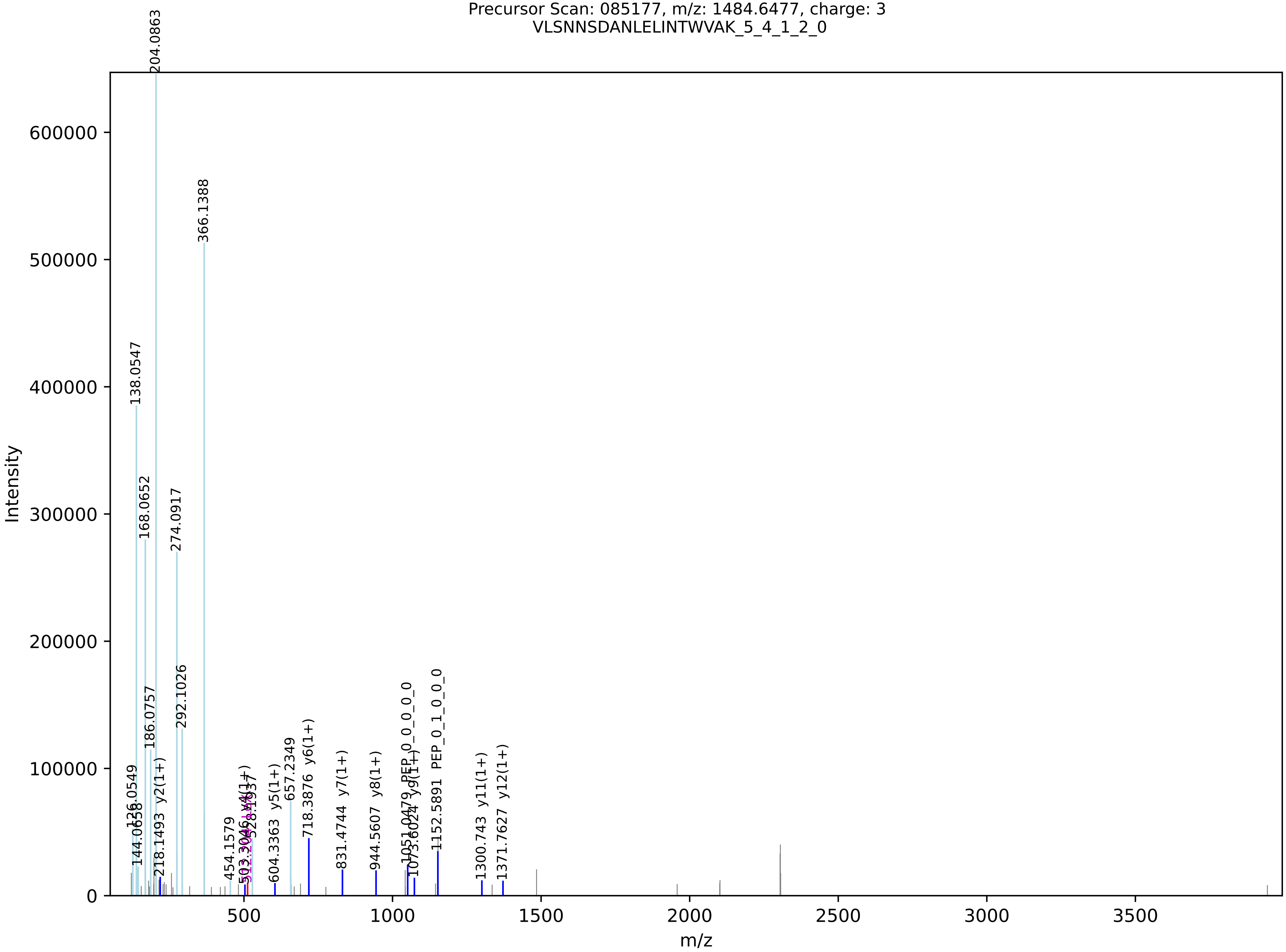

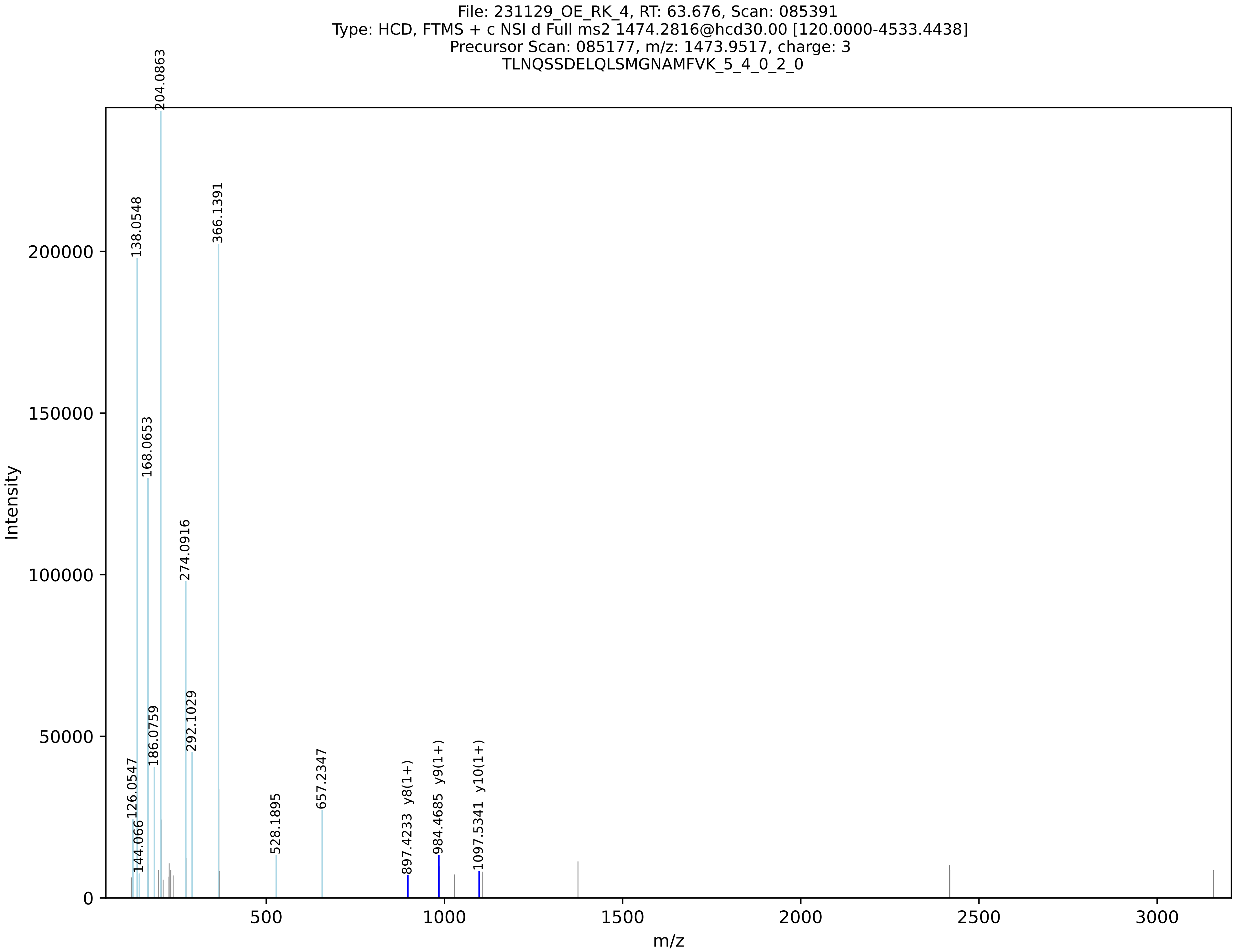

File: 231129_OE_RK_4, RT: 63.676, Scan: 085391
Type: HCD, FTMS + c NSI d Full ms2 1474.2816@hcd30.00 [120.0000-4533.4438]
Precursor Scan: 085177, m/z: 1473.9517, charge: 3
TLNQSSDELQLSMGNAMFVK_5_4_0_2_0
Intensity
m/z
204.0863
138.0548
366.1391
168.0653
274.0916
292.1029
186.0759
126.0547
144.066
528.1895
657.2347
897.4233 y8(1+)
984.4685 y9(1+)
1097.5341 y10(1+)
0
50000
100000
150000
200000
500
1000
1500
2000
2500
3000

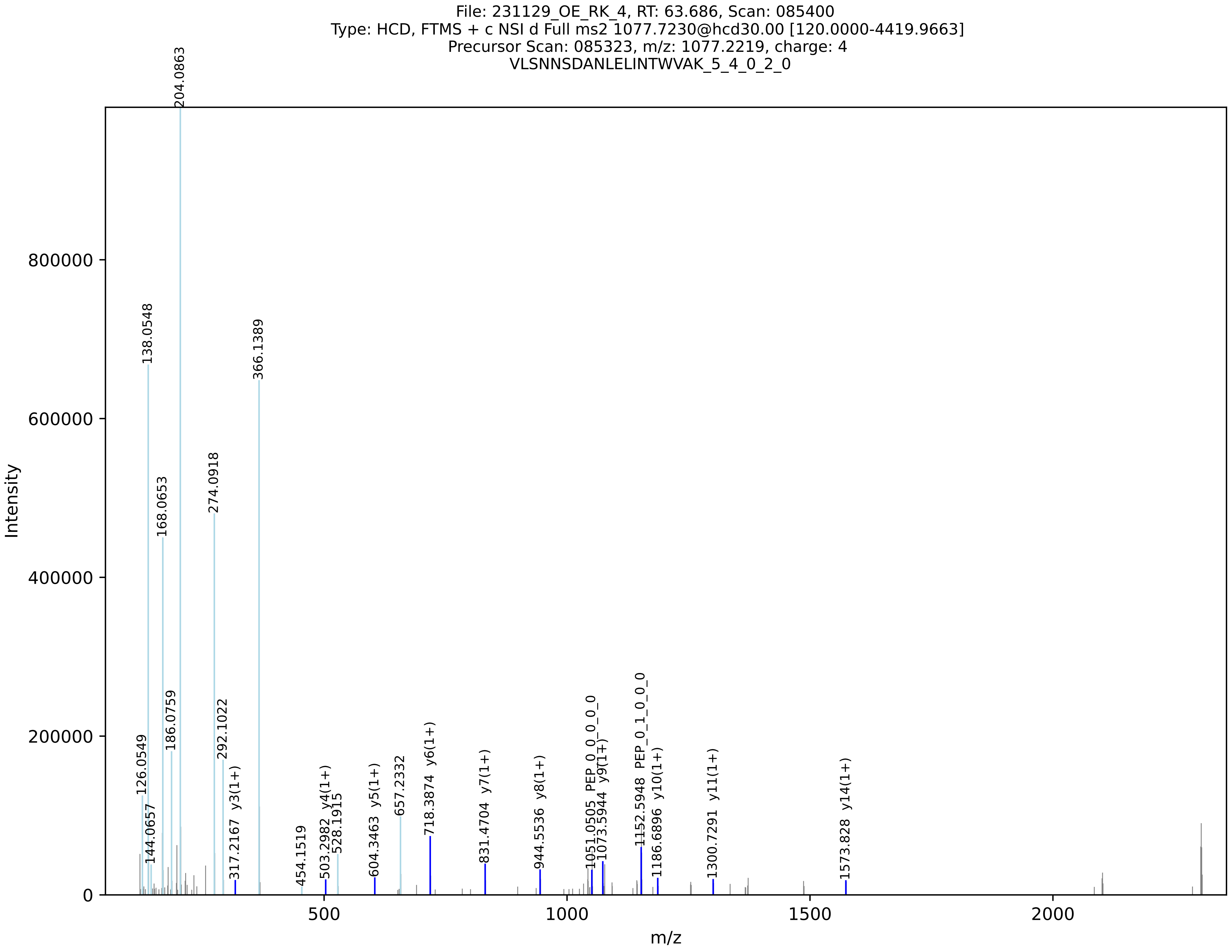

File: 231129_OE_RK_4, RT: 63.686, Scan: 085400
Type: HCD, FTMS + c NSI d Full ms2 1077.7230@hcd30.00 [120.0000-4419.9663]
Precursor Scan: 085323, m/z: 1077.2219, charge: 4
VLSNNSDANLELINTWVAK_5_4_0_2_0
Intensity
m/z
0
200000
400000
600000
800000
500
1000
1500
2000
204.0863
138.0548
366.1389
274.0918
168.0653
186.0759
292.1022
126.0549
144.0657
317.2167 y3(1+)
454.1519
503.2982 y4(1+)
528.1915
604.3463 y5(1+)
657.2332
718.3874 y6(1+)
831.4704 y7(1+)
944.5536 y8(1+)
1051.0505 PEP_0_0_0_0_0
1073.5944 y9(1+)
1152.5948 PEP_0_1_0_0_0
1186.6896 y10(1+)
1300.7291 y11(1+)
1573.828 y14(1+)

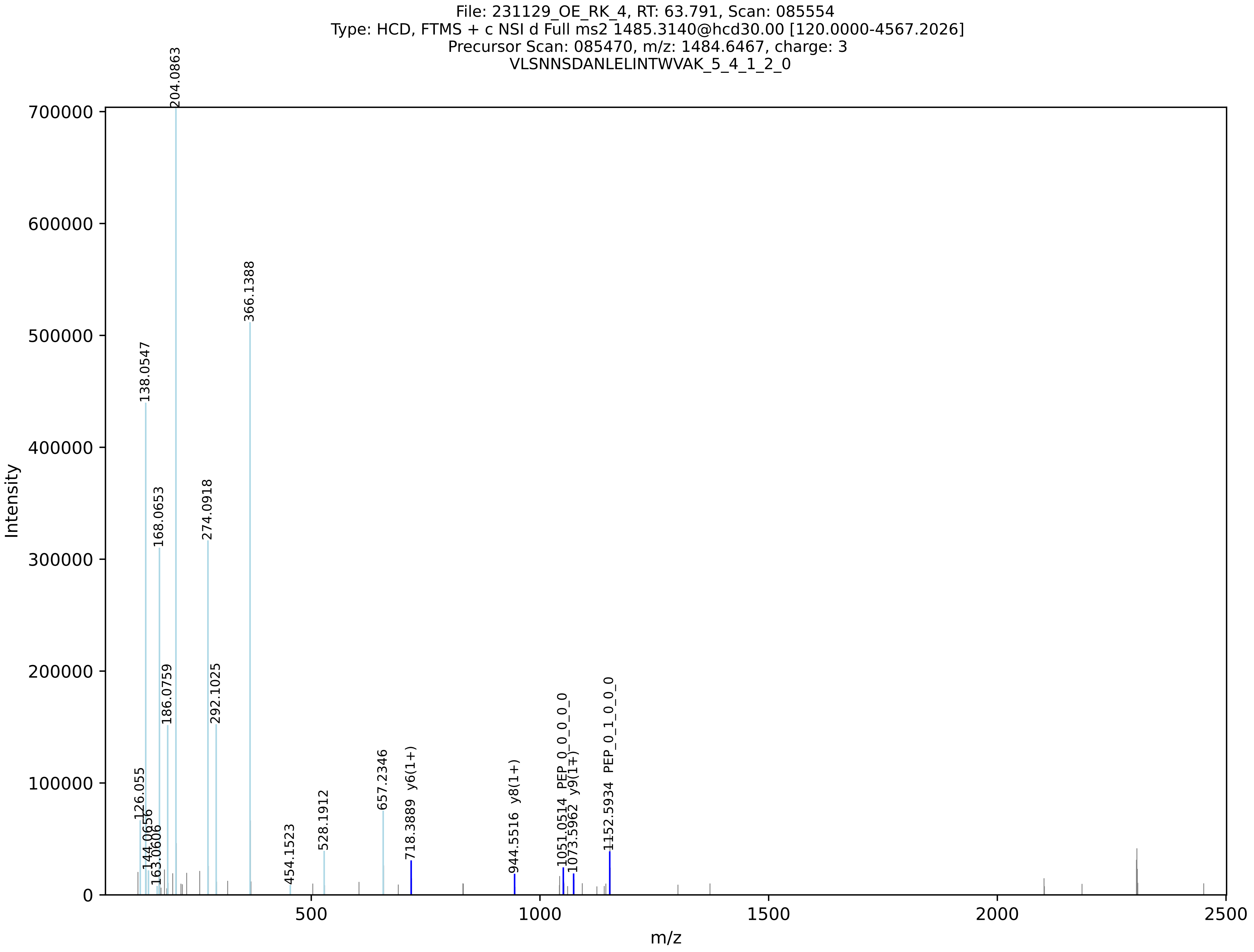

File: 231129_OE_RK_4, RT: 63.791, Scan: 085554
Type: HCD, FTMS + c NSI d Full ms2 1485.3140@hcd30.00 [120.0000-4567.2026]
Precursor Scan: 085470, m/z: 1484.6467, charge: 3
VLSNNSDANLELINTWVAK_5_4_1_2_0
Intensity
m/z
204.0863
366.1388
138.0547
168.0653
274.0918
186.0759
292.1025
126.055
144.0656
163.0606
454.1523
528.1912
657.2346
718.3889 y6(1+)
944.5516 y8(1+)
1051.0514 PEP_0_0_0_0_0
1073.5962 y9(1+)
1152.5934 PEP_0_1_0_0_0

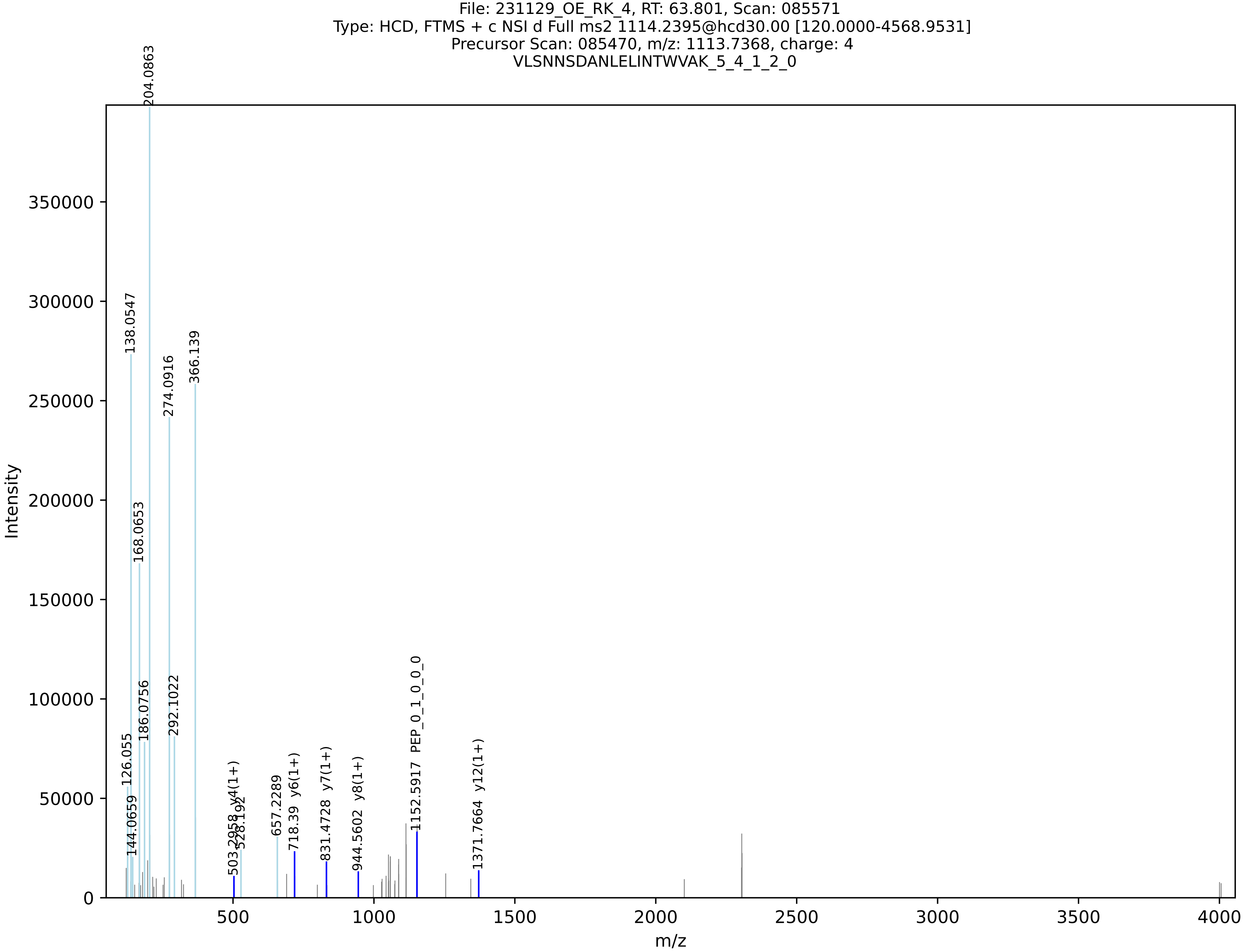

File: 231129_OE_RK_4, RT: 63.801, Scan: 085571
Type: HCD, FTMS + c NSI d Full ms2 1114.2395@hcd30.00 [120.0000-4568.9531]
Precursor Scan: 085470, m/z: 1113.7368, charge: 4
VLSNNSDANLELINTWVAK_5_4_1_2_0
204.0863
138.0547
366.139
274.0916
168.0653
186.0756
292.1022
126.055
144.0659
503.2958 y4(1+)
528.192
657.2289
718.39 y6(1+)
831.4728 y7(1+)
944.5602 y8(1+)
1152.5917 PEP_0_1_0_0_0
1371.7664 y12(1+)
Intensity
m/z
0
50000
100000
150000
200000
250000
300000
350000
500
1000
1500
2000
2500
3000
3500
4000

File: 231129_OE_RK_4, RT: 63.887, Scan: 085695
Type: HCD, FTMS + c NSI d Full ms2 1156.8856@hcd30.00 [120.0000-3562.2122]
Precursor Scan: 085618, m/z: 1156.2174, charge: 3
KLPPGLLANFTLLR_5_4_0_1_0

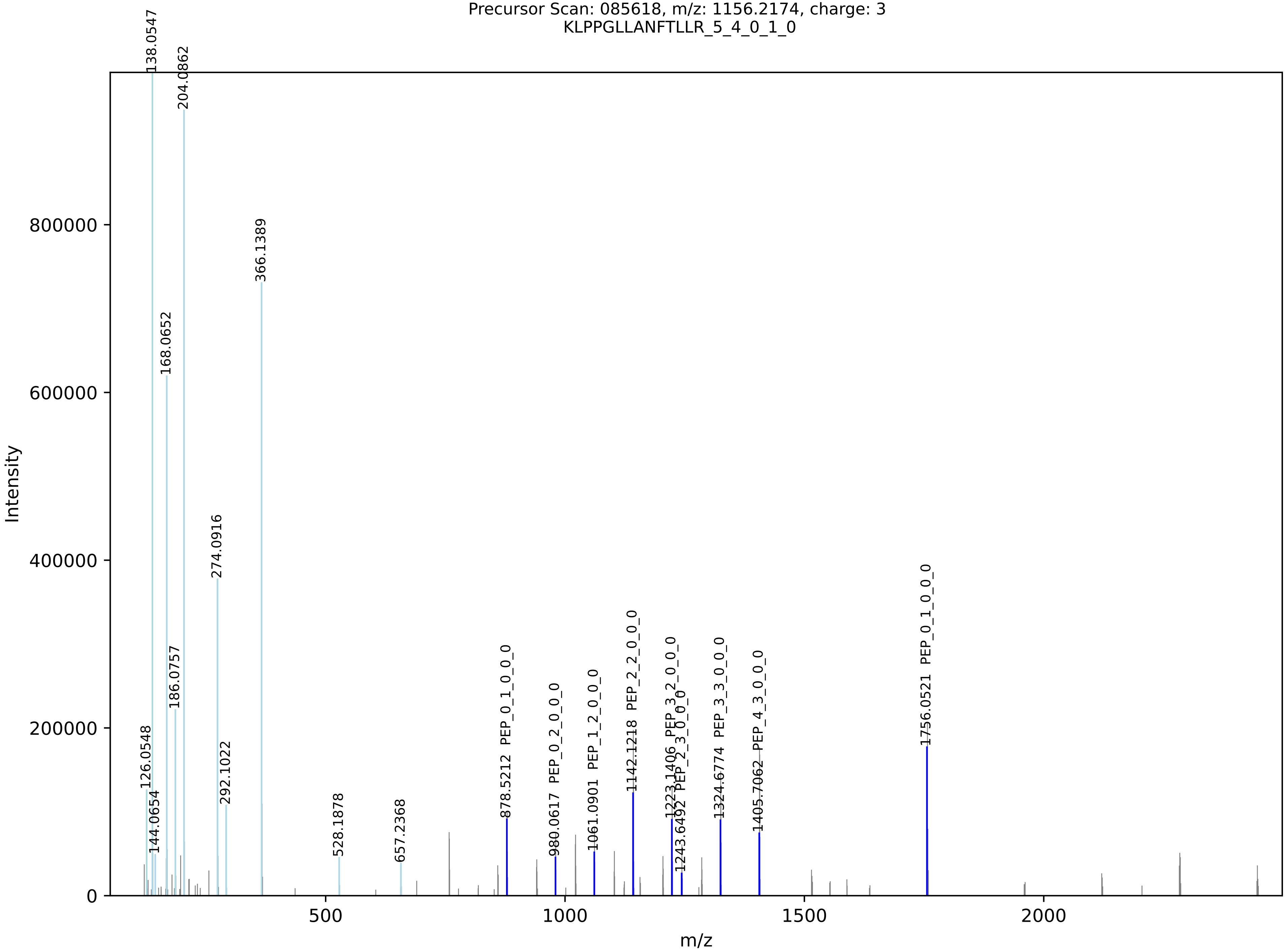

File: 231129_OE_RK_4, RT: 63.989, Scan: 085840
Type: HCD, FTMS + c NSI d Full ms2 1436.6289@hcd30.00 [120.0000-4418.2266]
Precursor Scan: 085765, m/z: 1435.9607, charge: 3
VLSNNSDANLELINTWVAK_5_4_0_2_0

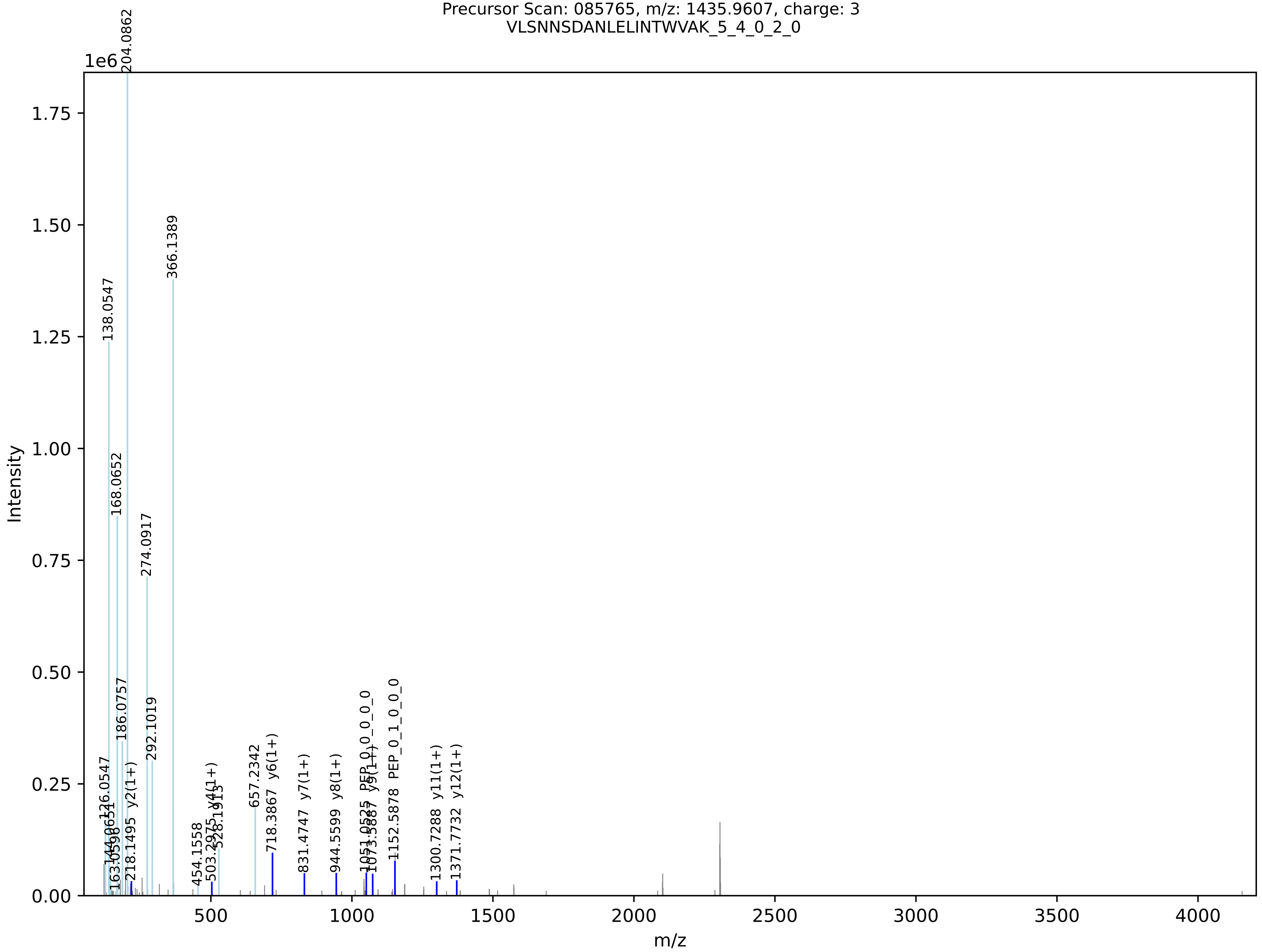

File: 231129_OE_RK_4, RT: 63.996, Scan: 085852
Type: HCD, FTMS + c NSI d Full ms2 1484.9819@hcd30.00 [120.0000-4566.1865]
Precursor Scan: 085765, m/z: 1484.6478, charge: 3
VLSNNSDANLELINTWVAK_5_4_1_2_0

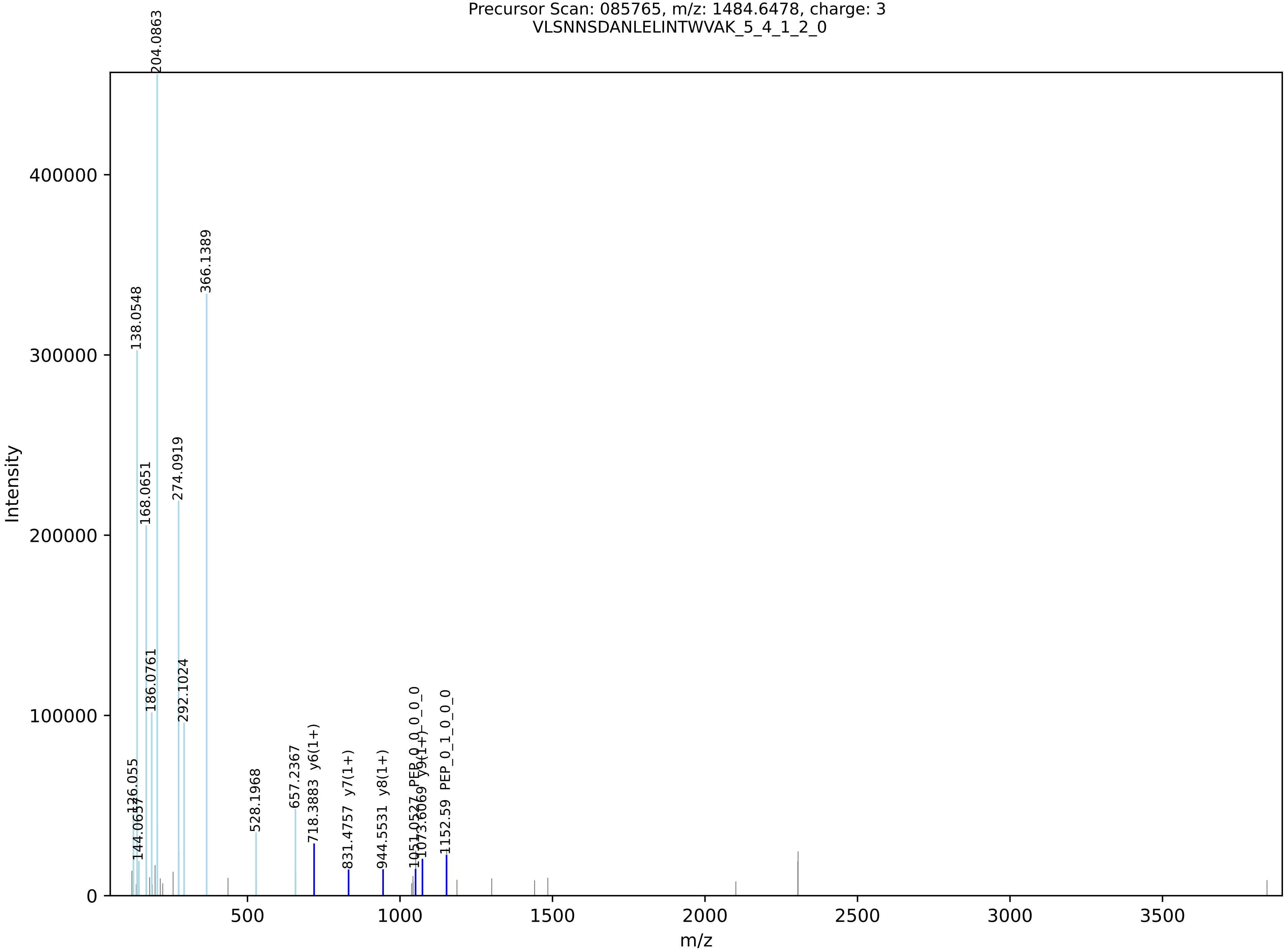

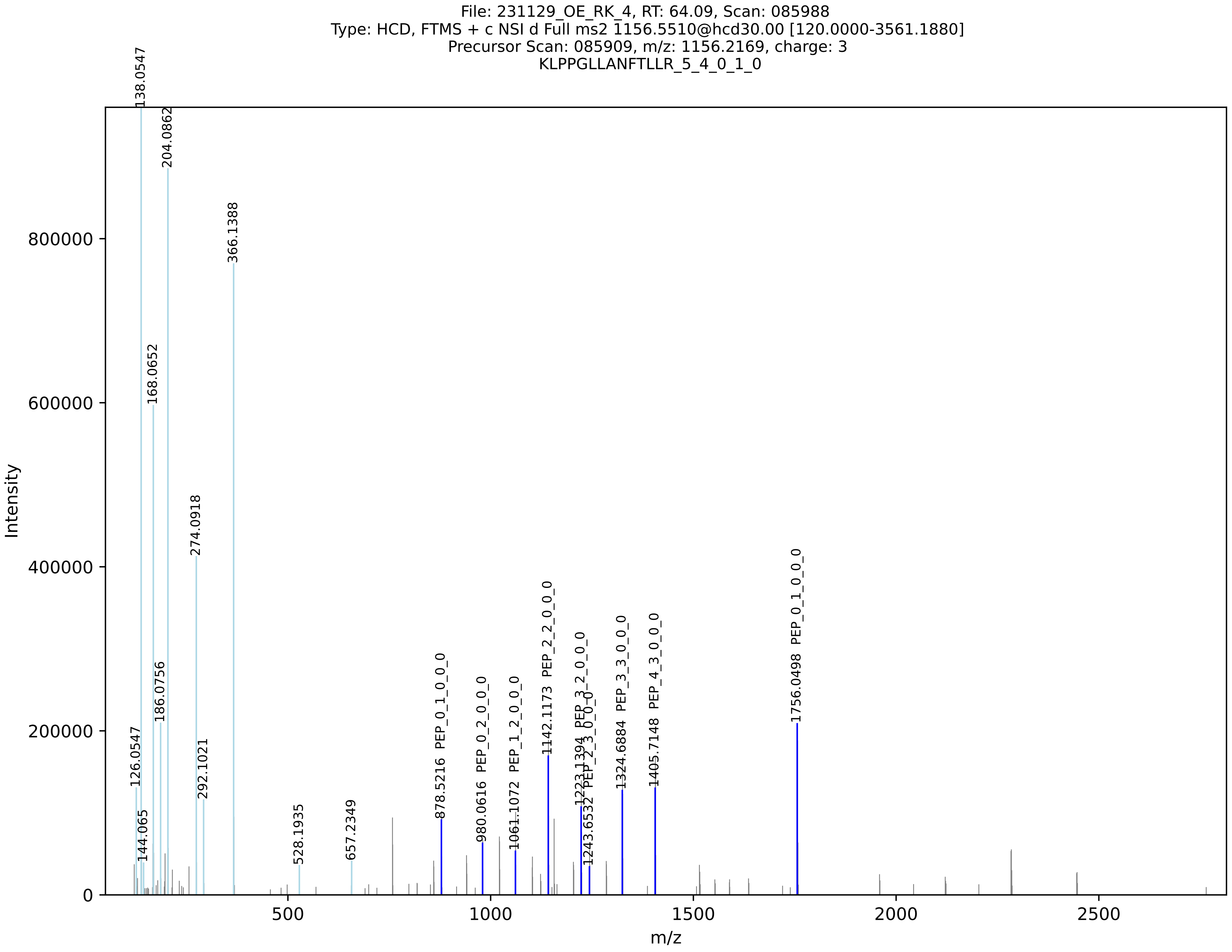
File: 231129_OE_RK_4, RT: 64.09, Scan: 085988
Type: HCD, FTMS + c NSI d Full ms2 1156.5510@hcd30.00 [120.0000-3561.1880]
Precursor Scan: 085909, m/z: 1156.2169, charge: 3
KLPPGLLANFTLLR_5_4_0_1_0
Intensity
m/z
0
200000
400000
600000
800000
500
1000
1500
2000
2500
138.0547
204.0862
366.1388
168.0652
274.0918
186.0756
126.0547
292.1021
144.065
528.1935
657.2349
878.5216 PEP_0_1_0_0_0
980.0616 PEP_0_2_0_0_0
1061.1072 PEP_1_2_0_0_0
1142.1173 PEP_2_2_0_0_0
1223.1394 PEP_3_2_0_0_0
1243.6532 PEP_2_3_0_0_0
1324.6884 PEP_3_3_0_0_0
1405.7148 PEP_4_3_0_0_0
1756.0498 PEP_0_1_0_0_0

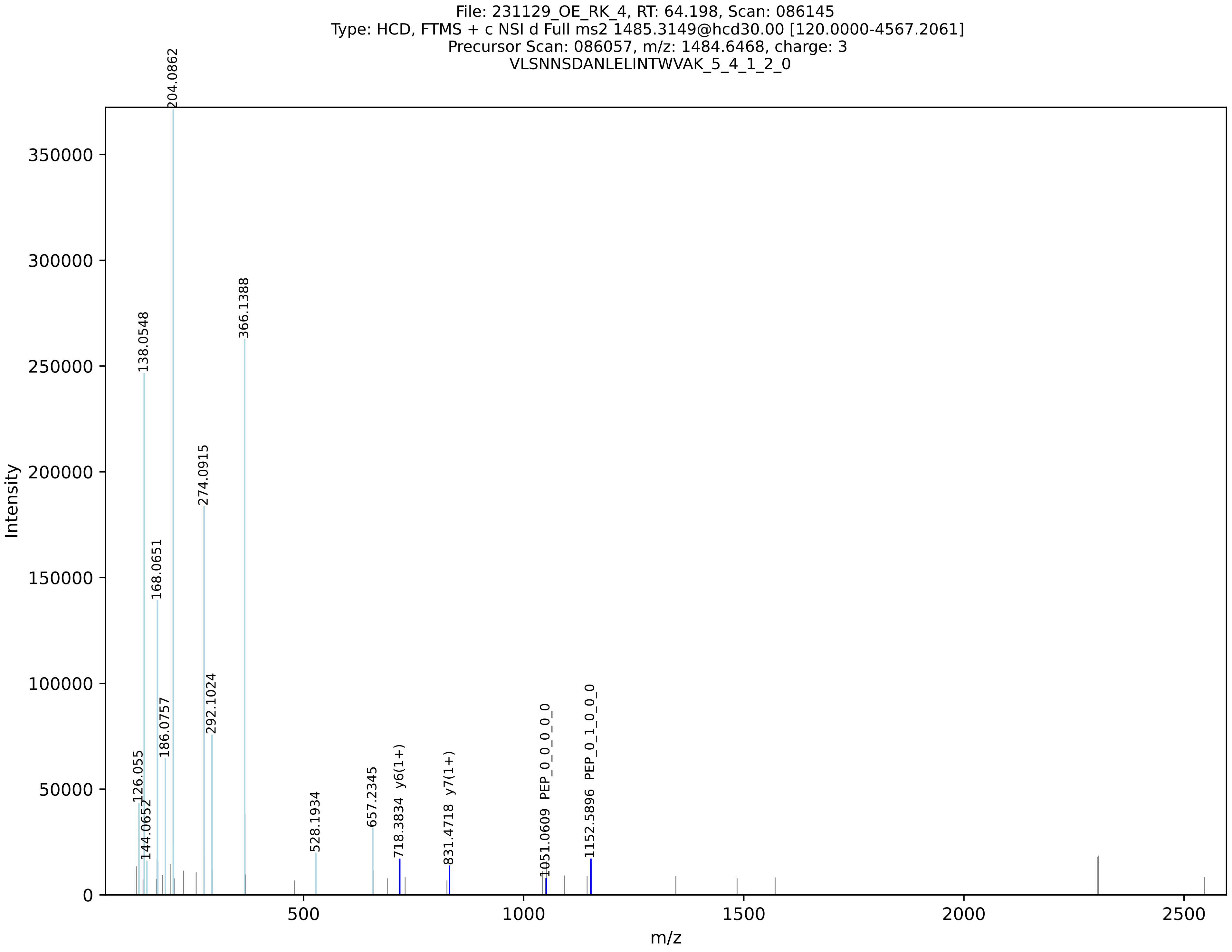
File: 231129_OE_RK_4, RT: 64.198, Scan: 086145
Type: HCD, FTMS + c NSI d Full ms2 1485.3149@hcd30.00 [120.0000-4567.2061]
Precursor Scan: 086057, m/z: 1484.6468, charge: 3
VLSNNSDANLELINTWVAK_5_4_1_2_0
204.0862
366.1388
138.0548
274.0915
168.0651
292.1024
186.0757
126.055
144.0652
528.1934
657.2345
718.3834 y6(1+)
831.4718 y7(1+)
1051.0609 PEP_0_0_0_0_0
1152.5896 PEP_0_1_0_0_0
Intensity
m/z

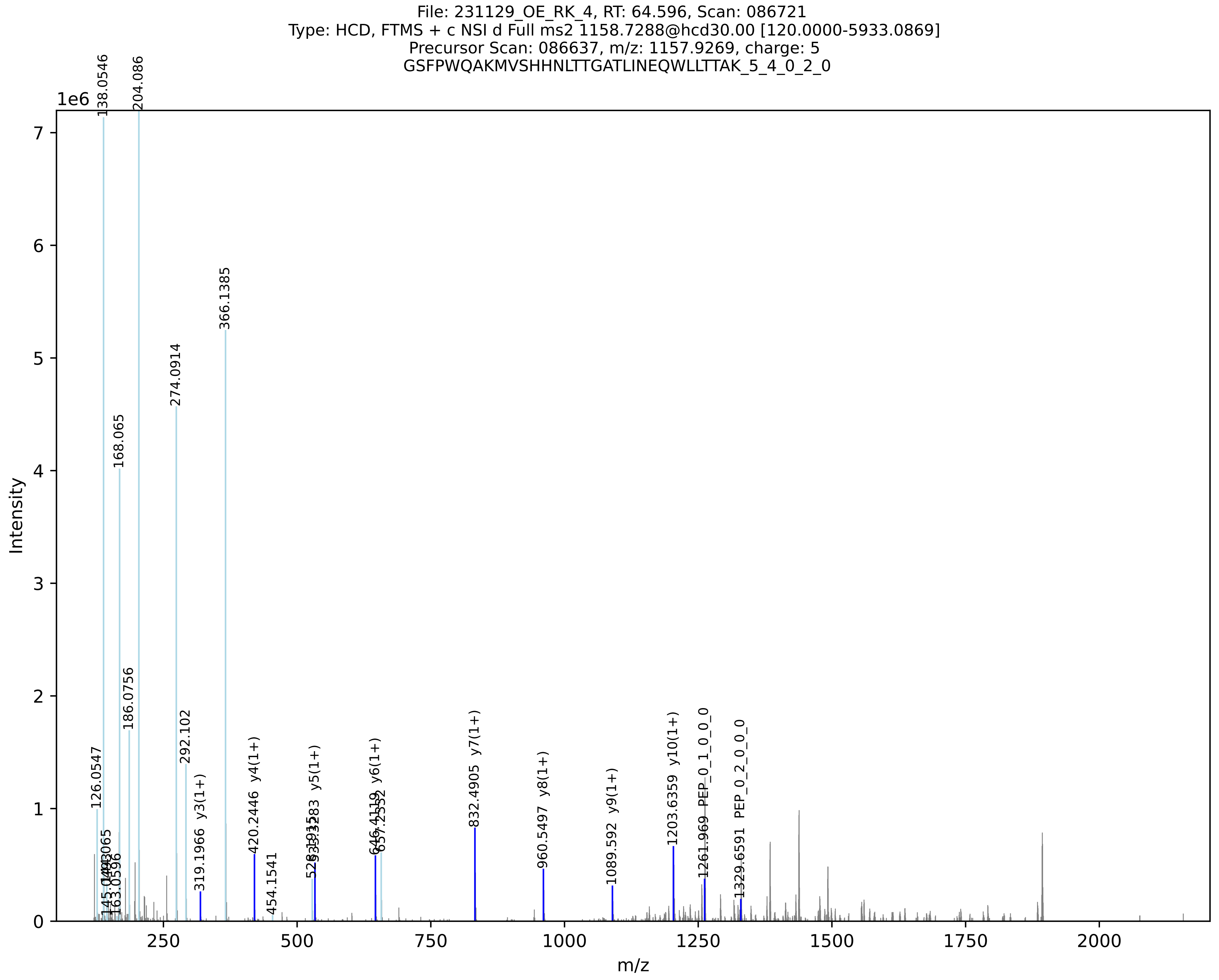

File: 231129_OE_RK_4, RT: 64.596, Scan: 086721
Type: HCD, FTMS + c NSI d Full ms2 1158.7288@hcd30.00 [120.0000-5933.0869]
Precursor Scan: 086637, m/z: 1157.9269, charge: 5
GSFPWQAKMVSHHNLTTGATLINEQWLLTTAK_5_4_0_2_0
1e6
Intensity
m/z
138.0546
204.086
366.1385
274.0914
168.065
186.0756
292.102
126.0547
319.1966 y3(1+)
420.2446 y4(1+)
454.1541
y5(1+)
y6(1+)
657.2532
832.4905 y7(1+)
960.5497 y8(1+)
1089.592 y9(1+)
1203.6359 y10(1+)
1261.969 PEP_0_1_0_0_0
1329.6591 PEP_0_2_0_0_0

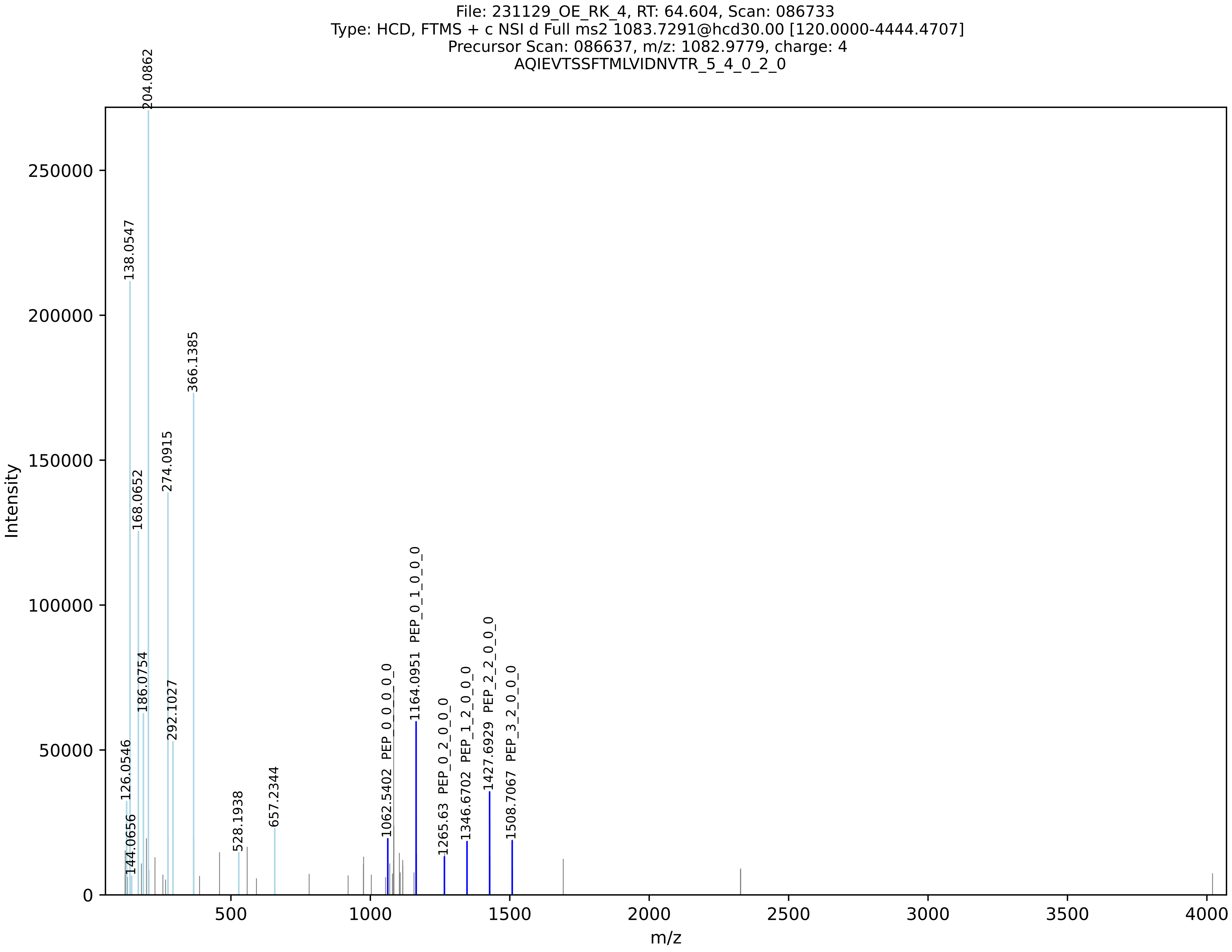

File: 231129_OE_RK_4, RT: 64.604, Scan: 086733
Type: HCD, FTMS + c NSI d Full ms2 1083.7291@hcd30.00 [120.0000-4444.4707]
Precursor Scan: 086637, m/z: 1082.9779, charge: 4
AQIEVTSSFTMLVIDNVTR_5_4_0_2_0
Intensity
m/z
204.0862
138.0547
366.1385
274.0915
168.0652
186.0754
292.1027
126.0546
144.0656
528.1938
657.2344
1062.5402 PEP_0_0_0_0_0
1164.0951 PEP_0_1_0_0_0
1265.63 PEP_0_2_0_0_0
1346.6702 PEP_1_2_0_0_0
1427.6929 PEP_2_2_0_0_0
1508.7067 PEP_3_2_0_0_0

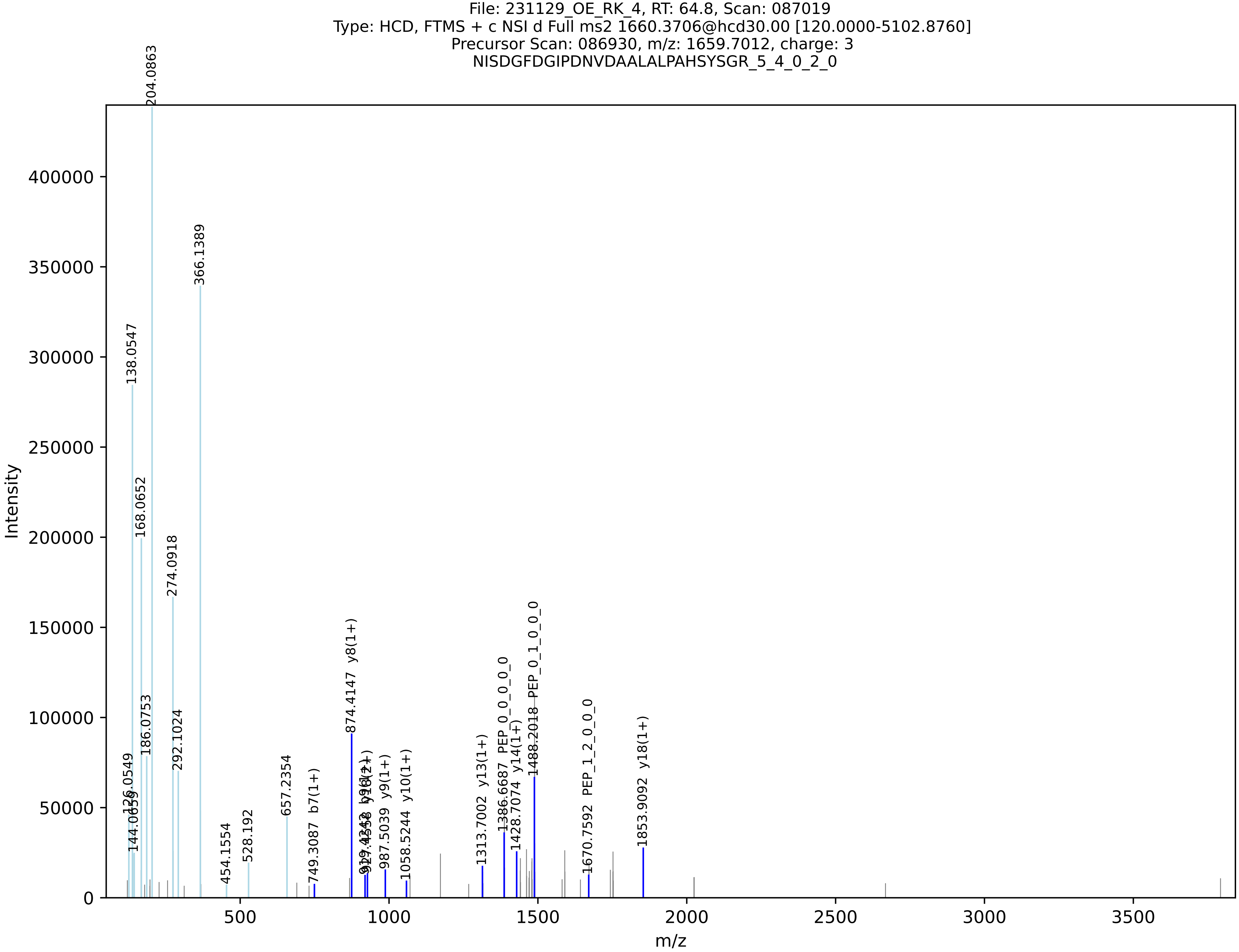

File: 231129_OE_RK_4, RT: 64.8, Scan: 087019
Type: HCD, FTMS + c NSI d Full ms2 1660.3706@hcd30.00 [120.0000-5102.8760]
Precursor Scan: 086930, m/z: 1659.7012, charge: 3
NISDGFDGIPDNVDAALALPAHSYSGR_5_4_0_2_0
Intensity
m/z
204.0863
366.1389
138.0547
168.0652
274.0918
186.0753
292.1024
126.0549
144.0659
454.1554
528.192
657.2354
749.3087 b7(1+)
874.4147 y8(1+)
987.5039 y9(1+)
1058.5244 y10(1+)
1313.7002 y13(1+)
1386.6687 PEP_0_0_0_0_0
1428.7074 y14(1+)
1488.2018 PEP_0_1_0_0_0
1670.7592 PEP_1_2_0_0_0
1853.9092 y18(1+)

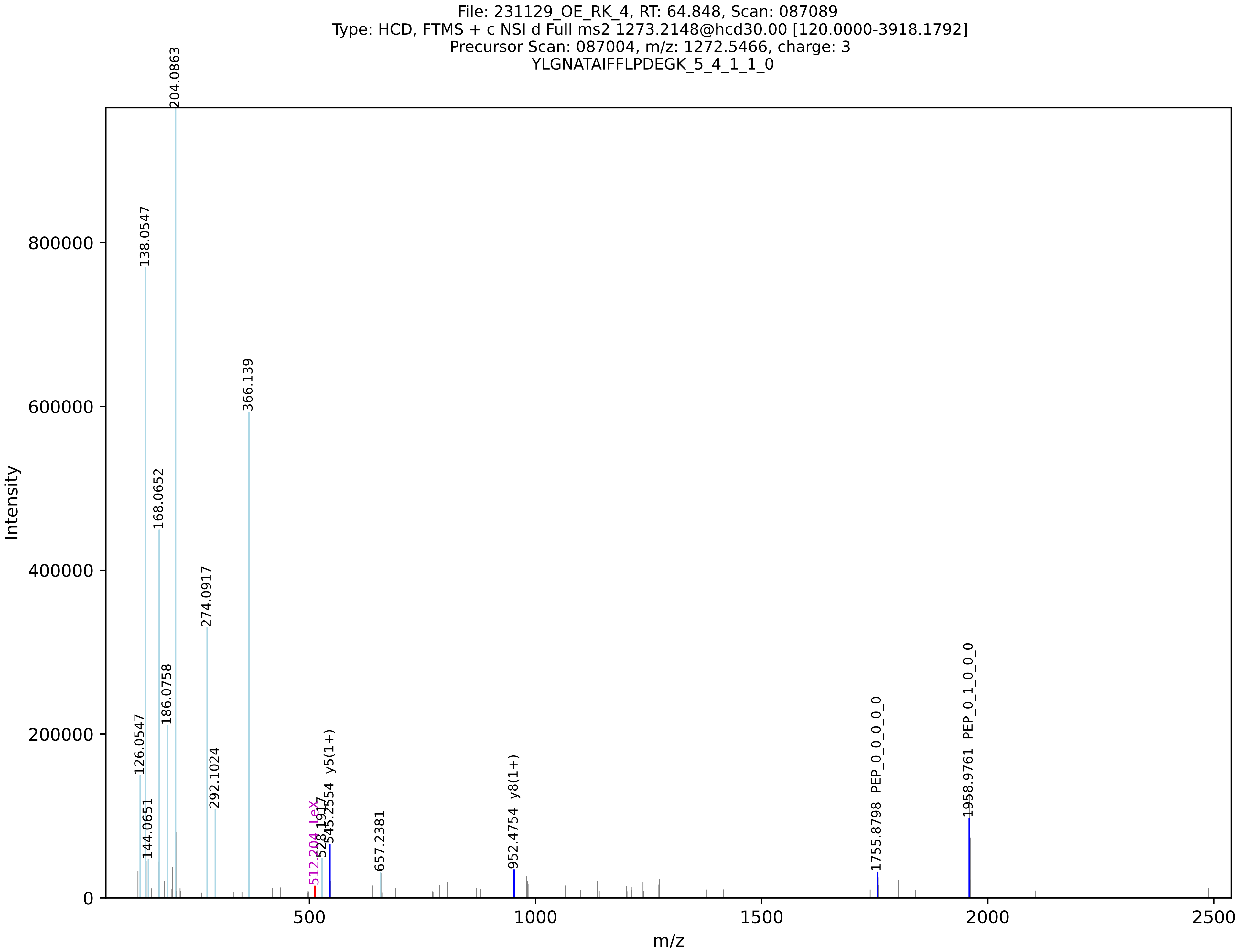
File: 231129_OE_RK_4, RT: 64.848, Scan: 087089
Type: HCD, FTMS + c NSI d Full ms2 1273.2148@hcd30.00 [120.0000-3918.1792]
Precursor Scan: 087004, m/z: 1272.5466, charge: 3
YLGNATAIFFLPDEGK_5_4_1_1_0
Intensity
m/z
204.0863
138.0547
168.0652
366.139
274.0917
186.0758
126.0547
292.1024
144.0651
512.204_LeX
528.1917
545.2554 y5(1+)
657.2381
952.4754 y8(1+)
1755.8798 PEP_0_0_0_0_0
1958.9761 PEP_0_1_0_0_0

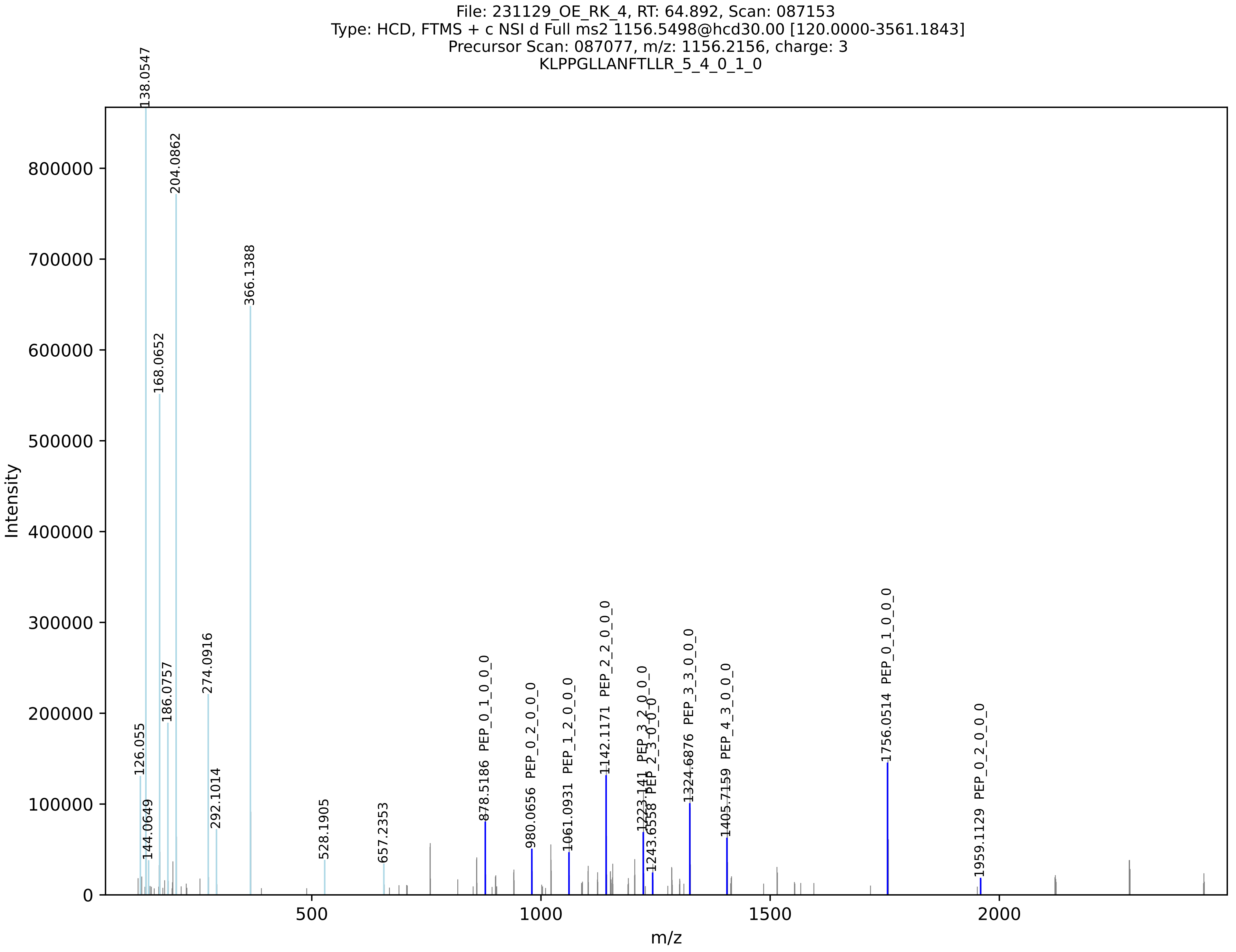
File: 231129_OE_RK_4, RT: 64.892, Scan: 087153
Type: HCD, FTMS + c NSI d Full ms2 1156.5498@hcd30.00 [120.0000-3561.1843]
Precursor Scan: 087077, m/z: 1156.2156, charge: 3
KLPPGLLANFTLLR_5_4_0_1_0
Intensity
m/z
138.0547
204.0862
366.1388
168.0652
274.0916
186.0757
126.055
144.0649
292.1014
528.1905
657.2353
878.5186 PEP_0_1_0_0_0
980.0656 PEP_0_2_0_0_0
1061.0931 PEP_1_2_0_0_0
1142.1171 PEP_2_2_0_0_0
1223.141 PEP_3_2_0_0_0
1243.6558 PEP_2_3_0_0_0
1324.6876 PEP_3_3_0_0_0
1405.7159 PEP_4_3_0_0_0
1756.0514 PEP_0_1_0_0_0
1959.1129 PEP_0_2_0_0_0

File: 231129_OE_RK_4, RT: 65.046, Scan: 087378
Type: HCD, FTMS + c NSI d Full ms2 1273.2148@hcd30.00 [120.0000-3918.1794]
Precursor Scan: 087296, m/z: 1272.5466, charge: 3
YLGNATAIFFLPDEGK_5_4_1_1_0

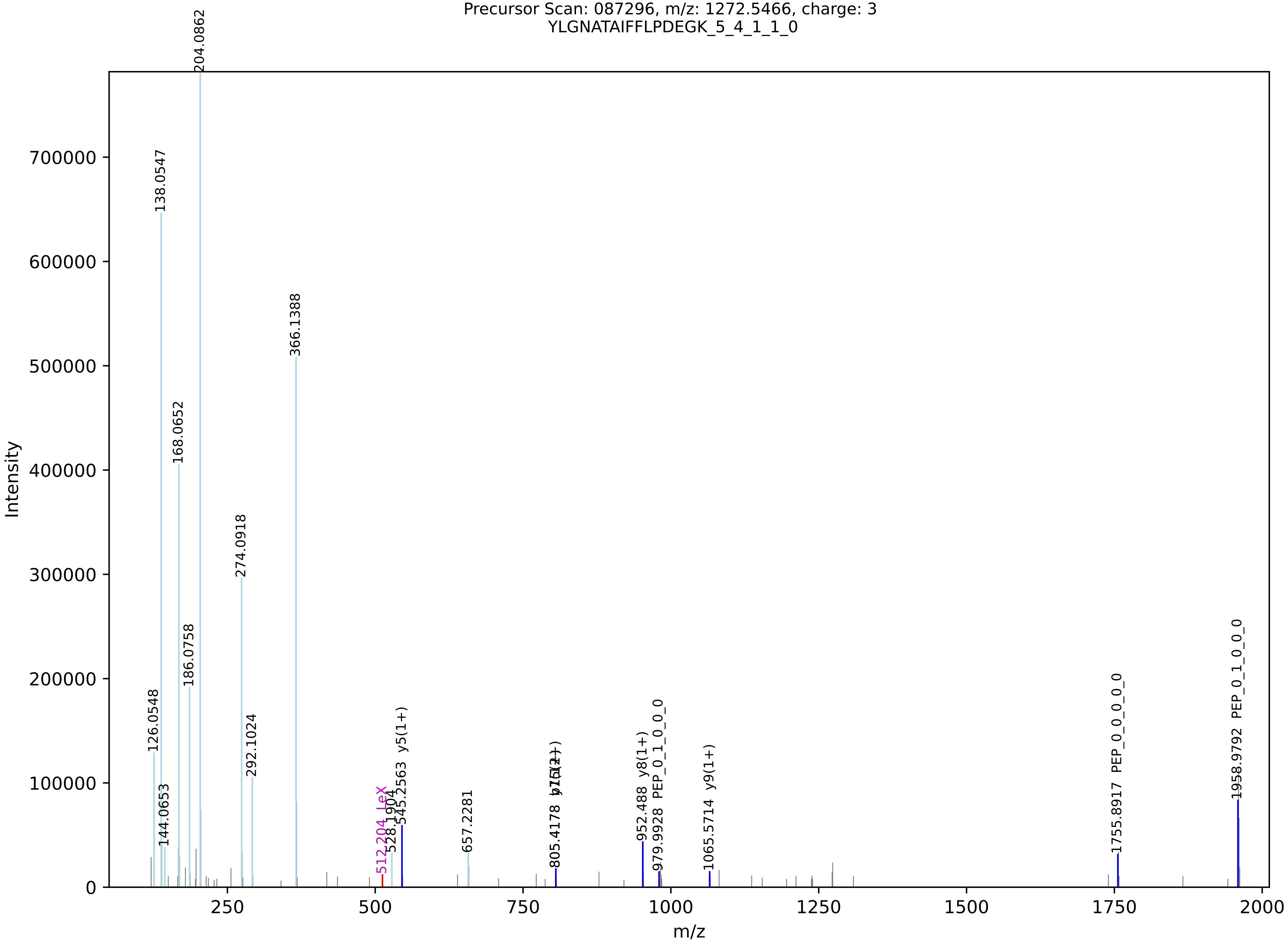

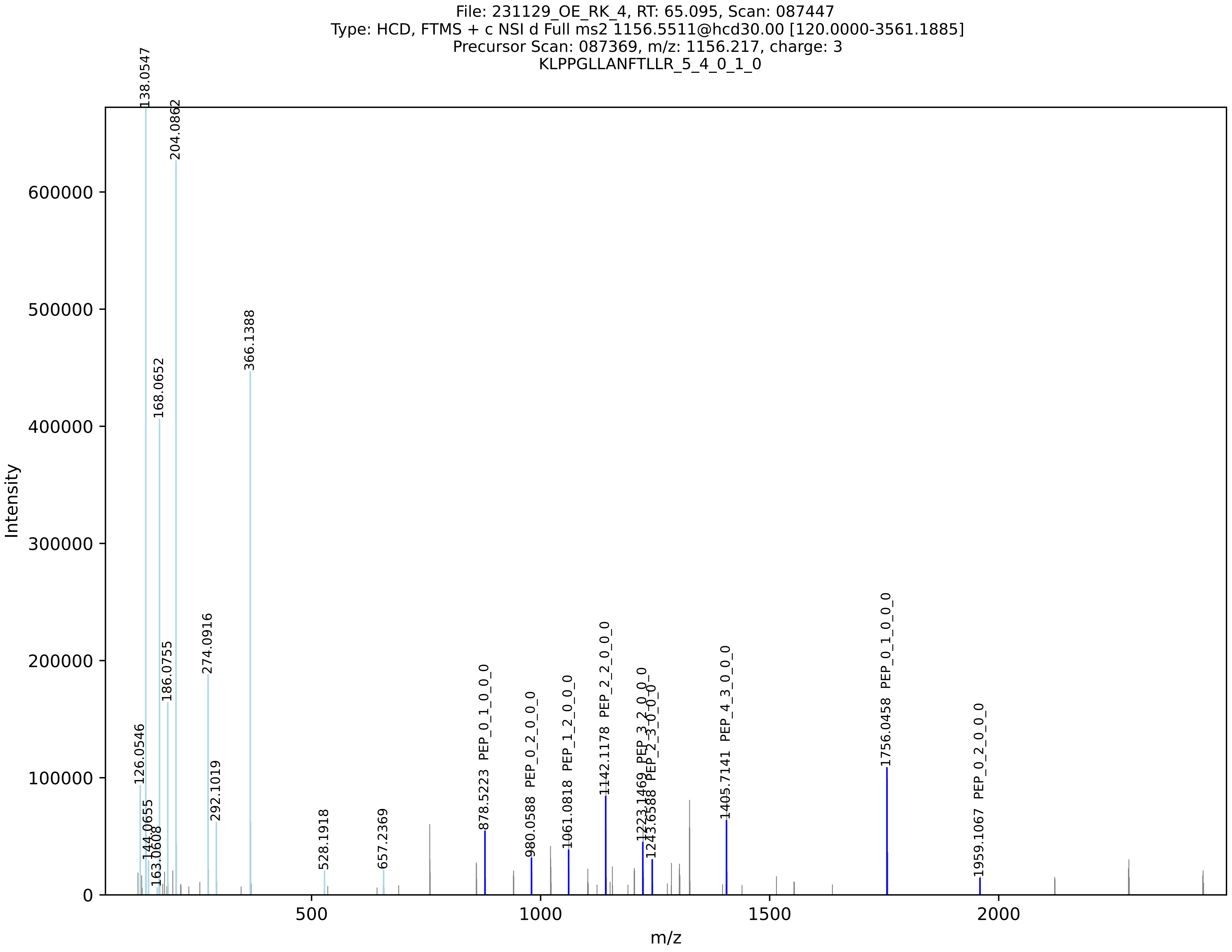
File: 231129_OE_RK_4, RT: 65.095, Scan: 087447
Type: HCD, FTMS + c NSI d Full ms2 1156.5511@hcd30.00 [120.0000-3561.1885]
Precursor Scan: 087369, m/z: 1156.217, charge: 3
KLPPGLLANFTLLR_5_4_0_1_0
Intensity
m/z
138.0547
204.0862
168.0652
366.1388
274.0916
186.0755
126.0546
292.1019
144.0655
163.0608
528.1918
657.2369
878.5223 PEP_0_1_0_0_0
980.0588 PEP_0_2_0_0_0
1061.0818 PEP_1_2_0_0_0
1142.1178 PEP_2_2_0_0_0
1223.1469 PEP_3_2_0_0_0
1243.6588 PEP_2_3_0_0_0
1405.7141 PEP_4_3_0_0_0
1756.0458 PEP_0_1_0_0_0
1959.1067 PEP_0_2_0_0_0

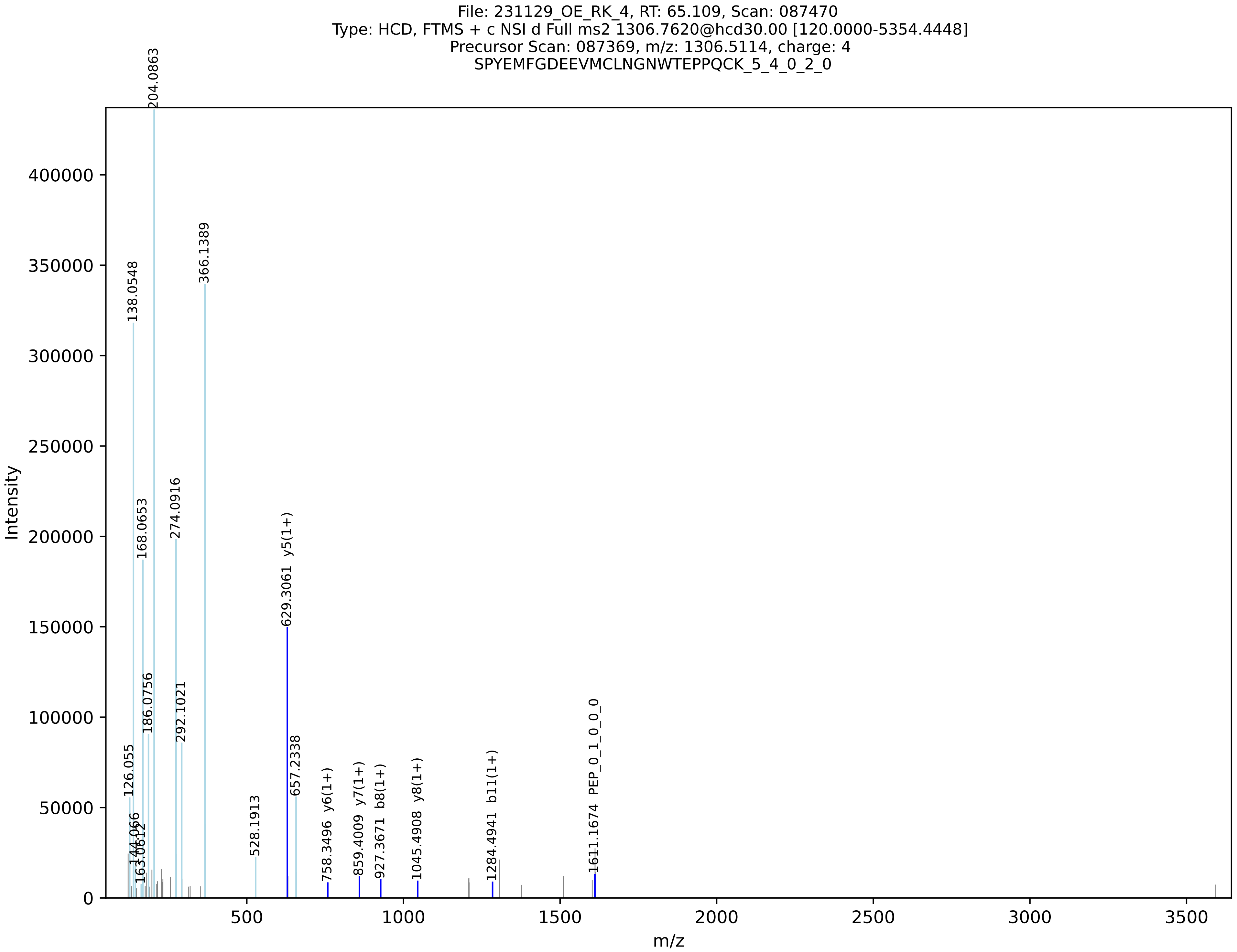

File: 231129_OE_RK_4, RT: 65.109, Scan: 087470
Type: HCD, FTMS + c NSI d Full ms2 1306.7620@hcd30.00 [120.0000-5354.4448]
Precursor Scan: 087369, m/z: 1306.5114, charge: 4
SPYEMFGDEEVMCLNGNWTEPPQCK_5_4_0_2_0
Intensity
m/z
204.0863
366.1389
138.0548
274.0916
168.0653
629.3061 y5(1+)
186.0756
292.1021
126.055
657.2338
528.1913
144.066
163.0612
758.3496 y6(1+)
859.4009 y7(1+)
927.3671 b8(1+)
1045.4908 y8(1+)
1284.4941 b11(1+)
1611.1674 PEP_0_1_0_0_0

File: 231129_OE_RK_4, RT: 65.195, Scan: 087593
Type: HCD, FTMS + c NSI d Full ms2 1285.0532@hcd30.00 [120.0000-5265.8730]
Precursor Scan: 087514, m/z: 1284.3014, charge: 4
ICDLLVANNHFAHFFAPQNLTNMNK_5_4_0_2_0

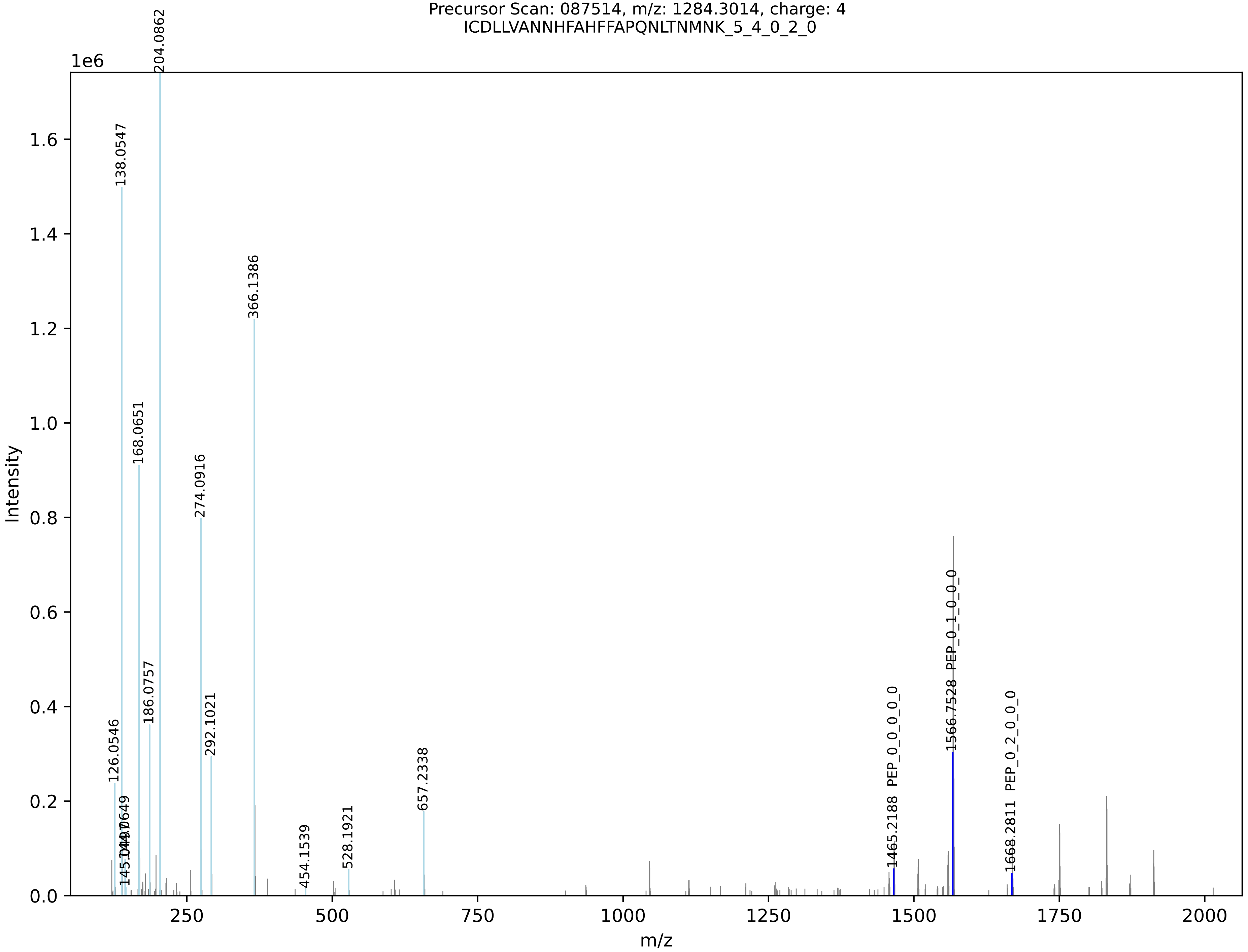

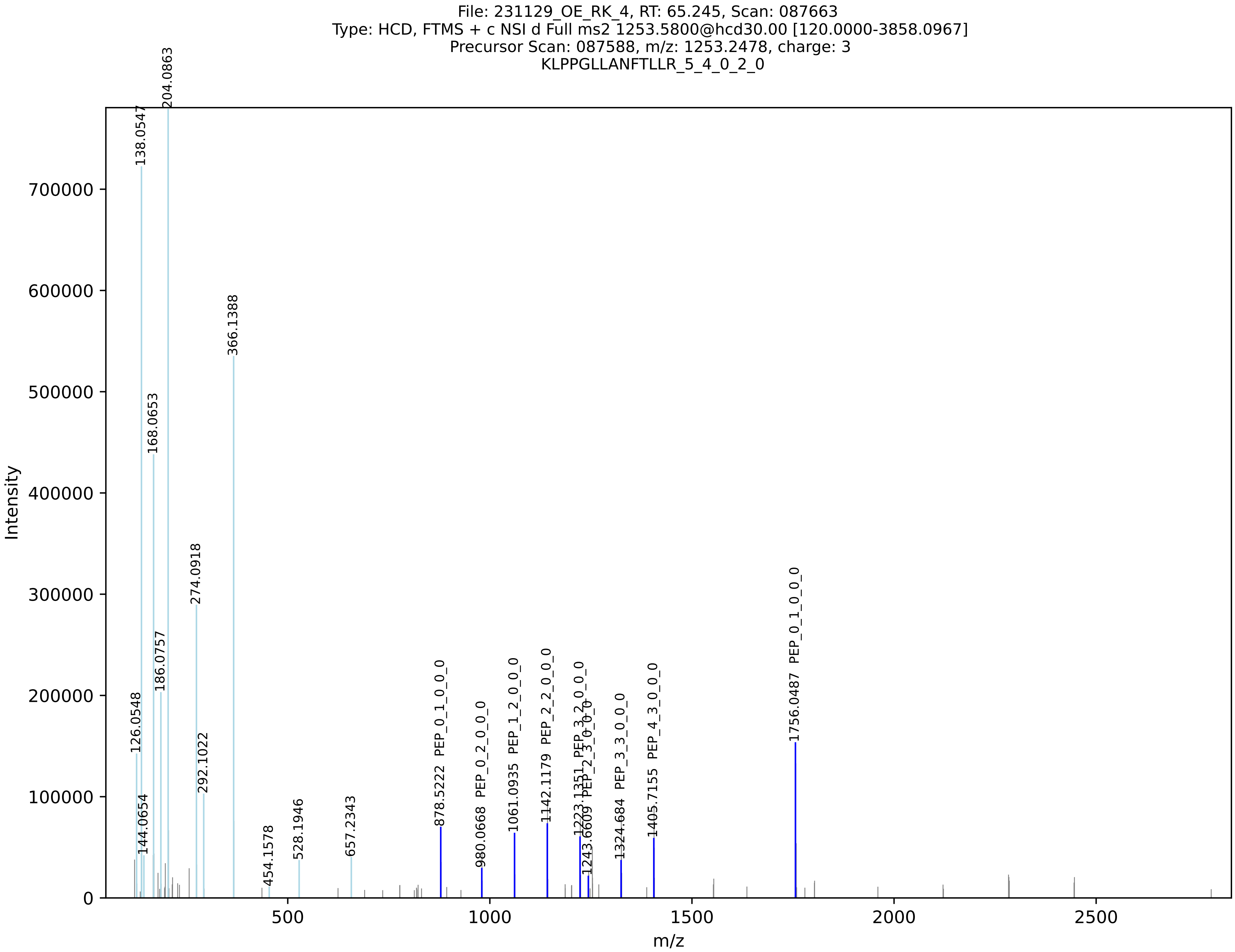
File: 231129_OE_RK_4, RT: 65.245, Scan: 087663
Type: HCD, FTMS + c NSI d Full ms2 1253.5800@hcd30.00 [120.0000-3858.0967]
Precursor Scan: 087588, m/z: 1253.2478, charge: 3
KLPPGLLANFTLLR_5_4_0_2_0
Intensity
m/z
204.0863
138.0547
168.0653
366.1388
274.0918
186.0757
126.0548
292.1022
144.0654
454.1578
528.1946
657.2343
878.5222 PEP_0_1_0_0_0
980.0668 PEP_0_2_0_0_0
1061.0935 PEP_1_2_0_0_0
1142.1179 PEP_2_2_0_0_0
1223.1351 PEP_3_2_0_0_0
1324.684 PEP_3_3_0_0_0
1405.7155 PEP_4_3_0_0_0
1756.0487 PEP_0_1_0_0_0

File: 231129_OE_RK_4, RT: 65.256, Scan: 087682
Type: HCD, FTMS + c NSI d Full ms2 940.4383@hcd30.00 [120.0000-3859.8442]
Precursor Scan: 087588, m/z: 940.1877, charge: 4
KLPPGLLANFTLLR_5_4_0_2_0

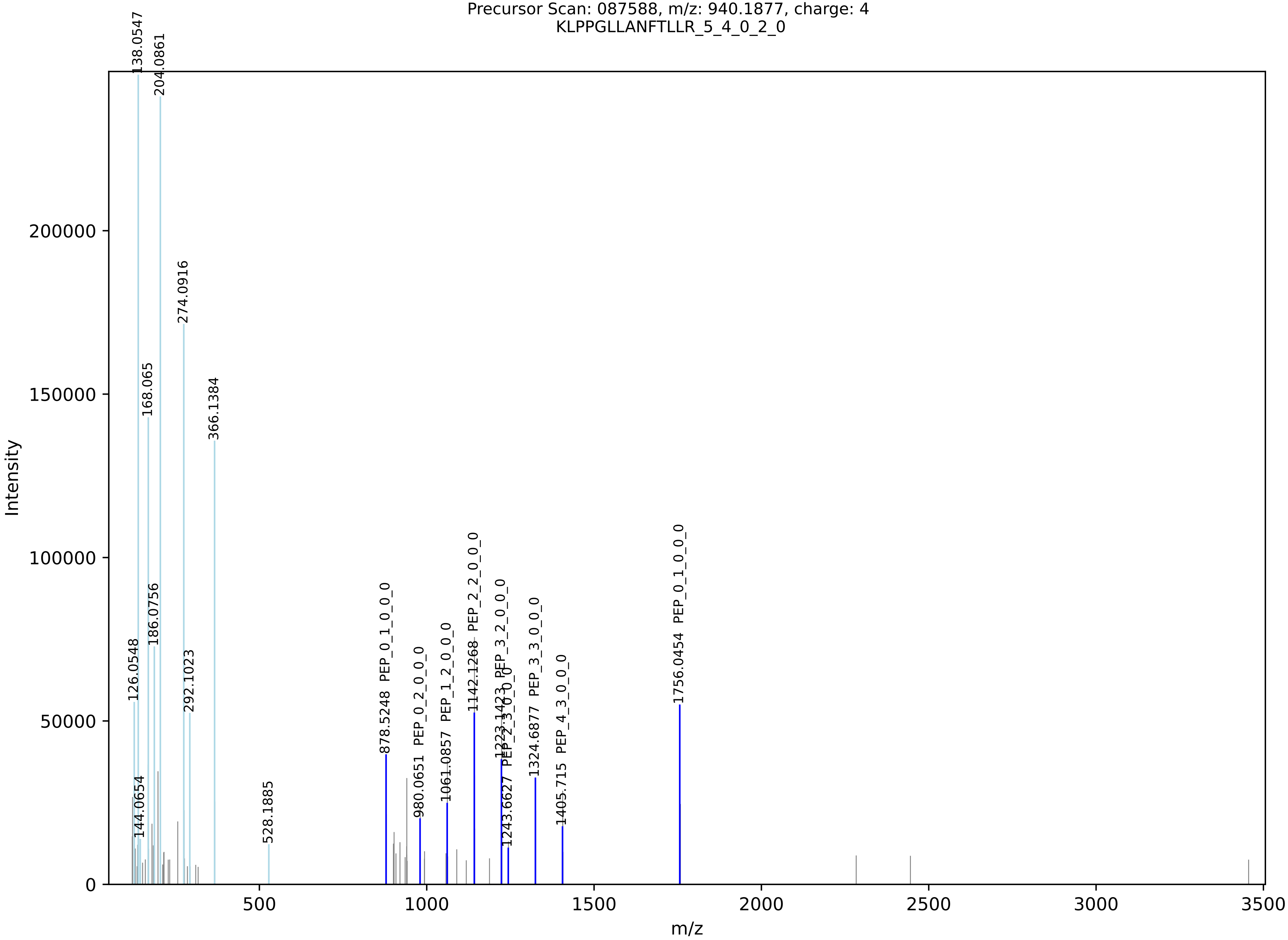

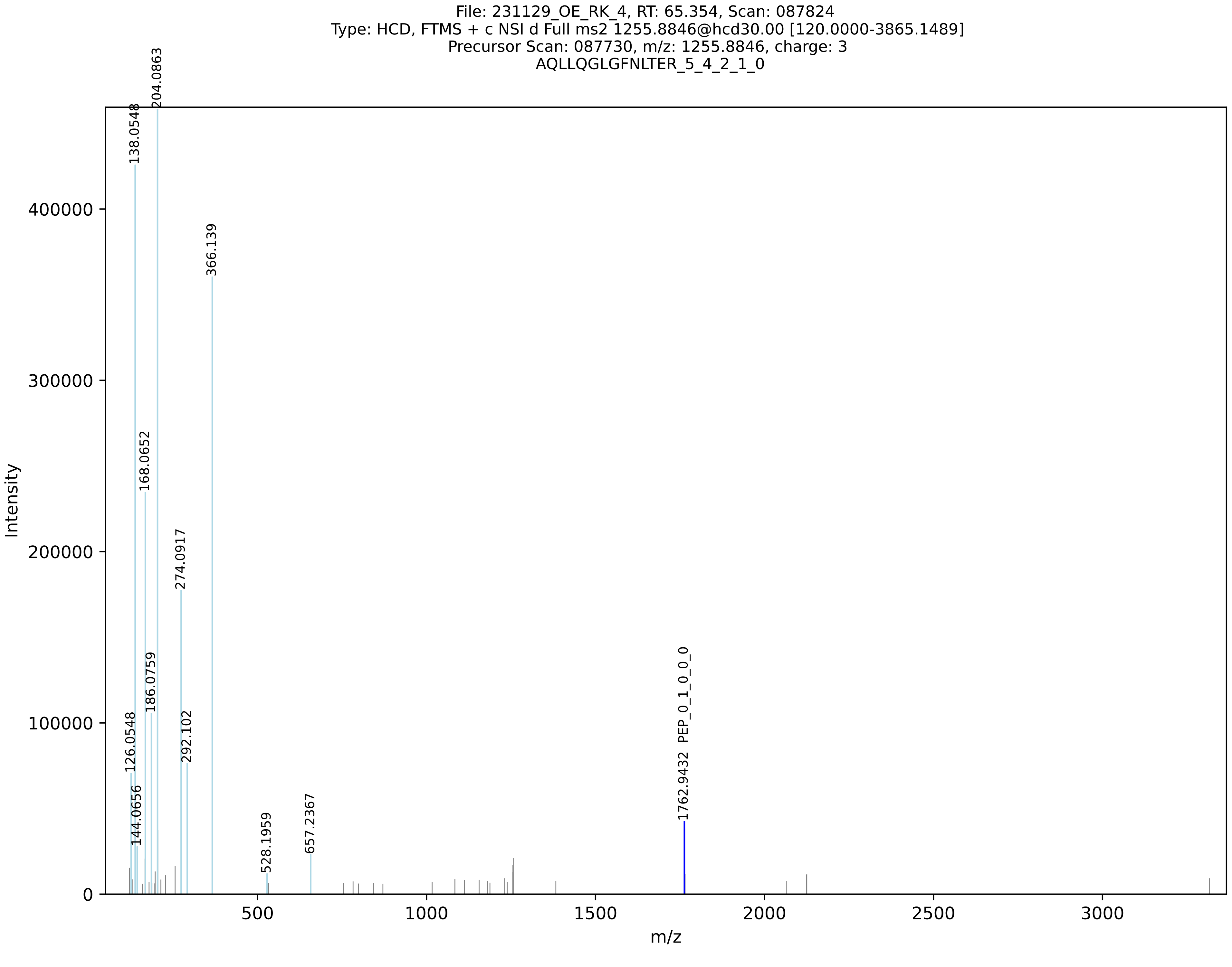
File: 231129_OE_RK_4, RT: 65.354, Scan: 087824
Type: HCD, FTMS + c NSI d Full ms2 1255.8846@hcd30.00 [120.0000-3865.1489]
Precursor Scan: 087730, m/z: 1255.8846, charge: 3
AQLLQGLGFNLTER_5_4_2_1_0
204.0863
138.0548
366.139
168.0652
274.0917
186.0759
292.102
126.0548
144.0656
528.1959
657.2367
1762.9432 PEP_0_1_0_0_0_
Intensity
m/z
0
100000
200000
300000
400000
500
1000
1500
2000
2500
3000

File: 231129_OE_RK_4, RT: 65.395, Scan: 087883
Type: HCD, FTMS + c NSI d Full ms2 1285.0558@hcd30.00 [120.0000-5265.8838]
Precursor Scan: 087803, m/z: 1284.304, charge: 4
YNFSFRYIYPLDSLTWIEYWPR_5_4_0_2_0

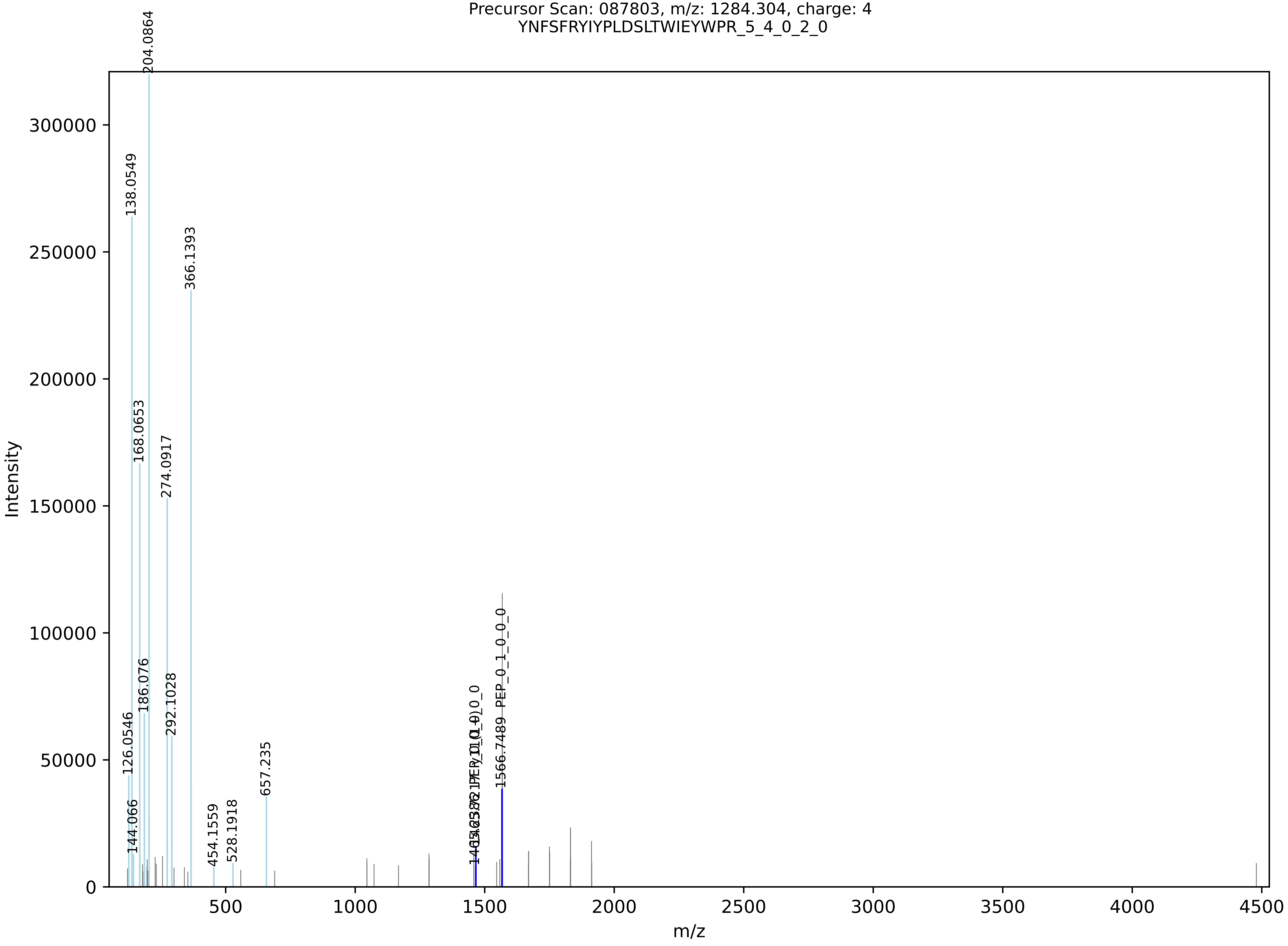

File: 231129_OE_RK_4, RT: 65.501, Scan: 088036
Type: HCD, FTMS + c NSI d Full ms2 1307.2651@hcd30.00 [120.0000-5356.4980]
Precursor Scan: 087951, m/z: 1306.5068, charge: 4
SPYEMFGDEEVMCLNGNWTEPPQCK_5_4_0_2_0

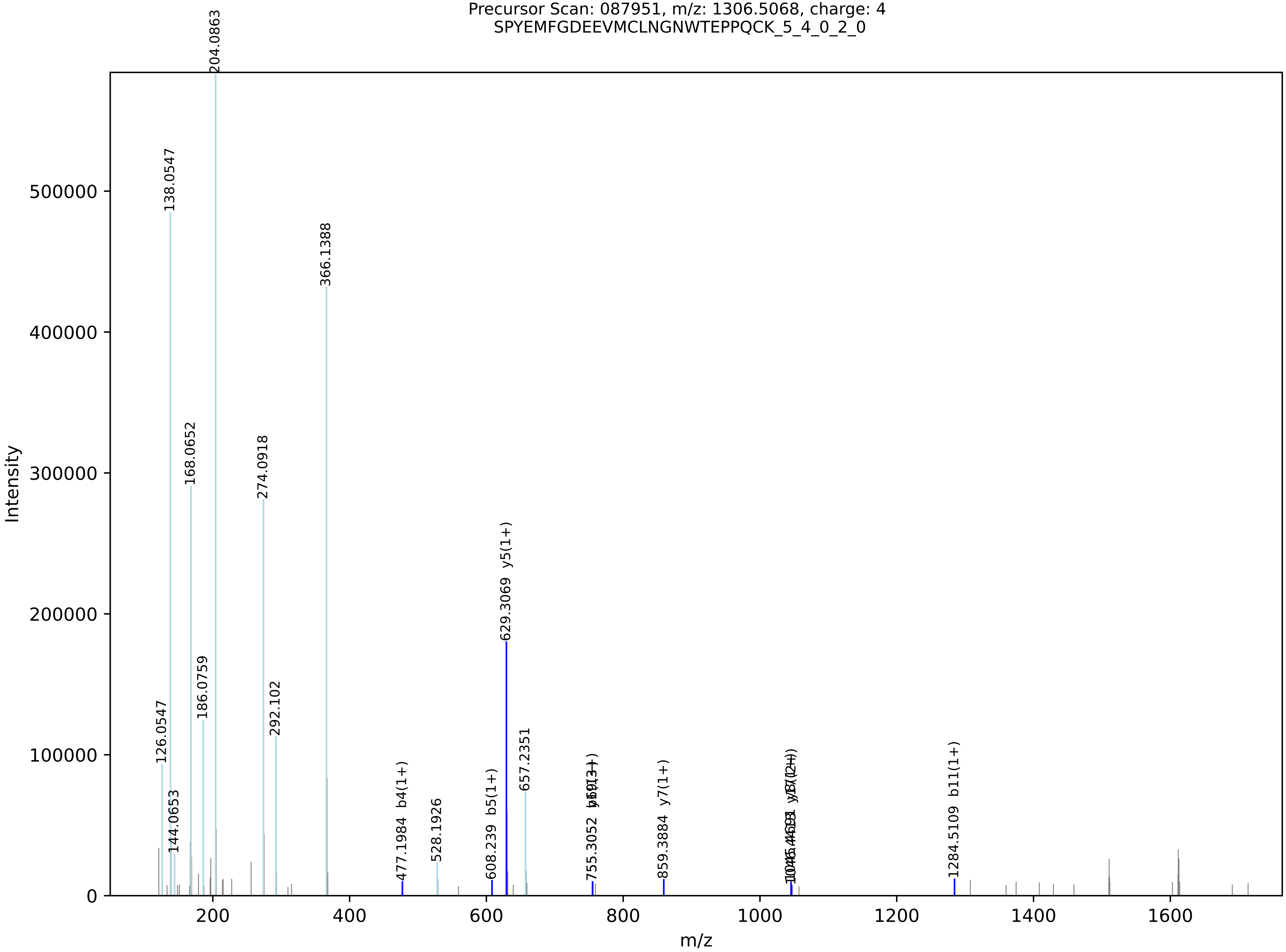

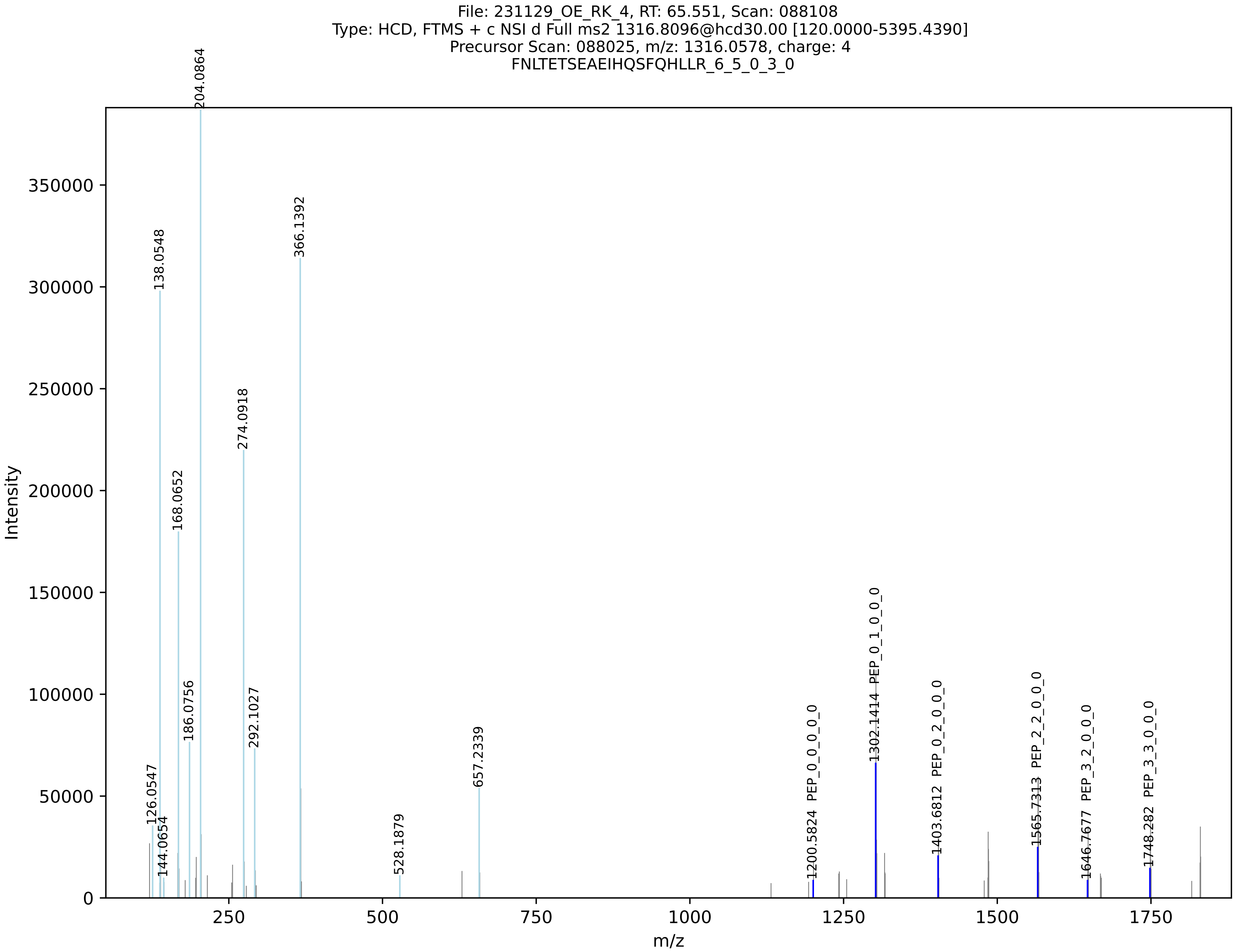
File: 231129_OE_RK_4, RT: 65.551, Scan: 088108
Type: HCD, FTMS + c NSI d Full ms2 1316.8096@hcd30.00 [120.0000-5395.4390]
Precursor Scan: 088025, m/z: 1316.0578, charge: 4
FNLTETSEAEIHQSFQHLLR_6_5_0_3_0
Intensity
m/z
204.0864
138.0548
366.1392
274.0918
168.0652
186.0756
292.1027
126.0547
144.0654
657.2339
528.1879
1200.5824 PEP_0_0_0_0_0
1302.1414 PEP_0_1_0_0_0
1403.6812 PEP_0_2_0_0_0
1565.7313 PEP_2_2_0_0_0
1646.7677 PEP_3_2_0_0_0
1748.282 PEP_3_3_0_0_0

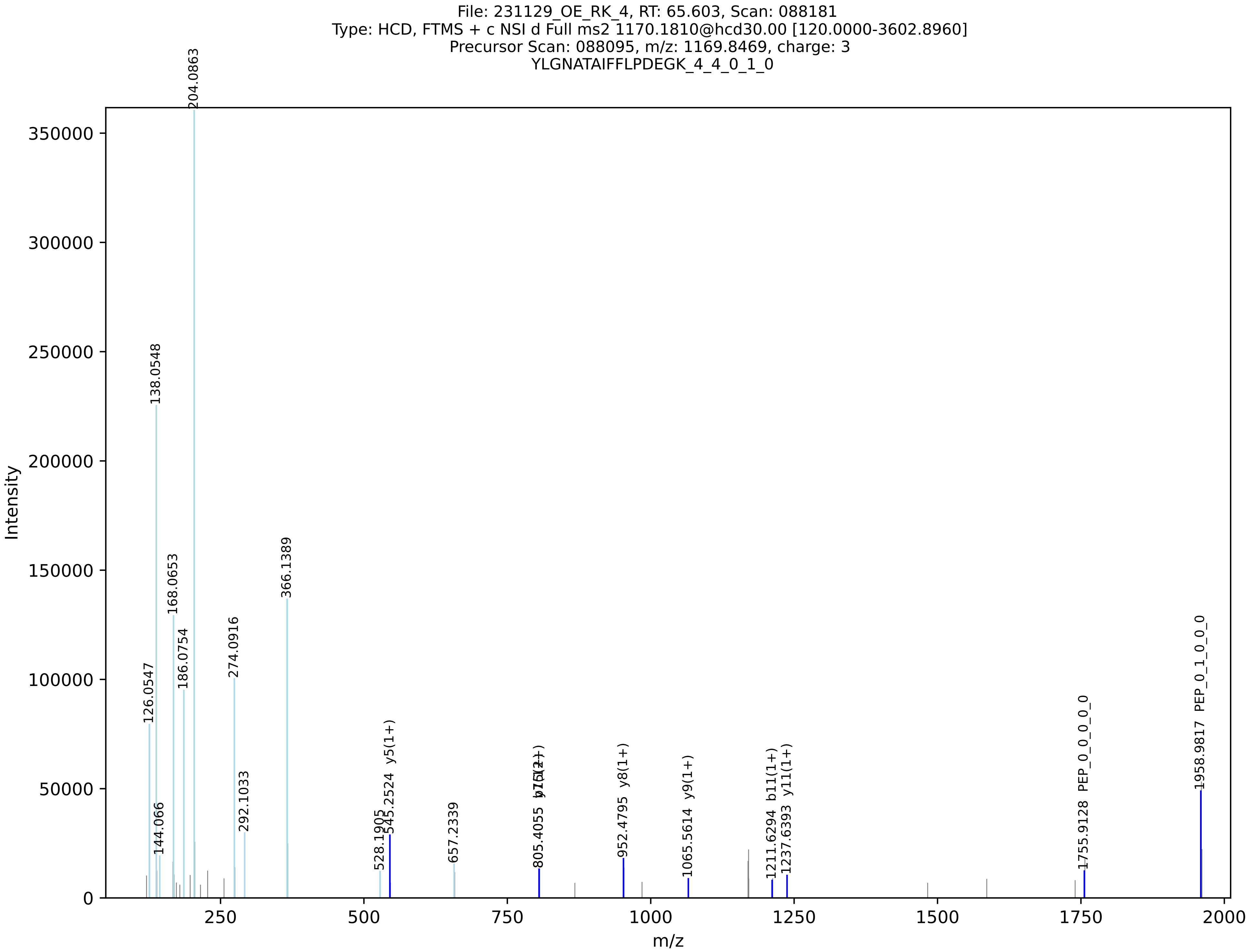
File: 231129_OE_RK_4, RT: 65.603, Scan: 088181
Type: HCD, FTMS + c NSI d Full ms2 1170.1810@hcd30.00 [120.0000-3602.8960]
Precursor Scan: 088095, m/z: 1169.8469, charge: 3
YLGNATAIFFLPDEGK_4_4_0_1_0
Intensity
m/z
204.0863
138.0548
168.0653
186.0754
126.0547
144.066
274.0916
292.1033
366.1389
528.1905
545.2524 y5(1+)
657.2339
805.4055
952.4795 y8(1+)
1065.5614 y9(1+)
1211.6294 b11(1+)
1237.6393 y11(1+)
1755.9128 PEP_0_0_0_0_0
1958.9817 PEP_0_1_0_0_0

File: 231129_OE_RK_4, RT: 65.798, Scan: 088465
Type: HCD, FTMS + c NSI d Full ms2 1316.5573@hcd30.00 [120.0000-5394.4097]
Precursor Scan: 088386, m/z: 1316.0581, charge: 4
FNLTETSEAEIHQSFQHLLR_6_5_0_3_0

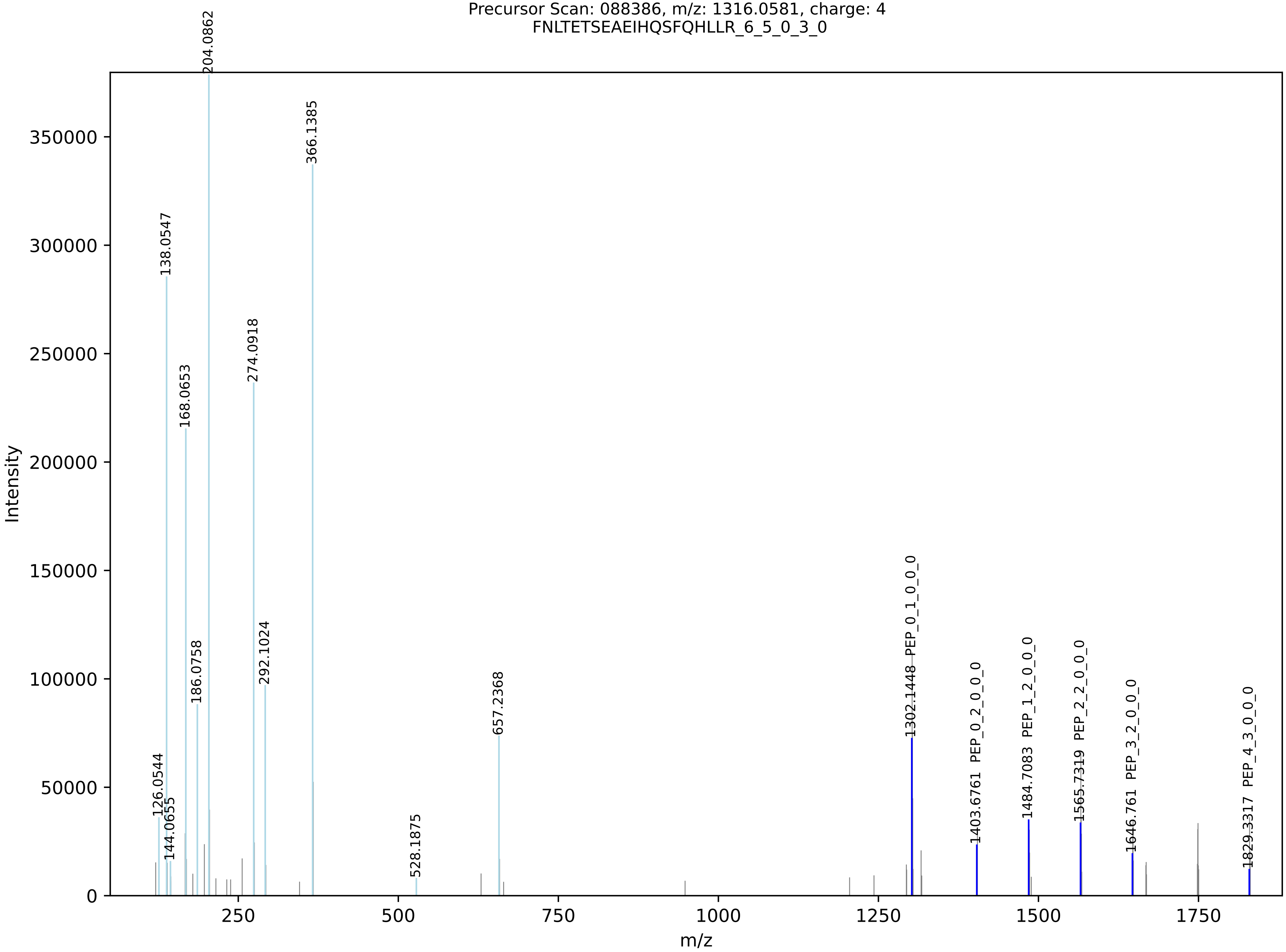

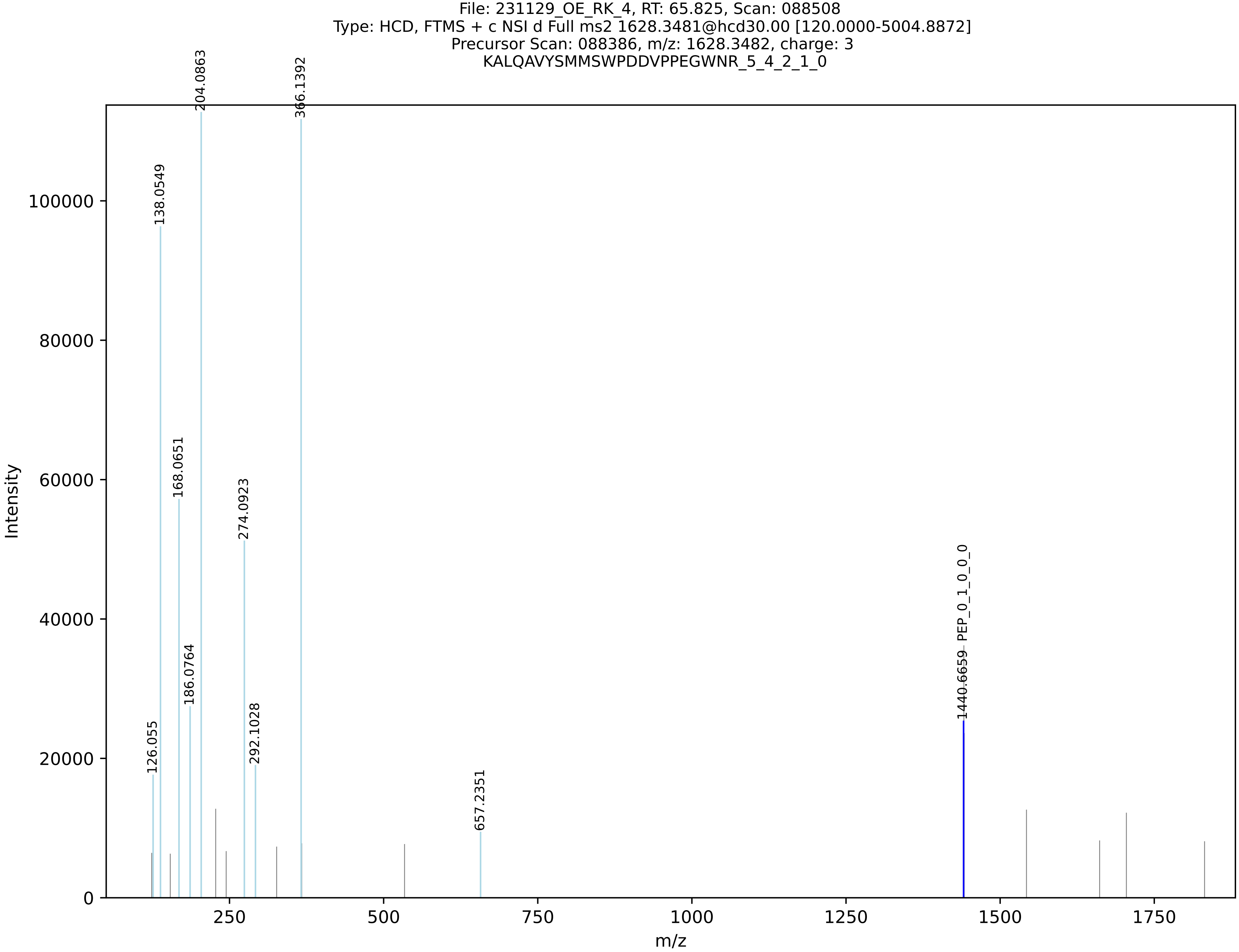
File: 231129_OE_RK_4, RT: 65.825, Scan: 088508
Type: HCD, FTMS + c NSI d Full ms2 1628.3481@hcd30.00 [120.0000-5004.8872]
Precursor Scan: 088386, m/z: 1628.3482, charge: 3
KALQAVYSMMSWPDDVPPEGWNR_5_4_2_1_0
204.0863
366.1392
138.0549
168.0651
274.0923
186.0764
126.055
292.1028
657.2351
1440.6659 PEP_0_1_0_0_0
Intensity
m/z
0
20000
40000
60000
80000
100000
250
500
750
1000
1250
1500
1750

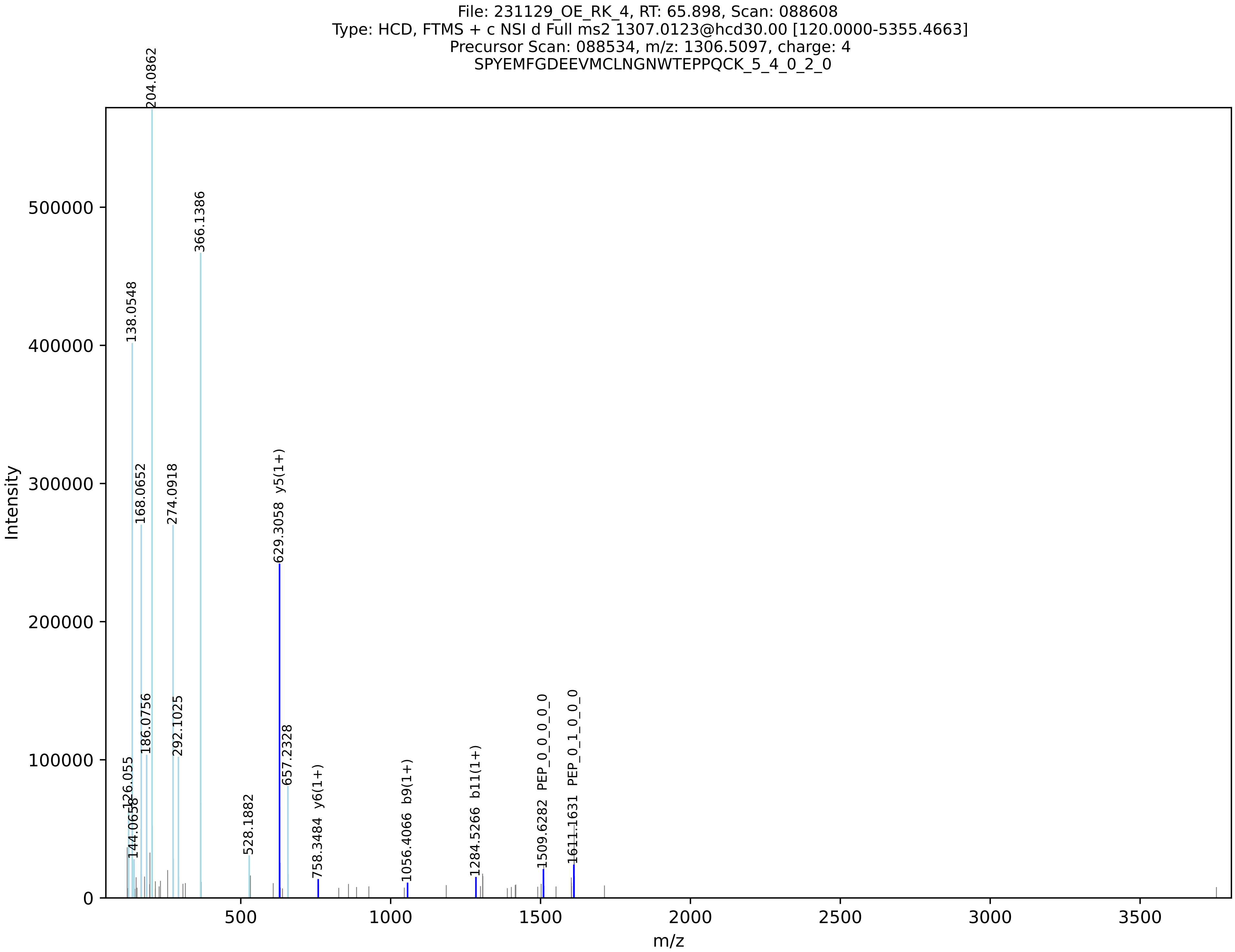
File: 231129_OE_RK_4, RT: 65.898, Scan: 088608
Type: HCD, FTMS + c NSI d Full ms2 1307.0123@hcd30.00 [120.0000-5355.4663]
Precursor Scan: 088534, m/z: 1306.5097, charge: 4
SPYEMFGDEEVMCLNGNWTEPPQCK_5_4_0_2_0
Intensity
m/z
204.0862
366.1386
138.0548
168.0652
274.0918
629.3058 y5(1+)
186.0756
292.1025
126.055
144.0658
528.1882
657.2328
758.3484 y6(1+)
1056.4066 b9(1+)
1284.5266 b11(1+)
1509.6282 PEP_0_0_0_0_0
1611.1631 PEP_0_1_0_0_0
0
100000
200000
300000
400000
500000
500
1000
1500
2000
2500
3000
3500

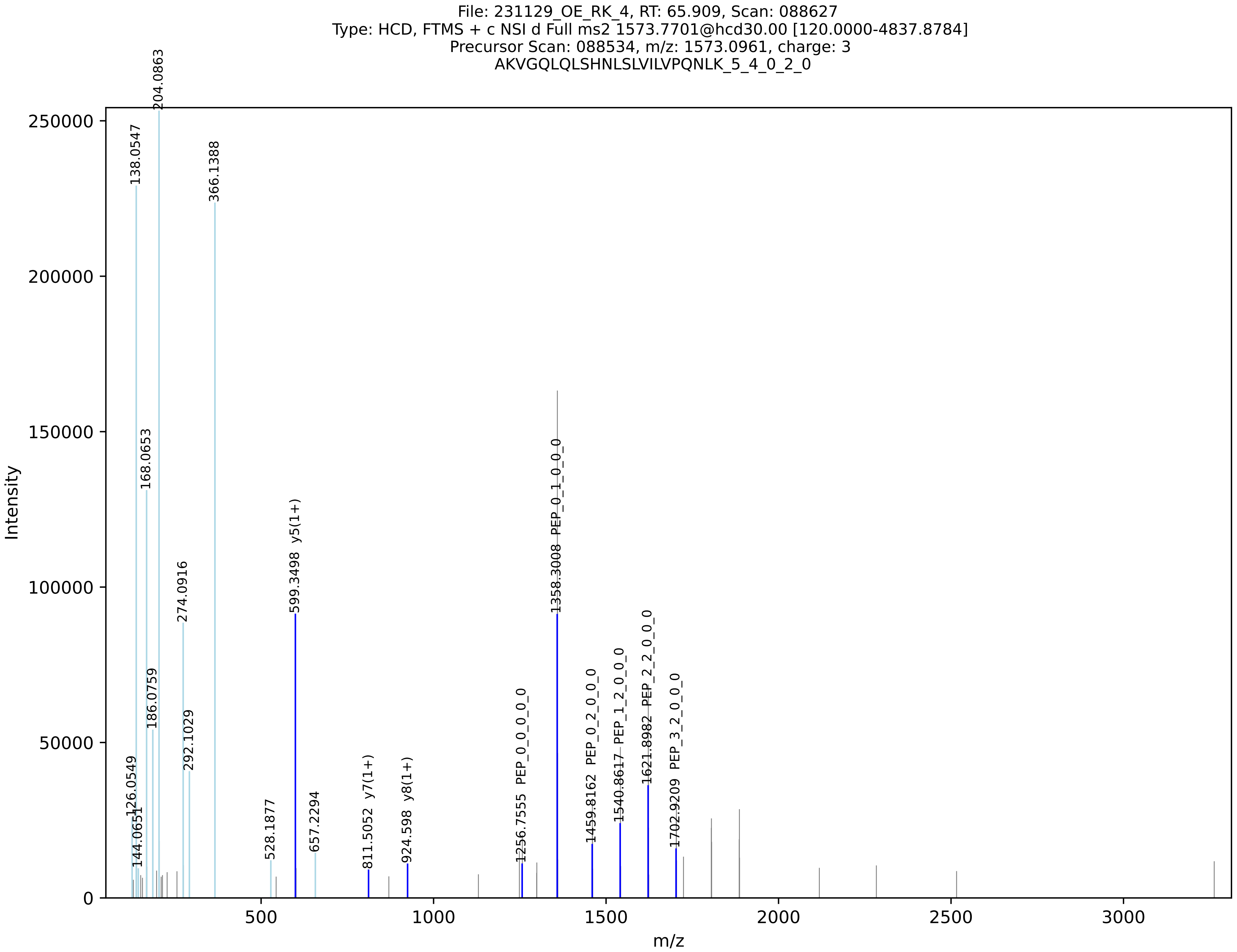

File: 231129_OE_RK_4, RT: 65.909, Scan: 088627
Type: HCD, FTMS + c NSI d Full ms2 1573.7701@hcd30.00 [120.0000-4837.8784]
Precursor Scan: 088534, m/z: 1573.0961, charge: 3
AKVGQLQLSHNLSLVILVPQNLK_5_4_0_2_0
Intensity
m/z
204.0863
138.0547
366.1388
168.0653
274.0916
186.0759
292.1029
126.0549
144.0651
528.1877
599.3498 y5(1+)
657.2294
811.5052 y7(1+)
924.598 y8(1+)
1256.7555 PEP_0_0_0_0_0
1358.3008 PEP_0_1_0_0_0
1459.8162 PEP_0_2_0_0_0
1540.8617 PEP_1_2_0_0_0
1621.8982 PEP_2_2_0_0_0
1702.9209 PEP_3_2_0_0_0

File: 231129_OE_RK_4, RT: 65.958, Scan: 088696
Type: HCD, FTMS + c NSI d Full ms2 1311.5172@hcd30.00 [120.0000-5373.8462]
Precursor Scan: 088605, m/z: 1310.7654, charge: 4
SPYEMFGDEEVMCLNGNWTEPPQCK_6_4_1_1_0

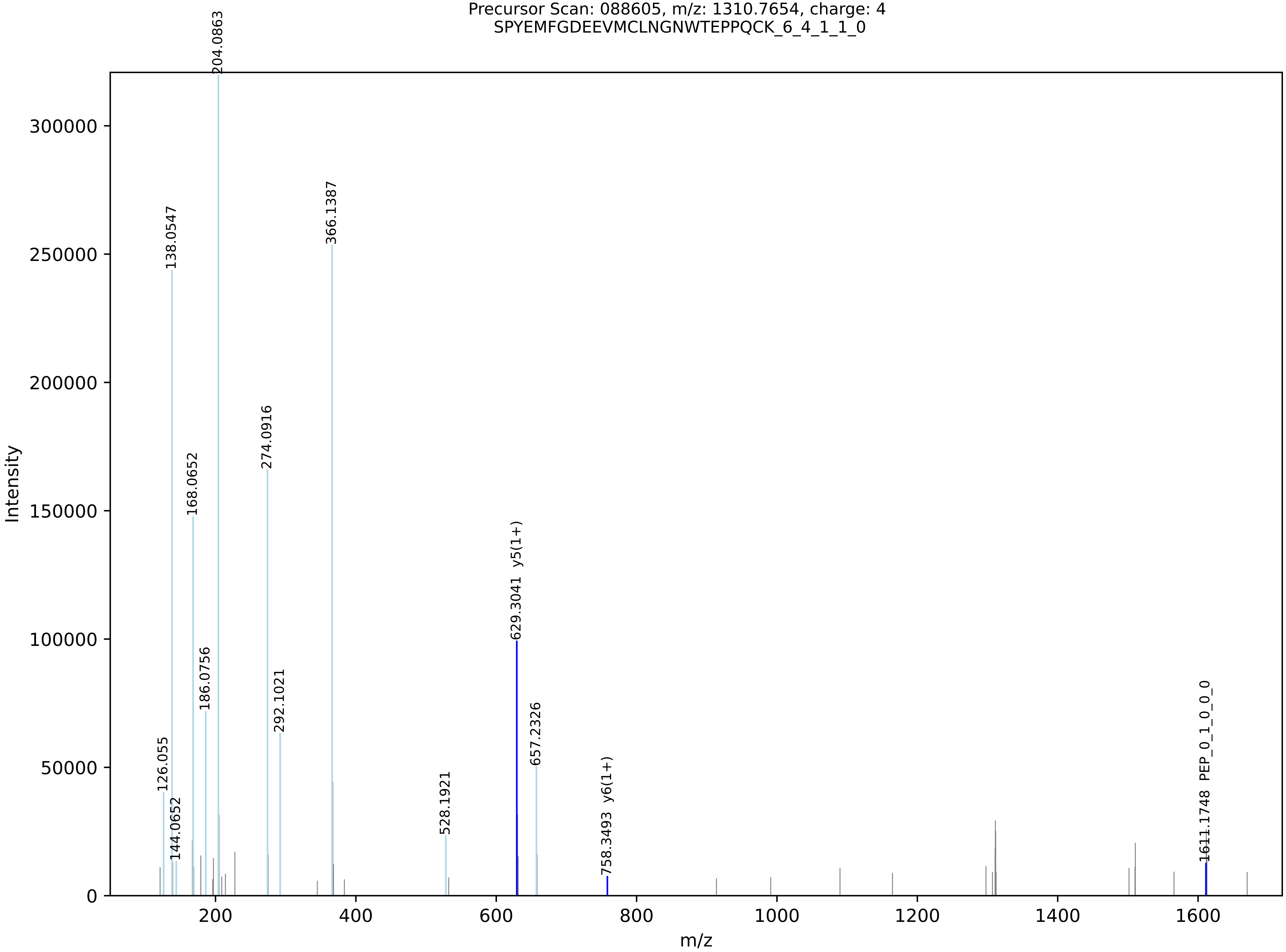

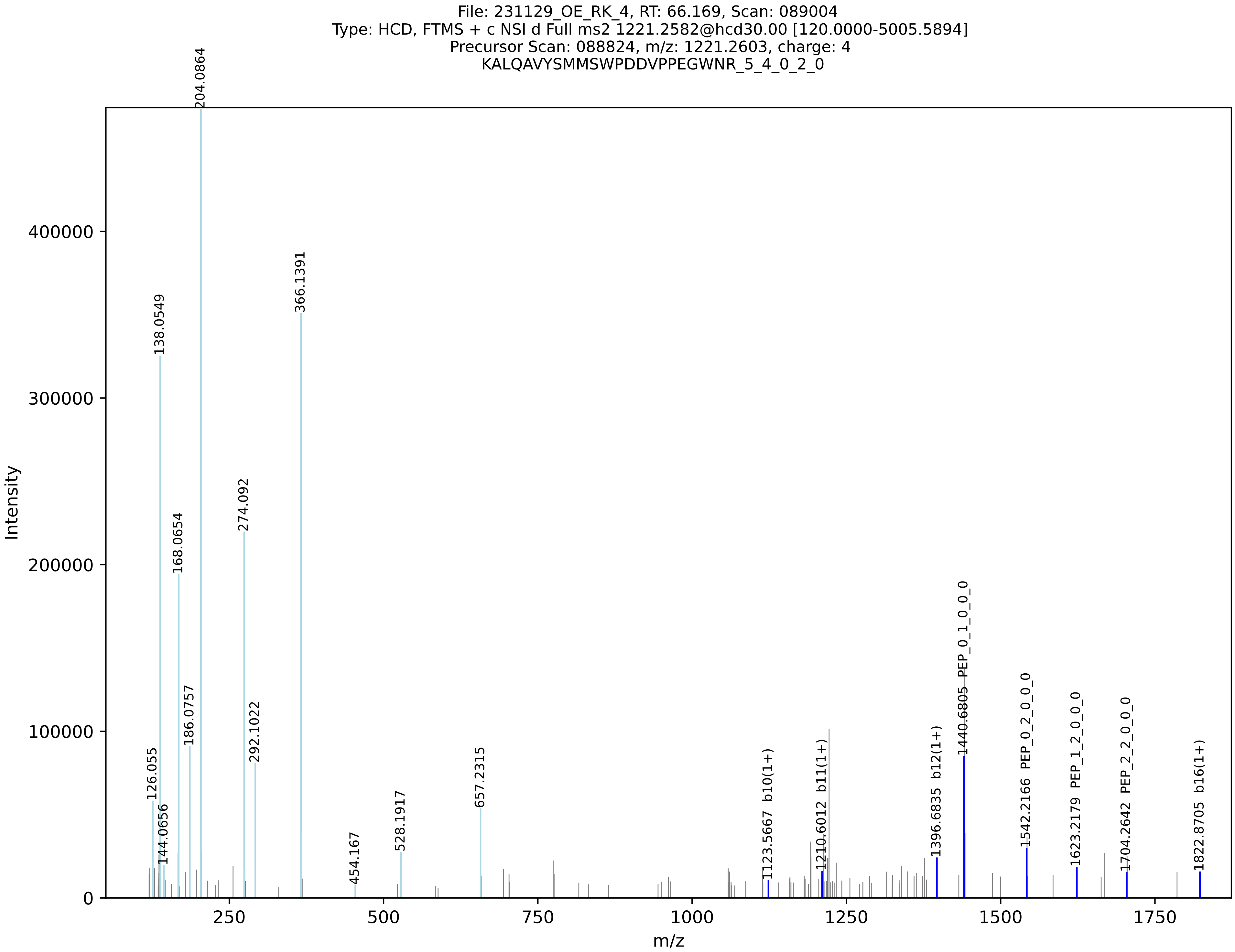
File: 231129_OE_RK_4, RT: 66.169, Scan: 089004
Type: HCD, FTMS + c NSI d Full ms2 1221.2582@hcd30.00 [120.0000-5005.5894]
Precursor Scan: 088824, m/z: 1221.2603, charge: 4
KALQAVYSMMSWPDDVPPEGWNR_5_4_0_2_0
Intensity
m/z
204.0864
366.1391
138.0549
274.092
168.0654
186.0757
292.1022
126.055
144.0656
657.2315
528.1917
454.167
1123.5667 b10(1+)
1210.6012 b11(1+)
1396.6835 b12(1+)
1440.6805 PEP_0_1_0_0_0
1542.2166 PEP_0_2_0_0_0
1623.2179 PEP_1_2_0_0_0
1704.2642 PEP_2_2_0_0_0
1822.8705 b16(1+)
0
100000
200000
300000
400000
250
500
750
1000
1250
1500
1750

File: 231129_OE_RK_4, RT: 66.218, Scan: 089069
Type: HCD, FTMS + c NSI d Full ms2 1148.2961@hcd30.00 [120.0000-5879.8799]
Precursor Scan: 089019, m/z: 1147.6919, charge: 5
CGLVPVLAENYNKSDNCEDTPEAGYFAVAVVK_5_4_0_2_0

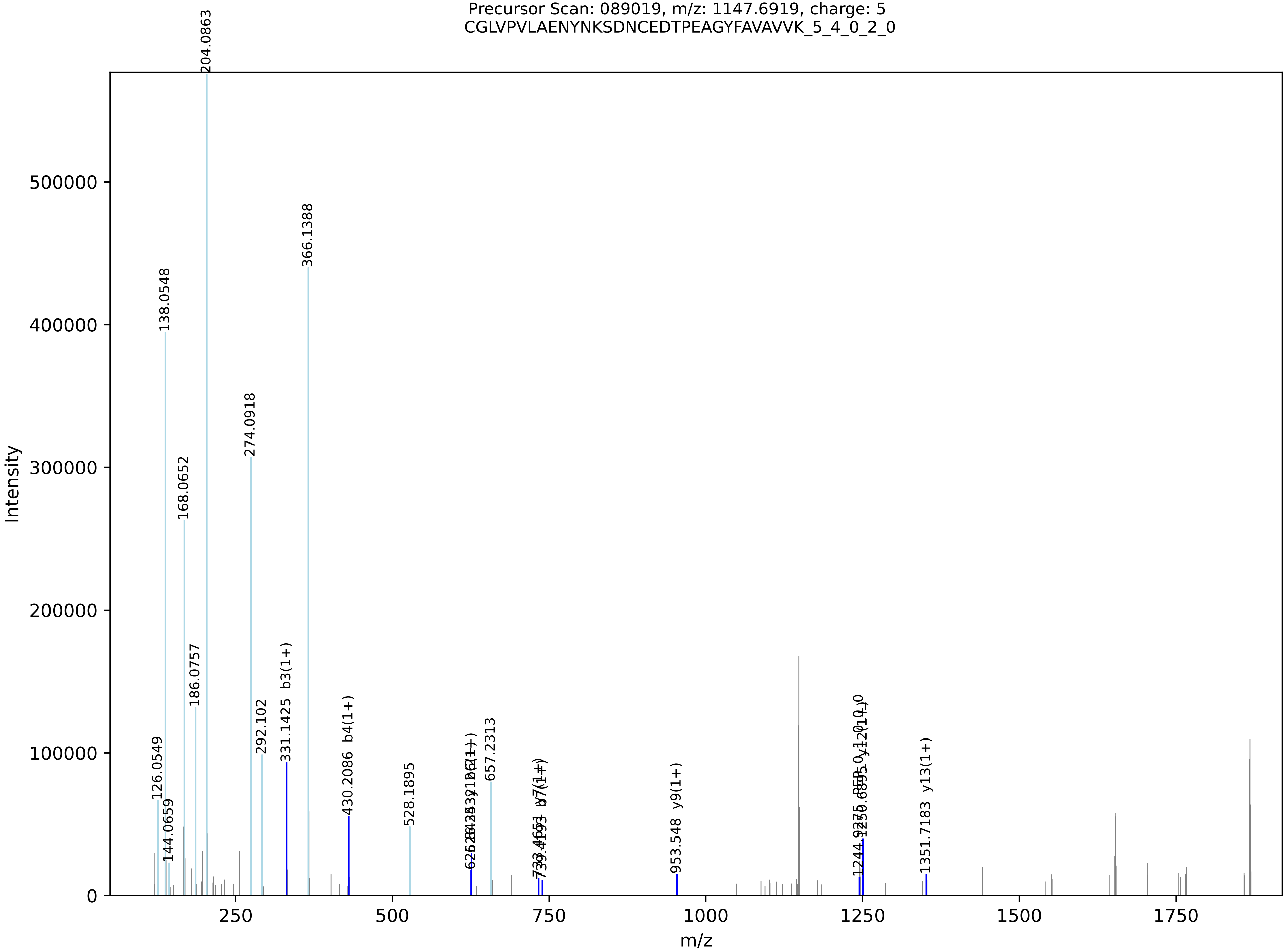

File: 231129_OE_RK_4, RT: 66.557, Scan: 089558
Type: HCD, FTMS + c NSI d Full ms2 1609.3977@hcd30.00 [120.0000-6589.1987]
Precursor Scan: 089468, m/z: 1608.6459, charge: 4
YTCEEPYYYMENGGGGEYHCAGNGSWVNEVLGPELPK_5_4_0_2_0

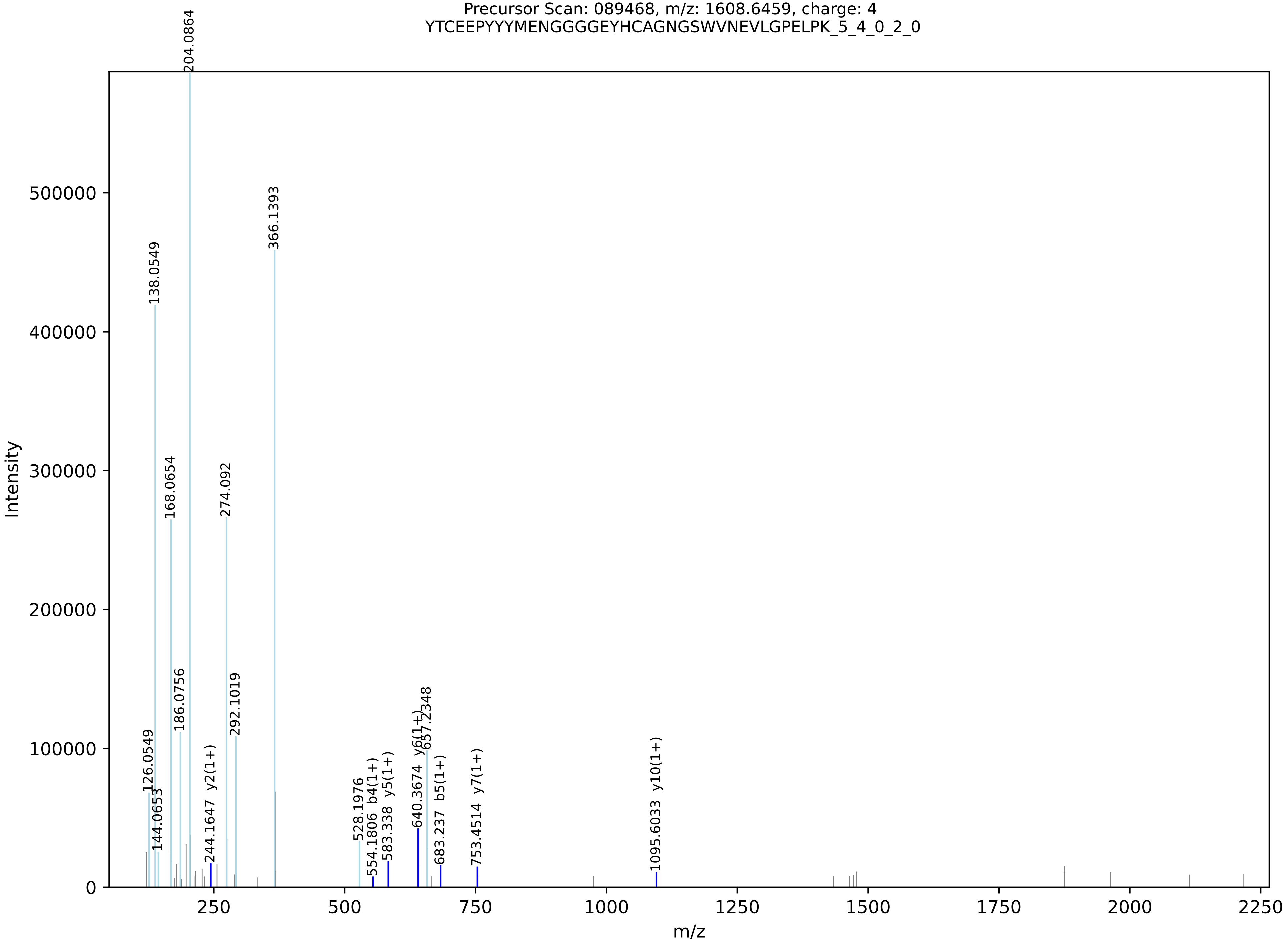

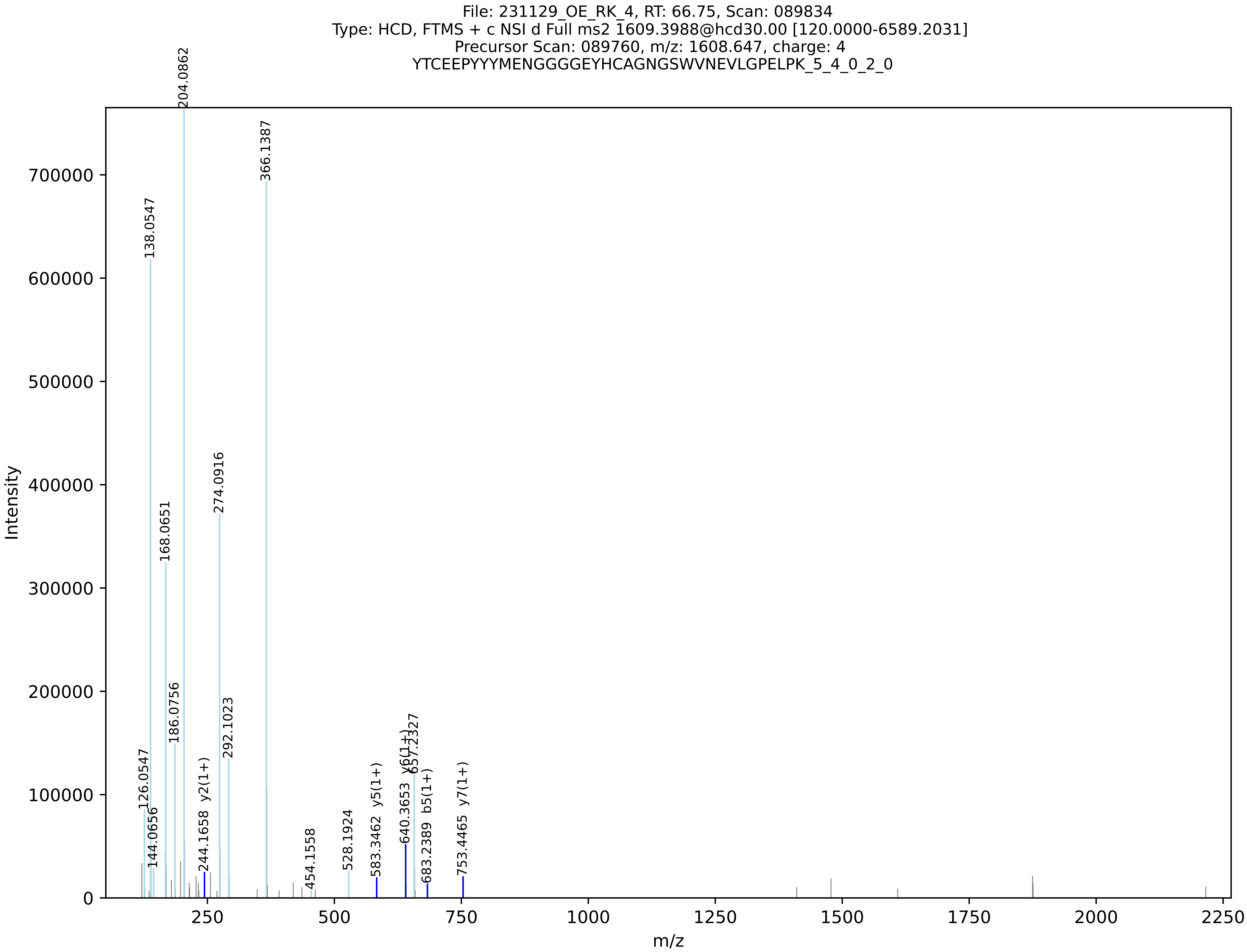
File: 231129_OE_RK_4, RT: 66.75, Scan: 089834
Type: HCD, FTMS + c NSI d Full ms2 1609.3988@hcd30.00 [120.0000-6589.2031]
Precursor Scan: 089760, m/z: 1608.647, charge: 4
YTCEEPYYYMENGGGGEYHCAGNGSWVNEVLGPELPK_5_4_0_2_0
Intensity
m/z
204.0862
366.1387
138.0547
274.0916
168.0651
186.0756
292.1023
126.0547
144.0656
244.1658 y2(1+)
454.1558
528.1924
583.3462 y5(1+)
640.3653 y6(1+)
657.2327
683.2389 b5(1+)
753.4465 y7(1+)
0
100000
200000
300000
400000
500000
600000
700000
250
500
750
1000
1250
1500
1750
2000
2250

File: 231129_OE_RK_4, RT: 66.755, Scan: 089841
Type: HCD, FTMS + c NSI d Full ms2 1307.2617@hcd30.00 [120.0000-5356.4839]
Precursor Scan: 089760, m/z: 1306.5094, charge: 4
SPYEMFGDEEVMCLNGNWTEPPQCK_5_4_0_2_0

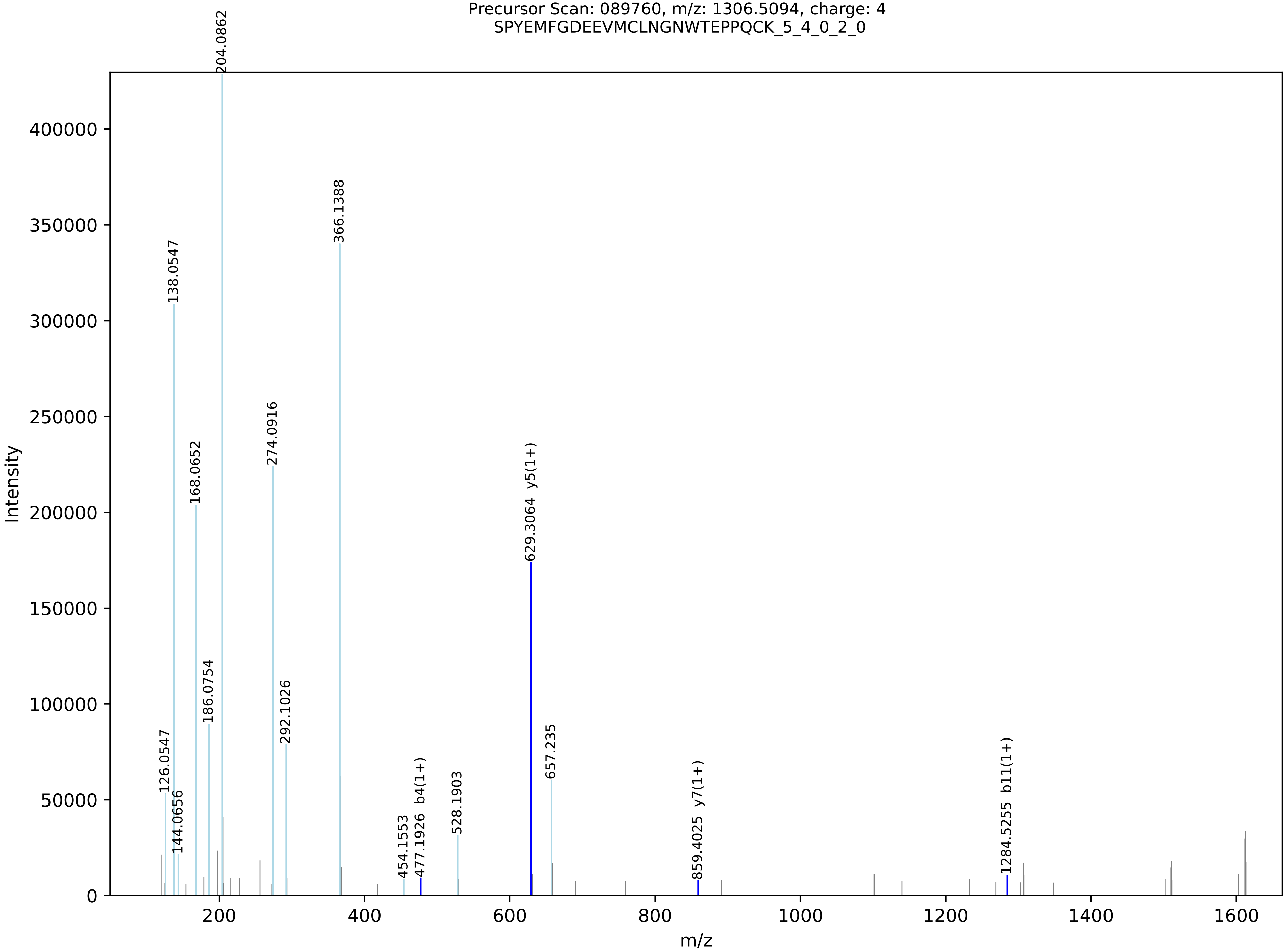

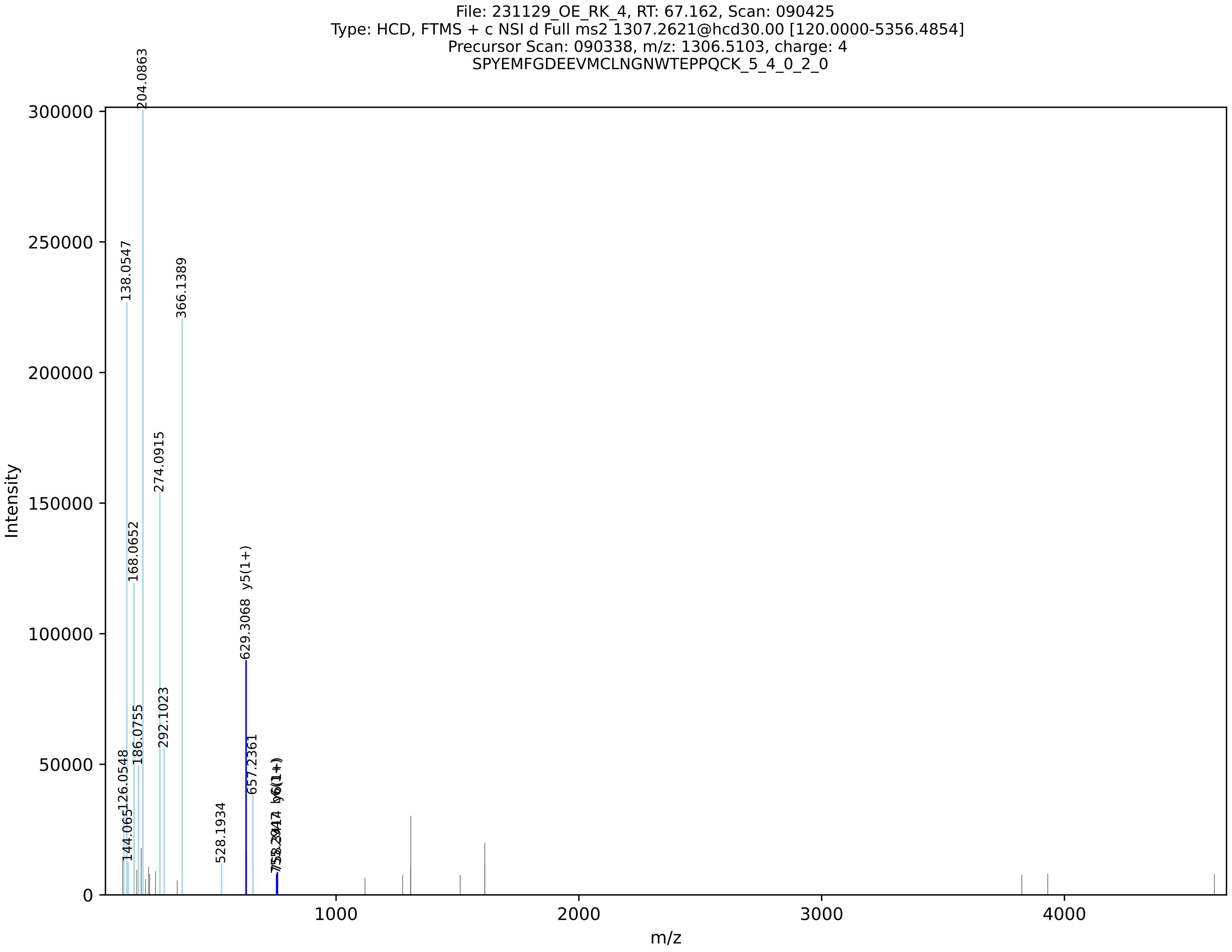

File: 231129_OE_RK_4, RT: 67.162, Scan: 090425
Type: HCD, FTMS + c NSI d Full ms2 1307.2621@hcd30.00 [120.0000-5356.4854]
Precursor Scan: 090338, m/z: 1306.5103, charge: 4
SPYEMFGDEEVMCLNGNWTEPPQCK_5_4_0_2_0
Intensity
m/z
0
50000
100000
150000
200000
250000
300000
1000
2000
3000
4000
204.0863
138.0547
366.1389
274.0915
168.0652
629.3068 y5(1+)
186.0755
292.1023
126.0548
144.065
528.1934
657.2361

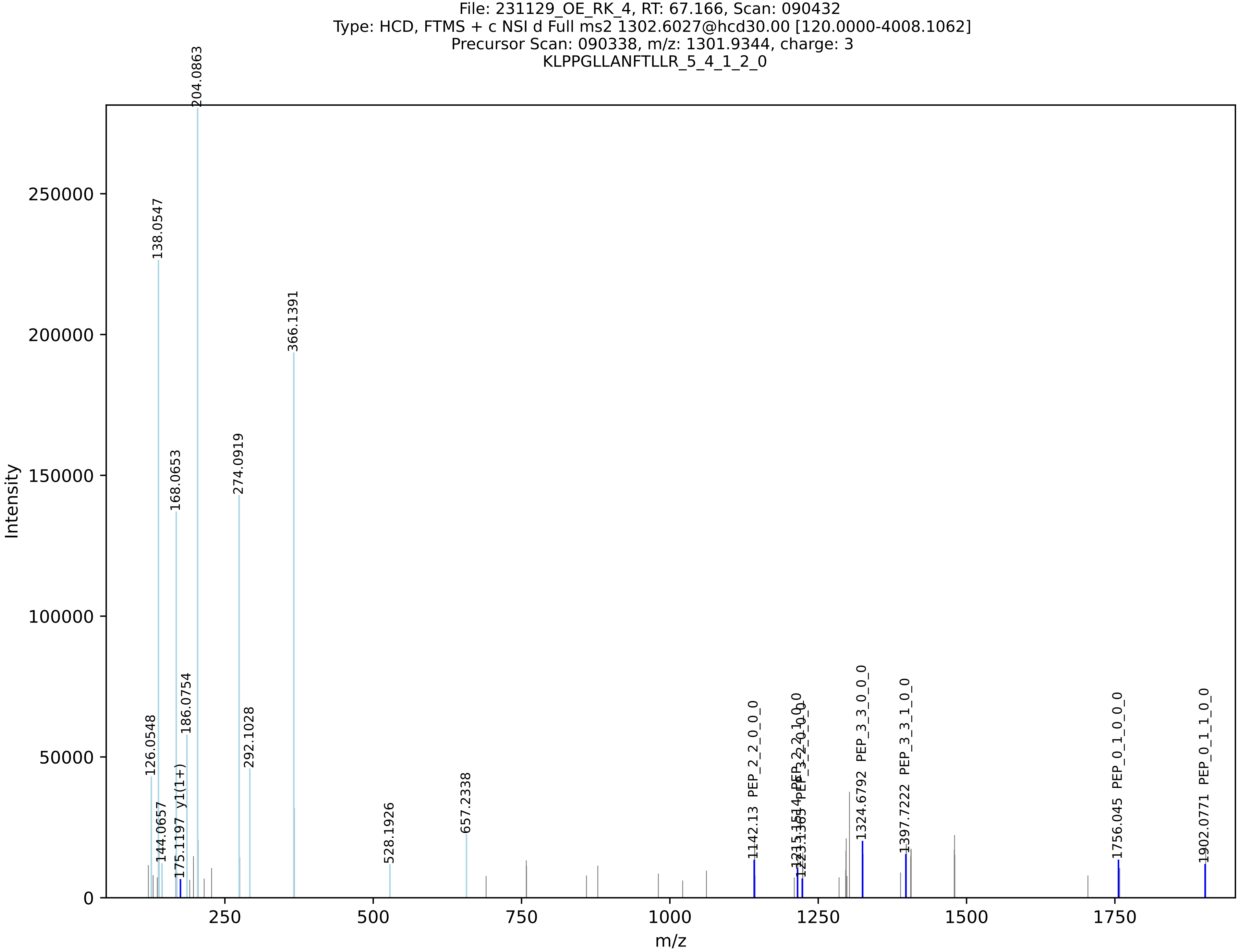

File: 231129_OE_RK_4, RT: 67.166, Scan: 090432
Type: HCD, FTMS + c NSI d Full ms2 1302.6027@hcd30.00 [120.0000-4008.1062]
Precursor Scan: 090338, m/z: 1301.9344, charge: 3
KLPPGLLANFTLLR_5_4_1_2_0
Intensity
m/z
0
50000
100000
150000
200000
250000
250
500
750
1000
1250
1500
1750
204.0863
138.0547
366.1391
168.0653
274.0919
186.0754
292.1028
126.0548
144.0657
175.1197 y1(1+)
528.1926
657.2338
1142.13 PEP_2_2_0_0_0
1215.1514 PEP_2_2_1_0_0
1324.6792 PEP_3_3_0_0_0
1397.7222 PEP_3_3_1_0_0
1756.045 PEP_0_1_0_0_0
1902.0771 PEP_0_1_1_0_0

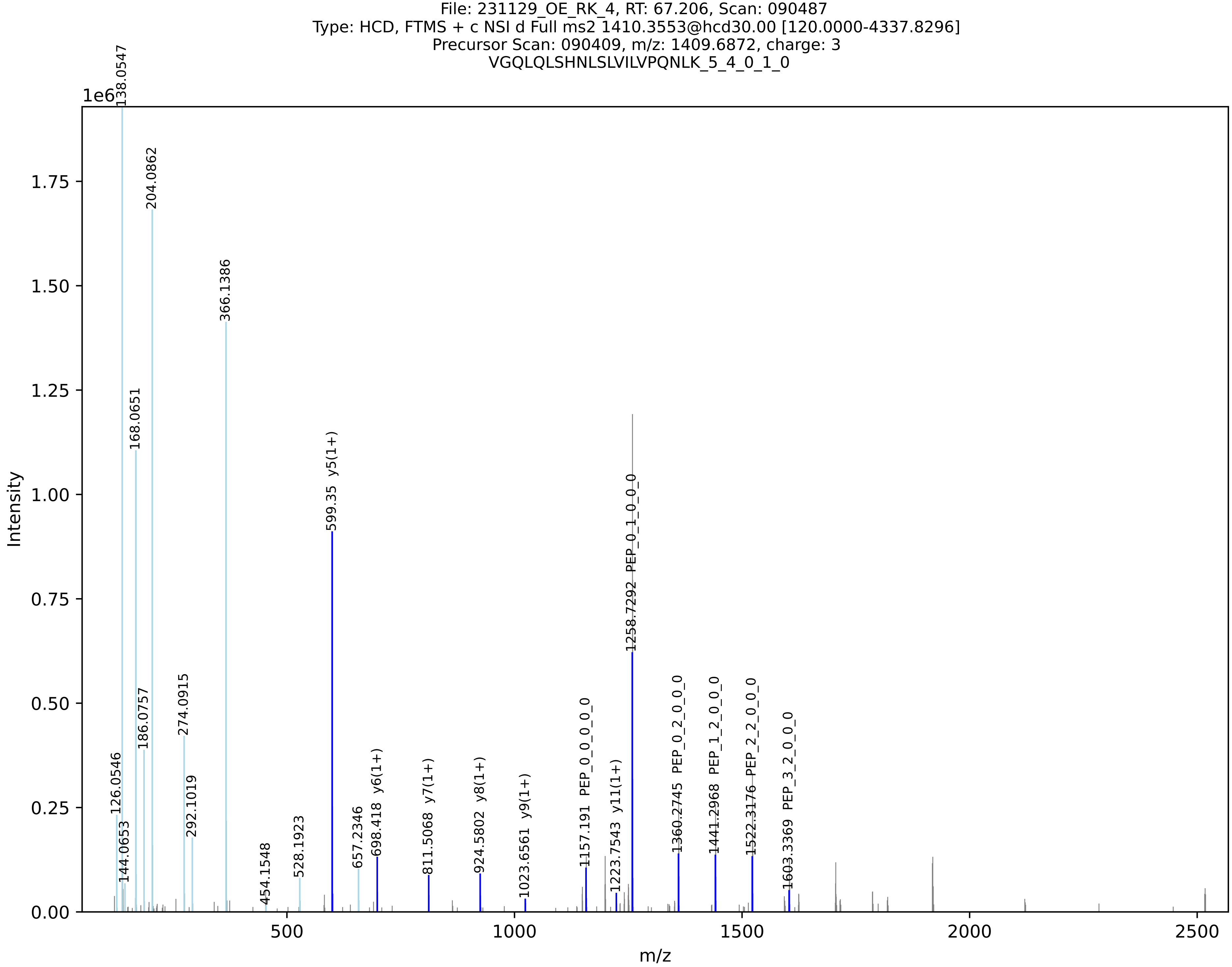
File: 231129_OE_RK_4, RT: 67.206, Scan: 090487
Type: HCD, FTMS + c NSI d Full ms2 1410.3553@hcd30.00 [120.0000-4337.8296]
Precursor Scan: 090409, m/z: 1409.6872, charge: 3
VGQLQLSHNLSLVILVPQNLK_5_4_0_1_0
1e6
Intensity
m/z
138.0547
204.0862
366.1386
168.0651
599.35 y5(1+)
1258.7292 PEP_0_1_0_0_0
274.0915
186.0757
126.0546
292.1019
144.0653
454.1548
528.1923
657.2346
698.418 y6(1+)
811.5068 y7(1+)
924.5802 y8(1+)
1023.6561 y9(1+)
1157.191 PEP_0_0_0_0_0
1223.7543 y11(1+)
1360.2745 PEP_0_2_0_0_0
1441.2968 PEP_1_2_0_0_0
1522.3176 PEP_2_2_0_0_0
1603.3369 PEP_3_2_0_0_0

File: 231129_OE_RK_4, RT: 67.411, Scan: 090787
Type: HCD, FTMS + c NSI d Full ms2 1302.2716@hcd30.00 [120.0000-4007.0930]
Precursor Scan: 090702, m/z: 1301.9374, charge: 3
KLPPGLLANFTLLR_5_4_1_2_0

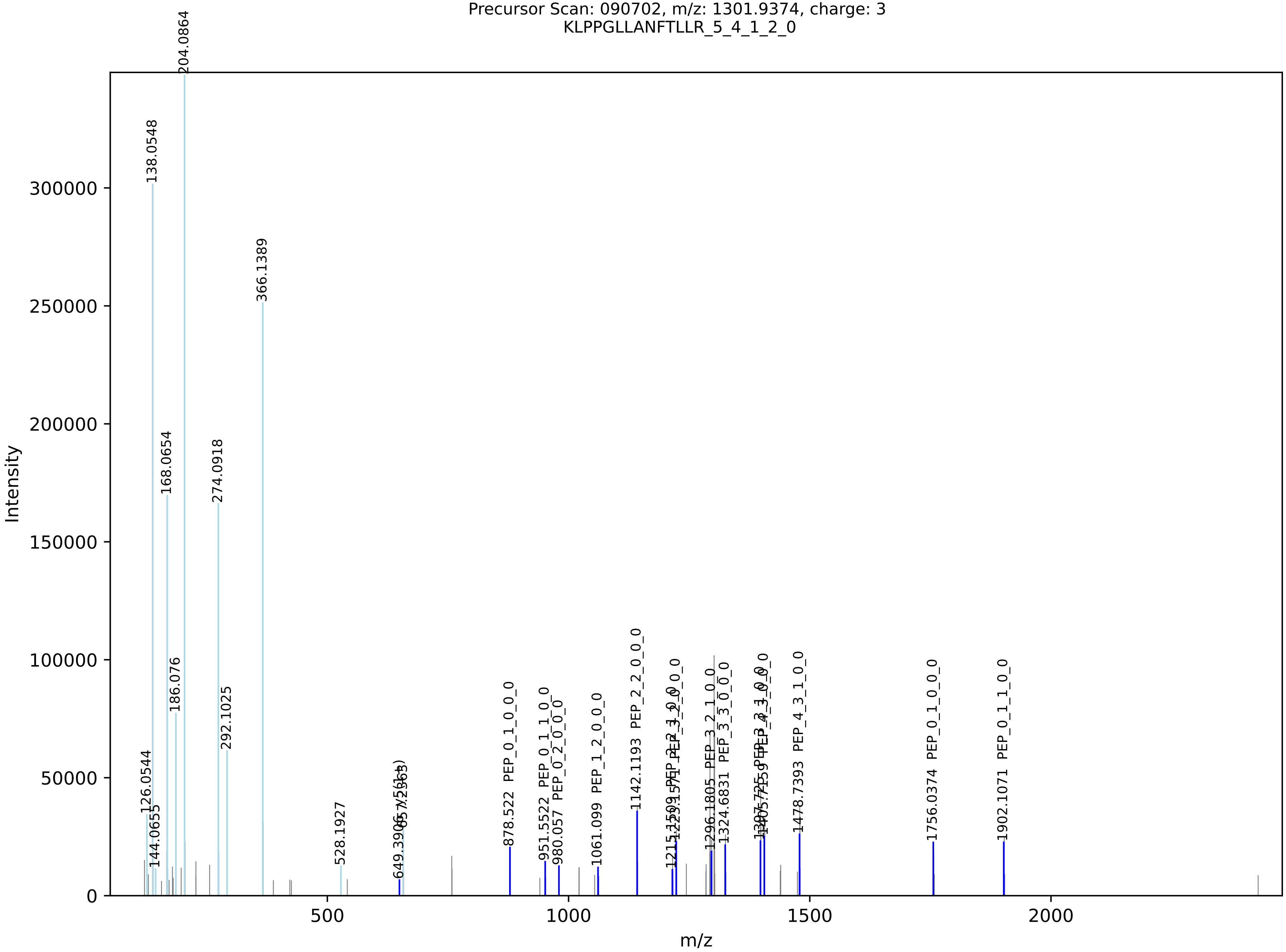

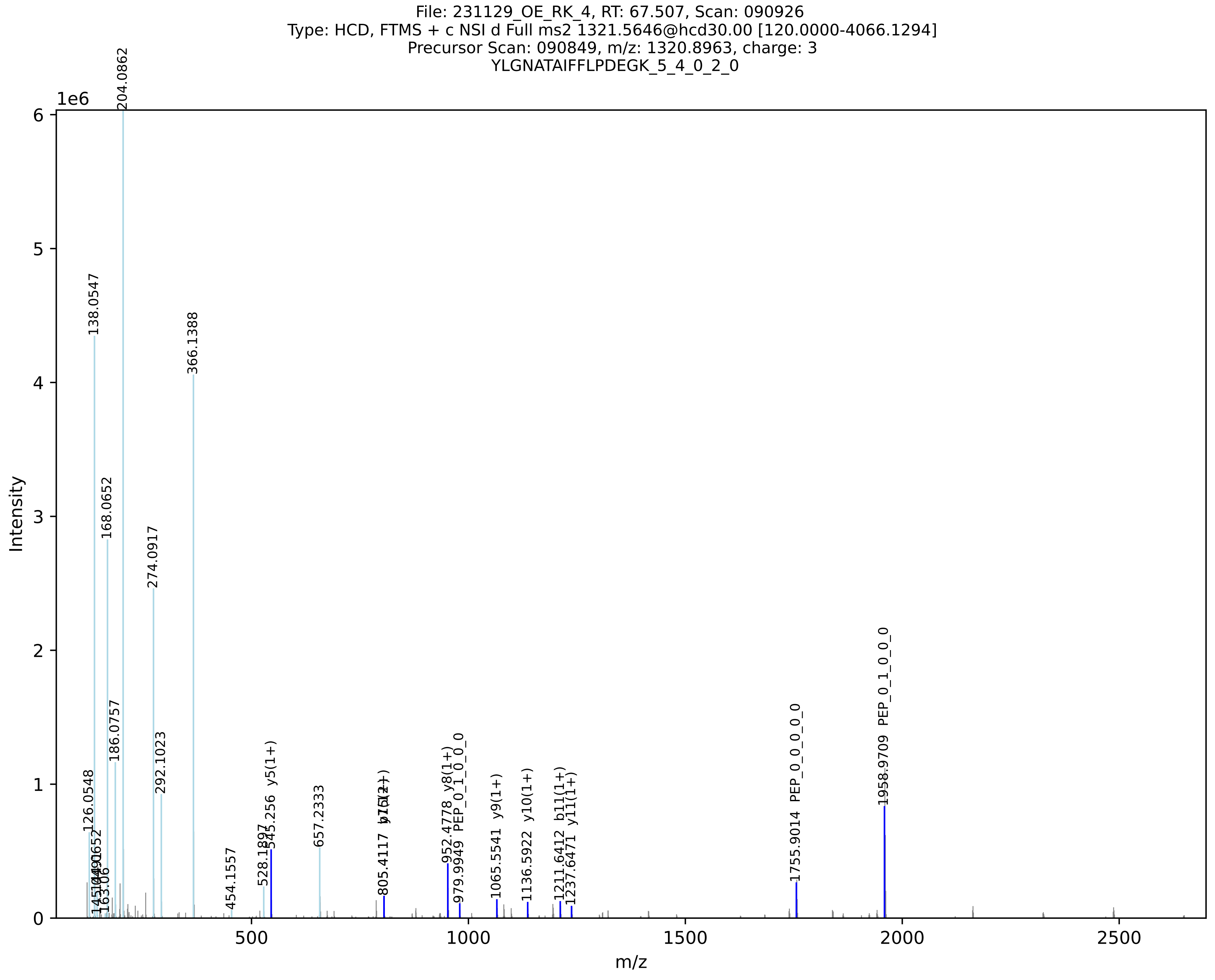
File: 231129_OE_RK_4, RT: 67.507, Scan: 090926
Type: HCD, FTMS + c NSI d Full ms2 1321.5646@hcd30.00 [120.0000-4066.1294]
Precursor Scan: 090849, m/z: 1320.8963, charge: 3
YLGNATAIFFLPDEGK_5_4_0_2_0
1e6
Intensity
m/z
204.0862
138.0547
366.1388
168.0652
274.0917
186.0757
292.1023
126.0548
545.256 y5(1+)
657.2333
528.1897
454.1557
805.4117
952.4778 y8(1+)
979.9949 PEP_0_1_0_0_0
1065.5541 y9(1+)
1136.5922 y10(1+)
1211.6412 b11(1+)
1237.6471 y11(1+)
1755.9014 PEP_0_0_0_0_0
1958.9709 PEP_0_1_0_0_0

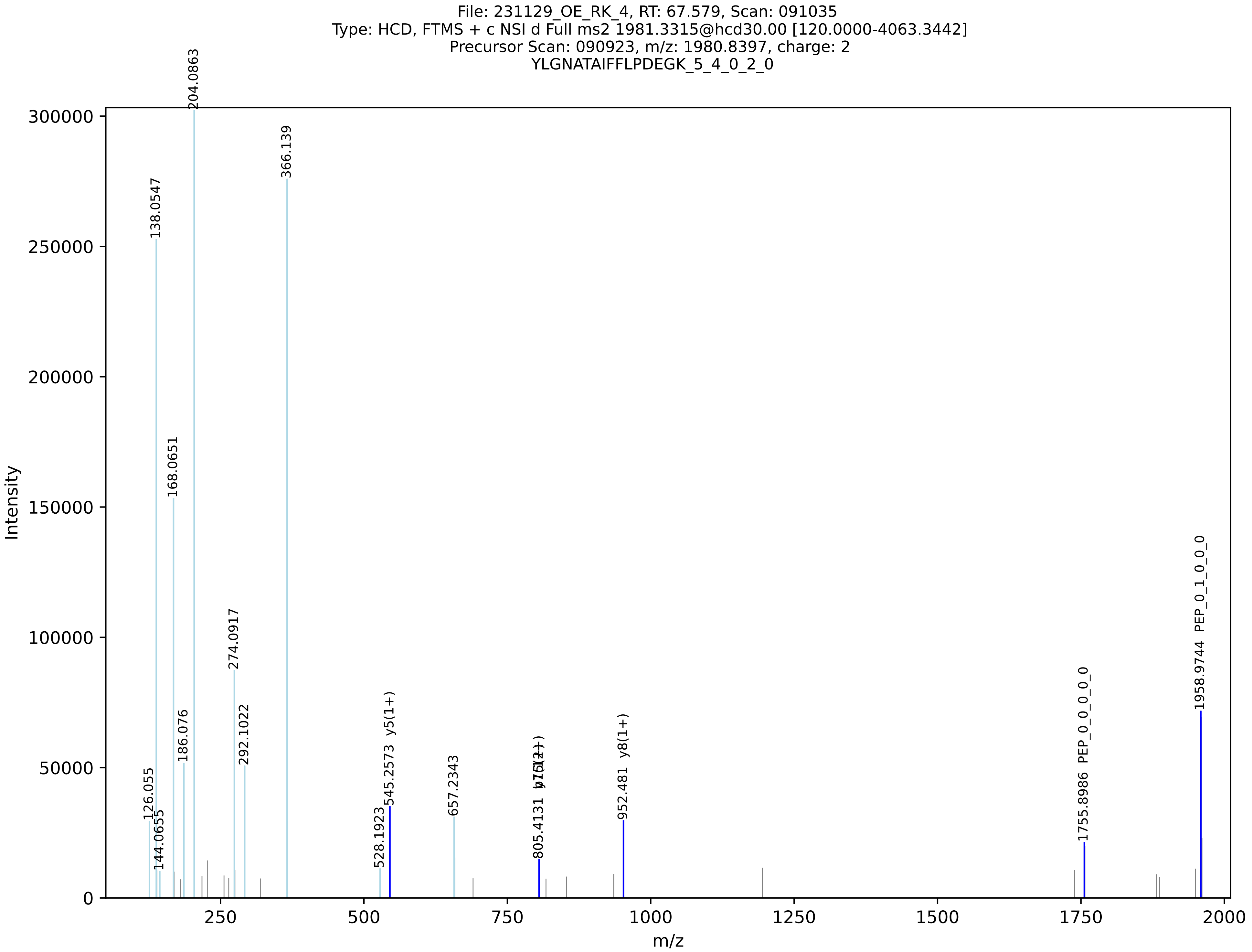
File: 231129_OE_RK_4, RT: 67.579, Scan: 091035
Type: HCD, FTMS + c NSI d Full ms2 1981.3315@hcd30.00 [120.0000-4063.3442]
Precursor Scan: 090923, m/z: 1980.8397, charge: 2
YLGNATAIFFLPDEGK_5_4_0_2_0
Intensity
m/z
204.0863
366.139
138.0547
168.0651
274.0917
186.076
292.1022
126.055
144.0655
528.1923
545.2573 y5(1+)
657.2343
805.4131
952.481 y8(1+)
1755.8986 PEP_0_0_0_0_0
1958.9744 PEP_0_1_0_0_0
0
50000
100000
150000
200000
250000
300000
250
500
750
1000
1250
1500
1750
2000

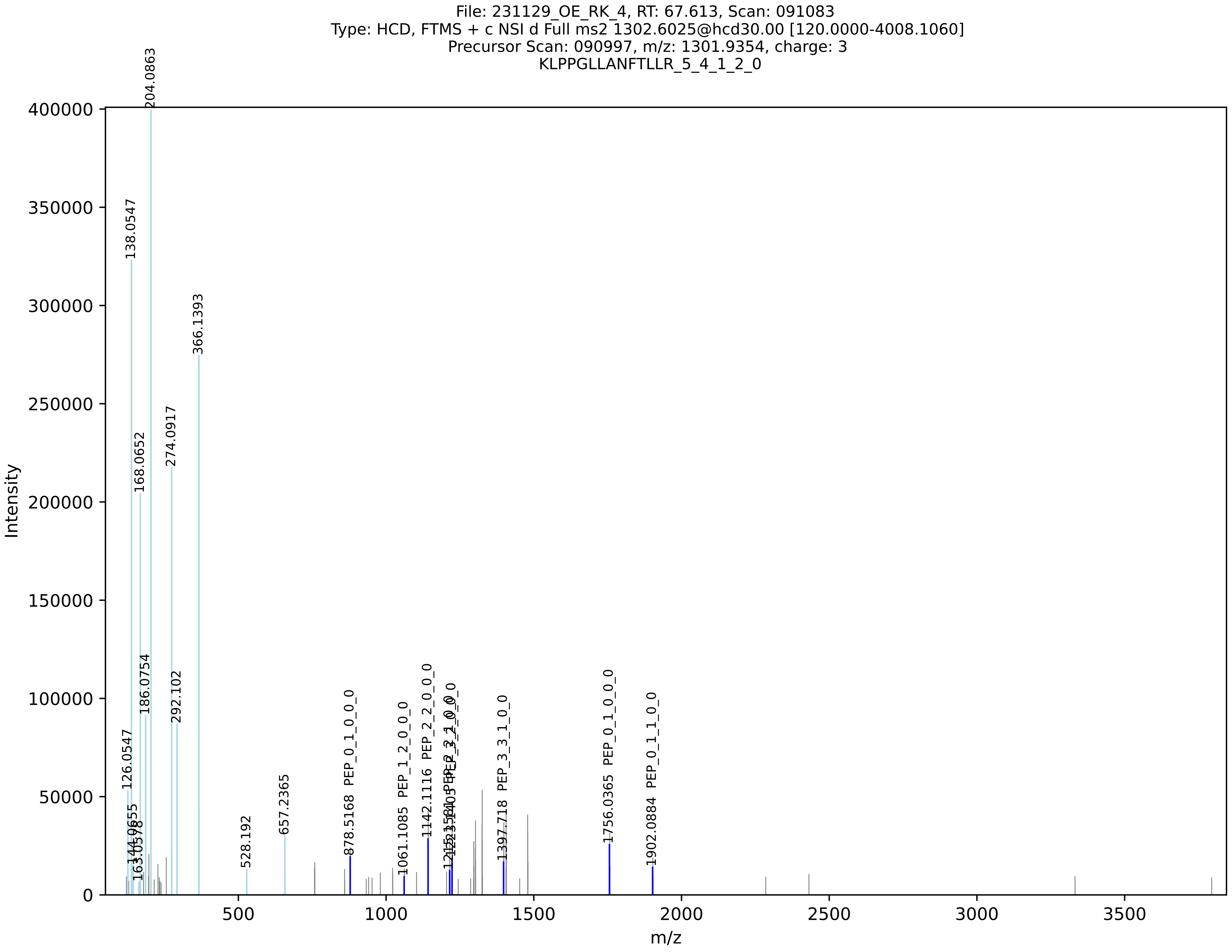

File: 231129_OE_RK_4, RT: 67.613, Scan: 091083
Type: HCD, FTMS + c NSI d Full ms2 1302.6025@hcd30.00 [120.0000-4008.1060]
Precursor Scan: 090997, m/z: 1301.9354, charge: 3
KLPPGLLANFTLLR_5_4_1_2_0
Intensity
m/z
204.0863
138.0547
366.1393
274.0917
168.0652
186.0754
292.102
126.0547
528.192
657.2365
878.5168 PEP_0_1_0_0_0_
1061.1085 PEP_1_2_0_0_0_
1142.1116 PEP_2_2_0_0_0_
1397.718 PEP_3_3_1_0_0_
1756.0365 PEP_0_1_0_0_0_
1902.0884 PEP_0_1_1_0_0_

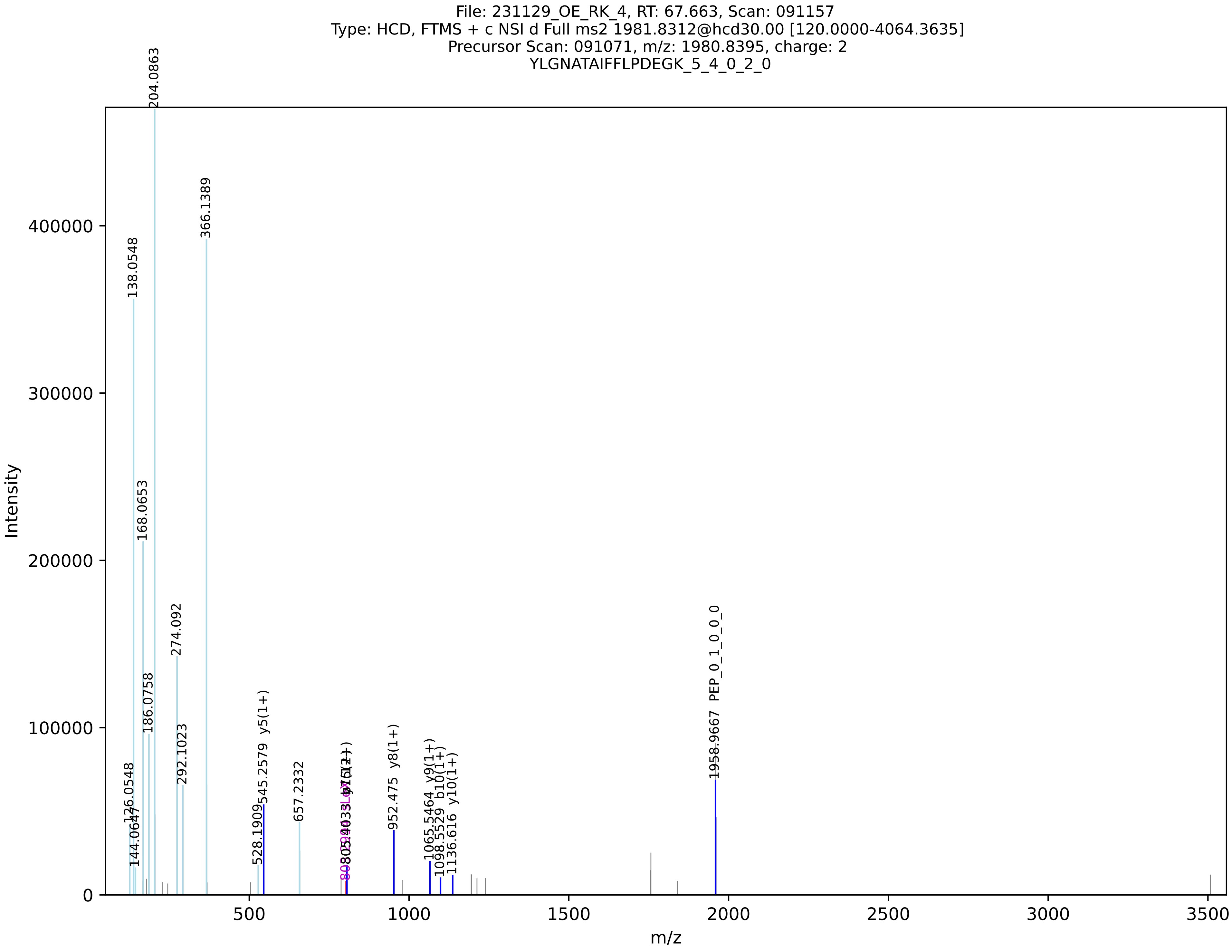
File: 231129_OE_RK_4, RT: 67.663, Scan: 091157
Type: HCD, FTMS + c NSI d Full ms2 1981.8312@hcd30.00 [120.0000-4064.3635]
Precursor Scan: 091071, m/z: 1980.8395, charge: 2
YLGNATAIFFLPDEGK_5_4_0_2_0
Intensity
m/z
204.0863
366.1389
138.0548
168.0653
274.092
186.0758
292.1023
126.0548
144.0647
528.1909
545.2579 y5(1+)
657.2332
952.475 y8(1+)
1065.5464 y9(1+)
1098.5529 b10(1+)
1136.616 y10(1+)
1958.9667 PEP_0_1_0_0_0
0
100000
200000
300000
400000
500
1000
1500
2000
2500
3000
3500

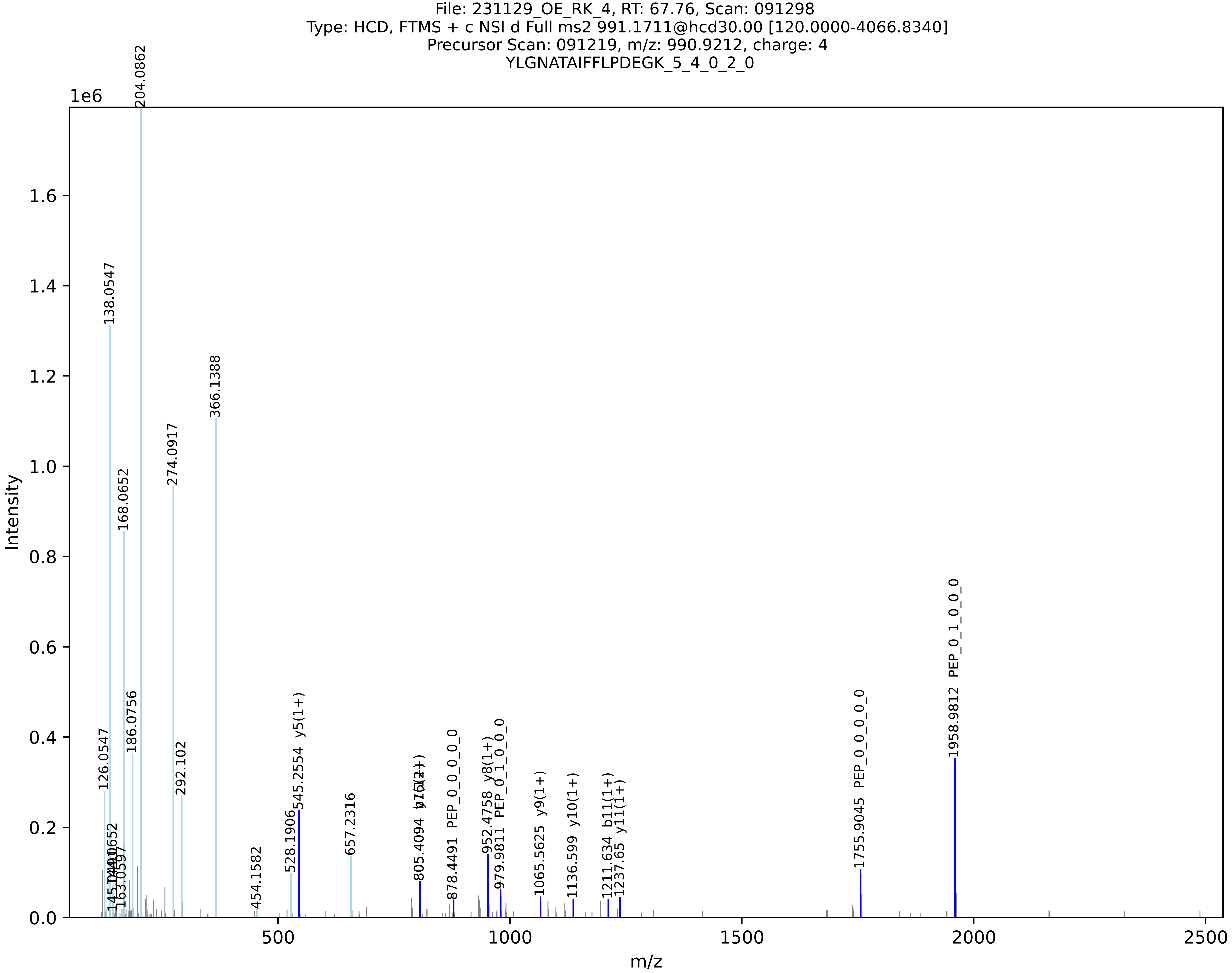
File: 231129_OE_RK_4, RT: 67.76, Scan: 091298
Type: HCD, FTMS + c NSI d Full ms2 991.1711@hcd30.00 [120.0000-4066.8340]
Precursor Scan: 091219, m/z: 990.9212, charge: 4
YLGNATAIFFLPDEGK_5_4_0_2_0
1e6
Intensity
m/z
0.0
0.2
0.4
0.6
0.8
1.0
1.2
1.4
1.6
500
1000
1500
2000
2500
204.0862
138.0547
168.0652
274.0917
366.1388
126.0547
186.0756
292.102
163.0597
454.1582
528.1906
545.2554 y5(1+)
657.2316
805.4094
878.4491 PEP_0_0_0_0_0
952.4758 y8(1+)
979.9811 PEP_0_1_0_0_0
1065.5625 y9(1+)
1136.599 y10(1+)
1211.634 b11(1+)
1237.65 y11(1+)
1755.9045 PEP_0_0_0_0_0
1958.9812 PEP_0_1_0_0_0

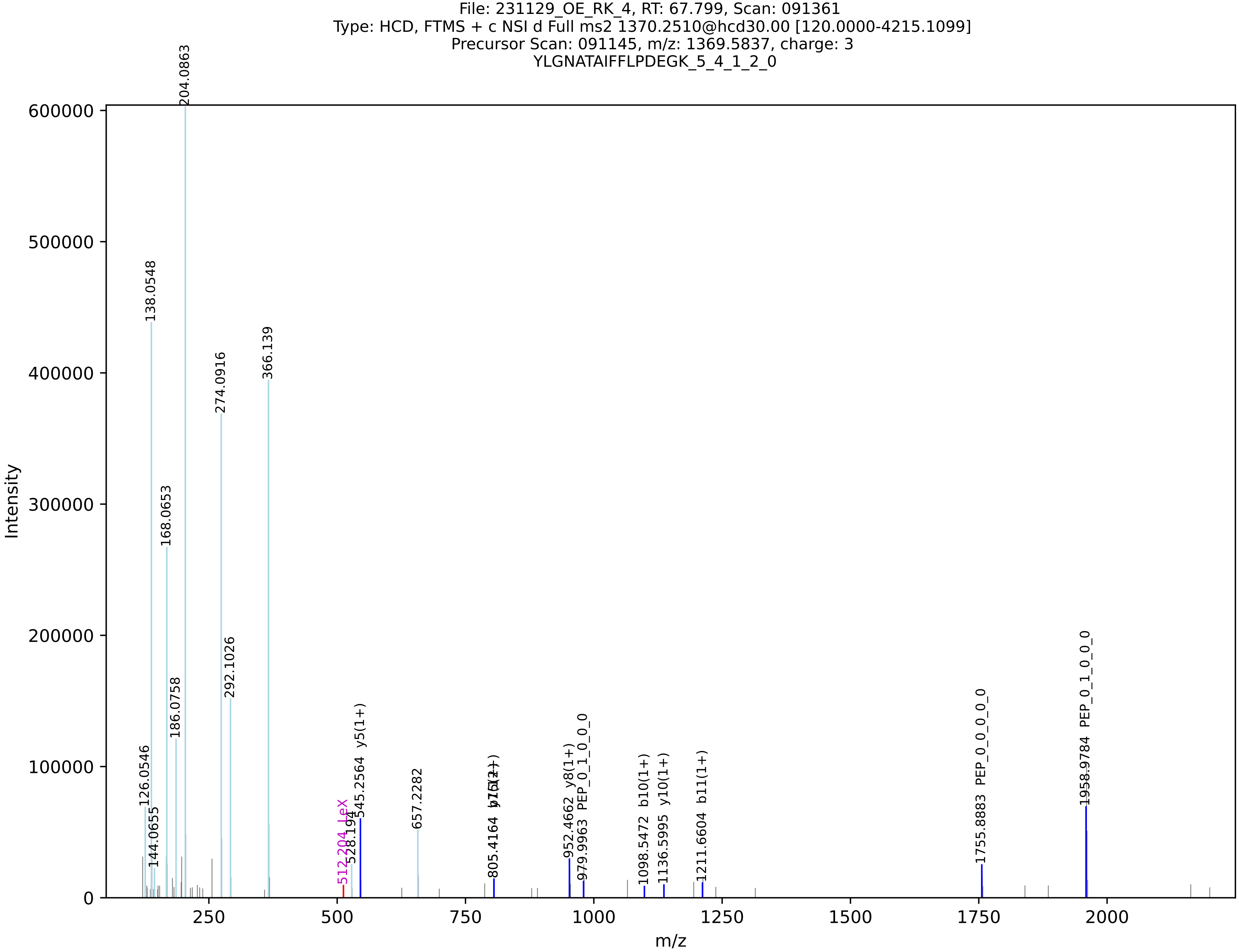
File: 231129_OE_RK_4, RT: 67.799, Scan: 091361
Type: HCD, FTMS + c NSI d Full ms2 1370.2510@hcd30.00 [120.0000-4215.1099]
Precursor Scan: 091145, m/z: 1369.5837, charge: 3
YLGNATAIFFLPDEGK_5_4_1_2_0
Intensity
m/z
204.0863
138.0548
366.139
274.0916
168.0653
292.1026
186.0758
126.0546
144.0655
512.204_LeX
528.194
545.2564 y5(1+)
657.2282
805.4164
952.4662 y8(1+)
979.9963 PEP_0_1_0_0_0
1098.5472 b10(1+)
1136.5995 y10(1+)
1211.6604 b11(1+)
1755.8883 PEP_0_0_0_0_0
1958.9784 PEP_0_1_0_0_0
0
100000
200000
300000
400000
500000
600000
250
500
750
1000
1250
1500
1750
2000

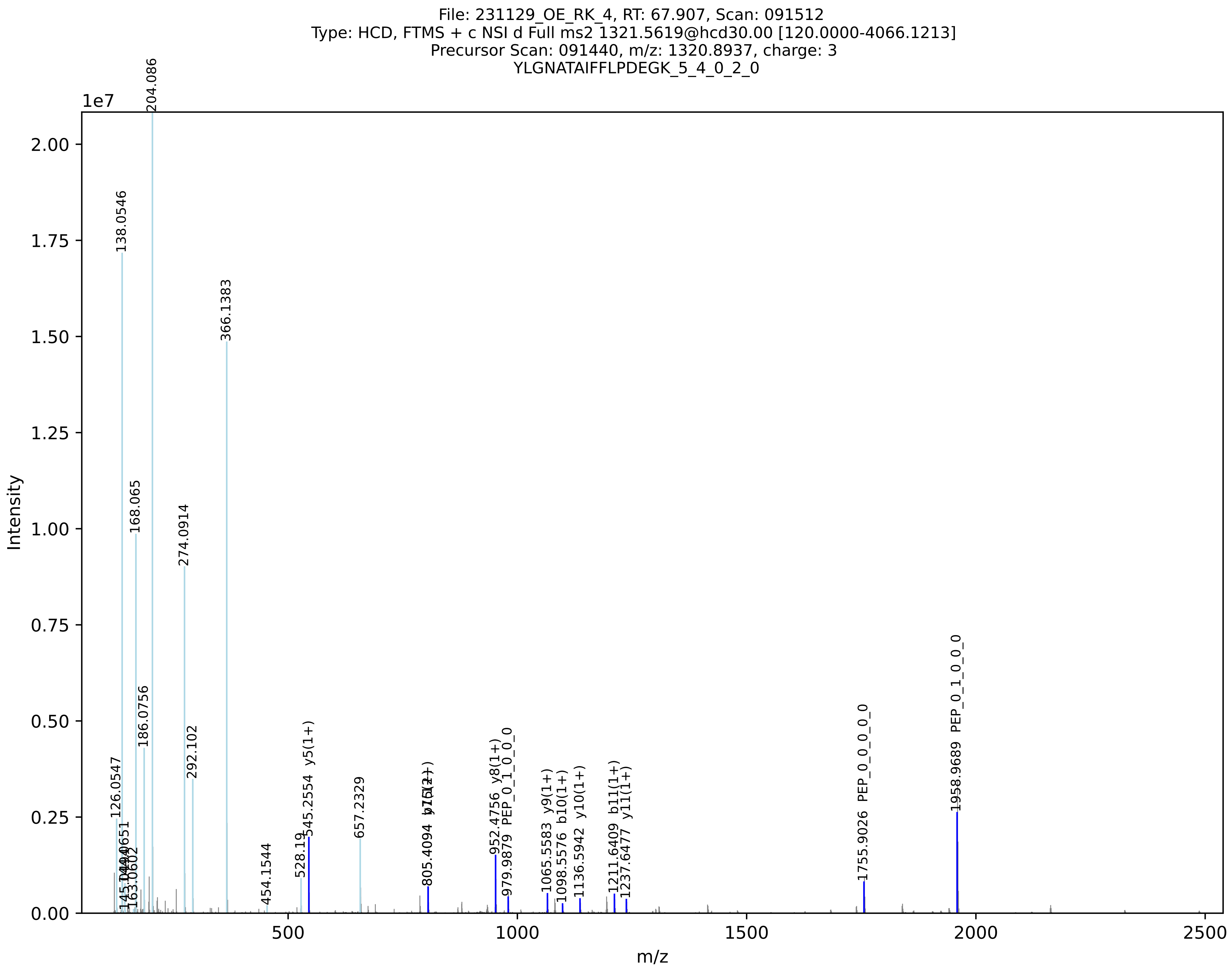
File: 231129_OE_RK_4, RT: 67.907, Scan: 091512
Type: HCD, FTMS + c NSI d Full ms2 1321.5619@hcd30.00 [120.0000-4066.1213]
Precursor Scan: 091440, m/z: 1320.8937, charge: 3
YLGNATAIFFLPDEGK_5_4_0_2_0
1e7
Intensity
m/z
204.086
138.0546
366.1383
168.065
274.0914
186.0756
292.102
126.0547
545.2554 y5(1+)
657.2329
528.19
454.1544
805.4094
952.4756 y8(1+)
979.9879 PEP_0_1_0_0_0
1065.5583 y9(1+)
1098.5576 b10(1+)
1136.5942 y10(1+)
1211.6409 b11(1+)
1237.6477 y11(1+)
1755.9026 PEP_0_0_0_0_0
1958.9689 PEP_0_1_0_0_0

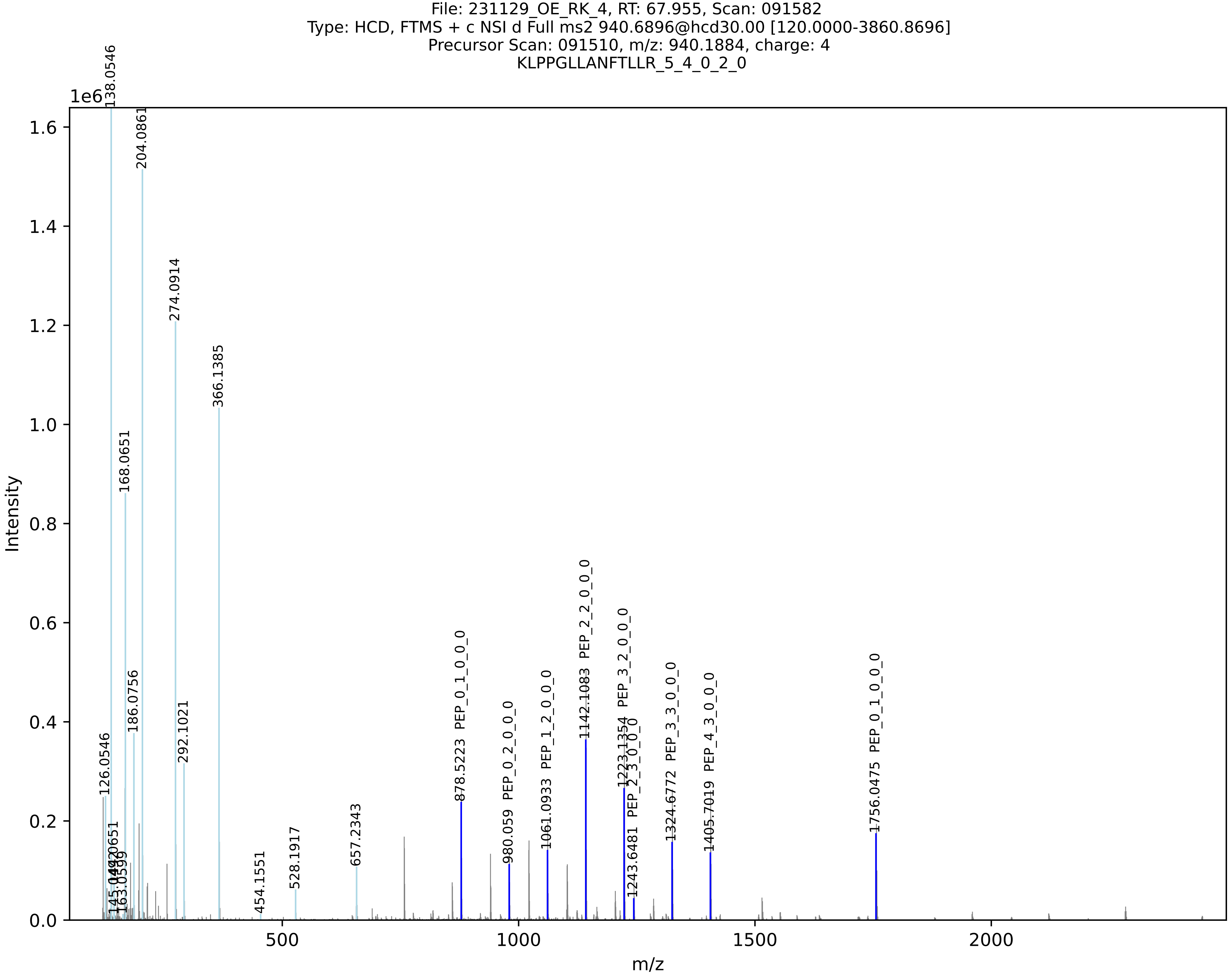

File: 231129_OE_RK_4, RT: 67.955, Scan: 091582
Type: HCD, FTMS + c NSI d Full ms2 940.6896@hcd30.00 [120.0000-3860.8696]
Precursor Scan: 091510, m/z: 940.1884, charge: 4
KLPPGLLANFTLLR_5_4_0_2_0
1e6
Intensity
m/z
138.0546
204.0861
274.0914
366.1385
168.0651
186.0756
292.1021
126.0546
163.0599
454.1551
528.1917
657.2343
878.5223 PEP_0_1_0_0_0
980.059 PEP_0_2_0_0_0
1061.0933 PEP_1_2_0_0_0
1142.1083 PEP_2_2_0_0_0
1223.1354 PEP_3_2_0_0_0
1243.6481 PEP_2_3_0_0_0
1324.6772 PEP_3_3_0_0_0
1405.7019 PEP_4_3_0_0_0
1756.0475 PEP_0_1_0_0_0

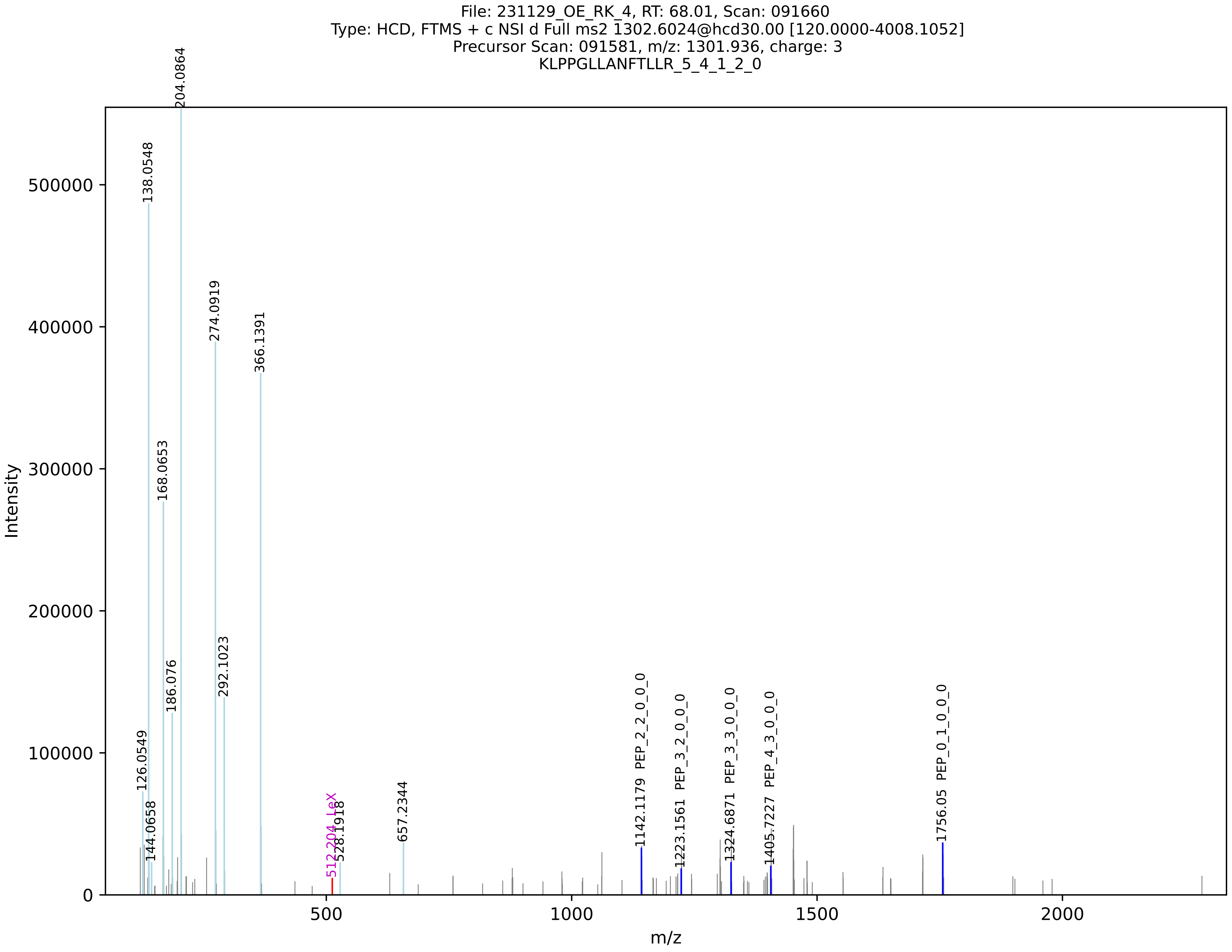
File: 231129_OE_RK_4, RT: 68.01, Scan: 091660
Type: HCD, FTMS + c NSI d Full ms2 1302.6024@hcd30.00 [120.0000-4008.1052]
Precursor Scan: 091581, m/z: 1301.936, charge: 3
KLPPGLLANFTLLR_5_4_1_2_0
Intensity
m/z
204.0864
138.0548
274.0919
366.1391
168.0653
292.1023
186.076
126.0549
144.0658
512.204 LeX
528.1918
657.2344
1142.1179 PEP_2_2_0_0_0
1223.1561 PEP_3_2_0_0_0
1324.6871 PEP_3_3_0_0_0
1405.7227 PEP_4_3_0_0_0
1756.05 PEP_0_1_0_0_0

File: 231129_OE_RK_4, RT: 68.063, Scan: 091739
Type: HCD, FTMS + c NSI d Full ms2 1188.0437@hcd30.00 [120.0000-4870.0747]
Precursor Scan: 091654, m/z: 1187.5426, charge: 4
EEQYNSTYRVVSVLTVLHQDWLNGK_5_4_1_0_0

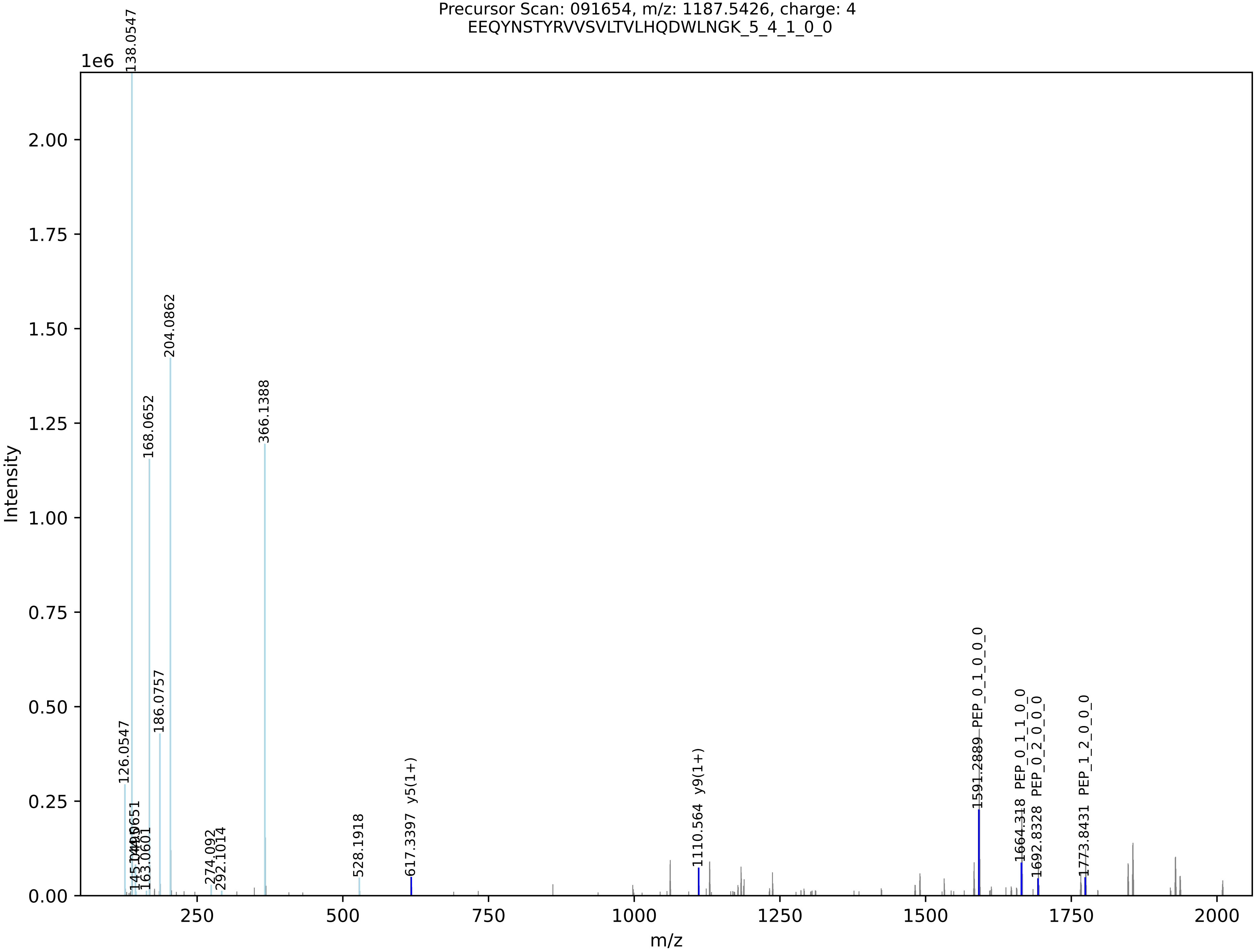

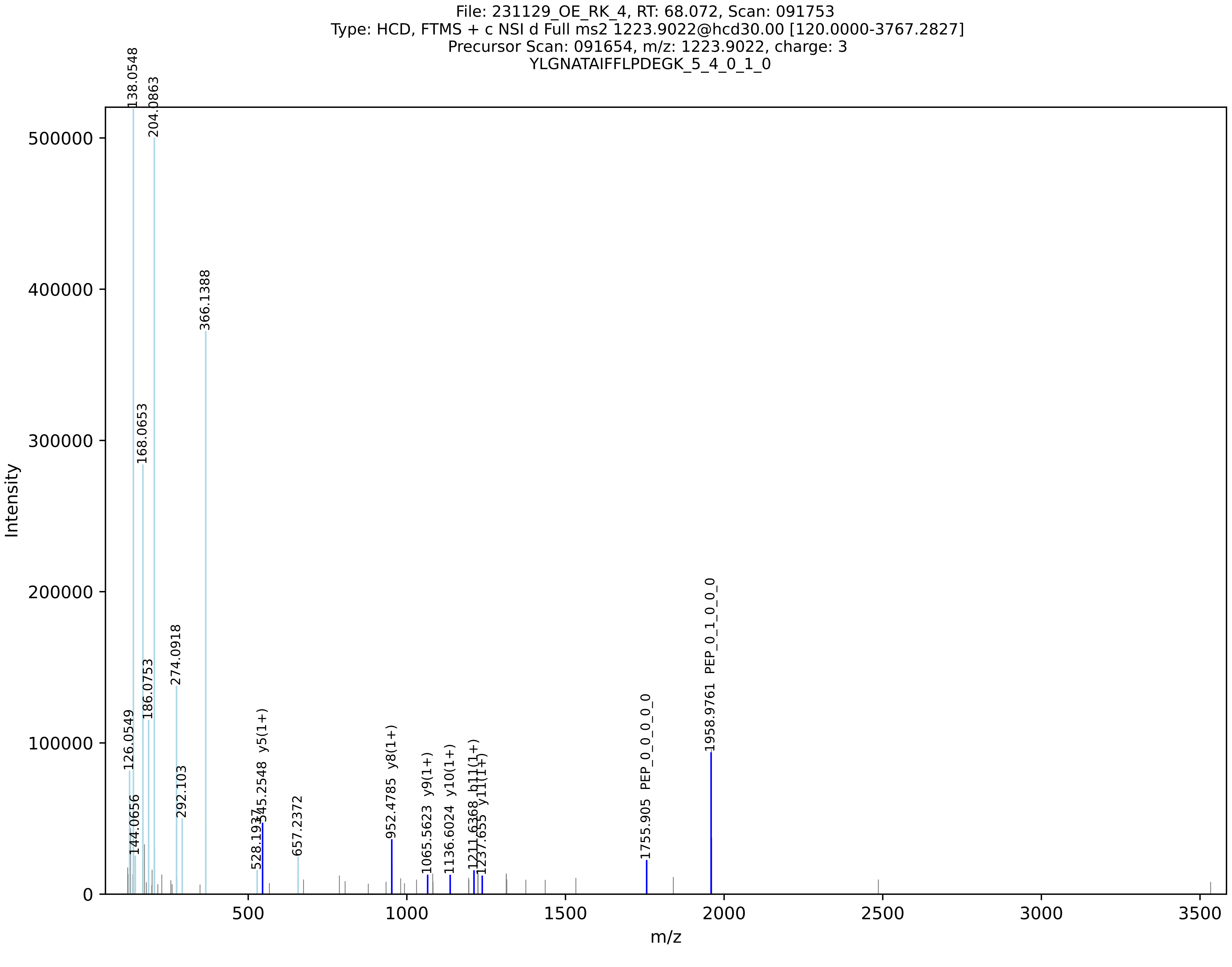
File: 231129_OE_RK_4, RT: 68.072, Scan: 091753
Type: HCD, FTMS + c NSI d Full ms2 1223.9022@hcd30.00 [120.0000-3767.2827]
Precursor Scan: 091654, m/z: 1223.9022, charge: 3
YLGNATAIFFLPDEGK_5_4_0_1_0
Intensity
m/z
138.0548
204.0863
366.1388
168.0653
274.0918
186.0753
126.0549
144.0656
292.103
528.1937
545.2548 y5(1+)
657.2372
952.4785 y8(1+)
1065.5623 y9(1+)
1136.6024 y10(1+)
1211.6368 b11(1+)
1237.655 y11(1+)
1755.905 PEP_0_0_0_0_0
1958.9761 PEP_0_1_0_0_0

File: 231129_OE_RK_4, RT: 68.119, Scan: 091818
Type: HCD, FTMS + c NSI d Full ms2 1051.4835@hcd30.00 [120.0000-5386.1362]
Precursor Scan: 091725, m/z: 1051.0841, charge: 5
LQFSDQAGVSVLYTVTLWVESWARNQTEK_4_4_1_1_0

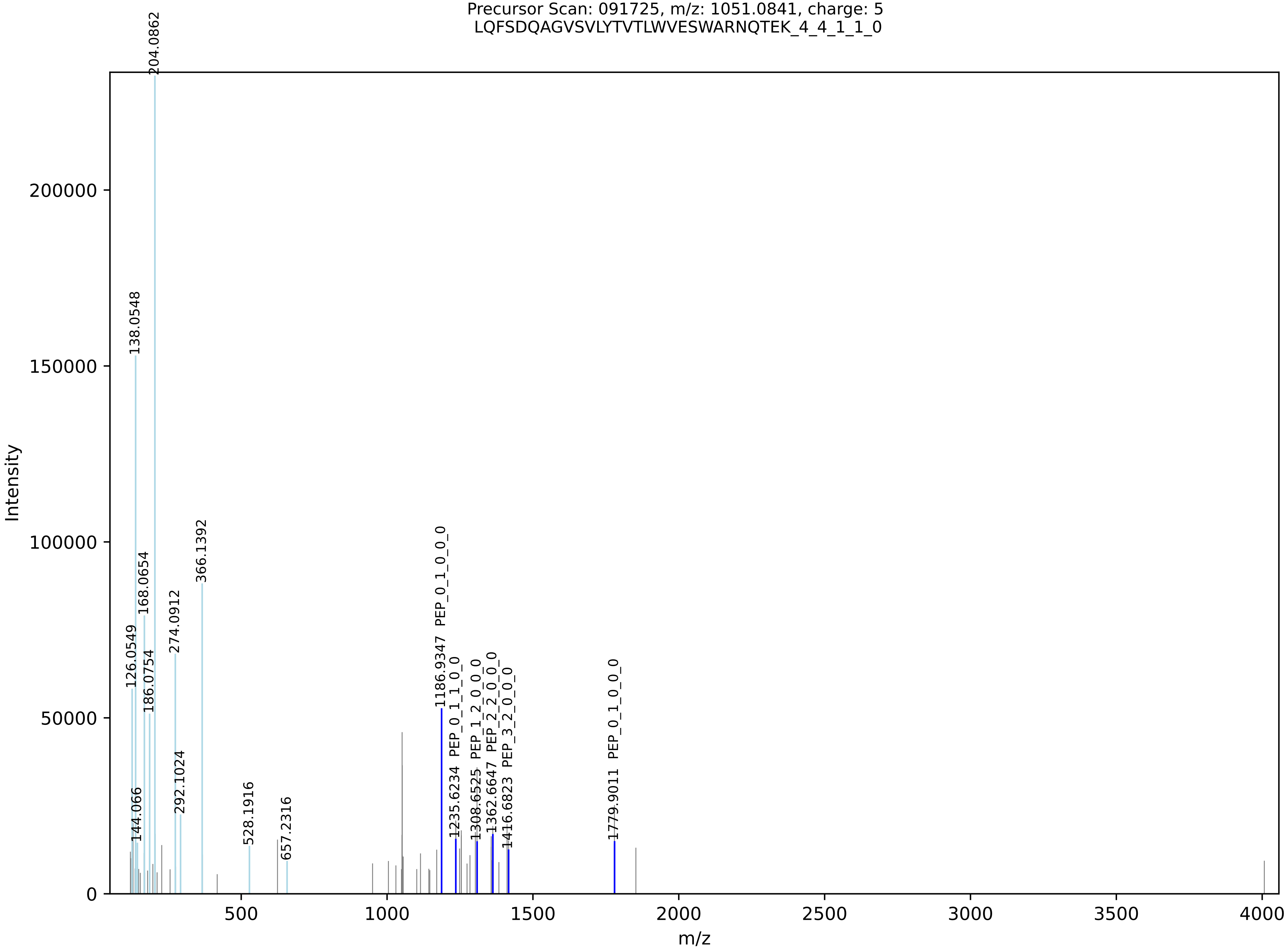

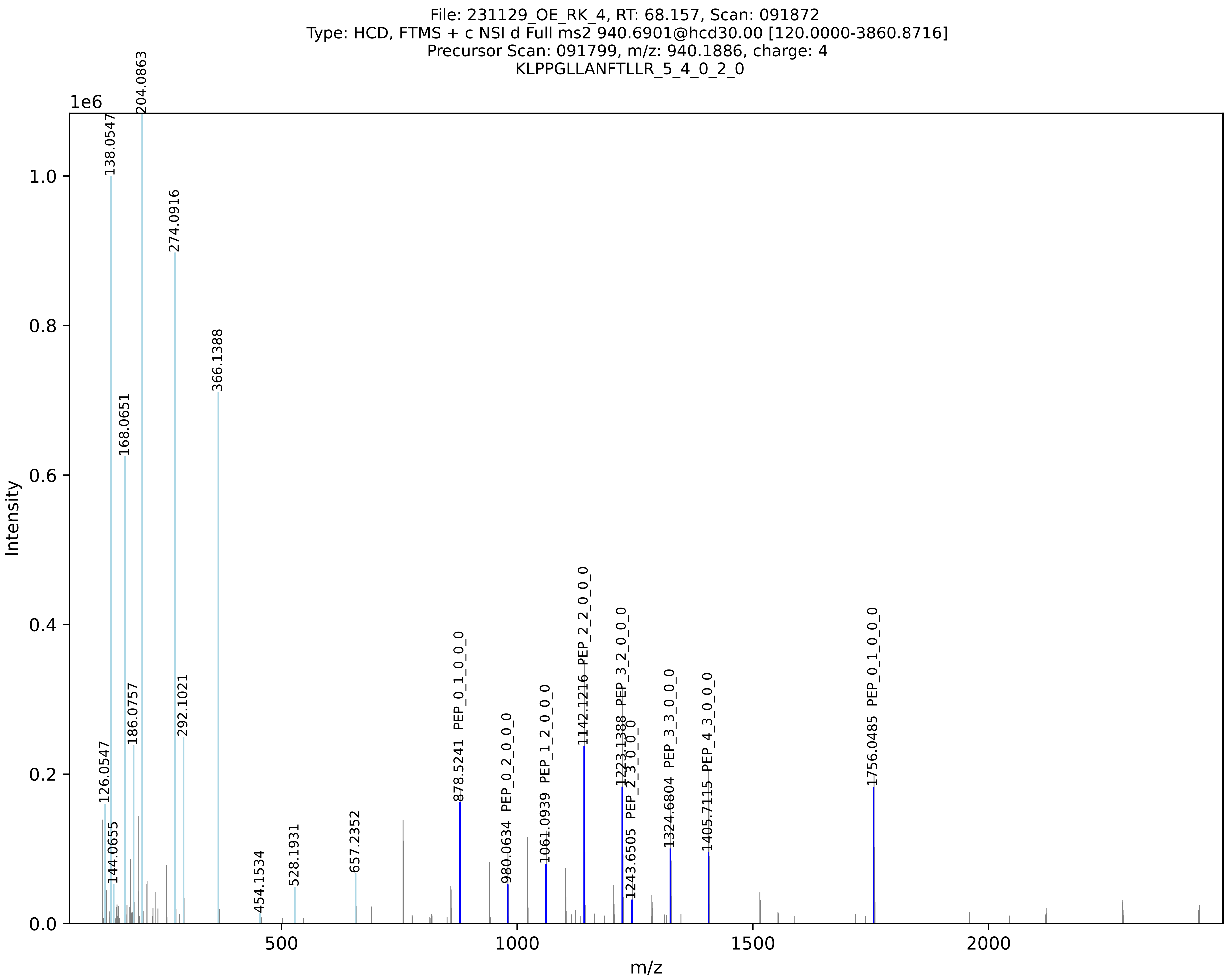
File: 231129_OE_RK_4, RT: 68.157, Scan: 091872
Type: HCD, FTMS + c NSI d Full ms2 940.6901@hcd30.00 [120.0000-3860.8716]
Precursor Scan: 091799, m/z: 940.1886, charge: 4
KLPPGLLANFTLLR_5_4_0_2_0
1e6
Intensity
m/z
204.0863
138.0547
274.0916
168.0651
366.1388
186.0757
292.1021
126.0547
144.0655
454.1534
528.1931
657.2352
878.5241 PEP_0_1_0_0_0
980.0634 PEP_0_2_0_0_0
1061.0939 PEP_1_2_0_0_0
1142.1216 PEP_2_2_0_0_0
1223.1388 PEP_3_2_0_0_0
1243.6505 PEP_2_3_0_0_0
1324.6804 PEP_3_3_0_0_0
1405.7115 PEP_4_3_0_0_0
1756.0485 PEP_0_1_0_0_0

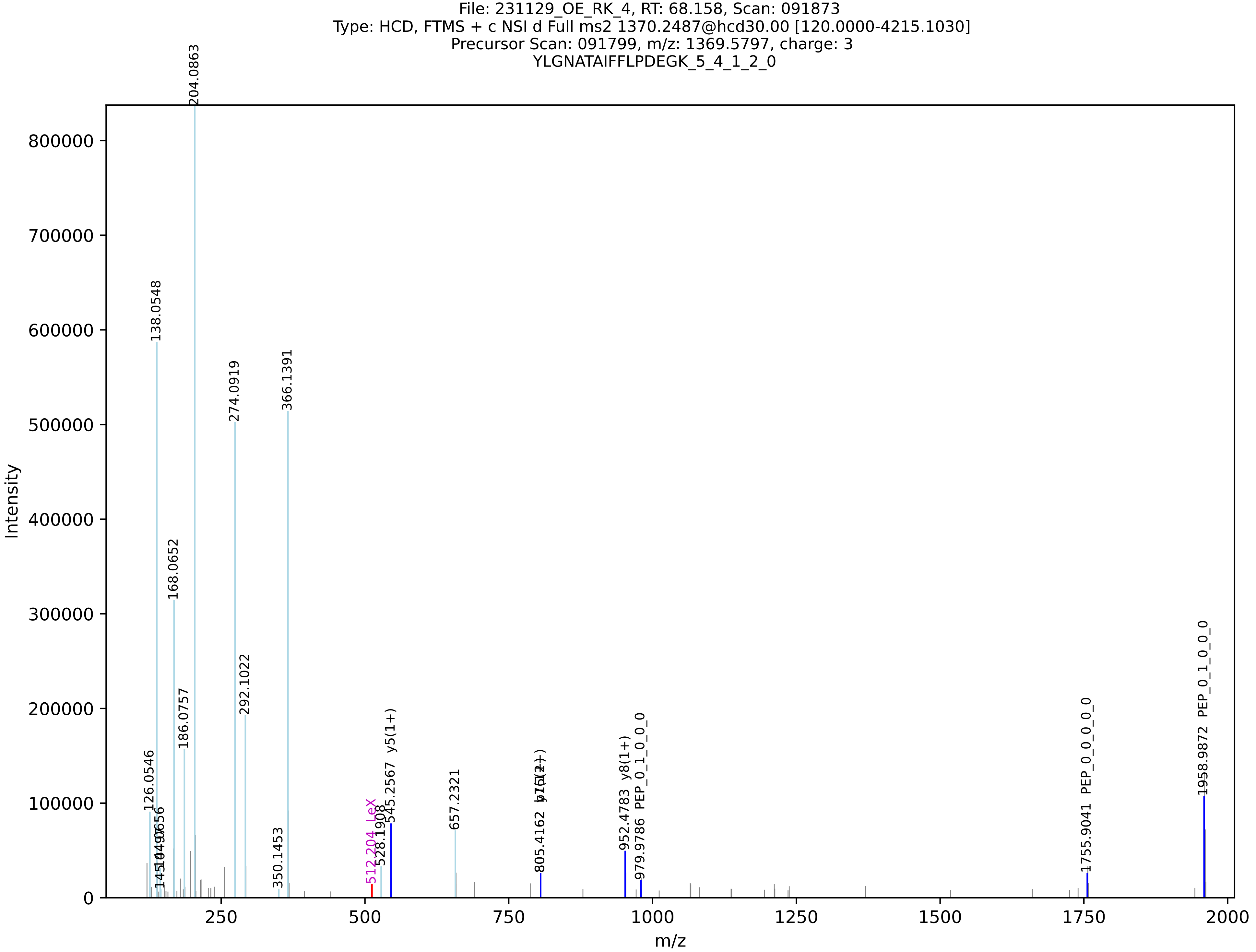

File: 231129_OE_RK_4, RT: 68.158, Scan: 091873
Type: HCD, FTMS + c NSI d Full ms2 1370.2487@hcd30.00 [120.0000-4215.1030]
Precursor Scan: 091799, m/z: 1369.5797, charge: 3
YLGNATAIFFLPDEGK_5_4_1_2_0
Intensity
m/z
204.0863
138.0548
274.0919
366.1391
168.0652
292.1022
186.0757
126.0546
350.1453
512.204_LeX
528.1908
545.2567 y5(1+)
657.2321
805.4162
952.4783 y8(1+)
979.9786 PEP_0_1_0_0_0
1755.9041 PEP_0_0_0_0_0
1958.9872 PEP_0_1_0_0_0

File: 231129_OE_RK_4, RT: 68.215, Scan: 091958
Type: HCD, FTMS + c NSI d Full ms2 1320.5602@hcd30.00 [120.0000-4063.0559]
Precursor Scan: 091870, m/z: 1320.5601, charge: 3
YLGNATAIFFLPDEGK_5_4_0_2_0

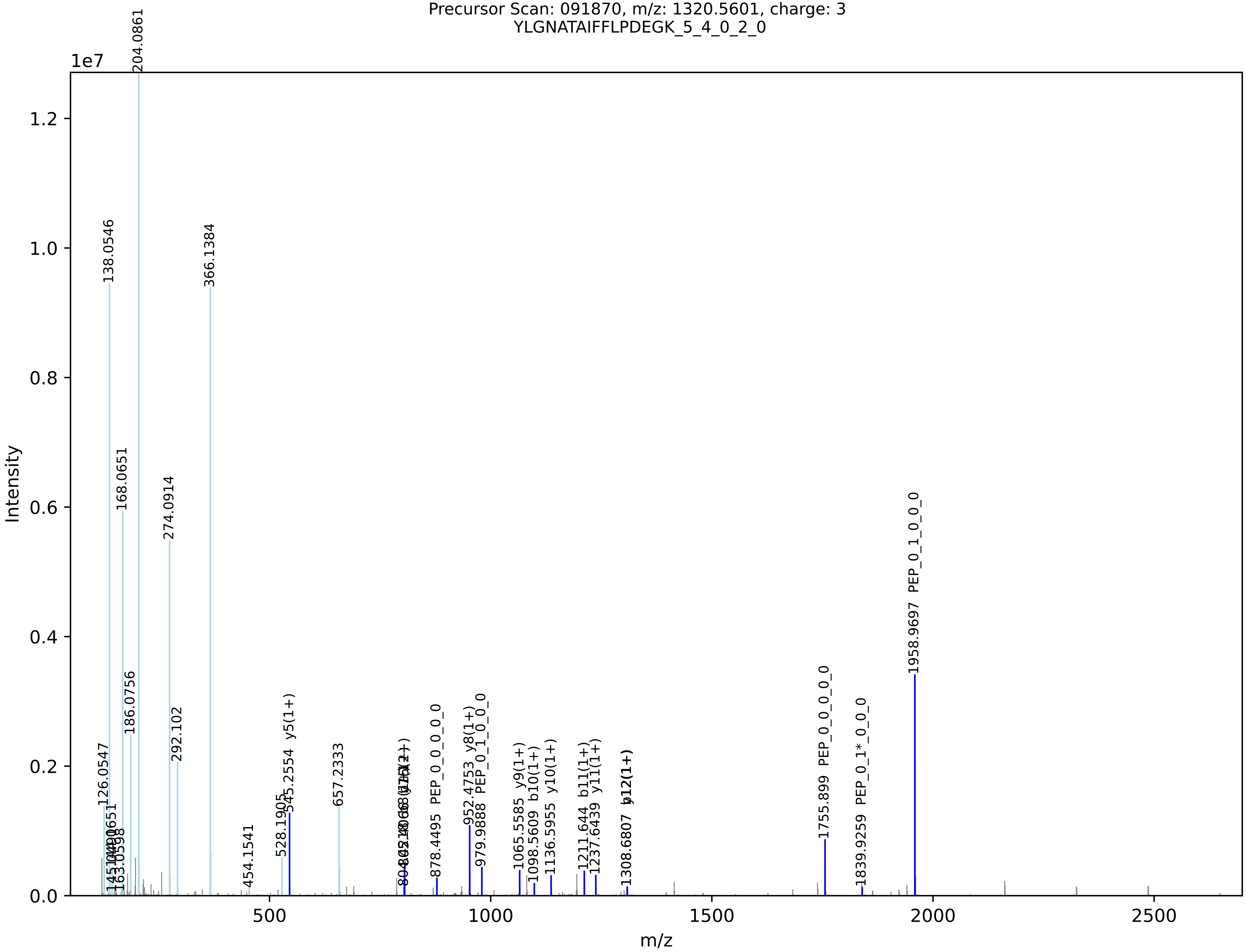

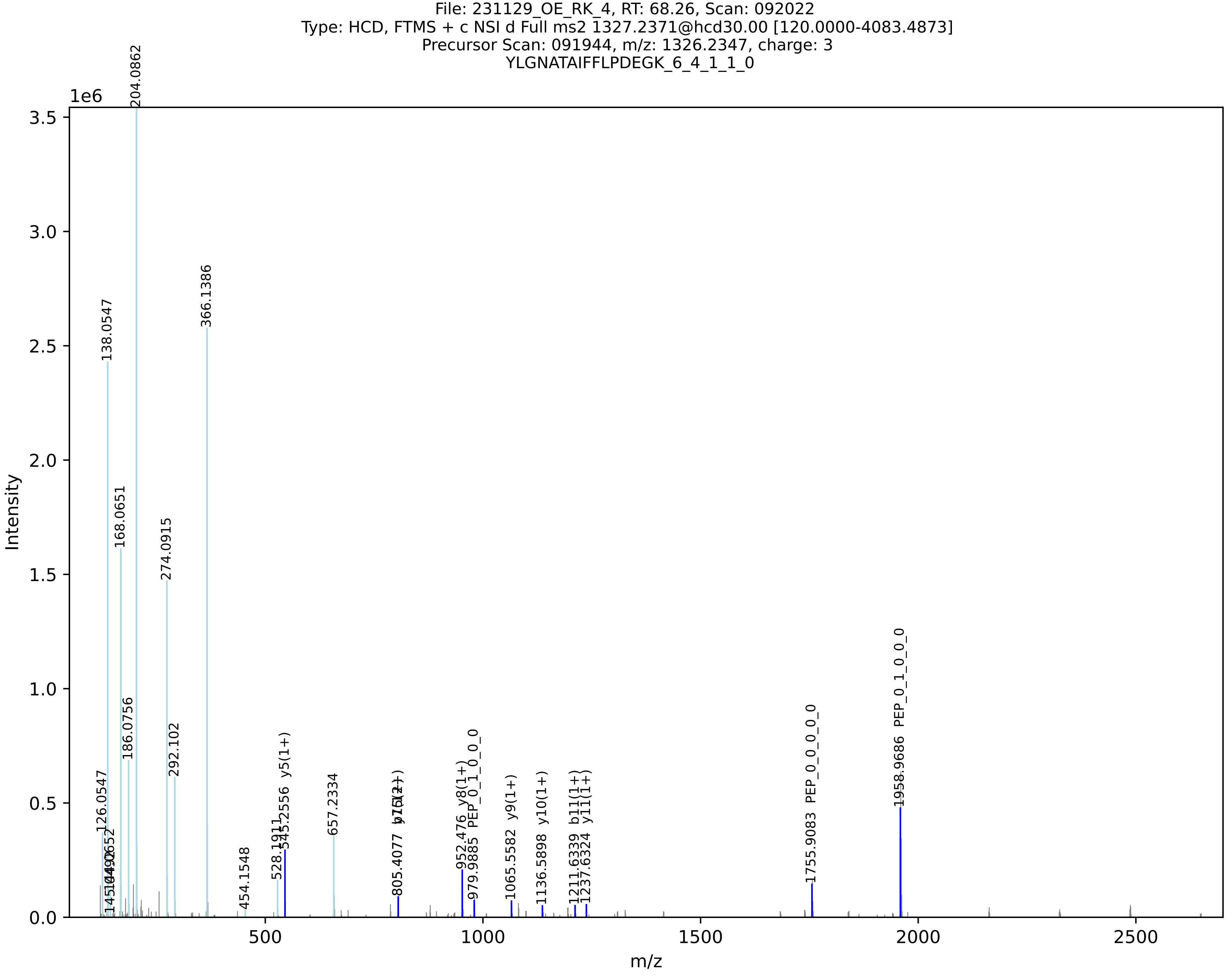

File: 231129_OE_RK_4, RT: 68.26, Scan: 092022
Type: HCD, FTMS + c NSI d Full ms2 1327.2371@hcd30.00 [120.0000-4083.4873]
Precursor Scan: 091944, m/z: 1326.2347, charge: 3
YLGNATAIFFLPDEGK_6_4_1_1_0
1e6
Intensity
m/z
204.0862
366.1386
138.0547
168.0651
274.0915
186.0756
292.102
126.0547
545.2556 y5(1+)
657.2334
528.1911
454.1548
805.4077
952.476 y8(1+)
979.9885 PEP_0_1_0_0_0
1065.5582 y9(1+)
1136.5898 y10(1+)
1211.6339 b11(1+)
1237.6324 y11(1+)
1755.9083 PEP_0_0_0_0_0
1958.9686 PEP_0_1_0_0_0

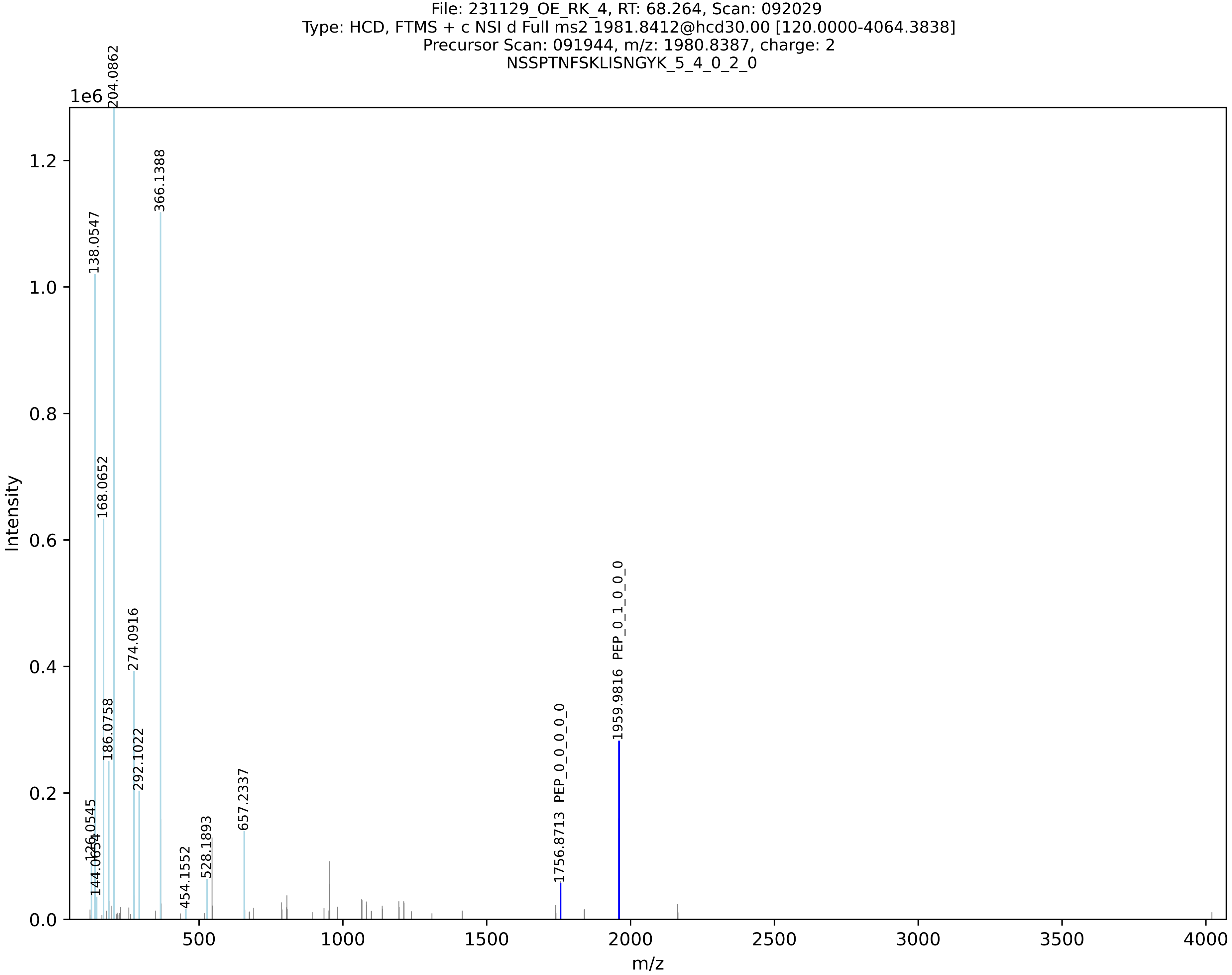
File: 231129_OE_RK_4, RT: 68.264, Scan: 092029
Type: HCD, FTMS + c NSI d Full ms2 1981.8412@hcd30.00 [120.0000-4064.3838]
Precursor Scan: 091944, m/z: 1980.8387, charge: 2
NSSPTNFSKLISNGYK_5_4_0_2_0
1e6
Intensity
m/z
204.0862
366.1388
138.0547
168.0652
274.0916
186.0758
292.1022
126.0545
144.0654
454.1552
528.1893
657.2337
1756.8713 PEP_0_0_0_0_0
1959.9816 PEP_0_1_0_0_0
0.0
0.2
0.4
0.6
0.8
1.0
1.2
500
1000
1500
2000
2500
3000
3500
4000

File: 231129_OE_RK_4, RT: 68.308, Scan: 092092
Type: HCD, FTMS + c NSI d Full ms2 1321.2295@hcd30.00 [120.0000-4065.1042]
Precursor Scan: 092018, m/z: 1320.8954, charge: 3
YLGNATAIFFLPDEGK_5_4_0_2_0

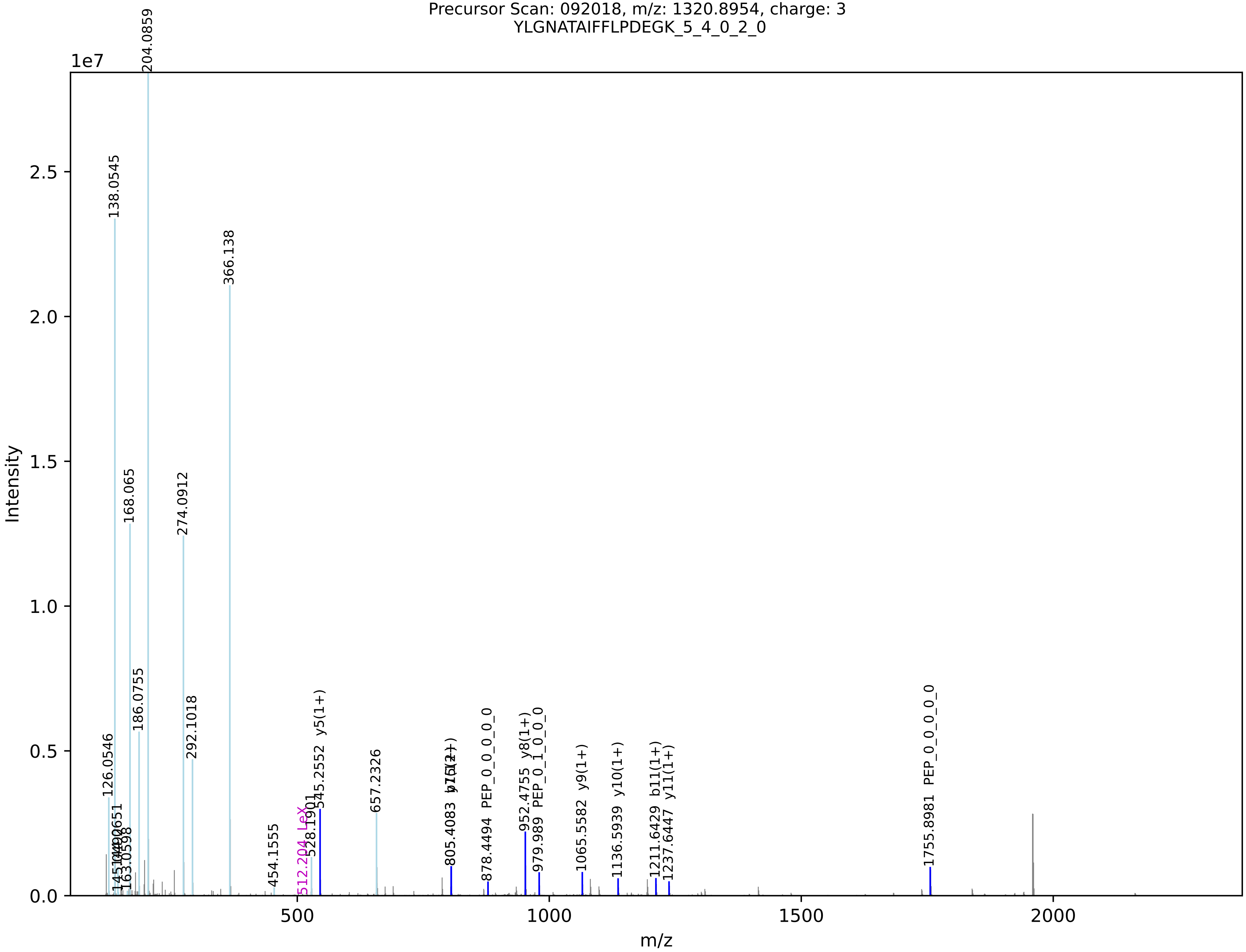

File: 231129_OE_RK_4, RT: 68.362, Scan: 092170
Type: HCD, FTMS + c NSI d Full ms2 940.4399@hcd30.00 [120.0000-3859.8511]
Precursor Scan: 092091, m/z: 940.1891, charge: 4
KLPPGLLANFTLLR_5_4_0_2_0

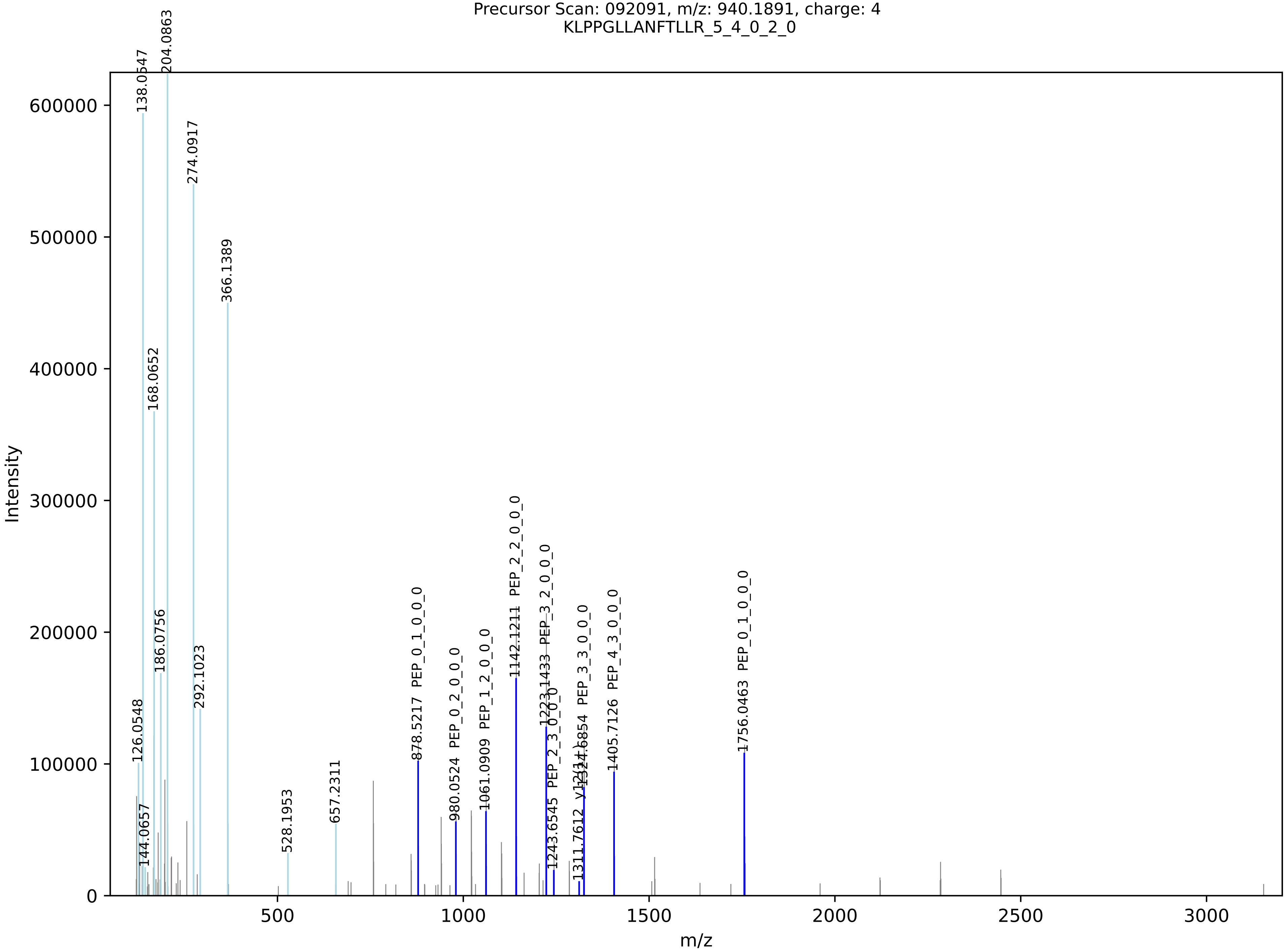

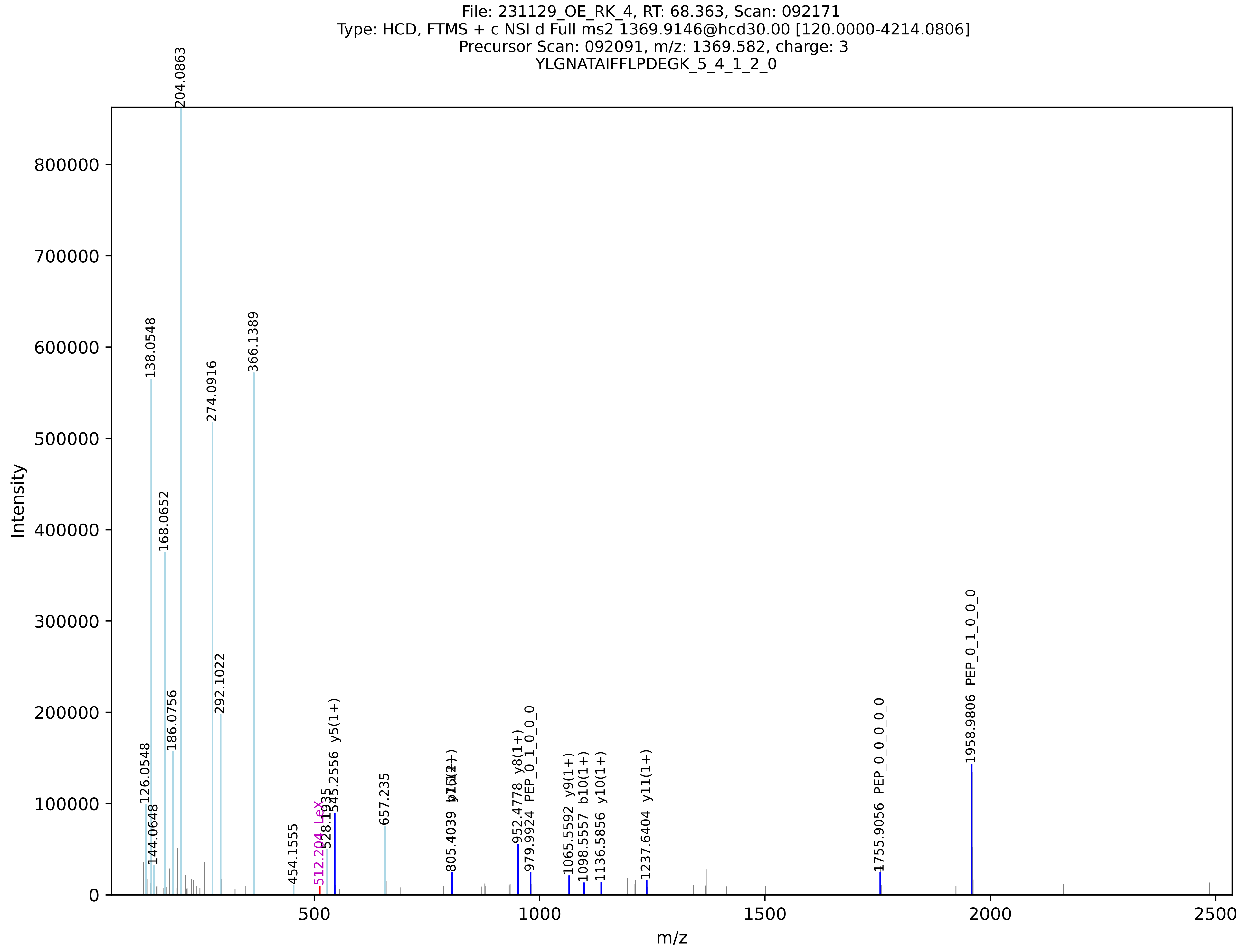
File: 231129_OE_RK_4, RT: 68.363, Scan: 092171
Type: HCD, FTMS + c NSI d Full ms2 1369.9146@hcd30.00 [120.0000-4214.0806]
Precursor Scan: 092091, m/z: 1369.582, charge: 3
YLGNATAIFFLPDEGK_5_4_1_2_0
Intensity
m/z
204.0863
138.0548
274.0916
366.1389
168.0652
292.1022
186.0756
126.0548
144.0648
454.1555
512.204 LeX
545.2556 y5(1+)
657.235
805.4039
952.4778 y8(1+)
979.9924 PEP_0_1_0_0_0
1065.5592 y9(1+)
1098.5557 b10(1+)
1136.5856 y10(1+)
1237.6404 y11(1+)
1755.9056 PEP_0_0_0_0_0
1958.9806 PEP_0_1_0_0_0
0
100000
200000
300000
400000
500000
600000
700000
800000
500
1000
1500
2000
2500

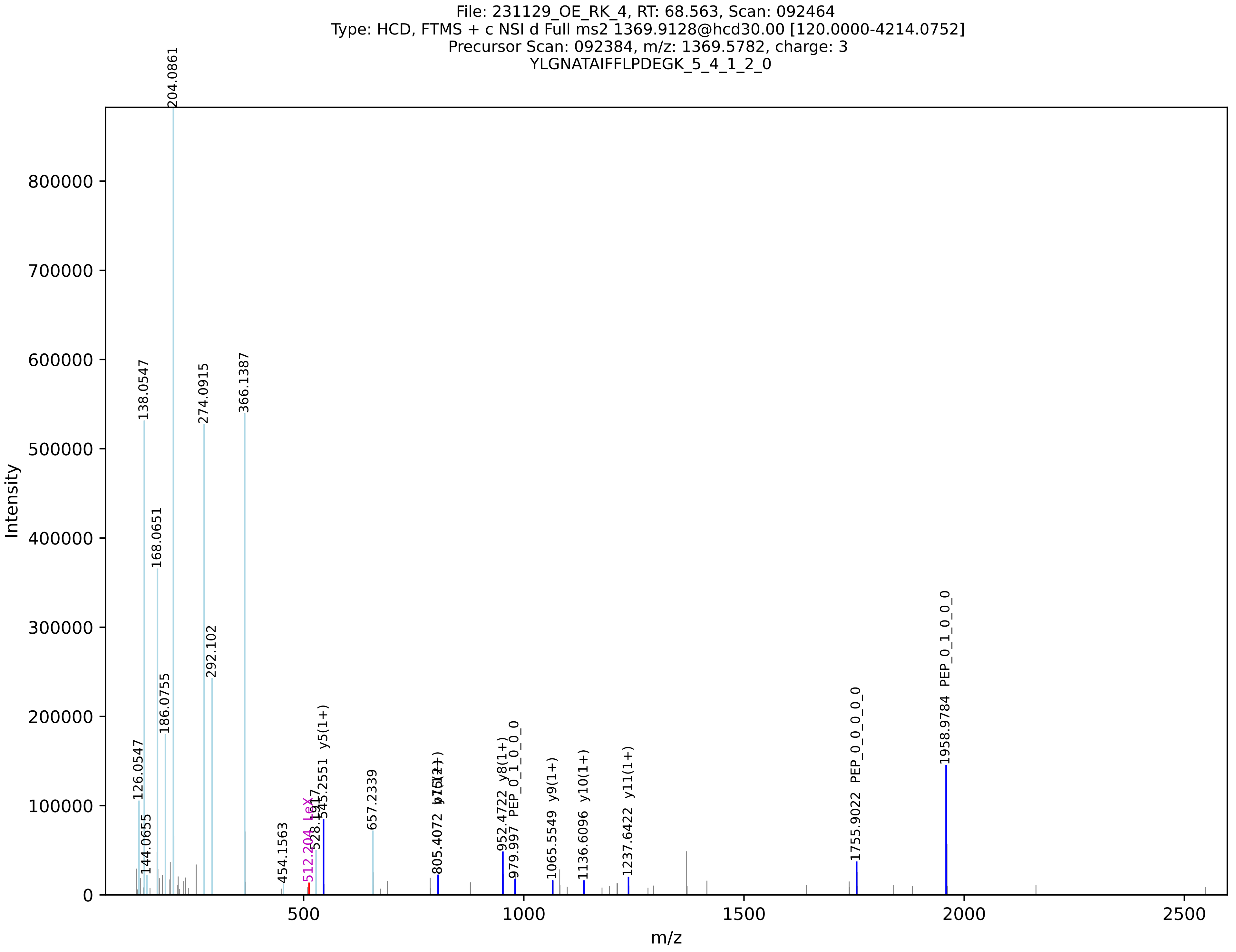

File: 231129_OE_RK_4, RT: 68.563, Scan: 092464
Type: HCD, FTMS + c NSI d Full ms2 1369.9128@hcd30.00 [120.0000-4214.0752]
Precursor Scan: 092384, m/z: 1369.5782, charge: 3
YLGNATAIFFLPDEGK_5_4_1_2_0
Intensity
m/z
204.0861
138.0547
274.0915
366.1387
168.0651
292.102
186.0755
126.0547
144.0655
454.1563
512.204_LeX
545.2551 y5(1+)
657.2339
805.4072
952.4722 y8(1+)
979.997 PEP_0_1_0_0_0
1065.5549 y9(1+)
1136.6096 y10(1+)
1237.6422 y11(1+)
1755.9022 PEP_0_0_0_0_0
1958.9784 PEP_0_1_0_0_0

File: 231129_OE_RK_4, RT: 68.663, Scan: 092609
Type: HCD, FTMS + c NSI d Full ms2 1253.5815@hcd30.00 [120.0000-3858.1016]
Precursor Scan: 092532, m/z: 1253.2475, charge: 3
KLPPGLLANFTLLR_5_4_0_2_0

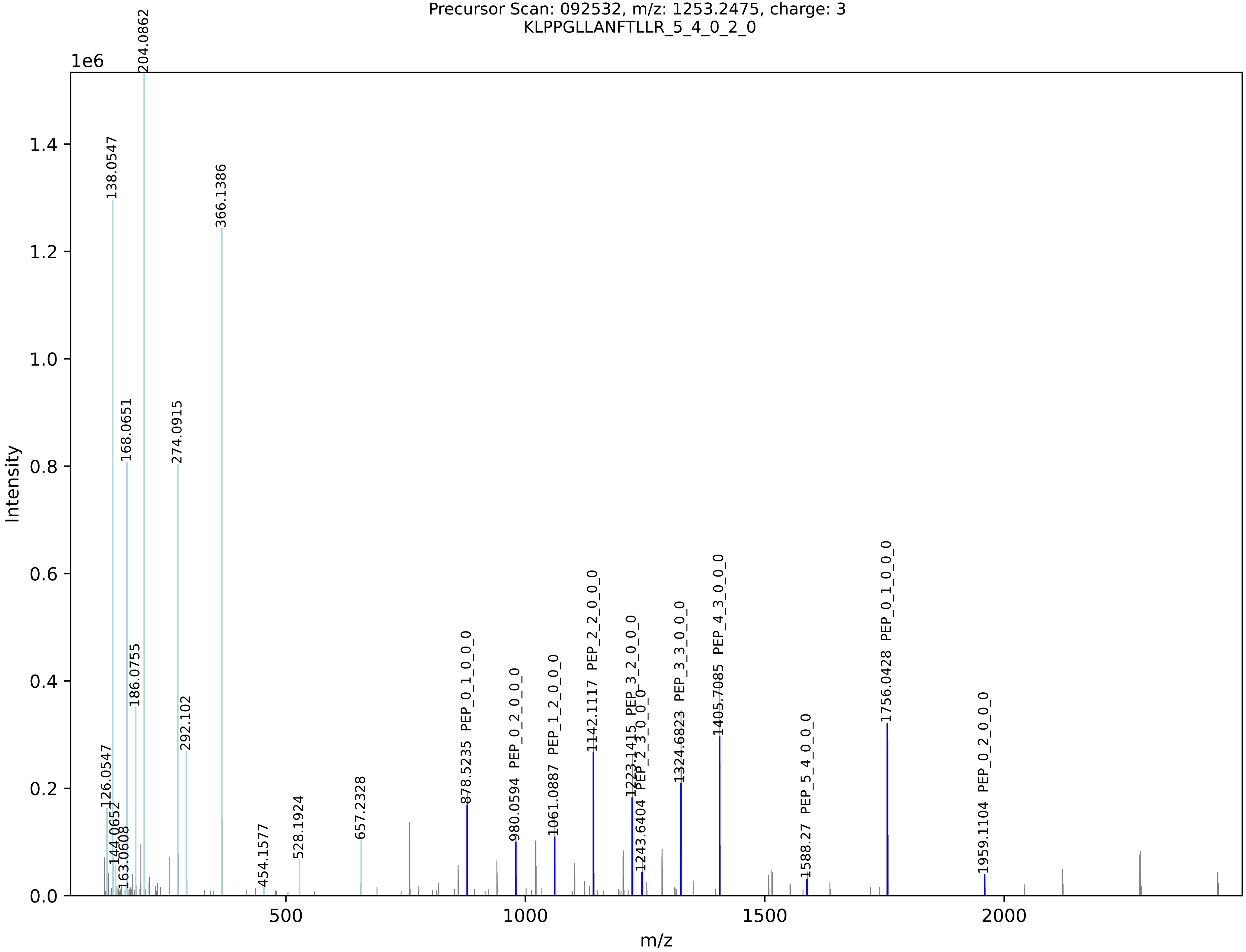

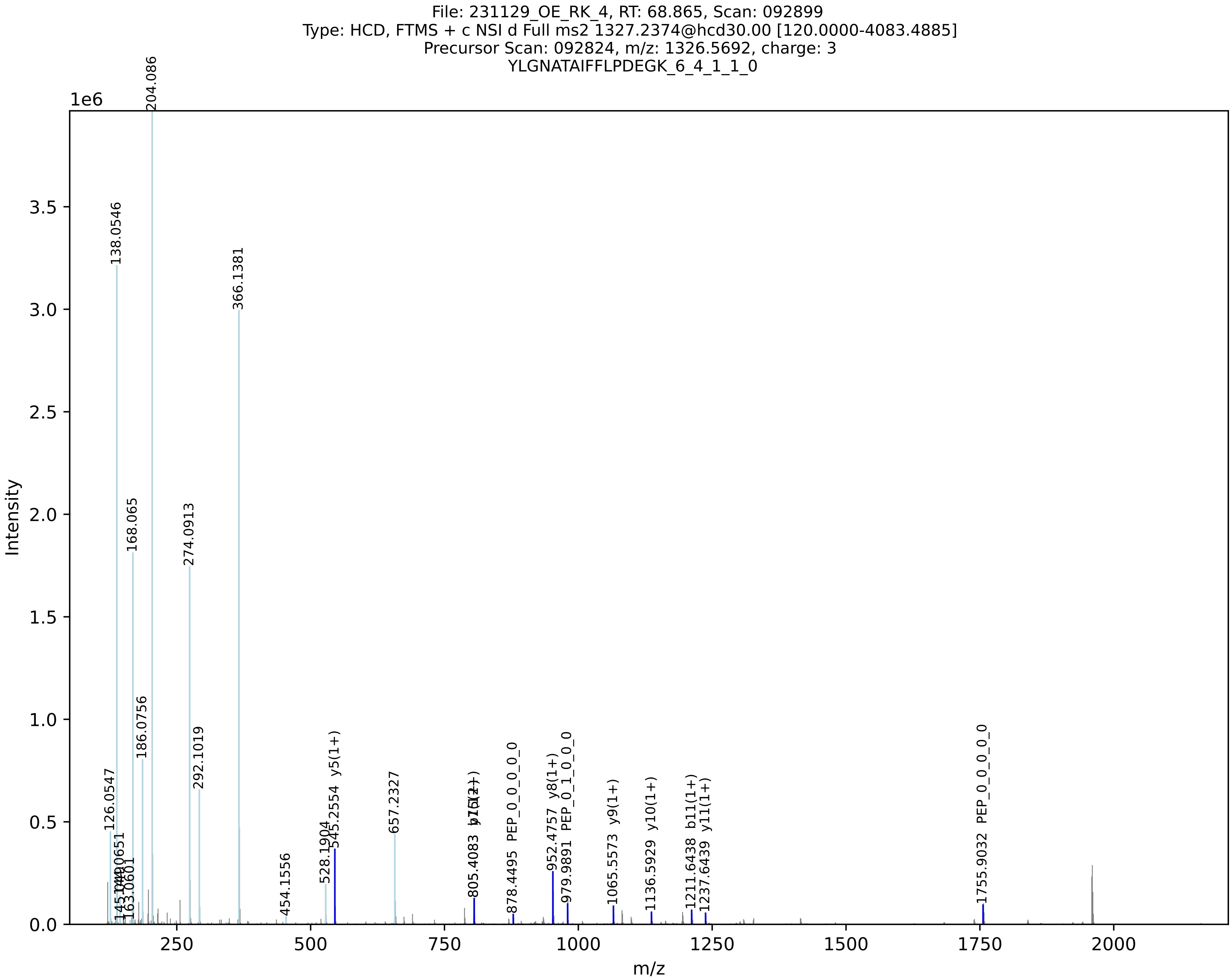
File: 231129_OE_RK_4, RT: 68.865, Scan: 092899
Type: HCD, FTMS + c NSI d Full ms2 1327.2374@hcd30.00 [120.0000-4083.4885]
Precursor Scan: 092824, m/z: 1326.5692, charge: 3
YLGNATAIFFLPDEGK_6_4_1_1_0
1e6
Intensity
m/z
204.086
138.0546
366.1381
168.065
274.0913
186.0756
292.1019
126.0547
163.0601
454.1556
528.1904
545.2554 y5(1+)
657.2327
805.4083
878.4495 PEP_0_0_0_0_0
952.4757 y8(1+)
979.9891 PEP_0_1_0_0_0
1065.5573 y9(1+)
1136.5929 y10(1+)
1211.6438 b11(1+)
1237.6439 y11(1+)
1755.9032 PEP_0_0_0_0_0
0.0
0.5
1.0
1.5
2.0
2.5
3.0
3.5
250
500
750
1000
1250
1500
1750
2000

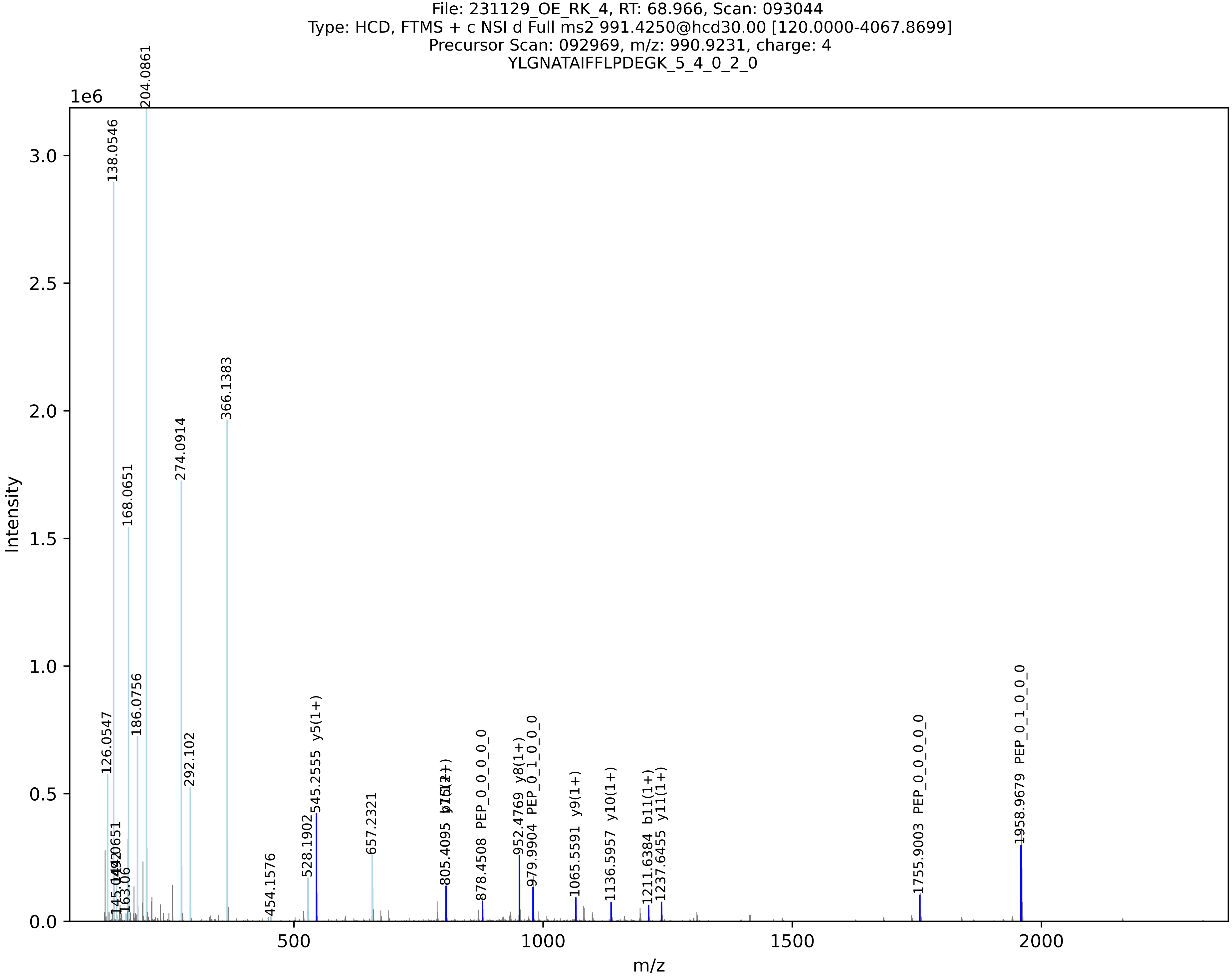
File: 231129_OE_RK_4, RT: 68.966, Scan: 093044
Type: HCD, FTMS + c NSI d Full ms2 991.4250@hcd30.00 [120.0000-4067.8699]
Precursor Scan: 092969, m/z: 990.9231, charge: 4
YLGNATAIFFLPDEGK_5_4_0_2_0
1e6
Intensity
m/z
0.0
0.5
1.0
1.5
2.0
2.5
3.0
500
1000
1500
2000
204.0861
138.0546
366.1383
274.0914
168.0651
186.0756
126.0547
292.102
163.06
454.1576
528.1902
545.2555 y5(1+)
657.2321
805.4095
878.4508 PEP_0_0_0_0_0
952.4769 y8(1+)
979.9904 PEP_0_1_0_0_0
1065.5591 y9(1+)
1136.5957 y10(1+)
1211.6384 b11(1+)
1237.6455 y11(1+)
1755.9003 PEP_0_0_0_0_0
1958.9679 PEP_0_1_0_0_0

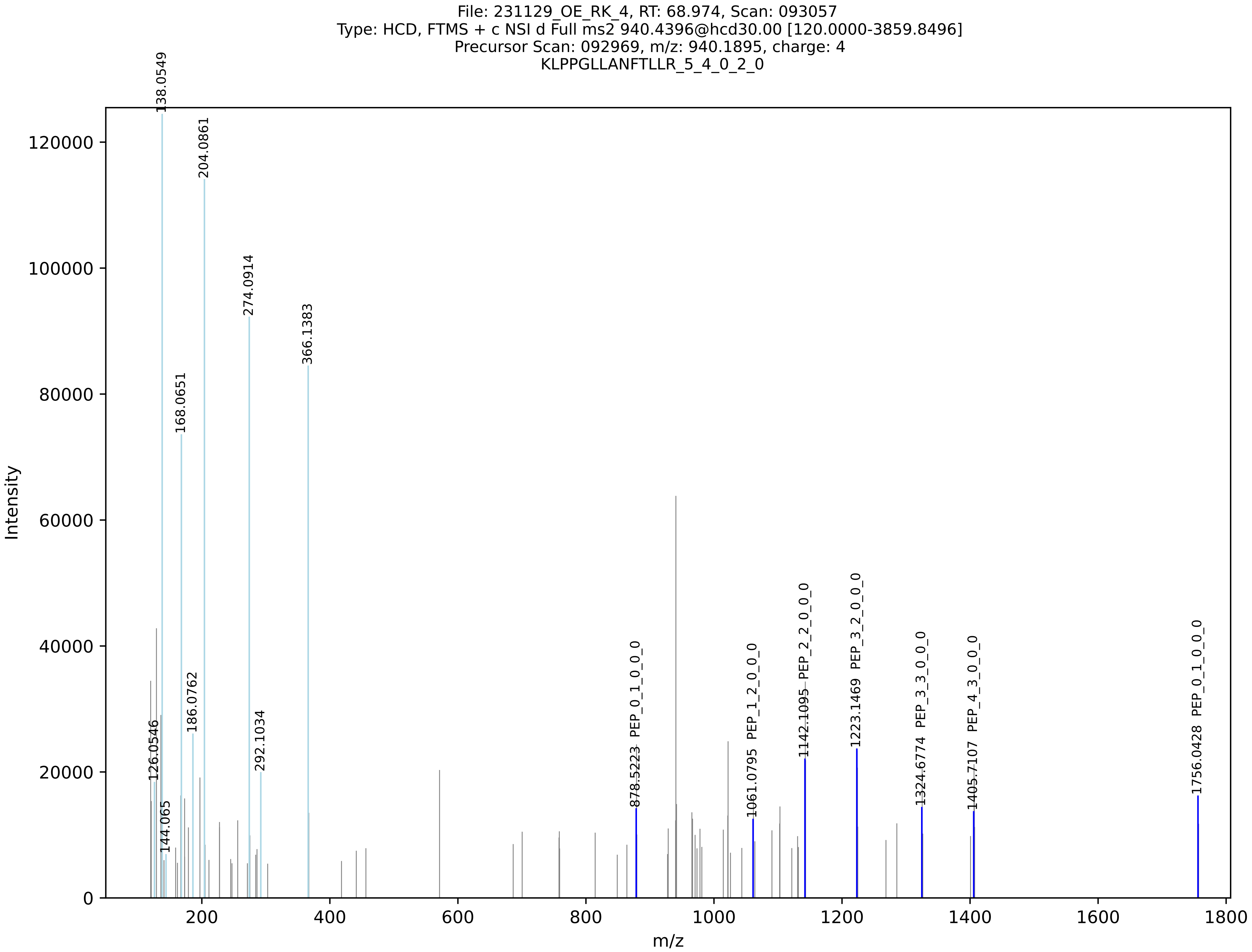
File: 231129_OE_RK_4, RT: 68.974, Scan: 093057
Type: HCD, FTMS + c NSI d Full ms2 940.4396@hcd30.00 [120.0000-3859.8496]
Precursor Scan: 092969, m/z: 940.1895, charge: 4
KLPPGLLANFTLLR_5_4_0_2_0
Intensity
m/z
138.0549
204.0861
274.0914
366.1383
168.0651
186.0762
126.0546
144.065
292.1034
878.5223 PEP_0_1_0_0_0
1061.0795 PEP_1_2_0_0_0
1142.1095 PEP_2_2_0_0_0
1223.1469 PEP_3_2_0_0_0
1324.6774 PEP_3_3_0_0_0
1405.7107 PEP_4_3_0_0_0
1756.0428 PEP_0_1_0_0_0

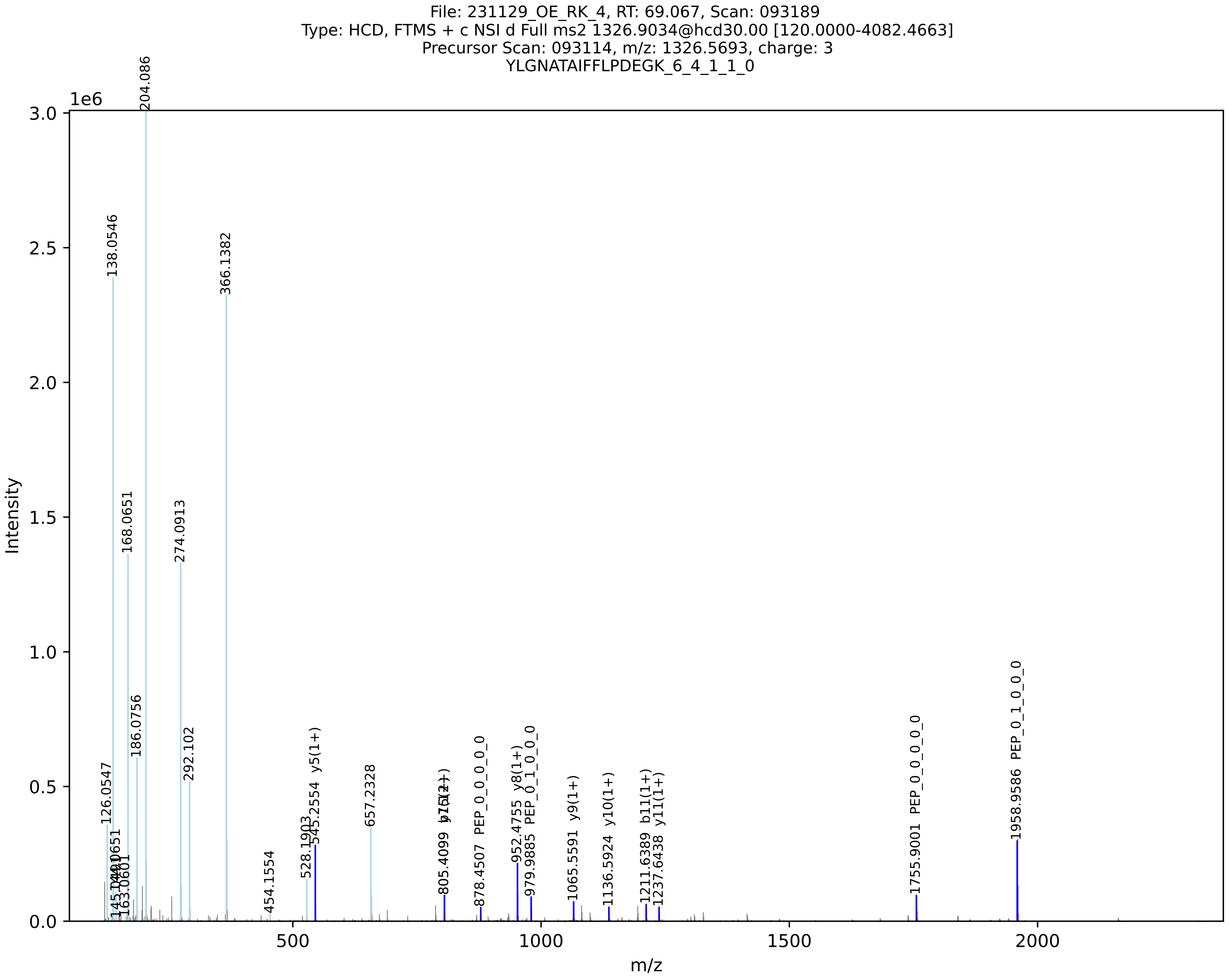
File: 231129_OE_RK_4, RT: 69.067, Scan: 093189
Type: HCD, FTMS + c NSI d Full ms2 1326.9034@hcd30.00 [120.0000-4082.4663]
Precursor Scan: 093114, m/z: 1326.5693, charge: 3
YLGNATAIFFLPDEGK_6_4_1_1_0
1e6
Intensity
m/z
204.086
138.0546
366.1382
168.0651
274.0913
186.0756
292.102
126.0547
545.2554 y5(1+)
657.2328
528.1903
454.1554
805.4099
878.4507 PEP_0_0_0_0_0
952.4755 y8(1+)
979.9885 PEP_0_1_0_0_0
1065.5591 y9(1+)
1136.5924 y10(1+)
1211.6389 b11(1+)
1237.6438 y11(1+)
1755.9001 PEP_0_0_0_0_0
1958.9586 PEP_0_1_0_0_0

File: 231129_OE_RK_4, RT: 69.115, Scan: 093260
Type: HCD, FTMS + c NSI d Full ms2 1321.5636@hcd30.00 [120.0000-4066.1267]
Precursor Scan: 093188, m/z: 1320.8954, charge: 3
YLGNATAIFFLPDEGK_5_4_0_2_0

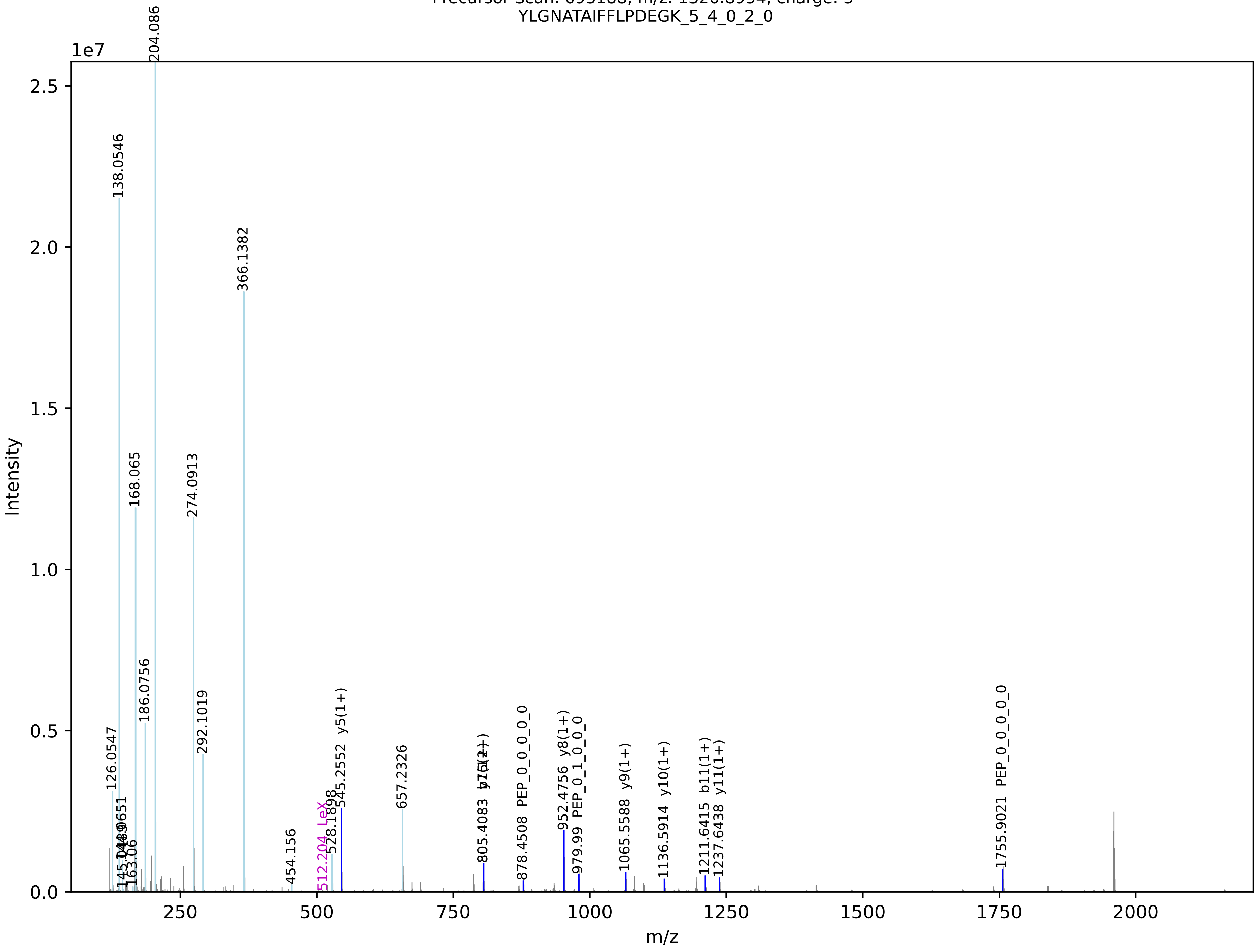

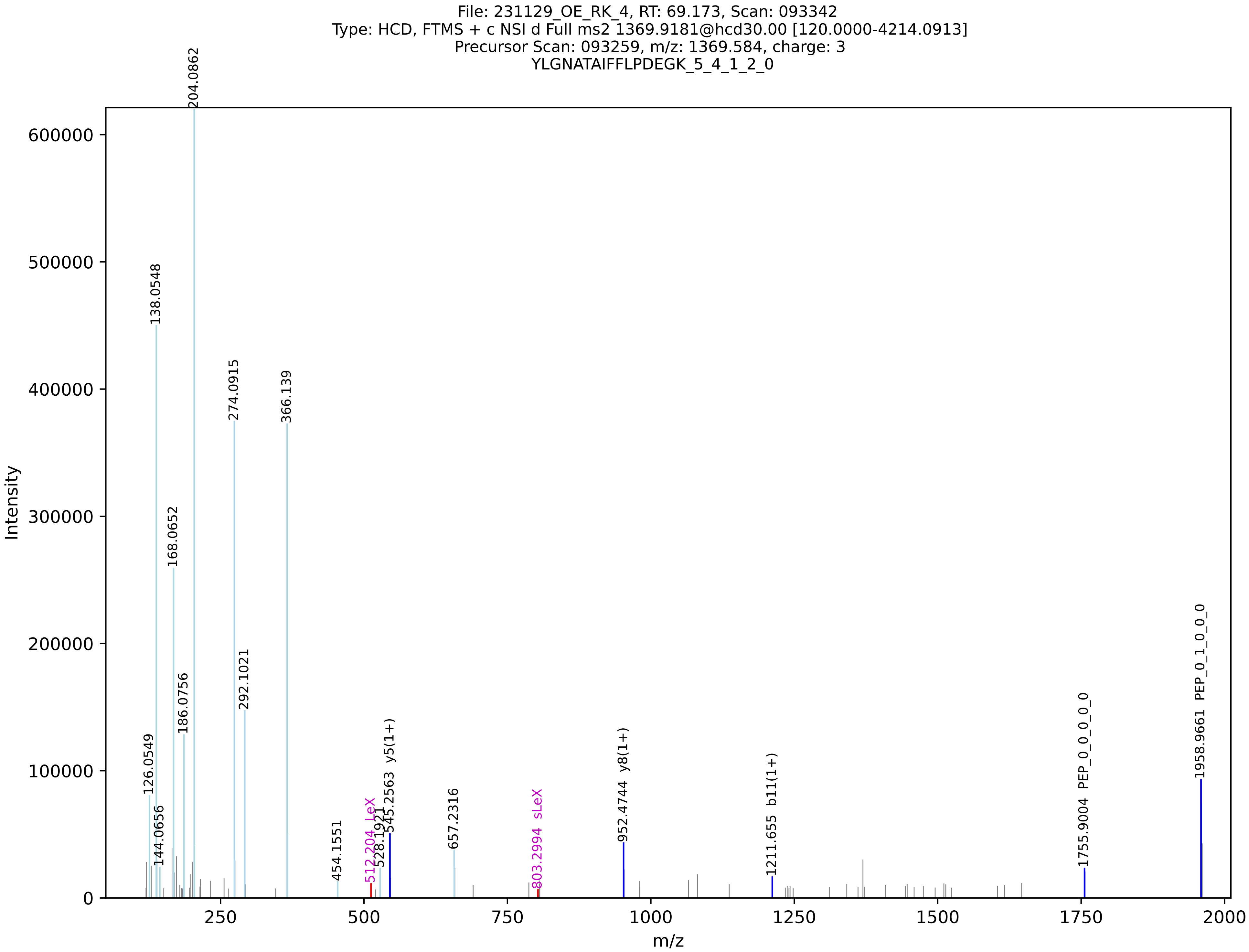
File: 231129_OE_RK_4, RT: 69.173, Scan: 093342
Type: HCD, FTMS + c NSI d Full ms2 1369.9181@hcd30.00 [120.0000-4214.0913]
Precursor Scan: 093259, m/z: 1369.584, charge: 3
YLGNATAIFFLPDEGK_5_4_1_2_0
Intensity
m/z
204.0862
138.0548
274.0915
366.139
168.0652
292.1021
186.0756
126.0549
144.0656
454.1551
512.204 LeX
528.1921
545.2563 y5(1+)
657.2316
803.2994 sLeX
952.4744 y8(1+)
1211.655 b11(1+)
1755.9004 PEP_0_0_0_0_0
1958.9661 PEP_0_1_0_0_0

File: 231129_OE_RK_4, RT: 69.369, Scan: 093628
Type: HCD, FTMS + c NSI d Full ms2 991.6725@hcd30.00 [120.0000-4068.8801]
Precursor Scan: 093552, m/z: 990.9219, charge: 4
YLGNATAIFFLPDEGK_5_4_0_2_0

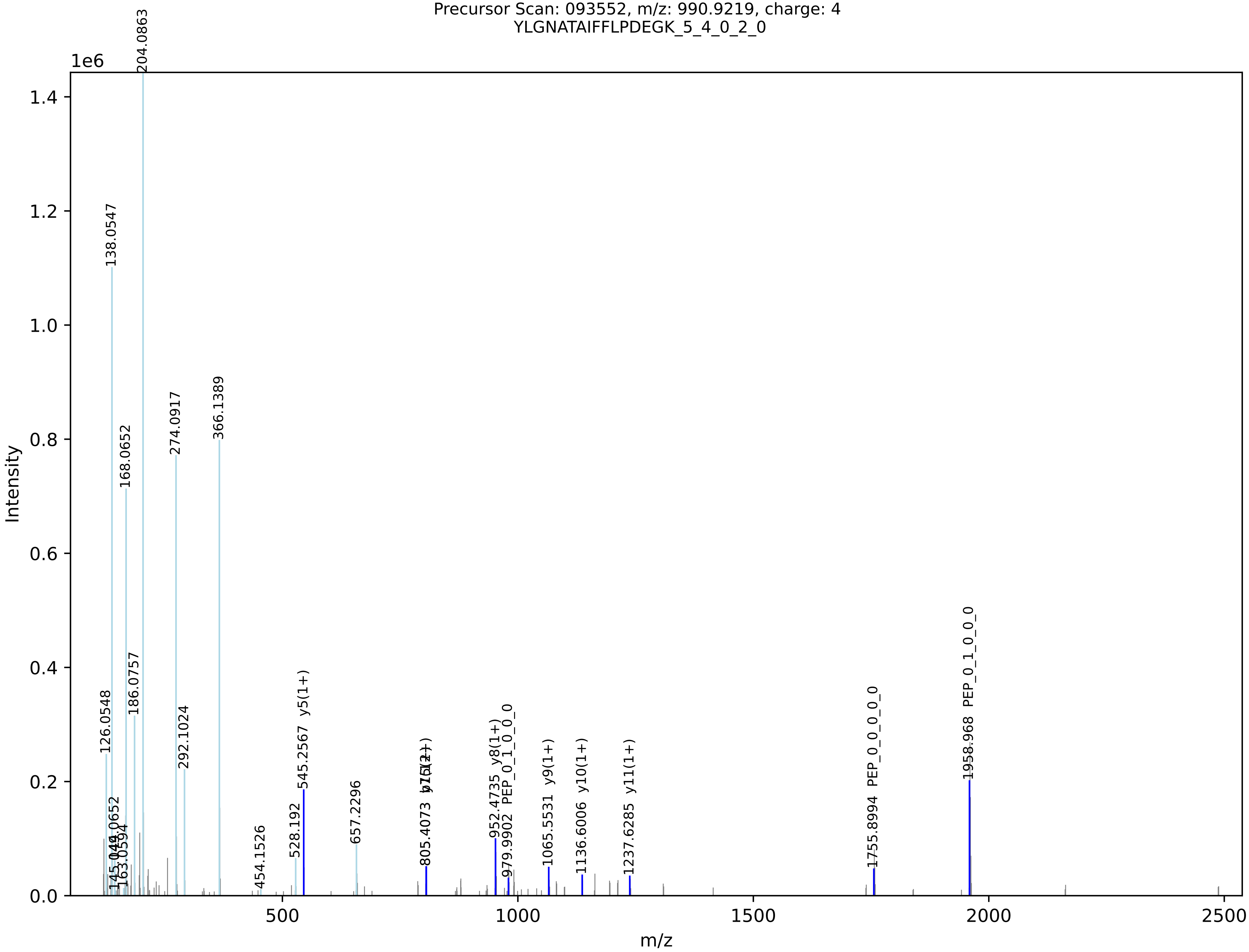

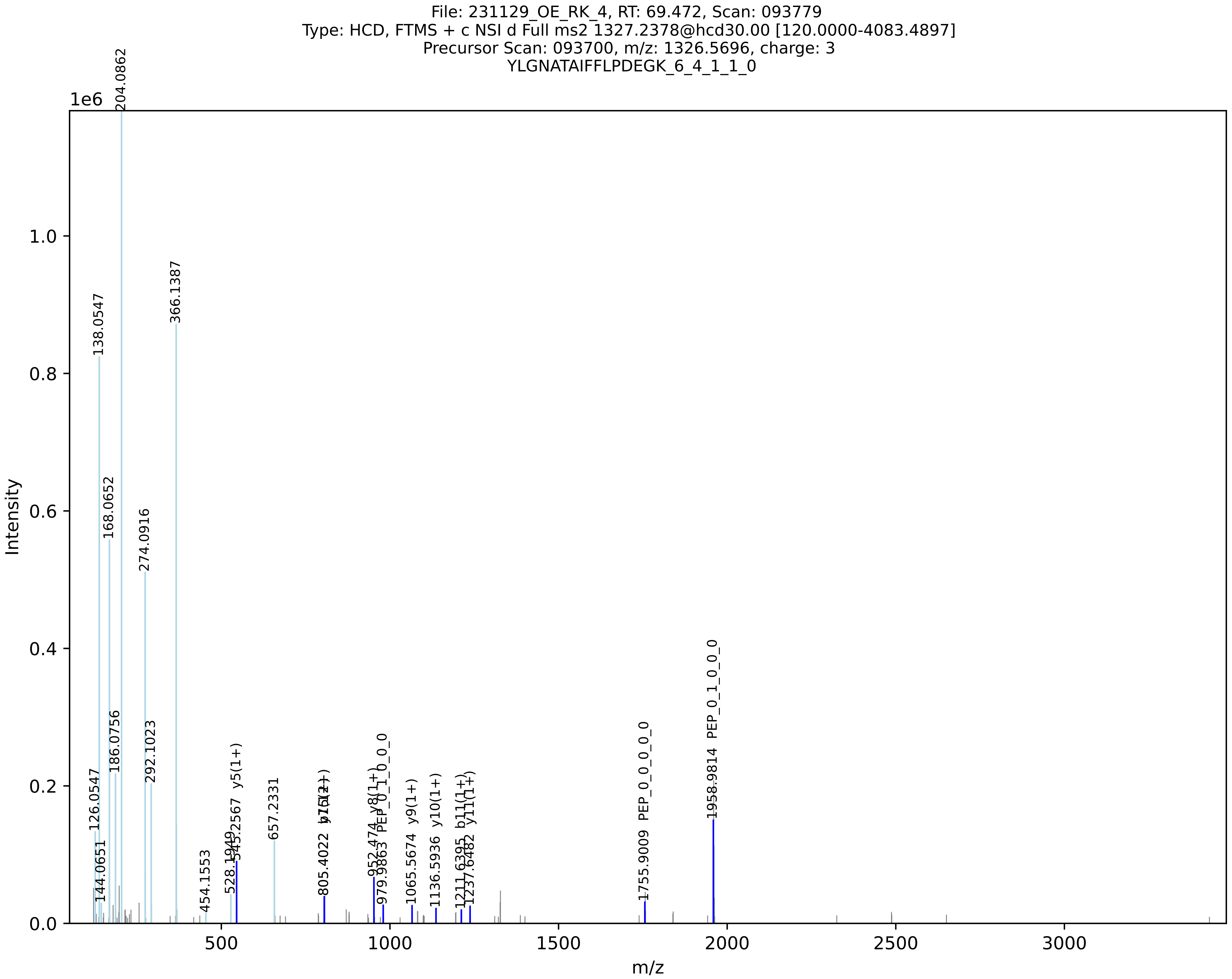
File: 231129_OE_RK_4, RT: 69.472, Scan: 093779
Type: HCD, FTMS + c NSI d Full ms2 1327.2378@hcd30.00 [120.0000-4083.4897]
Precursor Scan: 093700, m/z: 1326.5696, charge: 3
YLGNATAIFFLPDEGK_6_4_1_1_0
1e6
Intensity
m/z
204.0862
366.1387
138.0547
168.0652
274.0916
186.0756
292.1023
126.0547
144.0651
454.1553
528.1949
543.2567 y5(1+)
657.2331
805.4022
952.474 y8(1+)
979.9863 PEP_0_1_0_0_0
1065.5674 y9(1+)
1136.5936 y10(1+)
1211.6395 b11(1+)
1237.6482 y11(1+)
1755.9009 PEP_0_0_0_0_0
1958.9814 PEP_0_1_0_0_0
0.0
0.2
0.4
0.6
0.8
1.0
500
1000
1500
2000
2500
3000

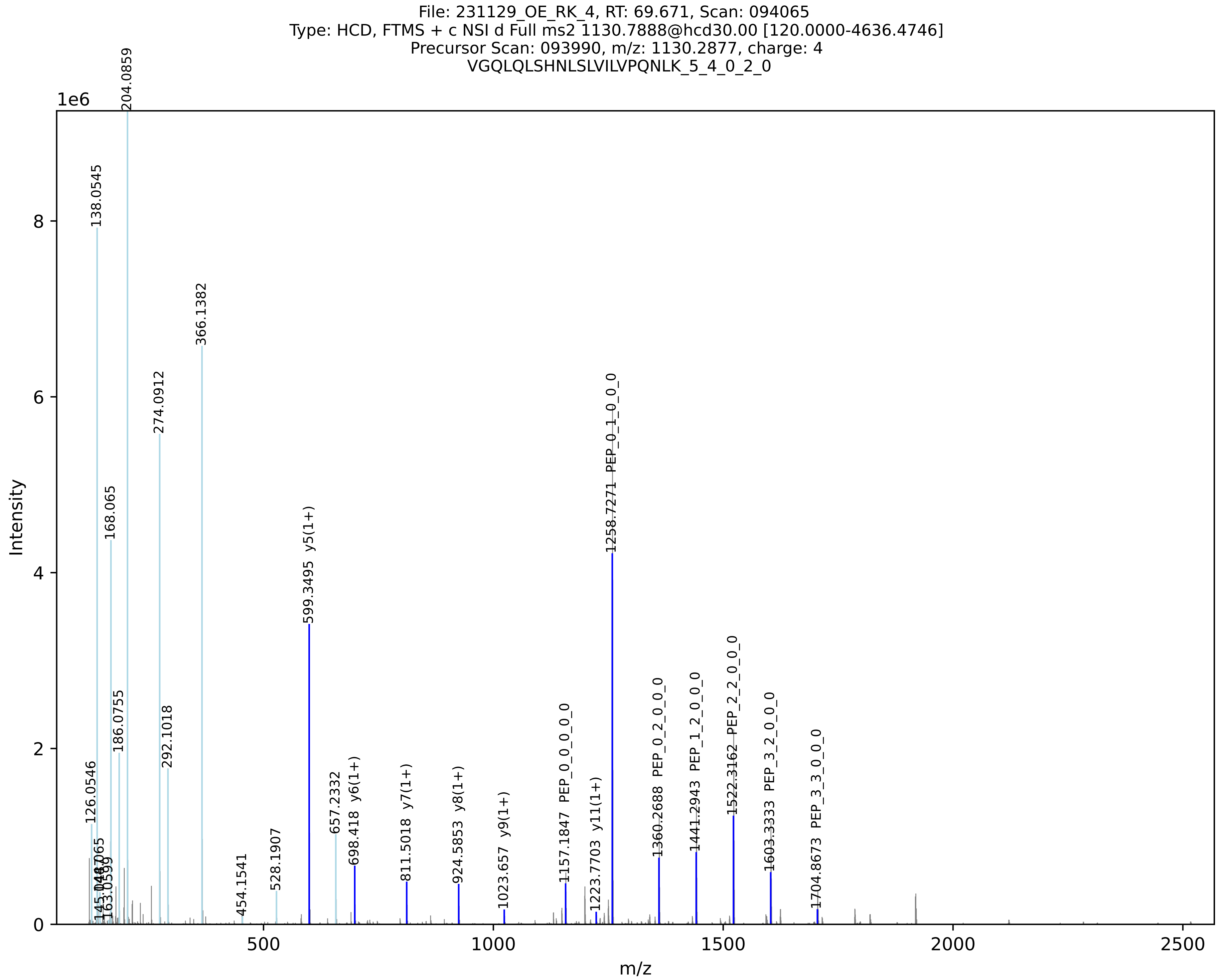
File: 231129_OE_RK_4, RT: 69.671, Scan: 094065
Type: HCD, FTMS + c NSI d Full ms2 1130.7888@hcd30.00 [120.0000-4636.4746]
Precursor Scan: 093990, m/z: 1130.2877, charge: 4
VGQLQLSHNLSLVILVPQNLK_5_4_0_2_0
1e6
Intensity
m/z
0
2
4
6
8
500
1000
1500
2000
2500
204.0859
138.0545
366.1382
274.0912
168.065
186.0755
292.1018
126.0546
163.0599
454.1541
528.1907
599.3495 y5(1+)
657.2332
698.418 y6(1+)
811.5018 y7(1+)
924.5853 y8(1+)
1023.657 y9(1+)
1157.1847 PEP_0_0_0_0_0
1223.7703 y11(1+)
1258.7271 PEP_0_1_0_0_0
1360.2688 PEP_0_2_0_0_0
1441.2943 PEP_1_2_0_0_0
1522.3162 PEP_2_2_0_0_0
1603.3333 PEP_3_2_0_0_0
1704.8673 PEP_3_3_0_0_0

File: 231129_OE_RK_4, RT: 69.722, Scan: 094139
Type: HCD, FTMS + c NSI d Full ms2 1321.2271@hcd30.00 [120.0000-4065.0967]
Precursor Scan: 094064, m/z: 1320.8929, charge: 3
YLGNATAIFFLPDEGK_5_4_0_2_0

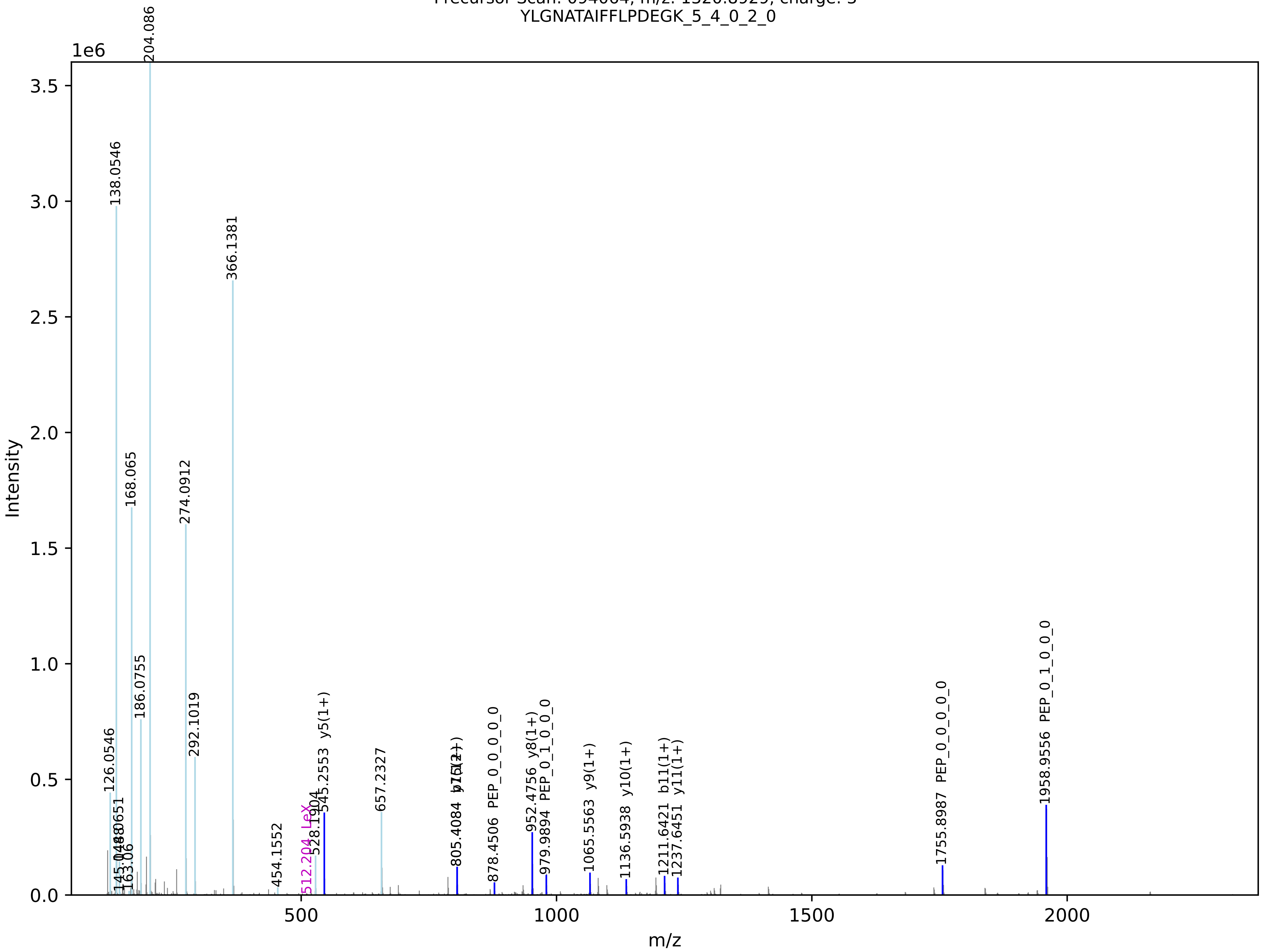

File: 231129_OE_RK_4, RT: 69.873, Scan: 094361
Type: HCD, FTMS + c NSI d Full ms2 1130.7892@hcd30.00 [120.0000-4636.4761]
Precursor Scan: 094286, m/z: 1130.2885, charge: 4
VGQLQLSHNLSLVILVPQNLK_5_4_0_2_0

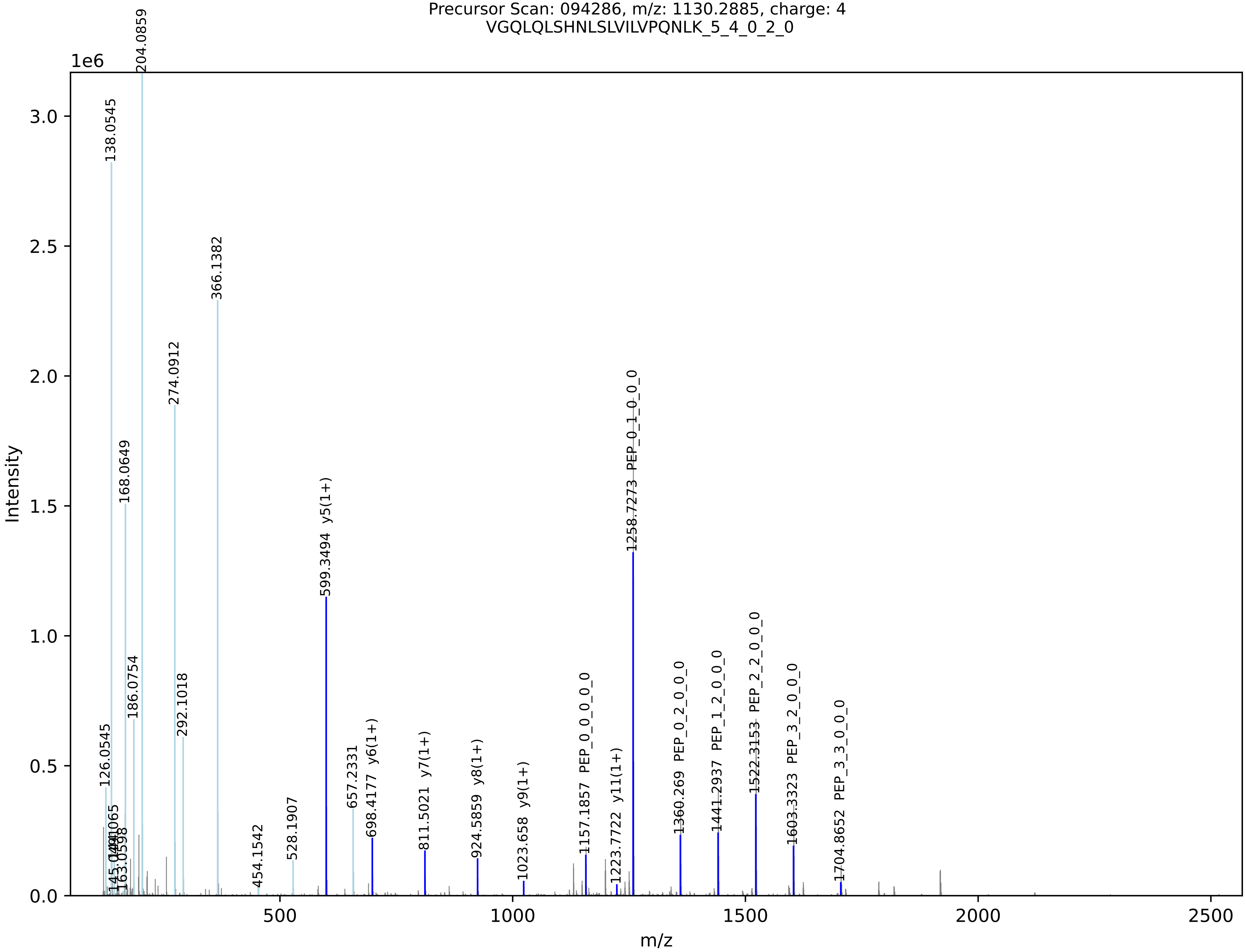

File: 231129_OE_RK_4, RT: 69.925, Scan: 094437
Type: HCD, FTMS + c NSI d Full ms2 1321.5619@hcd30.00 [120.0000-4066.1216]
Precursor Scan: 094360, m/z: 1320.8937, charge: 3
YLGNATAIFFLPDEGK_5_4_0_2_0

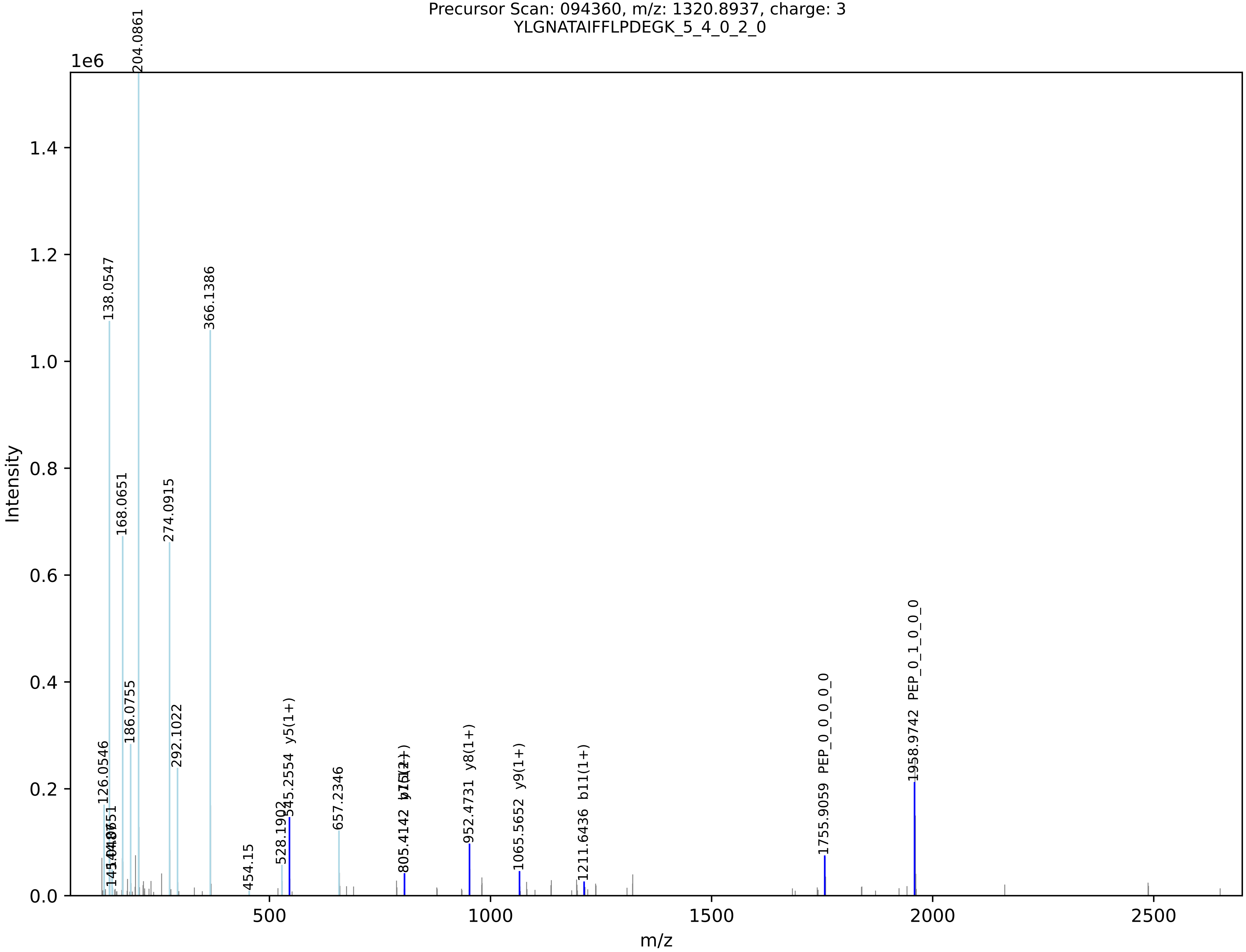

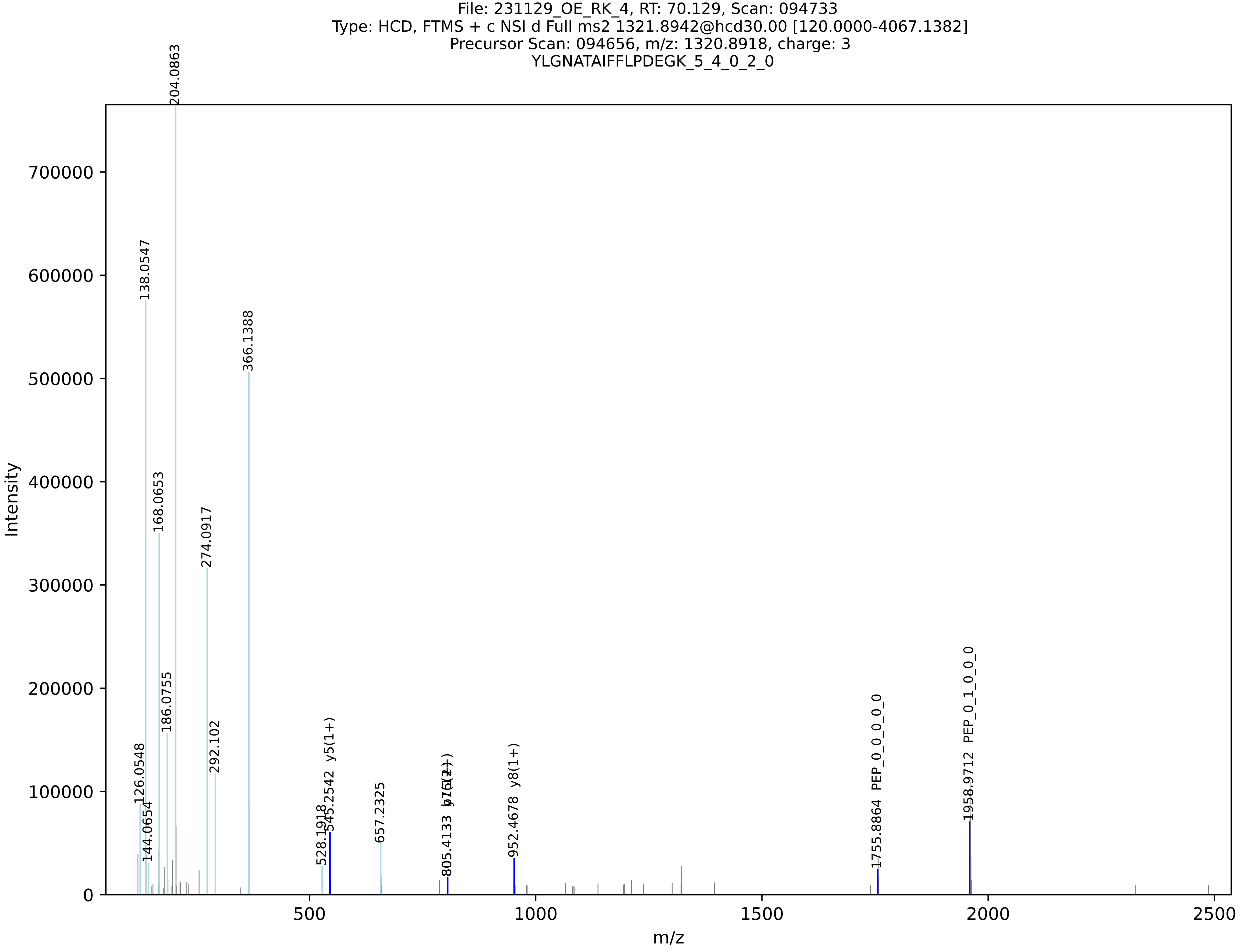

File: 231129_OE_RK_4, RT: 70.129, Scan: 094733
Type: HCD, FTMS + c NSI d Full ms2 1321.8942@hcd30.00 [120.0000-4067.1382]
Precursor Scan: 094656, m/z: 1320.8918, charge: 3
YLGNATAIFFLPDEGK_5_4_0_2_0
Intensity
m/z
0
100000
200000
300000
400000
500000
600000
700000
500
1000
1500
2000
2500
204.0863
138.0547
366.1388
168.0653
274.0917
186.0755
292.102
126.0548
144.0654
528.1918
545.2542 y5(1+)
657.2325
805.4133
952.4678 y8(1+)
1755.8864 PEP_0_0_0_0_0
1958.9712 PEP_0_1_0_0_0

File: 231129_OE_RK_4, RT: 70.373, Scan: 095094
Type: HCD, FTMS + c NSI d Full ms2 1320.8972@hcd30.00 [120.0000-4064.0874]
Precursor Scan: 094801, m/z: 1320.5594, charge: 3
YLGNATAIFFLPDEGK_5_4_0_2_0

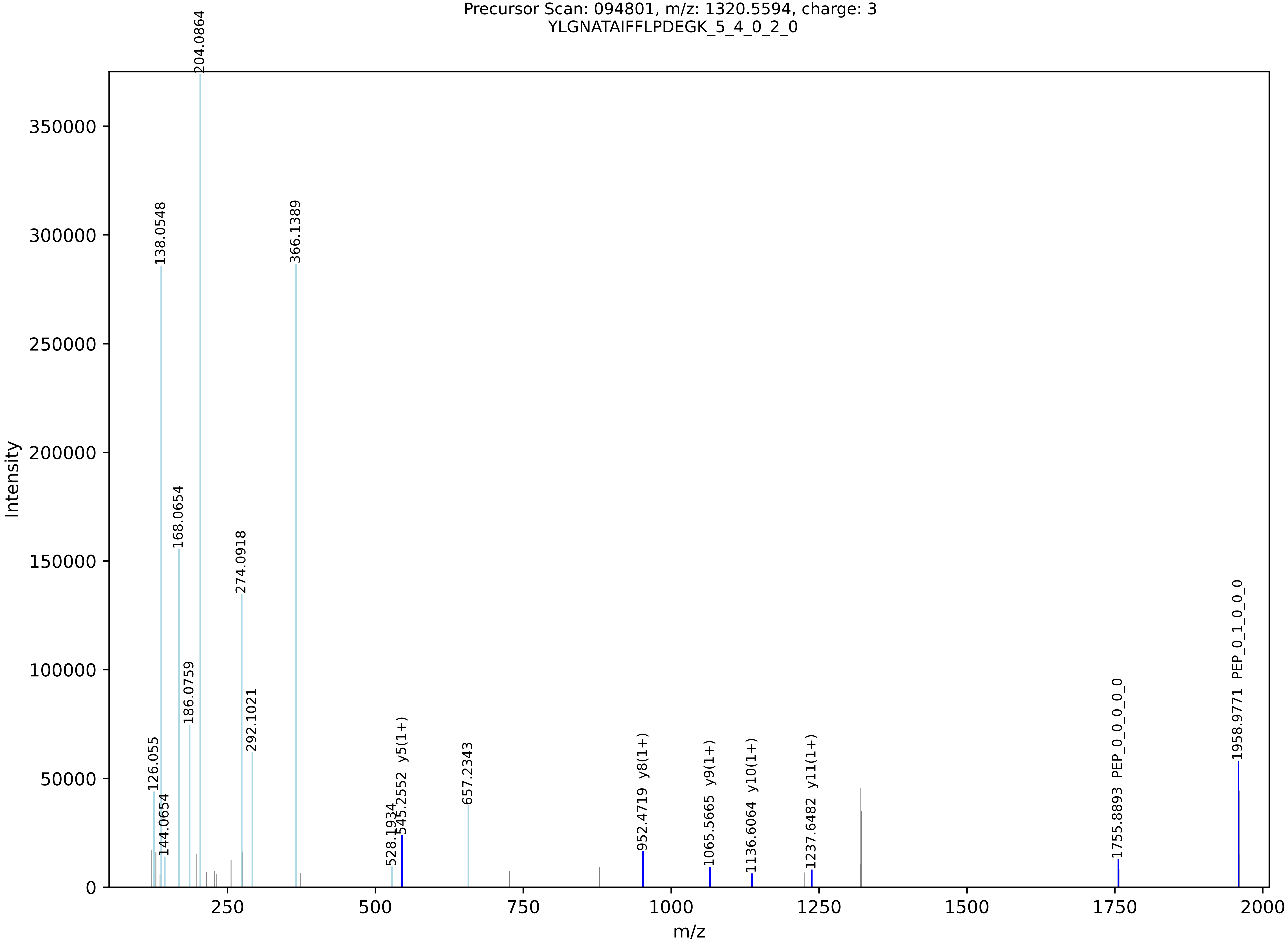

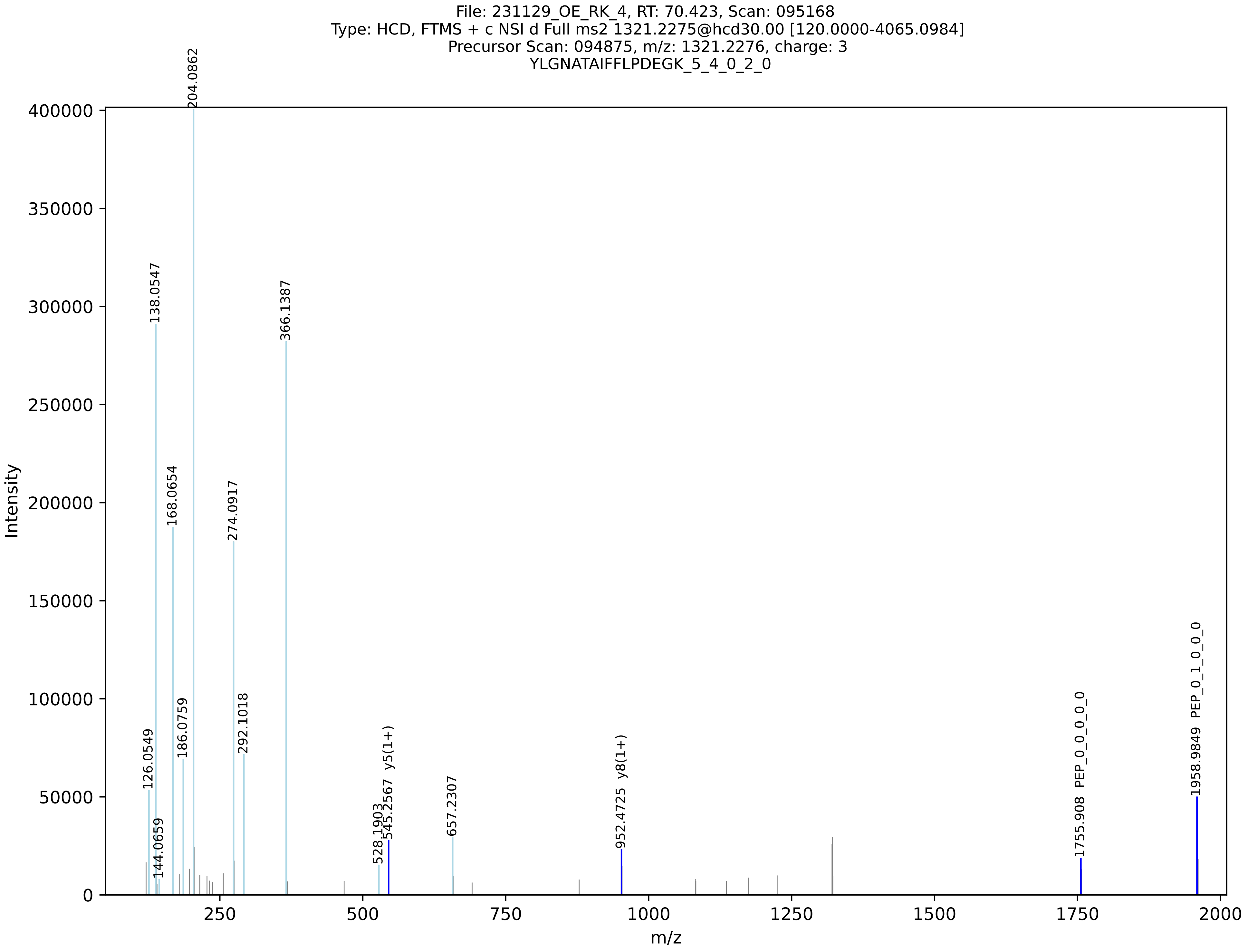

File: 231129_OE_RK_4, RT: 70.423, Scan: 095168
Type: HCD, FTMS + c NSI d Full ms2 1321.2275@hcd30.00 [120.0000-4065.0984]
Precursor Scan: 094875, m/z: 1321.2276, charge: 3
YLGNATAIFFLPDEGK_5_4_0_2_0
Intensity
m/z
204.0862
138.0547
366.1387
168.0654
274.0917
186.0759
292.1018
126.0549
144.0659
528.1903
545.2567 y5(1+)
657.2307
952.4725 y8(1+)
1755.908 PEP_0_0_0_0_0
1958.9849 PEP_0_1_0_0_0

File: 231129_OE_RK_4, RT: 71.791, Scan: 097155
Type: HCD, FTMS + c NSI d Full ms2 1147.8024@hcd30.00 [120.0000-4705.8896]
Precursor Scan: 097078, m/z: 1147.3011, charge: 4
LSLHRPALEDLLLGSEANLTCTLTGLR_5_4_0_0_0

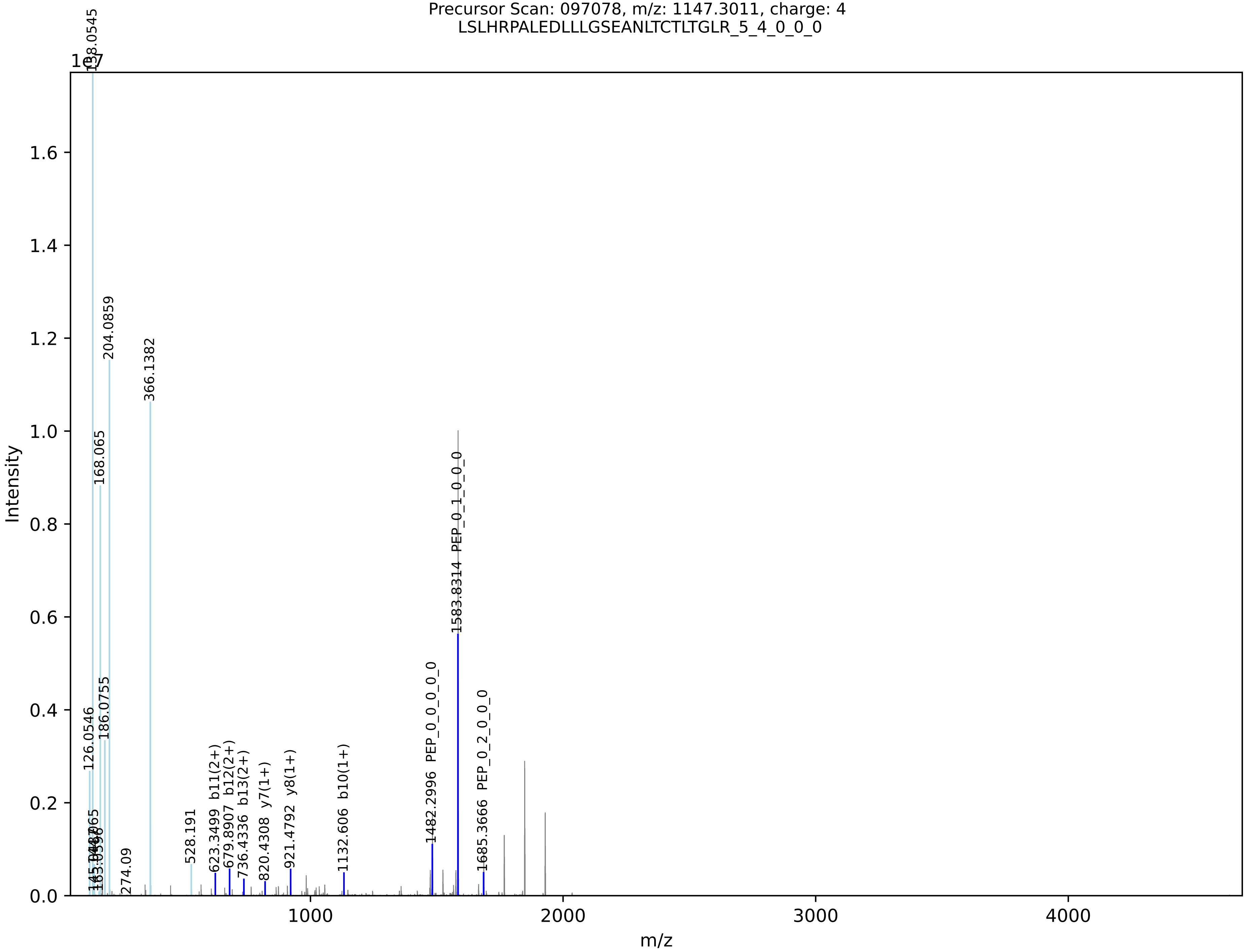

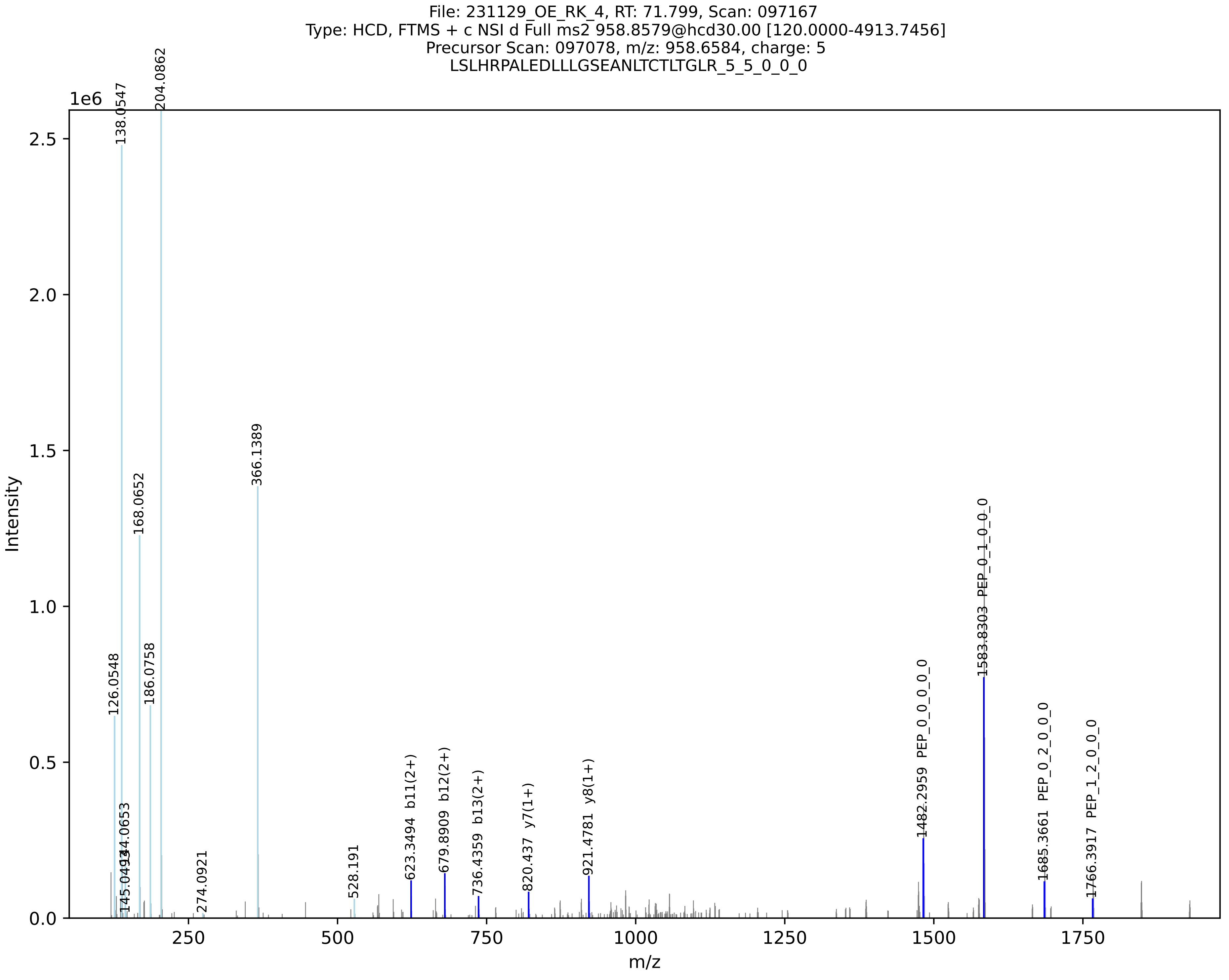
File: 231129_OE_RK_4, RT: 71.799, Scan: 097167
Type: HCD, FTMS + c NSI d Full ms2 958.8579@hcd30.00 [120.0000-4913.7456]
Precursor Scan: 097078, m/z: 958.6584, charge: 5
LSLHRPALEDLLLGSEANLTCTLTGLR_5_5_0_0_0
1e6
Intensity
m/z
204.0862
138.0547
366.1389
168.0652
126.0548
186.0758
145.0493
144.0653
274.0921
528.191
623.3494 b11(2+)
679.8909 b12(2+)
736.4359 b13(2+)
820.437 y7(1+)
921.4781 y8(1+)
1482.2959 PEP_0_0_0_0_0
1583.8303 PEP_0_1_0_0_0
1685.3661 PEP_0_2_0_0_0
1766.3917 PEP_1_2_0_0_0

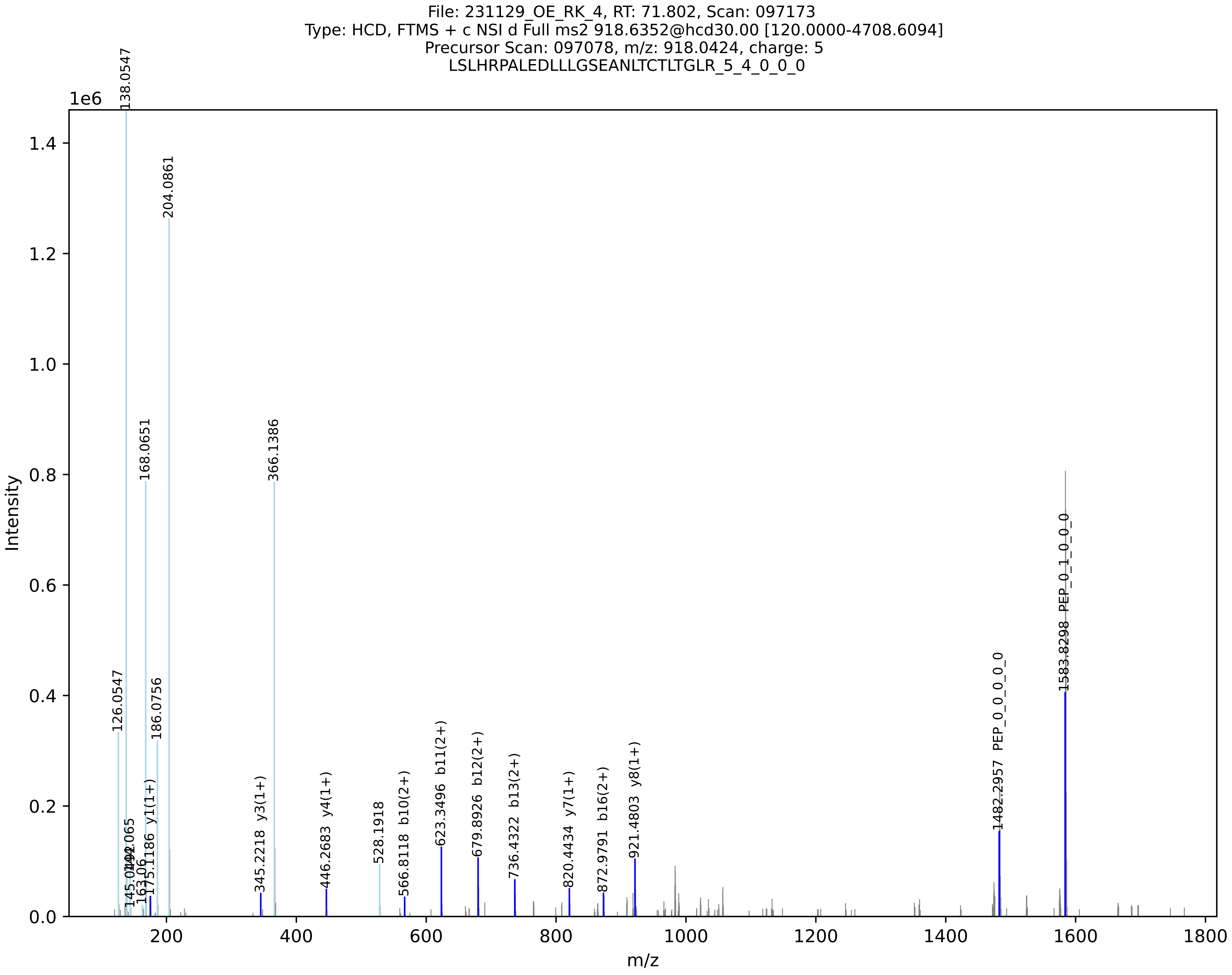

File: 231129_OE_RK_4, RT: 71.802, Scan: 097173
Type: HCD, FTMS + c NSI d Full ms2 918.6352@hcd30.00 [120.0000-4708.6094]
Precursor Scan: 097078, m/z: 918.0424, charge: 5
LSLHRPALEDLLLGSEANLTCTLTGLR_5_4_0_0_0
1e6
Intensity
m/z
138.0547
204.0861
168.0651
366.1386
126.0547
186.0756
145.0142
163.06
175.1186 y1(1+)
345.2218 y3(1+)
446.2683 y4(1+)
528.1918
566.8118 b10(2+)
623.3496 b11(2+)
679.8926 b12(2+)
736.4322 b13(2+)
820.4434 y7(1+)
872.9791 b16(2+)
921.4803 y8(1+)
1482.2957 PEP_0_0_0_0_0
1583.8298 PEP_0_1_0_0_0

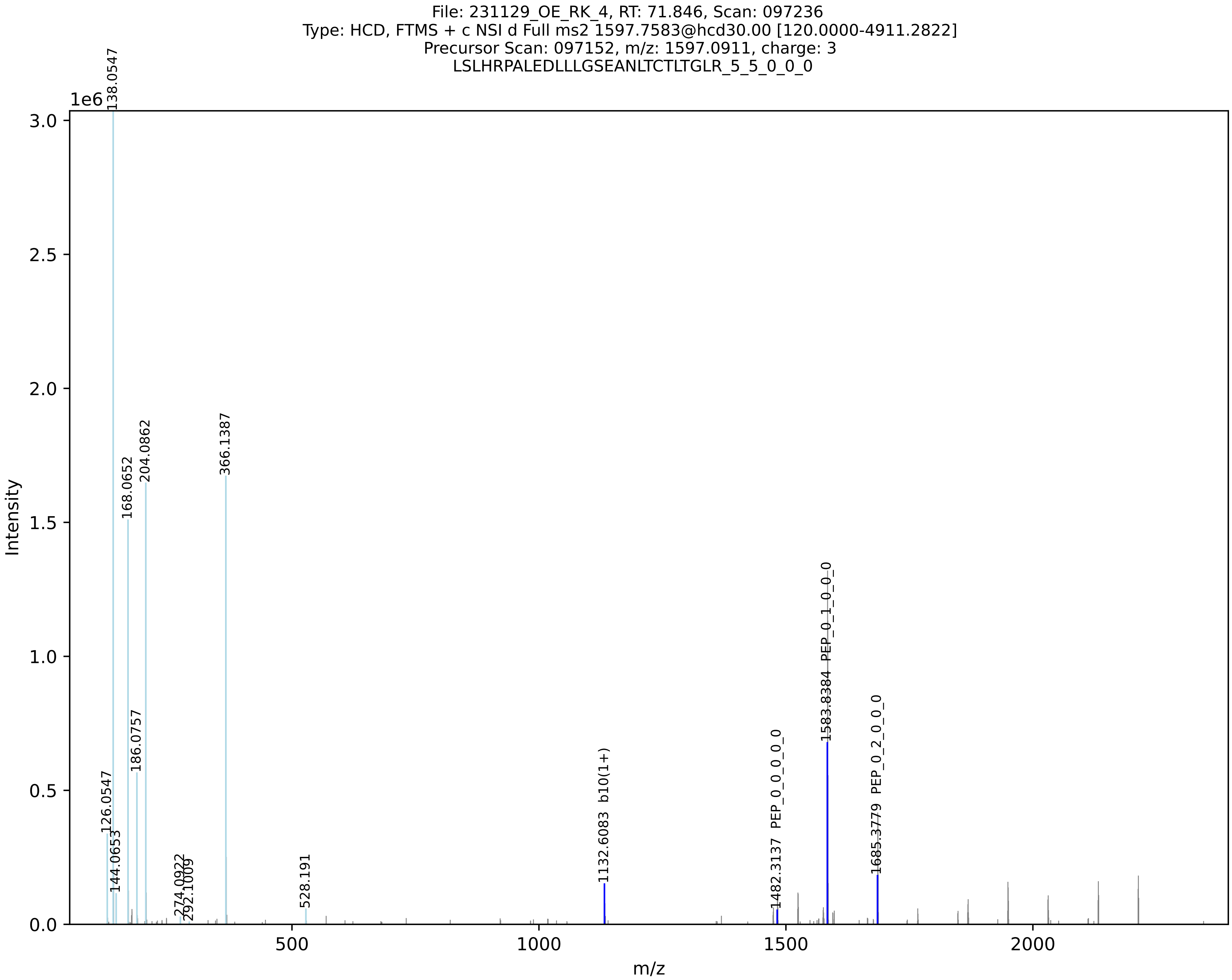
File: 231129_OE_RK_4, RT: 71.846, Scan: 097236
Type: HCD, FTMS + c NSI d Full ms2 1597.7583@hcd30.00 [120.0000-4911.2822]
Precursor Scan: 097152, m/z: 1597.0911, charge: 3
LSLHRPALEDLLLGSEANLTCTLTGLR_5_5_0_0_0
1e6
Intensity
m/z
138.0547
126.0547
144.0653
168.0652
186.0757
204.0862
274.0922
292.1009
366.1387
528.191
1132.6083 b10(1+)
1482.3137 PEP_0_0_0_0_0
1583.8384 PEP_0_1_0_0_0
1685.3779 PEP_0_2_0_0_0

File: 231129_OE_RK_4, RT: 71.854, Scan: 097248
Type: HCD, FTMS + c NSI d Full ms2 1544.0728@hcd30.00 [120.0000-4747.0049]
Precursor Scan: 097152, m/z: 1543.0735, charge: 3
LSLHRPALEDLLLGSEANLTCTLTGLR_4_5_0_0_0

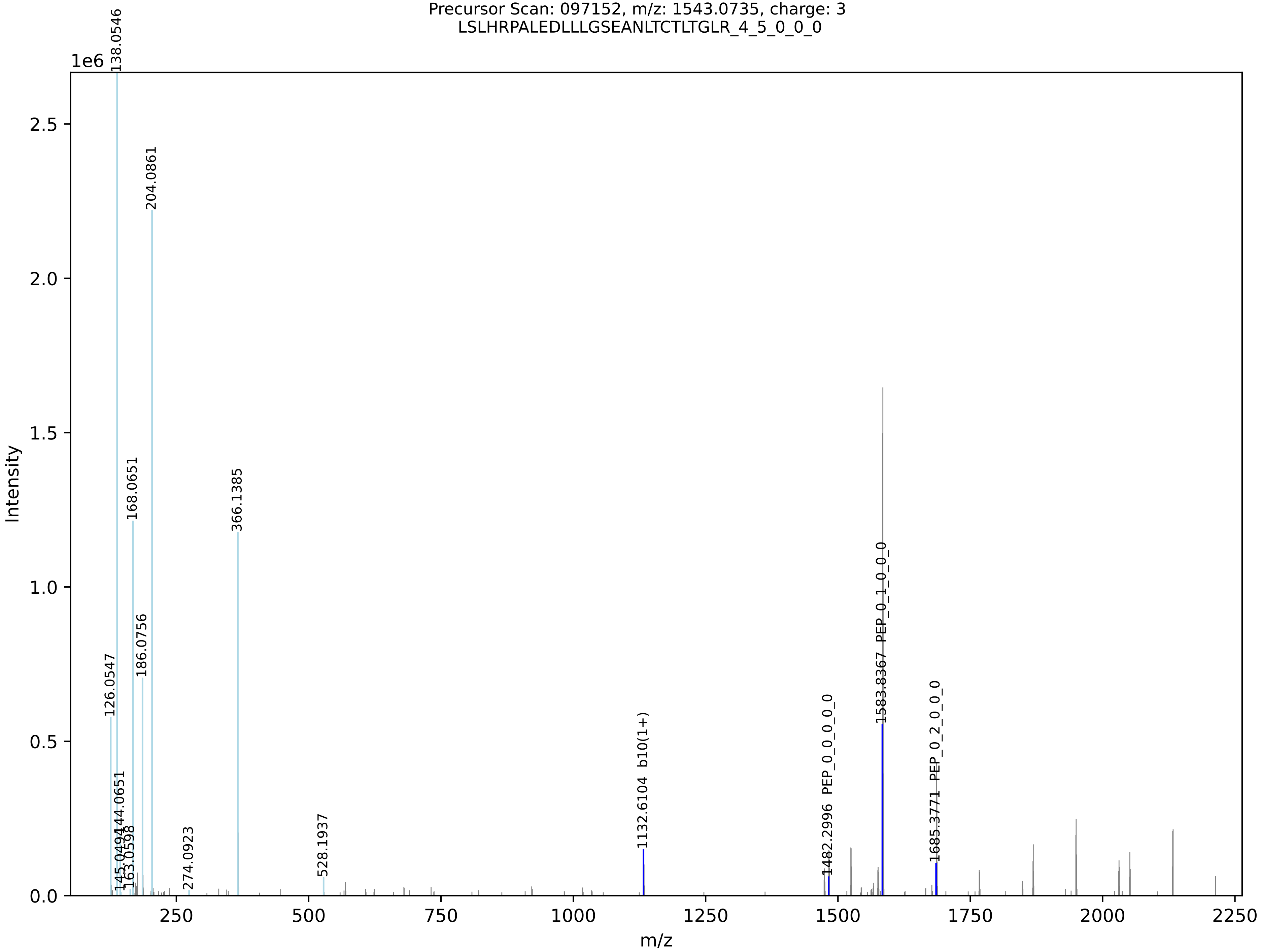

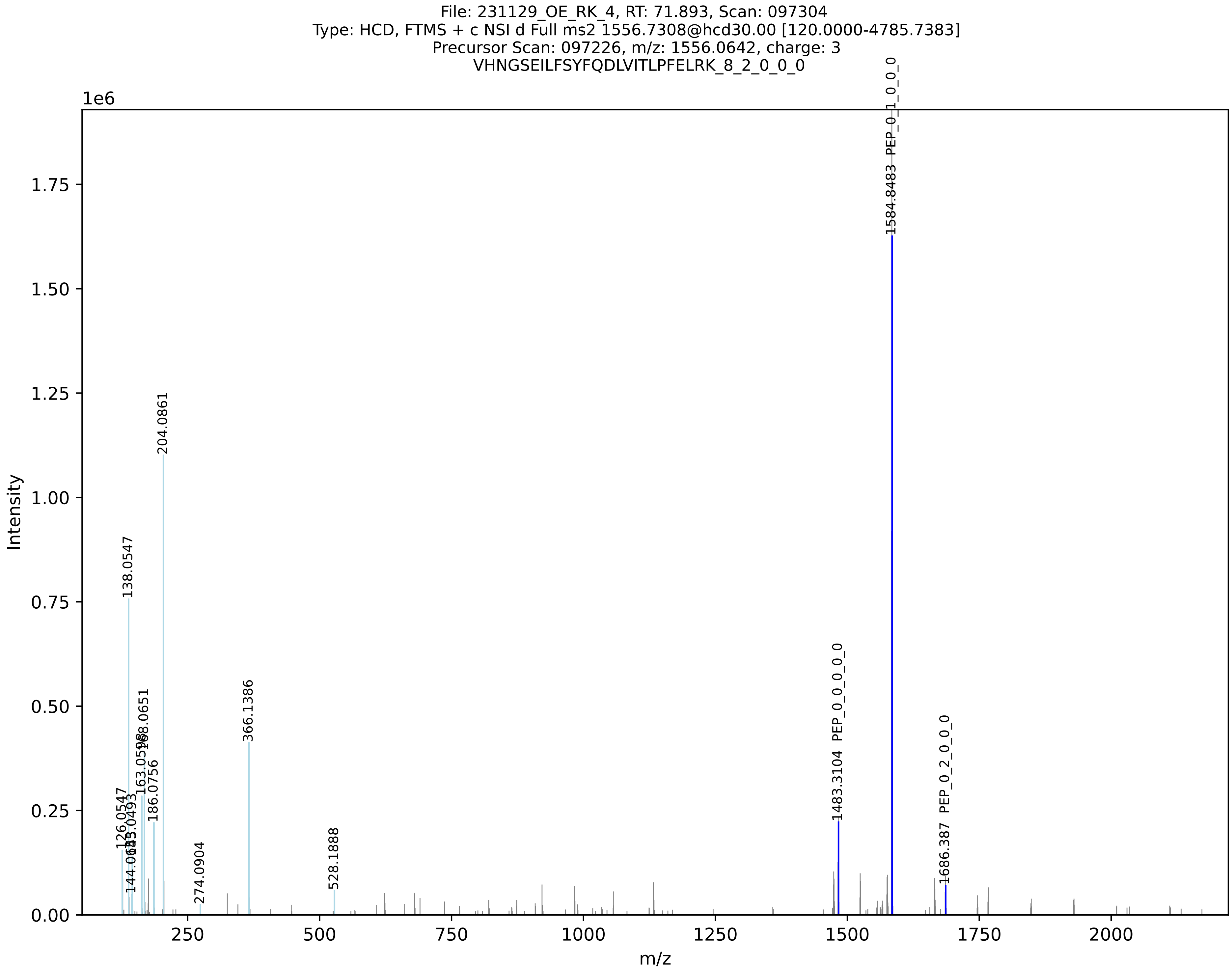
File: 231129_OE_RK_4, RT: 71.893, Scan: 097304
Type: HCD, FTMS + c NSI d Full ms2 1556.7308@hcd30.00 [120.0000-4785.7383]
Precursor Scan: 097226, m/z: 1556.0642, charge: 3
VHNGSEILFSYFQDLVITLPFELRK_8_2_0_0_0
1e6
Intensity
m/z
1584.8483 PEP_0_1_0_0_
1483.3104 PEP_0_0_0_0_
1686.387 PEP_0_2_0_0_
204.0861
138.0547
366.1386
168.0651
163.0598
186.0756
126.0547
138.0493
274.0904
528.1888

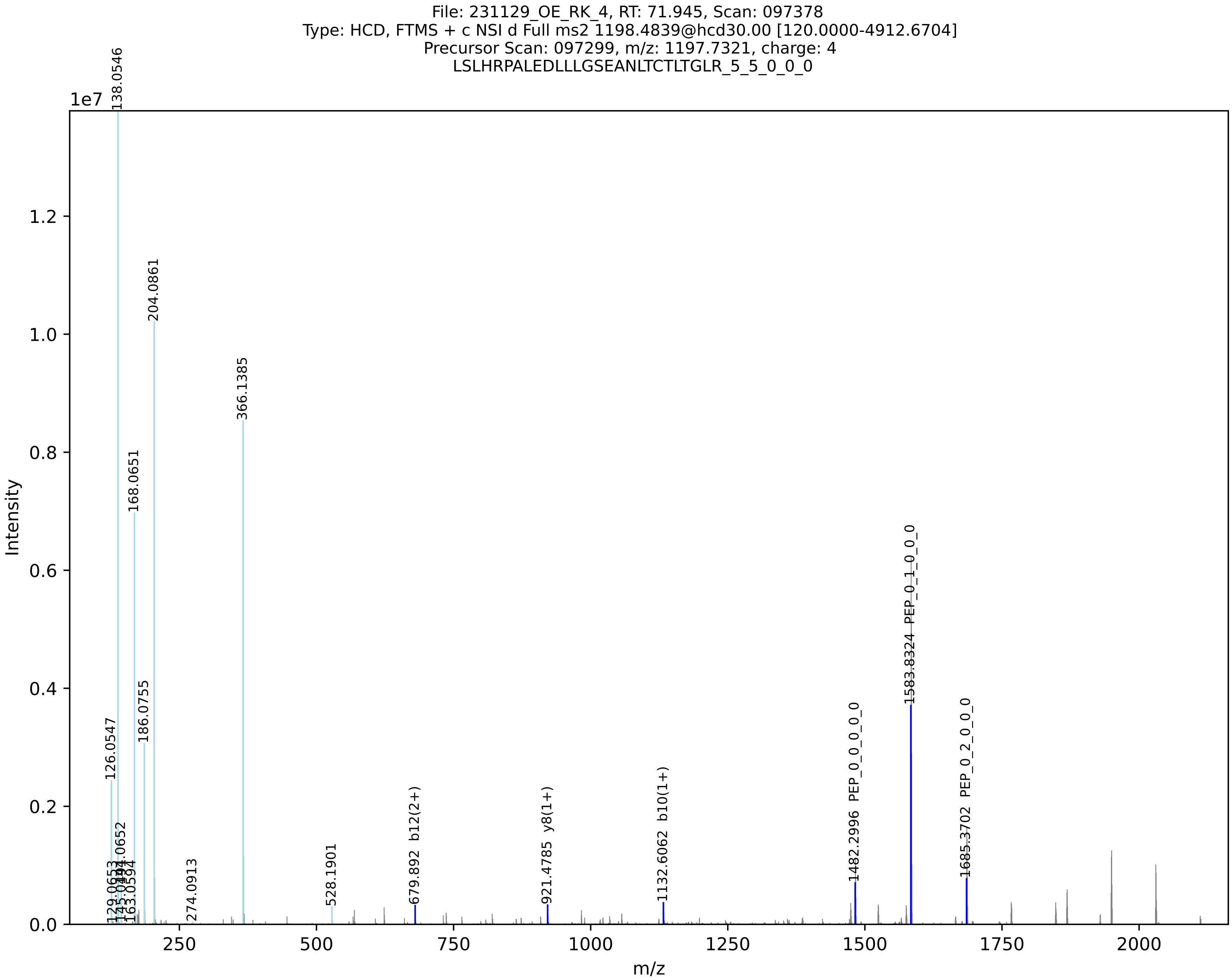
File: 231129_OE_RK_4, RT: 71.945, Scan: 097378
Type: HCD, FTMS + c NSI d Full ms2 1198.4839@hcd30.00 [120.0000-4912.6704]
Precursor Scan: 097299, m/z: 1197.7321, charge: 4
LSLHRPALEDLLLGSEANLTCTLTGLR_5_5_0_0_0
1e7
138.0546
204.0861
366.1385
168.0651
186.0755
126.0547
129.0653
163.0594
274.0913
528.1901
679.892 b12(2+)
921.4785 y8(1+)
1132.6062 b10(1+)
1482.2996 PEP_0_0_0_0_0
1583.8324 PEP_0_1_0_0_0
1685.3702 PEP_0_2_0_0_0
Intensity
m/z
0.0
0.2
0.4
0.6
0.8
1.0
1.2
250
500
750
1000
1250
1500
1750
2000

File: 231129_OE_RK_4, RT: 71.952, Scan: 097390
Type: HCD, FTMS + c NSI d Full ms2 1726.0938@hcd30.00 [120.0000-5303.9888]
Precursor Scan: 097299, m/z: 1725.4238, charge: 3
SVQEIQATFFYFTPNKTEDTIFLR_6_5_0_1_0

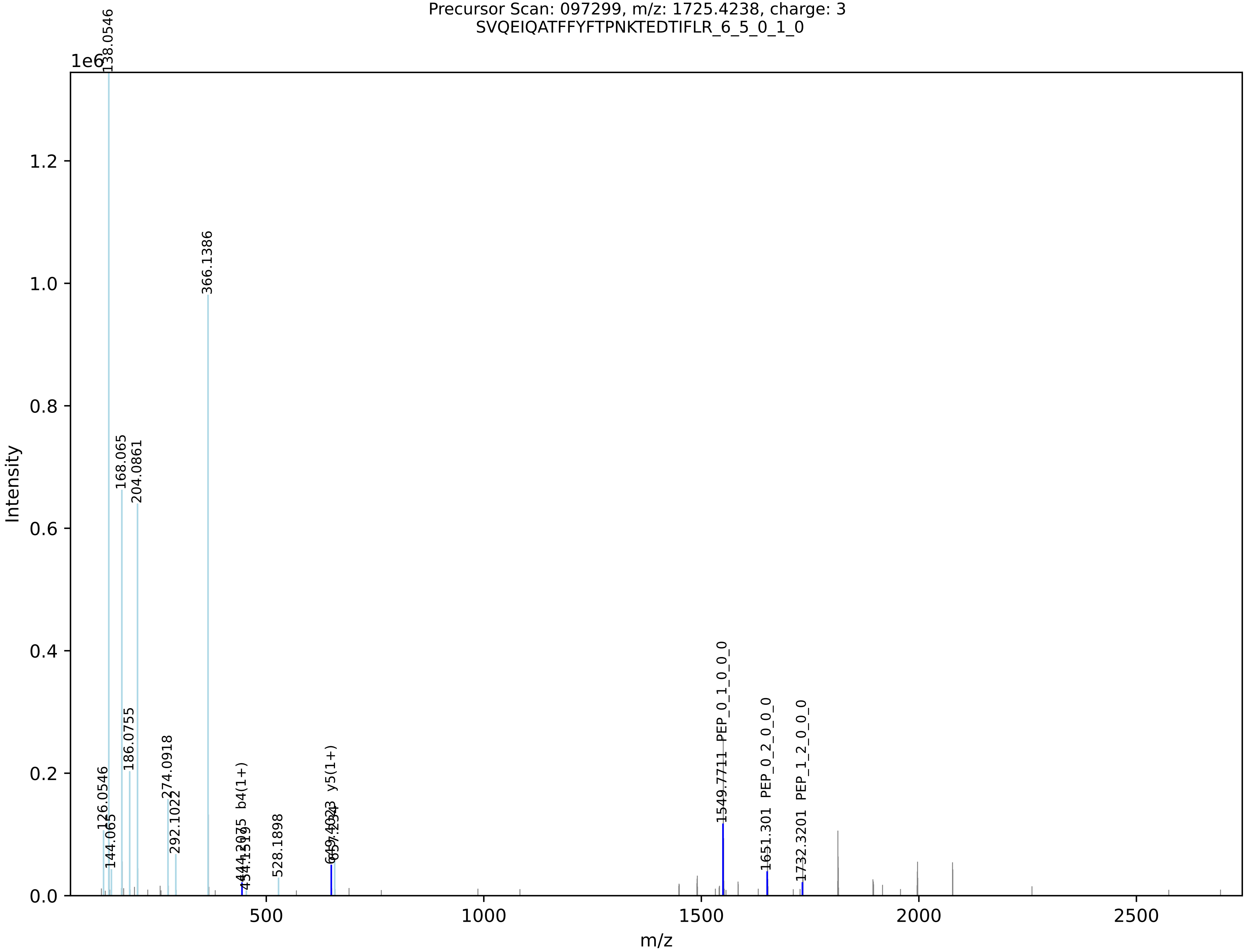

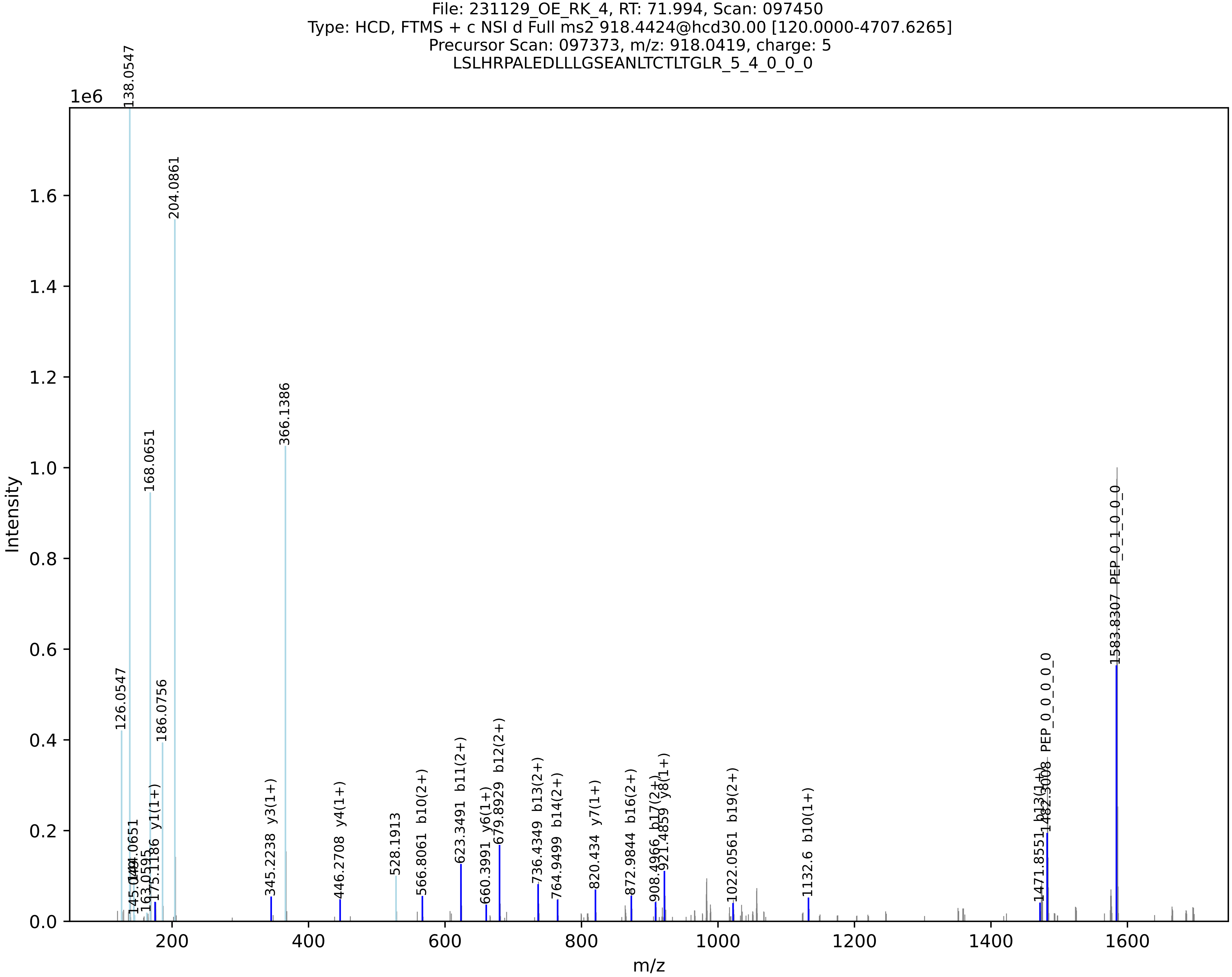
File: 231129_OE_RK_4, RT: 71.994, Scan: 097450
Type: HCD, FTMS + c NSI d Full ms2 918.4424@hcd30.00 [120.0000-4707.6265]
Precursor Scan: 097373, m/z: 918.0419, charge: 5
LSLHRPALEDLLLGSEANLTCTLTGLR_5_4_0_0_0
1e6
Intensity
m/z
138.0547
204.0861
366.1386
168.0651
126.0547
186.0756
145.0495
144.0651
163.0595
175.1186 y1(1+)
345.2238 y3(1+)
446.2708 y4(1+)
528.1913
566.8061 b10(2+)
623.3491 b11(2+)
660.3991 y6(1+)
679.8929 b12(2+)
736.4349 b13(2+)
764.9499 b14(2+)
820.434 y7(1+)
872.9844 b16(2+)
908.4966 b17(2+)
921.4859 y8(1+)
1022.0561 b19(2+)
1132.6 b10(1+)
1471.8551 b13(1+)
1482.3008 PEP_0_0_0_0_0
1583.8307 PEP_0_1_0_0_0

File: 231129_OE_RK_4, RT: 72.046, Scan: 097524
Type: HCD, FTMS + c NSI d Full ms2 1137.5436@hcd30.00 [120.0000-4664.0337]
Precursor Scan: 097445, m/z: 1137.0443, charge: 4
LSLHRPALEDLLLGSEANLTCTLTGLR_6_3_0_0_0

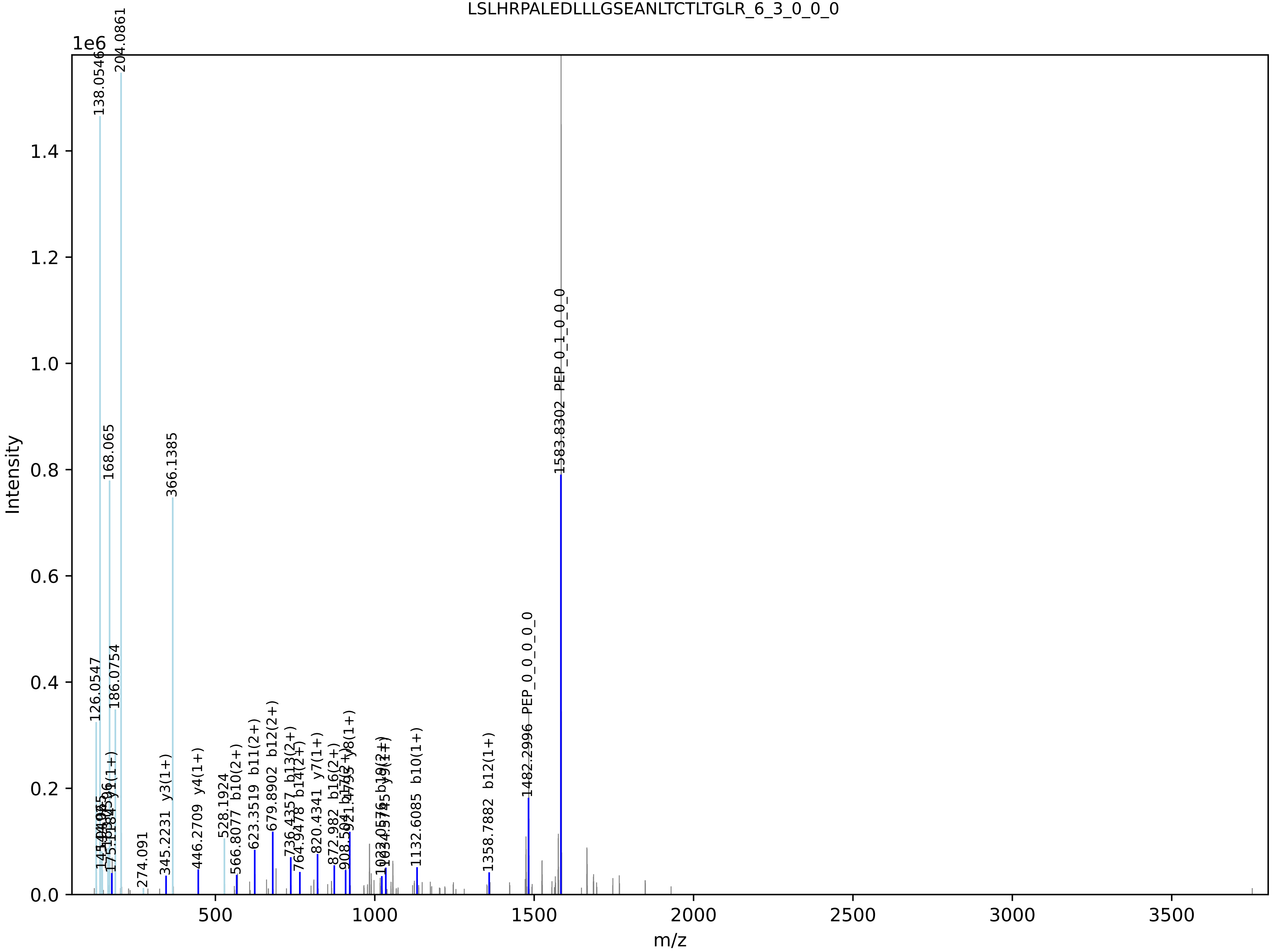

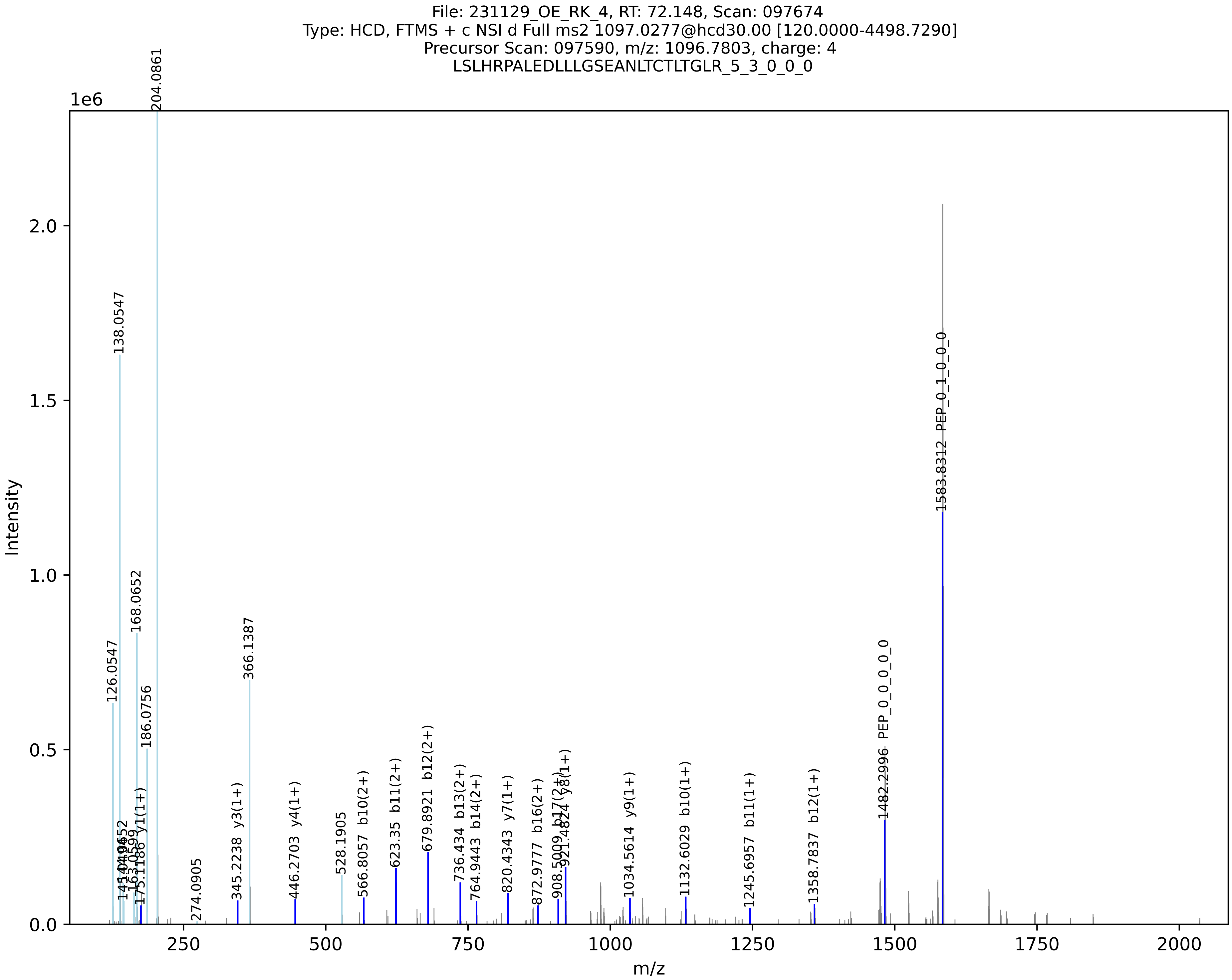

File: 231129_OE_RK_4, RT: 72.148, Scan: 097674
Type: HCD, FTMS + c NSI d Full ms2 1097.0277@hcd30.00 [120.0000-4498.7290]
Precursor Scan: 097590, m/z: 1096.7803, charge: 4
LSLHRPALEDLLLGSEANLTCTLTGLR_5_3_0_0_0
1e6
Intensity
m/z
204.0861
138.0547
168.0652
126.0547
186.0756
366.1387
175.1186 y1(1+)
274.0905
345.2238 y3(1+)
446.2703 y4(1+)
528.1905
566.8057 b10(2+)
623.35 b11(2+)
679.8921 b12(2+)
736.434 b13(2+)
764.9443 b14(2+)
820.4343 y7(1+)
872.9777 b16(2+)
921.4824 y8(1+)
1034.5614 y9(1+)
1132.6029 b10(1+)
1245.6957 b11(1+)
1358.7837 b12(1+)
1482.2996 PEP_0_0_0_0_0
1583.8312 PEP_0_1_0_0_0

File: 231129_OE_RK_4, RT: 72.196, Scan: 097743
Type: HCD, FTMS + c NSI d Full ms2 1198.5708@hcd30.00 [120.0000-4913.0249]
Precursor Scan: 097664, m/z: 1198.0697, charge: 4
LSLHRPALEDLLLGSEANLTCTLTGLR_5_5_0_0_0

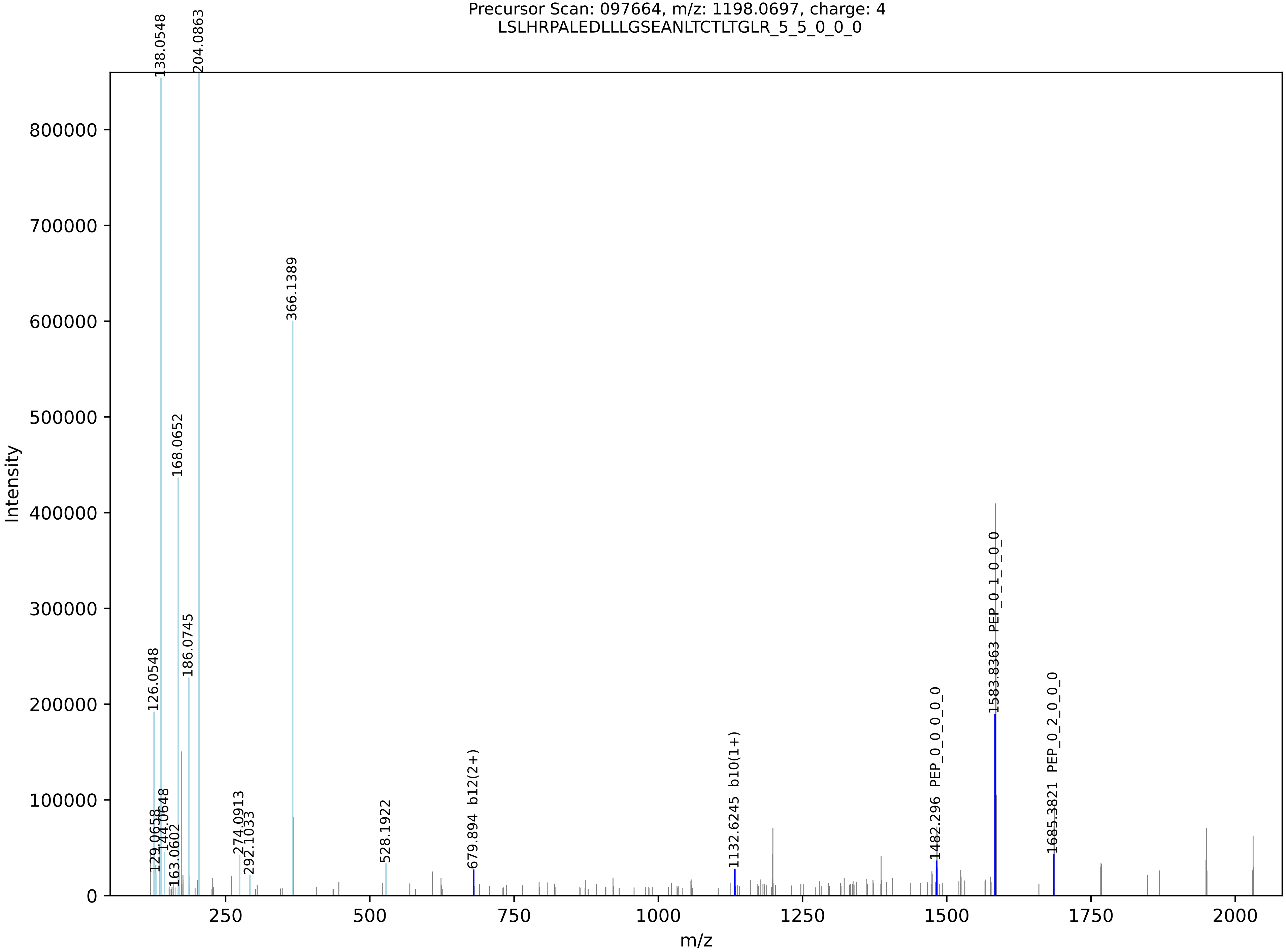

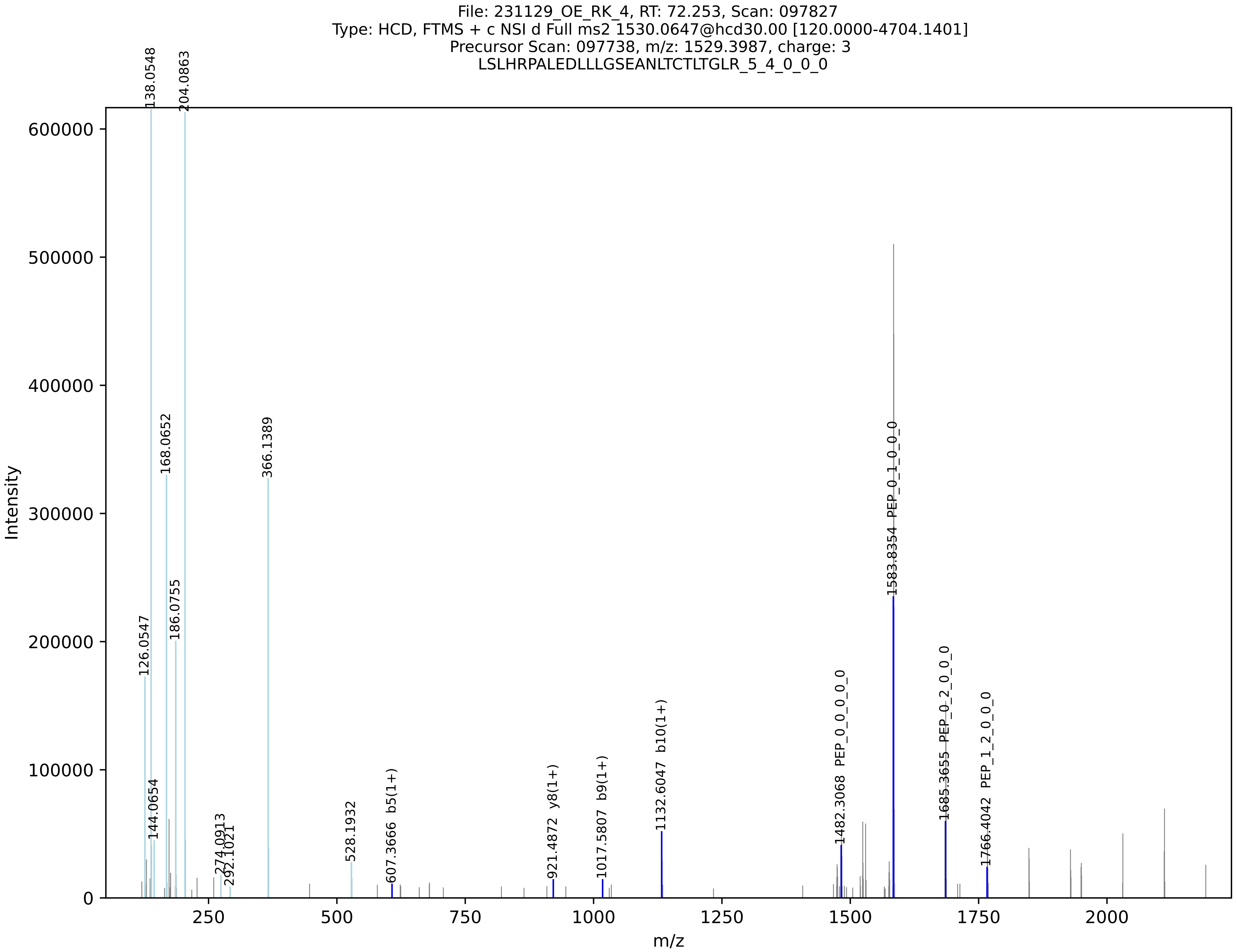

File: 231129_OE_RK_4, RT: 72.253, Scan: 097827
Type: HCD, FTMS + c NSI d Full ms2 1530.0647@hcd30.00 [120.0000-4704.1401]
Precursor Scan: 097738, m/z: 1529.3987, charge: 3
LSLHRPALEDLLLGSEANLTCTLTGLR_5_4_0_0_0
Intensity
m/z
138.0548
204.0863
168.0652
366.1389
186.0755
126.0547
144.0654
274.0913
292.1021
528.1932
607.3666 b5(1+)
921.4872 y8(1+)
1017.5807 b9(1+)
1132.6047 b10(1+)
1482.3068 PEP_0_0_0_0_0
1583.8354 PEP_0_1_0_0_0
1685.3655 PEP_0_2_0_0_0
1766.4042 PEP_1_2_0_0_0

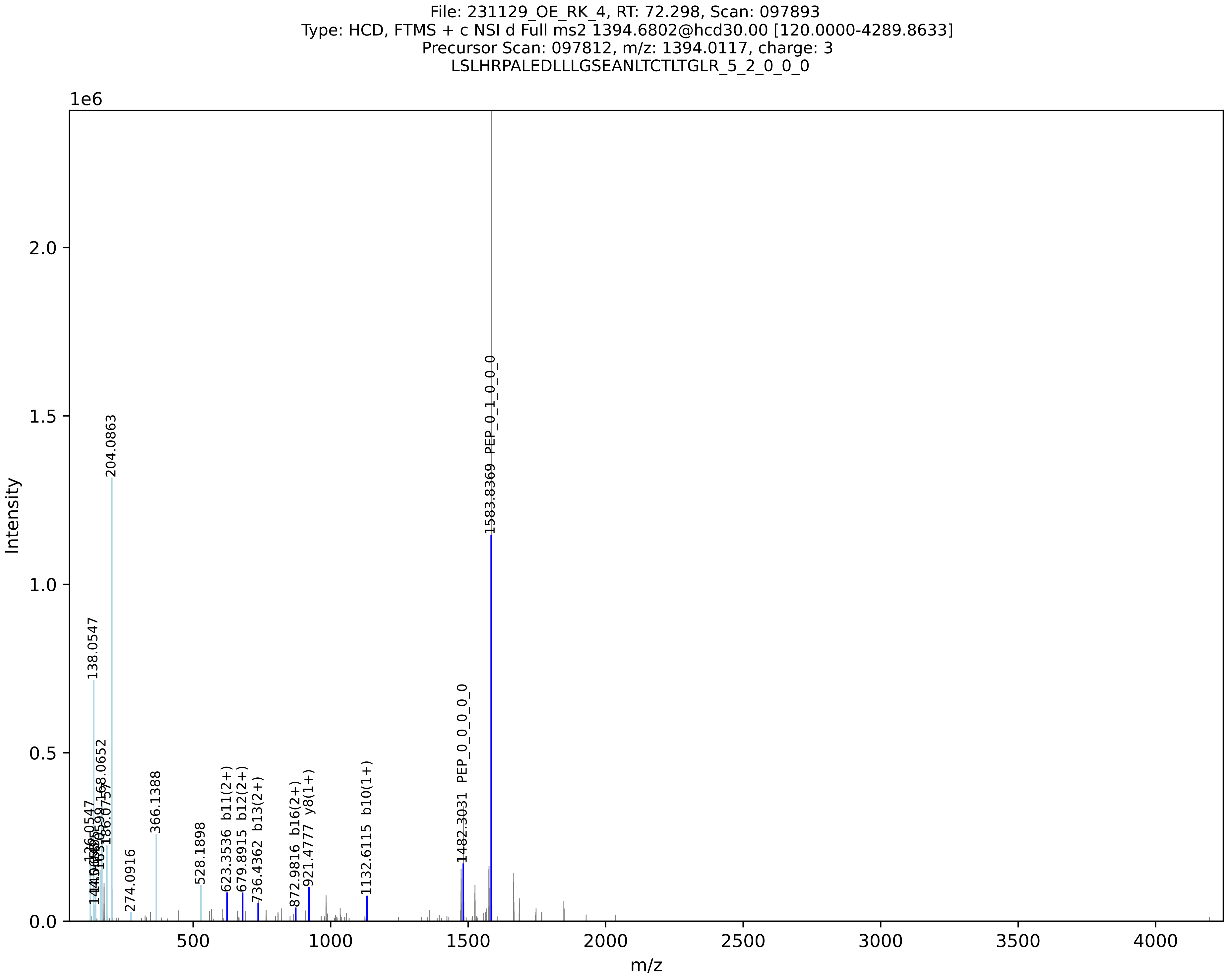
File: 231129_OE_RK_4, RT: 72.298, Scan: 097893
Type: HCD, FTMS + c NSI d Full ms2 1394.6802@hcd30.00 [120.0000-4289.8633]
Precursor Scan: 097812, m/z: 1394.0117, charge: 3
LSLHRPALEDLLLGSEANLTCTLTGLR_5_2_0_0_0
1e6
Intensity
m/z
204.0863
138.0547
168.0652
186.0757
126.0547
366.1388
274.0916
528.1898
623.3536 b11(2+)
679.8915 b12(2+)
736.4362 b13(2+)
872.9816 b16(2+)
921.4777 y8(1+)
1132.6115 b10(1+)
1482.3031 PEP_0_0_0_0_0
1583.8369 PEP_0_1_0_0_0

File: 231129_OE_RK_4, RT: 72.345, Scan: 097961
Type: HCD, FTMS + c NSI d Full ms2 1097.0323@hcd30.00 [120.0000-4498.7480]
Precursor Scan: 097886, m/z: 1096.5311, charge: 4
LSLHRPALEDLLLGSEANLTCTLTGLR_5_3_0_0_0

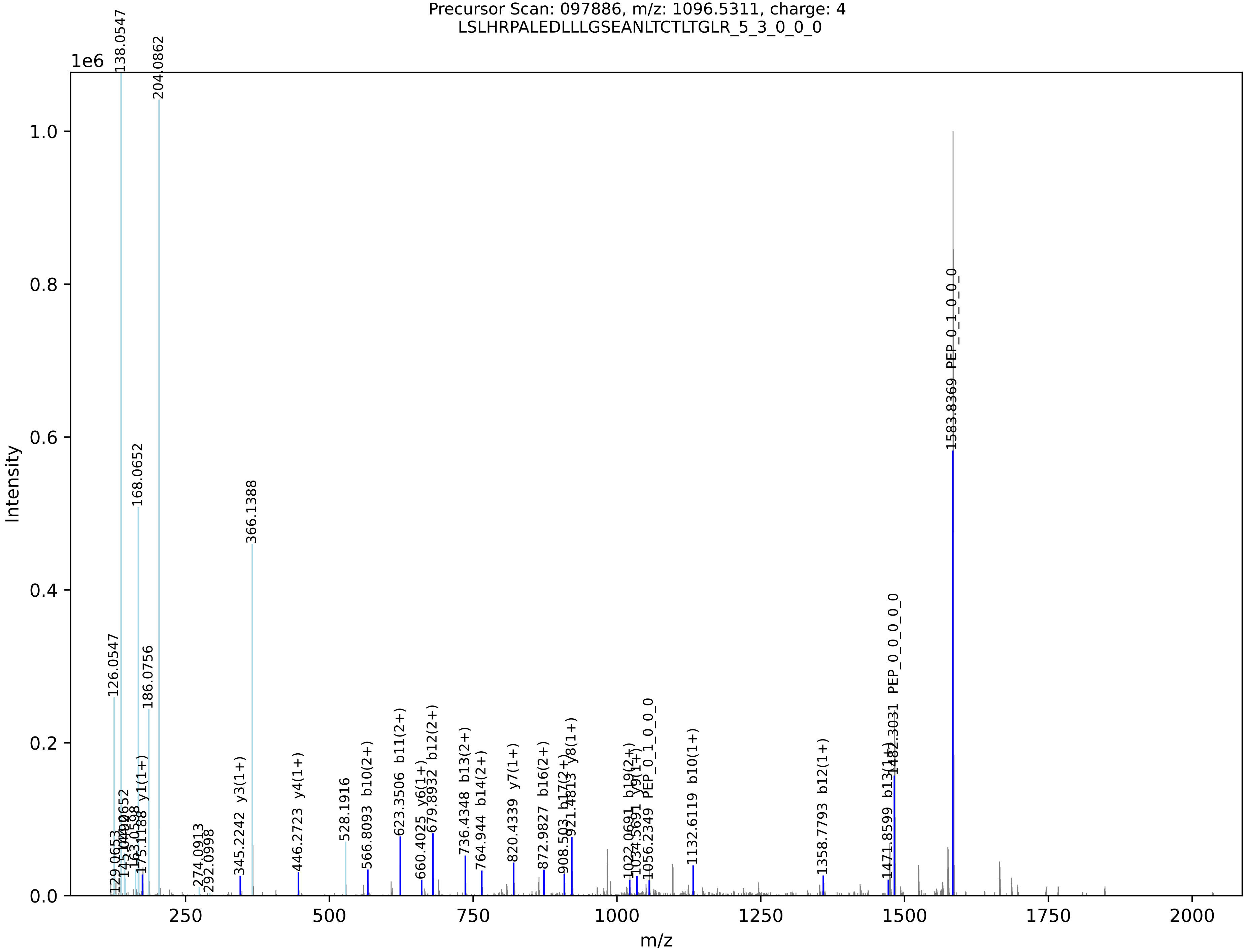

File: 231129_OE_RK_4, RT: 72.497, Scan: 098184
Type: HCD, FTMS + c NSI d Full ms2 1394.6782@hcd30.00 [120.0000-4289.8574]
Precursor Scan: 098108, m/z: 1394.0111, charge: 3
LSLHRPALEDLLLGSEANLTCTLTGLR_5_2_0_0_0

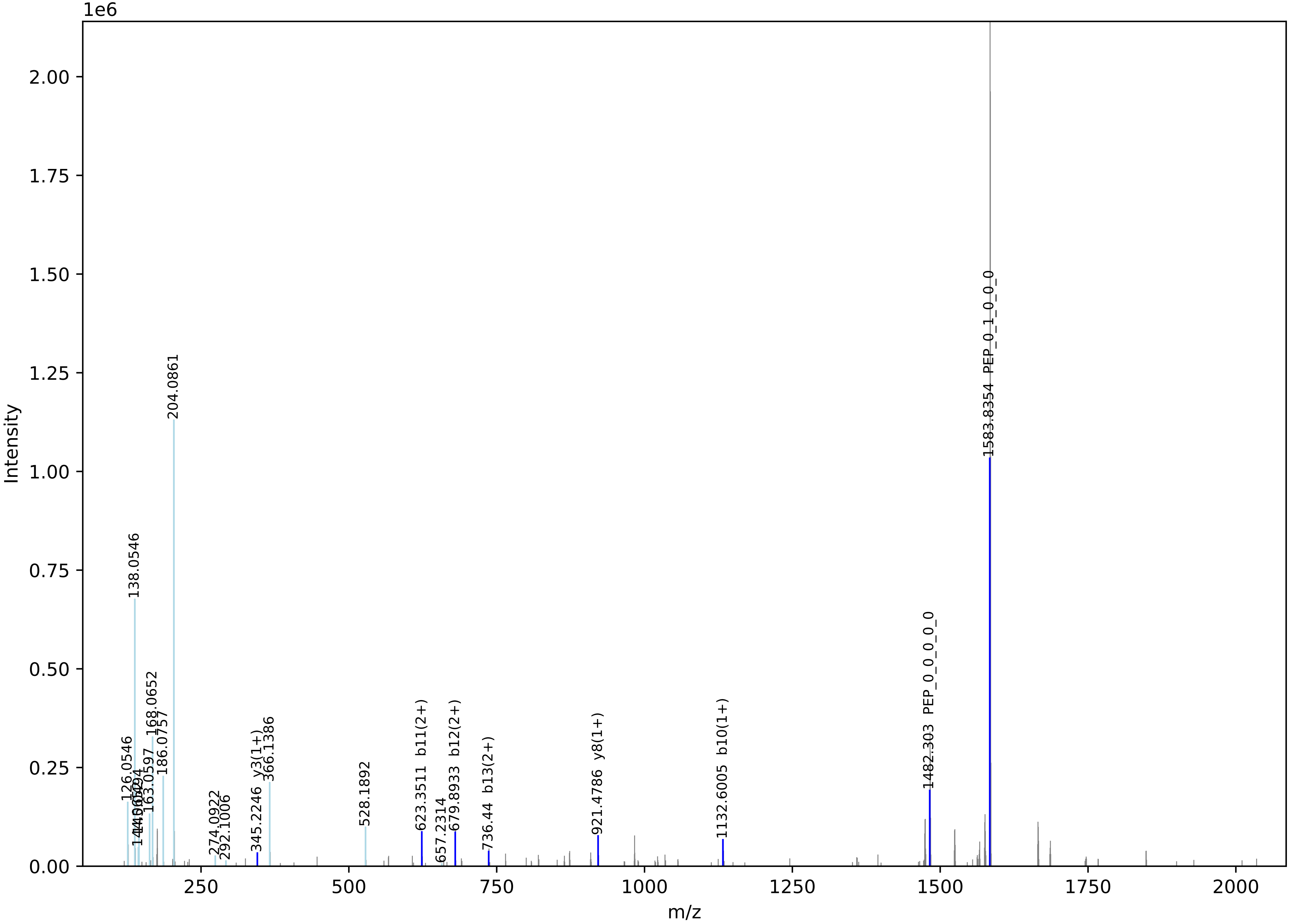

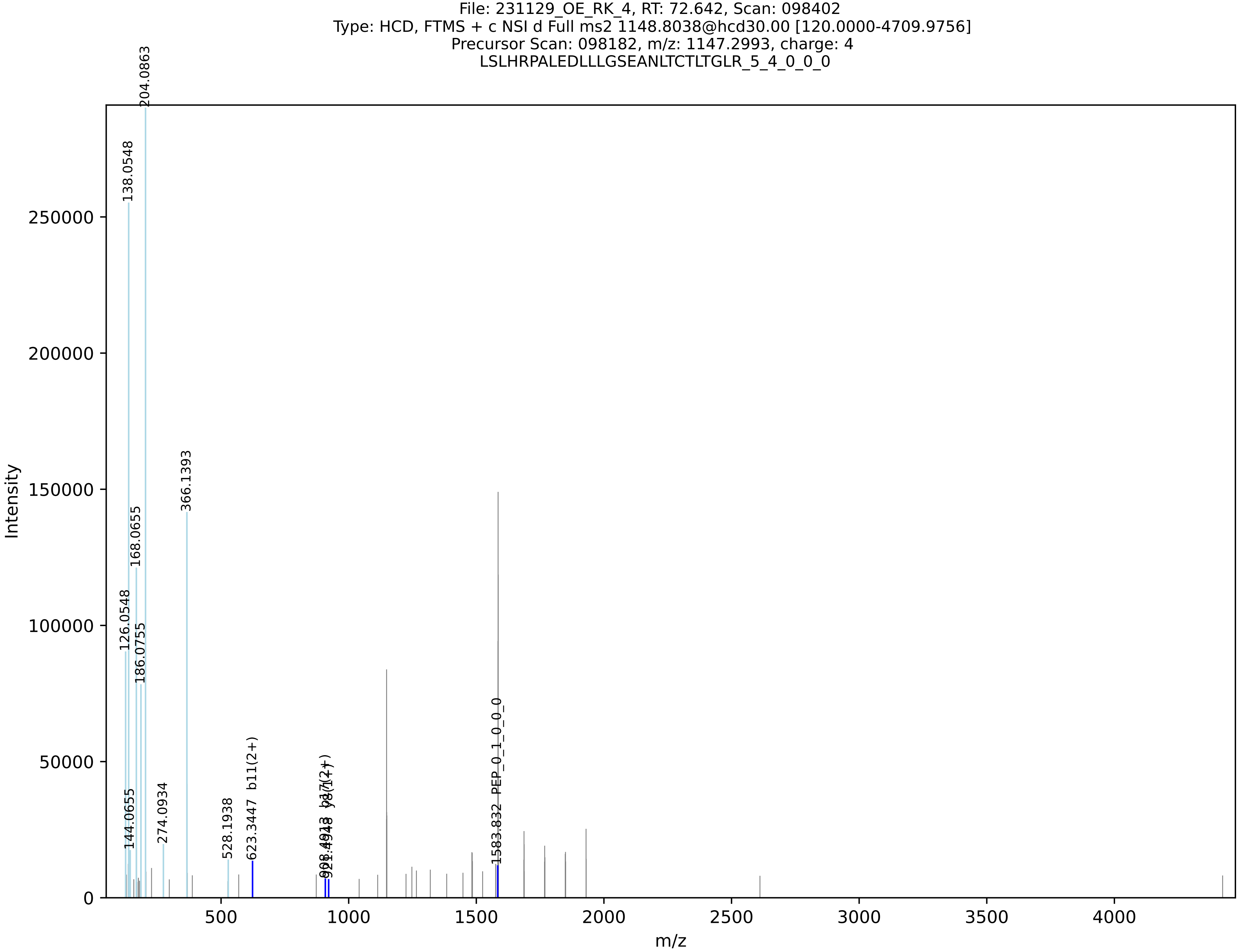
File: 231129_OE_RK_4, RT: 72.642, Scan: 098402
Type: HCD, FTMS + c NSI d Full ms2 1148.8038@hcd30.00 [120.0000-4709.9756]
Precursor Scan: 098182, m/z: 1147.2993, charge: 4
LSLHRPALEDLLLGSEANLTCTLTGLR_5_4_0_0_0
Intensity
m/z
204.0863
138.0548
366.1393
168.0655
126.0548
186.0755
144.0655
274.0934
528.1938
623.3447 b11(2+)
1583.832 PEP_0_1_0_0_0
0
50000
100000
150000
200000
250000
500
1000
1500
2000
2500
3000
3500
4000

File: 231129_OE_RK_4, RT: 72.749, Scan: 098554
Type: HCD, FTMS + c NSI d Full ms2 1203.7904@hcd30.00 [120.0000-4934.3208]
Precursor Scan: 098478, m/z: 1203.0387, charge: 4
SVQEIQATFFYFTPNKTEDTIFLR_5_4_0_1_0

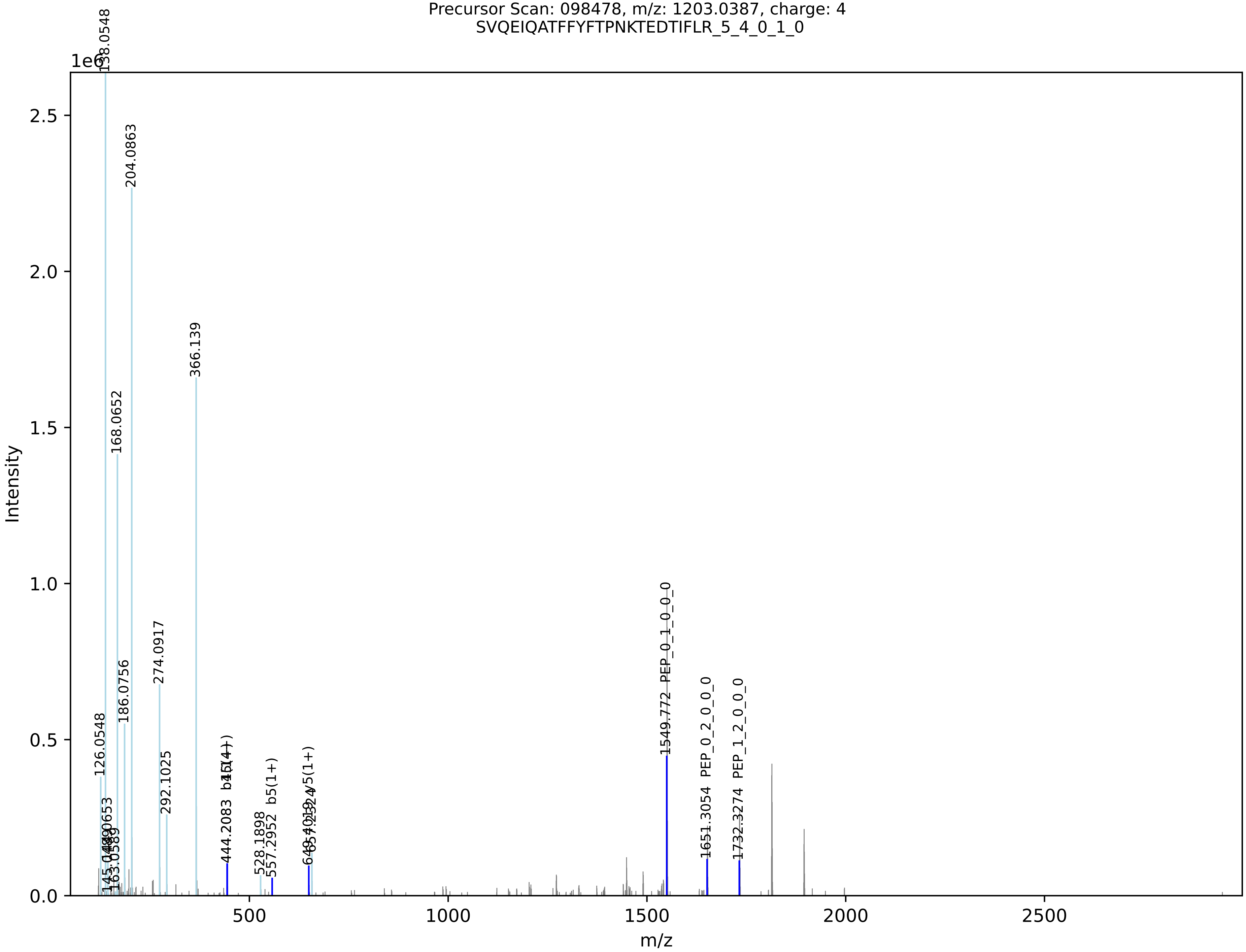

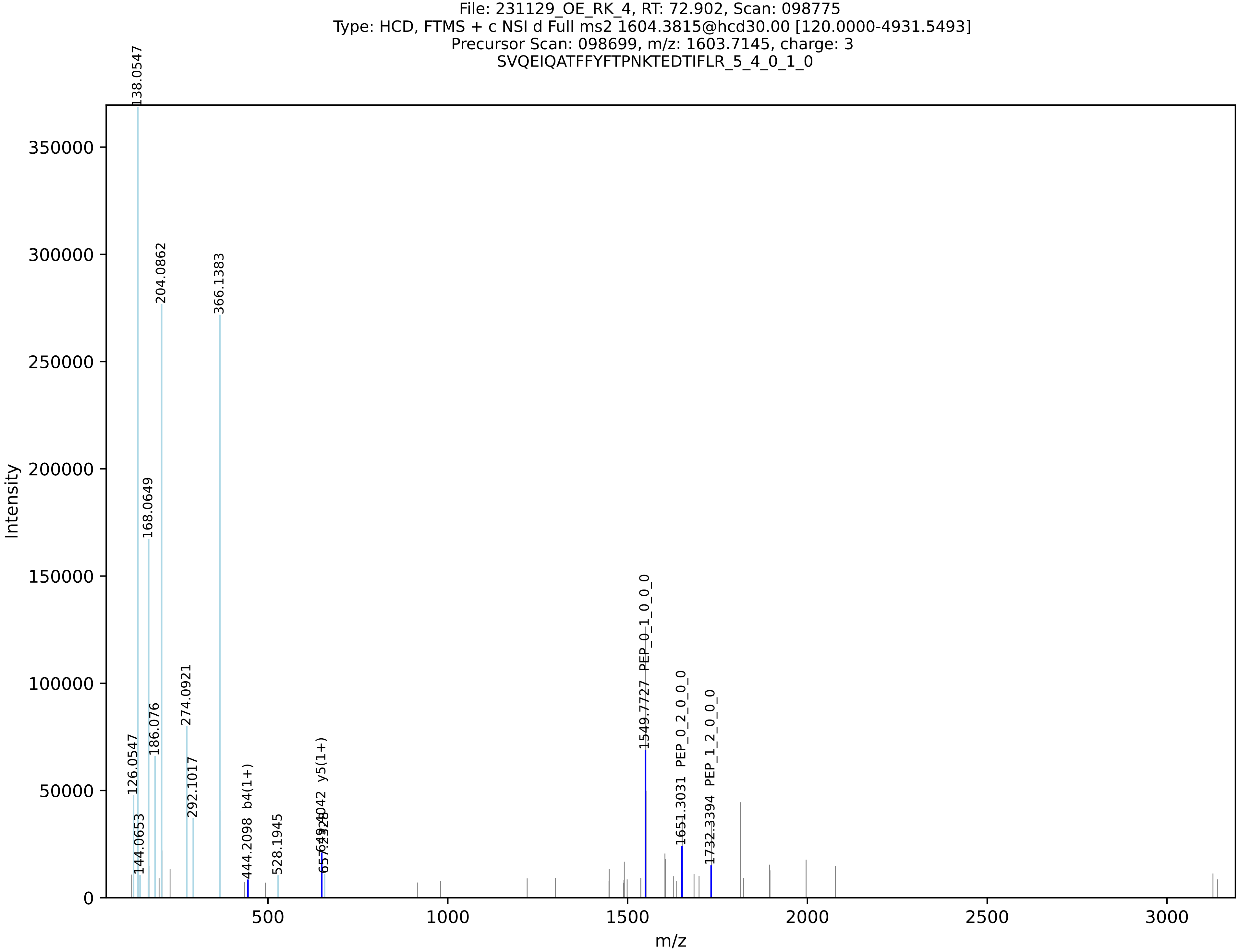
File: 231129_OE_RK_4, RT: 72.902, Scan: 098775
Type: HCD, FTMS + c NSI d Full ms2 1604.3815@hcd30.00 [120.0000-4931.5493]
Precursor Scan: 098699, m/z: 1603.7145, charge: 3
SVQEIQATFFYFTPNKTEDTIFLR_5_4_0_1_0
Intensity
m/z
138.0547
204.0862
366.1383
168.0649
274.0921
186.076
126.0547
292.1017
144.0653
444.2098 b4(1+)
528.1945
649.4042 y5(1+)
657.2320
1549.7727 PEP_0_1_0_0_0
1651.3031 PEP_0_2_0_0_0
1732.3394 PEP_1_2_0_0_0

File: 231129_OE_RK_4, RT: 73.055, Scan: 099000
Type: HCD, FTMS + c NSI d Full ms2 1481.9237@hcd30.00 [120.0000-4556.8286]
Precursor Scan: 098917, m/z: 1481.2555, charge: 3
CSDGWSFDATTLDDNGTMLFFK_5_4_0_1_0

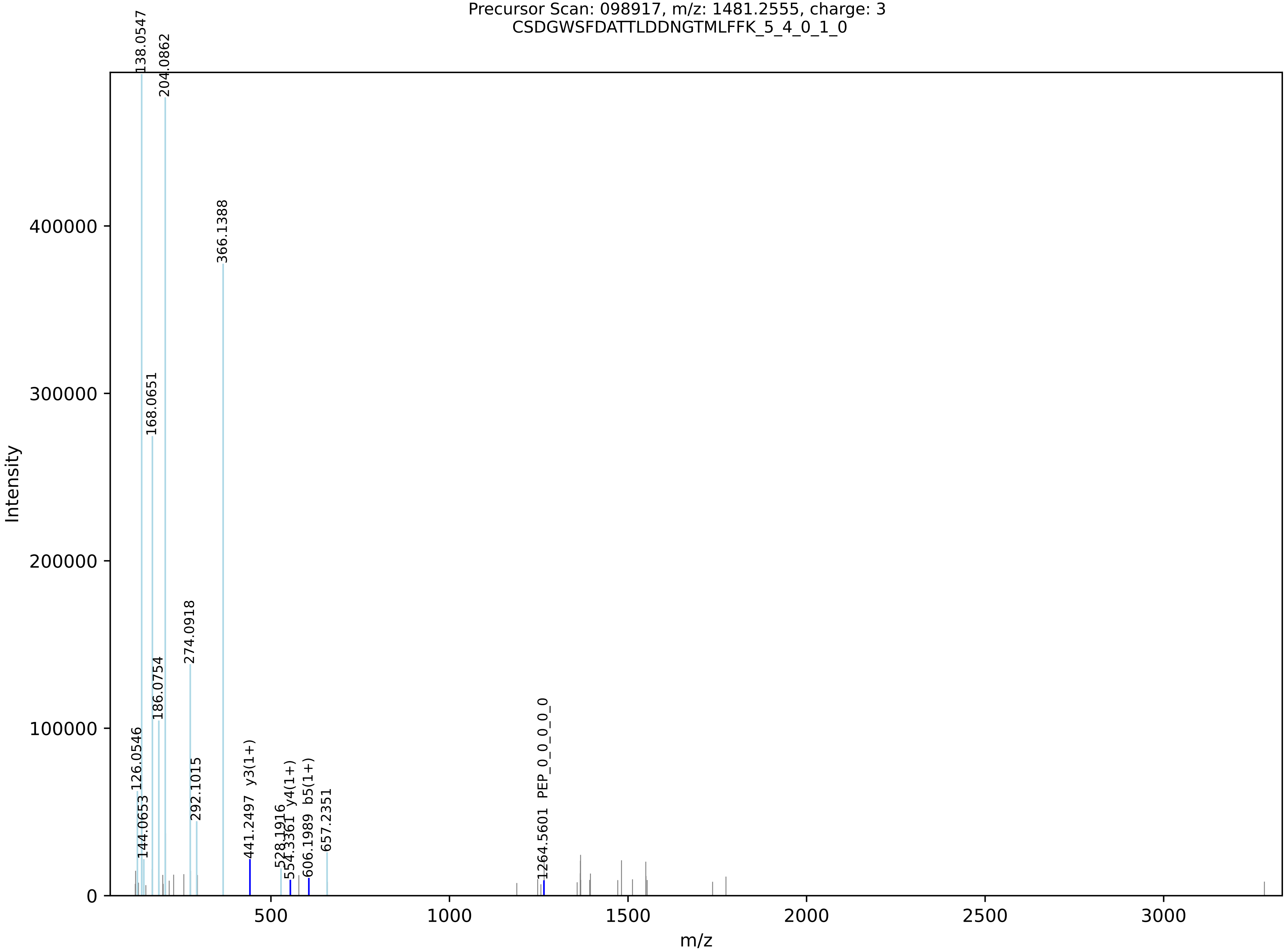

File: 231129_OE_RK_4, RT: 81.011, Scan: 110559
Type: HCD, FTMS + c NSI d Full ms2 1712.2698@hcd30.00 [120.0000-7008.9170]
Precursor Scan: 110481, m/z: 1711.5156, charge: 4
NQALNLSLAYSFVTPLTSMVVTKPDDQEQSQVAEKPMEGESR_5_4_2_1_0

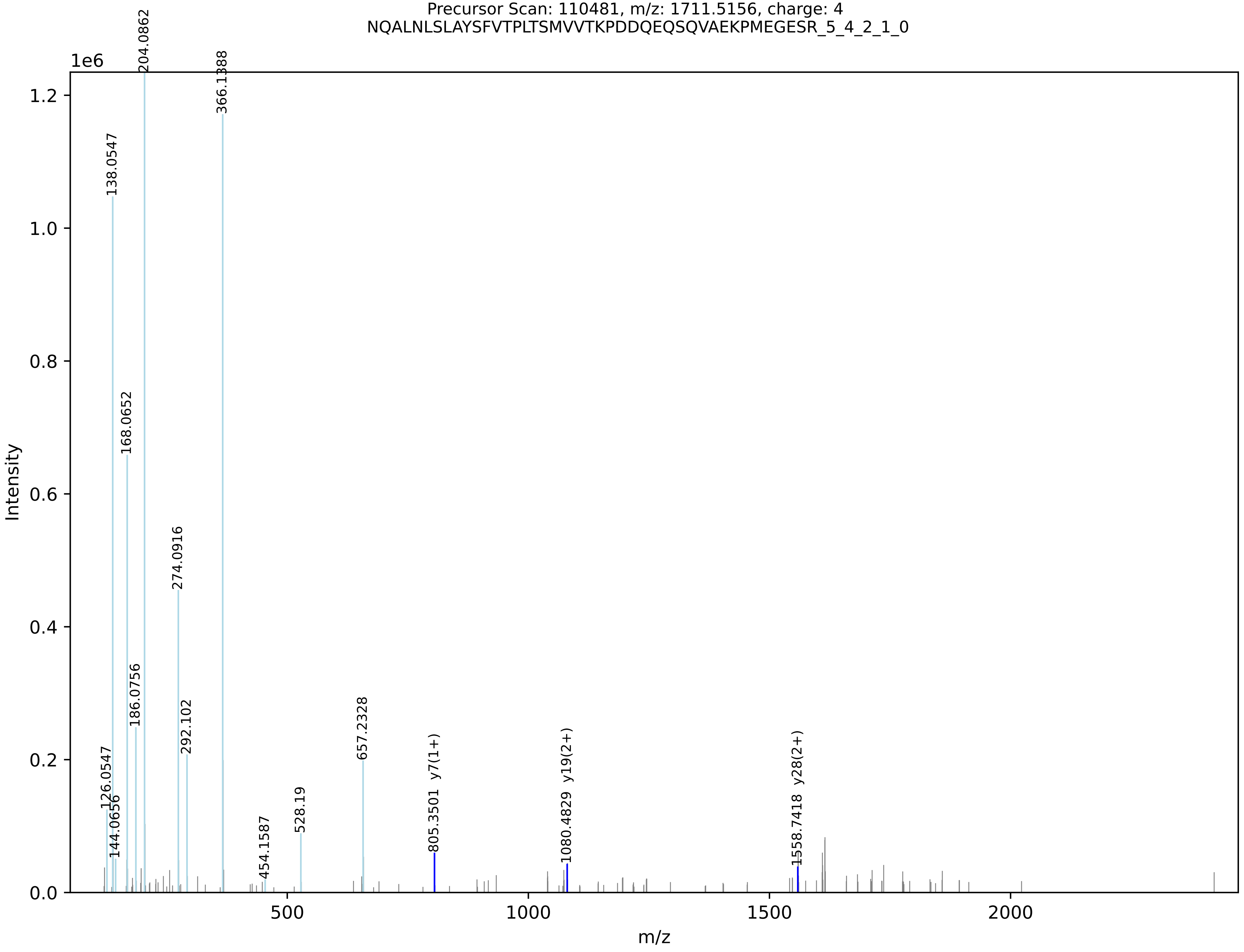

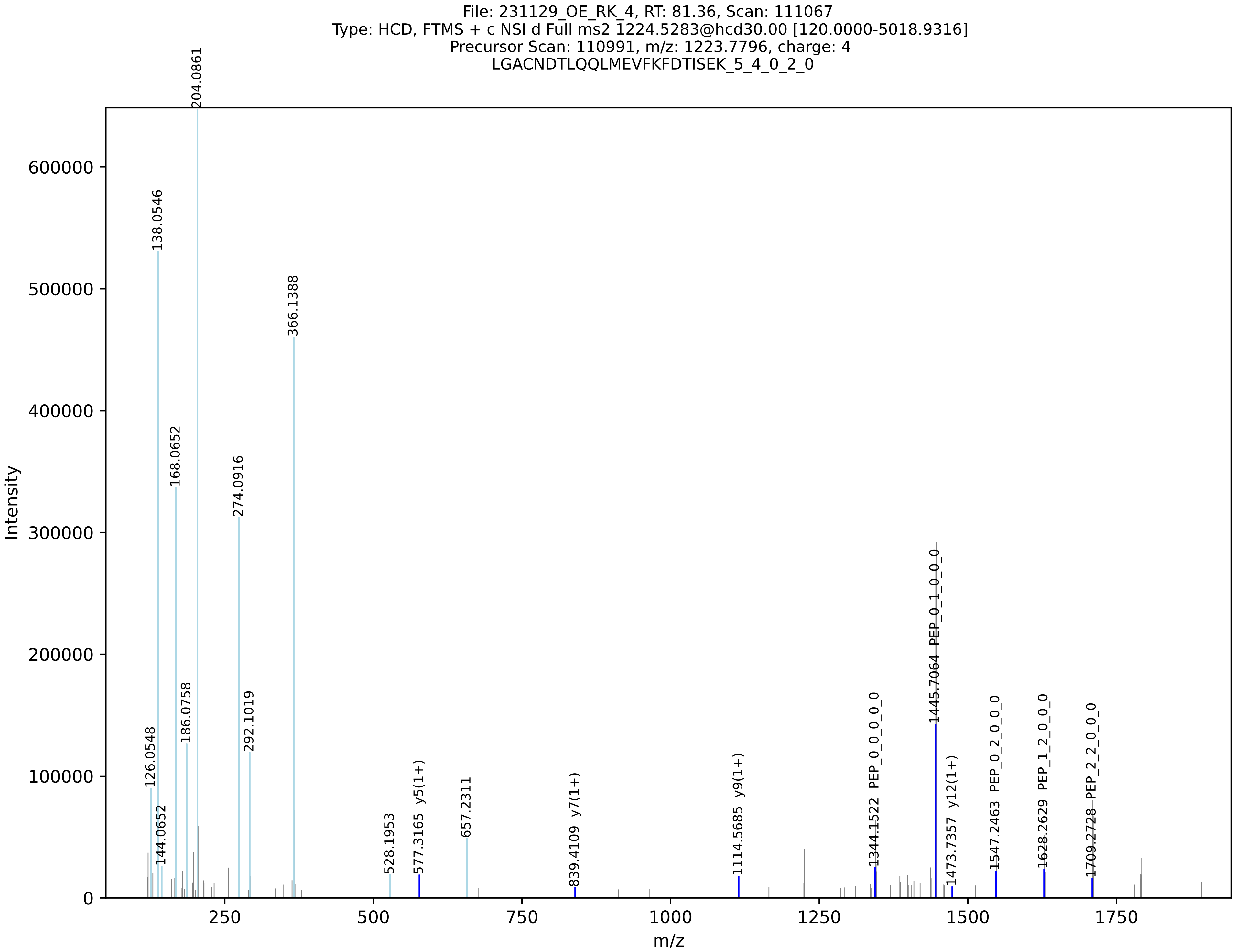
File: 231129_OE_RK_4, RT: 81.36, Scan: 111067
Type: HCD, FTMS + c NSI d Full ms2 1224.5283@hcd30.00 [120.0000-5018.9316]
Precursor Scan: 110991, m/z: 1223.7796, charge: 4
LGACNDTLQQLMEVFKFDTISEK_5_4_0_2_0
Intensity
m/z
204.0861
138.0546
366.1388
168.0652
274.0916
186.0758
292.1019
126.0548
144.0652
528.1953
577.3165 y5(1+)
657.2311
839.4109 y7(1+)
1114.5685 y9(1+)
1344.1522 PEP_0_0_0_0_0
1445.7064 PEP_0_1_0_0_0
1473.7357 y12(1+)
1547.2463 PEP_0_2_0_0_0
1628.2629 PEP_1_2_0_0_0
1709.2728 PEP_2_2_0_0_0

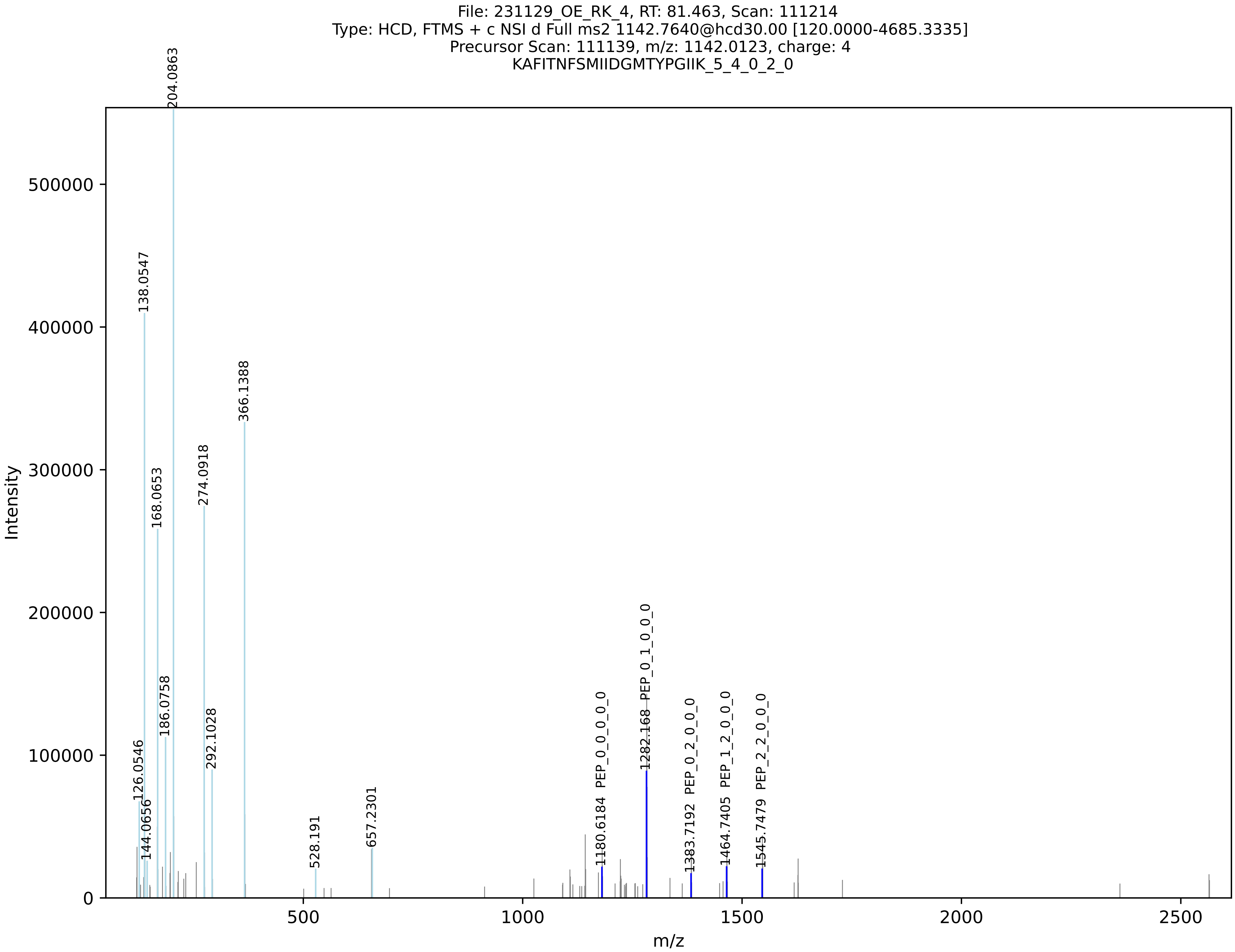

File: 231129_OE_RK_4, RT: 81.463, Scan: 111214
Type: HCD, FTMS + c NSI d Full ms2 1142.7640@hcd30.00 [120.0000-4685.3335]
Precursor Scan: 111139, m/z: 1142.0123, charge: 4
KAFITNFSMIIDGMTYPGIIK_5_4_0_2_0
Intensity
m/z
204.0863
138.0547
366.1388
274.0918
168.0653
186.0758
292.1028
126.0546
144.0656
528.191
657.2301
1180.6184 PEP_0_0_0_0_0
1282.168 PEP_0_1_0_0_0
1383.7192 PEP_0_2_0_0_0
1464.7405 PEP_1_2_0_0_0
1545.7479 PEP_2_2_0_0_0
0
100000
200000
300000
400000
500000
500
1000
1500
2000
2500

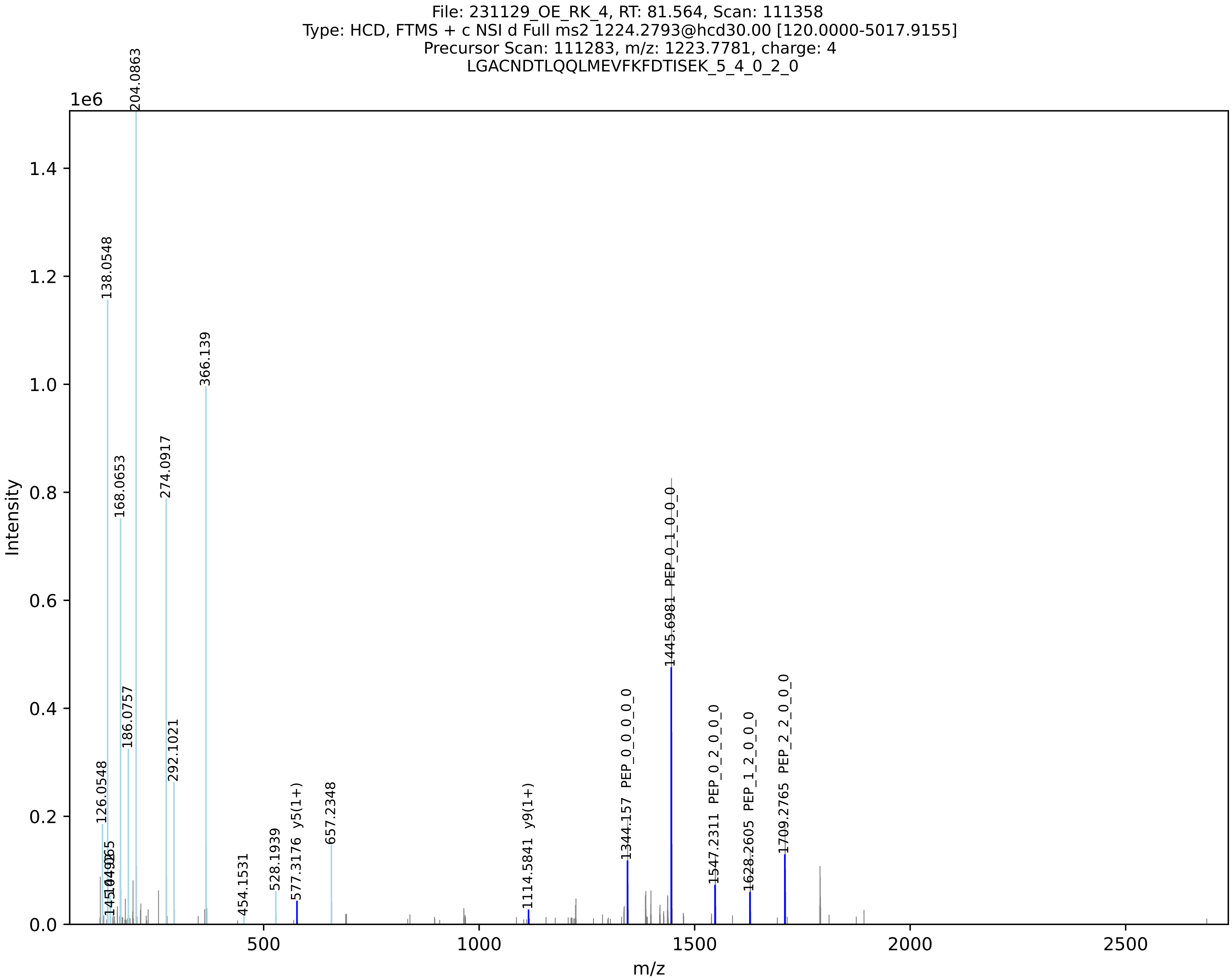
File: 231129_OE_RK_4, RT: 81.564, Scan: 111358
Type: HCD, FTMS + c NSI d Full ms2 1224.2793@hcd30.00 [120.0000-5017.9155]
Precursor Scan: 111283, m/z: 1223.7781, charge: 4
LGACNDTLQQLMEVFKFDTISEK_5_4_0_2_0
1e6
Intensity
m/z
204.0863
138.0548
366.139
168.0653
274.0917
186.0757
292.1021
126.0548
454.1531
528.1939
577.3176 y5(1+)
657.2348
1114.5841 y9(1+)
1344.157 PEP_0_0_0_0_0
1445.6981 PEP_0_1_0_0_0
1547.2311 PEP_0_2_0_0_0
1628.2605 PEP_1_2_0_0_0
1709.2765 PEP_2_2_0_0_0

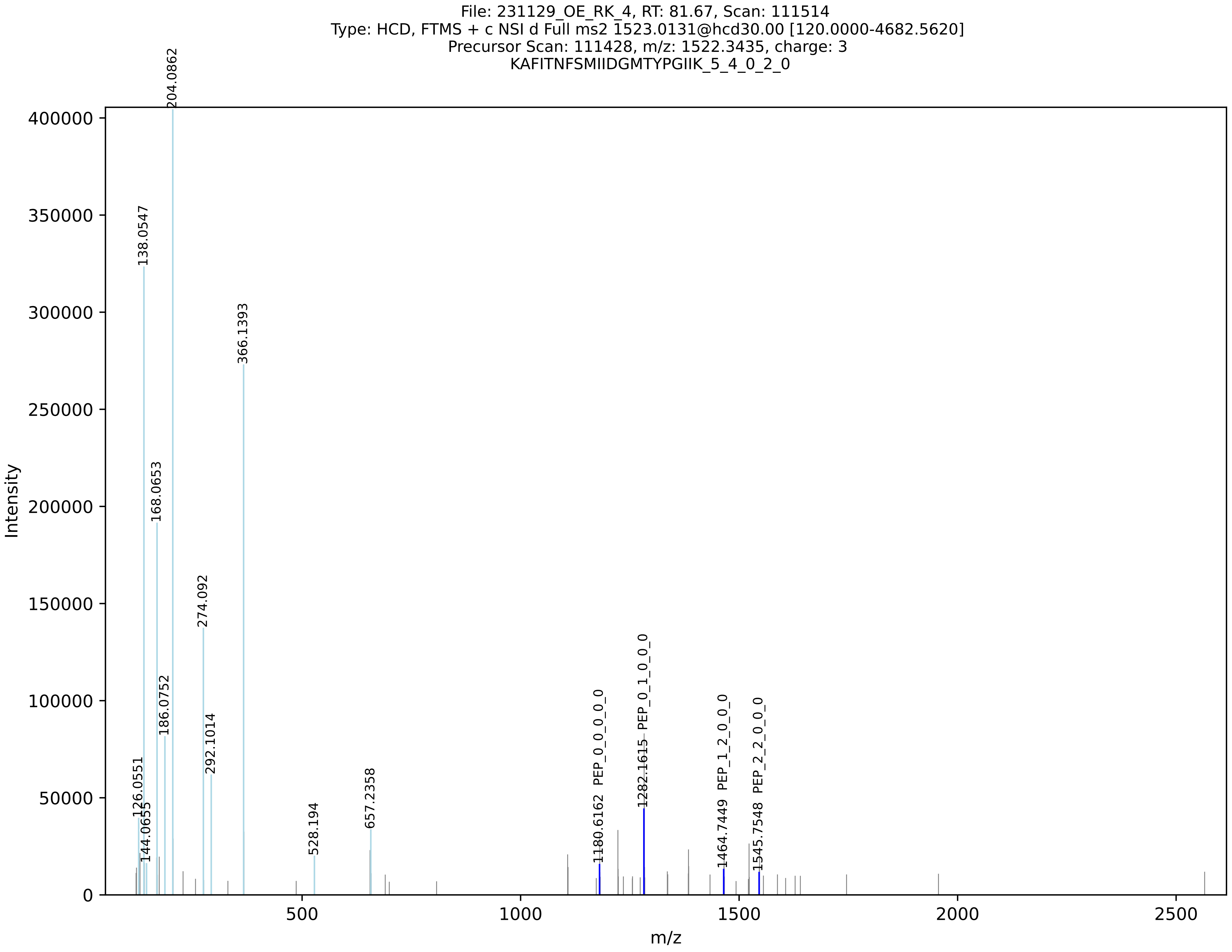
File: 231129_OE_RK_4, RT: 81.67, Scan: 111514
Type: HCD, FTMS + c NSI d Full ms2 1523.0131@hcd30.00 [120.0000-4682.5620]
Precursor Scan: 111428, m/z: 1522.3435, charge: 3
KAFITNFSMIIDGMTYPGIIK_5_4_0_2_0
Intensity
m/z
204.0862
138.0547
366.1393
168.0653
274.092
186.0752
292.1014
126.0551
144.0655
528.194
657.2358
1180.6162 PEP_0_0_0_0_0
1282.1615 PEP_0_1_0_0_0
1464.7449 PEP_1_2_0_0_0
1545.7548 PEP_2_2_0_0_0

File: 231129_OE_RK_4, RT: 81.767, Scan: 111656
Type: HCD, FTMS + c NSI d Full ms2 1224.5239@hcd30.00 [120.0000-5018.9136]
Precursor Scan: 111575, m/z: 1223.7722, charge: 4
LGACNDTLQQLMEVFKFDTISEK_5_4_0_2_0

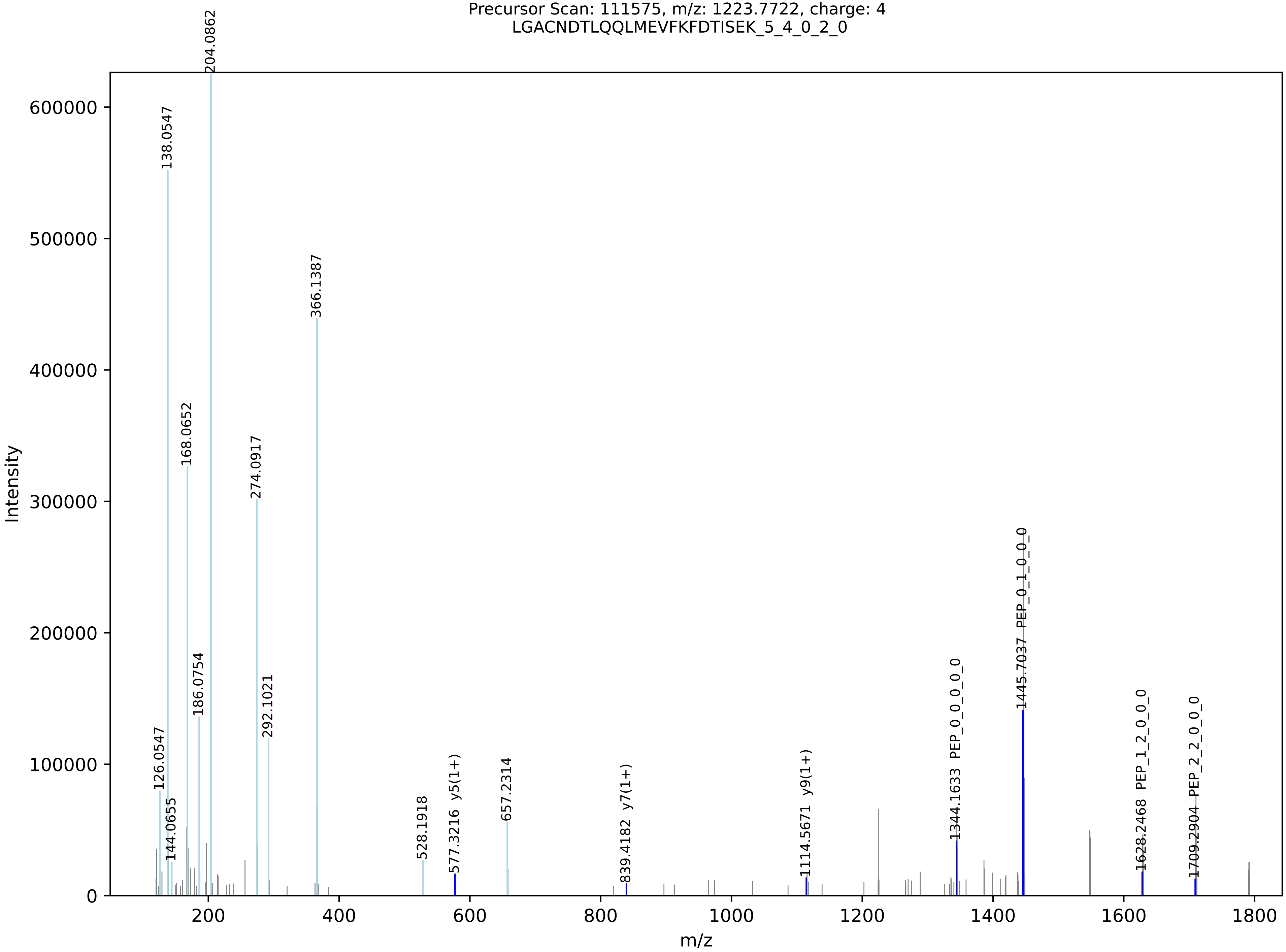

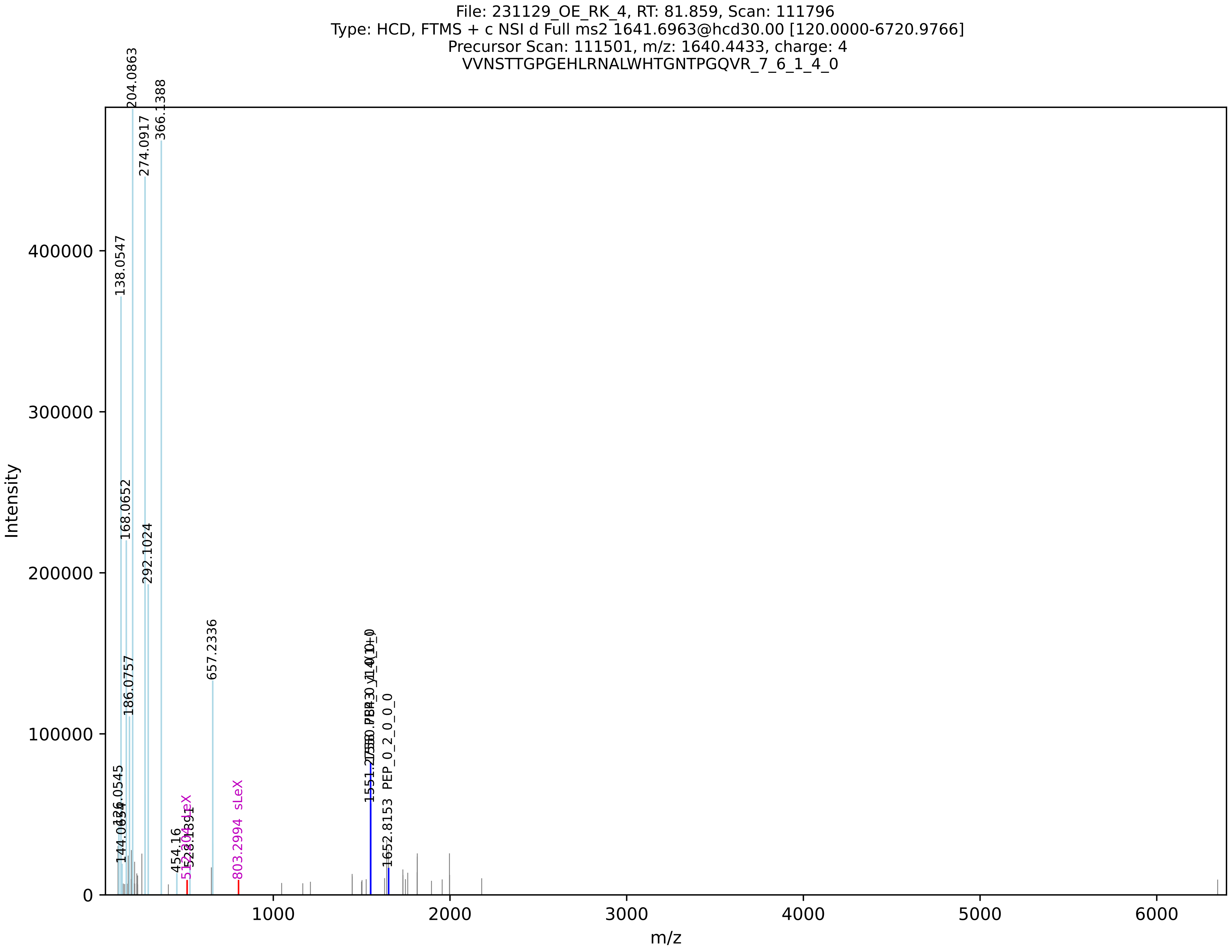
File: 231129_OE_RK_4, RT: 81.859, Scan: 111796
Type: HCD, FTMS + c NSI d Full ms2 1641.6963@hcd30.00 [120.0000-6720.9766]
Precursor Scan: 111501, m/z: 1640.4433, charge: 4
VVNSTTGPGEHLRNALWHTGNTPGQVR_7_6_1_4_0
204.0863
366.1388
274.0917
138.0547
168.0652
292.1024
657.2336
186.0757
144.0654
126.0545
454.16
528.1891
803.2994 sLeX
1652.8153 PEP 0_2_0_0_0
Intensity
m/z
0
100000
200000
300000
400000
1000
2000
3000
4000
5000
6000

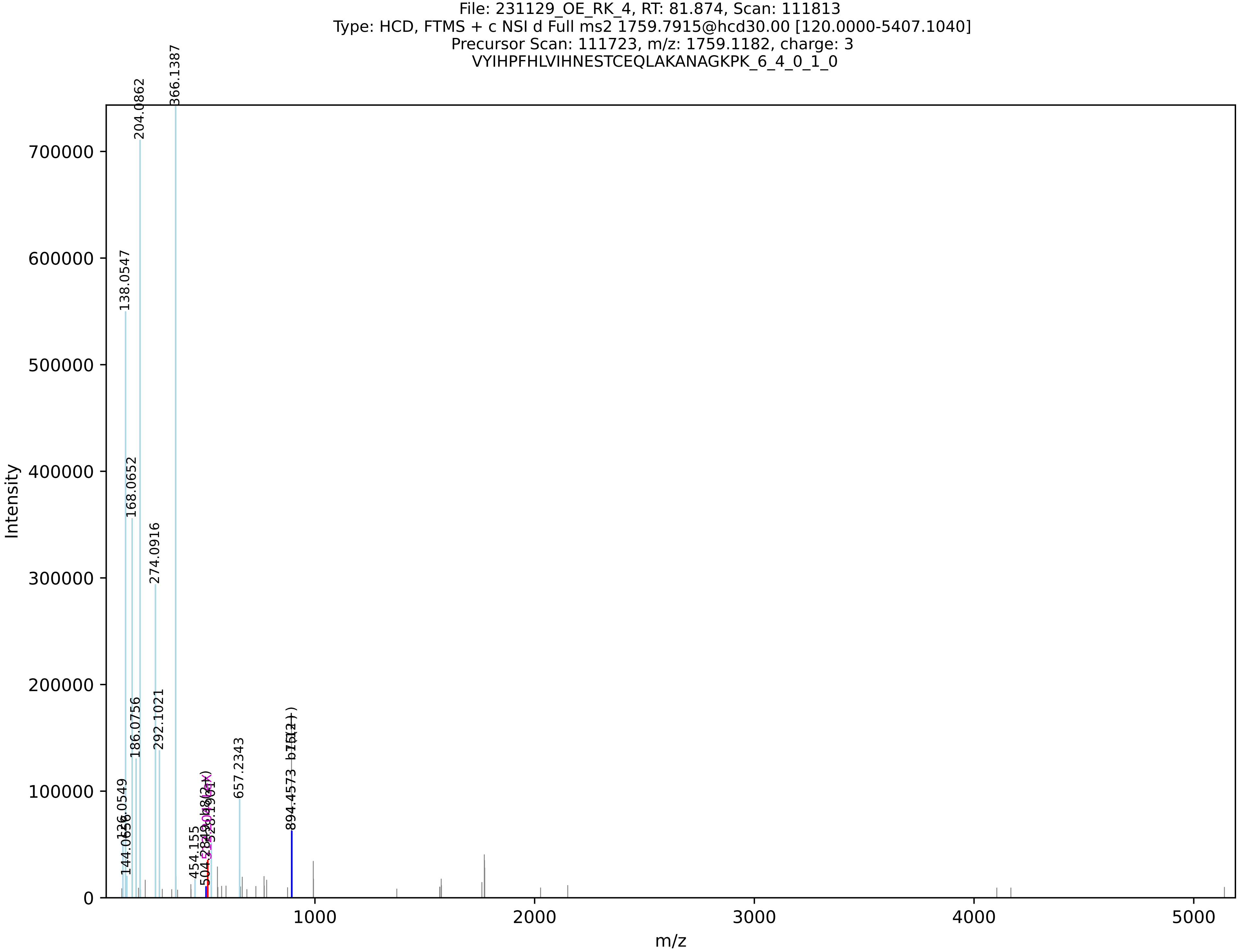

File: 231129_OE_RK_4, RT: 81.874, Scan: 111813
Type: HCD, FTMS + c NSI d Full ms2 1759.7915@hcd30.00 [120.0000-5407.1040]
Precursor Scan: 111723, m/z: 1759.1182, charge: 3
VYIHPFHLVIHNESTCEQLAKANAGKPK_6_4_0_1_0
Intensity
m/z
366.1387
204.0862
138.0547
168.0652
274.0916
186.0756
292.1021
126.0549
144.0656
454.155
504.2849
528.1901
657.2343
894.4573

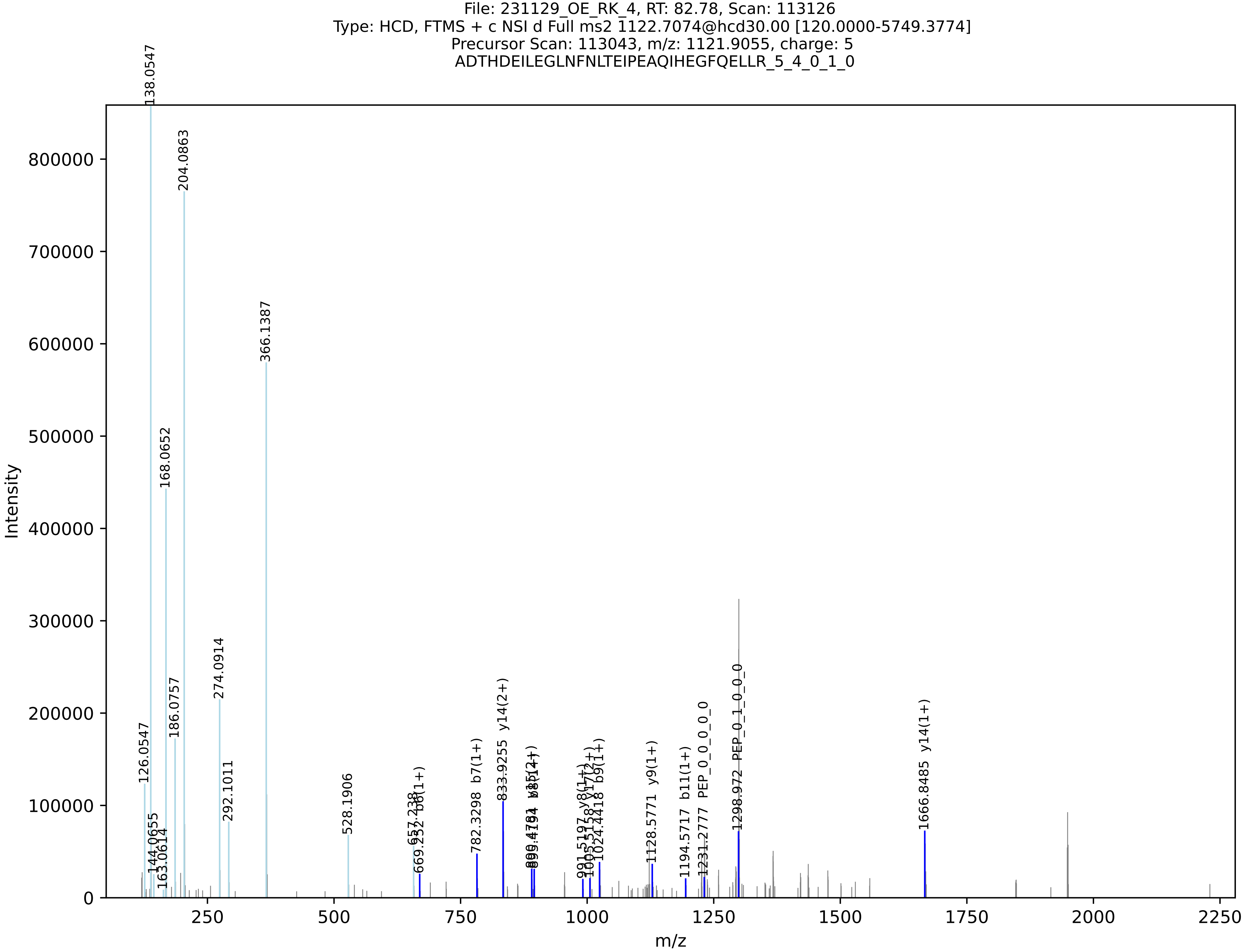

File: 231129_OE_RK_4, RT: 82.78, Scan: 113126
Type: HCD, FTMS + c NSI d Full ms2 1122.7074@hcd30.00 [120.0000-5749.3774]
Precursor Scan: 113043, m/z: 1121.9055, charge: 5
ADTHDEILEGLNFNLTEIPEAQIHEGFQELLR_5_4_0_1_0
Intensity
m/z
138.0547
204.0863
366.1387
168.0652
186.0757
274.0914
126.0547
292.1011
528.1906
144.0655
163.0614
657.238
669.252 b6(1+)
782.3298 b7(1+)
833.9255 y14(2+)
991.5197 y8(1+)
1024.4418 b9(1+)
1128.5771 y9(1+)
1194.5717 b11(1+)
1231.2777 PEP_0_0_0_0_0
1298.972 PEP_0_1_0_0_0
1666.8485 y14(1+)

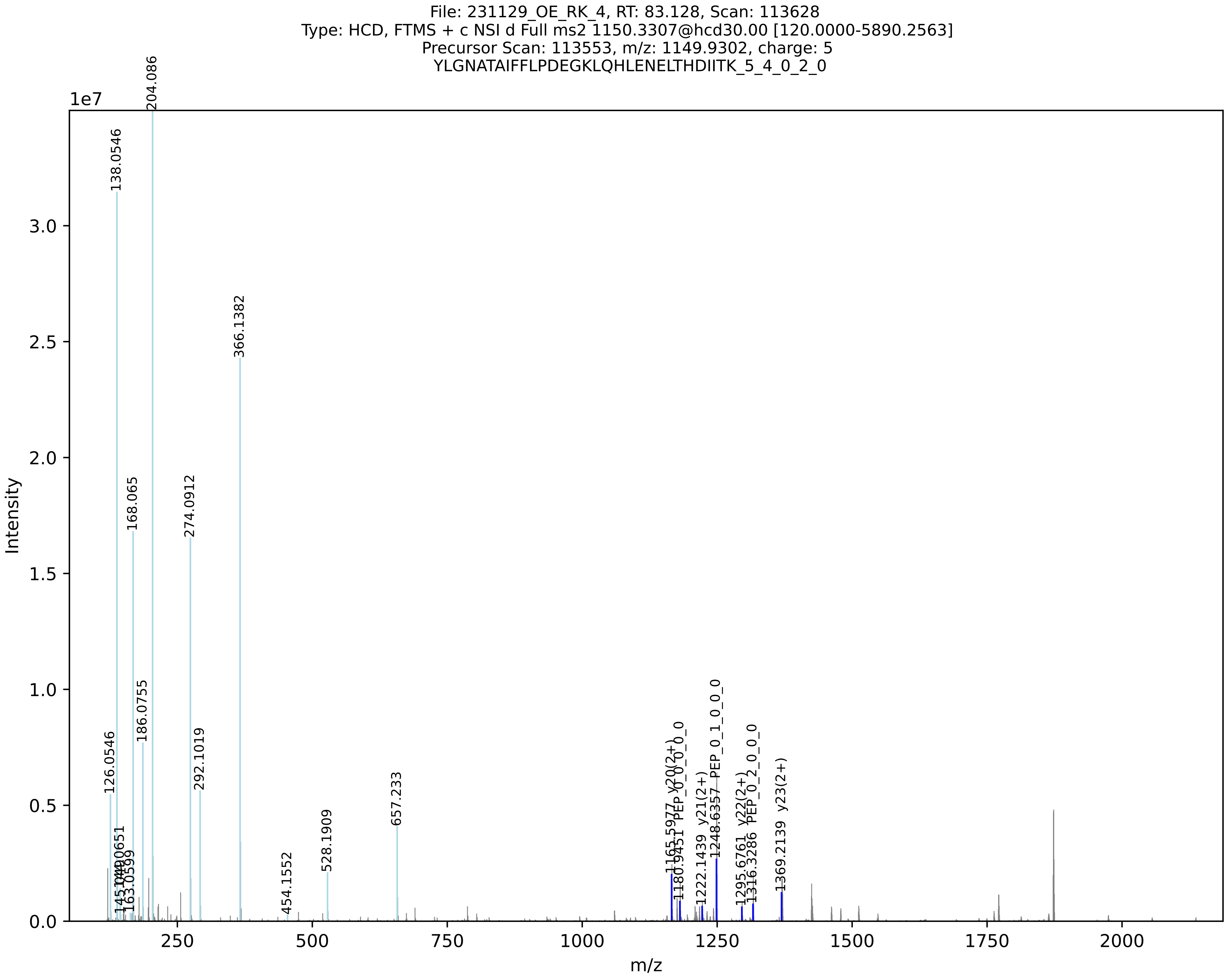

File: 231129_OE_RK_4, RT: 83.128, Scan: 113628
Type: HCD, FTMS + c NSI d Full ms2 1150.3307@hcd30.00 [120.0000-5890.2563]
Precursor Scan: 113553, m/z: 1149.9302, charge: 5
YLGNATAIFFLPDEGKLQHLENELTHDIITK_5_4_0_2_0
1e7
Intensity
m/z
204.086
138.0546
366.1382
168.065
274.0912
186.0755
126.0546
292.1019
657.233
528.1909
454.1552
163.0599
1165.5977 y20(2+)
1180.9451 PEP_0_0_0_0
1222.1439 y21(2+)
1248.6357 PEP_0_1_0_0_0
1295.6761 y22(2+)
1316.3286 PEP_0_2_0_0_0
1369.2139 y23(2+)

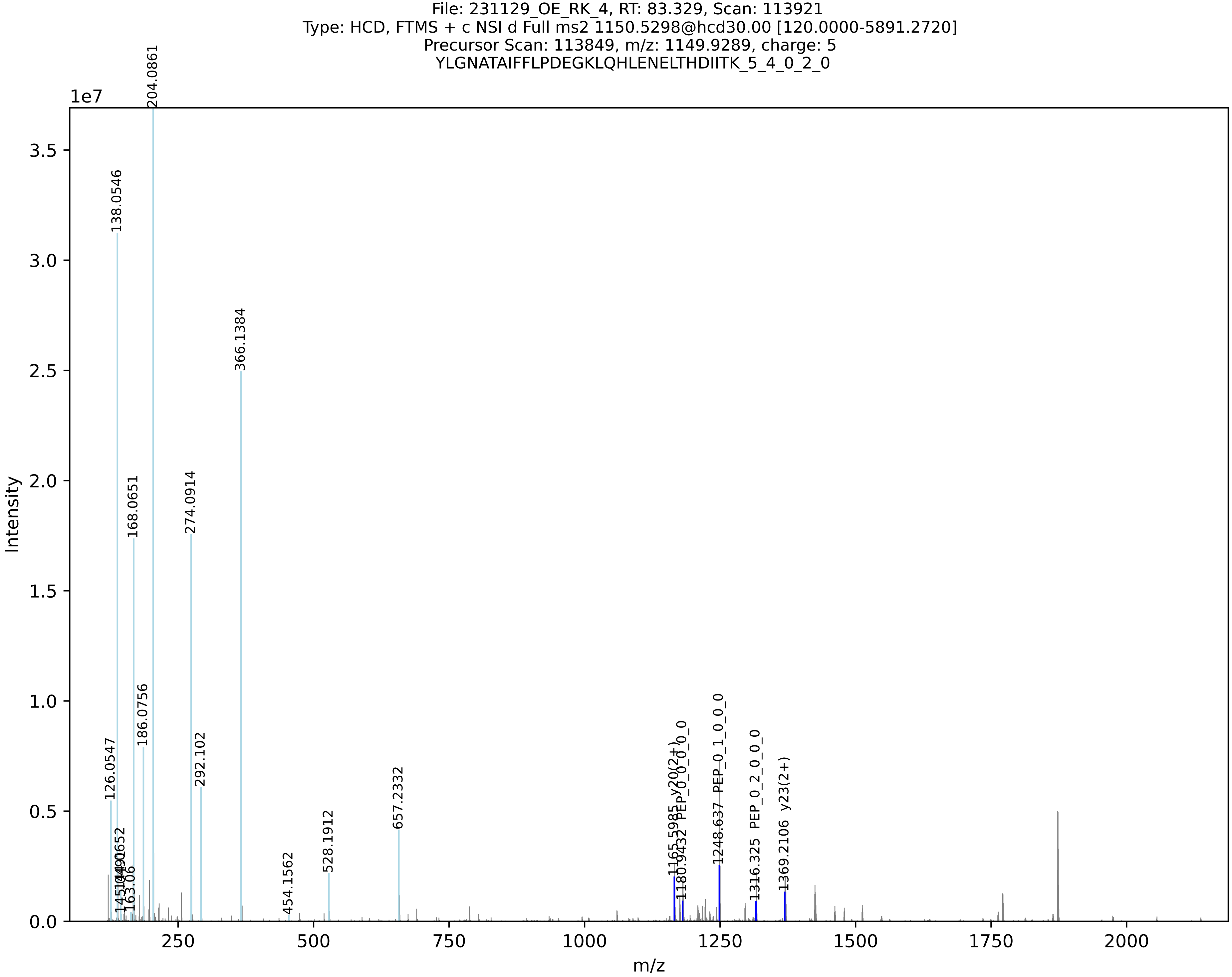
File: 231129_OE_RK_4, RT: 83.329, Scan: 113921
Type: HCD, FTMS + c NSI d Full ms2 1150.5298@hcd30.00 [120.0000-5891.2720]
Precursor Scan: 113849, m/z: 1149.9289, charge: 5
YLGNATAIFFLPDEGKLQHLENELTHDIITK_5_4_0_2_0
1e7
Intensity
m/z
204.0861
138.0546
366.1384
168.0651
274.0914
186.0756
292.102
126.0547
657.2332
528.1912
454.1562
163.06
1165.5985_y20(2+)
1180.9432_PEP_0_0_0_0
1248.637_PEP_0_1_0_0_0
1316.325_PEP_0_2_0_0_0
1369.2106_y23(2+)

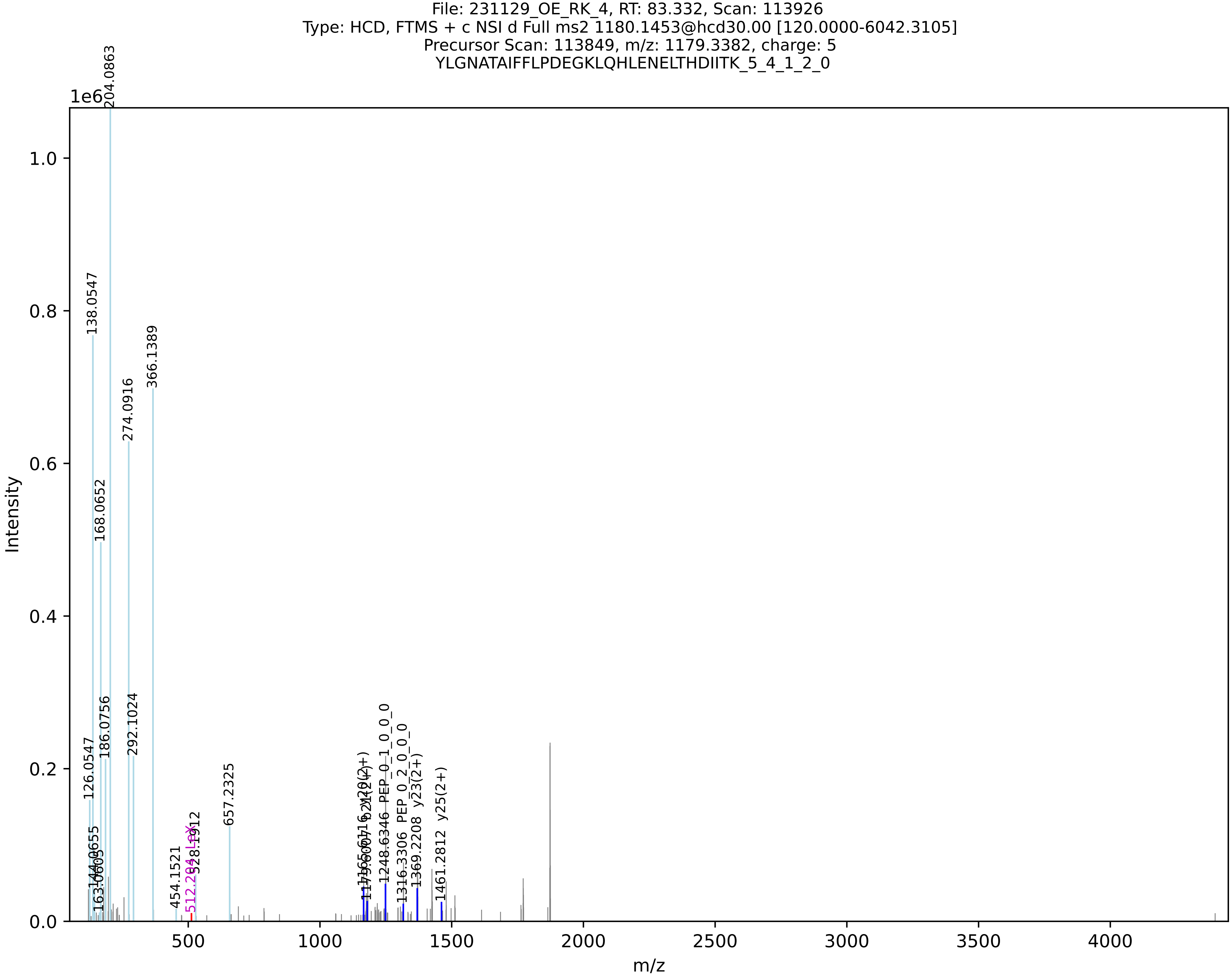
File: 231129_OE_RK_4, RT: 83.332, Scan: 113926
Type: HCD, FTMS + c NSI d Full ms2 1180.1453@hcd30.00 [120.0000-6042.3105]
Precursor Scan: 113849, m/z: 1179.3382, charge: 5
YLGNATAIFFLPDEGKLQHLENELTHDIITK_5_4_1_2_0
1e6
Intensity
m/z
204.0863
138.0547
366.1389
274.0916
168.0652
186.0756
292.1024
126.0547
144.0655
163.0603
454.1521
512.204
528.1912
657.2325
1248.6346 PEP_0_1_0_0_0_
1316.3306 PEP_0_2_0_0_0_
1369.2208 y23(2+)
1461.2812 y25(2+)
0.0
0.2
0.4
0.6
0.8
1.0
500
1000
1500
2000
2500
3000
3500
4000

File: 231129_OE_RK_4, RT: 83.381, Scan: 113997
Type: HCD, FTMS + c NSI d Full ms2 958.9437@hcd30.00 [120.0000-5893.0195]
Precursor Scan: 113920, m/z: 958.4423, charge: 6
YLGNATAIFFLPDEGKLQHLENELTHDIITK_5_4_0_2_0

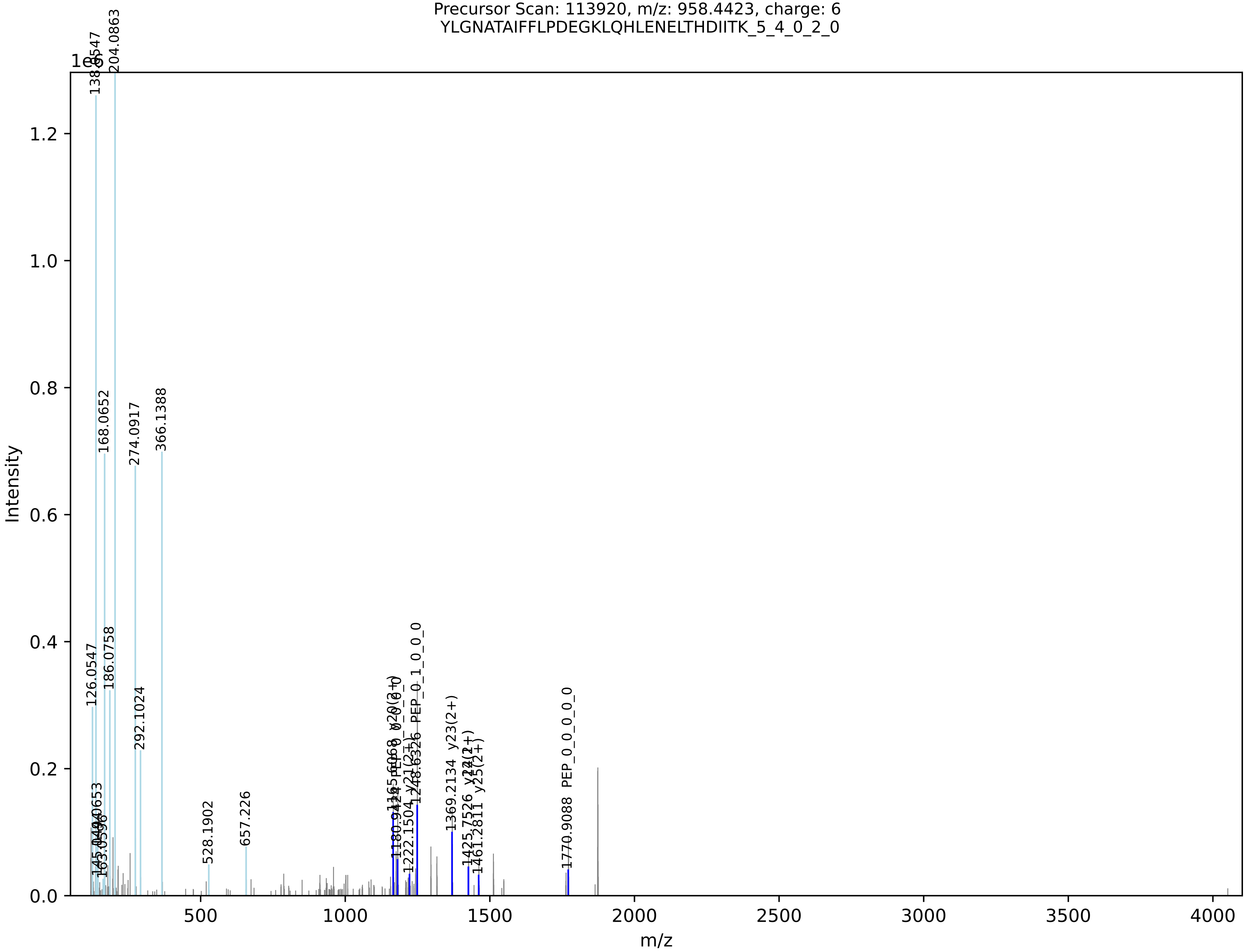

File: 231129_OE_RK_4, RT: 83.684, Scan: 114438
Type: HCD, FTMS + c NSI d Full ms2 1641.4446@hcd30.00 [120.0000-6719.9502]
Precursor Scan: 114360, m/z: 1640.4423, charge: 4
VVNSTTGPGEHLRNALWHTGNTPGQVR_7_6_1_4_0

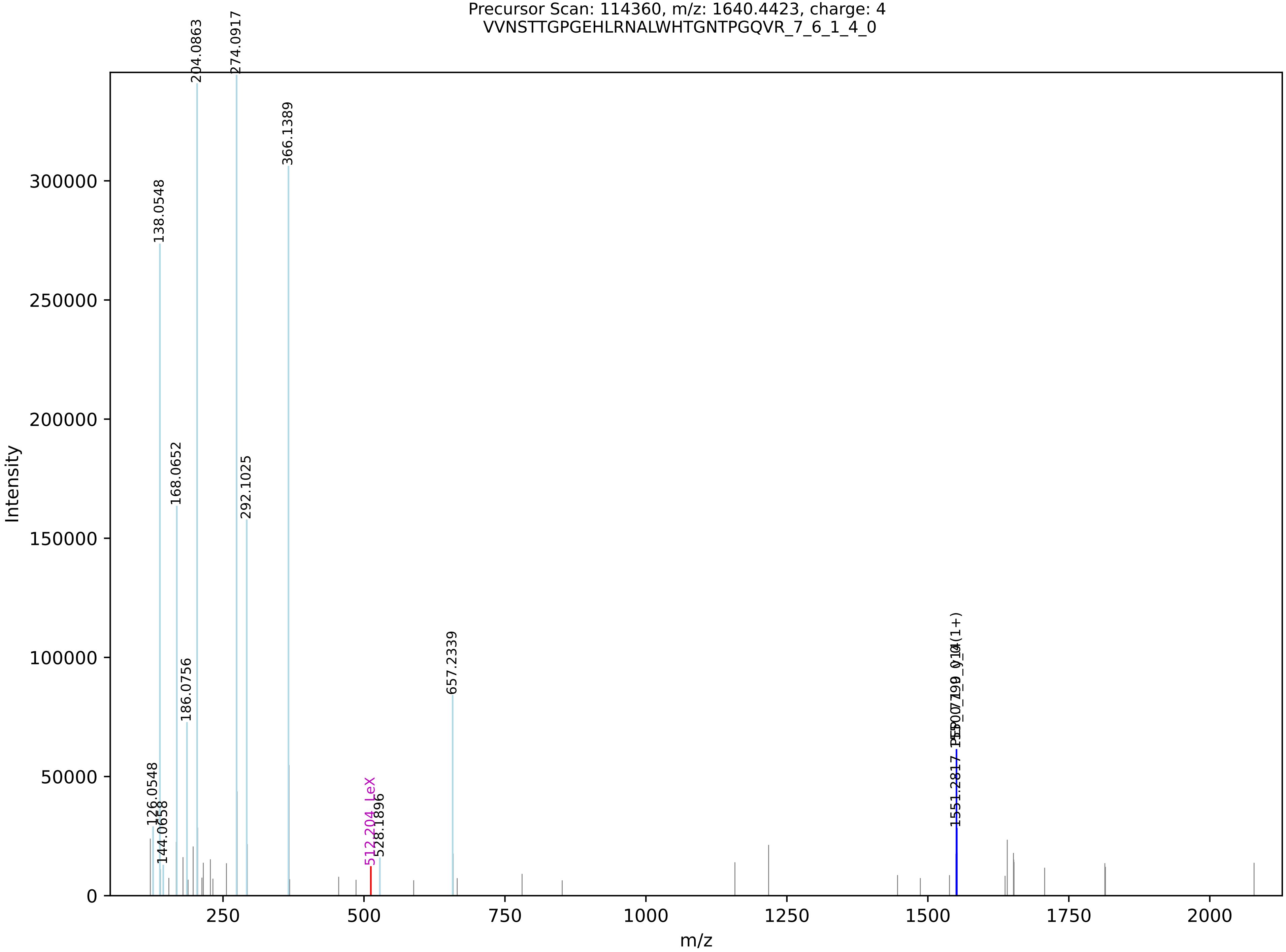

File: 231129_OE_RK_4, RT: 85.193, Scan: 116626
Type: HCD, FTMS + c NSI d Full ms2 1603.4568@hcd30.00 [120.0000-6564.9600]
Precursor Scan: 116556, m/z: 1602.9547, charge: 4
ADTHDEILEGLNFNLTEIPEAQIHEGFQELLR_6_5_1_2_0

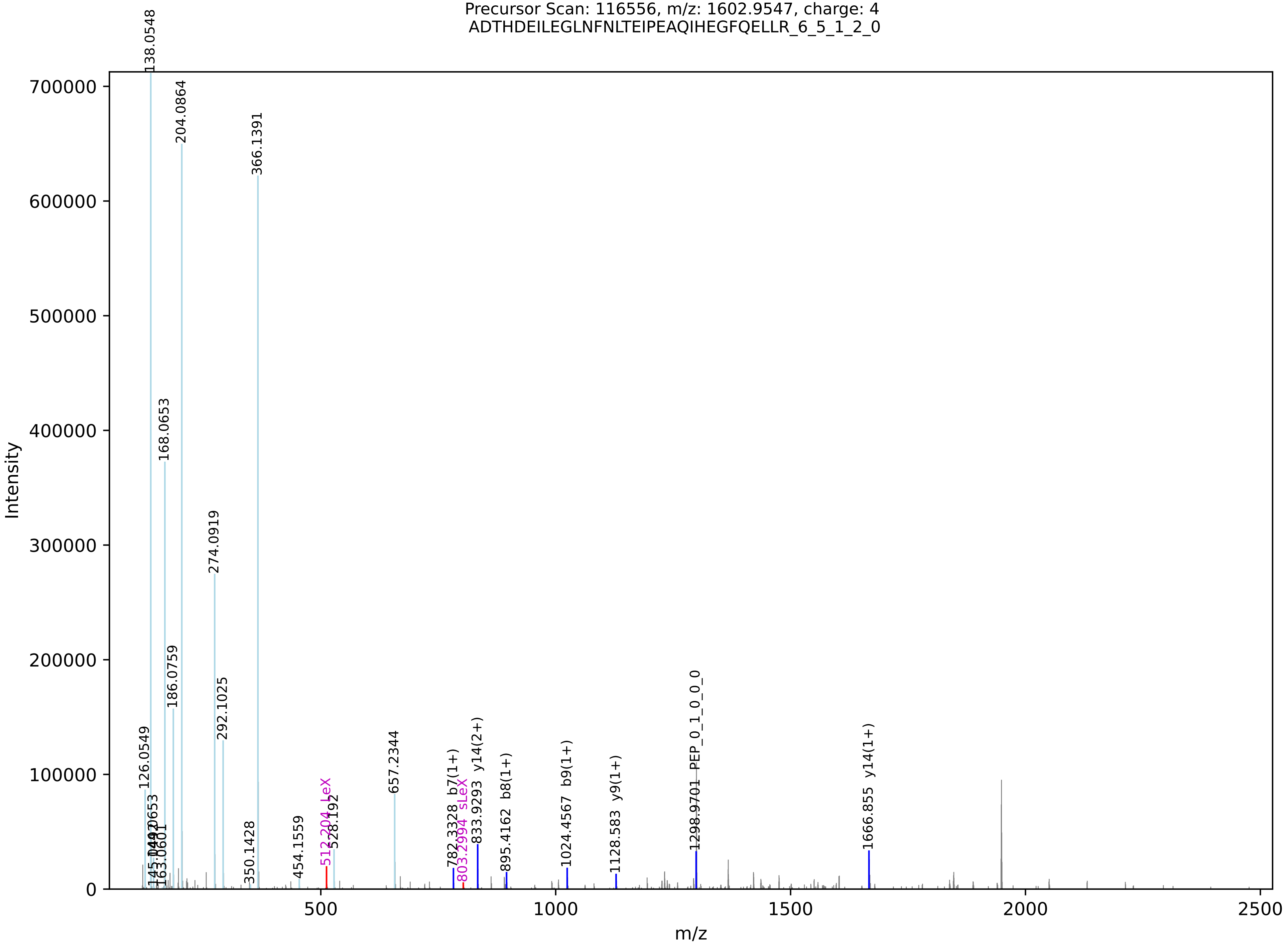

File: 231129_OE_RK_4, RT: 85.35, Scan: 116849
Type: HCD, FTMS + c NSI d Full ms2 1180.7292@hcd30.00 [120.0000-6045.2891]
Precursor Scan: 116769, m/z: 1180.1259, charge: 5
ADTHDEILEGLNFNLTEIPEAQIHEGFQELLR_5_4_0_2_0

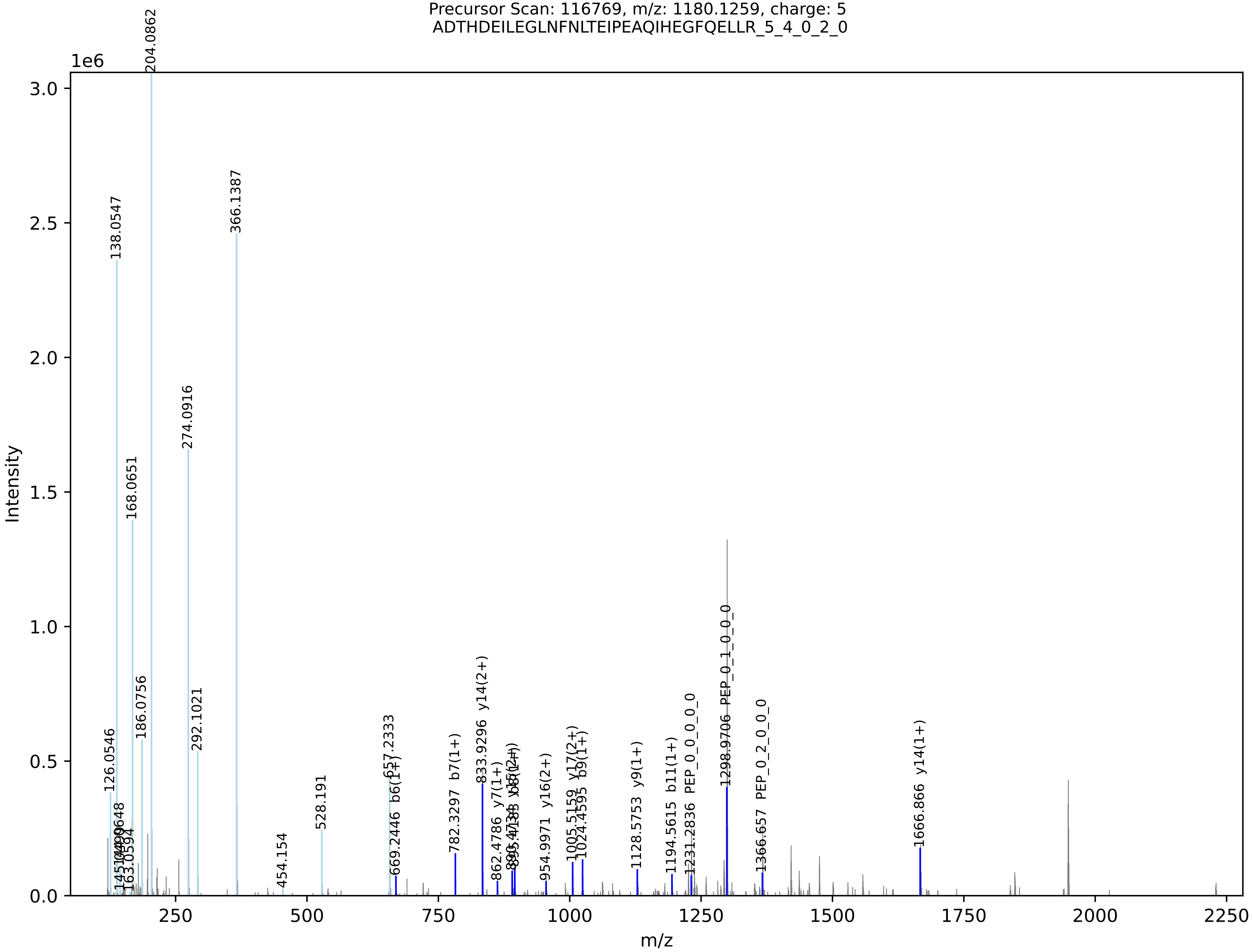

File: 231129_OE_RK_4, RT: 85.395, Scan: 116915
Type: HCD, FTMS + c NSI d Full ms2 1603.7056@hcd30.00 [120.0000-6565.9746]
Precursor Scan: 116843, m/z: 1602.7032, charge: 4
ADTHDEILEGLNFNLTEIPEAQIHEGFQELLR_6_5_1_2_0

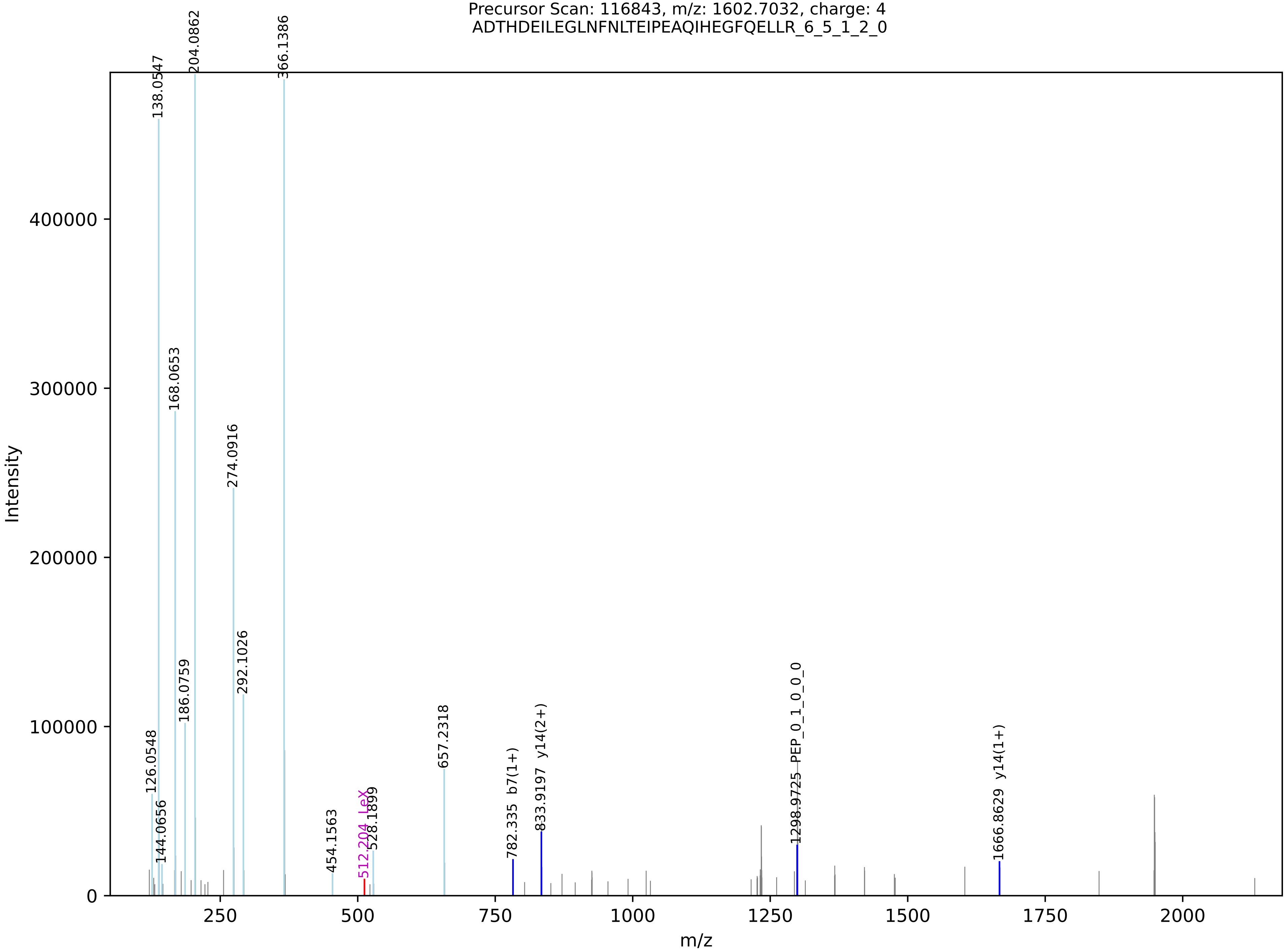

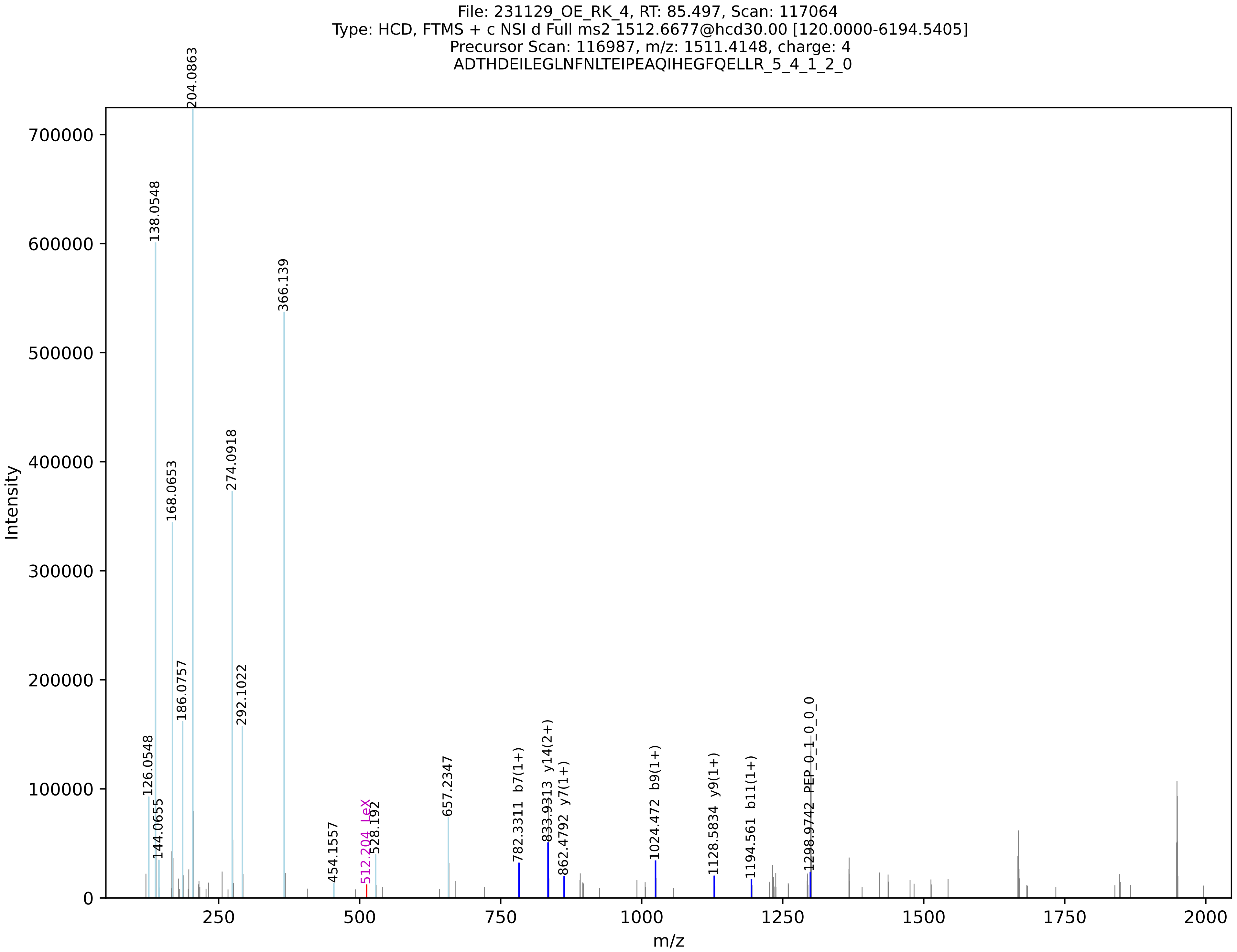

File: 231129_OE_RK_4, RT: 85.497, Scan: 117064
Type: HCD, FTMS + c NSI d Full ms2 1512.6677@hcd30.00 [120.0000-6194.5405]
Precursor Scan: 116987, m/z: 1511.4148, charge: 4
ADTHDEILEGLNFNLTEIPEAQIHEGFQELLR_5_4_1_2_0
Intensity
m/z
204.0863
138.0548
366.139
274.0918
168.0653
186.0757
292.1022
126.0548
144.0655
454.1557
512.204_LeX
528.192
657.2347
782.3311 b7(1+)
833.9313 y14(2+)
862.4792 y7(1+)
1024.472 b9(1+)
1128.5834 y9(1+)
1194.561 b11(1+)
1298.9742 PEP_0_1_0_0_0

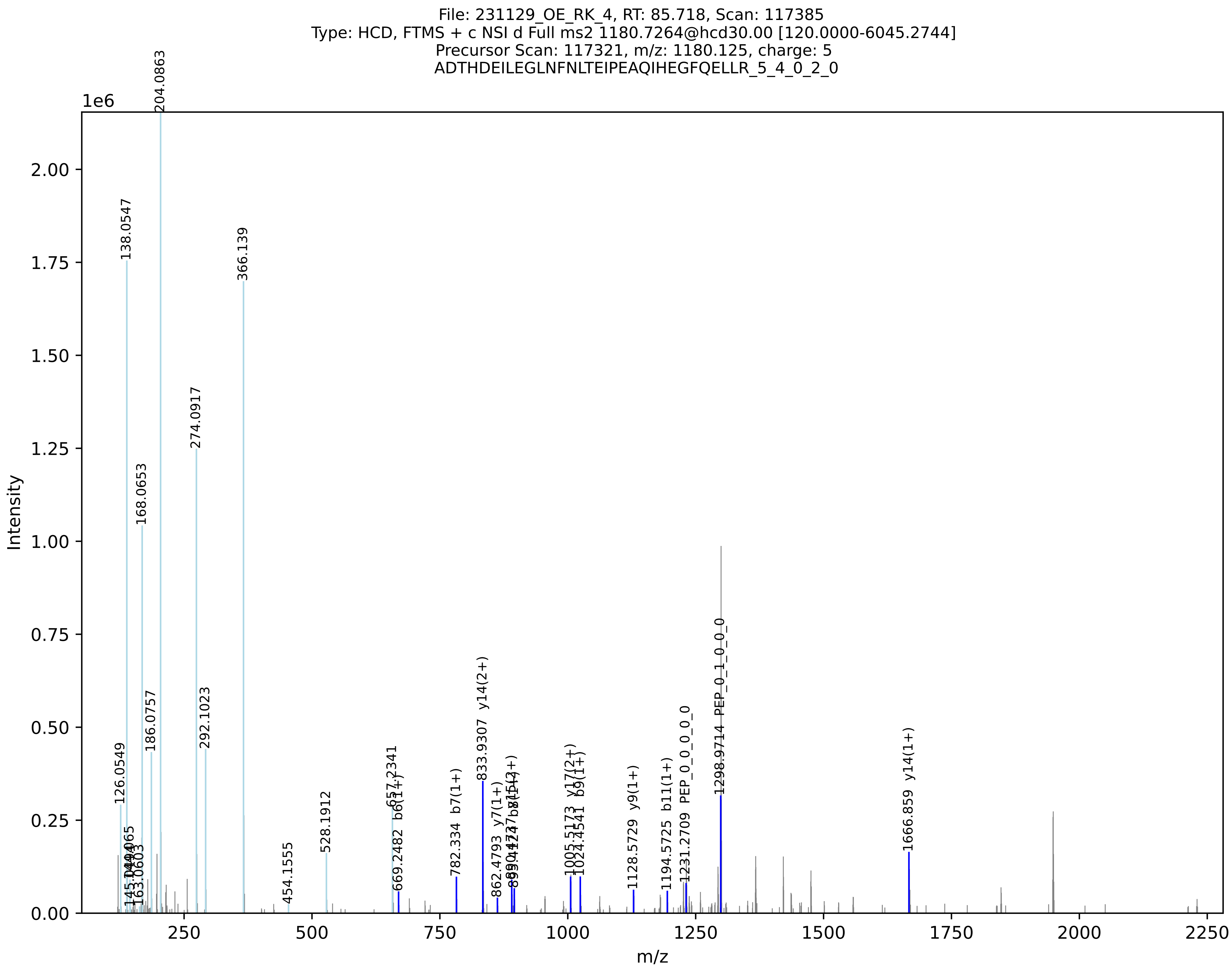
File: 231129_OE_RK_4, RT: 85.718, Scan: 117385
Type: HCD, FTMS + c NSI d Full ms2 1180.7264@hcd30.00 [120.0000-6045.2744]
Precursor Scan: 117321, m/z: 1180.125, charge: 5
ADTHDEILEGLNFNLTEIPEAQIHEGFQELLR_5_4_0_2_0
1e6
Intensity
m/z
204.0863
138.0547
366.139
274.0917
168.0653
186.0757
292.1023
126.0549
163.0603
454.1555
528.1912
657.2341
669.2482 b6(1+)
782.334 b7(1+)
833.9307 y14(2+)
862.4793 y7(1+)
1005.5173 y17(2+)
1024.4541 b9(1+)
1128.5729 y9(1+)
1194.5725 b11(1+)
1231.2709 PEP_0_0_0_0_0
1298.9714 PEP_0_1_0_0_0
1666.859 y14(1+)

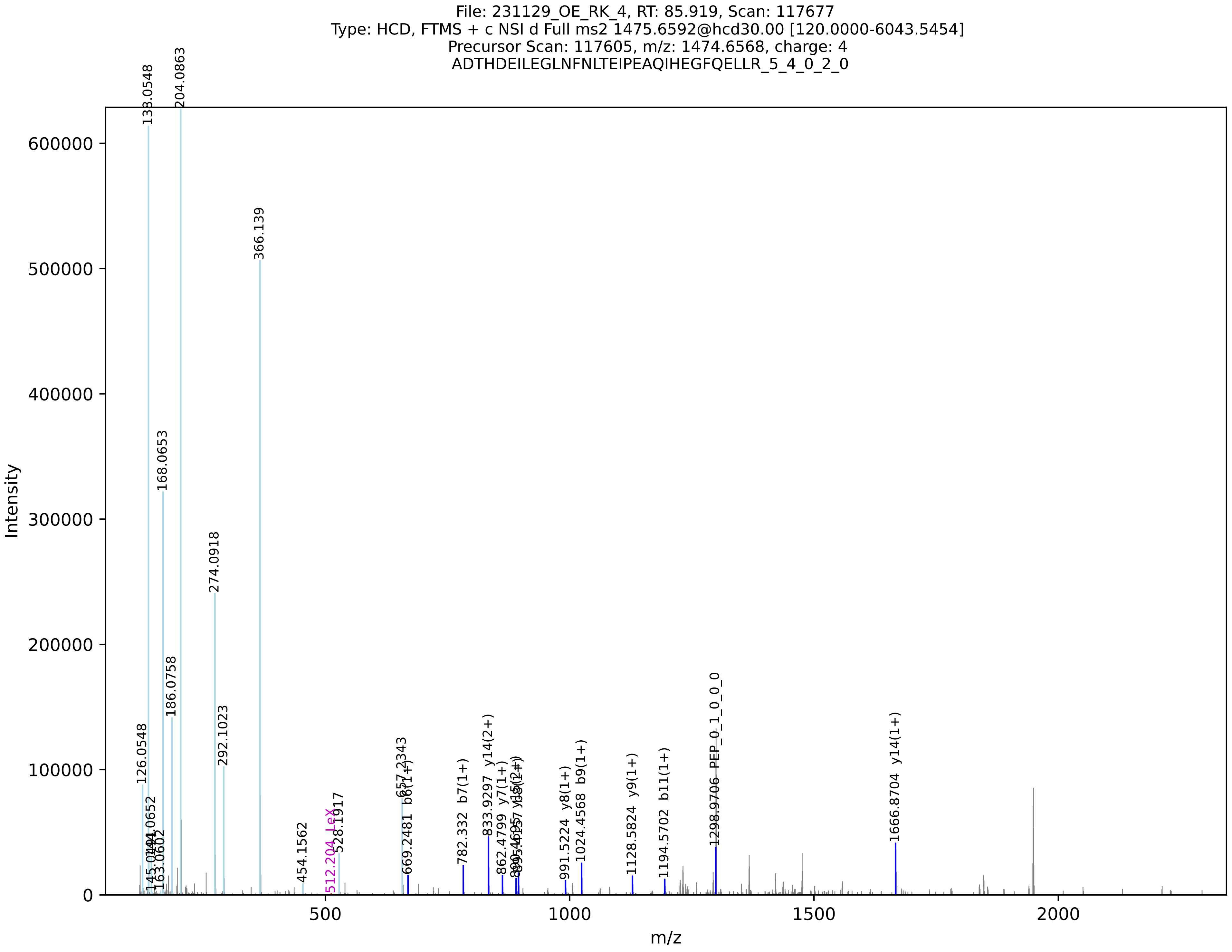
File: 231129_OE_RK_4, RT: 85.919, Scan: 117677
Type: HCD, FTMS + c NSI d Full ms2 1475.6592@hcd30.00 [120.0000-6043.5454]
Precursor Scan: 117605, m/z: 1474.6568, charge: 4
ADTHDEILEGLNFNLTEIPEAQIHEGFQELLR_5_4_0_2_0
Intensity
m/z
138.0548
204.0863
366.139
168.0653
274.0918
186.0758
292.1023
126.0548
454.1562
512.204_LeX
528.1917
657.2343
669.2481 b6(1+)
782.332 b7(1+)
833.9297 y14(2+)
862.4799 y7(1+)
991.5224 y8(1+)
1024.4568 b9(1+)
1128.5824 y9(1+)
1194.5702 b11(1+)
1298.9706 PEP_0_1_0_0_0
1666.8704 y14(1+)

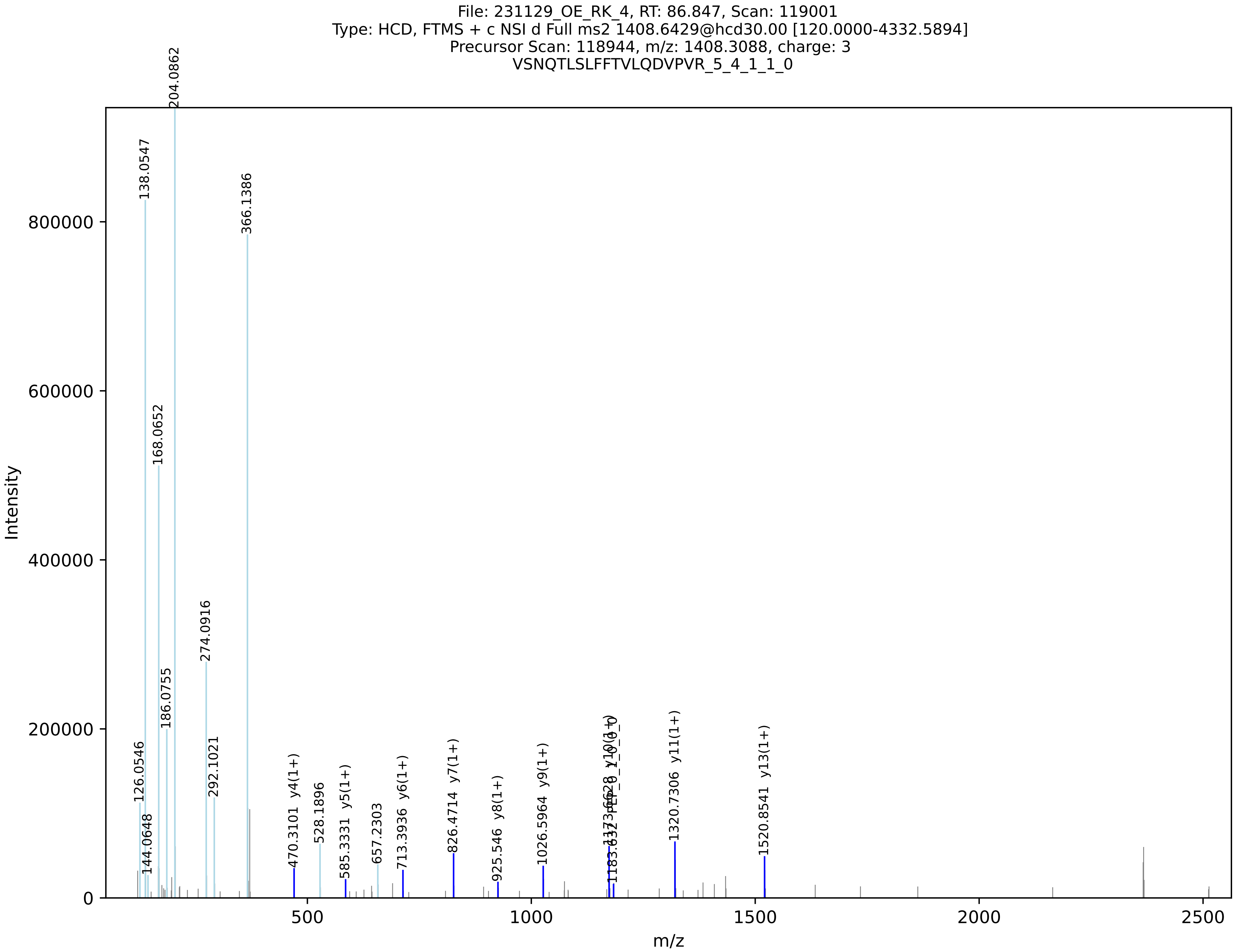
File: 231129_OE_RK_4, RT: 86.847, Scan: 119001
Type: HCD, FTMS + c NSI d Full ms2 1408.6429@hcd30.00 [120.0000-4332.5894]
Precursor Scan: 118944, m/z: 1408.3088, charge: 3
VSNQTLSLFFTVLQDVPVR_5_4_1_1_0
Intensity
m/z
0
200000
400000
600000
800000
500
1000
1500
2000
2500
204.0862
138.0547
366.1386
168.0652
274.0916
186.0755
126.0546
292.1021
144.0648
470.3101 y4(1+)
528.1896
585.3331 y5(1+)
657.2303
713.3936 y6(1+)
826.4714 y7(1+)
925.546 y8(1+)
1026.5964 y9(1+)
1173.6628 y10(1+)
1183.632 PEP_0_1_0_0
1320.7306 y11(1+)
1520.8541 y13(1+)

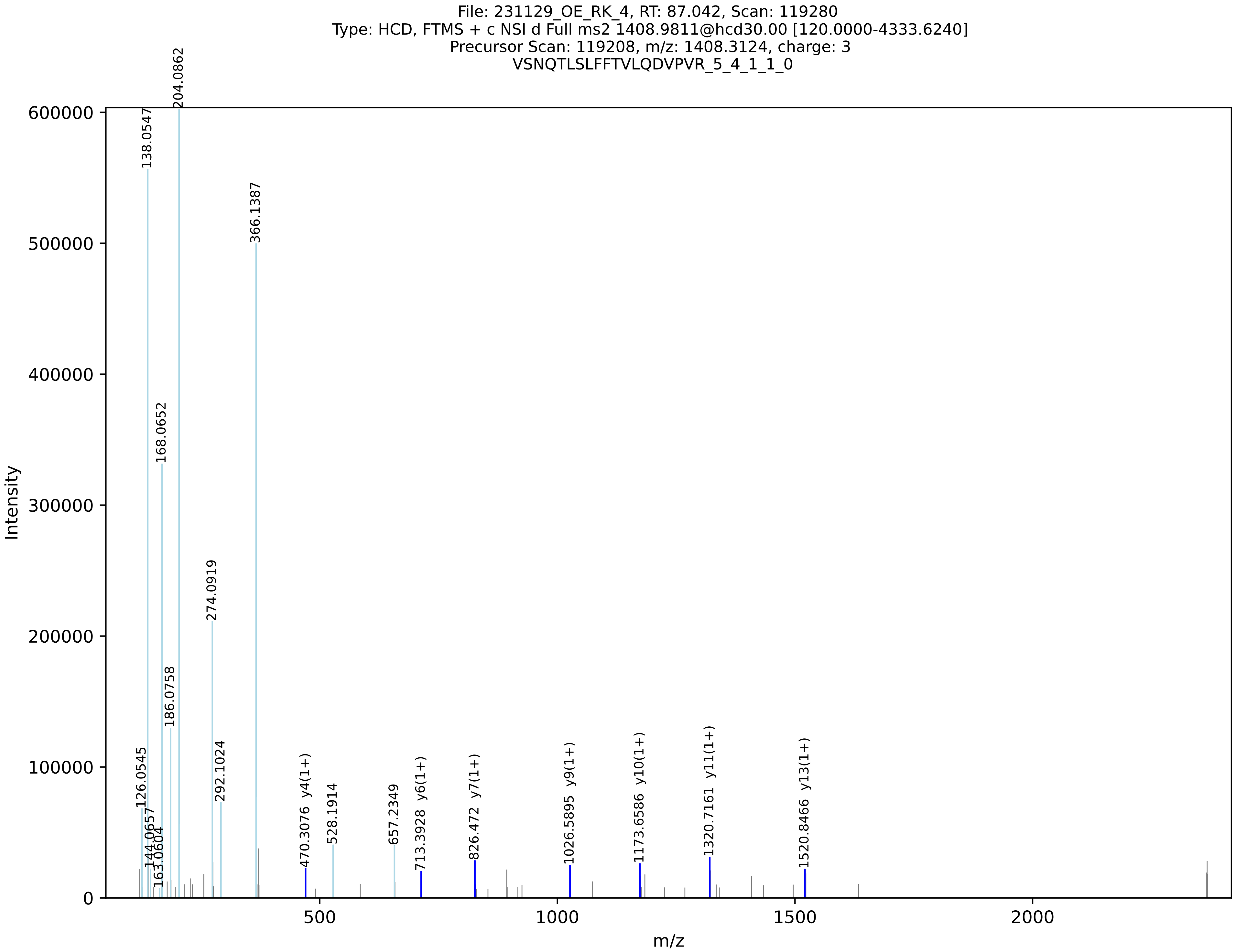
File: 231129_OE_RK_4, RT: 87.042, Scan: 119280
Type: HCD, FTMS + c NSI d Full ms2 1408.9811@hcd30.00 [120.0000-4333.6240]
Precursor Scan: 119208, m/z: 1408.3124, charge: 3
VSNQTLSLFFTVLQDVPVR_5_4_1_1_0
Intensity
m/z
204.0862
138.0547
366.1387
168.0652
274.0919
186.0758
126.0545
292.1024
144.0657
163.0604
470.3076 y4(1+)
528.1914
657.2349
713.3928 y6(1+)
826.472 y7(1+)
1026.5895 y9(1+)
1173.6586 y10(1+)
1320.7161 y11(1+)
1520.8466 y13(1+)
0
100000
200000
300000
400000
500000
600000
500
1000
1500
2000

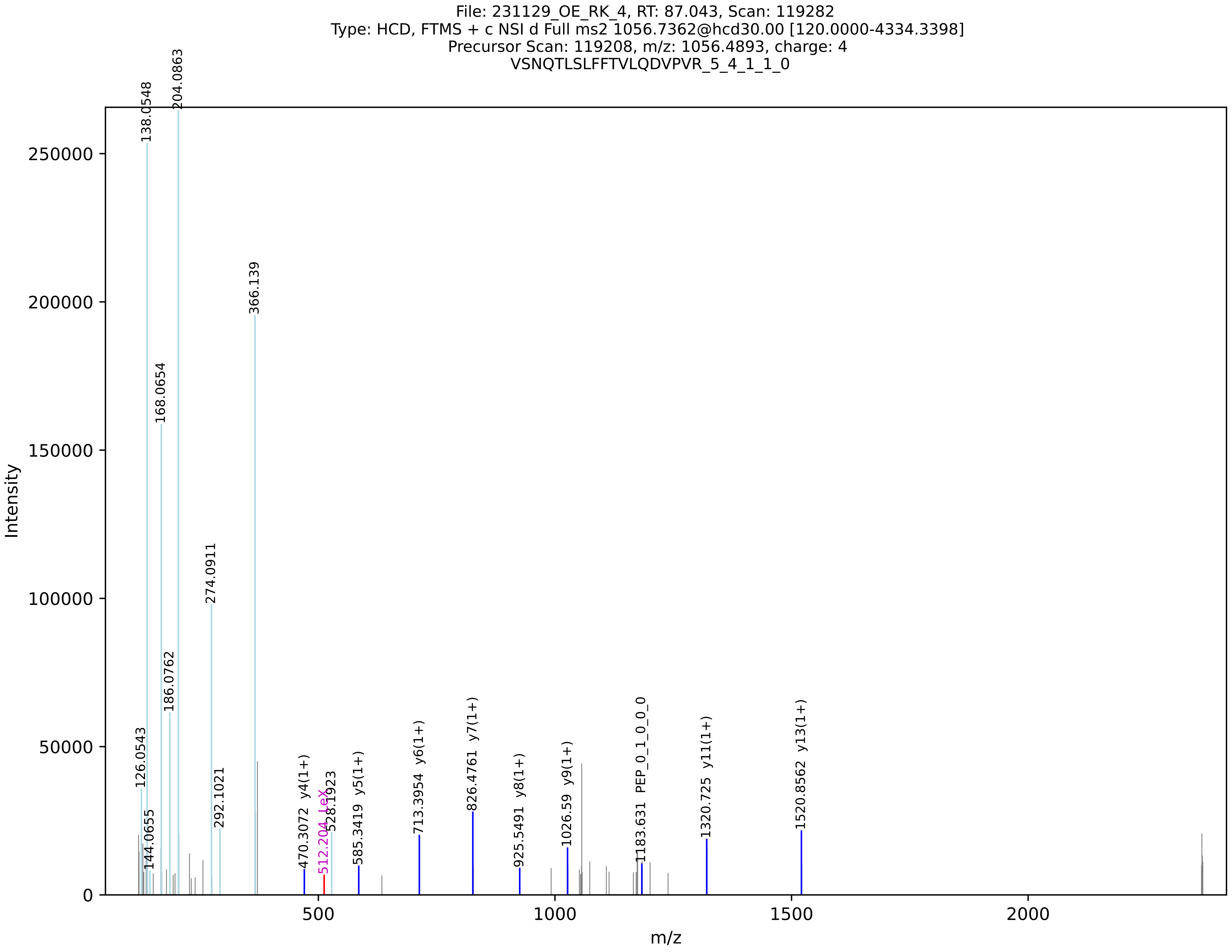

File: 231129_OE_RK_4, RT: 87.043, Scan: 119282
Type: HCD, FTMS + c NSI d Full ms2 1056.7362@hcd30.00 [120.0000-4334.3398]
Precursor Scan: 119208, m/z: 1056.4893, charge: 4
VSNQTLSLFFTVLQDVPVR_5_4_1_1_0
Intensity
m/z
0
50000
100000
150000
200000
250000
500
1000
1500
2000
126.0543
138.0548
144.0655
168.0654
186.0762
204.0863
274.0911
292.1021
366.139
470.3072 y4(1+)
512.204_LeX
528.1923
585.3419 y5(1+)
713.3954 y6(1+)
826.4761 y7(1+)
925.5491 y8(1+)
1026.59 y9(1+)
1183.631 PEP_0_1_0_0_0
1320.725 y11(1+)
1520.8562 y13(1+)

File: 231129_OE_RK_4, RT: 88.17, Scan: 120904
Type: HCD, FTMS + c NSI d Full ms2 1639.7142@hcd30.00 [120.0000-6712.8901]
Precursor Scan: 120832, m/z: 1638.9602, charge: 4
ADTHDEILEGLNFNLTEIPEAQIHEGFQELLR_6_5_0_3_0

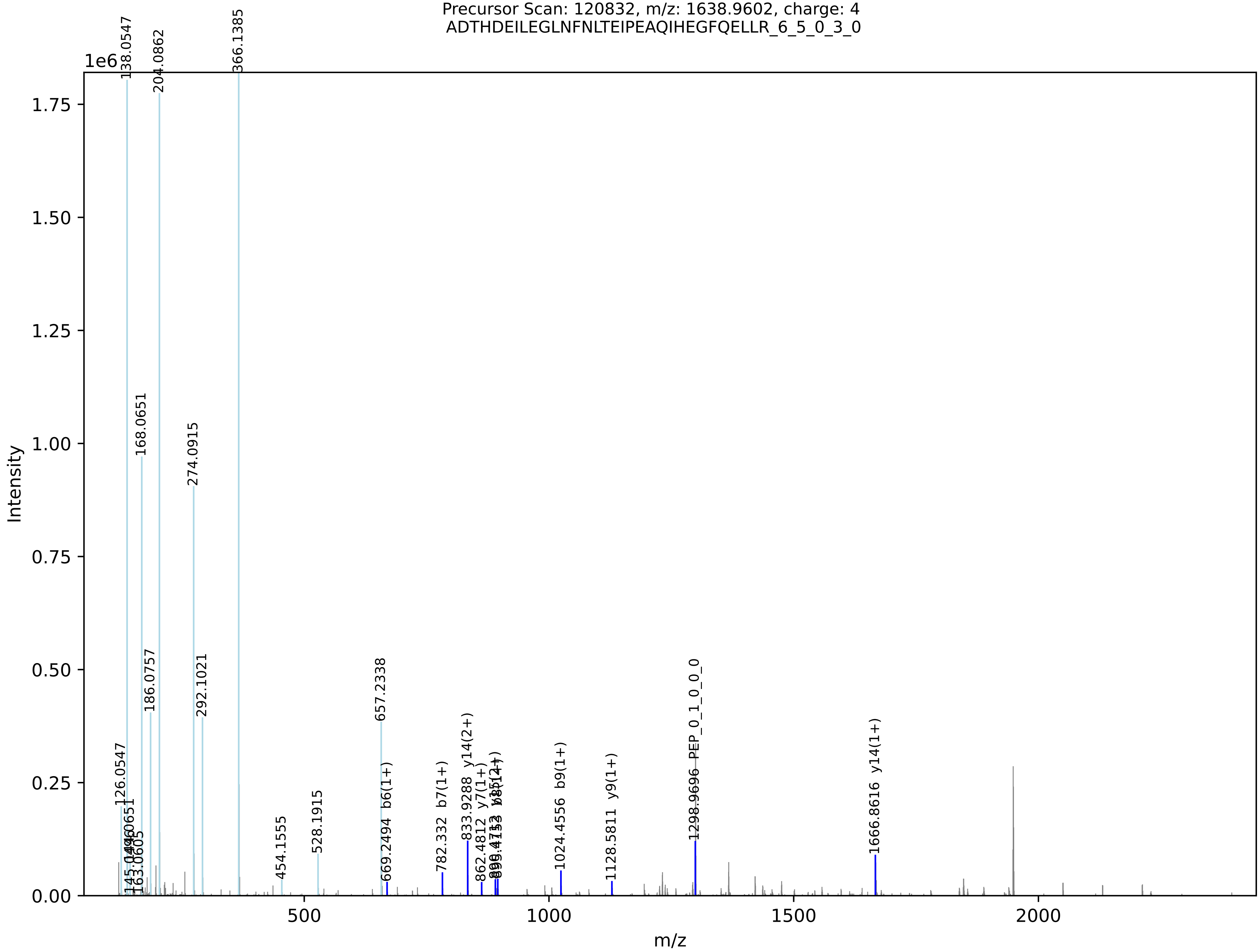

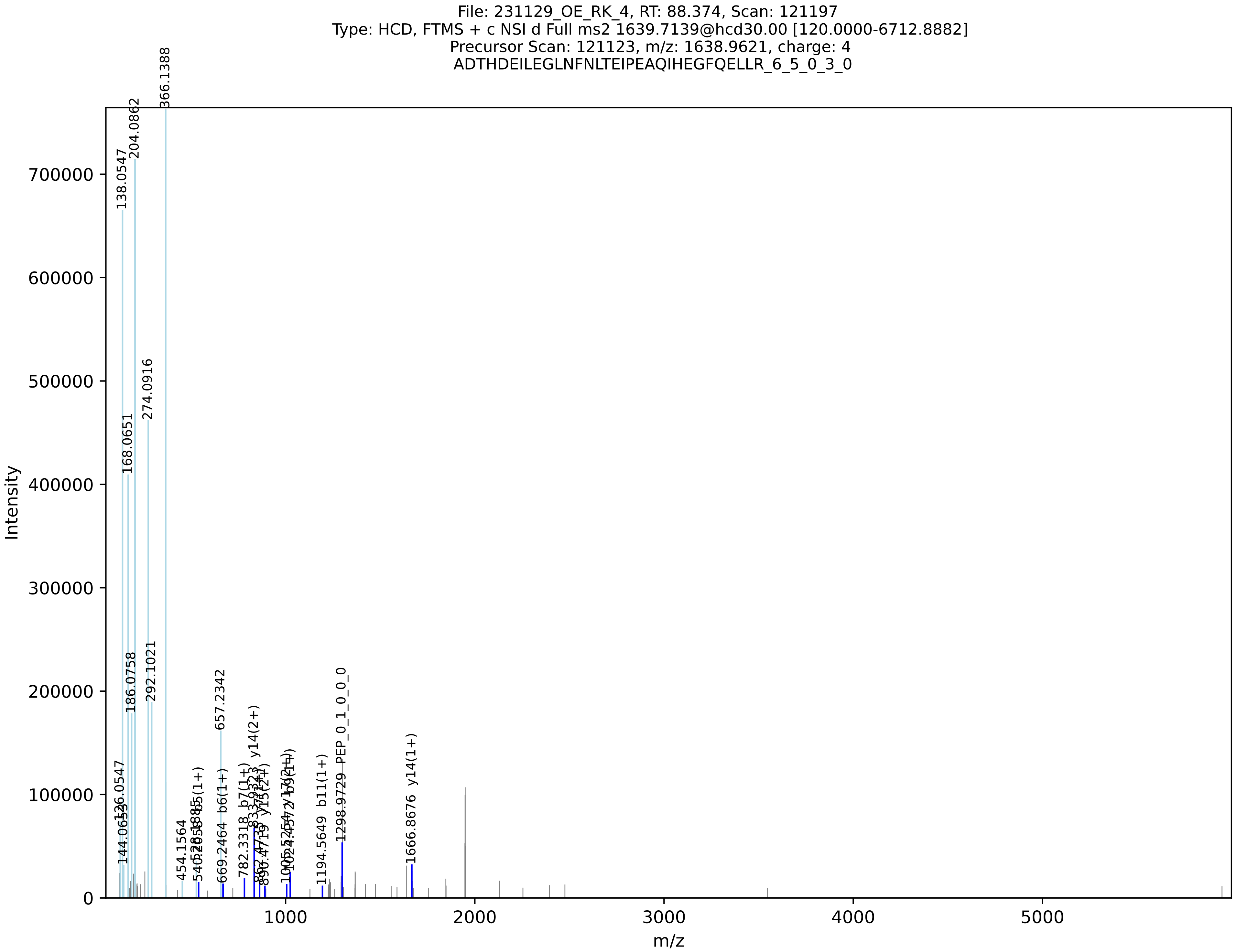
File: 231129_OE_RK_4, RT: 88.374, Scan: 121197
Type: HCD, FTMS + c NSI d Full ms2 1639.7139@hcd30.00 [120.0000-6712.8882]
Precursor Scan: 121123, m/z: 1638.9621, charge: 4
ADTHDEILEGLNFNLTEIPEAQIHEGFQELLR_6_5_0_3_0
Intensity
m/z
366.1388
204.0862
138.0547
274.0916
168.0651
186.0758
292.1021
657.2342
126.0547
144.0652
454.1564
669.2464 b6(1+)
782.3318 b7(1+)
y14(2+)
1194.5649 b11(1+)
1298.9729 PEP_0_1_0_0_0
1666.8676 y14(1+)
0
100000
200000
300000
400000
500000
600000
700000
1000
2000
3000
4000
5000

File: 231129_OE_RK_4, RT: 88.405, Scan: 121246
Type: HCD, FTMS + c NSI d Full ms2 1638.9589@hcd30.00 [120.0000-6709.8081]
Precursor Scan: 121050, m/z: 1638.9589, charge: 4
ADTHDEILEGLNFNLTEIPEAQIHEGFQELLR_6_5_0_3_0

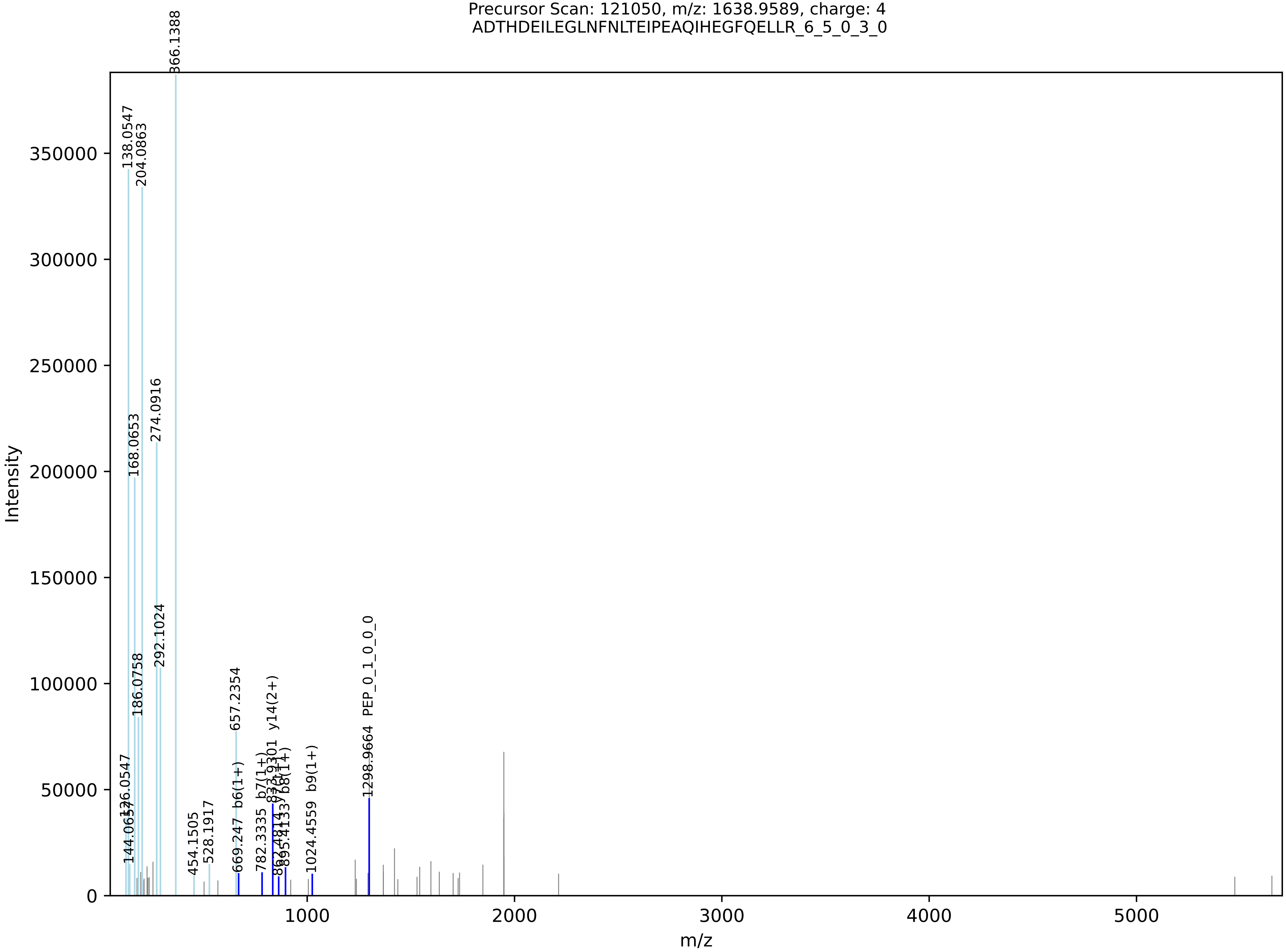

File: 231129_OE_RK_4, RT: 90.359, Scan: 124045
Type: HCD, FTMS + c NSI d Full ms2 1093.2491@hcd30.00 [120.0000-4483.3125]
Precursor Scan: 123972, m/z: 1092.7473, charge: 4
VSNQTLSLFFTVLQDVPVR_5_4_0_2_0

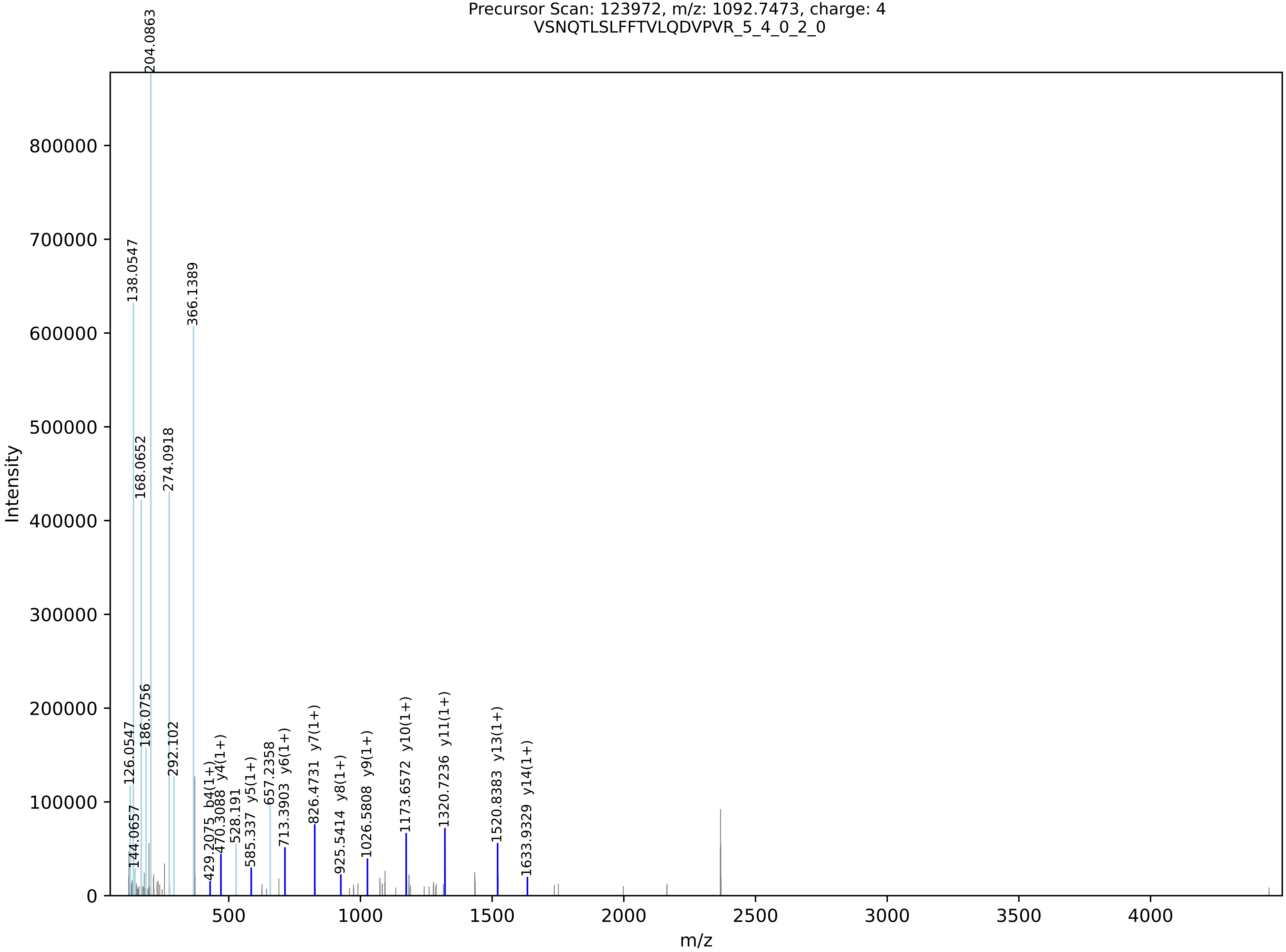

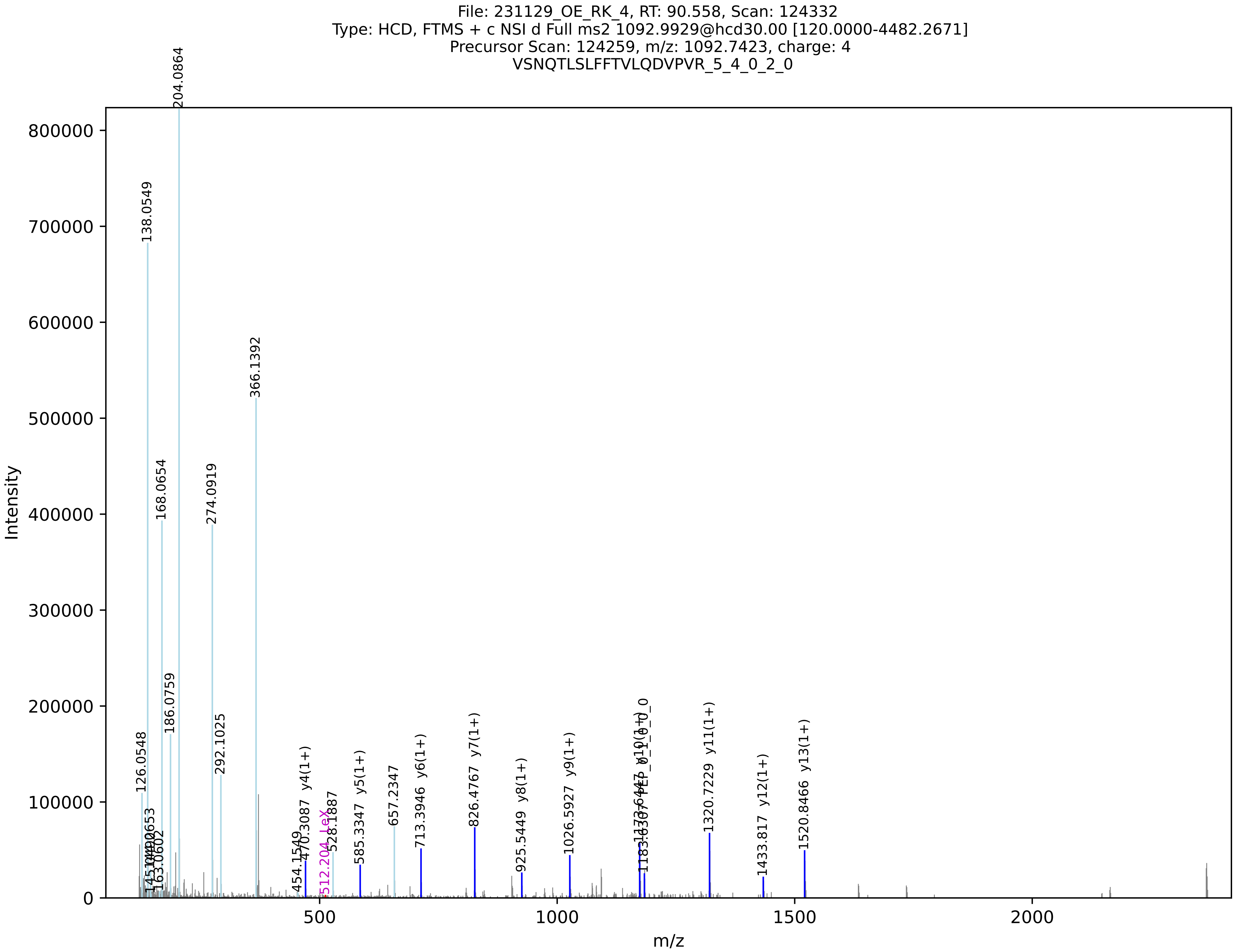
File: 231129_OE_RK_4, RT: 90.558, Scan: 124332
Type: HCD, FTMS + c NSI d Full ms2 1092.9929@hcd30.00 [120.0000-4482.2671]
Precursor Scan: 124259, m/z: 1092.7423, charge: 4
VSNQTLSLFFTVLQDVPVR_5_4_0_2_0
Intensity
m/z
204.0864
138.0549
366.1392
168.0654
274.0919
186.0759
292.1025
126.0548
163.0602
454.1549
470.3087 y4(1+)
512.204 LeX
528.1887
585.3347 y5(1+)
657.2347
713.3946 y6(1+)
826.4767 y7(1+)
925.5449 y8(1+)
1026.5927 y9(1+)
1320.7229 y11(1+)
1433.817 y12(1+)
1520.8466 y13(1+)

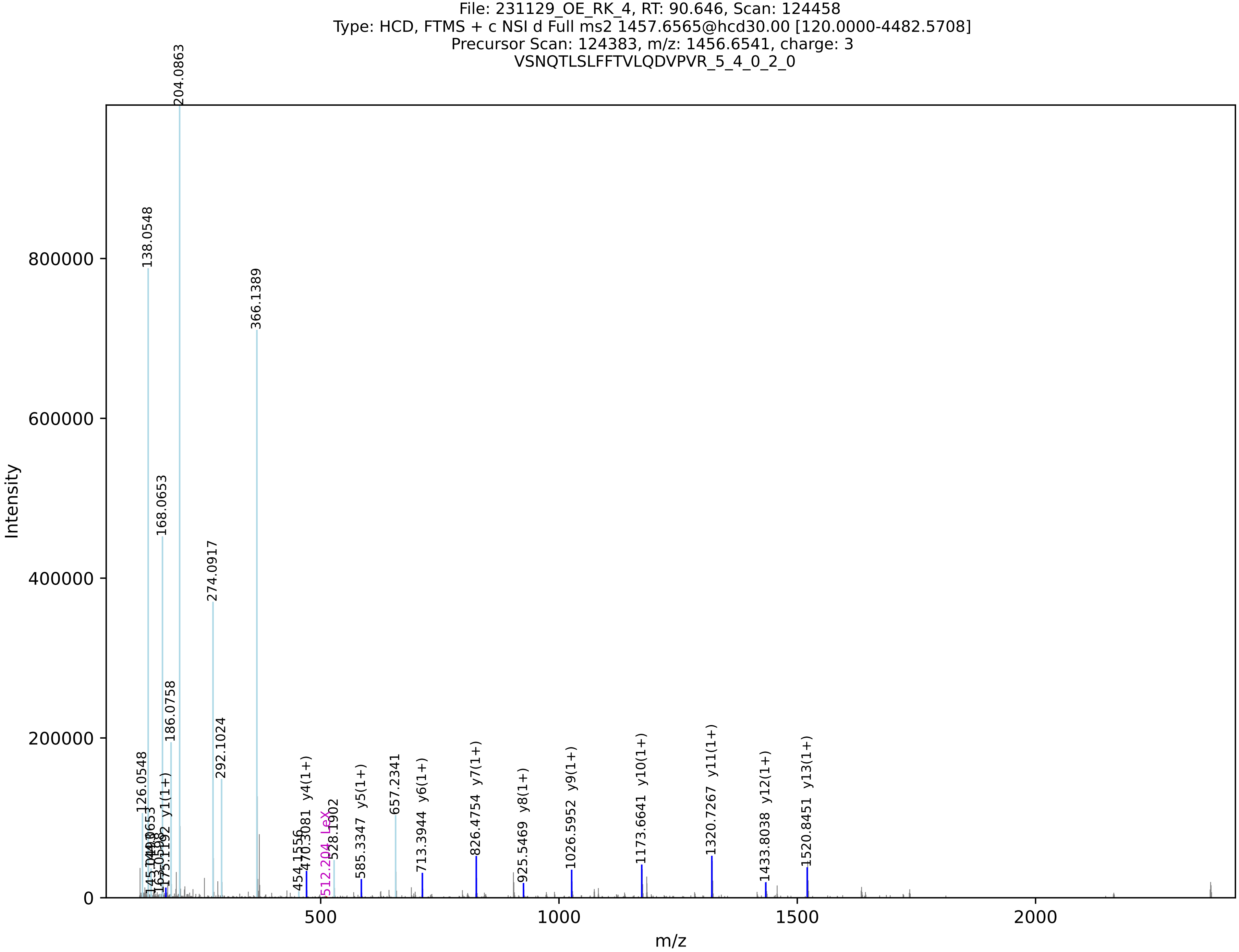
File: 231129_OE_RK_4, RT: 90.646, Scan: 124458
Type: HCD, FTMS + c NSI d Full ms2 1457.6565@hcd30.00 [120.0000-4482.5708]
Precursor Scan: 124383, m/z: 1456.6541, charge: 3
VSNQTLSLFFTVLQDVPVR_5_4_0_2_0
Intensity
m/z
204.0863
138.0548
366.1389
168.0653
274.0917
186.0758
292.1024
126.0548
175.1192 y1(1+)
454.1556
470.3081 y4(1+)
512.204 LeX
528.1902
585.3347 y5(1+)
657.2341
713.3944 y6(1+)
826.4754 y7(1+)
925.5469 y8(1+)
1026.5952 y9(1+)
1173.6641 y10(1+)
1320.7267 y11(1+)
1433.8038 y12(1+)
1520.8451 y13(1+)

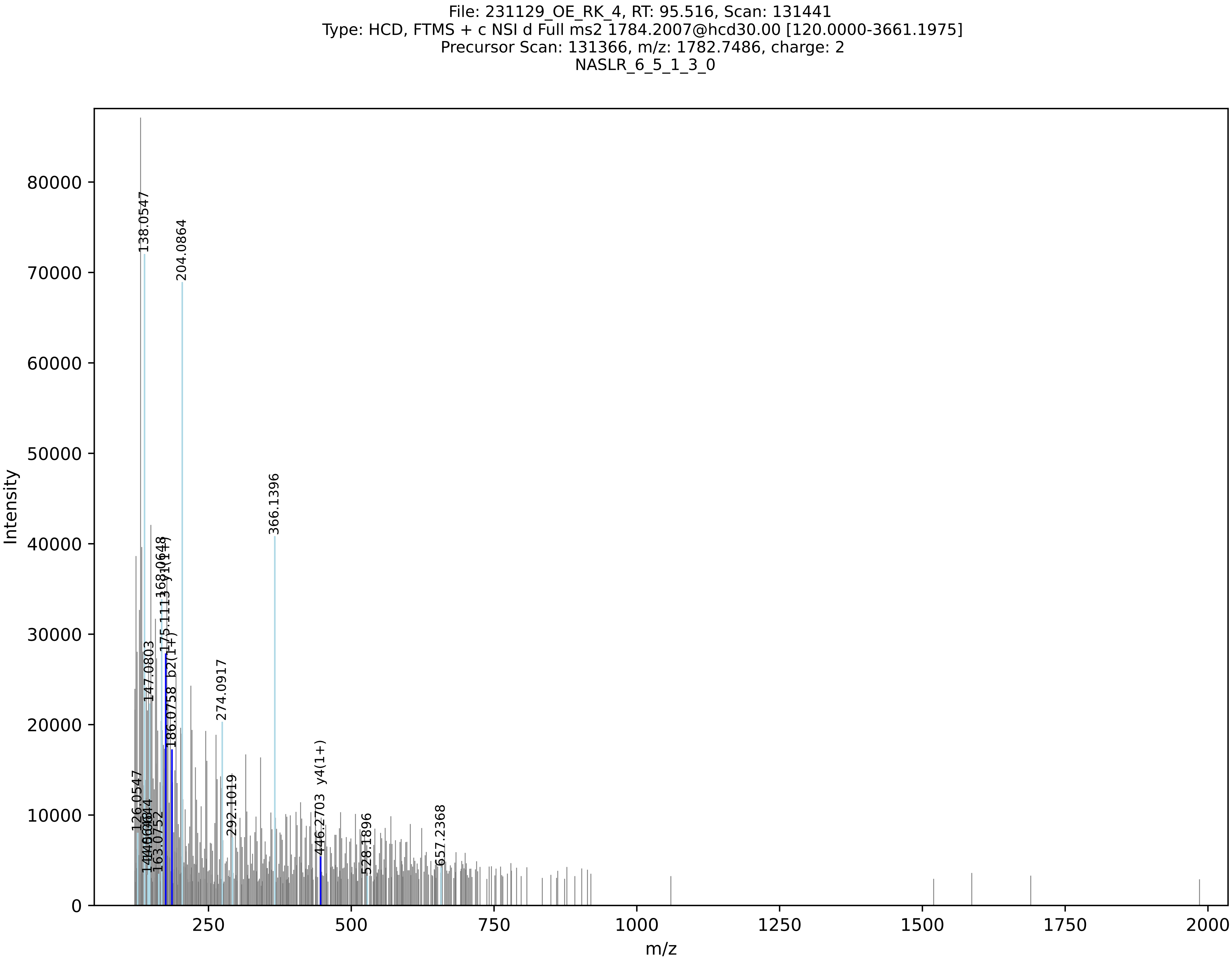

File: 231129_OE_RK_4, RT: 95.516, Scan: 131441
Type: HCD, FTMS + c NSI d Full ms2 1784.2007@hcd30.00 [120.0000-3661.1975]
Precursor Scan: 131366, m/z: 1782.7486, charge: 2
NASLR_6_5_1_3_0
Intensity
m/z
138.0547
204.0864
366.1396
168.0648
y1(1+)
175.1113
147.0803
b2(1+)
186.0758
274.0917
y4(1+)
126.0547
292.1019
446.2703
528.1896
657.2368
163.0752
0
10000
20000
30000
40000
50000
60000
70000
80000
250
500
750
1000
1250
1500
1750
2000

File: 231129_OE_RK_4, RT: 95.929, Scan: 132047
Type: HCD, FTMS + c NSI d Full ms2 1791.8668@hcd30.00 [120.0000-3676.8362]
Precursor Scan: 131958, m/z: 1791.8668, charge: 2
VERCDMDGQNR_5_4_2_1_0

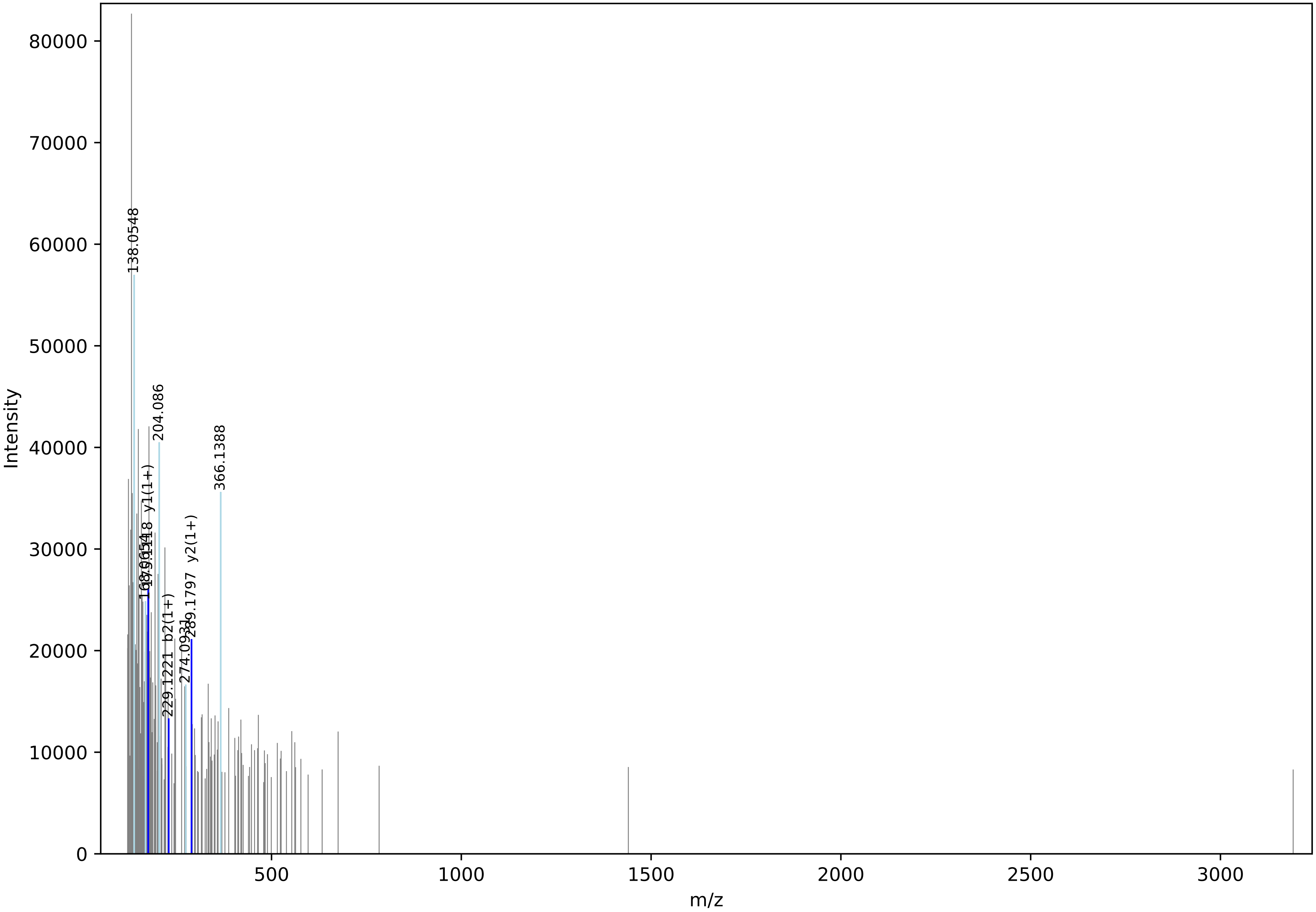

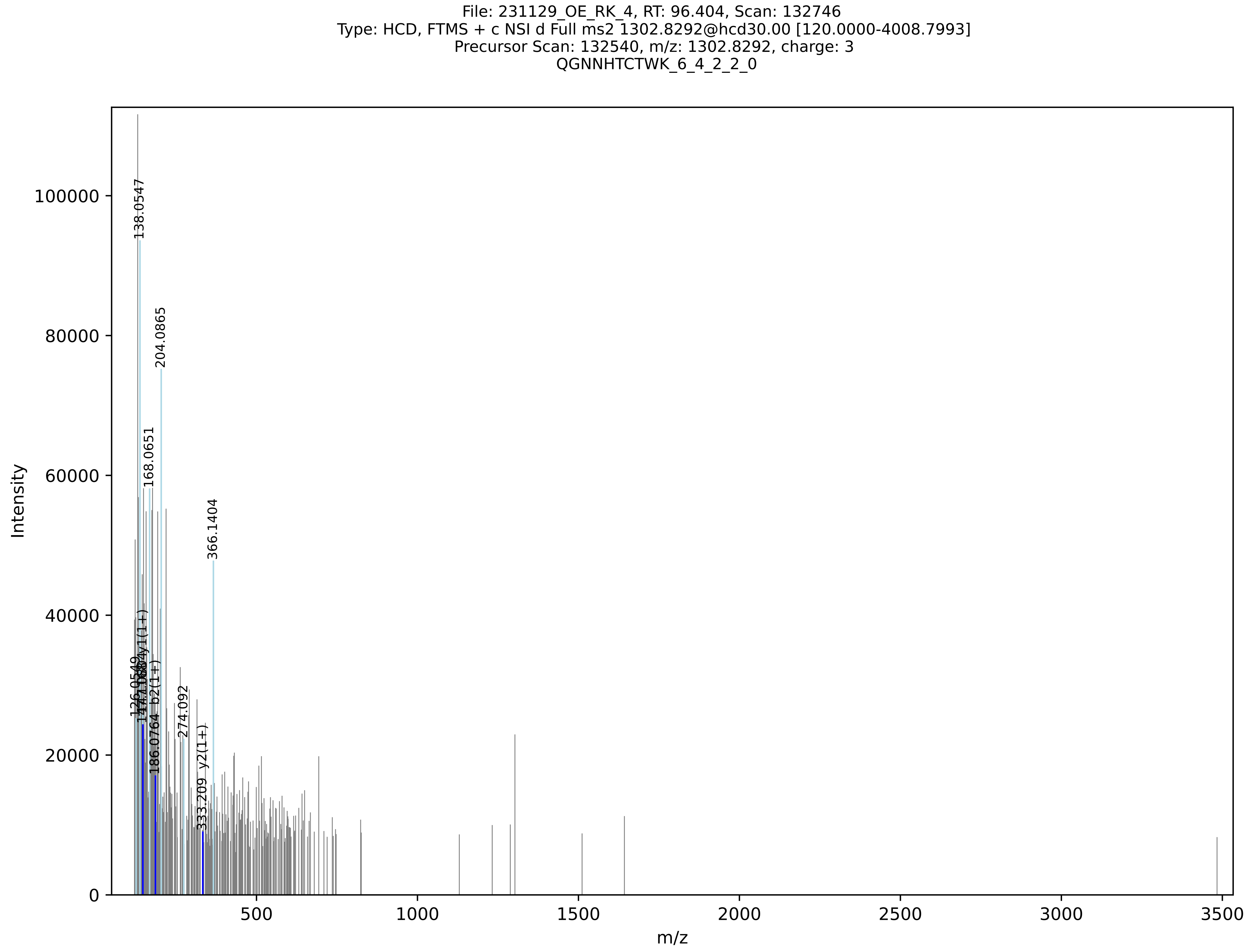
File: 231129_OE_RK_4, RT: 96.404, Scan: 132746
Type: HCD, FTMS + c NSI d Full ms2 1302.8292@hcd30.00 [120.0000-4008.7993]
Precursor Scan: 132540, m/z: 1302.8292, charge: 3
QGNNHTCTWK_6_4_2_2_0
138.0547
204.0865
168.0651
366.1404
126.0549
274.092
186.0764_b2(1+)
333.209_y2(1+)
Intensity
m/z

File: 231129_OE_RK_4, RT: 96.473, Scan: 132844
Type: HCD, FTMS + c NSI d Full ms2 1385.3518@hcd30.00 [120.0000-4261.3184]
Precursor Scan: 132762, m/z: 1385.3518, charge: 3
AKFVGTPEVNQTTLYQR_5_4_0_2_0

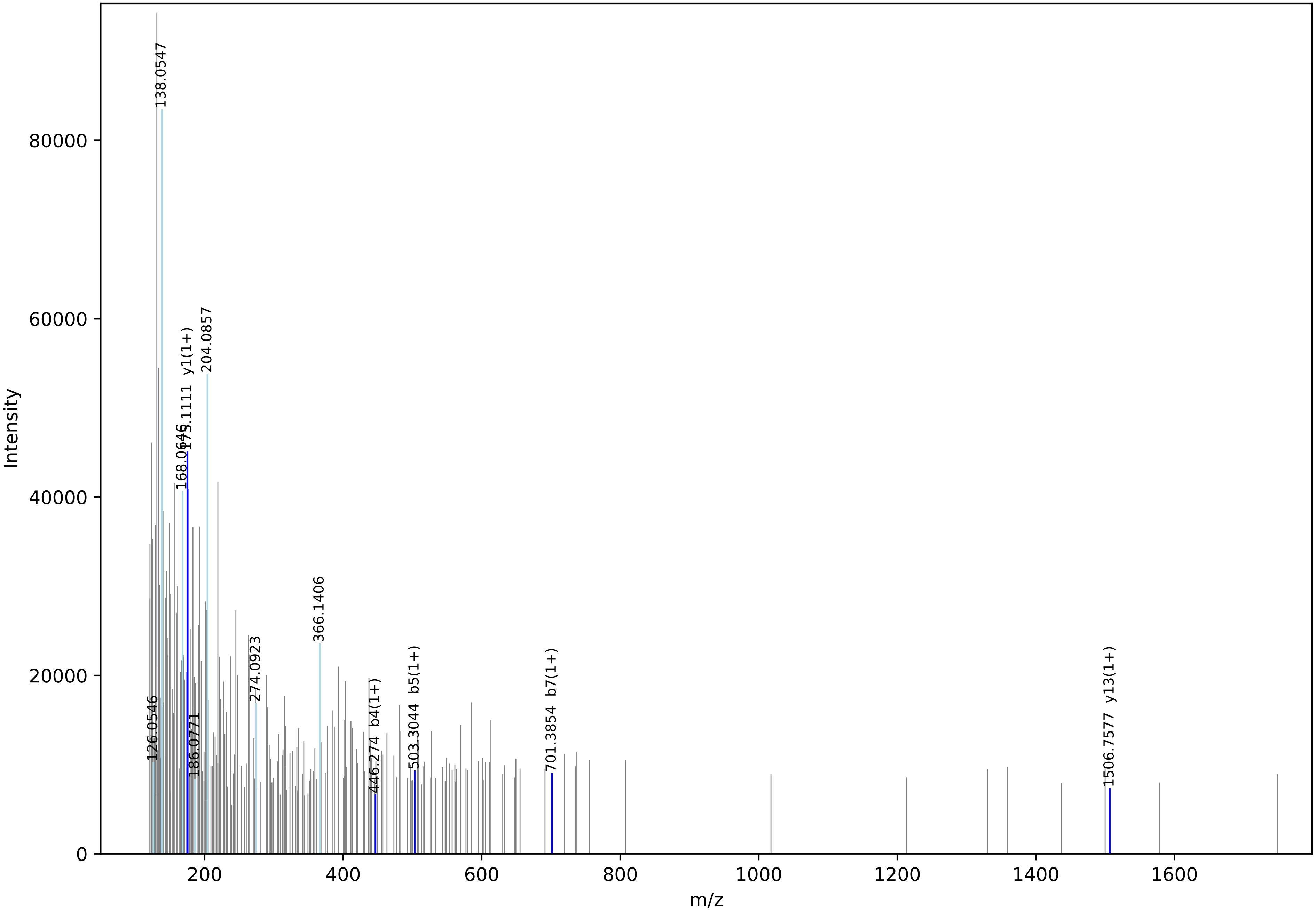

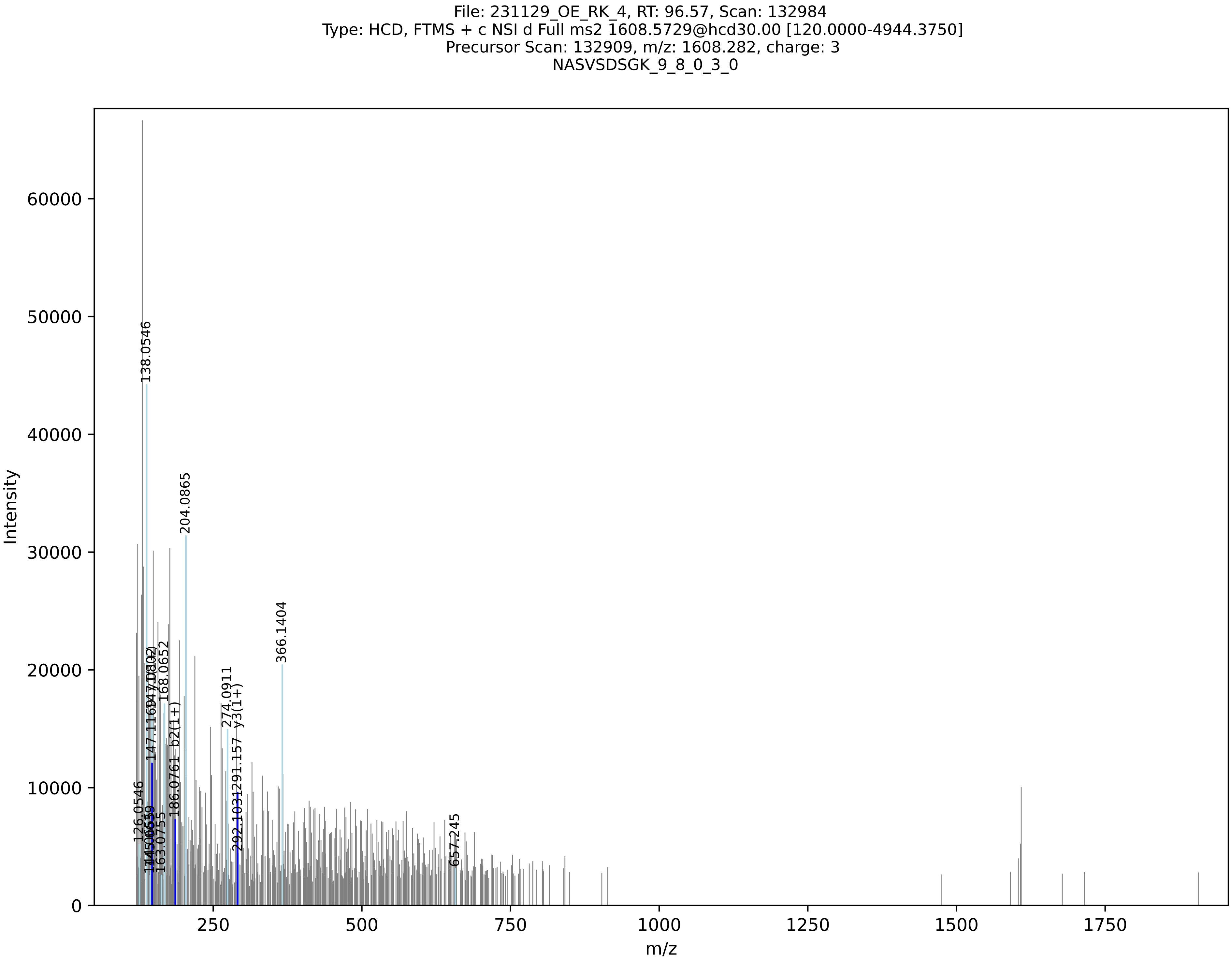
File: 231129_OE_RK_4, RT: 96.57, Scan: 132984
Type: HCD, FTMS + c NSI d Full ms2 1608.5729@hcd30.00 [120.0000-4944.3750]
Precursor Scan: 132909, m/z: 1608.282, charge: 3
NASVSDSGK_9_8_0_3_0
Intensity
m/z
138.0546
204.0865
366.1404
168.0652
274.0911
y3(1+)
291.157
b2(1+)
186.0761
292.1031
126.0546
163.0755
657.245

File: 231129_OE_RK_4, RT: 96.829, Scan: 133364
Type: HCD, FTMS + c NSI d Full ms2 1376.6025@hcd30.00 [120.0000-4234.5459]
Precursor Scan: 133277, m/z: 1376.6026, charge: 3
GLIGNLYLNDSPLK_5_6_0_2_0

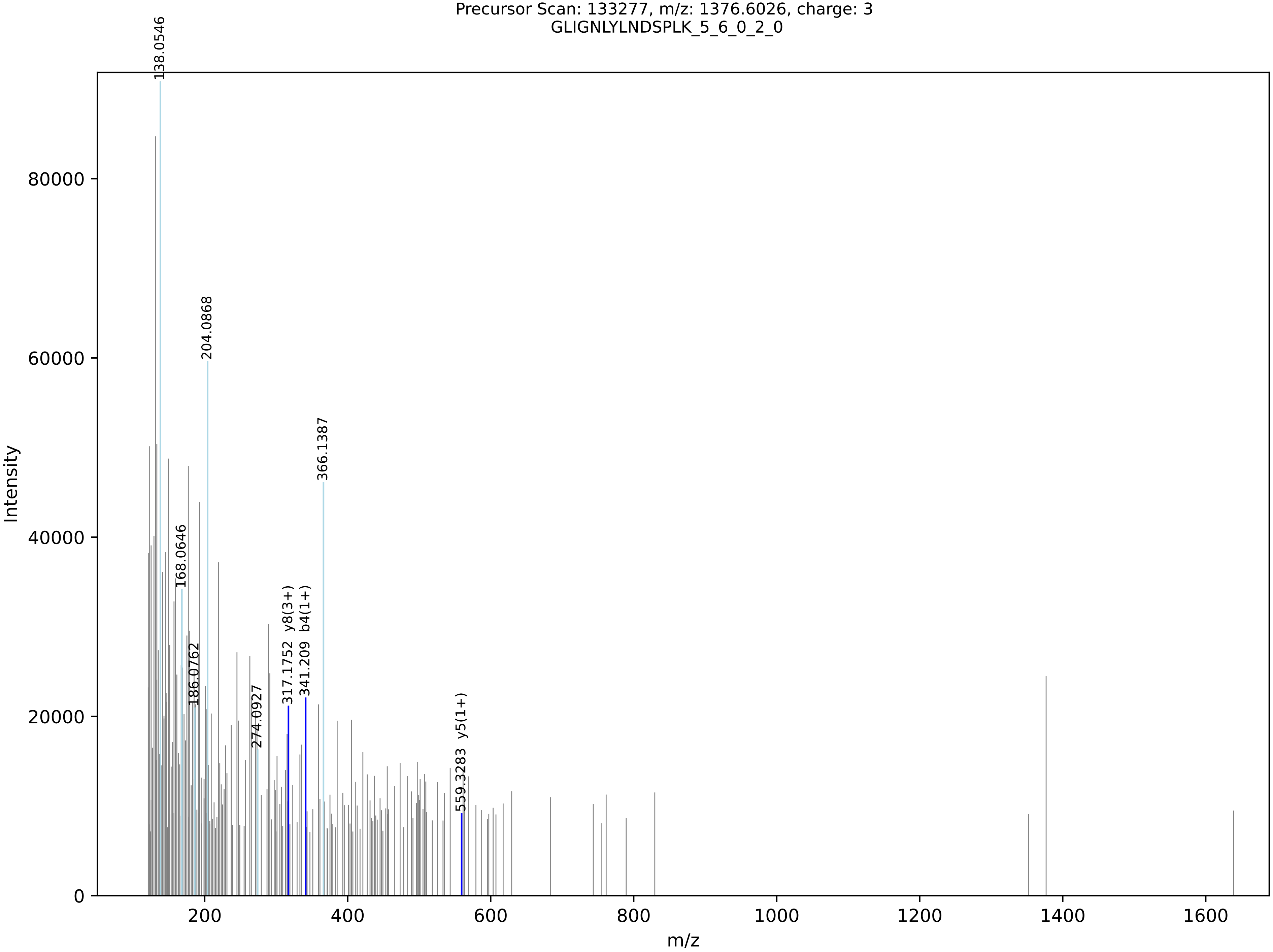

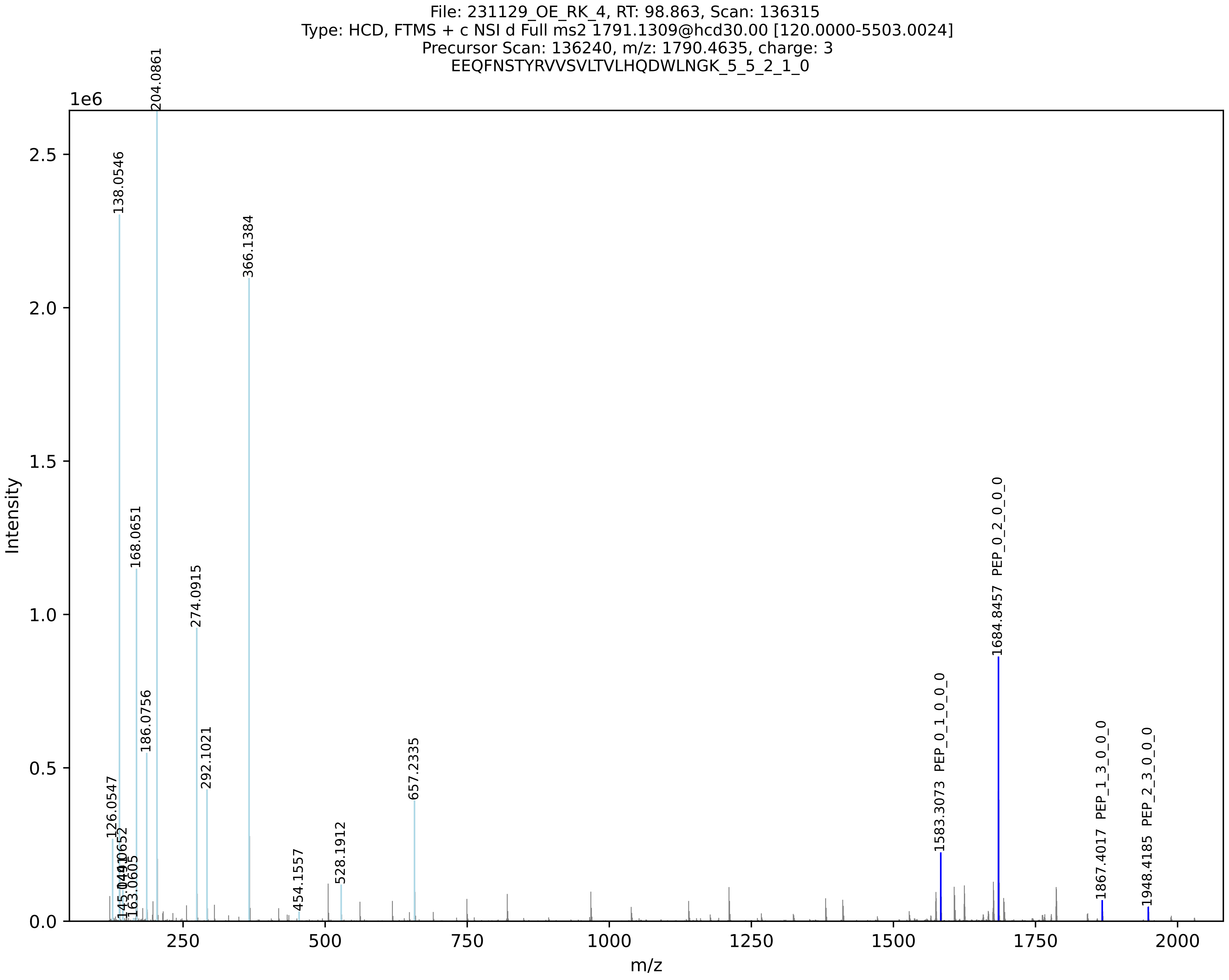

File: 231129_OE_RK_4, RT: 98.863, Scan: 136315
Type: HCD, FTMS + c NSI d Full ms2 1791.1309@hcd30.00 [120.0000-5503.0024]
Precursor Scan: 136240, m/z: 1790.4635, charge: 3
EEQFNSTYRVVSVLTVLHQDWLNGK_5_5_2_1_0
1e6
Intensity
m/z
204.0861
138.0546
366.1384
168.0651
274.0915
186.0756
292.1021
126.0547
163.0605
454.1557
528.1912
657.2335
1583.3073 PEP_0_1_0_0_0
1684.8457 PEP_0_2_0_0_0
1867.4017 PEP_1_3_0_0_0
1948.4185 PEP_2_3_0_0_0

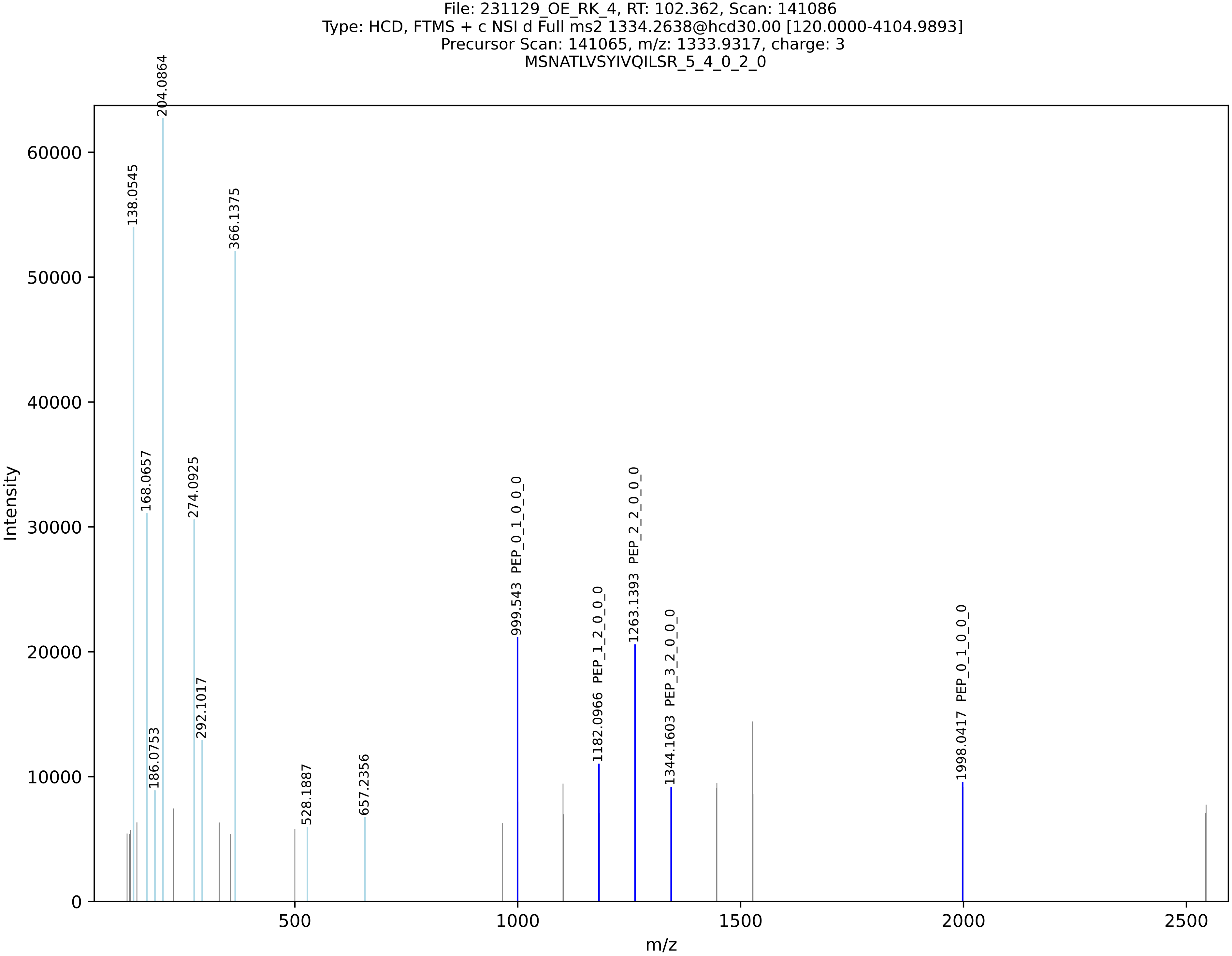
File: 231129_OE_RK_4, RT: 102.362, Scan: 141086
Type: HCD, FTMS + c NSI d Full ms2 1334.2638@hcd30.00 [120.0000-4104.9893]
Precursor Scan: 141065, m/z: 1333.9317, charge: 3
MSNATLVSYIVQILSR_5_4_0_2_0
Intensity
m/z
204.0864
138.0545
366.1375
168.0657
274.0925
292.1017
186.0753
528.1887
657.2356
999.543 PEP_0_1_0_0_0
1182.0966 PEP_1_2_0_0_0
1263.1393 PEP_2_2_0_0_0
1344.1603 PEP_3_2_0_0_0
1998.0417 PEP_0_1_0_0_0
0
10000
20000
30000
40000
50000
60000
500
1000
1500
2000
2500

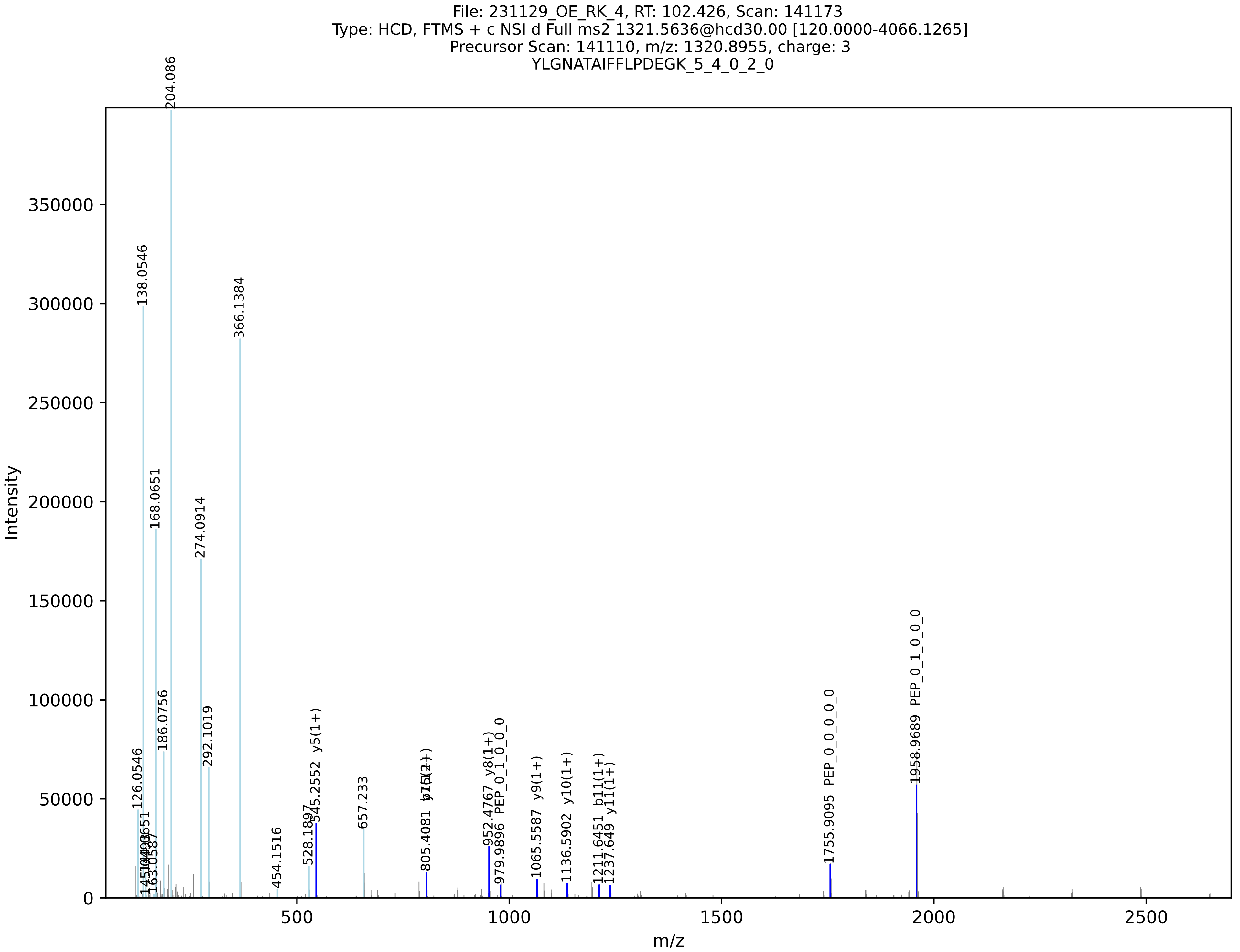
File: 231129_OE_RK_4, RT: 102.426, Scan: 141173
Type: HCD, FTMS + c NSI d Full ms2 1321.5636@hcd30.00 [120.0000-4066.1265]
Precursor Scan: 141110, m/z: 1320.8955, charge: 3
YLGNATAIFFLPDEGK_5_4_0_2_0
Intensity
m/z
204.086
138.0546
366.1384
168.0651
274.0914
186.0756
292.1019
126.0546
545.2552 y5(1+)
657.233
528.1897
454.1516
805.4081
952.4767 y8(1+)
979.9896 PEP_0_1_0_0_0
1065.5587 y9(1+)
1136.5902 y10(1+)
1211.6451 b11(1+)
1237.649 y11(1+)
1755.9095 PEP_0_0_0_0_0
1958.9689 PEP_0_1_0_0_0

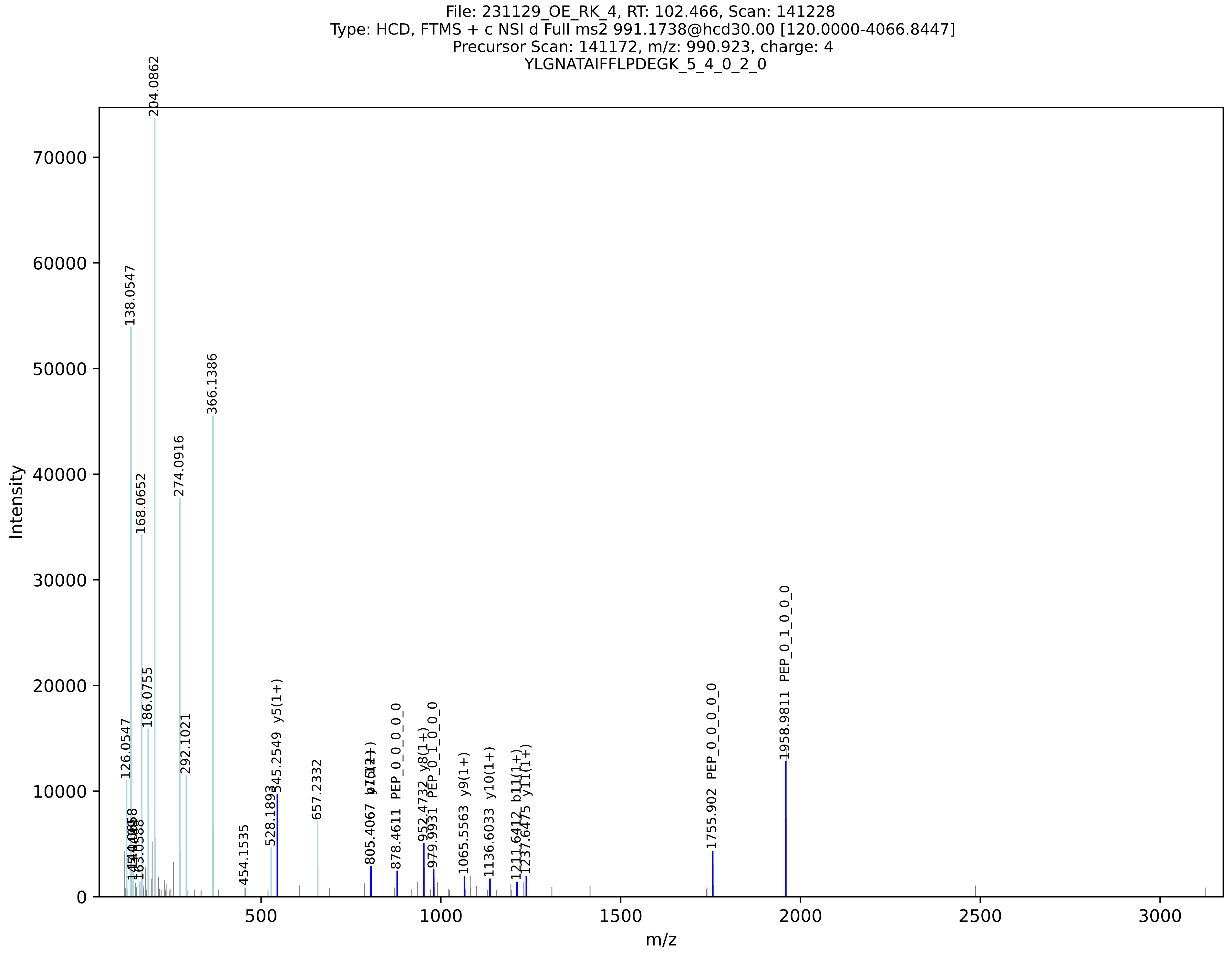
File: 231129_OE_RK_4, RT: 102.466, Scan: 141228
Type: HCD, FTMS + c NSI d Full ms2 991.1738@hcd30.00 [120.0000-4066.8447]
Precursor Scan: 141172, m/z: 990.923, charge: 4
YLGNATAIFFLPDEGK_5_4_0_2_0
Intensity
m/z
204.0862
138.0547
366.1386
274.0916
168.0652
186.0755
126.0547
292.1021
545.2549 y5(1+)
528.1893
657.2332
454.1535
805.4067
878.4611 PEP_0_0_0_0_0_
952.4732 y8(1+)
979.9931 PEP_0_1_0_0_0_
1065.5563 y9(1+)
1136.6033 y10(1+)
1211.6412 b11(1+)
1237.6475 y11(1+)
1755.902 PEP_0_0_0_0_0_
1958.9811 PEP_0_1_0_0_0_

File: 231129_OE_RK_4, RT: 102.77, Scan: 141540
Type: HCD, FTMS + c NSI d Full ms2 1321.2255@hcd30.00 [120.0000-4065.0920]
Precursor Scan: 141523, m/z: 1320.5571, charge: 3
YLGNATAIFFLPDEGK_5_4_0_2_0

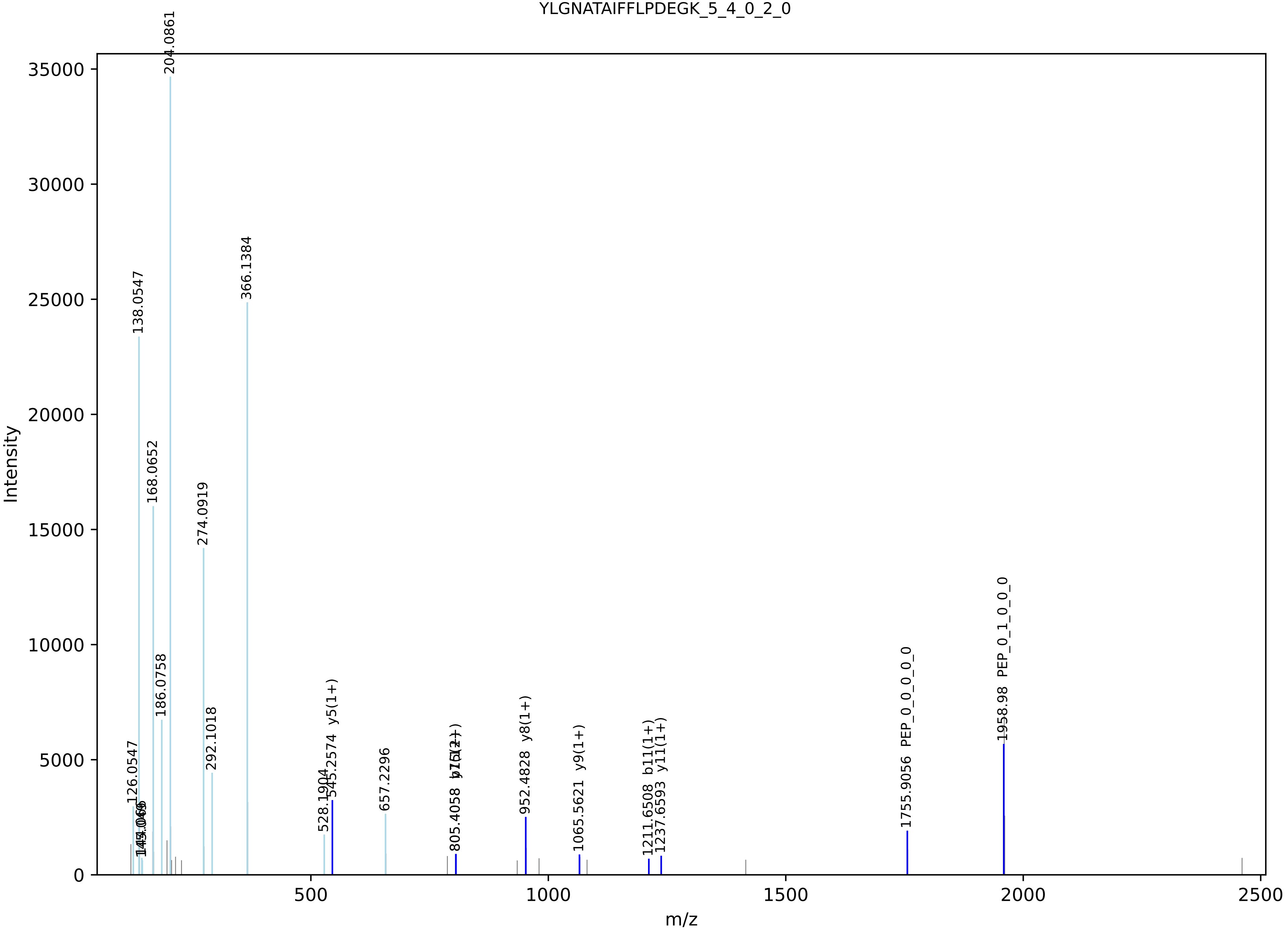

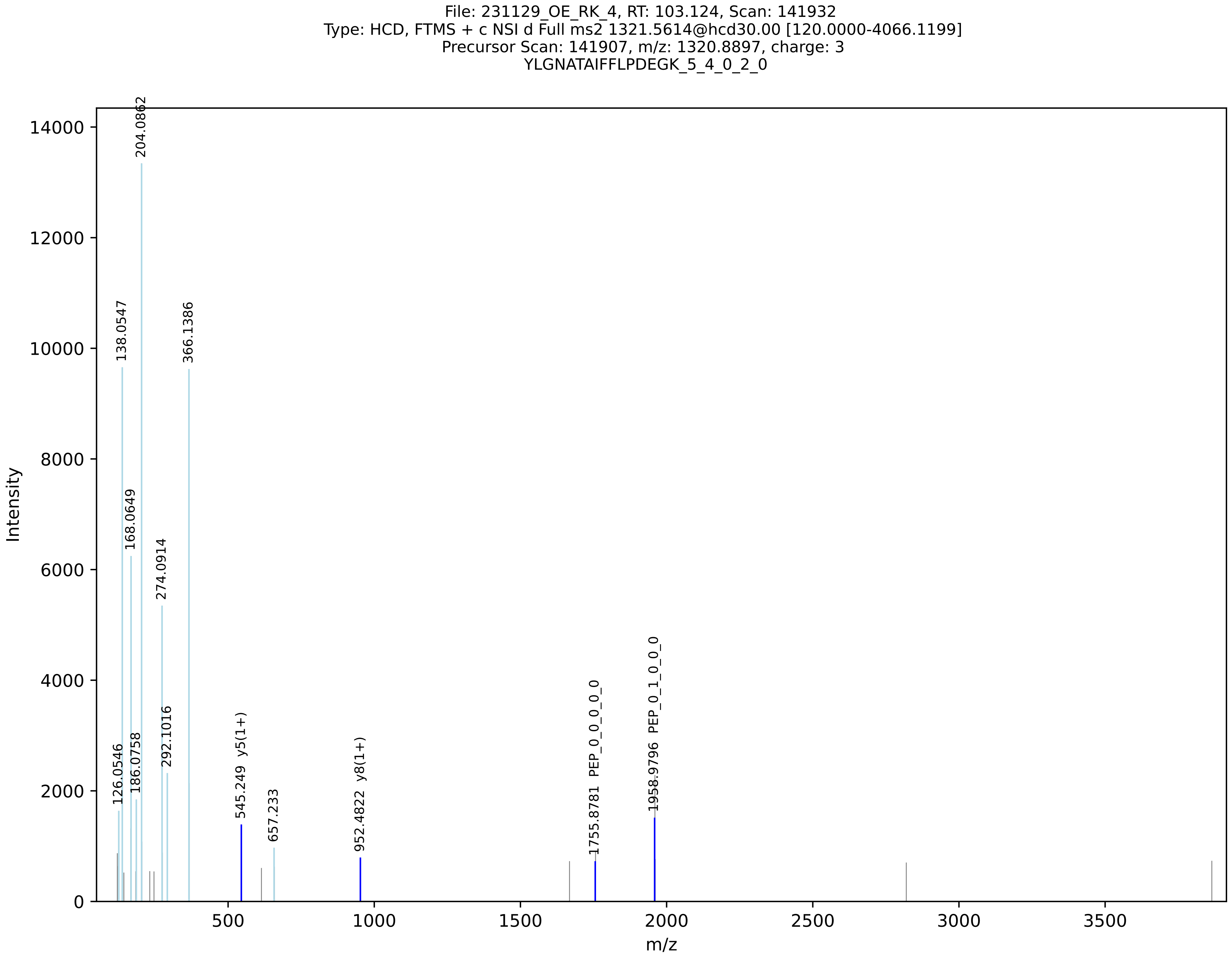
File: 231129_OE_RK_4, RT: 103.124, Scan: 141932
Type: HCD, FTMS + c NSI d Full ms2 1321.5614@hcd30.00 [120.0000-4066.1199]
Precursor Scan: 141907, m/z: 1320.8897, charge: 3
YLGNATAIFFLPDEGK_5_4_0_2_0
Intensity
m/z
204.0862
138.0547
366.1386
168.0649
274.0914
292.1016
126.0546
186.0758
545.249 y5(1+)
657.233
952.4822 y8(1+)
1755.8781 PEP_0_0_0_0_0
1958.9796 PEP_0_1_0_0_0
0
2000
4000
6000
8000
10000
12000
14000
500
1000
1500
2000
2500
3000
3500

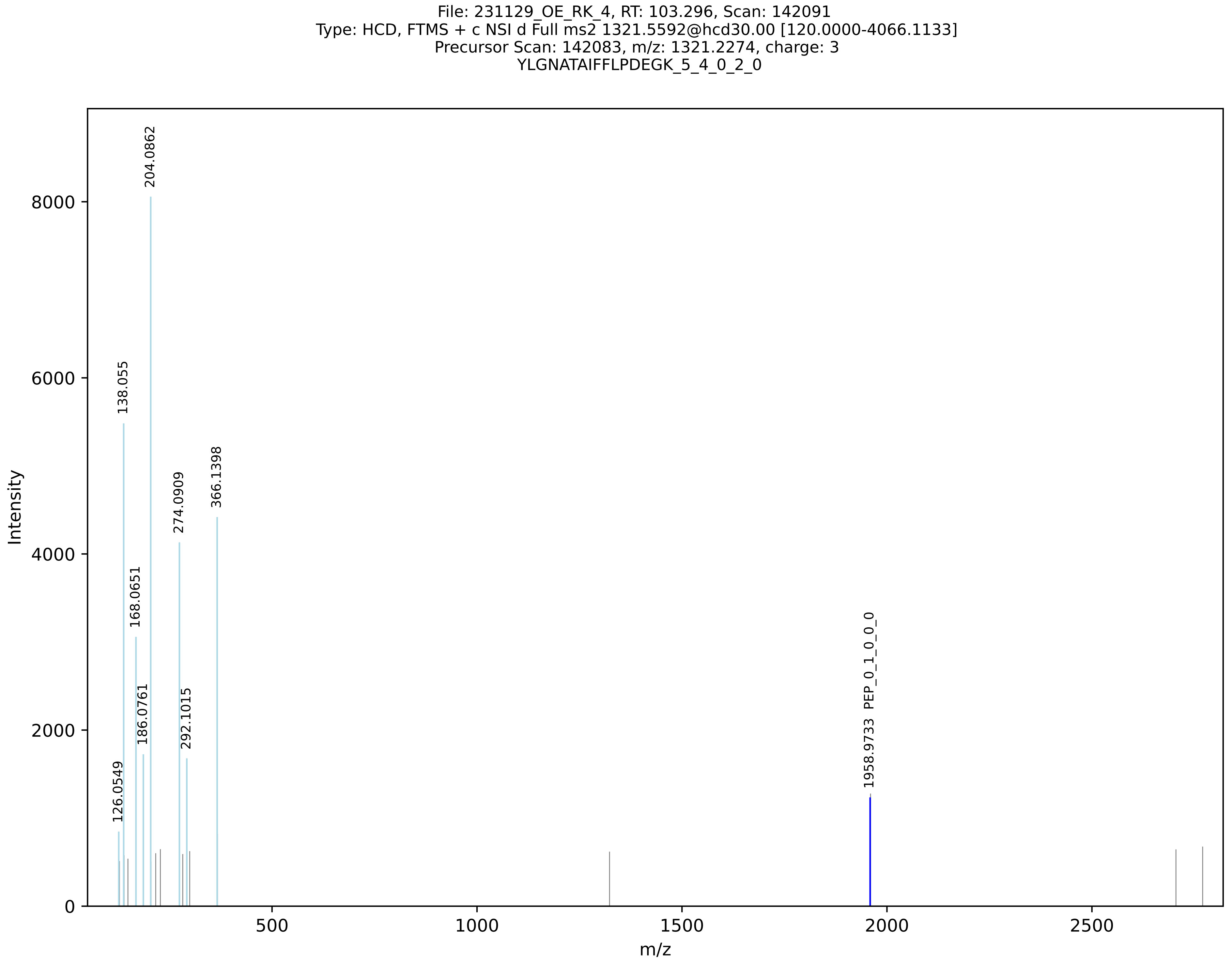
File: 231129_OE_RK_4, RT: 103.296, Scan: 142091
Type: HCD, FTMS + c NSI d Full ms2 1321.5592@hcd30.00 [120.0000-4066.1133]
Precursor Scan: 142083, m/z: 1321.2274, charge: 3
YLGNATAIFFLPDEGK_5_4_0_2_0
Intensity
m/z
204.0862
138.055
366.1398
274.0909
168.0651
186.0761
292.1015
126.0549
1958.9733 PEP_0_1_0_0_0
0
2000
4000
6000
8000
500
1000
1500
2000
2500

File: 231129_OE_RK_4, RT: 103.535, Scan: 142229
Type: HCD, FTMS + c NSI d Full ms2 1212.8838@hcd30.00 [120.0000-3733.5664]
Precursor Scan: 142224, m/z: 1211.88, charge: 3
DFVNASSKYEITTIHNLFR_6_2_0_0_0

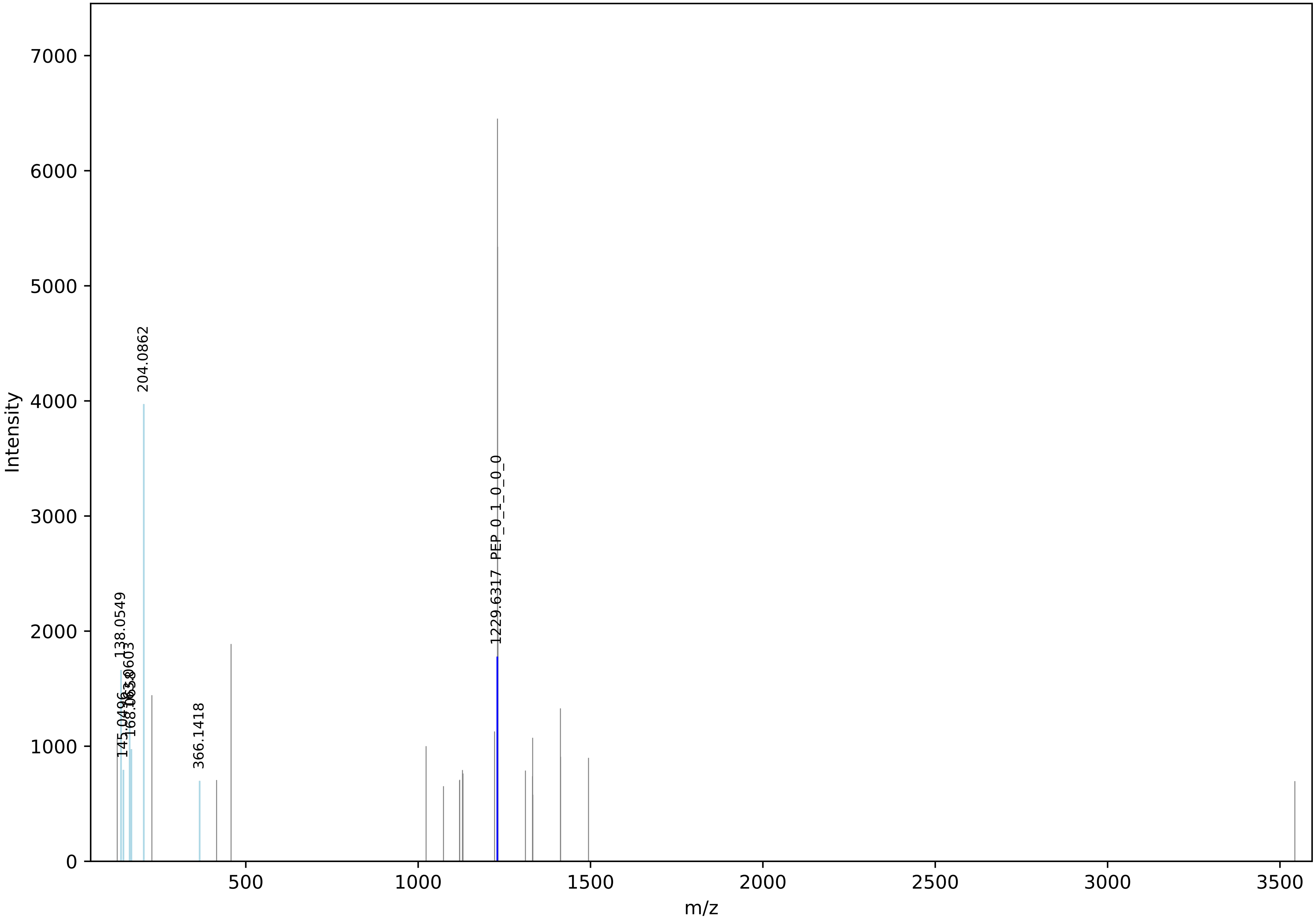

File: 231129_OE_RK_4, RT: 103.63, Scan: 142260
Type: HCD, FTMS + c NSI d Full ms2 991.6713@hcd30.00 [120.0000-4068.8748]
Precursor Scan: 142255, m/z: 990.6694, charge: 4
YLGNATAIFFLPDEGK_5_4_0_2_0

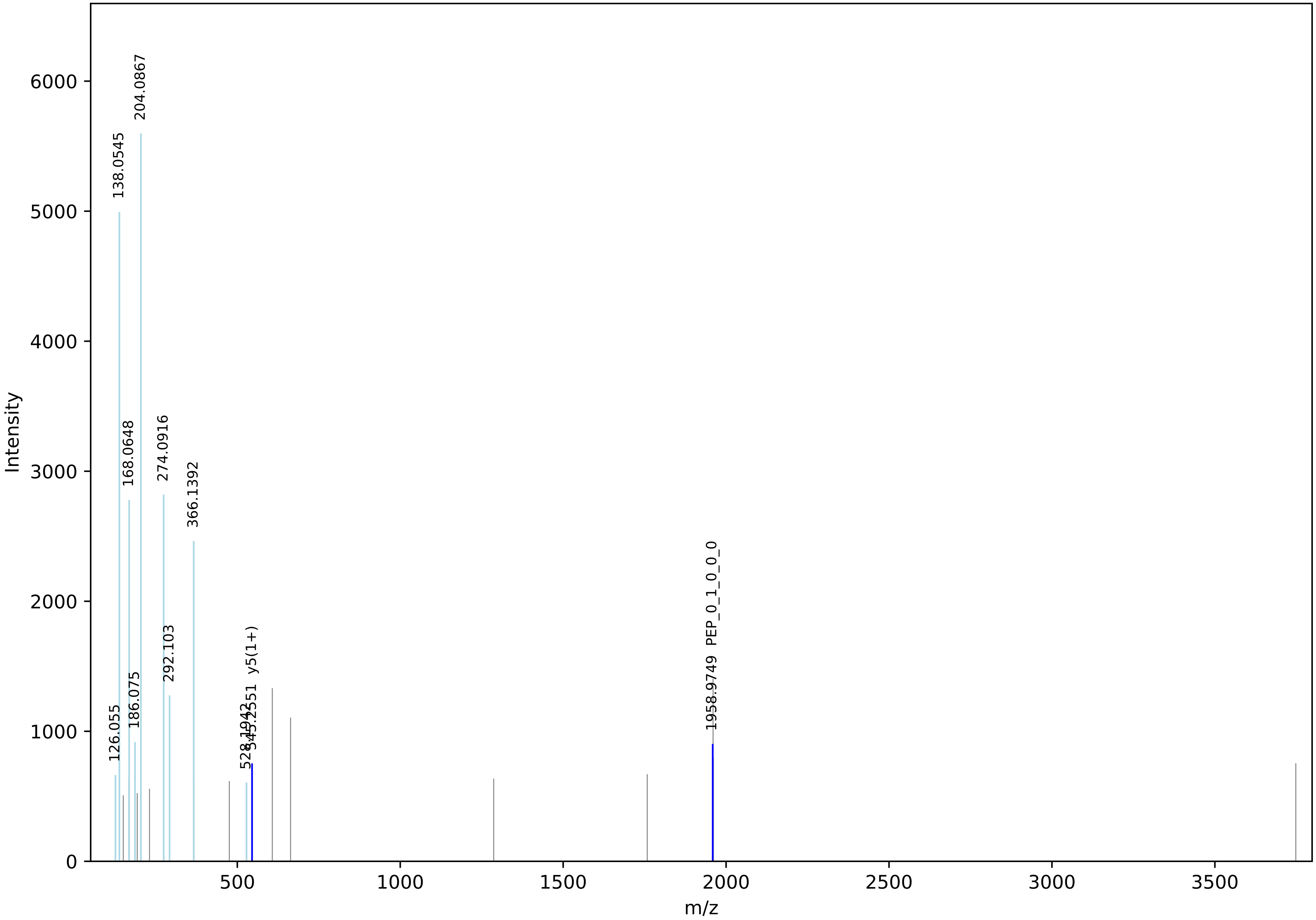

File: 231129_OE_RK_37, RT: 4.703, Scan: 001132
Type: HCD, FTMS + c NSI d Full ms2 1027.0918@hcd30.00 [120.0000-3165.0427]
Precursor Scan: 001075, m/z: 1026.7578, charge: 3
DIENQTVLK_6_3_1_1_0

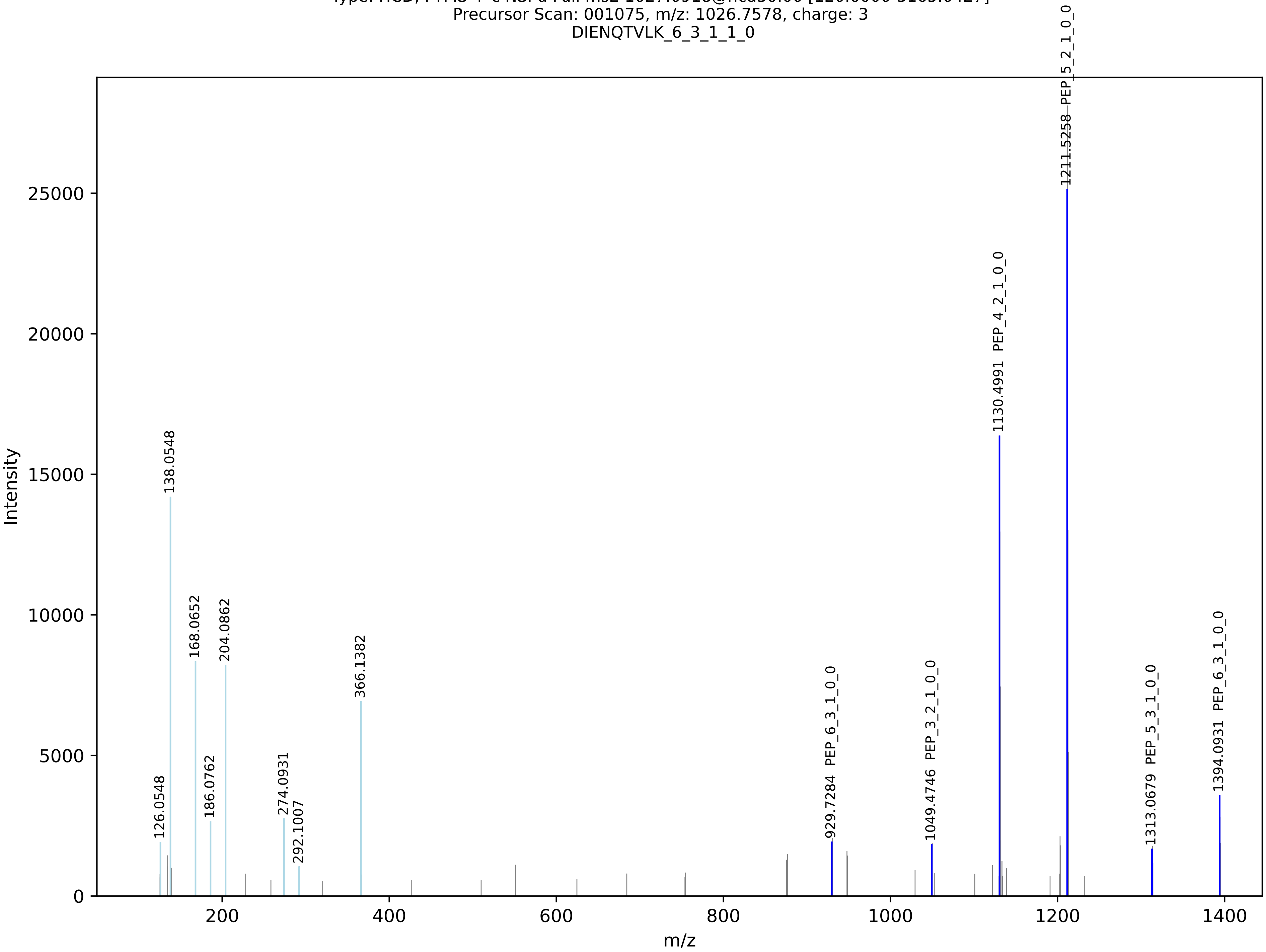

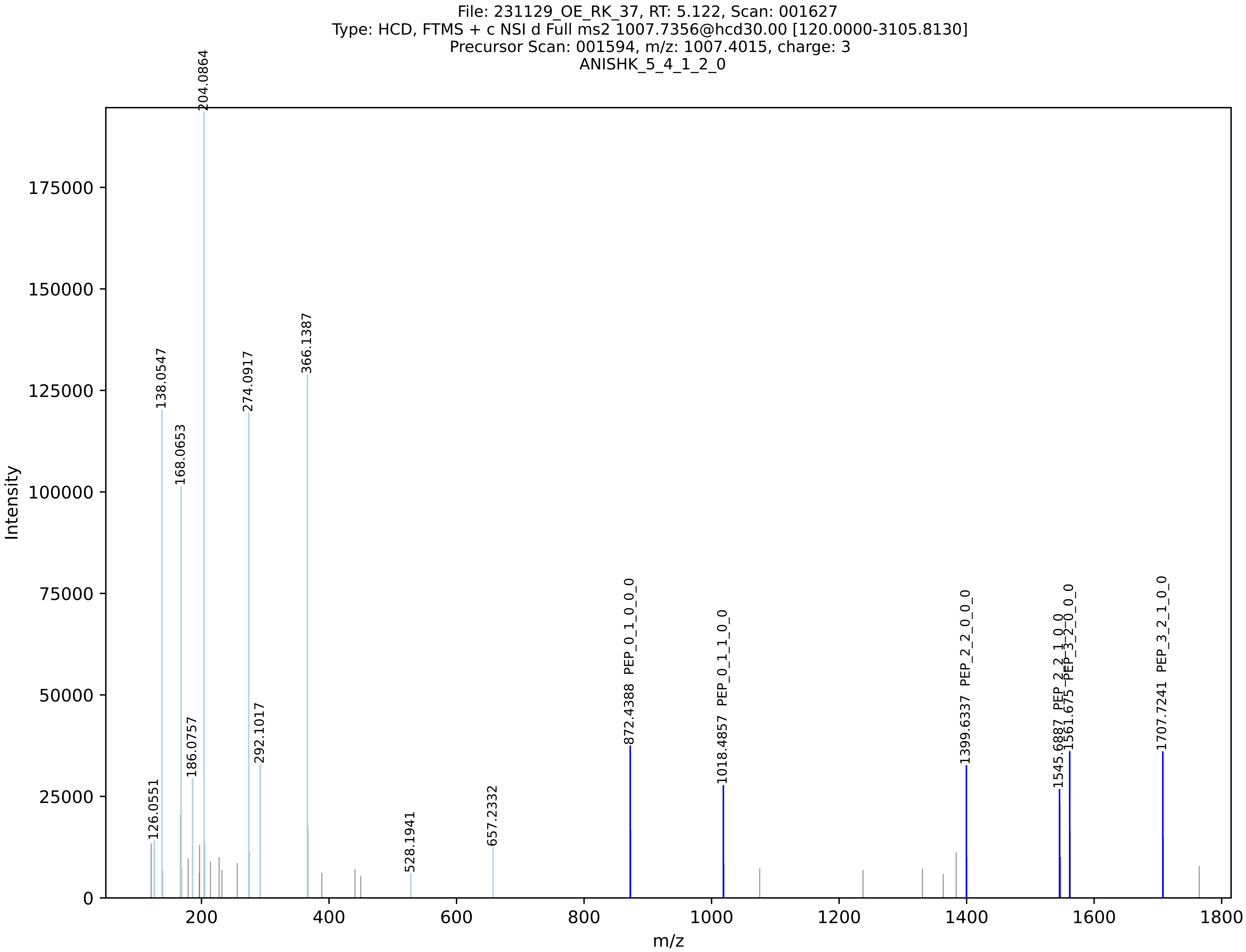

File: 231129_OE_RK_37, RT: 5.122, Scan: 001627
Type: HCD, FTMS + c NSI d Full ms2 1007.7356@hcd30.00 [120.0000-3105.8130]
Precursor Scan: 001594, m/z: 1007.4015, charge: 3
ANISHK_5_4_1_2_0
Intensity
m/z
204.0864
138.0547
168.0653
274.0917
366.1387
292.1017
186.0757
126.0551
528.1941
657.2332
872.4388 PEP_0_1_0_0_0
1018.4857 PEP_0_1_1_0_0
1399.6337 PEP_2_2_0_0_0
1545.6887 PEP_2_2_1_0_0
1561.675 PEP_3_2_0_0_0
1707.7241 PEP_3_2_1_0_0

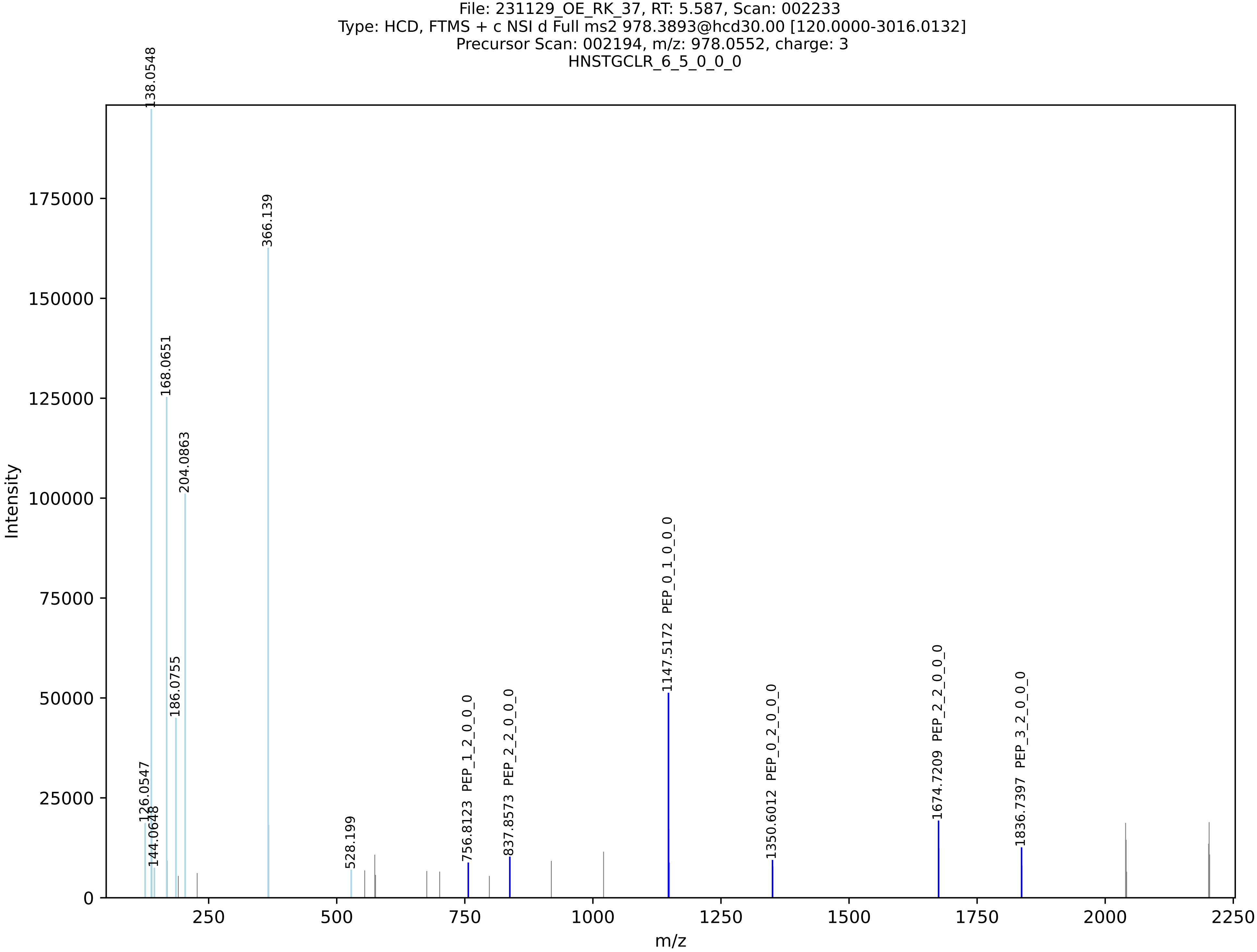
File: 231129_OE_RK_37, RT: 5.587, Scan: 002233
Type: HCD, FTMS + c NSI d Full ms2 978.3893@hcd30.00 [120.0000-3016.0132]
Precursor Scan: 002194, m/z: 978.0552, charge: 3
HNSTGCLR_6_5_0_0_0
Intensity
m/z
138.0548
366.139
168.0651
204.0863
186.0755
126.0547
144.0648
528.199
756.8123 PEP_1_2_0_0_0
837.8573 PEP_2_2_0_0_0
1147.5172 PEP_0_1_0_0_0
1350.6012 PEP_0_2_0_0_0
1674.7209 PEP_2_2_0_0_0
1836.7397 PEP_3_2_0_0_0
0
25000
50000
75000
100000
125000
150000
175000
250
500
750
1000
1250
1500
1750
2000
2250

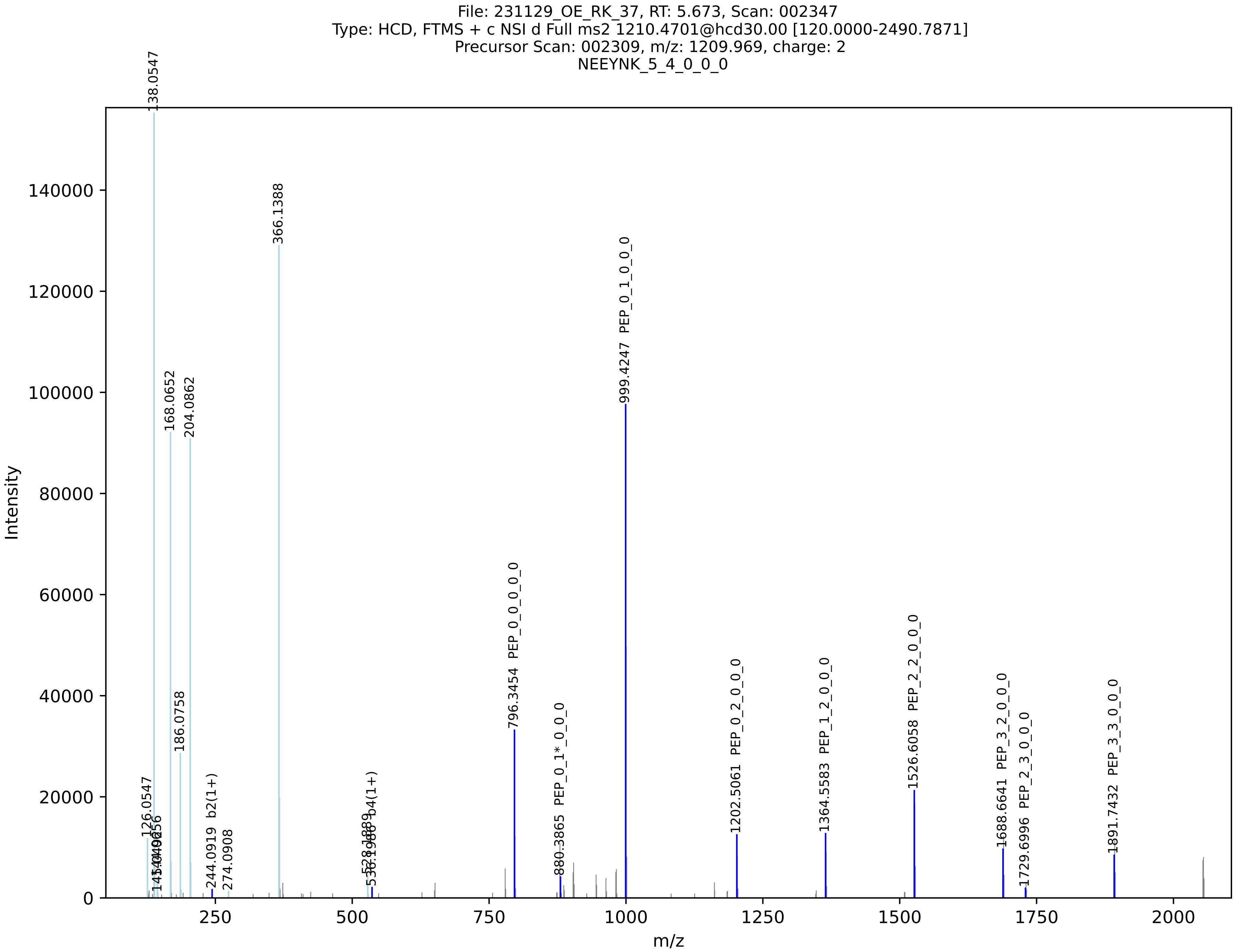
File: 231129_OE_RK_37, RT: 5.673, Scan: 002347
Type: HCD, FTMS + c NSI d Full ms2 1210.4701@hcd30.00 [120.0000-2490.7871]
Precursor Scan: 002309, m/z: 1209.969, charge: 2
NEEYNK_5_4_0_0_0
Intensity
m/z
0
20000
40000
60000
80000
100000
120000
140000
250
500
750
1000
1250
1500
1750
2000
138.0547
366.1388
168.0652
204.0862
186.0758
126.0547
244.0919 b2(1+)
274.0908
b4(1+)
796.3454 PEP_0_0_0_0_0_
880.3865 PEP_0_1*_0_0_0
999.4247 PEP_0_1_0_0_0
1202.5061 PEP_0_2_0_0_0
1364.5583 PEP_1_2_0_0_0
1526.6058 PEP_2_2_0_0_0
1688.6641 PEP_3_2_0_0_0
1729.6996 PEP_2_3_0_0_0
1891.7432 PEP_3_3_0_0_0

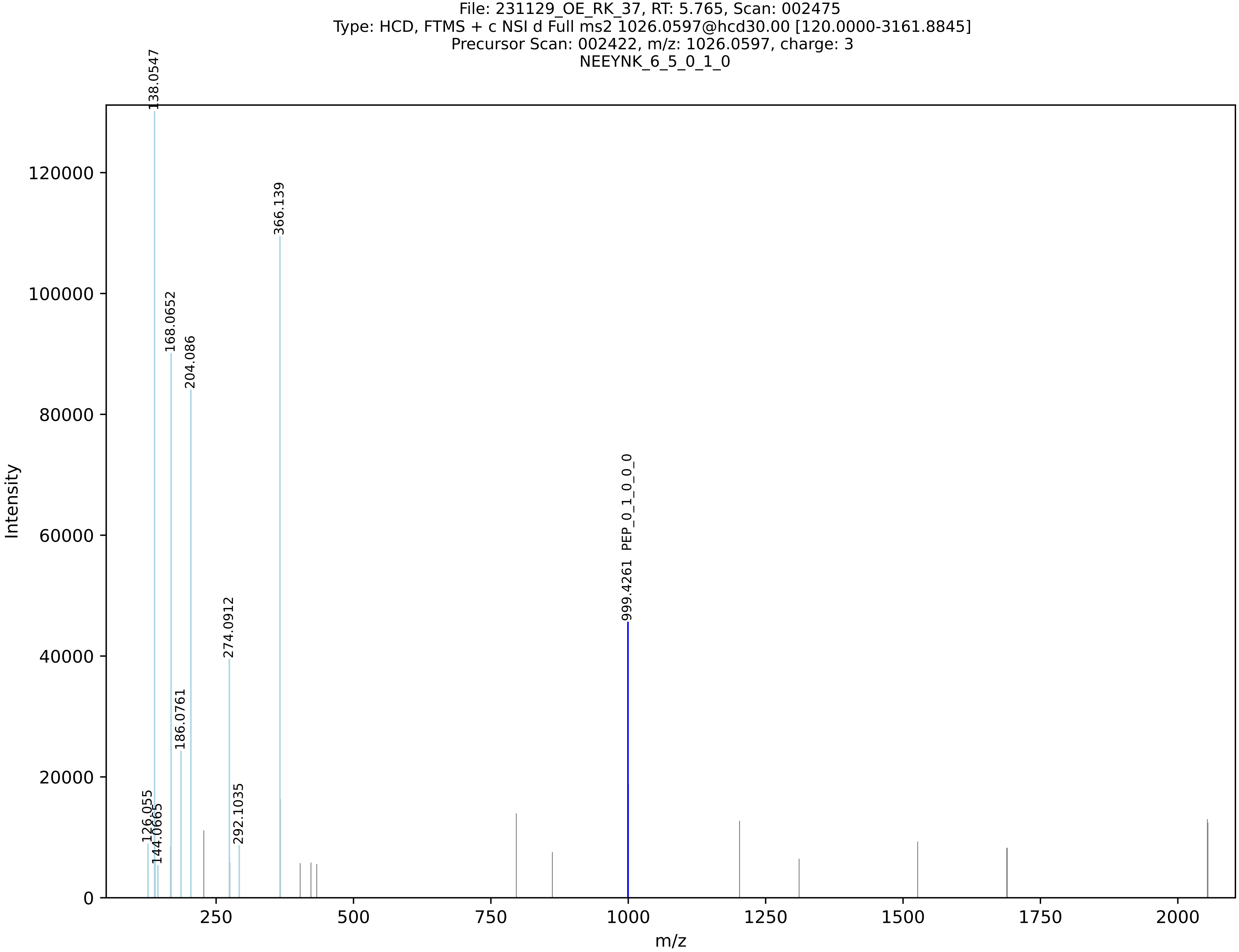
File: 231129_OE_RK_37, RT: 5.765, Scan: 002475
Type: HCD, FTMS + c NSI d Full ms2 1026.0597@hcd30.00 [120.0000-3161.8845]
Precursor Scan: 002422, m/z: 1026.0597, charge: 3
NEEYNK_6_5_0_1_0
138.0547
366.139
168.0652
204.086
999.4261 PEP_0_1_0_0_0
274.0912
186.0761
126.055
144.0665
292.1035
Intensity
m/z
0
20000
40000
60000
80000
100000
120000
250
500
750
1000
1250
1500
1750
2000

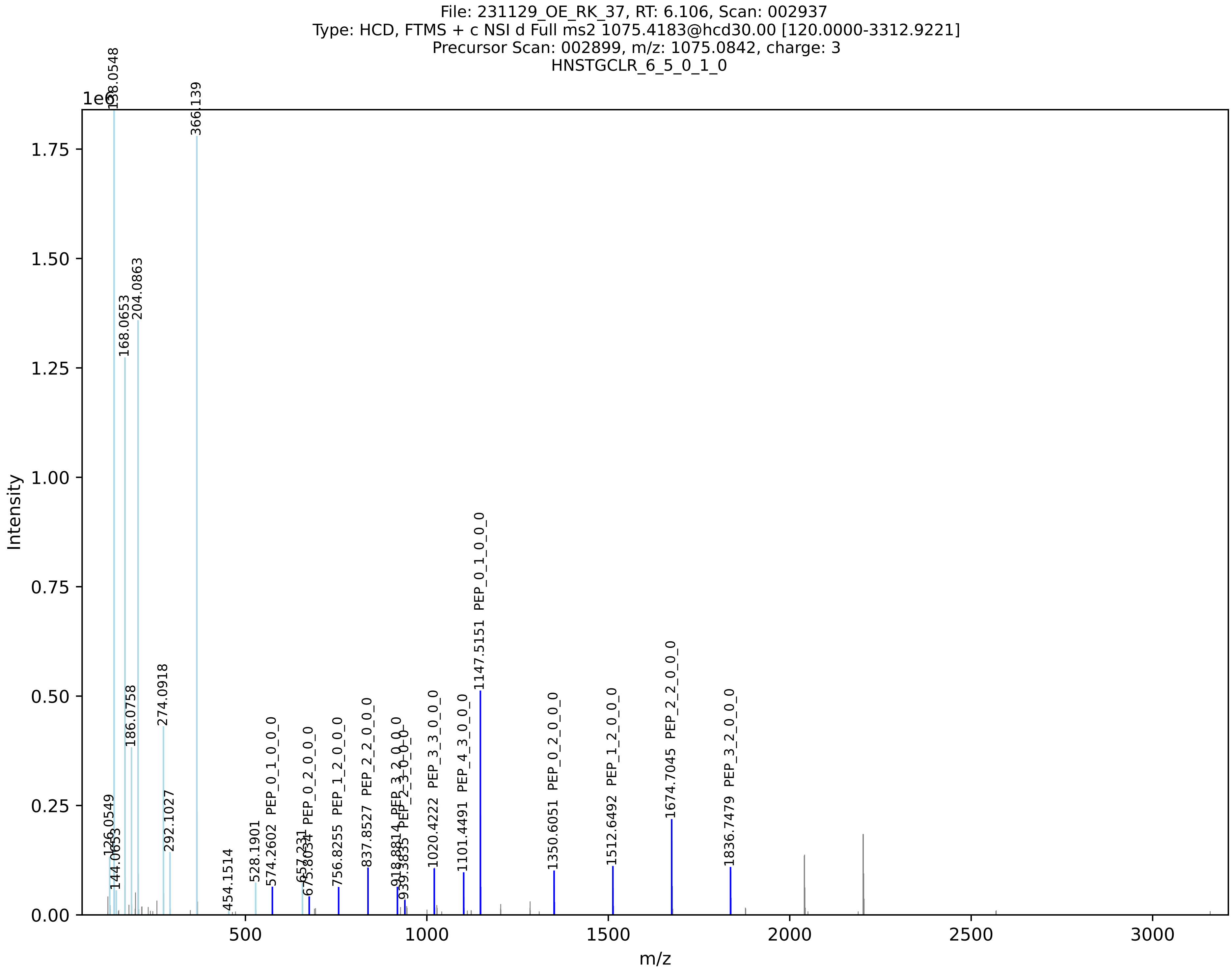

File: 231129_OE_RK_37, RT: 6.106, Scan: 002937
Type: HCD, FTMS + c NSI d Full ms2 1075.4183@hcd30.00 [120.0000-3312.9221]
Precursor Scan: 002899, m/z: 1075.0842, charge: 3
HNSTGCLR_6_5_0_1_0
1e6
Intensity
m/z
366.139
204.0863
168.0653
186.0758
274.0918
292.1027
126.0549
144.0653
454.1514
528.1901
574.2602 PEP_0_1_0_0_0
657.231
675.8034 PEP_0_2_0_0_0
756.8255 PEP_1_2_0_0_0
837.8527 PEP_2_2_0_0_0
918.8814 PEP_3_2_0_0_0
939.3835 PEP_2_3_0_0_0
1020.4222 PEP_3_3_0_0_0
1101.4491 PEP_4_3_0_0_0
1147.5151 PEP_0_1_0_0_0
1350.6051 PEP_0_2_0_0_0
1512.6492 PEP_1_2_0_0_0
1674.7045 PEP_2_2_0_0_0
1836.7479 PEP_3_2_0_0_0

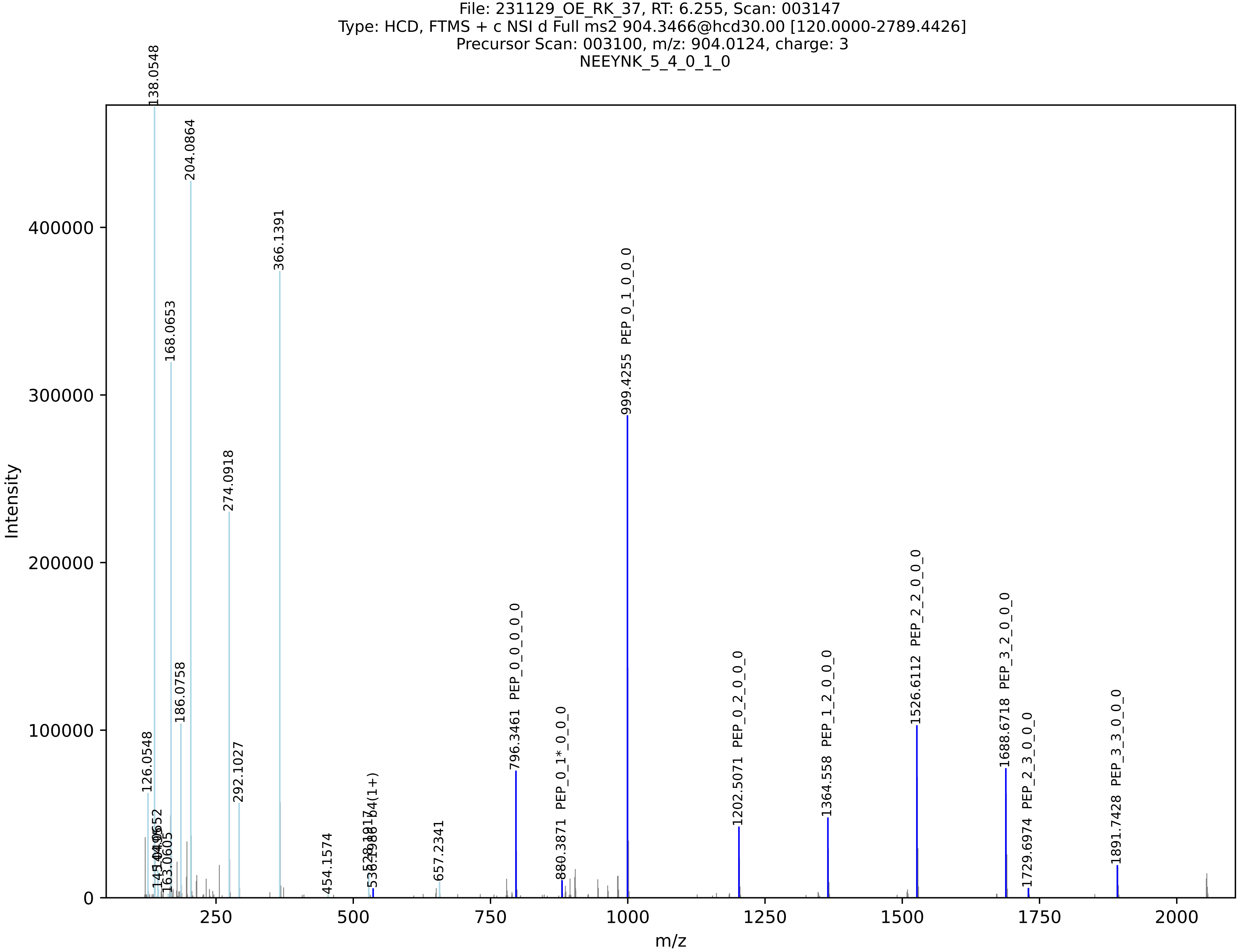
File: 231129_OE_RK_37, RT: 6.255, Scan: 003147
Type: HCD, FTMS + c NSI d Full ms2 904.3466@hcd30.00 [120.0000-2789.4426]
Precursor Scan: 003100, m/z: 904.0124, charge: 3
NEEYNK_5_4_0_1_0
Intensity
m/z
0
100000
200000
300000
400000
250
500
750
1000
1250
1500
1750
2000
138.0548
204.0864
366.1391
168.0653
274.0918
186.0758
126.0548
292.1027
163.0605
454.1574
657.2341
796.3461 PEP_0_0_0_0_0
880.3871 PEP_0_1*_0_0_0
999.4255 PEP_0_1_0_0_0
1202.5071 PEP_0_2_0_0_0
1364.558 PEP_1_2_0_0_0
1526.6112 PEP_2_2_0_0_0
1688.6718 PEP_3_2_0_0_0
1729.6974 PEP_2_3_0_0_0
1891.7428 PEP_3_3_0_0_0

File: 231129_OE_RK_37, RT: 6.294, Scan: 003201
Type: HCD, FTMS + c NSI d Full ms2 1356.0160@hcd30.00 [120.0000-2787.7004]
Precursor Scan: 003146, m/z: 1355.5154, charge: 2
NEEYNK_5_4_0_1_0

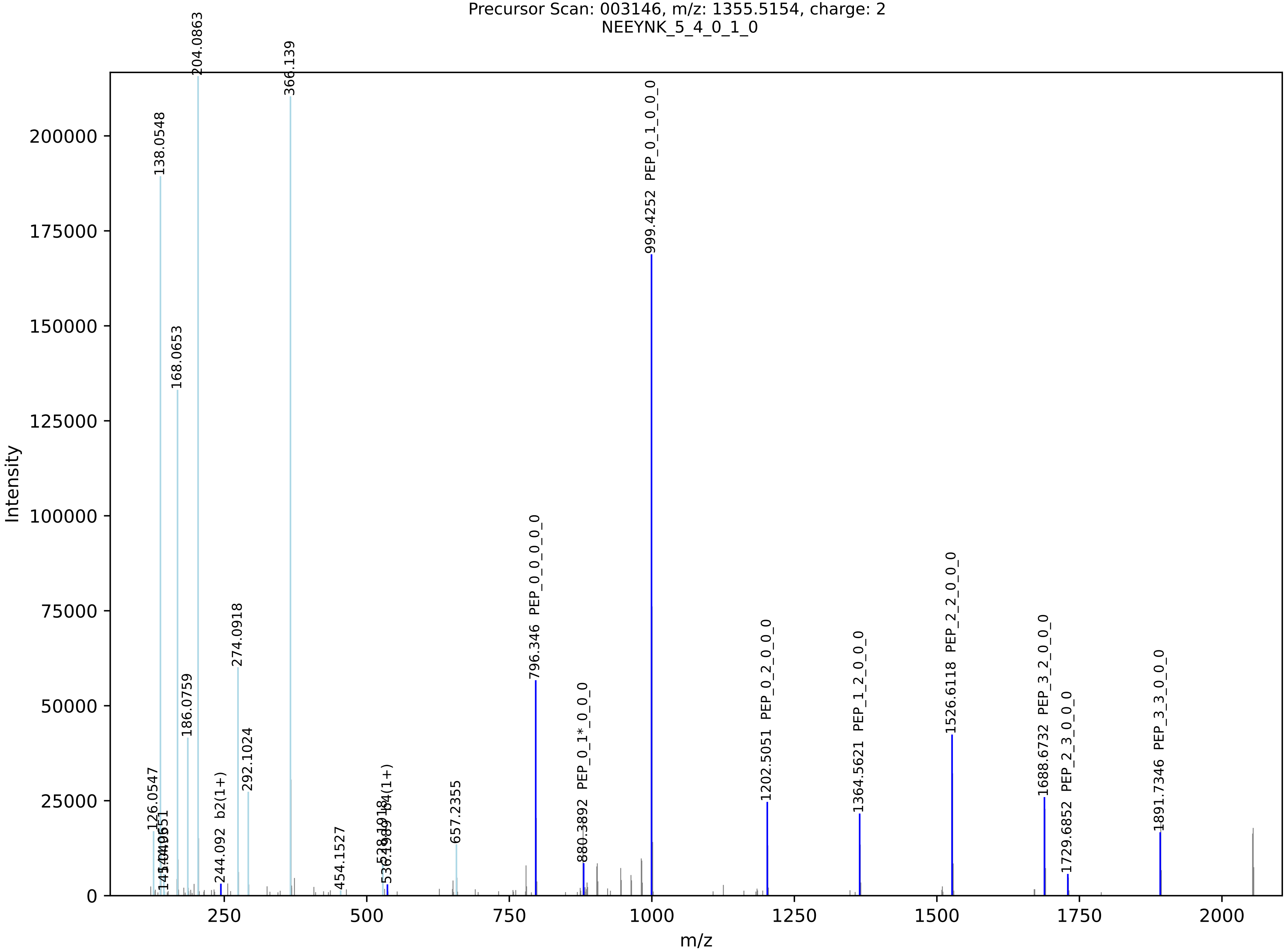

File: 231129_OE_RK_37, RT: 6.295, Scan: 003202
Type: HCD, FTMS + c NSI d Full ms2 1075.4193@hcd30.00 [120.0000-3312.9250]
Precursor Scan: 003146, m/z: 1075.0855, charge: 3
HNSTGCLR_6_5_0_1_0

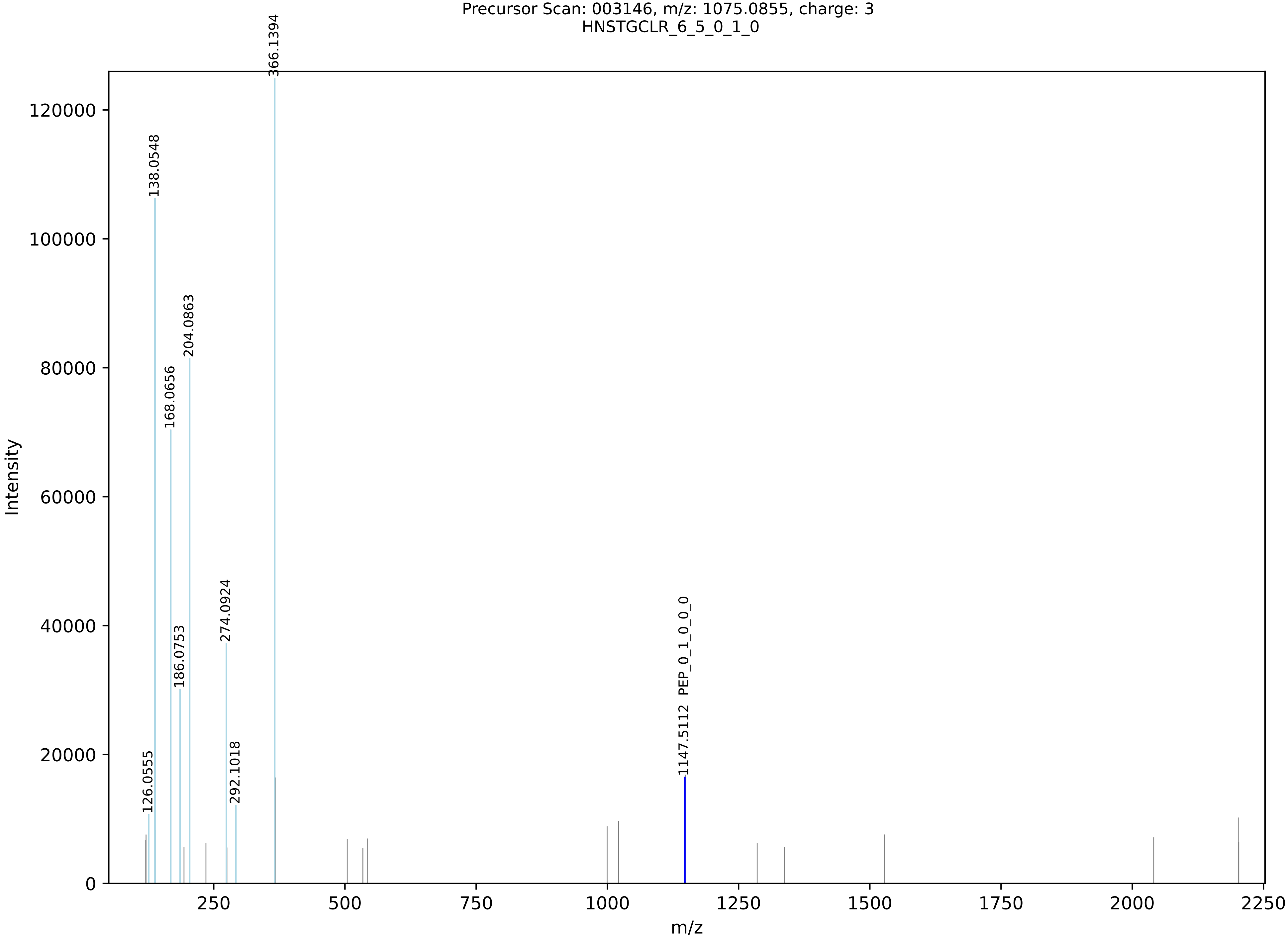

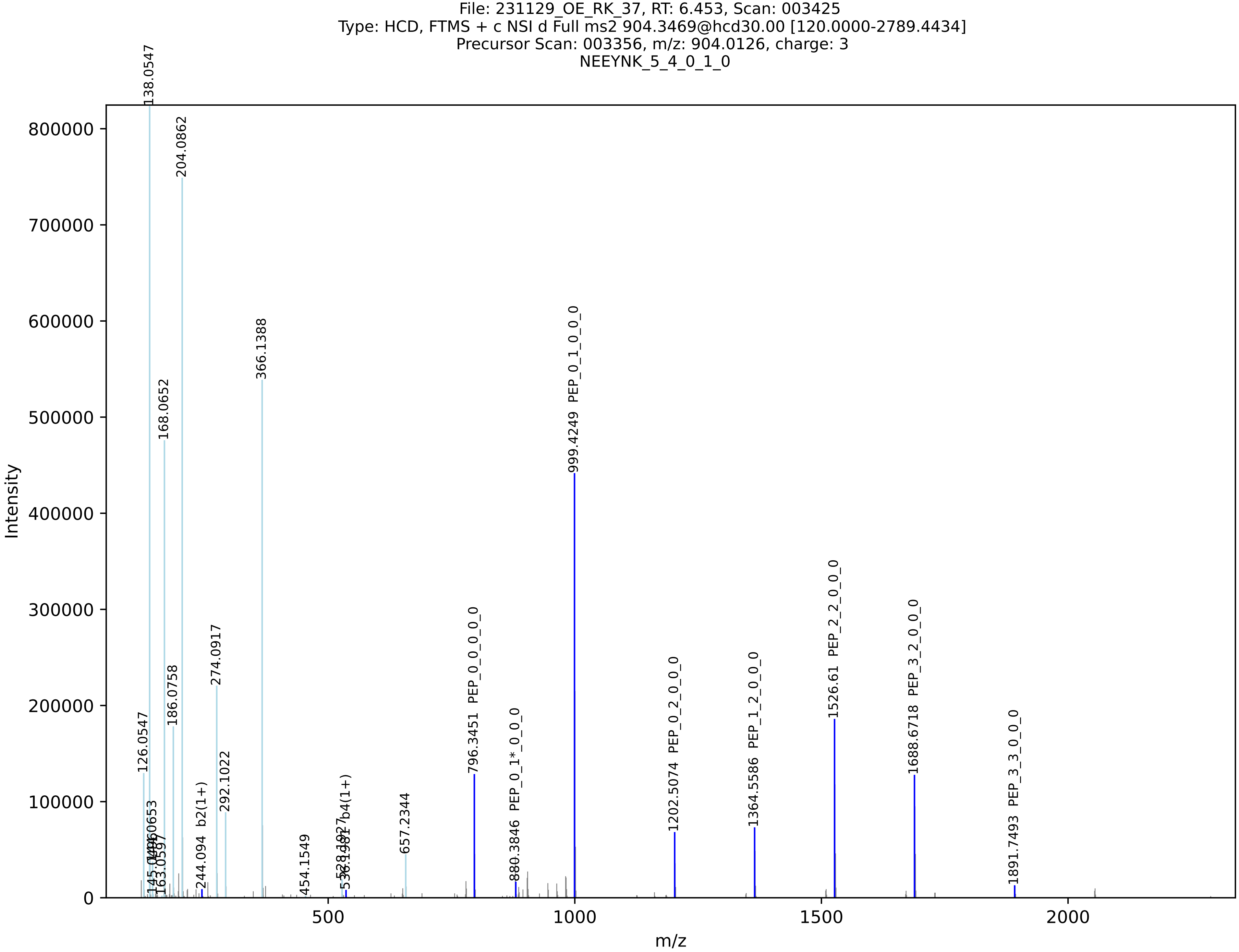

File: 231129_OE_RK_37, RT: 6.453, Scan: 003425
Type: HCD, FTMS + c NSI d Full ms2 904.3469@hcd30.00 [120.0000-2789.4434]
Precursor Scan: 003356, m/z: 904.0126, charge: 3
NEEYNK_5_4_0_1_0
Intensity
m/z
138.0547
204.0862
366.1388
168.0652
999.4249 PEP_0_1_0_0_0
1526.61 PEP_2_2_0_0_0
1688.6718 PEP_3_2_0_0_0
796.3451 PEP_0_0_0_0_0
274.0917
186.0758
126.0547
292.1022
880.3846 PEP_0_1* 0_0_0
1202.5074 PEP_0_2_0_0_0
1364.5586 PEP_1_2_0_0_0
1891.7493 PEP_3_3_0_0_0
244.094 b2(1+)
657.2344
454.1549
b4(1+)

File: 231129_OE_RK_37, RT: 6.499, Scan: 003492
Type: HCD, FTMS + c NSI d Full ms2 1128.4192@hcd30.00 [120.0000-3475.1050]
Precursor Scan: 003424, m/z: 1127.7515, charge: 3
HNFSHCCSK_5_4_0_2_0

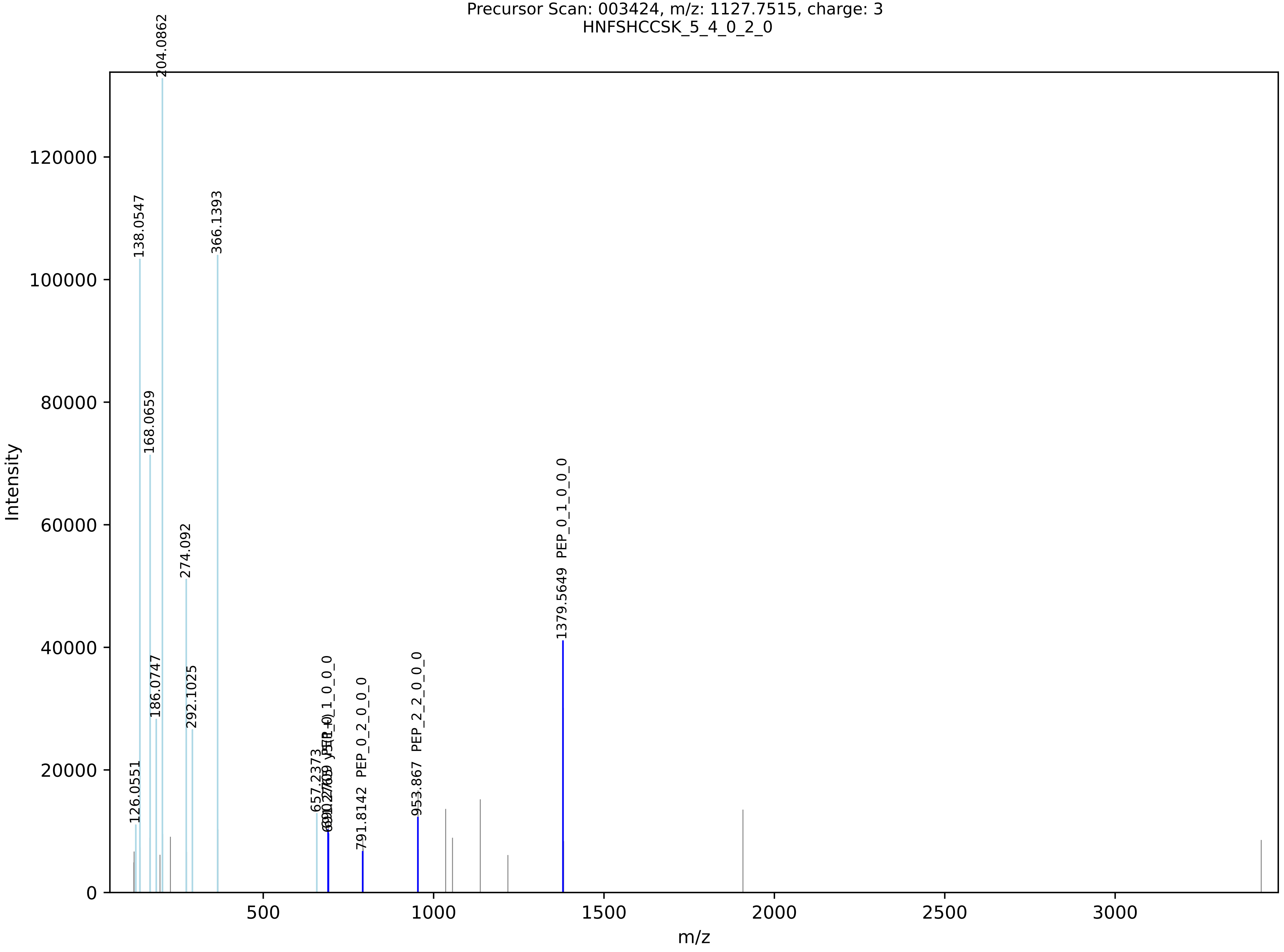

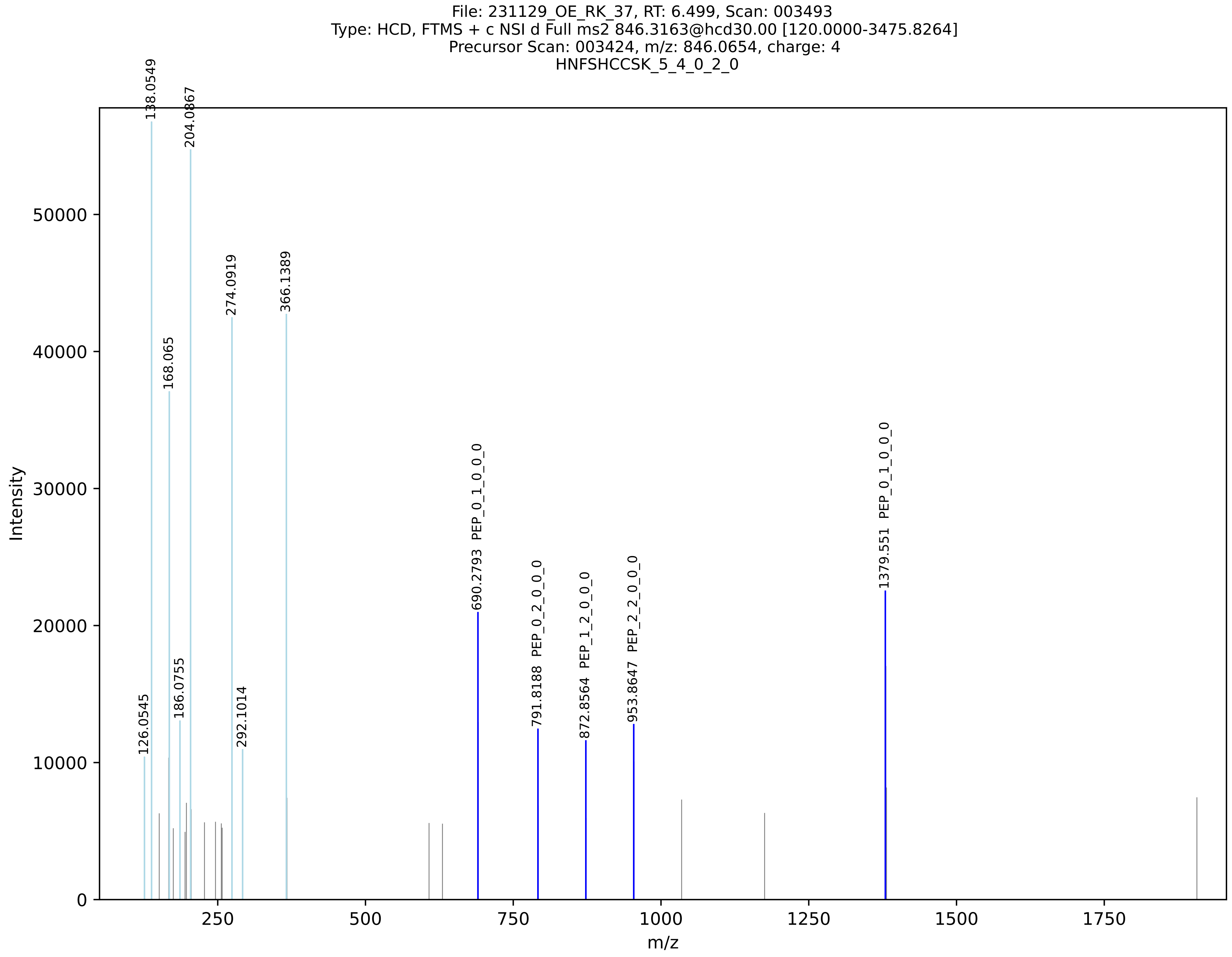
File: 231129_OE_RK_37, RT: 6.499, Scan: 003493
Type: HCD, FTMS + c NSI d Full ms2 846.3163@hcd30.00 [120.0000-3475.8264]
Precursor Scan: 003424, m/z: 846.0654, charge: 4
HNFSHCCSK_5_4_0_2_0
Intensity
m/z
138.0549
204.0867
168.065
274.0919
366.1389
126.0545
186.0755
292.1014
690.2793 PEP_0_1_0_0_0
791.8188 PEP_0_2_0_0_0
872.8564 PEP_1_2_0_0_0
953.8647 PEP_2_2_0_0_0
1379.551 PEP_0_1_0_0_0
0
10000
20000
30000
40000
50000
250
500
750
1000
1250
1500
1750

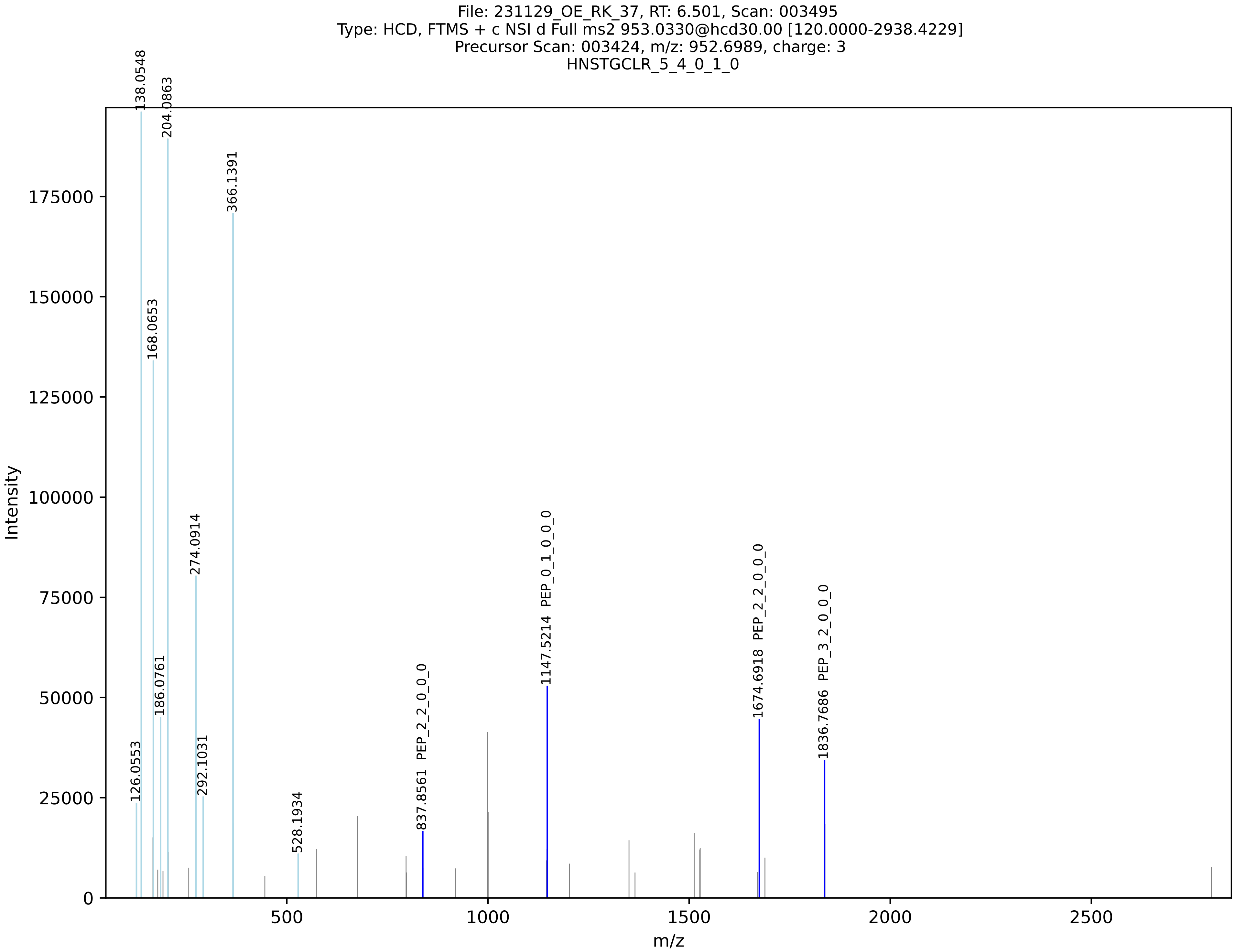

File: 231129_OE_RK_37, RT: 6.501, Scan: 003495
Type: HCD, FTMS + c NSI d Full ms2 953.0330@hcd30.00 [120.0000-2938.4229]
Precursor Scan: 003424, m/z: 952.6989, charge: 3
HNSTGCLR_5_4_0_1_0
Intensity
m/z
0
25000
50000
75000
100000
125000
150000
175000
500
1000
1500
2000
2500
138.0548
204.0863
366.1391
168.0653
274.0914
186.0761
126.0553
292.1031
528.1934
837.8561 PEP_2_2_0_0_0
1147.5214 PEP_0_1_0_0_0
1674.6918 PEP_2_2_0_0_0
1836.7686 PEP_3_2_0_0_0

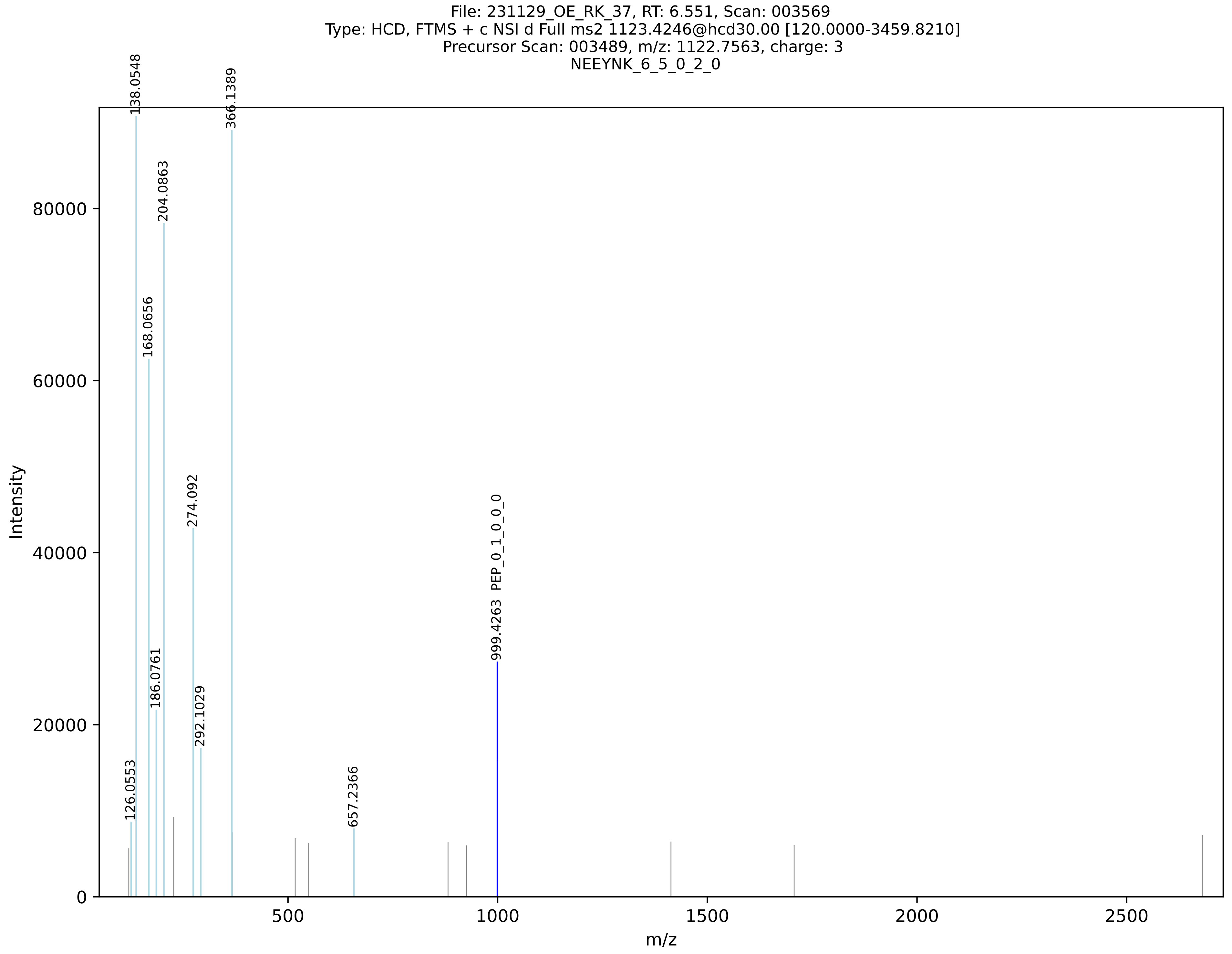
File: 231129_OE_RK_37, RT: 6.551, Scan: 003569
Type: HCD, FTMS + c NSI d Full ms2 1123.4246@hcd30.00 [120.0000-3459.8210]
Precursor Scan: 003489, m/z: 1122.7563, charge: 3
NEEYNK_6_5_0_2_0
138.0548
366.1389
204.0863
168.0656
274.092
186.0761
292.1029
126.0553
657.2366
999.4263 PEP_0_1_0_0_0
Intensity
m/z
0
20000
40000
60000
80000
500
1000
1500
2000
2500

File: 231129_OE_RK_37, RT: 6.556, Scan: 003576
Type: HCD, FTMS + c NSI d Full ms2 1429.0465@hcd30.00 [120.0000-2936.6831]
Precursor Scan: 003489, m/z: 1428.5454, charge: 2
NEEYNK_5_4_1_1_0

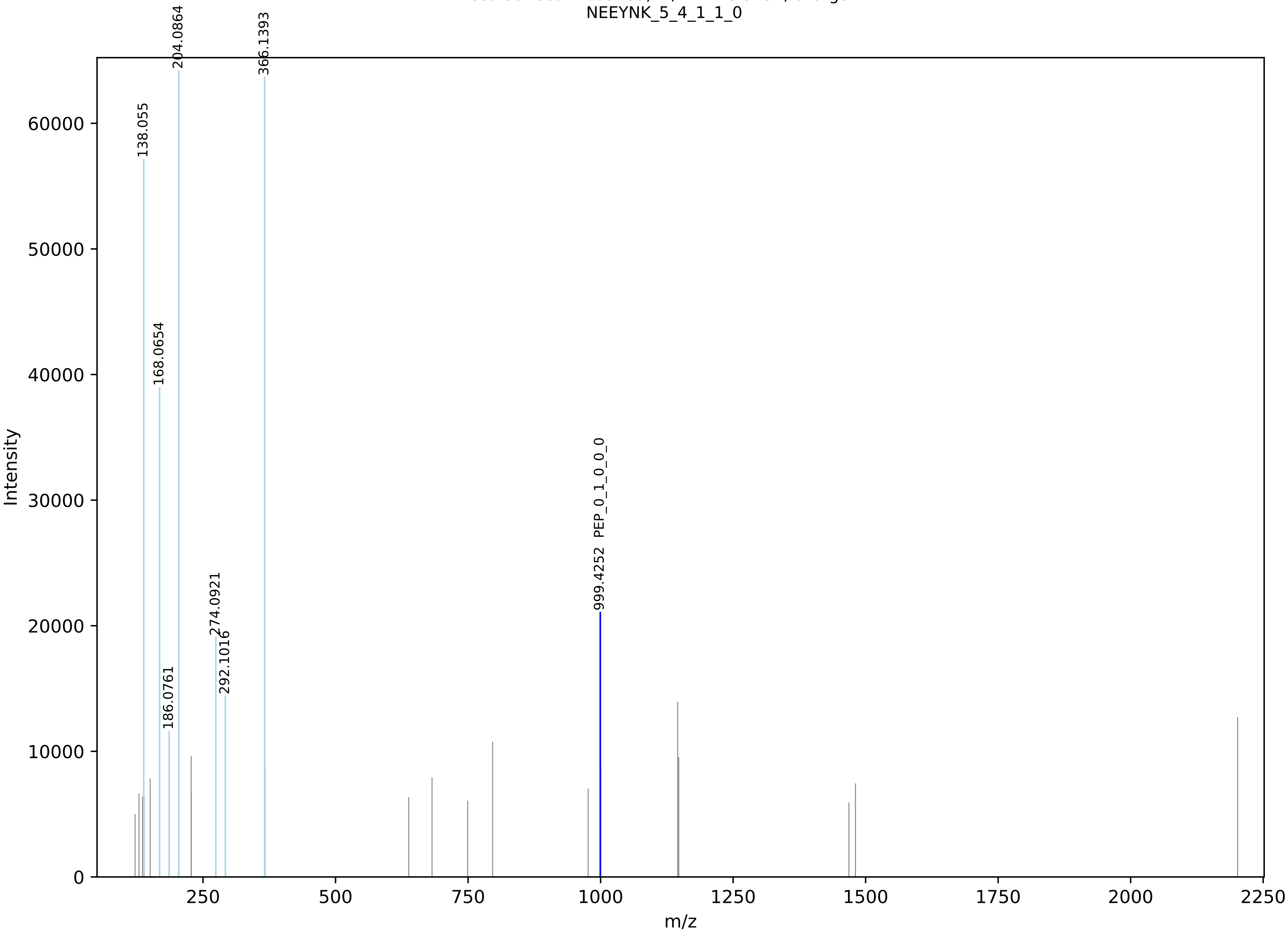

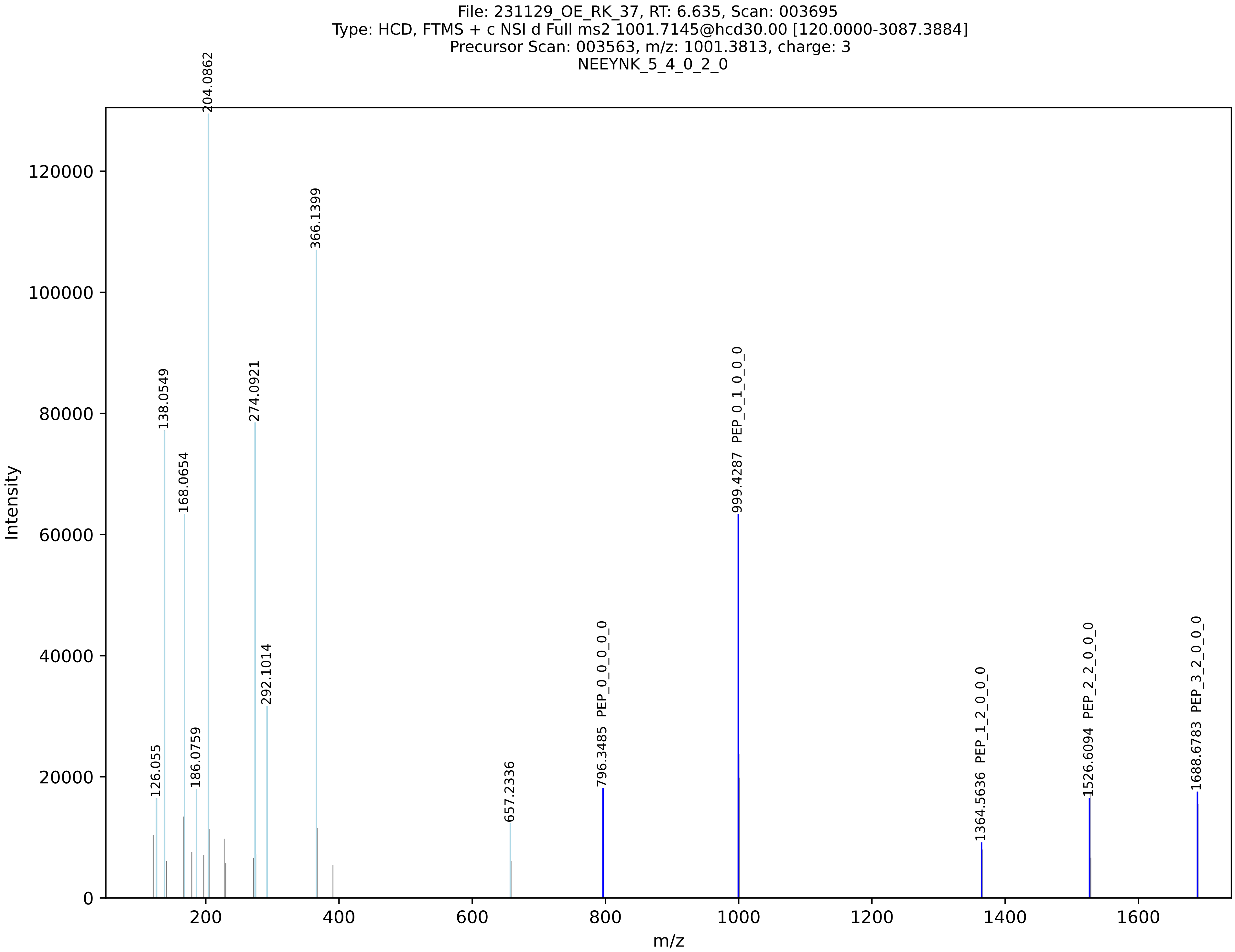
File: 231129_OE_RK_37, RT: 6.635, Scan: 003695
Type: HCD, FTMS + c NSI d Full ms2 1001.7145@hcd30.00 [120.0000-3087.3884]
Precursor Scan: 003563, m/z: 1001.3813, charge: 3
NEEYNK_5_4_0_2_0
Intensity
m/z
204.0862
366.1399
138.0549
274.0921
168.0654
292.1014
126.055
186.0759
657.2336
796.3485 PEP_0_0_0_0_0
999.4287 PEP_0_1_0_0_0
1364.5636 PEP_1_2_0_0_0
1526.6094 PEP_2_2_0_0_0
1688.6783 PEP_3_2_0_0_0
0
20000
40000
60000
80000
100000
120000
200
400
600
800
1000
1200
1400
1600

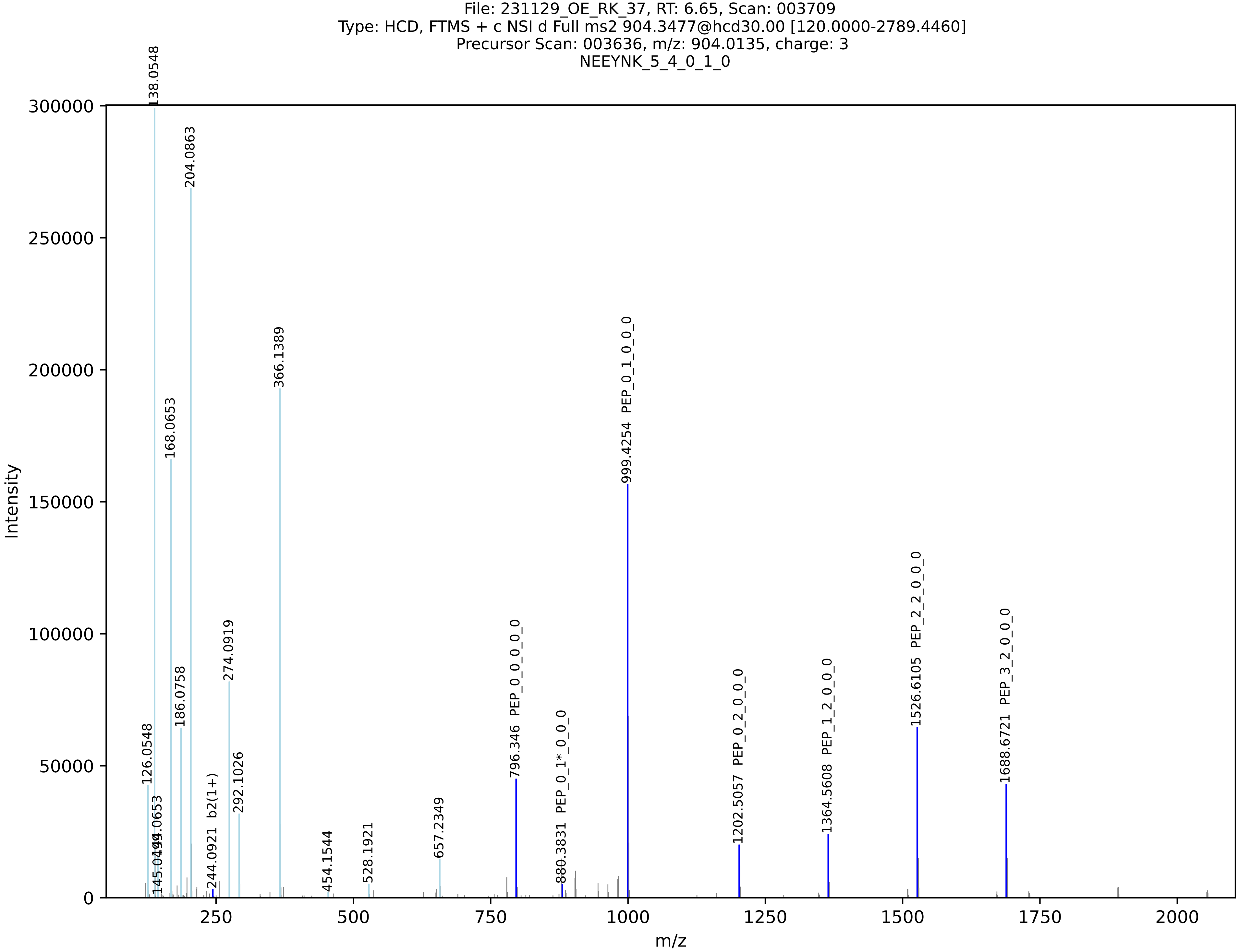

File: 231129_OE_RK_37, RT: 6.65, Scan: 003709
Type: HCD, FTMS + c NSI d Full ms2 904.3477@hcd30.00 [120.0000-2789.4460]
Precursor Scan: 003636, m/z: 904.0135, charge: 3
NEEYNK_5_4_0_1_0
Intensity
m/z
138.0548
204.0863
366.1389
168.0653
186.0758
126.0548
145.0653
244.0921 b2(1+)
274.0919
292.1026
454.1544
528.1921
657.2349
796.346 PEP_0_0_0_0_0
880.3831 PEP_0_1*_0_0_0
999.4254 PEP_0_1_0_0_0
1202.5057 PEP_0_2_0_0_0
1364.5608 PEP_1_2_0_0_0
1526.6105 PEP_2_2_0_0_0
1688.6721 PEP_3_2_0_0_0

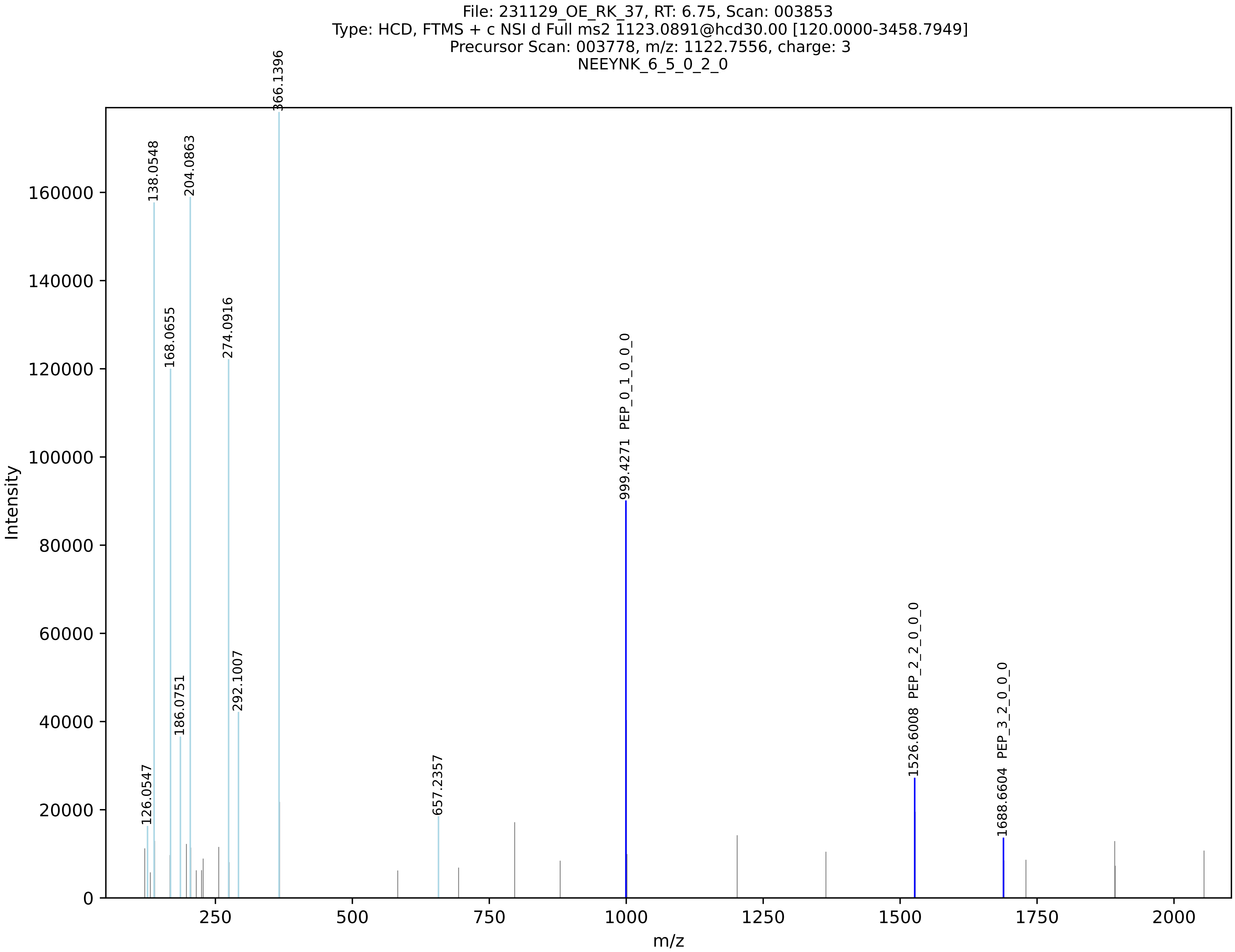
File: 231129_OE_RK_37, RT: 6.75, Scan: 003853
Type: HCD, FTMS + c NSI d Full ms2 1123.0891@hcd30.00 [120.0000-3458.7949]
Precursor Scan: 003778, m/z: 1122.7556, charge: 3
NEEYNK_6_5_0_2_0
366.1396
138.0548
204.0863
168.0655
274.0916
999.4271 PEP_0_1_0_0_0
1526.6008 PEP_2_2_0_0_0
1688.6604 PEP_3_2_0_0_0
292.1007
186.0751
657.2357
126.0547
Intensity
m/z
0
20000
40000
60000
80000
100000
120000
140000
160000
250
500
750
1000
1250
1500
1750
2000

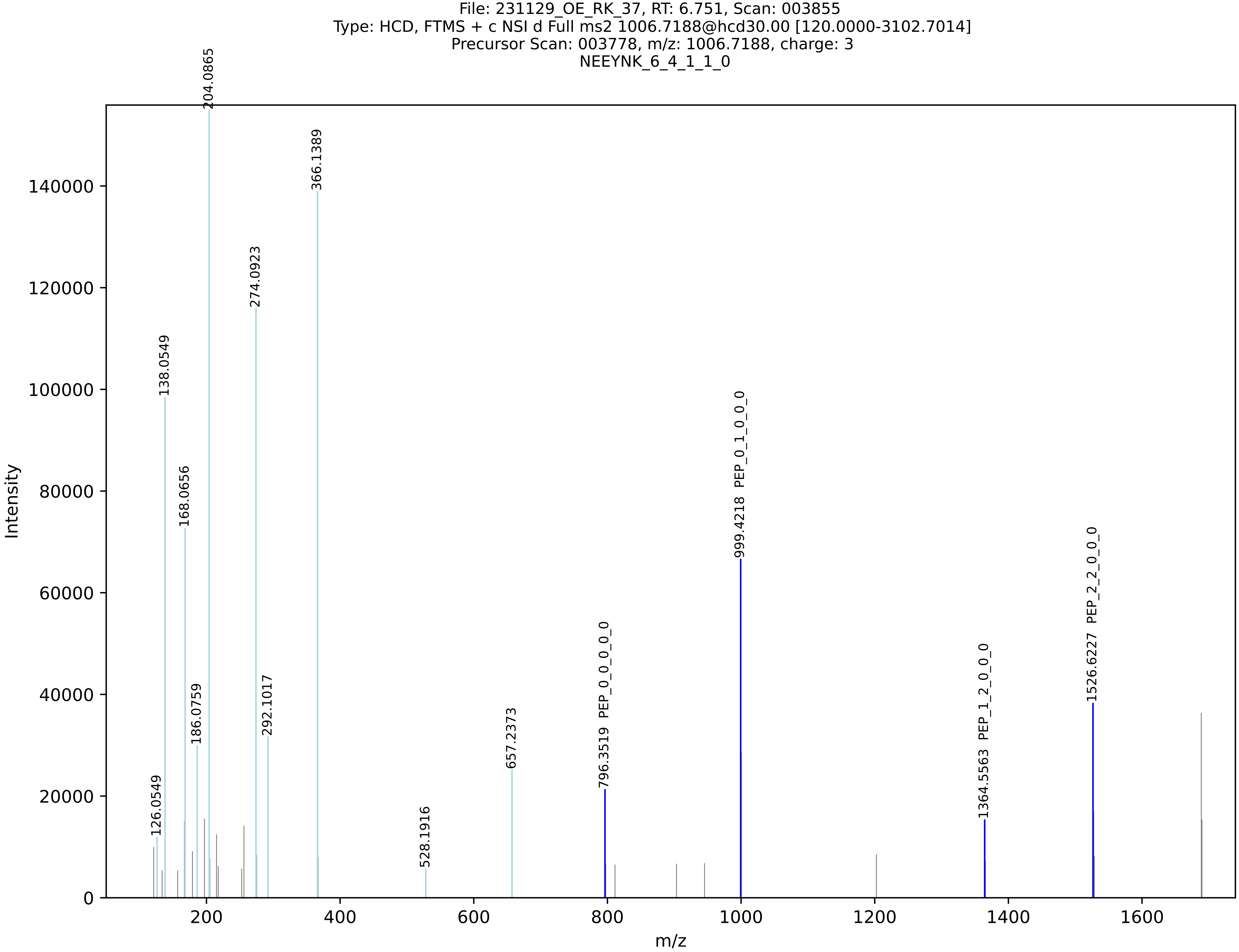
File: 231129_OE_RK_37, RT: 6.751, Scan: 003855
Type: HCD, FTMS + c NSI d Full ms2 1006.7188@hcd30.00 [120.0000-3102.7014]
Precursor Scan: 003778, m/z: 1006.7188, charge: 3
NEEYNK_6_4_1_1_0
Intensity
m/z
0
20000
40000
60000
80000
100000
120000
140000
200
400
600
800
1000
1200
1400
1600
204.0865
366.1389
274.0923
138.0549
168.0656
186.0759
292.1017
126.0549
528.1916
657.2373
796.3519 PEP_0_0_0_0_0
999.4218 PEP_0_1_0_0_0
1364.5563 PEP_1_2_0_0_0
1526.6227 PEP_2_2_0_0_0

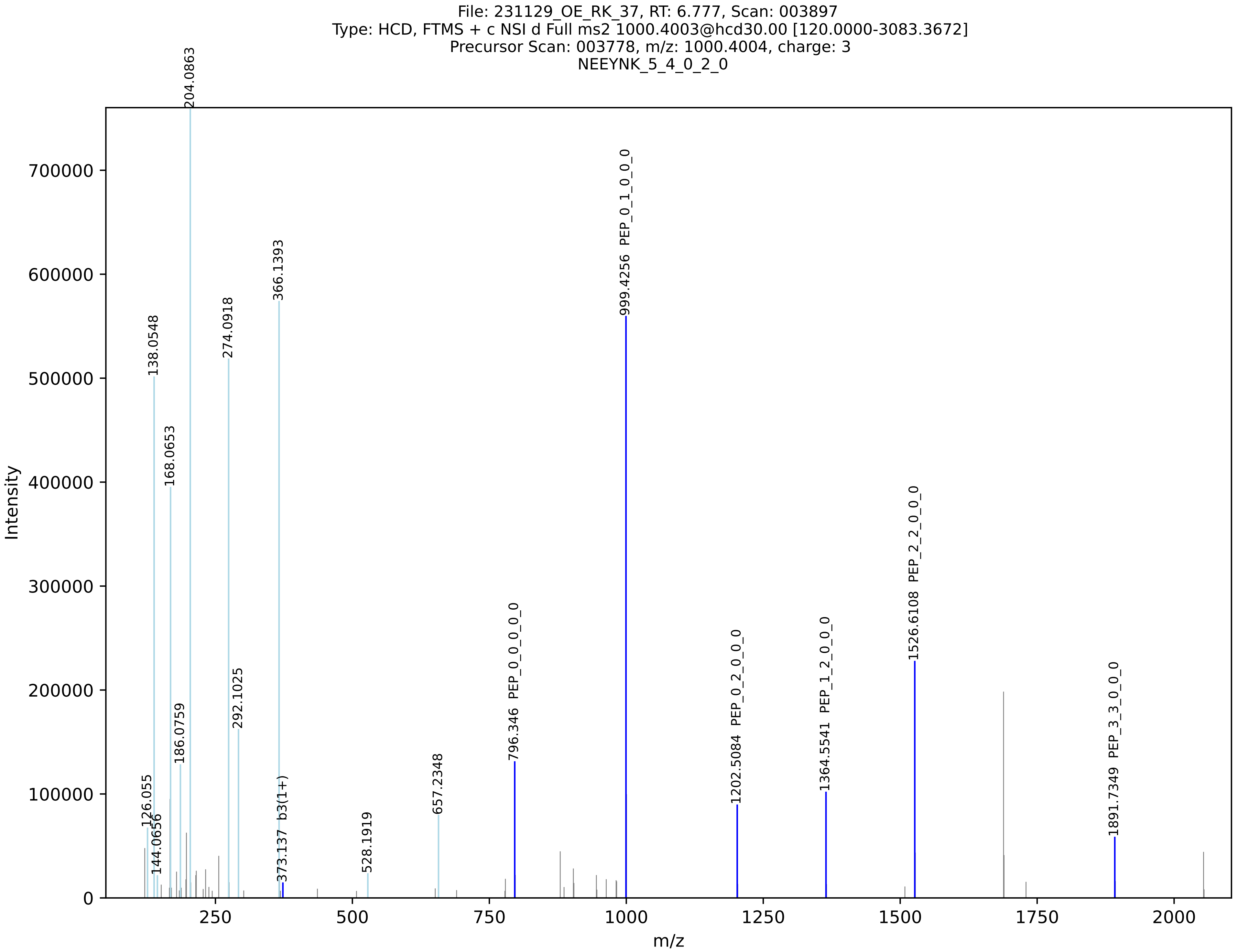

File: 231129_OE_RK_37, RT: 6.777, Scan: 003897
Type: HCD, FTMS + c NSI d Full ms2 1000.4003@hcd30.00 [120.0000-3083.3672]
Precursor Scan: 003778, m/z: 1000.4004, charge: 3
NEEYNK_5_4_0_2_0
Intensity
m/z
204.0863
138.0548
168.0653
274.0918
366.1393
999.4256 PEP_0_1_0_0_0
186.0759
292.1025
126.055
144.0656
373.137 b3(1+)
528.1919
657.2348
796.346 PEP_0_0_0_0_0
1202.5084 PEP_0_2_0_0_0
1364.5541 PEP_1_2_0_0_0
1526.6108 PEP_2_2_0_0_0
1891.7349 PEP_3_3_0_0_0

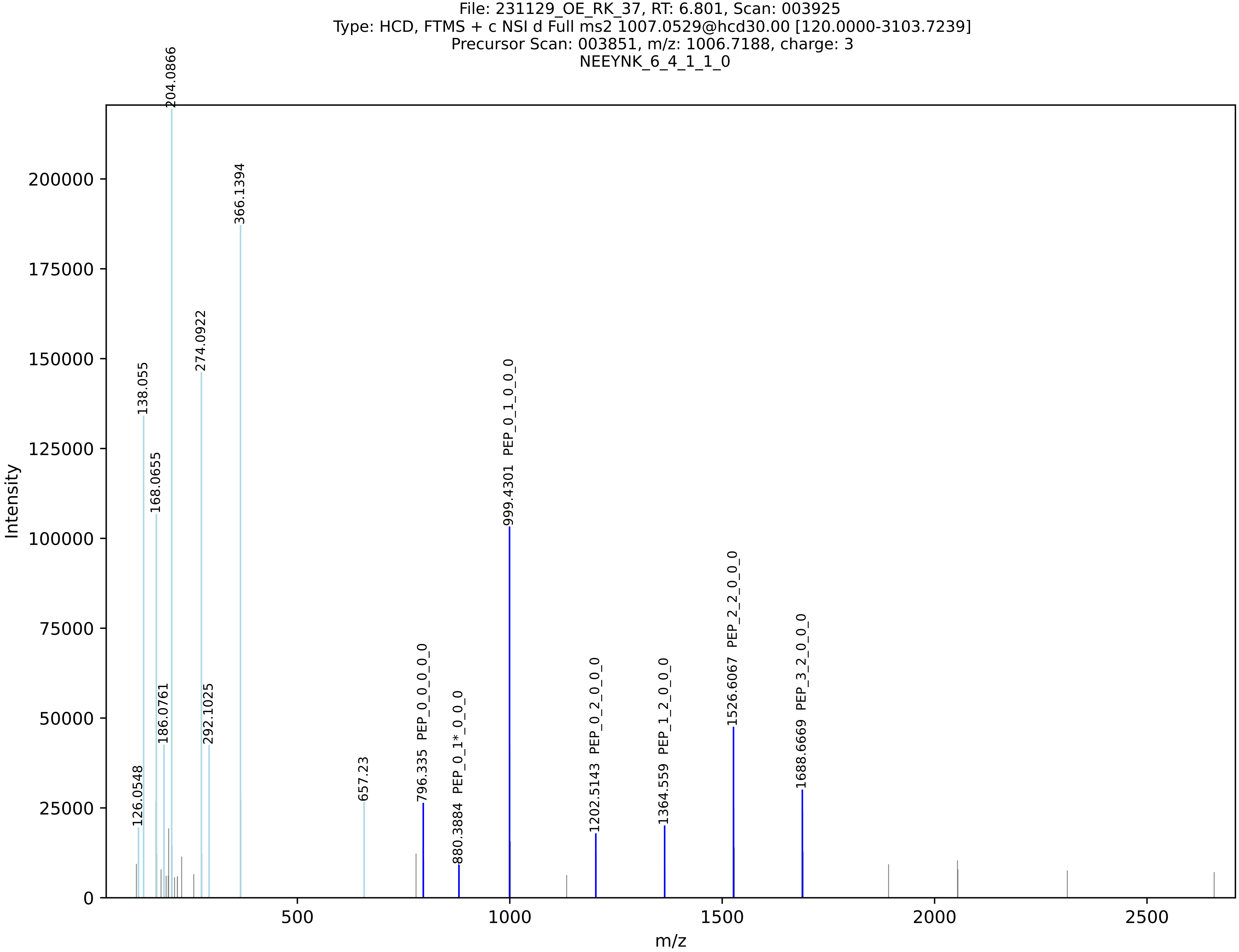
File: 231129_OE_RK_37, RT: 6.801, Scan: 003925
Type: HCD, FTMS + c NSI d Full ms2 1007.0529@hcd30.00 [120.0000-3103.7239]
Precursor Scan: 003851, m/z: 1006.7188, charge: 3
NEEYNK_6_4_1_1_0
Intensity
m/z
204.0866
366.1394
274.0922
138.055
168.0655
186.0761
292.1025
126.0548
657.23
796.335 PEP_0_0_0_0_0
880.3884 PEP_0_1*_0_0_0
999.4301 PEP_0_1_0_0_0
1202.5143 PEP_0_2_0_0_0
1364.559 PEP_1_2_0_0_0
1526.6067 PEP_2_2_0_0_0
1688.6669 PEP_3_2_0_0_0

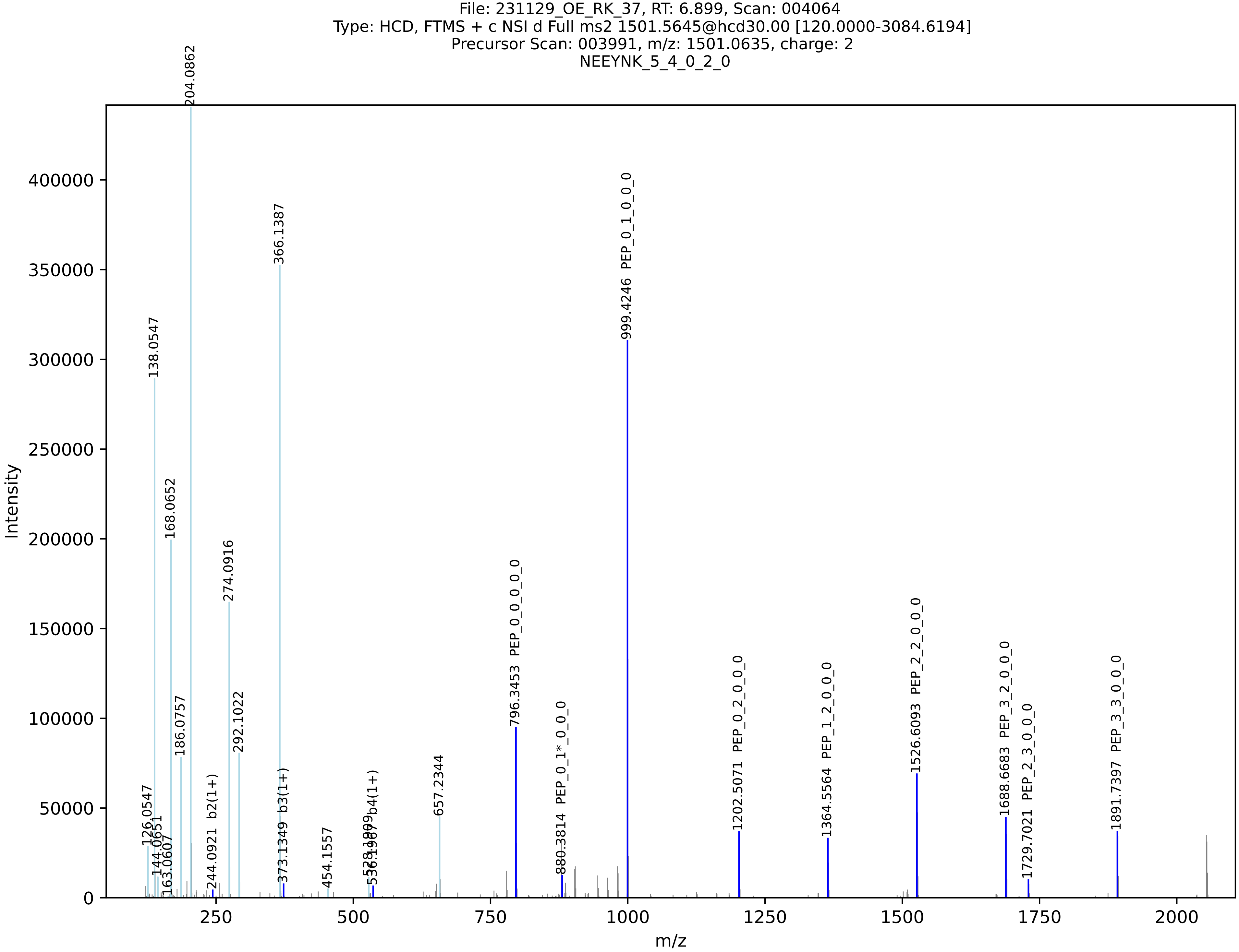
File: 231129_OE_RK_37, RT: 6.899, Scan: 004064
Type: HCD, FTMS + c NSI d Full ms2 1501.5645@hcd30.00 [120.0000-3084.6194]
Precursor Scan: 003991, m/z: 1501.0635, charge: 2
NEEYNK_5_4_0_2_0
Intensity
m/z
204.0862
366.1387
999.4246 PEP_0_1_0_0_0
138.0547
168.0652
274.0916
796.3453 PEP_0_0_0_0_0
1526.6093 PEP_2_2_0_0_0
1688.6683 PEP_3_2_0_0_0
1891.7397 PEP_3_3_0_0_0
1202.5071 PEP_0_2_0_0_0
1364.5564 PEP_1_2_0_0_0
880.3814 PEP_0_1*_0_0_0
1729.7021 PEP_2_3_0_0_0
292.1022
186.0757
657.2344
126.0547
144.0651
163.0607
244.0921 b2(1+)
373.1349 b3(1+)
b4(1+)
454.1557
528.1909

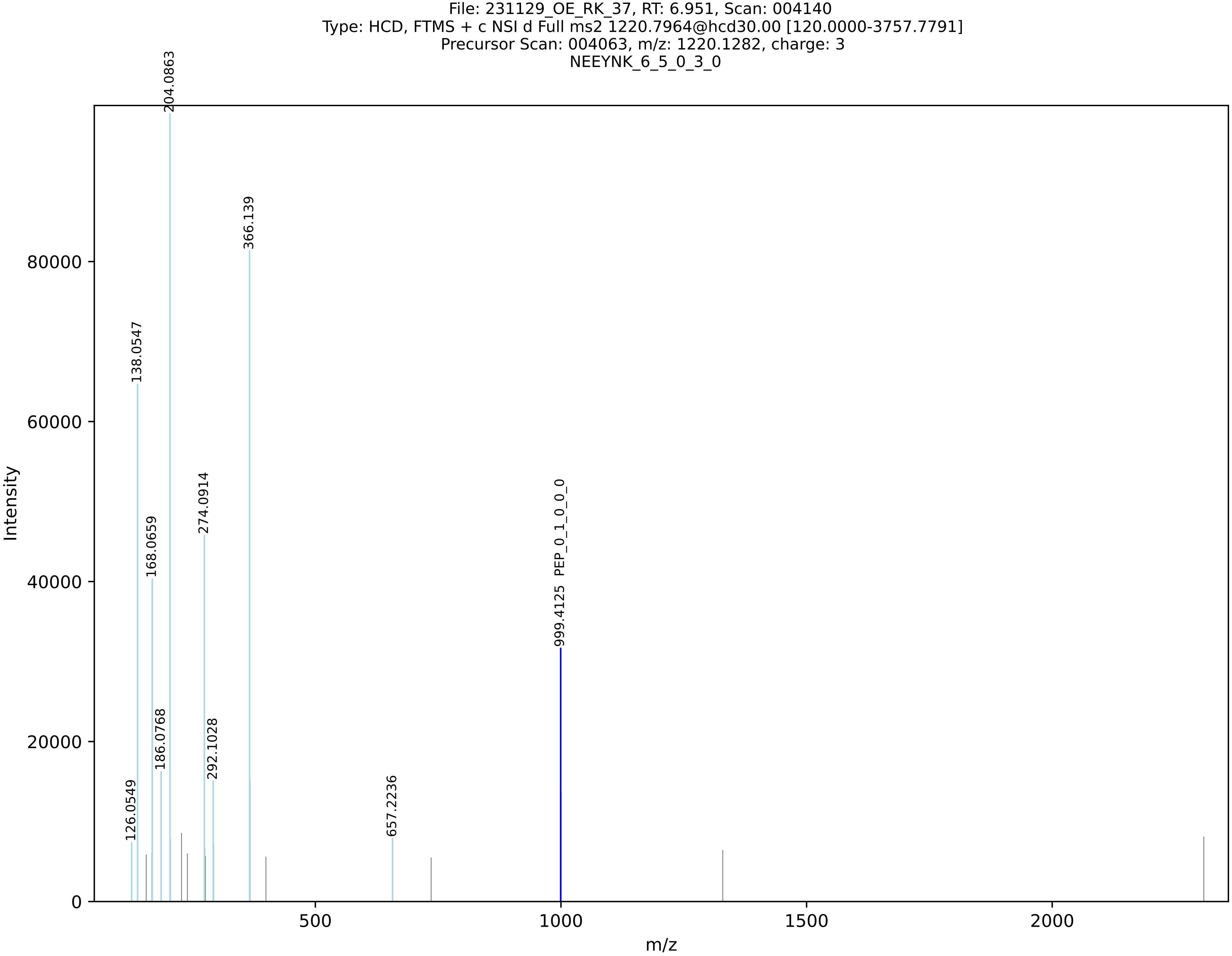

File: 231129_OE_RK_37, RT: 6.951, Scan: 004140
Type: HCD, FTMS + c NSI d Full ms2 1220.7964@hcd30.00 [120.0000-3757.7791]
Precursor Scan: 004063, m/z: 1220.1282, charge: 3
NEEYNK_6_5_0_3_0
204.0863
366.139
138.0547
274.0914
168.0659
999.4125 PEP_0_1_0_0_0
186.0768
292.1028
126.0549
657.2236
Intensity
m/z
0
20000
40000
60000
80000
500
1000
1500
2000

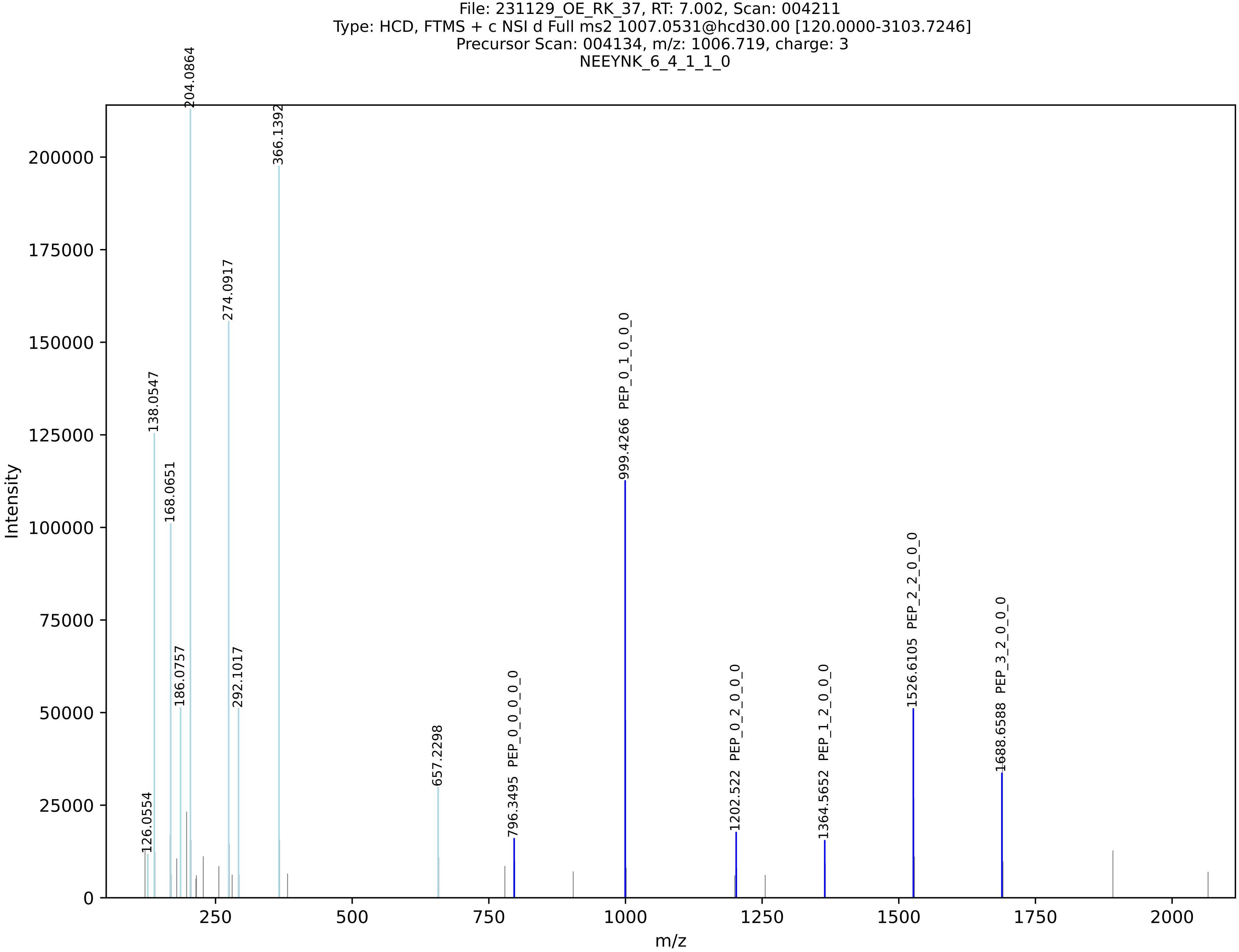
File: 231129_OE_RK_37, RT: 7.002, Scan: 004211
Type: HCD, FTMS + c NSI d Full ms2 1007.0531@hcd30.00 [120.0000-3103.7246]
Precursor Scan: 004134, m/z: 1006.719, charge: 3
NEEYNK_6_4_1_1_0
Intensity
m/z
0
25000
50000
75000
100000
125000
150000
175000
200000
250
500
750
1000
1250
1500
1750
2000
126.0554
138.0547
168.0651
186.0757
204.0864
274.0917
292.1017
366.1392
657.2298
796.3495 PEP_0_0_0_0_0
999.4266 PEP_0_1_0_0_0
1202.522 PEP_0_2_0_0_0
1364.5652 PEP_1_2_0_0_0
1526.6105 PEP_2_2_0_0_0
1688.6588 PEP_3_2_0_0_0

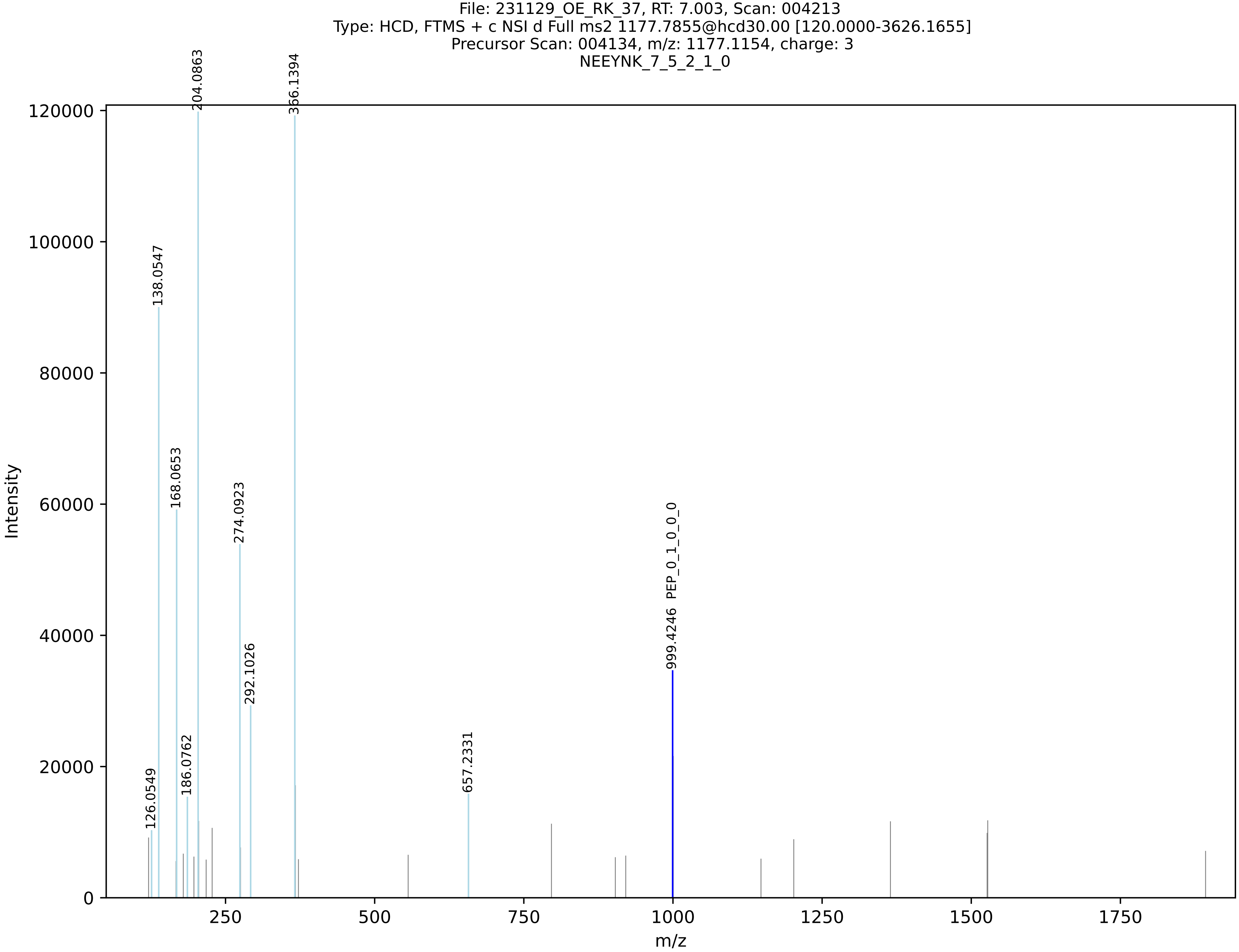
File: 231129_OE_RK_37, RT: 7.003, Scan: 004213
Type: HCD, FTMS + c NSI d Full ms2 1177.7855@hcd30.00 [120.0000-3626.1655]
Precursor Scan: 004134, m/z: 1177.1154, charge: 3
NEEYNK_7_5_2_1_0
204.0863
366.1394
138.0547
168.0653
274.0923
292.1026
186.0762
126.0549
657.2331
999.4246 PEP_0_1_0_0_0
Intensity
m/z
0
20000
40000
60000
80000
100000
120000
250
500
750
1000
1250
1500
1750

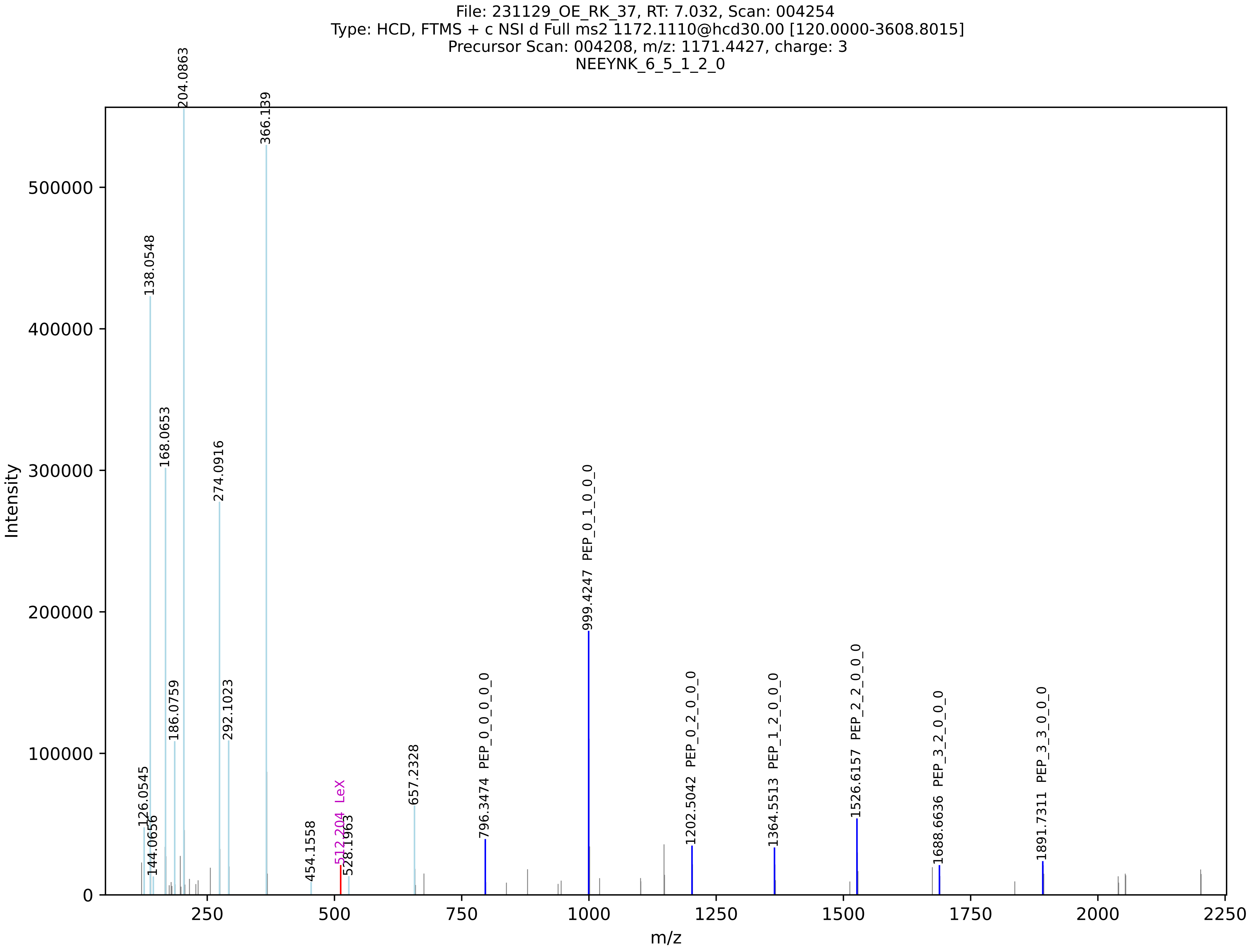
File: 231129_OE_RK_37, RT: 7.032, Scan: 004254
Type: HCD, FTMS + c NSI d Full ms2 1172.1110@hcd30.00 [120.0000-3608.8015]
Precursor Scan: 004208, m/z: 1171.4427, charge: 3
NEEYNK_6_5_1_2_0
Intensity
m/z
204.0863
366.139
138.0548
168.0653
274.0916
186.0759
292.1023
126.0545
144.0656
454.1558
512.204 LeX
528.1963
657.2328
796.3474 PEP_0_0_0_0_0
999.4247 PEP_0_1_0_0_0
1202.5042 PEP_0_2_0_0_0
1364.5513 PEP_1_2_0_0_0
1526.6157 PEP_2_2_0_0_0
1688.6636 PEP_3_2_0_0_0
1891.7311 PEP_3_3_0_0_0

File: 231129_OE_RK_37, RT: 7.034, Scan: 004256
Type: HCD, FTMS + c NSI d Full ms2 904.6802@hcd30.00 [120.0000-2790.4634]
Precursor Scan: 004208, m/z: 904.0119, charge: 3
NEEYNK_5_4_0_1_0

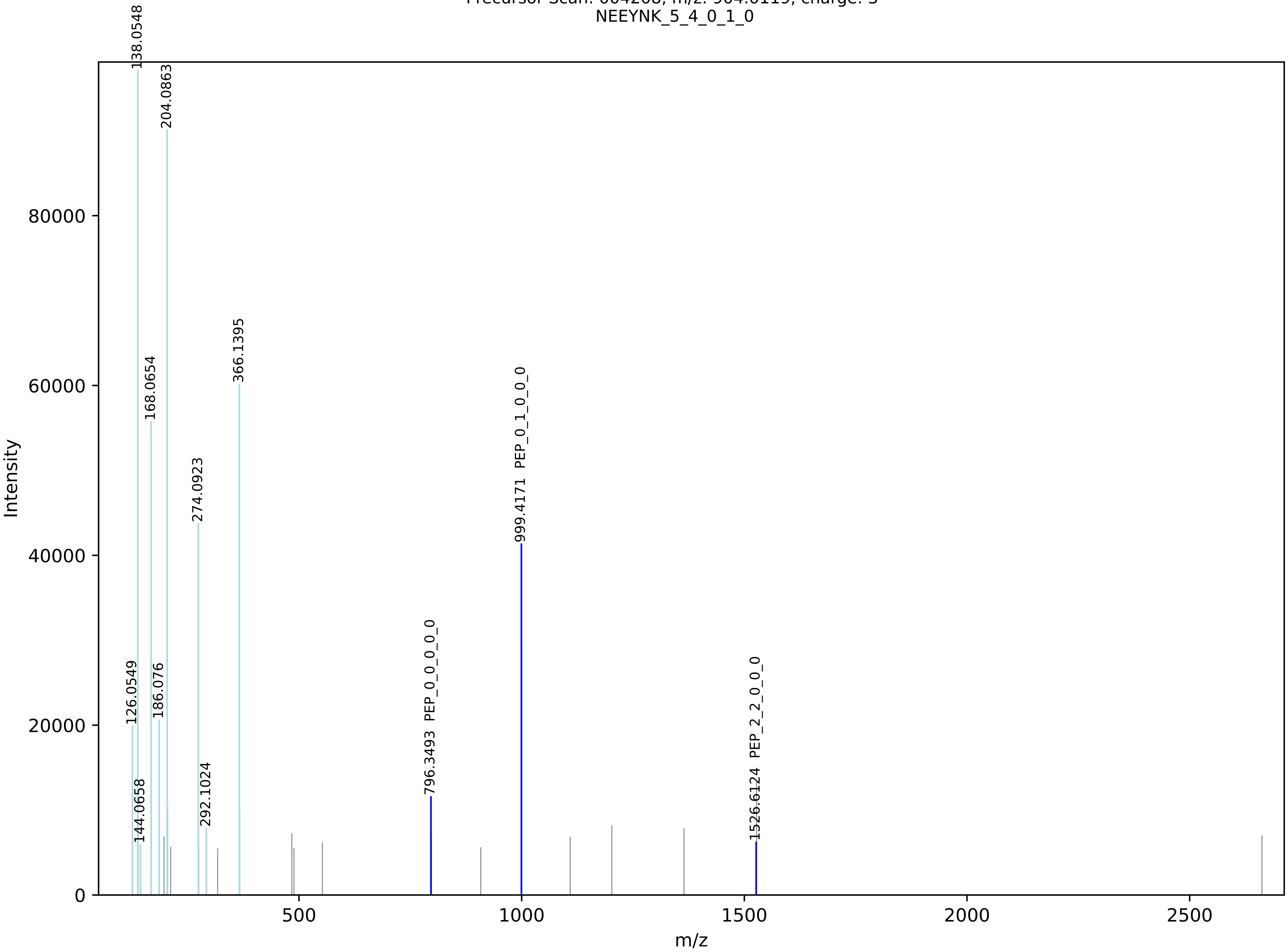

File: 231129_OE_RK_37, RT: 7.12, Scan: 004378
Type: HCD, FTMS + c NSI d Full ms2 1274.4830@hcd30.00 [120.0000-3922.0603]
Precursor Scan: 004325, m/z: 1274.1478, charge: 3
NEEYNK_7_5_2_2_0

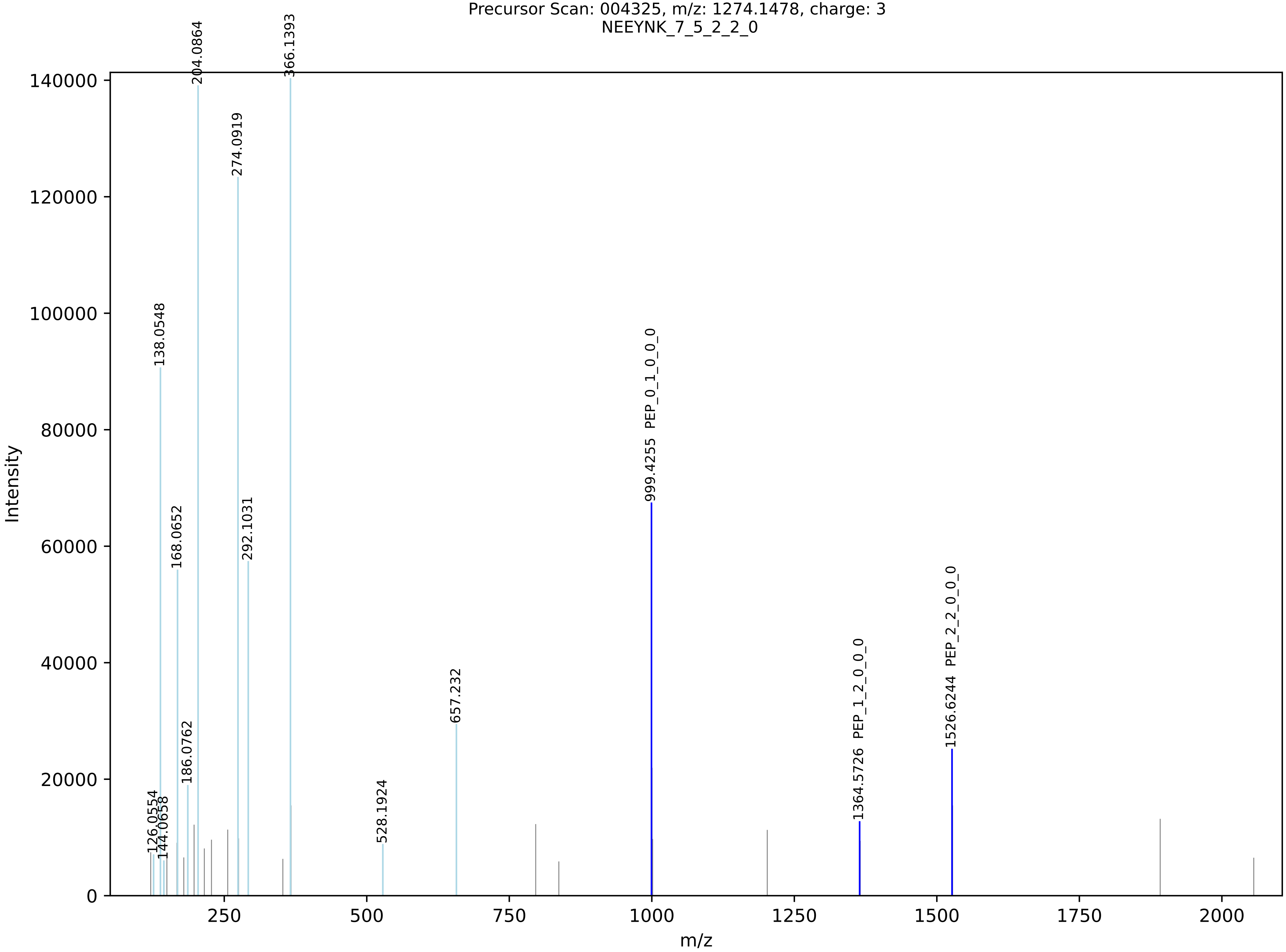

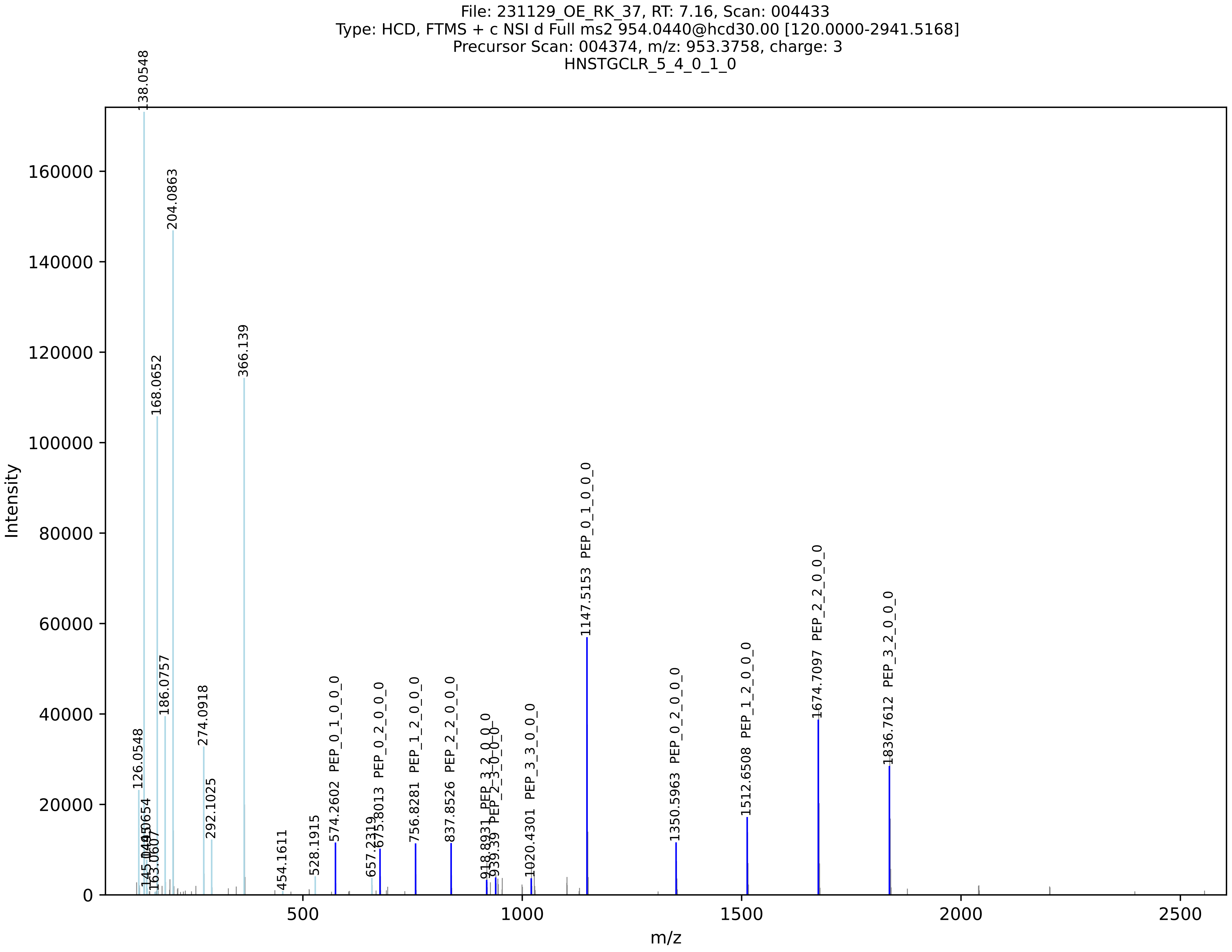

File: 231129_OE_RK_37, RT: 7.16, Scan: 004433
Type: HCD, FTMS + c NSI d Full ms2 954.0440@hcd30.00 [120.0000-2941.5168]
Precursor Scan: 004374, m/z: 953.3758, charge: 3
HNSTGCLR_5_4_0_1_0
Intensity
m/z
138.0548
204.0863
168.0652
366.139
186.0757
274.0918
126.0548
292.1025
163.0607
454.1611
528.1915
574.2602 PEP_0_1_0_0_0
657.2319
675.8013 PEP_0_2_0_0_0
756.8281 PEP_1_2_0_0_0
837.8526 PEP_2_2_0_0_0
918.8931 PEP_3_2_0_0_0
939.39 PEP_2_3_0_0_0
1020.4301 PEP_3_3_0_0_0
1147.5153 PEP_0_1_0_0_0
1350.5963 PEP_0_2_0_0_0
1512.6508 PEP_1_2_0_0_0
1674.7097 PEP_2_2_0_0_0
1836.7612 PEP_3_2_0_0_0

File: 231129_OE_RK_37, RT: 7.16, Scan: 004434
Type: HCD, FTMS + c NSI d Full ms2 1128.7645@hcd30.00 [120.0000-3476.1614]
Precursor Scan: 004374, m/z: 1128.4292, charge: 3
NEEYNK_7_5_1_1_0

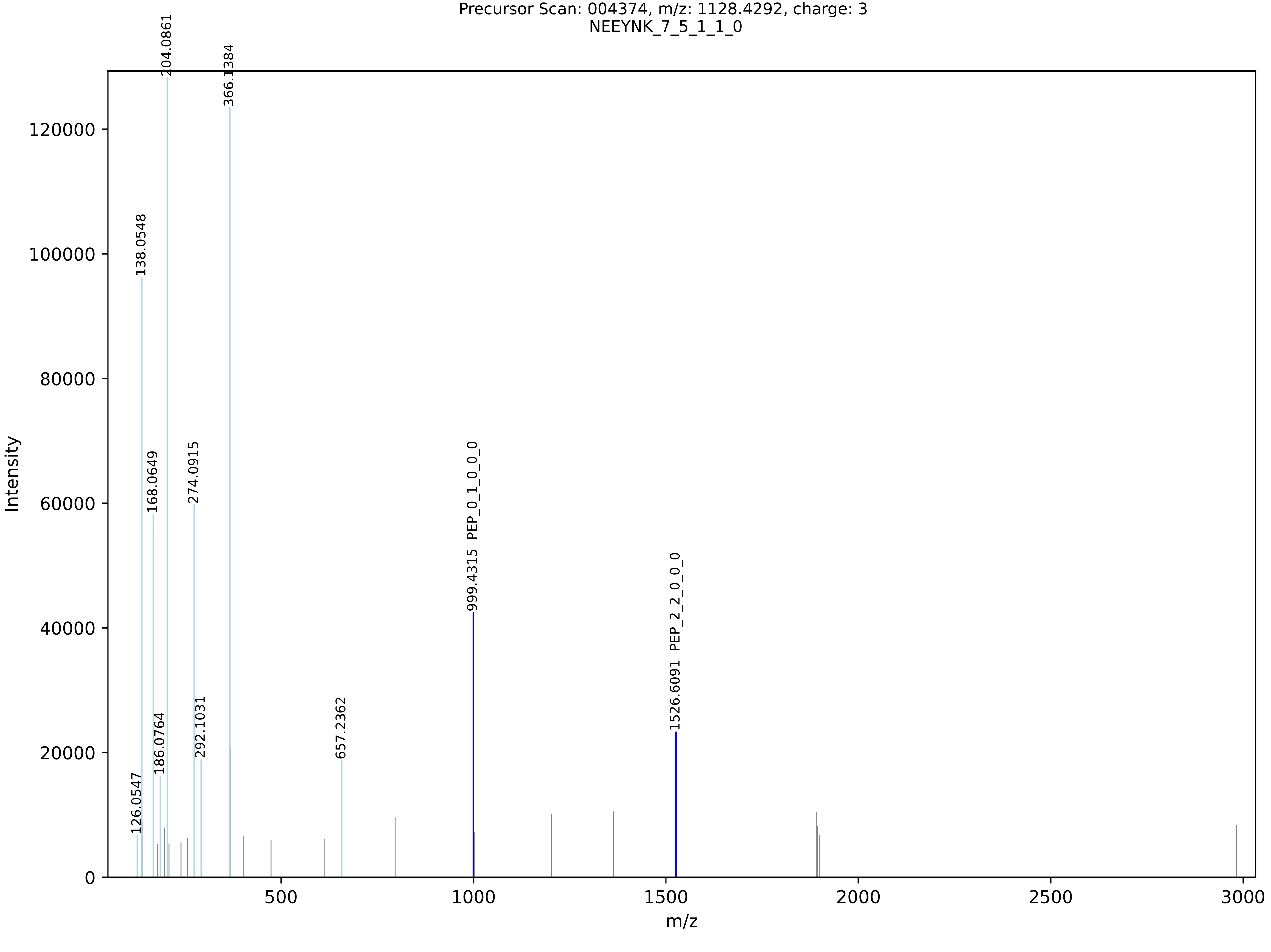

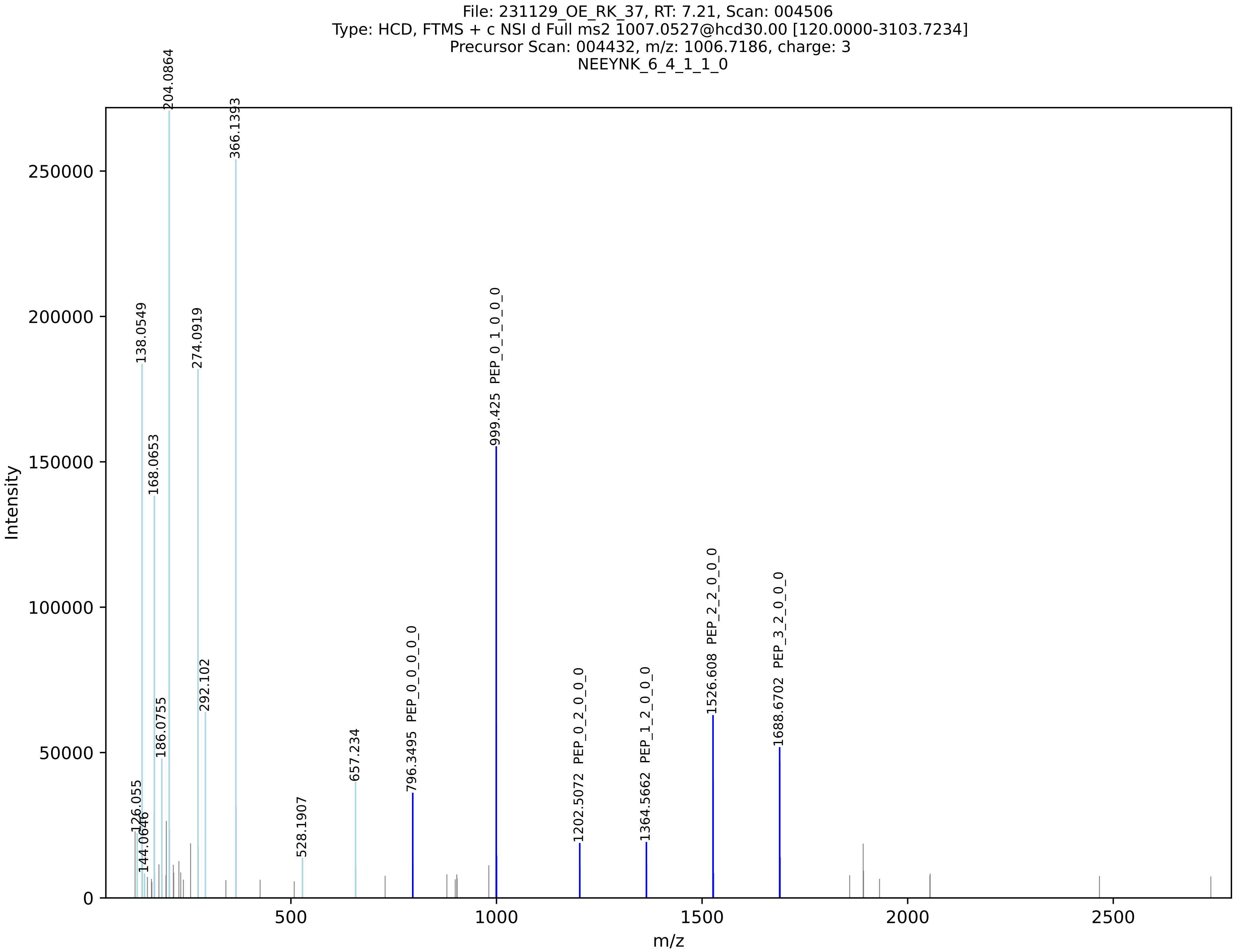
File: 231129_OE_RK_37, RT: 7.21, Scan: 004506
Type: HCD, FTMS + c NSI d Full ms2 1007.0527@hcd30.00 [120.0000-3103.7234]
Precursor Scan: 004432, m/z: 1006.7186, charge: 3
NEEYNK_6_4_1_1_0
Intensity
m/z
204.0864
366.1393
138.0549
274.0919
168.0653
186.0755
292.102
126.055
144.0646
528.1907
657.234
796.3495 PEP_0_0_0_0_0
999.425 PEP_0_1_0_0_0
1202.5072 PEP_0_2_0_0_0
1364.5662 PEP_1_2_0_0_0
1526.608 PEP_2_2_0_0_0
1688.6702 PEP_3_2_0_0_0
0
50000
100000
150000
200000
250000
500
1000
1500
2000
2500

File: 231129_OE_RK_37, RT: 7.315, Scan: 004657
Type: HCD, FTMS + c NSI d Full ms2 1094.4501@hcd30.00 [120.0000-3371.1592]
Precursor Scan: 004577, m/z: 1093.7818, charge: 3
NGSLFAFR_6_4_2_1_0

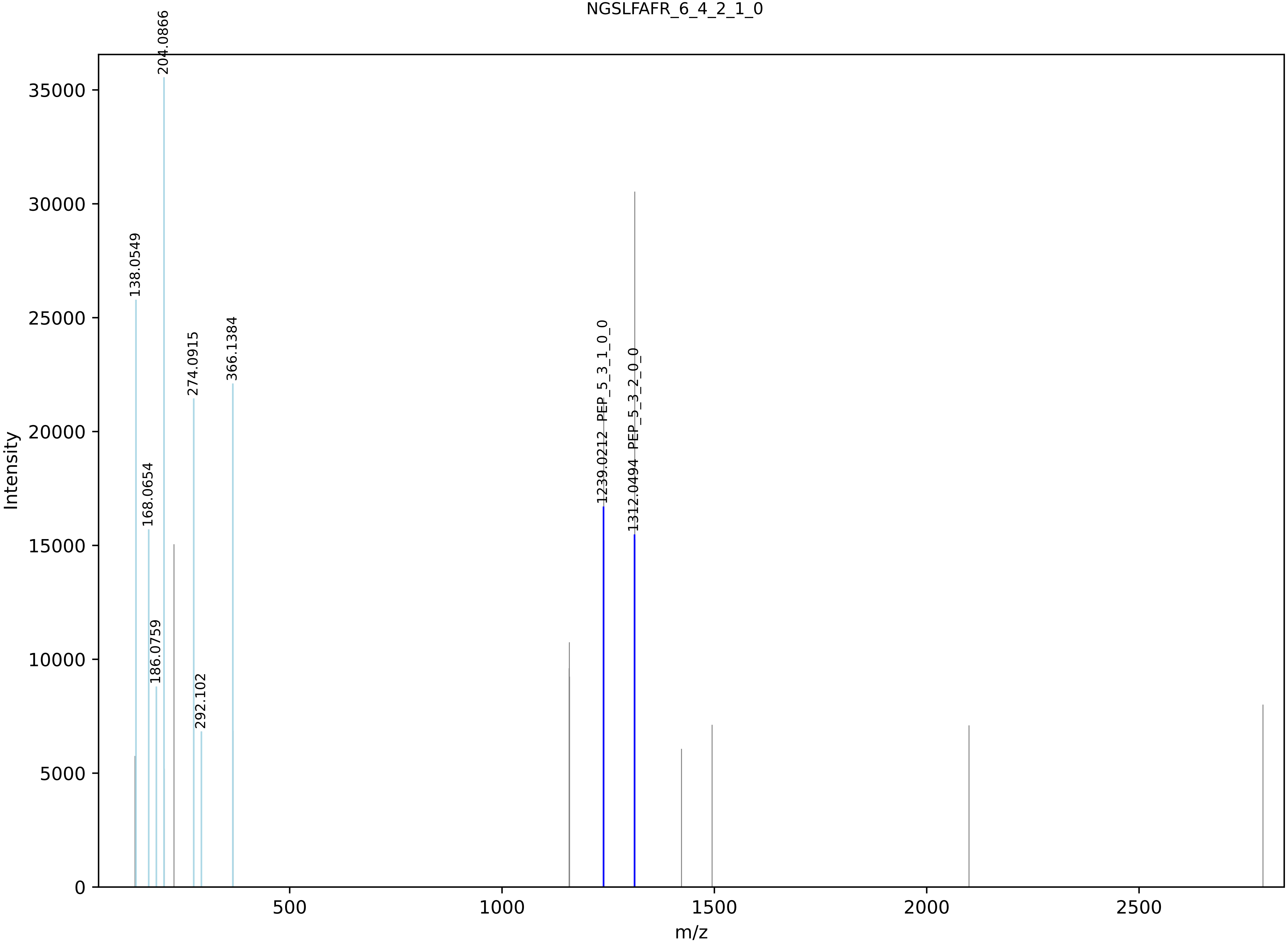

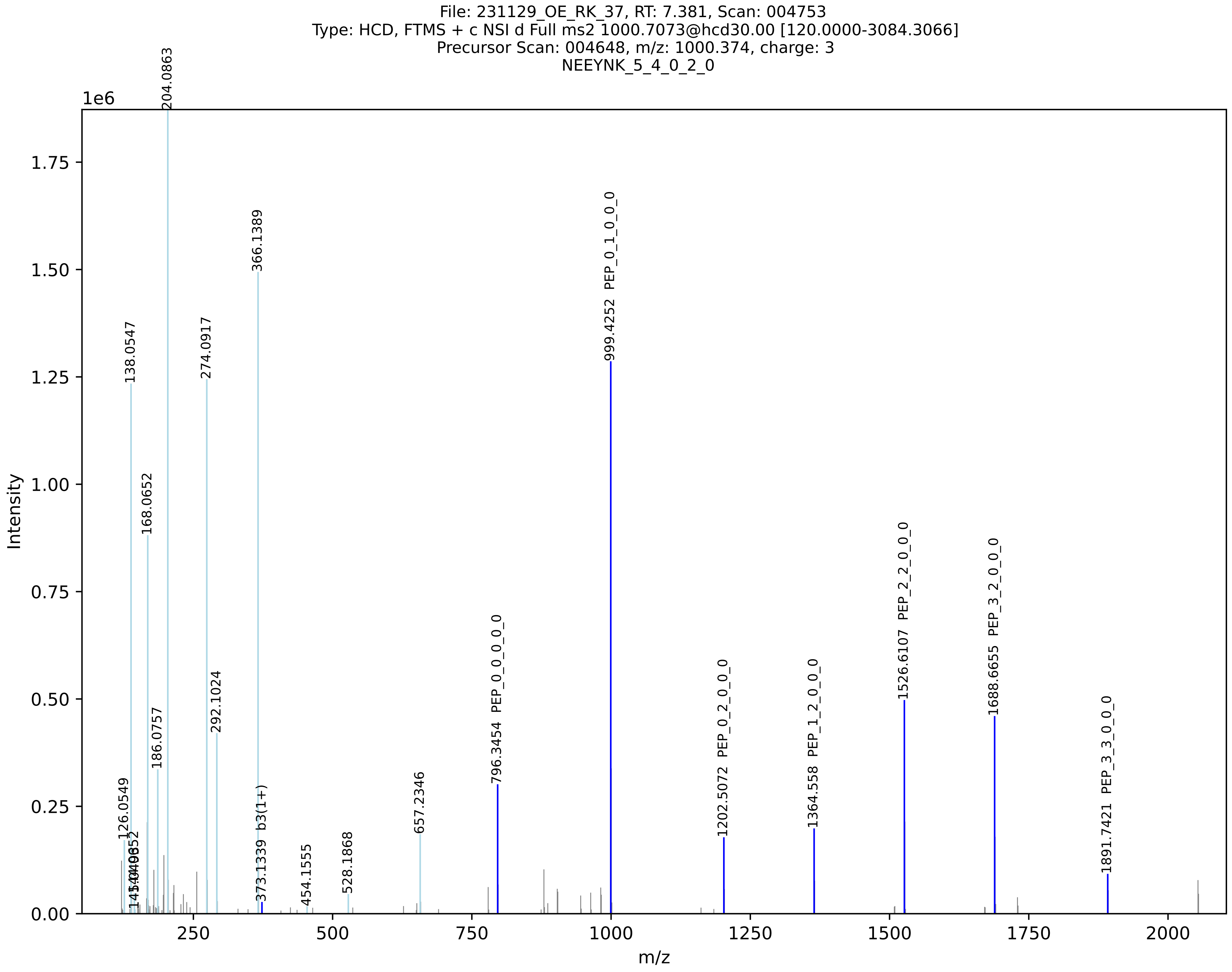
File: 231129_OE_RK_37, RT: 7.381, Scan: 004753
Type: HCD, FTMS + c NSI d Full ms2 1000.7073@hcd30.00 [120.0000-3084.3066]
Precursor Scan: 004648, m/z: 1000.374, charge: 3
NEEYNK_5_4_0_2_0
1e6
Intensity
m/z
204.0863
366.1389
138.0547
274.0917
168.0652
292.1024
186.0757
126.0549
373.1339 b3(1+)
454.1555
528.1868
657.2346
796.3454 PEP_0_0_0_0_0
999.4252 PEP_0_1_0_0_0
1202.5072 PEP_0_2_0_0_0
1364.558 PEP_1_2_0_0_0
1526.6107 PEP_2_2_0_0_0
1688.6655 PEP_3_2_0_0_0
1891.7421 PEP_3_3_0_0_0

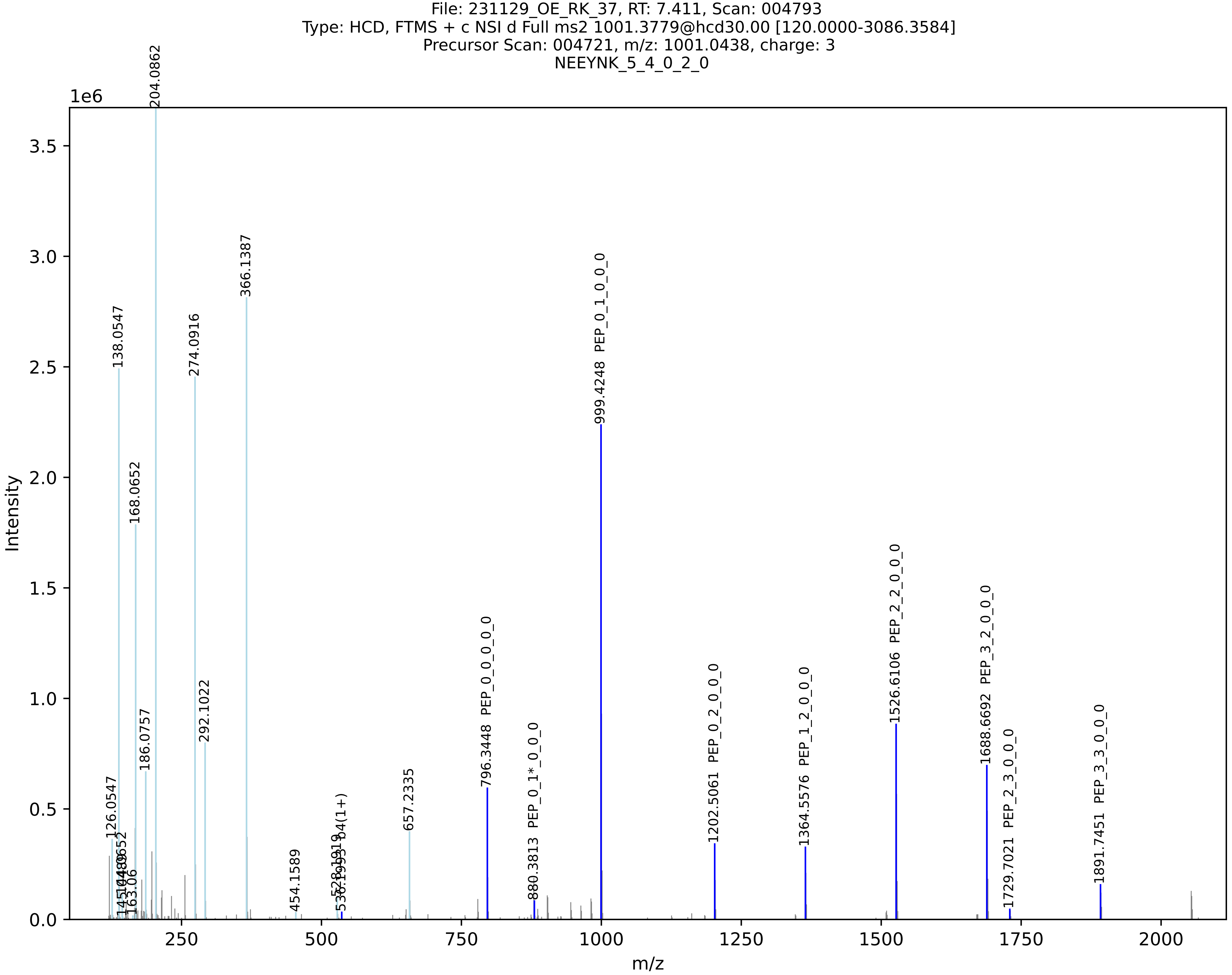
File: 231129_OE_RK_37, RT: 7.411, Scan: 004793
Type: HCD, FTMS + c NSI d Full ms2 1001.3779@hcd30.00 [120.0000-3086.3584]
Precursor Scan: 004721, m/z: 1001.0438, charge: 3
NEEYNK_5_4_0_2_0
1e6
Intensity
m/z
204.0862
366.1387
138.0547
274.0916
168.0652
186.0757
292.1022
126.0547
163.06
454.1589
657.2335
796.3448 PEP_0_0_0_0_0
880.3813 PEP_0_1*_0_0_0
999.4248 PEP_0_1_0_0_0
1202.5061 PEP_0_2_0_0_0
1364.5576 PEP_1_2_0_0_0
1526.6106 PEP_2_2_0_0_0
1688.6692 PEP_3_2_0_0_0
1729.7021 PEP_2_3_0_0_0
1891.7451 PEP_3_3_0_0_0

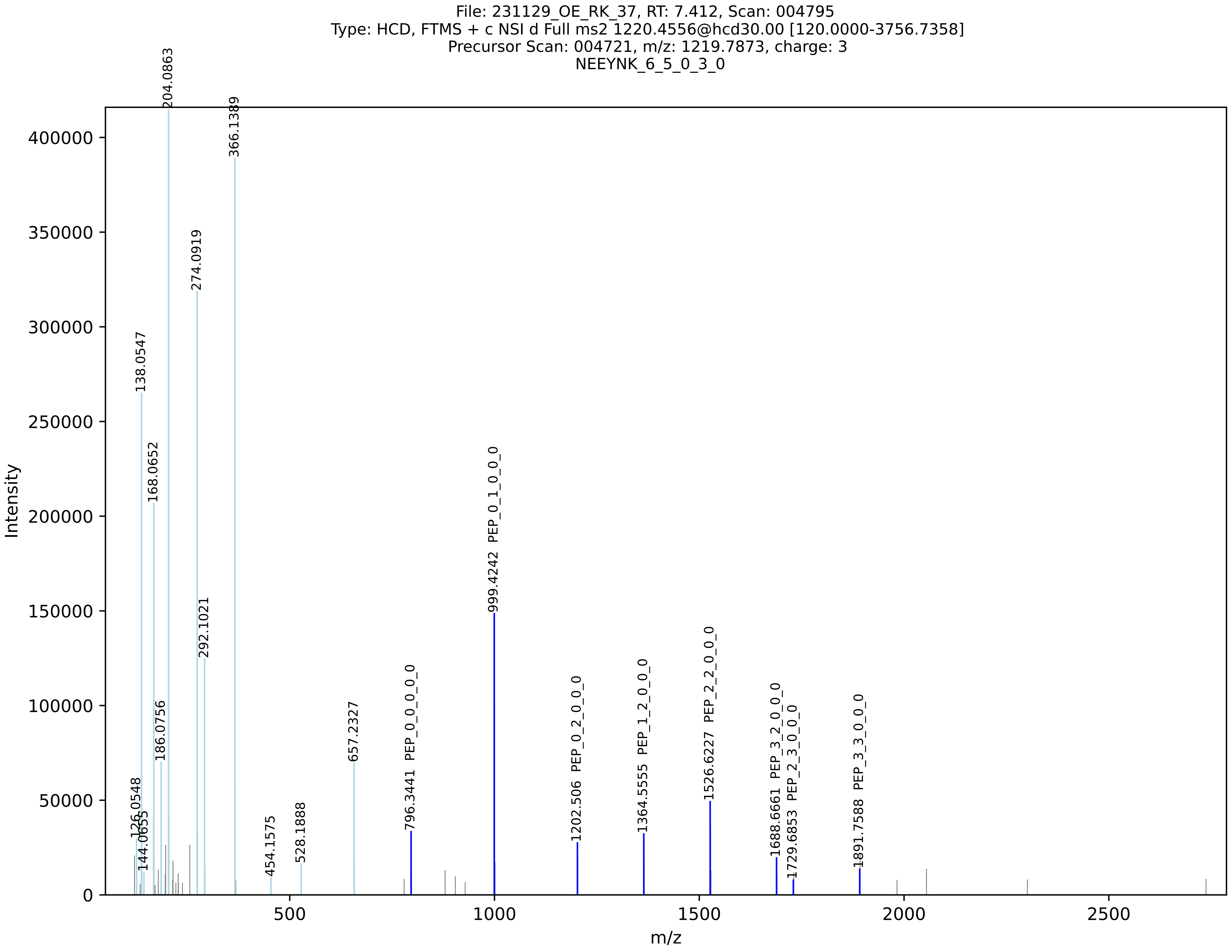
File: 231129_OE_RK_37, RT: 7.412, Scan: 004795
Type: HCD, FTMS + c NSI d Full ms2 1220.4556@hcd30.00 [120.0000-3756.7358]
Precursor Scan: 004721, m/z: 1219.7873, charge: 3
NEEYNK_6_5_0_3_0
Intensity
m/z
204.0863
366.1389
274.0919
138.0547
168.0652
292.1021
186.0756
126.0548
144.0655
454.1575
528.1888
657.2327
796.3441 PEP_0_0_0_0_0
999.4242 PEP_0_1_0_0_0
1202.506 PEP_0_2_0_0_0
1364.5555 PEP_1_2_0_0_0
1526.6227 PEP_2_2_0_0_0
1688.6661 PEP_3_2_0_0_0
1729.6853 PEP_2_3_0_0_0
1891.7588 PEP_3_3_0_0_0

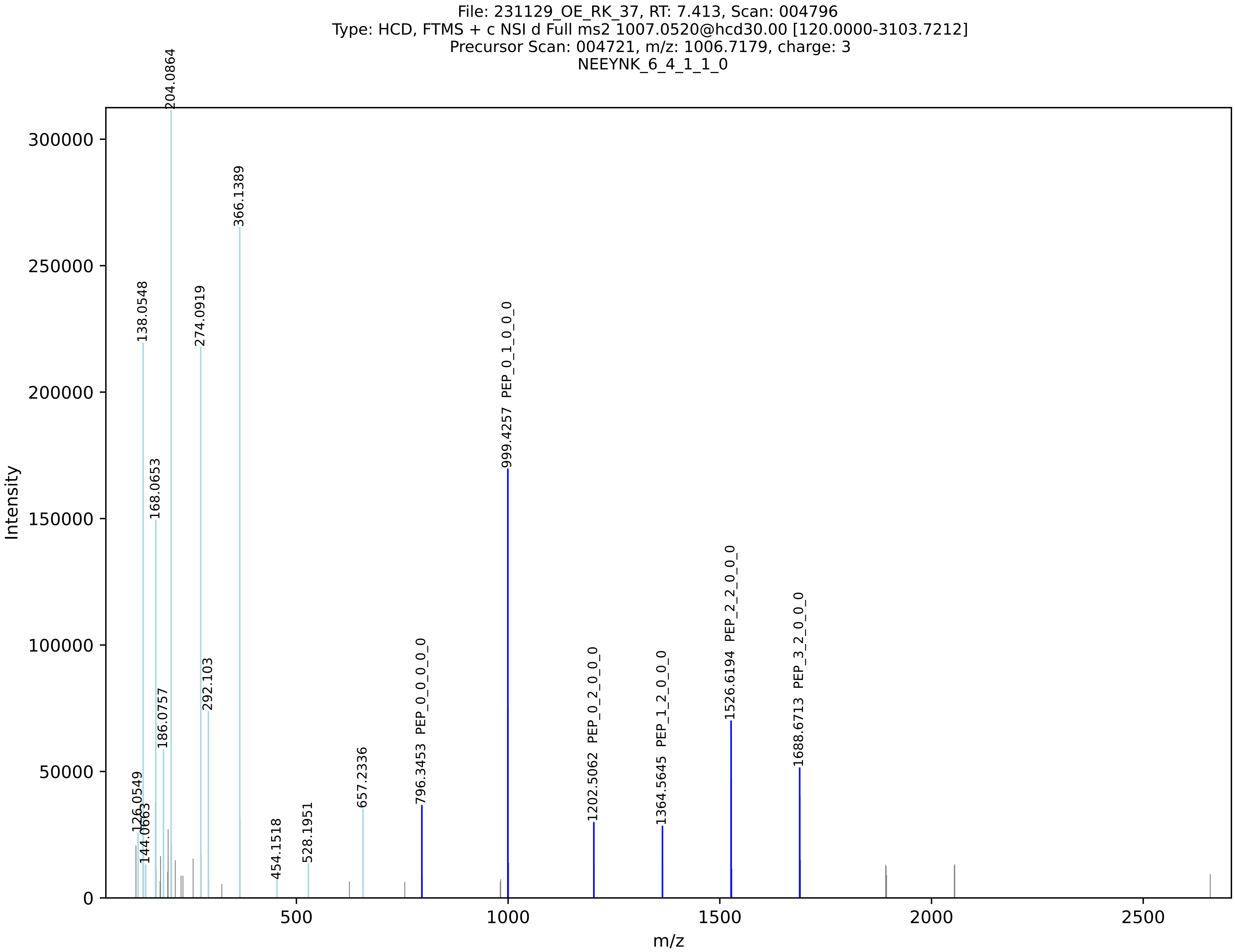
File: 231129_OE_RK_37, RT: 7.413, Scan: 004796
Type: HCD, FTMS + c NSI d Full ms2 1007.0520@hcd30.00 [120.0000-3103.7212]
Precursor Scan: 004721, m/z: 1006.7179, charge: 3
NEEYNK_6_4_1_1_0
Intensity
m/z
0
50000
100000
150000
200000
250000
300000
500
1000
1500
2000
2500
204.0864
366.1389
138.0548
274.0919
168.0653
186.0757
292.103
126.0549
144.0663
454.1518
528.1951
657.2336
796.3453 PEP_0_0_0_0_0
999.4257 PEP_0_1_0_0_0
1202.5062 PEP_0_2_0_0_0
1364.5645 PEP_1_2_0_0_0
1526.6194 PEP_2_2_0_0_0
1688.6713 PEP_3_2_0_0_0

File: 231129_OE_RK_37, RT: 7.429, Scan: 004823
Type: HCD, FTMS + c NSI d Full ms2 946.0309@hcd30.00 [120.0000-2916.9966]
Precursor Scan: 004721, m/z: 946.0319, charge: 3
NHSK_5_4_1_2_0

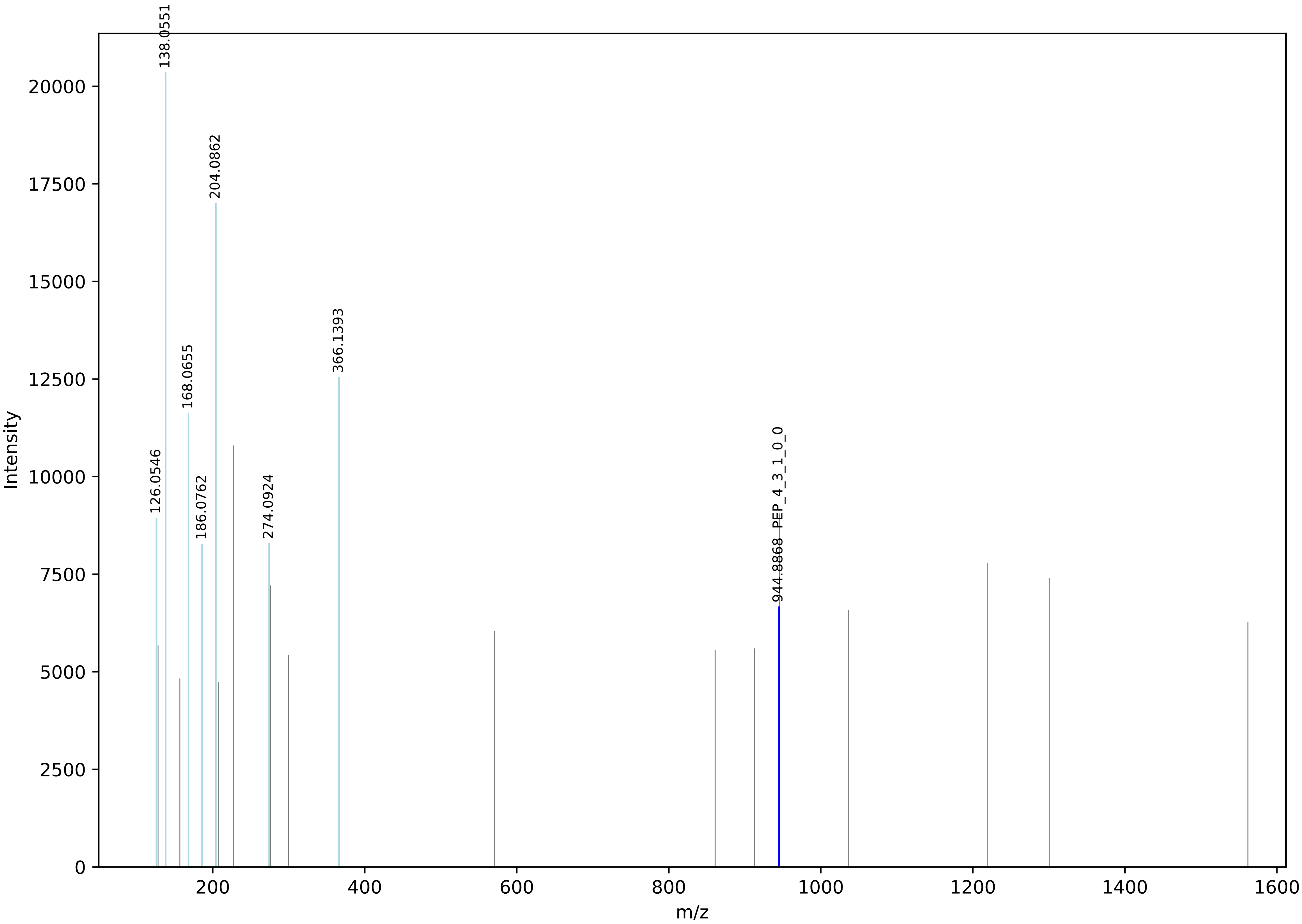

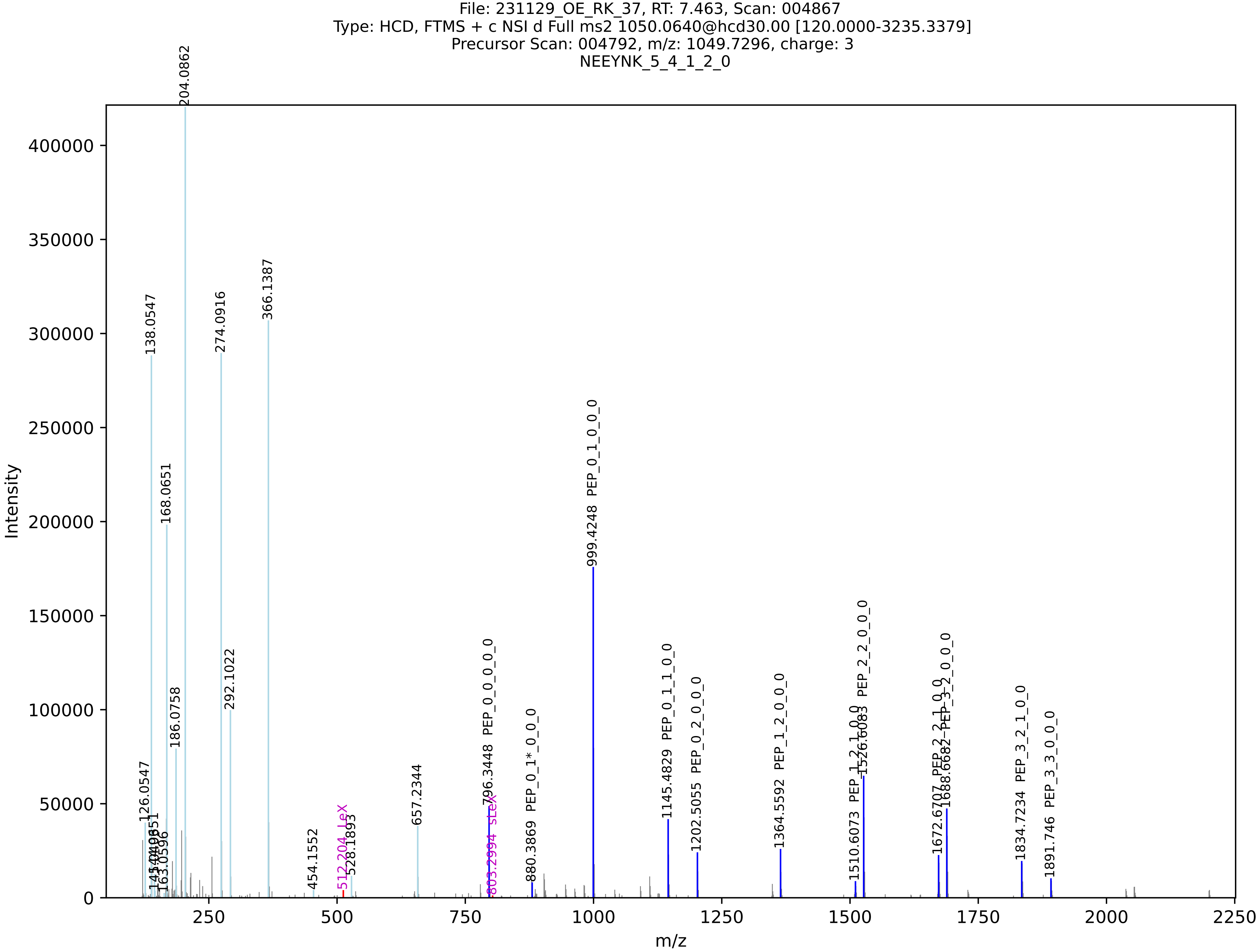
File: 231129_OE_RK_37, RT: 7.463, Scan: 004867
Type: HCD, FTMS + c NSI d Full ms2 1050.0640@hcd30.00 [120.0000-3235.3379]
Precursor Scan: 004792, m/z: 1049.7296, charge: 3
NEEYNK_5_4_1_2_0
Intensity
m/z
204.0862
138.0547
274.0916
366.1387
168.0651
186.0758
292.1022
126.0547
163.0596
454.1552
512.204 LeX
528.1893
657.2344
796.3448 PEP_0_0_0_0_0
803.2994 sLeX
880.3869 PEP_0_1* 0_0_0
999.4248 PEP_0_1_0_0_0
1145.4829 PEP_0_1_1_0_0
1202.5055 PEP_0_2_0_0_0
1364.5592 PEP_1_2_0_0_0
1510.6073 PEP_1_2_1_0_0
1526.6083 PEP_2_2_0_0_0
1672.6707 PEP_2_2_1_0_0
1688.6682 PEP_3_2_0_0_0
1834.7234 PEP_3_2_1_0_0
1891.746 PEP_3_3_0_0_0

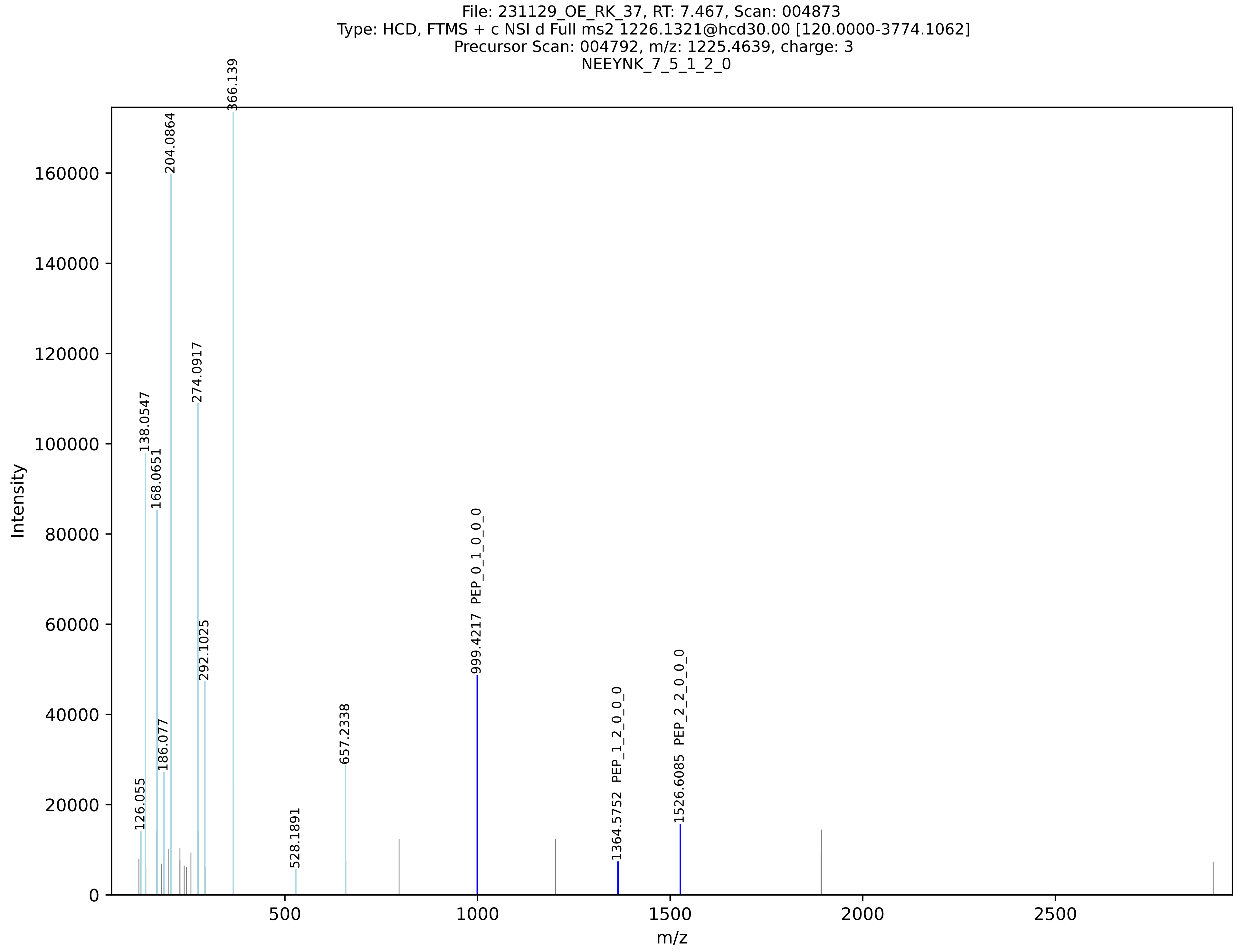
File: 231129_OE_RK_37, RT: 7.467, Scan: 004873
Type: HCD, FTMS + c NSI d Full ms2 1226.1321@hcd30.00 [120.0000-3774.1062]
Precursor Scan: 004792, m/z: 1225.4639, charge: 3
NEEYNK_7_5_1_2_0
366.139
204.0864
274.0917
138.0547
168.0651
999.4217 PEP_0_1_0_0_0
292.1025
1526.6085 PEP_2_2_0_0_0
1364.5752 PEP_1_2_0_0_0
657.2338
186.077
126.055
528.1891
Intensity
m/z
0
20000
40000
60000
80000
100000
120000
140000
160000
500
1000
1500
2000
2500

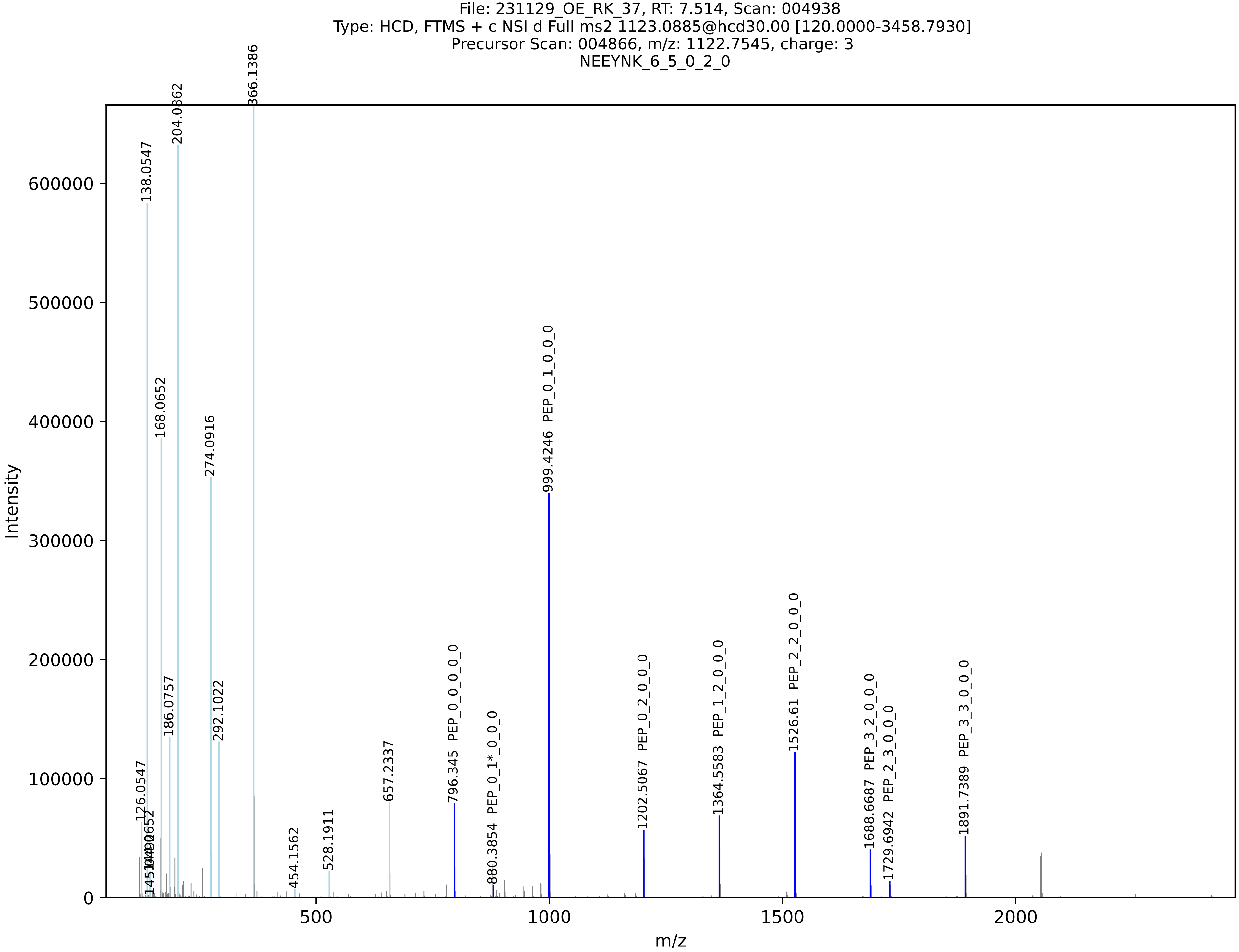
File: 231129_OE_RK_37, RT: 7.514, Scan: 004938
Type: HCD, FTMS + c NSI d Full ms2 1123.0885@hcd30.00 [120.0000-3458.7930]
Precursor Scan: 004866, m/z: 1122.7545, charge: 3
NEEYNK_6_5_0_2_0
Intensity
m/z
126.0547
138.0547
168.0652
186.0757
204.0862
274.0916
292.1022
366.1386
454.1562
528.1911
657.2337
796.345 PEP_0_0_0_0_0
880.3854 PEP_0_1*_0_0_0
999.4246 PEP_0_1_0_0_0
1202.5067 PEP_0_2_0_0_0
1364.5583 PEP_1_2_0_0_0
1526.61 PEP_2_2_0_0_0
1688.6687 PEP_3_2_0_0_0
1729.6942 PEP_2_3_0_0_0
1891.7389 PEP_3_3_0_0_0
0
100000
200000
300000
400000
500000
600000
500
1000
1500
2000

File: 231129_OE_RK_37, RT: 7.536, Scan: 004974
Type: HCD, FTMS + c NSI d Full ms2 1287.7795@hcd30.00 [120.0000-3962.7473]
Precursor Scan: 004866, m/z: 1287.4502, charge: 3
ENGTISR_7_6_1_2_0

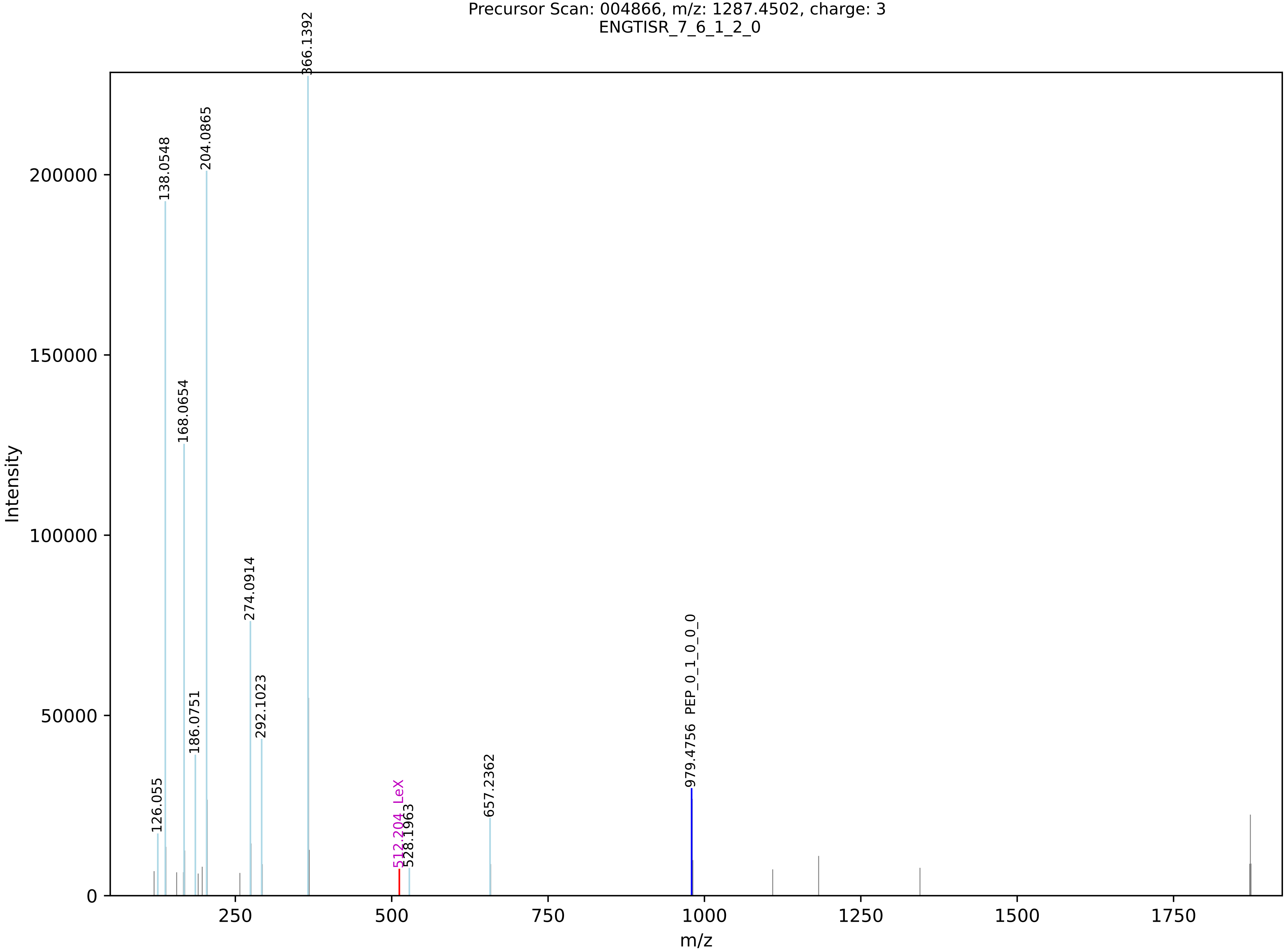

File: 231129_OE_RK_37, RT: 7.558, Scan: 005002
Type: HCD, FTMS + c NSI d Full ms2 1238.4807@hcd30.00 [120.0000-3811.8931]
Precursor Scan: 004937, m/z: 1237.8125, charge: 3
ENGTISR_7_6_0_2_0

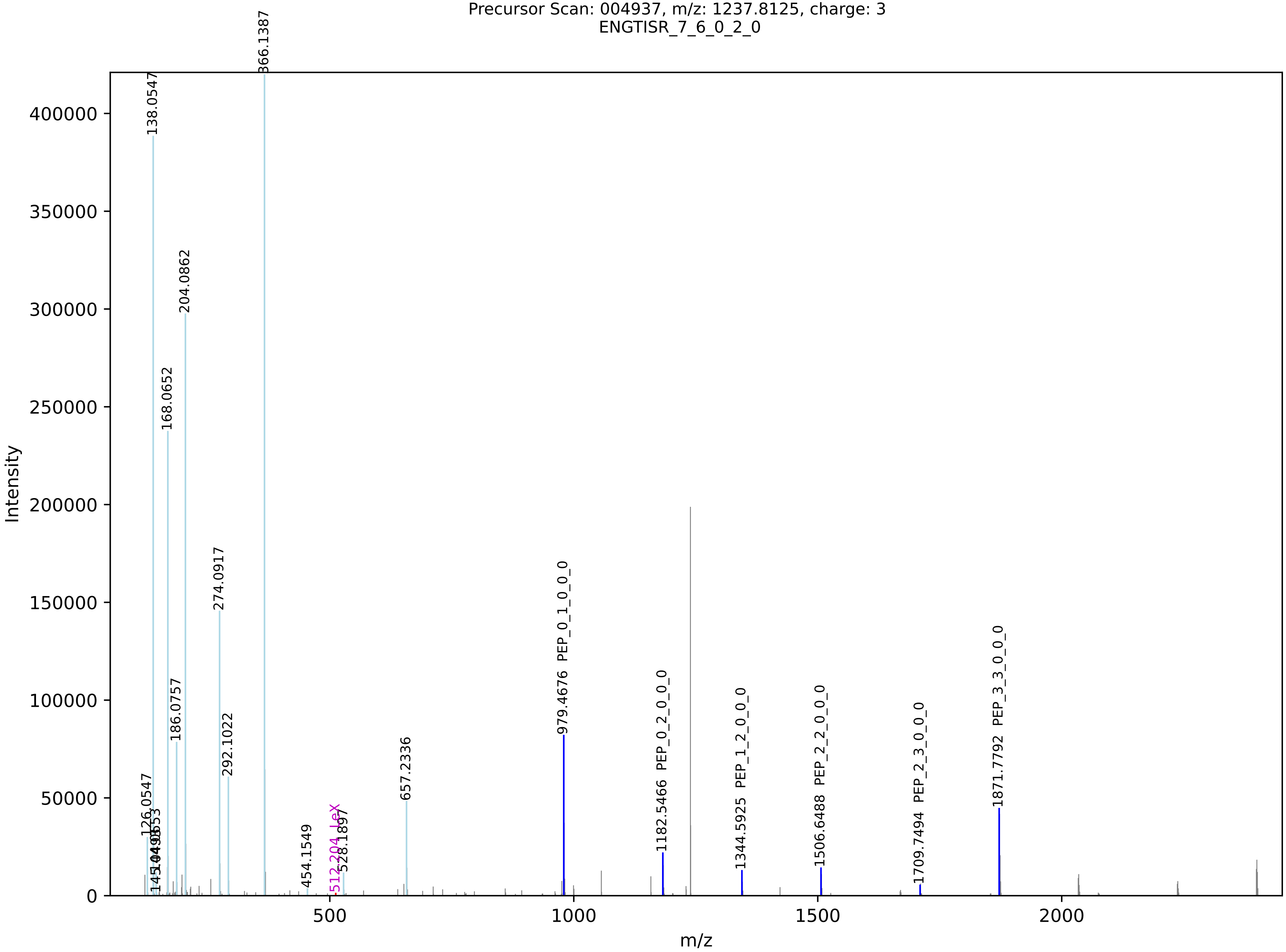

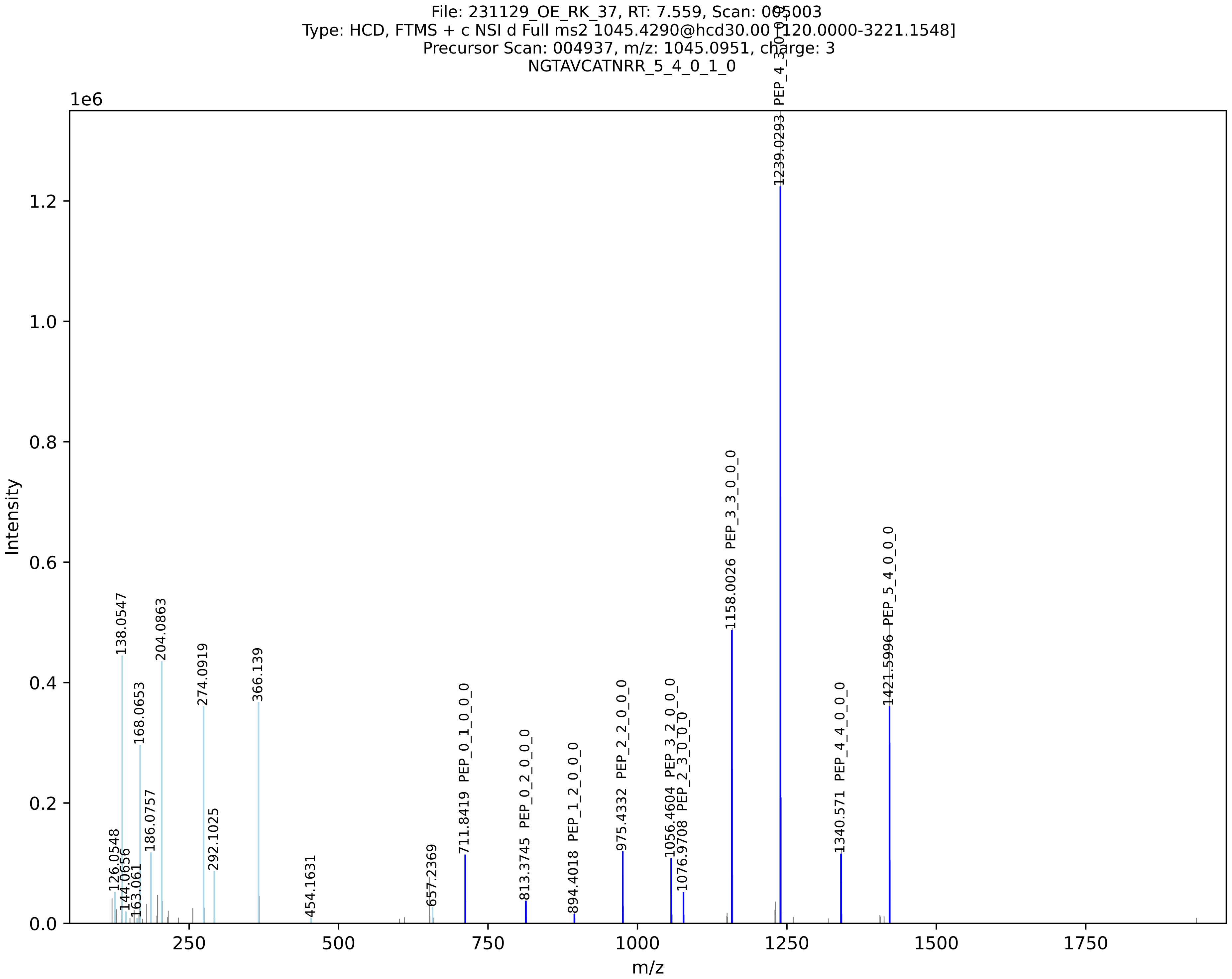
File: 231129_OE_RK_37, RT: 7.559, Scan: 005003
Type: HCD, FTMS + c NSI d Full ms2 1045.4290@hcd30.00 [120.0000-3221.1548]
Precursor Scan: 004937, m/z: 1045.0951, charge: 3
NGTAVCATNRR_5_4_0_1_0
1e6
Intensity
m/z
1239.0293 PEP_4_3_0_0_0
1158.0026 PEP_3_3_0_0_0
1421.5996 PEP_5_4_0_0_0
1340.571 PEP_4_4_0_0_0
711.8419 PEP_0_1_0_0_0
813.3745 PEP_0_2_0_0_0
894.4018 PEP_1_2_0_0_0
975.4332 PEP_2_2_0_0_0
1056.4604 PEP_3_2_0_0_0
1076.9708 PEP_2_3_0_0_0
138.0547
204.0863
274.0919
366.139
168.0653
186.0757
292.1025
126.0548
144.0656
163.061
454.1631
657.2369

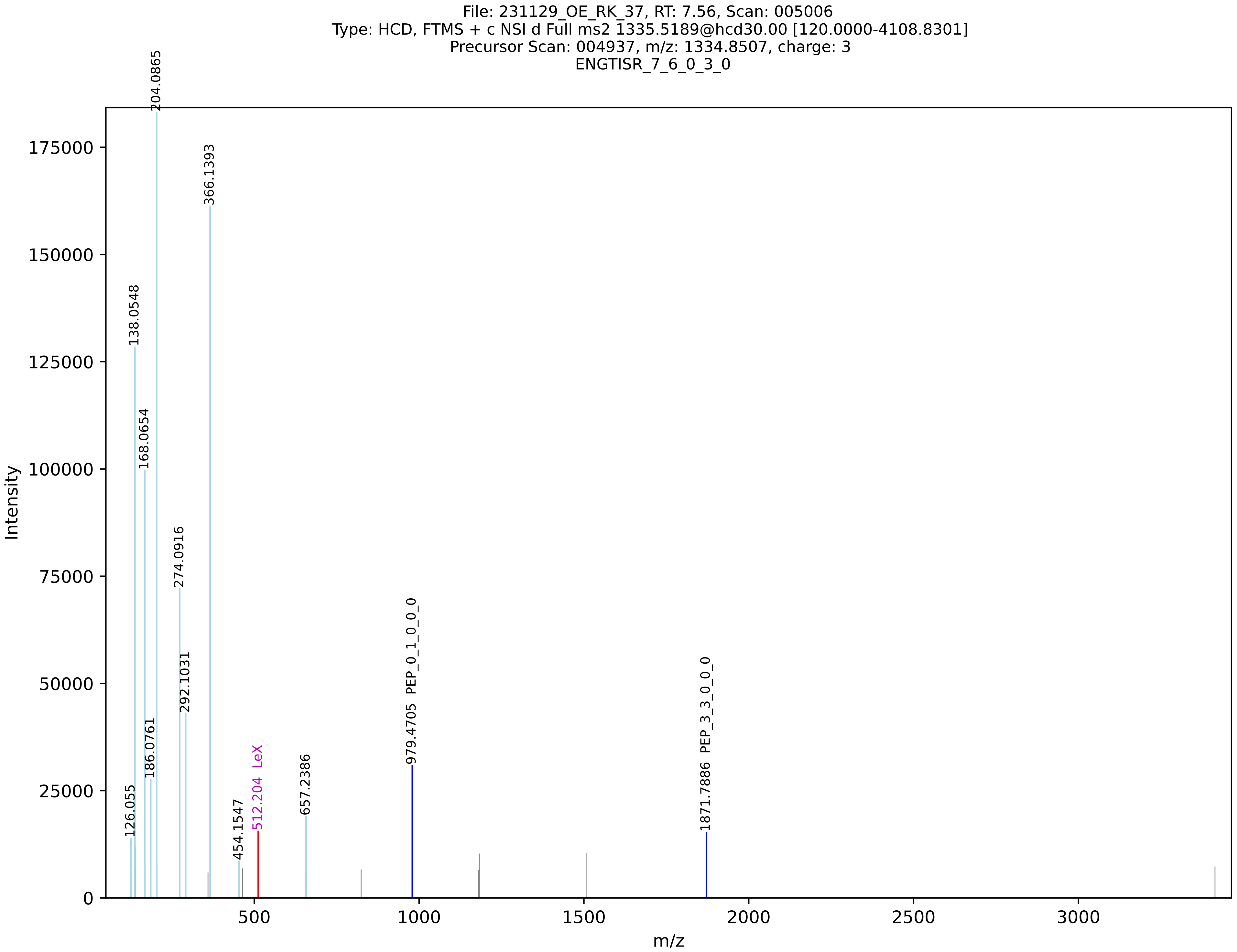

File: 231129_OE_RK_37, RT: 7.56, Scan: 005006
Type: HCD, FTMS + c NSI d Full ms2 1335.5189@hcd30.00 [120.0000-4108.8301]
Precursor Scan: 004937, m/z: 1334.8507, charge: 3
ENGTISR_7_6_0_3_0
Intensity
m/z
204.0865
366.1393
138.0548
168.0654
274.0916
292.1031
186.0761
126.055
454.1547
512.204 LeX
657.2386
979.4705 PEP_0_1_0_0_0
1871.7886 PEP_3_3_0_0_0

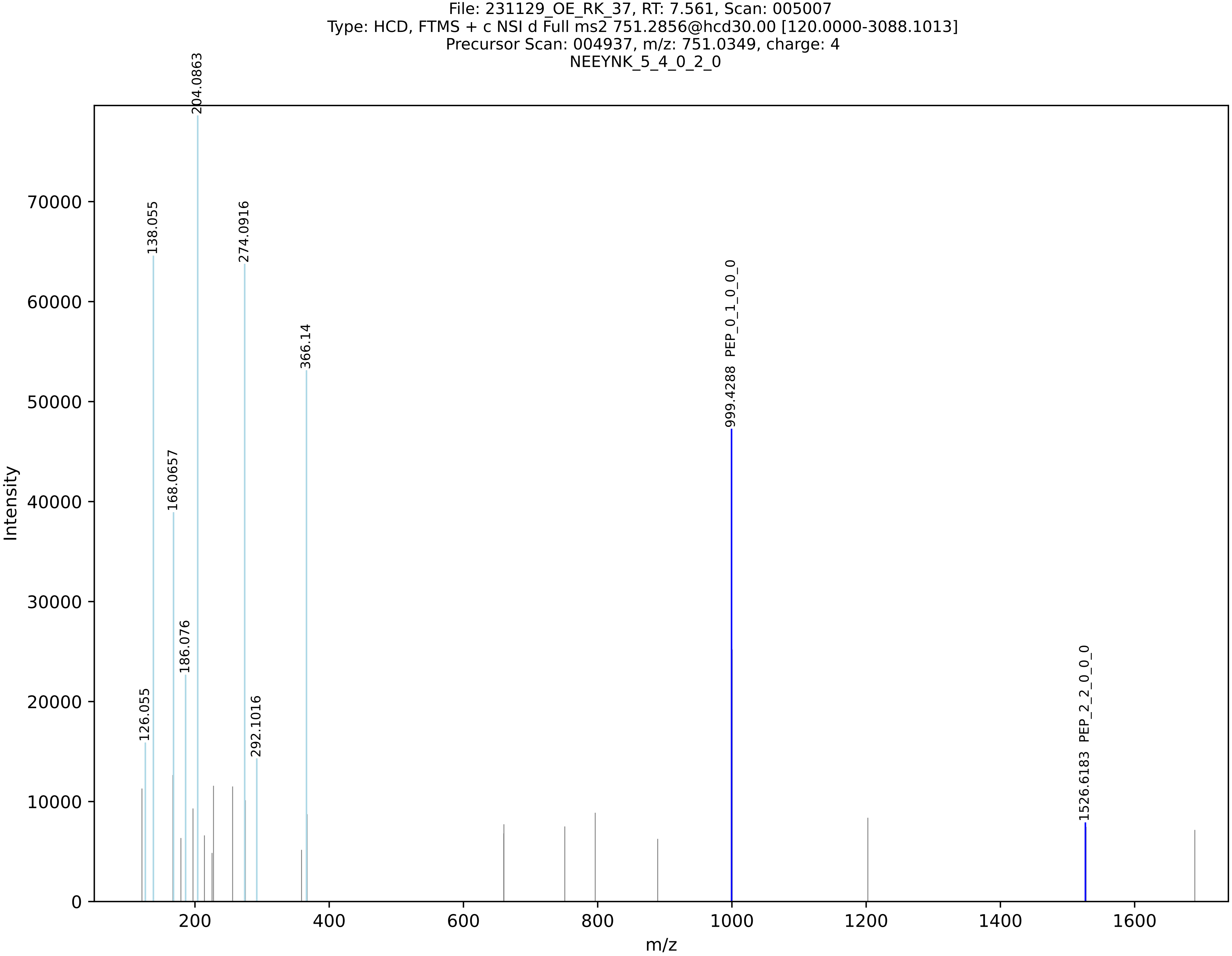
File: 231129_OE_RK_37, RT: 7.561, Scan: 005007
Type: HCD, FTMS + c NSI d Full ms2 751.2856@hcd30.00 [120.0000-3088.1013]
Precursor Scan: 004937, m/z: 751.0349, charge: 4
NEEYNK_5_4_0_2_0
204.0863
138.055
274.0916
366.14
168.0657
186.076
126.055
292.1016
999.4288 PEP_0_1_0_0_0
1526.6183 PEP_2_2_0_0_0
Intensity
m/z
0
10000
20000
30000
40000
50000
60000
70000
200
400
600
800
1000
1200
1400
1600

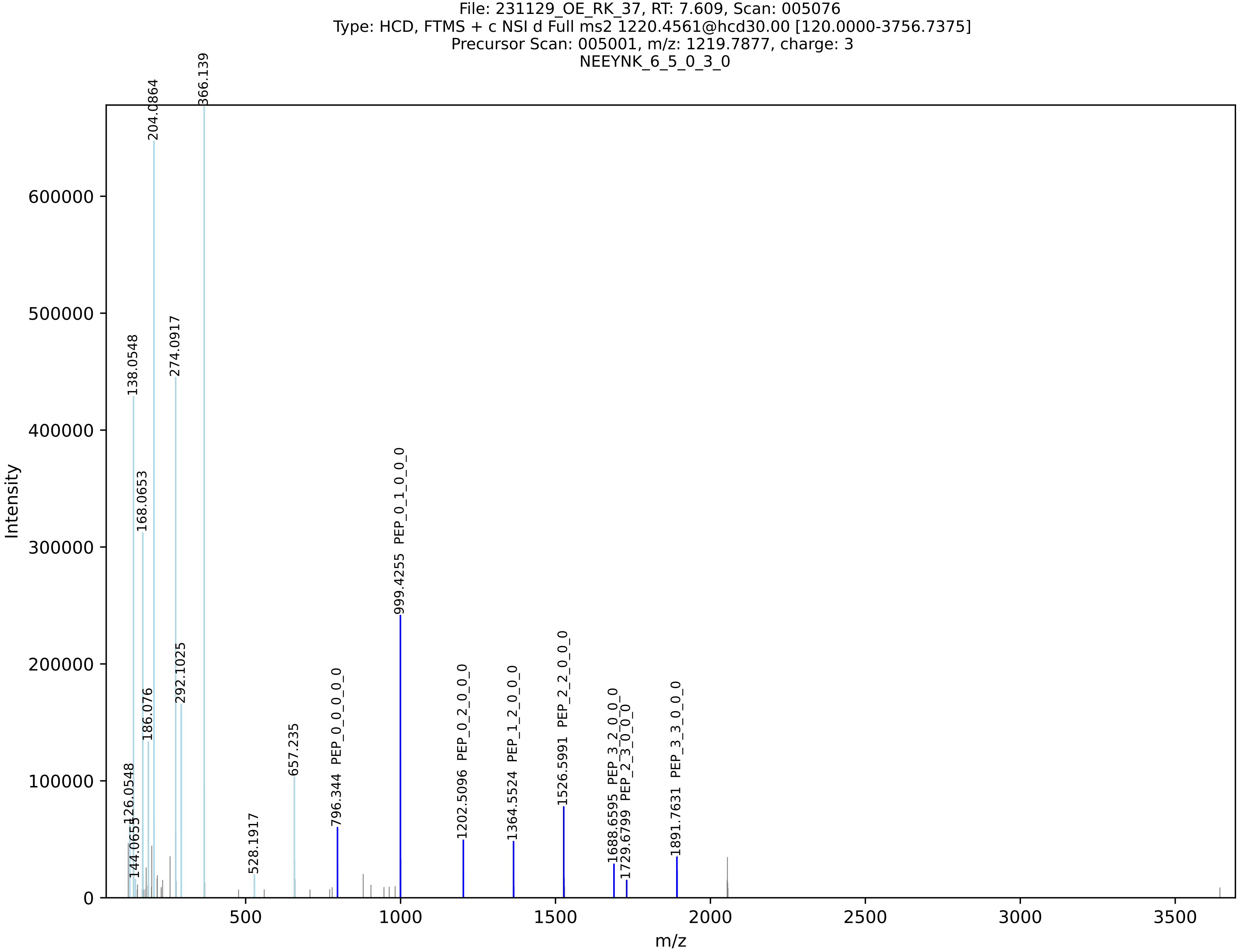
File: 231129_OE_RK_37, RT: 7.609, Scan: 005076
Type: HCD, FTMS + c NSI d Full ms2 1220.4561@hcd30.00 [120.0000-3756.7375]
Precursor Scan: 005001, m/z: 1219.7877, charge: 3
NEEYNK_6_5_0_3_0
Intensity
m/z
366.139
204.0864
274.0917
138.0548
168.0653
292.1025
186.076
126.0548
144.0655
528.1917
657.235
796.344 PEP_0_0_0_0_0_
999.4255 PEP_0_1_0_0_0
1202.5096 PEP_0_2_0_0_0
1364.5524 PEP_1_2_0_0_0
1526.5991 PEP_2_2_0_0_0
1688.6595 PEP_3_2_0_0_0
1729.6799 PEP_2_3_0_0_0
1891.7631 PEP_3_3_0_0_0

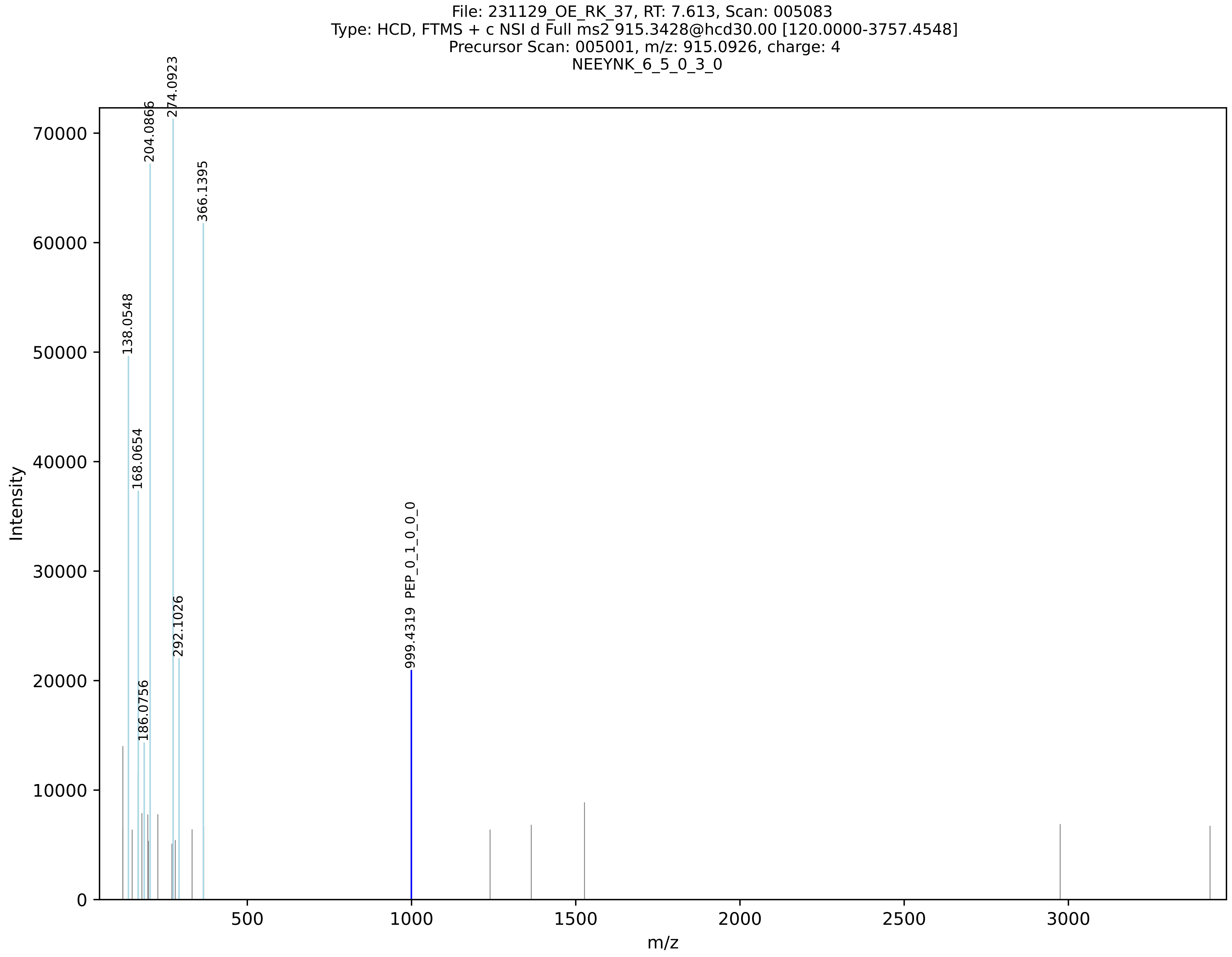

File: 231129_OE_RK_37, RT: 7.613, Scan: 005083
Type: HCD, FTMS + c NSI d Full ms2 915.3428@hcd30.00 [120.0000-3757.4548]
Precursor Scan: 005001, m/z: 915.0926, charge: 4
NEEYNK_6_5_0_3_0
274.0923
204.0866
366.1395
138.0548
168.0654
999.4319 PEP_0_1_0_0_0_
292.1026
186.0756
Intensity
m/z
0
10000
20000
30000
40000
50000
60000
70000
500
1000
1500
2000
2500
3000

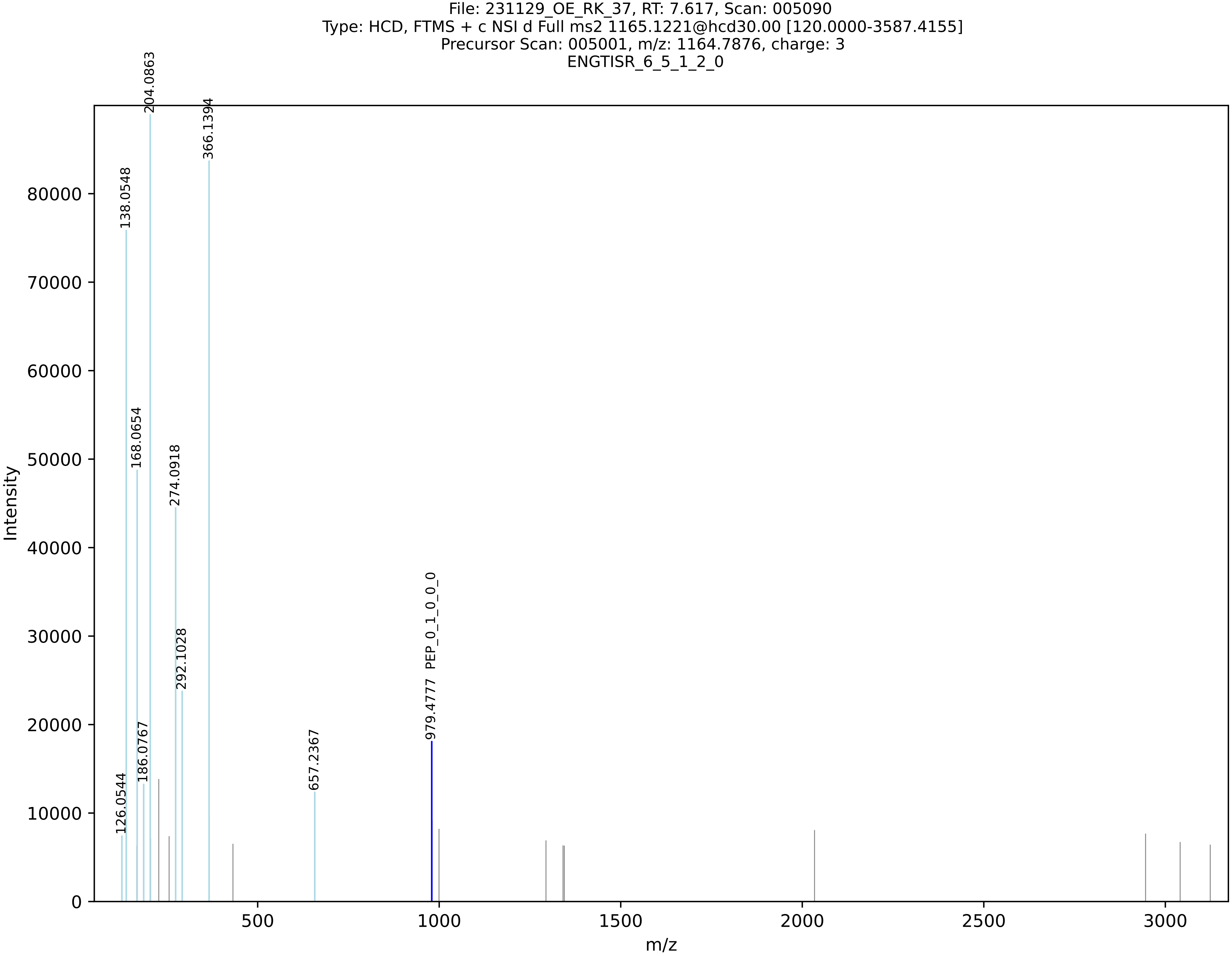

File: 231129_OE_RK_37, RT: 7.617, Scan: 005090
Type: HCD, FTMS + c NSI d Full ms2 1165.1221@hcd30.00 [120.0000-3587.4155]
Precursor Scan: 005001, m/z: 1164.7876, charge: 3
ENGTISR_6_5_1_2_0
Intensity
m/z
204.0863
366.1394
138.0548
168.0654
274.0918
292.1028
186.0767
126.0544
657.2367
979.4777 PEP_0_1_0_0_0_
0
10000
20000
30000
40000
50000
60000
70000
80000
500
1000
1500
2000
2500
3000

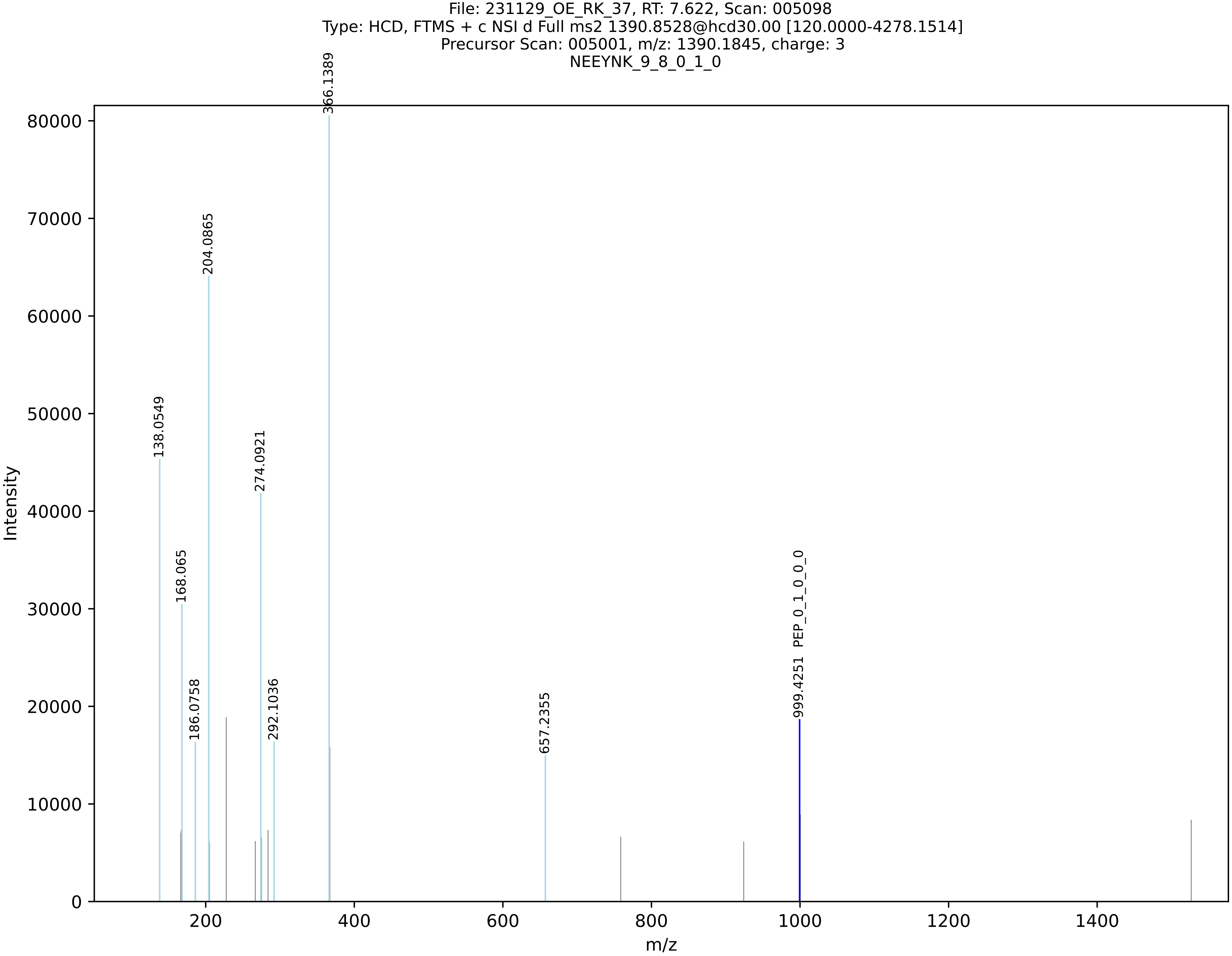
File: 231129_OE_RK_37, RT: 7.622, Scan: 005098
Type: HCD, FTMS + c NSI d Full ms2 1390.8528@hcd30.00 [120.0000-4278.1514]
Precursor Scan: 005001, m/z: 1390.1845, charge: 3
NEEYNK_9_8_0_1_0
366.1389
204.0865
138.0549
274.0921
168.065
186.0758
292.1036
657.2355
999.4251 PEP_0_1_0_0_0
Intensity
m/z
0
10000
20000
30000
40000
50000
60000
70000
80000
200
400
600
800
1000
1200
1400

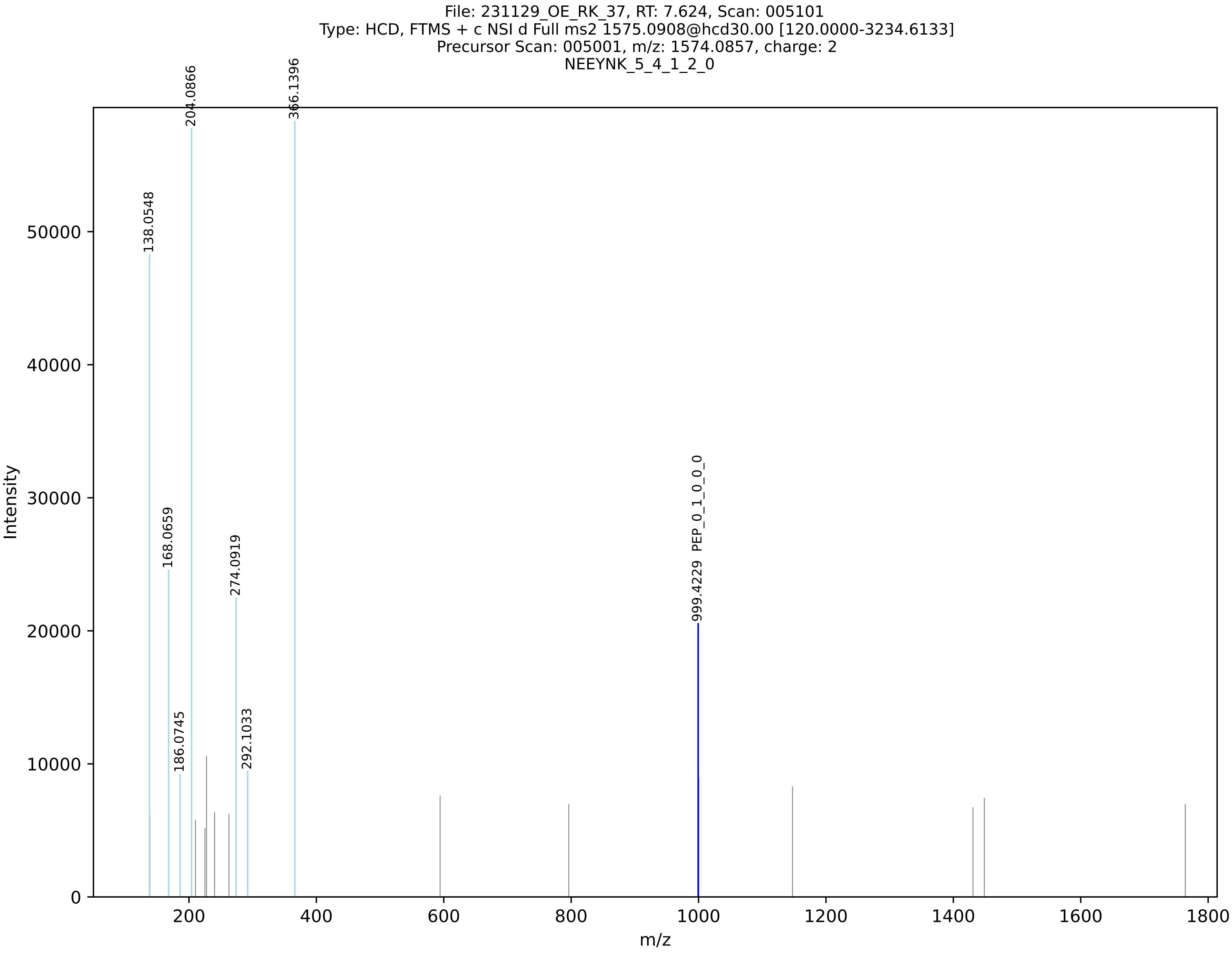
File: 231129_OE_RK_37, RT: 7.624, Scan: 005101
Type: HCD, FTMS + c NSI d Full ms2 1575.0908@hcd30.00 [120.0000-3234.6133]
Precursor Scan: 005001, m/z: 1574.0857, charge: 2
NEEYNK_5_4_1_2_0
Intensity
m/z
138.0548
168.0659
186.0745
204.0866
274.0919
292.1033
366.1396
999.4229 PEP_0_1_0_0_0
0
10000
20000
30000
40000
50000
200
400
600
800
1000
1200
1400
1600
1800

File: 231129_OE_RK_37, RT: 7.659, Scan: 005149
Type: HCD, FTMS + c NSI d Full ms2 1050.4015@hcd30.00 [120.0000-3236.3706]
Precursor Scan: 005074, m/z: 1050.0736, charge: 3
FNYSGSGGR_5_4_0_2_0

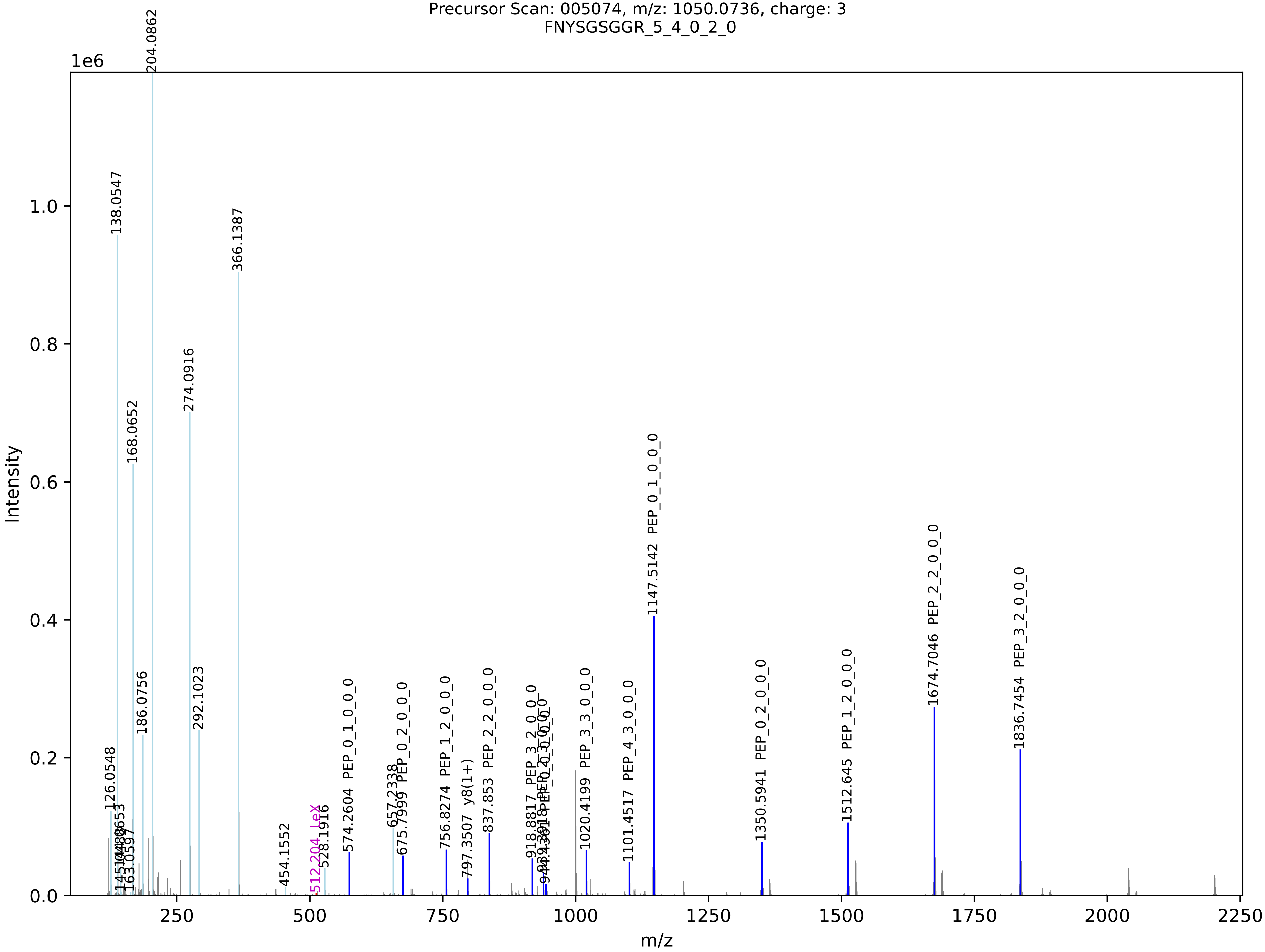

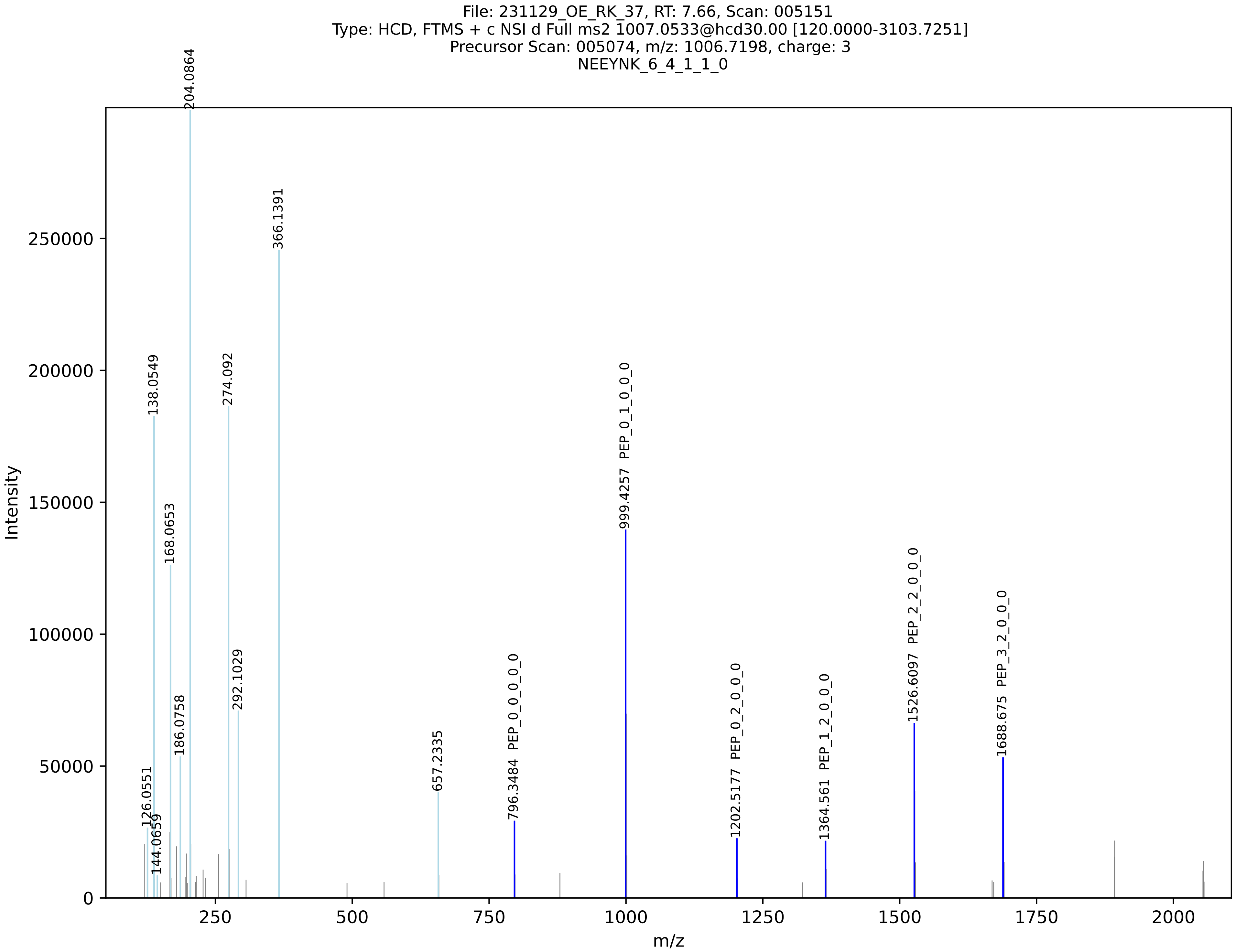
File: 231129_OE_RK_37, RT: 7.66, Scan: 005151
Type: HCD, FTMS + c NSI d Full ms2 1007.0533@hcd30.00 [120.0000-3103.7251]
Precursor Scan: 005074, m/z: 1006.7198, charge: 3
NEEYNK_6_4_1_1_0
Intensity
m/z
204.0864
366.1391
138.0549
274.092
168.0653
186.0758
292.1029
126.0551
144.0659
657.2335
796.3484 PEP_0_0_0_0_0
999.4257 PEP_0_1_0_0_0
1202.5177 PEP_0_2_0_0_0
1364.561 PEP_1_2_0_0_0
1526.6097 PEP_2_2_0_0_0
1688.675 PEP_3_2_0_0_0

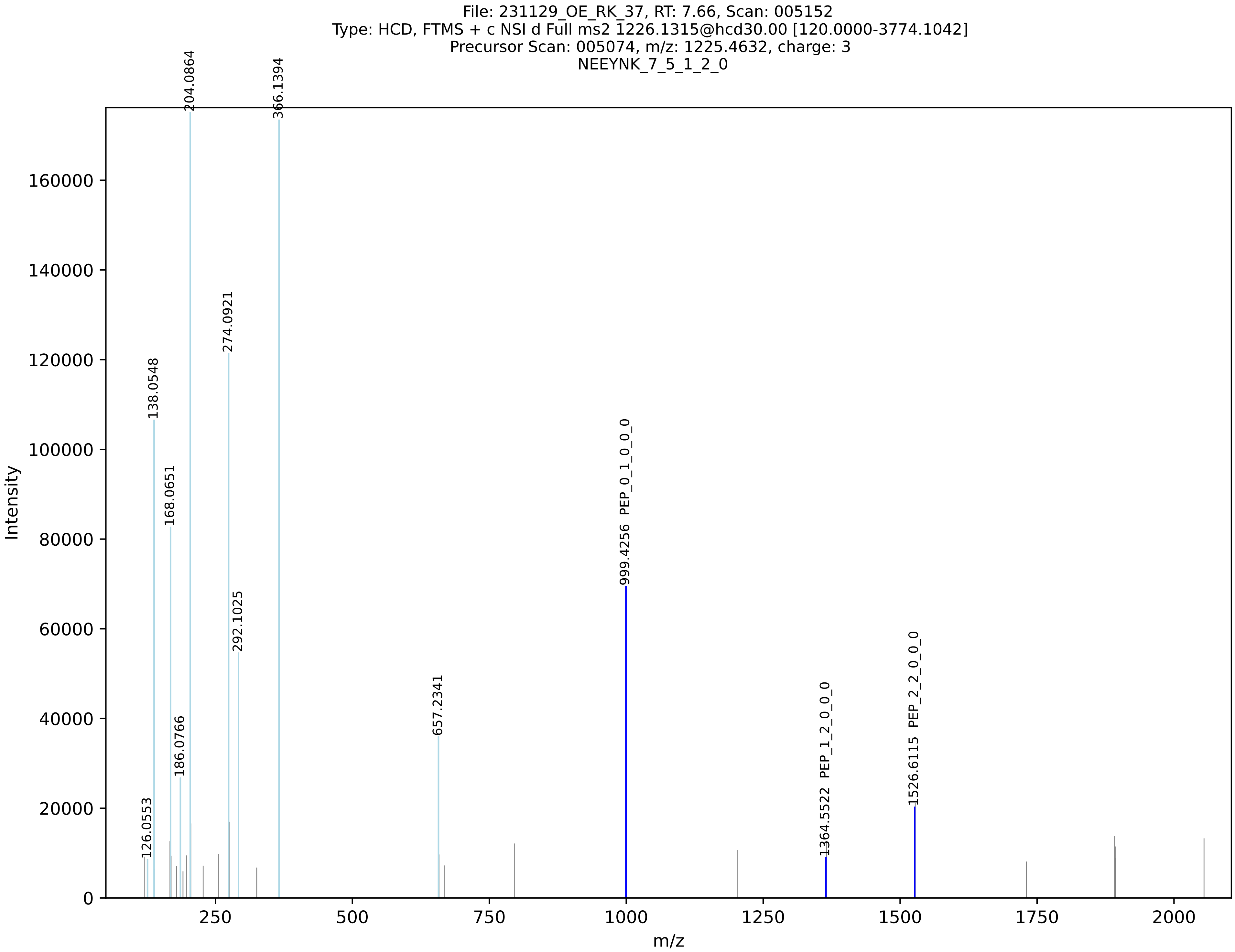
File: 231129_OE_RK_37, RT: 7.66, Scan: 005152
Type: HCD, FTMS + c NSI d Full ms2 1226.1315@hcd30.00 [120.0000-3774.1042]
Precursor Scan: 005074, m/z: 1225.4632, charge: 3
NEEYNK_7_5_1_2_0
204.0864
366.1394
274.0921
138.0548
168.0651
292.1025
186.0766
126.0553
657.2341
999.4256 PEP_0_1_0_0_0
1364.5522 PEP_1_2_0_0_0
1526.6115 PEP_2_2_0_0_0
Intensity
m/z
0
20000
40000
60000
80000
100000
120000
140000
160000
250
500
750
1000
1250
1500
1750
2000

File: 231129_OE_RK_37, RT: 7.71, Scan: 005222
Type: HCD, FTMS + c NSI d Full ms2 1123.0895@hcd30.00 [120.0000-3458.7957]
Precursor Scan: 005148, m/z: 1122.7553, charge: 3
NEEYNK_6_5_0_2_0

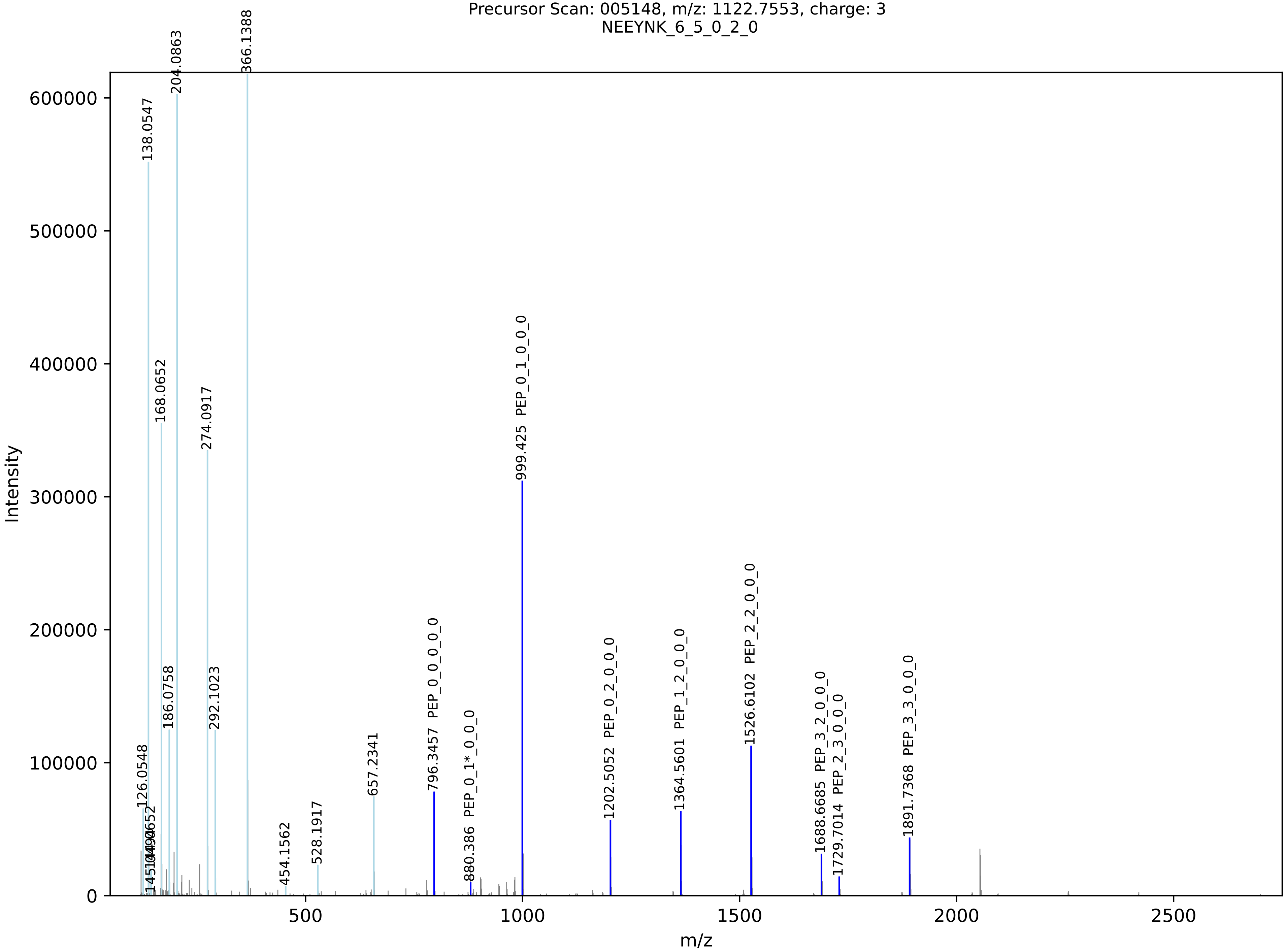

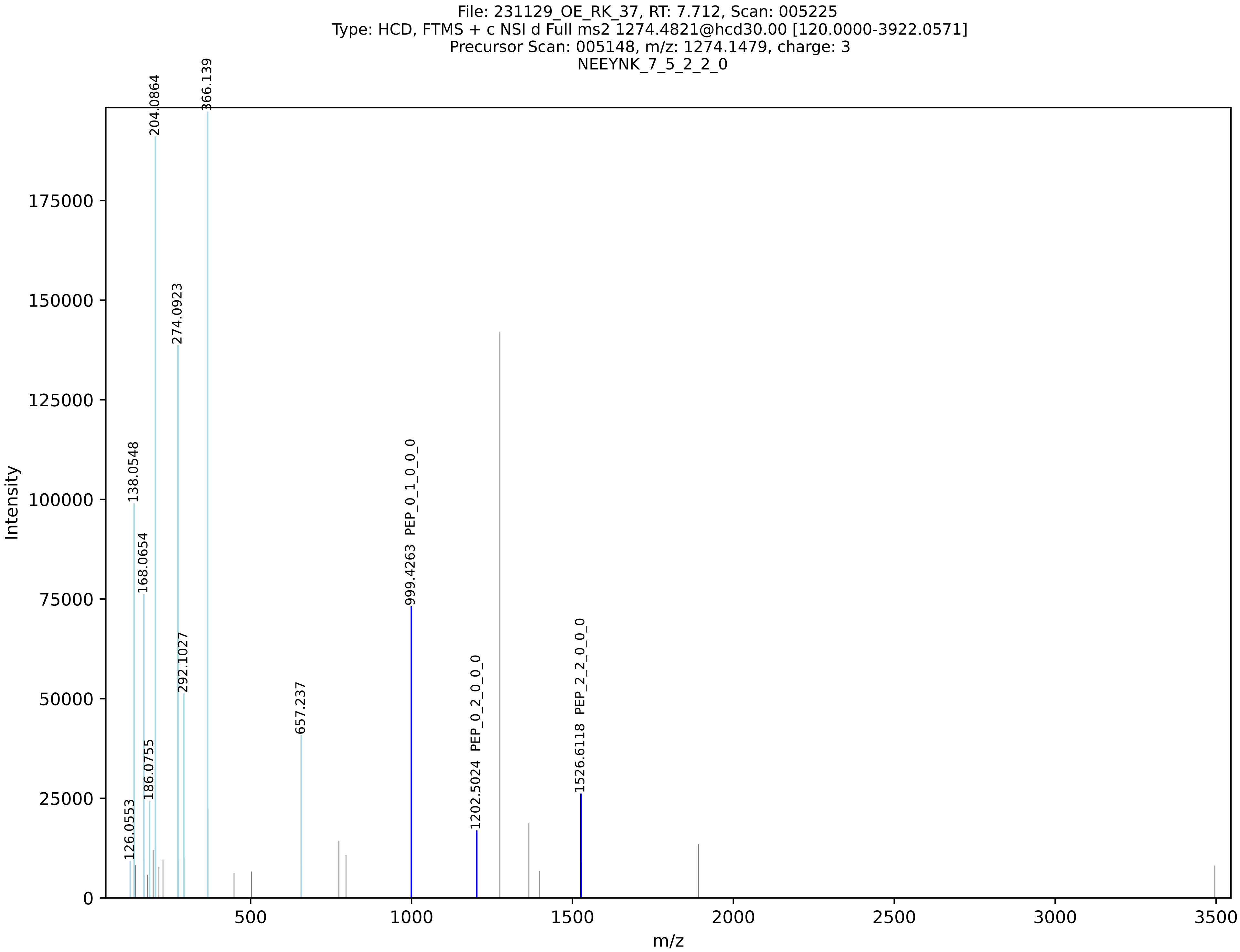

File: 231129_OE_RK_37, RT: 7.712, Scan: 005225
Type: HCD, FTMS + c NSI d Full ms2 1274.4821@hcd30.00 [120.0000-3922.0571]
Precursor Scan: 005148, m/z: 1274.1479, charge: 3
NEEYNK_7_5_2_2_0
Intensity
m/z
366.139
204.0864
274.0923
138.0548
168.0654
292.1027
186.0755
126.0553
657.237
999.4263 PEP_0_1_0_0_0
1202.5024 PEP_0_2_0_0_0
1526.6118 PEP_2_2_0_0_0

File: 231129_OE_RK_37, RT: 7.714, Scan: 005228
Type: HCD, FTMS + c NSI d Full ms2 951.8578@hcd30.00 [120.0000-3906.4360]
Precursor Scan: 005148, m/z: 951.6067, charge: 4
NEEYNK_6_5_1_3_0

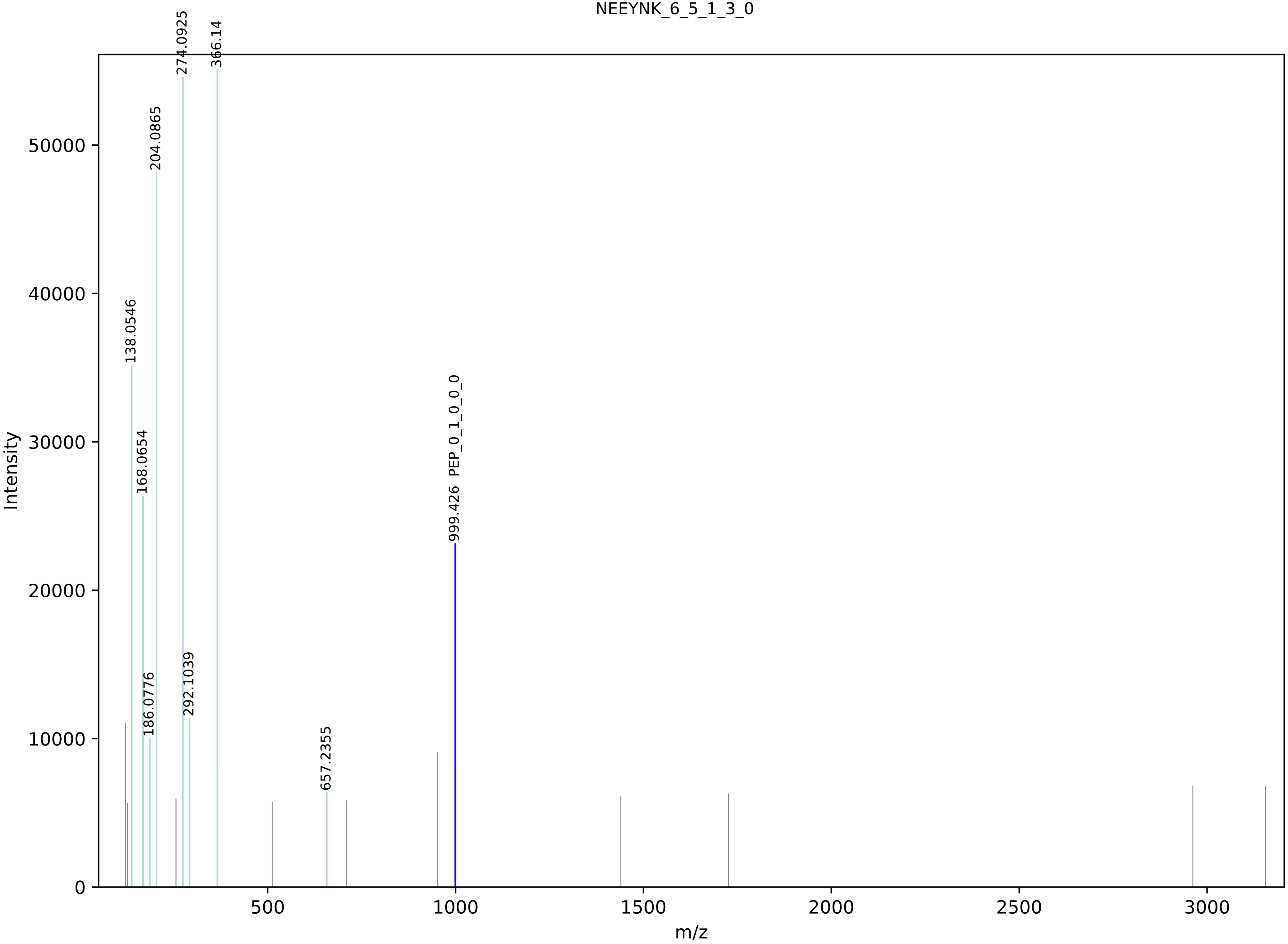

File: 231129_OE_RK_37, RT: 7.737, Scan: 005265
Type: HCD, FTMS + c NSI d Full ms2 1356.0133@hcd30.00 [120.0000-2787.6951]
Precursor Scan: 005148, m/z: 1355.5118, charge: 2
NEEYNK_5_4_0_1_0

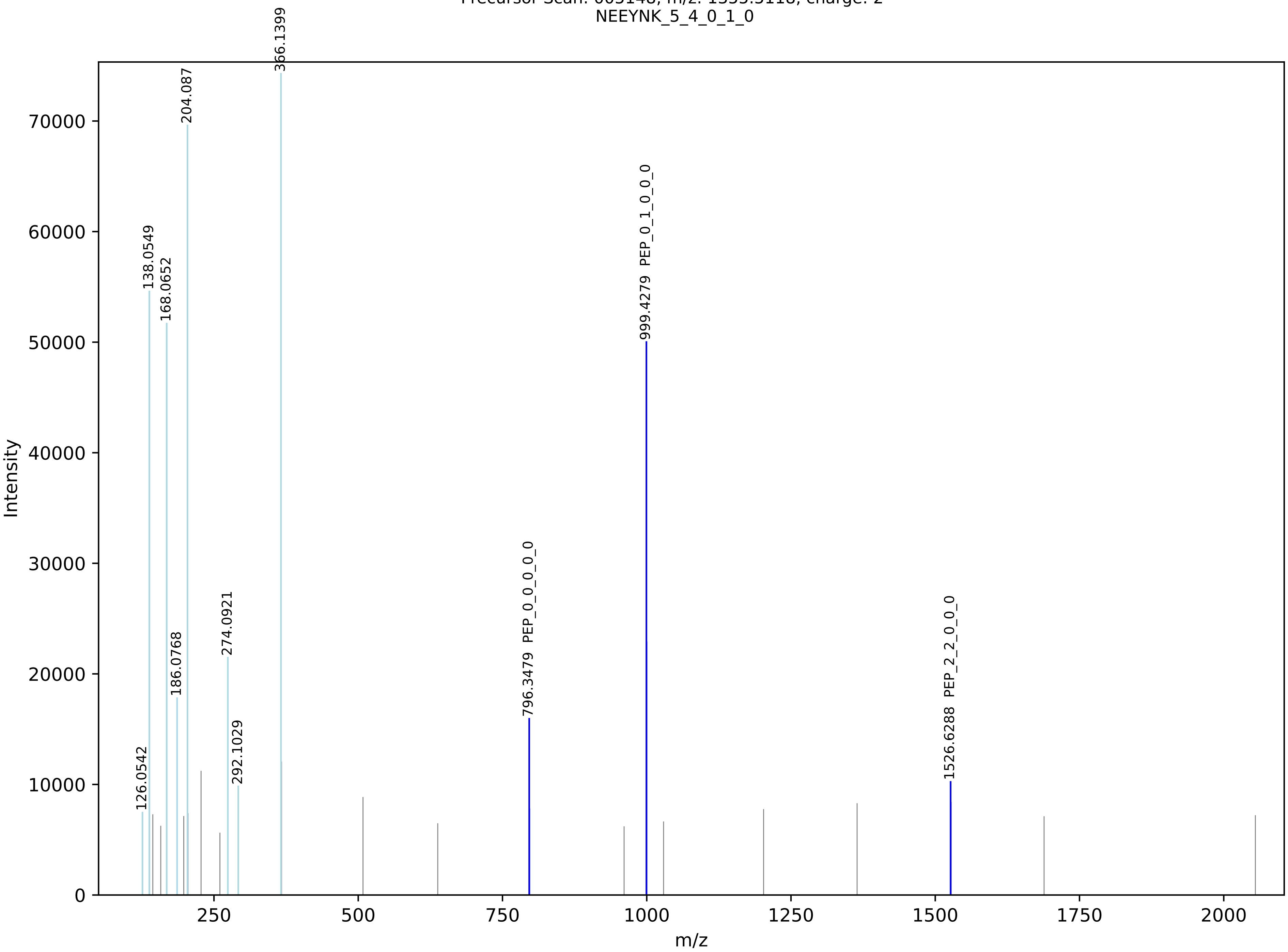

File: 231129_OE_RK_37, RT: 7.742, Scan: 005273
Type: HCD, FTMS + c NSI d Full ms2 1170.7975@hcd30.00 [120.0000-3604.7822]
Precursor Scan: 005148, m/z: 1170.7975, charge: 3
NEEYNK_6_5_1_2_0

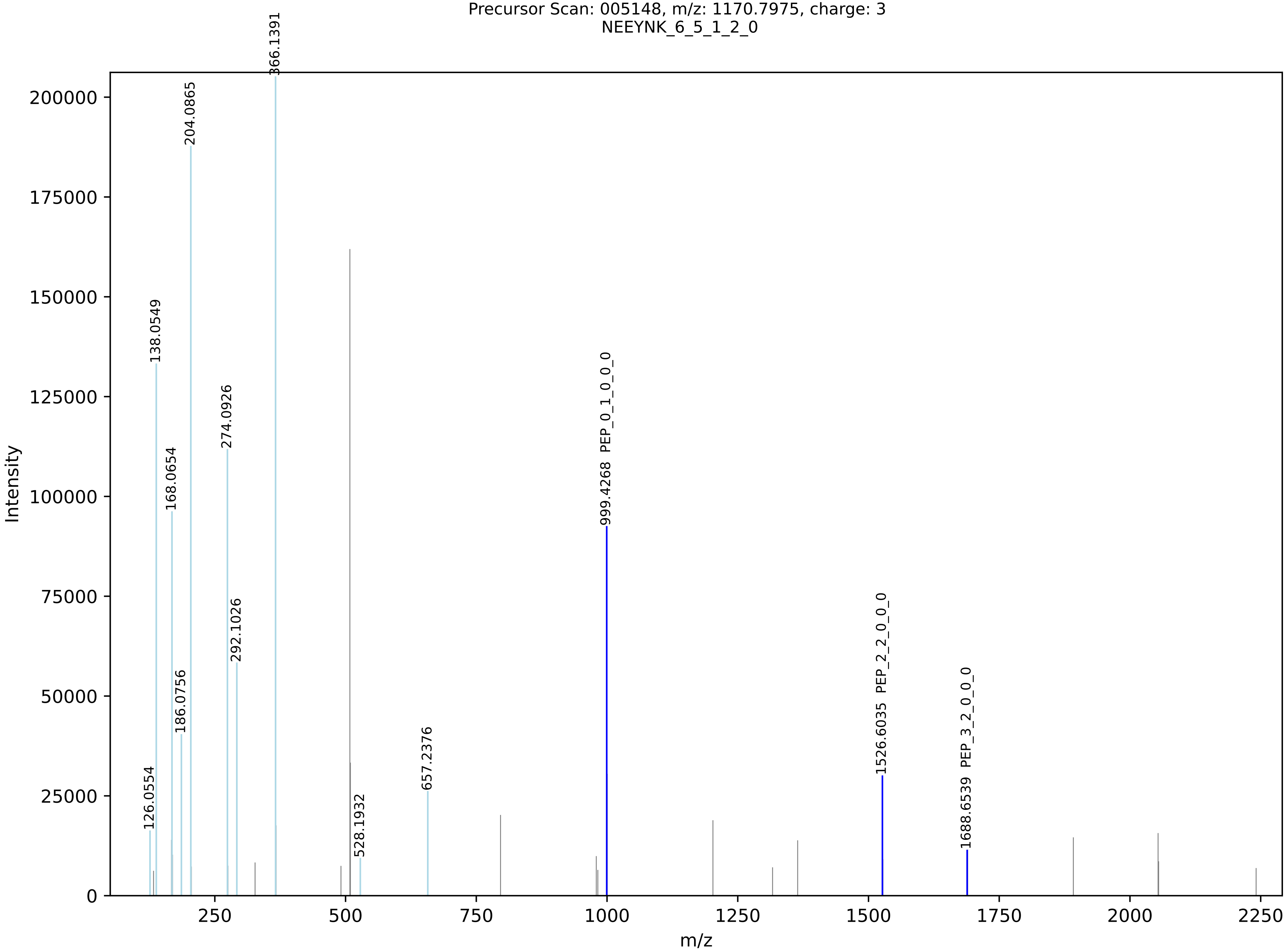

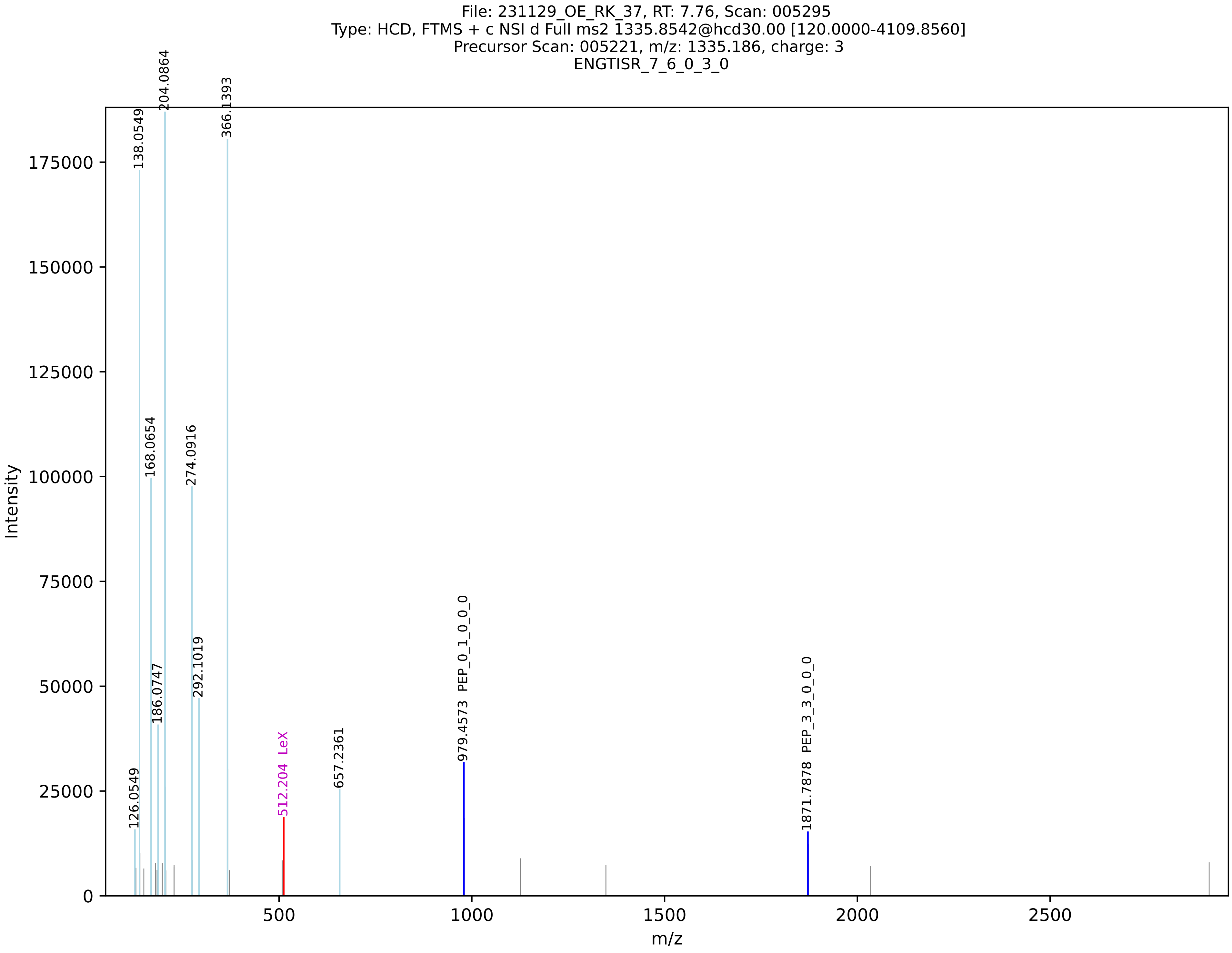
File: 231129_OE_RK_37, RT: 7.76, Scan: 005295
Type: HCD, FTMS + c NSI d Full ms2 1335.8542@hcd30.00 [120.0000-4109.8560]
Precursor Scan: 005221, m/z: 1335.186, charge: 3
ENGTISR_7_6_0_3_0
Intensity
m/z
204.0864
366.1393
138.0549
168.0654
274.0916
292.1019
186.0747
126.0549
512.204 LeX
657.2361
979.4573 PEP_0_1_0_0_0
1871.7878 PEP_3_3_0_0_0
0
25000
50000
75000
100000
125000
150000
175000
500
1000
1500
2000
2500

File: 231129_OE_RK_37, RT: 7.81, Scan: 005367
Type: HCD, FTMS + c NSI d Full ms2 1220.1210@hcd30.00 [120.0000-3755.7122]
Precursor Scan: 005291, m/z: 1219.7871, charge: 3
NEEYNK_6_5_0_3_0

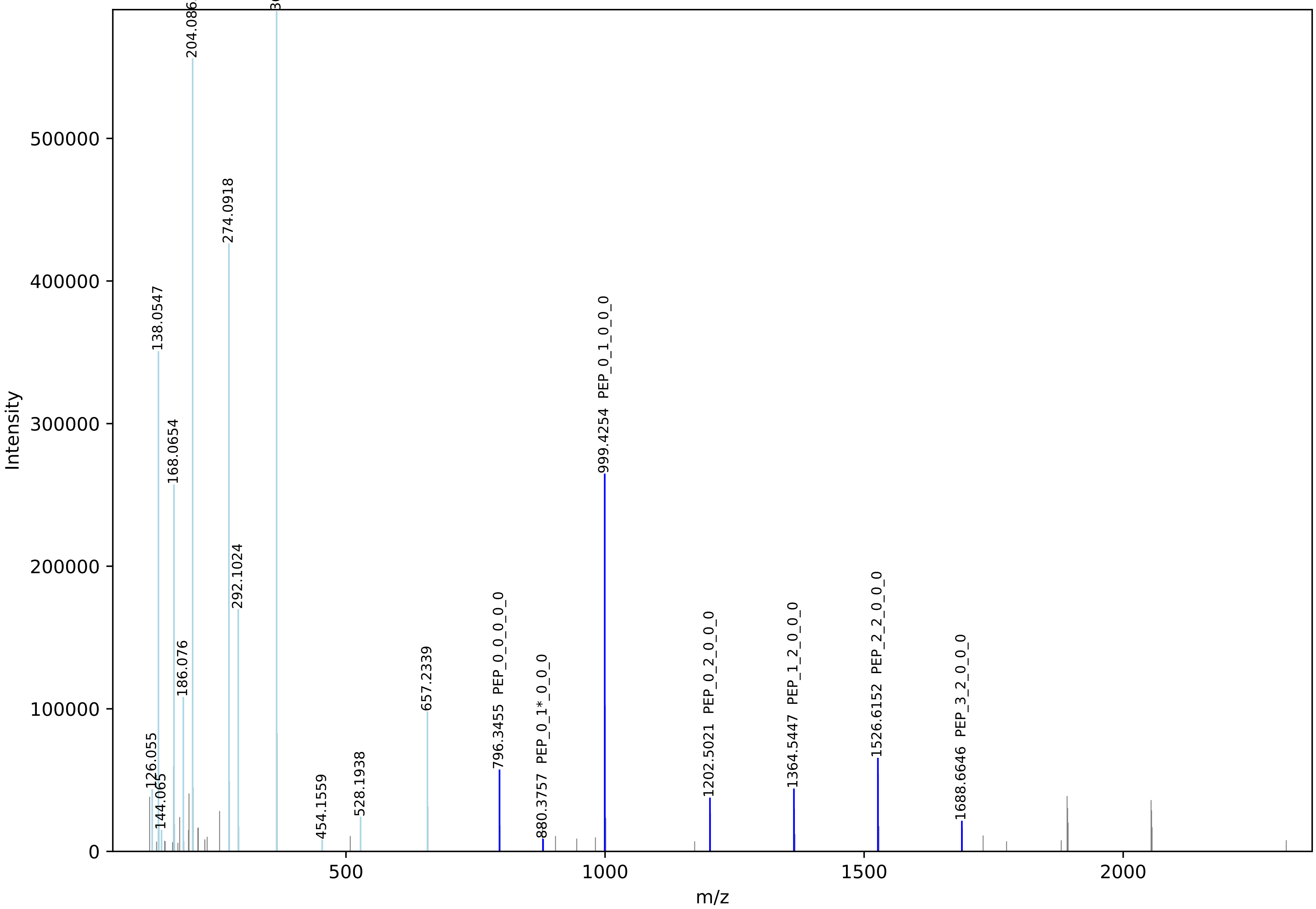

File: 231129_OE_RK_37, RT: 7.851, Scan: 005427
Type: HCD, FTMS + c NSI d Full ms2 1501.5643@hcd30.00 [120.0000-3084.6191]
Precursor Scan: 005365, m/z: 1501.0631, charge: 2
NEEYNK_5_4_0_2_0

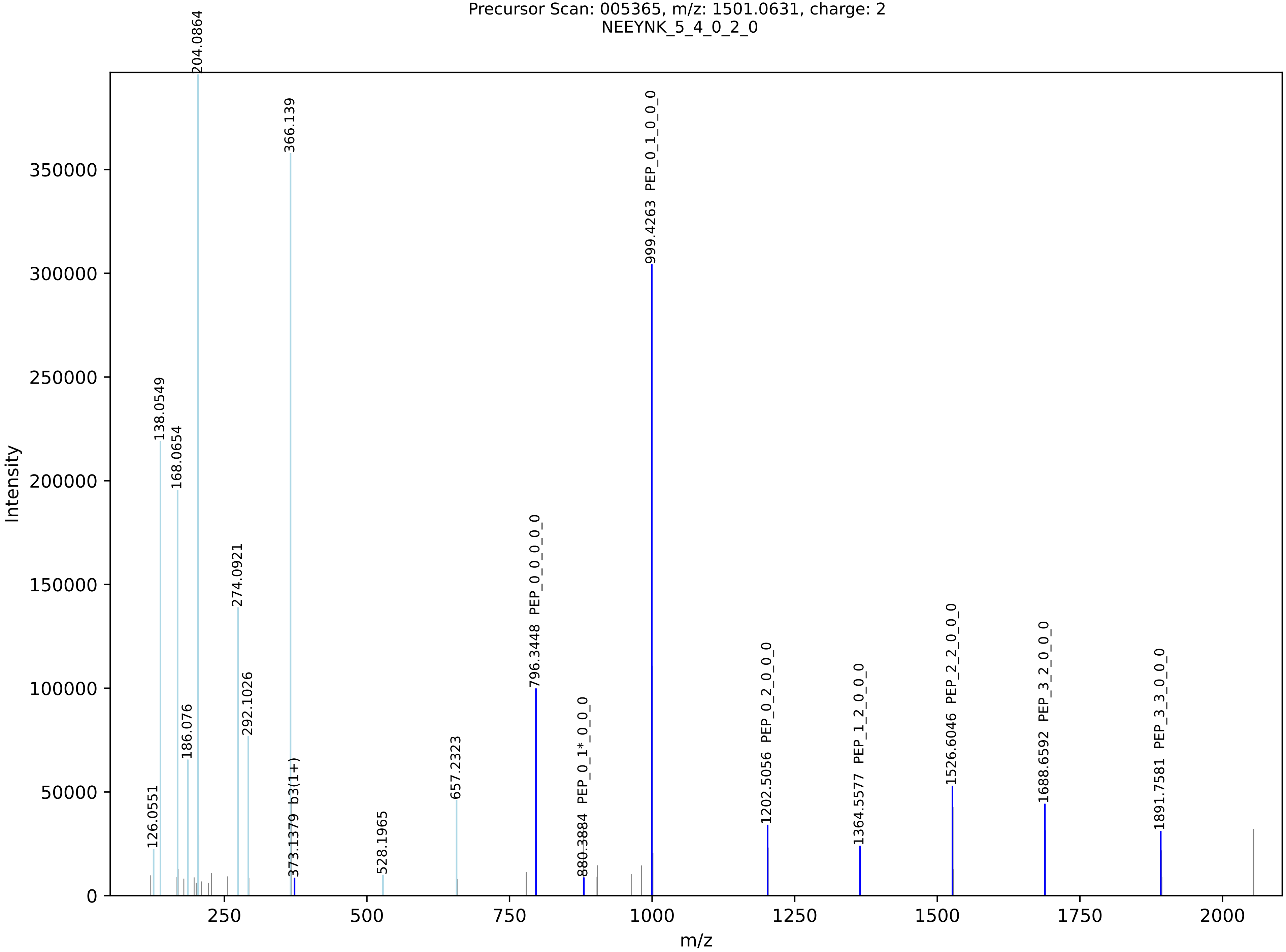

File: 231129_OE_RK_37, RT: 7.852, Scan: 005428
Type: HCD, FTMS + c NSI d Full ms2 1225.7982@hcd30.00 [120.0000-3773.0845]
Precursor Scan: 005365, m/z: 1225.4641, charge: 3
NEEYNK_7_5_1_2_0

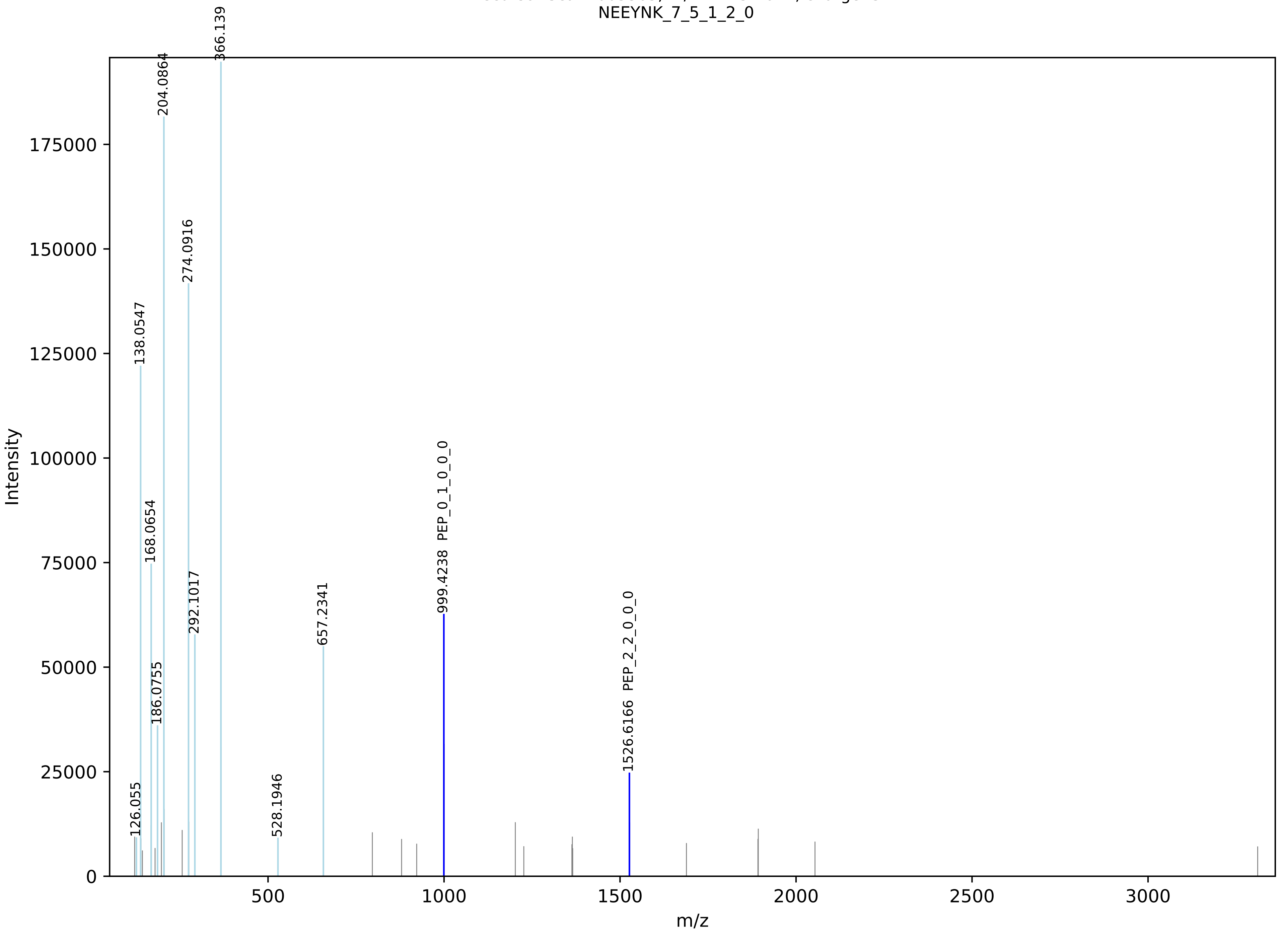

File: 231129_OE_RK_37, RT: 7.853, Scan: 005430
Type: HCD, FTMS + c NSI d Full ms2 1006.7195@hcd30.00 [120.0000-3102.7039]
Precursor Scan: 005365, m/z: 1006.7196, charge: 3
NEEYNK_6_4_1_1_0

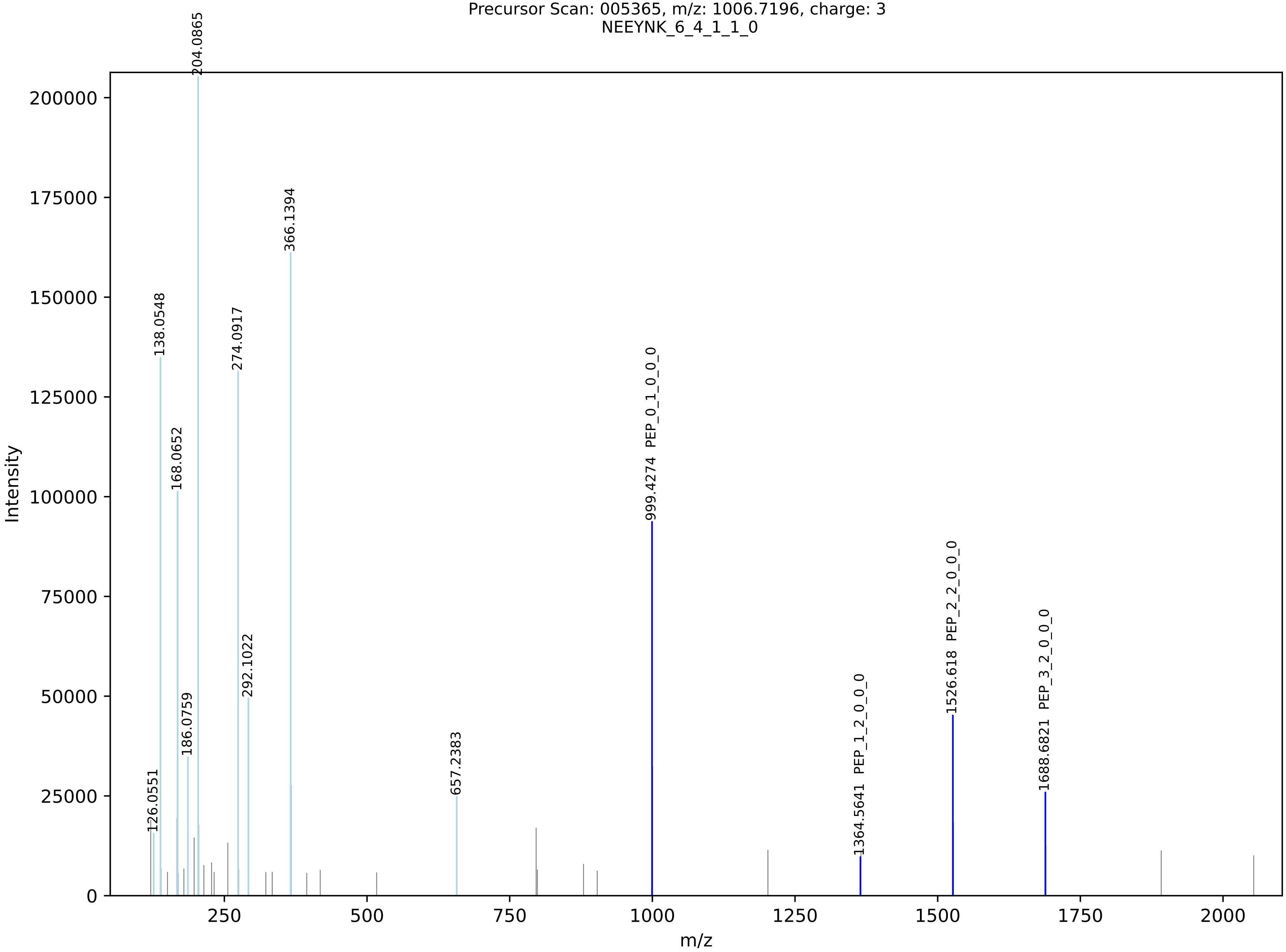

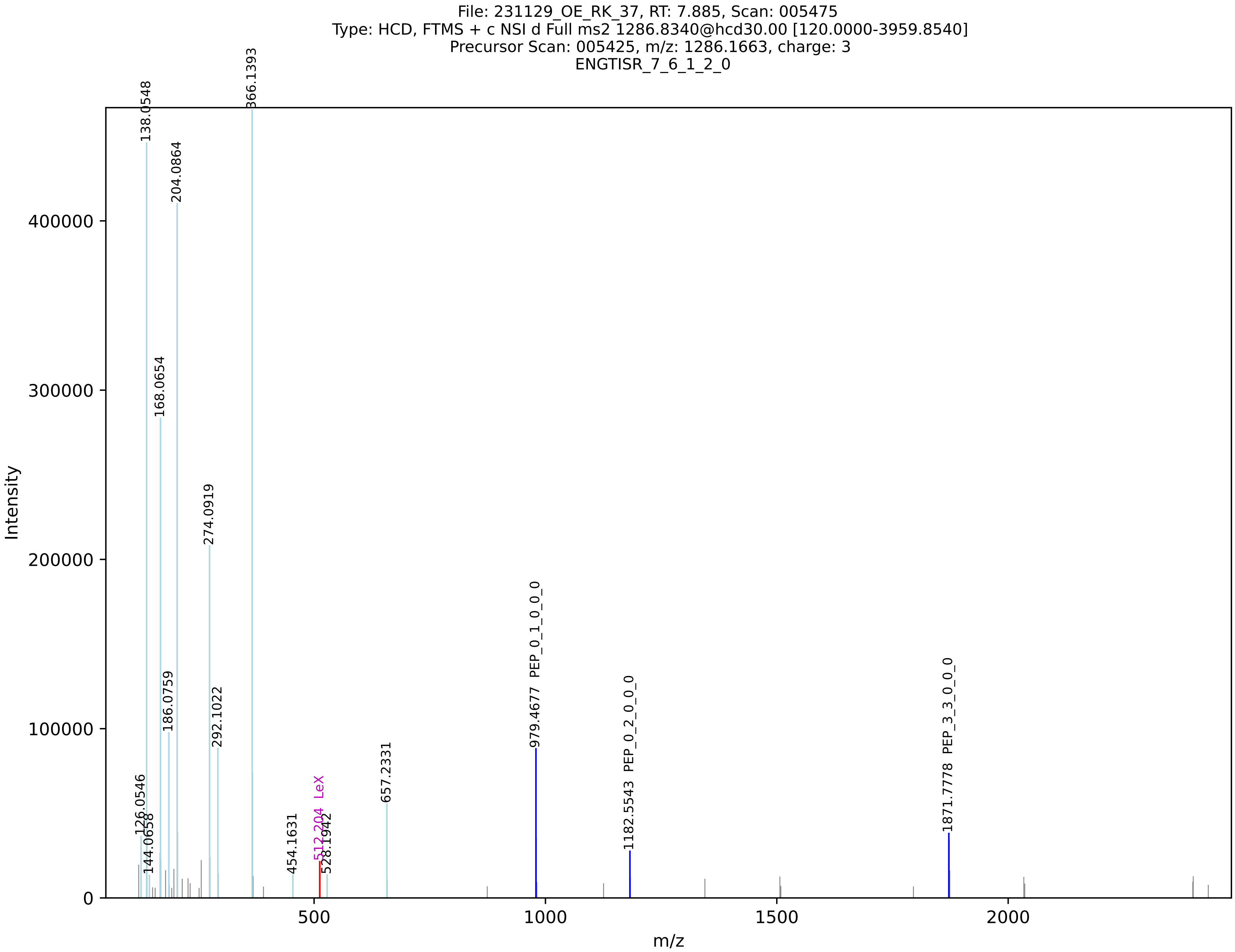
File: 231129_OE_RK_37, RT: 7.885, Scan: 005475
Type: HCD, FTMS + c NSI d Full ms2 1286.8340@hcd30.00 [120.0000-3959.8540]
Precursor Scan: 005425, m/z: 1286.1663, charge: 3
ENGTISR_7_6_1_2_0
Intensity
m/z
366.1393
138.0548
204.0864
168.0654
274.0919
186.0759
292.1022
126.0546
144.0658
454.1631
512.204 LeX
528.1942
657.2331
979.4677 PEP_0_1_0_0_0
1182.5543 PEP_0_2_0_0_0
1871.7778 PEP_3_3_0_0_0

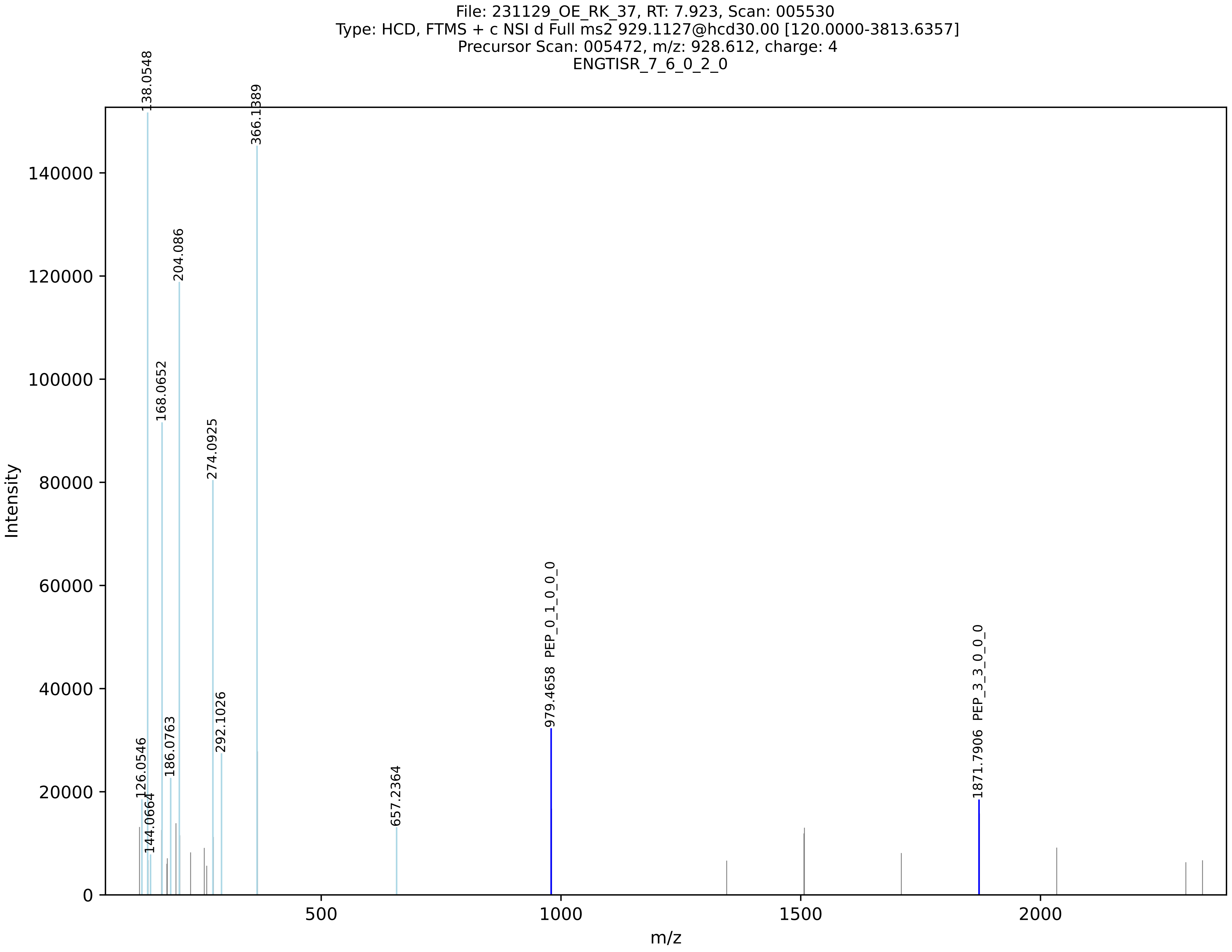

File: 231129_OE_RK_37, RT: 7.923, Scan: 005530
Type: HCD, FTMS + c NSI d Full ms2 929.1127@hcd30.00 [120.0000-3813.6357]
Precursor Scan: 005472, m/z: 928.612, charge: 4
ENGTISR_7_6_0_2_0
138.0548
366.1389
204.086
168.0652
274.0925
292.1026
186.0763
126.0546
144.0664
657.2364
979.4658 PEP_0_1_0_0_0
1871.7906 PEP_3_3_0_0_0
Intensity
m/z
0
20000
40000
60000
80000
100000
120000
140000
500
1000
1500
2000

File: 231129_OE_RK_37, RT: 7.925, Scan: 005533
Type: HCD, FTMS + c NSI d Full ms2 952.1077@hcd30.00 [120.0000-3907.4553]
Precursor Scan: 005472, m/z: 951.6065, charge: 4
NEEYNK_6_5_1_3_0

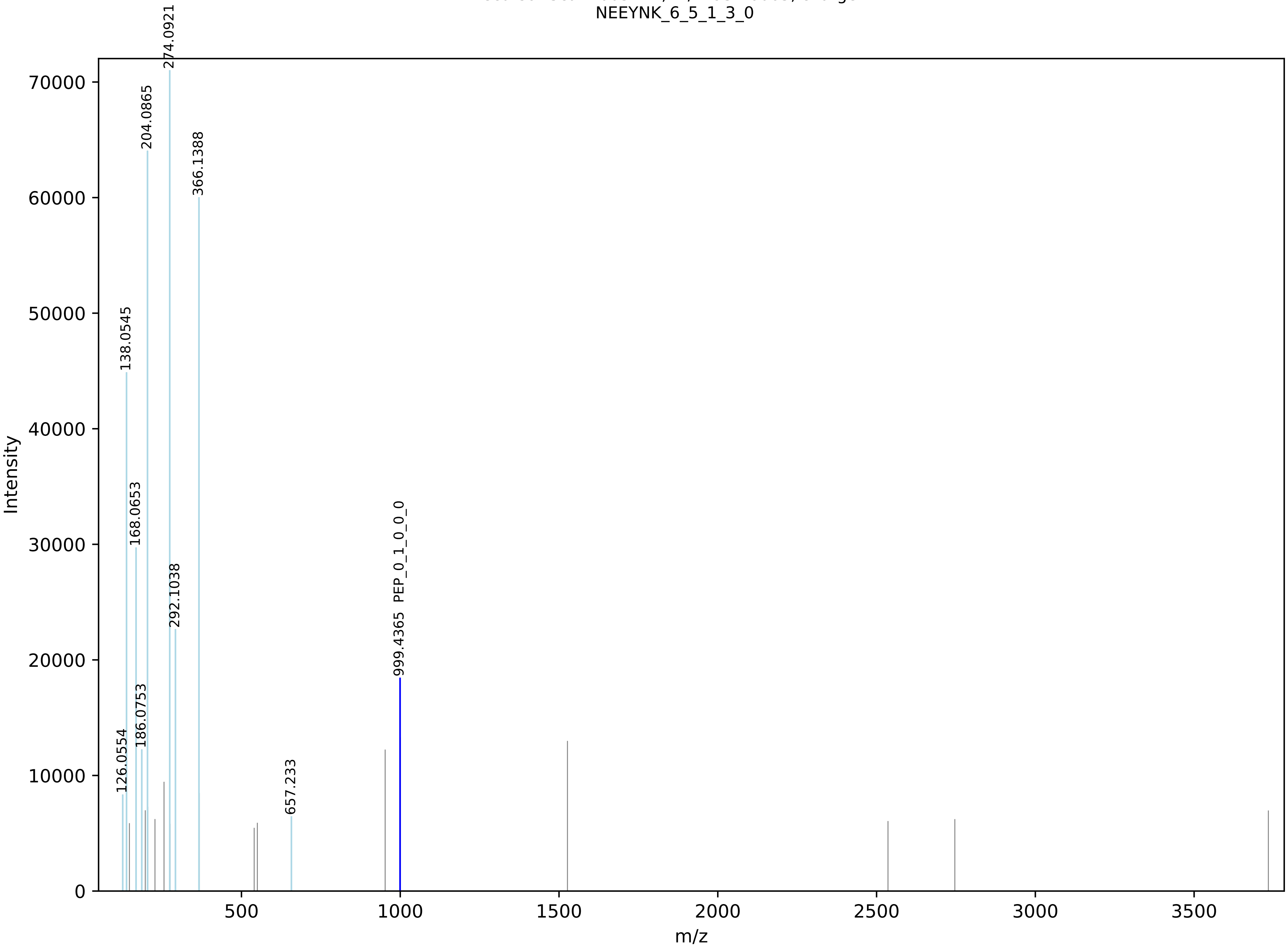

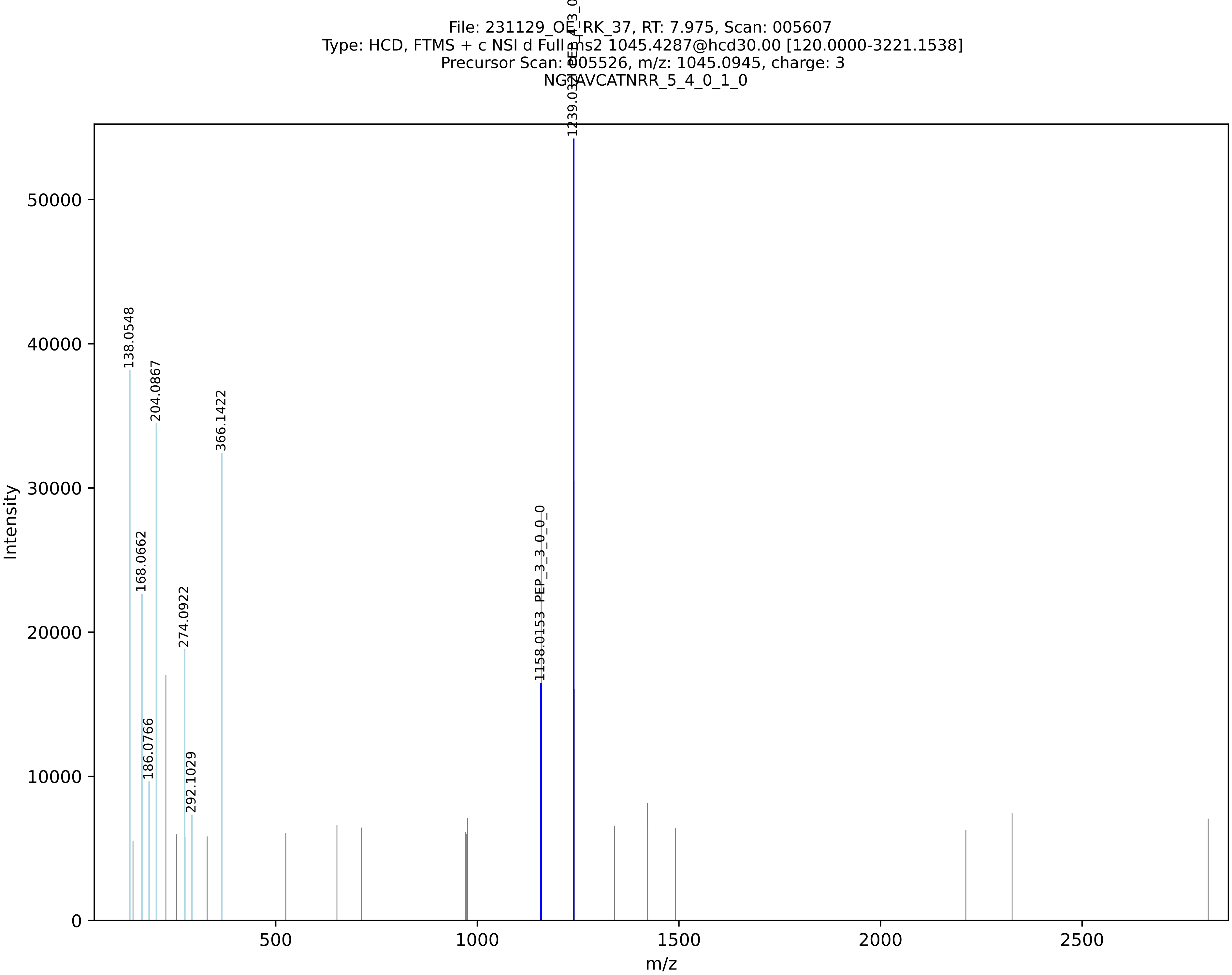
File: 231129_OE_RK_37, RT: 7.975, Scan: 005607
Type: HCD, FTMS + c NSI d Full ms2 1045.4287@hcd30.00 [120.0000-3221.1538]
Precursor Scan: 005526, m/z: 1045.0945, charge: 3
NGTAVCATNRR_5_4_0_1_0
138.0548
168.0662
186.0766
204.0867
274.0922
292.1029
366.1422
1158.0153_PEP_3_3_0_0_0
Intensity
m/z
0
10000
20000
30000
40000
50000
500
1000
1500
2000
2500

File: 231129_OE_RK_37, RT: 8.022, Scan: 005675
Type: HCD, FTMS + c NSI d Full ms2 1001.3790@hcd30.00 [120.0000-3086.3616]
Precursor Scan: 005600, m/z: 1001.0448, charge: 3
NEEYNK_5_4_0_2_0

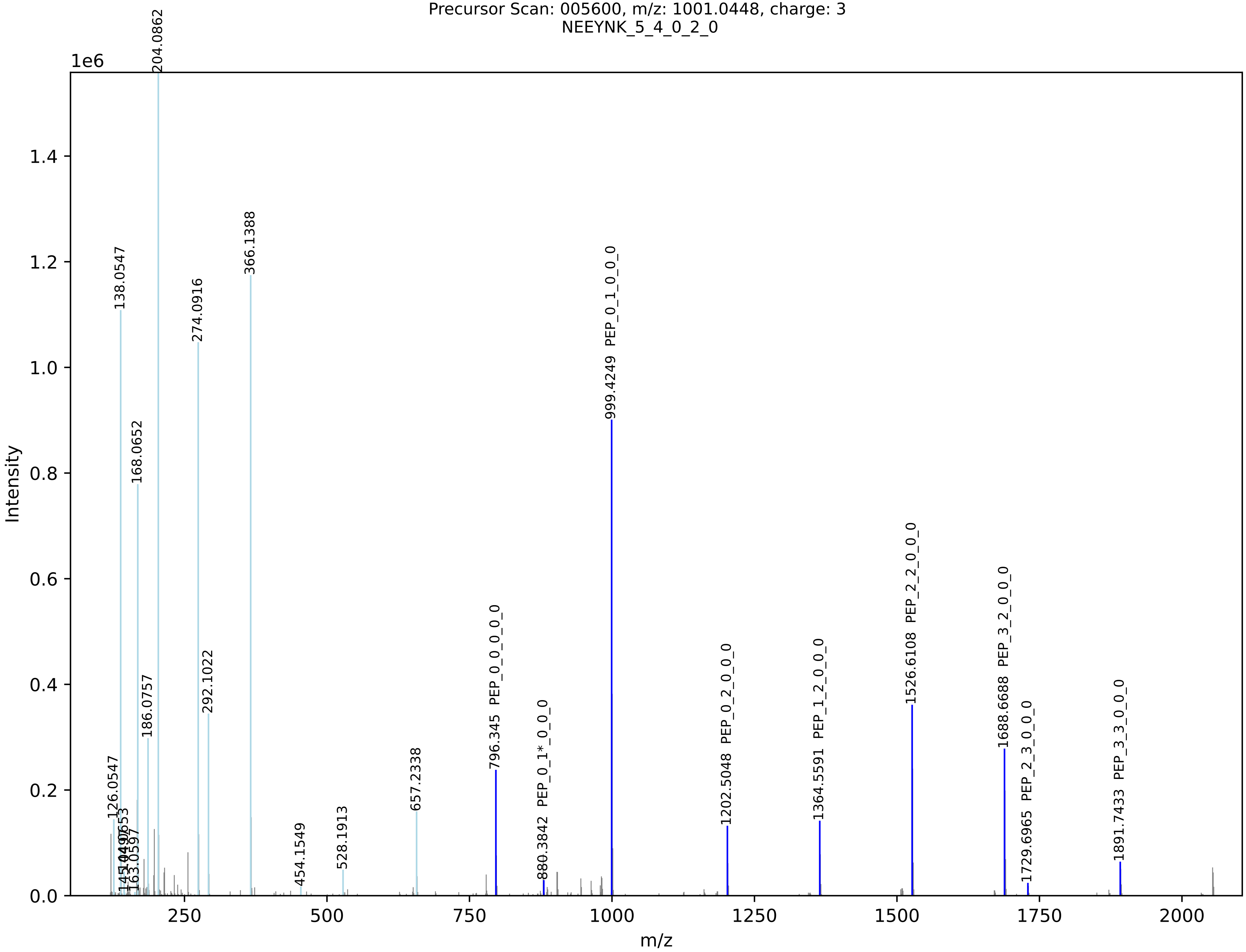

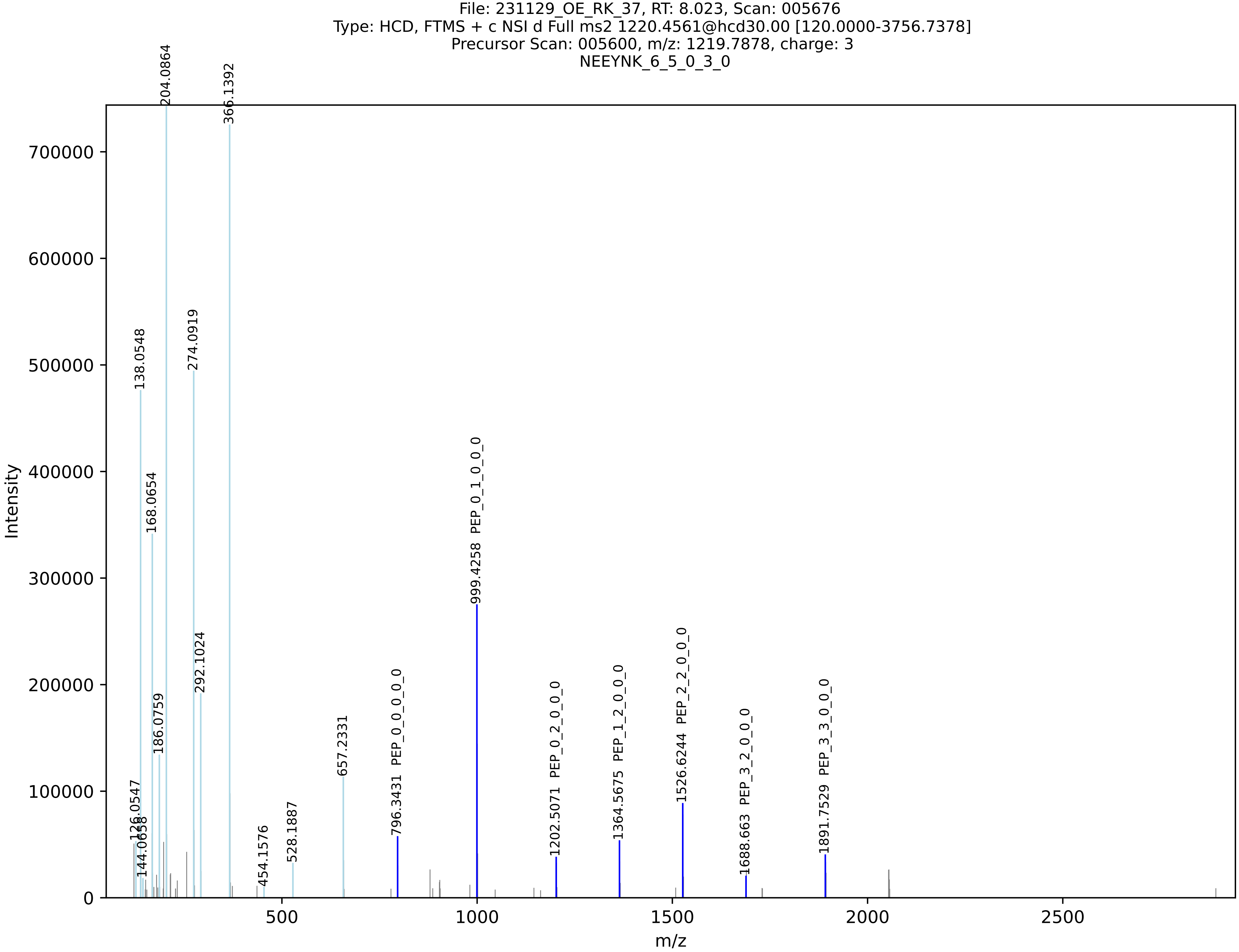
File: 231129_OE_RK_37, RT: 8.023, Scan: 005676
Type: HCD, FTMS + c NSI d Full ms2 1220.4561@hcd30.00 [120.0000-3756.7378]
Precursor Scan: 005600, m/z: 1219.7878, charge: 3
NEEYNK_6_5_0_3_0
Intensity
m/z
204.0864
366.1392
138.0548
274.0919
168.0654
292.1024
186.0759
126.0547
144.0658
454.1576
528.1887
657.2331
796.3431 PEP_0_0_0_0_0
999.4258 PEP_0_1_0_0_0
1202.5071 PEP_0_2_0_0_0
1364.5675 PEP_1_2_0_0_0
1526.6244 PEP_2_2_0_0_0
1688.663 PEP_3_2_0_0_0
1891.7529 PEP_3_3_0_0_0
0
100000
200000
300000
400000
500000
600000
700000
500
1000
1500
2000
2500

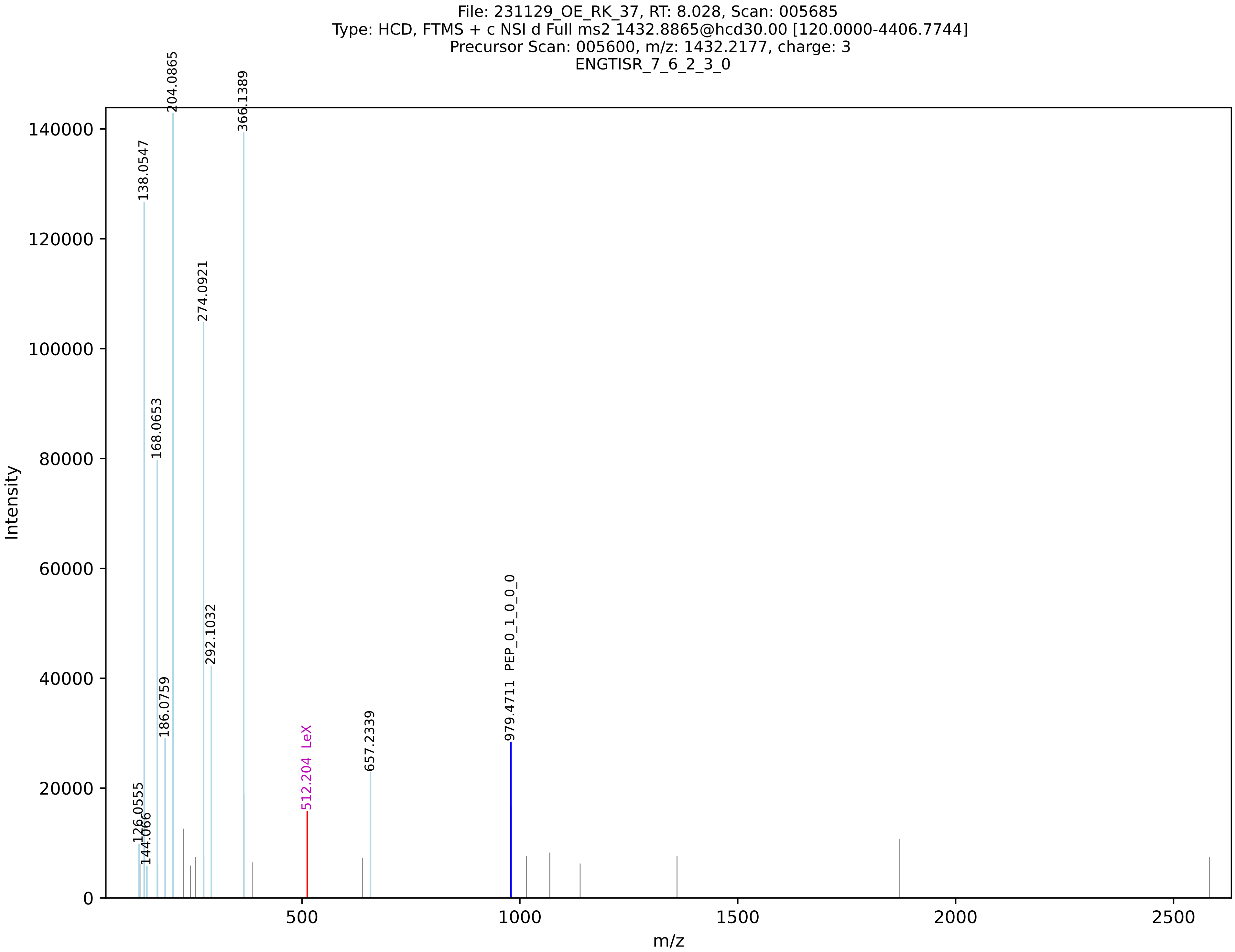

File: 231129_OE_RK_37, RT: 8.028, Scan: 005685
Type: HCD, FTMS + c NSI d Full ms2 1432.8865@hcd30.00 [120.0000-4406.7744]
Precursor Scan: 005600, m/z: 1432.2177, charge: 3
ENGTISR_7_6_2_3_0
Intensity
m/z
0
20000
40000
60000
80000
100000
120000
140000
500
1000
1500
2000
2500
204.0865
366.1389
138.0547
274.0921
168.0653
292.1032
186.0759
126.0555
144.066
512.204 LeX
657.2339
979.4711 PEP_0_1_0_0_0

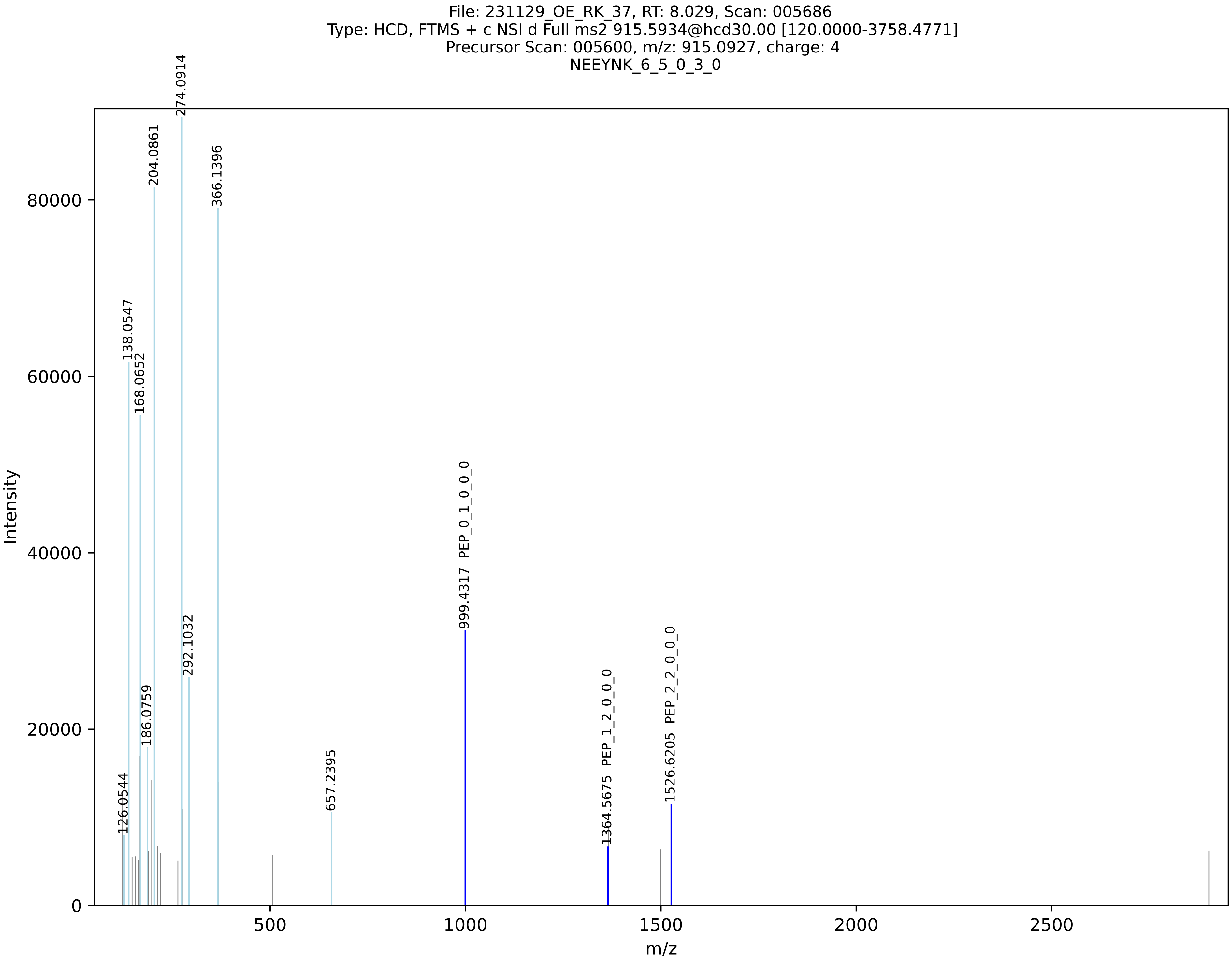

File: 231129_OE_RK_37, RT: 8.029, Scan: 005686
Type: HCD, FTMS + c NSI d Full ms2 915.5934@hcd30.00 [120.0000-3758.4771]
Precursor Scan: 005600, m/z: 915.0927, charge: 4
NEEYNK_6_5_0_3_0
Intensity
m/z
0
20000
40000
60000
80000
500
1000
1500
2000
2500
274.0914
204.0861
366.1396
138.0547
168.0652
186.0759
126.0544
292.1032
657.2395
999.4317 PEP_0_1_0_0_0
1364.5675 PEP_1_2_0_0_0
1526.6205 PEP_2_2_0_0_0

File: 231129_OE_RK_37, RT: 8.073, Scan: 005749
Type: HCD, FTMS + c NSI d Full ms2 1269.1421@hcd30.00 [120.0000-3905.7168]
Precursor Scan: 005674, m/z: 1268.474, charge: 3
NEEYNK_6_5_1_3_0

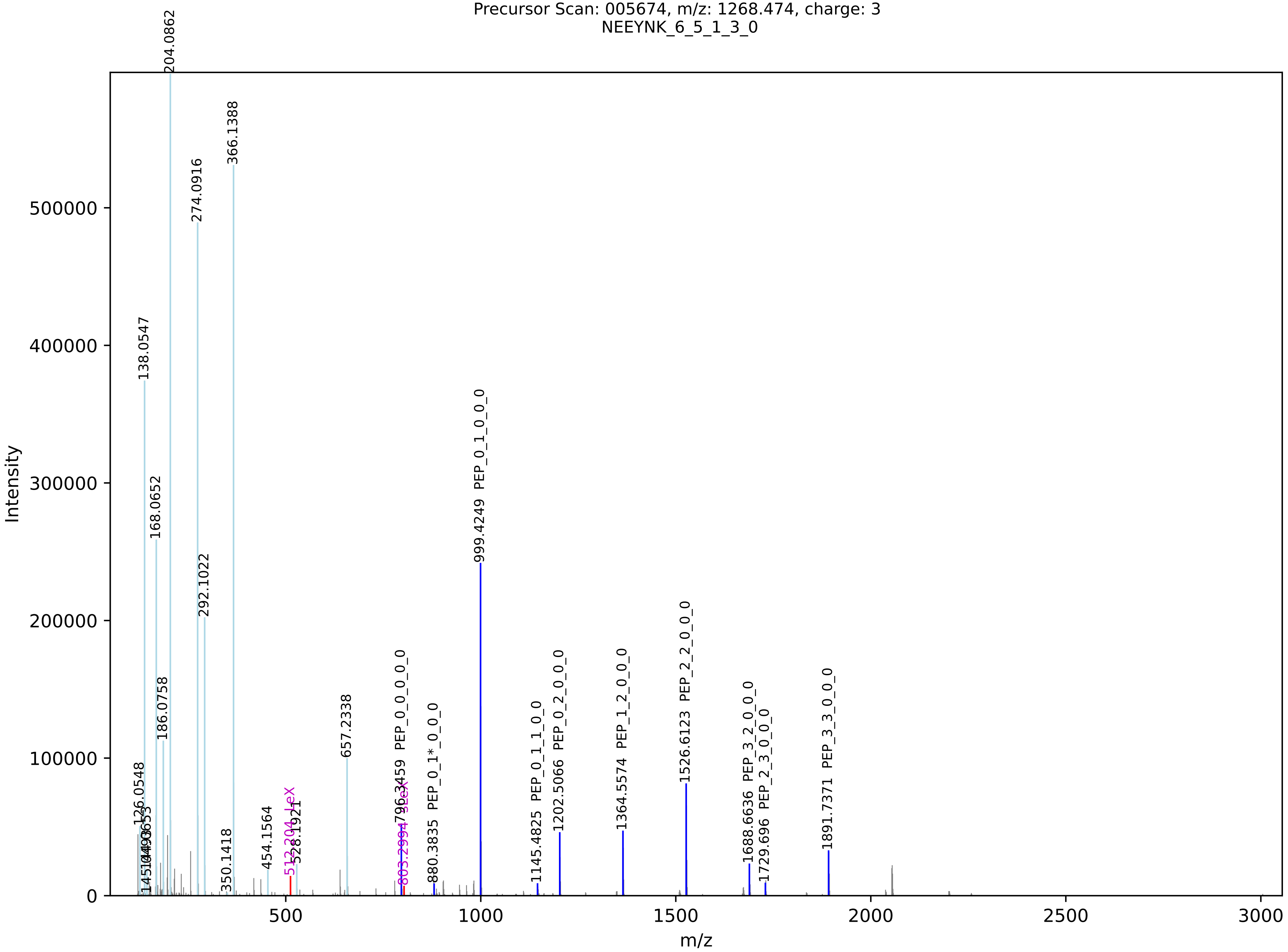

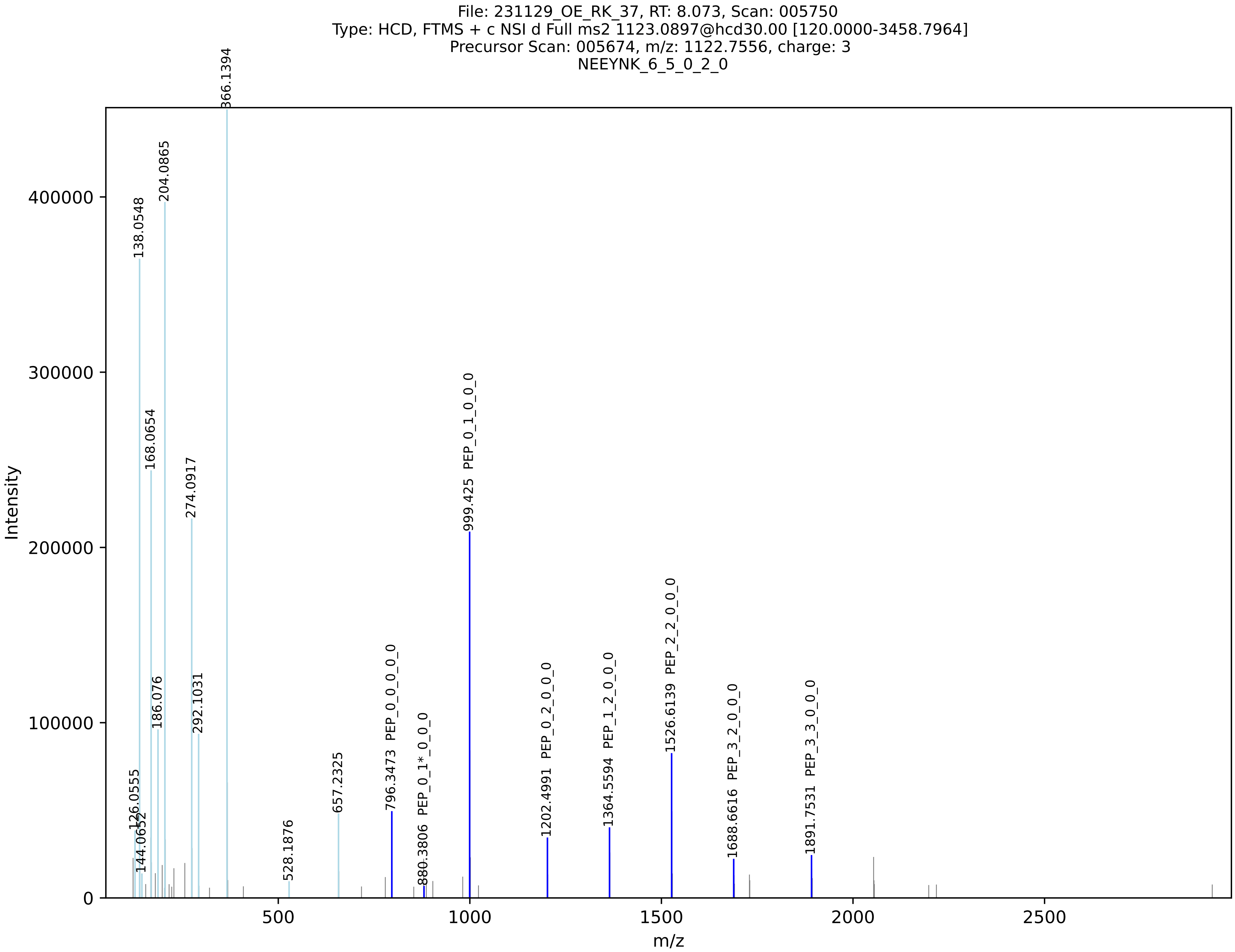
File: 231129_OE_RK_37, RT: 8.073, Scan: 005750
Type: HCD, FTMS + c NSI d Full ms2 1123.0897@hcd30.00 [120.0000-3458.7964]
Precursor Scan: 005674, m/z: 1122.7556, charge: 3
NEEYNK_6_5_0_2_0
366.1394
204.0865
138.0548
168.0654
274.0917
186.076
292.1031
126.0555
144.0652
528.1876
657.2325
796.3473 PEP_0_0_0_0_0
880.3806 PEP_0_1*_0_0_0
999.425 PEP_0_1_0_0_0
1202.4991 PEP_0_2_0_0_0
1364.5594 PEP_1_2_0_0_0
1526.6139 PEP_2_2_0_0_0
1688.6616 PEP_3_2_0_0_0
1891.7531 PEP_3_3_0_0_0
Intensity
m/z
0
100000
200000
300000
400000
500
1000
1500
2000
2500

File: 231129_OE_RK_37, RT: 8.078, Scan: 005757
Type: HCD, FTMS + c NSI d Full ms2 921.5688@hcd30.00 [120.0000-4723.5708]
Precursor Scan: 005674, m/z: 921.168, charge: 5
KANSSVGK_9_8_1_2_0

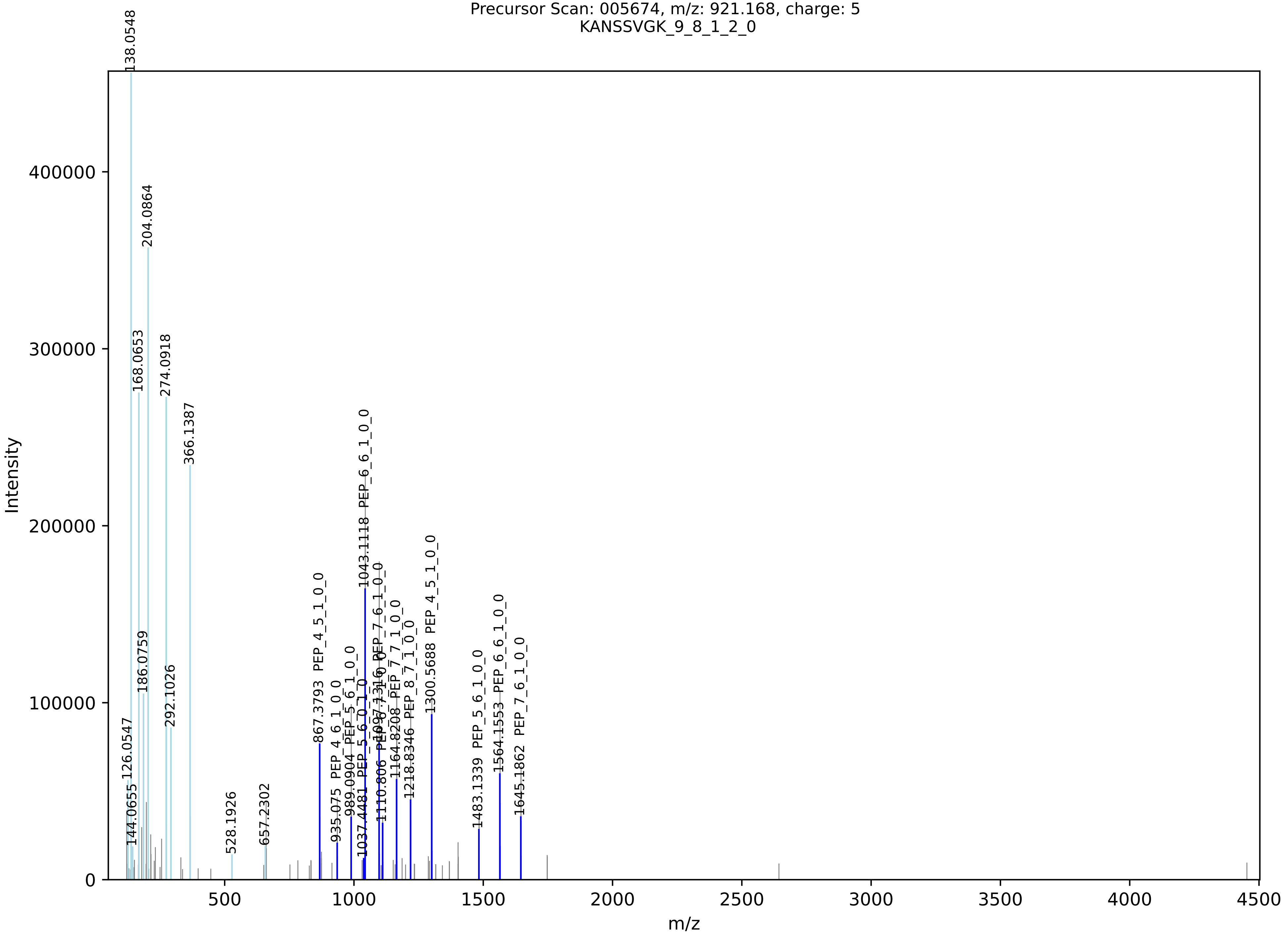

File: 231129_OE_RK_37, RT: 8.096, Scan: 005786
Type: HCD, FTMS + c NSI d Full ms2 1219.1481@hcd30.00 [120.0000-3752.7351]
Precursor Scan: 005674, m/z: 1219.1481, charge: 3
NEEYNK_6_5_0_3_0

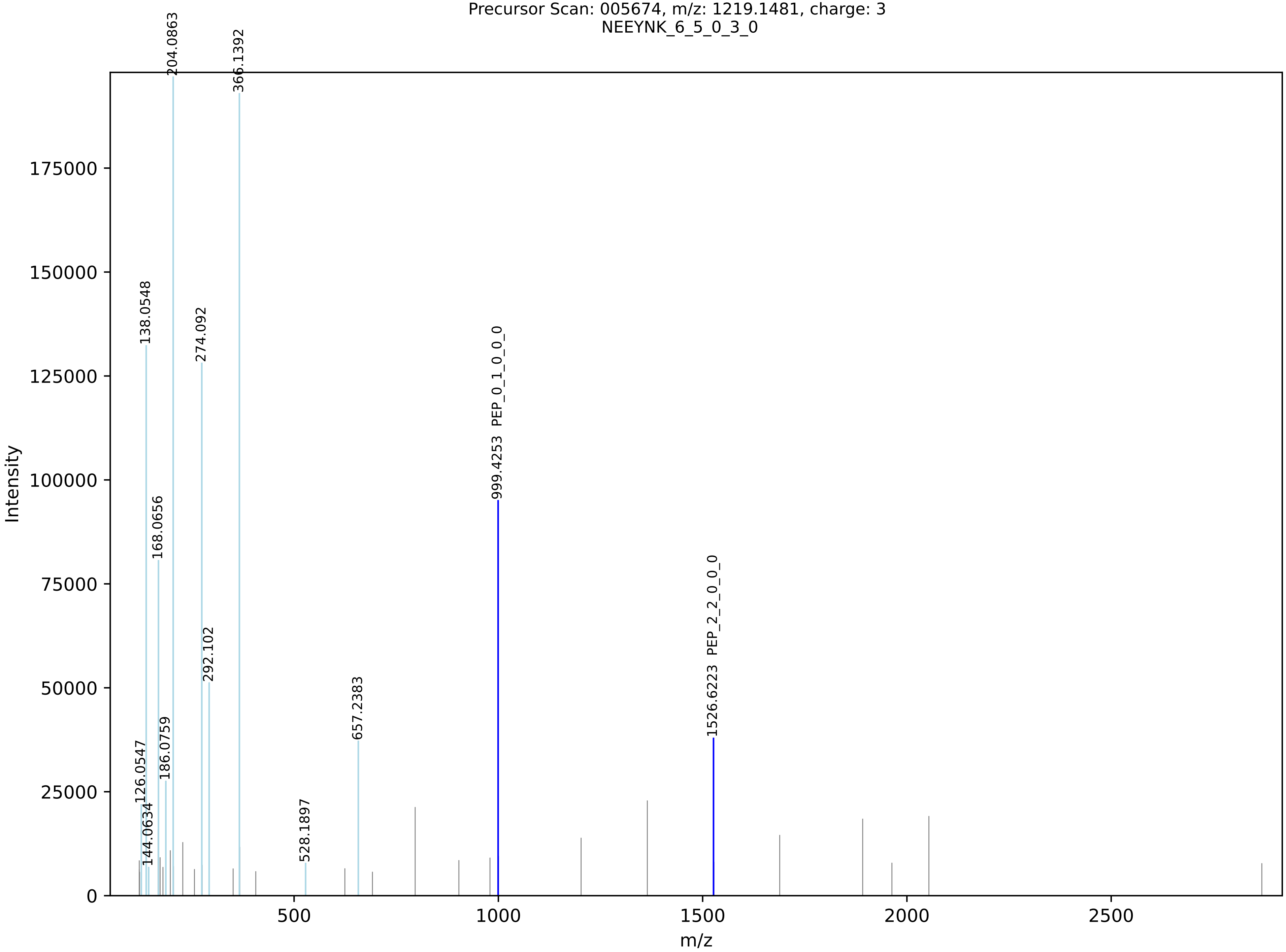

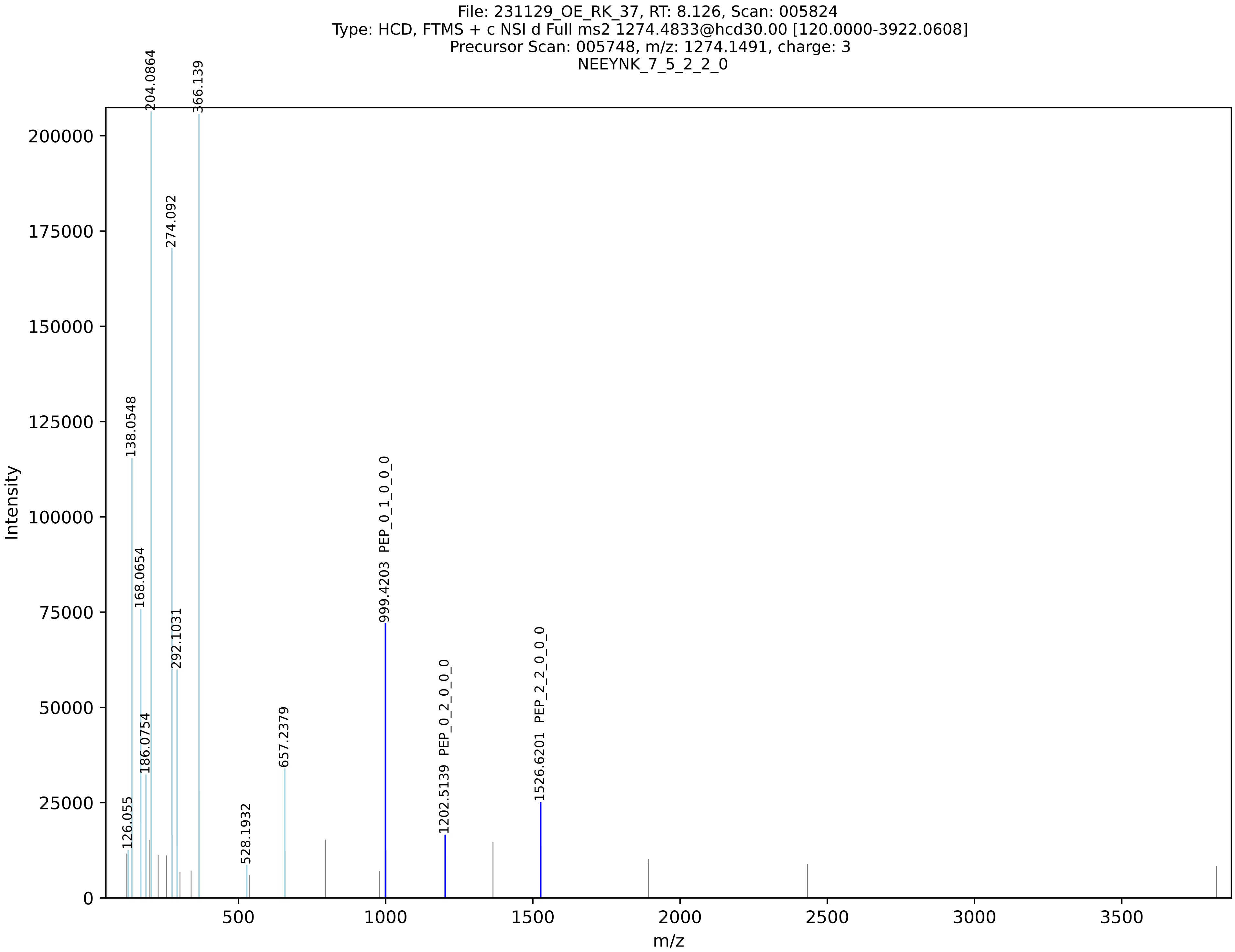
File: 231129_OE_RK_37, RT: 8.126, Scan: 005824
Type: HCD, FTMS + c NSI d Full ms2 1274.4833@hcd30.00 [120.0000-3922.0608]
Precursor Scan: 005748, m/z: 1274.1491, charge: 3
NEEYNK_7_5_2_2_0
204.0864
366.139
274.092
138.0548
168.0654
292.1031
186.0754
126.055
528.1932
657.2379
999.4203 PEP_0_1_0_0_0
1202.5139 PEP_0_2_0_0_0
1526.6201 PEP_2_2_0_0_0
Intensity
m/z
0
25000
50000
75000
100000
125000
150000
175000
200000
500
1000
1500
2000
2500
3000
3500

File: 231129_OE_RK_37, RT: 8.127, Scan: 005827
Type: HCD, FTMS + c NSI d Full ms2 928.8633@hcd30.00 [120.0000-3812.6187]
Precursor Scan: 005748, m/z: 928.6128, charge: 4
ENGTISR_7_6_0_2_0

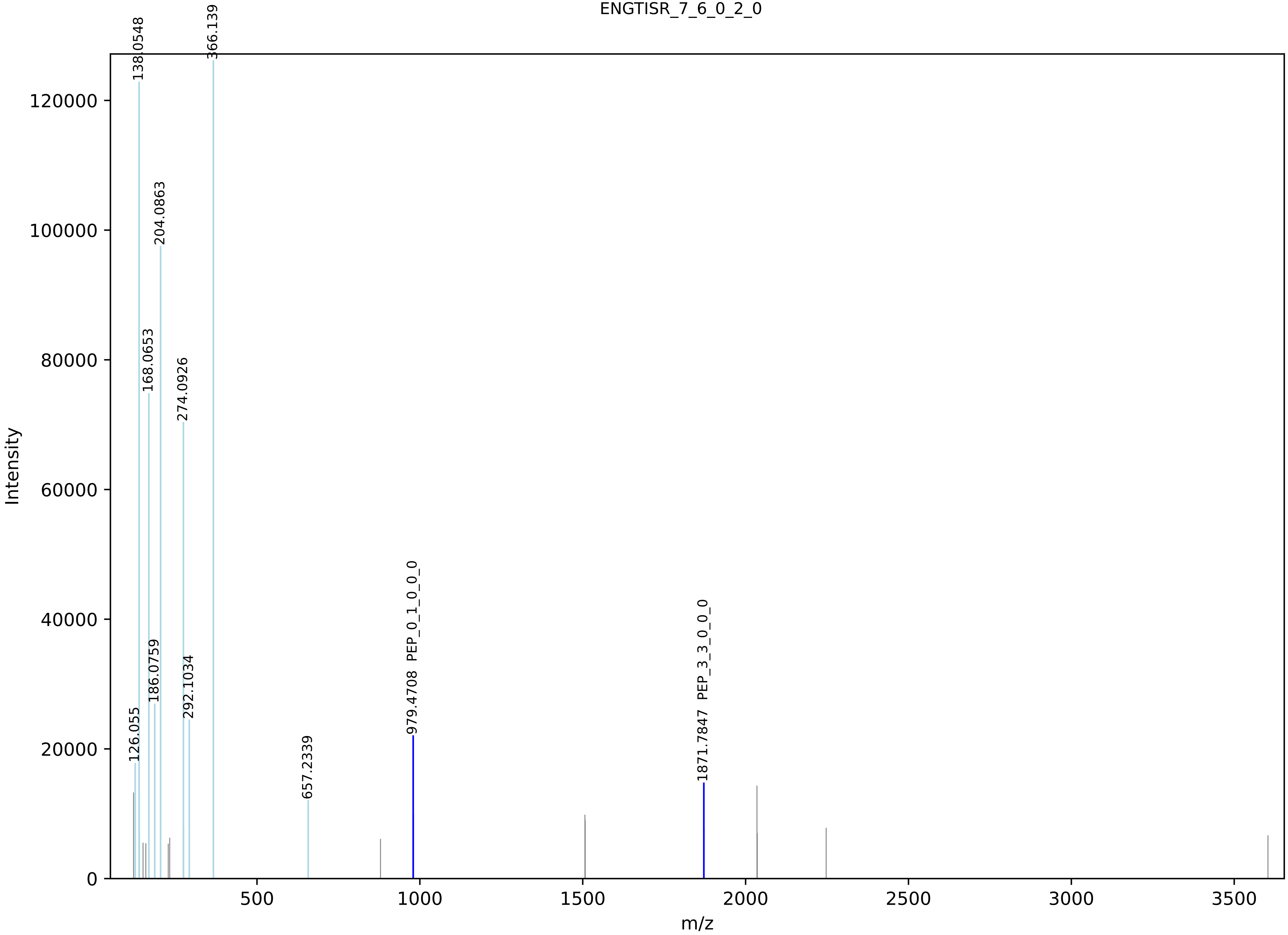

File: 231129_OE_RK_37, RT: 8.129, Scan: 005830
Type: HCD, FTMS + c NSI d Full ms2 951.8582@hcd30.00 [120.0000-3906.4375]
Precursor Scan: 005748, m/z: 951.6082, charge: 4
NEEYNK_6_5_1_3_0

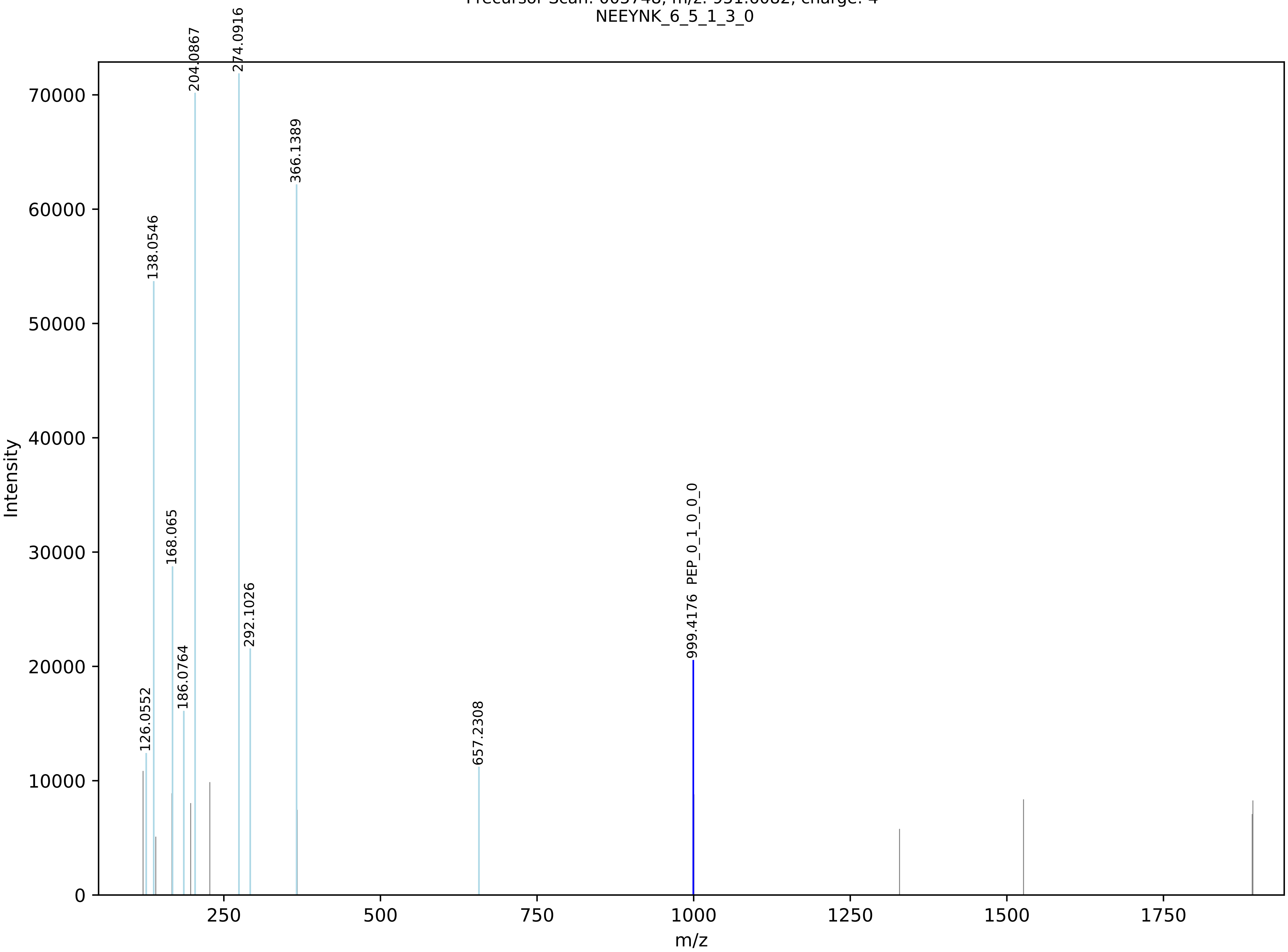

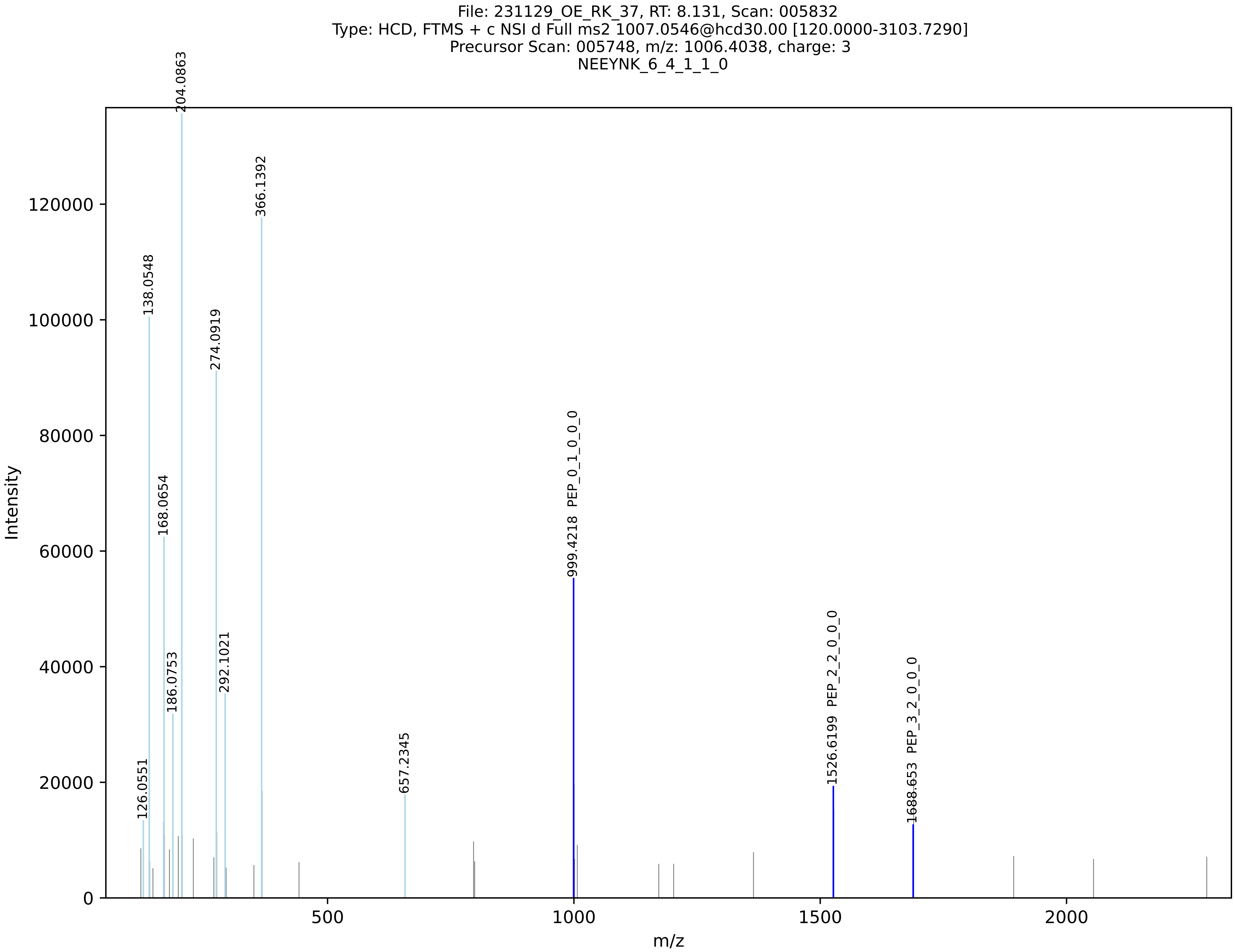
File: 231129_OE_RK_37, RT: 8.131, Scan: 005832
Type: HCD, FTMS + c NSI d Full ms2 1007.0546@hcd30.00 [120.0000-3103.7290]
Precursor Scan: 005748, m/z: 1006.4038, charge: 3
NEEYNK_6_4_1_1_0
Intensity
m/z
204.0863
366.1392
138.0548
274.0919
168.0654
186.0753
292.1021
126.0551
657.2345
999.4218 PEP_0_1_0_0_0
1526.6199 PEP_2_2_0_0_0
1688.653 PEP_3_2_0_0_0
0
20000
40000
60000
80000
100000
120000
500
1000
1500
2000

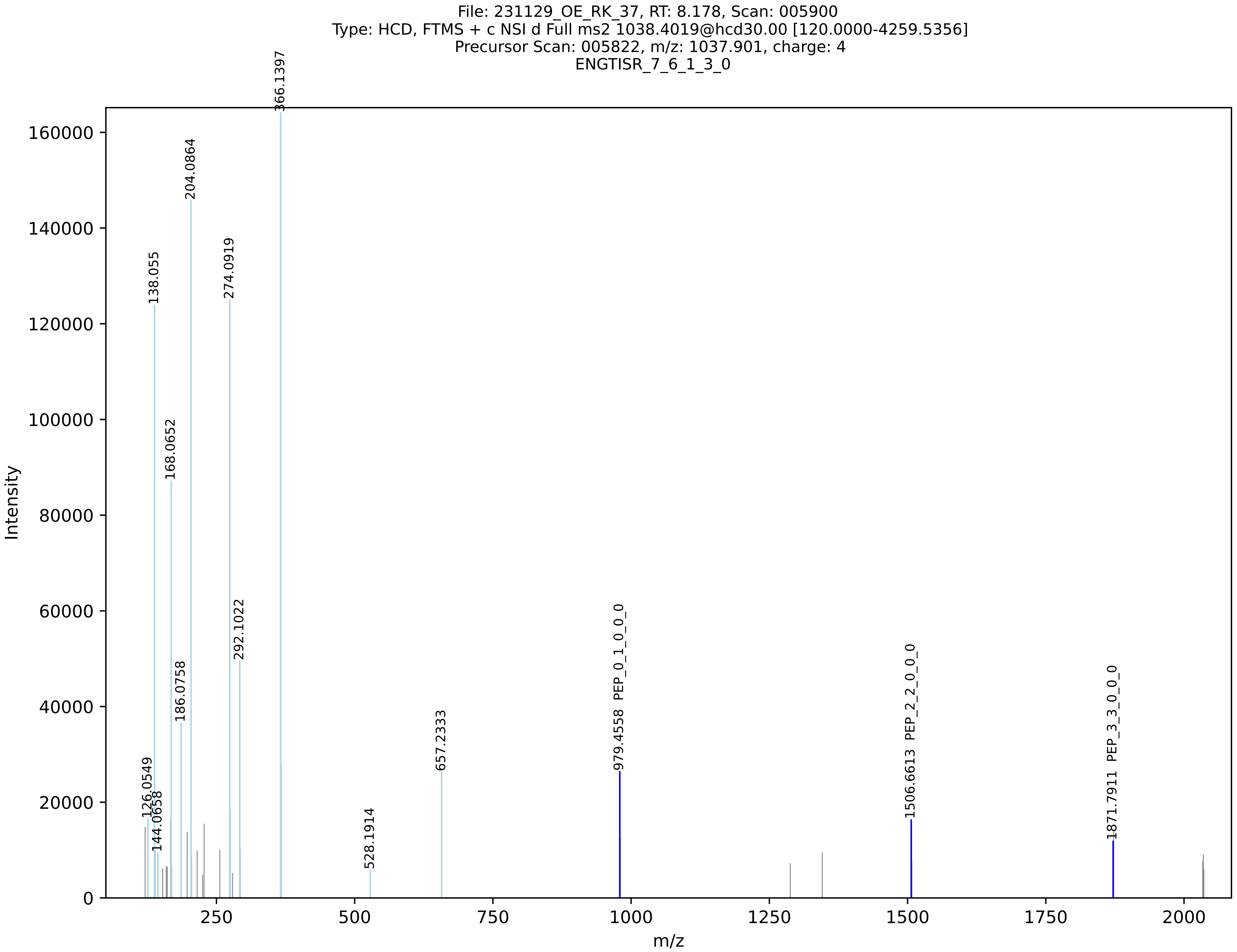

File: 231129_OE_RK_37, RT: 8.178, Scan: 005900
Type: HCD, FTMS + c NSI d Full ms2 1038.4019@hcd30.00 [120.0000-4259.5356]
Precursor Scan: 005822, m/z: 1037.901, charge: 4
ENGTISR_7_6_1_3_0
Intensity
m/z
366.1397
204.0864
138.055
274.0919
168.0652
292.1022
186.0758
126.0549
144.0658
528.1914
657.2333
979.4558 PEP_0_1_0_0_0
1506.6613 PEP_2_2_0_0_0
1871.7911 PEP_3_3_0_0_0

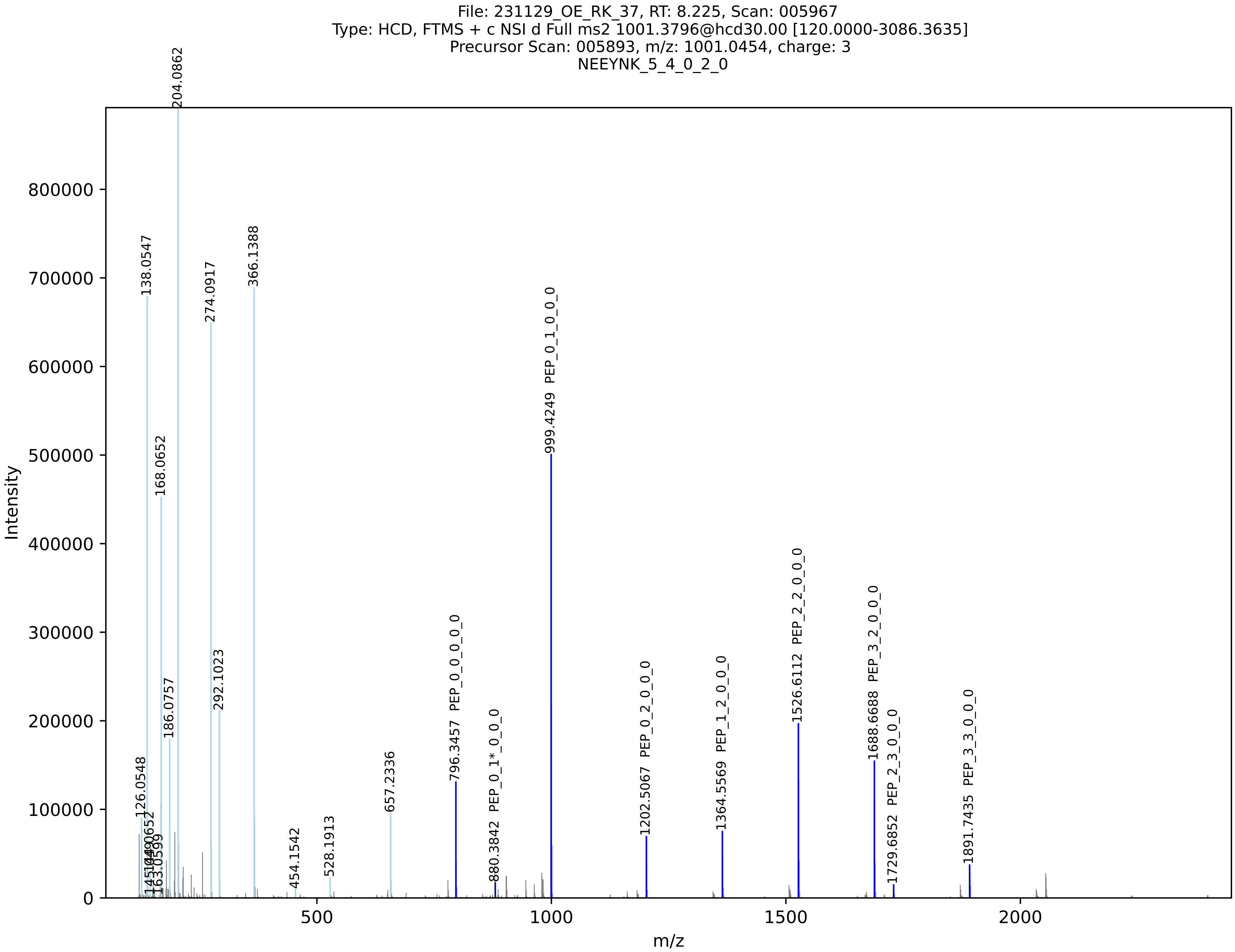
File: 231129_OE_RK_37, RT: 8.225, Scan: 005967
Type: HCD, FTMS + c NSI d Full ms2 1001.3796@hcd30.00 [120.0000-3086.3635]
Precursor Scan: 005893, m/z: 1001.0454, charge: 3
NEEYNK_5_4_0_2_0
Intensity
m/z
204.0862
138.0547
274.0917
366.1388
168.0652
186.0757
292.1023
126.0548
163.0599
454.1542
528.1913
657.2336
796.3457 PEP_0_0_0_0_0_
880.3842 PEP_0_1*_0_0_0
999.4249 PEP_0_1_0_0_0
1202.5067 PEP_0_2_0_0_0
1364.5569 PEP_1_2_0_0_0
1526.6112 PEP_2_2_0_0_0
1688.6688 PEP_3_2_0_0_0
1729.6852 PEP_2_3_0_0_0
1891.7435 PEP_3_3_0_0_0

File: 231129_OE_RK_37, RT: 8.23, Scan: 005975
Type: HCD, FTMS + c NSI d Full ms2 1432.8881@hcd30.00 [120.0000-4406.7793]
Precursor Scan: 005893, m/z: 1432.2188, charge: 3
ENGTISR_7_6_2_3_0

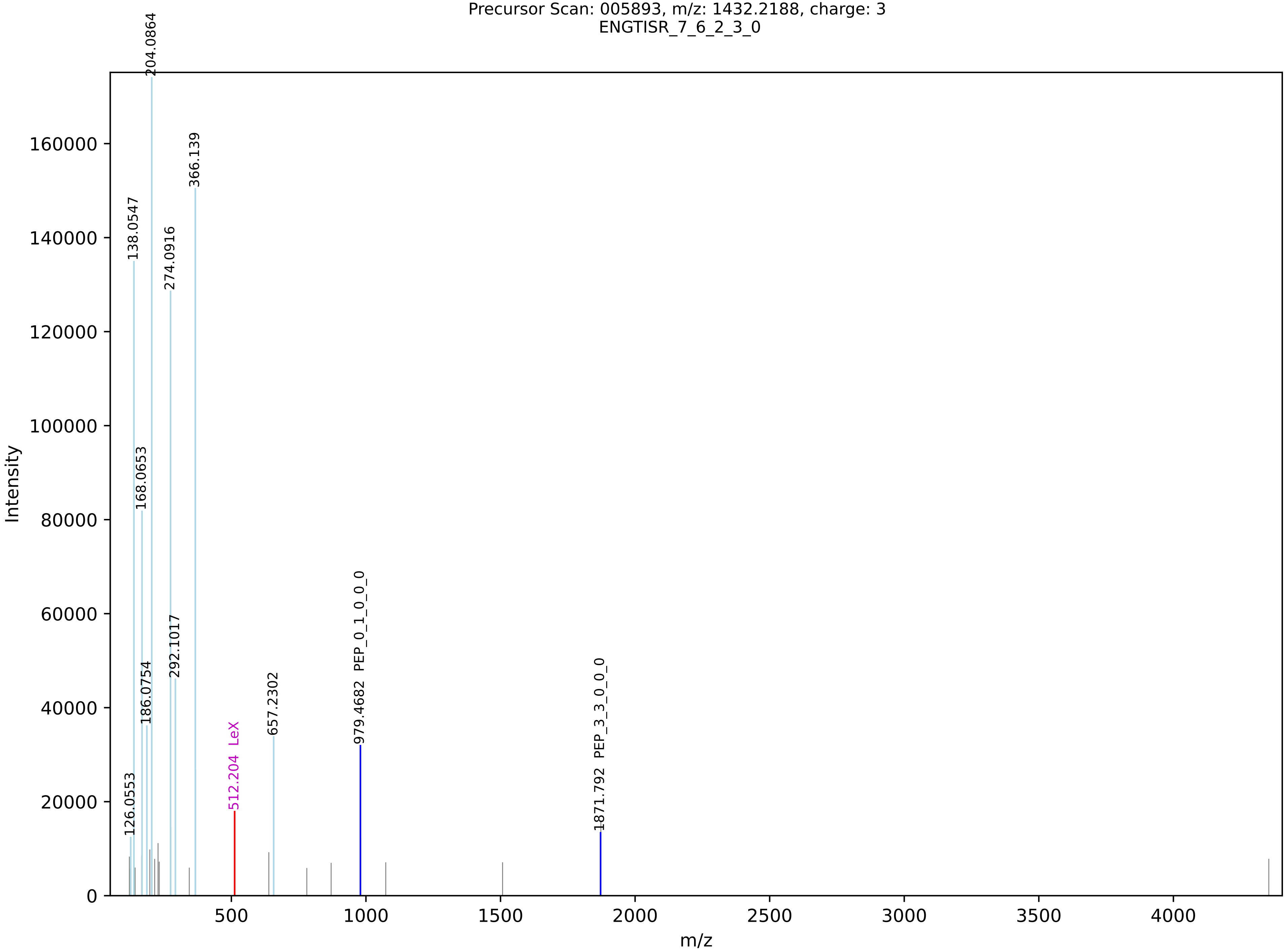

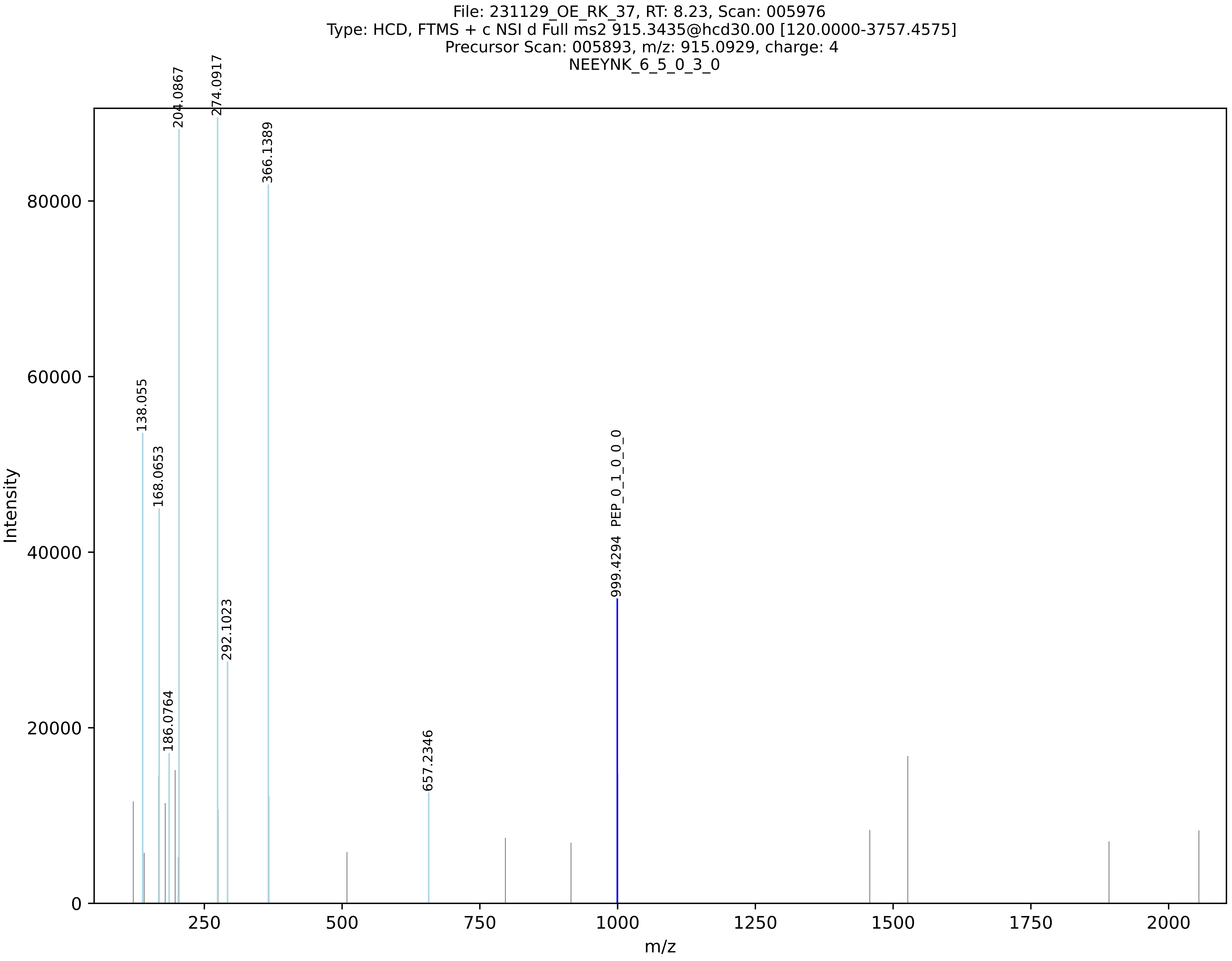
File: 231129_OE_RK_37, RT: 8.23, Scan: 005976
Type: HCD, FTMS + c NSI d Full ms2 915.3435@hcd30.00 [120.0000-3757.4575]
Precursor Scan: 005893, m/z: 915.0929, charge: 4
NEEYNK_6_5_0_3_0
204.0867
274.0917
366.1389
138.055
168.0653
186.0764
292.1023
657.2346
999.4294 PEP_0_1_0_0_0
Intensity
m/z
0
20000
40000
60000
80000
250
500
750
1000
1250
1500
1750
2000

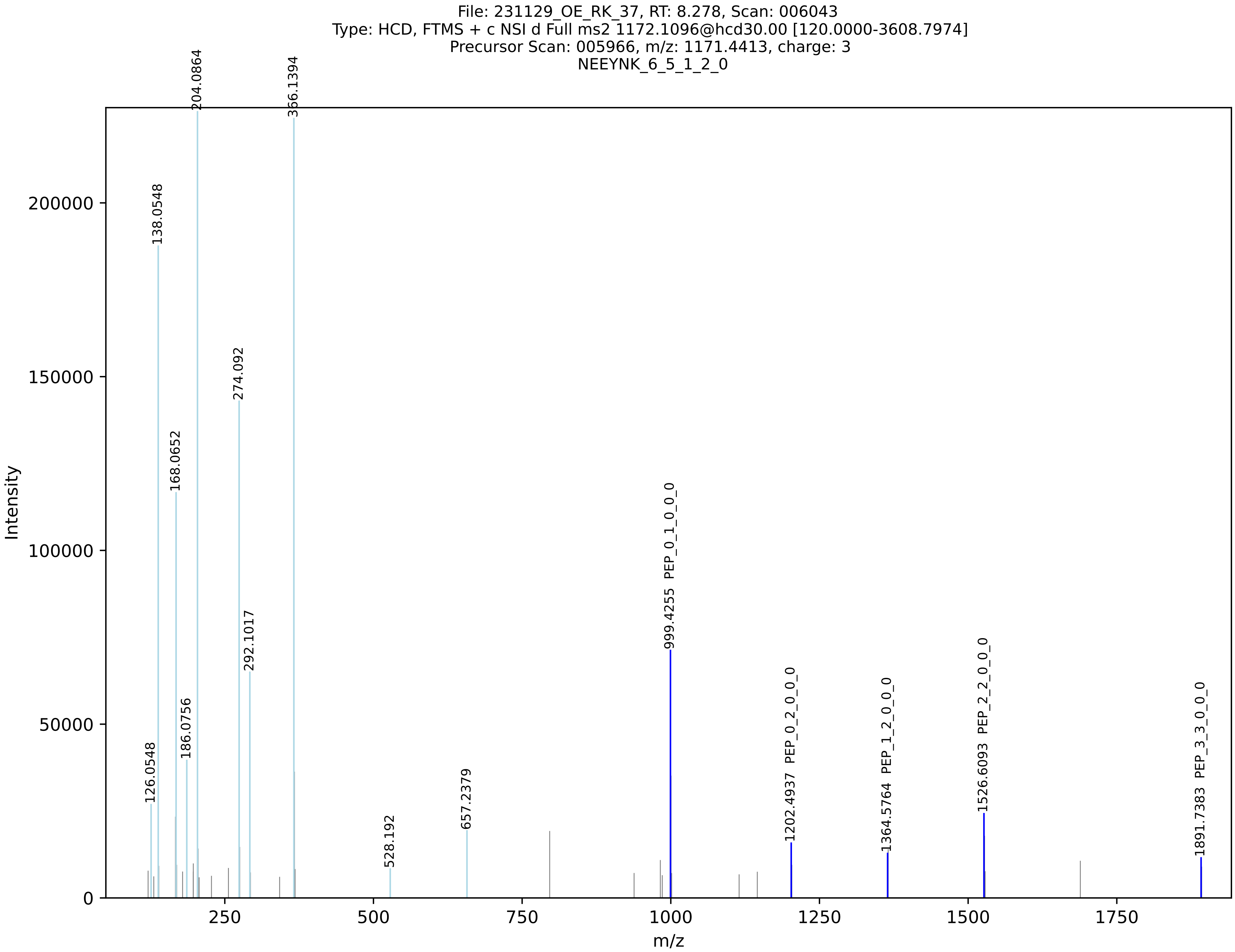
File: 231129_OE_RK_37, RT: 8.278, Scan: 006043
Type: HCD, FTMS + c NSI d Full ms2 1172.1096@hcd30.00 [120.0000-3608.7974]
Precursor Scan: 005966, m/z: 1171.4413, charge: 3
NEEYNK_6_5_1_2_0
Intensity
m/z
0
50000
100000
150000
200000
250
500
750
1000
1250
1500
1750
126.0548
138.0548
168.0652
186.0756
204.0864
274.092
292.1017
366.1394
528.192
657.2379
999.4255 PEP_0_1_0_0_
1202.4937 PEP_0_2_0_0_0
1364.5764 PEP_1_2_0_0_0
1526.6093 PEP_2_2_0_0_0
1891.7383 PEP_3_3_0_0_0

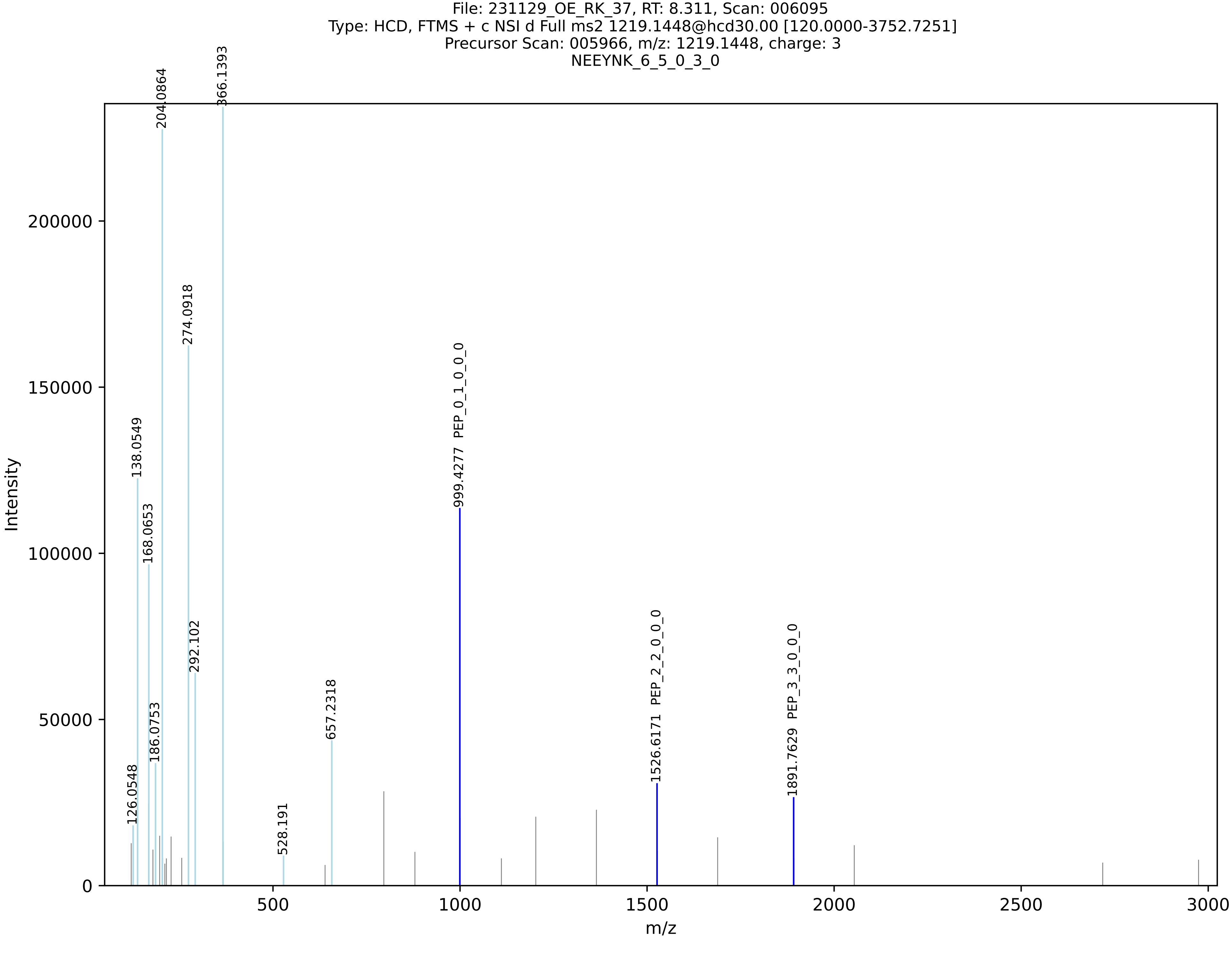
File: 231129_OE_RK_37, RT: 8.311, Scan: 006095
Type: HCD, FTMS + c NSI d Full ms2 1219.1448@hcd30.00 [120.0000-3752.7251]
Precursor Scan: 005966, m/z: 1219.1448, charge: 3
NEEYNK_6_5_0_3_0
Intensity
m/z
366.1393
204.0864
274.0918
138.0549
168.0653
292.102
186.0753
126.0548
528.191
657.2318
999.4277 PEP_0_1_0_0_0_
1526.6171 PEP_2_2_0_0_0
1891.7629 PEP_3_3_0_0_0_
0
50000
100000
150000
200000
500
1000
1500
2000
2500
3000

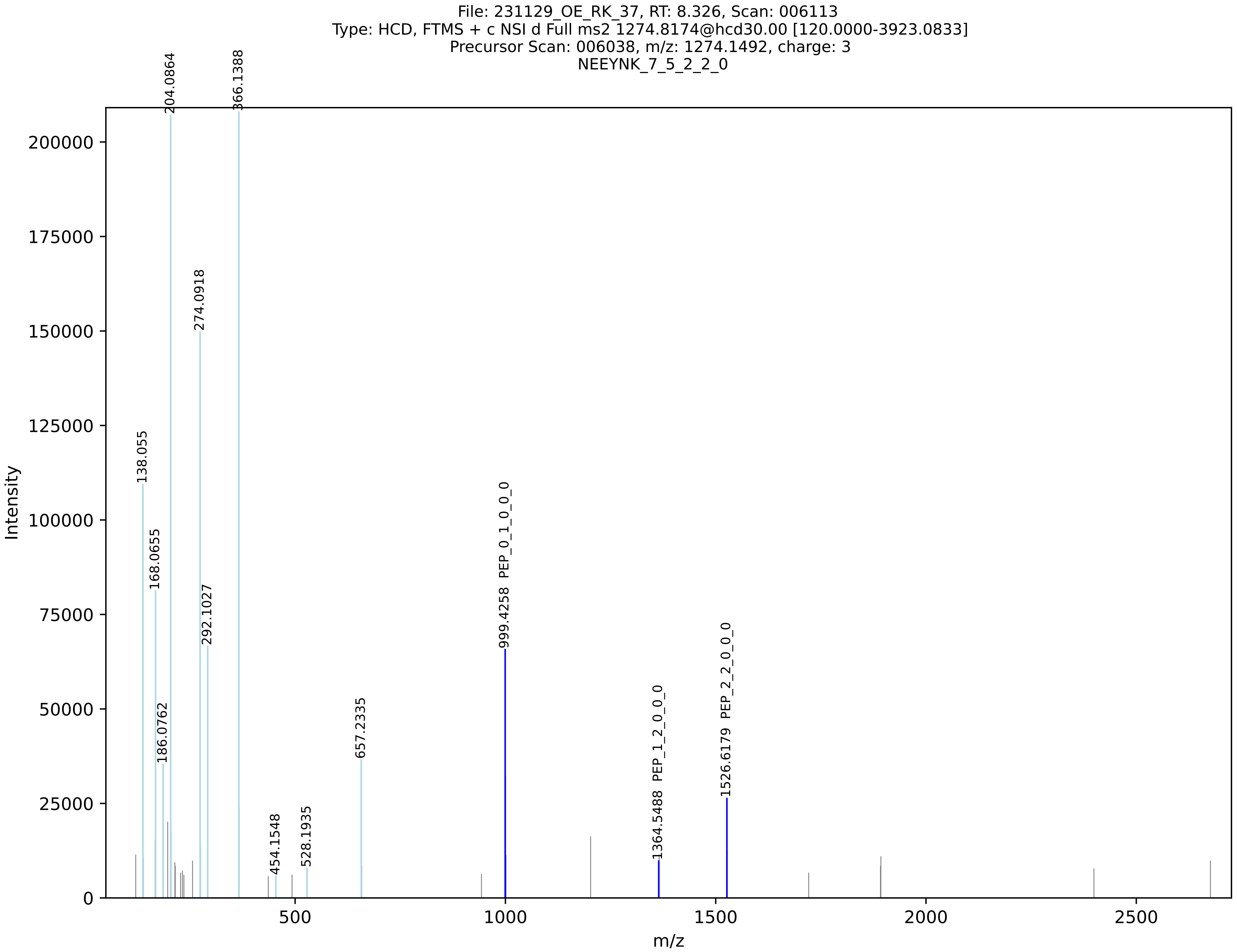
File: 231129_OE_RK_37, RT: 8.326, Scan: 006113
Type: HCD, FTMS + c NSI d Full ms2 1274.8174@hcd30.00 [120.0000-3923.0833]
Precursor Scan: 006038, m/z: 1274.1492, charge: 3
NEEYNK_7_5_2_2_0
Intensity
m/z
204.0864
366.1388
274.0918
138.055
168.0655
292.1027
186.0762
454.1548
528.1935
657.2335
999.4258 PEP_0_1_0_0_0
1364.5488 PEP_1_2_0_0_0
1526.6179 PEP_2_2_0_0_0
0
25000
50000
75000
100000
125000
150000
175000
200000
500
1000
1500
2000
2500

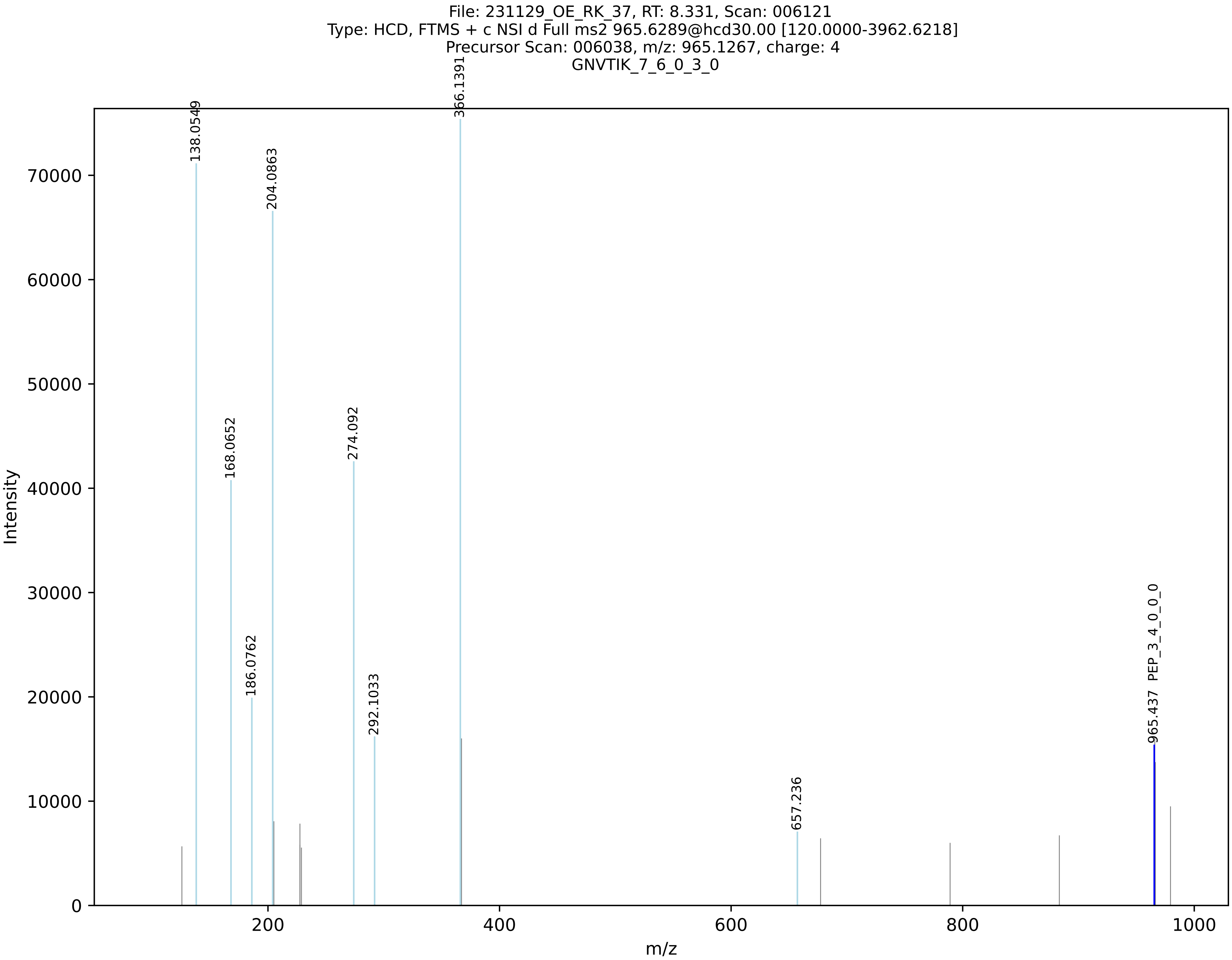
File: 231129_OE_RK_37, RT: 8.331, Scan: 006121
Type: HCD, FTMS + c NSI d Full ms2 965.6289@hcd30.00 [120.0000-3962.6218]
Precursor Scan: 006038, m/z: 965.1267, charge: 4
GNVTIK_7_6_0_3_0
138.0549
168.0652
186.0762
204.0863
274.092
292.1033
366.1391
657.236
965.437 PEP_3_4_0_0_0
Intensity
m/z
0
10000
20000
30000
40000
50000
60000
70000
200
400
600
800
1000

File: 231129_OE_RK_37, RT: 8.379, Scan: 006190
Type: HCD, FTMS + c NSI d Full ms2 1038.4014@hcd30.00 [120.0000-4259.5332]
Precursor Scan: 006109, m/z: 1037.9001, charge: 4
ENGTISR_7_6_1_3_0

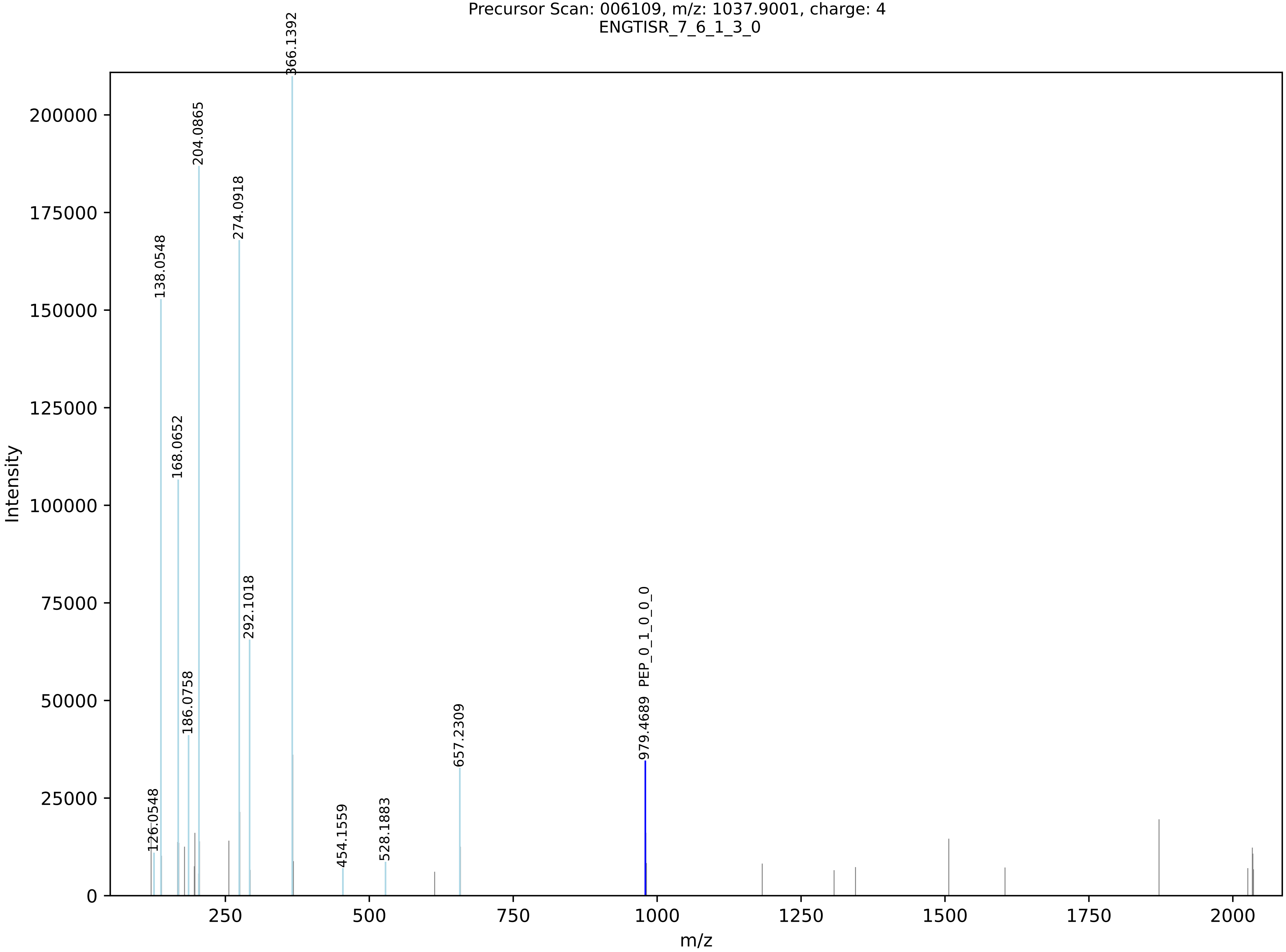

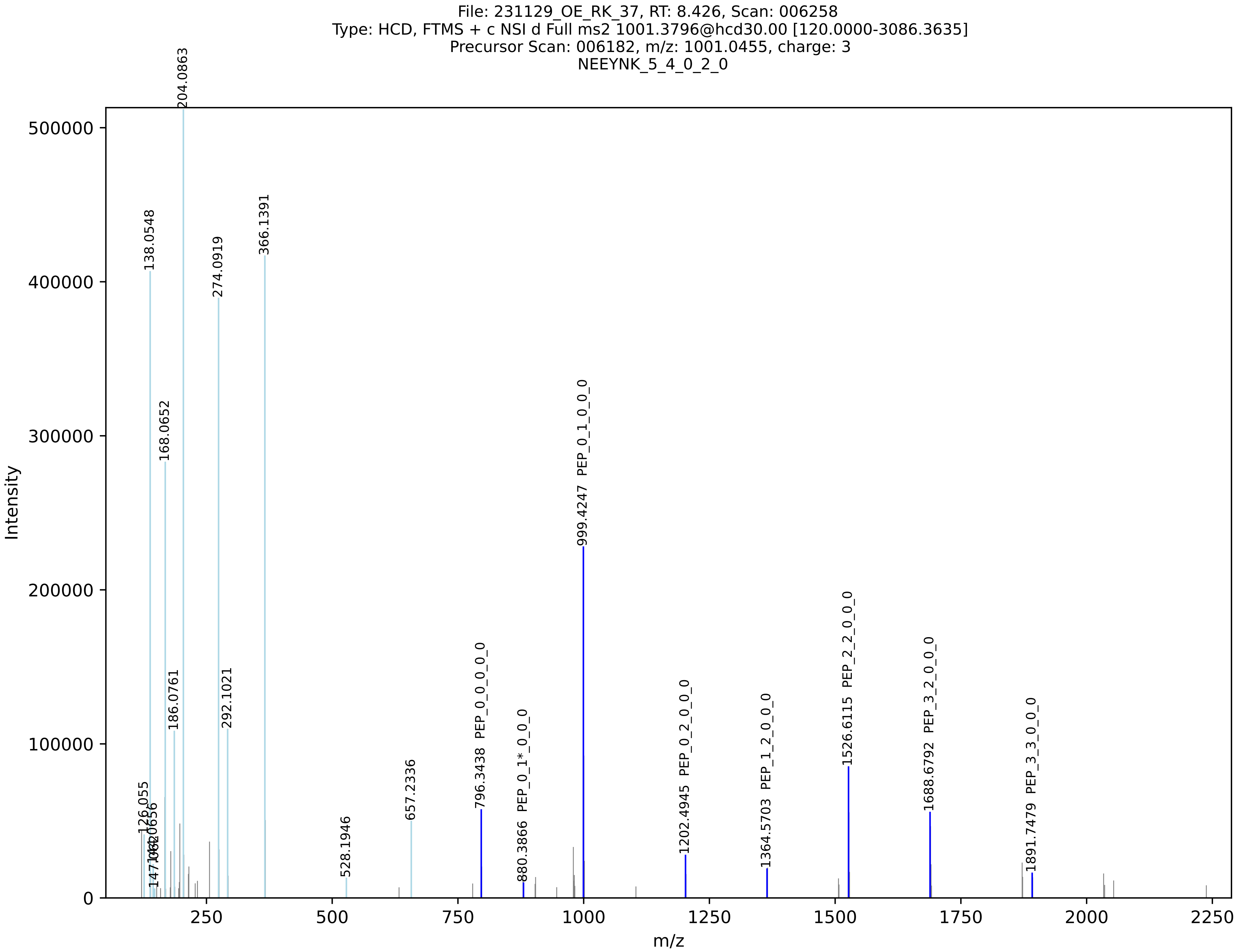
File: 231129_OE_RK_37, RT: 8.426, Scan: 006258
Type: HCD, FTMS + c NSI d Full ms2 1001.3796@hcd30.00 [120.0000-3086.3635]
Precursor Scan: 006182, m/z: 1001.0455, charge: 3
NEEYNK_5_4_0_2_0
Intensity
m/z
204.0863
138.0548
168.0652
186.0761
274.0919
292.1021
366.1391
528.1946
657.2336
796.3438 PEP_0_0_0_0_0
880.3866 PEP_0_1*_0_0_0
999.4247 PEP_0_1_0_0_0
1202.4945 PEP_0_2_0_0_0
1364.5703 PEP_1_2_0_0_0
1526.6115 PEP_2_2_0_0_0
1688.6792 PEP_3_2_0_0_0
1891.7479 PEP_3_3_0_0_0

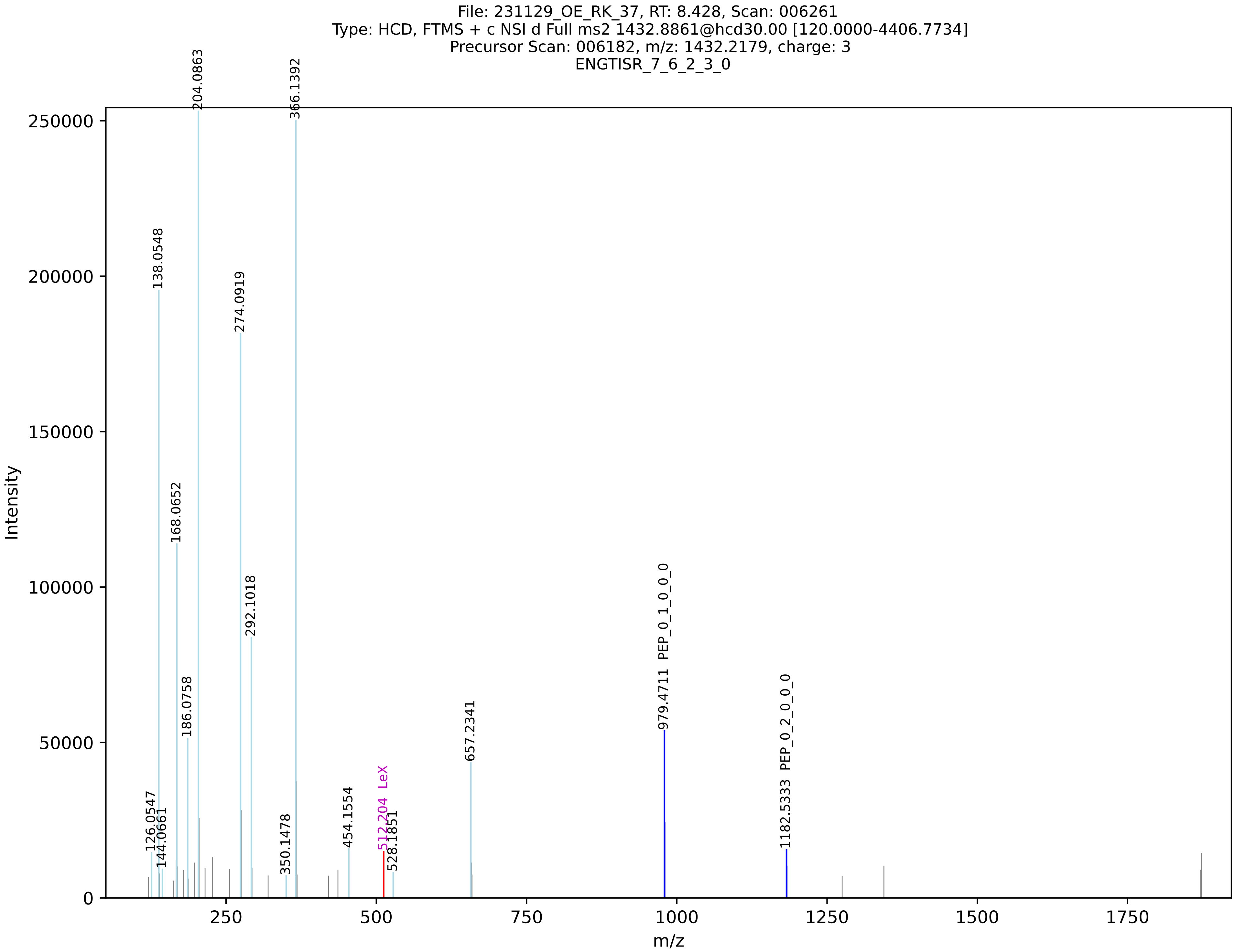
File: 231129_OE_RK_37, RT: 8.428, Scan: 006261
Type: HCD, FTMS + c NSI d Full ms2 1432.8861@hcd30.00 [120.0000-4406.7734]
Precursor Scan: 006182, m/z: 1432.2179, charge: 3
ENGTISR_7_6_2_3_0
Intensity
m/z
204.0863
366.1392
138.0548
274.0919
168.0652
292.1018
186.0758
126.0547
144.0661
350.1478
454.1554
512.204 LeX
528.1851
657.2341
979.4711 PEP_0_1_0_0_0
1182.5333 PEP_0_2_0_0_0

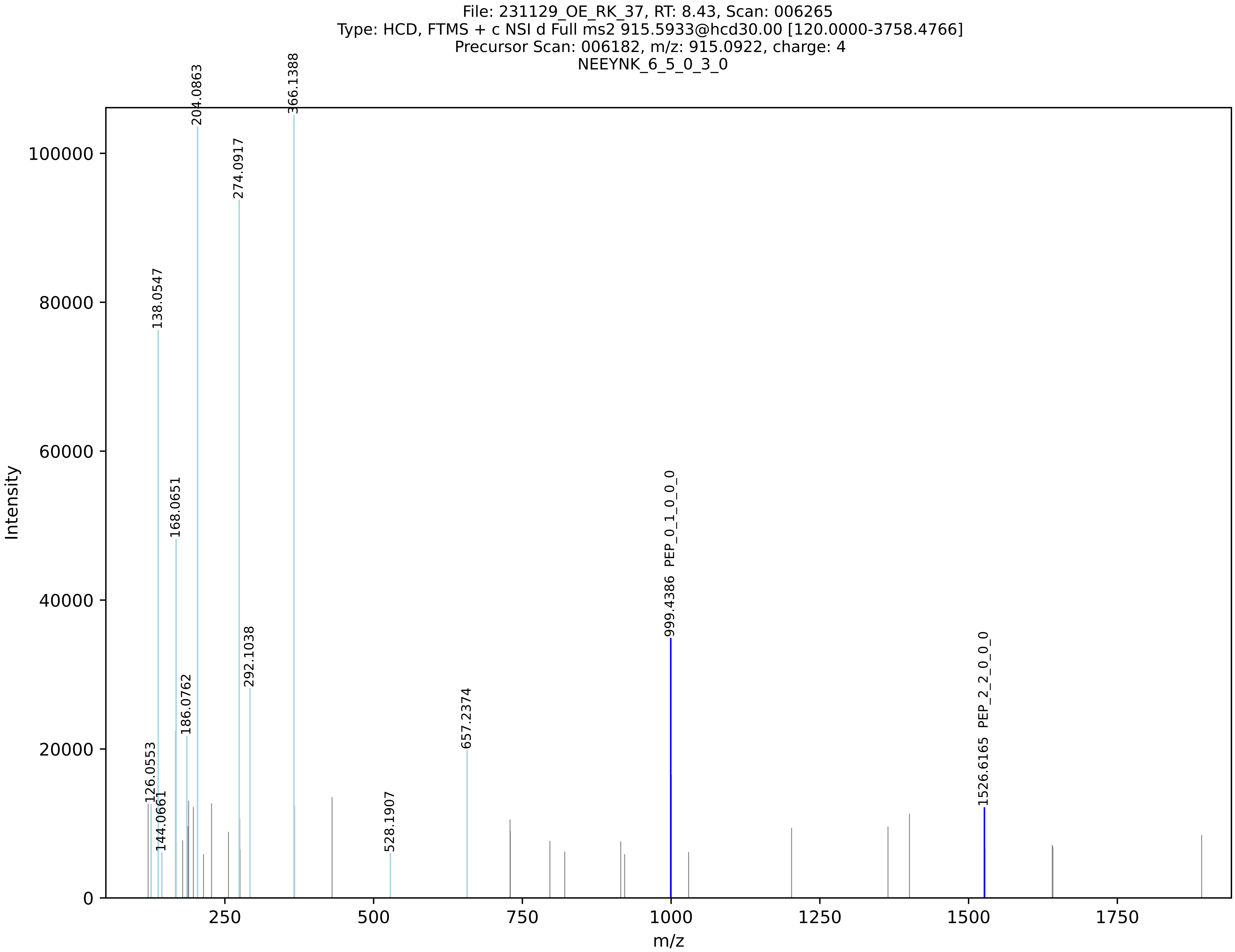
File: 231129_OE_RK_37, RT: 8.43, Scan: 006265
Type: HCD, FTMS + c NSI d Full ms2 915.5933@hcd30.00 [120.0000-3758.4766]
Precursor Scan: 006182, m/z: 915.0922, charge: 4
NEEYNK_6_5_0_3_0
Intensity
m/z
366.1388
204.0863
274.0917
138.0547
168.0651
292.1038
186.0762
126.0553
144.0661
528.1907
657.2374
999.4386 PEP_0_1_0_0_0
1526.6165 PEP_2_2_0_0_0
0
20000
40000
60000
80000
100000
250
500
750
1000
1250
1500
1750

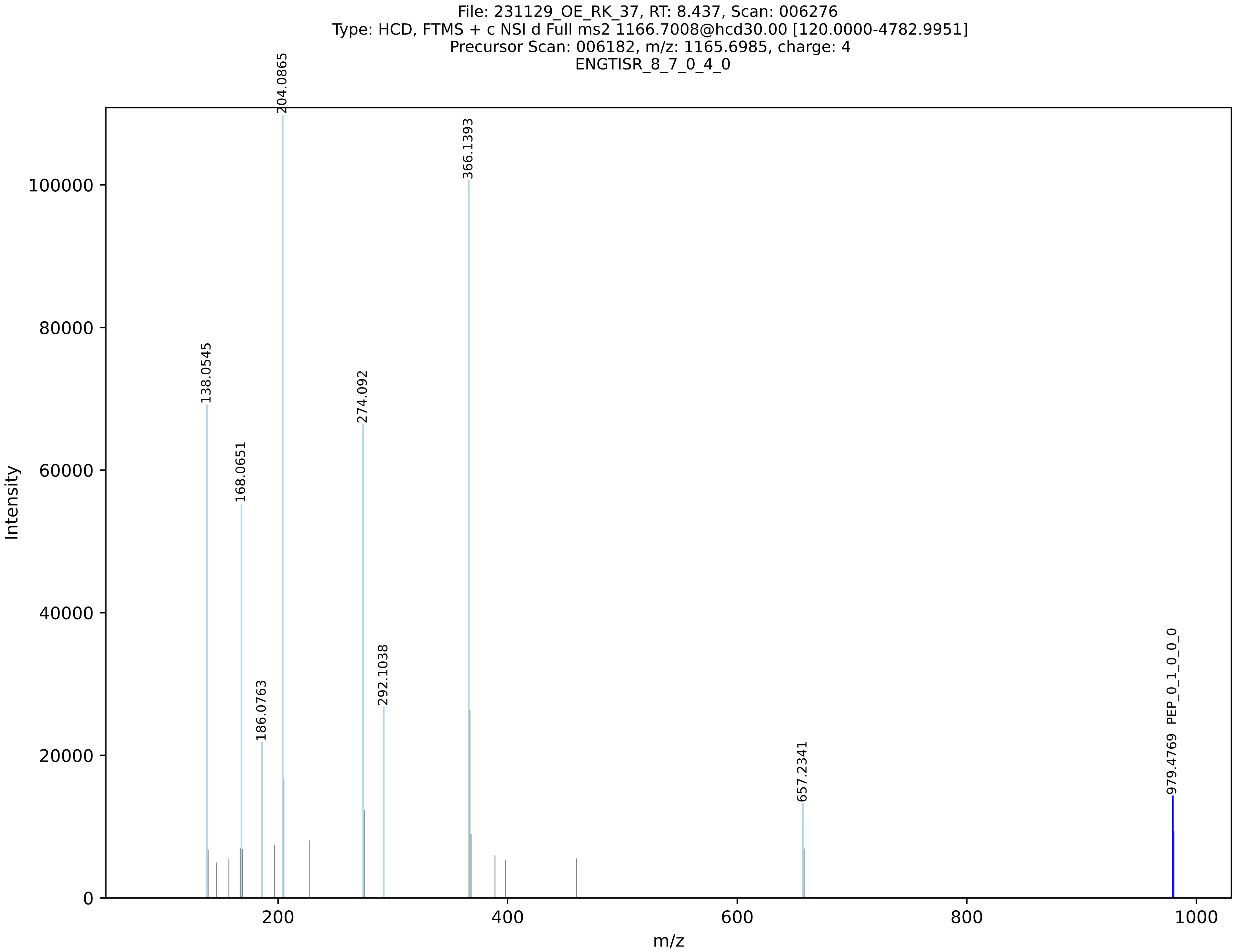
File: 231129_OE_RK_37, RT: 8.437, Scan: 006276
Type: HCD, FTMS + c NSI d Full ms2 1166.7008@hcd30.00 [120.0000-4782.9951]
Precursor Scan: 006182, m/z: 1165.6985, charge: 4
ENGTISR_8_7_0_4_0
204.0865
366.1393
138.0545
274.092
168.0651
292.1038
186.0763
657.2341
979.4769 PEP_0_1_0_0_0
Intensity
m/z
0
20000
40000
60000
80000
100000
200
400
600
800
1000

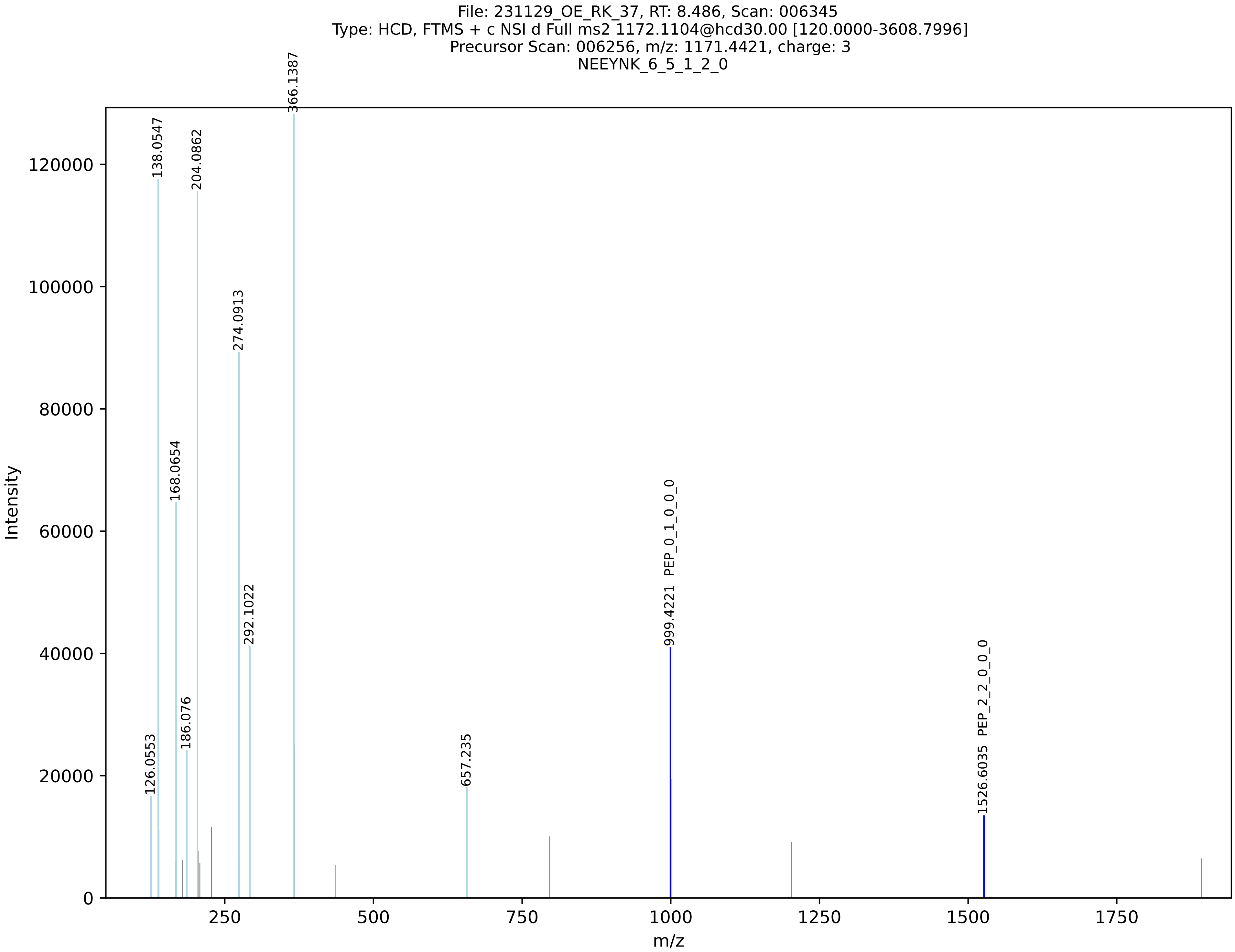
File: 231129_OE_RK_37, RT: 8.486, Scan: 006345
Type: HCD, FTMS + c NSI d Full ms2 1172.1104@hcd30.00 [120.0000-3608.7996]
Precursor Scan: 006256, m/z: 1171.4421, charge: 3
NEEYNK_6_5_1_2_0
366.1387
138.0547
204.0862
274.0913
168.0654
292.1022
186.076
126.0553
657.235
999.4221 PEP_0_1_0_0_0
1526.6035 PEP_2_2_0_0_0
Intensity
m/z
0
20000
40000
60000
80000
100000
120000
250
500
750
1000
1250
1500
1750

File: 231129_OE_RK_37, RT: 8.529, Scan: 006406
Type: HCD, FTMS + c NSI d Full ms2 952.3592@hcd30.00 [120.0000-3908.4814]
Precursor Scan: 006330, m/z: 951.6071, charge: 4
NEEYNK_6_5_1_3_0

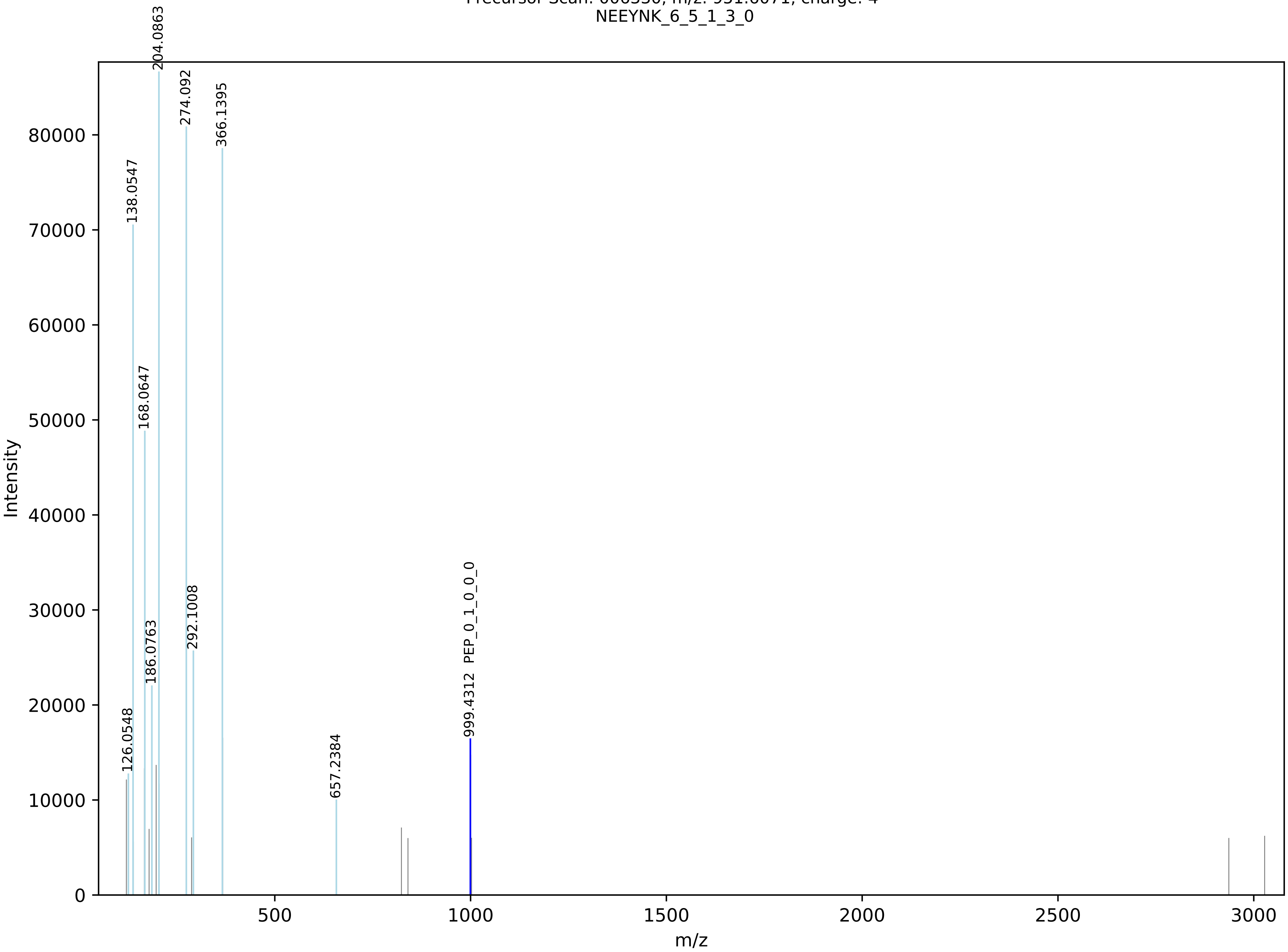

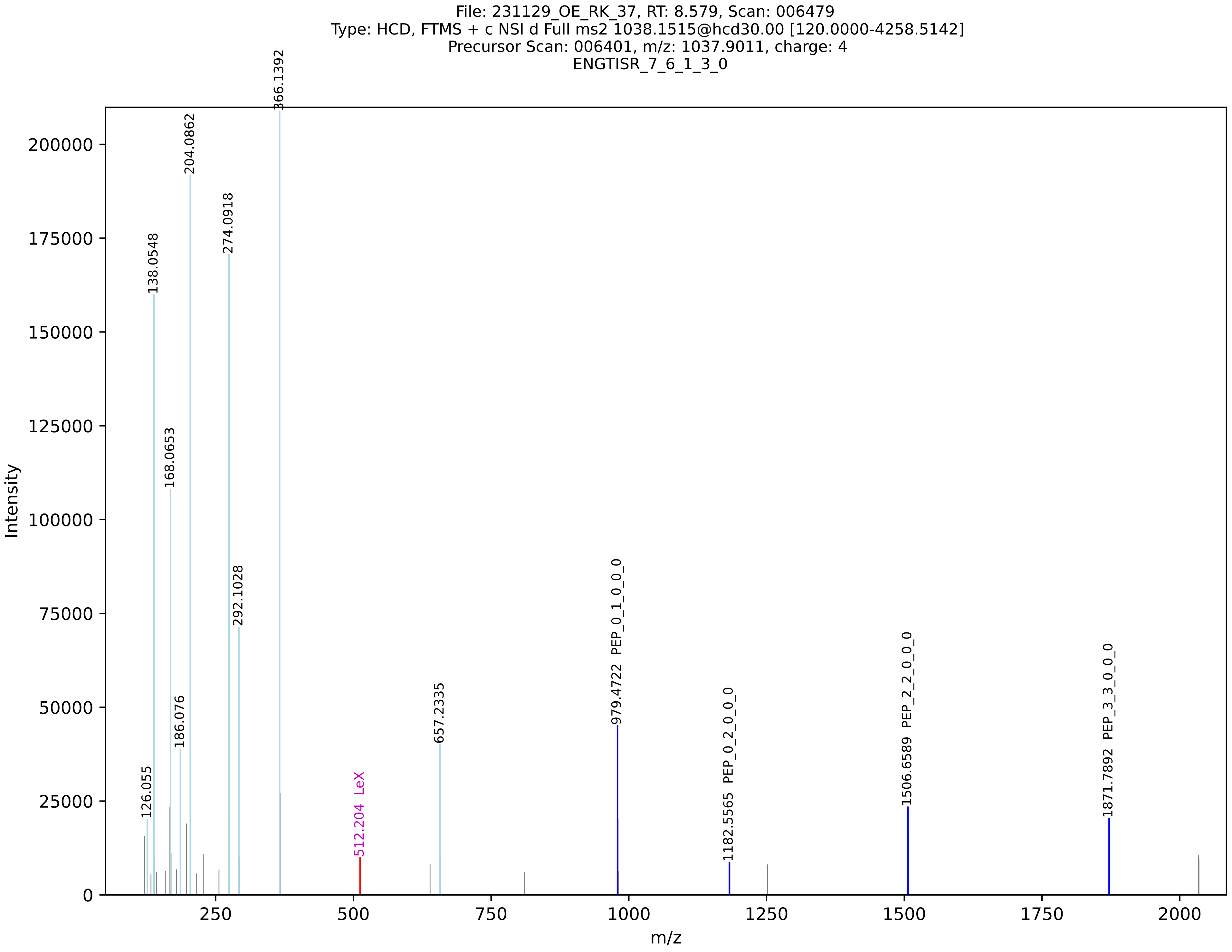
File: 231129_OE_RK_37, RT: 8.579, Scan: 006479
Type: HCD, FTMS + c NSI d Full ms2 1038.1515@hcd30.00 [120.0000-4258.5142]
Precursor Scan: 006401, m/z: 1037.9011, charge: 4
ENGTISR_7_6_1_3_0
Intensity
m/z
366.1392
204.0862
274.0918
138.0548
168.0653
292.1028
186.076
126.055
512.204 LeX
657.2335
979.4722 PEP_0_1_0_0_0
1182.5565 PEP_0_2_0_0_0
1506.6589 PEP_2_2_0_0_0
1871.7892 PEP_3_3_0_0_0
0
25000
50000
75000
100000
125000
150000
175000
200000
250
500
750
1000
1250
1500
1750
2000

File: 231129_OE_RK_37, RT: 8.629, Scan: 006549
Type: HCD, FTMS + c NSI d Full ms2 1220.4564@hcd30.00 [120.0000-3756.7385]
Precursor Scan: 006475, m/z: 1219.7881, charge: 3
NEEYNK_6_5_0_3_0

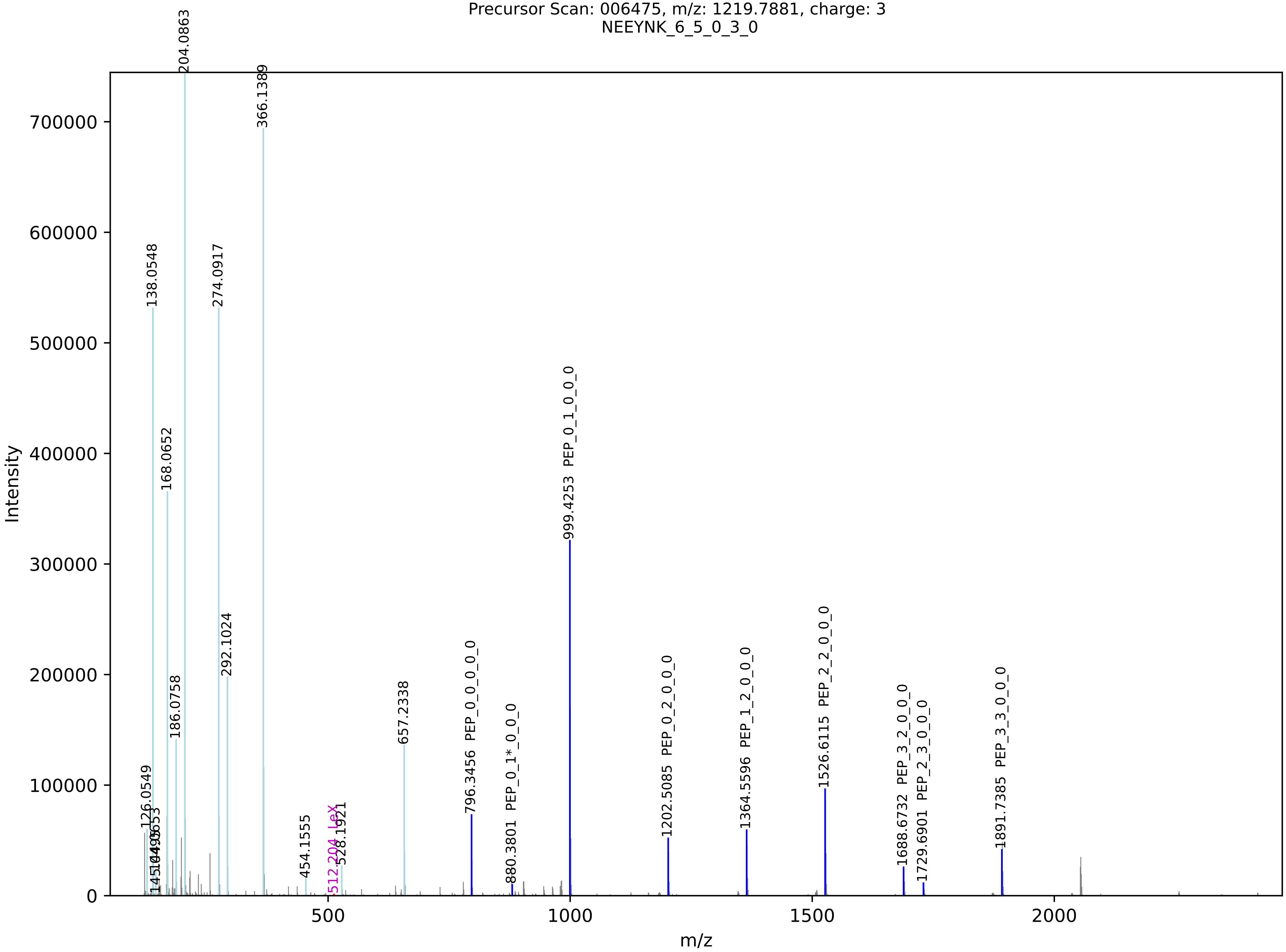

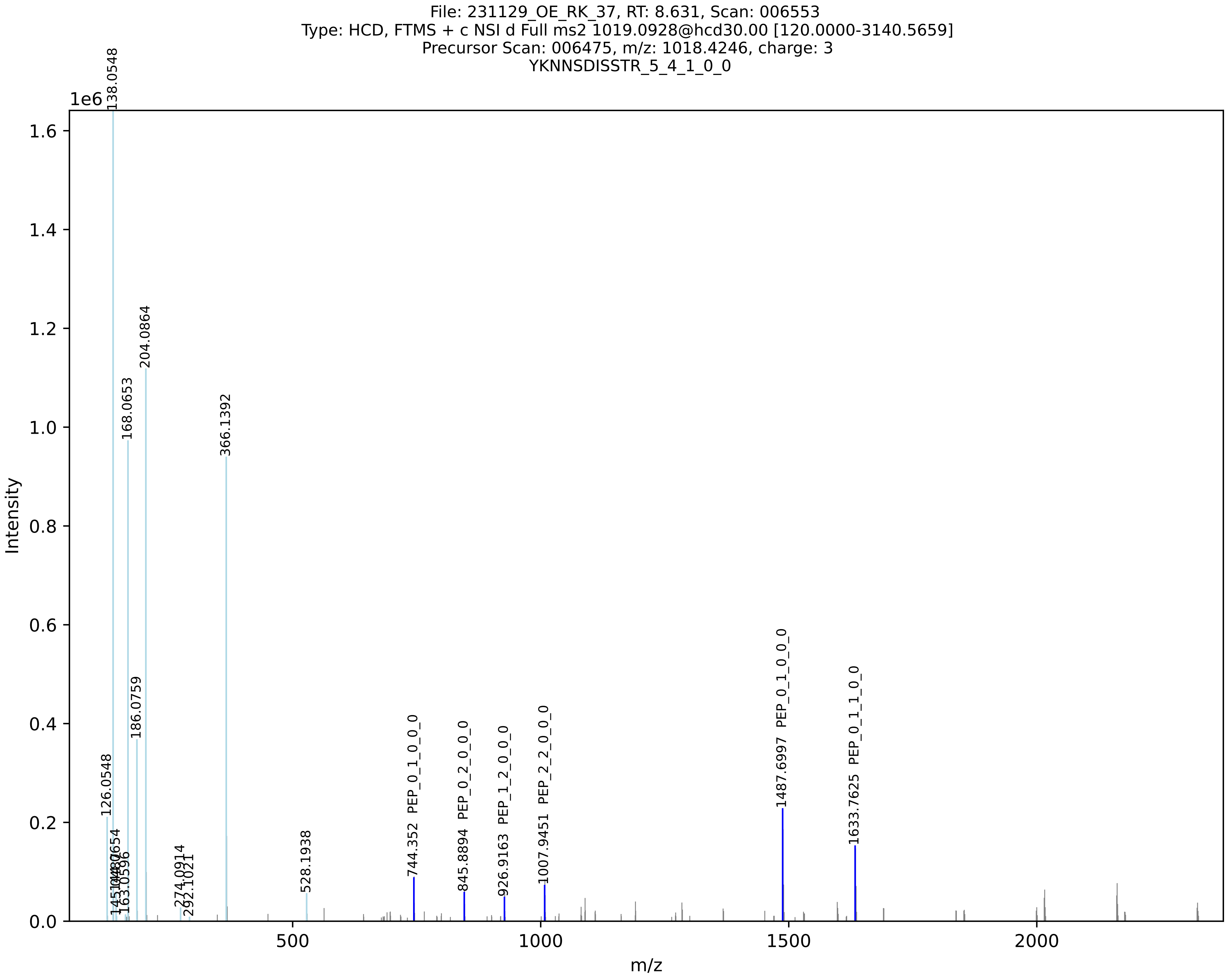

File: 231129_OE_RK_37, RT: 8.631, Scan: 006553
Type: HCD, FTMS + c NSI d Full ms2 1019.0928@hcd30.00 [120.0000-3140.5659]
Precursor Scan: 006475, m/z: 1018.4246, charge: 3
YKNNSDISSTR_5_4_1_0_0
1e6
Intensity
m/z
138.0548
204.0864
168.0653
366.1392
186.0759
126.0548
163.0596
274.0914
292.1021
528.1938
744.352 PEP_0_1_0_0_0
845.8894 PEP_0_2_0_0_0
926.9163 PEP_1_2_0_0_0
1007.9451 PEP_2_2_0_0_0
1487.6997 PEP_0_1_0_0_0
1633.7625 PEP_0_1_1_0_0

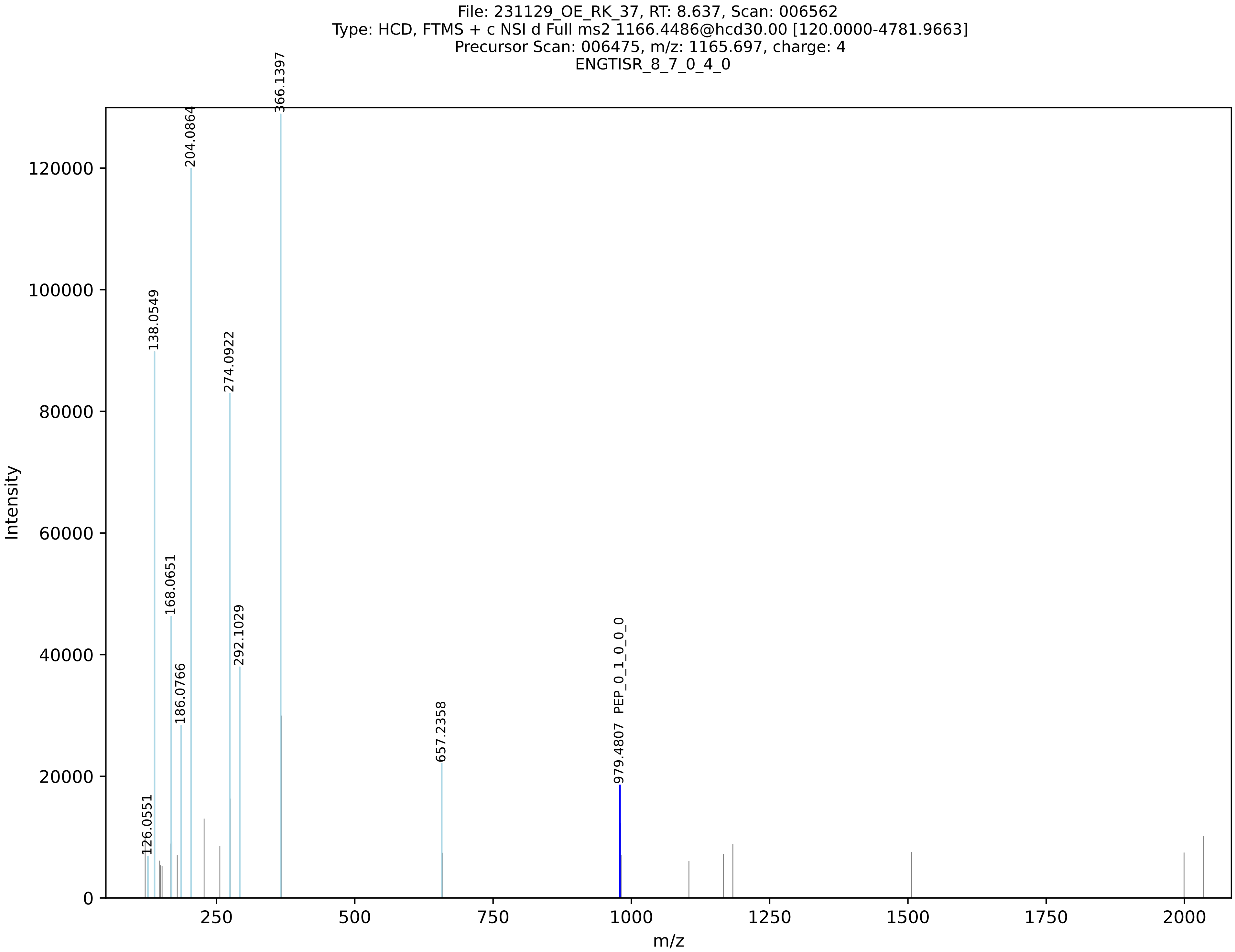

File: 231129_OE_RK_37, RT: 8.637, Scan: 006562
Type: HCD, FTMS + c NSI d Full ms2 1166.4486@hcd30.00 [120.0000-4781.9663]
Precursor Scan: 006475, m/z: 1165.697, charge: 4
ENGTISR_8_7_0_4_0
366.1397
204.0864
138.0549
274.0922
168.0651
292.1029
186.0766
657.2358
979.4807 PEP_0_1_0_0_0
126.0551
Intensity
m/z
0
20000
40000
60000
80000
100000
120000
250
500
750
1000
1250
1500
1750
2000

File: 231129_OE_RK_37, RT: 8.684, Scan: 006630
Type: HCD, FTMS + c NSI d Full ms2 1481.2344@hcd30.00 [120.0000-4554.7192]
Precursor Scan: 006548, m/z: 1480.5654, charge: 3
ENGTISR_7_6_1_4_0

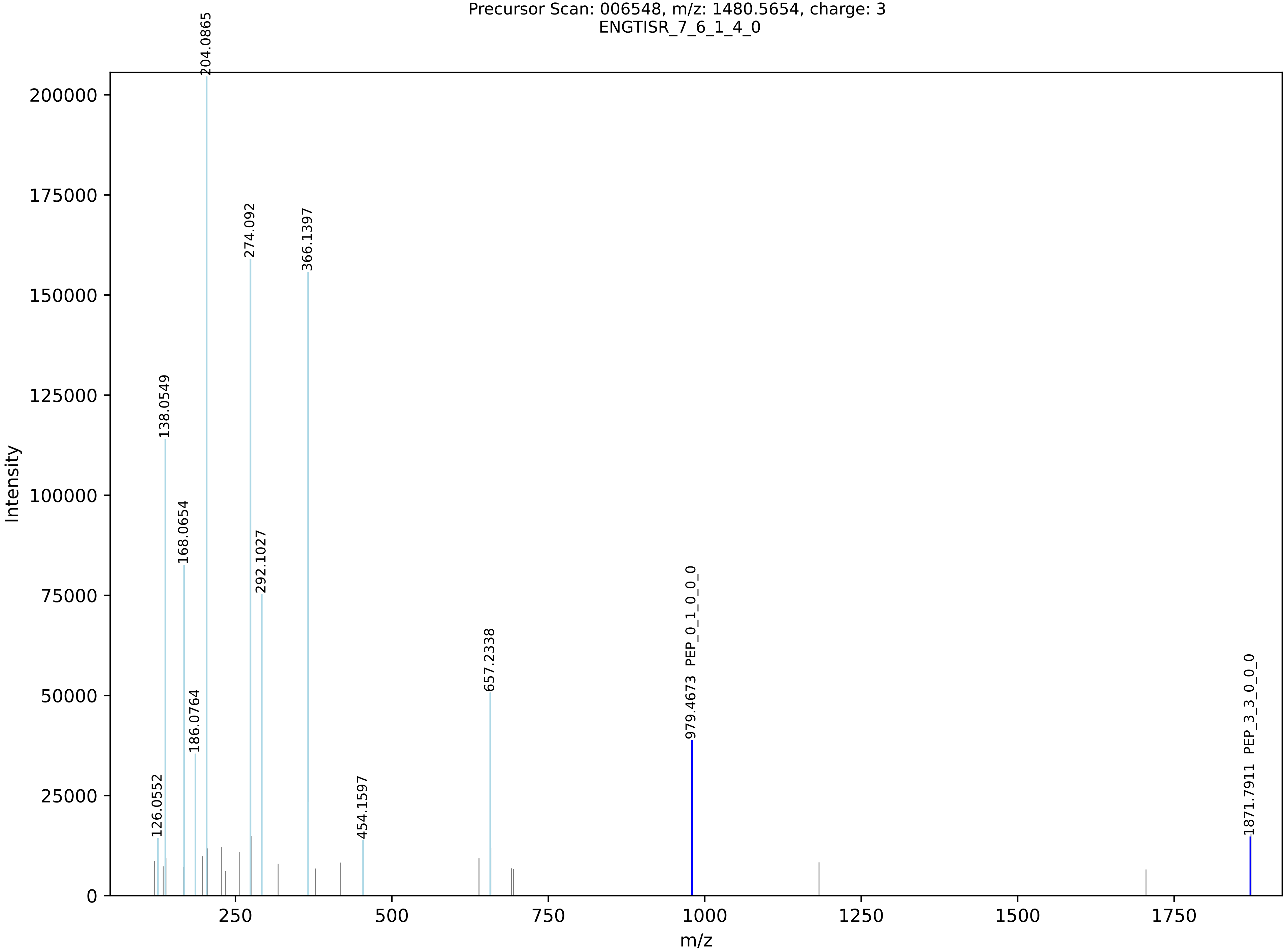

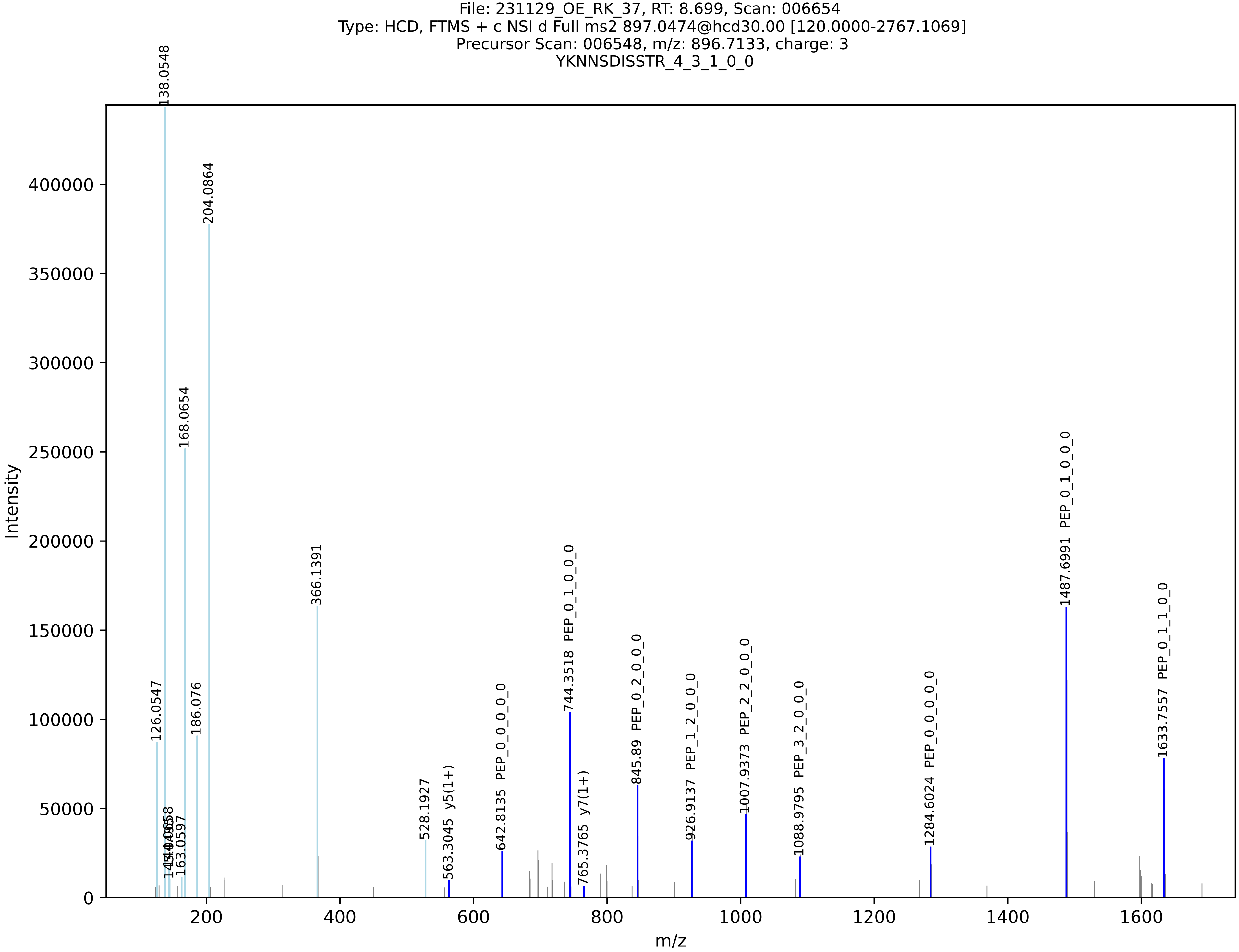
File: 231129_OE_RK_37, RT: 8.699, Scan: 006654
Type: HCD, FTMS + c NSI d Full ms2 897.0474@hcd30.00 [120.0000-2767.1069]
Precursor Scan: 006548, m/z: 896.7133, charge: 3
YKNNSDISSTR_4_3_1_0_0
Intensity
m/z
138.0548
204.0864
168.0654
366.1391
126.0547
186.076
163.0597
528.1927
563.3045 y5(1+)
642.8135 PEP_0_0_0_0_0
744.3518 PEP_0_1_0_0_0
765.3765 y7(1+)
845.89 PEP_0_2_0_0_0
926.9137 PEP_1_2_0_0_0
1007.9373 PEP_2_2_0_0_0
1088.9795 PEP_3_2_0_0_0
1284.6024 PEP_0_0_0_0_0
1487.6991 PEP_0_1_0_0_0
1633.7557 PEP_0_1_1_0_0

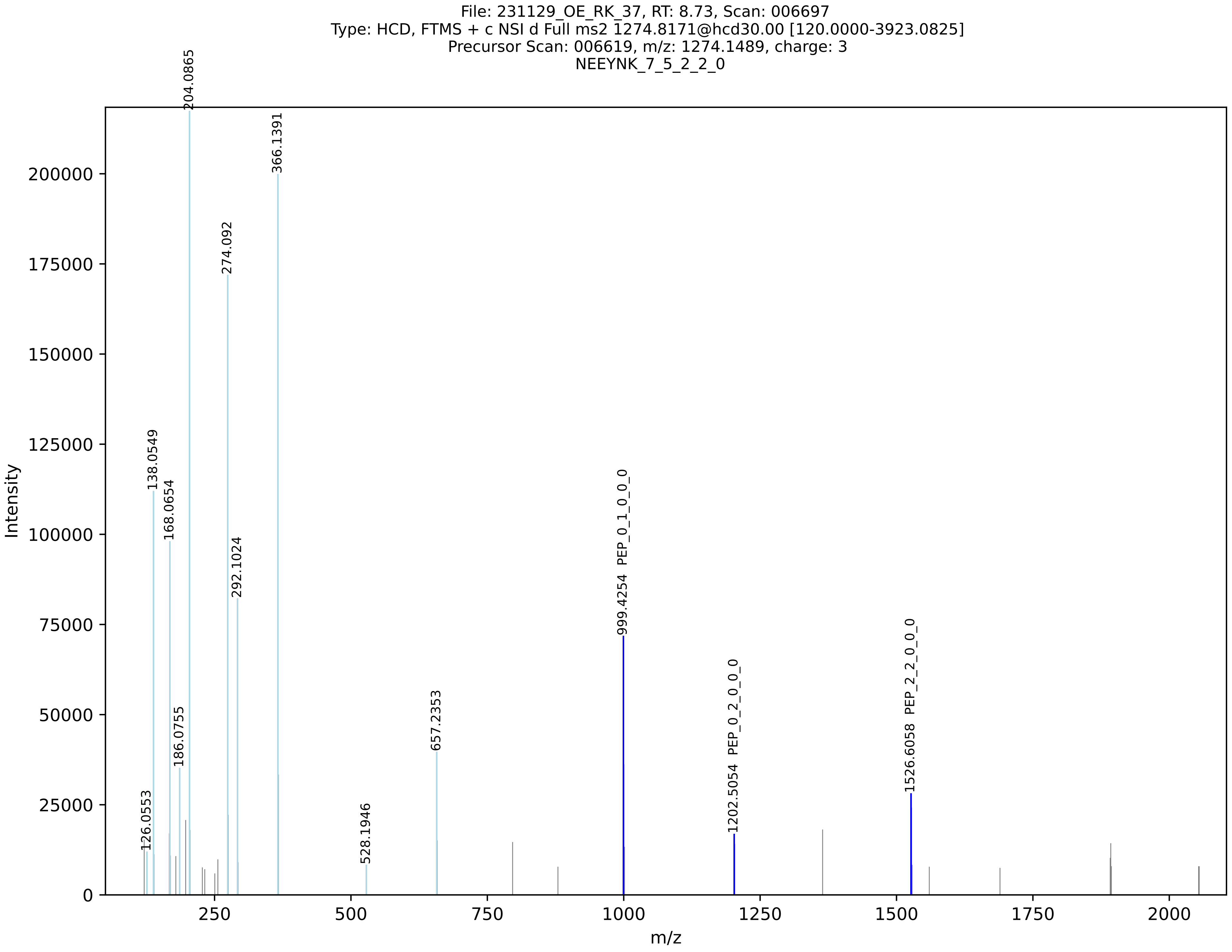
File: 231129_OE_RK_37, RT: 8.73, Scan: 006697
Type: HCD, FTMS + c NSI d Full ms2 1274.8171@hcd30.00 [120.0000-3923.0825]
Precursor Scan: 006619, m/z: 1274.1489, charge: 3
NEEYNK_7_5_2_2_0
Intensity
m/z
0
25000
50000
75000
100000
125000
150000
175000
200000
250
500
750
1000
1250
1500
1750
2000
126.0553
138.0549
168.0654
186.0755
204.0865
274.092
292.1024
366.1391
528.1946
657.2353
999.4254 PEP_0_1_0_0_0
1202.5054 PEP_0_2_0_0_0
1526.6058 PEP_2_2_0_0_0

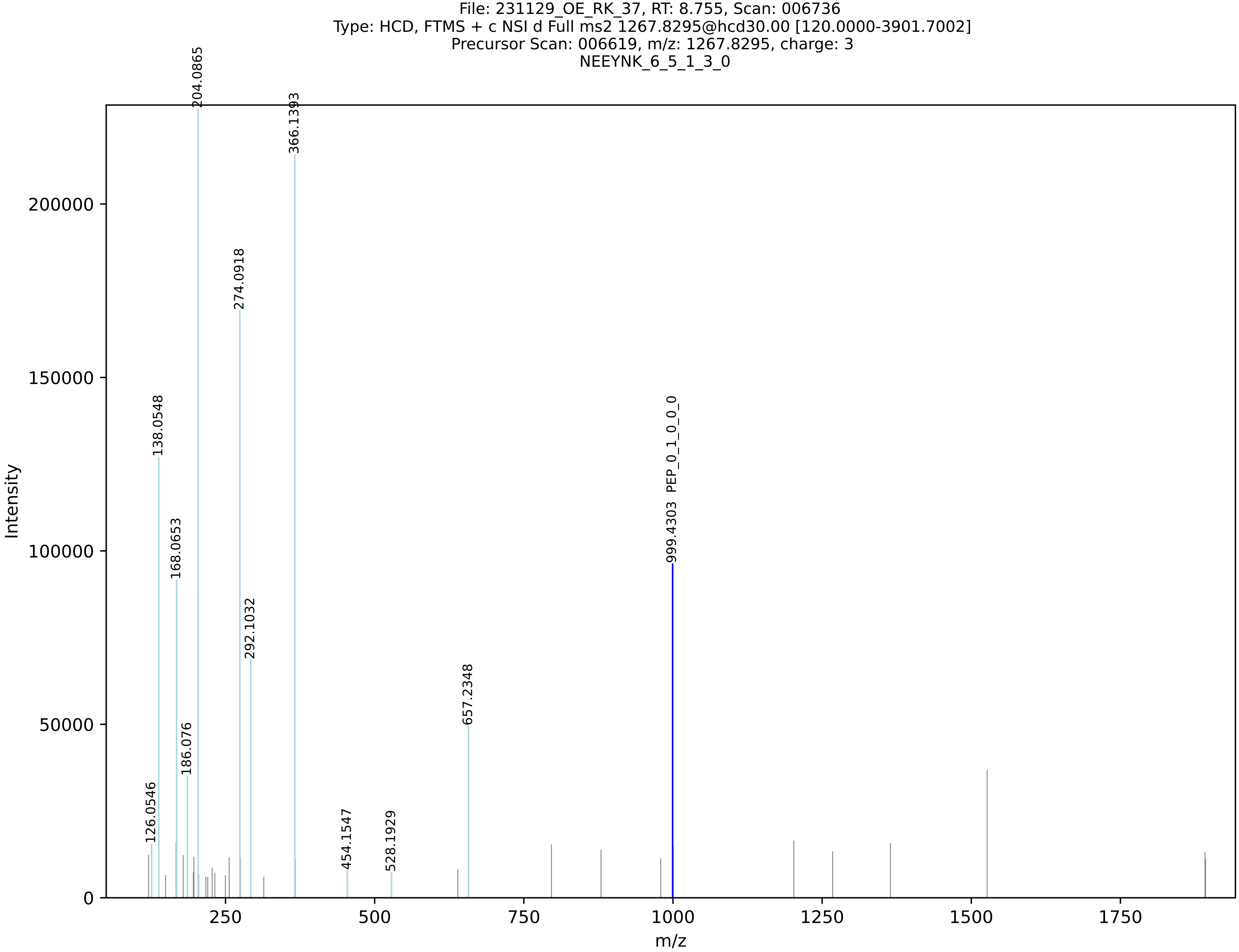
File: 231129_OE_RK_37, RT: 8.755, Scan: 006736
Type: HCD, FTMS + c NSI d Full ms2 1267.8295@hcd30.00 [120.0000-3901.7002]
Precursor Scan: 006619, m/z: 1267.8295, charge: 3
NEEYNK_6_5_1_3_0
204.0865
366.1393
274.0918
138.0548
168.0653
292.1032
186.076
126.0546
454.1547
528.1929
657.2348
999.4303 PEP_0_1_0_0_0
Intensity
m/z
0
50000
100000
150000
200000
250
500
750
1000
1250
1500
1750

File: 231129_OE_RK_37, RT: 8.779, Scan: 006769
Type: HCD, FTMS + c NSI d Full ms2 1053.4288@hcd30.00 [120.0000-3245.6343]
Precursor Scan: 006693, m/z: 1053.0948, charge: 3
YKNNSDISSTR_6_3_0_1_0

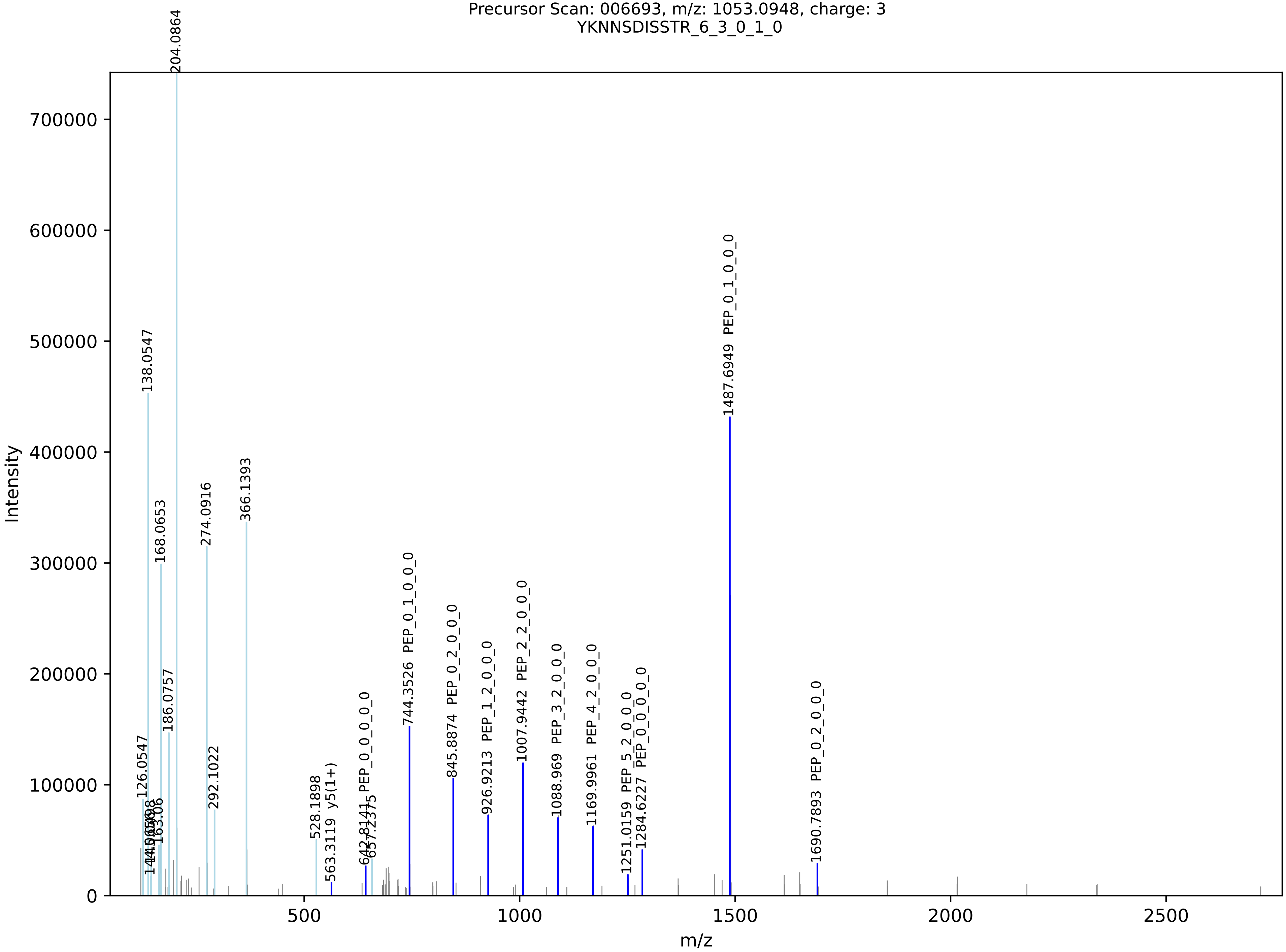

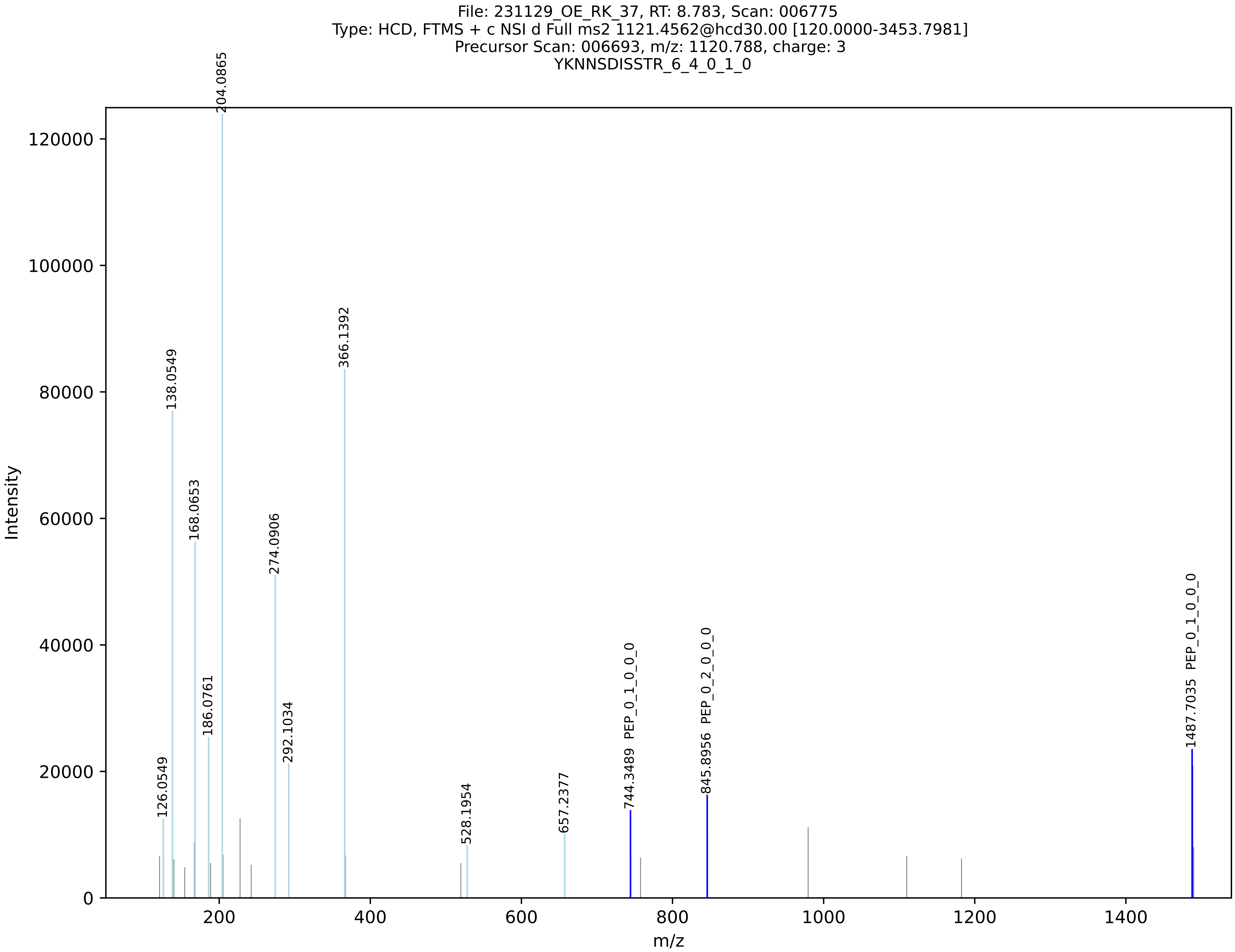
File: 231129_OE_RK_37, RT: 8.783, Scan: 006775
Type: HCD, FTMS + c NSI d Full ms2 1121.4562@hcd30.00 [120.0000-3453.7981]
Precursor Scan: 006693, m/z: 1120.788, charge: 3
YKNNSDISSTR_6_4_0_1_0
Intensity
m/z
204.0865
138.0549
168.0653
186.0761
126.0549
274.0906
292.1034
366.1392
528.1954
657.2377
744.3489 PEP_0_1_0_0_0
845.8956 PEP_0_2_0_0_0
1487.7035 PEP_0_1_0_0_0

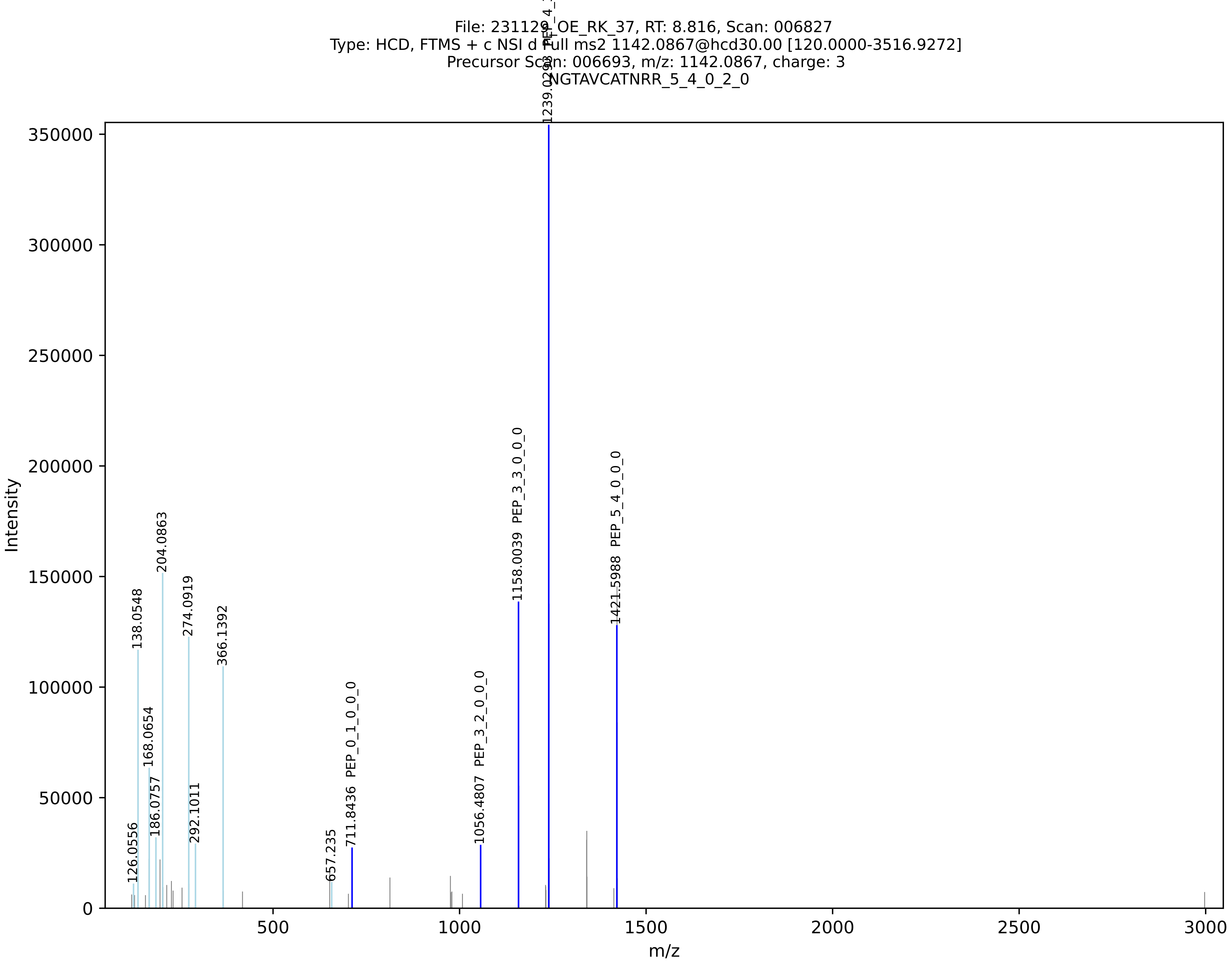
File: 231129_OE_RK_37, RT: 8.816, Scan: 006827
Type: HCD, FTMS + c NSI d Full ms2 1142.0867@hcd30.00 [120.0000-3516.9272]
Precursor Scan: 006693, m/z: 1142.0867, charge: 3
NGTAVCATNRR_5_4_0_2_0
Intensity
m/z
0
50000
100000
150000
200000
250000
300000
350000
500
1000
1500
2000
2500
3000
126.0556
138.0548
168.0654
186.0757
204.0863
274.0919
292.1011
366.1392
657.235
711.8436 PEP_0_1_0_0_0
1056.4807 PEP_3_2_0_0_0
1158.0039 PEP_3_3_0_0_0
1421.5988 PEP_5_4_0_0_0

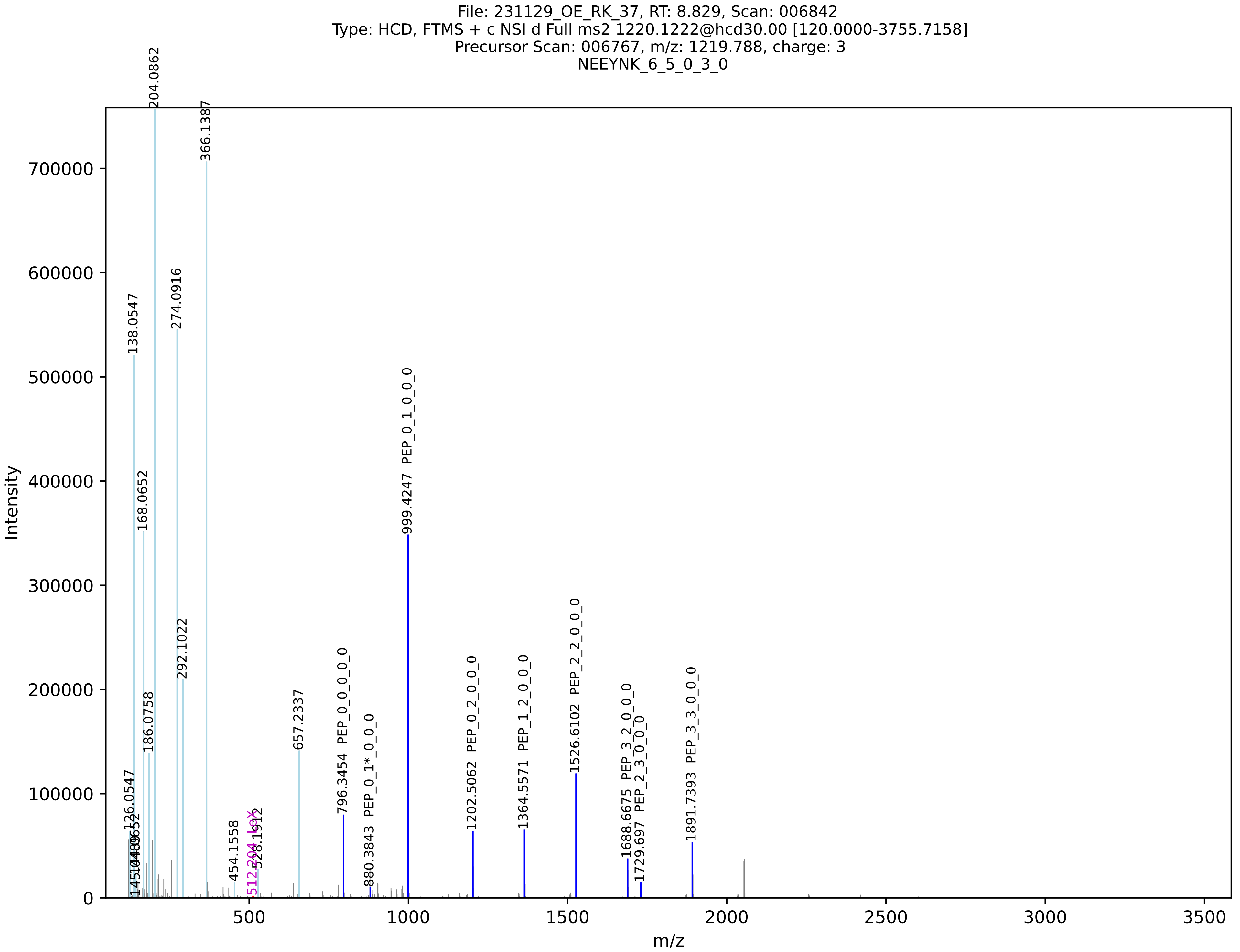

File: 231129_OE_RK_37, RT: 8.829, Scan: 006842
Type: HCD, FTMS + c NSI d Full ms2 1220.1222@hcd30.00 [120.0000-3755.7158]
Precursor Scan: 006767, m/z: 1219.788, charge: 3
NEEYNK_6_5_0_3_0
Intensity
m/z
204.0862
366.1387
138.0547
274.0916
168.0652
292.1022
186.0758
126.0547
657.2337
454.1558
512.204 LeX
528.1912
999.4247 PEP_0_1_0_0_0
796.3454 PEP_0_0_0_0_0
880.3843 PEP_0_1*_0_0_0
1202.5062 PEP_0_2_0_0_0
1364.5571 PEP_1_2_0_0_0
1526.6102 PEP_2_2_0_0_0
1688.6675 PEP_3_2_0_0_0
1729.697 PEP_2_3_0_0_0
1891.7393 PEP_3_3_0_0_0

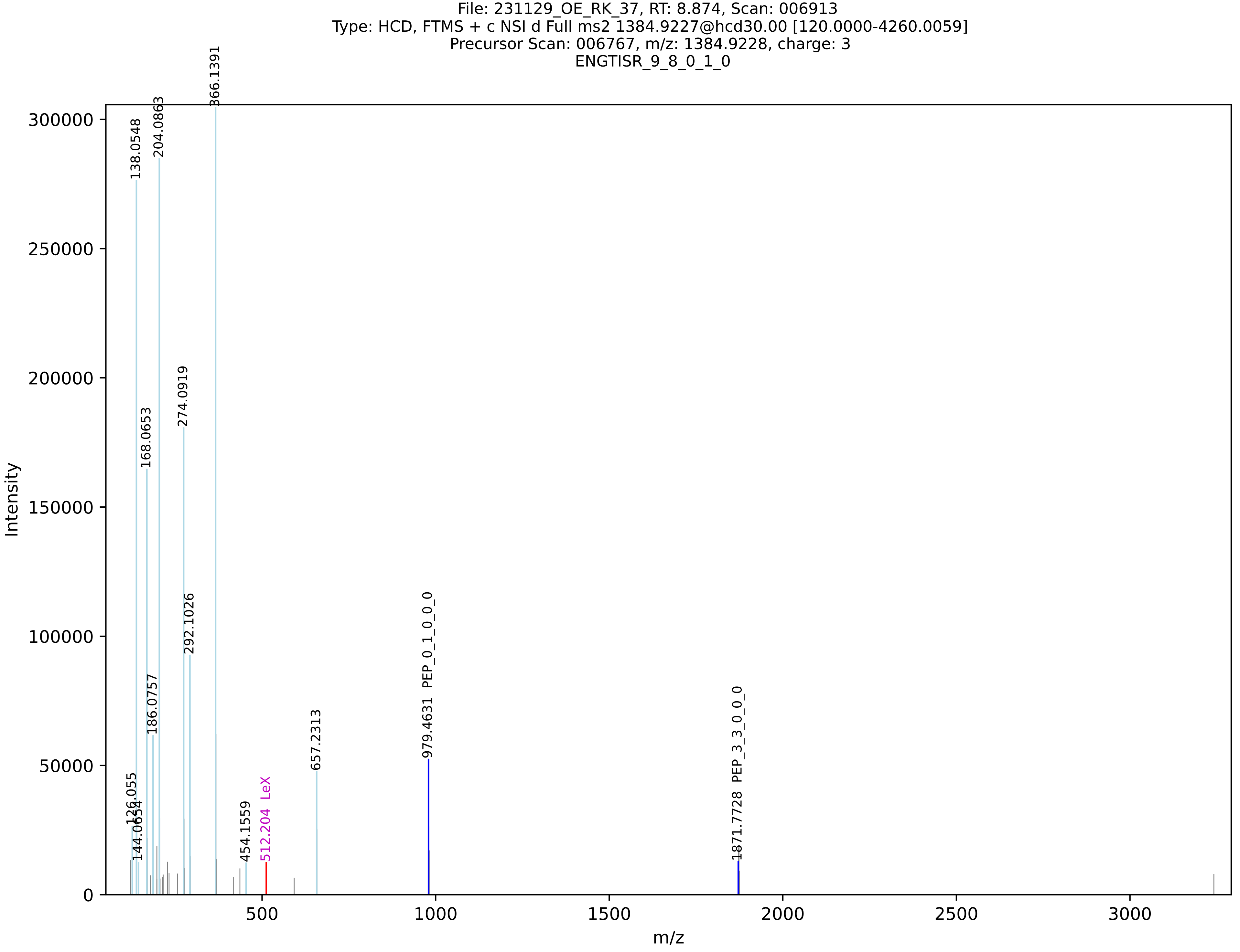

File: 231129_OE_RK_37, RT: 8.874, Scan: 006913
Type: HCD, FTMS + c NSI d Full ms2 1384.9227@hcd30.00 [120.0000-4260.0059]
Precursor Scan: 006767, m/z: 1384.9228, charge: 3
ENGTISR_9_8_0_1_0
Intensity
m/z
366.1391
204.0863
138.0548
274.0919
168.0653
292.1026
186.0757
126.055
144.0654
454.1559
512.204 LeX
657.2313
979.4631 PEP_0_1_0_0_0
1871.7728 PEP_3_3_0_0_0

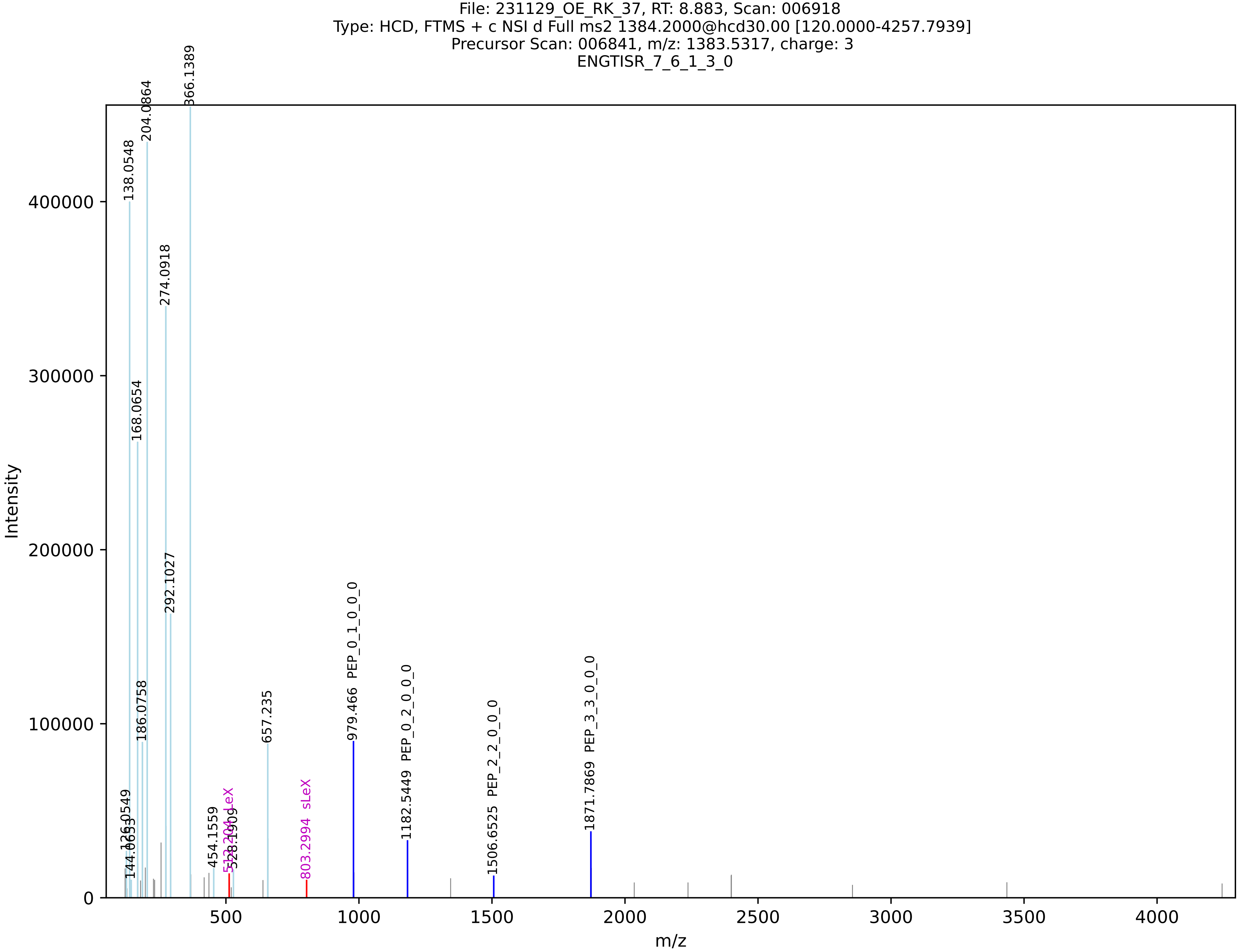

File: 231129_OE_RK_37, RT: 8.883, Scan: 006918
Type: HCD, FTMS + c NSI d Full ms2 1384.2000@hcd30.00 [120.0000-4257.7939]
Precursor Scan: 006841, m/z: 1383.5317, charge: 3
ENGTISR_7_6_1_3_0
Intensity
m/z
366.1389
204.0864
138.0548
274.0918
168.0654
292.1027
186.0758
657.235
979.466 PEP_0_1_0_0_0
1182.5449 PEP_0_2_0_0_0
1506.6525 PEP_2_2_0_0_0
1871.7869 PEP_3_3_0_0_0
126.0549
144.0653
454.1559
512.204 LeX
528.1909
803.2994 sLeX
0
100000
200000
300000
400000
500
1000
1500
2000
2500
3000
3500
4000

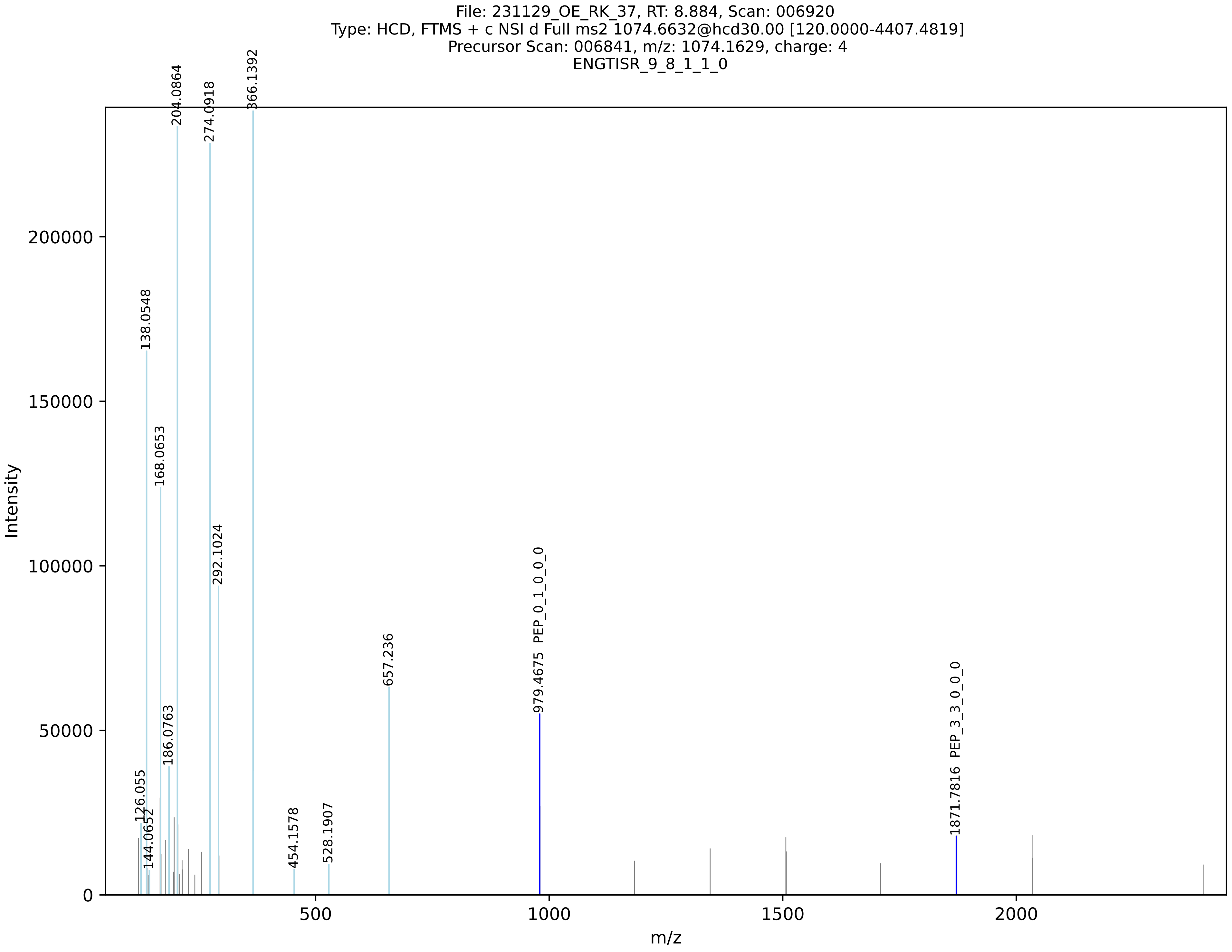
File: 231129_OE_RK_37, RT: 8.884, Scan: 006920
Type: HCD, FTMS + c NSI d Full ms2 1074.6632@hcd30.00 [120.0000-4407.4819]
Precursor Scan: 006841, m/z: 1074.1629, charge: 4
ENGTISR_9_8_1_1_0
Intensity
m/z
0
50000
100000
150000
200000
500
1000
1500
2000
204.0864
274.0918
366.1392
138.0548
168.0653
292.1024
186.0763
126.055
144.0652
454.1578
528.1907
657.236
979.4675 PEP_0_1_0_0_0
1871.7816 PEP_3_3_0_0_0

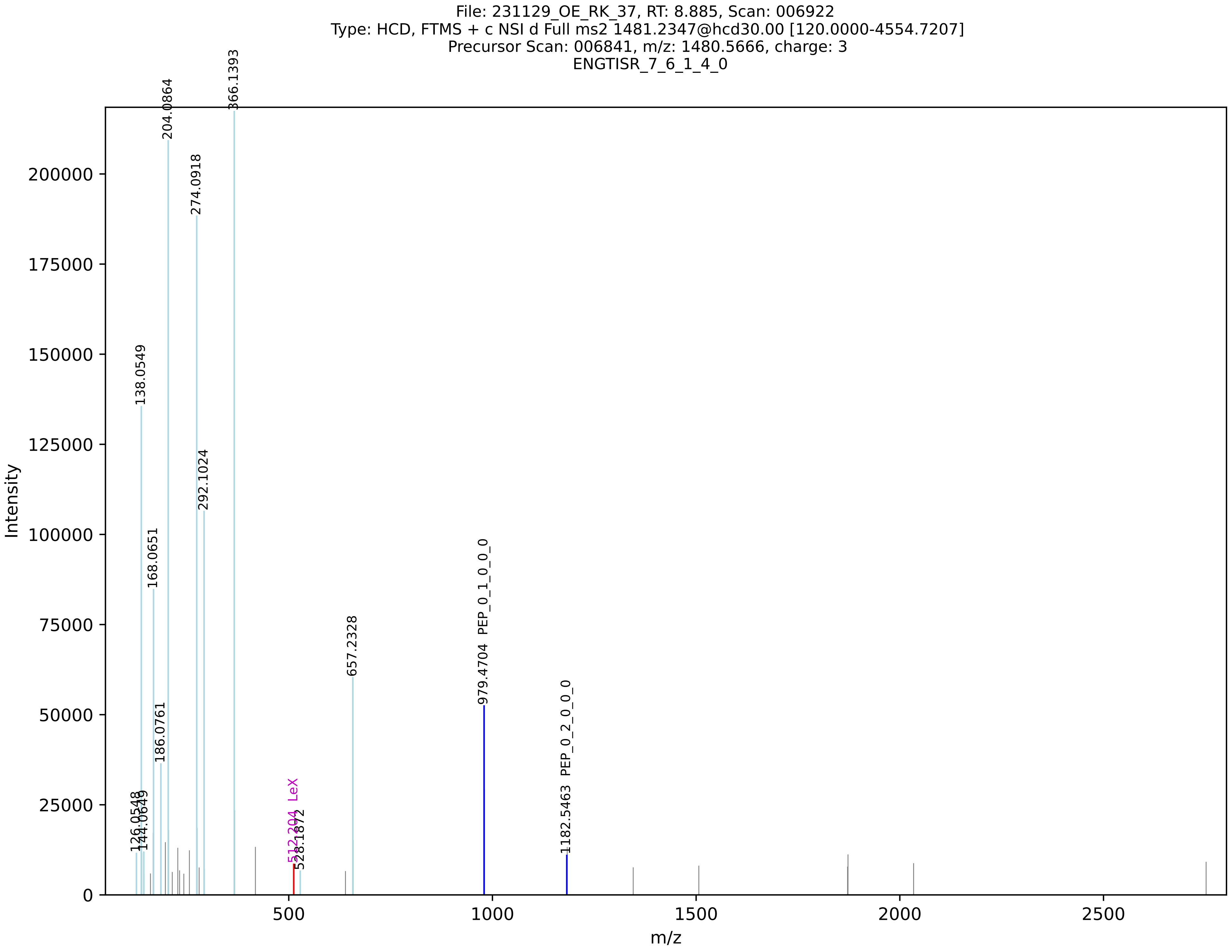
File: 231129_OE_RK_37, RT: 8.885, Scan: 006922
Type: HCD, FTMS + c NSI d Full ms2 1481.2347@hcd30.00 [120.0000-4554.7207]
Precursor Scan: 006841, m/z: 1480.5666, charge: 3
ENGTISR_7_6_1_4_0
Intensity
m/z
366.1393
204.0864
274.0918
138.0549
292.1024
168.0651
186.0761
126.0548
144.0649
512.204 LeX
528.1872
657.2328
979.4704 PEP_0_1_0_0_0
1182.5463 PEP_0_2_0_0_0
0
25000
50000
75000
100000
125000
150000
175000
200000
500
1000
1500
2000
2500

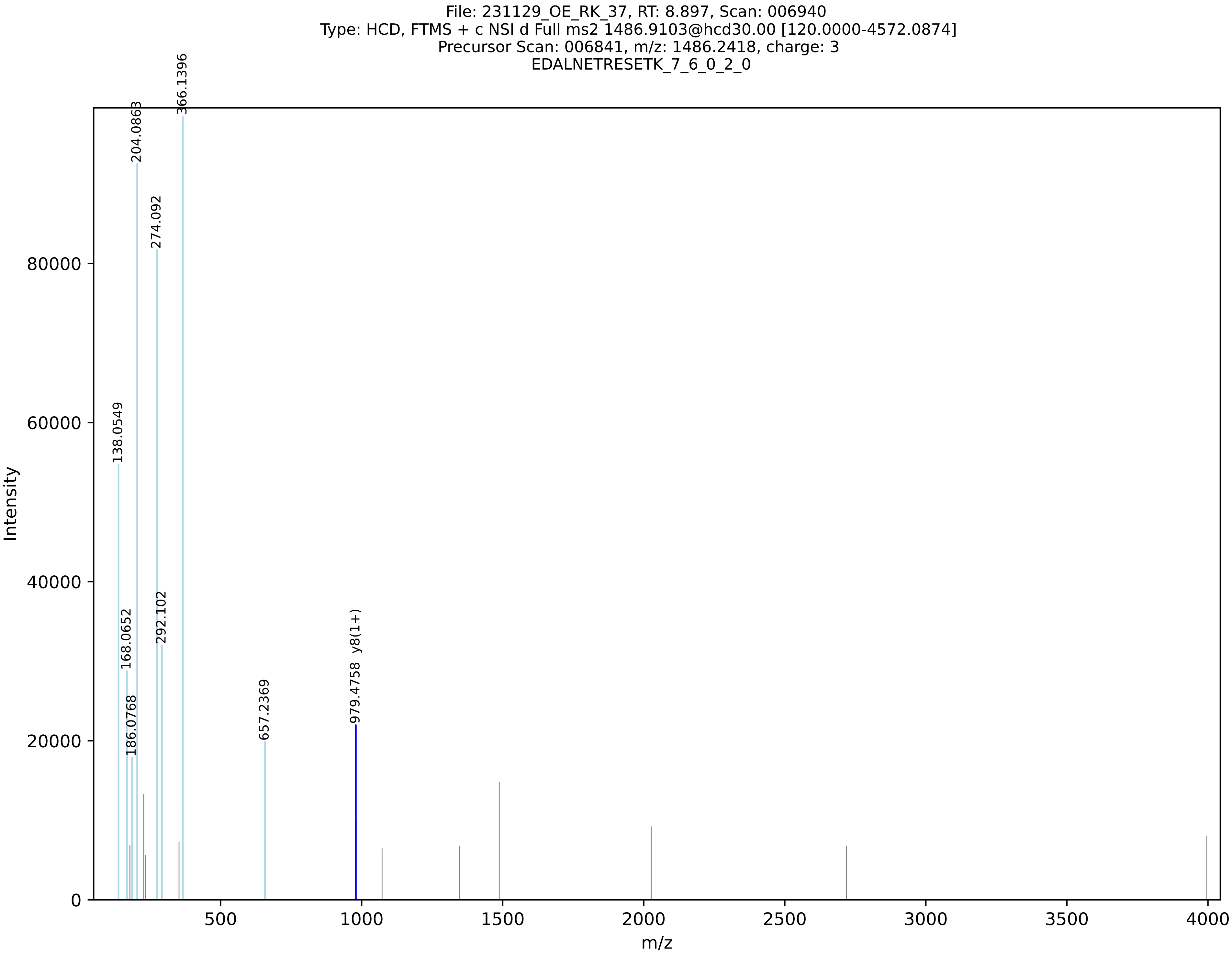
File: 231129_OE_RK_37, RT: 8.897, Scan: 006940
Type: HCD, FTMS + c NSI d Full ms2 1486.9103@hcd30.00 [120.0000-4572.0874]
Precursor Scan: 006841, m/z: 1486.2418, charge: 3
EDALNETRESETK_7_6_0_2_0
366.1396
204.0863
274.092
138.0549
168.0652
292.102
186.0768
657.2369
979.4758 y8(1+)
Intensity
m/z
0
20000
40000
60000
80000
500
1000
1500
2000
2500
3000
3500
4000

File: 231129_OE_RK_37, RT: 8.931, Scan: 006988
Type: HCD, FTMS + c NSI d Full ms2 1001.8881@hcd30.00 [120.0000-4110.5596]
Precursor Scan: 006915, m/z: 1001.3864, charge: 4
ENGTISR_7_6_0_3_0

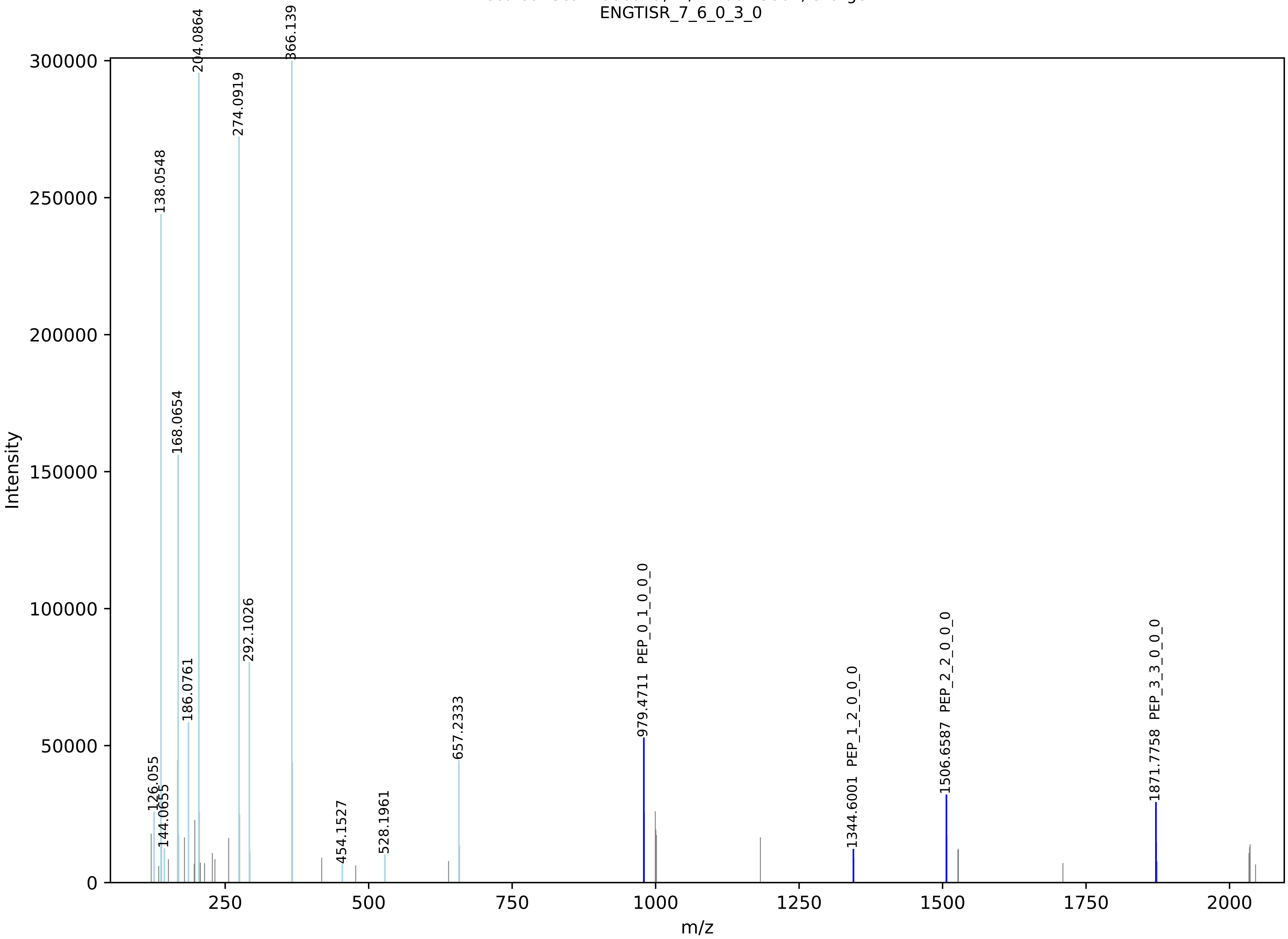

File: 231129_OE_RK_37, RT: 8.934, Scan: 006992
Type: HCD, FTMS + c NSI d Full ms2 1262.1558@hcd30.00 [120.0000-3884.3389]
Precursor Scan: 006915, m/z: 1261.4876, charge: 3
ENGTISR_6_5_1_3_0

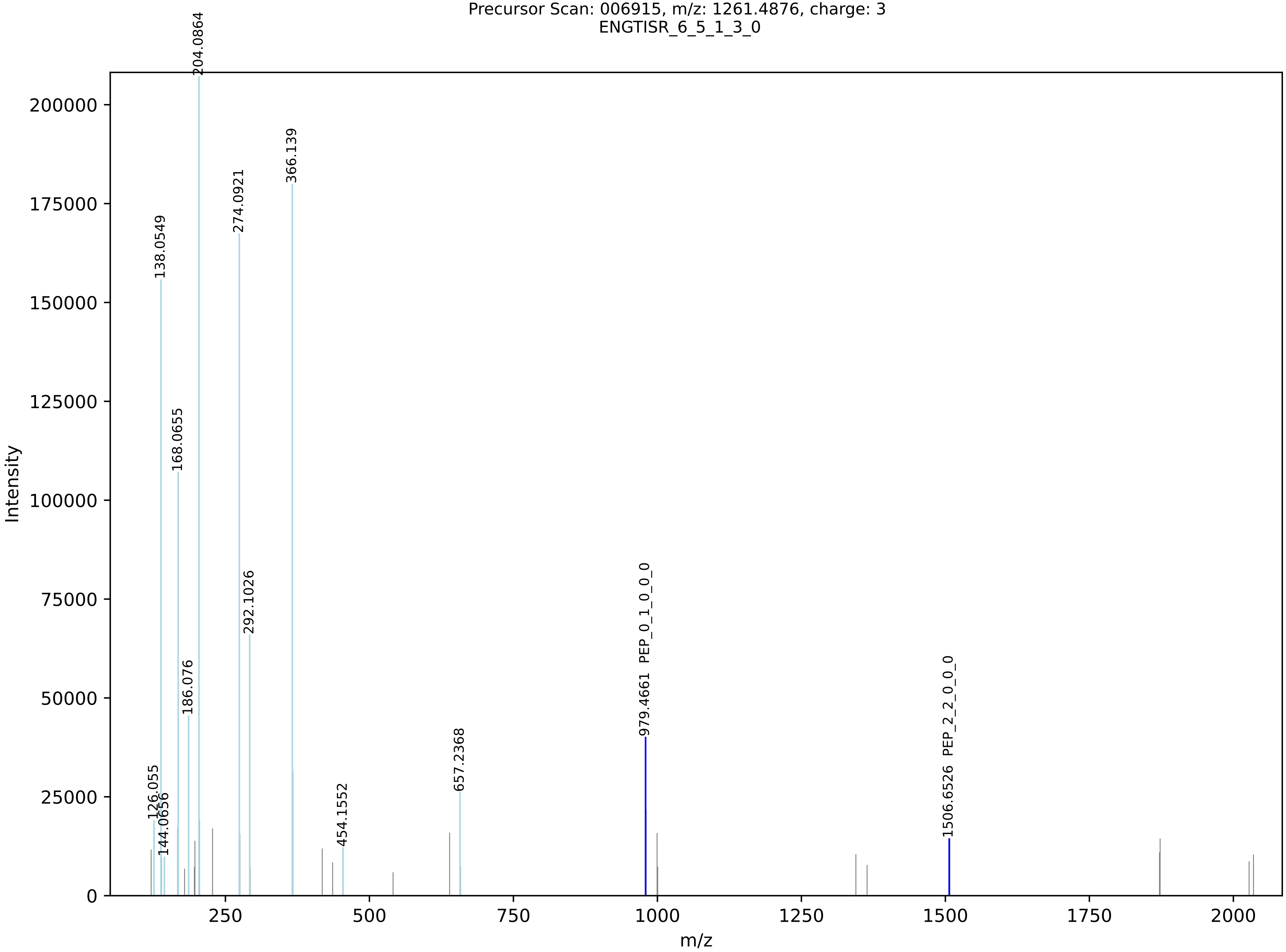

File: 231129_OE_RK_37, RT: 8.934, Scan: 006993
Type: HCD, FTMS + c NSI d Full ms2 1274.4833@hcd30.00 [120.0000-3922.0608]
Precursor Scan: 006915, m/z: 1274.1492, charge: 3
NEEYNK_7_5_2_2_0

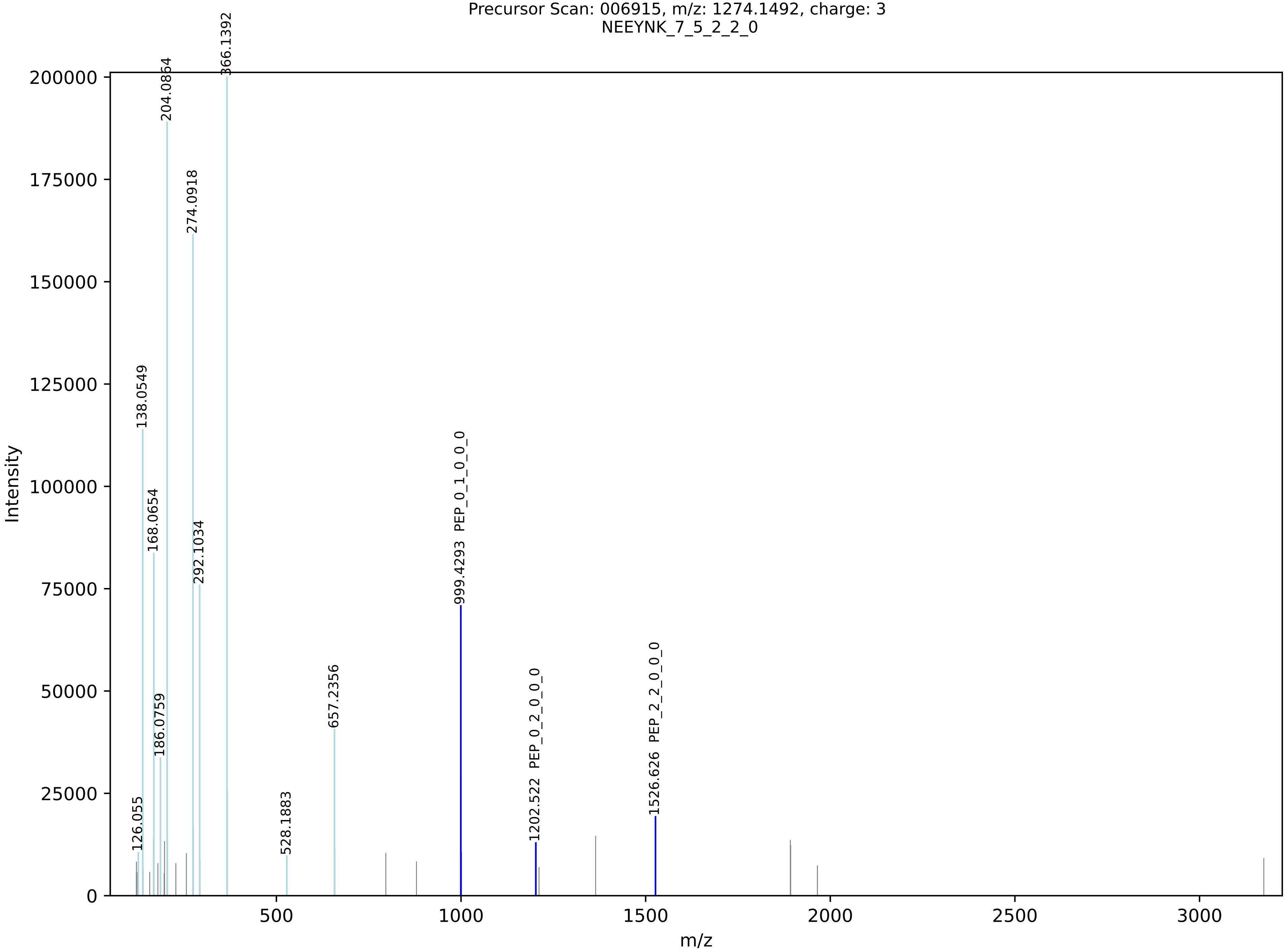

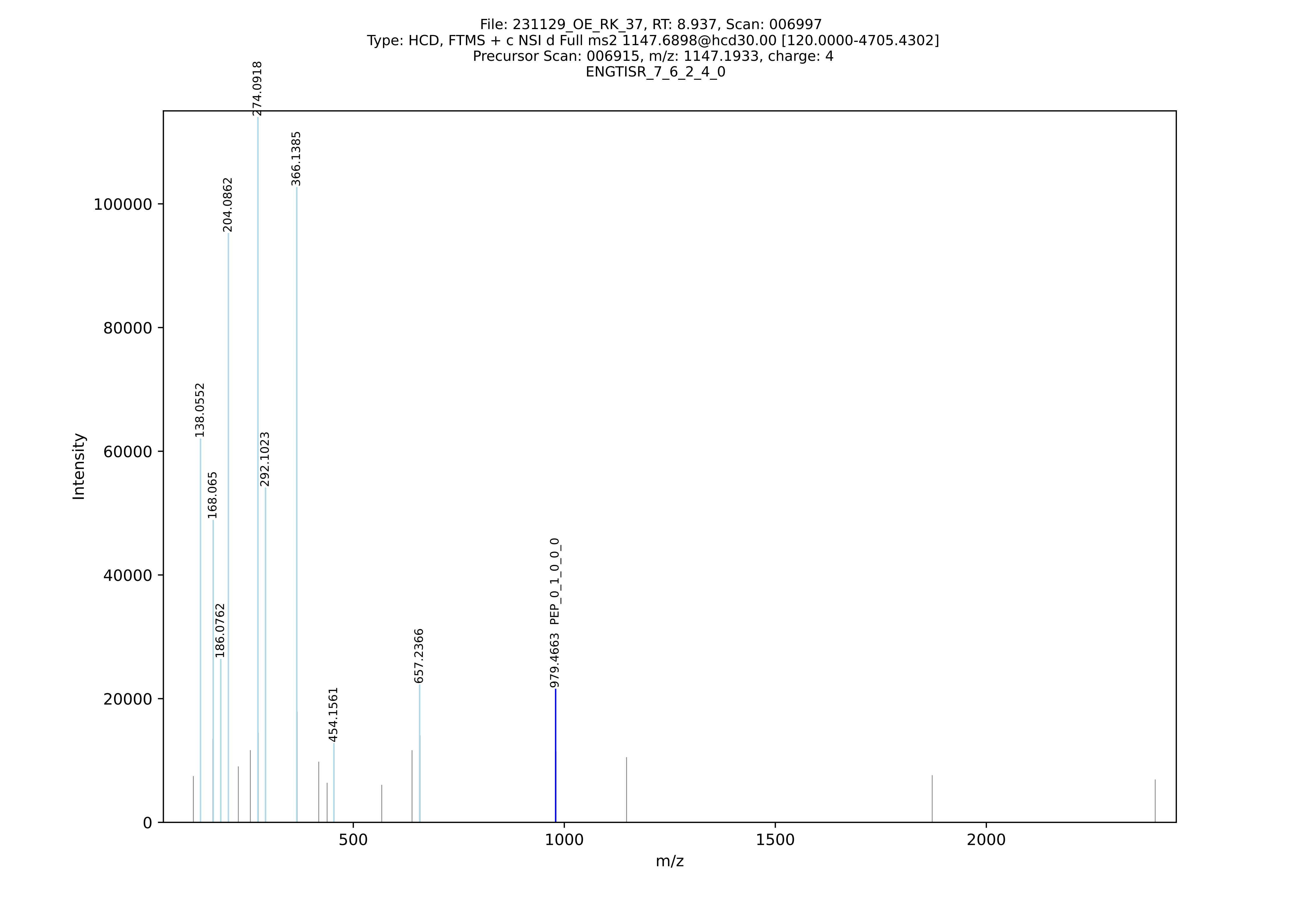
File: 231129_OE_RK_37, RT: 8.937, Scan: 006997
Type: HCD, FTMS + c NSI d Full ms2 1147.6898@hcd30.00 [120.0000-4705.4302]
Precursor Scan: 006915, m/z: 1147.1933, charge: 4
ENGTISR_7_6_2_4_0
274.0918
366.1385
204.0862
138.0552
292.1023
168.065
186.0762
454.1561
657.2366
979.4663 PEP_0_1_0_0_0
Intensity
m/z
0
20000
40000
60000
80000
100000
500
1000
1500
2000

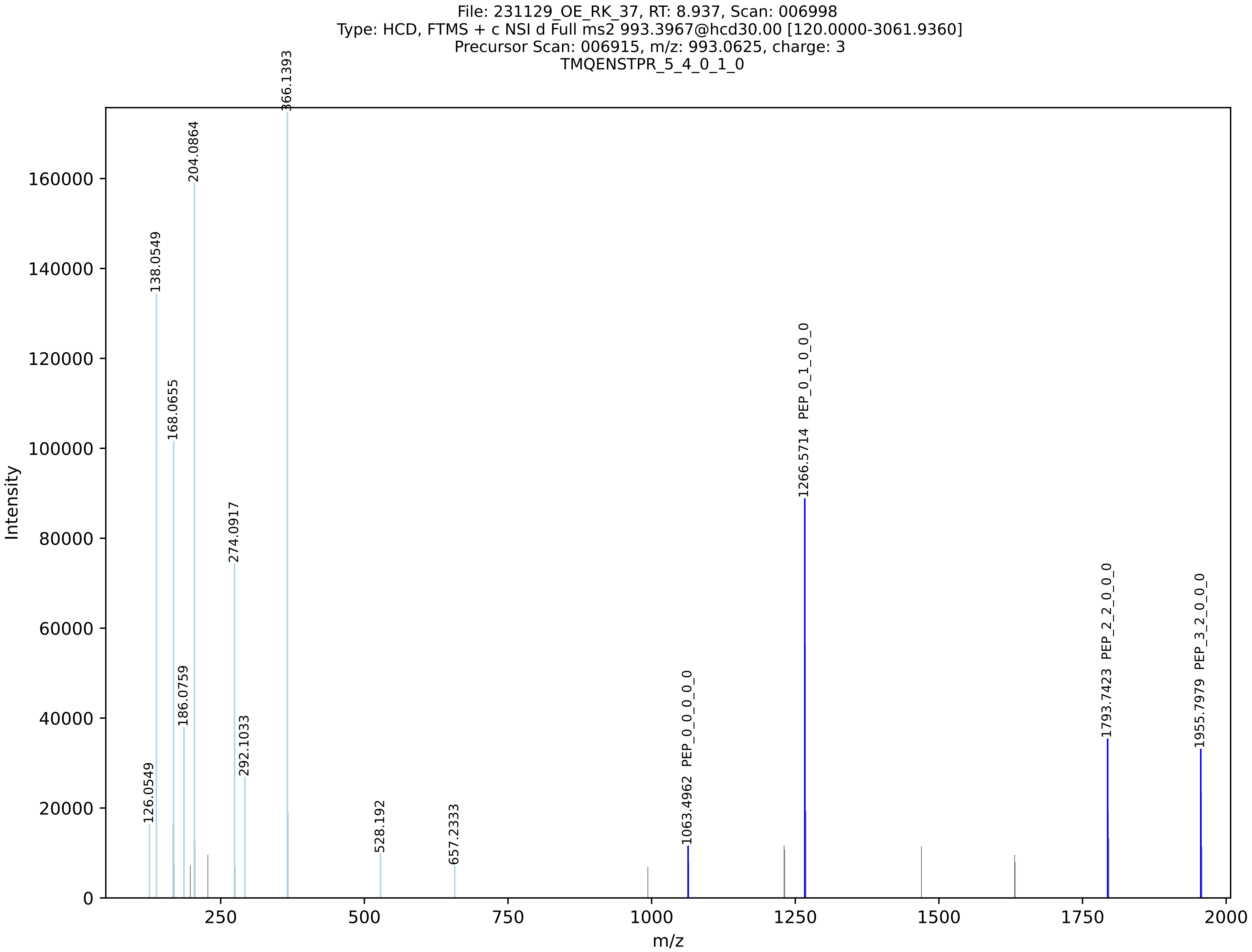

File: 231129_OE_RK_37, RT: 8.937, Scan: 006998
Type: HCD, FTMS + c NSI d Full ms2 993.3967@hcd30.00 [120.0000-3061.9360]
Precursor Scan: 006915, m/z: 993.0625, charge: 3
TMQENSTPR_5_4_0_1_0
Intensity
m/z
126.0549
138.0549
168.0655
186.0759
204.0864
274.0917
292.1033
366.1393
528.192
657.2333
1063.4962 PEP_0_0_0_0_0
1266.5714 PEP_0_1_0_0_0
1793.7423 PEP_2_2_0_0_0
1955.7979 PEP_3_2_0_0_0

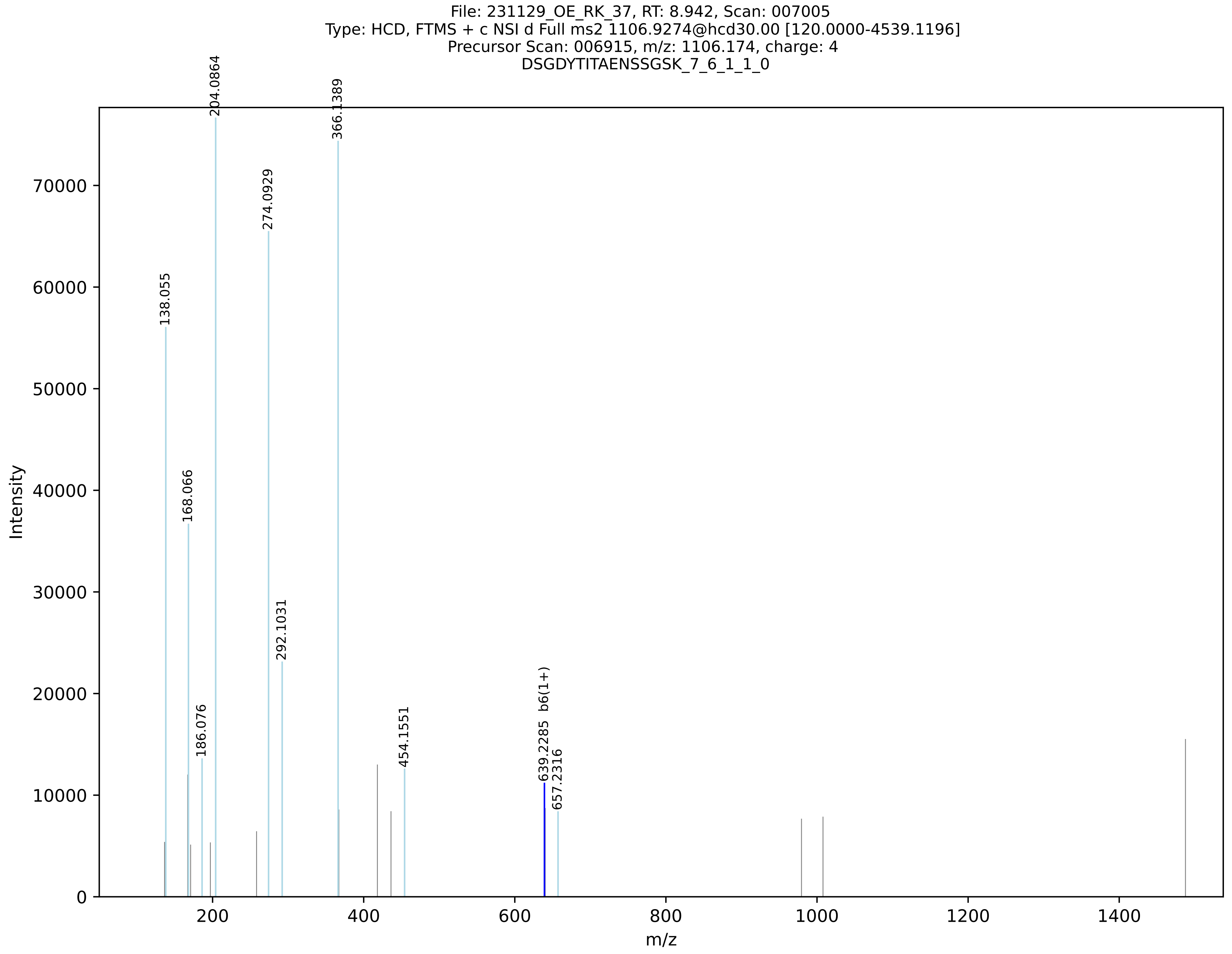
File: 231129_OE_RK_37, RT: 8.942, Scan: 007005
Type: HCD, FTMS + c NSI d Full ms2 1106.9274@hcd30.00 [120.0000-4539.1196]
Precursor Scan: 006915, m/z: 1106.174, charge: 4
DSGDYTITAENSSGSK_7_6_1_1_0
138.055
168.066
186.076
204.0864
274.0929
292.1031
366.1389
454.1551
639.2285 b6(1+)
657.2316
Intensity
m/z
0
10000
20000
30000
40000
50000
60000
70000
200
400
600
800
1000
1200
1400

File: 231129_OE_RK_37, RT: 8.955, Scan: 007026
Type: HCD, FTMS + c NSI d Full ms2 1172.1113@hcd30.00 [120.0000-3608.8027]
Precursor Scan: 006915, m/z: 1171.4457, charge: 3
NEEYNK_6_5_1_2_0

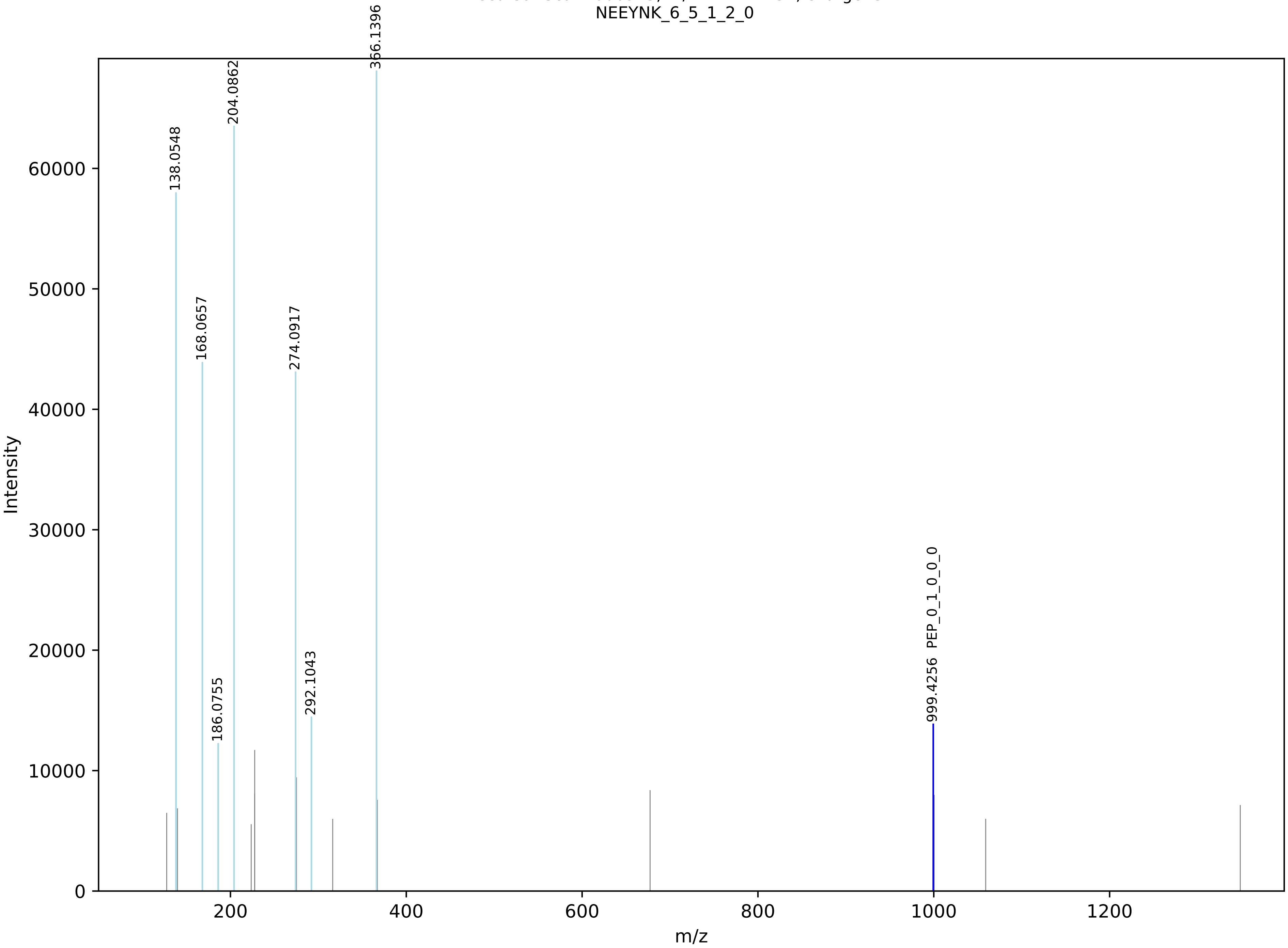

File: 231129_OE_RK_37, RT: 8.962, Scan: 007037
Type: HCD, FTMS + c NSI d Full ms2 1267.8298@hcd30.00 [120.0000-3901.7014]
Precursor Scan: 006915, m/z: 1267.8298, charge: 3
NEEYNK_6_5_1_3_0

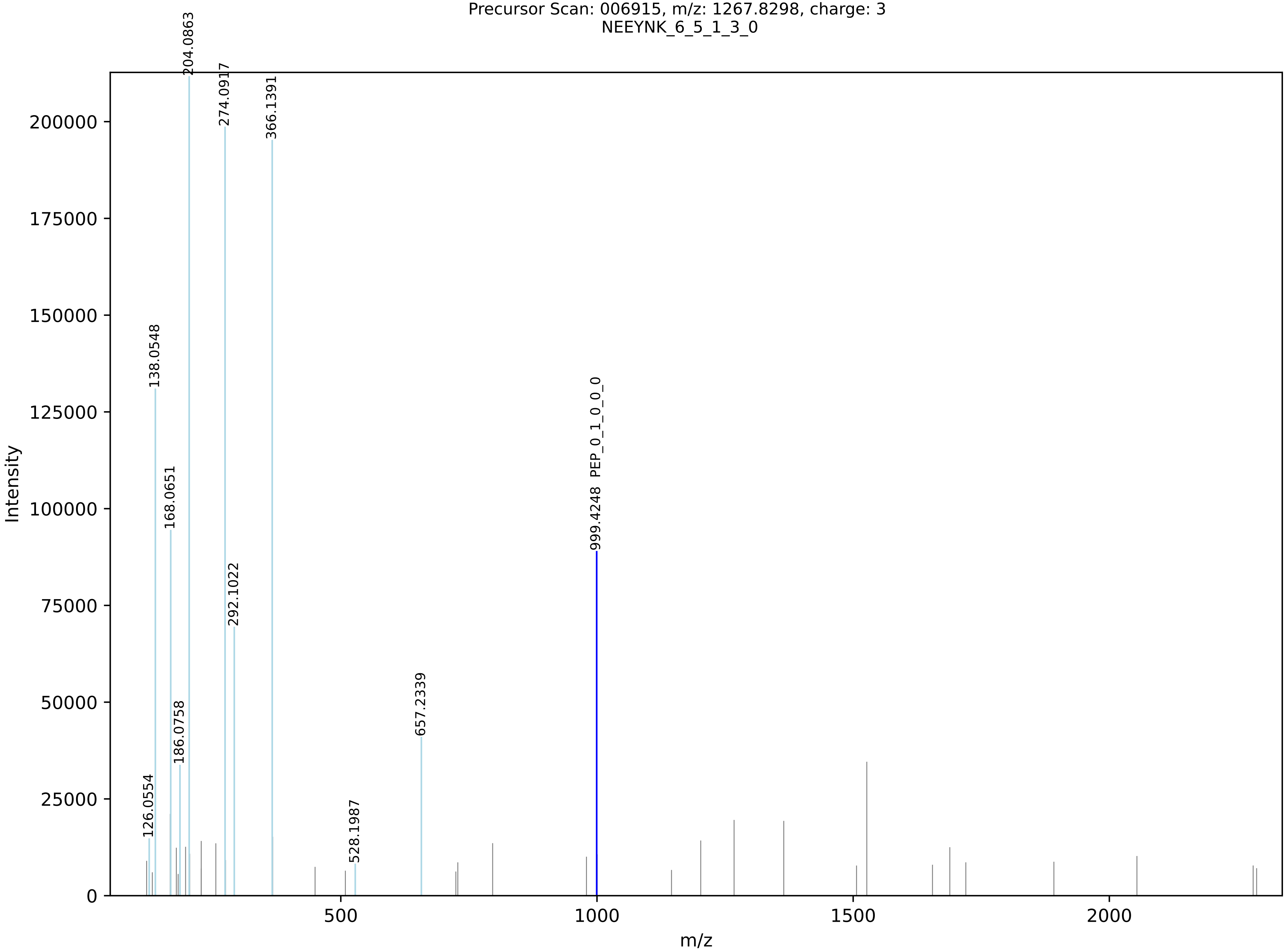

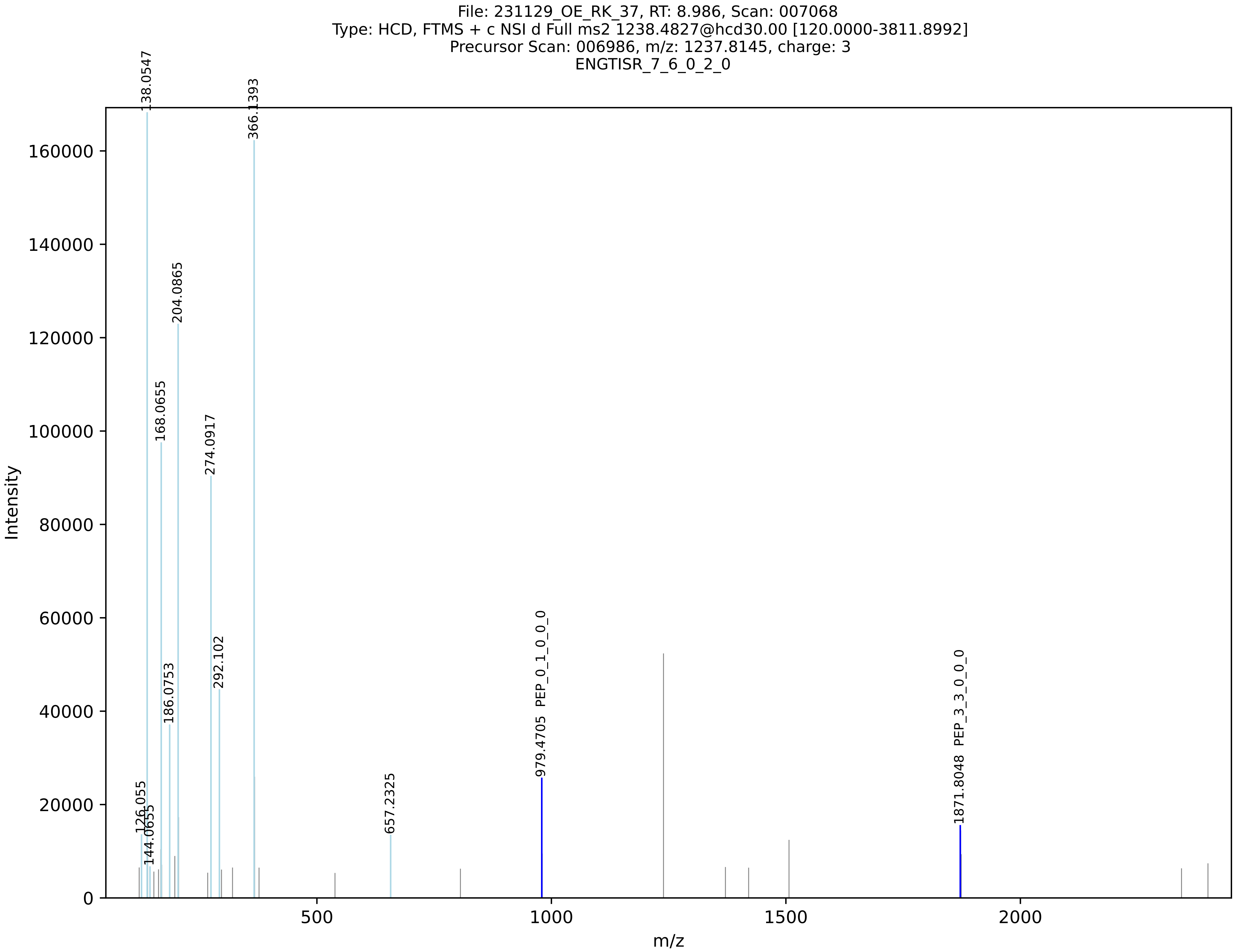
File: 231129_OE_RK_37, RT: 8.986, Scan: 007068
Type: HCD, FTMS + c NSI d Full ms2 1238.4827@hcd30.00 [120.0000-3811.8992]
Precursor Scan: 006986, m/z: 1237.8145, charge: 3
ENGTISR_7_6_0_2_0
138.0547
366.1393
204.0865
168.0655
274.0917
292.102
186.0753
126.055
144.0655
657.2325
979.4705 PEP_0_1_0_0_0
1871.8048 PEP_3_3_0_0_0
Intensity
0
20000
40000
60000
80000
100000
120000
140000
160000
500
1000
1500
2000
m/z

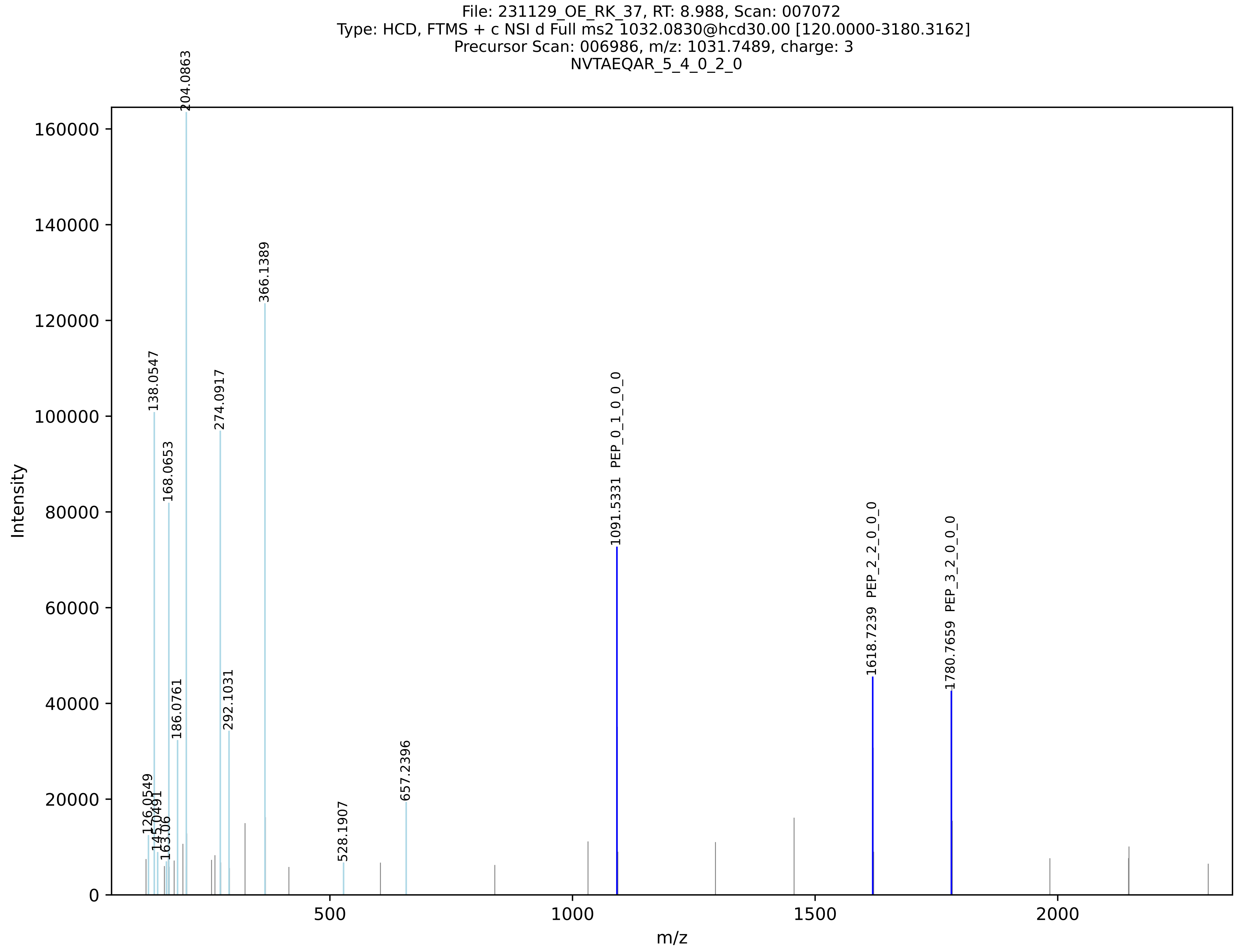

File: 231129_OE_RK_37, RT: 8.988, Scan: 007072
Type: HCD, FTMS + c NSI d Full ms2 1032.0830@hcd30.00 [120.0000-3180.3162]
Precursor Scan: 006986, m/z: 1031.7489, charge: 3
NVTAEQAR_5_4_0_2_0
204.0863
366.1389
138.0547
274.0917
168.0653
1091.5331 PEP_0_1_0_0_0
1618.7239 PEP_2_2_0_0_0
1780.7659 PEP_3_2_0_0_0
186.0761
292.1031
657.2396
126.0549
145.0491
163.06
528.1907
Intensity
m/z
0
20000
40000
60000
80000
100000
120000
140000
160000
500
1000
1500
2000

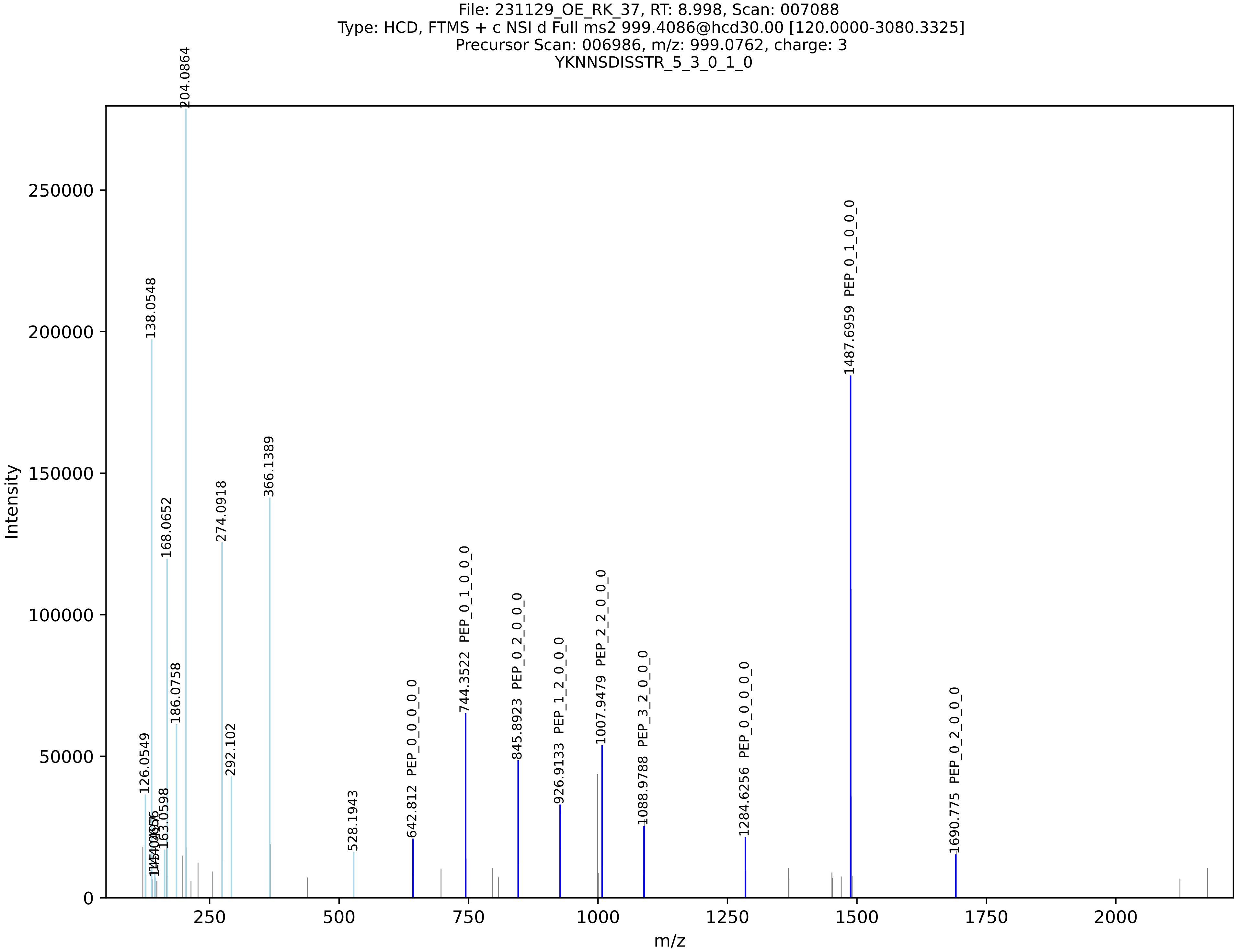
File: 231129_OE_RK_37, RT: 8.998, Scan: 007088
Type: HCD, FTMS + c NSI d Full ms2 999.4086@hcd30.00 [120.0000-3080.3325]
Precursor Scan: 006986, m/z: 999.0762, charge: 3
YKNNSDISSTR_5_3_0_1_0
Intensity
m/z
204.0864
138.0548
168.0652
274.0918
366.1389
186.0758
126.0549
292.102
163.0598
528.1943
642.812 PEP_0_0_0_0_0
744.3522 PEP_0_1_0_0_0
845.8923 PEP_0_2_0_0_0
926.9133 PEP_1_2_0_0_0
1007.9479 PEP_2_2_0_0_0
1088.9788 PEP_3_2_0_0_0
1284.6256 PEP_0_0_0_0_0
1487.6959 PEP_0_1_0_0_0
1690.775 PEP_0_2_0_0_0
0
50000
100000
150000
200000
250000
250
500
750
1000
1250
1500
1750
2000

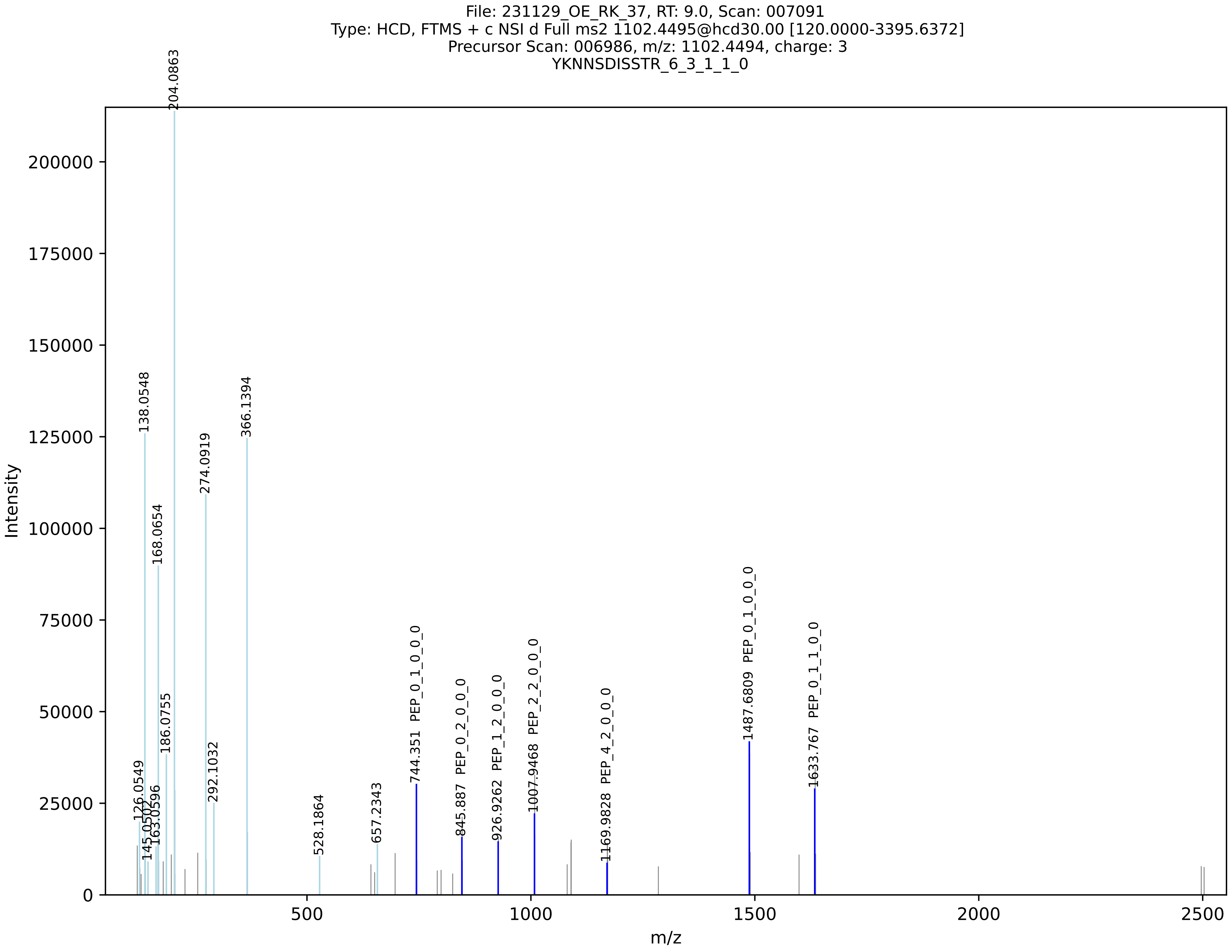
File: 231129_OE_RK_37, RT: 9.0, Scan: 007091
Type: HCD, FTMS + c NSI d Full ms2 1102.4495@hcd30.00 [120.0000-3395.6372]
Precursor Scan: 006986, m/z: 1102.4494, charge: 3
YKNNSDISSTR_6_3_1_1_0
Intensity
m/z
204.0863
138.0548
366.1394
274.0919
168.0654
186.0755
292.1032
126.0549
145.0502
163.0596
528.1864
657.2343
744.351 PEP_0_1_0_0_0
845.887 PEP_0_2_0_0_0
926.9262 PEP_1_2_0_0_0
1007.9468 PEP_2_2_0_0_0
1169.9828 PEP_4_2_0_0_0
1487.6809 PEP_0_1_0_0_0
1633.767 PEP_0_1_1_0_0

File: 231129_OE_RK_37, RT: 9.034, Scan: 007135
Type: HCD, FTMS + c NSI d Full ms2 1220.4570@hcd30.00 [120.0000-3756.7405]
Precursor Scan: 007060, m/z: 1219.7887, charge: 3
NEEYNK_6_5_0_3_0

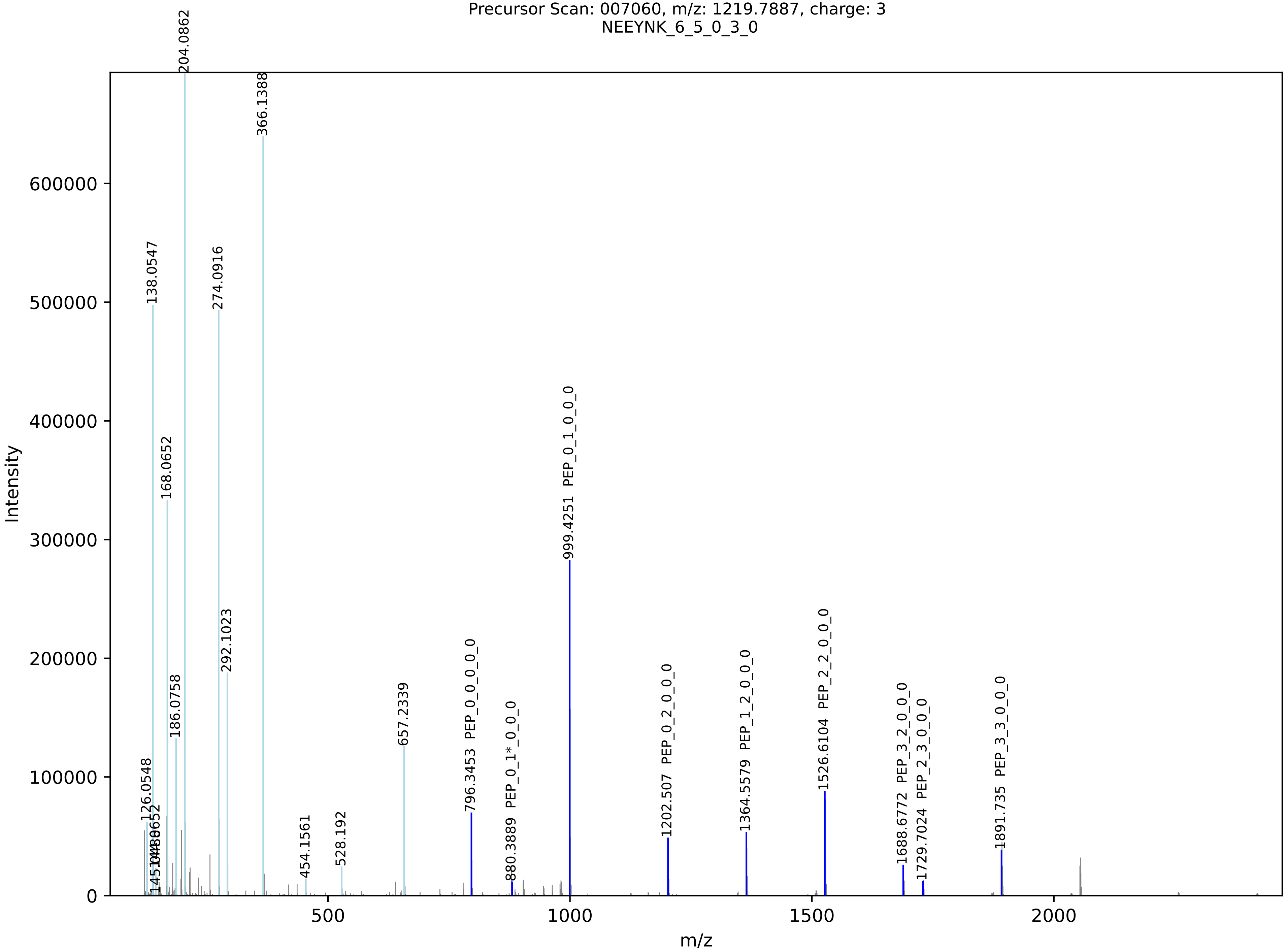

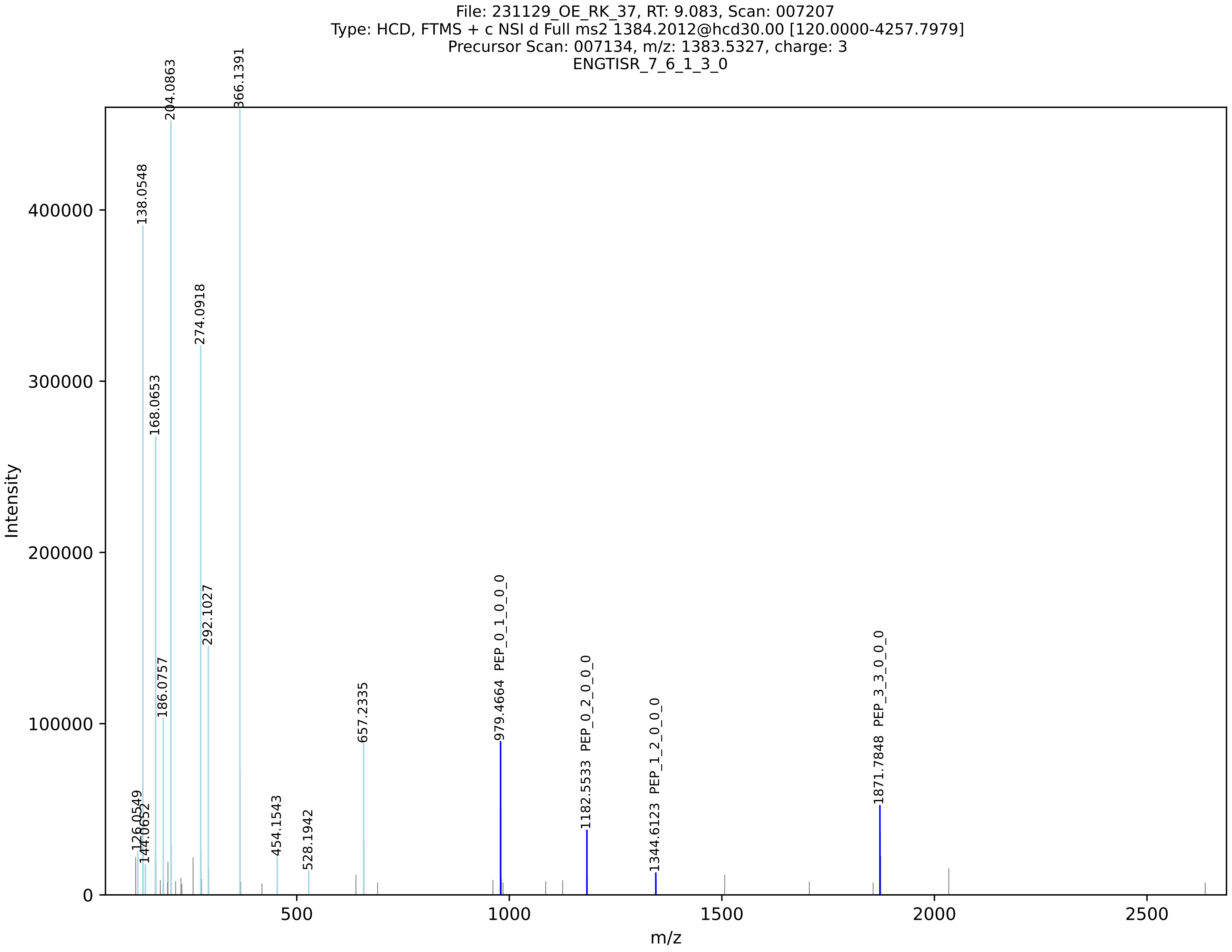
File: 231129_OE_RK_37, RT: 9.083, Scan: 007207
Type: HCD, FTMS + c NSI d Full ms2 1384.2012@hcd30.00 [120.0000-4257.7979]
Precursor Scan: 007134, m/z: 1383.5327, charge: 3
ENGTISR_7_6_1_3_0
Intensity
m/z
0
100000
200000
300000
400000
500
1000
1500
2000
2500
126.0549
144.0652
138.0548
168.0653
186.0757
204.0863
274.0918
292.1027
366.1391
454.1543
528.1942
657.2335
979.4664 PEP_0_1_0_0_0_
1182.5533 PEP_0_2_0_0_0_
1344.6123 PEP_1_2_0_0_0_
1871.7848 PEP_3_3_0_0_0_

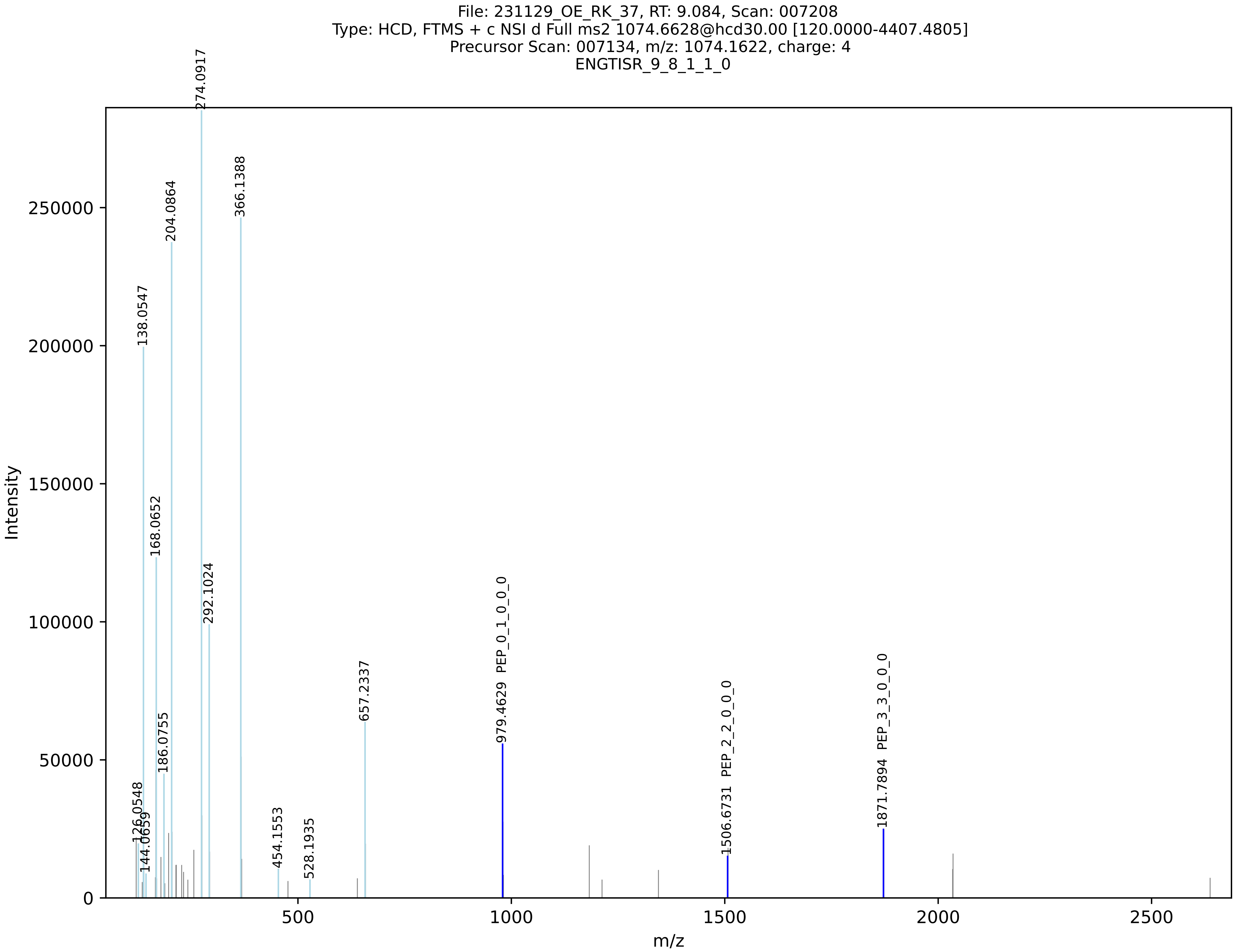

File: 231129_OE_RK_37, RT: 9.084, Scan: 007208
Type: HCD, FTMS + c NSI d Full ms2 1074.6628@hcd30.00 [120.0000-4407.4805]
Precursor Scan: 007134, m/z: 1074.1622, charge: 4
ENGTISR_9_8_1_1_0
Intensity
m/z
274.0917
366.1388
204.0864
138.0547
168.0652
292.1024
186.0755
126.0548
144.0659
454.1553
528.1935
657.2337
979.4629 PEP_0_1_0_0_0
1506.6731 PEP_2_2_0_0_0
1871.7894 PEP_3_3_0_0_0

File: 231129_OE_RK_37, RT: 9.085, Scan: 007210
Type: HCD, FTMS + c NSI d Full ms2 1067.1044@hcd30.00 [120.0000-3287.4812]
Precursor Scan: 007134, m/z: 1066.7702, charge: 3
YKNNSDISSTR_5_4_0_1_0

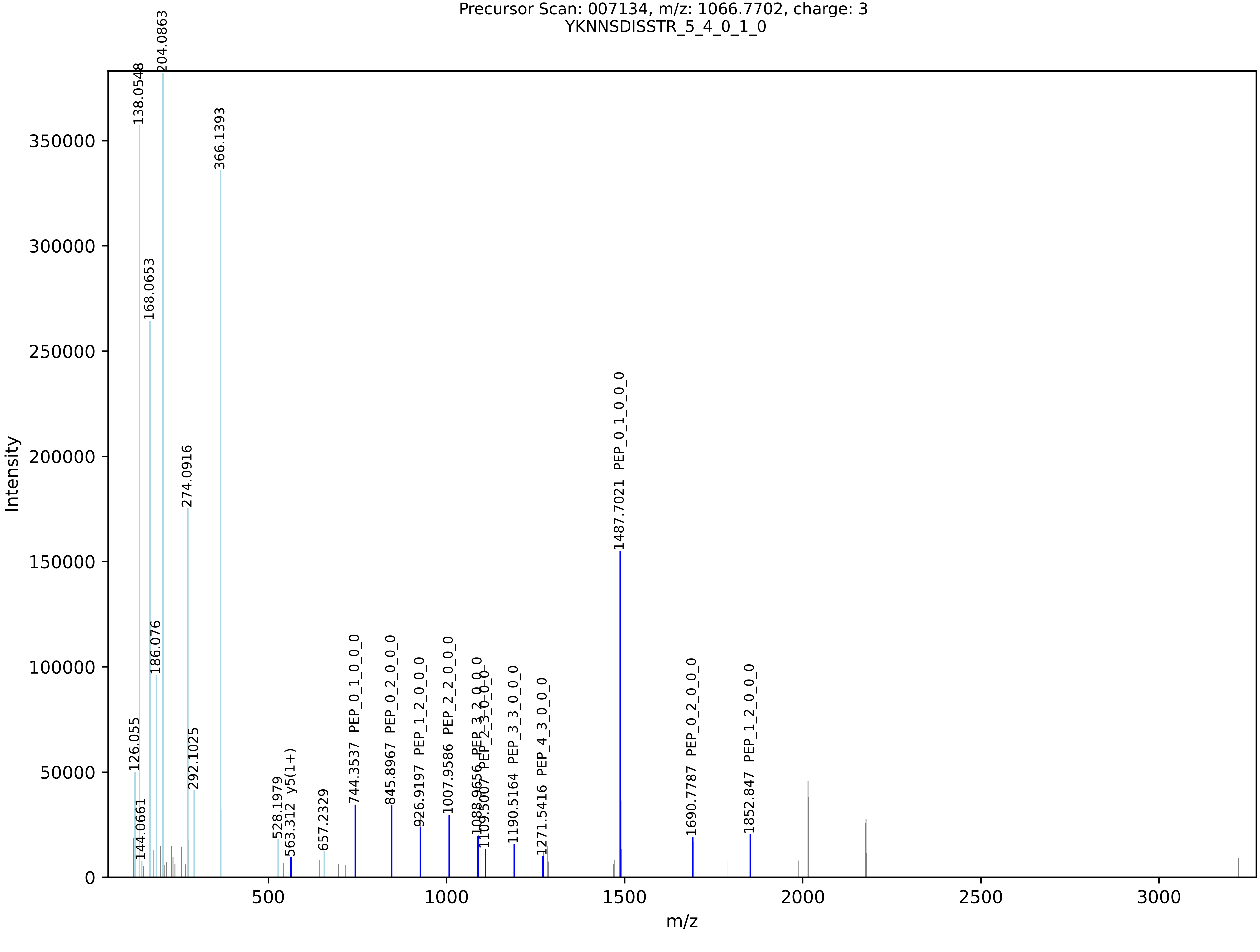

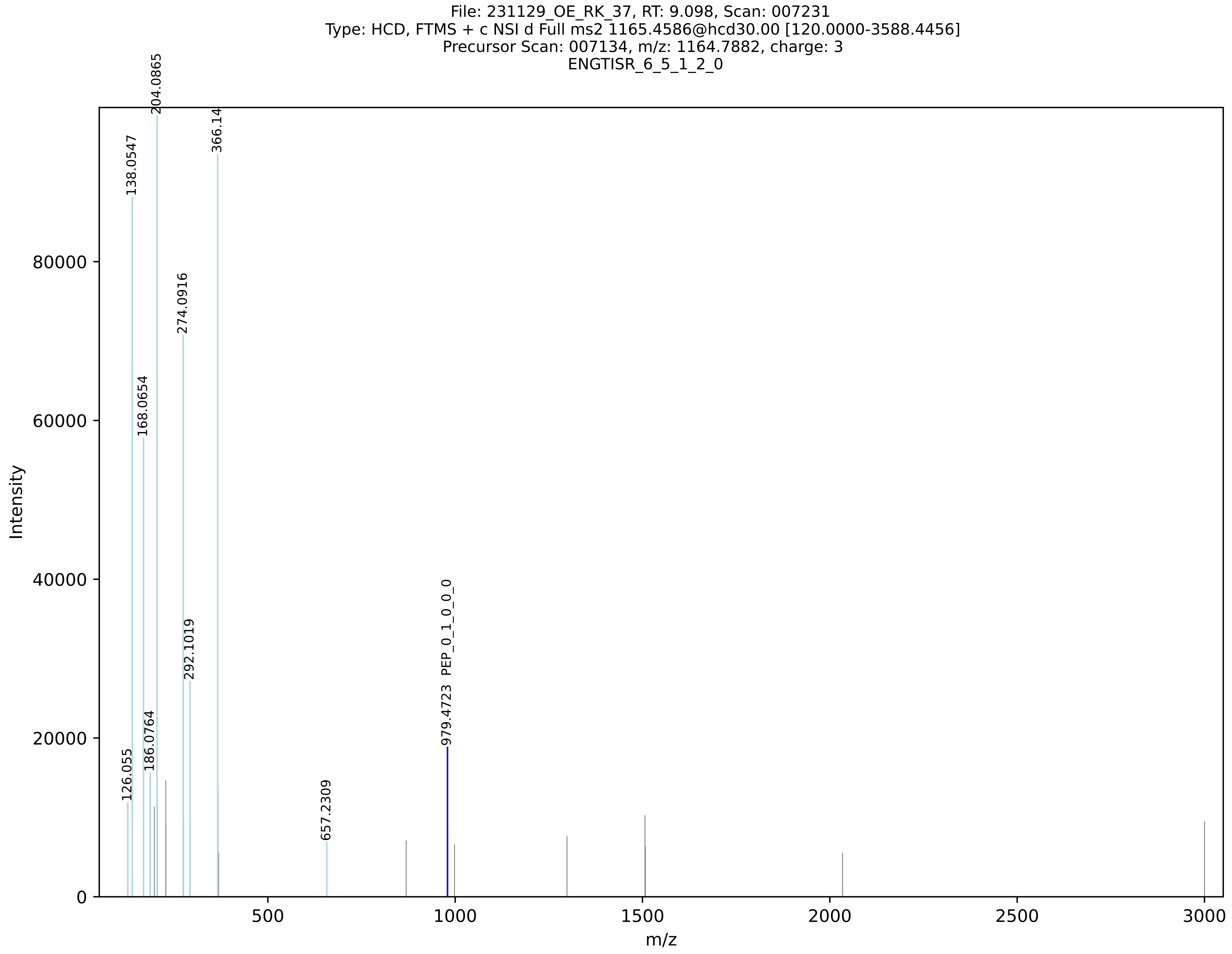

File: 231129_OE_RK_37, RT: 9.098, Scan: 007231
Type: HCD, FTMS + c NSI d Full ms2 1165.4586@hcd30.00 [120.0000-3588.4456]
Precursor Scan: 007134, m/z: 1164.7882, charge: 3
ENGTISR_6_5_1_2_0
Intensity
m/z
0
20000
40000
60000
80000
500
1000
1500
2000
2500
3000
126.055
138.0547
168.0654
186.0764
204.0865
274.0916
292.1019
366.14
657.2309
979.4723 PEP_0_1_0_0_0

File: 231129_OE_RK_37, RT: 9.138, Scan: 007287
Type: HCD, FTMS + c NSI d Full ms2 1274.8174@hcd30.00 [120.0000-3923.0833]
Precursor Scan: 007205, m/z: 1274.1473, charge: 3
NEEYNK_7_5_2_2_0

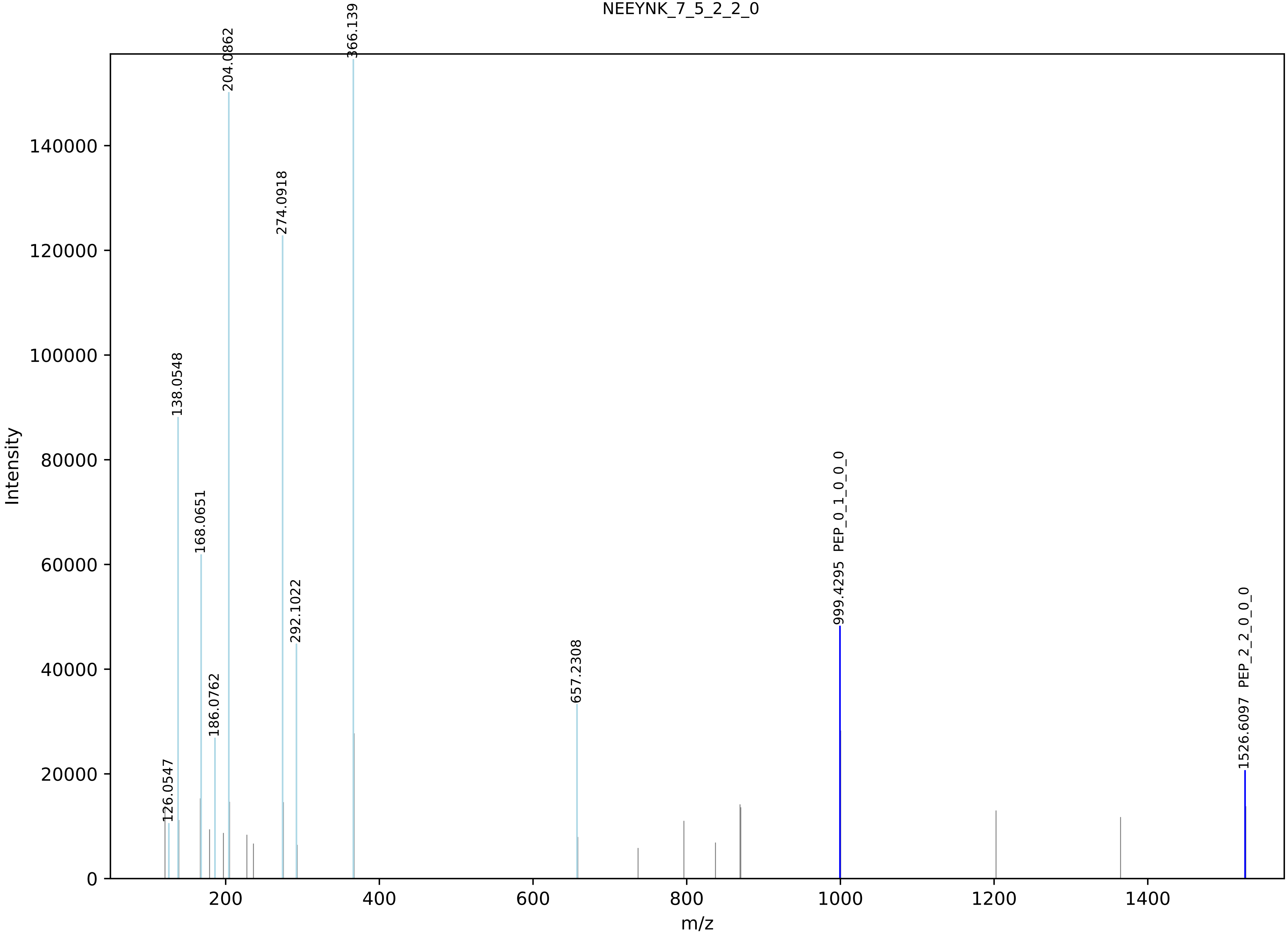

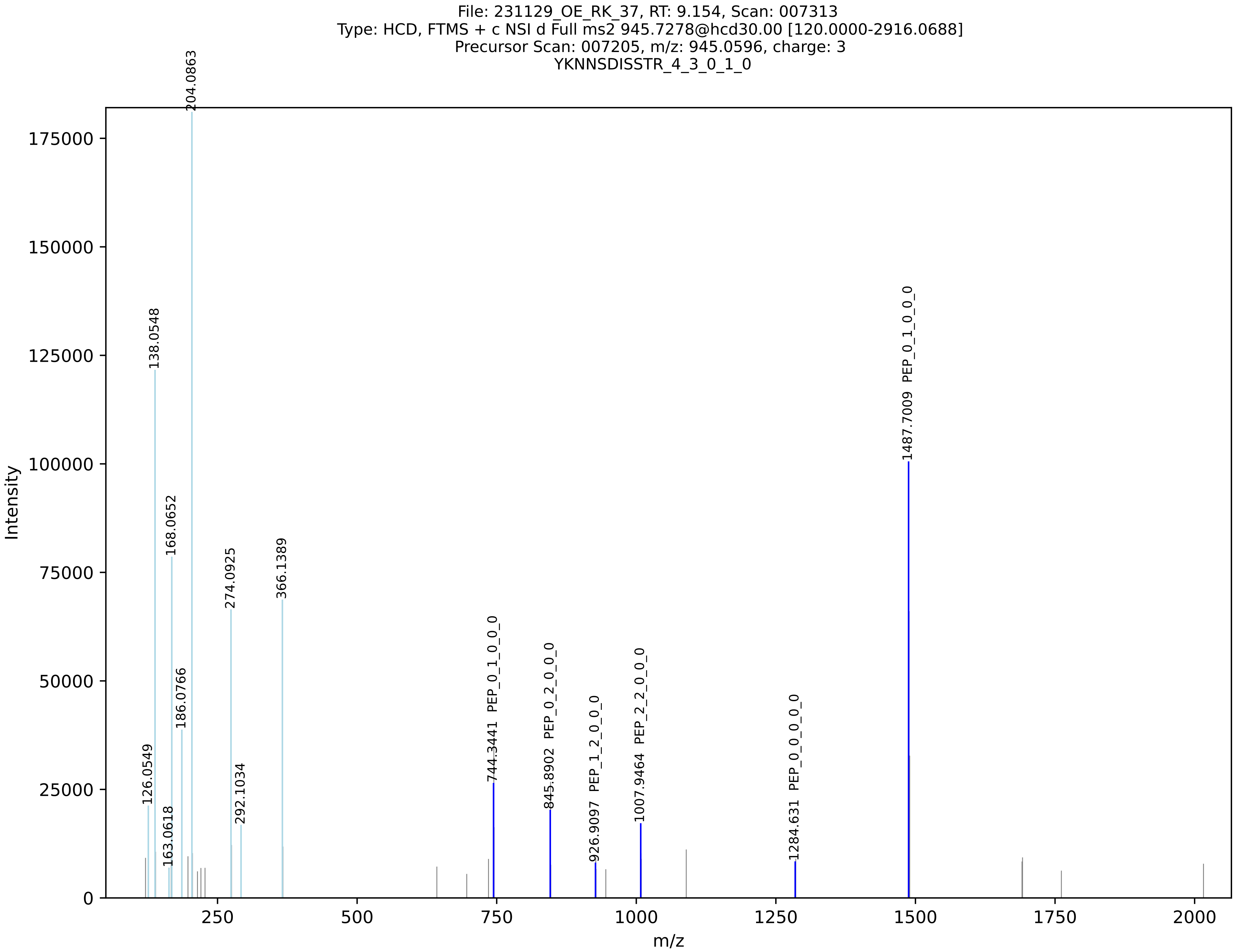

File: 231129_OE_RK_37, RT: 9.154, Scan: 007313
Type: HCD, FTMS + c NSI d Full ms2 945.7278@hcd30.00 [120.0000-2916.0688]
Precursor Scan: 007205, m/z: 945.0596, charge: 3
YKNNSDISSTR_4_3_0_1_0
Intensity
m/z
0
25000
50000
75000
100000
125000
150000
175000
250
500
750
1000
1250
1500
1750
2000
204.0863
138.0548
168.0652
186.0766
126.0549
163.0618
274.0925
292.1034
366.1389
744.3441 PEP_0_1_0_0_0
845.8902 PEP_0_2_0_0_0
926.9097 PEP_1_2_0_0_0
1007.9464 PEP_2_2_0_0_0
1284.631 PEP_0_0_0_0_0
1487.7009 PEP_0_1_0_0_0

File: 231129_OE_RK_37, RT: 9.186, Scan: 007357
Type: HCD, FTMS + c NSI d Full ms2 1032.0789@hcd30.00 [120.0000-3180.3032]
Precursor Scan: 007278, m/z: 1031.7454, charge: 3
NVTAEQAR_5_4_0_2_0

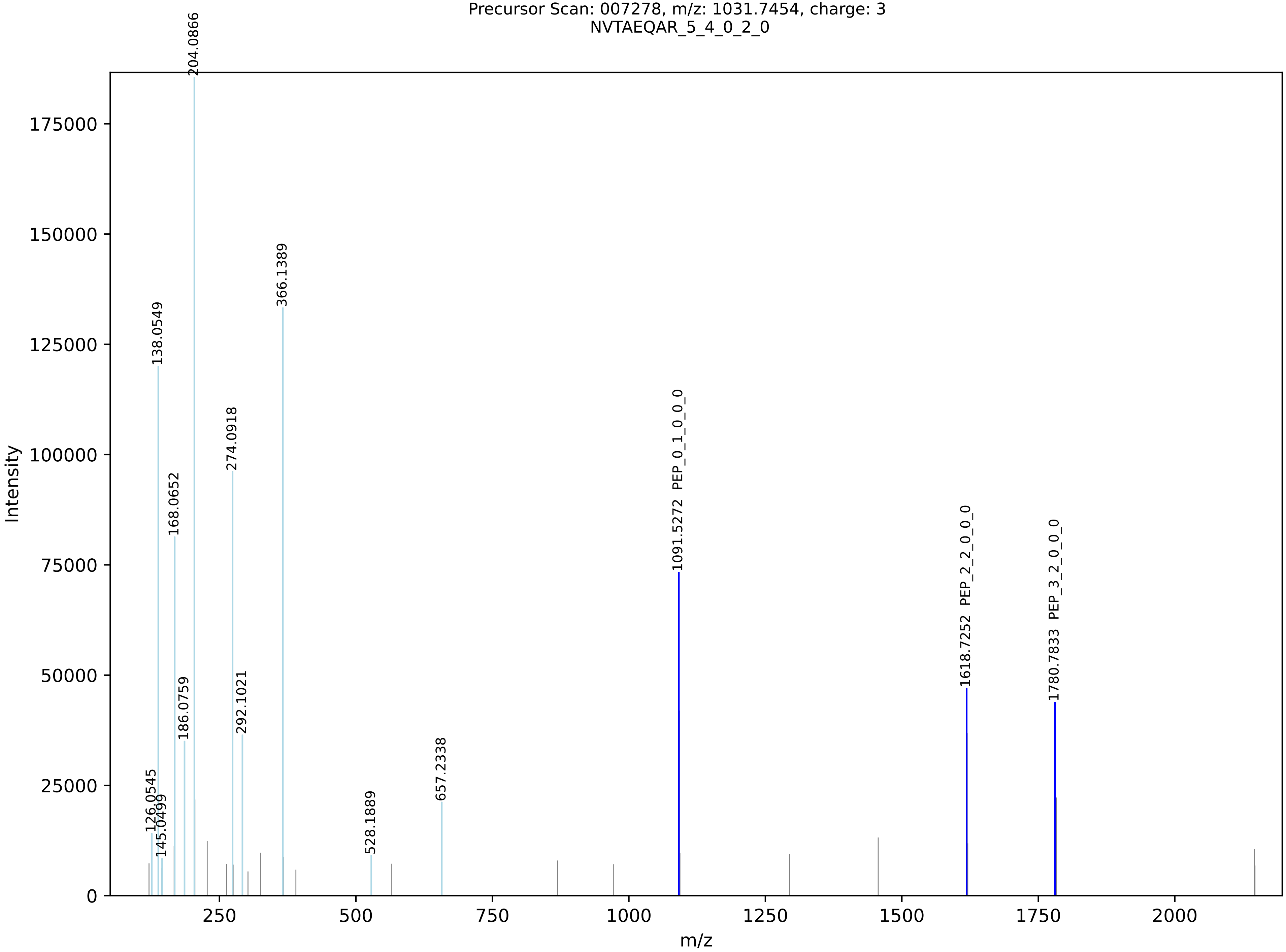

File: 231129_OE_RK_37, RT: 9.222, Scan: 007405
Type: HCD, FTMS + c NSI d Full ms2 1038.4012@hcd30.00 [120.0000-4259.5332]
Precursor Scan: 007352, m/z: 1037.9008, charge: 4
ENGTISR_7_6_1_3_0

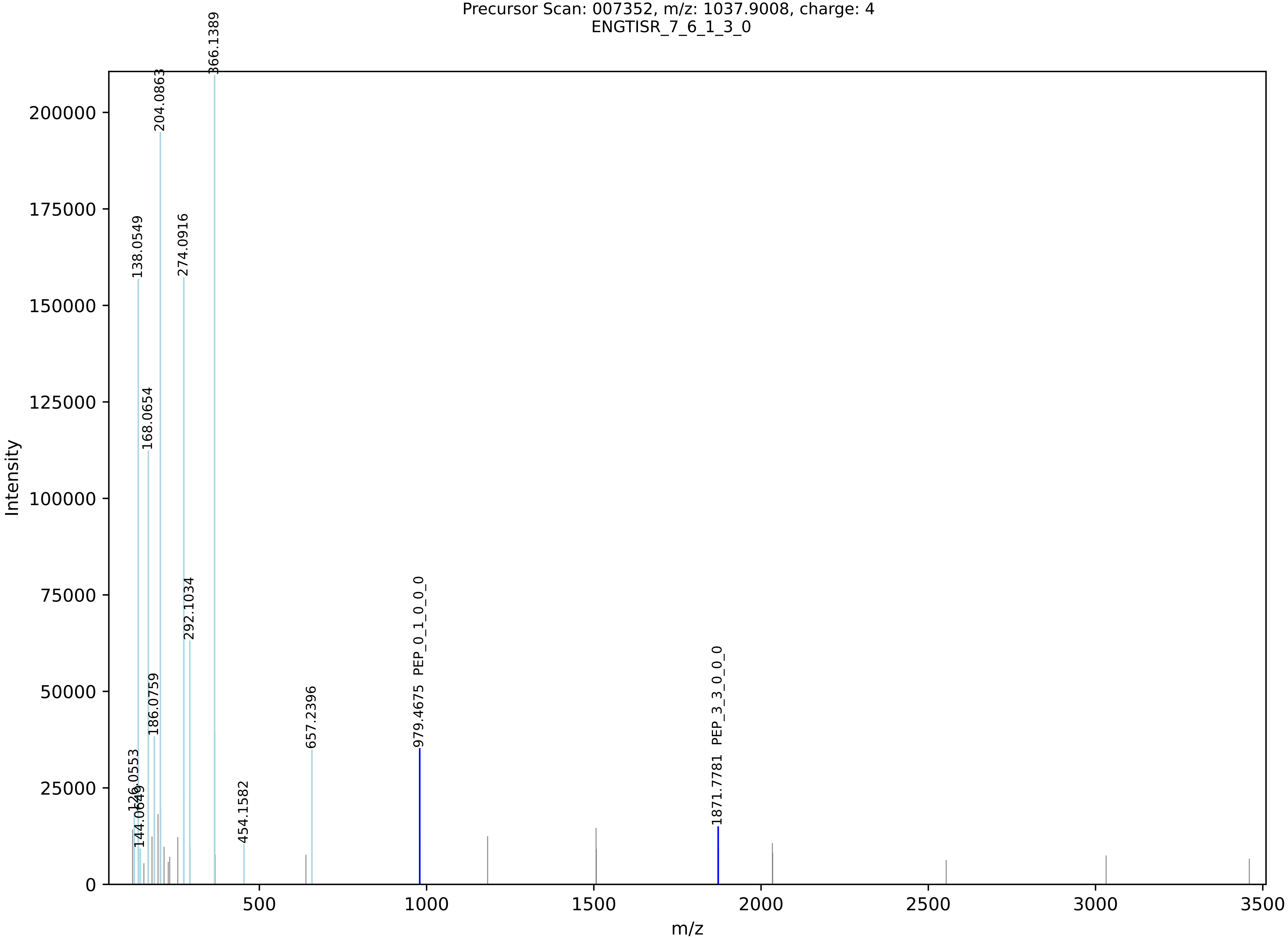

File: 231129_OE_RK_37, RT: 9.223, Scan: 007408
Type: HCD, FTMS + c NSI d Full ms2 1018.7582@hcd30.00 [120.0000-3139.5420]
Precursor Scan: 007352, m/z: 1018.4241, charge: 3
YKNNSDISSTR_5_4_1_0_0

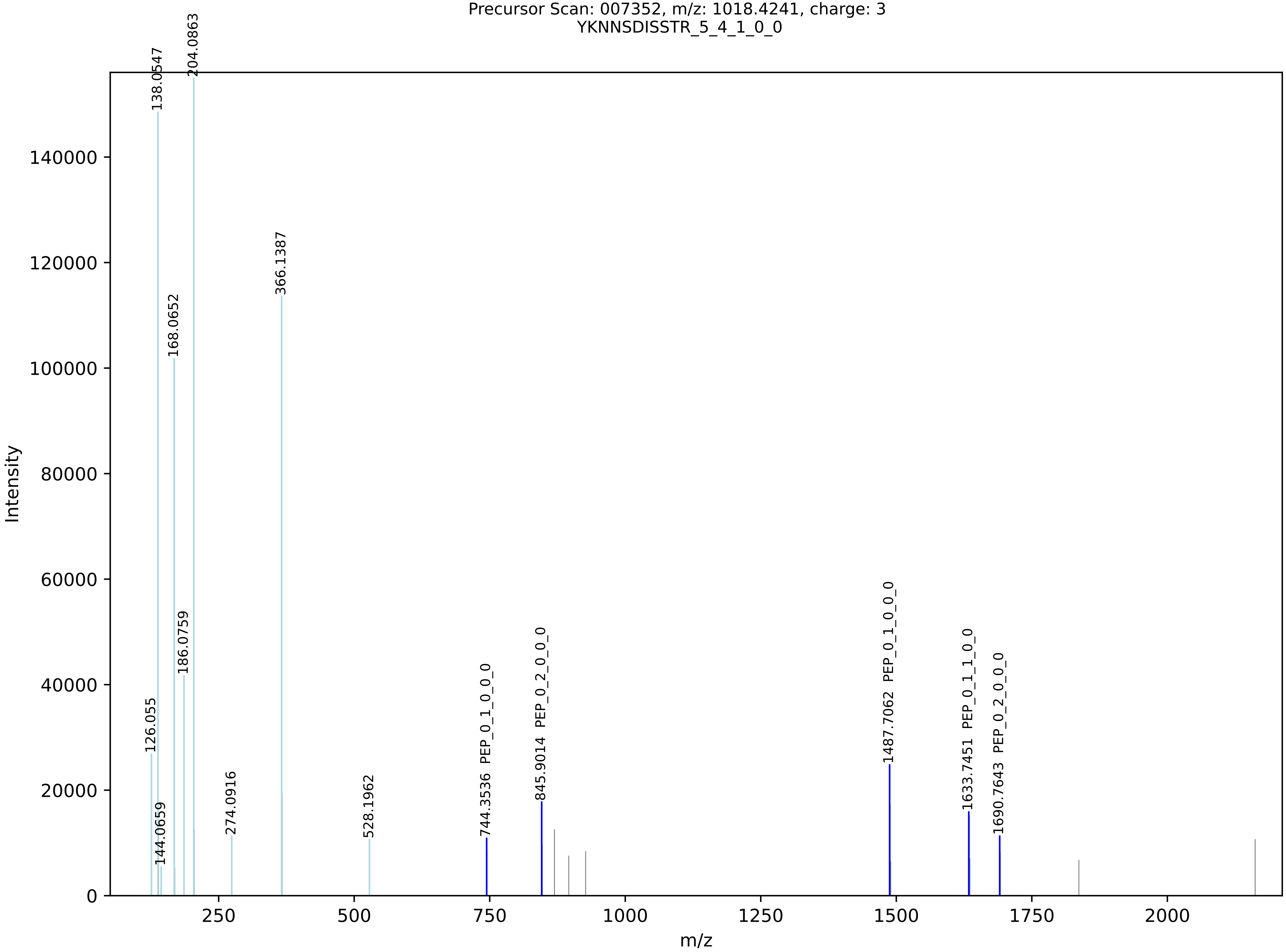

File: 231129_OE_RK_37, RT: 9.26, Scan: 007461
Type: HCD, FTMS + c NSI d Full ms2 1213.4688@hcd30.00 [120.0000-3735.3562]
Precursor Scan: 007401, m/z: 1213.1346, charge: 3
ENGTISR_6_5_0_3_0

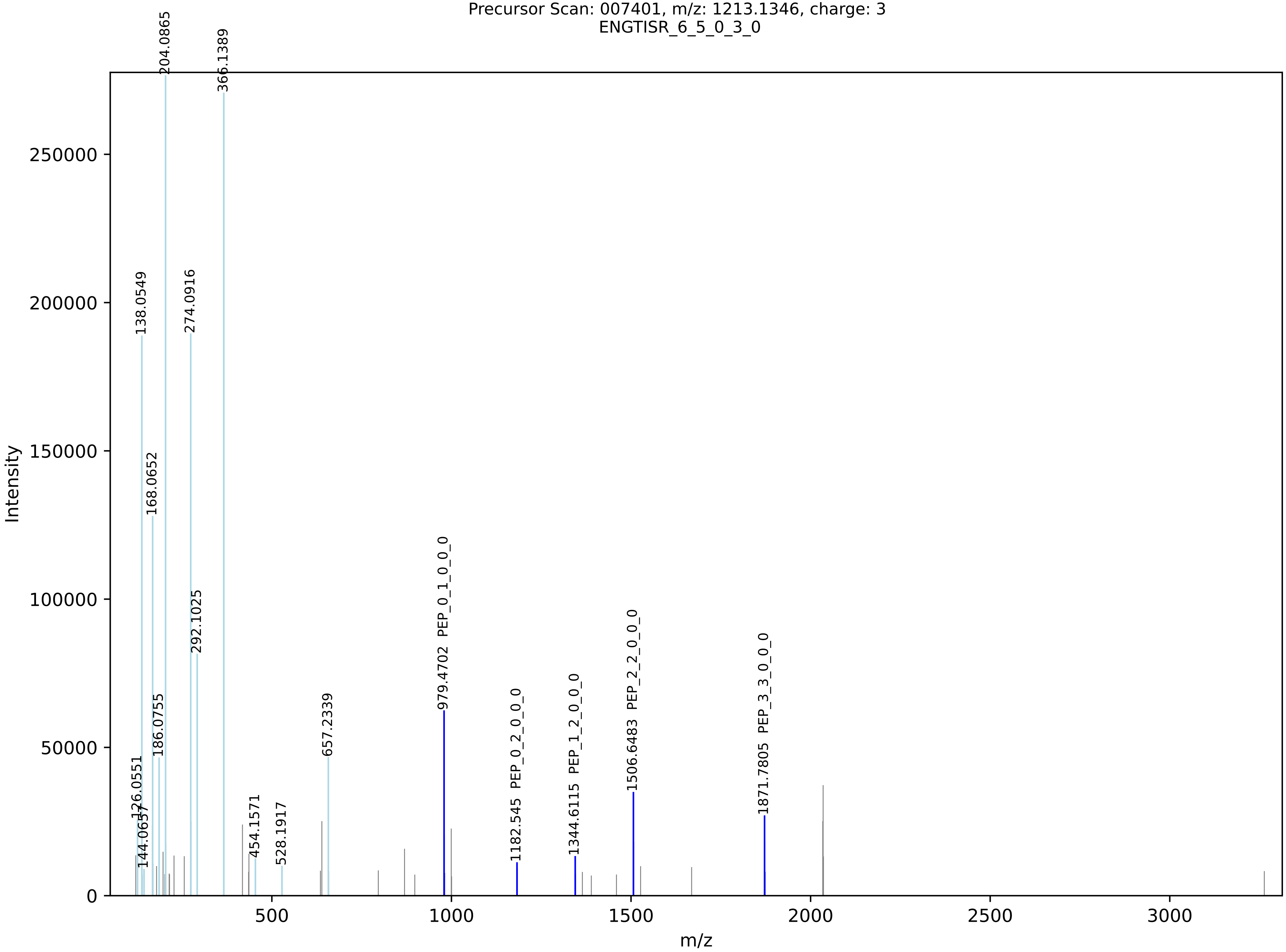

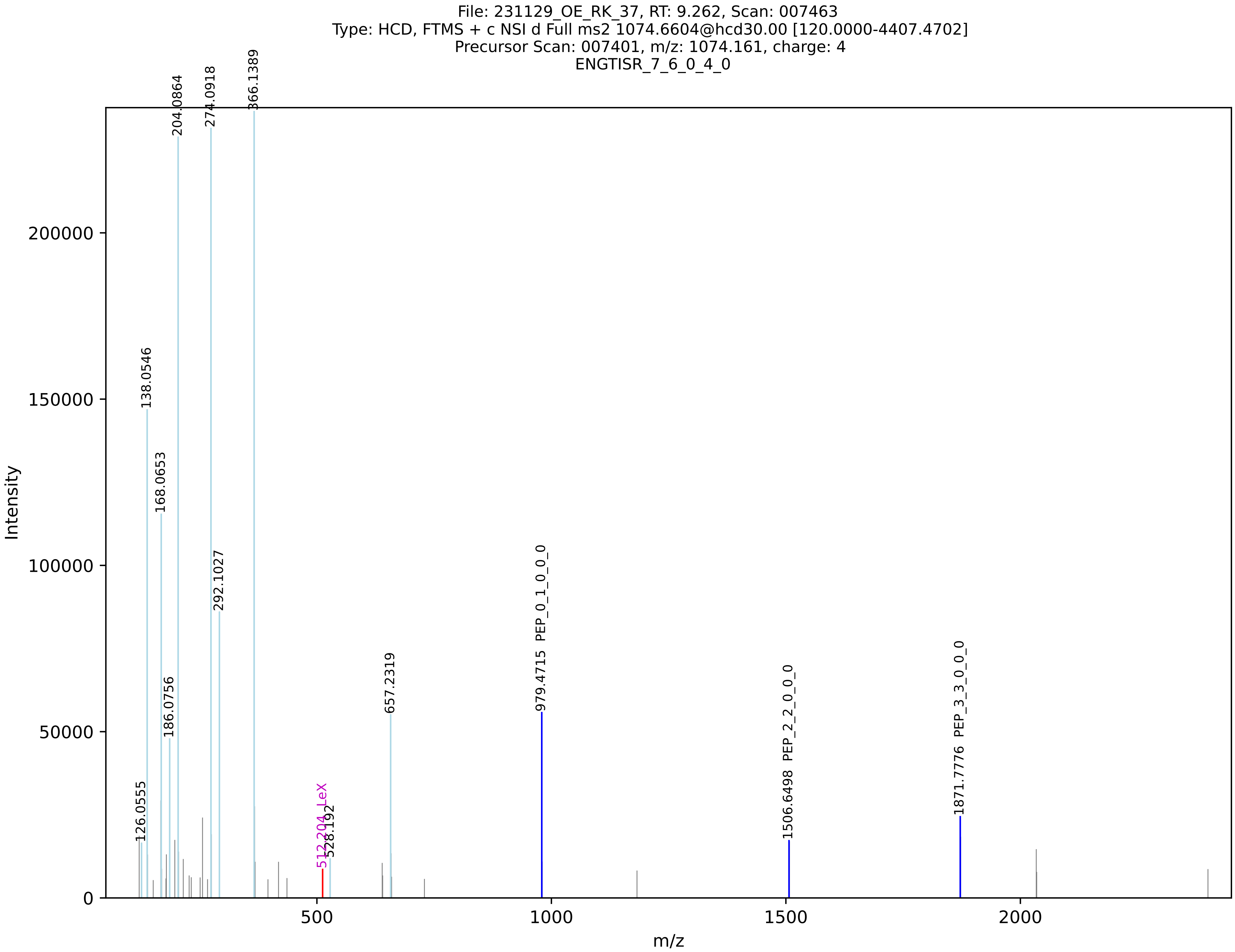

File: 231129_OE_RK_37, RT: 9.262, Scan: 007463
Type: HCD, FTMS + c NSI d Full ms2 1074.6604@hcd30.00 [120.0000-4407.4702]
Precursor Scan: 007401, m/z: 1074.161, charge: 4
ENGTISR_7_6_0_4_0
Intensity
m/z
126.0555
138.0546
168.0653
186.0756
204.0864
274.0918
292.1027
366.1389
512.204_LeX
528.192
657.2319
979.4715 PEP_0_1_0_0_0
1506.6498 PEP_2_2_0_0_0
1871.7776 PEP_3_3_0_0_0
0
50000
100000
150000
200000
500
1000
1500
2000

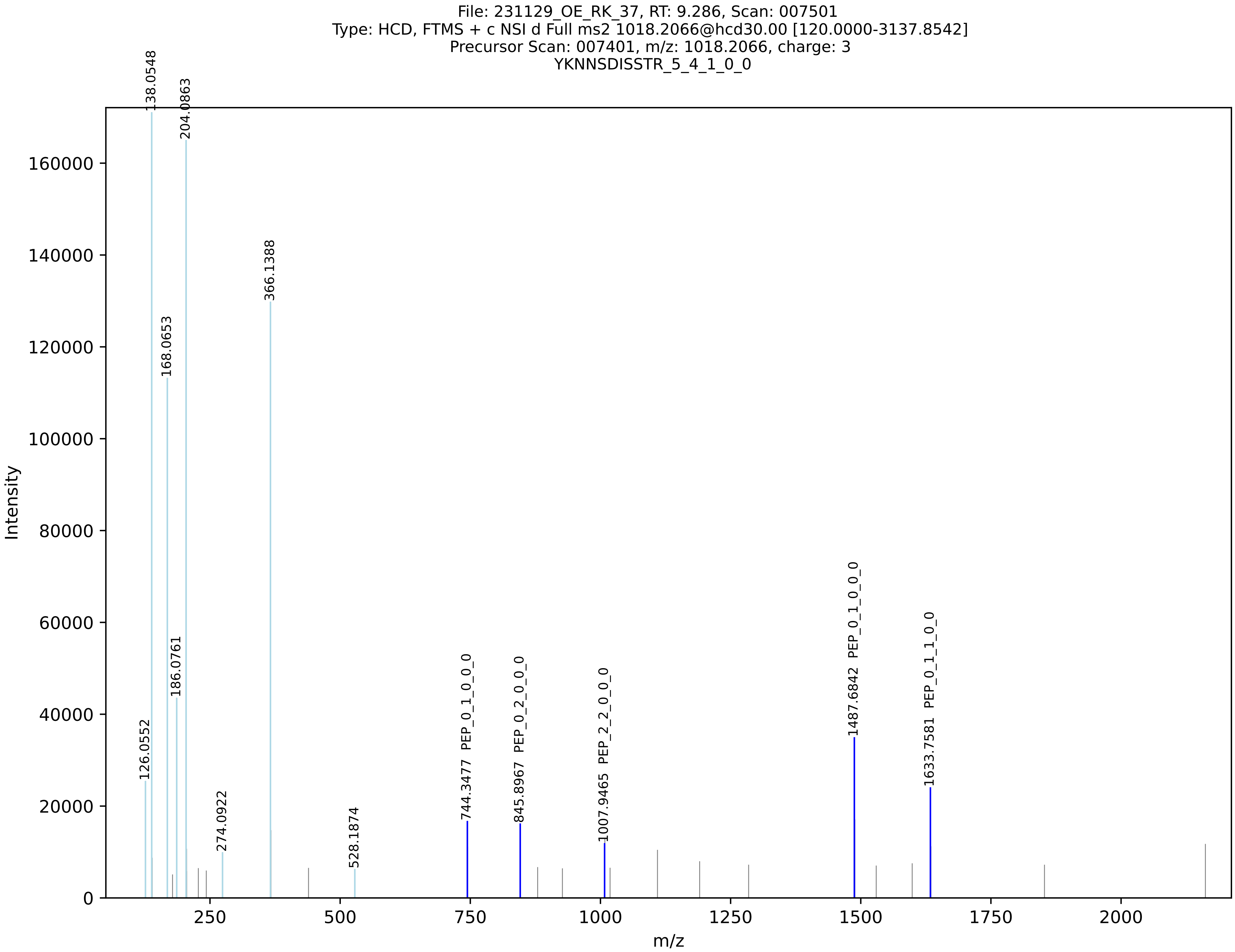
File: 231129_OE_RK_37, RT: 9.286, Scan: 007501
Type: HCD, FTMS + c NSI d Full ms2 1018.2066@hcd30.00 [120.0000-3137.8542]
Precursor Scan: 007401, m/z: 1018.2066, charge: 3
YKNNSDISSTR_5_4_1_0_0
Intensity
m/z
138.0548
204.0863
366.1388
168.0653
186.0761
126.0552
274.0922
528.1874
744.3477 PEP_0_1_0_0_0
845.8967 PEP_0_2_0_0_0
1007.9465 PEP_2_2_0_0_0
1487.6842 PEP_0_1_0_0_0
1633.7581 PEP_0_1_1_0_0

File: 231129_OE_RK_37, RT: 9.302, Scan: 007521
Type: HCD, FTMS + c NSI d Full ms2 1001.8876@hcd30.00 [120.0000-4110.5576]
Precursor Scan: 007456, m/z: 1001.3864, charge: 4
ENGTISR_7_6_0_3_0

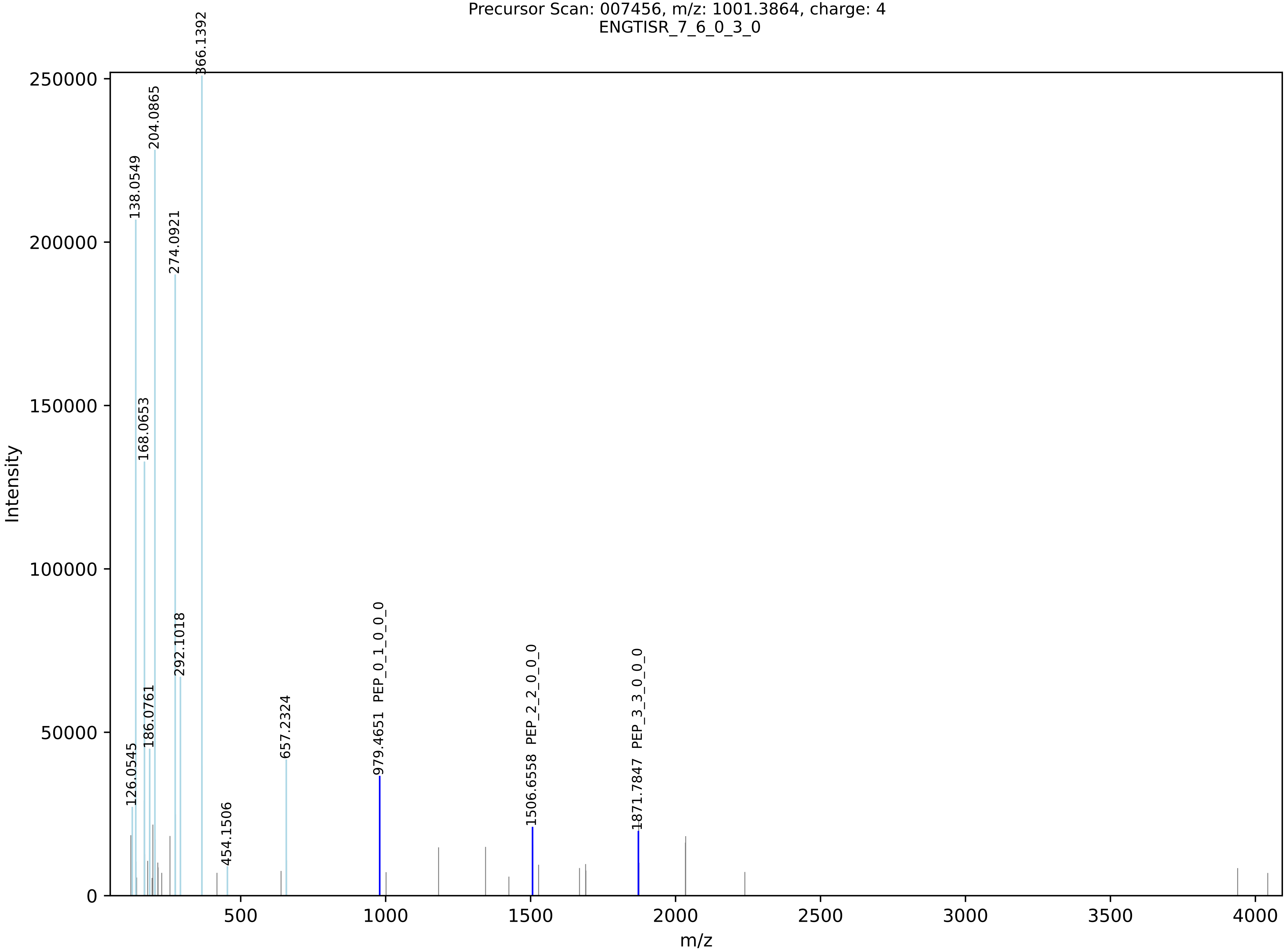

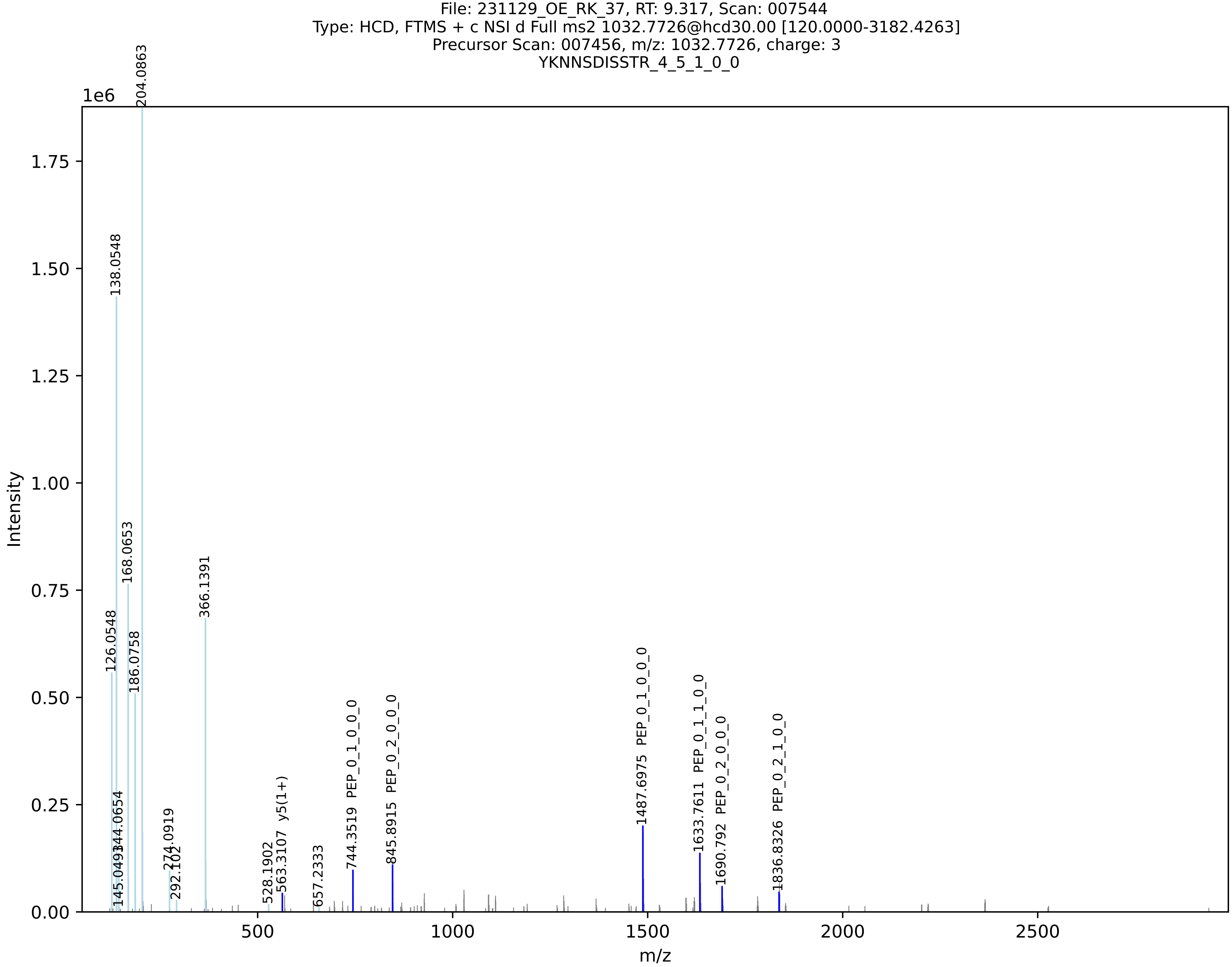
File: 231129_OE_RK_37, RT: 9.317, Scan: 007544
Type: HCD, FTMS + c NSI d Full ms2 1032.7726@hcd30.00 [120.0000-3182.4263]
Precursor Scan: 007456, m/z: 1032.7726, charge: 3
YKNNSDISSTR_4_5_1_0_0
1e6
Intensity
m/z
204.0863
138.0548
168.0653
366.1391
126.0548
186.0758
144.0654
145.0491
274.0919
292.102
528.1902
563.3107 y5(1+)
657.2333
744.3519 PEP_0_1_0_0_0
845.8915 PEP_0_2_0_0_0
1487.6975 PEP_0_1_0_0_0
1633.7611 PEP_0_1_1_0_0
1690.792 PEP_0_2_0_0_0
1836.8326 PEP_0_2_1_0_0

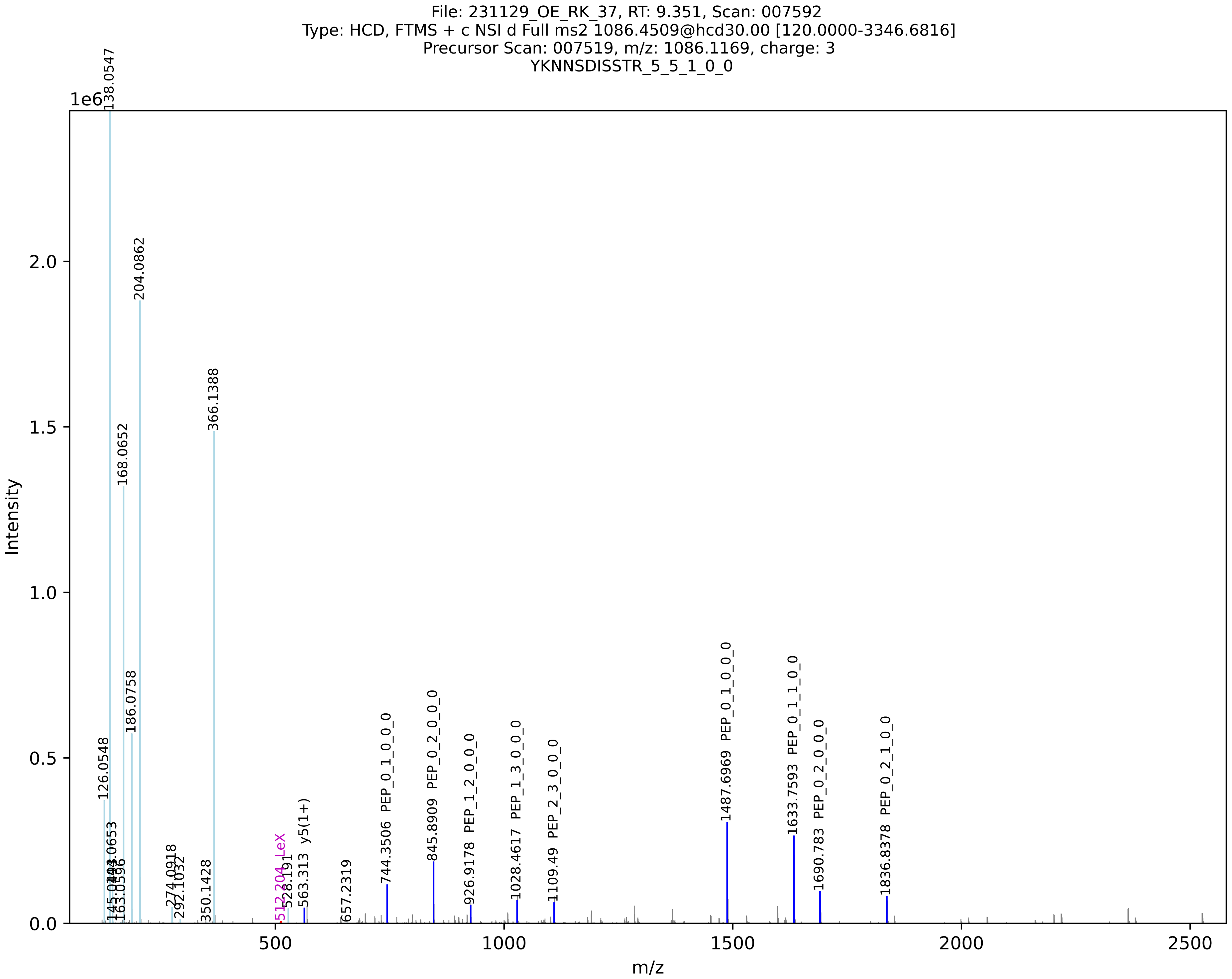

File: 231129_OE_RK_37, RT: 9.351, Scan: 007592
Type: HCD, FTMS + c NSI d Full ms2 1086.4509@hcd30.00 [120.0000-3346.6816]
Precursor Scan: 007519, m/z: 1086.1169, charge: 3
YKNNSDISSTR_5_5_1_0_0
1e6
Intensity
m/z
138.0547
204.0862
366.1388
168.0652
186.0758
126.0548
274.0918
292.1032
350.1428
512.204 LeX
528.191
563.313 y5(1+)
657.2319
744.3506 PEP_0_1_0_0_0
845.8909 PEP_0_2_0_0_0
926.9178 PEP_1_2_0_0_0
1028.4617 PEP_1_3_0_0_0
1109.49 PEP_2_3_0_0_0
1487.6969 PEP_0_1_0_0_0
1633.7593 PEP_0_1_1_0_0
1690.783 PEP_0_2_0_0_0
1836.8378 PEP_0_2_1_0_0

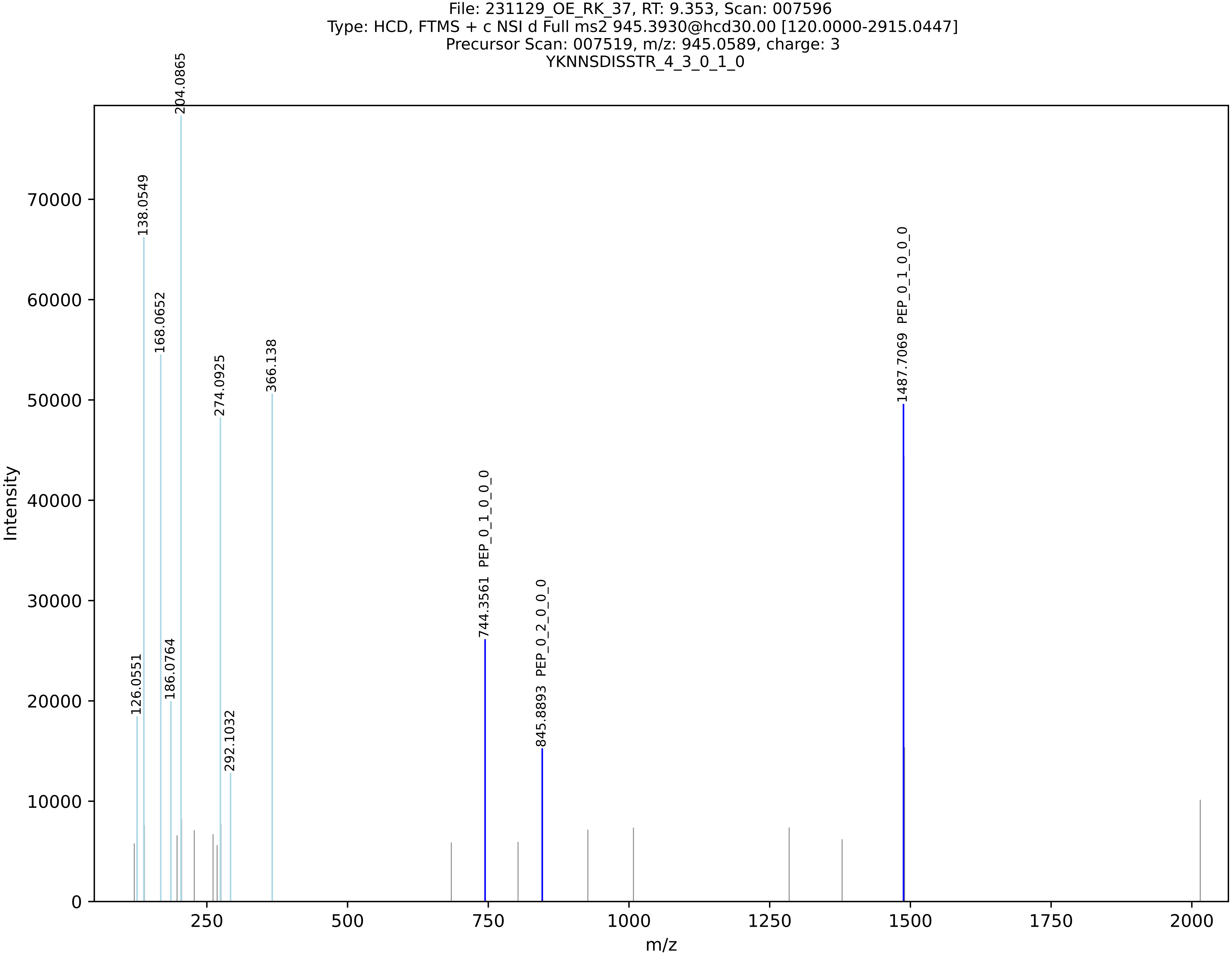
File: 231129_OE_RK_37, RT: 9.353, Scan: 007596
Type: HCD, FTMS + c NSI d Full ms2 945.3930@hcd30.00 [120.0000-2915.0447]
Precursor Scan: 007519, m/z: 945.0589, charge: 3
YKNNSDISSTR_4_3_0_1_0
204.0865
138.0549
168.0652
274.0925
366.138
1487.7069 PEP_0_1_0_0_0
744.3561 PEP_0_1_0_0_0
845.8893 PEP_0_2_0_0_0
126.0551
186.0764
292.1032
Intensity
m/z
0
10000
20000
30000
40000
50000
60000
70000
250
500
750
1000
1250
1500
1750
2000

File: 231129_OE_RK_37, RT: 9.357, Scan: 007601
Type: HCD, FTMS + c NSI d Full ms2 1262.4882@hcd30.00 [120.0000-3885.3560]
Precursor Scan: 007519, m/z: 1261.82, charge: 3
ENGTISR_6_5_1_3_0

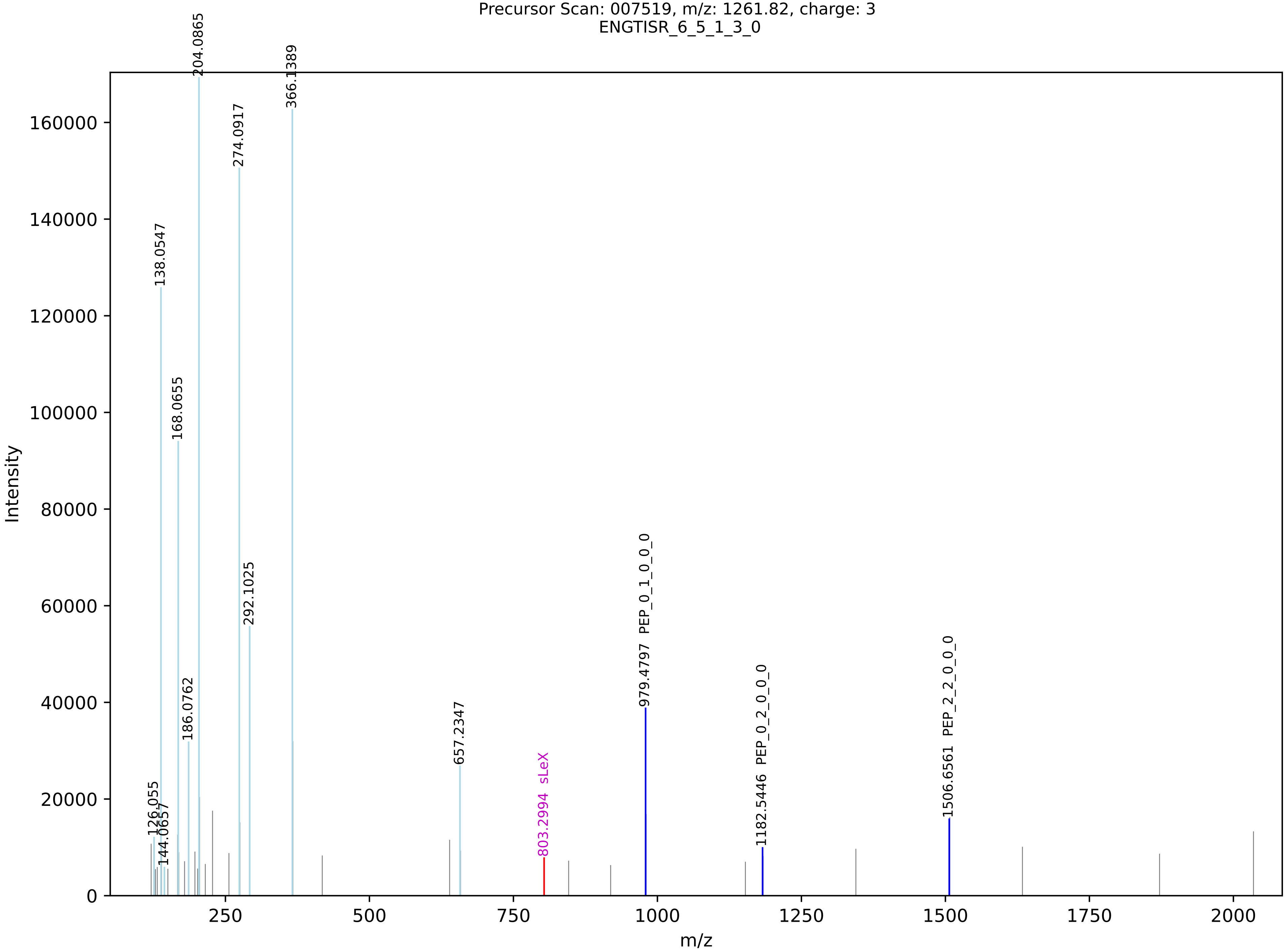

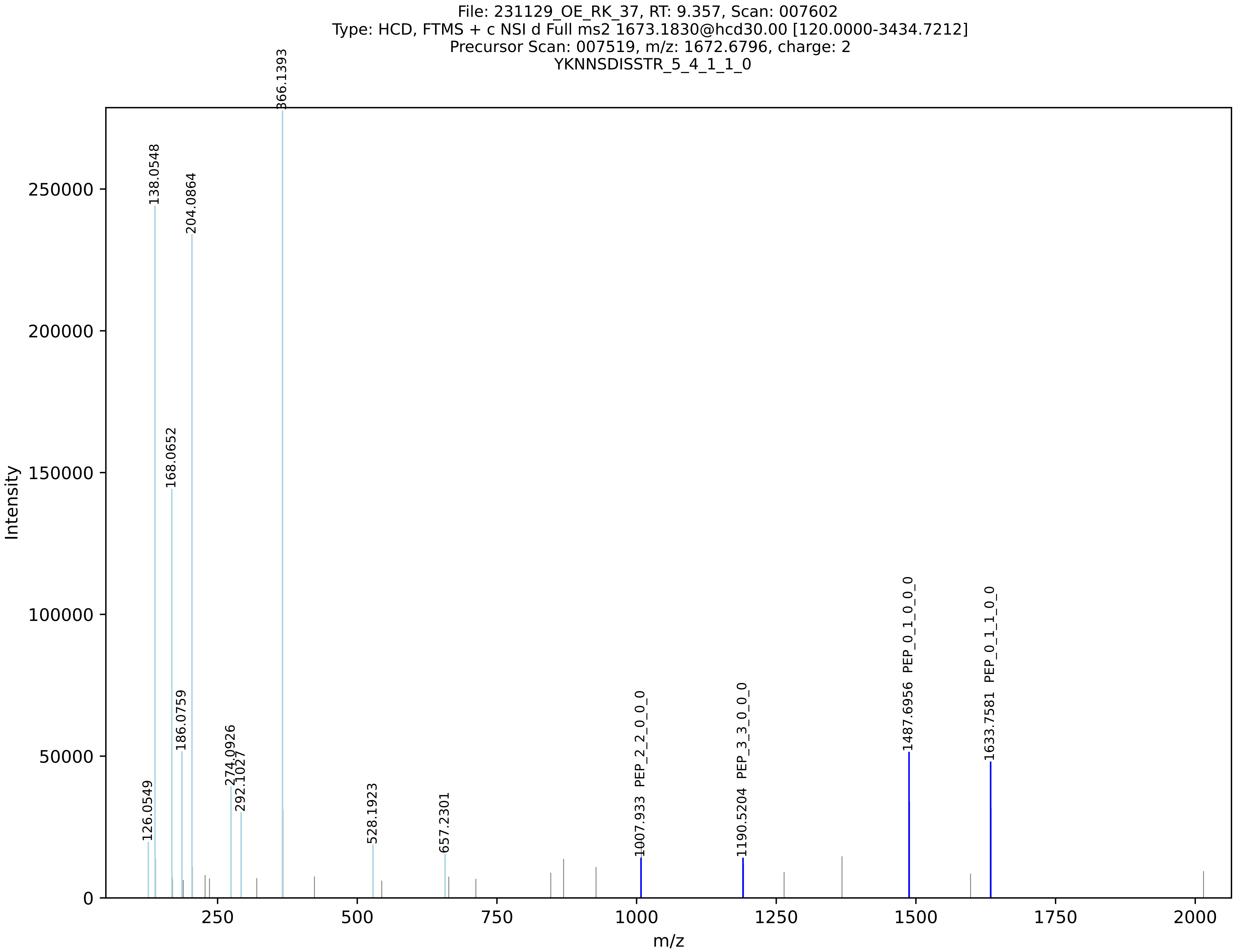
File: 231129_OE_RK_37, RT: 9.357, Scan: 007602
Type: HCD, FTMS + c NSI d Full ms2 1673.1830@hcd30.00 [120.0000-3434.7212]
Precursor Scan: 007519, m/z: 1672.6796, charge: 2
YKNNSDISSTR_5_4_1_1_0
Intensity
m/z
0
50000
100000
150000
200000
250000
250
500
750
1000
1250
1500
1750
2000
366.1393
138.0548
204.0864
168.0652
186.0759
126.0549
274.0926
292.1027
528.1923
657.2301
1007.933 PEP_2_2_0_0_0
1190.5204 PEP_3_3_0_0_0
1487.6956 PEP_0_1_0_0_0
1633.7581 PEP_0_1_1_0_0

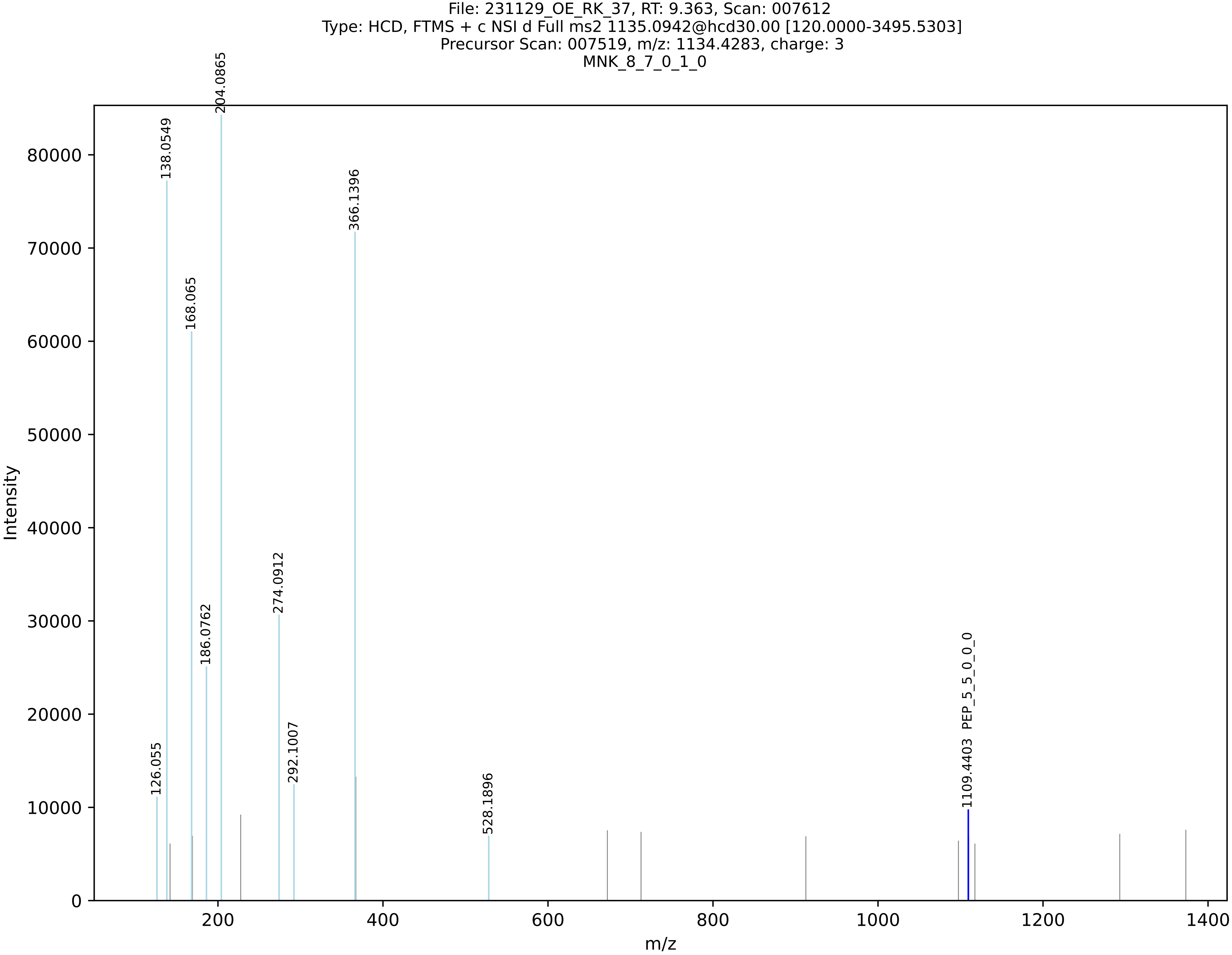
File: 231129_OE_RK_37, RT: 9.363, Scan: 007612
Type: HCD, FTMS + c NSI d Full ms2 1135.0942@hcd30.00 [120.0000-3495.5303]
Precursor Scan: 007519, m/z: 1134.4283, charge: 3
MNK_8_7_0_1_0
204.0865
138.0549
366.1396
168.065
274.0912
186.0762
PEP_5_5_0_0_0
1109.4403
292.1007
126.055
528.1896
Intensity
m/z
0
10000
20000
30000
40000
50000
60000
70000
80000
200
400
600
800
1000
1200
1400

File: 231129_OE_RK_37, RT: 9.369, Scan: 007622
Type: HCD, FTMS + c NSI d Full ms2 1147.1975@hcd30.00 [120.0000-4703.4219]
Precursor Scan: 007519, m/z: 1147.1975, charge: 4
EDALNETRESETK_6_6_0_3_0

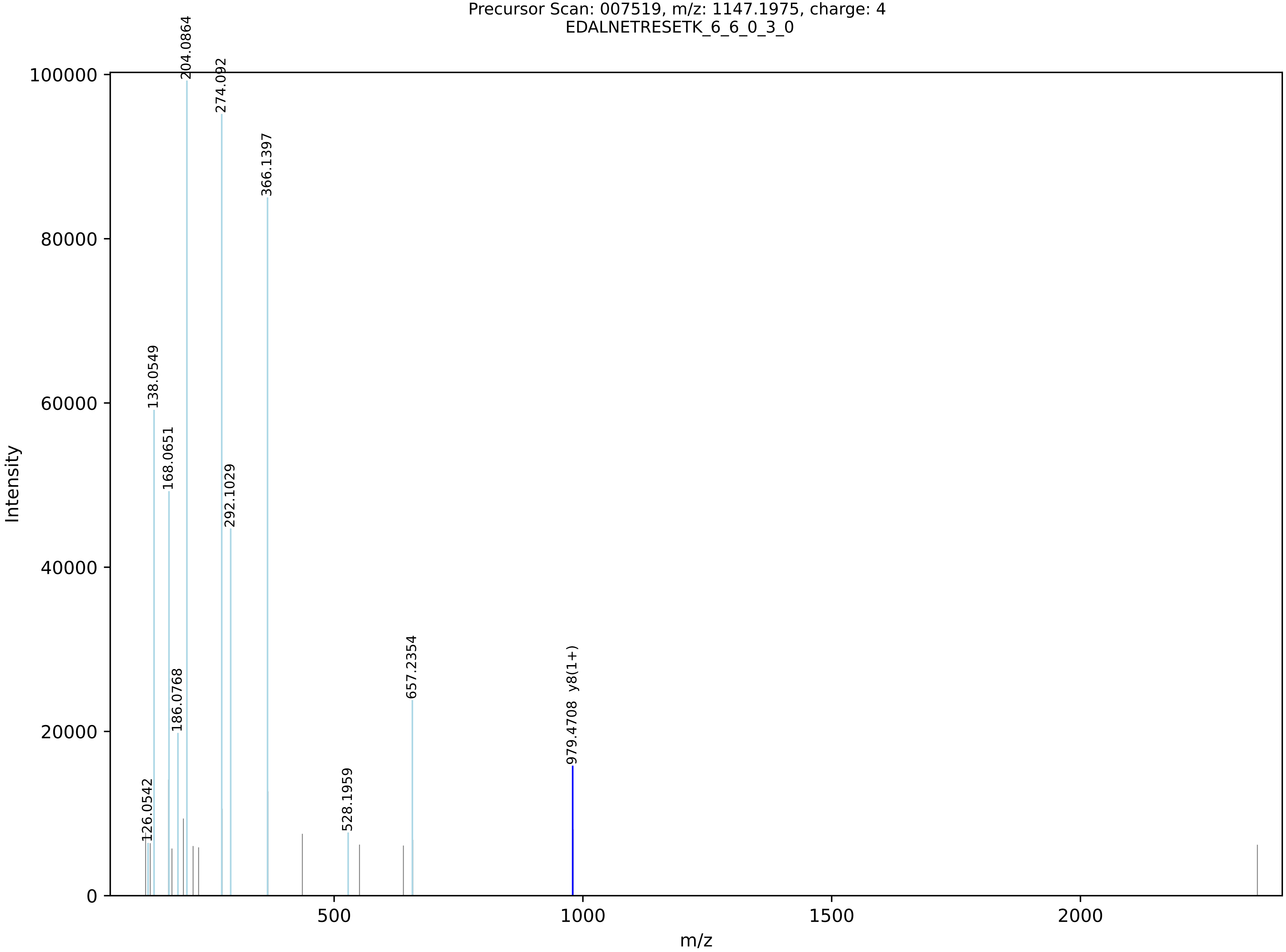

File: 231129_OE_RK_37, RT: 9.407, Scan: 007671
Type: HCD, FTMS + c NSI d Full ms2 1102.1141@hcd30.00 [120.0000-3394.6111]
Precursor Scan: 007591, m/z: 1101.7798, charge: 3
YKNNSDISSTR_6_3_1_1_0

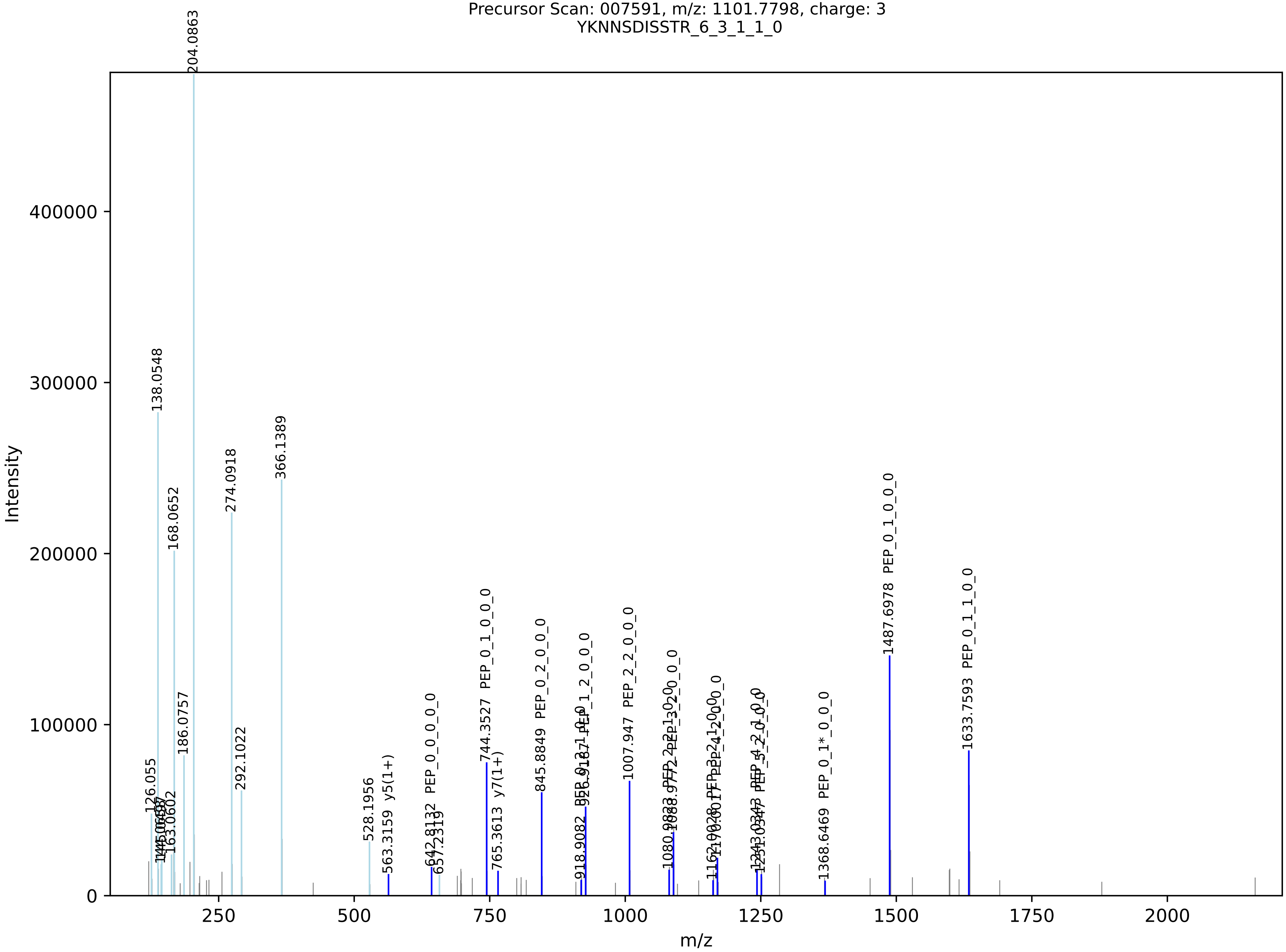

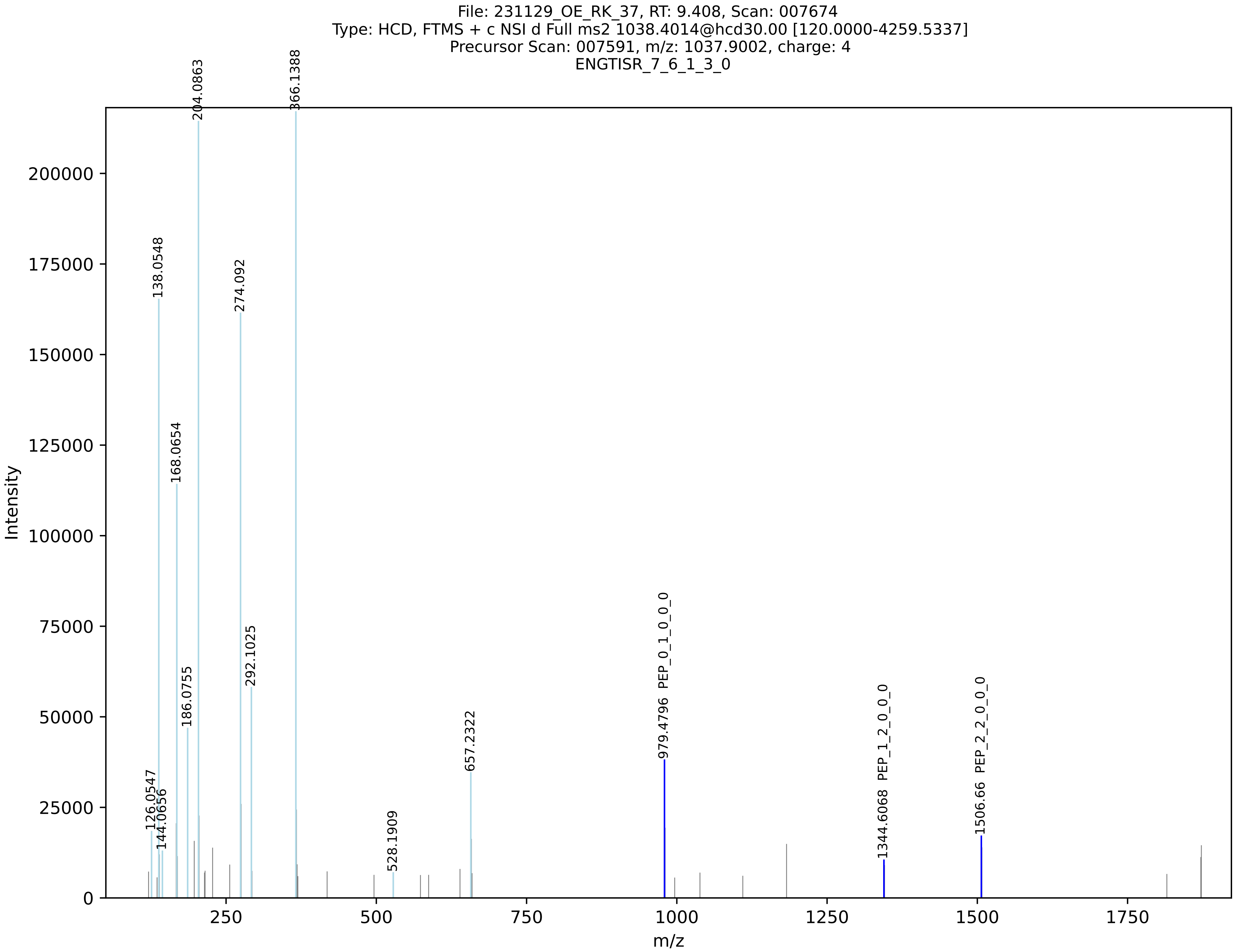

File: 231129_OE_RK_37, RT: 9.408, Scan: 007674
Type: HCD, FTMS + c NSI d Full ms2 1038.4014@hcd30.00 [120.0000-4259.5337]
Precursor Scan: 007591, m/z: 1037.9002, charge: 4
ENGTISR_7_6_1_3_0
Intensity
m/z
204.0863
366.1388
138.0548
274.092
168.0654
186.0755
292.1025
126.0547
144.0656
528.1909
657.2322
979.4796 PEP_0_1_0_0_0
1344.6068 PEP_1_2_0_0_0
1506.66 PEP_2_2_0_0_0

File: 231129_OE_RK_37, RT: 9.411, Scan: 007678
Type: HCD, FTMS + c NSI d Full ms2 1274.8199@hcd30.00 [120.0000-3923.0913]
Precursor Scan: 007591, m/z: 1274.1518, charge: 3
NEEYNK_7_5_2_2_0

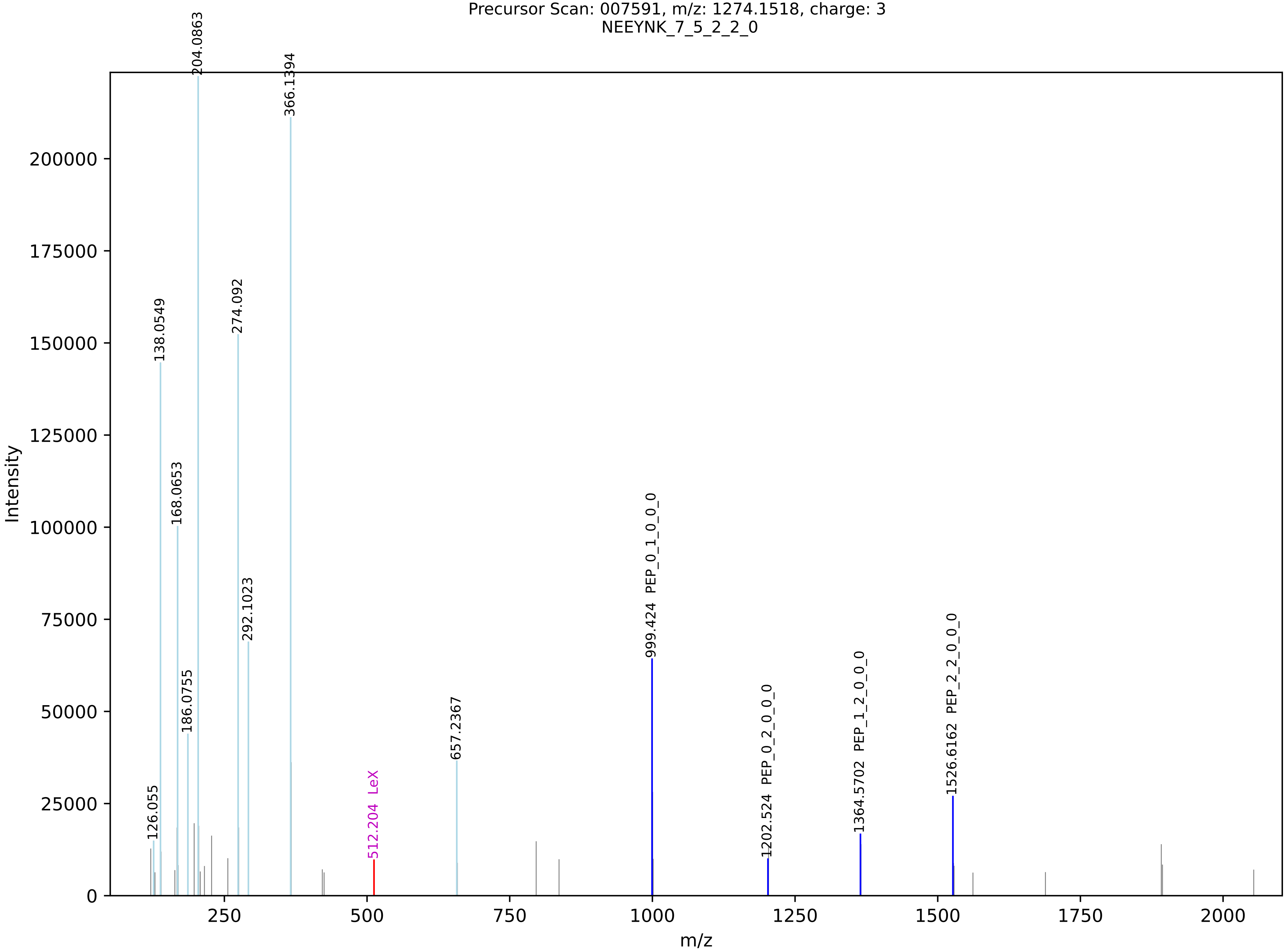

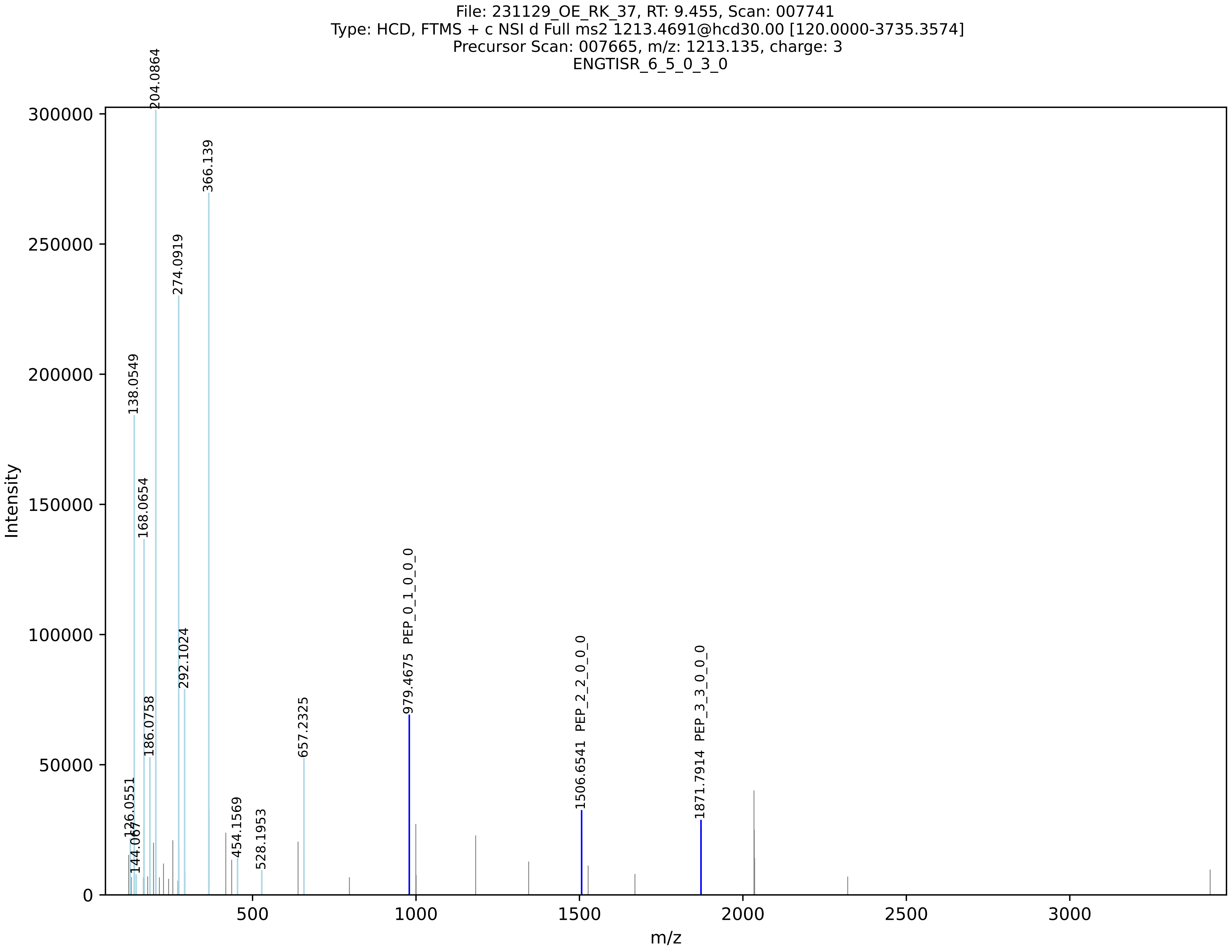
File: 231129_OE_RK_37, RT: 9.455, Scan: 007741
Type: HCD, FTMS + c NSI d Full ms2 1213.4691@hcd30.00 [120.0000-3735.3574]
Precursor Scan: 007665, m/z: 1213.135, charge: 3
ENGTISR_6_5_0_3_0
Intensity
m/z
204.0864
366.139
274.0919
138.0549
168.0654
292.1024
186.0758
126.0551
144.067
454.1569
528.1953
657.2325
979.4675 PEP_0_1_0_0_0
1506.6541 PEP_2_2_0_0_0
1871.7914 PEP_3_3_0_0_0
0
50000
100000
150000
200000
250000
300000
500
1000
1500
2000
2500
3000

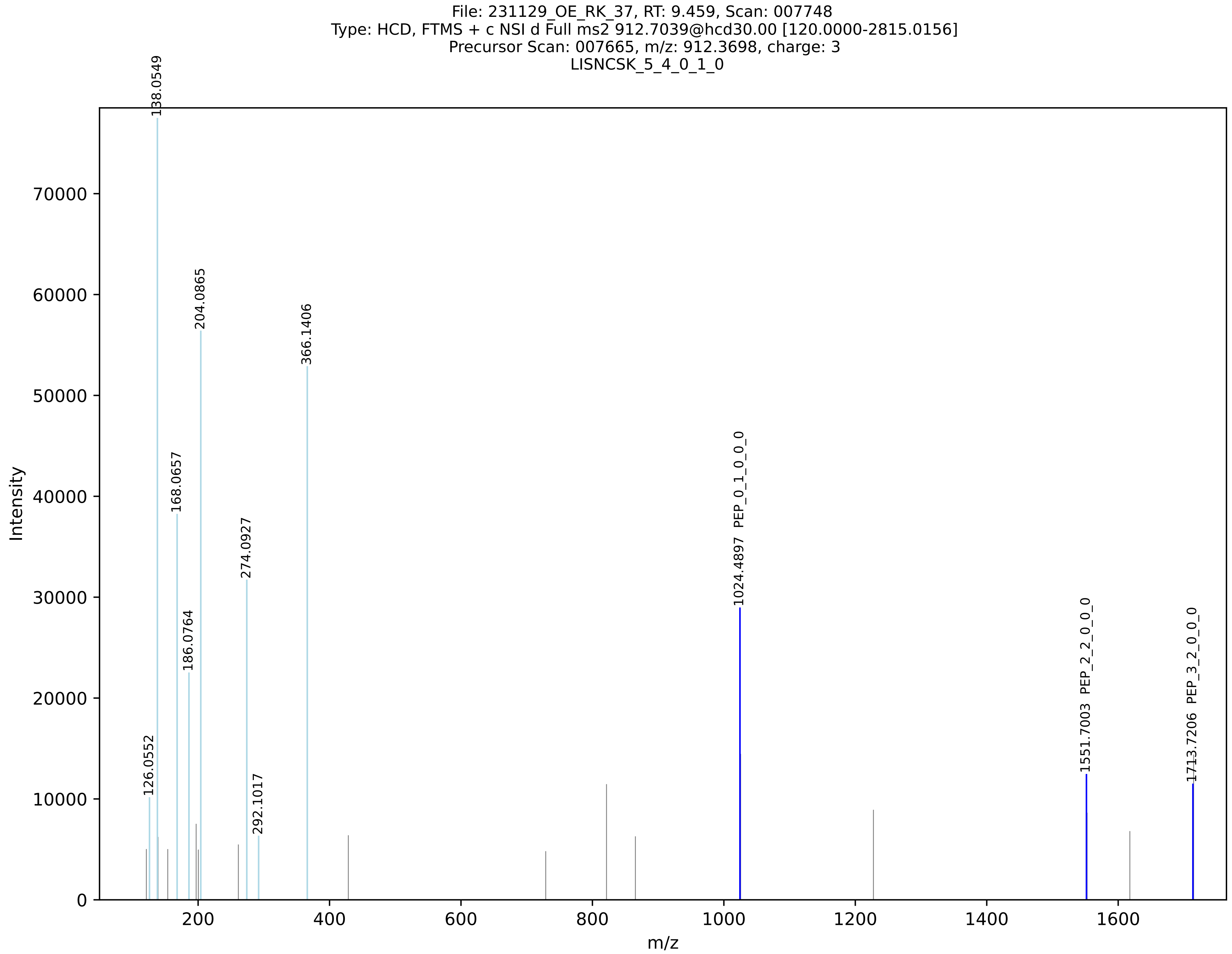

File: 231129_OE_RK_37, RT: 9.459, Scan: 007748
Type: HCD, FTMS + c NSI d Full ms2 912.7039@hcd30.00 [120.0000-2815.0156]
Precursor Scan: 007665, m/z: 912.3698, charge: 3
LISNCSK_5_4_0_1_0
138.0549
204.0865
366.1406
168.0657
274.0927
186.0764
126.0552
292.1017
1024.4897 PEP_0_1_0_0_0
1551.7003 PEP_2_2_0_0_0
1713.7206 PEP_3_2_0_0_0
Intensity
m/z
0
10000
20000
30000
40000
50000
60000
70000
200
400
600
800
1000
1200
1400
1600

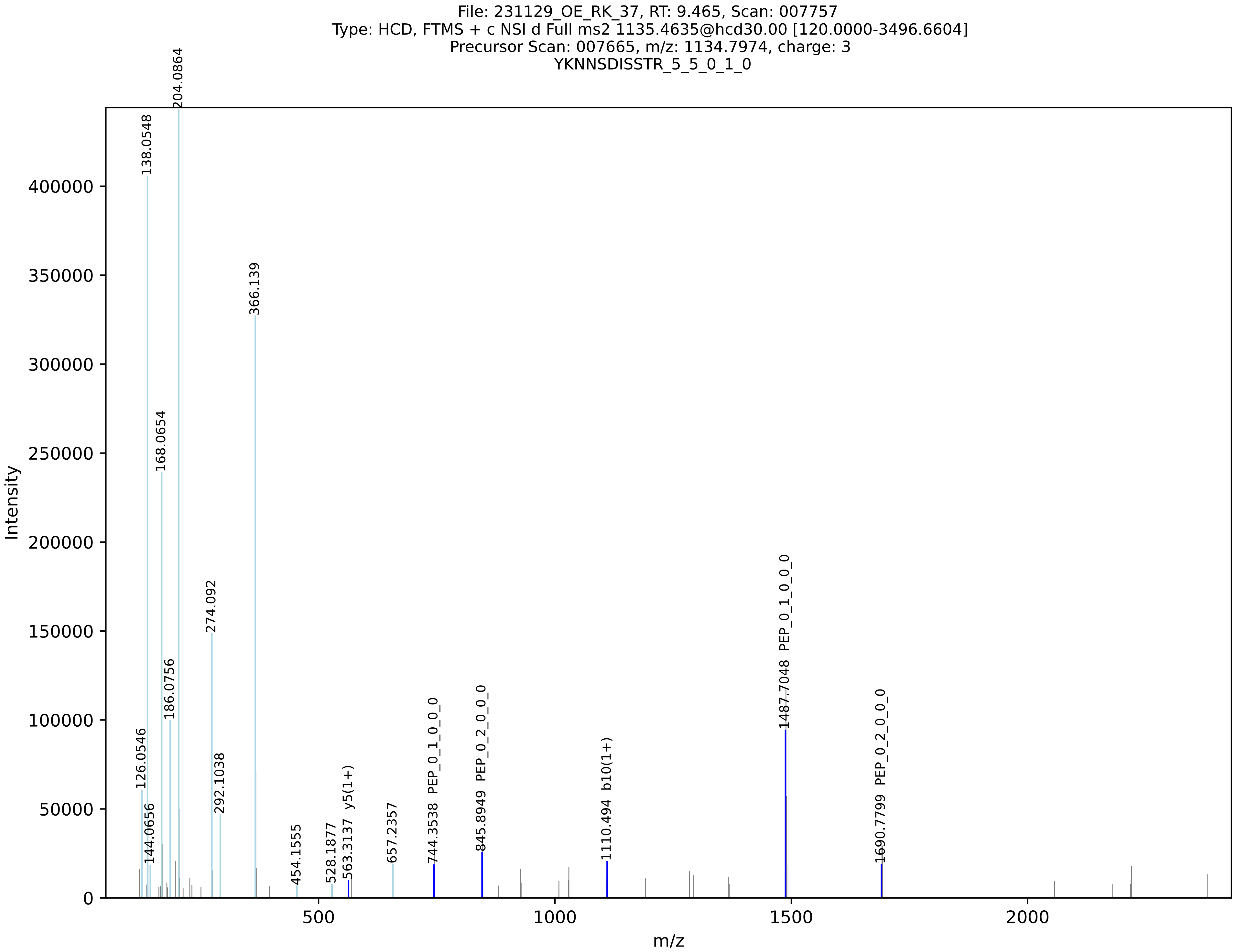

File: 231129_OE_RK_37, RT: 9.465, Scan: 007757
Type: HCD, FTMS + c NSI d Full ms2 1135.4635@hcd30.00 [120.0000-3496.6604]
Precursor Scan: 007665, m/z: 1134.7974, charge: 3
YKNNSDISSTR_5_5_0_1_0
Intensity
m/z
0
50000
100000
150000
200000
250000
300000
350000
400000
500
1000
1500
2000
204.0864
138.0548
366.139
168.0654
274.092
186.0756
126.0546
292.1038
144.0656
454.1555
528.1877
563.3137 y5(1+)
657.2357
744.3538 PEP_0_1_0_0_0
845.8949 PEP_0_2_0_0_0
1110.494 b10(1+)
1487.7048 PEP_0_1_0_0_0
1690.7799 PEP_0_2_0_0_0

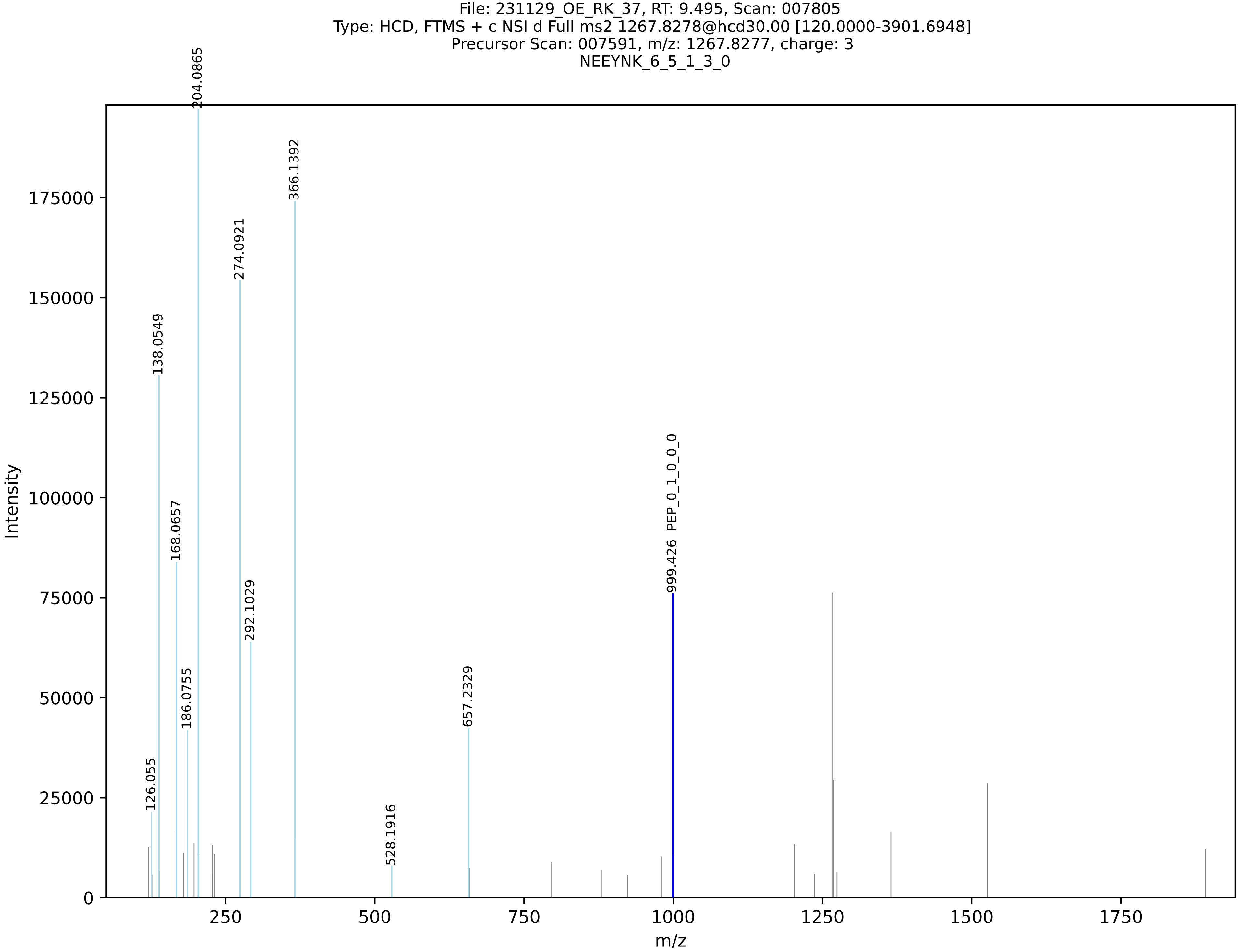
File: 231129_OE_RK_37, RT: 9.495, Scan: 007805
Type: HCD, FTMS + c NSI d Full ms2 1267.8278@hcd30.00 [120.0000-3901.6948]
Precursor Scan: 007591, m/z: 1267.8277, charge: 3
NEEYNK_6_5_1_3_0
Intensity
m/z
204.0865
366.1392
274.0921
138.0549
168.0657
292.1029
186.0755
126.055
528.1916
657.2329
999.426 PEP_0_1_0_0_0
0
25000
50000
75000
100000
125000
150000
175000
250
500
750
1000
1250
1500
1750

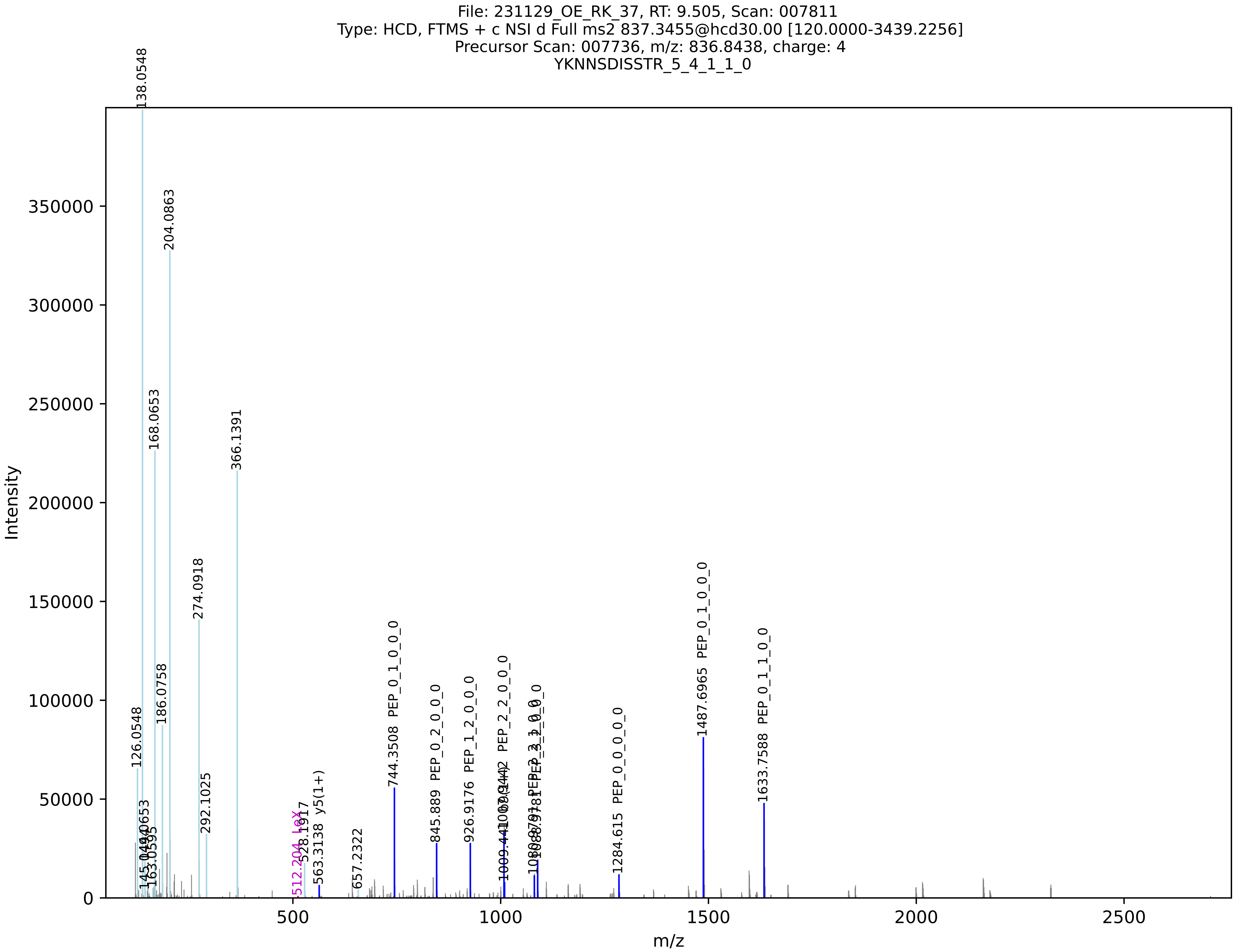
File: 231129_OE_RK_37, RT: 9.505, Scan: 007811
Type: HCD, FTMS + c NSI d Full ms2 837.3455@hcd30.00 [120.0000-3439.2256]
Precursor Scan: 007736, m/z: 836.8438, charge: 4
YKNNSDISSTR_5_4_1_1_0
Intensity
m/z
138.0548
204.0863
168.0653
366.1391
274.0918
186.0758
126.0548
292.1025
512.204_LeX
528.1917
563.3138 y5(1+)
657.2322
744.3508 PEP_0_1_0_0_0
845.889 PEP_0_2_0_0_0
926.9176 PEP_1_2_0_0_0
1284.615 PEP_0_0_0_0_0
1487.6965 PEP_0_1_0_0_0
1633.7588 PEP_0_1_1_0_0
0
50000
100000
150000
200000
250000
300000
350000
500
1000
1500
2000
2500

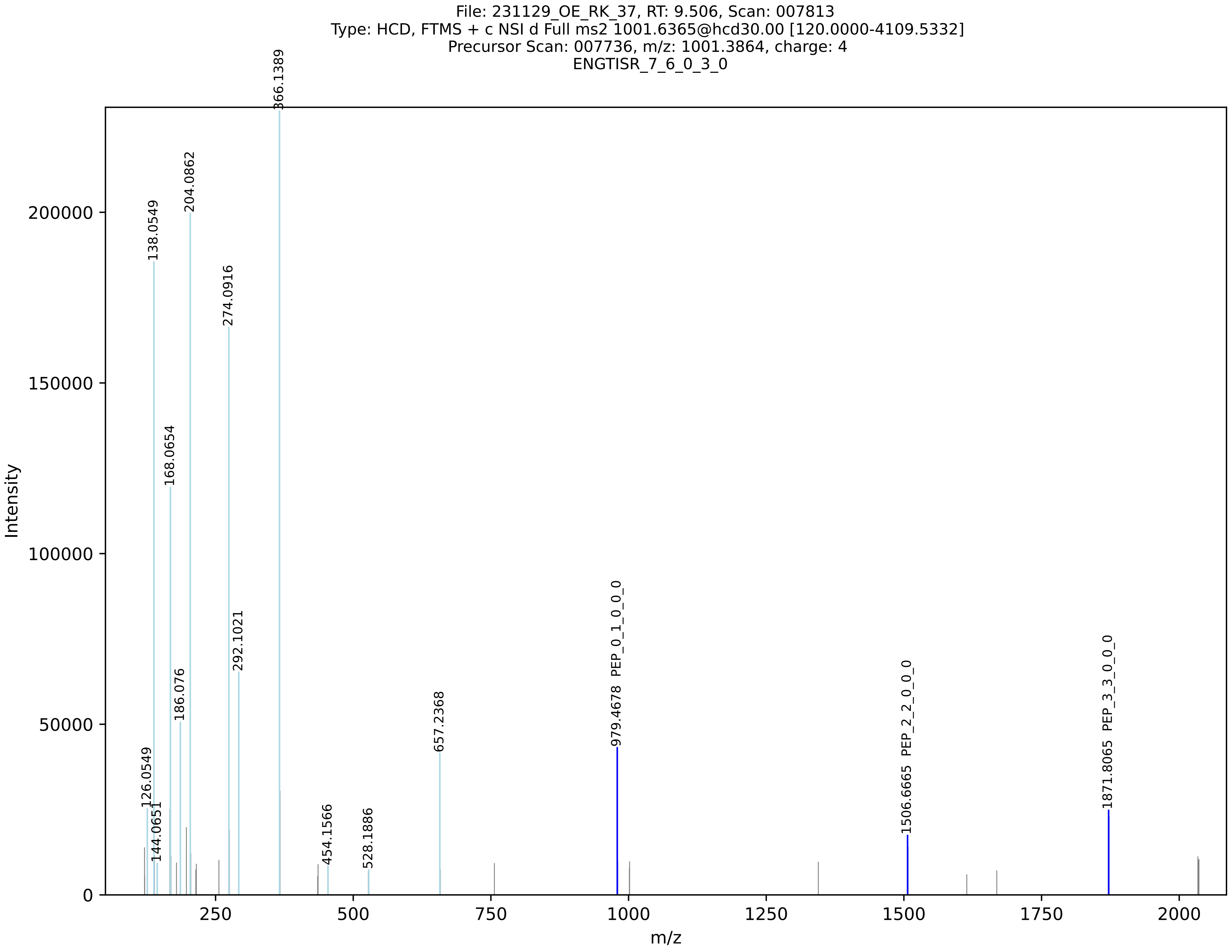
File: 231129_OE_RK_37, RT: 9.506, Scan: 007813
Type: HCD, FTMS + c NSI d Full ms2 1001.6365@hcd30.00 [120.0000-4109.5332]
Precursor Scan: 007736, m/z: 1001.3864, charge: 4
ENGTISR_7_6_0_3_0
Intensity
m/z
366.1389
204.0862
138.0549
274.0916
168.0654
292.1021
186.076
126.0549
144.0651
454.1566
528.1886
657.2368
979.4678 PEP_0_1_0_0_0
1506.6665 PEP_2_2_0_0_0
1871.8065 PEP_3_3_0_0_0
0
50000
100000
150000
200000
250
500
750
1000
1250
1500
1750
2000

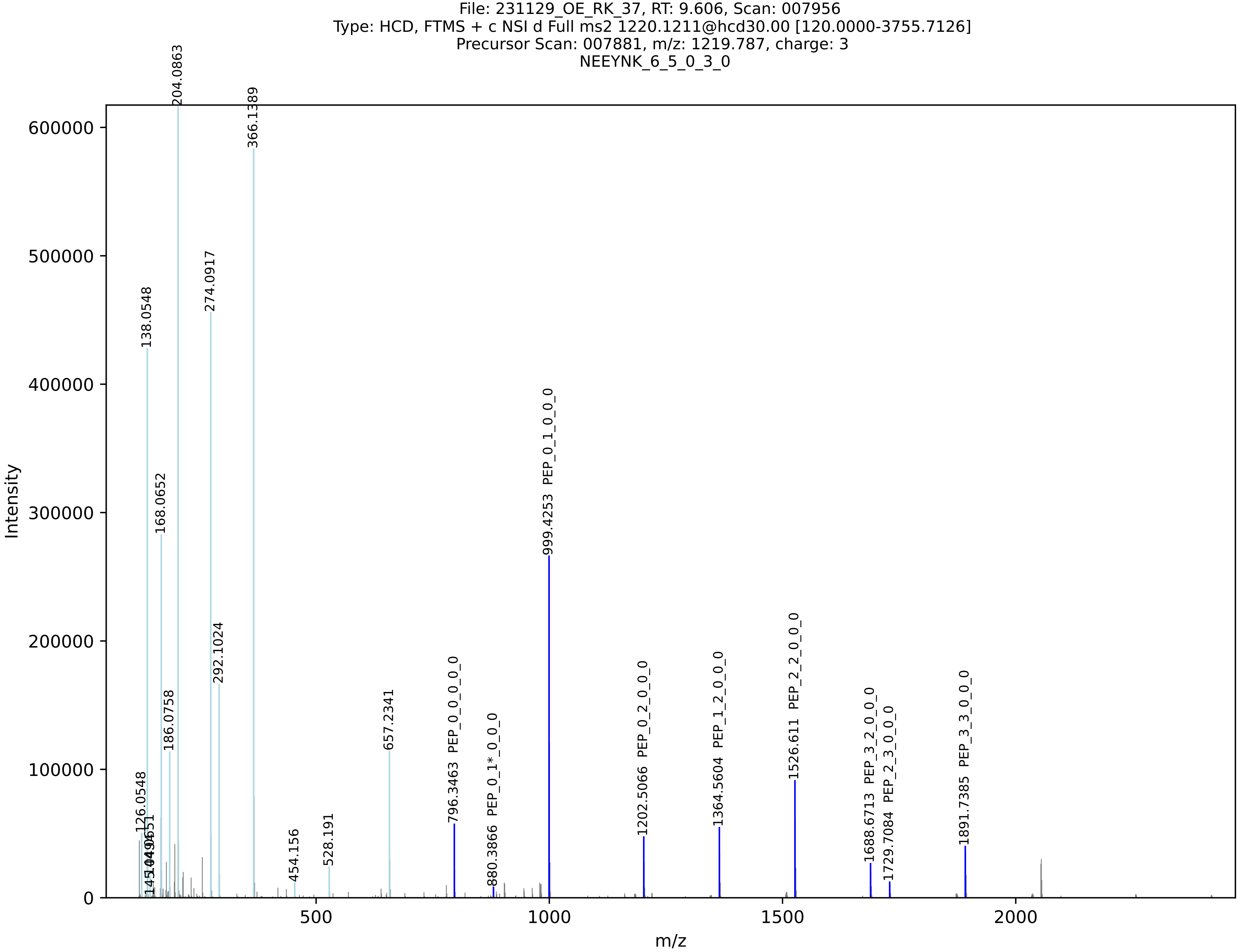
File: 231129_OE_RK_37, RT: 9.606, Scan: 007956
Type: HCD, FTMS + c NSI d Full ms2 1220.1211@hcd30.00 [120.0000-3755.7126]
Precursor Scan: 007881, m/z: 1219.787, charge: 3
NEEYNK_6_5_0_3_0
Intensity
m/z
204.0863
366.1389
274.0917
138.0548
168.0652
292.1024
186.0758
126.0548
657.2341
528.191
454.156
796.3463 PEP_0_0_0_0_0_
880.3866 PEP_0_1*_0_0_0
999.4253 PEP_0_1_0_0_0
1202.5066 PEP_0_2_0_0_0
1364.5604 PEP_1_2_0_0_0
1526.611 PEP_2_2_0_0_0
1688.6713 PEP_3_2_0_0_0
1729.7084 PEP_2_3_0_0_0
1891.7385 PEP_3_3_0_0_0

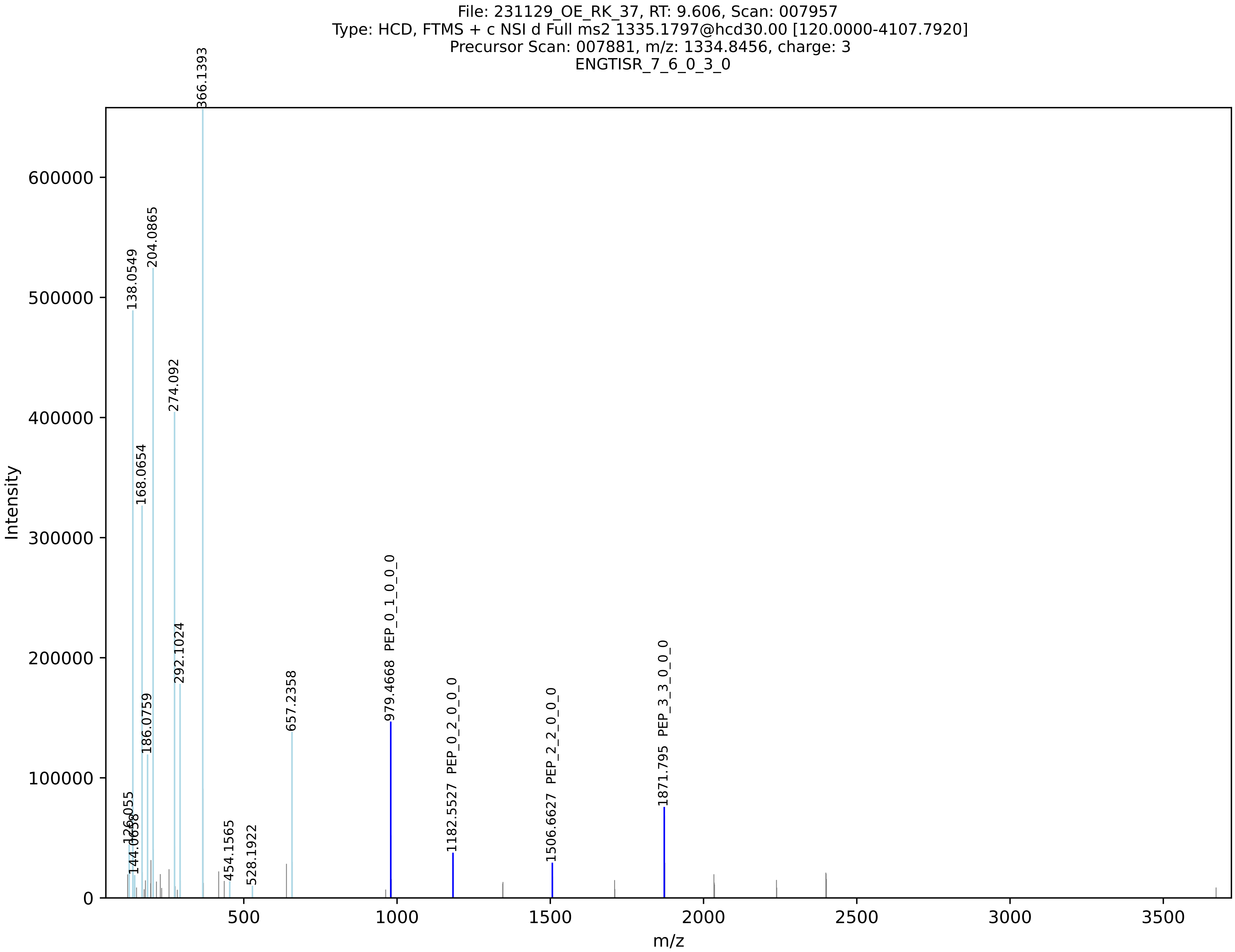

File: 231129_OE_RK_37, RT: 9.606, Scan: 007957
Type: HCD, FTMS + c NSI d Full ms2 1335.1797@hcd30.00 [120.0000-4107.7920]
Precursor Scan: 007881, m/z: 1334.8456, charge: 3
ENGTISR_7_6_0_3_0
366.1393
204.0865
138.0549
274.092
168.0654
292.1024
186.0759
126.055
144.0658
454.1565
528.1922
657.2358
979.4668 PEP_0_1_0_0_0
1182.5527 PEP_0_2_0_0_0
1506.6627 PEP_2_2_0_0_0
1871.795 PEP_3_3_0_0_0
Intensity
m/z

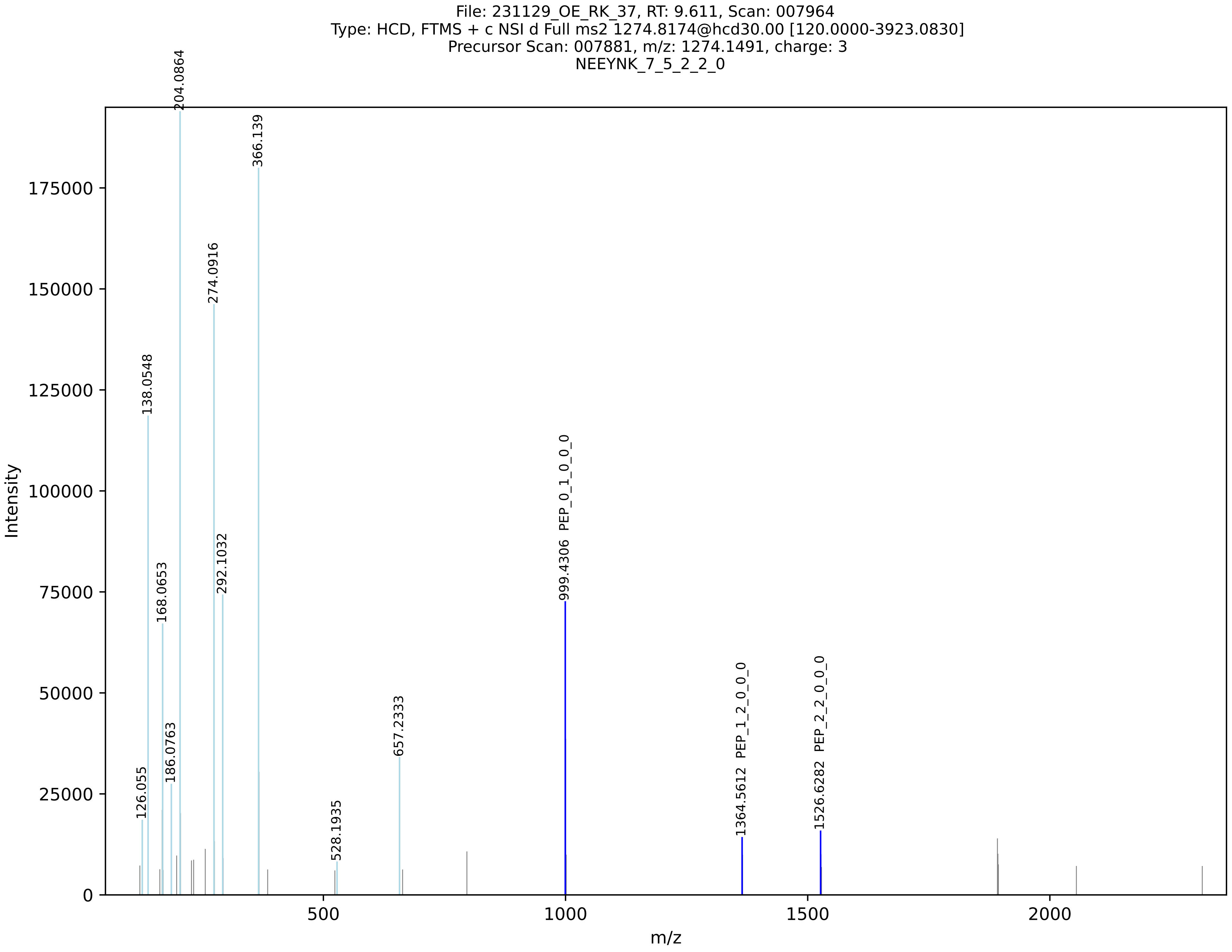
File: 231129_OE_RK_37, RT: 9.611, Scan: 007964
Type: HCD, FTMS + c NSI d Full ms2 1274.8174@hcd30.00 [120.0000-3923.0830]
Precursor Scan: 007881, m/z: 1274.1491, charge: 3
NEEYNK_7_5_2_2_0
Intensity
m/z
204.0864
366.139
274.0916
138.0548
292.1032
168.0653
186.0763
126.055
528.1935
657.2333
999.4306 PEP_0_1_0_0_0
1364.5612 PEP_1_2_0_0_0
1526.6282 PEP_2_2_0_0_0
175000
150000
125000
100000
75000
50000
25000
0
500
1000
1500
2000

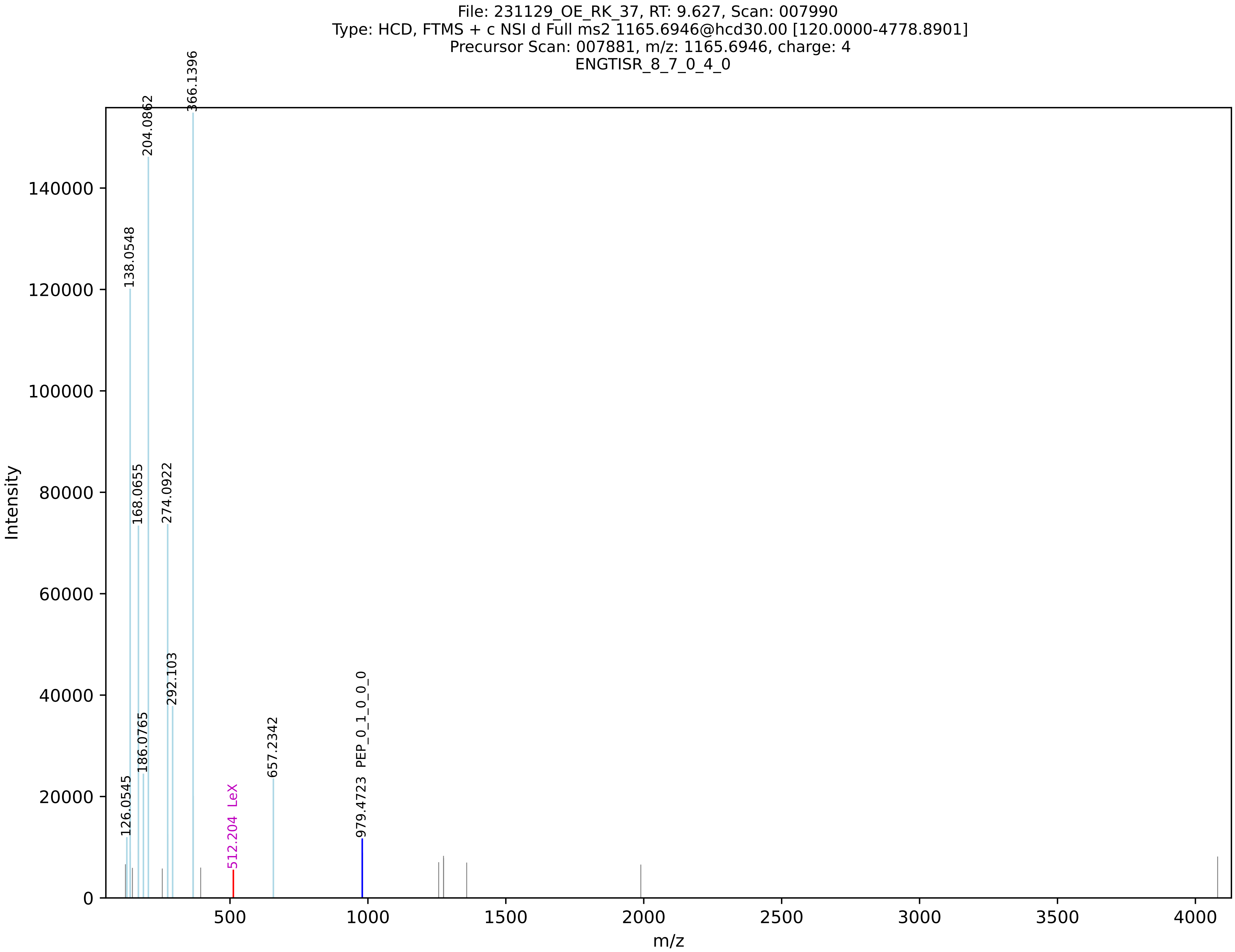

File: 231129_OE_RK_37, RT: 9.627, Scan: 007990
Type: HCD, FTMS + c NSI d Full ms2 1165.6946@hcd30.00 [120.0000-4778.8901]
Precursor Scan: 007881, m/z: 1165.6946, charge: 4
ENGTISR_8_7_0_4_0
366.1396
204.0862
138.0548
168.0655
274.0922
292.103
186.0765
126.0545
512.204 LeX
657.2342
979.4723 PEP_0_1_0_0_0
Intensity
m/z
0
20000
40000
60000
80000
100000
120000
140000
500
1000
1500
2000
2500
3000
3500
4000

File: 231129_OE_RK_37, RT: 9.655, Scan: 008028
Type: HCD, FTMS + c NSI d Full ms2 1115.7899@hcd30.00 [120.0000-3436.4592]
Precursor Scan: 007955, m/z: 1115.4561, charge: 3
YKNNSDISSTR_5_4_1_1_0

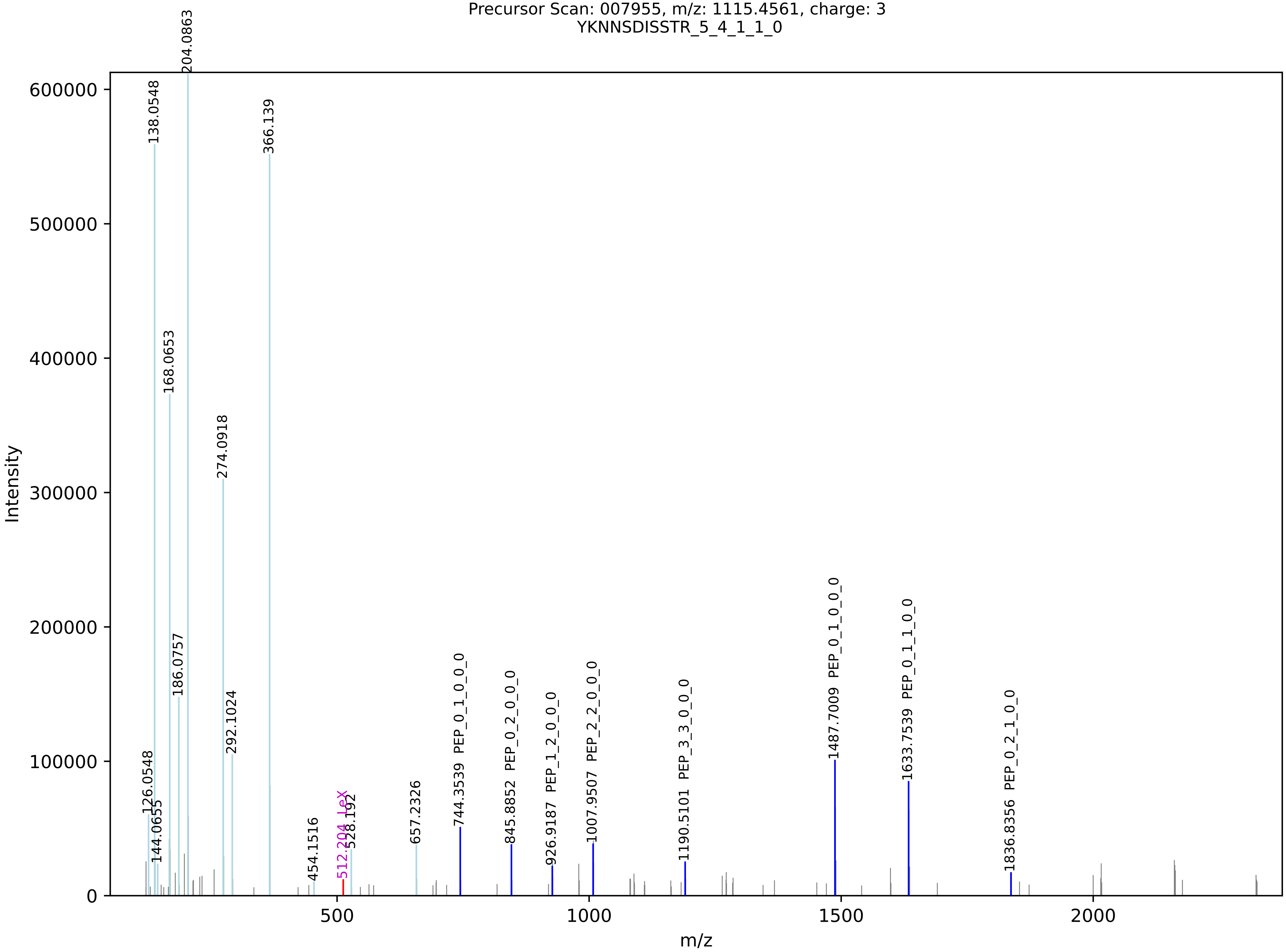

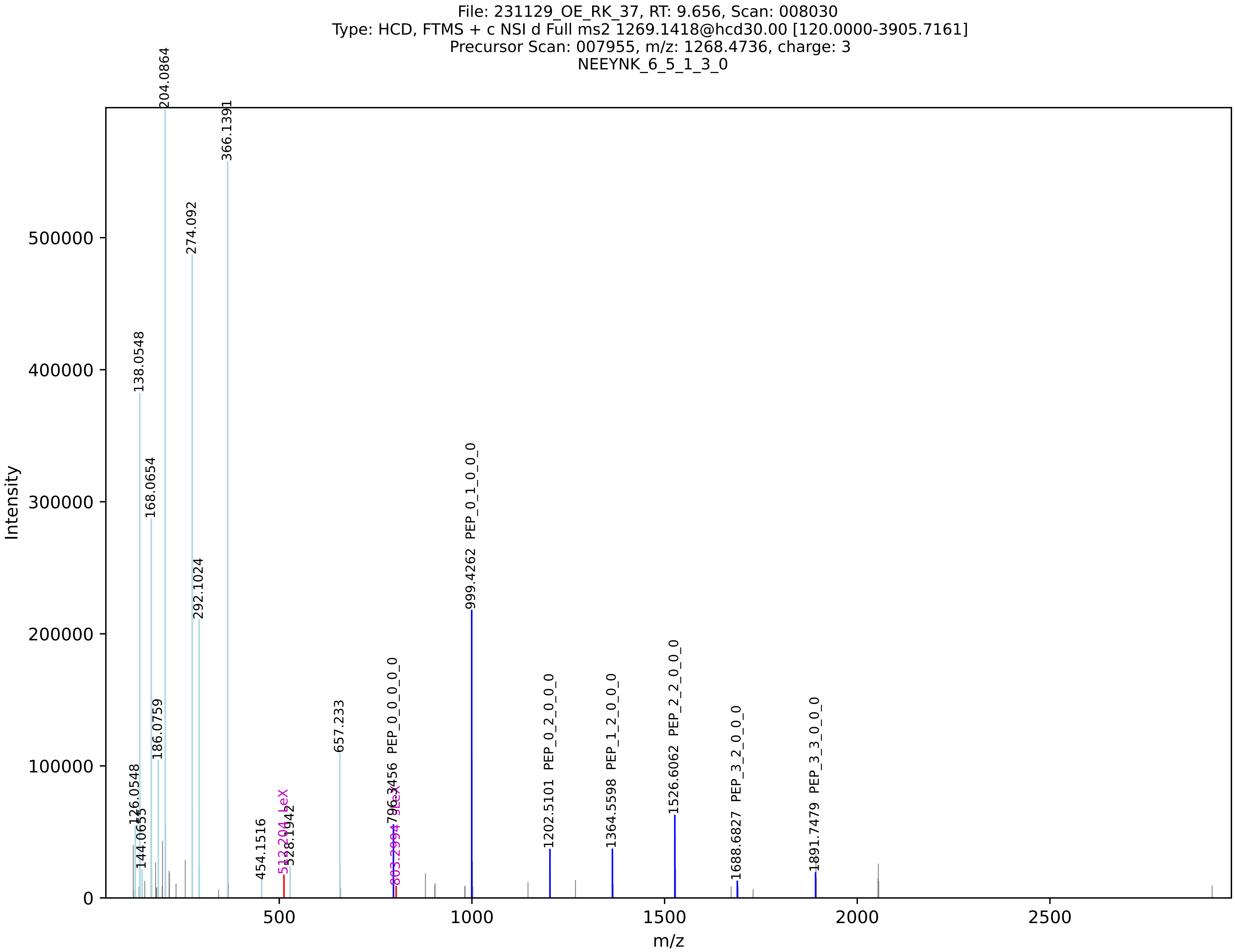
File: 231129_OE_RK_37, RT: 9.656, Scan: 008030
Type: HCD, FTMS + c NSI d Full ms2 1269.1418@hcd30.00 [120.0000-3905.7161]
Precursor Scan: 007955, m/z: 1268.4736, charge: 3
NEEYNK_6_5_1_3_0
204.0864
366.1391
274.092
138.0548
168.0654
292.1024
186.0759
126.0548
144.0655
454.1516
512.204 LeX
528.1942
657.233
796.3456 PEP_0_0_0_0_0
803.2994 sLeX
999.4262 PEP_0_1_0_0_0
1202.5101 PEP_0_2_0_0_0
1364.5598 PEP_1_2_0_0_0
1526.6062 PEP_2_2_0_0_0
1688.6827 PEP_3_2_0_0_0
1891.7479 PEP_3_3_0_0_0
Intensity
0
100000
200000
300000
400000
500000
500
1000
1500
2000
2500
m/z

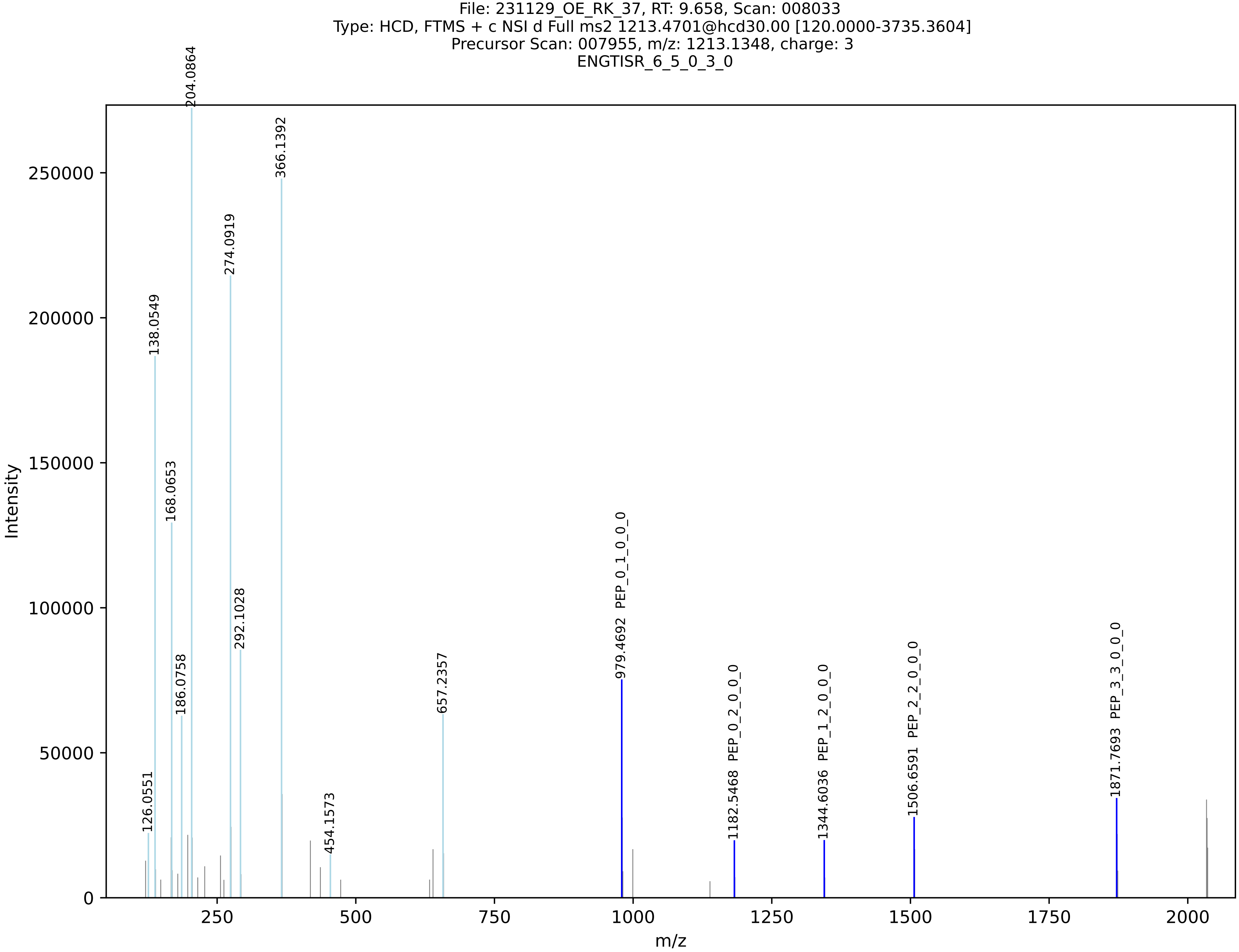

File: 231129_OE_RK_37, RT: 9.658, Scan: 008033
Type: HCD, FTMS + c NSI d Full ms2 1213.4701@hcd30.00 [120.0000-3735.3604]
Precursor Scan: 007955, m/z: 1213.1348, charge: 3
ENGTISR_6_5_0_3_0
Intensity
m/z
204.0864
366.1392
274.0919
138.0549
168.0653
292.1028
186.0758
126.0551
454.1573
657.2357
979.4692 PEP_0_1_0_0_0
1182.5468 PEP_0_2_0_0_0
1344.6036 PEP_1_2_0_0_0
1506.6591 PEP_2_2_0_0_0
1871.7693 PEP_3_3_0_0_0

File: 231129_OE_RK_37, RT: 9.659, Scan: 008034
Type: HCD, FTMS + c NSI d Full ms2 1074.6626@hcd30.00 [120.0000-4407.4795]
Precursor Scan: 007955, m/z: 1074.1622, charge: 4
ENGTISR_7_6_0_4_0

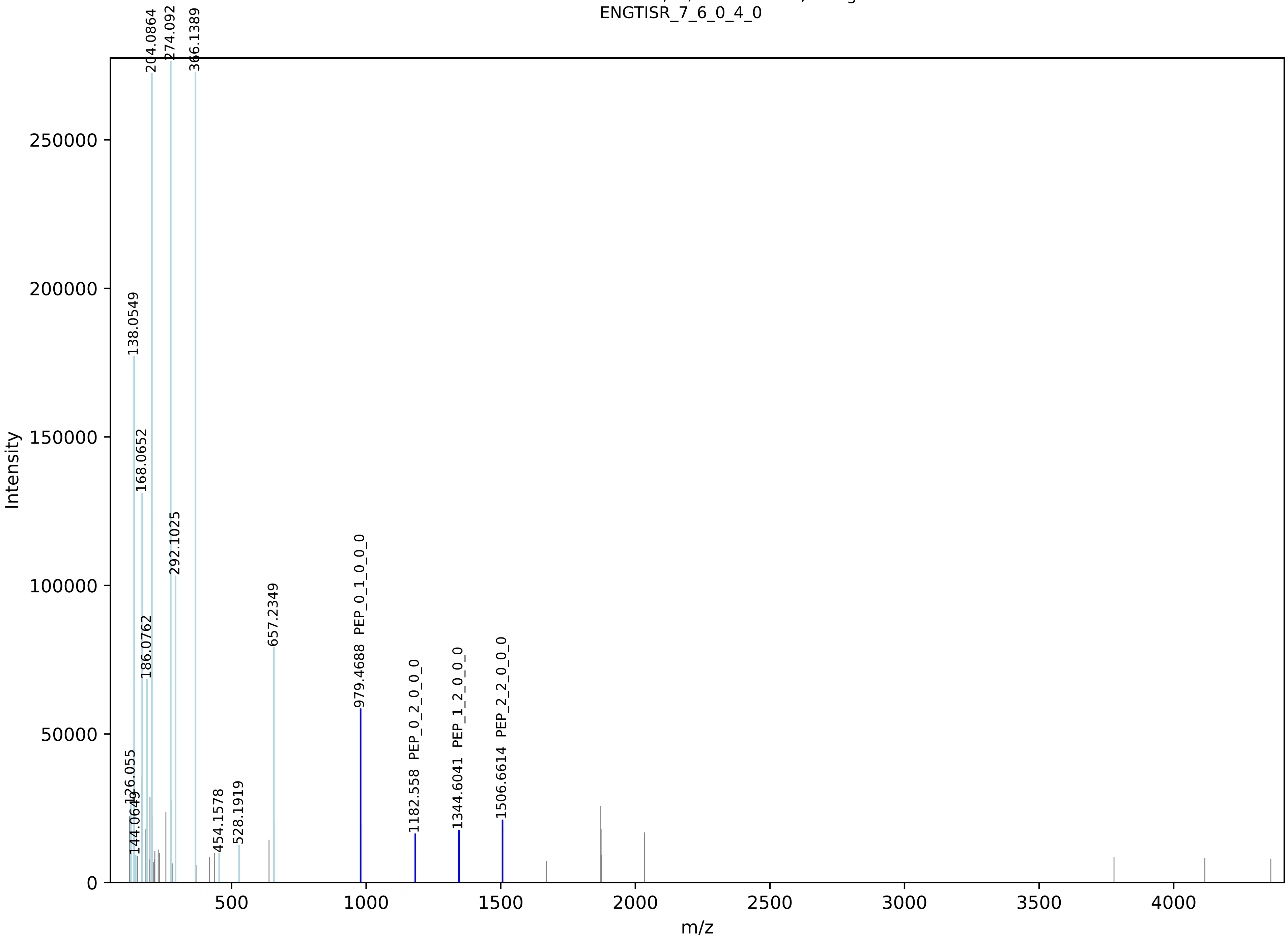

File: 231129_OE_RK_37, RT: 9.708, Scan: 008106
Type: HCD, FTMS + c NSI d Full ms2 1001.6370@hcd30.00 [120.0000-4109.5352]
Precursor Scan: 008026, m/z: 1001.3851, charge: 4
ENGTISR_7_6_0_3_0

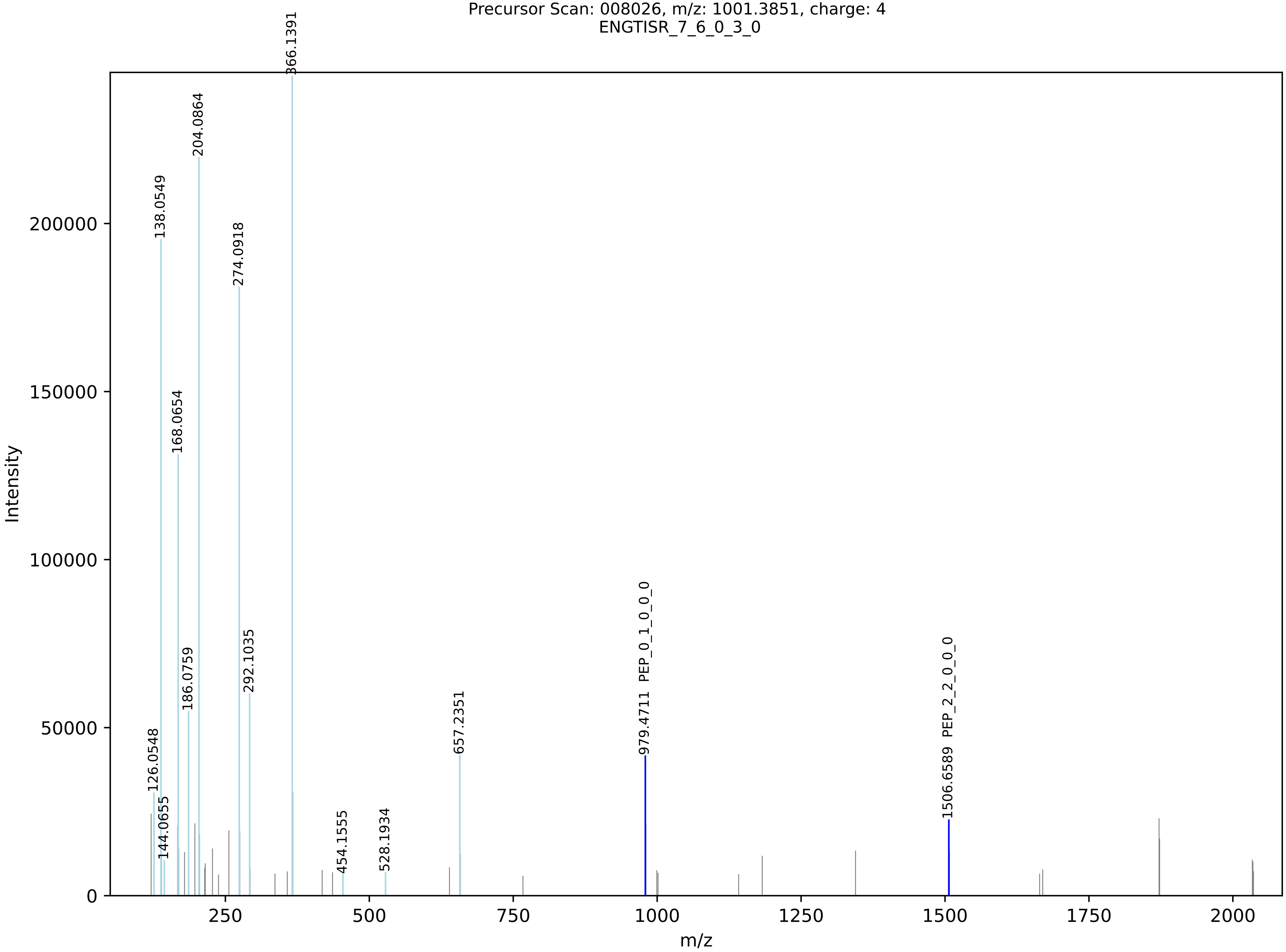

File: 231129_OE_RK_37, RT: 9.709, Scan: 008108
Type: HCD, FTMS + c NSI d Full ms2 837.0950@hcd30.00 [120.0000-3438.2034]
Precursor Scan: 008026, m/z: 836.844, charge: 4
YKNNSDISSTR_5_4_1_1_0

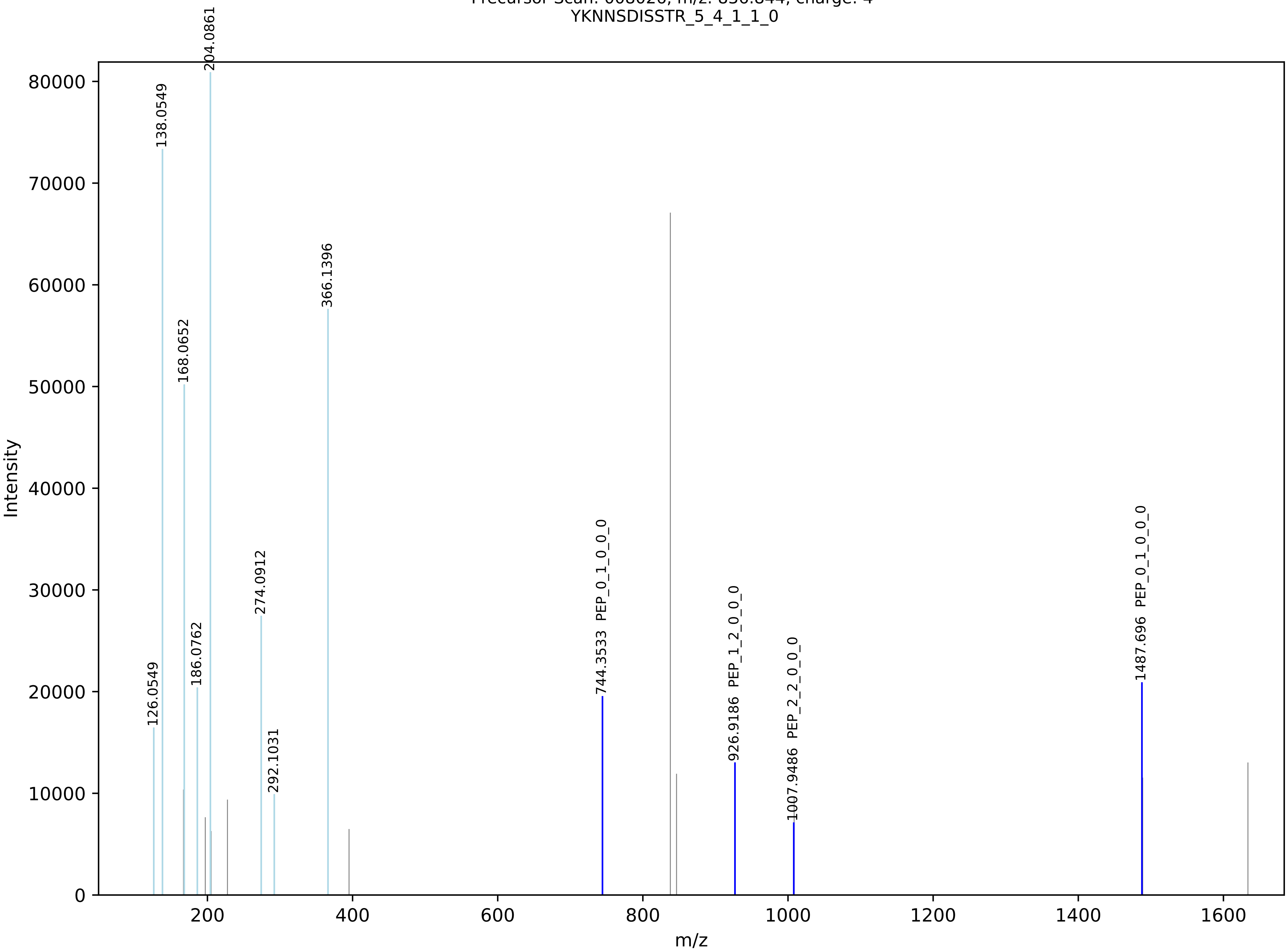

File: 231129_OE_RK_37, RT: 9.712, Scan: 008112
Type: HCD, FTMS + c NSI d Full ms2 1080.7798@hcd30.00 [120.0000-3329.3281]
Precursor Scan: 008026, m/z: 1080.4457, charge: 3
YKNNSDISSTR_4_5_0_1_0

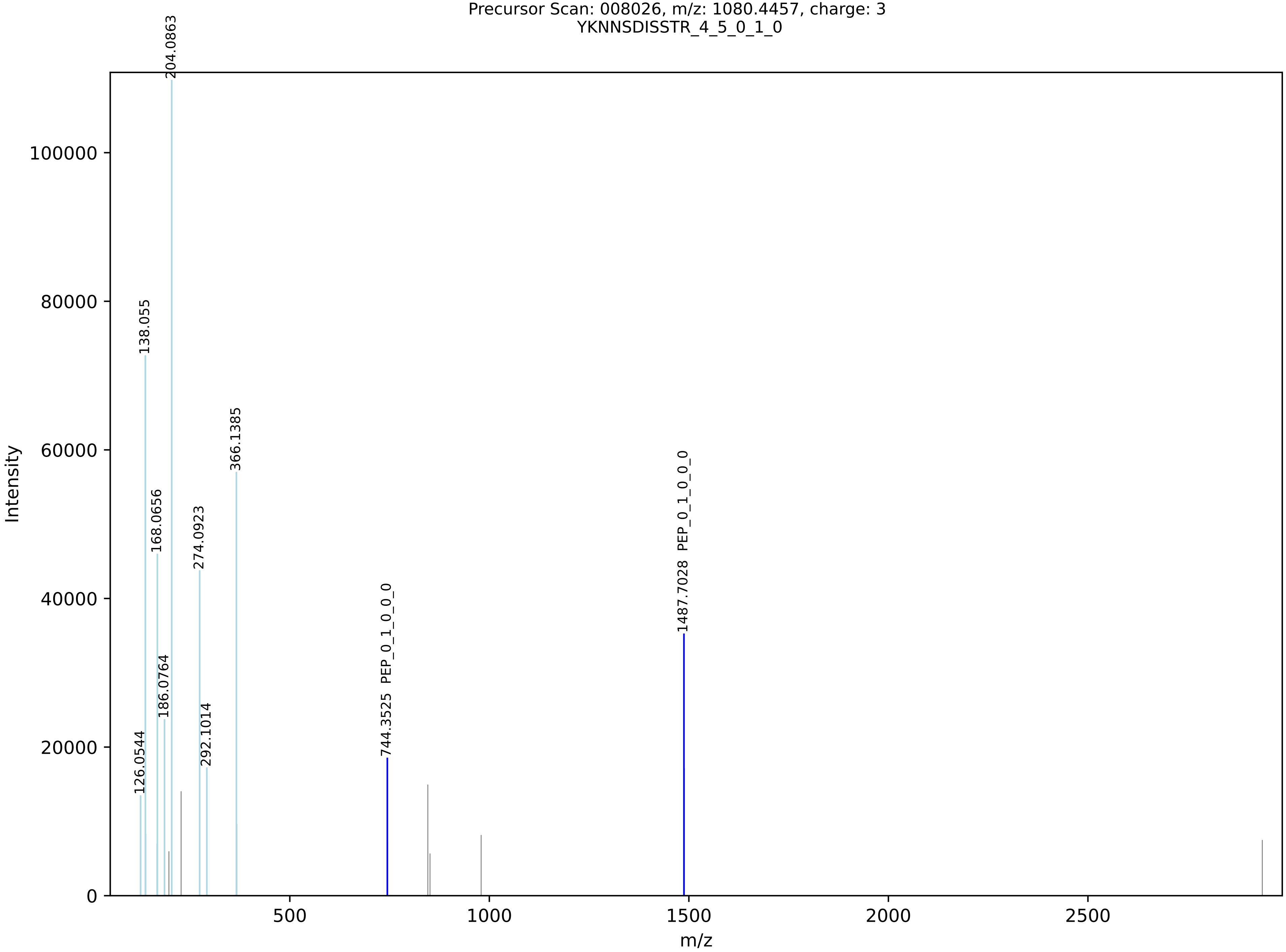

File: 231129_OE_RK_37, RT: 9.764, Scan: 008189
Type: HCD, FTMS + c NSI d Full ms2 951.8578@hcd30.00 [120.0000-3906.4360]
Precursor Scan: 008100, m/z: 951.6073, charge: 4
NGTR_6_6_2_3_0

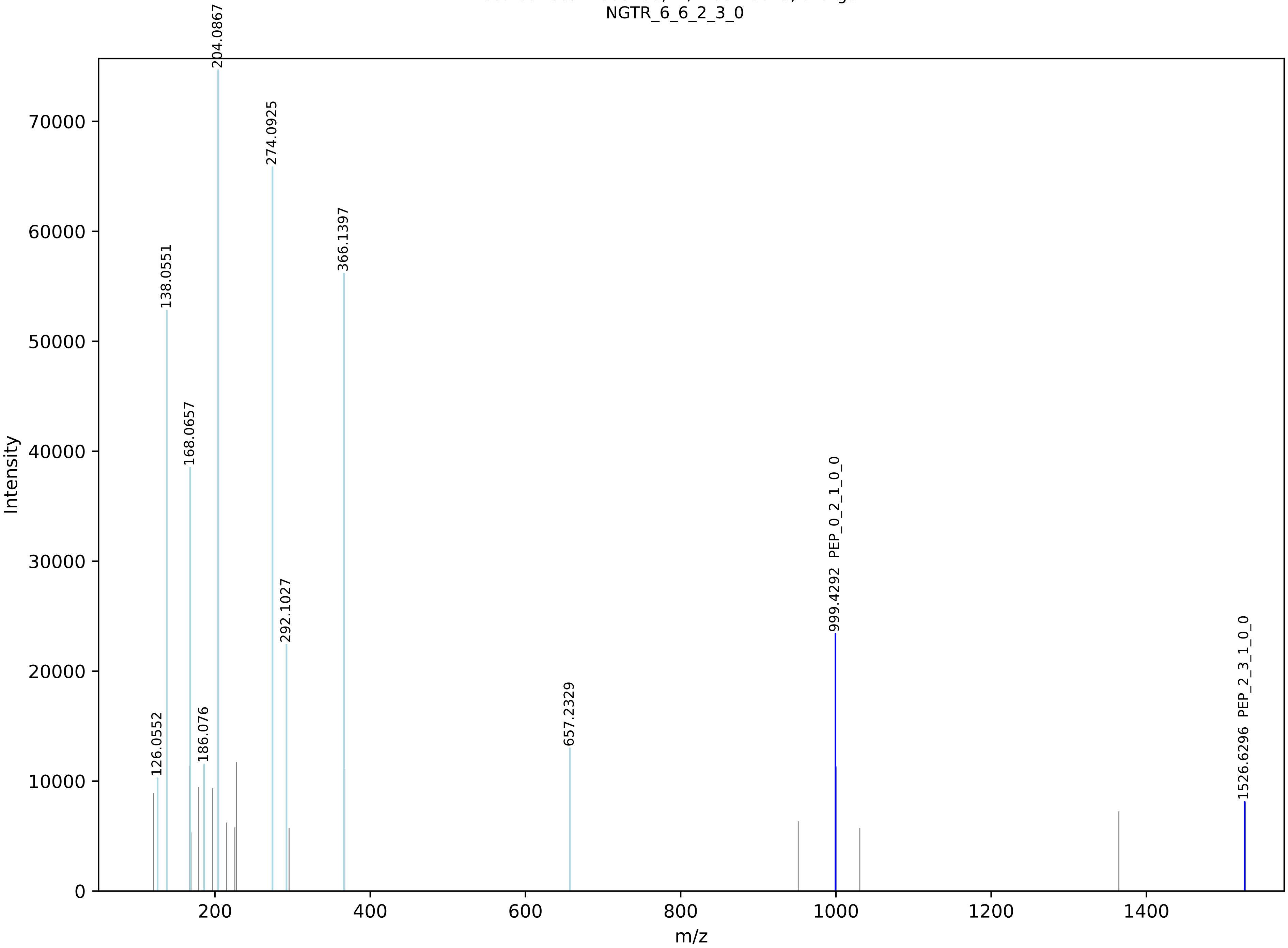

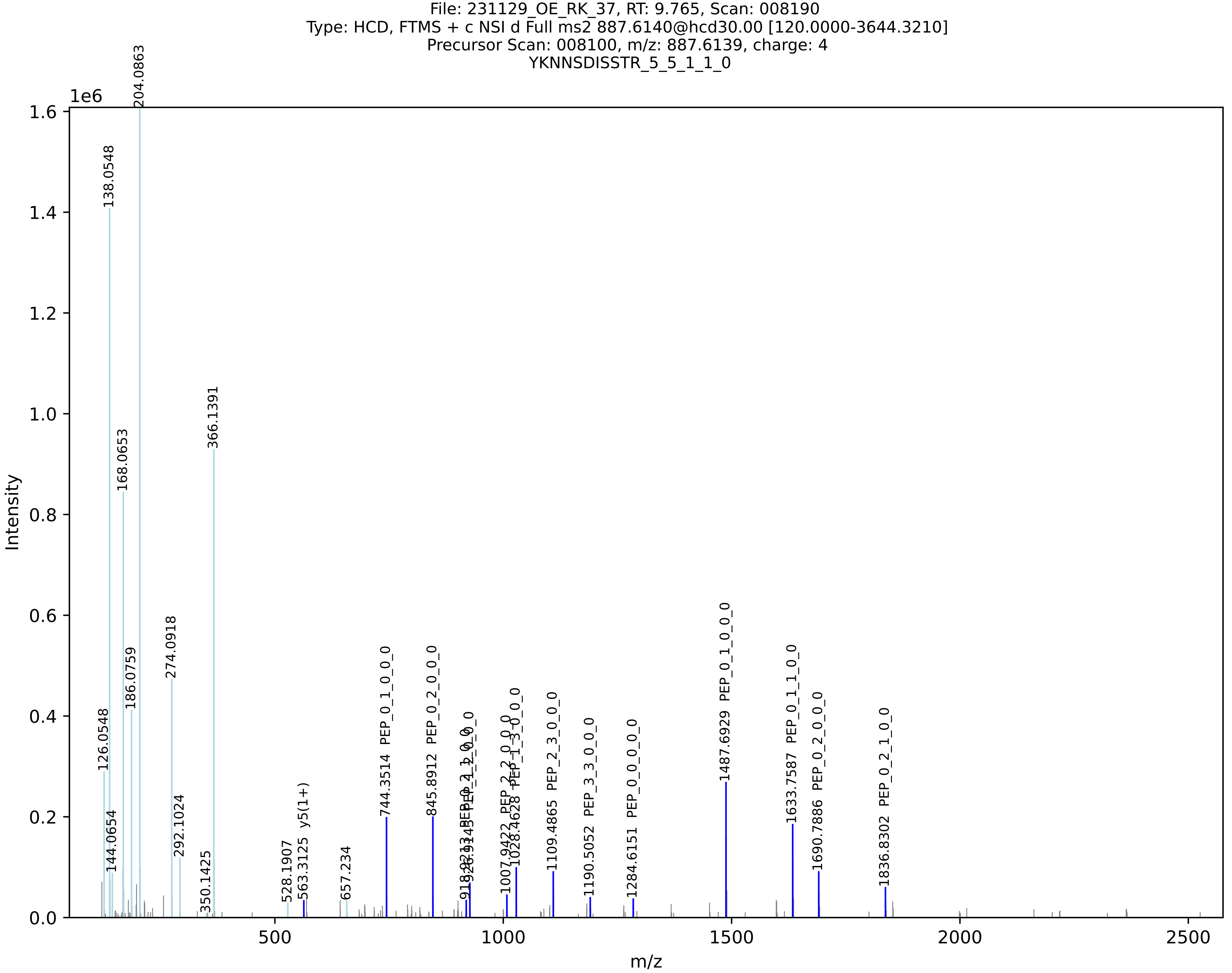
File: 231129_OE_RK_37, RT: 9.765, Scan: 008190
Type: HCD, FTMS + c NSI d Full ms2 887.6140@hcd30.00 [120.0000-3644.3210]
Precursor Scan: 008100, m/z: 887.6139, charge: 4
YKNNSDISSTR_5_5_1_1_0
1e6
Intensity
m/z
204.0863
138.0548
168.0653
366.1391
274.0918
186.0759
126.0548
144.0654
292.1024
350.1425
528.1907
563.3125 y5(1+)
657.234
744.3514 PEP_0_1_0_0_0
845.8912 PEP_0_2_0_0_0
1007.9422 PEP_2_2_0_0_0
1028.4628 PEP_1_3_0_0_0
1109.4865 PEP_2_3_0_0_0
1190.5052 PEP_3_3_0_0_0
1284.6151 PEP_0_0_0_0_0
1487.6929 PEP_0_1_0_0_0
1633.7587 PEP_0_1_1_0_0
1690.7886 PEP_0_2_0_0_0
1836.8302 PEP_0_2_1_0_0

File: 231129_OE_RK_37, RT: 9.816, Scan: 008265
Type: HCD, FTMS + c NSI d Full ms2 1166.1987@hcd30.00 [120.0000-4780.9468]
Precursor Scan: 008174, m/z: 1165.4473, charge: 4
ENGTISR_8_7_0_4_0

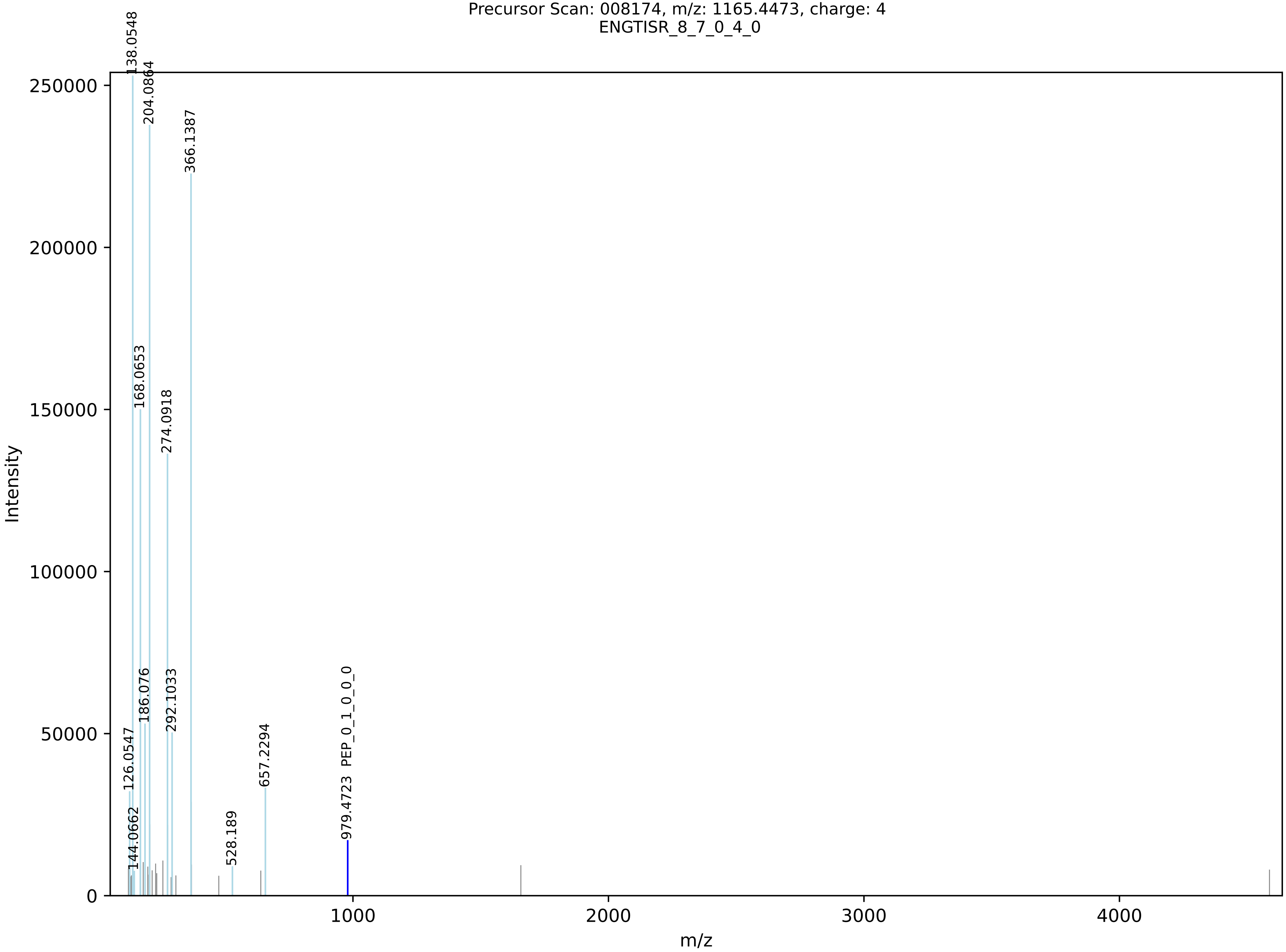

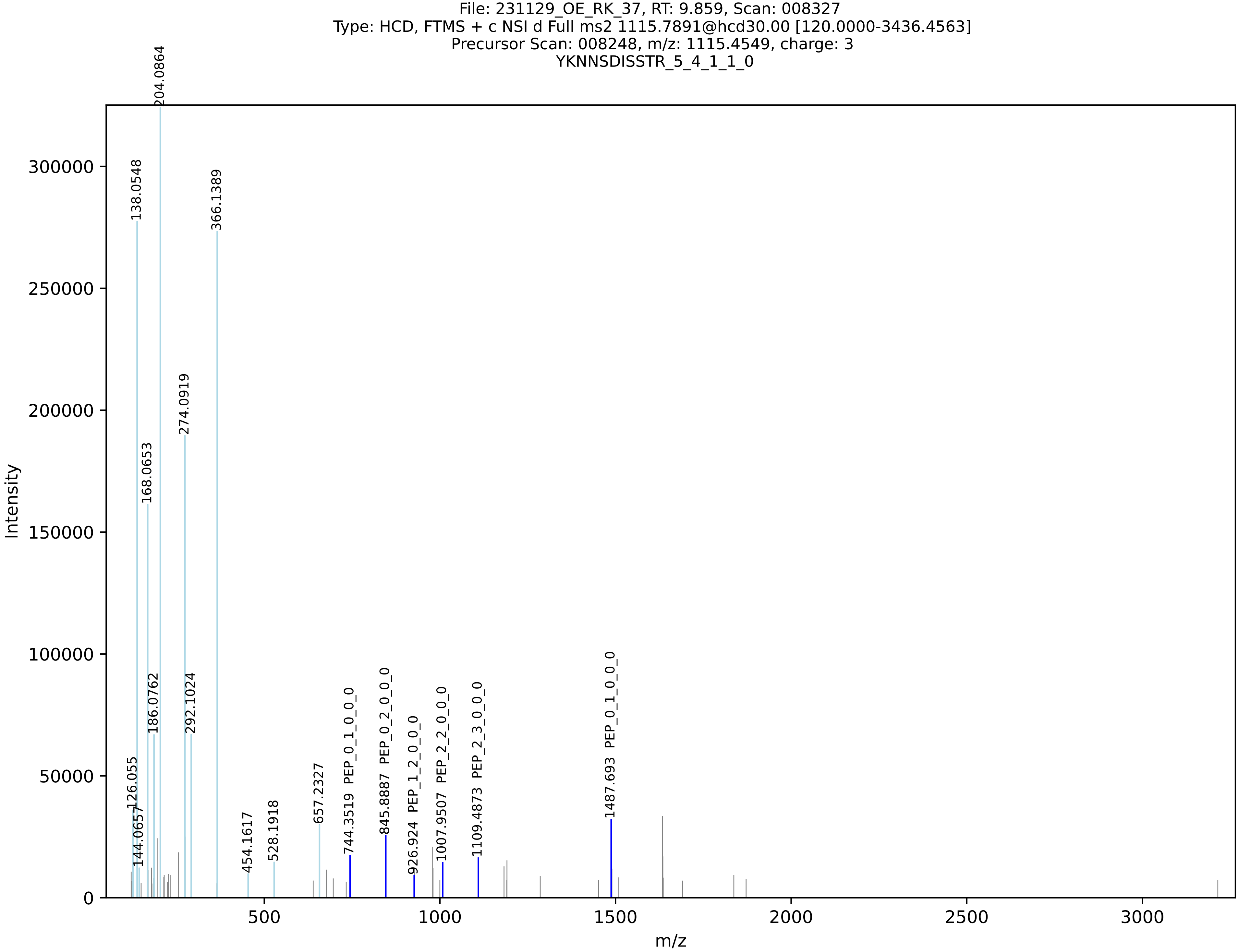
File: 231129_OE_RK_37, RT: 9.859, Scan: 008327
Type: HCD, FTMS + c NSI d Full ms2 1115.7891@hcd30.00 [120.0000-3436.4563]
Precursor Scan: 008248, m/z: 1115.4549, charge: 3
YKNNSDISSTR_5_4_1_1_0
204.0864
138.0548
366.1389
274.0919
168.0653
186.0762
292.1024
126.055
144.0657
454.1617
528.1918
657.2327
744.3519 PEP_0_1_0_0_0
845.8887 PEP_0_2_0_0_0
926.924 PEP_1_2_0_0_0
1007.9507 PEP_2_2_0_0_0
1109.4873 PEP_2_3_0_0_0
1487.693 PEP_0_1_0_0_0
Intensity
m/z
0
50000
100000
150000
200000
250000
300000
500
1000
1500
2000
2500
3000

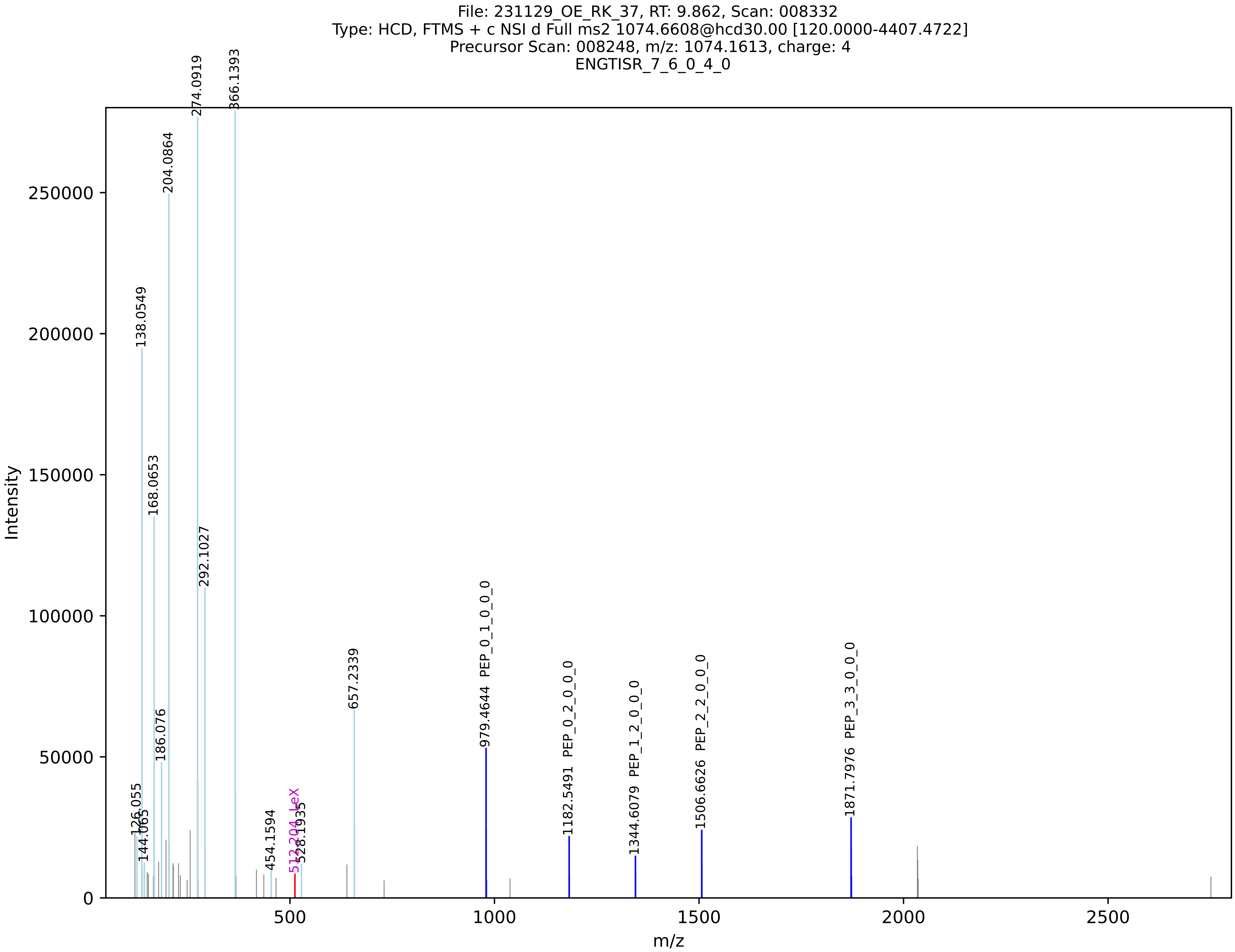
File: 231129_OE_RK_37, RT: 9.862, Scan: 008332
Type: HCD, FTMS + c NSI d Full ms2 1074.6608@hcd30.00 [120.0000-4407.4722]
Precursor Scan: 008248, m/z: 1074.1613, charge: 4
ENGTISR_7_6_0_4_0
Intensity
m/z
274.0919
366.1393
204.0864
138.0549
168.0653
292.1027
186.076
126.055
144.065
454.1594
512.204_LeX
528.1935
657.2339
979.4644 PEP_0_1_0_0_
1182.5491 PEP_0_2_0_0_0
1344.6079 PEP_1_2_0_0_0
1506.6626 PEP_2_2_0_0_0
1871.7976 PEP_3_3_0_0_0

File: 231129_OE_RK_37, RT: 9.864, Scan: 008334
Type: HCD, FTMS + c NSI d Full ms2 1038.4012@hcd30.00 [120.0000-4259.5327]
Precursor Scan: 008248, m/z: 1037.9001, charge: 4
ENGTISR_7_6_1_3_0

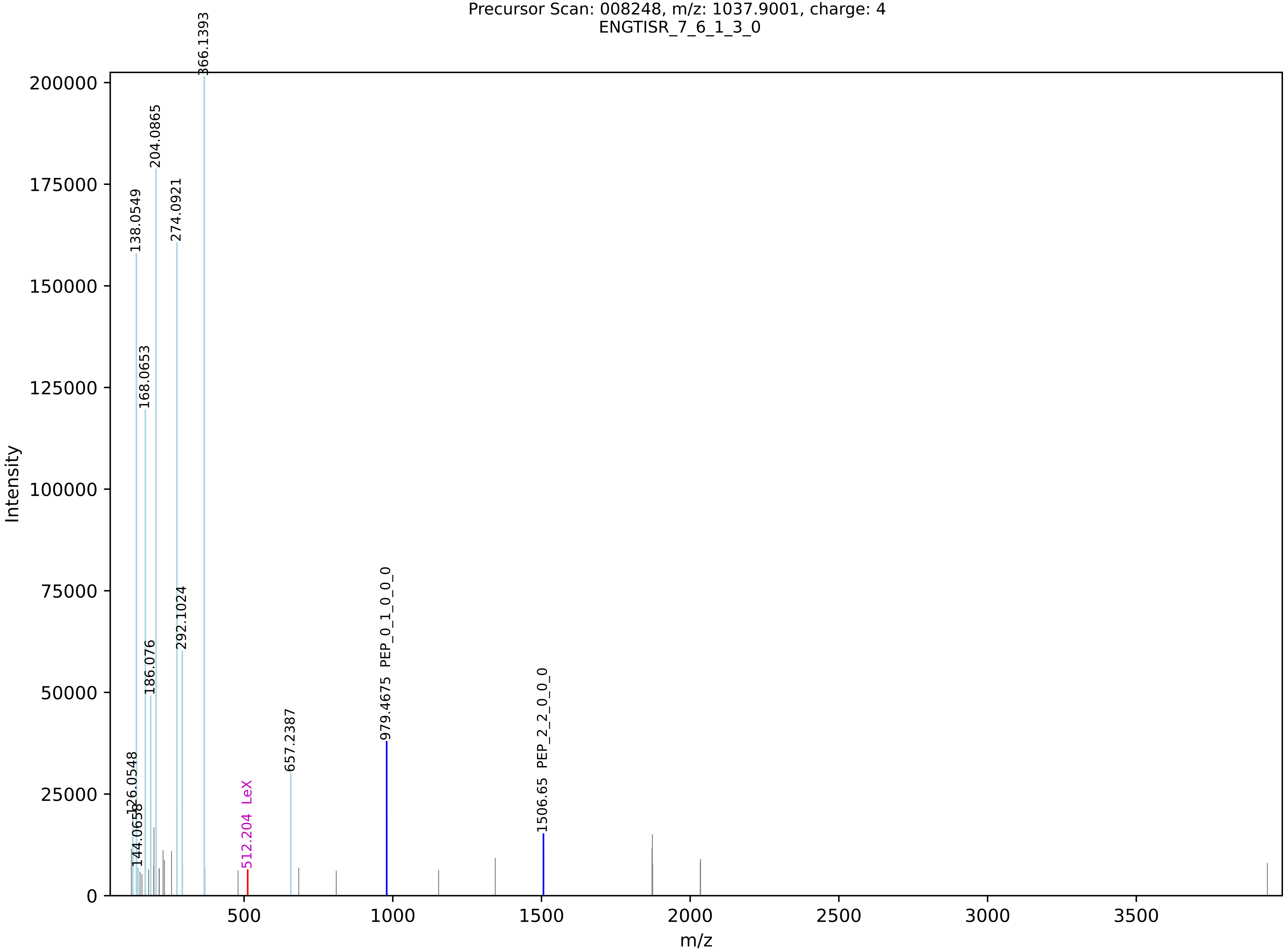

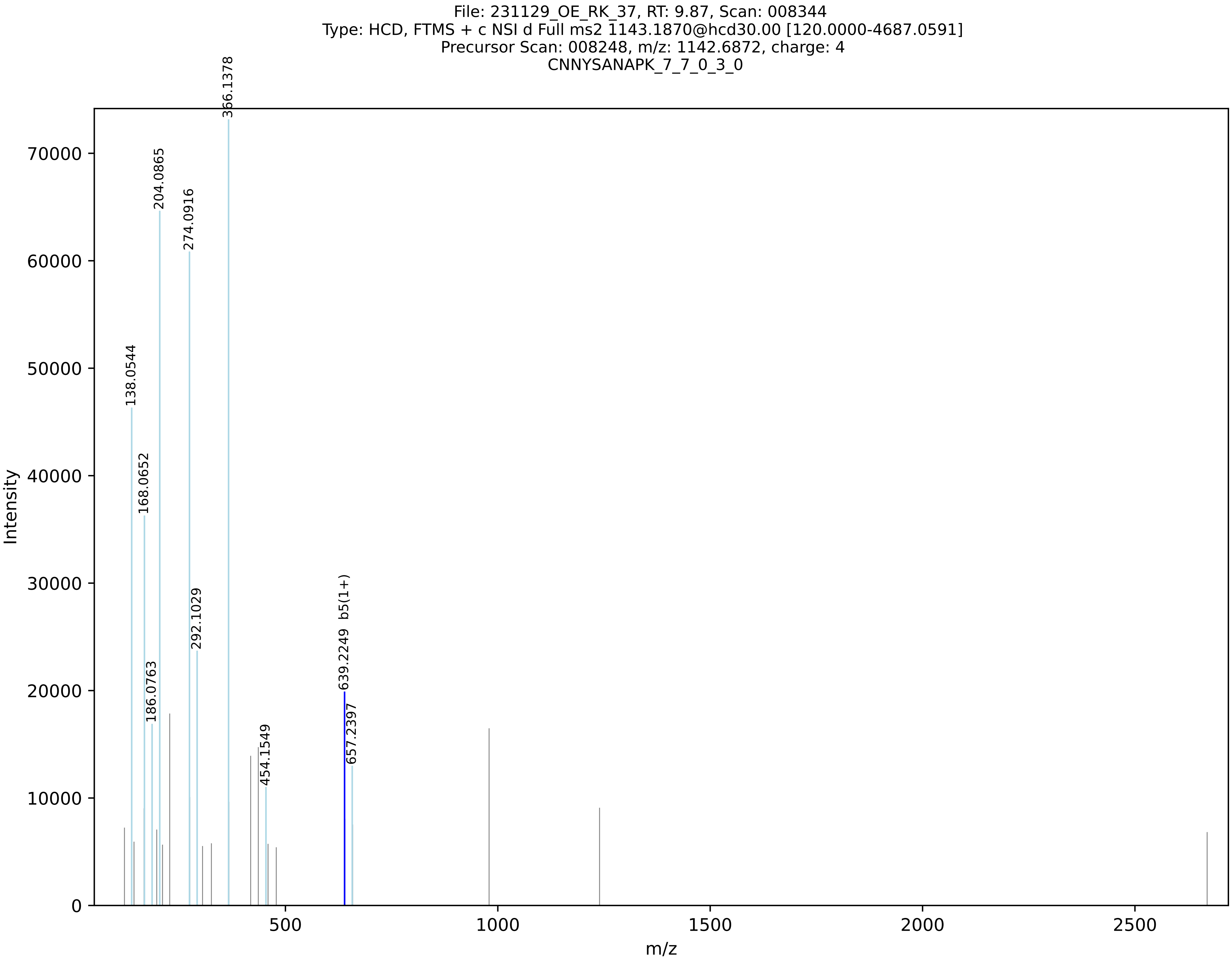

File: 231129_OE_RK_37, RT: 9.87, Scan: 008344
Type: HCD, FTMS + c NSI d Full ms2 1143.1870@hcd30.00 [120.0000-4687.0591]
Precursor Scan: 008248, m/z: 1142.6872, charge: 4
CNNYSANAPK_7_7_0_3_0
366.1378
204.0865
274.0916
138.0544
168.0652
292.1029
b5(1+)
639.2249
186.0763
657.2397
454.1549
Intensity
m/z
0
10000
20000
30000
40000
50000
60000
70000
500
1000
1500
2000
2500

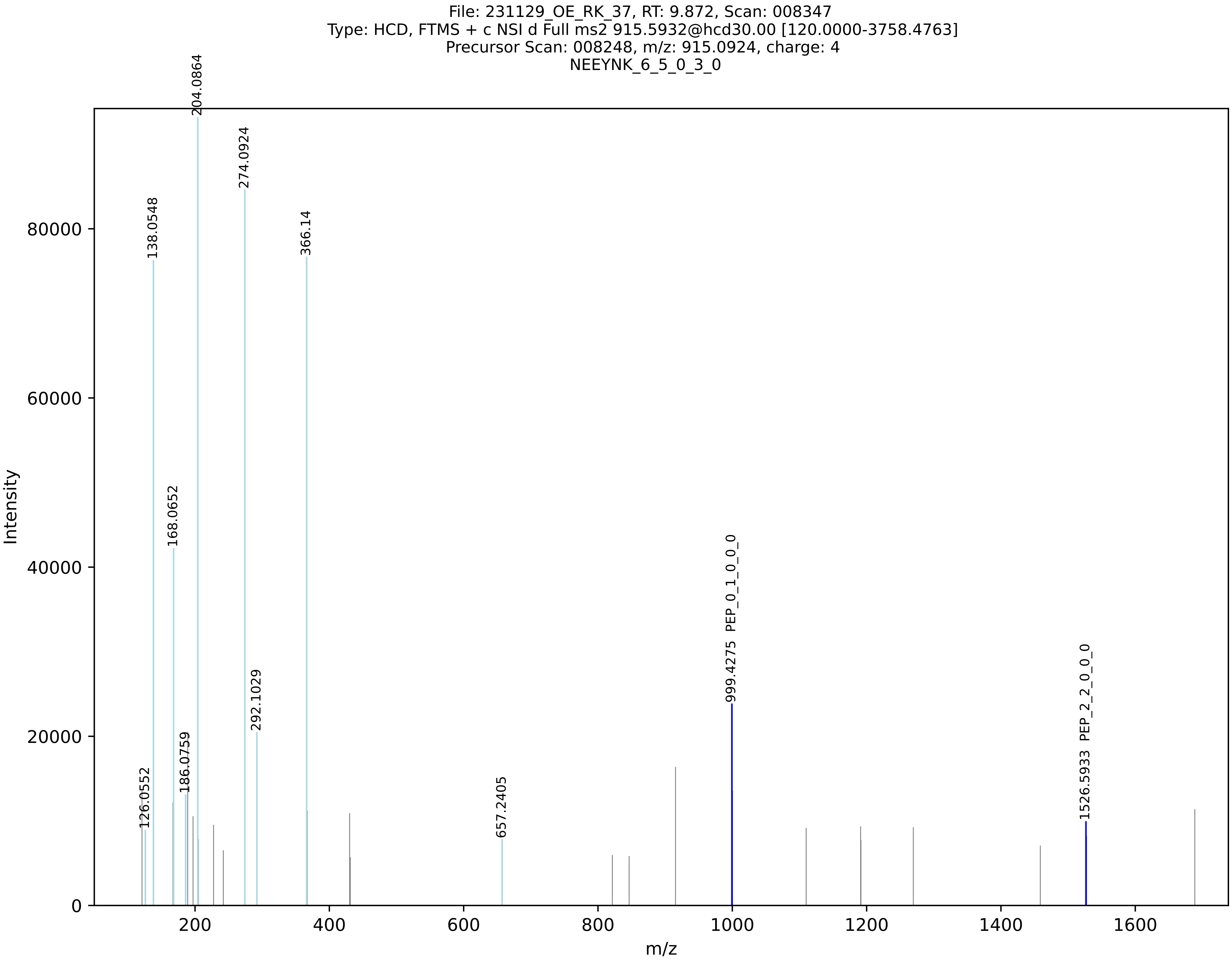

File: 231129_OE_RK_37, RT: 9.872, Scan: 008347
Type: HCD, FTMS + c NSI d Full ms2 915.5932@hcd30.00 [120.0000-3758.4763]
Precursor Scan: 008248, m/z: 915.0924, charge: 4
NEEYNK_6_5_0_3_0
204.0864
274.0924
138.0548
366.14
168.0652
292.1029
186.0759
126.0552
657.2405
999.4275 PEP_0_1_0_0_0_
1526.5933 PEP_2_2_0_0_0_
Intensity
m/z
0
20000
40000
60000
80000
200
400
600
800
1000
1200
1400
1600

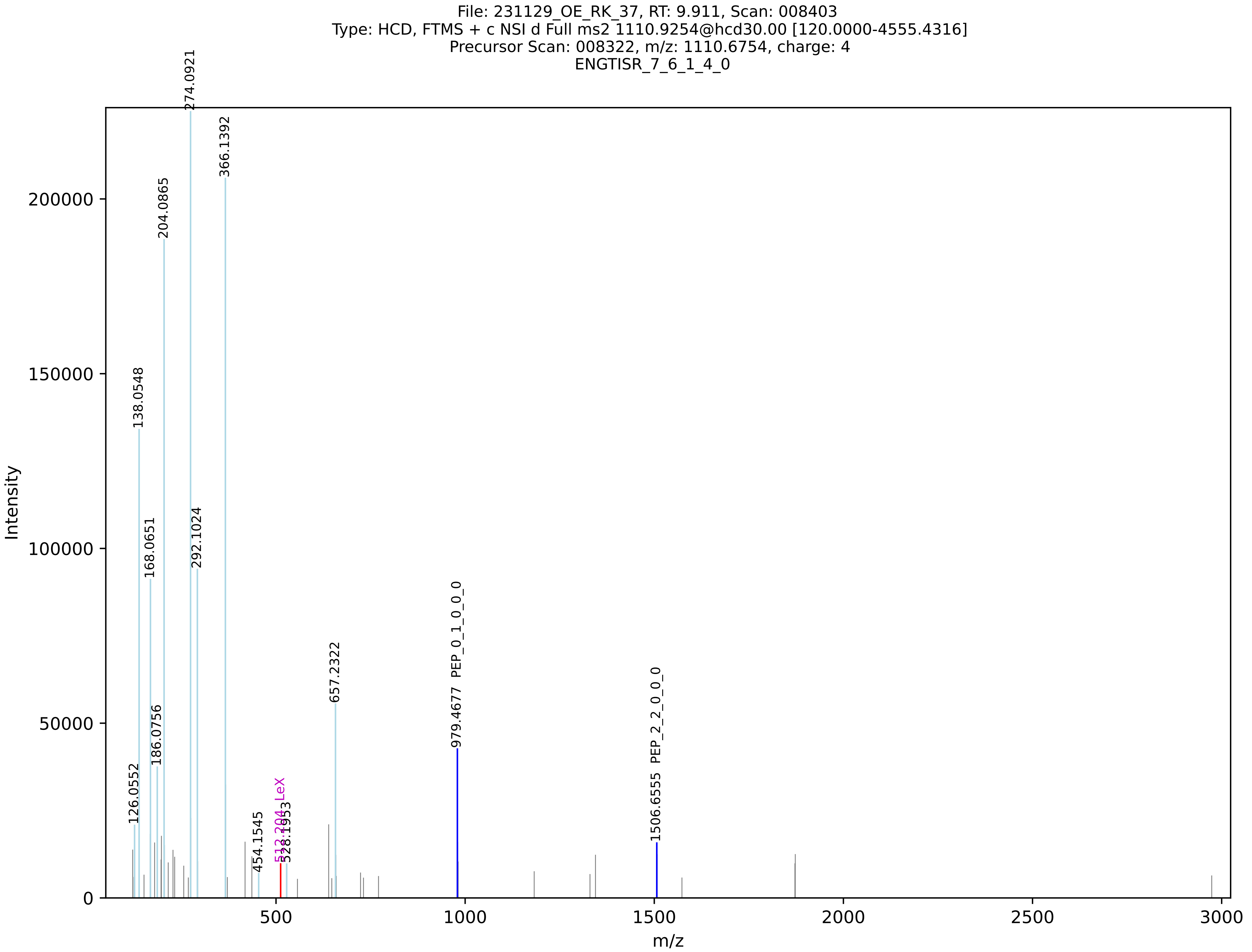
File: 231129_OE_RK_37, RT: 9.911, Scan: 008403
Type: HCD, FTMS + c NSI d Full ms2 1110.9254@hcd30.00 [120.0000-4555.4316]
Precursor Scan: 008322, m/z: 1110.6754, charge: 4
ENGTISR_7_6_1_4_0
274.0921
366.1392
204.0865
138.0548
168.0651
292.1024
186.0756
126.0552
454.1545
512.204 LeX
528.1953
657.2322
979.4677 PEP_0_1_0_0_0
1506.6555 PEP_2_2_0_0_0
Intensity
0
50000
100000
150000
200000
500
1000
1500
2000
2500
3000
m/z

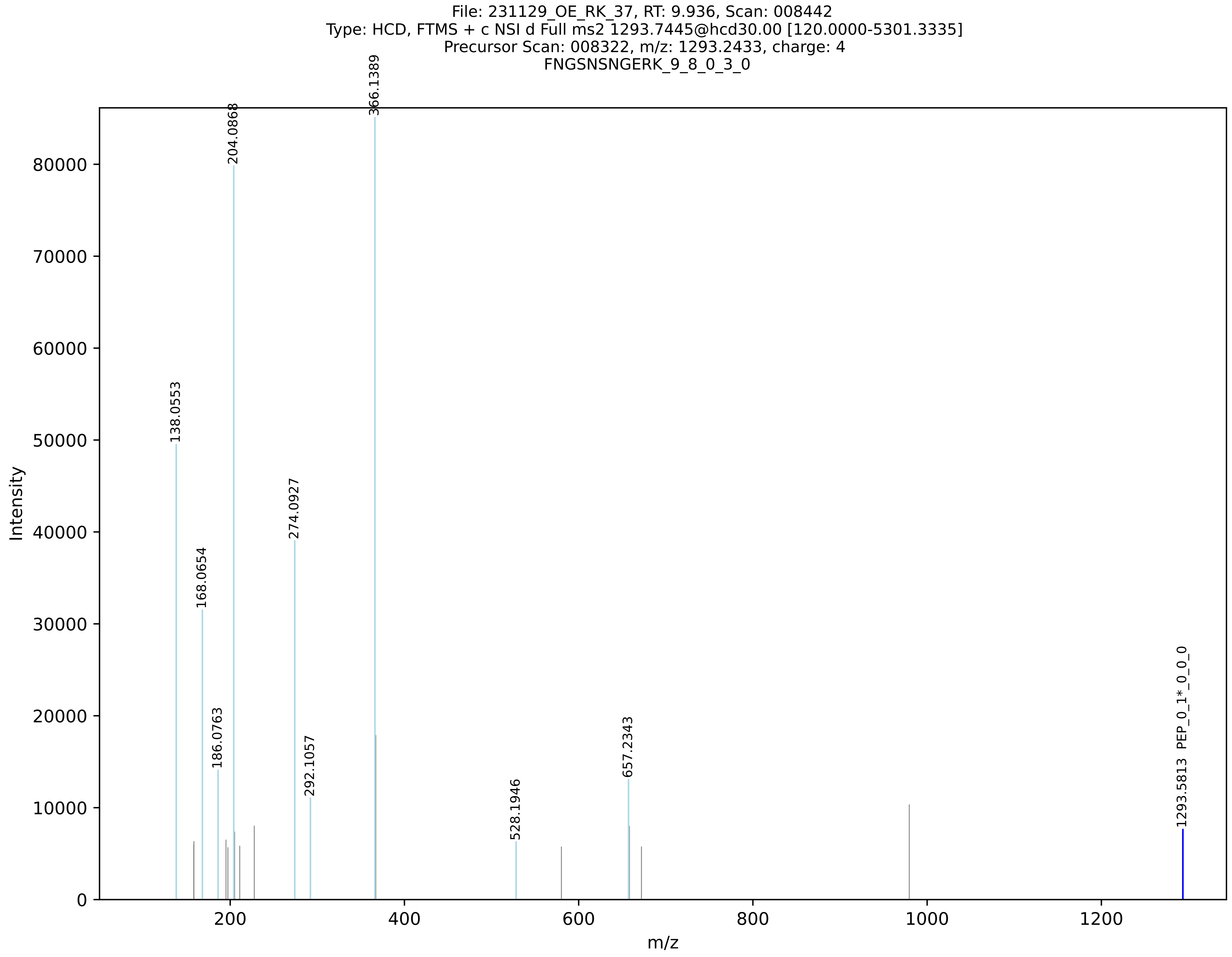

File: 231129_OE_RK_37, RT: 9.936, Scan: 008442
Type: HCD, FTMS + c NSI d Full ms2 1293.7445@hcd30.00 [120.0000-5301.3335]
Precursor Scan: 008322, m/z: 1293.2433, charge: 4
FNGSNSNGERK_9_8_0_3_0
Intensity
m/z
366.1389
204.0868
138.0553
168.0654
186.0763
274.0927
292.1057
528.1946
657.2343
1293.5813 PEP_0_1*_0_0_0
0
10000
20000
30000
40000
50000
60000
70000
80000
200
400
600
800
1000
1200

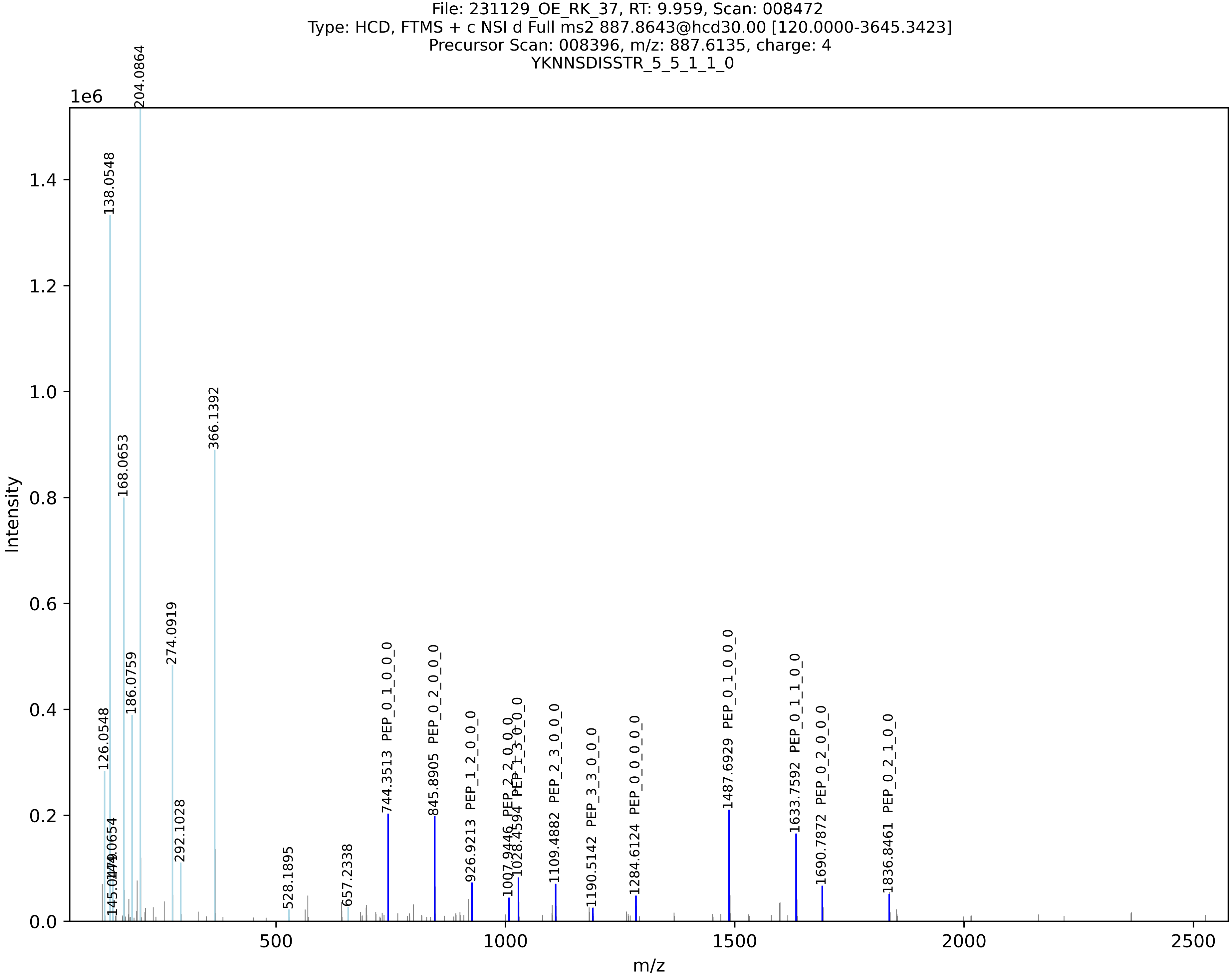

File: 231129_OE_RK_37, RT: 9.959, Scan: 008472
Type: HCD, FTMS + c NSI d Full ms2 887.8643@hcd30.00 [120.0000-3645.3423]
Precursor Scan: 008396, m/z: 887.6135, charge: 4
YKNNSDISSTR_5_5_1_1_0
1e6
Intensity
m/z
204.0864
138.0548
168.0653
366.1392
274.0919
186.0759
126.0548
292.1028
528.1895
657.2338
744.3513 PEP_0_1_0_0_0
845.8905 PEP_0_2_0_0_0
926.9213 PEP_1_2_0_0_0
1007.9446 PEP_2_2_0_0_0
1028.4594 PEP_1_3_0_0_0
1109.4882 PEP_2_3_0_0_0
1190.5142 PEP_3_3_0_0_0
1284.6124 PEP_0_0_0_0_0
1487.6929 PEP_0_1_0_0_0
1633.7592 PEP_0_1_1_0_0
1690.7872 PEP_0_2_0_0_0
1836.8461 PEP_0_2_1_0_0

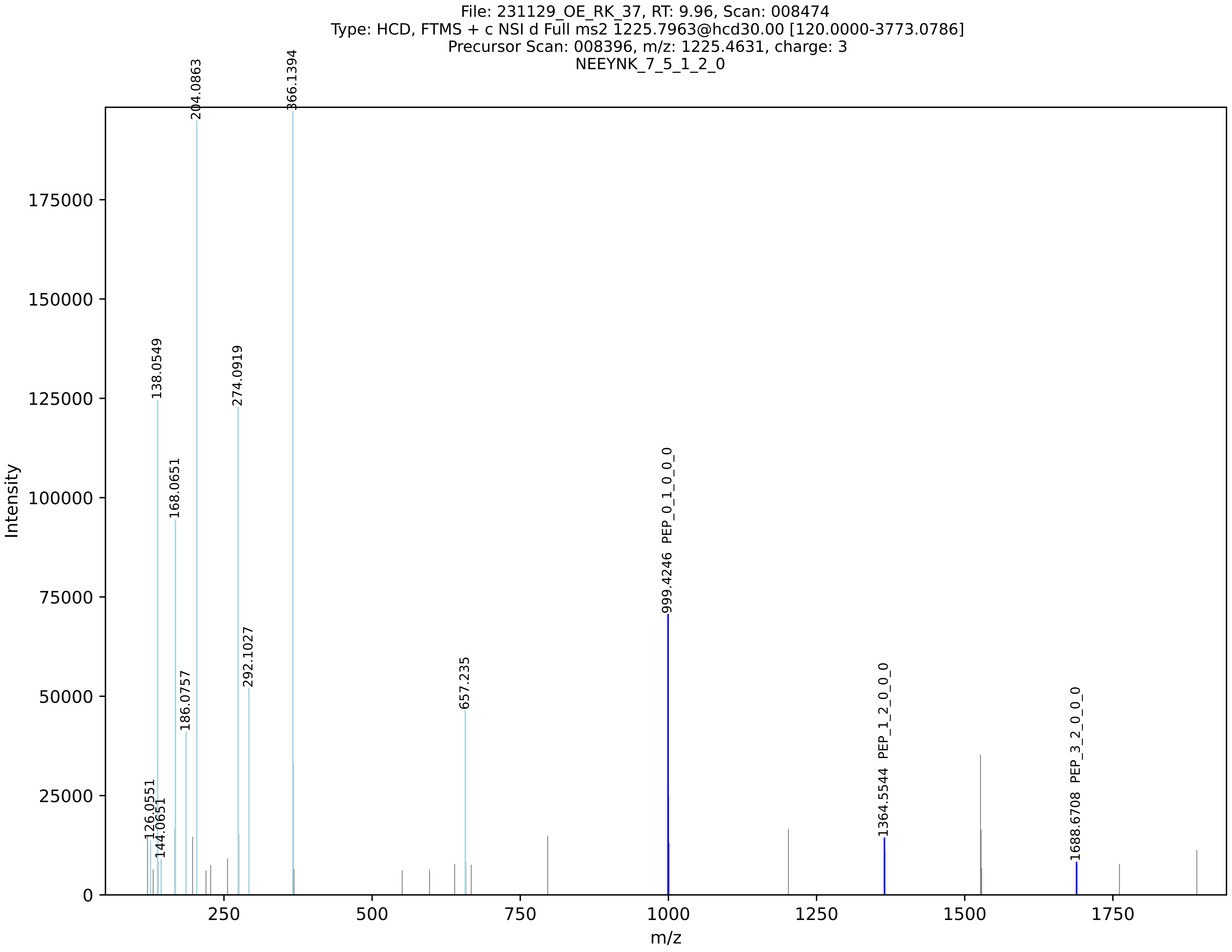

File: 231129_OE_RK_37, RT: 9.96, Scan: 008474
Type: HCD, FTMS + c NSI d Full ms2 1225.7963@hcd30.00 [120.0000-3773.0786]
Precursor Scan: 008396, m/z: 1225.4631, charge: 3
NEEYNK_7_5_1_2_0
Intensity
m/z
0
25000
50000
75000
100000
125000
150000
175000
250
500
750
1000
1250
1500
1750
126.0551
144.0651
138.0549
168.0651
186.0757
204.0863
274.0919
292.1027
366.1394
657.235
999.4246 PEP_0_1_0_0_
1364.5544 PEP_1_2_0_0_0
1688.6708 PEP_3_2_0_0_0

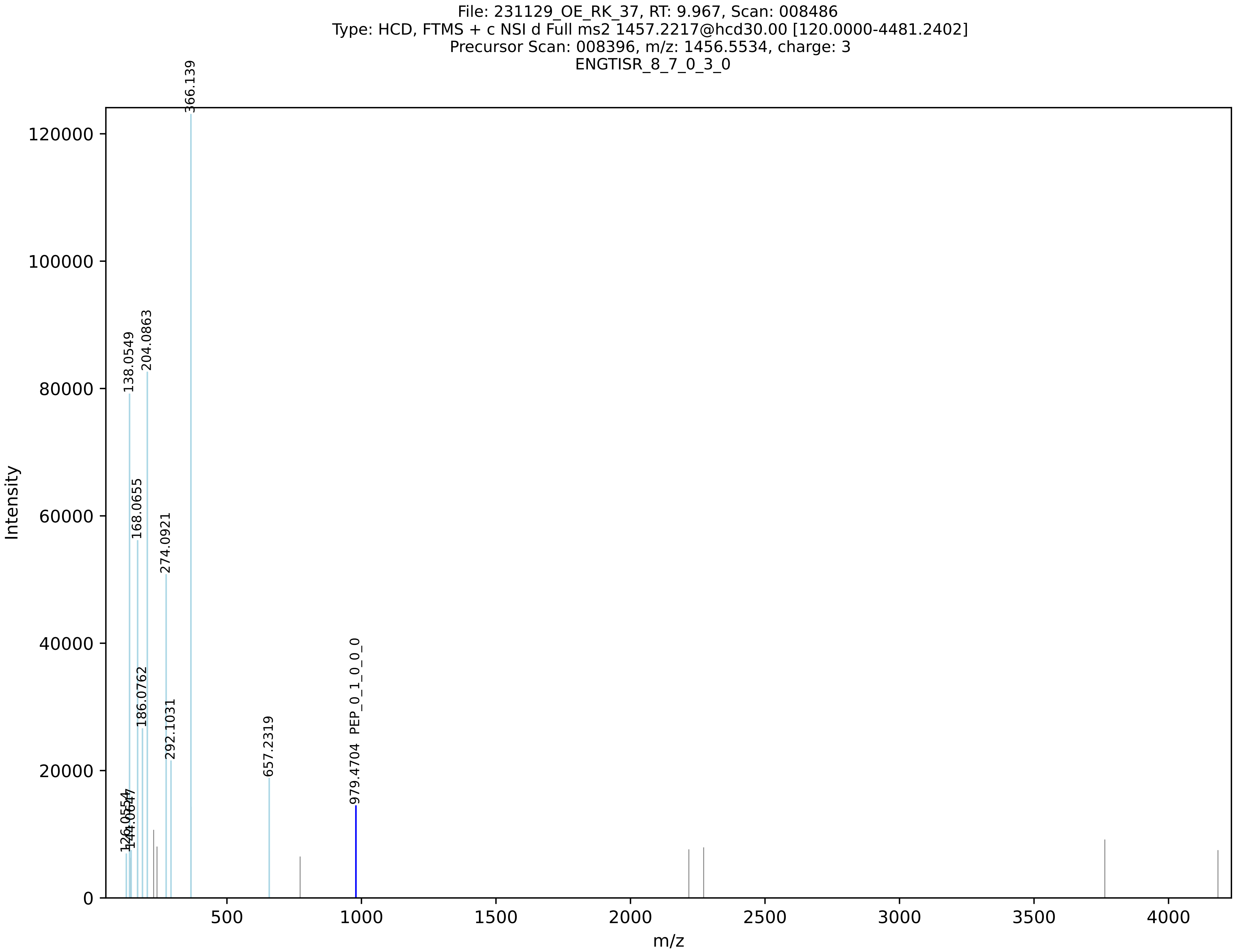

File: 231129_OE_RK_37, RT: 9.967, Scan: 008486
Type: HCD, FTMS + c NSI d Full ms2 1457.2217@hcd30.00 [120.0000-4481.2402]
Precursor Scan: 008396, m/z: 1456.5534, charge: 3
ENGTISR_8_7_0_3_0
Intensity
m/z
366.139
138.0549
204.0863
168.0655
274.0921
186.0762
292.1031
657.2319
979.4704 PEP_0_1_0_0_0_
126.0554
144.0647

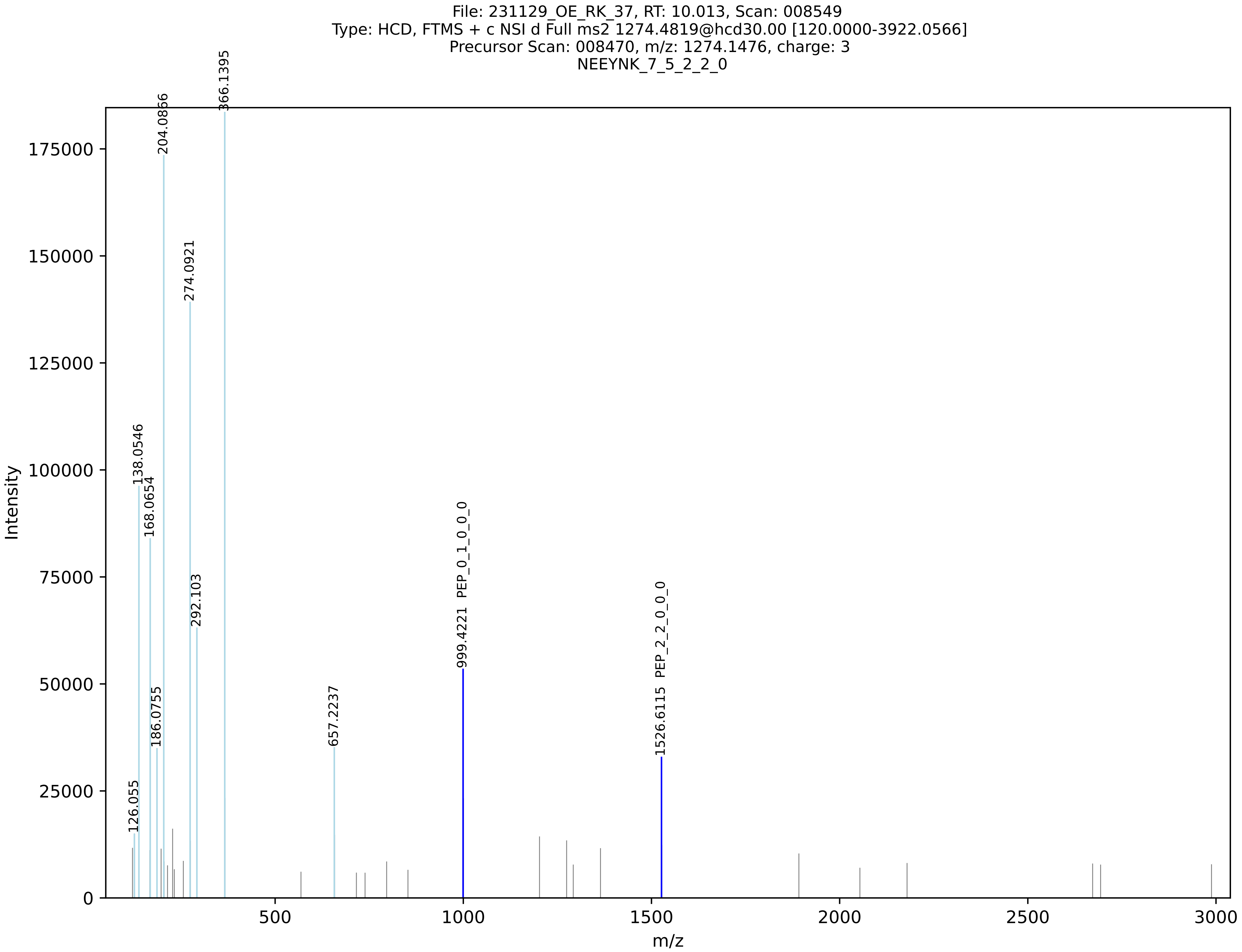
File: 231129_OE_RK_37, RT: 10.013, Scan: 008549
Type: HCD, FTMS + c NSI d Full ms2 1274.4819@hcd30.00 [120.0000-3922.0566]
Precursor Scan: 008470, m/z: 1274.1476, charge: 3
NEEYNK_7_5_2_2_0
Intensity
m/z
366.1395
204.0866
274.0921
138.0546
168.0654
292.103
186.0755
126.055
657.2237
999.4221 PEP_0_1_0_0_0
1526.6115 PEP_2_2_0_0_0
0
25000
50000
75000
100000
125000
150000
175000
500
1000
1500
2000
2500
3000

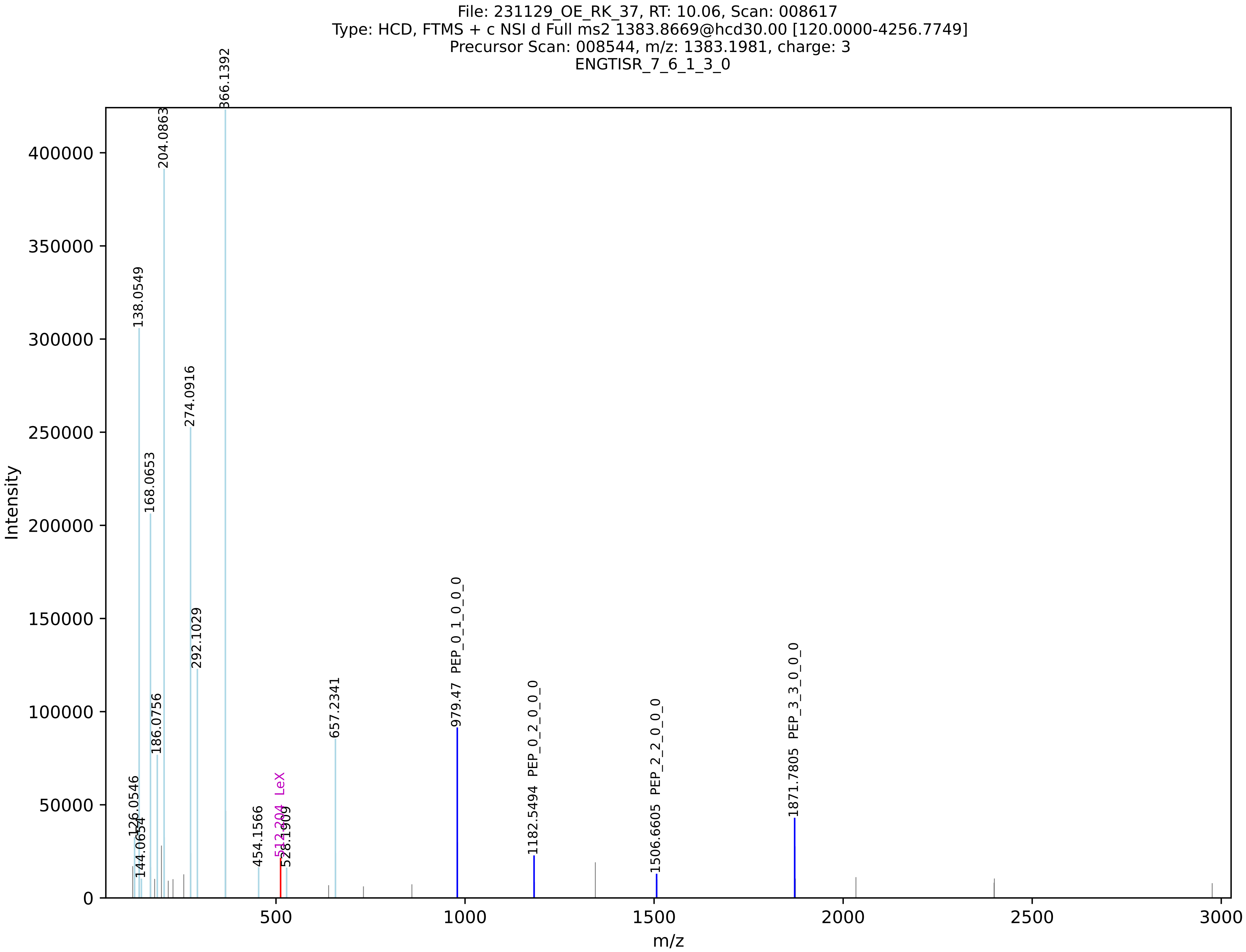

File: 231129_OE_RK_37, RT: 10.06, Scan: 008617
Type: HCD, FTMS + c NSI d Full ms2 1383.8669@hcd30.00 [120.0000-4256.7749]
Precursor Scan: 008544, m/z: 1383.1981, charge: 3
ENGTISR_7_6_1_3_0
366.1392
204.0863
138.0549
274.0916
168.0653
292.1029
186.0756
126.0546
144.0654
454.1566
512.204 LeX
528.1909
657.2341
979.47 PEP_0_1_0_0_0
1182.5494 PEP_0_2_0_0_0
1506.6605 PEP_2_2_0_0_0
1871.7805 PEP_3_3_0_0_0
Intensity
m/z
0
50000
100000
150000
200000
250000
300000
350000
400000
500
1000
1500
2000
2500
3000

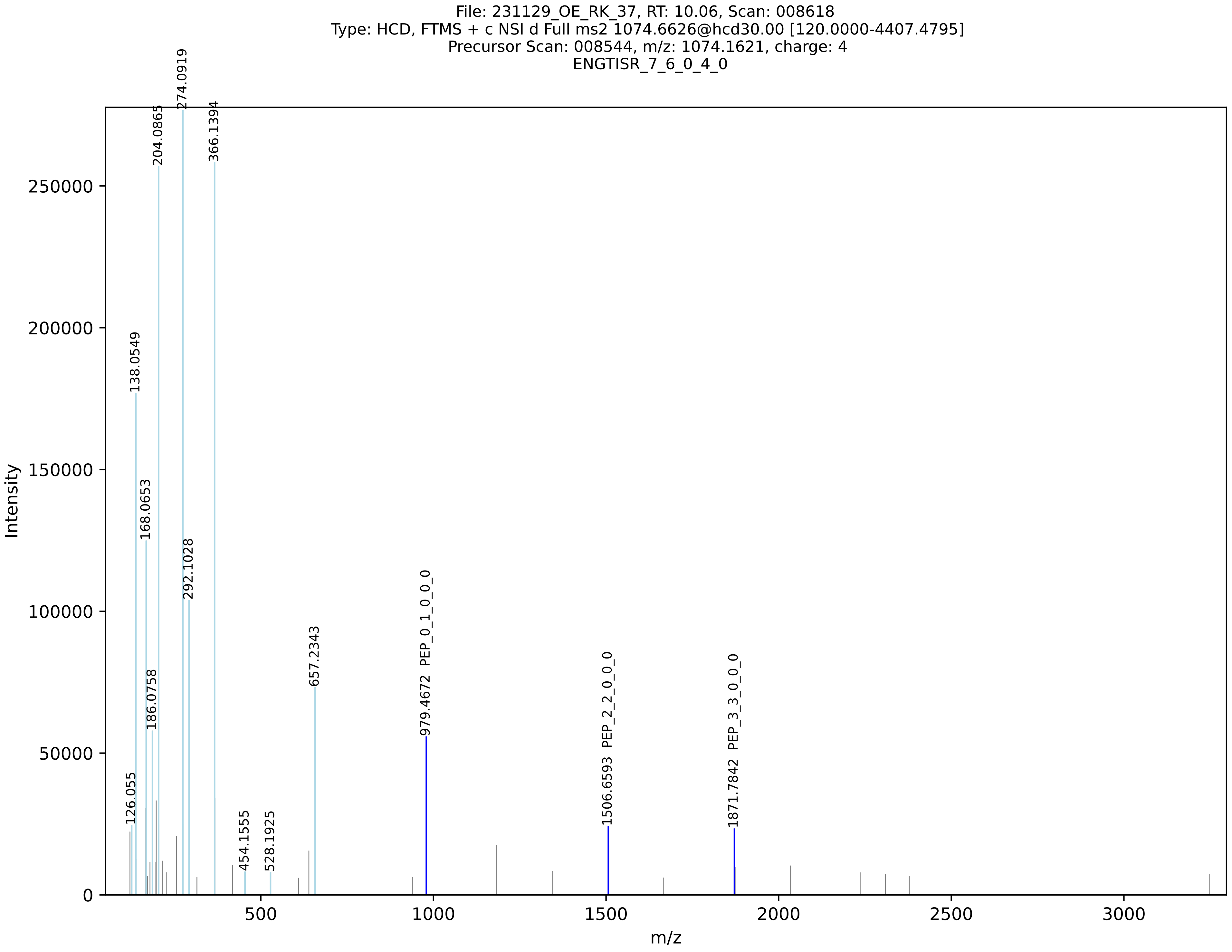
File: 231129_OE_RK_37, RT: 10.06, Scan: 008618
Type: HCD, FTMS + c NSI d Full ms2 1074.6626@hcd30.00 [120.0000-4407.4795]
Precursor Scan: 008544, m/z: 1074.1621, charge: 4
ENGTISR_7_6_0_4_0
274.0919
204.0865
366.1394
138.0549
168.0653
292.1028
186.0758
126.055
454.1555
528.1925
657.2343
979.4672 PEP_0_1_0_0_0
1506.6593 PEP_2_2_0_0_0
1871.7842 PEP_3_3_0_0_0
Intensity
m/z
0
50000
100000
150000
200000
250000
500
1000
1500
2000
2500
3000

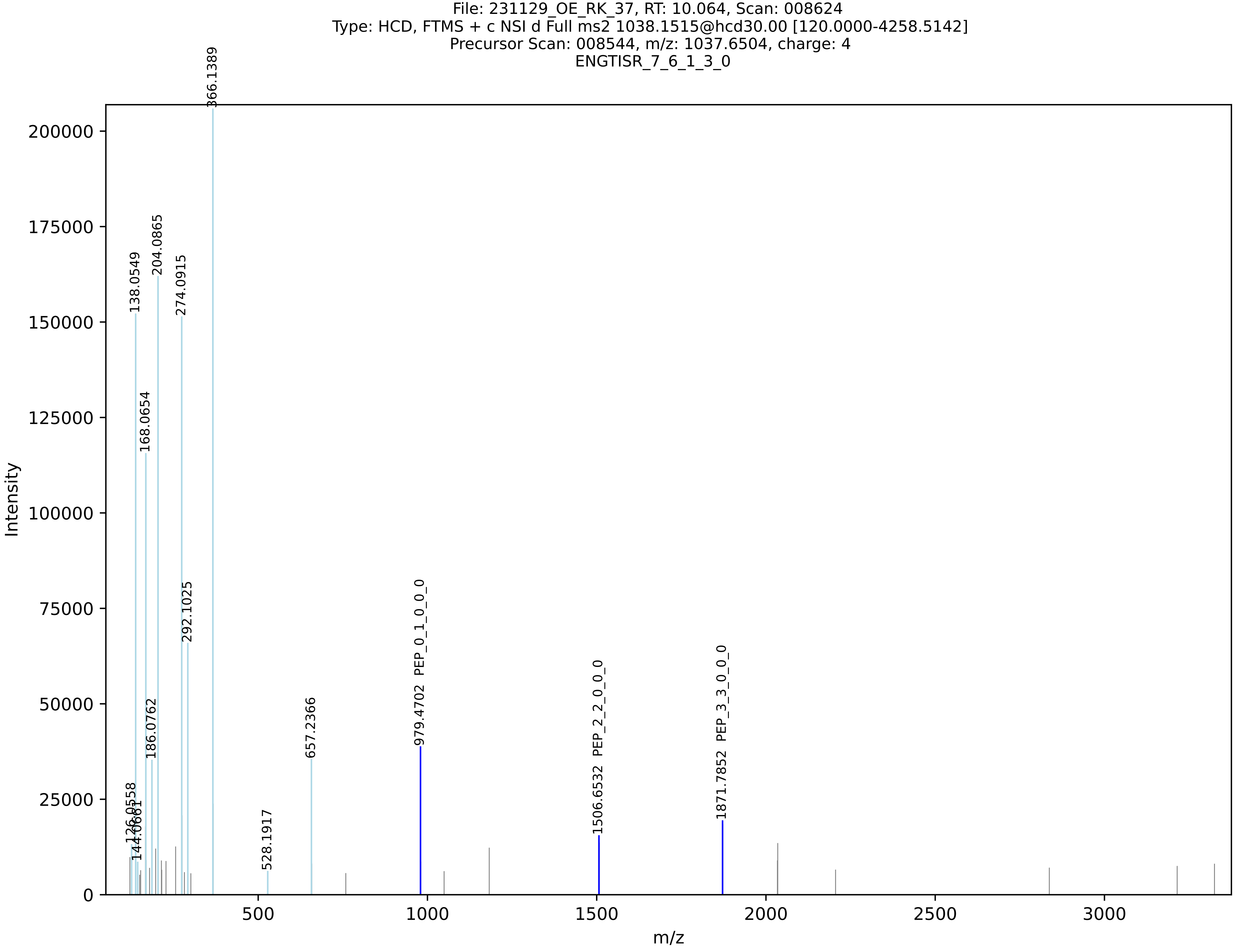

File: 231129_OE_RK_37, RT: 10.064, Scan: 008624
Type: HCD, FTMS + c NSI d Full ms2 1038.1515@hcd30.00 [120.0000-4258.5142]
Precursor Scan: 008544, m/z: 1037.6504, charge: 4
ENGTISR_7_6_1_3_0
366.1389
204.0865
138.0549
274.0915
168.0654
292.1025
186.0762
126.0558
144.0661
528.1917
657.2366
979.4702 PEP_0_1_0_0_0
1506.6532 PEP_2_2_0_0_0
1871.7852 PEP_3_3_0_0_0
Intensity
m/z

File: 231129_OE_RK_37, RT: 10.066, Scan: 008627
Type: HCD, FTMS + c NSI d Full ms2 915.3428@hcd30.00 [120.0000-3757.4543]
Precursor Scan: 008544, m/z: 915.0931, charge: 4
NEEYNK_6_5_0_3_0

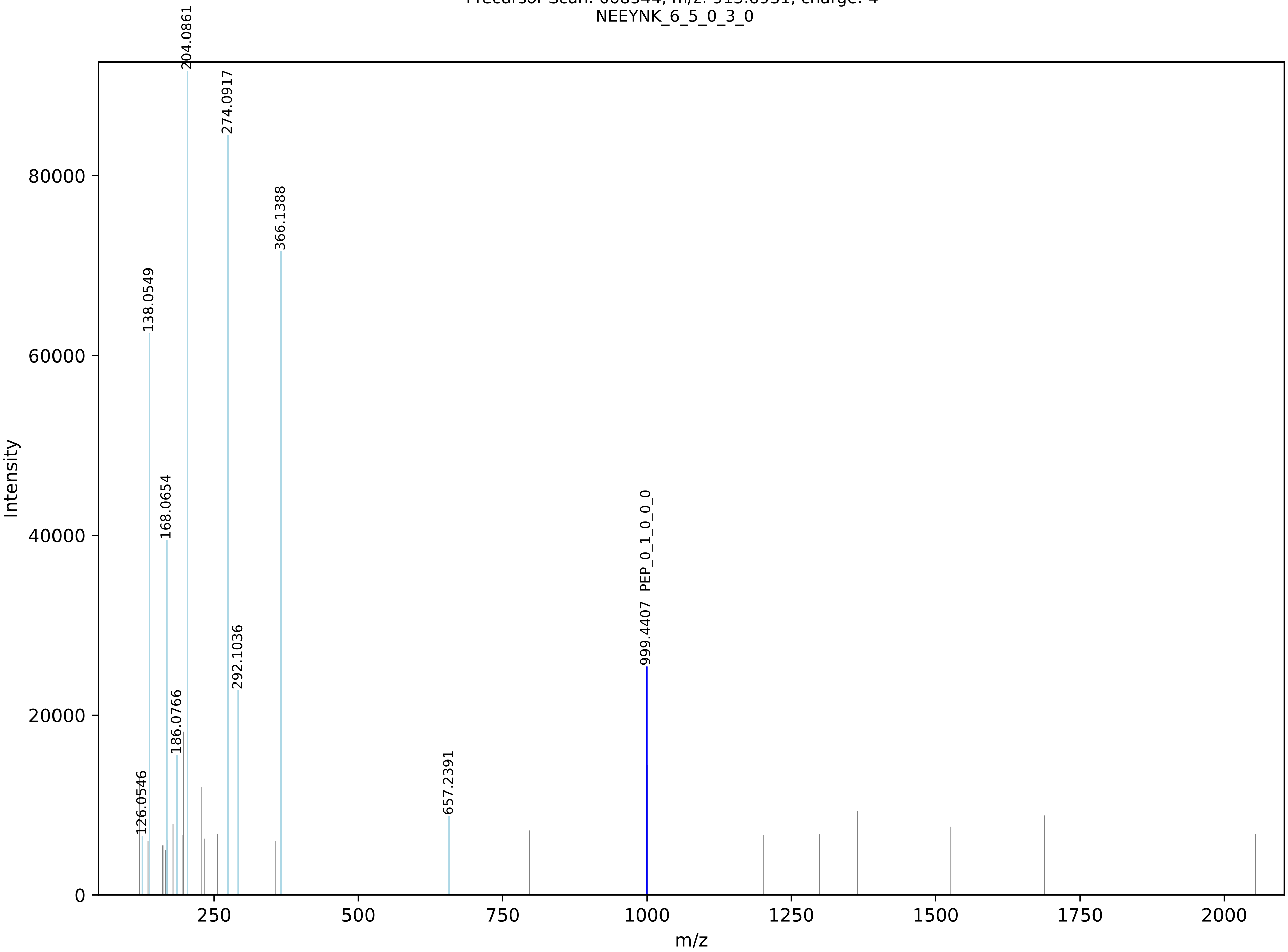

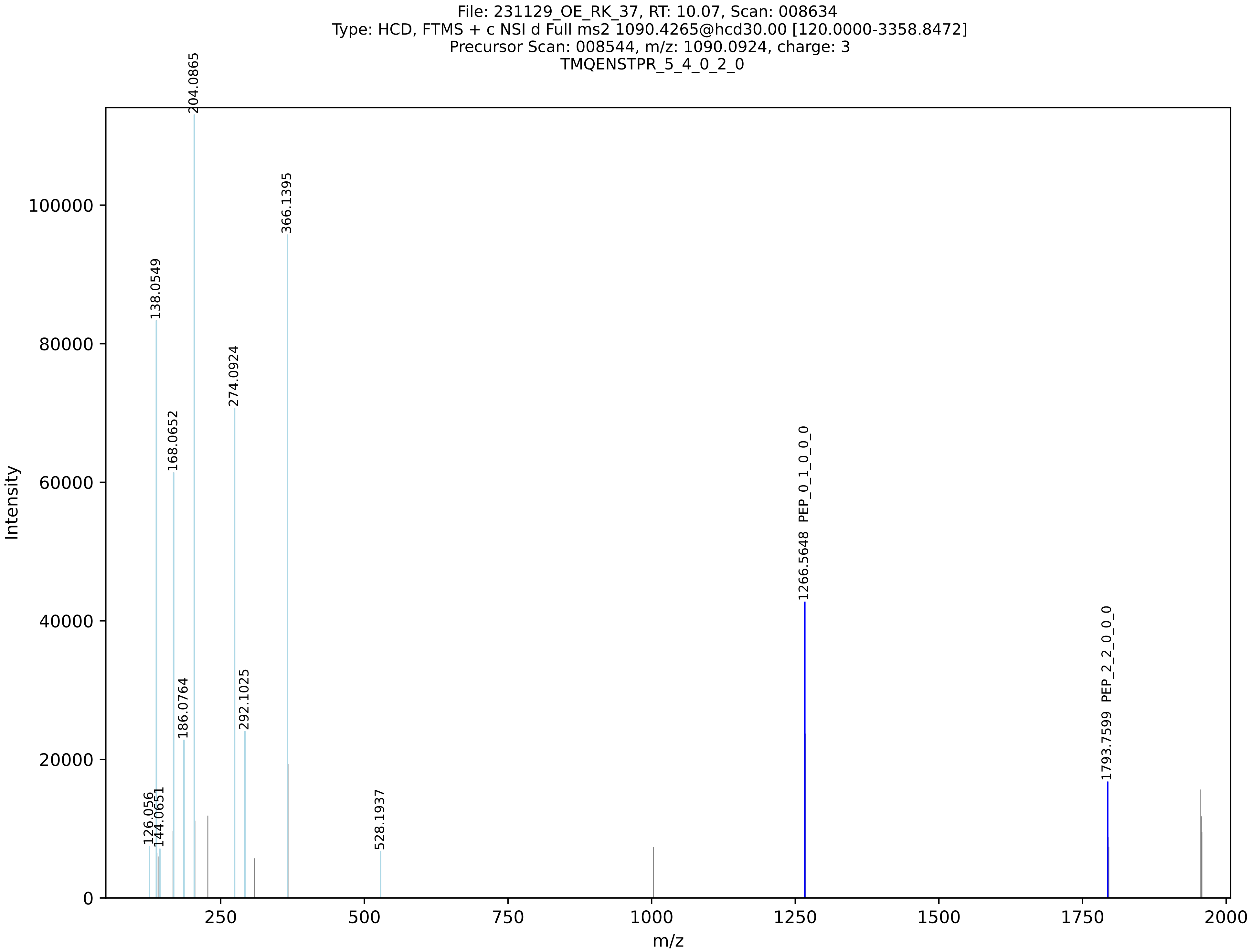
File: 231129_OE_RK_37, RT: 10.07, Scan: 008634
Type: HCD, FTMS + c NSI d Full ms2 1090.4265@hcd30.00 [120.0000-3358.8472]
Precursor Scan: 008544, m/z: 1090.0924, charge: 3
TMQENSTPR_5_4_0_2_0
Intensity
m/z
204.0865
366.1395
138.0549
274.0924
168.0652
186.0764
292.1025
126.056
144.0651
528.1937
1266.5648 PEP_0_1_0_0_0
1793.7599 PEP_2_2_0_0_0
0
20000
40000
60000
80000
100000
250
500
750
1000
1250
1500
1750
2000

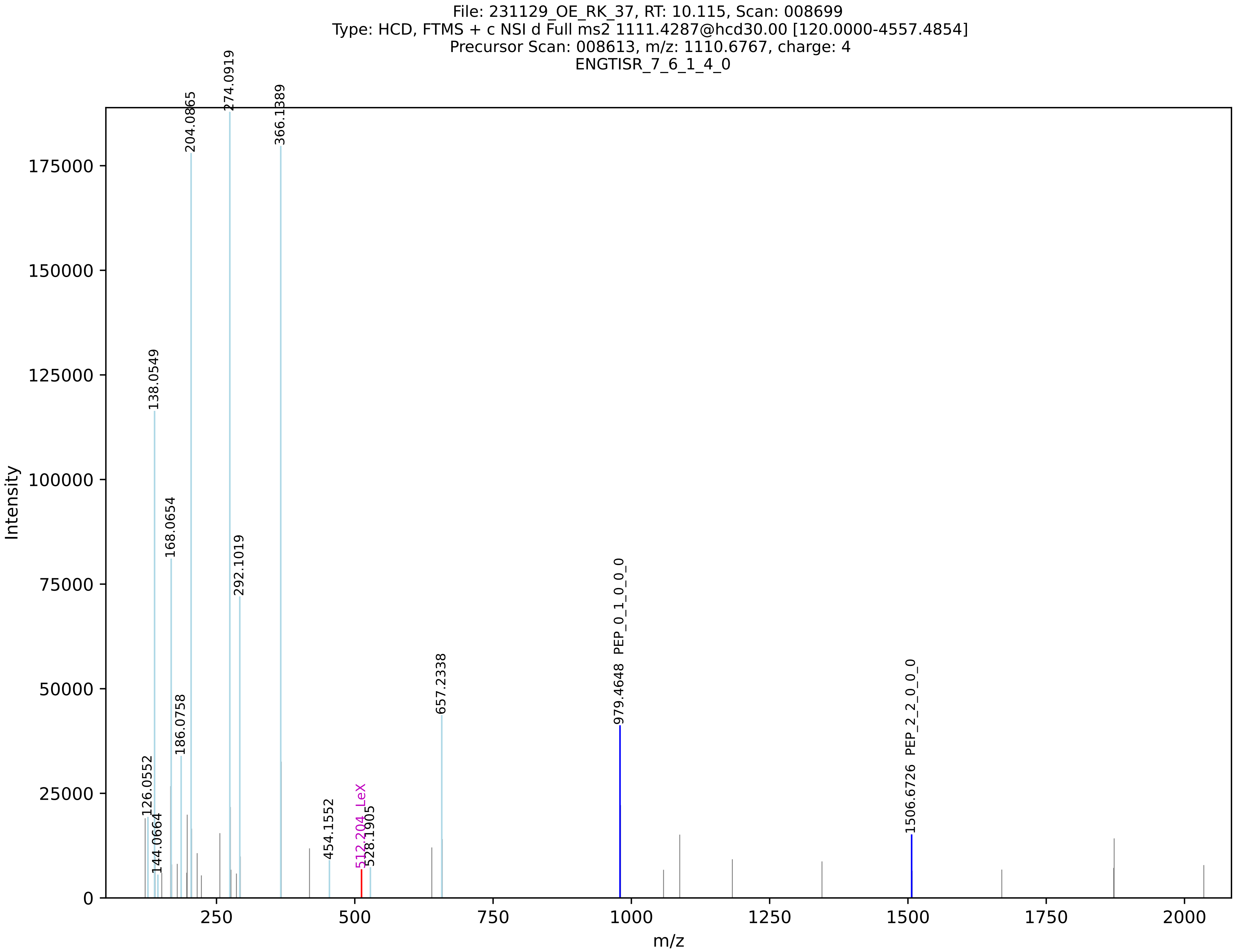
File: 231129_OE_RK_37, RT: 10.115, Scan: 008699
Type: HCD, FTMS + c NSI d Full ms2 1111.4287@hcd30.00 [120.0000-4557.4854]
Precursor Scan: 008613, m/z: 1110.6767, charge: 4
ENGTISR_7_6_1_4_0
Intensity
m/z
274.0919
366.1389
204.0865
138.0549
168.0654
292.1019
186.0758
126.0552
144.0664
454.1552
512.204_LeX
528.1905
657.2338
979.4648 PEP_0_1_0_0_0
1506.6726 PEP_2_2_0_0_0

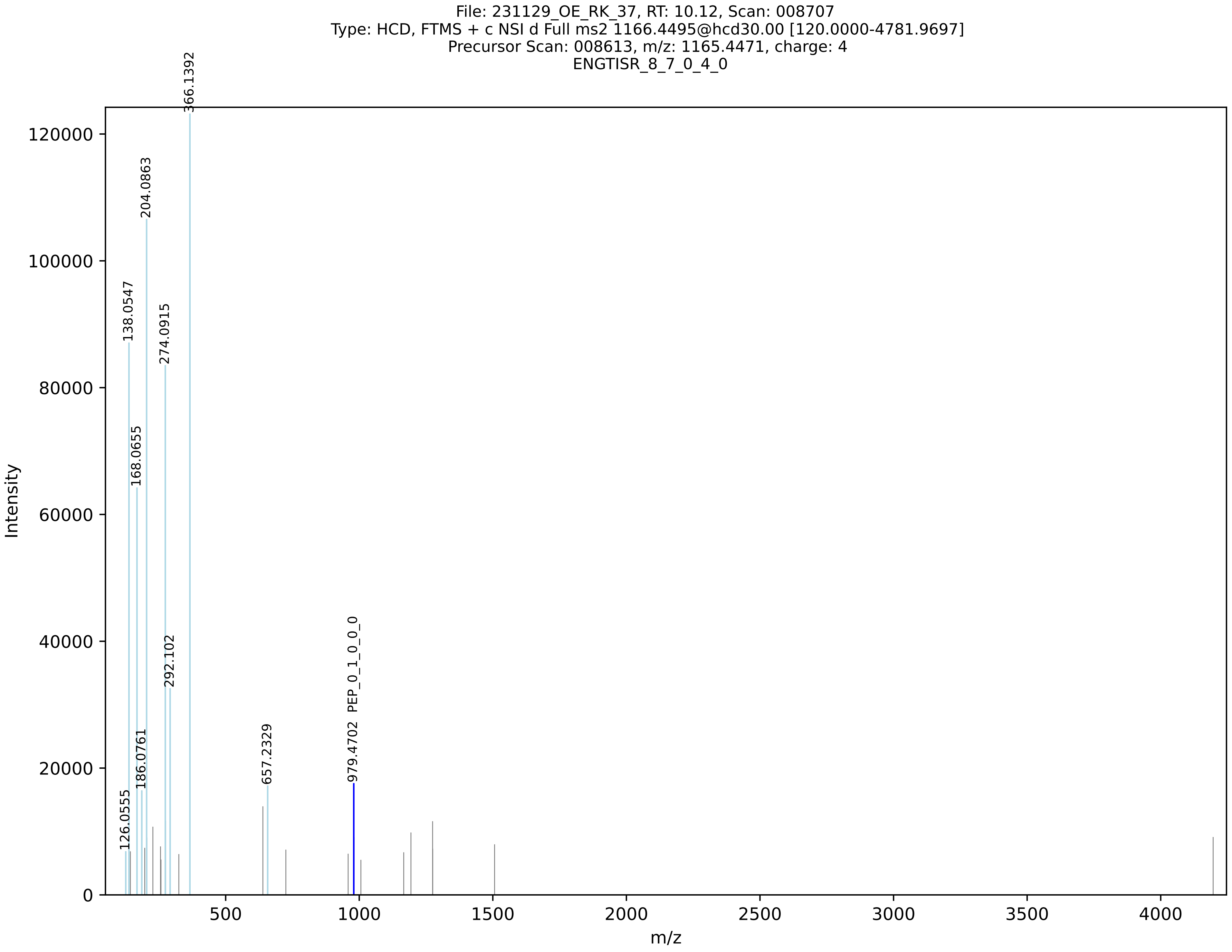
File: 231129_OE_RK_37, RT: 10.12, Scan: 008707
Type: HCD, FTMS + c NSI d Full ms2 1166.4495@hcd30.00 [120.0000-4781.9697]
Precursor Scan: 008613, m/z: 1165.4471, charge: 4
ENGTISR_8_7_0_4_0
366.1392
204.0863
138.0547
274.0915
168.0655
292.102
186.0761
126.0555
657.2329
979.4702 PEP_0_1_0_0_0
Intensity
m/z
0
20000
40000
60000
80000
100000
120000
500
1000
1500
2000
2500
3000
3500
4000

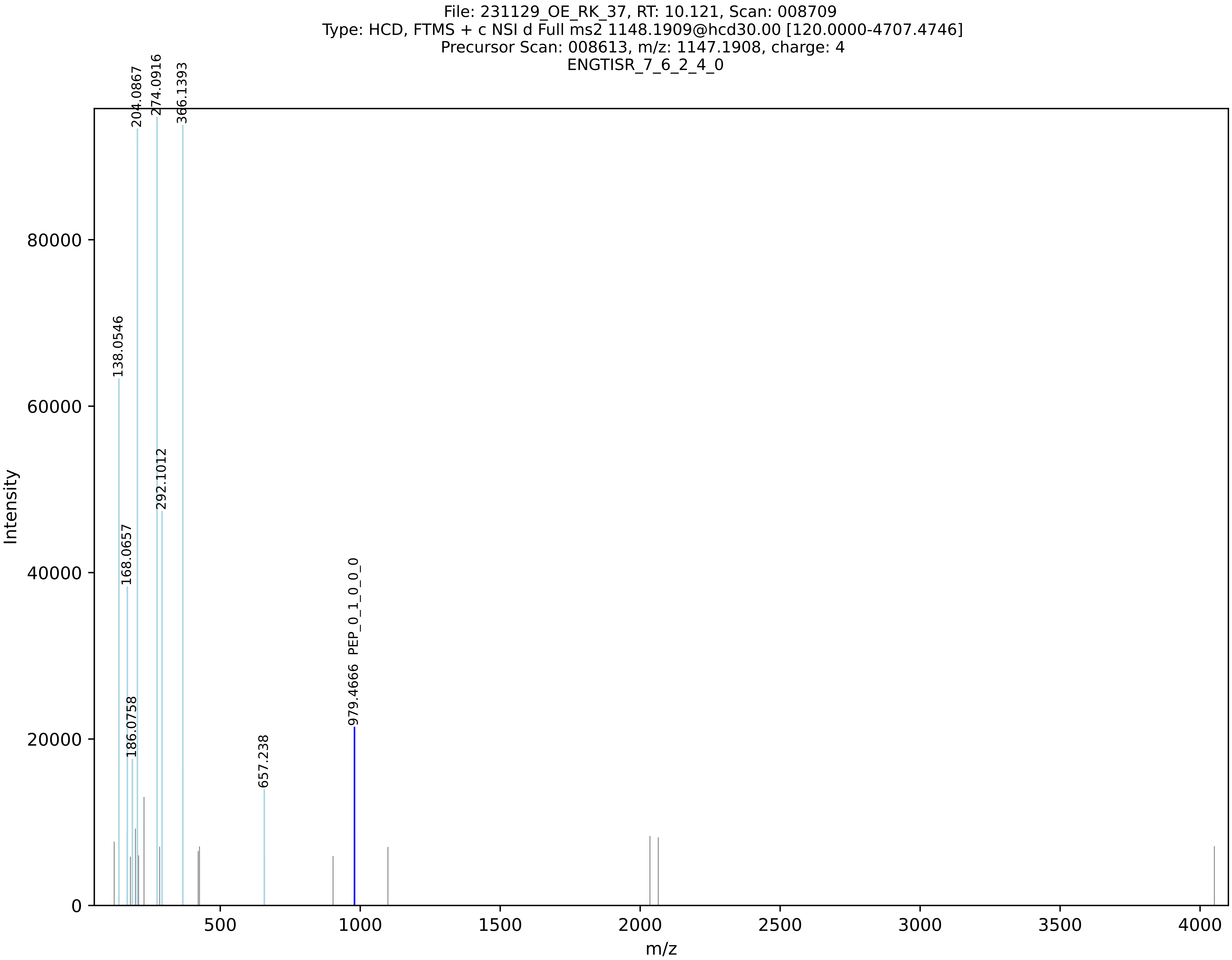
File: 231129_OE_RK_37, RT: 10.121, Scan: 008709
Type: HCD, FTMS + c NSI d Full ms2 1148.1909@hcd30.00 [120.0000-4707.4746]
Precursor Scan: 008613, m/z: 1147.1908, charge: 4
ENGTISR_7_6_2_4_0
204.0867
274.0916
366.1393
138.0546
292.1012
168.0657
186.0758
657.238
979.4666 PEP_0_1_0_0_0
Intensity
m/z
0
20000
40000
60000
80000
500
1000
1500
2000
2500
3000
3500
4000

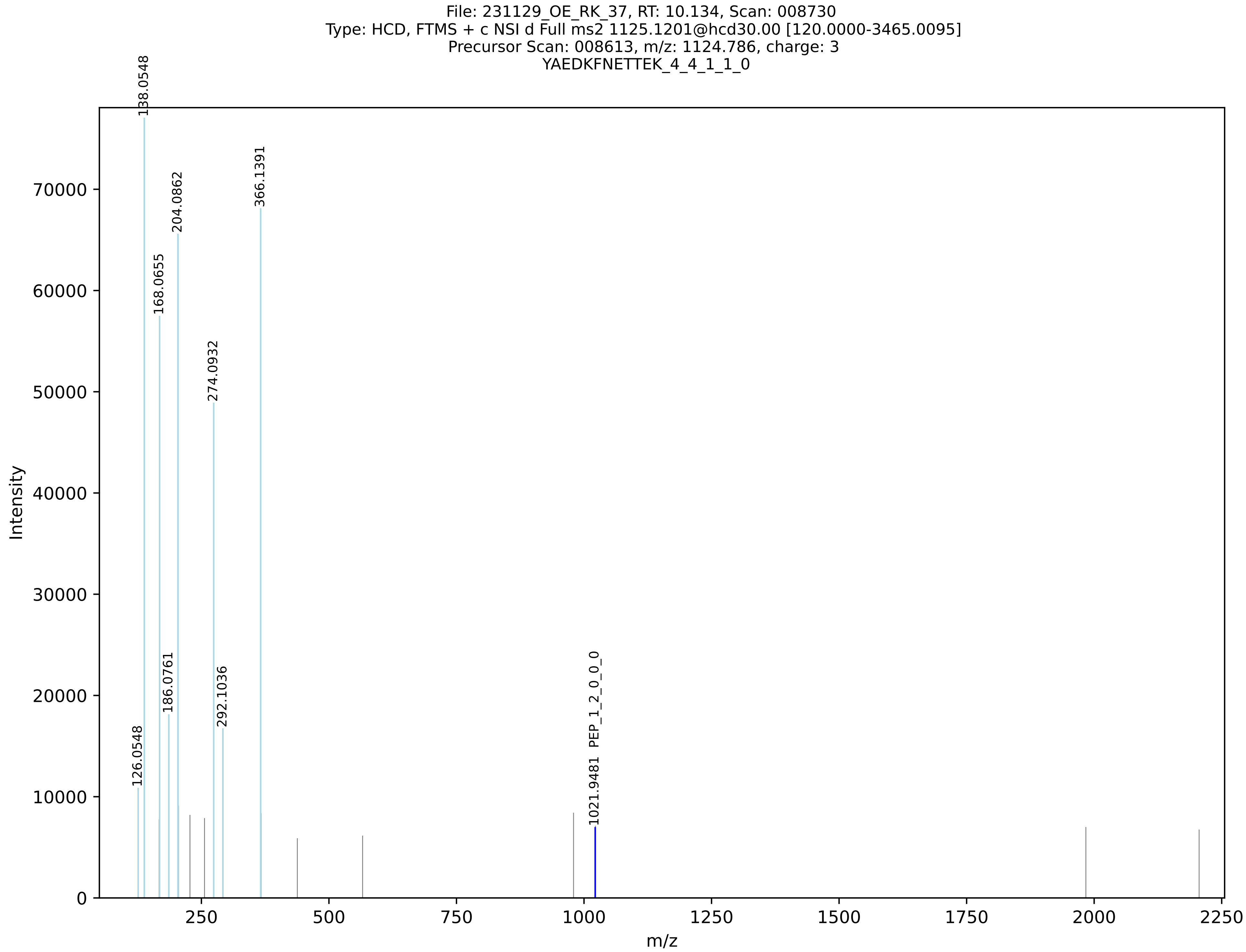
File: 231129_OE_RK_37, RT: 10.134, Scan: 008730
Type: HCD, FTMS + c NSI d Full ms2 1125.1201@hcd30.00 [120.0000-3465.0095]
Precursor Scan: 008613, m/z: 1124.786, charge: 3
YAEDKFNETTEK_4_4_1_1_0
Intensity
m/z
138.0548
366.1391
204.0862
168.0655
274.0932
186.0761
292.1036
126.0548
1021.9481 PEP_1_2_0_0_0
0
10000
20000
30000
40000
50000
60000
70000
250
500
750
1000
1250
1500
1750
2000
2250

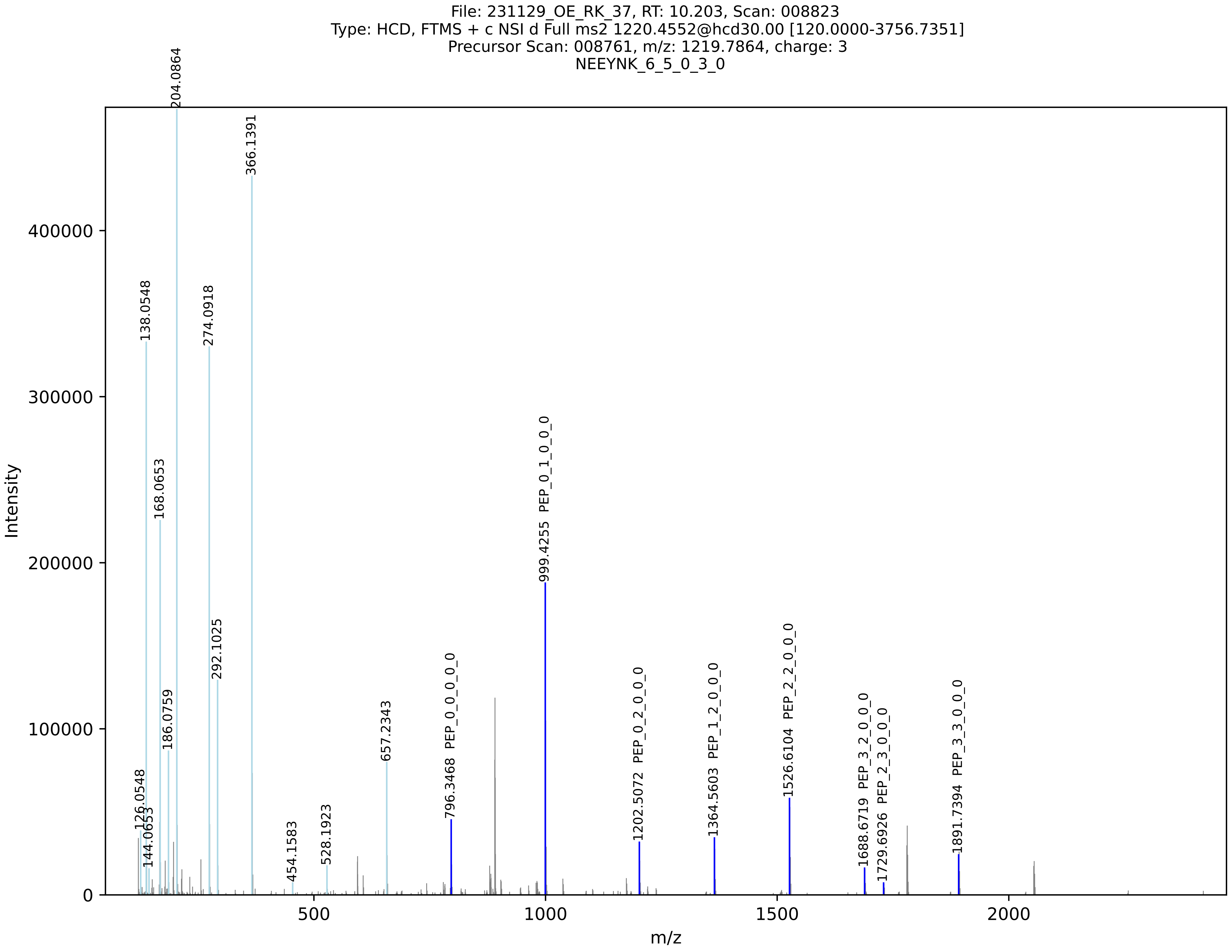

File: 231129_OE_RK_37, RT: 10.203, Scan: 008823
Type: HCD, FTMS + c NSI d Full ms2 1220.4552@hcd30.00 [120.0000-3756.7351]
Precursor Scan: 008761, m/z: 1219.7864, charge: 3
NEEYNK_6_5_0_3_0
204.0864
366.1391
138.0548
274.0918
168.0653
292.1025
186.0759
126.0548
144.0653
454.1583
528.1923
657.2343
796.3468 PEP_0_0_0_0_0
999.4255 PEP_0_1_0_0_0
1202.5072 PEP_0_2_0_0_0
1364.5603 PEP_1_2_0_0_0
1526.6104 PEP_2_2_0_0_0
1688.6719 PEP_3_2_0_0_0
1729.6926 PEP_2_3_0_0_0
1891.7394 PEP_3_3_0_0_0
Intensity
m/z
0
100000
200000
300000
400000
500
1000
1500
2000

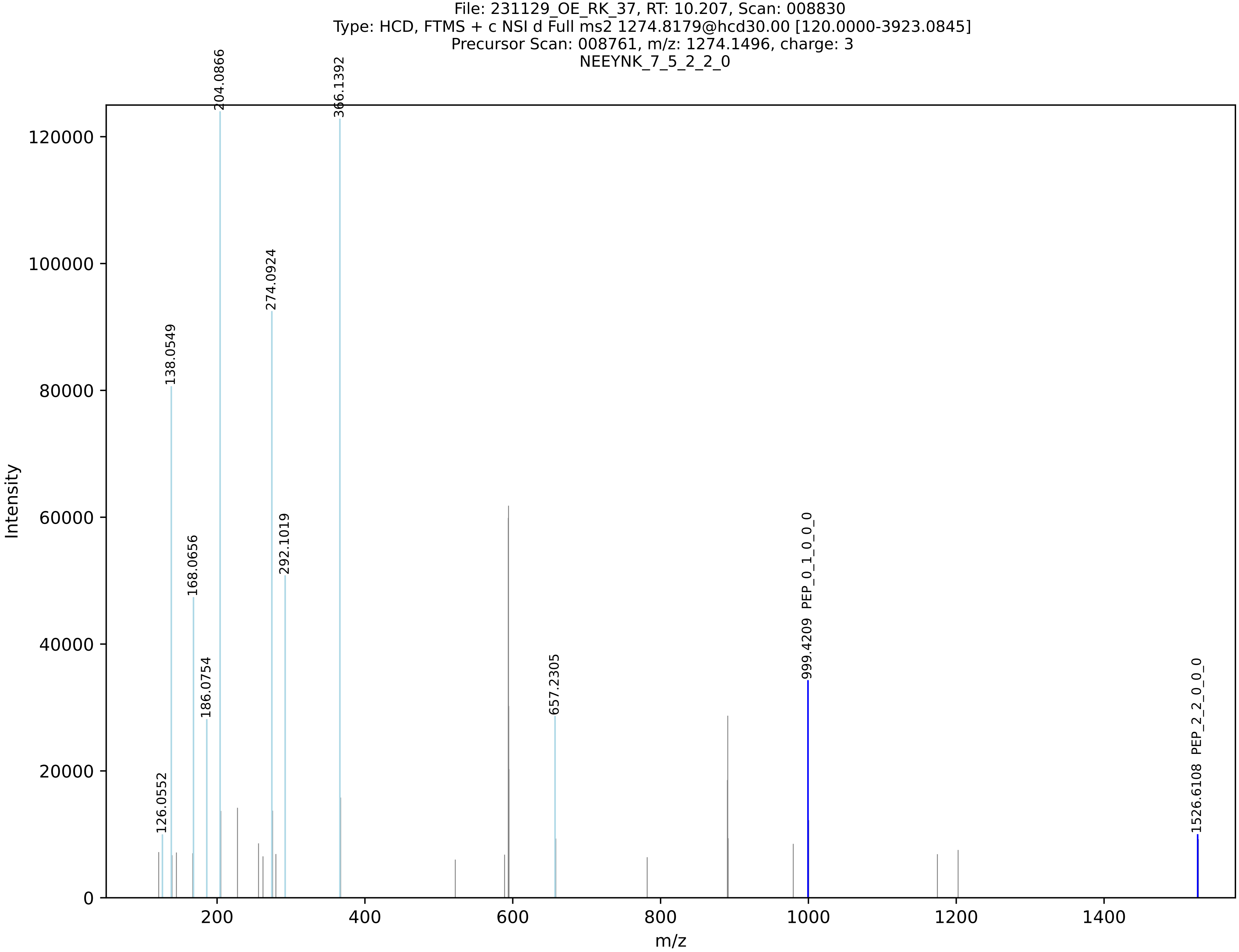
File: 231129_OE_RK_37, RT: 10.207, Scan: 008830
Type: HCD, FTMS + c NSI d Full ms2 1274.8179@hcd30.00 [120.0000-3923.0845]
Precursor Scan: 008761, m/z: 1274.1496, charge: 3
NEEYNK_7_5_2_2_0
Intensity
m/z
0
20000
40000
60000
80000
100000
120000
200
400
600
800
1000
1200
1400
126.0552
138.0549
168.0656
186.0754
204.0866
274.0924
292.1019
366.1392
657.2305
999.4209 PEP_0_1_0_0_0
1526.6108 PEP_2_2_0_0_0

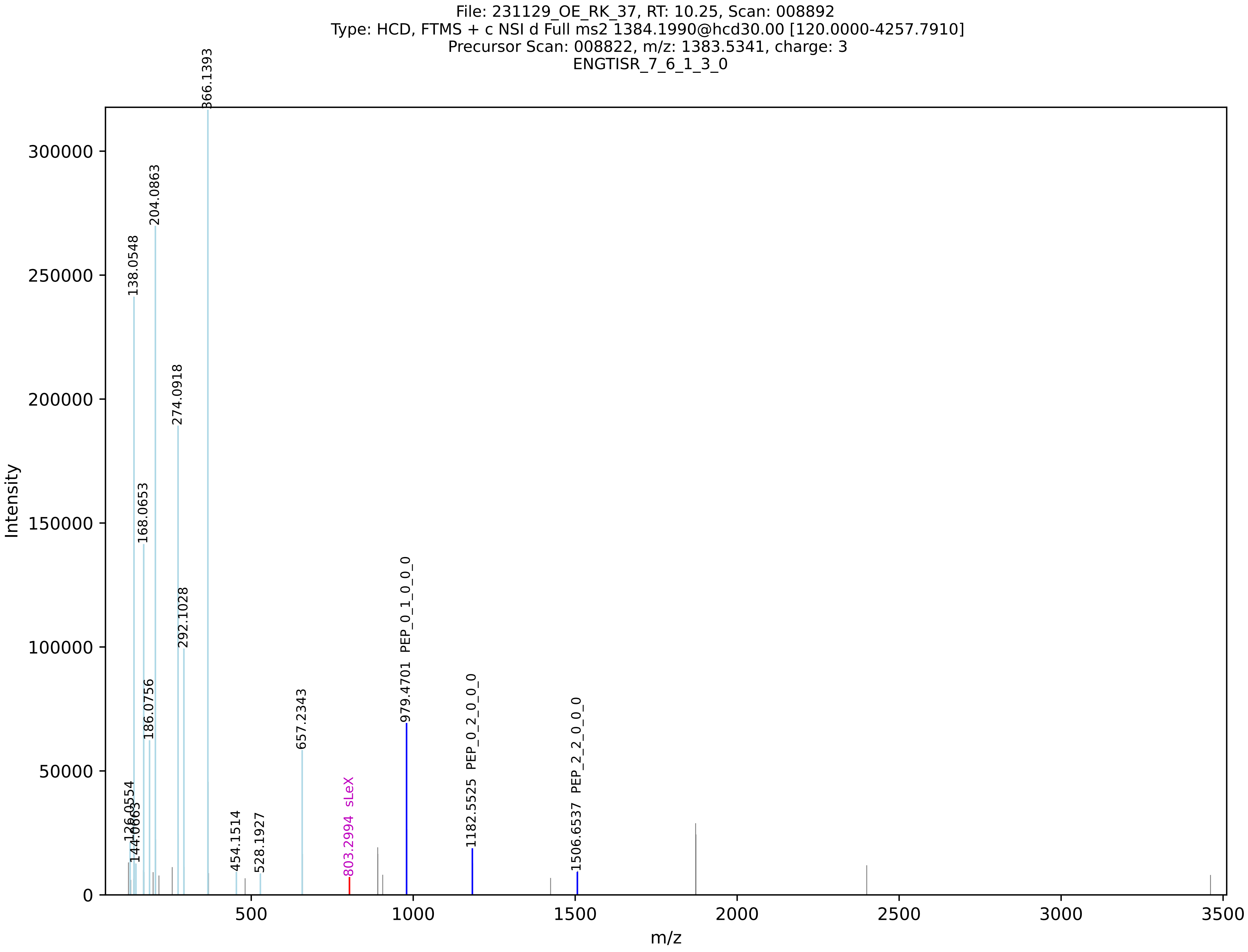
File: 231129_OE_RK_37, RT: 10.25, Scan: 008892
Type: HCD, FTMS + c NSI d Full ms2 1384.1990@hcd30.00 [120.0000-4257.7910]
Precursor Scan: 008822, m/z: 1383.5341, charge: 3
ENGTISR_7_6_1_3_0
366.1393
204.0863
138.0548
274.0918
168.0653
292.1028
186.0756
126.0554
144.0663
454.1514
528.1927
657.2343
803.2994 sLeX
979.4701 PEP_0_1_0_0_0
1182.5525 PEP_0_2_0_0_0
1506.6537 PEP_2_2_0_0_0
Intensity
m/z
0
50000
100000
150000
200000
250000
300000
500
1000
1500
2000
2500
3000
3500

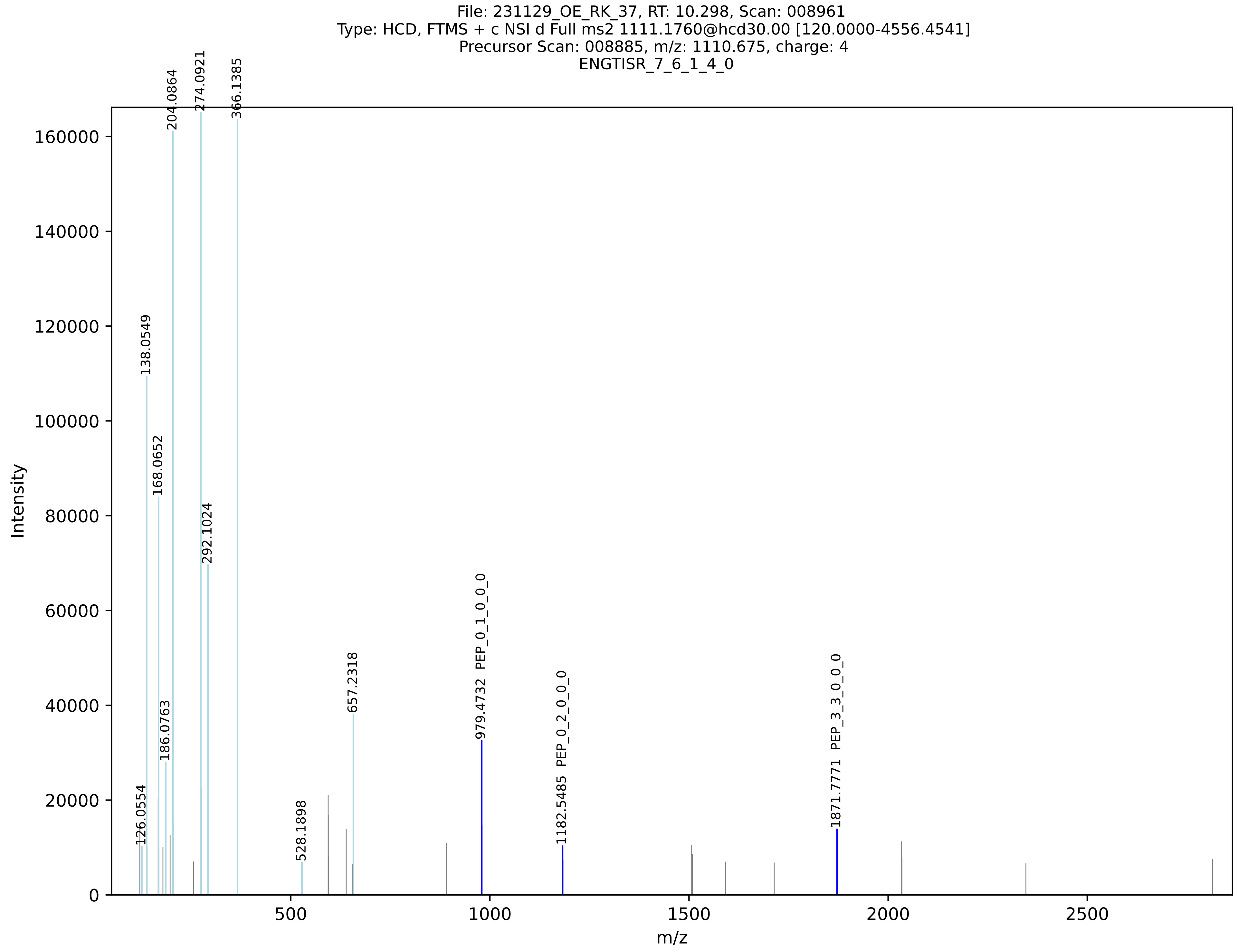
File: 231129_OE_RK_37, RT: 10.298, Scan: 008961
Type: HCD, FTMS + c NSI d Full ms2 1111.1760@hcd30.00 [120.0000-4556.4541]
Precursor Scan: 008885, m/z: 1110.675, charge: 4
ENGTISR_7_6_1_4_0
Intensity
m/z
0
20000
40000
60000
80000
100000
120000
140000
160000
500
1000
1500
2000
2500
126.0554
138.0549
168.0652
186.0763
204.0864
274.0921
292.1024
366.1385
528.1898
657.2318
979.4732 PEP_0_1_0_0_0
1182.5485 PEP_0_2_0_0_0
1871.7771 PEP_3_3_0_0_0

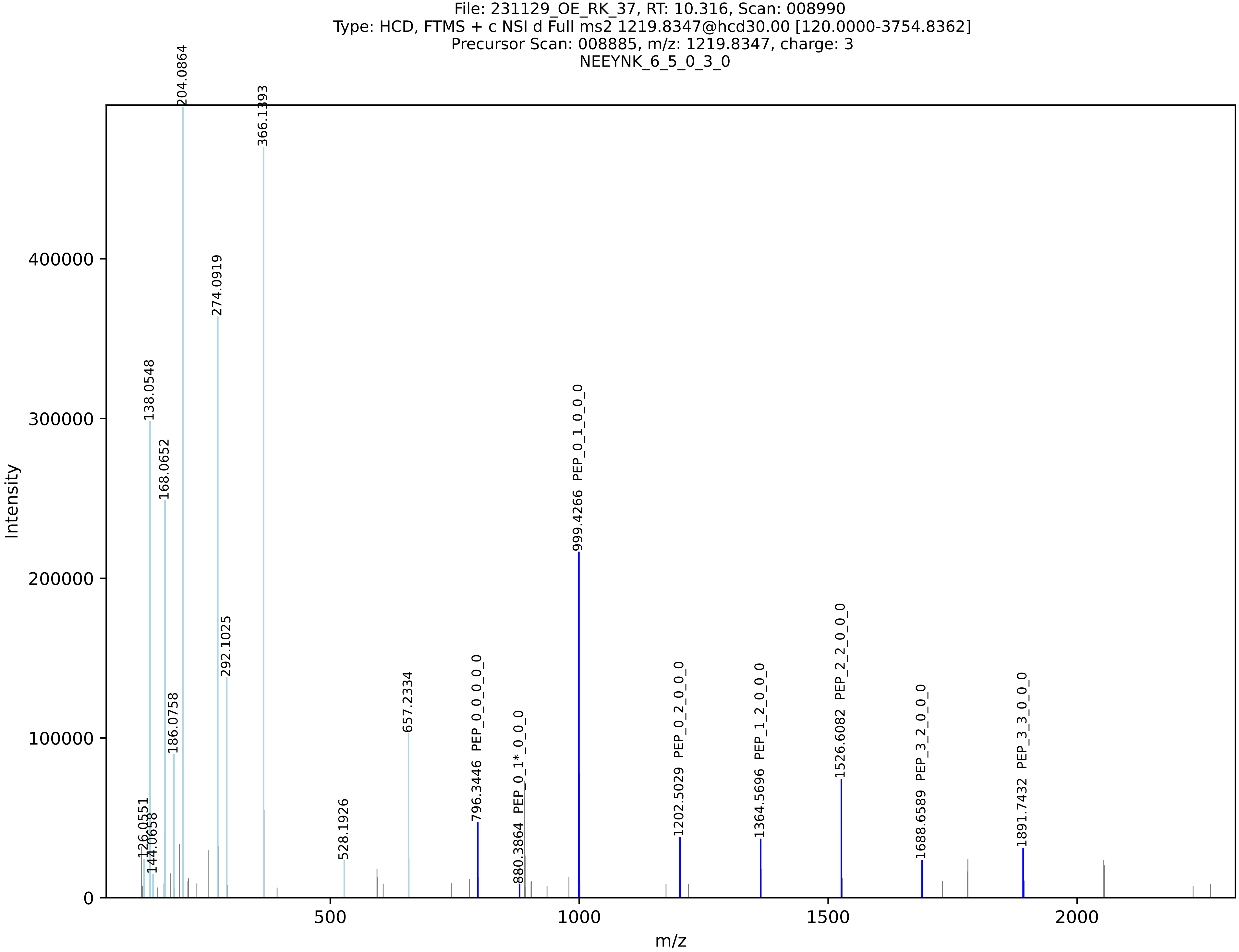
File: 231129_OE_RK_37, RT: 10.316, Scan: 008990
Type: HCD, FTMS + c NSI d Full ms2 1219.8347@hcd30.00 [120.0000-3754.8362]
Precursor Scan: 008885, m/z: 1219.8347, charge: 3
NEEYNK_6_5_0_3_0
Intensity
m/z
204.0864
366.1393
274.0919
138.0548
168.0652
186.0758
292.1025
126.0551
144.0658
528.1926
657.2334
796.3446 PEP_0_0_0_0_0
880.3864 PEP_0_1*_0_0_0
999.4266 PEP_0_1_0_0_0
1202.5029 PEP_0_2_0_0_0
1364.5696 PEP_1_2_0_0_0
1526.6082 PEP_2_2_0_0_0
1688.6589 PEP_3_2_0_0_0
1891.7432 PEP_3_3_0_0_0

File: 231129_OE_RK_37, RT: 10.339, Scan: 009020
Type: HCD, FTMS + c NSI d Full ms2 1335.1786@hcd30.00 [120.0000-4107.7886]
Precursor Scan: 008954, m/z: 1334.8445, charge: 3
ENGTISR_7_6_0_3_0

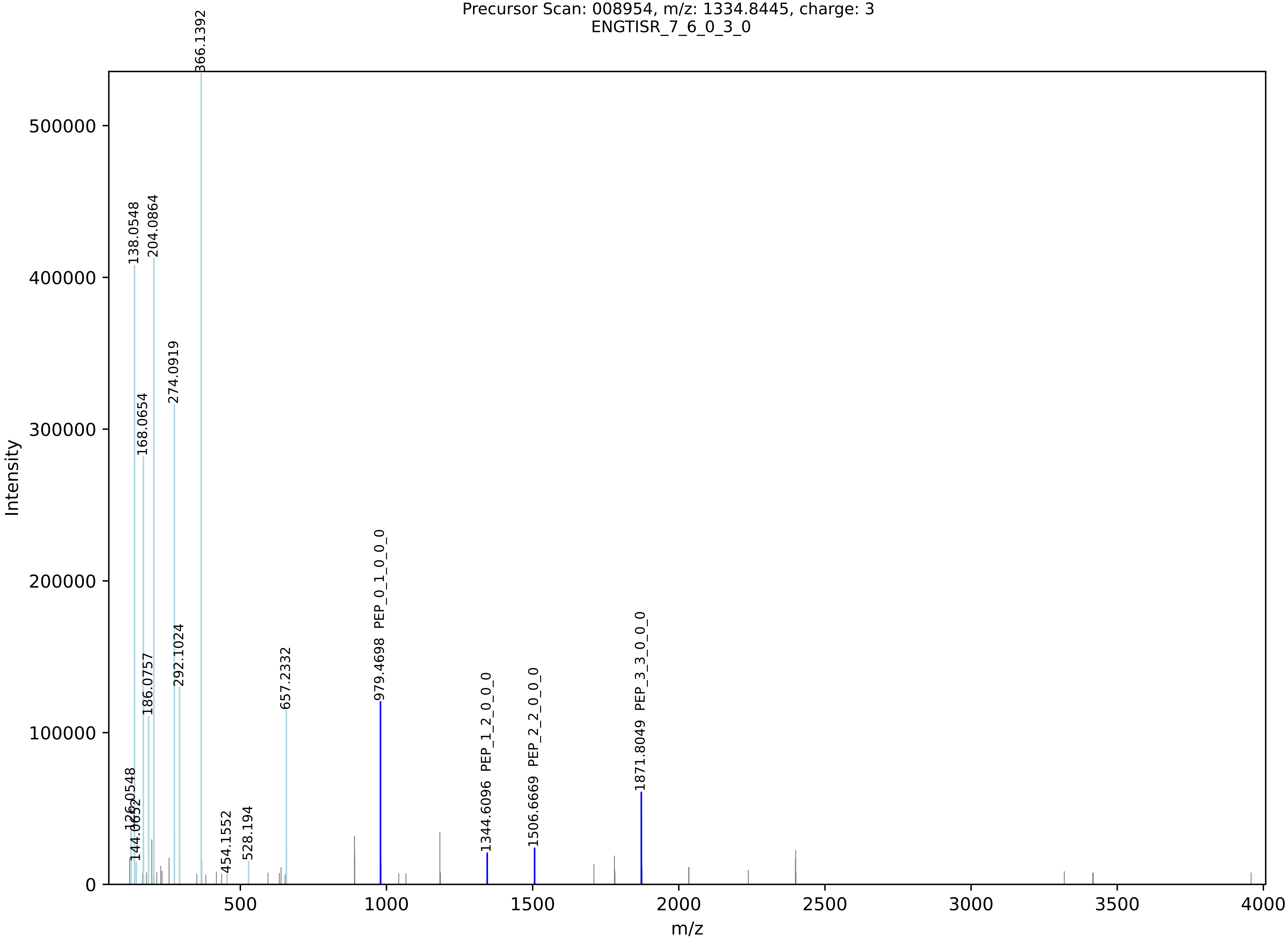

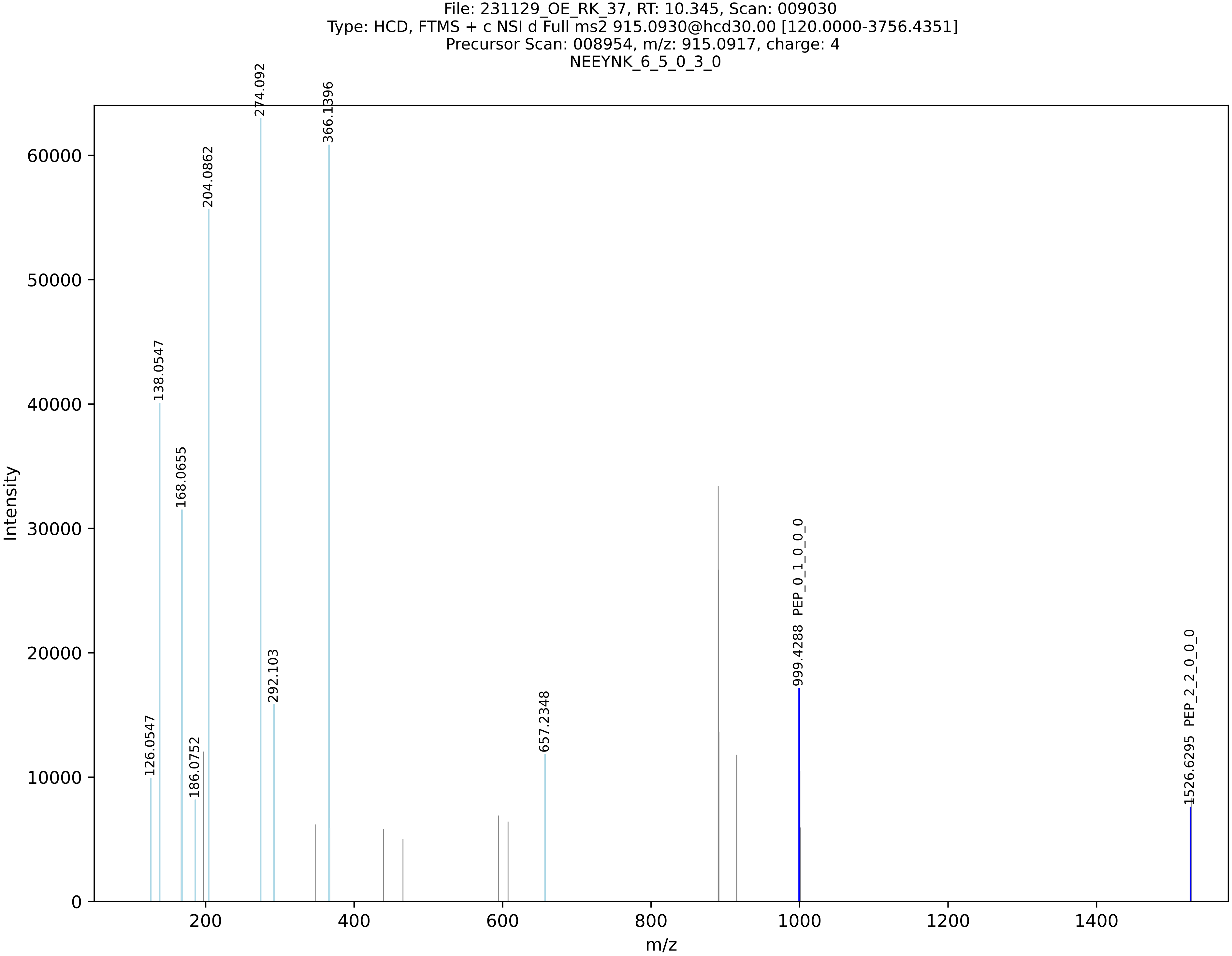

File: 231129_OE_RK_37, RT: 10.345, Scan: 009030
Type: HCD, FTMS + c NSI d Full ms2 915.0930@hcd30.00 [120.0000-3756.4351]
Precursor Scan: 008954, m/z: 915.0917, charge: 4
NEEYNK_6_5_0_3_0
Intensity
m/z
274.092
366.1396
204.0862
138.0547
168.0655
292.103
126.0547
186.0752
657.2348
999.4288 PEP_0_1_0_0_0
1526.6295 PEP_2_2_0_0_0

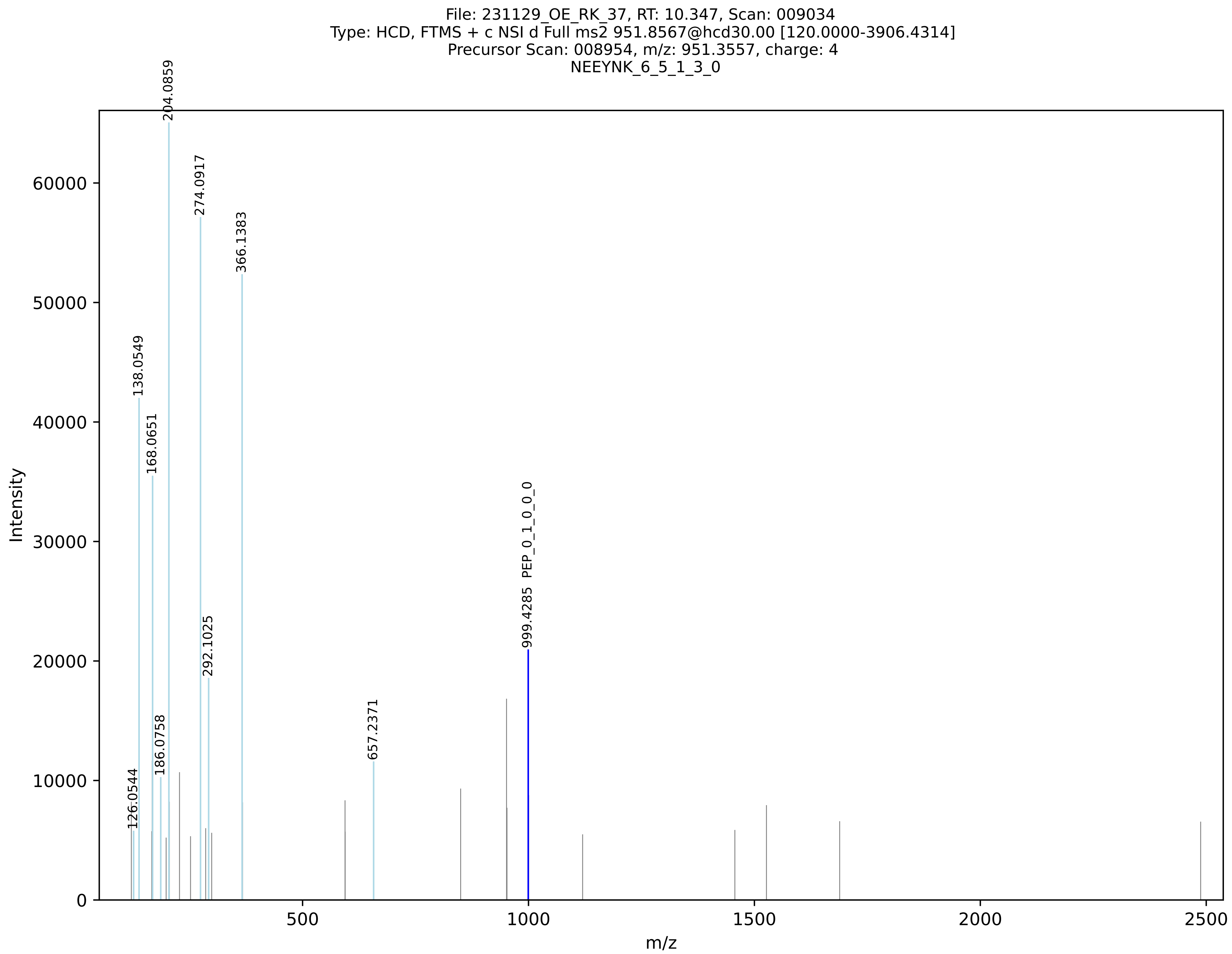
File: 231129_OE_RK_37, RT: 10.347, Scan: 009034
Type: HCD, FTMS + c NSI d Full ms2 951.8567@hcd30.00 [120.0000-3906.4314]
Precursor Scan: 008954, m/z: 951.3557, charge: 4
NEEYNK_6_5_1_3_0
Intensity
m/z
204.0859
274.0917
366.1383
138.0549
168.0651
292.1025
186.0758
126.0544
657.2371
999.4285 PEP_0_1_0_0_0_
0
10000
20000
30000
40000
50000
60000
500
1000
1500
2000
2500

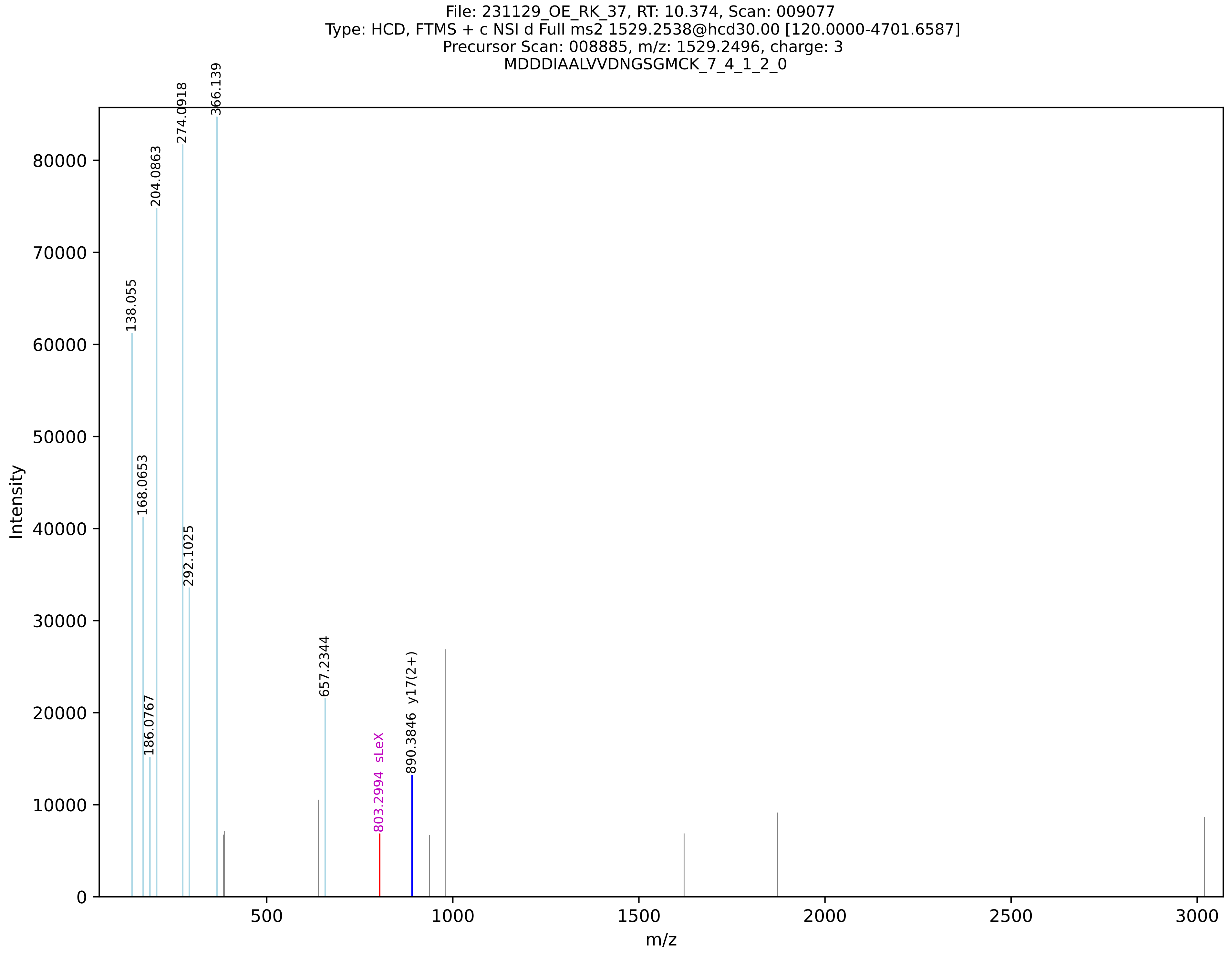
File: 231129_OE_RK_37, RT: 10.374, Scan: 009077
Type: HCD, FTMS + c NSI d Full ms2 1529.2538@hcd30.00 [120.0000-4701.6587]
Precursor Scan: 008885, m/z: 1529.2496, charge: 3
MDDDIAALVVDNGSGMCK_7_4_1_2_0
Intensity
m/z
138.055
168.0653
186.0767
204.0863
274.0918
292.1025
366.139
657.2344
803.2994 sLeX
890.3846 y17(2+)
0
10000
20000
30000
40000
50000
60000
70000
80000
500
1000
1500
2000
2500
3000

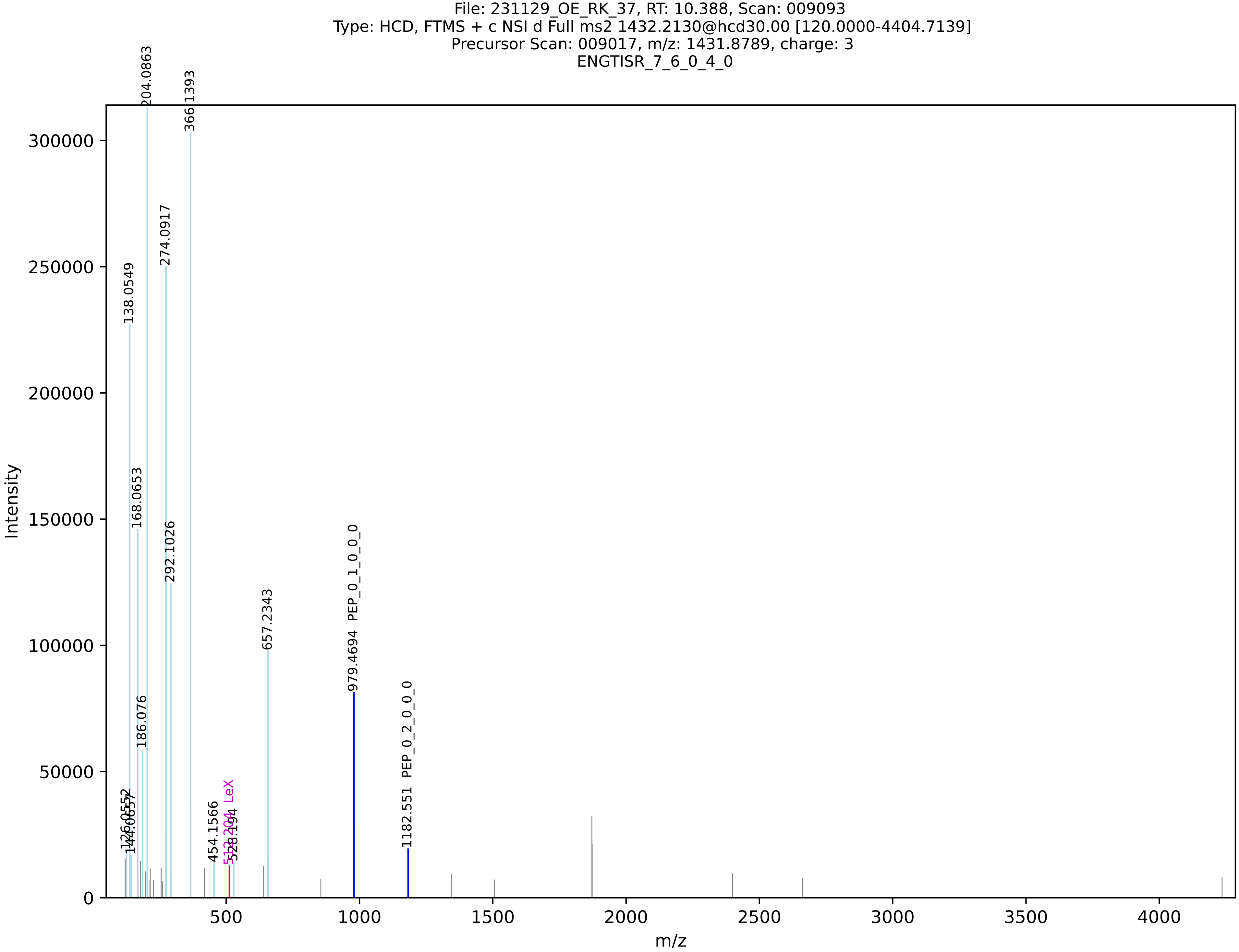
File: 231129_OE_RK_37, RT: 10.388, Scan: 009093
Type: HCD, FTMS + c NSI d Full ms2 1432.2130@hcd30.00 [120.0000-4404.7139]
Precursor Scan: 009017, m/z: 1431.8789, charge: 3
ENGTISR_7_6_0_4_0
Intensity
m/z
0
50000
100000
150000
200000
250000
300000
500
1000
1500
2000
2500
3000
3500
4000
204.0863
366.1393
274.0917
138.0549
168.0653
292.1026
657.2343
979.4694 PEP_0_1_0_0_0
1182.551 PEP_0_2_0_0_0
186.076
126.0552
144.0657
454.1566
512.204 LeX
528.194

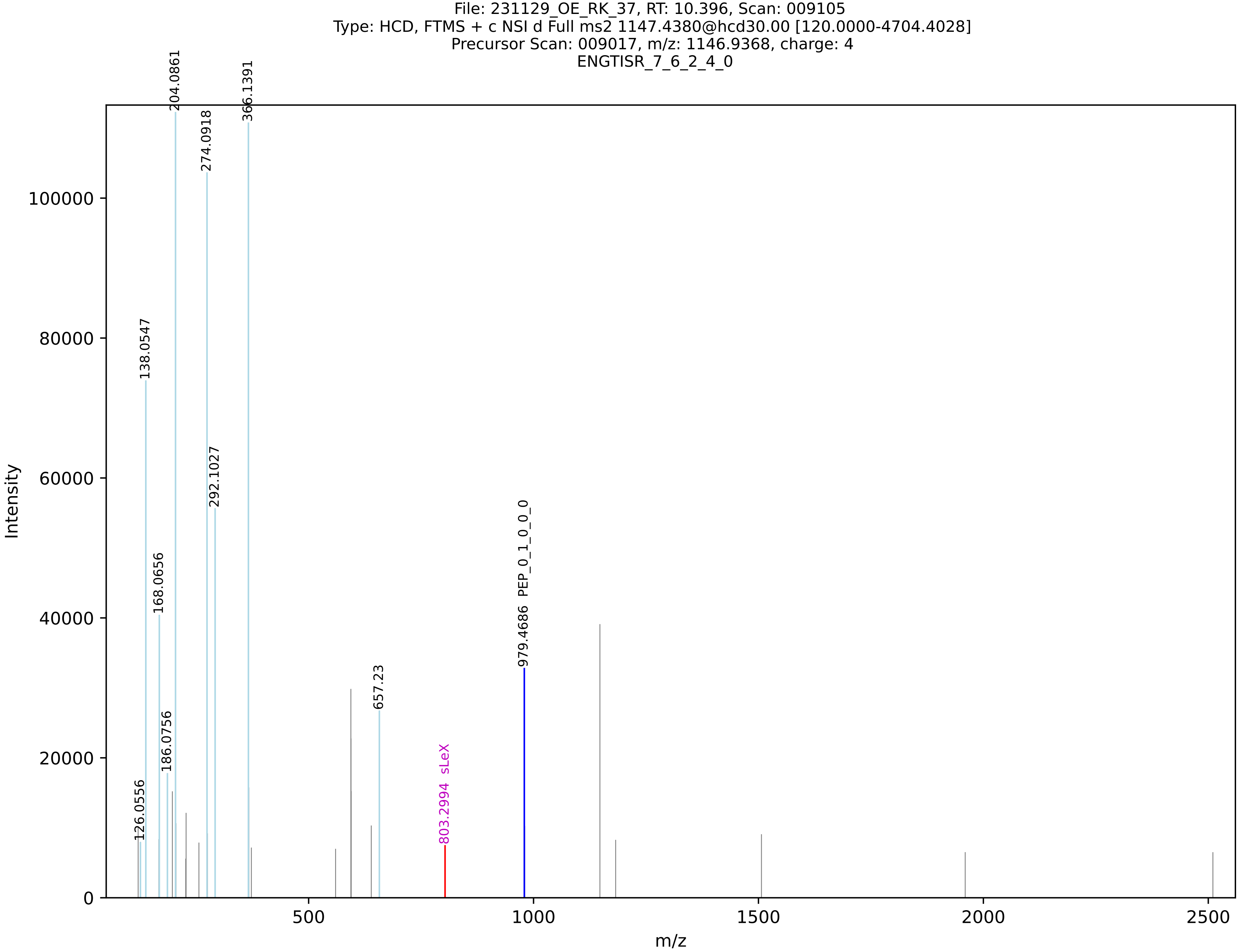
File: 231129_OE_RK_37, RT: 10.396, Scan: 009105
Type: HCD, FTMS + c NSI d Full ms2 1147.4380@hcd30.00 [120.0000-4704.4028]
Precursor Scan: 009017, m/z: 1146.9368, charge: 4
ENGTISR_7_6_2_4_0
Intensity
m/z
0
20000
40000
60000
80000
100000
500
1000
1500
2000
2500
204.0861
366.1391
274.0918
138.0547
292.1027
168.0656
186.0756
126.0556
657.23
803.2994 sLeX
979.4686 PEP_0_1_0_0_0

File: 231129_OE_RK_37, RT: 10.415, Scan: 009135
Type: HCD, FTMS + c NSI d Full ms2 1010.7399@hcd30.00 [120.0000-3115.0061]
Precursor Scan: 008954, m/z: 1009.4005, charge: 3
LENSSGSK_5_4_0_2_0

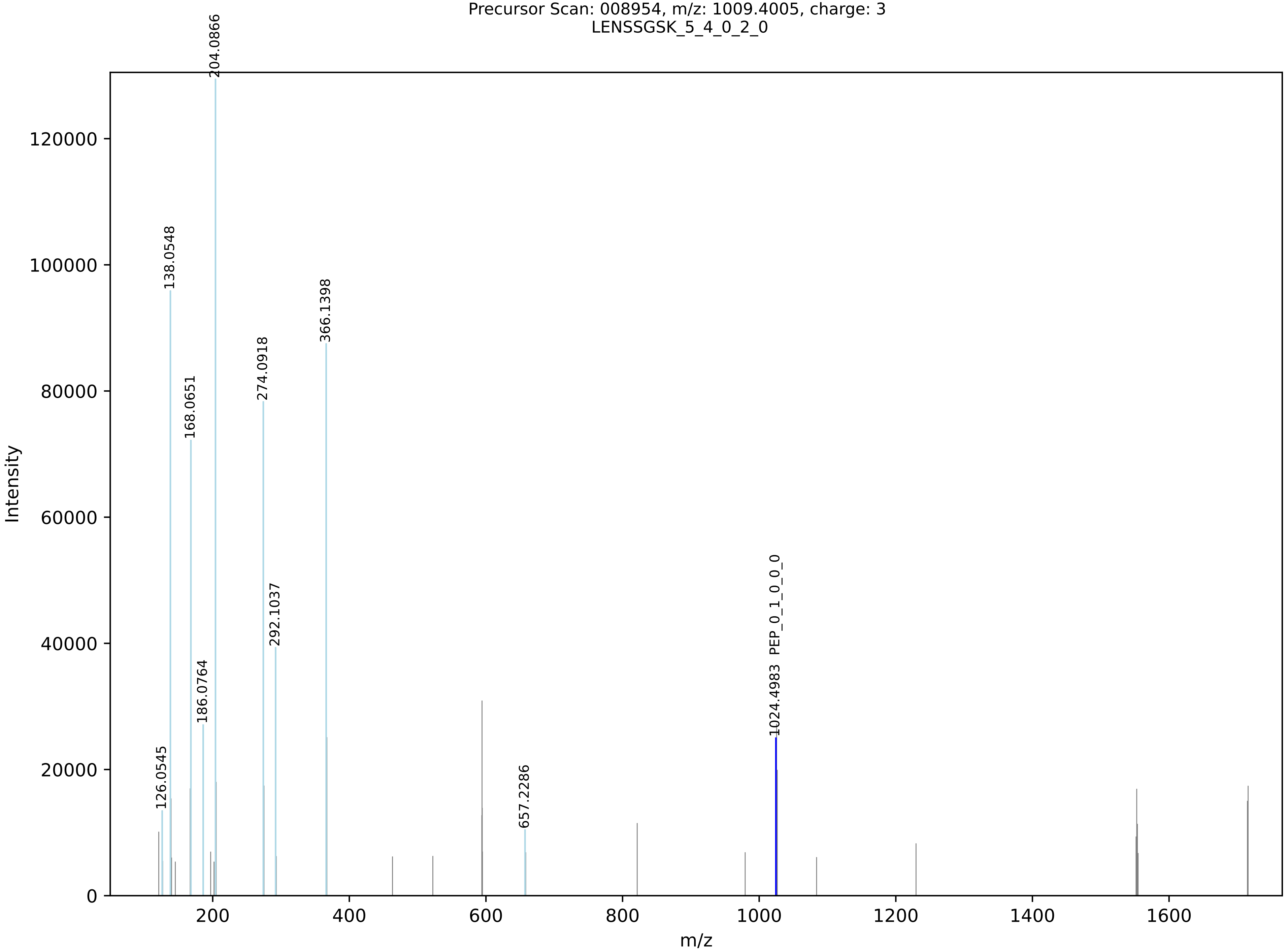

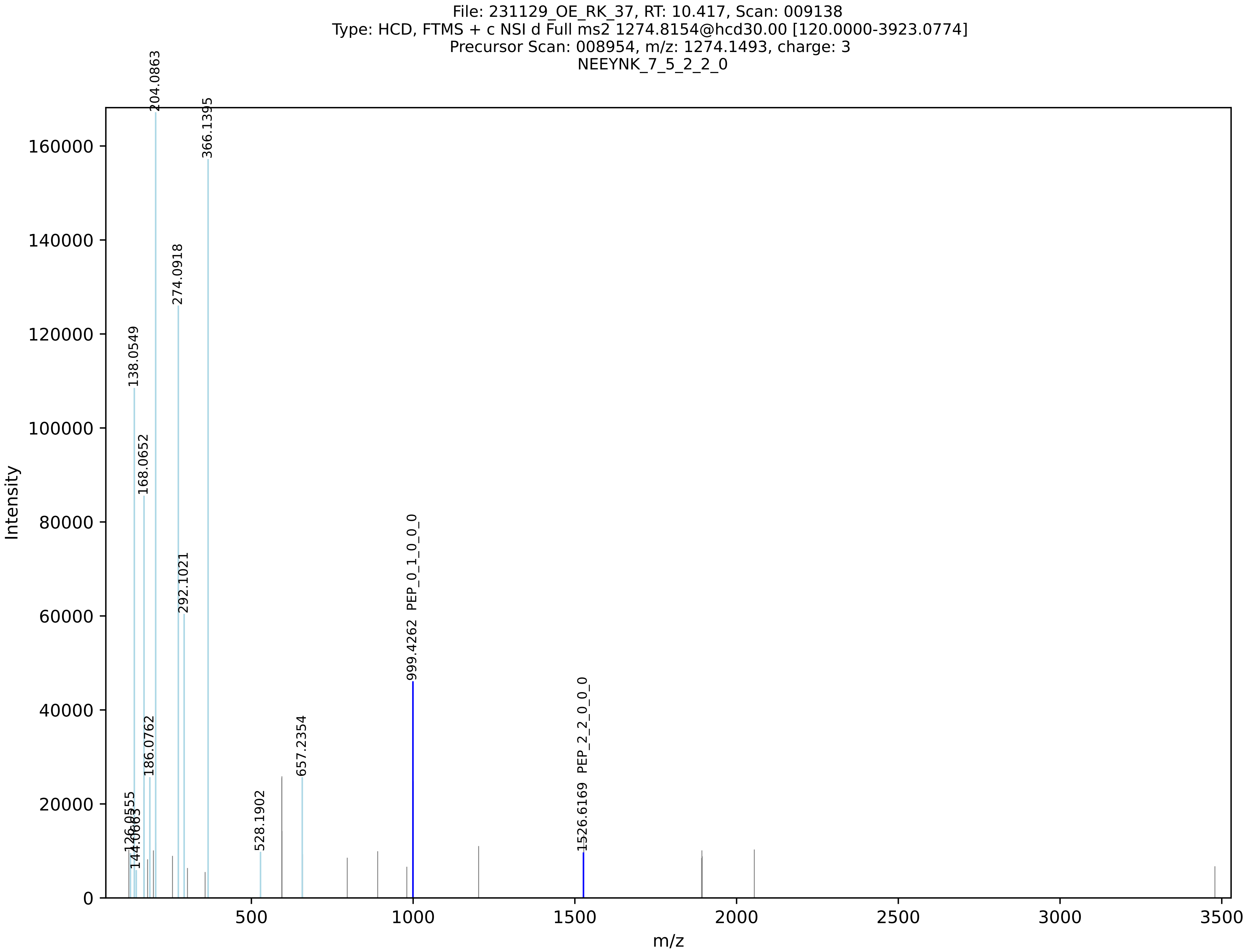
File: 231129_OE_RK_37, RT: 10.417, Scan: 009138
Type: HCD, FTMS + c NSI d Full ms2 1274.8154@hcd30.00 [120.0000-3923.0774]
Precursor Scan: 008954, m/z: 1274.1493, charge: 3
NEEYNK_7_5_2_2_0
Intensity
m/z
204.0863
366.1395
274.0918
138.0549
168.0652
292.1021
999.4262 PEP_0_1_0_0_0
186.0762
657.2354
PEP_2_2_0_0_0
1526.6169
528.1902
126.0555
144.0663
0
20000
40000
60000
80000
100000
120000
140000
160000
500
1000
1500
2000
2500
3000
3500

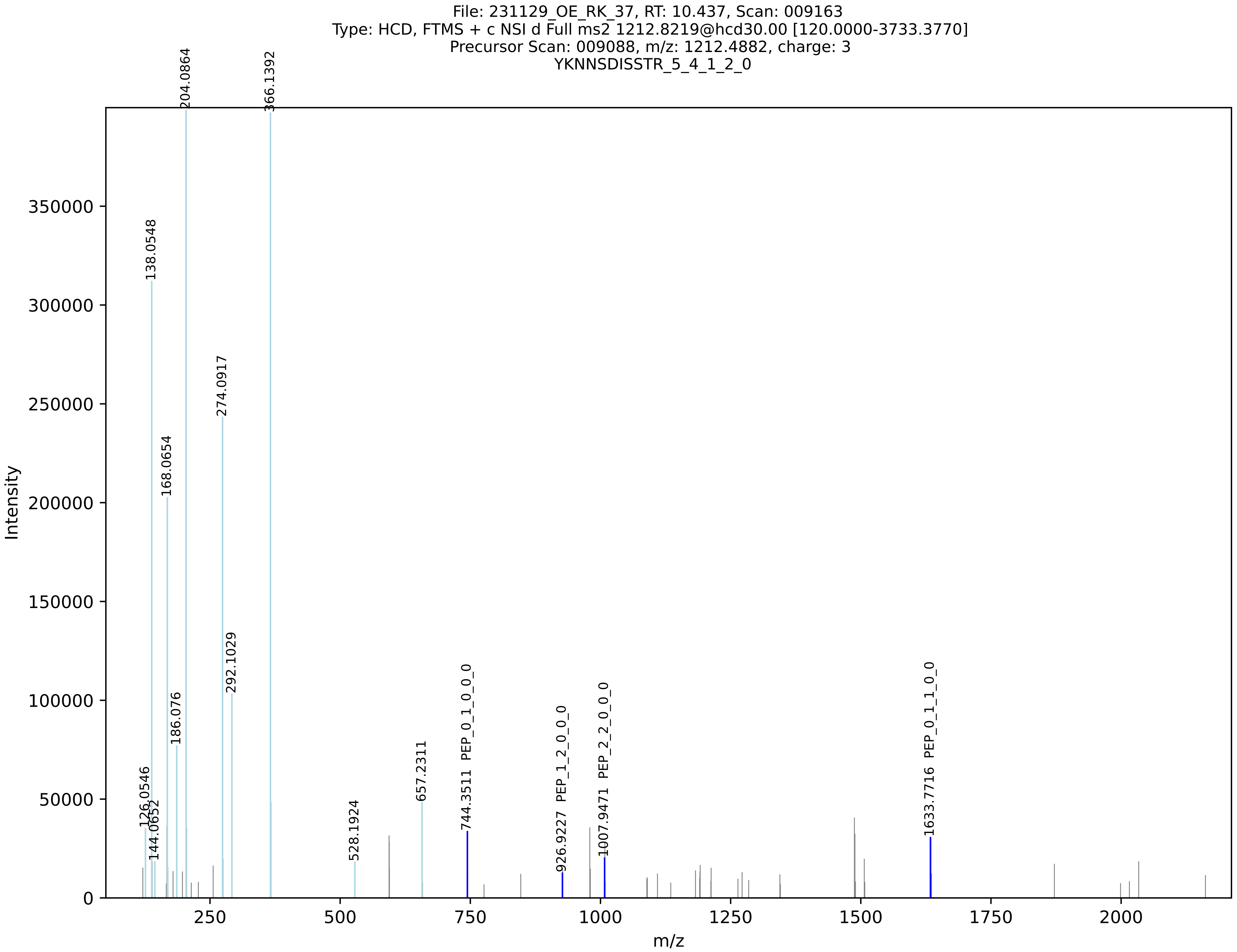
File: 231129_OE_RK_37, RT: 10.437, Scan: 009163
Type: HCD, FTMS + c NSI d Full ms2 1212.8219@hcd30.00 [120.0000-3733.3770]
Precursor Scan: 009088, m/z: 1212.4882, charge: 3
YKNNSDISSTR_5_4_1_2_0
Intensity
m/z
204.0864
366.1392
138.0548
274.0917
168.0654
292.1029
186.076
126.0546
144.0652
528.1924
657.2311
744.3511 PEP_0_1_0_0_0
926.9227 PEP_1_2_0_0_0
1007.9471 PEP_2_2_0_0_0
1633.7716 PEP_0_1_1_0_0

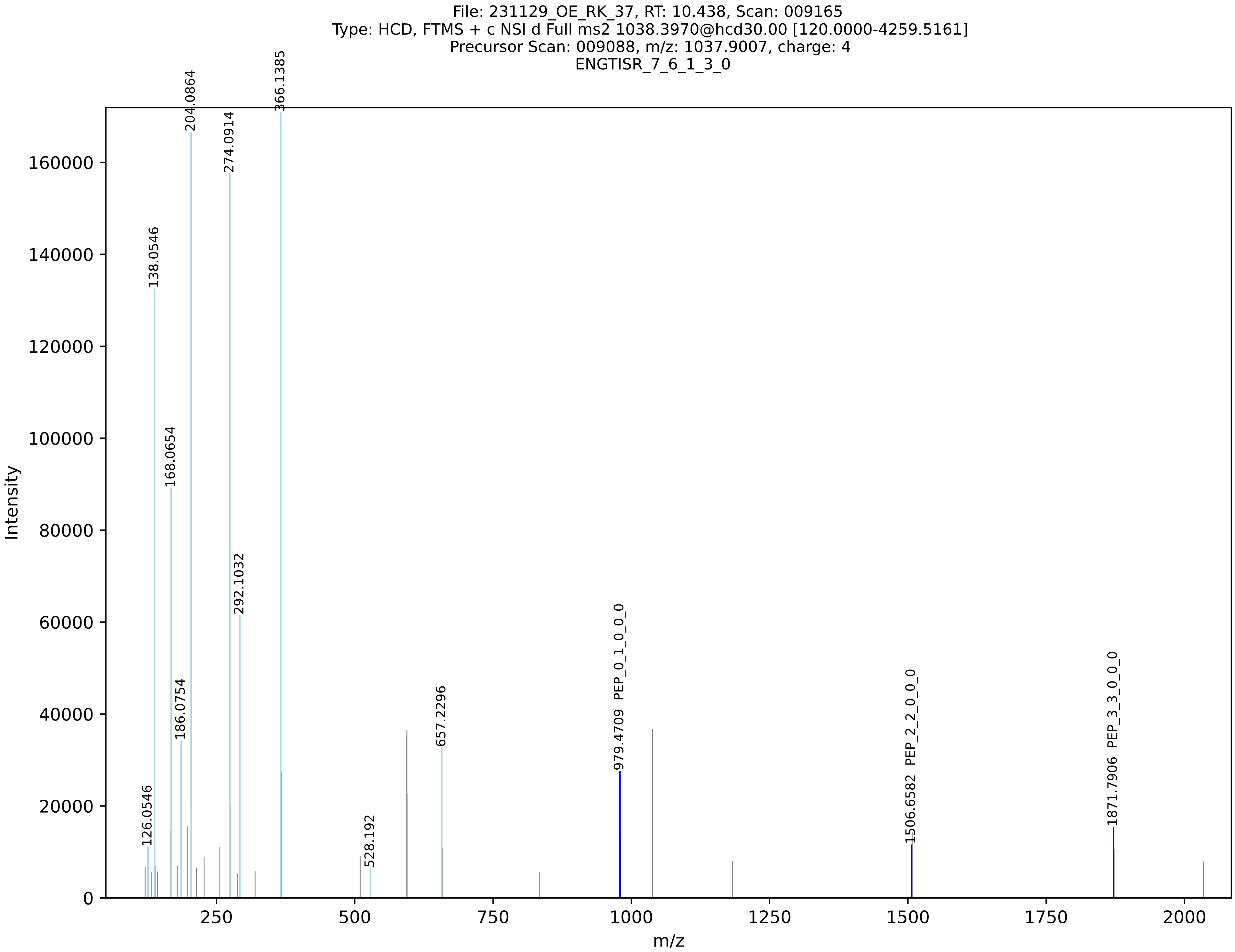

File: 231129_OE_RK_37, RT: 10.438, Scan: 009165
Type: HCD, FTMS + c NSI d Full ms2 1038.3970@hcd30.00 [120.0000-4259.5161]
Precursor Scan: 009088, m/z: 1037.9007, charge: 4
ENGTISR_7_6_1_3_0
Intensity
m/z
126.0546
138.0546
168.0654
186.0754
204.0864
274.0914
292.1032
366.1385
528.192
657.2296
979.4709 PEP_0_1_0_0_0
1506.6582 PEP_2_2_0_0_0
1871.7906 PEP_3_3_0_0_0

File: 231129_OE_RK_37, RT: 10.492, Scan: 009245
Type: HCD, FTMS + c NSI d Full ms2 1090.7631@hcd30.00 [120.0000-3359.8770]
Precursor Scan: 009158, m/z: 1090.0948, charge: 3
TMQENSTPR_5_4_0_2_0

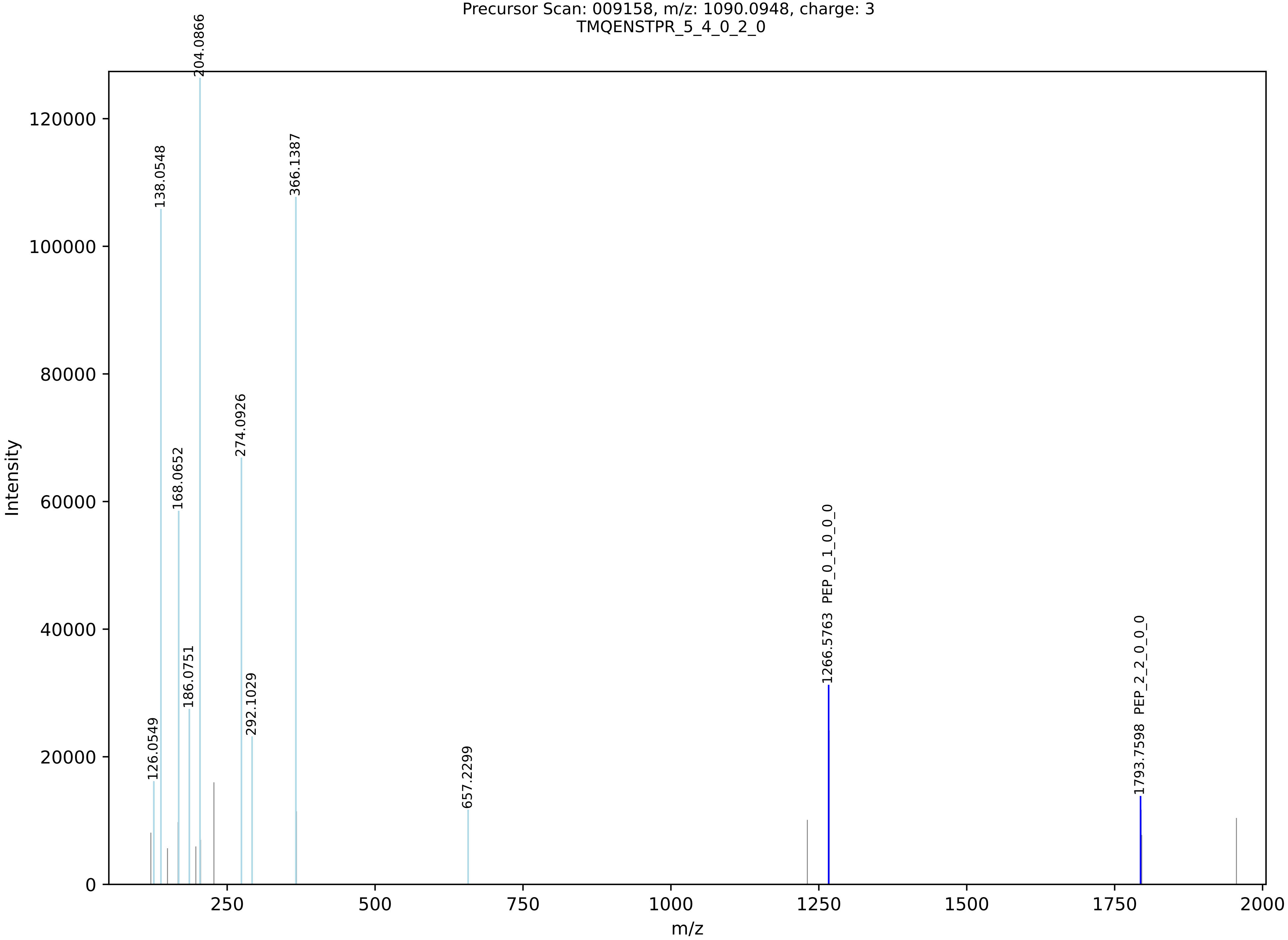

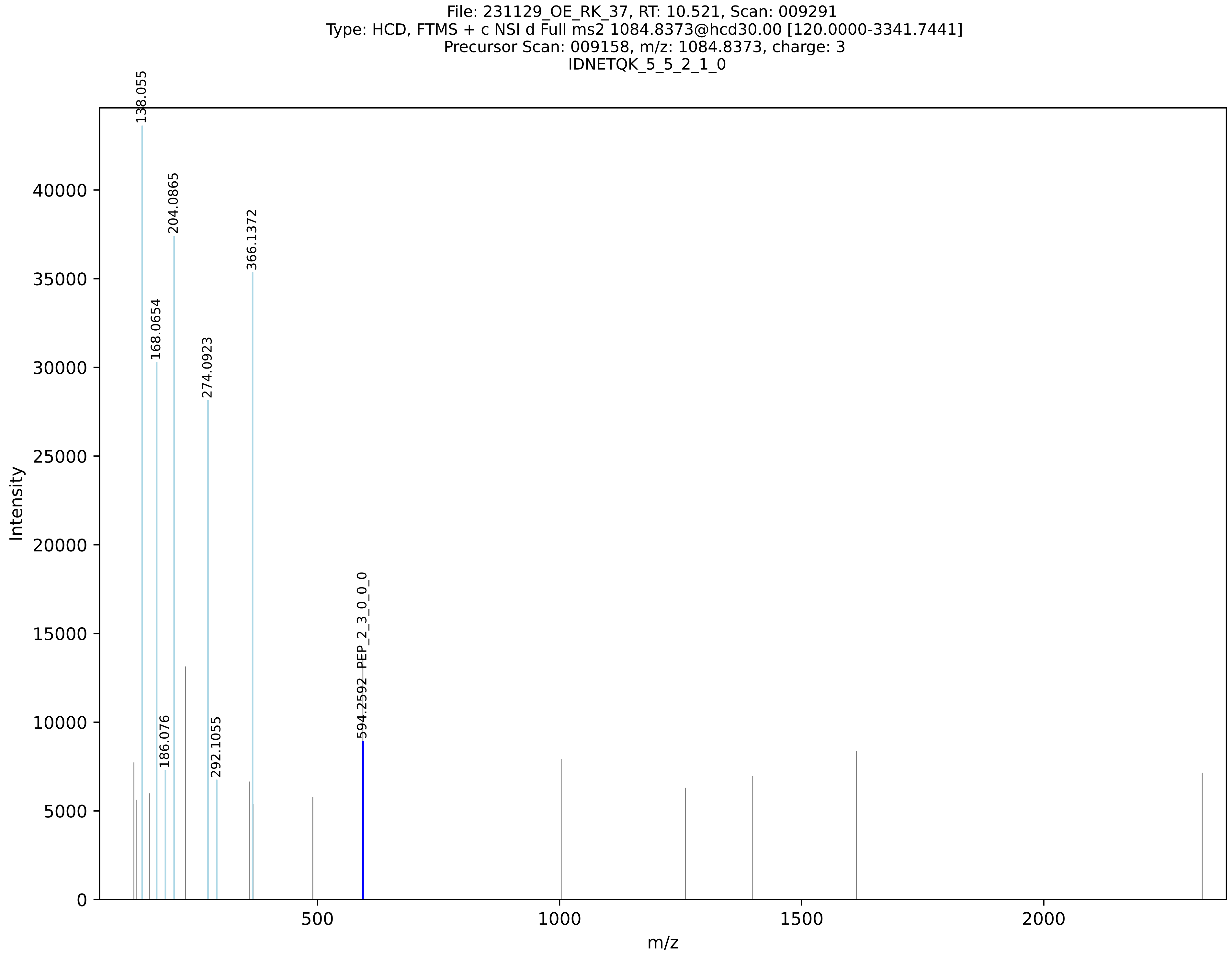
File: 231129_OE_RK_37, RT: 10.521, Scan: 009291
Type: HCD, FTMS + c NSI d Full ms2 1084.8373@hcd30.00 [120.0000-3341.7441]
Precursor Scan: 009158, m/z: 1084.8373, charge: 3
IDNETQK_5_5_2_1_0
138.055
204.0865
366.1372
168.0654
274.0923
594.2592_PEP_2_3_0_0_0
186.076
292.1055
Intensity
m/z
0
5000
10000
15000
20000
25000
30000
35000
40000
500
1000
1500
2000

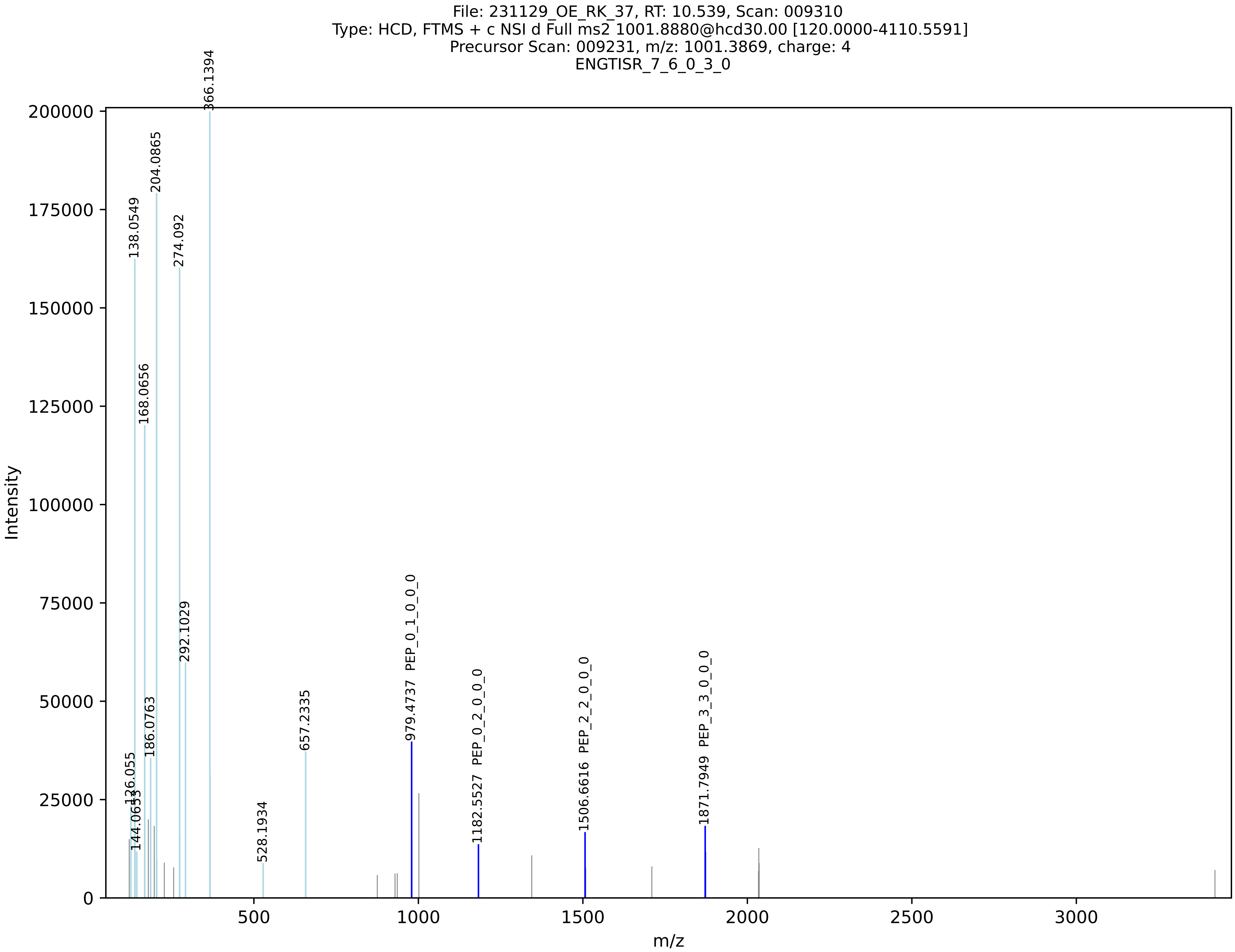
File: 231129_OE_RK_37, RT: 10.539, Scan: 009310
Type: HCD, FTMS + c NSI d Full ms2 1001.8880@hcd30.00 [120.0000-4110.5591]
Precursor Scan: 009231, m/z: 1001.3869, charge: 4
ENGTISR_7_6_0_3_0
Intensity
m/z
366.1394
204.0865
138.0549
274.092
168.0656
292.1029
186.0763
136.055
144.0655
528.1934
657.2335
979.4737 PEP_0_1_0_0_0
1182.5527 PEP_0_2_0_0_0
1506.6616 PEP_2_2_0_0_0
1871.7949 PEP_3_3_0_0_0
200000
175000
150000
125000
100000
75000
50000
25000
0
500
1000
1500
2000
2500
3000

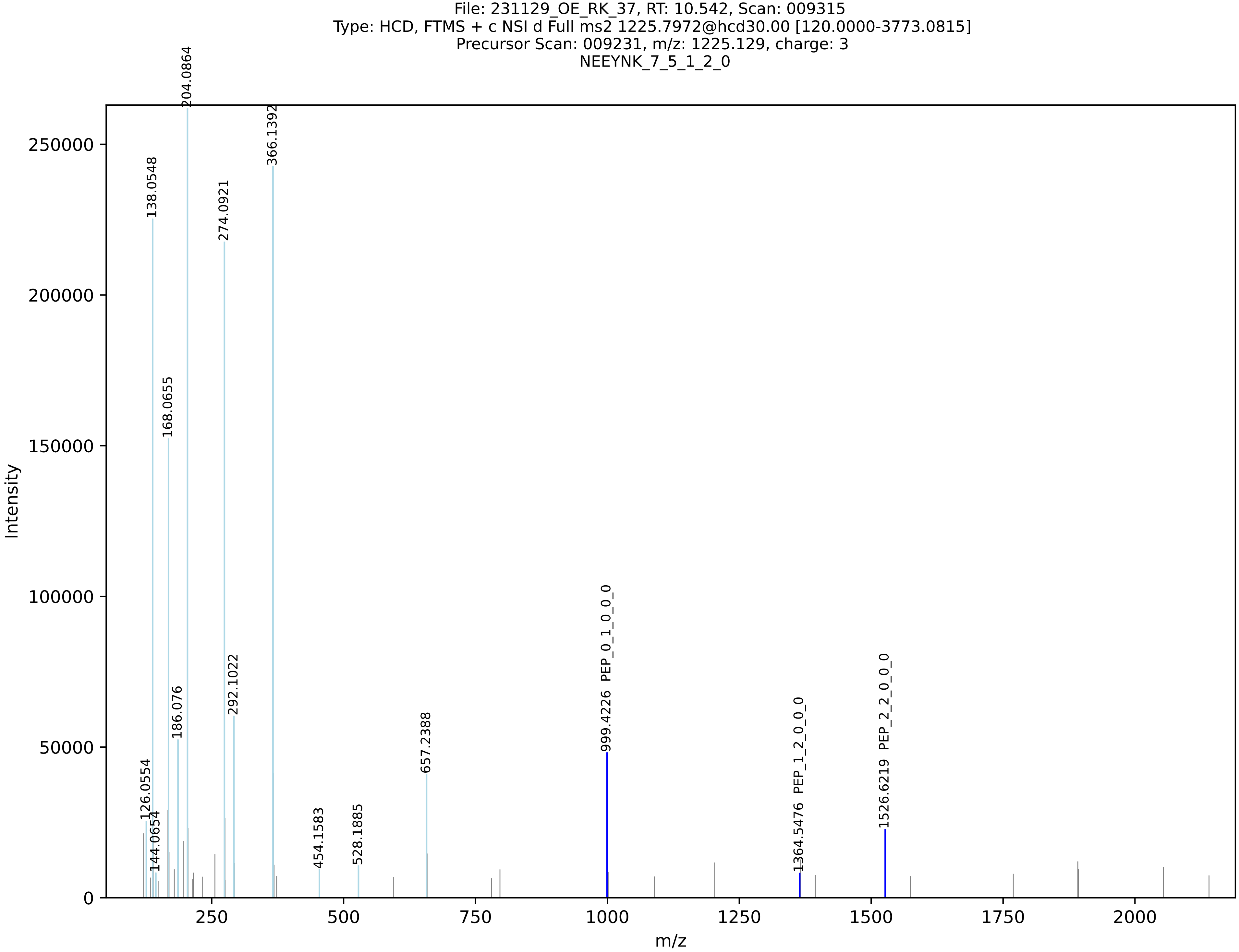
File: 231129_OE_RK_37, RT: 10.542, Scan: 009315
Type: HCD, FTMS + c NSI d Full ms2 1225.7972@hcd30.00 [120.0000-3773.0815]
Precursor Scan: 009231, m/z: 1225.129, charge: 3
NEEYNK_7_5_1_2_0
Intensity
m/z
204.0864
366.1392
138.0548
274.0921
168.0655
186.076
292.1022
126.0554
144.0654
454.1583
528.1885
657.2388
999.4226 PEP_0_1_0_0_0_
1364.5476 PEP_1_2_0_0_0_
1526.6219 PEP_2_2_0_0_0_
0
50000
100000
150000
200000
250000
250
500
750
1000
1250
1500
1750
2000

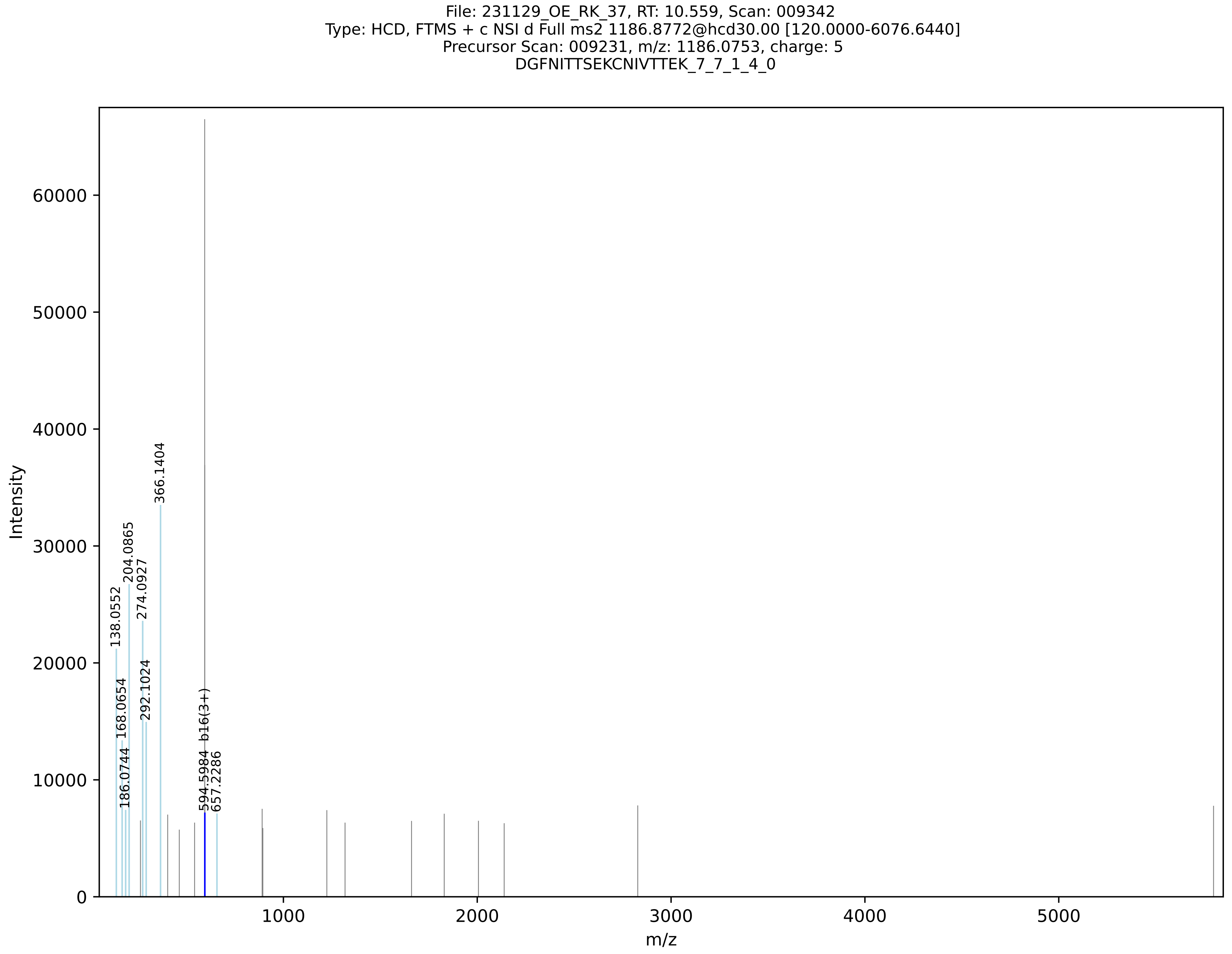
File: 231129_OE_RK_37, RT: 10.559, Scan: 009342
Type: HCD, FTMS + c NSI d Full ms2 1186.8772@hcd30.00 [120.0000-6076.6440]
Precursor Scan: 009231, m/z: 1186.0753, charge: 5
DGFNITTSEKCNIVTTEK_7_7_1_4_0
Intensity
m/z
0
10000
20000
30000
40000
50000
60000
1000
2000
3000
4000
5000
138.0552
168.0654
186.0744
204.0865
274.0927
292.1024
366.1404
594.5984 b16(3+)
657.2286

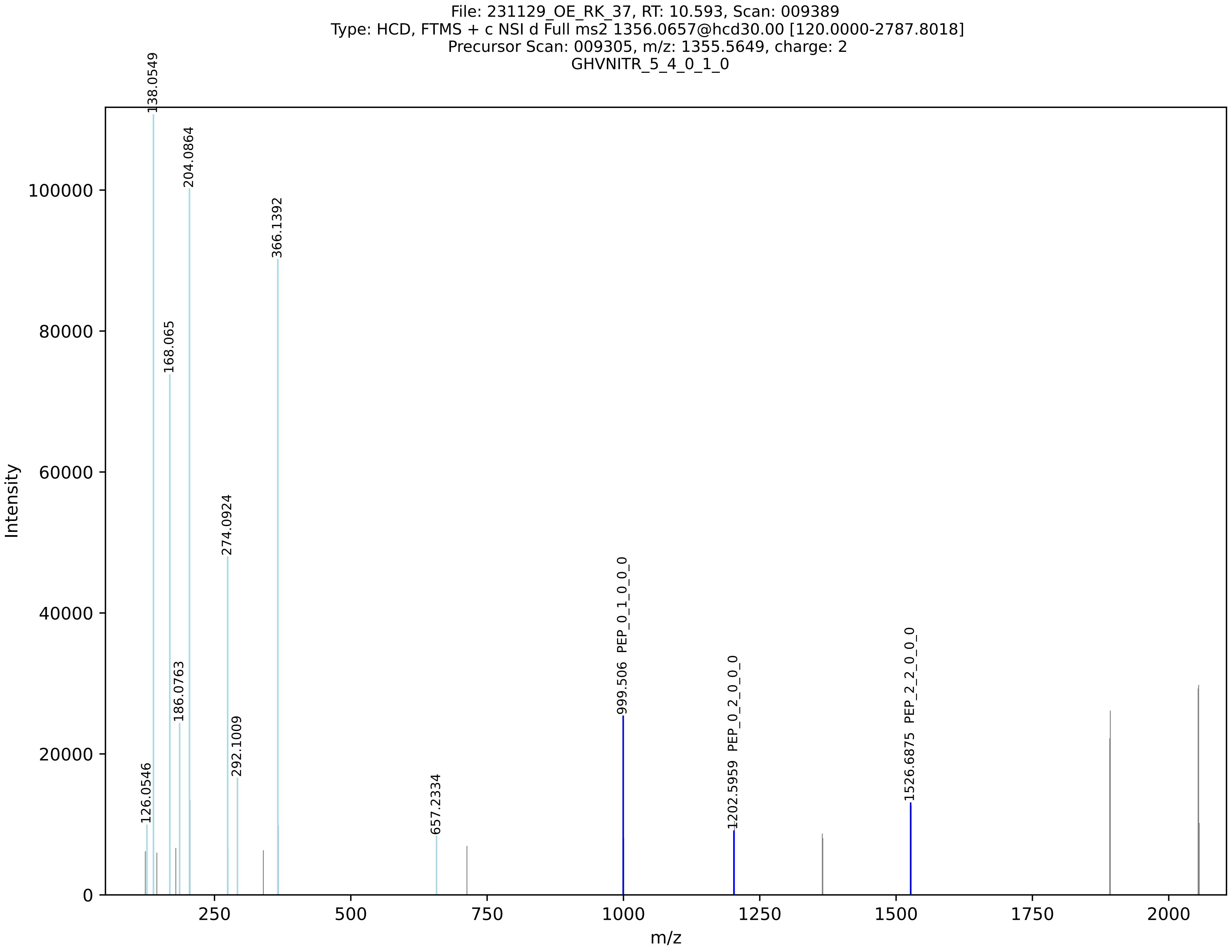
File: 231129_OE_RK_37, RT: 10.593, Scan: 009389
Type: HCD, FTMS + c NSI d Full ms2 1356.0657@hcd30.00 [120.0000-2787.8018]
Precursor Scan: 009305, m/z: 1355.5649, charge: 2
GHVNITR_5_4_0_1_0
138.0549
204.0864
366.1392
168.065
274.0924
186.0763
292.1009
126.0546
657.2334
999.506 PEP_0_1_0_0_0
1202.5959 PEP_0_2_0_0_0
1526.6875 PEP_2_2_0_0_0
Intensity
m/z
0
20000
40000
60000
80000
100000
250
500
750
1000
1250
1500
1750
2000

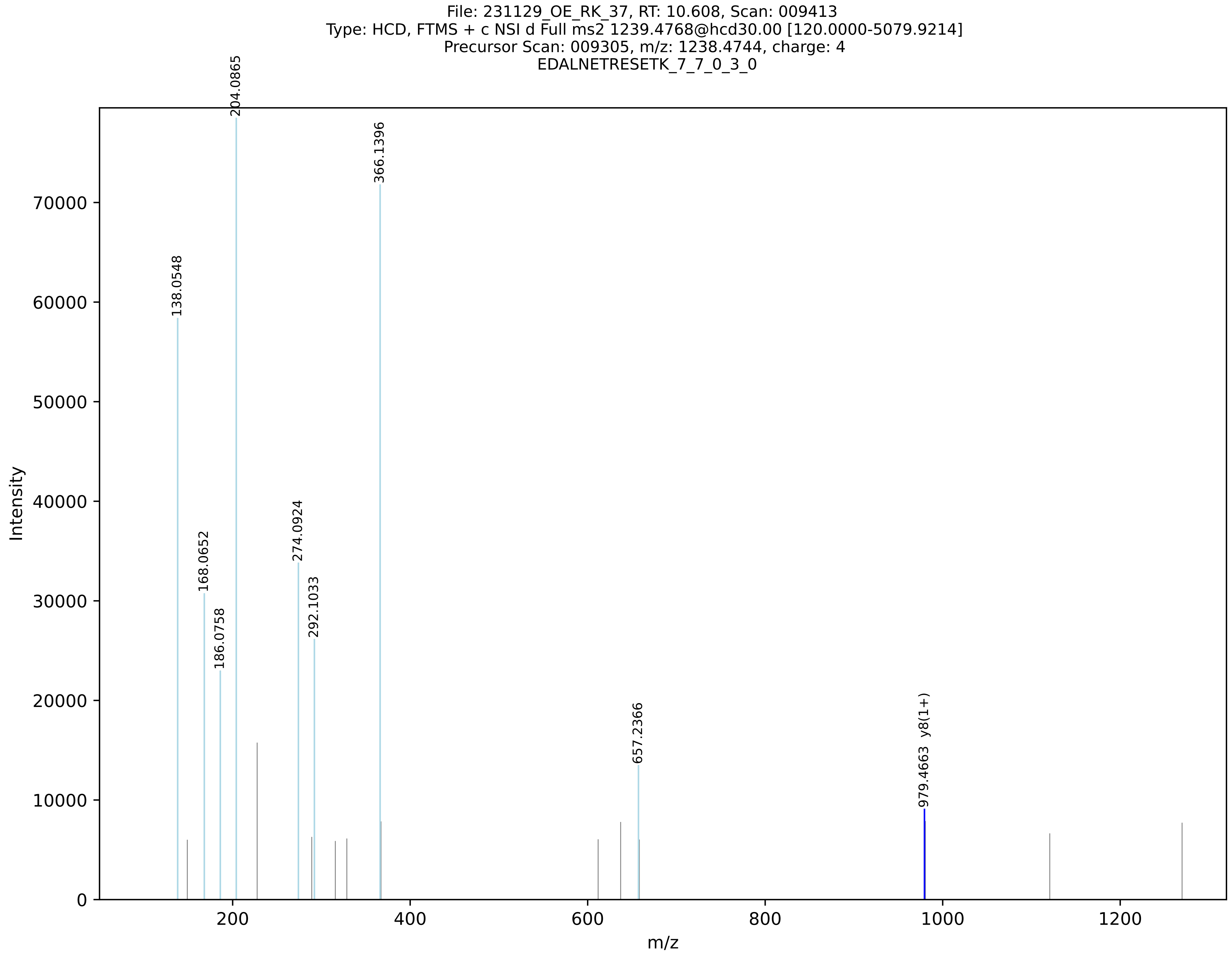
File: 231129_OE_RK_37, RT: 10.608, Scan: 009413
Type: HCD, FTMS + c NSI d Full ms2 1239.4768@hcd30.00 [120.0000-5079.9214]
Precursor Scan: 009305, m/z: 1238.4744, charge: 4
EDALNETRESETK_7_7_0_3_0
Intensity
m/z
138.0548
168.0652
186.0758
204.0865
274.0924
292.1033
366.1396
657.2366
979.4663 y8(1+)
0
10000
20000
30000
40000
50000
60000
70000
200
400
600
800
1000
1200

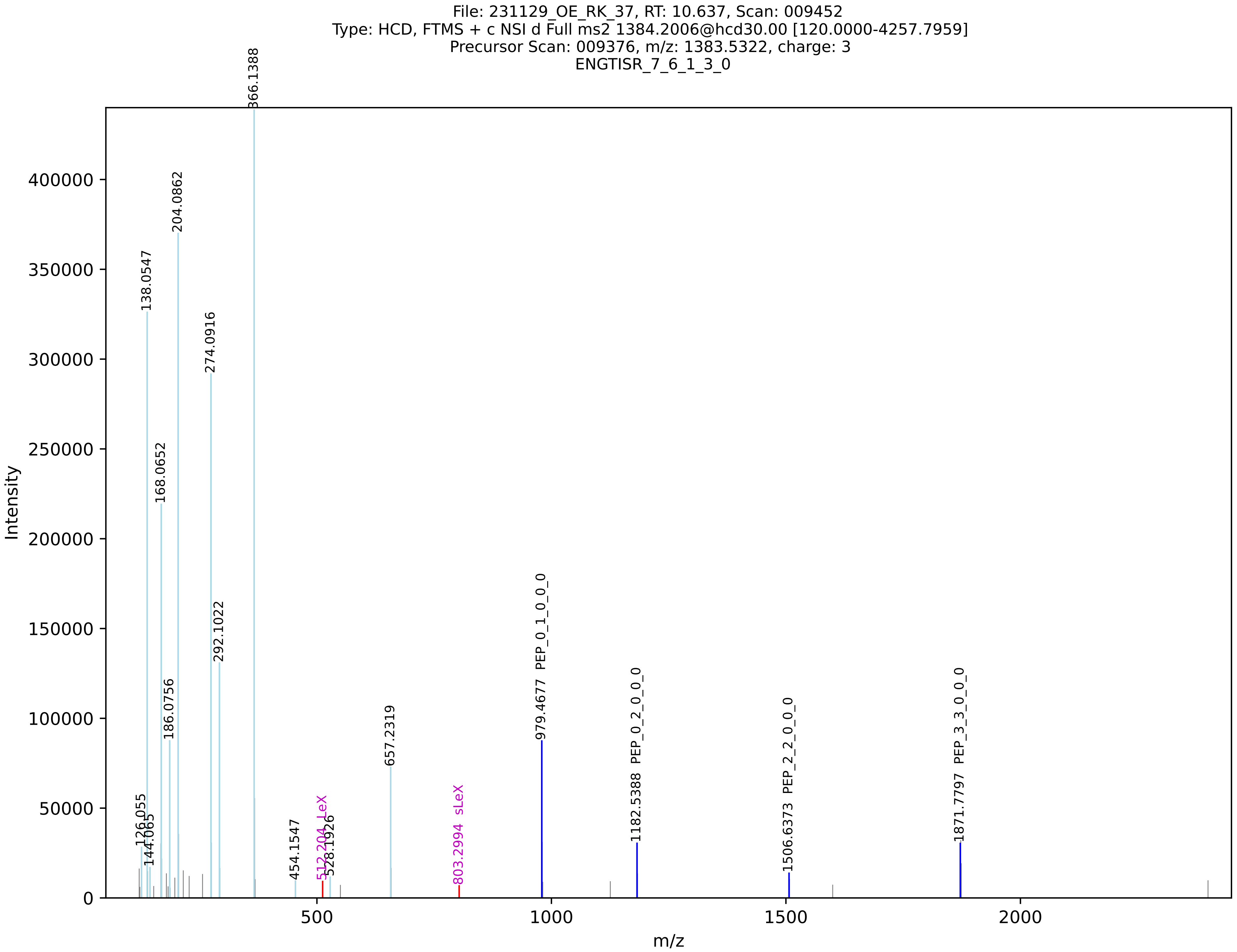

File: 231129_OE_RK_37, RT: 10.637, Scan: 009452
Type: HCD, FTMS + c NSI d Full ms2 1384.2006@hcd30.00 [120.0000-4257.7959]
Precursor Scan: 009376, m/z: 1383.5322, charge: 3
ENGTISR_7_6_1_3_0
Intensity
m/z
366.1388
204.0862
138.0547
274.0916
168.0652
292.1022
186.0756
126.055
144.065
454.1547
512.204_LeX
528.1926
657.2319
803.2994 sLeX
979.4677 PEP_0_1_0_0_0
1182.5388 PEP_0_2_0_0_0
1506.6373 PEP_2_2_0_0_0
1871.7797 PEP_3_3_0_0_0

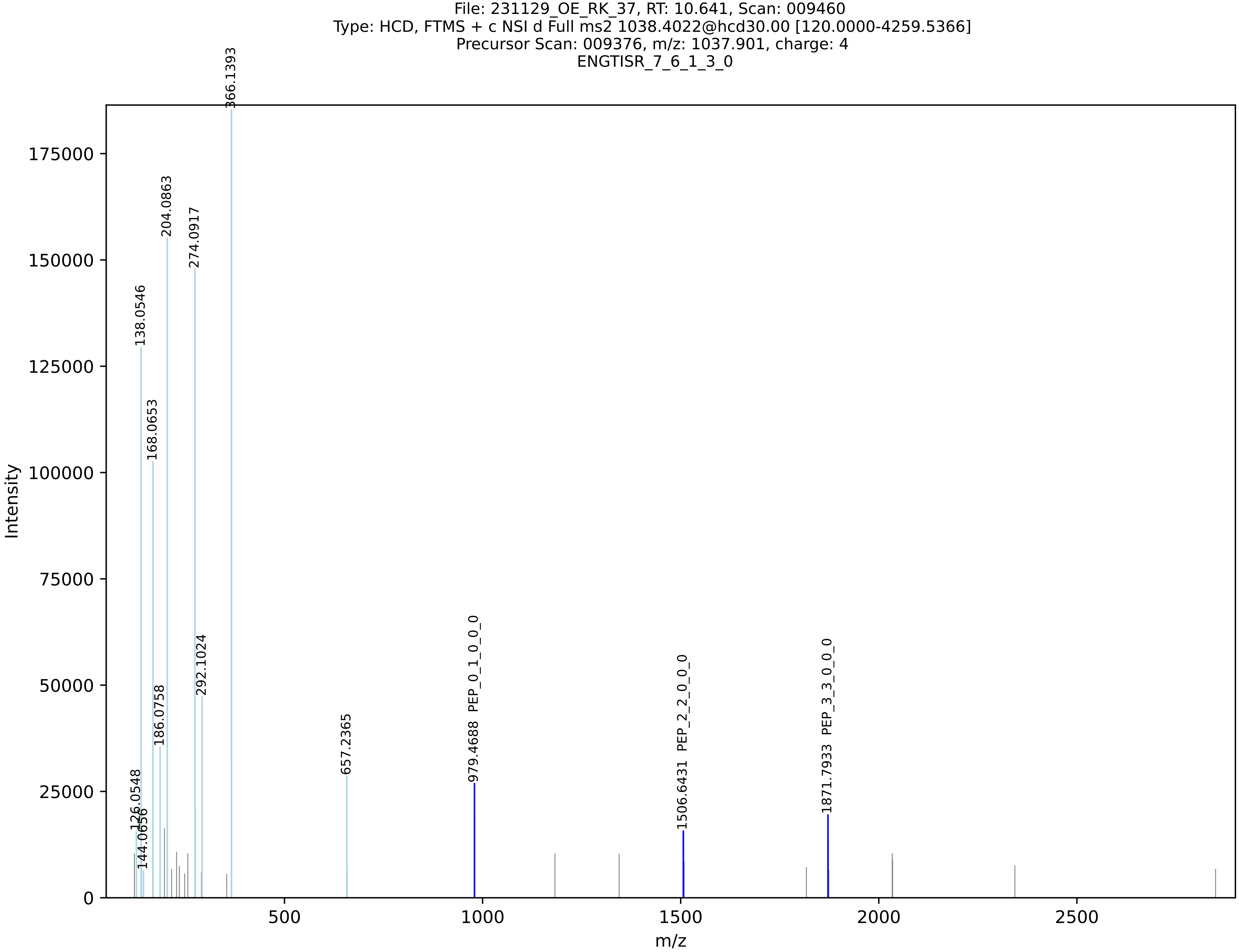
File: 231129_OE_RK_37, RT: 10.641, Scan: 009460
Type: HCD, FTMS + c NSI d Full ms2 1038.4022@hcd30.00 [120.0000-4259.5366]
Precursor Scan: 009376, m/z: 1037.901, charge: 4
ENGTISR_7_6_1_3_0
366.1393
204.0863
274.0917
138.0546
168.0653
292.1024
186.0758
126.0548
144.0656
657.2365
979.4688 PEP_0_1_0_0_0
1506.6431 PEP_2_2_0_0_0
1871.7933 PEP_3_3_0_0_0
Intensity
m/z
0
25000
50000
75000
100000
125000
150000
175000
500
1000
1500
2000
2500

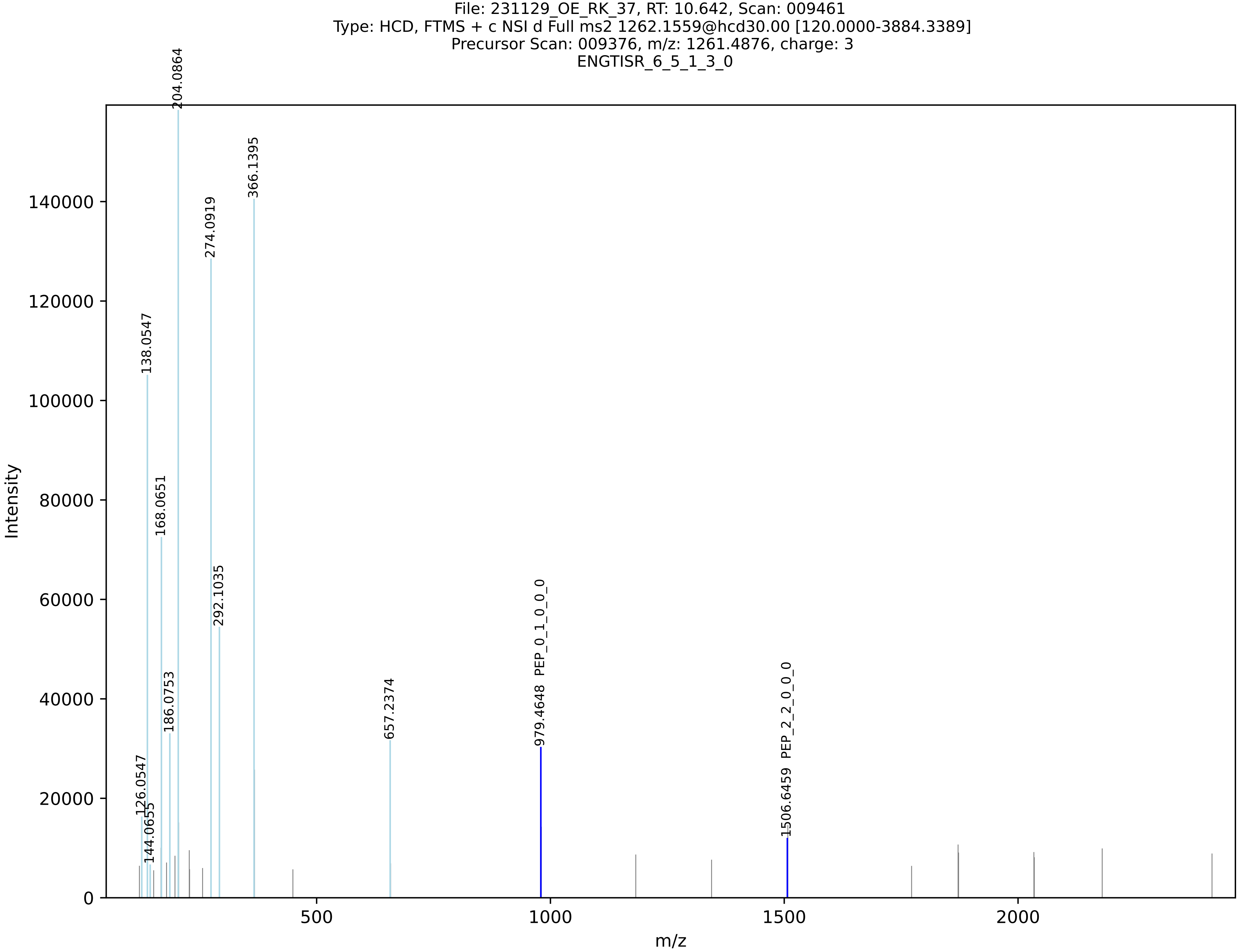
File: 231129_OE_RK_37, RT: 10.642, Scan: 009461
Type: HCD, FTMS + c NSI d Full ms2 1262.1559@hcd30.00 [120.0000-3884.3389]
Precursor Scan: 009376, m/z: 1261.4876, charge: 3
ENGTISR_6_5_1_3_0
204.0864
366.1395
274.0919
138.0547
168.0651
292.1035
186.0753
126.0547
144.0655
657.2374
979.4648 PEP_0_1_0_0_
1506.6459 PEP_2_2_0_0_
Intensity
m/z
0
20000
40000
60000
80000
100000
120000
140000
500
1000
1500
2000

File: 231129_OE_RK_37, RT: 10.644, Scan: 009464
Type: HCD, FTMS + c NSI d Full ms2 1274.8176@hcd30.00 [120.0000-3923.0840]
Precursor Scan: 009376, m/z: 1274.149, charge: 3
NEEYNK_7_5_2_2_0

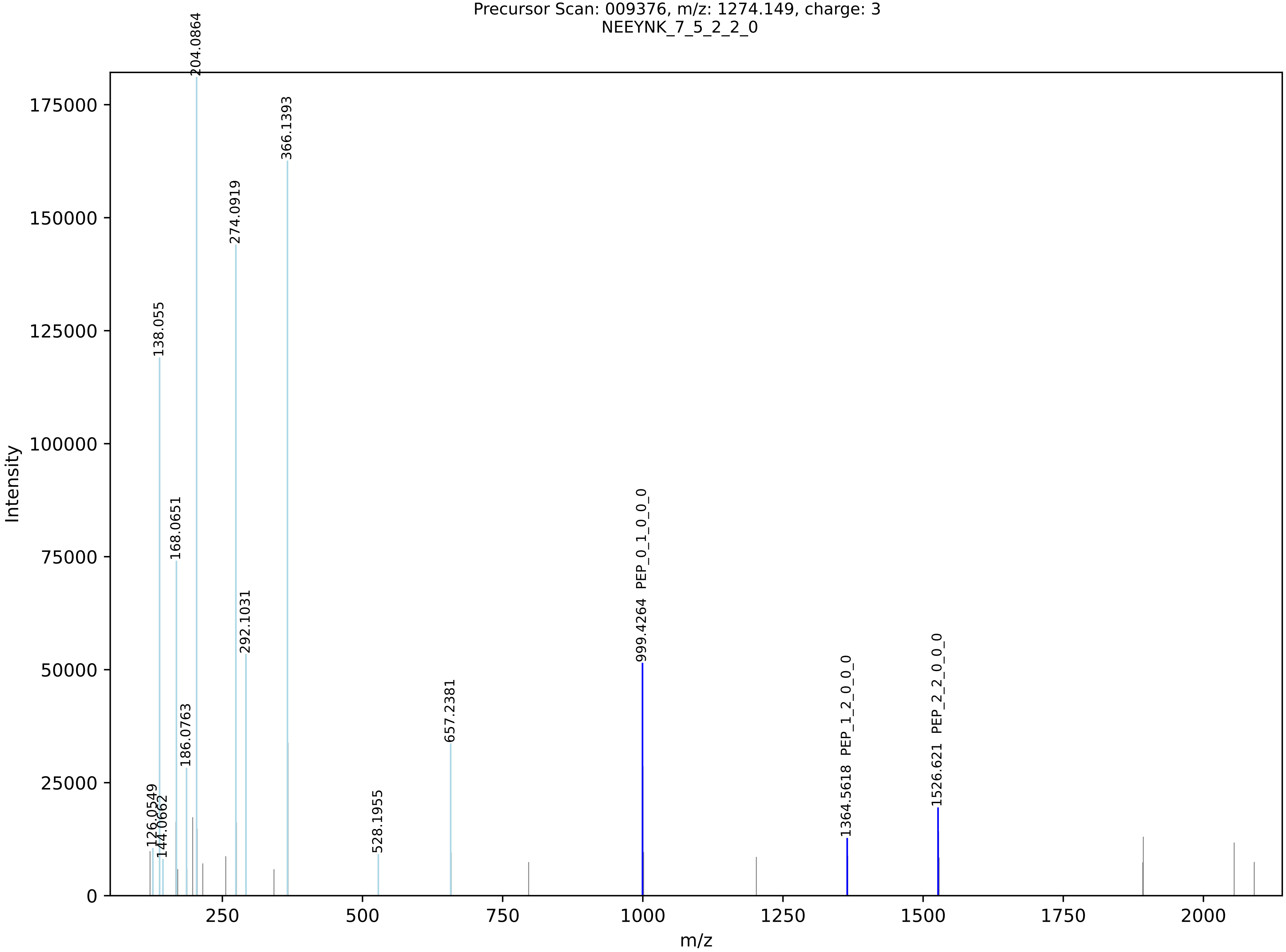

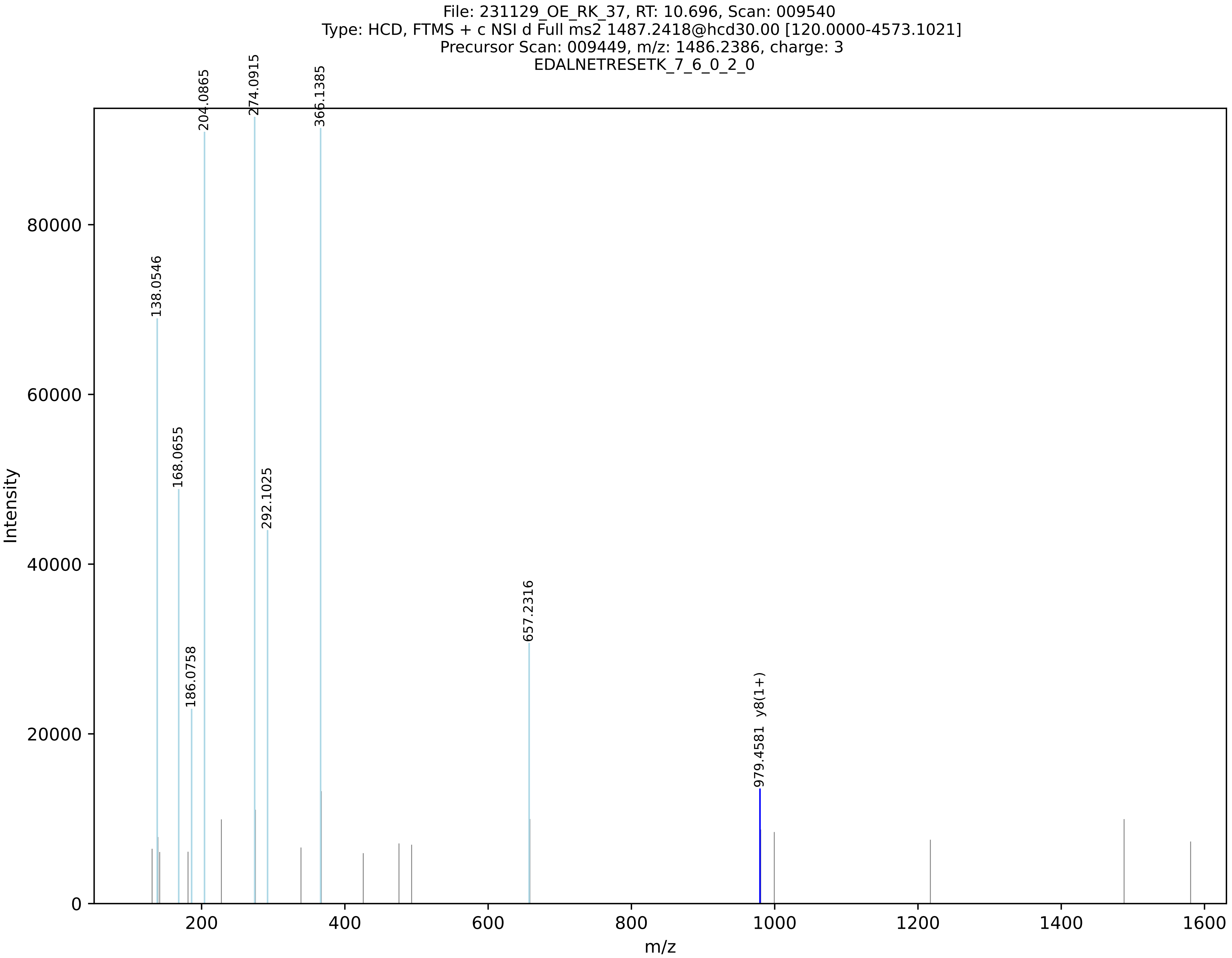
File: 231129_OE_RK_37, RT: 10.696, Scan: 009540
Type: HCD, FTMS + c NSI d Full ms2 1487.2418@hcd30.00 [120.0000-4573.1021]
Precursor Scan: 009449, m/z: 1486.2386, charge: 3
EDALNETRESETK_7_6_0_2_0
204.0865
274.0915
366.1385
138.0546
168.0655
292.1025
186.0758
657.2316
979.4581 y8(1+)
Intensity
m/z
0
20000
40000
60000
80000
200
400
600
800
1000
1200
1400
1600

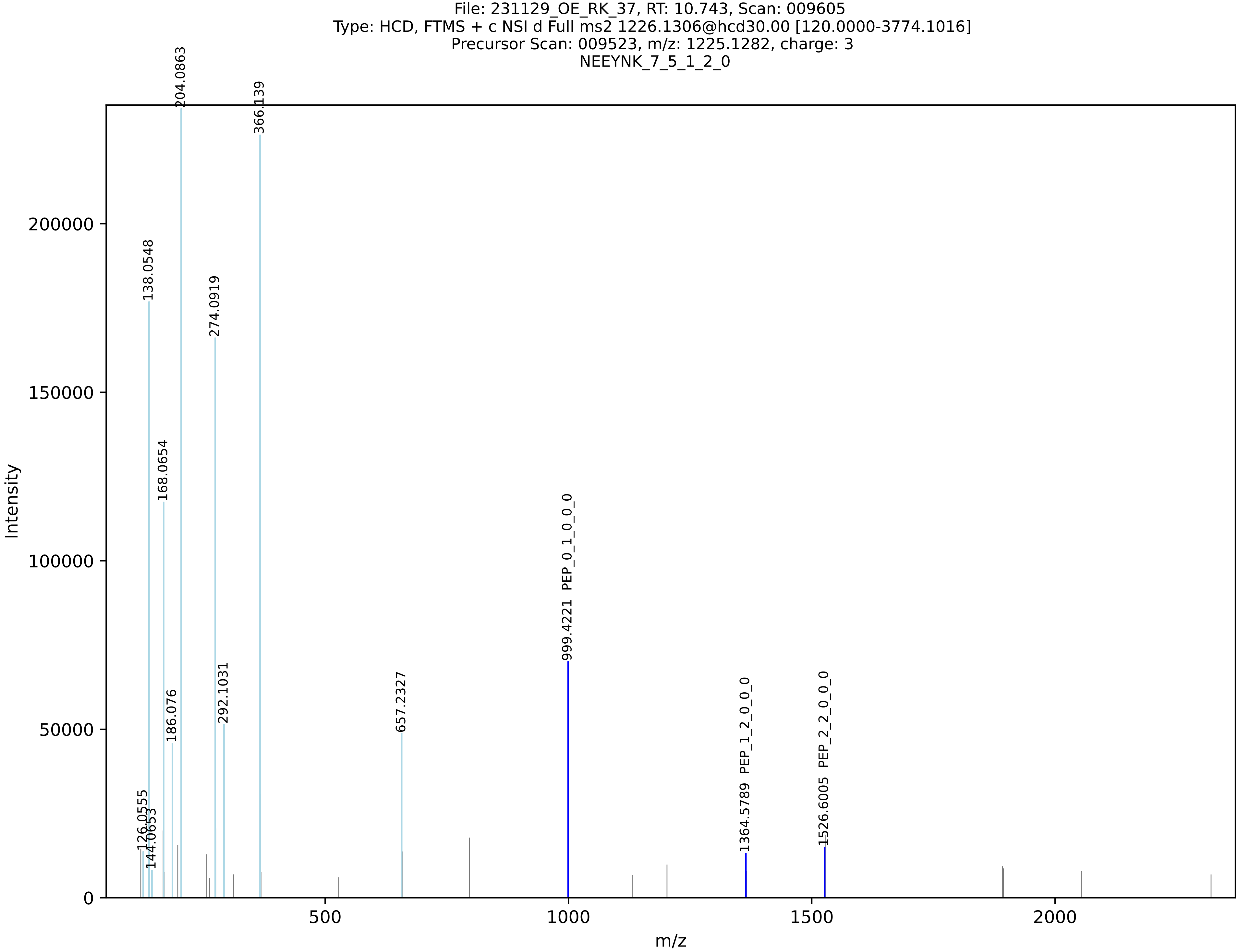
File: 231129_OE_RK_37, RT: 10.743, Scan: 009605
Type: HCD, FTMS + c NSI d Full ms2 1226.1306@hcd30.00 [120.0000-3774.1016]
Precursor Scan: 009523, m/z: 1225.1282, charge: 3
NEEYNK_7_5_1_2_0
Intensity
m/z
204.0863
366.139
138.0548
274.0919
168.0654
999.4221 PEP_0_1_0_0_0
657.2327
292.1031
186.076
1364.5789 PEP_1_2_0_0_0
1526.6005 PEP_2_2_0_0_0
126.0555
144.0653
0
50000
100000
150000
200000
500
1000
1500
2000

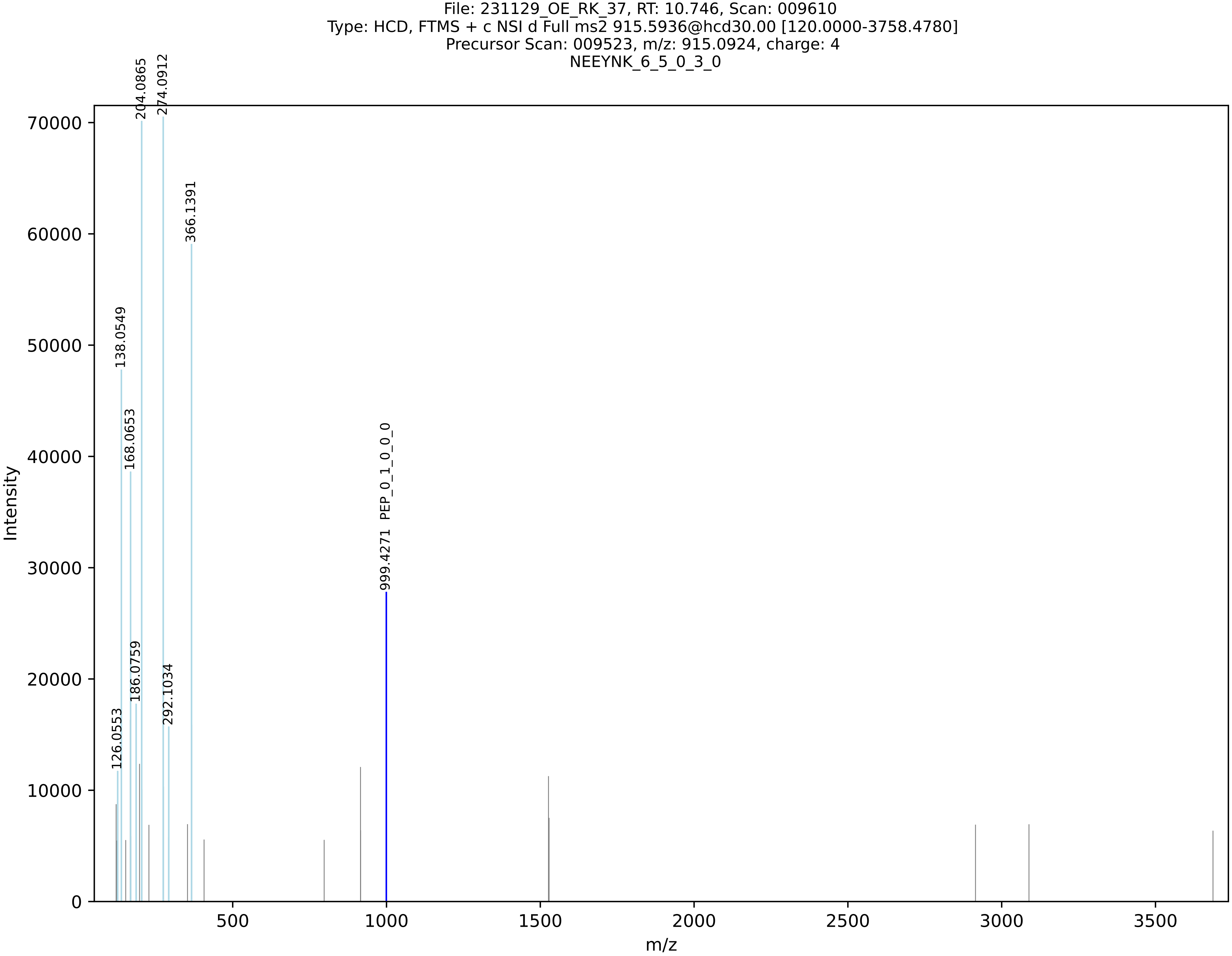
File: 231129_OE_RK_37, RT: 10.746, Scan: 009610
Type: HCD, FTMS + c NSI d Full ms2 915.5936@hcd30.00 [120.0000-3758.4780]
Precursor Scan: 009523, m/z: 915.0924, charge: 4
NEEYNK_6_5_0_3_0
204.0865
274.0912
366.1391
138.0549
168.0653
999.4271 PEP_0_1_0_0_0
186.0759
292.1034
126.0553
Intensity
m/z
0
10000
20000
30000
40000
50000
60000
70000
500
1000
1500
2000
2500
3000
3500

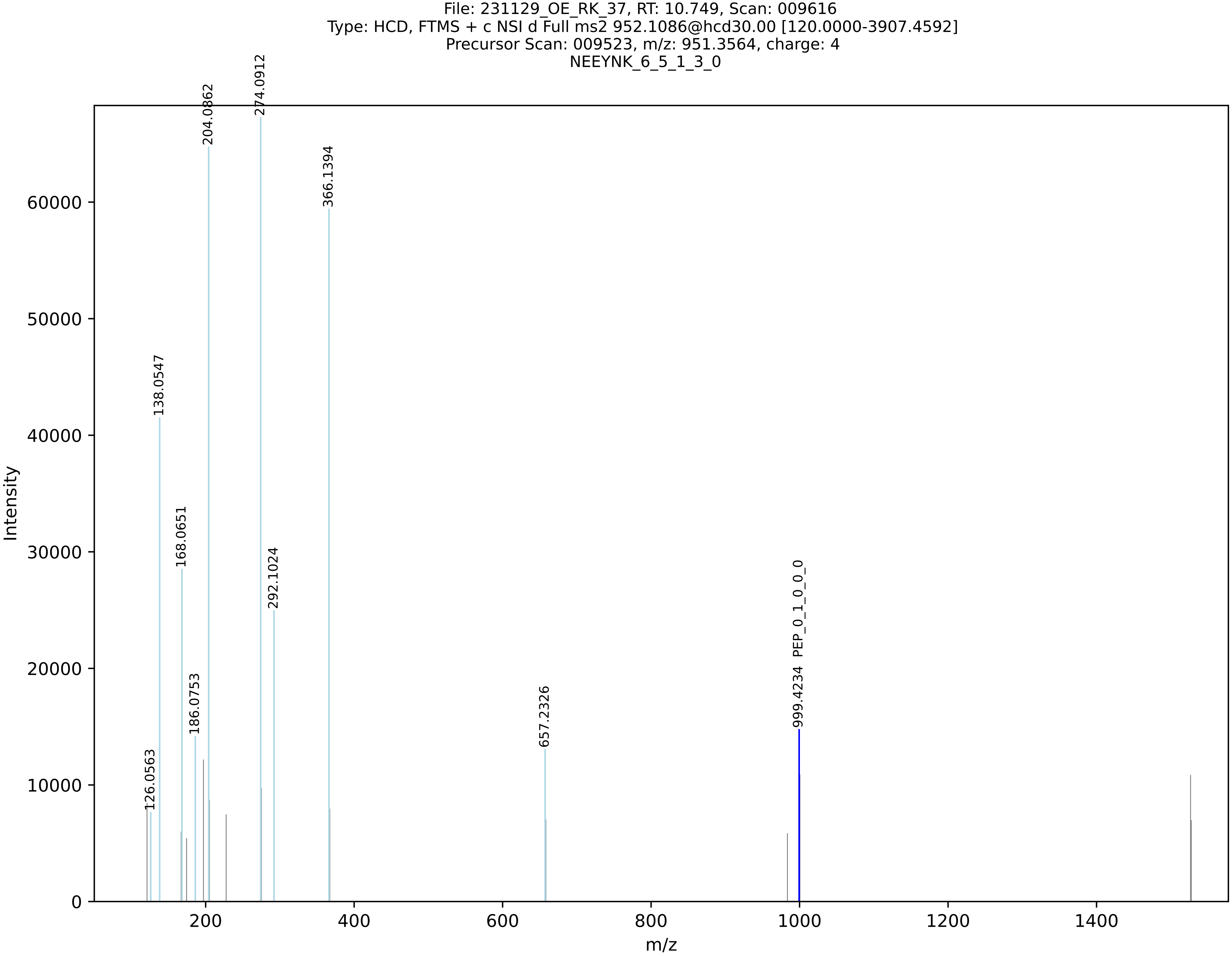
File: 231129_OE_RK_37, RT: 10.749, Scan: 009616
Type: HCD, FTMS + c NSI d Full ms2 952.1086@hcd30.00 [120.0000-3907.4592]
Precursor Scan: 009523, m/z: 951.3564, charge: 4
NEEYNK_6_5_1_3_0
274.0912
204.0862
366.1394
138.0547
168.0651
292.1024
PEP_0_1_0_0_0
999.4234
186.0753
657.2326
126.0563
Intensity
m/z
0
10000
20000
30000
40000
50000
60000
200
400
600
800
1000
1200
1400

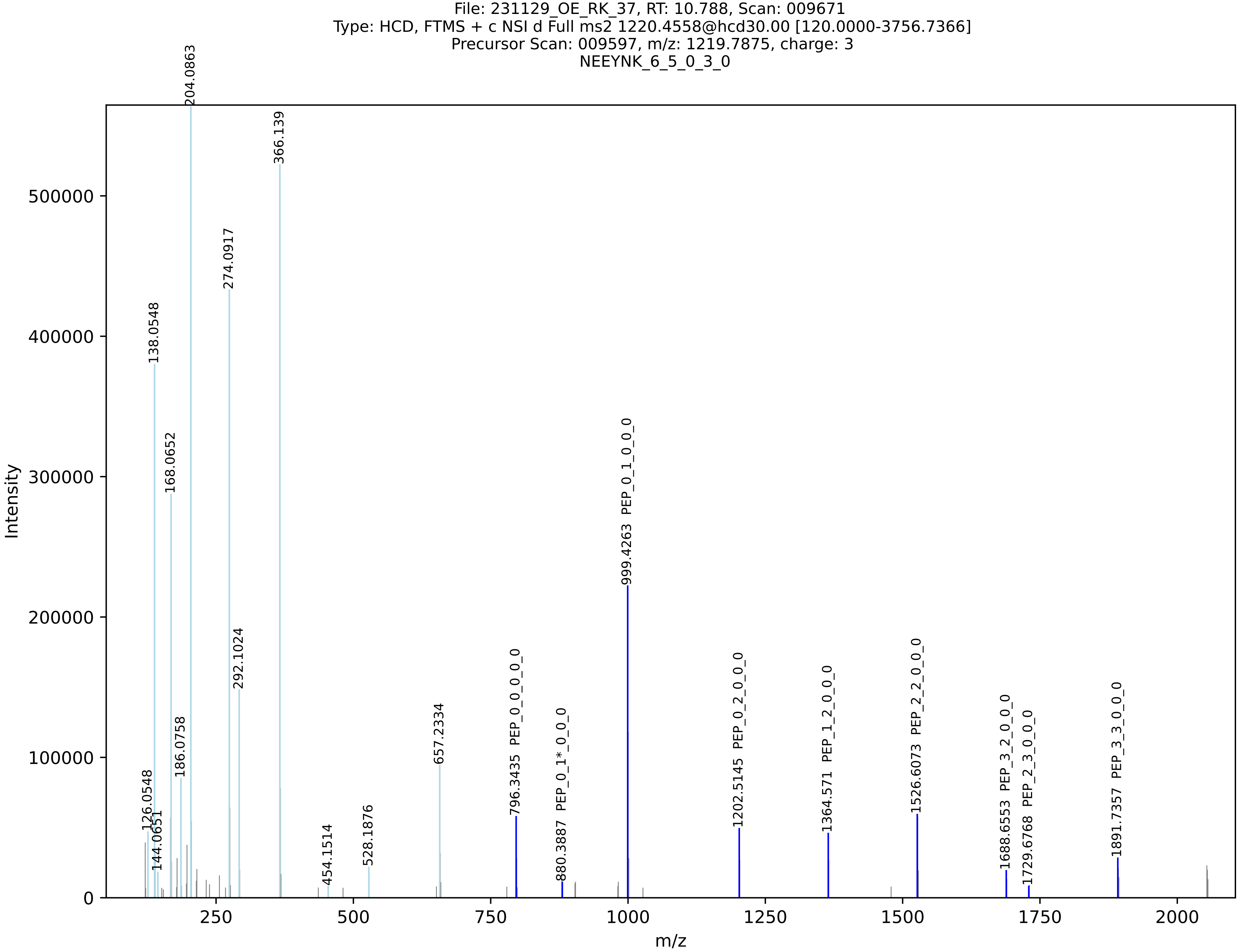
File: 231129_OE_RK_37, RT: 10.788, Scan: 009671
Type: HCD, FTMS + c NSI d Full ms2 1220.4558@hcd30.00 [120.0000-3756.7366]
Precursor Scan: 009597, m/z: 1219.7875, charge: 3
NEEYNK_6_5_0_3_0
Intensity
m/z
204.0863
366.139
274.0917
138.0548
168.0652
292.1024
186.0758
126.0548
144.0651
454.1514
528.1876
657.2334
796.3435 PEP_0_0_0_0_0
880.3887 PEP_0_1*_0_0_0
999.4263 PEP_0_1_0_0_0
1202.5145 PEP_0_2_0_0_0
1364.571 PEP_1_2_0_0_0
1526.6073 PEP_2_2_0_0_0
1688.6553 PEP_3_2_0_0_0
1729.6768 PEP_2_3_0_0_0
1891.7357 PEP_3_3_0_0_0

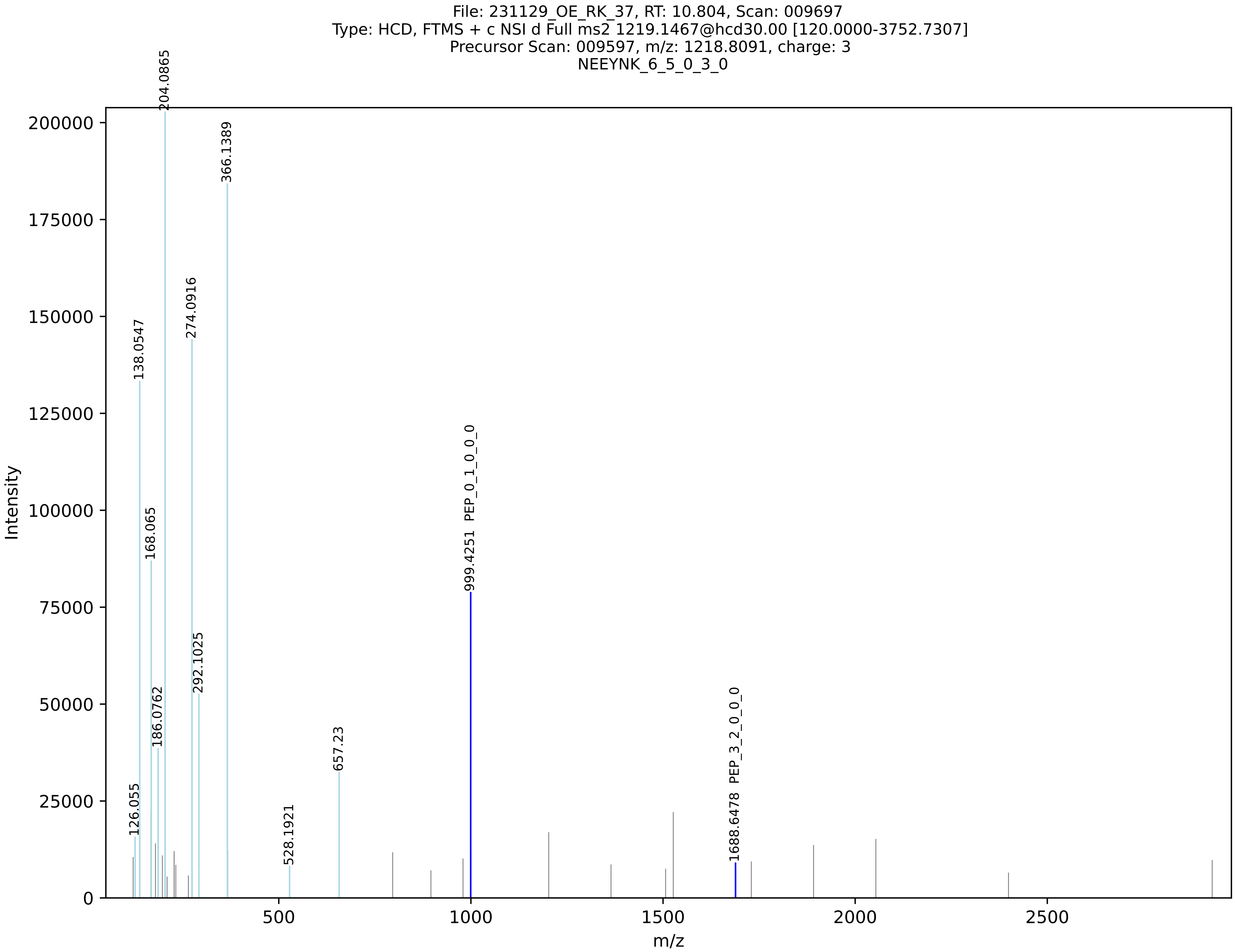
File: 231129_OE_RK_37, RT: 10.804, Scan: 009697
Type: HCD, FTMS + c NSI d Full ms2 1219.1467@hcd30.00 [120.0000-3752.7307]
Precursor Scan: 009597, m/z: 1218.8091, charge: 3
NEEYNK_6_5_0_3_0
Intensity
m/z
204.0865
366.1389
274.0916
138.0547
168.065
292.1025
186.0762
126.055
528.1921
657.23
999.4251 PEP_0_1_0_0_0
1688.6478 PEP_3_2_0_0_0
0
25000
50000
75000
100000
125000
150000
175000
200000
500
1000
1500
2000
2500

File: 231129_OE_RK_37, RT: 10.814, Scan: 009712
Type: HCD, FTMS + c NSI d Full ms2 1268.1663@hcd30.00 [120.0000-3902.7307]
Precursor Scan: 009597, m/z: 1267.4966, charge: 3
NEEYNK_6_5_1_3_0

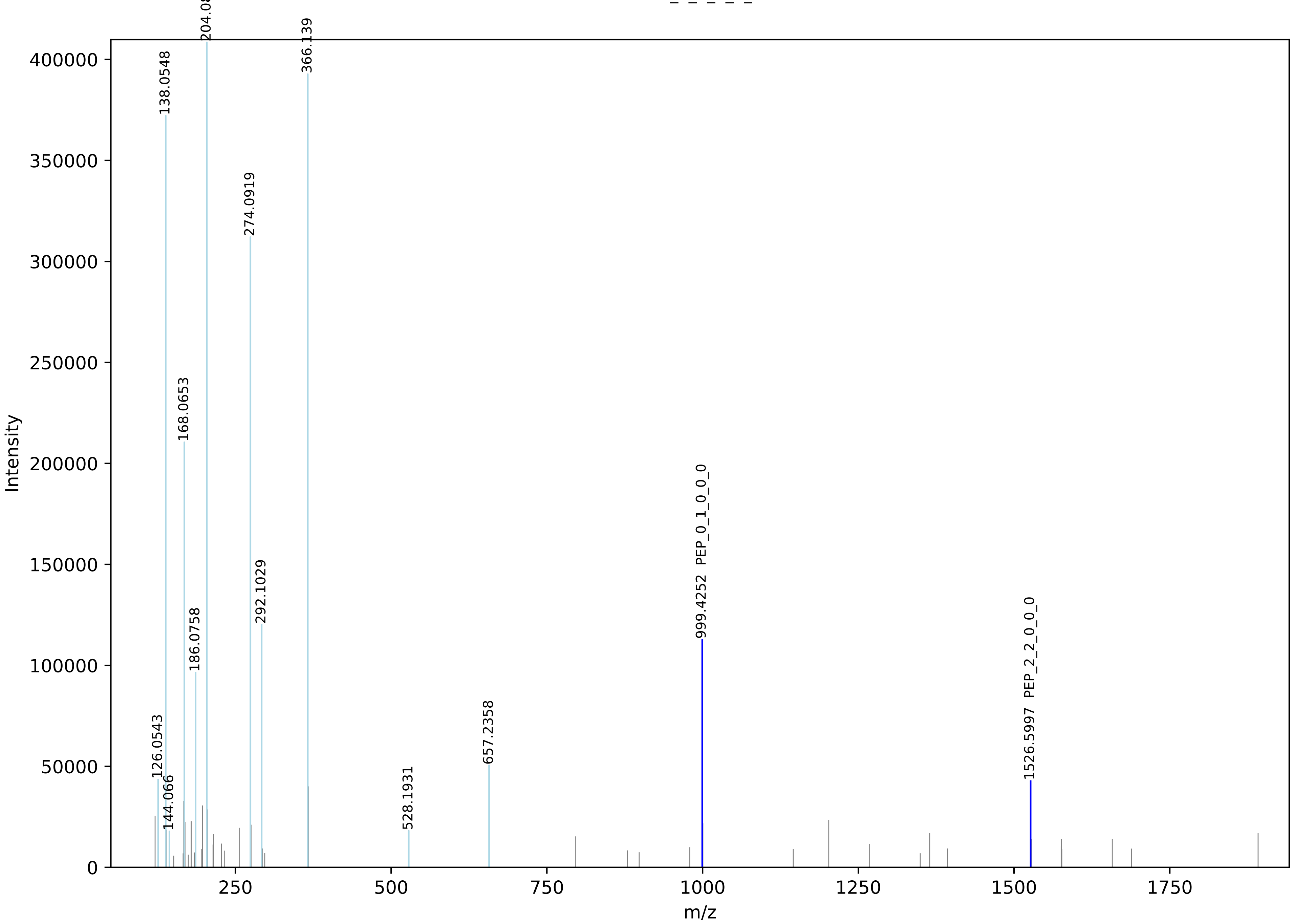

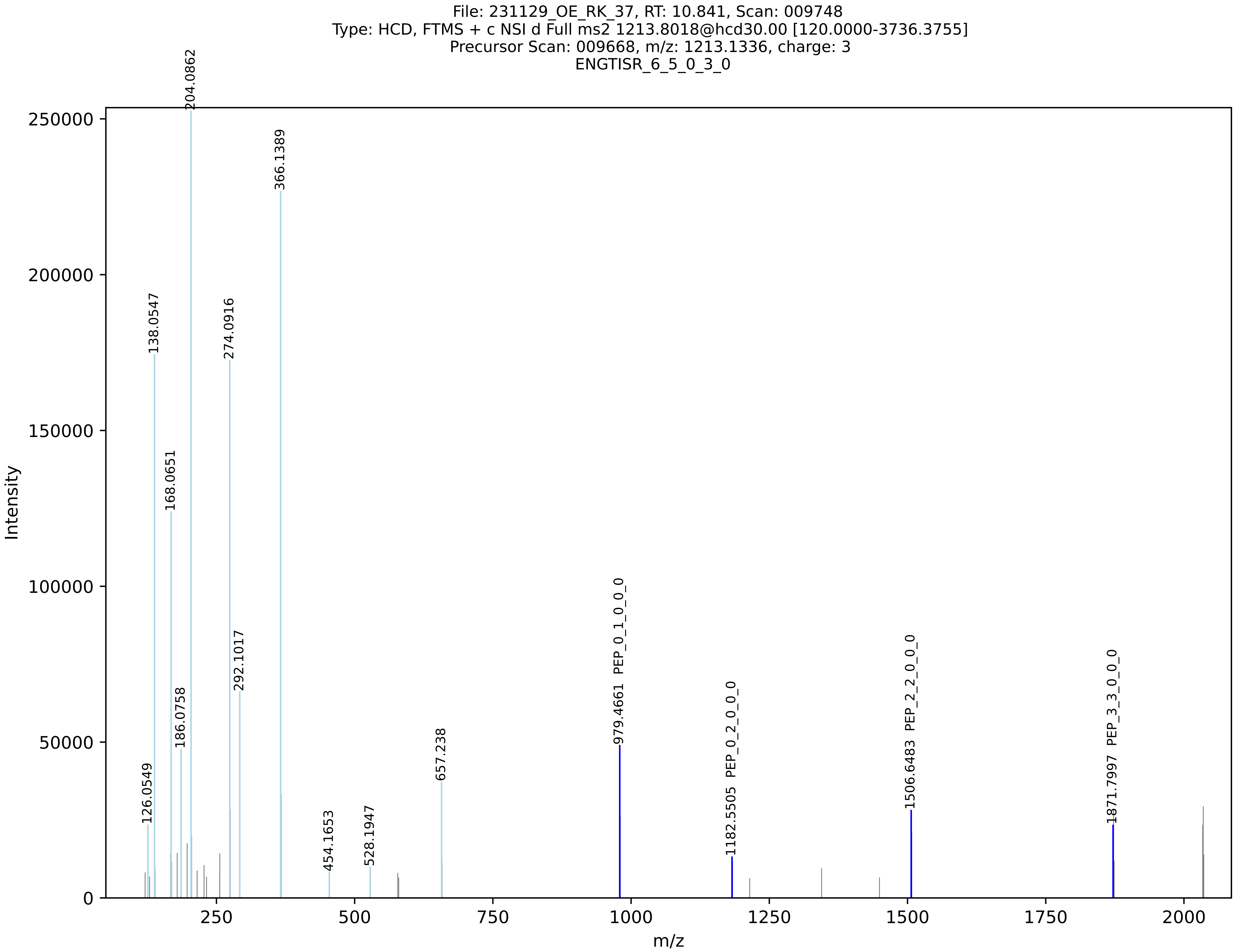
File: 231129_OE_RK_37, RT: 10.841, Scan: 009748
Type: HCD, FTMS + c NSI d Full ms2 1213.8018@hcd30.00 [120.0000-3736.3755]
Precursor Scan: 009668, m/z: 1213.1336, charge: 3
ENGTISR_6_5_0_3_0
Intensity
m/z
204.0862
366.1389
138.0547
274.0916
168.0651
292.1017
186.0758
126.0549
454.1653
528.1947
657.238
979.4661 PEP_0_1_0_0_0
1182.5505 PEP_0_2_0_0_0
1506.6483 PEP_2_2_0_0_0
1871.7997 PEP_3_3_0_0_0

File: 231129_OE_RK_37, RT: 10.841, Scan: 009749
Type: HCD, FTMS + c NSI d Full ms2 1038.6526@hcd30.00 [120.0000-4260.5586]
Precursor Scan: 009668, m/z: 1037.8997, charge: 4
ENGTISR_7_6_1_3_0

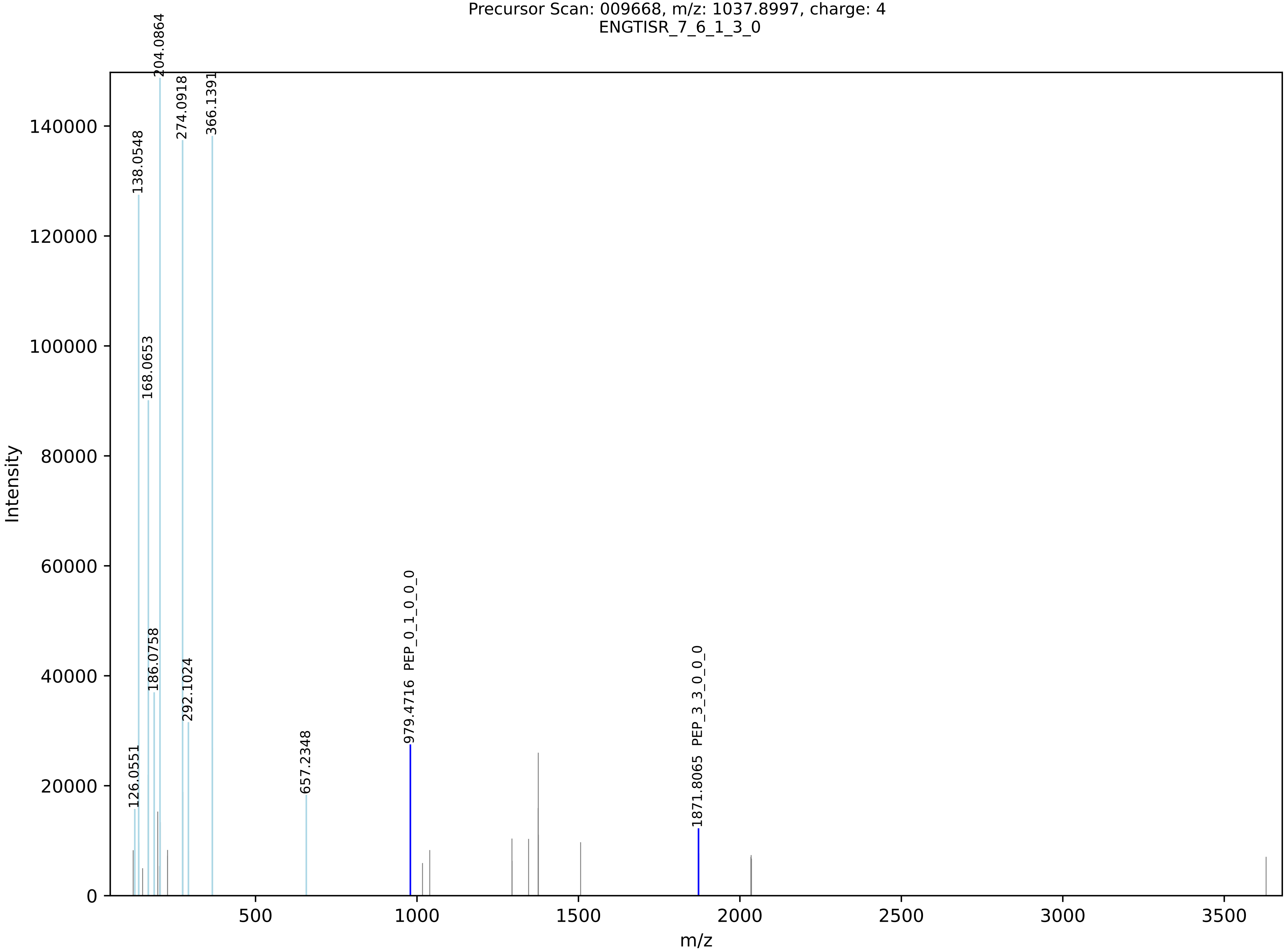

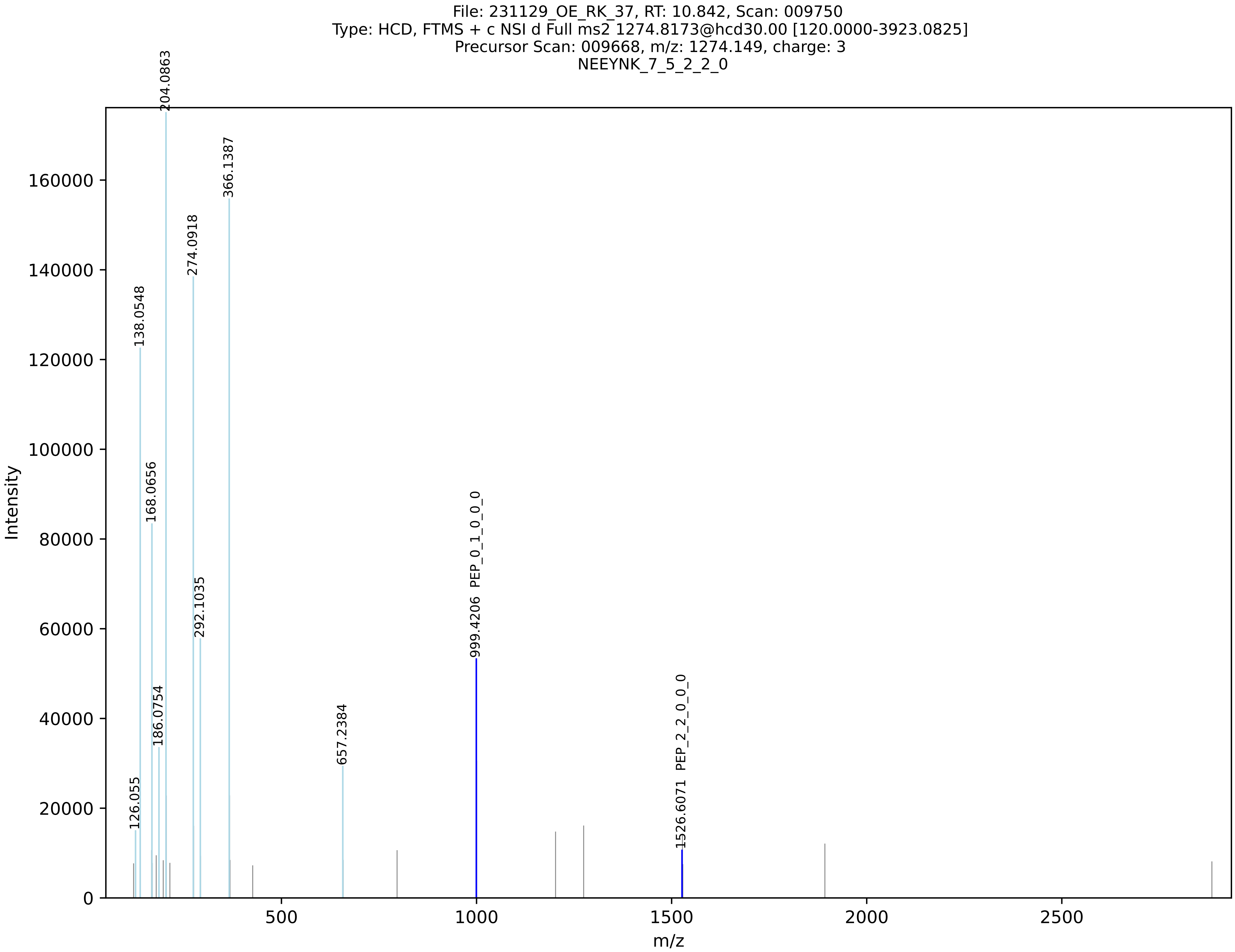
File: 231129_OE_RK_37, RT: 10.842, Scan: 009750
Type: HCD, FTMS + c NSI d Full ms2 1274.8173@hcd30.00 [120.0000-3923.0825]
Precursor Scan: 009668, m/z: 1274.149, charge: 3
NEEYNK_7_5_2_2_0
204.0863
366.1387
274.0918
138.0548
168.0656
292.1035
186.0754
126.055
657.2384
999.4206 PEP_0_1_0_0_0_
1526.6071 PEP_2_2_0_0_0_
Intensity
m/z
0
20000
40000
60000
80000
100000
120000
140000
160000
500
1000
1500
2000
2500

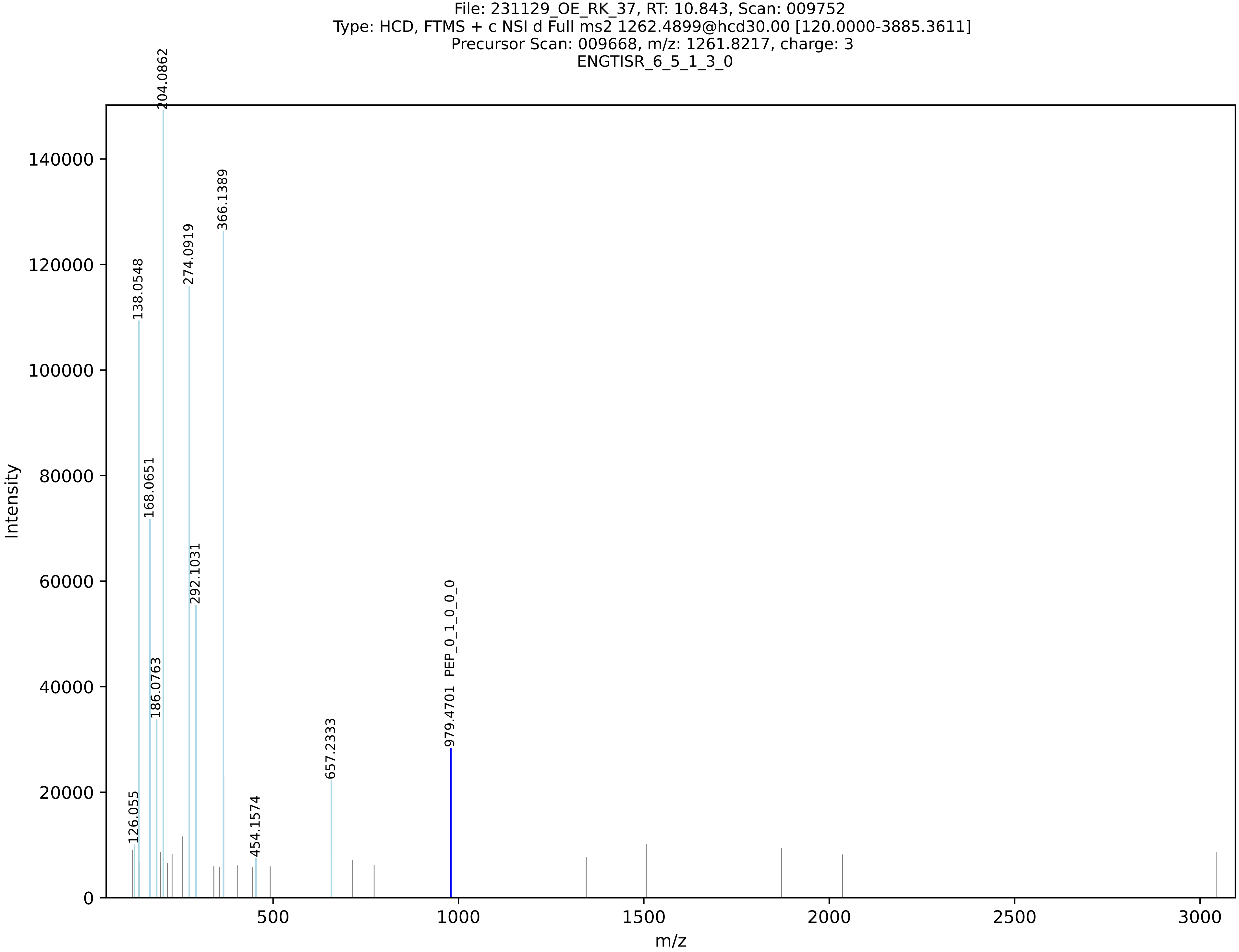
File: 231129_OE_RK_37, RT: 10.843, Scan: 009752
Type: HCD, FTMS + c NSI d Full ms2 1262.4899@hcd30.00 [120.0000-3885.3611]
Precursor Scan: 009668, m/z: 1261.8217, charge: 3
ENGTISR_6_5_1_3_0
204.0862
366.1389
274.0919
138.0548
168.0651
292.1031
186.0763
979.4701 PEP_0_1_0_0_0
657.2333
126.055
454.1574
Intensity
m/z
0
20000
40000
60000
80000
100000
120000
140000
500
1000
1500
2000
2500
3000

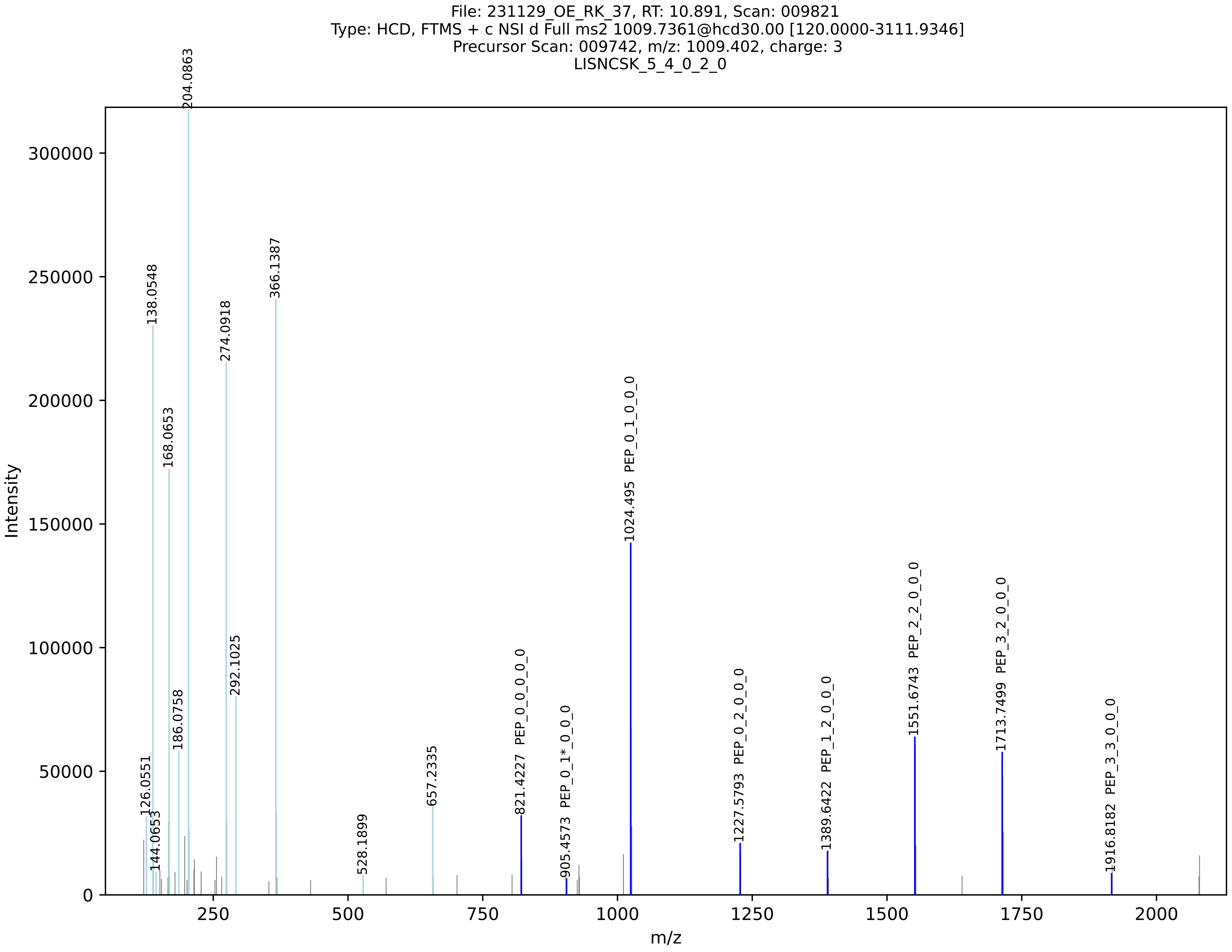
File: 231129_OE_RK_37, RT: 10.891, Scan: 009821
Type: HCD, FTMS + c NSI d Full ms2 1009.7361@hcd30.00 [120.0000-3111.9346]
Precursor Scan: 009742, m/z: 1009.402, charge: 3
LISNCSK_5_4_0_2_0
Intensity
m/z
204.0863
138.0548
366.1387
274.0918
168.0653
292.1025
186.0758
126.0551
144.0653
528.1899
657.2335
821.4227 PEP_0_0_0_0_0
905.4573 PEP_0_1*_0_0_0
1024.495 PEP_0_1_0_0_0
1227.5793 PEP_0_2_0_0_0
1389.6422 PEP_1_2_0_0_0
1551.6743 PEP_2_2_0_0_0
1713.7499 PEP_3_2_0_0_0
1916.8182 PEP_3_3_0_0_0

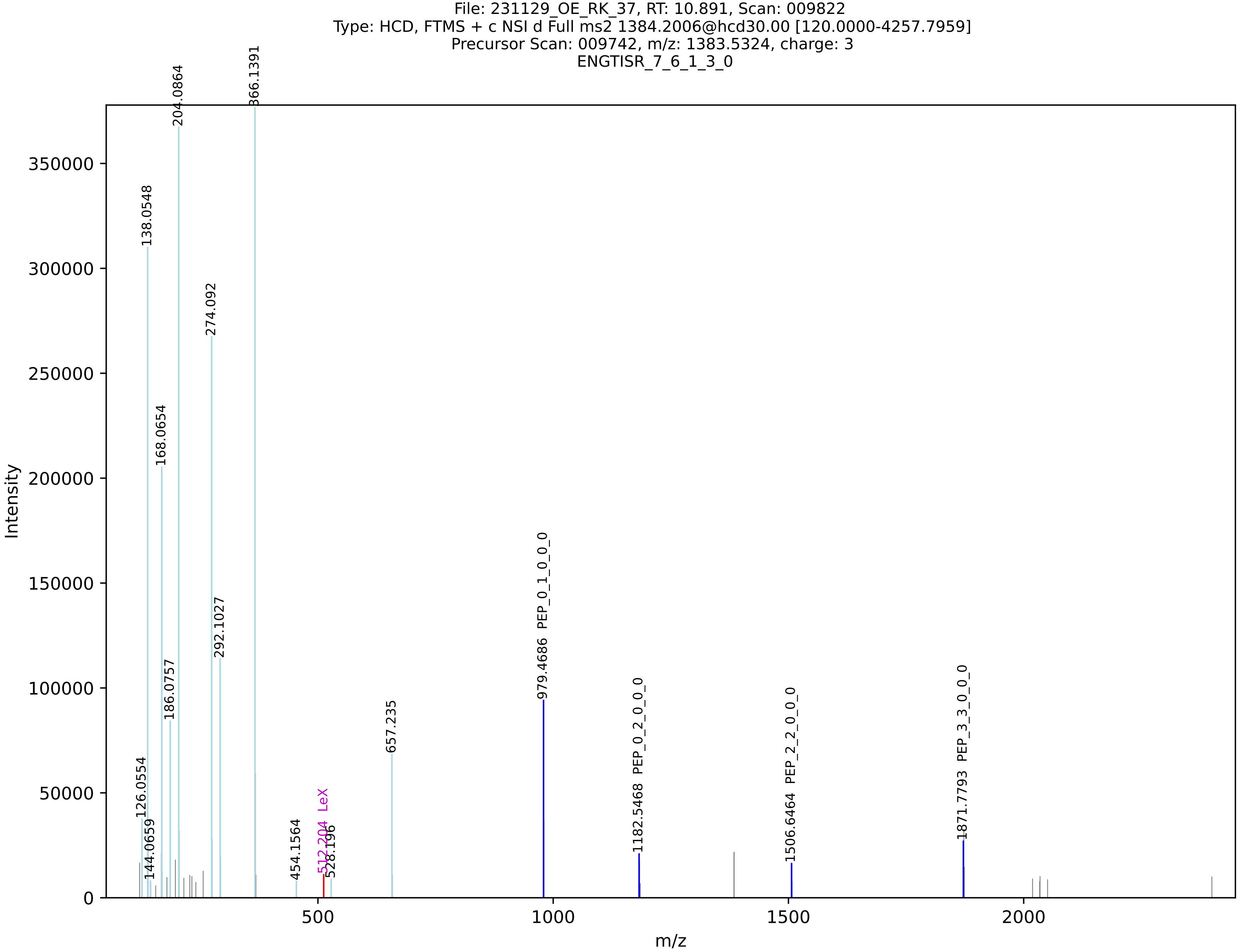
File: 231129_OE_RK_37, RT: 10.891, Scan: 009822
Type: HCD, FTMS + c NSI d Full ms2 1384.2006@hcd30.00 [120.0000-4257.7959]
Precursor Scan: 009742, m/z: 1383.5324, charge: 3
ENGTISR_7_6_1_3_0
Intensity
m/z
366.1391
204.0864
138.0548
274.092
168.0654
292.1027
186.0757
126.0554
144.0659
454.1564
512.204 LeX
528.196
657.235
979.4686 PEP_0_1_0_0_0
1182.5468 PEP_0_2_0_0_0
1506.6464 PEP_2_2_0_0_0
1871.7793 PEP_3_3_0_0_0

File: 231129_OE_RK_37, RT: 10.9, Scan: 009836
Type: HCD, FTMS + c NSI d Full ms2 1094.1394@hcd30.00 [120.0000-3370.2087]
Precursor Scan: 009742, m/z: 1093.4712, charge: 3
TKPREEQYNSTYR_4_4_1_0_0

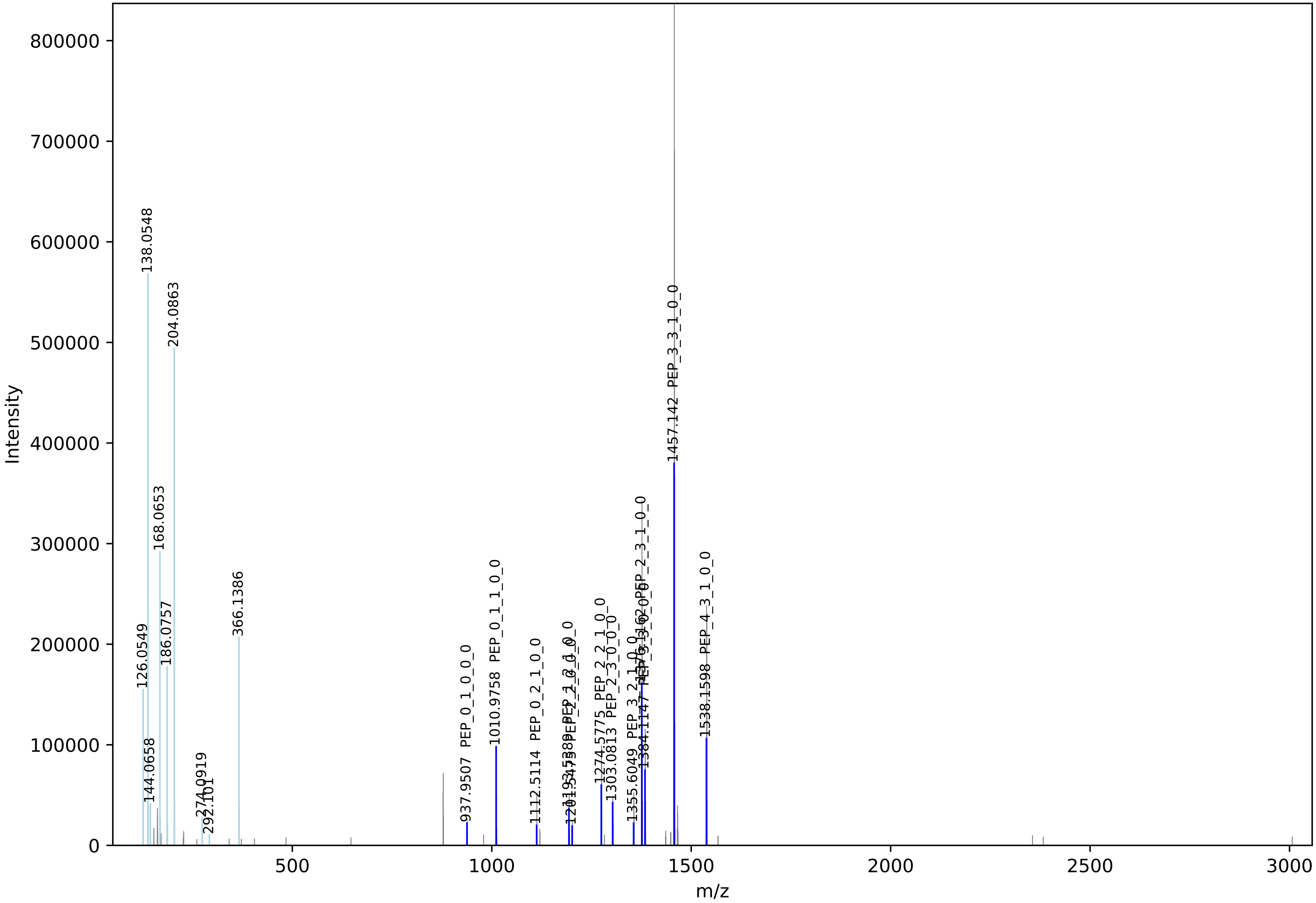

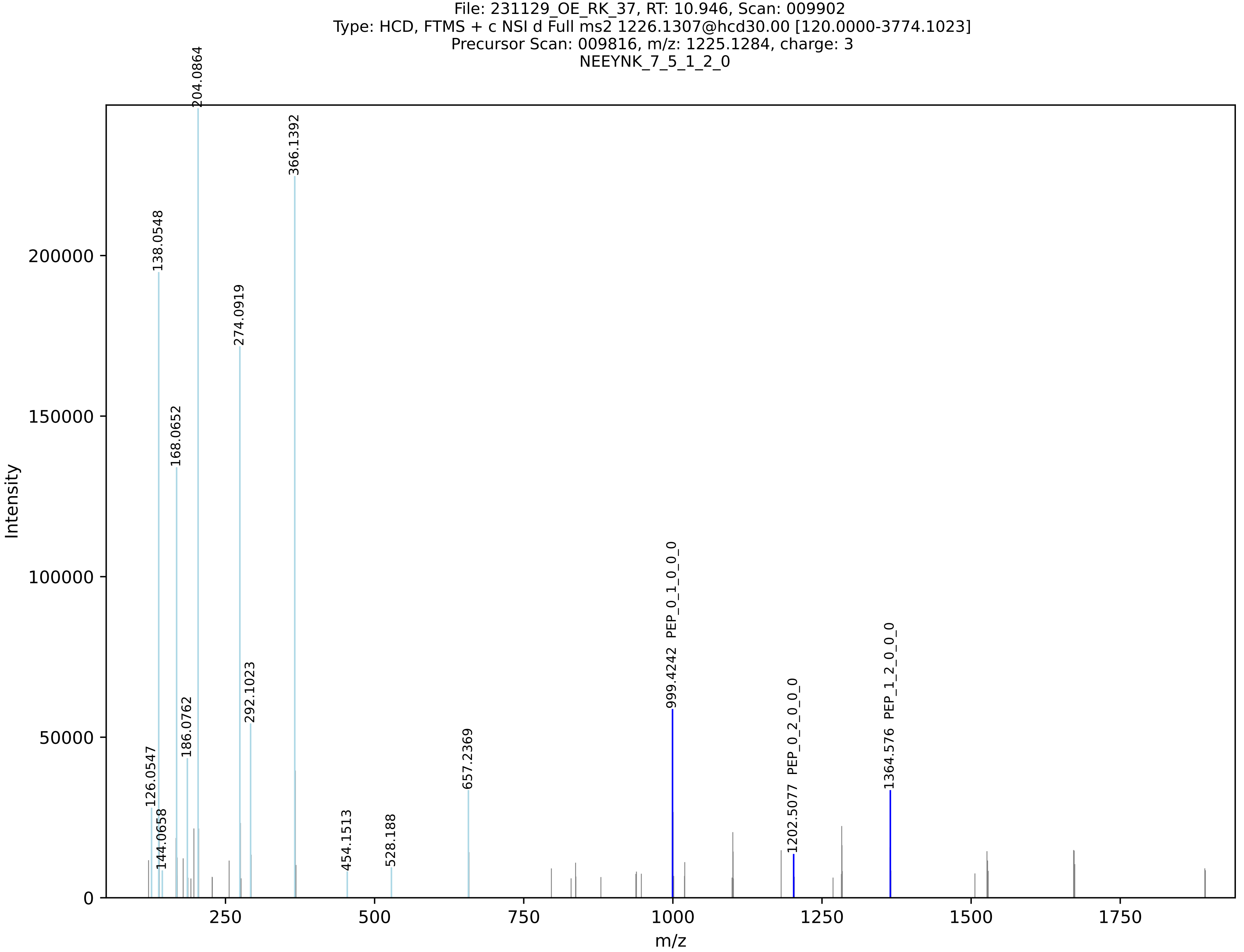
File: 231129_OE_RK_37, RT: 10.946, Scan: 009902
Type: HCD, FTMS + c NSI d Full ms2 1226.1307@hcd30.00 [120.0000-3774.1023]
Precursor Scan: 009816, m/z: 1225.1284, charge: 3
NEEYNK_7_5_1_2_0
Intensity
m/z
204.0864
366.1392
138.0548
274.0919
168.0652
292.1023
186.0762
126.0547
144.0658
454.1513
528.188
657.2369
999.4242 PEP_0_1_0_0_0
1202.5077 PEP_0_2_0_0_0
1364.576 PEP_1_2_0_0_0

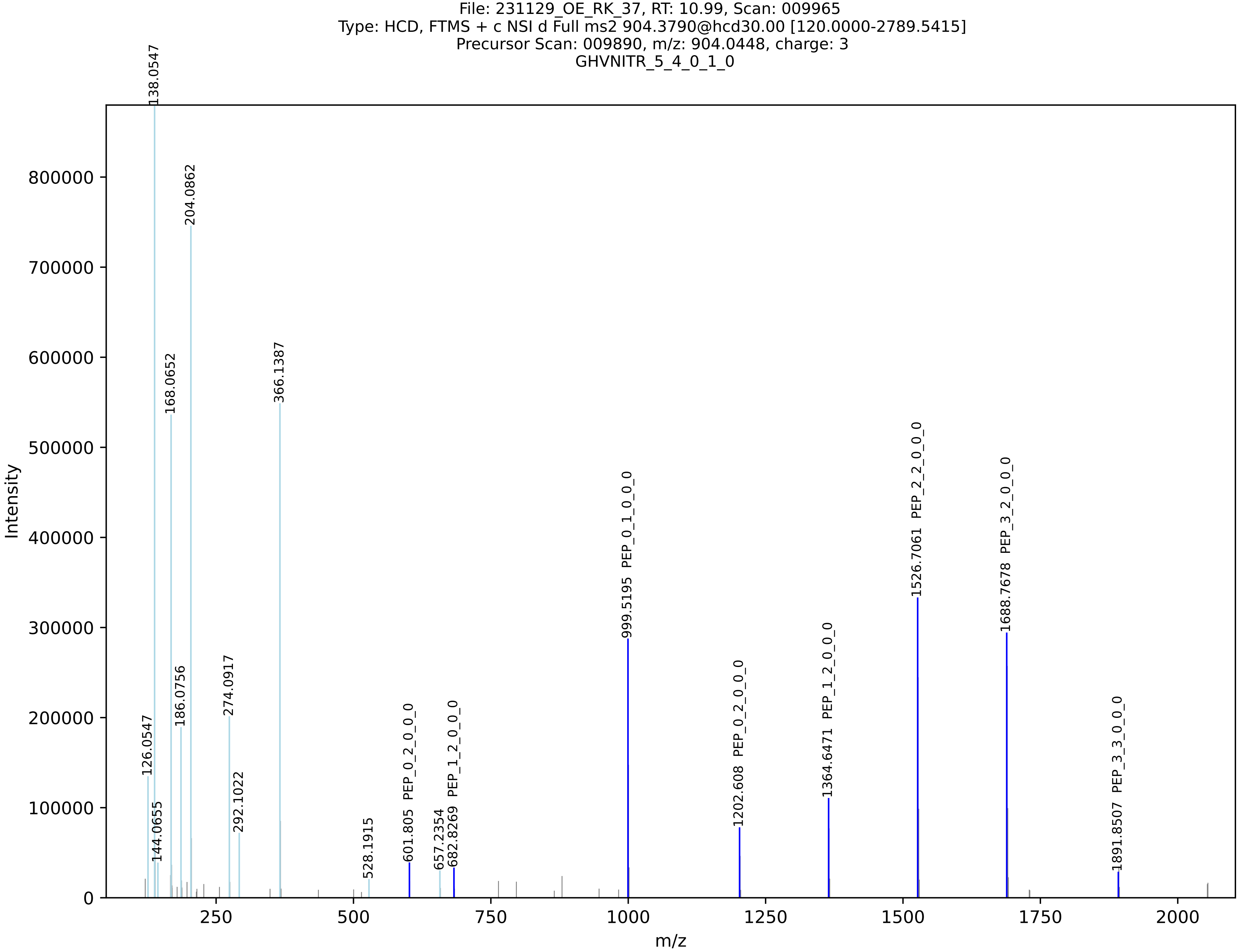

File: 231129_OE_RK_37, RT: 10.99, Scan: 009965
Type: HCD, FTMS + c NSI d Full ms2 904.3790@hcd30.00 [120.0000-2789.5415]
Precursor Scan: 009890, m/z: 904.0448, charge: 3
GHVNITR_5_4_0_1_0
Intensity
m/z
0
100000
200000
300000
400000
500000
600000
700000
800000
250
500
750
1000
1250
1500
1750
2000
138.0547
204.0862
168.0652
366.1387
126.0547
144.0655
186.0756
274.0917
292.1022
528.1915
601.805 PEP_0_2_0_0_0
657.2354
682.8269 PEP_1_2_0_0_0
999.5195 PEP_0_1_0_0_0
1202.608 PEP_0_2_0_0_0
1364.6471 PEP_1_2_0_0_0
1526.7061 PEP_2_2_0_0_0
1688.7678 PEP_3_2_0_0_0
1891.8507 PEP_3_3_0_0_0

File: 231129_OE_RK_37, RT: 10.99, Scan: 009966
Type: HCD, FTMS + c NSI d Full ms2 1220.4556@hcd30.00 [120.0000-3756.7361]
Precursor Scan: 009890, m/z: 1219.7873, charge: 3
NEEYNK_6_5_0_3_0

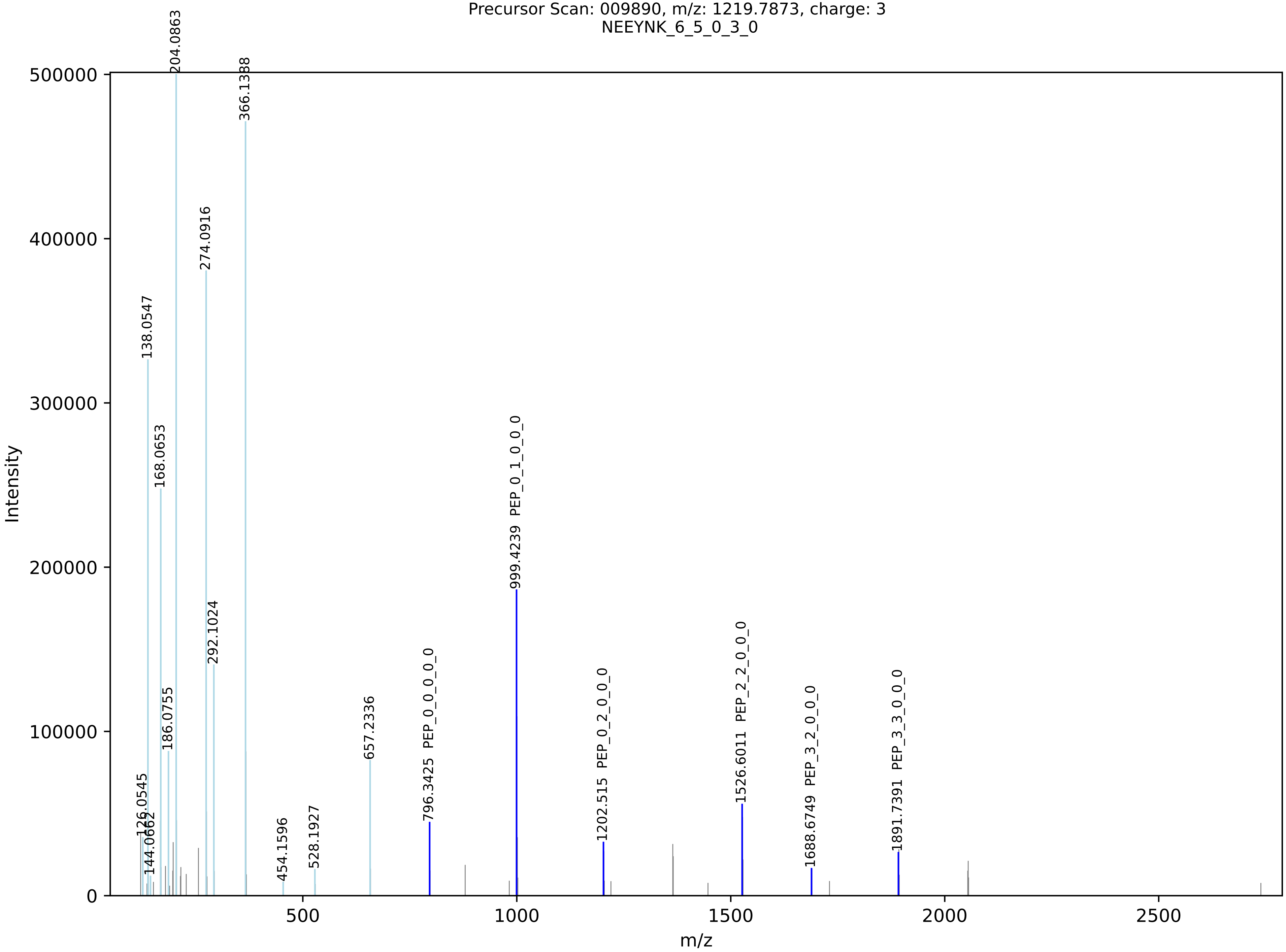

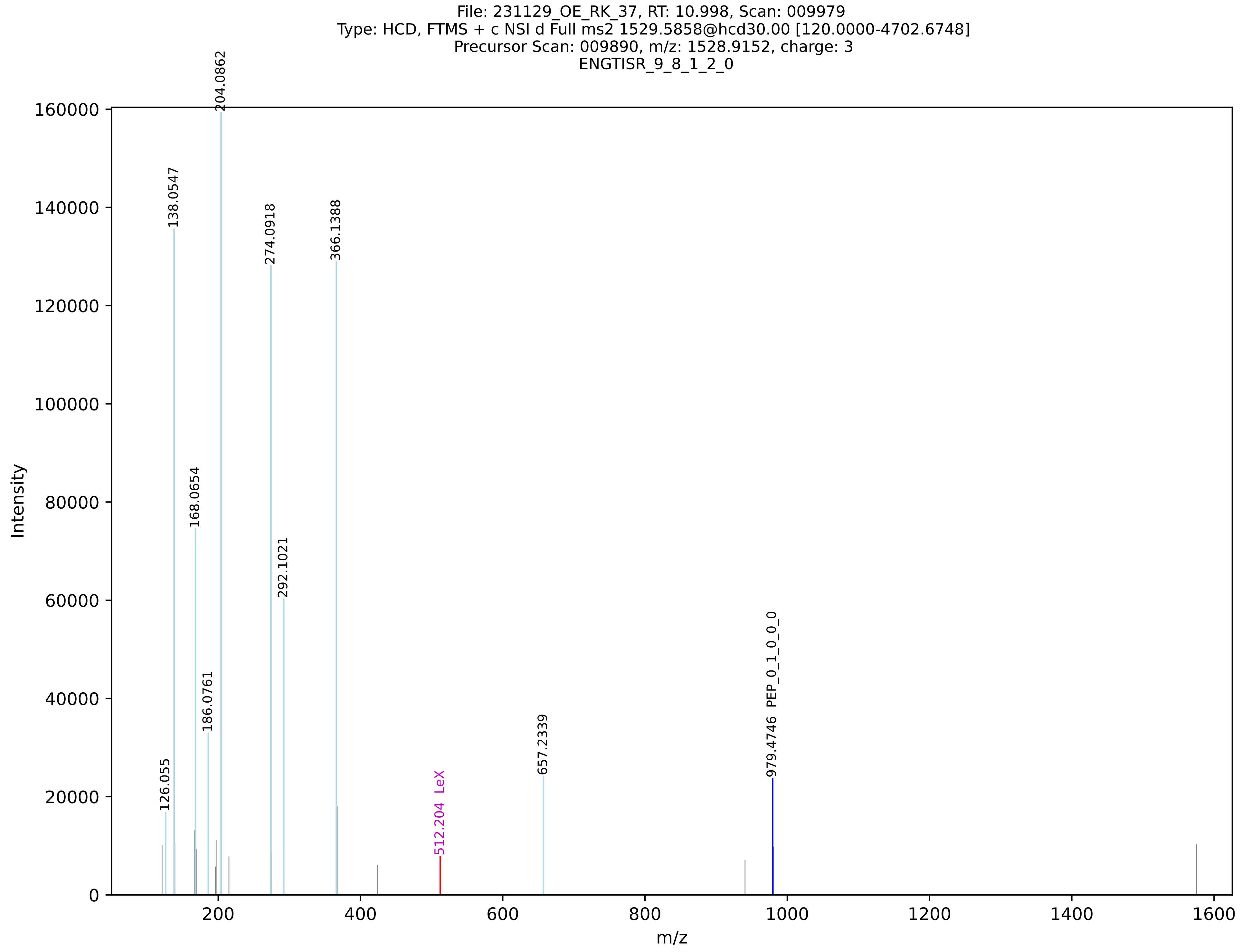
File: 231129_OE_RK_37, RT: 10.998, Scan: 009979
Type: HCD, FTMS + c NSI d Full ms2 1529.5858@hcd30.00 [120.0000-4702.6748]
Precursor Scan: 009890, m/z: 1528.9152, charge: 3
ENGTISR_9_8_1_2_0
Intensity
m/z
204.0862
138.0547
274.0918
366.1388
168.0654
292.1021
186.0761
126.055
512.204 LeX
657.2339
979.4746 PEP_0_1_0_0_0_
0
20000
40000
60000
80000
100000
120000
140000
160000
200
400
600
800
1000
1200
1400
1600

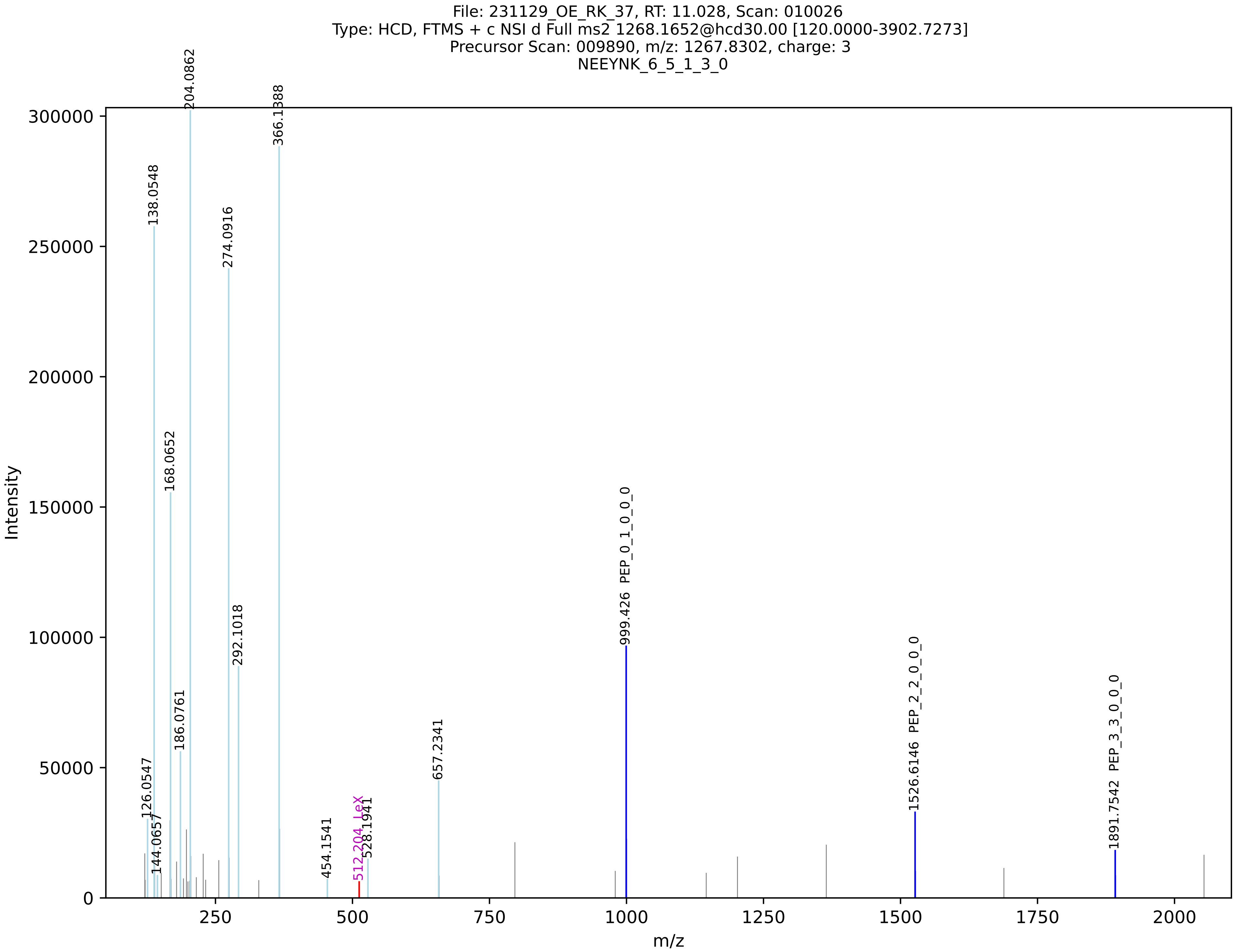
File: 231129_OE_RK_37, RT: 11.028, Scan: 010026
Type: HCD, FTMS + c NSI d Full ms2 1268.1652@hcd30.00 [120.0000-3902.7273]
Precursor Scan: 009890, m/z: 1267.8302, charge: 3
NEEYNK_6_5_1_3_0
Intensity
m/z
204.0862
366.1388
138.0548
274.0916
168.0652
292.1018
186.0761
126.0547
144.0657
454.1541
512.204_LeX
528.1941
657.2341
999.426 PEP_0_1_0_0_0
1526.6146 PEP_2_2_0_0_0
1891.7542 PEP_3_3_0_0_0

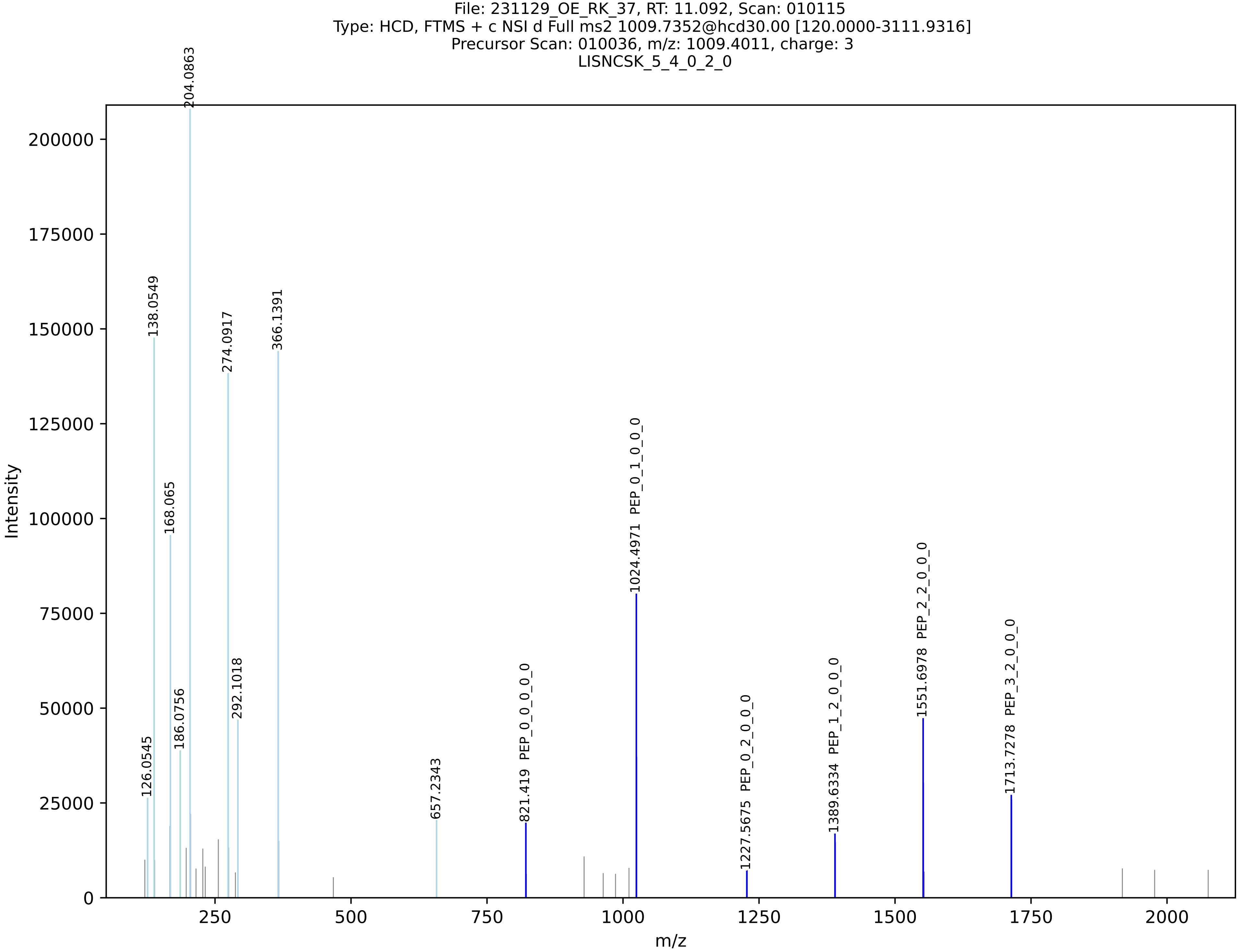
File: 231129_OE_RK_37, RT: 11.092, Scan: 010115
Type: HCD, FTMS + c NSI d Full ms2 1009.7352@hcd30.00 [120.0000-3111.9316]
Precursor Scan: 010036, m/z: 1009.4011, charge: 3
LISNCSK_5_4_0_2_0
Intensity
m/z
0
25000
50000
75000
100000
125000
150000
175000
200000
250
500
750
1000
1250
1500
1750
2000
204.0863
138.0549
274.0917
366.1391
168.065
186.0756
292.1018
126.0545
657.2343
821.419 PEP_0_0_0_0_0
1024.4971 PEP_0_1_0_0_0
1227.5675 PEP_0_2_0_0_0
1389.6334 PEP_1_2_0_0_0
1551.6978 PEP_2_2_0_0_0
1713.7278 PEP_3_2_0_0_0

File: 231129_OE_RK_37, RT: 11.093, Scan: 010116
Type: HCD, FTMS + c NSI d Full ms2 1384.1985@hcd30.00 [120.0000-4257.7896]
Precursor Scan: 010036, m/z: 1383.5303, charge: 3
ENGTISR_7_6_1_3_0

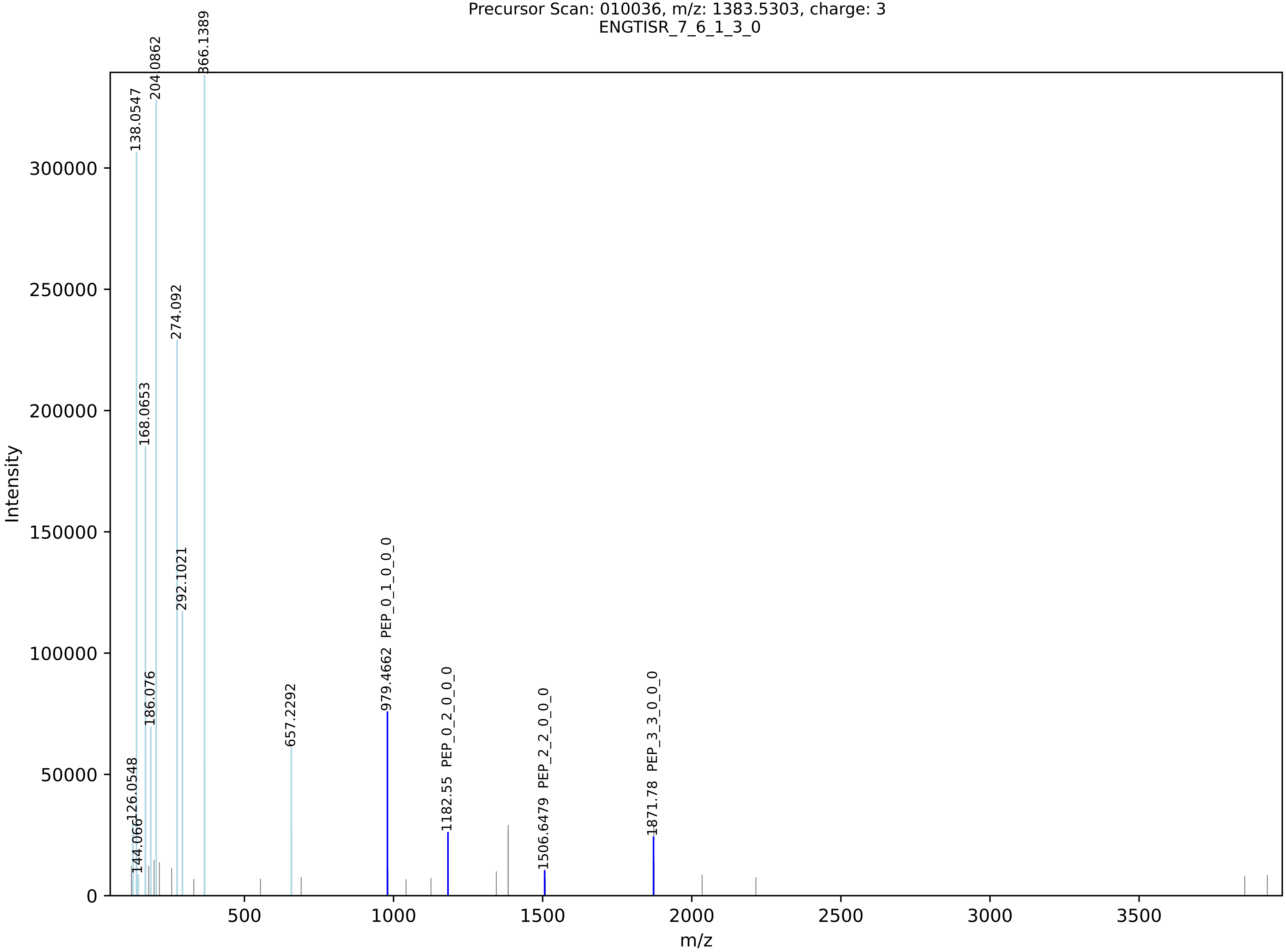

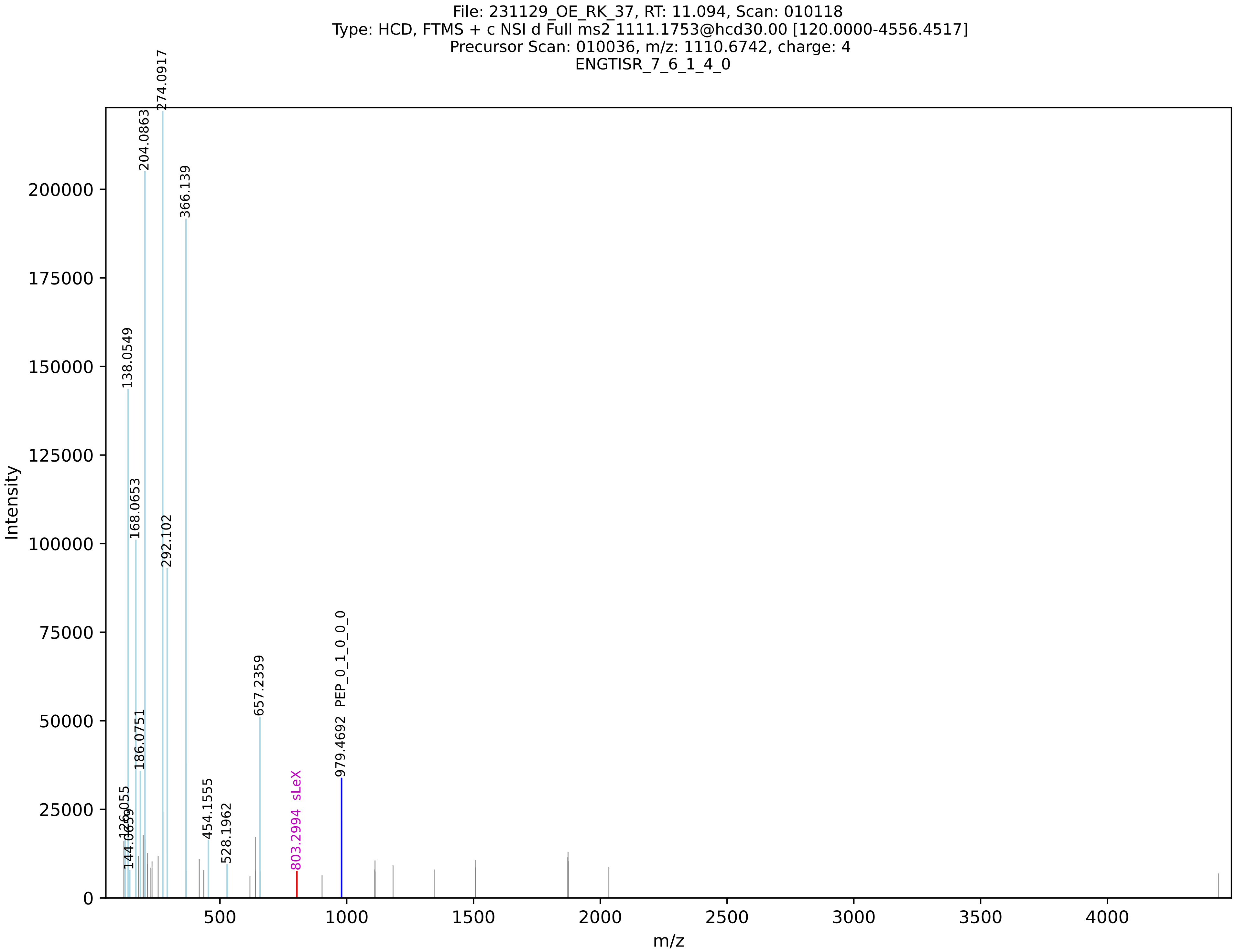

File: 231129_OE_RK_37, RT: 11.094, Scan: 010118
Type: HCD, FTMS + c NSI d Full ms2 1111.1753@hcd30.00 [120.0000-4556.4517]
Precursor Scan: 010036, m/z: 1110.6742, charge: 4
ENGTISR_7_6_1_4_0
Intensity
m/z
274.0917
204.0863
366.139
138.0549
168.0653
292.102
186.0751
126.055
144.0659
454.1555
528.1962
657.2359
803.2994 sLeX
979.4692 PEP_0_1_0_0_0

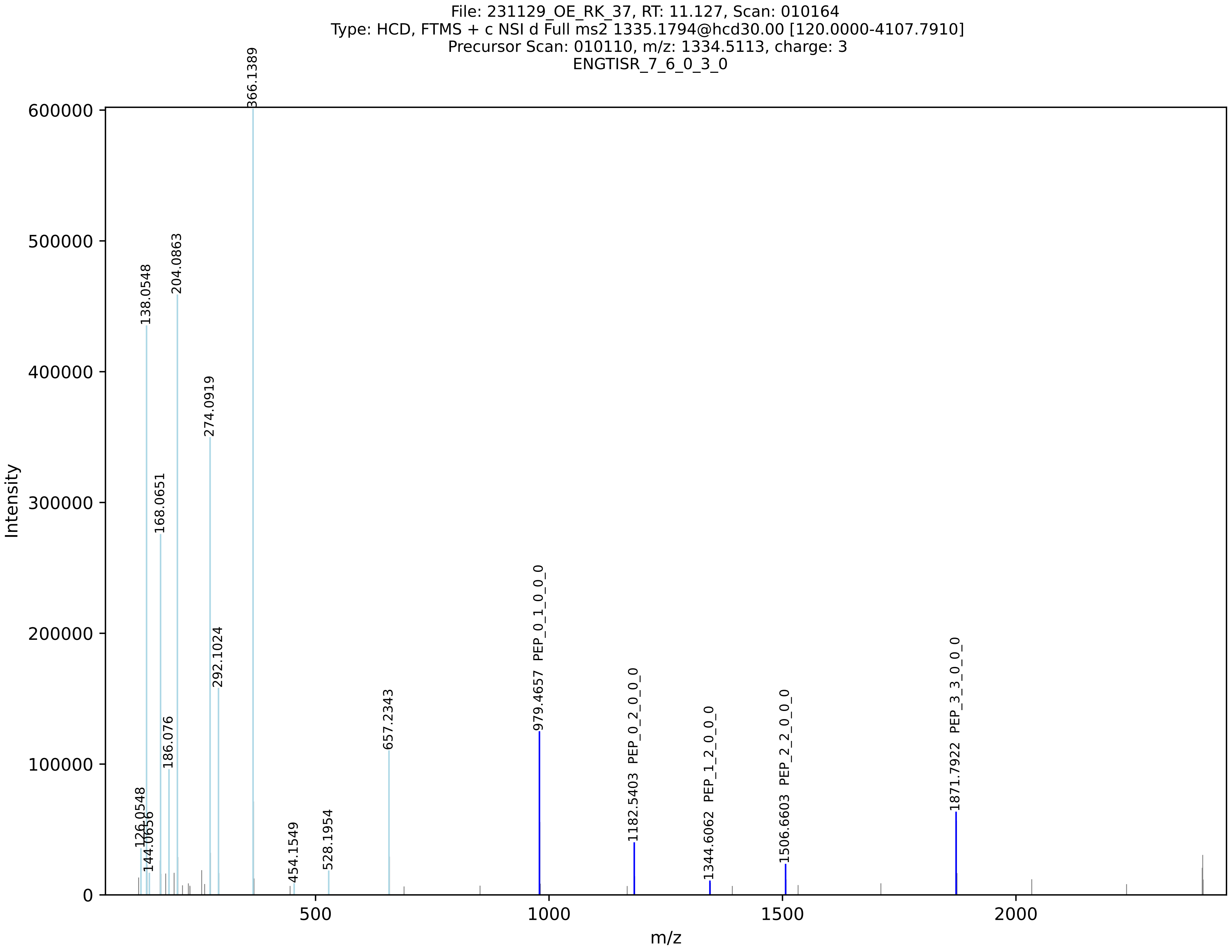

File: 231129_OE_RK_37, RT: 11.127, Scan: 010164
Type: HCD, FTMS + c NSI d Full ms2 1335.1794@hcd30.00 [120.0000-4107.7910]
Precursor Scan: 010110, m/z: 1334.5113, charge: 3
ENGTISR_7_6_0_3_0
Intensity
m/z
366.1389
138.0548
204.0863
274.0919
168.0651
292.1024
186.076
126.0548
144.0656
454.1549
528.1954
657.2343
979.4657 PEP_0_1_0_0_0
1182.5403 PEP_0_2_0_0_0
1344.6062 PEP_1_2_0_0_0
1506.6603 PEP_2_2_0_0_0
1871.7922 PEP_3_3_0_0_0

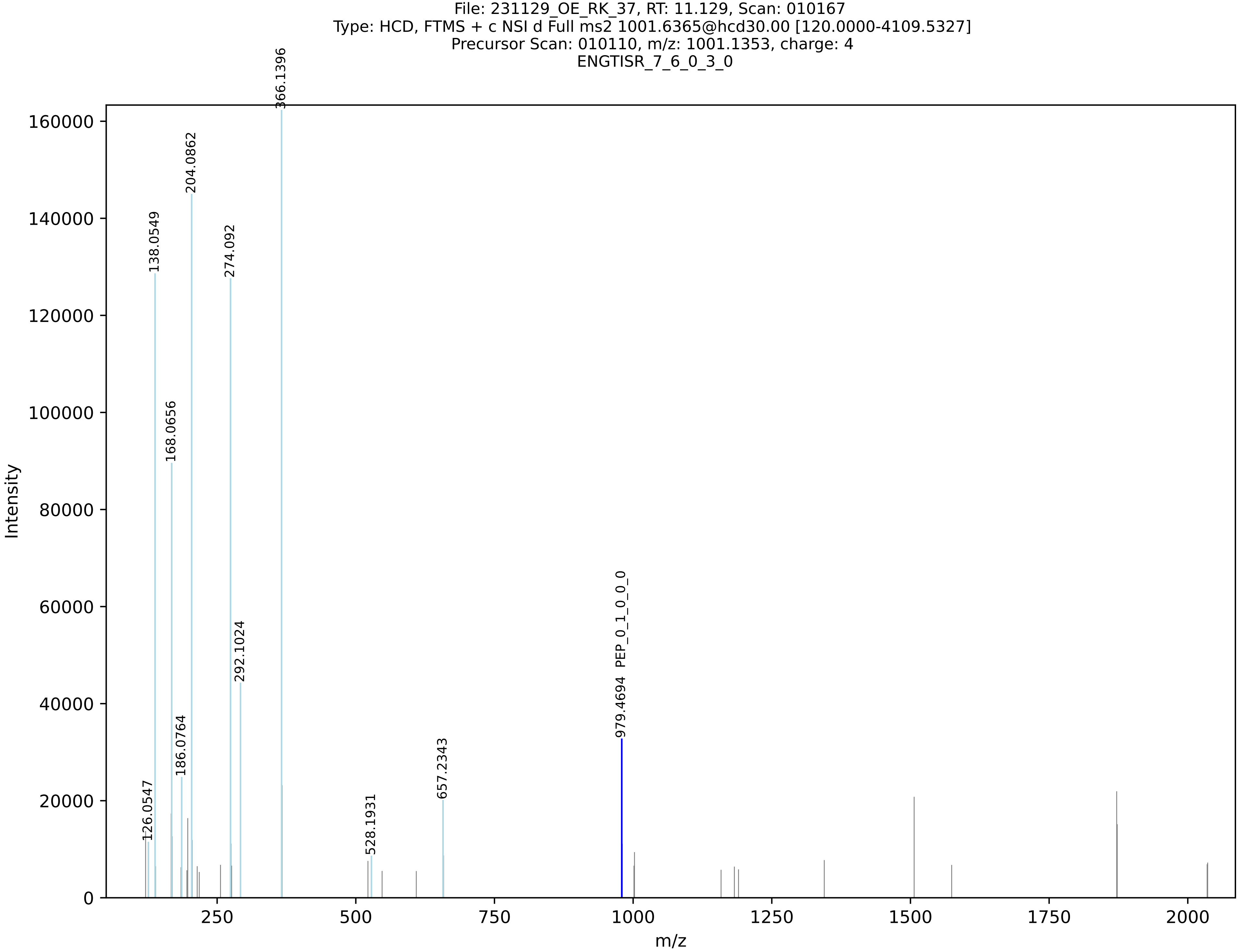
File: 231129_OE_RK_37, RT: 11.129, Scan: 010167
Type: HCD, FTMS + c NSI d Full ms2 1001.6365@hcd30.00 [120.0000-4109.5327]
Precursor Scan: 010110, m/z: 1001.1353, charge: 4
ENGTISR_7_6_0_3_0
Intensity
m/z
366.1396
204.0862
138.0549
274.092
168.0656
292.1024
186.0764
126.0547
528.1931
657.2343
979.4694 PEP_0_1_0_0_0
0
20000
40000
60000
80000
100000
120000
140000
160000
250
500
750
1000
1250
1500
1750
2000

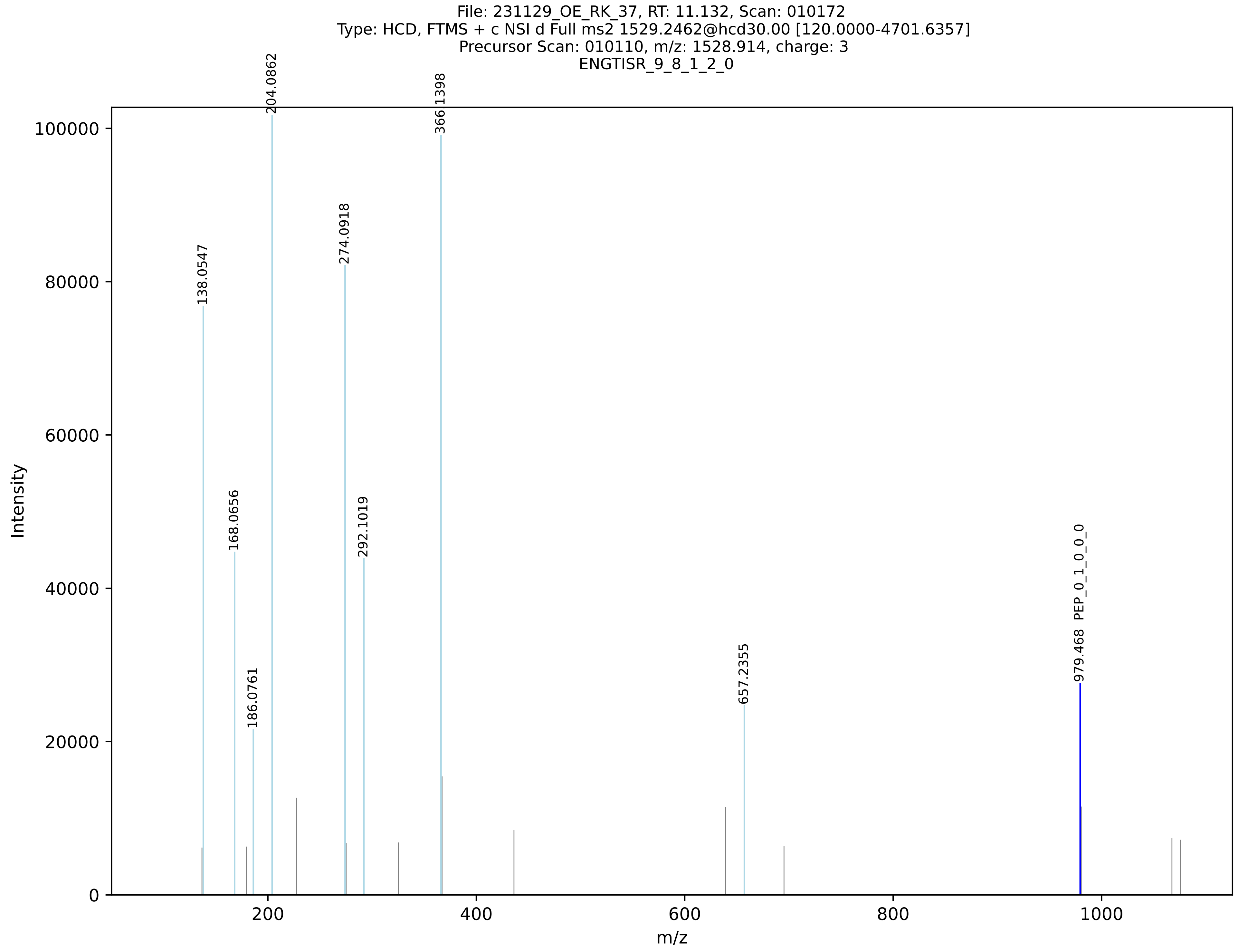

File: 231129_OE_RK_37, RT: 11.132, Scan: 010172
Type: HCD, FTMS + c NSI d Full ms2 1529.2462@hcd30.00 [120.0000-4701.6357]
Precursor Scan: 010110, m/z: 1528.914, charge: 3
ENGTISR_9_8_1_2_0
Intensity
m/z
0
20000
40000
60000
80000
100000
200
400
600
800
1000
138.0547
168.0656
186.0761
204.0862
274.0918
292.1019
366.1398
657.2355
979.468 PEP_0_1_0_0_0

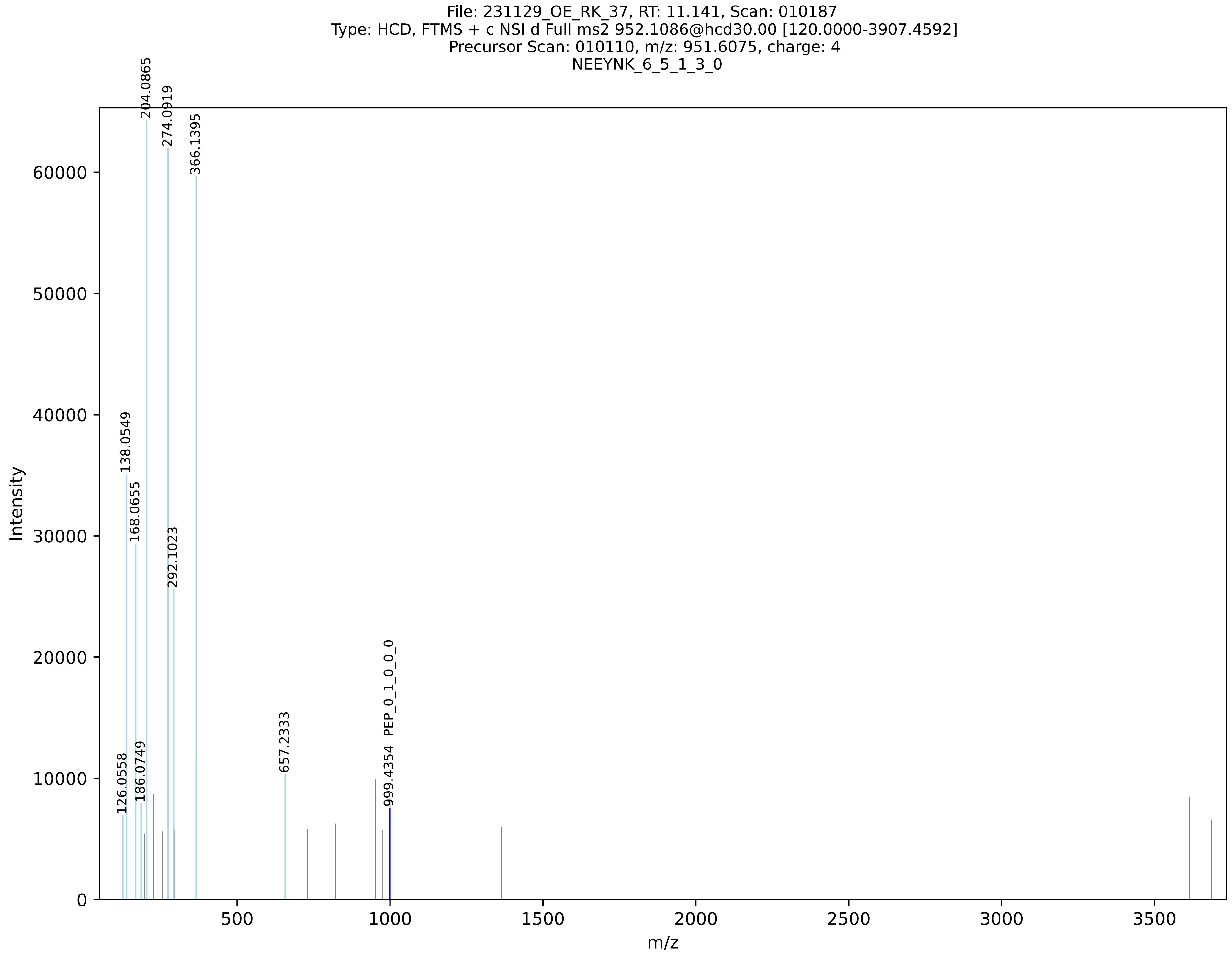

File: 231129_OE_RK_37, RT: 11.141, Scan: 010187
Type: HCD, FTMS + c NSI d Full ms2 952.1086@hcd30.00 [120.0000-3907.4592]
Precursor Scan: 010110, m/z: 951.6075, charge: 4
NEEYNK_6_5_1_3_0
204.0865
274.0919
366.1395
138.0549
168.0655
292.1023
657.2333
PEP_0_1_0_0_0
999.4354
126.0558
186.0749
Intensity
m/z
0
10000
20000
30000
40000
50000
60000
500
1000
1500
2000
2500
3000
3500

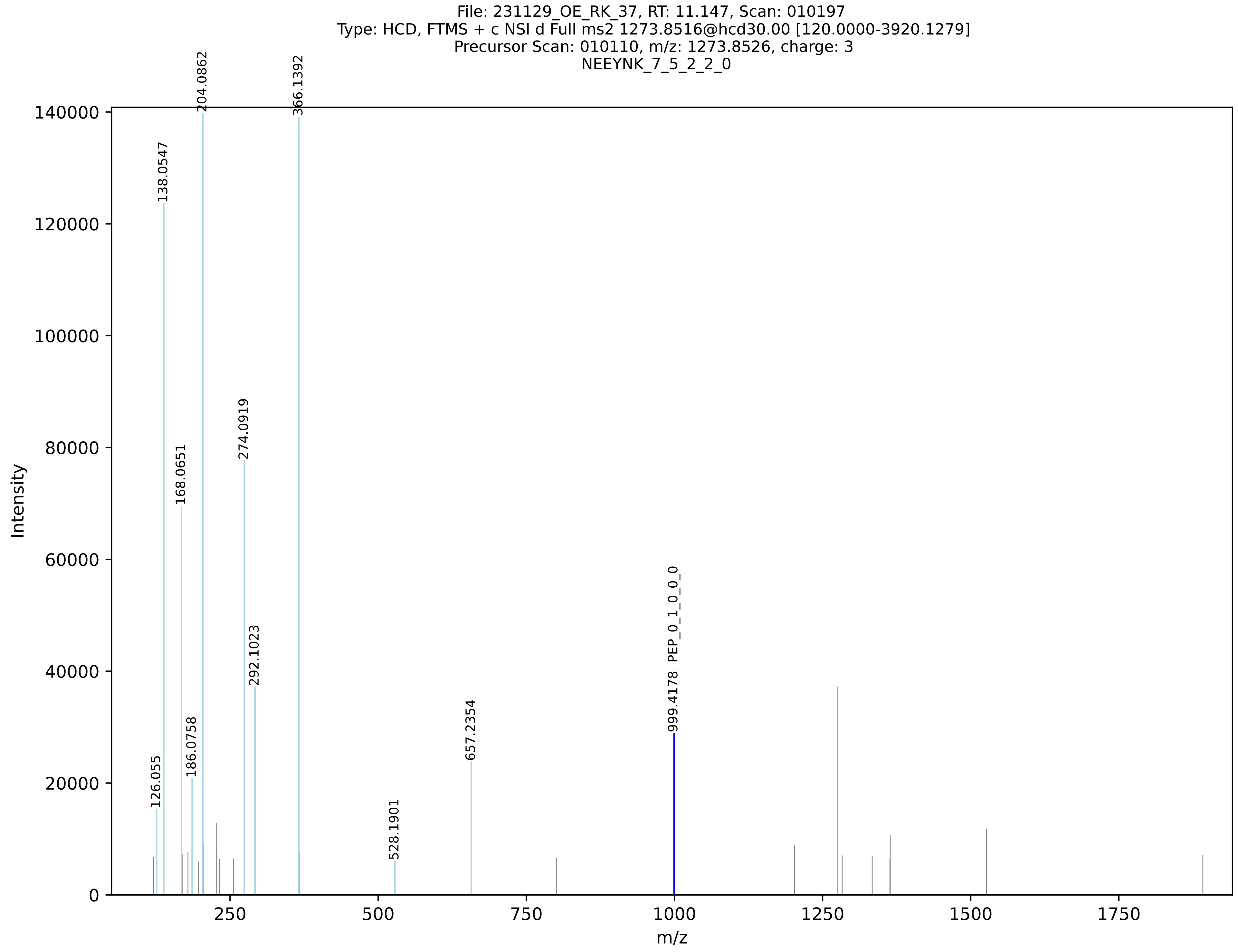
File: 231129_OE_RK_37, RT: 11.147, Scan: 010197
Type: HCD, FTMS + c NSI d Full ms2 1273.8516@hcd30.00 [120.0000-3920.1279]
Precursor Scan: 010110, m/z: 1273.8526, charge: 3
NEEYNK_7_5_2_2_0
Intensity
m/z
0
20000
40000
60000
80000
100000
120000
140000
250
500
750
1000
1250
1500
1750
126.055
138.0547
168.0651
186.0758
204.0862
274.0919
292.1023
366.1392
528.1901
657.2354
999.4178 PEP_0_1_0_0_0

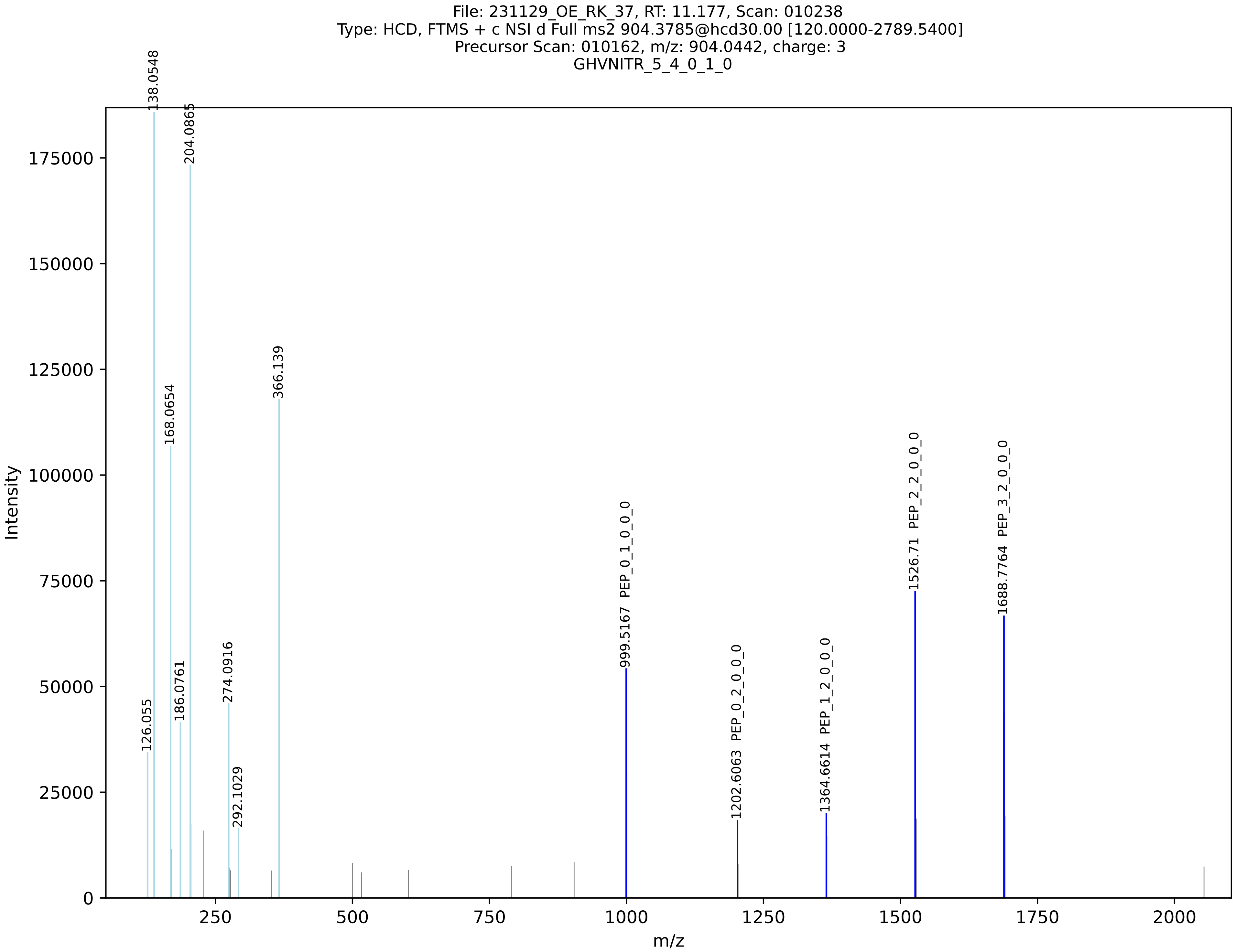
File: 231129_OE_RK_37, RT: 11.177, Scan: 010238
Type: HCD, FTMS + c NSI d Full ms2 904.3785@hcd30.00 [120.0000-2789.5400]
Precursor Scan: 010162, m/z: 904.0442, charge: 3
GHVNITR_5_4_0_1_0
138.0548
204.0865
168.0654
366.139
126.055
186.0761
274.0916
292.1029
999.5167 PEP_0_1_0_0_0
1202.6063 PEP_0_2_0_0_0
1364.6614 PEP_1_2_0_0_0
1526.71 PEP_2_2_0_0_0
1688.7764 PEP_3_2_0_0_0
Intensity
m/z
0
25000
50000
75000
100000
125000
150000
175000
250
500
750
1000
1250
1500
1750
2000

File: 231129_OE_RK_37, RT: 11.178, Scan: 010239
Type: HCD, FTMS + c NSI d Full ms2 1432.5491@hcd30.00 [120.0000-4405.7422]
Precursor Scan: 010162, m/z: 1431.8795, charge: 3
ENGTISR_7_6_0_4_0

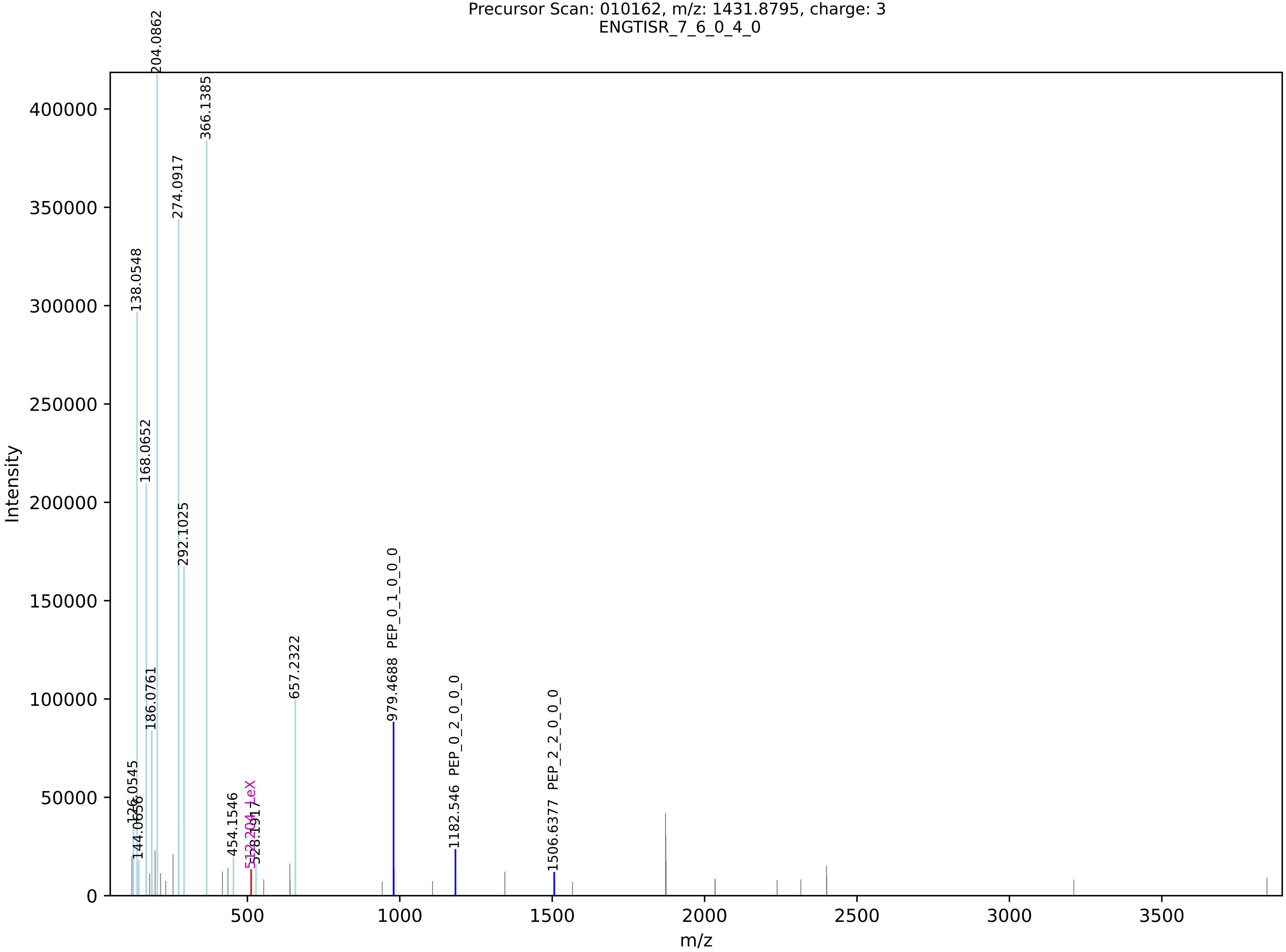

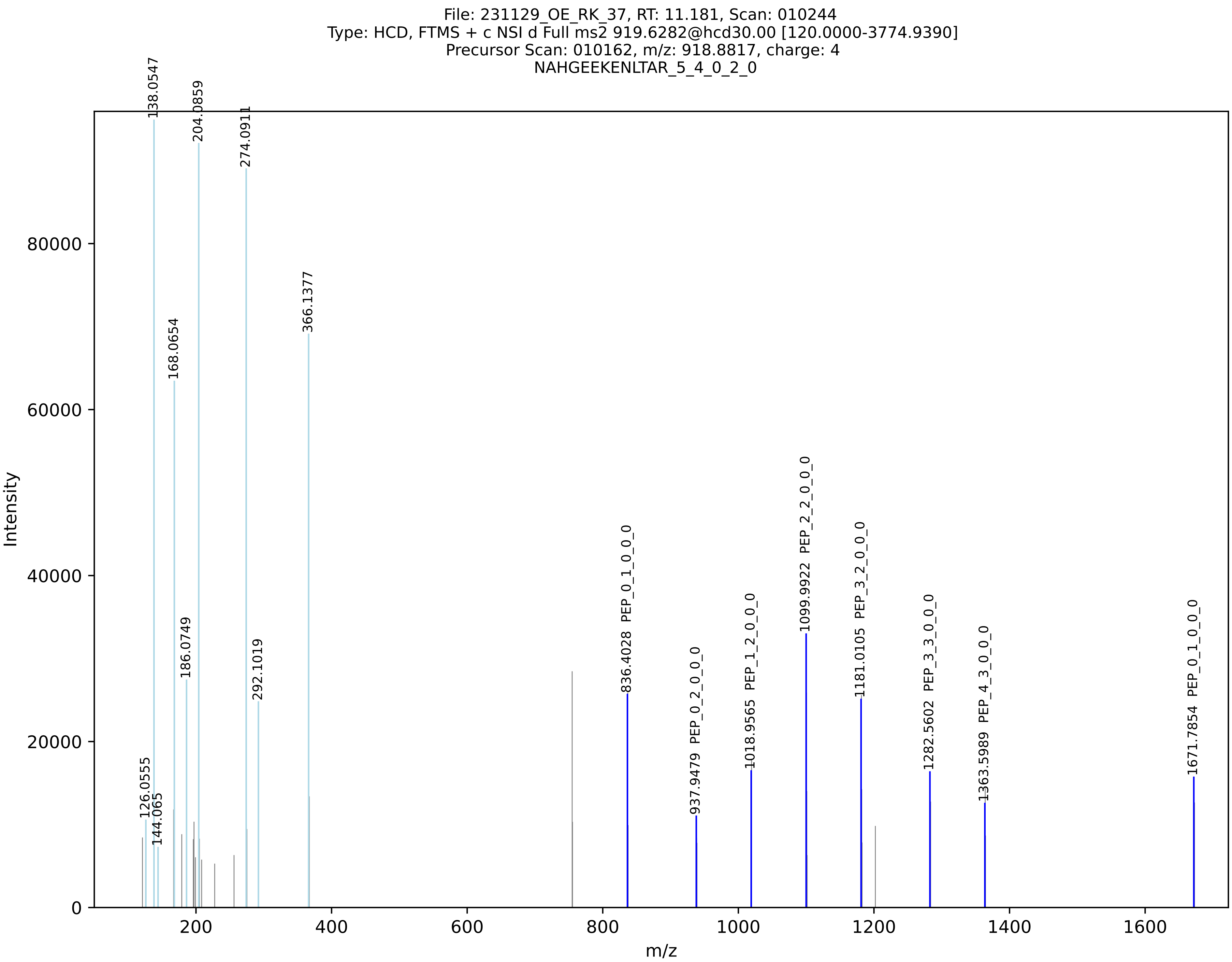
File: 231129_OE_RK_37, RT: 11.181, Scan: 010244
Type: HCD, FTMS + c NSI d Full ms2 919.6282@hcd30.00 [120.0000-3774.9390]
Precursor Scan: 010162, m/z: 918.8817, charge: 4
NAHGEEKENLTAR_5_4_0_2_0
138.0547
204.0859
274.0911
366.1377
168.0654
186.0749
292.1019
126.0555
144.065
836.4028 PEP_0_1_0_0_0
937.9479 PEP_0_2_0_0_0
1018.9565 PEP_1_2_0_0_0
1099.9922 PEP_2_2_0_0_0
1181.0105 PEP_3_2_0_0_0
1282.5602 PEP_3_3_0_0_0
1363.5989 PEP_4_3_0_0_0
1671.7854 PEP_0_1_0_0_0
Intensity
m/z
0
20000
40000
60000
80000
200
400
600
800
1000
1200
1400
1600

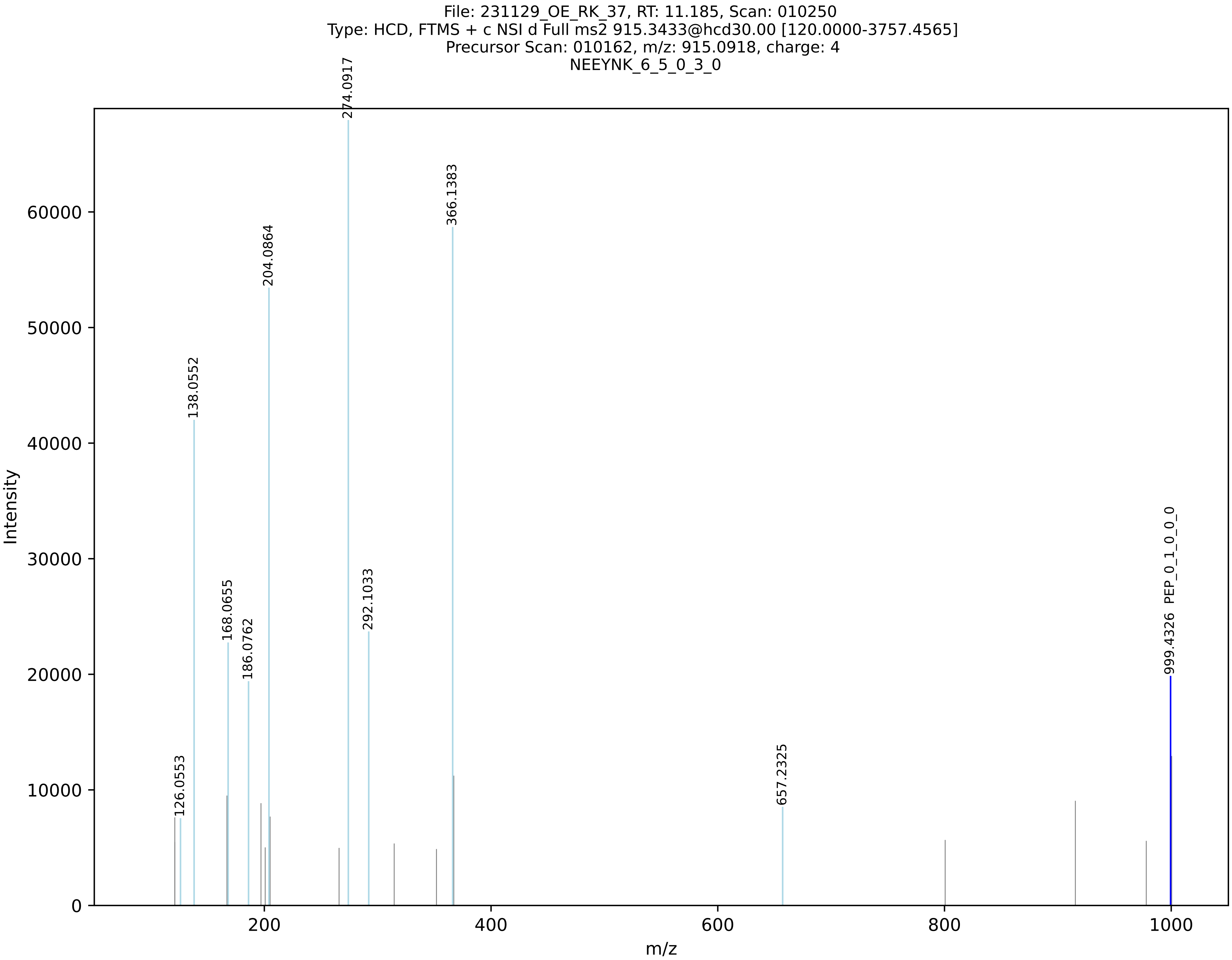
File: 231129_OE_RK_37, RT: 11.185, Scan: 010250
Type: HCD, FTMS + c NSI d Full ms2 915.3433@hcd30.00 [120.0000-3757.4565]
Precursor Scan: 010162, m/z: 915.0918, charge: 4
NEEYNK_6_5_0_3_0
Intensity
m/z
274.0917
366.1383
204.0864
138.0552
168.0655
186.0762
292.1033
126.0553
657.2325
999.4326 PEP_0_1_0_0_0
0
10000
20000
30000
40000
50000
60000
200
400
600
800
1000

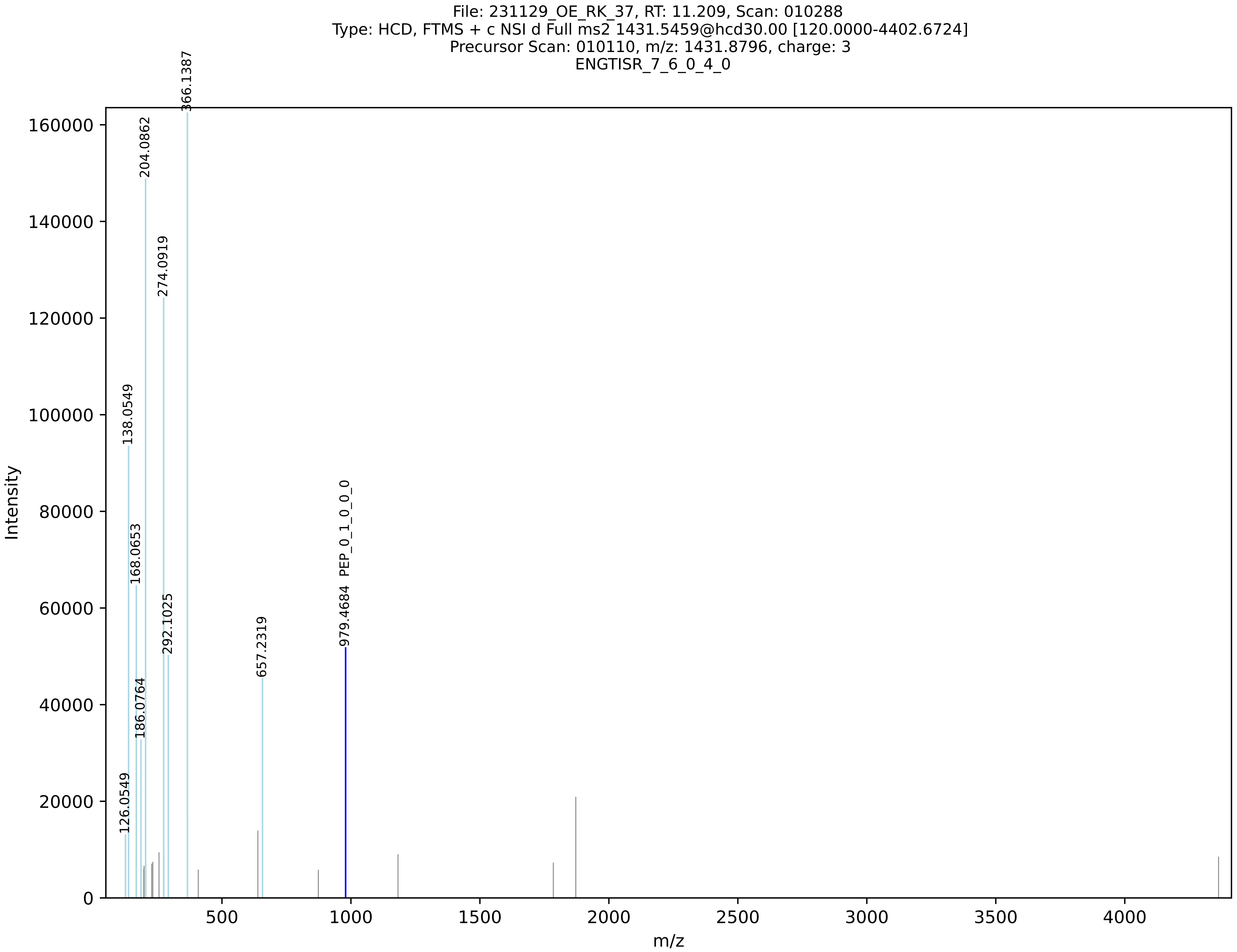
File: 231129_OE_RK_37, RT: 11.209, Scan: 010288
Type: HCD, FTMS + c NSI d Full ms2 1431.5459@hcd30.00 [120.0000-4402.6724]
Precursor Scan: 010110, m/z: 1431.8796, charge: 3
ENGTISR_7_6_0_4_0
366.1387
204.0862
274.0919
138.0549
168.0653
292.1025
186.0764
126.0549
657.2319
979.4684
PEP_0_1_0_0_0
Intensity
m/z
0
20000
40000
60000
80000
100000
120000
140000
160000
500
1000
1500
2000
2500
3000
3500
4000

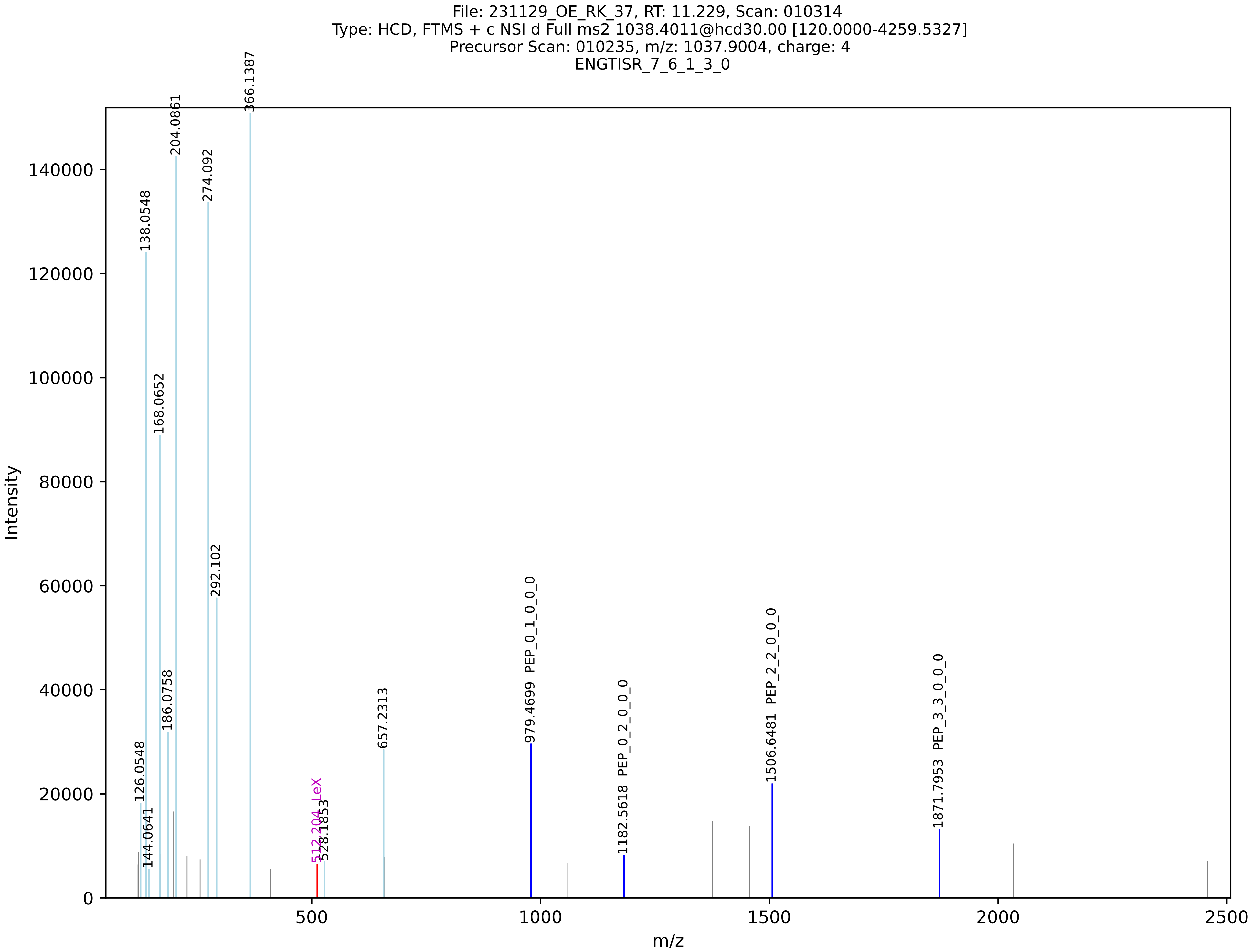

File: 231129_OE_RK_37, RT: 11.229, Scan: 010314
Type: HCD, FTMS + c NSI d Full ms2 1038.4011@hcd30.00 [120.0000-4259.5327]
Precursor Scan: 010235, m/z: 1037.9004, charge: 4
ENGTISR_7_6_1_3_0
366.1387
204.0861
274.092
138.0548
168.0652
292.102
979.4699 PEP_0_1_0_0_0
1506.6481 PEP_2_2_0_0_0
1871.7953 PEP_3_3_0_0_0
1182.5618 PEP_0_2_0_0_0
186.0758
657.2313
126.0548
512.204_LeX
528.1853
144.0641
Intensity
m/z
0
20000
40000
60000
80000
100000
120000
140000
500
1000
1500
2000
2500

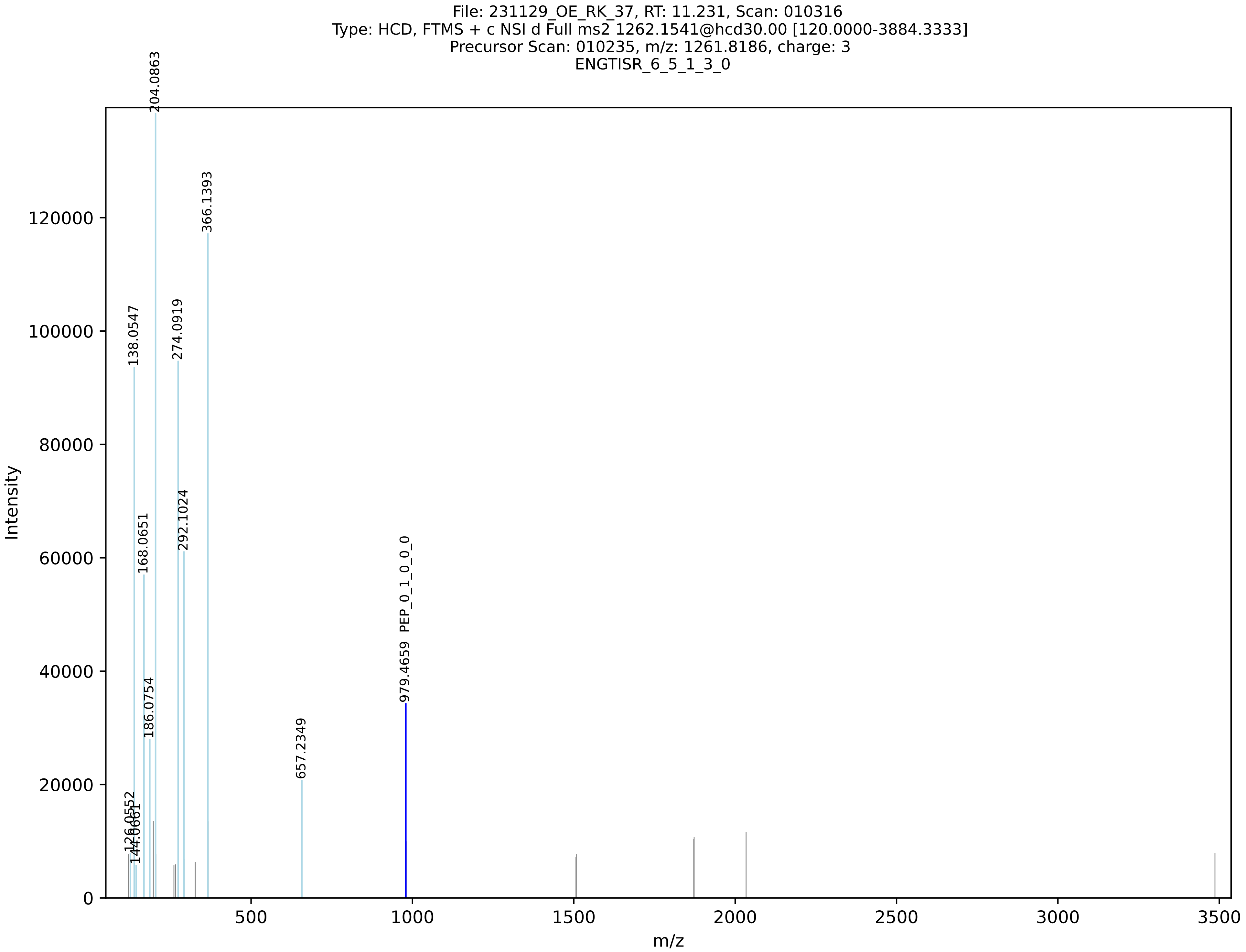
File: 231129_OE_RK_37, RT: 11.231, Scan: 010316
Type: HCD, FTMS + c NSI d Full ms2 1262.1541@hcd30.00 [120.0000-3884.3333]
Precursor Scan: 010235, m/z: 1261.8186, charge: 3
ENGTISR_6_5_1_3_0
204.0863
366.1393
138.0547
274.0919
292.1024
168.0651
186.0754
657.2349
979.4659 PEP_0_1_0_0_0
126.0552
144.0661
Intensity
m/z
0
20000
40000
60000
80000
100000
120000
500
1000
1500
2000
2500
3000
3500

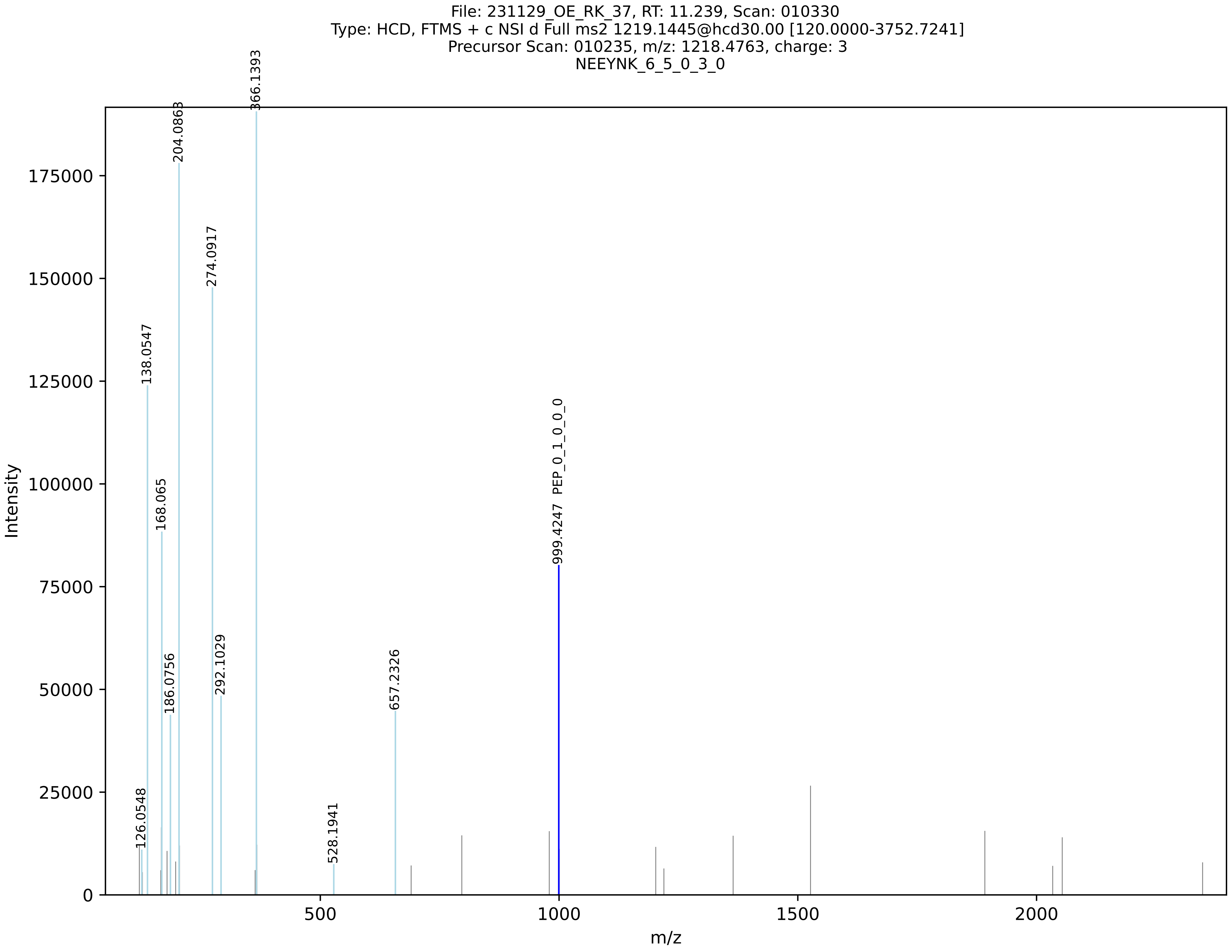

File: 231129_OE_RK_37, RT: 11.239, Scan: 010330
Type: HCD, FTMS + c NSI d Full ms2 1219.1445@hcd30.00 [120.0000-3752.7241]
Precursor Scan: 010235, m/z: 1218.4763, charge: 3
NEEYNK_6_5_0_3_0
366.1393
204.0863
274.0917
138.0547
168.065
186.0756
292.1029
657.2326
999.4247 PEP_0_1_0_0_0_
126.0548
528.1941
Intensity
m/z
0
25000
50000
75000
100000
125000
150000
175000
500
1000
1500
2000

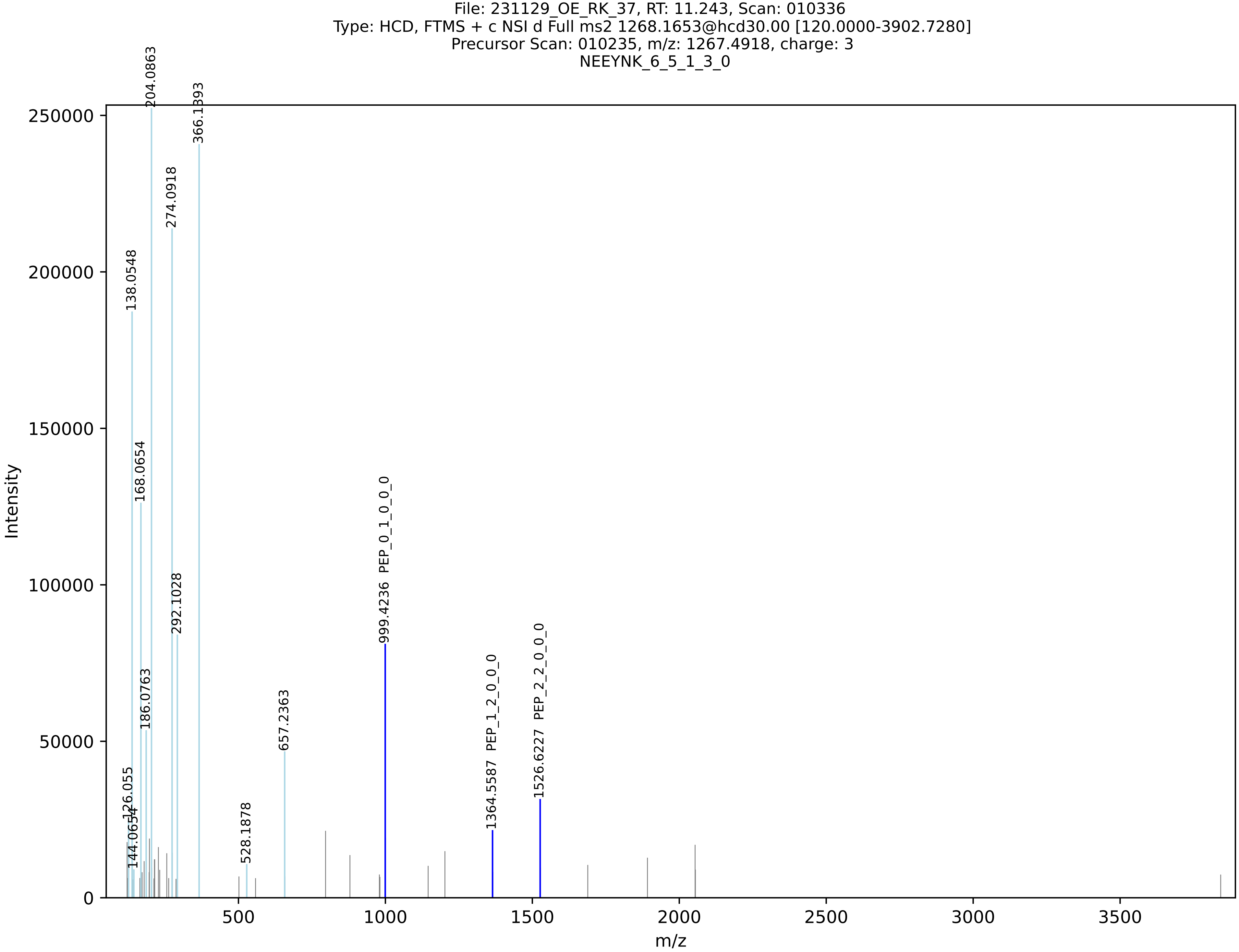

File: 231129_OE_RK_37, RT: 11.243, Scan: 010336
Type: HCD, FTMS + c NSI d Full ms2 1268.1653@hcd30.00 [120.0000-3902.7280]
Precursor Scan: 010235, m/z: 1267.4918, charge: 3
NEEYNK_6_5_1_3_0
Intensity
m/z
204.0863
366.1393
274.0918
138.0548
168.0654
292.1028
186.0763
126.055
144.0654
528.1878
657.2363
999.4236 PEP_0_1_0_0_0_
1364.5587 PEP_1_2_0_0_0_
1526.6227 PEP_2_2_0_0_0_

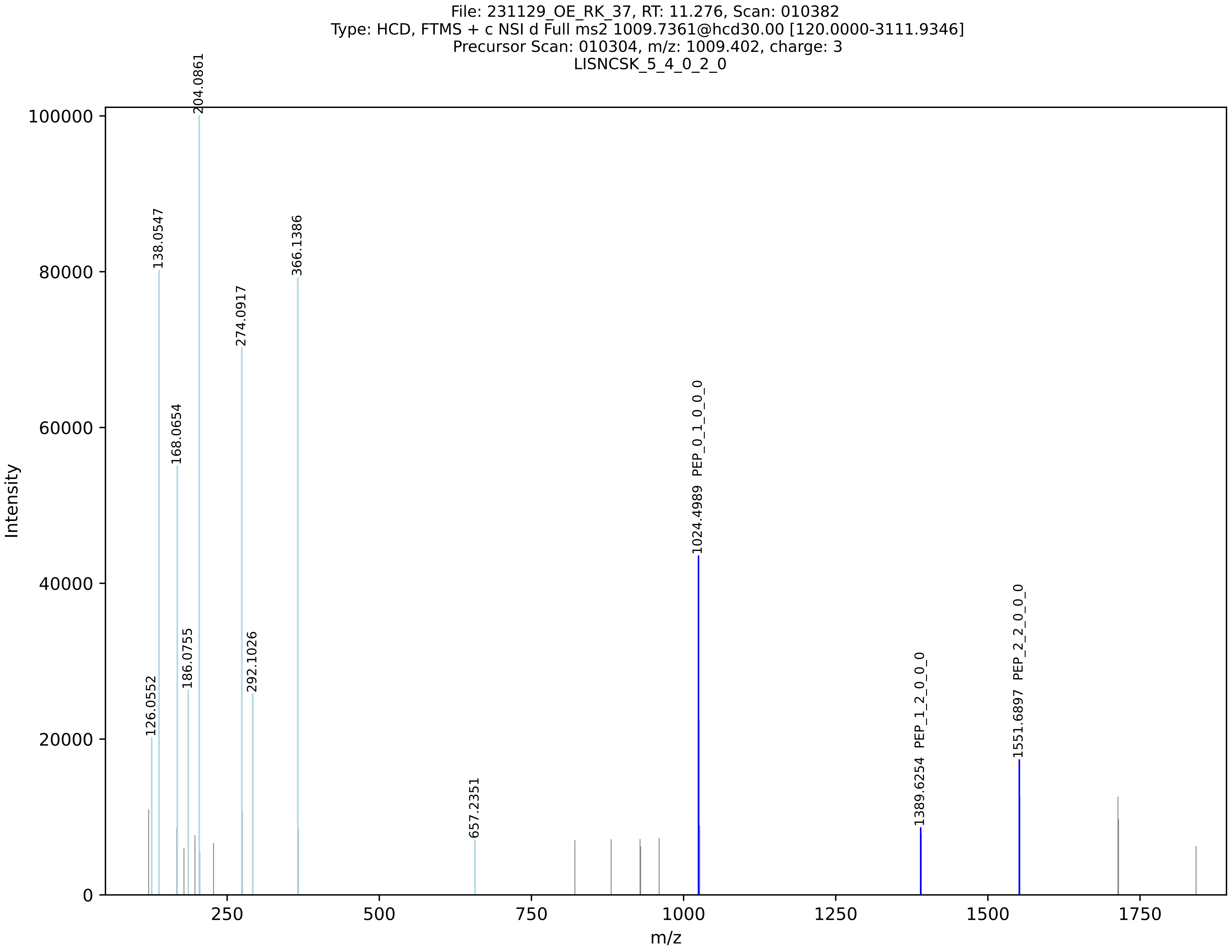
File: 231129_OE_RK_37, RT: 11.276, Scan: 010382
Type: HCD, FTMS + c NSI d Full ms2 1009.7361@hcd30.00 [120.0000-3111.9346]
Precursor Scan: 010304, m/z: 1009.402, charge: 3
LISNCSK_5_4_0_2_0
Intensity
m/z
0
20000
40000
60000
80000
100000
250
500
750
1000
1250
1500
1750
204.0861
138.0547
366.1386
274.0917
168.0654
186.0755
292.1026
126.0552
657.2351
1024.4989 PEP_0_1_0_0_0_
1389.6254 PEP_1_2_0_0_0_
1551.6897 PEP_2_2_0_0_0_

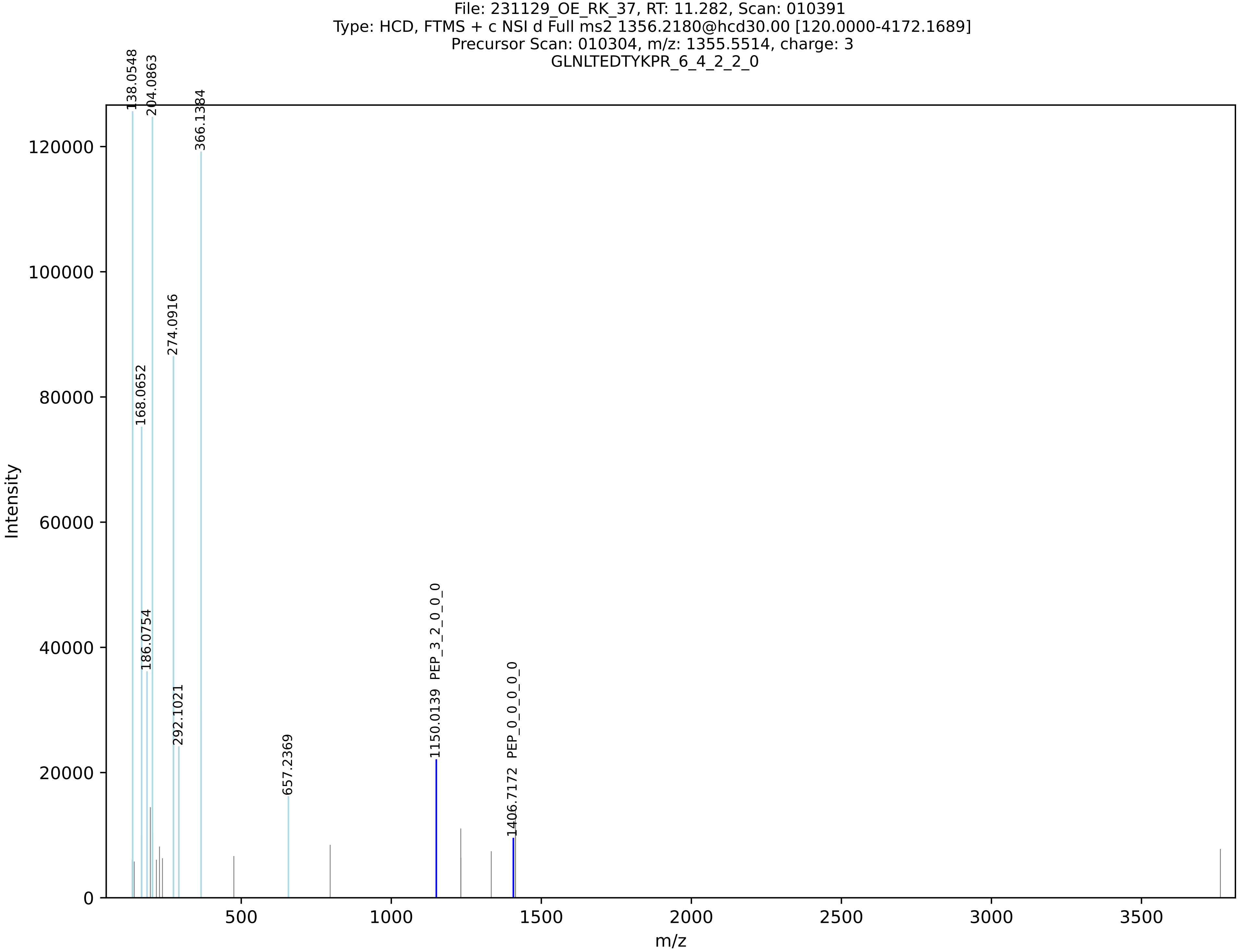

File: 231129_OE_RK_37, RT: 11.282, Scan: 010391
Type: HCD, FTMS + c NSI d Full ms2 1356.2180@hcd30.00 [120.0000-4172.1689]
Precursor Scan: 010304, m/z: 1355.5514, charge: 3
GLNLTEDTYKPR_6_4_2_2_0
Intensity
m/z
138.0548
204.0863
366.1384
274.0916
168.0652
186.0754
292.1021
657.2369
1150.0139 PEP_3_2_0_0_0
1406.7172 PEP_0_0_0_0_0
0
20000
40000
60000
80000
100000
120000
500
1000
1500
2000
2500
3000
3500

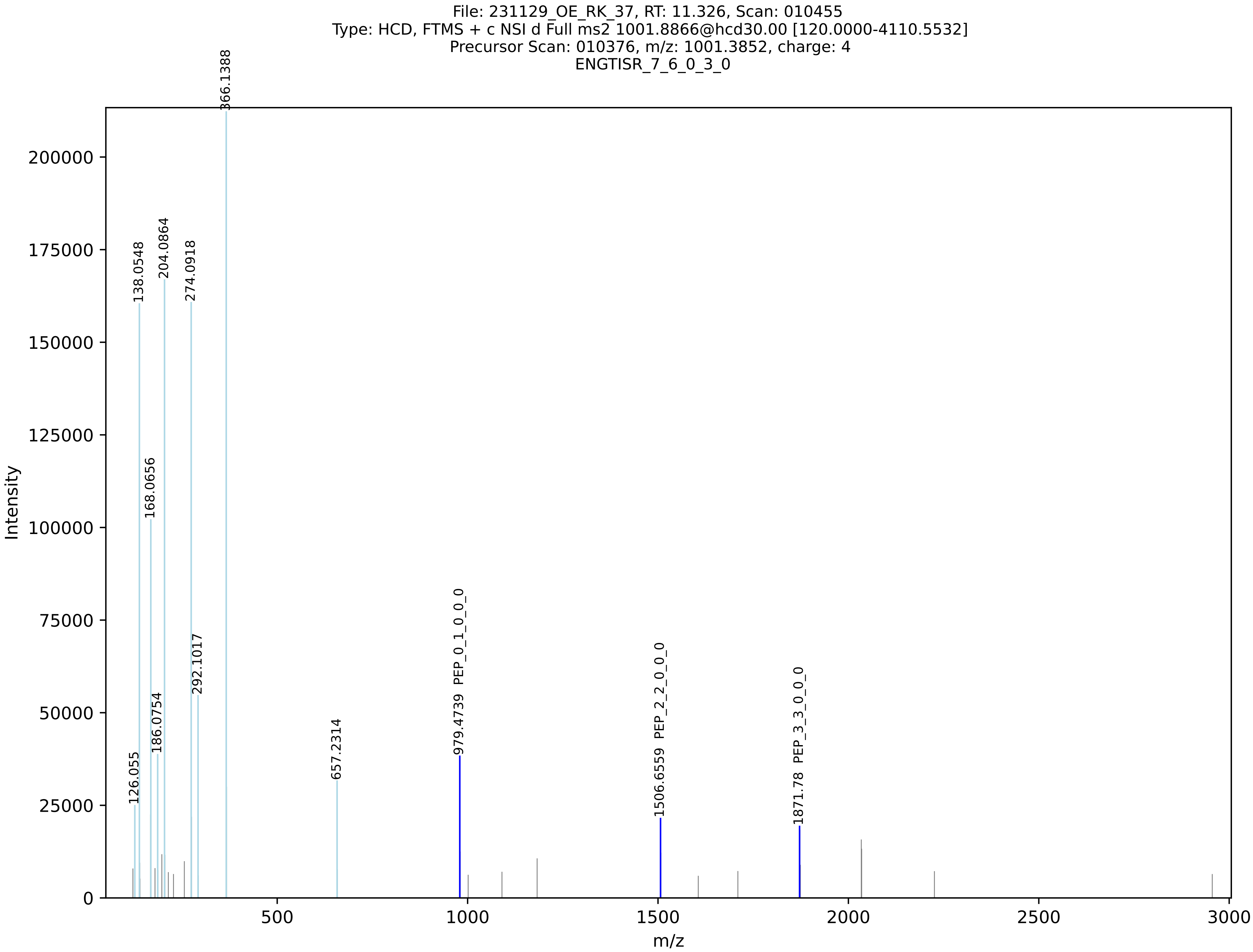

File: 231129_OE_RK_37, RT: 11.326, Scan: 010455
Type: HCD, FTMS + c NSI d Full ms2 1001.8866@hcd30.00 [120.0000-4110.5532]
Precursor Scan: 010376, m/z: 1001.3852, charge: 4
ENGTISR_7_6_0_3_0
366.1388
138.0548
204.0864
274.0918
168.0656
292.1017
186.0754
126.055
657.2314
979.4739 PEP_0_1_0_0_0
1506.6559 PEP_2_2_0_0_0
1871.78 PEP_3_3_0_0_0
Intensity
m/z
0
25000
50000
75000
100000
125000
150000
175000
200000
500
1000
1500
2000
2500
3000

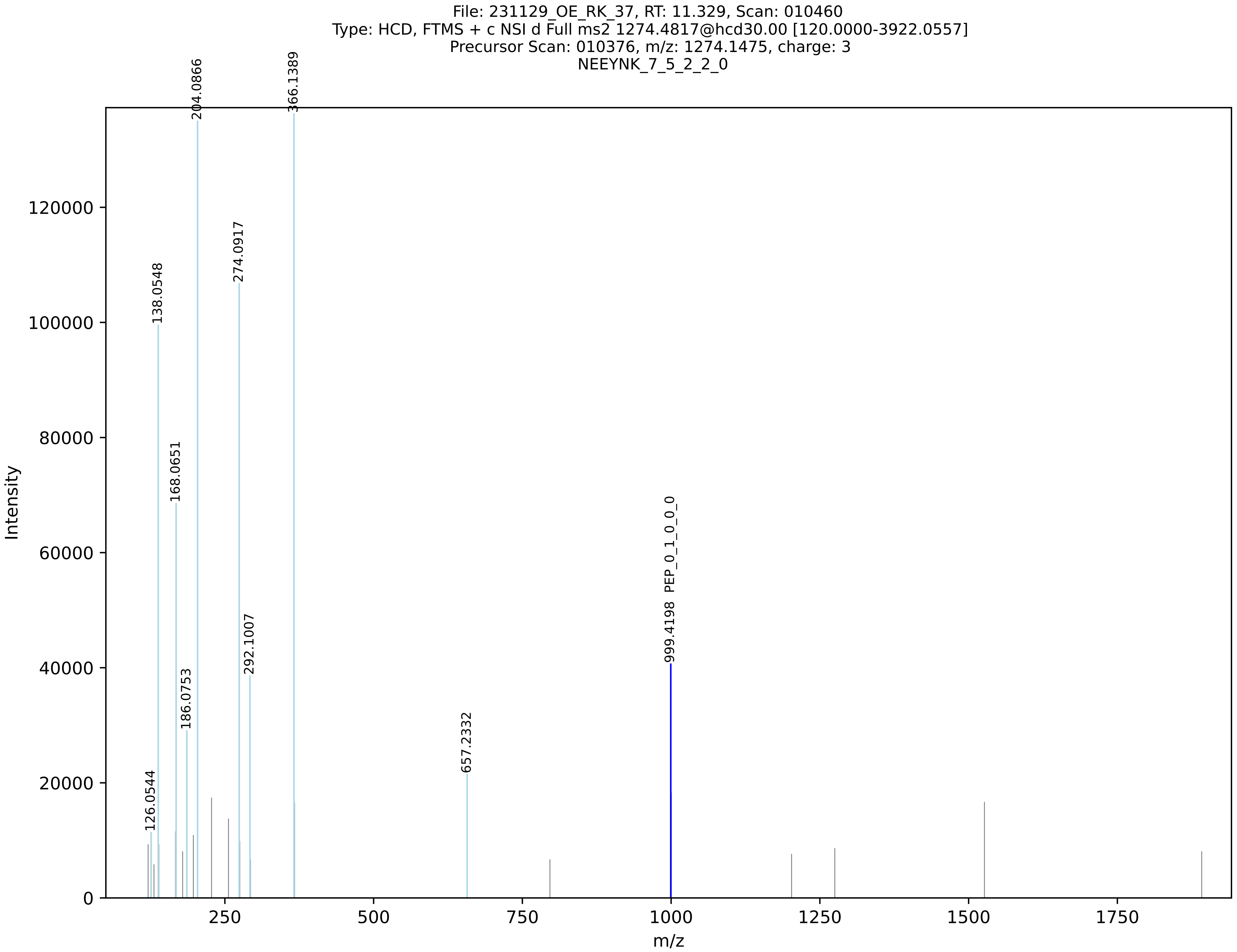
File: 231129_OE_RK_37, RT: 11.329, Scan: 010460
Type: HCD, FTMS + c NSI d Full ms2 1274.4817@hcd30.00 [120.0000-3922.0557]
Precursor Scan: 010376, m/z: 1274.1475, charge: 3
NEEYNK_7_5_2_2_0
Intensity
m/z
0
20000
40000
60000
80000
100000
120000
250
500
750
1000
1250
1500
1750
126.0544
138.0548
168.0651
186.0753
204.0866
274.0917
292.1007
366.1389
657.2332
999.4198 PEP_0_1_0_0_0

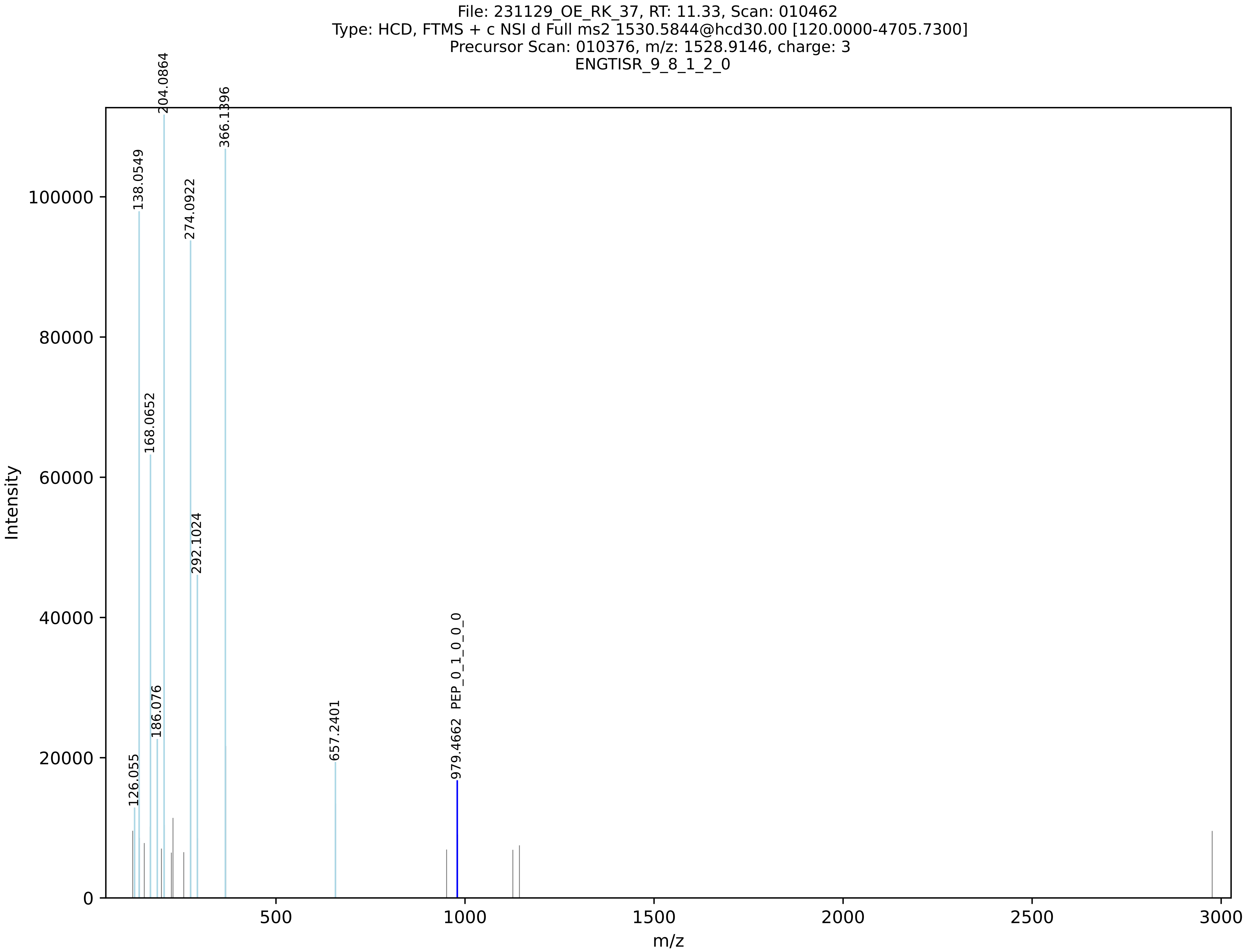
File: 231129_OE_RK_37, RT: 11.33, Scan: 010462
Type: HCD, FTMS + c NSI d Full ms2 1530.5844@hcd30.00 [120.0000-4705.7300]
Precursor Scan: 010376, m/z: 1528.9146, charge: 3
ENGTISR_9_8_1_2_0
204.0864
366.1396
138.0549
274.0922
168.0652
292.1024
186.076
126.055
657.2401
979.4662 PEP_0_1_0_0_0
Intensity
0
20000
40000
60000
80000
100000
500
1000
1500
2000
2500
3000
m/z

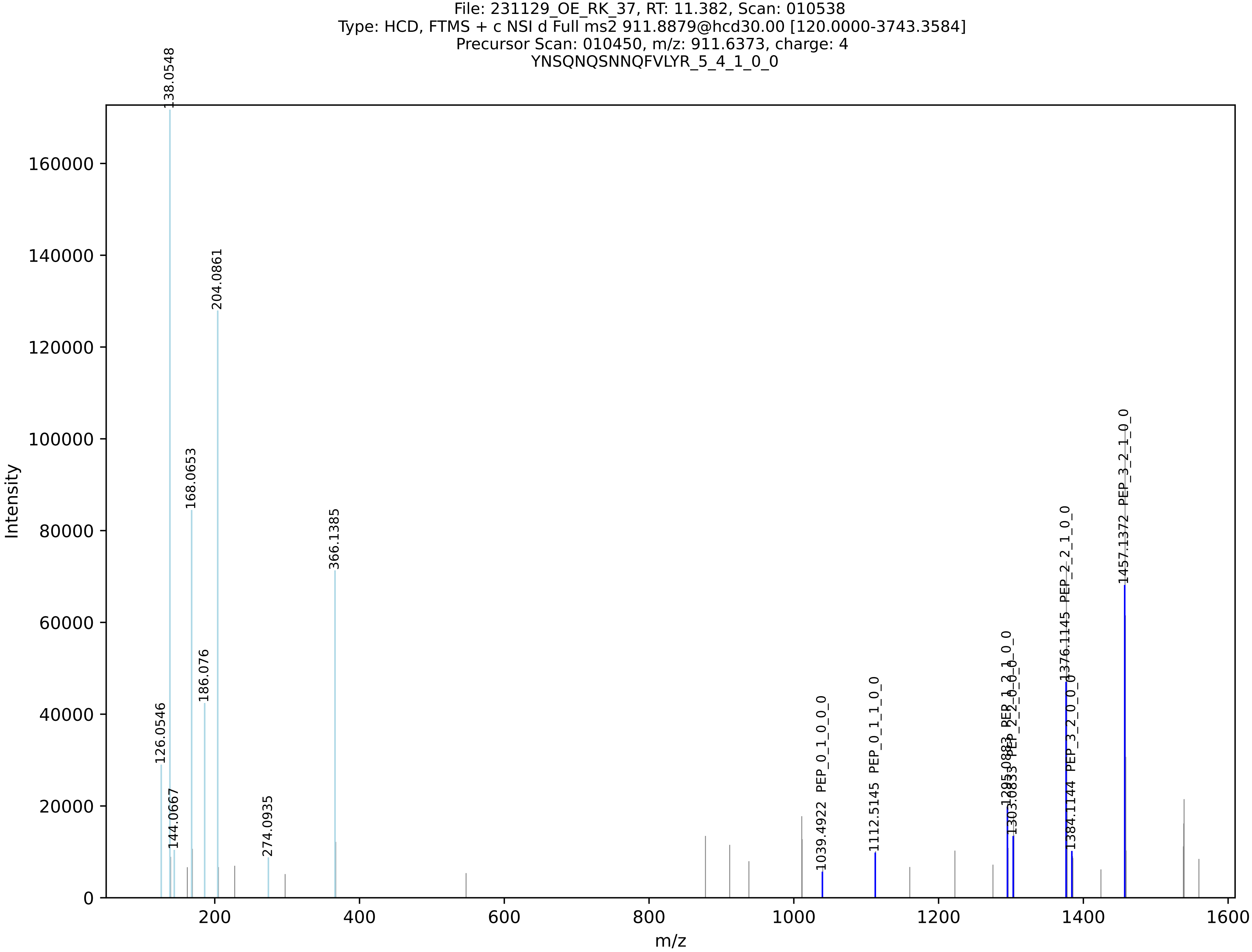
File: 231129_OE_RK_37, RT: 11.382, Scan: 010538
Type: HCD, FTMS + c NSI d Full ms2 911.8879@hcd30.00 [120.0000-3743.3584]
Precursor Scan: 010450, m/z: 911.6373, charge: 4
YNSQNQSNNQFVLYR_5_4_1_0_0
138.0548
204.0861
168.0653
366.1385
186.076
126.0546
144.0667
274.0935
1039.4922 PEP_0_1_0_0_0
1112.5145 PEP_0_1_1_0_0
1295.0883 PEP_1_2_1_0_0
1303.0833 PEP_2_2_0_0_0
1376.1145 PEP_2_2_1_0_0
1384.1144 PEP_3_2_0_0_0
1457.1372 PEP_3_2_1_0_0
Intensity
m/z
0
20000
40000
60000
80000
100000
120000
140000
160000
200
400
600
800
1000
1200
1400
1600

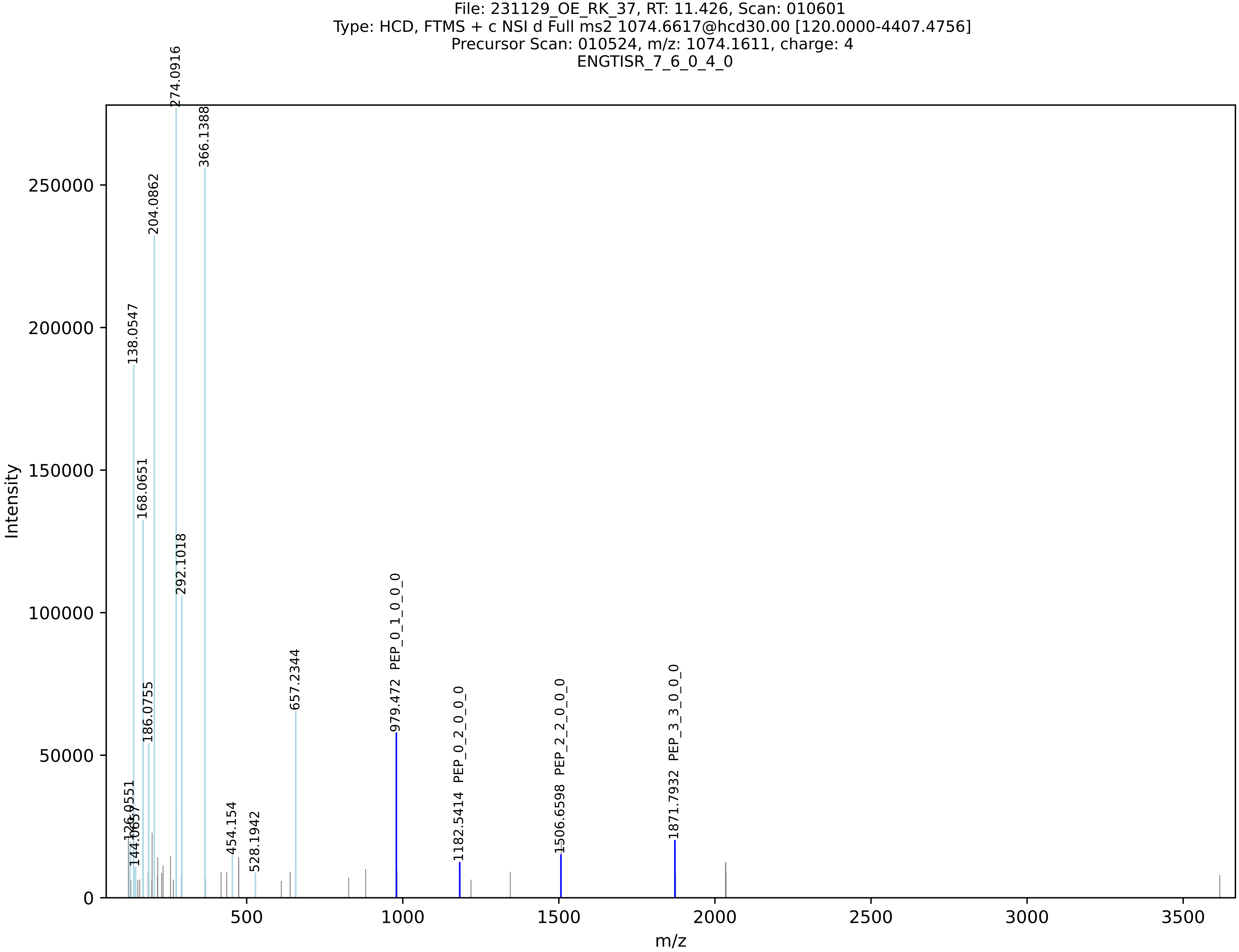
File: 231129_OE_RK_37, RT: 11.426, Scan: 010601
Type: HCD, FTMS + c NSI d Full ms2 1074.6617@hcd30.00 [120.0000-4407.4756]
Precursor Scan: 010524, m/z: 1074.1611, charge: 4
ENGTISR_7_6_0_4_0
274.0916
366.1388
204.0862
138.0547
168.0651
292.1018
186.0755
657.2344
979.472 PEP_0_1_0_0_0
1182.5414 PEP_0_2_0_0_0
1506.6598 PEP_2_2_0_0_0
1871.7932 PEP_3_3_0_0_0
126.0551
144.0657
454.154
528.1942
Intensity
m/z
0
50000
100000
150000
200000
250000
500
1000
1500
2000
2500
3000
3500

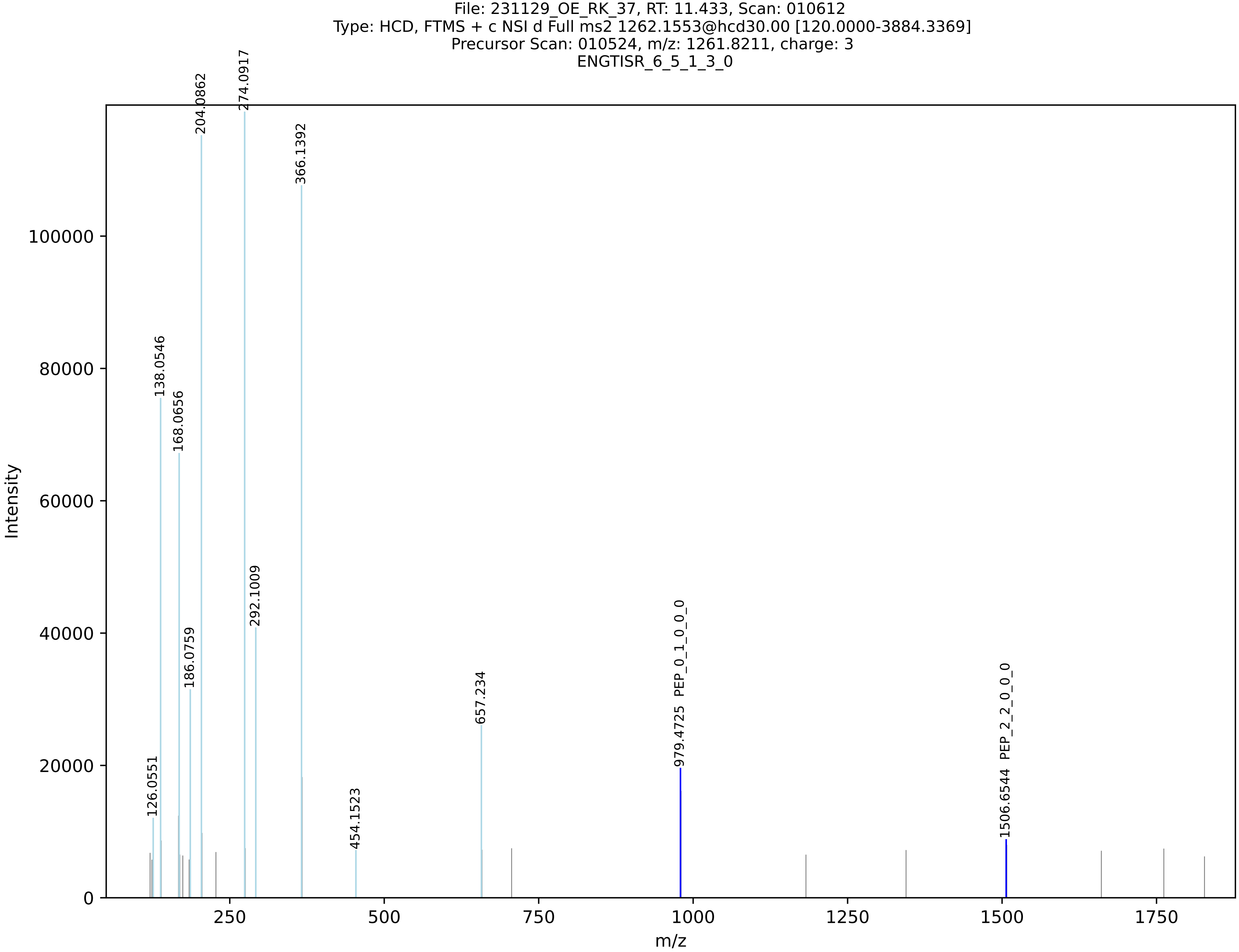
File: 231129_OE_RK_37, RT: 11.433, Scan: 010612
Type: HCD, FTMS + c NSI d Full ms2 1262.1553@hcd30.00 [120.0000-3884.3369]
Precursor Scan: 010524, m/z: 1261.8211, charge: 3
ENGTISR_6_5_1_3_0
Intensity
m/z
0
20000
40000
60000
80000
100000
250
500
750
1000
1250
1500
1750
126.0551
138.0546
168.0656
186.0759
204.0862
274.0917
292.1009
366.1392
454.1523
657.234
979.4725 PEP_0_1_0_0_0
1506.6544 PEP_2_2_0_0_0

File: 231129_OE_RK_37, RT: 11.448, Scan: 010636
Type: HCD, FTMS + c NSI d Full ms2 1216.1801@hcd30.00 [120.0000-3743.6528]
Precursor Scan: 010524, m/z: 1215.8475, charge: 3
NTSKELQGATHR_4_6_1_1_0

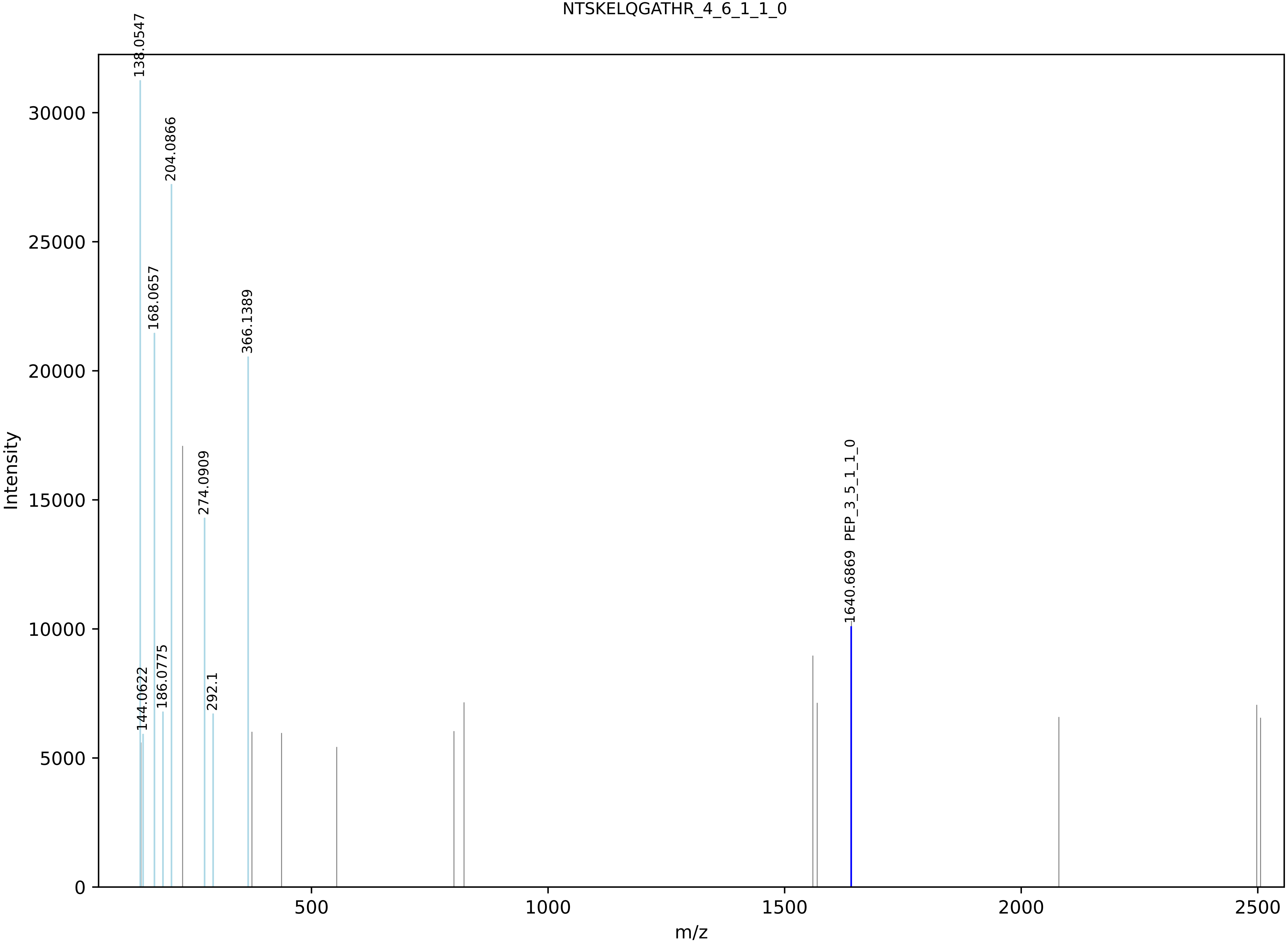

File: 231129_OE_RK_37, RT: 11.491, Scan: 010695
Type: HCD, FTMS + c NSI d Full ms2 1040.1206@hcd30.00 [120.0000-3204.9109]
Precursor Scan: 010598, m/z: 1039.1526, charge: 3
YNSQNQSNNQFVLYR_3_3_1_0_0

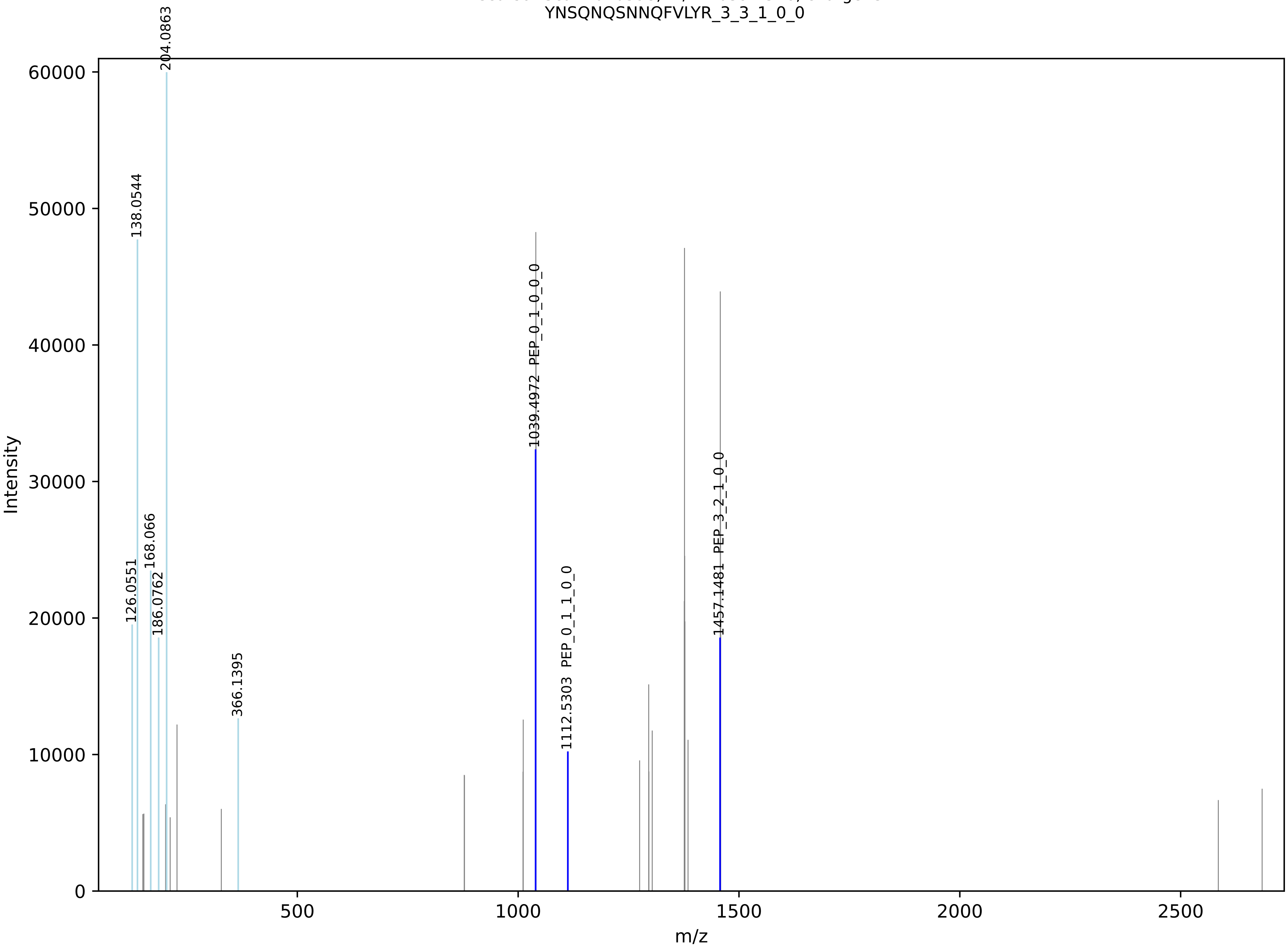

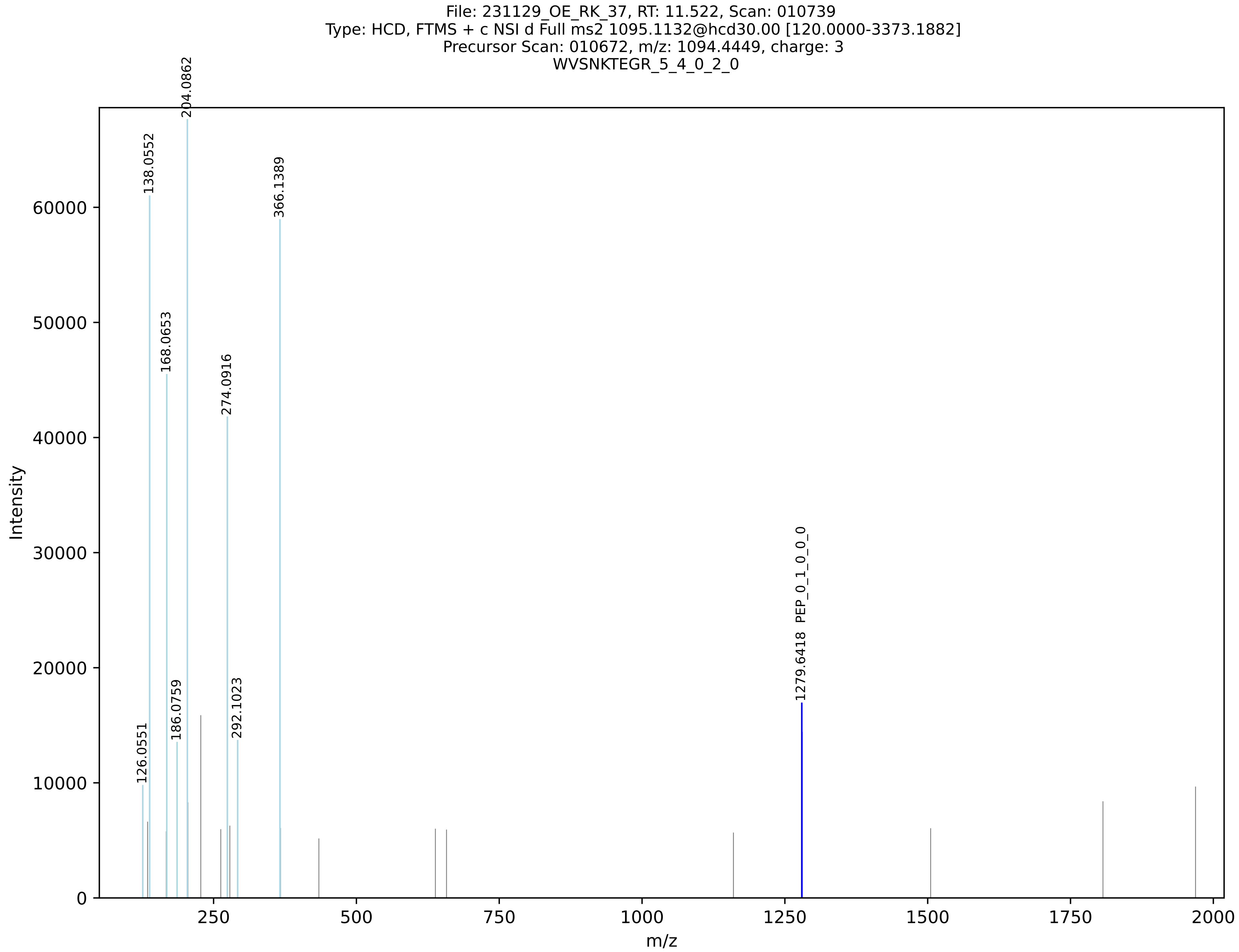

File: 231129_OE_RK_37, RT: 11.522, Scan: 010739
Type: HCD, FTMS + c NSI d Full ms2 1095.1132@hcd30.00 [120.0000-3373.1882]
Precursor Scan: 010672, m/z: 1094.4449, charge: 3
WVSNKTEGR_5_4_0_2_0
Intensity
m/z
204.0862
138.0552
366.1389
168.0653
274.0916
PEP_0_1_0_0_0
1279.6418
186.0759
292.1023
126.0551
0
10000
20000
30000
40000
50000
60000
250
500
750
1000
1250
1500
1750
2000

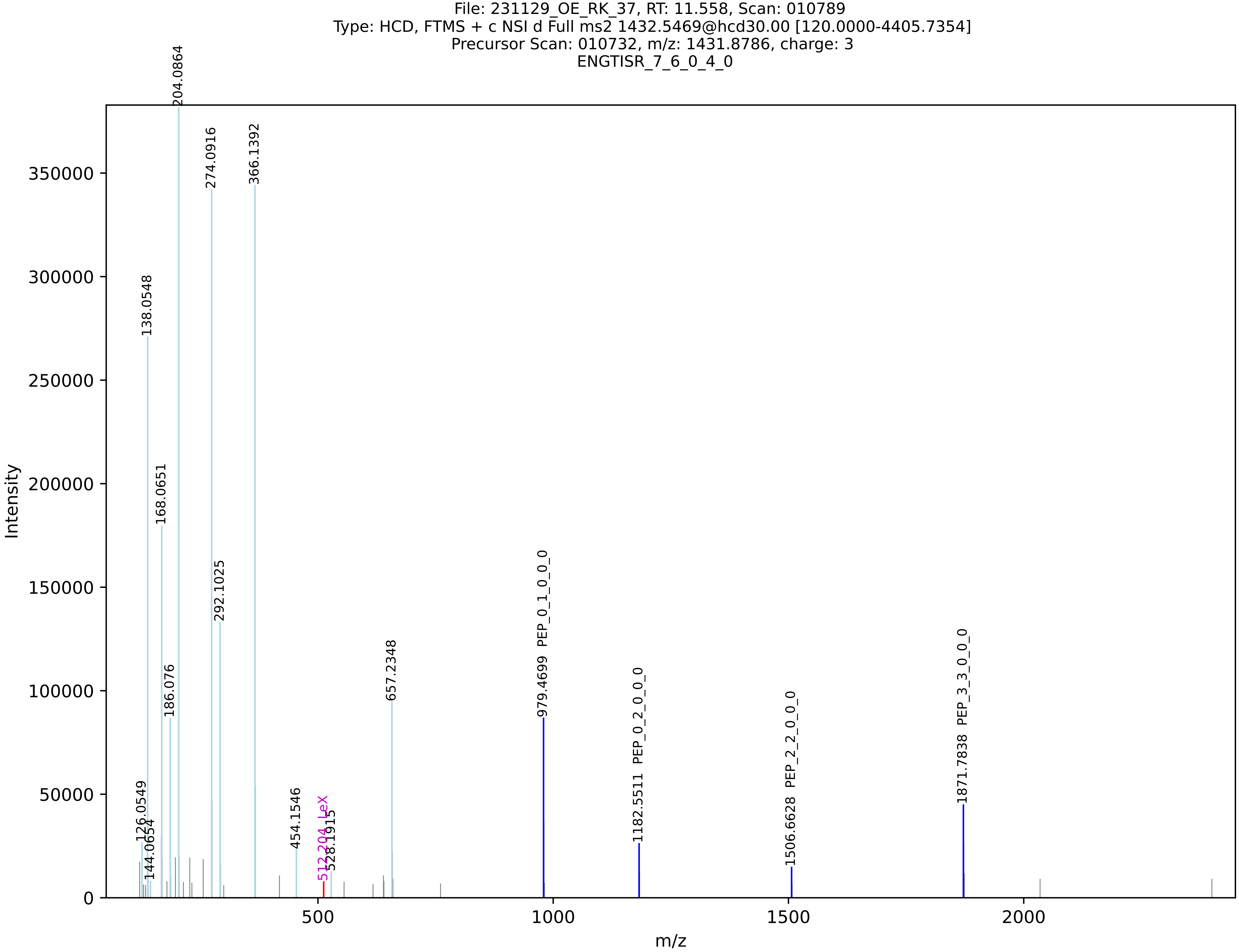

File: 231129_OE_RK_37, RT: 11.558, Scan: 010789
Type: HCD, FTMS + c NSI d Full ms2 1432.5469@hcd30.00 [120.0000-4405.7354]
Precursor Scan: 010732, m/z: 1431.8786, charge: 3
ENGTISR_7_6_0_4_0
Intensity
m/z
204.0864
274.0916
366.1392
138.0548
168.0651
292.1025
186.076
126.0549
144.0654
454.1546
512.204_LeX
528.1915
657.2348
979.4699 PEP_0_1_0_0_0
1182.5511 PEP_0_2_0_0_0
1506.6628 PEP_2_2_0_0_0
1871.7838 PEP_3_3_0_0_0

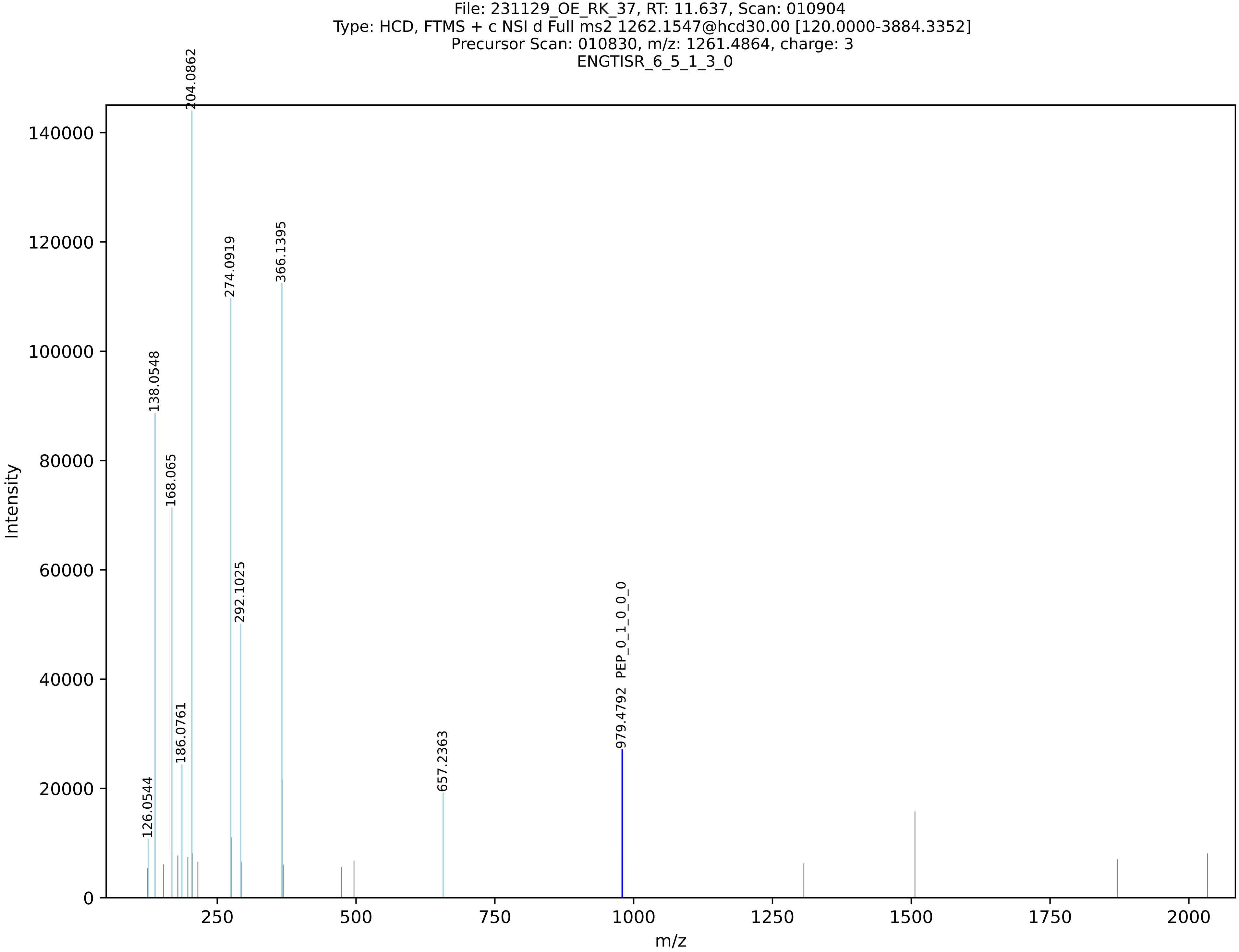
File: 231129_OE_RK_37, RT: 11.637, Scan: 010904
Type: HCD, FTMS + c NSI d Full ms2 1262.1547@hcd30.00 [120.0000-3884.3352]
Precursor Scan: 010830, m/z: 1261.4864, charge: 3
ENGTISR_6_5_1_3_0
204.0862
366.1395
274.0919
138.0548
168.065
292.1025
979.4792 PEP_0_1_0_0_0
186.0761
657.2363
126.0544
Intensity
m/z
0
20000
40000
60000
80000
100000
120000
140000
250
500
750
1000
1250
1500
1750
2000

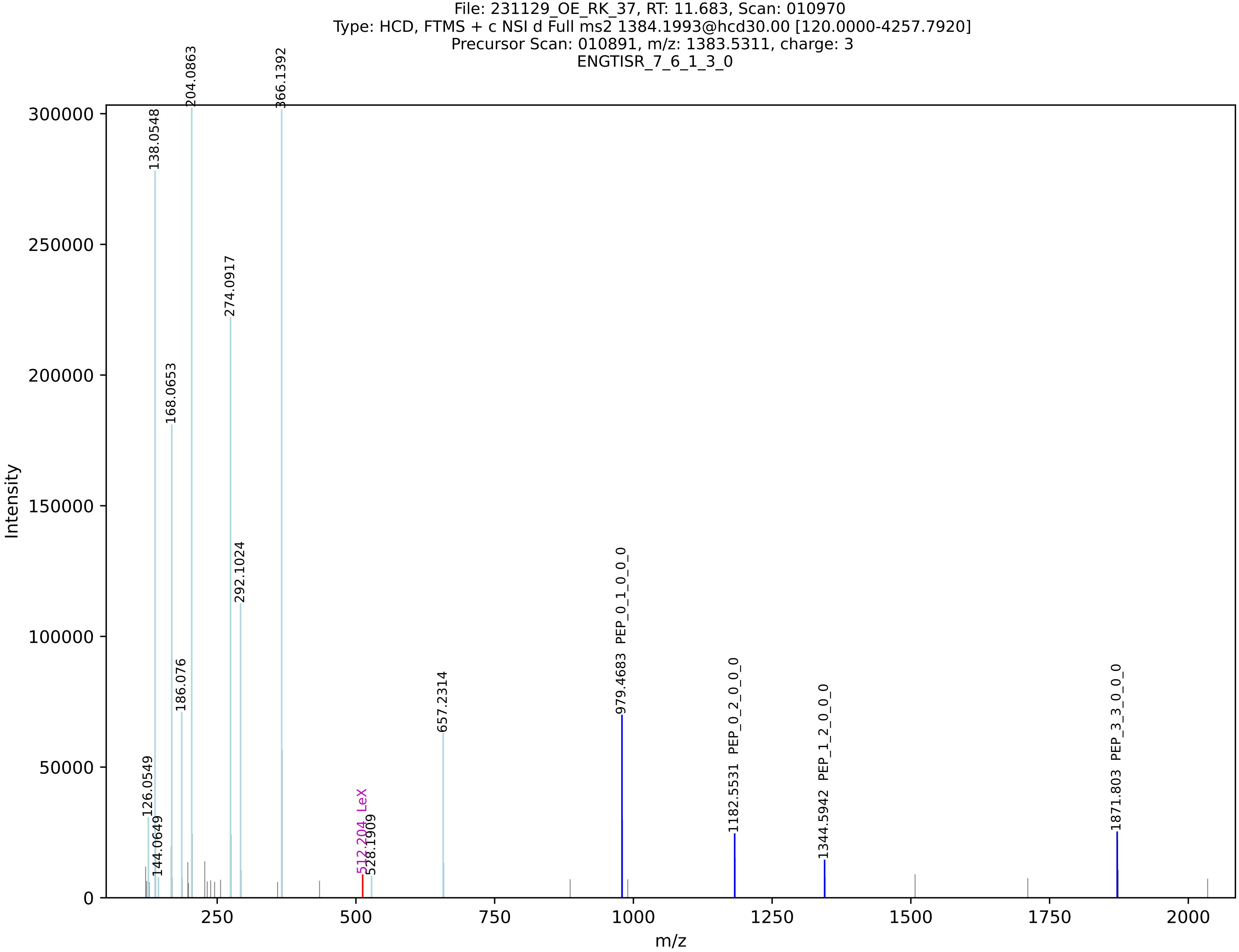
File: 231129_OE_RK_37, RT: 11.683, Scan: 010970
Type: HCD, FTMS + c NSI d Full ms2 1384.1993@hcd30.00 [120.0000-4257.7920]
Precursor Scan: 010891, m/z: 1383.5311, charge: 3
ENGTISR_7_6_1_3_0
Intensity
m/z
0
50000
100000
150000
200000
250000
300000
250
500
750
1000
1250
1500
1750
2000
204.0863
366.1392
138.0548
274.0917
168.0653
292.1024
186.076
126.0549
144.0649
512.204 LeX
528.1909
657.2314
979.4683 PEP_0_1_0_0_0
1182.5531 PEP_0_2_0_0_0
1344.5942 PEP_1_2_0_0_0
1871.803 PEP_3_3_0_0_0

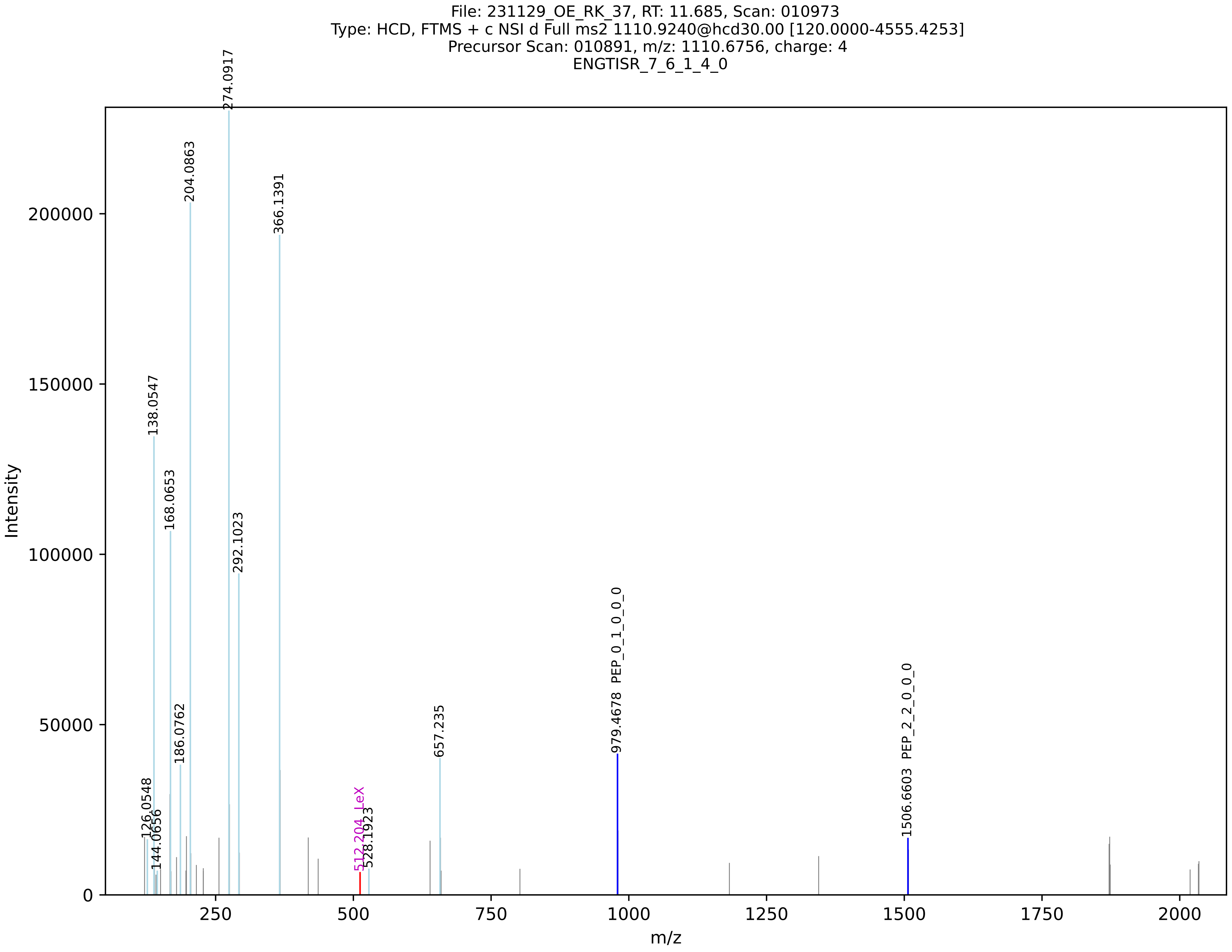
File: 231129_OE_RK_37, RT: 11.685, Scan: 010973
Type: HCD, FTMS + c NSI d Full ms2 1110.9240@hcd30.00 [120.0000-4555.4253]
Precursor Scan: 010891, m/z: 1110.6756, charge: 4
ENGTISR_7_6_1_4_0
274.0917
204.0863
366.1391
138.0547
168.0653
292.1023
186.0762
126.0548
144.0656
512.204_LeX
528.1923
657.235
979.4678 PEP_0_1_0_0_
1506.6603 PEP_2_2_0_0_0
Intensity
m/z
0
50000
100000
150000
200000
250
500
750
1000
1250
1500
1750
2000

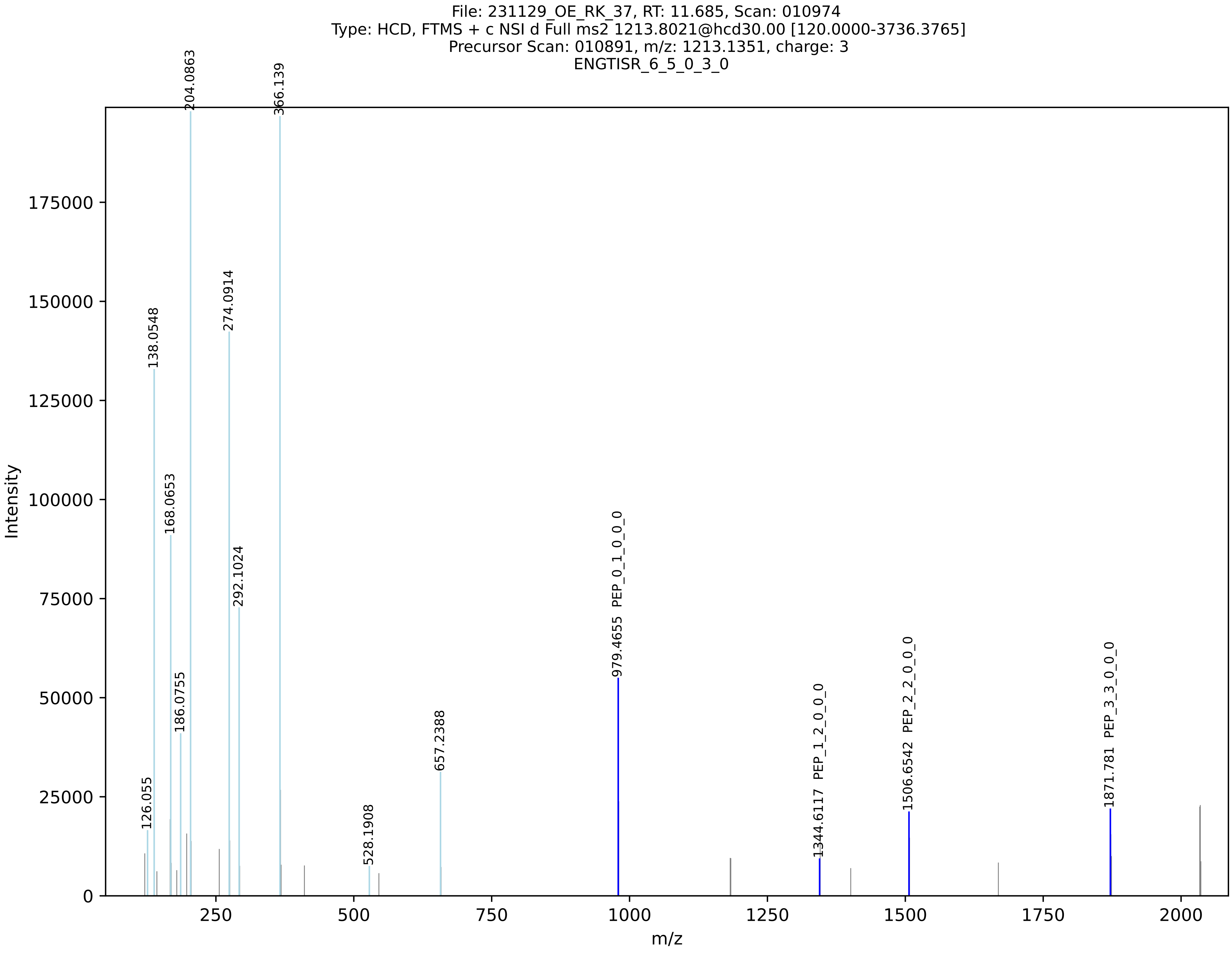

File: 231129_OE_RK_37, RT: 11.685, Scan: 010974
Type: HCD, FTMS + c NSI d Full ms2 1213.8021@hcd30.00 [120.0000-3736.3765]
Precursor Scan: 010891, m/z: 1213.1351, charge: 3
ENGTISR_6_5_0_3_0
Intensity
m/z
0
25000
50000
75000
100000
125000
150000
175000
250
500
750
1000
1250
1500
1750
2000
126.055
138.0548
168.0653
186.0755
204.0863
274.0914
292.1024
366.139
528.1908
657.2388
979.4655 PEP_0_1_0_0_0
1344.6117 PEP_1_2_0_0_0
1506.6542 PEP_2_2_0_0_0
1871.781 PEP_3_3_0_0_0

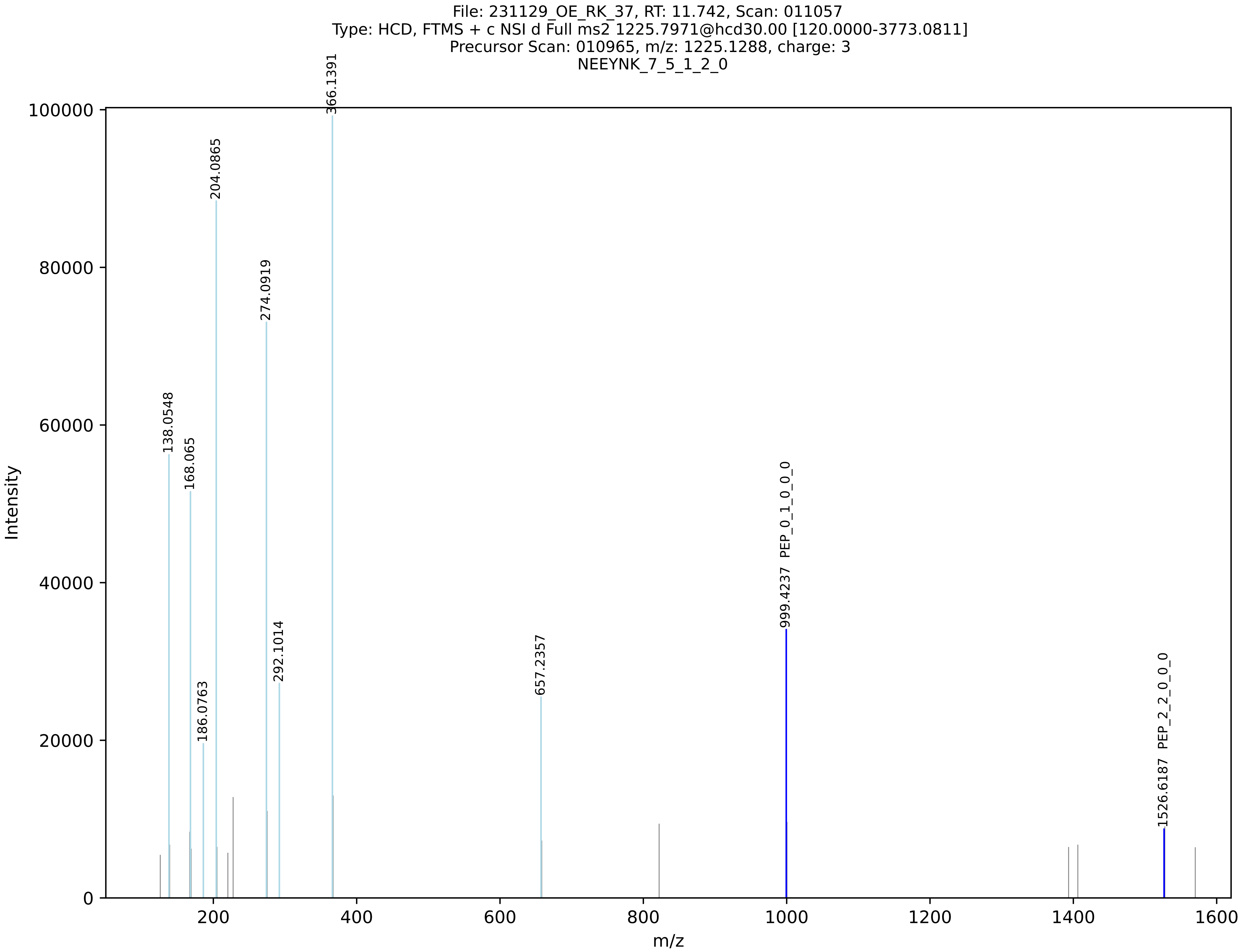
File: 231129_OE_RK_37, RT: 11.742, Scan: 011057
Type: HCD, FTMS + c NSI d Full ms2 1225.7971@hcd30.00 [120.0000-3773.0811]
Precursor Scan: 010965, m/z: 1225.1288, charge: 3
NEEYNK_7_5_1_2_0
366.1391
204.0865
274.0919
138.0548
168.065
186.0763
292.1014
657.2357
999.4237 PEP_0_1_0_0_0
1526.6187 PEP_2_2_0_0_0
Intensity
m/z
0
20000
40000
60000
80000
100000
200
400
600
800
1000
1200
1400
1600

File: 231129_OE_RK_37, RT: 11.835, Scan: 011193
Type: HCD, FTMS + c NSI d Full ms2 1038.4019@hcd30.00 [120.0000-4259.5356]
Precursor Scan: 011105, m/z: 1037.9005, charge: 4
ENGTISR_9_8_0_1_0

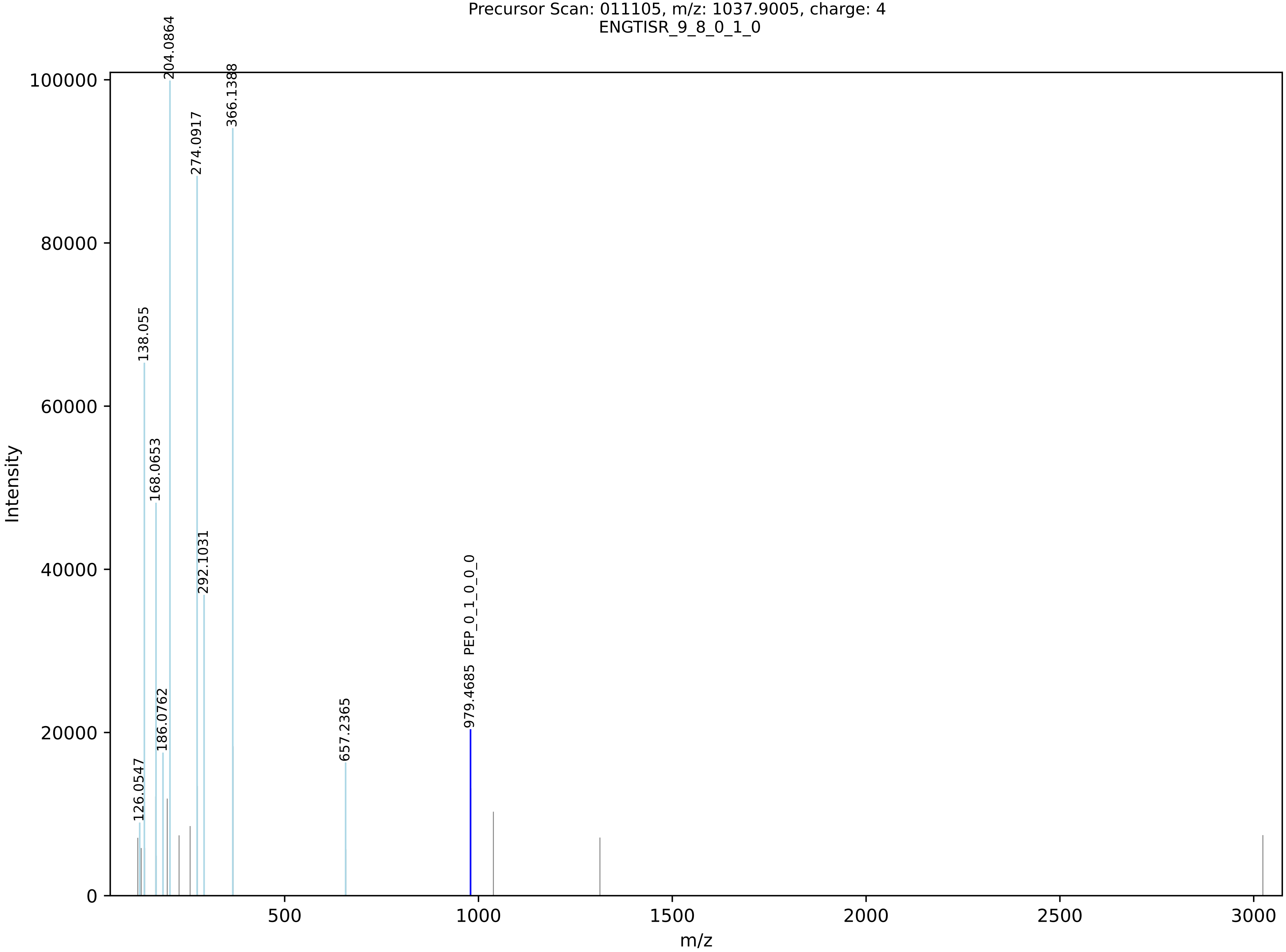

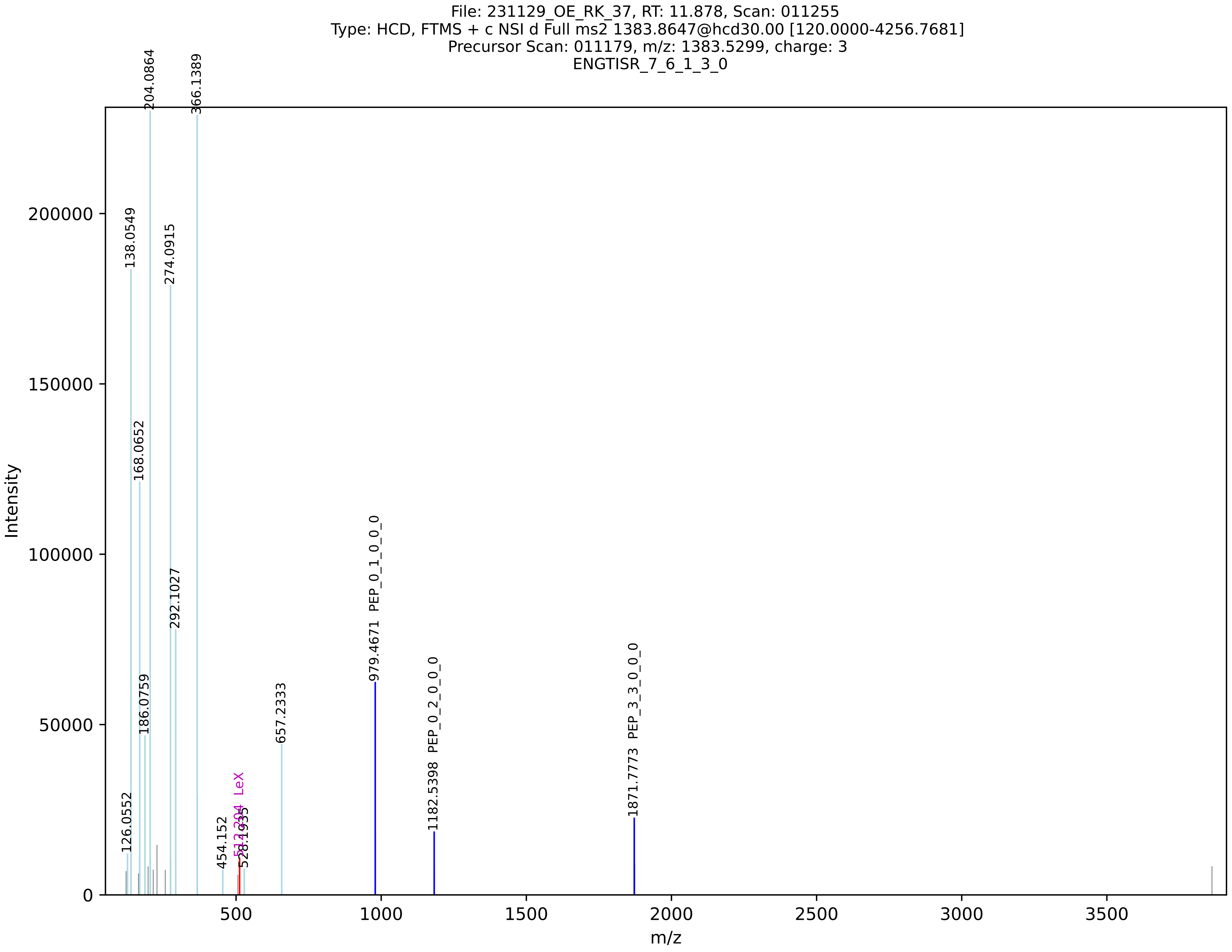
File: 231129_OE_RK_37, RT: 11.878, Scan: 011255
Type: HCD, FTMS + c NSI d Full ms2 1383.8647@hcd30.00 [120.0000-4256.7681]
Precursor Scan: 011179, m/z: 1383.5299, charge: 3
ENGTISR_7_6_1_3_0
204.0864
366.1389
138.0549
274.0915
168.0652
292.1027
186.0759
126.0552
454.152
512.204 LeX
528.1935
657.2333
979.4671 PEP_0_1_0_0_0
1182.5398 PEP_0_2_0_0_0
1871.7773 PEP_3_3_0_0_0
Intensity
0
50000
100000
150000
200000
500
1000
1500
2000
2500
3000
3500
m/z

File: 231129_OE_RK_37, RT: 11.879, Scan: 011256
Type: HCD, FTMS + c NSI d Full ms2 1481.2329@hcd30.00 [120.0000-4554.7148]
Precursor Scan: 011179, m/z: 1480.2305, charge: 3
ENGTISR_7_6_1_4_0

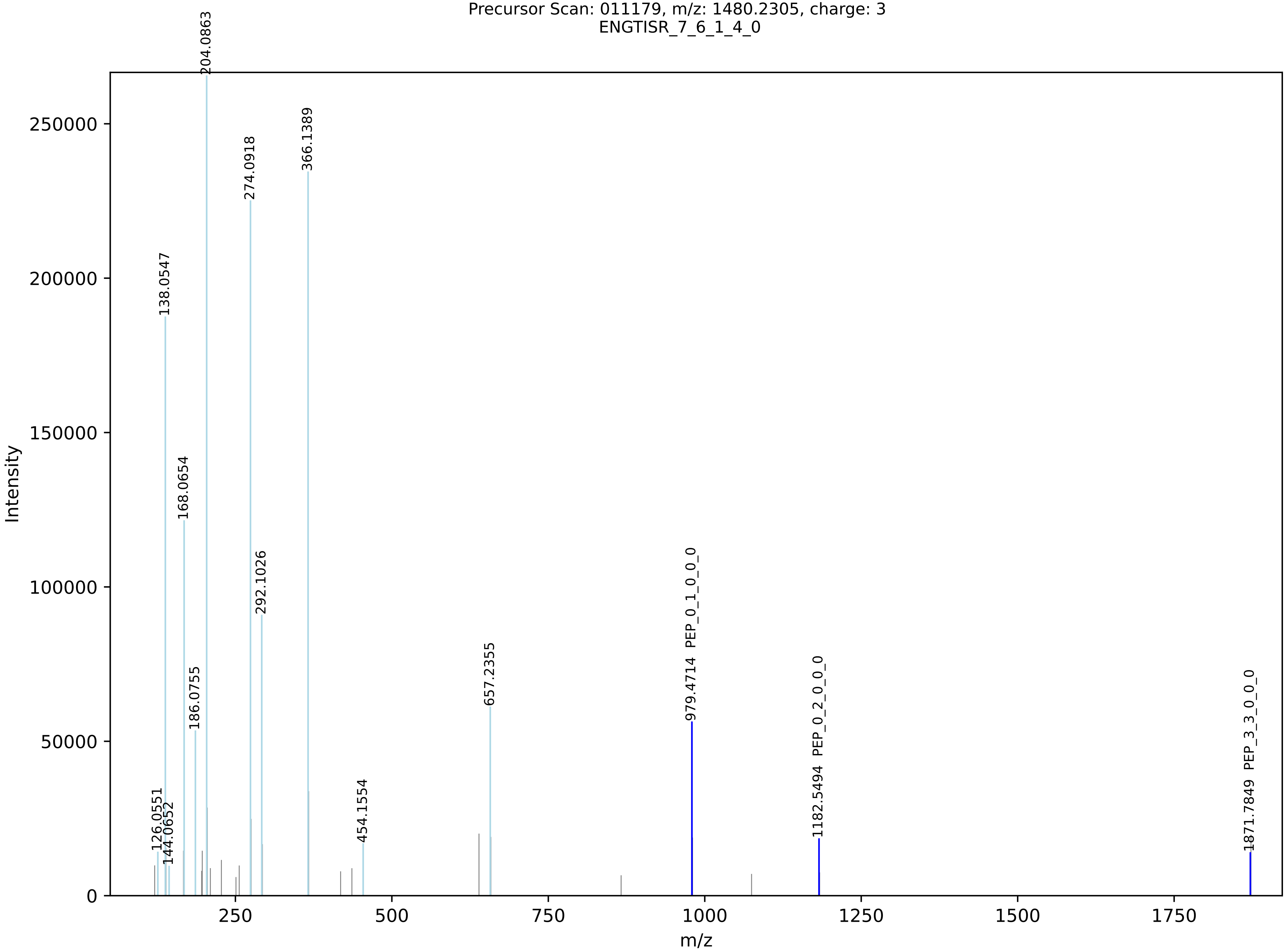

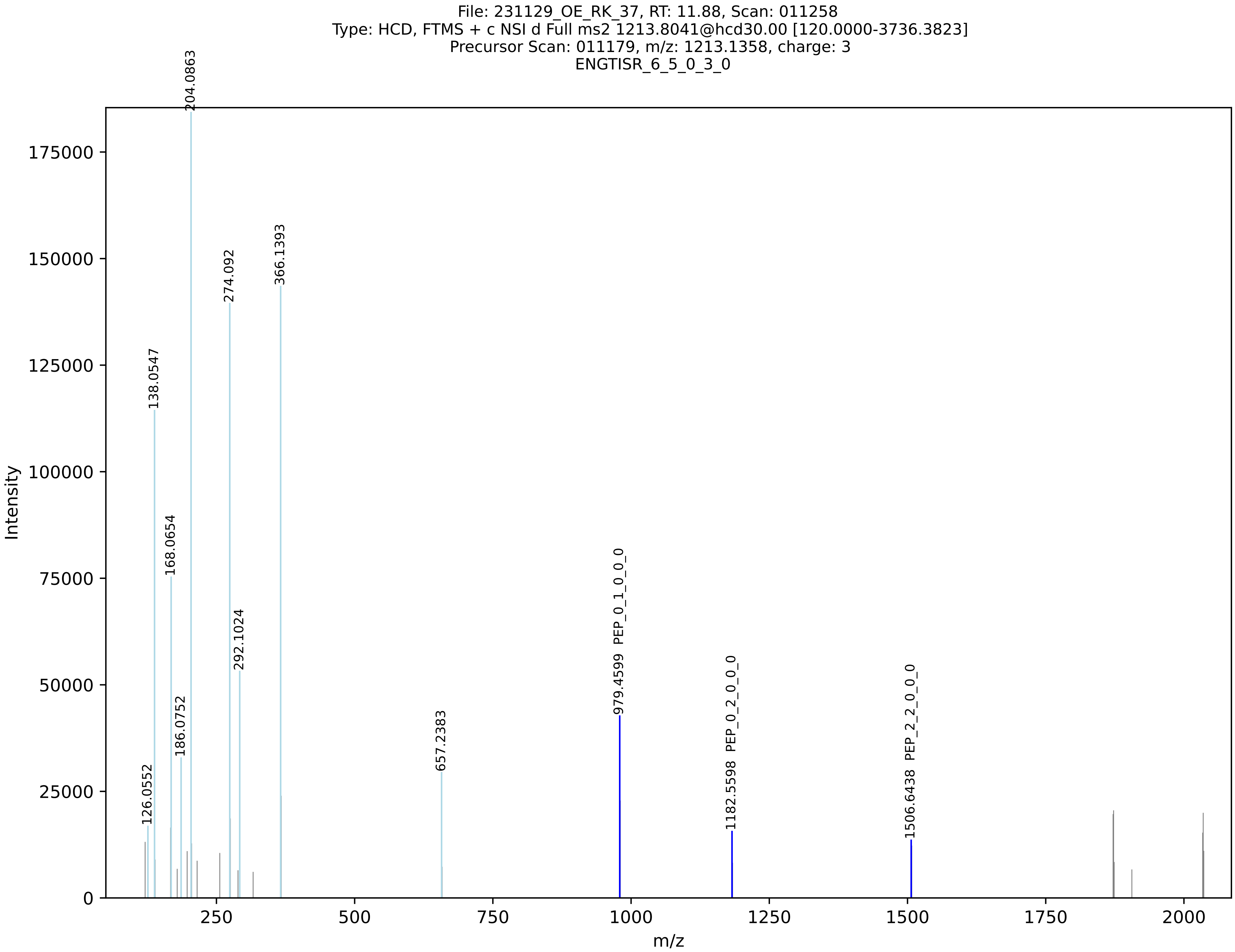
File: 231129_OE_RK_37, RT: 11.88, Scan: 011258
Type: HCD, FTMS + c NSI d Full ms2 1213.8041@hcd30.00 [120.0000-3736.3823]
Precursor Scan: 011179, m/z: 1213.1358, charge: 3
ENGTISR_6_5_0_3_0
Intensity
m/z
204.0863
366.1393
274.092
138.0547
168.0654
292.1024
186.0752
126.0552
657.2383
979.4599 PEP_0_1_0_0_0
1182.5598 PEP_0_2_0_0_0
1506.6438 PEP_2_2_0_0_0
0
25000
50000
75000
100000
125000
150000
175000
250
500
750
1000
1250
1500
1750
2000

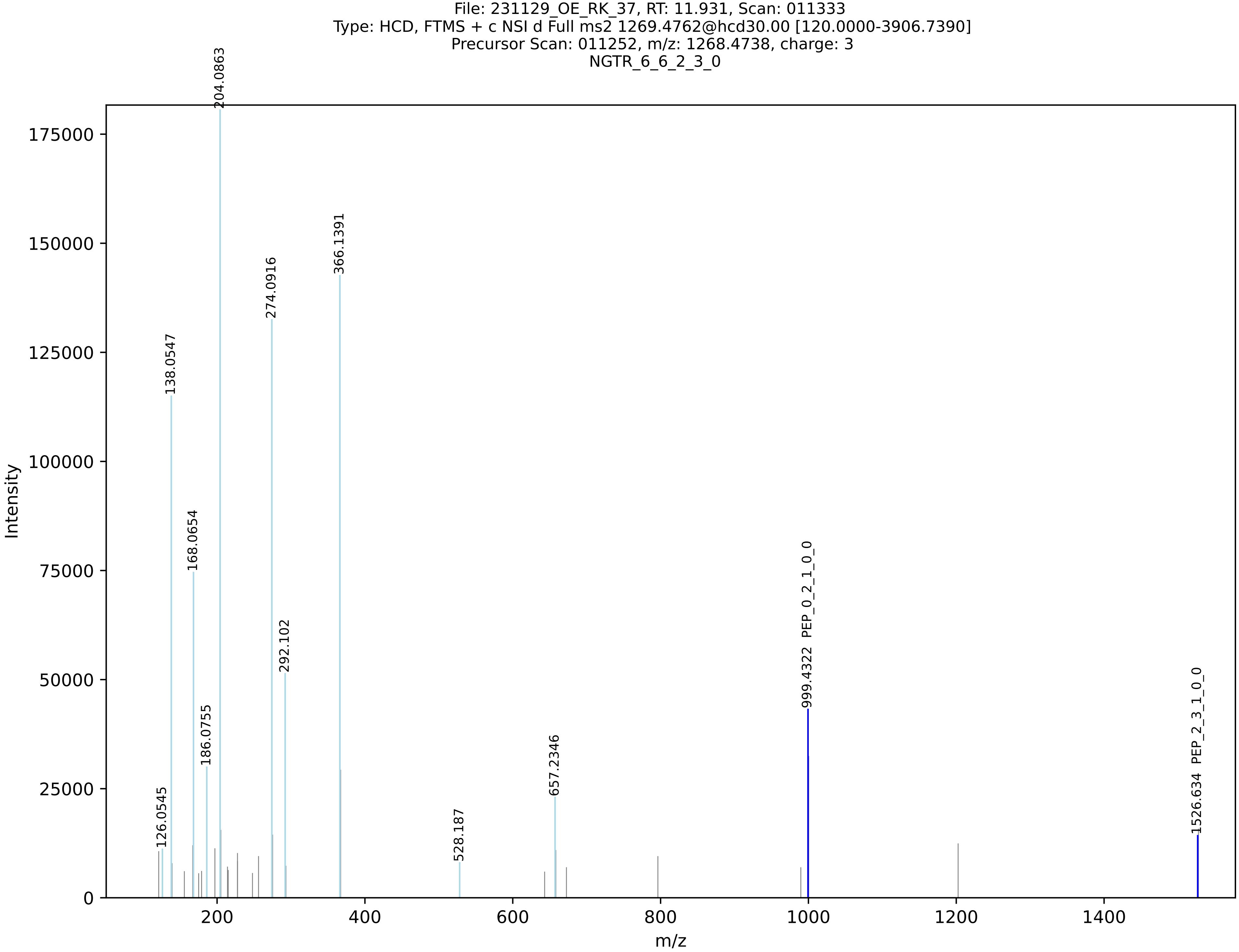
File: 231129_OE_RK_37, RT: 11.931, Scan: 011333
Type: HCD, FTMS + c NSI d Full ms2 1269.4762@hcd30.00 [120.0000-3906.7390]
Precursor Scan: 011252, m/z: 1268.4738, charge: 3
NGTR_6_6_2_3_0
Intensity
m/z
204.0863
366.1391
274.0916
138.0547
168.0654
292.102
186.0755
126.0545
528.187
657.2346
999.4322 PEP_0_2_1_0_0_
1526.634 PEP_2_3_1_0_0_
175000
150000
125000
100000
75000
50000
25000
0
200
400
600
800
1000
1200
1400

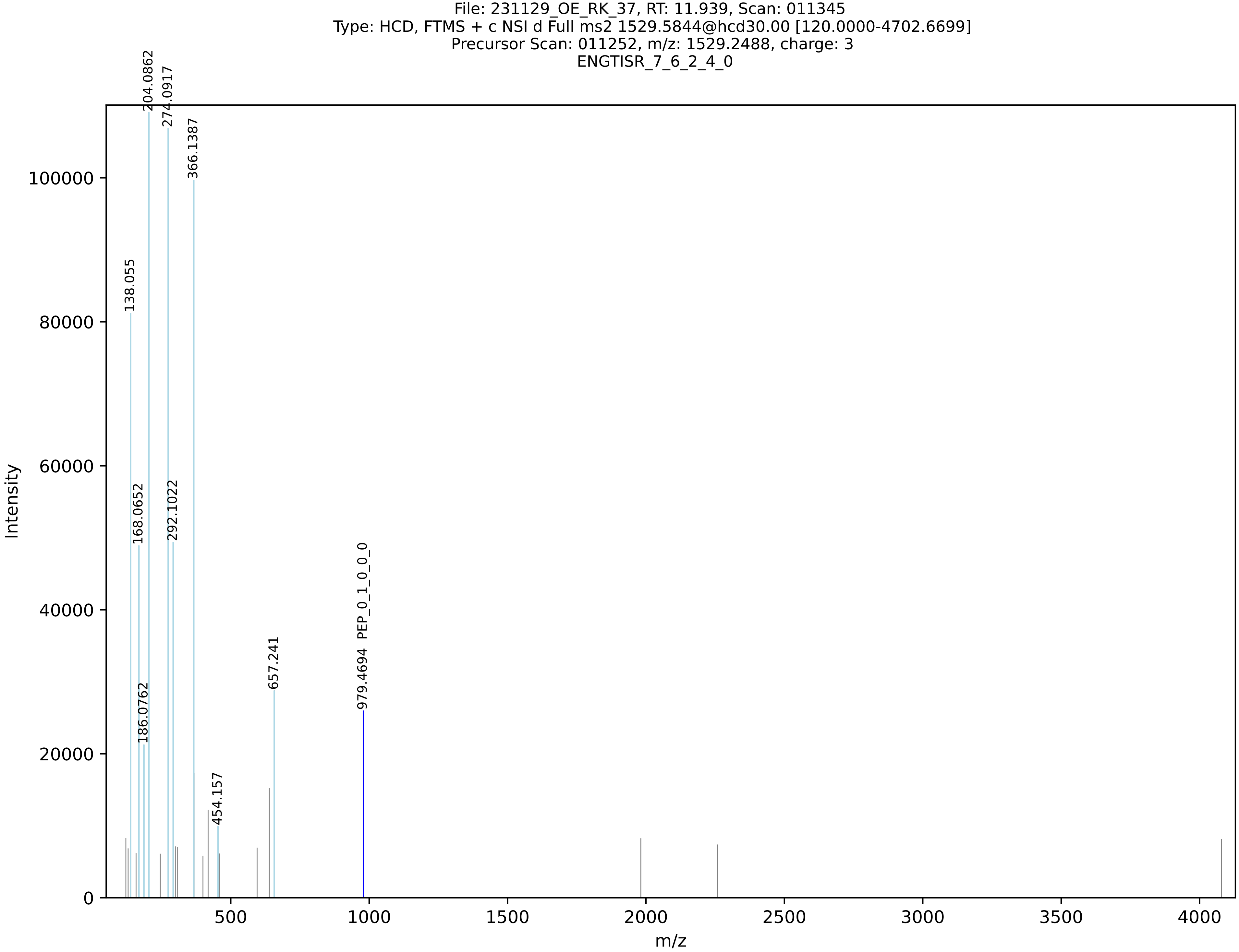
File: 231129_OE_RK_37, RT: 11.939, Scan: 011345
Type: HCD, FTMS + c NSI d Full ms2 1529.5844@hcd30.00 [120.0000-4702.6699]
Precursor Scan: 011252, m/z: 1529.2488, charge: 3
ENGTISR_7_6_2_4_0
204.0862
274.0917
366.1387
138.055
168.0652
292.1022
186.0762
454.157
657.241
979.4694 PEP_0_1_0_0_0_
Intensity
m/z
0
20000
40000
60000
80000
100000
500
1000
1500
2000
2500
3000
3500
4000

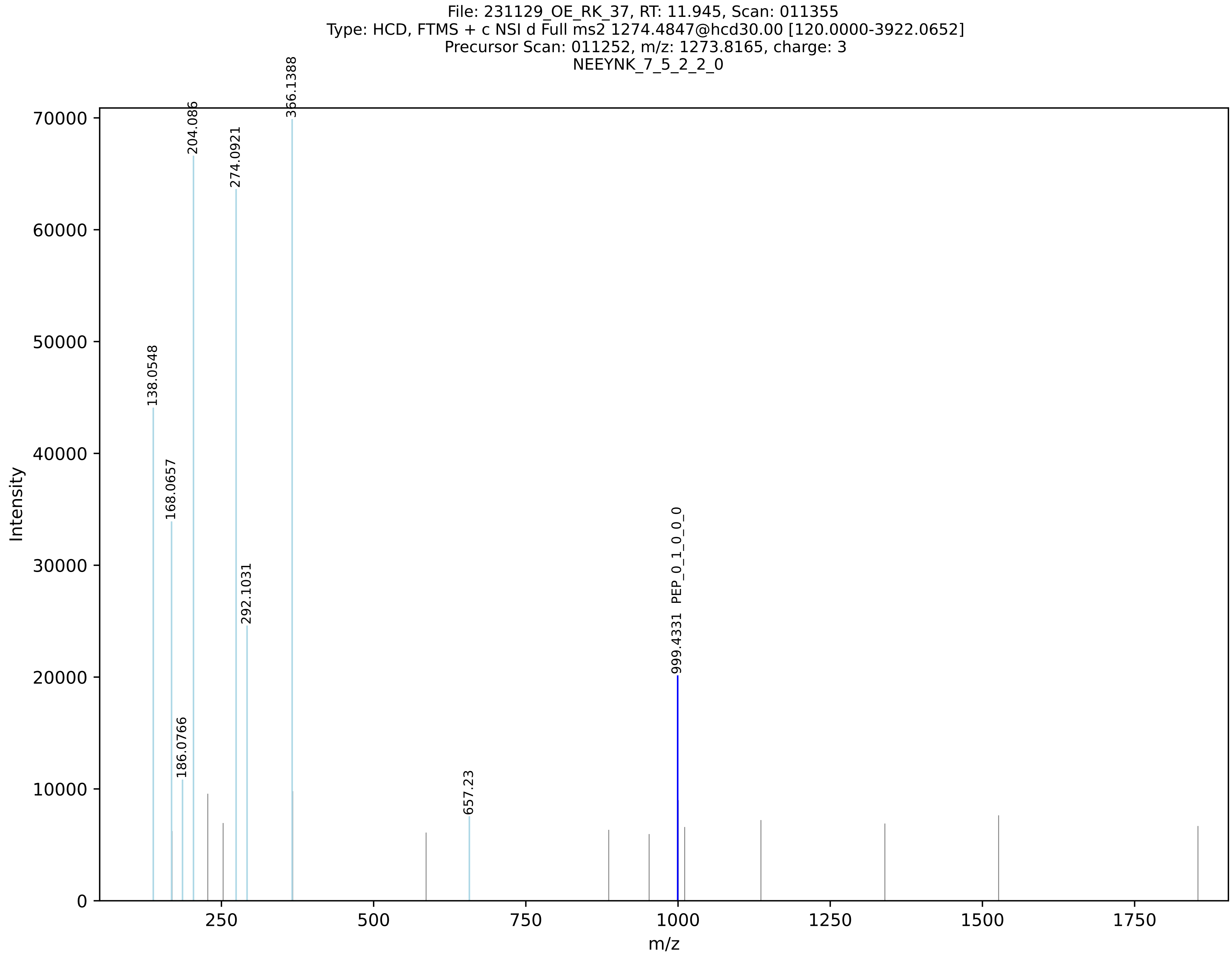
File: 231129_OE_RK_37, RT: 11.945, Scan: 011355
Type: HCD, FTMS + c NSI d Full ms2 1274.4847@hcd30.00 [120.0000-3922.0652]
Precursor Scan: 011252, m/z: 1273.8165, charge: 3
NEEYNK_7_5_2_2_0
Intensity
m/z
138.0548
168.0657
186.0766
204.086
274.0921
292.1031
366.1388
657.23
999.4331 PEP_0_1_0_0_0
0
10000
20000
30000
40000
50000
60000
70000
250
500
750
1000
1250
1500
1750

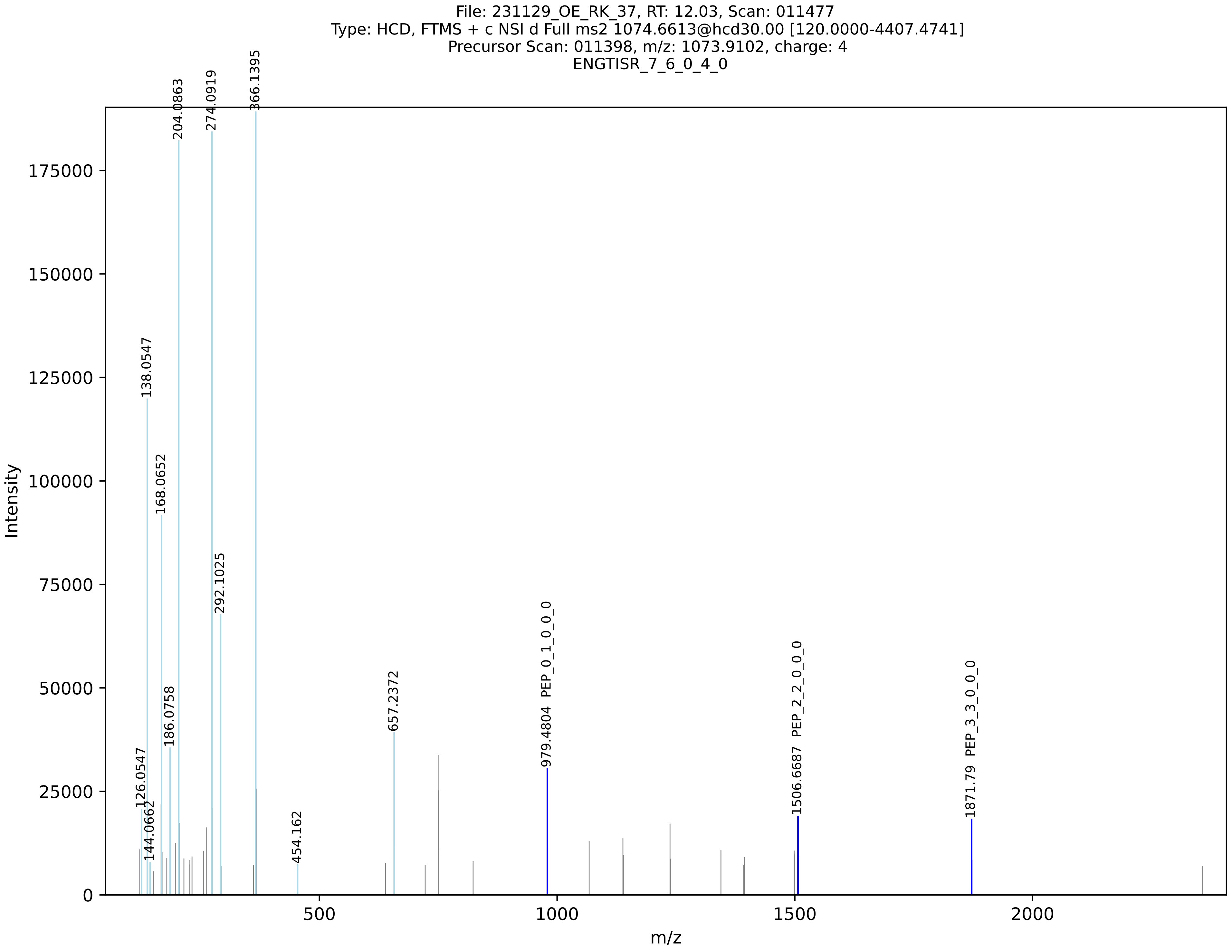

File: 231129_OE_RK_37, RT: 12.03, Scan: 011477
Type: HCD, FTMS + c NSI d Full ms2 1074.6613@hcd30.00 [120.0000-4407.4741]
Precursor Scan: 011398, m/z: 1073.9102, charge: 4
ENGTISR_7_6_0_4_0
Intensity
m/z
0
25000
50000
75000
100000
125000
150000
175000
500
1000
1500
2000
126.0547
138.0547
144.0662
168.0652
186.0758
204.0863
274.0919
292.1025
366.1395
454.162
657.2372
979.4804 PEP_0_1_0_0_0
1506.6687 PEP_2_2_0_0_0
1871.79 PEP_3_3_0_0_0

File: 231129_OE_RK_37, RT: 12.068, Scan: 011531
Type: HCD, FTMS + c NSI d Full ms2 1213.4708@hcd30.00 [120.0000-3735.3628]
Precursor Scan: 011398, m/z: 1213.1384, charge: 3
ENGTISR_6_5_0_3_0

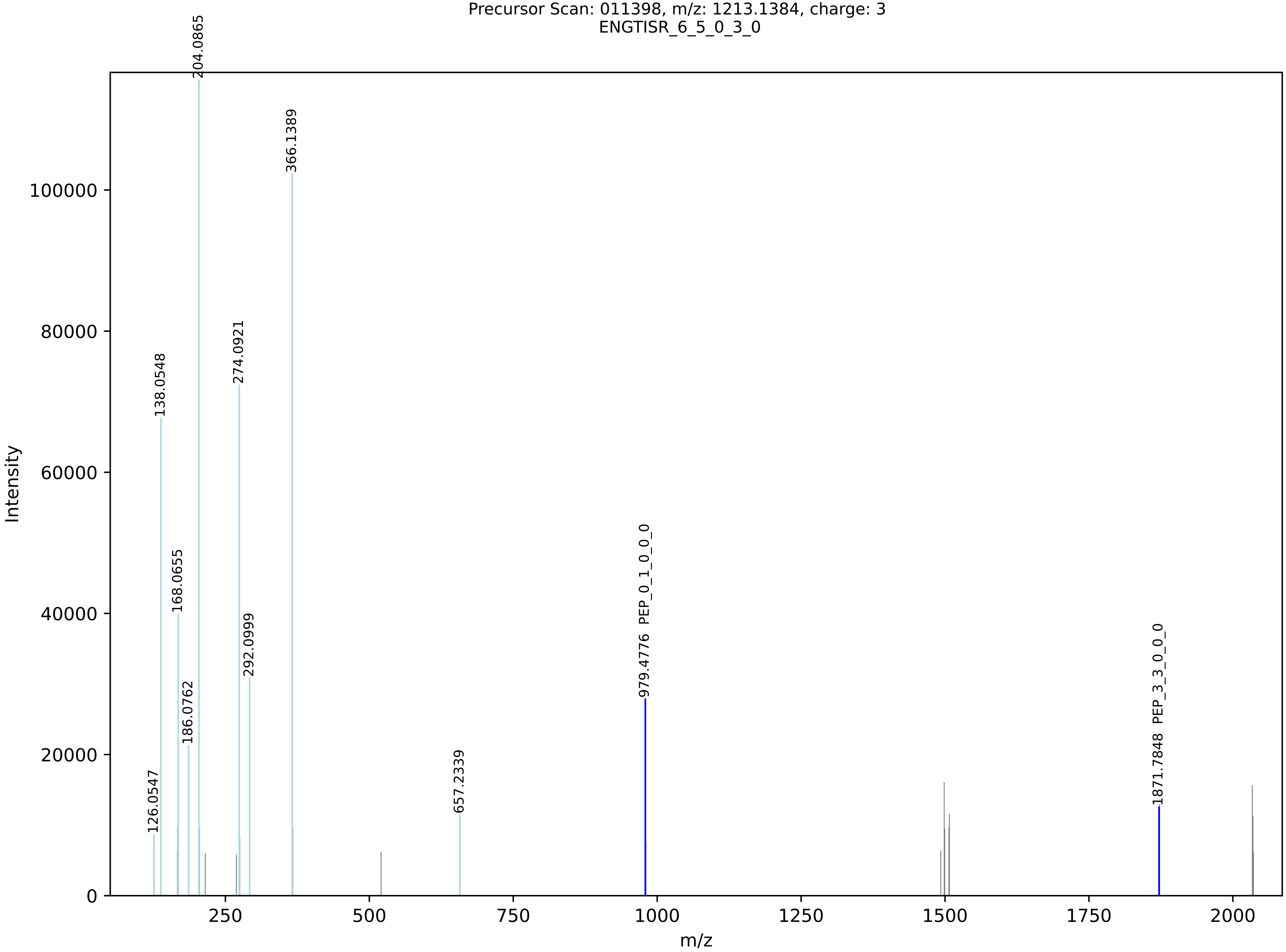

File: 231129_OE_RK_37, RT: 12.146, Scan: 011620
Type: HCD, FTMS + c NSI d Full ms2 1335.5109@hcd30.00 [120.0000-4108.8057]
Precursor Scan: 011599, m/z: 1334.8443, charge: 3
ENGTISR_7_6_0_3_0

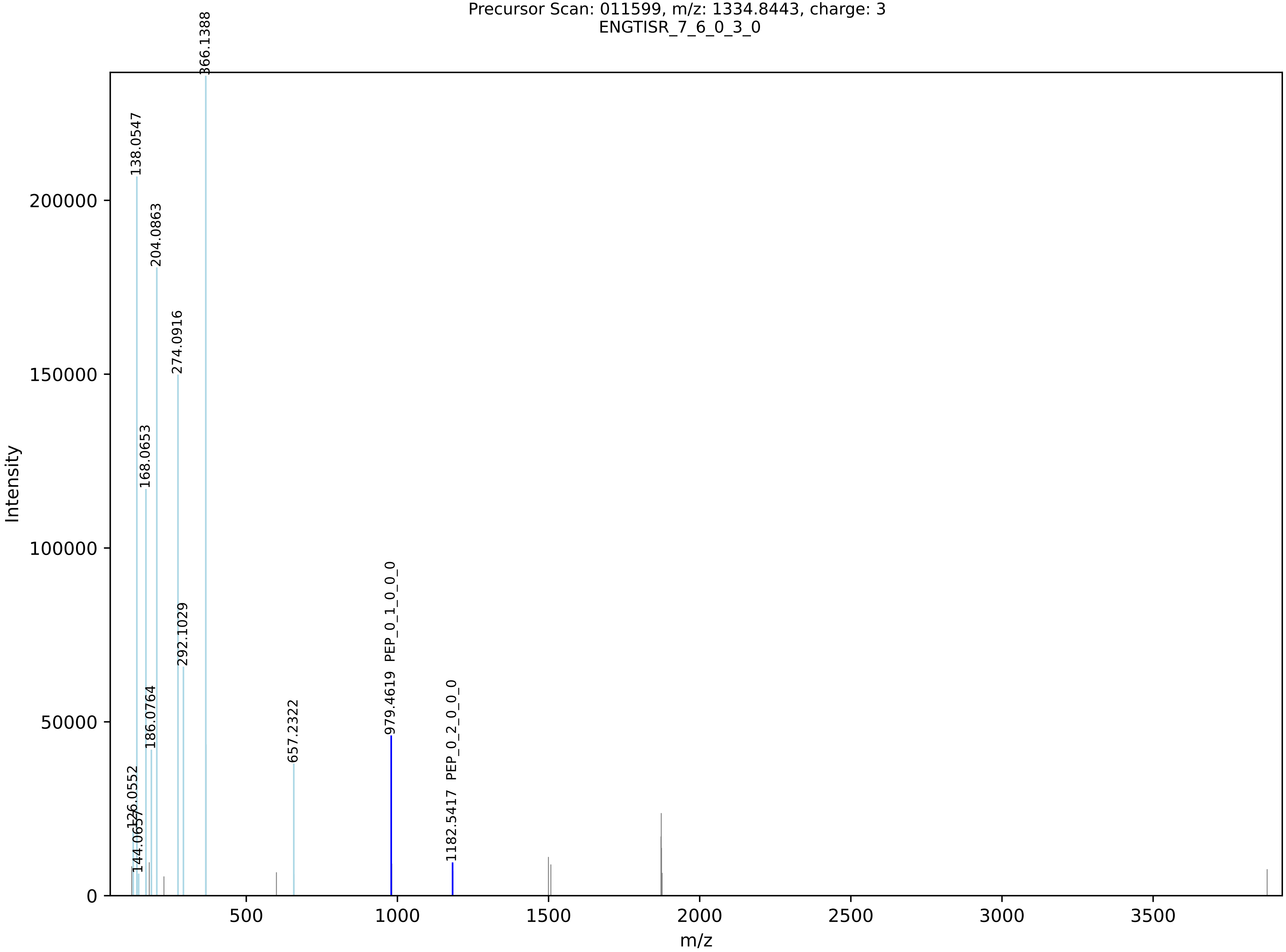

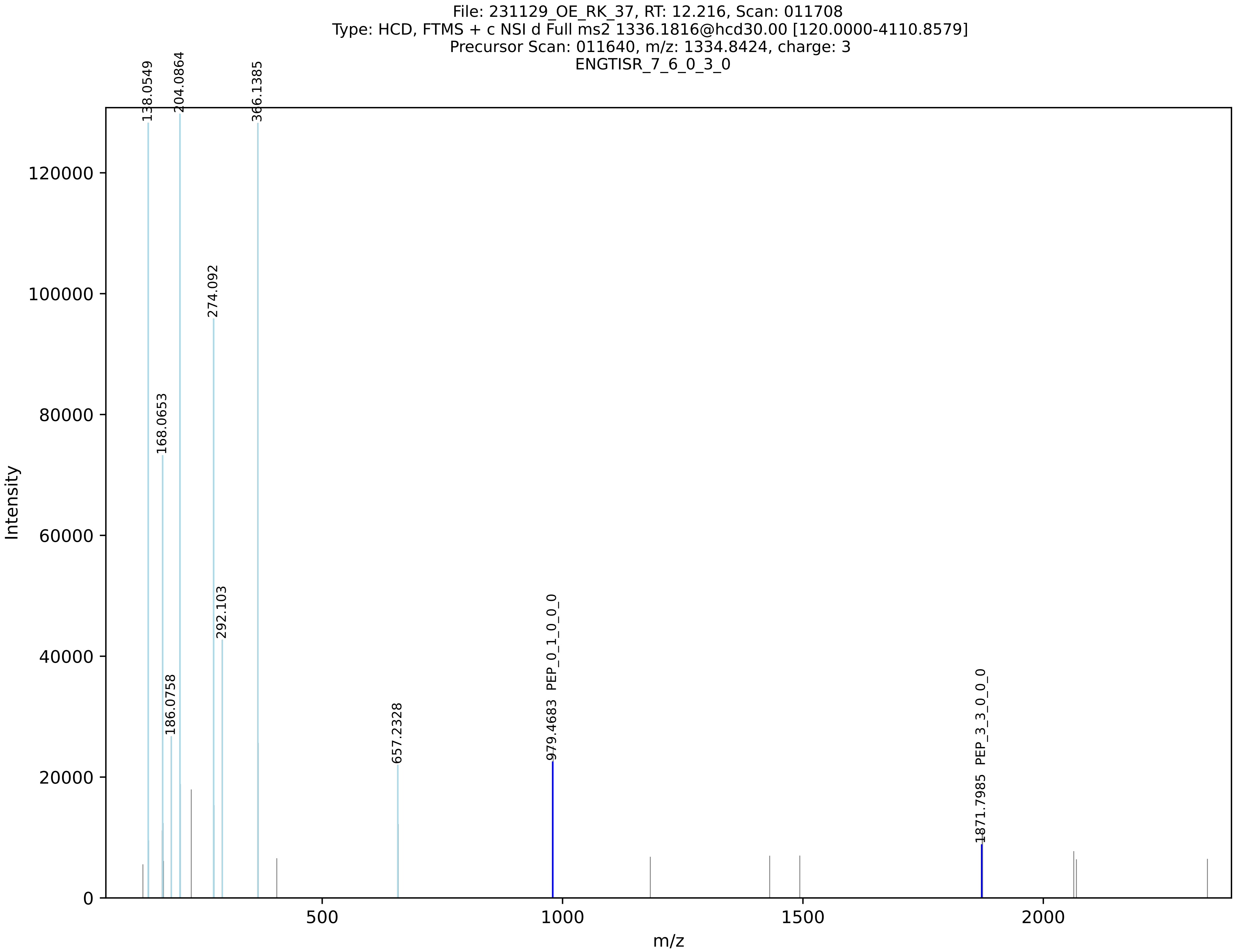

File: 231129_OE_RK_37, RT: 12.216, Scan: 011708
Type: HCD, FTMS + c NSI d Full ms2 1336.1816@hcd30.00 [120.0000-4110.8579]
Precursor Scan: 011640, m/z: 1334.8424, charge: 3
ENGTISR_7_6_0_3_0
138.0549
204.0864
366.1385
274.092
168.0653
292.103
186.0758
657.2328
979.4683 PEP_0_1_0_0_0_
1871.7985 PEP_3_3_0_0_0_
Intensity
m/z
0
20000
40000
60000
80000
100000
120000
500
1000
1500
2000

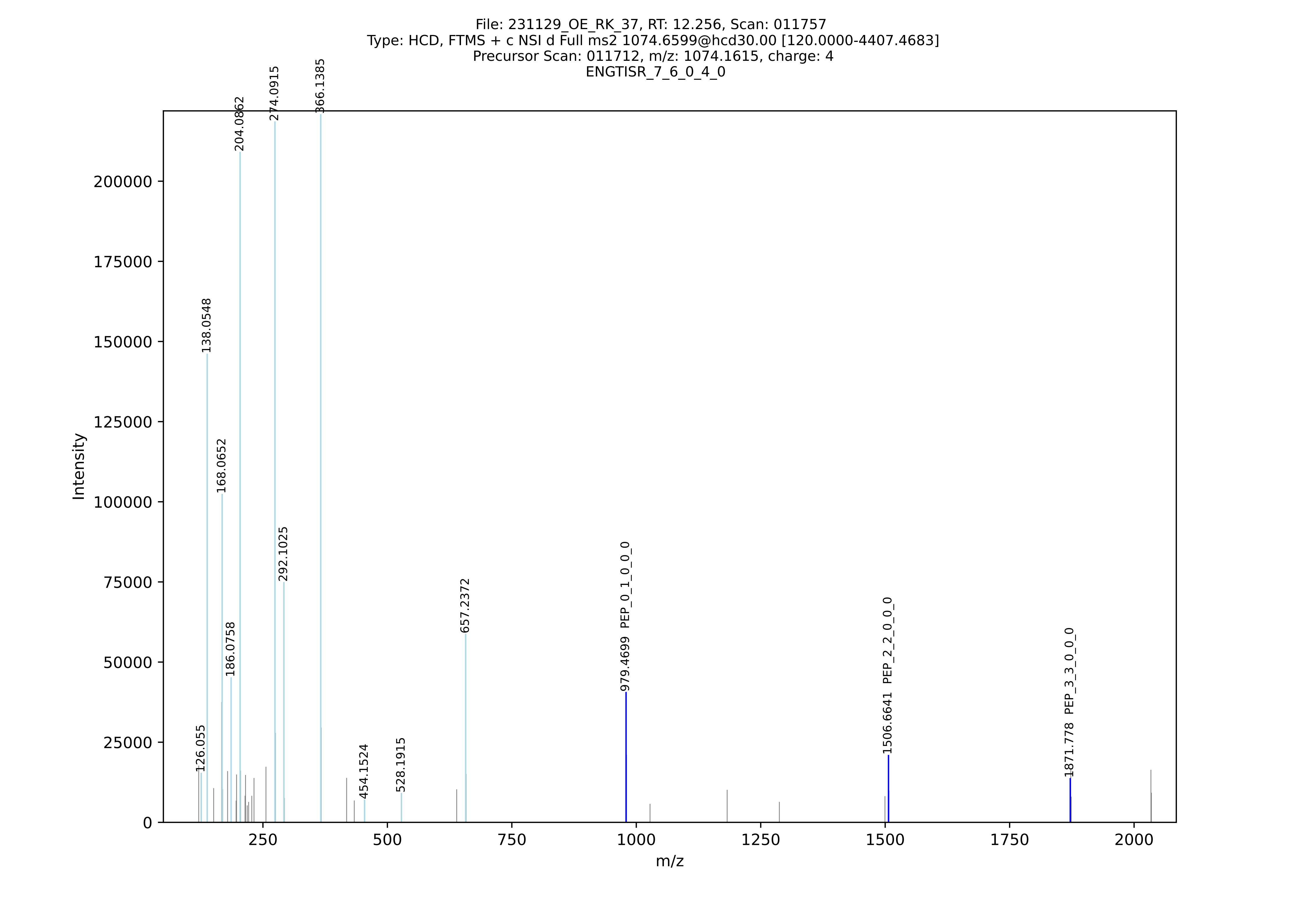
File: 231129_OE_RK_37, RT: 12.256, Scan: 011757
Type: HCD, FTMS + c NSI d Full ms2 1074.6599@hcd30.00 [120.0000-4407.4683]
Precursor Scan: 011712, m/z: 1074.1615, charge: 4
ENGTISR_7_6_0_4_0
Intensity
m/z
0
25000
50000
75000
100000
125000
150000
175000
200000
250
500
750
1000
1250
1500
1750
2000
126.055
138.0548
168.0652
186.0758
204.0862
274.0915
292.1025
366.1385
454.1524
528.1915
657.2372
979.4699 PEP_0_1_0_0_0
1506.6641 PEP_2_2_0_0_0
1871.778 PEP_3_3_0_0_0

File: 231129_OE_RK_37, RT: 12.28, Scan: 011795
Type: HCD, FTMS + c NSI d Full ms2 1214.4697@hcd30.00 [120.0000-3738.4194]
Precursor Scan: 011685, m/z: 1213.472, charge: 3
ENGTISR_6_5_0_3_0

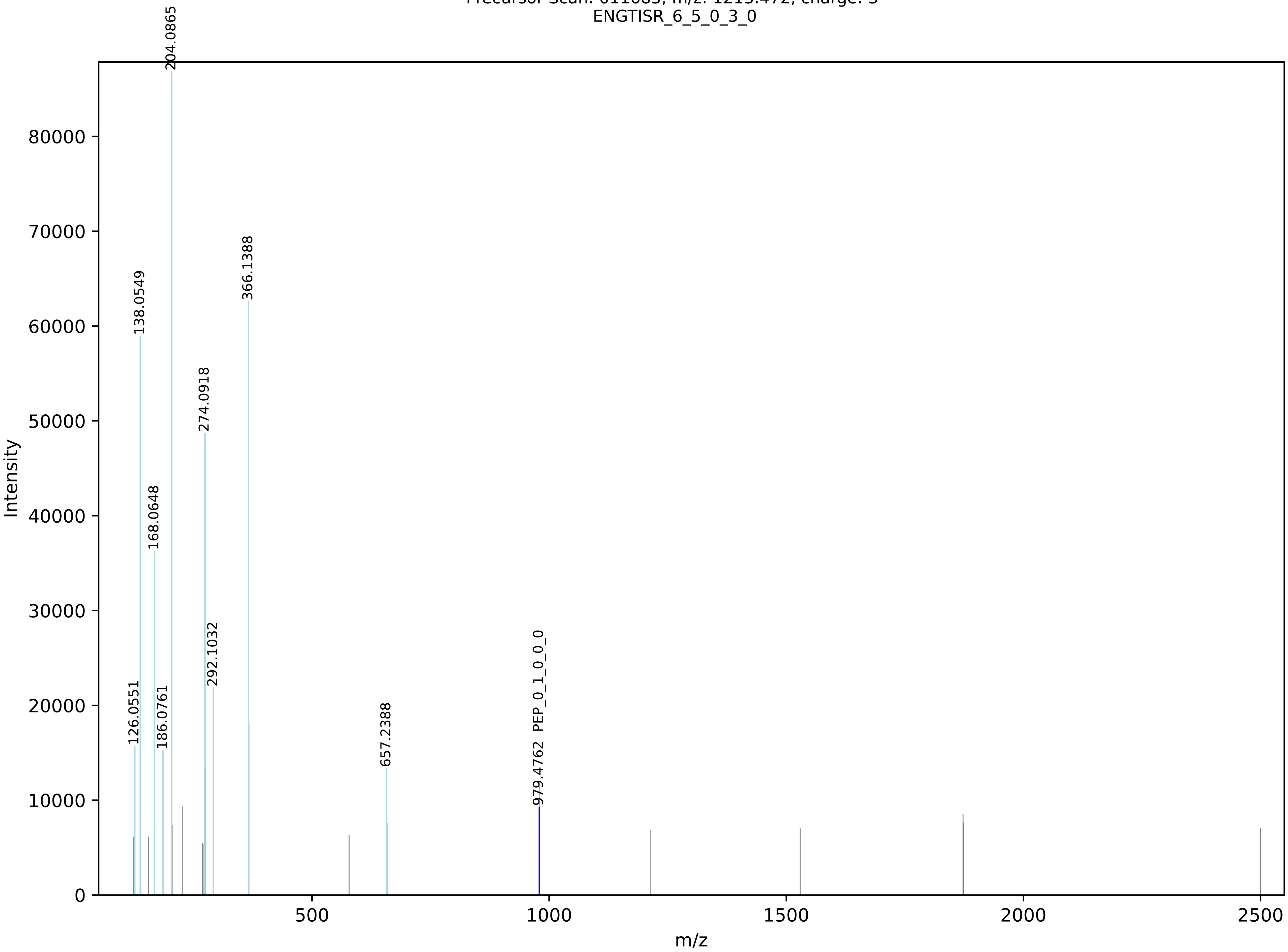

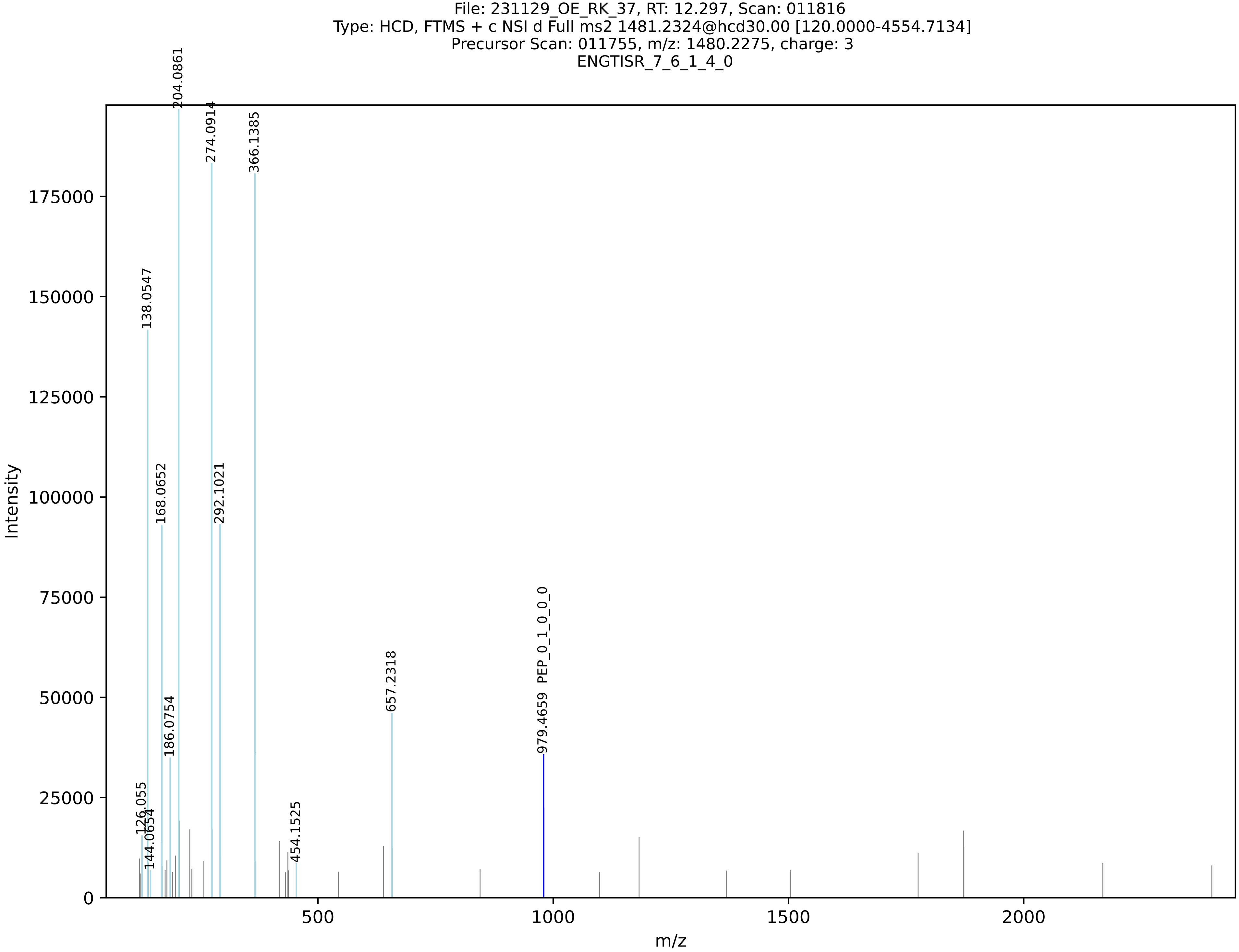

File: 231129_OE_RK_37, RT: 12.297, Scan: 011816
Type: HCD, FTMS + c NSI d Full ms2 1481.2324@hcd30.00 [120.0000-4554.7134]
Precursor Scan: 011755, m/z: 1480.2275, charge: 3
ENGTISR_7_6_1_4_0
204.0861
274.0914
366.1385
138.0547
168.0652
292.1021
186.0754
126.055
144.0654
454.1525
657.2318
979.4659 PEP_0_1_0_0_0
Intensity
m/z
0
25000
50000
75000
100000
125000
150000
175000
500
1000
1500
2000

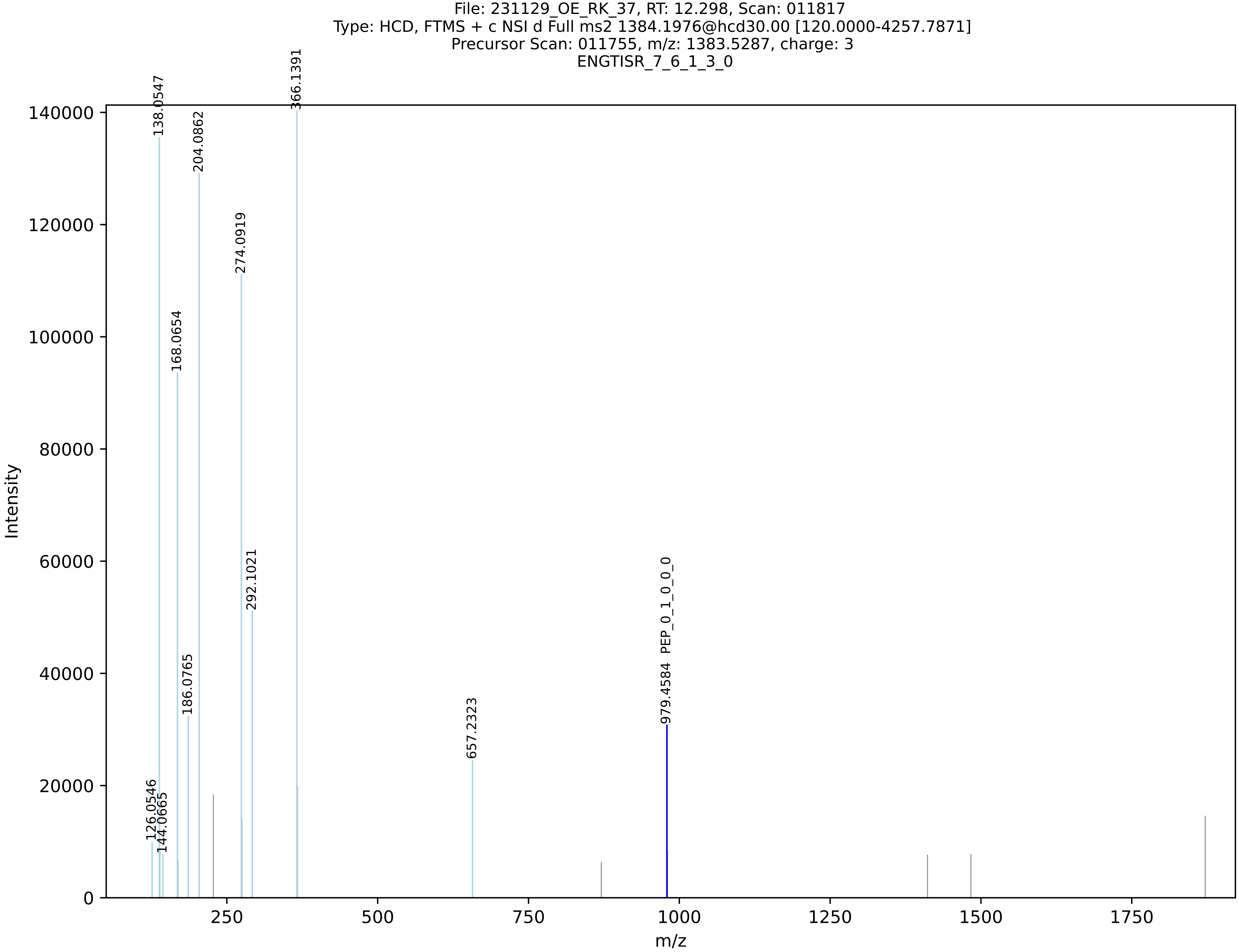
File: 231129_OE_RK_37, RT: 12.298, Scan: 011817
Type: HCD, FTMS + c NSI d Full ms2 1384.1976@hcd30.00 [120.0000-4257.7871]
Precursor Scan: 011755, m/z: 1383.5287, charge: 3
ENGTISR_7_6_1_3_0
Intensity
m/z
0
20000
40000
60000
80000
100000
120000
140000
250
500
750
1000
1250
1500
1750
126.0546
138.0547
144.0665
168.0654
186.0765
204.0862
274.0919
292.1021
366.1391
657.2323
979.4584 PEP_0_1_0_0_0

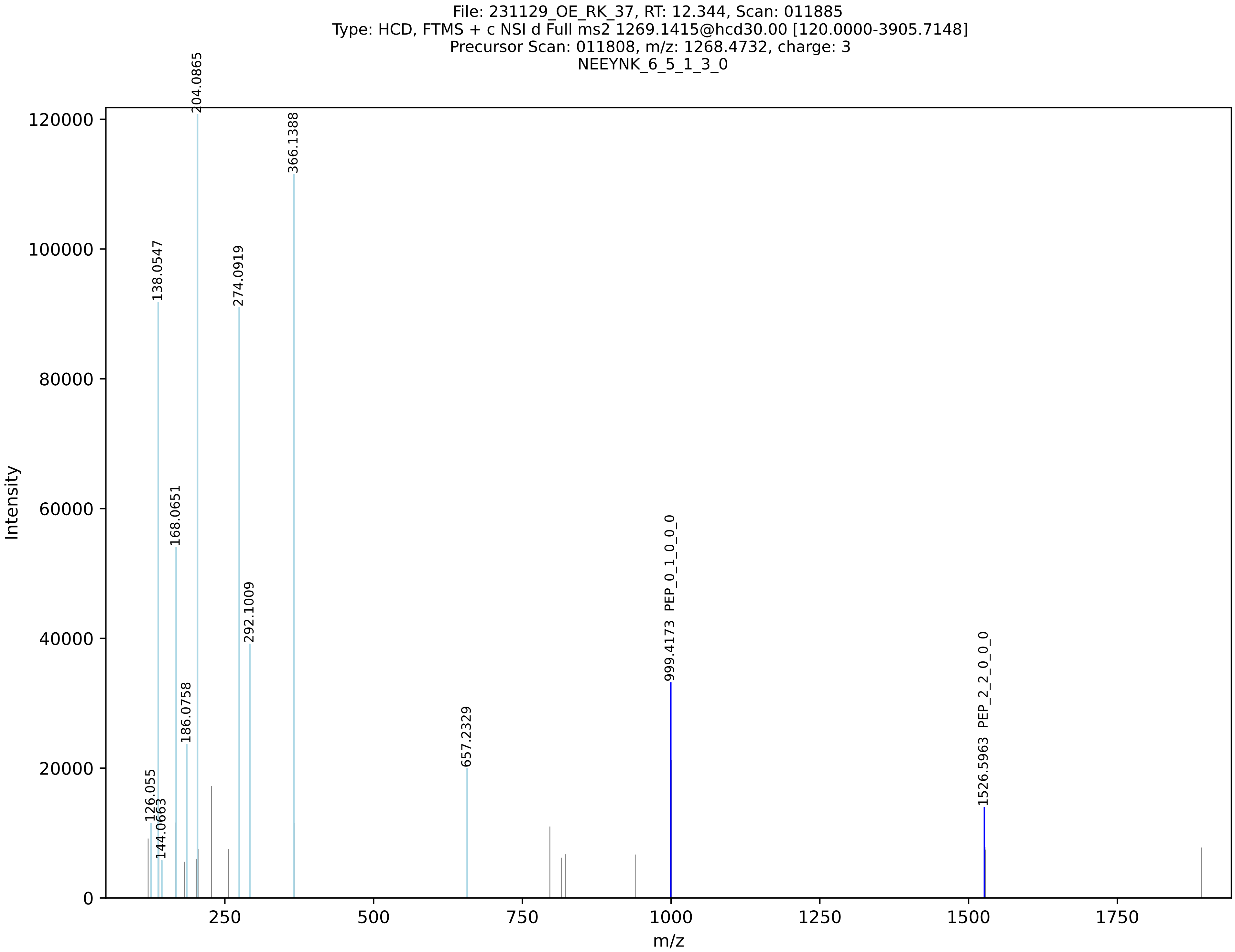
File: 231129_OE_RK_37, RT: 12.344, Scan: 011885
Type: HCD, FTMS + c NSI d Full ms2 1269.1415@hcd30.00 [120.0000-3905.7148]
Precursor Scan: 011808, m/z: 1268.4732, charge: 3
NEEYNK_6_5_1_3_0
Intensity
m/z
204.0865
366.1388
138.0547
274.0919
168.0651
292.1009
186.0758
126.055
144.0663
657.2329
999.4173 PEP_0_1_0_0_0_
1526.5963 PEP_2_2_0_0_0_
0
20000
40000
60000
80000
100000
120000
250
500
750
1000
1250
1500
1750

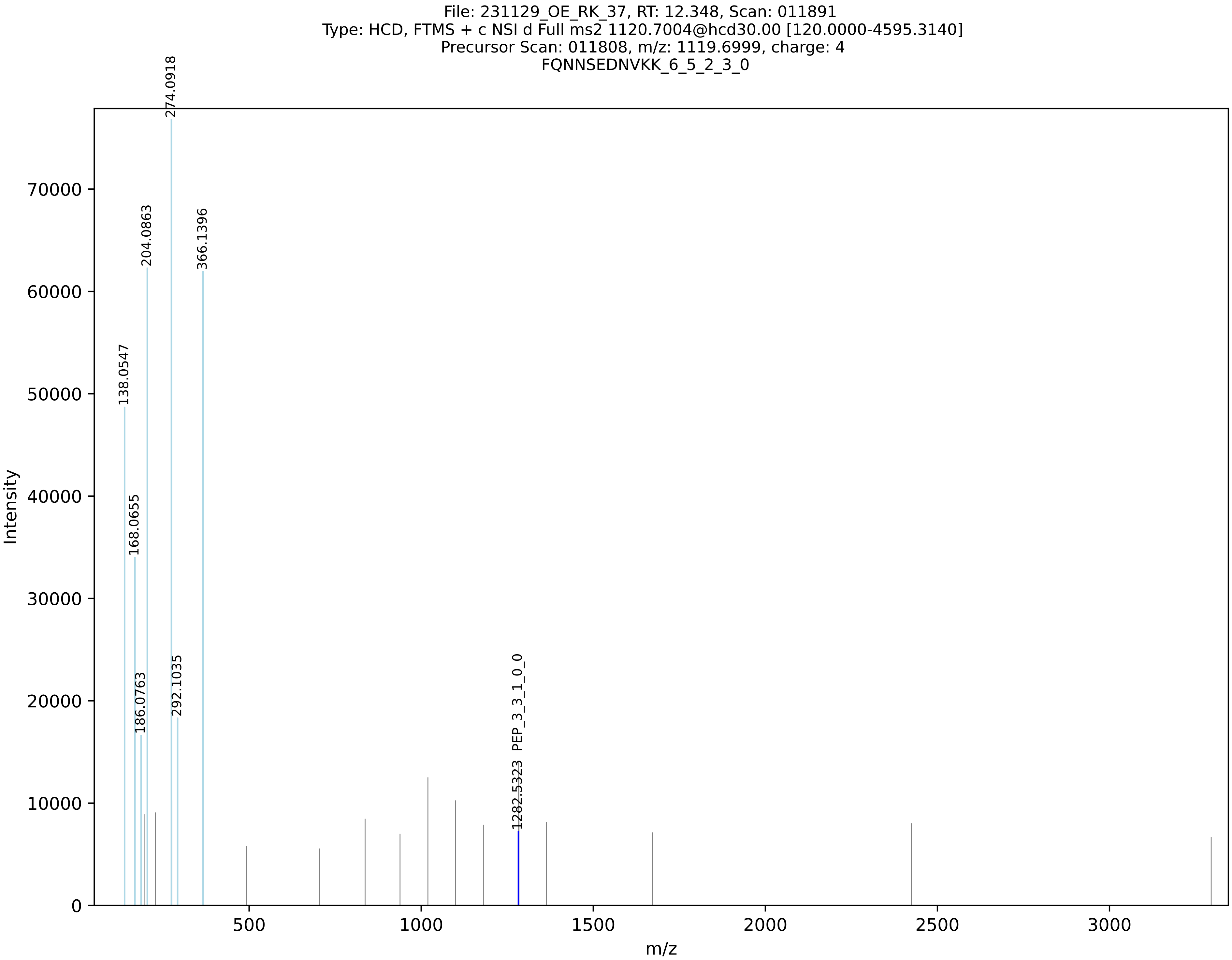
File: 231129_OE_RK_37, RT: 12.348, Scan: 011891
Type: HCD, FTMS + c NSI d Full ms2 1120.7004@hcd30.00 [120.0000-4595.3140]
Precursor Scan: 011808, m/z: 1119.6999, charge: 4
FQNNSEDNVKK_6_5_2_3_0
274.0918
204.0863
366.1396
138.0547
168.0655
186.0763
292.1035
1282.5323 PEP_3_3_1_0_0
Intensity
m/z
0
10000
20000
30000
40000
50000
60000
70000
500
1000
1500
2000
2500
3000

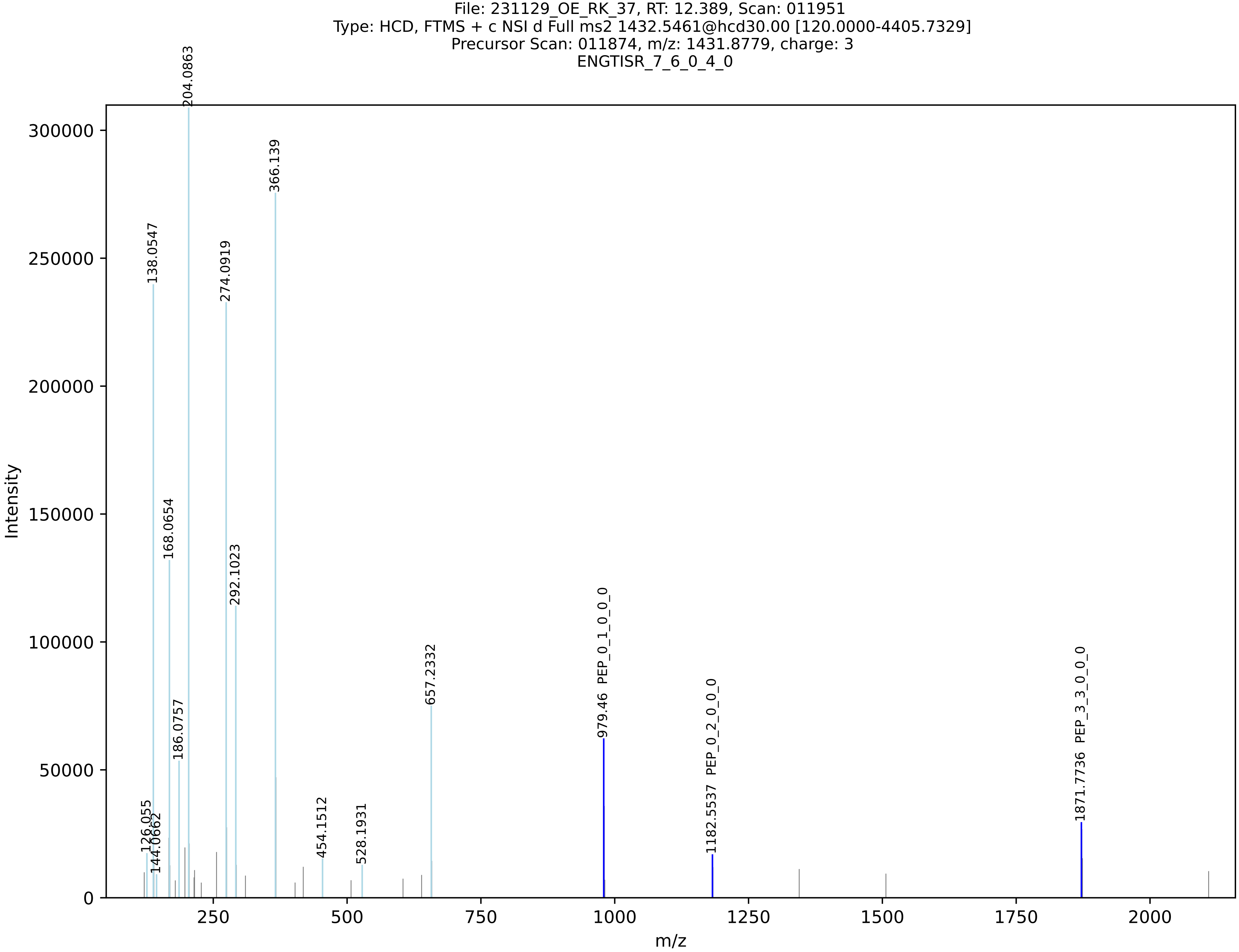

File: 231129_OE_RK_37, RT: 12.389, Scan: 011951
Type: HCD, FTMS + c NSI d Full ms2 1432.5461@hcd30.00 [120.0000-4405.7329]
Precursor Scan: 011874, m/z: 1431.8779, charge: 3
ENGTISR_7_6_0_4_0
Intensity
m/z
204.0863
366.139
138.0547
274.0919
168.0654
292.1023
186.0757
126.055
144.0662
454.1512
528.1931
657.2332
979.46 PEP_0_1_0_0_0
1182.5537 PEP_0_2_0_0_0
1871.7736 PEP_3_3_0_0_0

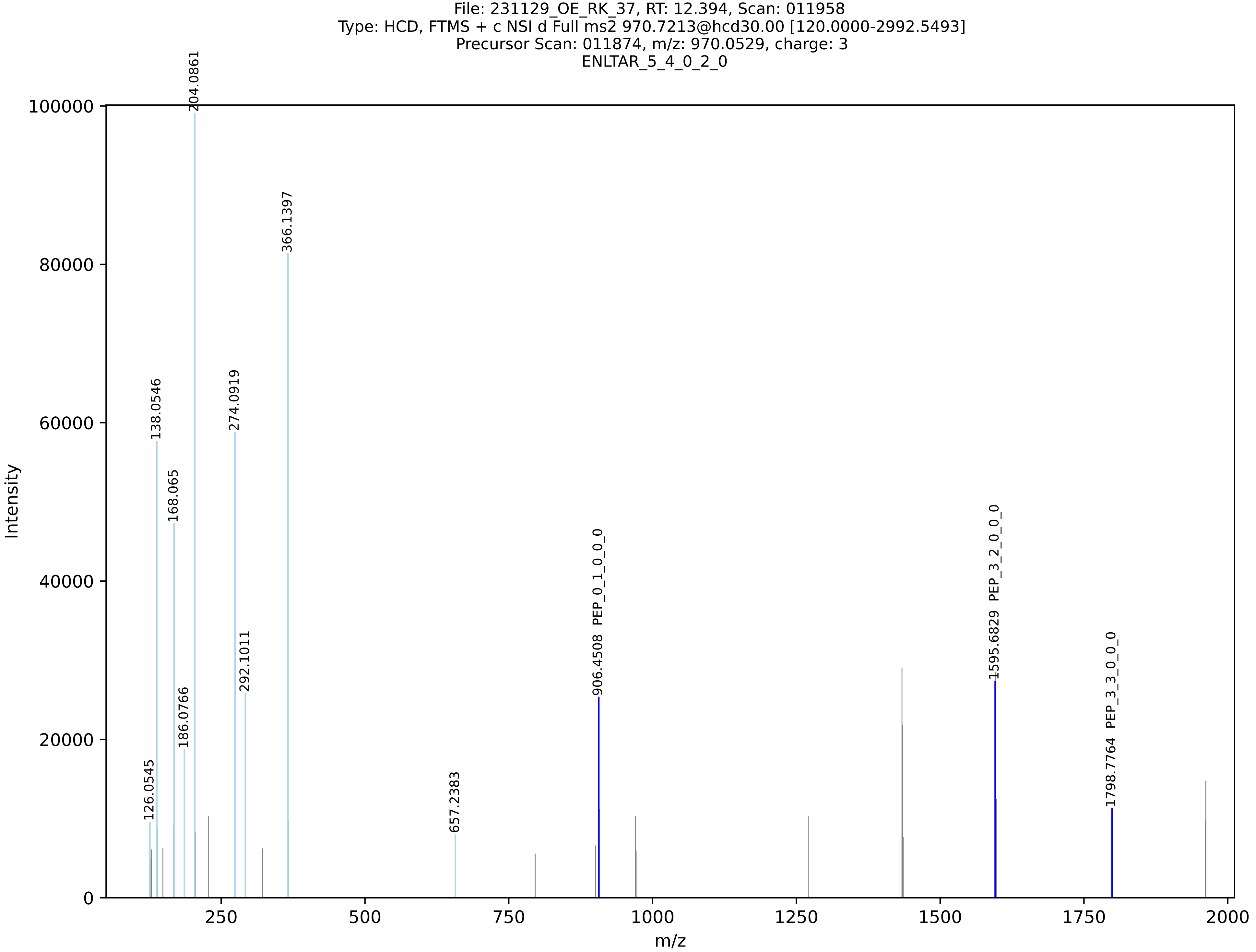

File: 231129_OE_RK_37, RT: 12.394, Scan: 011958
Type: HCD, FTMS + c NSI d Full ms2 970.7213@hcd30.00 [120.0000-2992.5493]
Precursor Scan: 011874, m/z: 970.0529, charge: 3
ENLTAR_5_4_0_2_0
Intensity
m/z
0
20000
40000
60000
80000
100000
250
500
750
1000
1250
1500
1750
2000
204.0861
366.1397
138.0546
274.0919
168.065
186.0766
292.1011
126.0545
657.2383
906.4508 PEP_0_1_0_0_0
1595.6829 PEP_3_2_0_0_0
1798.7764 PEP_3_3_0_0_0

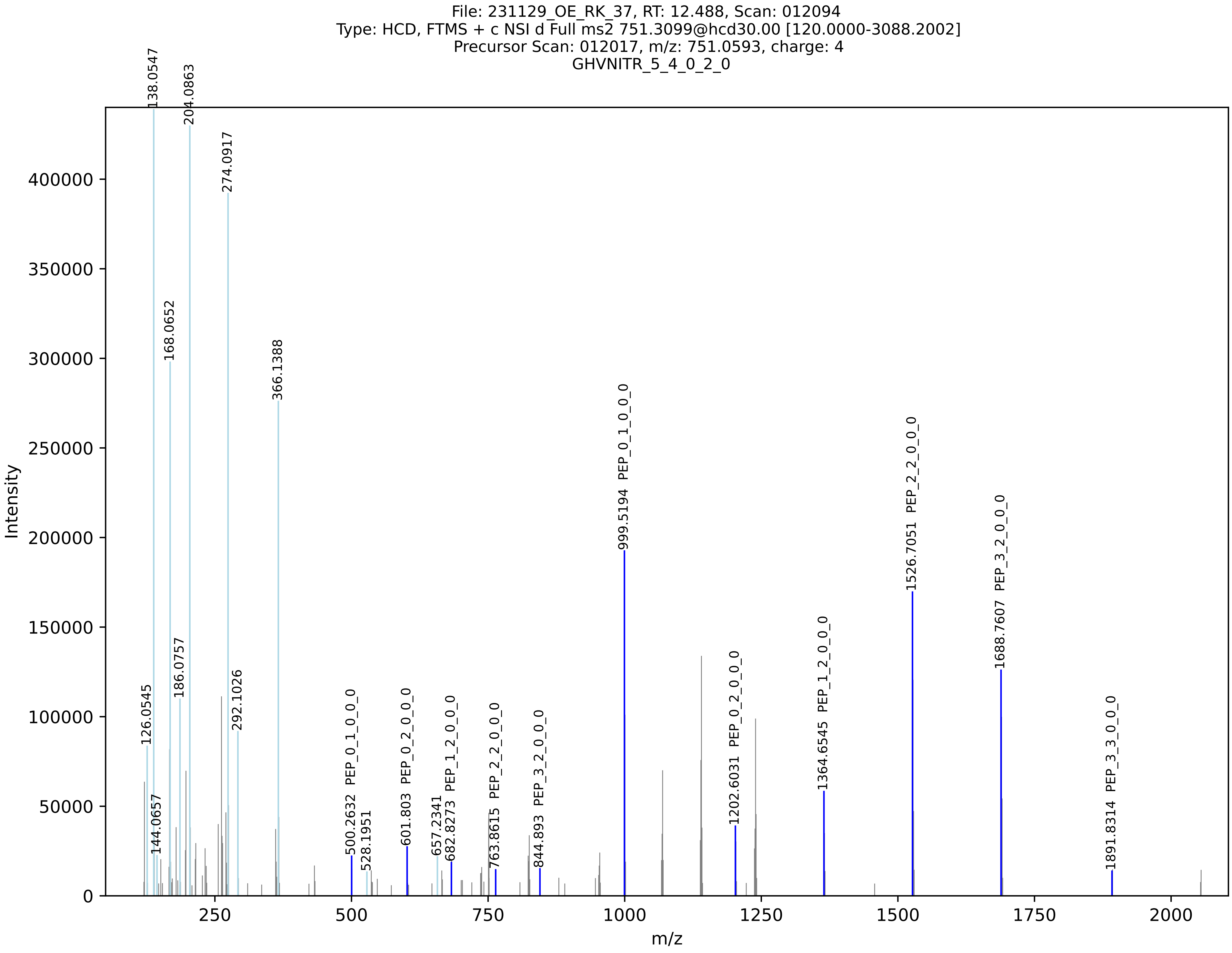
File: 231129_OE_RK_37, RT: 12.488, Scan: 012094
Type: HCD, FTMS + c NSI d Full ms2 751.3099@hcd30.00 [120.0000-3088.2002]
Precursor Scan: 012017, m/z: 751.0593, charge: 4
GHVNITR_5_4_0_2_0
Intensity
m/z
138.0547
204.0863
274.0917
168.0652
366.1388
186.0757
126.0545
292.1026
144.0657
500.2632 PEP_0_1_0_0_0
528.1951
601.803 PEP_0_2_0_0_0
657.2341
682.8273 PEP_1_2_0_0_0
763.8615 PEP_2_2_0_0_0
844.893 PEP_3_2_0_0_0
999.5194 PEP_0_1_0_0_
1202.6031 PEP_0_2_0_0_0
1364.6545 PEP_1_2_0_0_0
1526.7051 PEP_2_2_0_0_0
1688.7607 PEP_3_2_0_0_0
1891.8314 PEP_3_3_0_0_0

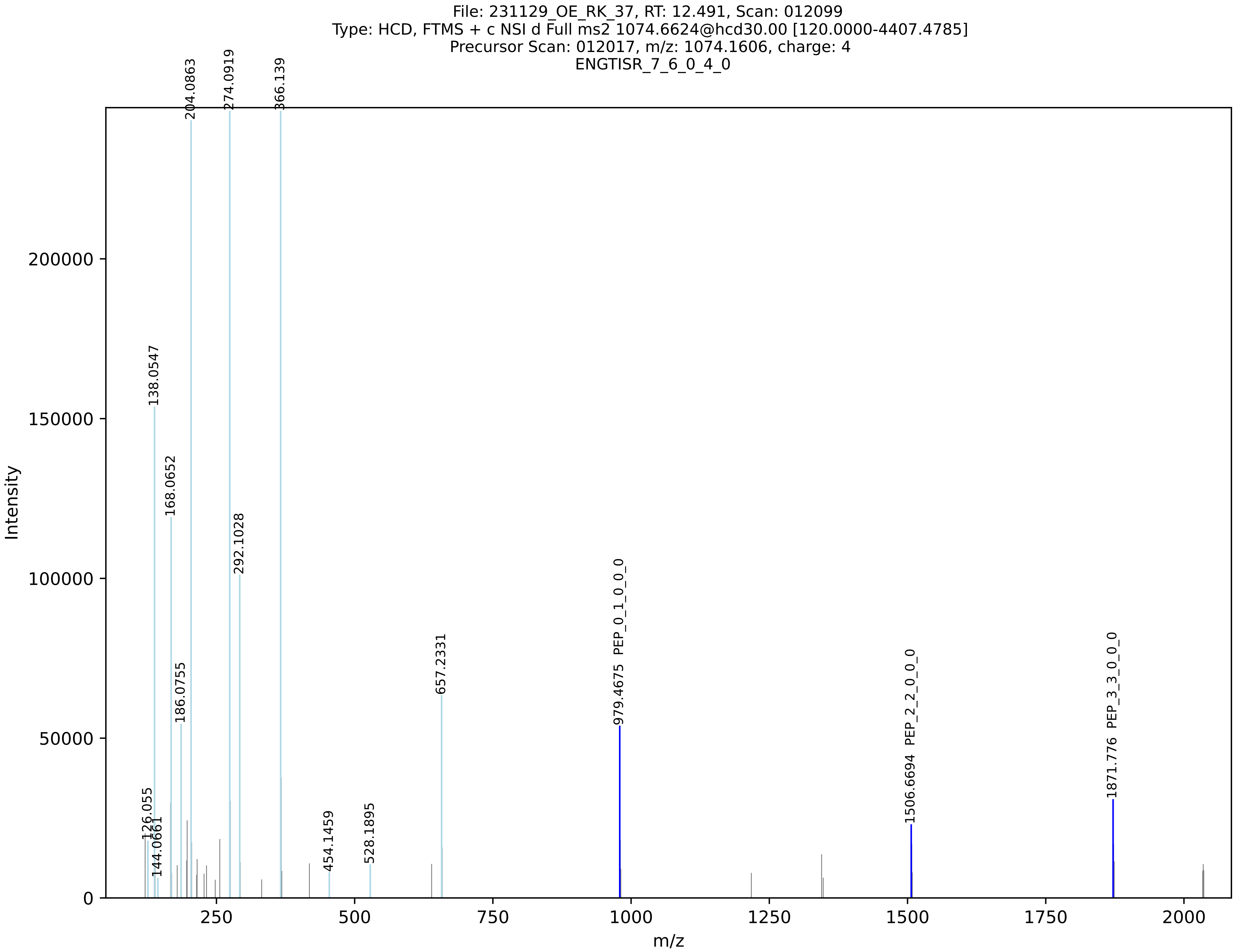
File: 231129_OE_RK_37, RT: 12.491, Scan: 012099
Type: HCD, FTMS + c NSI d Full ms2 1074.6624@hcd30.00 [120.0000-4407.4785]
Precursor Scan: 012017, m/z: 1074.1606, charge: 4
ENGTISR_7_6_0_4_0
Intensity
m/z
0
50000
100000
150000
200000
250
500
750
1000
1250
1500
1750
2000
126.055
144.0661
138.0547
168.0652
186.0755
204.0863
274.0919
292.1028
366.139
454.1459
528.1895
657.2331
979.4675 PEP_0_1_0_0_0
1506.6694 PEP_2_2_0_0_0
1871.776 PEP_3_3_0_0_0

File: 231129_OE_RK_37, RT: 12.497, Scan: 012109
Type: HCD, FTMS + c NSI d Full ms2 1384.5328@hcd30.00 [120.0000-4258.8125]
Precursor Scan: 012017, m/z: 1383.5338, charge: 3
ENGTISR_7_6_1_3_0

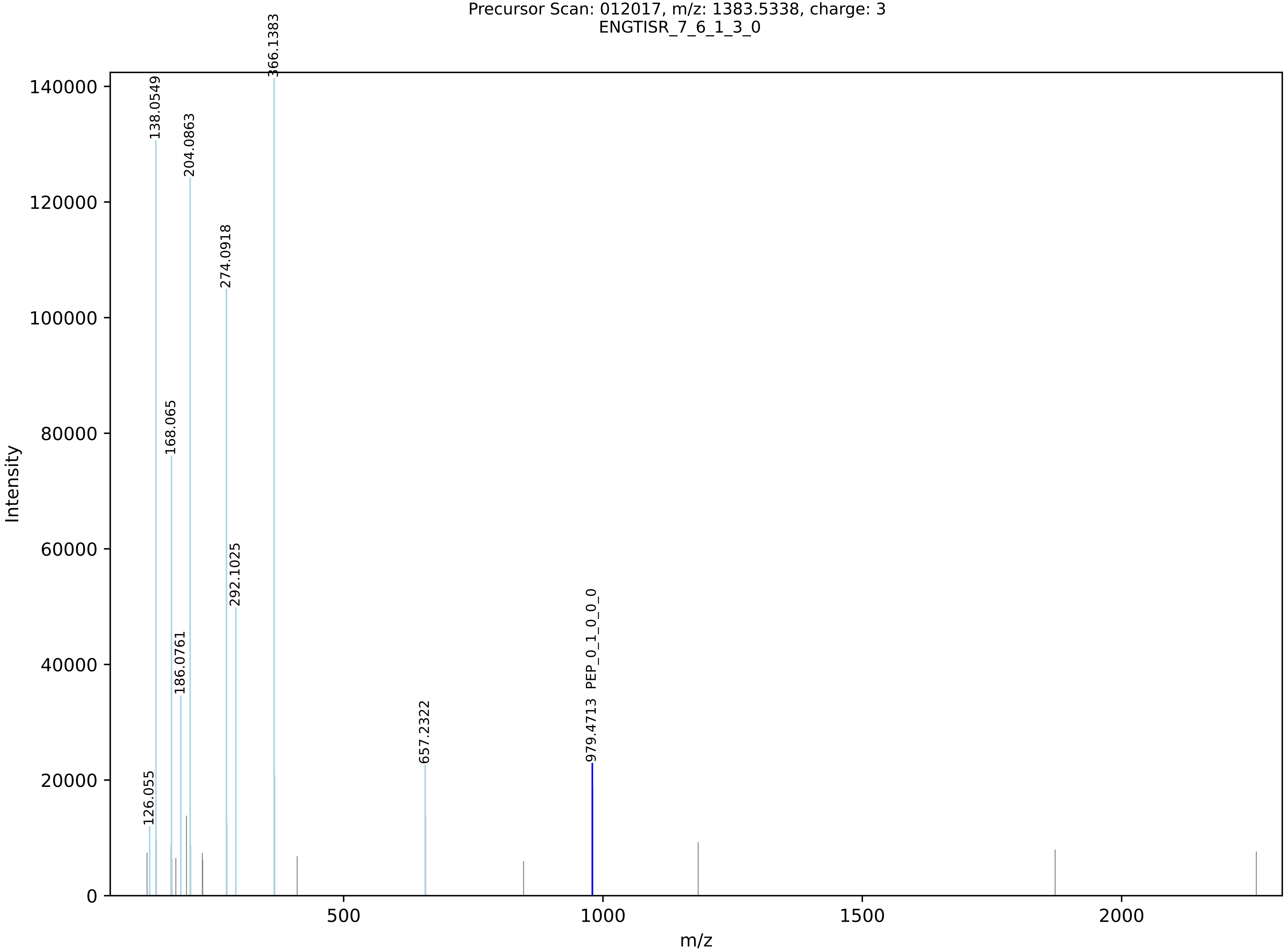

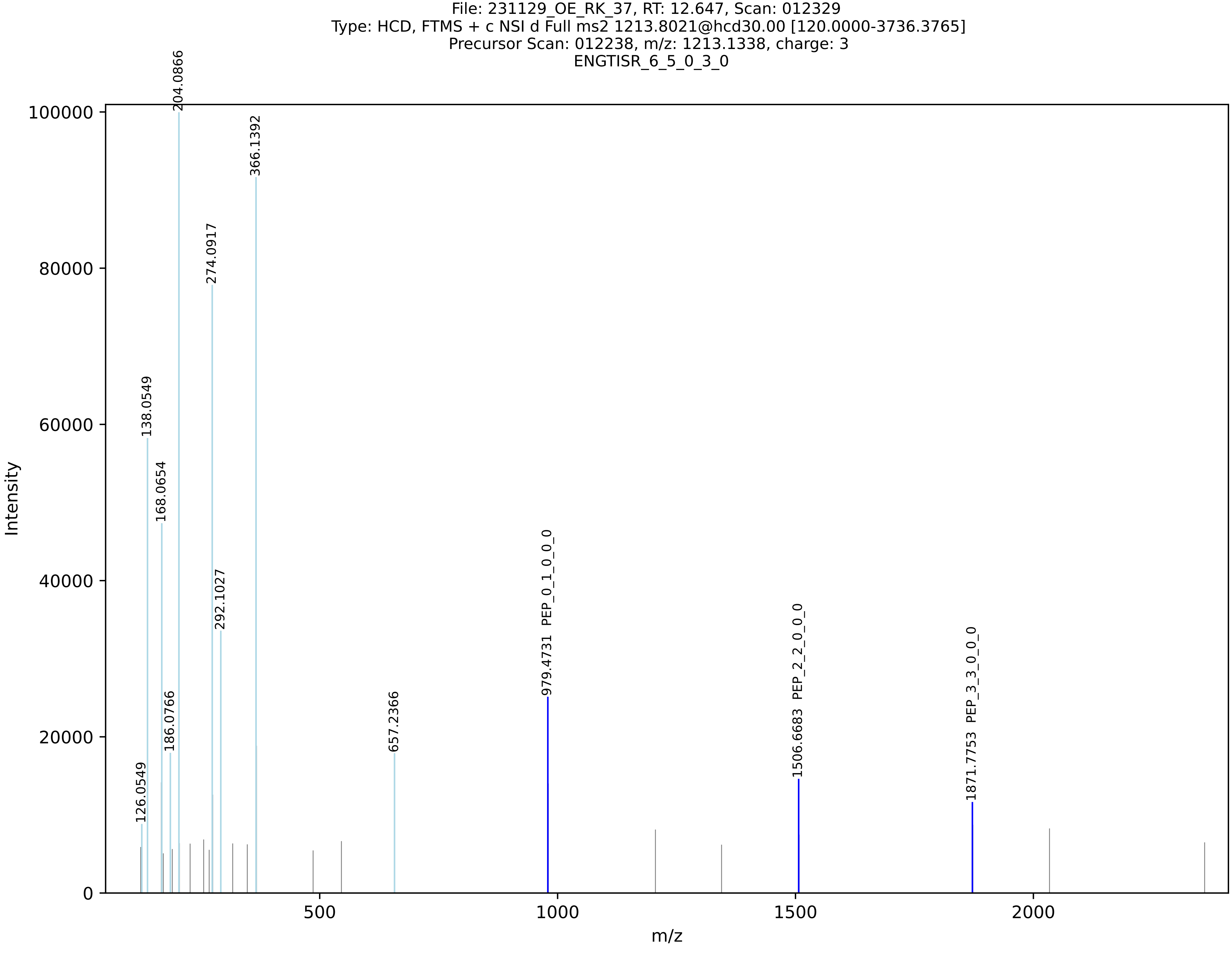
File: 231129_OE_RK_37, RT: 12.647, Scan: 012329
Type: HCD, FTMS + c NSI d Full ms2 1213.8021@hcd30.00 [120.0000-3736.3765]
Precursor Scan: 012238, m/z: 1213.1338, charge: 3
ENGTISR_6_5_0_3_0
Intensity
m/z
204.0866
366.1392
274.0917
138.0549
168.0654
292.1027
186.0766
126.0549
657.2366
979.4731 PEP_0_1_0_0_0
1506.6683 PEP_2_2_0_0_0
1871.7753 PEP_3_3_0_0_0

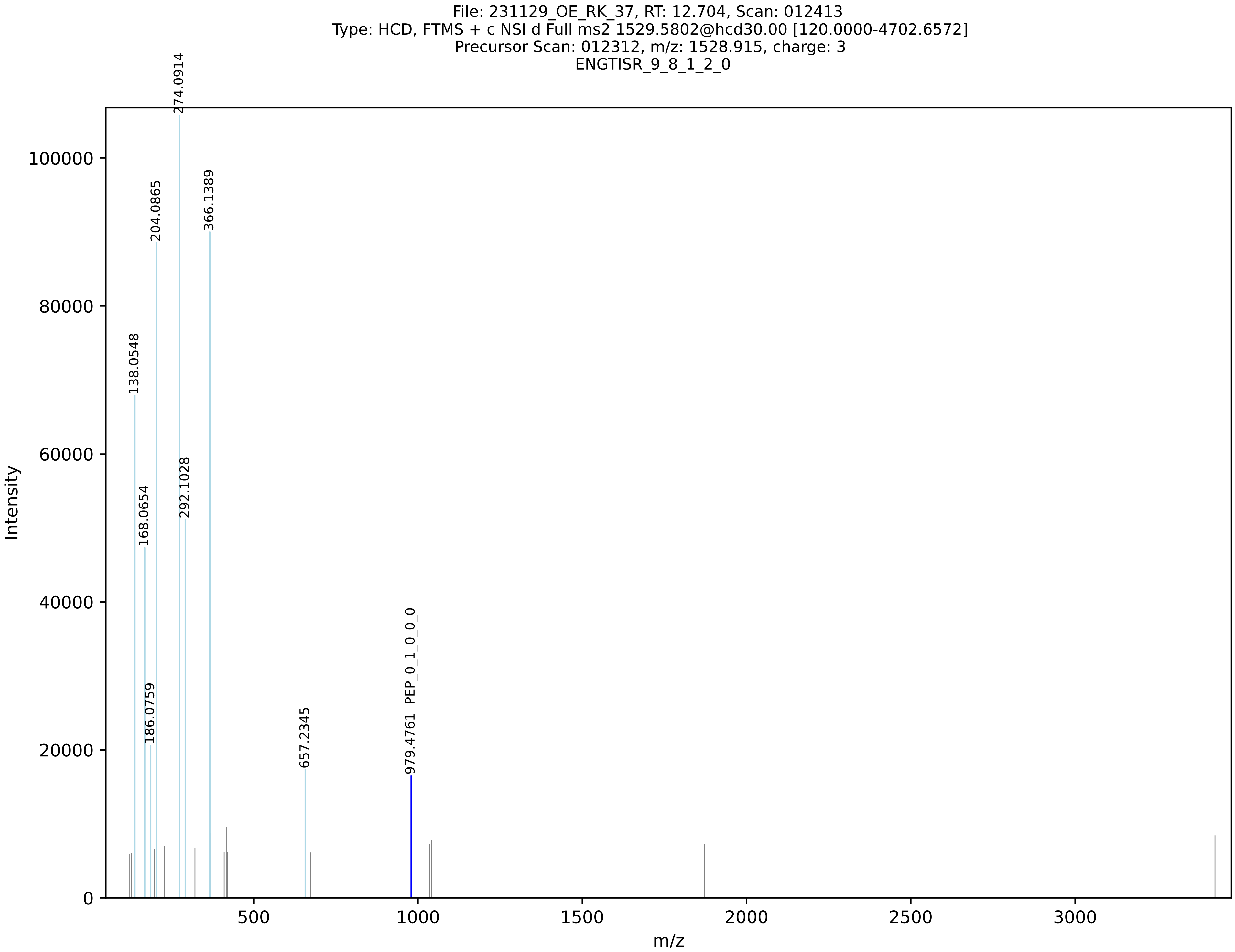
File: 231129_OE_RK_37, RT: 12.704, Scan: 012413
Type: HCD, FTMS + c NSI d Full ms2 1529.5802@hcd30.00 [120.0000-4702.6572]
Precursor Scan: 012312, m/z: 1528.915, charge: 3
ENGTISR_9_8_1_2_0
274.0914
204.0865
366.1389
138.0548
292.1028
168.0654
186.0759
657.2345
979.4761 PEP_0_1_0_0_0
Intensity
m/z
0
20000
40000
60000
80000
100000
500
1000
1500
2000
2500
3000

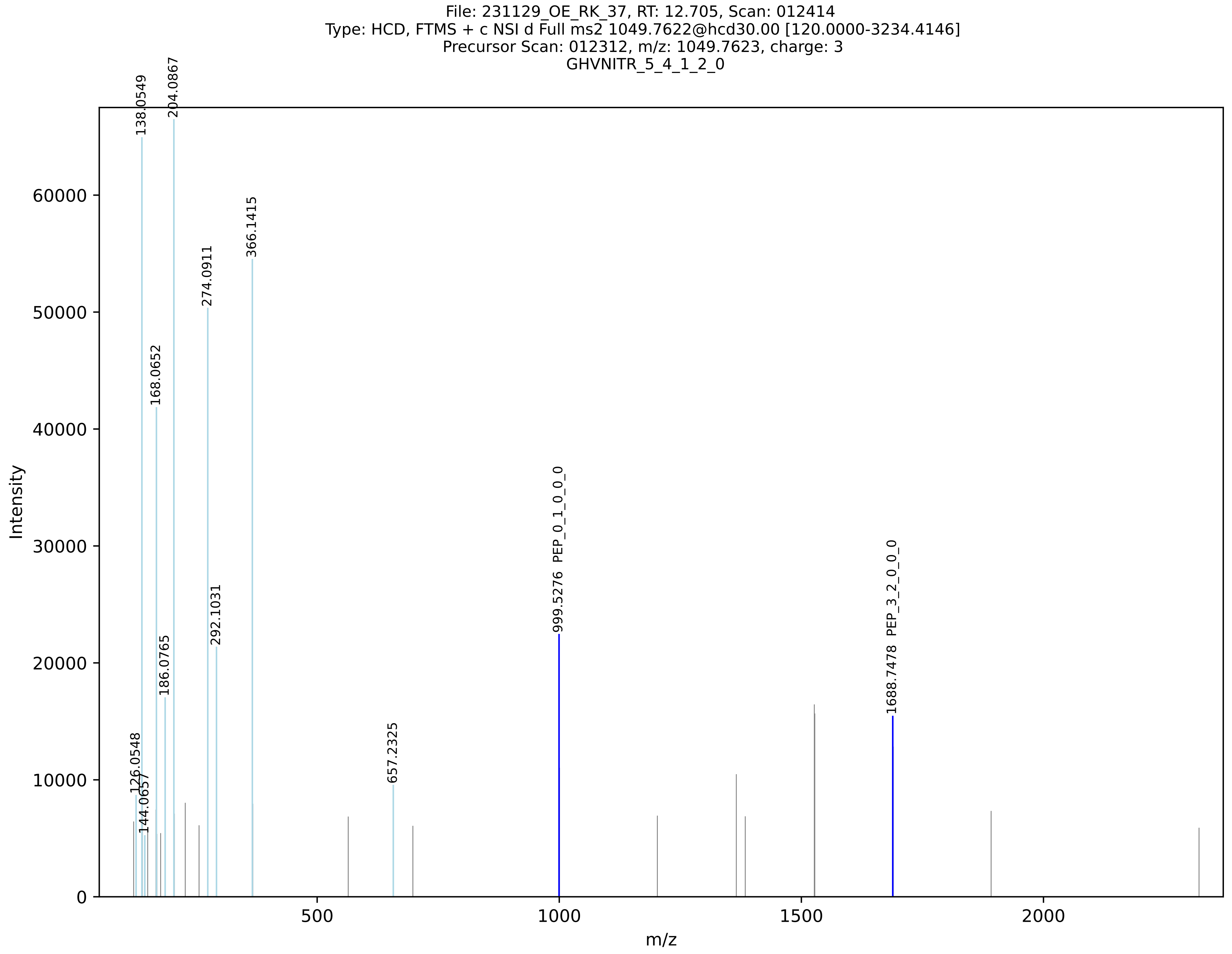

File: 231129_OE_RK_37, RT: 12.705, Scan: 012414
Type: HCD, FTMS + c NSI d Full ms2 1049.7622@hcd30.00 [120.0000-3234.4146]
Precursor Scan: 012312, m/z: 1049.7623, charge: 3
GHVNITR_5_4_1_2_0
Intensity
m/z
0
10000
20000
30000
40000
50000
60000
500
1000
1500
2000
138.0549
204.0867
366.1415
274.0911
168.0652
292.1031
186.0765
126.0548
144.0657
657.2325
999.5276 PEP_0_1_0_0_0
1688.7478 PEP_3_2_0_0_0

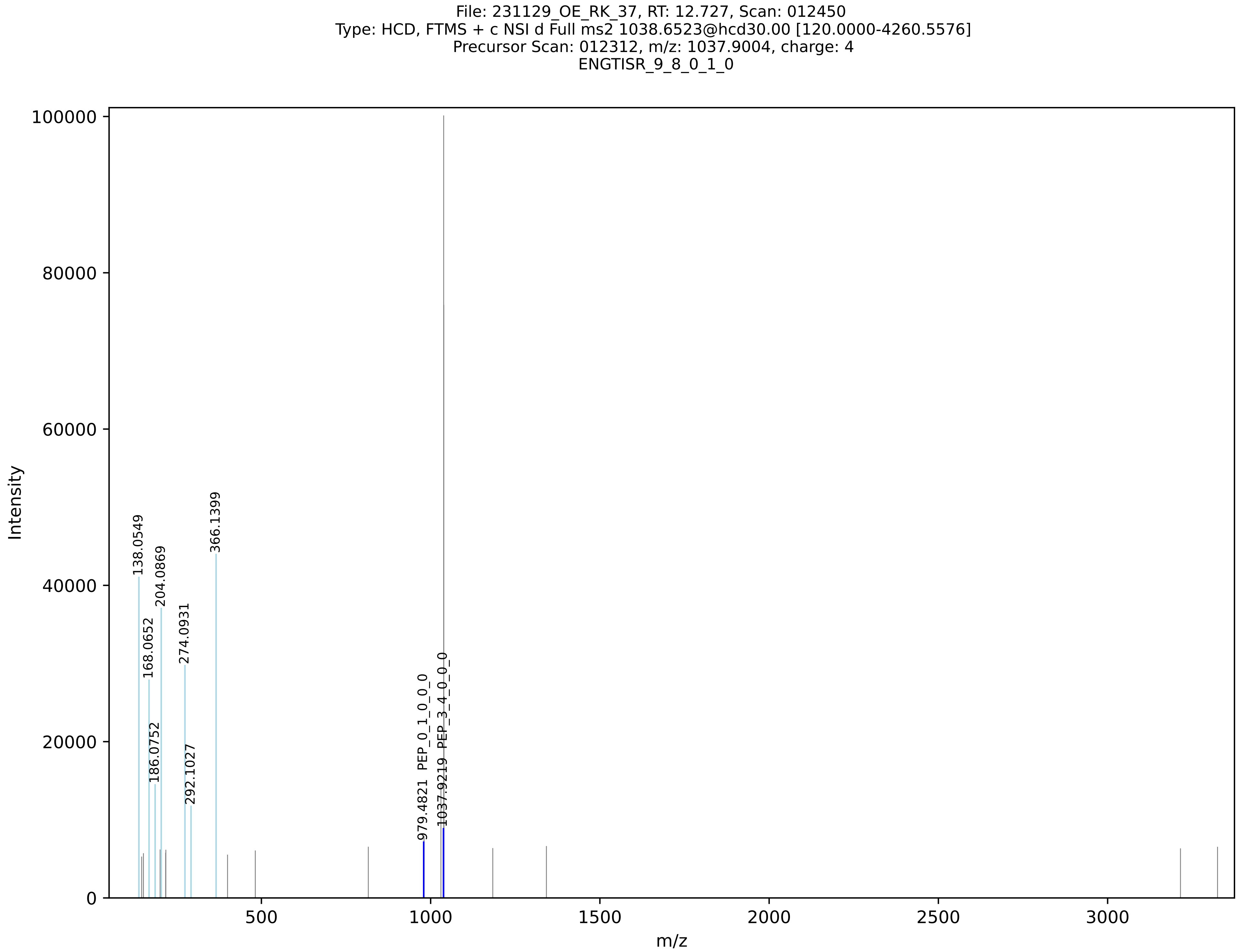
File: 231129_OE_RK_37, RT: 12.727, Scan: 012450
Type: HCD, FTMS + c NSI d Full ms2 1038.6523@hcd30.00 [120.0000-4260.5576]
Precursor Scan: 012312, m/z: 1037.9004, charge: 4
ENGTISR_9_8_0_1_0
Intensity
m/z
138.0549
168.0652
186.0752
204.0869
274.0931
292.1027
366.1399
979.4821 PEP_0_1_0_0_0
1037.9219 PEP_3_4_0_0_0
0
20000
40000
60000
80000
100000
500
1000
1500
2000
2500
3000

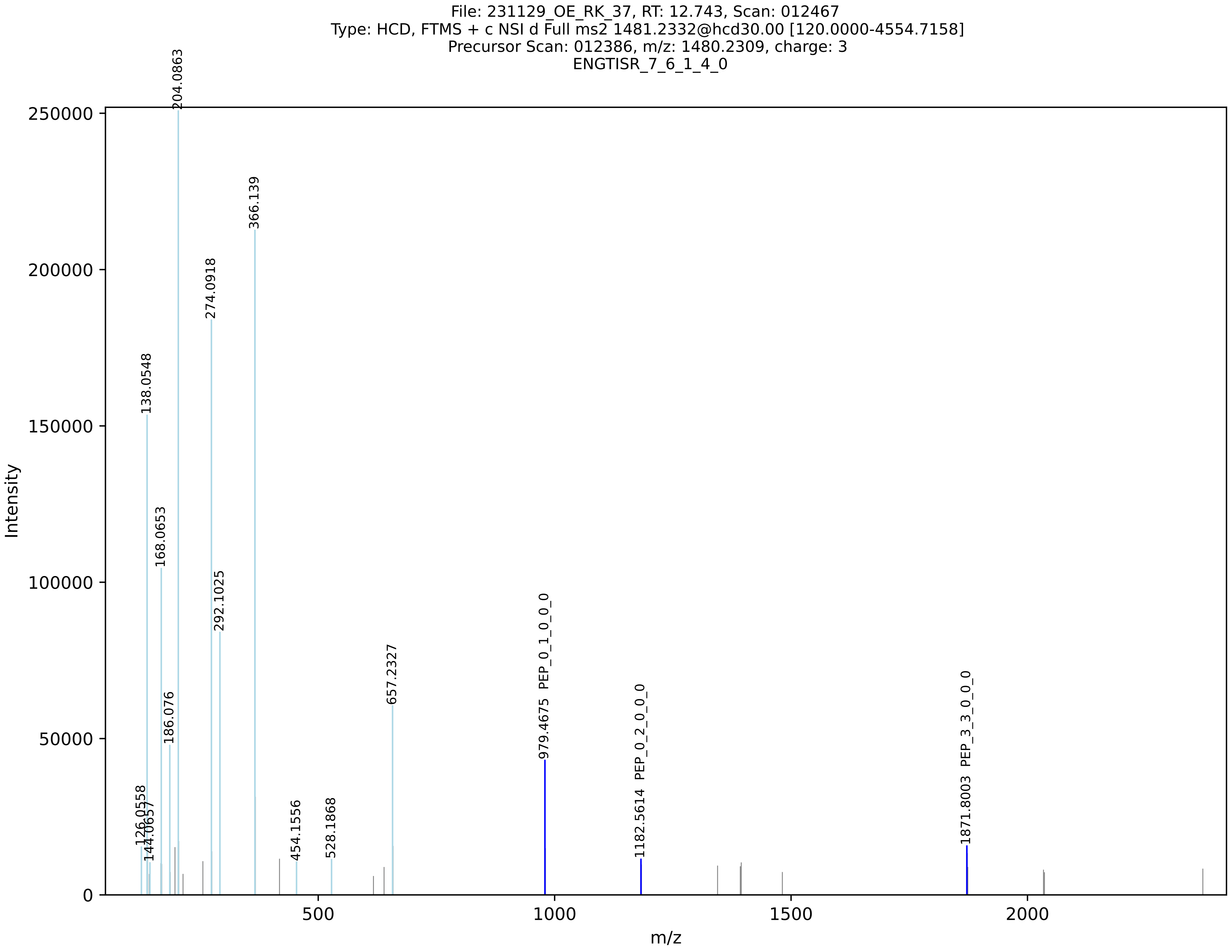
File: 231129_OE_RK_37, RT: 12.743, Scan: 012467
Type: HCD, FTMS + c NSI d Full ms2 1481.2332@hcd30.00 [120.0000-4554.7158]
Precursor Scan: 012386, m/z: 1480.2309, charge: 3
ENGTISR_7_6_1_4_0
Intensity
m/z
250000
200000
150000
100000
50000
0
500
1000
1500
2000
204.0863
366.139
274.0918
138.0548
168.0653
292.1025
186.076
126.0558
144.0657
454.1556
528.1868
657.2327
979.4675 PEP_0_1_0_0_0_
1182.5614 PEP_0_2_0_0_0_
1871.8003 PEP_3_3_0_0_0_

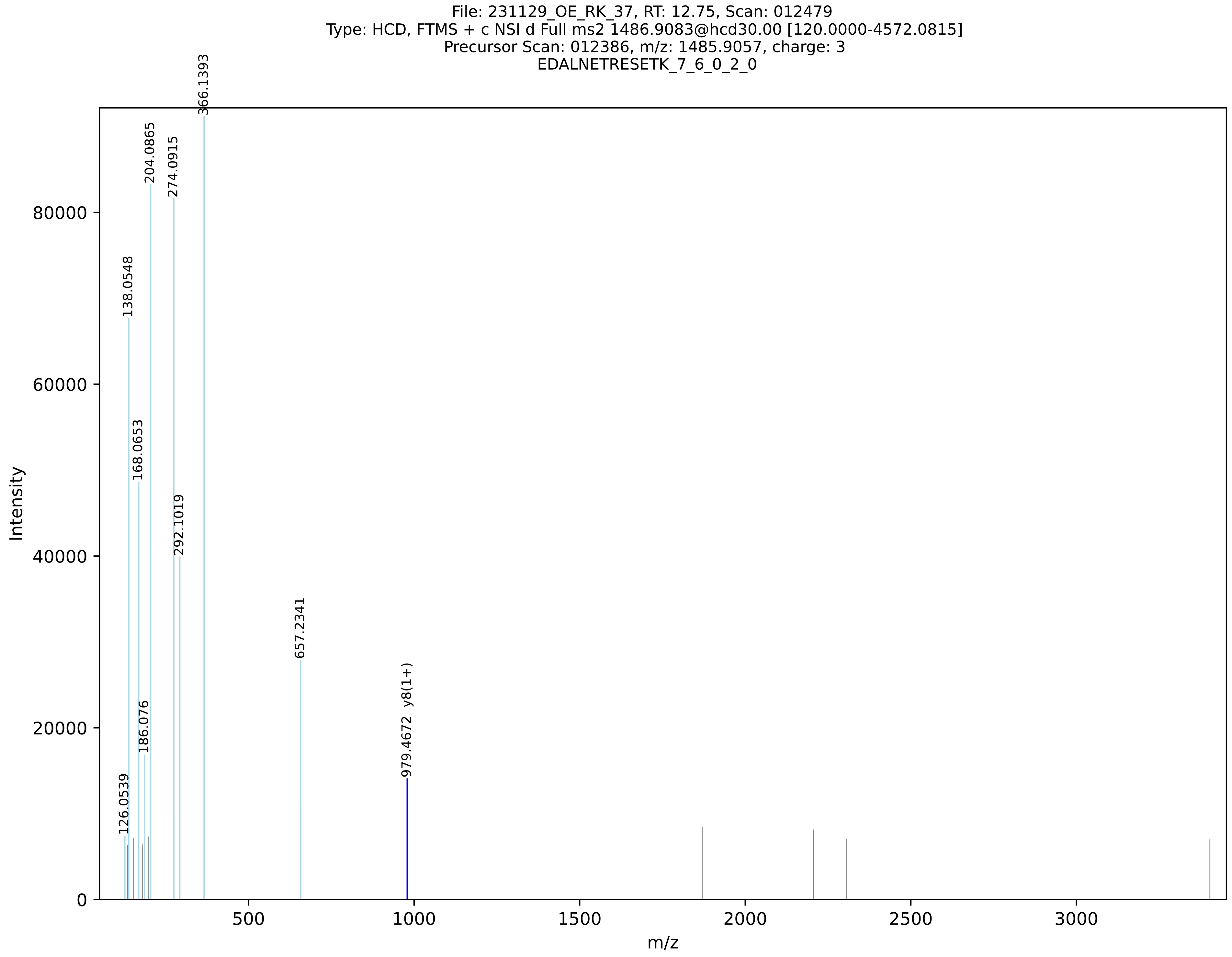

File: 231129_OE_RK_37, RT: 12.75, Scan: 012479
Type: HCD, FTMS + c NSI d Full ms2 1486.9083@hcd30.00 [120.0000-4572.0815]
Precursor Scan: 012386, m/z: 1485.9057, charge: 3
EDALNETRESETK_7_6_0_2_0
366.1393
204.0865
274.0915
138.0548
168.0653
292.1019
657.2341
979.4672 y8(1+)
186.076
126.0539
Intensity
0
20000
40000
60000
80000
500
1000
1500
2000
2500
3000
m/z

File: 231129_OE_RK_37, RT: 12.761, Scan: 012497
Type: HCD, FTMS + c NSI d Full ms2 1038.3994@hcd30.00 [120.0000-4259.5254]
Precursor Scan: 012386, m/z: 1037.9015, charge: 4
WEYCNLTR_8_7_0_1_0

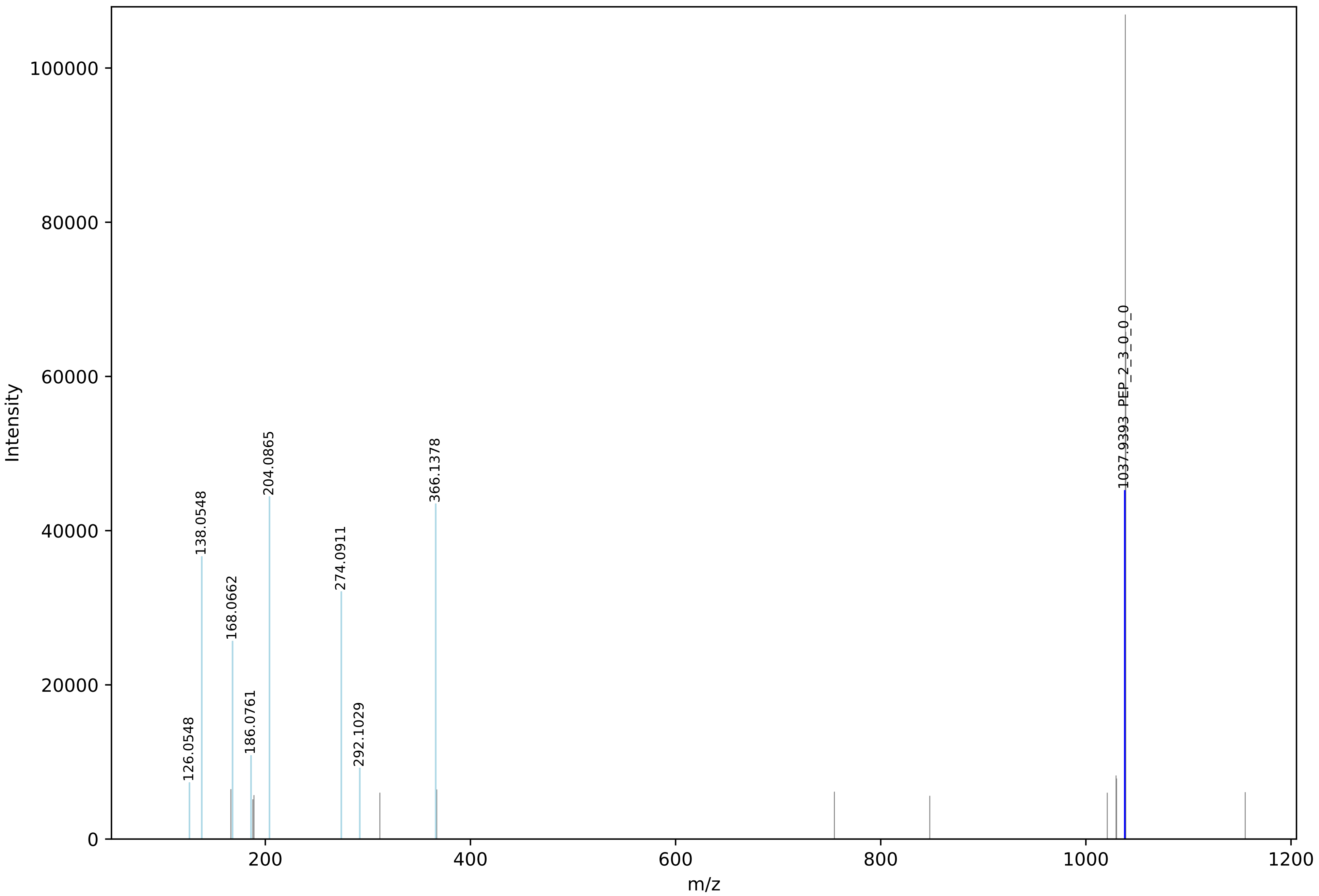

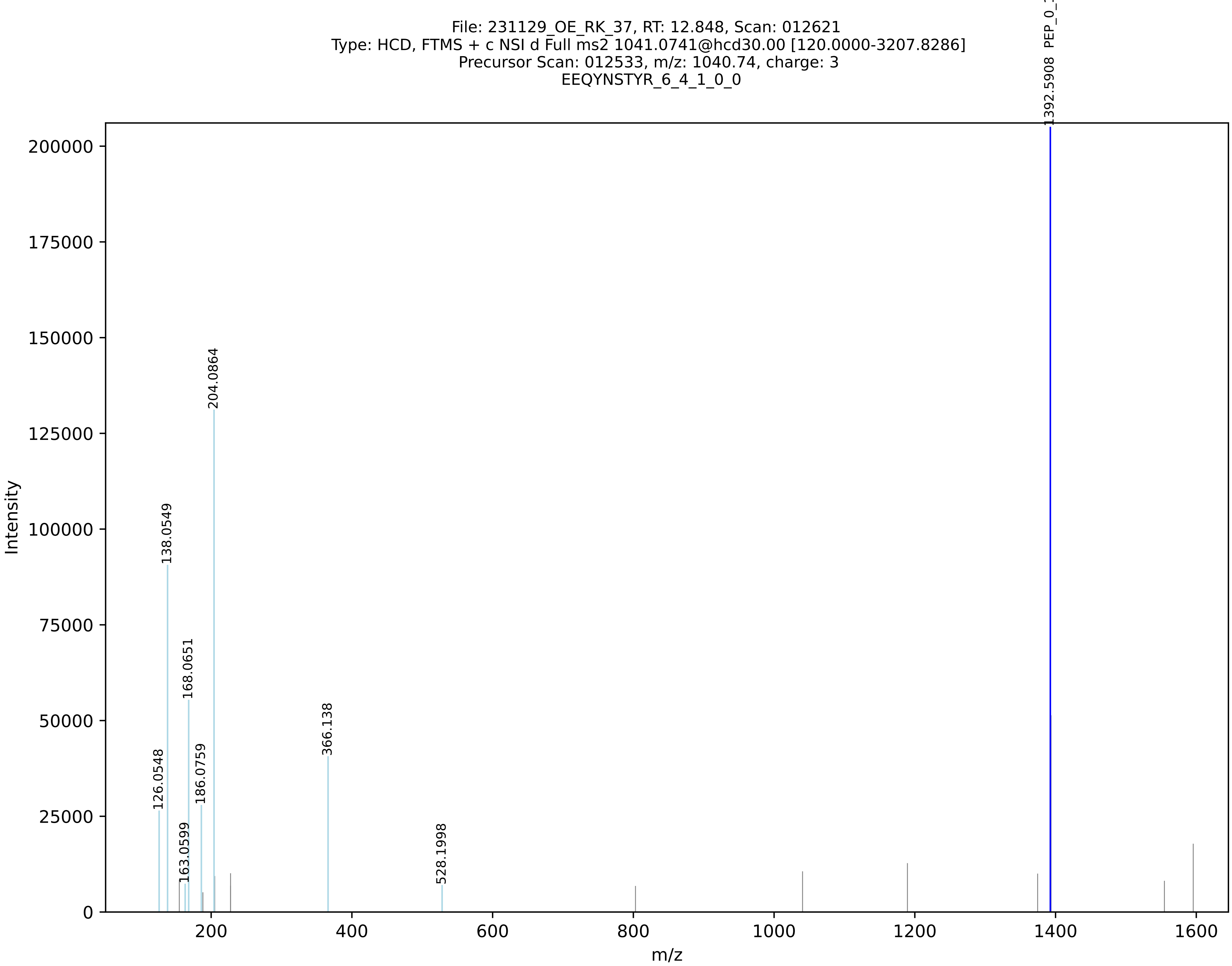
File: 231129_OE_RK_37, RT: 12.848, Scan: 012621
Type: HCD, FTMS + c NSI d Full ms2 1041.0741@hcd30.00 [120.0000-3207.8286]
Precursor Scan: 012533, m/z: 1040.74, charge: 3
EEQYNSTYR_6_4_1_0_0
1392.5908 PEP_0_
204.0864
138.0549
168.0651
366.138
126.0548
186.0759
163.0599
528.1998
Intensity
m/z
0
25000
50000
75000
100000
125000
150000
175000
200000
200
400
600
800
1000
1200
1400
1600

File: 231129_OE_RK_37, RT: 12.863, Scan: 012646
Type: HCD, FTMS + c NSI d Full ms2 1326.0282@hcd30.00 [120.0000-2726.5256]
Precursor Scan: 012533, m/z: 1325.5264, charge: 2
EEQYNSTYR_4_4_0_0_0

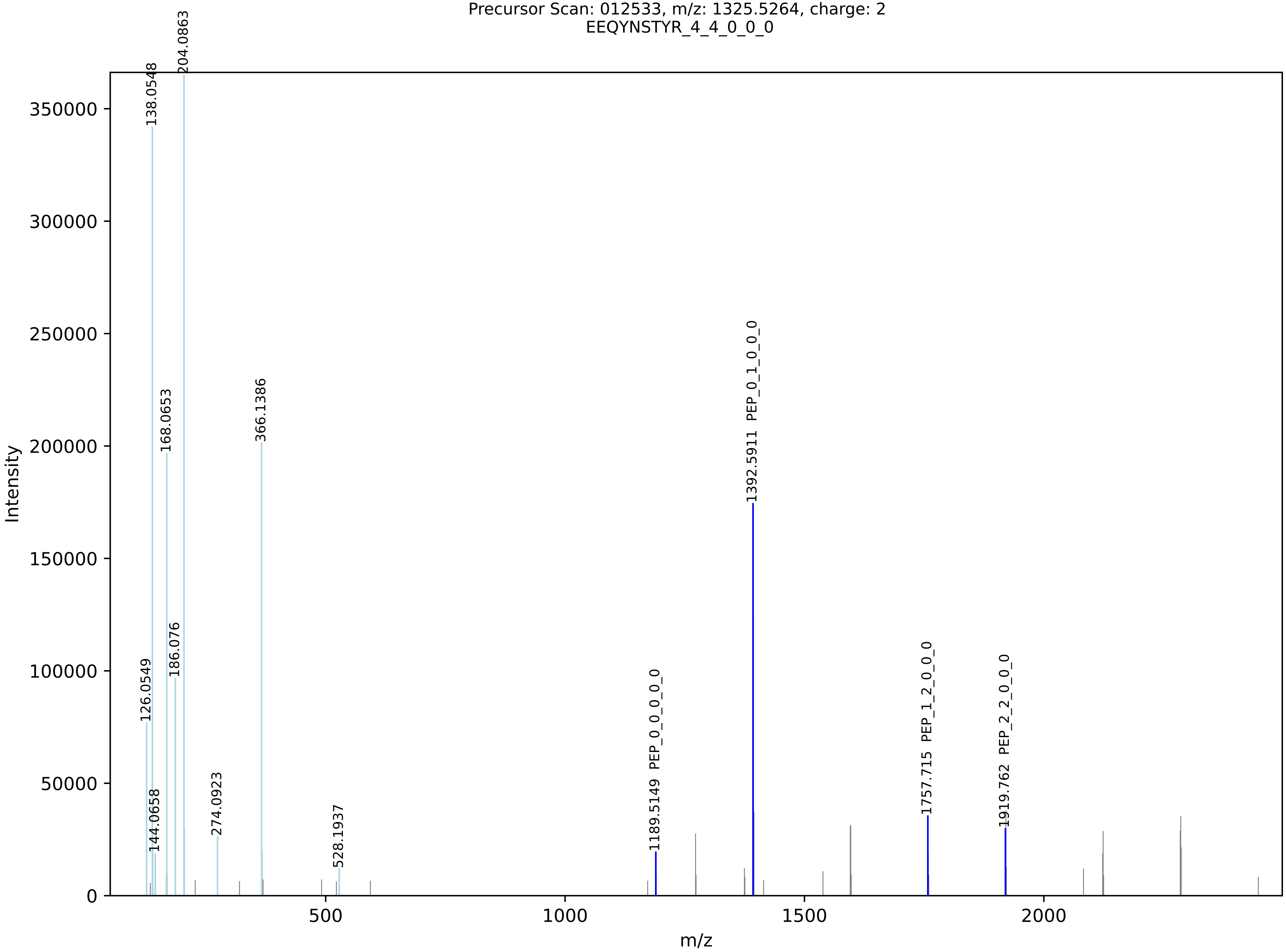

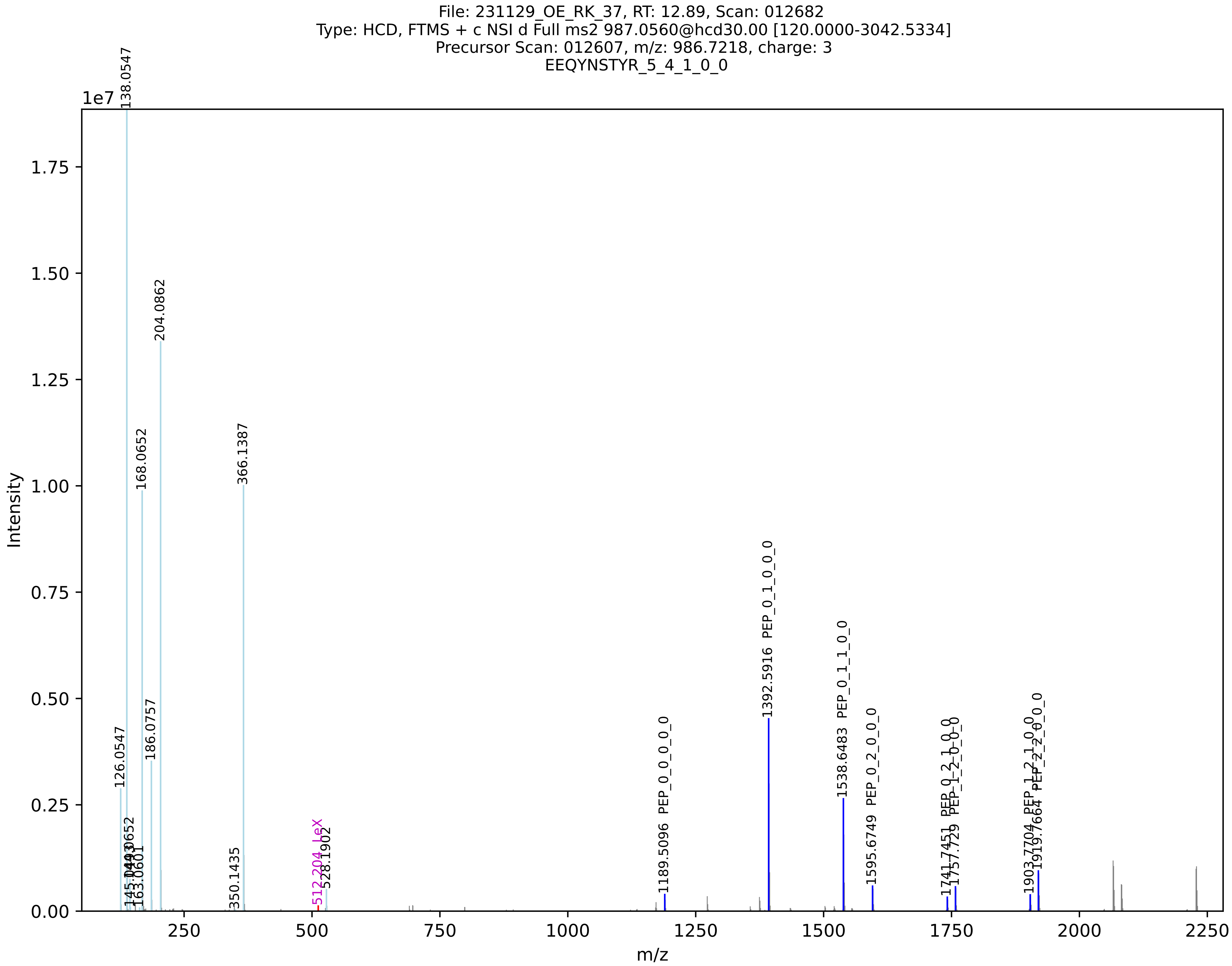

File: 231129_OE_RK_37, RT: 12.89, Scan: 012682
Type: HCD, FTMS + c NSI d Full ms2 987.0560@hcd30.00 [120.0000-3042.5334]
Precursor Scan: 012607, m/z: 986.7218, charge: 3
EEQYNSTYR_5_4_1_0_0
1e7
Intensity
m/z
138.0547
204.0862
168.0652
366.1387
186.0757
126.0547
163.0601
350.1435
512.204_LeX
528.1902
1189.5096 PEP_0_0_0_0_0
1392.5916 PEP_0_1_0_0_0
1538.6483 PEP_0_1_1_0_0
1595.6749 PEP_0_2_0_0_0
1741.7451 PEP_0_2_1_0_0
1757.729 PEP_1_2_0_0_0
1903.7704 PEP_1_2_1_0_0
1919.7664 PEP_2_2_0_0_0

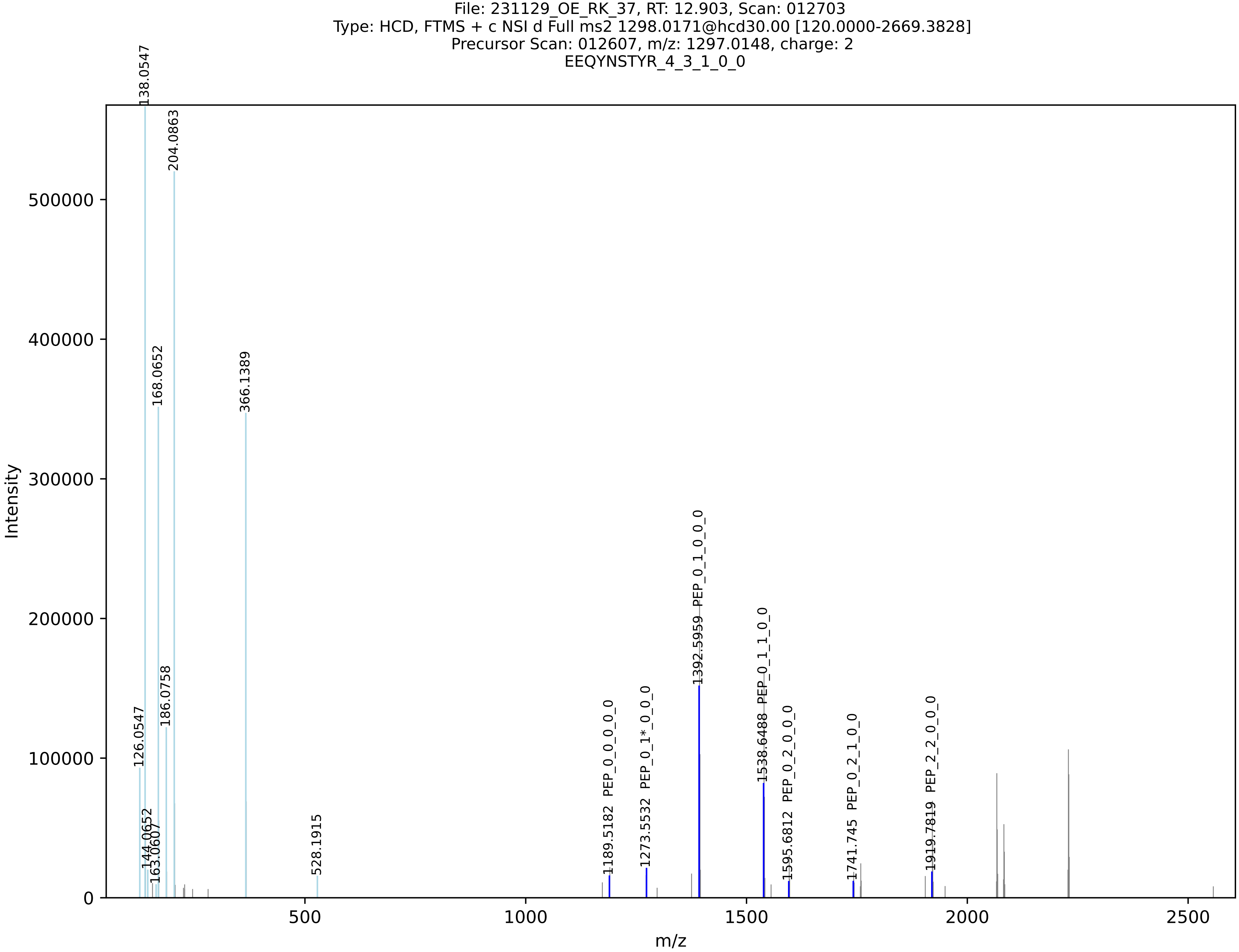
File: 231129_OE_RK_37, RT: 12.903, Scan: 012703
Type: HCD, FTMS + c NSI d Full ms2 1298.0171@hcd30.00 [120.0000-2669.3828]
Precursor Scan: 012607, m/z: 1297.0148, charge: 2
EEQYNSTYR_4_3_1_0_0
Intensity
m/z
138.0547
204.0863
168.0652
366.1389
186.0758
126.0547
144.0652
163.0607
528.1915
1189.5182 PEP_0_0_0_0_0
1273.5532 PEP_0_1*_0_0_0
1392.5959 PEP_0_1_0_0_0
1538.6488 PEP_0_1_1_0_0
1595.6812 PEP_0_2_0_0_0
1741.745 PEP_0_2_1_0_0
1919.7819 PEP_2_2_0_0_0
0
100000
200000
300000
400000
500000
500
1000
1500
2000
2500

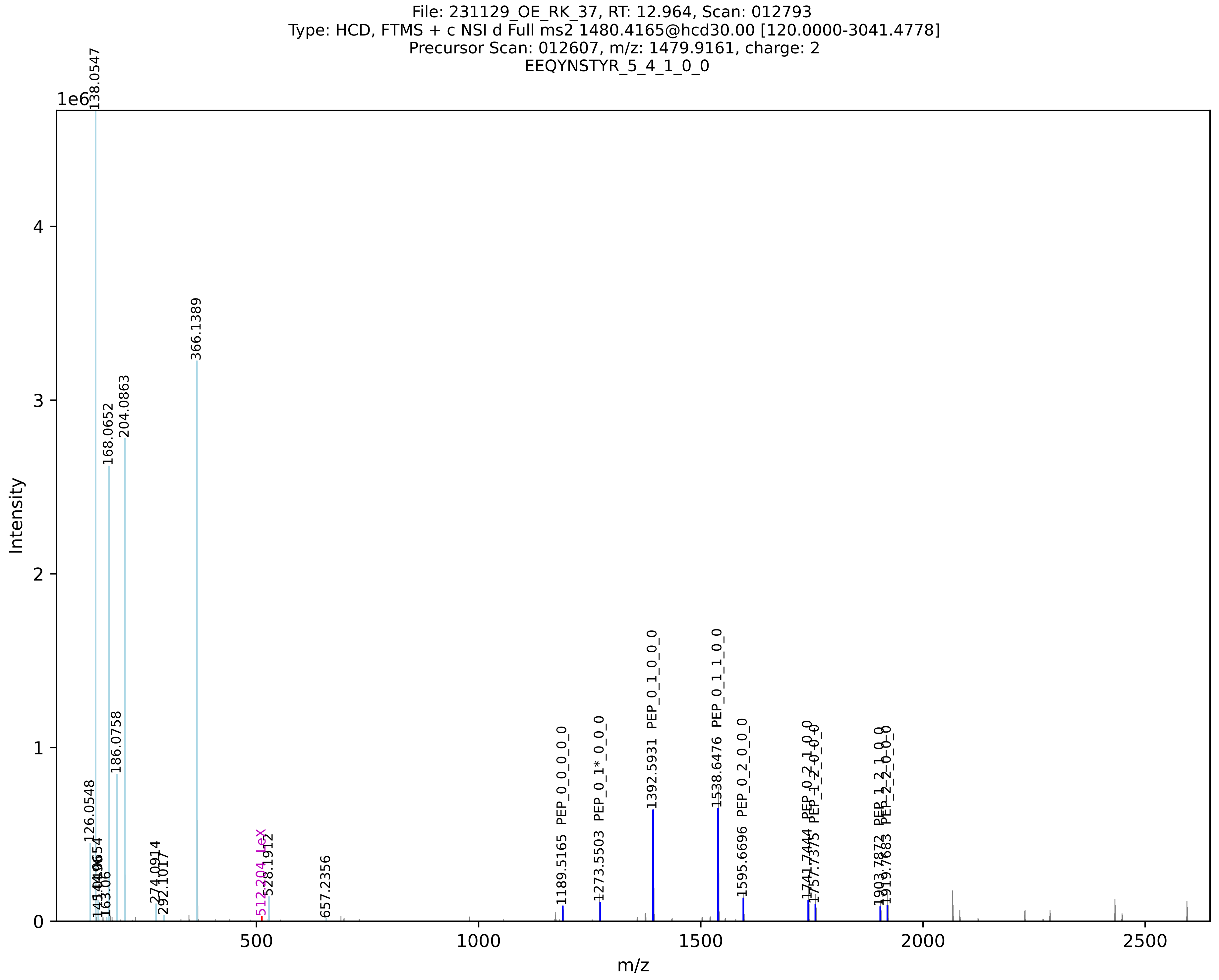
File: 231129_OE_RK_37, RT: 12.964, Scan: 012793
Type: HCD, FTMS + c NSI d Full ms2 1480.4165@hcd30.00 [120.0000-3041.4778]
Precursor Scan: 012607, m/z: 1479.9161, charge: 2
EEQYNSTYR_5_4_1_0_0
1e6
Intensity
m/z
138.0547
126.0548
163.06
168.0652
186.0758
204.0863
274.0914
292.1017
366.1389
512.204_LeX
528.1912
657.2356
1189.5165 PEP_0_0_0_0_0
1273.5503 PEP_0_1*_0_0_0
1392.5931 PEP_0_1_0_0_0
1538.6476 PEP_0_1_1_0_0
1595.6696 PEP_0_2_0_0_0
1741.7444 PEP_0_2_1_0_0
1757.7375 PEP_1_2_0_0_0
1903.7872 PEP_1_2_1_0_0
1919.7683 PEP_2_2_0_0_0
0
1
2
3
4
500
1000
1500
2000
2500

File: 231129_OE_RK_37, RT: 13.01, Scan: 012860
Type: HCD, FTMS + c NSI d Full ms2 1406.2118@hcd30.00 [120.0000-2890.1001]
Precursor Scan: 012755, m/z: 1406.2118, charge: 2
EEQYNSTYR_5_4_0_0_0

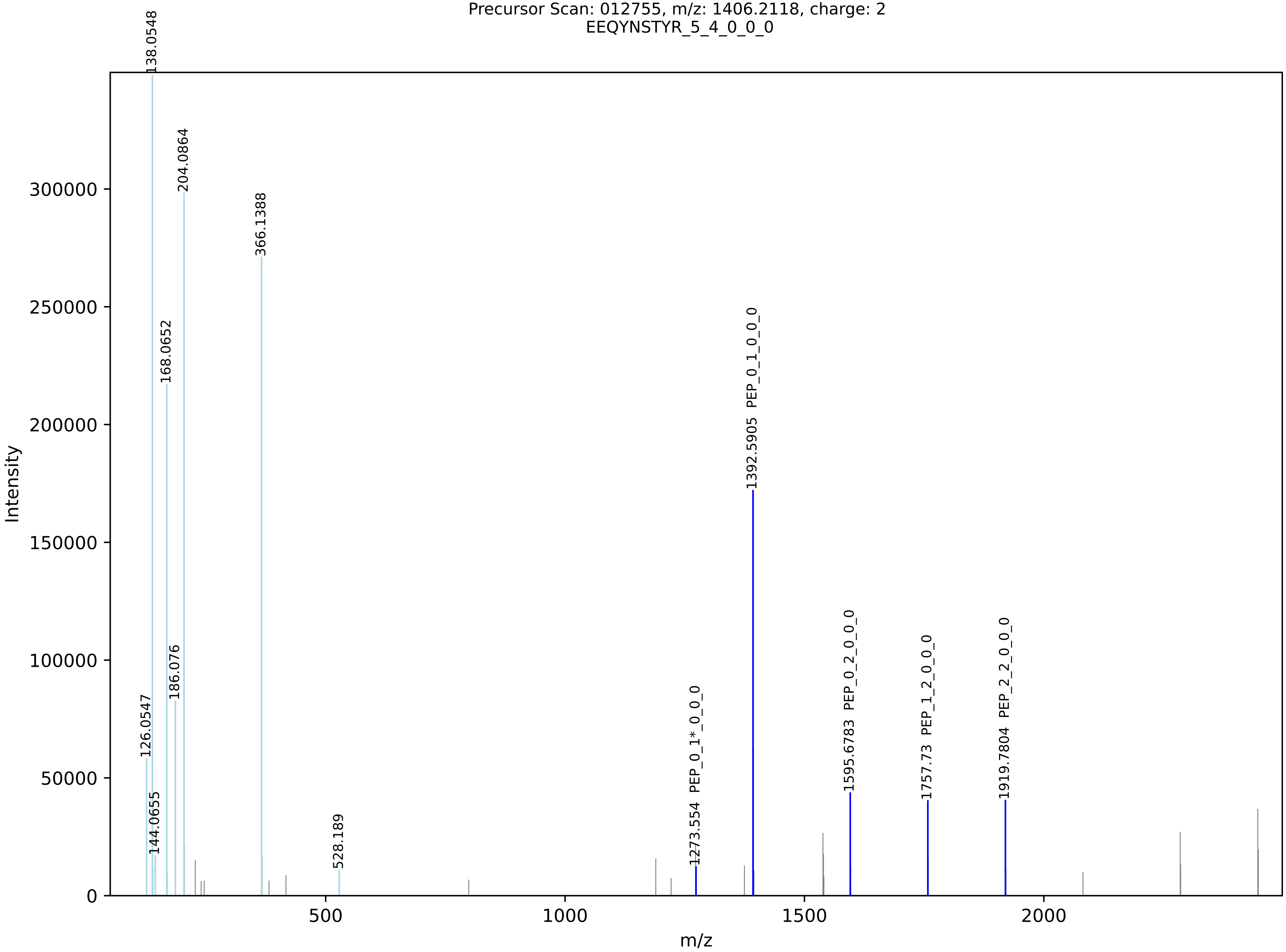

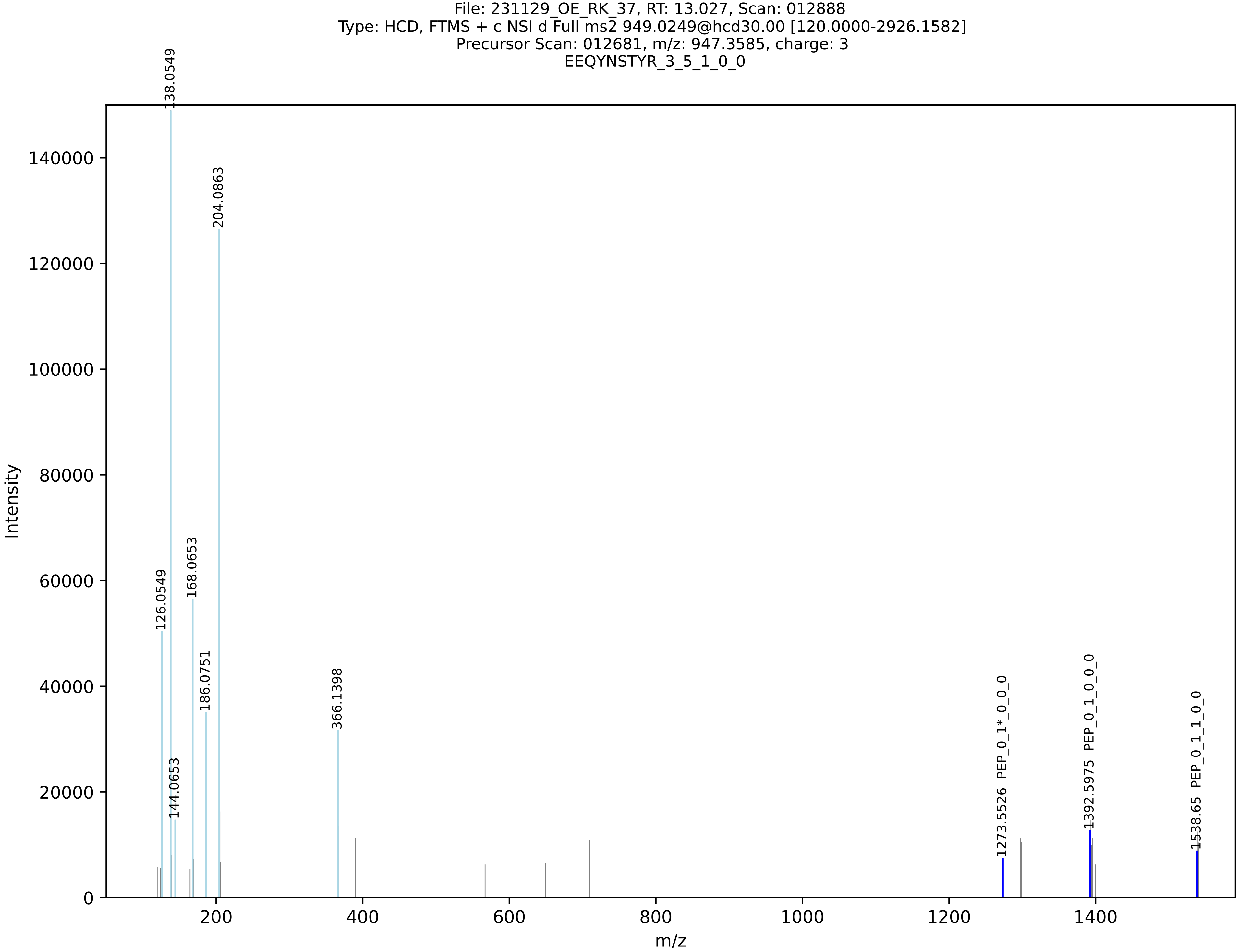
File: 231129_OE_RK_37, RT: 13.027, Scan: 012888
Type: HCD, FTMS + c NSI d Full ms2 949.0249@hcd30.00 [120.0000-2926.1582]
Precursor Scan: 012681, m/z: 947.3585, charge: 3
EEQYNSTYR_3_5_1_0_0
138.0549
204.0863
126.0549
168.0653
186.0751
144.0653
366.1398
1273.5526 PEP_0_1*_0_0_0
1392.5975 PEP_0_1_0_0_0
1538.65 PEP_0_1_1_0_0
Intensity
m/z
0
20000
40000
60000
80000
100000
120000
140000
200
400
600
800
1000
1200
1400

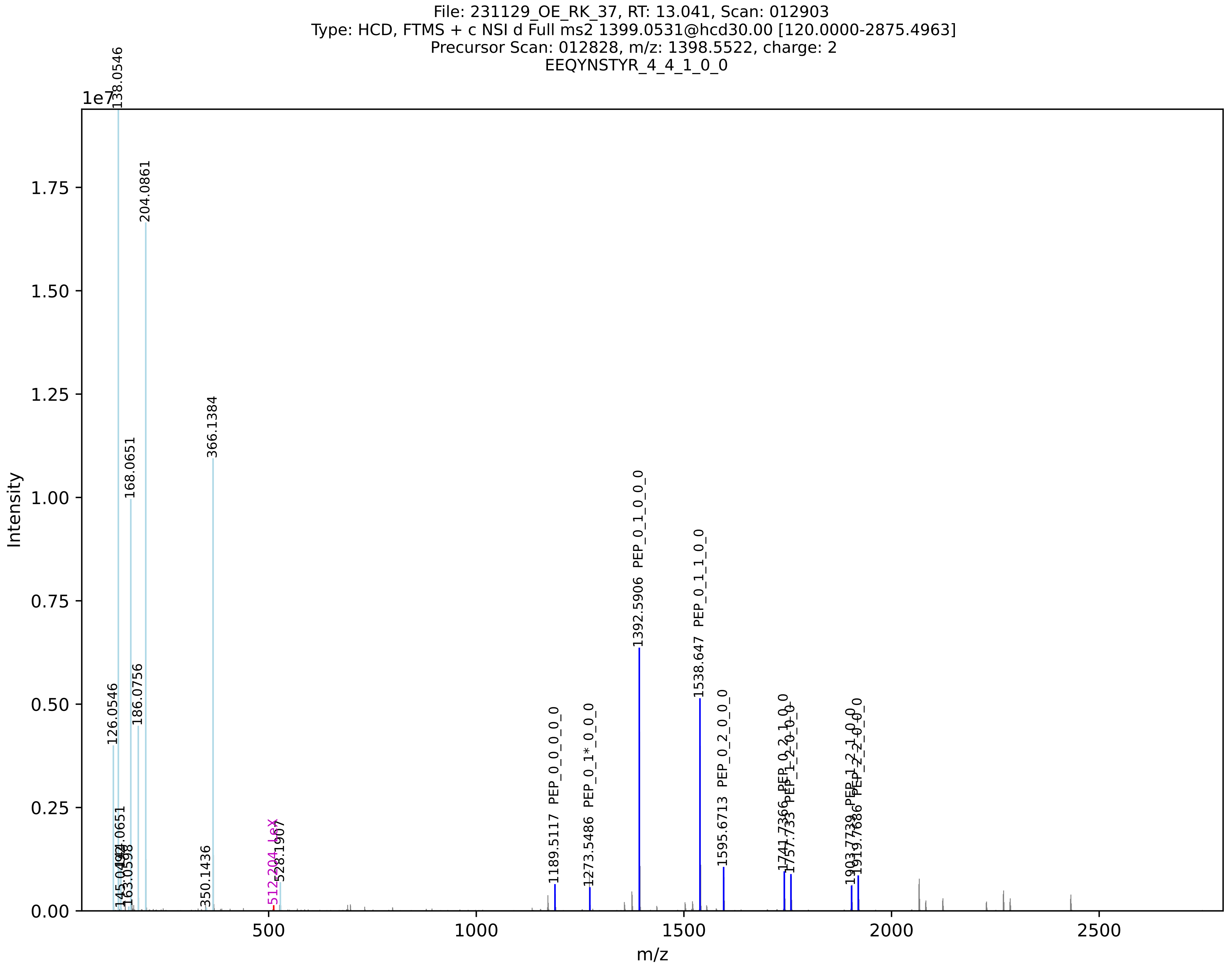
File: 231129_OE_RK_37, RT: 13.041, Scan: 012903
Type: HCD, FTMS + c NSI d Full ms2 1399.0531@hcd30.00 [120.0000-2875.4963]
Precursor Scan: 012828, m/z: 1398.5522, charge: 2
EEQYNSTYR_4_4_1_0_0
1e7
Intensity
m/z
138.0546
204.0861
366.1384
168.0651
186.0756
126.0546
350.1436
512.204_LeX
528.1907
1189.5117 PEP_0_0_0_0_0
1273.5486 PEP_0_1*_0_0_0
1392.5906 PEP_0_1_0_0_0
1538.647 PEP_0_1_1_0_0
1595.6713 PEP_0_2_0_0_0
1741.7366 PEP_0_2_1_0_0
1757.733 PEP_1_2_0_0_0
1903.7739 PEP_1_2_1_0_0
1919.7686 PEP_2_2_0_0_0

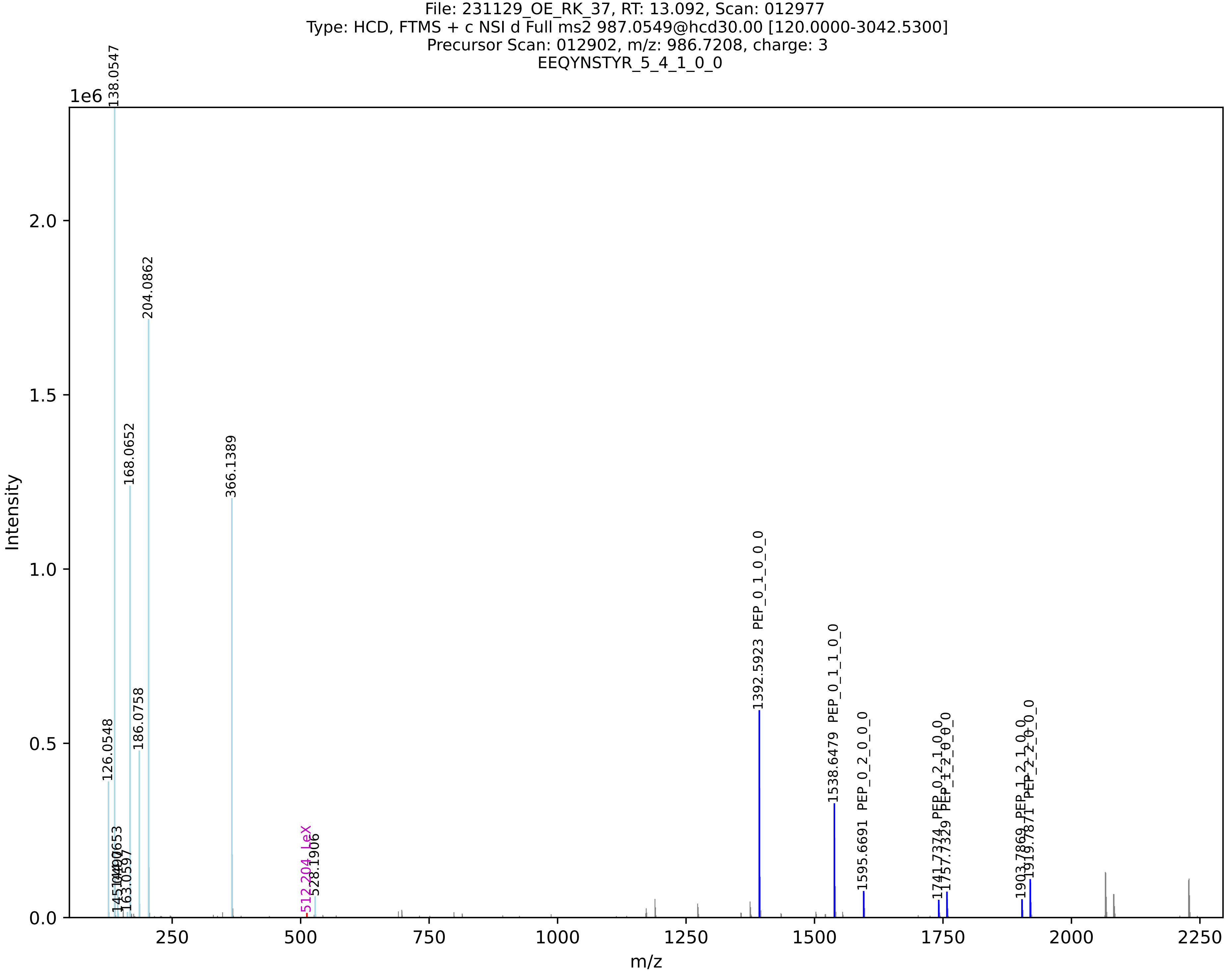

File: 231129_OE_RK_37, RT: 13.092, Scan: 012977
Type: HCD, FTMS + c NSI d Full ms2 987.0549@hcd30.00 [120.0000-3042.5300]
Precursor Scan: 012902, m/z: 986.7208, charge: 3
EEQYNSTYR_5_4_1_0_0
1e6
Intensity
m/z
138.0547
204.0862
168.0652
366.1389
186.0758
126.0548
163.0597
512.204_LeX
528.1906
1392.5923 PEP_0_1_0_0_0
1538.6479 PEP_0_1_1_0_0
1595.6691 PEP_0_2_0_0_0
1741.7374 PEP_0_2_1_0_0
1757.7329 PEP_1_2_0_0_0
1903.7869 PEP_1_2_1_0_0
1919.7871 PEP_2_2_0_0_0

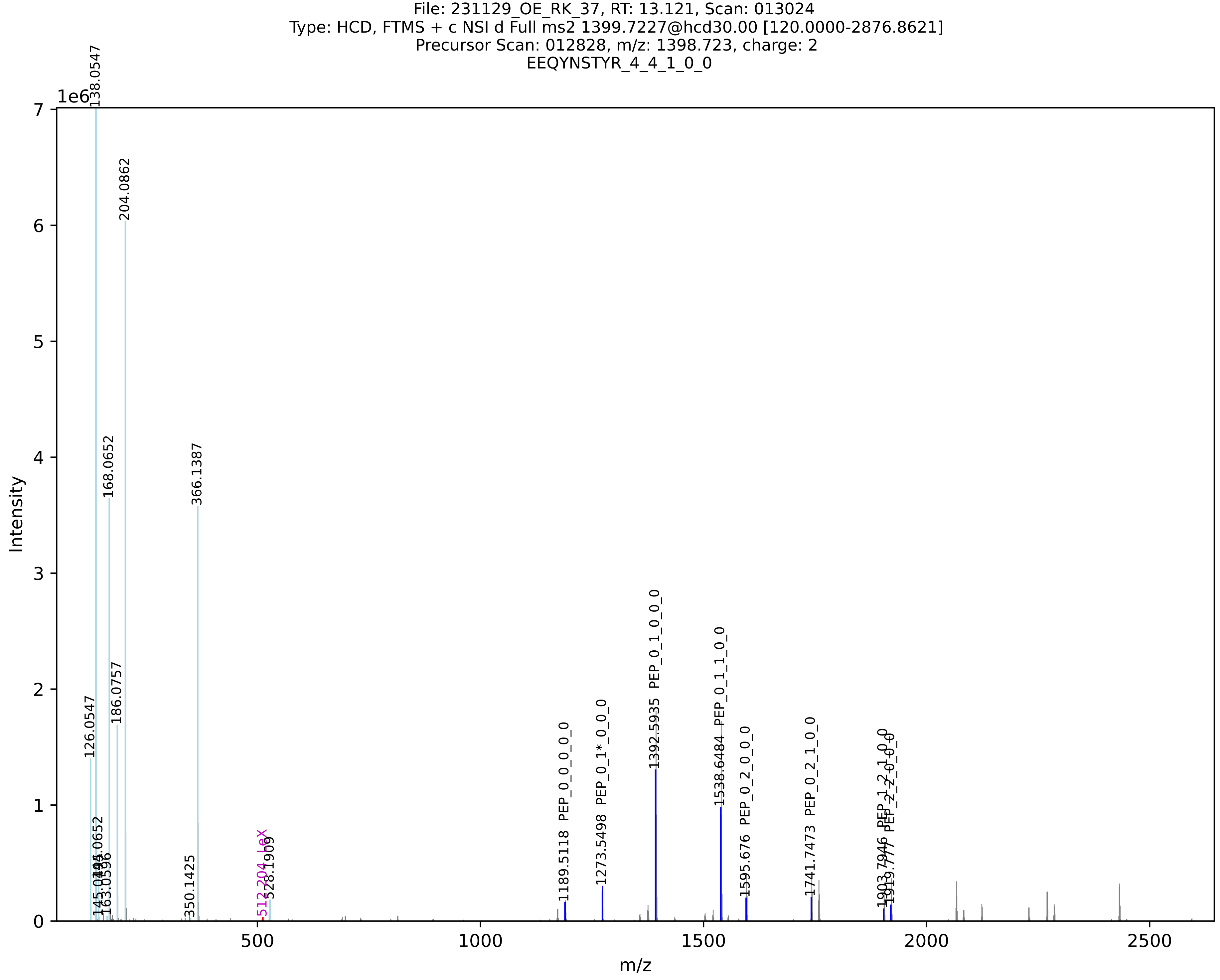

File: 231129_OE_RK_37, RT: 13.121, Scan: 013024
Type: HCD, FTMS + c NSI d Full ms2 1399.7227@hcd30.00 [120.0000-2876.8621]
Precursor Scan: 012828, m/z: 1398.723, charge: 2
EEQYNSTYR_4_4_1_0_0
1e6
Intensity
m/z
138.0547
204.0862
168.0652
366.1387
186.0757
126.0547
350.1425
512.204_LeX
528.1909
1189.5118 PEP_0_0_0_0_0
1273.5498 PEP_0_1*_0_0_0
1392.5935 PEP_0_1_0_0_0
1538.6484 PEP_0_1_1_0_0
1595.676 PEP_0_2_0_0_0
1741.7473 PEP_0_2_1_0_0
1903.7946 PEP_1_2_1_0_0
1919.777 PEP_2_2_0_0_0

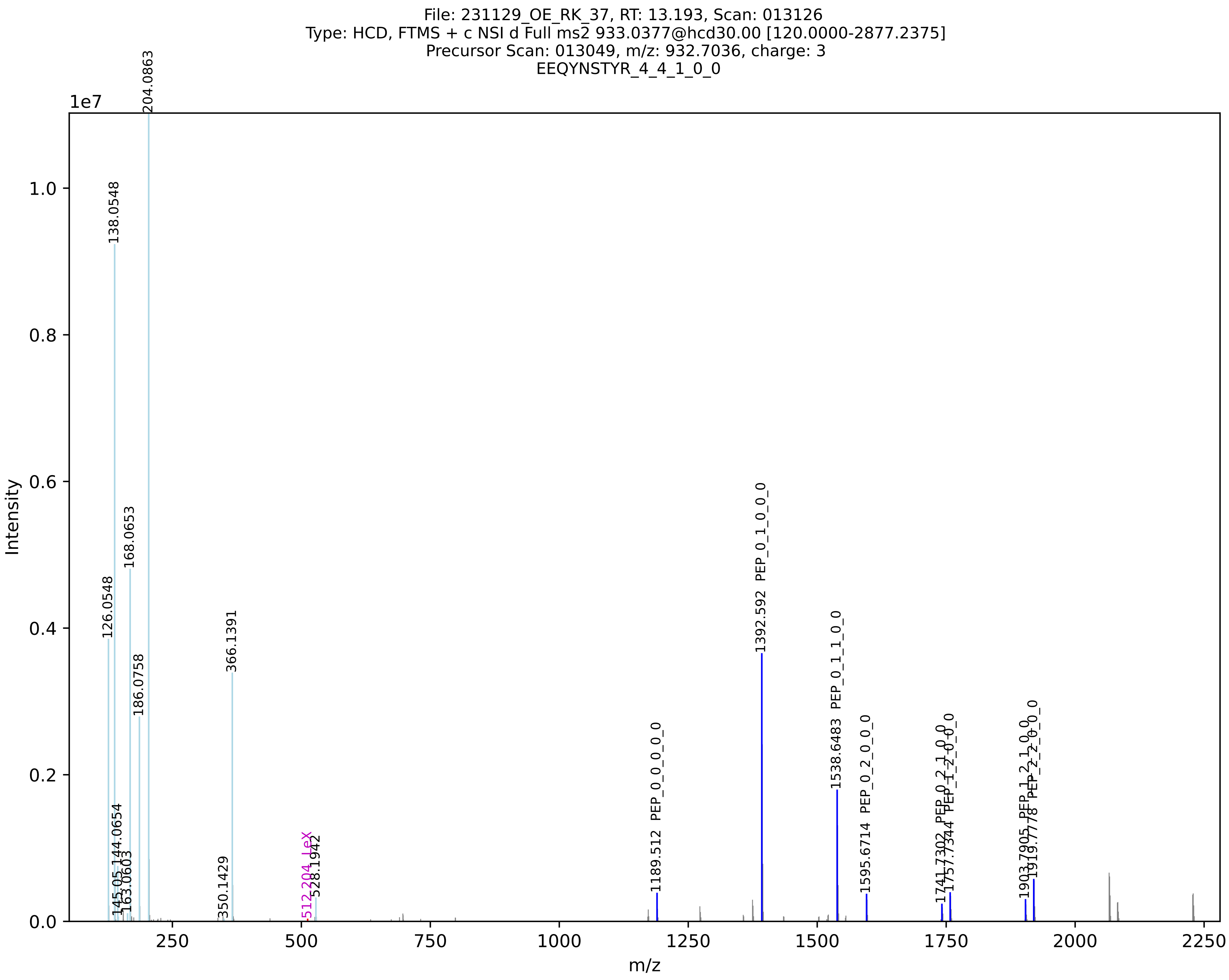

File: 231129_OE_RK_37, RT: 13.193, Scan: 013126
Type: HCD, FTMS + c NSI d Full ms2 933.0377@hcd30.00 [120.0000-2877.2375]
Precursor Scan: 013049, m/z: 932.7036, charge: 3
EEQYNSTYR_4_4_1_0_0
1e7
Intensity
m/z
204.0863
138.0548
168.0653
126.0548
186.0758
366.1391
144.0654
145.05
163.0603
350.1429
512.204_LeX
528.1942
1189.512 PEP_0_0_0_0_0
1392.592 PEP_0_1_0_0_0
1538.6483 PEP_0_1_1_0_0
1595.6714 PEP_0_2_0_0_0
1741.7302 PEP_0_2_1_0_0
1757.7344 PEP_1_2_0_0_0
1903.7905 PEP_1_2_1_0_0
1919.7778 PEP_2_2_0_0_0

File: 231129_OE_RK_37, RT: 13.226, Scan: 013173
Type: HCD, FTMS + c NSI d Full ms2 1399.0529@hcd30.00 [120.0000-2875.4958]
Precursor Scan: 013123, m/z: 1398.5518, charge: 2
EEQYNSTYR_4_4_1_0_0

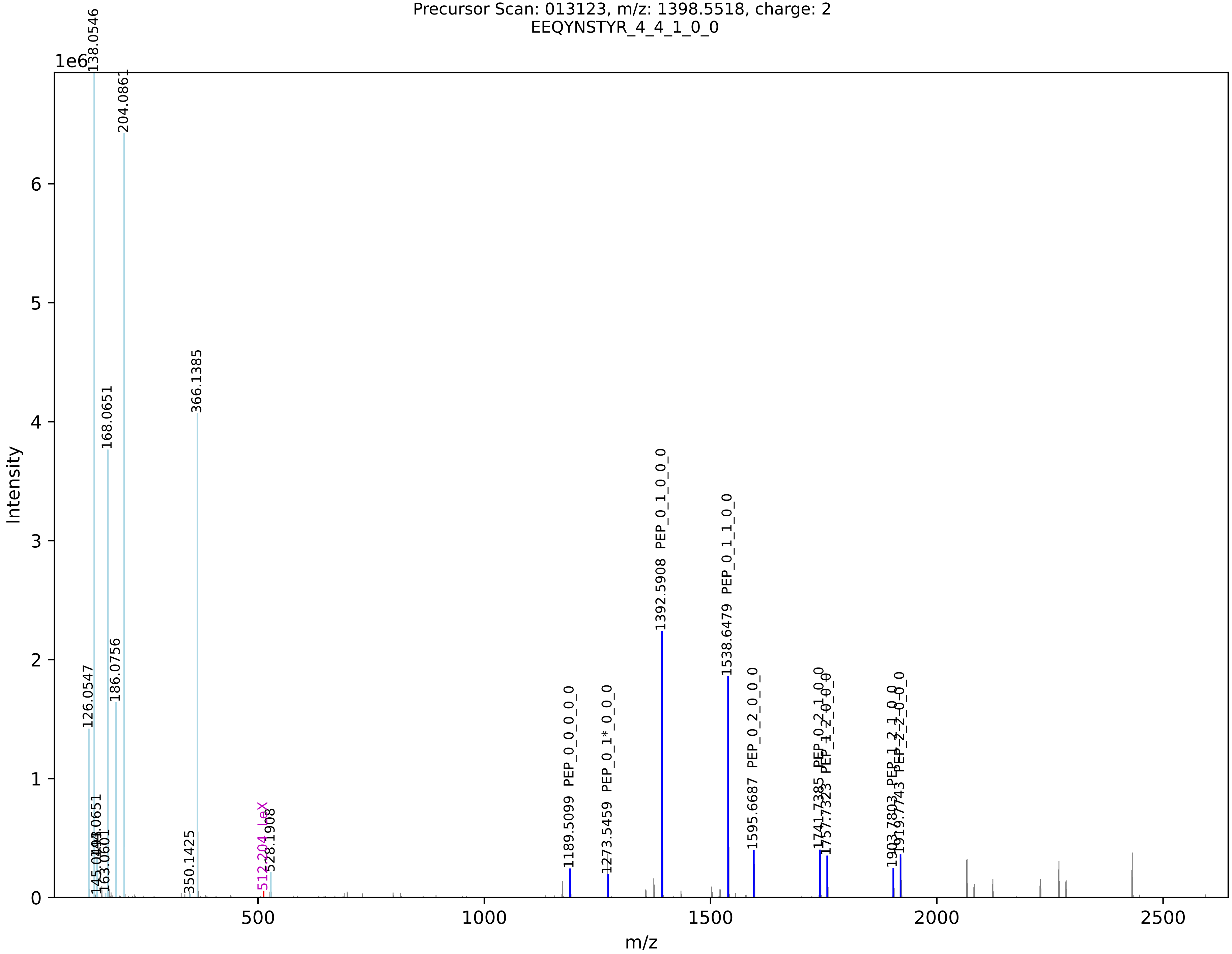

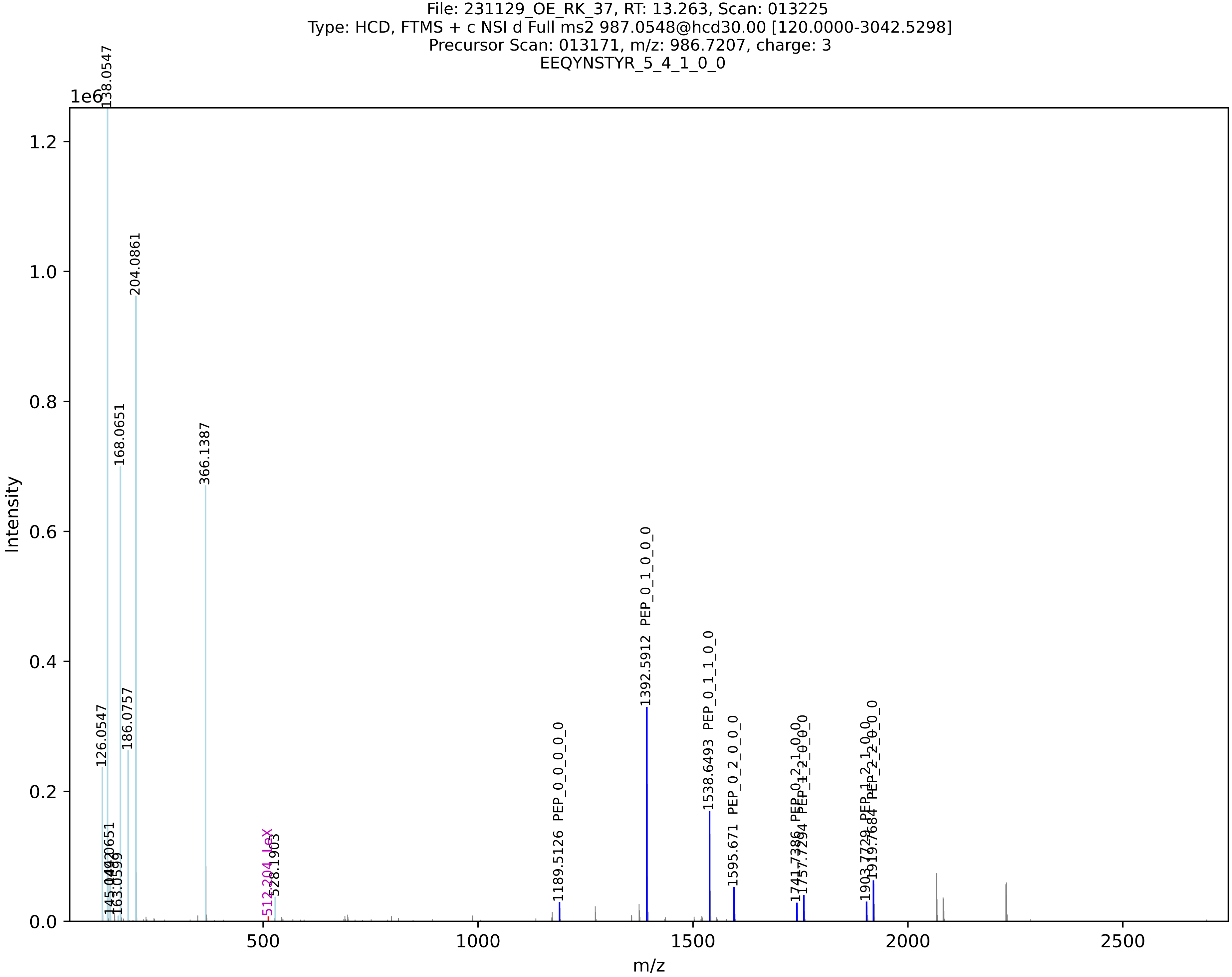
File: 231129_OE_RK_37, RT: 13.263, Scan: 013225
Type: HCD, FTMS + c NSI d Full ms2 987.0548@hcd30.00 [120.0000-3042.5298]
Precursor Scan: 013171, m/z: 986.7207, charge: 3
EEQYNSTYR_5_4_1_0_0
1e6
Intensity
m/z
138.0547
204.0861
168.0651
366.1387
126.0547
186.0757
528.1903
512.204_LeX
1189.5126 PEP_0_0_0_0_0
1392.5912 PEP_0_1_0_0_0
1538.6493 PEP_0_1_1_0_0
1595.671 PEP_0_2_0_0_0
1903.7729 PEP_1_2_1_0_0
1919.7684 PEP_2_2_0_0_0

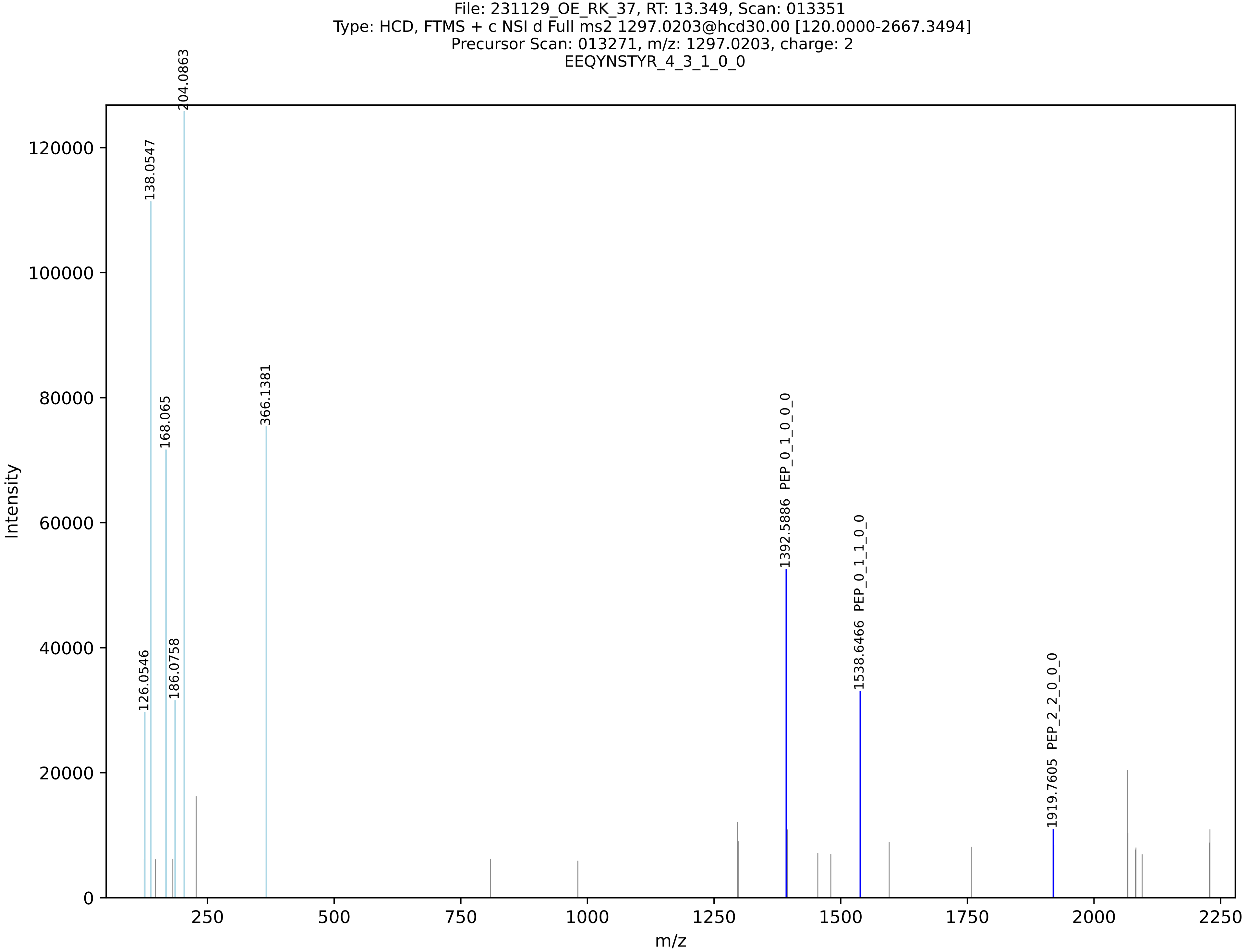
File: 231129_OE_RK_37, RT: 13.349, Scan: 013351
Type: HCD, FTMS + c NSI d Full ms2 1297.0203@hcd30.00 [120.0000-2667.3494]
Precursor Scan: 013271, m/z: 1297.0203, charge: 2
EEQYNSTYR_4_3_1_0_0
Intensity
m/z
204.0863
138.0547
168.065
366.1381
126.0546
186.0758
1392.5886 PEP_0_1_0_0_0
1538.6466 PEP_0_1_1_0_0
1919.7605 PEP_2_2_0_0_0
0
20000
40000
60000
80000
100000
120000
250
500
750
1000
1250
1500
1750
2000
2250

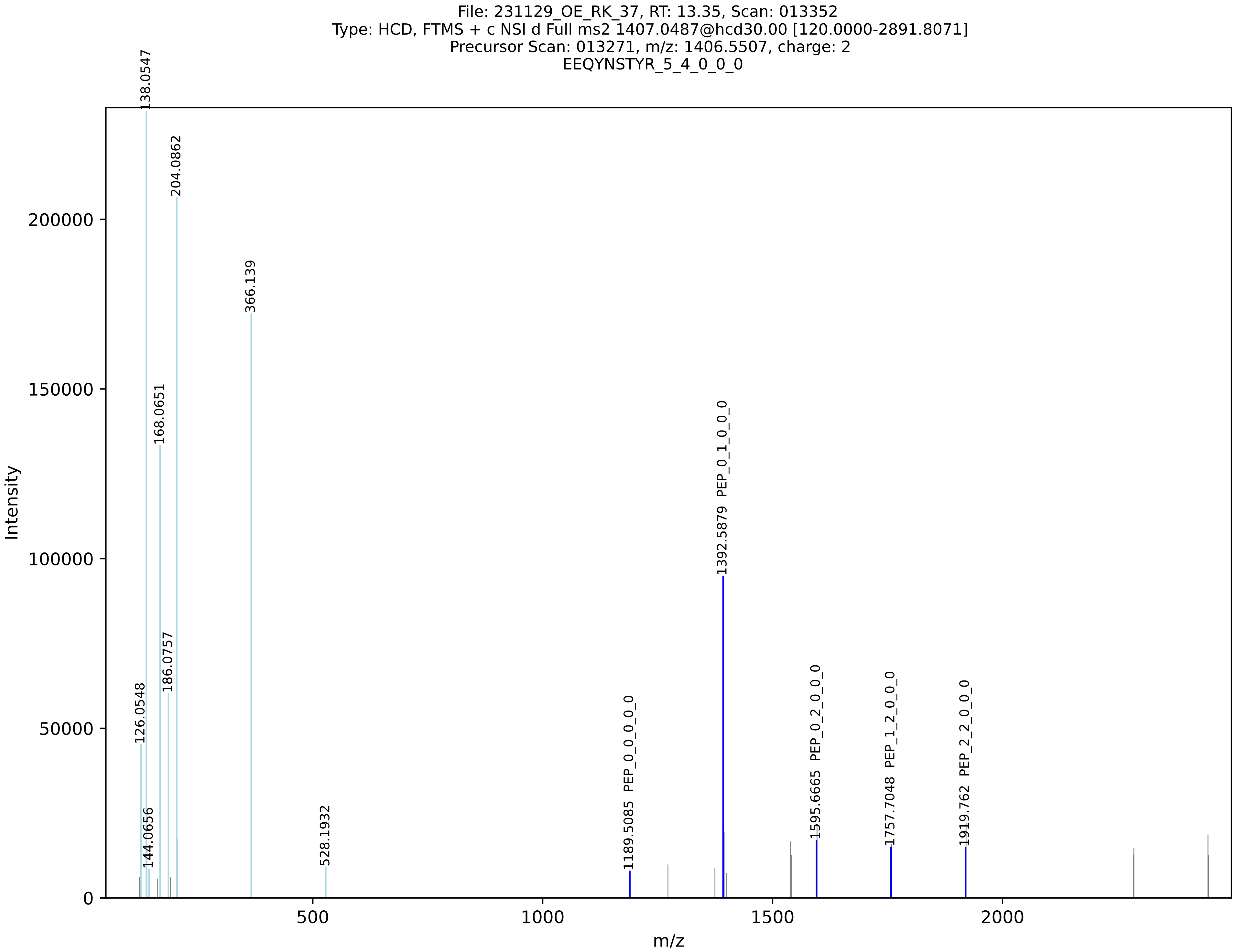
File: 231129_OE_RK_37, RT: 13.35, Scan: 013352
Type: HCD, FTMS + c NSI d Full ms2 1407.0487@hcd30.00 [120.0000-2891.8071]
Precursor Scan: 013271, m/z: 1406.5507, charge: 2
EEQYNSTYR_5_4_0_0_0
Intensity
m/z
138.0547
204.0862
366.139
168.0651
186.0757
126.0548
144.0656
528.1932
1189.5085 PEP_0_0_0_0_0
1392.5879 PEP_0_1_0_0_0
1595.6665 PEP_0_2_0_0_0
1757.7048 PEP_1_2_0_0_0
1919.762 PEP_2_2_0_0_0
0
50000
100000
150000
200000
500
1000
1500
2000

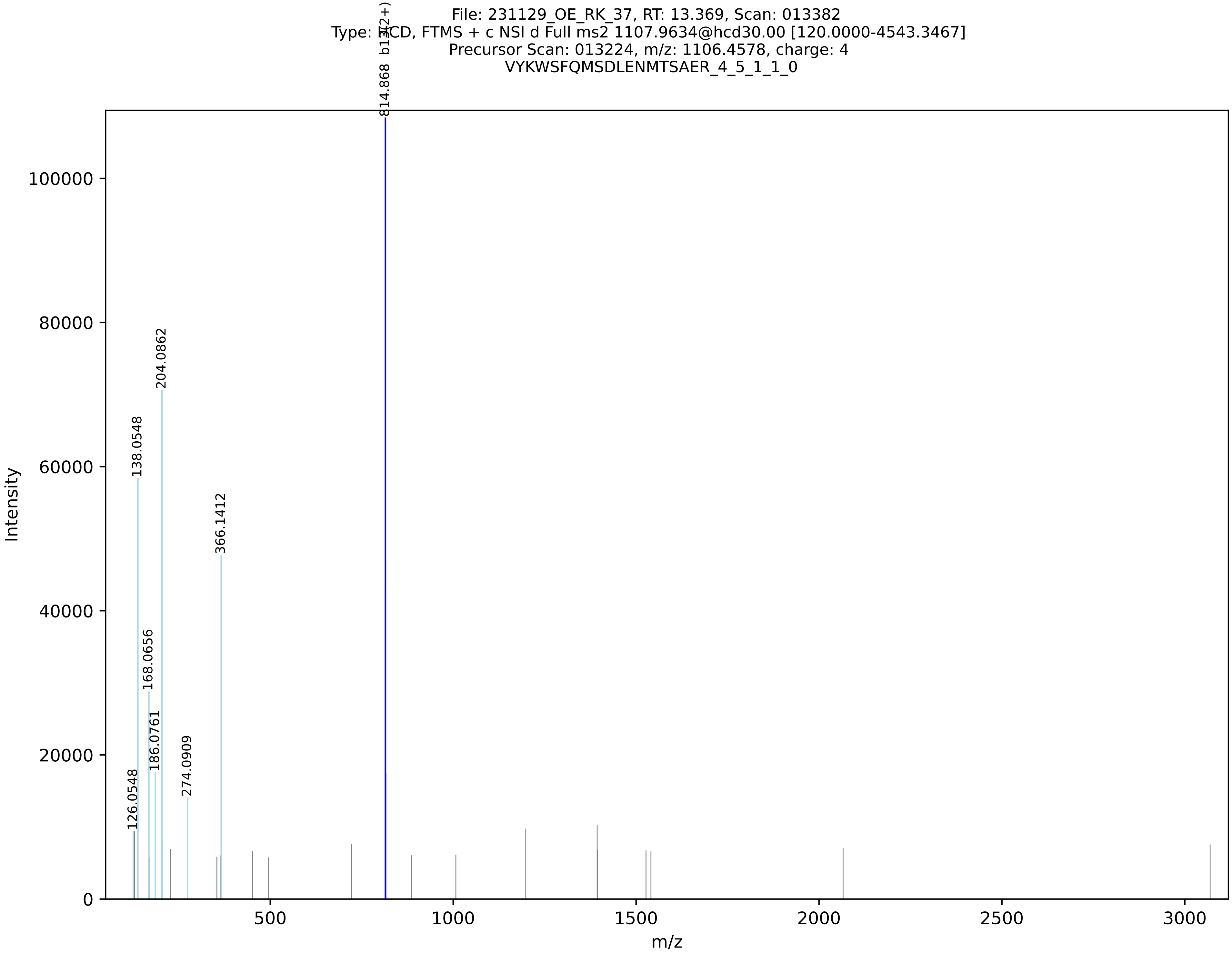
File: 231129_OE_RK_37, RT: 13.369, Scan: 013382
Type: HCD, FTMS + c NSI d Full ms2 1107.9634@hcd30.00 [120.0000-4543.3467]
Precursor Scan: 013224, m/z: 1106.4578, charge: 4
VYKWSFQMSDLENMTSAER_4_5_1_1_0
814.868 b13(2+)
204.0862
138.0548
366.1412
168.0656
186.0761
274.0909
126.0548
Intensity
m/z
0
20000
40000
60000
80000
100000
500
1000
1500
2000
2500
3000

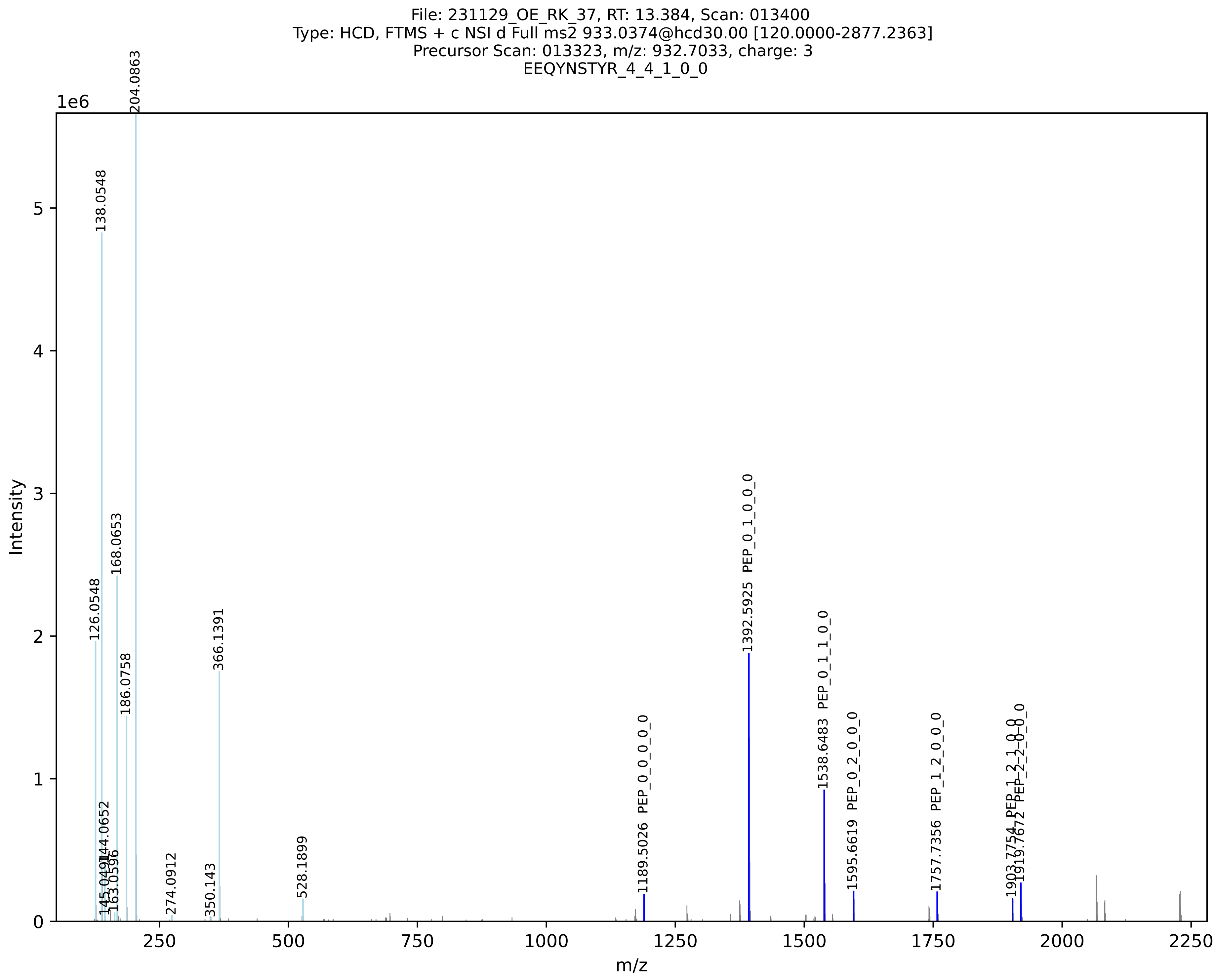
File: 231129_OE_RK_37, RT: 13.384, Scan: 013400
Type: HCD, FTMS + c NSI d Full ms2 933.0374@hcd30.00 [120.0000-2877.2363]
Precursor Scan: 013323, m/z: 932.7033, charge: 3
EEQYNSTYR_4_4_1_0_0
1e6
Intensity
m/z
204.0863
138.0548
168.0653
126.0548
186.0758
366.1391
144.0652
145.0491
163.0596
274.0912
350.143
528.1899
1189.5026 PEP_0_0_0_0_0
1392.5925 PEP_0_1_0_0_0
1538.6483 PEP_0_1_1_0_0
1595.6619 PEP_0_2_0_0_0
1757.7356 PEP_1_2_0_0_0
1903.7754 PEP_1_2_1_0_0
1919.7672 PEP_2_2_0_0_0

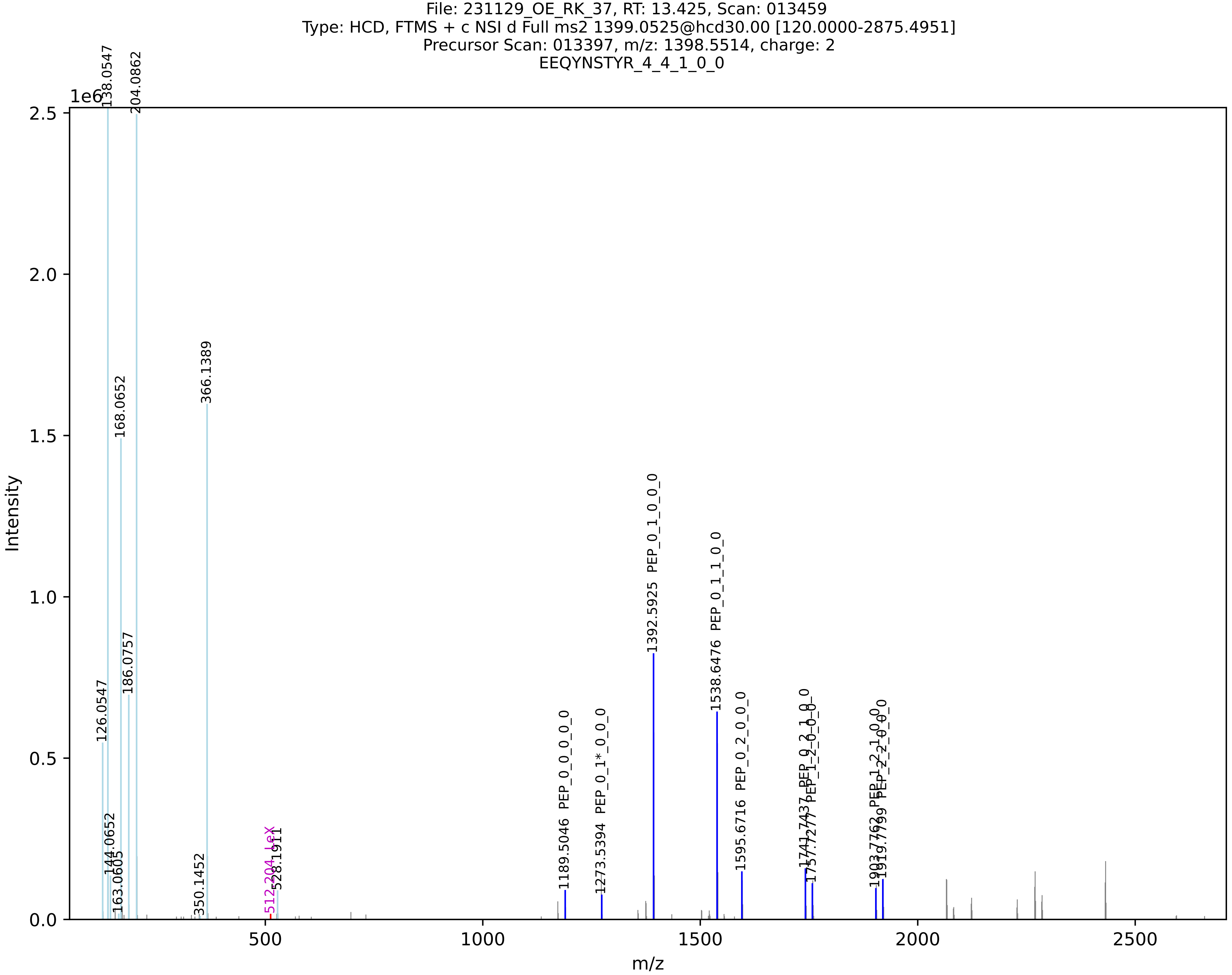
File: 231129_OE_RK_37, RT: 13.425, Scan: 013459
Type: HCD, FTMS + c NSI d Full ms2 1399.0525@hcd30.00 [120.0000-2875.4951]
Precursor Scan: 013397, m/z: 1398.5514, charge: 2
EEQYNSTYR_4_4_1_0_0
1e6
Intensity
m/z
138.0547
204.0862
366.1389
168.0652
186.0757
126.0547
144.0652
163.0605
350.1452
512.204_LeX
528.1911
1189.5046 PEP_0_0_0_0_0
1273.5394 PEP_0_1*_0_0_0
1392.5925 PEP_0_1_0_0_0
1538.6476 PEP_0_1_1_0_0
1595.6716 PEP_0_2_0_0_0
1741.7437 PEP_0_2_1_0_0
1757.7277 PEP_1_2_0_0_0
1903.7762 PEP_1_2_1_0_0
1919.7799 PEP_2_2_0_0_0

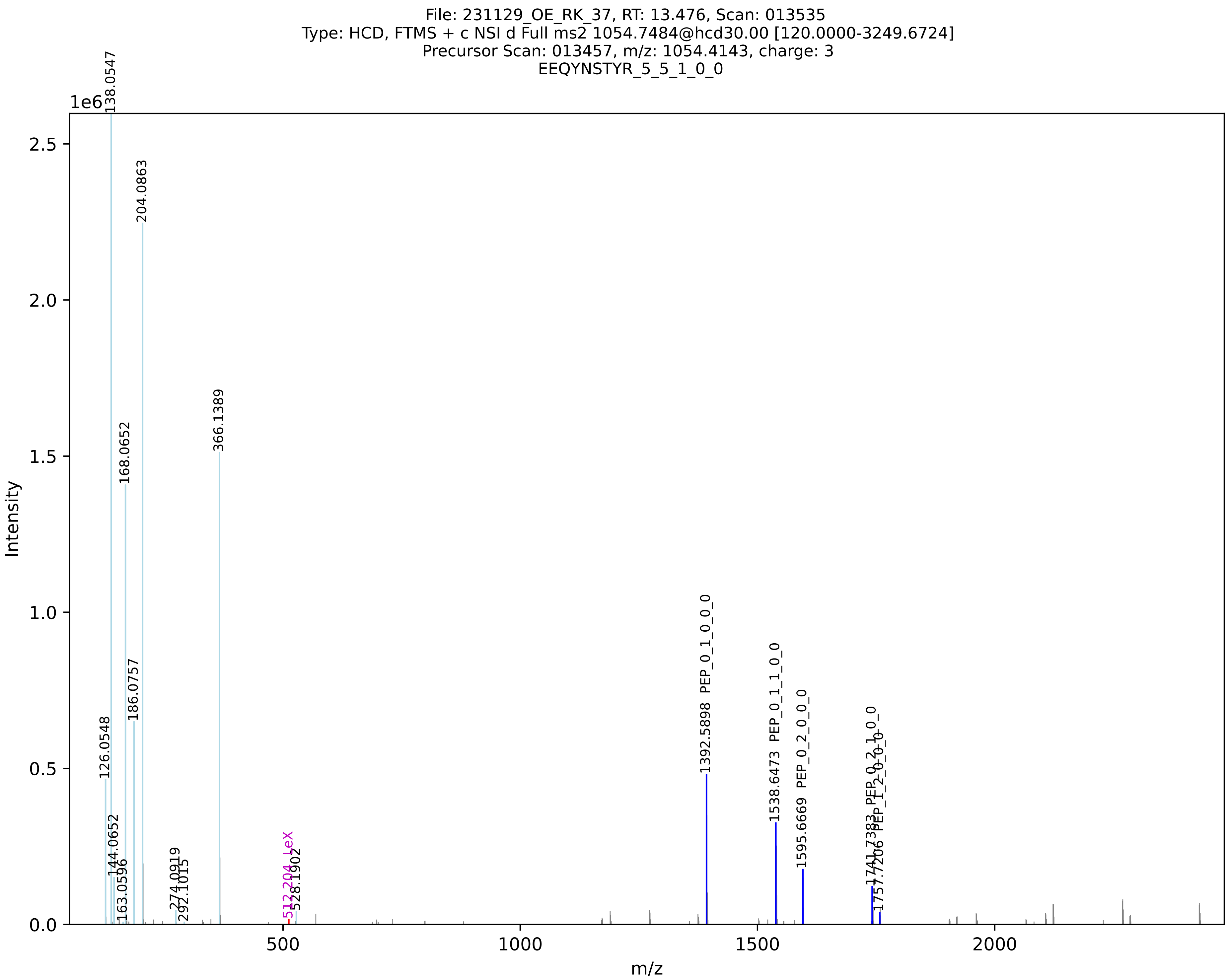
File: 231129_OE_RK_37, RT: 13.476, Scan: 013535
Type: HCD, FTMS + c NSI d Full ms2 1054.7484@hcd30.00 [120.0000-3249.6724]
Precursor Scan: 013457, m/z: 1054.4143, charge: 3
EEQYNSTYR_5_5_1_0_0
1e6
Intensity
m/z
138.0547
204.0863
366.1389
168.0652
186.0757
126.0548
144.0652
163.0596
274.0919
292.1015
512.204 LeX
528.1902
1392.5898 PEP_0_1_0_0_0
1538.6473 PEP_0_1_1_0_0
1595.6669 PEP_0_2_0_0_0
1741.7383 PEP_0_2_1_0_0
1757.7206 PEP_1_2_0_0_0

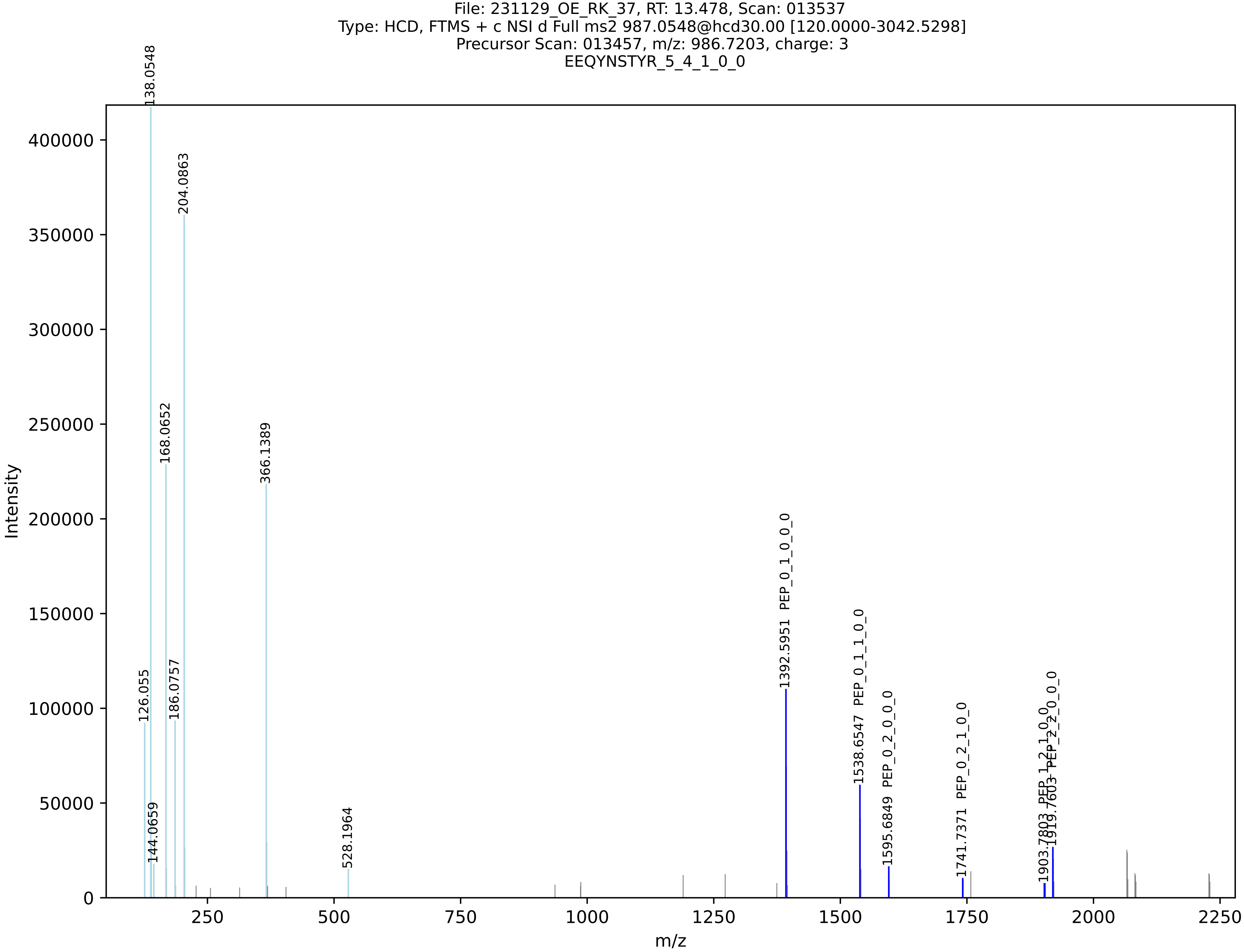
File: 231129_OE_RK_37, RT: 13.478, Scan: 013537
Type: HCD, FTMS + c NSI d Full ms2 987.0548@hcd30.00 [120.0000-3042.5298]
Precursor Scan: 013457, m/z: 986.7203, charge: 3
EEQYNSTYR_5_4_1_0_0
Intensity
m/z
138.0548
204.0863
168.0652
366.1389
126.055
186.0757
144.0659
528.1964
1392.5951 PEP_0_1_0_0_0
1538.6547 PEP_0_1_1_0_0
1595.6849 PEP_0_2_0_0_0
1741.7371 PEP_0_2_1_0_0
1903.7803 PEP_1_2_1_0_0
1919.7603 PEP_2_2_0_0_0
0
50000
100000
150000
200000
250000
300000
350000
400000
250
500
750
1000
1250
1500
1750
2000
2250

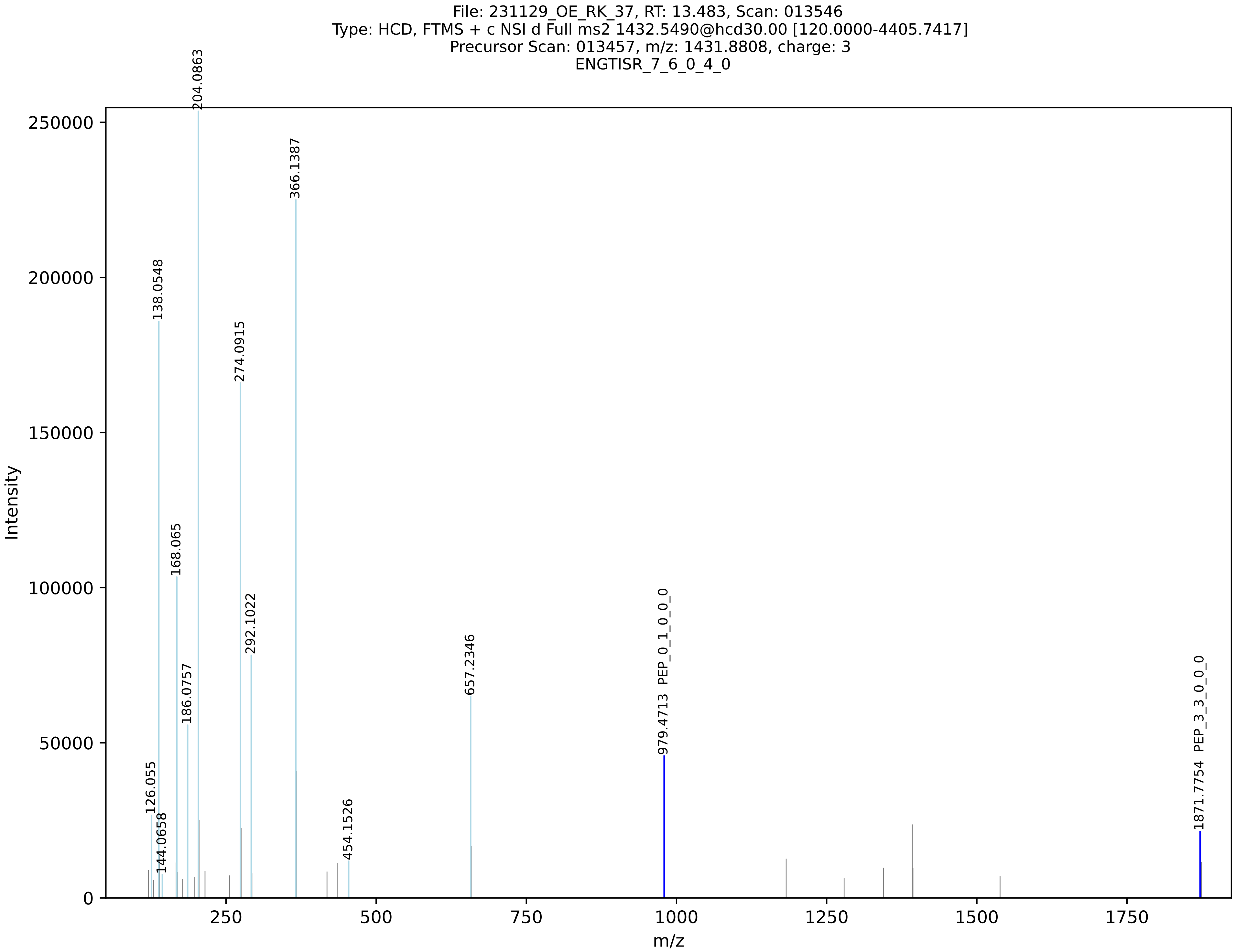
File: 231129_OE_RK_37, RT: 13.483, Scan: 013546
Type: HCD, FTMS + c NSI d Full ms2 1432.5490@hcd30.00 [120.0000-4405.7417]
Precursor Scan: 013457, m/z: 1431.8808, charge: 3
ENGTISR_7_6_0_4_0
Intensity
m/z
204.0863
366.1387
138.0548
274.0915
168.065
292.1022
186.0757
657.2346
979.4713 PEP_0_1_0_0_0
1871.7754 PEP_3_3_0_0_0
126.055
144.0658
454.1526
0
50000
100000
150000
200000
250000
250
500
750
1000
1250
1500
1750

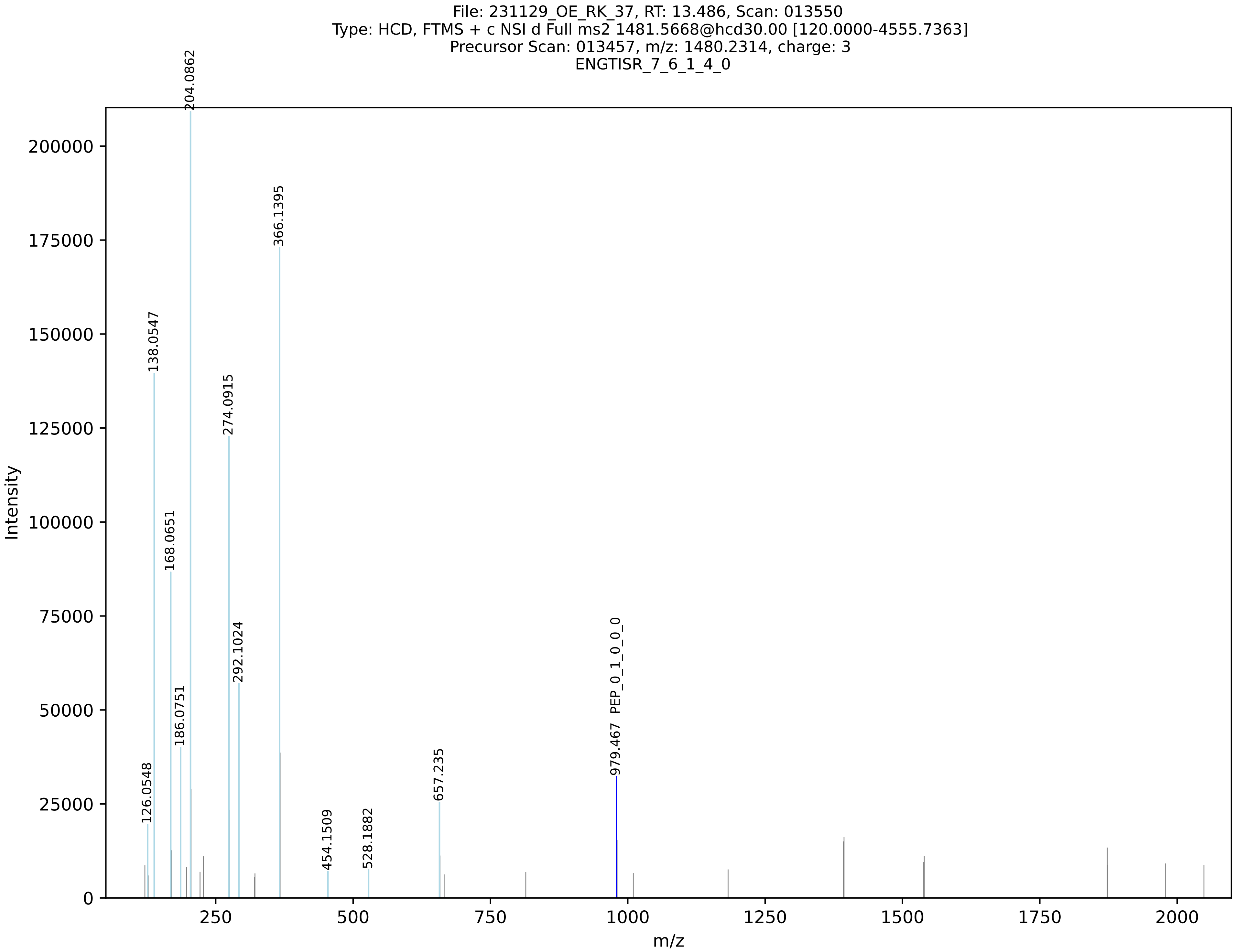
File: 231129_OE_RK_37, RT: 13.486, Scan: 013550
Type: HCD, FTMS + c NSI d Full ms2 1481.5668@hcd30.00 [120.0000-4555.7363]
Precursor Scan: 013457, m/z: 1480.2314, charge: 3
ENGTISR_7_6_1_4_0
204.0862
366.1395
138.0547
274.0915
168.0651
292.1024
186.0751
126.0548
454.1509
528.1882
657.235
979.467 PEP_0_1_0_0_0_
Intensity
m/z
0
25000
50000
75000
100000
125000
150000
175000
200000
250
500
750
1000
1250
1500
1750
2000

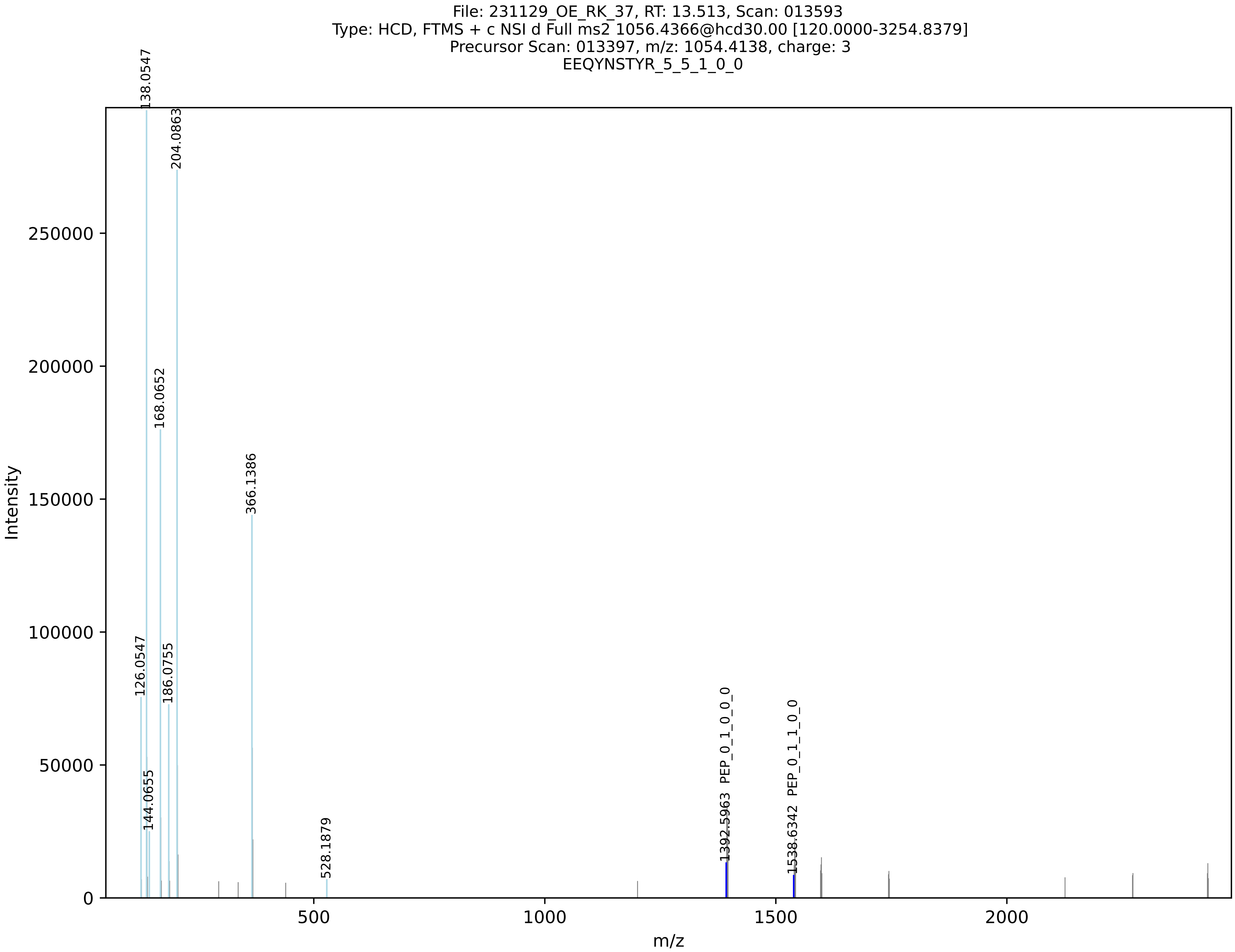
File: 231129_OE_RK_37, RT: 13.513, Scan: 013593
Type: HCD, FTMS + c NSI d Full ms2 1056.4366@hcd30.00 [120.0000-3254.8379]
Precursor Scan: 013397, m/z: 1054.4138, charge: 3
EEQYNSTYR_5_5_1_0_0
Intensity
m/z
138.0547
204.0863
168.0652
366.1386
126.0547
186.0755
144.0655
528.1879
1392.5963 PEP_0_1_0_0_0
1538.6342 PEP_0_1_1_0_0
0
50000
100000
150000
200000
250000
500
1000
1500
2000

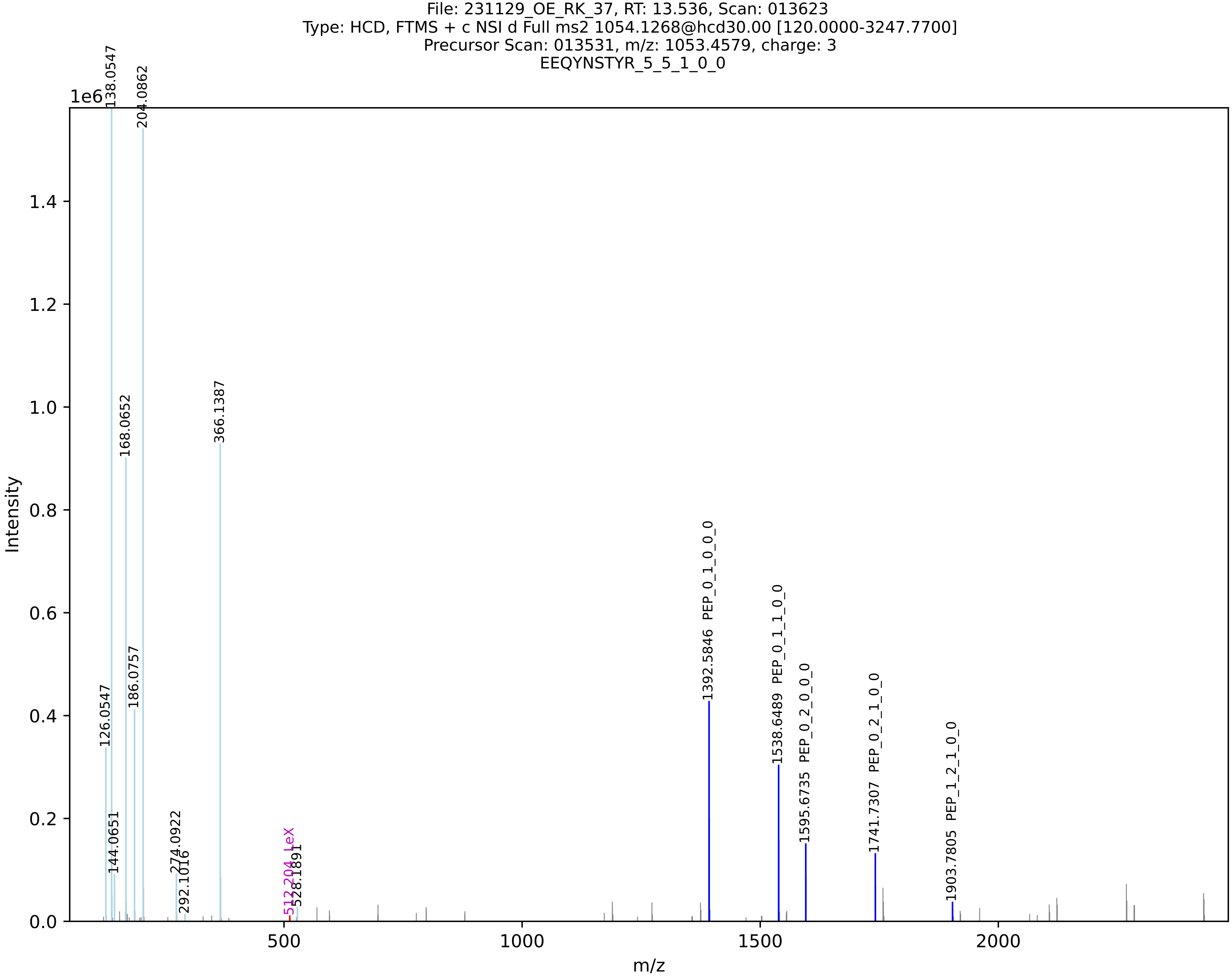

File: 231129_OE_RK_37, RT: 13.536, Scan: 013623
Type: HCD, FTMS + c NSI d Full ms2 1054.1268@hcd30.00 [120.0000-3247.7700]
Precursor Scan: 013531, m/z: 1053.4579, charge: 3
EEQYNSTYR_5_5_1_0_0
1e6
Intensity
m/z
138.0547
204.0862
168.0652
366.1387
186.0757
126.0547
144.0651
274.0922
292.1016
512.204_LeX
528.1891
1392.5846 PEP_0_1_0_0_0
1538.6489 PEP_0_1_1_0_0
1595.6735 PEP_0_2_0_0_0
1741.7307 PEP_0_2_1_0_0
1903.7805 PEP_1_2_1_0_0

File: 231129_OE_RK_37, RT: 13.552, Scan: 013649
Type: HCD, FTMS + c NSI d Full ms2 1070.4081@hcd30.00 [120.0000-3297.5908]
Precursor Scan: 013531, m/z: 1070.4081, charge: 3
EEQYNSTYR_6_3_1_1_0

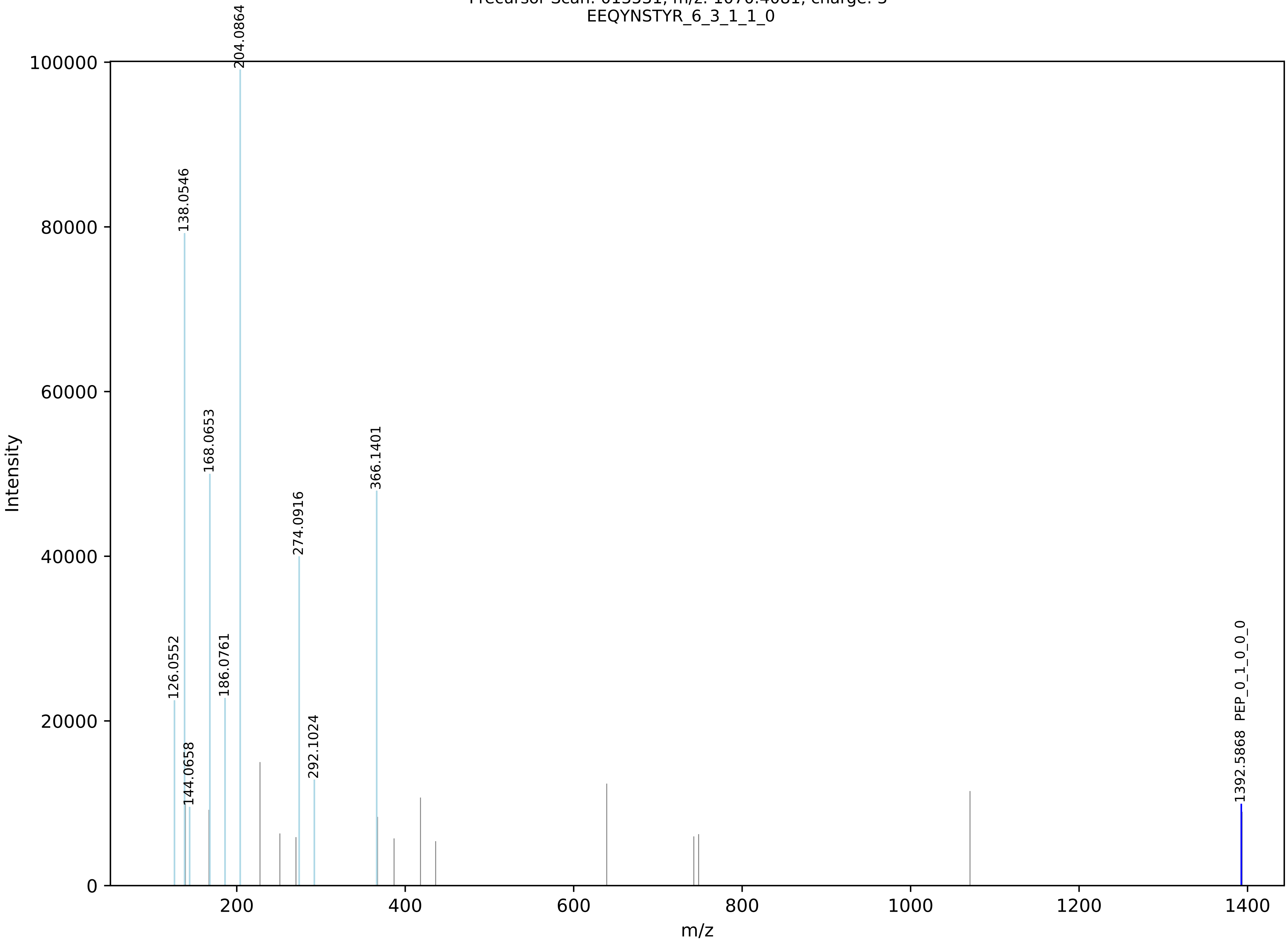

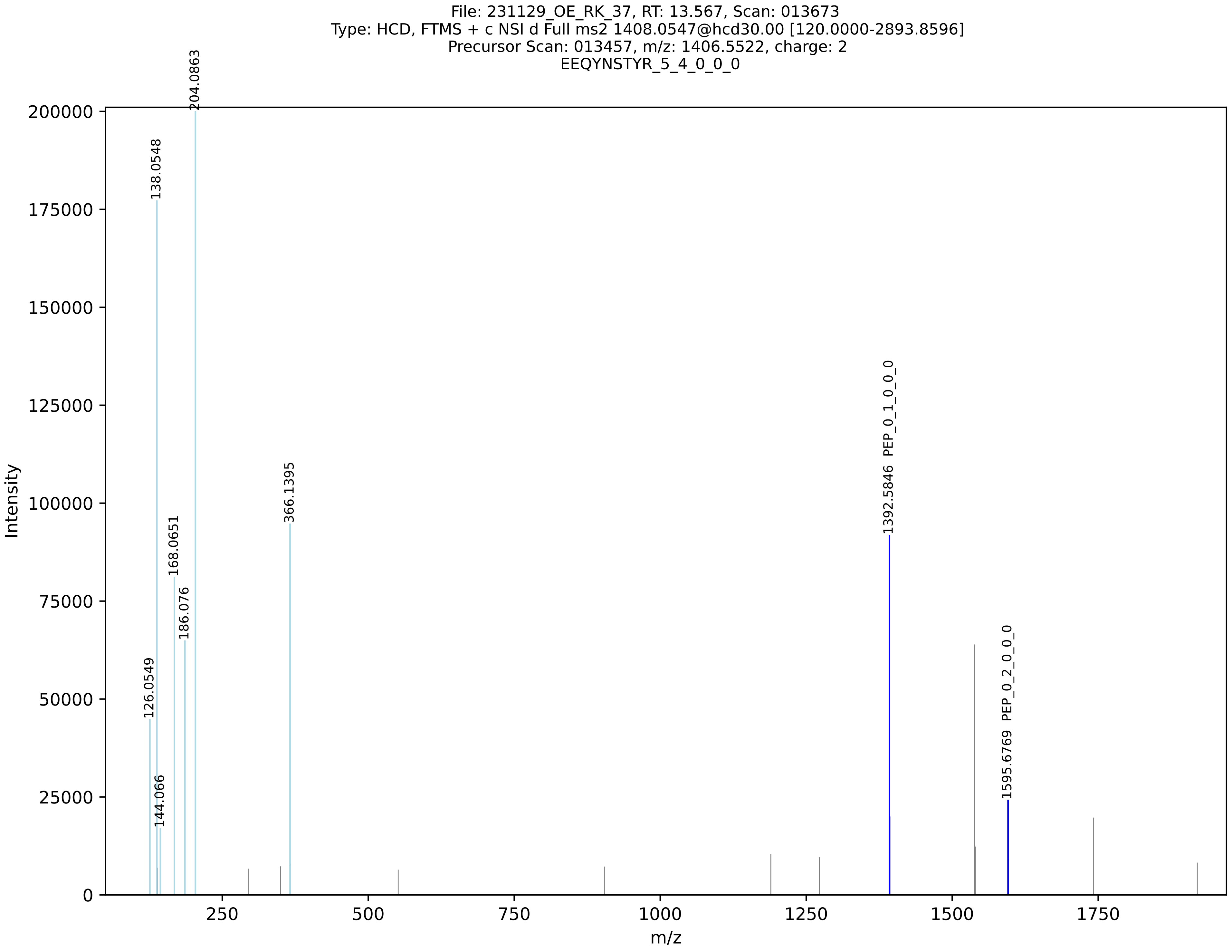

File: 231129_OE_RK_37, RT: 13.567, Scan: 013673
Type: HCD, FTMS + c NSI d Full ms2 1408.0547@hcd30.00 [120.0000-2893.8596]
Precursor Scan: 013457, m/z: 1406.5522, charge: 2
EEQYNSTYR_5_4_0_0_0
Intensity
m/z
204.0863
138.0548
126.0549
144.066
168.0651
186.076
366.1395
1392.5846 PEP_0_1_0_0_0
1595.6769 PEP_0_2_0_0_0
0
25000
50000
75000
100000
125000
150000
175000
200000
250
500
750
1000
1250
1500
1750

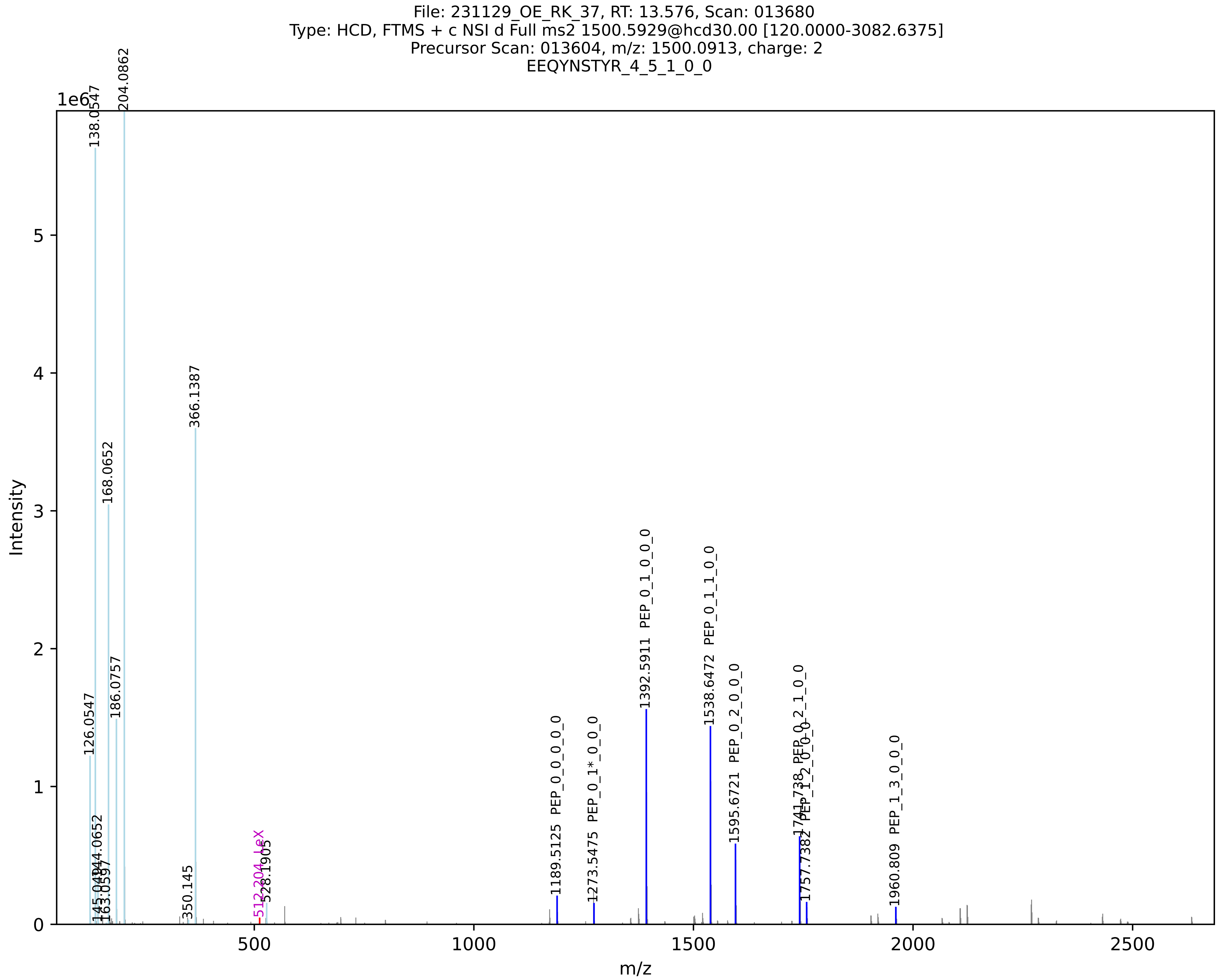

File: 231129_OE_RK_37, RT: 13.576, Scan: 013680
Type: HCD, FTMS + c NSI d Full ms2 1500.5929@hcd30.00 [120.0000-3082.6375]
Precursor Scan: 013604, m/z: 1500.0913, charge: 2
EEQYNSTYR_4_5_1_0_0
1e6
Intensity
m/z
204.0862
138.0547
168.0652
186.0757
126.0547
144.0652
145.0494
163.0597
366.1387
350.145
512.204_LeX
528.1905
1189.5125 PEP_0_0_0_0_0
1273.5475 PEP_0_1*_0_0_0
1392.5911 PEP_0_1_0_0_0
1538.6472 PEP_0_1_1_0_0
1595.6721 PEP_0_2_0_0_0
1741.738 PEP_0_2_1_0_0
1757.7382 PEP_1_2_0_0_0
1960.809 PEP_1_3_0_0_0

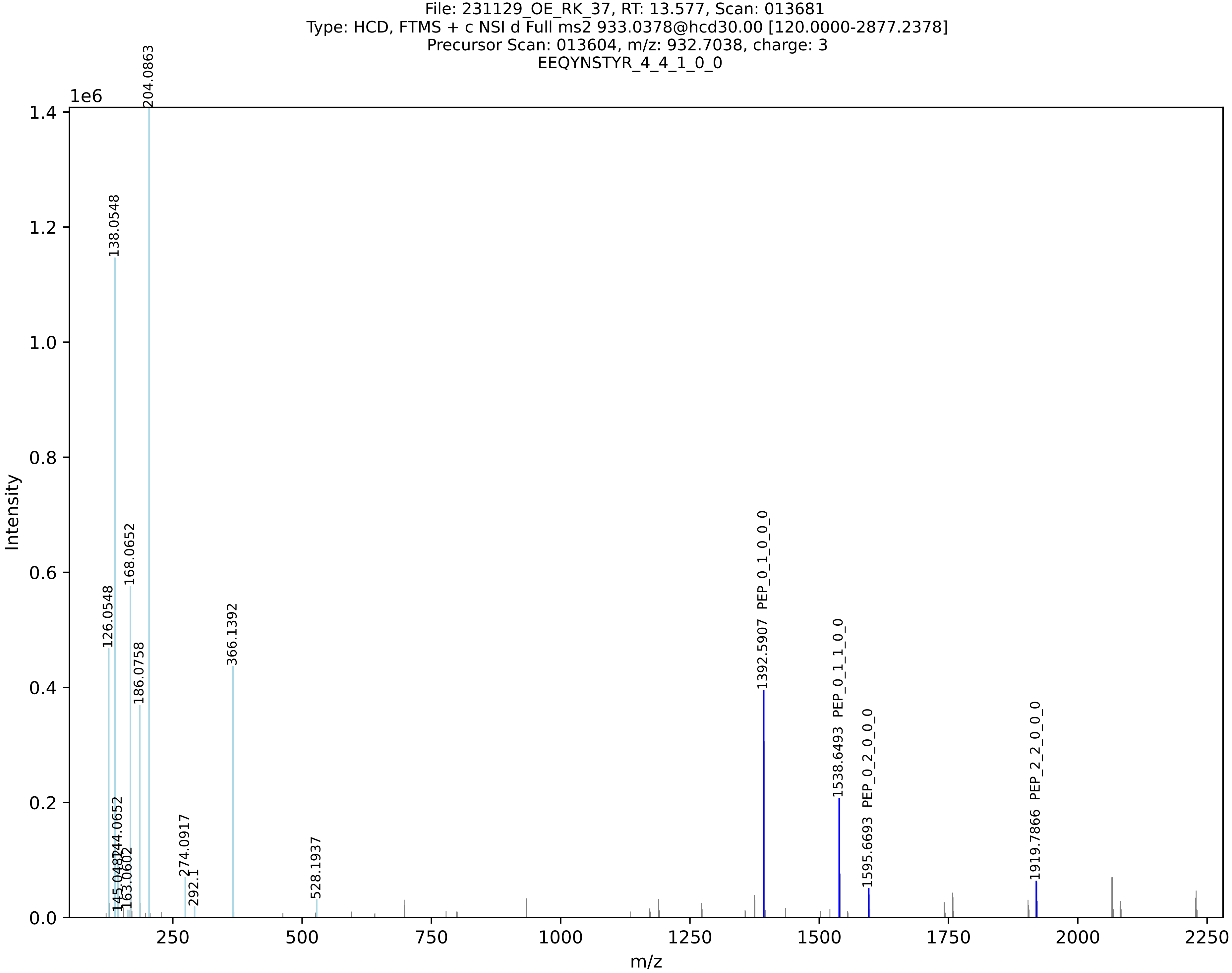
File: 231129_OE_RK_37, RT: 13.577, Scan: 013681
Type: HCD, FTMS + c NSI d Full ms2 933.0378@hcd30.00 [120.0000-2877.2378]
Precursor Scan: 013604, m/z: 932.7038, charge: 3
EEQYNSTYR_4_4_1_0_0
1e6
Intensity
m/z
204.0863
138.0548
168.0652
126.0548
186.0758
366.1392
144.0652
145.0481
163.0602
274.0917
292.1
528.1937
1392.5907 PEP_0_1_0_0_0
1538.6493 PEP_0_1_1_0_0
1595.6693 PEP_0_2_0_0_0
1919.7866 PEP_2_2_0_0_0

File: 231129_OE_RK_37, RT: 13.626, Scan: 013754
Type: HCD, FTMS + c NSI d Full ms2 1399.0540@hcd30.00 [120.0000-2875.4980]
Precursor Scan: 013677, m/z: 1398.5525, charge: 2
EEQYNSTYR_4_4_1_0_0

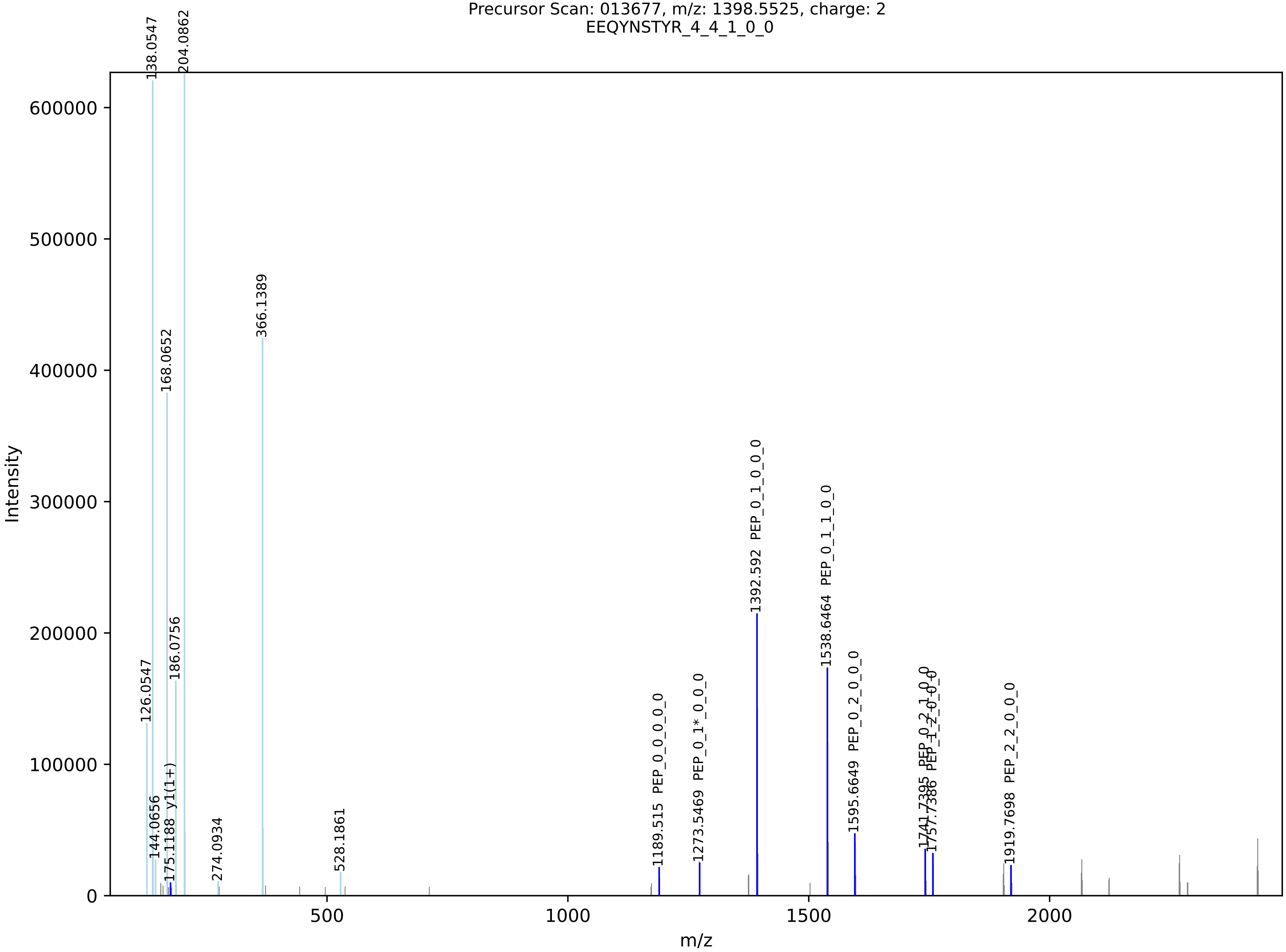

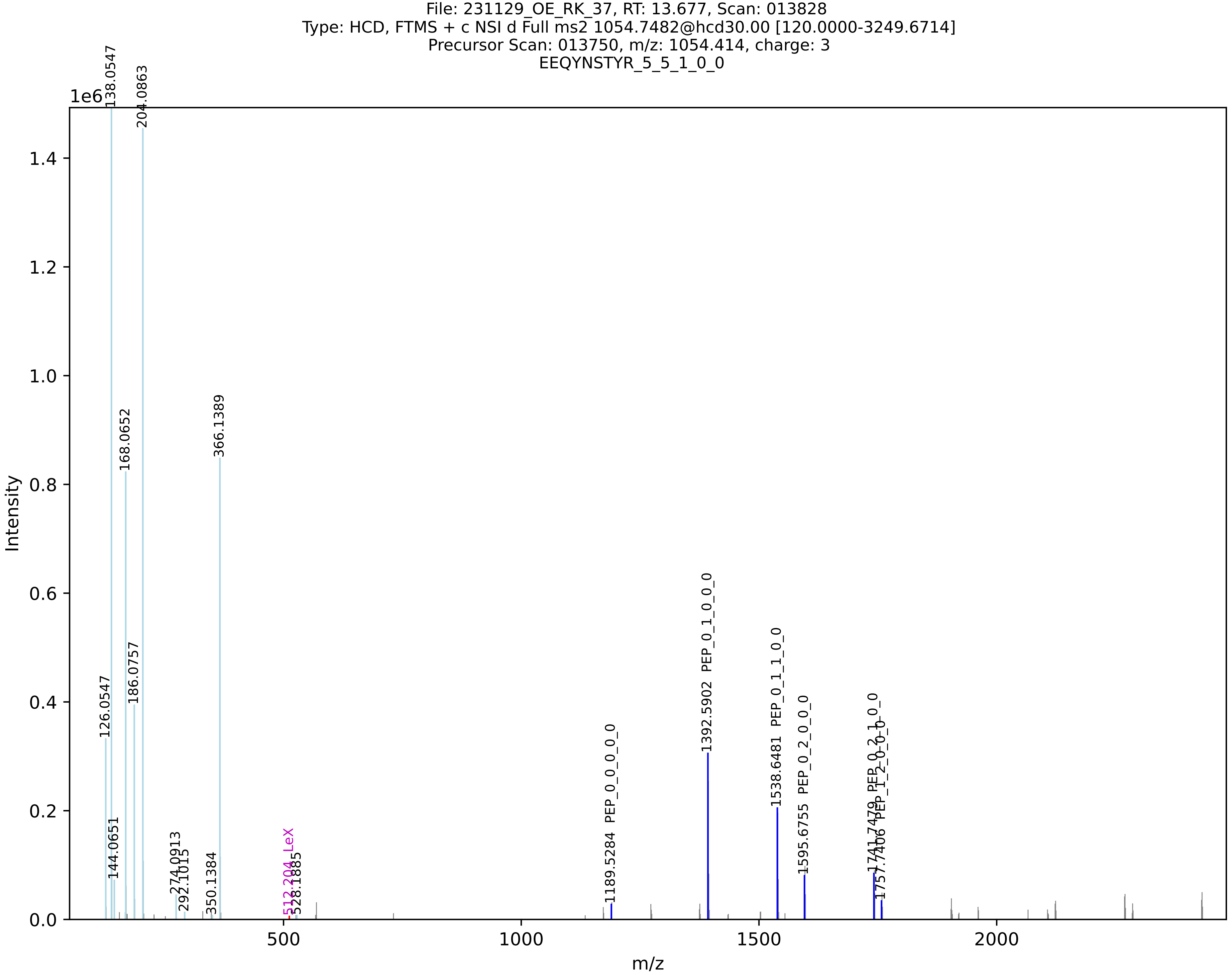

File: 231129_OE_RK_37, RT: 13.677, Scan: 013828
Type: HCD, FTMS + c NSI d Full ms2 1054.7482@hcd30.00 [120.0000-3249.6714]
Precursor Scan: 013750, m/z: 1054.414, charge: 3
EEQYNSTYR_5_5_1_0_0
1e6
138.0547
204.0863
366.1389
168.0652
186.0757
126.0547
144.0651
274.0913
292.1015
350.1384
512.204 LeX
528.1885
1189.5284 PEP_0_0_0_0_0
1392.5902 PEP_0_1_0_0_0
1538.6481 PEP_0_1_1_0_0
1595.6755 PEP_0_2_0_0_0
1741.7479 PEP_0_2_1_0_0
1757.7406 PEP_1_2_0_0_0
Intensity
m/z

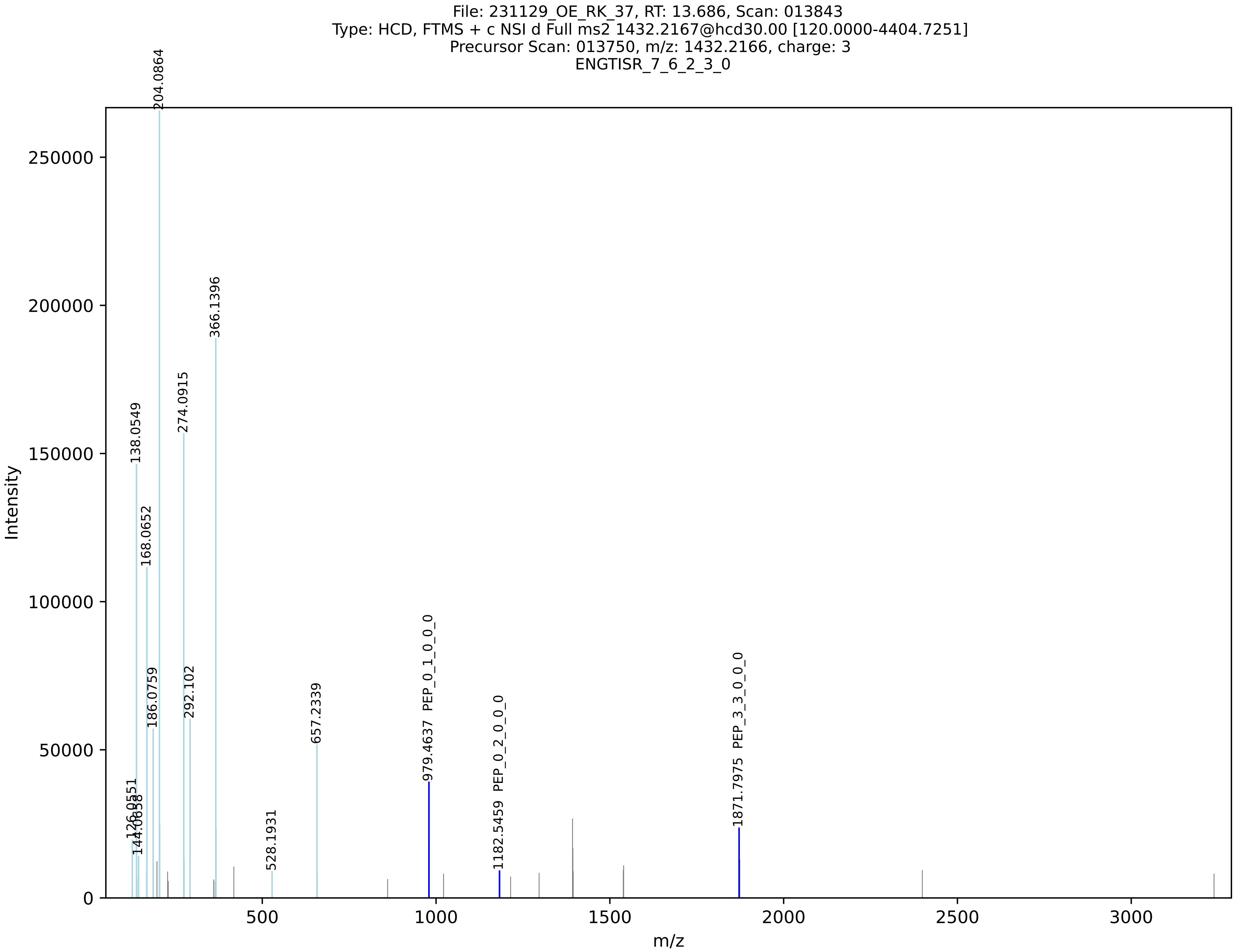
File: 231129_OE_RK_37, RT: 13.686, Scan: 013843
Type: HCD, FTMS + c NSI d Full ms2 1432.2167@hcd30.00 [120.0000-4404.7251]
Precursor Scan: 013750, m/z: 1432.2166, charge: 3
ENGTISR_7_6_2_3_0
Intensity
m/z
204.0864
366.1396
274.0915
138.0549
168.0652
186.0759
292.102
657.2339
528.1931
126.0551
144.0658
979.4637 PEP_0_1_0_0_0
1182.5459 PEP_0_2_0_0_0
1871.7975 PEP_3_3_0_0_0
0
50000
100000
150000
200000
250000
500
1000
1500
2000
2500
3000

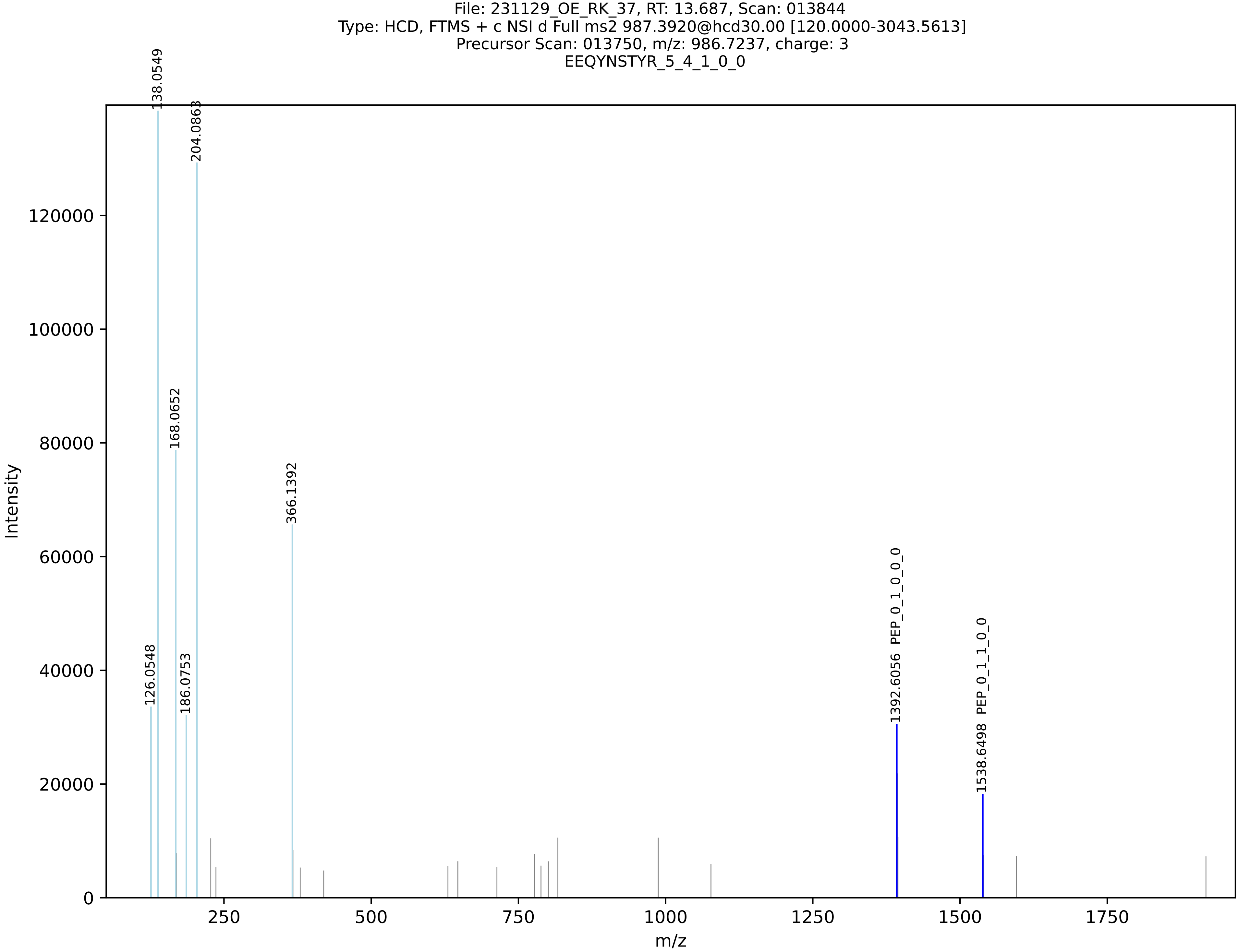
File: 231129_OE_RK_37, RT: 13.687, Scan: 013844
Type: HCD, FTMS + c NSI d Full ms2 987.3920@hcd30.00 [120.0000-3043.5613]
Precursor Scan: 013750, m/z: 986.7237, charge: 3
EEQYNSTYR_5_4_1_0_0
138.0549
204.0863
168.0652
366.1392
126.0548
186.0753
1392.6056 PEP_0_1_0_0_0
1538.6498 PEP_0_1_1_0_0
Intensity
m/z
0
20000
40000
60000
80000
100000
120000
250
500
750
1000
1250
1500
1750

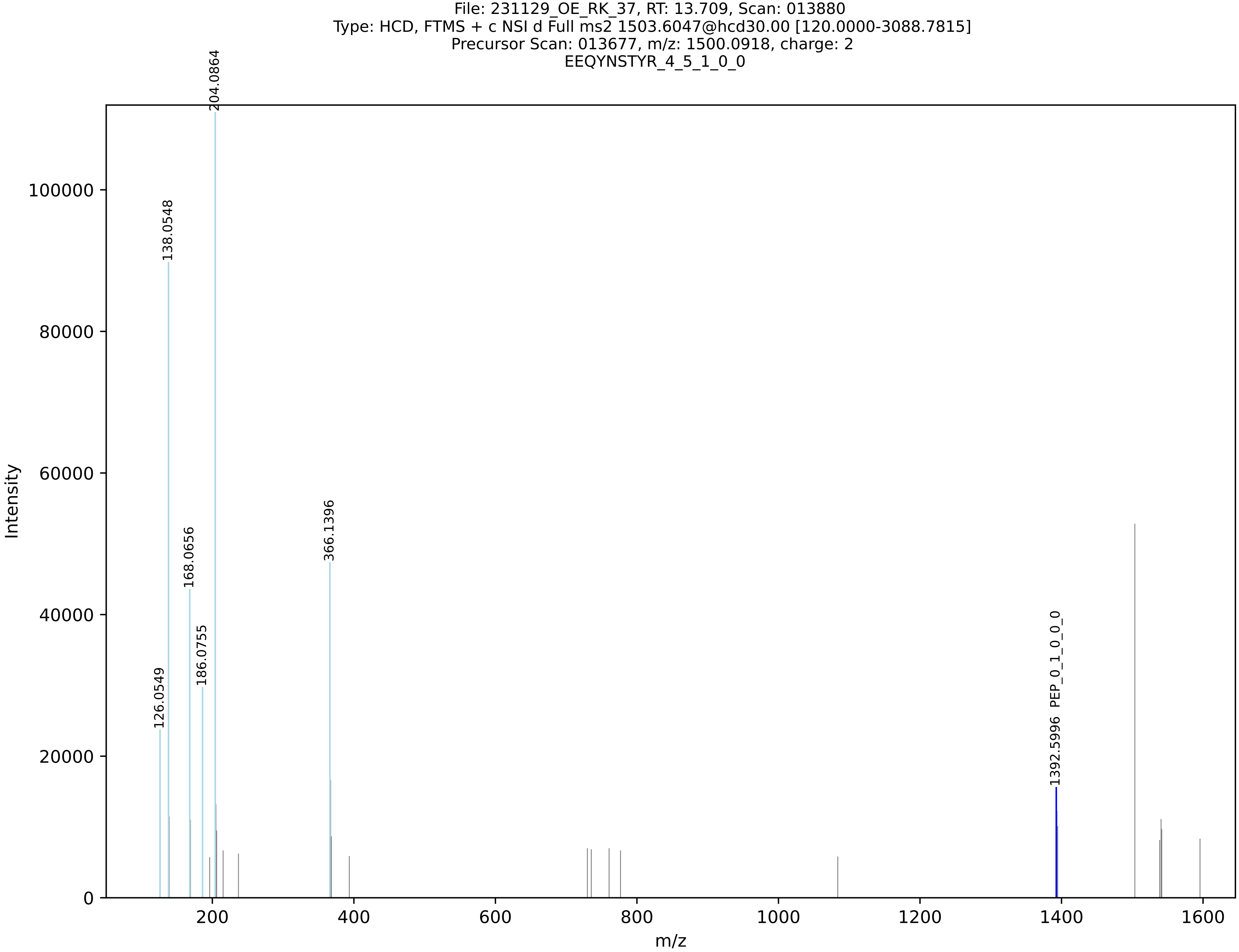
File: 231129_OE_RK_37, RT: 13.709, Scan: 013880
Type: HCD, FTMS + c NSI d Full ms2 1503.6047@hcd30.00 [120.0000-3088.7815]
Precursor Scan: 013677, m/z: 1500.0918, charge: 2
EEQYNSTYR_4_5_1_0_0
204.0864
138.0548
168.0656
186.0755
126.0549
366.1396
1392.5996 PEP_0_1_0_0_0
Intensity
m/z
0
20000
40000
60000
80000
100000
200
400
600
800
1000
1200
1400
1600

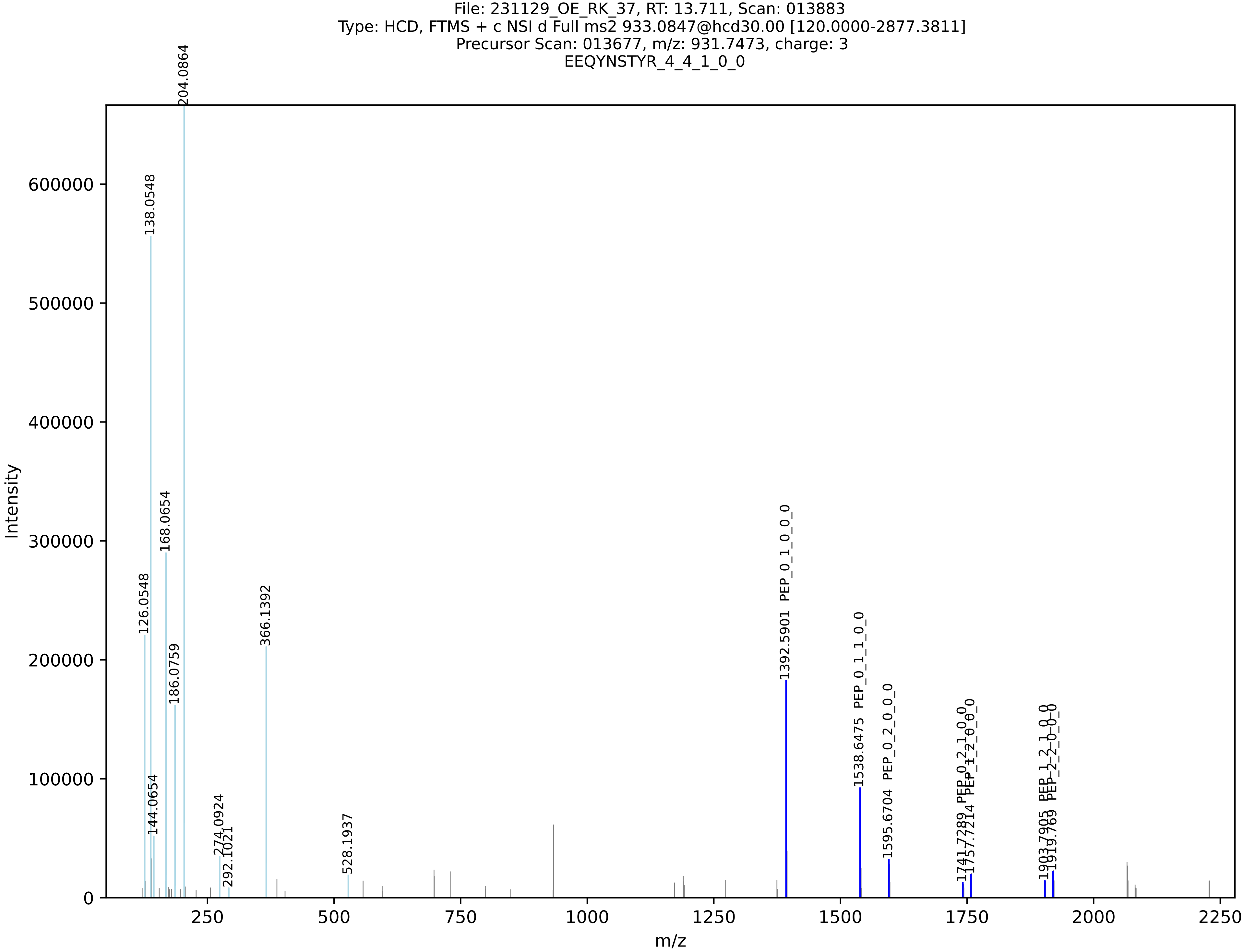
File: 231129_OE_RK_37, RT: 13.711, Scan: 013883
Type: HCD, FTMS + c NSI d Full ms2 933.0847@hcd30.00 [120.0000-2877.3811]
Precursor Scan: 013677, m/z: 931.7473, charge: 3
EEQYNSTYR_4_4_1_0_0
Intensity
m/z
204.0864
138.0548
168.0654
126.0548
186.0759
366.1392
144.0654
274.0924
292.1021
528.1937
1392.5901 PEP_0_1_0_0_0
1538.6475 PEP_0_1_1_0_0
1595.6704 PEP_0_2_0_0_0
1741.7289 PEP_0_2_1_0_0
1757.7214 PEP_1_2_0_0_0
1903.7905 PEP_1_2_1_0_0
1919.769 PEP_2_2_0_0_0

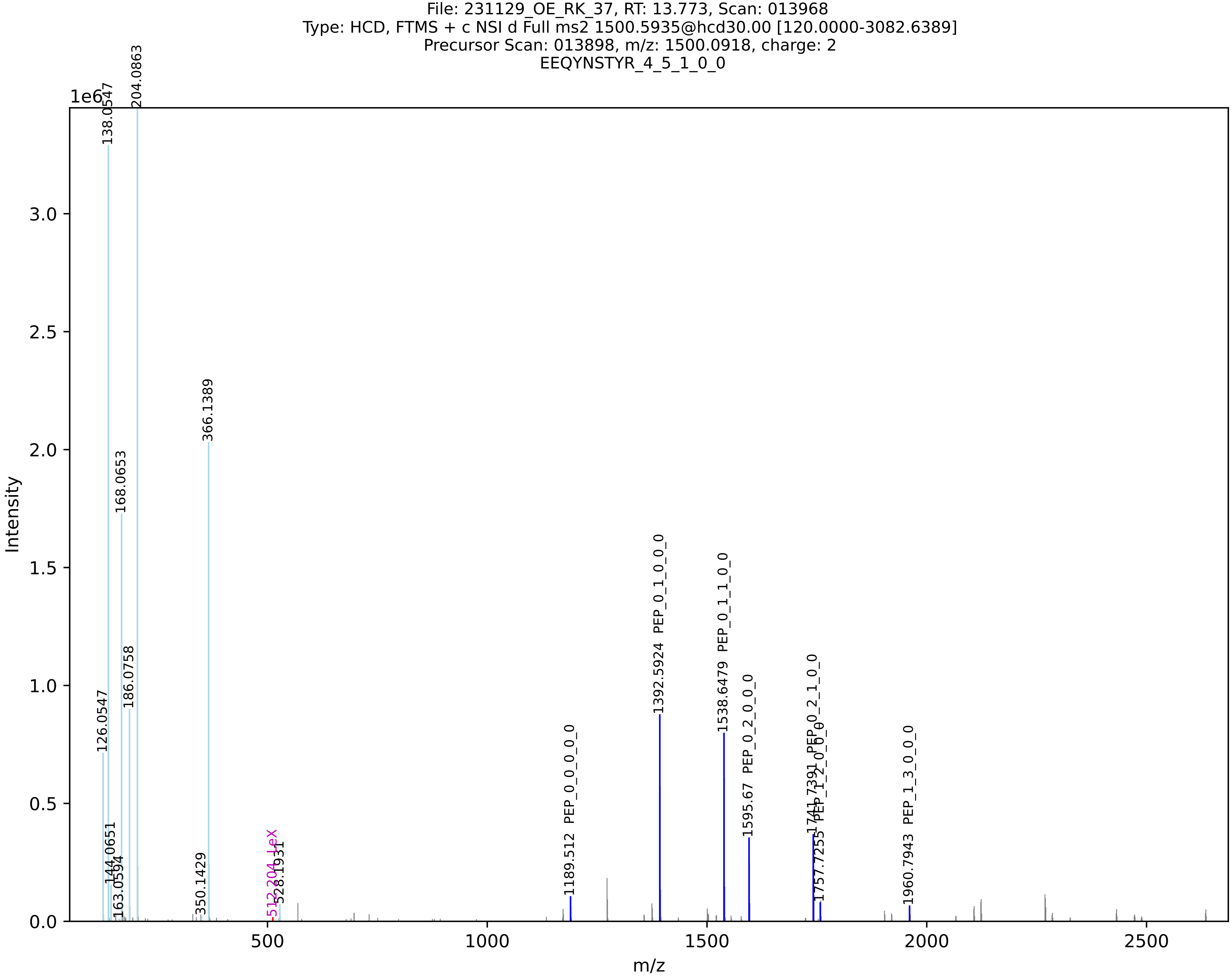

File: 231129_OE_RK_37, RT: 13.773, Scan: 013968
Type: HCD, FTMS + c NSI d Full ms2 1500.5935@hcd30.00 [120.0000-3082.6389]
Precursor Scan: 013898, m/z: 1500.0918, charge: 2
EEQYNSTYR_4_5_1_0_0
1e6
Intensity
m/z
204.0863
138.0547
366.1389
168.0653
186.0758
126.0547
144.0651
163.0594
350.1429
512.204_LeX
528.1931
1189.512 PEP_0_0_0_0_0
1392.5924 PEP_0_1_0_0_0
1538.6479 PEP_0_1_1_0_0
1595.67 PEP_0_2_0_0_0
1741.7391 PEP_0_2_1_0_0
1757.7255 PEP_1_2_0_0_0
1960.7943 PEP_1_3_0_0_0

File: 231129_OE_RK_37, RT: 13.801, Scan: 014013
Type: HCD, FTMS + c NSI d Full ms2 934.3758@hcd30.00 [120.0000-2881.3318]
Precursor Scan: 013824, m/z: 932.7041, charge: 3
EEQYNSTYR_4_4_1_0_0

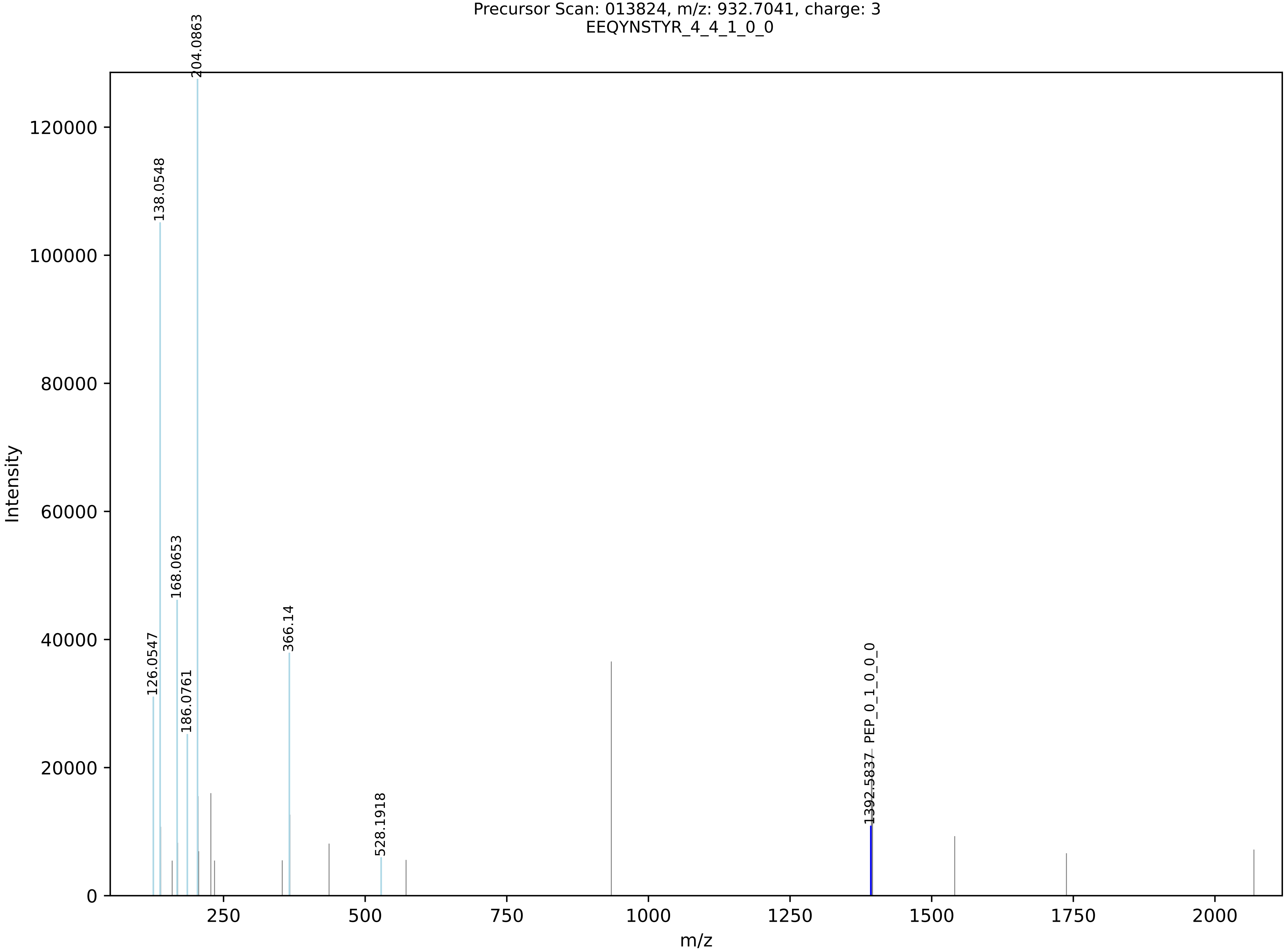

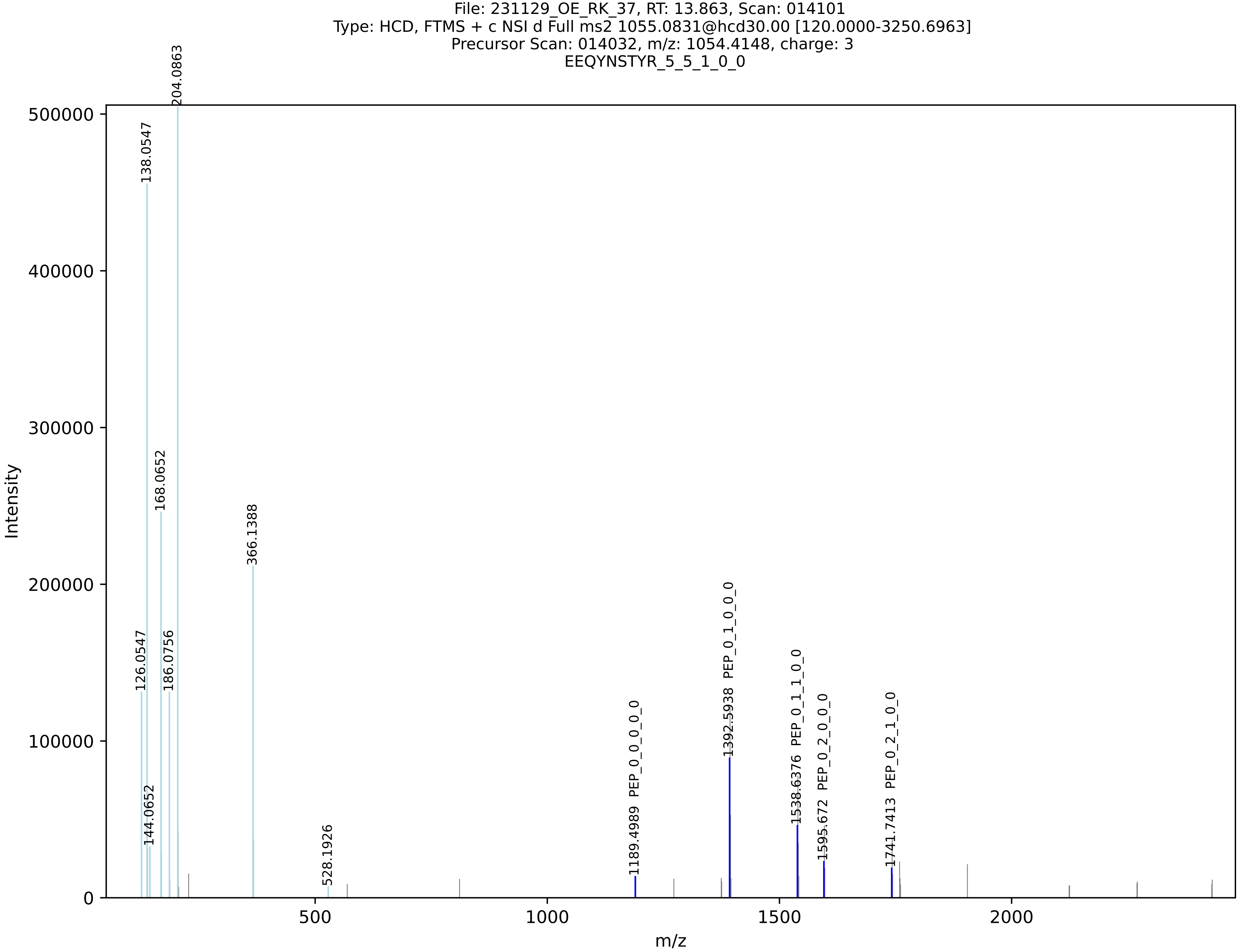

File: 231129_OE_RK_37, RT: 13.863, Scan: 014101
Type: HCD, FTMS + c NSI d Full ms2 1055.0831@hcd30.00 [120.0000-3250.6963]
Precursor Scan: 014032, m/z: 1054.4148, charge: 3
EEQYNSTYR_5_5_1_0_0
Intensity
m/z
204.0863
138.0547
168.0652
366.1388
126.0547
186.0756
144.0652
528.1926
1189.4989 PEP_0_0_0_0_0
1392.5938 PEP_0_1_0_0_0
1538.6376 PEP_0_1_1_0_0
1595.672 PEP_0_2_0_0_0
1741.7413 PEP_0_2_1_0_0

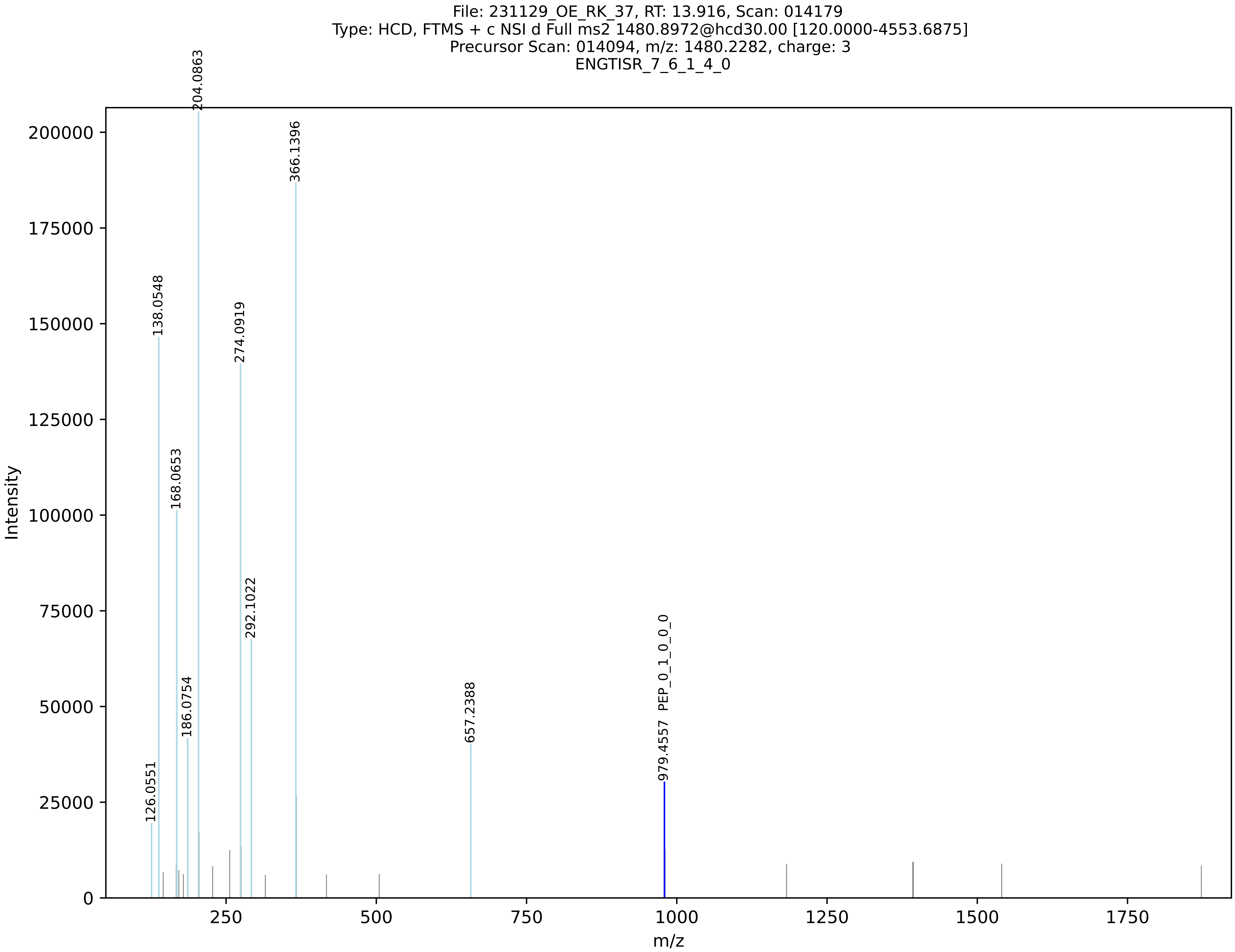
File: 231129_OE_RK_37, RT: 13.916, Scan: 014179
Type: HCD, FTMS + c NSI d Full ms2 1480.8972@hcd30.00 [120.0000-4553.6875]
Precursor Scan: 014094, m/z: 1480.2282, charge: 3
ENGTISR_7_6_1_4_0
204.0863
366.1396
138.0548
274.0919
168.0653
292.1022
186.0754
657.2388
PEP_0_1_0_0_0
979.4557
126.0551
Intensity
m/z
0
25000
50000
75000
100000
125000
150000
175000
200000
250
500
750
1000
1250
1500
1750

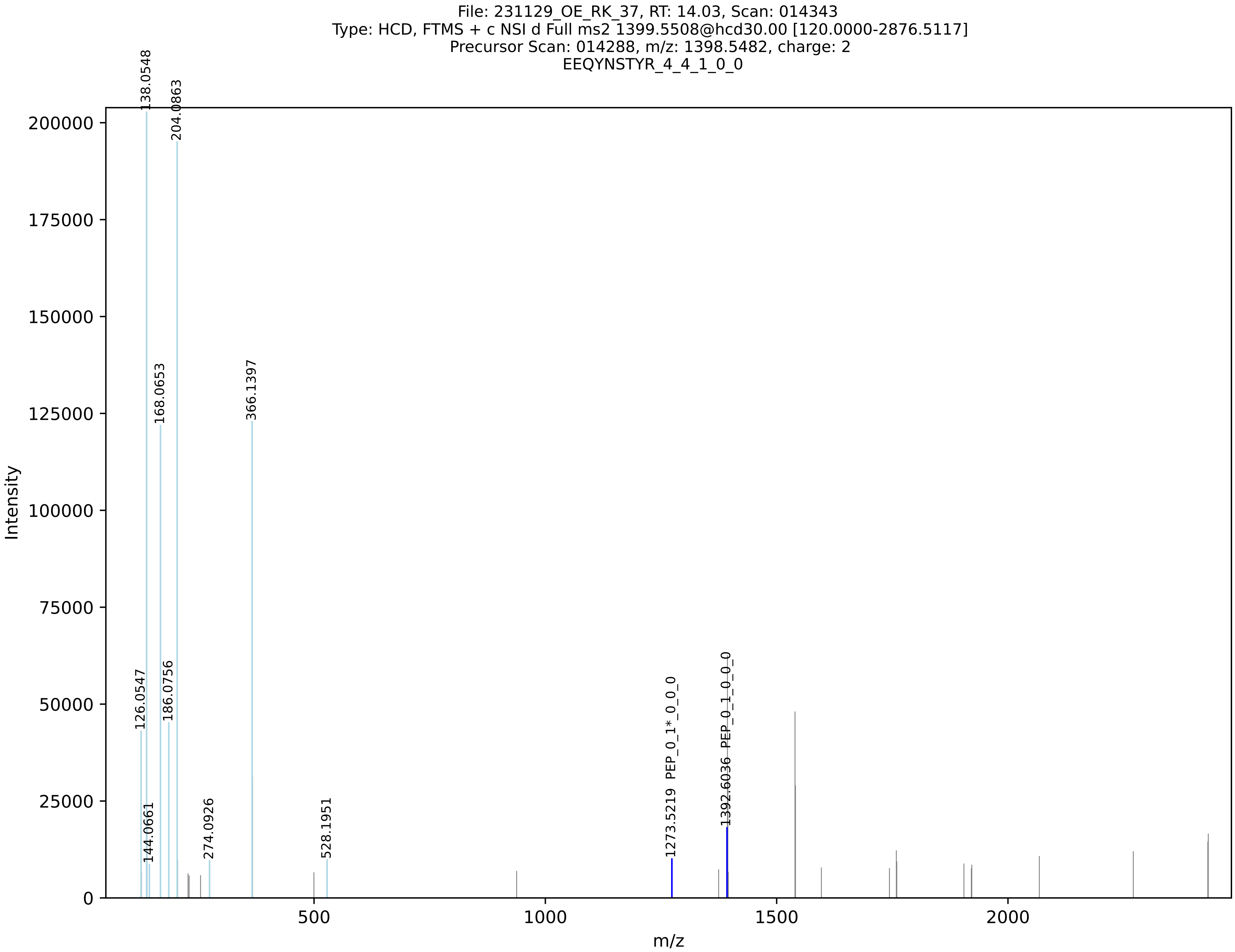
File: 231129_OE_RK_37, RT: 14.03, Scan: 014343
Type: HCD, FTMS + c NSI d Full ms2 1399.5508@hcd30.00 [120.0000-2876.5117]
Precursor Scan: 014288, m/z: 1398.5482, charge: 2
EEQYNSTYR_4_4_1_0_0
Intensity
m/z
138.0548
204.0863
168.0653
366.1397
126.0547
186.0756
144.0661
274.0926
528.1951
1273.5219 PEP_0_1*_0_0_0
1392.6036 PEP_0_1_0_0_
0
25000
50000
75000
100000
125000
150000
175000
200000
500
1000
1500
2000

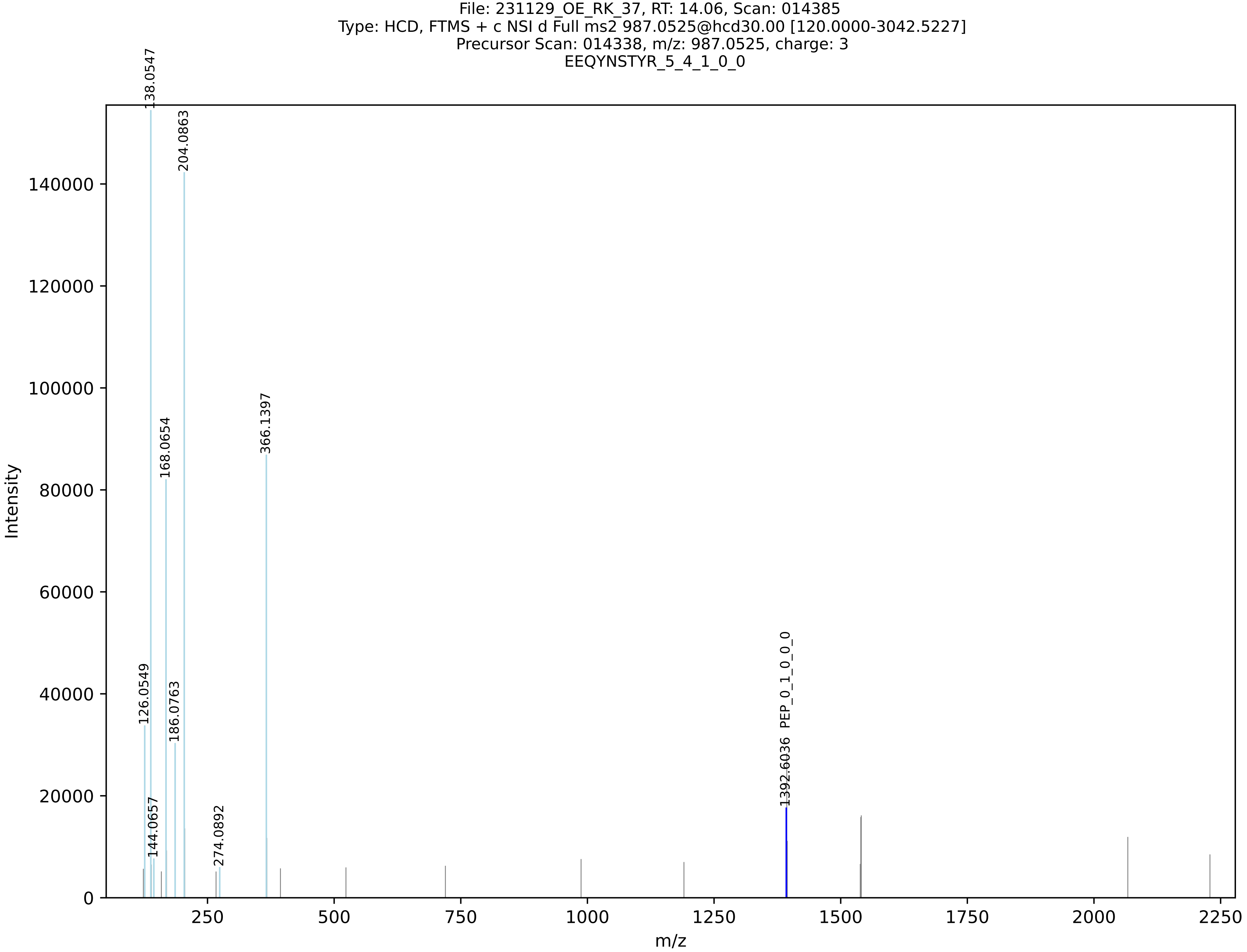
File: 231129_OE_RK_37, RT: 14.06, Scan: 014385
Type: HCD, FTMS + c NSI d Full ms2 987.0525@hcd30.00 [120.0000-3042.5227]
Precursor Scan: 014338, m/z: 987.0525, charge: 3
EEQYNSTYR_5_4_1_0_0
138.0547
204.0863
168.0654
366.1397
126.0549
186.0763
144.0657
274.0892
1392.6036 PEP_0_1_0_0_0
Intensity
m/z
0
20000
40000
60000
80000
100000
120000
140000
250
500
750
1000
1250
1500
1750
2000
2250

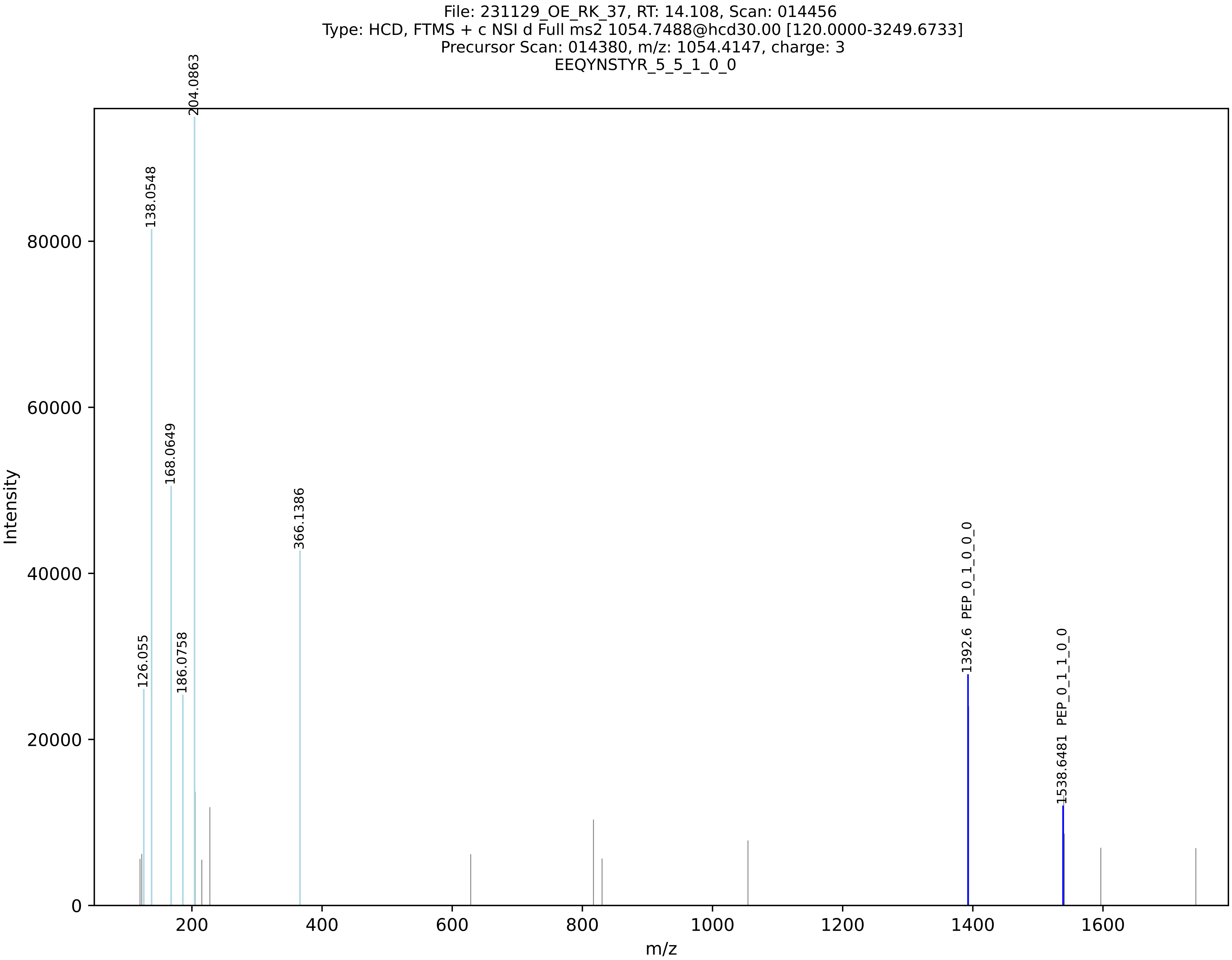
File: 231129_OE_RK_37, RT: 14.108, Scan: 014456
Type: HCD, FTMS + c NSI d Full ms2 1054.7488@hcd30.00 [120.0000-3249.6733]
Precursor Scan: 014380, m/z: 1054.4147, charge: 3
EEQYNSTYR_5_5_1_0_0
204.0863
138.0548
168.0649
366.1386
126.055
186.0758
1392.6 PEP_0_1_0_0_
1538.6481 PEP_0_1_1_0_0
Intensity
m/z
0
20000
40000
60000
80000
200
400
600
800
1000
1200
1400
1600

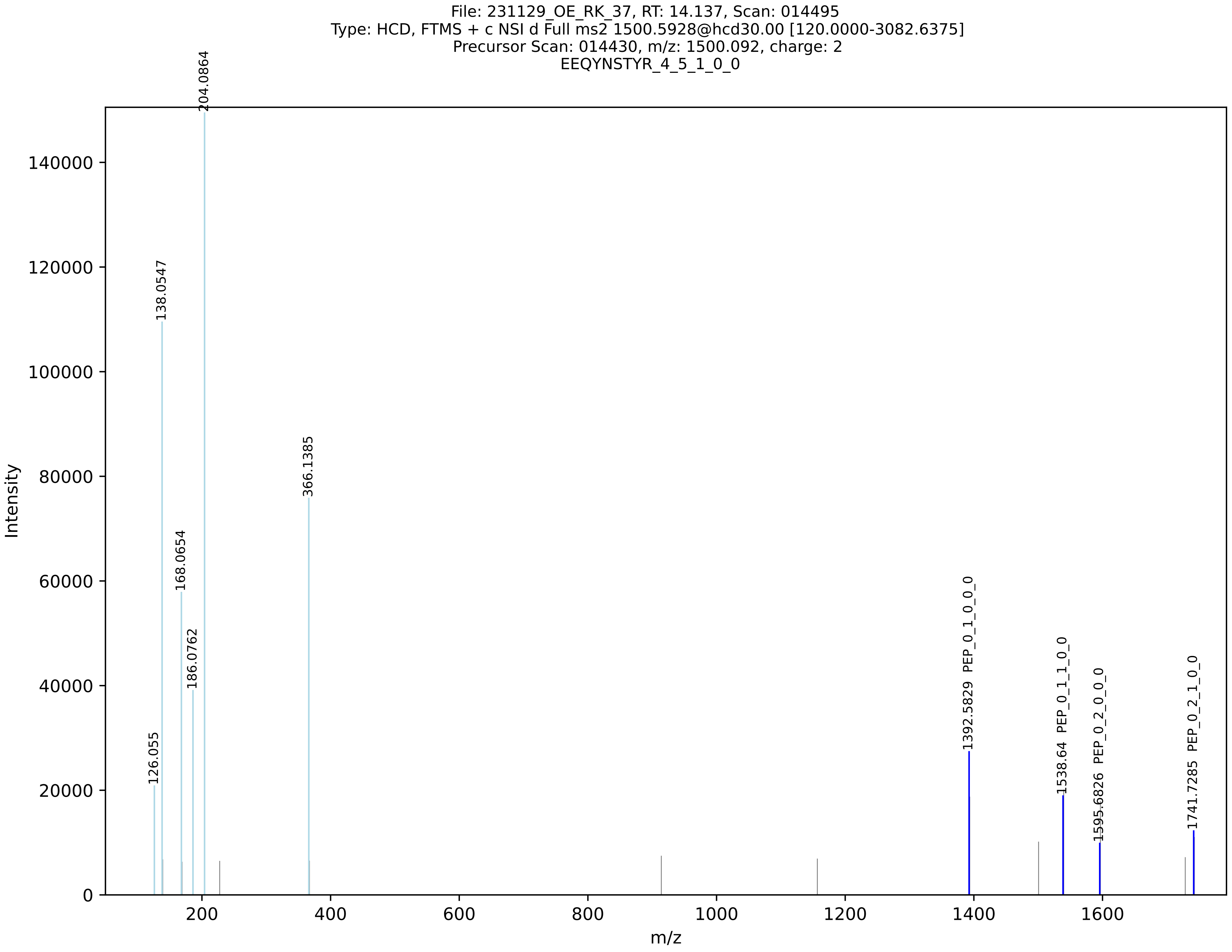
File: 231129_OE_RK_37, RT: 14.137, Scan: 014495
Type: HCD, FTMS + c NSI d Full ms2 1500.5928@hcd30.00 [120.0000-3082.6375]
Precursor Scan: 014430, m/z: 1500.092, charge: 2
EEQYNSTYR_4_5_1_0_0
Intensity
m/z
204.0864
138.0547
168.0654
186.0762
126.055
366.1385
1392.5829 PEP_0_1_0_0_0
1538.64 PEP_0_1_1_0_0
1595.6826 PEP_0_2_0_0_0
1741.7285 PEP_0_2_1_0_0
0
20000
40000
60000
80000
100000
120000
140000
200
400
600
800
1000
1200
1400
1600

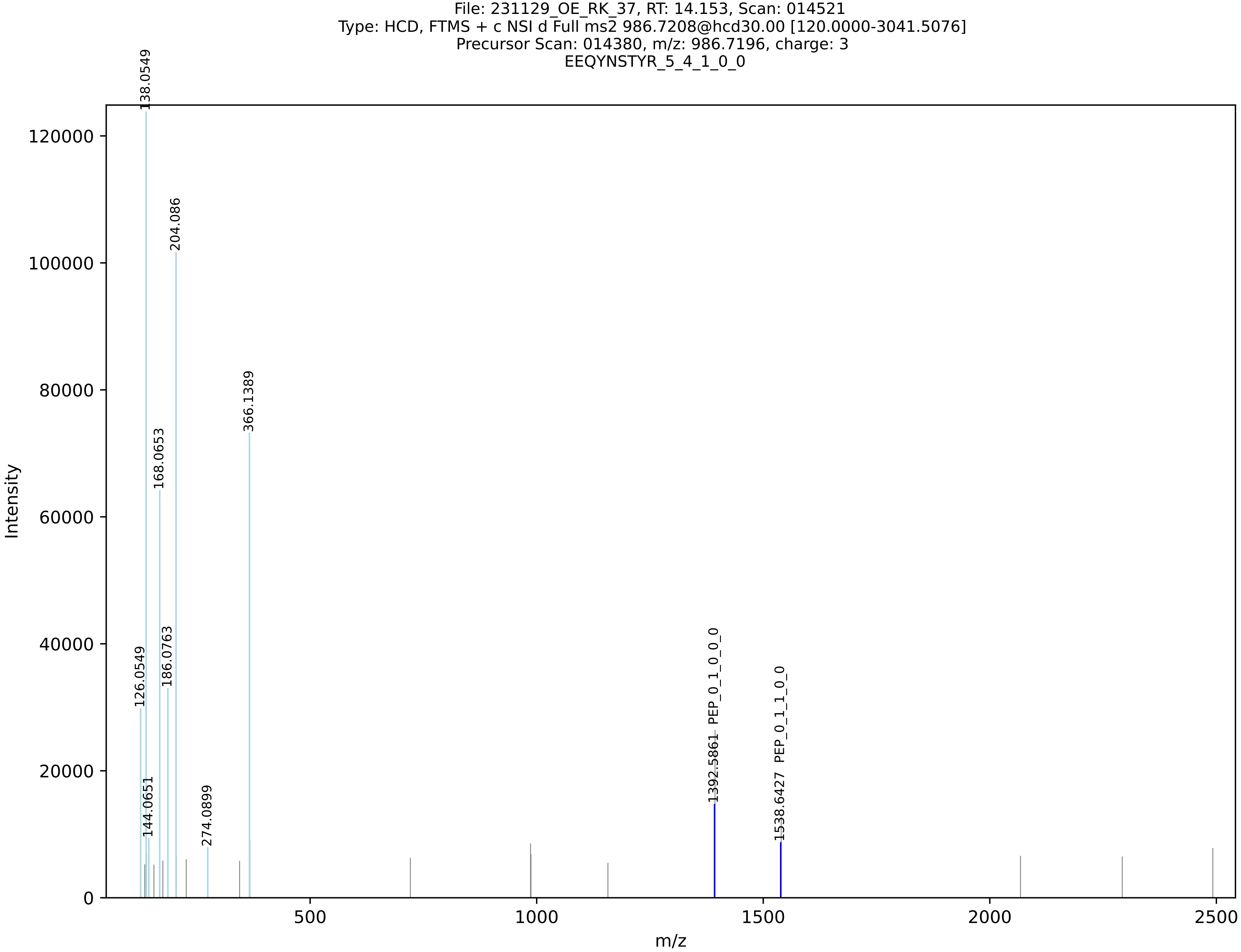
File: 231129_OE_RK_37, RT: 14.153, Scan: 014521
Type: HCD, FTMS + c NSI d Full ms2 986.7208@hcd30.00 [120.0000-3041.5076]
Precursor Scan: 014380, m/z: 986.7196, charge: 3
EEQYNSTYR_5_4_1_0_0
138.0549
204.086
366.1389
168.0653
126.0549
186.0763
144.0651
274.0899
1392.5861 PEP_0_1_0_0_0
1538.6427 PEP_0_1_1_0_0
Intensity
m/z
0
20000
40000
60000
80000
100000
120000
500
1000
1500
2000
2500

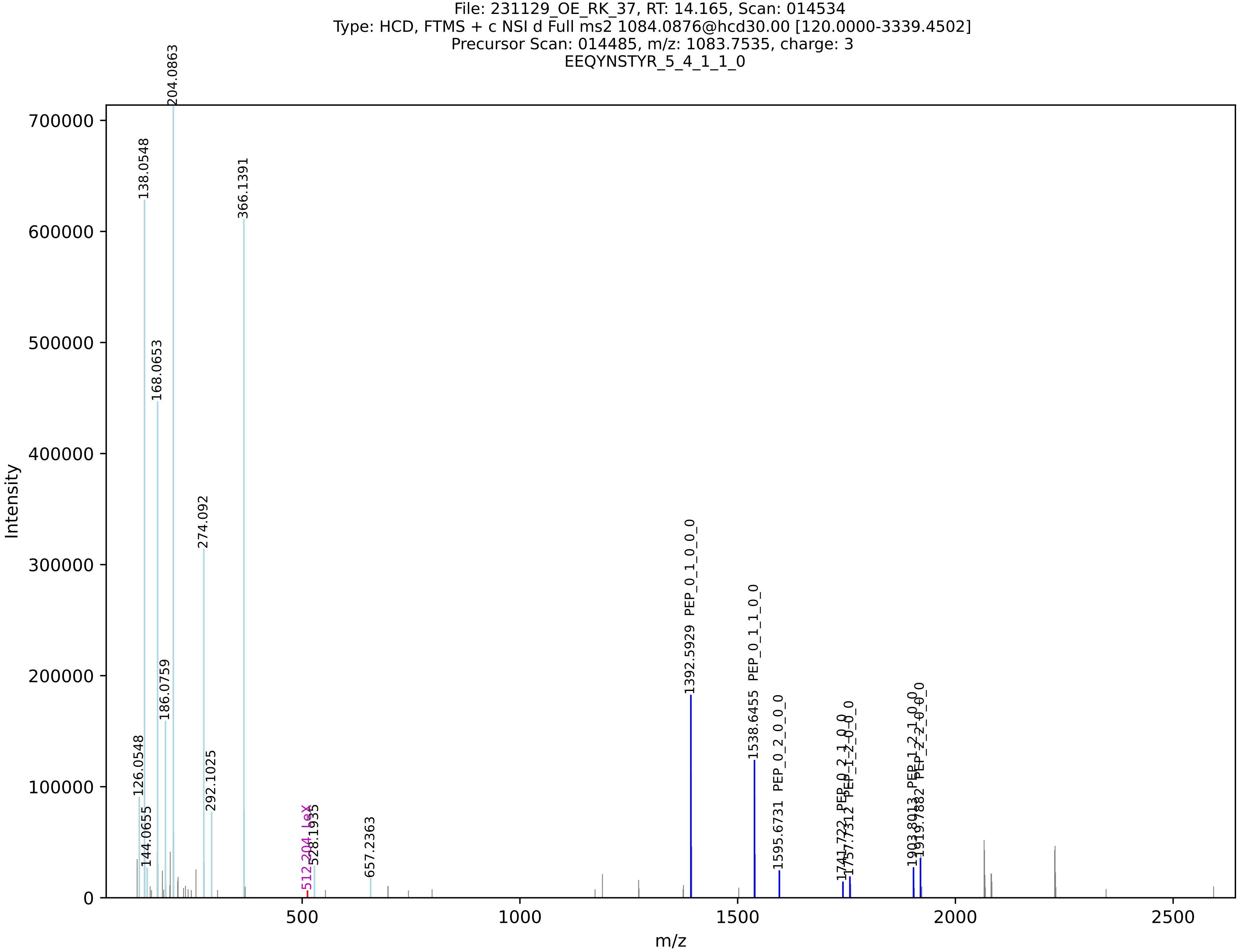
File: 231129_OE_RK_37, RT: 14.165, Scan: 014534
Type: HCD, FTMS + c NSI d Full ms2 1084.0876@hcd30.00 [120.0000-3339.4502]
Precursor Scan: 014485, m/z: 1083.7535, charge: 3
EEQYNSTYR_5_4_1_1_0
Intensity
m/z
204.0863
138.0548
366.1391
168.0653
274.092
186.0759
126.0548
292.1025
144.0655
512.204+ LeX
528.1935
657.2363
1392.5929 PEP_0_1_0_0_0
1538.6455 PEP_0_1_1_0_0
1595.6731 PEP_0_2_0_0_0
1741.722 PEP_0_2_1_0_0
1757.7312 PEP_1_2_0_0_0
1903.8013 PEP_1_2_1_0_0
1919.7882 PEP_2_2_0_0_0

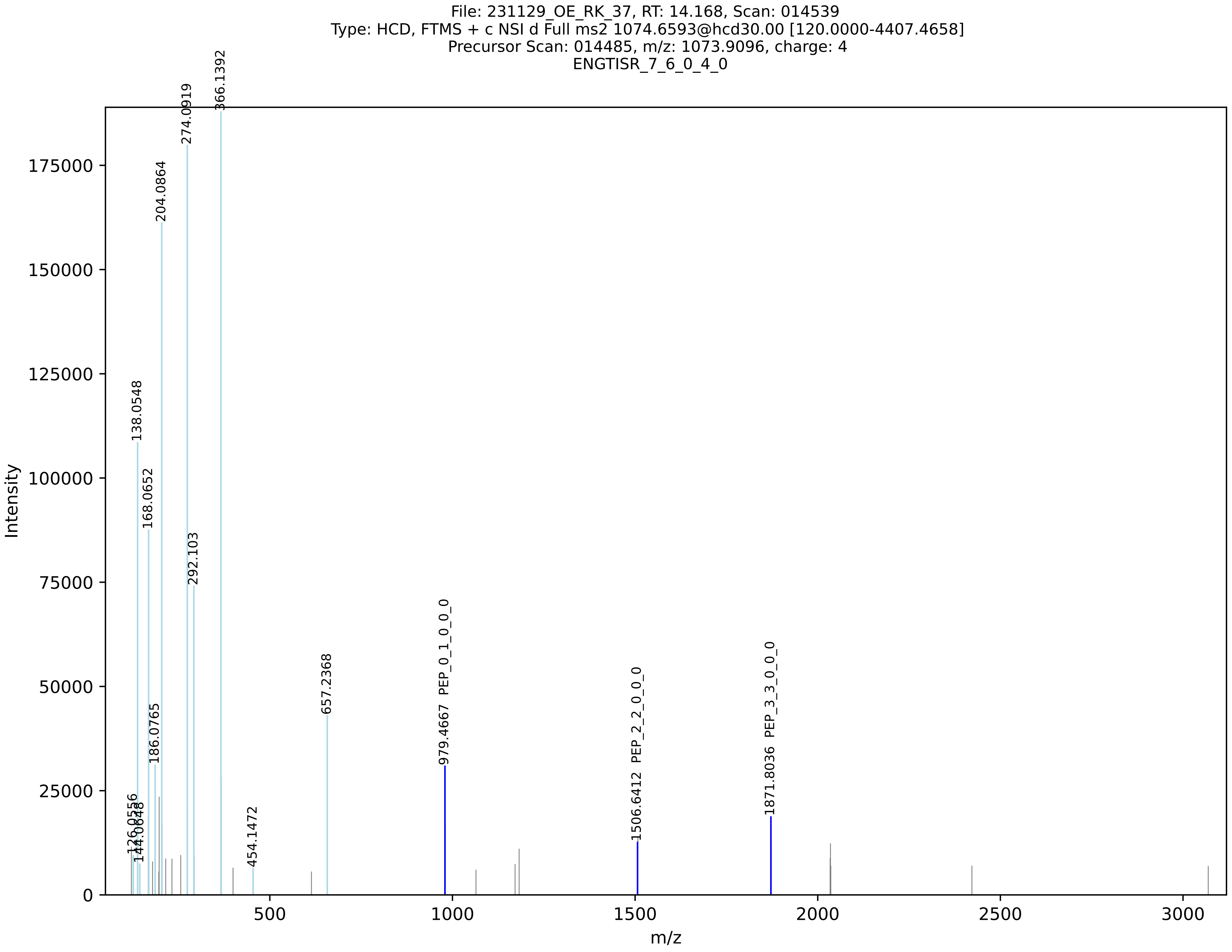
File: 231129_OE_RK_37, RT: 14.168, Scan: 014539
Type: HCD, FTMS + c NSI d Full ms2 1074.6593@hcd30.00 [120.0000-4407.4658]
Precursor Scan: 014485, m/z: 1073.9096, charge: 4
ENGTISR_7_6_0_4_0
Intensity
m/z
366.1392
274.0919
204.0864
138.0548
168.0652
292.103
186.0765
126.0556
144.0648
454.1472
657.2368
979.4667 PEP_0_1_0_0_0
1506.6412 PEP_2_2_0_0_0
1871.8036 PEP_3_3_0_0_0
0
25000
50000
75000
100000
125000
150000
175000
500
1000
1500
2000
2500
3000

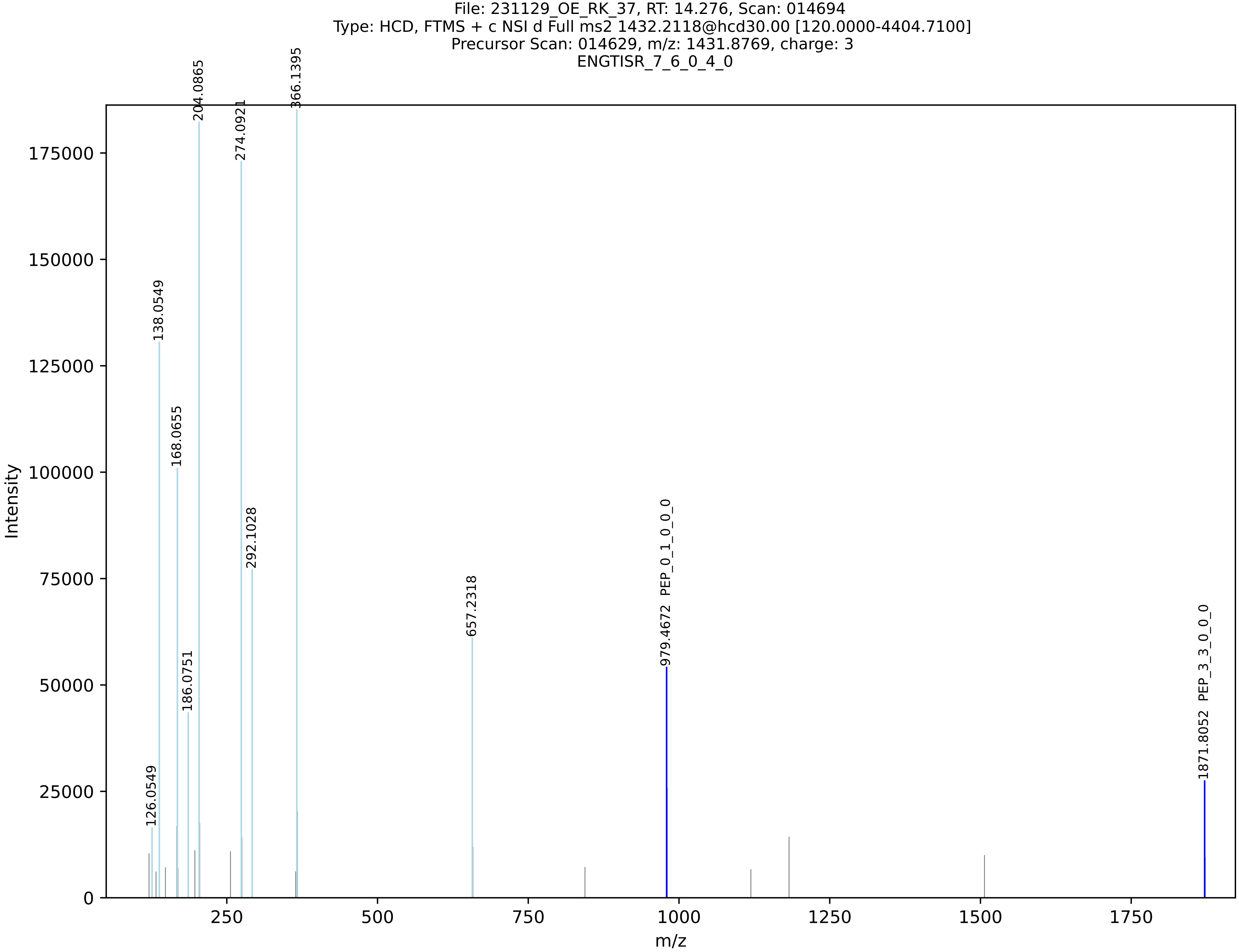

File: 231129_OE_RK_37, RT: 14.276, Scan: 014694
Type: HCD, FTMS + c NSI d Full ms2 1432.2118@hcd30.00 [120.0000-4404.7100]
Precursor Scan: 014629, m/z: 1431.8769, charge: 3
ENGTISR_7_6_0_4_0
Intensity
m/z
0
25000
50000
75000
100000
125000
150000
175000
250
500
750
1000
1250
1500
1750
126.0549
138.0549
168.0655
186.0751
204.0865
274.0921
292.1028
366.1395
657.2318
979.4672 PEP_0_1_0_0_0
1871.8052 PEP_3_3_0_0_0

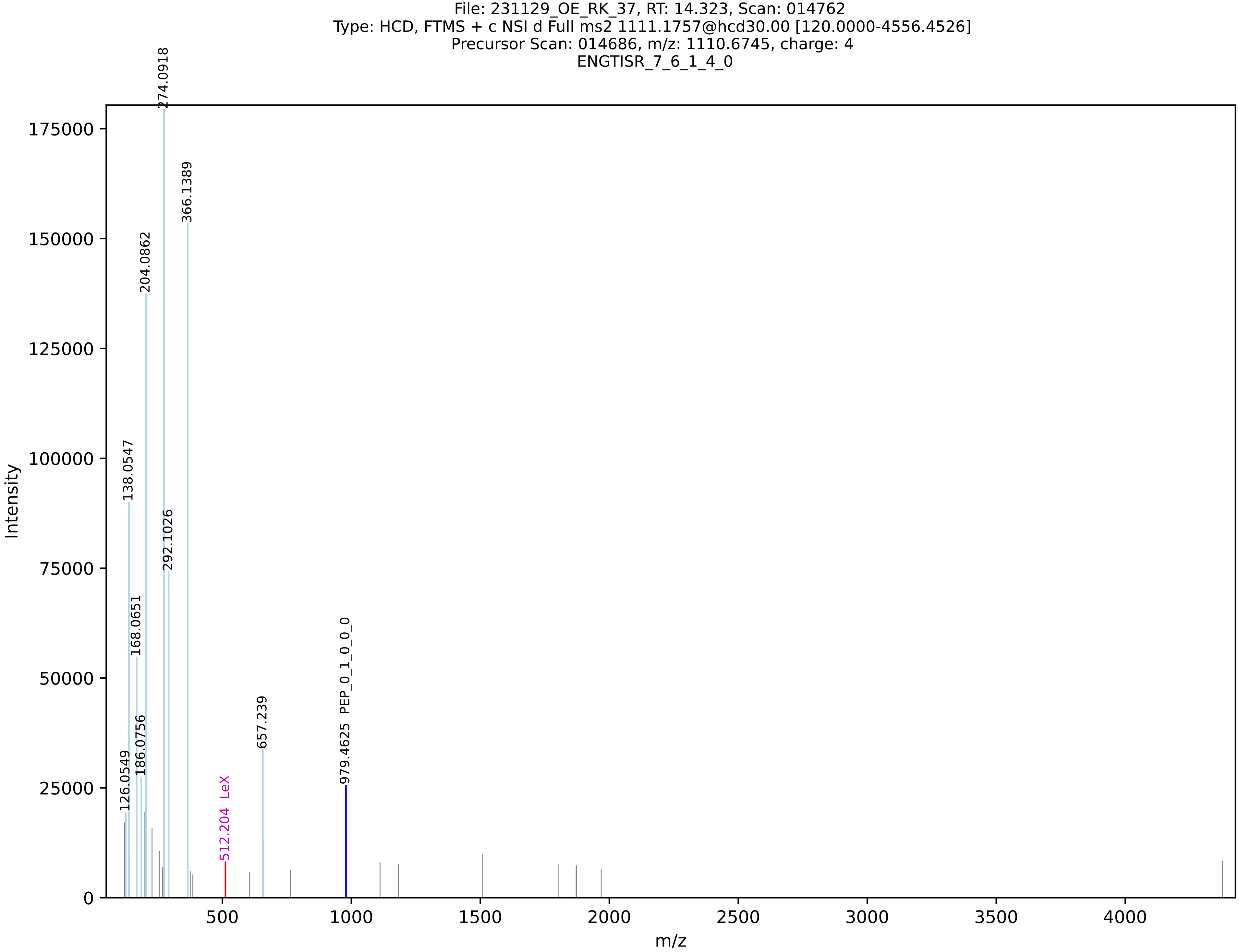
File: 231129_OE_RK_37, RT: 14.323, Scan: 014762
Type: HCD, FTMS + c NSI d Full ms2 1111.1757@hcd30.00 [120.0000-4556.4526]
Precursor Scan: 014686, m/z: 1110.6745, charge: 4
ENGTISR_7_6_1_4_0
Intensity
m/z
274.0918
366.1389
204.0862
138.0547
292.1026
168.0651
186.0756
126.0549
512.204 LeX
657.239
979.4625 PEP_0_1_0_0_0
0
25000
50000
75000
100000
125000
150000
175000
500
1000
1500
2000
2500
3000
3500
4000

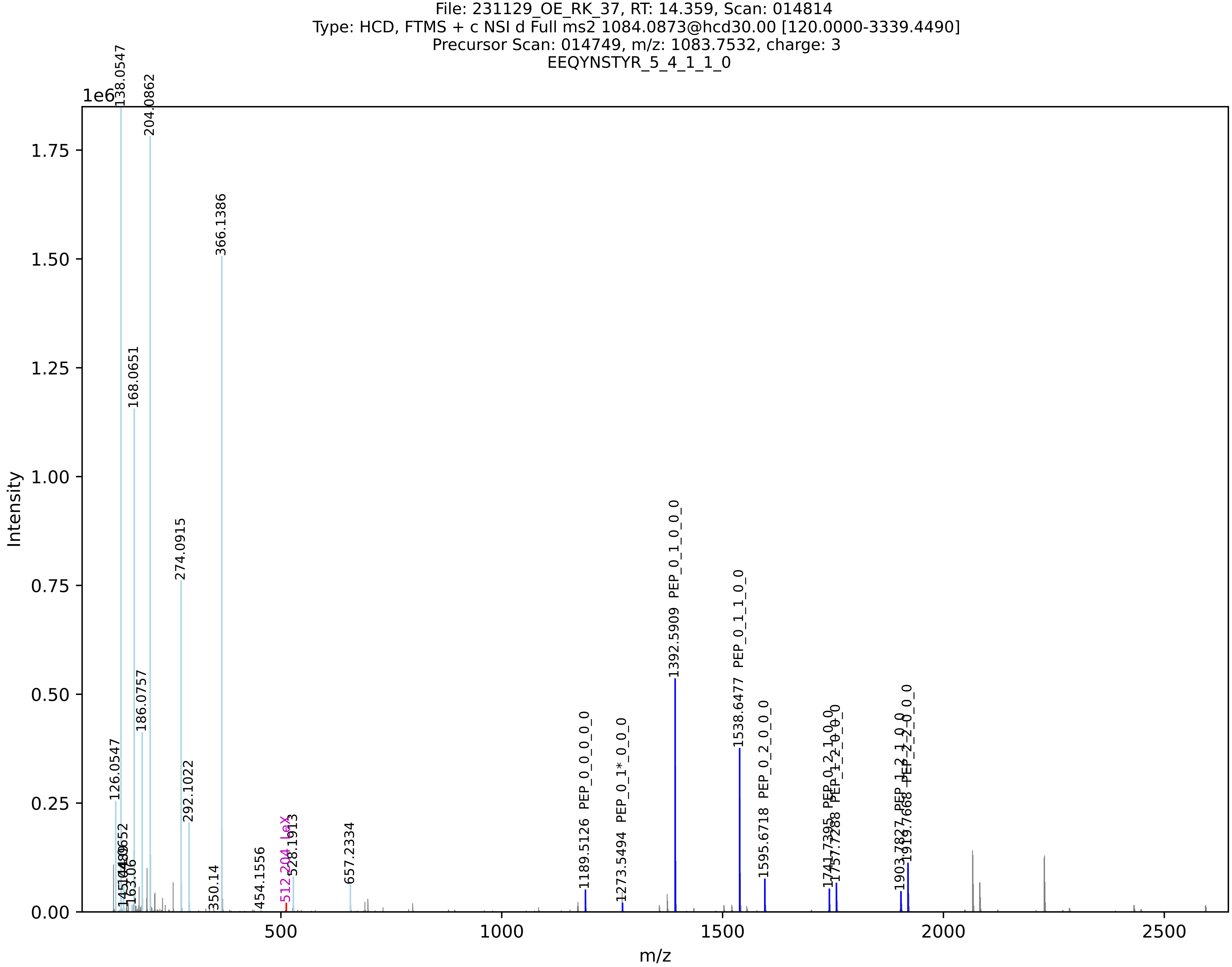
File: 231129_OE_RK_37, RT: 14.359, Scan: 014814
Type: HCD, FTMS + c NSI d Full ms2 1084.0873@hcd30.00 [120.0000-3339.4490]
Precursor Scan: 014749, m/z: 1083.7532, charge: 3
EEQYNSTYR_5_4_1_1_0
1e6
Intensity
m/z
138.0547
204.0862
366.1386
168.0651
274.0915
186.0757
126.0547
292.1022
163.06
350.14
454.1556
512.204 LeX
528.1913
657.2334
1189.5126 PEP_0_0_0_0_0
1273.5494 PEP_0_1* 0_0_0
1392.5909 PEP_0_1_0_0_0
1538.6477 PEP_0_1_1_0_0
1595.6718 PEP_0_2_0_0_0
1741.7395 PEP_0_2_1_0_0
1757.7288 PEP_1_2_0_0_0
1903.7827 PEP_1_2_1_0_0
1919.7668 PEP_2_2_0_0_0

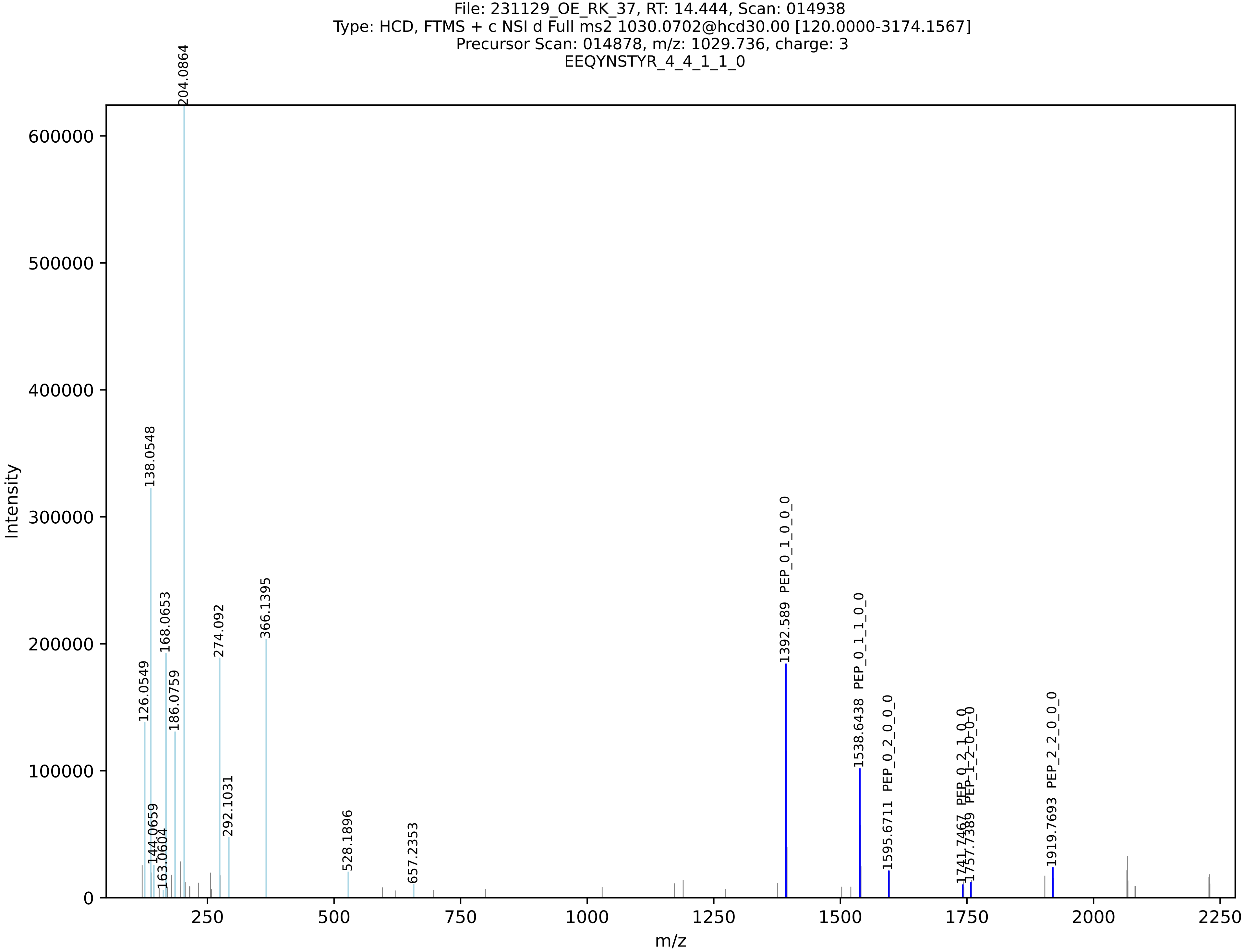
File: 231129_OE_RK_37, RT: 14.444, Scan: 014938
Type: HCD, FTMS + c NSI d Full ms2 1030.0702@hcd30.00 [120.0000-3174.1567]
Precursor Scan: 014878, m/z: 1029.736, charge: 3
EEQYNSTYR_4_4_1_1_0
Intensity
m/z
204.0864
138.0548
126.0549
168.0653
186.0759
144.0659
163.0604
274.092
292.1031
366.1395
528.1896
657.2353
1392.589 PEP_0_1_0_0_0
1538.6438 PEP_0_1_1_0_0
1595.6711 PEP_0_2_0_0_0
1741.7467 PEP_0_2_1_0_0
1757.7389 PEP_1_2_0_0_0
1919.7693 PEP_2_2_0_0_0

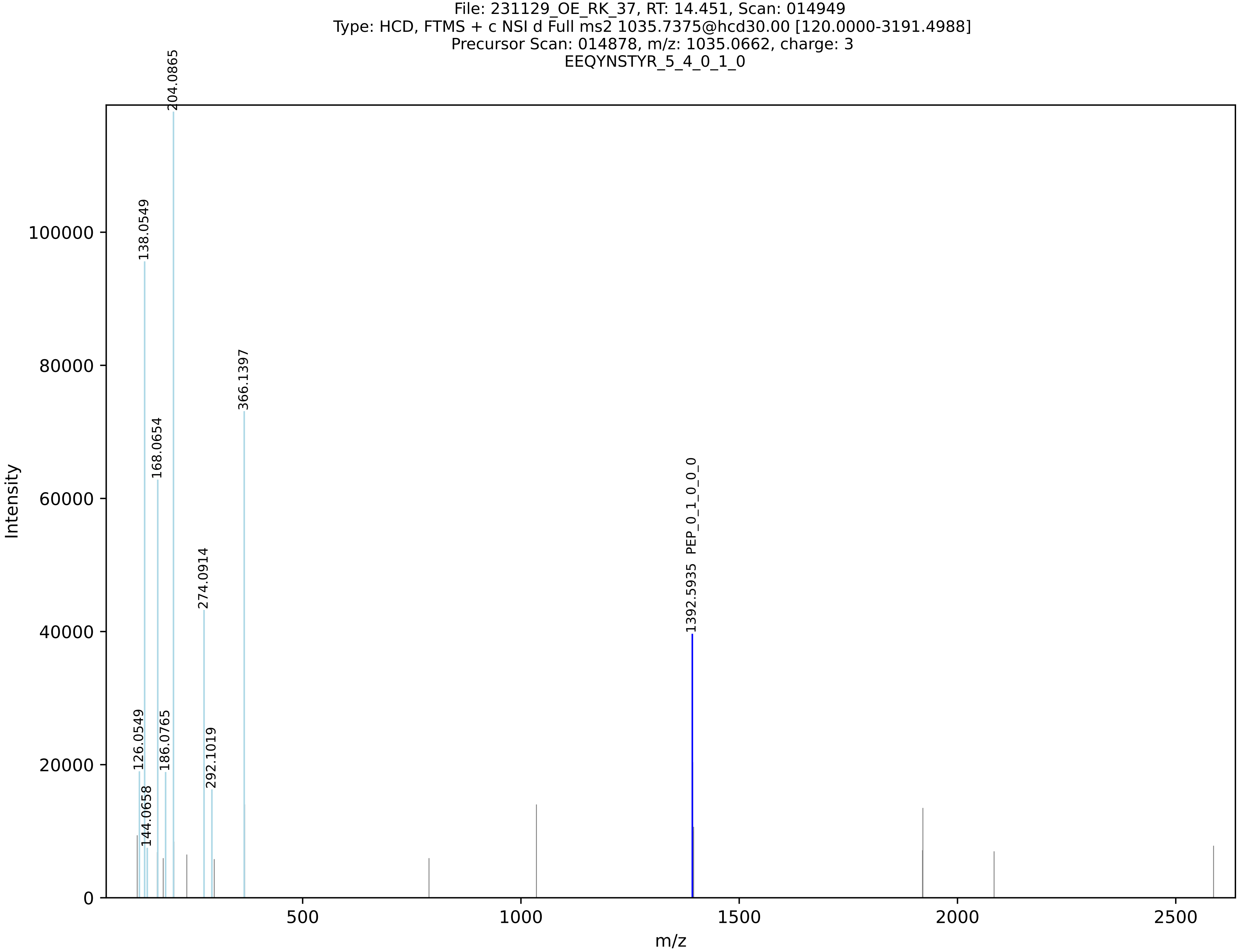

File: 231129_OE_RK_37, RT: 14.451, Scan: 014949
Type: HCD, FTMS + c NSI d Full ms2 1035.7375@hcd30.00 [120.0000-3191.4988]
Precursor Scan: 014878, m/z: 1035.0662, charge: 3
EEQYNSTYR_5_4_0_1_0
204.0865
138.0549
366.1397
168.0654
1392.5935 PEP_0_1_0_0_0
274.0914
126.0549
186.0765
292.1019
144.0658
Intensity
100000
80000
60000
40000
20000
0
500
1000
1500
2000
2500
m/z

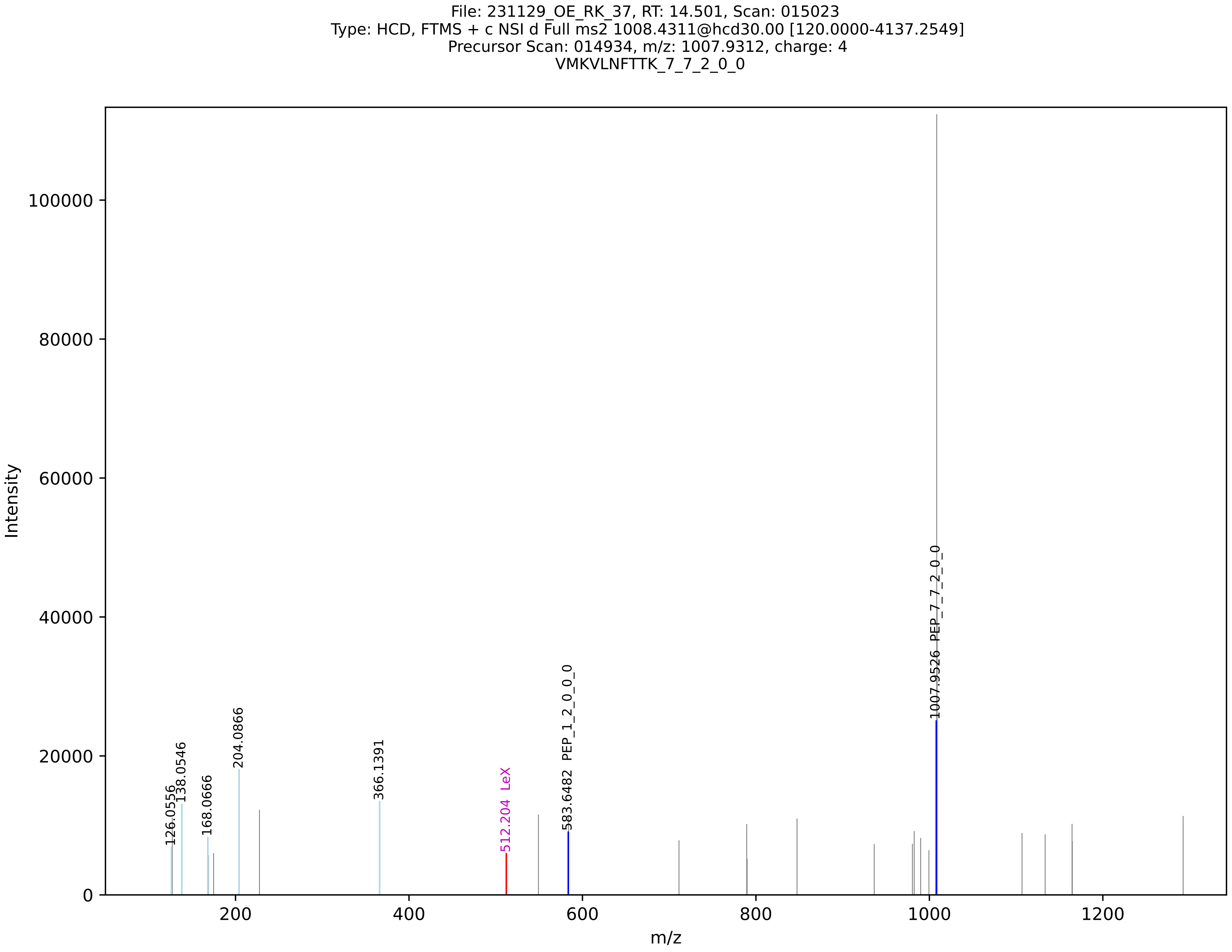
File: 231129_OE_RK_37, RT: 14.501, Scan: 015023
Type: HCD, FTMS + c NSI d Full ms2 1008.4311@hcd30.00 [120.0000-4137.2549]
Precursor Scan: 014934, m/z: 1007.9312, charge: 4
VMKVLNFTTK_7_7_2_0_0
Intensity
m/z
0
20000
40000
60000
80000
100000
200
400
600
800
1000
1200
126.0556
138.0546
168.0666
204.0866
366.1391
512.204 LeX
583.6482 PEP_1_2_0_0_0
1007.9526 PEP_7_7_2_0_0

File: 231129_OE_RK_37, RT: 14.542, Scan: 015082
Type: HCD, FTMS + c NSI d Full ms2 1480.8994@hcd30.00 [120.0000-4553.6943]
Precursor Scan: 014997, m/z: 1480.2312, charge: 3
ENGTISR_7_6_1_4_0

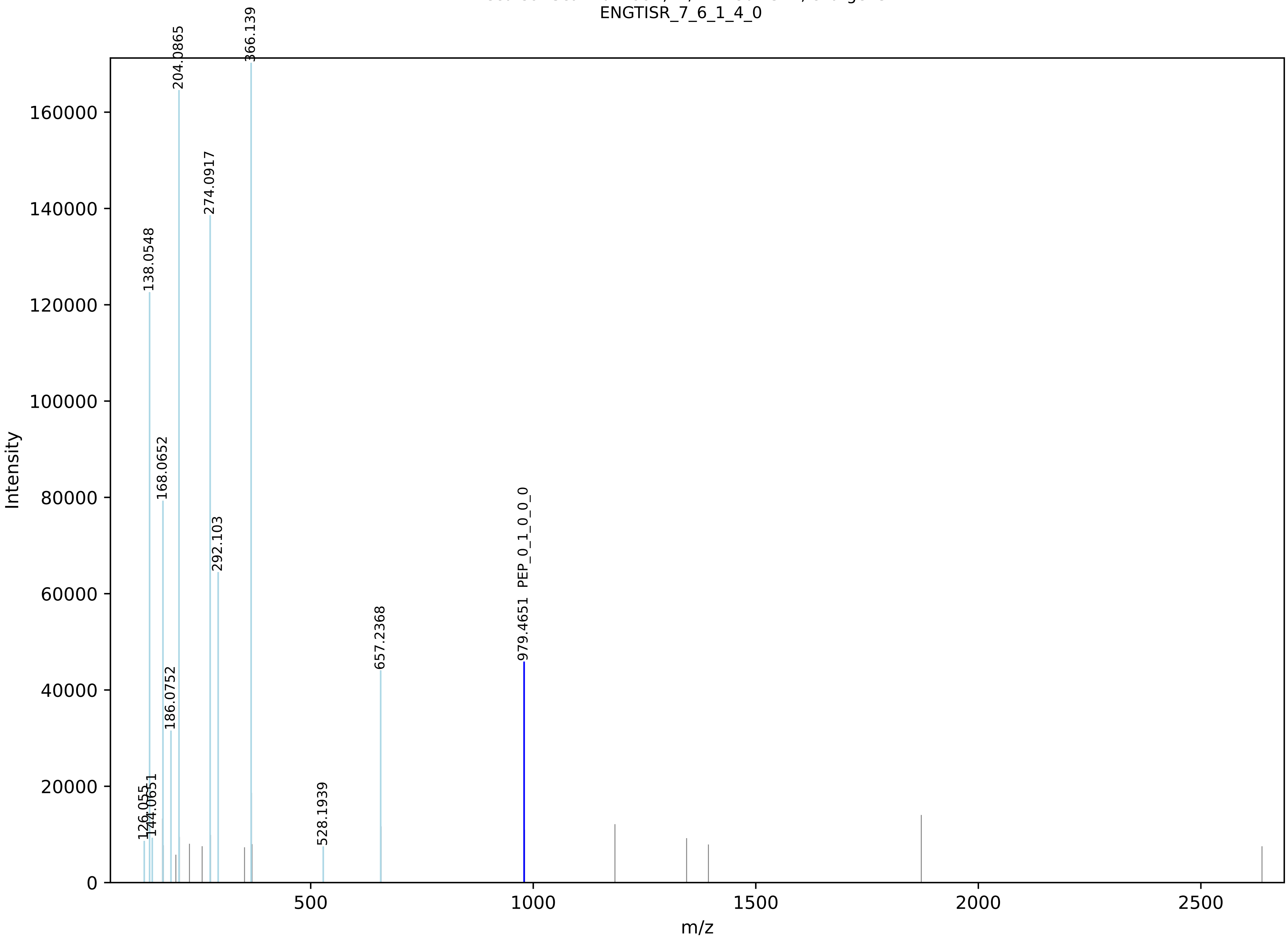

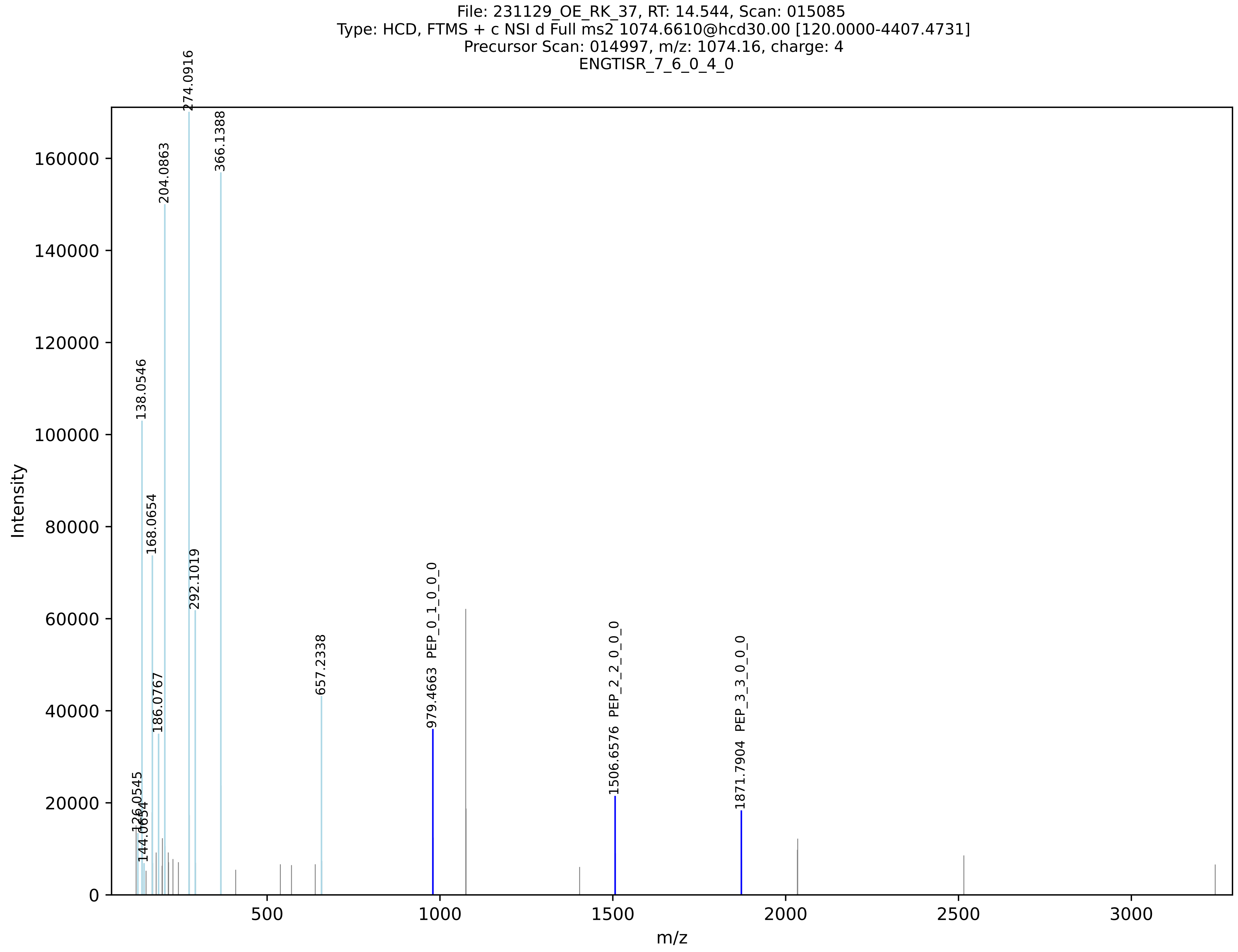
File: 231129_OE_RK_37, RT: 14.544, Scan: 015085
Type: HCD, FTMS + c NSI d Full ms2 1074.6610@hcd30.00 [120.0000-4407.4731]
Precursor Scan: 014997, m/z: 1074.16, charge: 4
ENGTISR_7_6_0_4_0
Intensity
m/z
274.0916
366.1388
204.0863
138.0546
168.0654
292.1019
186.0767
126.0545
144.0654
657.2338
979.4663 PEP_0_1_0_0_0
1506.6576 PEP_2_2_0_0_0
1871.7904 PEP_3_3_0_0_0

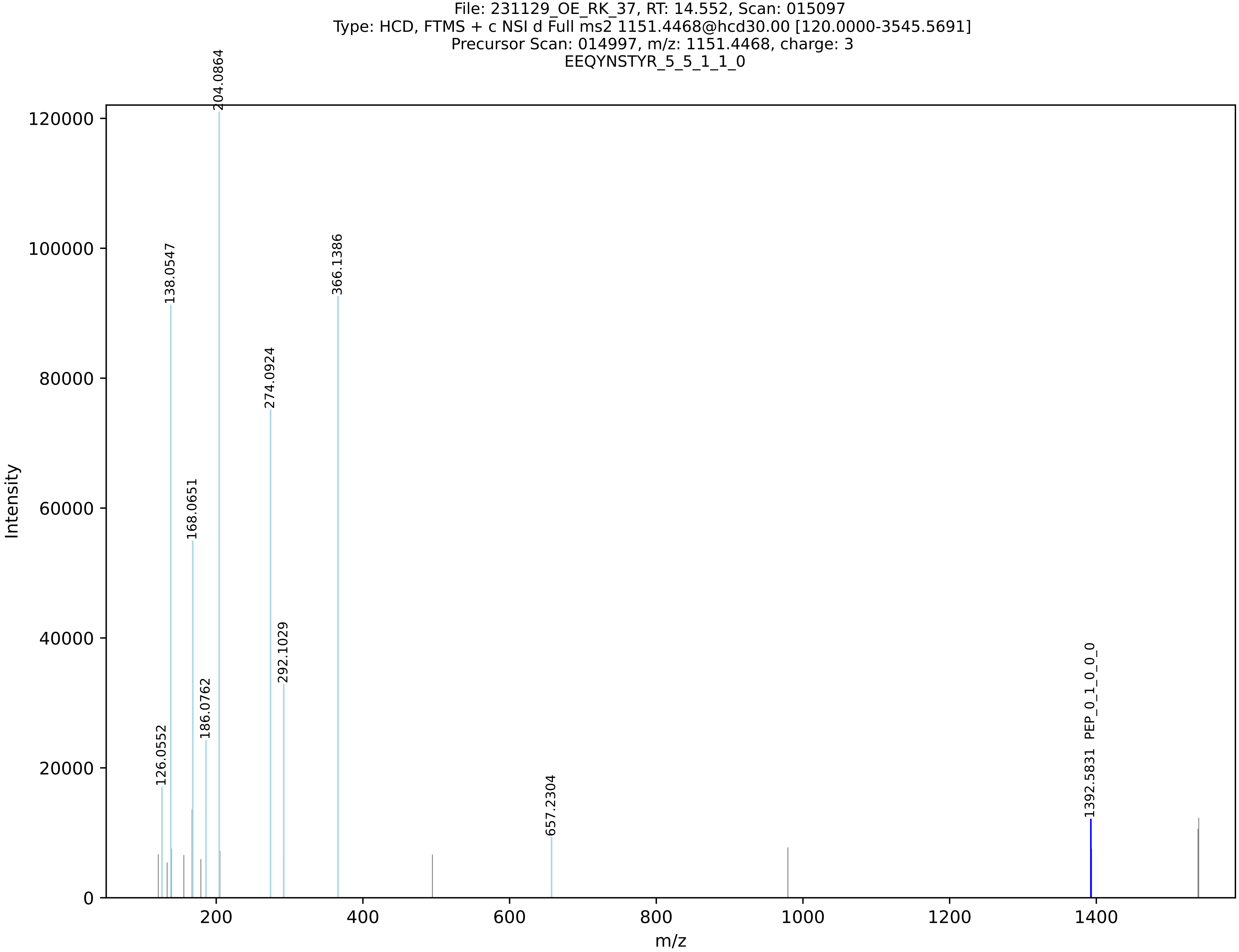
File: 231129_OE_RK_37, RT: 14.552, Scan: 015097
Type: HCD, FTMS + c NSI d Full ms2 1151.4468@hcd30.00 [120.0000-3545.5691]
Precursor Scan: 014997, m/z: 1151.4468, charge: 3
EEQYNSTYR_5_5_1_1_0
Intensity
m/z
204.0864
138.0547
366.1386
274.0924
168.0651
292.1029
186.0762
126.0552
657.2304
1392.5831 PEP_0_1_0_0_0
0
20000
40000
60000
80000
100000
120000
200
400
600
800
1000
1200
1400

File: 231129_OE_RK_37, RT: 14.593, Scan: 015157
Type: HCD, FTMS + c NSI d Full ms2 1545.1034@hcd30.00 [120.0000-3173.4390]
Precursor Scan: 015070, m/z: 1544.1011, charge: 2
EEQYNSTYR_4_4_1_1_0

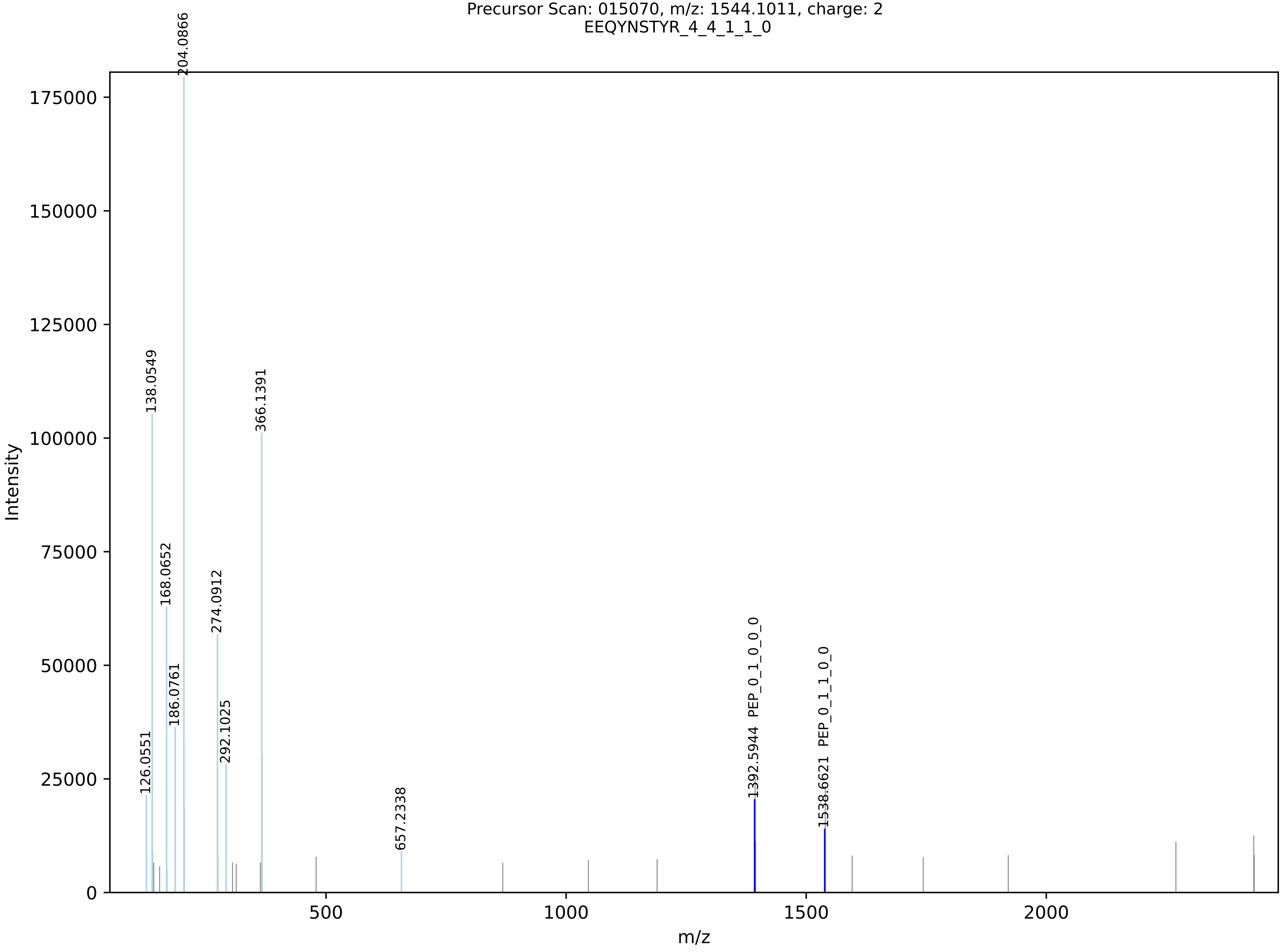

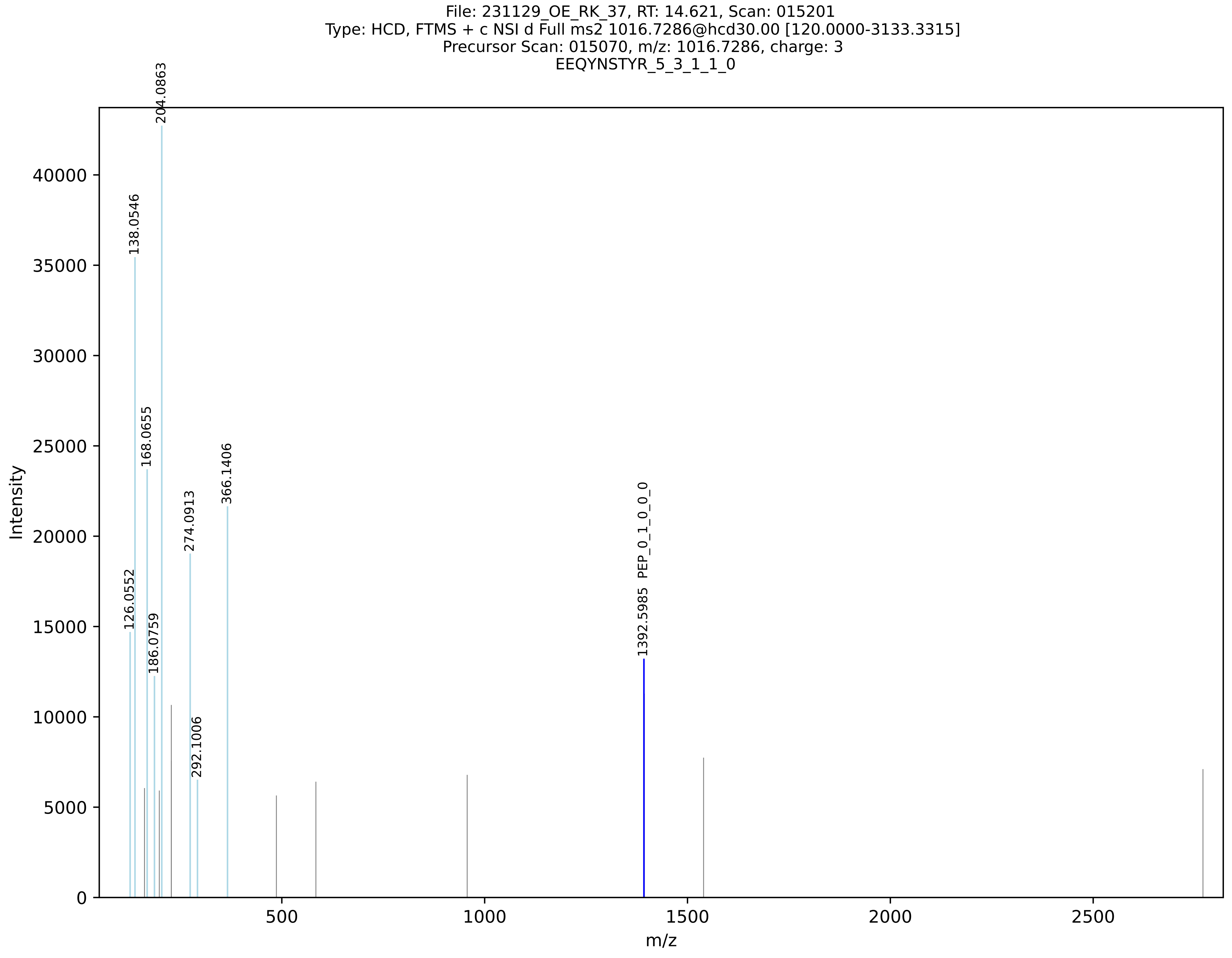
File: 231129_OE_RK_37, RT: 14.621, Scan: 015201
Type: HCD, FTMS + c NSI d Full ms2 1016.7286@hcd30.00 [120.0000-3133.3315]
Precursor Scan: 015070, m/z: 1016.7286, charge: 3
EEQYNSTYR_5_3_1_1_0
Intensity
m/z
0
5000
10000
15000
20000
25000
30000
35000
40000
500
1000
1500
2000
2500
204.0863
138.0546
168.0655
366.1406
274.0913
126.0552
186.0759
292.1006
1392.5985 PEP_0_1_0_0_0

File: 231129_OE_RK_37, RT: 14.642, Scan: 015228
Type: HCD, FTMS + c NSI d Full ms2 1035.4008@hcd30.00 [120.0000-3190.4683]
Precursor Scan: 015144, m/z: 1035.0677, charge: 3
EEQYNSTYR_5_4_0_1_0

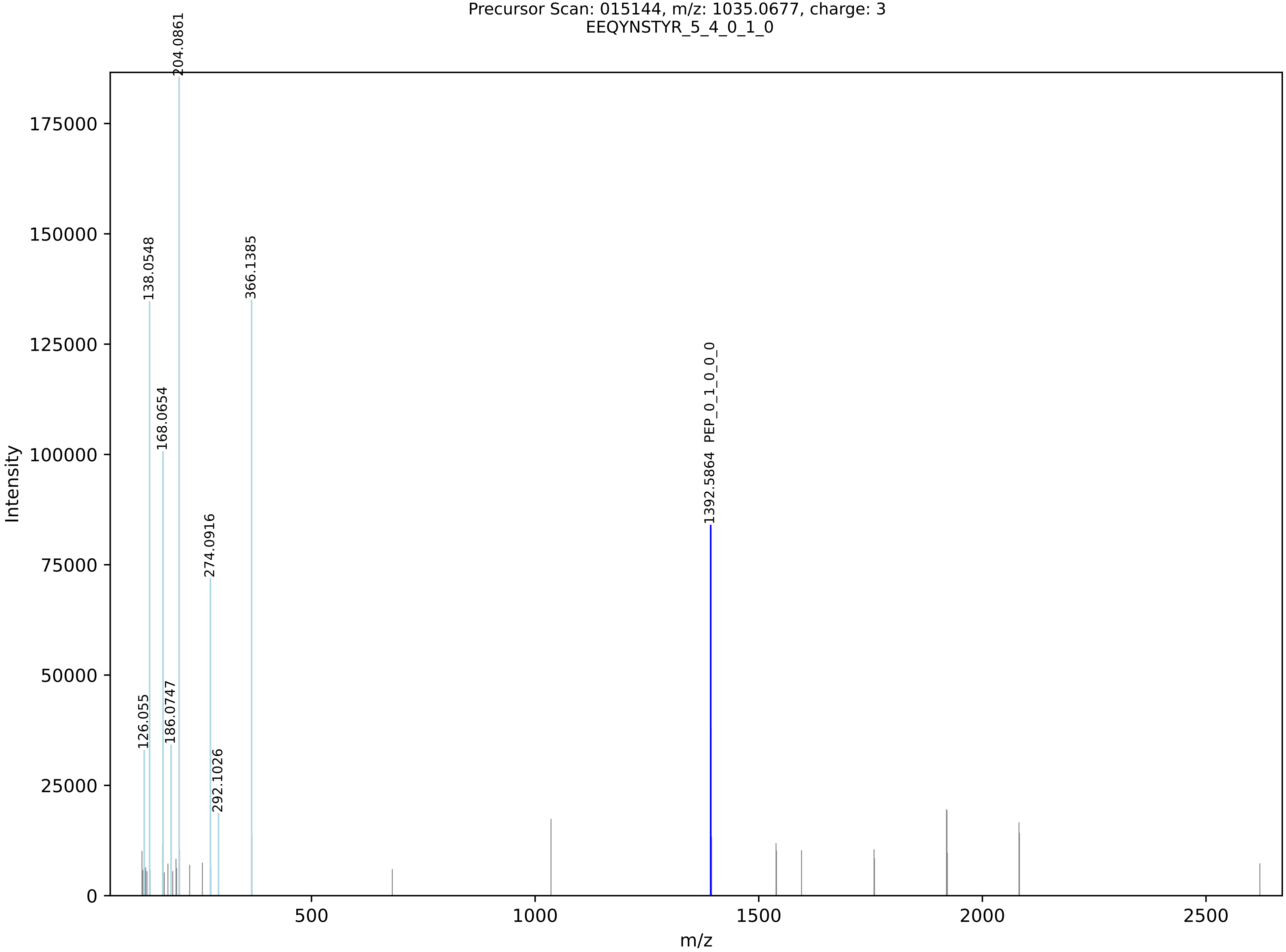

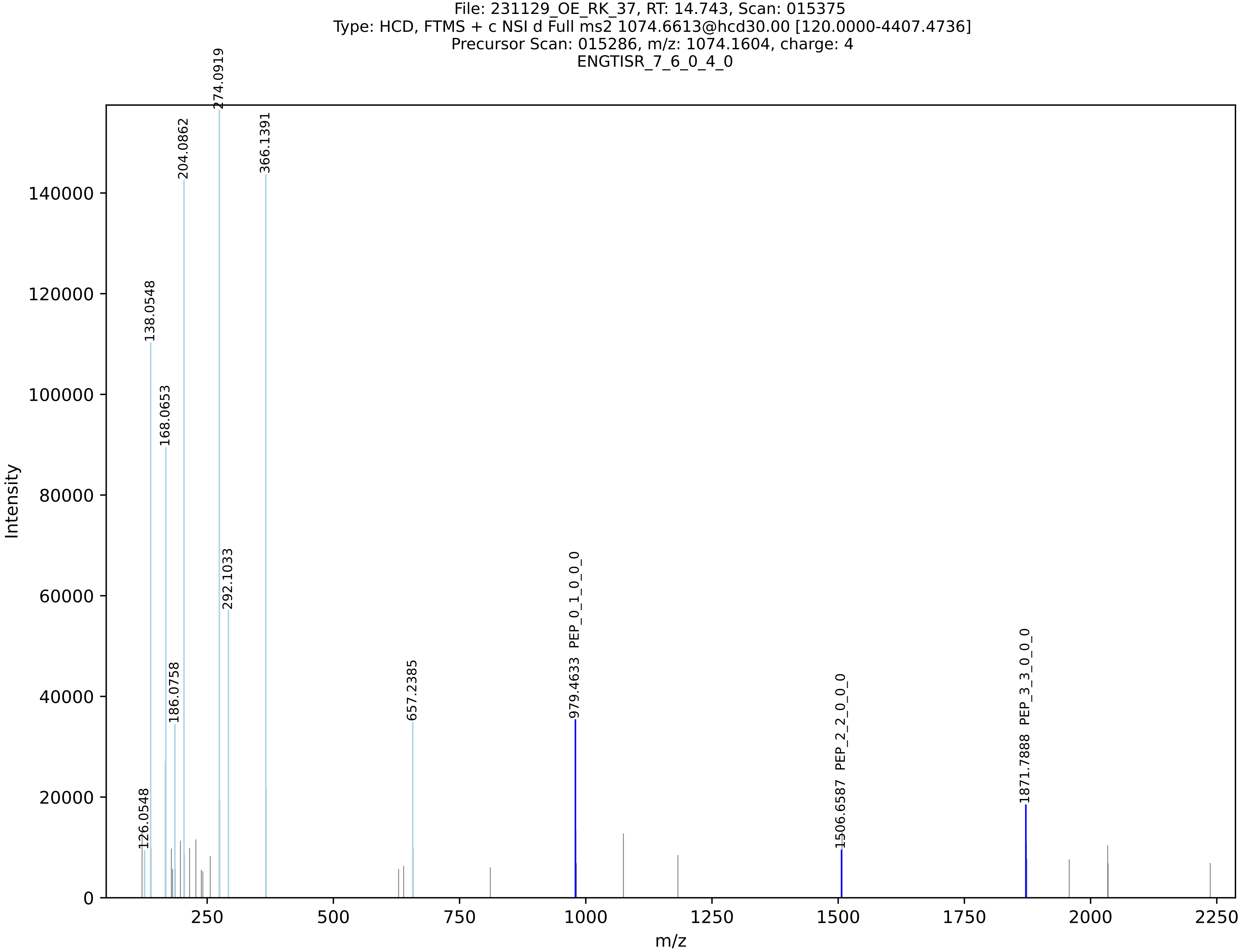
File: 231129_OE_RK_37, RT: 14.743, Scan: 015375
Type: HCD, FTMS + c NSI d Full ms2 1074.6613@hcd30.00 [120.0000-4407.4736]
Precursor Scan: 015286, m/z: 1074.1604, charge: 4
ENGTISR_7_6_0_4_0
Intensity
m/z
274.0919
366.1391
204.0862
138.0548
168.0653
292.1033
186.0758
126.0548
657.2385
979.4633 PEP_0_1_0_0_0
1506.6587 PEP_2_2_0_0_0
1871.7888 PEP_3_3_0_0_0
0
20000
40000
60000
80000
100000
120000
140000
250
500
750
1000
1250
1500
1750
2000
2250

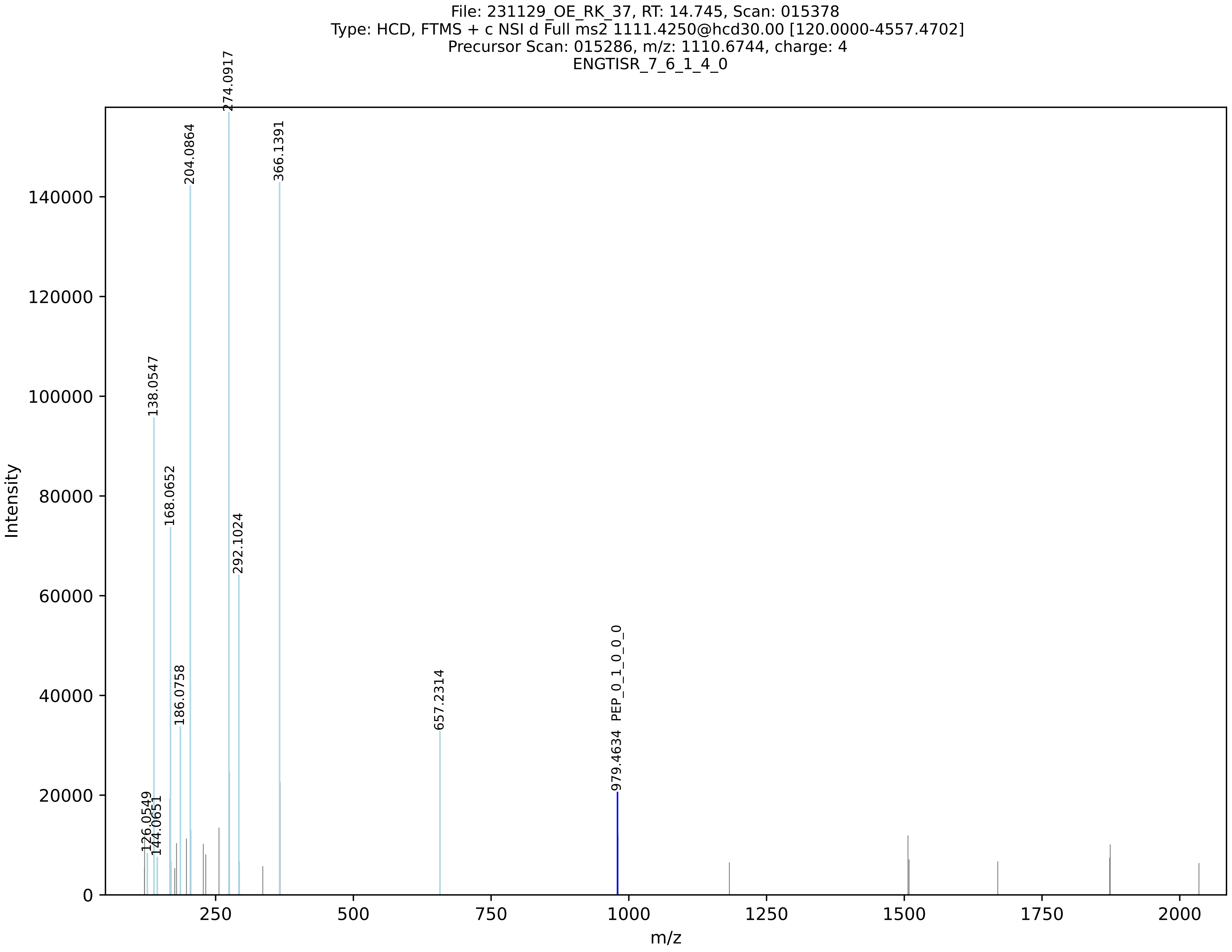
File: 231129_OE_RK_37, RT: 14.745, Scan: 015378
Type: HCD, FTMS + c NSI d Full ms2 1111.4250@hcd30.00 [120.0000-4557.4702]
Precursor Scan: 015286, m/z: 1110.6744, charge: 4
ENGTISR_7_6_1_4_0
Intensity
m/z
274.0917
204.0864
366.1391
138.0547
168.0652
292.1024
186.0758
657.2314
979.4634 PEP_0_1_0_0_0
126.0549
144.0651
0
20000
40000
60000
80000
100000
120000
140000
250
500
750
1000
1250
1500
1750
2000

File: 231129_OE_RK_37, RT: 14.8, Scan: 015460
Type: HCD, FTMS + c NSI d Full ms2 825.3851@hcd30.00 [120.0000-3390.4270]
Precursor Scan: 015358, m/z: 825.1332, charge: 4
LPPGLLANFTLLR_6_3_0_1_0

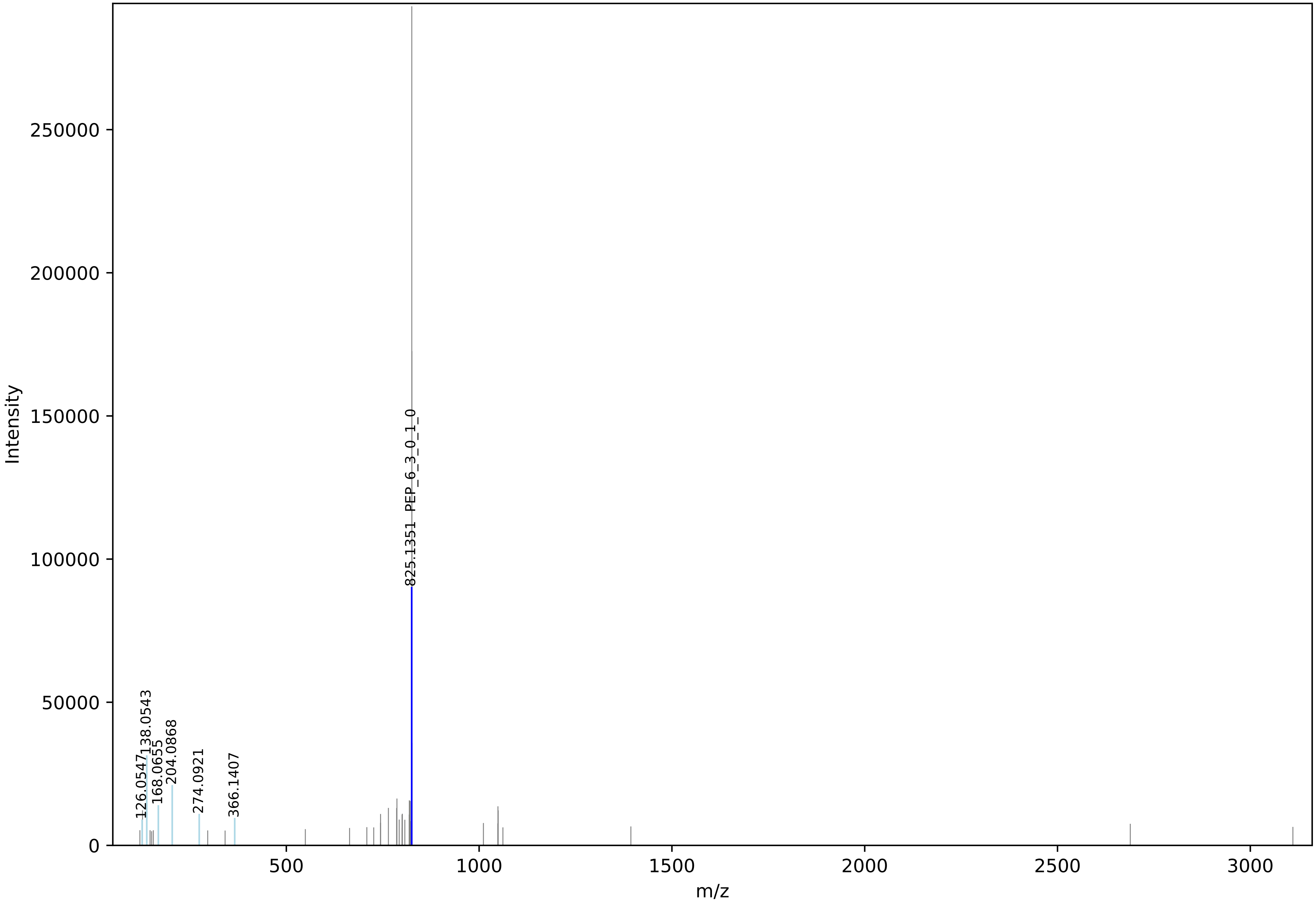

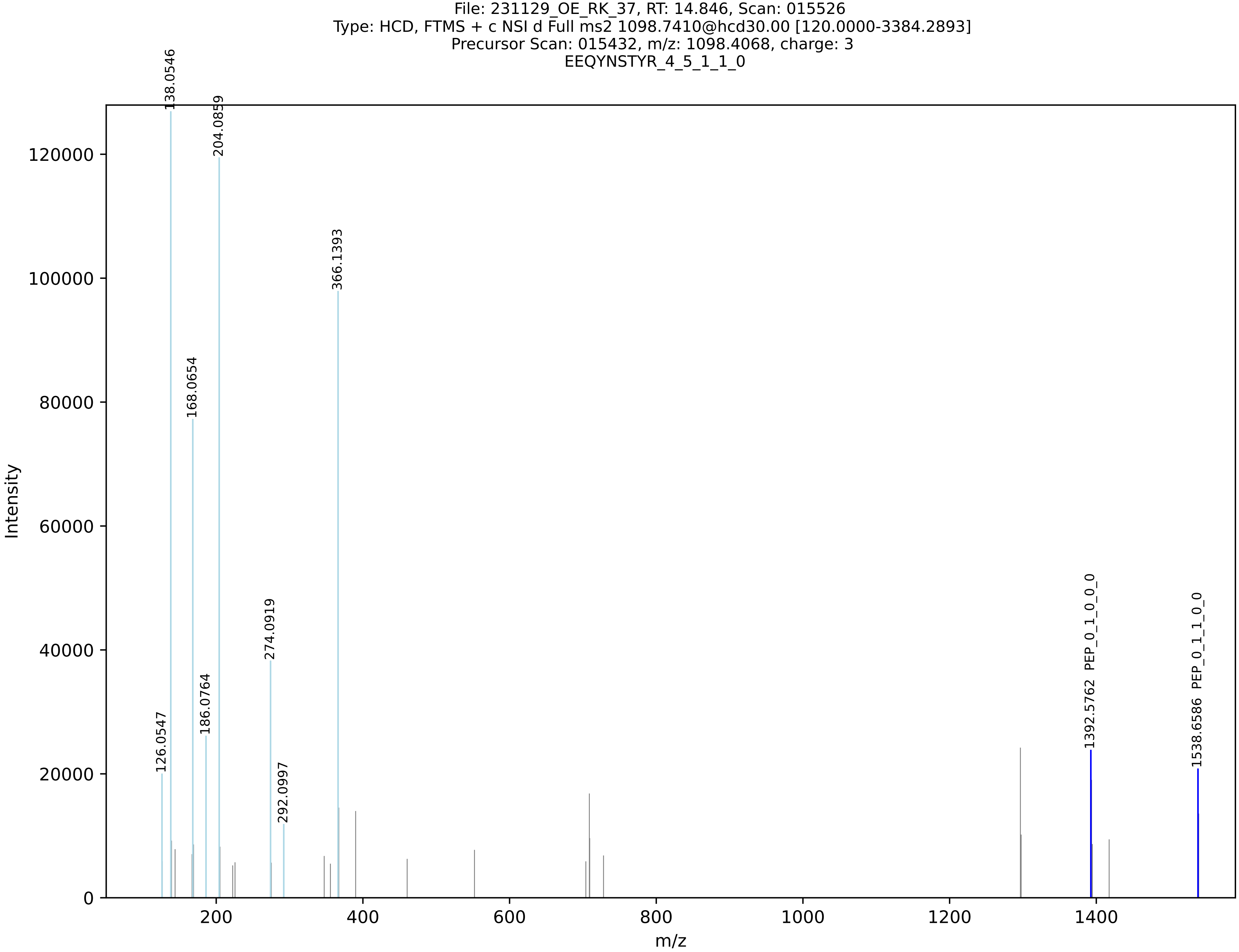
File: 231129_OE_RK_37, RT: 14.846, Scan: 015526
Type: HCD, FTMS + c NSI d Full ms2 1098.7410@hcd30.00 [120.0000-3384.2893]
Precursor Scan: 015432, m/z: 1098.4068, charge: 3
EEQYNSTYR_4_5_1_1_0
138.0546
204.0859
366.1393
168.0654
274.0919
186.0764
126.0547
292.0997
1392.5762 PEP_0_1_0_0_0
1538.6586 PEP_0_1_1_0_0
Intensity
m/z
0
20000
40000
60000
80000
100000
120000
200
400
600
800
1000
1200
1400

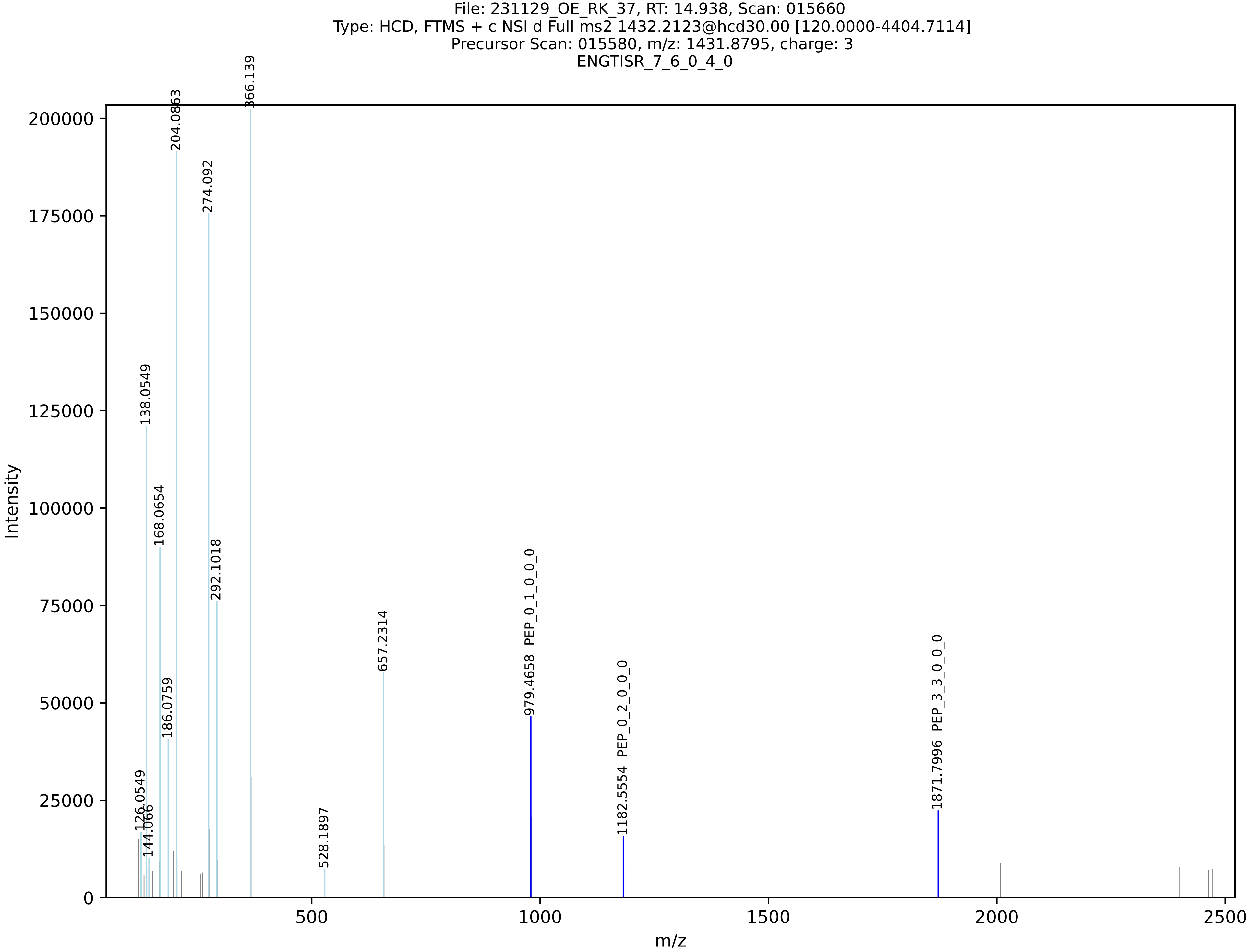

File: 231129_OE_RK_37, RT: 14.938, Scan: 015660
Type: HCD, FTMS + c NSI d Full ms2 1432.2123@hcd30.00 [120.0000-4404.7114]
Precursor Scan: 015580, m/z: 1431.8795, charge: 3
ENGTISR_7_6_0_4_0
Intensity
m/z
366.139
204.0863
274.092
138.0549
168.0654
292.1018
186.0759
126.0549
144.066
528.1897
657.2314
979.4658 PEP_0_1_0_0_0
1182.5554 PEP_0_2_0_0_0
1871.7996 PEP_3_3_0_0_0

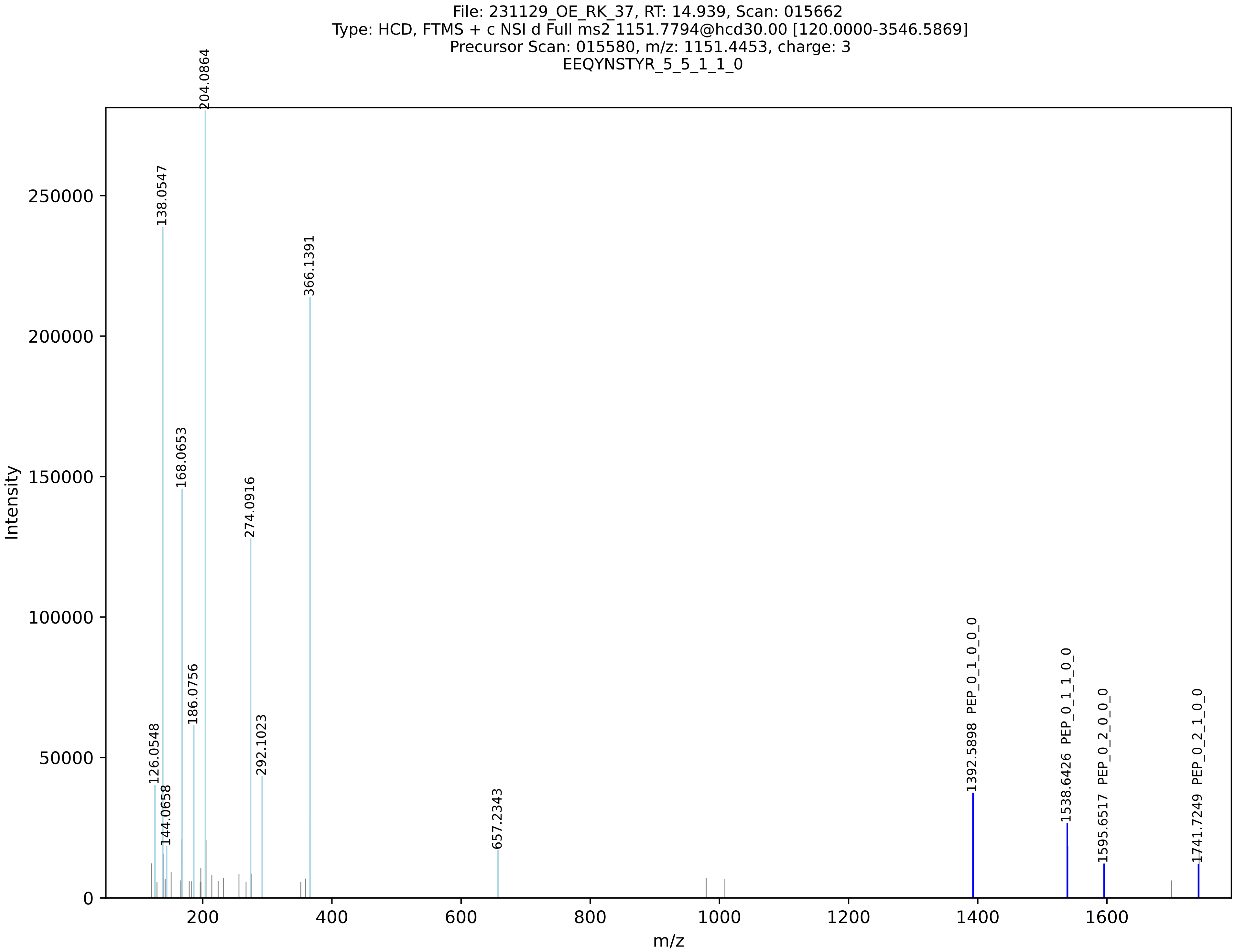
File: 231129_OE_RK_37, RT: 14.939, Scan: 015662
Type: HCD, FTMS + c NSI d Full ms2 1151.7794@hcd30.00 [120.0000-3546.5869]
Precursor Scan: 015580, m/z: 1151.4453, charge: 3
EEQYNSTYR_5_5_1_1_0
204.0864
138.0547
366.1391
168.0653
274.0916
186.0756
292.1023
126.0548
144.0658
657.2343
1392.5898 PEP_0_1_0_0_
1538.6426 PEP_0_1_1_0_0
1595.6517 PEP_0_2_0_0_0
1741.7249 PEP_0_2_1_0_0
Intensity
m/z
250000
200000
150000
100000
50000
0
200
400
600
800
1000
1200
1400
1600

File: 231129_OE_RK_37, RT: 14.941, Scan: 015664
Type: HCD, FTMS + c NSI d Full ms2 1481.2317@hcd30.00 [120.0000-4554.7109]
Precursor Scan: 015580, m/z: 1480.2294, charge: 3
ENGTISR_7_6_1_4_0

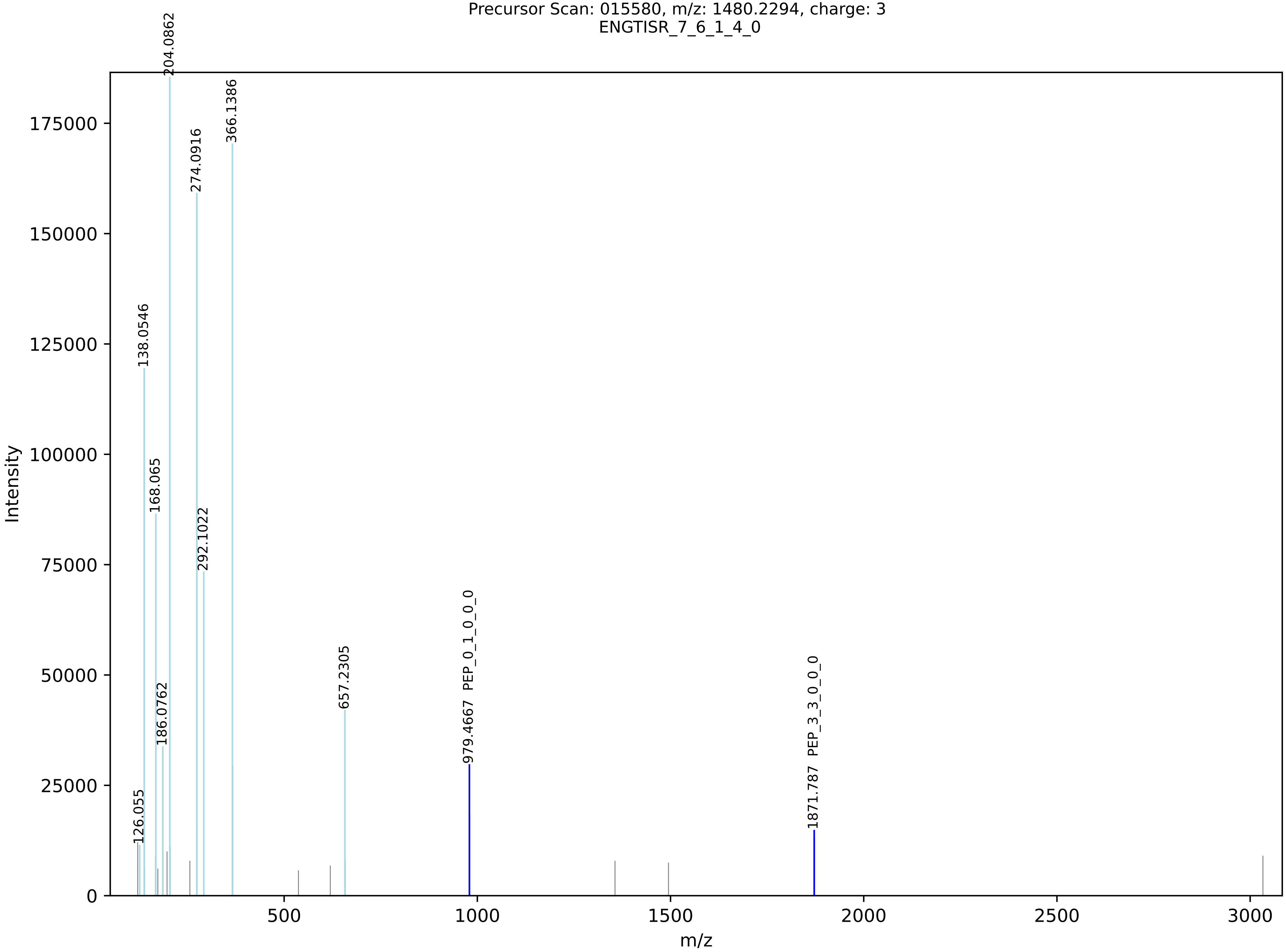

File: 231129_OE_RK_37, RT: 14.97, Scan: 015710
Type: HCD, FTMS + c NSI d Full ms2 1433.5544@hcd30.00 [120.0000-4408.8188]
Precursor Scan: 015506, m/z: 1431.8778, charge: 3
ENGTISR_7_6_0_4_0

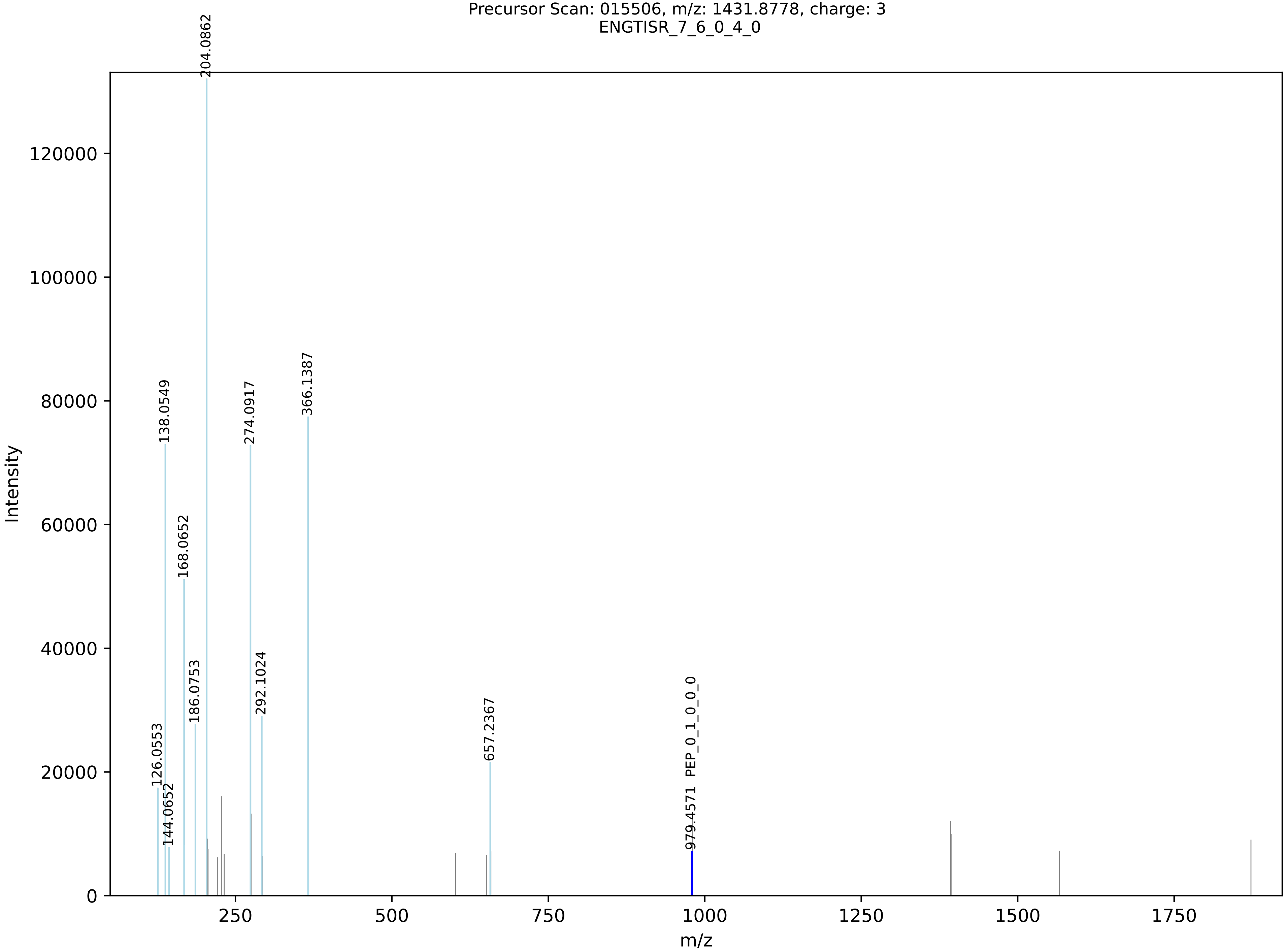

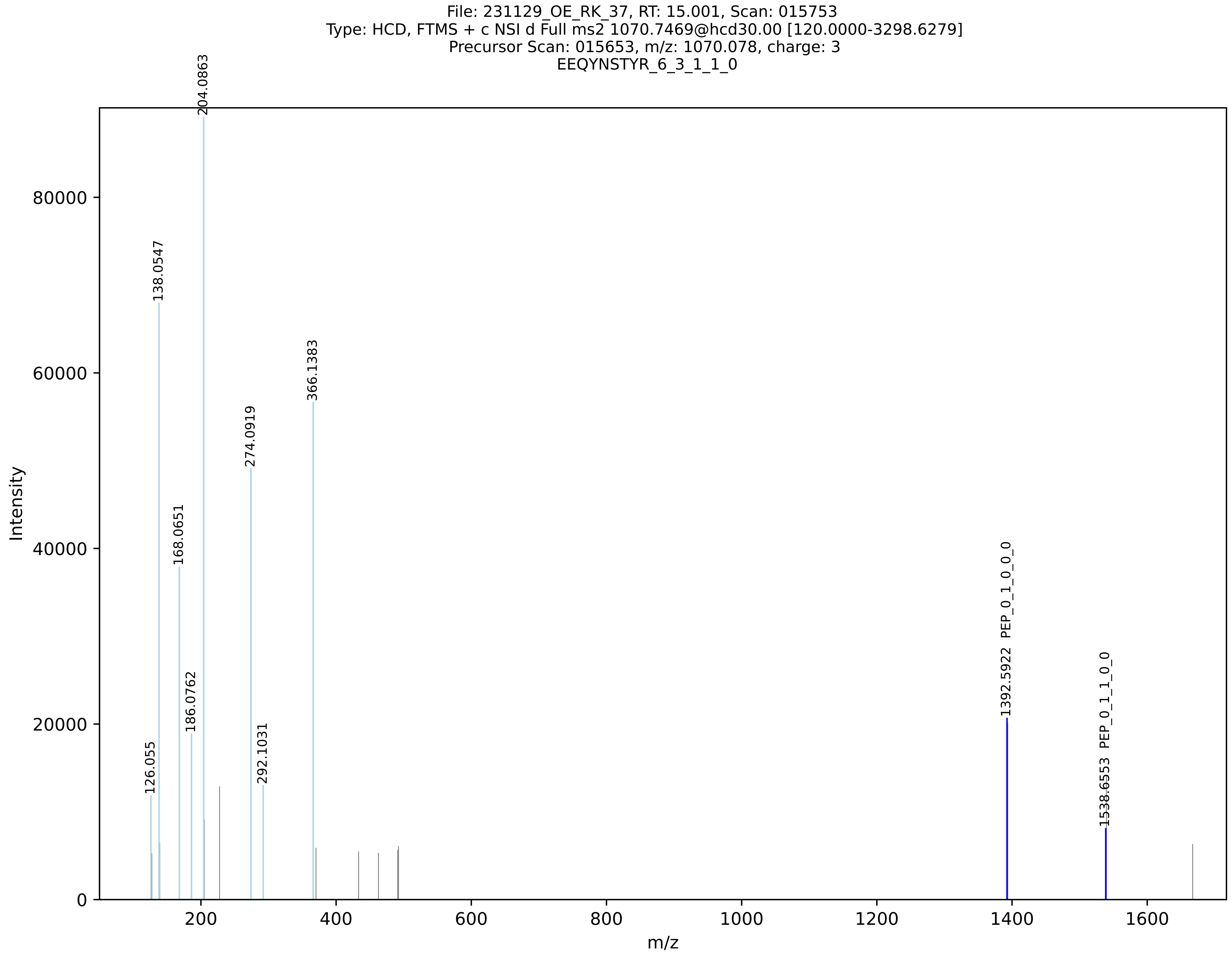
File: 231129_OE_RK_37, RT: 15.001, Scan: 015753
Type: HCD, FTMS + c NSI d Full ms2 1070.7469@hcd30.00 [120.0000-3298.6279]
Precursor Scan: 015653, m/z: 1070.078, charge: 3
EEQYNSTYR_6_3_1_1_0
Intensity
m/z
0
20000
40000
60000
80000
200
400
600
800
1000
1200
1400
1600
204.0863
138.0547
168.0651
186.0762
126.055
274.0919
292.1031
366.1383
1392.5922 PEP_0_1_0_0_0
1538.6553 PEP_0_1_1_0_0

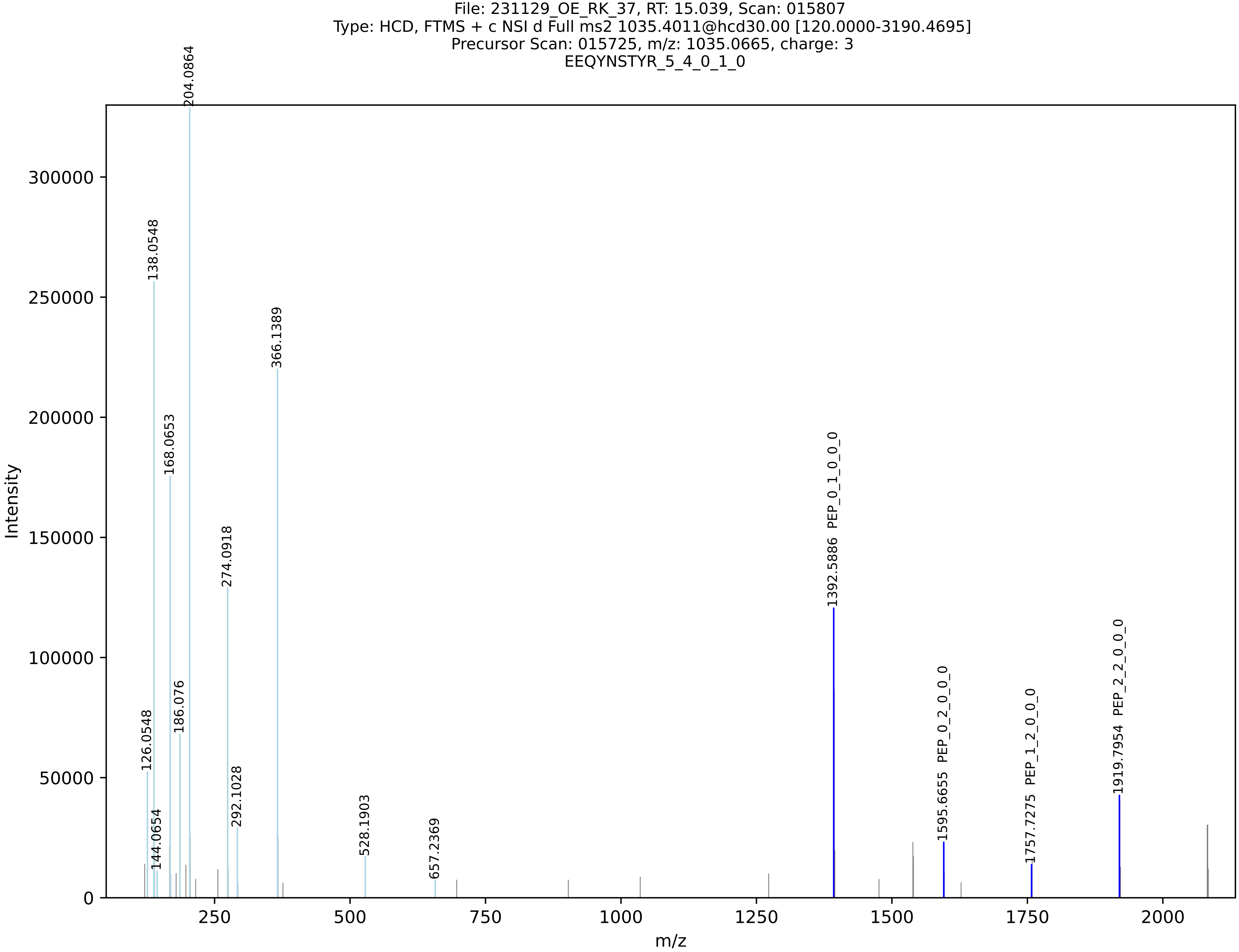
File: 231129_OE_RK_37, RT: 15.039, Scan: 015807
Type: HCD, FTMS + c NSI d Full ms2 1035.4011@hcd30.00 [120.0000-3190.4695]
Precursor Scan: 015725, m/z: 1035.0665, charge: 3
EEQYNSTYR_5_4_0_1_0
Intensity
m/z
204.0864
138.0548
366.1389
168.0653
274.0918
186.076
126.0548
144.0654
292.1028
528.1903
657.2369
1392.5886 PEP_0_1_0_0_0
1595.6655 PEP_0_2_0_0_0
1757.7275 PEP_1_2_0_0_0
1919.7954 PEP_2_2_0_0_0

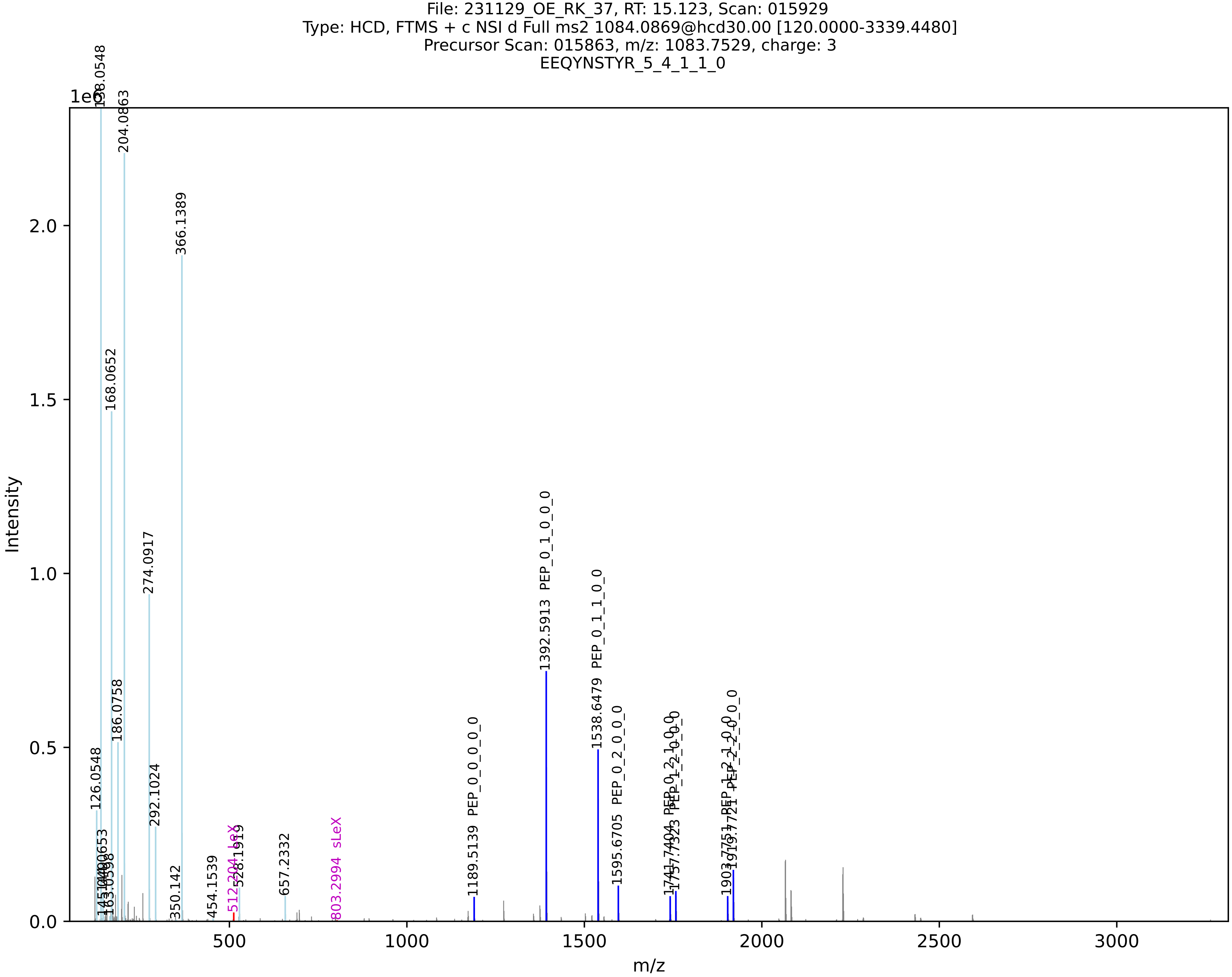
File: 231129_OE_RK_37, RT: 15.123, Scan: 015929
Type: HCD, FTMS + c NSI d Full ms2 1084.0869@hcd30.00 [120.0000-3339.4480]
Precursor Scan: 015863, m/z: 1083.7529, charge: 3
EEQYNSTYR_5_4_1_1_0
1e6
Intensity
m/z
138.0548
204.0863
366.1389
168.0652
274.0917
186.0758
126.0548
292.1024
350.142
454.1539
512.204 LeX
528.1919
657.2332
803.2994 sLeX
1189.5139 PEP_0_0_0_0_0
1392.5913 PEP_0_1_0_0_0
1538.6479 PEP_0_1_1_0_0
1595.6705 PEP_0_2_0_0_0
0.0
0.5
1.0
1.5
2.0
500
1000
1500
2000
2500
3000

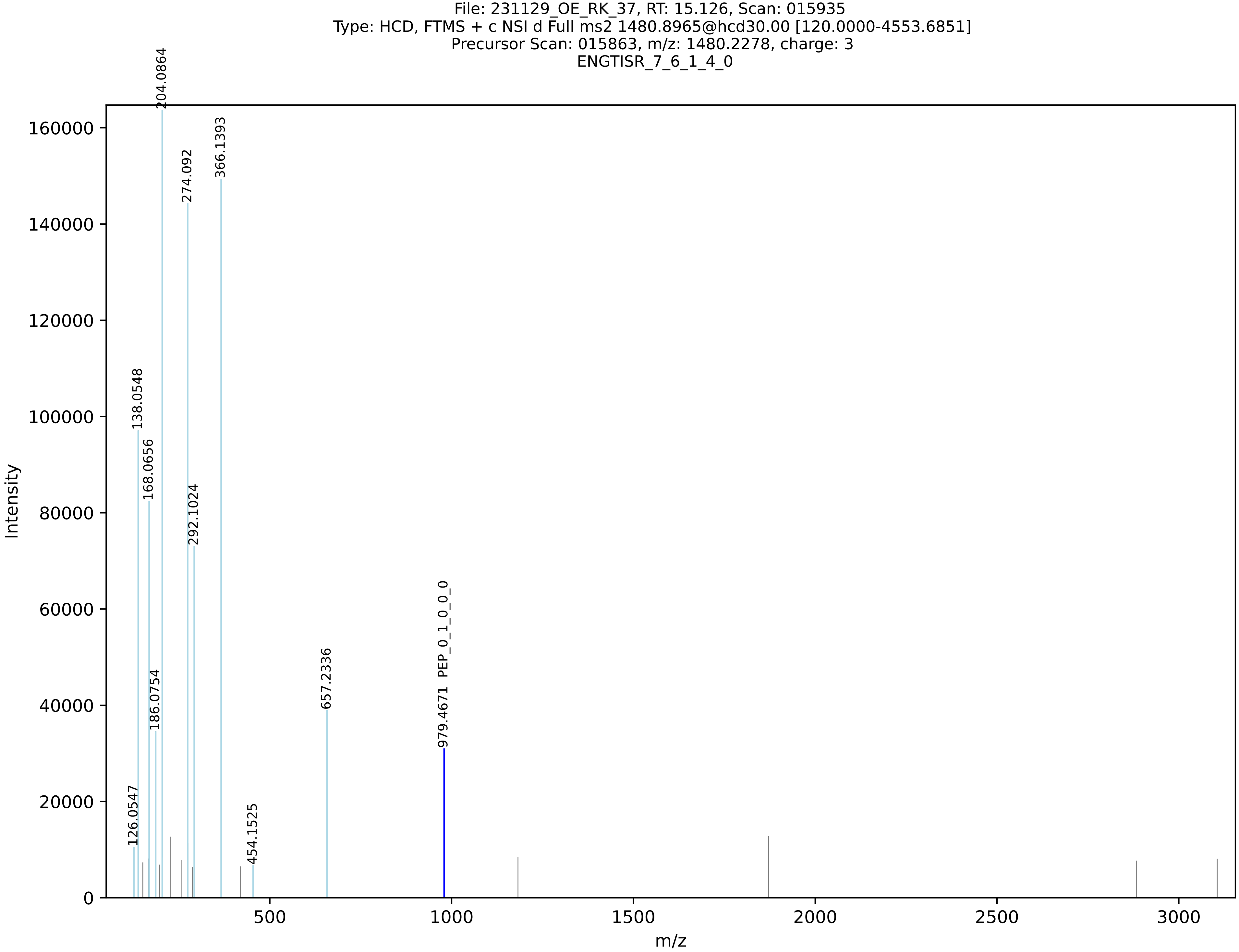

File: 231129_OE_RK_37, RT: 15.126, Scan: 015935
Type: HCD, FTMS + c NSI d Full ms2 1480.8965@hcd30.00 [120.0000-4553.6851]
Precursor Scan: 015863, m/z: 1480.2278, charge: 3
ENGTISR_7_6_1_4_0
Intensity
m/z
204.0864
366.1393
274.092
138.0548
168.0656
292.1024
186.0754
126.0547
454.1525
657.2336
979.4671 PEP_0_1_0_0_0_
0
20000
40000
60000
80000
100000
120000
140000
160000
500
1000
1500
2000
2500
3000

File: 231129_OE_RK_37, RT: 15.163, Scan: 015988
Type: HCD, FTMS + c NSI d Full ms2 1544.6003@hcd30.00 [120.0000-3172.4128]
Precursor Scan: 015928, m/z: 1544.0996, charge: 2
EEQYNSTYR_4_4_1_1_0

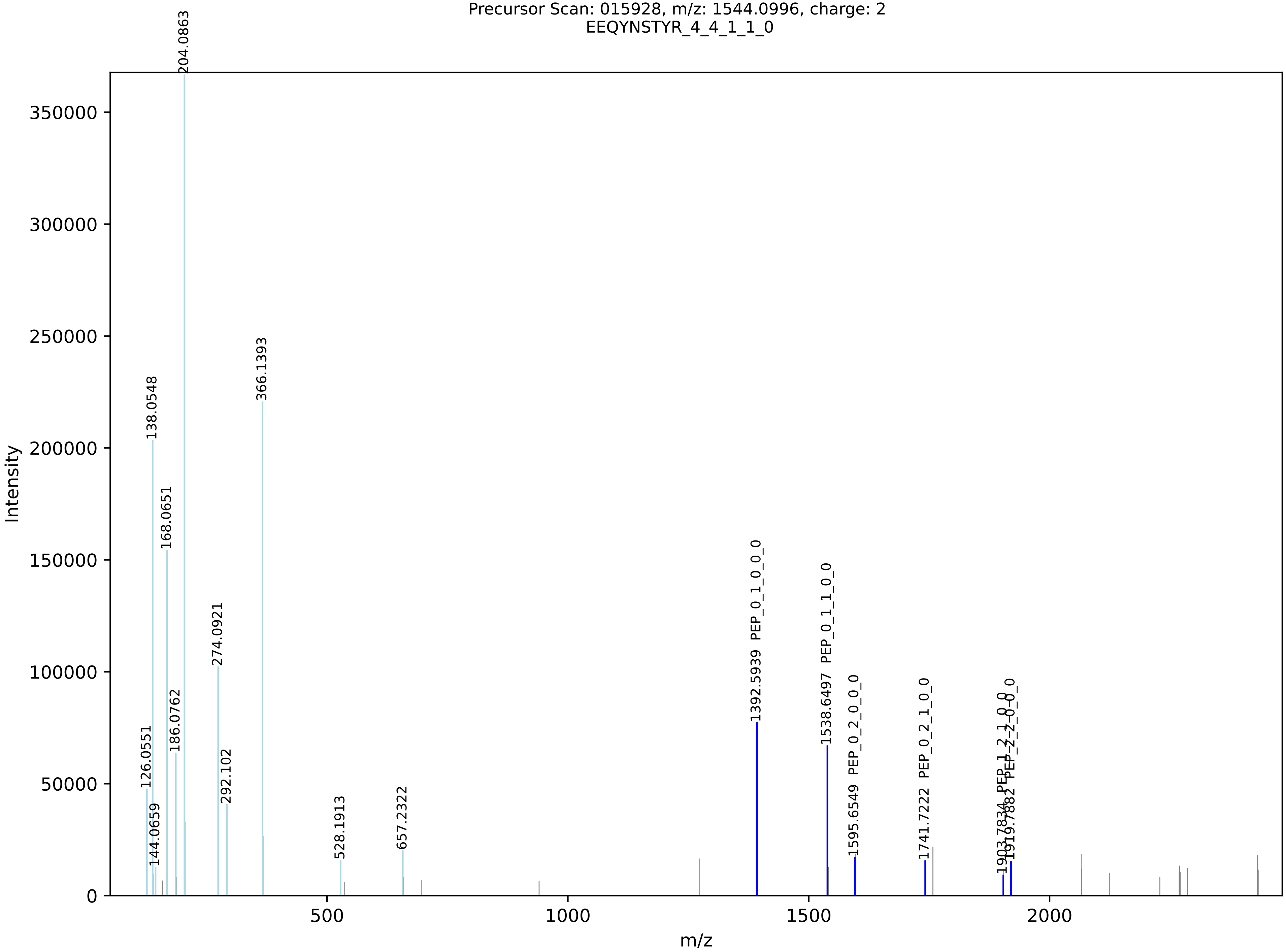

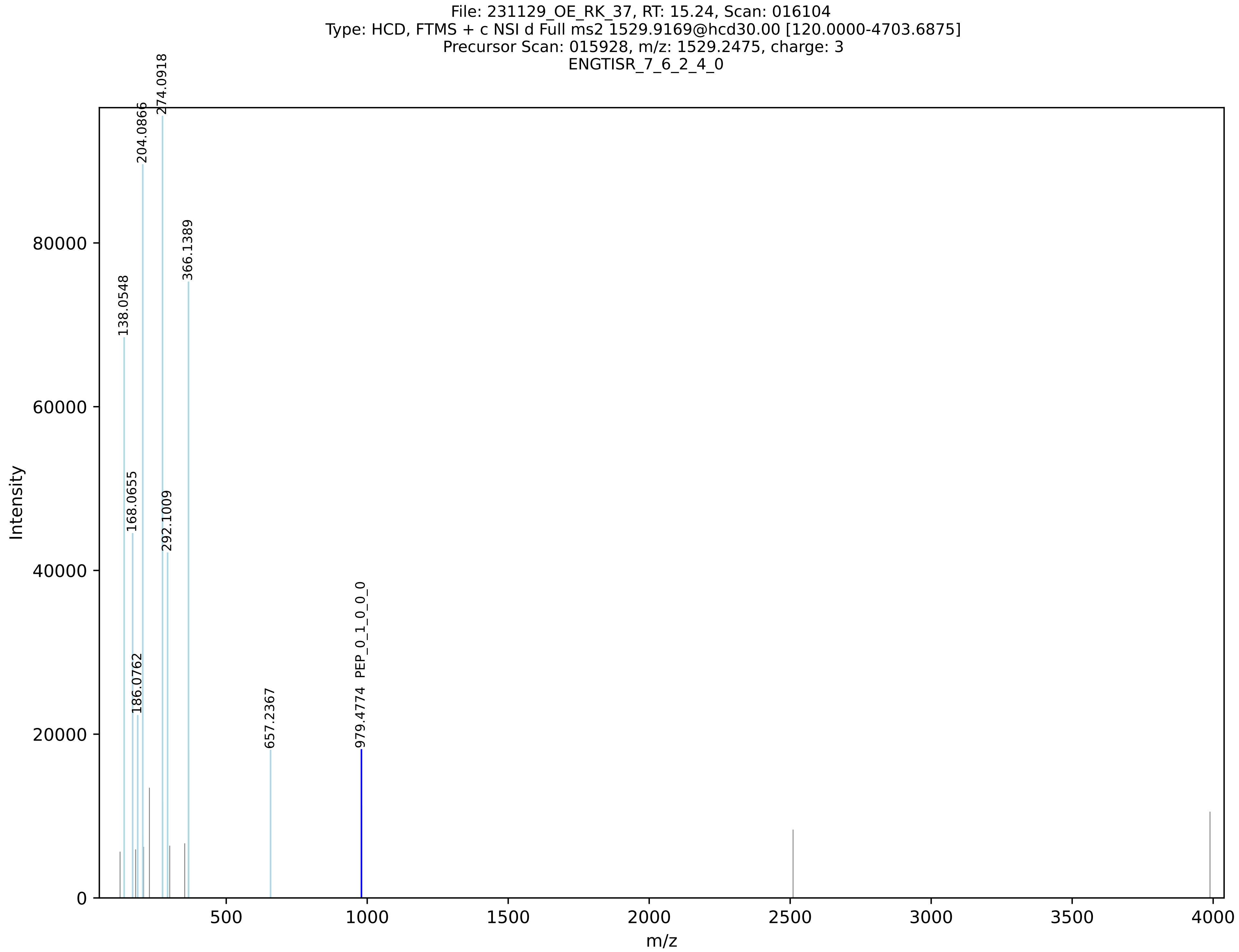
File: 231129_OE_RK_37, RT: 15.24, Scan: 016104
Type: HCD, FTMS + c NSI d Full ms2 1529.9169@hcd30.00 [120.0000-4703.6875]
Precursor Scan: 015928, m/z: 1529.2475, charge: 3
ENGTISR_7_6_2_4_0
274.0918
204.0866
366.1389
138.0548
168.0655
292.1009
PEP_0_1_0_0_0
979.4774
186.0762
657.2367
Intensity
m/z
0
20000
40000
60000
80000
500
1000
1500
2000
2500
3000
3500
4000

File: 231129_OE_RK_37, RT: 15.261, Scan: 016132
Type: HCD, FTMS + c NSI d Full ms2 1035.7345@hcd30.00 [120.0000-3191.4895]
Precursor Scan: 016046, m/z: 1035.0662, charge: 3
EEQYNSTYR_5_4_0_1_0

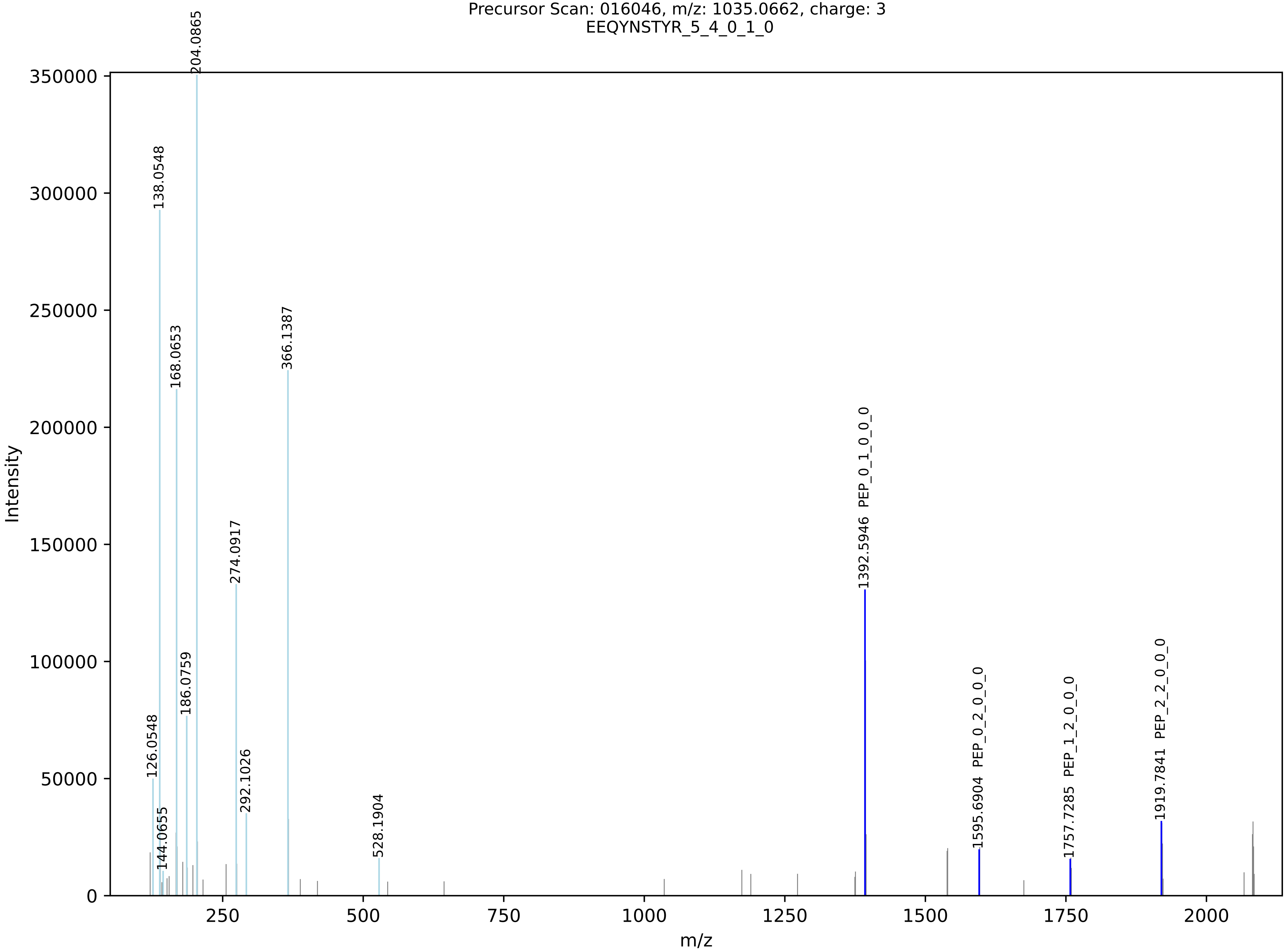

File: 231129_OE_RK_37, RT: 15.3, Scan: 016188
Type: HCD, FTMS + c NSI d Full ms2 1084.0874@hcd30.00 [120.0000-3339.4495]
Precursor Scan: 016120, m/z: 1083.7533, charge: 3
EEQYNSTYR_5_4_1_1_0

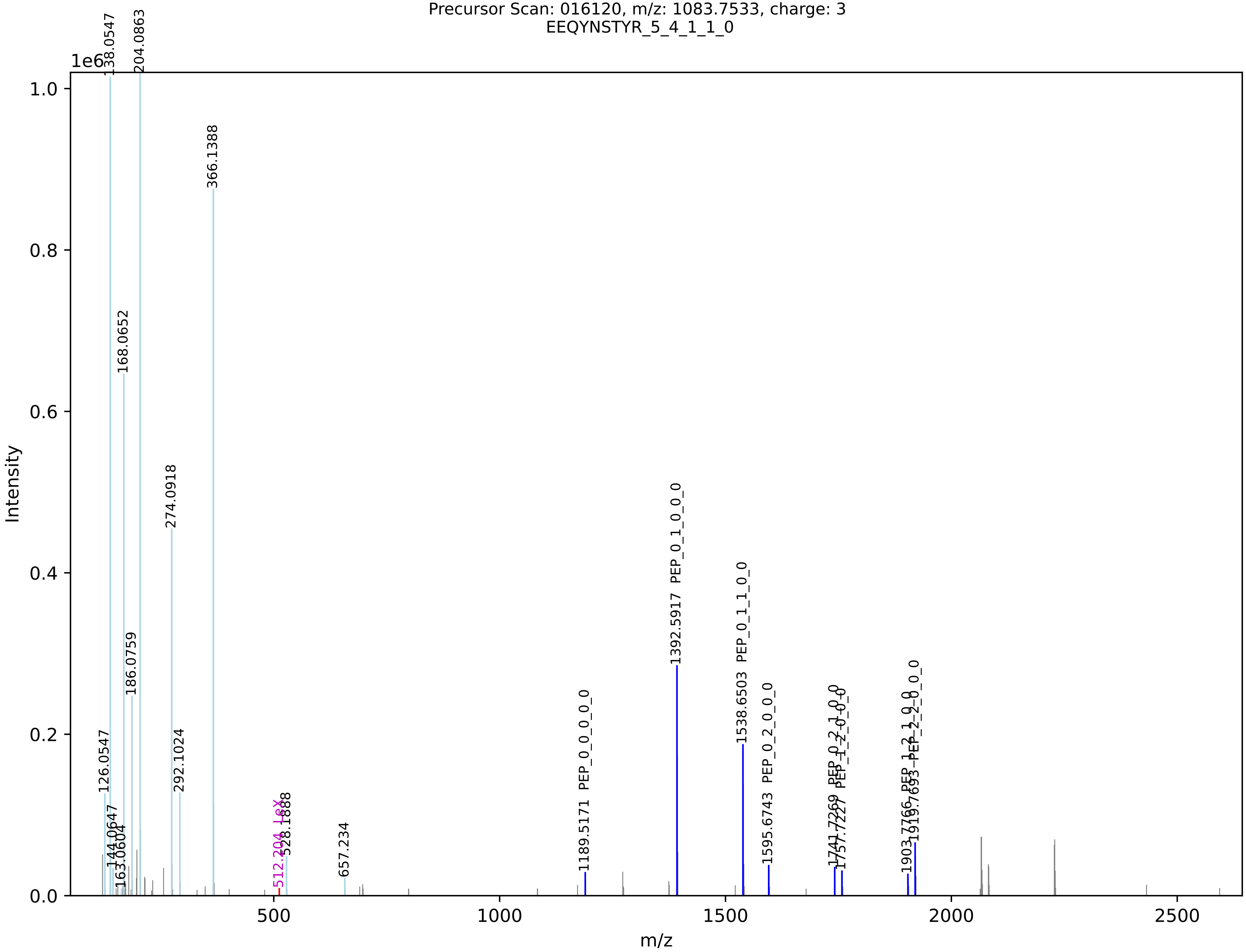

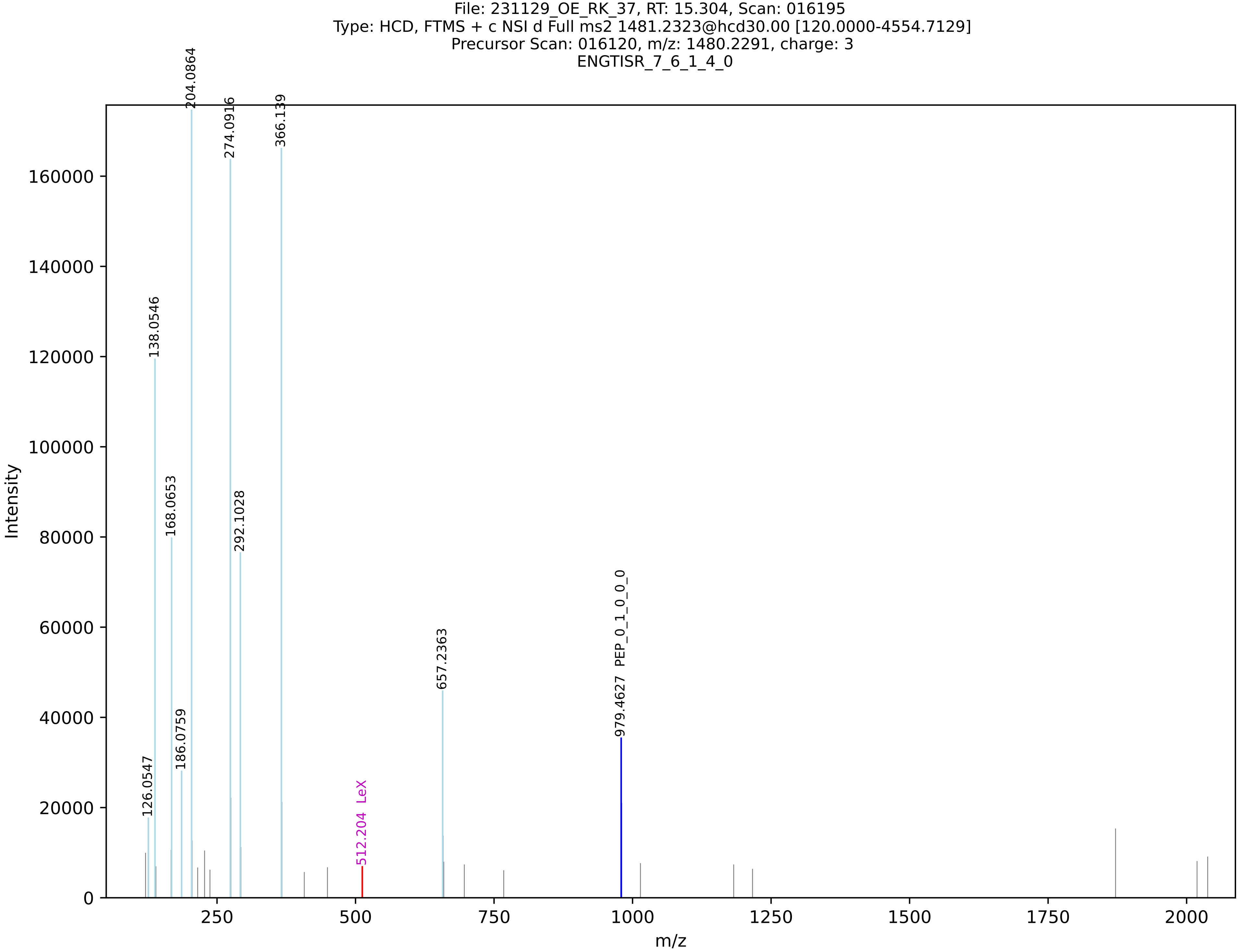
File: 231129_OE_RK_37, RT: 15.304, Scan: 016195
Type: HCD, FTMS + c NSI d Full ms2 1481.2323@hcd30.00 [120.0000-4554.7129]
Precursor Scan: 016120, m/z: 1480.2291, charge: 3
ENGTISR_7_6_1_4_0
Intensity
m/z
204.0864
274.0916
366.139
138.0546
168.0653
292.1028
186.0759
126.0547
512.204 LeX
657.2363
979.4627 PEP_0_1_0_0_0
0
20000
40000
60000
80000
100000
120000
140000
160000
250
500
750
1000
1250
1500
1750
2000

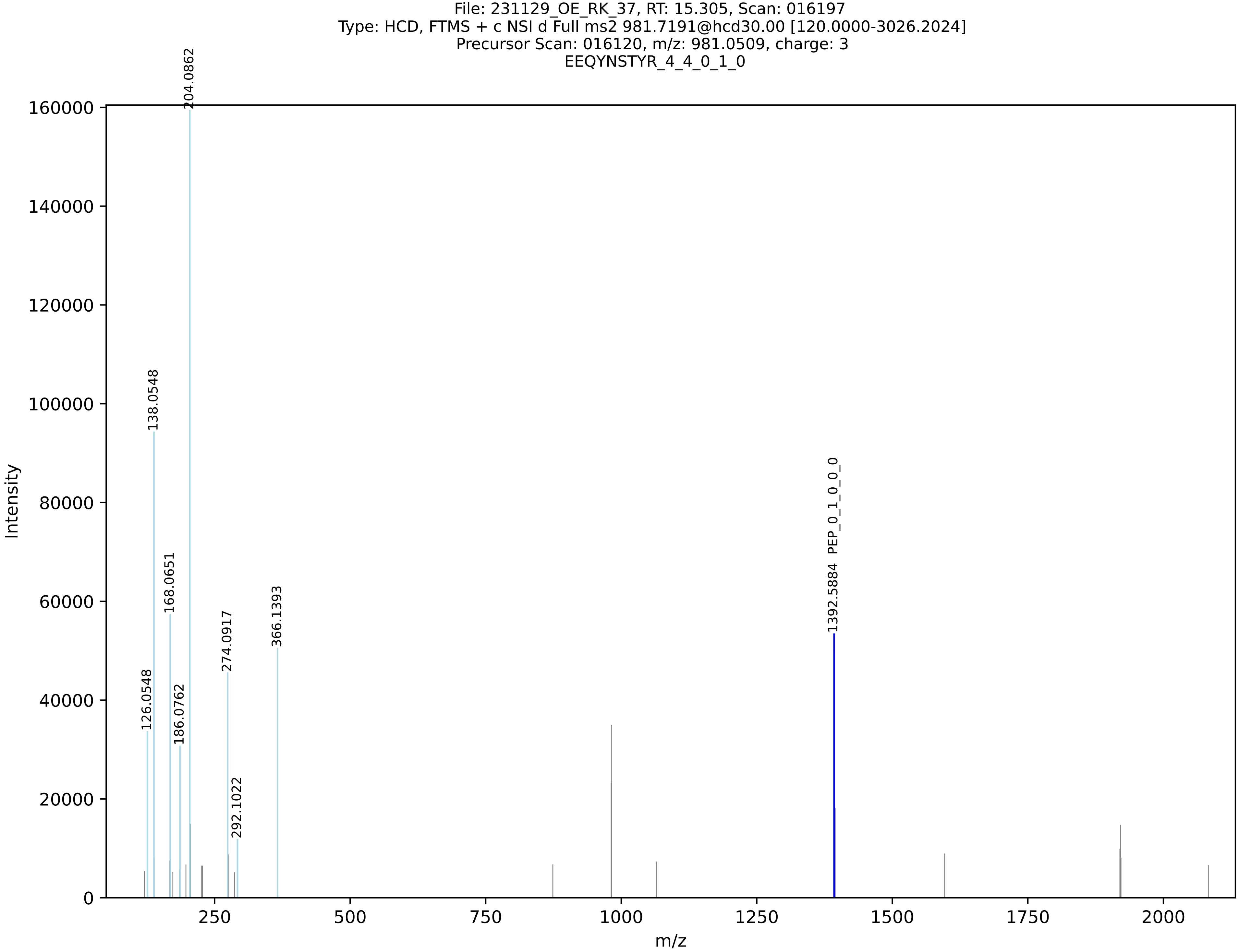

File: 231129_OE_RK_37, RT: 15.305, Scan: 016197
Type: HCD, FTMS + c NSI d Full ms2 981.7191@hcd30.00 [120.0000-3026.2024]
Precursor Scan: 016120, m/z: 981.0509, charge: 3
EEQYNSTYR_4_4_0_1_0
204.0862
138.0548
168.0651
126.0548
186.0762
274.0917
292.1022
366.1393
1392.5884 PEP_0_1_0_0_0
Intensity
m/z

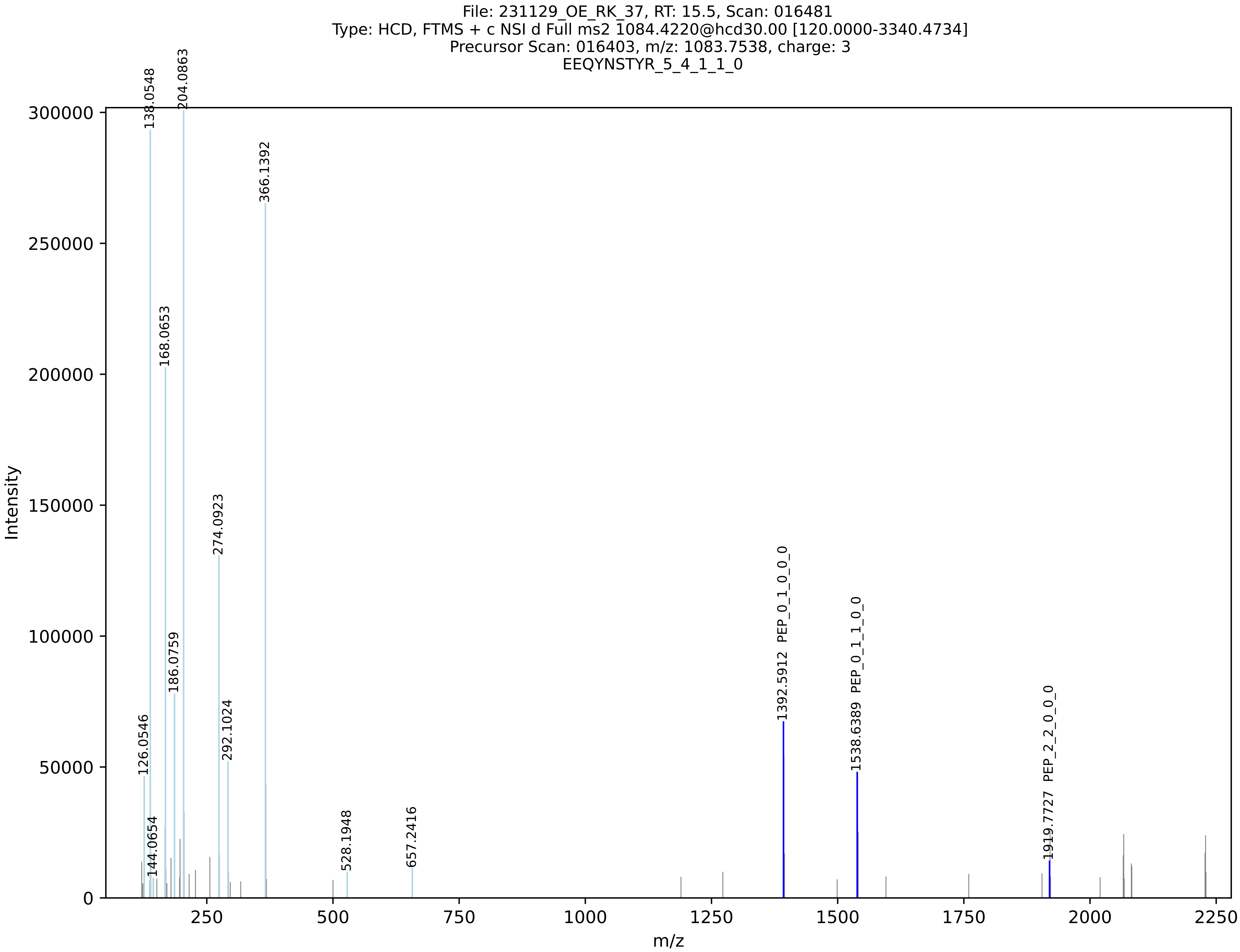

File: 231129_OE_RK_37, RT: 15.5, Scan: 016481
Type: HCD, FTMS + c NSI d Full ms2 1084.4220@hcd30.00 [120.0000-3340.4734]
Precursor Scan: 016403, m/z: 1083.7538, charge: 3
EEQYNSTYR_5_4_1_1_0
Intensity
m/z
138.0548
204.0863
366.1392
168.0653
274.0923
186.0759
126.0546
292.1024
144.0654
528.1948
657.2416
1392.5912 PEP_0_1_0_0_0
1538.6389 PEP_0_1_1_0_0
1919.7727 PEP_2_2_0_0_0

File: 231129_OE_RK_37, RT: 15.503, Scan: 016485
Type: HCD, FTMS + c NSI d Full ms2 981.3846@hcd30.00 [120.0000-3025.1787]
Precursor Scan: 016403, m/z: 981.0503, charge: 3
EEQYNSTYR_4_4_0_1_0

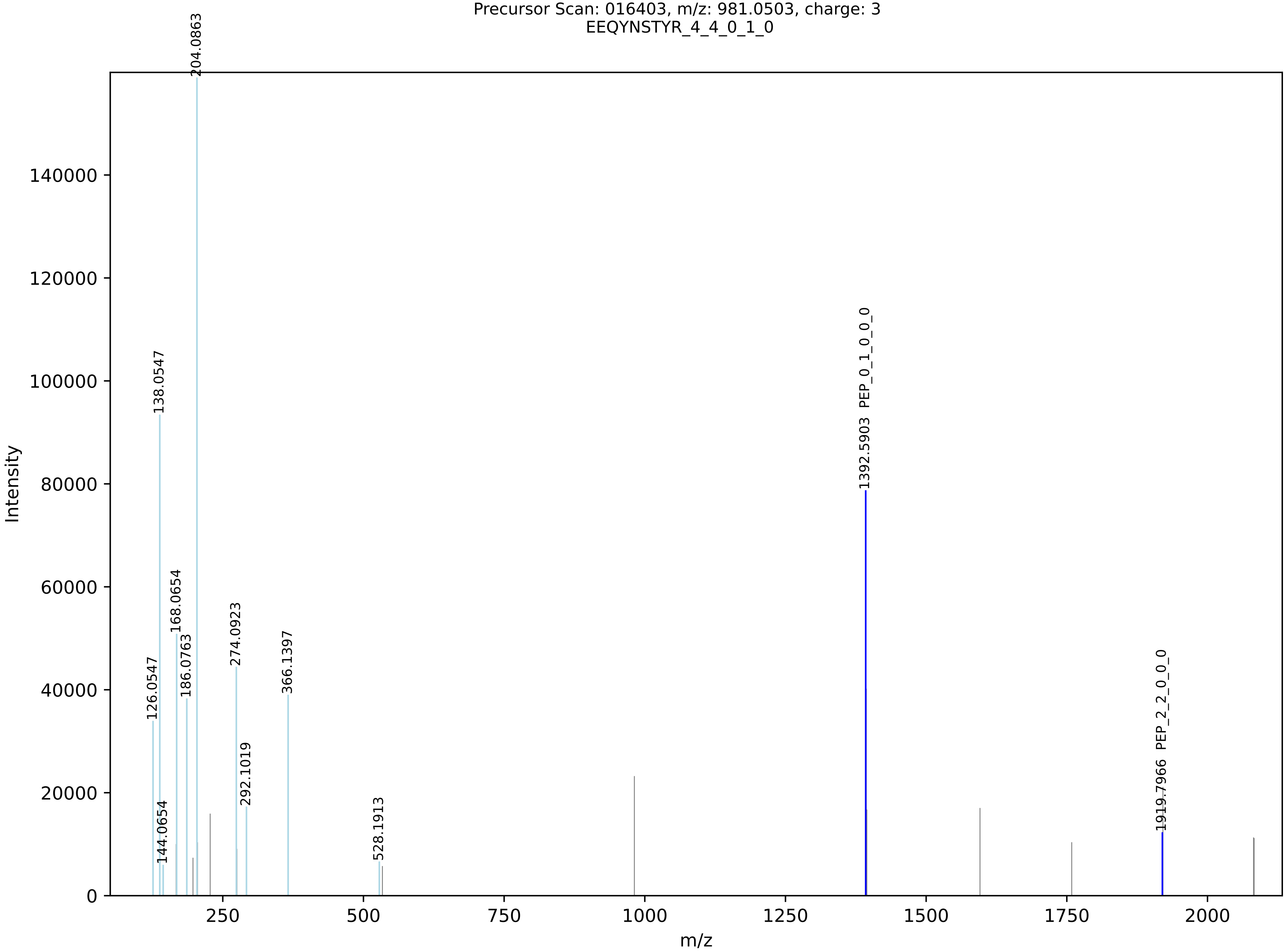

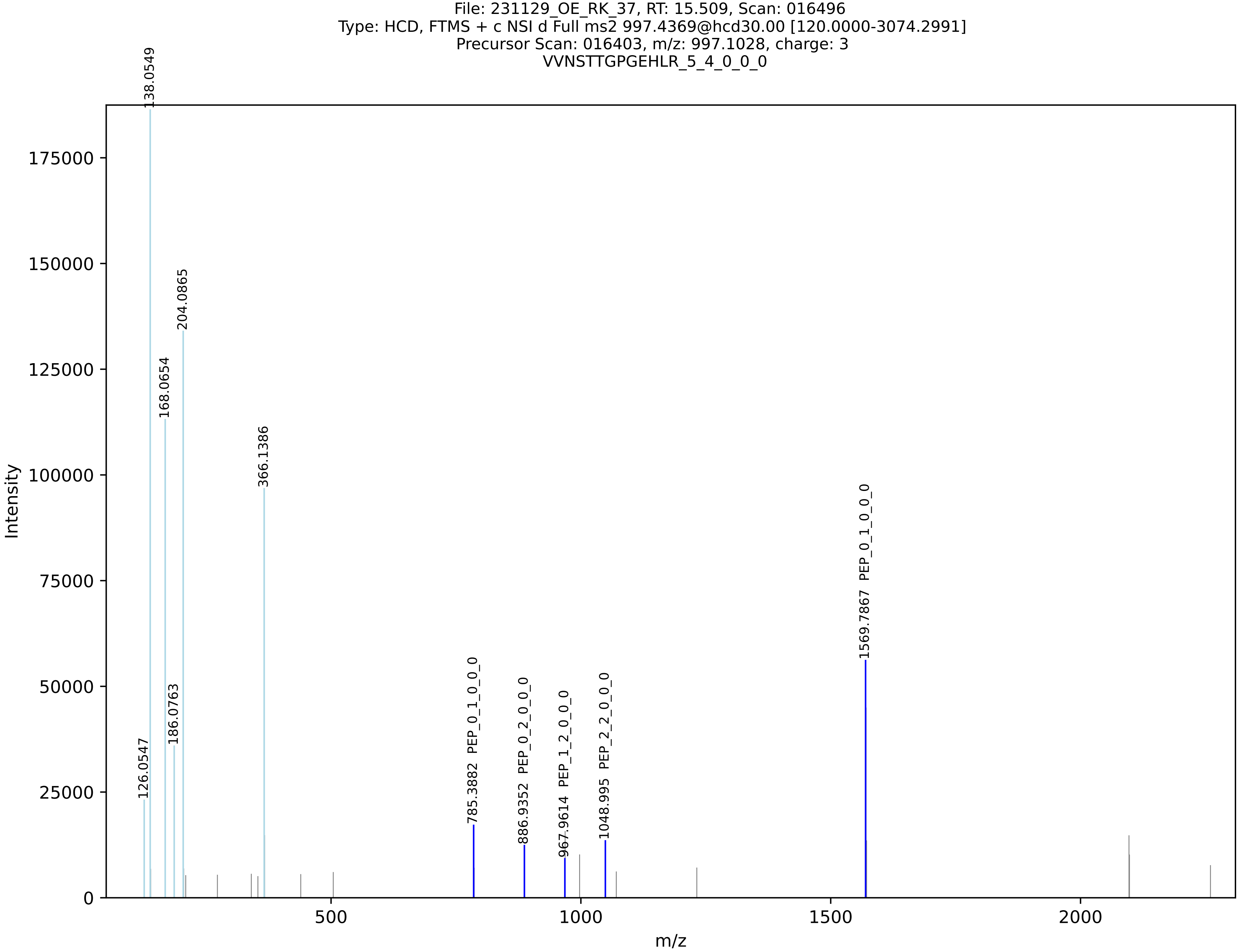
File: 231129_OE_RK_37, RT: 15.509, Scan: 016496
Type: HCD, FTMS + c NSI d Full ms2 997.4369@hcd30.00 [120.0000-3074.2991]
Precursor Scan: 016403, m/z: 997.1028, charge: 3
VVNSTTGPGEHLR_5_4_0_0_0
Intensity
m/z
126.0547
138.0549
168.0654
186.0763
204.0865
366.1386
785.3882 PEP_0_1_0_0_0
886.9352 PEP_0_2_0_0_0
967.9614 PEP_1_2_0_0_0
1048.995 PEP_2_2_0_0_0
1569.7867 PEP_0_1_0_0_0
0
25000
50000
75000
100000
125000
150000
175000
500
1000
1500
2000

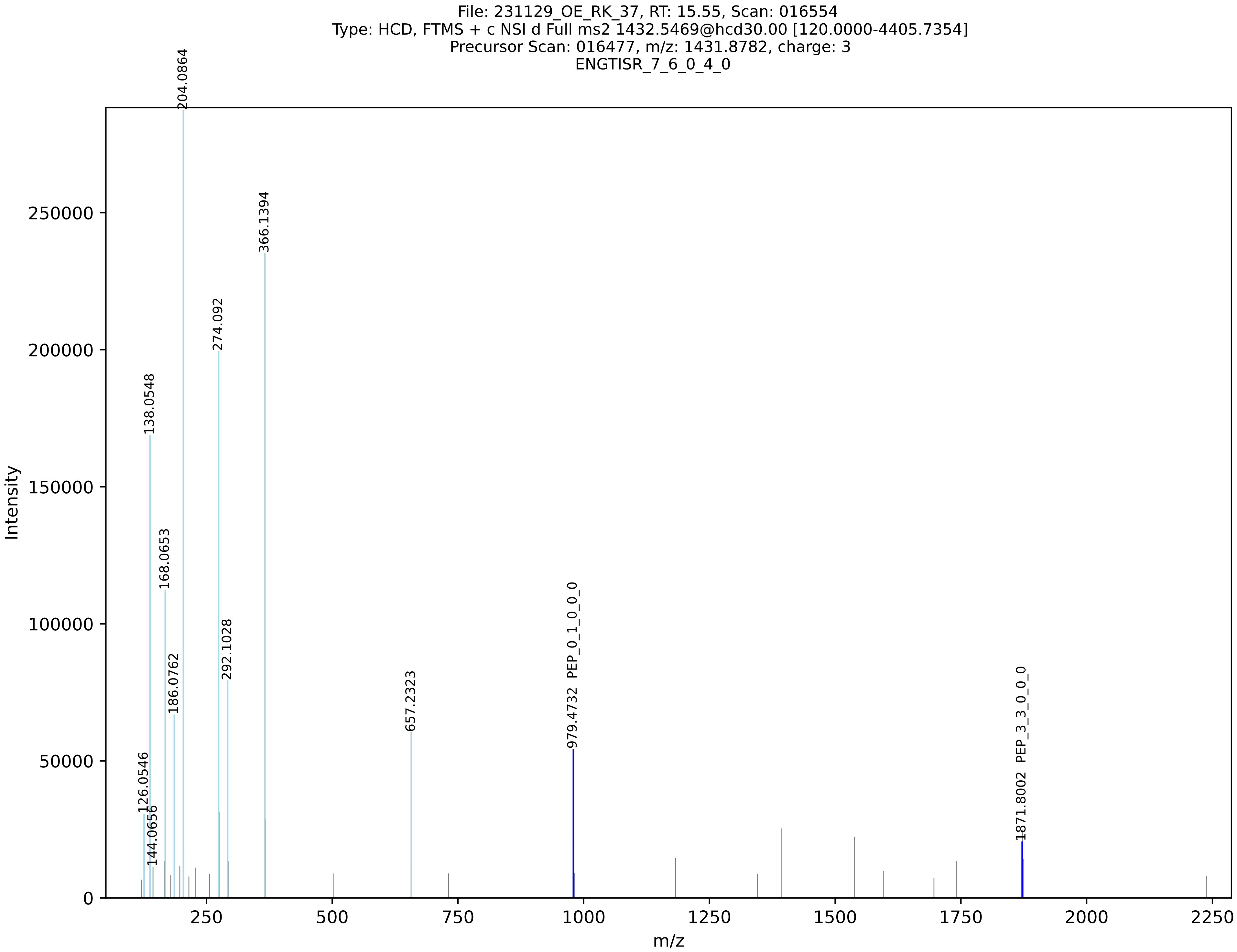
File: 231129_OE_RK_37, RT: 15.55, Scan: 016554
Type: HCD, FTMS + c NSI d Full ms2 1432.5469@hcd30.00 [120.0000-4405.7354]
Precursor Scan: 016477, m/z: 1431.8782, charge: 3
ENGTISR_7_6_0_4_0
Intensity
m/z
204.0864
366.1394
274.092
138.0548
168.0653
292.1028
186.0762
657.2323
979.4732 PEP_0_1_0_0_0_
1871.8002 PEP_3_3_0_0_0_
126.0546
144.0656
0
50000
100000
150000
200000
250000
250
500
750
1000
1250
1500
1750
2000
2250

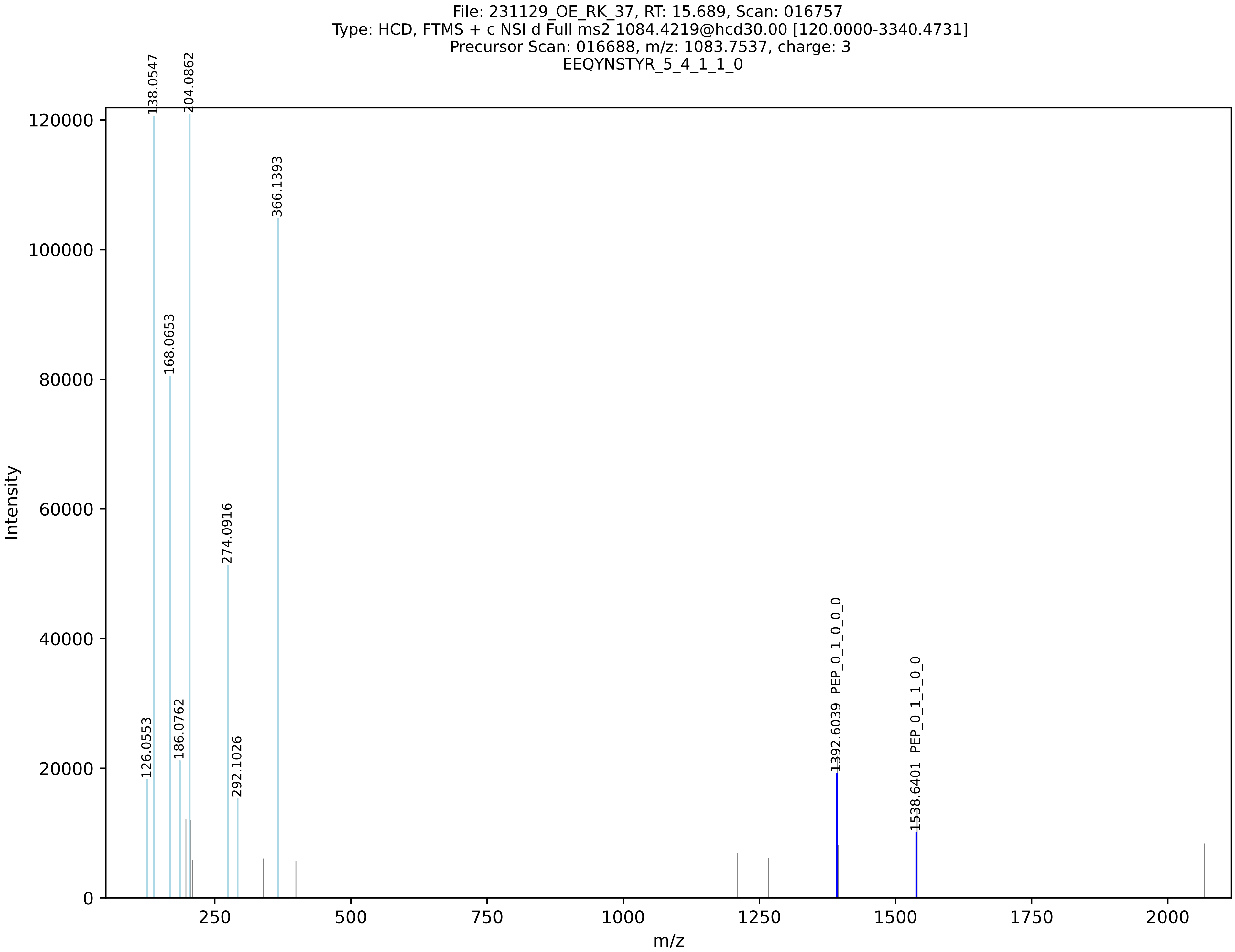

File: 231129_OE_RK_37, RT: 15.689, Scan: 016757
Type: HCD, FTMS + c NSI d Full ms2 1084.4219@hcd30.00 [120.0000-3340.4731]
Precursor Scan: 016688, m/z: 1083.7537, charge: 3
EEQYNSTYR_5_4_1_1_0
138.0547
204.0862
366.1393
168.0653
274.0916
126.0553
186.0762
292.1026
1392.6039 PEP_0_1_0_0_0
1538.6401 PEP_0_1_1_0_0
Intensity
m/z

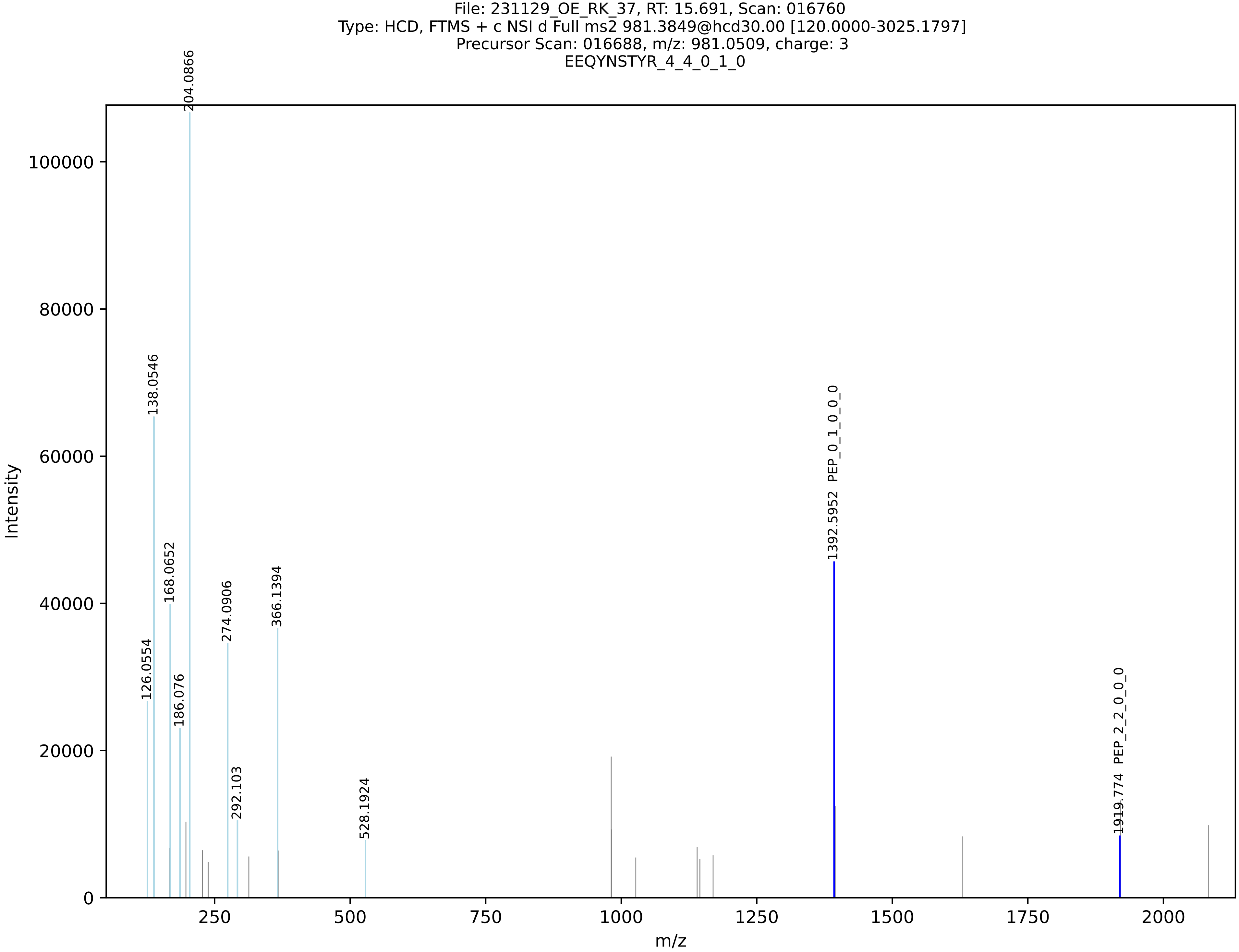
File: 231129_OE_RK_37, RT: 15.691, Scan: 016760
Type: HCD, FTMS + c NSI d Full ms2 981.3849@hcd30.00 [120.0000-3025.1797]
Precursor Scan: 016688, m/z: 981.0509, charge: 3
EEQYNSTYR_4_4_0_1_0
204.0866
138.0546
168.0652
126.0554
186.076
274.0906
292.103
366.1394
528.1924
1392.5952 PEP_0_1_0_0_0
1919.774 PEP_2_2_0_0_0
Intensity
m/z
0
20000
40000
60000
80000
100000
250
500
750
1000
1250
1500
1750
2000

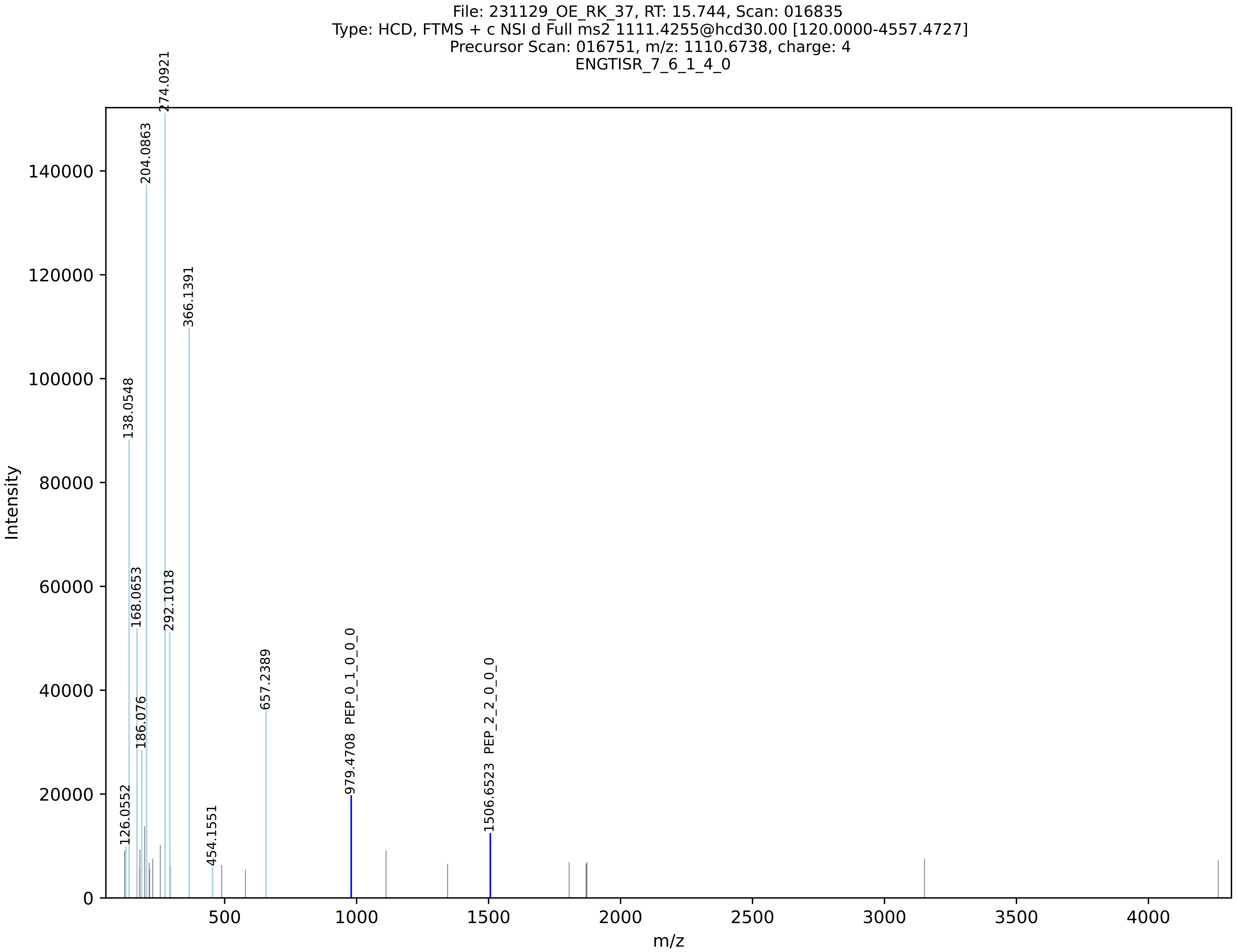
File: 231129_OE_RK_37, RT: 15.744, Scan: 016835
Type: HCD, FTMS + c NSI d Full ms2 1111.4255@hcd30.00 [120.0000-4557.4727]
Precursor Scan: 016751, m/z: 1110.6738, charge: 4
ENGTISR_7_6_1_4_0
Intensity
m/z
274.0921
204.0863
366.1391
138.0548
168.0653
292.1018
657.2389
186.076
979.4708 PEP_0_1_0_0_0
1506.6523 PEP_2_2_0_0_0
126.0552
454.1551
0
20000
40000
60000
80000
100000
120000
140000
500
1000
1500
2000
2500
3000
3500
4000

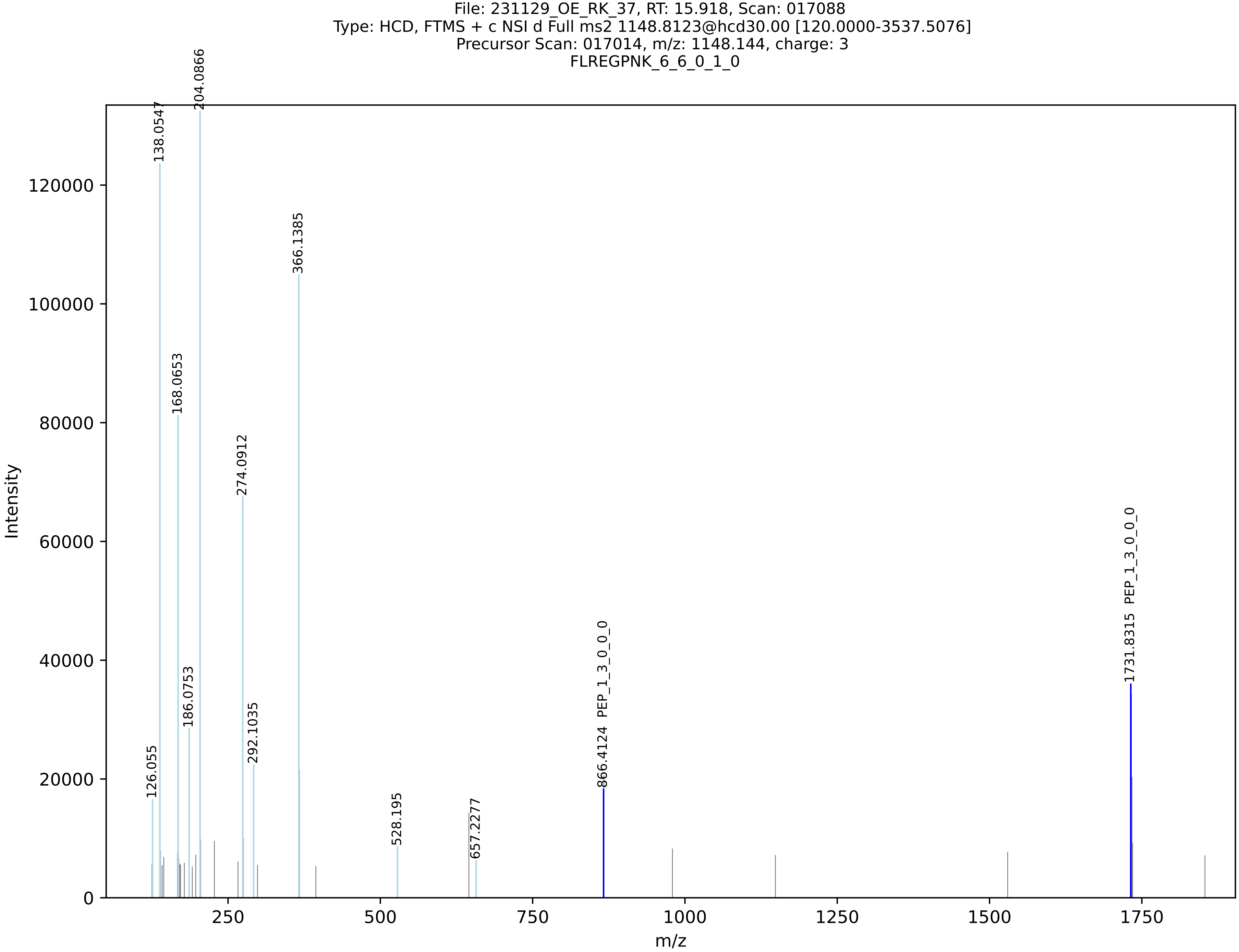
File: 231129_OE_RK_37, RT: 15.918, Scan: 017088
Type: HCD, FTMS + c NSI d Full ms2 1148.8123@hcd30.00 [120.0000-3537.5076]
Precursor Scan: 017014, m/z: 1148.144, charge: 3
FLREGPNK_6_6_0_1_0
Intensity
m/z
204.0866
138.0547
366.1385
168.0653
274.0912
186.0753
292.1035
126.055
528.195
657.2277
866.4124 PEP_1_3_0_0_0
1731.8315 PEP_1_3_0_0_0
0
20000
40000
60000
80000
100000
120000
250
500
750
1000
1250
1500
1750

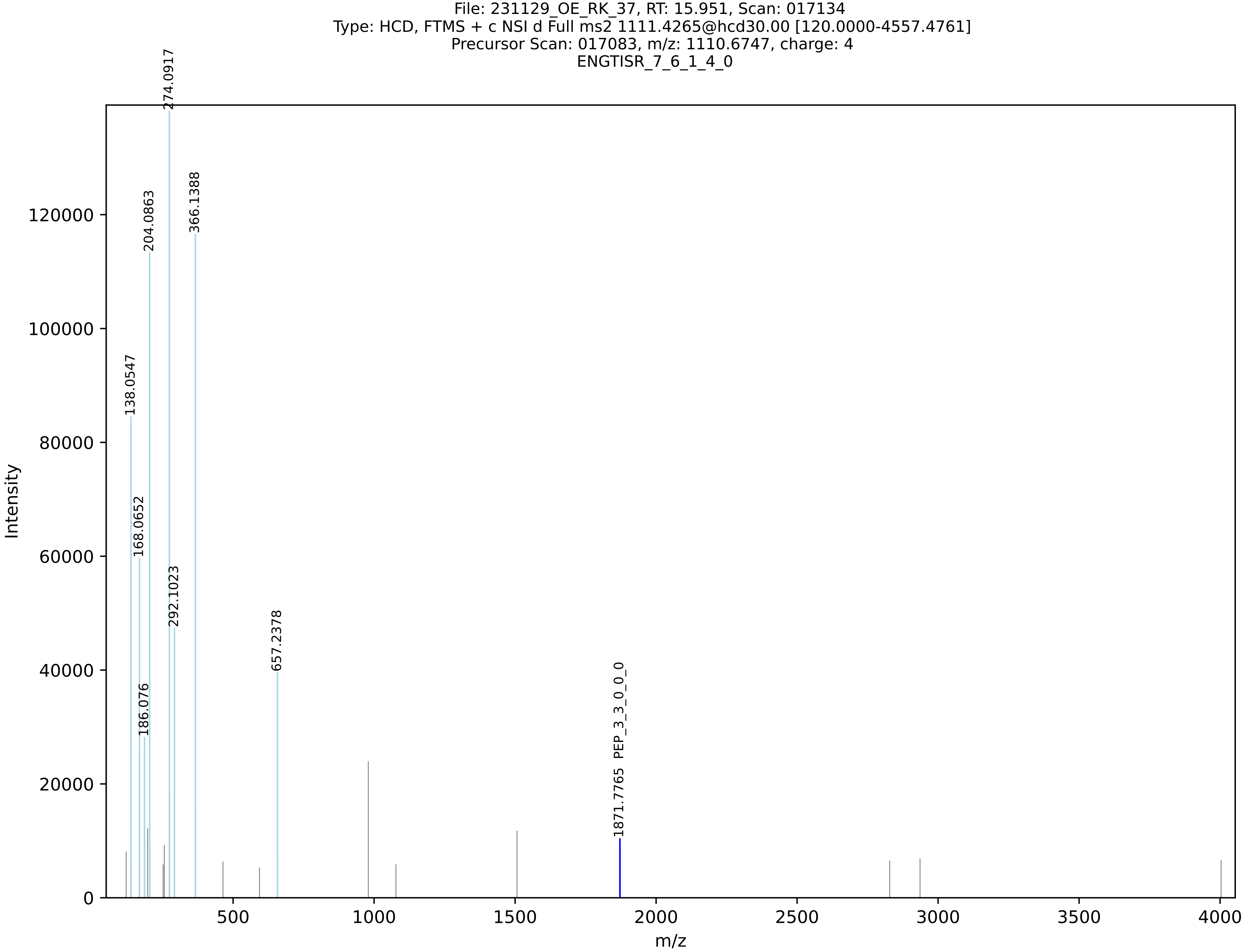
File: 231129_OE_RK_37, RT: 15.951, Scan: 017134
Type: HCD, FTMS + c NSI d Full ms2 1111.4265@hcd30.00 [120.0000-4557.4761]
Precursor Scan: 017083, m/z: 1110.6747, charge: 4
ENGTISR_7_6_1_4_0
274.0917
366.1388
204.0863
138.0547
168.0652
292.1023
657.2378
186.076
1871.7765 PEP_3_3_0_0_0
Intensity
m/z
0
20000
40000
60000
80000
100000
120000
500
1000
1500
2000
2500
3000
3500
4000

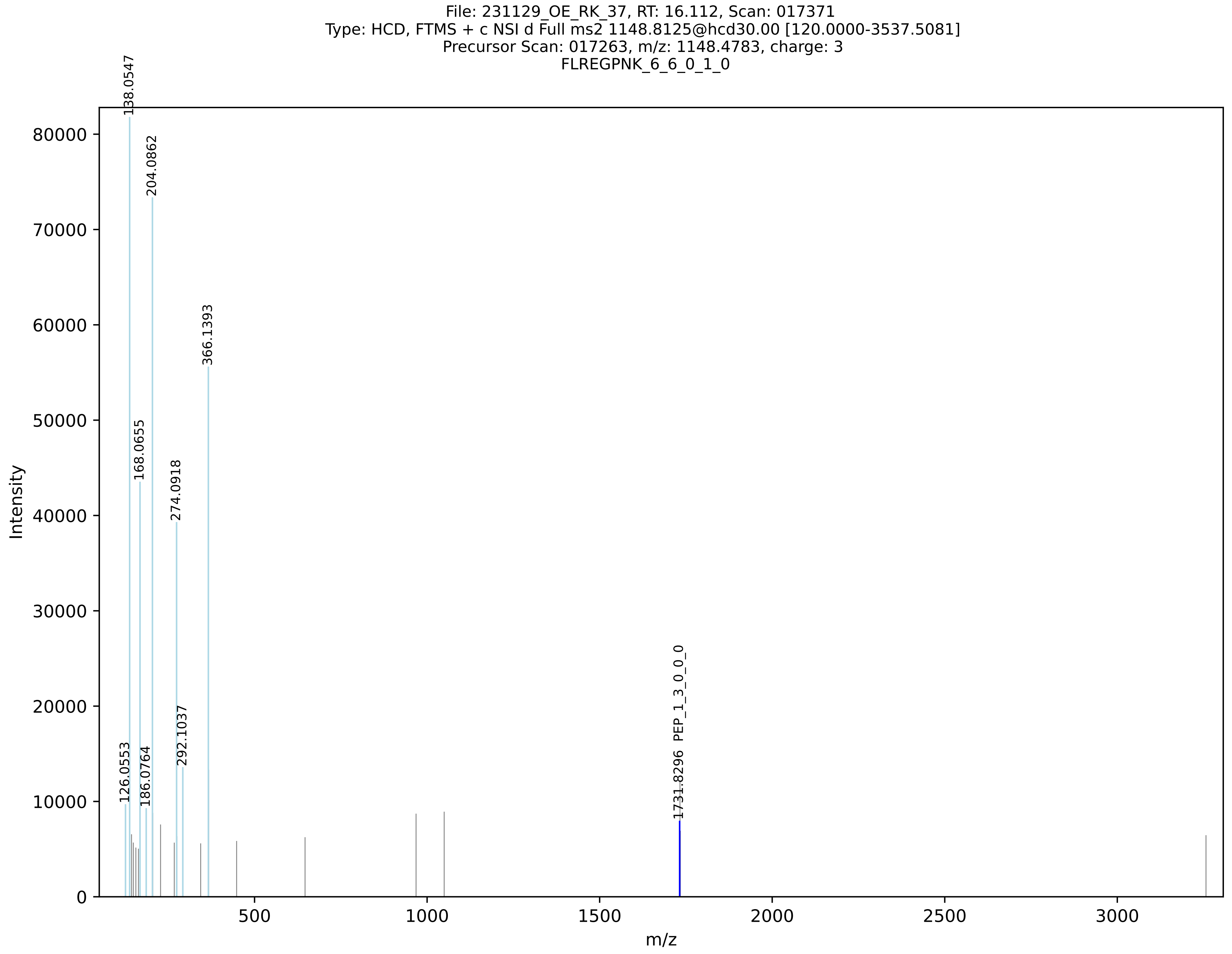
File: 231129_OE_RK_37, RT: 16.112, Scan: 017371
Type: HCD, FTMS + c NSI d Full ms2 1148.8125@hcd30.00 [120.0000-3537.5081]
Precursor Scan: 017263, m/z: 1148.4783, charge: 3
FLREGPNK_6_6_0_1_0
Intensity
m/z
138.0547
204.0862
366.1393
168.0655
274.0918
292.1037
126.0553
186.0764
1731.8296 PEP_1_3_0_0_0

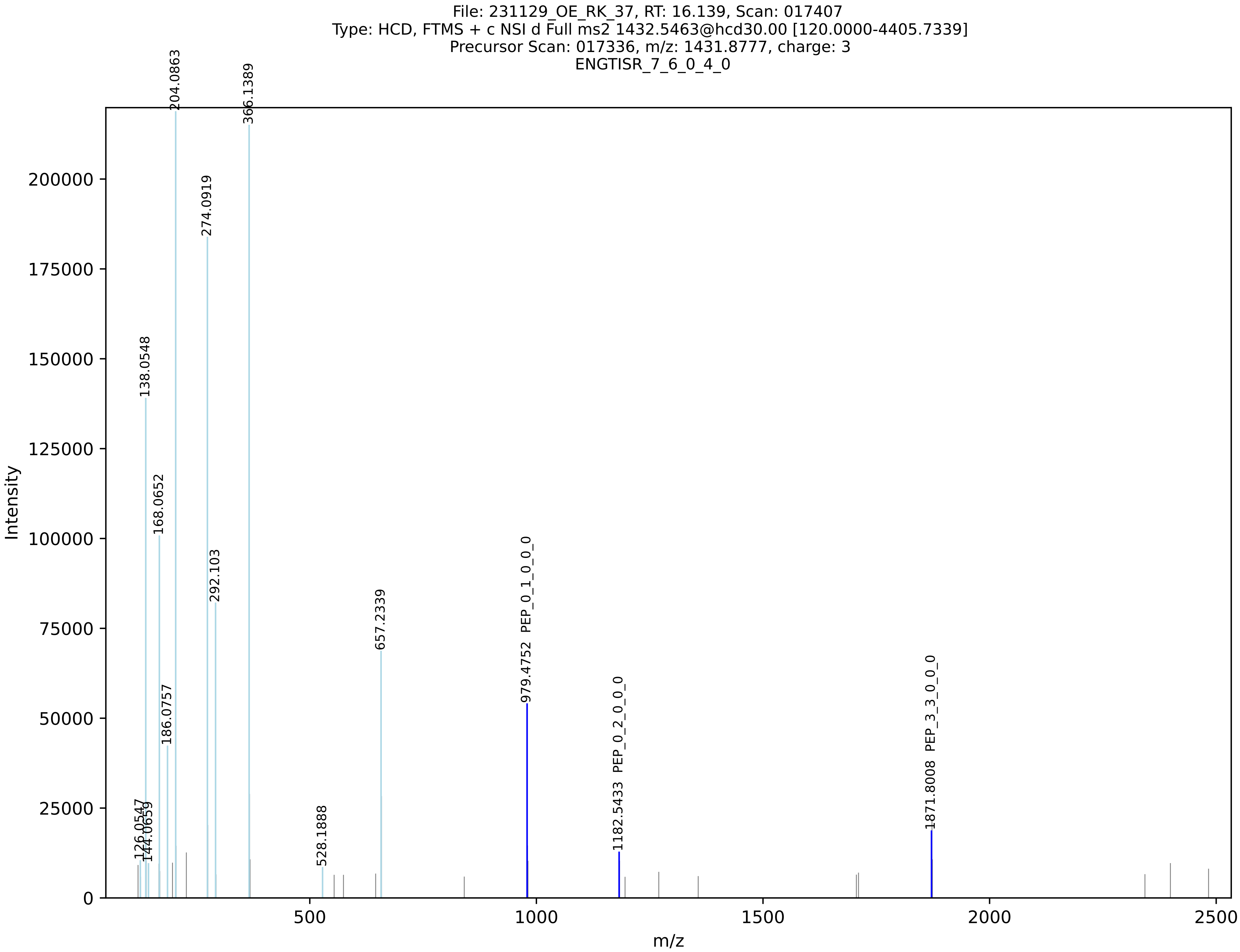

File: 231129_OE_RK_37, RT: 16.139, Scan: 017407
Type: HCD, FTMS + c NSI d Full ms2 1432.5463@hcd30.00 [120.0000-4405.7339]
Precursor Scan: 017336, m/z: 1431.8777, charge: 3
ENGTISR_7_6_0_4_0
Intensity
m/z
204.0863
366.1389
274.0919
138.0548
168.0652
292.103
186.0757
126.0547
144.0659
528.1888
657.2339
979.4752 PEP_0_1_0_0_0
1182.5433 PEP_0_2_0_0_0
1871.8008 PEP_3_3_0_0_0

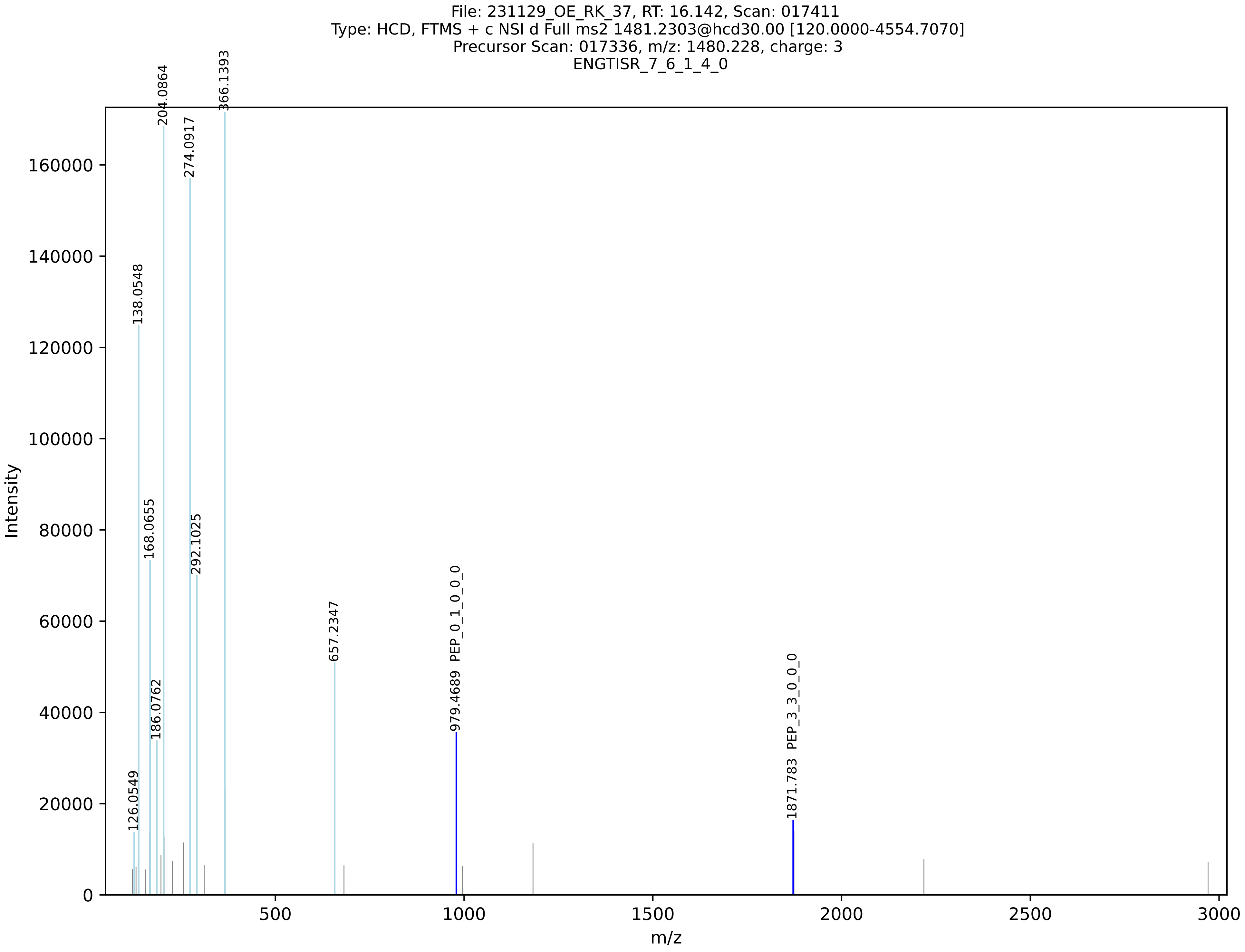
File: 231129_OE_RK_37, RT: 16.142, Scan: 017411
Type: HCD, FTMS + c NSI d Full ms2 1481.2303@hcd30.00 [120.0000-4554.7070]
Precursor Scan: 017336, m/z: 1480.228, charge: 3
ENGTISR_7_6_1_4_0
366.1393
204.0864
274.0917
138.0548
168.0655
292.1025
186.0762
126.0549
657.2347
979.4689 PEP_0_1_0_0_0
1871.783 PEP_3_3_0_0_0
Intensity
m/z
0
20000
40000
60000
80000
100000
120000
140000
160000
500
1000
1500
2000
2500
3000

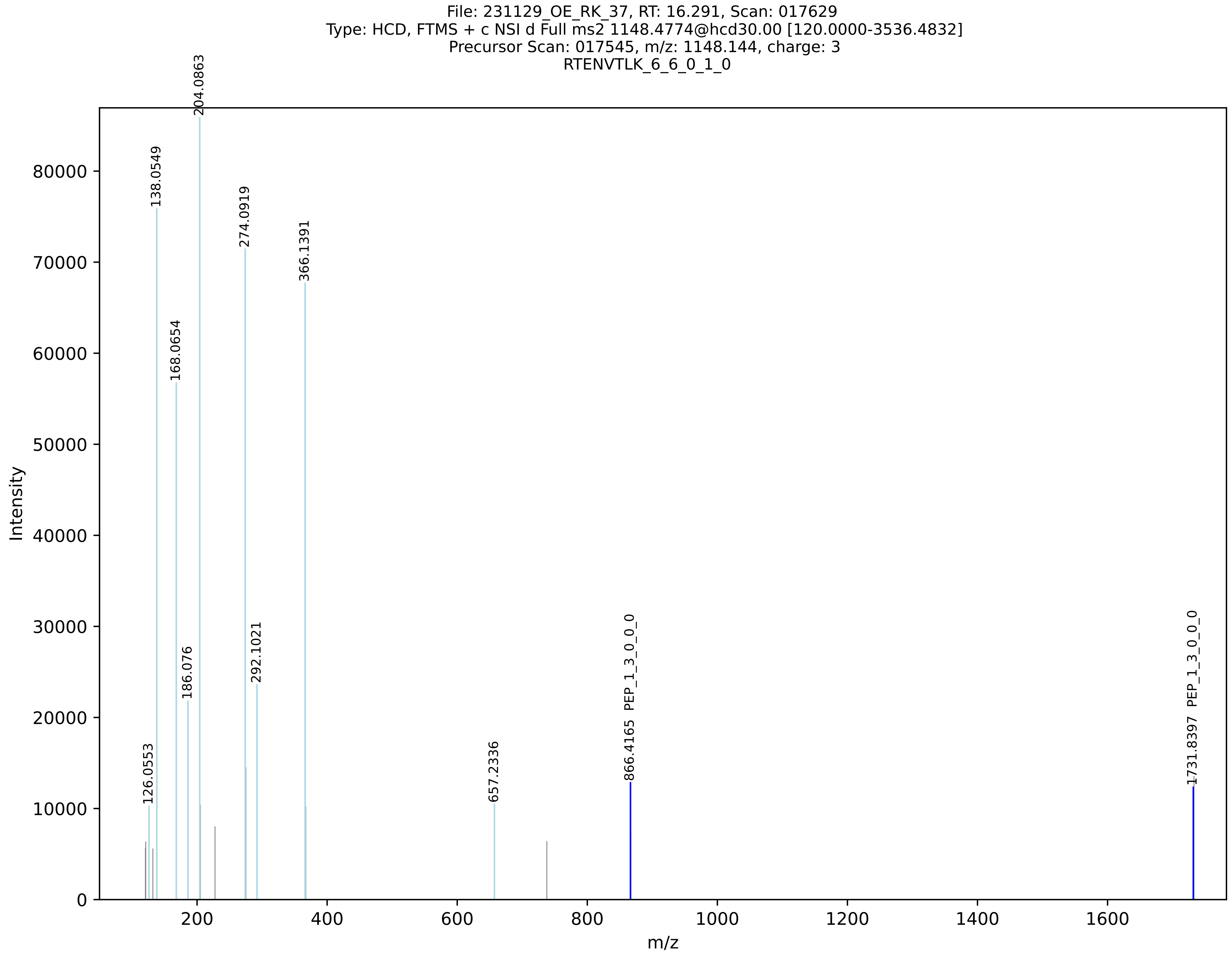
File: 231129_OE_RK_37, RT: 16.291, Scan: 017629
Type: HCD, FTMS + c NSI d Full ms2 1148.4774@hcd30.00 [120.0000-3536.4832]
Precursor Scan: 017545, m/z: 1148.144, charge: 3
RTENVTLK_6_6_0_1_0
Intensity
m/z
204.0863
138.0549
274.0919
366.1391
168.0654
186.076
292.1021
126.0553
657.2336
866.4165 PEP_1_3_0_0_0
1731.8397 PEP_1_3_0_0_0
0
10000
20000
30000
40000
50000
60000
70000
80000
200
400
600
800
1000
1200
1400
1600

File: 231129_OE_RK_37, RT: 16.339, Scan: 017695
Type: HCD, FTMS + c NSI d Full ms2 1432.5444@hcd30.00 [120.0000-4405.7280]
Precursor Scan: 017618, m/z: 1431.8764, charge: 3
ENGTISR_7_6_0_4_0

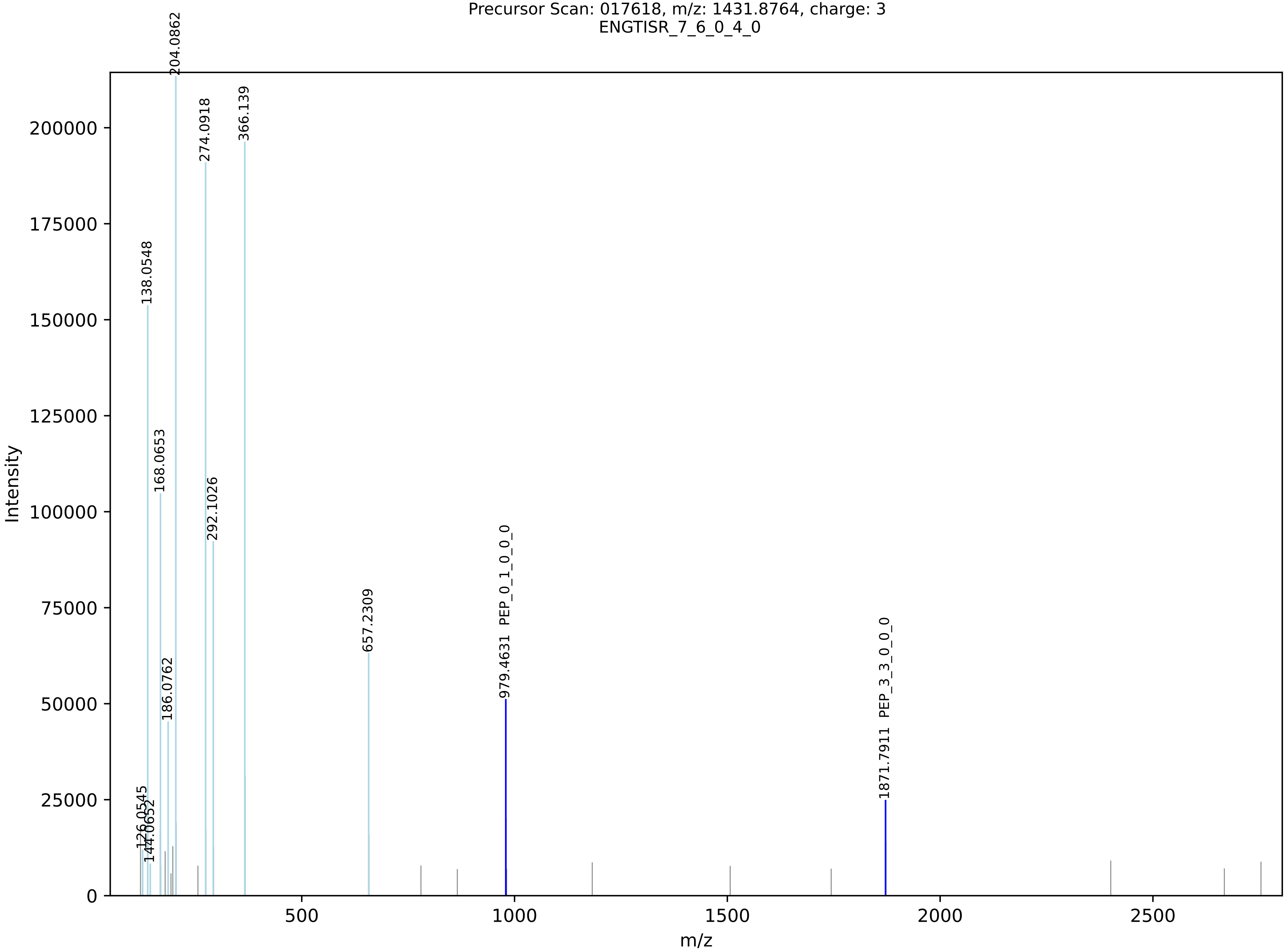

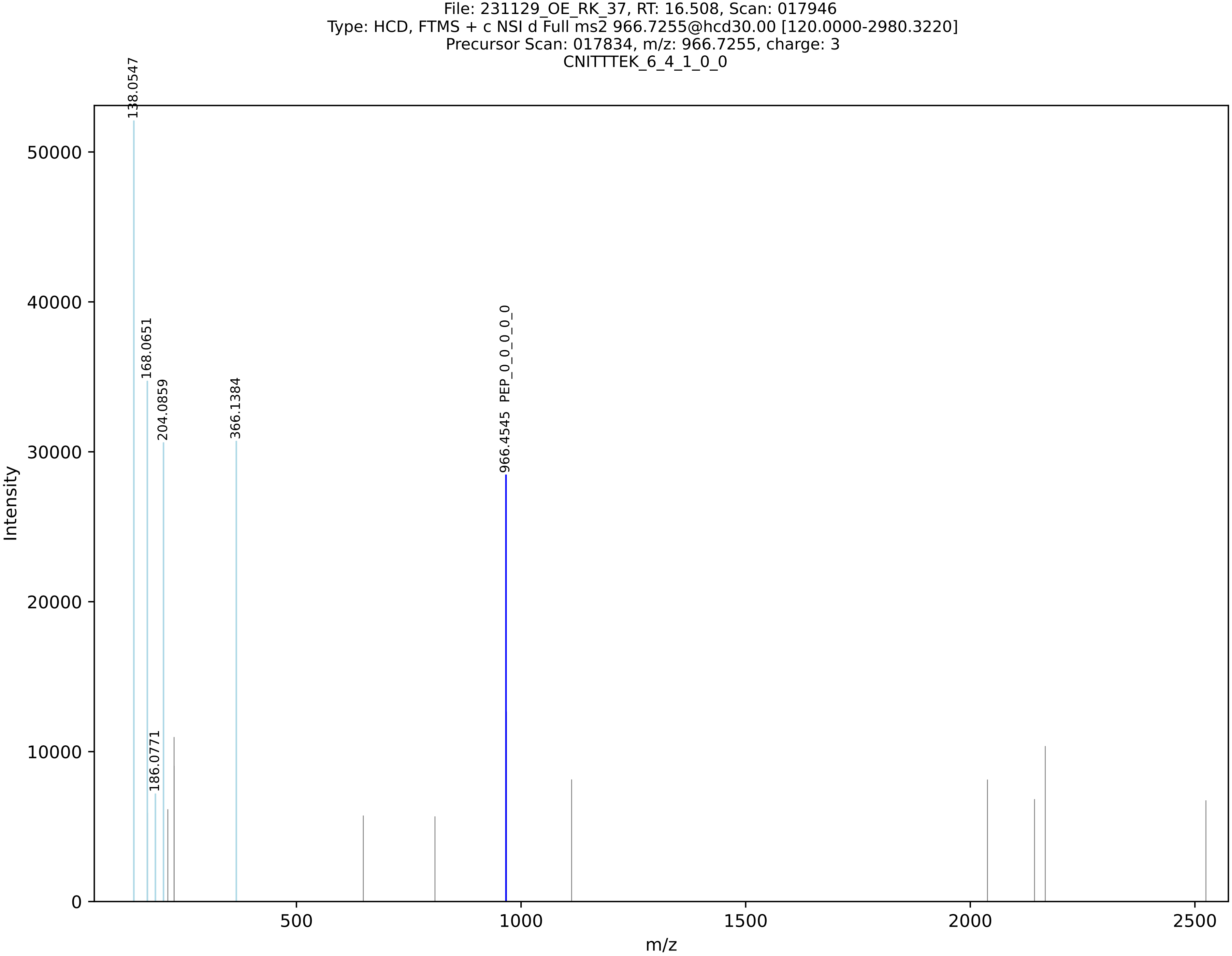

File: 231129_OE_RK_37, RT: 16.508, Scan: 017946
Type: HCD, FTMS + c NSI d Full ms2 966.7255@hcd30.00 [120.0000-2980.3220]
Precursor Scan: 017834, m/z: 966.7255, charge: 3
CNITTTEK_6_4_1_0_0
138.0547
168.0651
204.0859
366.1384
966.4545 PEP_0_0_0_0_0
186.0771
Intensity
m/z
0
10000
20000
30000
40000
50000
500
1000
1500
2000
2500

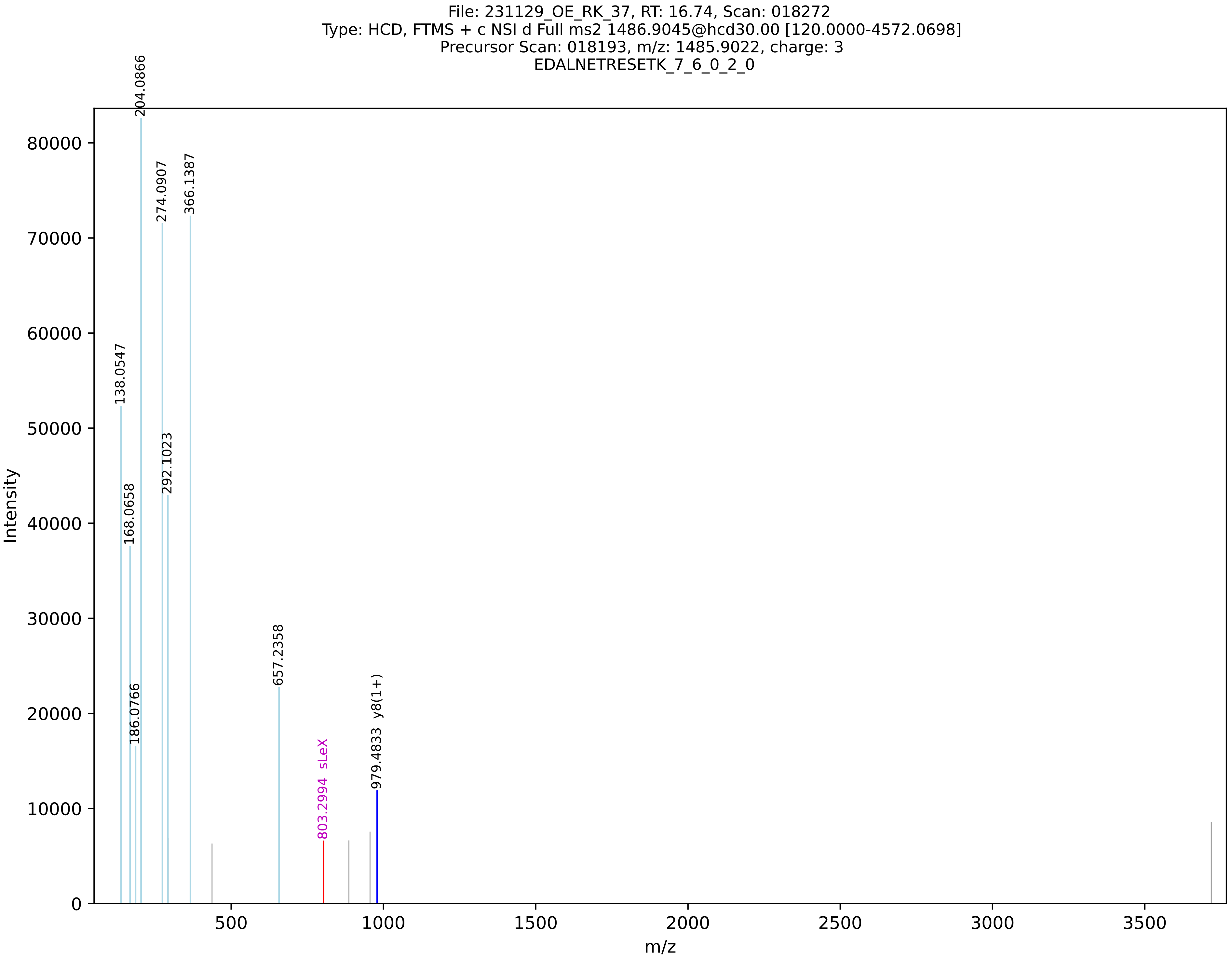
File: 231129_OE_RK_37, RT: 16.74, Scan: 018272
Type: HCD, FTMS + c NSI d Full ms2 1486.9045@hcd30.00 [120.0000-4572.0698]
Precursor Scan: 018193, m/z: 1485.9022, charge: 3
EDALNETRESETK_7_6_0_2_0
204.0866
274.0907
366.1387
138.0547
292.1023
168.0658
657.2358
186.0766
y8(1+)
979.4833
sLeX
803.2994
Intensity
m/z
0
10000
20000
30000
40000
50000
60000
70000
80000
500
1000
1500
2000
2500
3000
3500

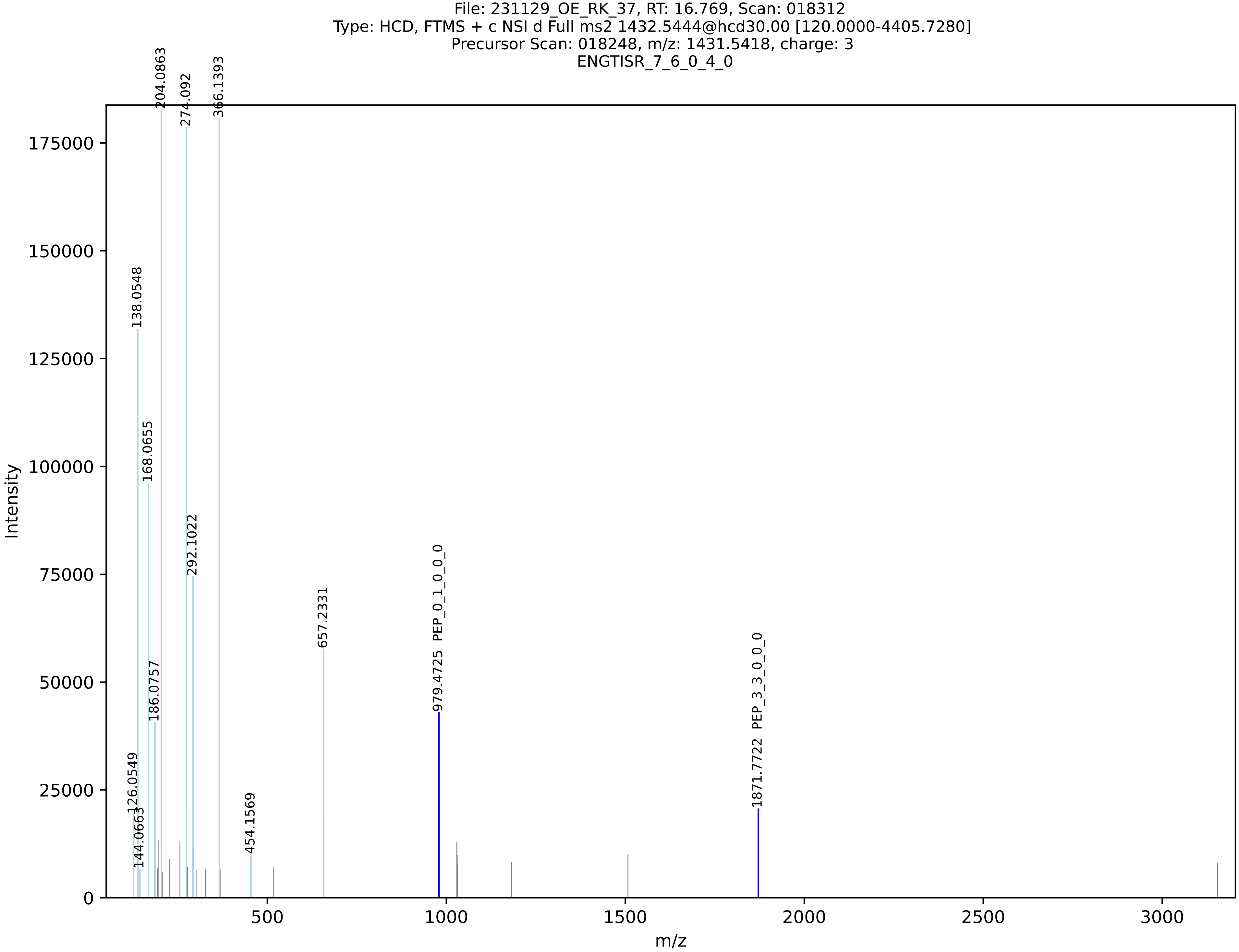
File: 231129_OE_RK_37, RT: 16.769, Scan: 018312
Type: HCD, FTMS + c NSI d Full ms2 1432.5444@hcd30.00 [120.0000-4405.7280]
Precursor Scan: 018248, m/z: 1431.5418, charge: 3
ENGTISR_7_6_0_4_0
Intensity
m/z
204.0863
274.092
366.1393
138.0548
168.0655
292.1022
657.2331
PEP_0_1_0_0_0
979.4725
PEP_3_3_0_0_0
1871.7722
186.0757
126.0549
144.0663
454.1569
0
25000
50000
75000
100000
125000
150000
175000
500
1000
1500
2000
2500
3000

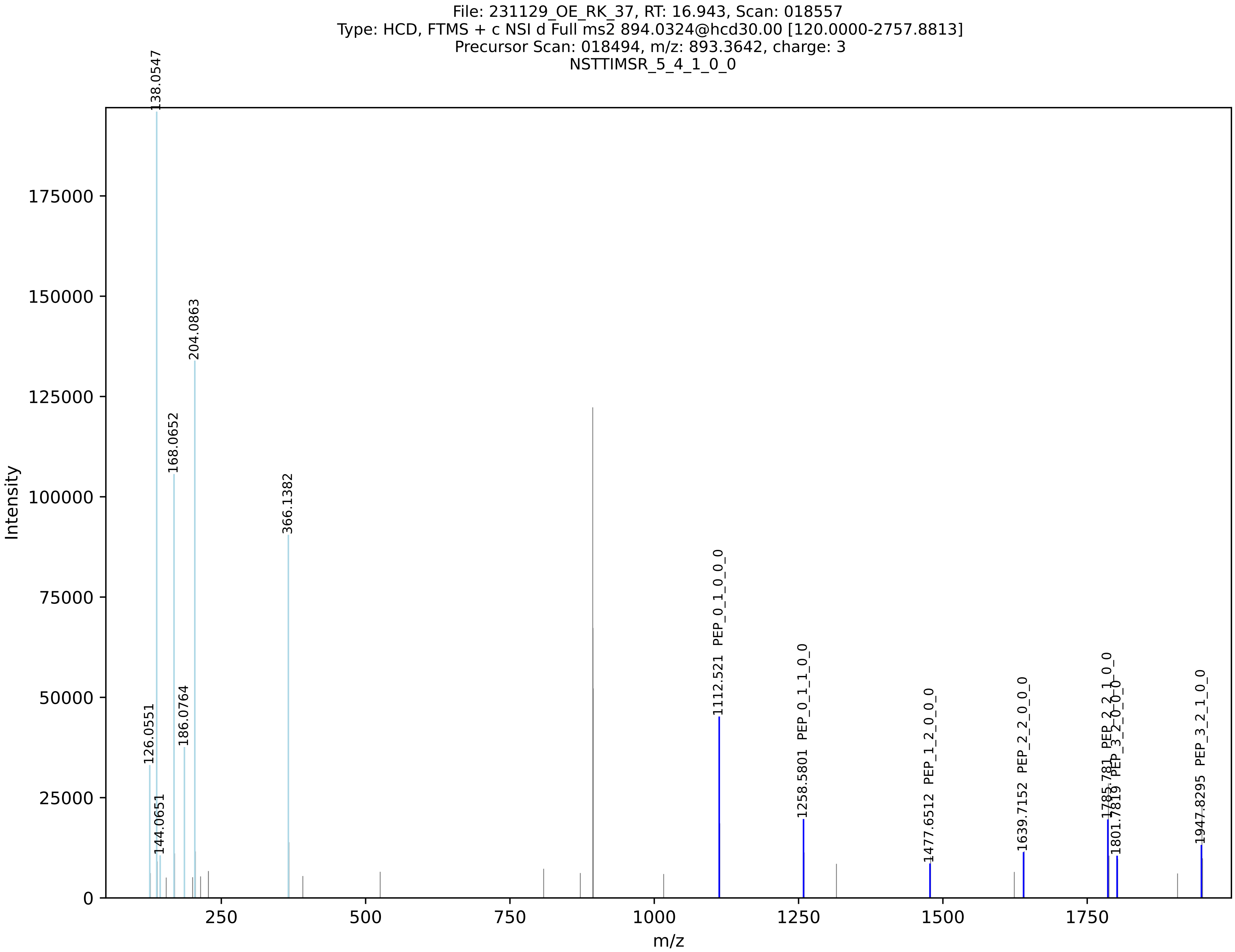
File: 231129_OE_RK_37, RT: 16.943, Scan: 018557
Type: HCD, FTMS + c NSI d Full ms2 894.0324@hcd30.00 [120.0000-2757.8813]
Precursor Scan: 018494, m/z: 893.3642, charge: 3
NSTTIMSR_5_4_1_0_0
138.0547
204.0863
168.0652
366.1382
126.0551
186.0764
144.0651
1112.521 PEP_0_1_0_0_0
1258.5801 PEP_0_1_1_0_0
1477.6512 PEP_1_2_0_0_0
1639.7152 PEP_2_2_0_0_0
1785.781 PEP_2_2_1_0_0
1801.7819 PEP_3_2_0_0_0
1947.8295 PEP_3_2_1_0_0
Intensity
m/z
0
25000
50000
75000
100000
125000
150000
175000
250
500
750
1000
1250
1500
1750

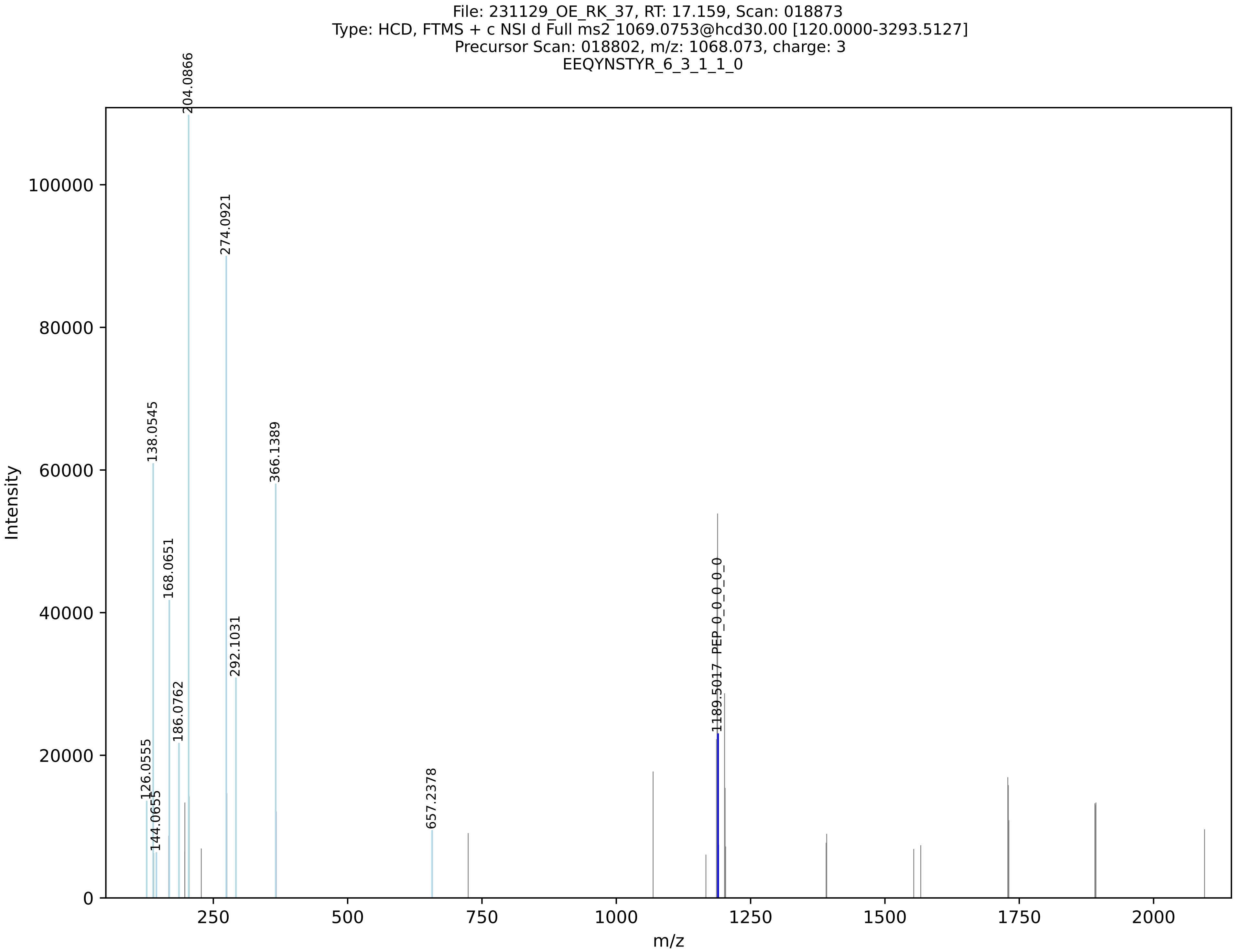
File: 231129_OE_RK_37, RT: 17.159, Scan: 018873
Type: HCD, FTMS + c NSI d Full ms2 1069.0753@hcd30.00 [120.0000-3293.5127]
Precursor Scan: 018802, m/z: 1068.073, charge: 3
EEQYNSTYR_6_3_1_1_0
204.0866
274.0921
138.0545
366.1389
168.0651
292.1031
186.0762
126.0555
144.0655
657.2378
1189.5017_PEP_0_0_0_0_
Intensity
m/z
0
20000
40000
60000
80000
100000
250
500
750
1000
1250
1500
1750
2000

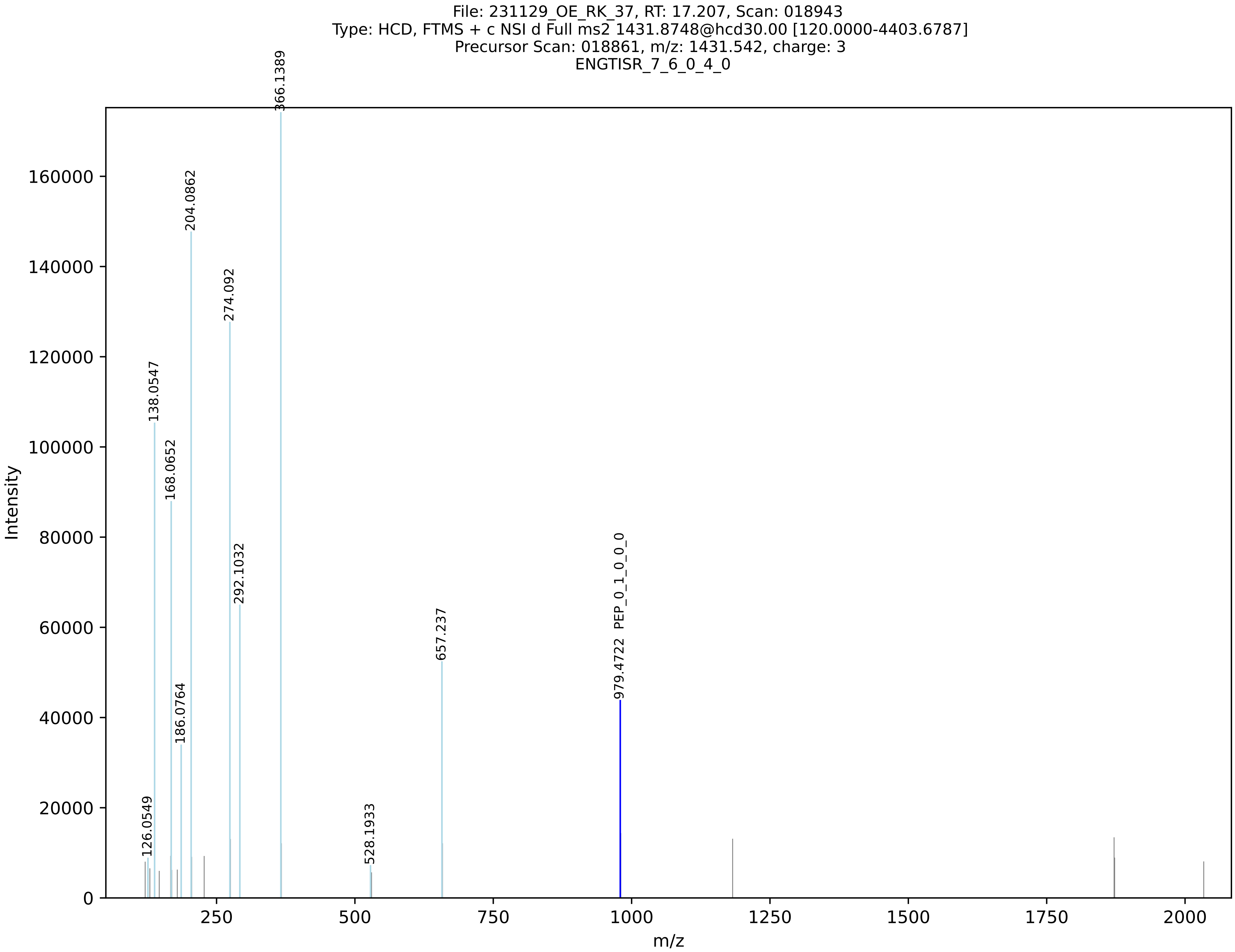

File: 231129_OE_RK_37, RT: 17.207, Scan: 018943
Type: HCD, FTMS + c NSI d Full ms2 1431.8748@hcd30.00 [120.0000-4403.6787]
Precursor Scan: 018861, m/z: 1431.542, charge: 3
ENGTISR_7_6_0_4_0
366.1389
204.0862
274.092
138.0547
168.0652
292.1032
186.0764
126.0549
528.1933
657.237
979.4722 PEP_0_1_0_0_0
Intensity
m/z
0
20000
40000
60000
80000
100000
120000
140000
160000
250
500
750
1000
1250
1500
1750
2000

File: 231129_OE_RK_37, RT: 17.235, Scan: 018987
Type: HCD, FTMS + c NSI d Full ms2 1433.5496@hcd30.00 [120.0000-4408.8037]
Precursor Scan: 018802, m/z: 1431.8773, charge: 3
ENGTISR_7_6_0_4_0

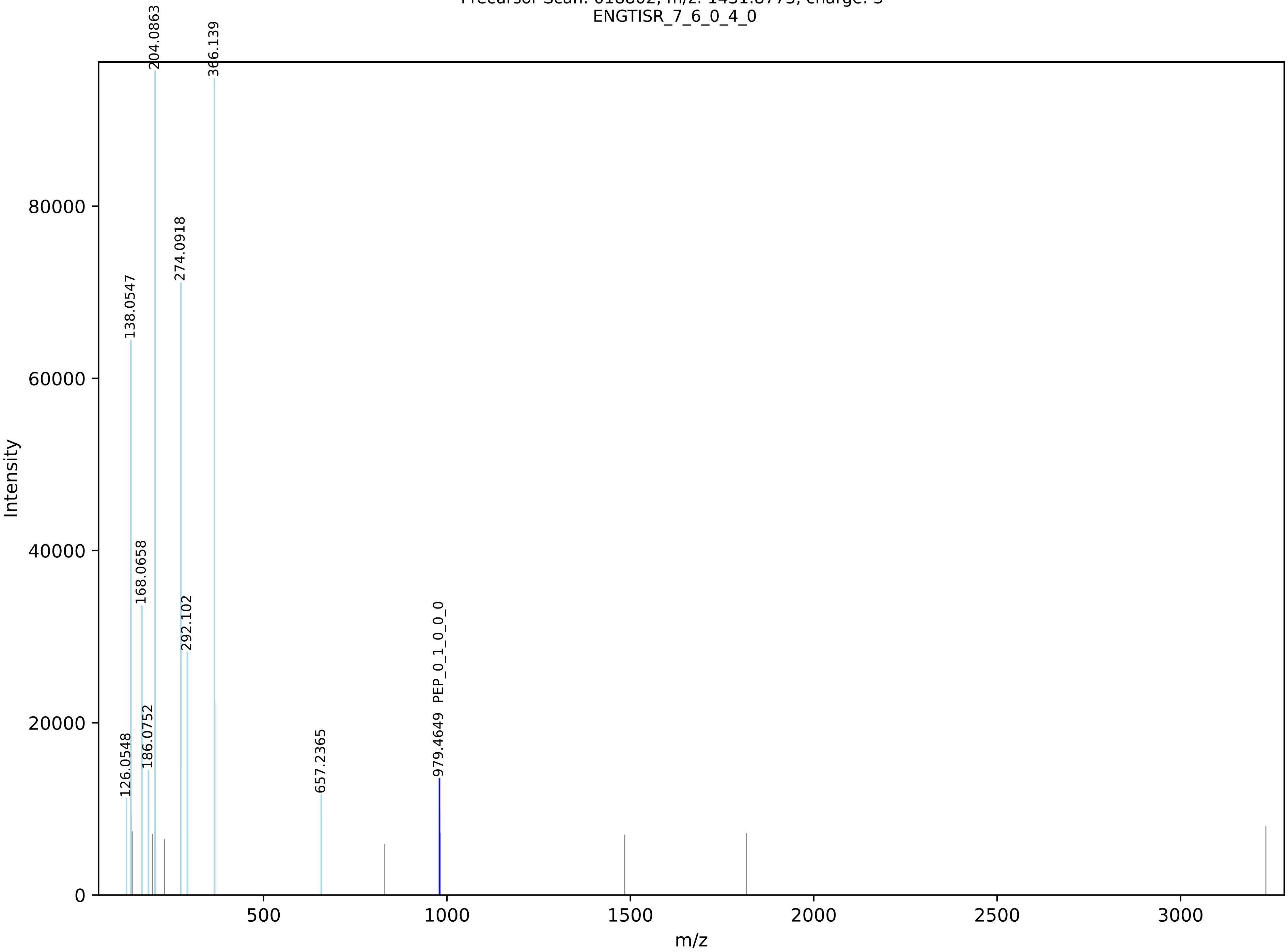

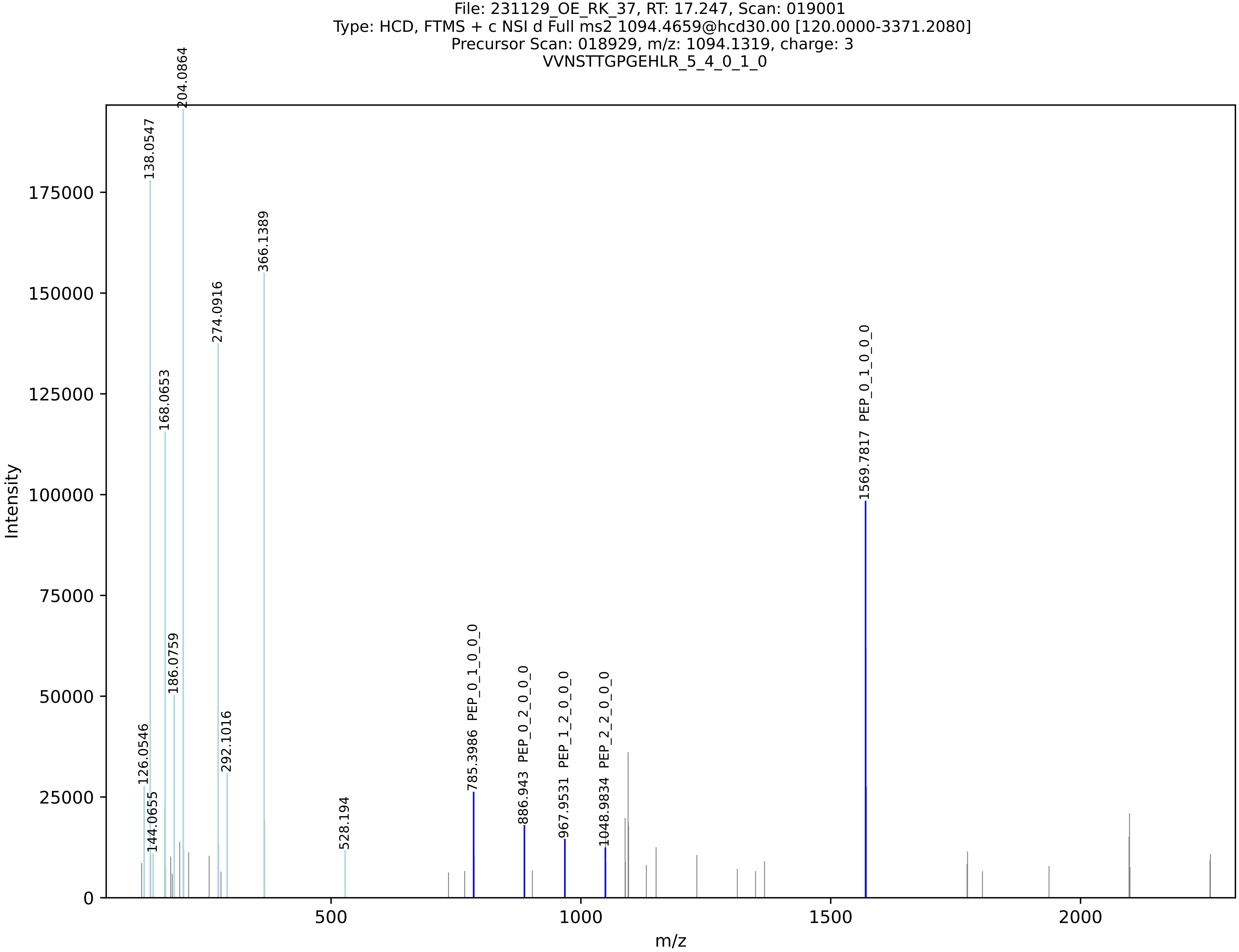

File: 231129_OE_RK_37, RT: 17.247, Scan: 019001
Type: HCD, FTMS + c NSI d Full ms2 1094.4659@hcd30.00 [120.0000-3371.2080]
Precursor Scan: 018929, m/z: 1094.1319, charge: 3
VVNSTTGPGEHLR_5_4_0_1_0
Intensity
m/z
0
25000
50000
75000
100000
125000
150000
175000
500
1000
1500
2000
204.0864
138.0547
366.1389
274.0916
168.0653
186.0759
126.0546
292.1016
144.0655
528.194
785.3986 PEP_0_1_0_0_0
886.943 PEP_0_2_0_0_0
967.9531 PEP_1_2_0_0_0
1048.9834 PEP_2_2_0_0_0
1569.7817 PEP_0_1_0_0_0

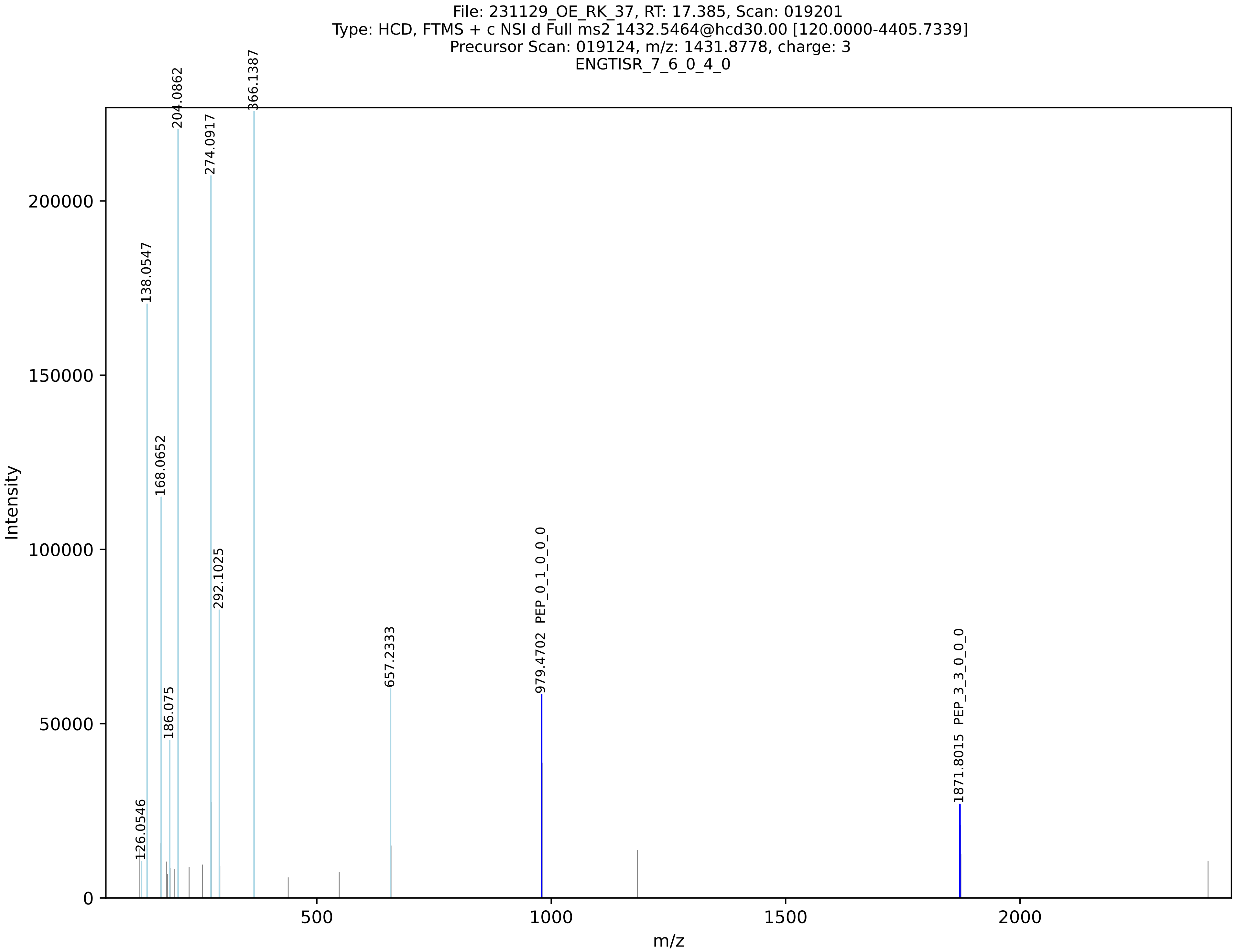
File: 231129_OE_RK_37, RT: 17.385, Scan: 019201
Type: HCD, FTMS + c NSI d Full ms2 1432.5464@hcd30.00 [120.0000-4405.7339]
Precursor Scan: 019124, m/z: 1431.8778, charge: 3
ENGTISR_7_6_0_4_0
Intensity
m/z
0
50000
100000
150000
200000
500
1000
1500
2000
126.0546
138.0547
168.0652
186.075
204.0862
274.0917
292.1025
366.1387
657.2333
979.4702 PEP_0_1_0_0_0
1871.8015 PEP_3_3_0_0_0

File: 231129_OE_RK_37, RT: 17.385, Scan: 019202
Type: HCD, FTMS + c NSI d Full ms2 934.6356@hcd30.00 [120.0000-3836.1692]
Precursor Scan: 019124, m/z: 934.1342, charge: 4
THTCQVIIENVSK_5_4_0_2_0

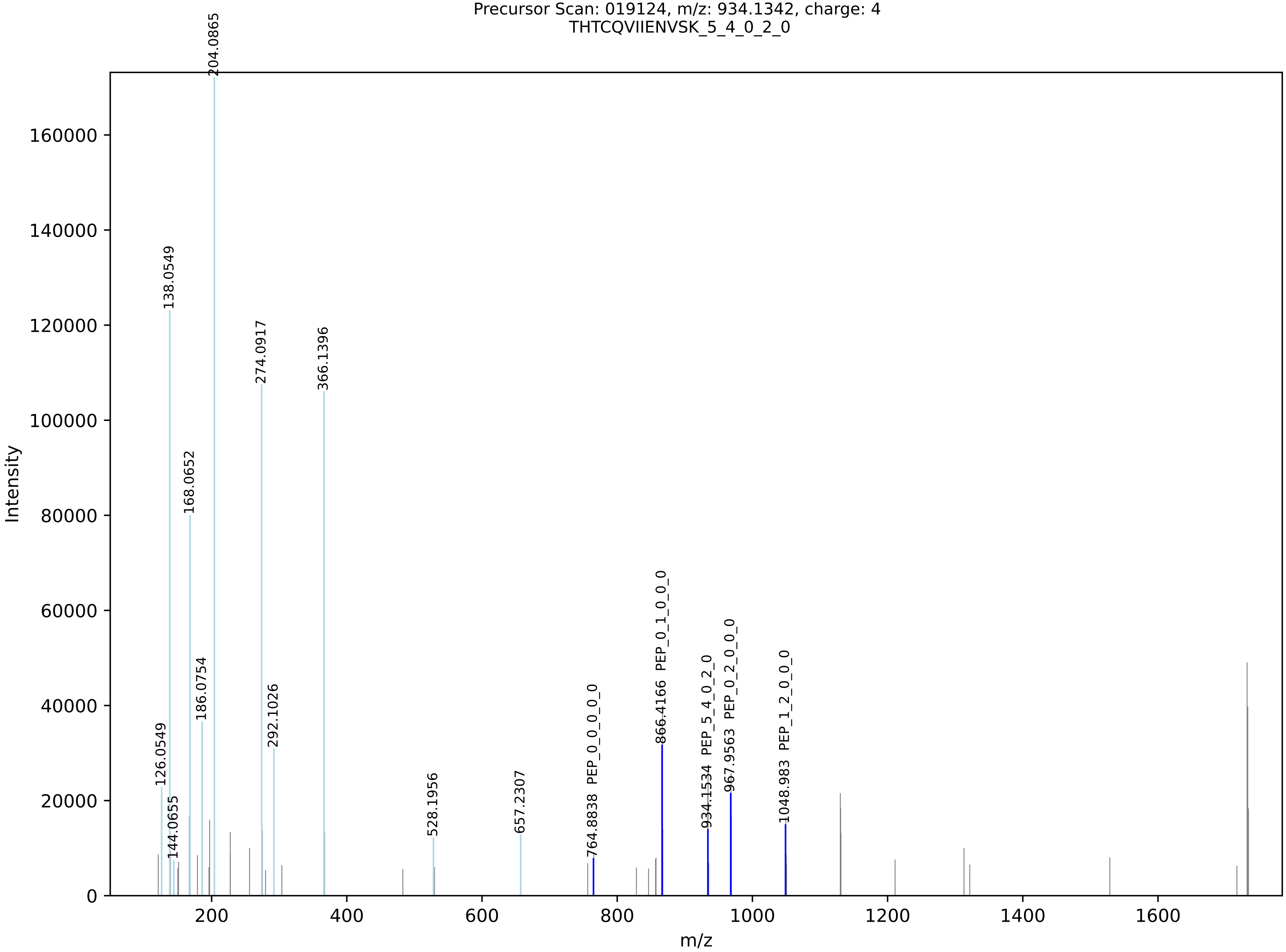

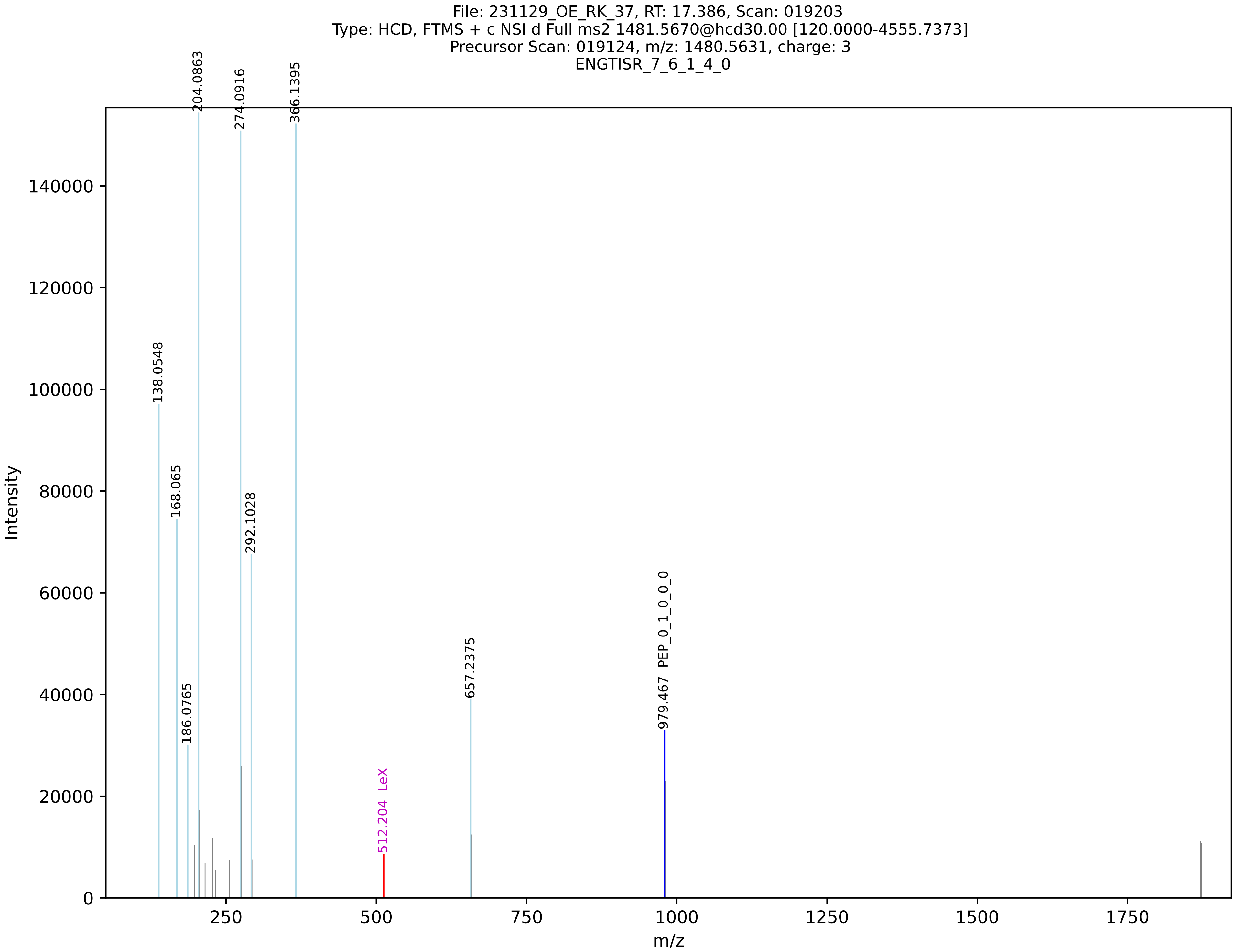
File: 231129_OE_RK_37, RT: 17.386, Scan: 019203
Type: HCD, FTMS + c NSI d Full ms2 1481.5670@hcd30.00 [120.0000-4555.7373]
Precursor Scan: 019124, m/z: 1480.5631, charge: 3
ENGTISR_7_6_1_4_0
Intensity
m/z
204.0863
274.0916
366.1395
138.0548
168.065
292.1028
186.0765
657.2375
979.467 PEP_0_1_0_0_0
512.204 LeX
0
20000
40000
60000
80000
100000
120000
140000
250
500
750
1000
1250
1500
1750

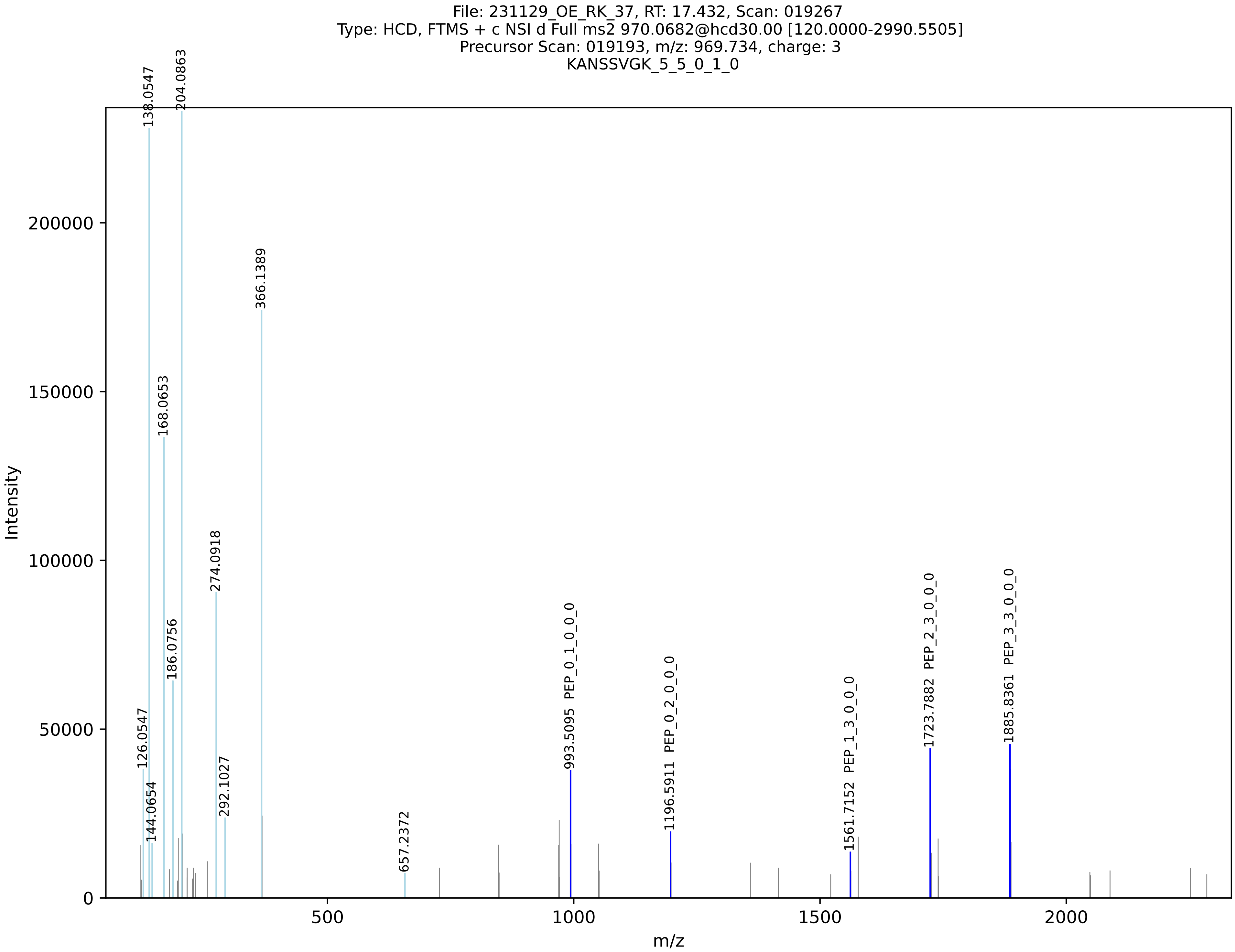
File: 231129_OE_RK_37, RT: 17.432, Scan: 019267
Type: HCD, FTMS + c NSI d Full ms2 970.0682@hcd30.00 [120.0000-2990.5505]
Precursor Scan: 019193, m/z: 969.734, charge: 3
KANSSVGK_5_5_0_1_0
Intensity
m/z
138.0547
204.0863
366.1389
168.0653
274.0918
186.0756
126.0547
144.0654
292.1027
657.2372
993.5095 PEP_0_1_0_0_0
1196.5911 PEP_0_2_0_0_0
1561.7152 PEP_1_3_0_0_0
1723.7882 PEP_2_3_0_0_0
1885.8361 PEP_3_3_0_0_0
0
50000
100000
150000
200000
500
1000
1500
2000

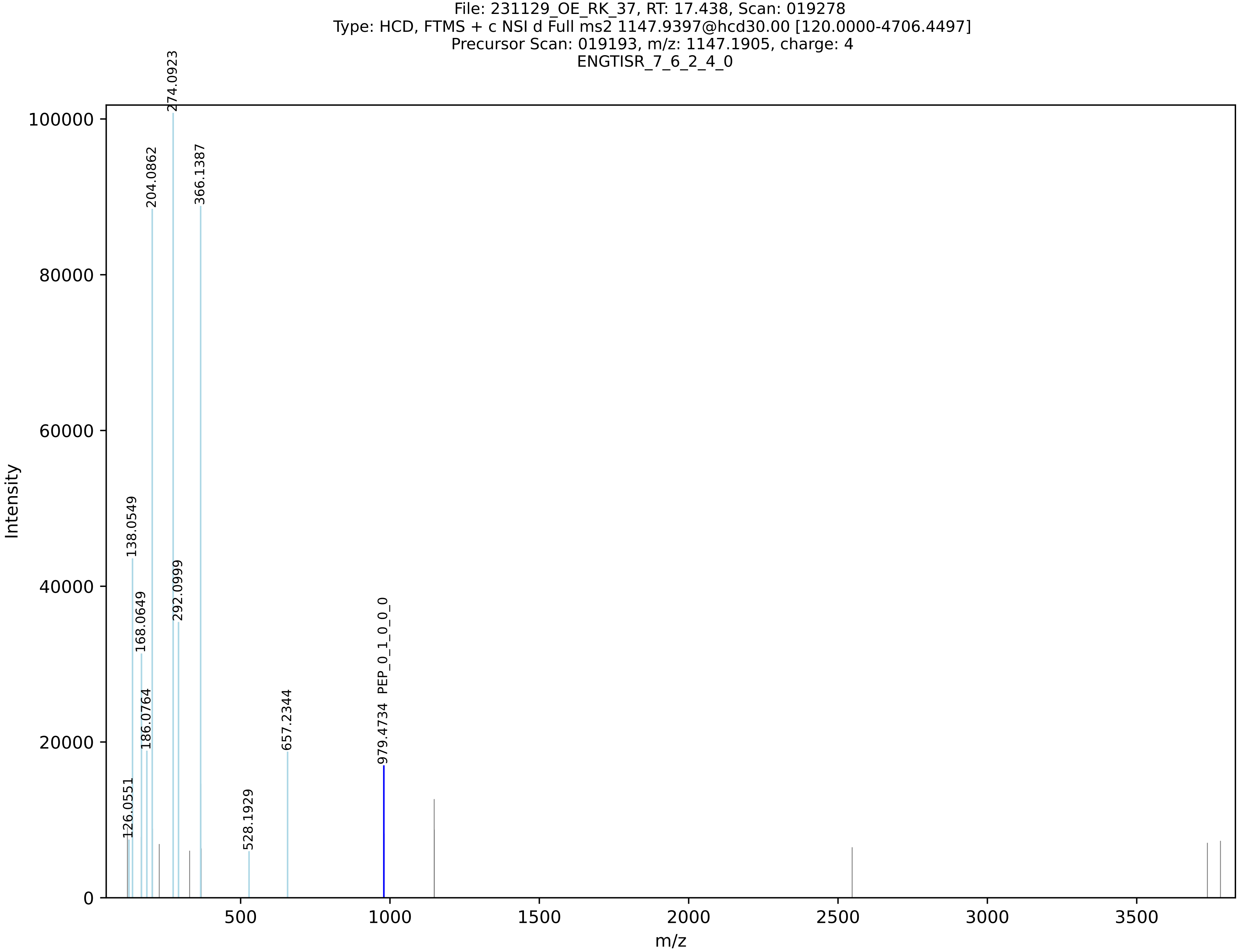
File: 231129_OE_RK_37, RT: 17.438, Scan: 019278
Type: HCD, FTMS + c NSI d Full ms2 1147.9397@hcd30.00 [120.0000-4706.4497]
Precursor Scan: 019193, m/z: 1147.1905, charge: 4
ENGTISR_7_6_2_4_0
274.0923
204.0862
366.1387
138.0549
292.0999
168.0649
186.0764
126.0551
528.1929
657.2344
979.4734 PEP_0_1_0_0_0
Intensity
m/z
0
20000
40000
60000
80000
100000
500
1000
1500
2000
2500
3000
3500

File: 231129_OE_RK_37, RT: 17.485, Scan: 019346
Type: HCD, FTMS + c NSI d Full ms2 1214.1609@hcd30.00 [120.0000-3737.4744]
Precursor Scan: 019261, m/z: 1213.4927, charge: 3
HEQARNSSR_5_5_1_2_0

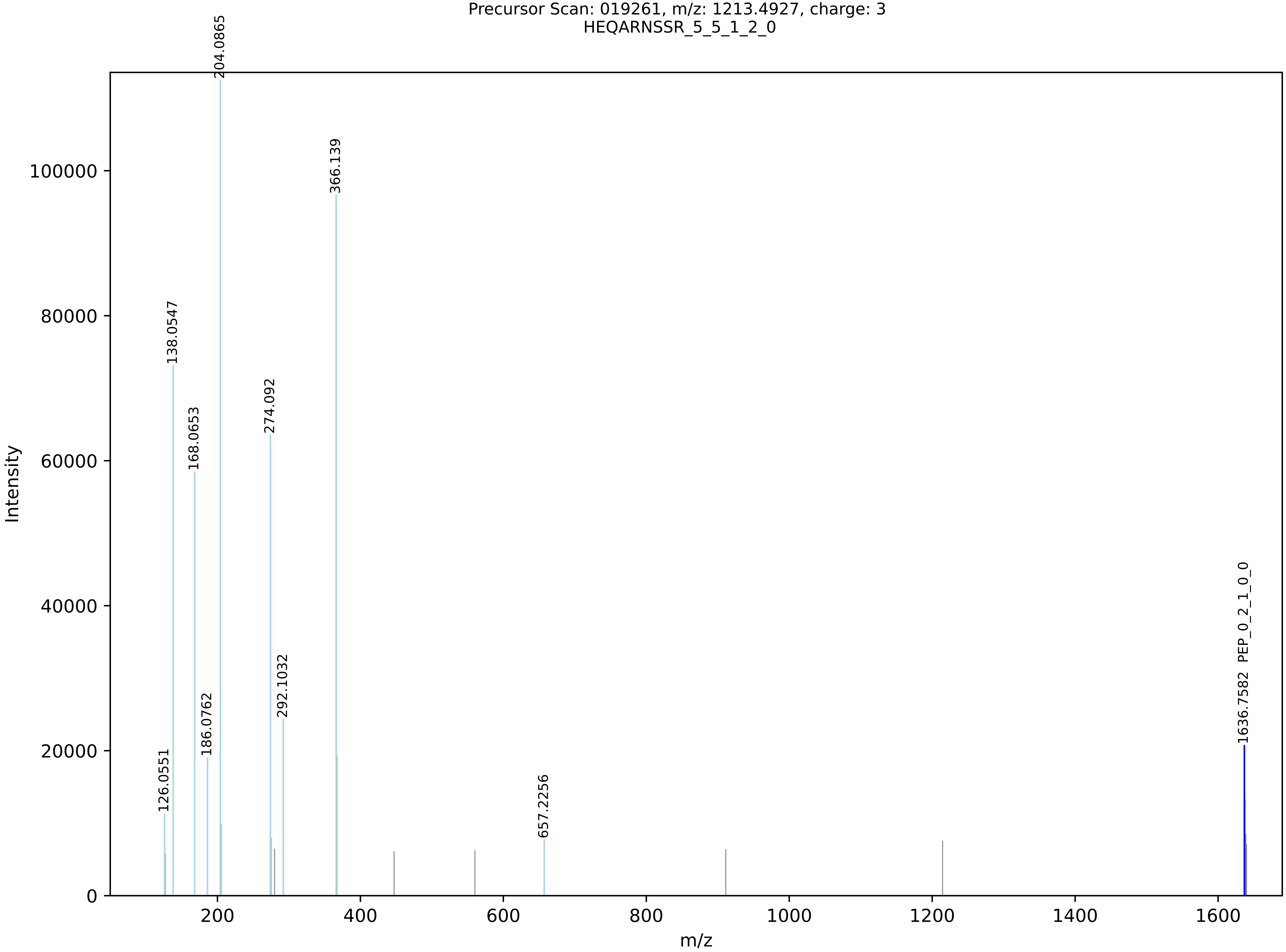

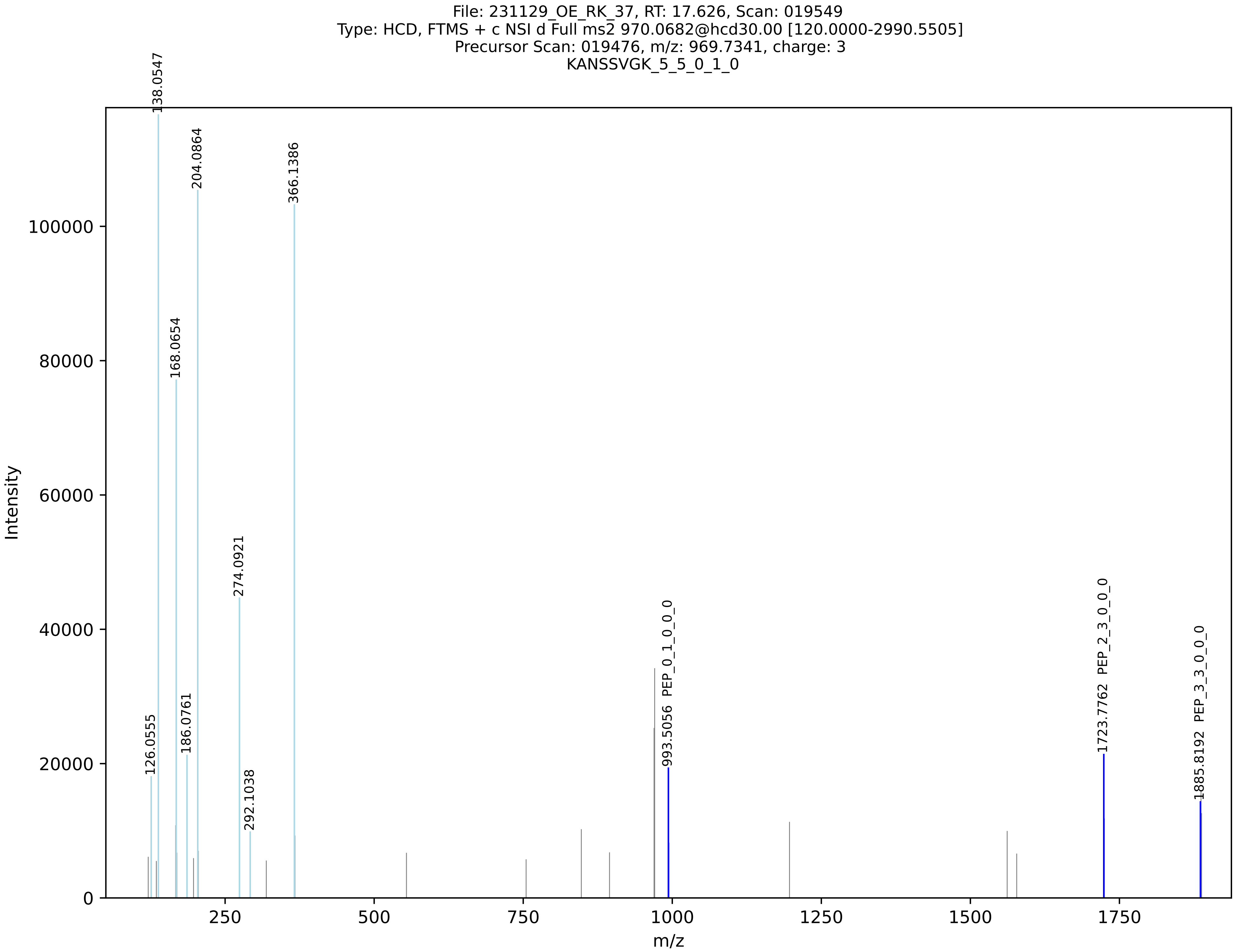
File: 231129_OE_RK_37, RT: 17.626, Scan: 019549
Type: HCD, FTMS + c NSI d Full ms2 970.0682@hcd30.00 [120.0000-2990.5505]
Precursor Scan: 019476, m/z: 969.7341, charge: 3
KANSSVGK_5_5_0_1_0
138.0547
204.0864
366.1386
168.0654
274.0921
126.0555
186.0761
292.1038
993.5056 PEP_0_1_0_0_0
1723.7762 PEP_2_3_0_0_0
1885.8192 PEP_3_3_0_0_0
Intensity
m/z
0
20000
40000
60000
80000
100000
250
500
750
1000
1250
1500
1750

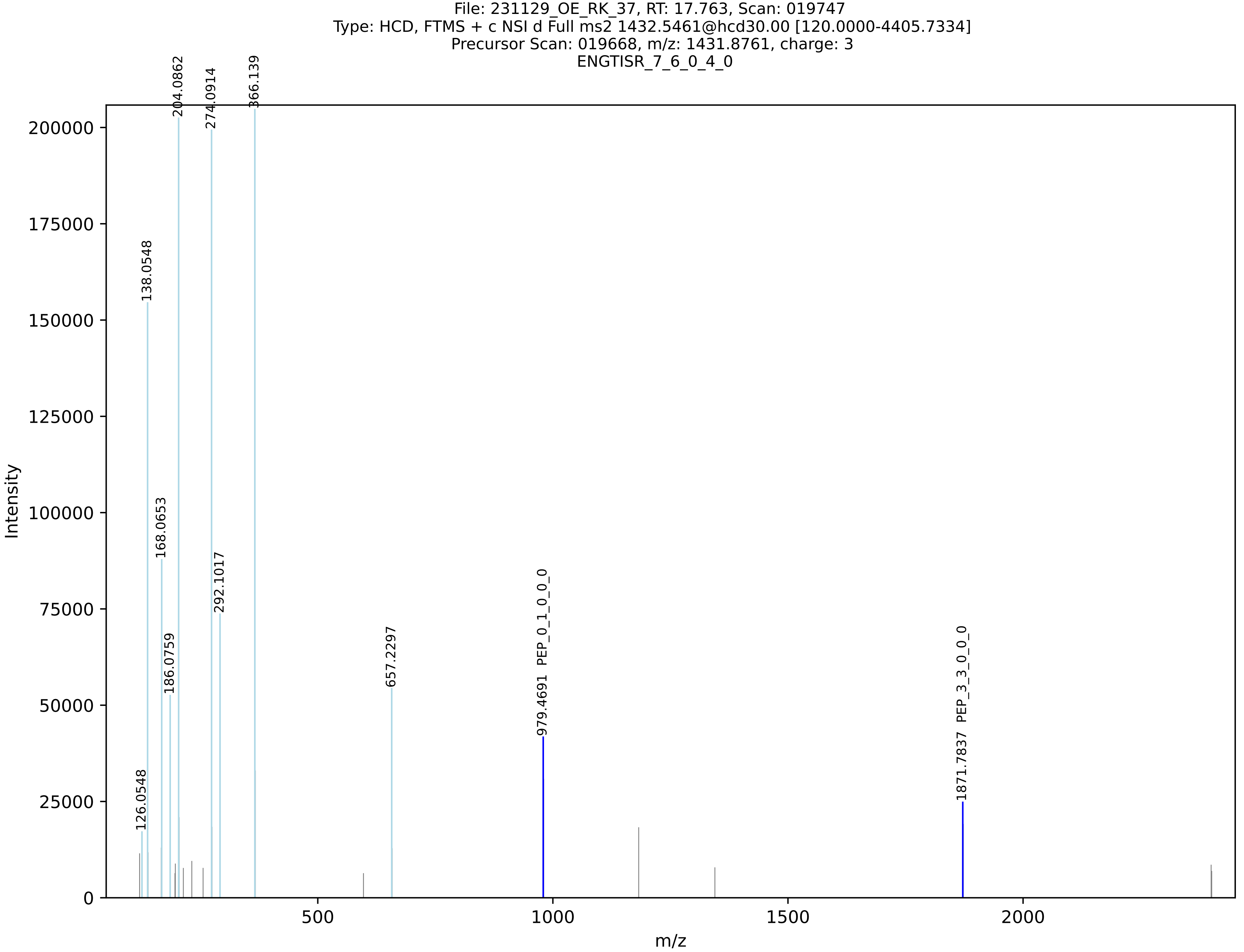
File: 231129_OE_RK_37, RT: 17.763, Scan: 019747
Type: HCD, FTMS + c NSI d Full ms2 1432.5461@hcd30.00 [120.0000-4405.7334]
Precursor Scan: 019668, m/z: 1431.8761, charge: 3
ENGTISR_7_6_0_4_0
Intensity
m/z
0
25000
50000
75000
100000
125000
150000
175000
200000
500
1000
1500
2000
126.0548
138.0548
168.0653
186.0759
204.0862
274.0914
292.1017
366.139
657.2297
979.4691 PEP_0_1_0_0_0
1871.7837 PEP_3_3_0_0_0

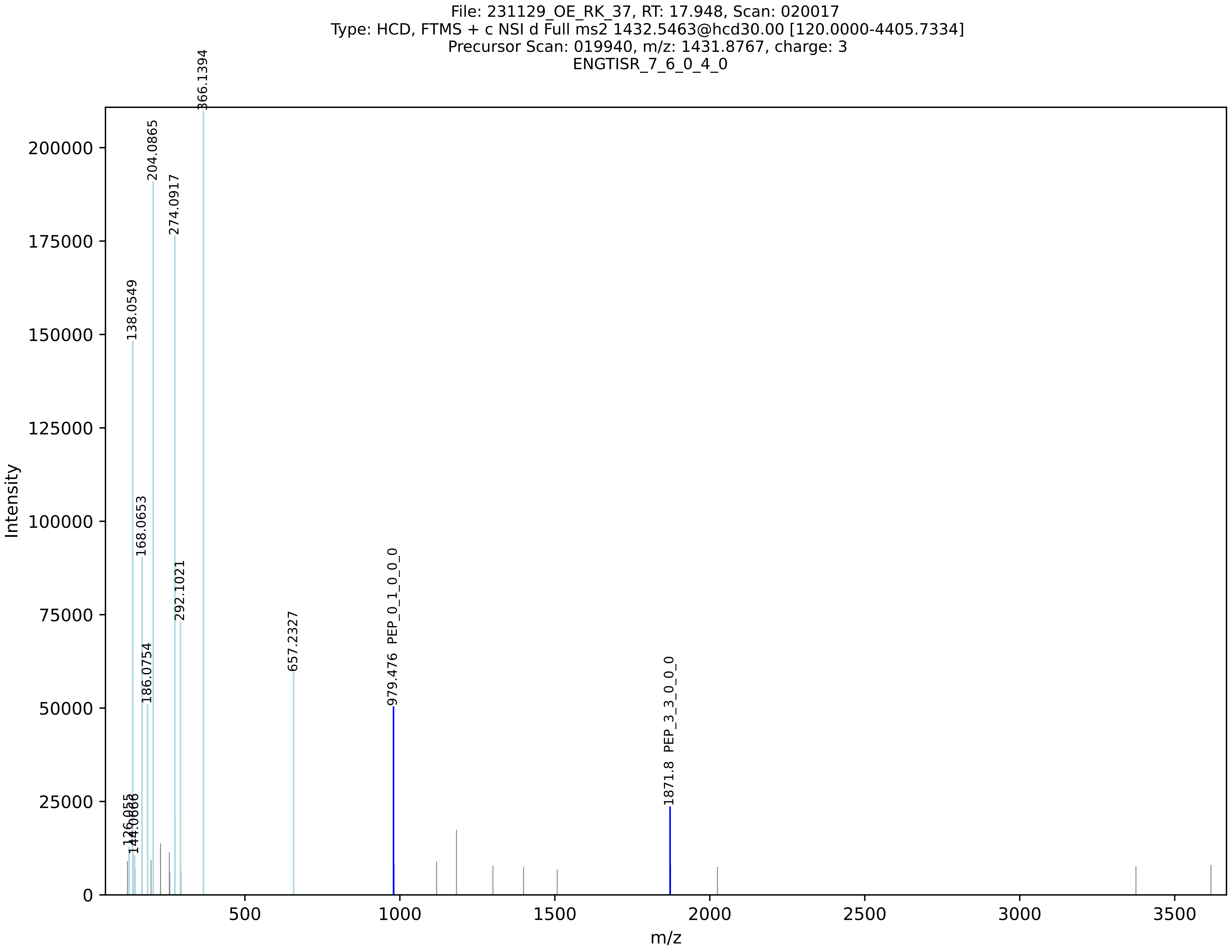

File: 231129_OE_RK_37, RT: 17.948, Scan: 020017
Type: HCD, FTMS + c NSI d Full ms2 1432.5463@hcd30.00 [120.0000-4405.7334]
Precursor Scan: 019940, m/z: 1431.8767, charge: 3
ENGTISR_7_6_0_4_0
366.1394
204.0865
274.0917
138.0549
168.0653
292.1021
657.2327
979.476 PEP_0_1_0_0_0
186.0754
1871.8 PEP_3_3_0_0_0
126.055
144.0808
Intensity
m/z

File: 231129_OE_RK_37, RT: 17.953, Scan: 020024
Type: HCD, FTMS + c NSI d Full ms2 1068.7419@hcd30.00 [120.0000-3292.4924]
Precursor Scan: 019940, m/z: 1068.4081, charge: 3
MFSQNDTR_5_4_0_2_0

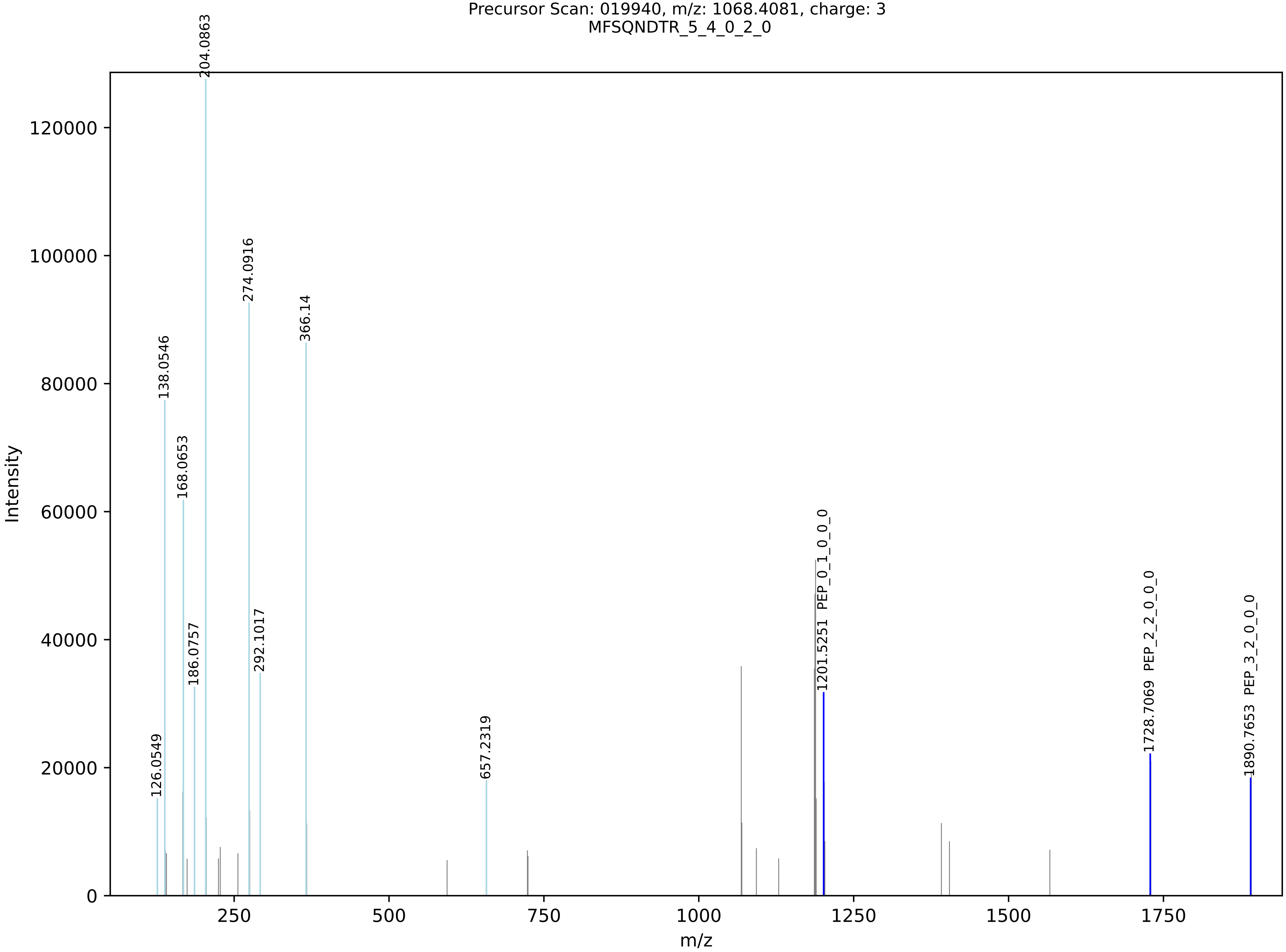

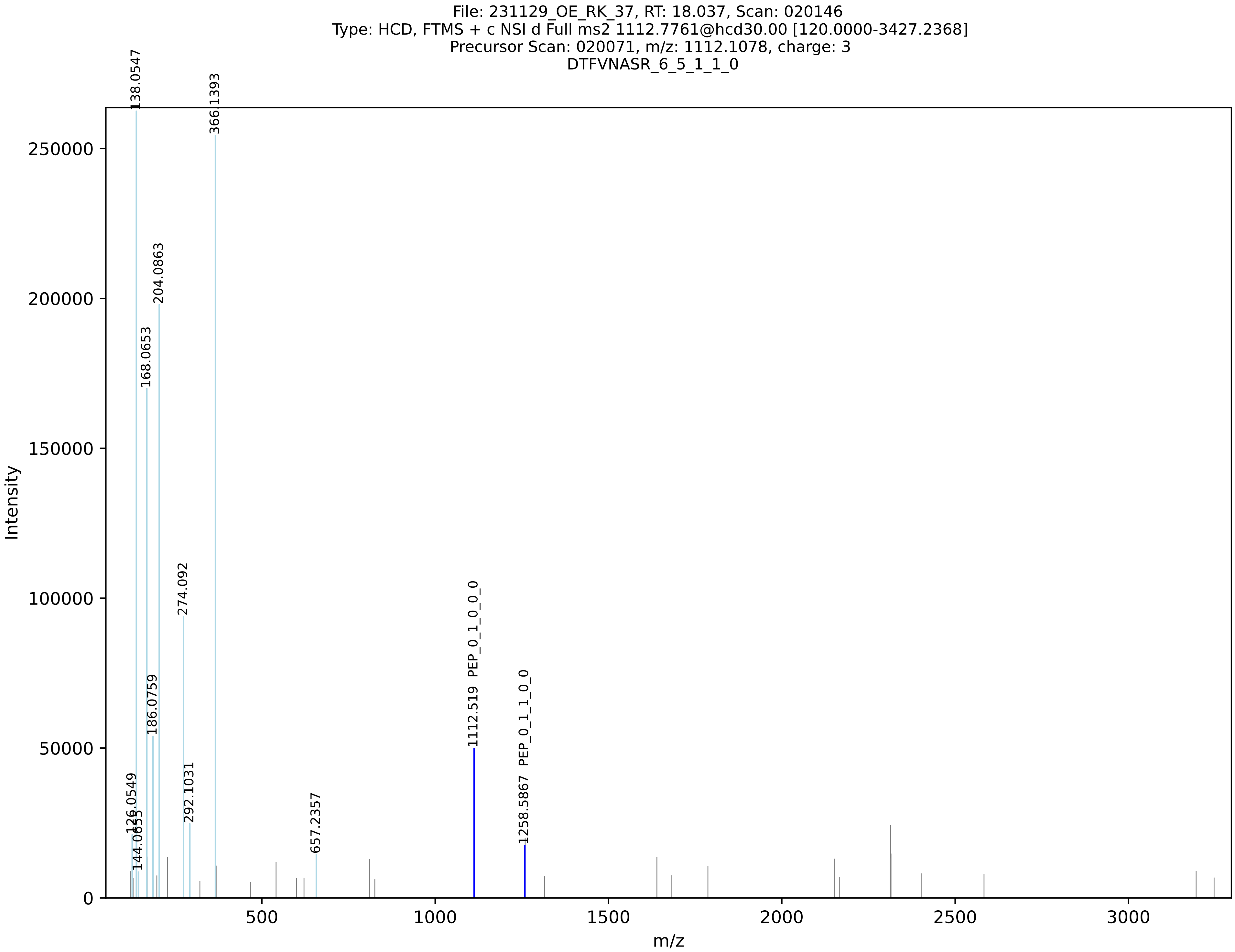
File: 231129_OE_RK_37, RT: 18.037, Scan: 020146
Type: HCD, FTMS + c NSI d Full ms2 1112.7761@hcd30.00 [120.0000-3427.2368]
Precursor Scan: 020071, m/z: 1112.1078, charge: 3
DTFVNASR_6_5_1_1_0
138.0547
366.1393
204.0863
168.0653
274.092
186.0759
126.0549
144.0653
292.1031
657.2357
1112.519 PEP_0_1_0_0_0
1258.5867 PEP_0_1_1_0_0
Intensity
m/z
0
50000
100000
150000
200000
250000
500
1000
1500
2000
2500
3000

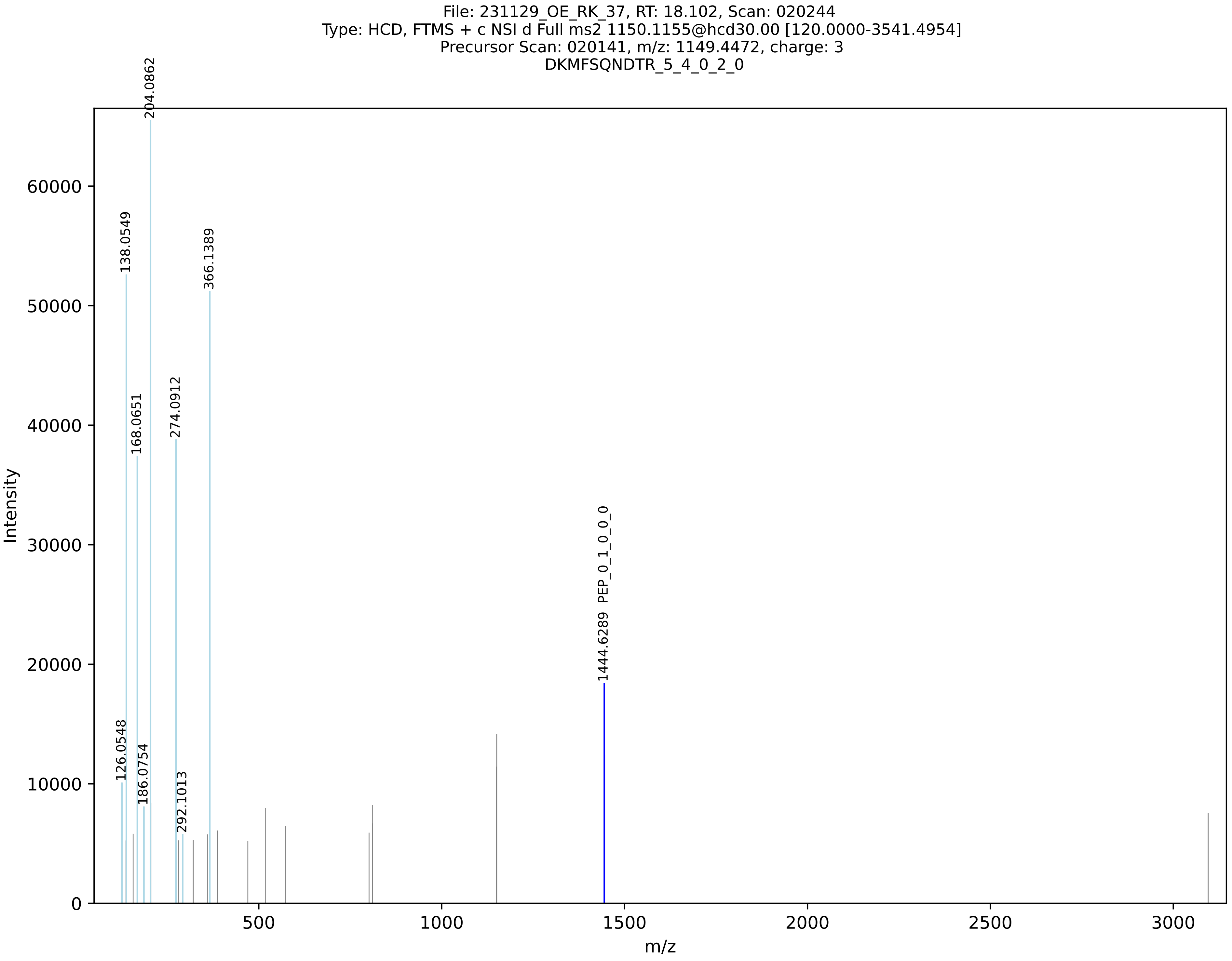
File: 231129_OE_RK_37, RT: 18.102, Scan: 020244
Type: HCD, FTMS + c NSI d Full ms2 1150.1155@hcd30.00 [120.0000-3541.4954]
Precursor Scan: 020141, m/z: 1149.4472, charge: 3
DKMFSQNDTR_5_4_0_2_0
204.0862
138.0549
366.1389
168.0651
274.0912
PEP_0_1_0_0_0
1444.6289
126.0548
186.0754
292.1013
Intensity
m/z
0
10000
20000
30000
40000
50000
60000
500
1000
1500
2000
2500
3000

File: 231129_OE_RK_37, RT: 18.143, Scan: 020302
Type: HCD, FTMS + c NSI d Full ms2 1074.6603@hcd30.00 [120.0000-4407.4697]
Precursor Scan: 020214, m/z: 1074.1591, charge: 4
ENGTISR_7_6_0_4_0

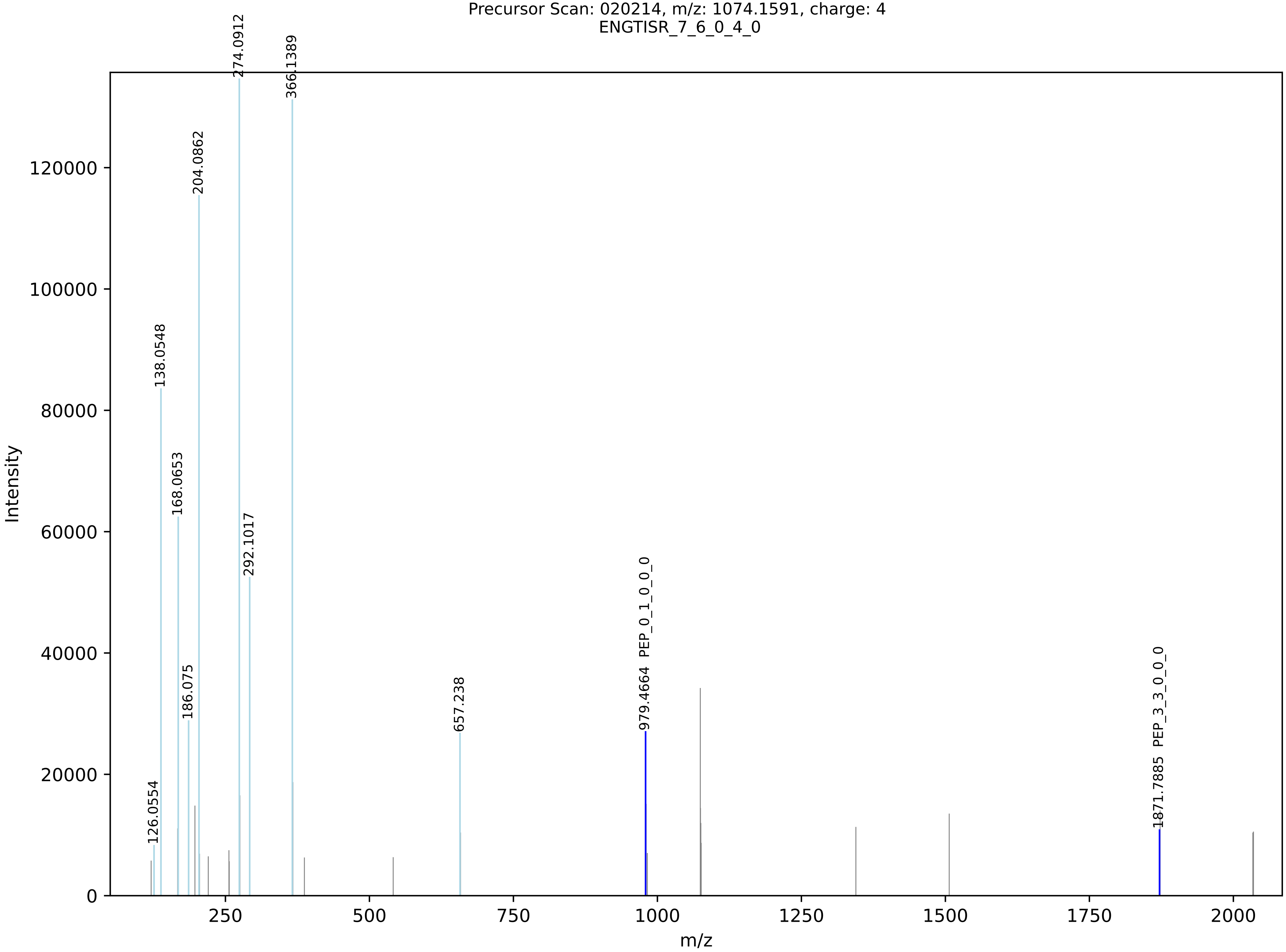

File: 231129_OE_RK_37, RT: 18.19, Scan: 020369
Type: HCD, FTMS + c NSI d Full ms2 1130.4542@hcd30.00 [120.0000-3481.3320]
Precursor Scan: 020288, m/z: 1130.1202, charge: 3
YAEDKFNETTEK_5_4_0_1_0

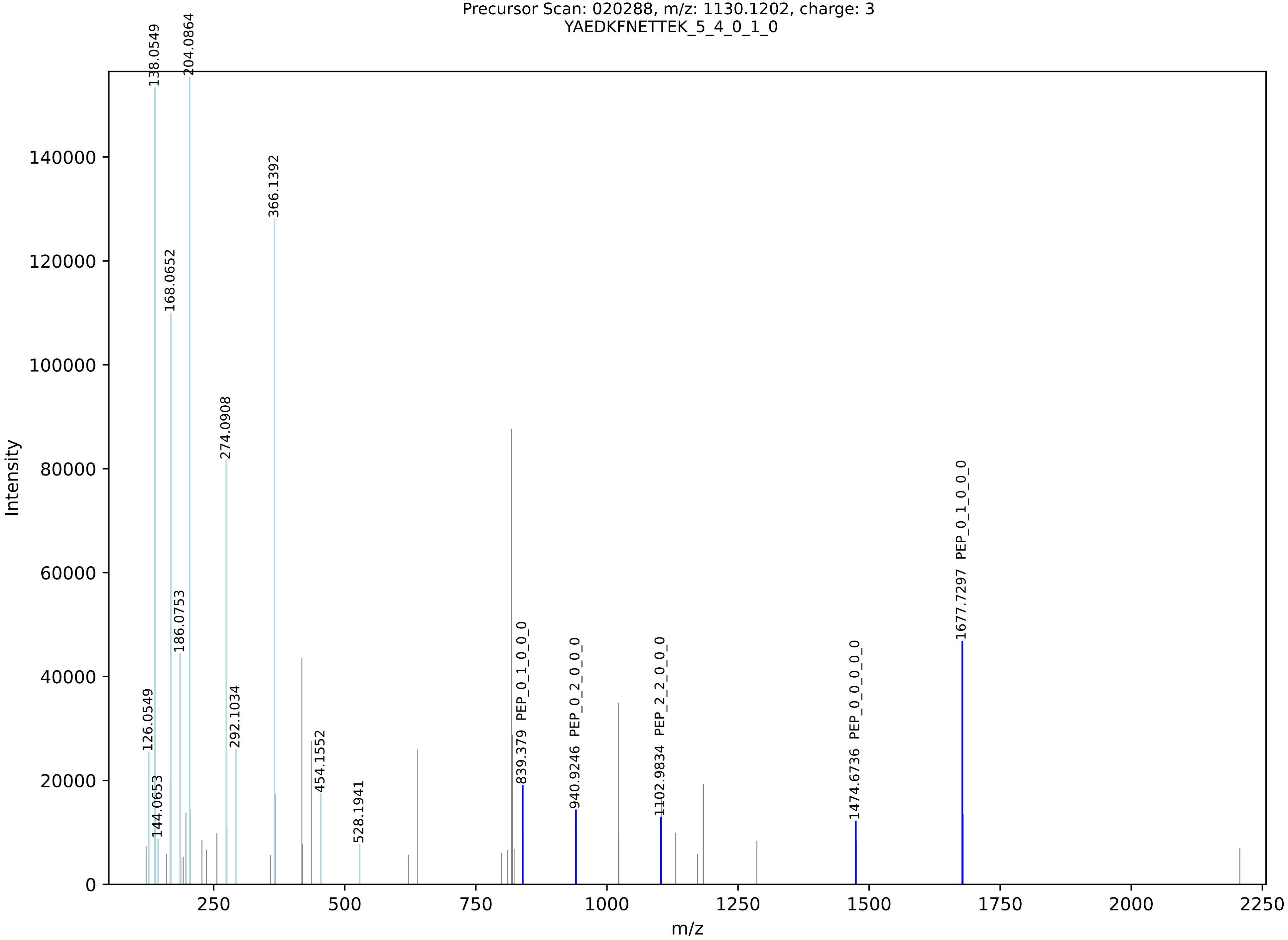

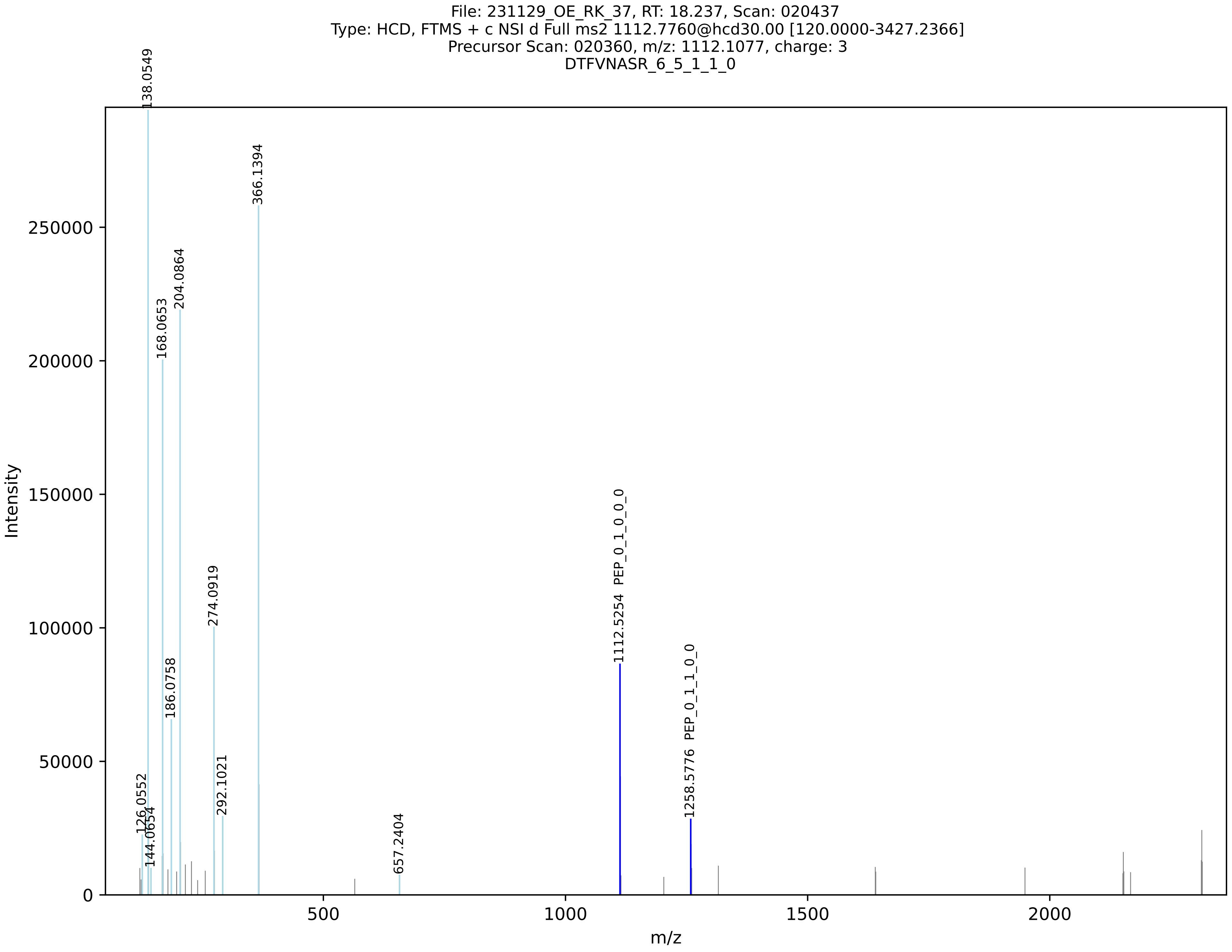

File: 231129_OE_RK_37, RT: 18.237, Scan: 020437
Type: HCD, FTMS + c NSI d Full ms2 1112.7760@hcd30.00 [120.0000-3427.2366]
Precursor Scan: 020360, m/z: 1112.1077, charge: 3
DTFVNASR_6_5_1_1_0
Intensity
m/z
0
50000
100000
150000
200000
250000
500
1000
1500
2000
138.0549
366.1394
204.0864
168.0653
274.0919
186.0758
126.0552
144.0654
292.1021
657.2404
1112.5254 PEP_0_1_0_0_0
1258.5776 PEP_0_1_1_0_0

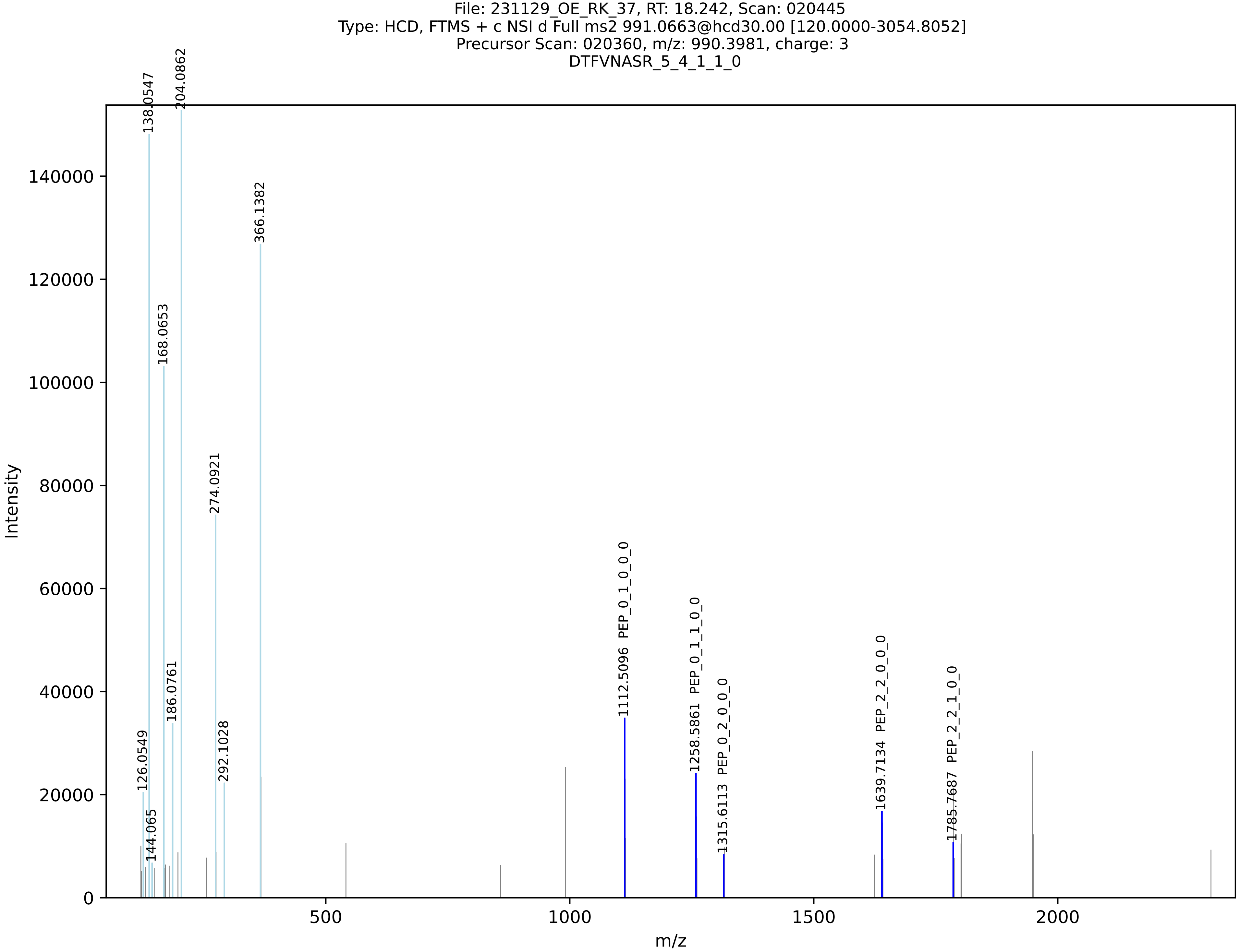
File: 231129_OE_RK_37, RT: 18.242, Scan: 020445
Type: HCD, FTMS + c NSI d Full ms2 991.0663@hcd30.00 [120.0000-3054.8052]
Precursor Scan: 020360, m/z: 990.3981, charge: 3
DTFVNASR_5_4_1_1_0
Intensity
m/z
204.0862
138.0547
366.1382
168.0653
274.0921
186.0761
292.1028
126.0549
144.065
1112.5096 PEP_0_1_0_0_0
1258.5861 PEP_0_1_1_0_0
1315.6113 PEP_0_2_0_0_0
1639.7134 PEP_2_2_0_0_0
1785.7687 PEP_2_2_1_0_0

File: 231129_OE_RK_37, RT: 18.249, Scan: 020457
Type: HCD, FTMS + c NSI d Full ms2 848.4150@hcd30.00 [120.0000-2618.2917]
Precursor Scan: 020360, m/z: 848.0817, charge: 3
DNGSPILGYWLEK_4_2_0_0_0

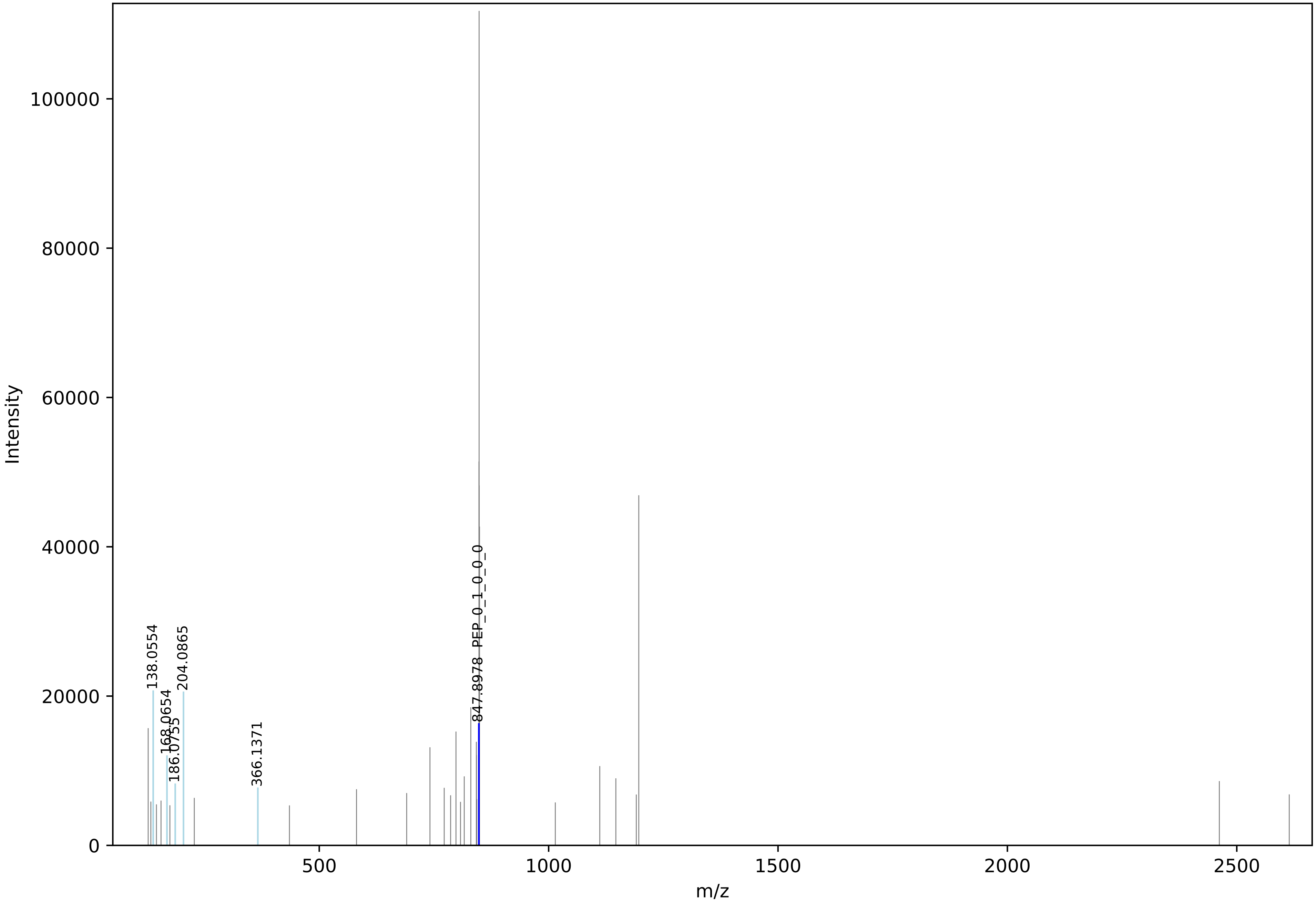

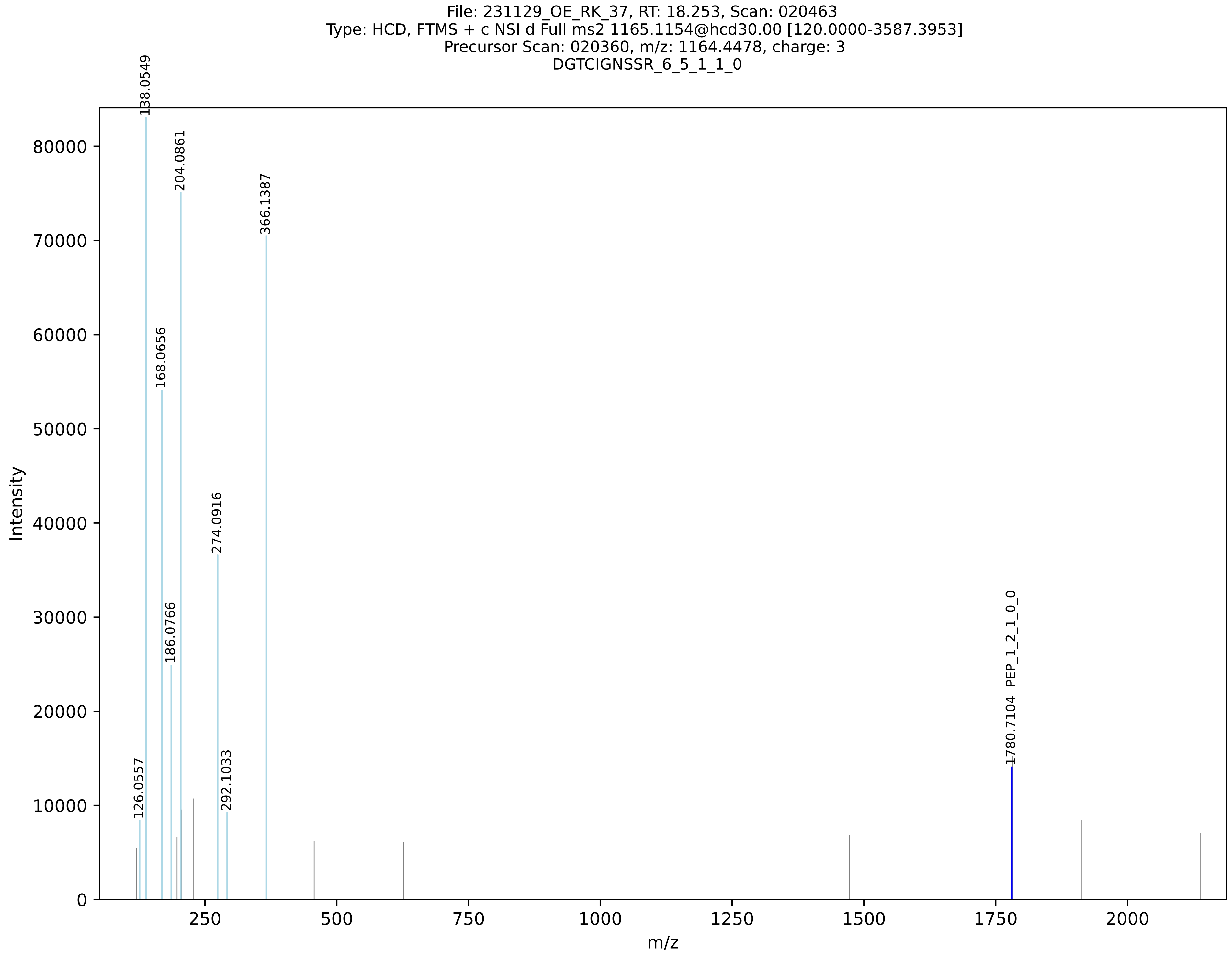
File: 231129_OE_RK_37, RT: 18.253, Scan: 020463
Type: HCD, FTMS + c NSI d Full ms2 1165.1154@hcd30.00 [120.0000-3587.3953]
Precursor Scan: 020360, m/z: 1164.4478, charge: 3
DGTCIGNSSR_6_5_1_1_0
138.0549
204.0861
366.1387
168.0656
274.0916
186.0766
126.0557
292.1033
1780.7104 PEP_1_2_1_0_0
Intensity
m/z
0
10000
20000
30000
40000
50000
60000
70000
80000
250
500
750
1000
1250
1500
1750
2000

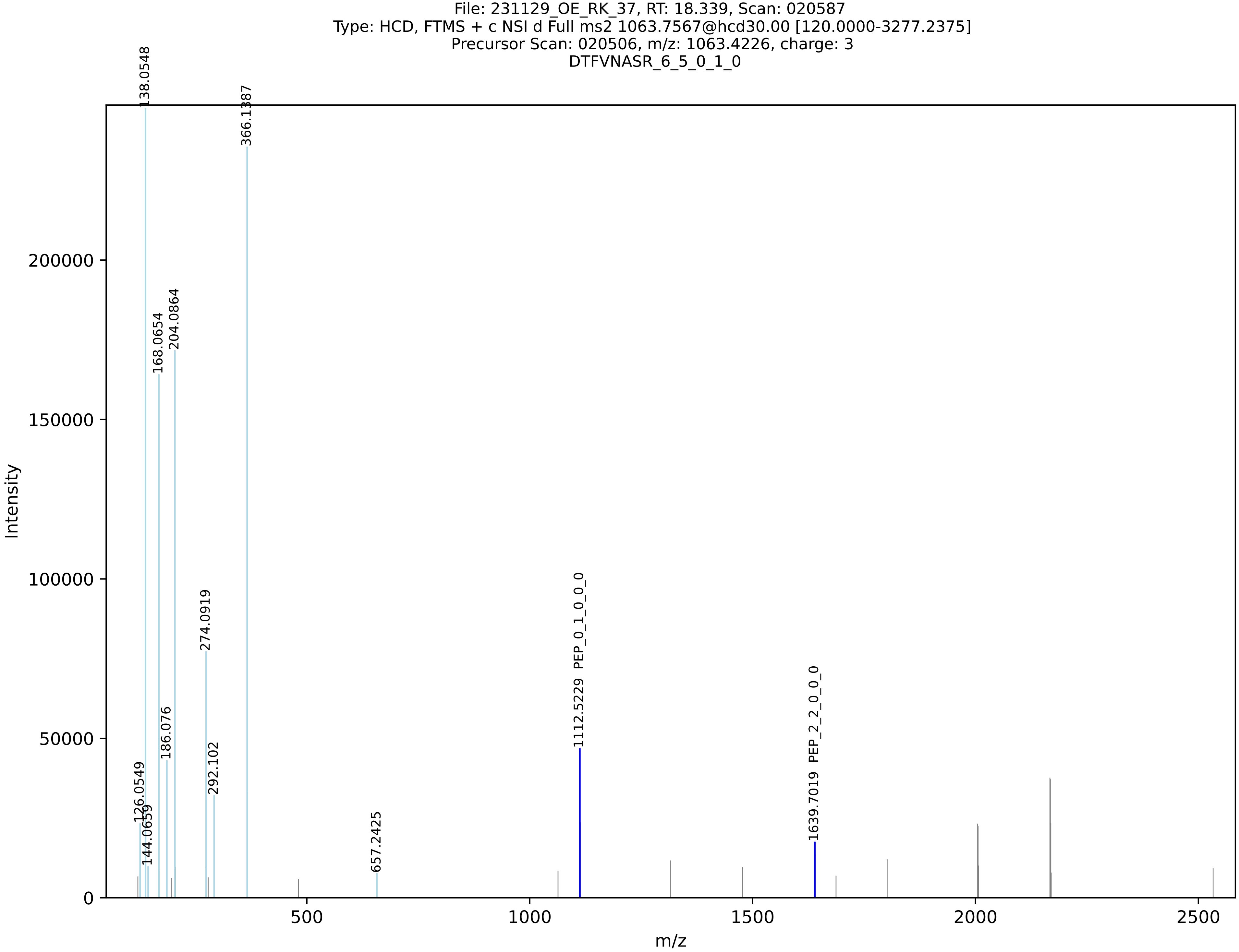

File: 231129_OE_RK_37, RT: 18.339, Scan: 020587
Type: HCD, FTMS + c NSI d Full ms2 1063.7567@hcd30.00 [120.0000-3277.2375]
Precursor Scan: 020506, m/z: 1063.4226, charge: 3
DTFVNASR_6_5_0_1_0
138.0548
366.1387
168.0654
204.0864
274.0919
186.076
292.102
126.0549
144.0659
657.2425
1112.5229 PEP_0_1_0_0_0
1639.7019 PEP_2_2_0_0_0
Intensity
m/z
0
50000
100000
150000
200000
500
1000
1500
2000
2500

File: 231129_OE_RK_37, RT: 18.342, Scan: 020591
Type: HCD, FTMS + c NSI d Full ms2 1074.6606@hcd30.00 [120.0000-4407.4712]
Precursor Scan: 020506, m/z: 1074.1592, charge: 4
ENGTISR_7_6_0_4_0

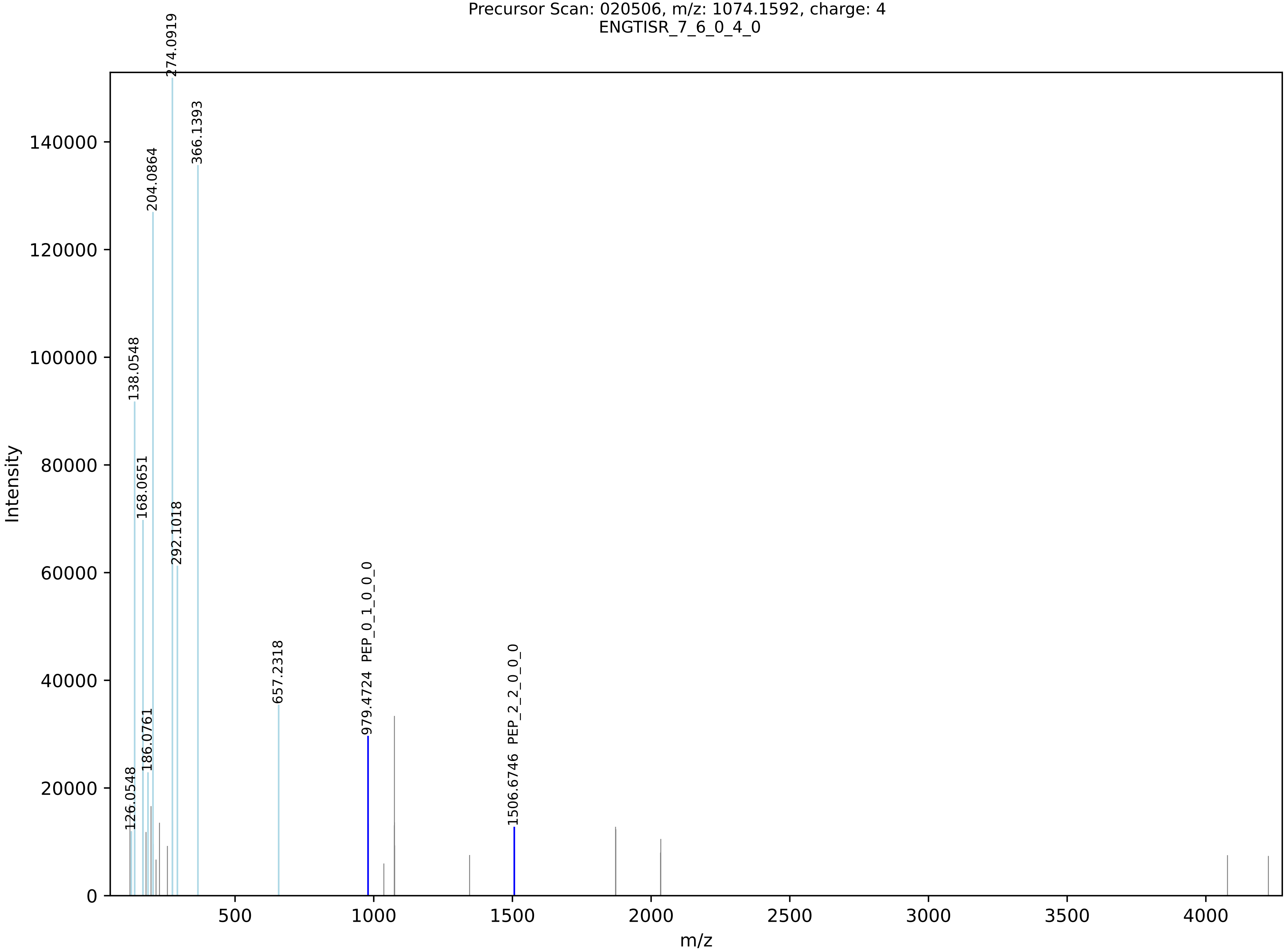

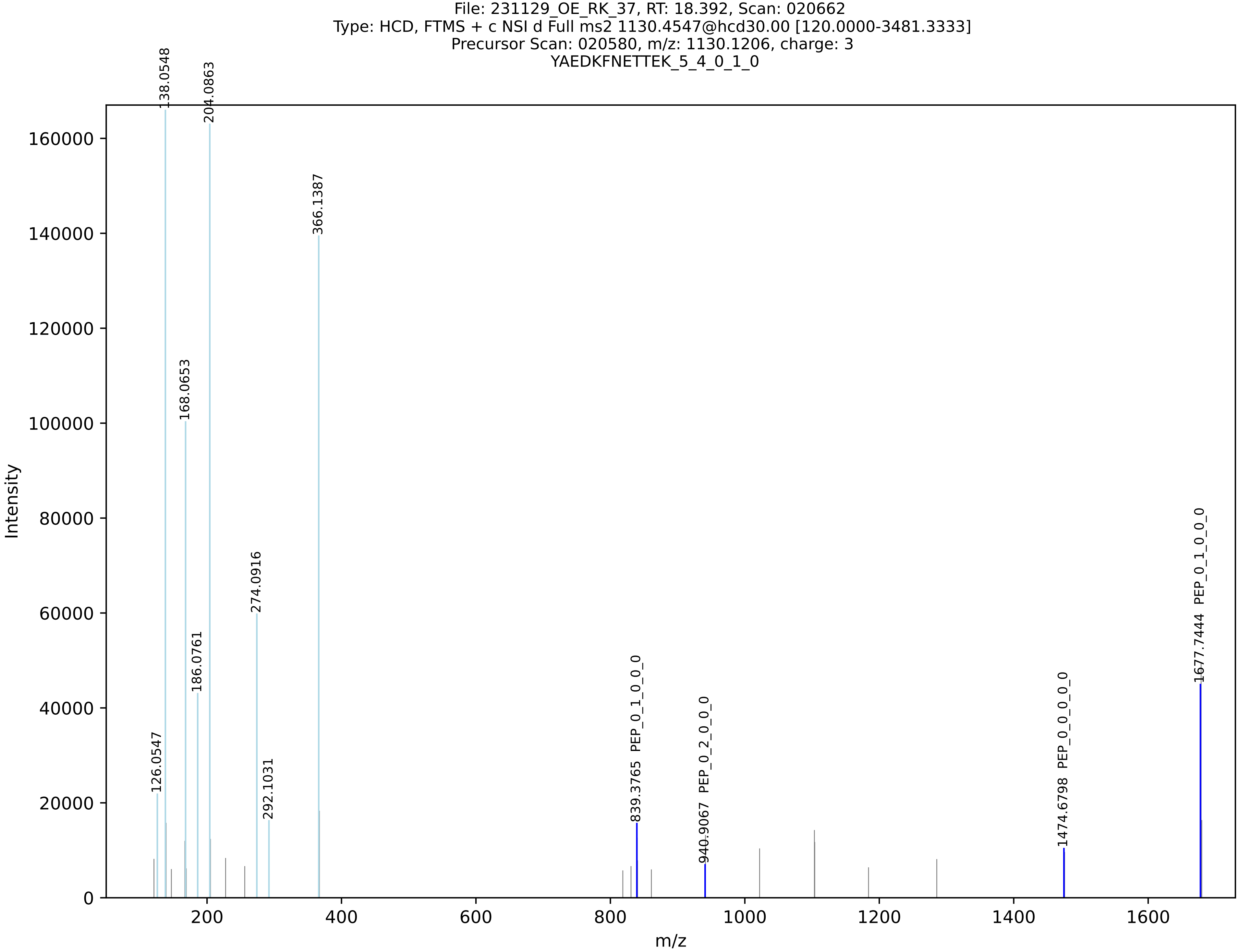

File: 231129_OE_RK_37, RT: 18.392, Scan: 020662
Type: HCD, FTMS + c NSI d Full ms2 1130.4547@hcd30.00 [120.0000-3481.3333]
Precursor Scan: 020580, m/z: 1130.1206, charge: 3
YAEDKFNETTEK_5_4_0_1_0
138.0548
204.0863
366.1387
168.0653
274.0916
186.0761
126.0547
292.1031
839.3765 PEP_0_1_0_0_0
940.9067 PEP_0_2_0_0_0
1474.6798 PEP_0_0_0_0_0
1677.7444 PEP_0_1_0_0_0
Intensity
m/z
0
20000
40000
60000
80000
100000
120000
140000
160000
200
400
600
800
1000
1200
1400
1600

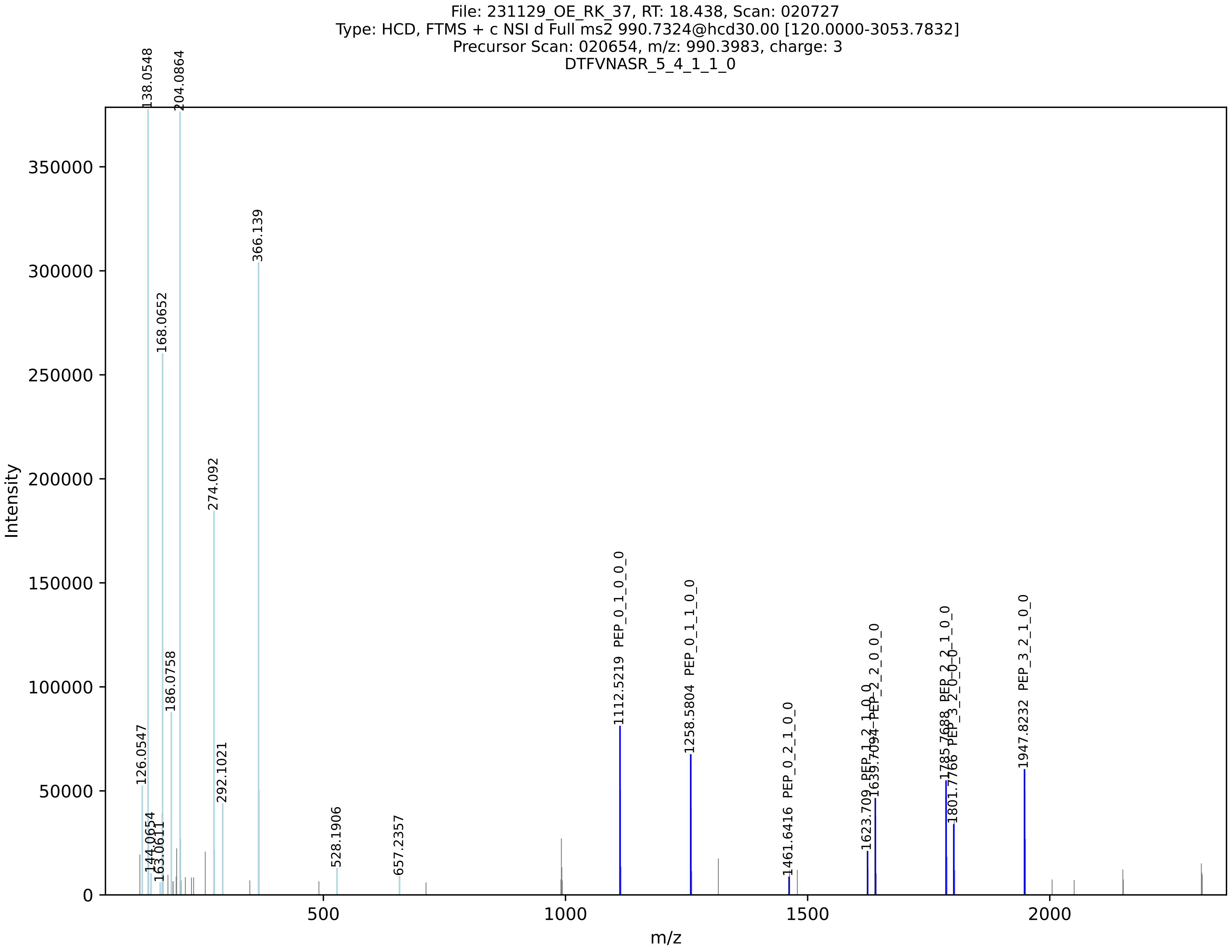
File: 231129_OE_RK_37, RT: 18.438, Scan: 020727
Type: HCD, FTMS + c NSI d Full ms2 990.7324@hcd30.00 [120.0000-3053.7832]
Precursor Scan: 020654, m/z: 990.3983, charge: 3
DTFVNASR_5_4_1_1_0
Intensity
m/z
138.0548
204.0864
366.139
168.0652
274.092
186.0758
126.0547
292.1021
144.0654
163.0611
528.1906
657.2357
1112.5219 PEP_0_1_0_0_0
1258.5804 PEP_0_1_1_0_0
1461.6416 PEP_0_2_1_0_0
1623.709 PEP_1_2_1_0_0
1639.7094 PEP_2_2_0_0_0
1785.7688 PEP_2_2_1_0_0
1801.7766 PEP_3_2_0_0_0
1947.8232 PEP_3_2_1_0_0

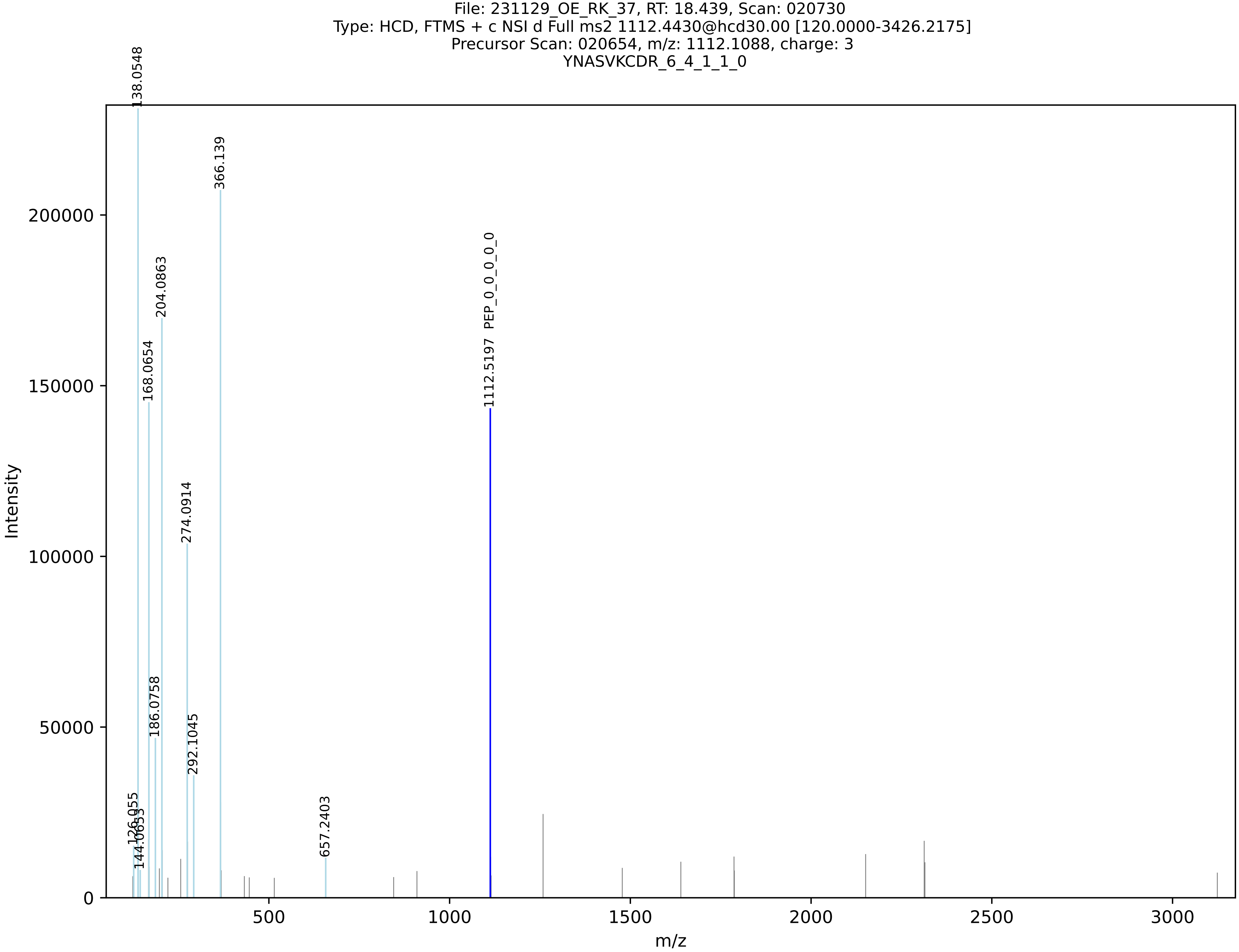
File: 231129_OE_RK_37, RT: 18.439, Scan: 020730
Type: HCD, FTMS + c NSI d Full ms2 1112.4430@hcd30.00 [120.0000-3426.2175]
Precursor Scan: 020654, m/z: 1112.1088, charge: 3
YNASVKCDR_6_4_1_1_0
138.0548
366.139
204.0863
168.0654
1112.5197 PEP_0_0_0_0_0
274.0914
186.0758
292.1045
126.055
144.0653
657.2403
Intensity
m/z
0
50000
100000
150000
200000
500
1000
1500
2000
2500
3000

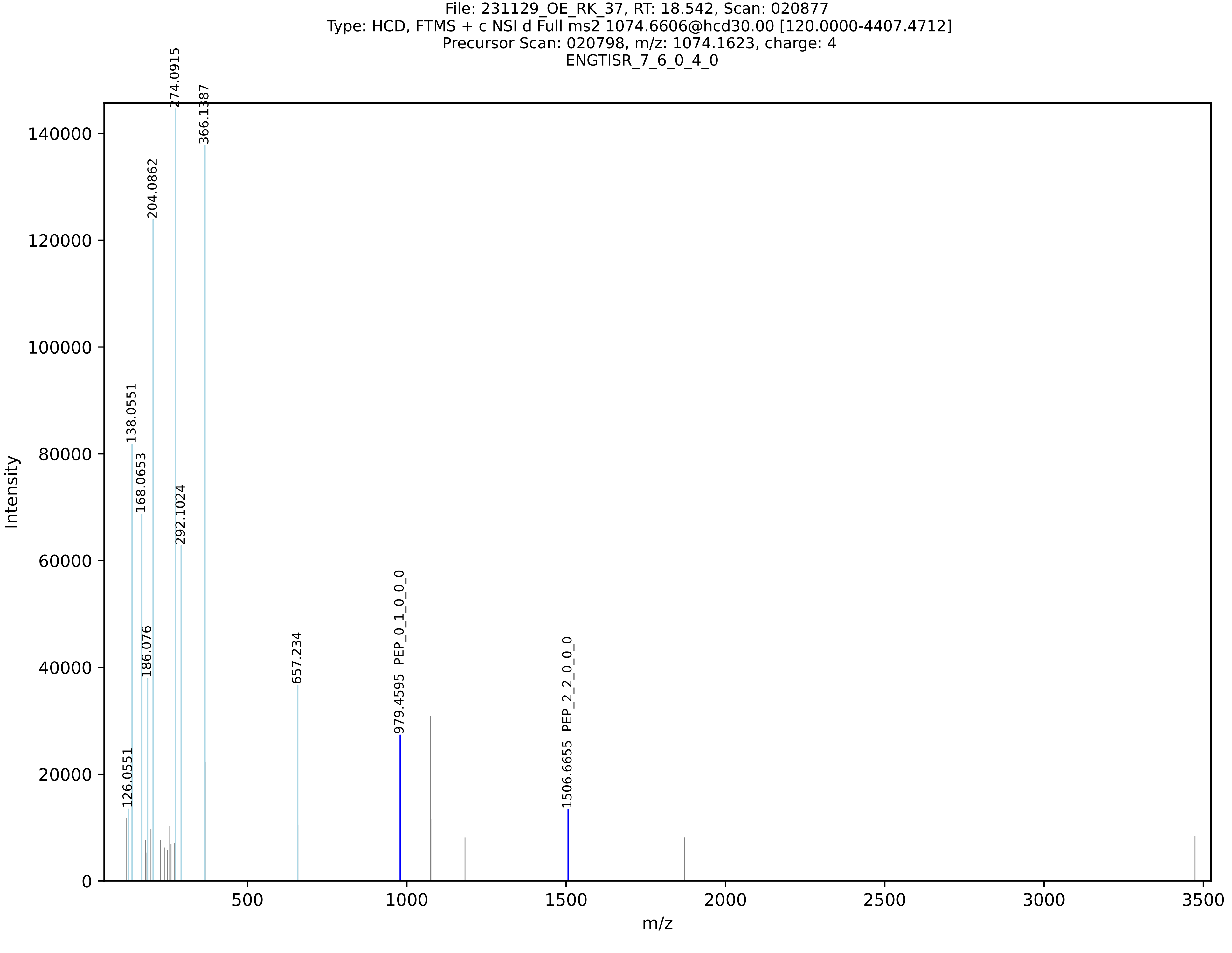

File: 231129_OE_RK_37, RT: 18.542, Scan: 020877
Type: HCD, FTMS + c NSI d Full ms2 1074.6606@hcd30.00 [120.0000-4407.4712]
Precursor Scan: 020798, m/z: 1074.1623, charge: 4
ENGTISR_7_6_0_4_0
274.0915
366.1387
204.0862
138.0551
168.0653
292.1024
186.076
657.234
979.4595 PEP_0_1_0_0_0_
1506.6655 PEP_2_2_0_0_0_
126.0551
Intensity
m/z
0
20000
40000
60000
80000
100000
120000
140000
500
1000
1500
2000
2500
3000
3500

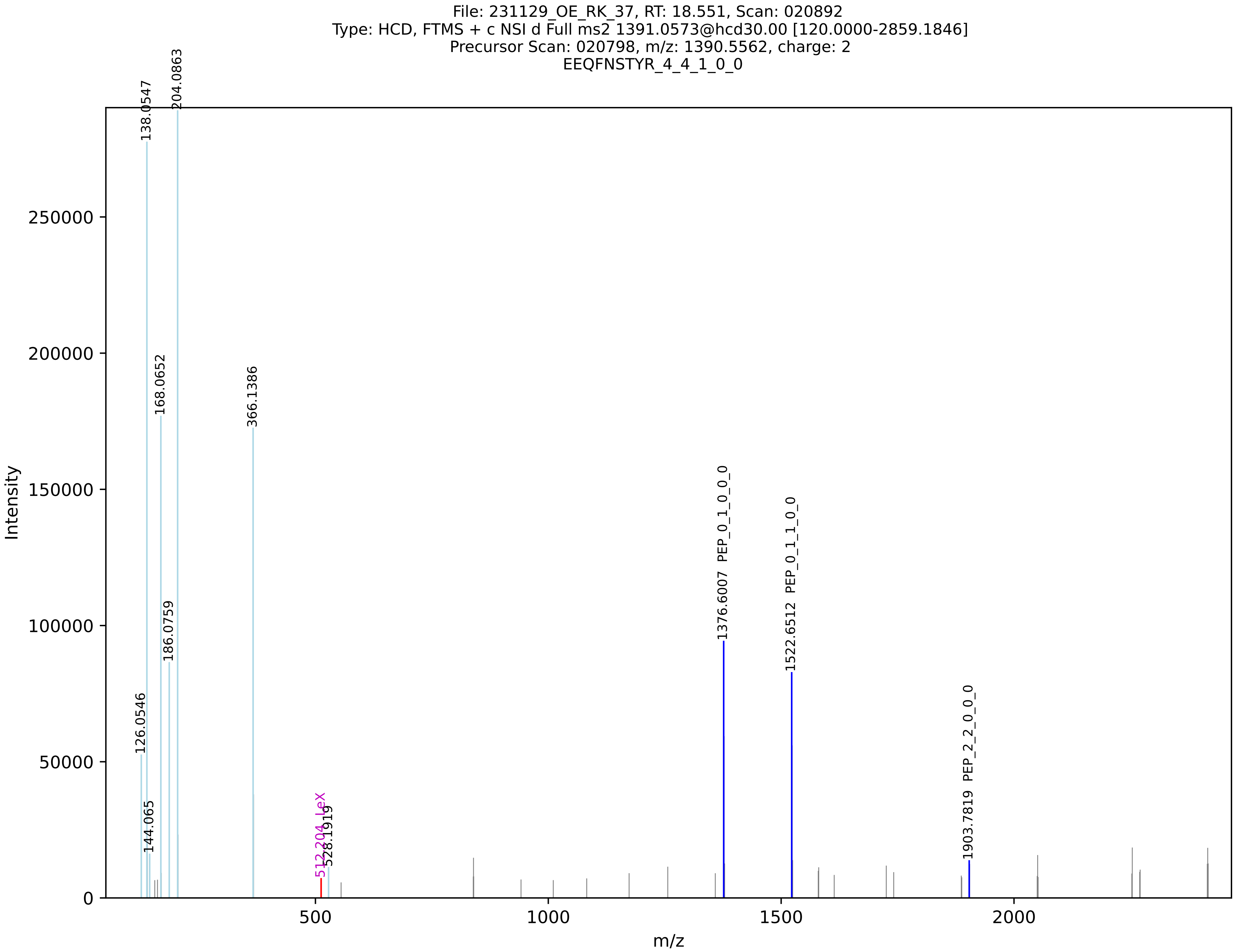
File: 231129_OE_RK_37, RT: 18.551, Scan: 020892
Type: HCD, FTMS + c NSI d Full ms2 1391.0573@hcd30.00 [120.0000-2859.1846]
Precursor Scan: 020798, m/z: 1390.5562, charge: 2
EEQFNSTYR_4_4_1_0_0
Intensity
m/z
0
50000
100000
150000
200000
250000
500
1000
1500
2000
126.0546
138.0547
144.065
168.0652
186.0759
204.0863
366.1386
512.204_LeX
528.1919
1376.6007 PEP_0_1_0_0_0
1522.6512 PEP_0_1_1_0_0
1903.7819 PEP_2_2_0_0_0

File: 231129_OE_RK_37, RT: 18.591, Scan: 020949
Type: HCD, FTMS + c NSI d Full ms2 1130.7886@hcd30.00 [120.0000-3482.3552]
Precursor Scan: 020869, m/z: 1130.1204, charge: 3
YAEDKFNETTEK_5_4_0_1_0

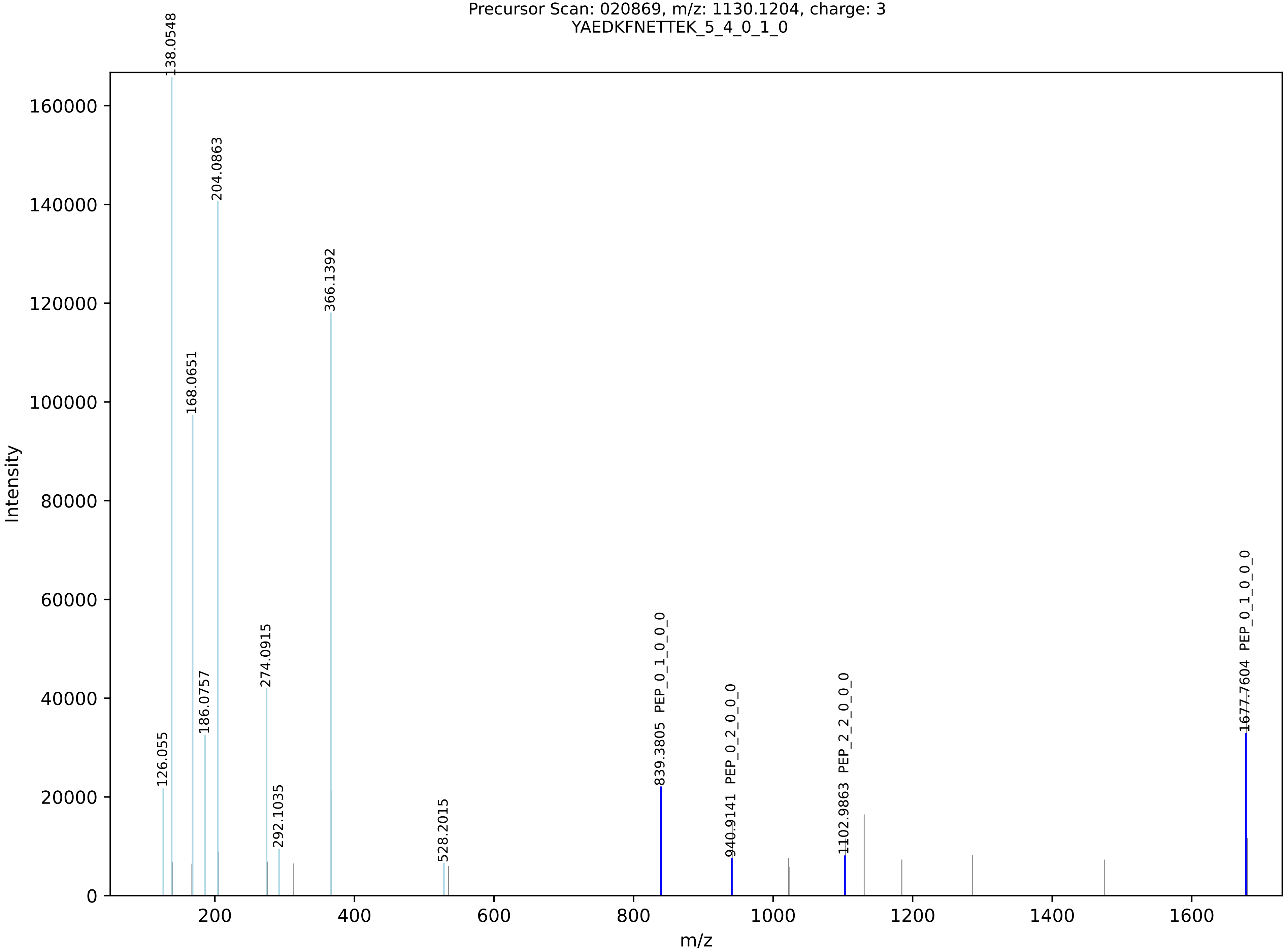

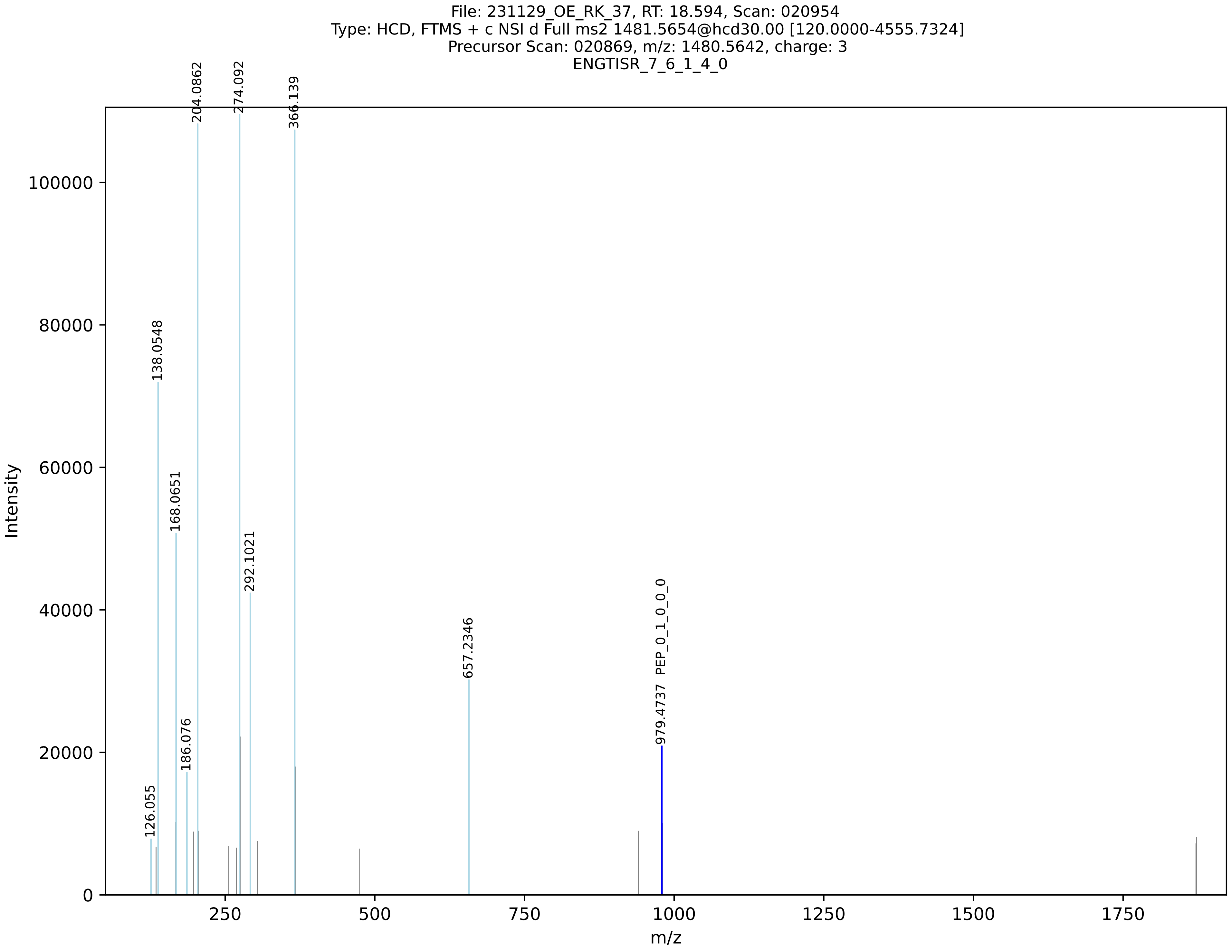
File: 231129_OE_RK_37, RT: 18.594, Scan: 020954
Type: HCD, FTMS + c NSI d Full ms2 1481.5654@hcd30.00 [120.0000-4555.7324]
Precursor Scan: 020869, m/z: 1480.5642, charge: 3
ENGTISR_7_6_1_4_0
Intensity
m/z
204.0862
274.092
366.139
138.0548
168.0651
292.1021
657.2346
979.4737 PEP_0_1_0_0_0
186.076
126.055
0
20000
40000
60000
80000
100000
250
500
750
1000
1250
1500
1750

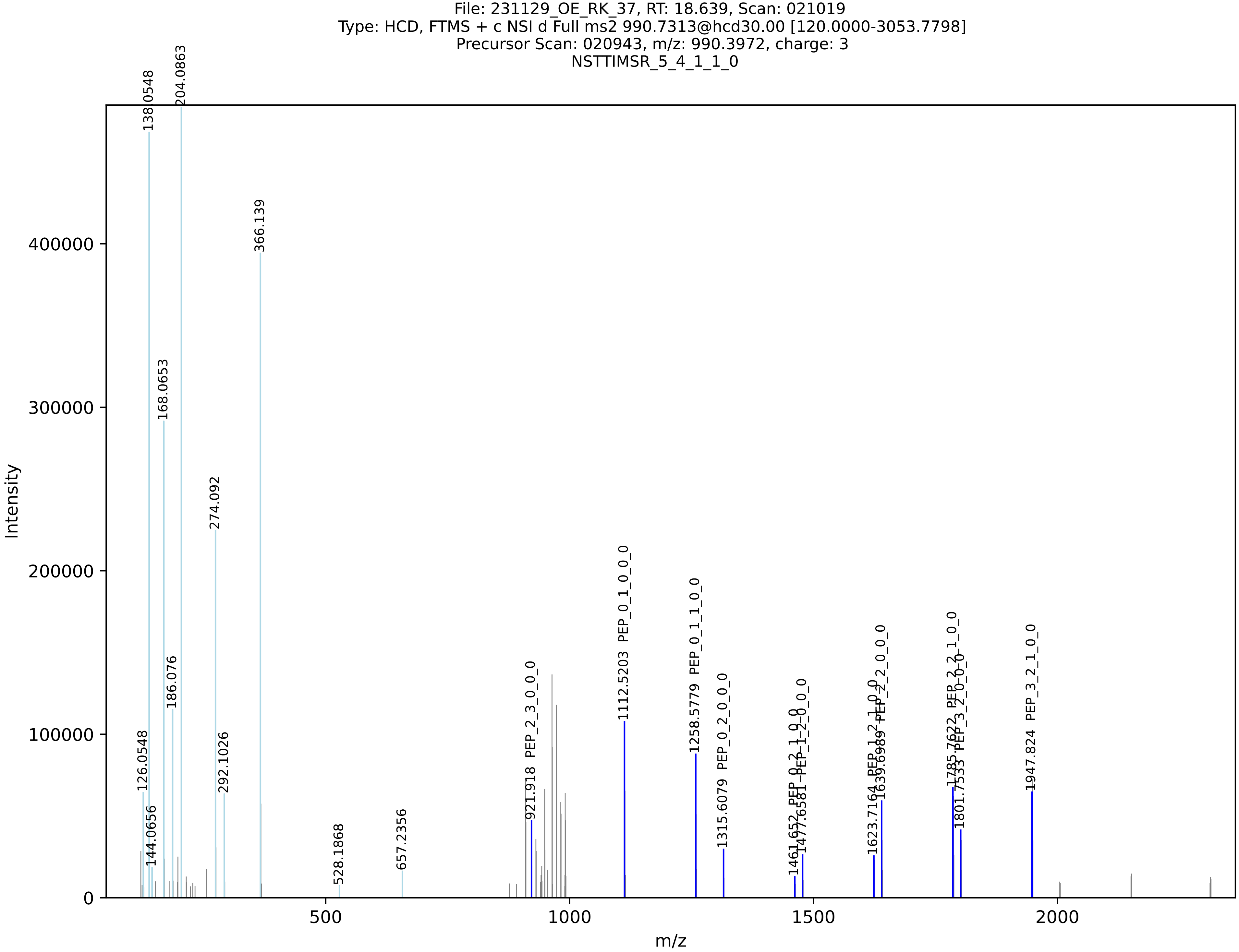
File: 231129_OE_RK_37, RT: 18.639, Scan: 021019
Type: HCD, FTMS + c NSI d Full ms2 990.7313@hcd30.00 [120.0000-3053.7798]
Precursor Scan: 020943, m/z: 990.3972, charge: 3
NSTTIMSR_5_4_1_1_0
Intensity
m/z
138.0548
204.0863
366.139
168.0653
274.092
186.076
126.0548
292.1026
144.0656
528.1868
657.2356
921.918 PEP_2_3_0_0_
1112.5203 PEP_0_1_0_0_0_
1258.5779 PEP_0_1_1_0_0_
1315.6079 PEP_0_2_0_0_0_
1947.824 PEP_3_2_1_0_0_

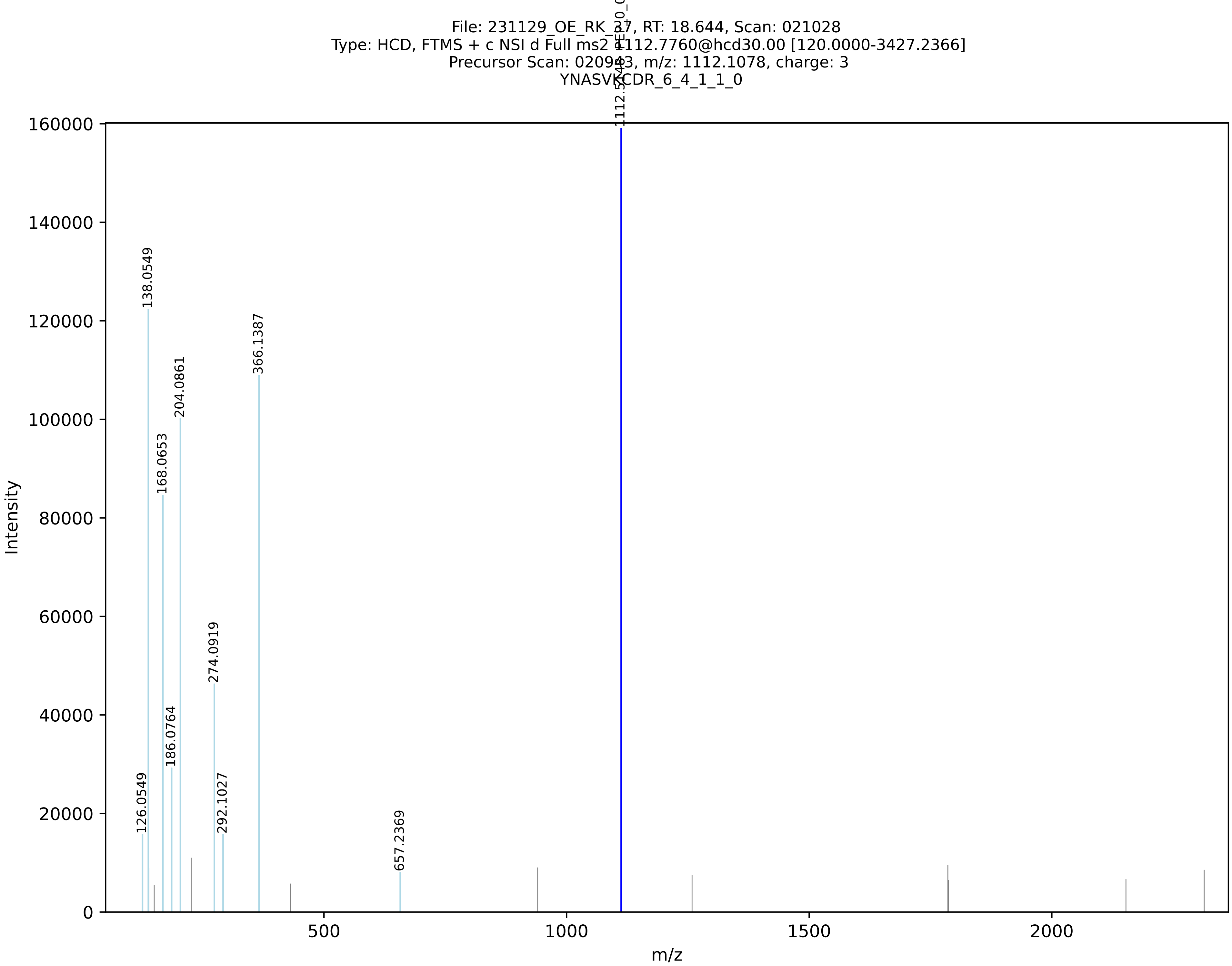

File: 231129_OE_RK_37, RT: 18.644, Scan: 021028
Type: HCD, FTMS + c NSI d Full ms2 1112.7760@hcd30.00 [120.0000-3427.2366]
Precursor Scan: 020943, m/z: 1112.1078, charge: 3
YNASVKCDR_6_4_1_1_0
1112.5448_PEP_0_
138.0549
366.1387
204.0861
168.0653
274.0919
186.0764
126.0549
292.1027
657.2369
Intensity
0
20000
40000
60000
80000
100000
120000
140000
160000
500
1000
1500
2000
m/z

File: 231129_OE_RK_37, RT: 18.748, Scan: 021181
Type: HCD, FTMS + c NSI d Full ms2 1074.4086@hcd30.00 [120.0000-4406.4429]
Precursor Scan: 021090, m/z: 1074.1597, charge: 4
ENGTISR_7_6_0_4_0

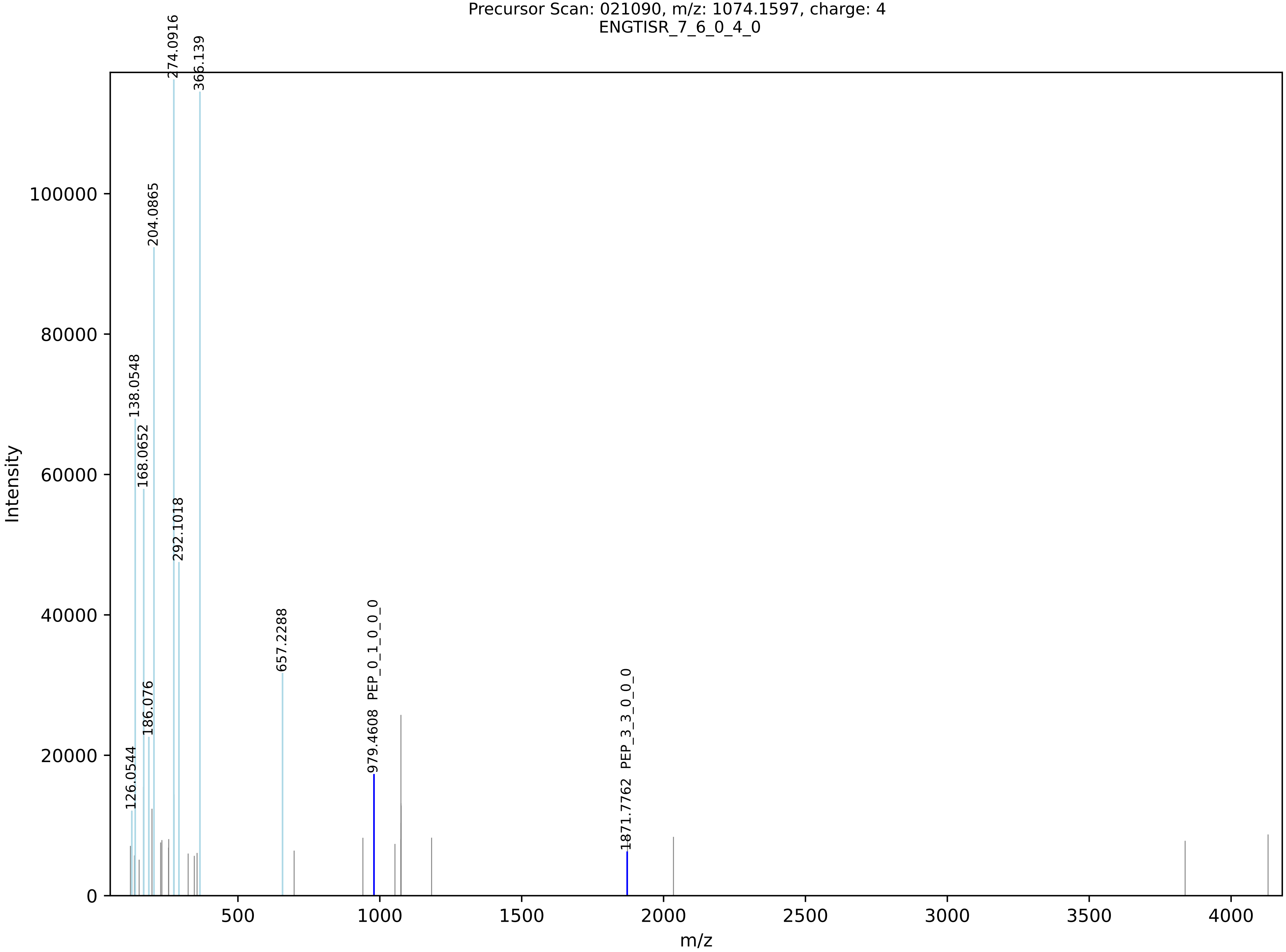

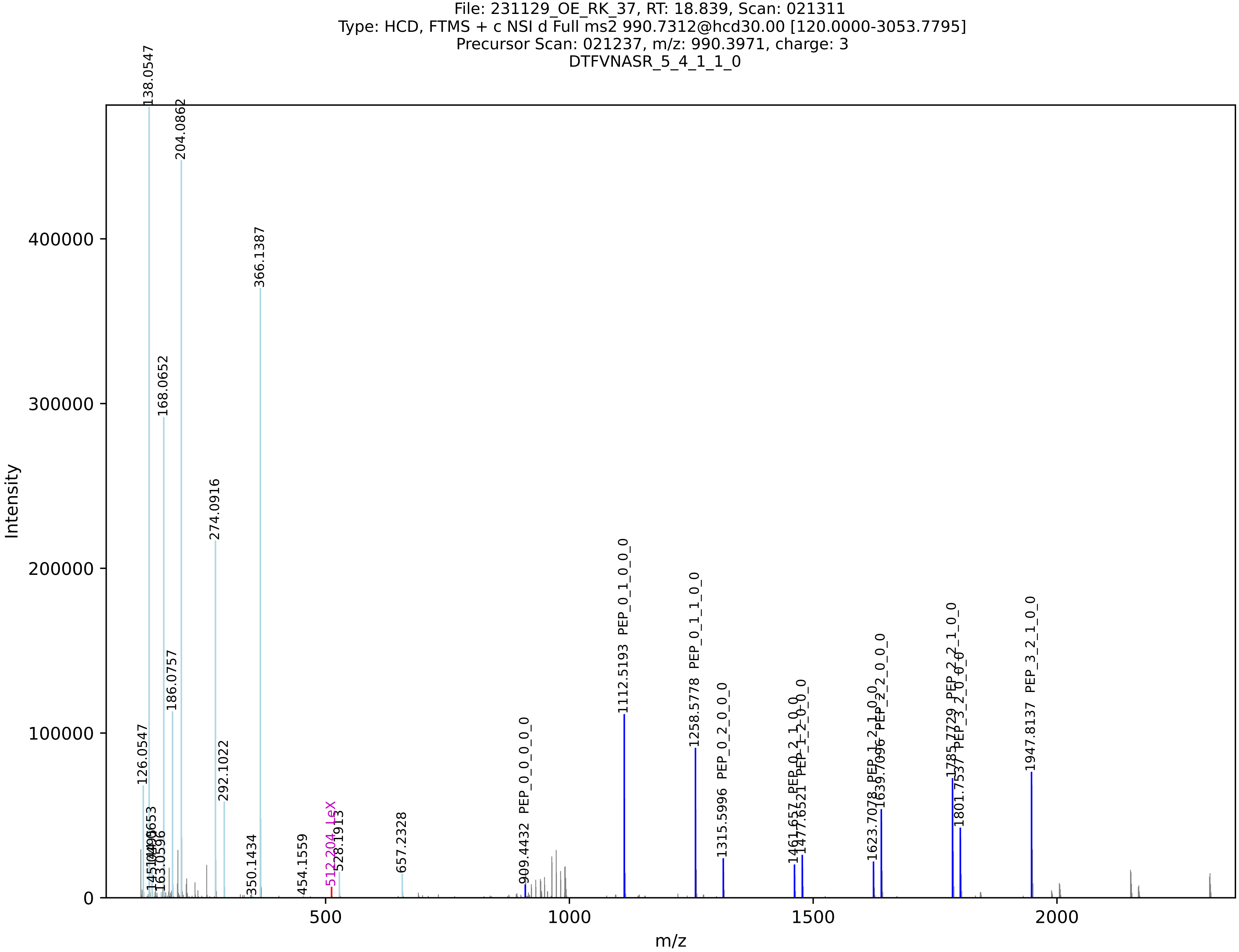
File: 231129_OE_RK_37, RT: 18.839, Scan: 021311
Type: HCD, FTMS + c NSI d Full ms2 990.7312@hcd30.00 [120.0000-3053.7795]
Precursor Scan: 021237, m/z: 990.3971, charge: 3
DTFVNASR_5_4_1_1_0
Intensity
m/z
138.0547
204.0862
366.1387
168.0652
274.0916
186.0757
126.0547
292.1022
163.0598
350.1434
454.1559
512.204_LeX
528.1913
657.2328
909.4432 PEP_0_0_0_0_0
1112.5193 PEP_0_1_0_0_0
1258.5778 PEP_0_1_1_0_0
1315.5996 PEP_0_2_0_0_0
1461.657 PEP_0_2_1_0_0
1477.6521 PEP_1_2_0_0_0
1623.7078 PEP_1_2_1_0_0
1639.7096 PEP_2_2_0_0_0
1785.7729 PEP_2_2_1_0_0
1801.7537 PEP_3_2_0_0_0
1947.8137 PEP_3_2_1_0_0

File: 231129_OE_RK_37, RT: 18.844, Scan: 021320
Type: HCD, FTMS + c NSI d Full ms2 1112.7776@hcd30.00 [120.0000-3427.2412]
Precursor Scan: 021237, m/z: 1112.1074, charge: 3
YNASVKCDR_6_4_1_1_0

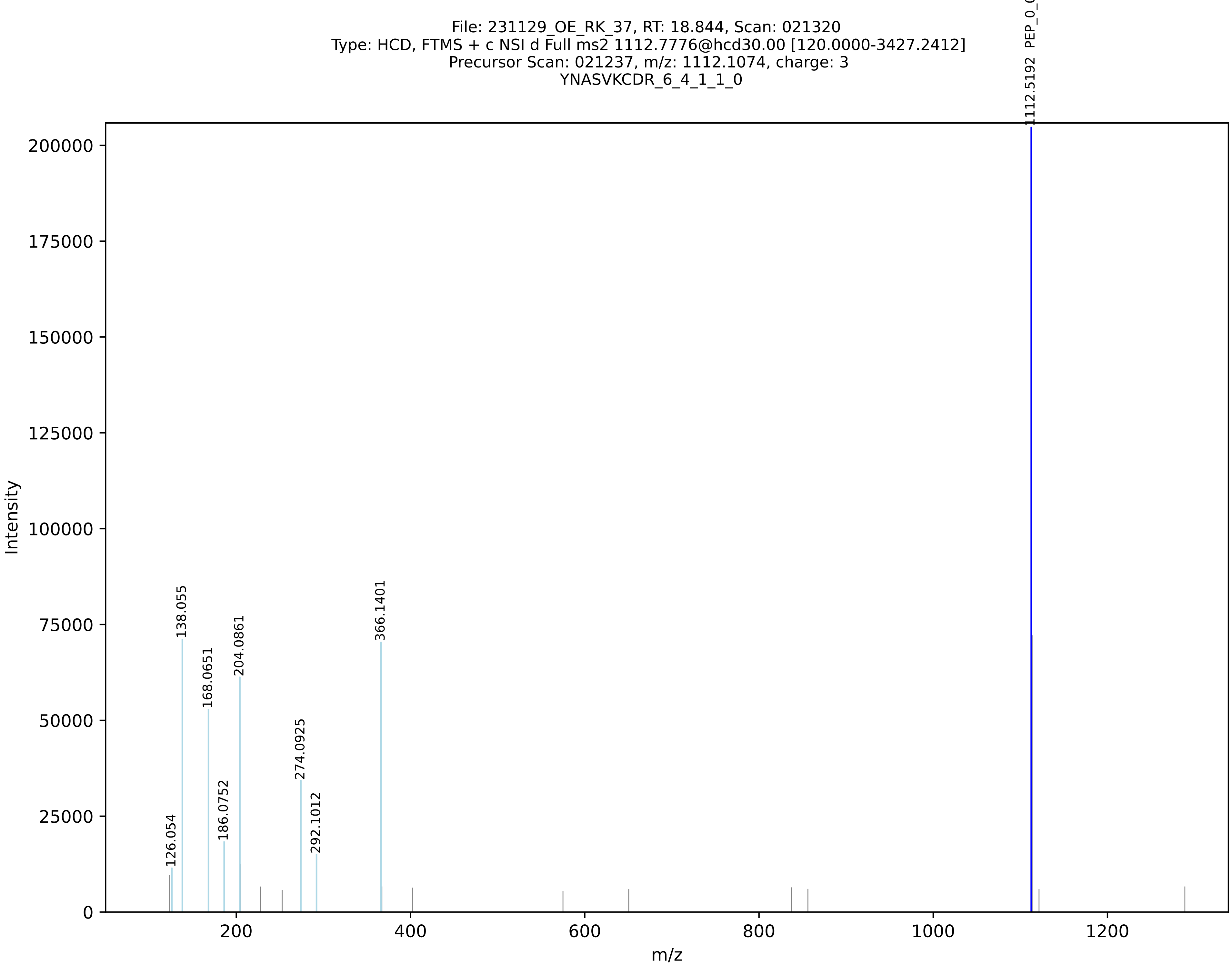

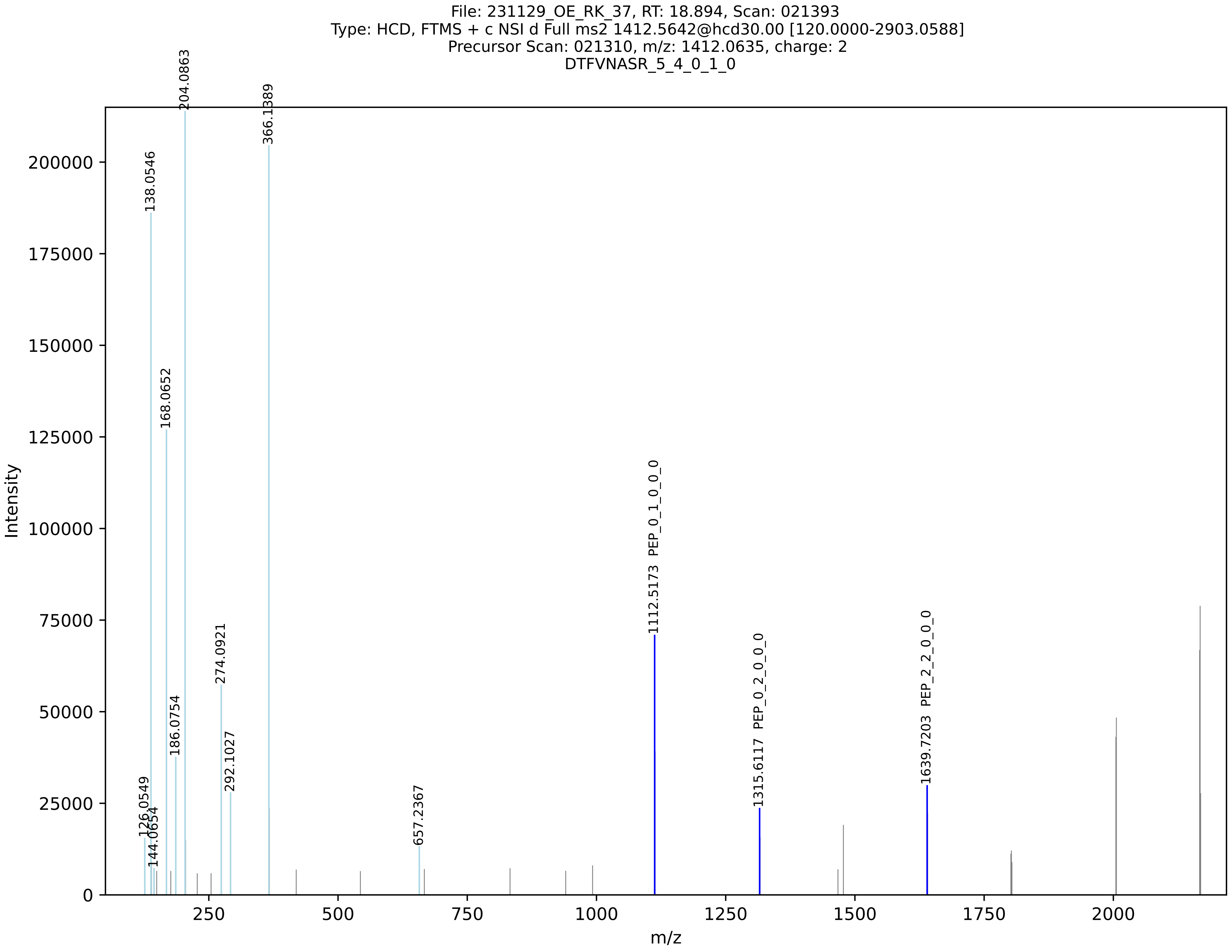
File: 231129_OE_RK_37, RT: 18.894, Scan: 021393
Type: HCD, FTMS + c NSI d Full ms2 1412.5642@hcd30.00 [120.0000-2903.0588]
Precursor Scan: 021310, m/z: 1412.0635, charge: 2
DTFVNASR_5_4_0_1_0
Intensity
m/z
204.0863
366.1389
138.0546
168.0652
186.0754
274.0921
292.1027
126.0549
144.0654
657.2367
1112.5173 PEP_0_1_0_0_0
1315.6117 PEP_0_2_0_0_0
1639.7203 PEP_2_2_0_0_0

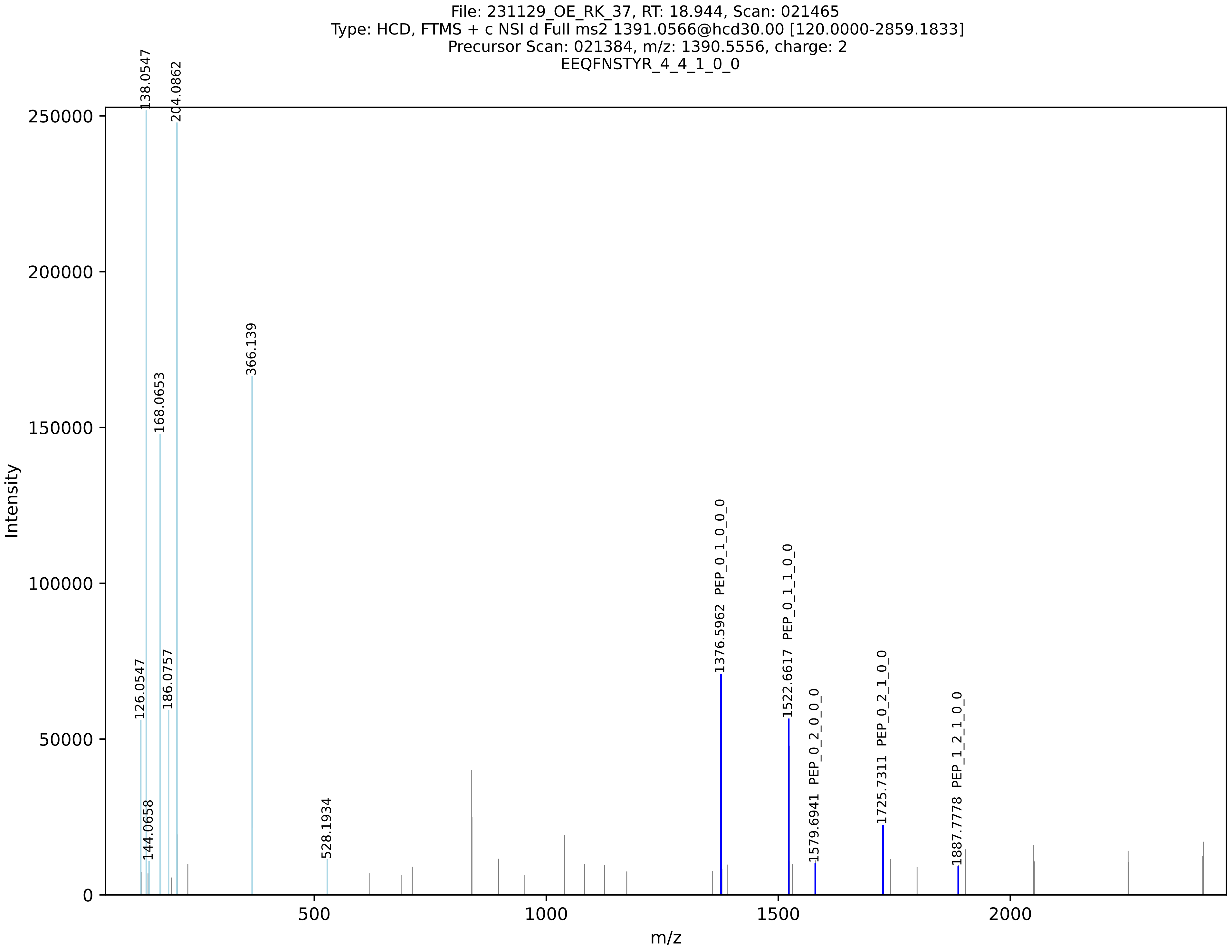

File: 231129_OE_RK_37, RT: 18.944, Scan: 021465
Type: HCD, FTMS + c NSI d Full ms2 1391.0566@hcd30.00 [120.0000-2859.1833]
Precursor Scan: 021384, m/z: 1390.5556, charge: 2
EEQFNSTYR_4_4_1_0_0
138.0547
204.0862
366.139
168.0653
126.0547
186.0757
144.0658
528.1934
1376.5962 PEP_0_1_0_0_0
1522.6617 PEP_0_1_1_0_0
1579.6941 PEP_0_2_0_0_0
1725.7311 PEP_0_2_1_0_0
1887.7778 PEP_1_2_1_0_0
Intensity
m/z
0
50000
100000
150000
200000
250000
500
1000
1500
2000

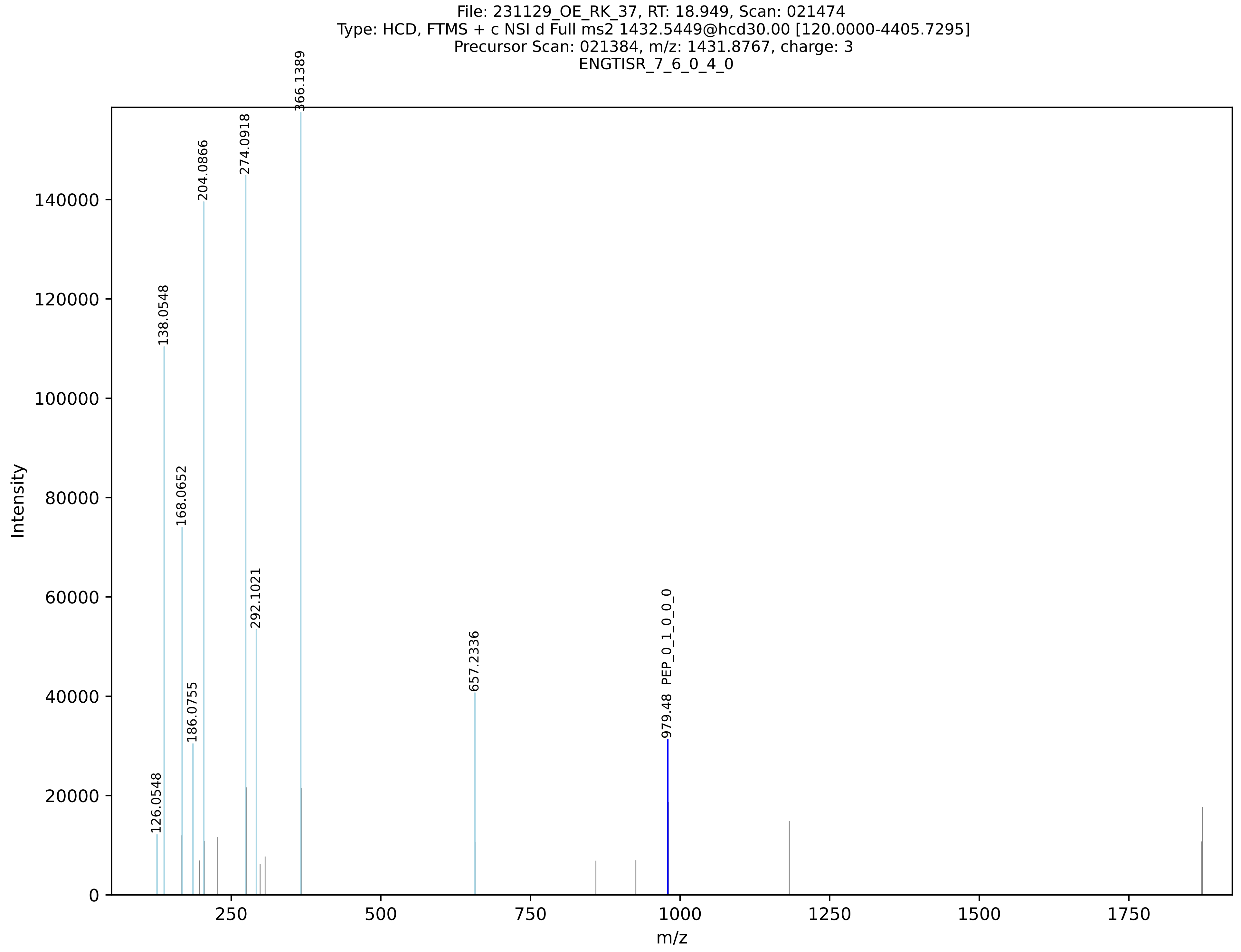
File: 231129_OE_RK_37, RT: 18.949, Scan: 021474
Type: HCD, FTMS + c NSI d Full ms2 1432.5449@hcd30.00 [120.0000-4405.7295]
Precursor Scan: 021384, m/z: 1431.8767, charge: 3
ENGTISR_7_6_0_4_0
Intensity
m/z
366.1389
274.0918
204.0866
138.0548
168.0652
292.1021
186.0755
126.0548
657.2336
979.48 PEP_0_1_0_0_0
0
20000
40000
60000
80000
100000
120000
140000
250
500
750
1000
1250
1500
1750

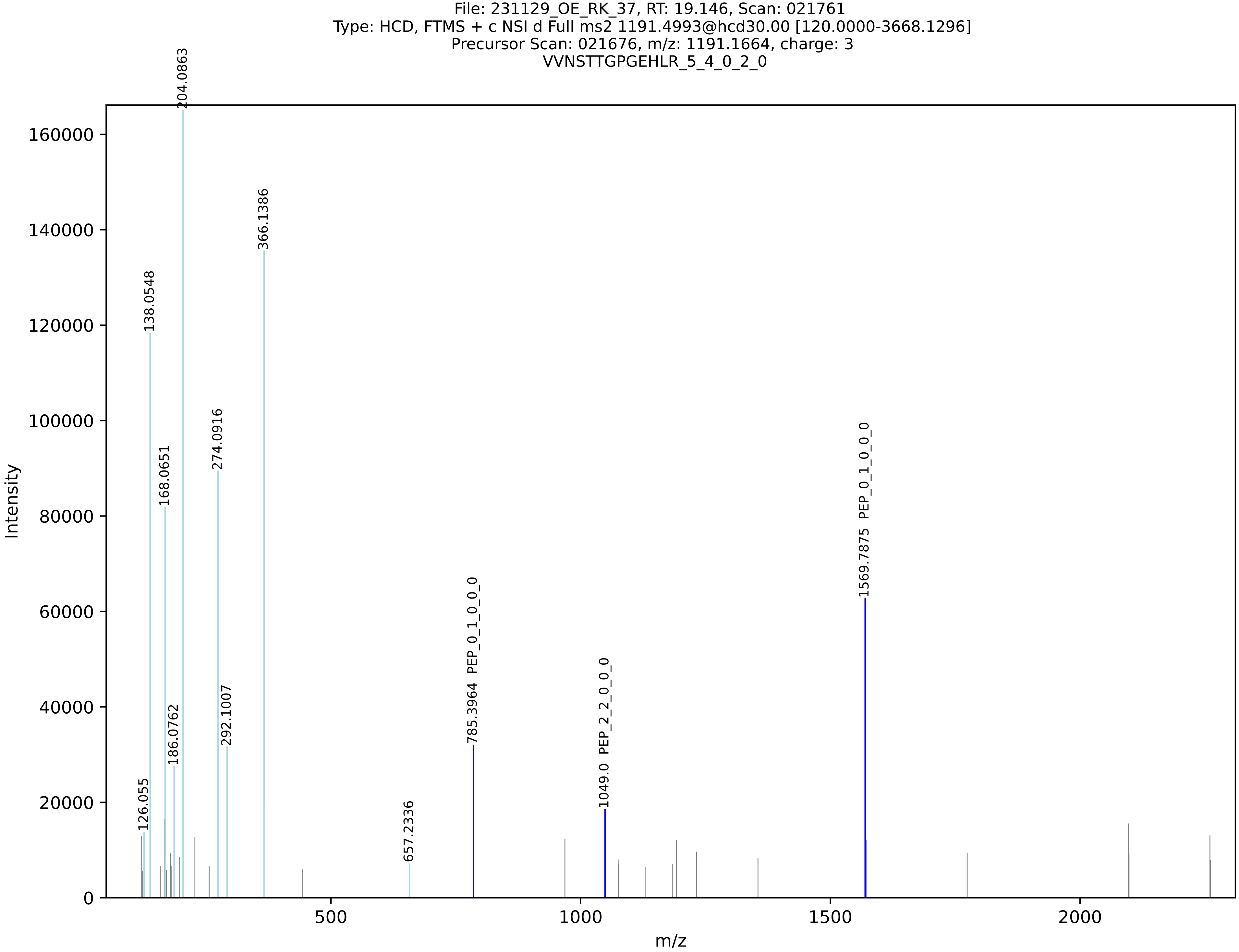

File: 231129_OE_RK_37, RT: 19.146, Scan: 021761
Type: HCD, FTMS + c NSI d Full ms2 1191.4993@hcd30.00 [120.0000-3668.1296]
Precursor Scan: 021676, m/z: 1191.1664, charge: 3
VVNSTTGPGEHLR_5_4_0_2_0
204.0863
366.1386
138.0548
274.0916
168.0651
1569.7875 PEP_0_1_0_0_0
785.3964 PEP_0_1_0_0_0
1049.0 PEP_2_2_0_0_0
186.0762
292.1007
126.055
657.2336
Intensity
m/z
0
20000
40000
60000
80000
100000
120000
140000
160000
500
1000
1500
2000

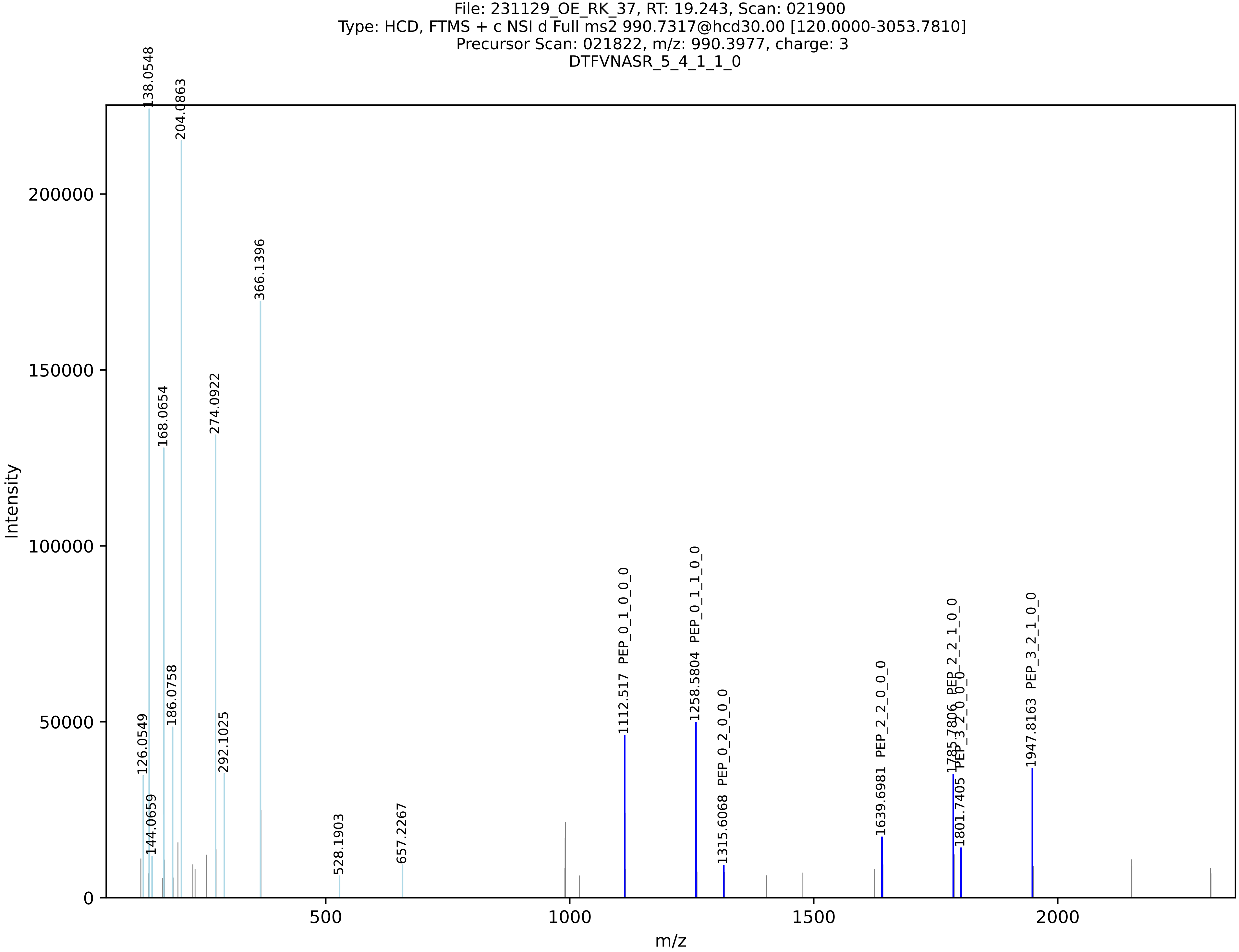
File: 231129_OE_RK_37, RT: 19.243, Scan: 021900
Type: HCD, FTMS + c NSI d Full ms2 990.7317@hcd30.00 [120.0000-3053.7810]
Precursor Scan: 021822, m/z: 990.3977, charge: 3
DTFVNASR_5_4_1_1_0
Intensity
m/z
138.0548
204.0863
366.1396
168.0654
274.0922
186.0758
126.0549
292.1025
144.0659
528.1903
657.2267
1112.517 PEP_0_1_0_0_0
1258.5804 PEP_0_1_1_0_0
1315.6068 PEP_0_2_0_0_0
1639.6981 PEP_2_2_0_0_0
1785.7806 PEP_2_2_1_0_0
1801.7405 PEP_3_2_0_0_0
1947.8163 PEP_3_2_1_0_0

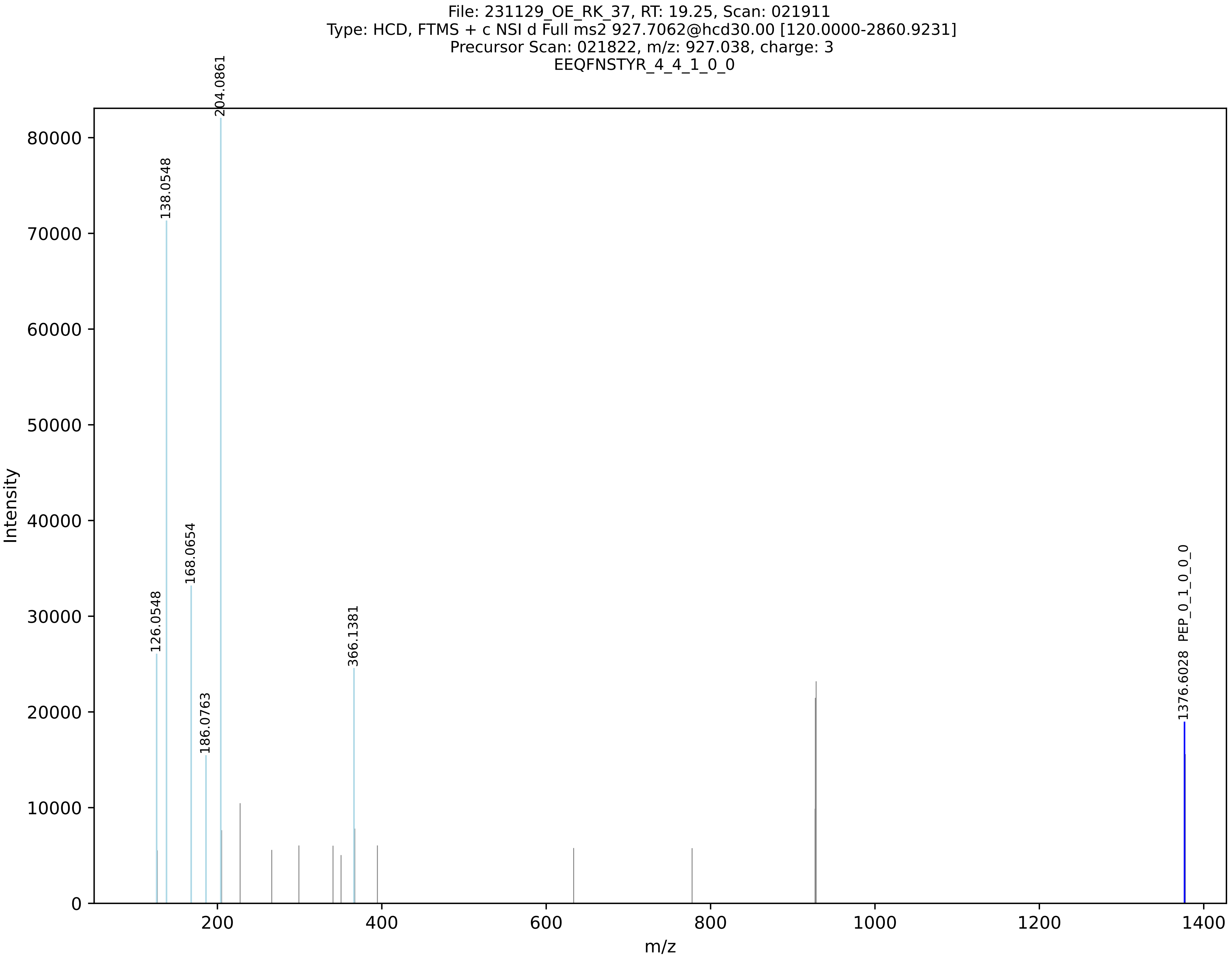
File: 231129_OE_RK_37, RT: 19.25, Scan: 021911
Type: HCD, FTMS + c NSI d Full ms2 927.7062@hcd30.00 [120.0000-2860.9231]
Precursor Scan: 021822, m/z: 927.038, charge: 3
EEQFNSTYR_4_4_1_0_0
Intensity
m/z
204.0861
138.0548
168.0654
126.0548
186.0763
366.1381
1376.6028 PEP_0_1_0_0_0
0
10000
20000
30000
40000
50000
60000
70000
80000
200
400
600
800
1000
1200
1400

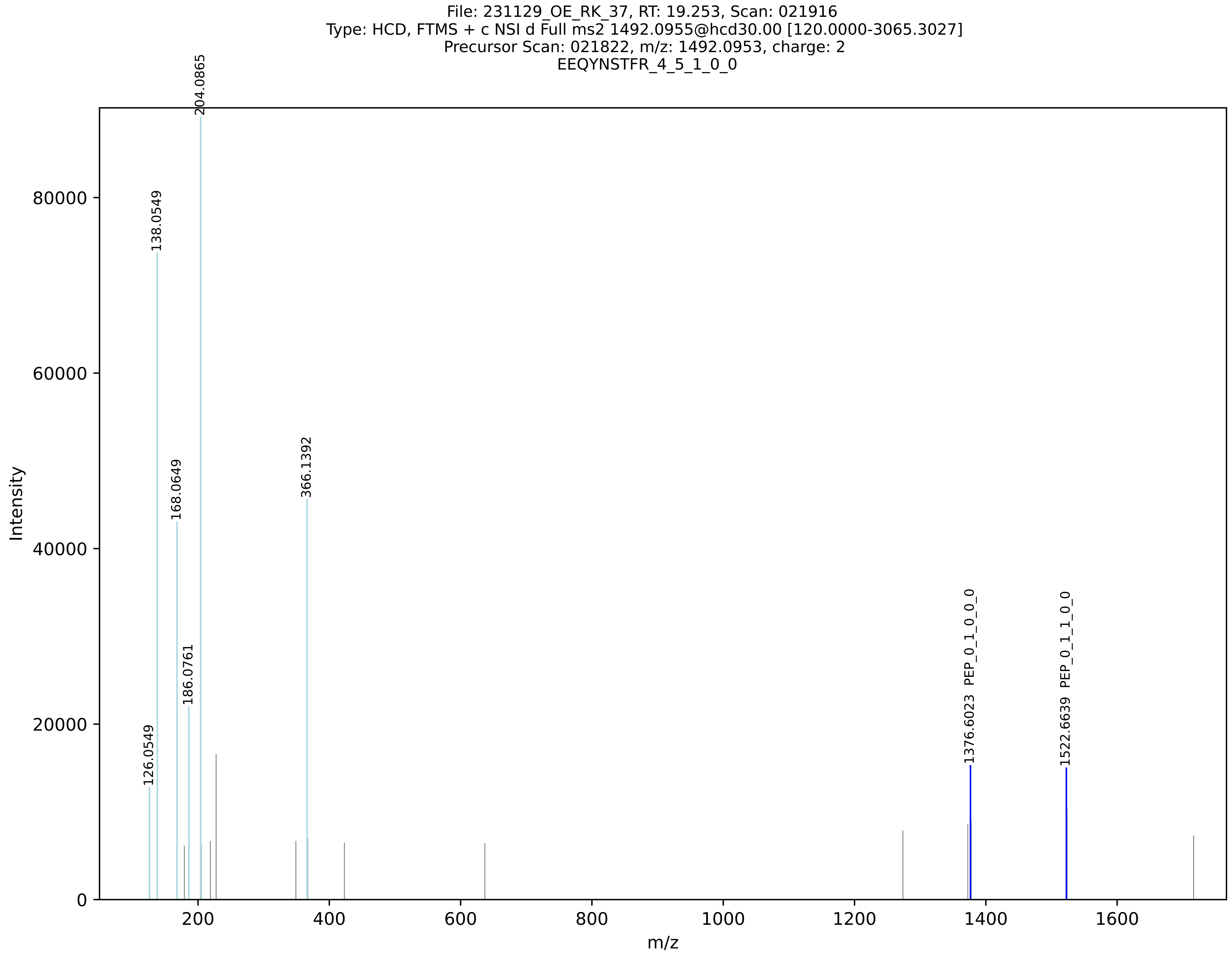
File: 231129_OE_RK_37, RT: 19.253, Scan: 021916
Type: HCD, FTMS + c NSI d Full ms2 1492.0955@hcd30.00 [120.0000-3065.3027]
Precursor Scan: 021822, m/z: 1492.0953, charge: 2
EEQYNSTFR_4_5_1_0_0
204.0865
138.0549
168.0649
366.1392
186.0761
126.0549
1376.6023 PEP_0_1_0_0_0
1522.6639 PEP_0_1_1_0_0
Intensity
m/z
0
20000
40000
60000
80000
200
400
600
800
1000
1200
1400
1600

File: 231129_OE_RK_37, RT: 19.259, Scan: 021926
Type: HCD, FTMS + c NSI d Full ms2 1517.6832@hcd30.00 [120.0000-3117.5017]
Precursor Scan: 021822, m/z: 1517.1856, charge: 2
ETKDQVANSTIVQR_3_4_1_0_0

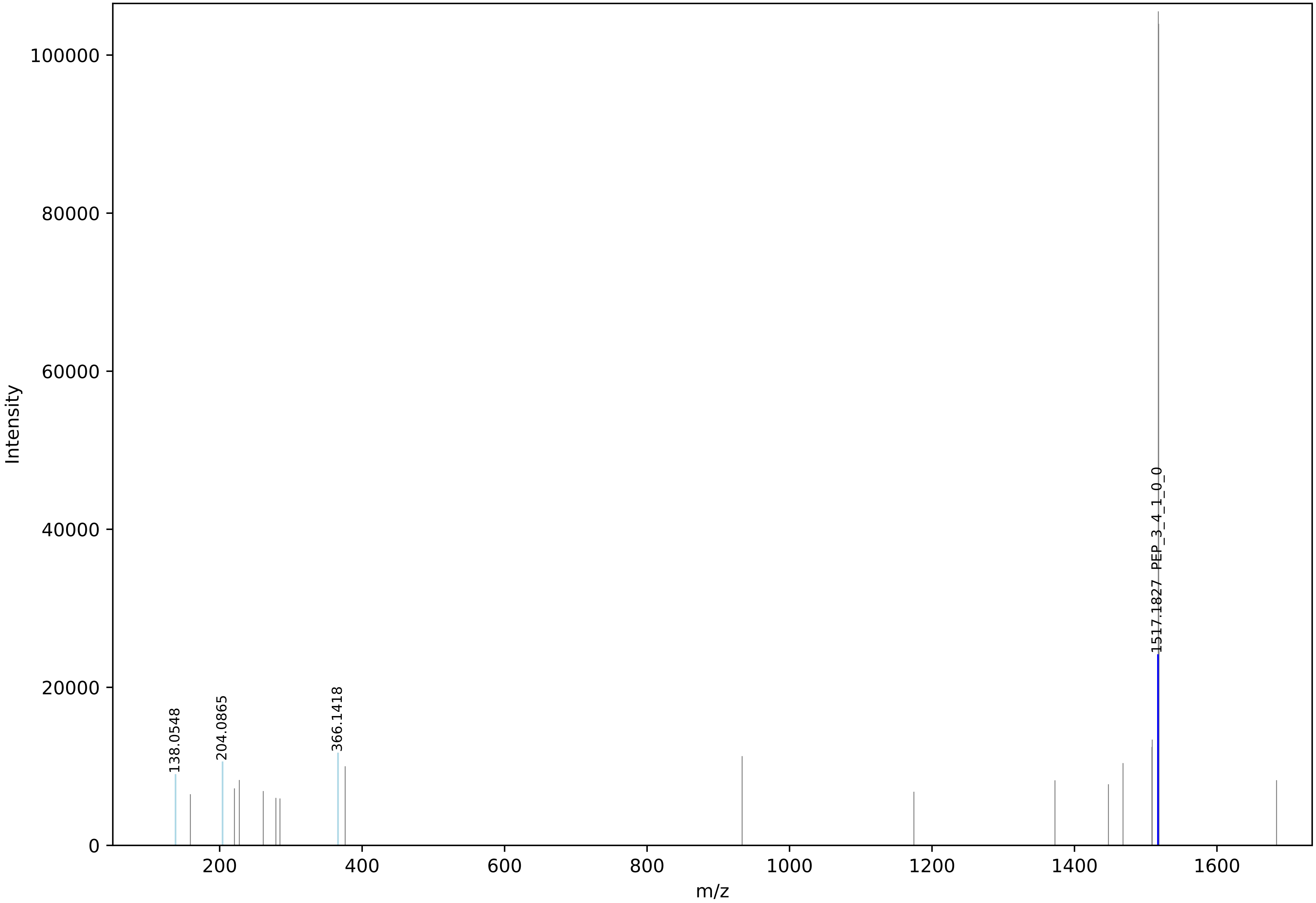

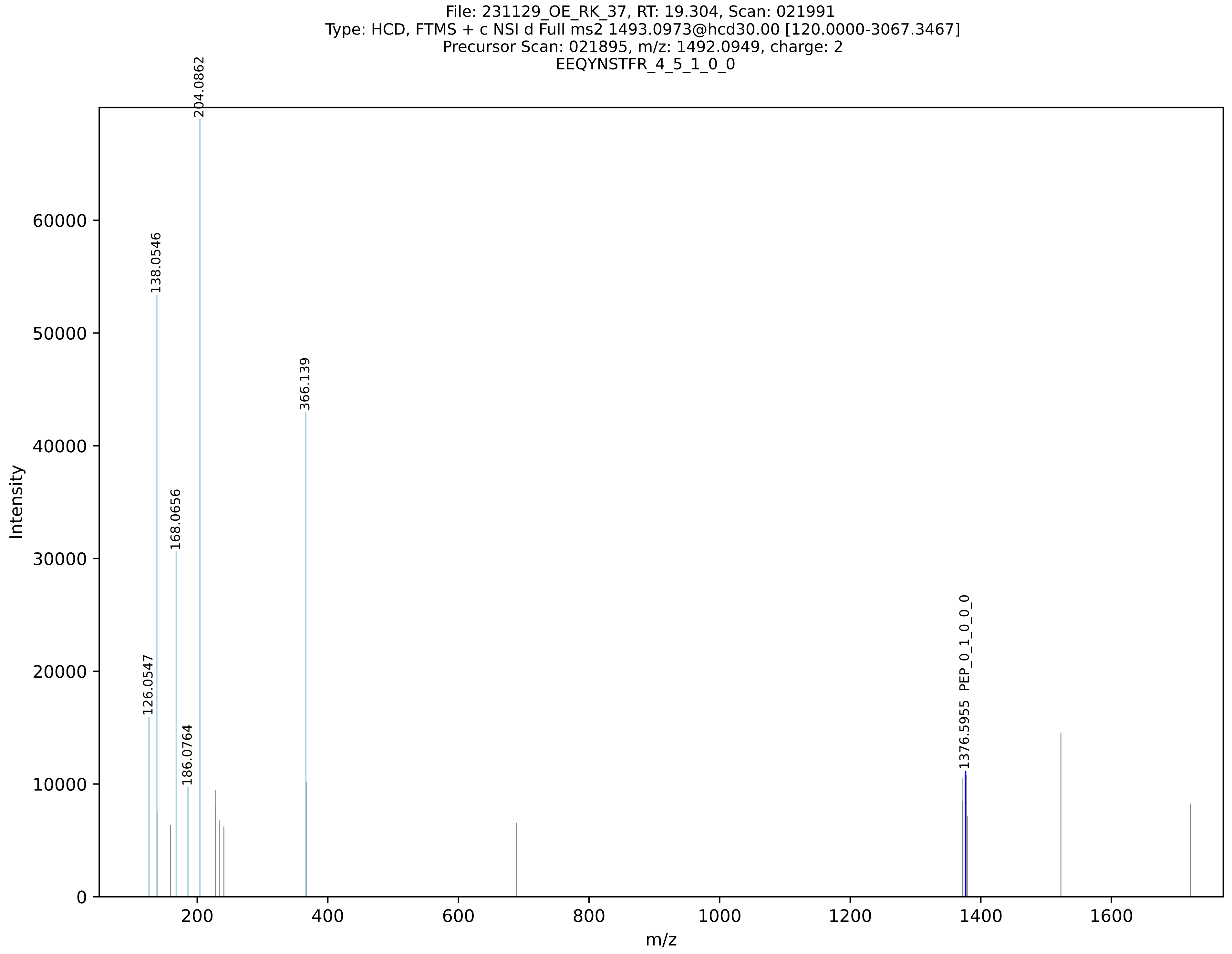

File: 231129_OE_RK_37, RT: 19.304, Scan: 021991
Type: HCD, FTMS + c NSI d Full ms2 1493.0973@hcd30.00 [120.0000-3067.3467]
Precursor Scan: 021895, m/z: 1492.0949, charge: 2
EEQYNSTFR_4_5_1_0_0
Intensity
m/z
204.0862
138.0546
366.139
168.0656
126.0547
186.0764
1376.5955 PEP_0_1_0_0_0
0
10000
20000
30000
40000
50000
60000
200
400
600
800
1000
1200
1400
1600

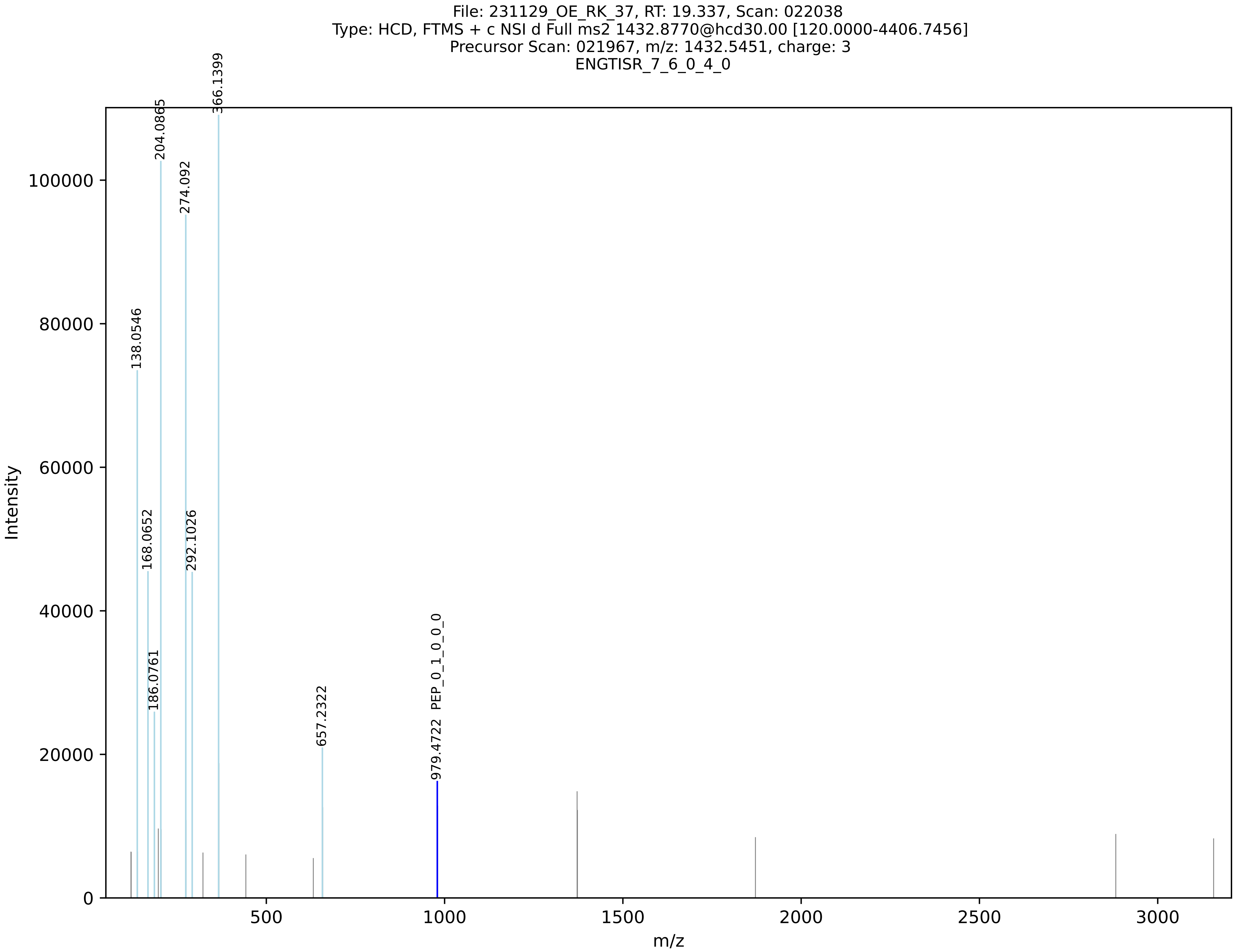
File: 231129_OE_RK_37, RT: 19.337, Scan: 022038
Type: HCD, FTMS + c NSI d Full ms2 1432.8770@hcd30.00 [120.0000-4406.7456]
Precursor Scan: 021967, m/z: 1432.5451, charge: 3
ENGTISR_7_6_0_4_0
366.1399
204.0865
274.092
138.0546
168.0652
292.1026
186.0761
657.2322
979.4722 PEP_0_1_0_0_0
Intensity
m/z
0
20000
40000
60000
80000
100000
500
1000
1500
2000
2500
3000

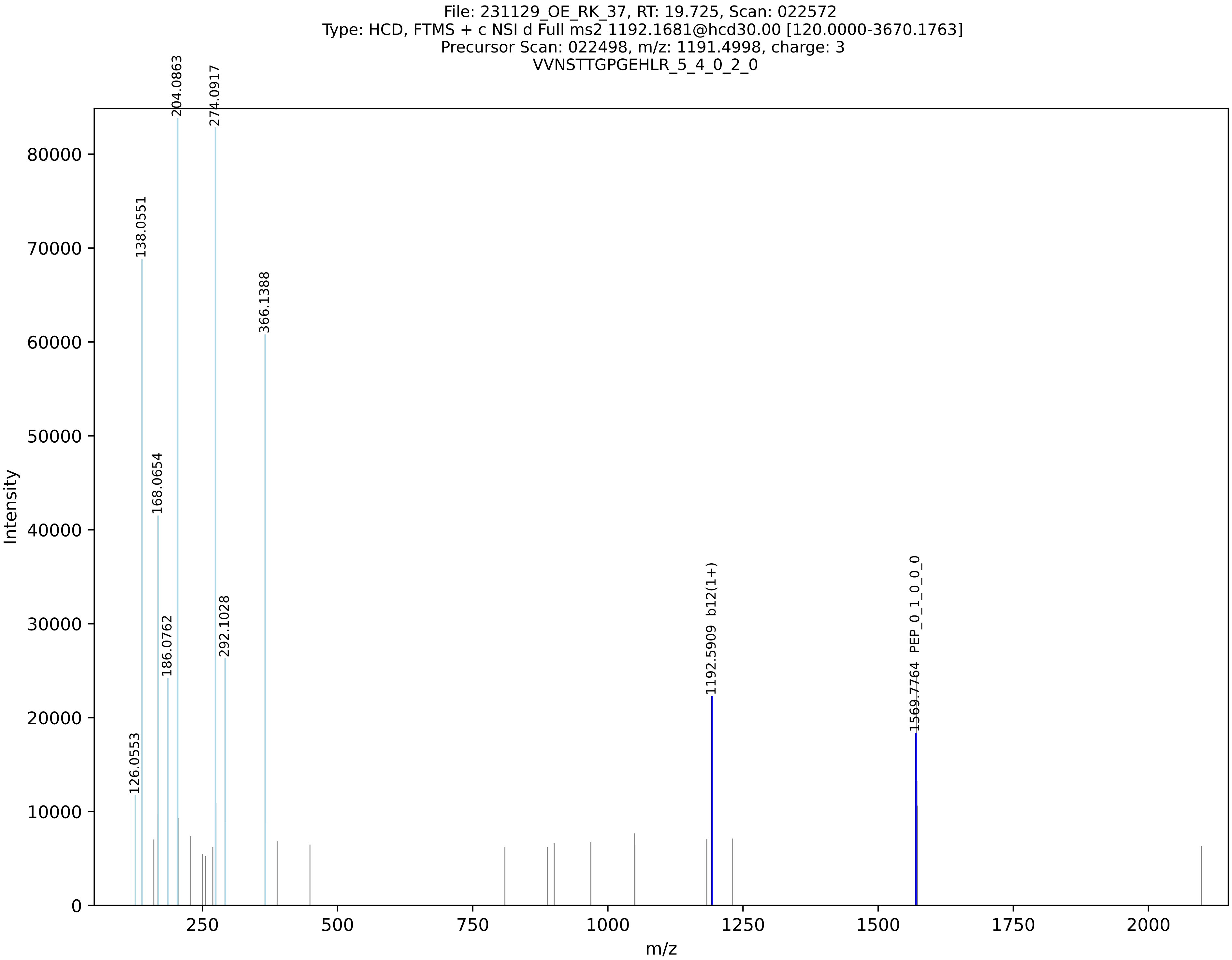

File: 231129_OE_RK_37, RT: 19.725, Scan: 022572
Type: HCD, FTMS + c NSI d Full ms2 1192.1681@hcd30.00 [120.0000-3670.1763]
Precursor Scan: 022498, m/z: 1191.4998, charge: 3
VVNSTTGPGEHLR_5_4_0_2_0
204.0863
274.0917
138.0551
366.1388
168.0654
292.1028
186.0762
126.0553
1192.5909 b12(1+)
1569.7764 PEP_0_1_0_0_0
Intensity
m/z
0
10000
20000
30000
40000
50000
60000
70000
80000
250
500
750
1000
1250
1500
1750
2000

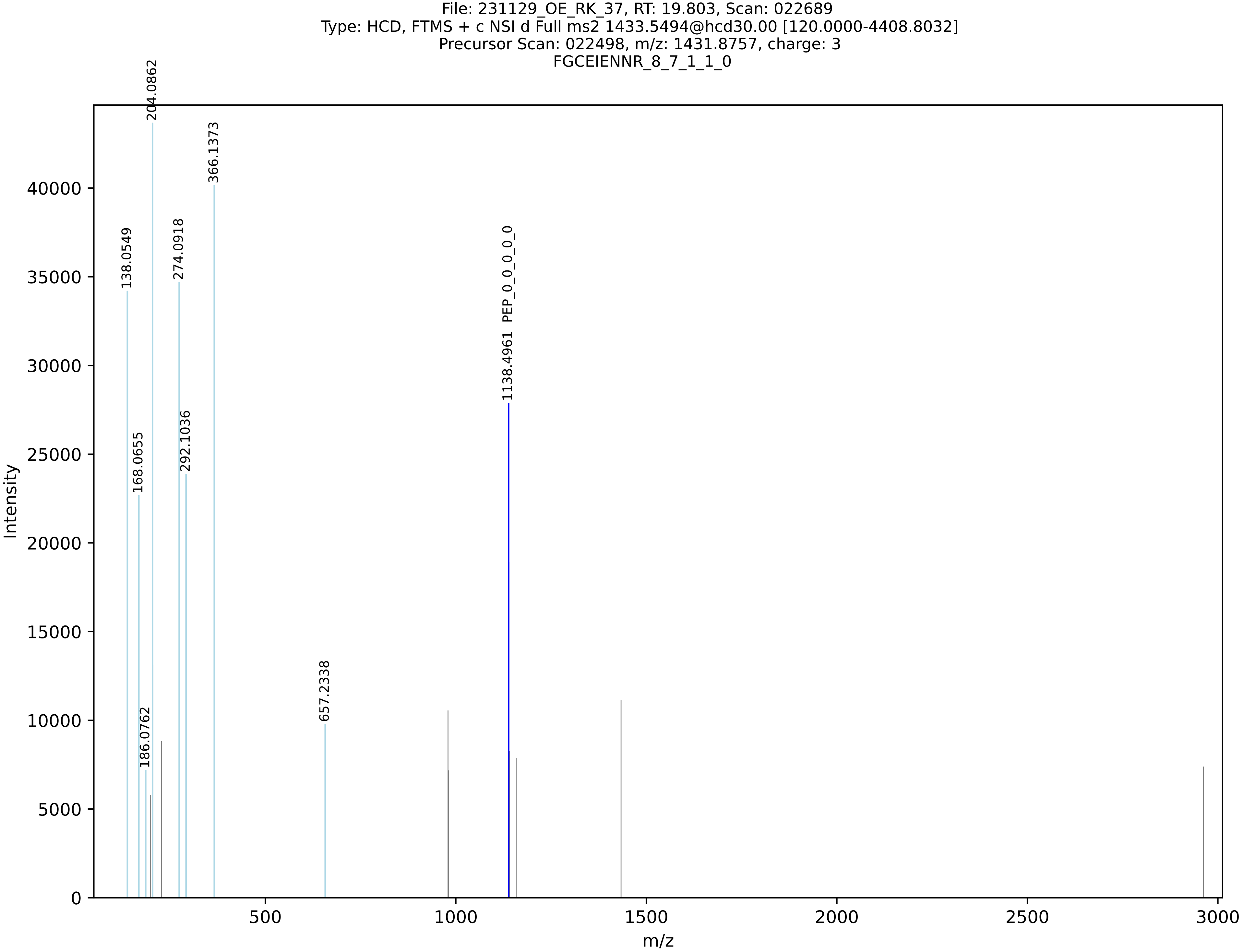

File: 231129_OE_RK_37, RT: 19.803, Scan: 022689
Type: HCD, FTMS + c NSI d Full ms2 1433.5494@hcd30.00 [120.0000-4408.8032]
Precursor Scan: 022498, m/z: 1431.8757, charge: 3
FGCEIENNR_8_7_1_1_0
204.0862
366.1373
138.0549
274.0918
1138.4961 PEP_0_0_0_0_0
168.0655
292.1036
186.0762
657.2338
Intensity
m/z
0
5000
10000
15000
20000
25000
30000
35000
40000
500
1000
1500
2000
2500
3000

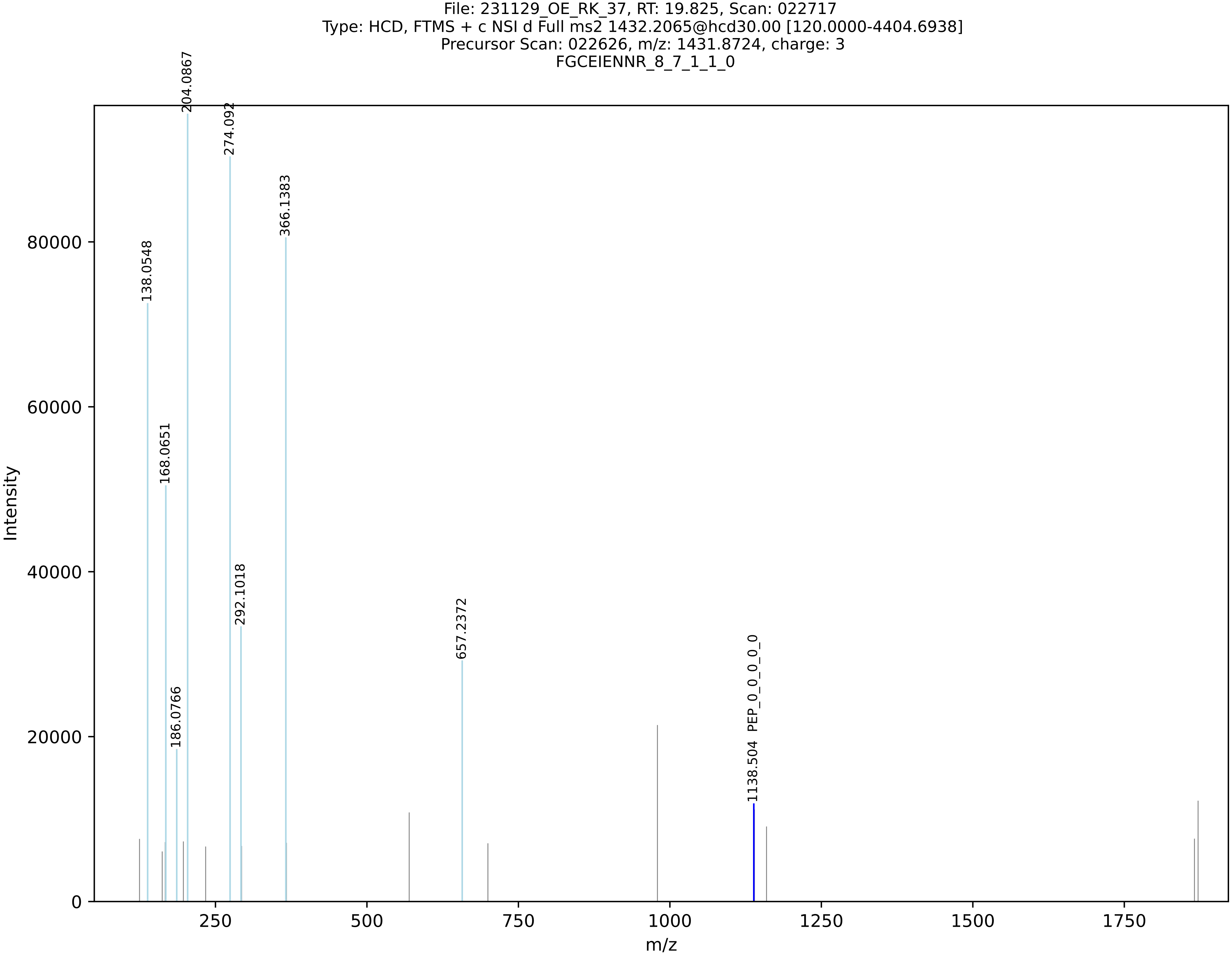

File: 231129_OE_RK_37, RT: 19.825, Scan: 022717
Type: HCD, FTMS + c NSI d Full ms2 1432.2065@hcd30.00 [120.0000-4404.6938]
Precursor Scan: 022626, m/z: 1431.8724, charge: 3
FGCEIENNR_8_7_1_1_0
204.0867
274.092
366.1383
138.0548
168.0651
292.1018
186.0766
657.2372
1138.504 PEP_0_0_0_0_0
Intensity
m/z
0
20000
40000
60000
80000
250
500
750
1000
1250
1500
1750

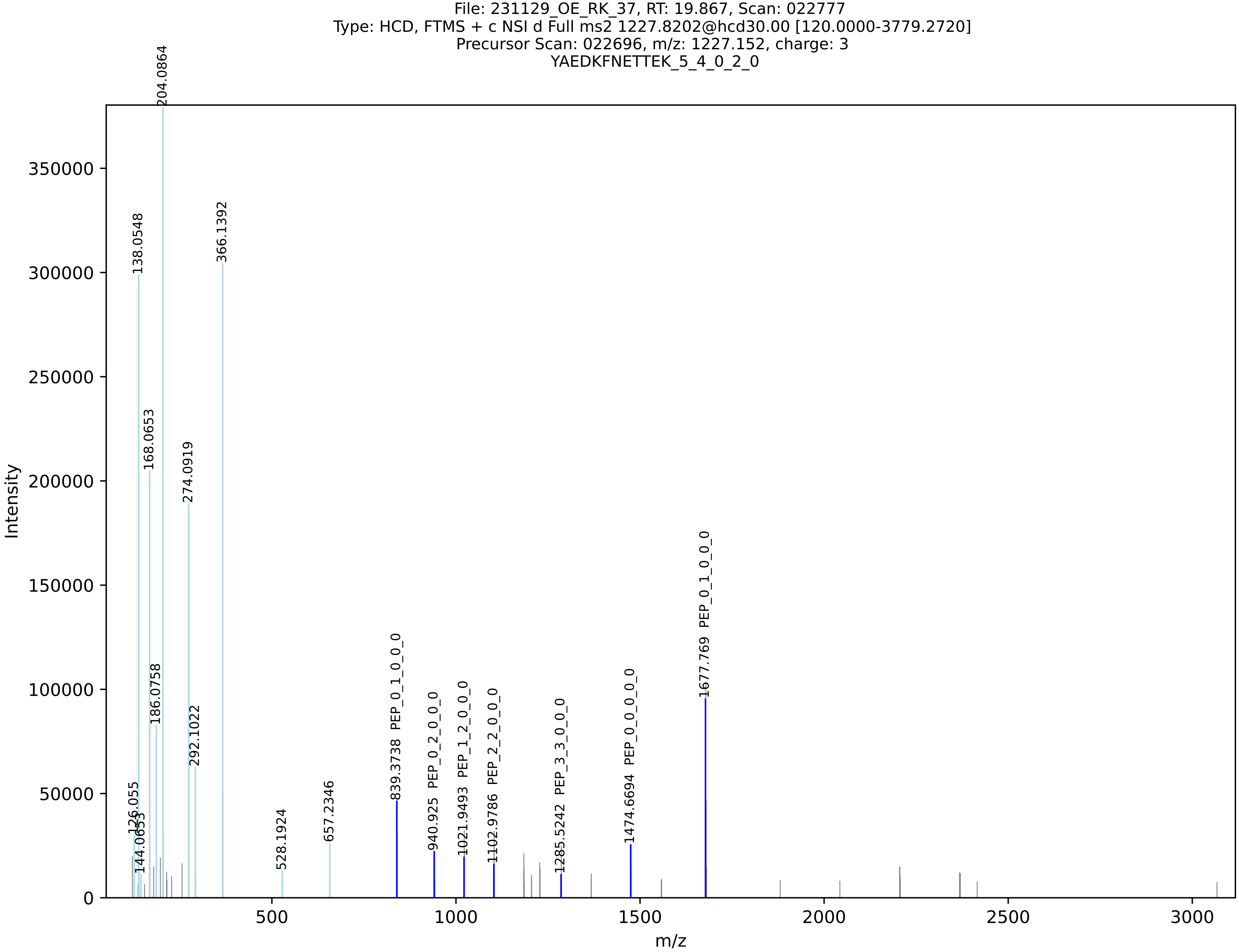
File: 231129_OE_RK_37, RT: 19.867, Scan: 022777
Type: HCD, FTMS + c NSI d Full ms2 1227.8202@hcd30.00 [120.0000-3779.2720]
Precursor Scan: 022696, m/z: 1227.152, charge: 3
YAEDKFNETTEK_5_4_0_2_0
Intensity
m/z
204.0864
138.0548
366.1392
168.0653
274.0919
186.0758
292.1022
126.055
144.0653
528.1924
657.2346
839.3738 PEP_0_1_0_0_0
940.925 PEP_0_2_0_0_0
1021.9493 PEP_1_2_0_0_0
1102.9786 PEP_2_2_0_0_0
1285.5242 PEP_3_3_0_0_0
1474.6694 PEP_0_0_0_0_0
1677.769 PEP_0_1_0_0_0
0
50000
100000
150000
200000
250000
300000
350000
500
1000
1500
2000
2500
3000

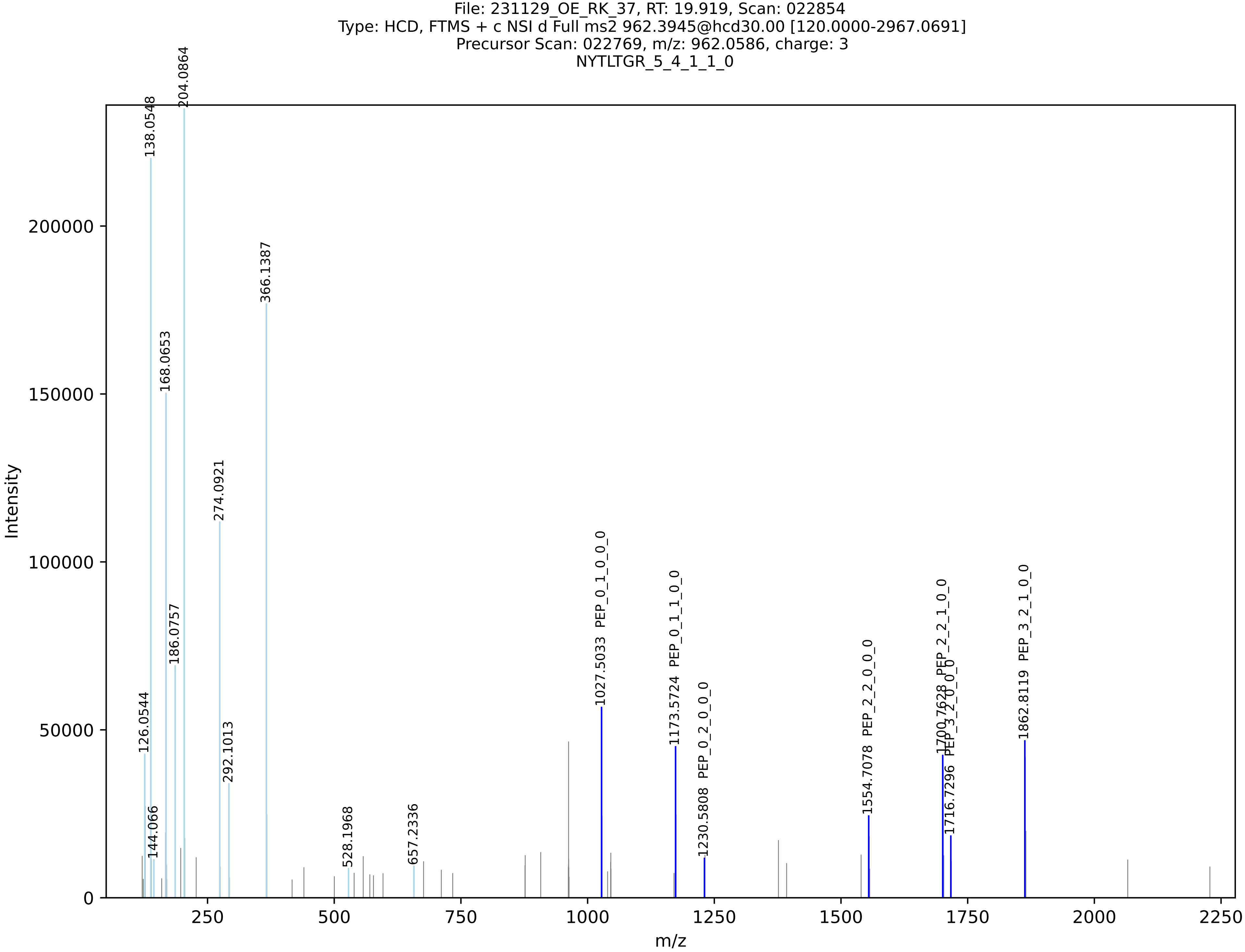
File: 231129_OE_RK_37, RT: 19.919, Scan: 022854
Type: HCD, FTMS + c NSI d Full ms2 962.3945@hcd30.00 [120.0000-2967.0691]
Precursor Scan: 022769, m/z: 962.0586, charge: 3
NYTLTGR_5_4_1_1_0
Intensity
m/z
204.0864
138.0548
366.1387
168.0653
274.0921
186.0757
126.0544
292.1013
144.066
528.1968
657.2336
1027.5033 PEP_0_1_0_0_0
1173.5724 PEP_0_1_1_0_0
1230.5808 PEP_0_2_0_0_0
1554.7078 PEP_2_2_0_0_0
1700.7628 PEP_2_2_1_0_0
1716.7296 PEP_3_2_0_0_0
1862.8119 PEP_3_2_1_0_0

File: 231129_OE_RK_37, RT: 20.022, Scan: 023005
Type: HCD, FTMS + c NSI d Full ms2 1432.8788@hcd30.00 [120.0000-4406.7515]
Precursor Scan: 022917, m/z: 1431.8765, charge: 3
ENGTISR_7_6_0_4_0

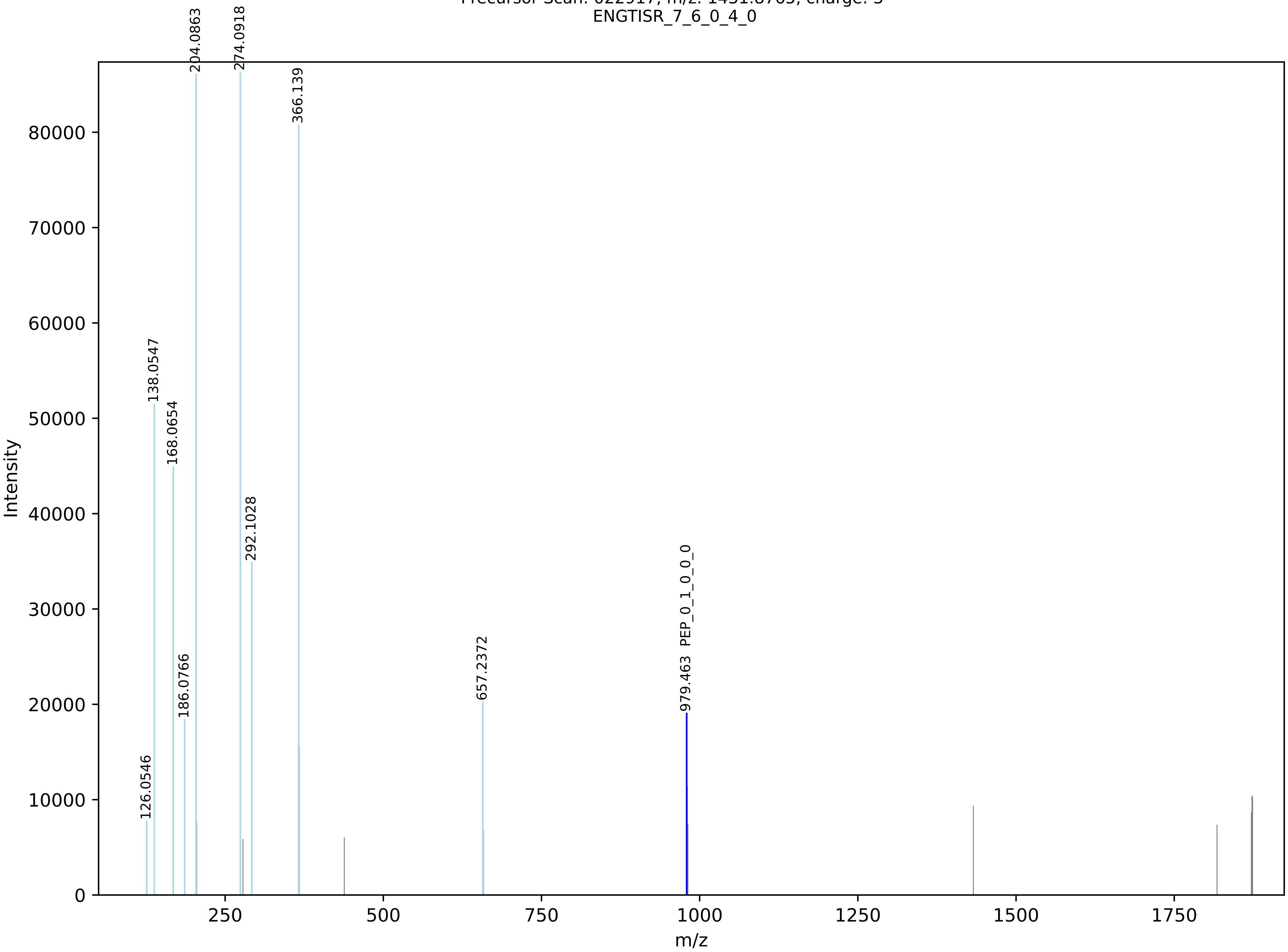

File: 231129_OE_RK_37, RT: 20.038, Scan: 023031
Type: HCD, FTMS + c NSI d Full ms2 1018.7319@hcd30.00 [120.0000-3139.4614]
Precursor Scan: 022917, m/z: 1018.3974, charge: 3
CNNYSANAPK_5_4_0_1_0

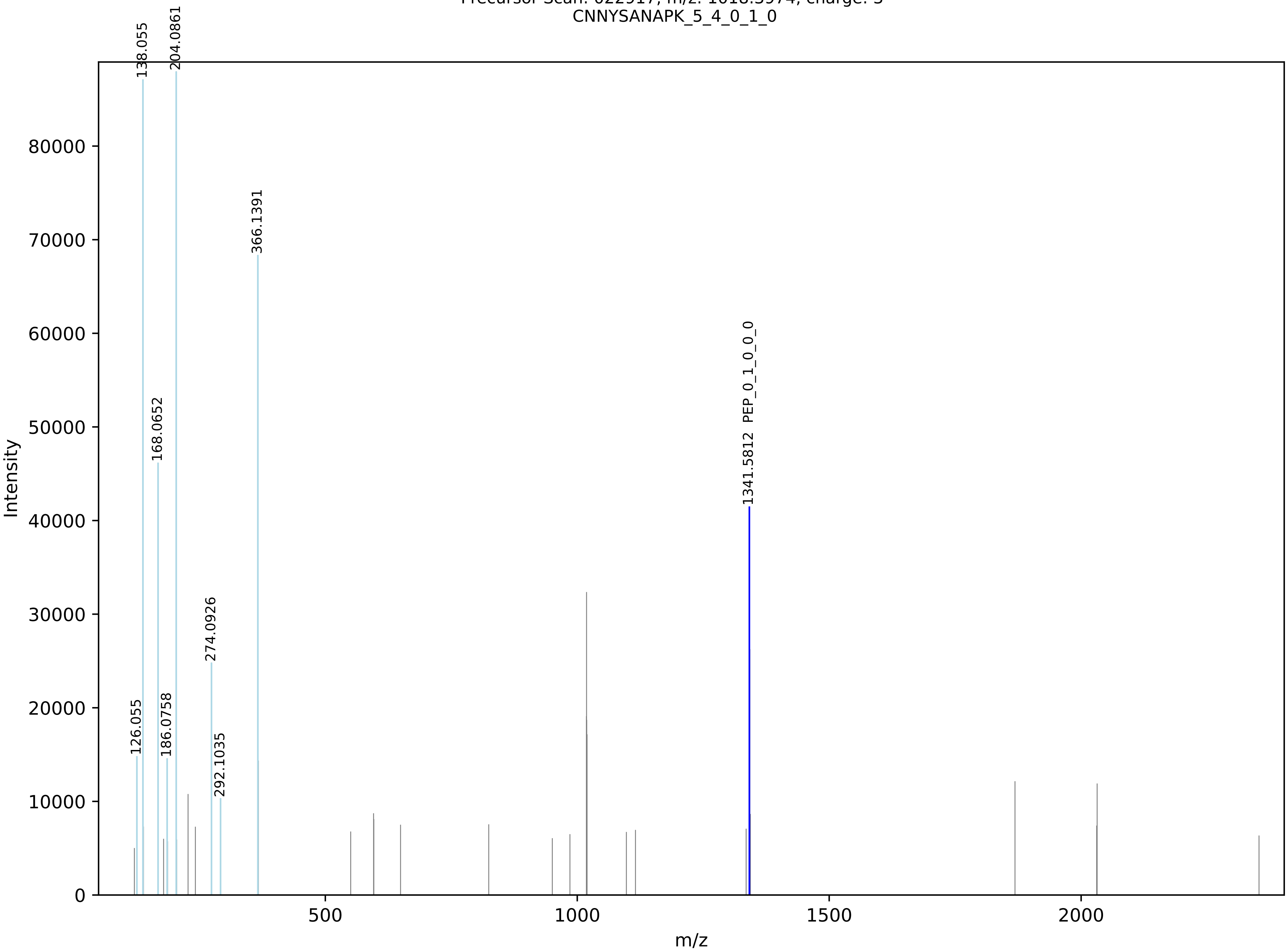

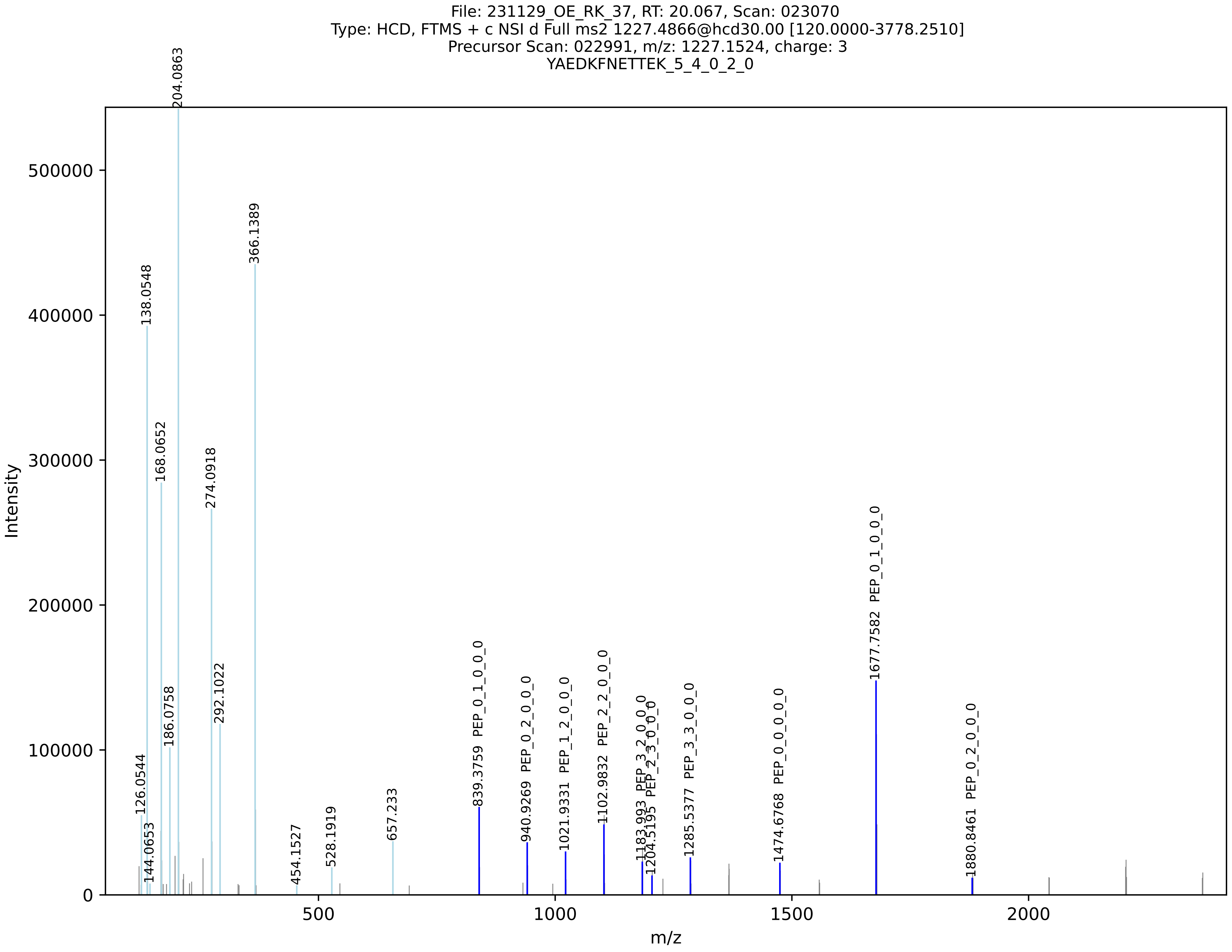

File: 231129_OE_RK_37, RT: 20.067, Scan: 023070
Type: HCD, FTMS + c NSI d Full ms2 1227.4866@hcd30.00 [120.0000-3778.2510]
Precursor Scan: 022991, m/z: 1227.1524, charge: 3
YAEDKFNETTEK_5_4_0_2_0
Intensity
m/z
204.0863
366.1389
138.0548
168.0652
274.0918
292.1022
186.0758
126.0544
144.0653
454.1527
528.1919
657.233
839.3759 PEP_0_1_0_0_0
940.9269 PEP_0_2_0_0_0
1021.9331 PEP_1_2_0_0_0
1102.9832 PEP_2_2_0_0_0
1183.993 PEP_3_2_0_0_0
1204.5195 PEP_2_3_0_0_0
1285.5377 PEP_3_3_0_0_0
1474.6768 PEP_0_0_0_0_0
1677.7582 PEP_0_1_0_0_0
1880.8461 PEP_0_2_0_0_0

File: 231129_OE_RK_37, RT: 20.08, Scan: 023091
Type: HCD, FTMS + c NSI d Full ms2 1443.0848@hcd30.00 [120.0000-2965.3210]
Precursor Scan: 022991, m/z: 1442.5859, charge: 2
NYTLTGR_5_4_1_1_0

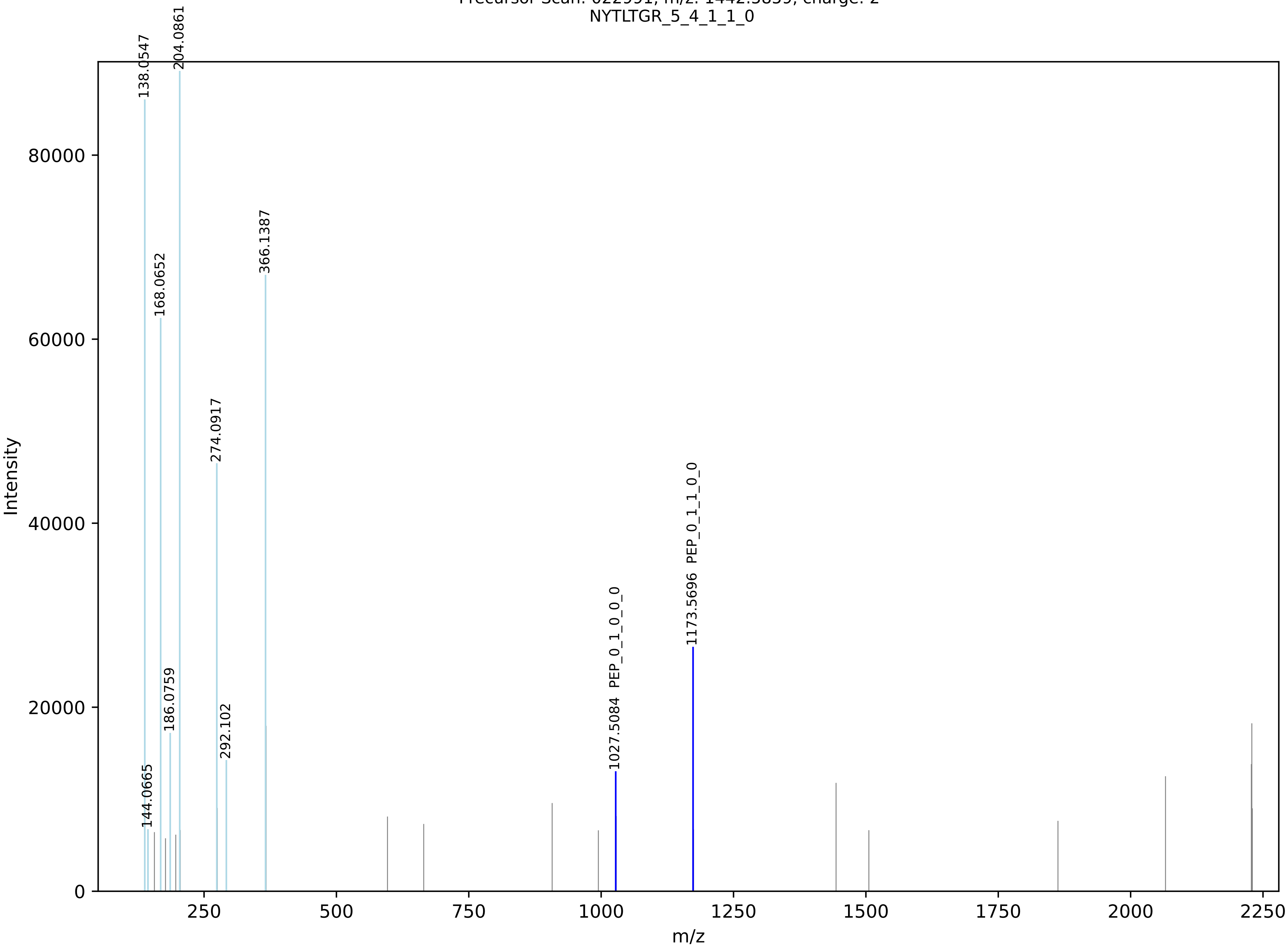

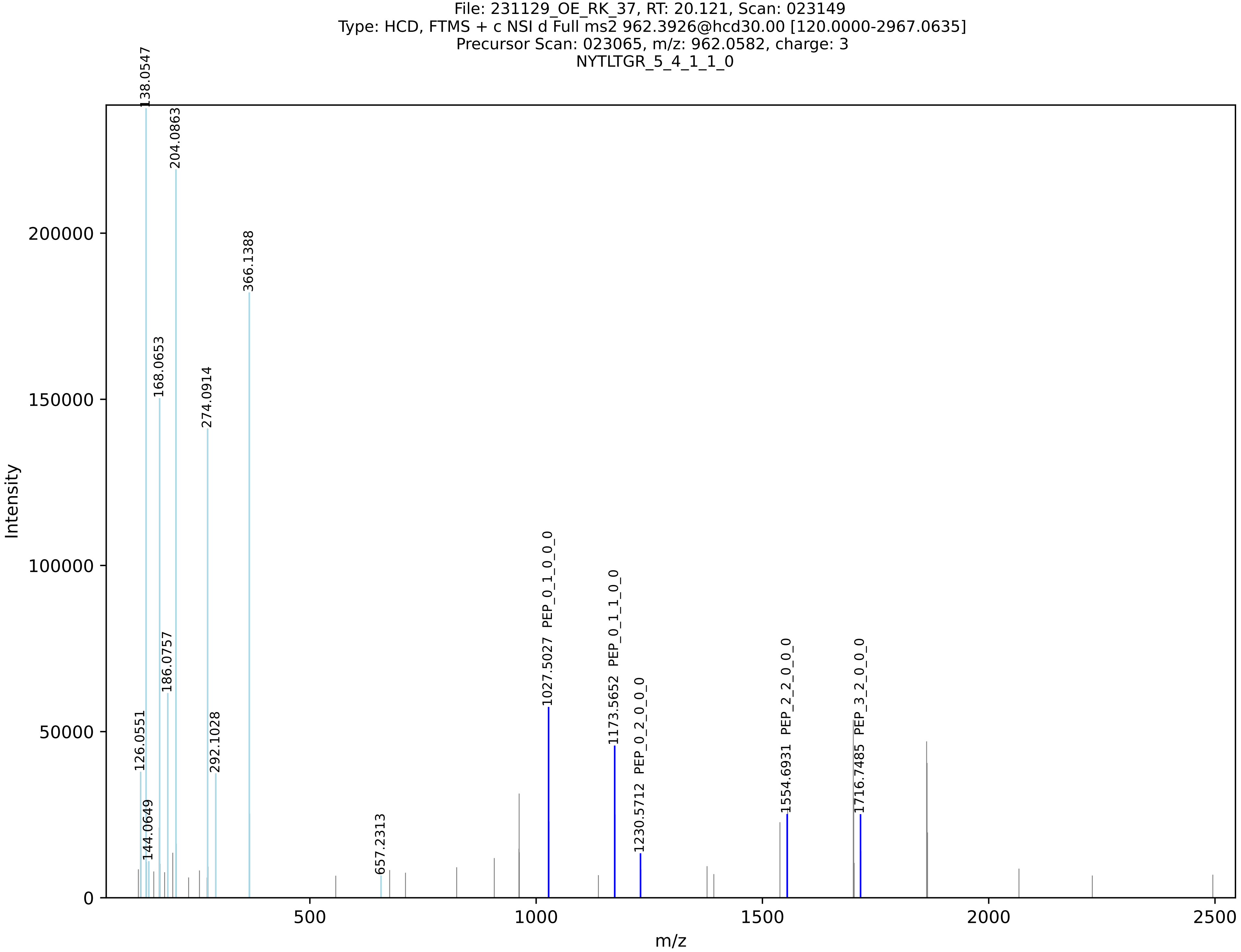
File: 231129_OE_RK_37, RT: 20.121, Scan: 023149
Type: HCD, FTMS + c NSI d Full ms2 962.3926@hcd30.00 [120.0000-2967.0635]
Precursor Scan: 023065, m/z: 962.0582, charge: 3
NYTLTGR_5_4_1_1_0
138.0547
204.0863
366.1388
168.0653
274.0914
186.0757
126.0551
292.1028
144.0649
657.2313
1027.5027 PEP_0_1_0_0_0
1173.5652 PEP_0_1_1_0_0
1230.5712 PEP_0_2_0_0_0
1554.6931 PEP_2_2_0_0_0
1716.7485 PEP_3_2_0_0_0
Intensity
m/z
0
50000
100000
150000
200000
500
1000
1500
2000
2500

File: 231129_OE_RK_37, RT: 20.217, Scan: 023288
Type: HCD, FTMS + c NSI d Full ms2 1039.0775@hcd30.00 [120.0000-3201.7192]
Precursor Scan: 023213, m/z: 1038.7434, charge: 3
DTFVNASR_5_4_0_2_0

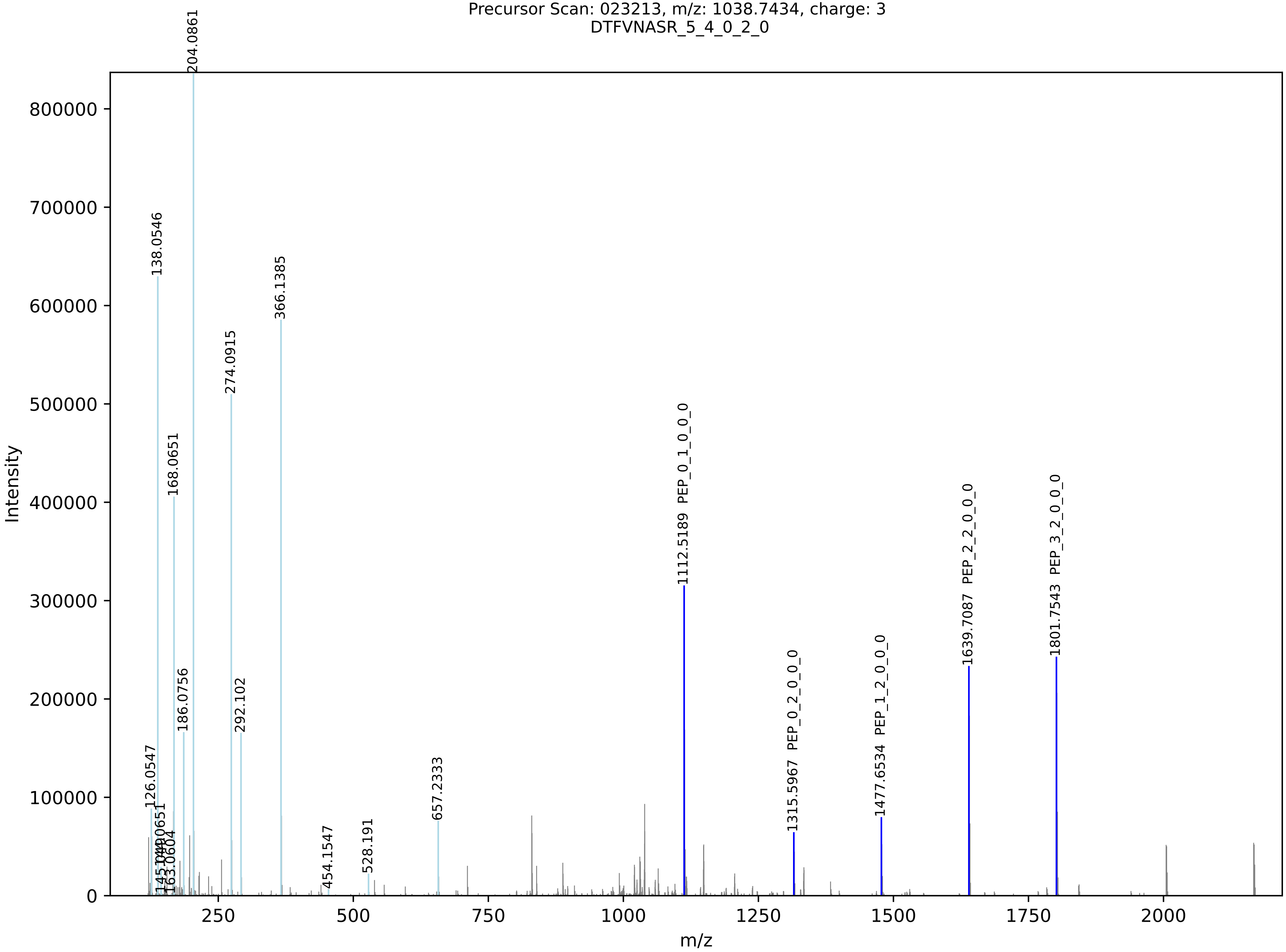

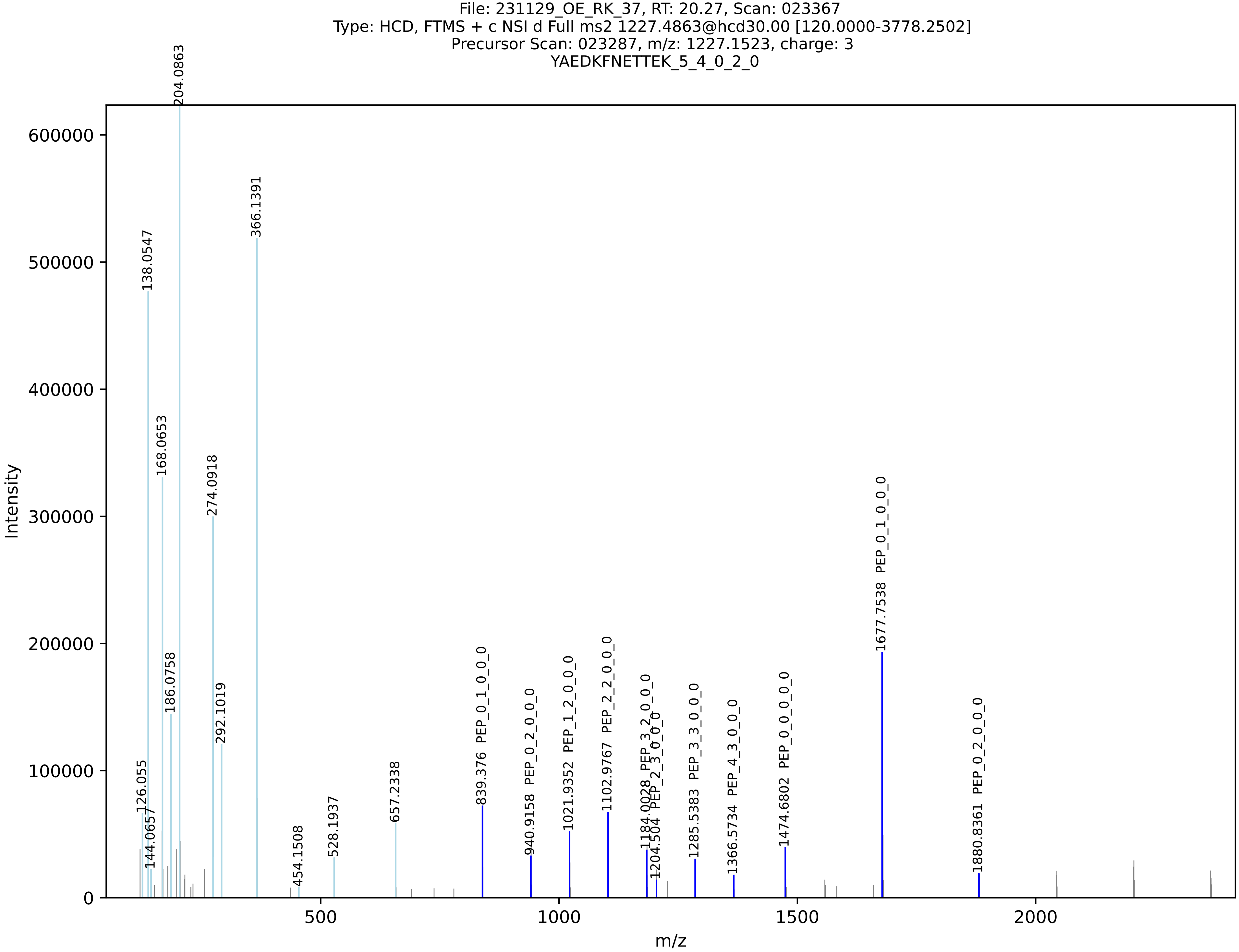
File: 231129_OE_RK_37, RT: 20.27, Scan: 023367
Type: HCD, FTMS + c NSI d Full ms2 1227.4863@hcd30.00 [120.0000-3778.2502]
Precursor Scan: 023287, m/z: 1227.1523, charge: 3
YAEDKFNETTEK_5_4_0_2_0
Intensity
m/z
204.0863
366.1391
138.0547
168.0653
274.0918
186.0758
292.1019
126.055
144.0657
454.1508
528.1937
657.2338
839.376 PEP_0_1_0_0_0
940.9158 PEP_0_2_0_0_0
1021.9352 PEP_1_2_0_0_0
1102.9767 PEP_2_2_0_0_0
1184.0028 PEP_3_2_0_0_0
1204.504 PEP_2_3_0_0_0
1285.5383 PEP_3_3_0_0_0
1366.5734 PEP_4_3_0_0_0
1474.6802 PEP_0_0_0_0_0
1677.7538 PEP_0_1_0_0_0
1880.8361 PEP_0_2_0_0_0

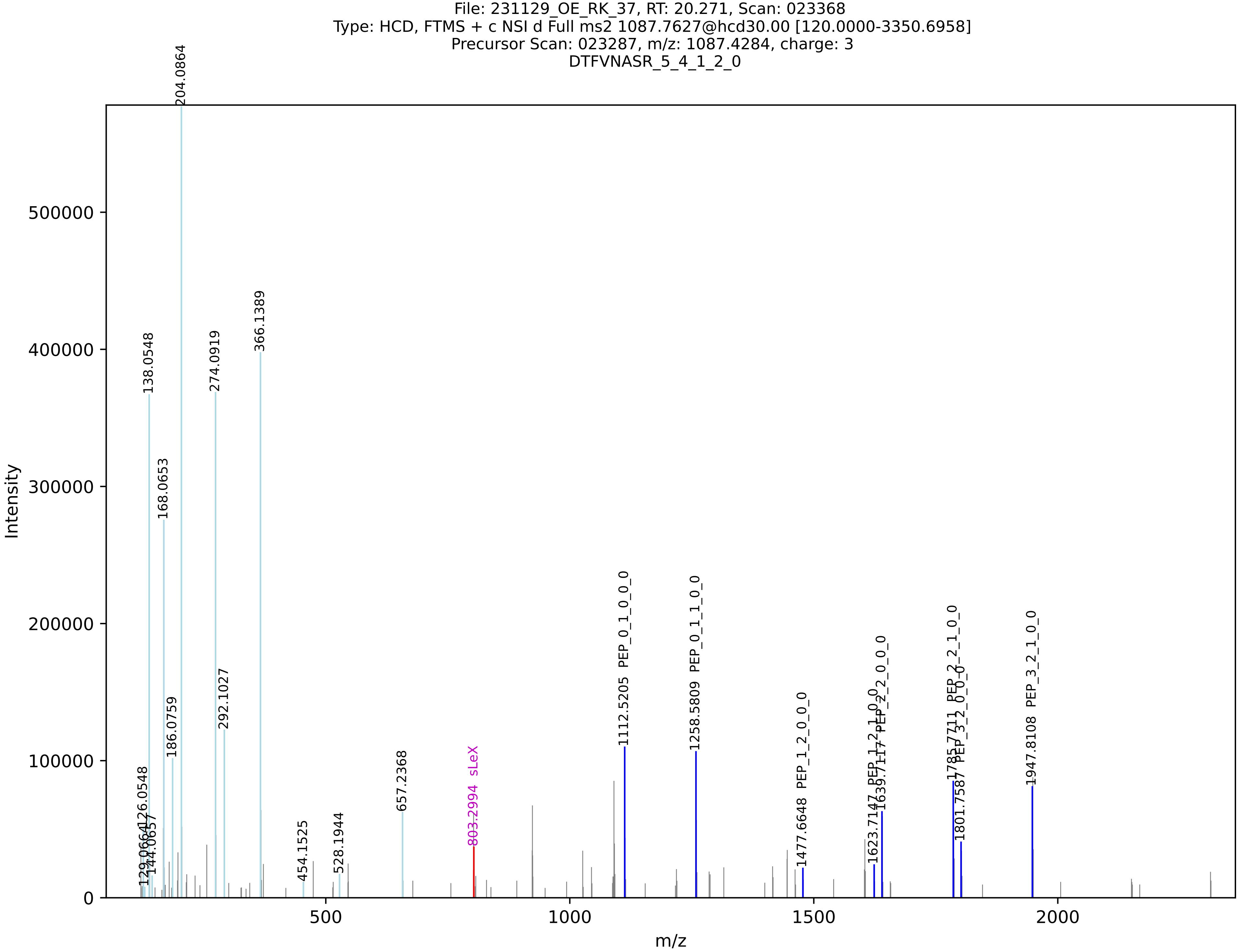
File: 231129_OE_RK_37, RT: 20.271, Scan: 023368
Type: HCD, FTMS + c NSI d Full ms2 1087.7627@hcd30.00 [120.0000-3350.6958]
Precursor Scan: 023287, m/z: 1087.4284, charge: 3
DTFVNASR_5_4_1_2_0
Intensity
m/z
204.0864
138.0548
274.0919
366.1389
168.0653
186.0759
292.1027
126.0548
129.0664
144.0657
454.1525
528.1944
657.2368
803.2994 sLeX
1112.5205 PEP_0_1_0_0_0
1258.5809 PEP_0_1_1_0_0
1477.6648 PEP_1_2_0_0_0
1623.7147 PEP_1_2_1_0_0
1639.7117 PEP_2_2_0_0_0
1785.7711 PEP_2_2_1_0_0
1801.7587 PEP_3_2_0_0_0
1947.8108 PEP_3_2_1_0_0

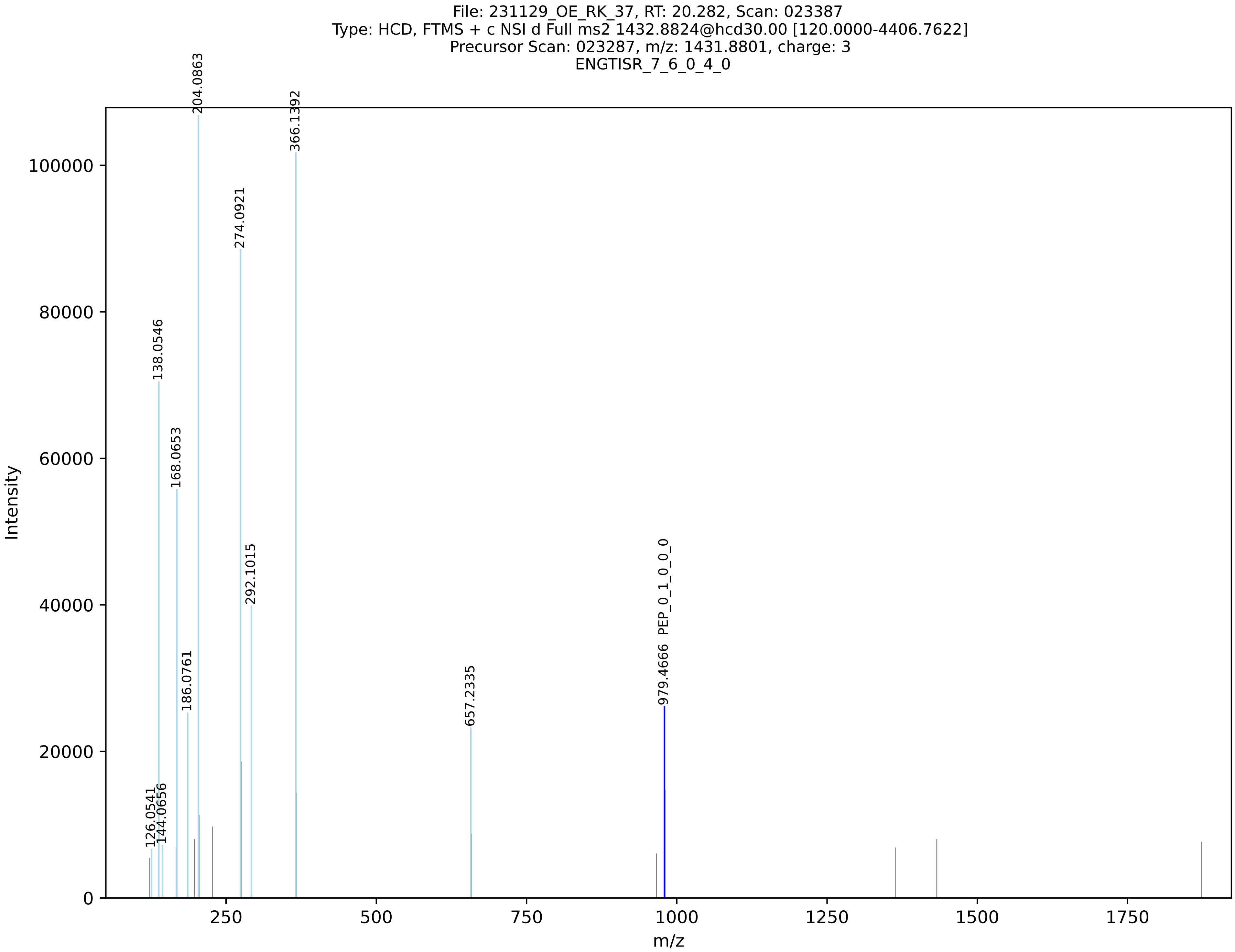

File: 231129_OE_RK_37, RT: 20.282, Scan: 023387
Type: HCD, FTMS + c NSI d Full ms2 1432.8824@hcd30.00 [120.0000-4406.7622]
Precursor Scan: 023287, m/z: 1431.8801, charge: 3
ENGTISR_7_6_0_4_0
Intensity
m/z
204.0863
366.1392
274.0921
138.0546
168.0653
292.1015
186.0761
657.2335
979.4666 PEP_0_1_0_0_0
126.0541
144.0656
0
20000
40000
60000
80000
100000
250
500
750
1000
1250
1500
1750

File: 231129_OE_RK_37, RT: 20.32, Scan: 023440
Type: HCD, FTMS + c NSI d Full ms2 962.3942@hcd30.00 [120.0000-2967.0684]
Precursor Scan: 023361, m/z: 962.0601, charge: 3
NYTLTGR_5_4_1_1_0

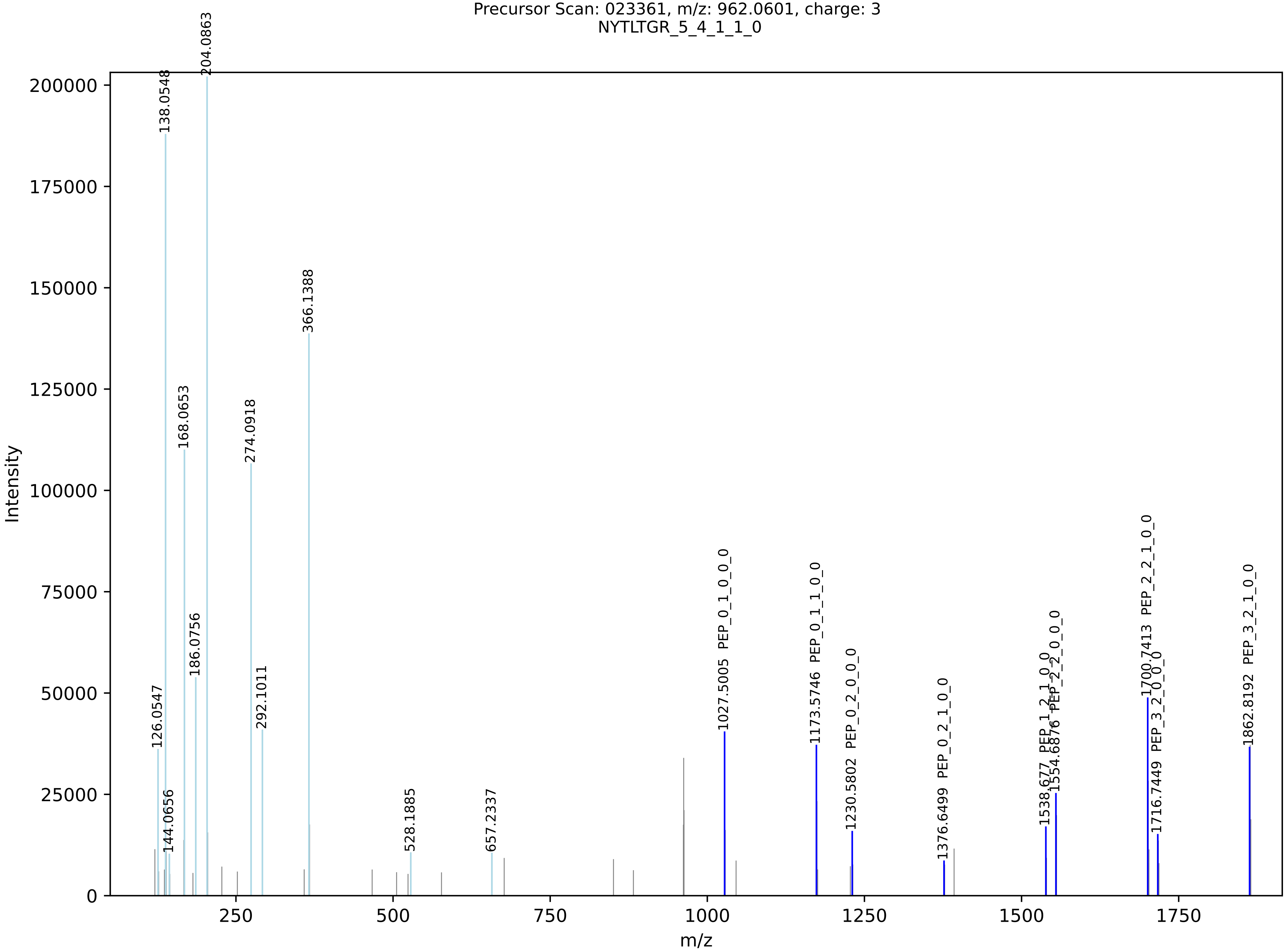

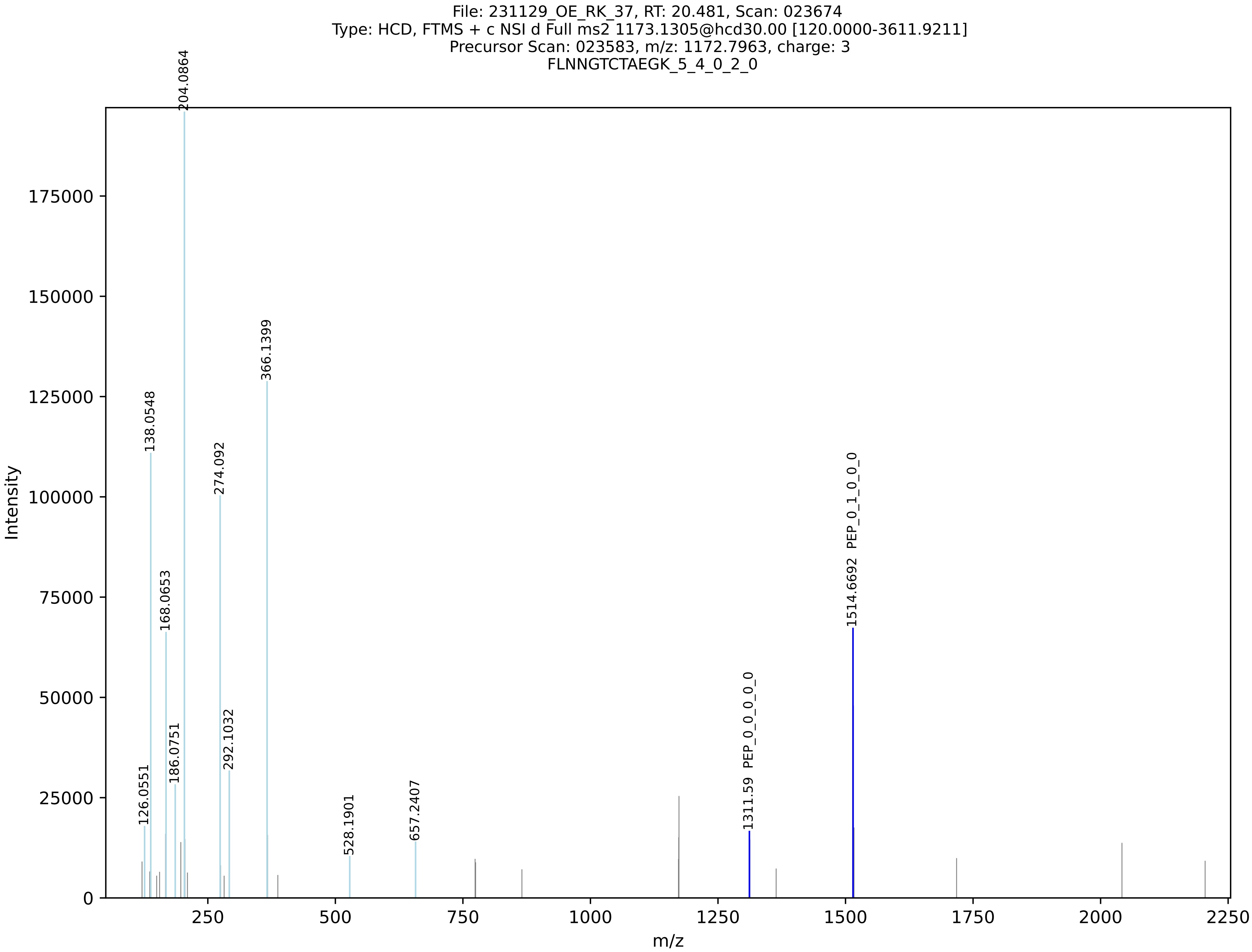

File: 231129_OE_RK_37, RT: 20.481, Scan: 023674
Type: HCD, FTMS + c NSI d Full ms2 1173.1305@hcd30.00 [120.0000-3611.9211]
Precursor Scan: 023583, m/z: 1172.7963, charge: 3
FLNNGTCTAEGK_5_4_0_2_0
Intensity
m/z
204.0864
366.1399
138.0548
274.092
168.0653
186.0751
292.1032
126.0551
528.1901
657.2407
1311.59 PEP_0_0_0_0_0
1514.6692 PEP_0_1_0_0_0
0
25000
50000
75000
100000
125000
150000
175000
250
500
750
1000
1250
1500
1750
2000
2250

File: 231129_OE_RK_37, RT: 20.527, Scan: 023740
Type: HCD, FTMS + c NSI d Full ms2 1078.7554@hcd30.00 [120.0000-3323.1333]
Precursor Scan: 023654, m/z: 1078.4212, charge: 3
EEQYNSTFR_5_4_1_1_0

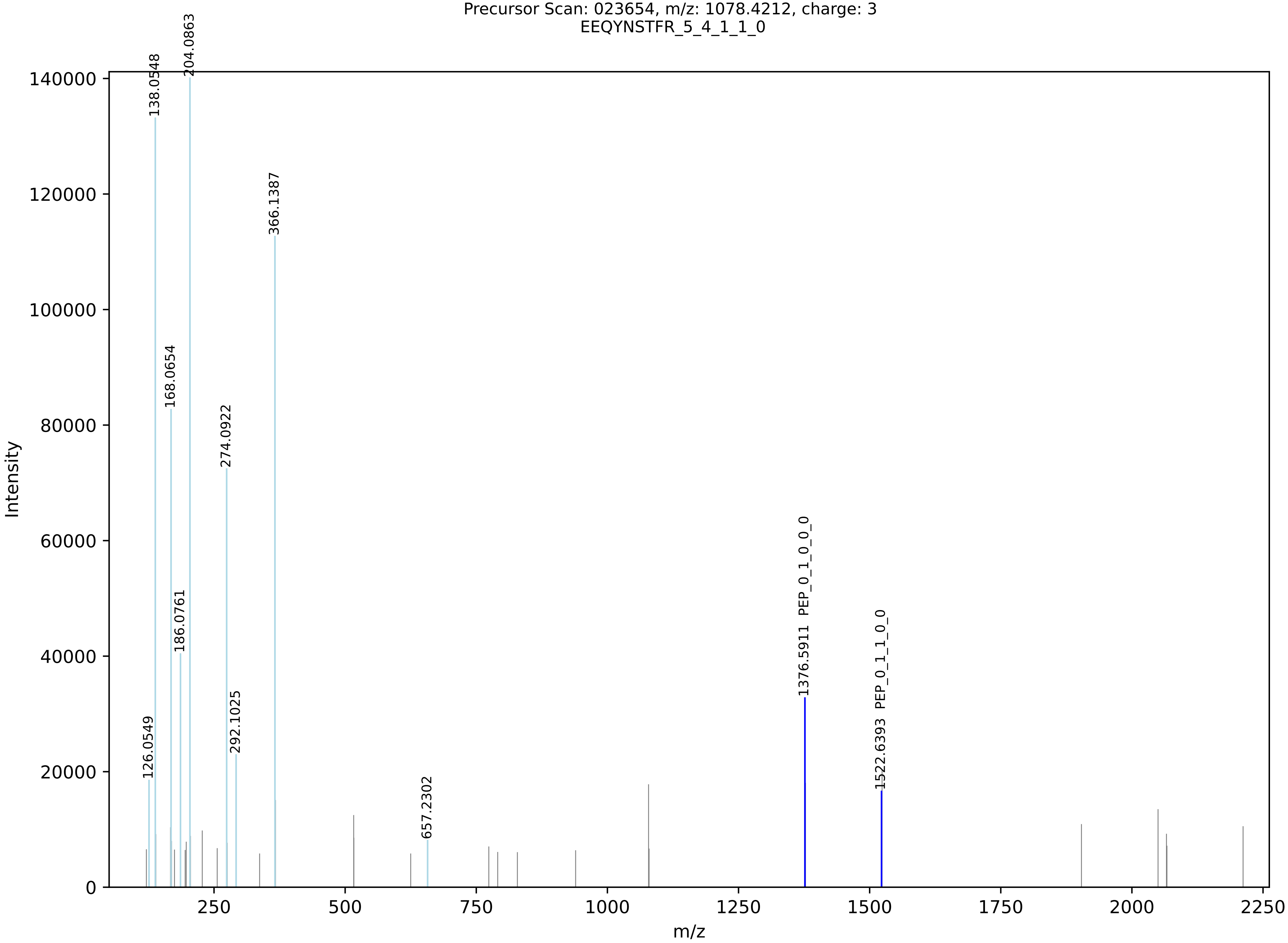

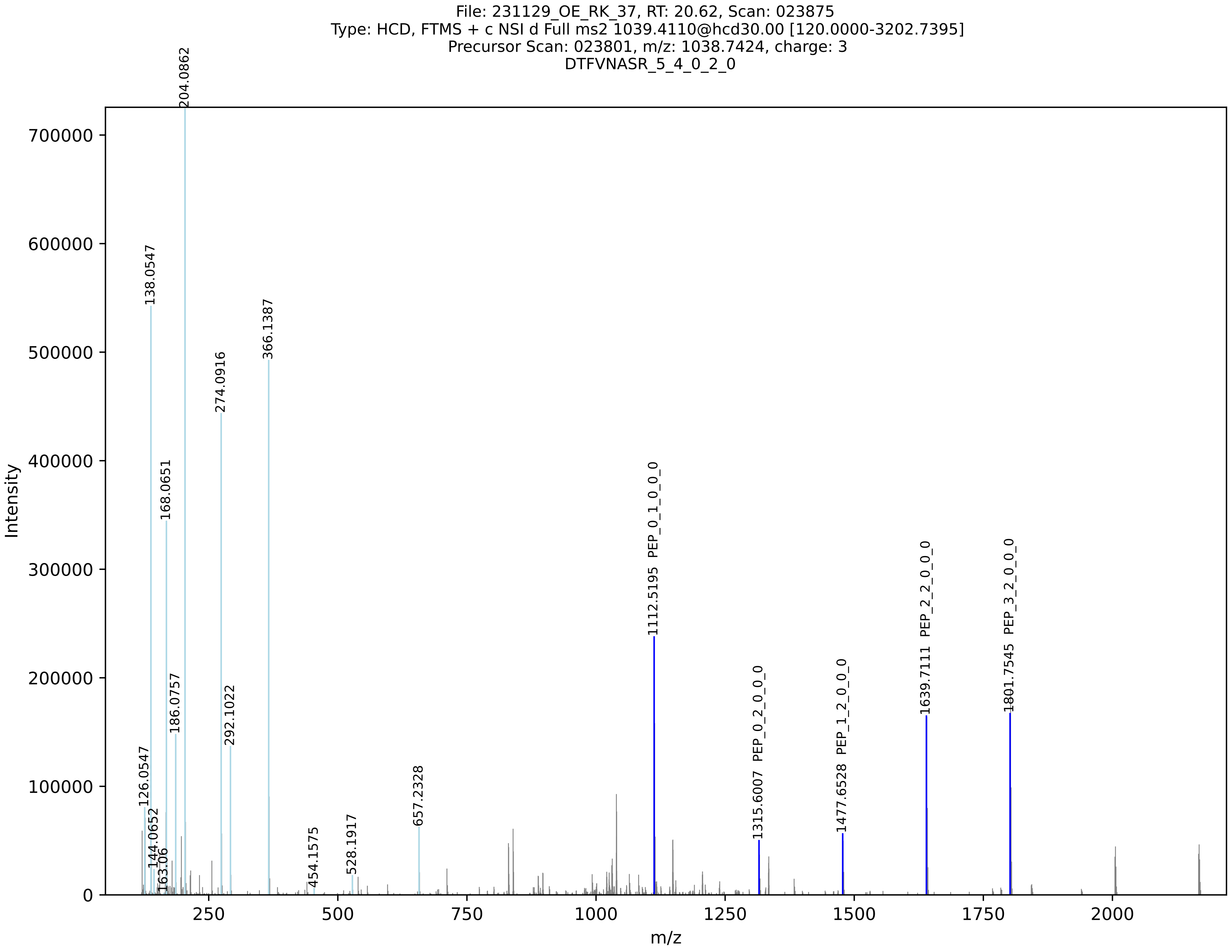
File: 231129_OE_RK_37, RT: 20.62, Scan: 023875
Type: HCD, FTMS + c NSI d Full ms2 1039.4110@hcd30.00 [120.0000-3202.7395]
Precursor Scan: 023801, m/z: 1038.7424, charge: 3
DTFVNASR_5_4_0_2_0
Intensity
m/z
204.0862
138.0547
366.1387
274.0916
168.0651
186.0757
292.1022
126.0547
144.0652
163.06
454.1575
528.1917
657.2328
1112.5195 PEP_0_1_0_0_0
1315.6007 PEP_0_2_0_0_0
1477.6528 PEP_1_2_0_0_0
1639.7111 PEP_2_2_0_0_0
1801.7545 PEP_3_2_0_0_0

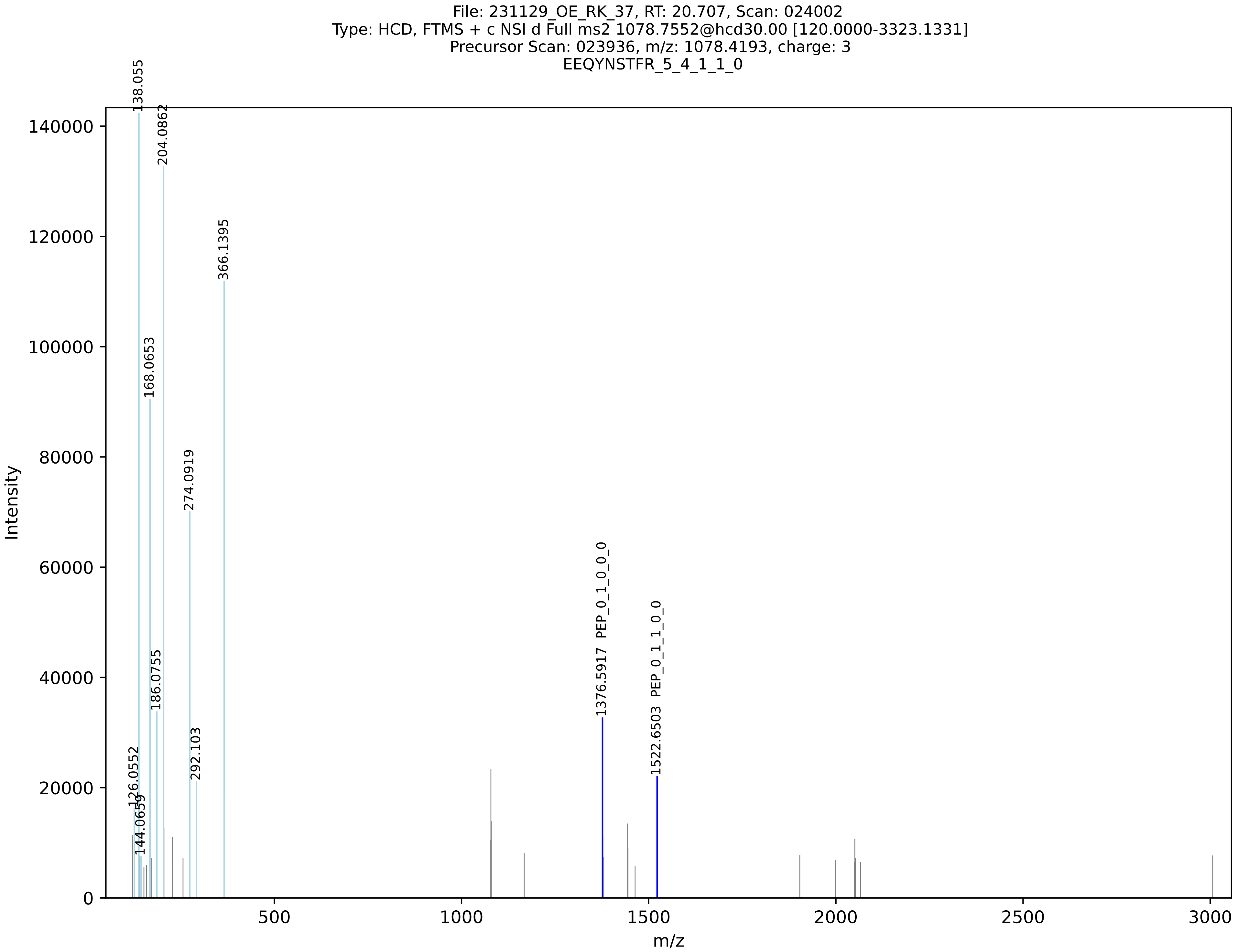

File: 231129_OE_RK_37, RT: 20.707, Scan: 024002
Type: HCD, FTMS + c NSI d Full ms2 1078.7552@hcd30.00 [120.0000-3323.1331]
Precursor Scan: 023936, m/z: 1078.4193, charge: 3
EEQYNSTFR_5_4_1_1_0
Intensity
m/z
138.055
204.0862
366.1395
168.0653
274.0919
186.0755
292.103
126.0552
144.0659
1376.5917 PEP_0_1_0_0_0
1522.6503 PEP_0_1_1_0_0

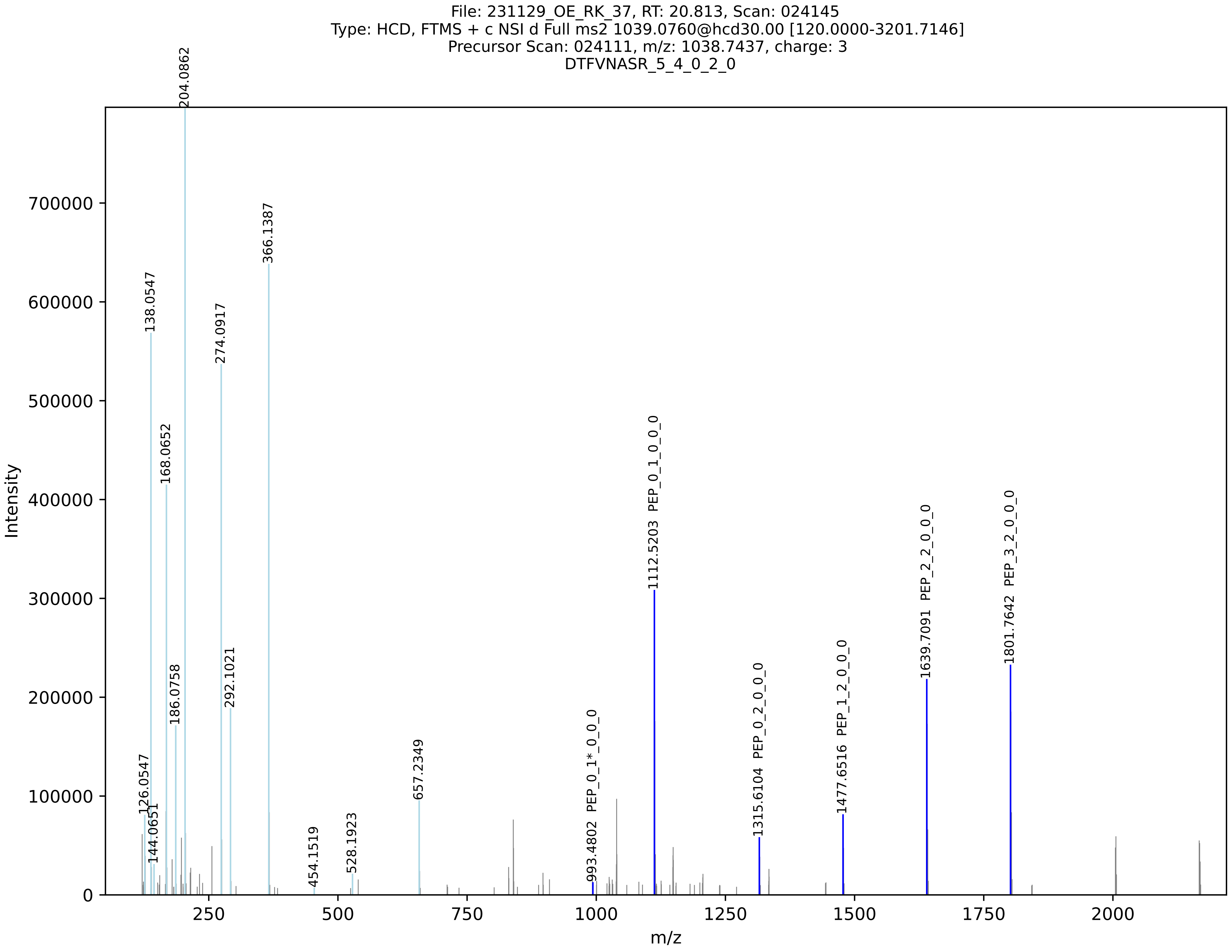
File: 231129_OE_RK_37, RT: 20.813, Scan: 024145
Type: HCD, FTMS + c NSI d Full ms2 1039.0760@hcd30.00 [120.0000-3201.7146]
Precursor Scan: 024111, m/z: 1038.7437, charge: 3
DTFVNASR_5_4_0_2_0
Intensity
m/z
126.0547
138.0547
144.0651
168.0652
186.0758
204.0862
274.0917
292.1021
366.1387
454.1519
528.1923
657.2349
993.4802 PEP_0_1*_0_0_0
1112.5203 PEP_0_1_0_0_0
1315.6104 PEP_0_2_0_0_0
1477.6516 PEP_1_2_0_0_0
1639.7091 PEP_2_2_0_0_0
1801.7642 PEP_3_2_0_0_0

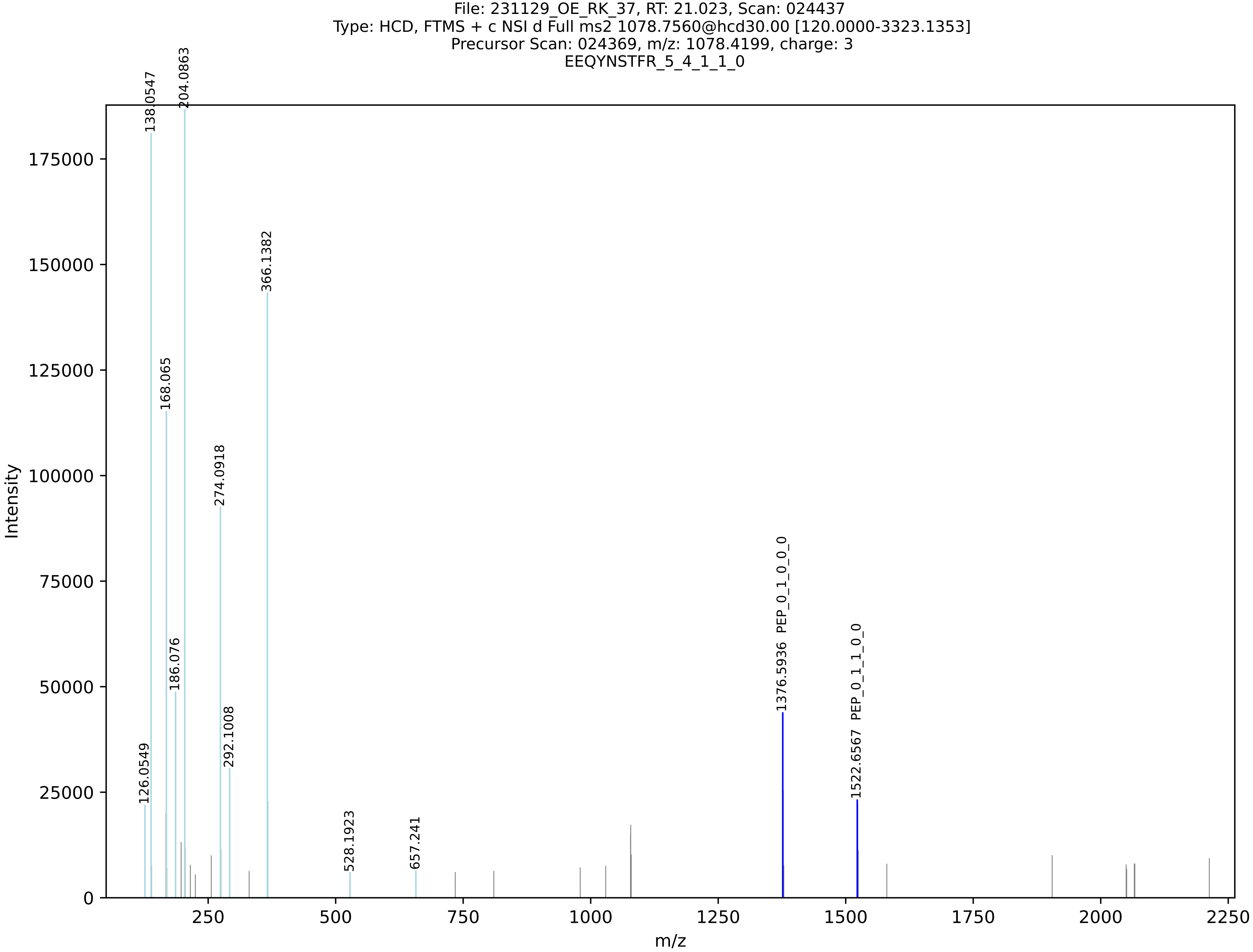
File: 231129_OE_RK_37, RT: 21.023, Scan: 024437
Type: HCD, FTMS + c NSI d Full ms2 1078.7560@hcd30.00 [120.0000-3323.1353]
Precursor Scan: 024369, m/z: 1078.4199, charge: 3
EEQYNSTFR_5_4_1_1_0
Intensity
m/z
138.0547
204.0863
366.1382
168.065
274.0918
186.076
292.1008
126.0549
528.1923
657.241
1376.5936 PEP_0_1_0_0_
1522.6567 PEP_0_1_1_0_
0
25000
50000
75000
100000
125000
150000
175000
250
500
750
1000
1250
1500
1750
2000
2250

File: 231129_OE_RK_37, RT: 21.08, Scan: 024522
Type: HCD, FTMS + c NSI d Full ms2 1312.1960@hcd30.00 [120.0000-4037.4619]
Precursor Scan: 024428, m/z: 1311.5292, charge: 3
RNHSCEPCQTLAVR_5_4_0_2_0

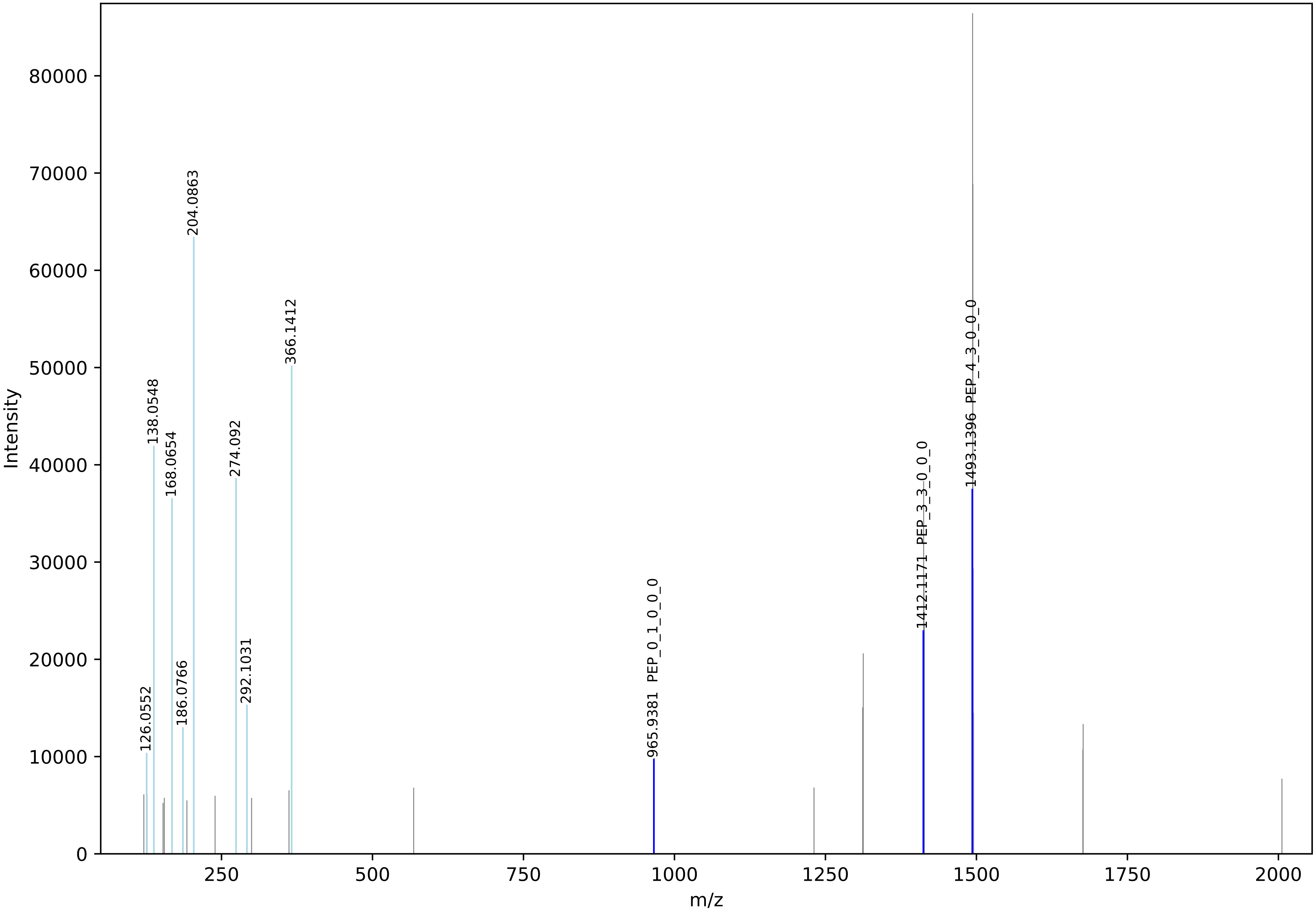

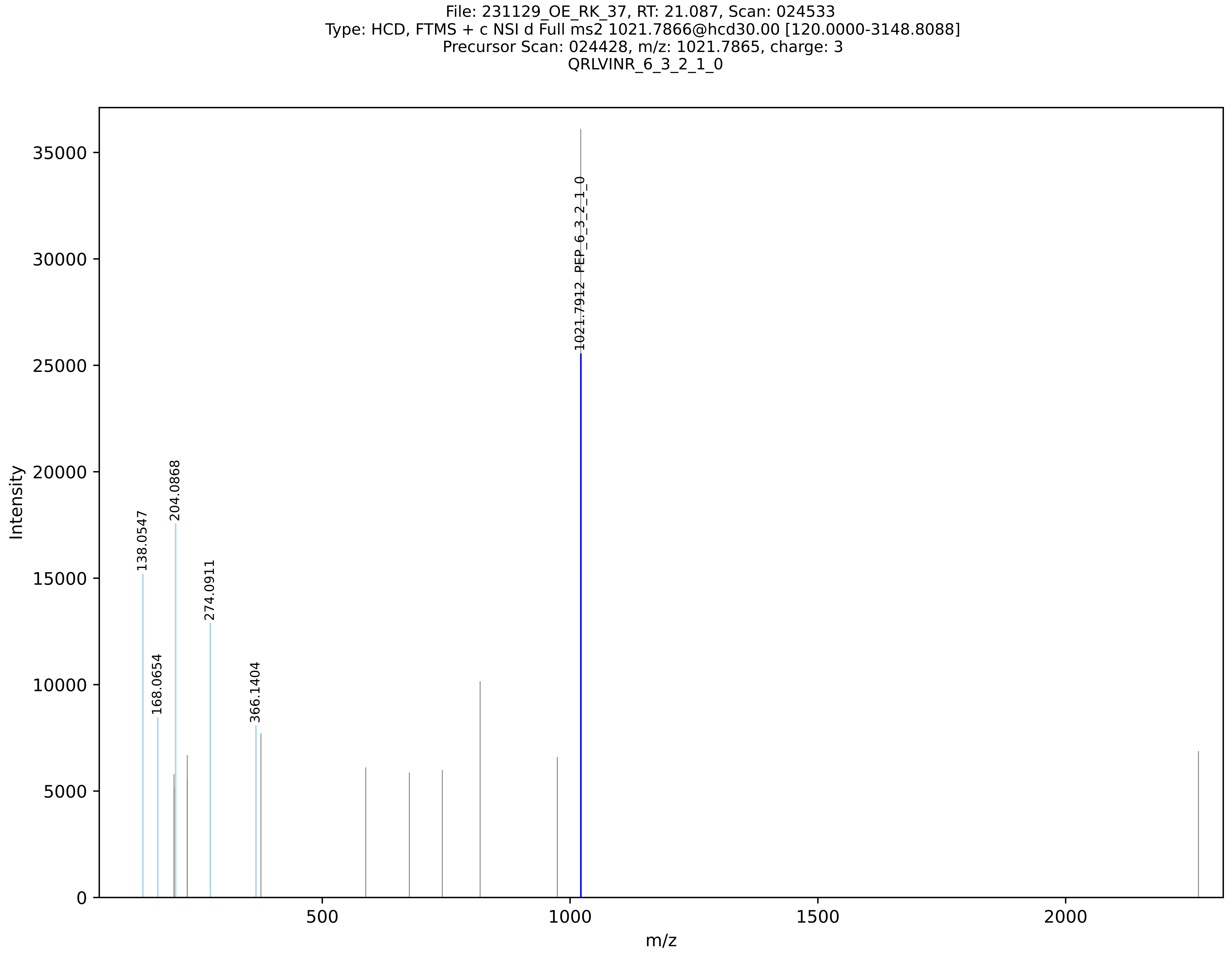
File: 231129_OE_RK_37, RT: 21.087, Scan: 024533
Type: HCD, FTMS + c NSI d Full ms2 1021.7866@hcd30.00 [120.0000-3148.8088]
Precursor Scan: 024428, m/z: 1021.7865, charge: 3
QRLVINR_6_3_2_1_0
Intensity
m/z
138.0547
168.0654
204.0868
274.0911
366.1404
1021.7912_PEP_6_3_2_1_0
0
5000
10000
15000
20000
25000
30000
35000
500
1000
1500
2000

File: 231129_OE_RK_37, RT: 21.232, Scan: 024744
Type: HCD, FTMS + c NSI d Full ms2 1429.5687@hcd30.00 [120.0000-4396.6226]
Precursor Scan: 024647, m/z: 1428.5707, charge: 3
GLMSNNLSELDTK_6_5_2_2_0

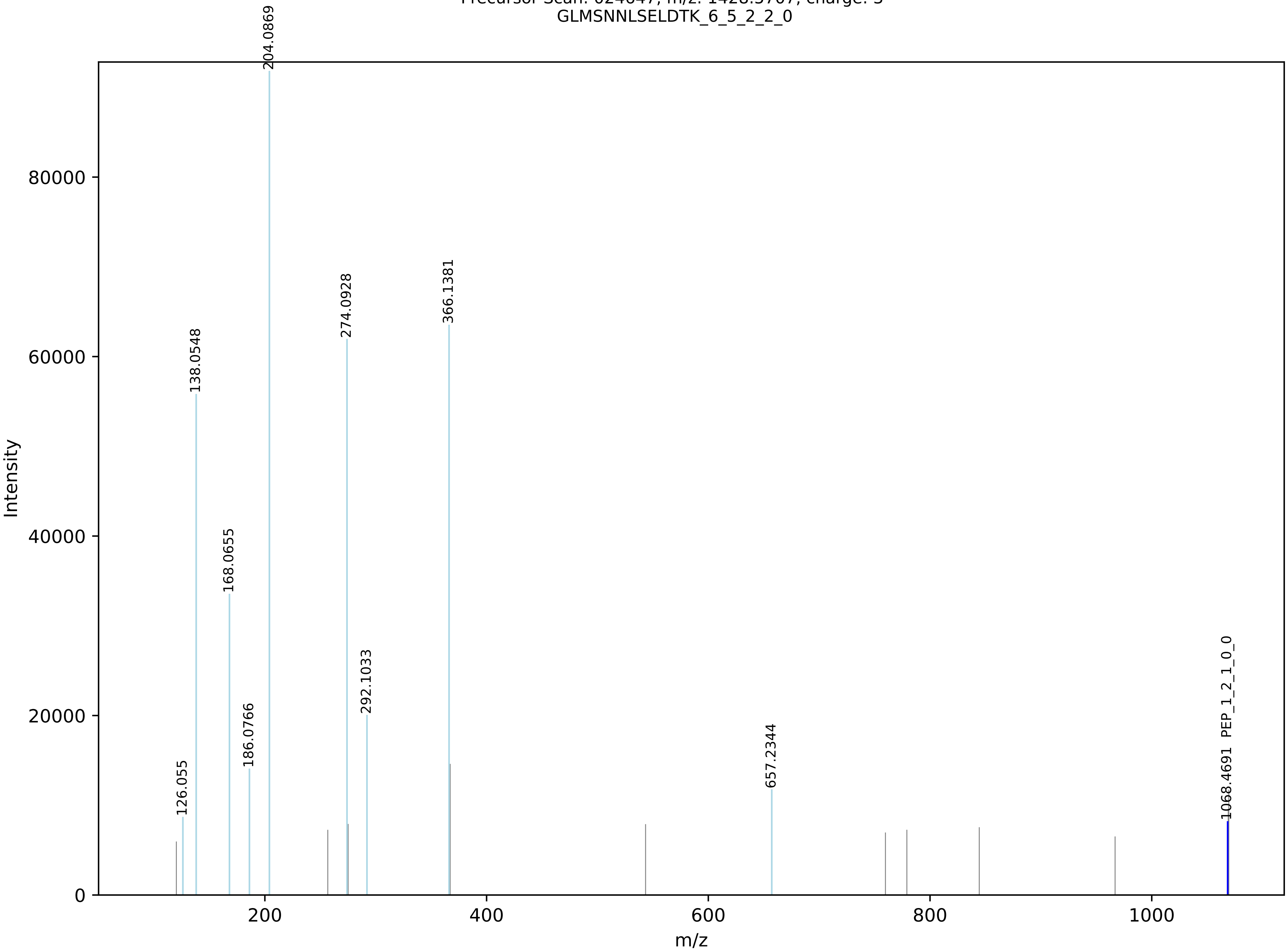

File: 231129_OE_RK_37, RT: 21.323, Scan: 024876
Type: HCD, FTMS + c NSI d Full ms2 1173.1302@hcd30.00 [120.0000-3611.9207]
Precursor Scan: 024793, m/z: 1172.7962, charge: 3
FLNNGTCTAEGK_5_4_0_2_0

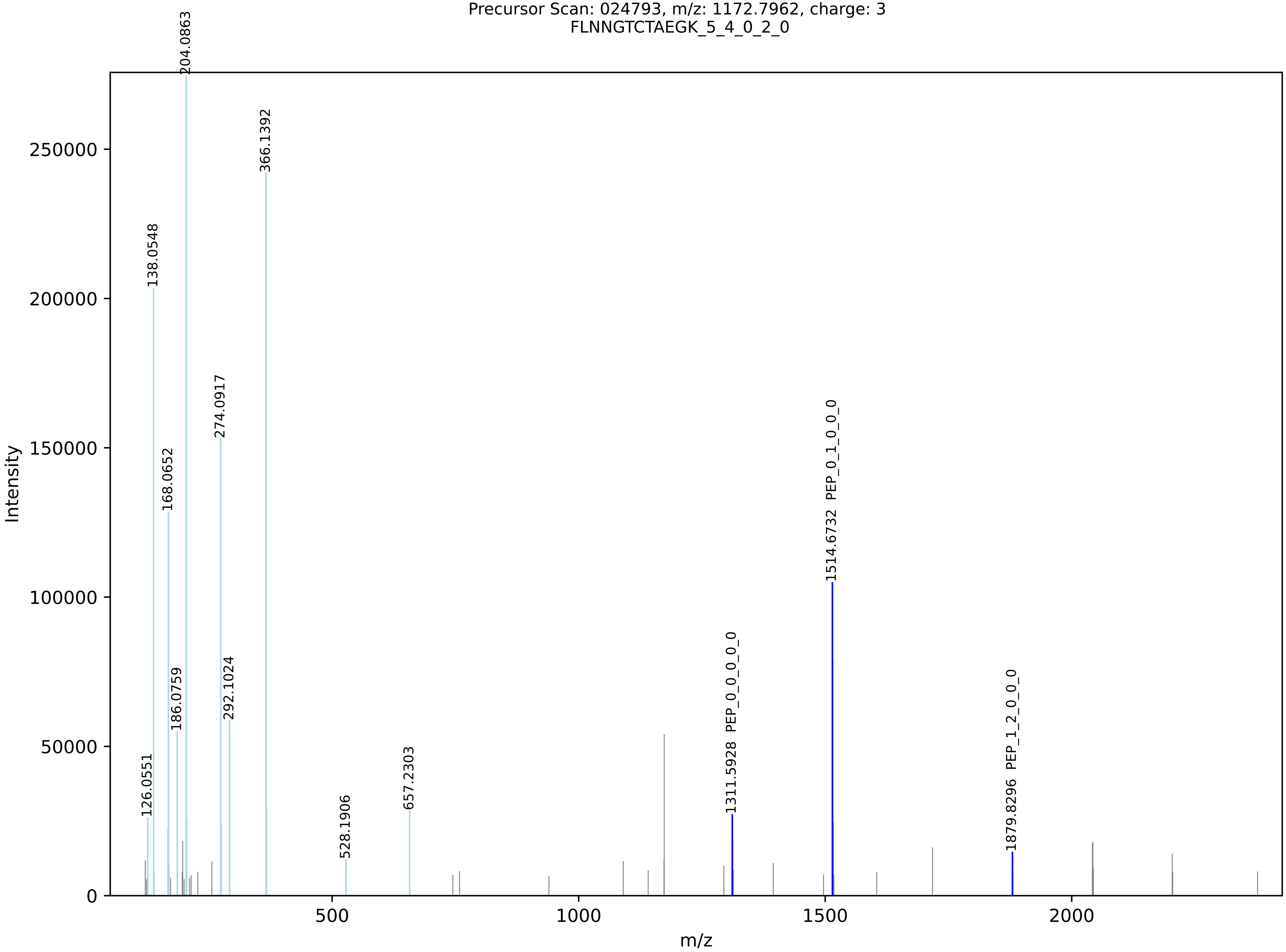

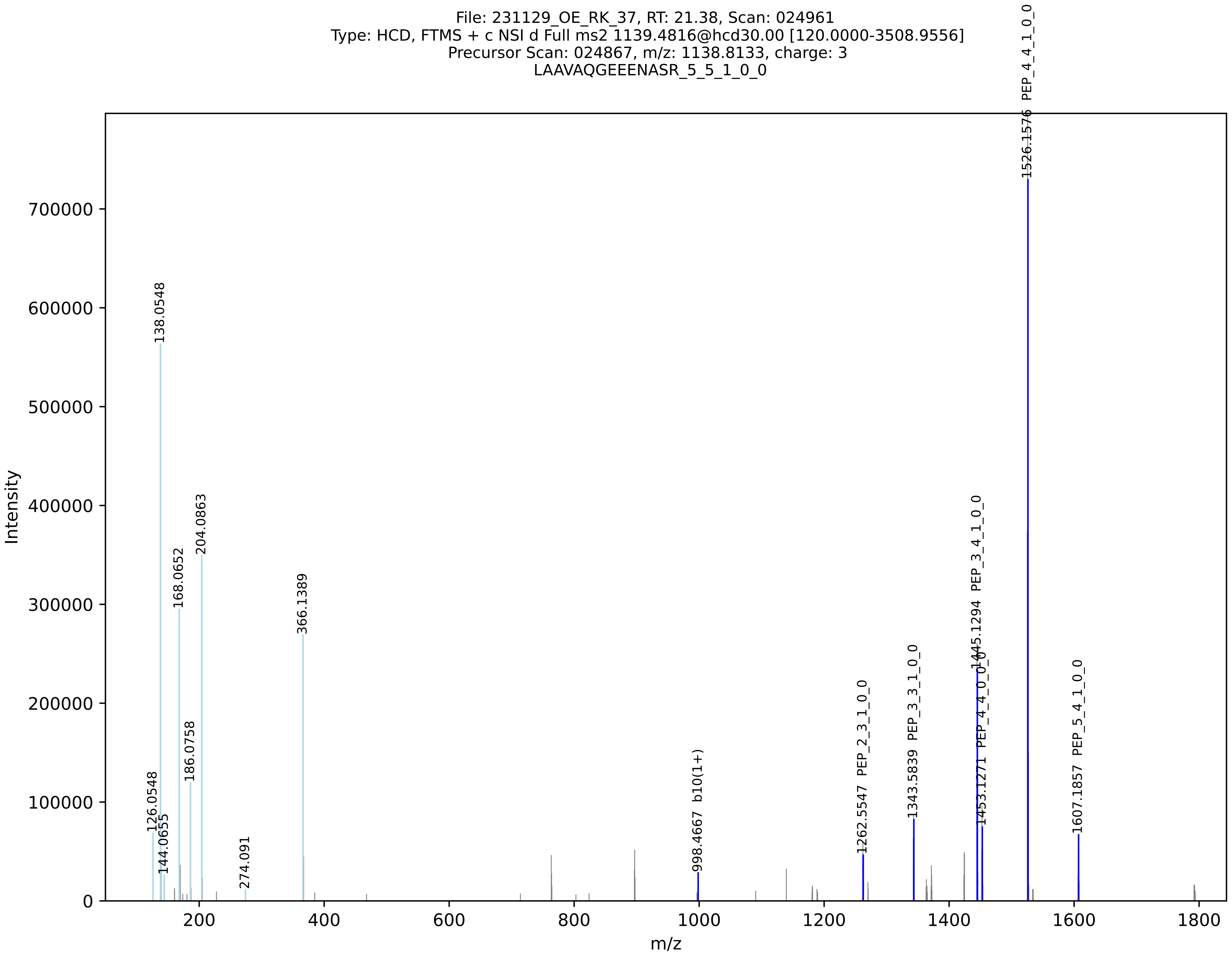

File: 231129_OE_RK_37, RT: 21.38, Scan: 024961
Type: HCD, FTMS + c NSI d Full ms2 1139.4816@hcd30.00 [120.0000-3508.9556]
Precursor Scan: 024867, m/z: 1138.8133, charge: 3
LAAVAQGEEENASR_5_5_1_0_0
Intensity
m/z
126.0548
138.0548
144.0655
168.0652
186.0758
204.0863
274.091
366.1389
998.4667 b10(1+)
1262.5547 PEP_2_3_1_0_0
1343.5839 PEP_3_3_1_0_0
1445.1294 PEP_3_4_1_0_0_
1453.1271 PEP_4_4_0_0_0_
1526.1576 PEP_4_4_1_0_0_
1607.1857 PEP_5_4_1_0_0_

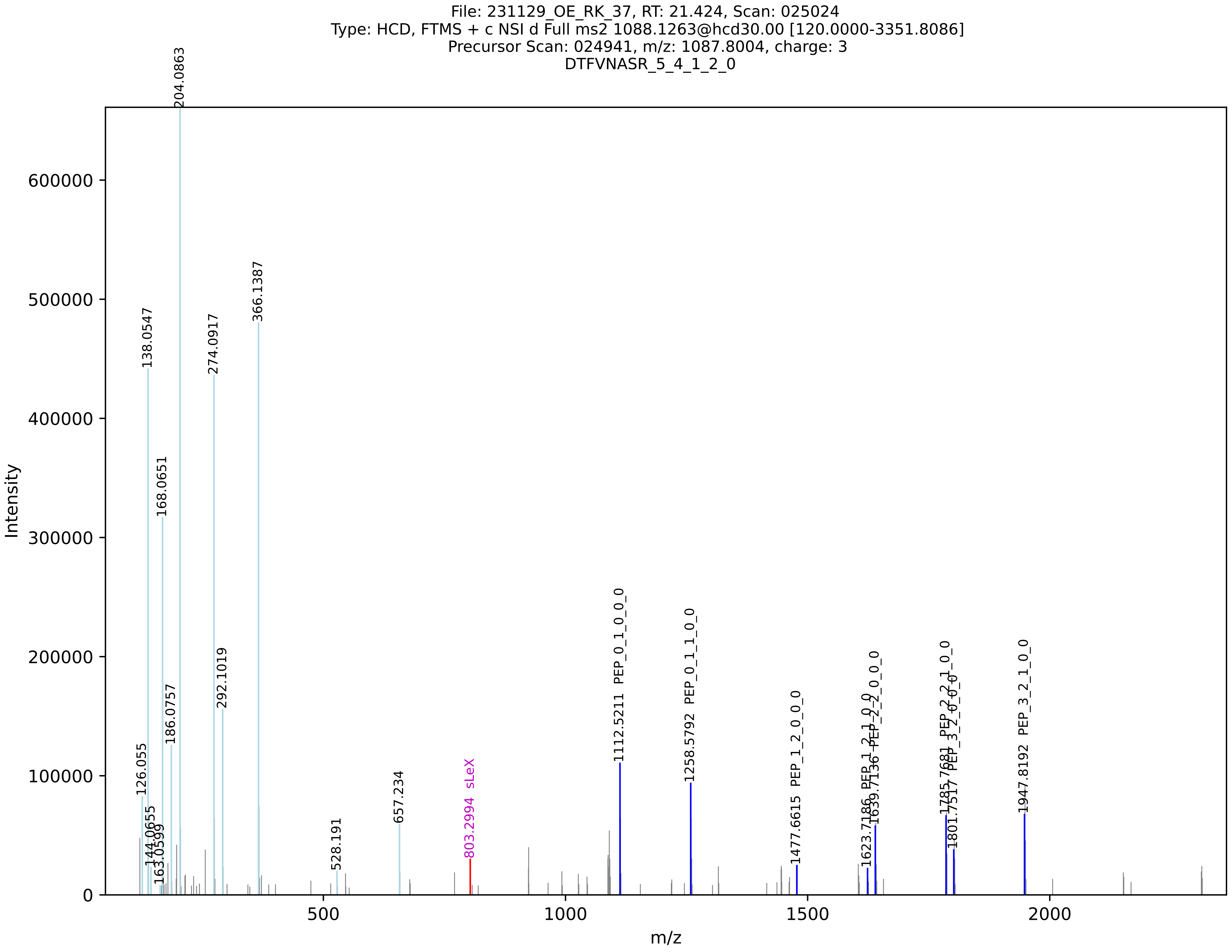

File: 231129_OE_RK_37, RT: 21.424, Scan: 025024
Type: HCD, FTMS + c NSI d Full ms2 1088.1263@hcd30.00 [120.0000-3351.8086]
Precursor Scan: 024941, m/z: 1087.8004, charge: 3
DTFVNASR_5_4_1_2_0
Intensity
m/z
204.0863
366.1387
138.0547
274.0917
168.0651
292.1019
186.0757
126.055
144.0655
163.0599
528.191
657.234
803.2994 sLeX
1112.5211 PEP_0_1_0_0_0
1258.5792 PEP_0_1_1_0_0
1477.6615 PEP_1_2_0_0_0
1623.7186 PEP_1_2_1_0_0
1639.7136 PEP_2_2_0_0_0
1785.7681 PEP_2_2_1_0_0
1801.7517 PEP_3_2_0_0_0
1947.8192 PEP_3_2_1_0_0

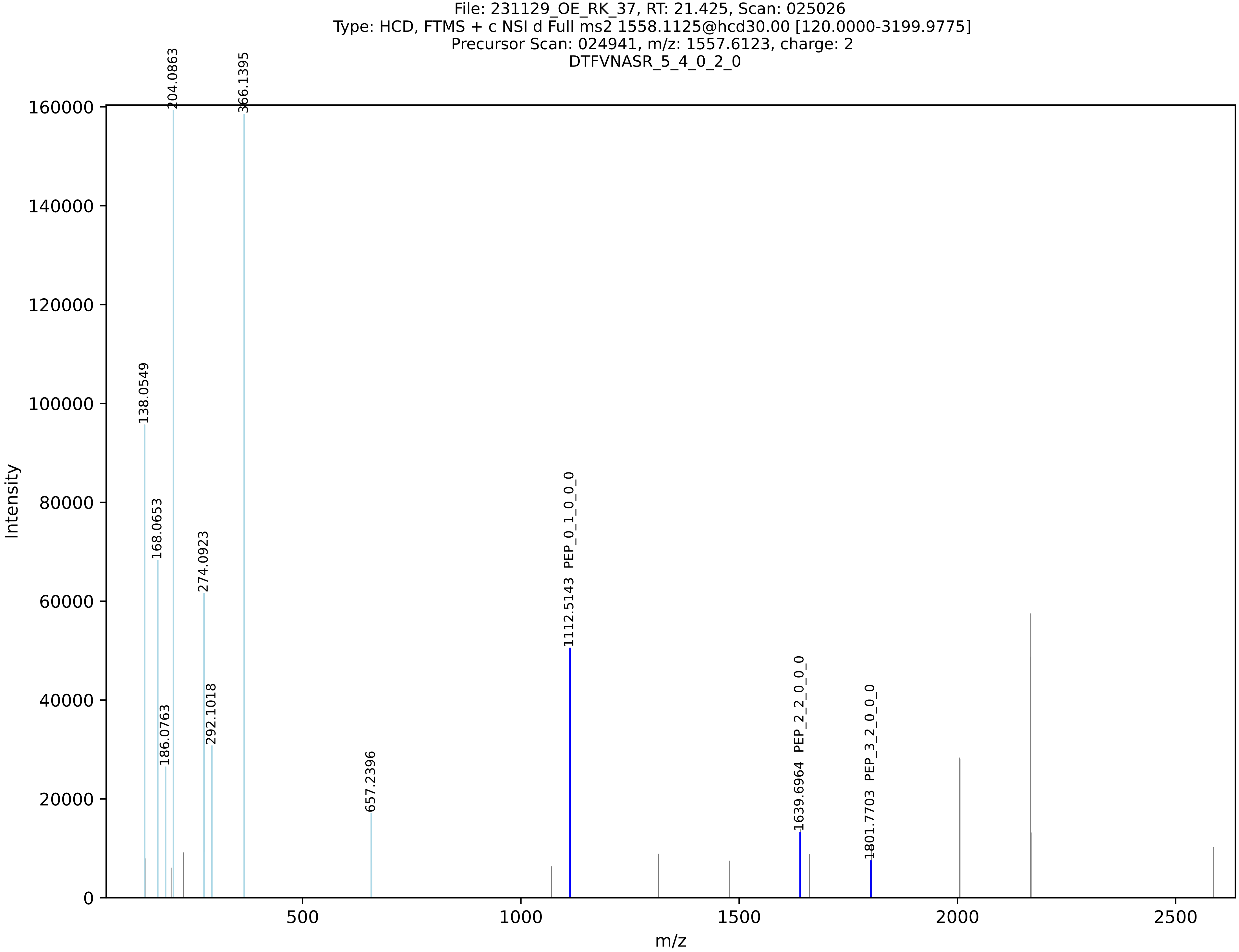

File: 231129_OE_RK_37, RT: 21.425, Scan: 025026
Type: HCD, FTMS + c NSI d Full ms2 1558.1125@hcd30.00 [120.0000-3199.9775]
Precursor Scan: 024941, m/z: 1557.6123, charge: 2
DTFVNASR_5_4_0_2_0
Intensity
m/z
204.0863
366.1395
138.0549
168.0653
274.0923
186.0763
292.1018
657.2396
1112.5143 PEP_0_1_0_0_0
1639.6964 PEP_2_2_0_0_0
1801.7703 PEP_3_2_0_0_0

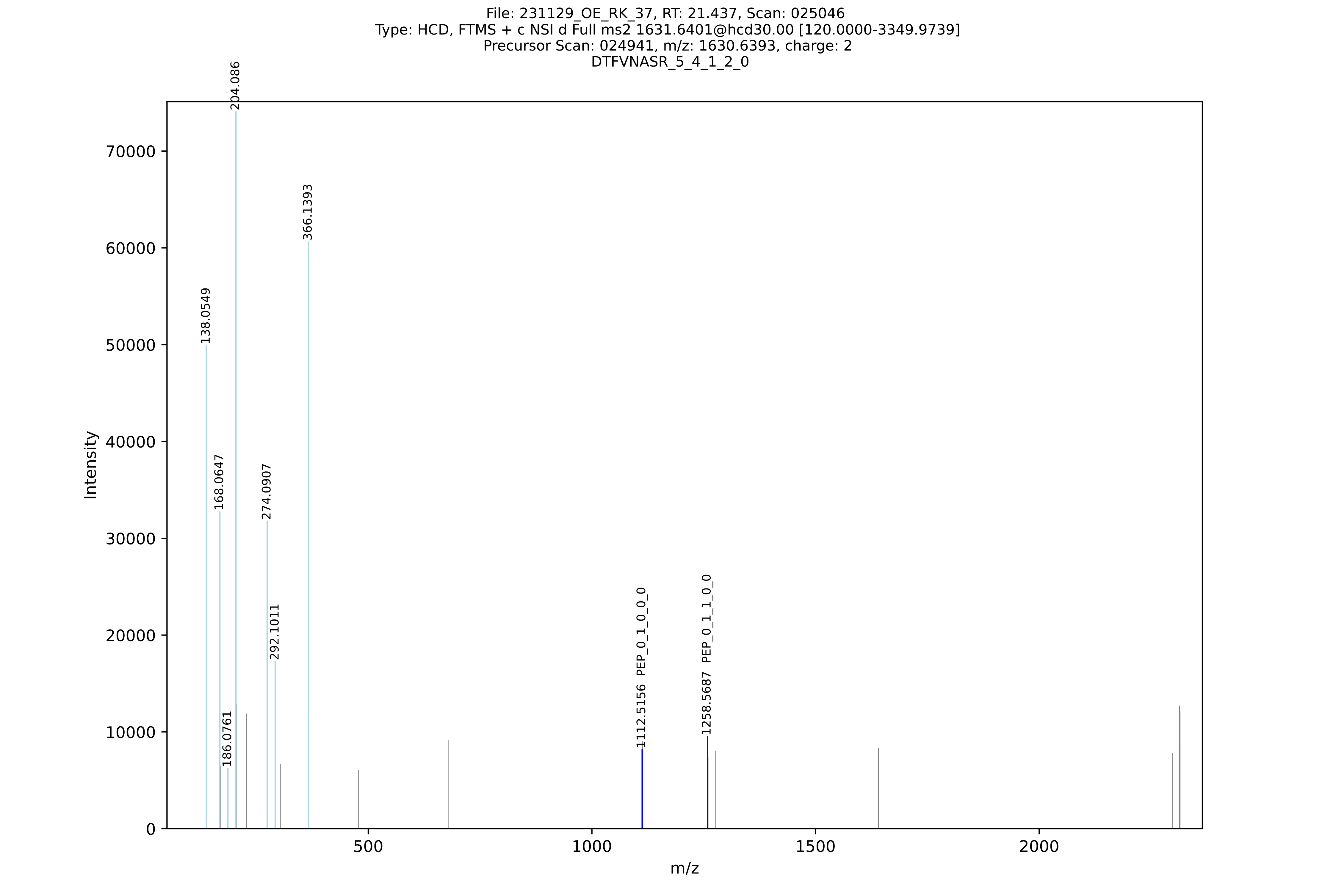
File: 231129_OE_RK_37, RT: 21.437, Scan: 025046
Type: HCD, FTMS + c NSI d Full ms2 1631.6401@hcd30.00 [120.0000-3349.9739]
Precursor Scan: 024941, m/z: 1630.6393, charge: 2
DTFVNASR_5_4_1_2_0
204.086
366.1393
138.0549
168.0647
274.0907
292.1011
186.0761
1112.5156 PEP_0_1_0_0_0
1258.5687 PEP_0_1_1_0_0
Intensity
m/z
0
10000
20000
30000
40000
50000
60000
70000
500
1000
1500
2000

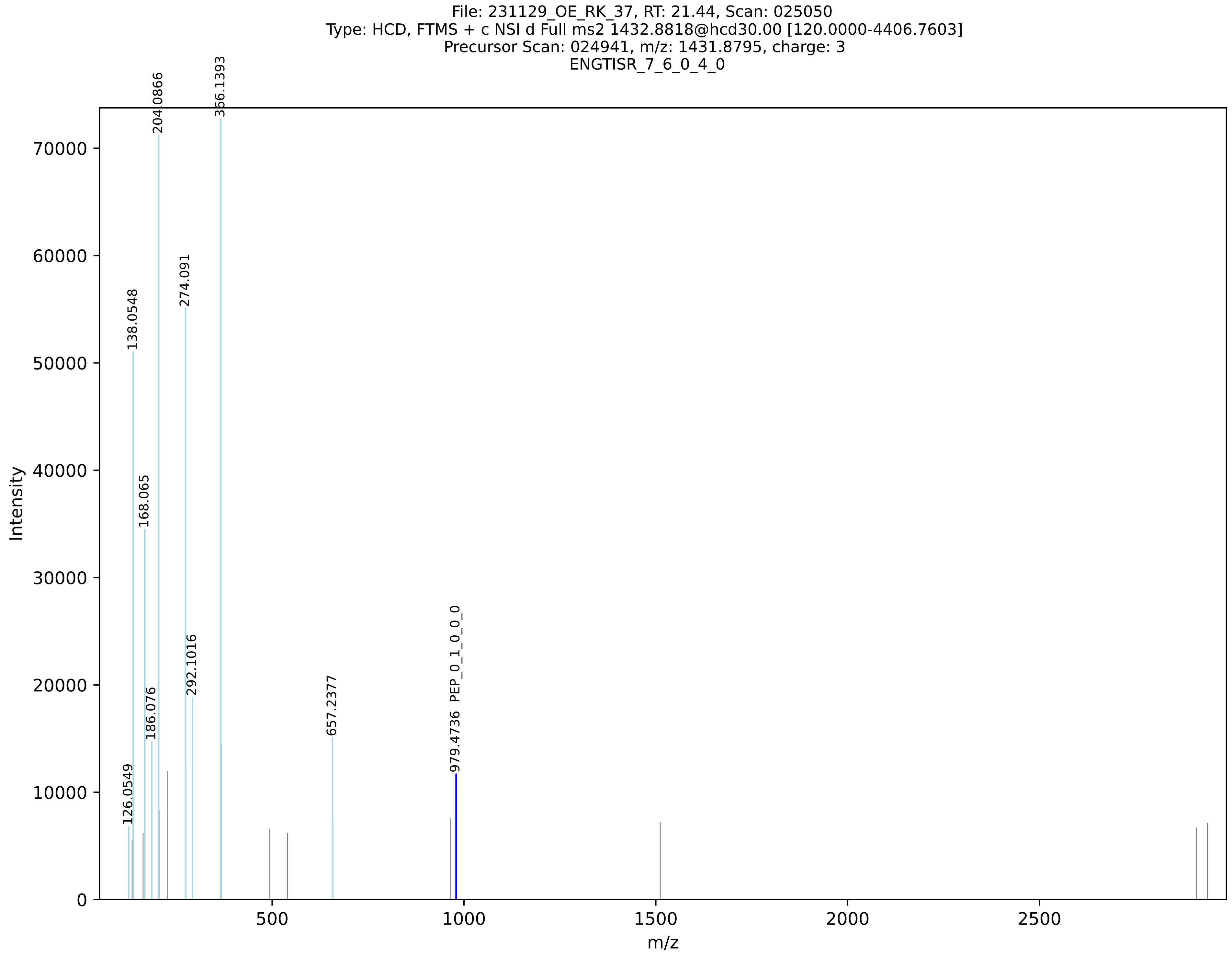
File: 231129_OE_RK_37, RT: 21.44, Scan: 025050
Type: HCD, FTMS + c NSI d Full ms2 1432.8818@hcd30.00 [120.0000-4406.7603]
Precursor Scan: 024941, m/z: 1431.8795, charge: 3
ENGTISR_7_6_0_4_0
Intensity
m/z
0
10000
20000
30000
40000
50000
60000
70000
500
1000
1500
2000
2500
126.0549
138.0548
168.065
186.076
204.0866
274.091
292.1016
366.1393
657.2377
979.4736 PEP_0_1_0_0_0

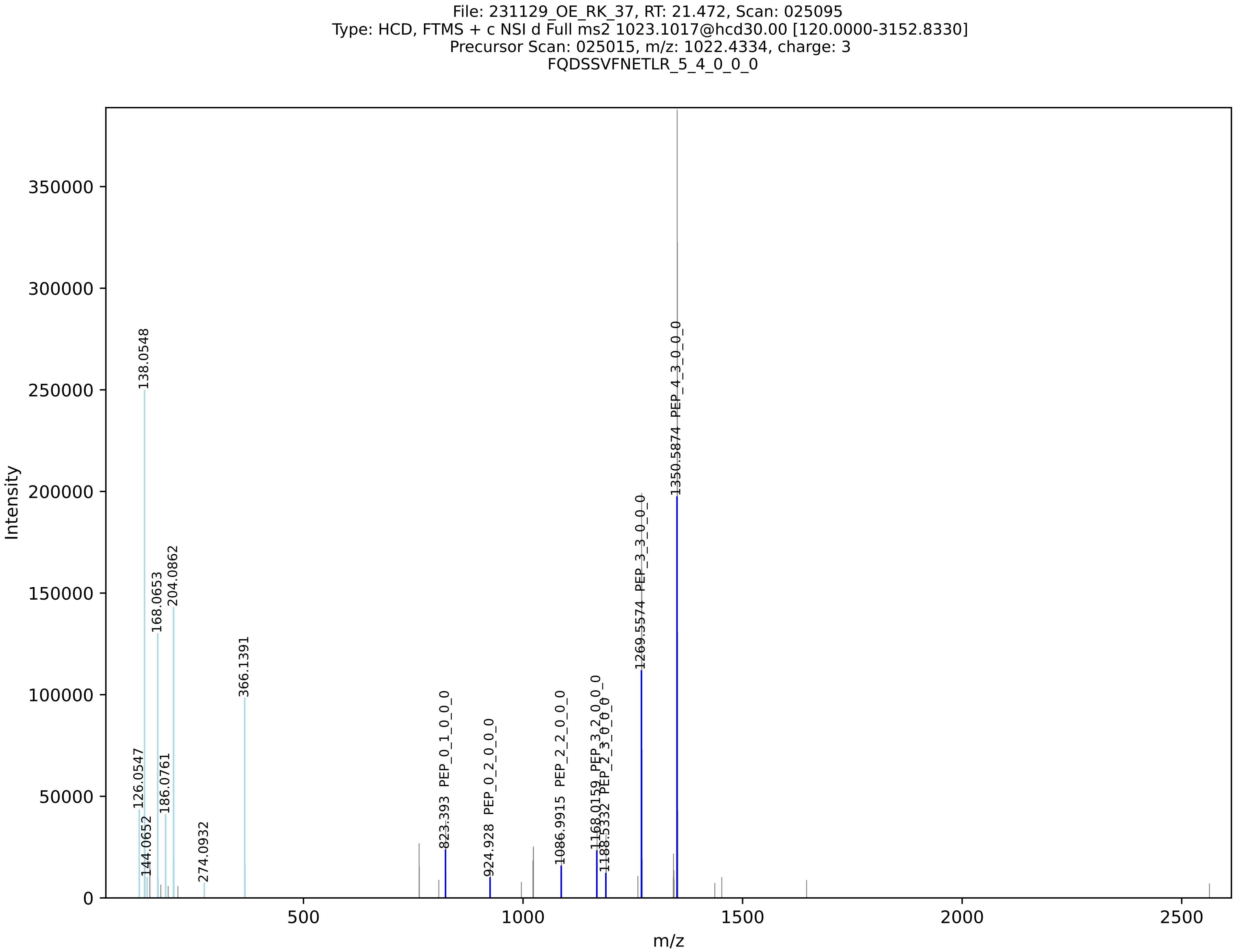
File: 231129_OE_RK_37, RT: 21.472, Scan: 025095
Type: HCD, FTMS + c NSI d Full ms2 1023.1017@hcd30.00 [120.0000-3152.8330]
Precursor Scan: 025015, m/z: 1022.4334, charge: 3
FQDSSVFNETLR_5_4_0_0_0
Intensity
m/z
138.0548
168.0653
204.0862
366.1391
126.0547
186.0761
144.0652
274.0932
823.393 PEP_0_1_0_0_0
924.928 PEP_0_2_0_0_0
1086.9915 PEP_2_2_0_0_0
1168.0159 PEP_3_2_0_0_0
1188.5332 PEP_2_3_0_0_0
1269.5574 PEP_3_3_0_0_0
1350.5874 PEP_4_3_0_0_0

File: 231129_OE_RK_37, RT: 21.496, Scan: 025133
Type: HCD, FTMS + c NSI d Full ms2 1525.6497@hcd30.00 [120.0000-3133.7534]
Precursor Scan: 025015, m/z: 1525.1507, charge: 2
HWNGSFHSRK_3_5_2_0_0

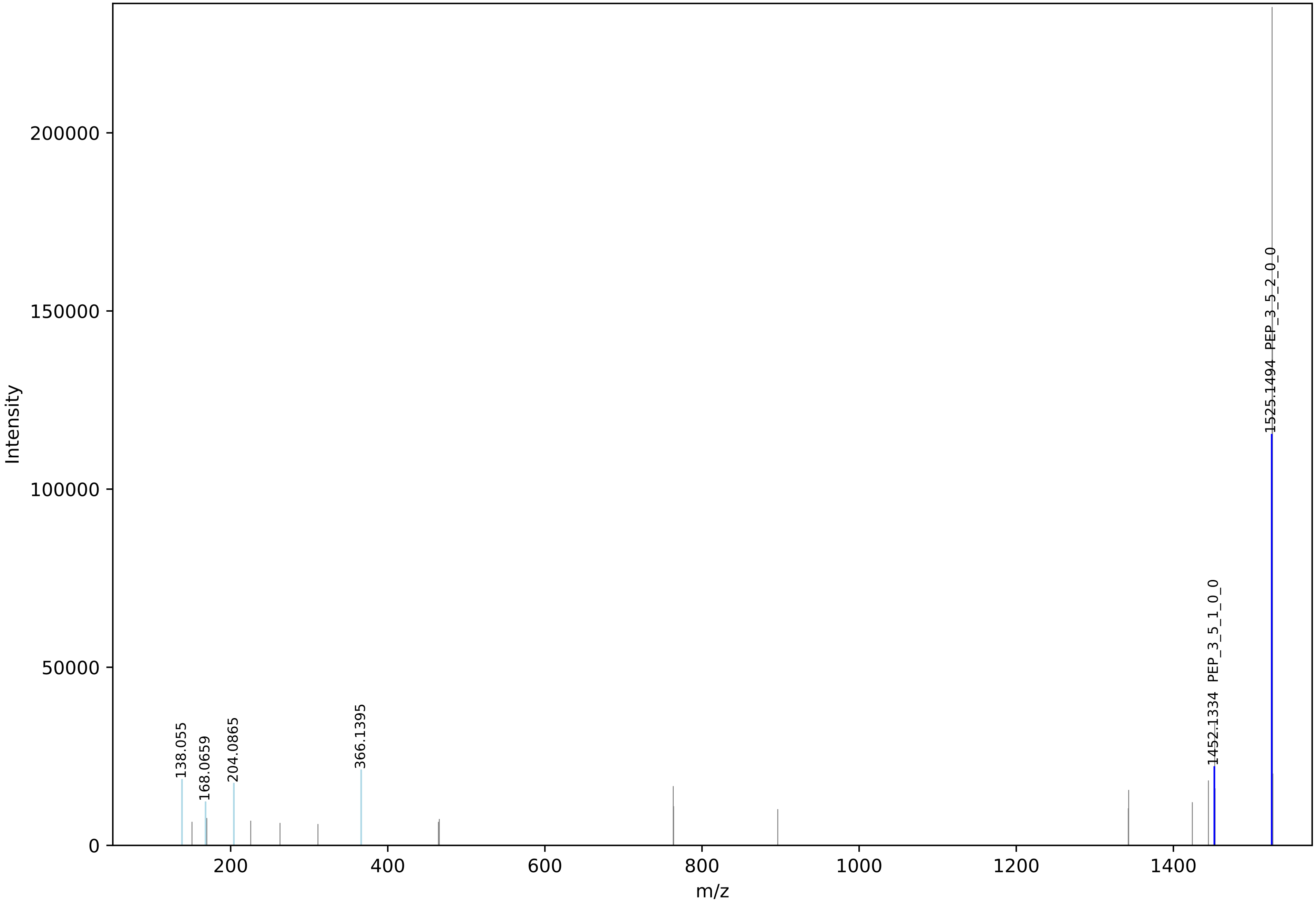

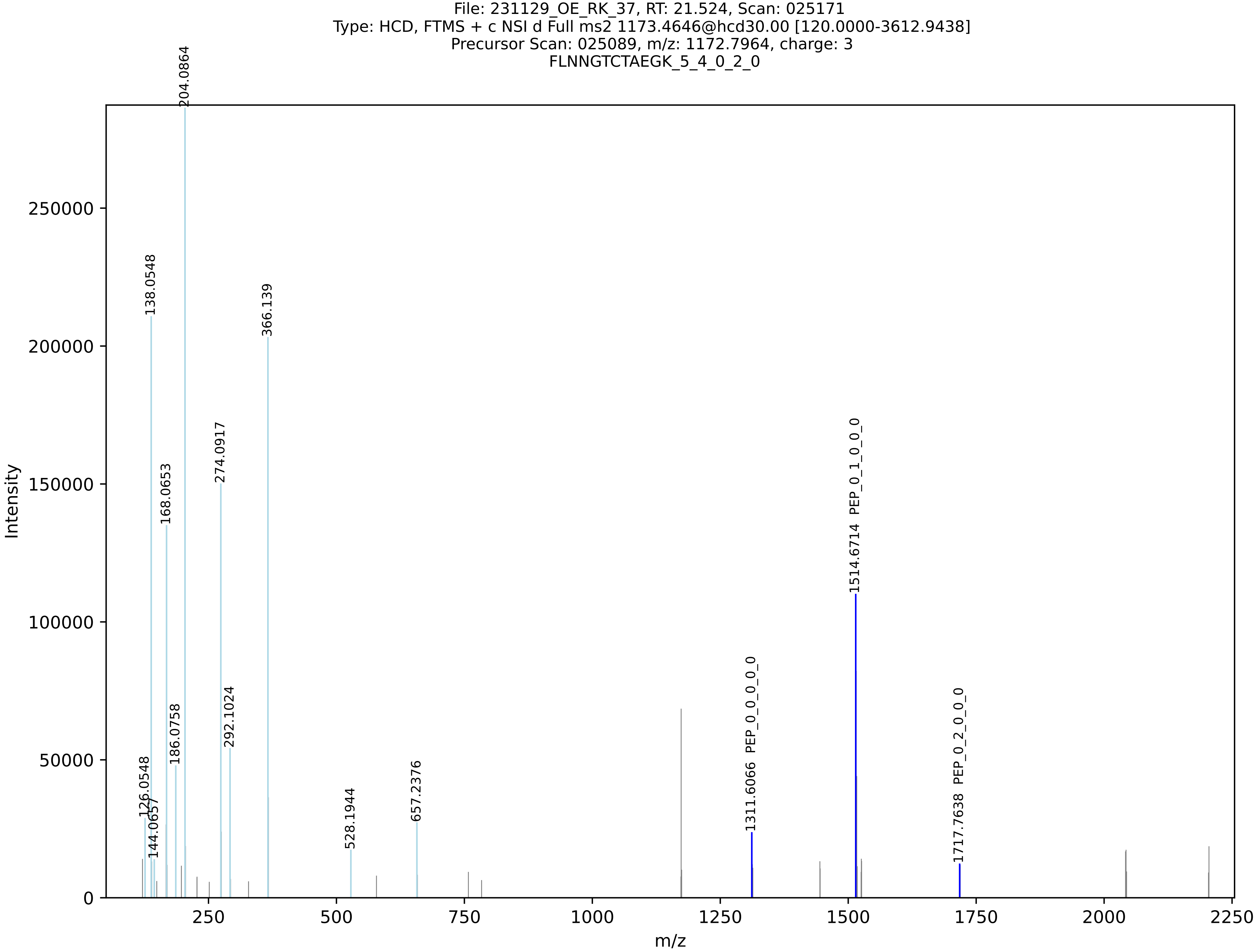

File: 231129_OE_RK_37, RT: 21.524, Scan: 025171
Type: HCD, FTMS + c NSI d Full ms2 1173.4646@hcd30.00 [120.0000-3612.9438]
Precursor Scan: 025089, m/z: 1172.7964, charge: 3
FLNNGTCTAEGK_5_4_0_2_0
204.0864
138.0548
366.139
274.0917
168.0653
186.0758
292.1024
126.0548
144.0657
528.1944
657.2376
1311.6066 PEP_0_0_0_0_0
1514.6714 PEP_0_1_0_0_0
1717.7638 PEP_0_2_0_0_0
Intensity
m/z
0
50000
100000
150000
200000
250000
250
500
750
1000
1250
1500
1750
2000
2250

File: 231129_OE_RK_37, RT: 21.572, Scan: 025240
Type: HCD, FTMS + c NSI d Full ms2 1087.7629@hcd30.00 [120.0000-3350.6965]
Precursor Scan: 025163, m/z: 1087.4288, charge: 3
DTFVNASR_5_4_1_2_0

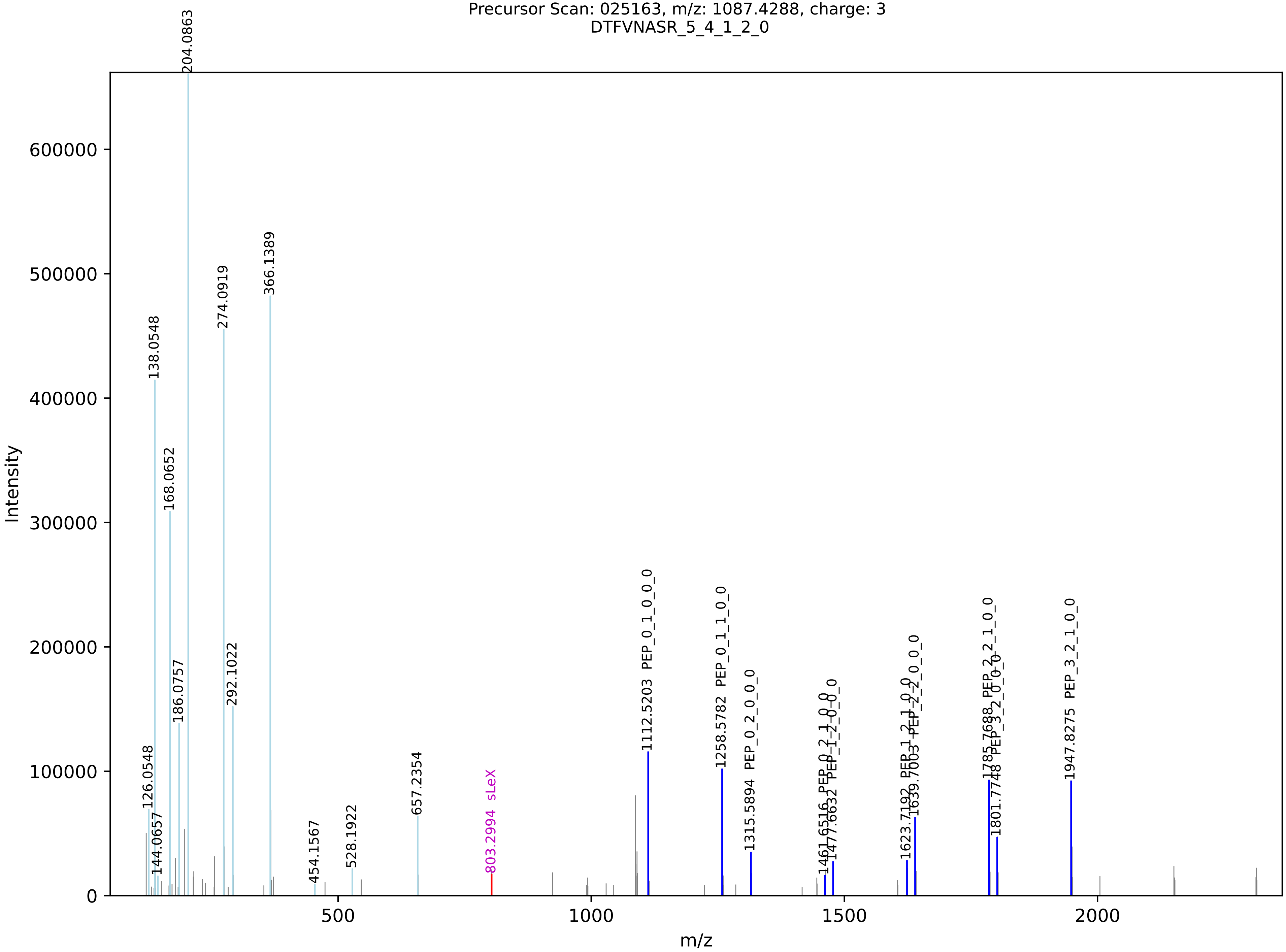

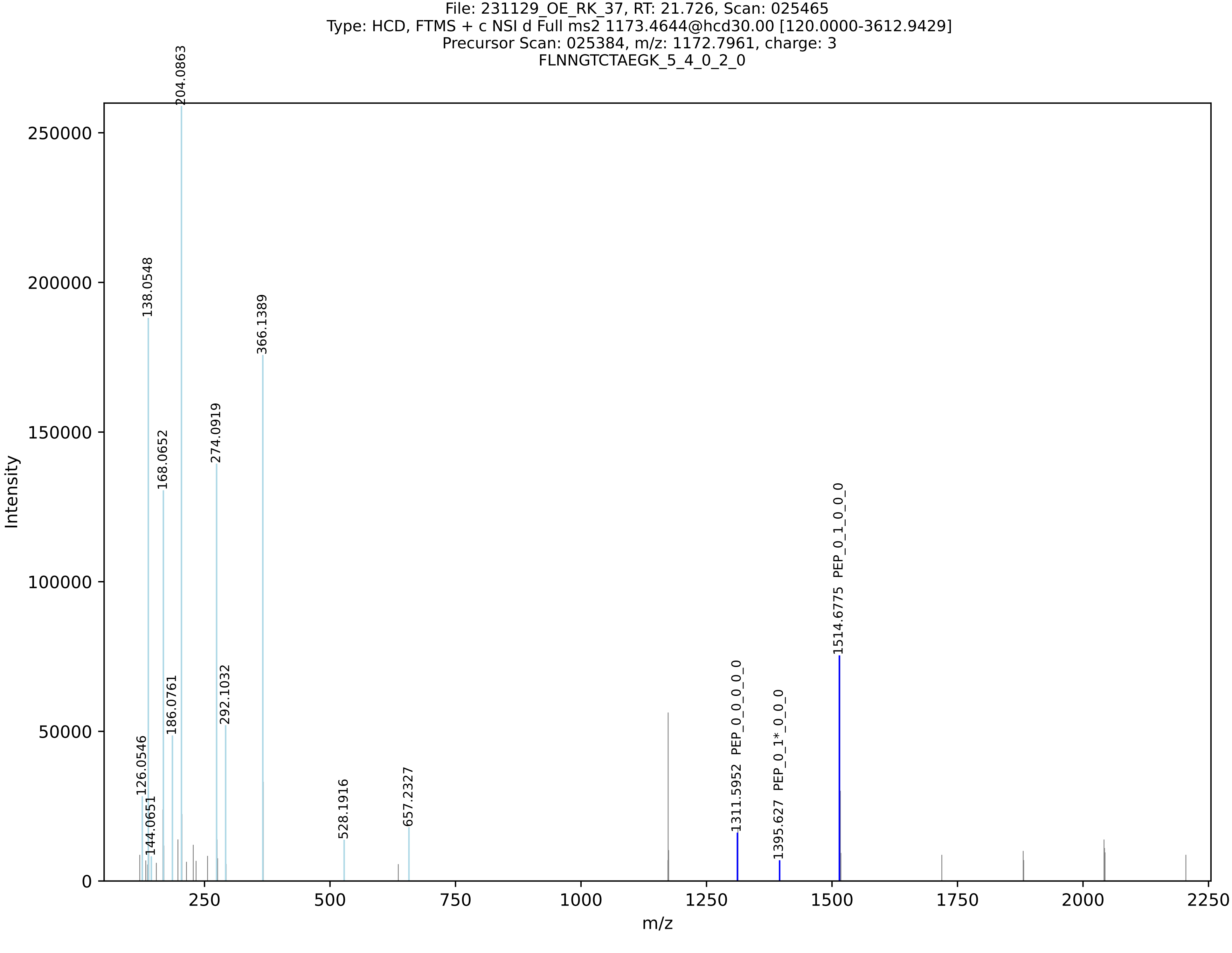
File: 231129_OE_RK_37, RT: 21.726, Scan: 025465
Type: HCD, FTMS + c NSI d Full ms2 1173.4644@hcd30.00 [120.0000-3612.9429]
Precursor Scan: 025384, m/z: 1172.7961, charge: 3
FLNNGTCTAEGK_5_4_0_2_0
Intensity
m/z
204.0863
138.0548
366.1389
274.0919
168.0652
186.0761
292.1032
126.0546
144.0651
528.1916
657.2327
1311.5952 PEP_0_0_0_0_0
1395.627 PEP_0_1*_0_0_0
1514.6775 PEP_0_1_0_0_0

File: 231129_OE_RK_37, RT: 21.75, Scan: 025503
Type: HCD, FTMS + c NSI d Full ms2 1272.5118@hcd30.00 [120.0000-3916.0281]
Precursor Scan: 025384, m/z: 1272.5118, charge: 3
NTTSYVLR_6_5_2_2_0

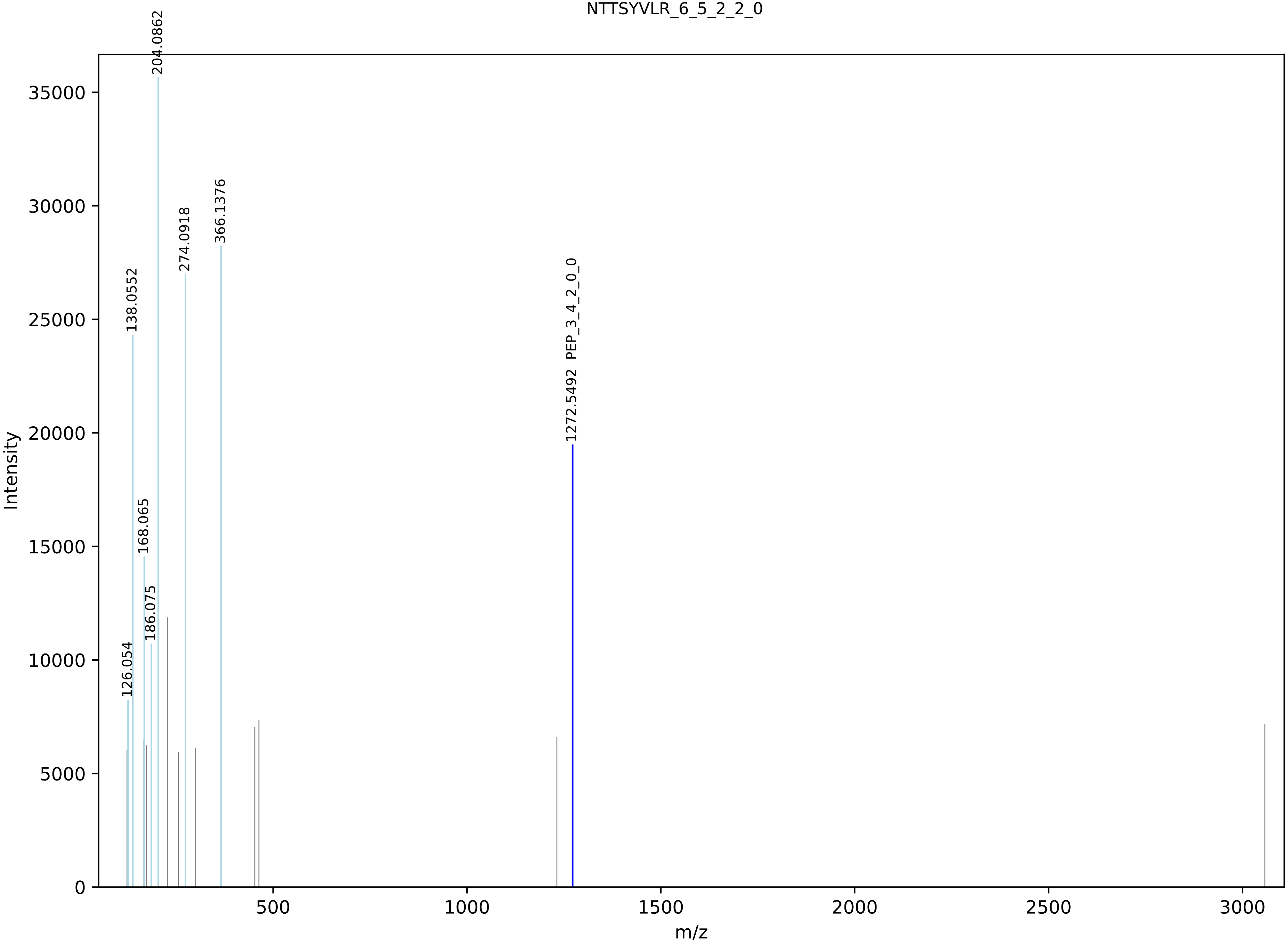

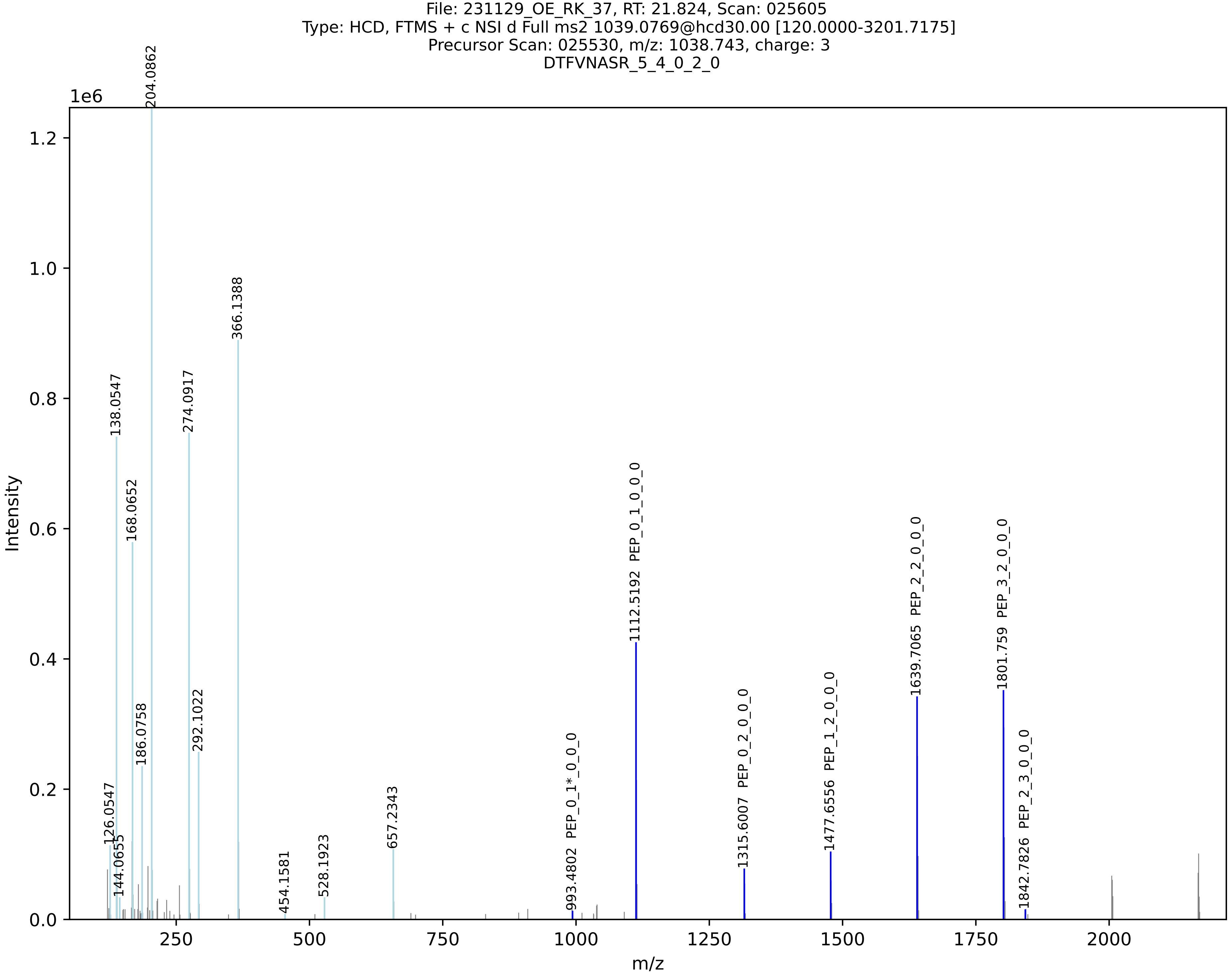

File: 231129_OE_RK_37, RT: 21.824, Scan: 025605
Type: HCD, FTMS + c NSI d Full ms2 1039.0769@hcd30.00 [120.0000-3201.7175]
Precursor Scan: 025530, m/z: 1038.743, charge: 3
DTFVNASR_5_4_0_2_0
1e6
Intensity
m/z
204.0862
366.1388
138.0547
274.0917
168.0652
186.0758
292.1022
126.0547
144.0655
454.1581
528.1923
657.2343
993.4802 PEP_0_1*_0_0_0
1112.5192 PEP_0_1_0_0_0
1315.6007 PEP_0_2_0_0_0
1477.6556 PEP_1_2_0_0_0
1639.7065 PEP_2_2_0_0_0
1801.759 PEP_3_2_0_0_0
1842.7826 PEP_2_3_0_0_0

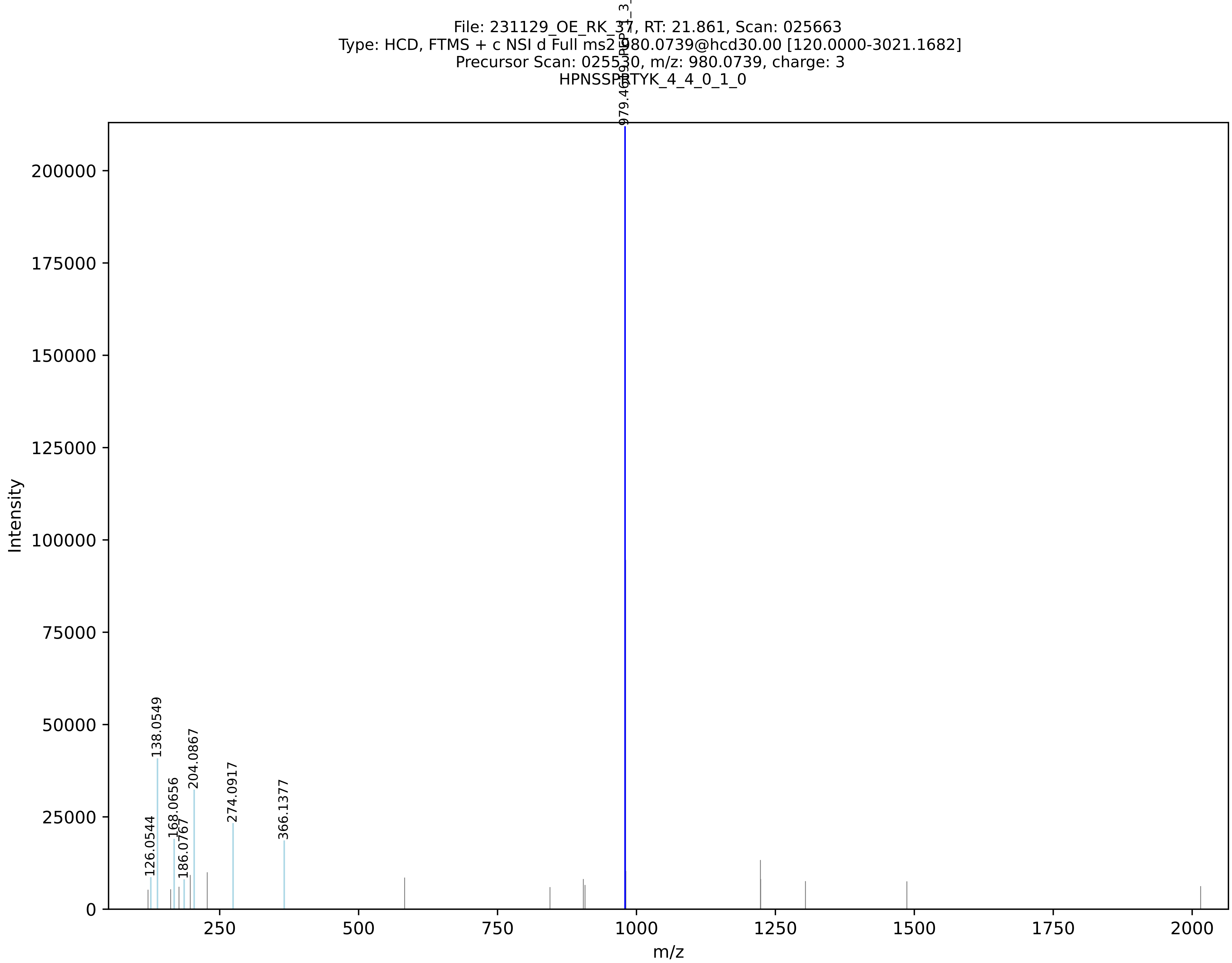
File: 231129_OE_RK_37, RT: 21.861, Scan: 025663
Type: HCD, FTMS + c NSI d Full ms2 980.0739@hcd30.00 [120.0000-3021.1682]
Precursor Scan: 025530, m/z: 980.0739, charge: 3
HPNSSPRTYK_4_4_0_1_0
Intensity
m/z
126.0544
138.0549
168.0656
186.0767
204.0867
274.0917
366.1377

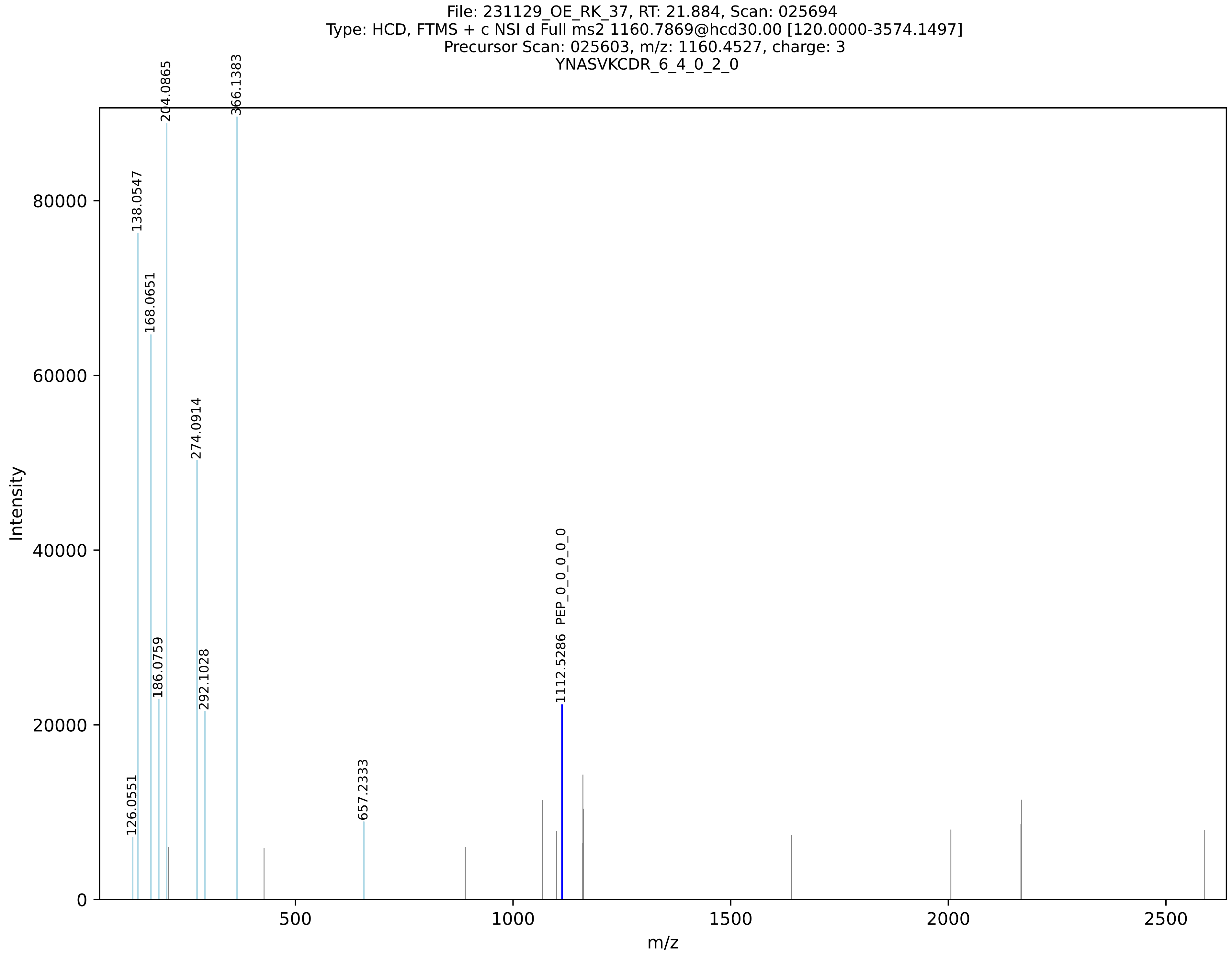
File: 231129_OE_RK_37, RT: 21.884, Scan: 025694
Type: HCD, FTMS + c NSI d Full ms2 1160.7869@hcd30.00 [120.0000-3574.1497]
Precursor Scan: 025603, m/z: 1160.4527, charge: 3
YNASVKCDR_6_4_0_2_0
Intensity
m/z
0
20000
40000
60000
80000
500
1000
1500
2000
2500
126.0551
138.0547
168.0651
186.0759
204.0865
274.0914
292.1028
366.1383
657.2333
1112.5286 PEP_0_0_0_0_0

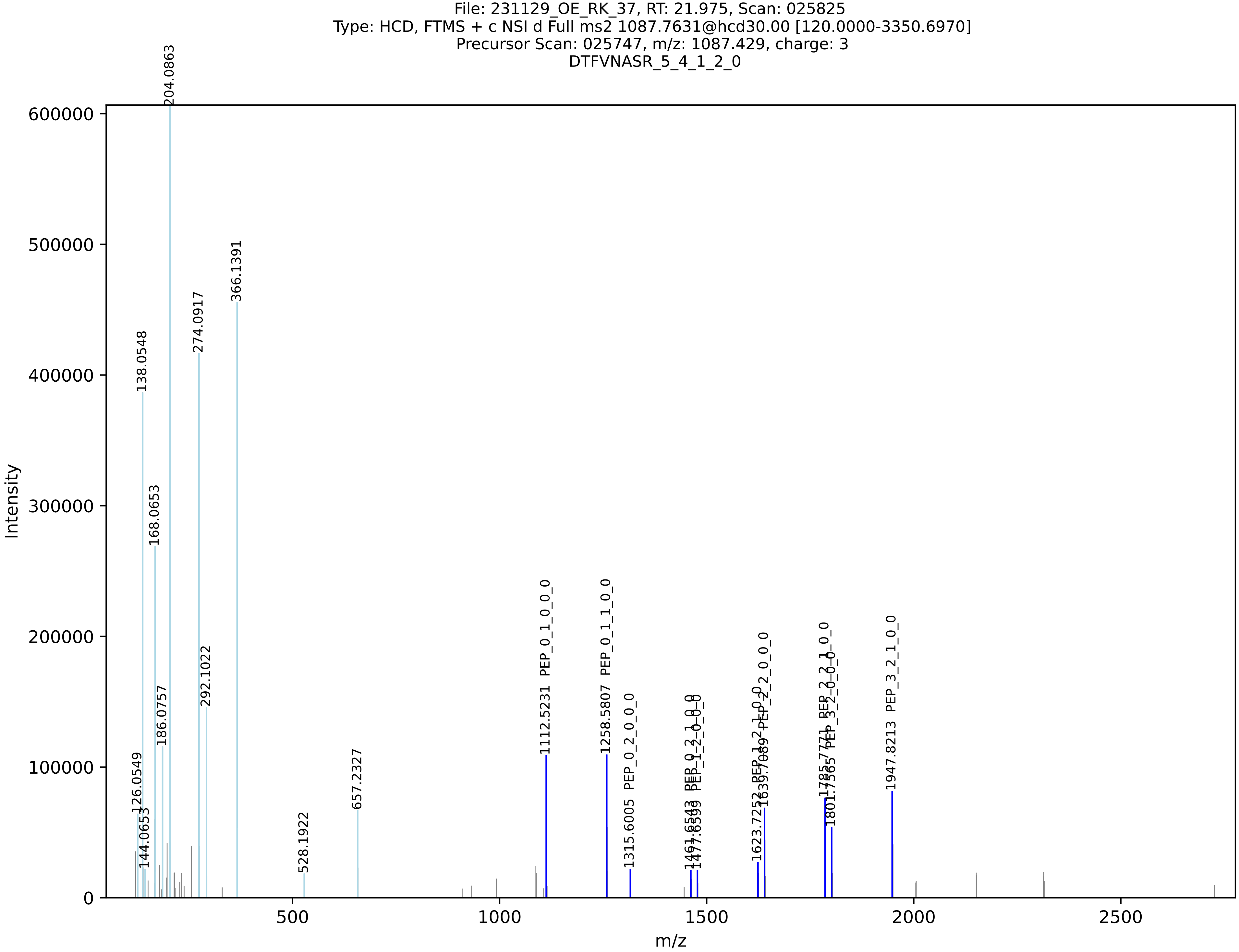
File: 231129_OE_RK_37, RT: 21.975, Scan: 025825
Type: HCD, FTMS + c NSI d Full ms2 1087.7631@hcd30.00 [120.0000-3350.6970]
Precursor Scan: 025747, m/z: 1087.429, charge: 3
DTFVNASR_5_4_1_2_0
Intensity
m/z
204.0863
366.1391
274.0917
138.0548
168.0653
292.1022
186.0757
126.0549
144.0653
528.1922
657.2327
1112.5231 PEP_0_1_0_0_0
1258.5807 PEP_0_1_1_0_0
1315.6005 PEP_0_2_0_0_0
1947.8213 PEP_3_2_1_0_0

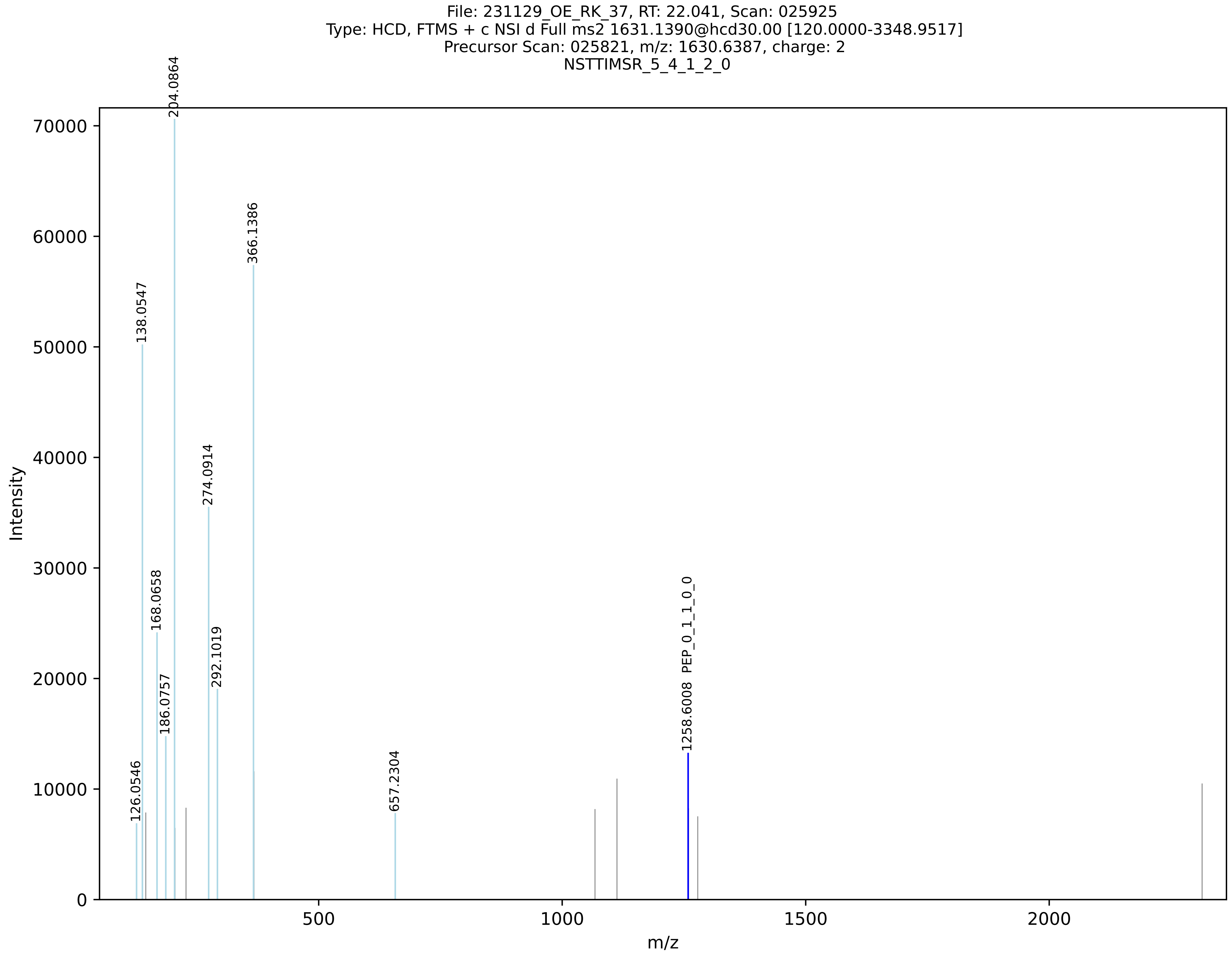
File: 231129_OE_RK_37, RT: 22.041, Scan: 025925
Type: HCD, FTMS + c NSI d Full ms2 1631.1390@hcd30.00 [120.0000-3348.9517]
Precursor Scan: 025821, m/z: 1630.6387, charge: 2
NSTTIMSR_5_4_1_2_0
Intensity
m/z
204.0864
366.1386
138.0547
274.0914
168.0658
292.1019
186.0757
126.0546
657.2304
1258.6008 PEP_0_1_1_0_0
0
10000
20000
30000
40000
50000
60000
70000
500
1000
1500
2000

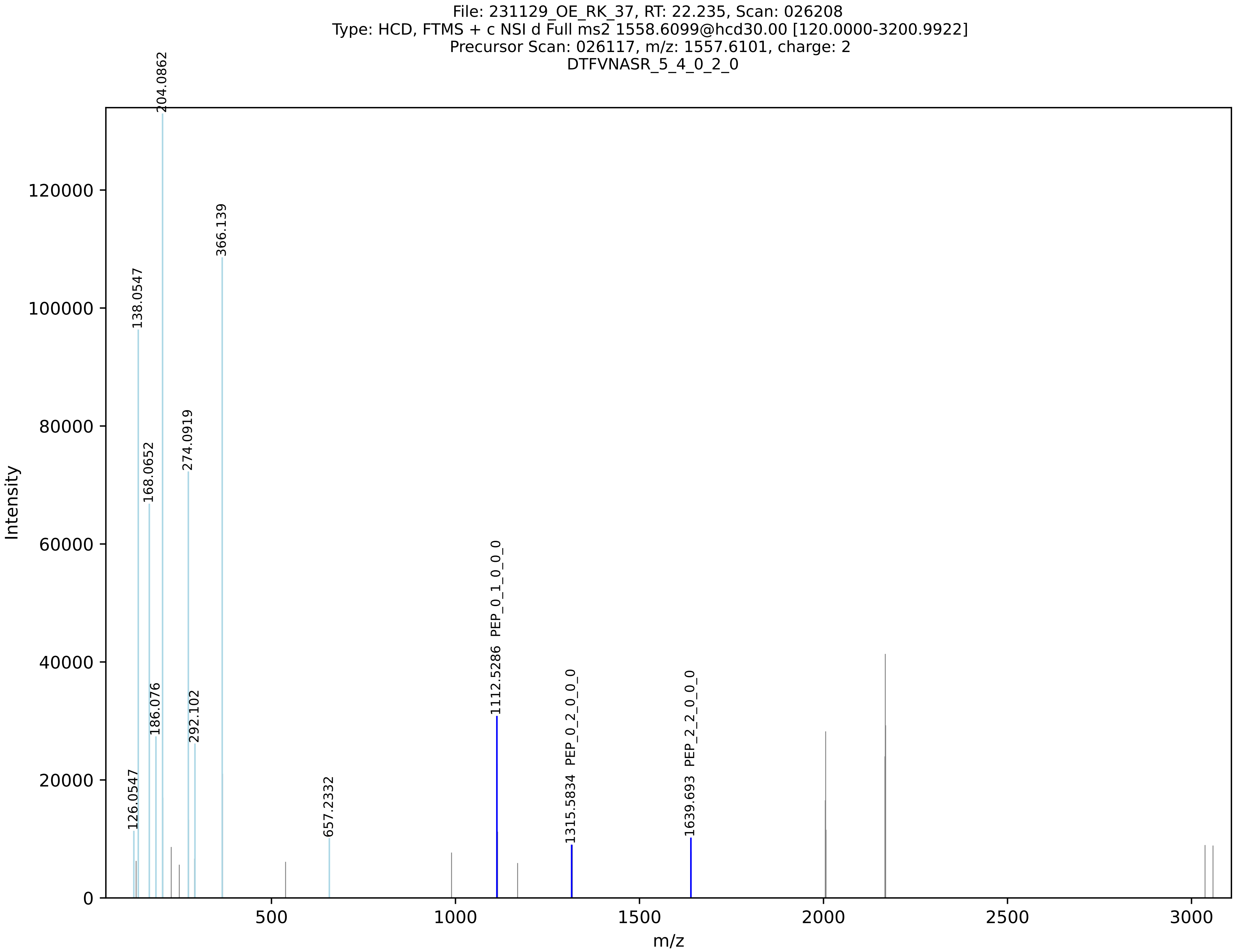
File: 231129_OE_RK_37, RT: 22.235, Scan: 026208
Type: HCD, FTMS + c NSI d Full ms2 1558.6099@hcd30.00 [120.0000-3200.9922]
Precursor Scan: 026117, m/z: 1557.6101, charge: 2
DTFVNASR_5_4_0_2_0
Intensity
m/z
0
20000
40000
60000
80000
100000
120000
500
1000
1500
2000
2500
3000
204.0862
366.139
138.0547
274.0919
168.0652
186.076
292.102
126.0547
657.2332
1112.5286 PEP_0_1_0_0_0
1315.5834 PEP_0_2_0_0_0
1639.693 PEP_2_2_0_0_0

File: 231129_OE_RK_37, RT: 22.286, Scan: 026283
Type: HCD, FTMS + c NSI d Full ms2 1295.5328@hcd30.00 [120.0000-3986.4724]
Precursor Scan: 026191, m/z: 1294.5305, charge: 3
VVNCSDPGFVENAIR_5_4_2_1_0

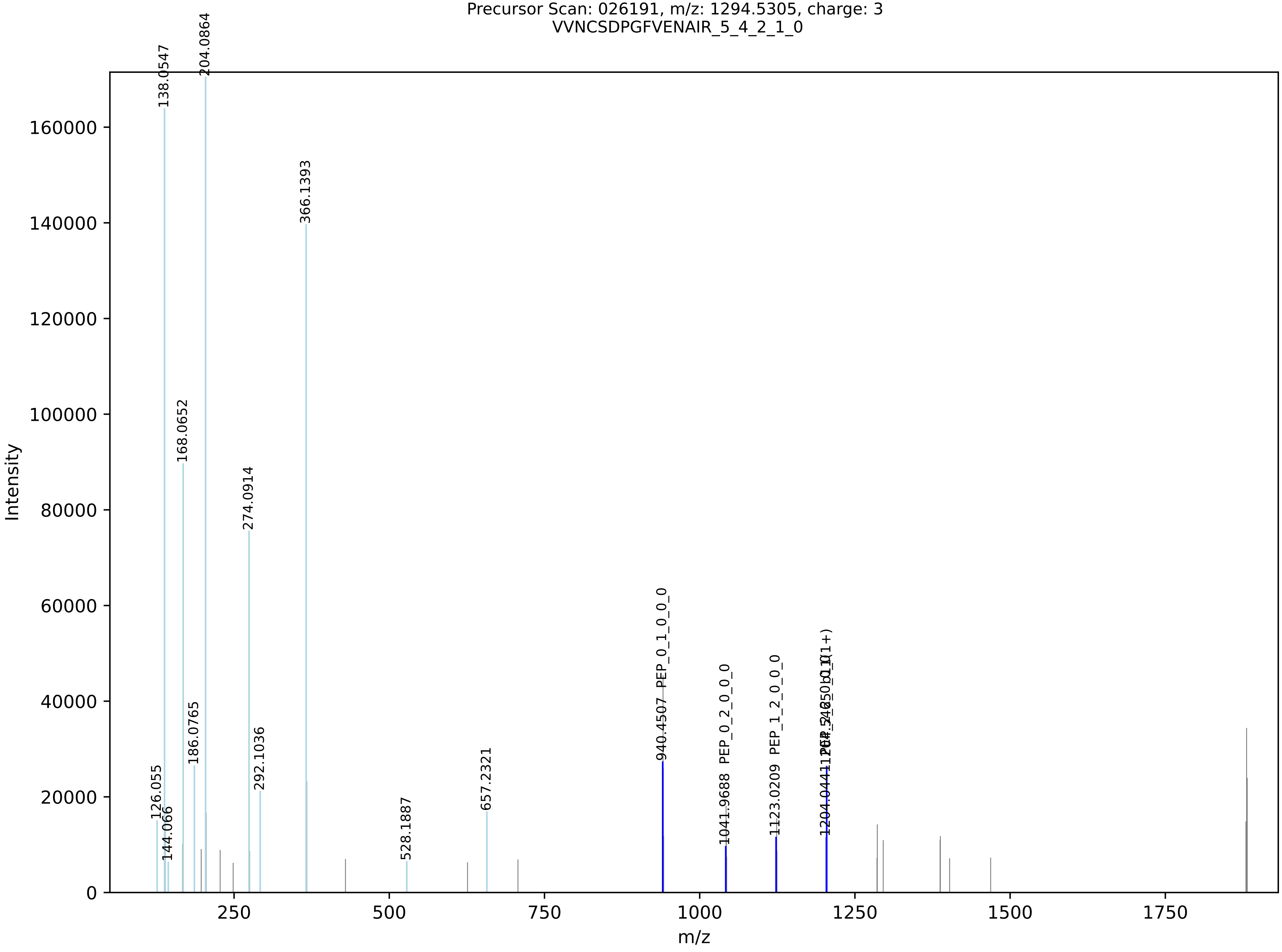

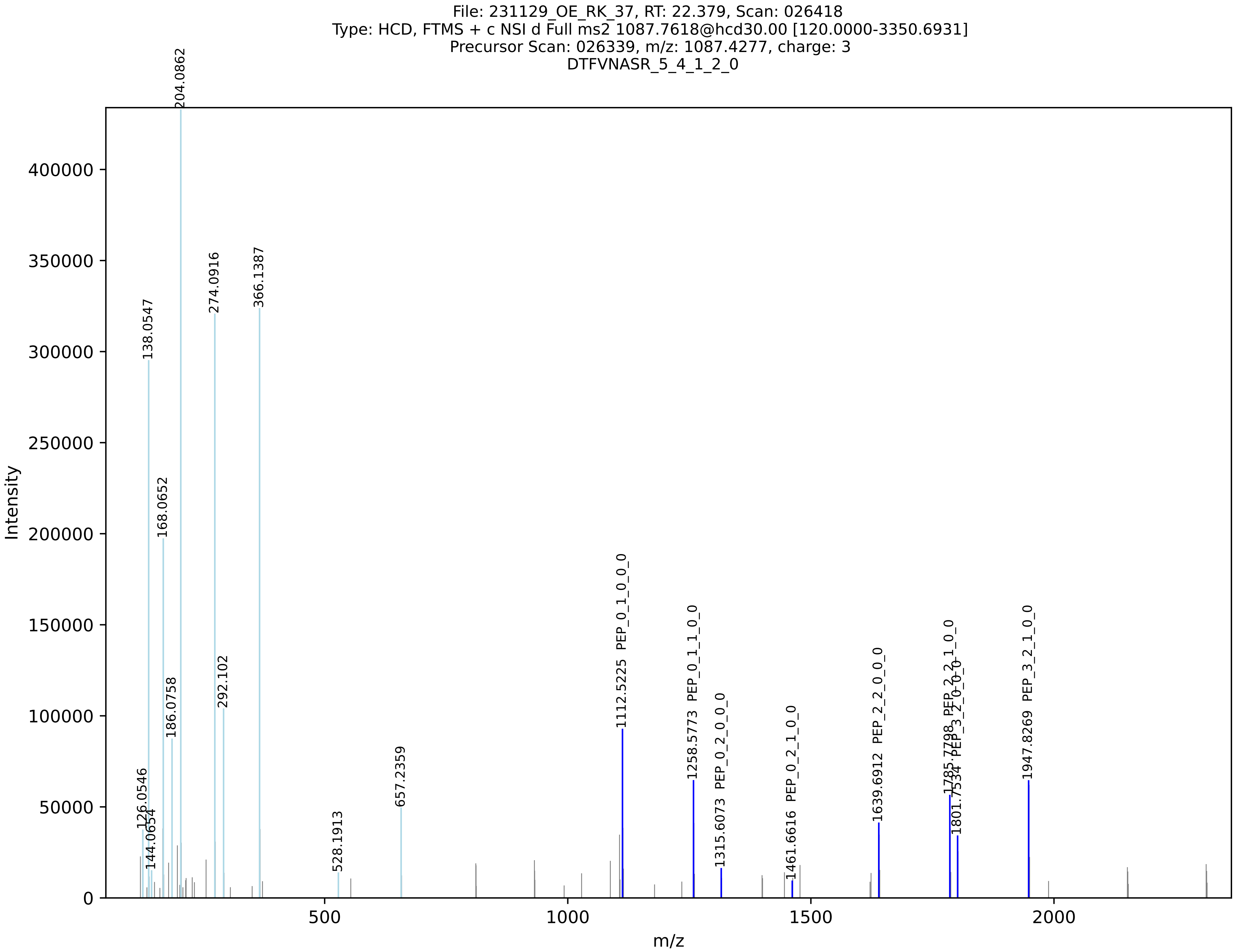

File: 231129_OE_RK_37, RT: 22.379, Scan: 026418
Type: HCD, FTMS + c NSI d Full ms2 1087.7618@hcd30.00 [120.0000-3350.6931]
Precursor Scan: 026339, m/z: 1087.4277, charge: 3
DTFVNASR_5_4_1_2_0
Intensity
m/z
204.0862
274.0916
366.1387
138.0547
168.0652
186.0758
292.102
126.0546
144.0654
528.1913
657.2359
1112.5225 PEP_0_1_0_0_0
1258.5773 PEP_0_1_1_0_0
1315.6073 PEP_0_2_0_0_0
1461.6616 PEP_0_2_1_0_0
1639.6912 PEP_2_2_0_0_0
1785.7798 PEP_2_2_1_0_0
1801.7534 PEP_3_2_0_0_0
1947.8269 PEP_3_2_1_0_0

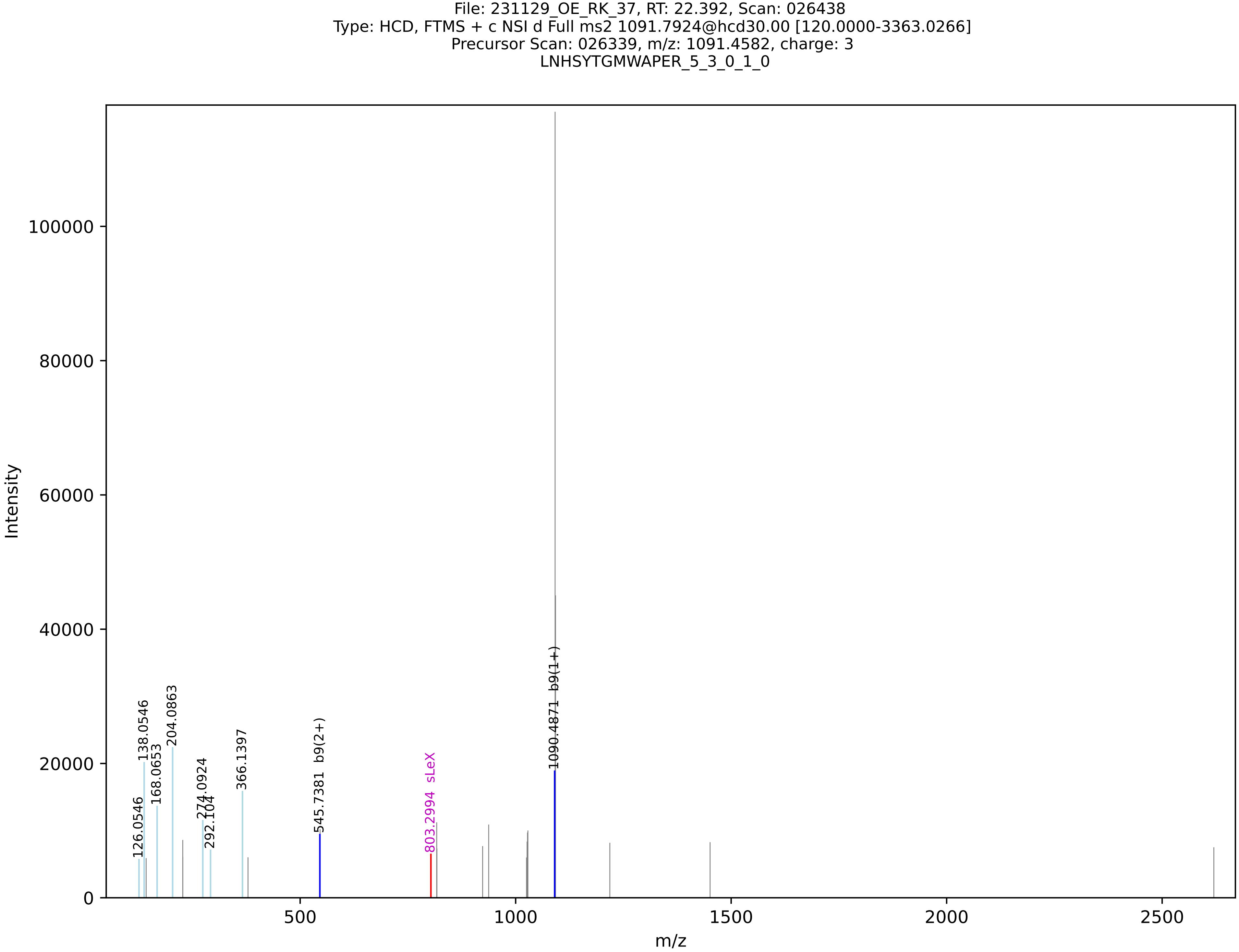
File: 231129_OE_RK_37, RT: 22.392, Scan: 026438
Type: HCD, FTMS + c NSI d Full ms2 1091.7924@hcd30.00 [120.0000-3363.0266]
Precursor Scan: 026339, m/z: 1091.4582, charge: 3
LNHSYTGMWAPER_5_3_0_1_0
Intensity
m/z
126.0546
138.0546
168.0653
204.0863
274.0924
292.104
366.1397
545.7381 b9(2+)
803.2994 sLeX
1090.4871 b9(1+)
0
20000
40000
60000
80000
100000
500
1000
1500
2000
2500

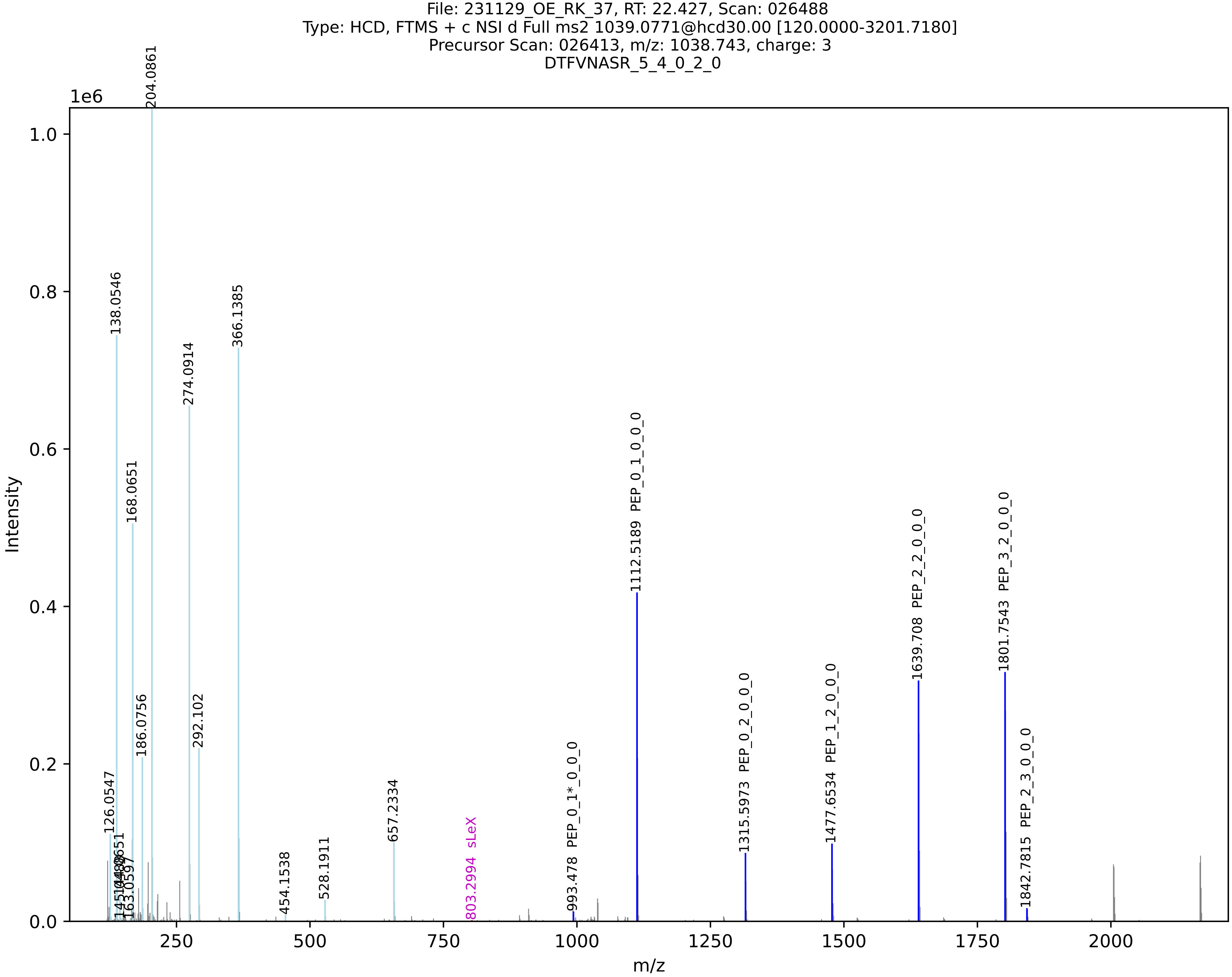
File: 231129_OE_RK_37, RT: 22.427, Scan: 026488
Type: HCD, FTMS + c NSI d Full ms2 1039.0771@hcd30.00 [120.0000-3201.7180]
Precursor Scan: 026413, m/z: 1038.743, charge: 3
DTFVNASR_5_4_0_2_0
1e6
Intensity
m/z
204.0861
138.0546
366.1385
274.0914
168.0651
186.0756
292.102
126.0547
163.0597
454.1538
528.1911
657.2334
803.2994 sLeX
993.478 PEP_0_1*_0_0_0
1112.5189 PEP_0_1_0_0_0
1315.5973 PEP_0_2_0_0_0
1477.6534 PEP_1_2_0_0_0
1639.708 PEP_2_2_0_0_0
1801.7543 PEP_3_2_0_0_0
1842.7815 PEP_2_3_0_0_0

File: 231129_OE_RK_37, RT: 22.941, Scan: 027230
Type: HCD, FTMS + c NSI d Full ms2 1088.0969@hcd30.00 [120.0000-3351.7185]
Precursor Scan: 027168, m/z: 1087.4286, charge: 3
DTFVNASR_5_4_1_2_0

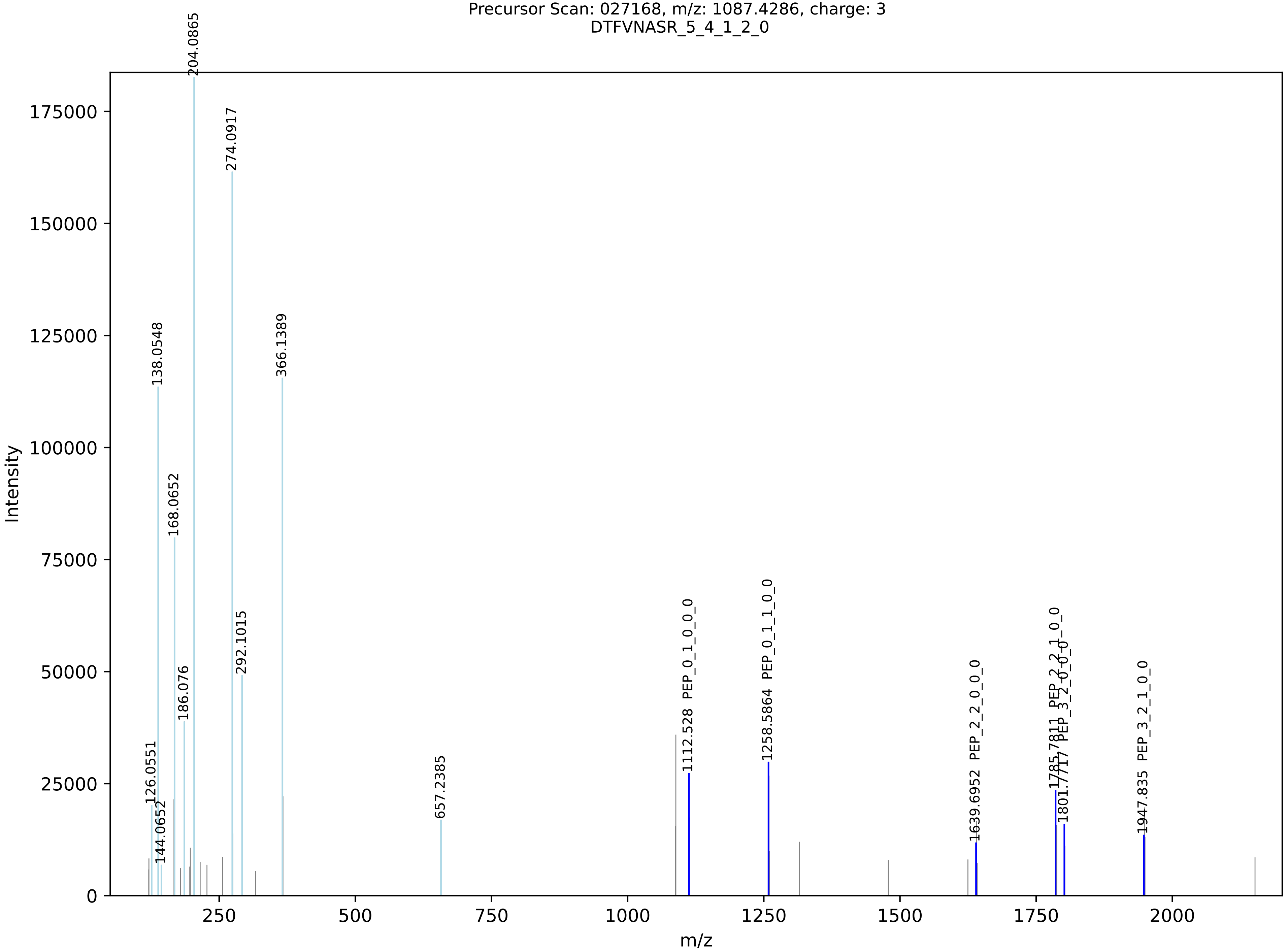

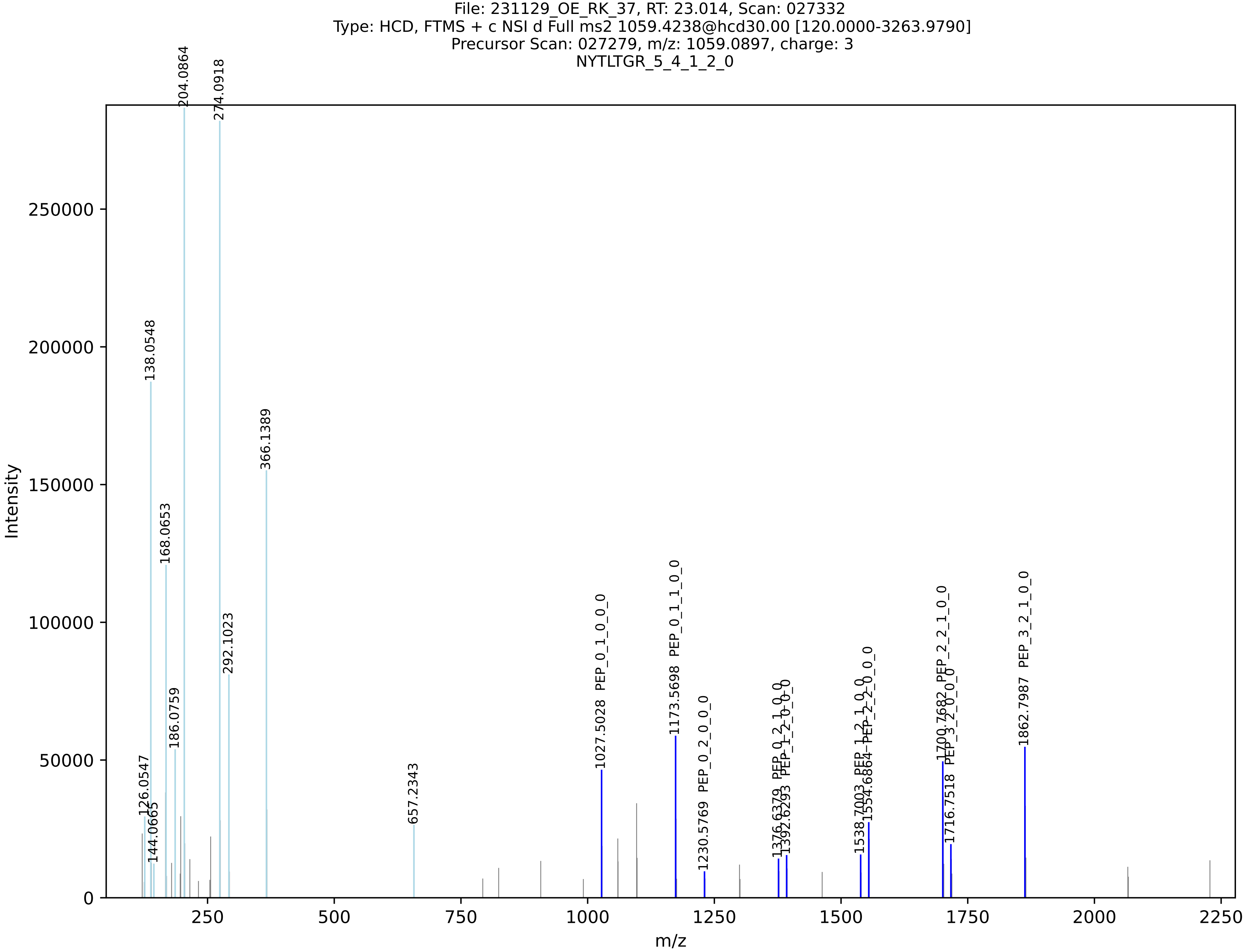

File: 231129_OE_RK_37, RT: 23.014, Scan: 027332
Type: HCD, FTMS + c NSI d Full ms2 1059.4238@hcd30.00 [120.0000-3263.9790]
Precursor Scan: 027279, m/z: 1059.0897, charge: 3
NYTLTGR_5_4_1_2_0
Intensity
m/z
0
50000
100000
150000
200000
250000
250
500
750
1000
1250
1500
1750
2000
2250
204.0864
274.0918
138.0548
366.1389
168.0653
292.1023
186.0759
126.0547
144.0665
657.2343
1027.5028 PEP_0_1_0_0_0
1173.5698 PEP_0_1_1_0_0
1230.5769 PEP_0_2_0_0_0
1376.6379 PEP_0_2_1_0_0
1392.6293 PEP_1_2_0_0_0
1538.7003 PEP_1_2_1_0_0
1554.6864 PEP_2_2_0_0_0
1700.7682 PEP_2_2_1_0_0
1716.7518 PEP_3_2_0_0_0
1862.7987 PEP_3_2_1_0_0

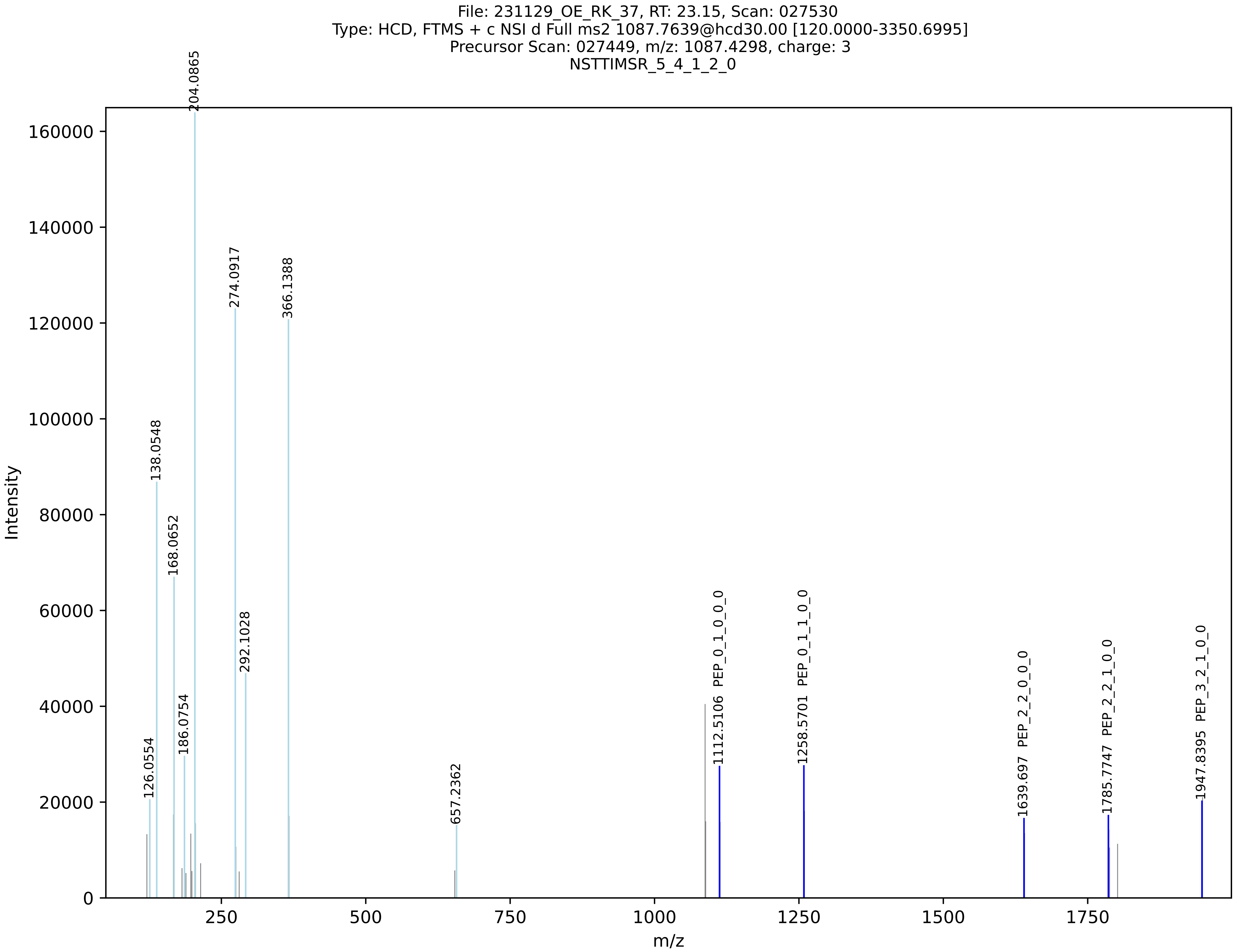
File: 231129_OE_RK_37, RT: 23.15, Scan: 027530
Type: HCD, FTMS + c NSI d Full ms2 1087.7639@hcd30.00 [120.0000-3350.6995]
Precursor Scan: 027449, m/z: 1087.4298, charge: 3
NSTTIMSR_5_4_1_2_0
Intensity
m/z
204.0865
274.0917
366.1388
138.0548
168.0652
292.1028
186.0754
126.0554
657.2362
1112.5106 PEP_0_1_0_0_0
1258.5701 PEP_0_1_1_0_0
1639.697 PEP_2_2_0_0_0
1785.7747 PEP_2_2_1_0_0
1947.8395 PEP_3_2_1_0_0

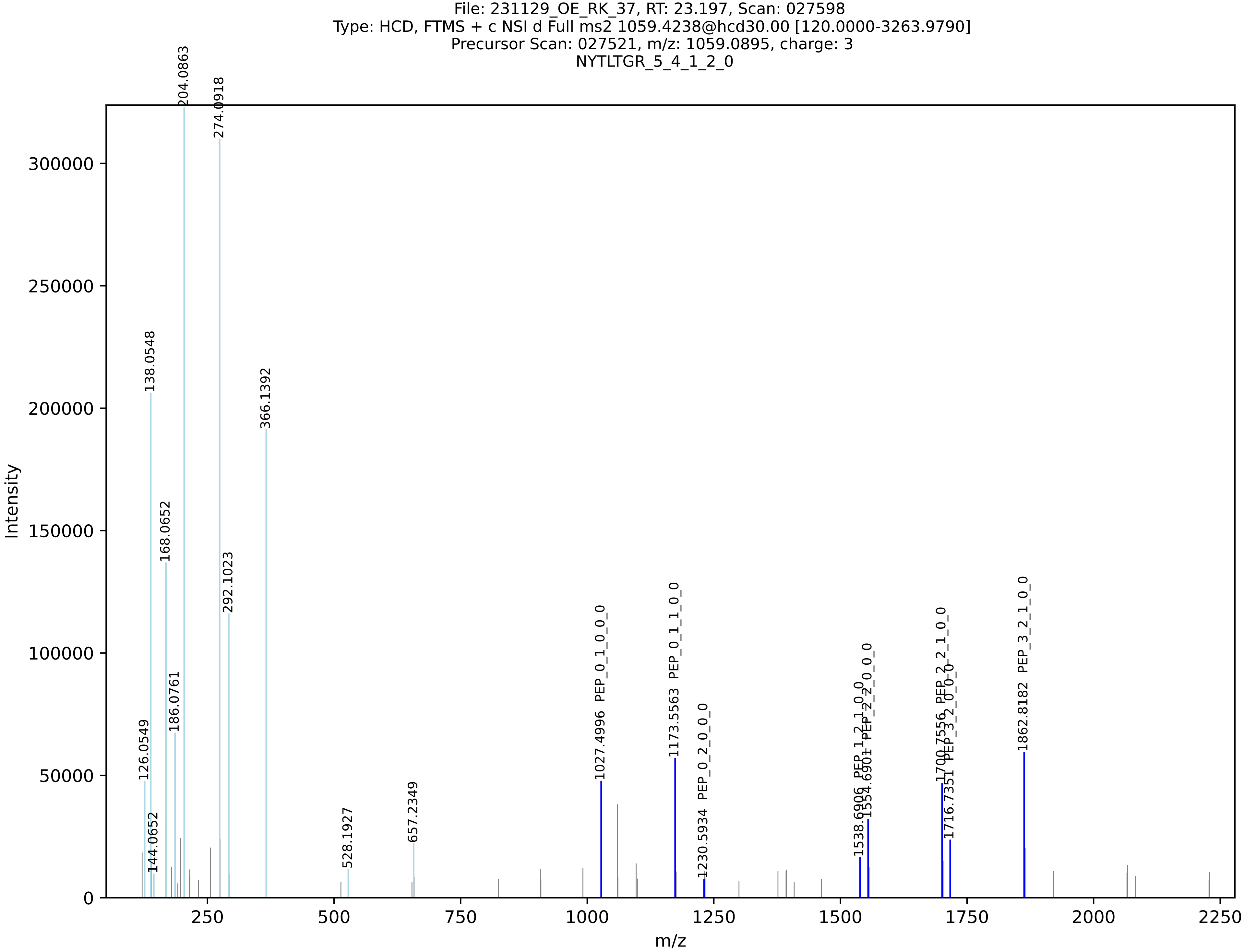
File: 231129_OE_RK_37, RT: 23.197, Scan: 027598
Type: HCD, FTMS + c NSI d Full ms2 1059.4238@hcd30.00 [120.0000-3263.9790]
Precursor Scan: 027521, m/z: 1059.0895, charge: 3
NYTLTGR_5_4_1_2_0
Intensity
m/z
204.0863
274.0918
138.0548
366.1392
168.0652
292.1023
186.0761
126.0549
144.0652
528.1927
657.2349
1027.4996 PEP_0_1_0_0_0
1173.5563 PEP_0_1_1_0_0
1230.5934 PEP_0_2_0_0_0
1538.6906 PEP_1_2_1_0_0
1554.6901 PEP_2_2_0_0_0
1700.7556 PEP_2_2_1_0_0
1716.7351 PEP_3_2_0_0_0
1862.8182 PEP_3_2_1_0_0

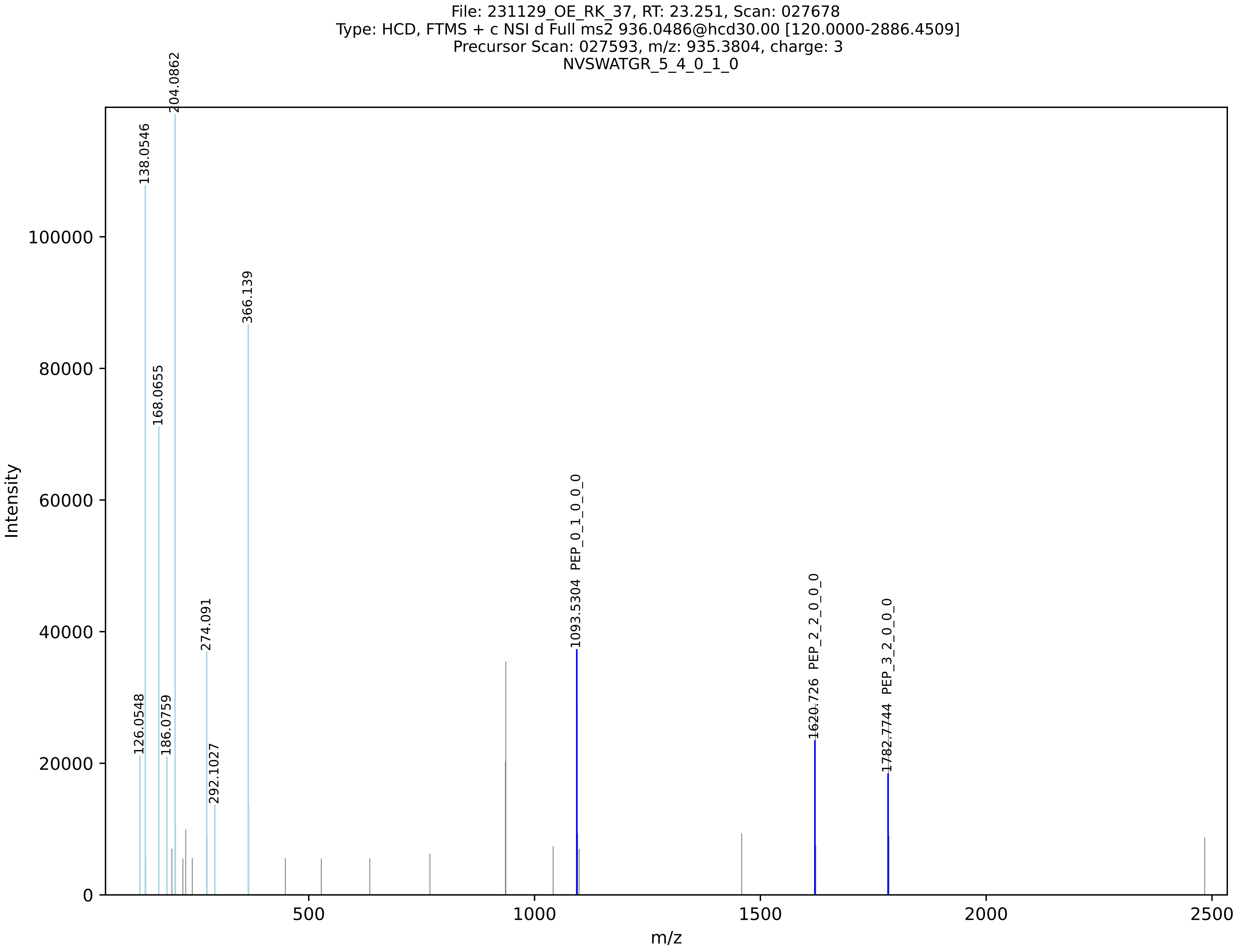
File: 231129_OE_RK_37, RT: 23.251, Scan: 027678
Type: HCD, FTMS + c NSI d Full ms2 936.0486@hcd30.00 [120.0000-2886.4509]
Precursor Scan: 027593, m/z: 935.3804, charge: 3
NVSWATGR_5_4_0_1_0
204.0862
138.0546
168.0655
366.139
274.091
126.0548
186.0759
292.1027
1093.5304 PEP_0_1_0_0_0
1620.726 PEP_2_2_0_0_0
1782.7744 PEP_3_2_0_0_0
Intensity
m/z
0
20000
40000
60000
80000
100000
500
1000
1500
2000
2500

File: 231129_OE_RK_37, RT: 23.266, Scan: 027702
Type: HCD, FTMS + c NSI d Full ms2 1040.1151@hcd30.00 [120.0000-3204.8945]
Precursor Scan: 027593, m/z: 1039.7788, charge: 3
DTFVNASR_5_4_2_1_0

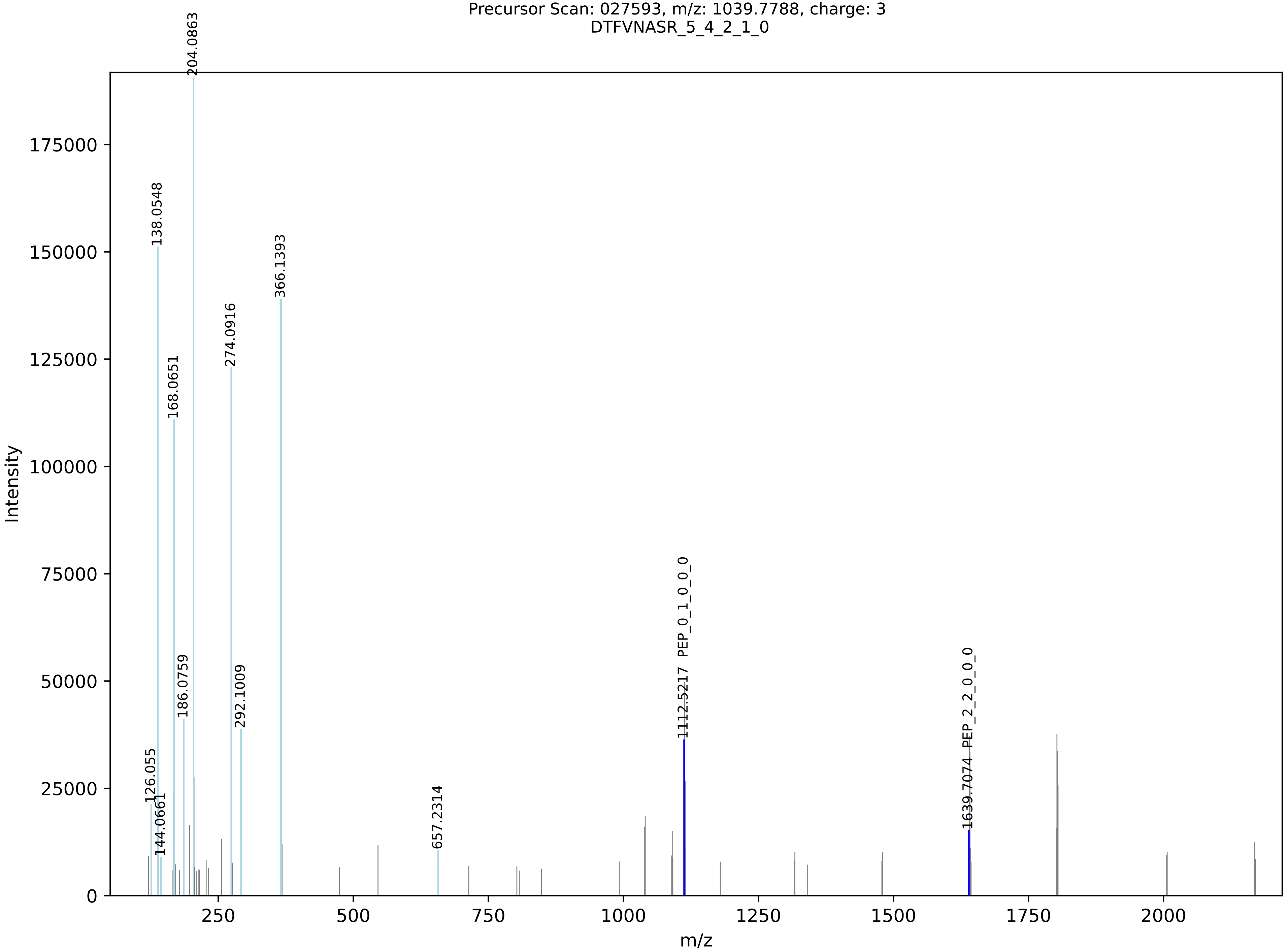

File: 231129_OE_RK_37, RT: 23.353, Scan: 027828
Type: HCD, FTMS + c NSI d Full ms2 1087.7651@hcd30.00 [120.0000-3350.7031]
Precursor Scan: 027740, m/z: 1087.431, charge: 3
DTFVNASR_5_4_1_2_0

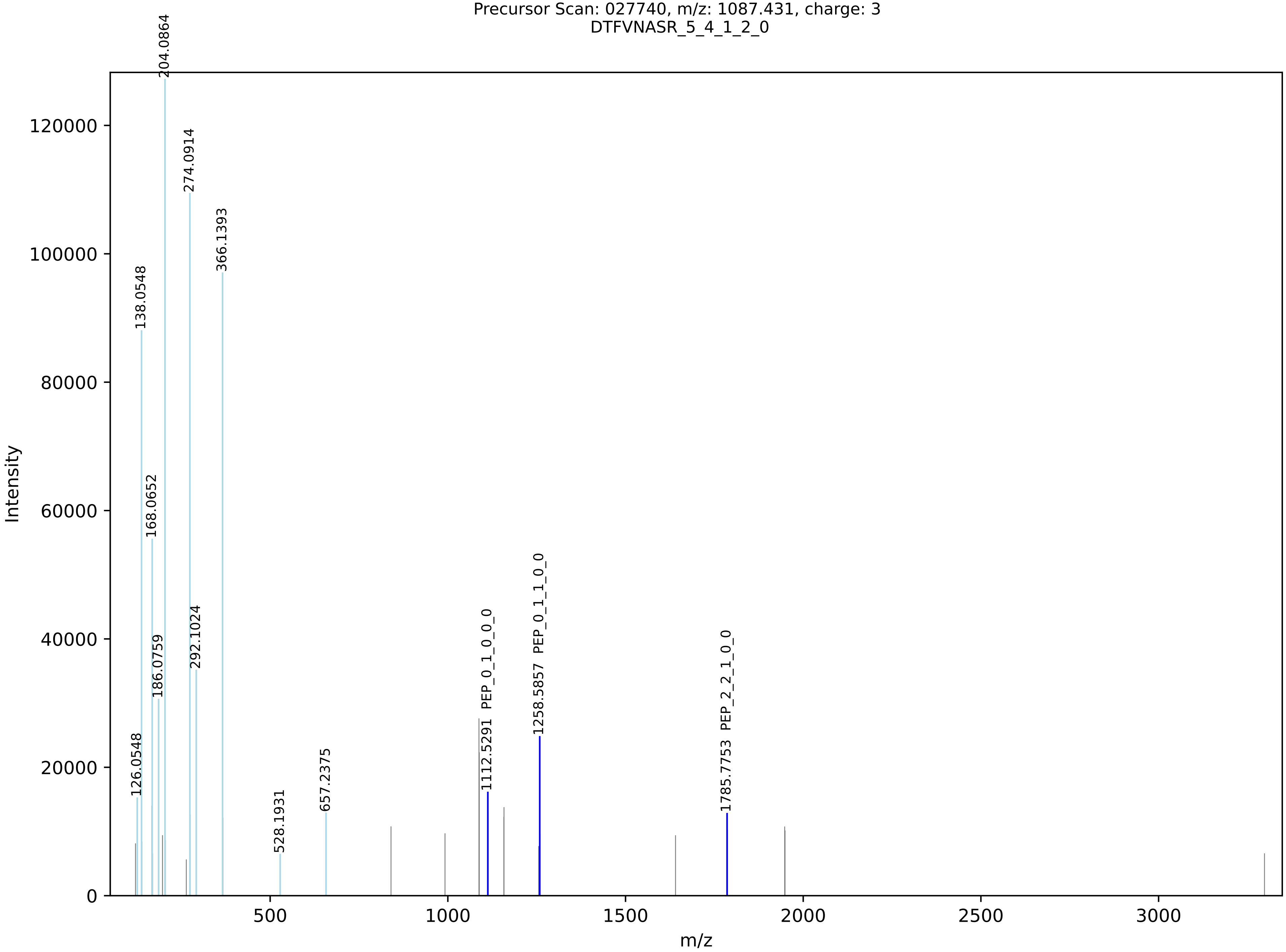

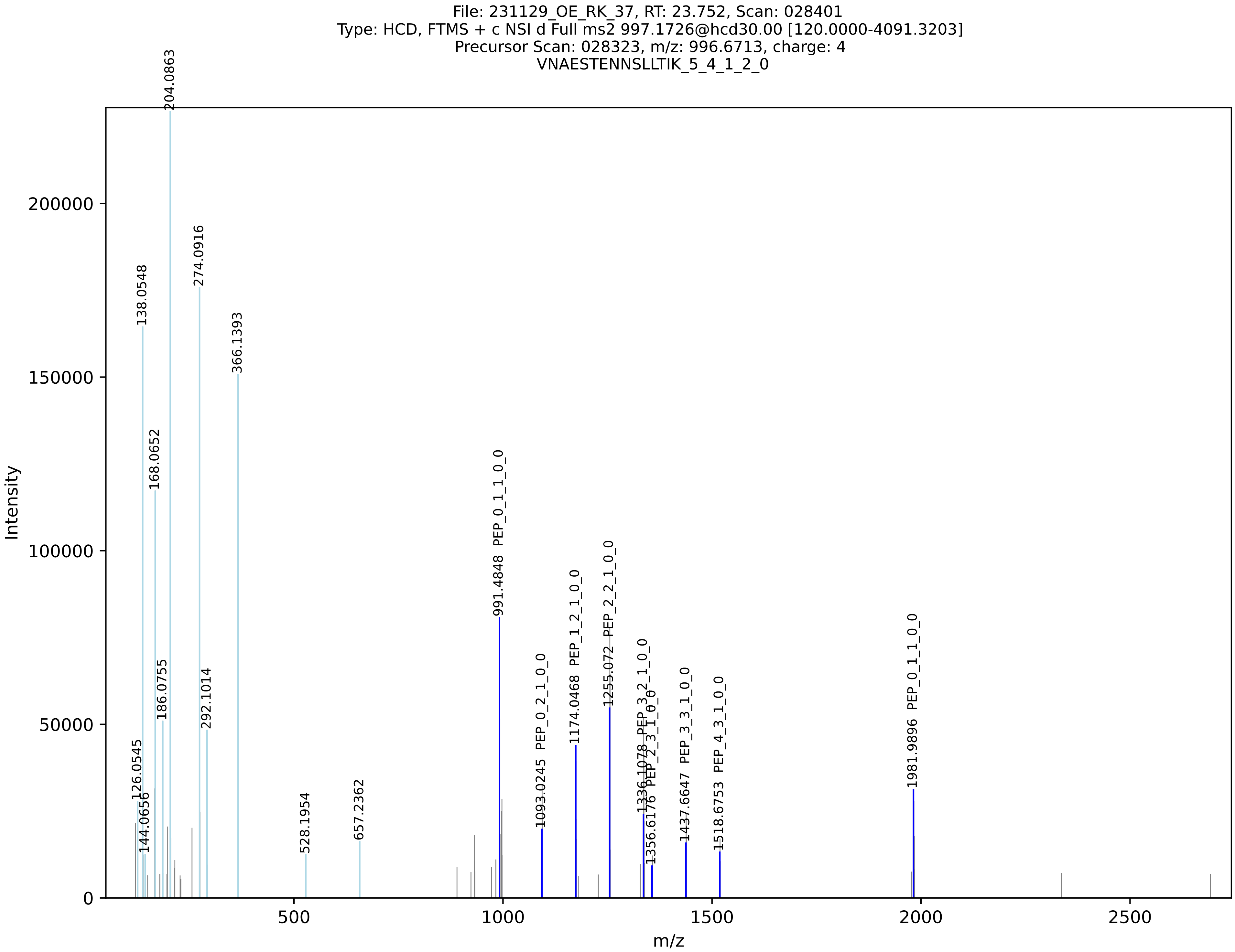
File: 231129_OE_RK_37, RT: 23.752, Scan: 028401
Type: HCD, FTMS + c NSI d Full ms2 997.1726@hcd30.00 [120.0000-4091.3203]
Precursor Scan: 028323, m/z: 996.6713, charge: 4
VNAESTENNSLLTIK_5_4_1_2_0
Intensity
m/z
204.0863
274.0916
138.0548
366.1393
168.0652
186.0755
292.1014
126.0545
144.0656
528.1954
657.2362
991.4848 PEP_0_1_1_0_0
1093.0245 PEP_0_2_1_0_0
1174.0468 PEP_1_2_1_0_0
1255.072 PEP_2_2_1_0_0
1336.1078 PEP_3_2_1_0_0
1356.6176 PEP_2_3_1_0_0
1437.6647 PEP_3_3_1_0_0
1518.6753 PEP_4_3_1_0_0
1981.9896 PEP_0_1_1_0_0

File: 231129_OE_RK_37, RT: 23.853, Scan: 028547
Type: HCD, FTMS + c NSI d Full ms2 1262.2184@hcd30.00 [120.0000-5172.7070]
Precursor Scan: 028470, m/z: 1261.7172, charge: 4
HCDGNVSSCGDHPSEGCFCPPDK_6_5_1_1_0

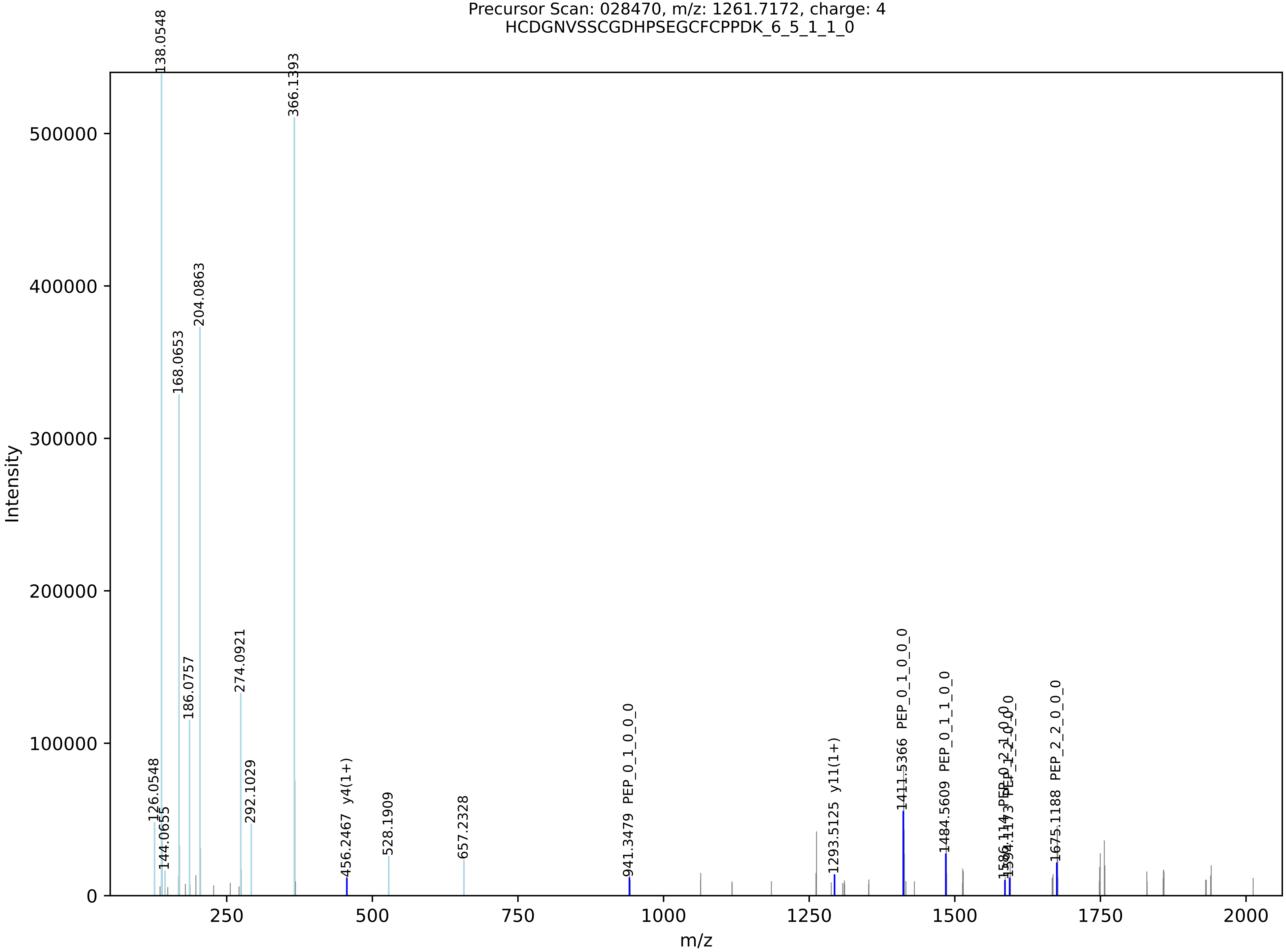

File: 231129_OE_RK_37, RT: 23.882, Scan: 028592
Type: HCD, FTMS + c NSI d Full ms2 1100.5132@hcd30.00 [120.0000-3389.7124]
Precursor Scan: 028470, m/z: 1099.8515, charge: 3
LLNYLLDNLSGTAKR_4_4_1_0_0

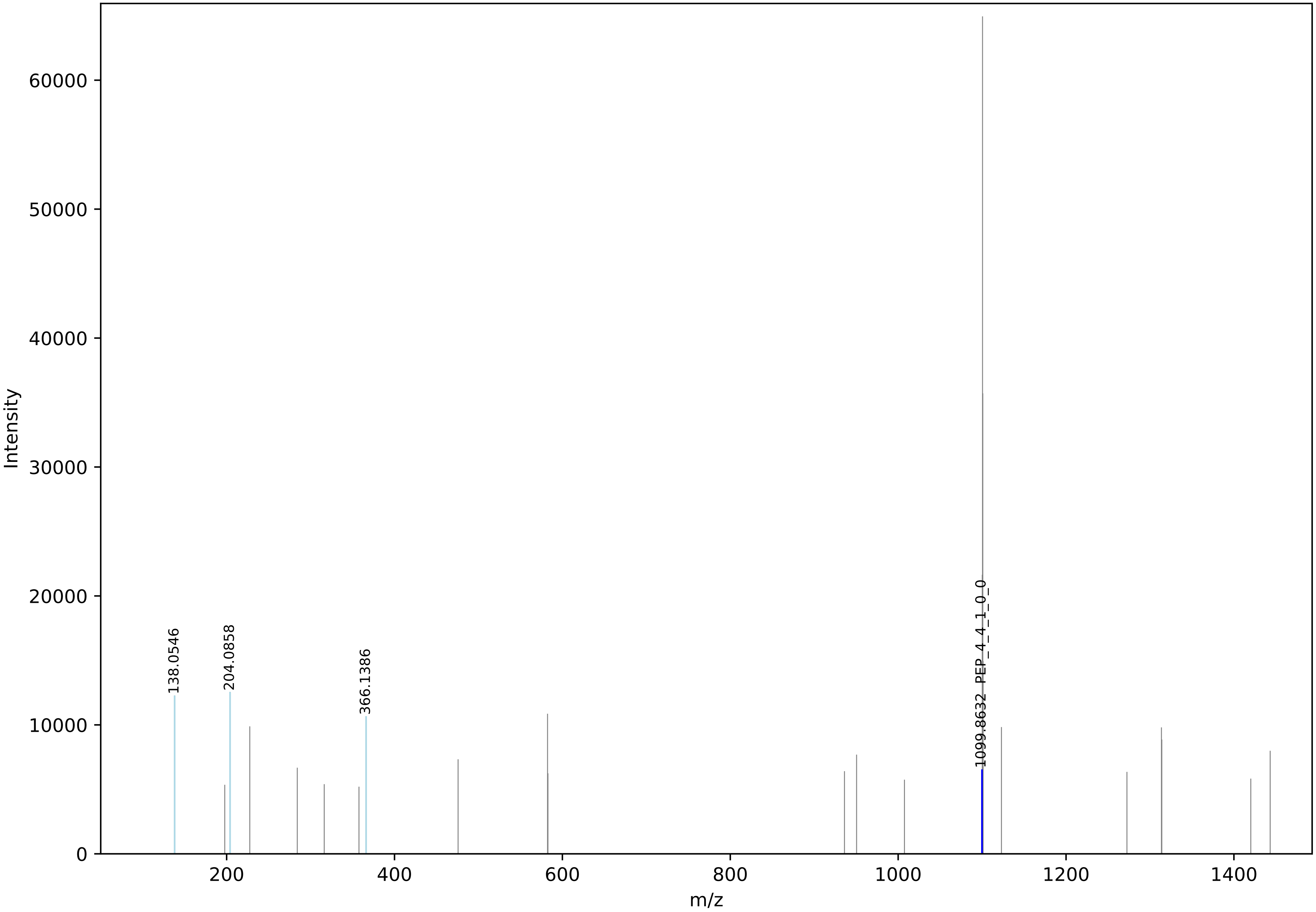

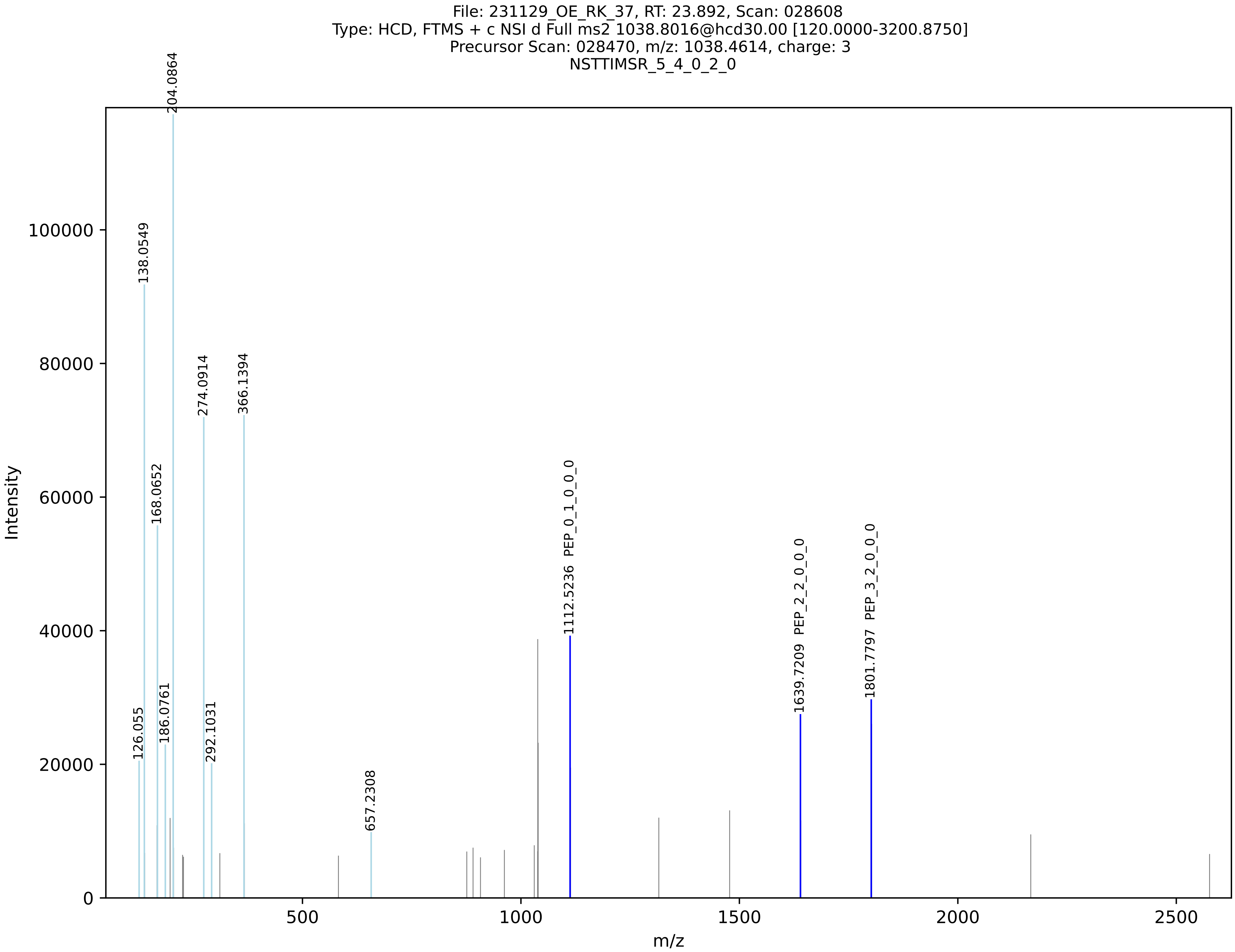
File: 231129_OE_RK_37, RT: 23.892, Scan: 028608
Type: HCD, FTMS + c NSI d Full ms2 1038.8016@hcd30.00 [120.0000-3200.8750]
Precursor Scan: 028470, m/z: 1038.4614, charge: 3
NSTTIMSR_5_4_0_2_0
Intensity
m/z
204.0864
138.0549
168.0652
274.0914
366.1394
126.055
186.0761
292.1031
657.2308
1112.5236 PEP_0_1_0_0_0
1639.7209 PEP_2_2_0_0_0
1801.7797 PEP_3_2_0_0_0
0
20000
40000
60000
80000
100000
500
1000
1500
2000
2500

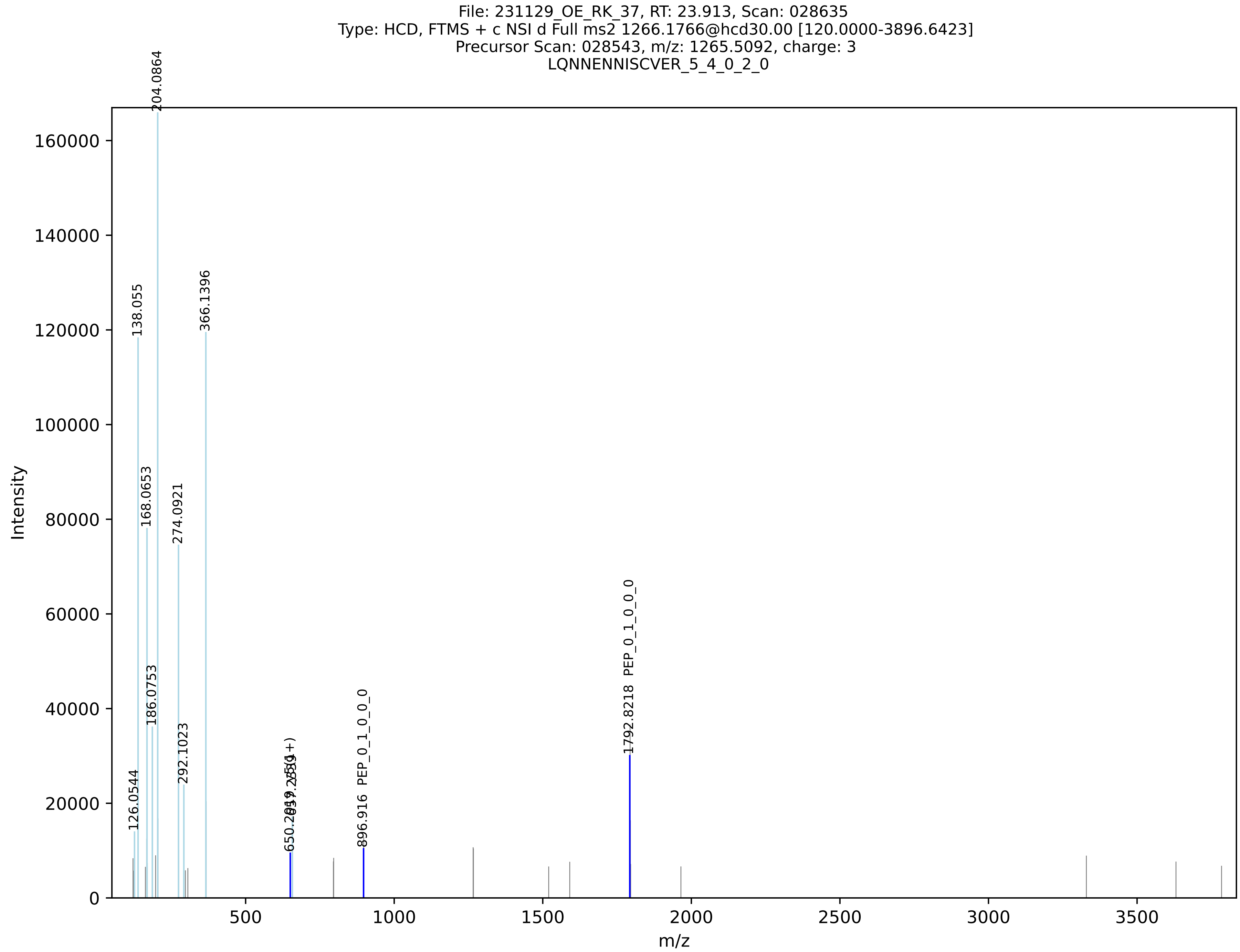
File: 231129_OE_RK_37, RT: 23.913, Scan: 028635
Type: HCD, FTMS + c NSI d Full ms2 1266.1766@hcd30.00 [120.0000-3896.6423]
Precursor Scan: 028543, m/z: 1265.5092, charge: 3
LQNNENNISCVER_5_4_0_2_0
204.0864
138.055
366.1396
168.0653
274.0921
186.0753
292.1023
126.0544
896.916 PEP_0_1_0_0_0
1792.8218 PEP_0_1_0_0_
Intensity
m/z

File: 231129_OE_RK_37, RT: 23.994, Scan: 028758
Type: HCD, FTMS + c NSI d Full ms2 928.4366@hcd30.00 [120.0000-1915.4387]
Precursor Scan: 028614, m/z: 928.4366, charge: 2
LVINR_3_3_1_0_0

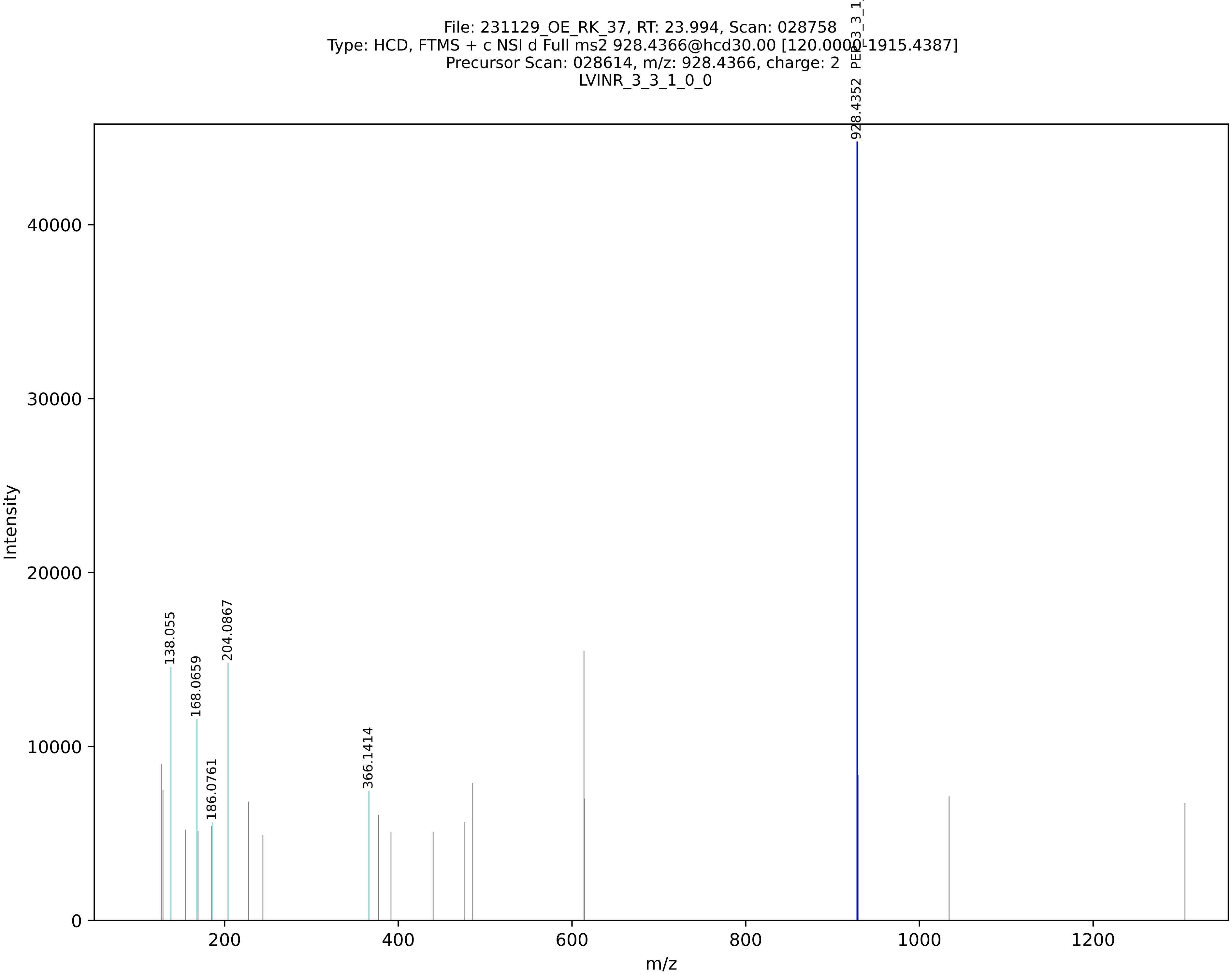

File: 231129_OE_RK_37, RT: 24.002, Scan: 028764
Type: HCD, FTMS + c NSI d Full ms2 1177.1692@hcd30.00 [120.0000-3624.2795]
Precursor Scan: 028688, m/z: 1176.835, charge: 3
VYKPSAGNNSLYR_5_4_1_1_0

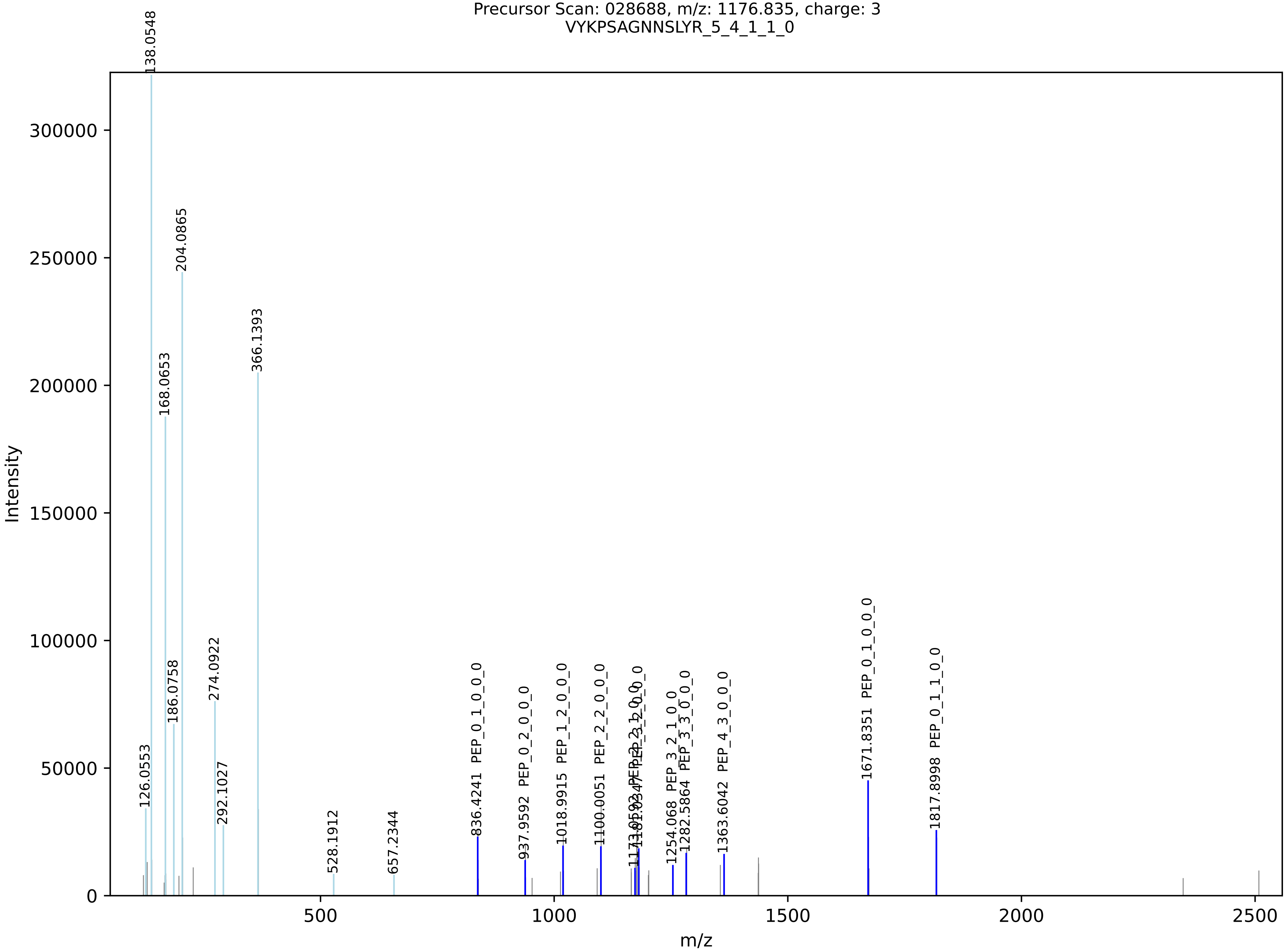

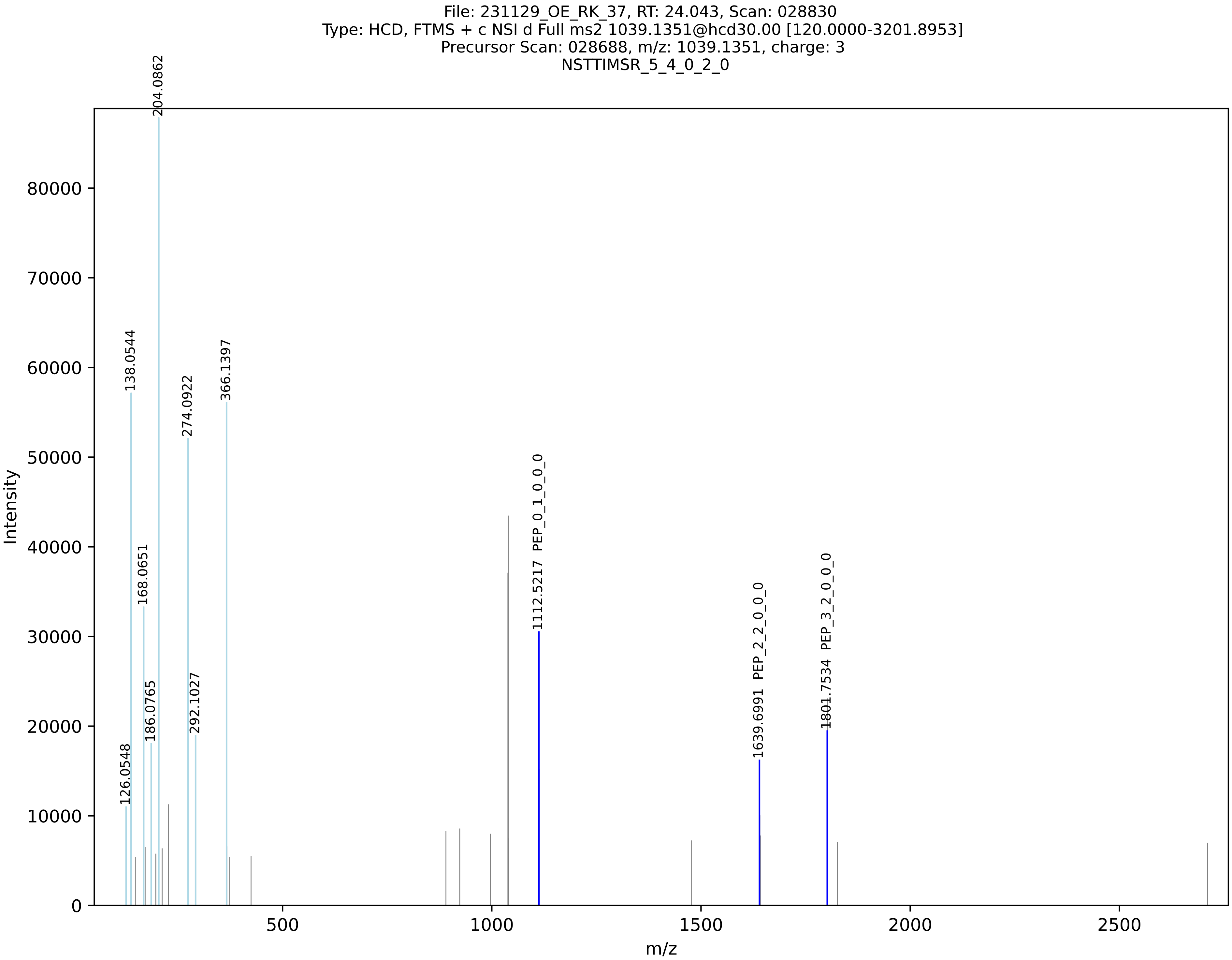
File: 231129_OE_RK_37, RT: 24.043, Scan: 028830
Type: HCD, FTMS + c NSI d Full ms2 1039.1351@hcd30.00 [120.0000-3201.8953]
Precursor Scan: 028688, m/z: 1039.1351, charge: 3
NSTTIMSR_5_4_0_2_0
Intensity
m/z
204.0862
138.0544
366.1397
274.0922
168.0651
186.0765
292.1027
126.0548
1112.5217 PEP_0_1_0_0_0
1639.6991 PEP_2_2_0_0_0
1801.7534 PEP_3_2_0_0_0
0
10000
20000
30000
40000
50000
60000
70000
80000
500
1000
1500
2000
2500

File: 231129_OE_RK_37, RT: 24.052, Scan: 028837
Type: HCD, FTMS + c NSI d Full ms2 1170.9346@hcd30.00 [120.0000-4800.2690]
Precursor Scan: 028762, m/z: 1170.4336, charge: 4
HCDGNVSSCGDHPSEGCFCPPDK_5_4_1_1_0

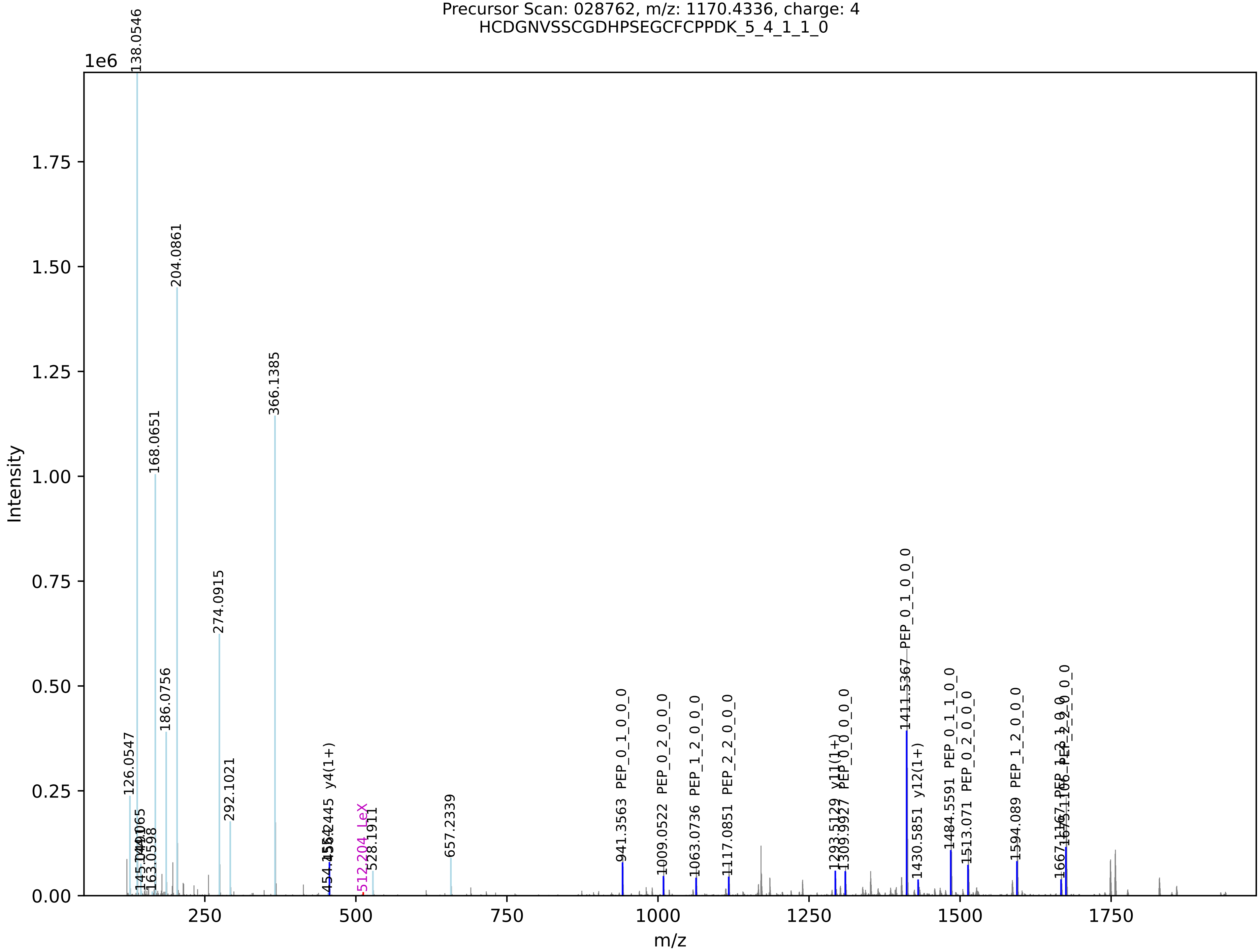

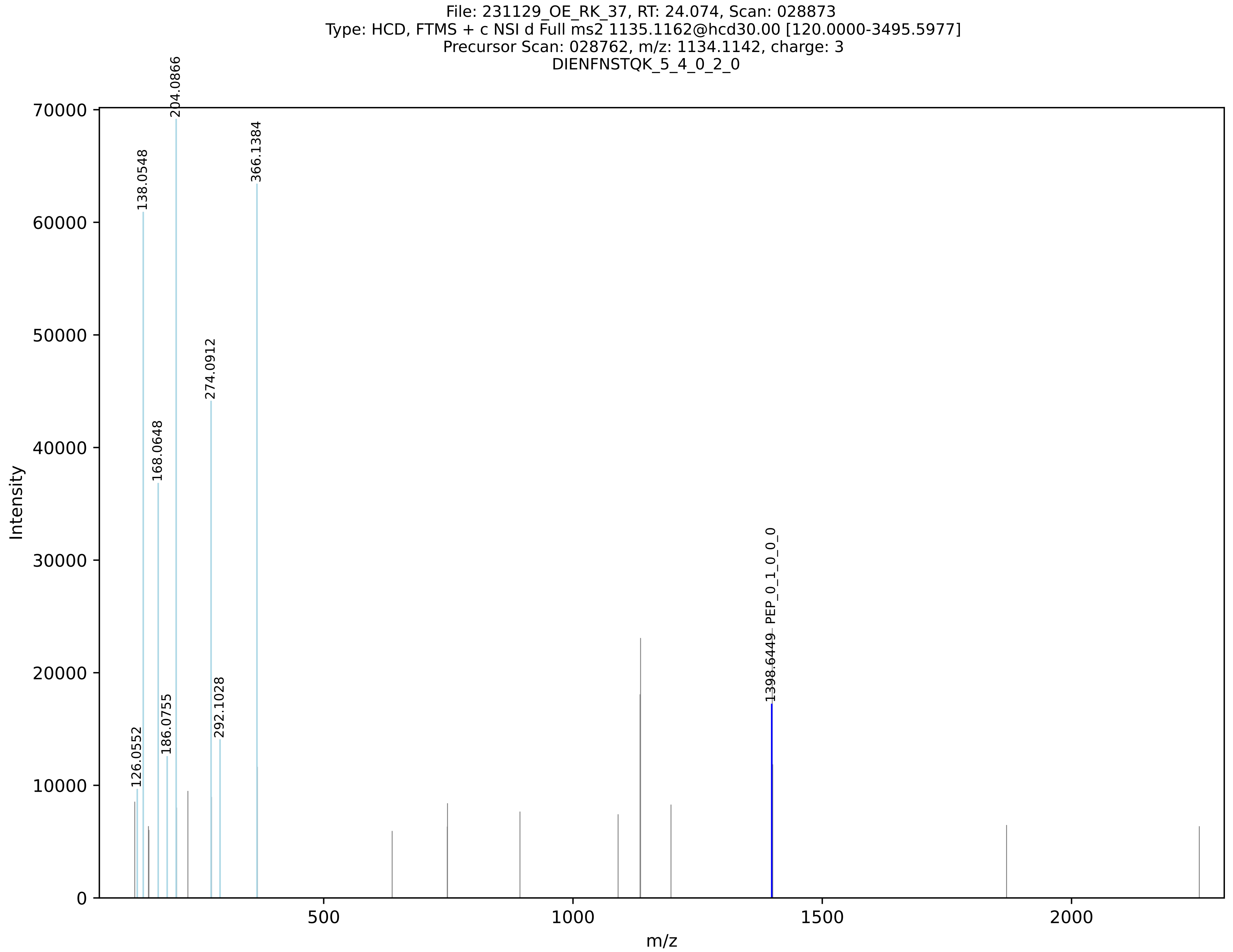

File: 231129_OE_RK_37, RT: 24.074, Scan: 028873
Type: HCD, FTMS + c NSI d Full ms2 1135.1162@hcd30.00 [120.0000-3495.5977]
Precursor Scan: 028762, m/z: 1134.1142, charge: 3
DIENFNSTQK_5_4_0_2_0
Intensity
m/z
204.0866
366.1384
138.0548
274.0912
168.0648
1398.6449 PEP_0_1_0_0_0
292.1028
186.0755
126.0552

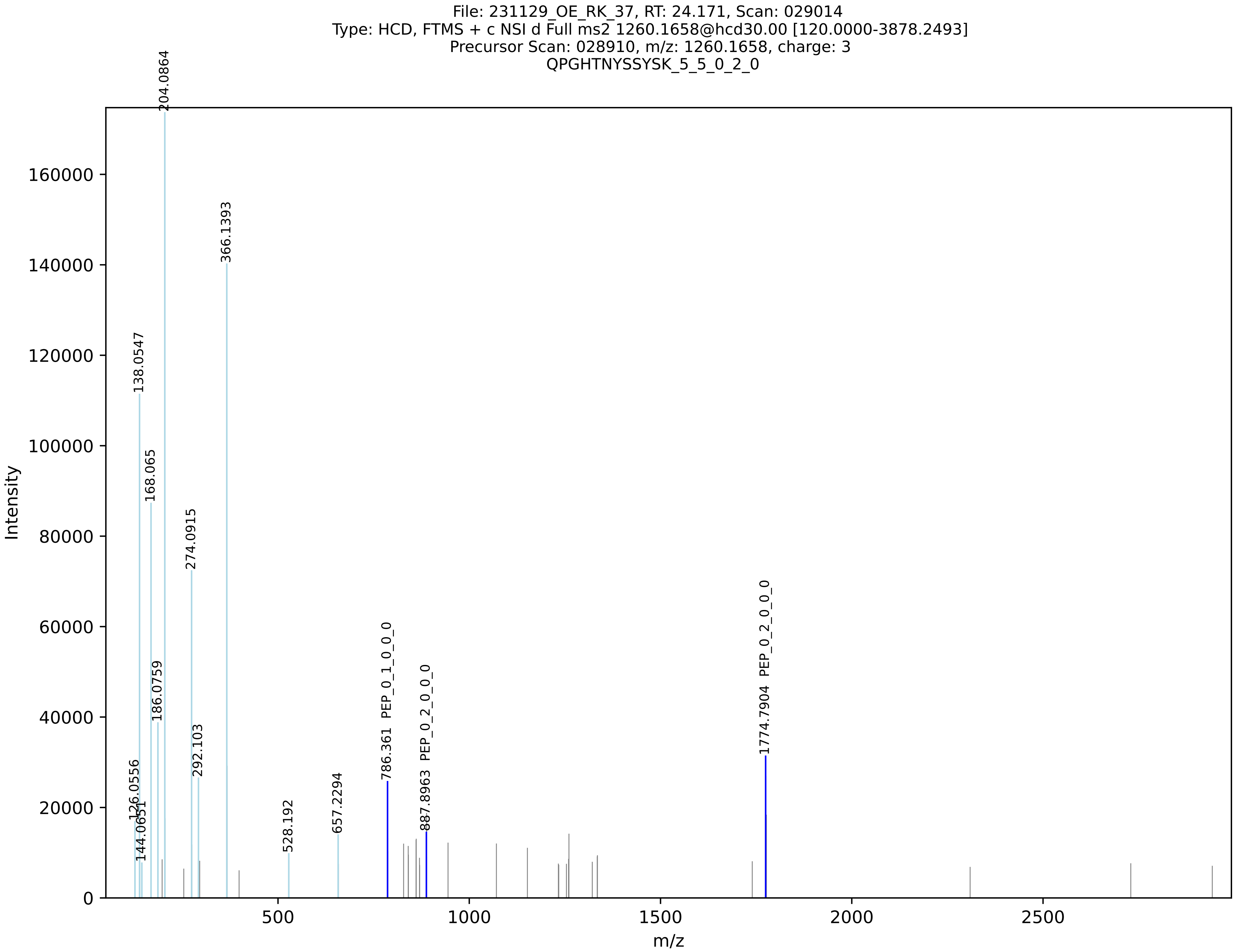
File: 231129_OE_RK_37, RT: 24.171, Scan: 029014
Type: HCD, FTMS + c NSI d Full ms2 1260.1658@hcd30.00 [120.0000-3878.2493]
Precursor Scan: 028910, m/z: 1260.1658, charge: 3
QPGHTNYSSYSK_5_5_0_2_0
Intensity
m/z
204.0864
366.1393
138.0547
168.065
274.0915
186.0759
292.103
126.0556
144.0651
528.192
657.2294
786.361 PEP_0_1_0_0_0
887.8963 PEP_0_2_0_0_0
1774.7904 PEP_0_2_0_0_0

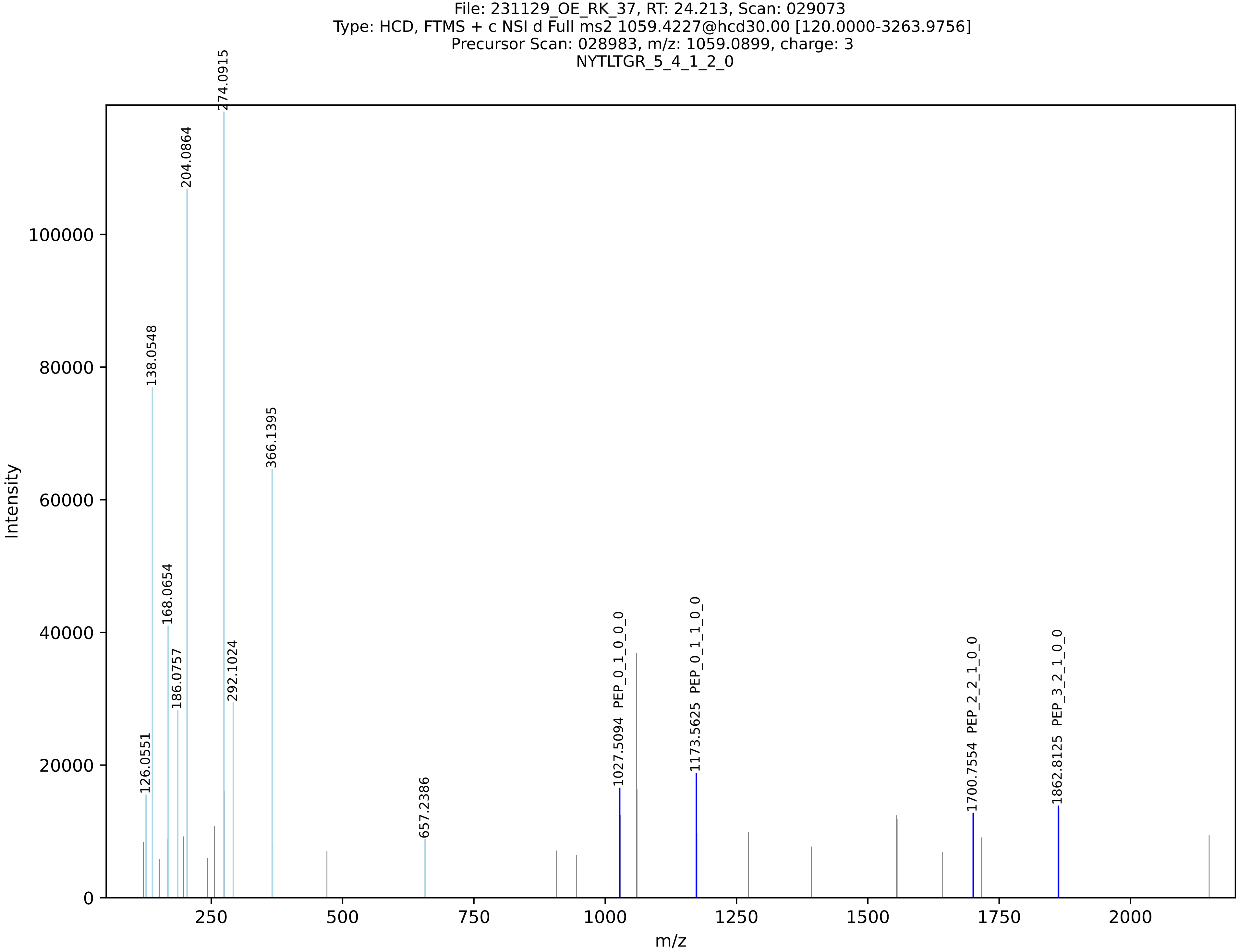
File: 231129_OE_RK_37, RT: 24.213, Scan: 029073
Type: HCD, FTMS + c NSI d Full ms2 1059.4227@hcd30.00 [120.0000-3263.9756]
Precursor Scan: 028983, m/z: 1059.0899, charge: 3
NYTLTGR_5_4_1_2_0
Intensity
m/z
126.0551
138.0548
168.0654
186.0757
204.0864
274.0915
292.1024
366.1395
657.2386
1027.5094 PEP_0_1_0_0_0
1173.5625 PEP_0_1_1_0_0
1700.7554 PEP_2_2_1_0_0
1862.8125 PEP_3_2_1_0_0
0
20000
40000
60000
80000
100000
250
500
750
1000
1250
1500
1750
2000

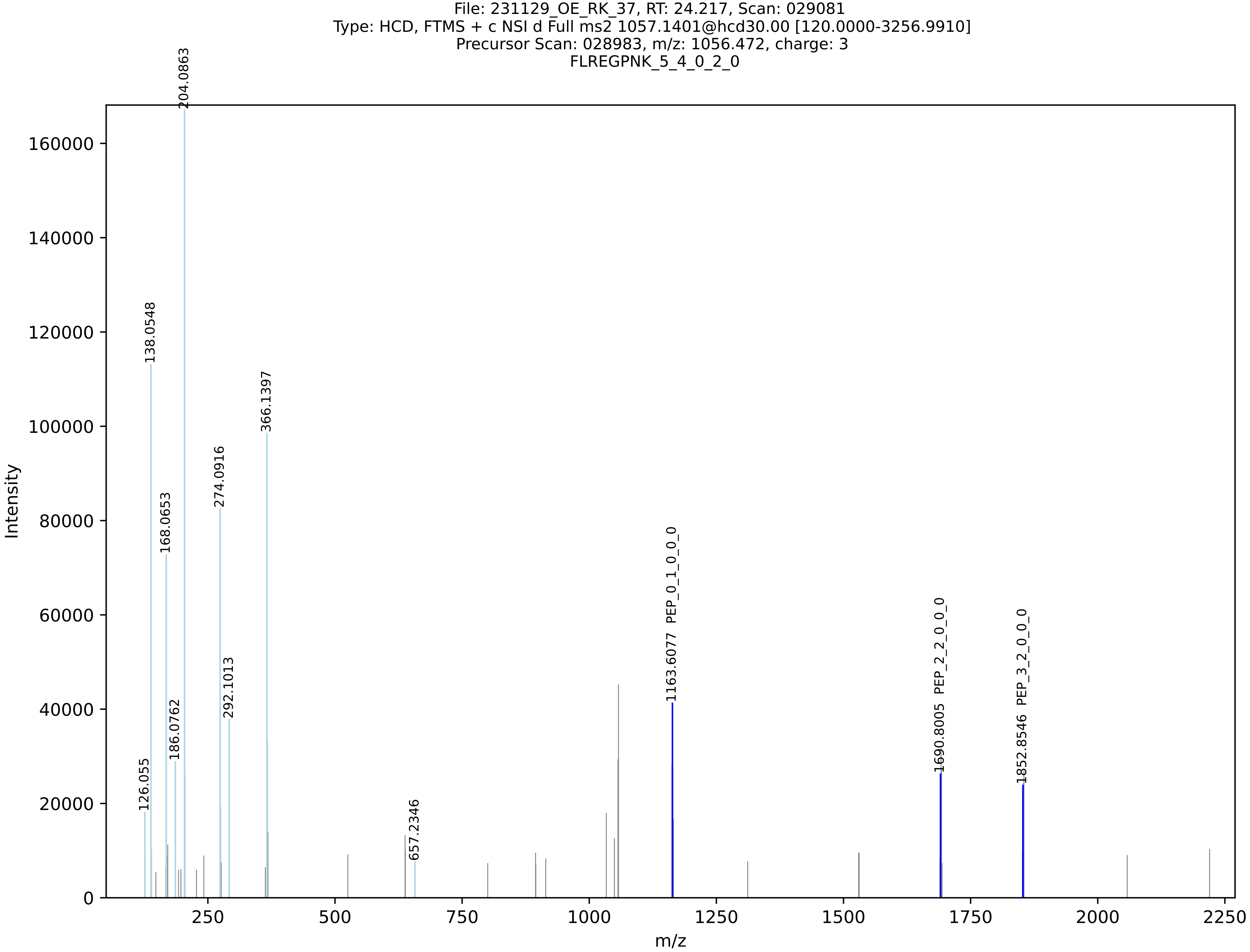

File: 231129_OE_RK_37, RT: 24.217, Scan: 029081
Type: HCD, FTMS + c NSI d Full ms2 1057.1401@hcd30.00 [120.0000-3256.9910]
Precursor Scan: 028983, m/z: 1056.472, charge: 3
FLREGPNK_5_4_0_2_0
Intensity
m/z
126.055
138.0548
168.0653
186.0762
204.0863
274.0916
292.1013
366.1397
657.2346
1163.6077 PEP_0_1_0_0_0
1690.8005 PEP_2_2_0_0_0
1852.8546 PEP_3_2_0_0_0

File: 231129_OE_RK_37, RT: 24.256, Scan: 029132
Type: HCD, FTMS + c NSI d Full ms2 1170.9340@hcd30.00 [120.0000-4800.2666]
Precursor Scan: 029057, m/z: 1170.4328, charge: 4
HCDGNVSSCGDHPSEGCFCPPDK_5_4_1_1_0

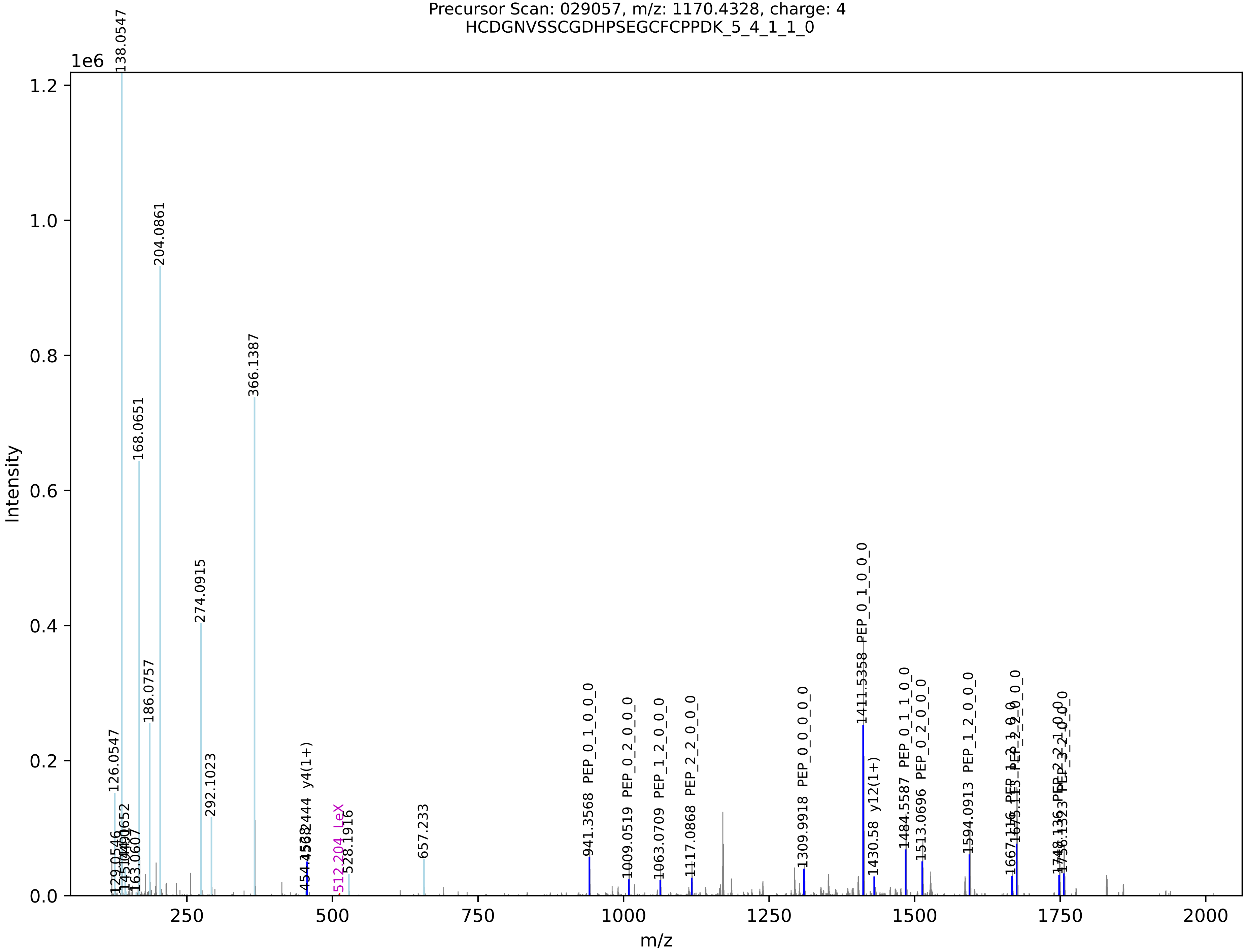

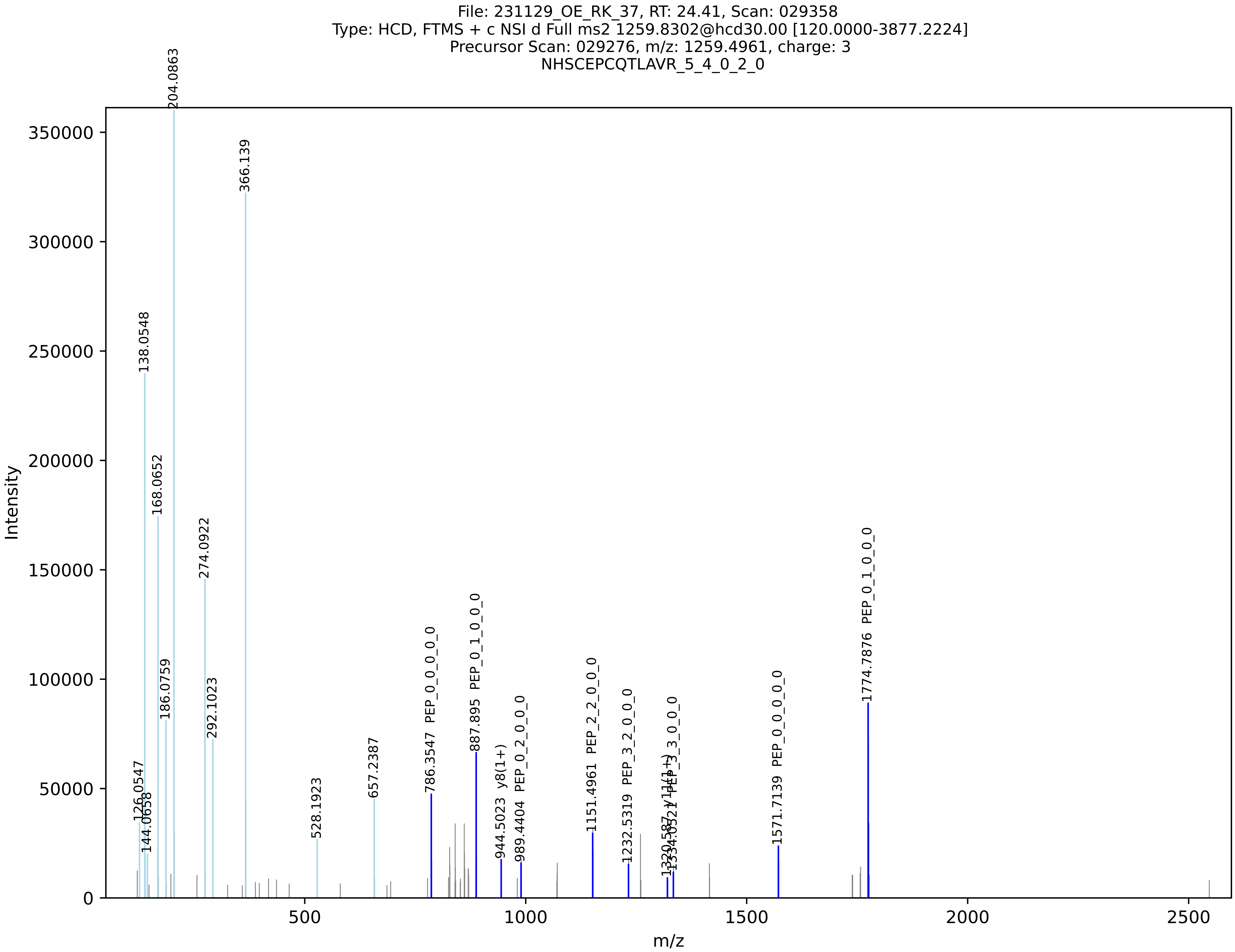
File: 231129_OE_RK_37, RT: 24.41, Scan: 029358
Type: HCD, FTMS + c NSI d Full ms2 1259.8302@hcd30.00 [120.0000-3877.2224]
Precursor Scan: 029276, m/z: 1259.4961, charge: 3
NHSCEPCQTLAVR_5_4_0_2_0
Intensity
m/z
204.0863
366.139
138.0548
168.0652
274.0922
186.0759
292.1023
126.0547
144.0658
528.1923
657.2387
786.3547 PEP_0_0_0_0_0
887.895 PEP_0_1_0_0_0
944.5023 y8(1+)
989.4404 PEP_0_2_0_0_0
1151.4961 PEP_2_2_0_0_0
1232.5319 PEP_3_2_0_0_0
1320.587 y11(1+)
1334.0521 PEP_3_0_0_0
1571.7139 PEP_0_0_0_0_0
1774.7876 PEP_0_1_0_0_0

File: 231129_OE_RK_37, RT: 24.412, Scan: 029361
Type: HCD, FTMS + c NSI d Full ms2 945.1251@hcd30.00 [120.0000-3878.9663]
Precursor Scan: 029276, m/z: 944.8739, charge: 4
NHSCEPCQTLAVR_5_4_0_2_0

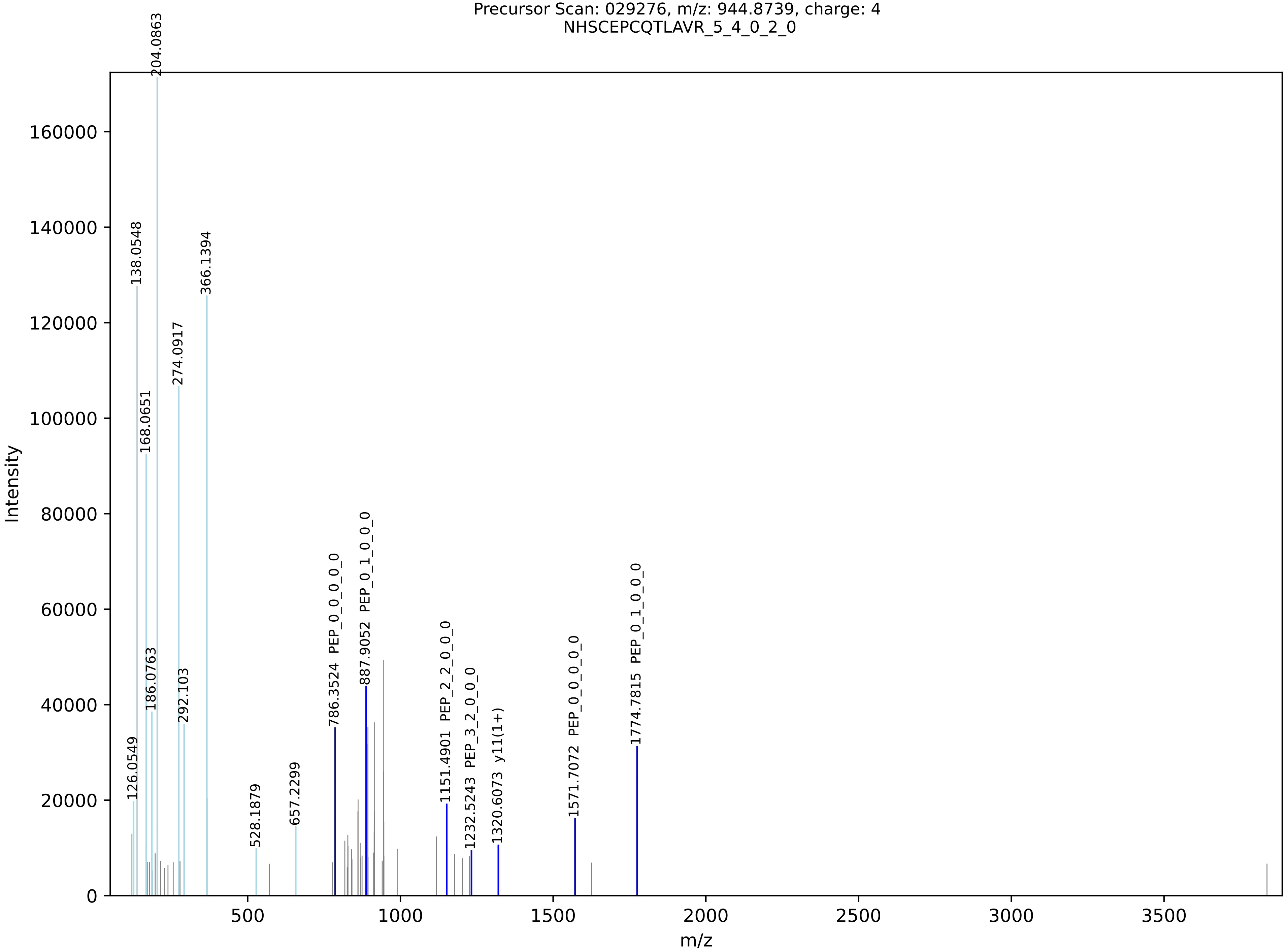

File: 231129_OE_RK_37, RT: 24.615, Scan: 029659
Type: HCD, FTMS + c NSI d Full ms2 1260.1654@hcd30.00 [120.0000-3878.2480]
Precursor Scan: 029572, m/z: 1259.4966, charge: 3
NHSCEPCQTLAVR_5_4_0_2_0

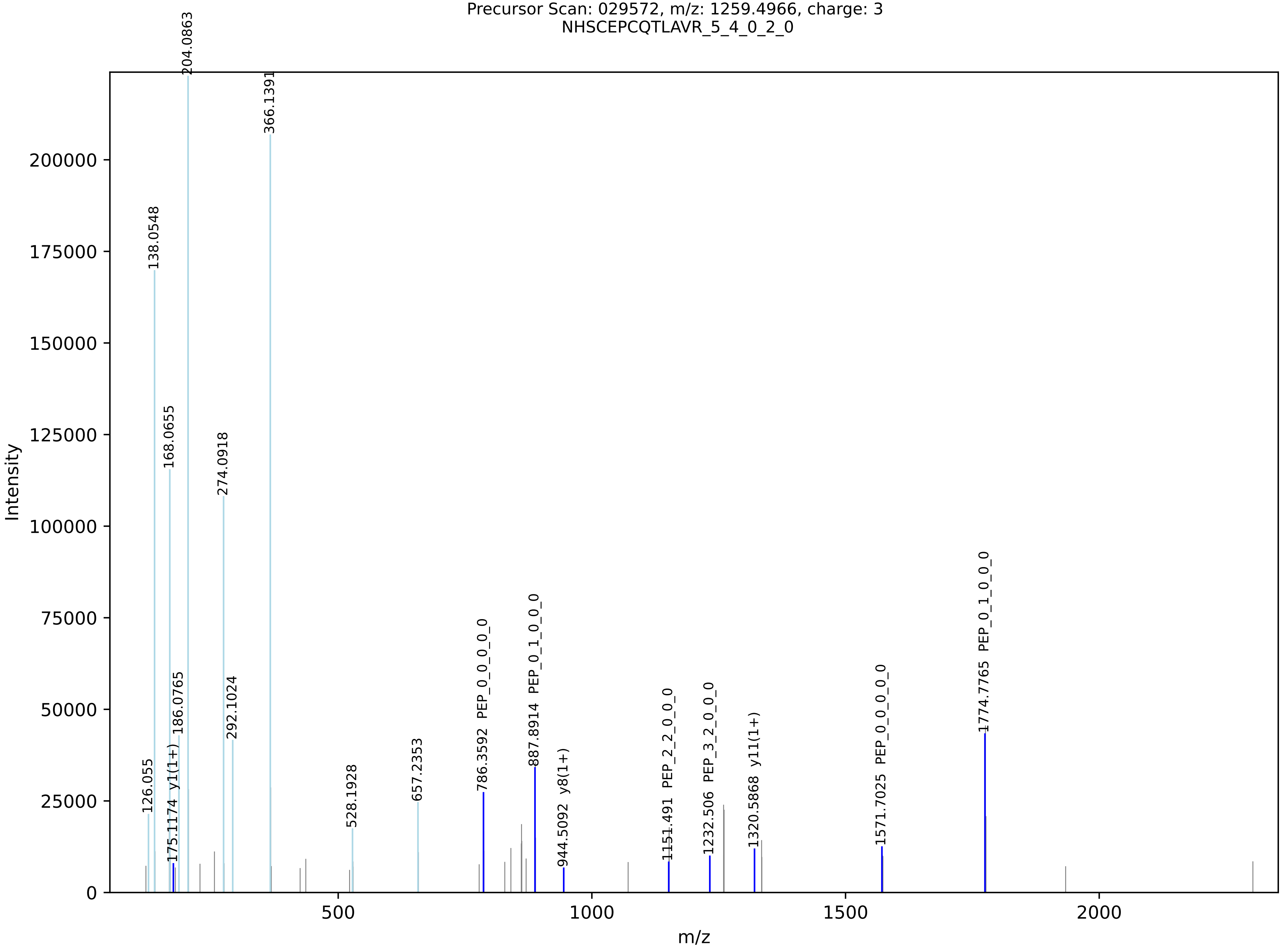

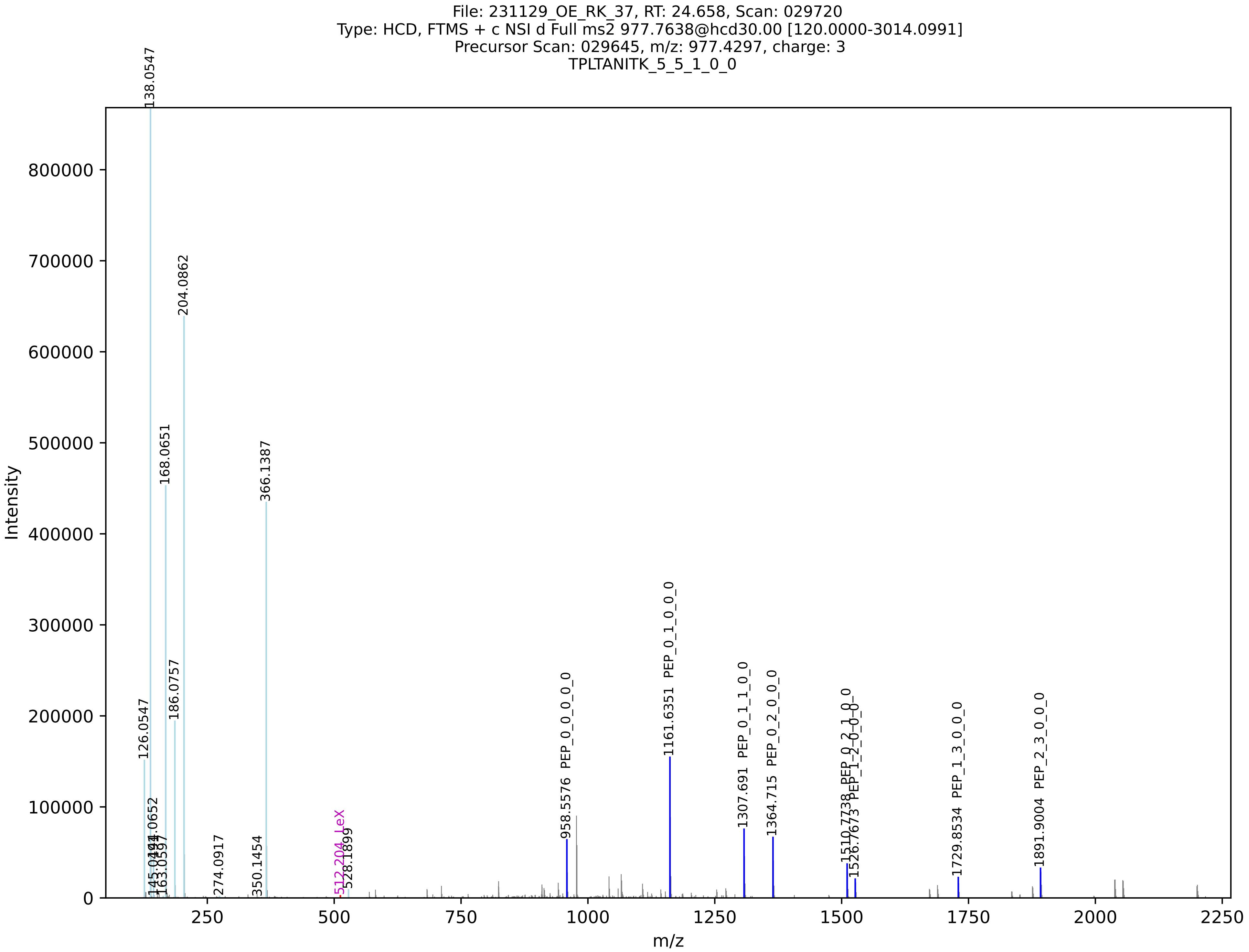
File: 231129_OE_RK_37, RT: 24.658, Scan: 029720
Type: HCD, FTMS + c NSI d Full ms2 977.7638@hcd30.00 [120.0000-3014.0991]
Precursor Scan: 029645, m/z: 977.4297, charge: 3
TPLTANITK_5_5_1_0_0
Intensity
m/z
0
100000
200000
300000
400000
500000
600000
700000
800000
250
500
750
1000
1250
1500
1750
2000
2250
138.0547
204.0862
168.0651
366.1387
186.0757
126.0547
163.0597
274.0917
350.1454
512.204_LeX
528.1899
958.5576 PEP_0_0_0_0_0
1161.6351 PEP_0_1_0_0_0
1307.691 PEP_0_1_1_0_0
1364.715 PEP_0_2_0_0_0
1510.7738 PEP_0_2_1_0_0
1526.7673 PEP_1_2_0_0_0
1729.8534 PEP_1_3_0_0_0
1891.9004 PEP_2_3_0_0_0

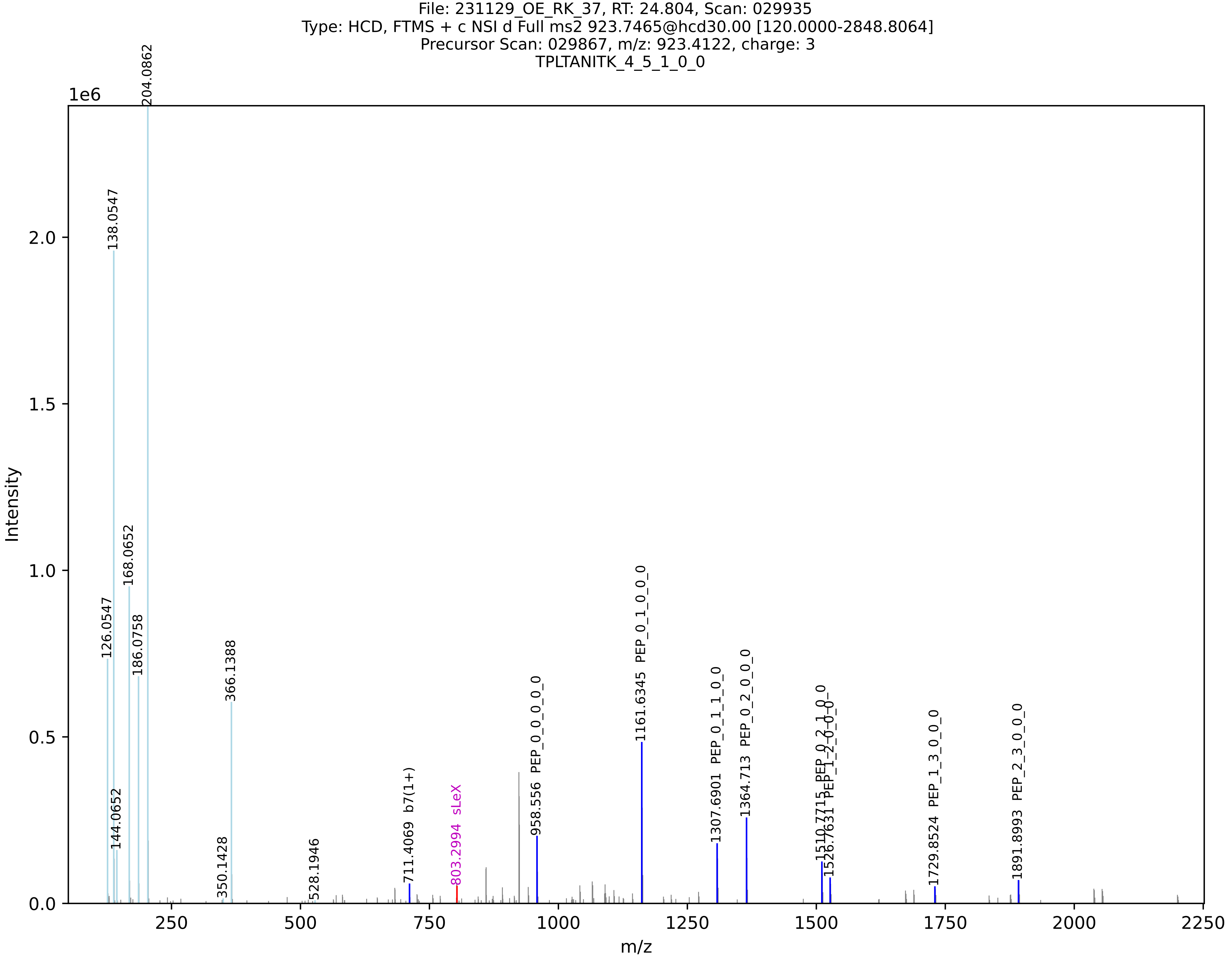
File: 231129_OE_RK_37, RT: 24.804, Scan: 029935
Type: HCD, FTMS + c NSI d Full ms2 923.7465@hcd30.00 [120.0000-2848.8064]
Precursor Scan: 029867, m/z: 923.4122, charge: 3
TPLTANITK_4_5_1_0_0
1e6
Intensity
m/z
204.0862
138.0547
168.0652
126.0547
186.0758
366.1388
144.0652
350.1428
528.1946
711.4069 b7(1+)
803.2994 sLeX
958.556 PEP_0_0_0_0_0
1161.6345 PEP_0_1_0_0_0
1307.6901 PEP_0_1_1_0_0
1364.713 PEP_0_2_0_0_0
1510.7715 PEP_0_2_1_0_0
1526.7631 PEP_1_2_0_0_0
1729.8524 PEP_1_3_0_0_0
1891.8993 PEP_2_3_0_0_0

File: 231129_OE_RK_37, RT: 24.81, Scan: 029944
Type: HCD, FTMS + c NSI d Full ms2 1159.4465@hcd30.00 [120.0000-3570.0483]
Precursor Scan: 029867, m/z: 1159.1124, charge: 3
ISEENETTCYMGK_5_4_0_1_0

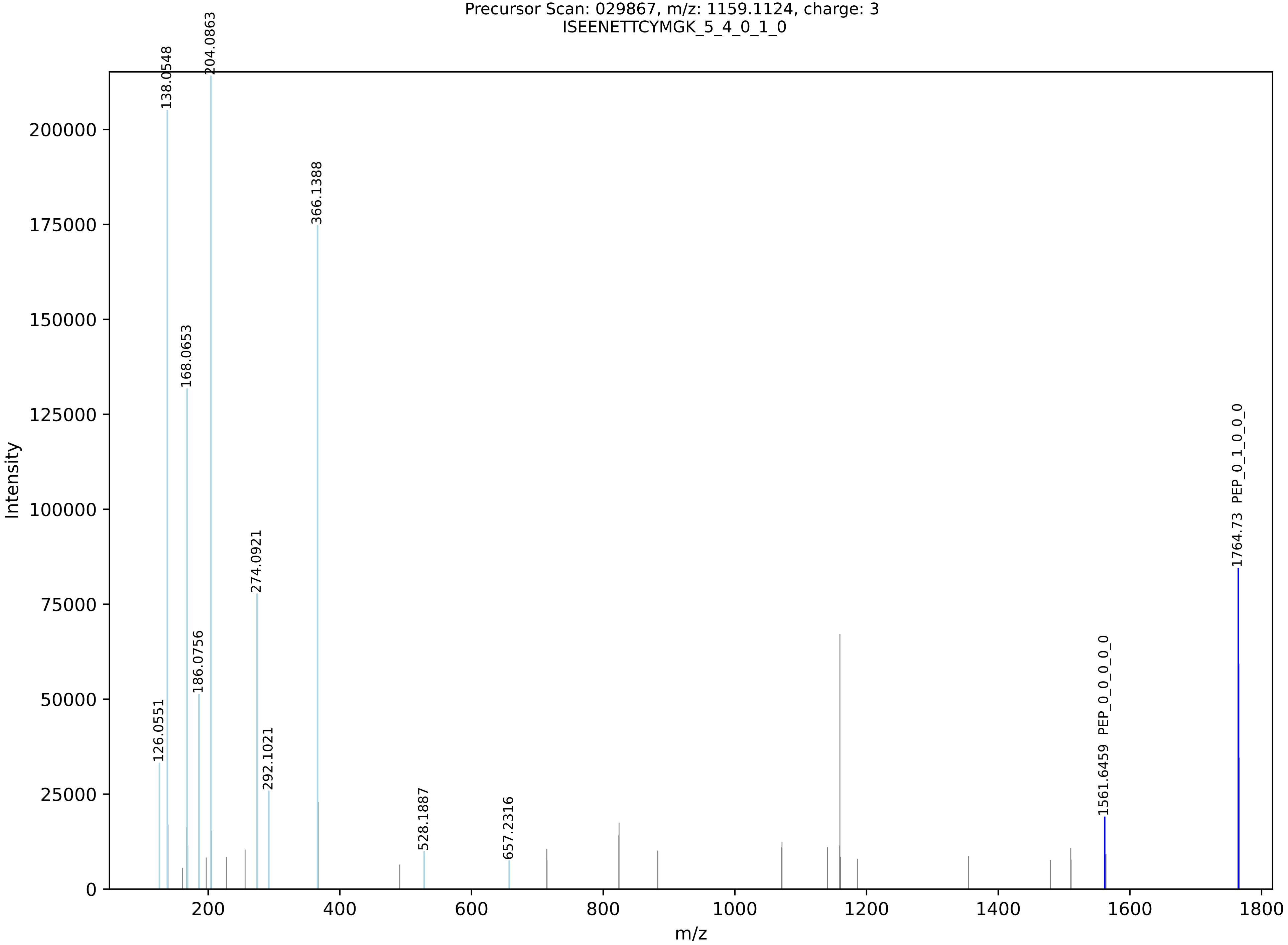

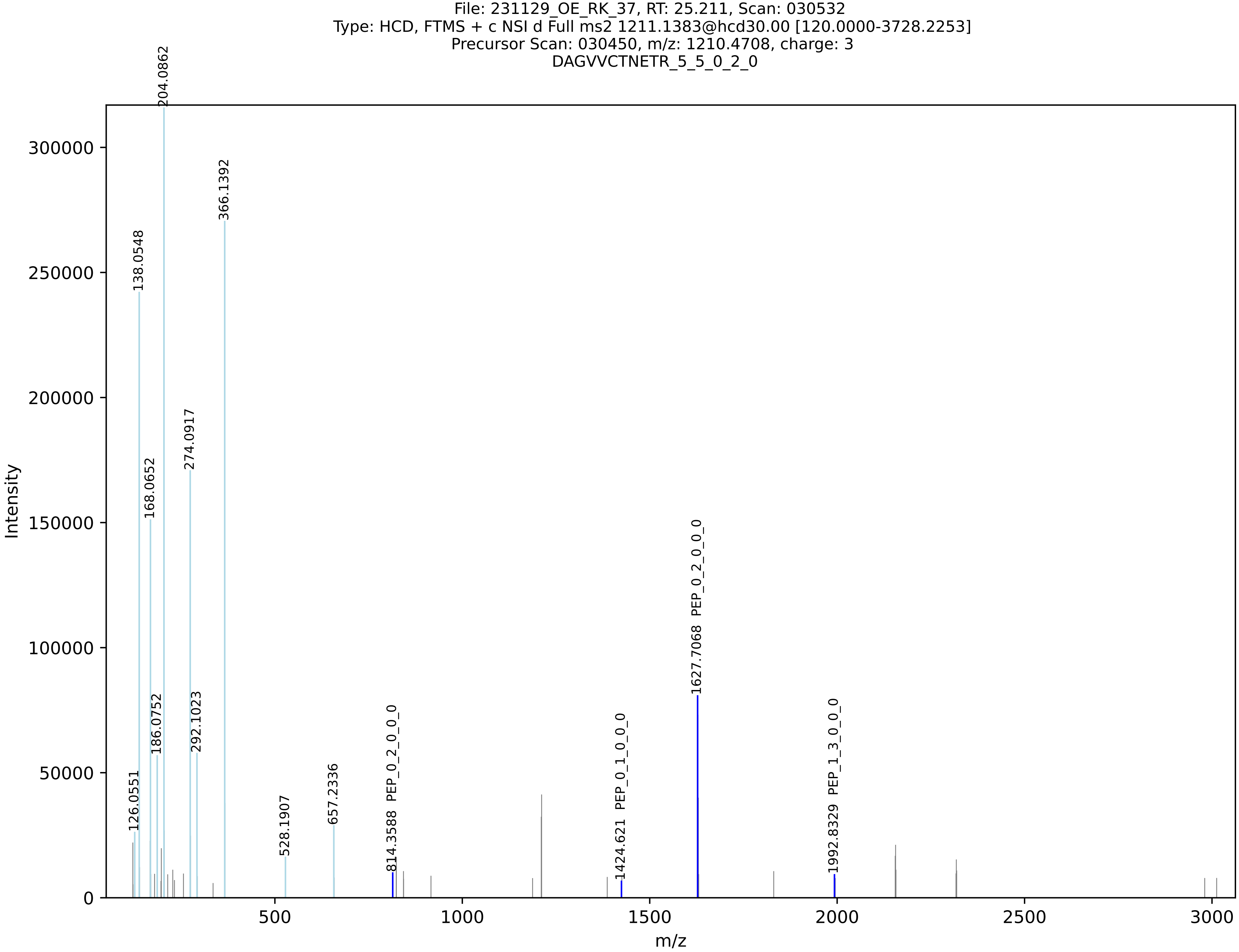
File: 231129_OE_RK_37, RT: 25.211, Scan: 030532
Type: HCD, FTMS + c NSI d Full ms2 1211.1383@hcd30.00 [120.0000-3728.2253]
Precursor Scan: 030450, m/z: 1210.4708, charge: 3
DAGVVCTNETR_5_5_0_2_0
204.0862
366.1392
138.0548
274.0917
168.0652
186.0752
292.1023
126.0551
528.1907
657.2336
814.3588 PEP_0_2_0_0_
1424.621 PEP_0_1_0_0_
1627.7068 PEP_0_2_0_0_
1992.8329 PEP_1_3_0_0_
Intensity
m/z
0
50000
100000
150000
200000
250000
300000
500
1000
1500
2000
2500
3000

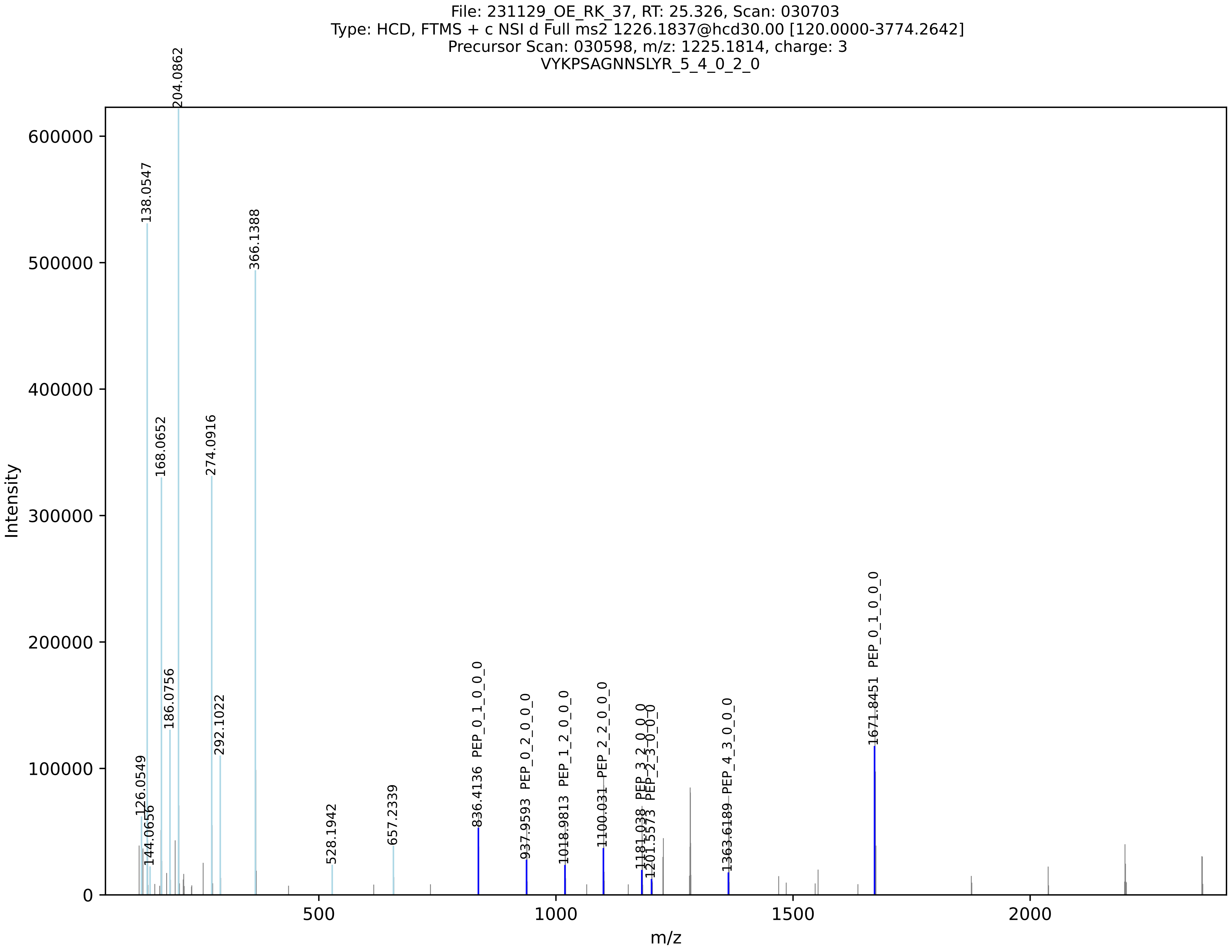
File: 231129_OE_RK_37, RT: 25.326, Scan: 030703
Type: HCD, FTMS + c NSI d Full ms2 1226.1837@hcd30.00 [120.0000-3774.2642]
Precursor Scan: 030598, m/z: 1225.1814, charge: 3
VYKPSAGNNSLYR_5_4_0_2_0
Intensity
m/z
204.0862
138.0547
366.1388
168.0652
274.0916
186.0756
292.1022
126.0549
144.0656
528.1942
657.2339
836.4136 PEP_0_1_0_0_0
937.9593 PEP_0_2_0_0_0
1018.9813 PEP_1_2_0_0_0
1100.031 PEP_2_2_0_0_0
1181.038 PEP_3_2_0_0_0
1201.5573 PEP_2_3_0_0_0
1363.6189 PEP_4_3_0_0_0
1671.8451 PEP_0_1_0_0_0

File: 231129_OE_RK_37, RT: 25.362, Scan: 030753
Type: HCD, FTMS + c NSI d Full ms2 1274.5350@hcd30.00 [120.0000-3922.2192]
Precursor Scan: 030672, m/z: 1273.8671, charge: 3
VYKPSAGNNSLYR_5_4_1_2_0

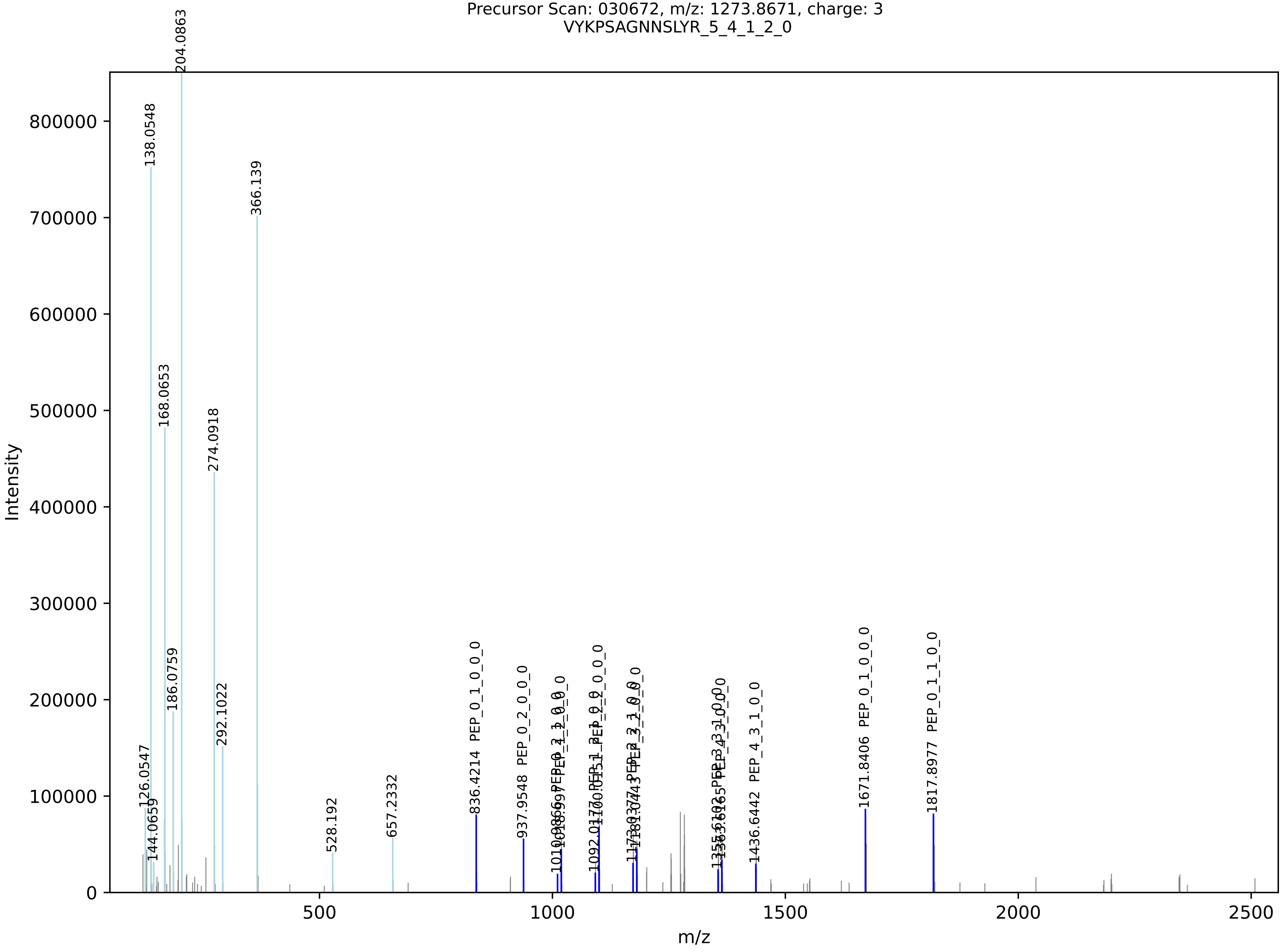

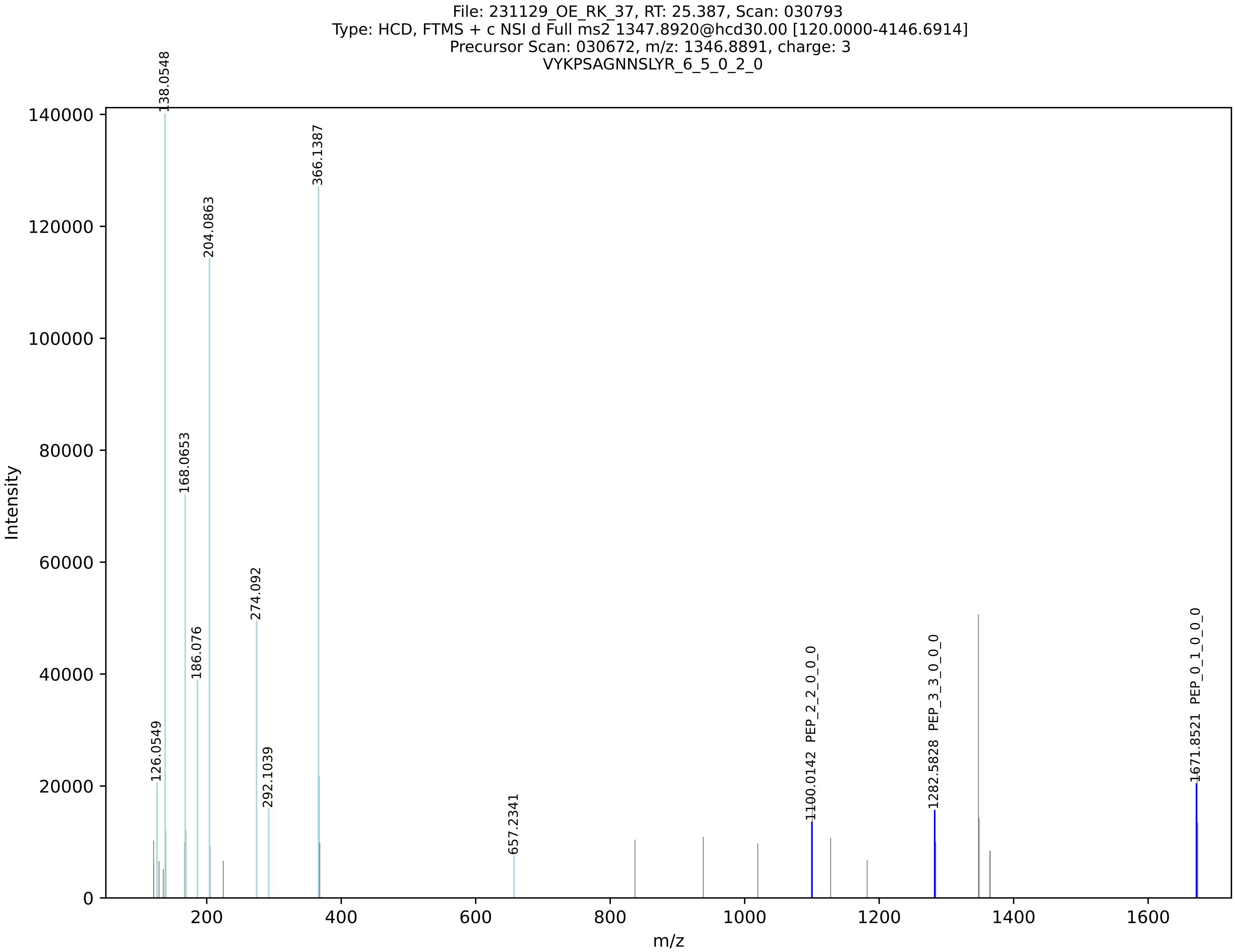

File: 231129_OE_RK_37, RT: 25.387, Scan: 030793
Type: HCD, FTMS + c NSI d Full ms2 1347.8920@hcd30.00 [120.0000-4146.6914]
Precursor Scan: 030672, m/z: 1346.8891, charge: 3
VYKPSAGNNSLYR_6_5_0_2_0
Intensity
m/z
138.0548
366.1387
204.0863
168.0653
186.076
274.092
126.0549
292.1039
657.2341
1100.0142 PEP_2_2_0_0_0
1282.5828 PEP_3_3_0_0_0
1671.8521 PEP_0_1_0_0_0

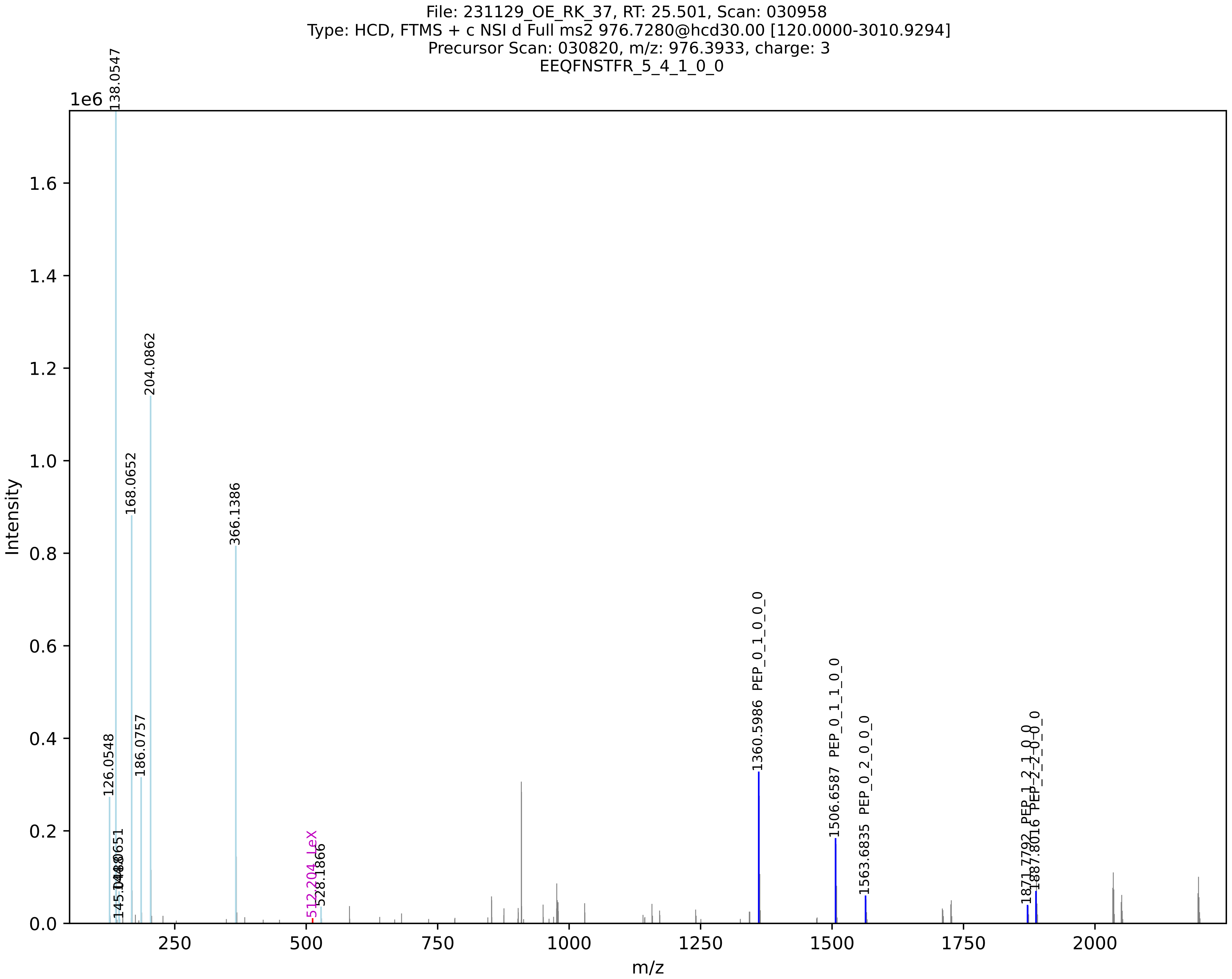

File: 231129_OE_RK_37, RT: 25.501, Scan: 030958
Type: HCD, FTMS + c NSI d Full ms2 976.7280@hcd30.00 [120.0000-3010.9294]
Precursor Scan: 030820, m/z: 976.3933, charge: 3
EEQFNSTFR_5_4_1_0_0
1e6
Intensity
m/z
138.0547
204.0862
168.0652
366.1386
186.0757
126.0548
512.204_LeX
528.1866
1360.5986 PEP_0_1_0_0_0
1506.6587 PEP_0_1_1_0_0
1563.6835 PEP_0_2_0_0_0
1871.7792 PEP_1_2_1_0_0
1887.8016 PEP_2_2_0_0_0

File: 231129_OE_RK_37, RT: 25.531, Scan: 031000
Type: HCD, FTMS + c NSI d Full ms2 1464.0837@hcd30.00 [120.0000-3008.1587]
Precursor Scan: 030891, m/z: 1463.5855, charge: 2
EEQFNSTFR_5_4_1_0_0

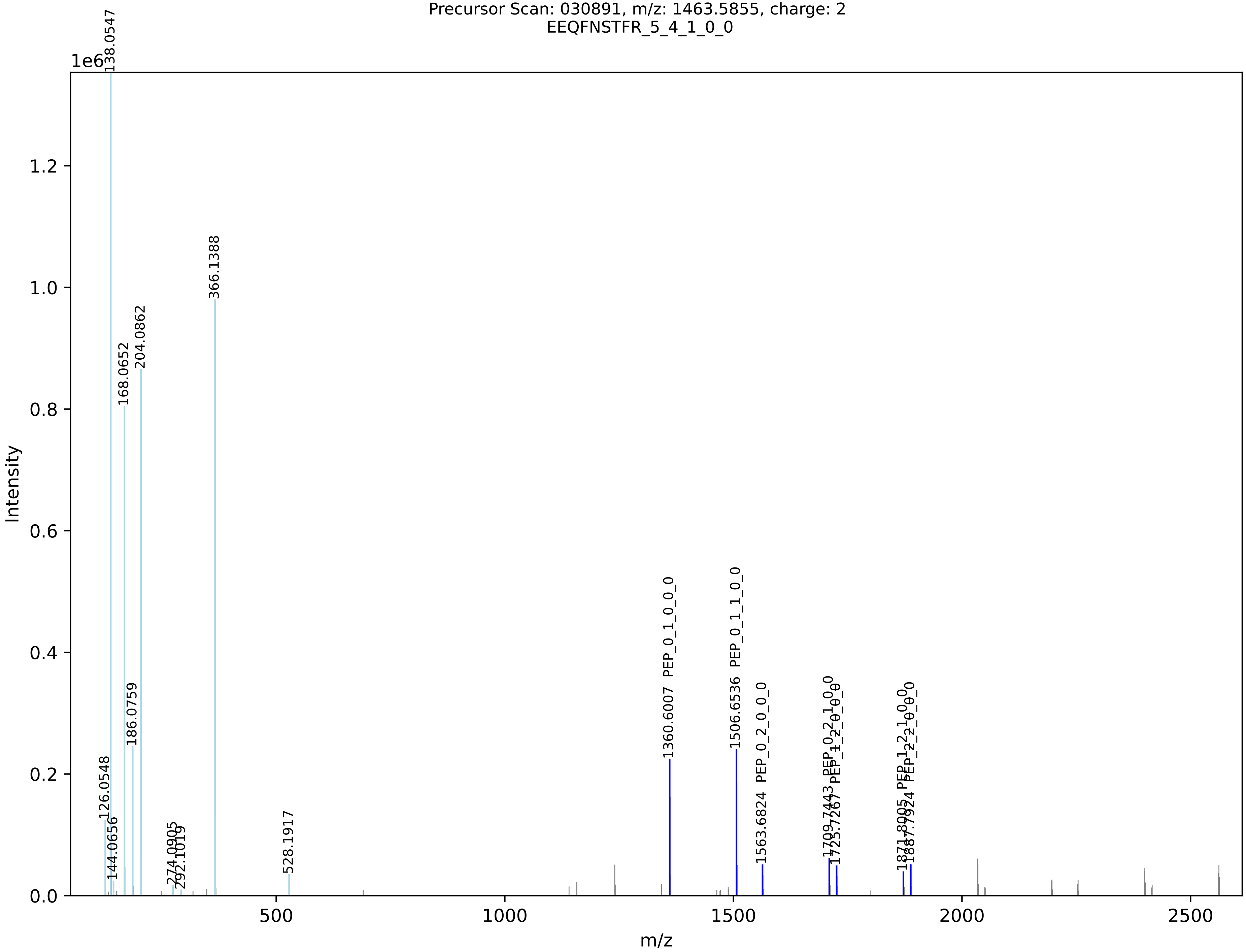

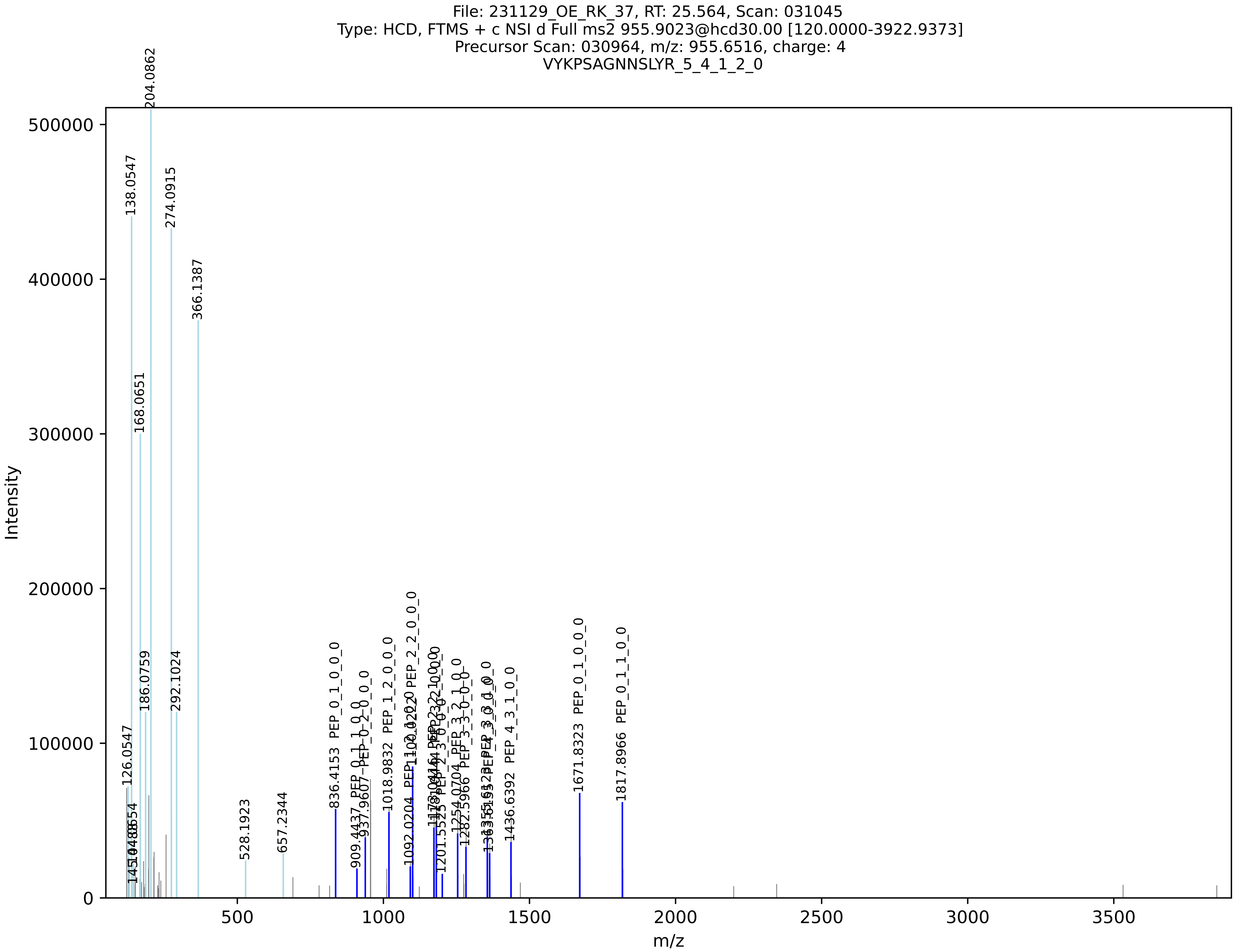
File: 231129_OE_RK_37, RT: 25.564, Scan: 031045
Type: HCD, FTMS + c NSI d Full ms2 955.9023@hcd30.00 [120.0000-3922.9373]
Precursor Scan: 030964, m/z: 955.6516, charge: 4
VYKPSAGNNSLYR_5_4_1_2_0
Intensity
m/z
204.0862
138.0547
274.0915
366.1387
168.0651
186.0759
292.1024
126.0547
528.1923
657.2344
836.4153 PEP_0_1_0_0_0
1018.9832 PEP_1_2_0_0_0
1671.8323 PEP_0_1_0_0_0
1817.8966 PEP_0_1_1_0_0
1436.6392 PEP_4_3_1_0_0

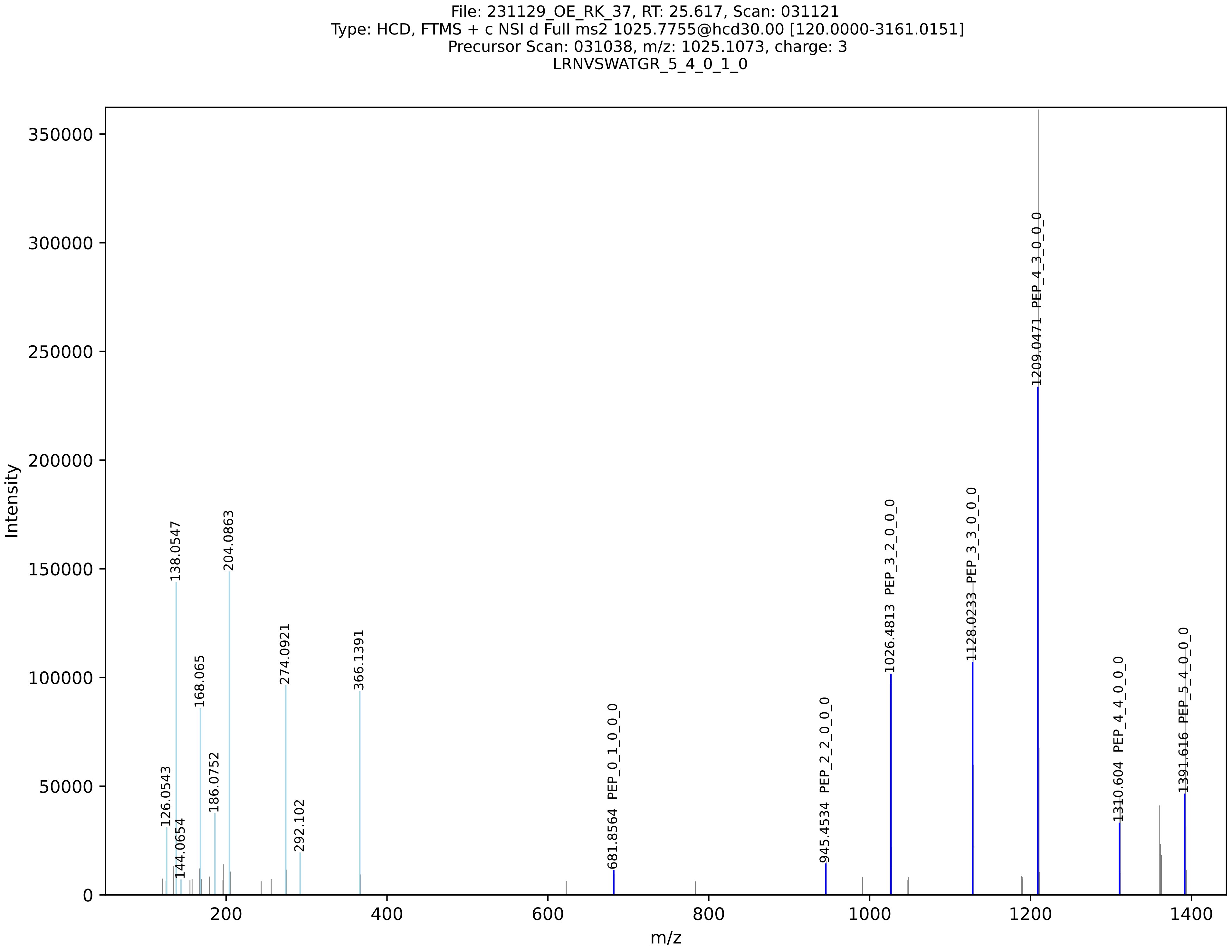
File: 231129_OE_RK_37, RT: 25.617, Scan: 031121
Type: HCD, FTMS + c NSI d Full ms2 1025.7755@hcd30.00 [120.0000-3161.0151]
Precursor Scan: 031038, m/z: 1025.1073, charge: 3
LRNVSWATGR_5_4_0_1_0
Intensity
m/z
126.0543
138.0547
144.0654
168.065
186.0752
204.0863
274.0921
292.102
366.1391
681.8564 PEP_0_1_0_0_0
945.4534 PEP_2_2_0_0_0
1026.4813 PEP_3_2_0_0_0
1128.0233 PEP_3_3_0_0_0
1209.0471 PEP_4_3_0_0_0
1310.604 PEP_4_4_0_0_0
1391.616 PEP_5_4_0_0_0

File: 231129_OE_RK_37, RT: 25.713, Scan: 031259
Type: HCD, FTMS + c NSI d Full ms2 976.3930@hcd30.00 [120.0000-3009.9045]
Precursor Scan: 031183, m/z: 976.0589, charge: 3
EEQFNSTFR_5_4_1_0_0

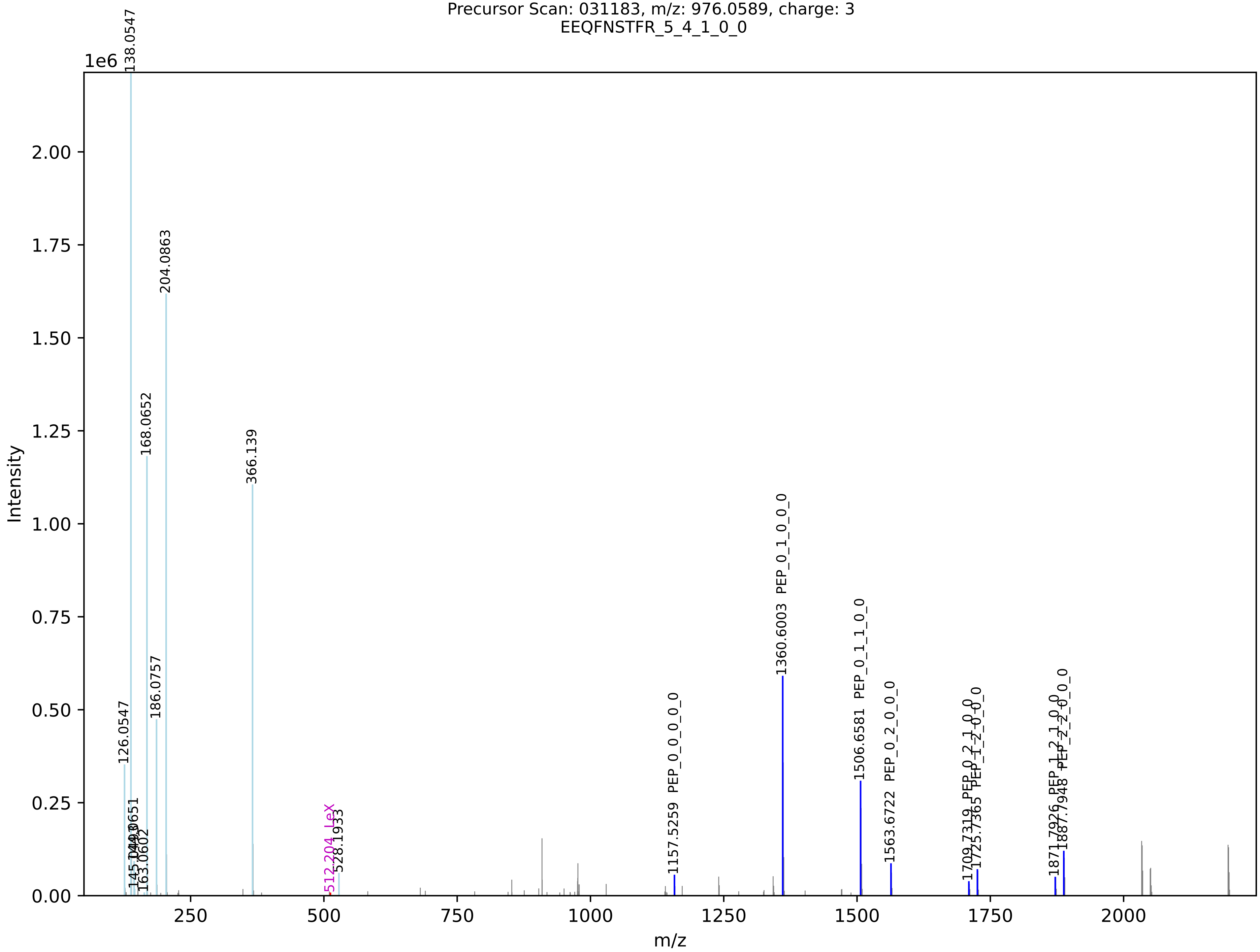

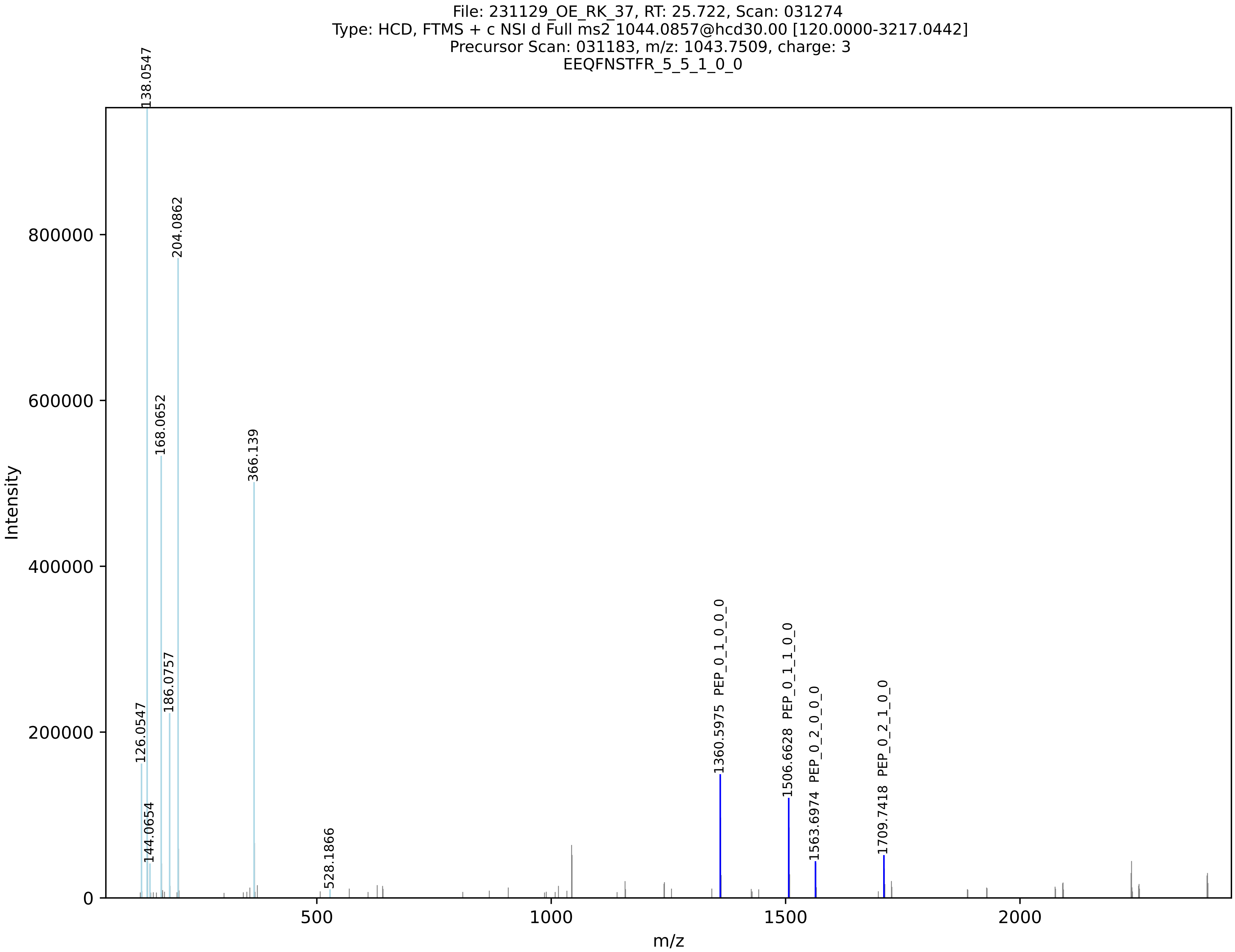

File: 231129_OE_RK_37, RT: 25.722, Scan: 031274
Type: HCD, FTMS + c NSI d Full ms2 1044.0857@hcd30.00 [120.0000-3217.0442]
Precursor Scan: 031183, m/z: 1043.7509, charge: 3
EEQFNSTFR_5_5_1_0_0
Intensity
m/z
138.0547
204.0862
168.0652
366.139
186.0757
126.0547
144.0654
528.1866
1360.5975 PEP_0_1_0_0_0
1506.6628 PEP_0_1_1_0_0
1563.6974 PEP_0_2_0_0_0
1709.7418 PEP_0_2_1_0_0

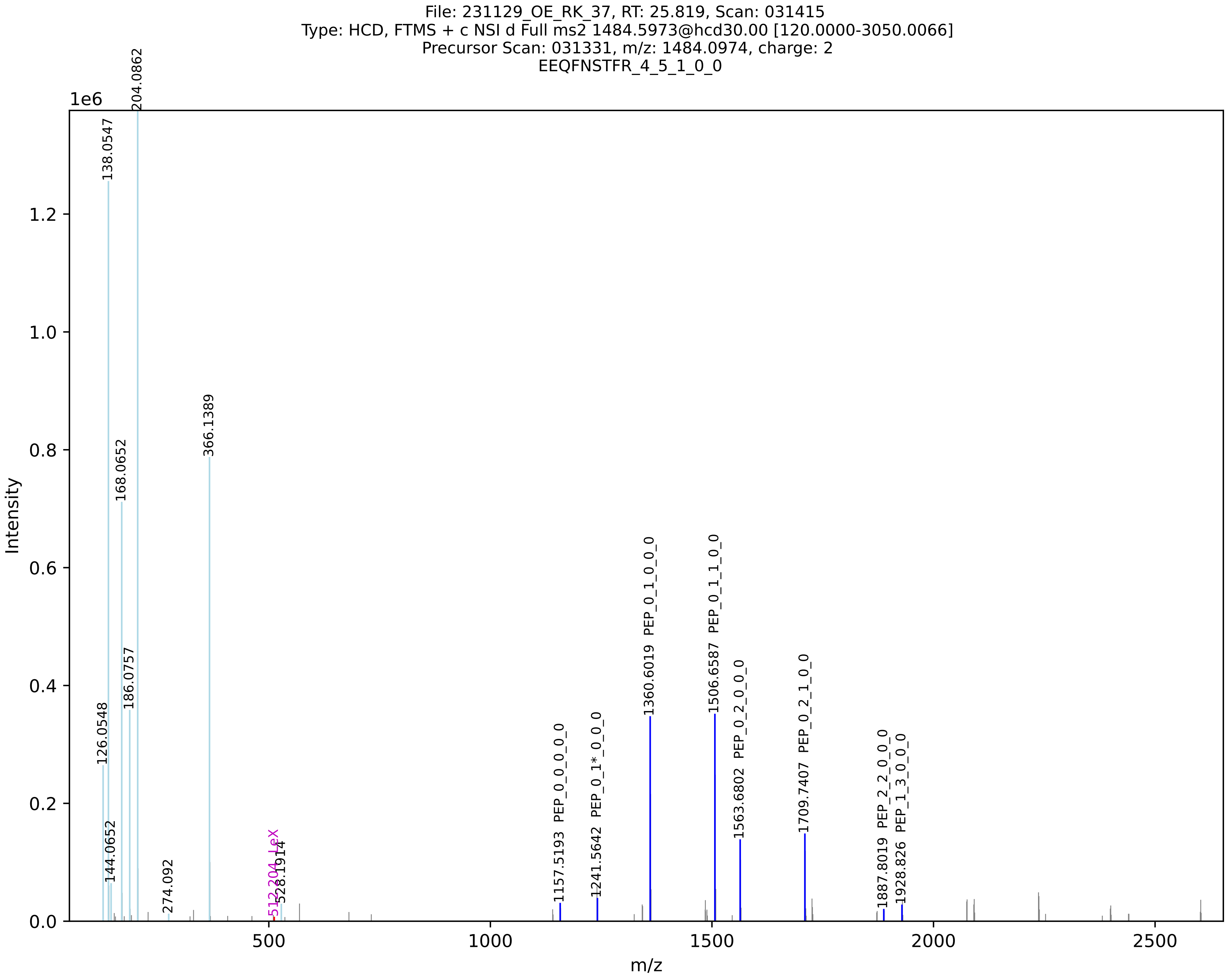
File: 231129_OE_RK_37, RT: 25.819, Scan: 031415
Type: HCD, FTMS + c NSI d Full ms2 1484.5973@hcd30.00 [120.0000-3050.0066]
Precursor Scan: 031331, m/z: 1484.0974, charge: 2
EEQFNSTFR_4_5_1_0_0
1e6
Intensity
m/z
204.0862
138.0547
366.1389
168.0652
186.0757
126.0548
144.0652
274.092
512.204_LeX
528.1914
1157.5193 PEP_0_0_0_0_
1241.5642 PEP_0_1*_0_0_0
1360.6019 PEP_0_1_0_0_0
1506.6587 PEP_0_1_1_0_0
1563.6802 PEP_0_2_0_0_0
1709.7407 PEP_0_2_1_0_0
1887.8019 PEP_2_2_0_0_0
1928.826 PEP_1_3_0_0_0

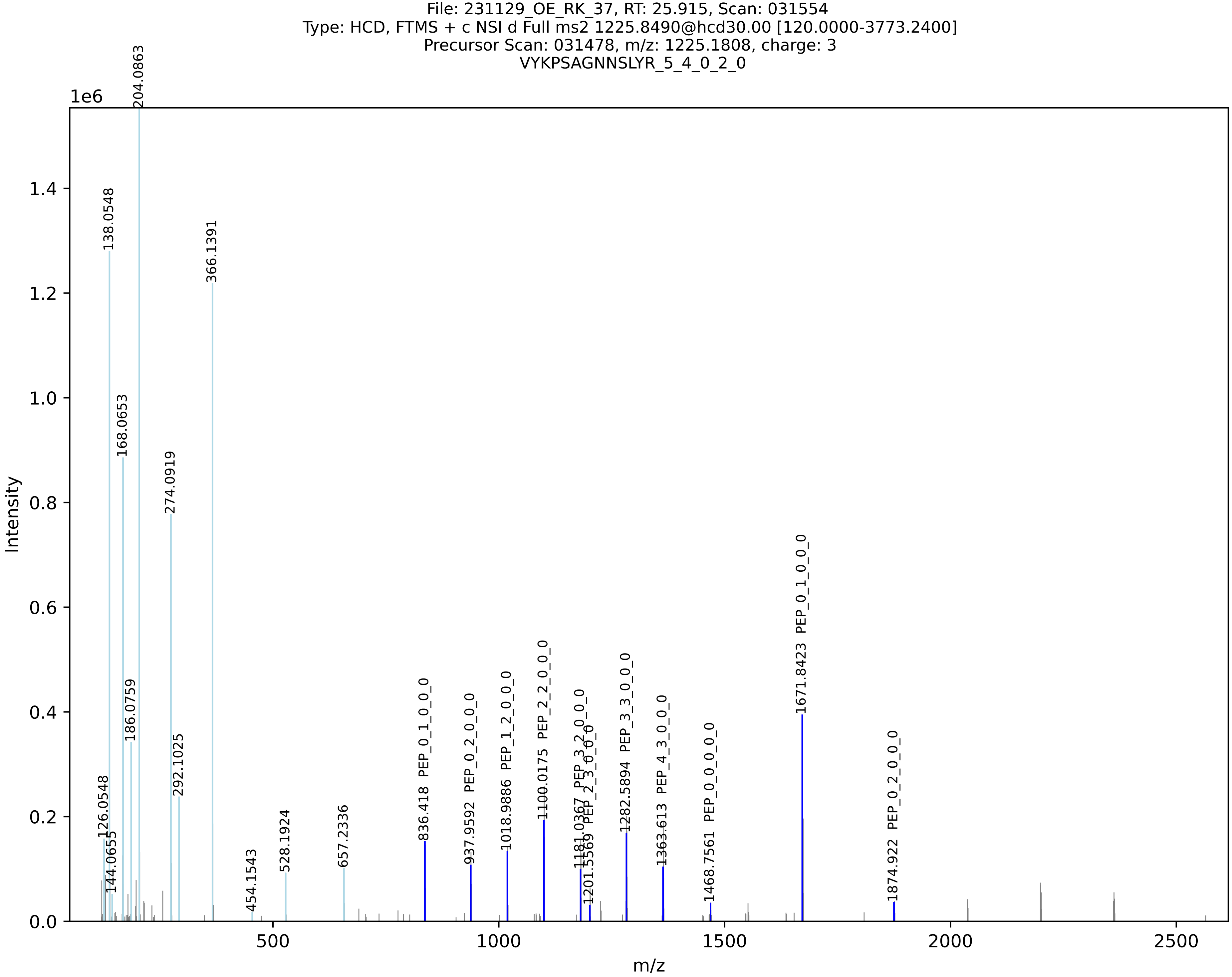
File: 231129_OE_RK_37, RT: 25.915, Scan: 031554
Type: HCD, FTMS + c NSI d Full ms2 1225.8490@hcd30.00 [120.0000-3773.2400]
Precursor Scan: 031478, m/z: 1225.1808, charge: 3
VYKPSAGNNSLYR_5_4_0_2_0
1e6
Intensity
m/z
204.0863
138.0548
366.1391
168.0653
274.0919
186.0759
292.1025
126.0548
144.0655
454.1543
528.1924
657.2336
836.418 PEP_0_1_0_0_0
937.9592 PEP_0_2_0_0_0
1018.9886 PEP_1_2_0_0_0
1100.0175 PEP_2_2_0_0_0
1181.0367 PEP_3_2_0_0_0
1201.5569 PEP_2_3_0_0_0
1282.5894 PEP_3_3_0_0_0
1363.613 PEP_4_3_0_0_0
1468.7561 PEP_0_0_0_0_0
1671.8423 PEP_0_1_0_0_0
1874.922 PEP_0_2_0_0_0

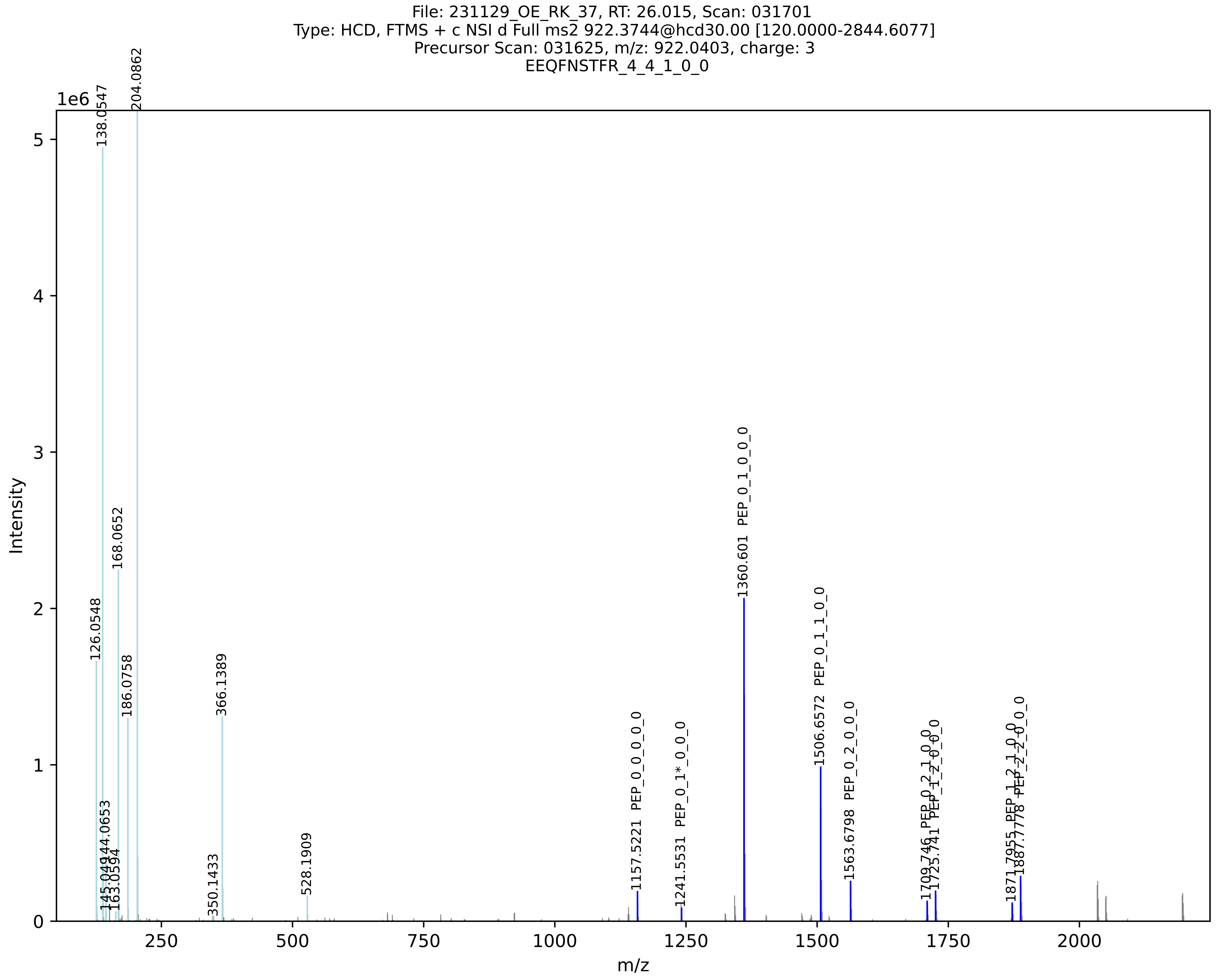
File: 231129_OE_RK_37, RT: 26.015, Scan: 031701
Type: HCD, FTMS + c NSI d Full ms2 922.3744@hcd30.00 [120.0000-2844.6077]
Precursor Scan: 031625, m/z: 922.0403, charge: 3
EEQFNSTFR_4_4_1_0_0
1e6
Intensity
m/z
204.0862
138.0547
168.0652
126.0548
186.0758
366.1389
144.0653
145.0494
163.0594
350.1433
528.1909
1157.5221 PEP_0_0_0_0_0
1241.5531 PEP_0_1*_0_0_0
1360.601 PEP_0_1_0_0_0
1506.6572 PEP_0_1_1_0_0
1563.6798 PEP_0_2_0_0_0
1709.746 PEP_0_2_1_0_0
1725.741 PEP_1_2_0_0_0
1871.7955 PEP_1_2_1_0_0
1887.7778 PEP_2_2_0_0_0

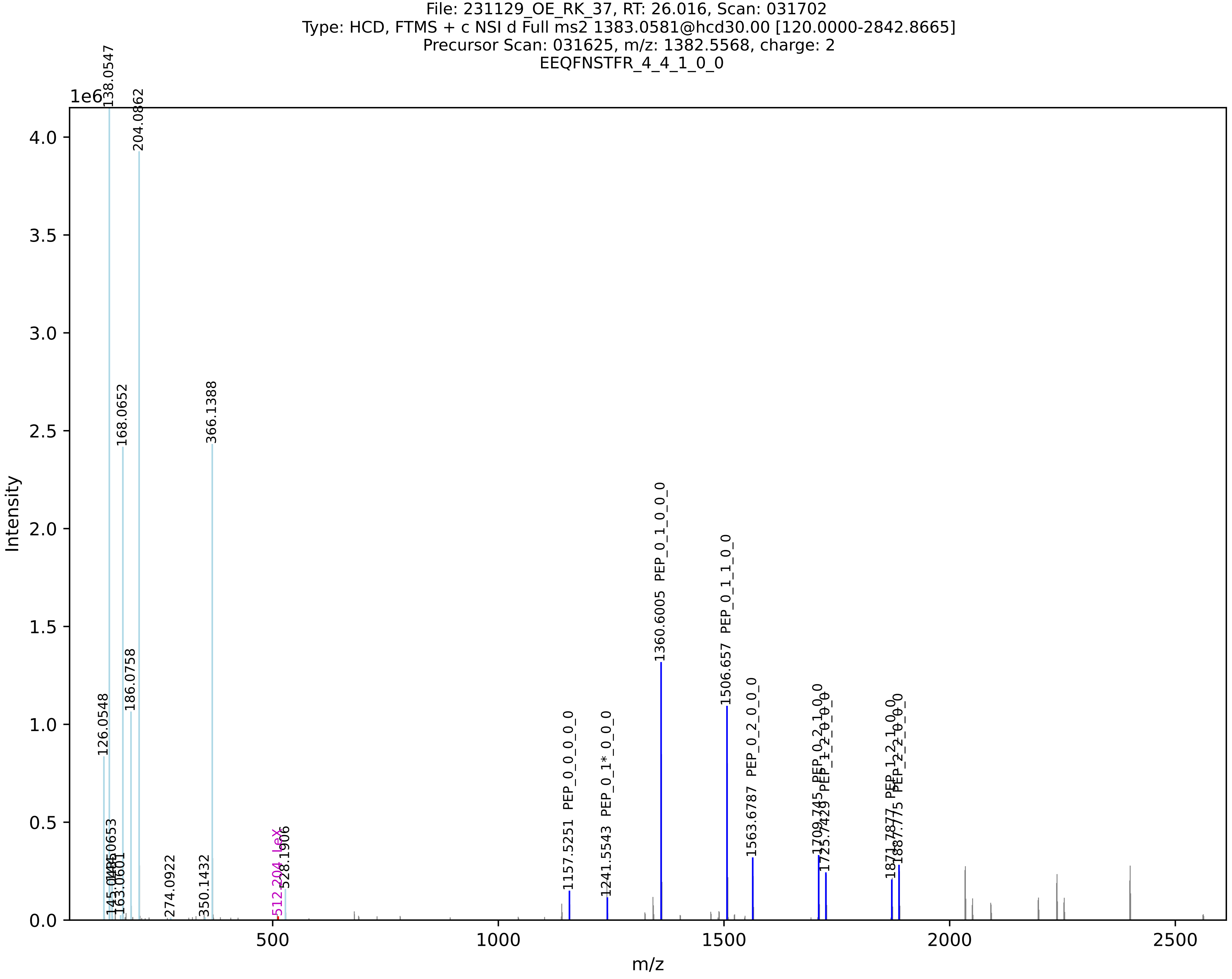
File: 231129_OE_RK_37, RT: 26.016, Scan: 031702
Type: HCD, FTMS + c NSI d Full ms2 1383.0581@hcd30.00 [120.0000-2842.8665]
Precursor Scan: 031625, m/z: 1382.5568, charge: 2
EEQFNSTFR_4_4_1_0_0
1e6
Intensity
m/z
138.0547
204.0862
168.0652
366.1388
186.0758
126.0548
274.0922
350.1432
512.204_LeX
528.1906
1157.5251 PEP_0_0_0_0_0
1241.5543 PEP_0_1*_0_0_0
1360.6005 PEP_0_1_0_0_0
1506.657 PEP_0_1_1_0_0
1563.6787 PEP_0_2_0_0_0
1709.745 PEP_0_2_1_0_0
1725.7429 PEP_1_2_0_0_0
1871.7877 PEP_1_2_1_0_0
1887.775 PEP_2_2_0_0_0

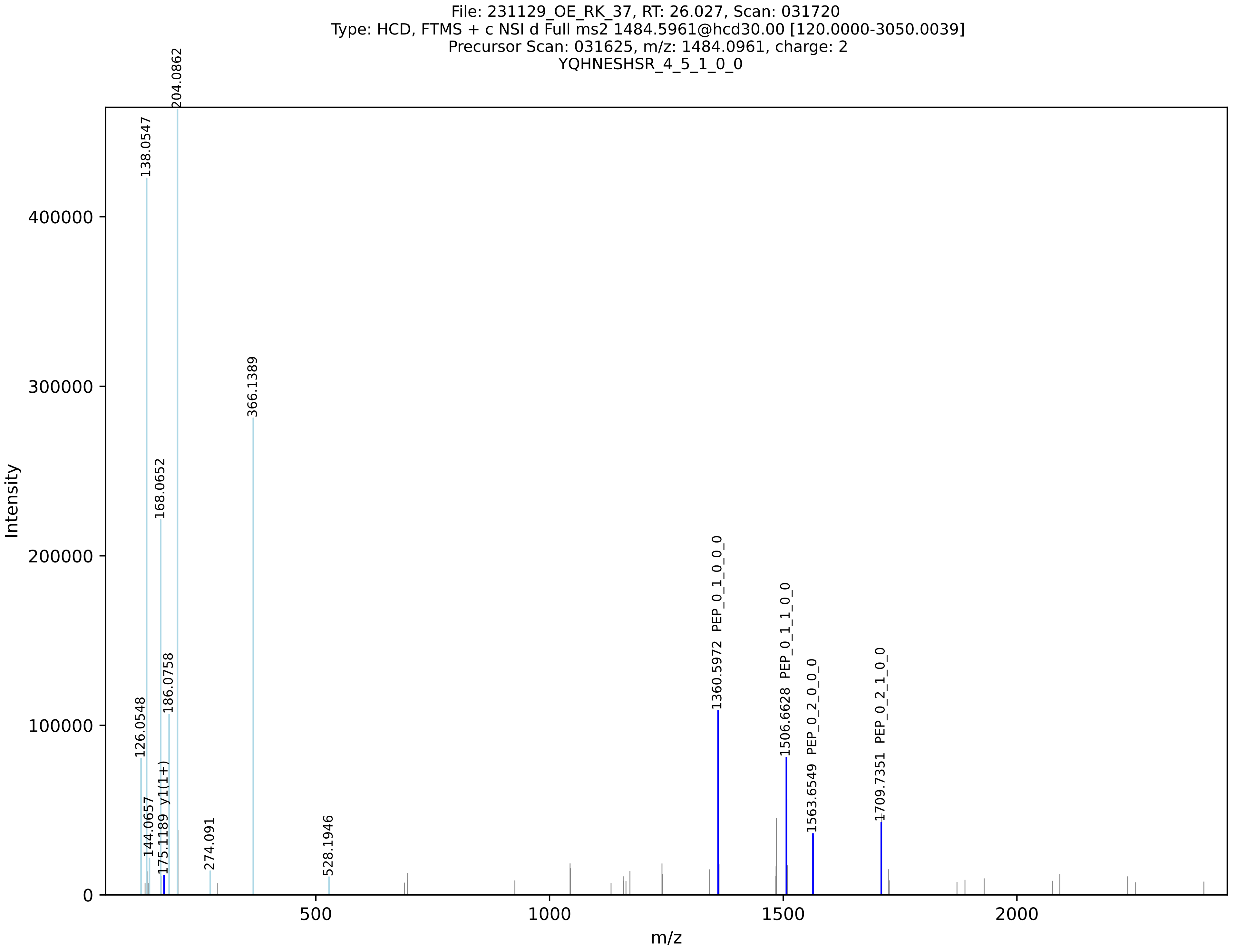

File: 231129_OE_RK_37, RT: 26.027, Scan: 031720
Type: HCD, FTMS + c NSI d Full ms2 1484.5961@hcd30.00 [120.0000-3050.0039]
Precursor Scan: 031625, m/z: 1484.0961, charge: 2
YQHNESHSR_4_5_1_0_0
204.0862
138.0547
366.1389
168.0652
186.0758
126.0548
144.0657
175.1189 y1(1+)
274.091
528.1946
1360.5972 PEP_0_1_0_0_0
1506.6628 PEP_0_1_1_0_0
1563.6549 PEP_0_2_0_0_0
1709.7351 PEP_0_2_1_0_0
Intensity
m/z
0
100000
200000
300000
400000
500
1000
1500
2000

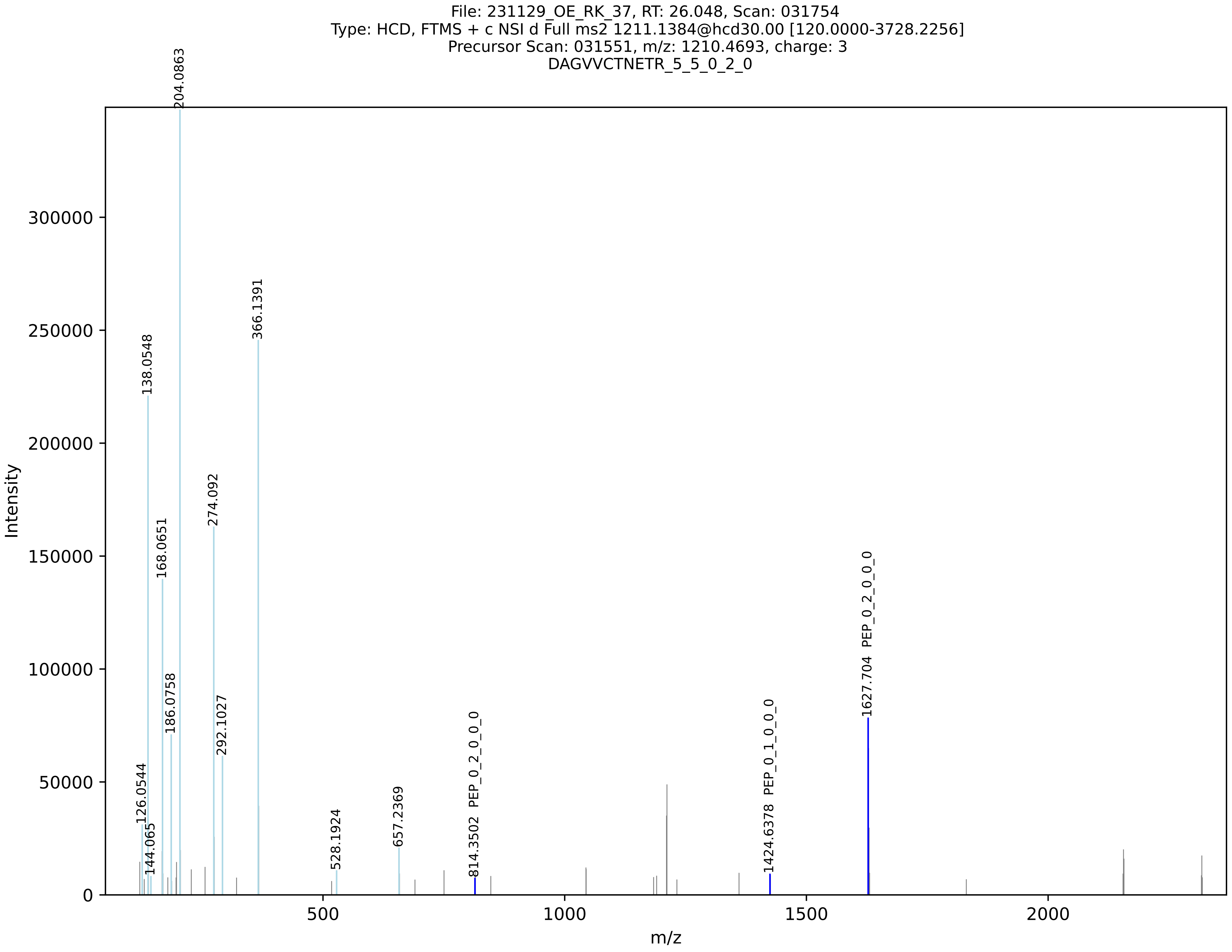
File: 231129_OE_RK_37, RT: 26.048, Scan: 031754
Type: HCD, FTMS + c NSI d Full ms2 1211.1384@hcd30.00 [120.0000-3728.2256]
Precursor Scan: 031551, m/z: 1210.4693, charge: 3
DAGVVCTNETR_5_5_0_2_0
Intensity
m/z
0
50000
100000
150000
200000
250000
300000
500
1000
1500
2000
204.0863
366.1391
138.0548
274.092
168.0651
186.0758
292.1027
126.0544
144.065
528.1924
657.2369
814.3502 PEP_0_2_0_0_
1424.6378 PEP_0_1_0_0_
1627.704 PEP_0_2_0_0_

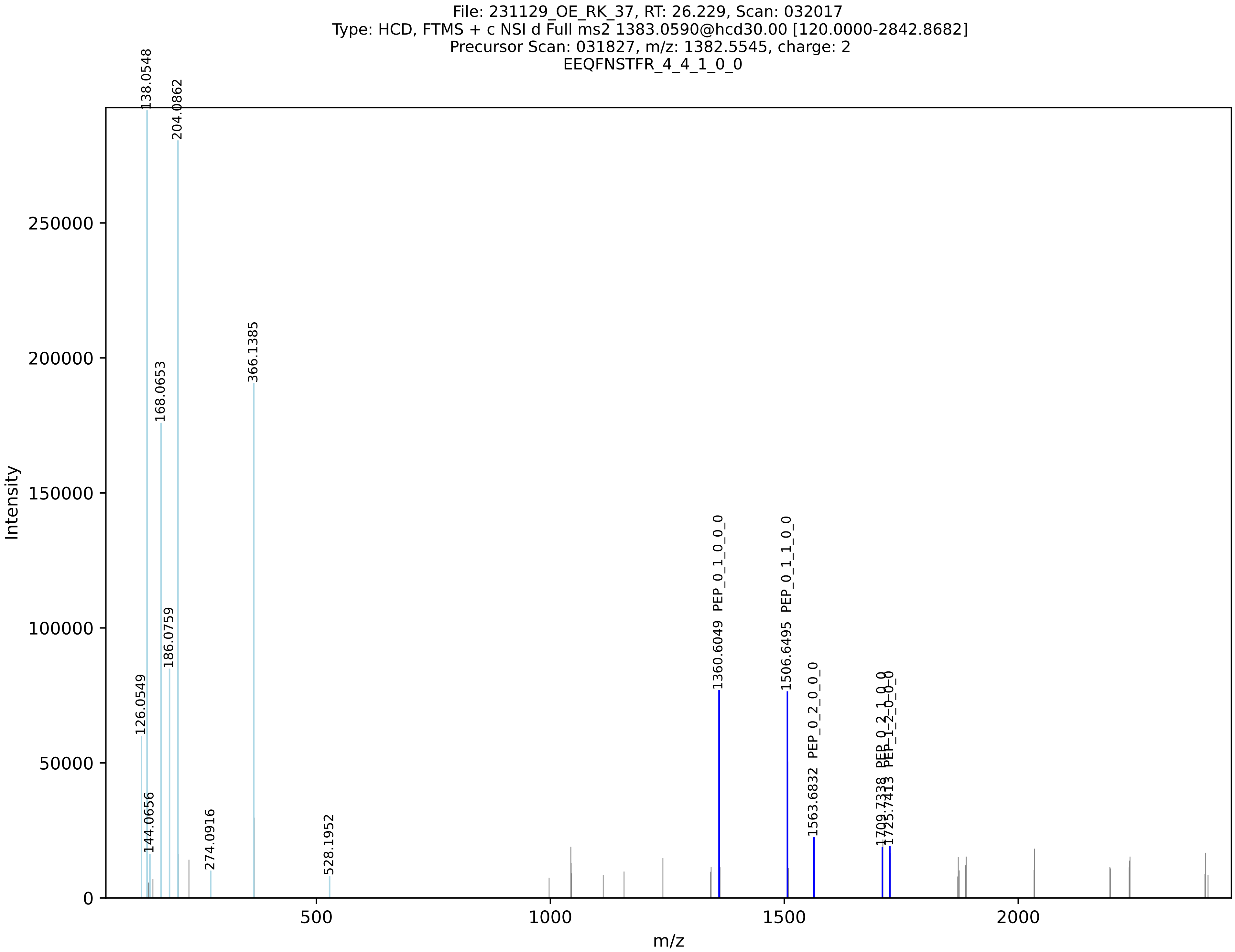

File: 231129_OE_RK_37, RT: 26.229, Scan: 032017
Type: HCD, FTMS + c NSI d Full ms2 1383.0590@hcd30.00 [120.0000-2842.8682]
Precursor Scan: 031827, m/z: 1382.5545, charge: 2
EEQFNSTFR_4_4_1_0_0
Intensity
m/z
138.0548
204.0862
366.1385
168.0653
186.0759
126.0549
144.0656
274.0916
528.1952
1360.6049 PEP_0_1_0_0_0
1506.6495 PEP_0_1_1_0_0
1563.6832 PEP_0_2_0_0_0
1709.7338 PEP_0_2_1_0_0
1725.7413 PEP_1_2_0_0_0

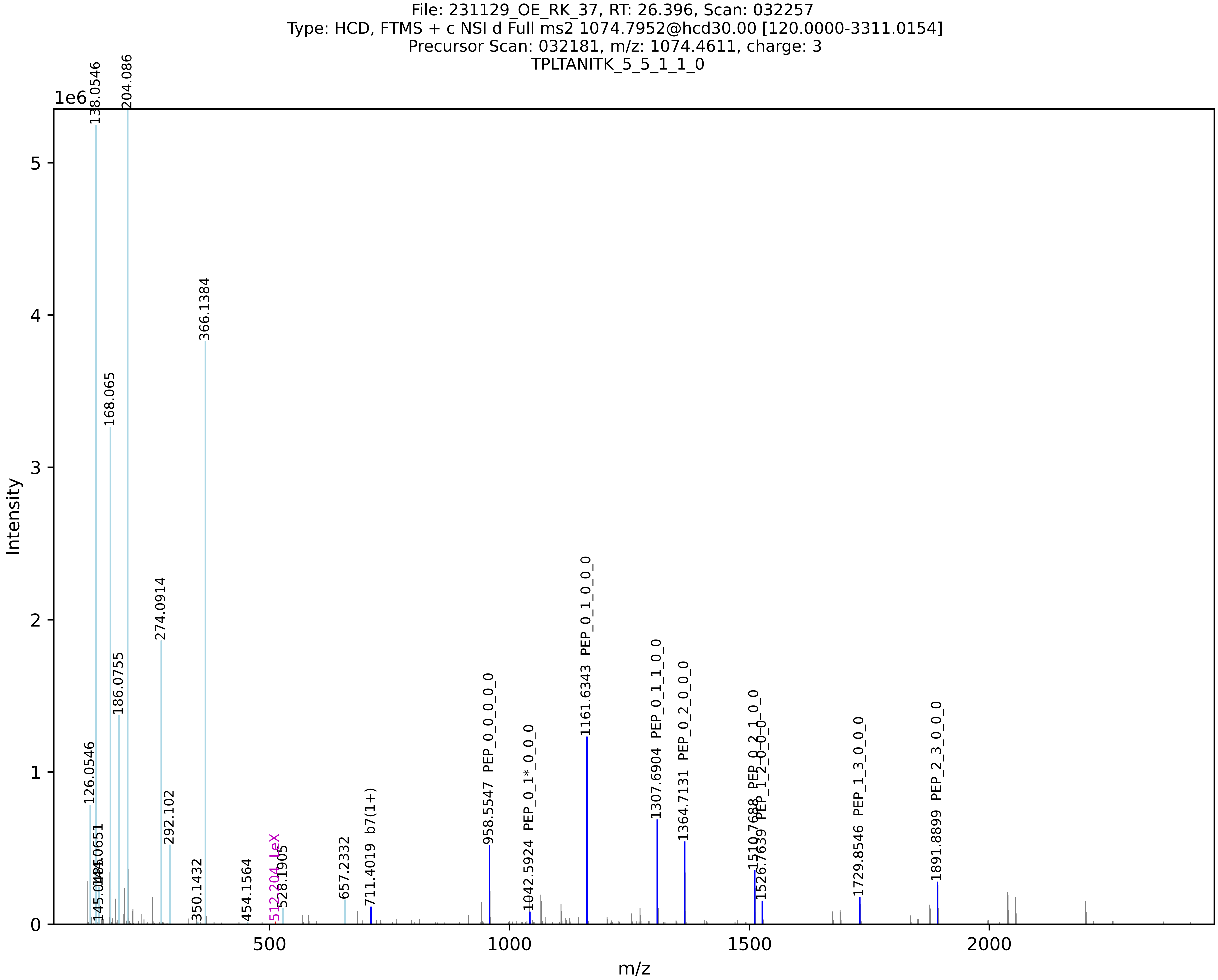
File: 231129_OE_RK_37, RT: 26.396, Scan: 032257
Type: HCD, FTMS + c NSI d Full ms2 1074.7952@hcd30.00 [120.0000-3311.0154]
Precursor Scan: 032181, m/z: 1074.4611, charge: 3
TPLTANITK_5_5_1_1_0
1e6
Intensity
m/z
138.0546
204.086
366.1384
168.065
274.0914
186.0755
126.0546
292.102
350.1432
454.1564
512.204_LeX
528.1905
657.2332
711.4019 b7(1+)
958.5547 PEP_0_0_0_0_0
1042.5924 PEP_0_1*_0_0_0
1161.6343 PEP_0_1_0_0_0
1307.6904 PEP_0_1_1_0_0
1364.7131 PEP_0_2_0_0_0
1510.7688 PEP_0_2_1_0_0
1526.7639 PEP_1_2_0_0_0
1729.8546 PEP_1_3_0_0_0
1891.8899 PEP_2_3_0_0_0
0
1
2
3
4
5
500
1000
1500
2000

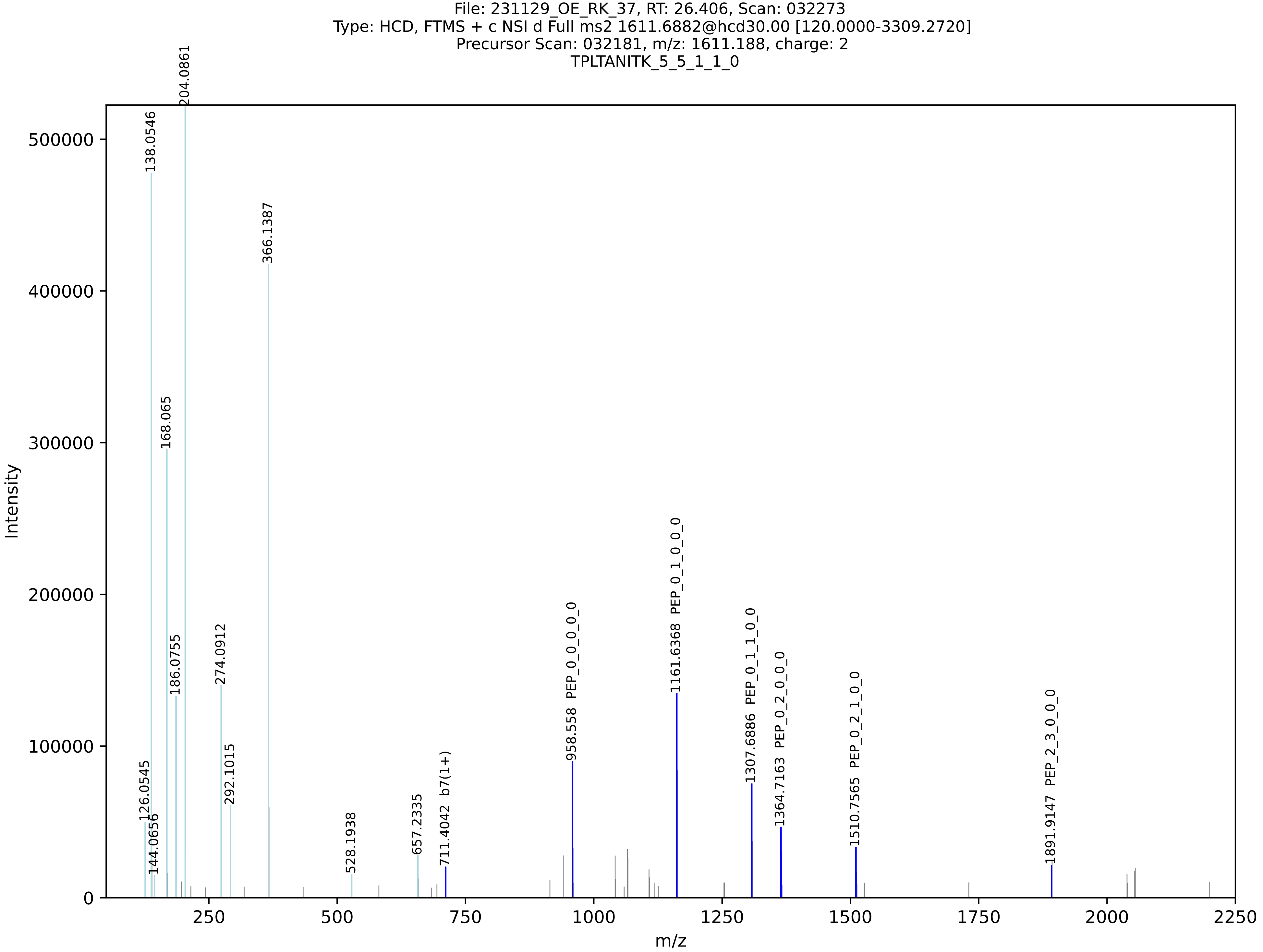
File: 231129_OE_RK_37, RT: 26.406, Scan: 032273
Type: HCD, FTMS + c NSI d Full ms2 1611.6882@hcd30.00 [120.0000-3309.2720]
Precursor Scan: 032181, m/z: 1611.188, charge: 2
TPLTANITK_5_5_1_1_0
Intensity
m/z
204.0861
138.0546
366.1387
168.065
186.0755
274.0912
292.1015
126.0545
144.0656
528.1938
657.2335
711.4042 b7(1+)
958.558 PEP_0_0_0_0_0
1161.6368 PEP_0_1_0_0_0
1307.6886 PEP_0_1_1_0_0
1364.7163 PEP_0_2_0_0_0
1510.7565 PEP_0_2_1_0_0
1891.9147 PEP_2_3_0_0_0

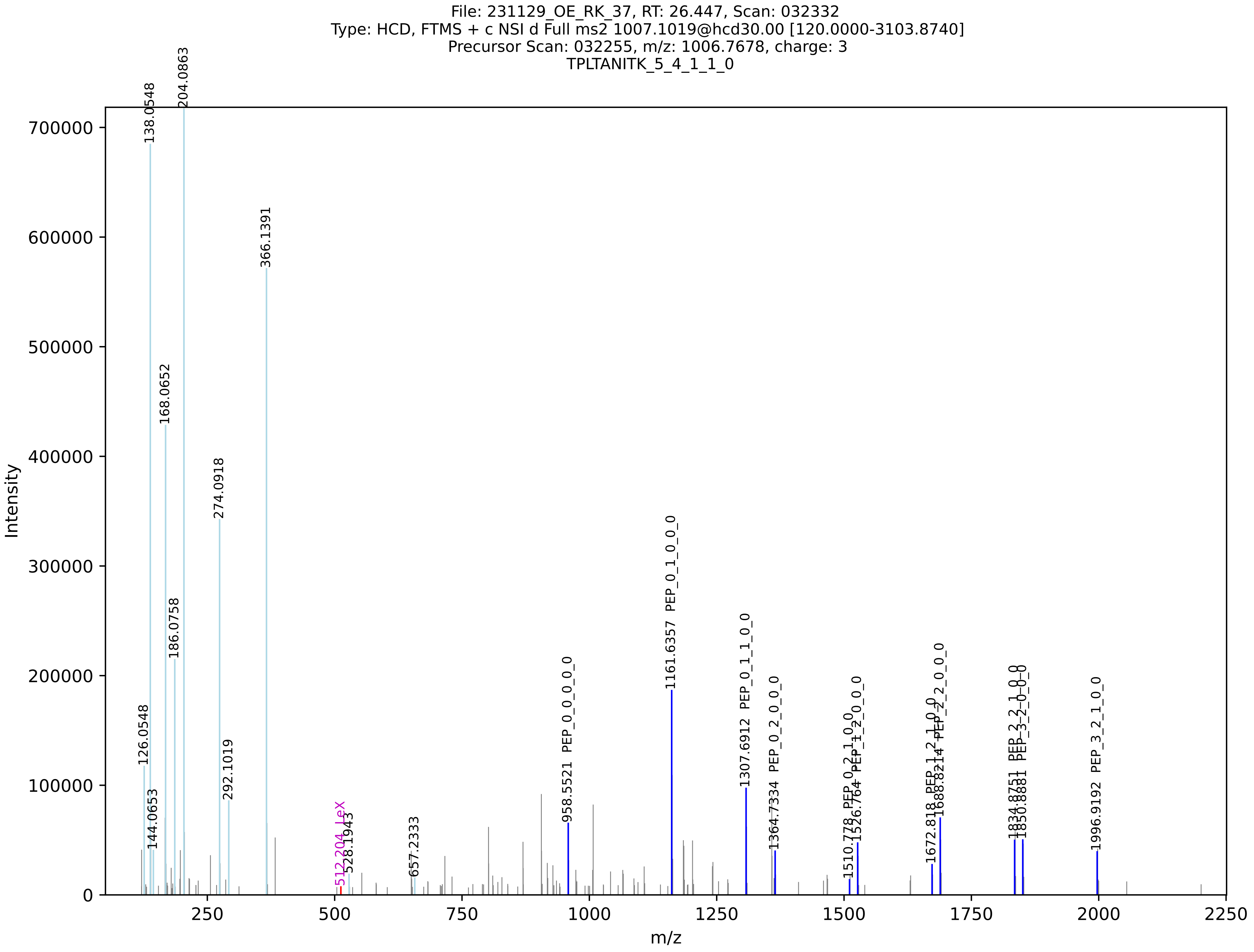

File: 231129_OE_RK_37, RT: 26.447, Scan: 032332
Type: HCD, FTMS + c NSI d Full ms2 1007.1019@hcd30.00 [120.0000-3103.8740]
Precursor Scan: 032255, m/z: 1006.7678, charge: 3
TPLTANITK_5_4_1_1_0
Intensity
m/z
204.0863
138.0548
366.1391
168.0652
274.0918
186.0758
126.0548
292.1019
144.0653
512.204_LeX
528.1943
657.2333
958.5521 PEP_0_0_0_0_0
1161.6357 PEP_0_1_0_0_0
1307.6912 PEP_0_1_1_0_0
1364.7334 PEP_0_2_0_0_0
1510.778 PEP_0_2_1_0_0
1526.764 PEP_1_2_0_0_0
1672.818 PEP_1_2_1_0_0
1688.8214 PEP_2_2_0_0_0
1834.8751 PEP_2_2_1_0_0
1850.8881 PEP_3_2_0_0_0
1996.9192 PEP_3_2_1_0_0

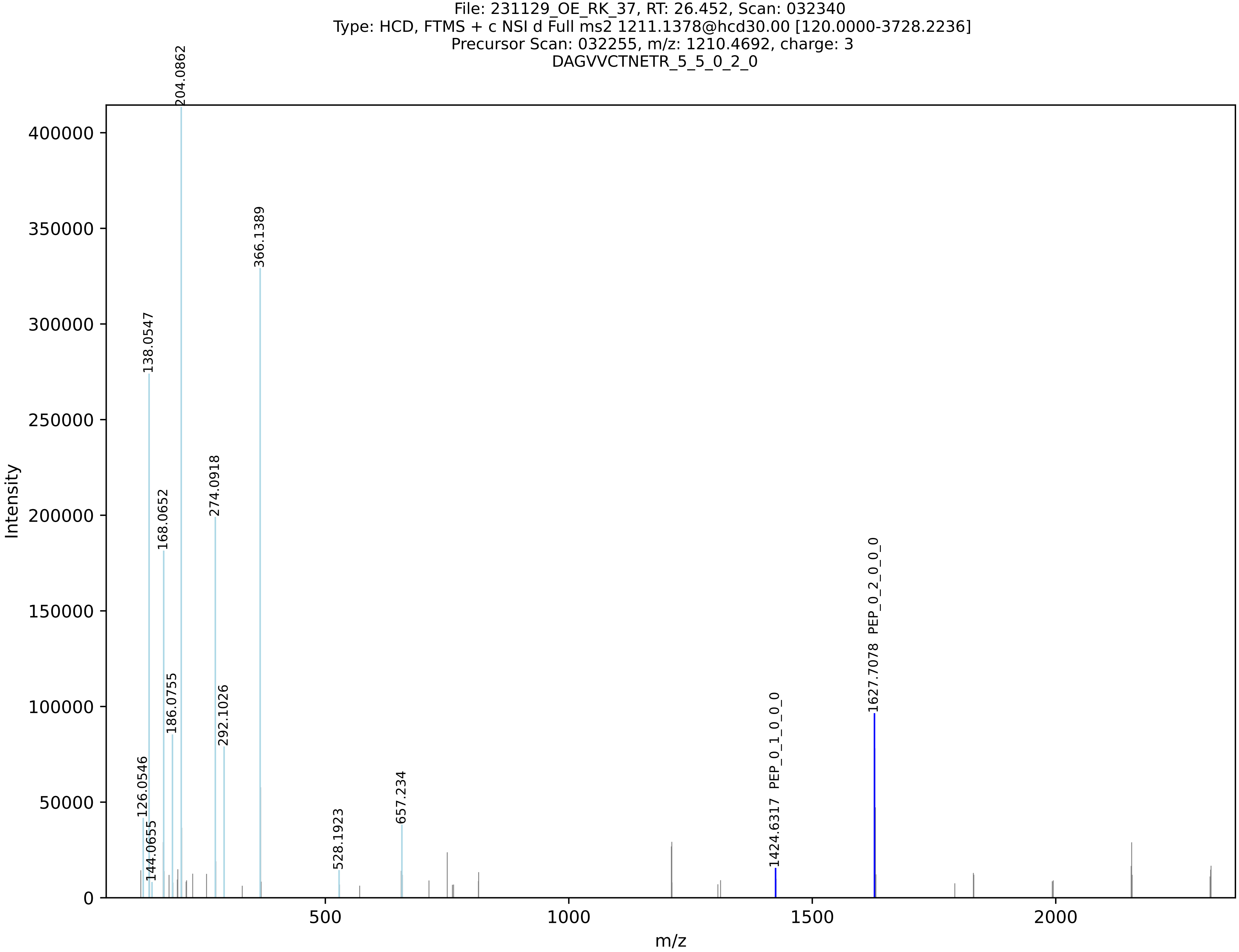
File: 231129_OE_RK_37, RT: 26.452, Scan: 032340
Type: HCD, FTMS + c NSI d Full ms2 1211.1378@hcd30.00 [120.0000-3728.2236]
Precursor Scan: 032255, m/z: 1210.4692, charge: 3
DAGVVCTNETR_5_5_0_2_0
Intensity
m/z
0
50000
100000
150000
200000
250000
300000
350000
400000
500
1000
1500
2000
204.0862
366.1389
138.0547
274.0918
168.0652
186.0755
292.1026
126.0546
144.0655
528.1923
657.234
1424.6317 PEP_0_1_0_0_0
1627.7078 PEP_0_2_0_0_0

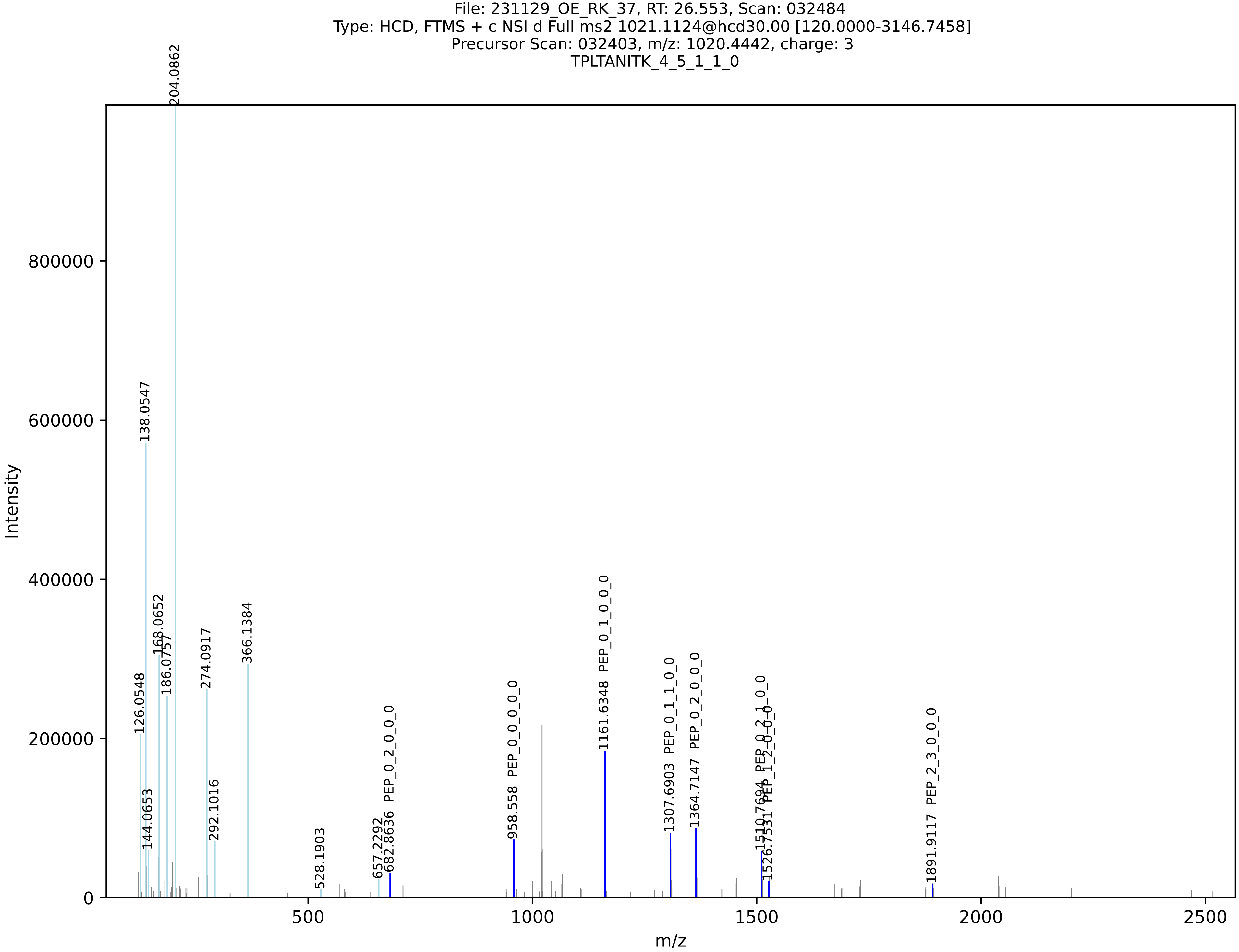
File: 231129_OE_RK_37, RT: 26.553, Scan: 032484
Type: HCD, FTMS + c NSI d Full ms2 1021.1124@hcd30.00 [120.0000-3146.7458]
Precursor Scan: 032403, m/z: 1020.4442, charge: 3
TPLTANITK_4_5_1_1_0
204.0862
138.0547
168.0652
186.0757
126.0548
144.0653
274.0917
292.1016
366.1384
528.1903
657.2292
682.8636 PEP_0_2_0_0_0
958.558 PEP_0_0_0_0_0
1161.6348 PEP_0_1_0_0_0
1307.6903 PEP_0_1_1_0_0
1364.7147 PEP_0_2_0_0_0
1510.7694 PEP_0_2_1_0_0
1526.7531 PEP_1_2_0_0_0
1891.9117 PEP_2_3_0_0_0
Intensity
m/z
0
200000
400000
600000
800000
500
1000
1500
2000
2500

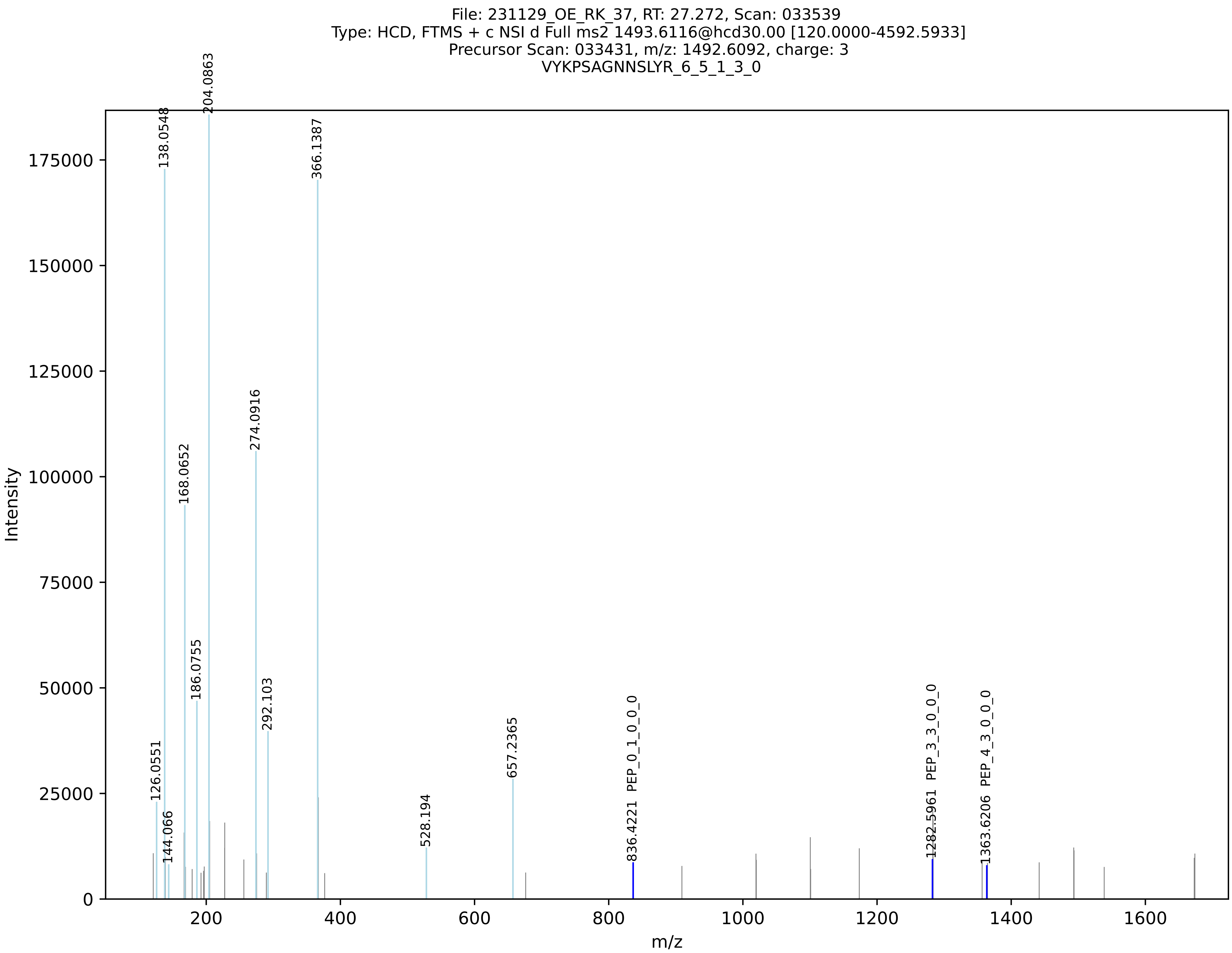

File: 231129_OE_RK_37, RT: 27.272, Scan: 033539
Type: HCD, FTMS + c NSI d Full ms2 1493.6116@hcd30.00 [120.0000-4592.5933]
Precursor Scan: 033431, m/z: 1492.6092, charge: 3
VYKPSAGNNSLYR_6_5_1_3_0
Intensity
m/z
204.0863
138.0548
366.1387
274.0916
168.0652
186.0755
292.103
126.0551
144.066
528.194
657.2365
836.4221 PEP_0_1_0_0_0
1282.5961 PEP_3_3_0_0_0
1363.6206 PEP_4_3_0_0_0

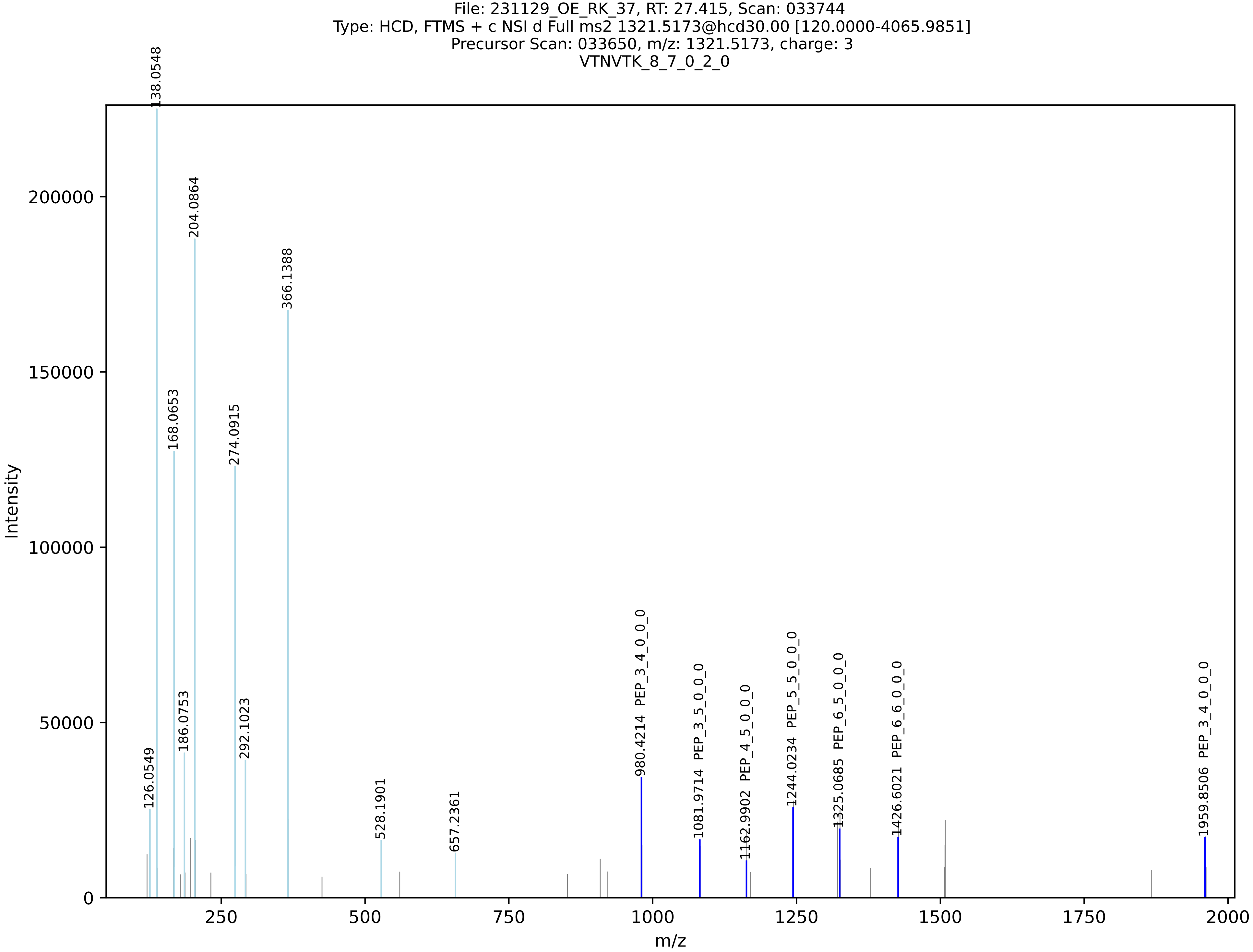

File: 231129_OE_RK_37, RT: 27.415, Scan: 033744
Type: HCD, FTMS + c NSI d Full ms2 1321.5173@hcd30.00 [120.0000-4065.9851]
Precursor Scan: 033650, m/z: 1321.5173, charge: 3
VTNVTK_8_7_0_2_0
Intensity
m/z
138.0548
204.0864
366.1388
168.0653
274.0915
186.0753
292.1023
126.0549
528.1901
657.2361
980.4214 PEP_3_4_0_0_0
1081.9714 PEP_3_5_0_0_0
1162.9902 PEP_4_5_0_0_0
1244.0234 PEP_5_5_0_0_0
1325.0685 PEP_6_5_0_0_0
1426.6021 PEP_6_6_0_0_0
1959.8506 PEP_3_4_0_0_0

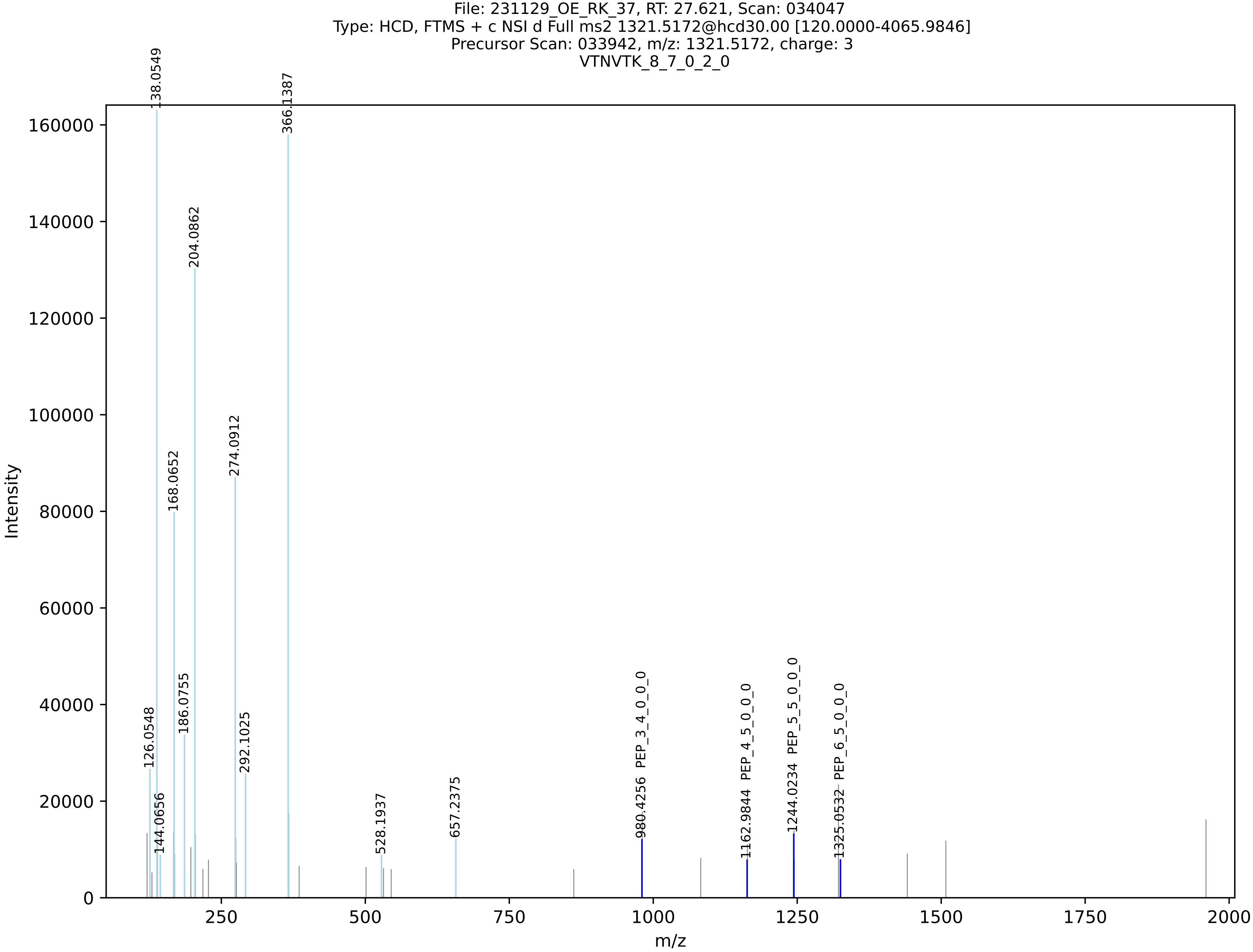
File: 231129_OE_RK_37, RT: 27.621, Scan: 034047
Type: HCD, FTMS + c NSI d Full ms2 1321.5172@hcd30.00 [120.0000-4065.9846]
Precursor Scan: 033942, m/z: 1321.5172, charge: 3
VTNVTK_8_7_0_2_0
Intensity
m/z
138.0549
366.1387
204.0862
274.0912
168.0652
186.0755
126.0548
292.1025
144.0656
528.1937
657.2375
980.4256 PEP_3_4_0_0_0
1162.9844 PEP_4_5_0_0_0
1244.0234 PEP_5_5_0_0_0
1325.0532 PEP_6_5_0_0_0

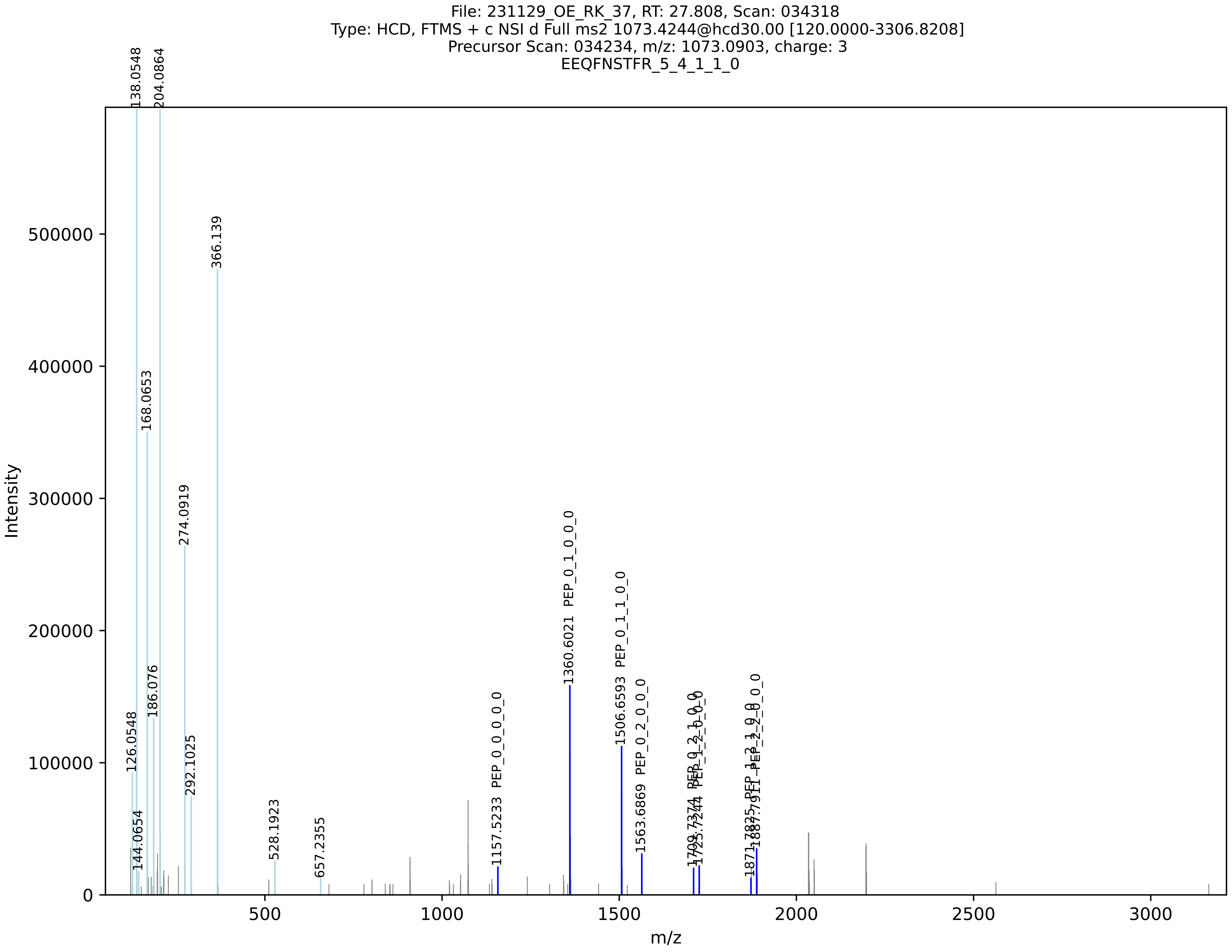
File: 231129_OE_RK_37, RT: 27.808, Scan: 034318
Type: HCD, FTMS + c NSI d Full ms2 1073.4244@hcd30.00 [120.0000-3306.8208]
Precursor Scan: 034234, m/z: 1073.0903, charge: 3
EEQFNSTFR_5_4_1_1_0
Intensity
m/z
138.0548
204.0864
366.139
168.0653
274.0919
186.076
126.0548
292.1025
144.0654
528.1923
657.2355
1157.5233 PEP_0_0_0_0_0
1360.6021 PEP_0_1_0_0_0
1506.6593 PEP_0_1_1_0_0
1563.6869 PEP_0_2_0_0_0

File: 231129_OE_RK_37, RT: 28.015, Scan: 034622
Type: HCD, FTMS + c NSI d Full ms2 1019.4054@hcd30.00 [120.0000-3141.5225]
Precursor Scan: 034530, m/z: 1019.4054, charge: 3
EEQFNSTFR_4_4_1_1_0

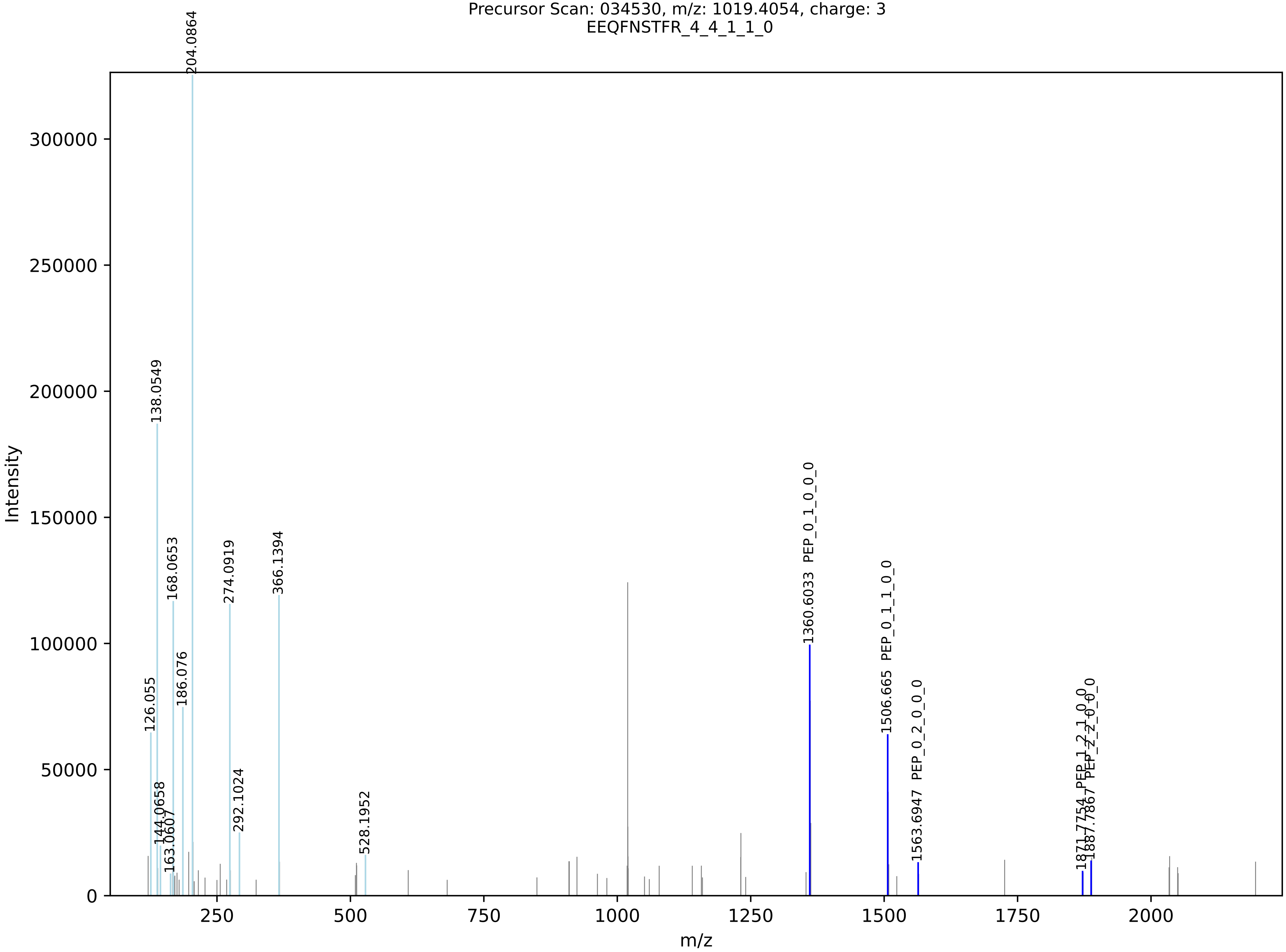

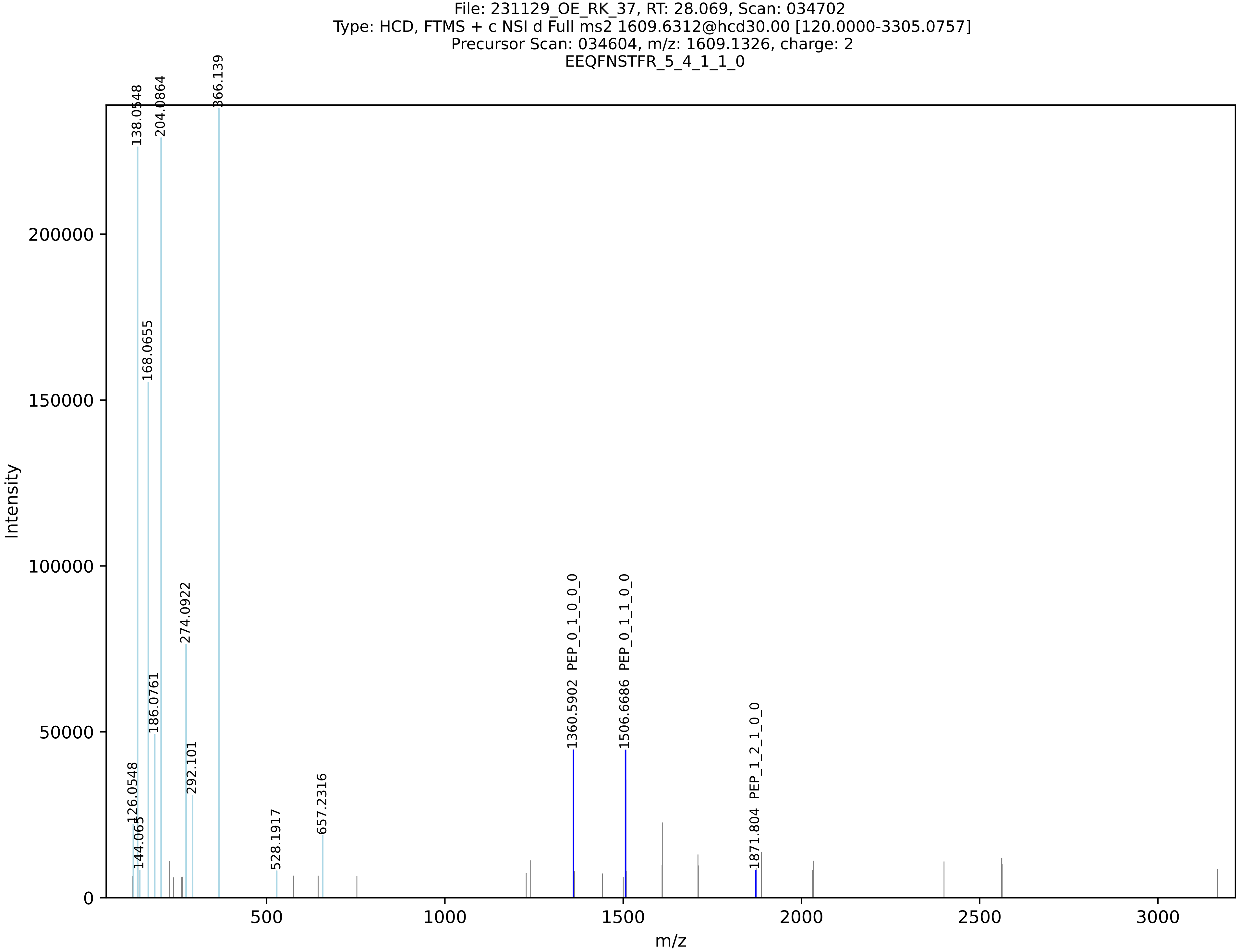
File: 231129_OE_RK_37, RT: 28.069, Scan: 034702
Type: HCD, FTMS + c NSI d Full ms2 1609.6312@hcd30.00 [120.0000-3305.0757]
Precursor Scan: 034604, m/z: 1609.1326, charge: 2
EEQFNSTFR_5_4_1_1_0
Intensity
m/z
0
50000
100000
150000
200000
500
1000
1500
2000
2500
3000
138.0548
204.0864
366.139
168.0655
274.0922
186.0761
292.101
126.0548
144.065
528.1917
657.2316
1360.5902 PEP_0_1_0_0_0
1506.6686 PEP_0_1_1_0_0
1871.804 PEP_1_2_1_0_0

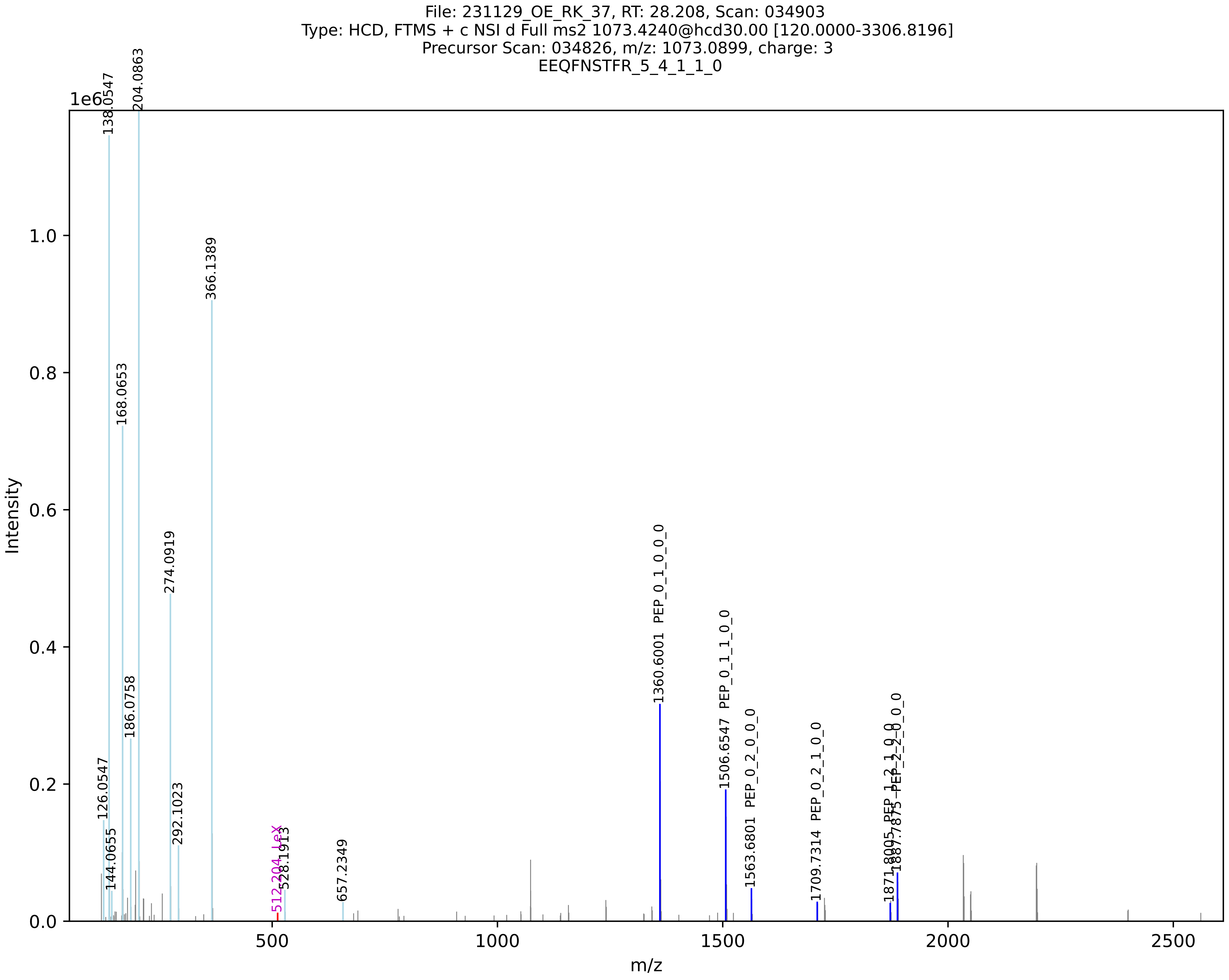

File: 231129_OE_RK_37, RT: 28.208, Scan: 034903
Type: HCD, FTMS + c NSI d Full ms2 1073.4240@hcd30.00 [120.0000-3306.8196]
Precursor Scan: 034826, m/z: 1073.0899, charge: 3
EEQFNSTFR_5_4_1_1_0
1e6
138.0547
204.0863
168.0653
366.1389
274.0919
186.0758
126.0547
144.0655
292.1023
512.204_LeX
528.1913
657.2349
1360.6001 PEP_0_1_0_0_0
1506.6547 PEP_0_1_1_0_0
1563.6801 PEP_0_2_0_0_0
1709.7314 PEP_0_2_1_0_0
1871.8005 PEP_1_2_1_0_0
1887.7875 PEP_2_2_0_0_0
Intensity
0.0
0.2
0.4
0.6
0.8
1.0
500
1000
1500
2000
2500
m/z

File: 231129_OE_RK_37, RT: 28.233, Scan: 034942
Type: HCD, FTMS + c NSI d Full ms2 1122.8076@hcd30.00 [120.0000-3457.9333]
Precursor Scan: 034826, m/z: 1122.8076, charge: 3
VNTSKPRGR_5_4_1_2_0

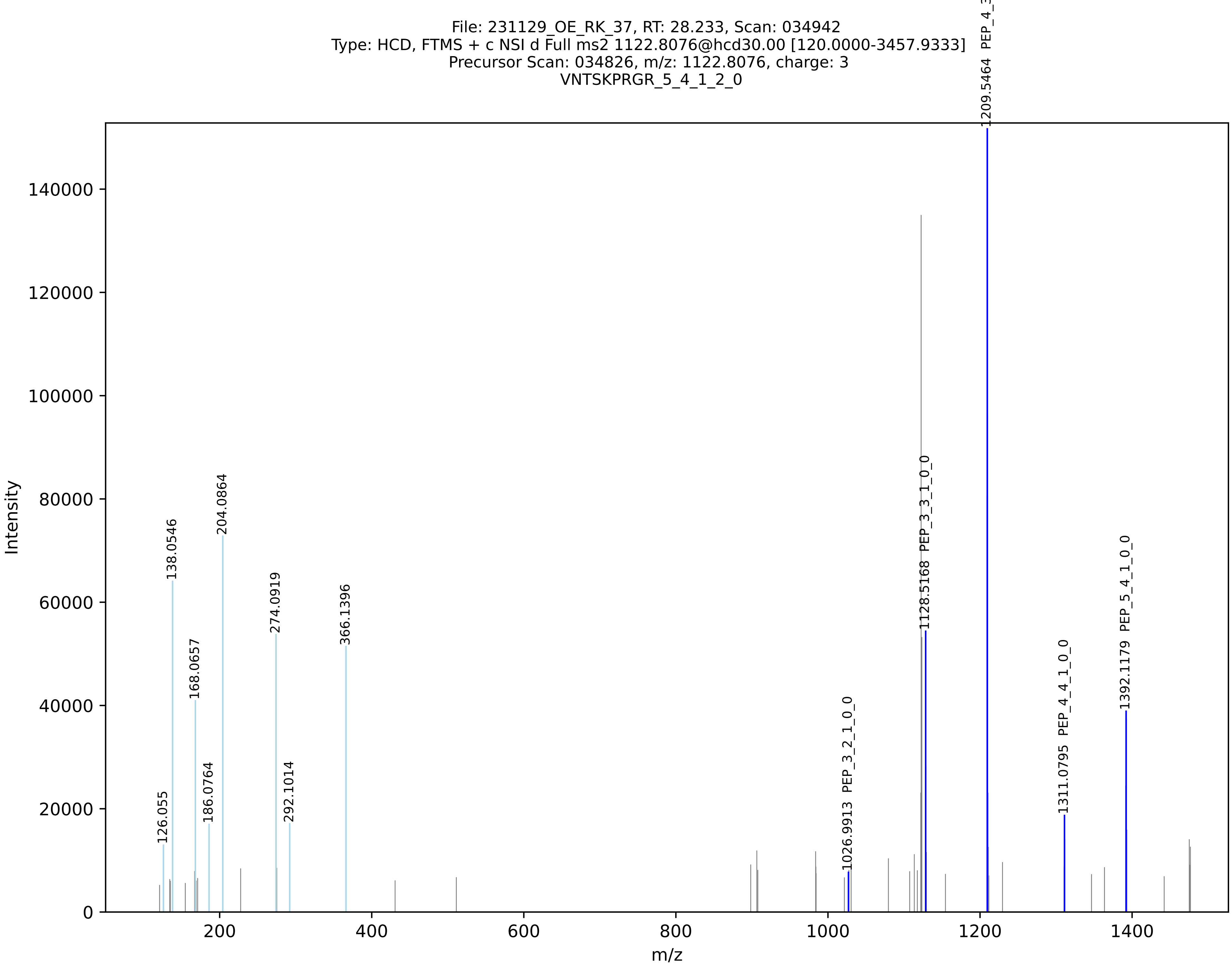

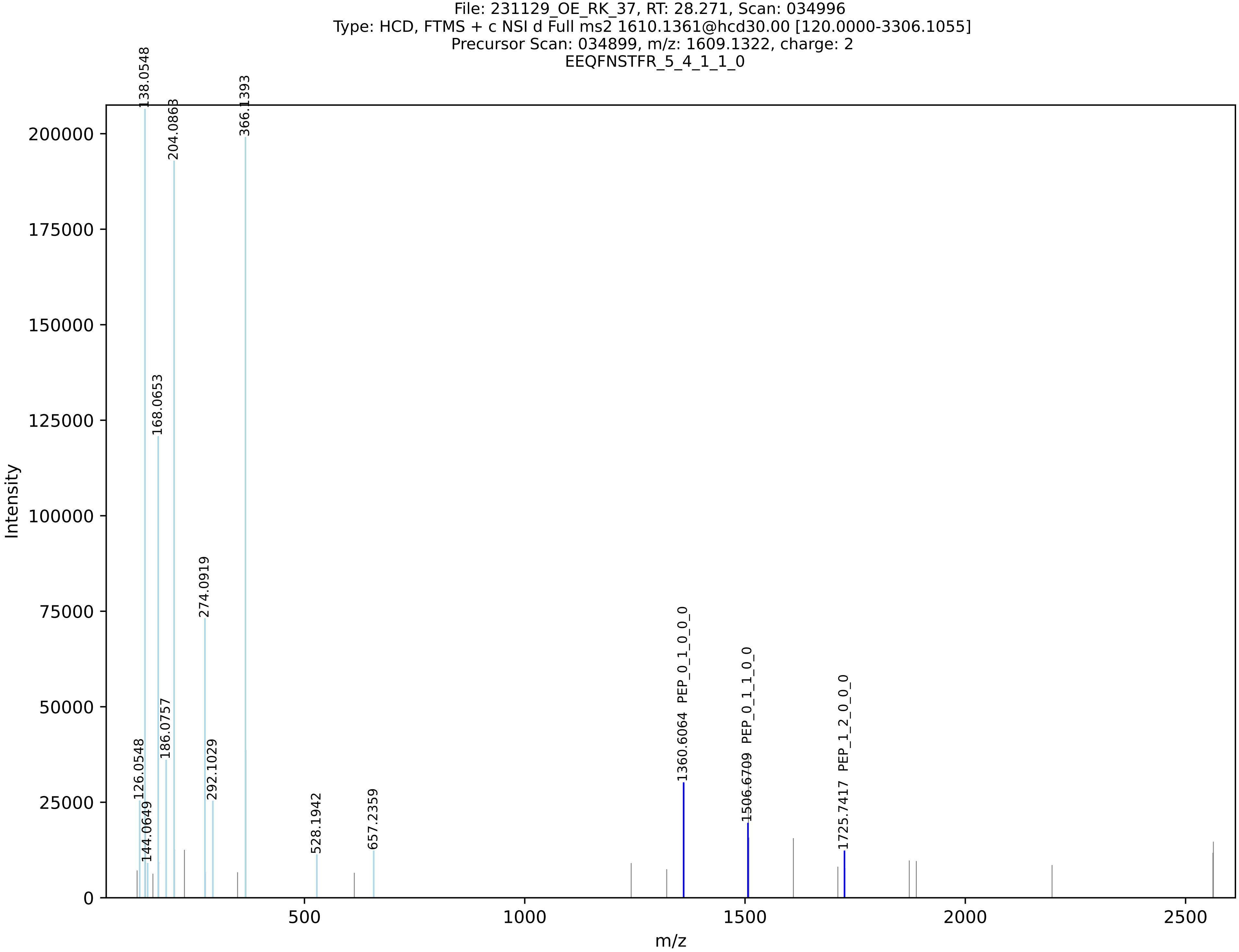

File: 231129_OE_RK_37, RT: 28.271, Scan: 034996
Type: HCD, FTMS + c NSI d Full ms2 1610.1361@hcd30.00 [120.0000-3306.1055]
Precursor Scan: 034899, m/z: 1609.1322, charge: 2
EEQFNSTFR_5_4_1_1_0
Intensity
m/z
138.0548
204.0863
366.1393
168.0653
274.0919
186.0757
126.0548
292.1029
144.0649
528.1942
657.2359
1360.6064 PEP_0_1_0_0_0
1506.6709 PEP_0_1_1_0_0
1725.7417 PEP_1_2_0_0_0

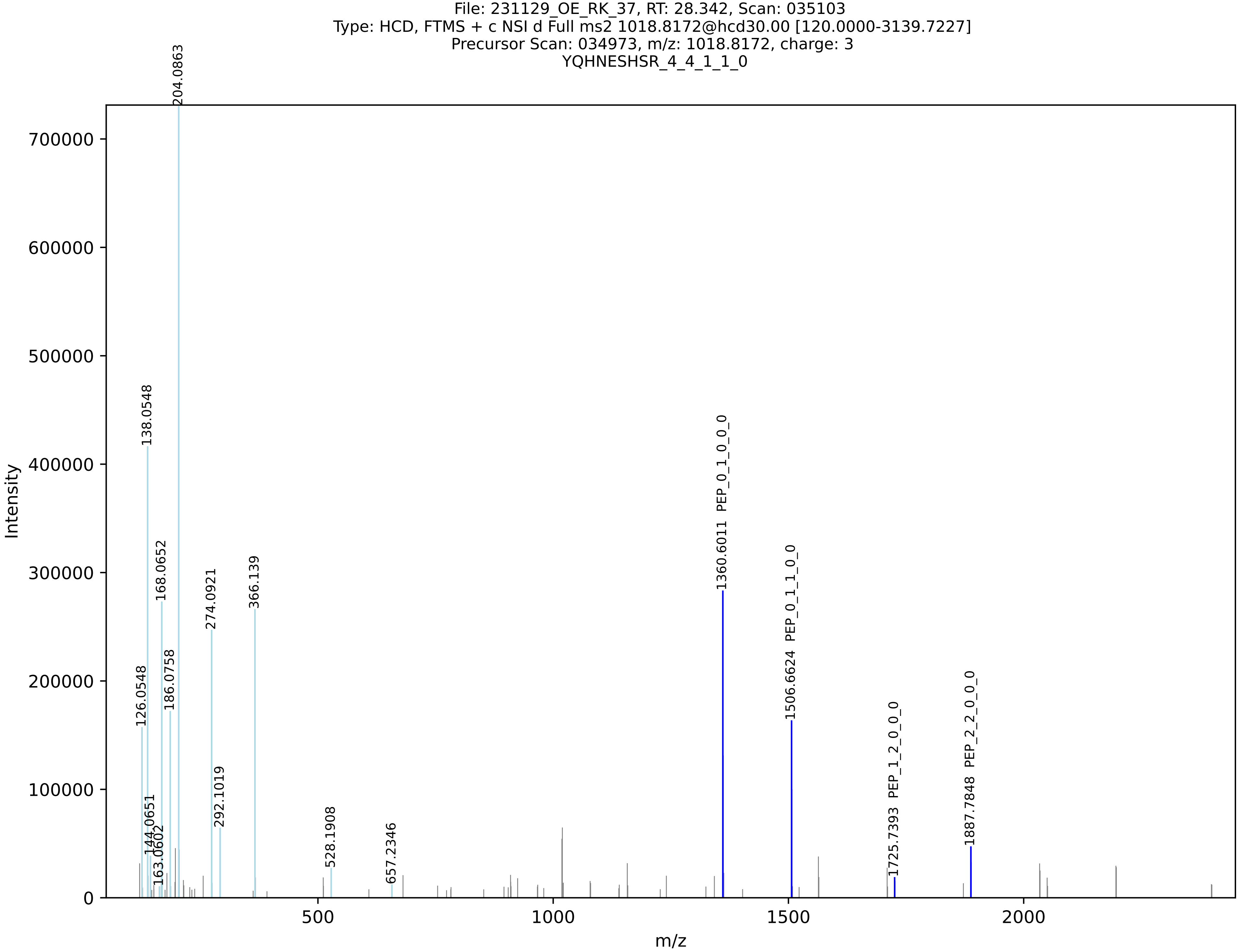
File: 231129_OE_RK_37, RT: 28.342, Scan: 035103
Type: HCD, FTMS + c NSI d Full ms2 1018.8172@hcd30.00 [120.0000-3139.7227]
Precursor Scan: 034973, m/z: 1018.8172, charge: 3
YQHNESHSR_4_4_1_1_0
Intensity
m/z
204.0863
138.0548
168.0652
186.0758
126.0548
274.0921
366.139
292.1019
144.0651
163.0602
528.1908
657.2346
1360.6011 PEP_0_1_0_0_0
1506.6624 PEP_0_1_1_0_0
1725.7393 PEP_1_2_0_0_0
1887.7848 PEP_2_2_0_0_0

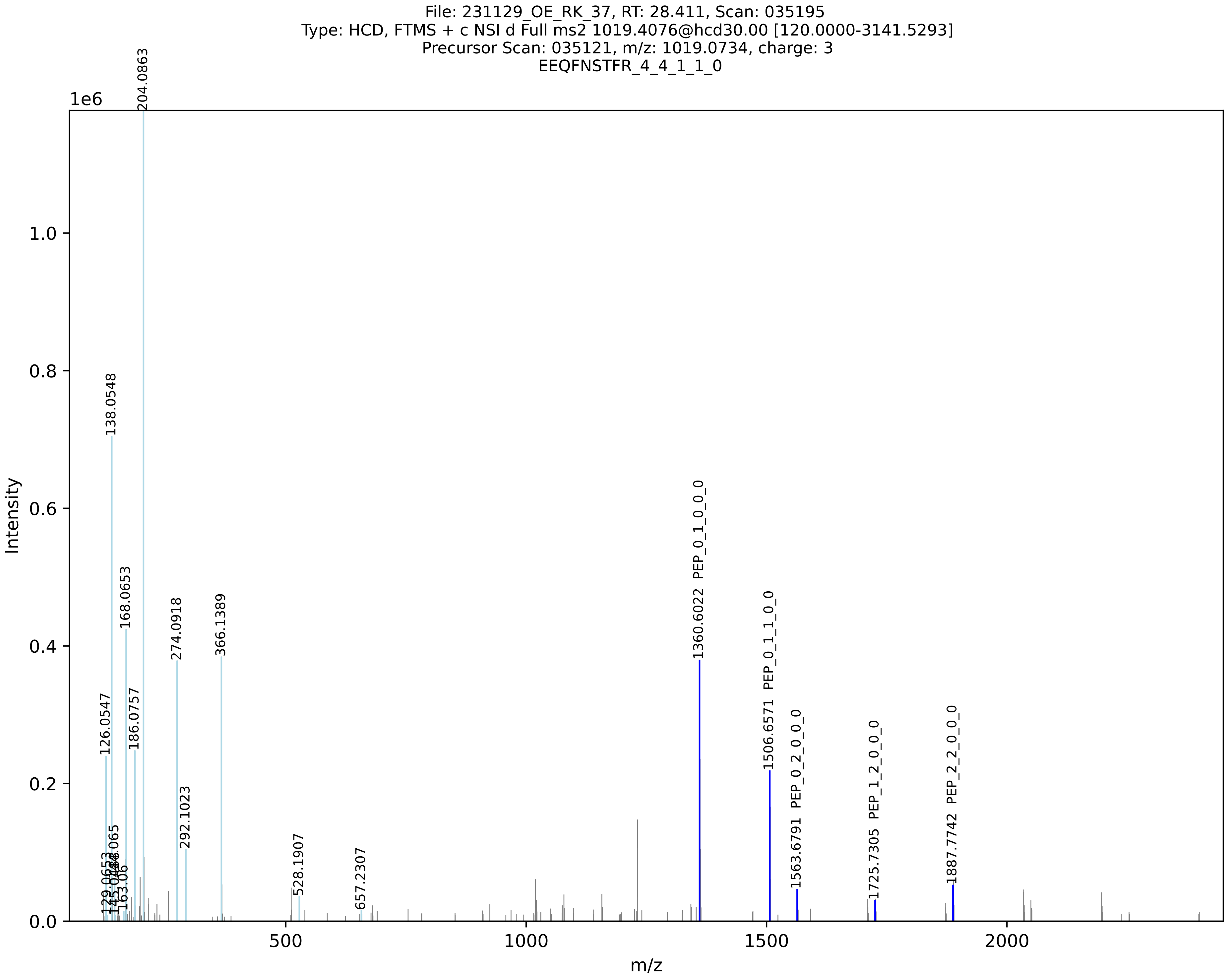
File: 231129_OE_RK_37, RT: 28.411, Scan: 035195
Type: HCD, FTMS + c NSI d Full ms2 1019.4076@hcd30.00 [120.0000-3141.5293]
Precursor Scan: 035121, m/z: 1019.0734, charge: 3
EEQFNSTFR_4_4_1_1_0
1e6
Intensity
m/z
204.0863
138.0548
168.0653
126.0547
186.0757
274.0918
366.1389
292.1023
528.1907
657.2307
1360.6022 PEP_0_1_0_0_0
1506.6571 PEP_0_1_1_0_0
1563.6791 PEP_0_2_0_0_0
1725.7305 PEP_1_2_0_0_0
1887.7742 PEP_2_2_0_0_0
163.06
0.0
0.2
0.4
0.6
0.8
1.0
500
1000
1500
2000

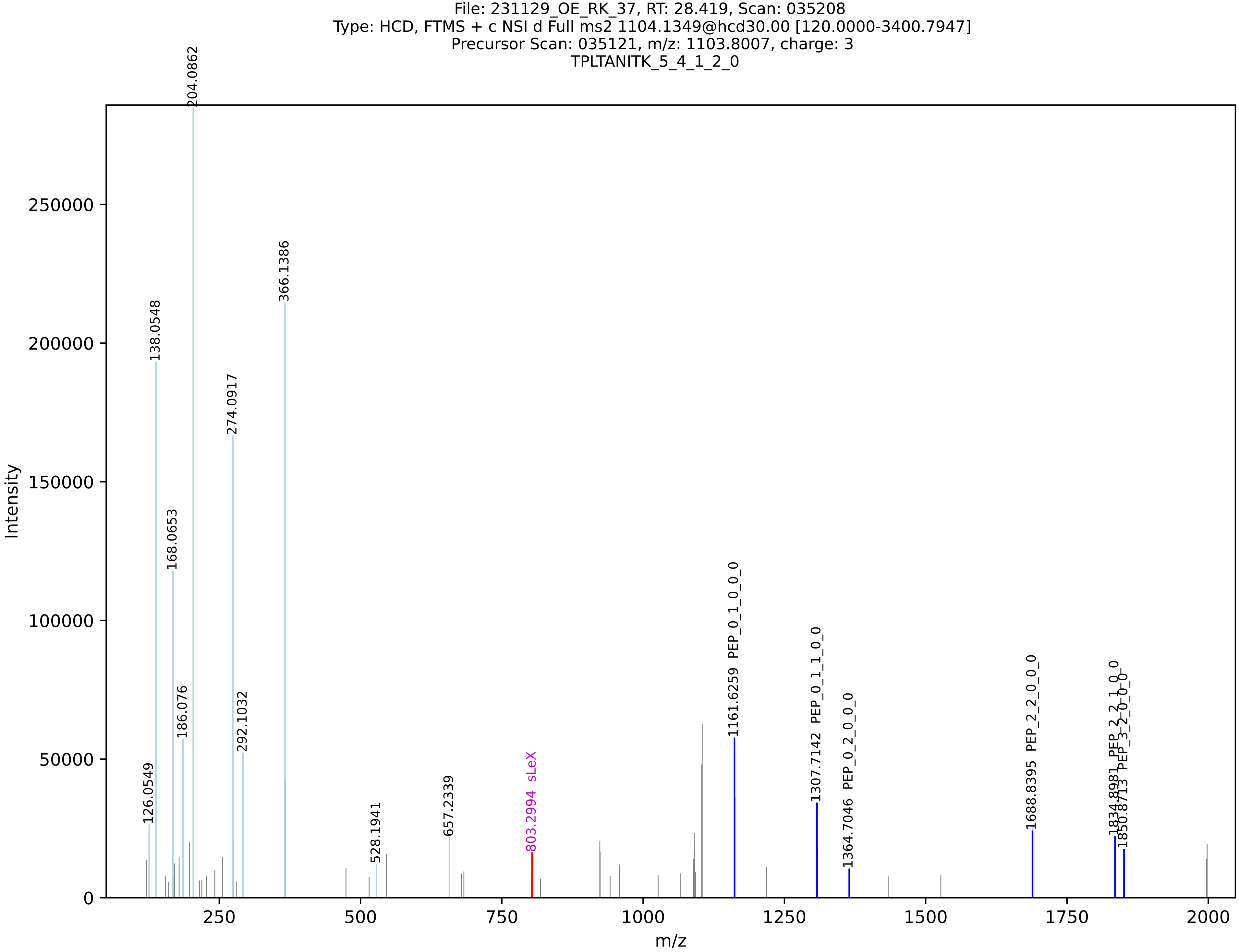

File: 231129_OE_RK_37, RT: 28.419, Scan: 035208
Type: HCD, FTMS + c NSI d Full ms2 1104.1349@hcd30.00 [120.0000-3400.7947]
Precursor Scan: 035121, m/z: 1103.8007, charge: 3
TPLTANITK_5_4_1_2_0
Intensity
m/z
204.0862
366.1386
138.0548
274.0917
168.0653
186.076
292.1032
126.0549
528.1941
657.2339
803.2994 sLeX
1161.6259 PEP_0_1_0_0_0
1307.7142 PEP_0_1_1_0_0
1364.7046 PEP_0_2_0_0_0
1688.8395 PEP_2_2_0_0_0
1834.8981 PEP_2_2_1_0_0
1850.8713 PEP_3_2_0_0_0

File: 231129_OE_RK_37, RT: 28.42, Scan: 035211
Type: HCD, FTMS + c NSI d Full ms2 1256.8141@hcd30.00 [120.0000-3867.9929]
Precursor Scan: 035121, m/z: 1256.1458, charge: 3
ISEENETTCYMGK_5_4_0_2_0

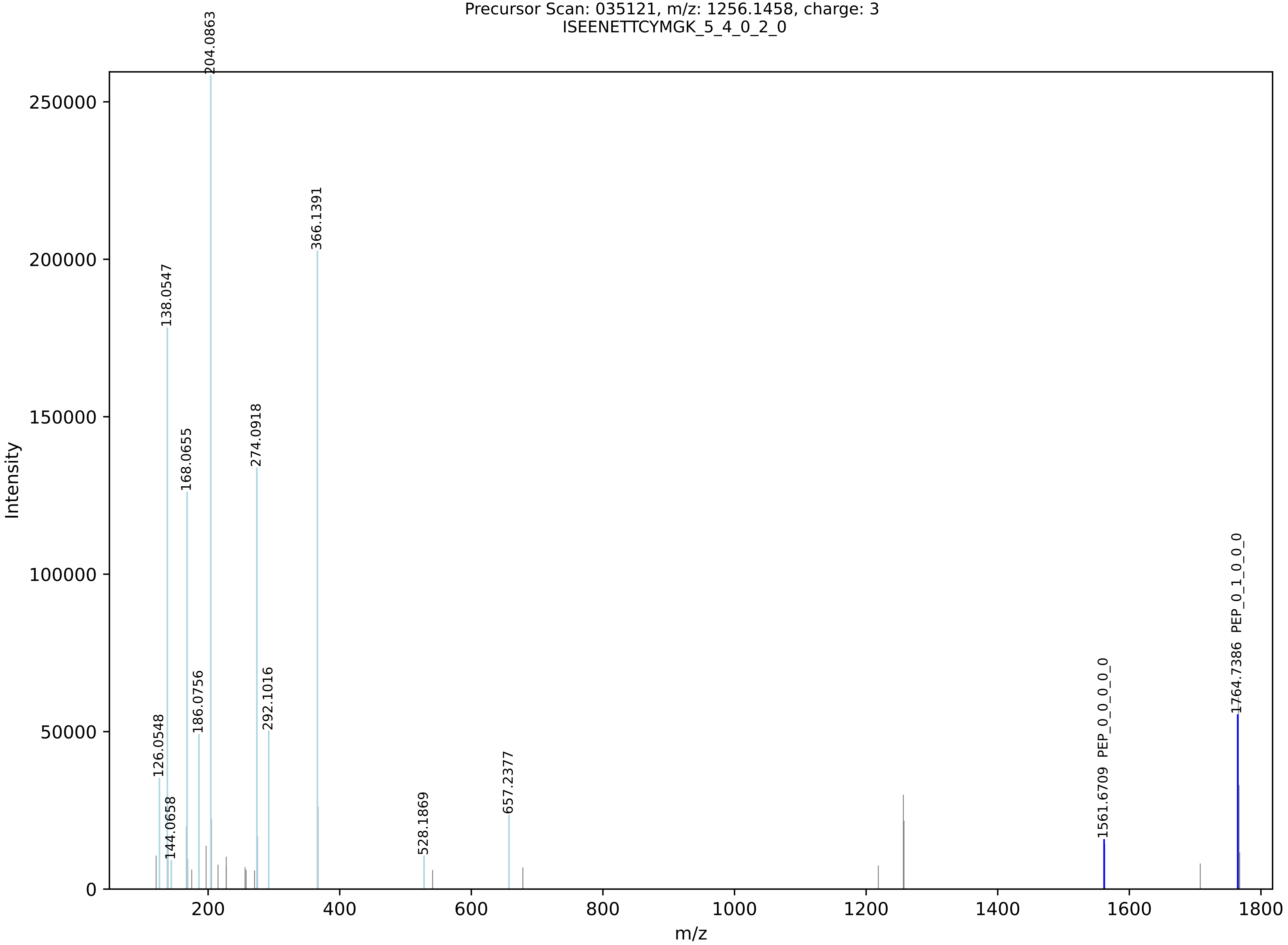

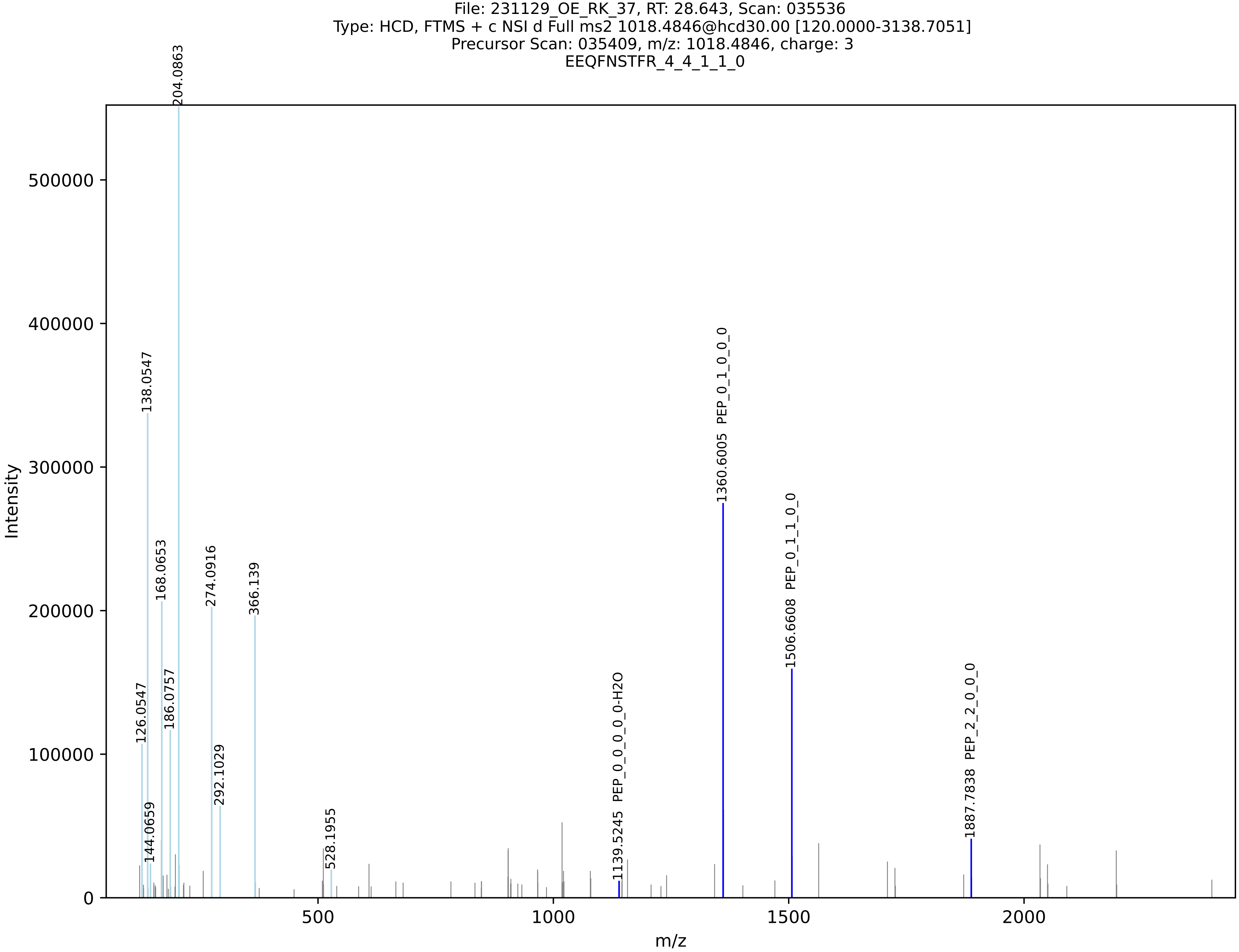

File: 231129_OE_RK_37, RT: 28.643, Scan: 035536
Type: HCD, FTMS + c NSI d Full ms2 1018.4846@hcd30.00 [120.0000-3138.7051]
Precursor Scan: 035409, m/z: 1018.4846, charge: 3
EEQFNSTFR_4_4_1_1_0
Intensity
m/z
204.0863
138.0547
168.0653
274.0916
366.139
126.0547
186.0757
292.1029
144.0659
528.1955
1139.5245 PEP_0_0_0_0_0-H2O
1360.6005 PEP_0_1_0_0_0
1506.6608 PEP_0_1_1_0_0
1887.7838 PEP_2_2_0_0_0
0
100000
200000
300000
400000
500000
500
1000
1500
2000

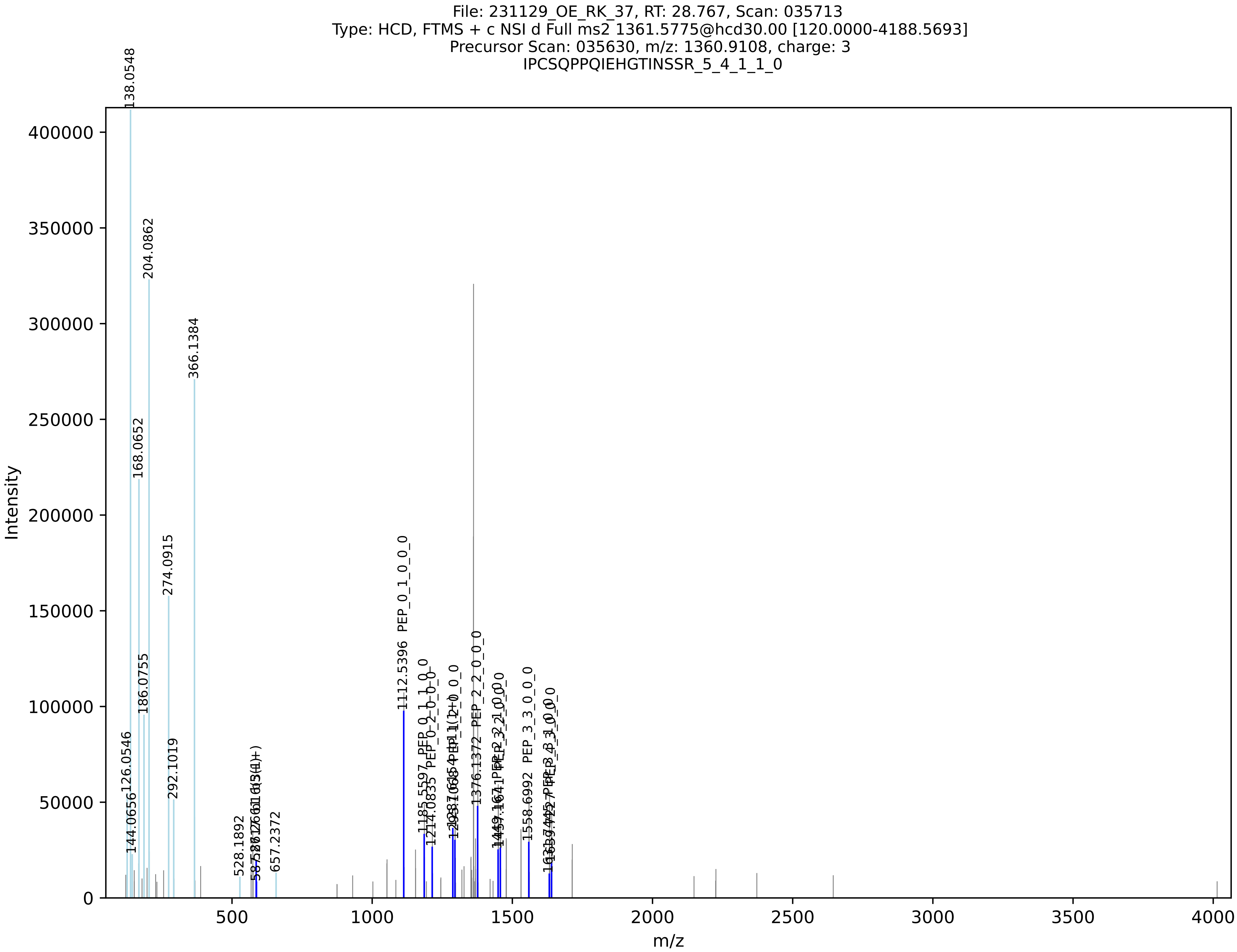
File: 231129_OE_RK_37, RT: 28.767, Scan: 035713
Type: HCD, FTMS + c NSI d Full ms2 1361.5775@hcd30.00 [120.0000-4188.5693]
Precursor Scan: 035630, m/z: 1360.9108, charge: 3
IPCSQPPQIEHGTINSSR_5_4_1_1_0
Intensity
m/z
138.0548
204.0862
366.1384
168.0652
274.0915
186.0755
126.0546
292.1019
144.0656
528.1892
657.2372
1112.5396 PEP_0_1_0_0_0
1376.1372 PEP_2_2_0_0_0
1558.6992 PEP_3_3_0_0_0
1214.0835 PEP_0_2_0_0_0

File: 231129_OE_RK_37, RT: 28.82, Scan: 035792
Type: HCD, FTMS + c NSI d Full ms2 1104.4694@hcd30.00 [120.0000-3401.8184]
Precursor Scan: 035704, m/z: 1103.467, charge: 3
TPLTANITK_5_4_1_2_0

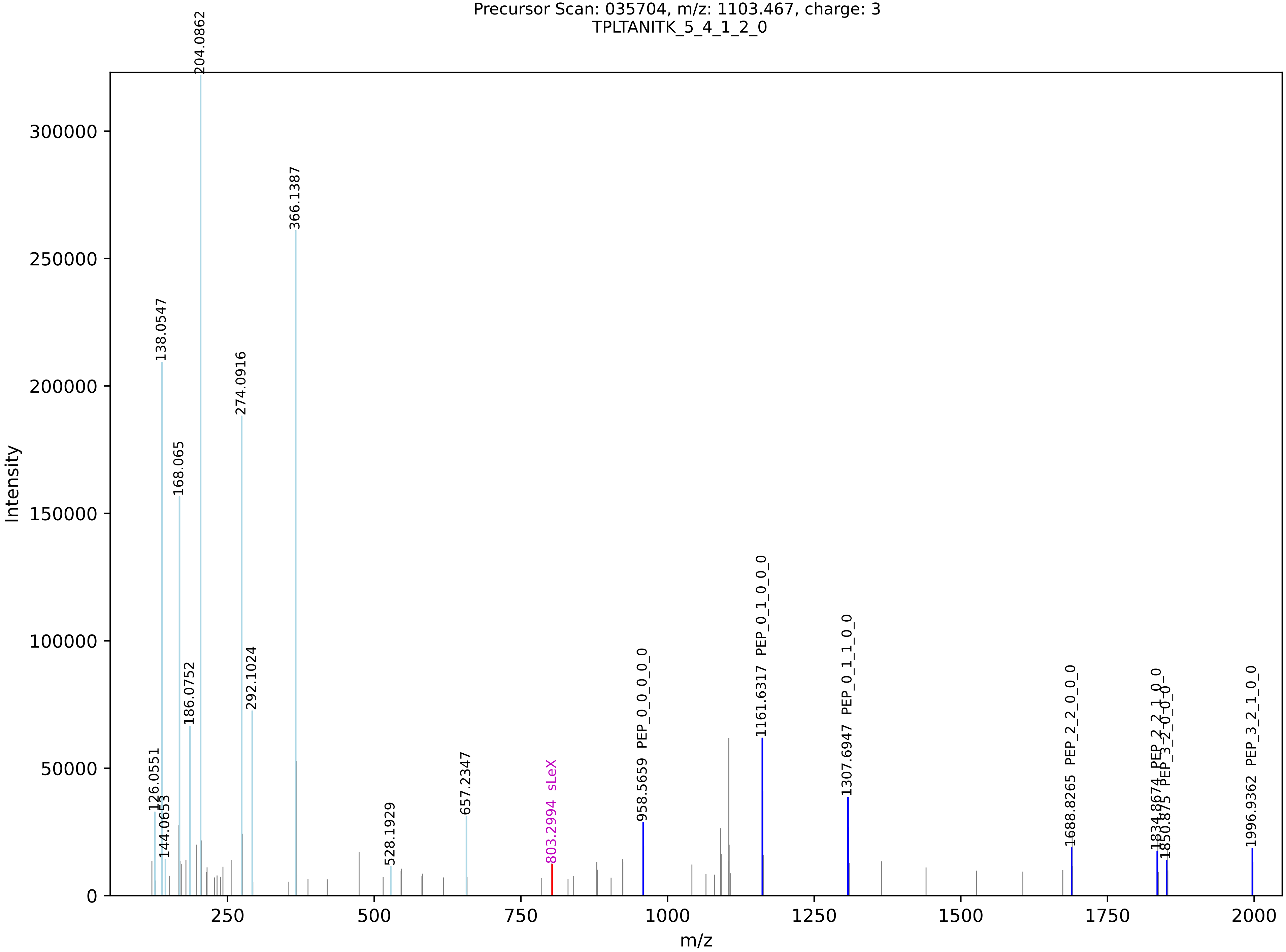

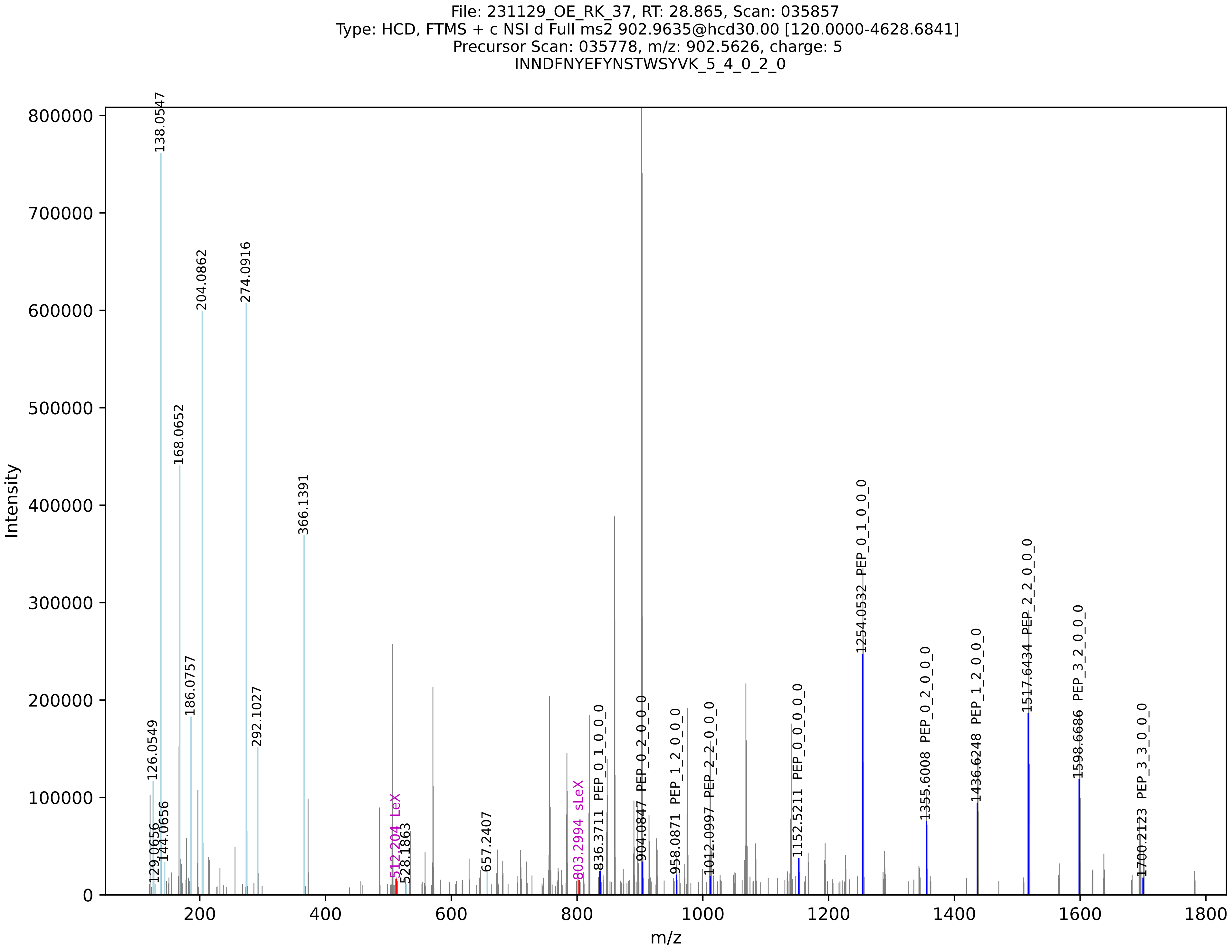
File: 231129_OE_RK_37, RT: 28.865, Scan: 035857
Type: HCD, FTMS + c NSI d Full ms2 902.9635@hcd30.00 [120.0000-4628.6841]
Precursor Scan: 035778, m/z: 902.5626, charge: 5
INNDFNYEFYNSTWSYVK_5_4_0_2_0
Intensity
m/z
138.0547
204.0862
274.0916
168.0652
366.1391
186.0757
292.1027
126.0549
144.0656
129.0656
512.204 LeX
528.1863
657.2407
803.2994 sLeX
836.3711 PEP_0_1_0_0_0
904.0847 PEP_0_2_0_0_0
958.0871 PEP_1_2_0_0_0
1012.0997 PEP_2_2_0_0_0
1152.5211 PEP_0_0_0_0_0
1254.0532 PEP_0_1_0_0_0
1355.6008 PEP_0_2_0_0_0
1436.6248 PEP_1_2_0_0_0
1517.6434 PEP_2_2_0_0_0
1598.6686 PEP_3_2_0_0_0
1700.2123 PEP_3_3_0_0_0

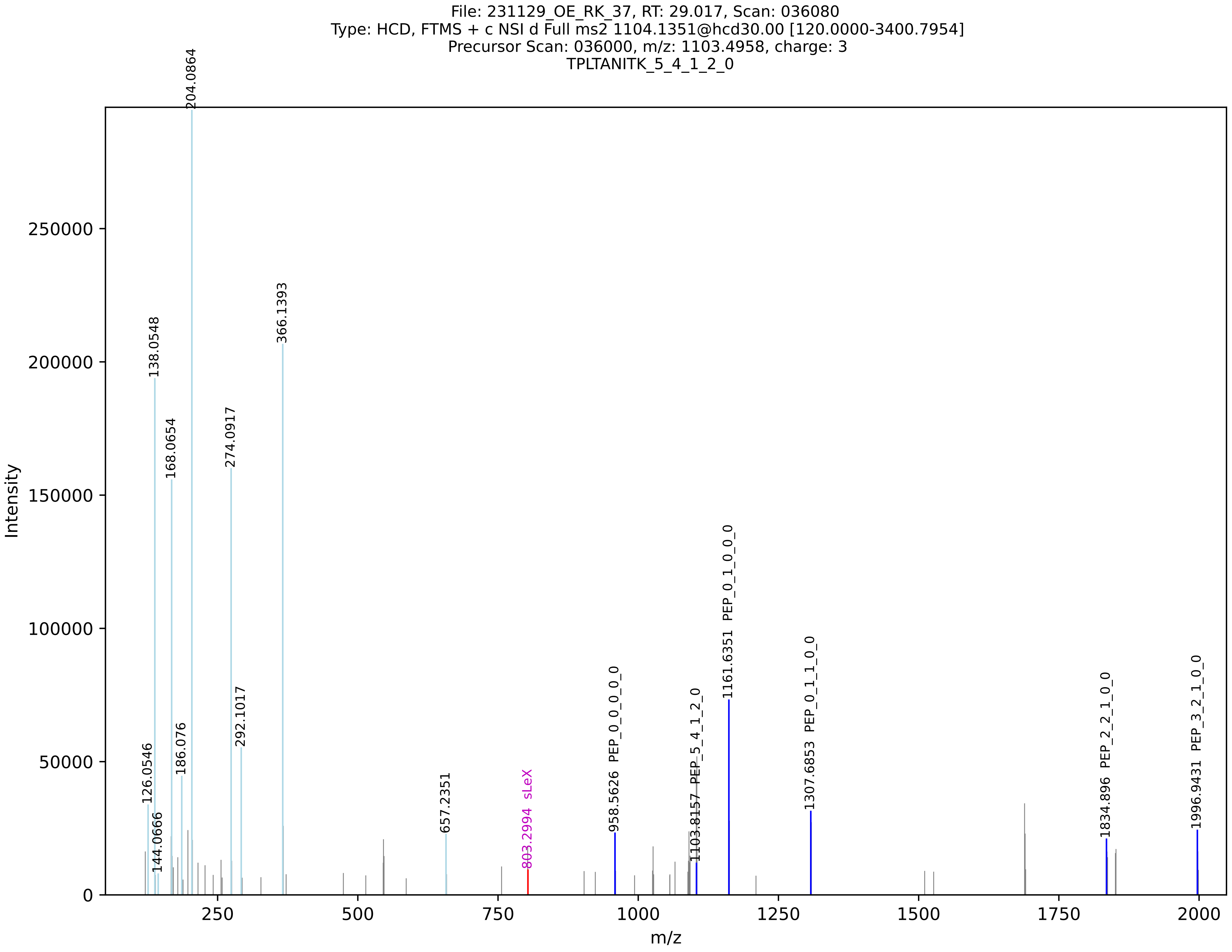
File: 231129_OE_RK_37, RT: 29.017, Scan: 036080
Type: HCD, FTMS + c NSI d Full ms2 1104.1351@hcd30.00 [120.0000-3400.7954]
Precursor Scan: 036000, m/z: 1103.4958, charge: 3
TPLTANITK_5_4_1_2_0
Intensity
m/z
0
50000
100000
150000
200000
250000
250
500
750
1000
1250
1500
1750
2000
204.0864
138.0548
366.1393
168.0654
274.0917
292.1017
186.076
126.0546
144.0666
657.2351
803.2994 sLeX
958.5626 PEP_0_0_0_0_0
1103.8157 PEP_5_4_1_2_0
1161.6351 PEP_0_1_0_0_0
1307.6853 PEP_0_1_1_0_0
1834.896 PEP_2_2_1_0_0
1996.9431 PEP_3_2_1_0_0

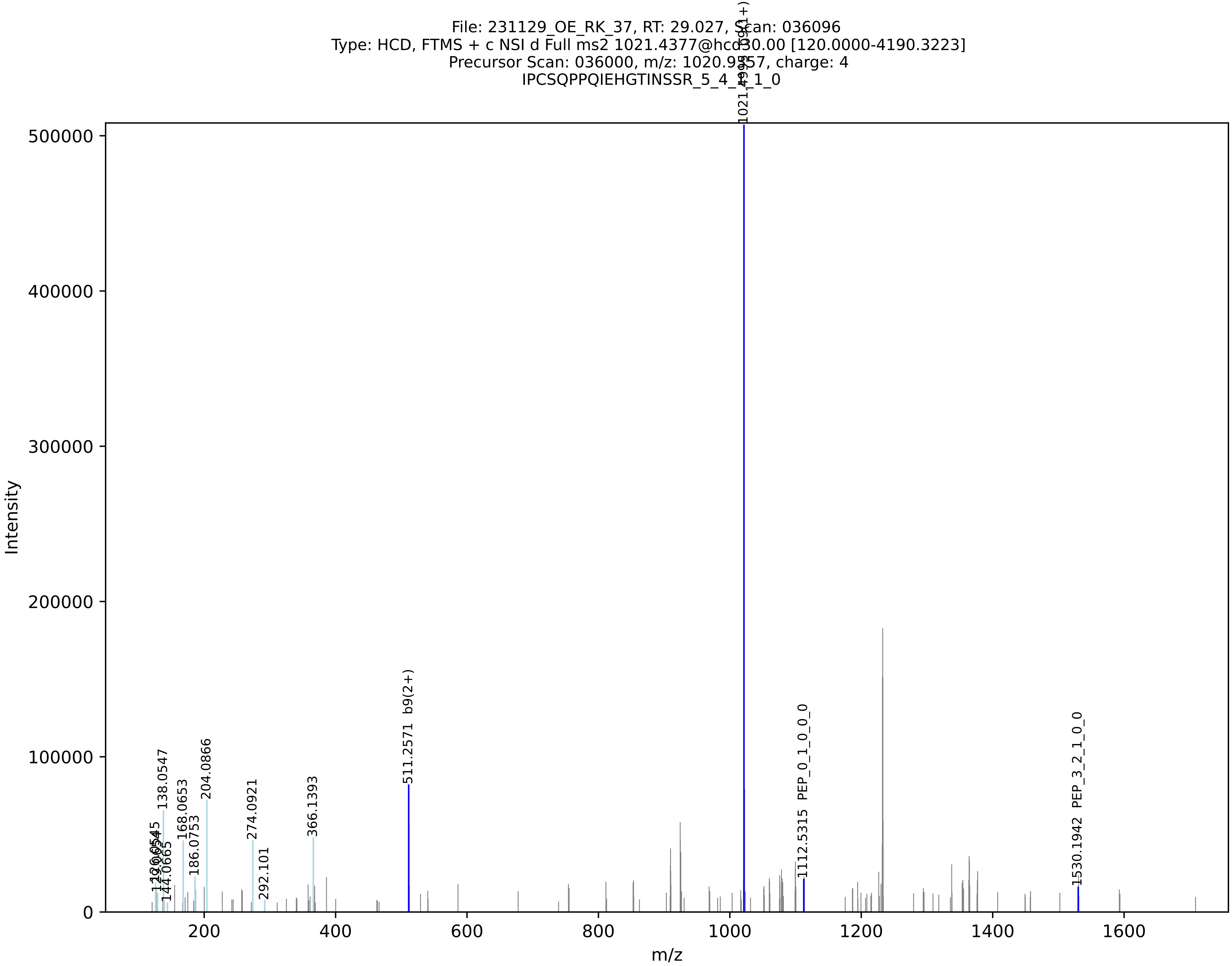

File: 231129_OE_RK_37, RT: 29.027, Scan: 036096
Type: HCD, FTMS + c NSI d Full ms2 1021.4377@hcd30.00 [120.0000-4190.3223]
Precursor Scan: 036000, m/z: 1020.9957, charge: 4
IPCSQPPQIEHGTINSSR_5_4_1_1_0
Intensity
m/z
138.0547
168.0653
186.0753
204.0866
144.0665
274.0921
292.101
366.1393
511.2571 b9(2+)
1112.5315 PEP_0_1_0_0_0
1530.1942 PEP_3_2_1_0_0

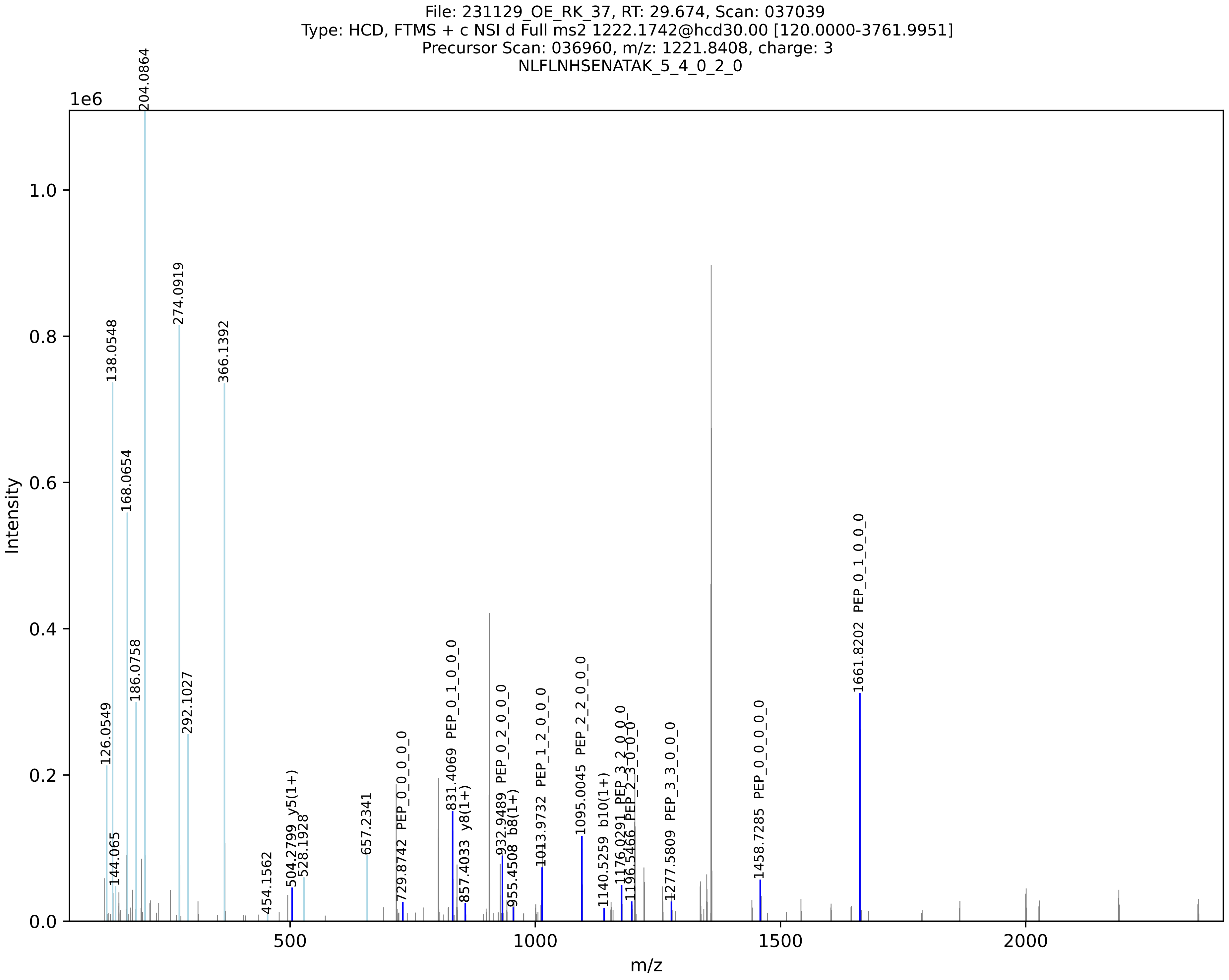
File: 231129_OE_RK_37, RT: 29.674, Scan: 037039
Type: HCD, FTMS + c NSI d Full ms2 1222.1742@hcd30.00 [120.0000-3761.9951]
Precursor Scan: 036960, m/z: 1221.8408, charge: 3
NLFLNHSENATAK_5_4_0_2_0
1e6
Intensity
m/z
0.0
0.2
0.4
0.6
0.8
1.0
500
1000
1500
2000
204.0864
274.0919
138.0548
366.1392
168.0654
186.0758
292.1027
126.0549
144.065
454.1562
504.2799 y5(1+)
528.1928
657.2341
729.8742 PEP_0_0_0_0_0
831.4069 PEP_0_1_0_0_0
857.4033 y8(1+)
932.9489 PEP_0_2_0_0_0
955.4508 b8(1+)
1013.9732 PEP_1_2_0_0_0
1095.0045 PEP_2_2_0_0_0
1140.5259 b10(1+)
1176.0291 PEP_3_2_0_0_0
1196.5466 PEP_2_3_0_0_0
1277.5809 PEP_3_3_0_0_0
1458.7285 PEP_0_0_0_0_0
1661.8202 PEP_0_1_0_0_0

File: 231129_OE_RK_37, RT: 29.826, Scan: 037261
Type: HCD, FTMS + c NSI d Full ms2 1221.5535@hcd30.00 [120.0000-3760.0955]
Precursor Scan: 037179, m/z: 1221.5514, charge: 3
NLFLNHSENATAK_5_4_0_2_0

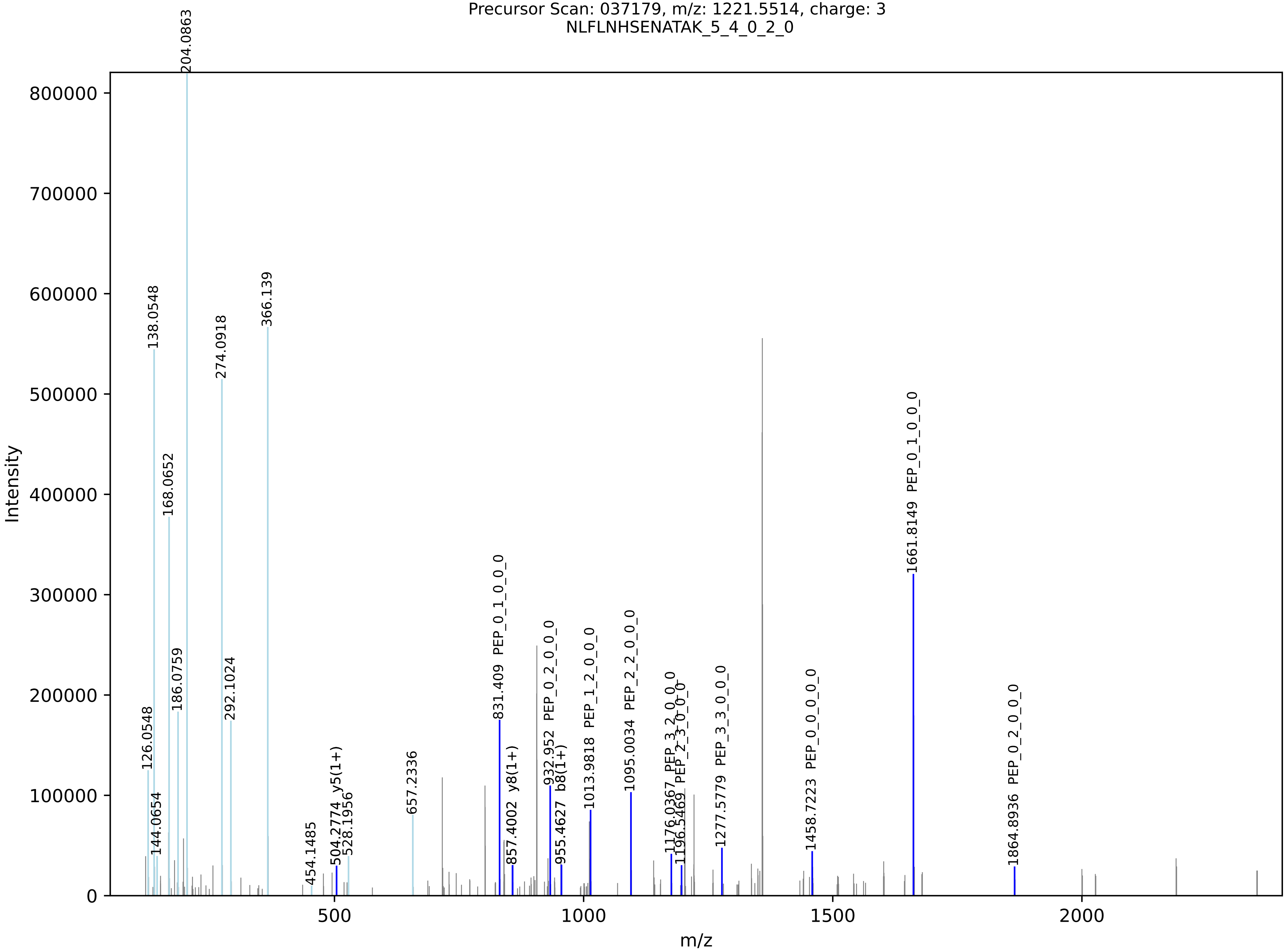

File: 231129_OE_RK_37, RT: 30.03, Scan: 037561
Type: HCD, FTMS + c NSI d Full ms2 1221.5533@hcd30.00 [120.0000-3760.0955]
Precursor Scan: 037474, m/z: 1221.5534, charge: 3
NLFLNHSENATAK_5_4_0_2_0

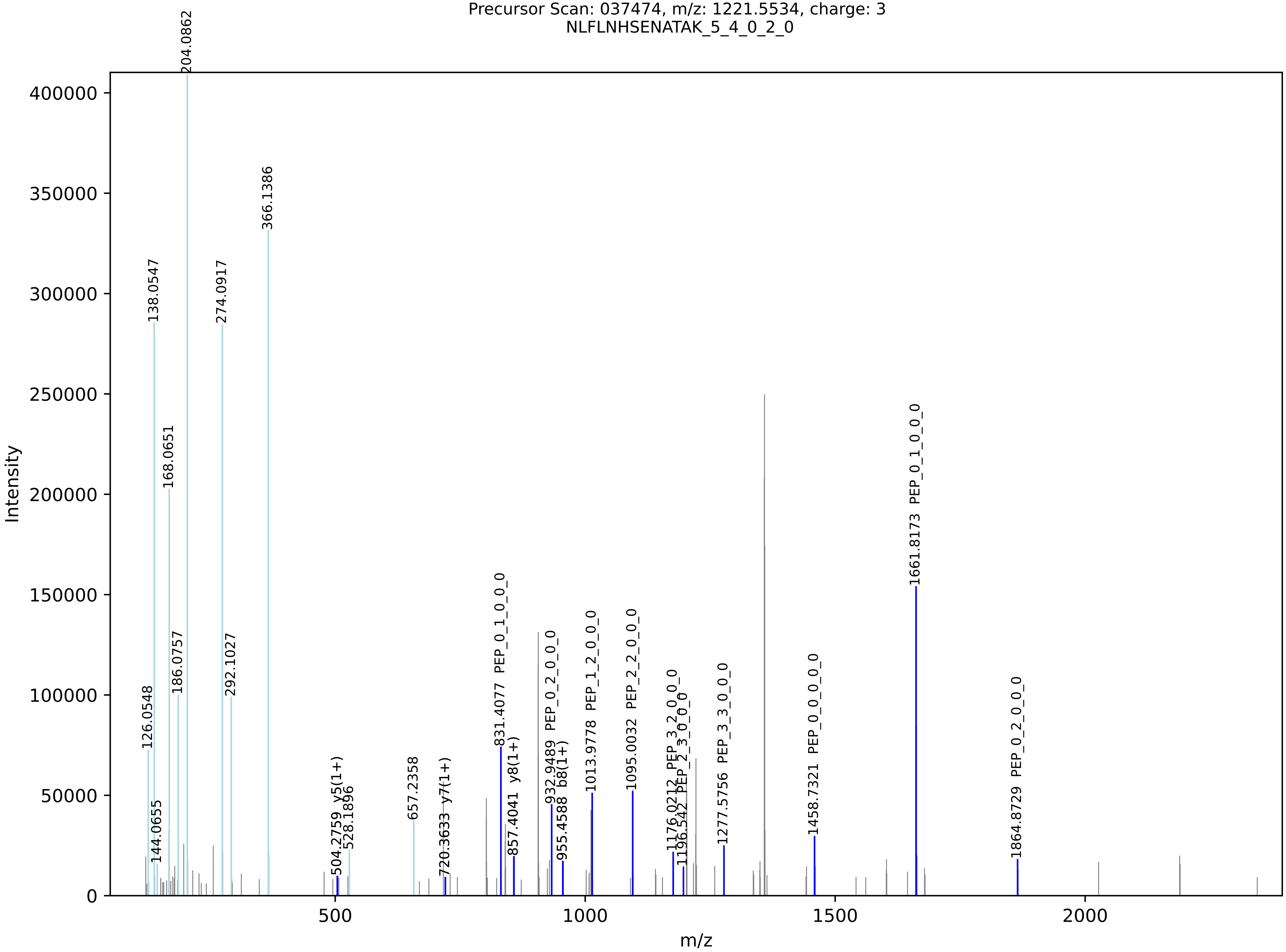

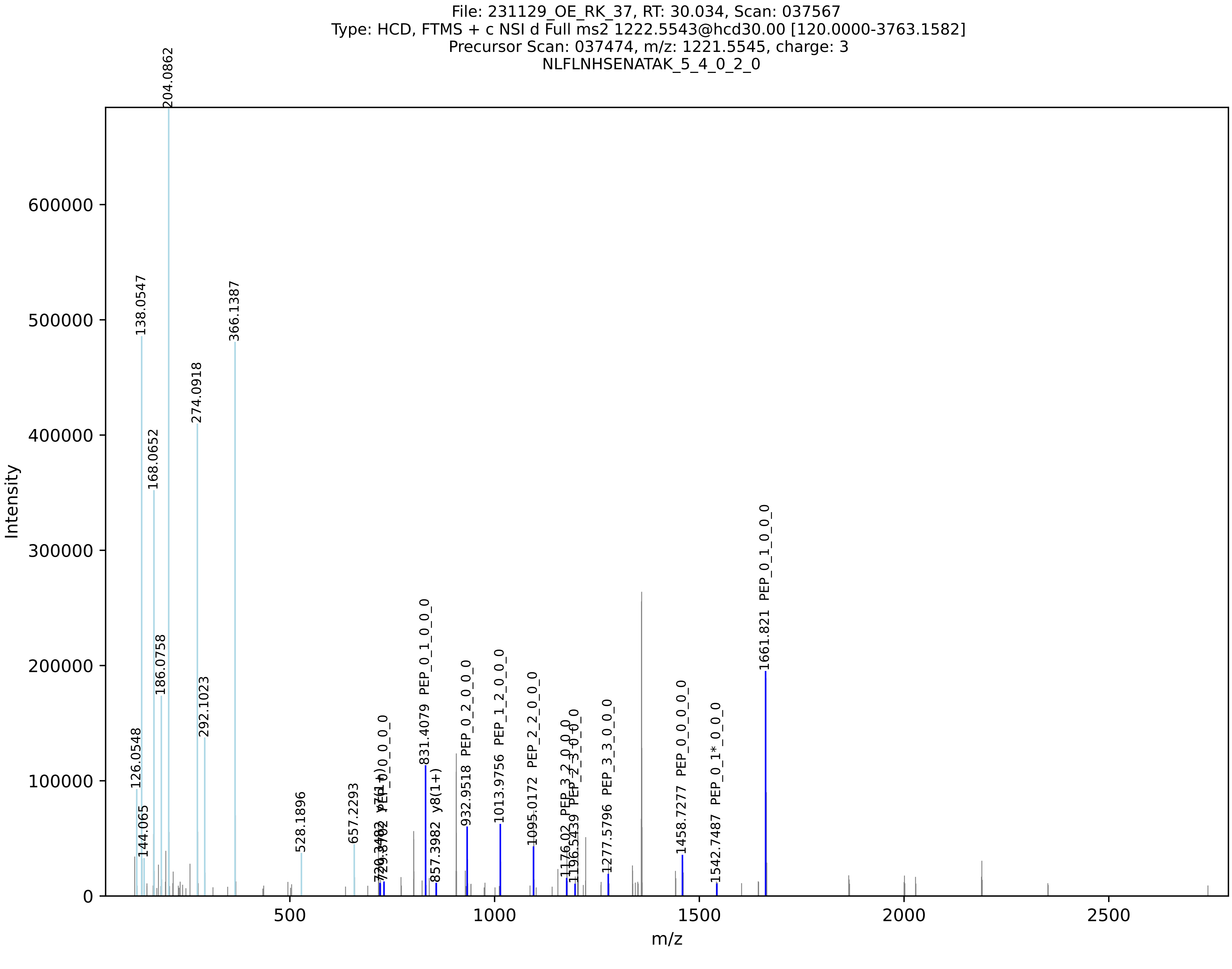
File: 231129_OE_RK_37, RT: 30.034, Scan: 037567
Type: HCD, FTMS + c NSI d Full ms2 1222.5543@hcd30.00 [120.0000-3763.1582]
Precursor Scan: 037474, m/z: 1221.5545, charge: 3
NLFLNHSENATAK_5_4_0_2_0
Intensity
m/z
204.0862
138.0547
366.1387
274.0918
168.0652
186.0758
292.1023
126.0548
144.065
528.1896
657.2293
831.4079 PEP_0_1_0_0_0
857.3982 y8(1+)
932.9518 PEP_0_2_0_0_0
1013.9756 PEP_1_2_0_0_0
1095.0172 PEP_2_2_0_0_0
1277.5796 PEP_3_3_0_0_0
1458.7277 PEP_0_0_0_0_0
1542.7487 PEP_0_1*_0_0_0
1661.821 PEP_0_1_0_0_0

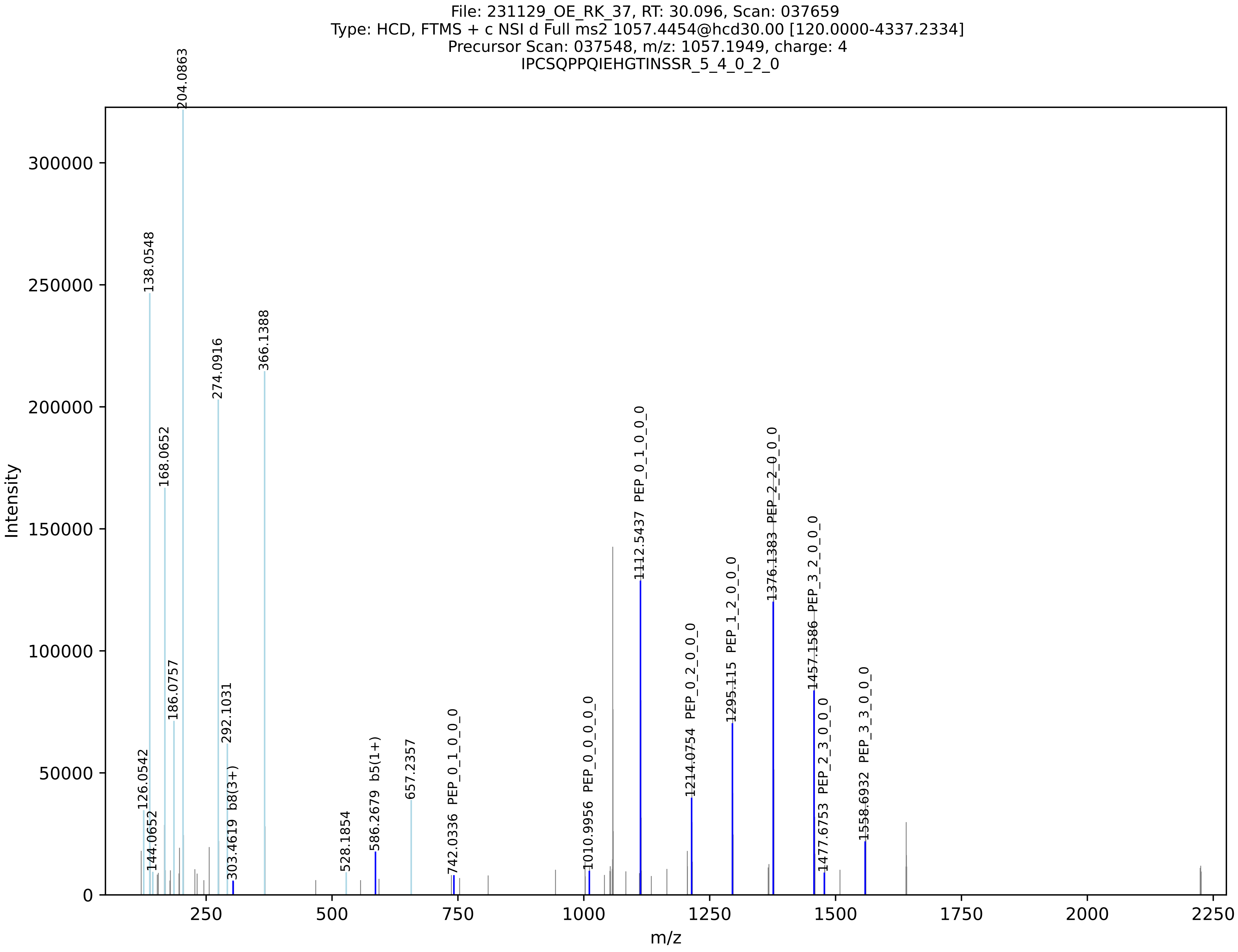

File: 231129_OE_RK_37, RT: 30.096, Scan: 037659
Type: HCD, FTMS + c NSI d Full ms2 1057.4454@hcd30.00 [120.0000-4337.2334]
Precursor Scan: 037548, m/z: 1057.1949, charge: 4
IPCSQPPQIEHGTINSSR_5_4_0_2_0
Intensity
m/z
204.0863
138.0548
366.1388
274.0916
168.0652
186.0757
292.1031
126.0542
144.0652
303.4619 b8(3+)
528.1854
586.2679 b5(1+)
657.2357
742.0336 PEP_0_1_0_0_0
1010.9956 PEP_0_0_0_0_0
1112.5437 PEP_0_1_0_0_0
1214.0754 PEP_0_2_0_0_0
1295.115 PEP_1_2_0_0_0
1376.1383 PEP_2_2_0_0_0
1457.1586 PEP_3_2_0_0_0
1477.6753 PEP_2_3_0_0_0
1558.6932 PEP_3_3_0_0_0

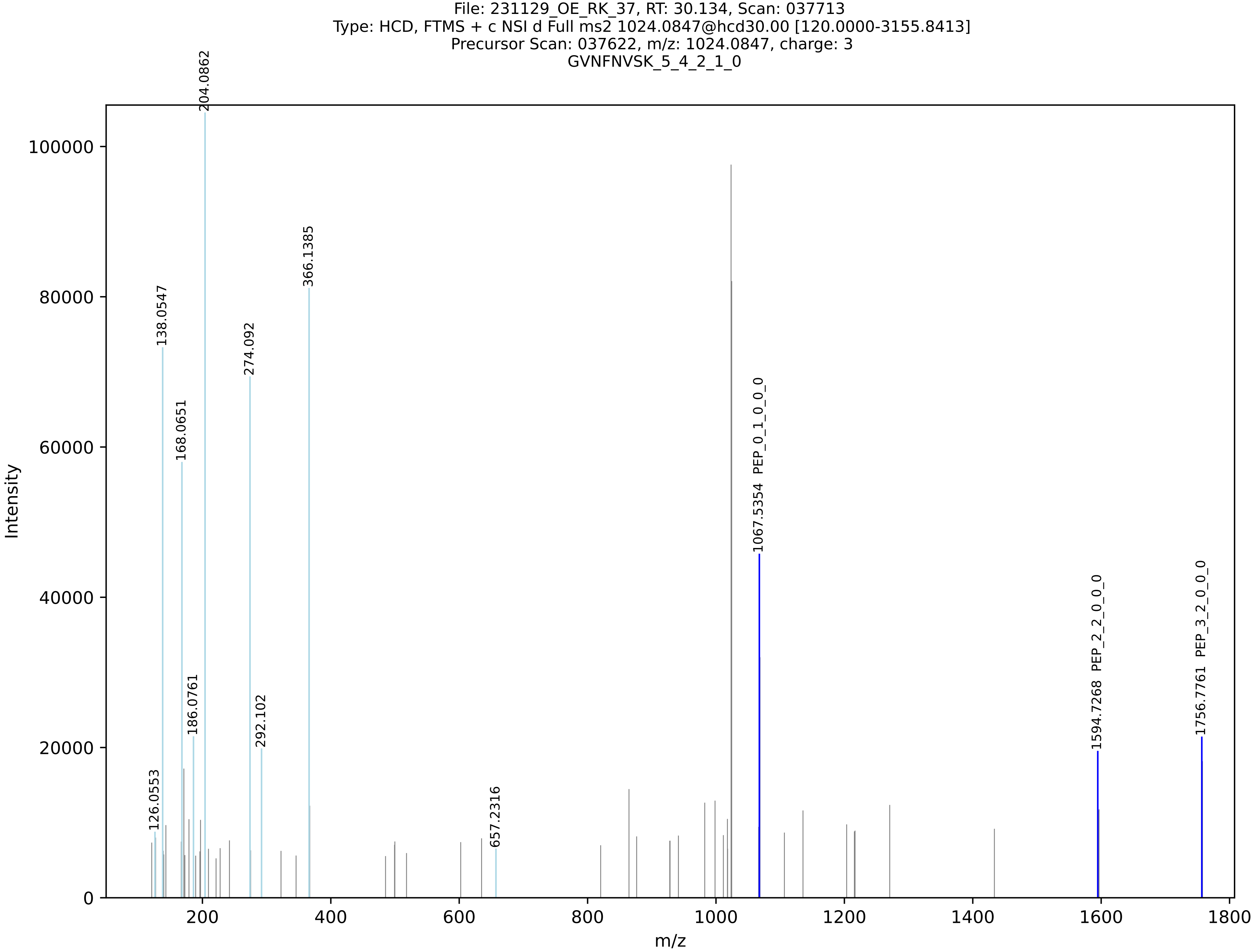
File: 231129_OE_RK_37, RT: 30.134, Scan: 037713
Type: HCD, FTMS + c NSI d Full ms2 1024.0847@hcd30.00 [120.0000-3155.8413]
Precursor Scan: 037622, m/z: 1024.0847, charge: 3
GVNFNVSK_5_4_2_1_0
Intensity
m/z
126.0553
138.0547
168.0651
186.0761
204.0862
274.092
292.102
366.1385
657.2316
1067.5354 PEP_0_1_0_0_0
1594.7268 PEP_2_2_0_0_0
1756.7761 PEP_3_2_0_0_0

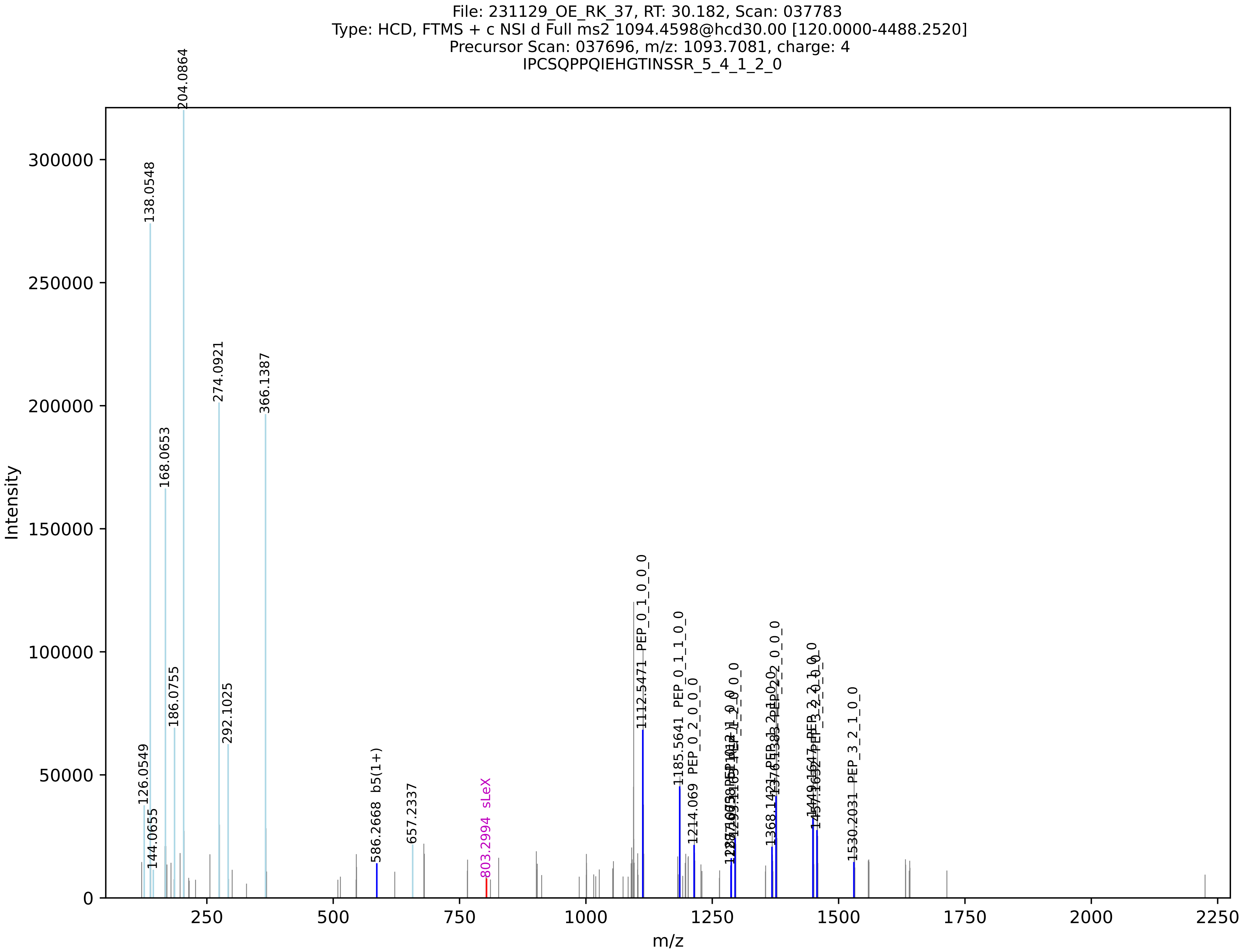
File: 231129_OE_RK_37, RT: 30.182, Scan: 037783
Type: HCD, FTMS + c NSI d Full ms2 1094.4598@hcd30.00 [120.0000-4488.2520]
Precursor Scan: 037696, m/z: 1093.7081, charge: 4
IPCSQPPQIEHGTINSSR_5_4_1_2_0
Intensity
m/z
204.0864
138.0548
168.0653
186.0755
126.0549
144.0655
274.0921
292.1025
366.1387
586.2668 b5(1+)
657.2337
803.2994 sLeX
1112.5471 PEP_0_1_0_0_0
1185.5641 PEP_0_1_1_0_0
1214.069 PEP_0_2_0_0_0
1530.2031 PEP_3_2_1_0_0

File: 231129_OE_RK_37, RT: 30.227, Scan: 037849
Type: HCD, FTMS + c NSI d Full ms2 1409.5933@hcd30.00 [120.0000-4335.4976]
Precursor Scan: 037770, m/z: 1409.2589, charge: 3
IPCSQPPQIEHGTINSSR_5_4_0_2_0

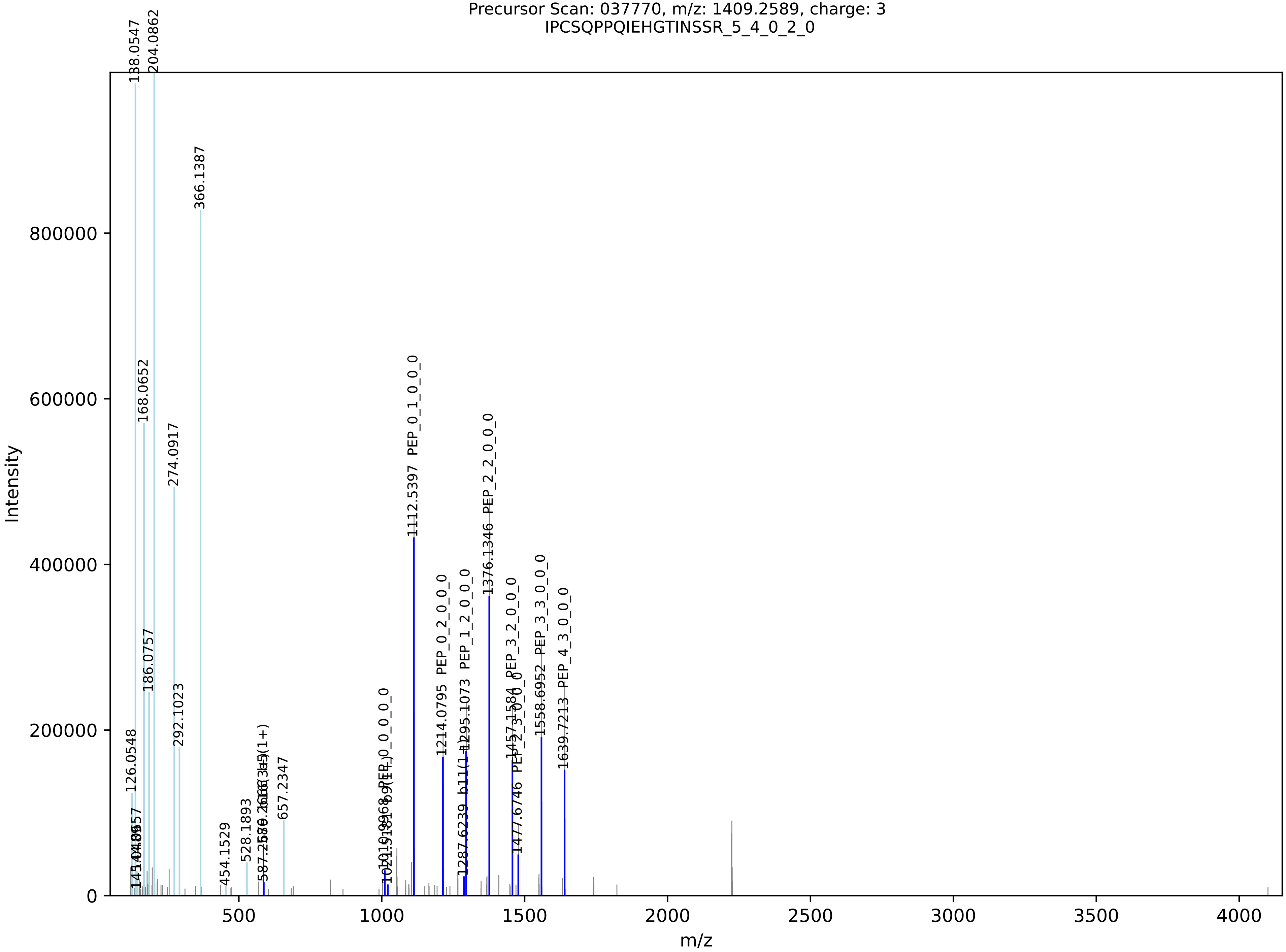

File: 231129_OE_RK_37, RT: 30.275, Scan: 037919
Type: HCD, FTMS + c NSI d Full ms2 1057.6968@hcd30.00 [120.0000-4338.2588]
Precursor Scan: 037844, m/z: 1057.1956, charge: 4
IPCSQPPQIEHGTINSSR_5_4_0_2_0

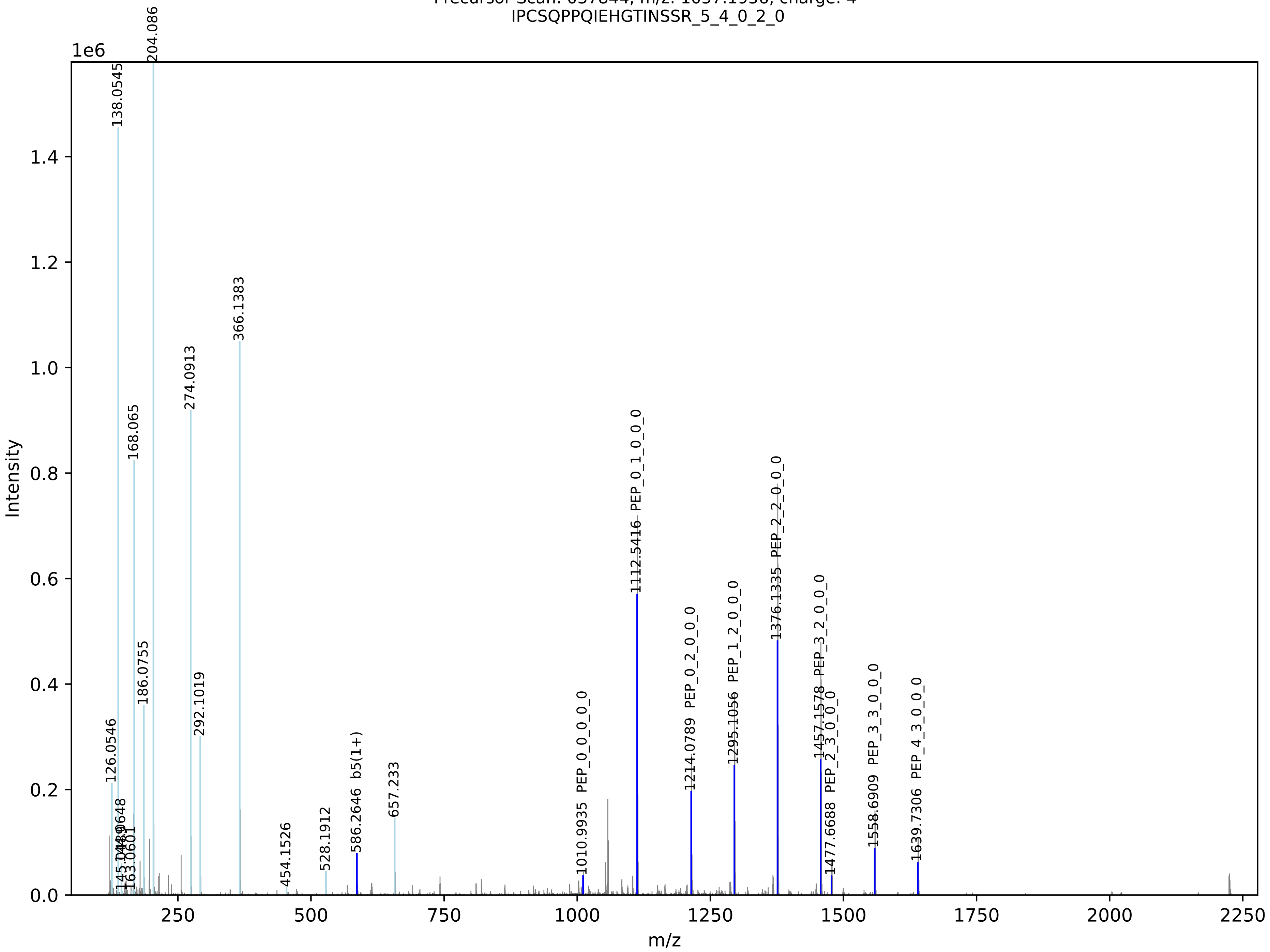

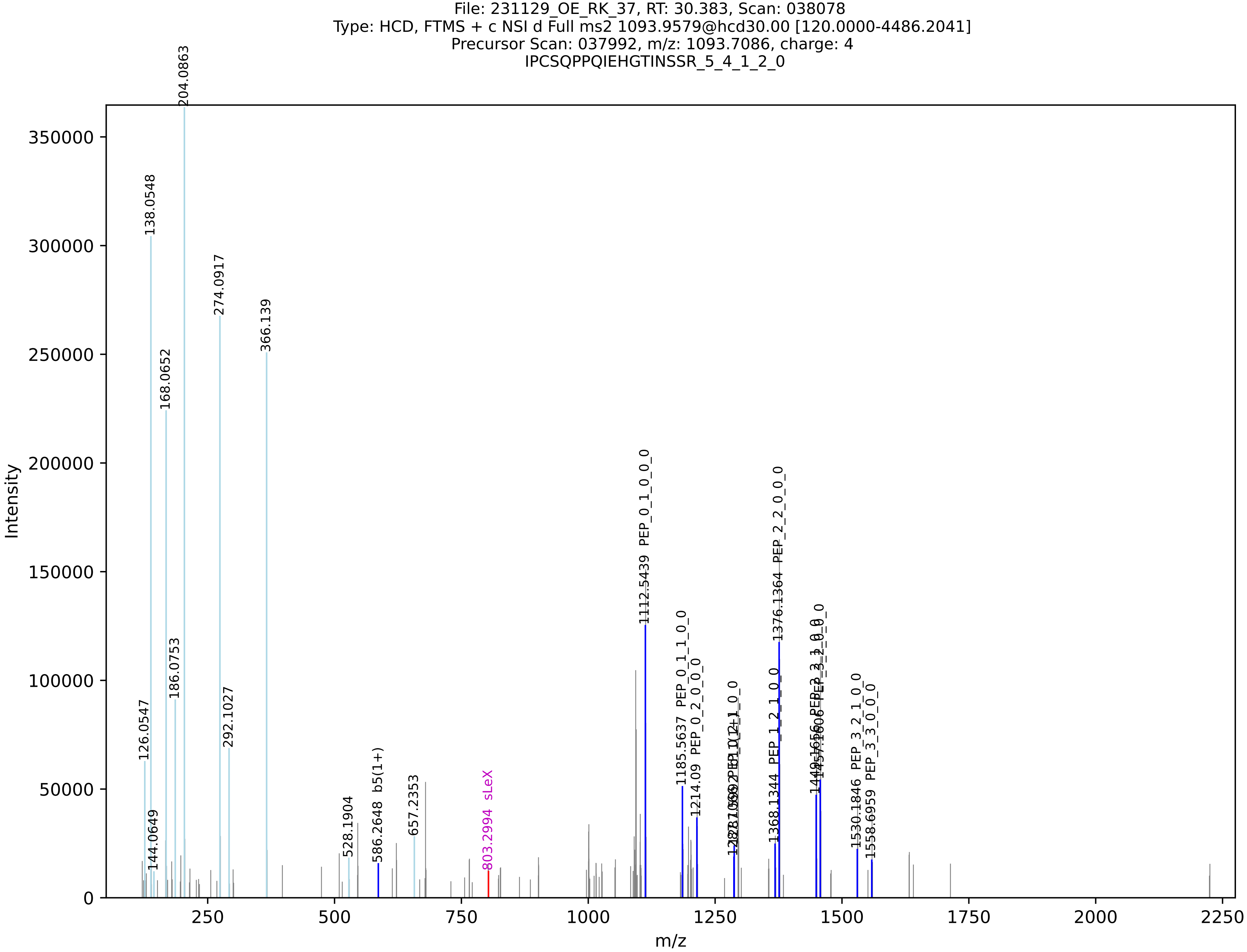

File: 231129_OE_RK_37, RT: 30.383, Scan: 038078
Type: HCD, FTMS + c NSI d Full ms2 1093.9579@hcd30.00 [120.0000-4486.2041]
Precursor Scan: 037992, m/z: 1093.7086, charge: 4
IPCSQPPQIEHGTINSSR_5_4_1_2_0
Intensity
m/z
0
50000
100000
150000
200000
250000
300000
350000
250
500
750
1000
1250
1500
1750
2000
2250
204.0863
138.0548
168.0652
274.0917
366.139
186.0753
126.0547
292.1027
144.0649
528.1904
586.2648 b5(1+)
657.2353
803.2994 sLeX
1112.5439 PEP_0_1_0_0_0
1185.5637 PEP_0_1_1_0_0
1214.09 PEP_0_2_0_0_0
1376.1364 PEP_2_2_0_0_0
1368.1344 PEP_1_2_1_0_0
1530.1846 PEP_3_2_1_0_0
1558.6959 PEP_3_3_0_0_0

File: 231129_OE_RK_37, RT: 30.585, Scan: 038374
Type: HCD, FTMS + c NSI d Full ms2 1458.6133@hcd30.00 [120.0000-4485.4985]
Precursor Scan: 038286, m/z: 1457.945, charge: 3
IPCSQPPQIEHGTINSSR_5_4_1_2_0

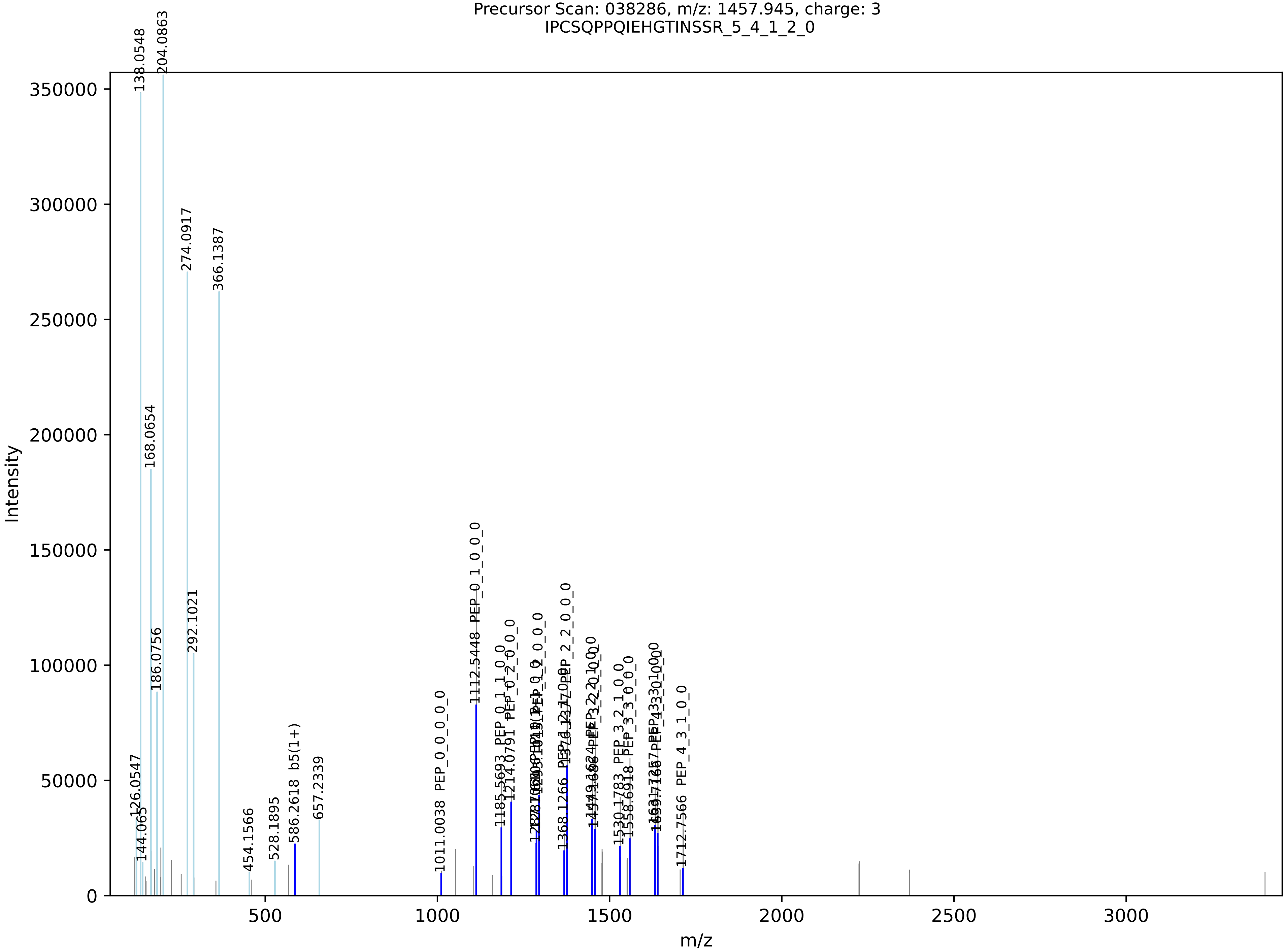

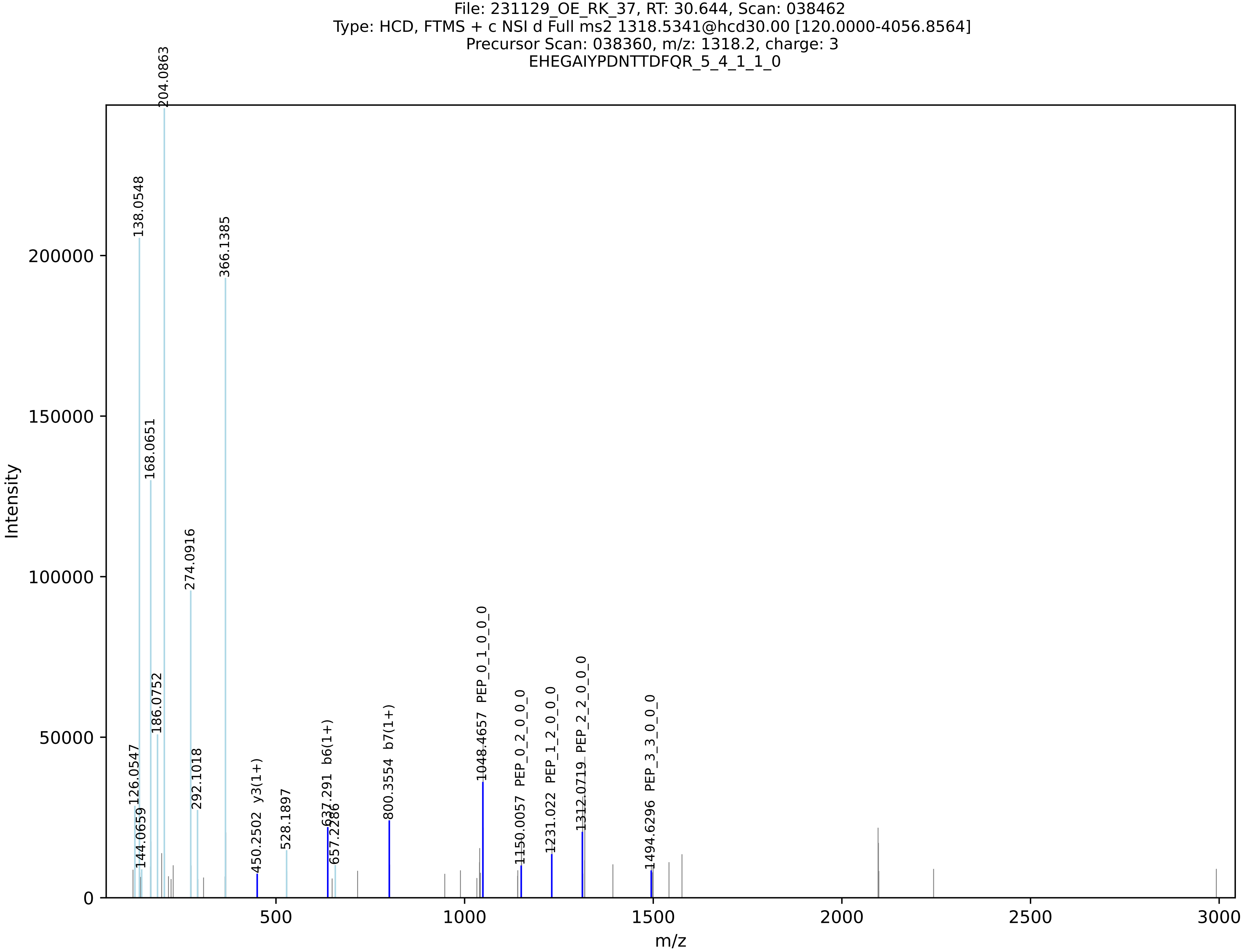
File: 231129_OE_RK_37, RT: 30.644, Scan: 038462
Type: HCD, FTMS + c NSI d Full ms2 1318.5341@hcd30.00 [120.0000-4056.8564]
Precursor Scan: 038360, m/z: 1318.2, charge: 3
EHEGAIYPDNTTDFQR_5_4_1_1_0
204.0863
138.0548
366.1385
168.0651
274.0916
186.0752
126.0547
144.0659
292.1018
450.2502 y3(1+)
528.1897
637.291 b6(1+)
657.2286
800.3554 b7(1+)
1048.4657 PEP_0_1_0_0_0
1150.0057 PEP_0_2_0_0_0
1231.022 PEP_1_2_0_0_0
1312.0719 PEP_2_2_0_0_0
1494.6296 PEP_3_3_0_0_0
Intensity
m/z
0
50000
100000
150000
200000
500
1000
1500
2000
2500
3000

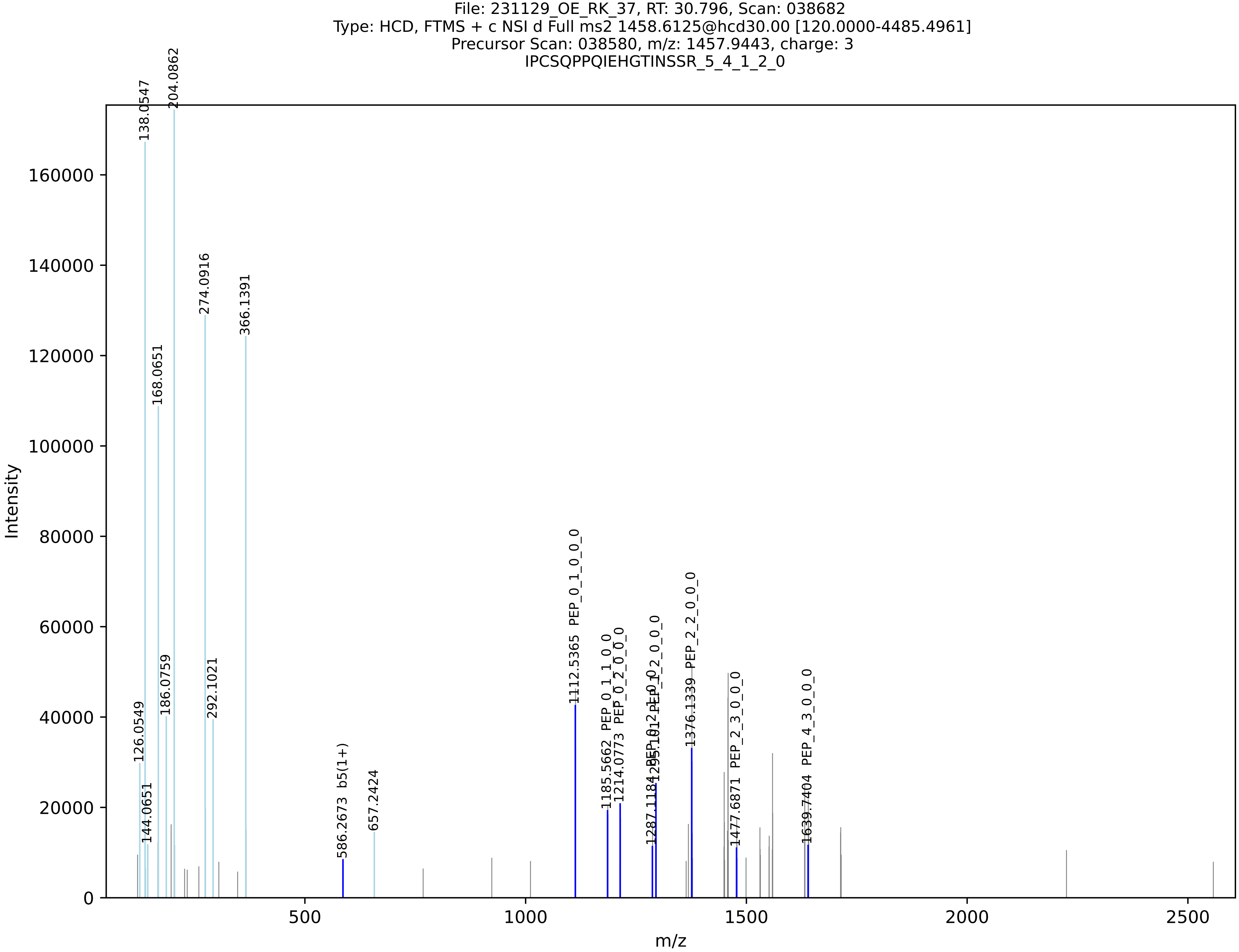
File: 231129_OE_RK_37, RT: 30.796, Scan: 038682
Type: HCD, FTMS + c NSI d Full ms2 1458.6125@hcd30.00 [120.0000-4485.4961]
Precursor Scan: 038580, m/z: 1457.9443, charge: 3
IPCSQPPQIEHGTINSSR_5_4_1_2_0
Intensity
m/z
138.0547
204.0862
274.0916
366.1391
168.0651
186.0759
292.1021
126.0549
144.0651
586.2673 b5(1+)
657.2424
1112.5365 PEP_0_1_0_0_0
1185.5662 PEP_0_1_1_0_0
1214.0773 PEP_0_2_0_0_0
1376.1339 PEP_2_2_0_0_0
1477.6871 PEP_2_3_0_0_0
1639.7404 PEP_4_3_0_0_0

File: 231129_OE_RK_37, RT: 30.799, Scan: 038687
Type: HCD, FTMS + c NSI d Full ms2 1094.2091@hcd30.00 [120.0000-4487.2290]
Precursor Scan: 038580, m/z: 1093.71, charge: 4
IPCSQPPQIEHGTINSSR_5_4_1_2_0

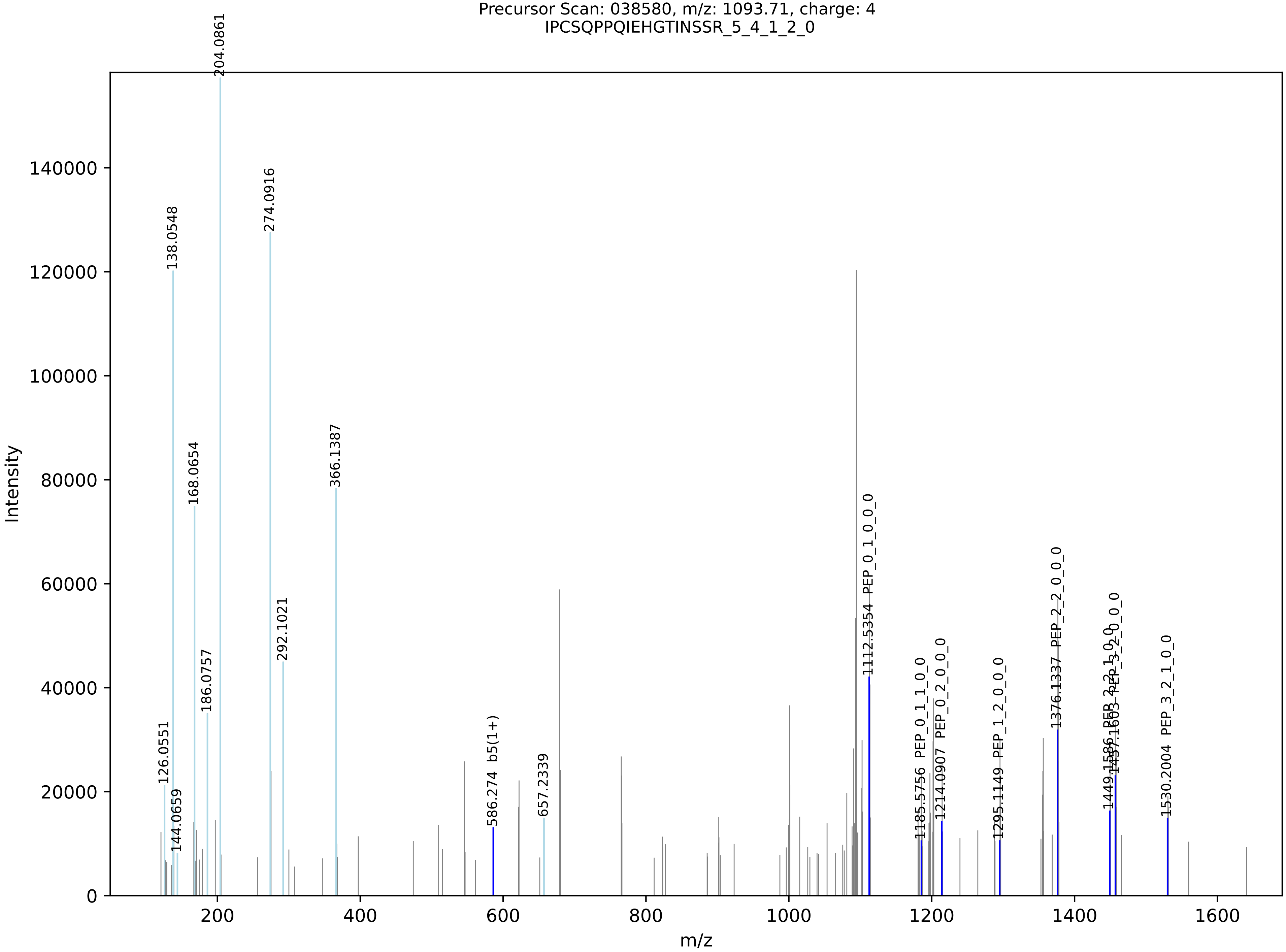

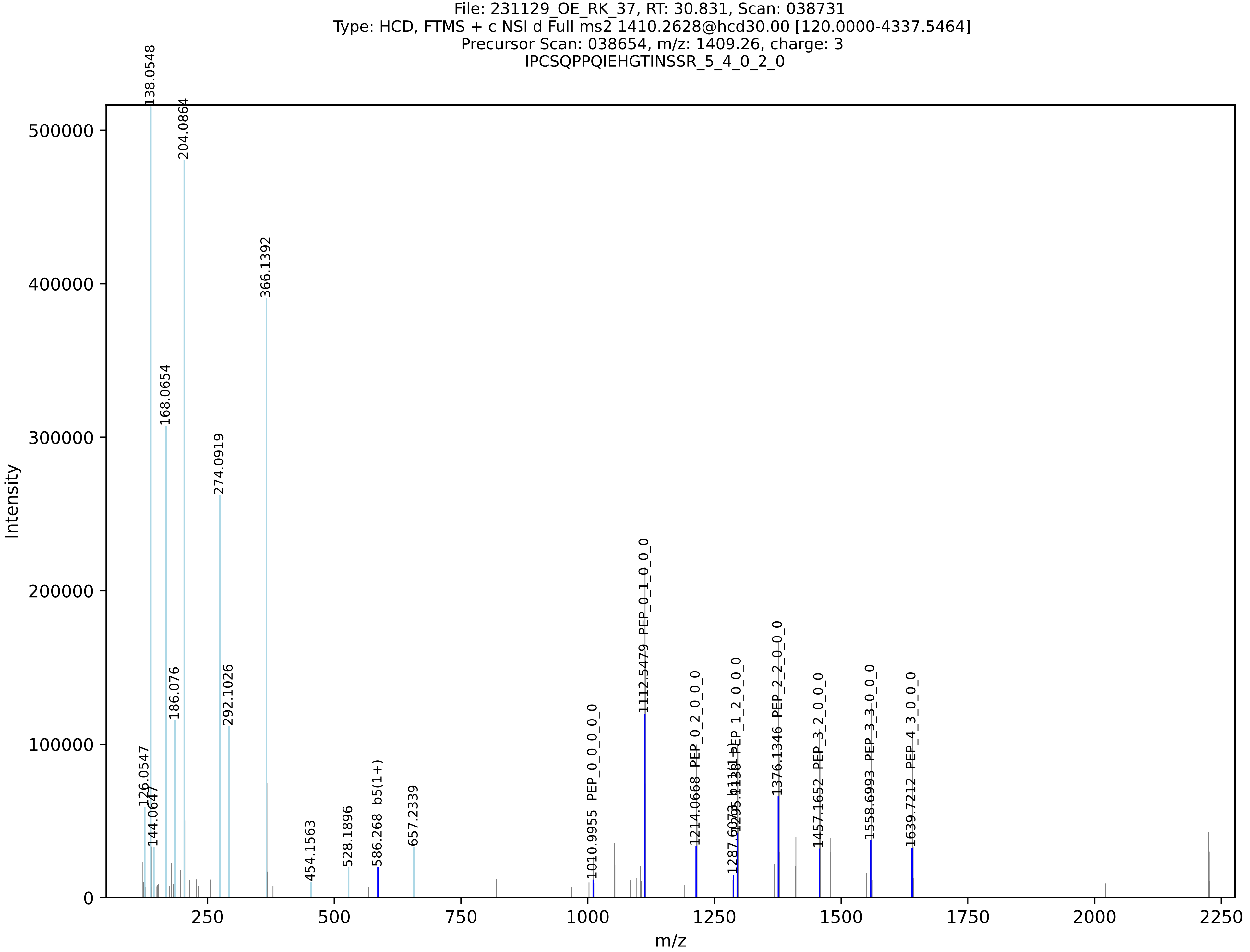
File: 231129_OE_RK_37, RT: 30.831, Scan: 038731
Type: HCD, FTMS + c NSI d Full ms2 1410.2628@hcd30.00 [120.0000-4337.5464]
Precursor Scan: 038654, m/z: 1409.26, charge: 3
IPCSQPPQIEHGTINSSR_5_4_0_2_0
Intensity
m/z
138.0548
204.0864
366.1392
168.0654
274.0919
186.076
292.1026
126.0547
144.0647
454.1563
528.1896
586.268 b5(1+)
657.2339
1010.9955 PEP_0_0_0_0_0
1112.5479 PEP_0_1_0_0_0
1214.0668 PEP_0_2_0_0_0
1376.1346 PEP_2_2_0_0_0
1457.1652 PEP_3_2_0_0_0
1558.6993 PEP_3_3_0_0_0
1639.7212 PEP_4_3_0_0_0

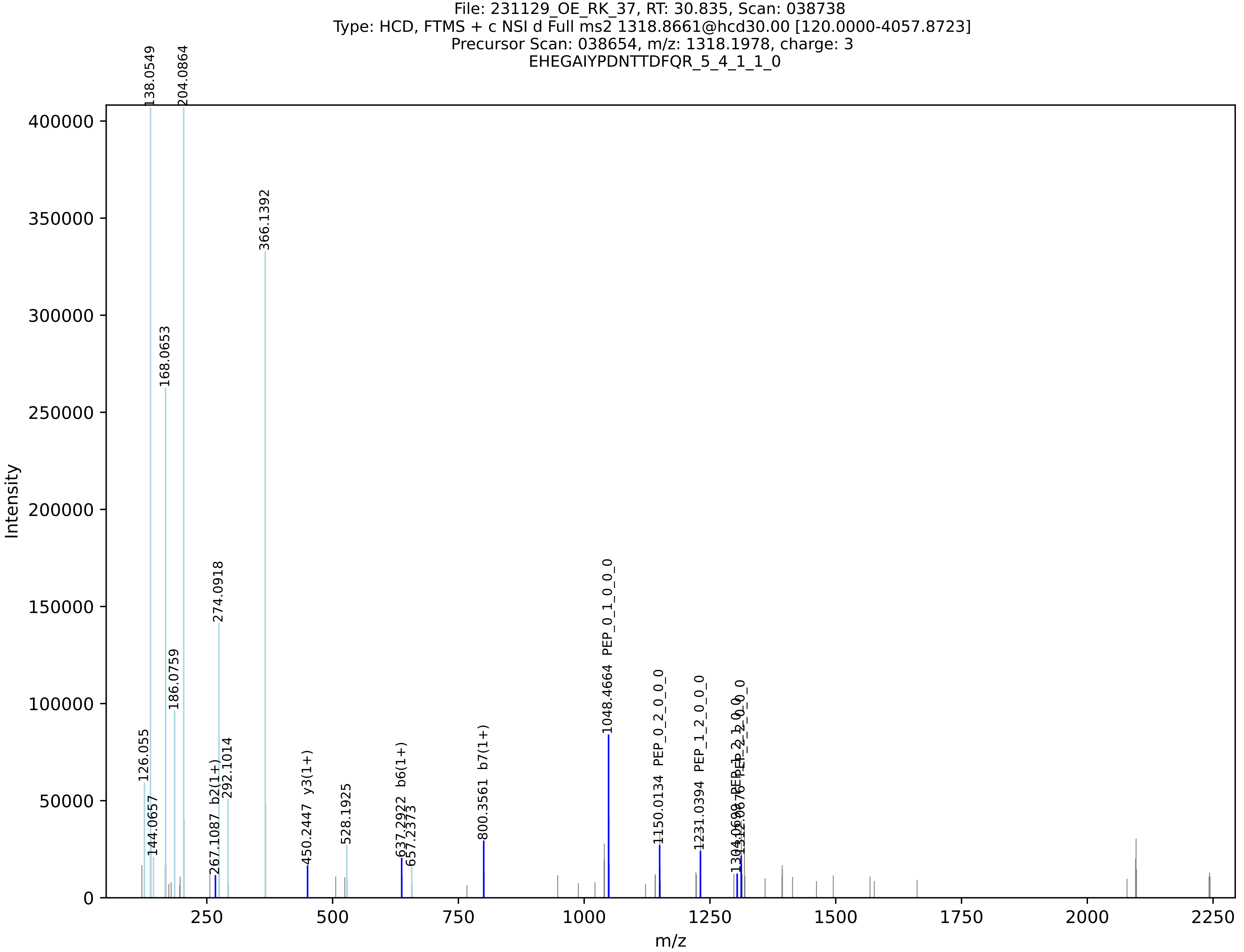
File: 231129_OE_RK_37, RT: 30.835, Scan: 038738
Type: HCD, FTMS + c NSI d Full ms2 1318.8661@hcd30.00 [120.0000-4057.8723]
Precursor Scan: 038654, m/z: 1318.1978, charge: 3
EHEGAIYPDNTTDFQR_5_4_1_1_0
Intensity
m/z
138.0549
204.0864
366.1392
168.0653
274.0918
186.0759
126.055
144.0657
267.1087 b2(1+)
292.1014
450.2447 y3(1+)
528.1925
637.2922 b6(1+)
657.2373
800.3561 b7(1+)
1048.4664 PEP_0_1_0_0_0
1150.0134 PEP_0_2_0_0_0
1231.0394 PEP_1_2_0_0_0

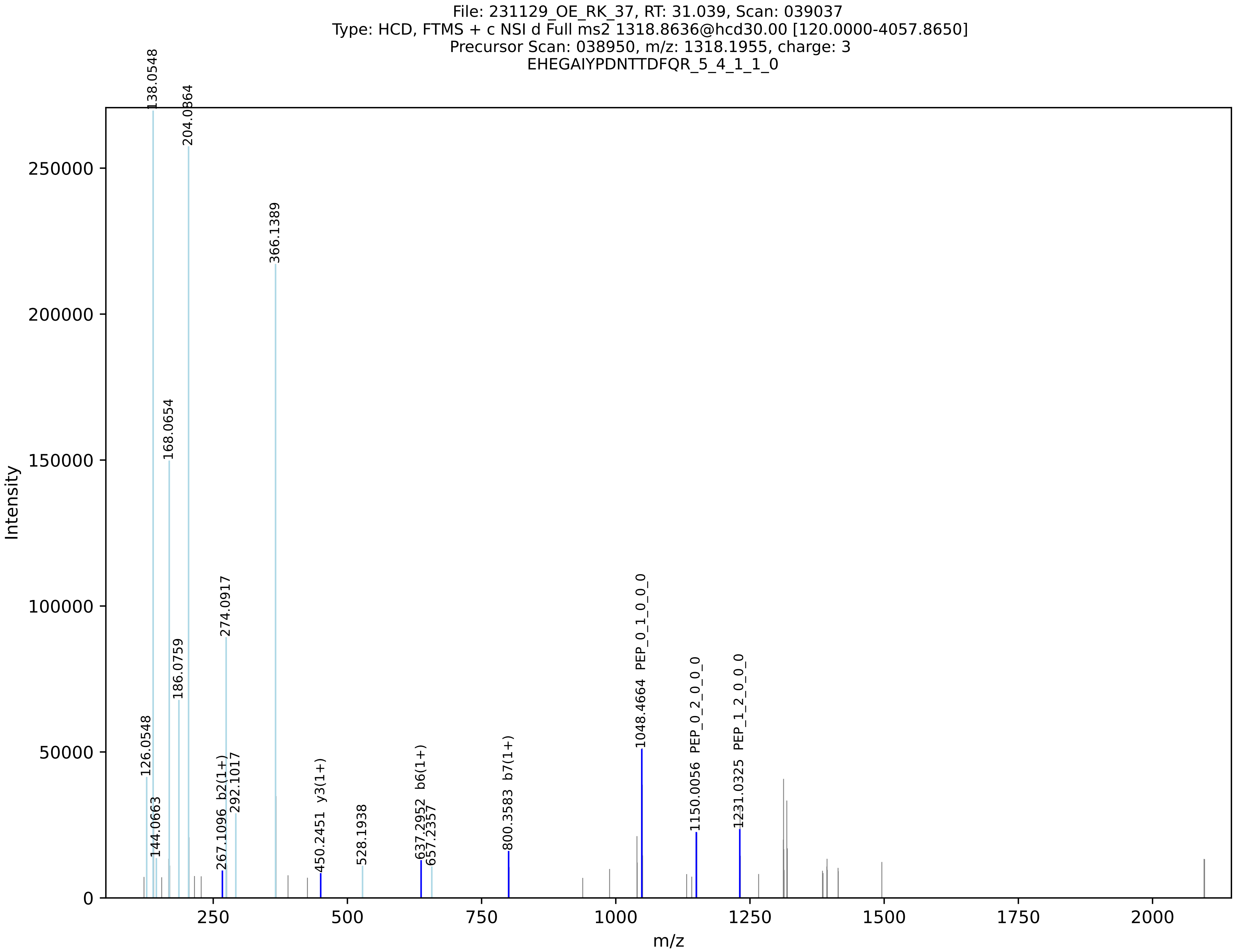

File: 231129_OE_RK_37, RT: 31.039, Scan: 039037
Type: HCD, FTMS + c NSI d Full ms2 1318.8636@hcd30.00 [120.0000-4057.8650]
Precursor Scan: 038950, m/z: 1318.1955, charge: 3
EHEGAIYPDNTTDFQR_5_4_1_1_0
Intensity
m/z
138.0548
204.0864
366.1389
168.0654
186.0759
274.0917
126.0548
144.0663
267.1096 b2(1+)
292.1017
450.2451 y3(1+)
528.1938
637.2952 b6(1+)
657.2357
800.3583 b7(1+)
1048.4664 PEP_0_1_0_0_0
1150.0056 PEP_0_2_0_0_0
1231.0325 PEP_1_2_0_0_0

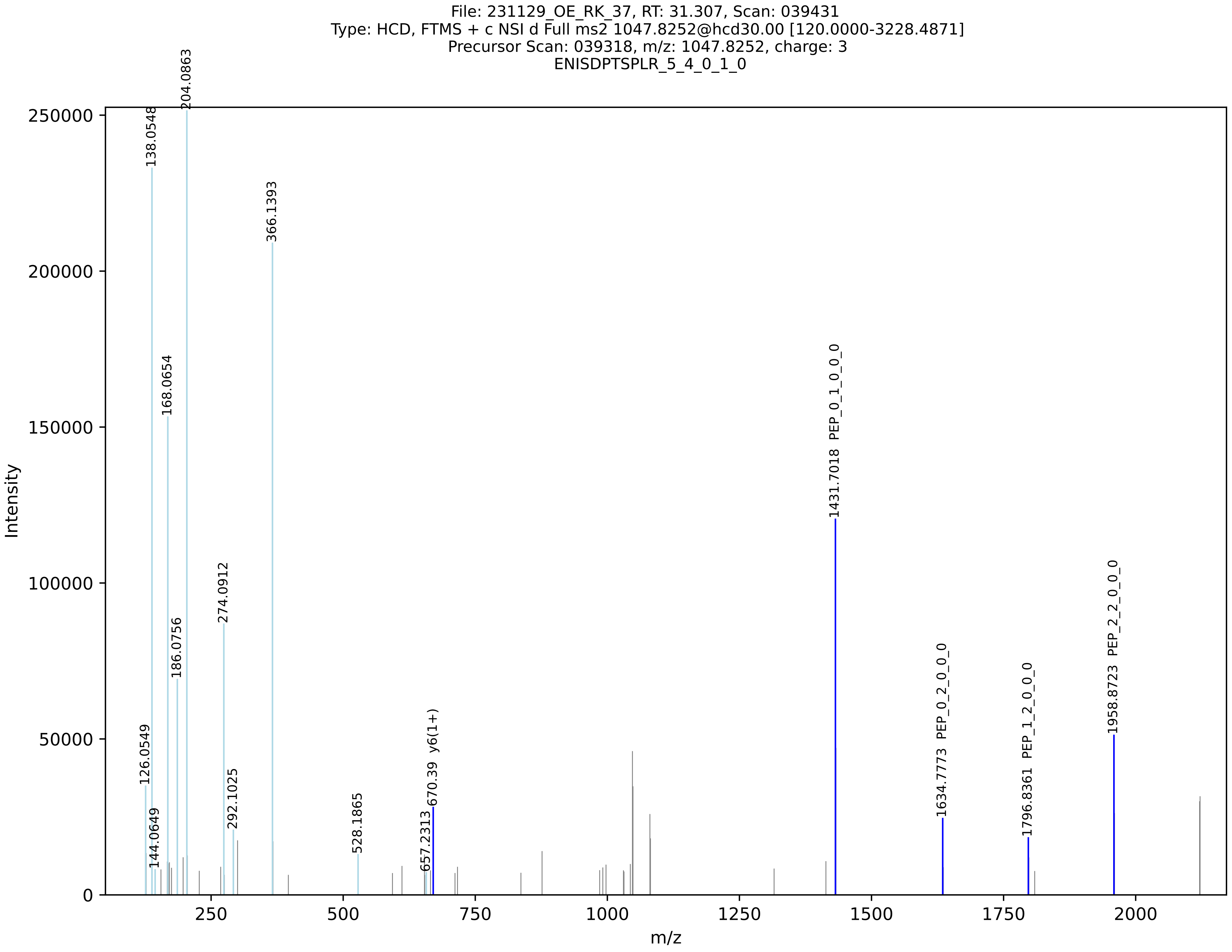
File: 231129_OE_RK_37, RT: 31.307, Scan: 039431
Type: HCD, FTMS + c NSI d Full ms2 1047.8252@hcd30.00 [120.0000-3228.4871]
Precursor Scan: 039318, m/z: 1047.8252, charge: 3
ENISDPTSPLR_5_4_0_1_0
Intensity
m/z
250000
200000
150000
100000
50000
0
250
500
750
1000
1250
1500
1750
2000
204.0863
138.0548
366.1393
168.0654
186.0756
274.0912
126.0549
144.0649
292.1025
528.1865
657.2313
670.39 y6(1+)
1431.7018 PEP_0_1_0_0_0
1634.7773 PEP_0_2_0_0_0
1796.8361 PEP_1_2_0_0_0
1958.8723 PEP_2_2_0_0_0

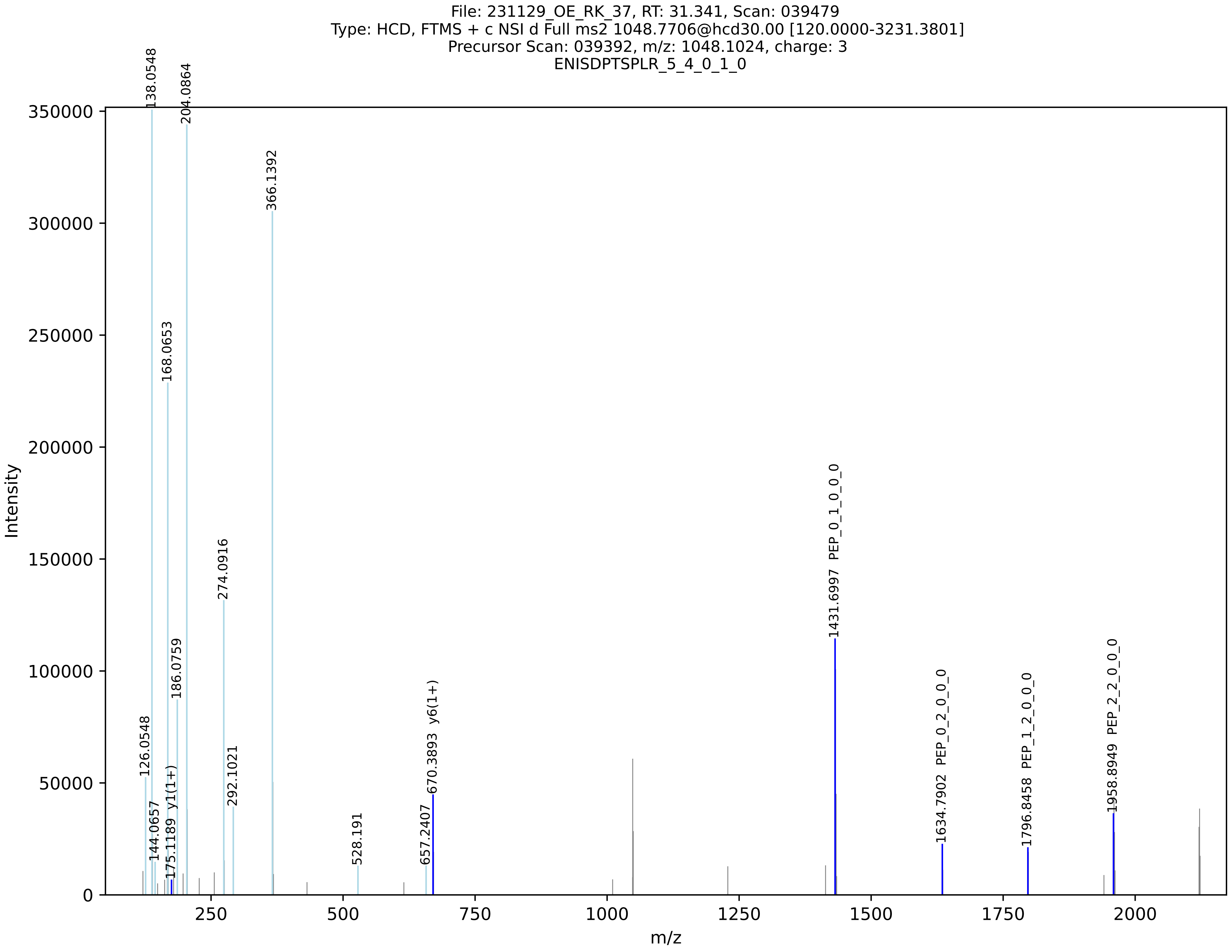

File: 231129_OE_RK_37, RT: 31.341, Scan: 039479
Type: HCD, FTMS + c NSI d Full ms2 1048.7706@hcd30.00 [120.0000-3231.3801]
Precursor Scan: 039392, m/z: 1048.1024, charge: 3
ENISDPTSPLR_5_4_0_1_0
Intensity
m/z
138.0548
204.0864
366.1392
168.0653
274.0916
186.0759
126.0548
292.1021
144.0657
175.1189 y1(1+)
528.191
657.2407
670.3893 y6(1+)
1431.6997 PEP_0_1_0_0_0
1634.7902 PEP_0_2_0_0_0
1796.8458 PEP_1_2_0_0_0
1958.8949 PEP_2_2_0_0_0

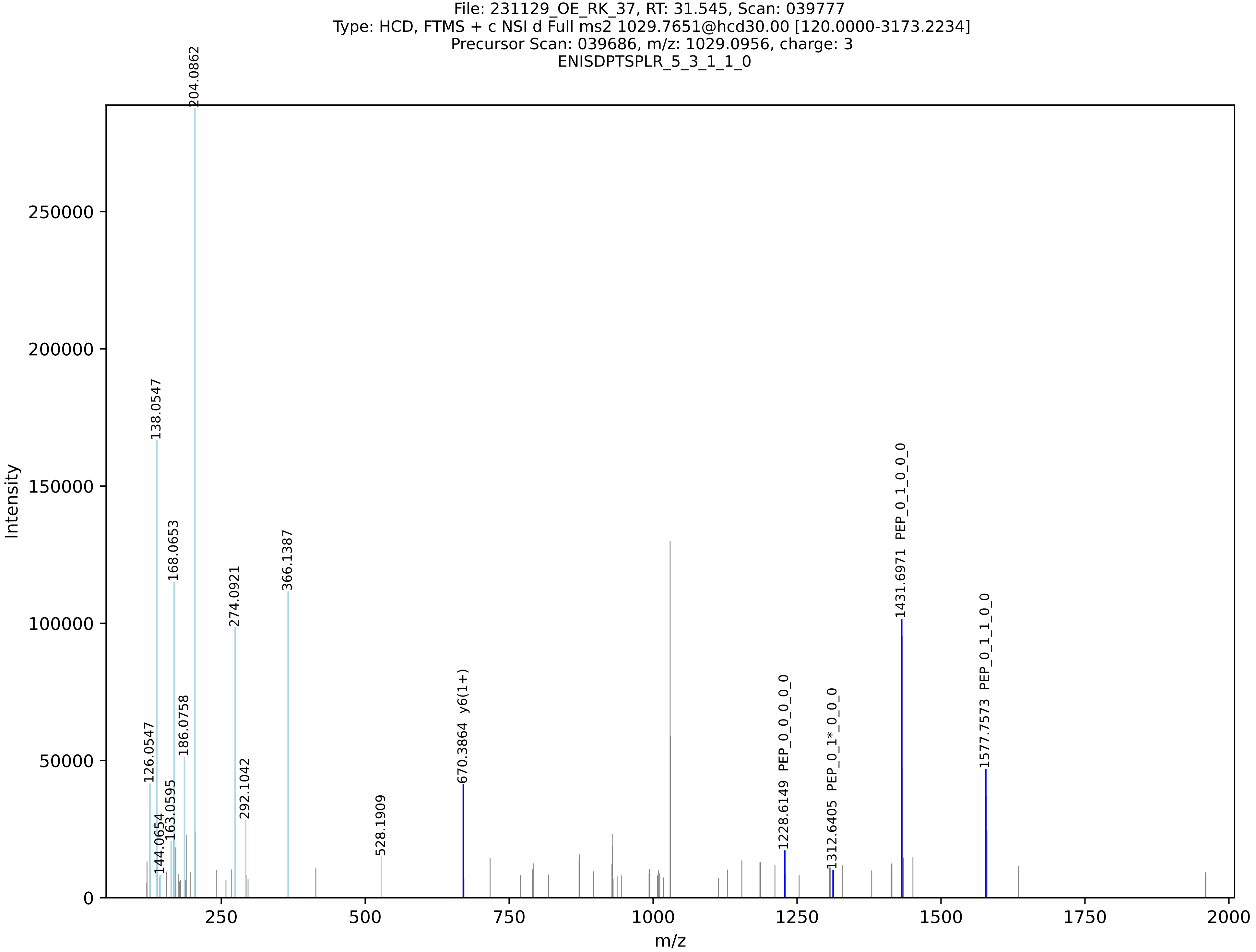

File: 231129_OE_RK_37, RT: 31.545, Scan: 039777
Type: HCD, FTMS + c NSI d Full ms2 1029.7651@hcd30.00 [120.0000-3173.2234]
Precursor Scan: 039686, m/z: 1029.0956, charge: 3
ENISDPTSPLR_5_3_1_1_0
Intensity
m/z
204.0862
138.0547
168.0653
366.1387
274.0921
186.0758
126.0547
292.1042
163.0595
144.0654
528.1909
670.3864 y6(1+)
1228.6149 PEP_0_0_0_0_0
1312.6405 PEP_0_1*_0_0_0
1431.6971 PEP_0_1_0_0_0
1577.7573 PEP_0_1_1_0_0
0
50000
100000
150000
200000
250000
250
500
750
1000
1250
1500
1750
2000

File: 231129_OE_RK_37, RT: 31.59, Scan: 039843
Type: HCD, FTMS + c NSI d Full ms2 1311.2050@hcd30.00 [120.0000-4034.4292]
Precursor Scan: 039760, m/z: 1310.5367, charge: 3
FSDGLESNSSTQFEVKK_10_2_0_0_0

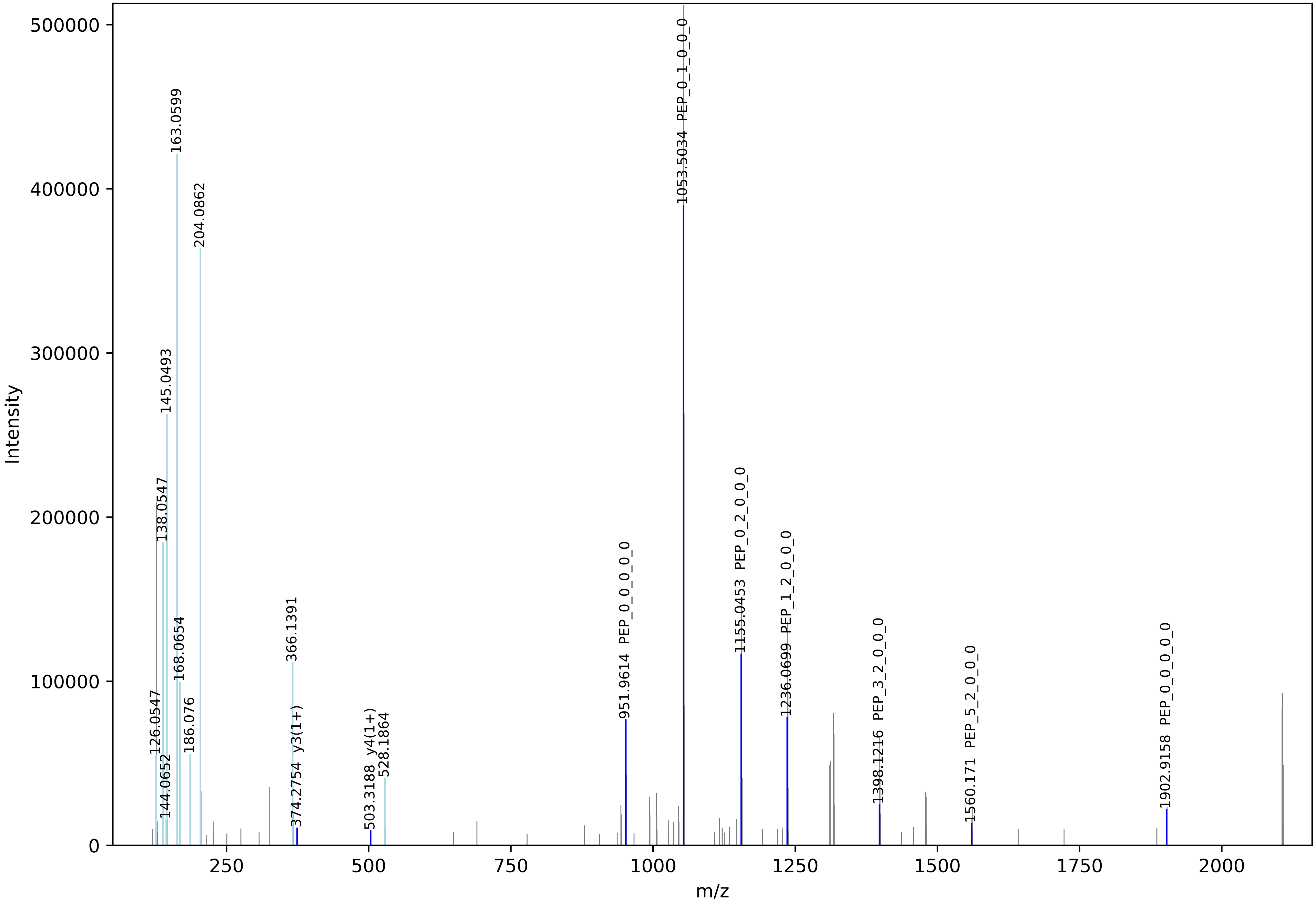

File: 231129_OE_RK_37, RT: 32.452, Scan: 041104
Type: HCD, FTMS + c NSI d Full ms2 1145.4696@hcd30.00 [120.0000-3527.2791]
Precursor Scan: 041016, m/z: 1145.1355, charge: 3
ENISDPTSPLR_5_4_0_2_0

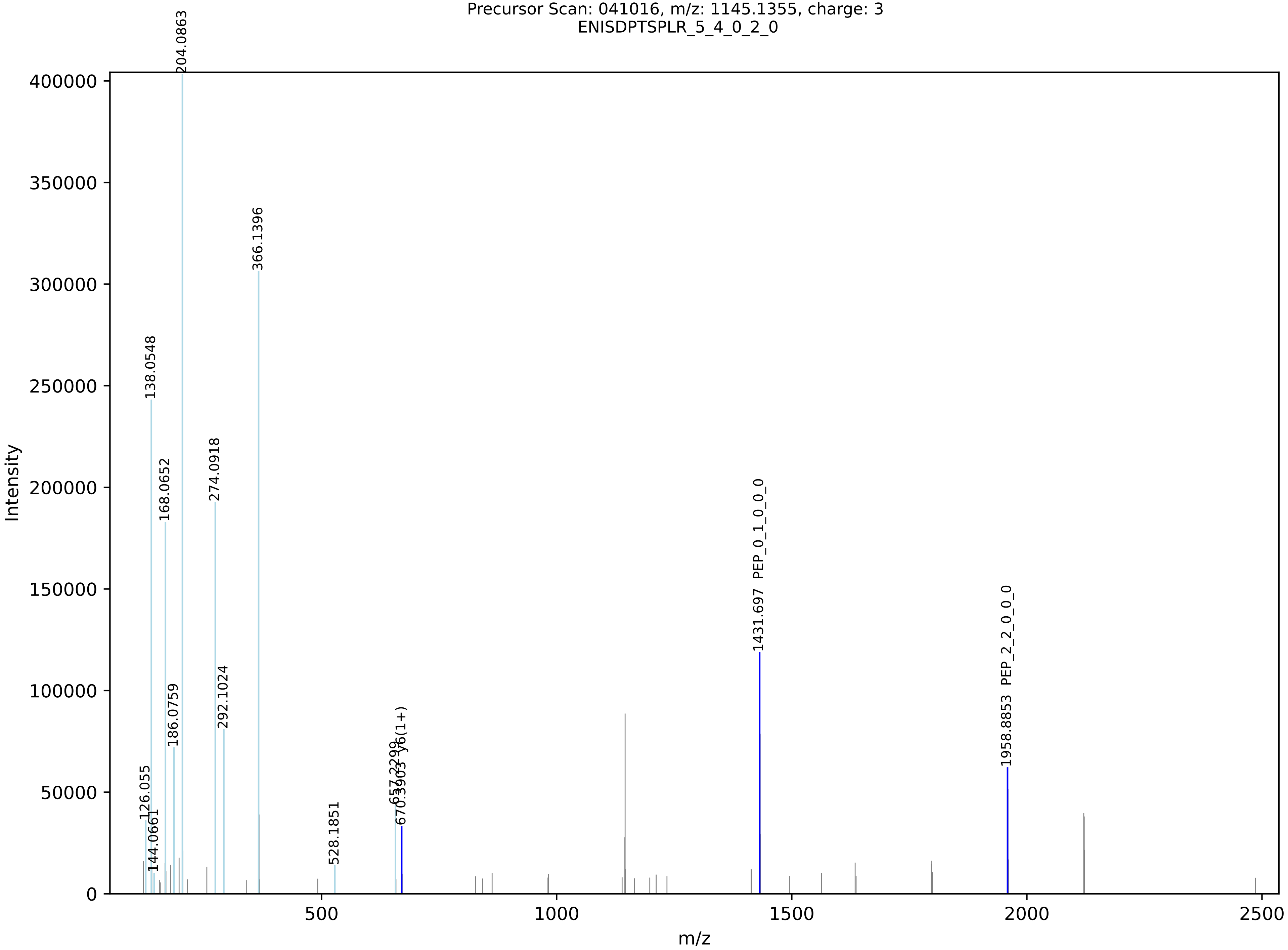

File: 231129_OE_RK_37, RT: 32.658, Scan: 041400
Type: HCD, FTMS + c NSI d Full ms2 1062.4235@hcd30.00 [120.0000-4357.5435]
Precursor Scan: 041349, m/z: 1061.4226, charge: 4
GTAGNALMDGASQLMGENR_5_4_1_2_0

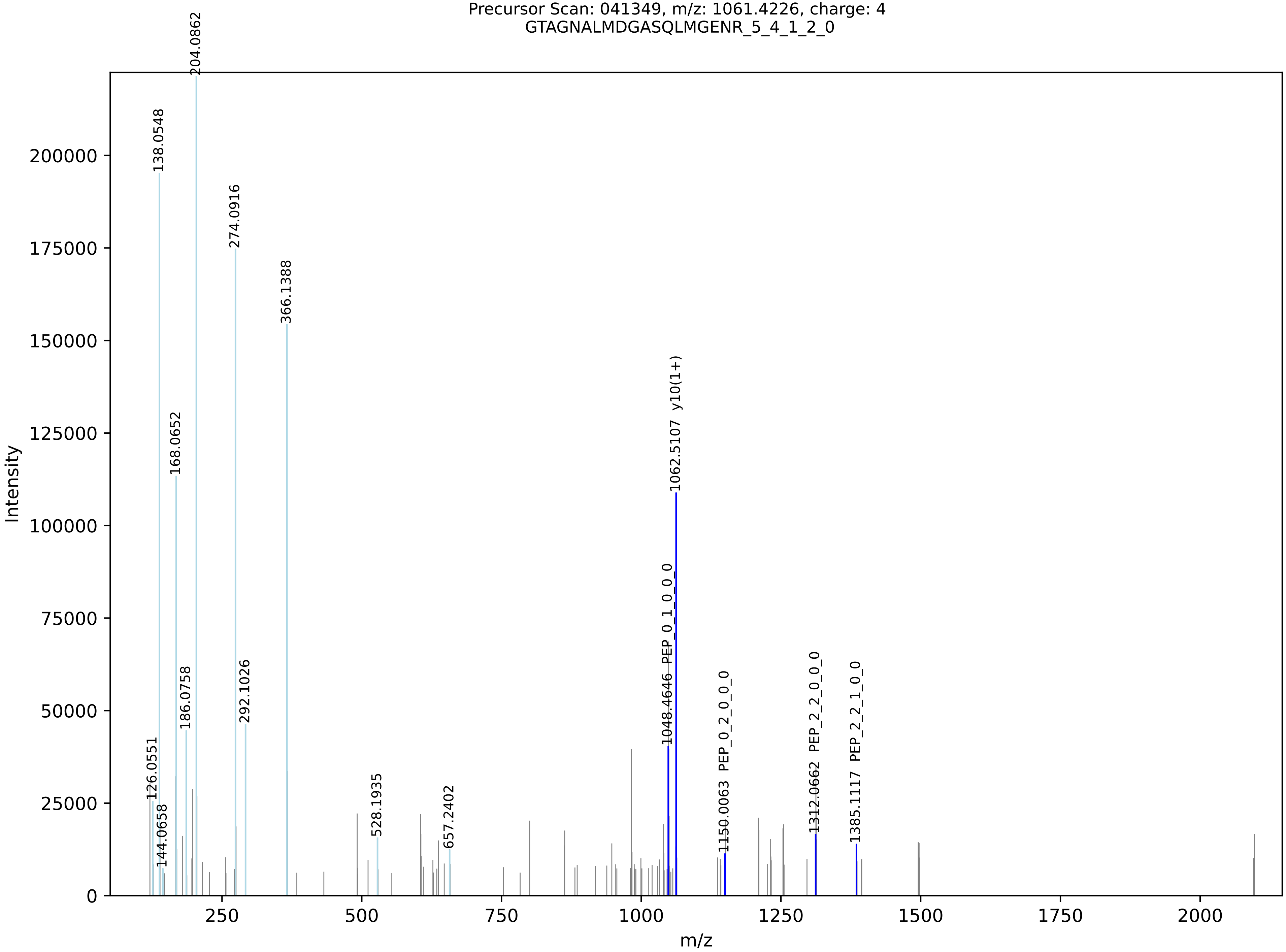

File: 231129_OE_RK_37, RT: 32.745, Scan: 041525
Type: HCD, FTMS + c NSI d Full ms2 1415.8959@hcd30.00 [120.0000-4354.7837]
Precursor Scan: 041457, m/z: 1415.2283, charge: 3
EHEGAIYPDNTTDFQR_5_4_1_2_0

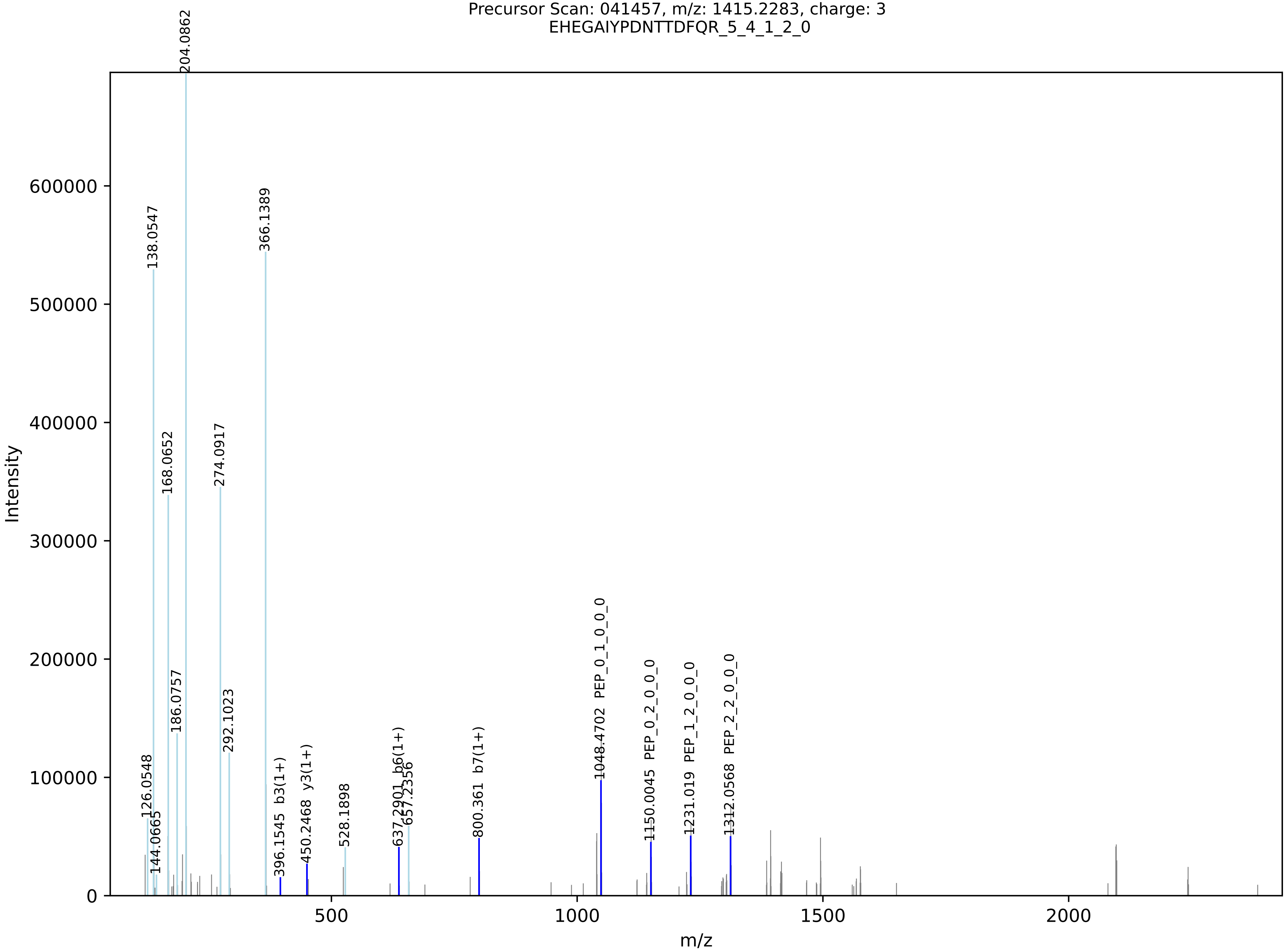

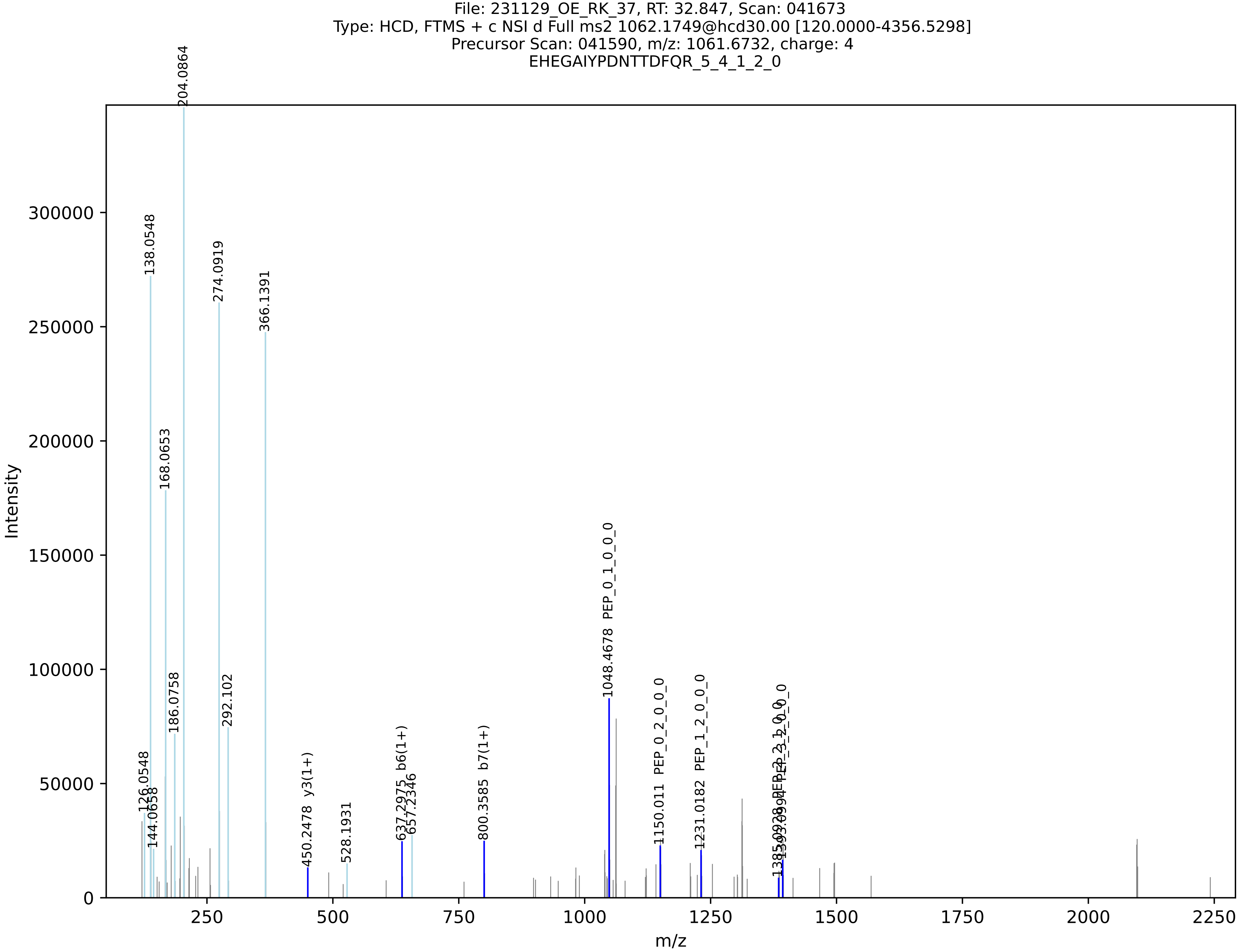

File: 231129_OE_RK_37, RT: 32.847, Scan: 041673
Type: HCD, FTMS + c NSI d Full ms2 1062.1749@hcd30.00 [120.0000-4356.5298]
Precursor Scan: 041590, m/z: 1061.6732, charge: 4
EHEGAIYPDNTTDFQR_5_4_1_2_0
204.0864
138.0548
274.0919
366.1391
168.0653
186.0758
292.102
126.0548
144.0658
450.2478 y3(1+)
528.1931
637.2975 b6(1+)
657.2346
800.3585 b7(1+)
1048.4678 PEP_0_1_0_0_0
1150.011 PEP_0_2_0_0_0
1231.0182 PEP_1_2_0_0_0
Intensity
m/z
0
50000
100000
150000
200000
250000
300000
250
500
750
1000
1250
1500
1750
2000
2250

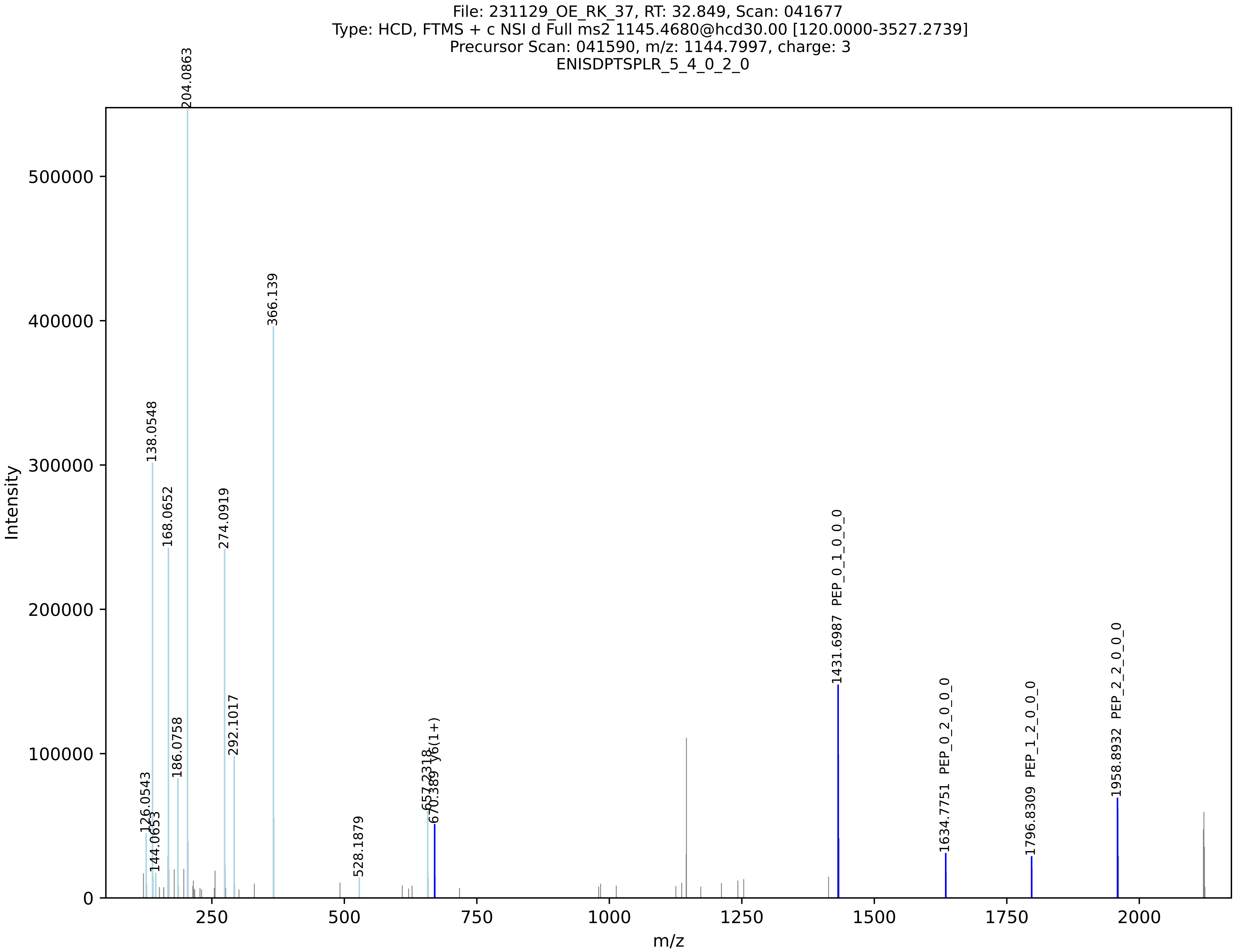

File: 231129_OE_RK_37, RT: 32.849, Scan: 041677
Type: HCD, FTMS + c NSI d Full ms2 1145.4680@hcd30.00 [120.0000-3527.2739]
Precursor Scan: 041590, m/z: 1144.7997, charge: 3
ENISDPTSPLR_5_4_0_2_0
204.0863
366.139
138.0548
168.0652
274.0919
186.0758
292.1017
126.0543
144.0653
528.1879
657.2318
670.389 y6(1+)
1431.6987 PEP_0_1_0_0_0
1634.7751 PEP_0_2_0_0_
1796.8309 PEP_1_2_0_0_0
1958.8932 PEP_2_2_0_0_0
Intensity
500000
400000
300000
200000
100000
0
250
500
750
1000
1250
1500
1750
2000
m/z

File: 231129_OE_RK_37, RT: 33.145, Scan: 042103
Type: HCD, FTMS + c NSI d Full ms2 1415.8987@hcd30.00 [120.0000-4354.7920]
Precursor Scan: 042029, m/z: 1415.2307, charge: 3
EHEGAIYPDNTTDFQR_5_4_1_2_0

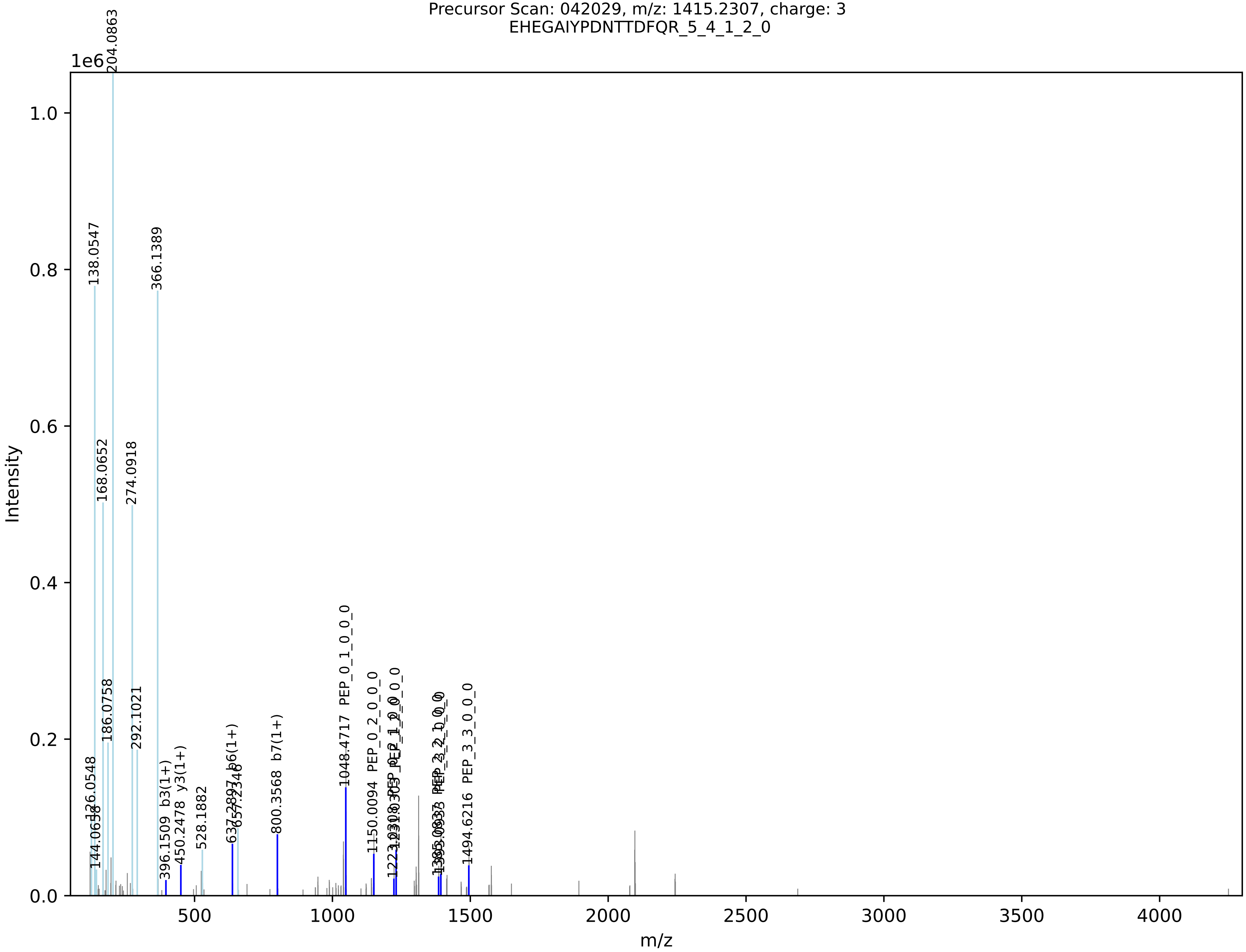

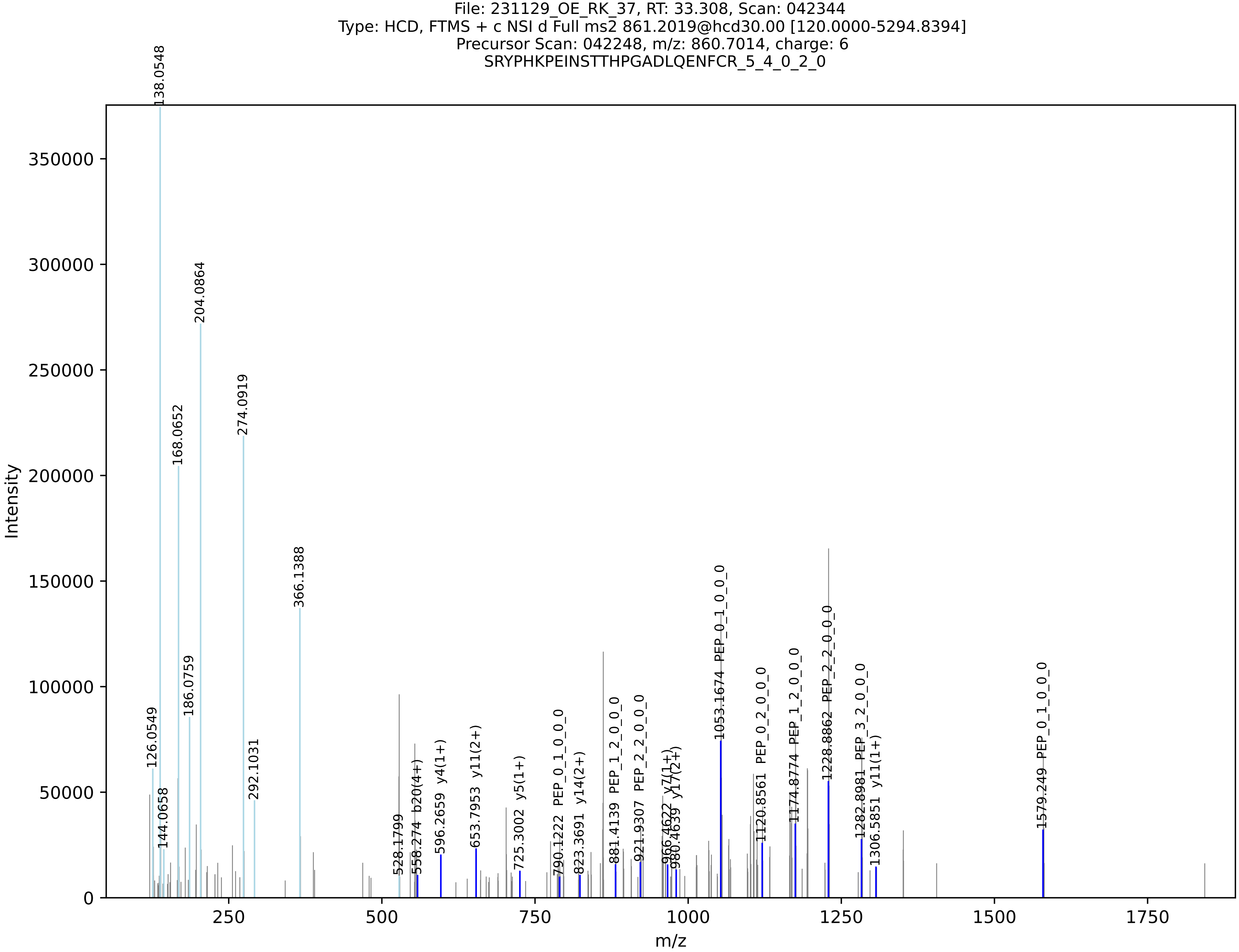
File: 231129_OE_RK_37, RT: 33.308, Scan: 042344
Type: HCD, FTMS + c NSI d Full ms2 861.2019@hcd30.00 [120.0000-5294.8394]
Precursor Scan: 042248, m/z: 860.7014, charge: 6
SRYPHKPEINSTTHPGADLQENFCR_5_4_0_2_0
Intensity
m/z
138.0548
204.0864
274.0919
168.0652
366.1388
186.0759
126.0549
292.1031
144.0658
528.1799
558.274 b20(4+)
596.2659 y4(1+)
653.7953 y11(2+)
725.3002 y5(1+)
790.1222 PEP_0_1_0_0_0
823.3691 y14(2+)
881.4139 PEP_1_2_0_0_0
921.9307 PEP_2_2_0_0_0
966.4622 y7(1+)
980.4639 y17(2+)
1053.1674 PEP_0_1_0_0_0
1120.8561 PEP_0_2_0_0_0
1174.8774 PEP_1_2_0_0_0
1228.8862 PEP_2_2_0_0_0
1282.8981 PEP_3_2_0_0_0
1306.5851 y11(1+)
1579.249 PEP_0_1_0_0_0

File: 231129_OE_RK_37, RT: 33.348, Scan: 042401
Type: HCD, FTMS + c NSI d Full ms2 1415.8987@hcd30.00 [120.0000-4354.7920]
Precursor Scan: 042322, m/z: 1415.2305, charge: 3
EHEGAIYPDNTTDFQR_5_4_1_2_0

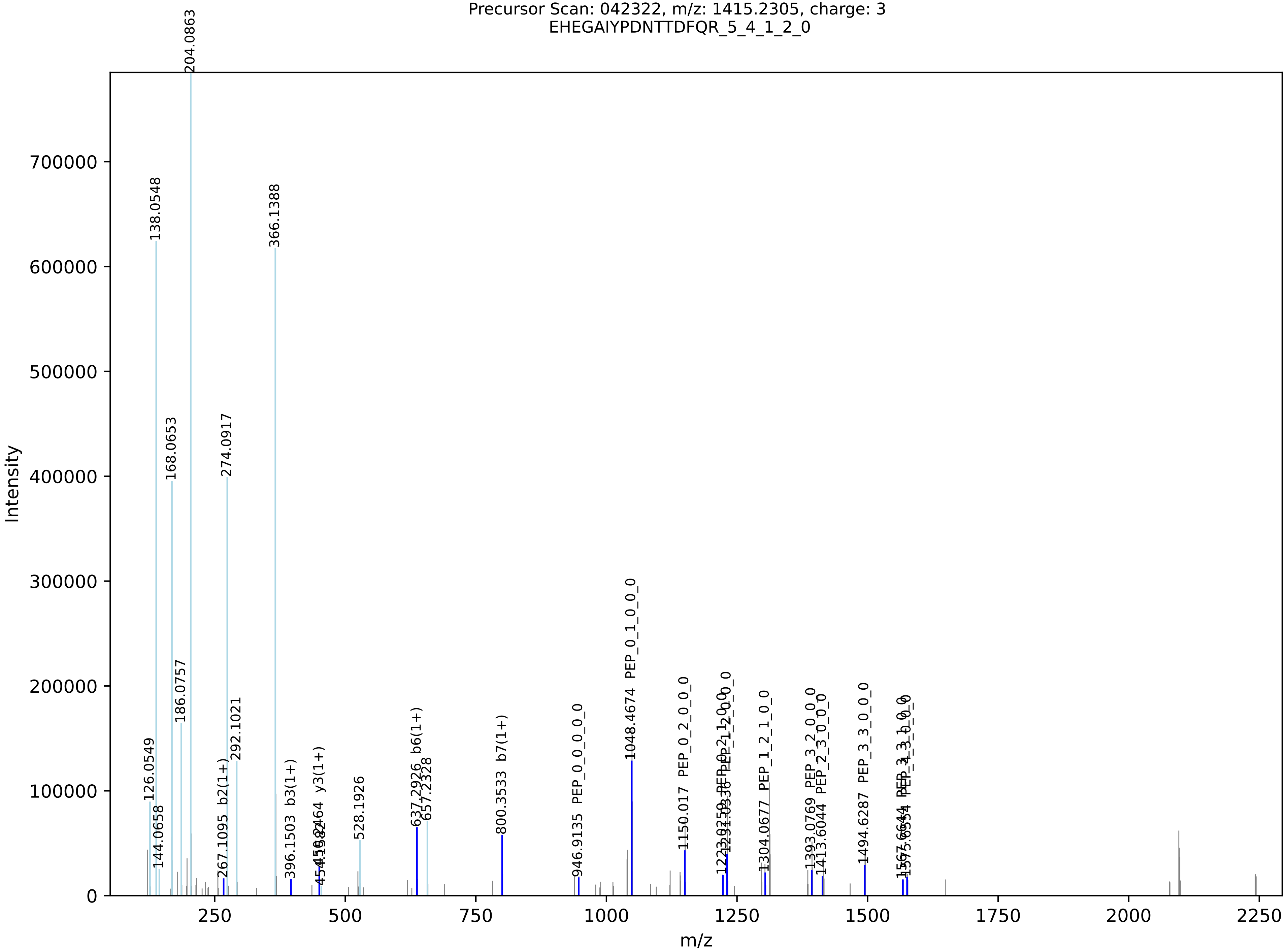

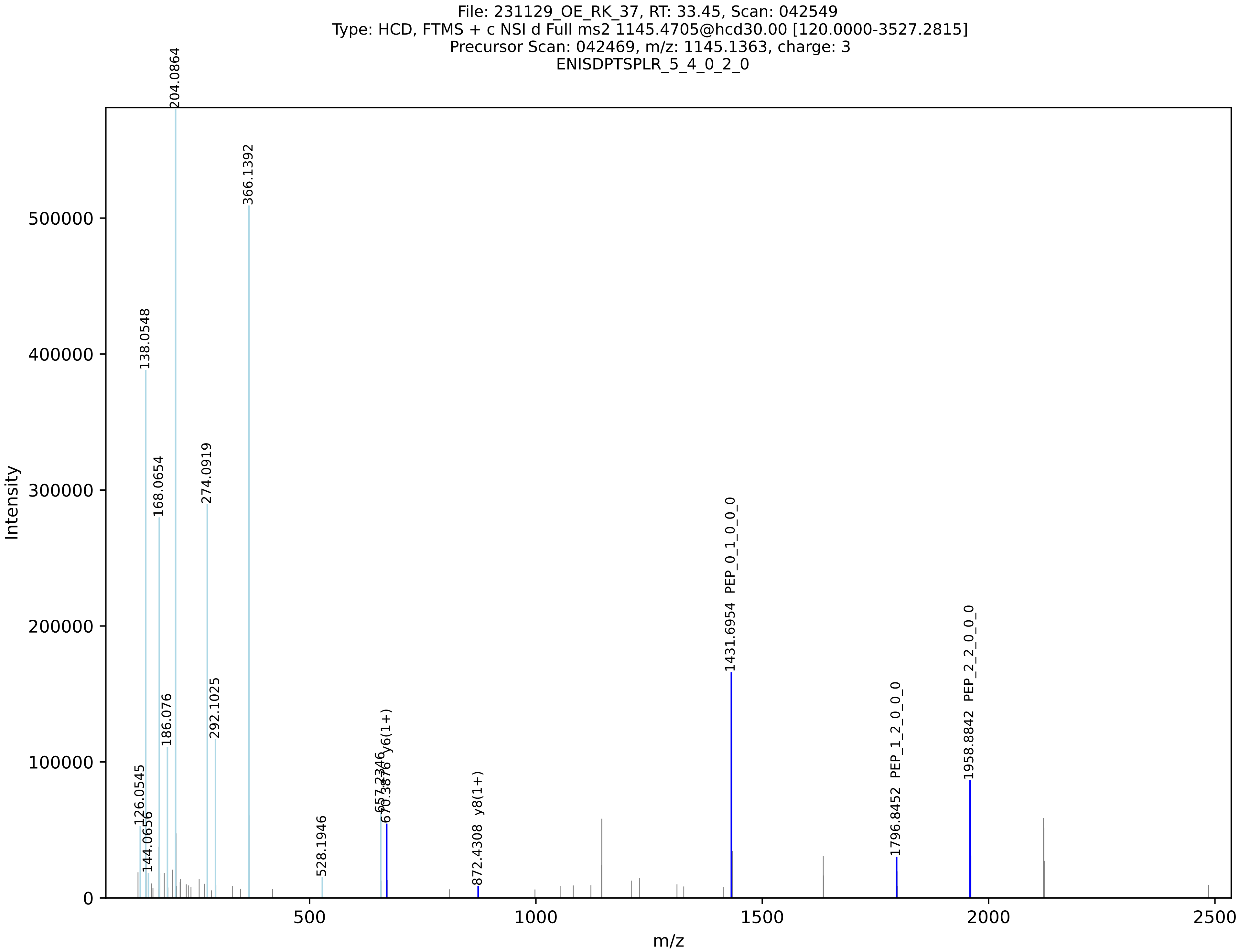
File: 231129_OE_RK_37, RT: 33.45, Scan: 042549
Type: HCD, FTMS + c NSI d Full ms2 1145.4705@hcd30.00 [120.0000-3527.2815]
Precursor Scan: 042469, m/z: 1145.1363, charge: 3
ENISDPTSPLR_5_4_0_2_0
Intensity
m/z
204.0864
366.1392
138.0548
168.0654
274.0919
186.076
292.1025
126.0545
144.0656
528.1946
657.2346
670.3876 y6(1+)
872.4308 y8(1+)
1431.6954 PEP_0_1_0_0_0
1796.8452 PEP_1_2_0_0_0
1958.8842 PEP_2_2_0_0_0
0
100000
200000
300000
400000
500000
500
1000
1500
2000
2500

File: 231129_OE_RK_37, RT: 33.651, Scan: 042841
Type: HCD, FTMS + c NSI d Full ms2 1145.8049@hcd30.00 [120.0000-3528.3052]
Precursor Scan: 042765, m/z: 1145.1367, charge: 3
ENISDPTSPLR_5_4_0_2_0

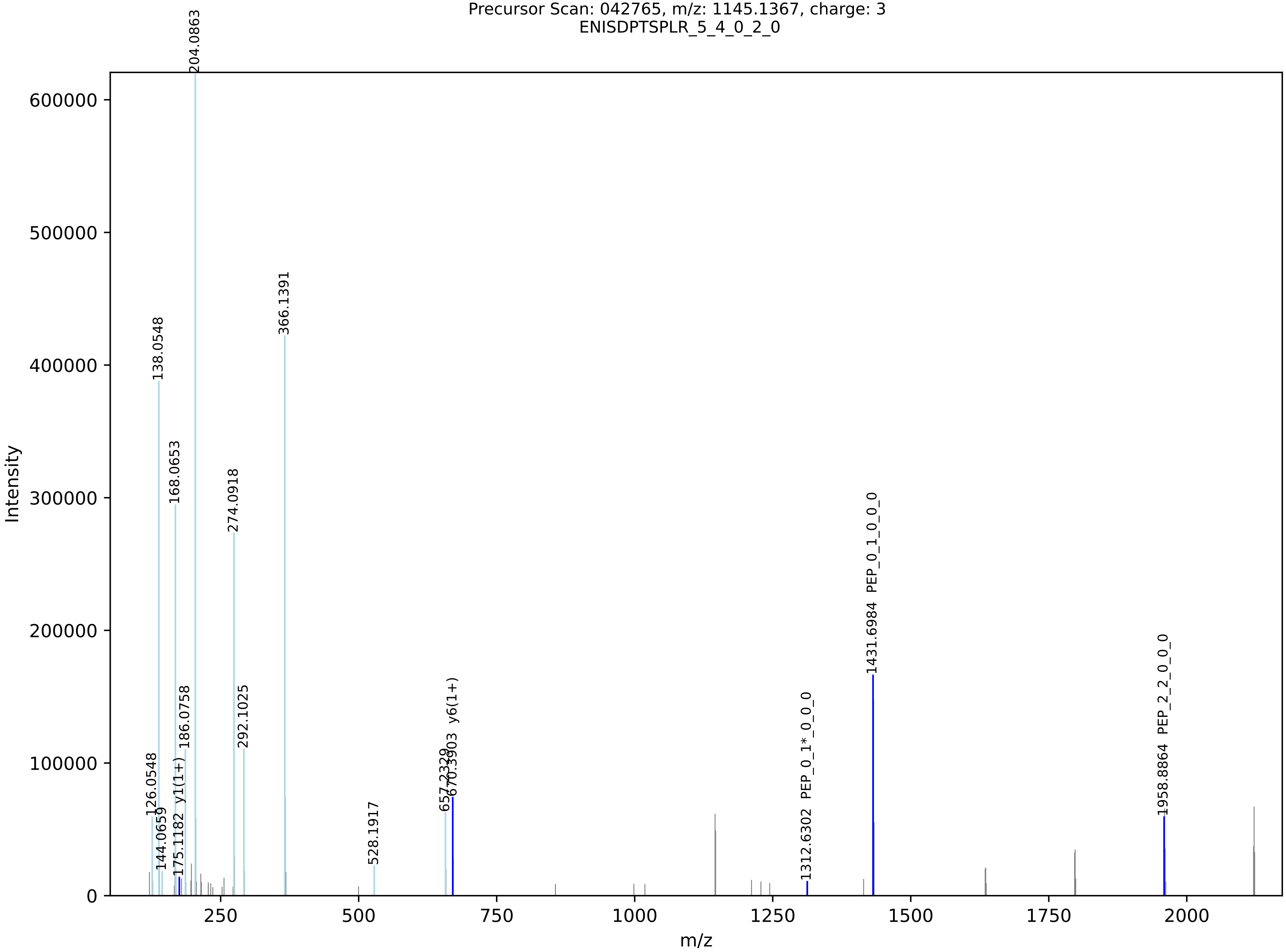

File: 231129_OE_RK_37, RT: 33.655, Scan: 042847
Type: HCD, FTMS + c NSI d Full ms2 1367.2129@hcd30.00 [120.0000-4205.8135]
Precursor Scan: 042765, m/z: 1366.5447, charge: 3
EHEGAIYPDNTTDFQR_5_4_0_2_0

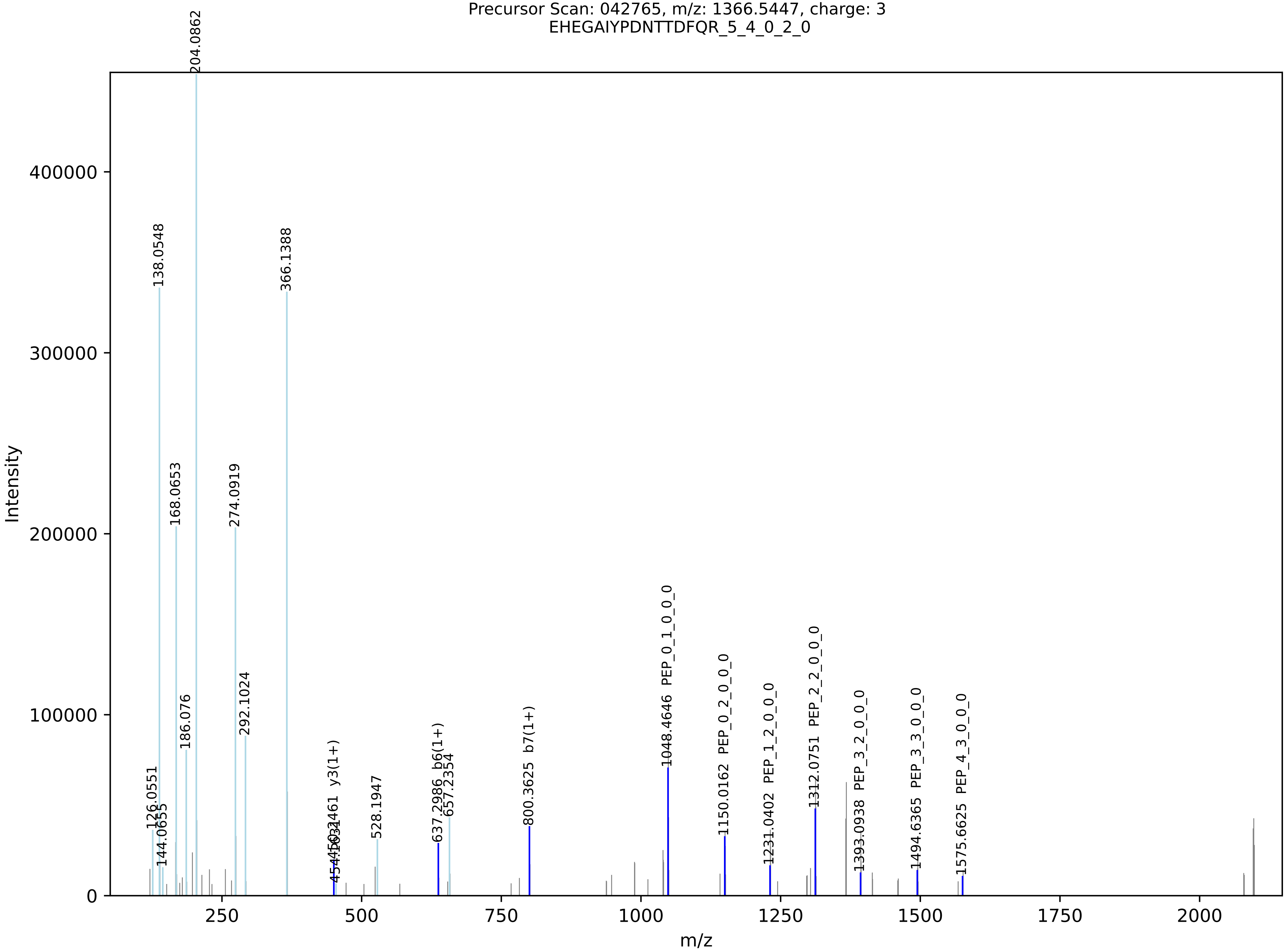

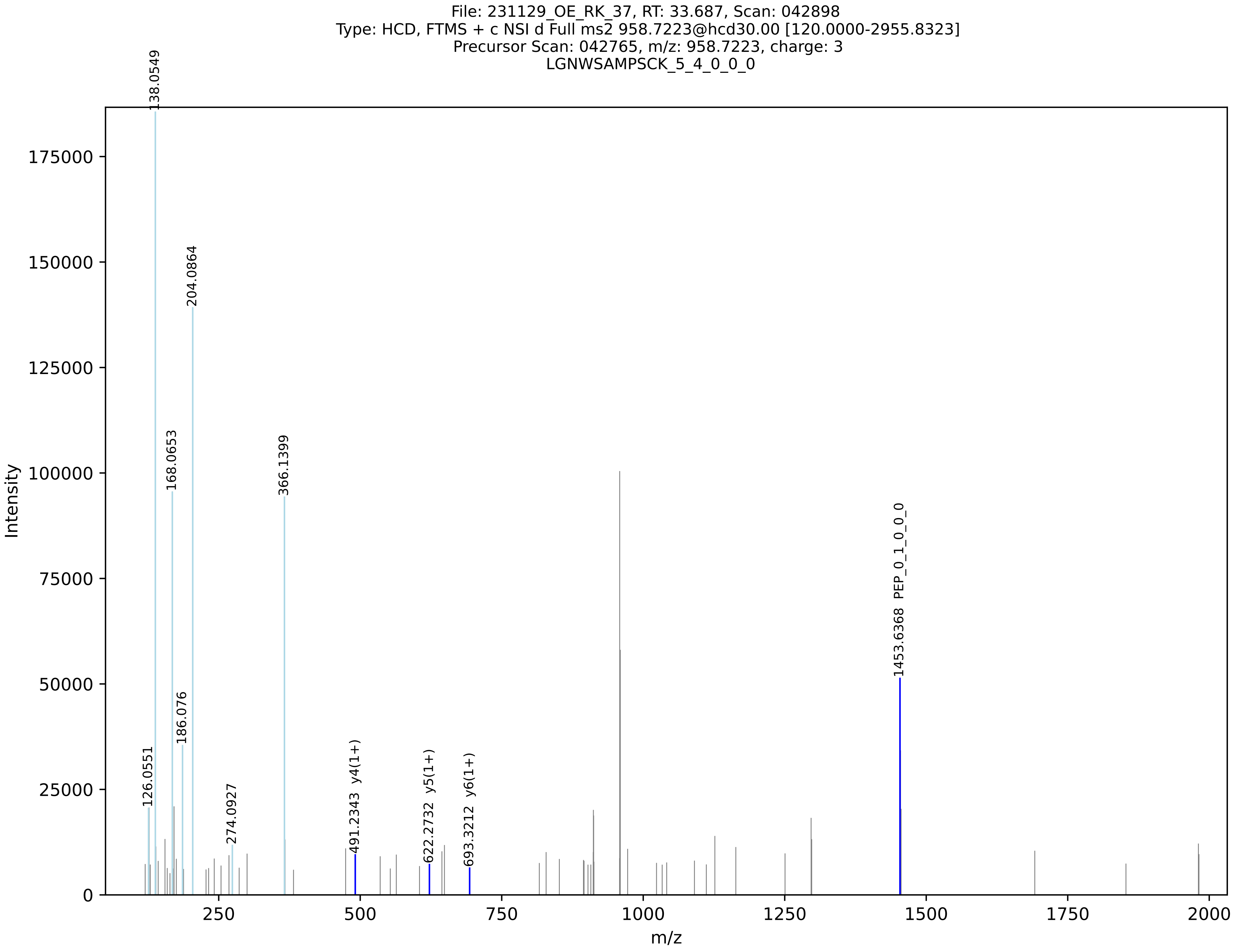
File: 231129_OE_RK_37, RT: 33.687, Scan: 042898
Type: HCD, FTMS + c NSI d Full ms2 958.7223@hcd30.00 [120.0000-2955.8323]
Precursor Scan: 042765, m/z: 958.7223, charge: 3
LGNWSAMPSCK_5_4_0_0_0
138.0549
204.0864
168.0653
366.1399
186.076
126.0551
274.0927
491.2343 y4(1+)
622.2732 y5(1+)
693.3212 y6(1+)
1453.6368 PEP_0_1_0_0_0
Intensity
m/z
0
25000
50000
75000
100000
125000
150000
175000
250
500
750
1000
1250
1500
1750
2000

File: 231129_OE_RK_37, RT: 33.769, Scan: 043012
Type: HCD, FTMS + c NSI d Full ms2 1415.8989@hcd30.00 [120.0000-4354.7930]
Precursor Scan: 042910, m/z: 1415.2329, charge: 3
EHEGAIYPDNTTDFQR_5_4_1_2_0

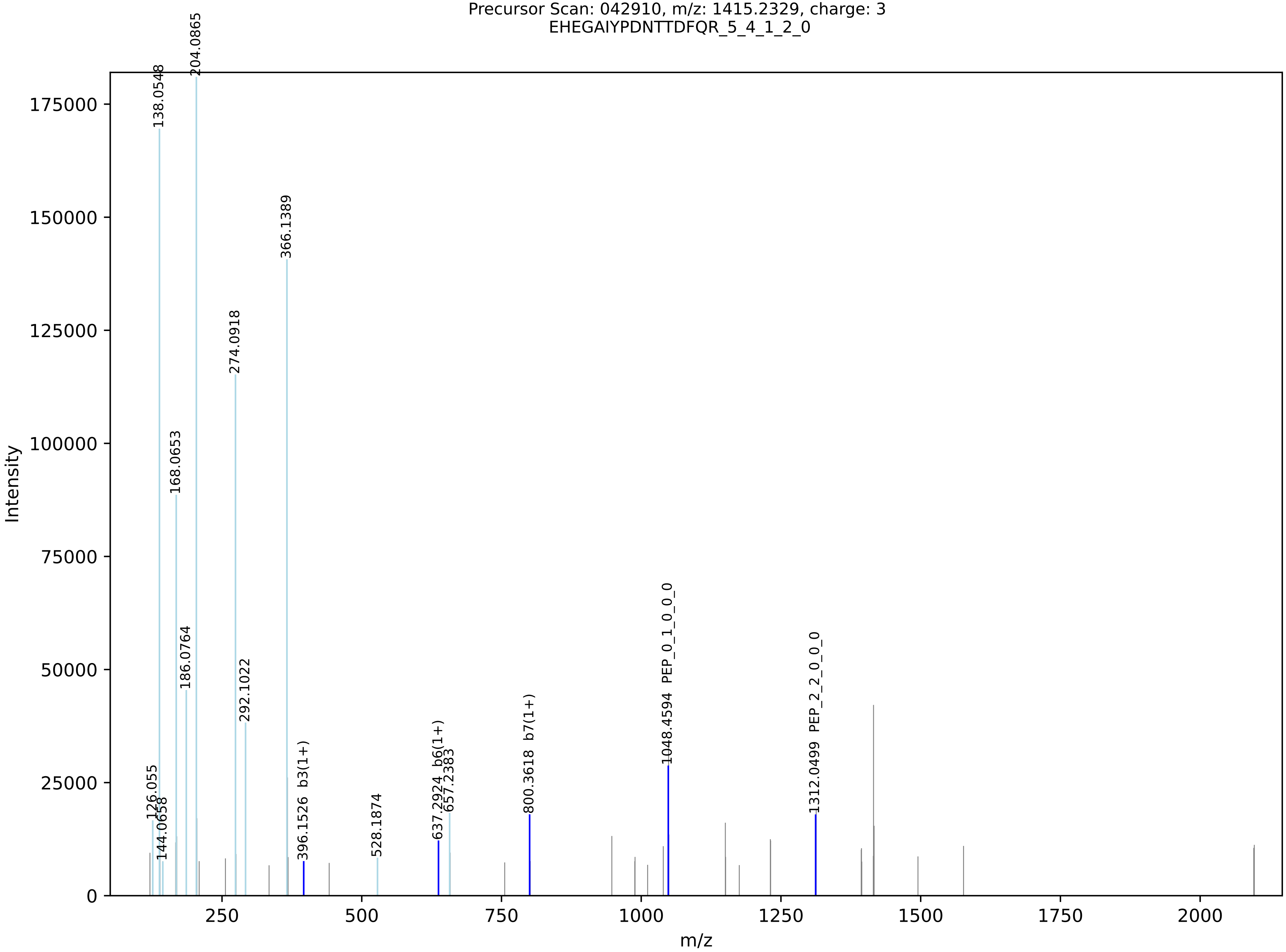

File: 231129_OE_RK_37, RT: 33.977, Scan: 043314
Type: HCD, FTMS + c NSI d Full ms2 1257.1906@hcd30.00 [120.0000-3869.1453]
Precursor Scan: 043202, m/z: 1256.5252, charge: 3
FSDGLESNSSTQFEVKK_9_2_0_0_0

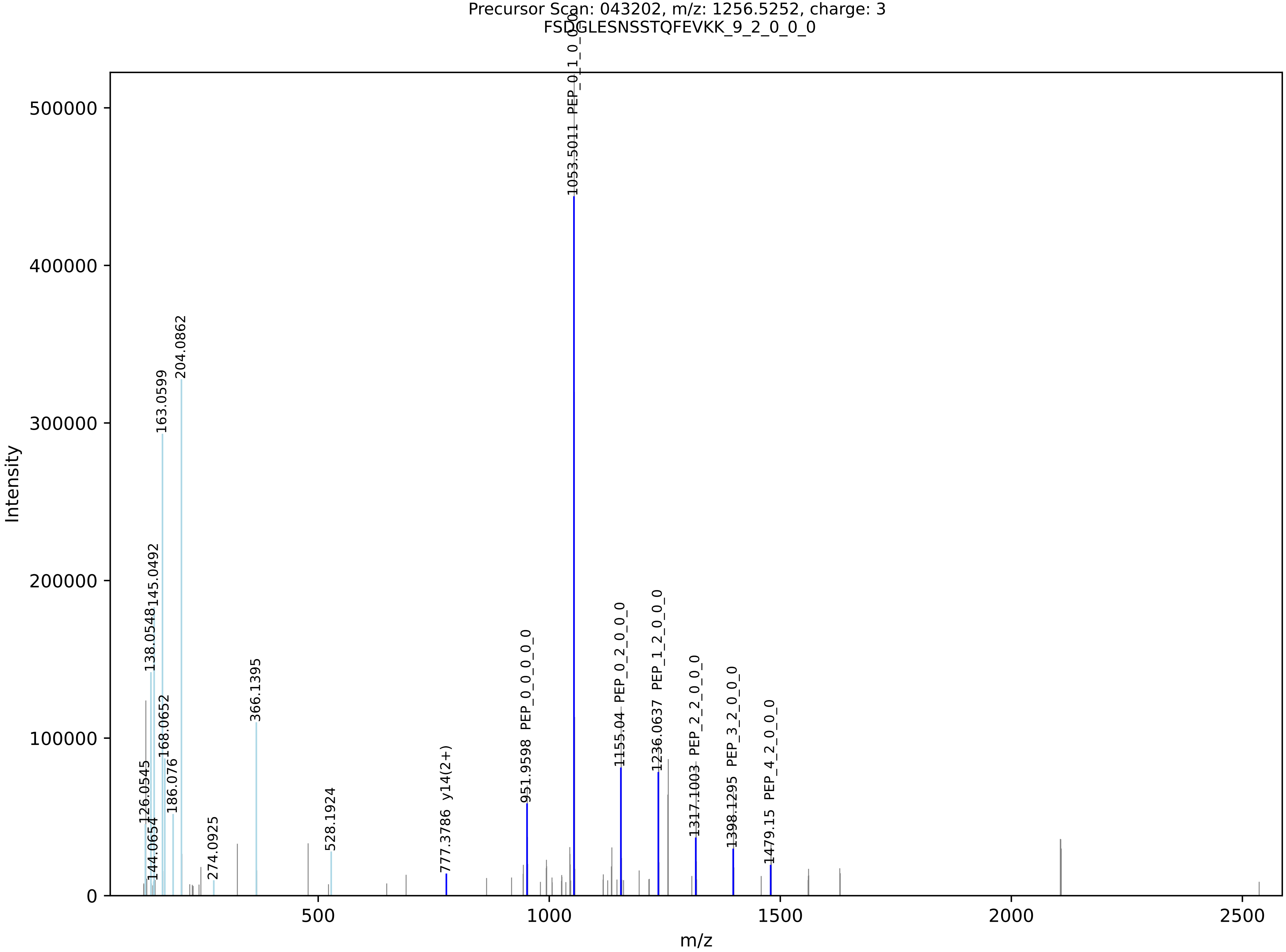

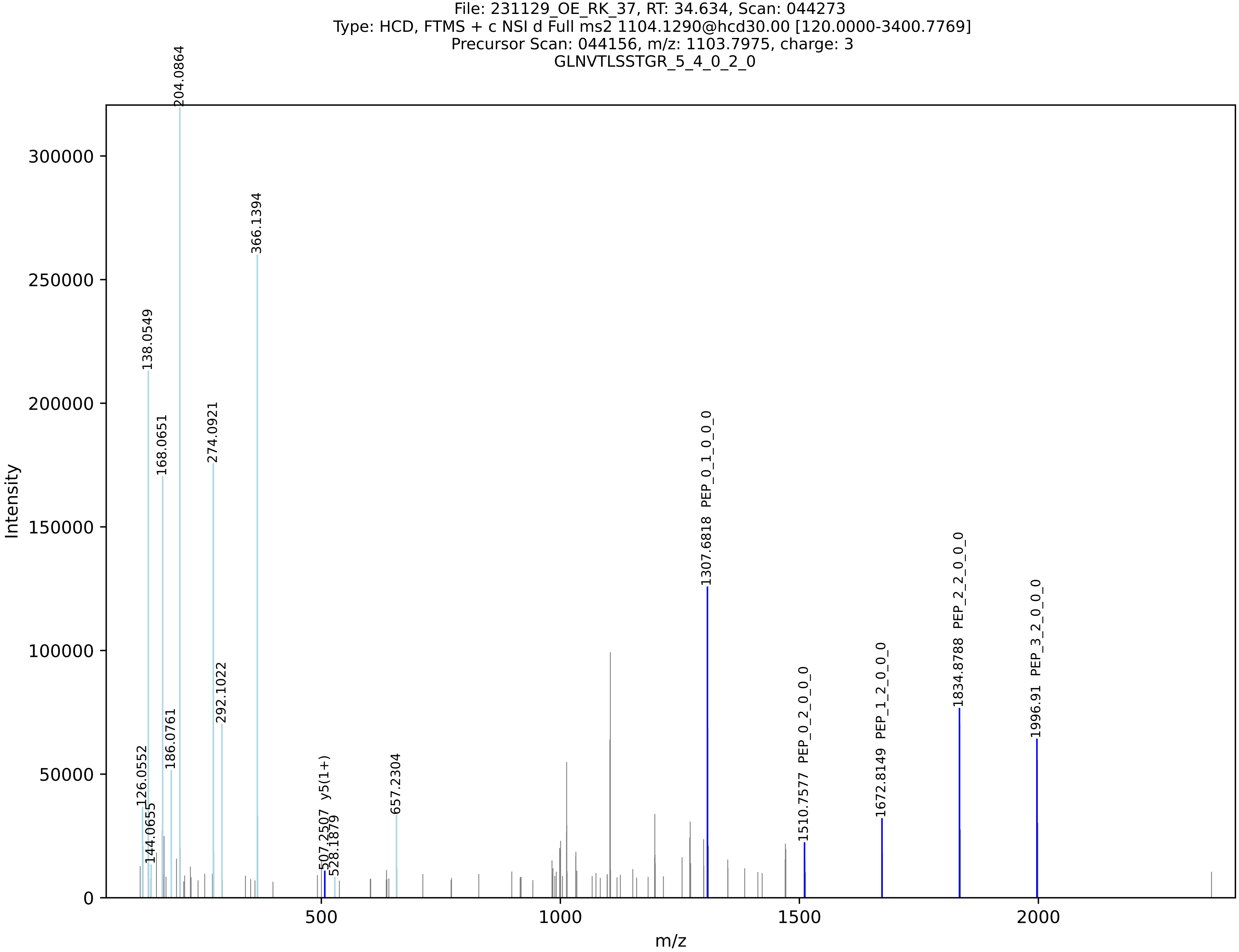
File: 231129_OE_RK_37, RT: 34.634, Scan: 044273
Type: HCD, FTMS + c NSI d Full ms2 1104.1290@hcd30.00 [120.0000-3400.7769]
Precursor Scan: 044156, m/z: 1103.7975, charge: 3
GLNVTLSSTGR_5_4_0_2_0
Intensity
m/z
204.0864
366.1394
138.0549
168.0651
274.0921
292.1022
186.0761
126.0552
144.0655
507.2507 y5(1+)
528.1879
657.2304
1307.6818 PEP_0_1_0_0_0
1510.7577 PEP_0_2_0_0_0
1672.8149 PEP_1_2_0_0_0
1834.8788 PEP_2_2_0_0_0
1996.91 PEP_3_2_0_0_0

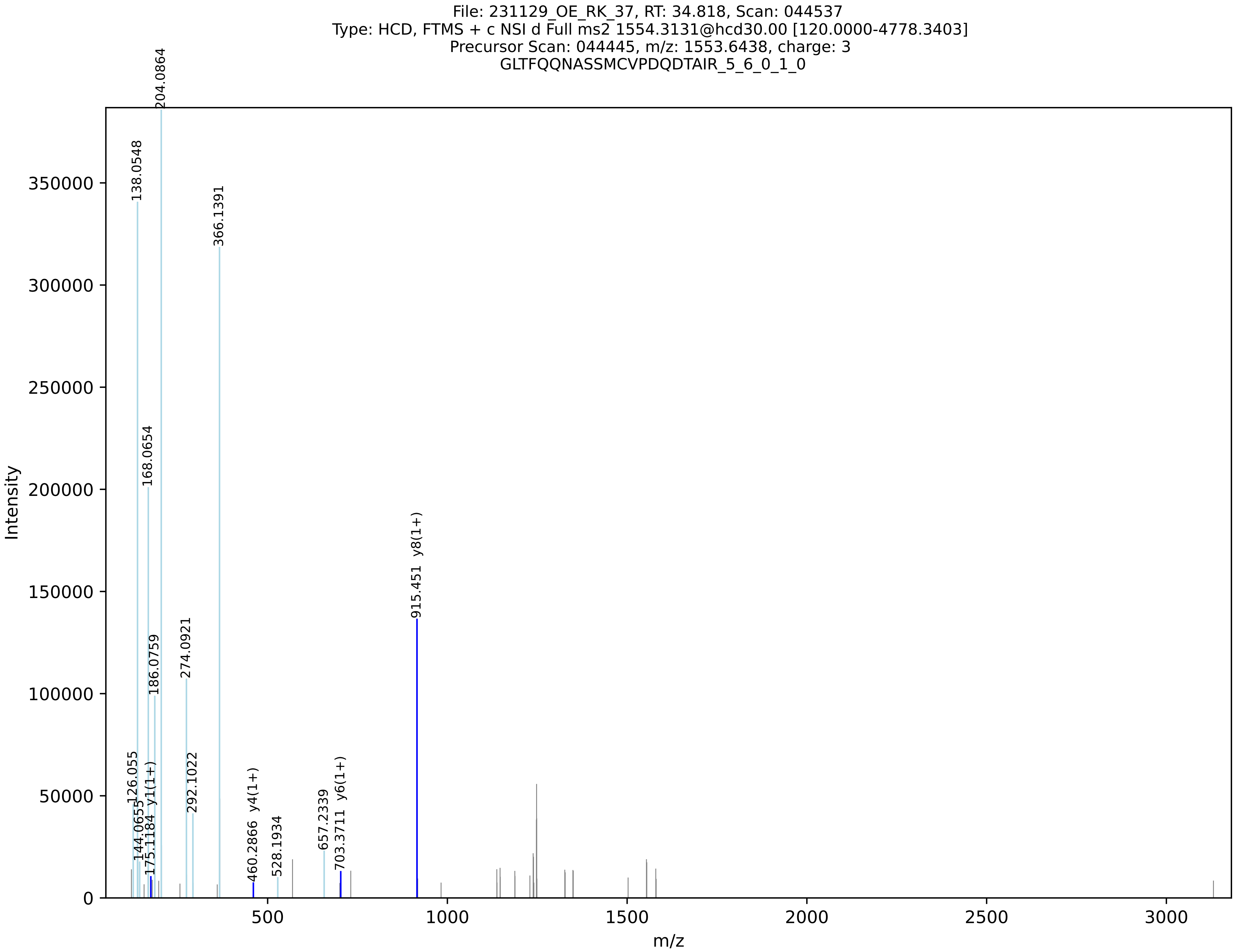
File: 231129_OE_RK_37, RT: 34.818, Scan: 044537
Type: HCD, FTMS + c NSI d Full ms2 1554.3131@hcd30.00 [120.0000-4778.3403]
Precursor Scan: 044445, m/z: 1553.6438, charge: 3
GLTFQQNASSMCVPDQDTAIR_5_6_0_1_0
204.0864
138.0548
366.1391
168.0654
915.451 y8(1+)
274.0921
186.0759
126.055
292.1022
144.0655 y1(1+)
175.1184
460.2866 y4(1+)
528.1934
657.2339
703.3711 y6(1+)
Intensity
m/z
0
50000
100000
150000
200000
250000
300000
350000
500
1000
1500
2000
2500
3000

File: 231129_OE_RK_37, RT: 34.888, Scan: 044642
Type: HCD, FTMS + c NSI d Full ms2 1104.8606@hcd30.00 [120.0000-3403.0154]
Precursor Scan: 044519, m/z: 1104.5268, charge: 3
GLNVTLSSTGR_5_4_0_2_0

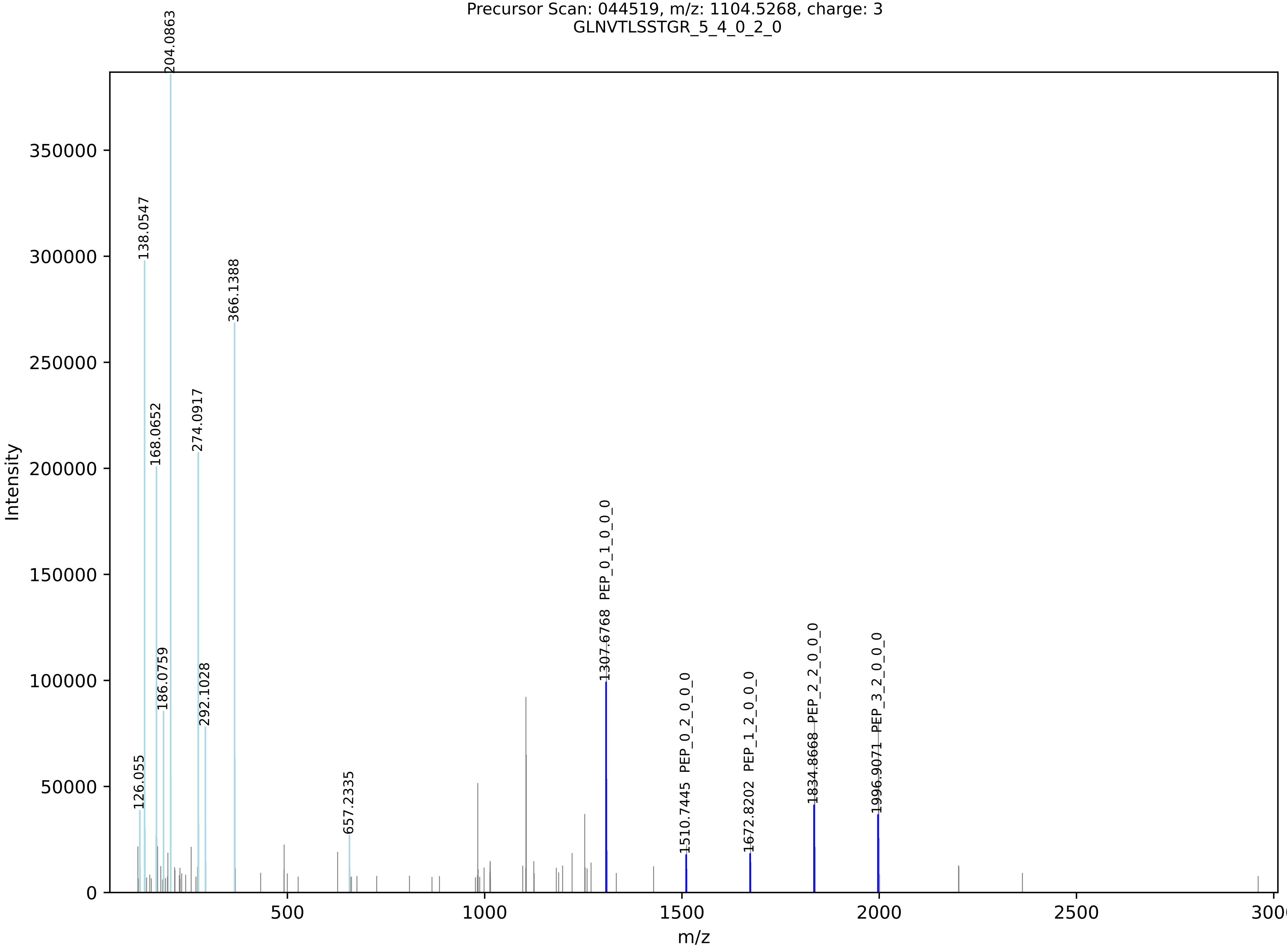

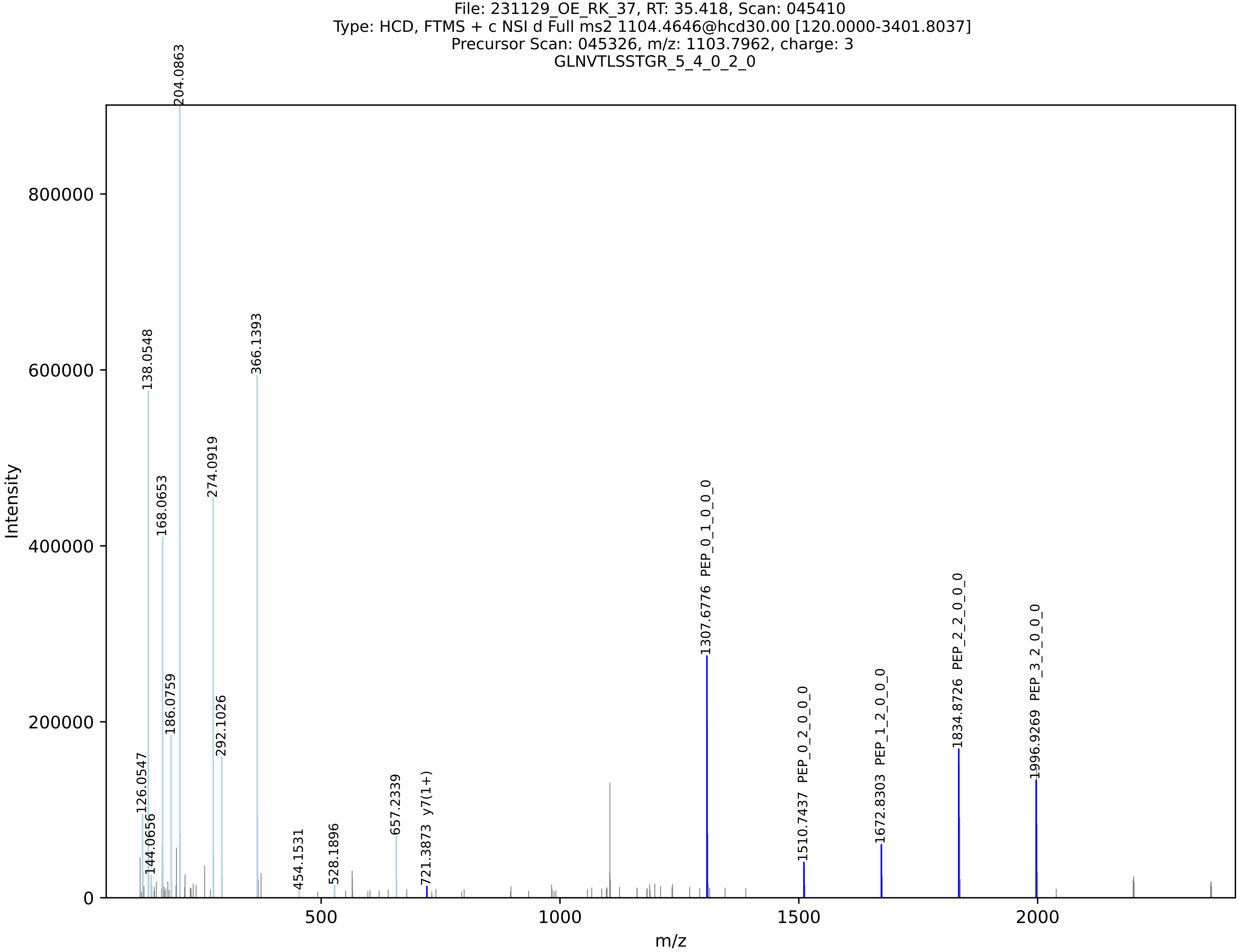

File: 231129_OE_RK_37, RT: 35.418, Scan: 045410
Type: HCD, FTMS + c NSI d Full ms2 1104.4646@hcd30.00 [120.0000-3401.8037]
Precursor Scan: 045326, m/z: 1103.7962, charge: 3
GLNVTLSSTGR_5_4_0_2_0
Intensity
m/z
204.0863
138.0548
366.1393
274.0919
168.0653
186.0759
292.1026
126.0547
144.0656
454.1531
528.1896
657.2339
721.3873 y7(1+)
1307.6776 PEP_0_1_0_0_0
1510.7437 PEP_0_2_0_0_0
1672.8303 PEP_1_2_0_0_0
1834.8726 PEP_2_2_0_0_0
1996.9269 PEP_3_2_0_0_0
0
200000
400000
600000
800000
500
1000
1500
2000

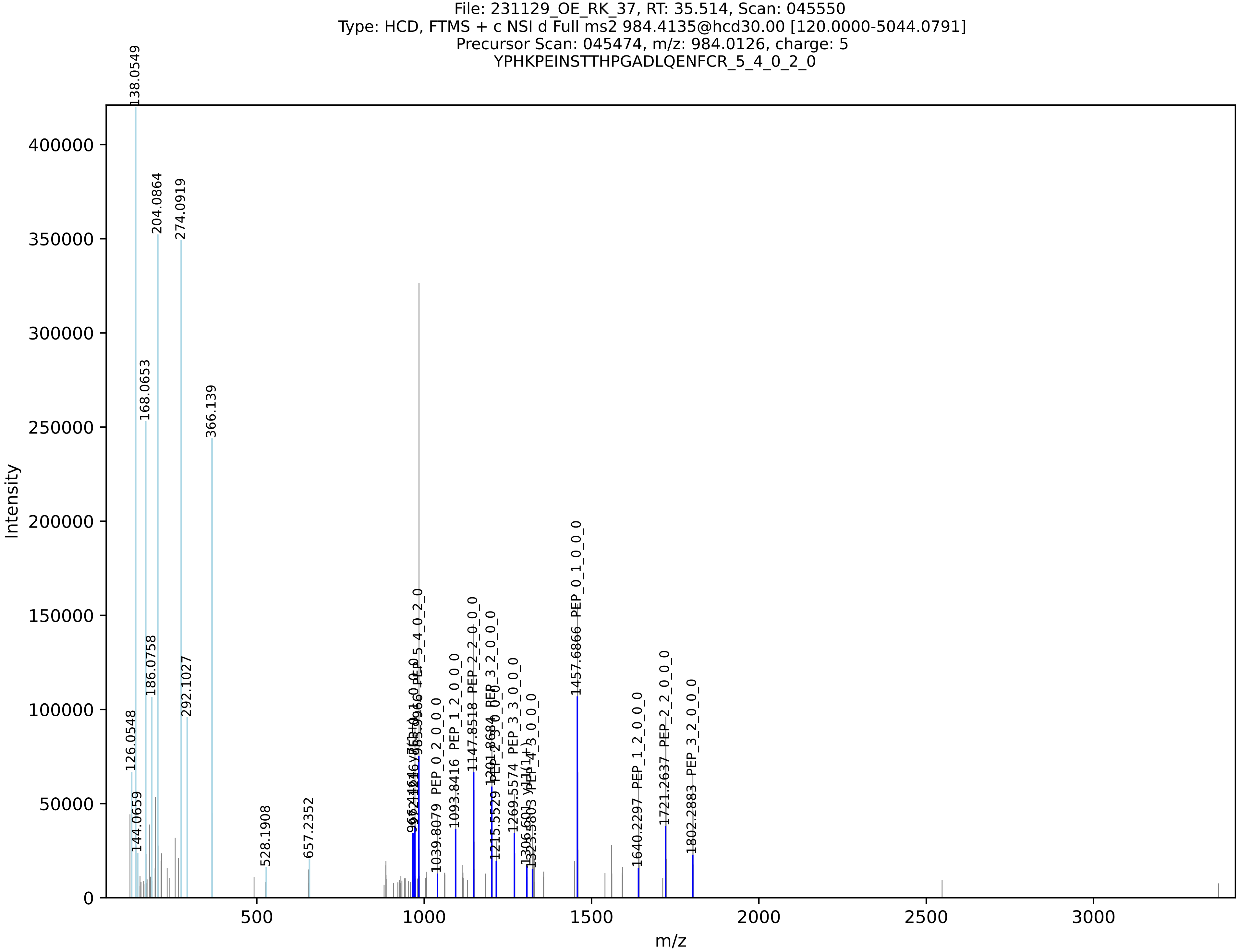
File: 231129_OE_RK_37, RT: 35.514, Scan: 045550
Type: HCD, FTMS + c NSI d Full ms2 984.4135@hcd30.00 [120.0000-5044.0791]
Precursor Scan: 045474, m/z: 984.0126, charge: 5
YPHKPEINSTTHPGADLQENFCR_5_4_0_2_0
Intensity
m/z
138.0549
204.0864
274.0919
168.0653
366.139
186.0758
292.1027
126.0548
144.0659
528.1908
657.2352
1039.8079_PEP_0_2_0_0_0
1093.8416_PEP_1_2_0_0_0
1147.8518_PEP_2_2_0_0_0
1269.5574_PEP_3_3_0_0_0
1457.6866_PEP_0_1_0_0_0
1640.2297_PEP_1_2_0_0_0
1721.2637_PEP_2_2_0_0_0
1802.2883_PEP_3_2_0_0_0

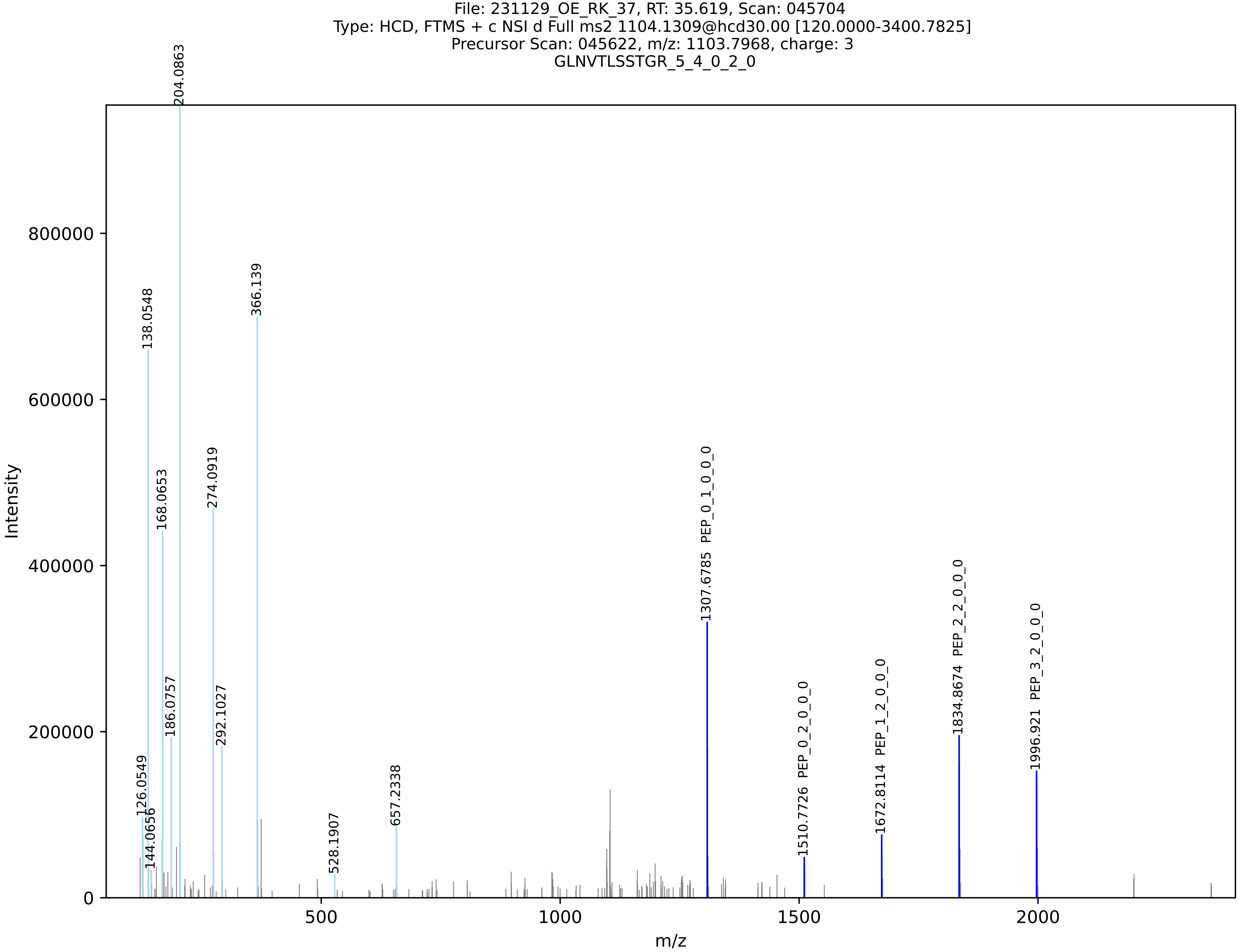

File: 231129_OE_RK_37, RT: 35.619, Scan: 045704
Type: HCD, FTMS + c NSI d Full ms2 1104.1309@hcd30.00 [120.0000-3400.7825]
Precursor Scan: 045622, m/z: 1103.7968, charge: 3
GLNVTLSSTGR_5_4_0_2_0
Intensity
m/z
0
200000
400000
600000
800000
500
1000
1500
2000
204.0863
138.0548
366.139
168.0653
274.0919
186.0757
292.1027
126.0549
144.0656
528.1907
657.2338
1307.6785 PEP_0_1_0_0_0
1510.7726 PEP_0_2_0_0_0
1672.8114 PEP_1_2_0_0_0
1834.8674 PEP_2_2_0_0_0
1996.921 PEP_3_2_0_0_0

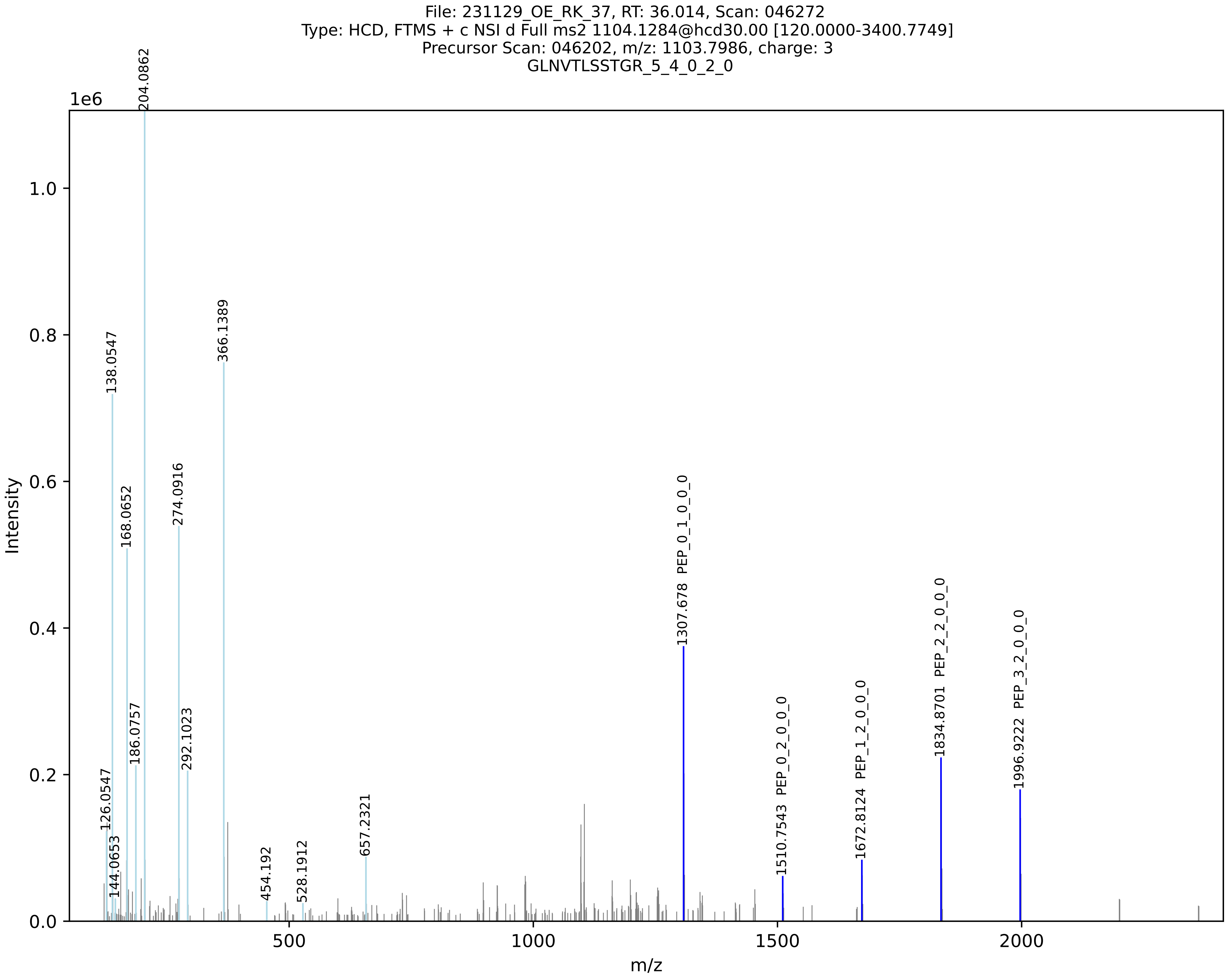
File: 231129_OE_RK_37, RT: 36.014, Scan: 046272
Type: HCD, FTMS + c NSI d Full ms2 1104.1284@hcd30.00 [120.0000-3400.7749]
Precursor Scan: 046202, m/z: 1103.7986, charge: 3
GLNVTLSSTGR_5_4_0_2_0
1e6
Intensity
m/z
204.0862
138.0547
366.1389
168.0652
274.0916
186.0757
292.1023
126.0547
144.0653
454.192
528.1912
657.2321
1307.678 PEP_0_1_0_0_0
1510.7543 PEP_0_2_0_0_0
1672.8124 PEP_1_2_0_0_0
1834.8701 PEP_2_2_0_0_0
1996.9222 PEP_3_2_0_0_0
0.0
0.2
0.4
0.6
0.8
1.0
500
1000
1500
2000

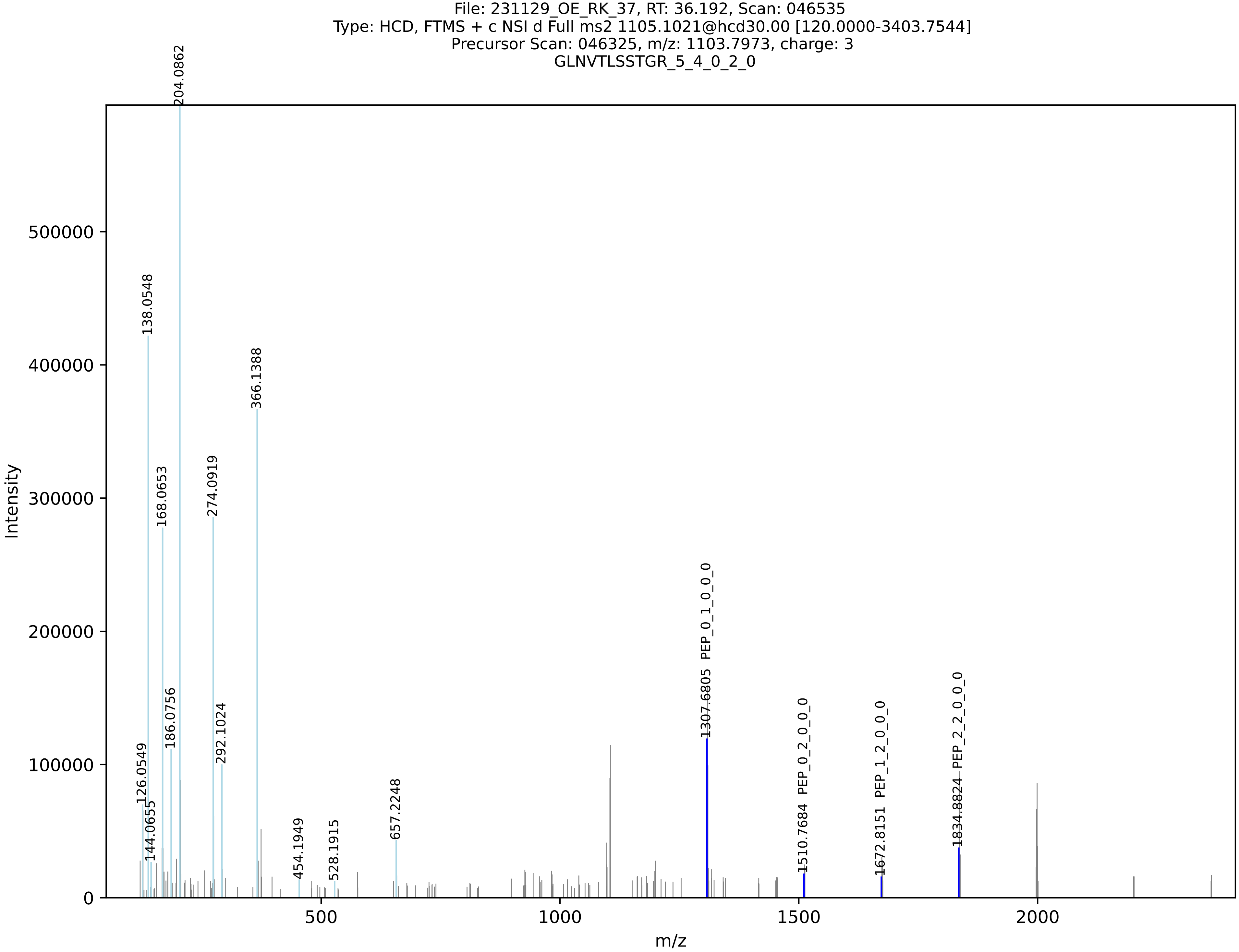

File: 231129_OE_RK_37, RT: 36.192, Scan: 046535
Type: HCD, FTMS + c NSI d Full ms2 1105.1021@hcd30.00 [120.0000-3403.7544]
Precursor Scan: 046325, m/z: 1103.7973, charge: 3
GLNVTLSSTGR_5_4_0_2_0
204.0862
138.0548
366.1388
274.0919
168.0653
186.0756
292.1024
126.0549
144.0655
454.1949
528.1915
657.2248
1307.6805 PEP_0_1_0_0_0
1510.7684 PEP_0_2_0_0_0
1672.8151 PEP_1_2_0_0_0
1834.8824 PEP_2_2_0_0_0
Intensity
m/z
0
100000
200000
300000
400000
500000
500
1000
1500
2000

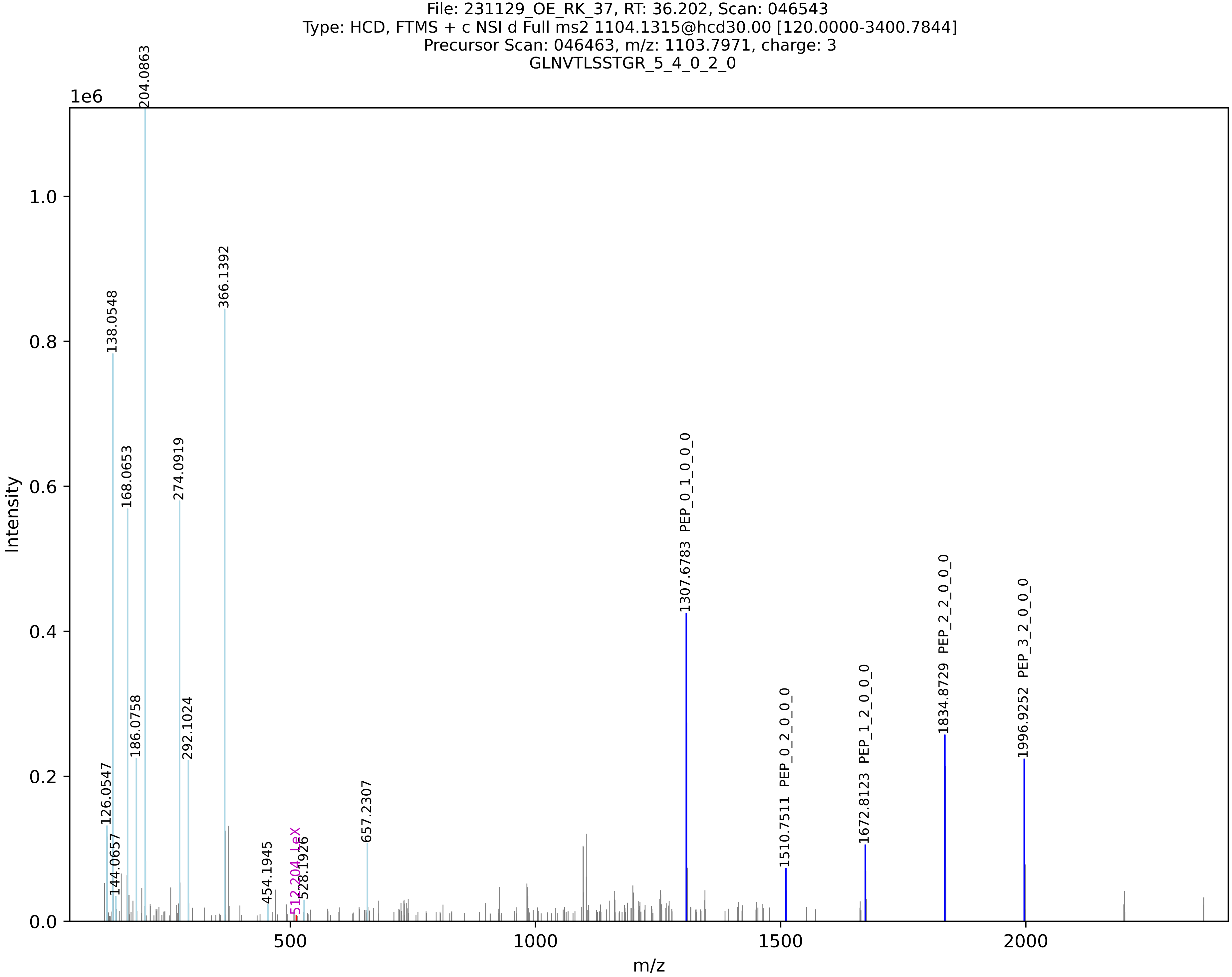
File: 231129_OE_RK_37, RT: 36.202, Scan: 046543
Type: HCD, FTMS + c NSI d Full ms2 1104.1315@hcd30.00 [120.0000-3400.7844]
Precursor Scan: 046463, m/z: 1103.7971, charge: 3
GLNVTLSSTGR_5_4_0_2_0
1e6
Intensity
m/z
204.0863
138.0548
366.1392
168.0653
274.0919
186.0758
292.1024
126.0547
144.0657
454.1945
512.204_LeX
528.1926
657.2307
1307.6783 PEP_0_1_0_0_0
1510.7511 PEP_0_2_0_0_0
1672.8123 PEP_1_2_0_0_0
1834.8729 PEP_2_2_0_0_0
1996.9252 PEP_3_2_0_0_0

File: 231129_OE_RK_37, RT: 36.205, Scan: 046548
Type: HCD, FTMS + c NSI d Full ms2 1055.7540@hcd30.00 [120.0000-3252.7493]
Precursor Scan: 046463, m/z: 1055.4199, charge: 3
LGNWSAMPSCK_5_4_0_1_0

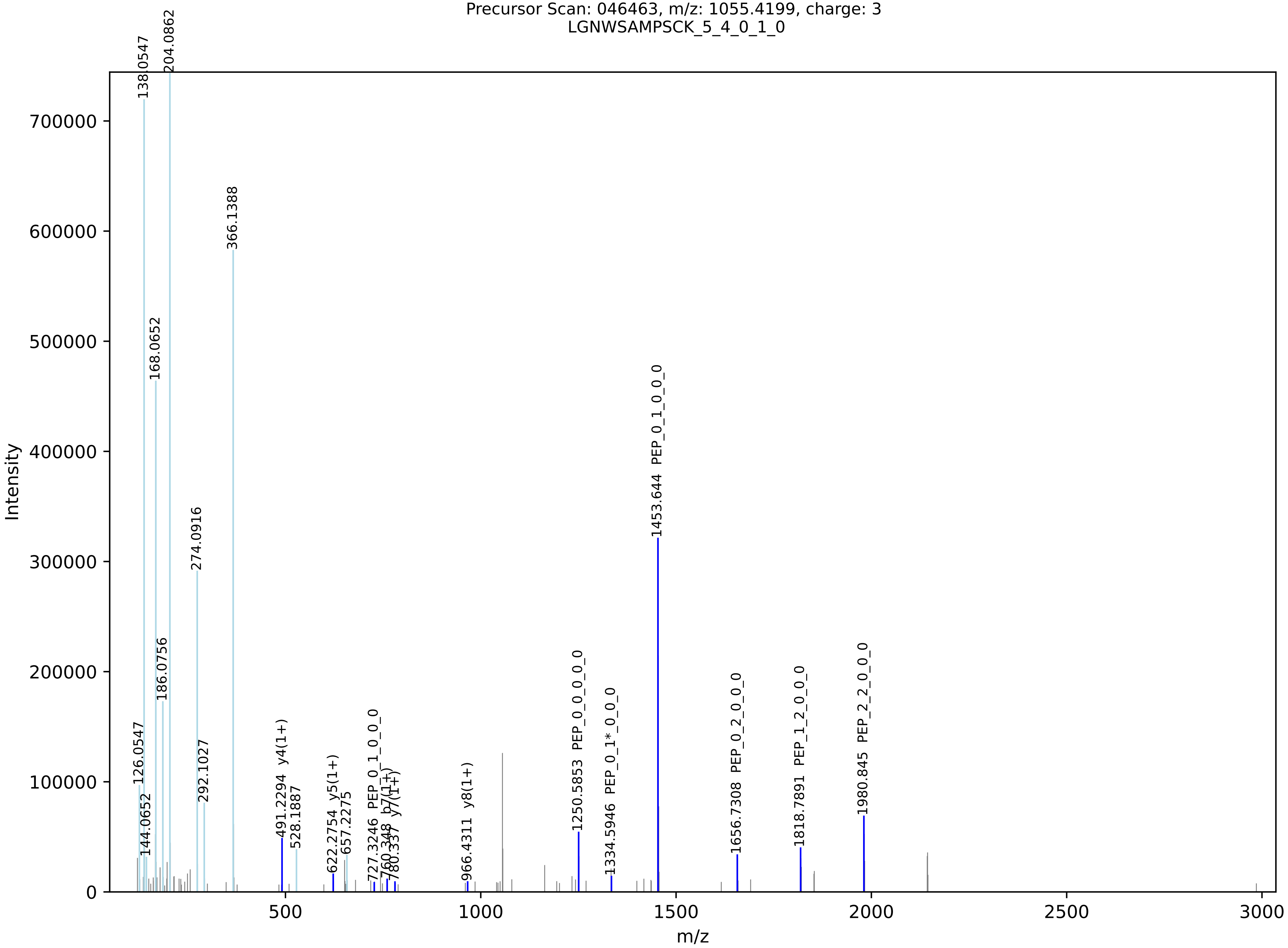

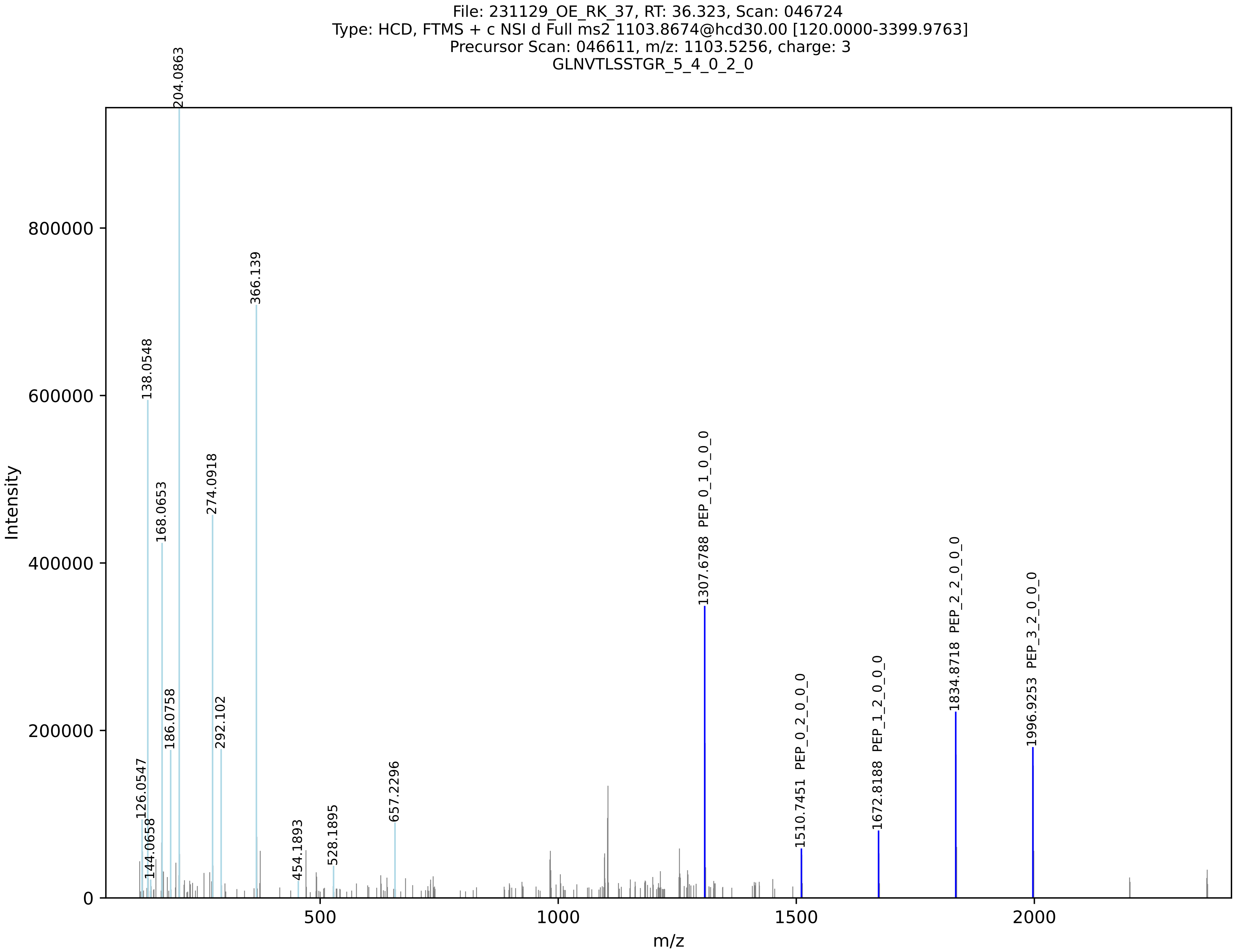
File: 231129_OE_RK_37, RT: 36.323, Scan: 046724
Type: HCD, FTMS + c NSI d Full ms2 1103.8674@hcd30.00 [120.0000-3399.9763]
Precursor Scan: 046611, m/z: 1103.5256, charge: 3
GLNVTLSSTGR_5_4_0_2_0
Intensity
m/z
204.0863
366.139
138.0548
168.0653
274.0918
186.0758
292.102
126.0547
144.0658
454.1893
528.1895
657.2296
1307.6788 PEP_0_1_0_0_0
1510.7451 PEP_0_2_0_0_0
1672.8188 PEP_1_2_0_0_0
1834.8718 PEP_2_2_0_0_0
1996.9253 PEP_3_2_0_0_0
0
200000
400000
600000
800000
500
1000
1500
2000

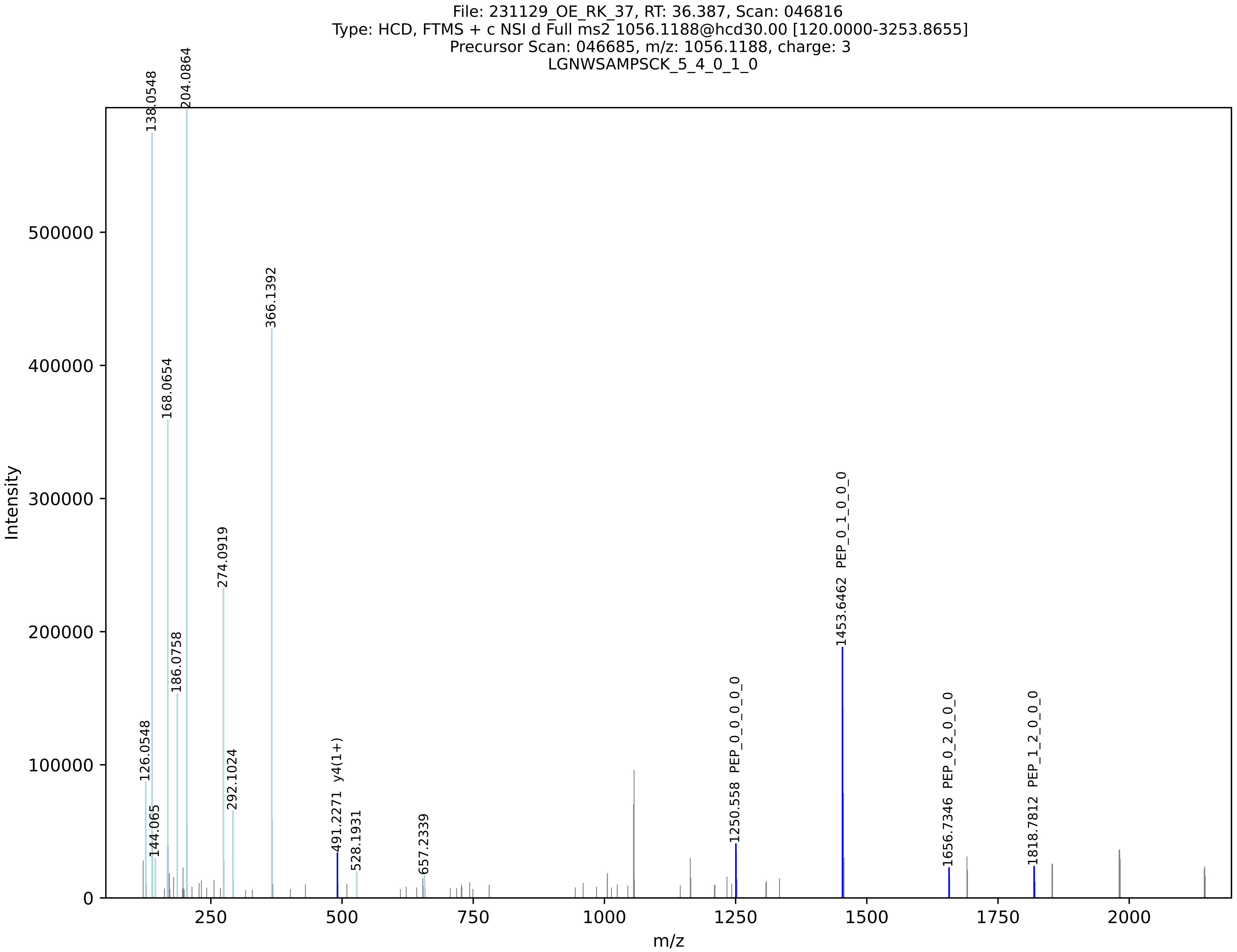

File: 231129_OE_RK_37, RT: 36.387, Scan: 046816
Type: HCD, FTMS + c NSI d Full ms2 1056.1188@hcd30.00 [120.0000-3253.8655]
Precursor Scan: 046685, m/z: 1056.1188, charge: 3
LGNWSAMPSCK_5_4_0_1_0
Intensity
m/z
0
100000
200000
300000
400000
500000
250
500
750
1000
1250
1500
1750
2000
138.0548
204.0864
366.1392
168.0654
274.0919
186.0758
126.0548
144.065
292.1024
491.2271 y4(1+)
528.1931
657.2339
1250.558 PEP_0_0_0_0_0
1453.6462 PEP_0_1_0_0_0
1656.7346 PEP_0_2_0_0_0
1818.7812 PEP_1_2_0_0_0

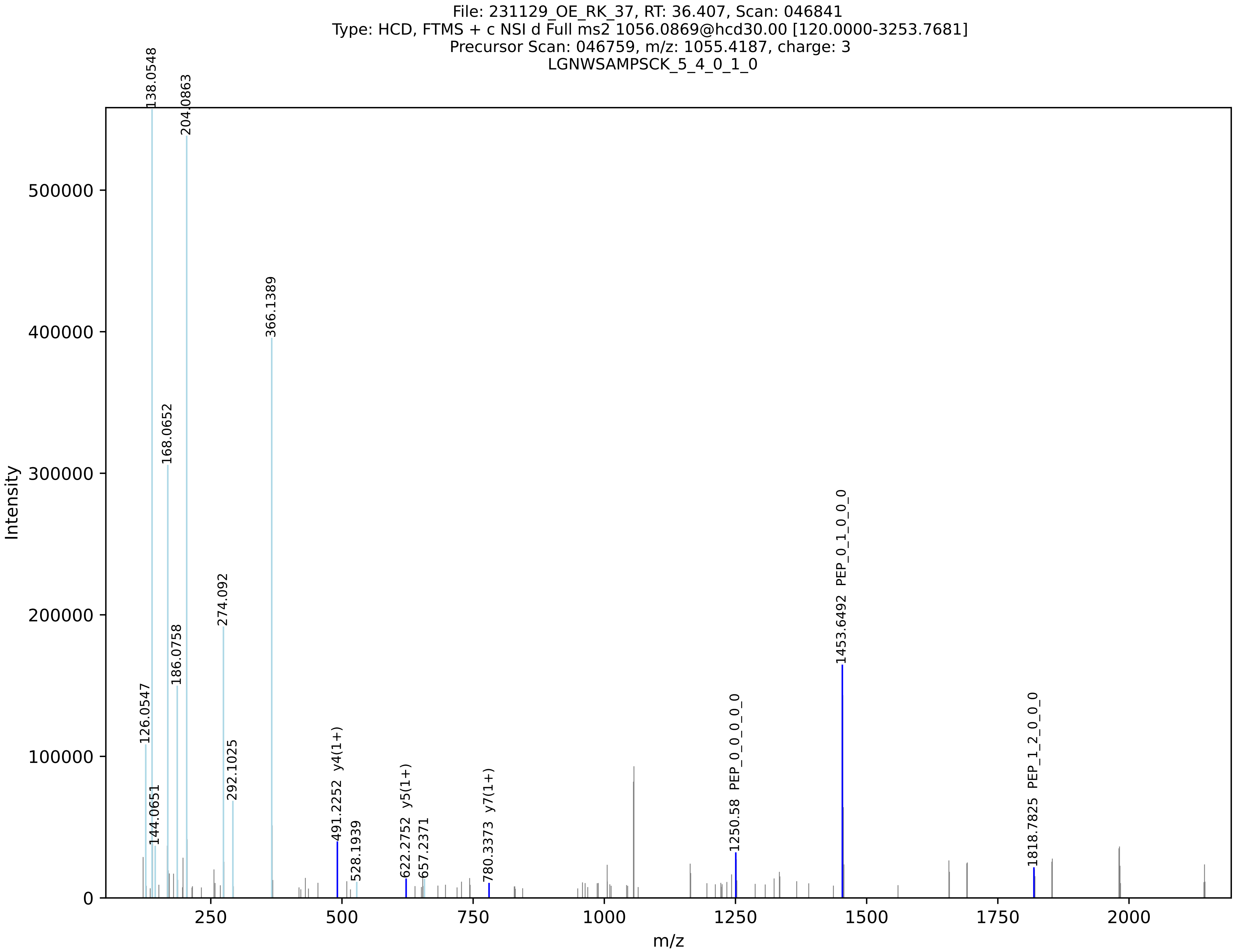

File: 231129_OE_RK_37, RT: 36.407, Scan: 046841
Type: HCD, FTMS + c NSI d Full ms2 1056.0869@hcd30.00 [120.0000-3253.7681]
Precursor Scan: 046759, m/z: 1055.4187, charge: 3
LGNWSAMPSCK_5_4_0_1_0
Intensity
m/z
0
100000
200000
300000
400000
500000
250
500
750
1000
1250
1500
1750
2000
138.0548
204.0863
366.1389
168.0652
186.0758
274.092
126.0547
144.0651
292.1025
491.2252 y4(1+)
528.1939
622.2752 y5(1+)
657.2371
780.3373 y7(1+)
1250.58 PEP_0_0_0_0_0
1453.6492 PEP_0_1_0_0_0
1818.7825 PEP_1_2_0_0_0

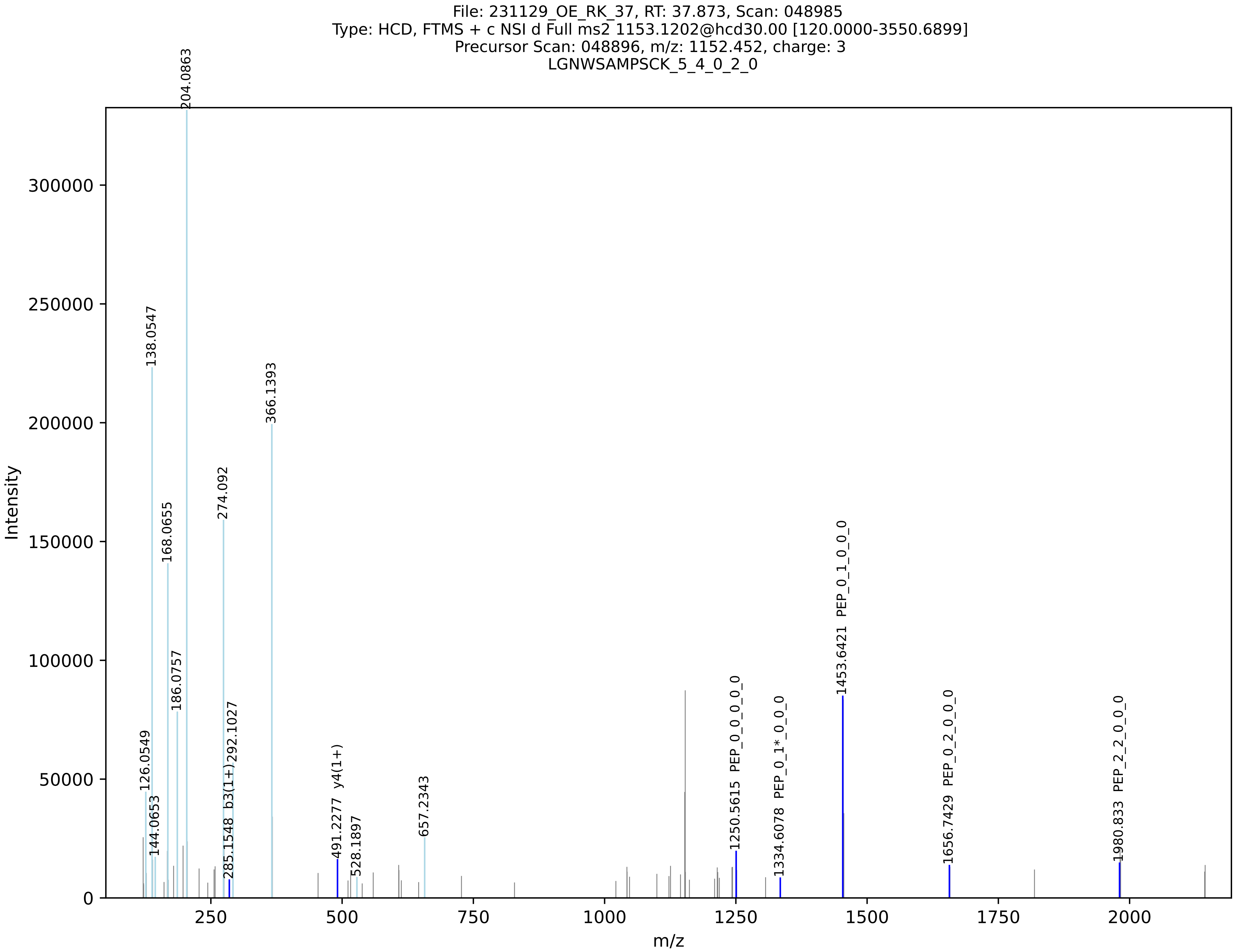
File: 231129_OE_RK_37, RT: 37.873, Scan: 048985
Type: HCD, FTMS + c NSI d Full ms2 1153.1202@hcd30.00 [120.0000-3550.6899]
Precursor Scan: 048896, m/z: 1152.452, charge: 3
LGNWSAMPSCK_5_4_0_2_0
Intensity
m/z
204.0863
138.0547
366.1393
274.092
168.0655
186.0757
126.0549
144.0653
292.1027
285.1548 b3(1+)
491.2277 y4(1+)
528.1897
657.2343
1250.5615 PEP_0_0_0_0_0
1334.6078 PEP_0_1*_0_0_0
1453.6421 PEP_0_1_0_0_0
1656.7429 PEP_0_2_0_0_0
1980.833 PEP_2_2_0_0_0

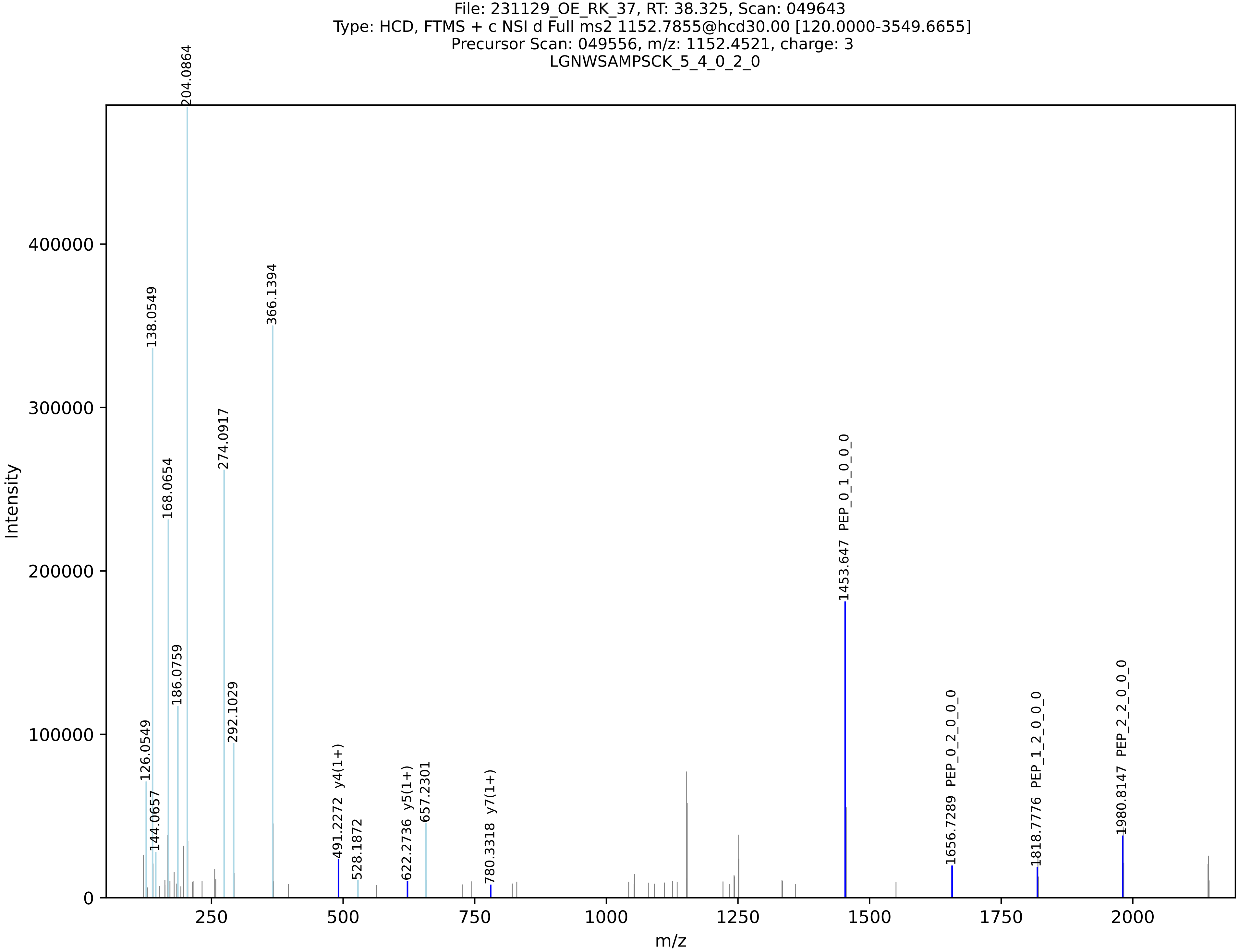
File: 231129_OE_RK_37, RT: 38.325, Scan: 049643
Type: HCD, FTMS + c NSI d Full ms2 1152.7855@hcd30.00 [120.0000-3549.6655]
Precursor Scan: 049556, m/z: 1152.4521, charge: 3
LGNWSAMPSCK_5_4_0_2_0
204.0864
138.0549
366.1394
274.0917
168.0654
186.0759
292.1029
126.0549
144.0657
491.2272 y4(1+)
528.1872
622.2736 y5(1+)
657.2301
780.3318 y7(1+)
1453.647 PEP_0_1_0_0_0
1656.7289 PEP_0_2_0_0_0
1818.7776 PEP_1_2_0_0_0
1980.8147 PEP_2_2_0_0_0
Intensity
0
100000
200000
300000
400000
m/z
250
500
750
1000
1250
1500
1750
2000

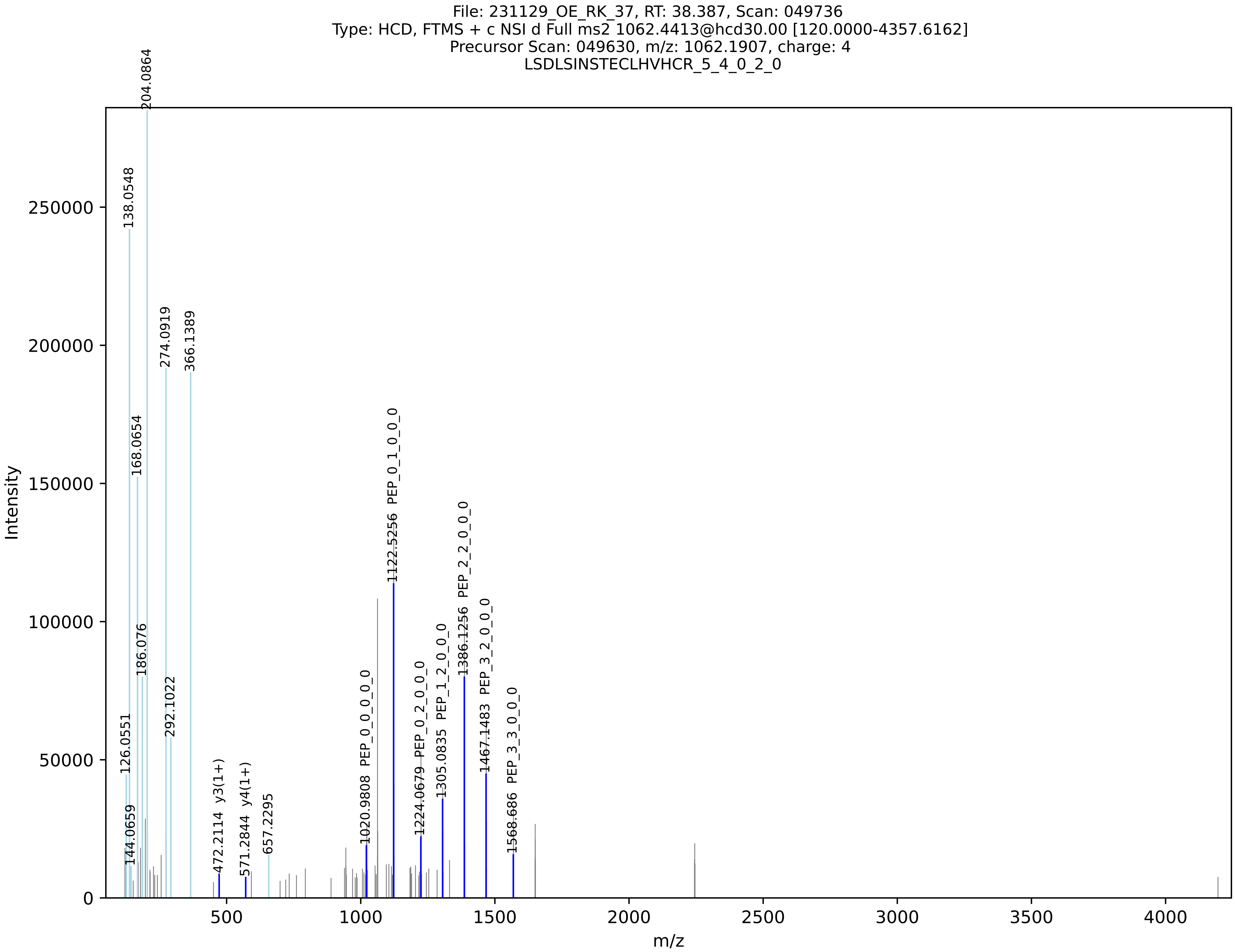
File: 231129_OE_RK_37, RT: 38.387, Scan: 049736
Type: HCD, FTMS + c NSI d Full ms2 1062.4413@hcd30.00 [120.0000-4357.6162]
Precursor Scan: 049630, m/z: 1062.1907, charge: 4
LSDLSINSTECLHVHCR_5_4_0_2_0
Intensity
m/z
204.0864
138.0548
274.0919
366.1389
168.0654
186.076
292.1022
126.0551
144.0659
472.2114 y3(1+)
571.2844 y4(1+)
657.2295
1020.9808 PEP_0_0_0_0_0_
1122.5256 PEP_0_1_0_0_0_
1224.0679 PEP_0_2_0_0_0_
1305.0835 PEP_1_2_0_0_0_
1386.1256 PEP_2_2_0_0_0_
1467.1483 PEP_3_2_0_0_0_
1568.686 PEP_3_3_0_0_0_
0
50000
100000
150000
200000
250000
500
1000
1500
2000
2500
3000
3500
4000

File: 231129_OE_RK_37, RT: 38.521, Scan: 049928
Type: HCD, FTMS + c NSI d Full ms2 1250.2263@hcd30.00 [120.0000-3847.8345]
Precursor Scan: 049850, m/z: 1249.5581, charge: 3
VTVRPGESVMVNISAK_5_4_1_1_0

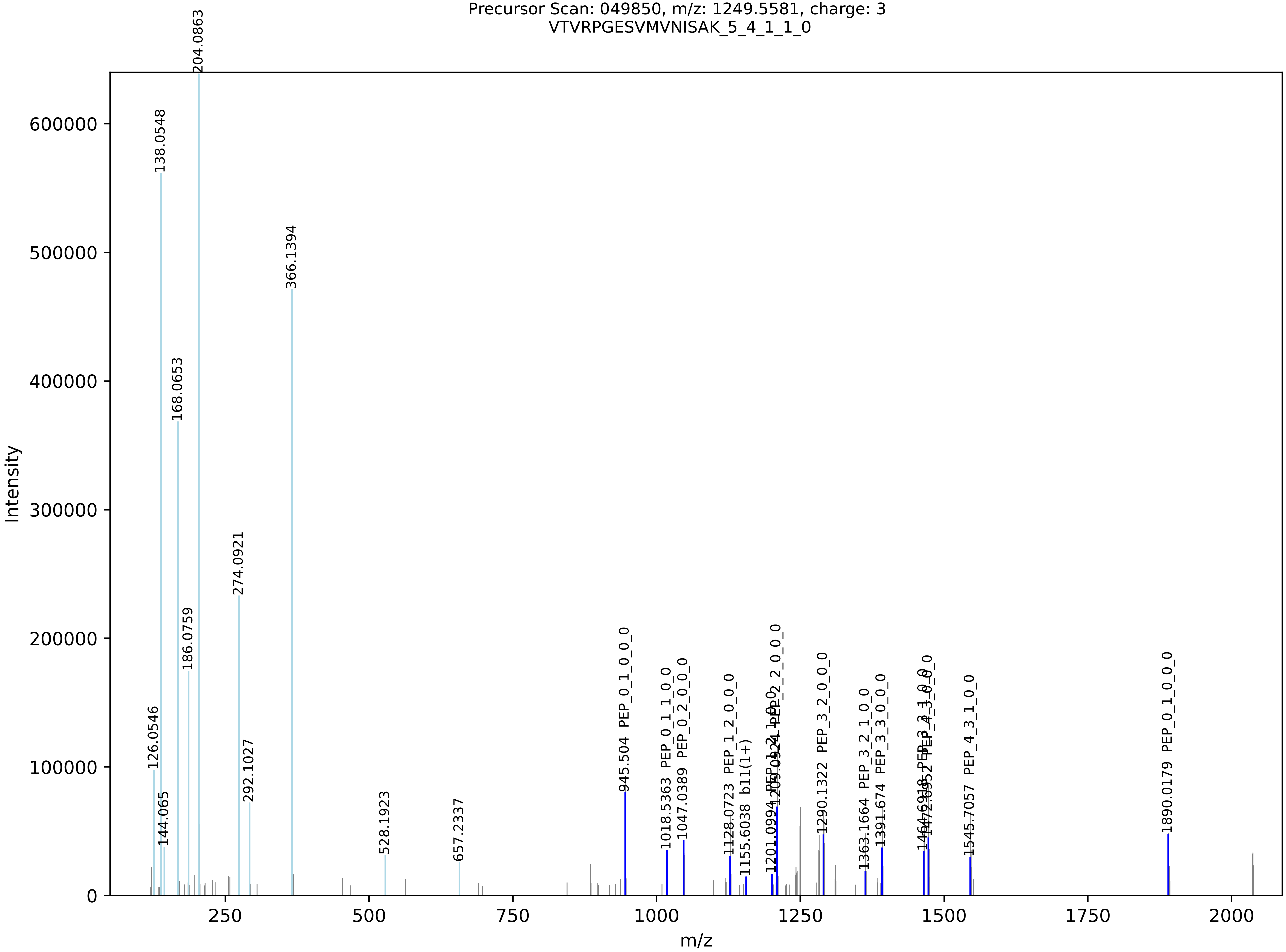

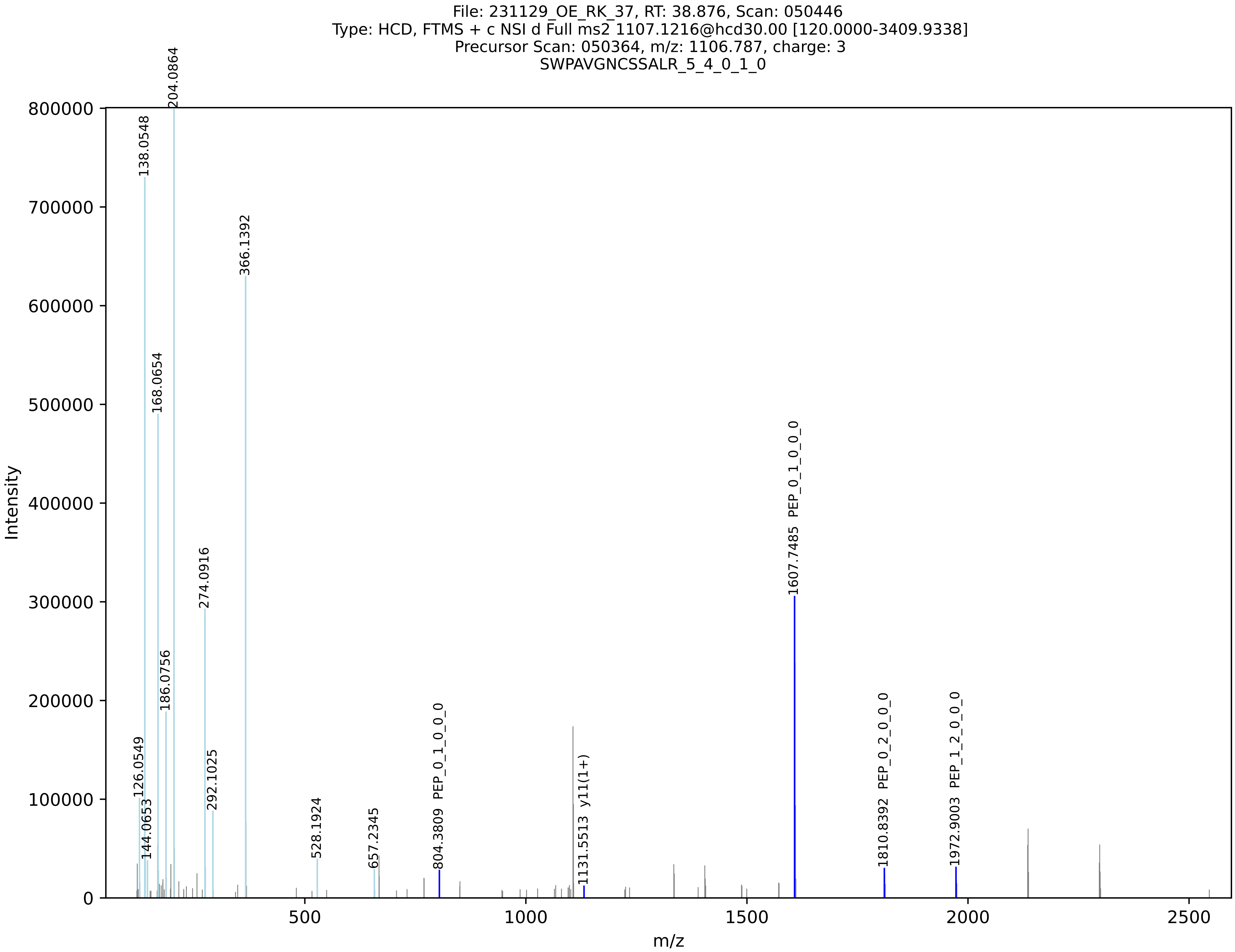
File: 231129_OE_RK_37, RT: 38.876, Scan: 050446
Type: HCD, FTMS + c NSI d Full ms2 1107.1216@hcd30.00 [120.0000-3409.9338]
Precursor Scan: 050364, m/z: 1106.787, charge: 3
SWPAVGNCSSALR_5_4_0_1_0
Intensity
m/z
204.0864
138.0548
366.1392
168.0654
274.0916
186.0756
126.0549
292.1025
144.0653
528.1924
657.2345
804.3809 PEP_0_1_0_0_0_
1131.5513 y11(1+)
1607.7485 PEP_0_1_0_0_0_
1810.8392 PEP_0_2_0_0_0_
1972.9003 PEP_1_2_0_0_0_

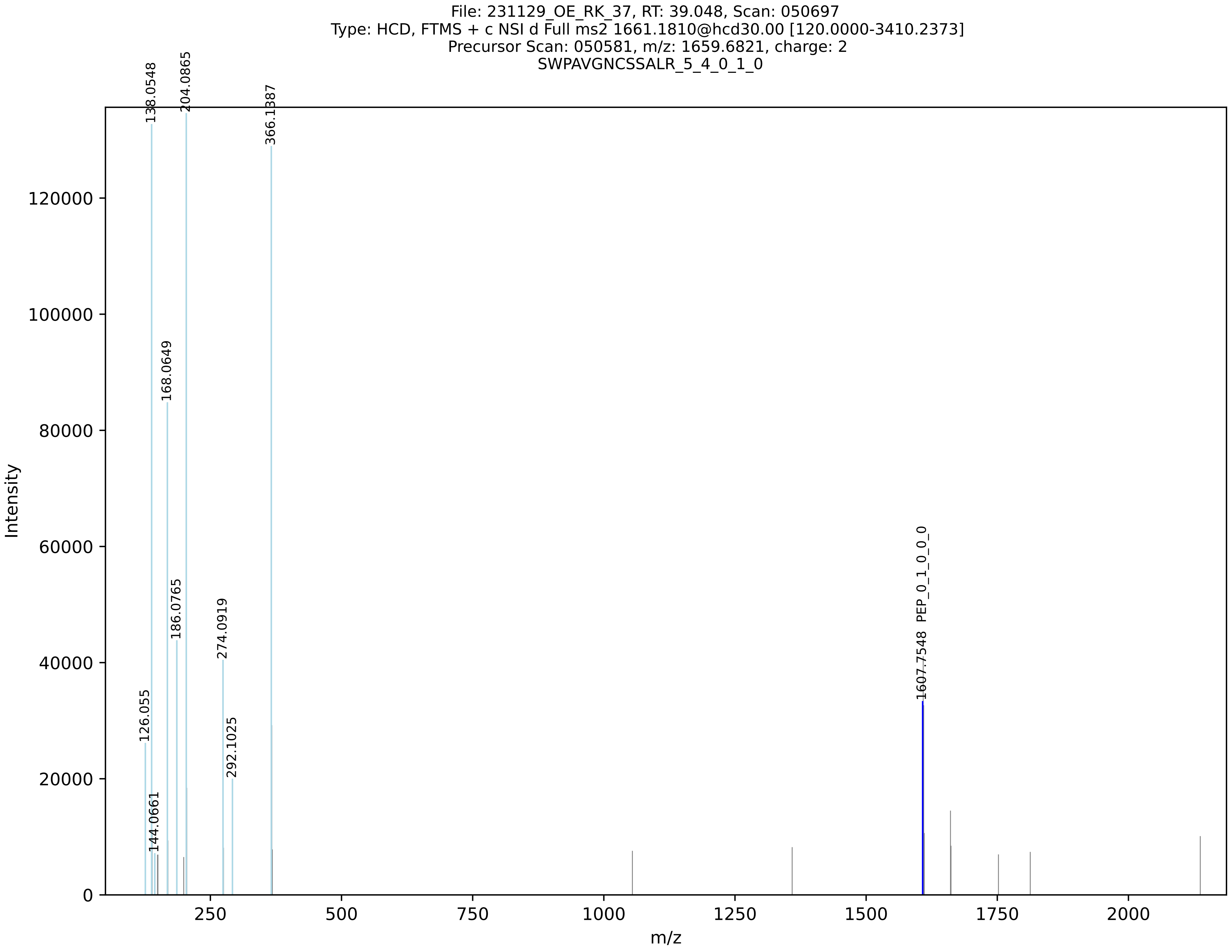
File: 231129_OE_RK_37, RT: 39.048, Scan: 050697
Type: HCD, FTMS + c NSI d Full ms2 1661.1810@hcd30.00 [120.0000-3410.2373]
Precursor Scan: 050581, m/z: 1659.6821, charge: 2
SWPAVGNCSSALR_5_4_0_1_0
138.0548
204.0865
366.1387
168.0649
186.0765
274.0919
126.055
292.1025
144.0661
1607.7548 PEP_0_1_0_0_0
Intensity
m/z
0
20000
40000
60000
80000
100000
120000
250
500
750
1000
1250
1500
1750
2000

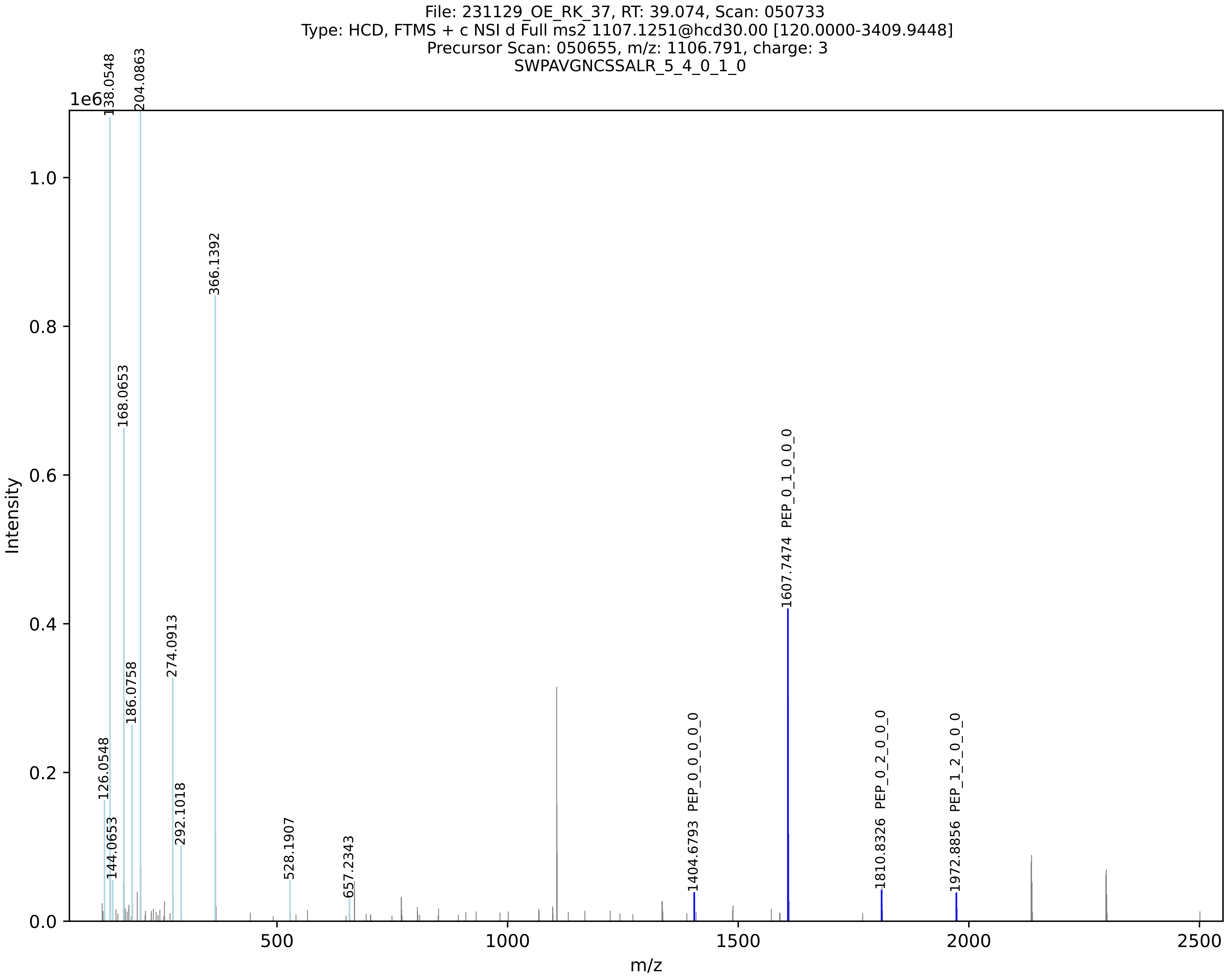
File: 231129_OE_RK_37, RT: 39.074, Scan: 050733
Type: HCD, FTMS + c NSI d Full ms2 1107.1251@hcd30.00 [120.0000-3409.9448]
Precursor Scan: 050655, m/z: 1106.791, charge: 3
SWPAVGNCSSALR_5_4_0_1_0
1e6
Intensity
m/z
138.0548
204.0863
366.1392
168.0653
186.0758
274.0913
126.0548
144.0653
292.1018
528.1907
657.2343
1404.6793 PEP_0_0_0_0_0_
1607.7474 PEP_0_1_0_0_0_
1810.8326 PEP_0_2_0_0_0_
1972.8856 PEP_1_2_0_0_0_

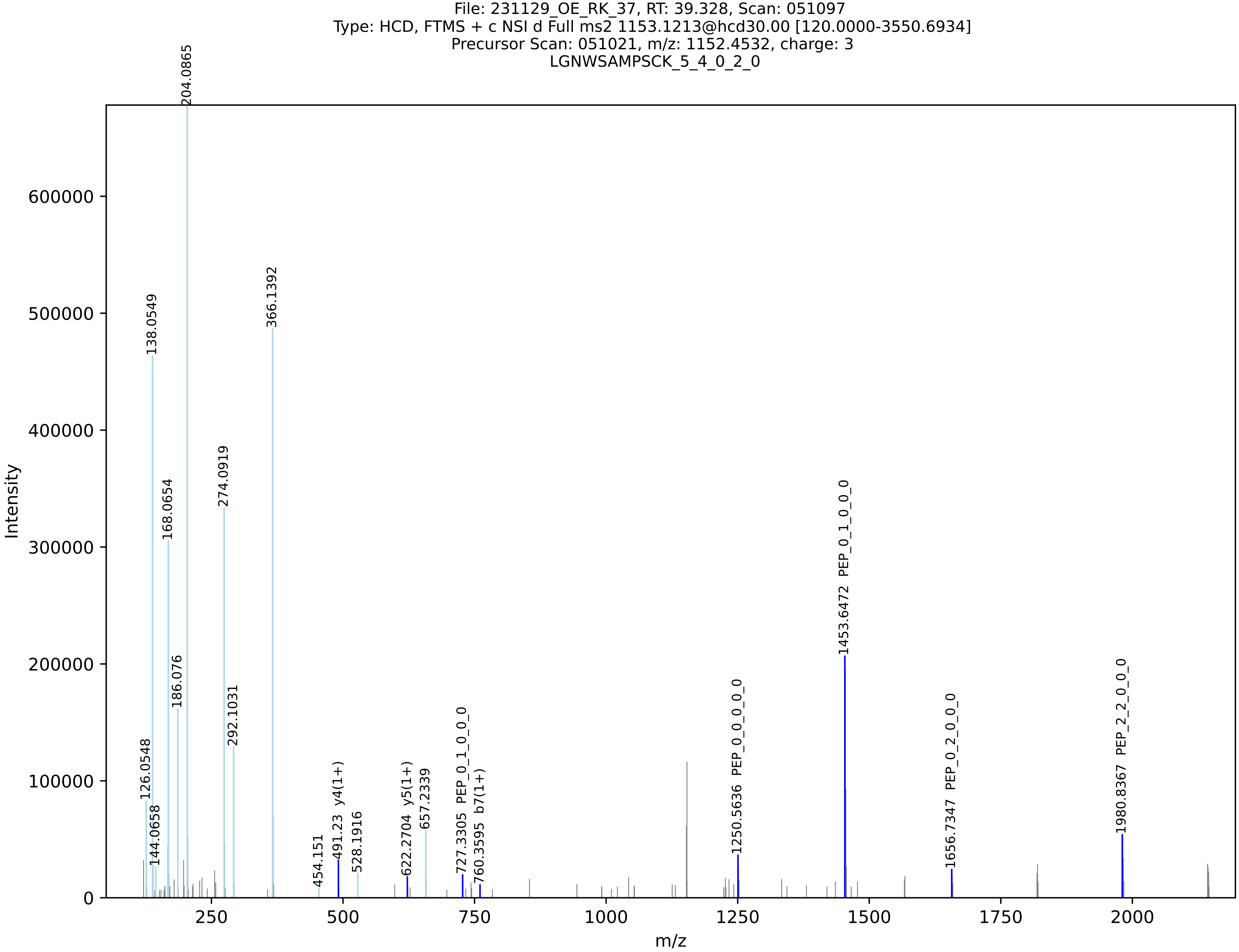

File: 231129_OE_RK_37, RT: 39.328, Scan: 051097
Type: HCD, FTMS + c NSI d Full ms2 1153.1213@hcd30.00 [120.0000-3550.6934]
Precursor Scan: 051021, m/z: 1152.4532, charge: 3
LGNWSAMPSCK_5_4_0_2_0
Intensity
m/z
204.0865
138.0549
366.1392
274.0919
168.0654
186.076
292.1031
126.0548
144.0658
454.151
491.23 y4(1+)
528.1916
622.2704 y5(1+)
657.2339
727.3305 PEP_0_1_0_0_0
760.3595 b7(1+)
1250.5636 PEP_0_0_0_0_0
1453.6472 PEP_0_1_0_0_0
1656.7347 PEP_0_2_0_0_0
1980.8367 PEP_2_2_0_0_0

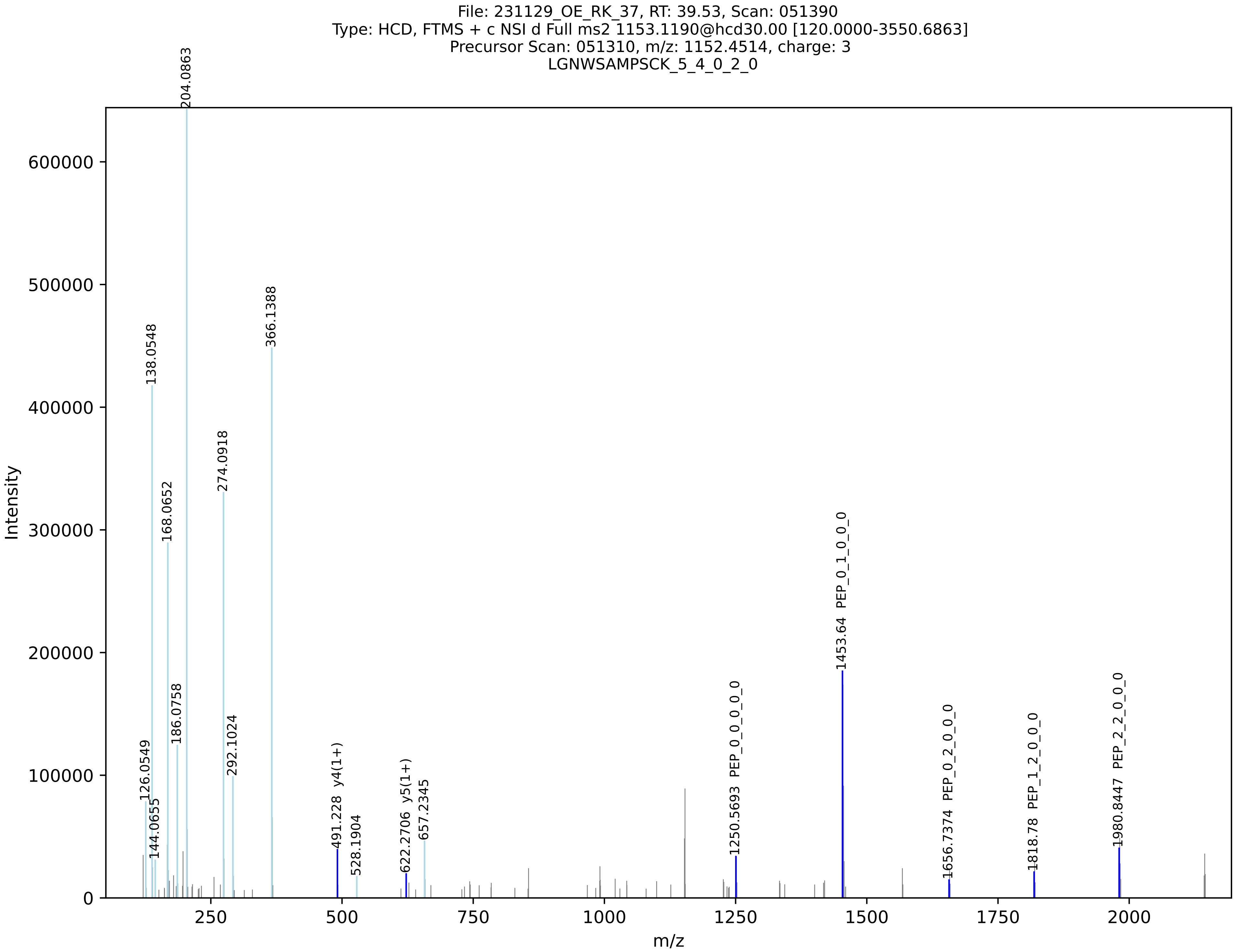

File: 231129_OE_RK_37, RT: 39.53, Scan: 051390
Type: HCD, FTMS + c NSI d Full ms2 1153.1190@hcd30.00 [120.0000-3550.6863]
Precursor Scan: 051310, m/z: 1152.4514, charge: 3
LGNWSAMPSCK_5_4_0_2_0
Intensity
m/z
204.0863
138.0548
366.1388
274.0918
168.0652
186.0758
292.1024
126.0549
144.0655
491.228 y4(1+)
528.1904
622.2706 y5(1+)
657.2345
1250.5693 PEP_0_0_0_0_0
1453.64 PEP_0_1_0_0_0
1656.7374 PEP_0_2_0_0_0
1818.78 PEP_1_2_0_0_0
1980.8447 PEP_2_2_0_0_0

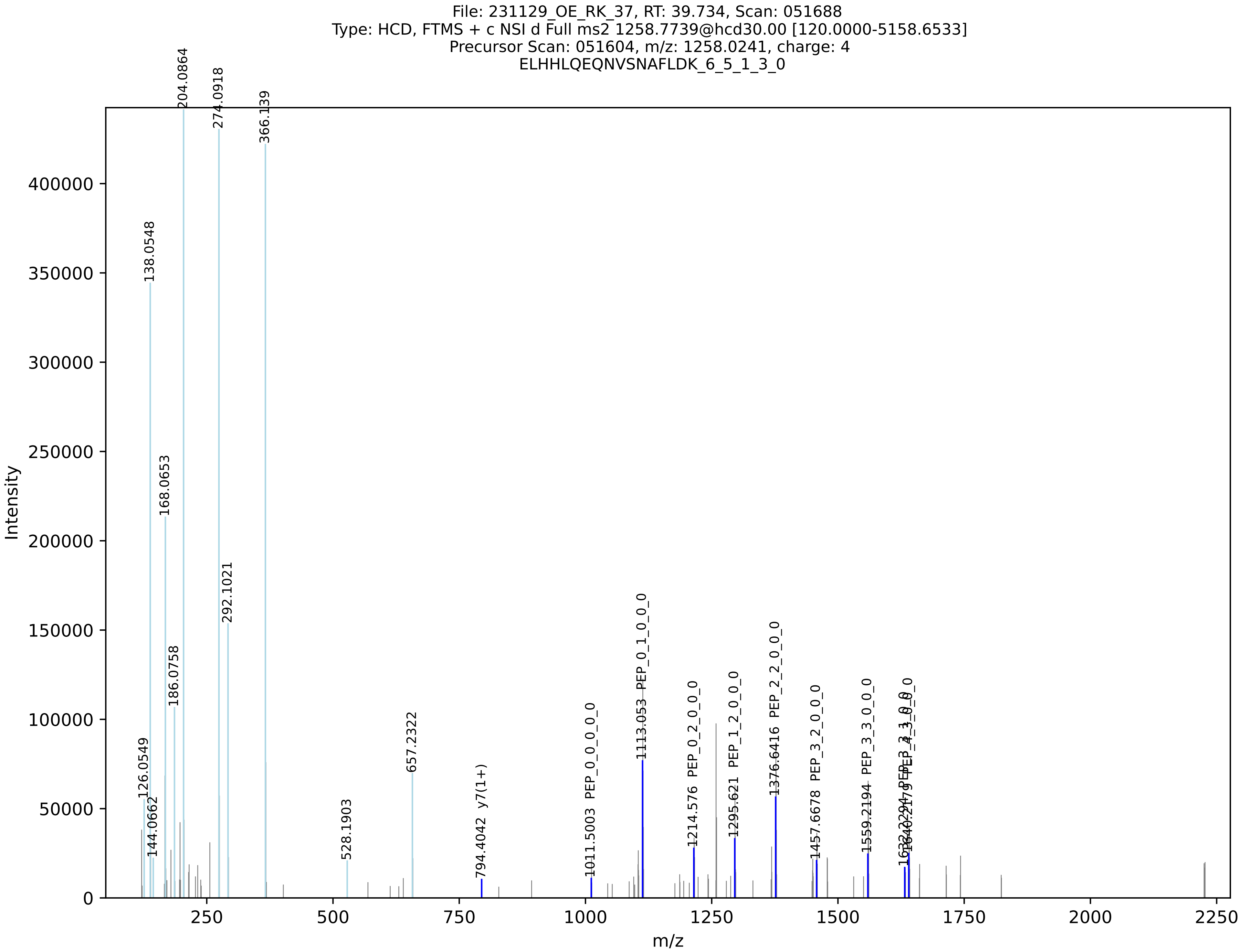

File: 231129_OE_RK_37, RT: 39.734, Scan: 051688
Type: HCD, FTMS + c NSI d Full ms2 1258.7739@hcd30.00 [120.0000-5158.6533]
Precursor Scan: 051604, m/z: 1258.0241, charge: 4
ELHHLQEQNVSNAFLDK_6_5_1_3_0
Intensity
m/z
204.0864
274.0918
366.139
138.0548
168.0653
292.1021
186.0758
126.0549
144.0662
528.1903
657.2322
794.4042 y7(1+)
1011.5003 PEP_0_0_0_0_0
1113.053 PEP_0_1_0_0_0
1214.576 PEP_0_2_0_0_0
1295.621 PEP_1_2_0_0_0
1376.6416 PEP_2_2_0_0_0
1457.6678 PEP_3_2_0_0_0
1559.2194 PEP_3_3_0_0_0

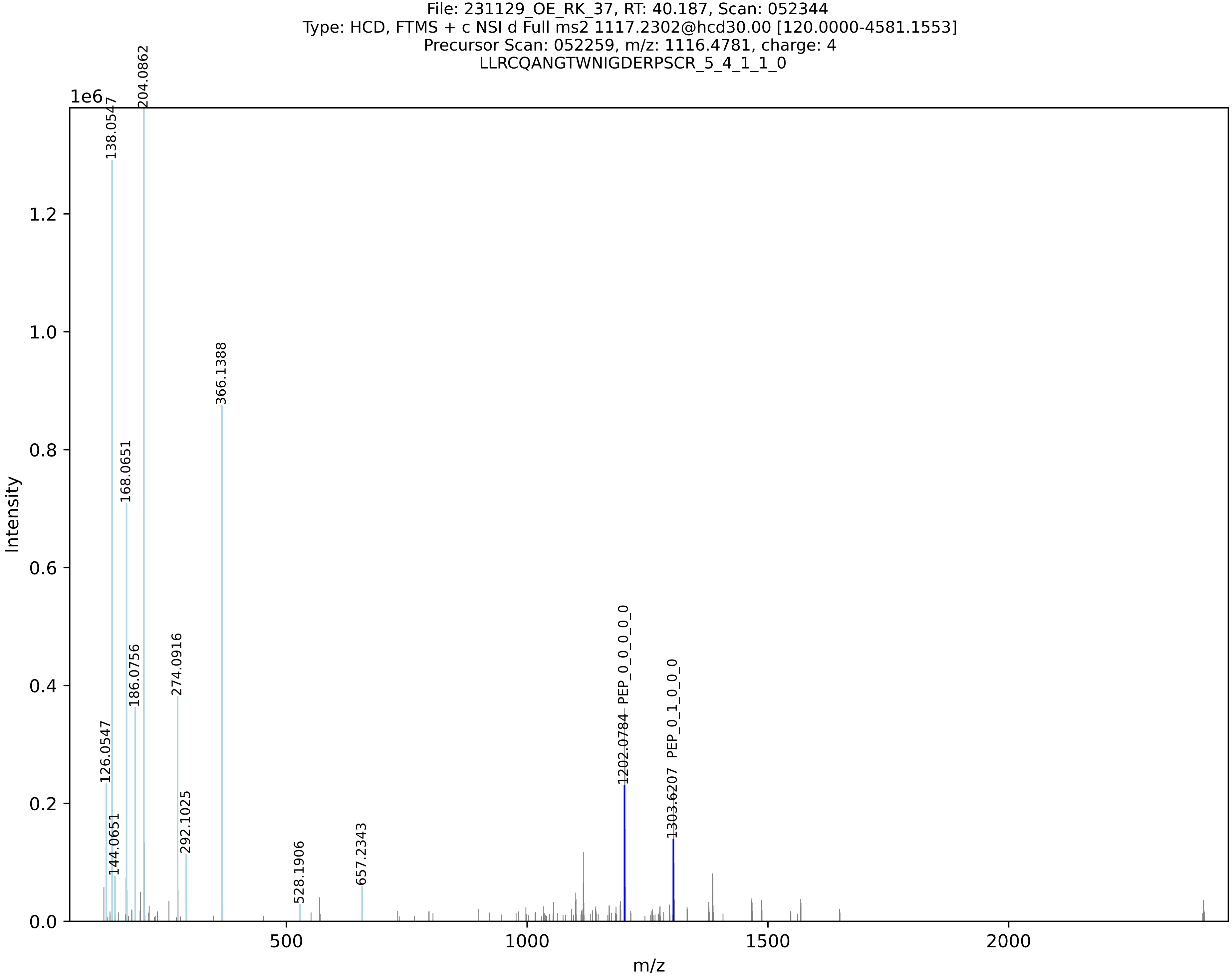
File: 231129_OE_RK_37, RT: 40.187, Scan: 052344
Type: HCD, FTMS + c NSI d Full ms2 1117.2302@hcd30.00 [120.0000-4581.1553]
Precursor Scan: 052259, m/z: 1116.4781, charge: 4
LLRCQANGTWNIGDERPSCR_5_4_1_1_0
1e6
Intensity
m/z
204.0862
138.0547
366.1388
168.0651
186.0756
274.0916
126.0547
292.1025
144.0651
528.1906
657.2343
1202.0784 PEP_0_0_0_0_0
1303.6207 PEP_0_1_0_0_0
0.0
0.2
0.4
0.6
0.8
1.0
1.2
500
1000
1500
2000

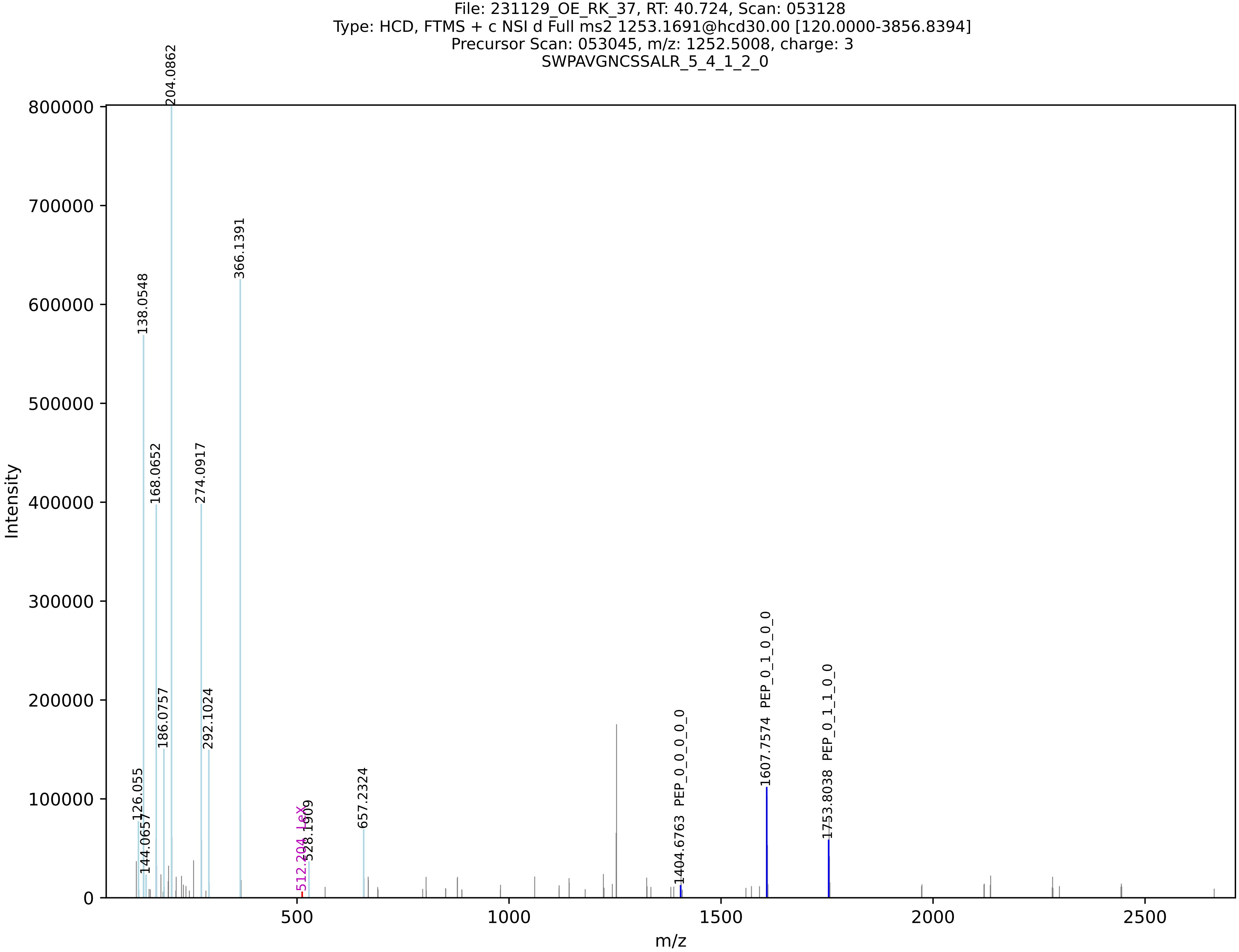

File: 231129_OE_RK_37, RT: 40.724, Scan: 053128
Type: HCD, FTMS + c NSI d Full ms2 1253.1691@hcd30.00 [120.0000-3856.8394]
Precursor Scan: 053045, m/z: 1252.5008, charge: 3
SWPAVGNCSSALR_5_4_1_2_0
Intensity
m/z
204.0862
366.1391
138.0548
168.0652
274.0917
186.0757
292.1024
126.055
144.0657
512.204_LeX
528.1909
657.2324
1404.6763 PEP_0_0_0_0_0
1607.7574 PEP_0_1_0_0_0
1753.8038 PEP_0_1_1_0_0

File: 231129_OE_RK_37, RT: 40.862, Scan: 053328
Type: HCD, FTMS + c NSI d Full ms2 1203.9015@hcd30.00 [120.0000-3706.0803]
Precursor Scan: 053267, m/z: 1203.9014, charge: 3
SWPAVGNCSSALR_5_4_0_2_0

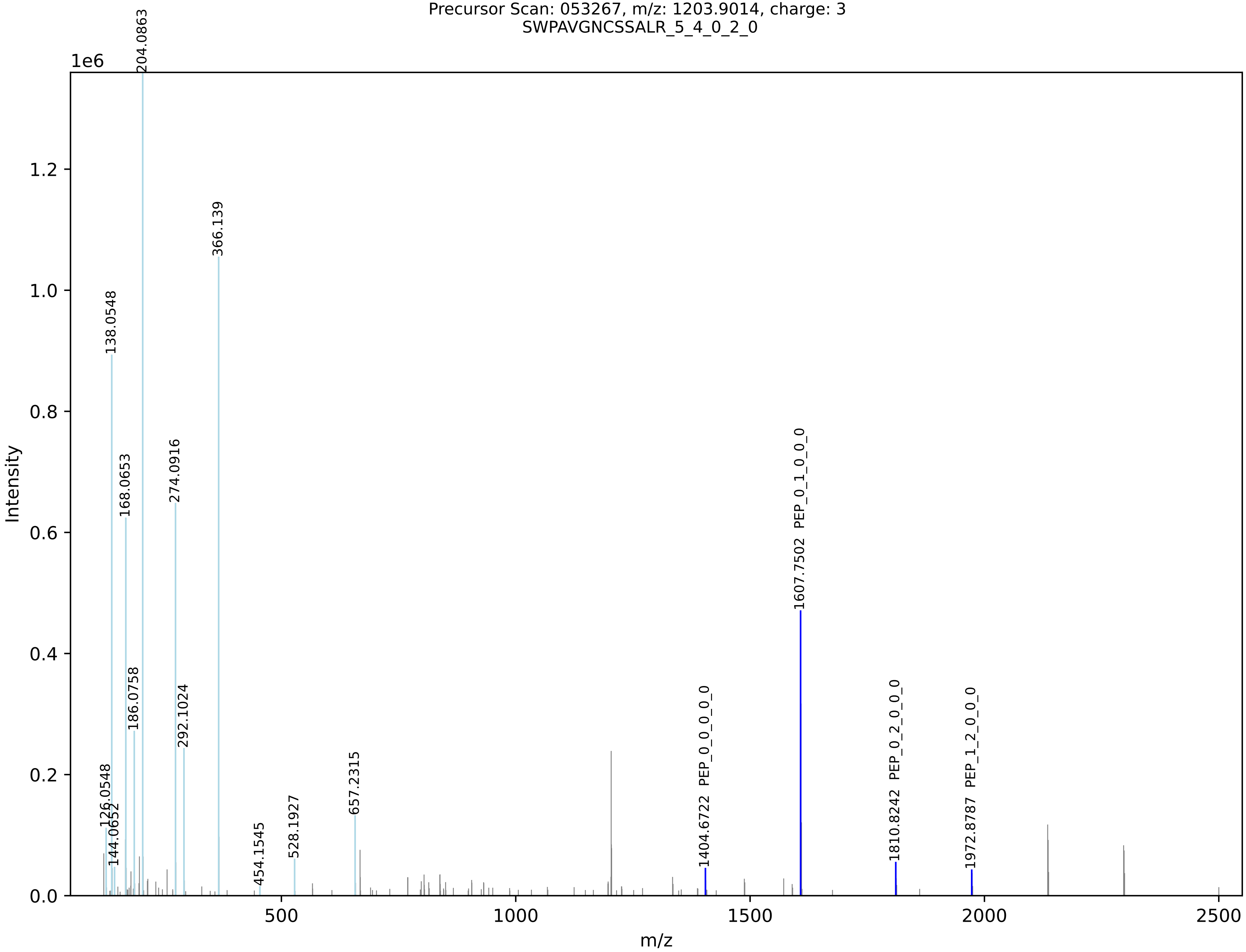

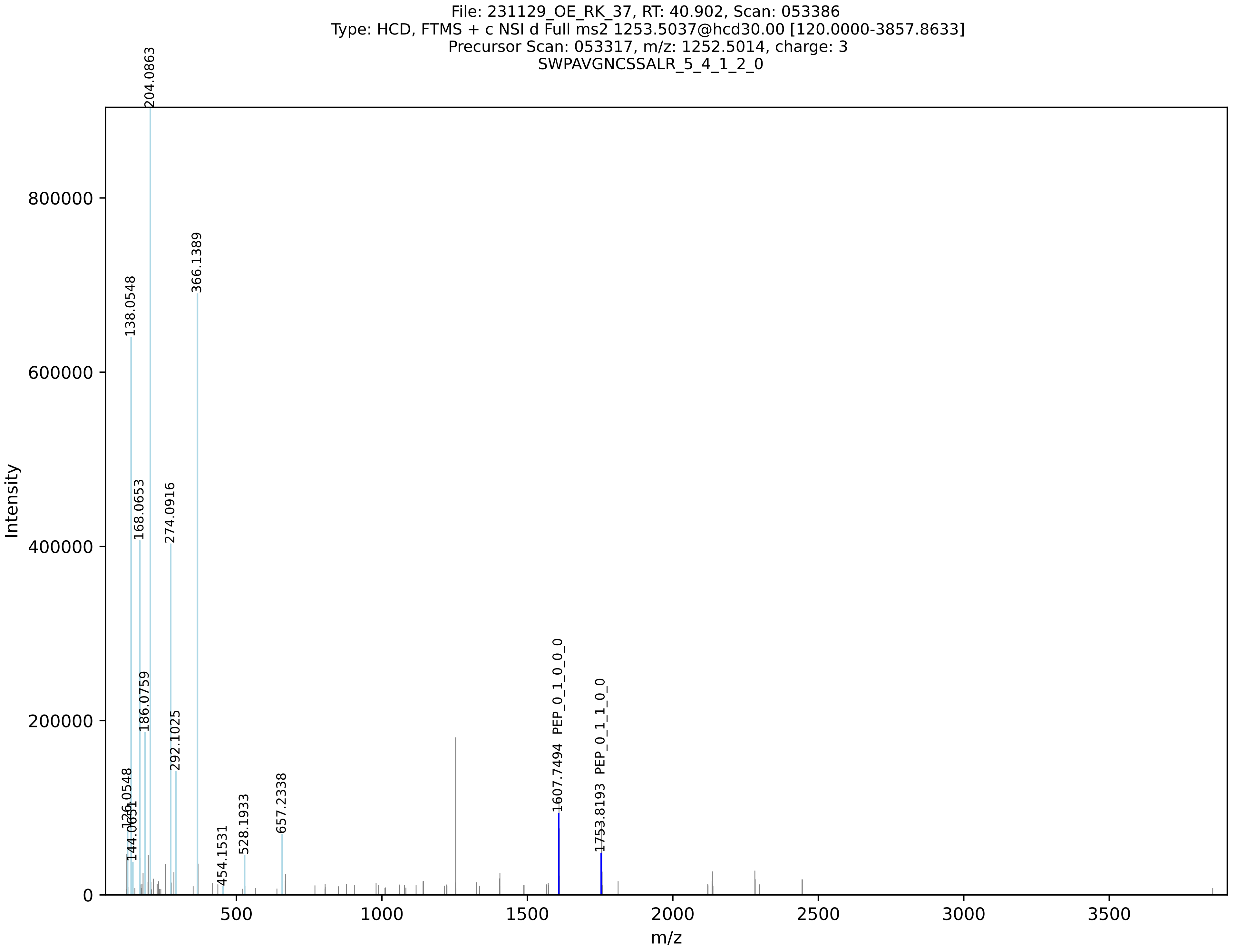

File: 231129_OE_RK_37, RT: 40.902, Scan: 053386
Type: HCD, FTMS + c NSI d Full ms2 1253.5037@hcd30.00 [120.0000-3857.8633]
Precursor Scan: 053317, m/z: 1252.5014, charge: 3
SWPAVGNCSSALR_5_4_1_2_0
Intensity
m/z
204.0863
366.1389
138.0548
168.0653
274.0916
186.0759
292.1025
126.0548
144.0651
454.1531
528.1933
657.2338
1607.7494 PEP_0_1_0_0_0
1753.8193 PEP_0_1_1_0_0
0
200000
400000
600000
800000
500
1000
1500
2000
2500
3000
3500

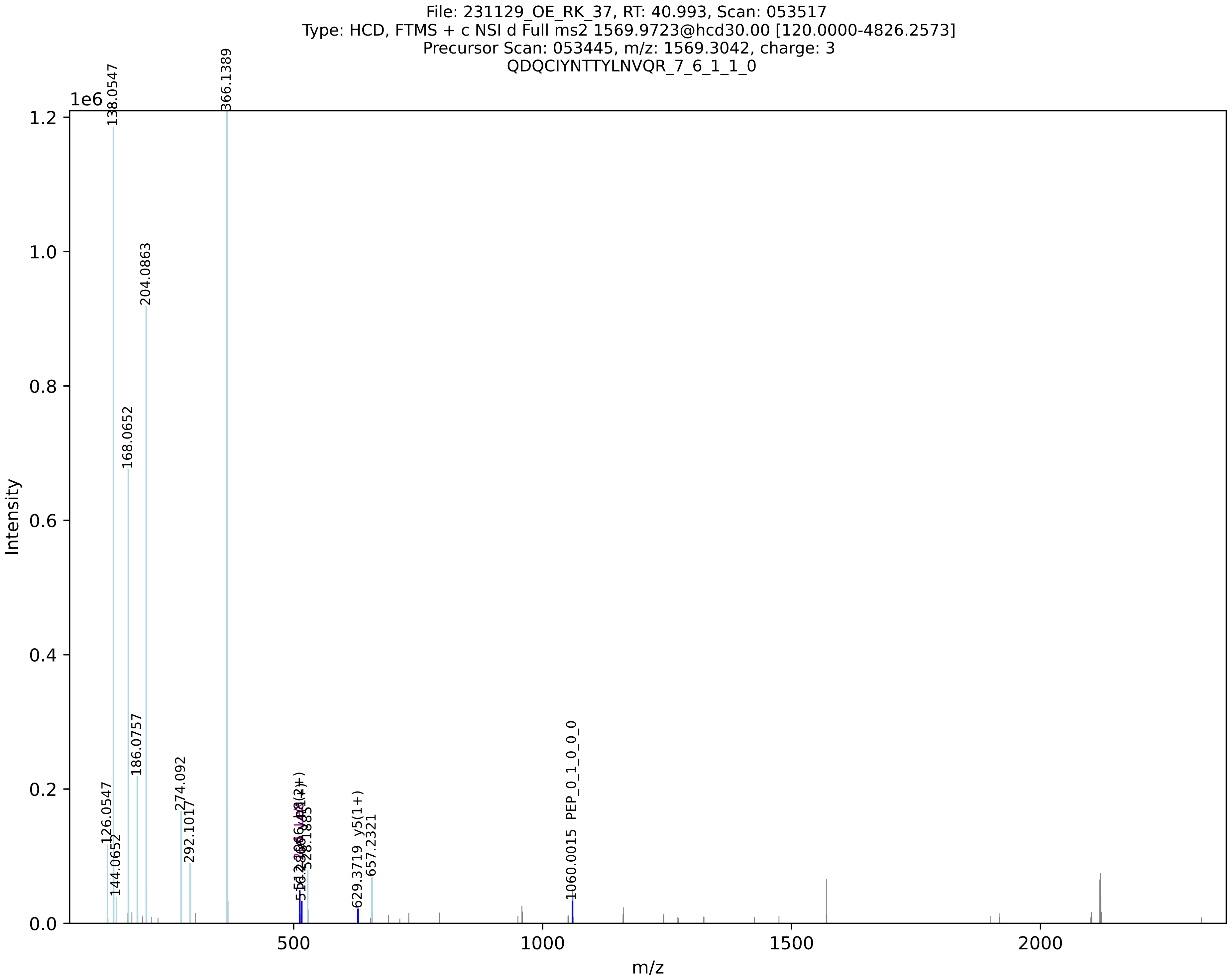
File: 231129_OE_RK_37, RT: 40.993, Scan: 053517
Type: HCD, FTMS + c NSI d Full ms2 1569.9723@hcd30.00 [120.0000-4826.2573]
Precursor Scan: 053445, m/z: 1569.3042, charge: 3
QDQCIYNTTYLNVQR_7_6_1_1_0
1e6
Intensity
m/z
138.0547
366.1389
204.0863
168.0652
186.0757
126.0547
144.0652
274.092
292.1017
629.3719 y5(1+)
657.2321
1060.0015 PEP_0_1_0_0_0_
528.1885

File: 231129_OE_RK_37, RT: 41.041, Scan: 053588
Type: HCD, FTMS + c NSI d Full ms2 1204.4874@hcd30.00 [120.0000-3707.8735]
Precursor Scan: 053511, m/z: 1203.8192, charge: 3
SWPAVGNCSSALR_5_4_0_2_0

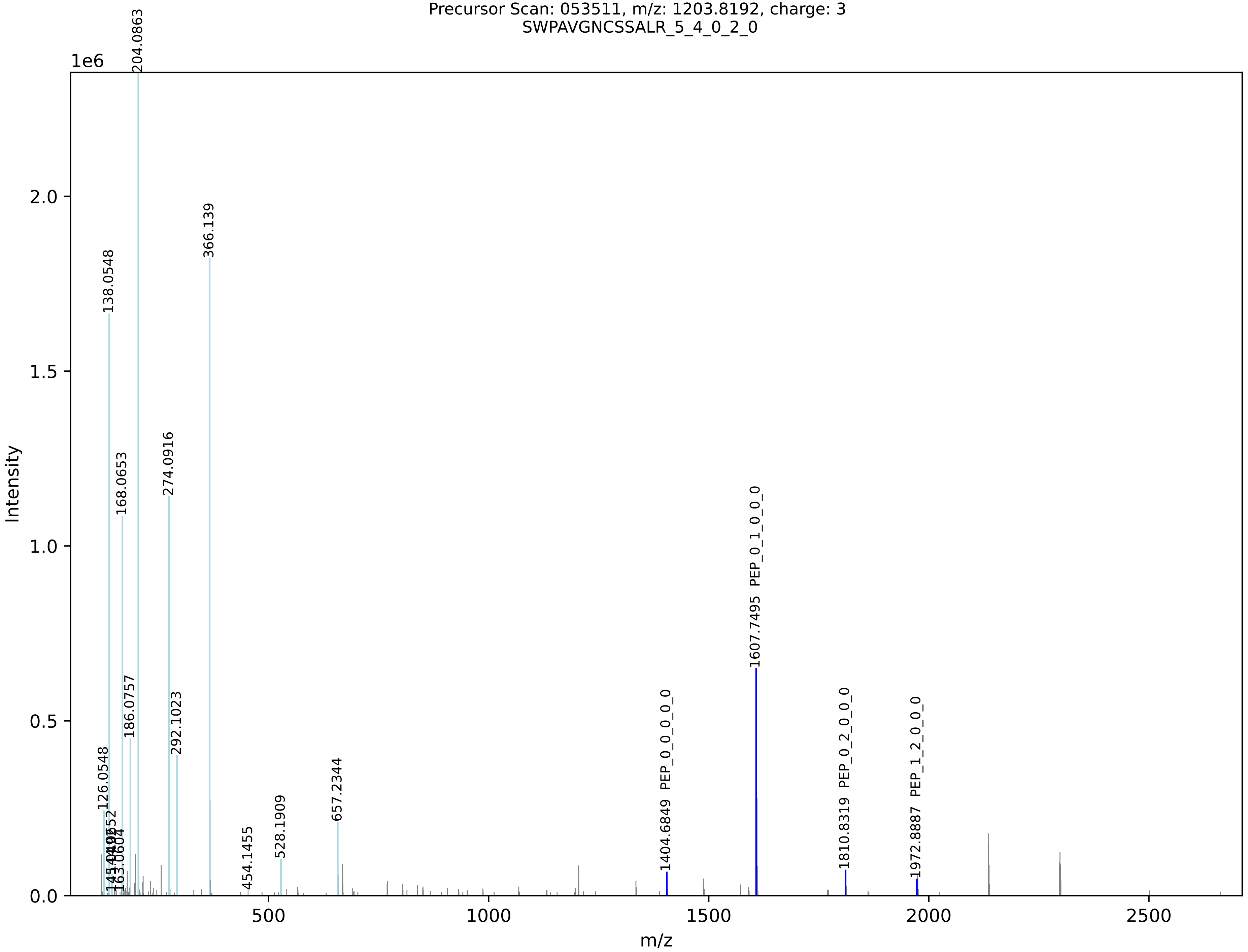

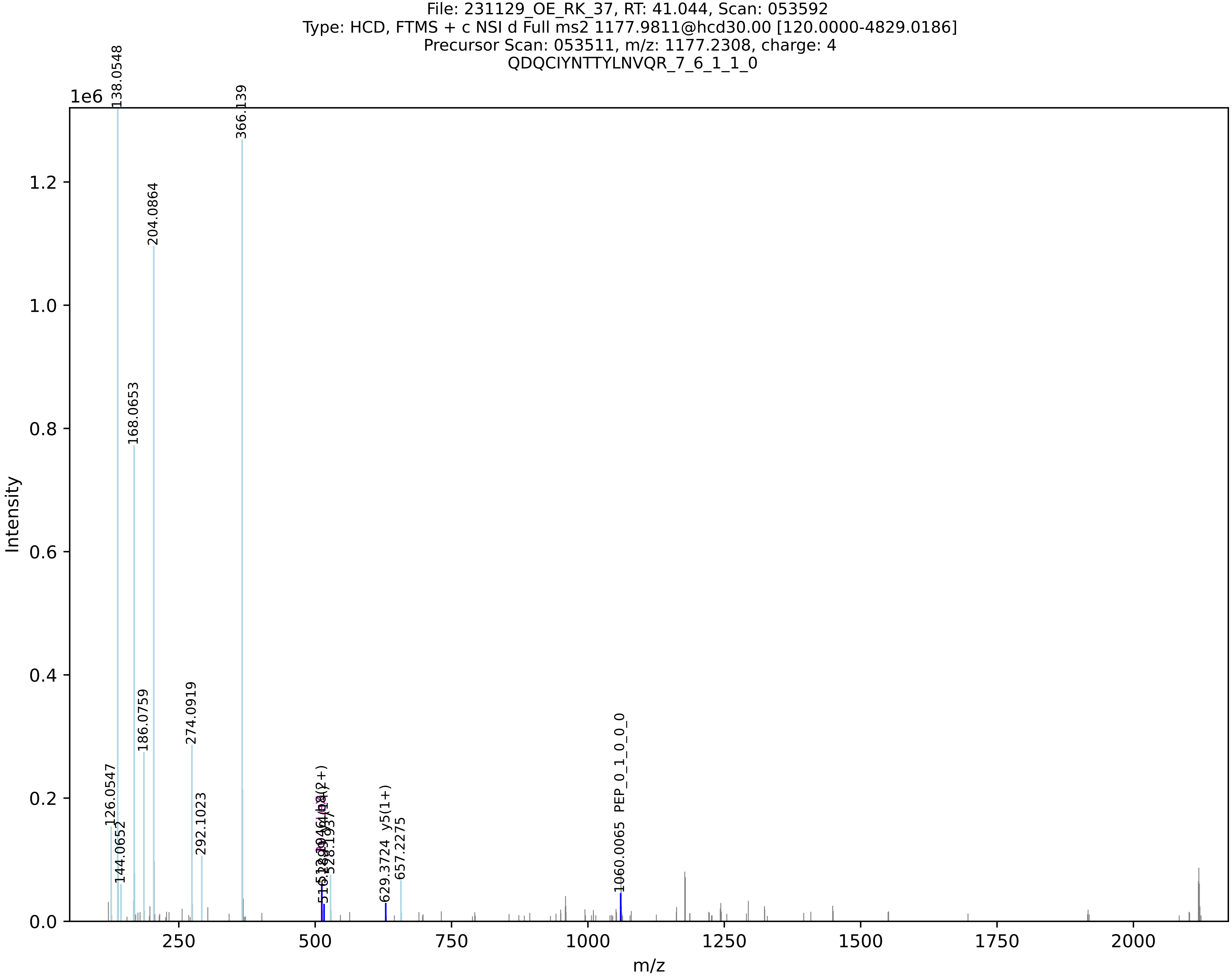
File: 231129_OE_RK_37, RT: 41.044, Scan: 053592
Type: HCD, FTMS + c NSI d Full ms2 1177.9811@hcd30.00 [120.0000-4829.0186]
Precursor Scan: 053511, m/z: 1177.2308, charge: 4
QDQCIYNTTYLNVQR_7_6_1_1_0
1e6
138.0548
366.139
204.0864
168.0653
186.0759
274.0919
292.1023
126.0547
144.0652
528.1937
629.3724 y5(1+)
657.2275
1060.0065 PEP_0_1_0_0_0
Intensity
m/z
0.0
0.2
0.4
0.6
0.8
1.0
1.2
250
500
750
1000
1250
1500
1750
2000

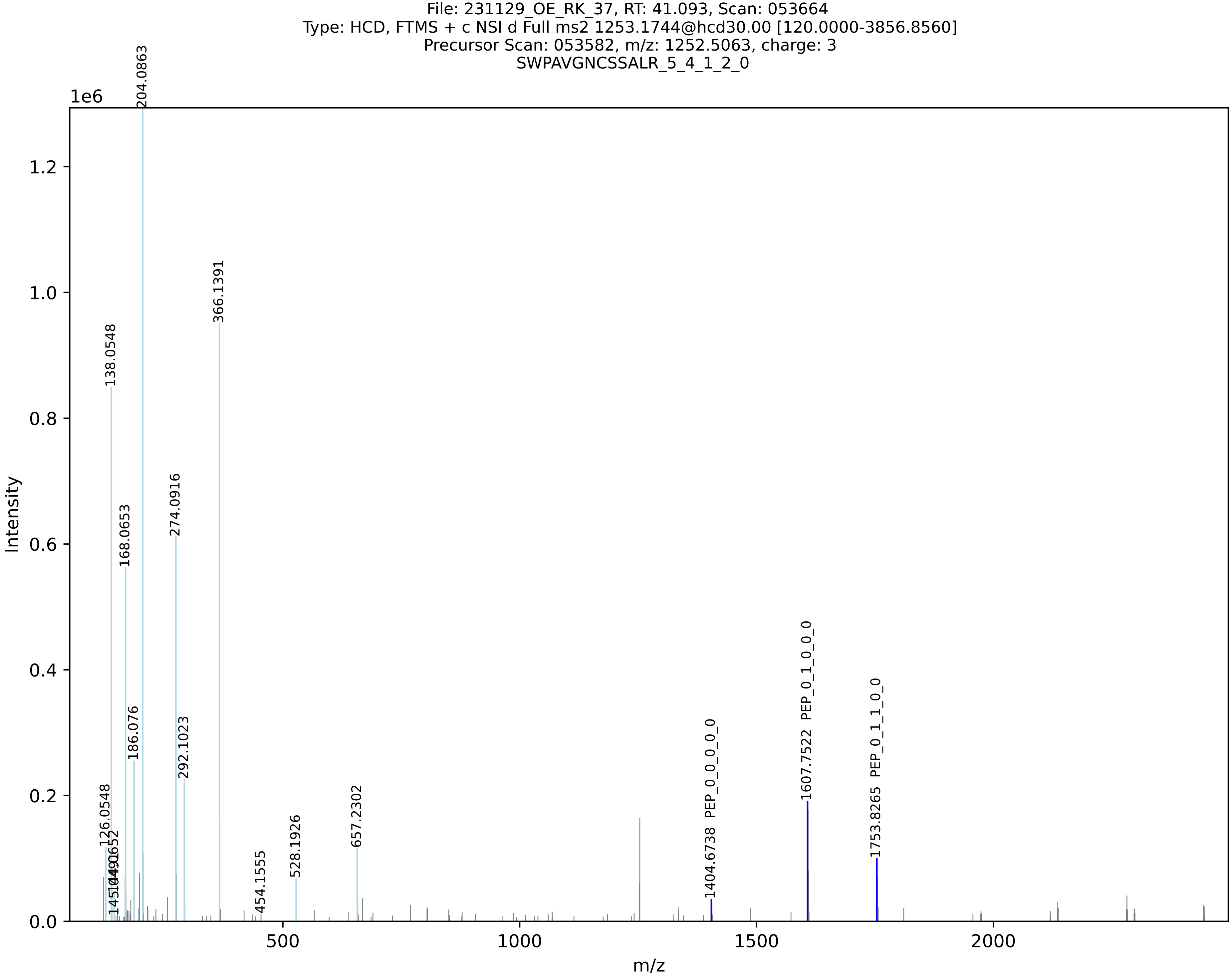
File: 231129_OE_RK_37, RT: 41.093, Scan: 053664
Type: HCD, FTMS + c NSI d Full ms2 1253.1744@hcd30.00 [120.0000-3856.8560]
Precursor Scan: 053582, m/z: 1252.5063, charge: 3
SWPAVGNCSSALR_5_4_1_2_0
1e6
204.0863
366.1391
138.0548
274.0916
168.0653
186.076
292.1023
126.0548
454.1555
528.1926
657.2302
1404.6738 PEP_0_0_0_0_0
1607.7522 PEP_0_1_0_0_0
1753.8265 PEP_0_1_1_0_0
Intensity
m/z
0.0
0.2
0.4
0.6
0.8
1.0
1.2
500
1000
1500
2000

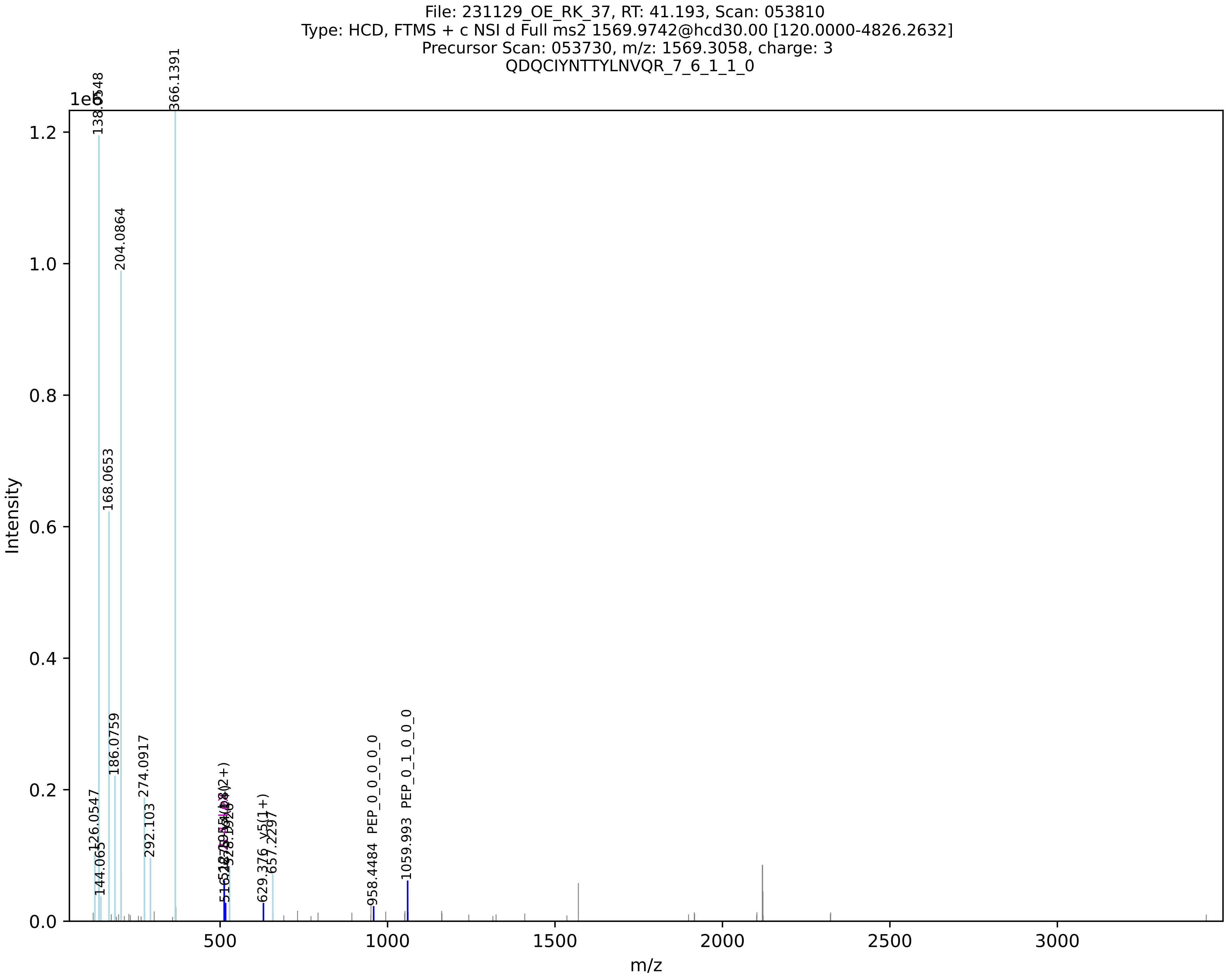
File: 231129_OE_RK_37, RT: 41.193, Scan: 053810
Type: HCD, FTMS + c NSI d Full ms2 1569.9742@hcd30.00 [120.0000-4826.2632]
Precursor Scan: 053730, m/z: 1569.3058, charge: 3
QDQCIYNTTYLNVQR_7_6_1_1_0
1e6
Intensity
m/z
366.1391
204.0864
168.0653
186.0759
274.0917
292.103
126.0547
144.065
629.376 y5(1+)
657.2297
958.4484 PEP_0_0_0_0_0_
1059.993 PEP_0_1_0_0_0_

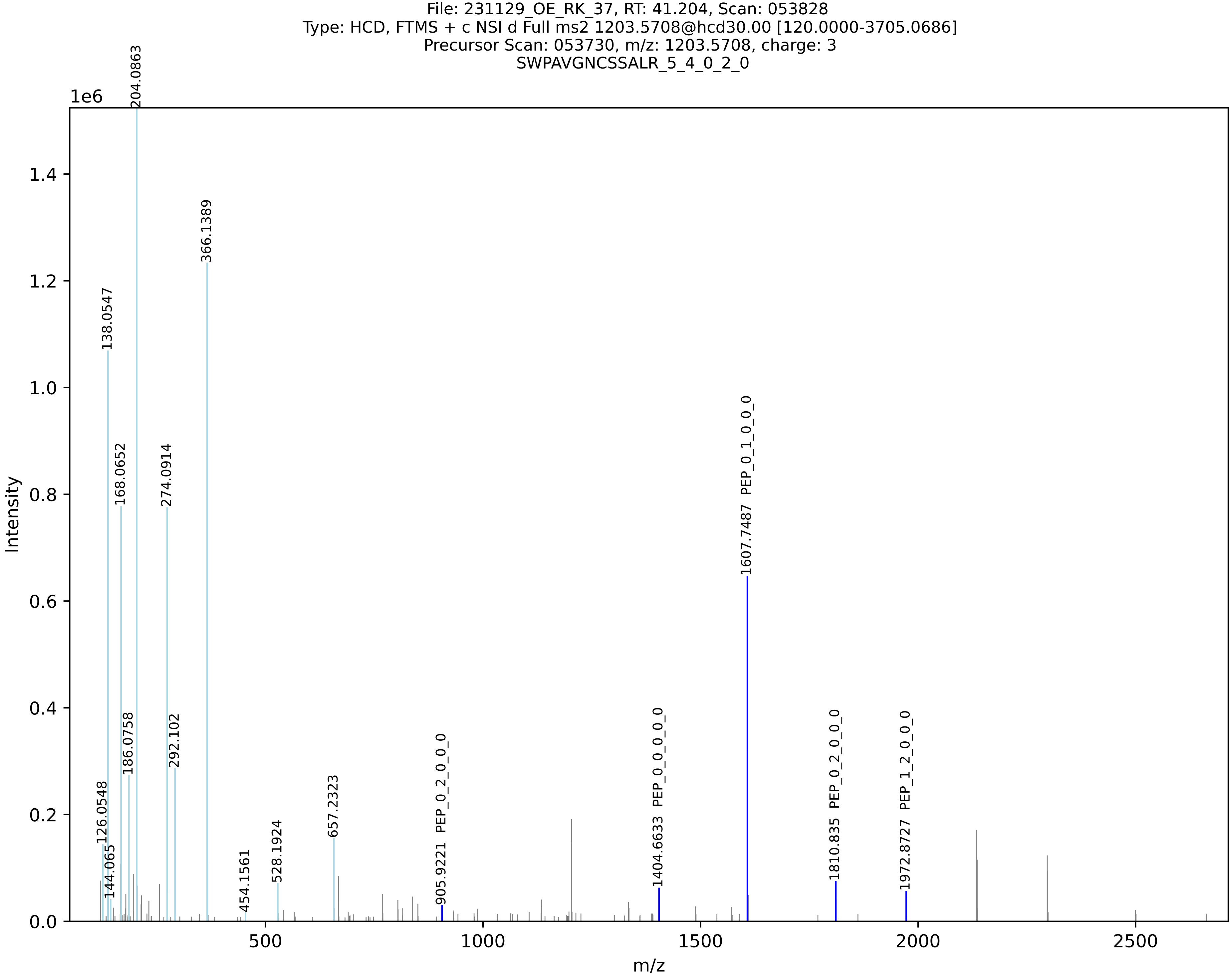
File: 231129_OE_RK_37, RT: 41.204, Scan: 053828
Type: HCD, FTMS + c NSI d Full ms2 1203.5708@hcd30.00 [120.0000-3705.0686]
Precursor Scan: 053730, m/z: 1203.5708, charge: 3
SWPAVGNCSSALR_5_4_0_2_0
1e6
Intensity
m/z
204.0863
366.1389
138.0547
168.0652
274.0914
186.0758
292.102
126.0548
144.065
454.1561
528.1924
657.2323
905.9221 PEP_0_2_0_0_0
1404.6633 PEP_0_0_0_0_0
1607.7487 PEP_0_1_0_0_0
1810.835 PEP_0_2_0_0_0
1972.8727 PEP_1_2_0_0_0

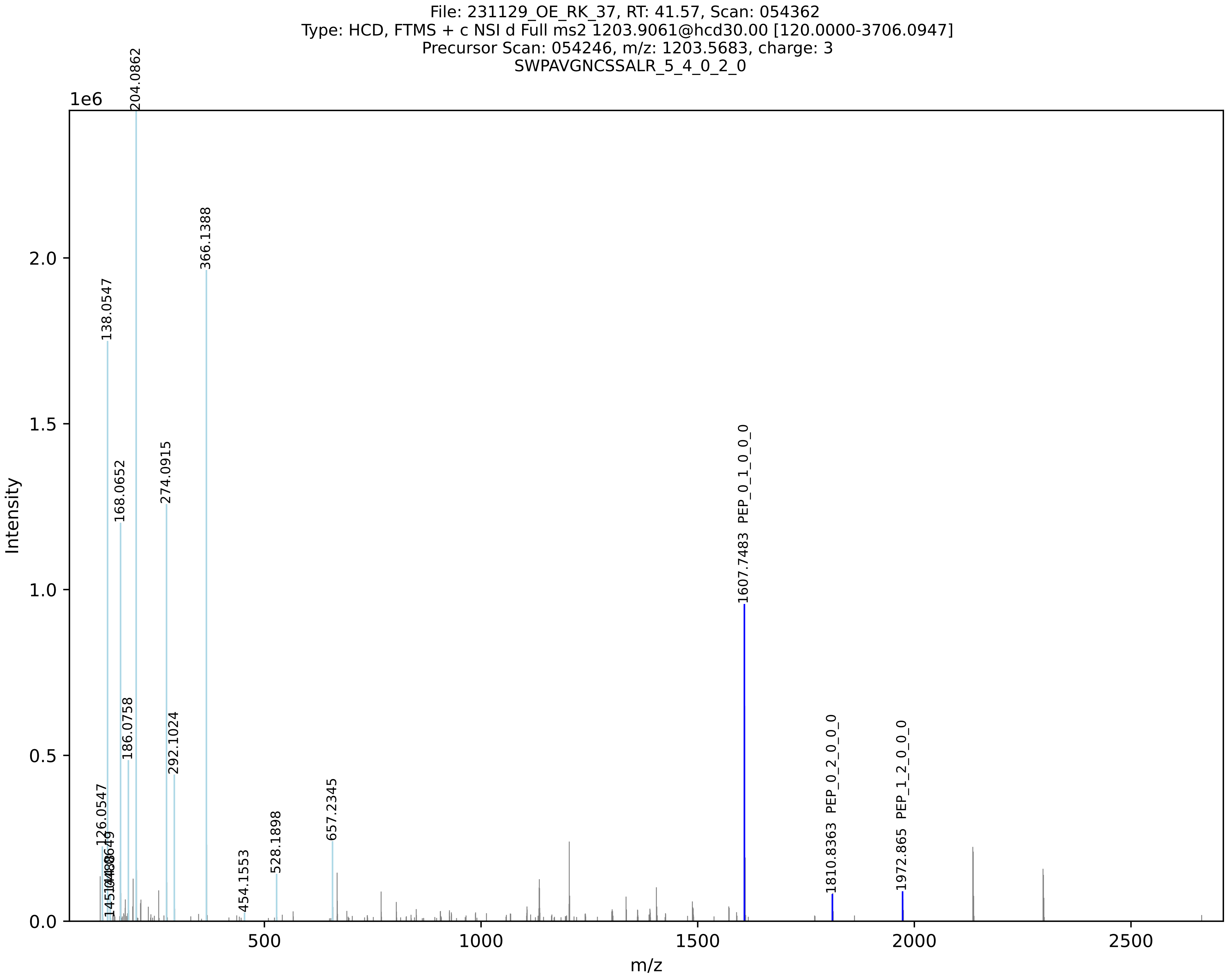
File: 231129_OE_RK_37, RT: 41.57, Scan: 054362
Type: HCD, FTMS + c NSI d Full ms2 1203.9061@hcd30.00 [120.0000-3706.0947]
Precursor Scan: 054246, m/z: 1203.5683, charge: 3
SWPAVGNCSSALR_5_4_0_2_0
1e6
204.0862
366.1388
138.0547
168.0652
274.0915
186.0758
292.1024
126.0547
454.1553
528.1898
657.2345
1607.7483 PEP_0_1_0_0_0
1810.8363 PEP_0_2_0_0_0
1972.865 PEP_1_2_0_0_0
Intensity
m/z
0.0
0.5
1.0
1.5
2.0
500
1000
1500
2000
2500

File: 231129_OE_RK_37, RT: 41.646, Scan: 054471
Type: HCD, FTMS + c NSI d Full ms2 1189.7535@hcd30.00 [120.0000-4877.0508]
Precursor Scan: 054391, m/z: 1189.2523, charge: 4
LLRCQANGTWNIGDERPSCR_5_4_1_2_0

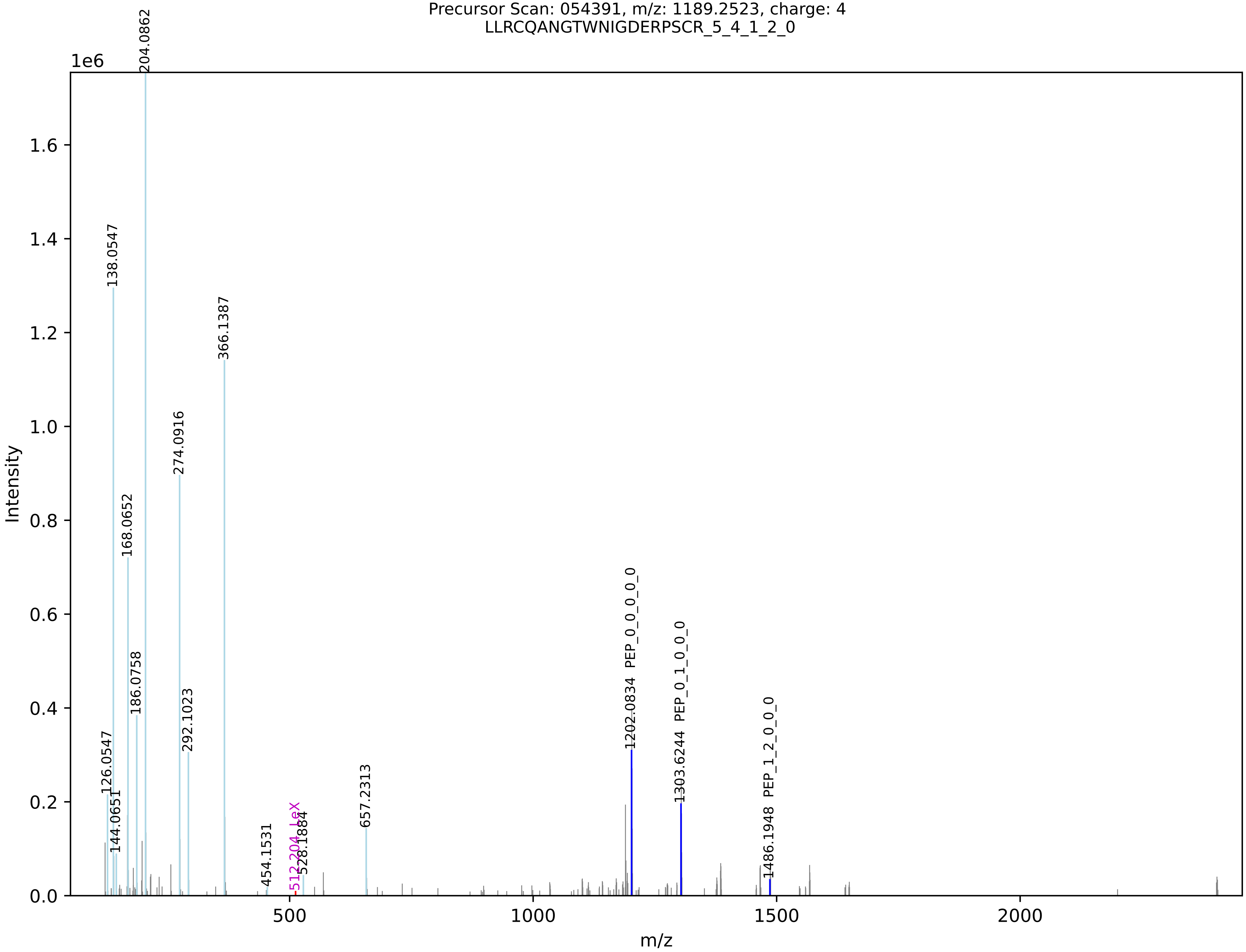

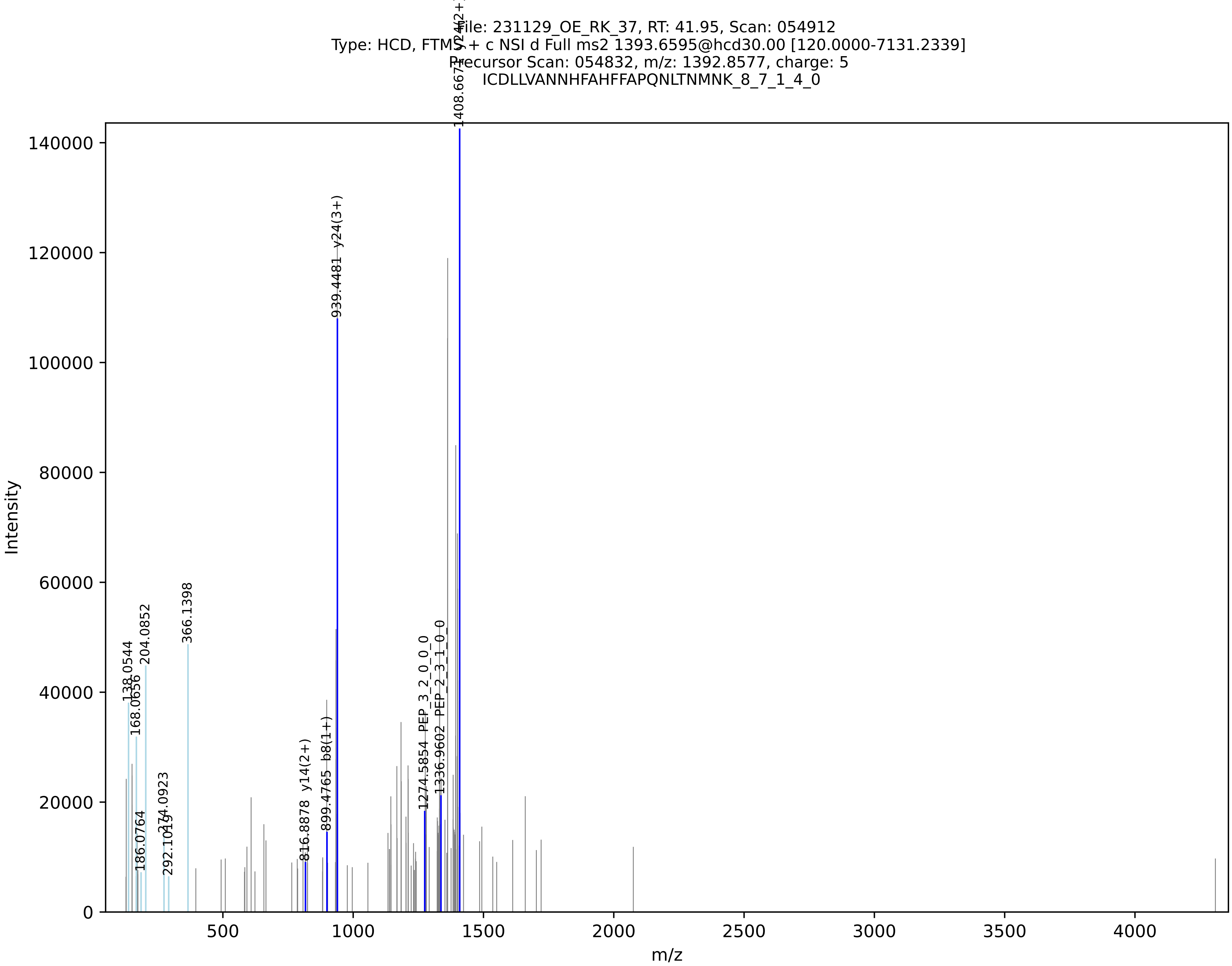

File: 231129_OE_RK_37, RT: 41.95, Scan: 054912
Type: HCD, FTMS + c NSI d Full ms2 1393.6595@hcd30.00 [120.0000-7131.2339]
Precursor Scan: 054832, m/z: 1392.8577, charge: 5
ICDLLVANNHFAHFFAPQNLTNMNK_8_7_1_4_0
Intensity
m/z
1408.6677 y24(2+)
939.4481 y24(3+)
366.1398
204.0852
138.0544
168.0656
186.0764
274.0923
292.1019
816.8878 y14(2+)
899.4765 b8(1+)
1274.5854 PEP_3_2_0_0_0
1336.9602 PEP_2_3_1_0_0

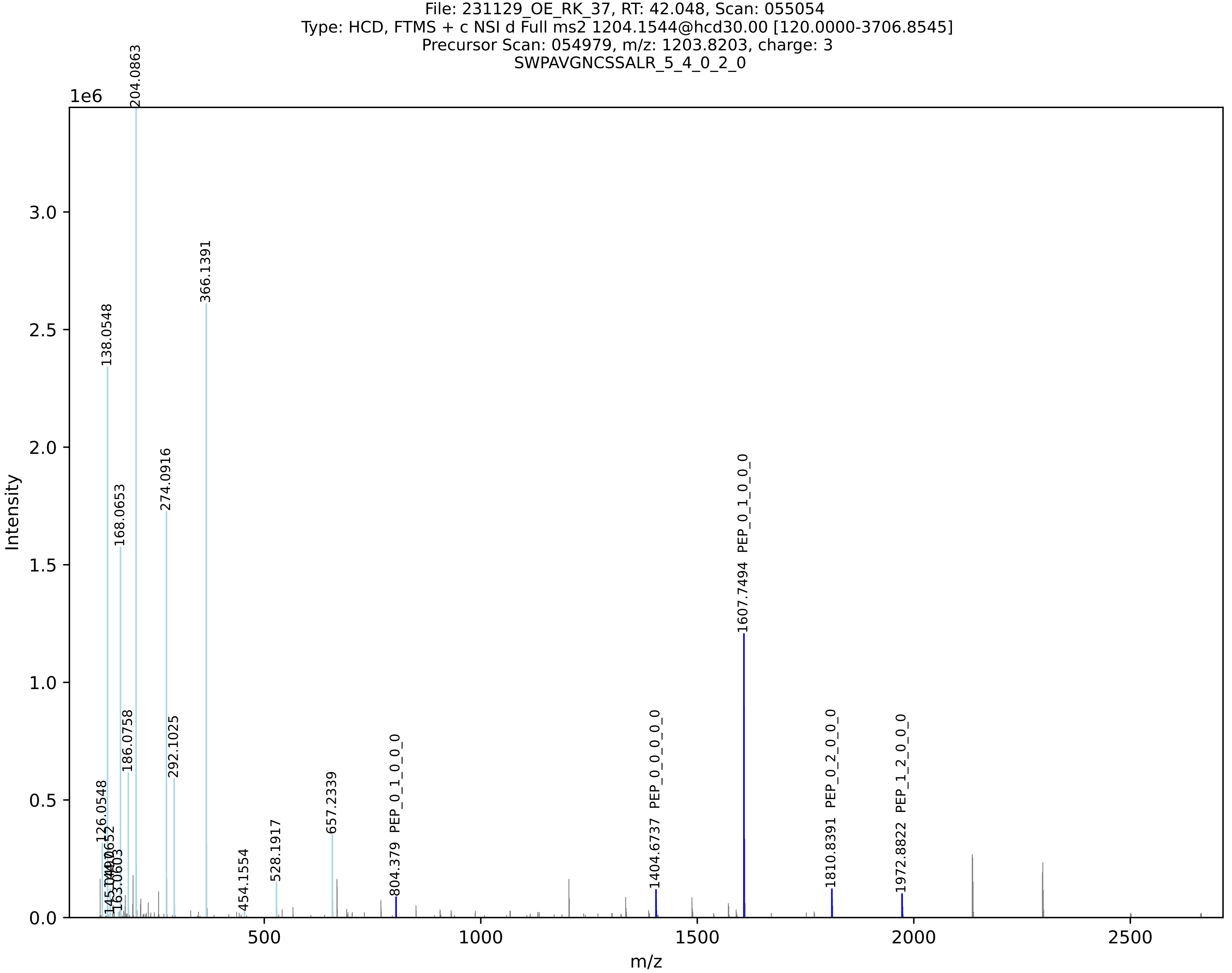
File: 231129_OE_RK_37, RT: 42.048, Scan: 055054
Type: HCD, FTMS + c NSI d Full ms2 1204.1544@hcd30.00 [120.0000-3706.8545]
Precursor Scan: 054979, m/z: 1203.8203, charge: 3
SWPAVGNCSSALR_5_4_0_2_0
1e6
Intensity
m/z
204.0863
366.1391
138.0548
274.0916
168.0653
186.0758
292.1025
126.0548
454.1554
528.1917
657.2339
804.379 PEP_0_1_0_0_0
1404.6737 PEP_0_0_0_0_0
1607.7494 PEP_0_1_0_0_0
1810.8391 PEP_0_2_0_0_0
1972.8822 PEP_1_2_0_0_0

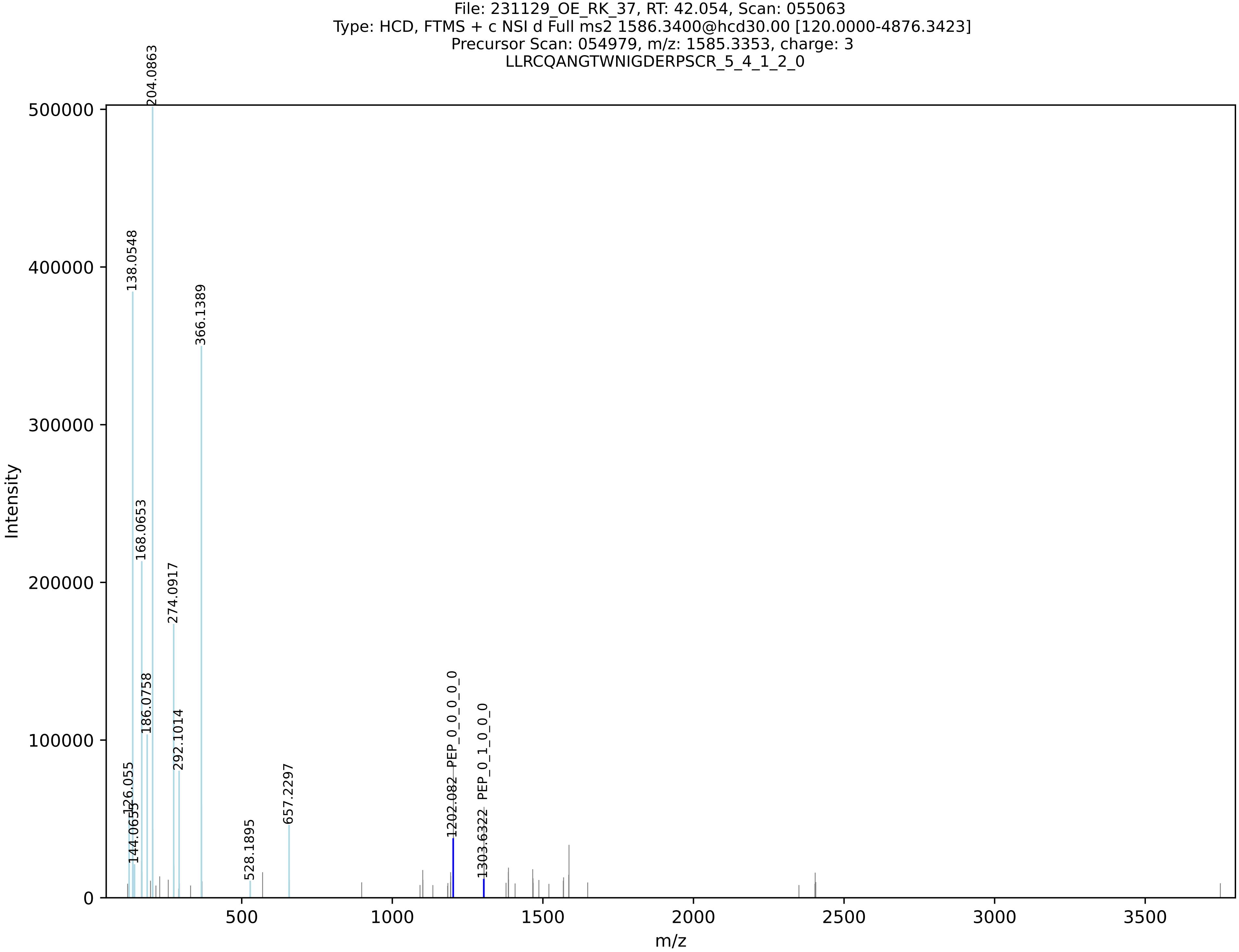
File: 231129_OE_RK_37, RT: 42.054, Scan: 055063
Type: HCD, FTMS + c NSI d Full ms2 1586.3400@hcd30.00 [120.0000-4876.3423]
Precursor Scan: 054979, m/z: 1585.3353, charge: 3
LLRCQANGTWNIGDERPSCR_5_4_1_2_0
Intensity
m/z
204.0863
138.0548
366.1389
168.0653
274.0917
186.0758
292.1014
126.055
144.0655
528.1895
657.2297
1202.082_PEP_0_0_0_0_0
1303.6322_PEP_0_1_0_0_0
0
100000
200000
300000
400000
500000
500
1000
1500
2000
2500
3000
3500

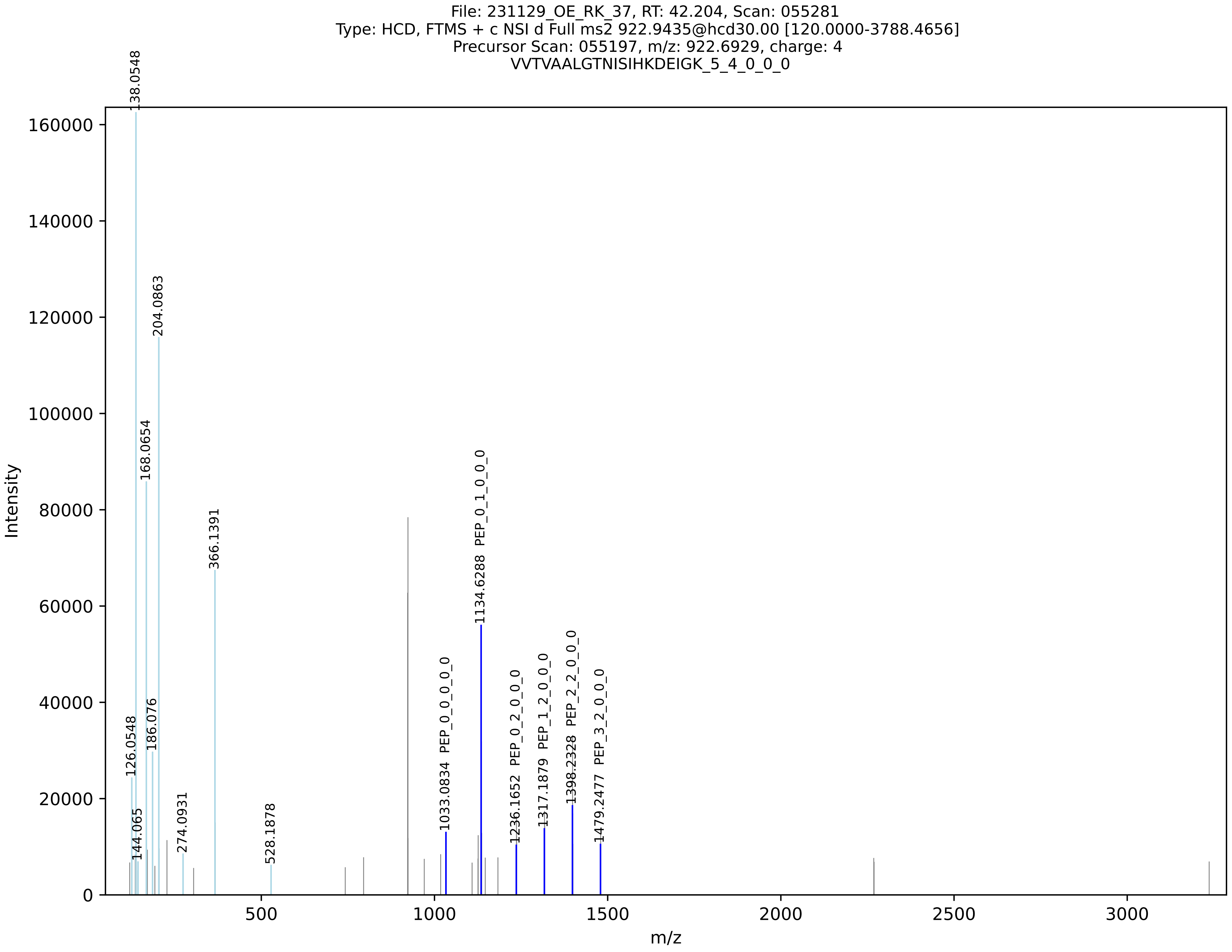
File: 231129_OE_RK_37, RT: 42.204, Scan: 055281
Type: HCD, FTMS + c NSI d Full ms2 922.9435@hcd30.00 [120.0000-3788.4656]
Precursor Scan: 055197, m/z: 922.6929, charge: 4
VVTVAALGTNISIHKDEIGK_5_4_0_0_0
Intensity
m/z
138.0548
204.0863
168.0654
366.1391
126.0548
186.076
144.065
274.0931
528.1878
1033.0834 PEP_0_0_0_0_0
1134.6288 PEP_0_1_0_0_0
1236.1652 PEP_0_2_0_0_0
1317.1879 PEP_1_2_0_0_0
1398.2328 PEP_2_2_0_0_0
1479.2477 PEP_3_2_0_0_0
0
20000
40000
60000
80000
100000
120000
140000
160000
500
1000
1500
2000
2500
3000

File: 231129_OE_RK_37, RT: 42.303, Scan: 055425
Type: HCD, FTMS + c NSI d Full ms2 1253.1738@hcd30.00 [120.0000-3856.8540]
Precursor Scan: 055345, m/z: 1252.5056, charge: 3
SWPAVGNCSSALR_5_4_1_2_0

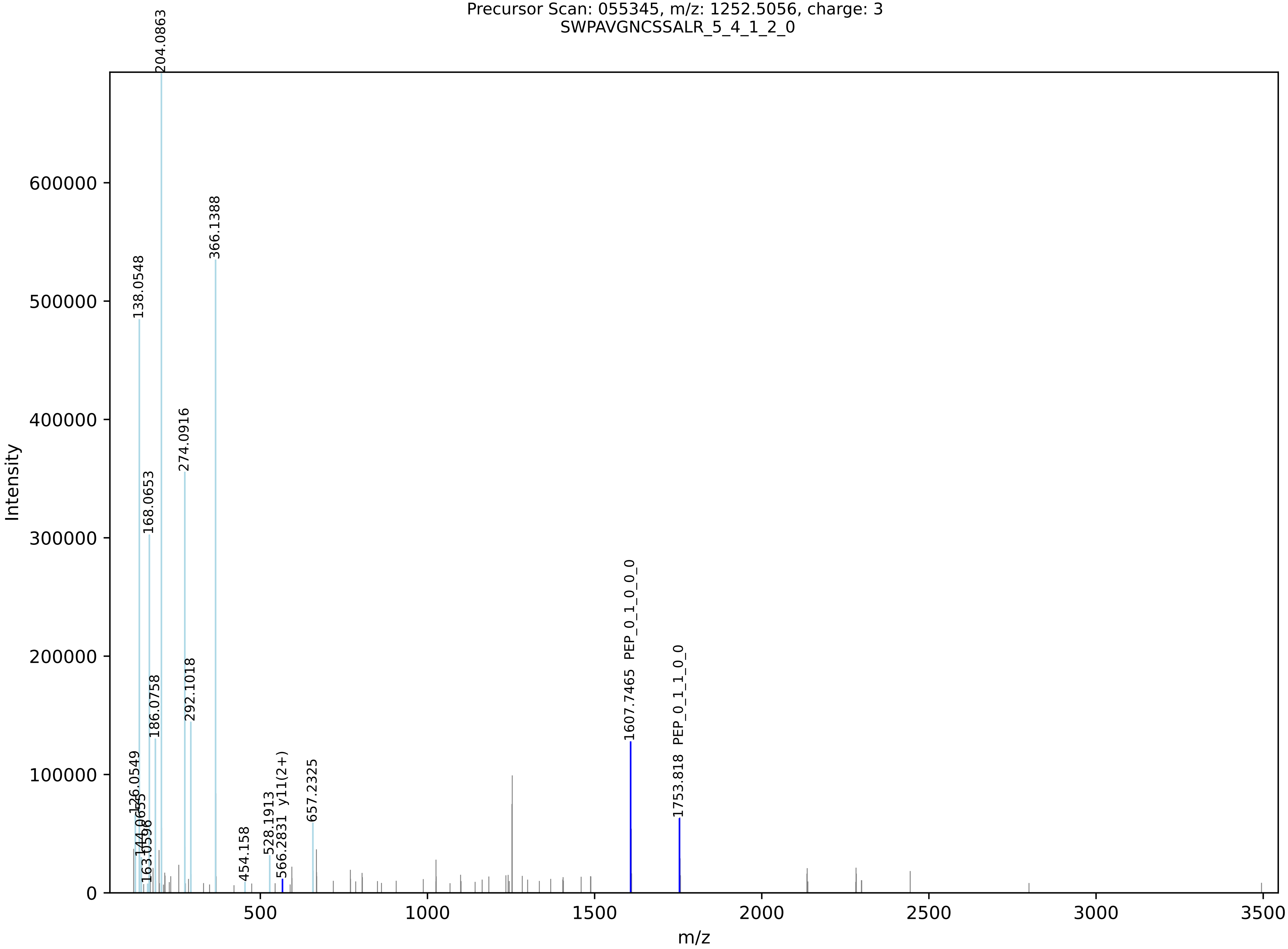

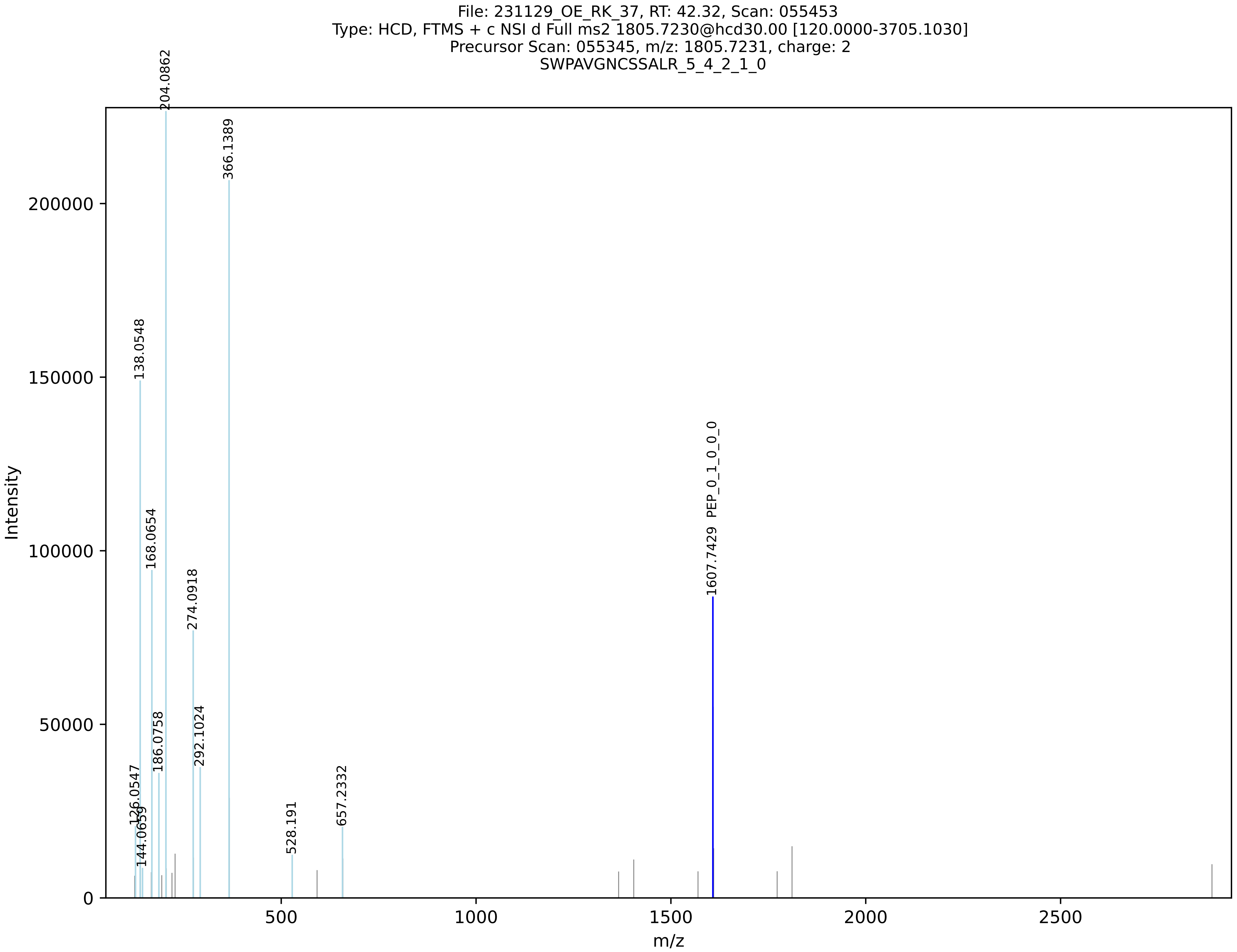

File: 231129_OE_RK_37, RT: 42.32, Scan: 055453
Type: HCD, FTMS + c NSI d Full ms2 1805.7230@hcd30.00 [120.0000-3705.1030]
Precursor Scan: 055345, m/z: 1805.7231, charge: 2
SWPAVGNCSSALR_5_4_2_1_0
204.0862
366.1389
138.0548
168.0654
274.0918
186.0758
292.1024
126.0547
144.0659
528.191
657.2332
1607.7429 PEP_0_1_0_0_0
Intensity
m/z
0
50000
100000
150000
200000
500
1000
1500
2000
2500

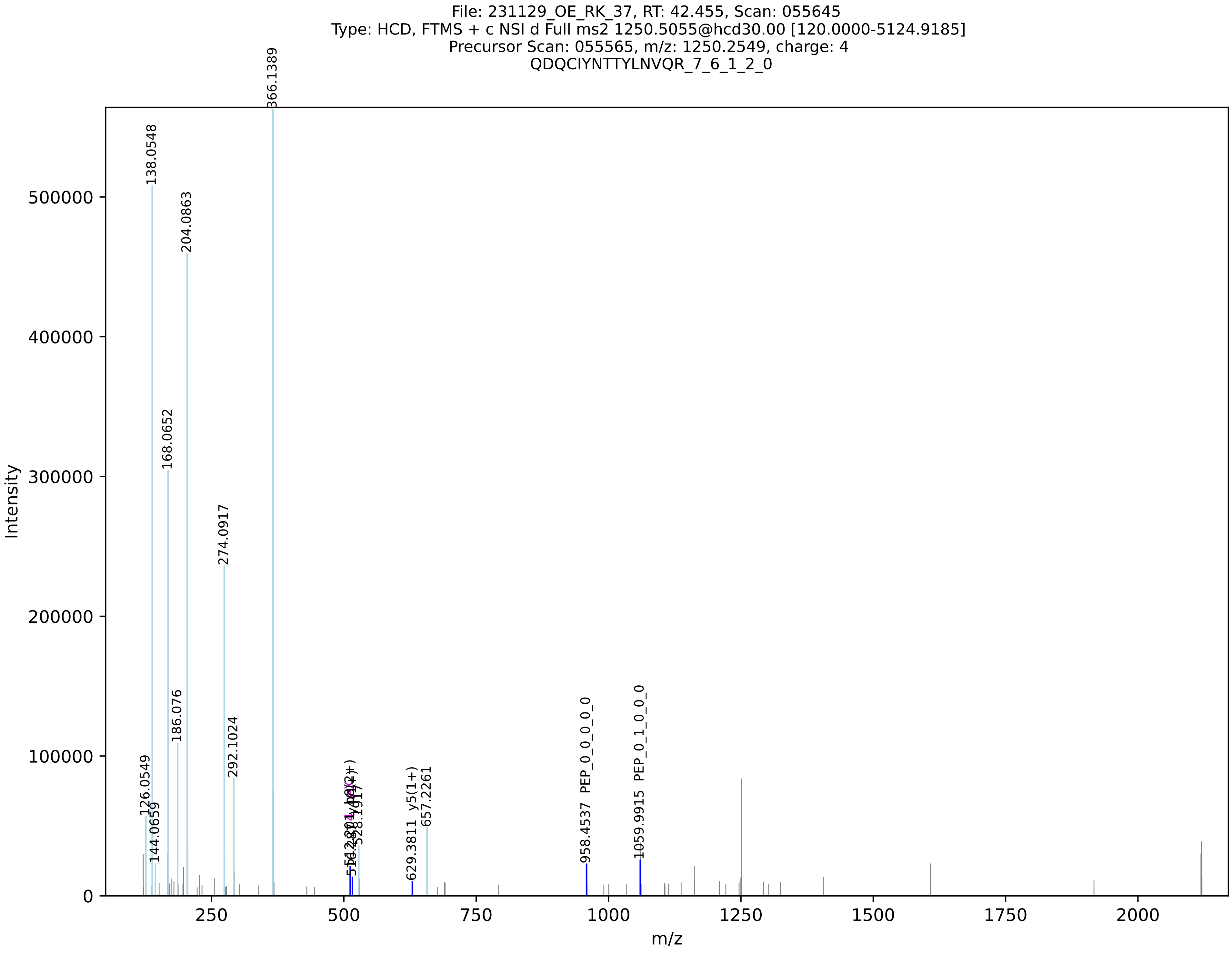
File: 231129_OE_RK_37, RT: 42.455, Scan: 055645
Type: HCD, FTMS + c NSI d Full ms2 1250.5055@hcd30.00 [120.0000-5124.9185]
Precursor Scan: 055565, m/z: 1250.2549, charge: 4
QDQCIYNTTYLNVQR_7_6_1_2_0
366.1389
138.0548
204.0863
168.0652
274.0917
186.076
292.1024
126.0549
144.0659
528.1917
629.3811 y5(1+)
657.2261
958.4537 PEP_0_0_0_0_0
1059.9915 PEP_0_1_0_0_0
Intensity
m/z
0
100000
200000
300000
400000
500000
250
500
750
1000
1250
1500
1750
2000

File: 231129_OE_RK_37, RT: 42.654, Scan: 055929
Type: HCD, FTMS + c NSI d Full ms2 1204.1559@hcd30.00 [120.0000-3706.8591]
Precursor Scan: 055855, m/z: 1203.8218, charge: 3
SWPAVGNCSSALR_5_4_0_2_0

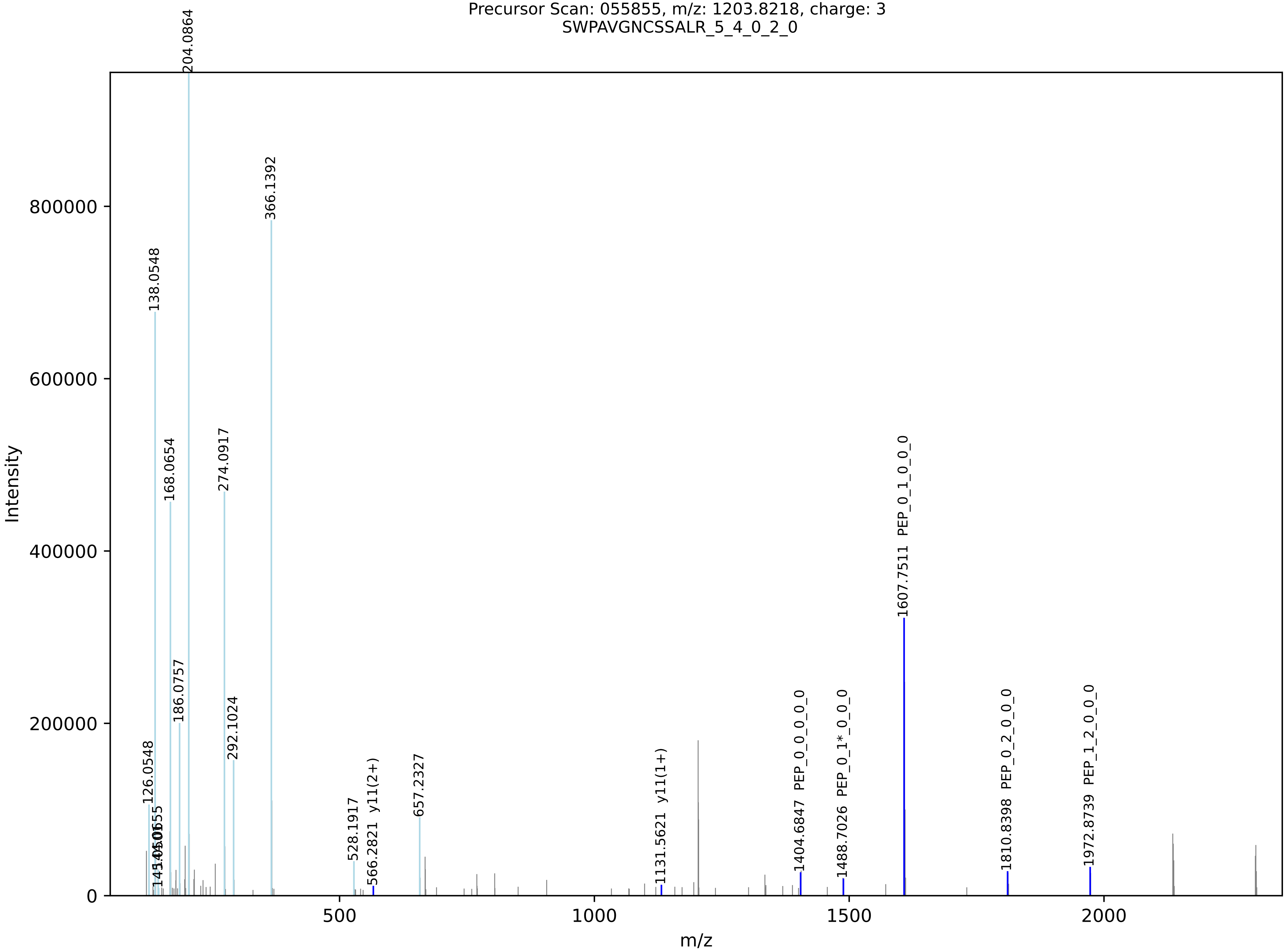

File: 231129_OE_RK_37, RT: 42.656, Scan: 055933
Type: HCD, FTMS + c NSI d Full ms2 1189.7559@hcd30.00 [120.0000-4877.0601]
Precursor Scan: 055855, m/z: 1189.255, charge: 4
LLRCQANGTWNIGDERPSCR_5_4_1_2_0

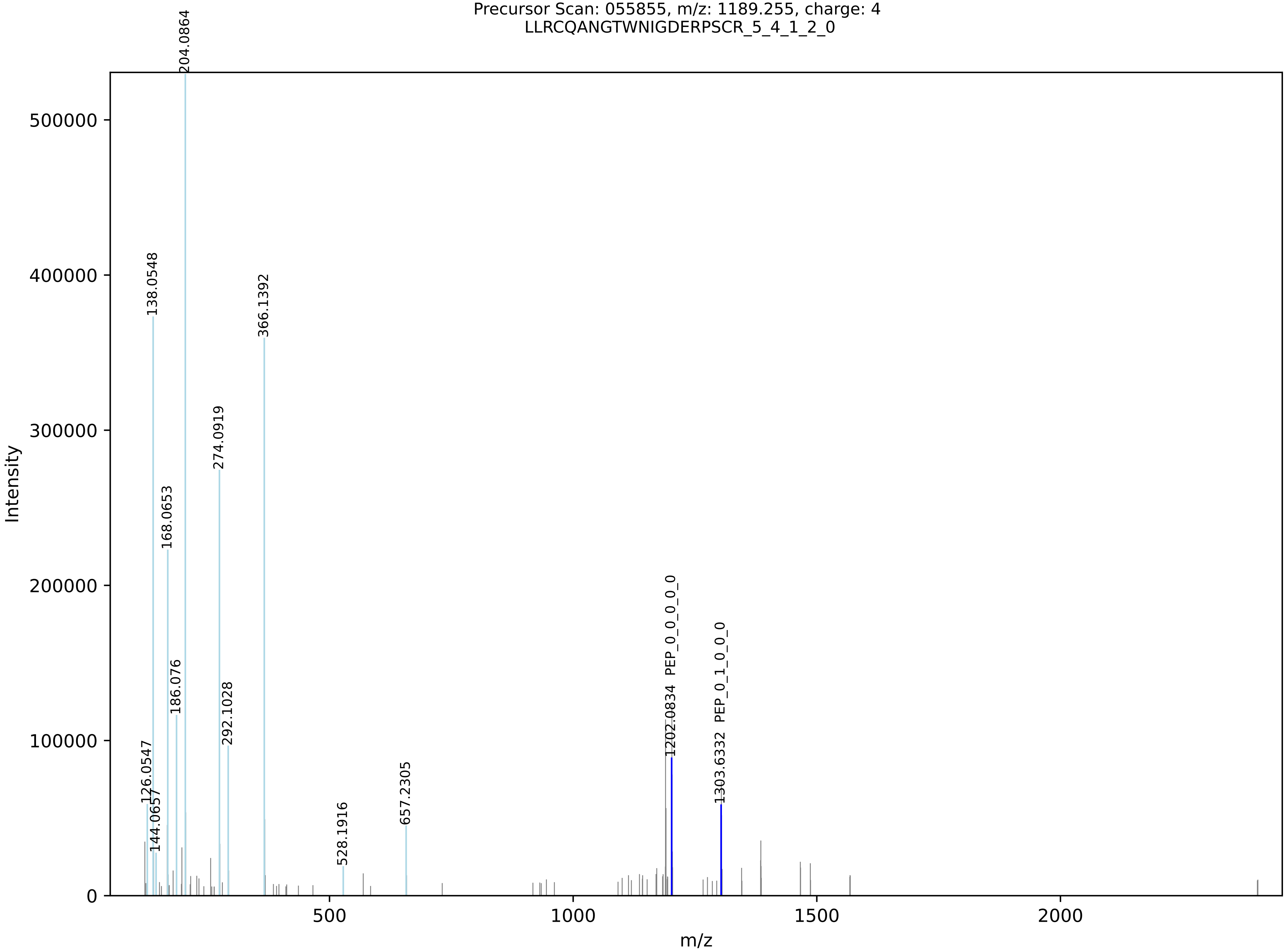

File: 231129_OE_RK_37, RT: 43.017, Scan: 056456
Type: HCD, FTMS + c NSI d Full ms2 1287.2695@hcd30.00 [120.0000-5274.9155]
Precursor Scan: 056362, m/z: 1286.7724, charge: 4
QDQCIYNTTYLNVQR_7_6_0_3_0

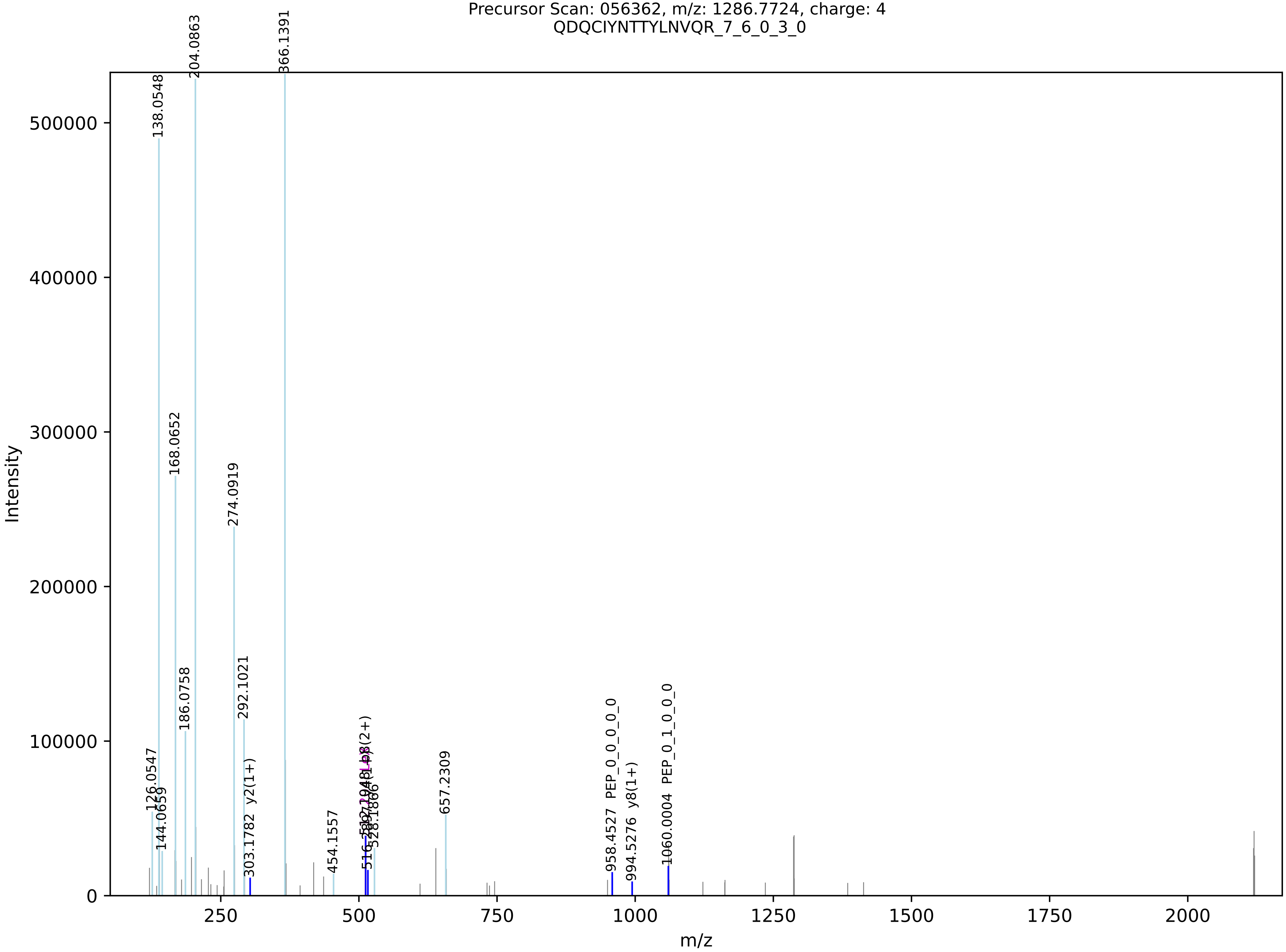

File: 231129_OE_RK_37, RT: 43.058, Scan: 056511
Type: HCD, FTMS + c NSI d Full ms2 1250.5065@hcd30.00 [120.0000-5124.9224]
Precursor Scan: 056435, m/z: 1250.0053, charge: 4
QDQCIYNTTYLNVQR_7_6_1_2_0

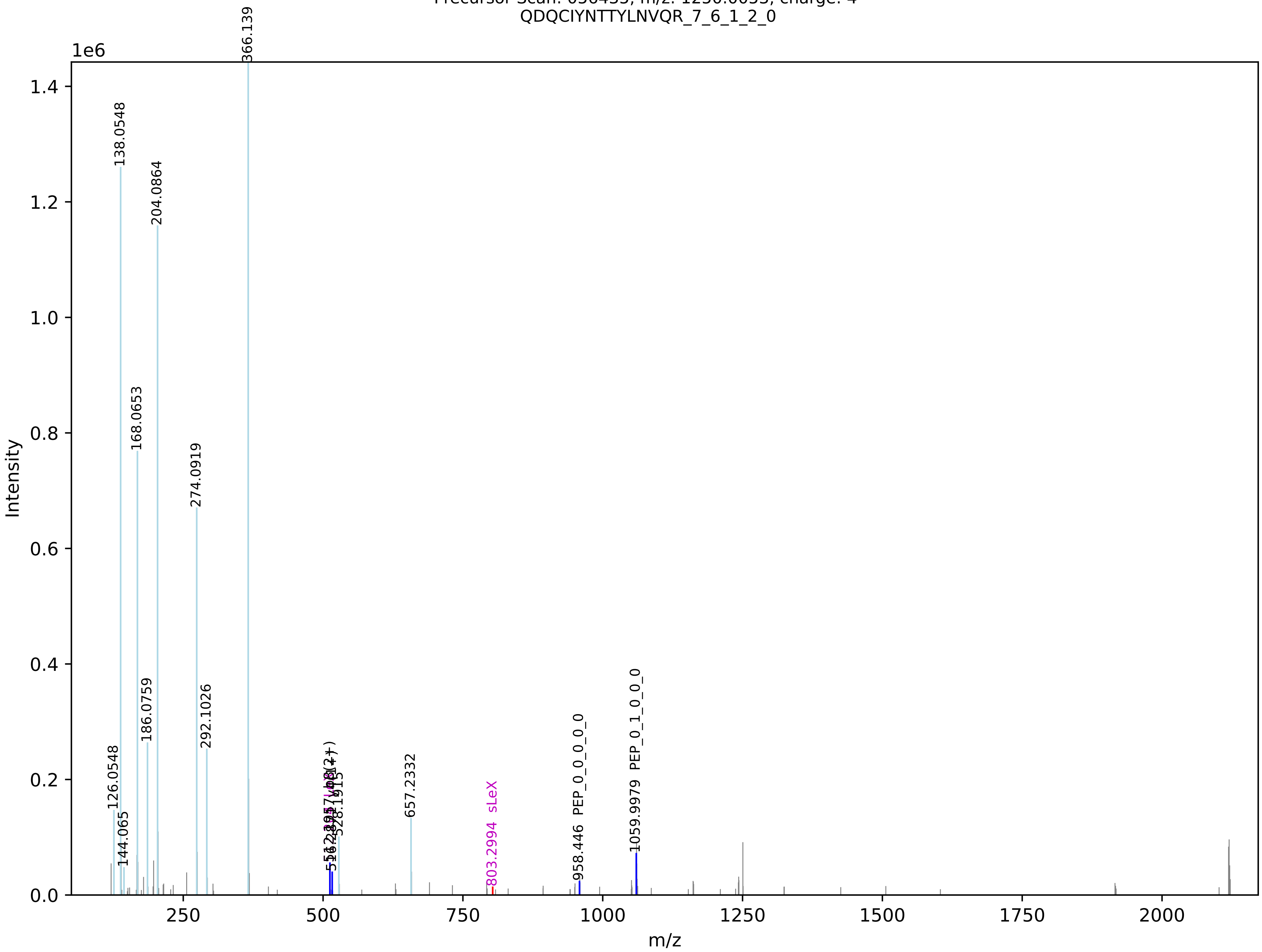

File: 231129_OE_RK_37, RT: 43.219, Scan: 056748
Type: HCD, FTMS + c NSI d Full ms2 1287.2732@hcd30.00 [120.0000-5274.9302]
Precursor Scan: 056654, m/z: 1286.5214, charge: 4
QDQCIYNTTYLNVQR_7_6_0_3_0

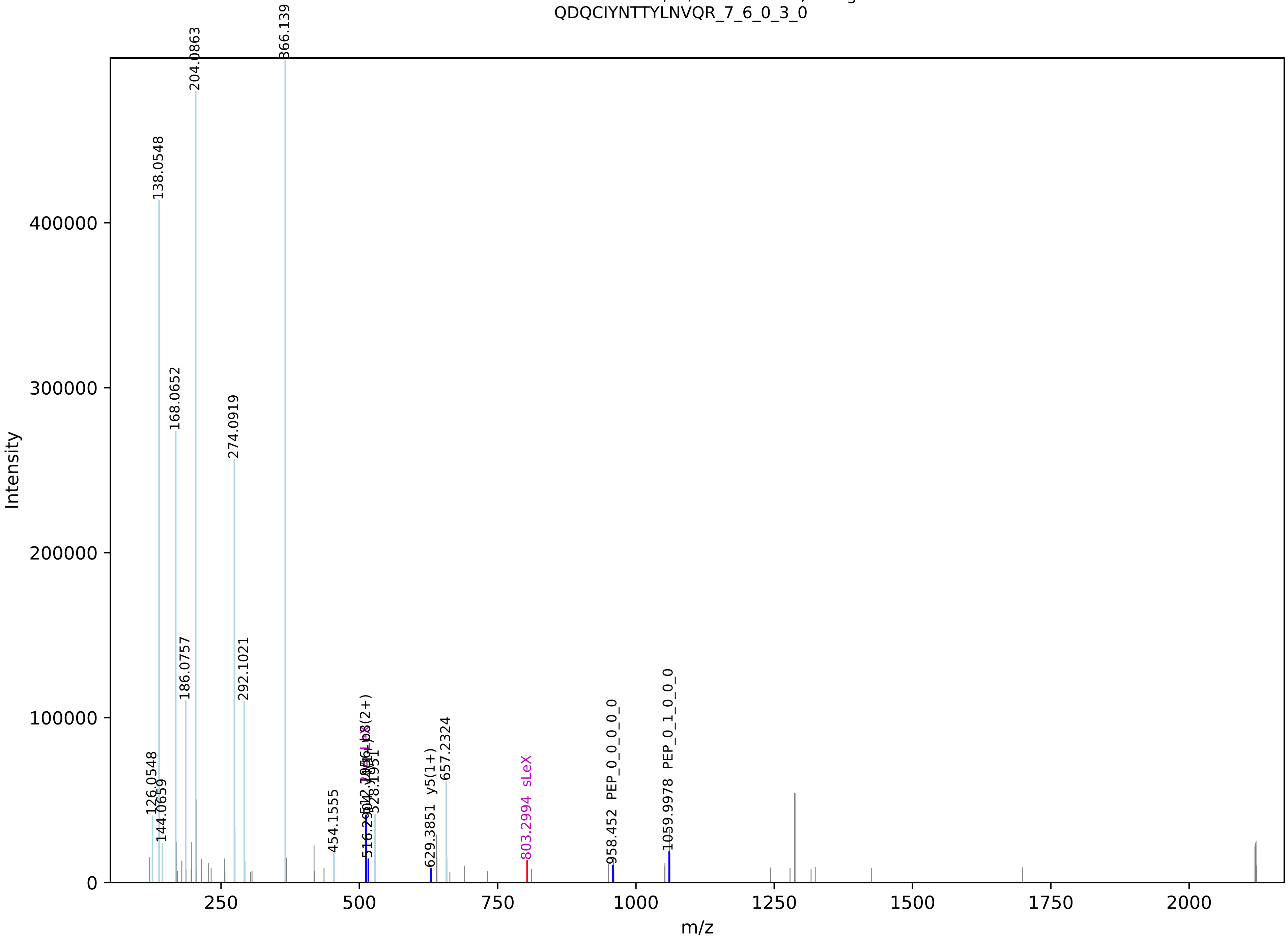

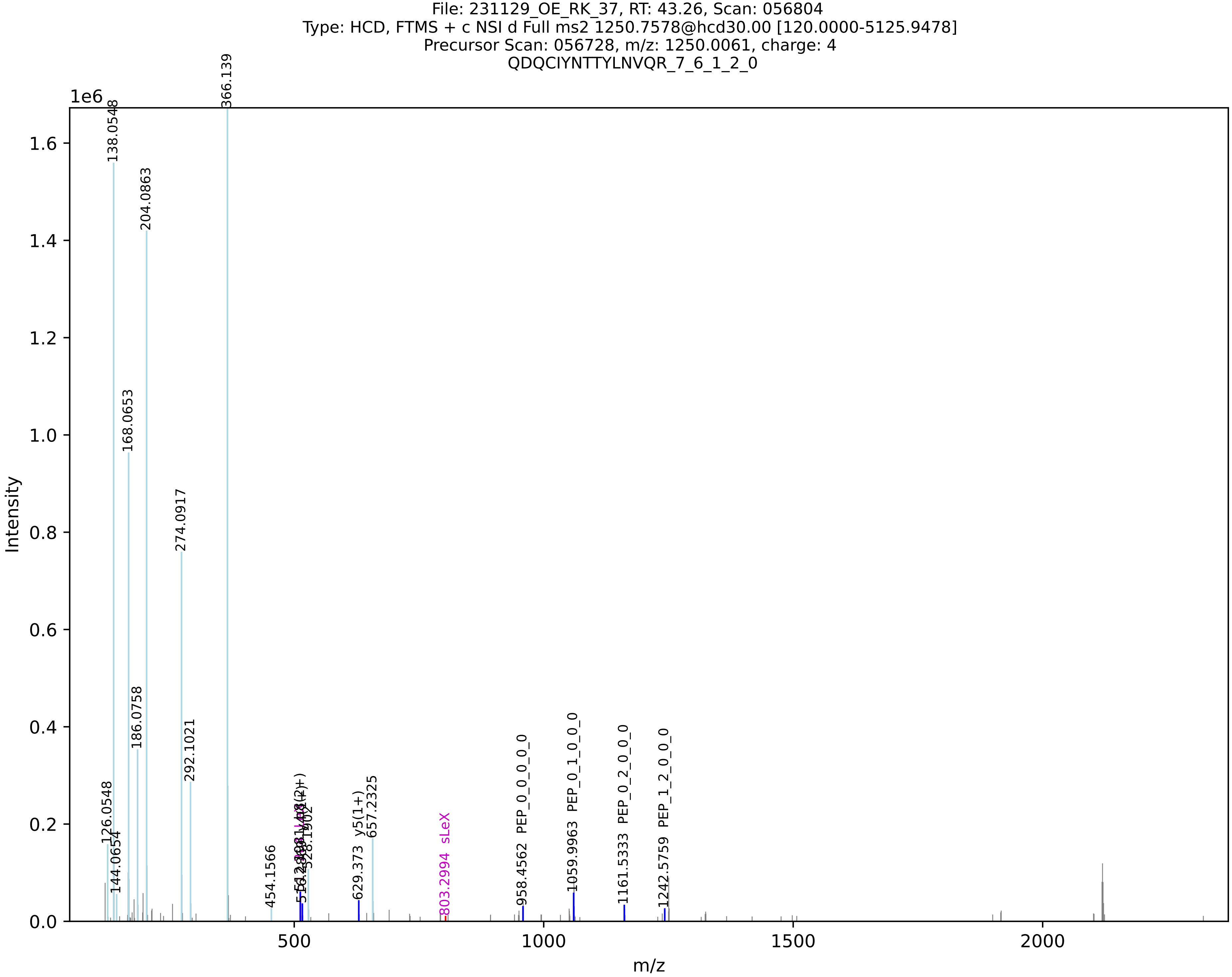

File: 231129_OE_RK_37, RT: 43.26, Scan: 056804
Type: HCD, FTMS + c NSI d Full ms2 1250.7578@hcd30.00 [120.0000-5125.9478]
Precursor Scan: 056728, m/z: 1250.0061, charge: 4
QDQCIYNTTYLNVQR_7_6_1_2_0
1e6
Intensity
m/z
366.139
138.0548
204.0863
168.0653
274.0917
186.0758
292.1021
126.0548
144.0654
454.1566
629.373 y5(1+)
657.2325
803.2994 sLeX
958.4562 PEP_0_0_0_0_0
1059.9963 PEP_0_1_0_0_0
1161.5333 PEP_0_2_0_0_0
1242.5759 PEP_1_2_0_0_0

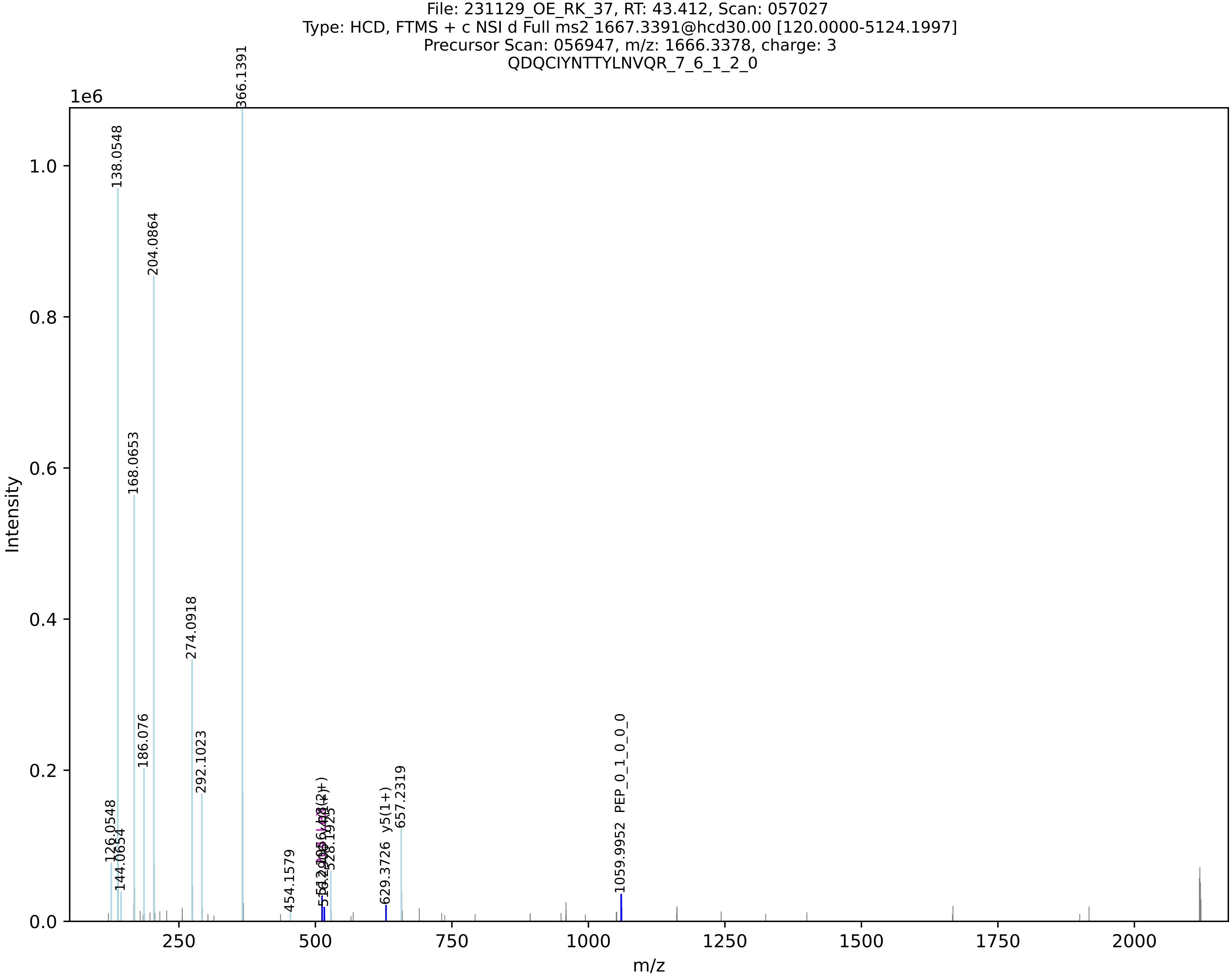
File: 231129_OE_RK_37, RT: 43.412, Scan: 057027
Type: HCD, FTMS + c NSI d Full ms2 1667.3391@hcd30.00 [120.0000-5124.1997]
Precursor Scan: 056947, m/z: 1666.3378, charge: 3
QDQCIYNTTYLNVQR_7_6_1_2_0
1e6
Intensity
m/z
366.1391
138.0548
204.0864
168.0653
274.0918
186.076
292.1023
126.0548
144.0654
454.1579
528.1925
629.3726
y5(1+)
657.2319
PEP_0_1_0_0_0
1059.9952

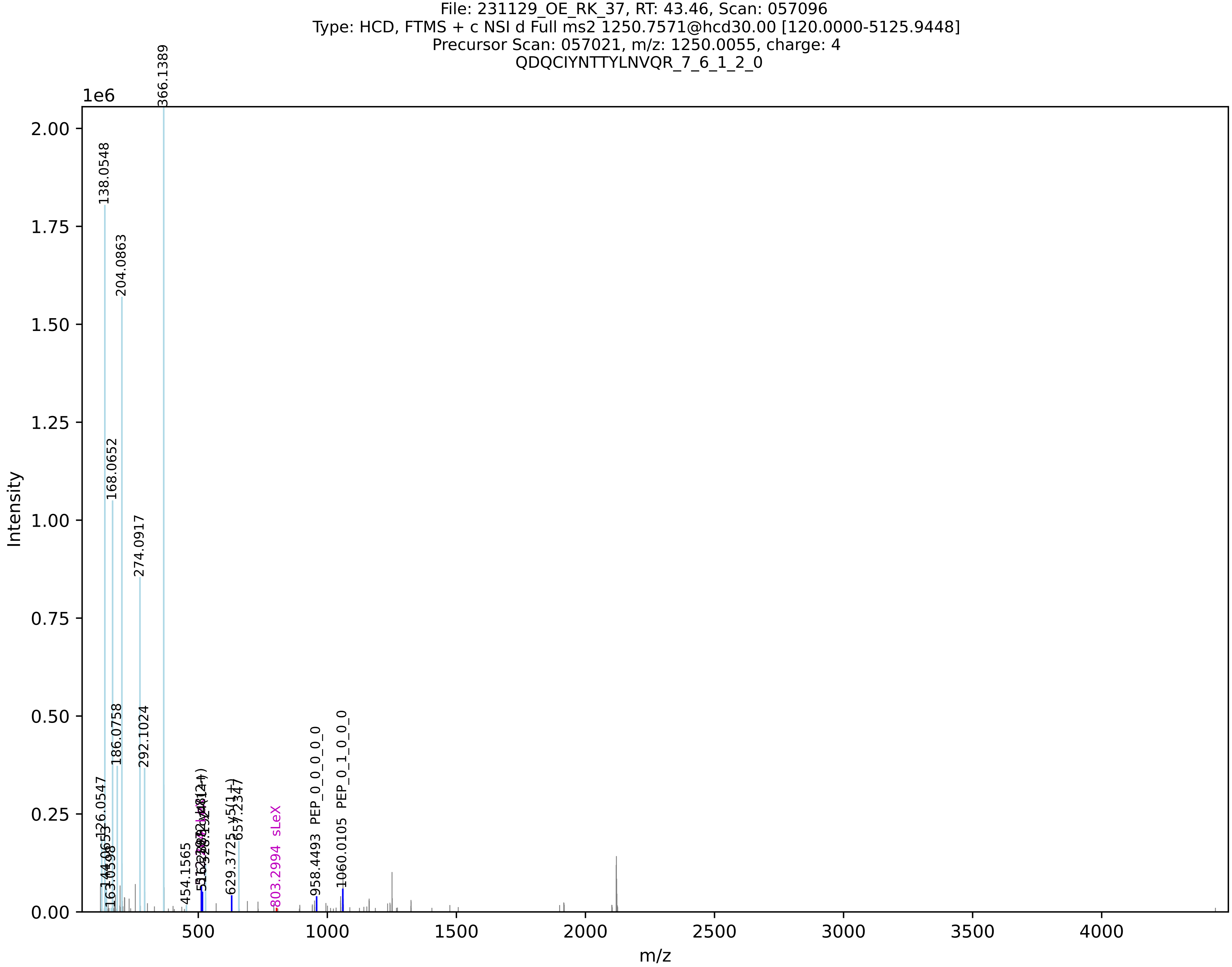
File: 231129_OE_RK_37, RT: 43.46, Scan: 057096
Type: HCD, FTMS + c NSI d Full ms2 1250.7571@hcd30.00 [120.0000-5125.9448]
Precursor Scan: 057021, m/z: 1250.0055, charge: 4
QDQCIYNTTYLNVQR_7_6_1_2_0
1e6
Intensity
m/z
366.1389
138.0548
204.0863
168.0652
274.0917
186.0758
292.1024
126.0547
144.0653
163.0598
454.1565
629.3725_y5(1+)
657.2347
803.2994 sLeX
958.4493 PEP_0_0_0_0_0
1060.0105 PEP_0_1_0_0_0

File: 231129_OE_RK_37, RT: 43.509, Scan: 057168
Type: HCD, FTMS + c NSI d Full ms2 1213.9941@hcd30.00 [120.0000-4975.9521]
Precursor Scan: 057094, m/z: 1213.4928, charge: 4
QDQCIYNTTYLNVQR_7_6_0_2_0

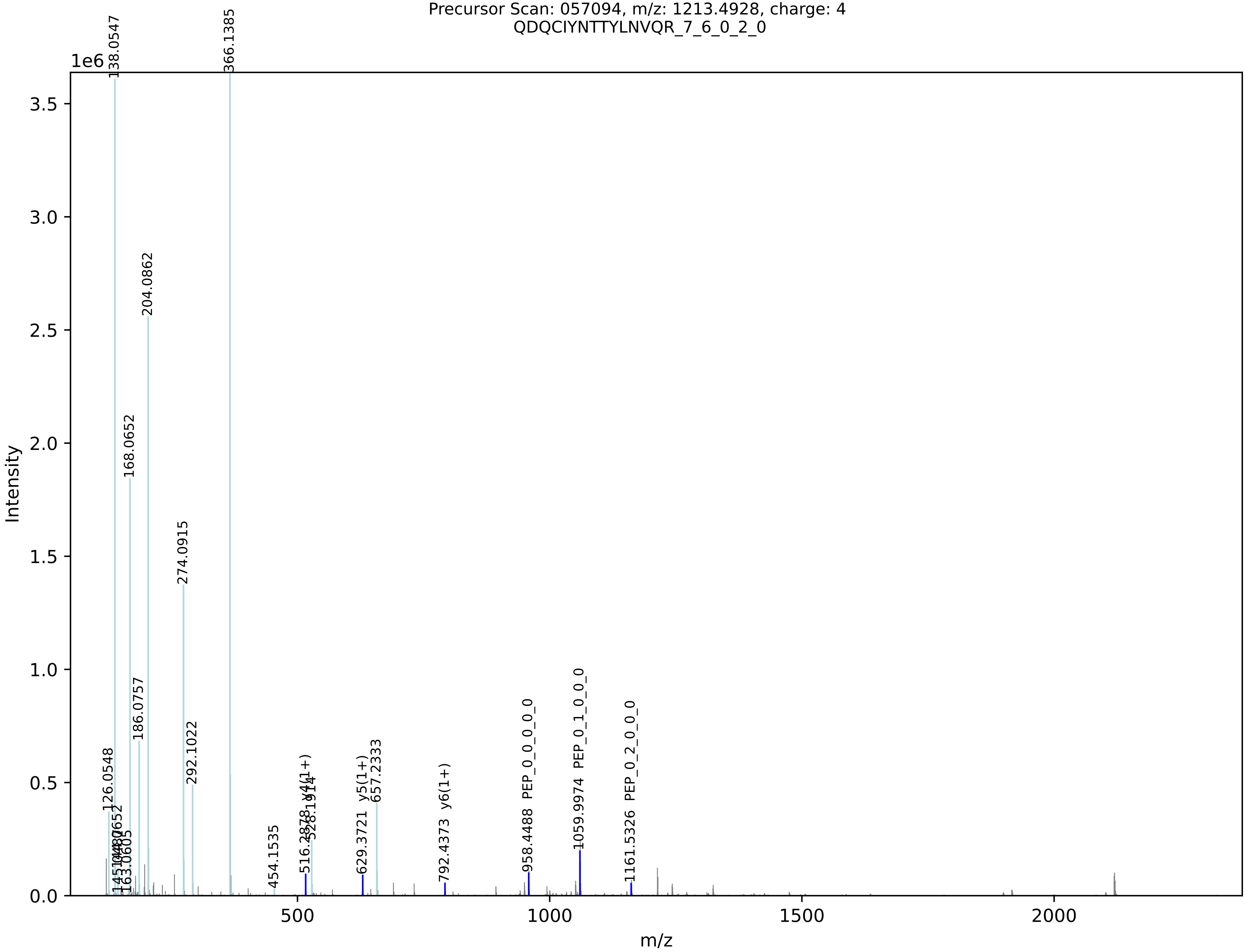

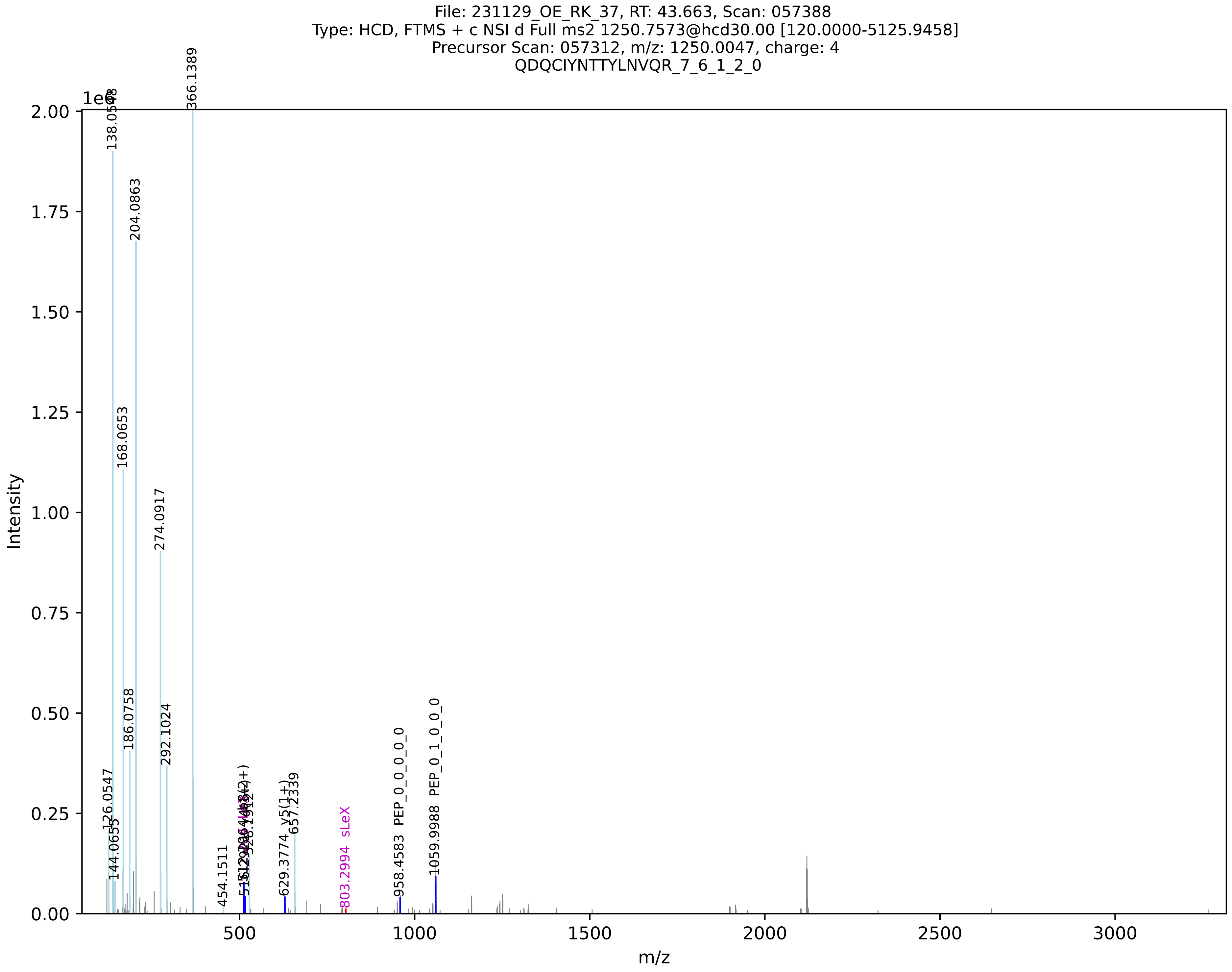
File: 231129_OE_RK_37, RT: 43.663, Scan: 057388
Type: HCD, FTMS + c NSI d Full ms2 1250.7573@hcd30.00 [120.0000-5125.9458]
Precursor Scan: 057312, m/z: 1250.0047, charge: 4
QDQCIYNTTYLNVQR_7_6_1_2_0
1e6
Intensity
m/z
366.1389
204.0863
168.0653
274.0917
186.0758
292.1024
126.0547
144.0655
454.1511
528.1912
629.3774 y5(1+)
657.2339
803.2994 sLeX
958.4583 PEP_0_0_0_0_0
1059.9988 PEP_0_1_0_0_0
0.00
0.25
0.50
0.75
1.00
1.25
1.50
1.75
2.00
500
1000
1500
2000
2500
3000

File: 231129_OE_RK_37, RT: 43.711, Scan: 057457
Type: HCD, FTMS + c NSI d Full ms2 1213.9926@hcd30.00 [120.0000-4975.9453]
Precursor Scan: 057386, m/z: 1213.4912, charge: 4
QDQCIYNTTYLNVQR_7_6_0_2_0

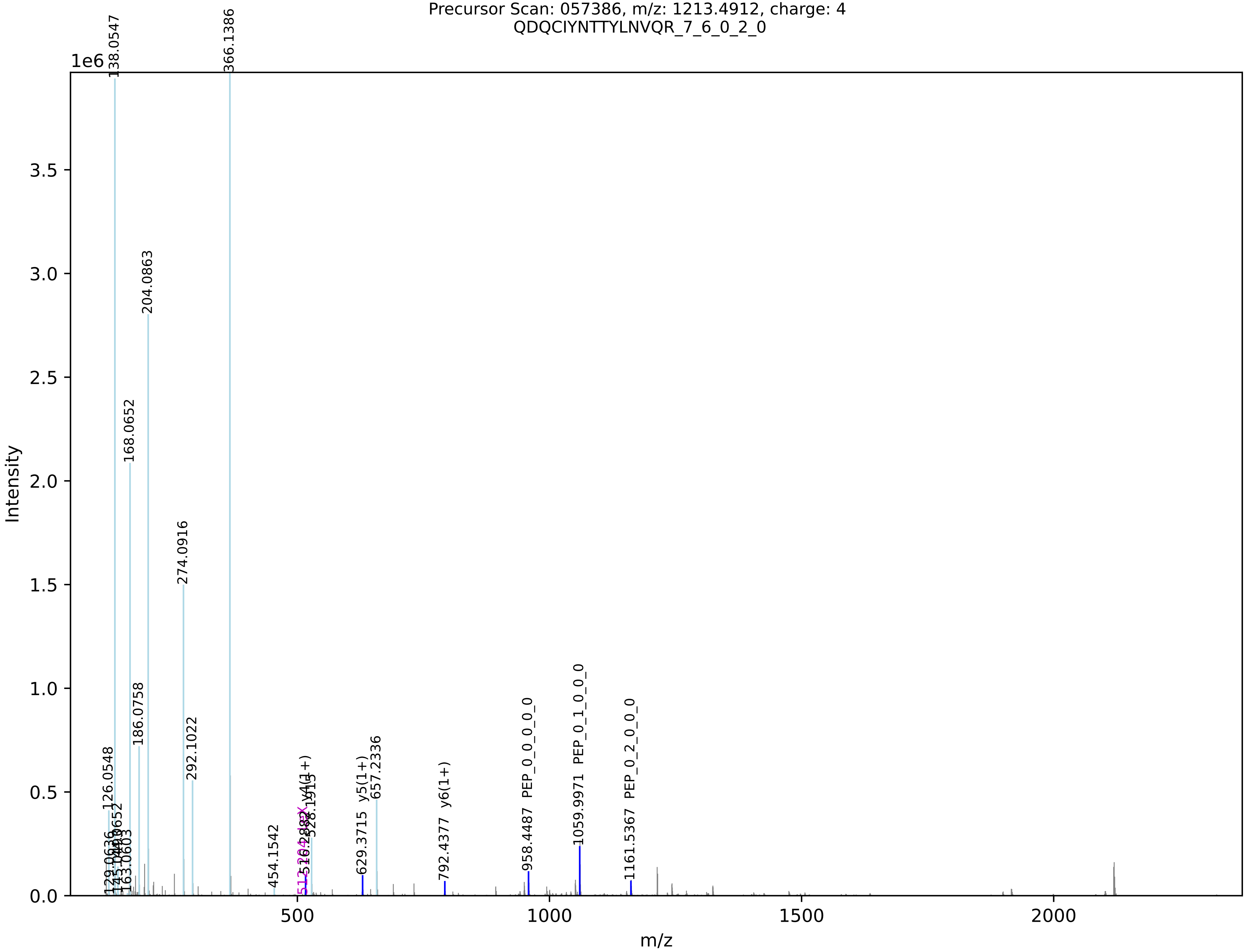

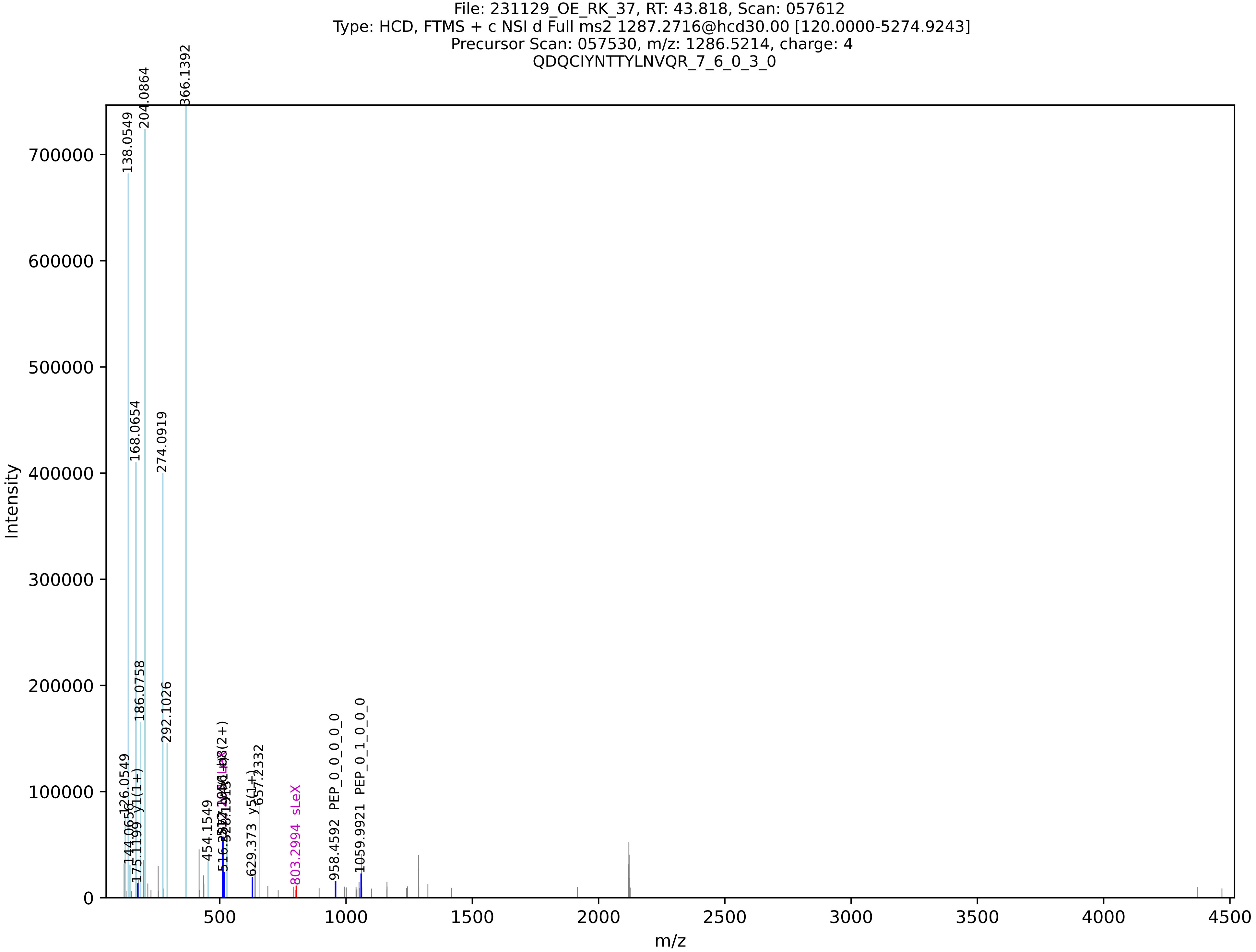
File: 231129_OE_RK_37, RT: 43.818, Scan: 057612
Type: HCD, FTMS + c NSI d Full ms2 1287.2716@hcd30.00 [120.0000-5274.9243]
Precursor Scan: 057530, m/z: 1286.5214, charge: 4
QDQCIYNTTYLNVQR_7_6_0_3_0
366.1392
204.0864
138.0549
168.0654
274.0919
186.0758
292.1026
126.0549
144.0655
175.1199 y1(1+)
454.1549
629.373 y5(1+)
657.2332
803.2994 sLeX
958.4592 PEP_0_0_0_0_0
1059.9921 PEP_0_1_0_0_0
Intensity
m/z
0
100000
200000
300000
400000
500000
600000
700000
500
1000
1500
2000
2500
3000
3500
4000
4500

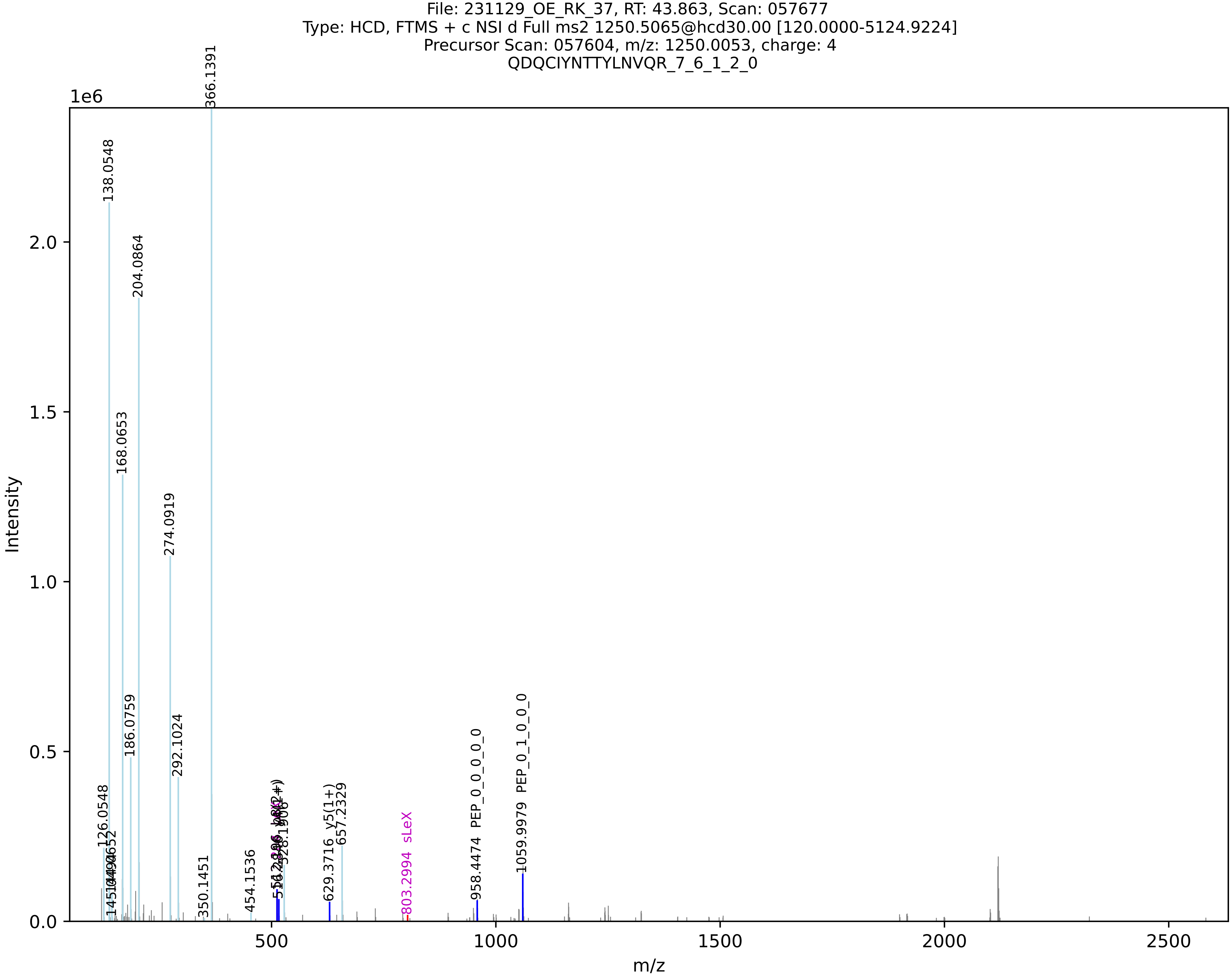
File: 231129_OE_RK_37, RT: 43.863, Scan: 057677
Type: HCD, FTMS + c NSI d Full ms2 1250.5065@hcd30.00 [120.0000-5124.9224]
Precursor Scan: 057604, m/z: 1250.0053, charge: 4
QDQCIYNTTYLNVQR_7_6_1_2_0
1e6
Intensity
m/z
366.1391
138.0548
204.0864
168.0653
274.0919
186.0759
292.1024
126.0548
350.1451
454.1536
528.1906
629.3716 y5(1+)
657.2329
803.2994 sLeX
958.4474 PEP_0_0_0_0_0
1059.9979 PEP_0_1_0_0_0

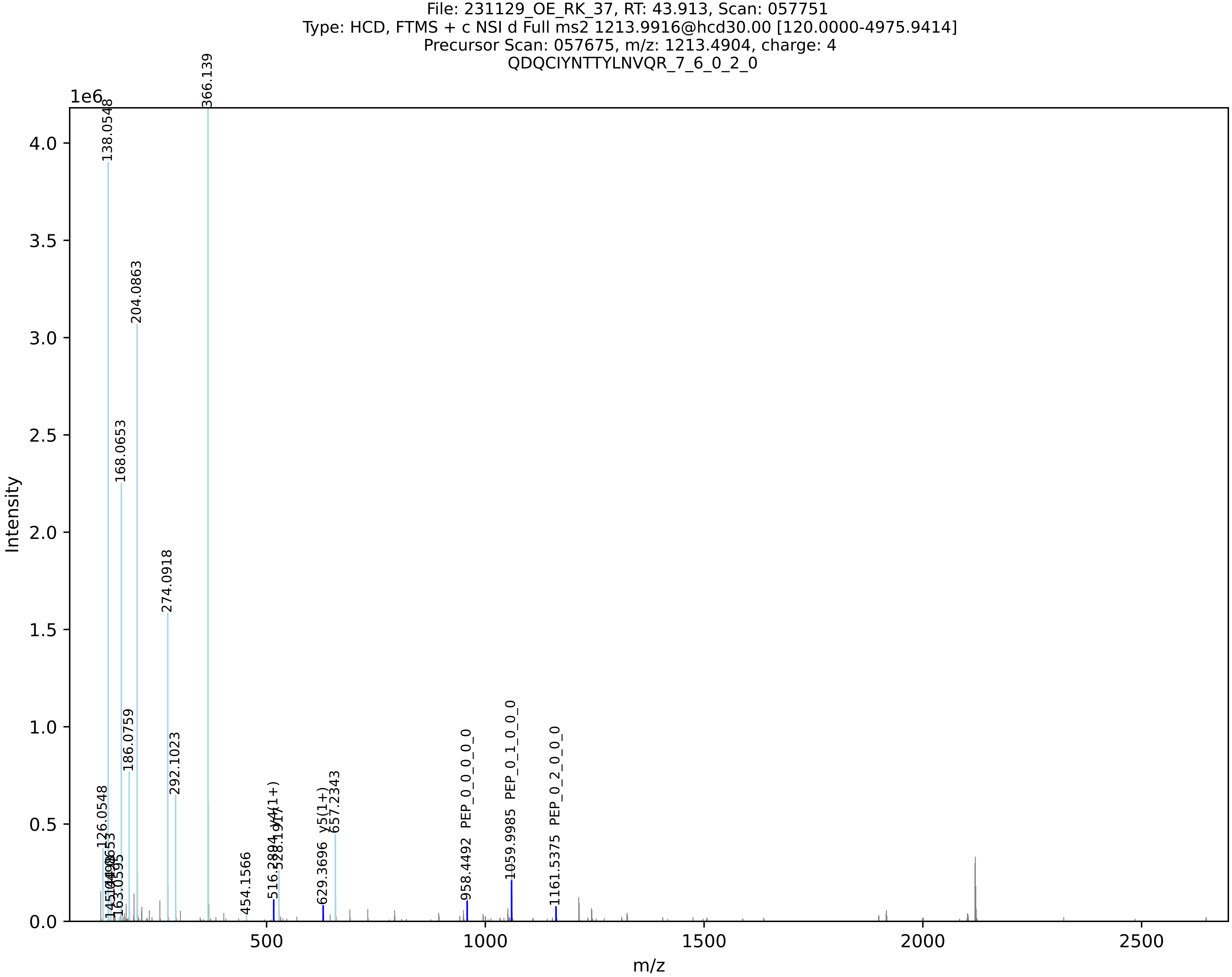
File: 231129_OE_RK_37, RT: 43.913, Scan: 057751
Type: HCD, FTMS + c NSI d Full ms2 1213.9916@hcd30.00 [120.0000-4975.9414]
Precursor Scan: 057675, m/z: 1213.4904, charge: 4
QDQCIYNTTYLNVQR_7_6_0_2_0
1e6
Intensity
m/z
0.0
0.5
1.0
1.5
2.0
2.5
3.0
3.5
4.0
500
1000
1500
2000
2500
366.139
138.0548
204.0863
168.0653
274.0918
186.0759
292.1023
126.0548
163.0595
454.1566
y4(1+)
629.3696
y5(1+)
657.2343
958.4492
PEP_0_0_0_0_0_
1059.9985
PEP_0_1_0_0_0_
1161.5375
PEP_0_2_0_0_0_

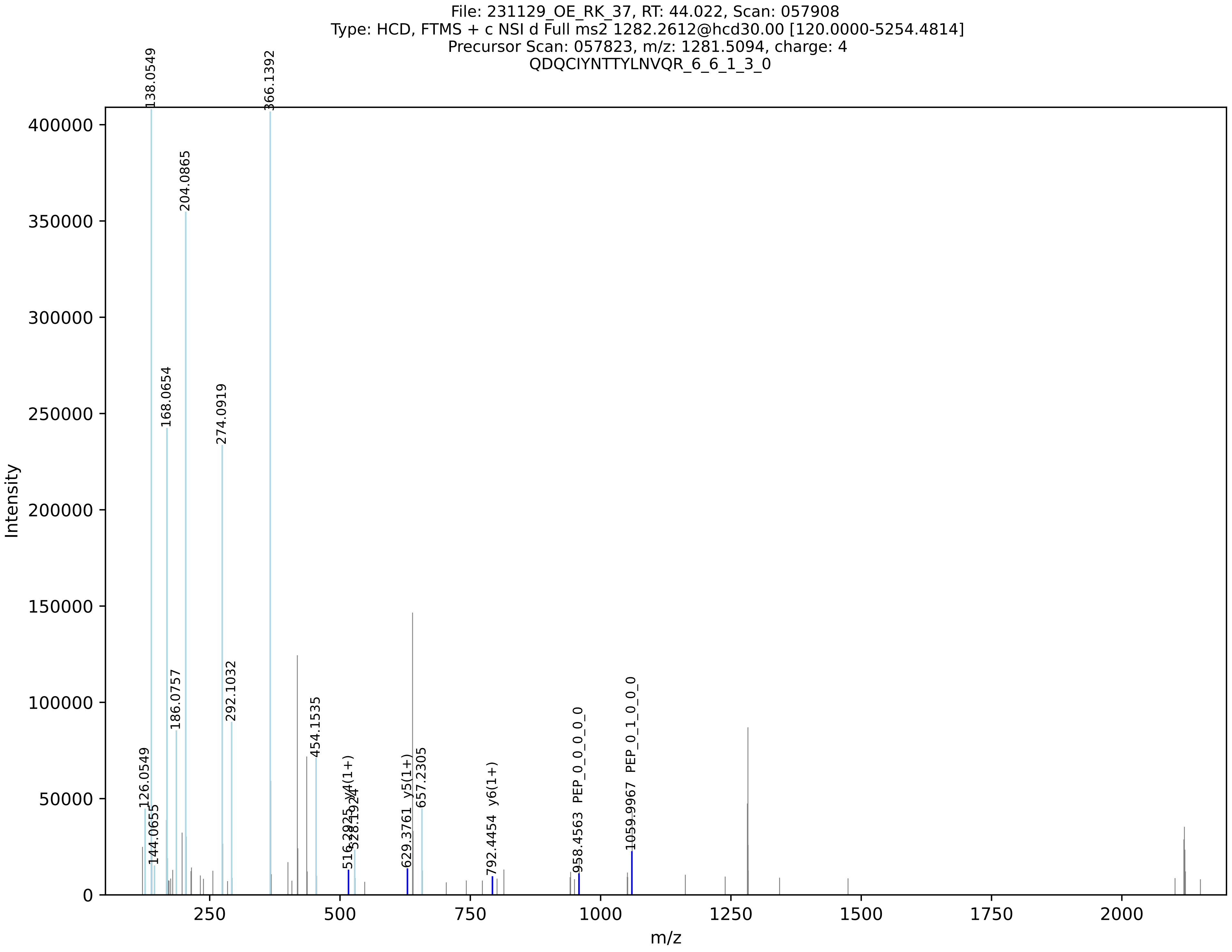

File: 231129_OE_RK_37, RT: 44.022, Scan: 057908
Type: HCD, FTMS + c NSI d Full ms2 1282.2612@hcd30.00 [120.0000-5254.4814]
Precursor Scan: 057823, m/z: 1281.5094, charge: 4
QDQCIYNTTYLNVQR_6_6_1_3_0
Intensity
m/z
138.0549
366.1392
204.0865
168.0654
274.0919
186.0757
292.1032
454.1535
126.0549
144.0655
516.2925 y4(1+)
528.1924
629.3761 y5(1+)
657.2305
792.4454 y6(1+)
958.4563 PEP_0_0_0_0_0
1059.9967 PEP_0_1_0_0_0

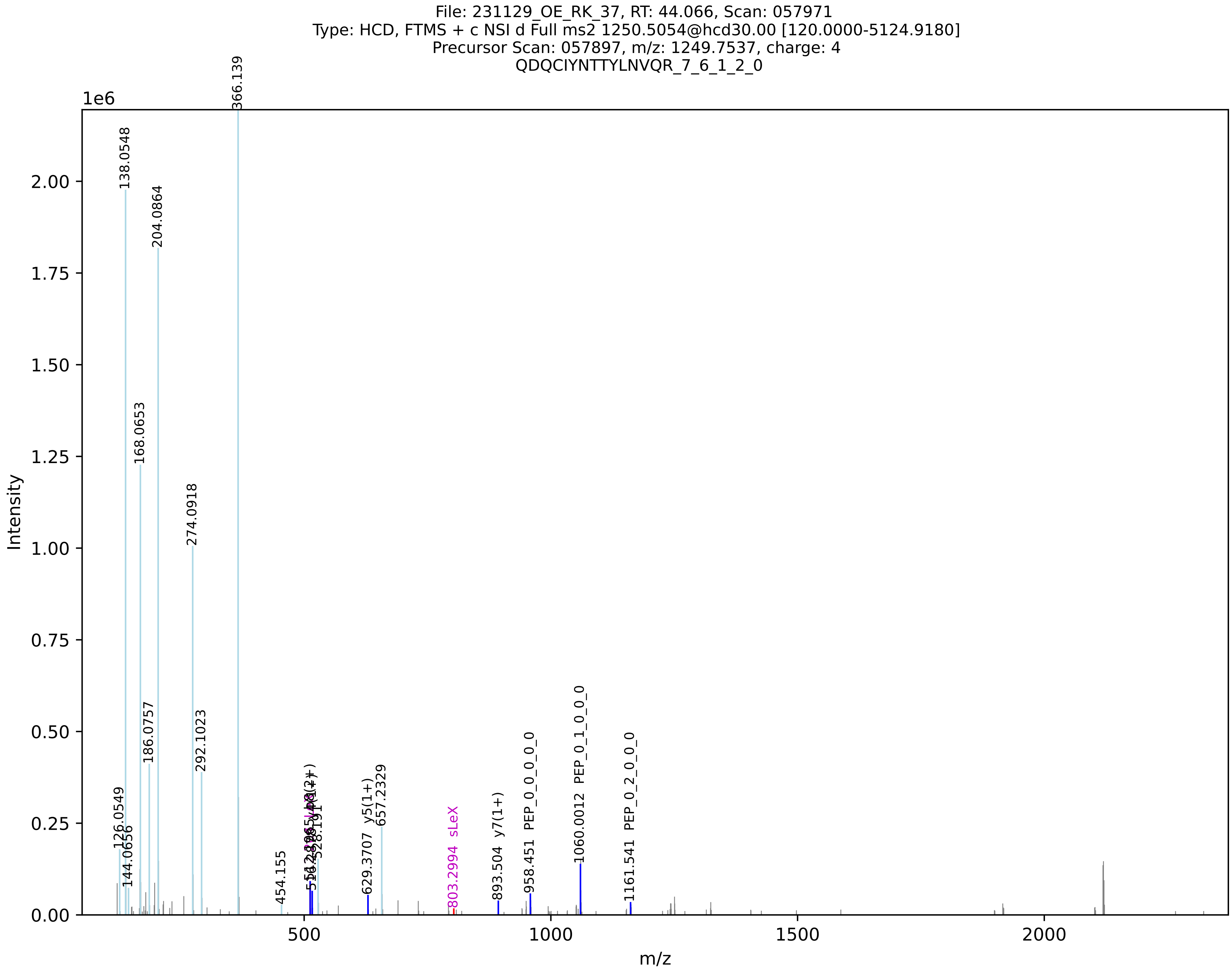

File: 231129_OE_RK_37, RT: 44.066, Scan: 057971
Type: HCD, FTMS + c NSI d Full ms2 1250.5054@hcd30.00 [120.0000-5124.9180]
Precursor Scan: 057897, m/z: 1249.7537, charge: 4
QDQCIYNTTYLNVQR_7_6_1_2_0
1e6
Intensity
m/z
0.00
0.25
0.50
0.75
1.00
1.25
1.50
1.75
2.00
500
1000
1500
2000
126.0549
144.0656
138.0548
168.0653
186.0757
204.0864
274.0918
292.1023
366.139
454.155
528.191
629.3707 y5(1+)
657.2329
803.2994 sLeX
893.504 y7(1+)
958.451 PEP_0_0_0_0_0
1060.0012 PEP_0_1_0_0_0
1161.541 PEP_0_2_0_0_0

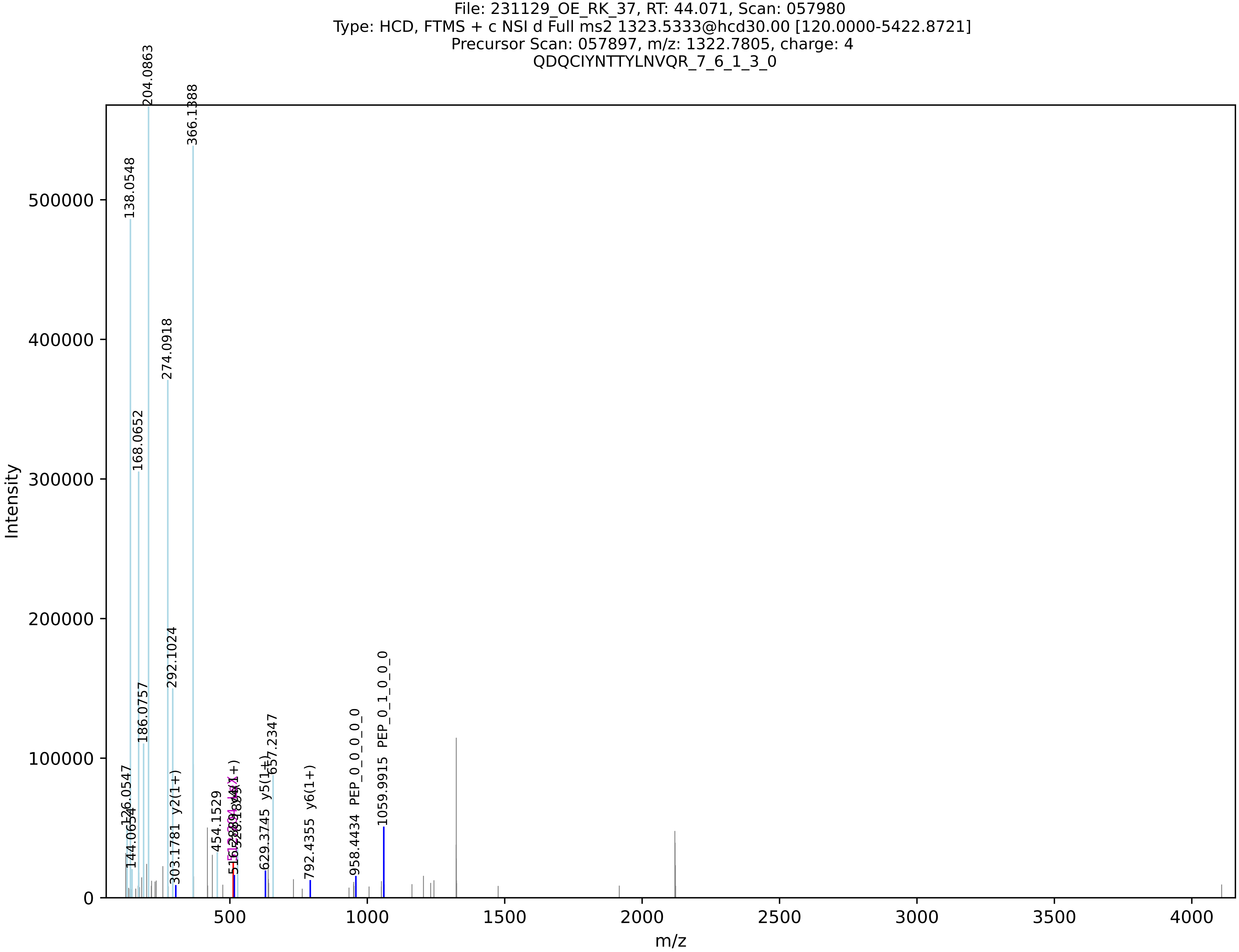

File: 231129_OE_RK_37, RT: 44.071, Scan: 057980
Type: HCD, FTMS + c NSI d Full ms2 1323.5333@hcd30.00 [120.0000-5422.8721]
Precursor Scan: 057897, m/z: 1322.7805, charge: 4
QDQCIYNTTYLNVQR_7_6_1_3_0
204.0863
366.1388
138.0548
274.0918
168.0652
292.1024
186.0757
126.0547
144.0654
303.1781 y2(1+)
454.1529
629.3745 y5(1+)
657.2347
792.4355 y6(1+)
958.4434 PEP_0_0_0_0_0
1059.9915 PEP_0_1_0_0_0
Intensity
m/z

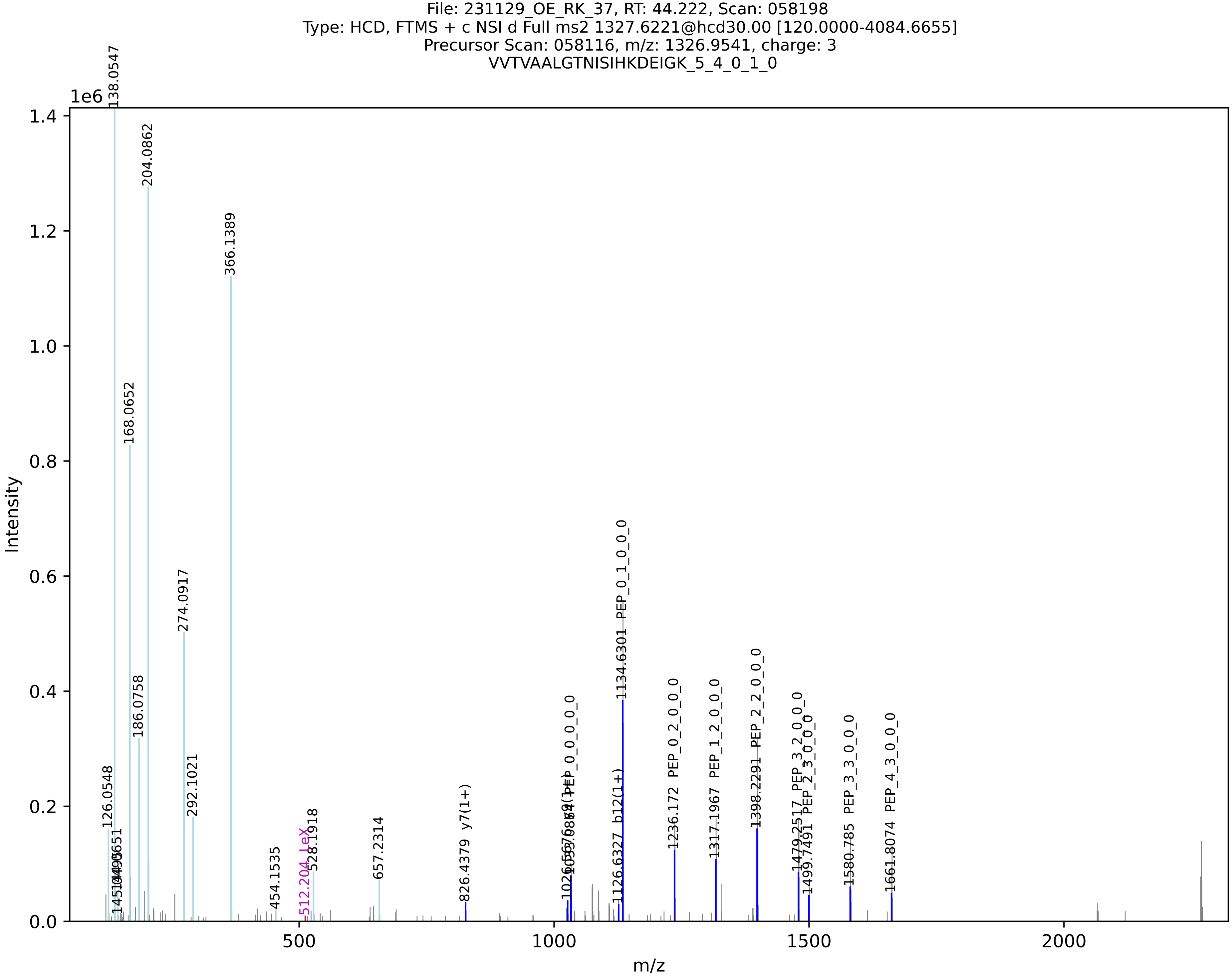

File: 231129_OE_RK_37, RT: 44.222, Scan: 058198
Type: HCD, FTMS + c NSI d Full ms2 1327.6221@hcd30.00 [120.0000-4084.6655]
Precursor Scan: 058116, m/z: 1326.9541, charge: 3
VVTVAALGTNISIHKDEIGK_5_4_0_1_0
1e6
Intensity
m/z
138.0547
204.0862
366.1389
168.0652
274.0917
186.0758
292.1021
126.0548
454.1535
512.204_LeX
528.1918
657.2314
826.4379 y7(1+)
1134.6301 PEP_0_1_0_0_0
1126.6327 b12(1+)
1236.172 PEP_0_2_0_0_0
1317.1967 PEP_1_2_0_0_0
1398.2291 PEP_2_2_0_0_0
1479.2517 PEP_3_2_0_0_0
1499.7491 PEP_2_3_0_0_0
1580.785 PEP_3_3_0_0_0
1661.8074 PEP_4_3_0_0_0

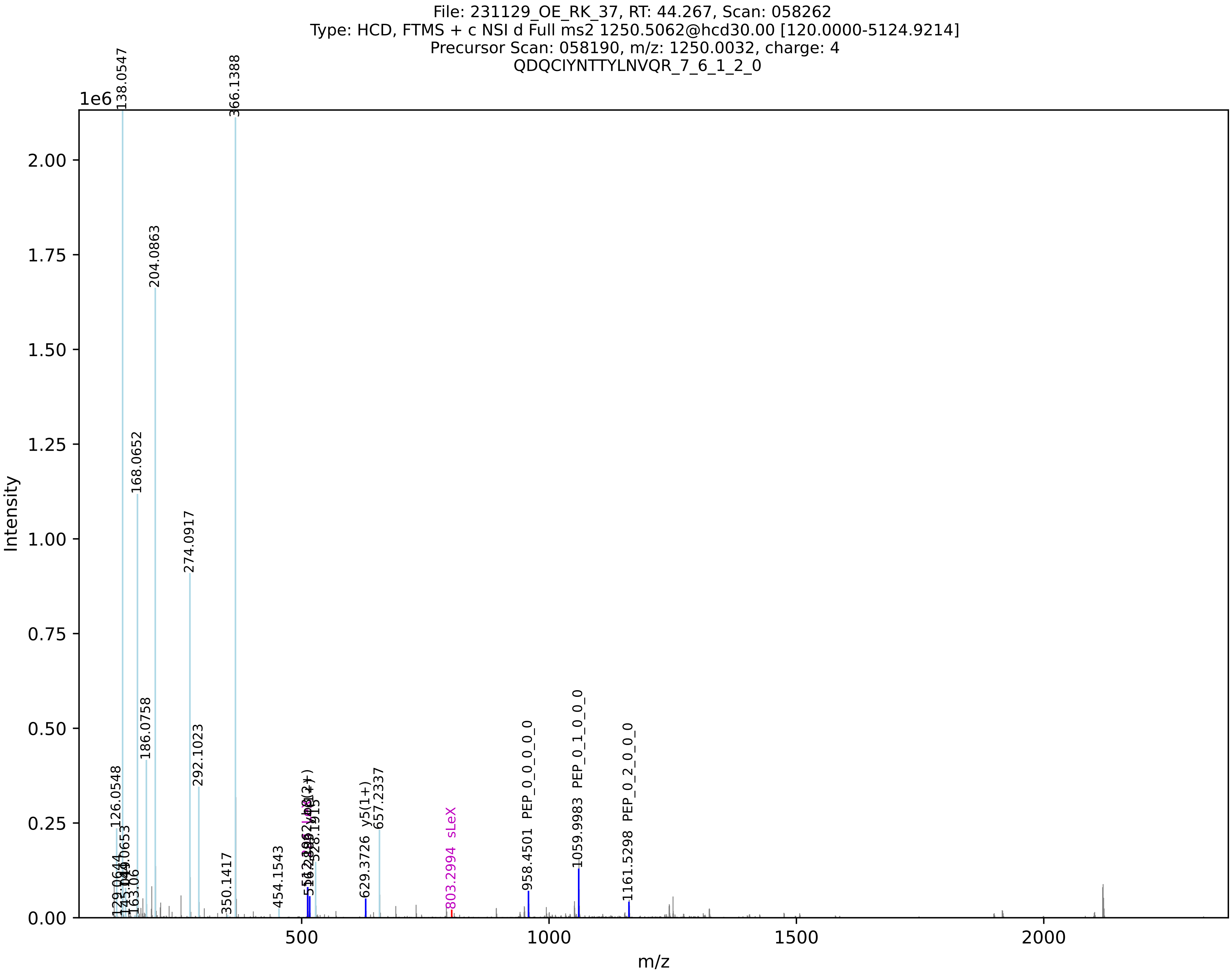
File: 231129_OE_RK_37, RT: 44.267, Scan: 058262
Type: HCD, FTMS + c NSI d Full ms2 1250.5062@hcd30.00 [120.0000-5124.9214]
Precursor Scan: 058190, m/z: 1250.0032, charge: 4
QDQCIYNTTYLNVQR_7_6_1_2_0
1e6
Intensity
m/z
138.0547
366.1388
204.0863
168.0652
274.0917
186.0758
292.1023
126.0548
350.1417
454.1543
629.3726 y5(1+)
657.2337
803.2994 sLeX
958.4501 PEP_0_0_0_0_0
1059.9983 PEP_0_1_0_0_0
1161.5298 PEP_0_2_0_0_0
0.00
0.25
0.50
0.75
1.00
1.25
1.50
1.75
2.00
500
1000
1500
2000

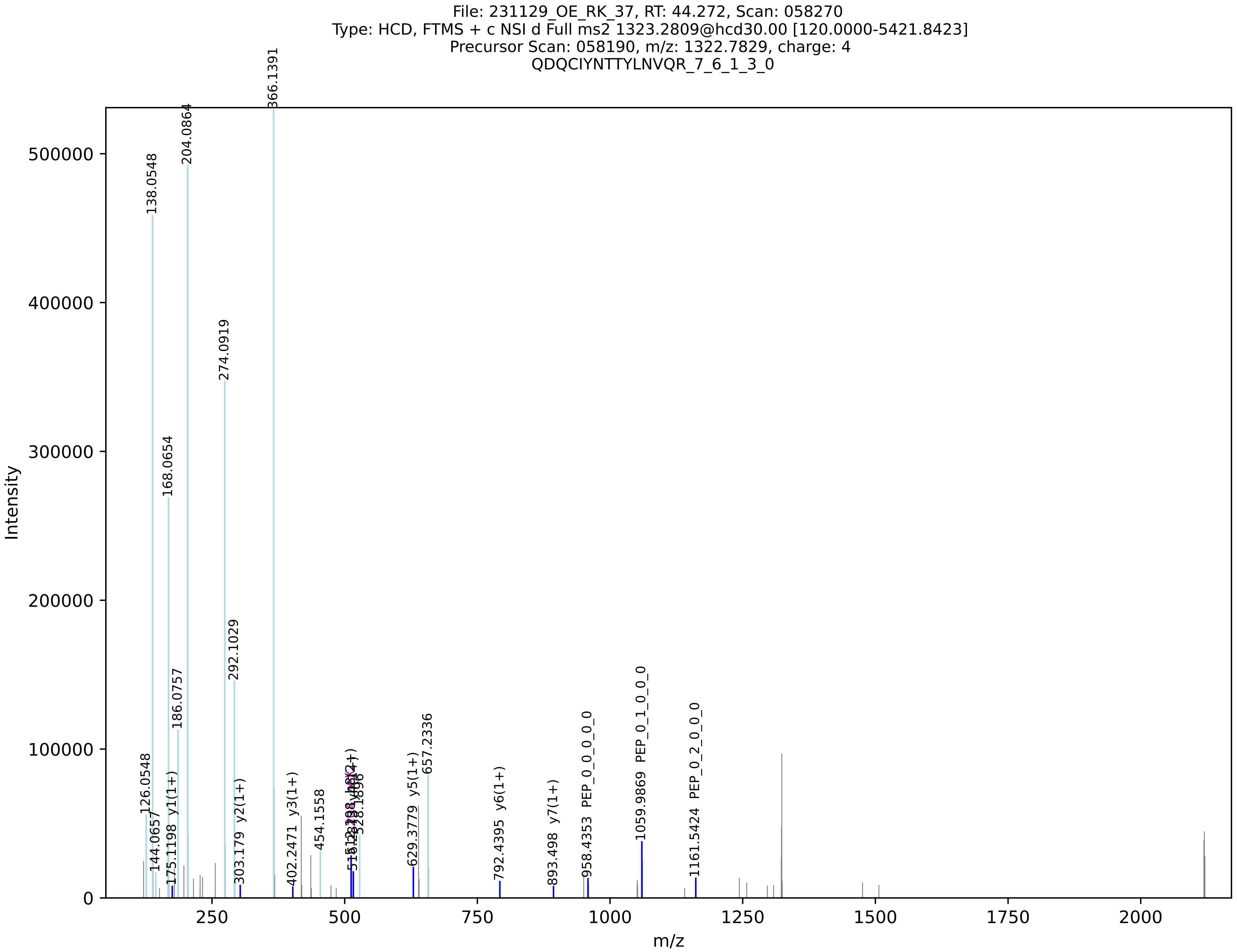
File: 231129_OE_RK_37, RT: 44.272, Scan: 058270
Type: HCD, FTMS + c NSI d Full ms2 1323.2809@hcd30.00 [120.0000-5421.8423]
Precursor Scan: 058190, m/z: 1322.7829, charge: 4
QDQCIYNTTYLNVQR_7_6_1_3_0
Intensity
m/z
366.1391
204.0864
138.0548
274.0919
168.0654
292.1029
186.0757
126.0548
144.0657
175.1198 y1(1+)
303.179 y2(1+)
402.2471 y3(1+)
454.1558
528.1896
657.2336
629.3779 y5(1+)
792.4395 y6(1+)
893.498 y7(1+)
958.4353 PEP_0_0_0_0_0
1059.9869 PEP_0_1_0_0_0
1161.5424 PEP_0_2_0_0_0

File: 231129_OE_RK_37, RT: 44.52, Scan: 058629
Type: HCD, FTMS + c NSI d Full ms2 1213.9904@hcd30.00 [120.0000-4975.9365]
Precursor Scan: 058553, m/z: 1213.4892, charge: 4
QDQCIYNTTYLNVQR_7_6_0_2_0

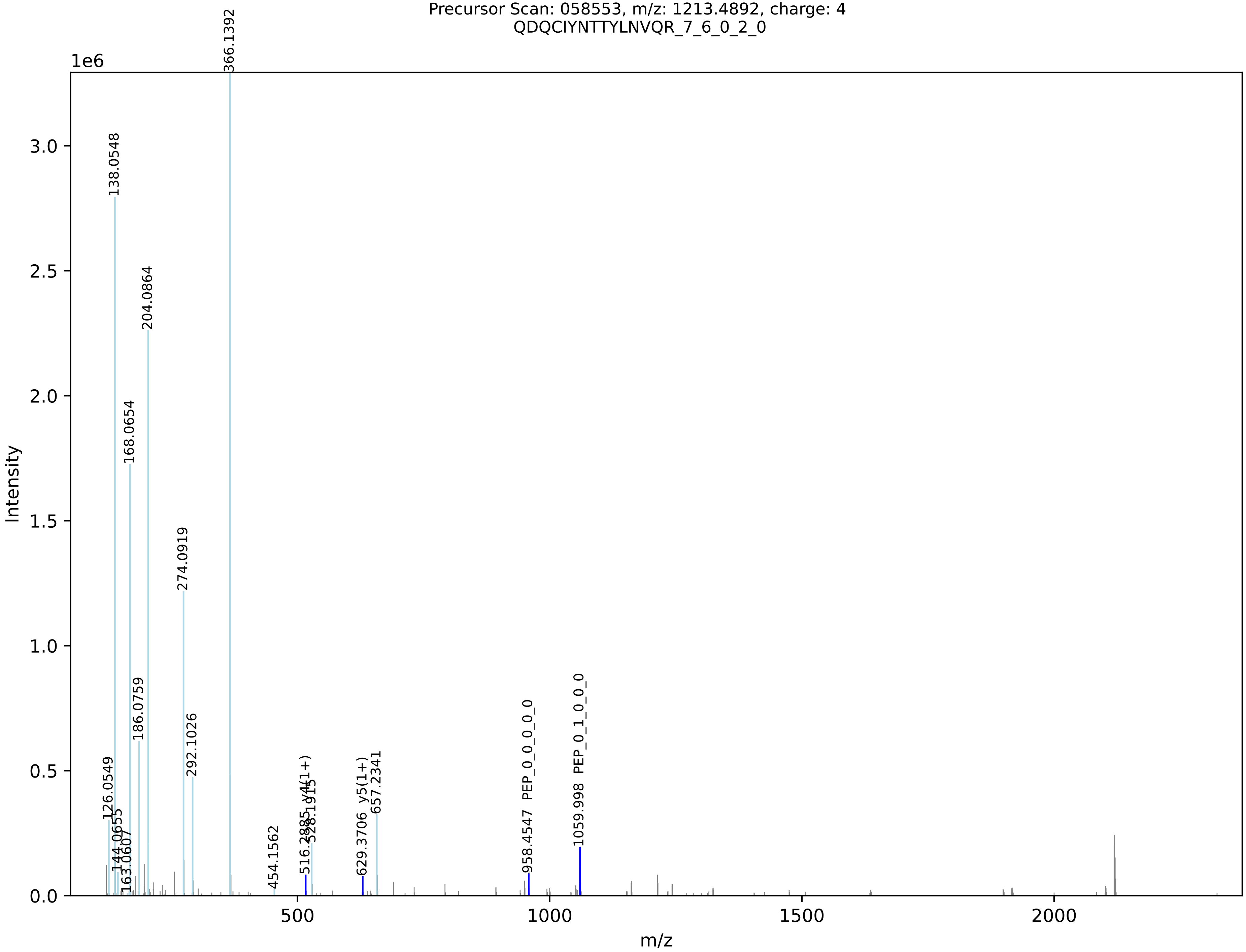

File: 231129_OE_RK_37, RT: 44.551, Scan: 058679
Type: HCD, FTMS + c NSI d Full ms2 955.2758@hcd30.00 [120.0000-5870.5718]
Precursor Scan: 058553, m/z: 954.609, charge: 6
WFVVFPGFAYDNVSAVYIYNCNSWVREYTK_10_2_0_0_0

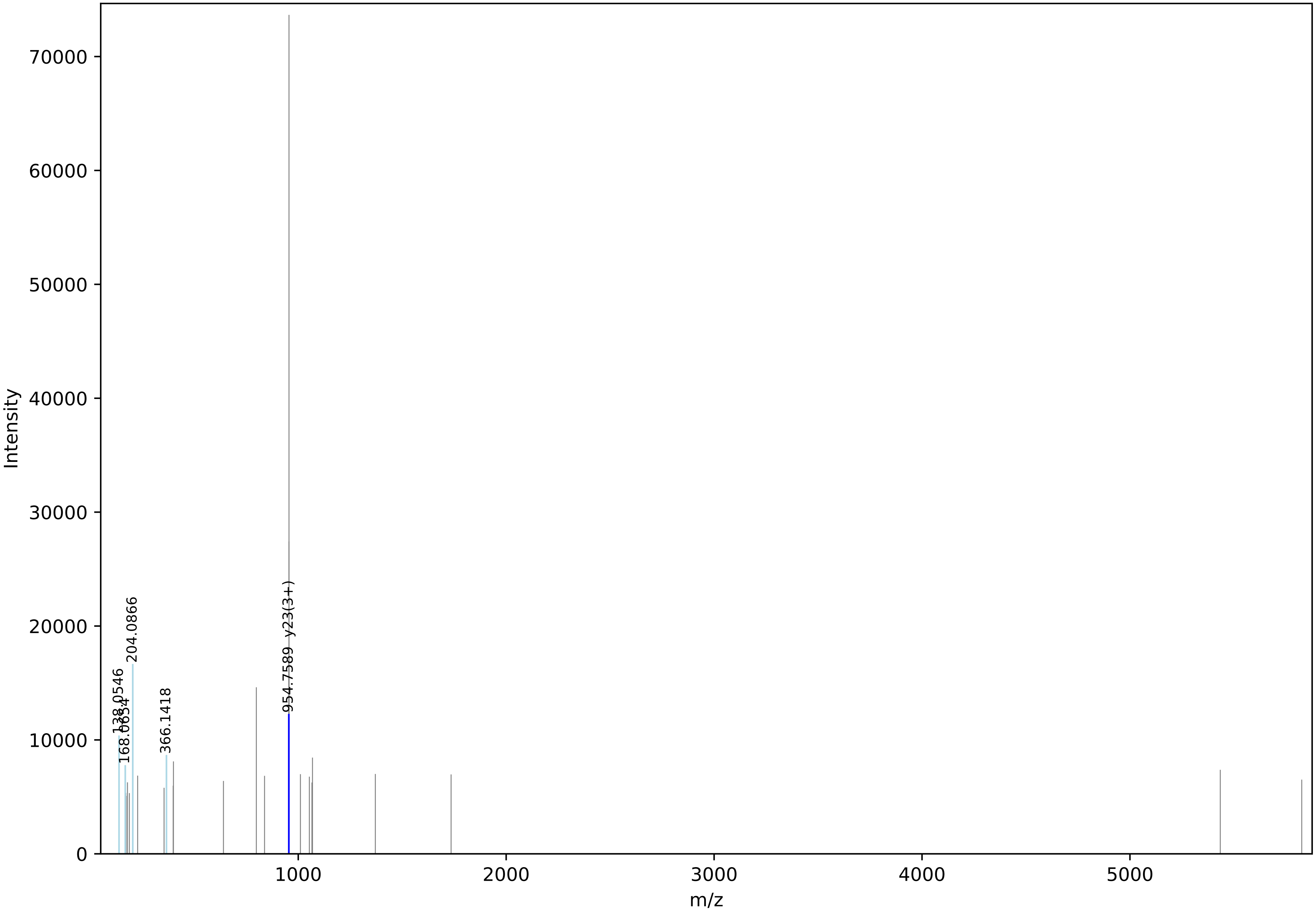

File: 231129_OE_RK_37, RT: 44.57, Scan: 058702
Type: HCD, FTMS + c NSI d Full ms2 1146.5306@hcd30.00 [120.0000-3530.5256]
Precursor Scan: 058627, m/z: 1146.1965, charge: 3
VVTVAALGTNISIHK_5_4_0_1_0

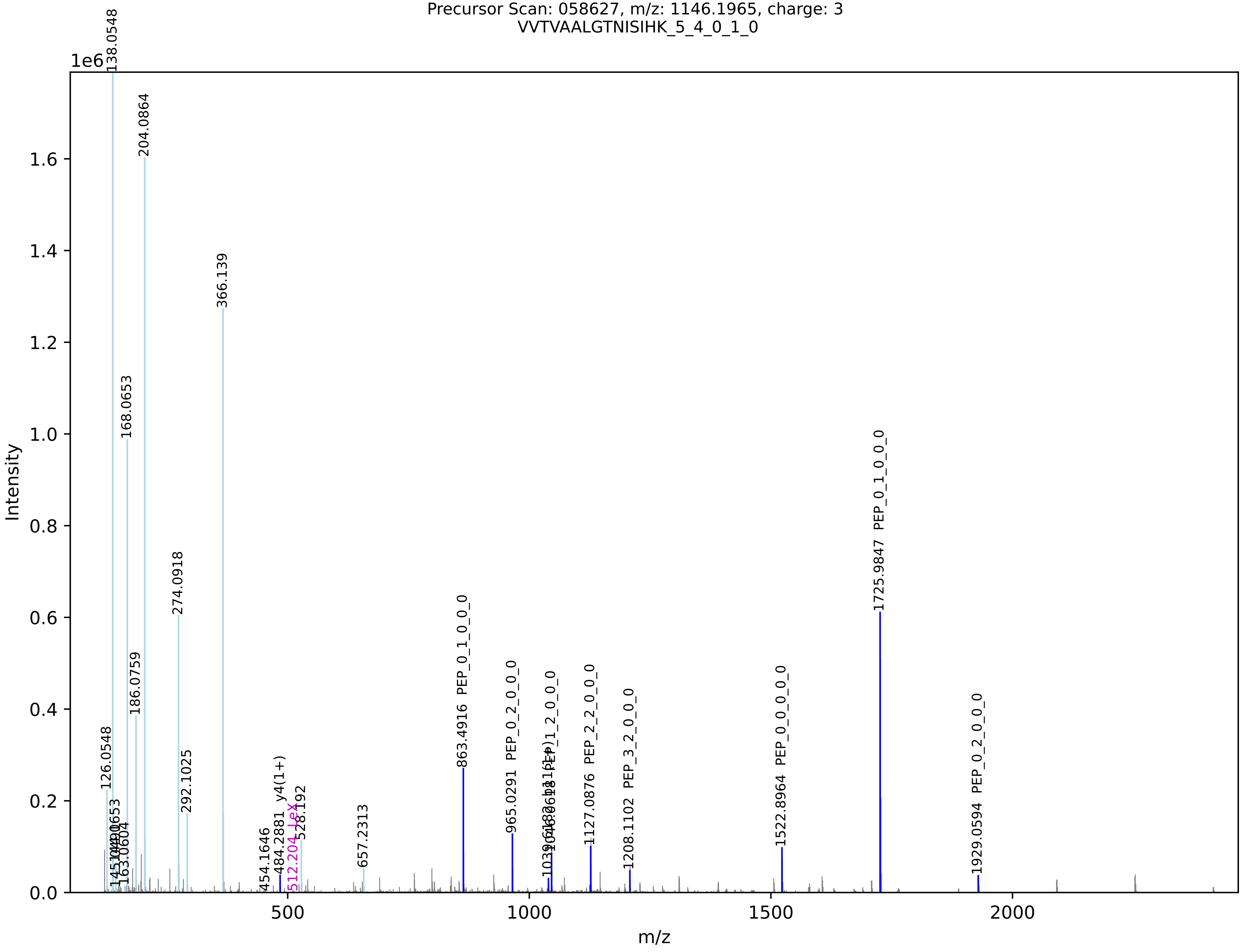

File: 231129_OE_RK_37, RT: 44.57, Scan: 058703
Type: HCD, FTMS + c NSI d Full ms2 1618.3182@hcd30.00 [120.0000-4974.1958]
Precursor Scan: 058627, m/z: 1617.6505, charge: 3
QDQCIYNTTYLNVQR_7_6_0_2_0

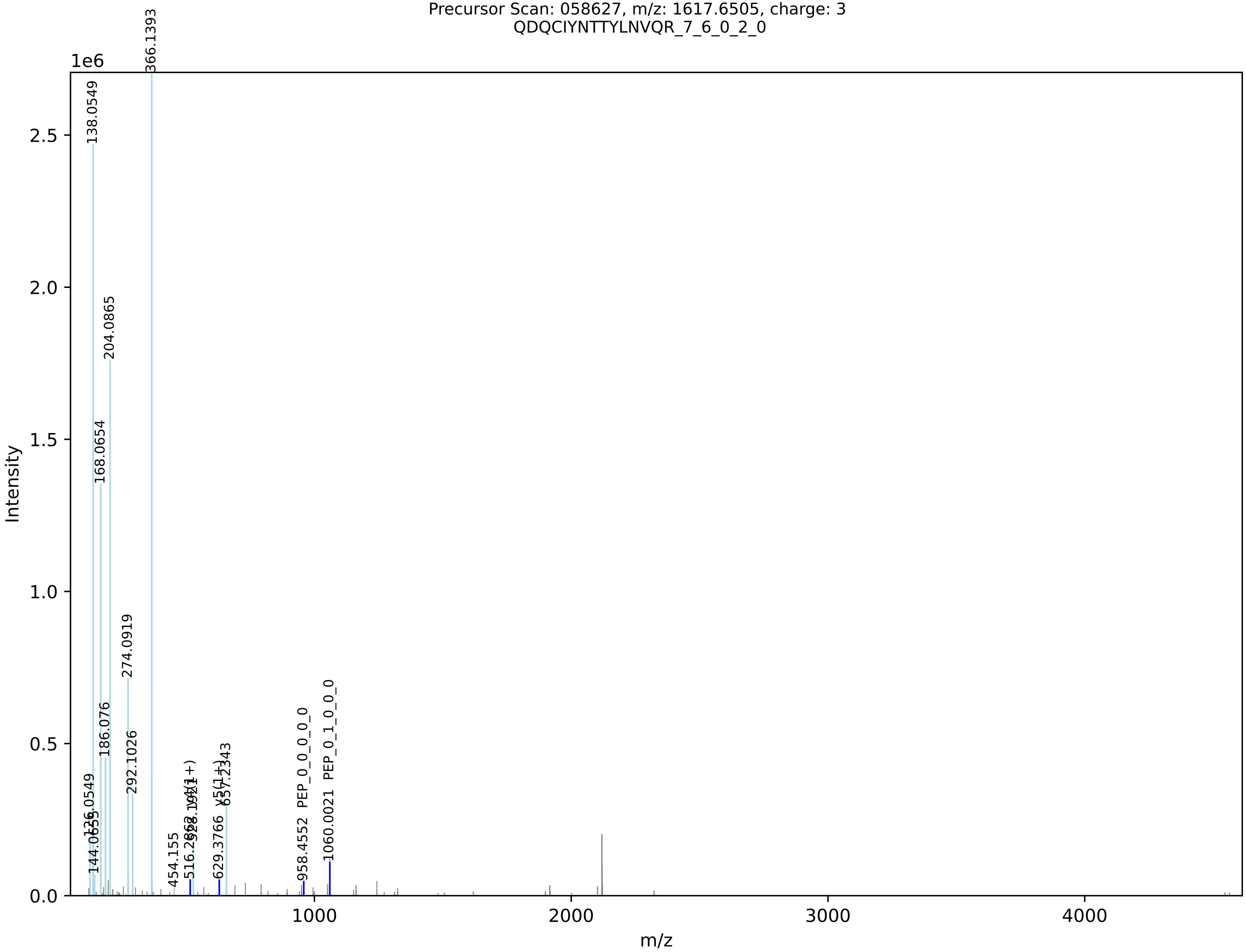

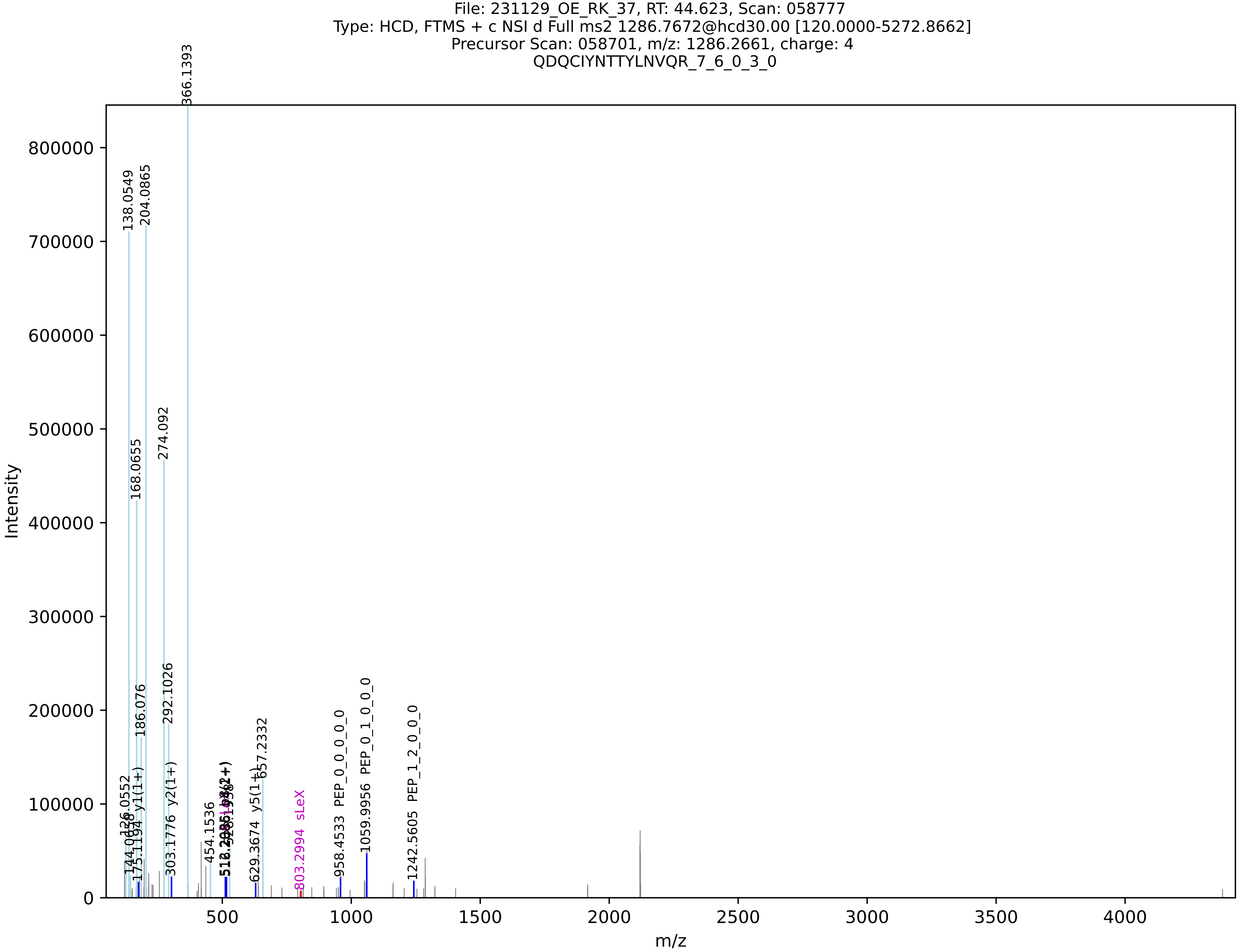
File: 231129_OE_RK_37, RT: 44.623, Scan: 058777
Type: HCD, FTMS + c NSI d Full ms2 1286.7672@hcd30.00 [120.0000-5272.8662]
Precursor Scan: 058701, m/z: 1286.2661, charge: 4
QDQCIYNTTYLNVQR_7_6_0_3_0
366.1393
138.0549
204.0865
274.092
168.0655
292.1026
186.076
657.2332
126.0552
144.0658
175.1194 y1(1+)
303.1776 y2(1+)
454.1536
629.3674 y5(1+)
803.2994 sLeX
958.4533 PEP_0_0_0_0_0
1059.9956 PEP_0_1_0_0_0
1242.5605 PEP_1_2_0_0_0
Intensity
m/z
0
100000
200000
300000
400000
500000
600000
700000
800000
500
1000
1500
2000
2500
3000
3500
4000

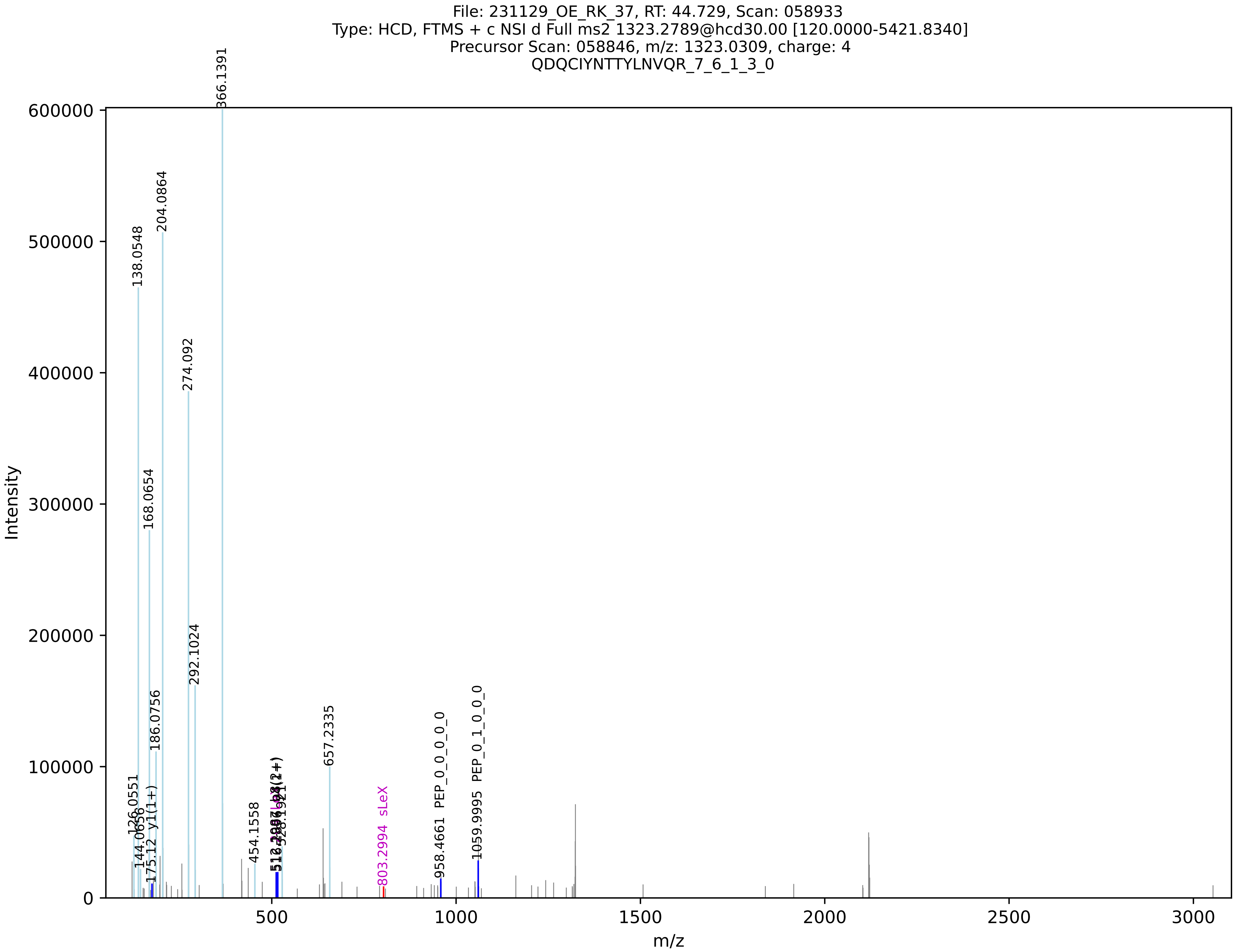

File: 231129_OE_RK_37, RT: 44.729, Scan: 058933
Type: HCD, FTMS + c NSI d Full ms2 1323.2789@hcd30.00 [120.0000-5421.8340]
Precursor Scan: 058846, m/z: 1323.0309, charge: 4
QDQCIYNTTYLNVQR_7_6_1_3_0
366.1391
204.0864
138.0548
274.092
168.0654
292.1024
186.0756
657.2335
126.0551
144.0656
175.12 y1(1+)
454.1558
803.2994 sLeX
958.4661 PEP_0_0_0_0_0
1059.9995 PEP_0_1_0_0_0
Intensity
m/z

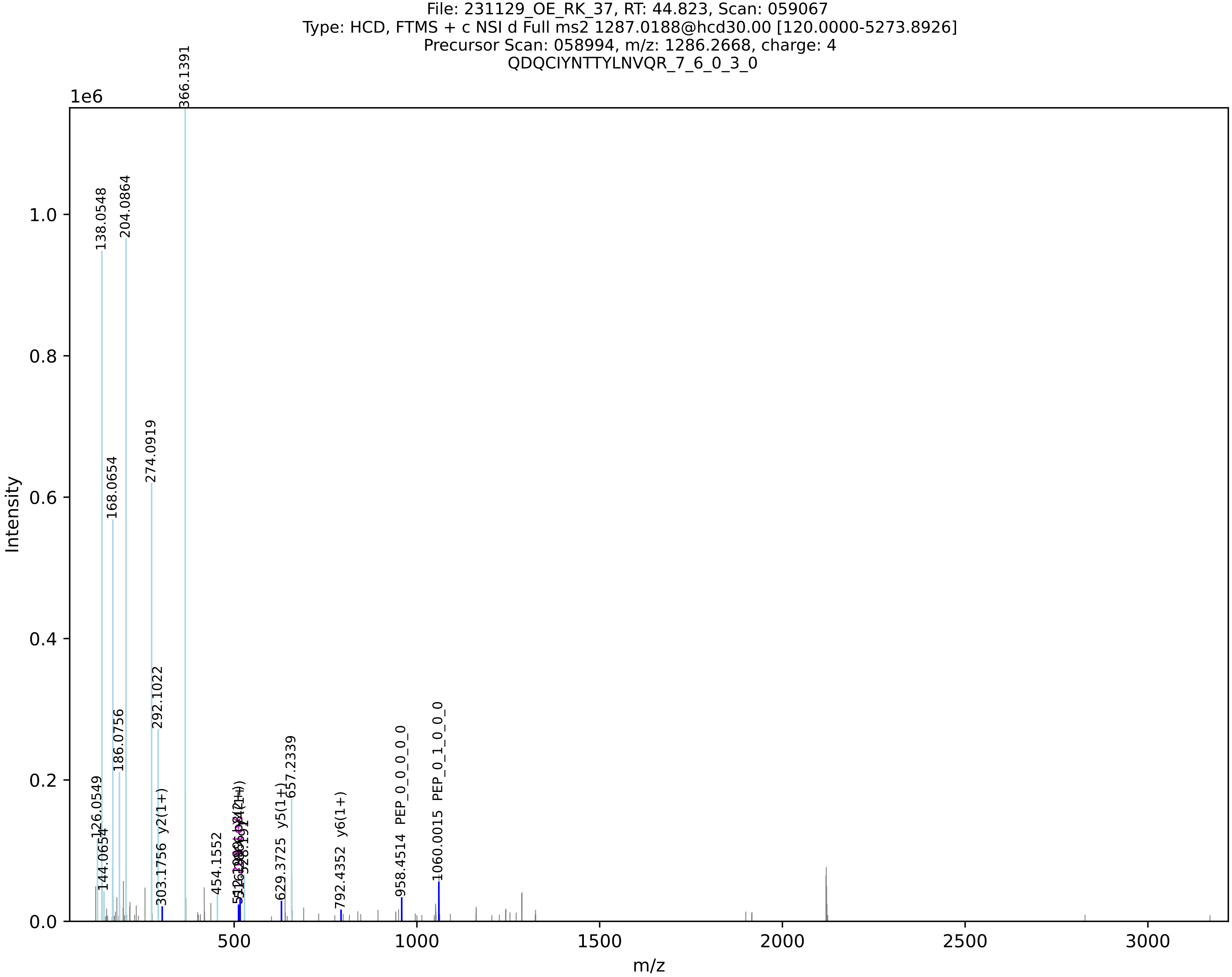
File: 231129_OE_RK_37, RT: 44.823, Scan: 059067
Type: HCD, FTMS + c NSI d Full ms2 1287.0188@hcd30.00 [120.0000-5273.8926]
Precursor Scan: 058994, m/z: 1286.2668, charge: 4
QDQCIYNTTYLNVQR_7_6_0_3_0
1e6
Intensity
m/z
0.0
0.2
0.4
0.6
0.8
1.0
500
1000
1500
2000
2500
3000
366.1391
138.0548
204.0864
274.0919
168.0654
292.1022
186.0756
126.0549
144.0654
303.1756 y2(1+)
454.1552
657.2339
629.3725 y5(1+)
792.4352 y6(1+)
958.4514 PEP_0_0_0_0_0
1060.0015 PEP_0_1_0_0_0

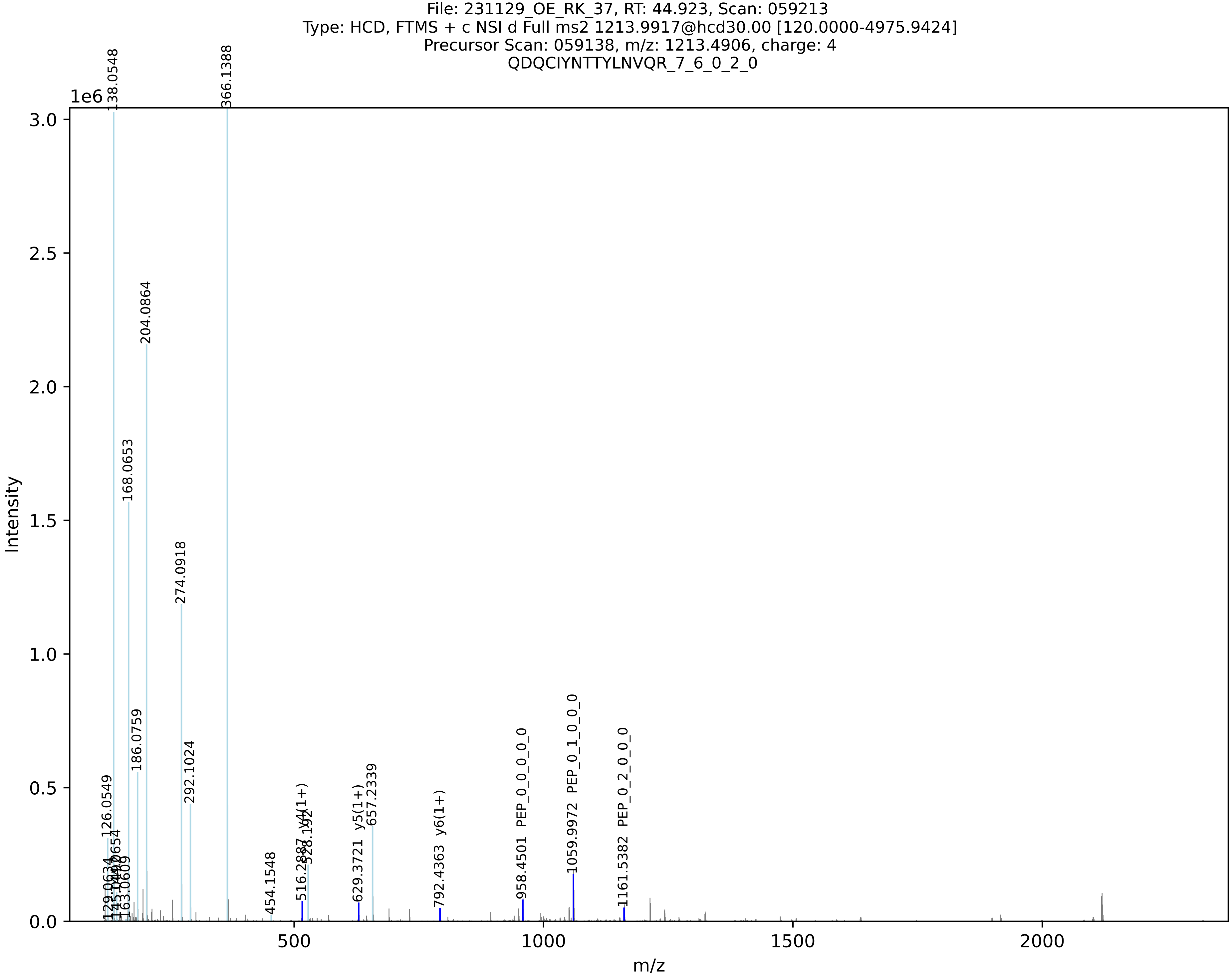
File: 231129_OE_RK_37, RT: 44.923, Scan: 059213
Type: HCD, FTMS + c NSI d Full ms2 1213.9917@hcd30.00 [120.0000-4975.9424]
Precursor Scan: 059138, m/z: 1213.4906, charge: 4
QDQCIYNTTYLNVQR_7_6_0_2_0
1e6
Intensity
m/z
138.0548
366.1388
204.0864
168.0653
274.0918
186.0759
292.1024
126.0549
129.0634
163.0609
454.1548
516.2887 y4(1+)
528.192
629.3721 y5(1+)
657.2339
792.4363 y6(1+)
958.4501 PEP_0_0_0_0_0
1059.9972 PEP_0_1_0_0_0
1161.5382 PEP_0_2_0_0_0

File: 231129_OE_RK_37, RT: 44.925, Scan: 059216
Type: HCD, FTMS + c NSI d Full ms2 1250.7571@hcd30.00 [120.0000-5125.9448]
Precursor Scan: 059138, m/z: 1250.0053, charge: 4
QDQCIYNTTYLNVQR_7_6_1_2_0

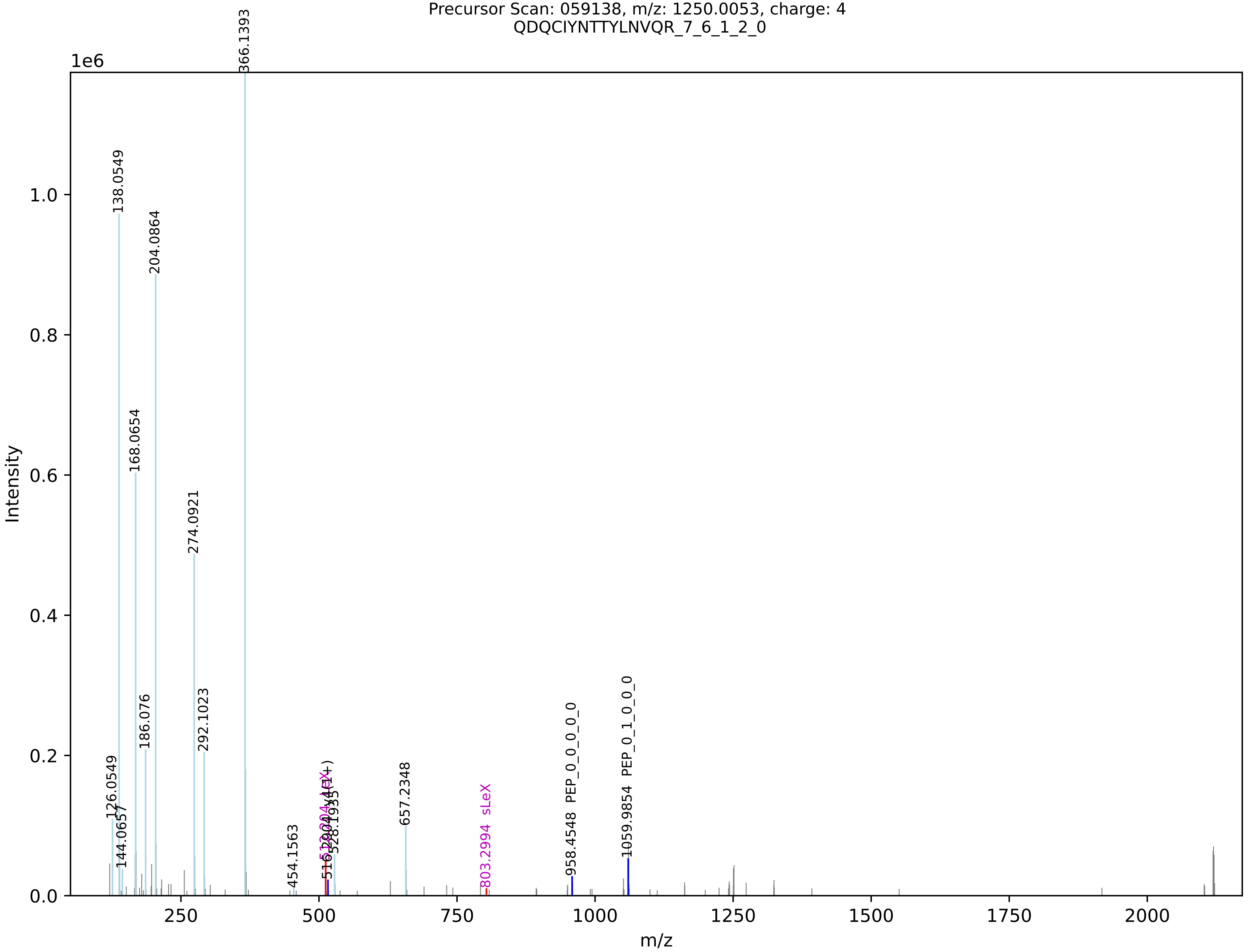

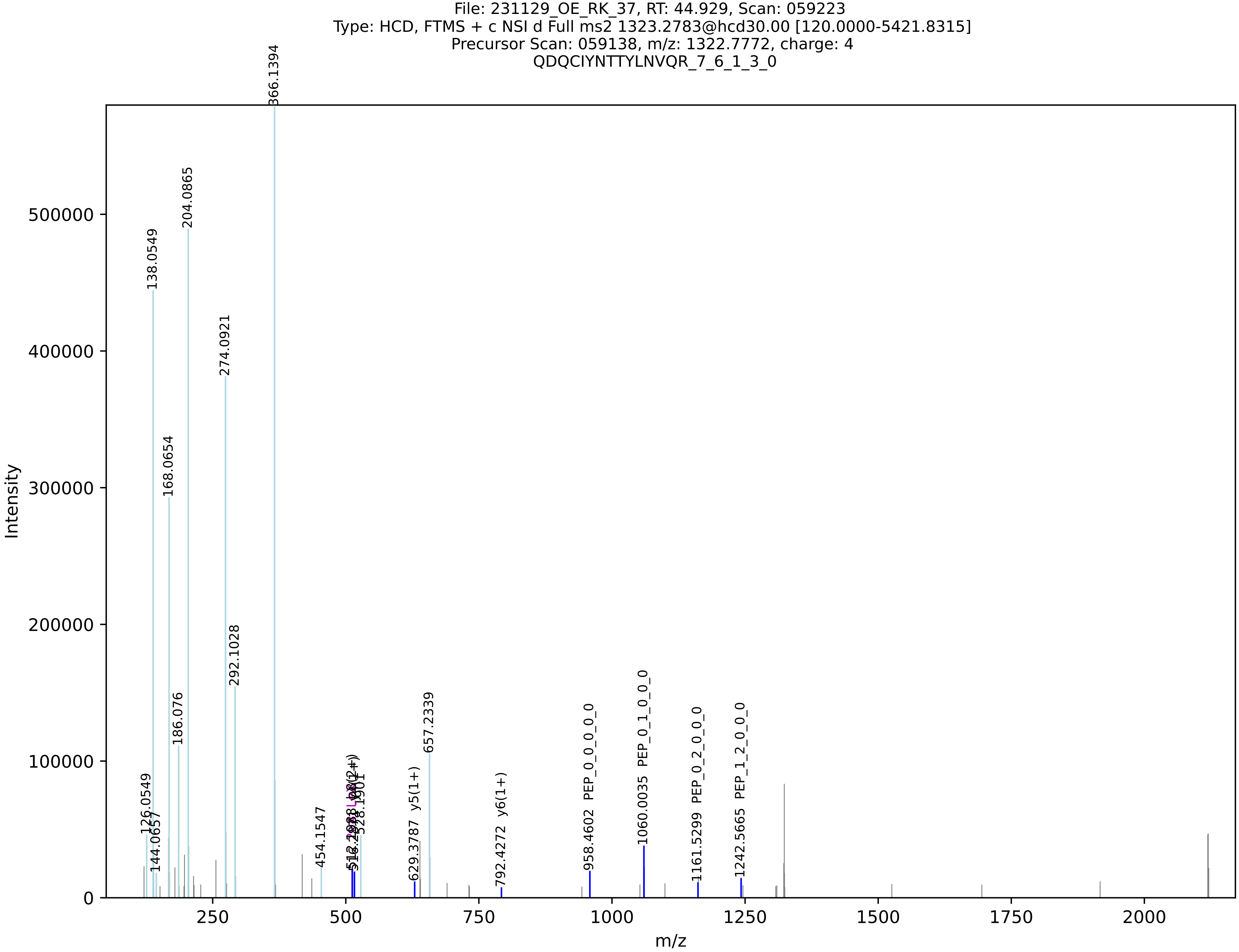
File: 231129_OE_RK_37, RT: 44.929, Scan: 059223
Type: HCD, FTMS + c NSI d Full ms2 1323.2783@hcd30.00 [120.0000-5421.8315]
Precursor Scan: 059138, m/z: 1322.7772, charge: 4
QDQCIYNTTYLNVQR_7_6_1_3_0
366.1394
204.0865
138.0549
274.0921
168.0654
292.1028
186.076
126.0549
144.0657
454.1547
528.1901
657.2339
629.3787 y5(1+)
792.4272 y6(1+)
958.4602 PEP_0_0_0_0_0
1060.0035 PEP_0_1_0_0_0
1161.5299 PEP_0_2_0_0_0
1242.5665 PEP_1_2_0_0_0
Intensity
m/z
0
100000
200000
300000
400000
500000
250
500
750
1000
1250
1500
1750
2000

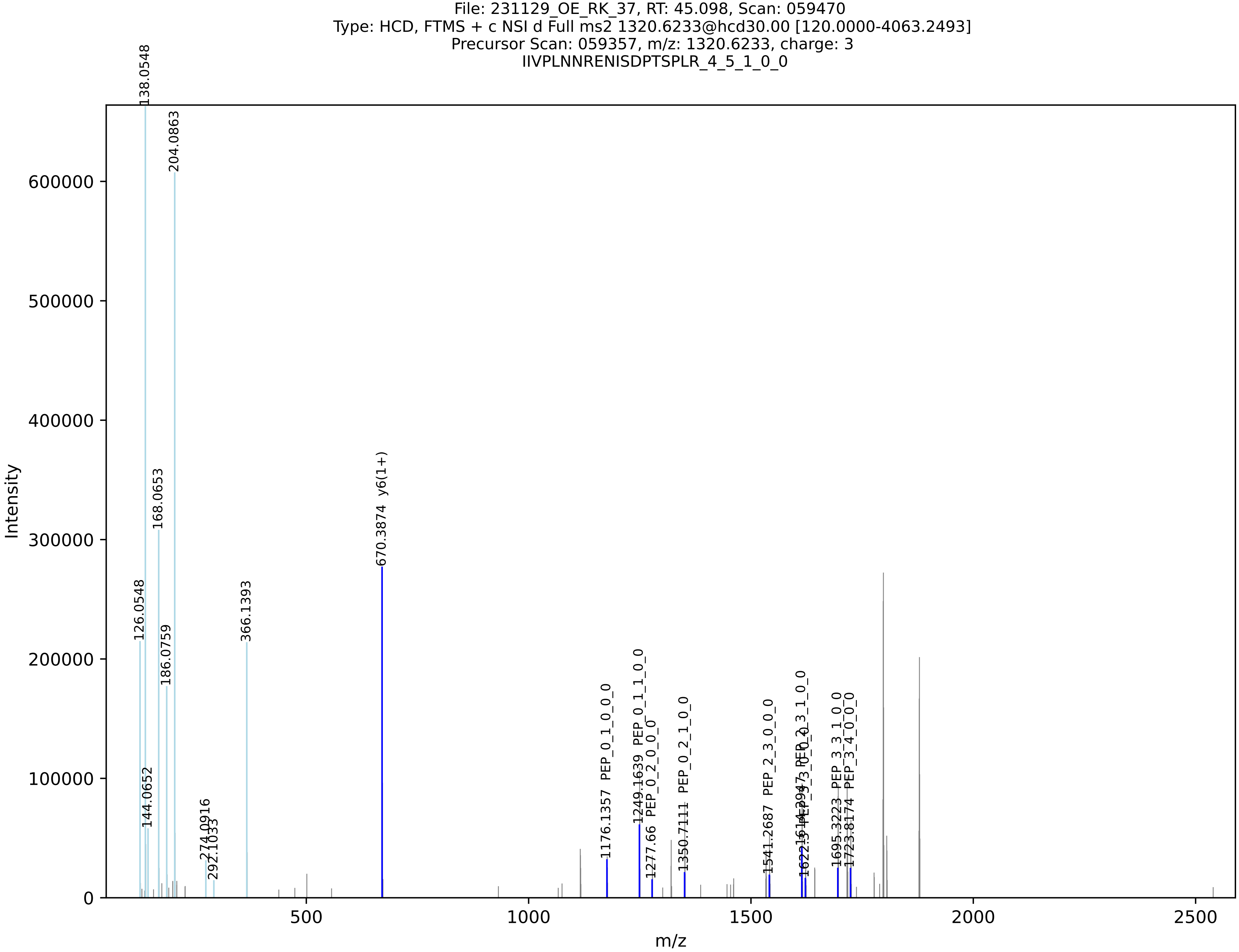
File: 231129_OE_RK_37, RT: 45.098, Scan: 059470
Type: HCD, FTMS + c NSI d Full ms2 1320.6233@hcd30.00 [120.0000-4063.2493]
Precursor Scan: 059357, m/z: 1320.6233, charge: 3
IIVPLNNRENISDPTSPLR_4_5_1_0_0
Intensity
m/z
0
100000
200000
300000
400000
500000
600000
500
1000
1500
2000
2500
138.0548
204.0863
168.0653
126.0548
186.0759
366.1393
670.3874 y6(1+)
144.0652
274.0916
292.1033
1176.1357 PEP_0_1_0_0_0
1249.1639 PEP_0_1_1_0_0
1277.66 PEP_0_2_0_0_0
1350.7111 PEP_0_2_1_0_0
1541.2687 PEP_2_3_0_0_0
1695.3223 PEP_3_3_1_0_0
1723.8174 PEP_3_4_0_0_0

File: 231129_OE_RK_37, RT: 45.126, Scan: 059508
Type: HCD, FTMS + c NSI d Full ms2 949.4200@hcd30.00 [120.0000-4865.6123]
Precursor Scan: 059431, m/z: 948.8187, charge: 5
VYIHPFHLVIHNESTCEQLAK_5_4_0_2_0

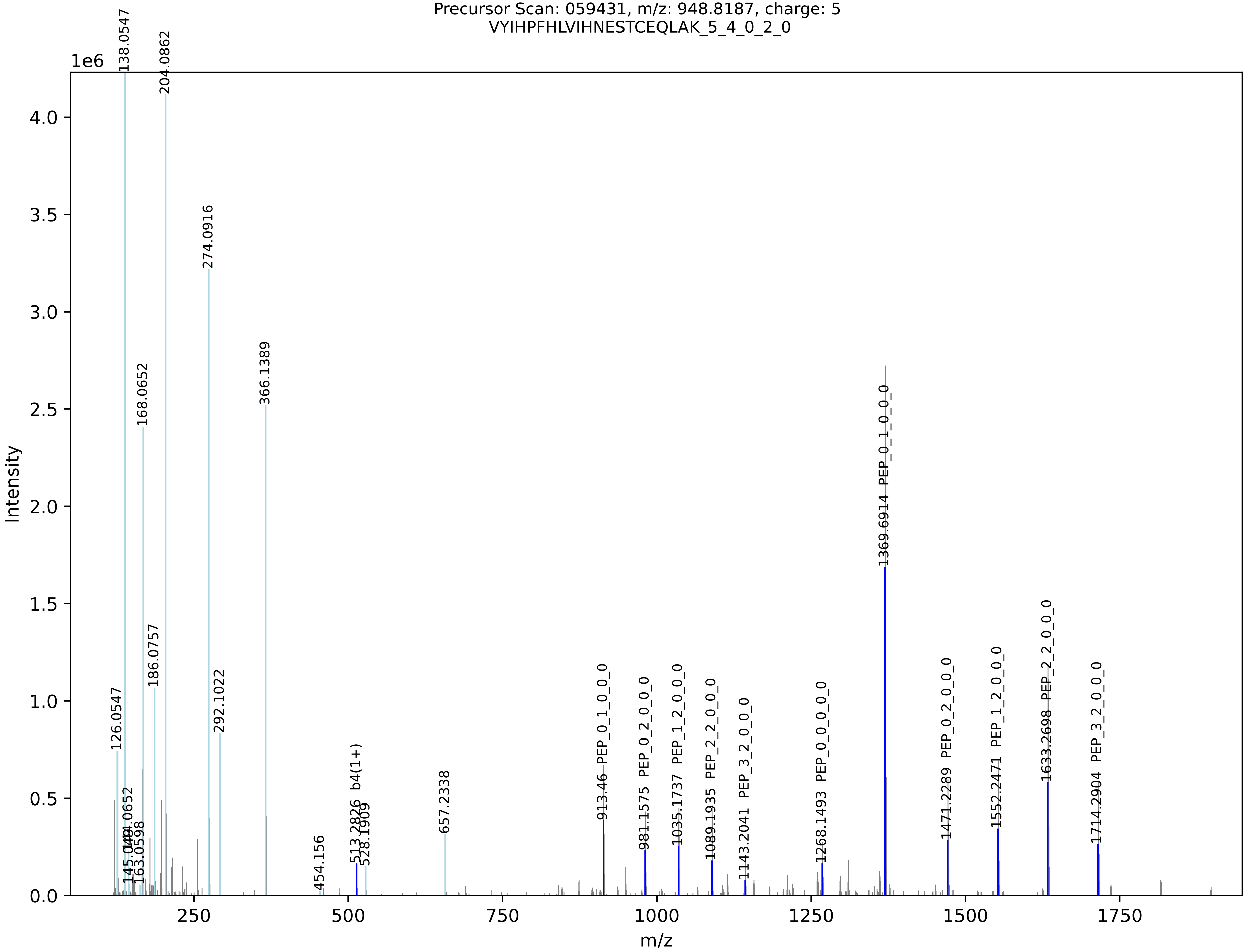

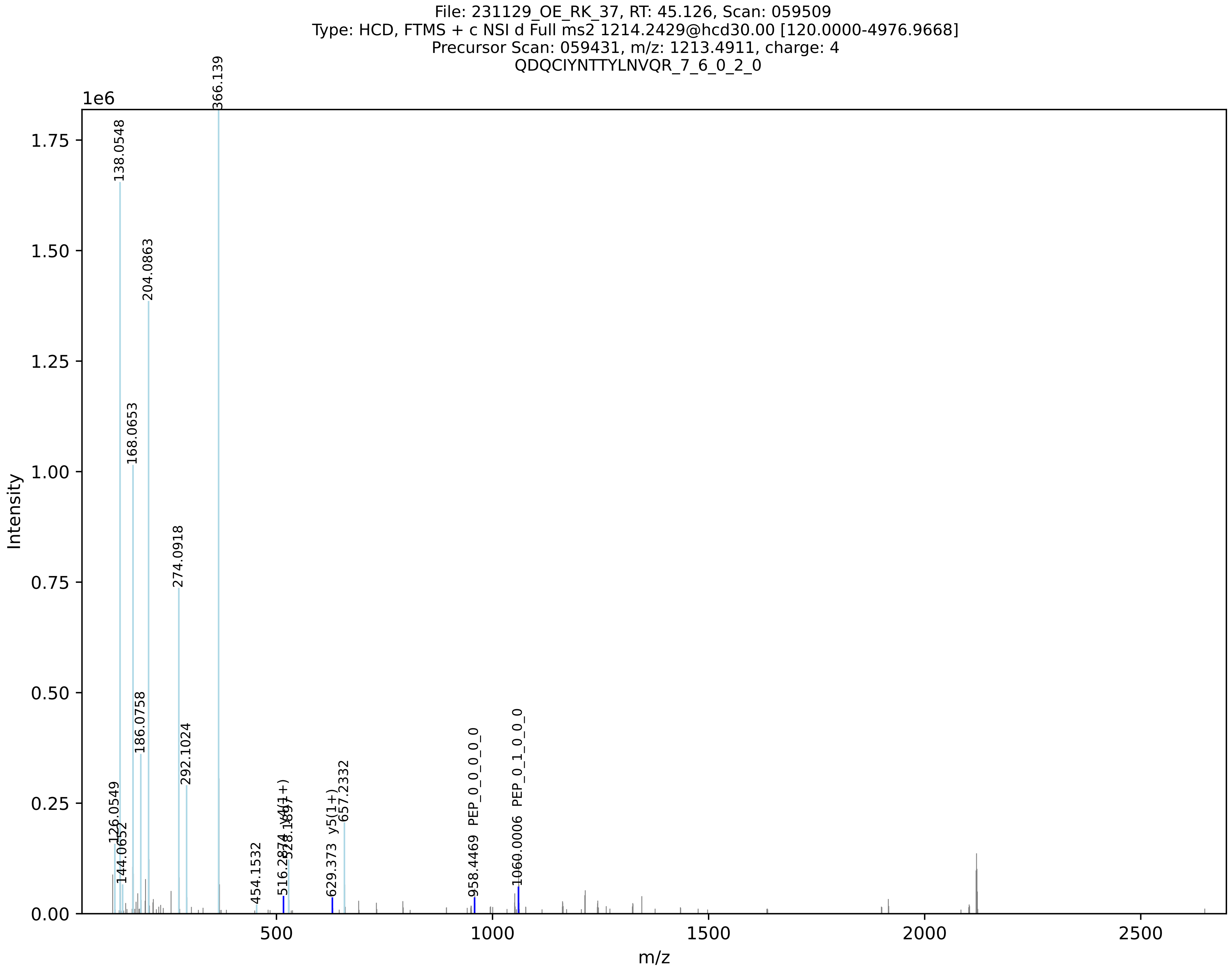

File: 231129_OE_RK_37, RT: 45.126, Scan: 059509
Type: HCD, FTMS + c NSI d Full ms2 1214.2429@hcd30.00 [120.0000-4976.9668]
Precursor Scan: 059431, m/z: 1213.4911, charge: 4
QDQCIYNTTYLNVQR_7_6_0_2_0
1e6
Intensity
m/z
366.139
138.0548
204.0863
168.0653
274.0918
186.0758
292.1024
126.0549
144.0652
454.1532
516.2874
y4(1+)
528.1897
629.373
y5(1+)
657.2332
958.4469
PEP_0_0_0_0_0
1060.0006
PEP_0_1_0_0_0

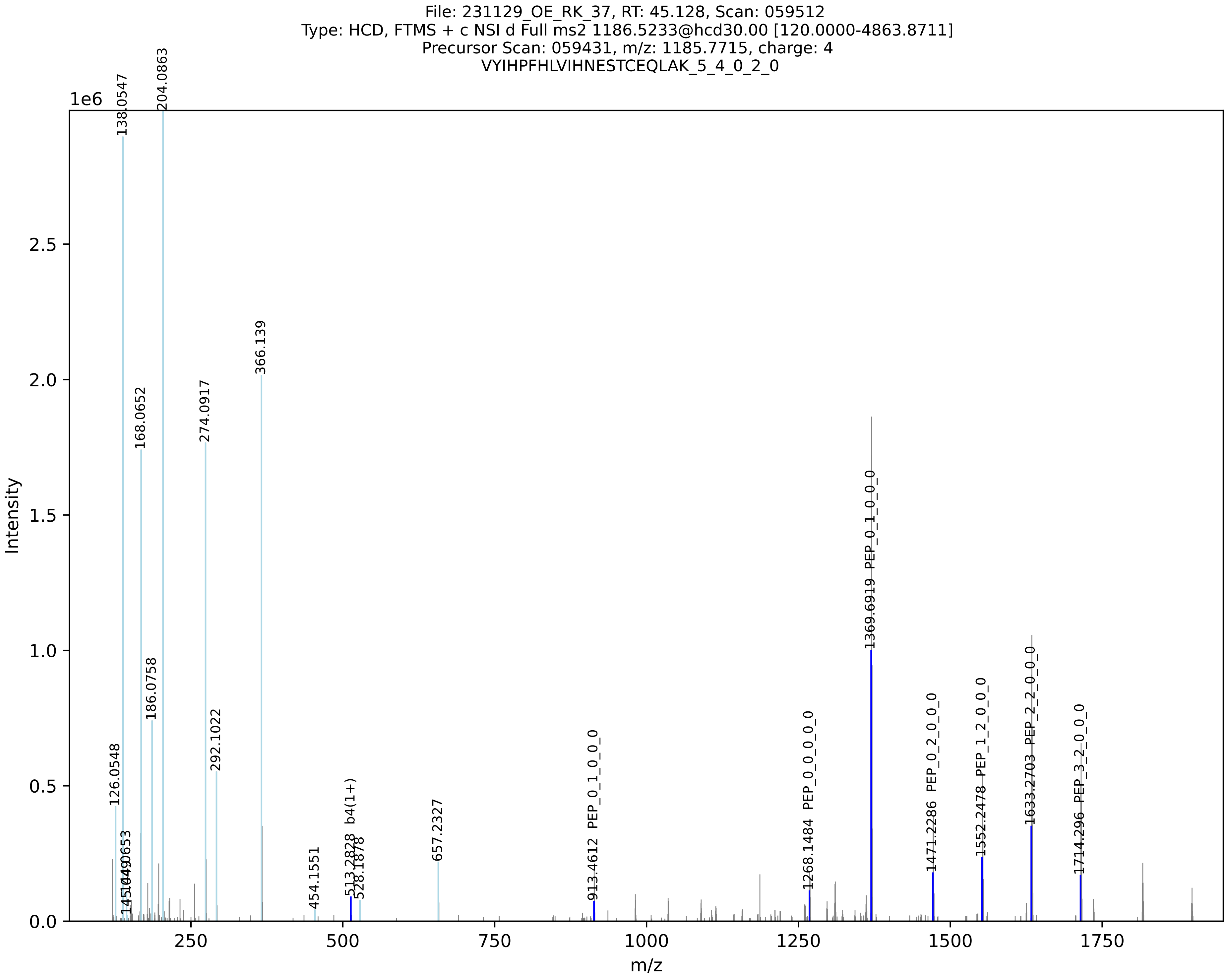

File: 231129_OE_RK_37, RT: 45.128, Scan: 059512
Type: HCD, FTMS + c NSI d Full ms2 1186.5233@hcd30.00 [120.0000-4863.8711]
Precursor Scan: 059431, m/z: 1185.7715, charge: 4
VYIHPFHLVIHNESTCEQLAK_5_4_0_2_0
1e6
Intensity
m/z
204.0863
138.0547
366.139
274.0917
168.0652
186.0758
292.1022
126.0548
454.1551
513.2828 b4(1+)
528.1878
657.2327
913.4612 PEP_0_1_0_0_0
1268.1484 PEP_0_0_0_0_0
1369.6919 PEP_0_1_0_0_
1471.2286 PEP_0_2_0_0_0
1552.2478 PEP_1_2_0_0_0
1633.2703 PEP_2_2_0_0_0
1714.296 PEP_3_2_0_0_0

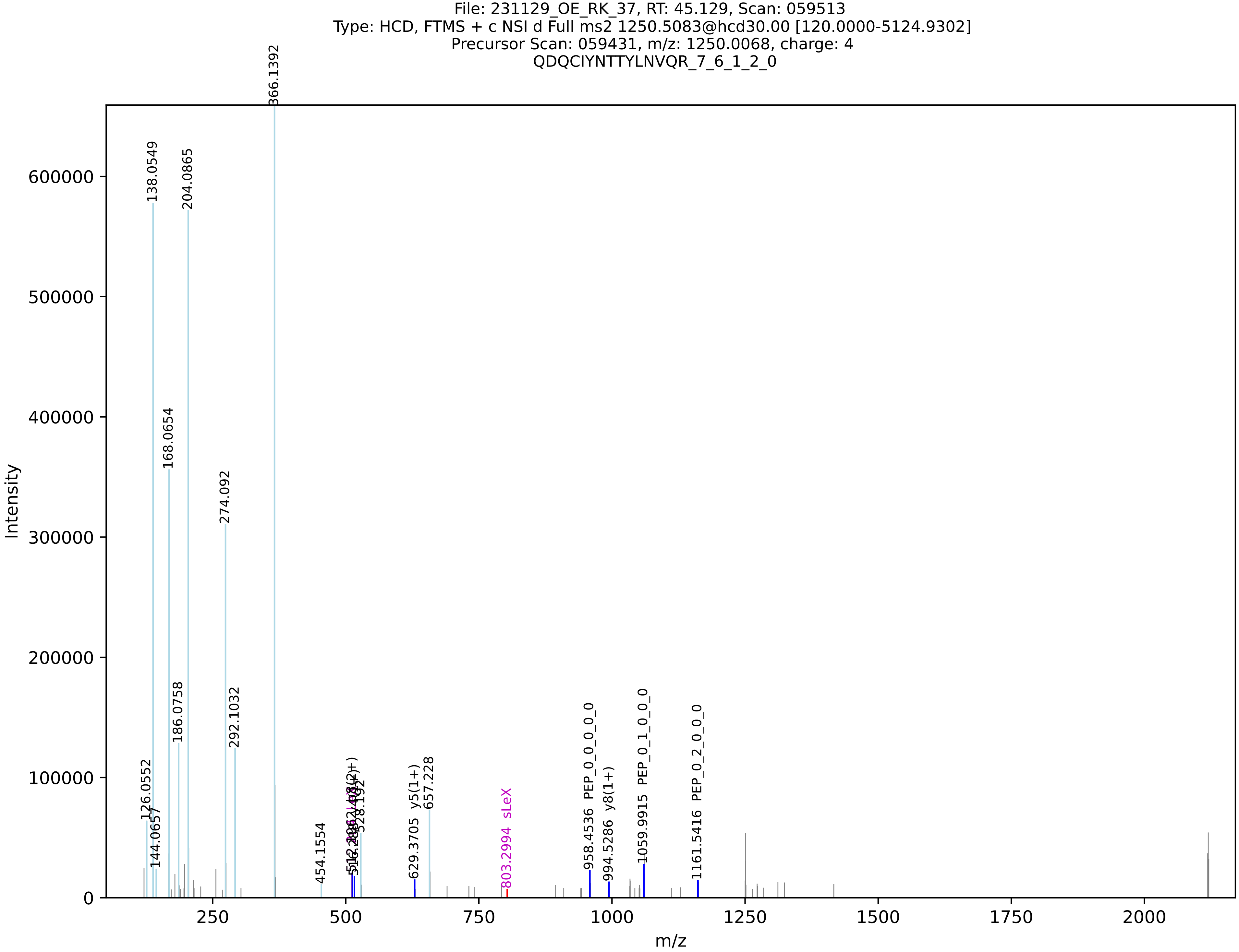

File: 231129_OE_RK_37, RT: 45.129, Scan: 059513
Type: HCD, FTMS + c NSI d Full ms2 1250.5083@hcd30.00 [120.0000-5124.9302]
Precursor Scan: 059431, m/z: 1250.0068, charge: 4
QDQCIYNTTYLNVQR_7_6_1_2_0
Intensity
m/z
366.1392
138.0549
204.0865
168.0654
274.092
186.0758
292.1032
126.0552
144.0657
454.1554
528.192
629.3705 y5(1+)
657.228
803.2994 sLeX
958.4536 PEP_0_0_0_0_0_
994.5286 y8(1+)
1059.9915 PEP_0_1_0_0_0_
1161.5416 PEP_0_2_0_0_0_
0
100000
200000
300000
400000
500000
600000
250
500
750
1000
1250
1500
1750
2000

File: 231129_OE_RK_37, RT: 45.19, Scan: 059602
Type: HCD, FTMS + c NSI d Full ms2 791.3512@hcd30.00 [120.0000-4867.3535]
Precursor Scan: 059505, m/z: 790.8494, charge: 6
VYIHPFHLVIHNESTCEQLAK_5_4_0_2_0

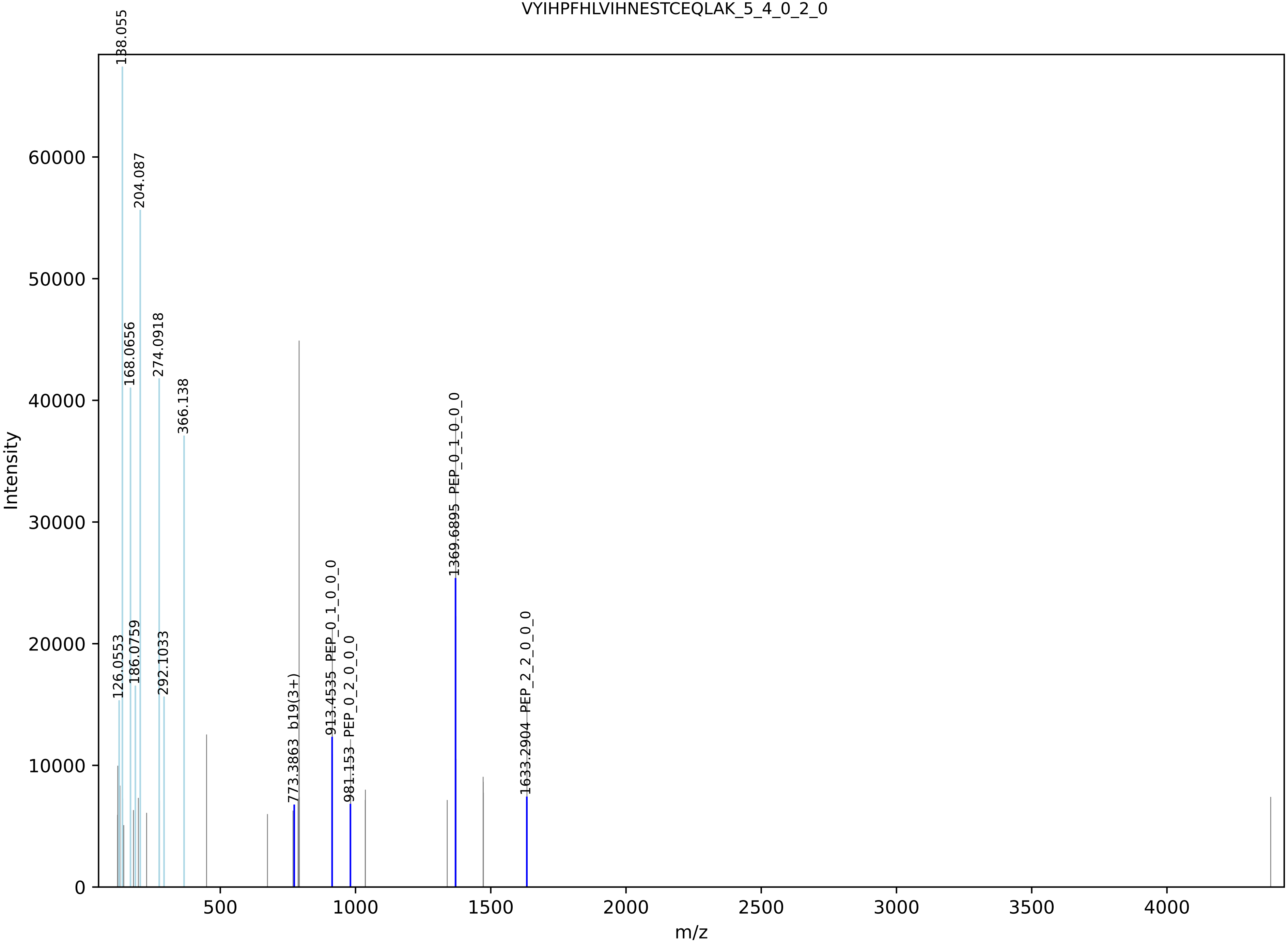

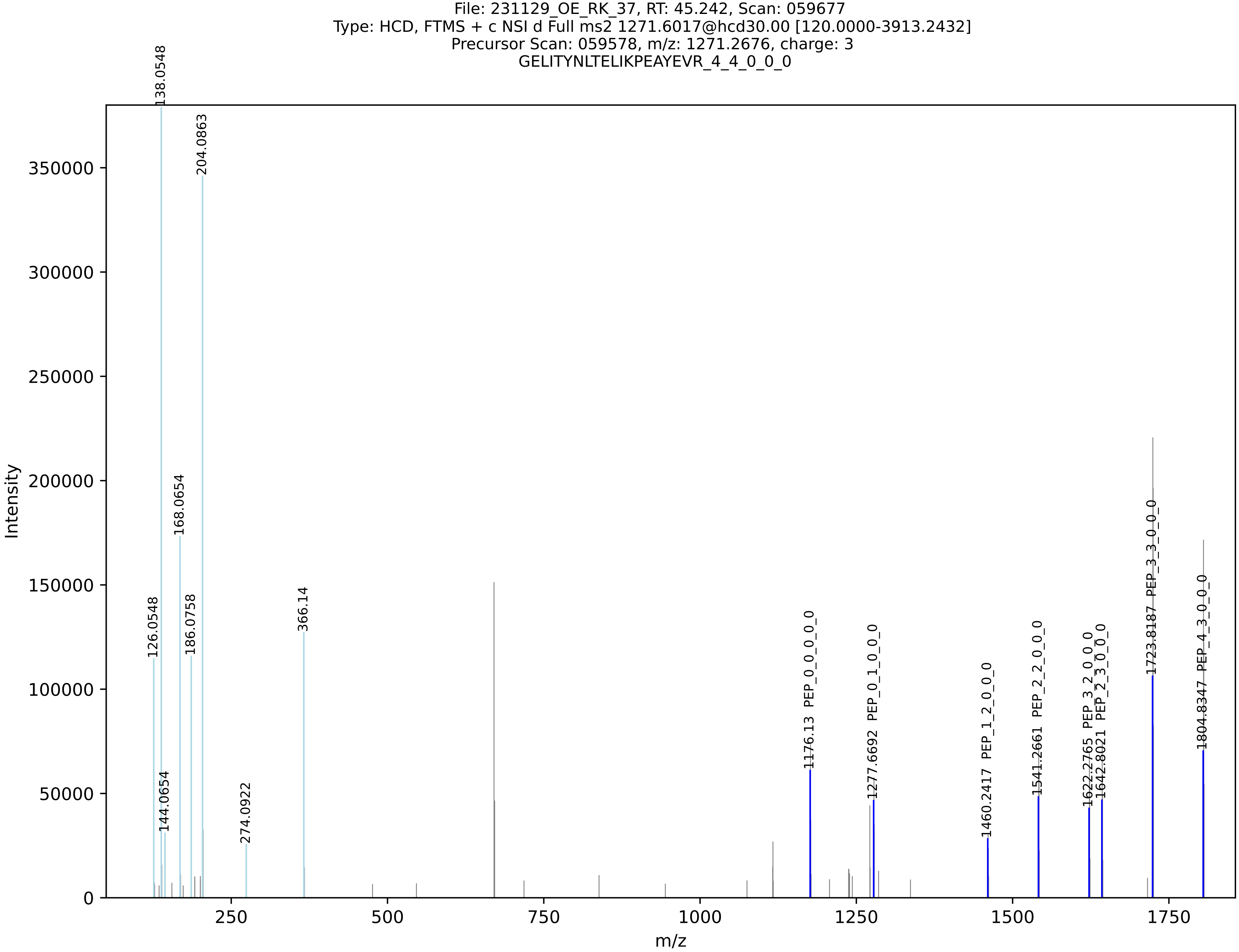
File: 231129_OE_RK_37, RT: 45.242, Scan: 059677
Type: HCD, FTMS + c NSI d Full ms2 1271.6017@hcd30.00 [120.0000-3913.2432]
Precursor Scan: 059578, m/z: 1271.2676, charge: 3
GELITYNLTELIKPEAYEVR_4_4_0_0_0
Intensity
m/z
138.0548
204.0863
168.0654
126.0548
186.0758
366.14
144.0654
274.0922
1176.13 PEP_0_0_0_0_0
1277.6692 PEP_0_1_0_0_0
1460.2417 PEP_1_2_0_0_0
1541.2661 PEP_2_2_0_0_0
1622.2765 PEP_3_2_0_0_0
1642.8021 PEP_2_3_0_0_0
1723.8187 PEP_3_3_0_0_0
1804.8347 PEP_4_3_0_0_0

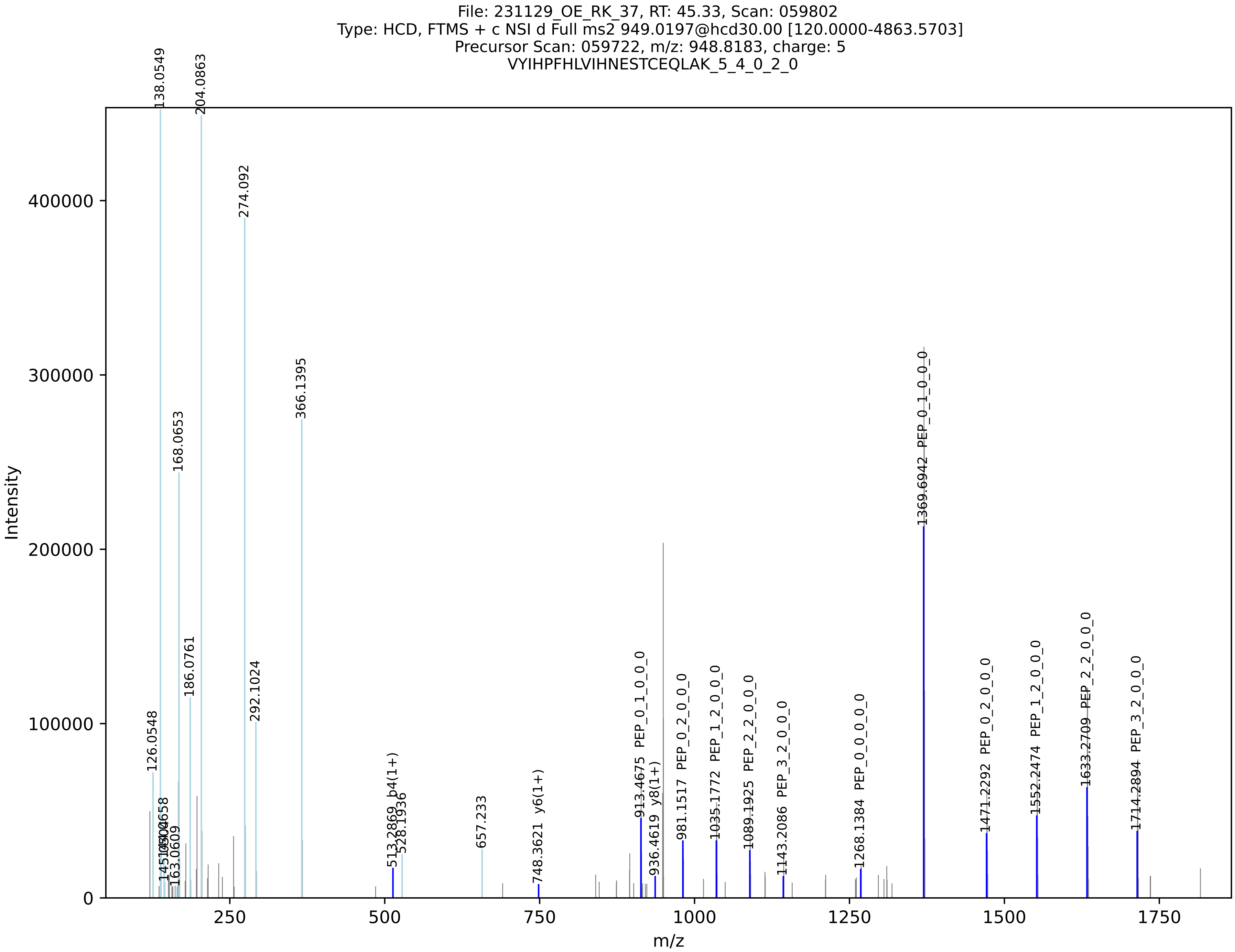

File: 231129_OE_RK_37, RT: 45.33, Scan: 059802
Type: HCD, FTMS + c NSI d Full ms2 949.0197@hcd30.00 [120.0000-4863.5703]
Precursor Scan: 059722, m/z: 948.8183, charge: 5
VYIHPFHLVIHNESTCEQLAK_5_4_0_2_0
Intensity
m/z
138.0549
204.0863
274.092
366.1395
168.0653
186.0761
292.1024
126.0548
163.0609
513.2869 b4(1+)
528.1936
657.233
748.3621 y6(1+)
913.4675 PEP_0_1_0_0_0
936.4619 y8(1+)
981.1517 PEP_0_2_0_0_0
1035.1772 PEP_1_2_0_0_0
1089.1925 PEP_2_2_0_0_0
1143.2086 PEP_3_2_0_0_0
1268.1384 PEP_0_0_0_0_0
1369.6942 PEP_0_1_0_0_
1471.2292 PEP_0_2_0_0_0
1552.2474 PEP_1_2_0_0_0
1633.2709 PEP_2_2_0_0_0
1714.2894 PEP_3_2_0_0_0

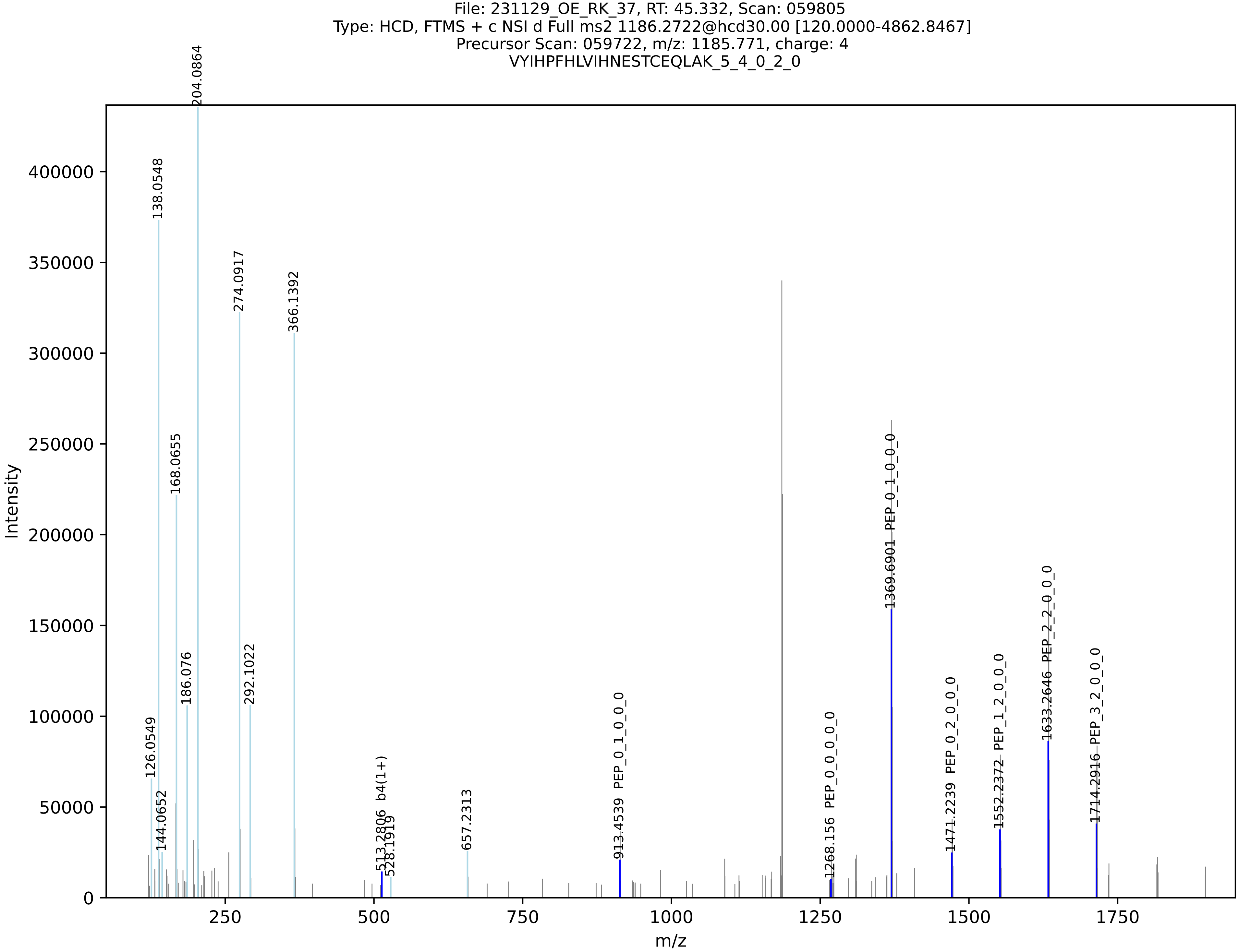
File: 231129_OE_RK_37, RT: 45.332, Scan: 059805
Type: HCD, FTMS + c NSI d Full ms2 1186.2722@hcd30.00 [120.0000-4862.8467]
Precursor Scan: 059722, m/z: 1185.771, charge: 4
VYIHPFHLVIHNESTCEQLAK_5_4_0_2_0
Intensity
m/z
204.0864
138.0548
274.0917
366.1392
168.0655
186.076
292.1022
126.0549
144.0652
513.2806 b4(1+)
528.1919
657.2313
913.4539 PEP_0_1_0_0_0
1268.156 PEP_0_0_0_0_0
1369.6901 PEP_0_1_0_0_0
1471.2239 PEP_0_2_0_0_0
1552.2372 PEP_1_2_0_0_0
1633.2646 PEP_2_2_0_0_0
1714.2916 PEP_3_2_0_0_0

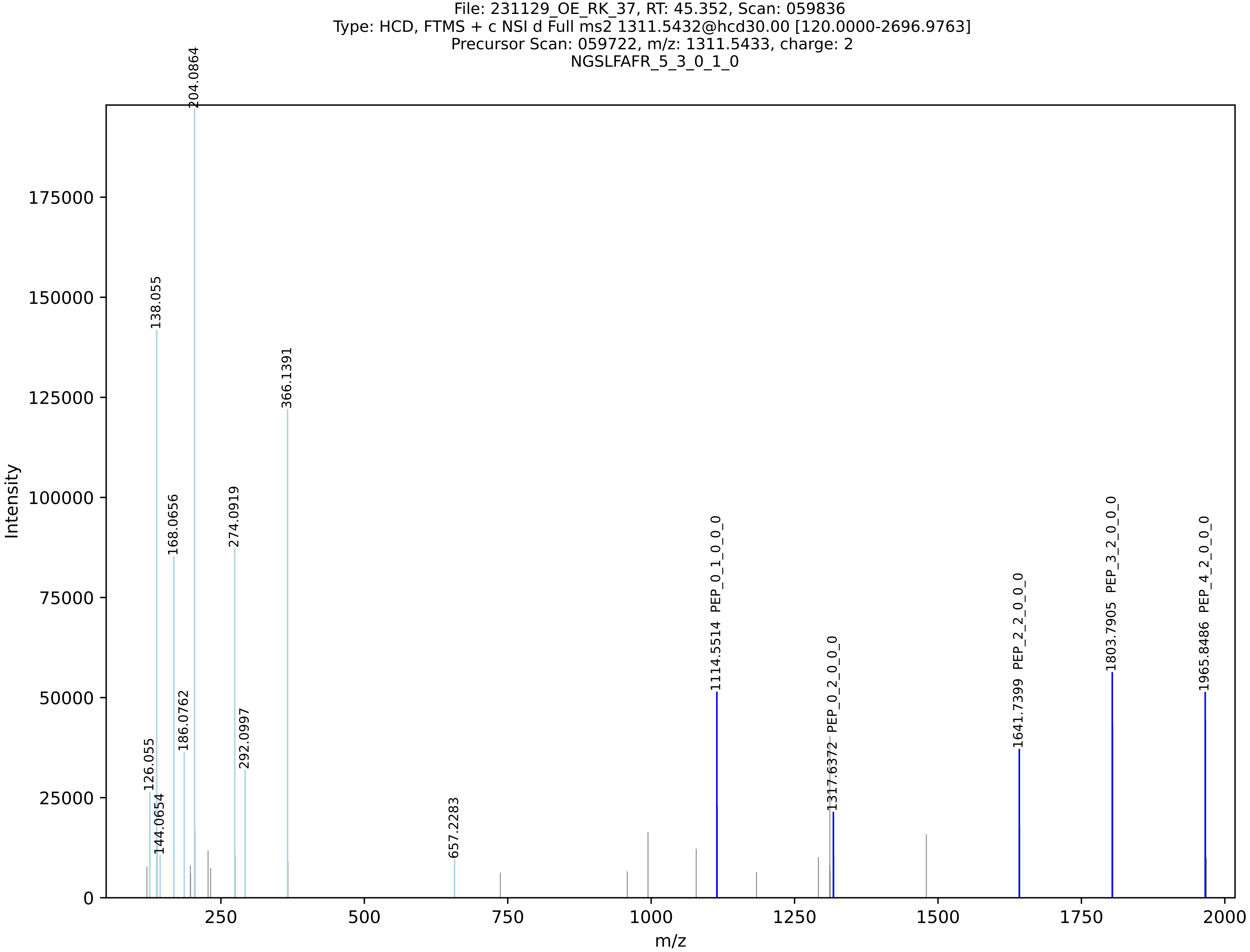
File: 231129_OE_RK_37, RT: 45.352, Scan: 059836
Type: HCD, FTMS + c NSI d Full ms2 1311.5432@hcd30.00 [120.0000-2696.9763]
Precursor Scan: 059722, m/z: 1311.5433, charge: 2
NGSLFAFR_5_3_0_1_0
204.0864
138.055
366.1391
168.0656
274.0919
186.0762
292.0997
126.055
144.0654
657.2283
1114.5514 PEP_0_1_0_0_0
1317.6372 PEP_0_2_0_0_0
1641.7399 PEP_2_2_0_0_0
1803.7905 PEP_3_2_0_0_0
1965.8486 PEP_4_2_0_0_0
Intensity
m/z
0
25000
50000
75000
100000
125000
150000
175000
250
500
750
1000
1250
1500
1750
2000

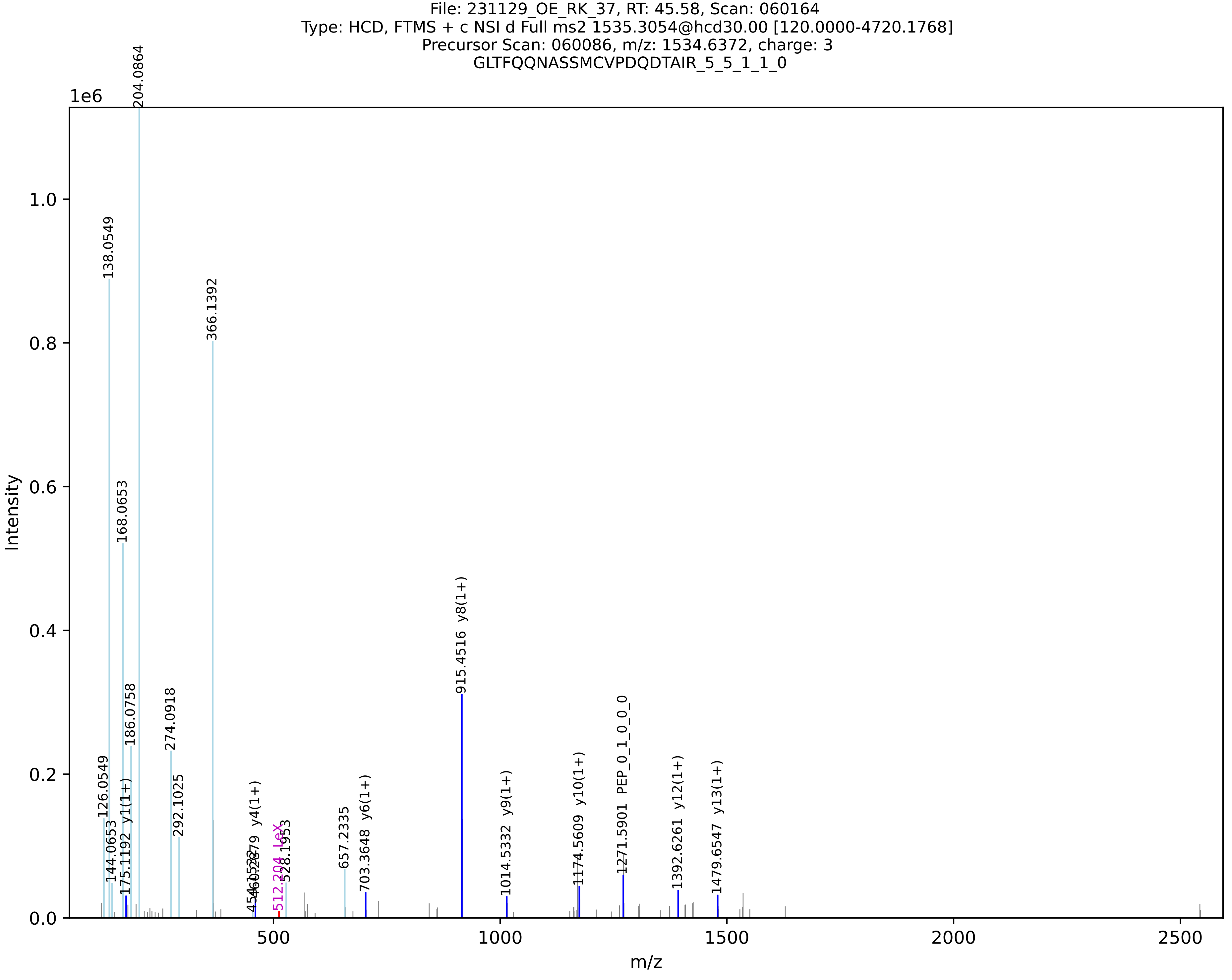

File: 231129_OE_RK_37, RT: 45.58, Scan: 060164
Type: HCD, FTMS + c NSI d Full ms2 1535.3054@hcd30.00 [120.0000-4720.1768]
Precursor Scan: 060086, m/z: 1534.6372, charge: 3
GLTFQQNASSMCVPDQDTAIR_5_5_1_1_0
1e6
Intensity
m/z
204.0864
138.0549
366.1392
168.0653
186.0758
274.0918
292.1025
126.0549
144.0653
175.1192 y1(1+)
y4(1+)
512.204 LeX
528.1953
657.2335
703.3648 y6(1+)
915.4516 y8(1+)
1014.5332 y9(1+)
1174.5609 y10(1+)
1271.5901 PEP_0_1_0_0_0
1392.6261 y12(1+)
1479.6547 y13(1+)

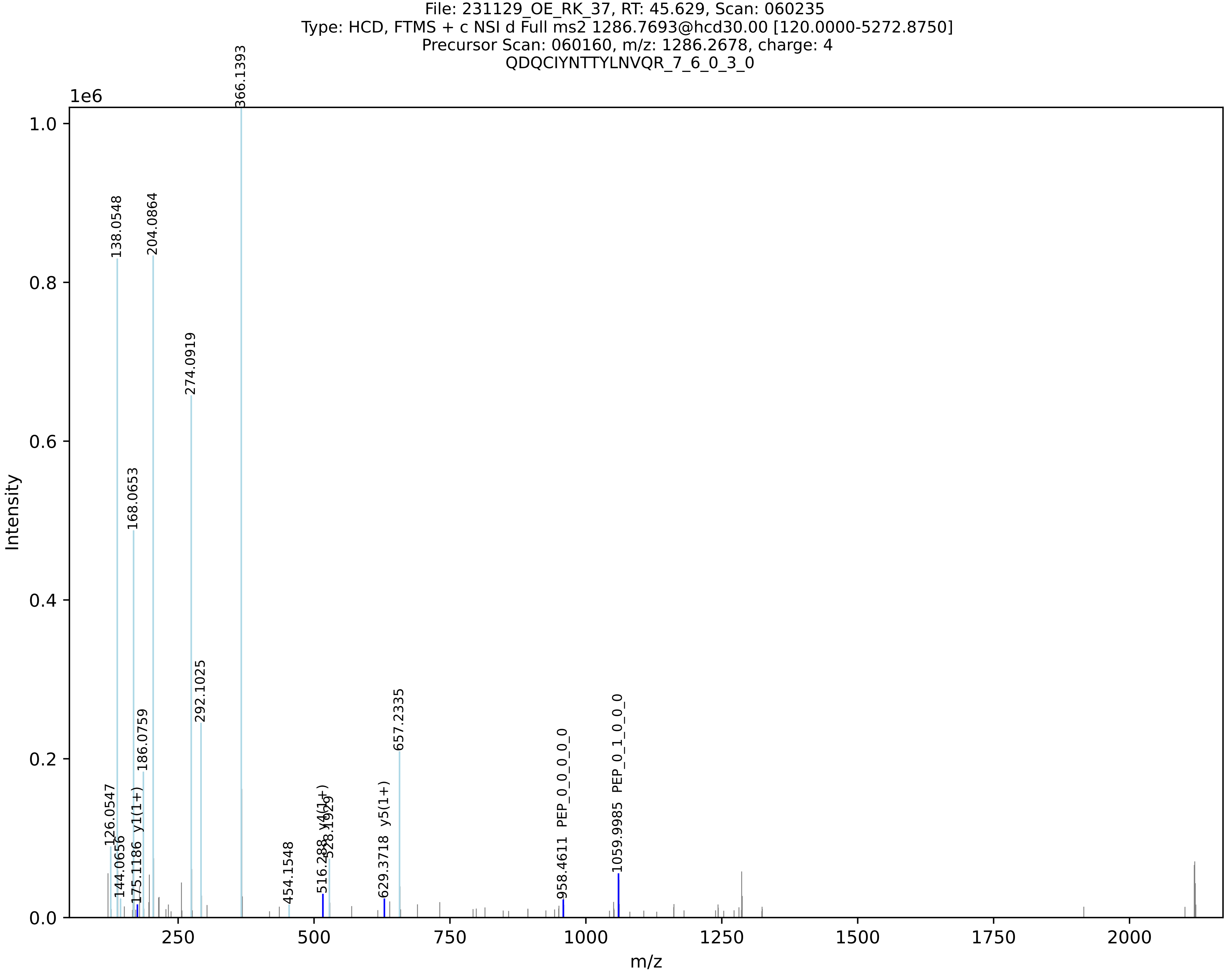

File: 231129_OE_RK_37, RT: 45.629, Scan: 060235
Type: HCD, FTMS + c NSI d Full ms2 1286.7693@hcd30.00 [120.0000-5272.8750]
Precursor Scan: 060160, m/z: 1286.2678, charge: 4
QDQCIYNTTYLNVQR_7_6_0_3_0
1e6
Intensity
m/z
366.1393
138.0548
204.0864
274.0919
168.0653
292.1025
186.0759
657.2335
126.0547
144.0656
175.1186 y1(1+)
454.1548
516.2888 y4(1+)
528.1929
629.3718 y5(1+)
958.4611 PEP_0_0_0_0_0
1059.9985 PEP_0_1_0_0_0

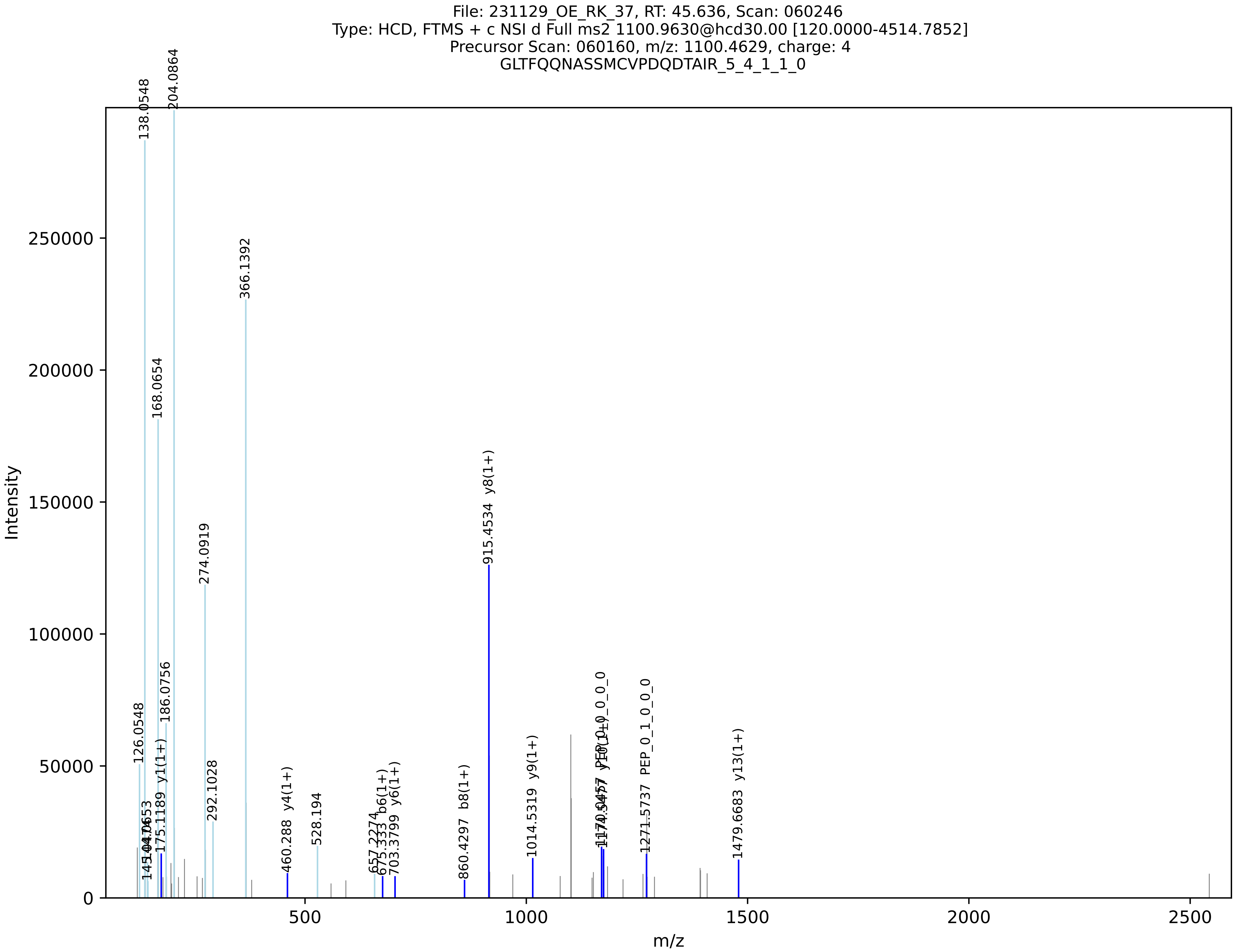
File: 231129_OE_RK_37, RT: 45.636, Scan: 060246
Type: HCD, FTMS + c NSI d Full ms2 1100.9630@hcd30.00 [120.0000-4514.7852]
Precursor Scan: 060160, m/z: 1100.4629, charge: 4
GLTFQQNASSMCVPDQDTAIR_5_4_1_1_0
Intensity
m/z
138.0548
204.0864
366.1392
168.0654
274.0919
126.0548
186.0756
175.1189 y1(1+)
292.1028
460.288 y4(1+)
528.194
657.2274
675.333 b6(1+)
703.3799 y6(1+)
860.4297 b8(1+)
915.4534 y8(1+)
1014.5319 y9(1+)
1271.5737 PEP_0_1_0_0_0
1479.6683 y13(1+)

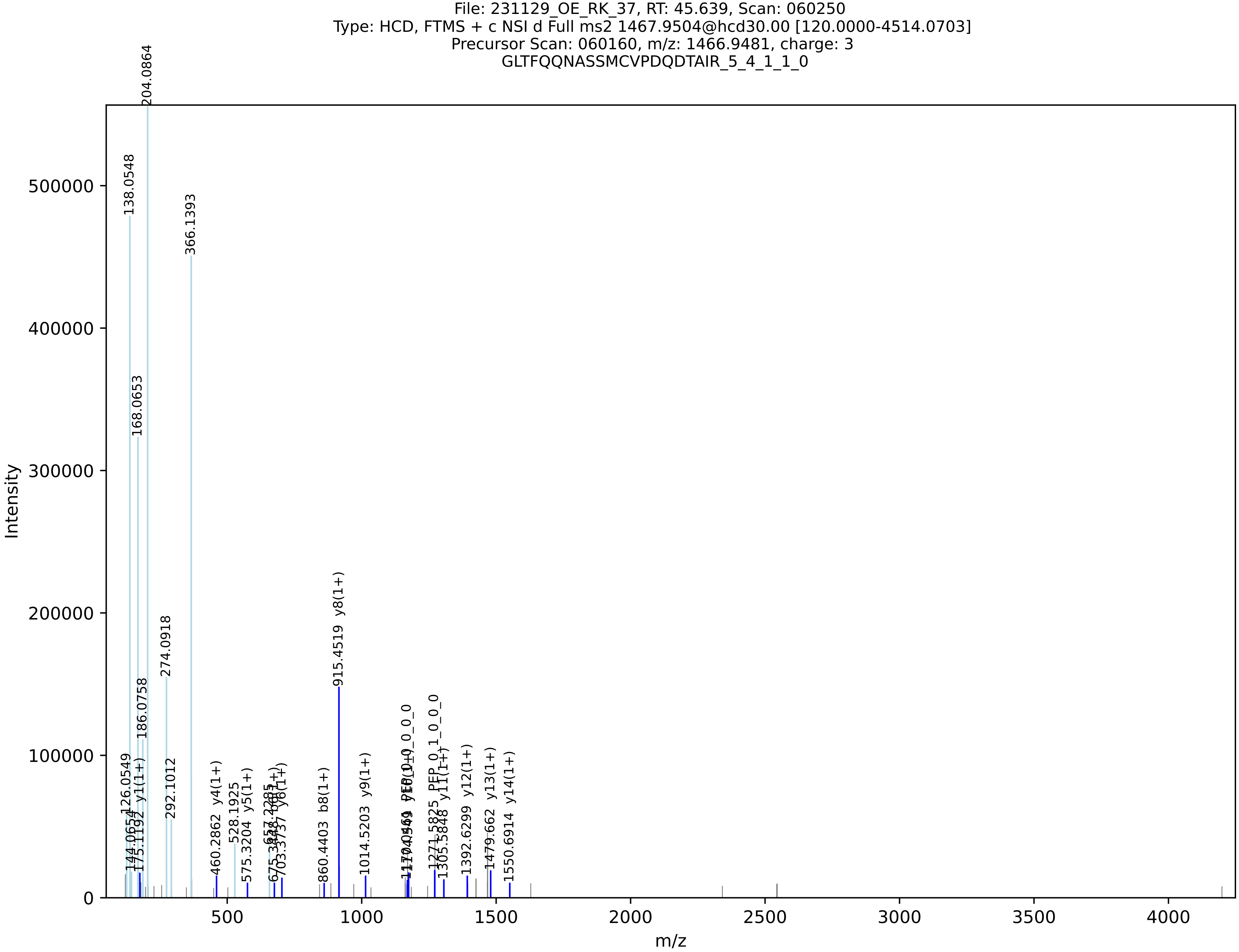
File: 231129_OE_RK_37, RT: 45.639, Scan: 060250
Type: HCD, FTMS + c NSI d Full ms2 1467.9504@hcd30.00 [120.0000-4514.0703]
Precursor Scan: 060160, m/z: 1466.9481, charge: 3
GLTFQQNASSMCVPDQDTAIR_5_4_1_1_0
Intensity
m/z
204.0864
138.0548
366.1393
168.0653
274.0918
186.0758
292.1012
126.0549
144.0654
175.1192 y1(1+)
460.2862 y4(1+)
528.1925
575.3204 y5(1+)
703.3737 y6(1+)
860.4403 b8(1+)
915.4519 y8(1+)
1014.5203 y9(1+)
1271.5825 PEP_0_1_0_0_0
1305.5848 y11(1+)
1392.6299 y12(1+)
1479.662 y13(1+)
1550.6914 y14(1+)

File: 231129_OE_RK_37, RT: 45.796, Scan: 060476
Type: HCD, FTMS + c NSI d Full ms2 1593.6416@hcd30.00 [120.0000-4898.6855]
Precursor Scan: 060377, m/z: 1592.9745, charge: 3
QDQCIYNTTYLNVQR_6_5_0_3_0

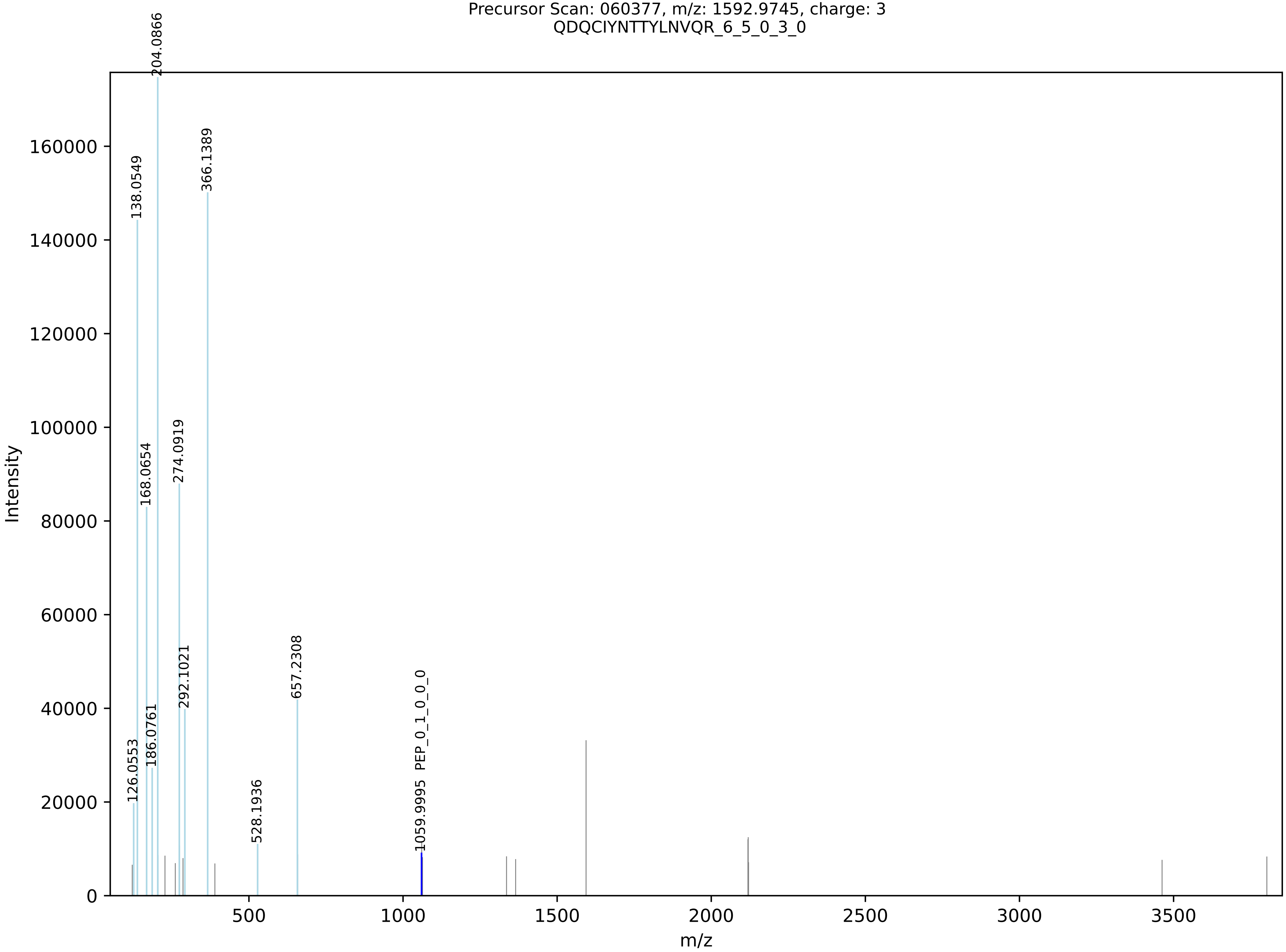

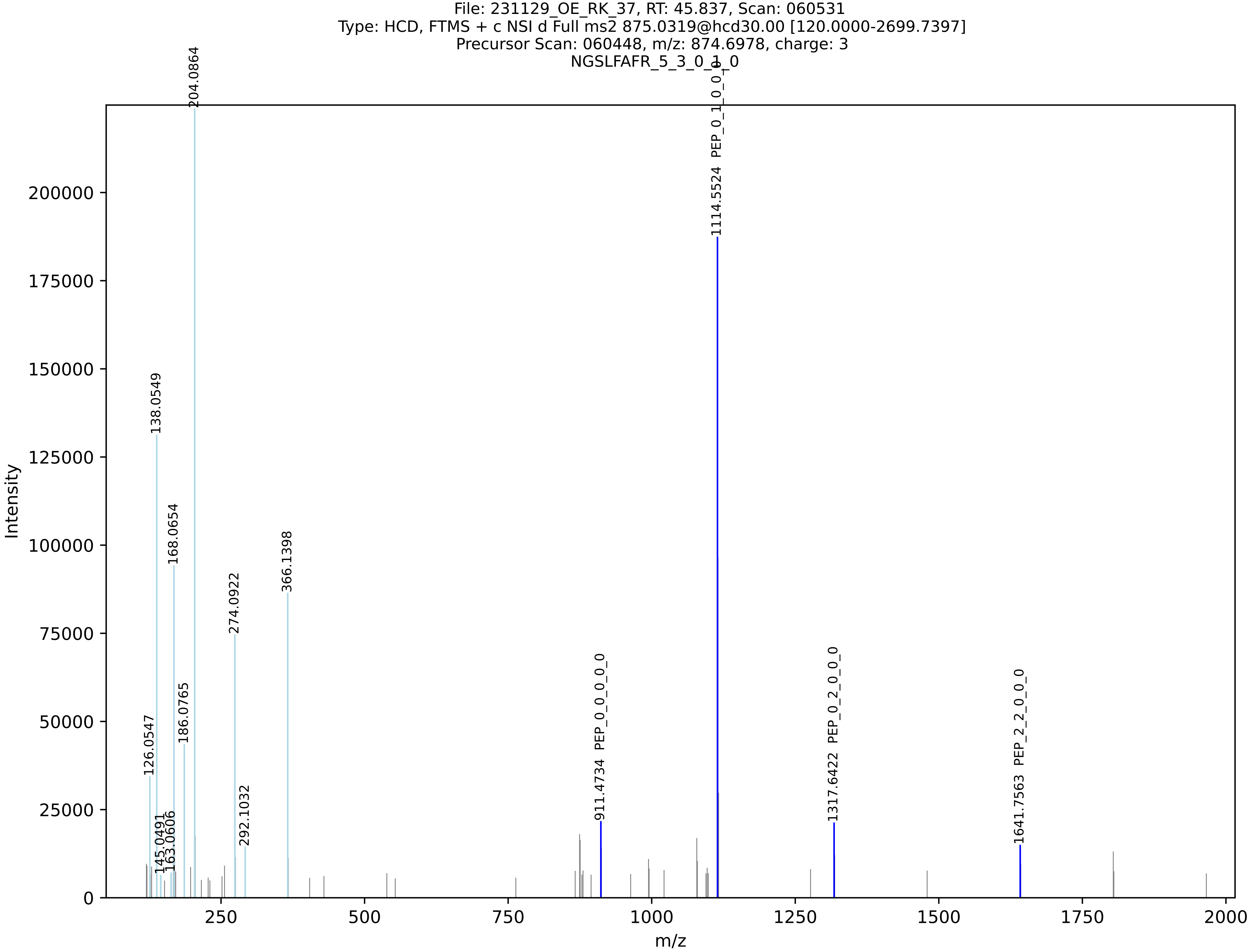
File: 231129_OE_RK_37, RT: 45.837, Scan: 060531
Type: HCD, FTMS + c NSI d Full ms2 875.0319@hcd30.00 [120.0000-2699.7397]
Precursor Scan: 060448, m/z: 874.6978, charge: 3
NGSLFAFR_5_3_0_1_0
204.0864
138.0549
168.0654
186.0765
126.0547
145.0491
163.0606
274.0922
292.1032
366.1398
911.4734 PEP_0_0_0_0_0
1114.5524 PEP_0_1_0_0_0
1317.6422 PEP_0_2_0_0_0
1641.7563 PEP_2_2_0_0_0
Intensity
m/z
0
25000
50000
75000
100000
125000
150000
175000
200000
250
500
750
1000
1250
1500
1750
2000

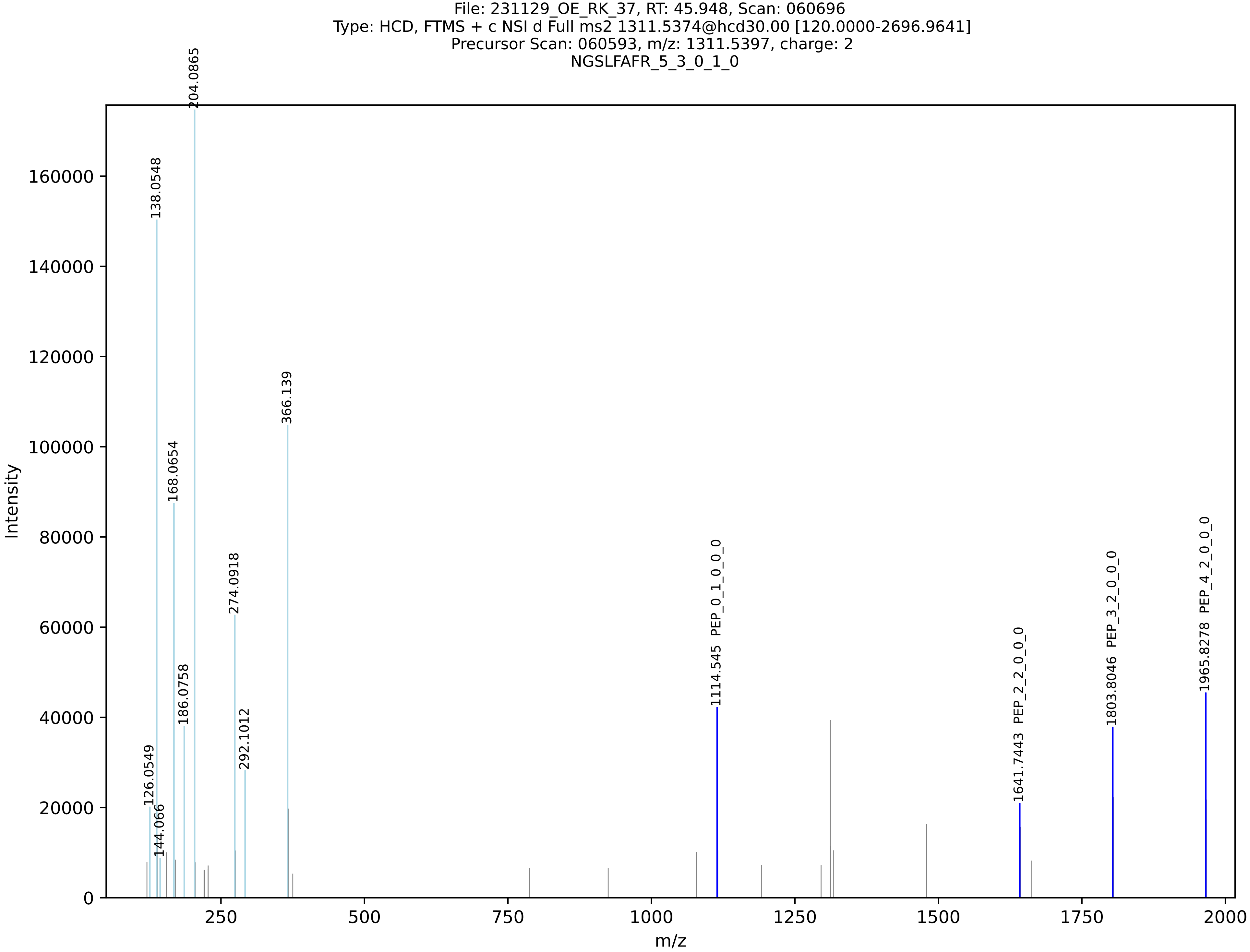
File: 231129_OE_RK_37, RT: 45.948, Scan: 060696
Type: HCD, FTMS + c NSI d Full ms2 1311.5374@hcd30.00 [120.0000-2696.9641]
Precursor Scan: 060593, m/z: 1311.5397, charge: 2
NGSLFAFR_5_3_0_1_0
204.0865
138.0548
168.0654
186.0758
126.0549
144.066
274.0918
292.1012
366.139
1114.545 PEP_0_1_0_0_0
1641.7443 PEP_2_2_0_0_0
1803.8046 PEP_3_2_0_0_0
1965.8278 PEP_4_2_0_0_0
Intensity
m/z

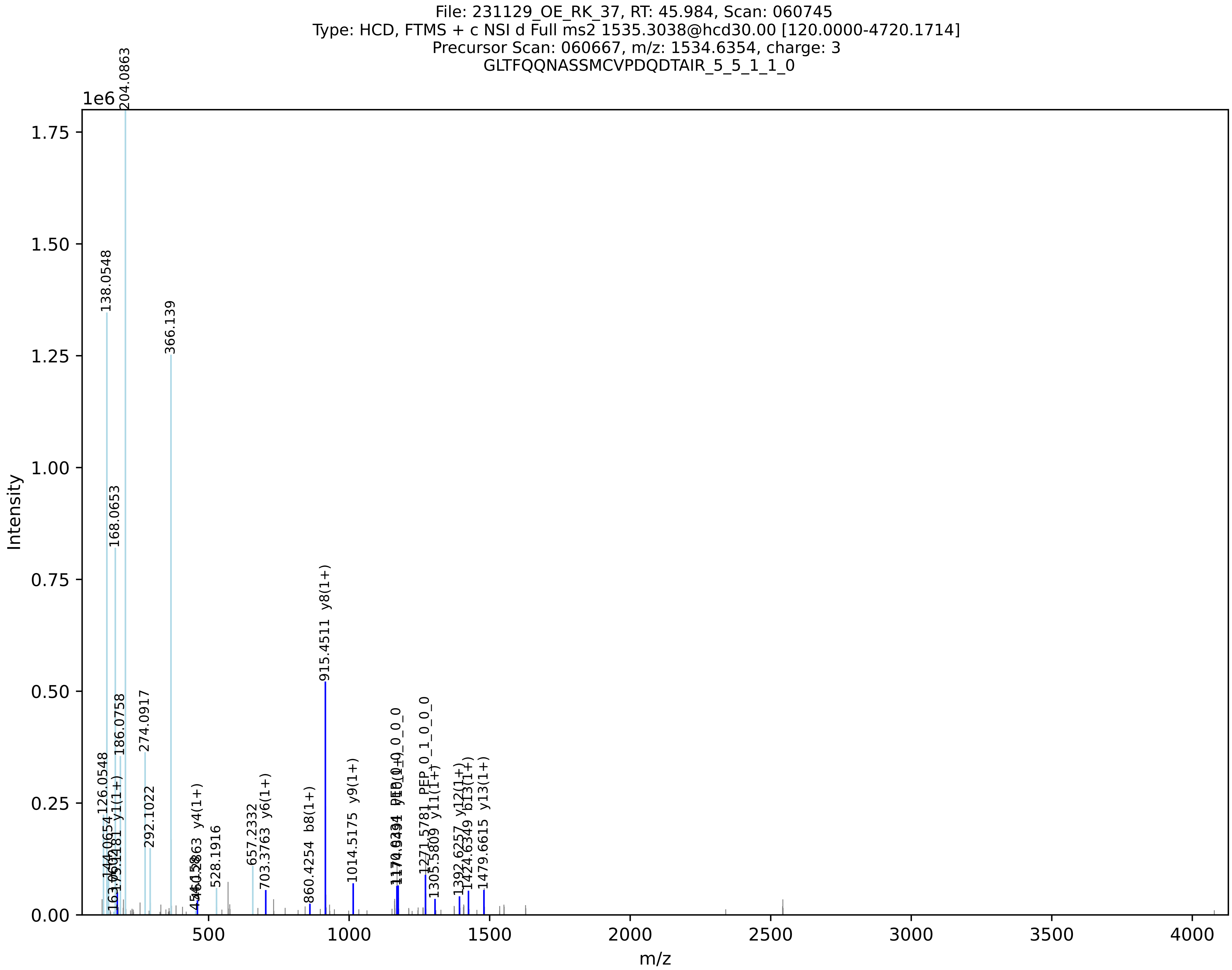
File: 231129_OE_RK_37, RT: 45.984, Scan: 060745
Type: HCD, FTMS + c NSI d Full ms2 1535.3038@hcd30.00 [120.0000-4720.1714]
Precursor Scan: 060667, m/z: 1534.6354, charge: 3
GLTFQQNASSMCVPDQDTAIR_5_5_1_1_0
1e6
Intensity
m/z
204.0863
138.0548
366.139
168.0653
915.4511 y8(1+)
186.0758
274.0917
126.0548
y1(1+)
292.1022
y4(1+)
528.1916
657.2332
703.3763 y6(1+)
860.4254 b8(1+)
1014.5175 y9(1+)
1271.5781 PEP_0_1_0_0_0
1305.5809 y11(1+)
1392.6257 y12(1+)
1424.6349 b13(1+)
1479.6615 y13(1+)

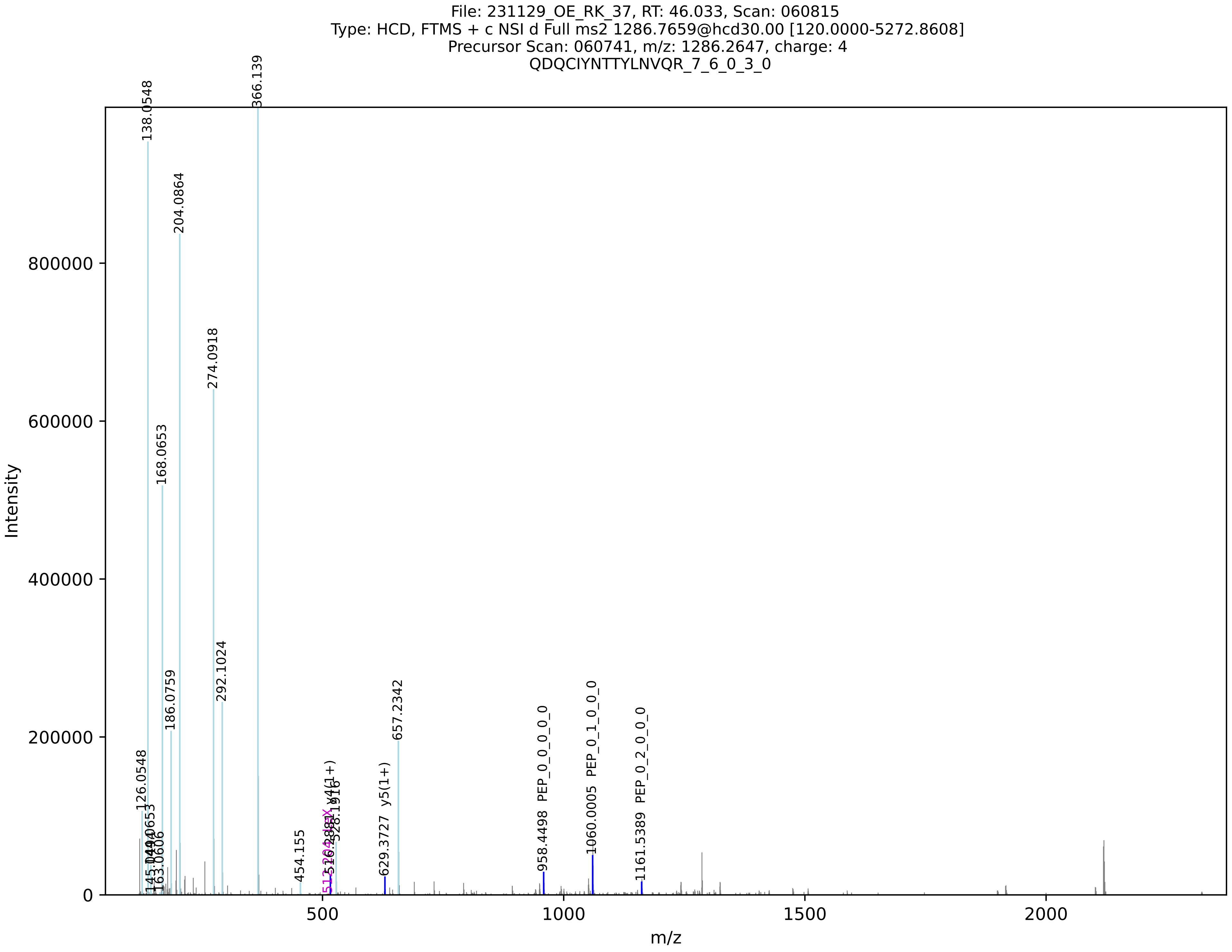
File: 231129_OE_RK_37, RT: 46.033, Scan: 060815
Type: HCD, FTMS + c NSI d Full ms2 1286.7659@hcd30.00 [120.0000-5272.8608]
Precursor Scan: 060741, m/z: 1286.2647, charge: 4
QDQCIYNTTYLNVQR_7_6_0_3_0
Intensity
m/z
138.0548
366.139
204.0864
274.0918
168.0653
186.0759
292.1024
126.0548
163.0606
657.2342
454.155
y4(1+)
528.1916
629.3727 y5(1+)
958.4498 PEP_0_0_0_0_0
1060.0005 PEP_0_1_0_0_0
1161.5389 PEP_0_2_0_0_0

File: 231129_OE_RK_37, RT: 46.035, Scan: 060819
Type: HCD, FTMS + c NSI d Full ms2 1101.2111@hcd30.00 [120.0000-4515.7969]
Precursor Scan: 060741, m/z: 1100.4594, charge: 4
GLTFQQNASSMCVPDQDTAIR_5_4_1_1_0

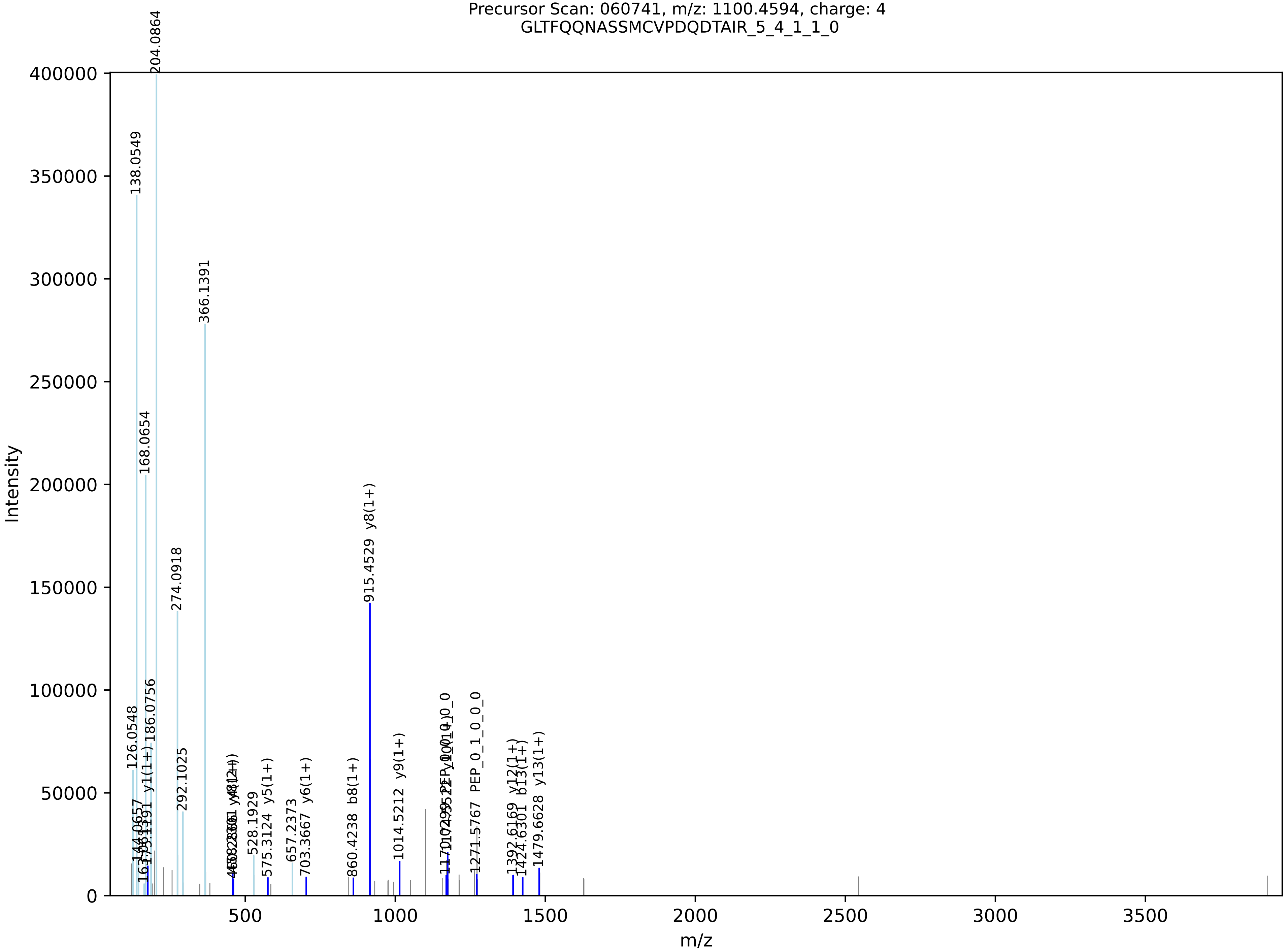

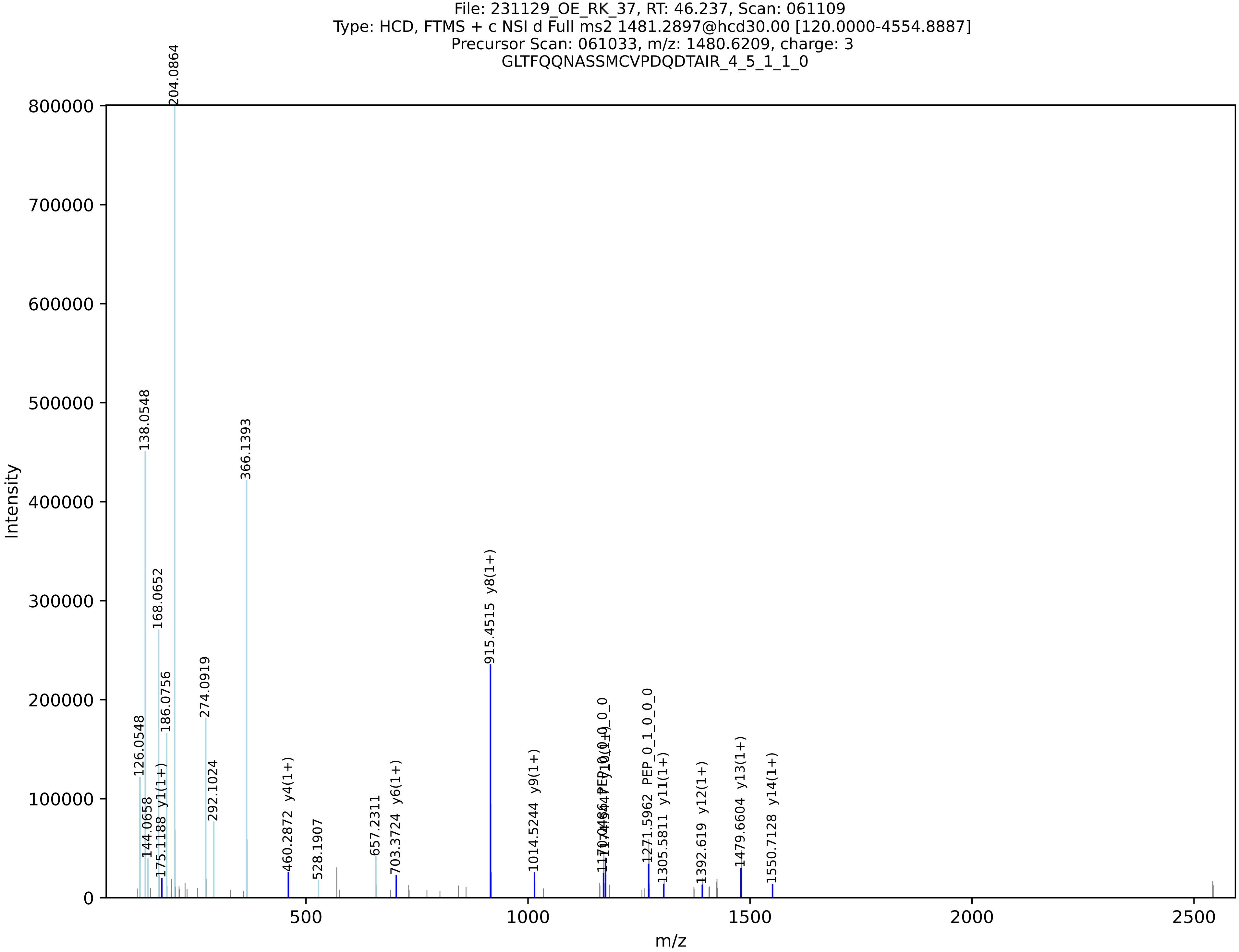

File: 231129_OE_RK_37, RT: 46.237, Scan: 061109
Type: HCD, FTMS + c NSI d Full ms2 1481.2897@hcd30.00 [120.0000-4554.8887]
Precursor Scan: 061033, m/z: 1480.6209, charge: 3
GLTFQQNASSMCVPDQDTAIR_4_5_1_1_0
Intensity
m/z
204.0864
138.0548
366.1393
168.0652
274.0919
186.0756
126.0548
292.1024
144.0658
175.1188 y1(1+)
460.2872 y4(1+)
528.1907
657.2311
703.3724 y6(1+)
915.4515 y8(1+)
1014.5244 y9(1+)
1271.5962 PEP_0_1_0_0_0
1305.5811 y11(1+)
1392.619 y12(1+)
1479.6604 y13(1+)
1550.7128 y14(1+)

File: 231129_OE_RK_37, RT: 46.238, Scan: 061110
Type: HCD, FTMS + c NSI d Full ms2 1111.2184@hcd30.00 [120.0000-4556.6270]
Precursor Scan: 061033, m/z: 1110.7175, charge: 4
GLTFQQNASSMCVPDQDTAIR_4_5_1_1_0

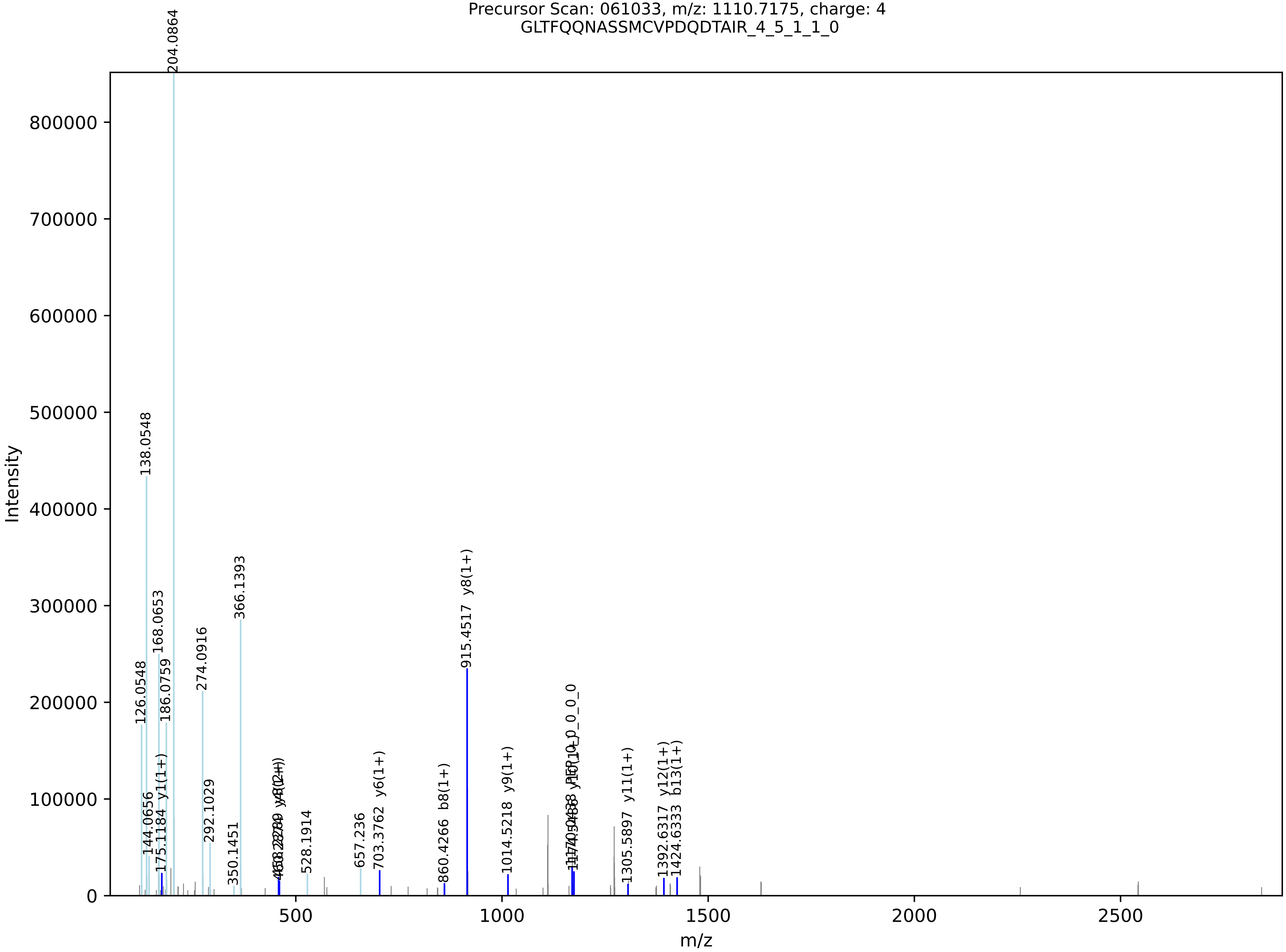

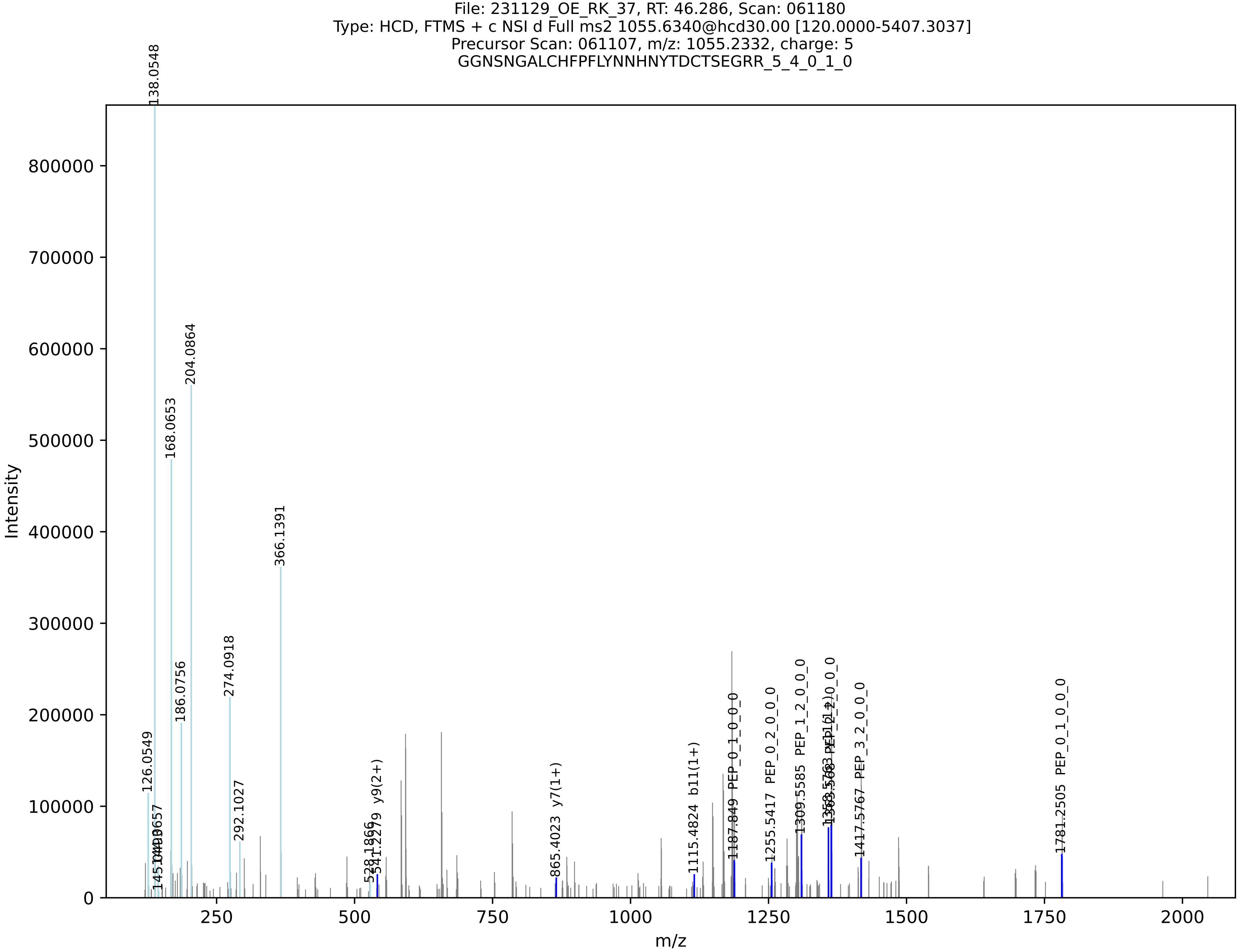
File: 231129_OE_RK_37, RT: 46.286, Scan: 061180
Type: HCD, FTMS + c NSI d Full ms2 1055.6340@hcd30.00 [120.0000-5407.3037]
Precursor Scan: 061107, m/z: 1055.2332, charge: 5
GGNSNGALCHFPFLYNNHNYTDCTSEGRR_5_4_0_1_0
Intensity
m/z
0
100000
200000
300000
400000
500000
600000
700000
800000
250
500
750
1000
1250
1500
1750
2000
138.0548
204.0864
168.0653
366.1391
186.0756
274.0918
126.0549
292.1027
y9(2+)
y7(1+)
865.4023
b11(1+)
1115.4824
PEP_0_1_0_0_0
1187.849
PEP_0_2_0_0_0
1255.5417
PEP_1_2_0_0_0
1309.5585
PEP_3_2_0_0_0
1417.5767
PEP_0_1_0_0_0
1781.2505

File: 231129_OE_RK_37, RT: 46.489, Scan: 061476
Type: HCD, FTMS + c NSI d Full ms2 1359.7938@hcd30.00 [120.0000-5570.8149]
Precursor Scan: 061398, m/z: 1359.0421, charge: 4
QDQCIYNTTYLNVQR_7_6_0_4_0

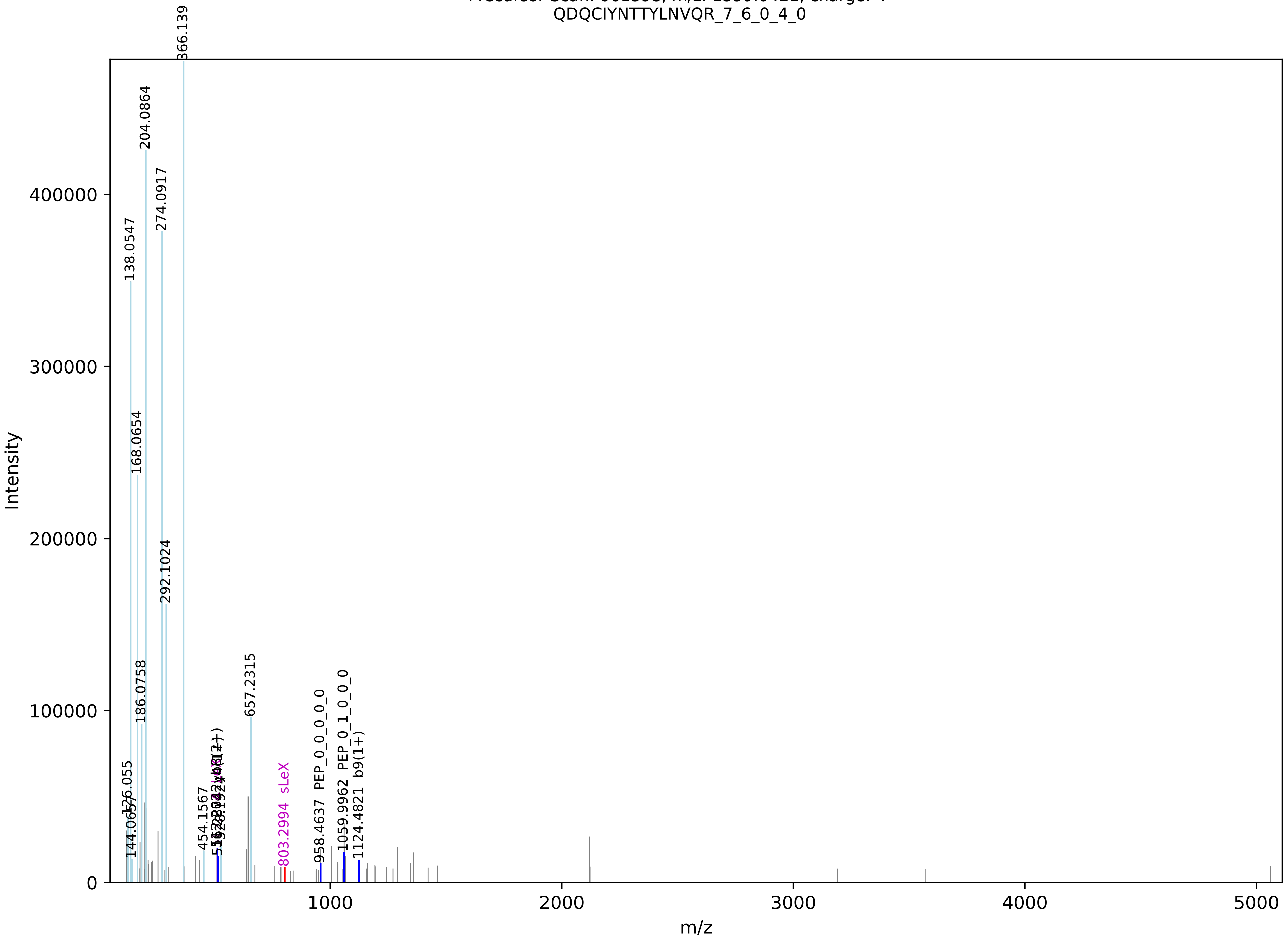

File: 231129_OE_RK_37, RT: 46.588, Scan: 061617
Type: HCD, FTMS + c NSI d Full ms2 1323.2813@hcd30.00 [120.0000-5421.8433]
Precursor Scan: 061545, m/z: 1322.7802, charge: 4
QDQCIYNTTYLNVQR_7_6_1_3_0

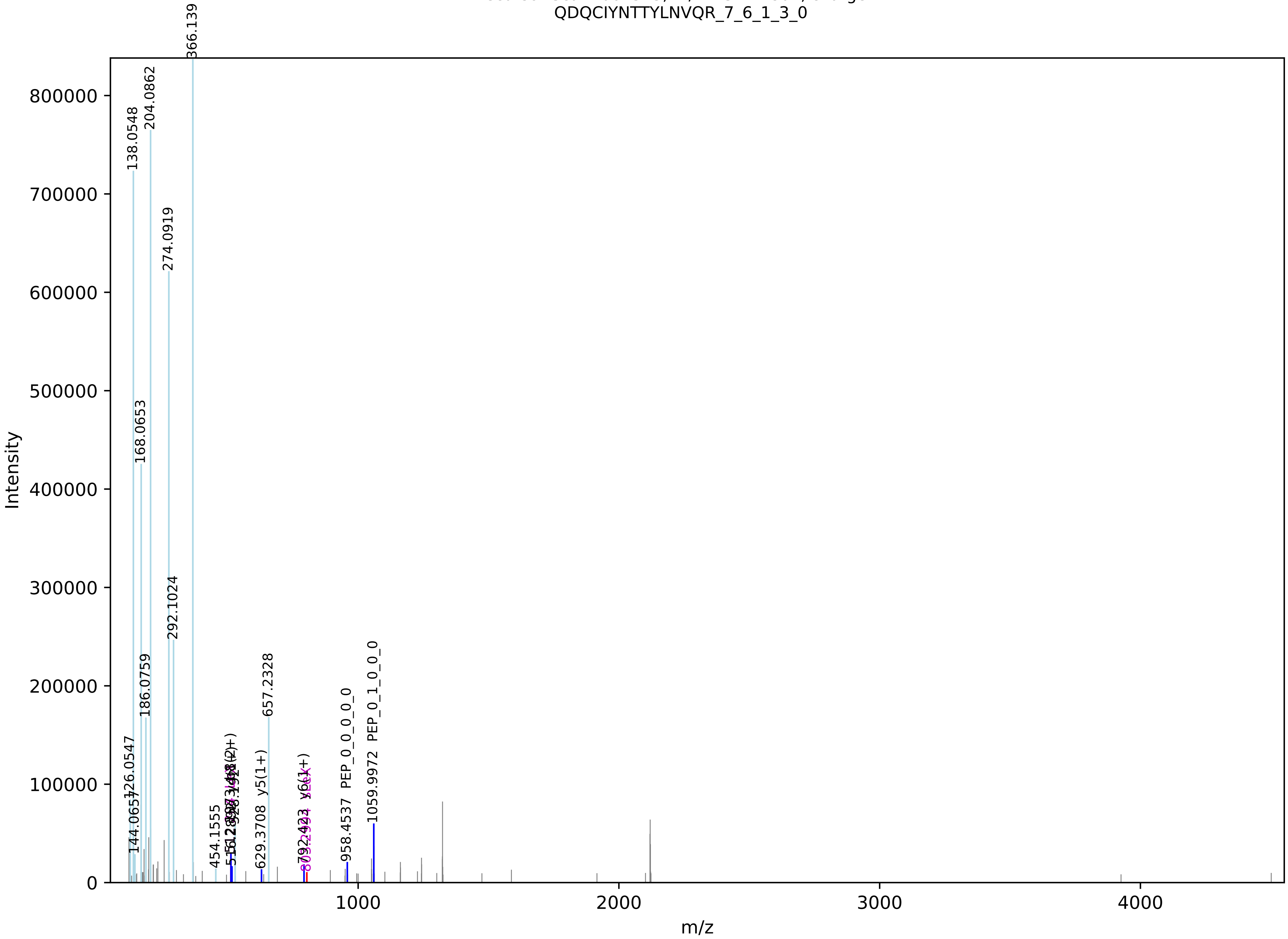

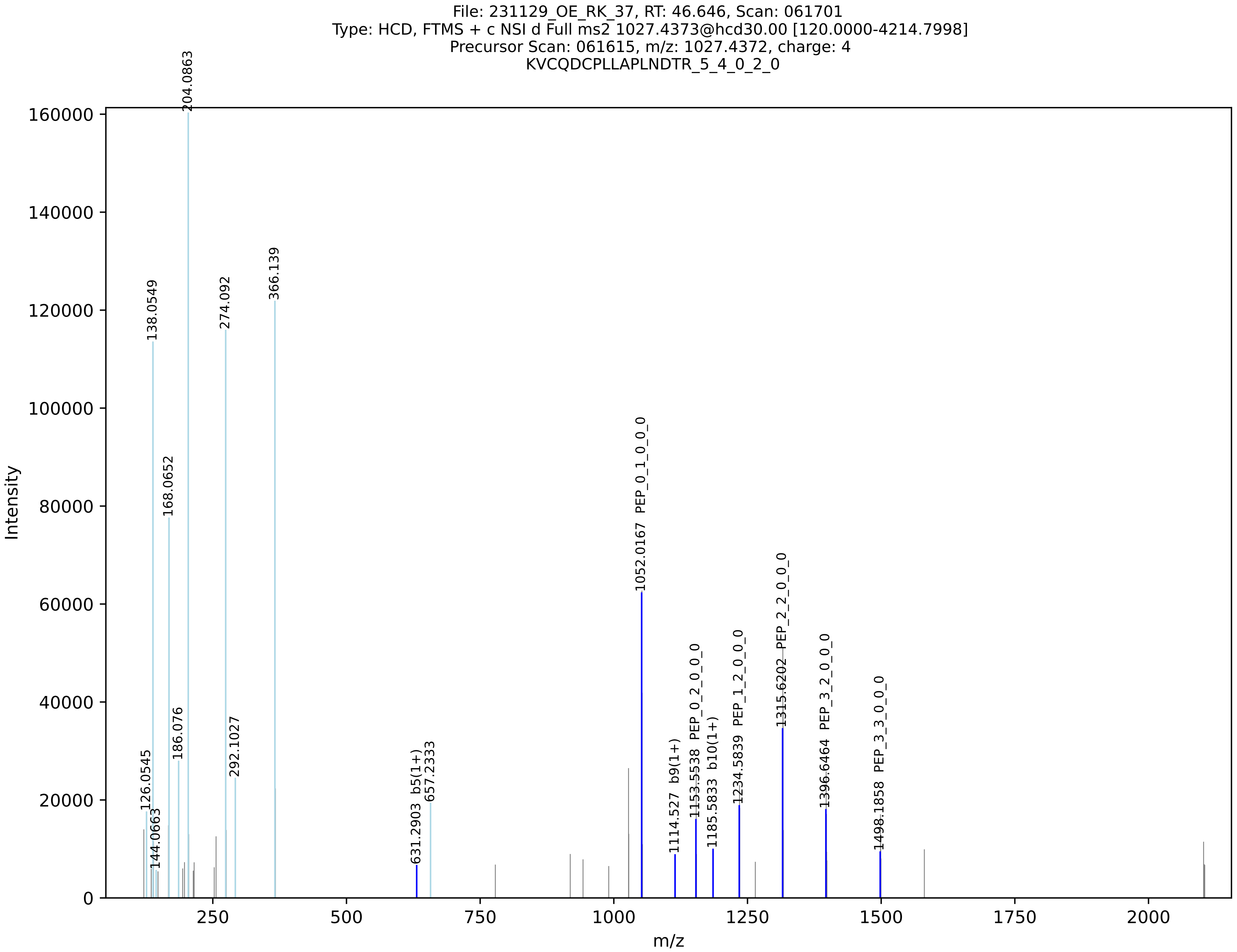
File: 231129_OE_RK_37, RT: 46.646, Scan: 061701
Type: HCD, FTMS + c NSI d Full ms2 1027.4373@hcd30.00 [120.0000-4214.7998]
Precursor Scan: 061615, m/z: 1027.4372, charge: 4
KVCQDCPLLAPLNDTR_5_4_0_2_0
Intensity
m/z
204.0863
138.0549
274.092
366.139
168.0652
186.076
292.1027
126.0545
144.0663
631.2903 b5(1+)
657.2333
1052.0167 PEP_0_1_0_0_0
1114.527 b9(1+)
1153.5538 PEP_0_2_0_0_0
1185.5833 b10(1+)
1234.5839 PEP_1_2_0_0_0
1315.6202 PEP_2_2_0_0_0
1396.6464 PEP_3_2_0_0_0
1498.1858 PEP_3_3_0_0_0

File: 231129_OE_RK_37, RT: 46.841, Scan: 061982
Type: HCD, FTMS + c NSI d Full ms2 1403.6429@hcd30.00 [120.0000-4317.2896]
Precursor Scan: 061908, m/z: 1403.3128, charge: 3
IIVPLNNRENISDPTSPLR_5_4_1_1_0

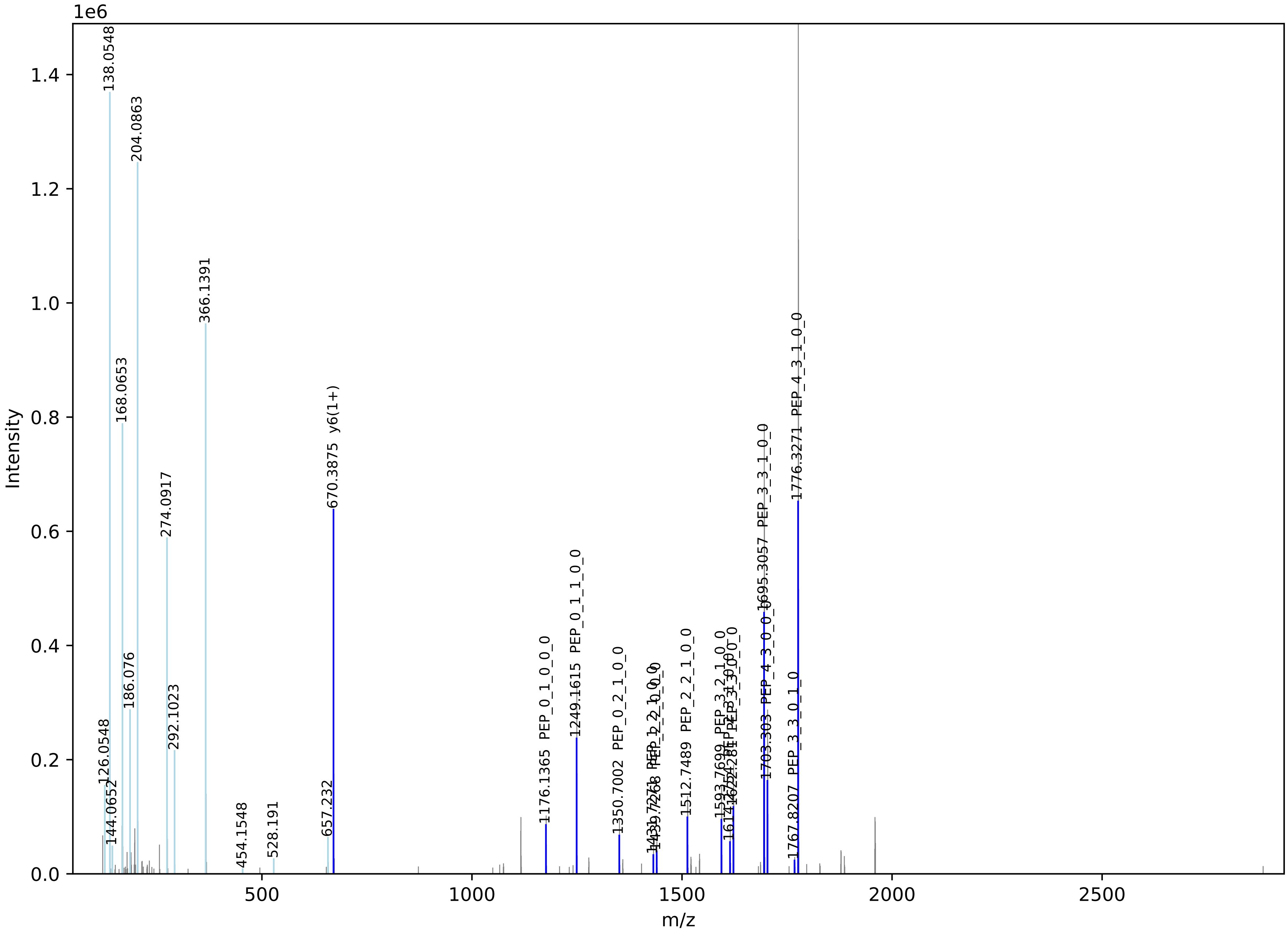

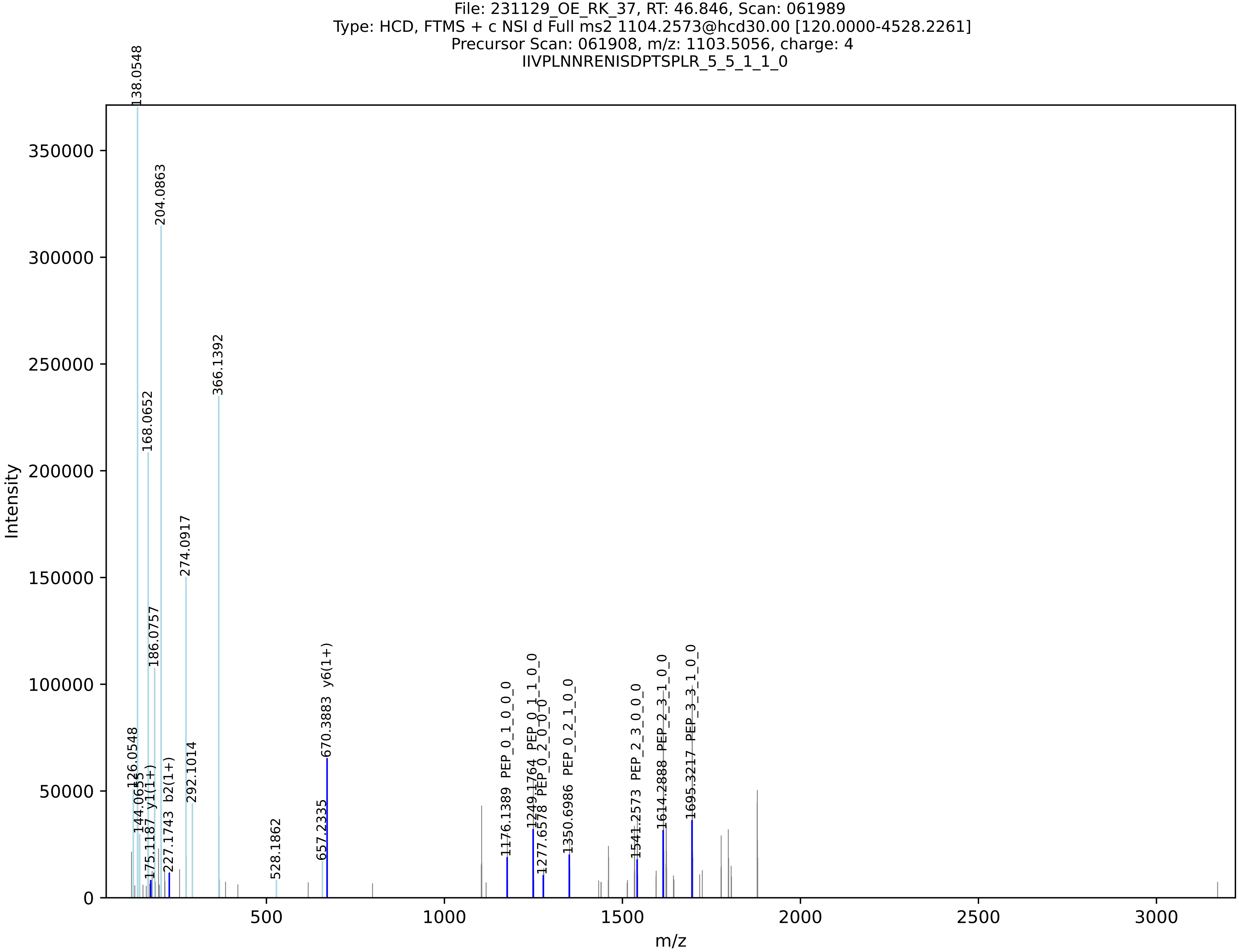

File: 231129_OE_RK_37, RT: 46.846, Scan: 061989
Type: HCD, FTMS + c NSI d Full ms2 1104.2573@hcd30.00 [120.0000-4528.2261]
Precursor Scan: 061908, m/z: 1103.5056, charge: 4
IIVPLNNRENISDPTSPLR_5_5_1_1_0
Intensity
m/z
138.0548
204.0863
366.1392
168.0652
274.0917
186.0757
670.3883 y6(1+)
126.0548
144.0655
175.1187 y1(1+)
227.1743 b2(1+)
292.1014
528.1862
657.2335
1176.1389 PEP_0_1_0_0_0_
1249.1764 PEP_0_1_1_0_0_
1277.6578 PEP_0_2_0_0_0_
1350.6986 PEP_0_2_1_0_0_
1541.2573 PEP_2_3_0_0_0_
1614.2888 PEP_2_3_1_0_0_
1695.3217 PEP_3_3_1_0_0_

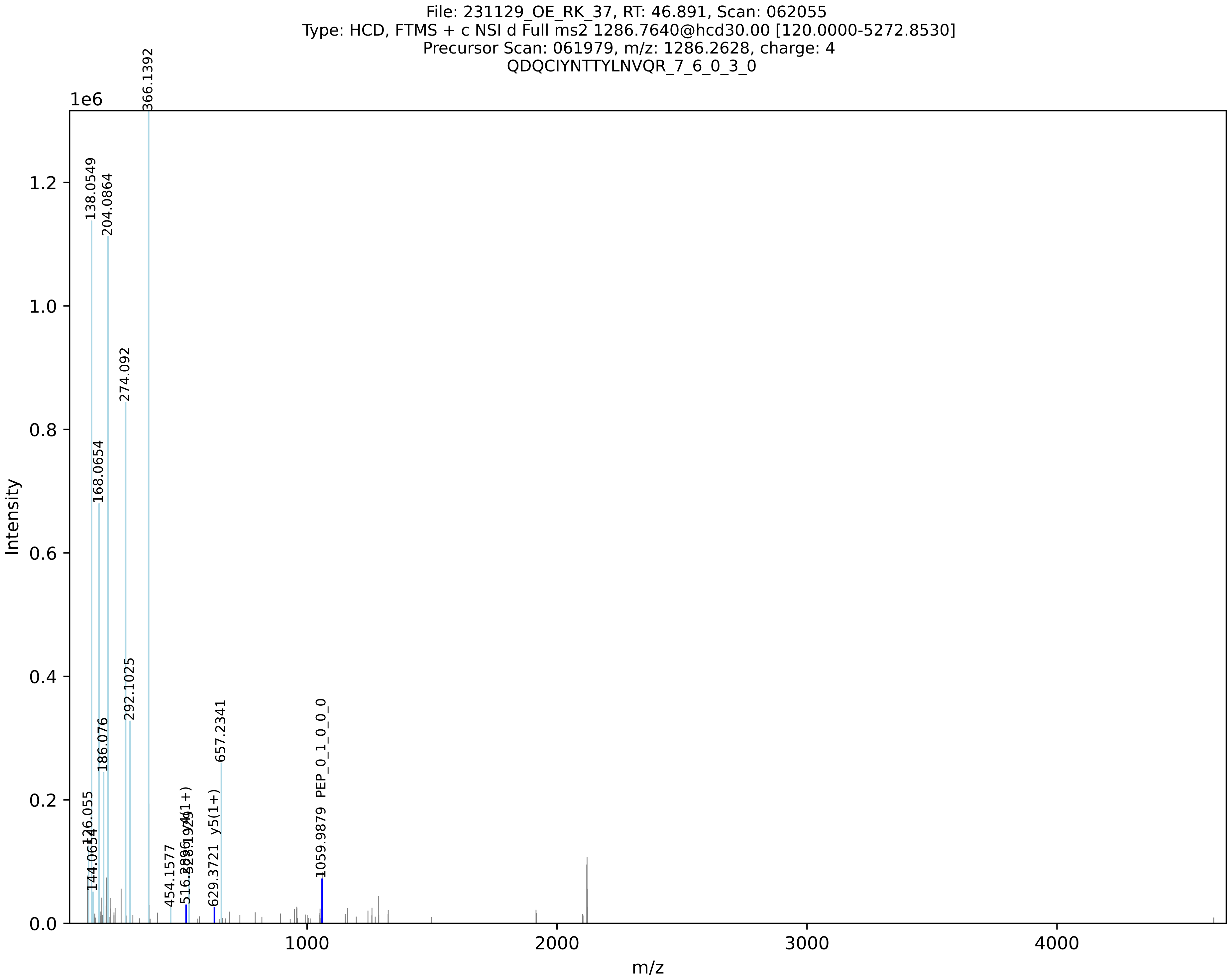

File: 231129_OE_RK_37, RT: 46.891, Scan: 062055
Type: HCD, FTMS + c NSI d Full ms2 1286.7640@hcd30.00 [120.0000-5272.8530]
Precursor Scan: 061979, m/z: 1286.2628, charge: 4
QDQCIYNTTYLNVQR_7_6_0_3_0
1e6
366.1392
138.0549
204.0864
274.092
168.0654
292.1025
186.076
657.2341
126.055
144.0654
454.1577
629.3721 y5(1+)
1059.9879 PEP_0_1_0_0_0
Intensity
m/z

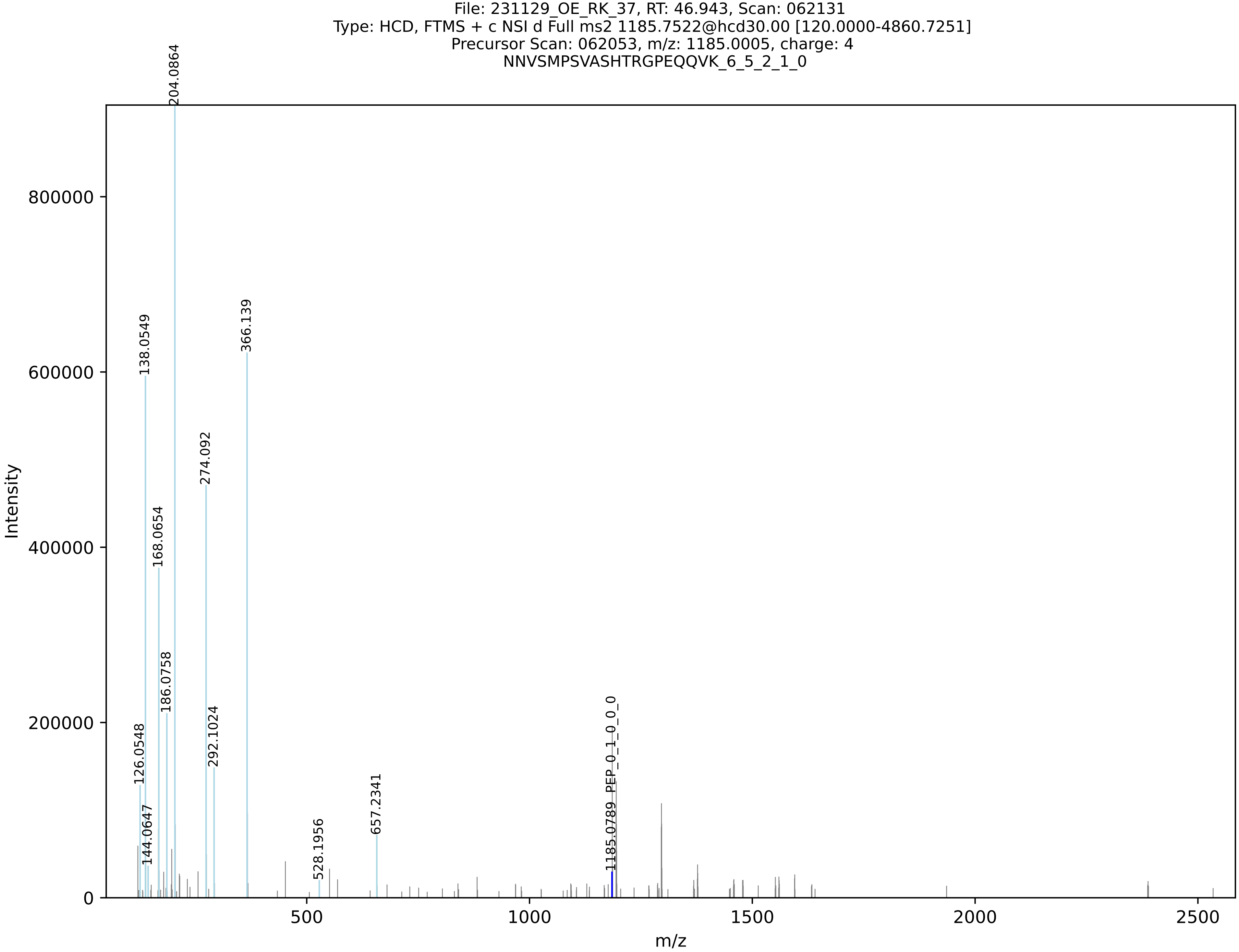

File: 231129_OE_RK_37, RT: 46.943, Scan: 062131
Type: HCD, FTMS + c NSI d Full ms2 1185.7522@hcd30.00 [120.0000-4860.7251]
Precursor Scan: 062053, m/z: 1185.0005, charge: 4
NNVSMPSVASHTRGPEQQVK_6_5_2_1_0
204.0864
138.0549
366.139
274.092
168.0654
186.0758
292.1024
126.0548
144.0647
528.1956
657.2341
1185.0789_PEP_0_1_0_0_0_
Intensity
m/z
800000
600000
400000
200000
0
500
1000
1500
2000
2500

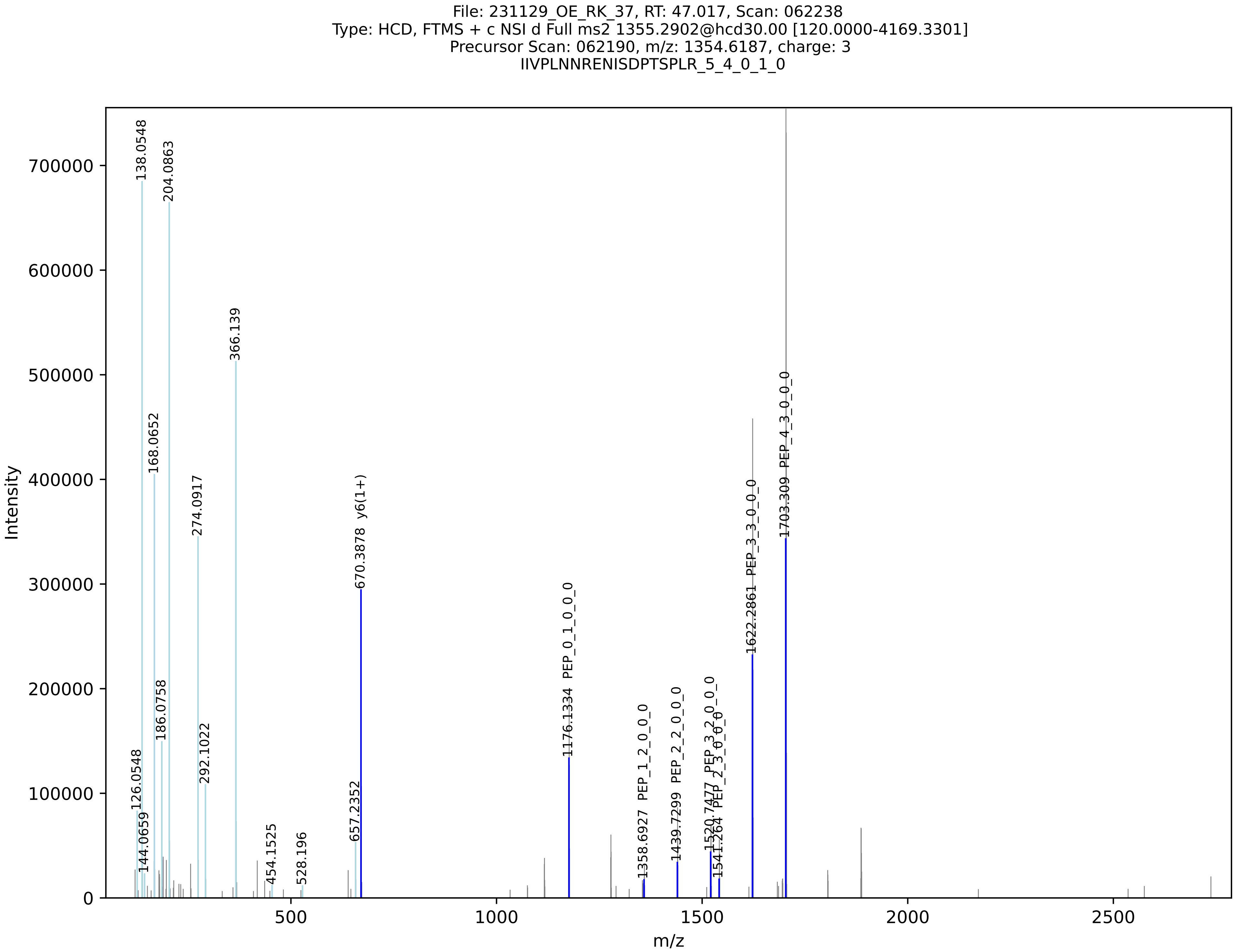

File: 231129_OE_RK_37, RT: 47.017, Scan: 062238
Type: HCD, FTMS + c NSI d Full ms2 1355.2902@hcd30.00 [120.0000-4169.3301]
Precursor Scan: 062190, m/z: 1354.6187, charge: 3
IIVPLNNRENISDPTSPLR_5_4_0_1_0
Intensity
m/z
138.0548
204.0863
168.0652
366.139
274.0917
186.0758
292.1022
126.0548
144.0659
454.1525
528.196
657.2352
670.3878 y6(1+)
1176.1334 PEP_0_1_0_0_0
1358.6927 PEP_1_2_0_0_0
1439.7299 PEP_2_2_0_0_0
1520.7477 PEP_3_2_0_0_0
1541.264 PEP_2_3_0_0_0
1622.2861 PEP_3_3_0_0_0
1703.309 PEP_4_3_0_0_0

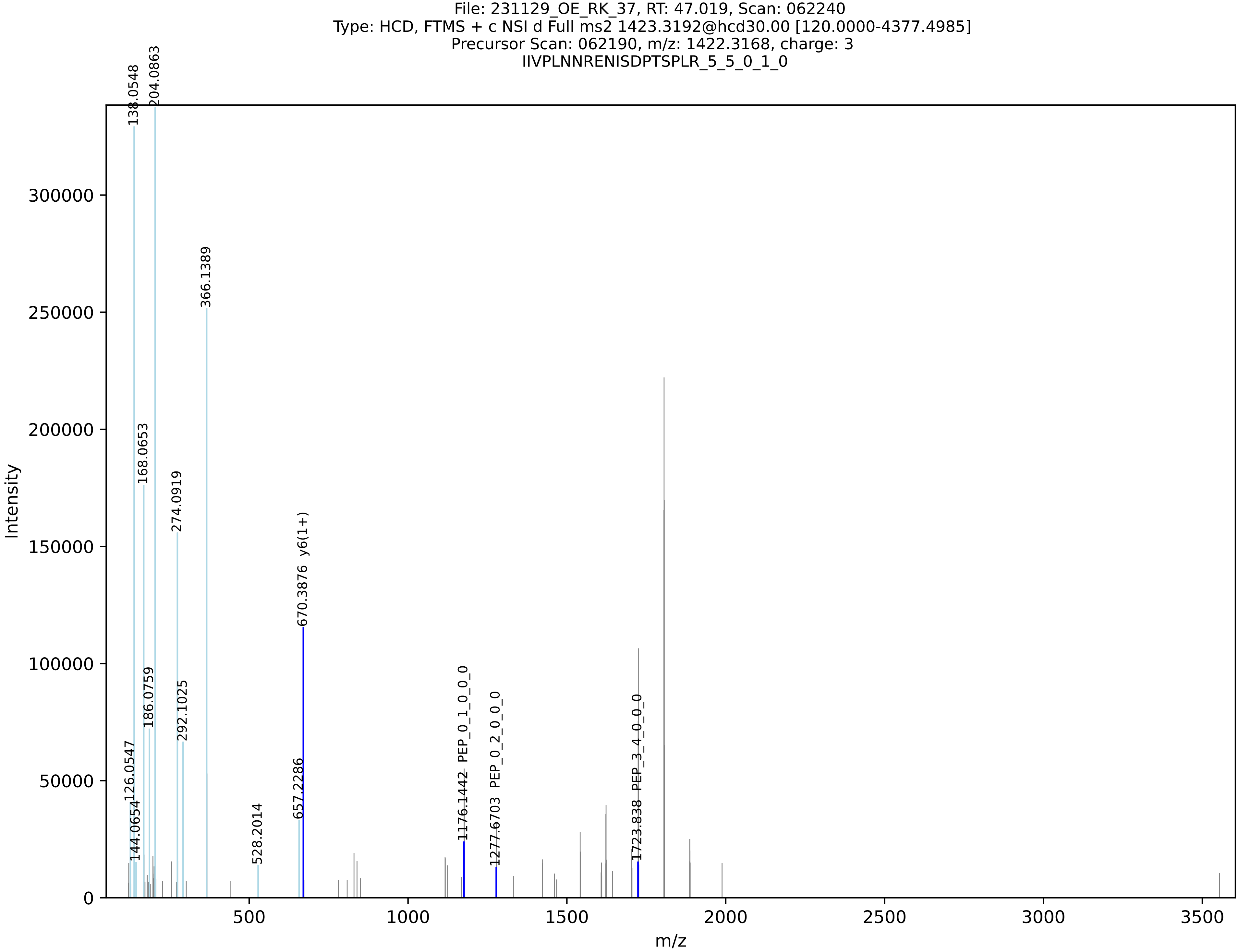
File: 231129_OE_RK_37, RT: 47.019, Scan: 062240
Type: HCD, FTMS + c NSI d Full ms2 1423.3192@hcd30.00 [120.0000-4377.4985]
Precursor Scan: 062190, m/z: 1422.3168, charge: 3
IIVPLNNRENISDPTSPLR_5_5_0_1_0
Intensity
m/z
138.0548
204.0863
366.1389
168.0653
274.0919
670.3876 y6(1+)
186.0759
292.1025
126.0547
144.0654
528.2014
657.2286
1176.1442 PEP_0_1_0_0_0
1277.6703 PEP_0_2_0_0_0
1723.838 PEP_3_4_0_0_0

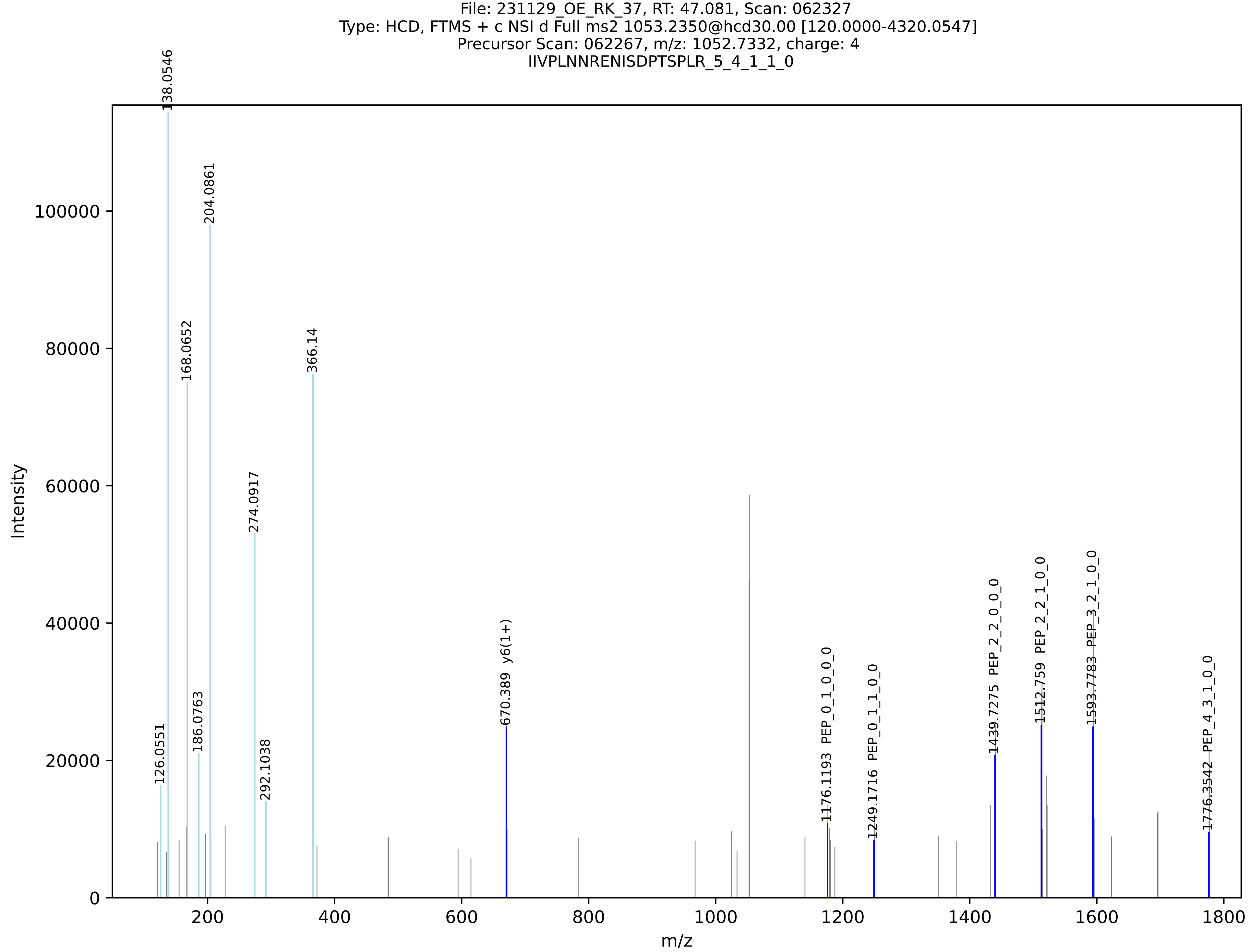
File: 231129_OE_RK_37, RT: 47.081, Scan: 062327
Type: HCD, FTMS + c NSI d Full ms2 1053.2350@hcd30.00 [120.0000-4320.0547]
Precursor Scan: 062267, m/z: 1052.7332, charge: 4
IIVPLNNRENISDPTSPLR_5_4_1_1_0
Intensity
m/z
138.0546
204.0861
168.0652
366.14
274.0917
126.0551
186.0763
292.1038
670.389 y6(1+)
1176.1193 PEP_0_1_0_0_0
1249.1716 PEP_0_1_1_0_0
1439.7275 PEP_2_2_0_0_0
1512.759 PEP_2_2_1_0_0
1593.7783 PEP_3_2_1_0_0
1776.3542 PEP_4_3_1_0_0

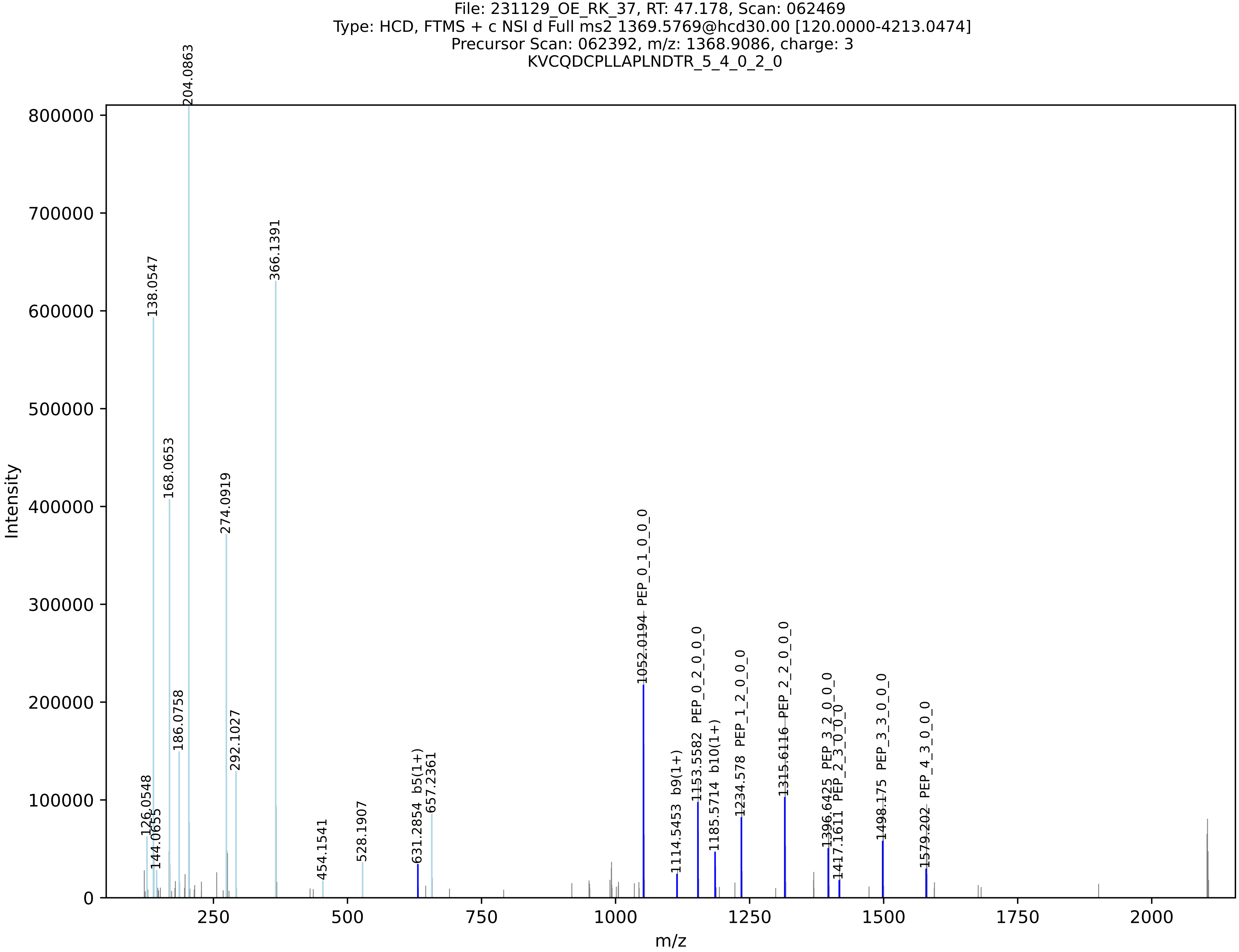
File: 231129_OE_RK_37, RT: 47.178, Scan: 062469
Type: HCD, FTMS + c NSI d Full ms2 1369.5769@hcd30.00 [120.0000-4213.0474]
Precursor Scan: 062392, m/z: 1368.9086, charge: 3
KVCQDCPLLAPLNDTR_5_4_0_2_0
Intensity
m/z
204.0863
366.1391
138.0547
168.0653
274.0919
186.0758
292.1027
126.0548
144.0655
454.1541
528.1907
631.2854 b5(1+)
657.2361
1052.0194 PEP_0_1_0_0_0_
1114.5453 b9(1+)
1153.5582 PEP_0_2_0_0_0_
1185.5714 b10(1+)
1234.578 PEP_1_2_0_0_0_
1315.6116 PEP_2_2_0_0_0_
1396.6425 PEP_3_2_0_0_0_
1417.1611 PEP_2_3_0_0_0_
1498.175 PEP_3_3_0_0_0_
1579.202 PEP_4_3_0_0_0_

File: 231129_OE_RK_37, RT: 47.228, Scan: 062539
Type: HCD, FTMS + c NSI d Full ms2 1002.4630@hcd30.00 [120.0000-4112.9053]
Precursor Scan: 062466, m/z: 1001.9624, charge: 4
IIVPLNNRENISDPTSPLR_5_3_1_1_0

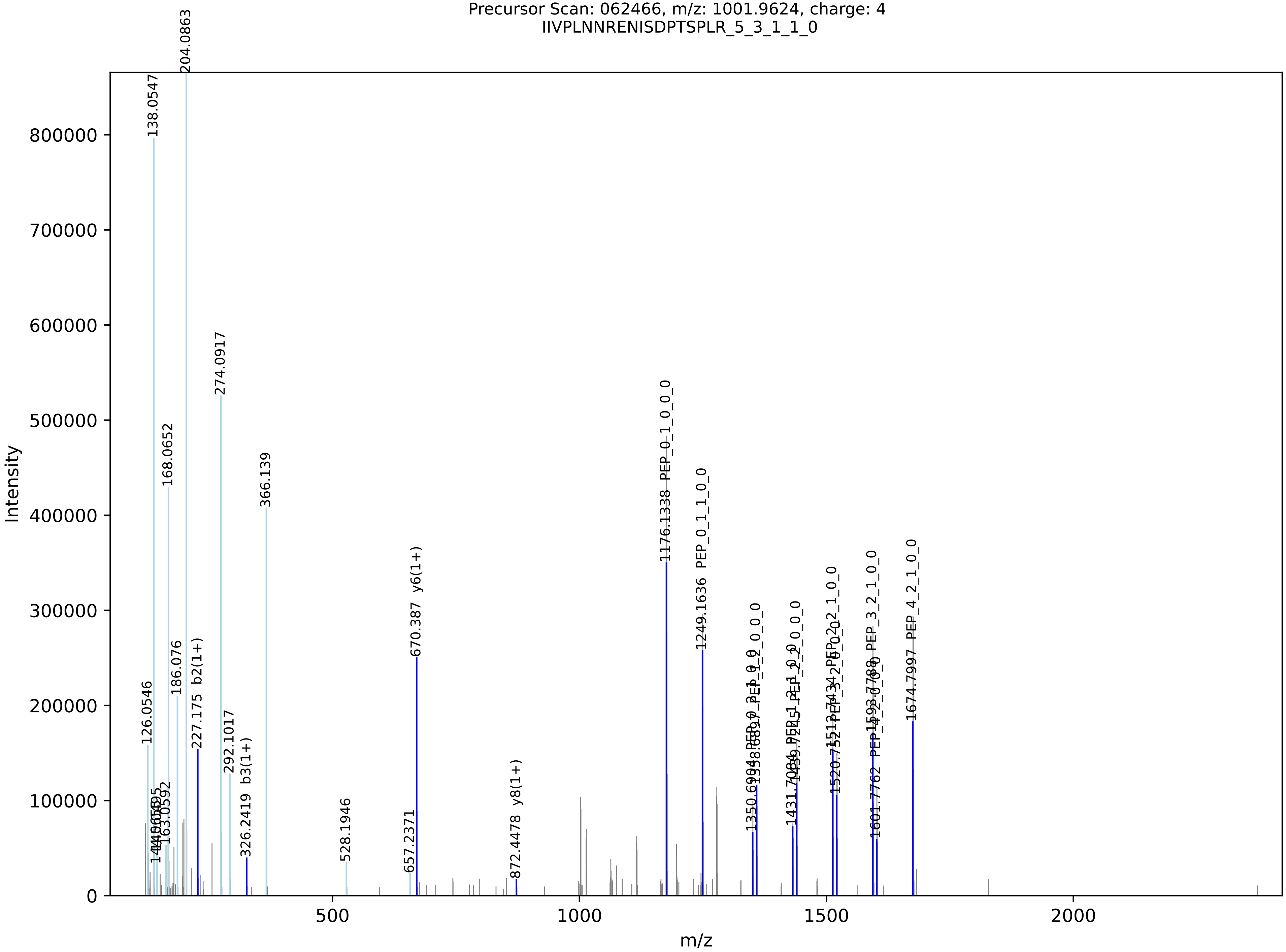

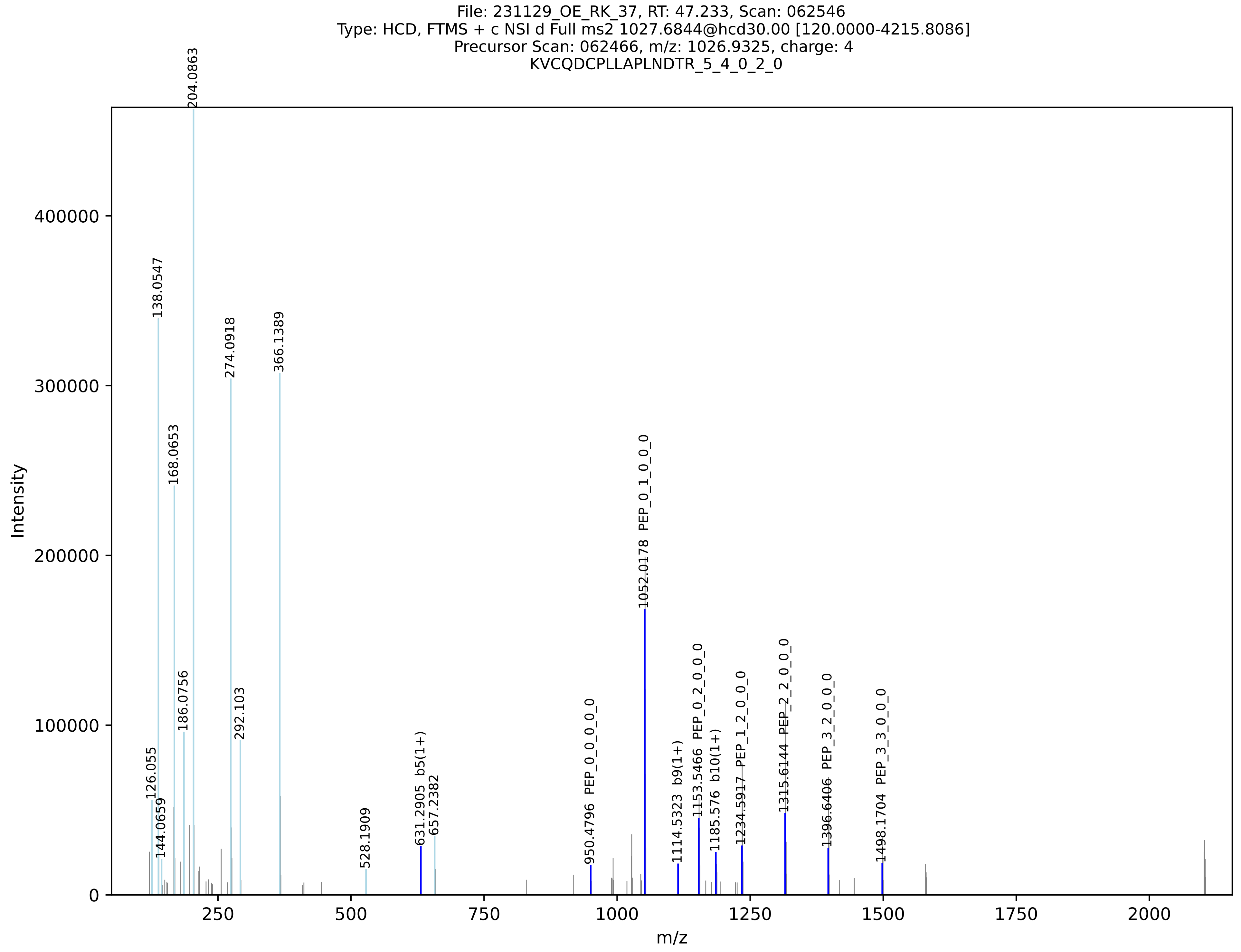
File: 231129_OE_RK_37, RT: 47.233, Scan: 062546
Type: HCD, FTMS + c NSI d Full ms2 1027.6844@hcd30.00 [120.0000-4215.8086]
Precursor Scan: 062466, m/z: 1026.9325, charge: 4
KVCQDCPLLAPLNDTR_5_4_0_2_0
Intensity
m/z
204.0863
138.0547
168.0653
274.0918
366.1389
186.0756
292.103
126.055
144.0659
528.1909
631.2905 b5(1+)
657.2382
950.4796 PEP_0_0_0_0_0
1052.0178 PEP_0_1_0_0_0
1114.5323 b9(1+)
1153.5466 PEP_0_2_0_0_0
1185.576 b10(1+)
1234.5917 PEP_1_2_0_0_0
1315.6144 PEP_2_2_0_0_0
1396.6406 PEP_3_2_0_0_0
1498.1704 PEP_3_3_0_0_0

File: 231129_OE_RK_37, RT: 47.317, Scan: 062668
Type: HCD, FTMS + c NSI d Full ms2 1282.2654@hcd30.00 [120.0000-3945.8740]
Precursor Scan: 062607, m/z: 1281.5971, charge: 3
IIVPLNNRENISDPTSPLR_4_3_1_1_0

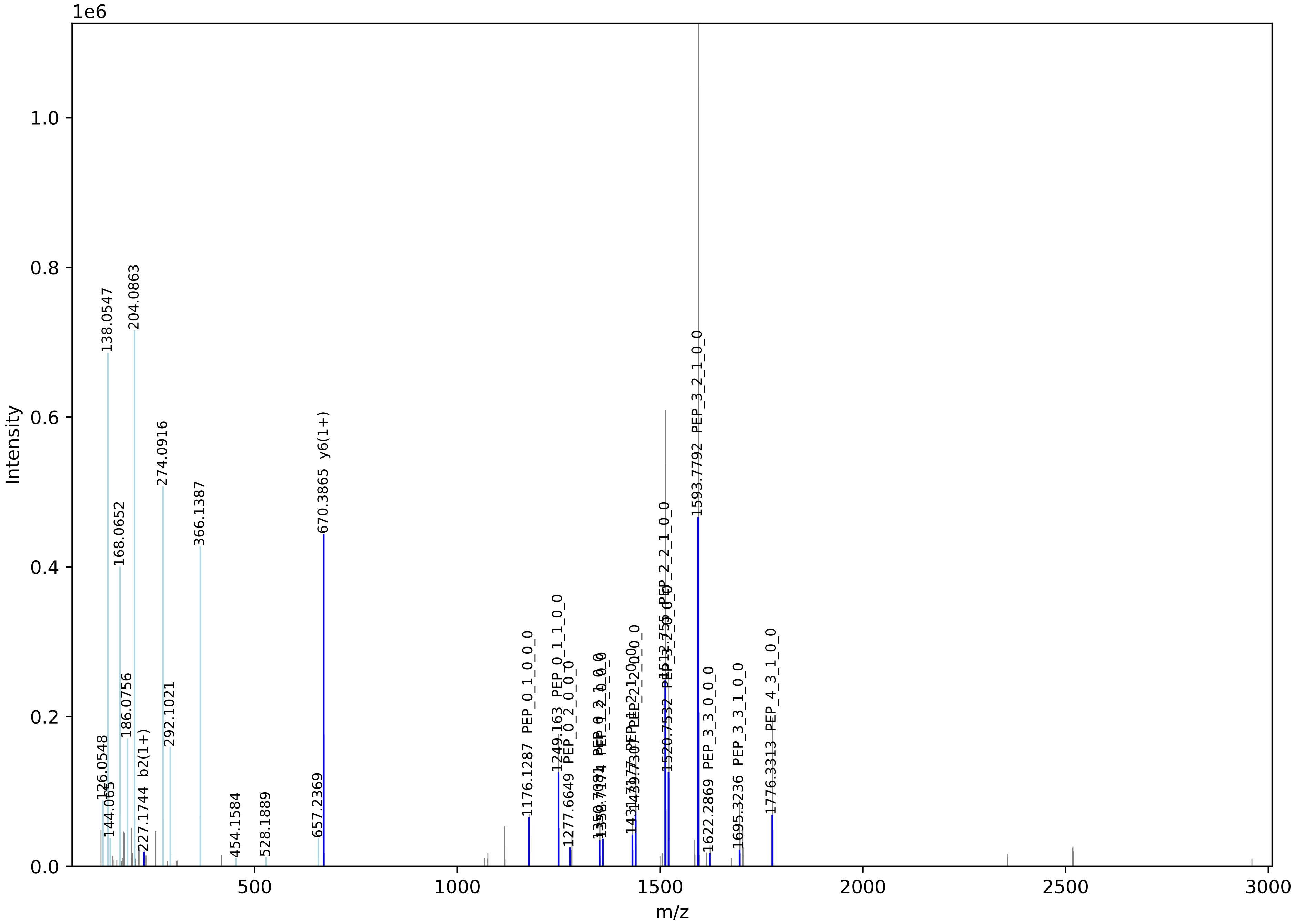

File: 231129_OE_RK_37, RT: 47.421, Scan: 062817
Type: HCD, FTMS + c NSI d Full ms2 1764.0343@hcd30.00 [120.0000-5420.0869]
Precursor Scan: 062729, m/z: 1763.3672, charge: 3
QDQCIYNTTYLNVQR_7_6_1_3_0

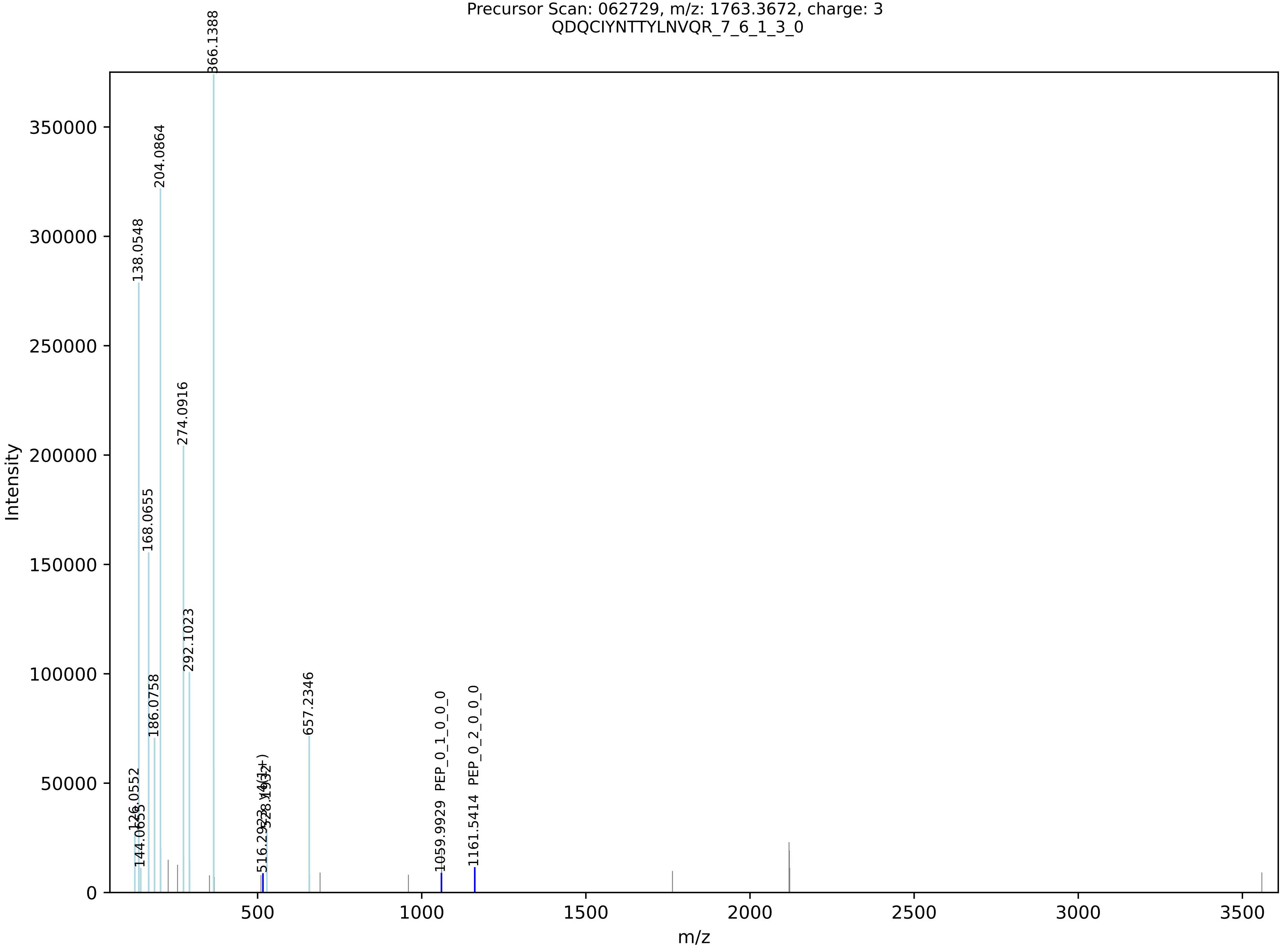

File: 231129_OE_RK_37, RT: 47.463, Scan: 062877
Type: HCD, FTMS + c NSI d Full ms2 1266.2365@hcd30.00 [120.0000-3896.8254]
Precursor Scan: 062799, m/z: 1265.9023, charge: 3
TVLTPATNHMGNVTFTIPANR_7_2_0_0_0

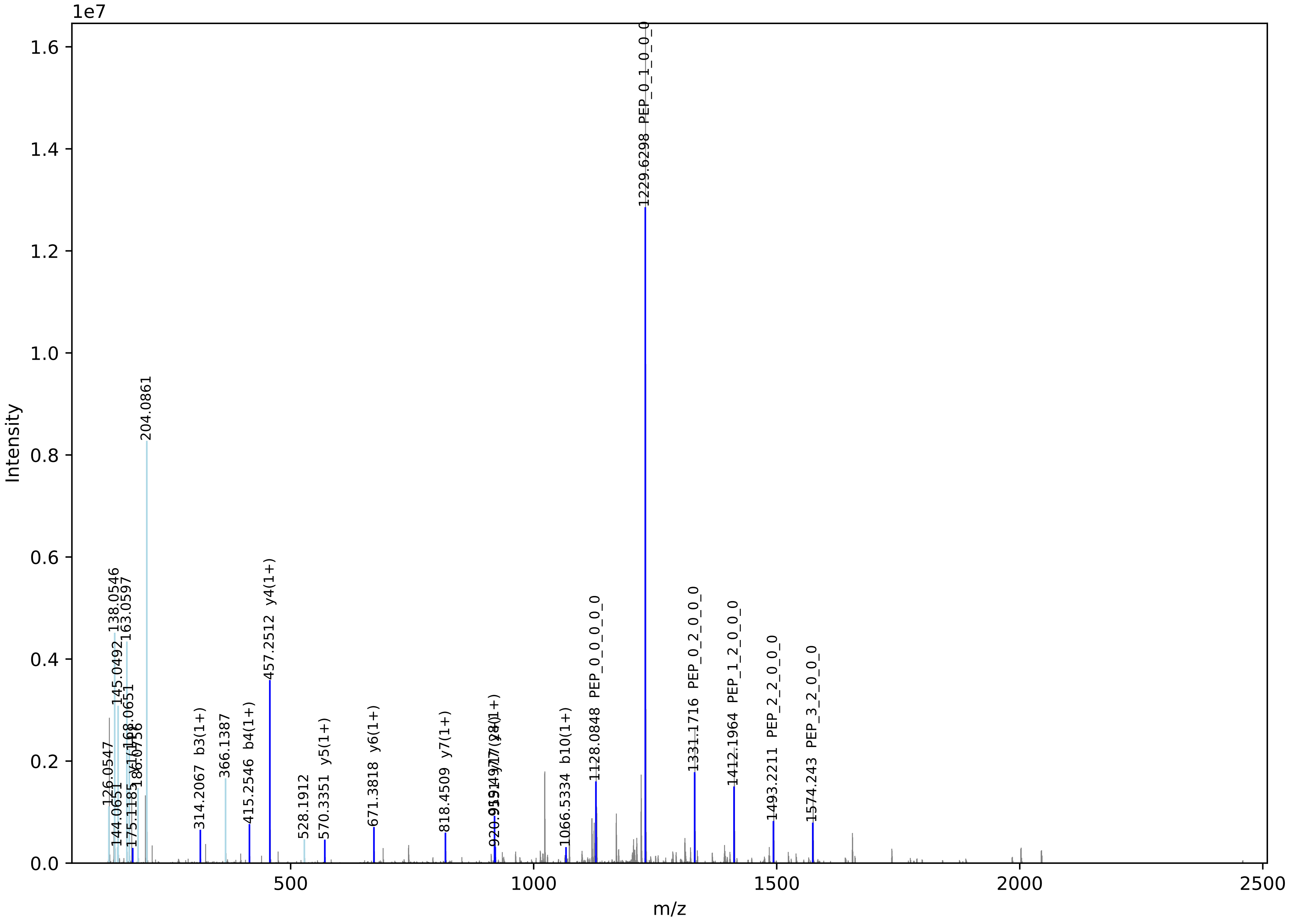

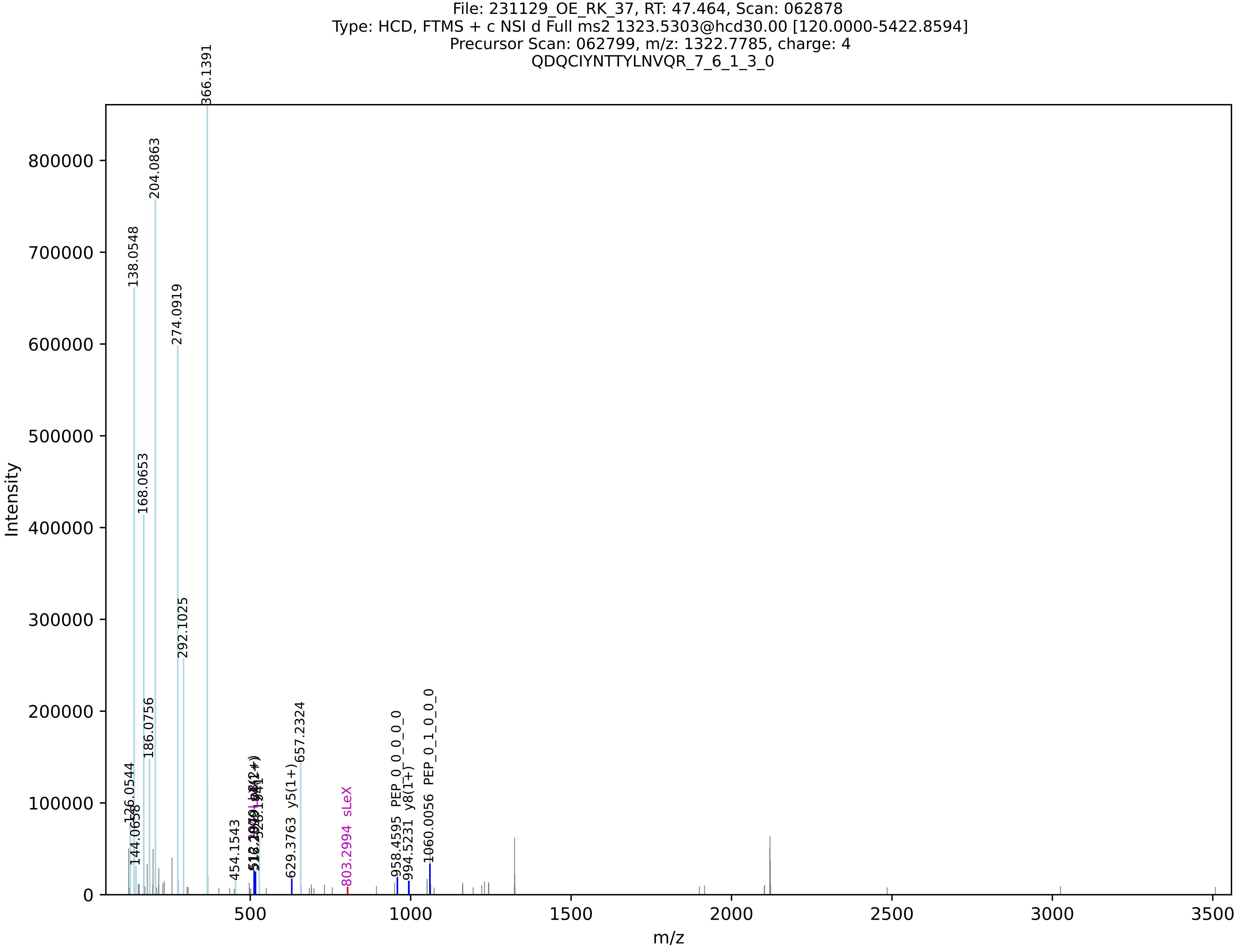
File: 231129_OE_RK_37, RT: 47.464, Scan: 062878
Type: HCD, FTMS + c NSI d Full ms2 1323.5303@hcd30.00 [120.0000-5422.8594]
Precursor Scan: 062799, m/z: 1322.7785, charge: 4
QDQCIYNTTYLNVQR_7_6_1_3_0
Intensity
m/z
366.1391
204.0863
138.0548
274.0919
168.0653
292.1025
186.0756
126.0544
144.0658
454.1543
657.2324
629.3763 y5(1+)
803.2994 sLeX
958.4595 PEP_0_0_0_0_0_
994.5231 y8(1+)
1060.0056 PEP_0_1_0_0_0_

File: 231129_OE_RK_37, RT: 47.477, Scan: 062900
Type: HCD, FTMS + c NSI d Full ms2 949.6774@hcd30.00 [120.0000-3897.5398]
Precursor Scan: 062799, m/z: 949.6786, charge: 4
TVLTPATNHMGNVTFTIPANR_7_2_0_0_0

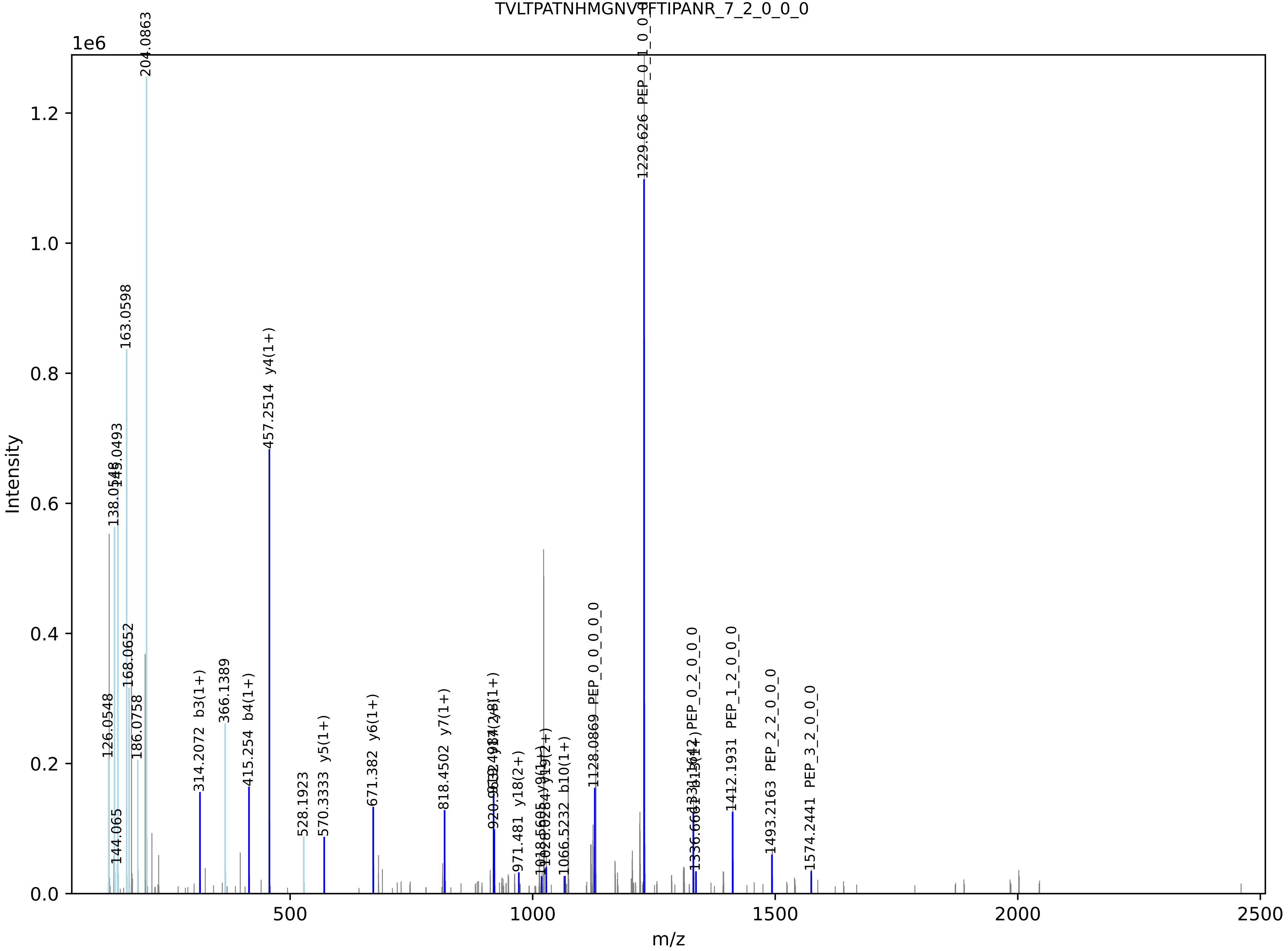

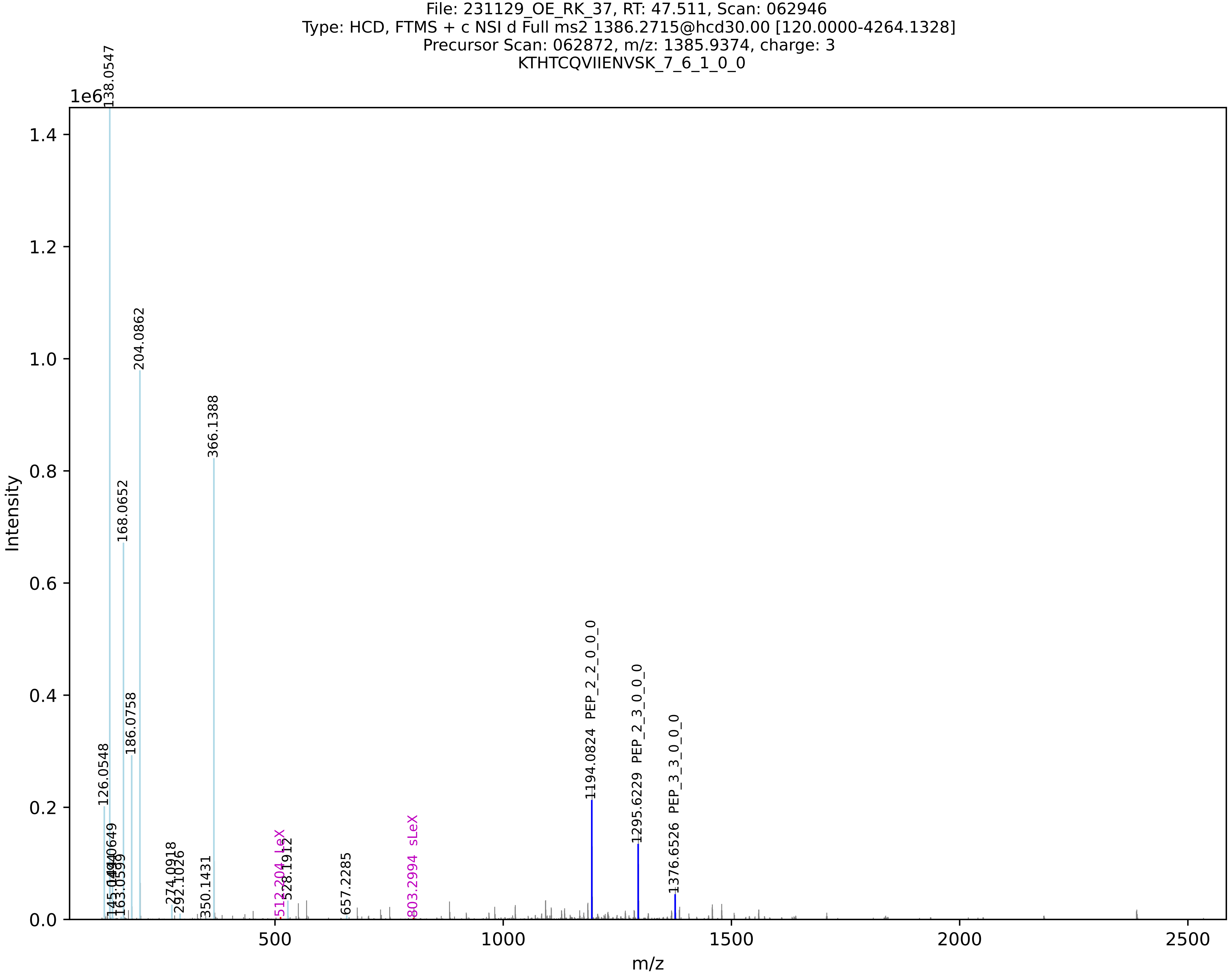

File: 231129_OE_RK_37, RT: 47.511, Scan: 062946
Type: HCD, FTMS + c NSI d Full ms2 1386.2715@hcd30.00 [120.0000-4264.1328]
Precursor Scan: 062872, m/z: 1385.9374, charge: 3
KTHTCQVIIENVSK_7_6_1_0_0
1e6
Intensity
m/z
138.0547
204.0862
366.1388
168.0652
186.0758
126.0548
274.0918
292.1026
350.1431
512.204 LeX
528.1912
657.2285
803.2994 sLeX
1194.0824 PEP_2_2_0_0_0
1295.6229 PEP_2_3_0_0_0
1376.6526 PEP_3_3_0_0_0

File: 231129_OE_RK_37, RT: 47.513, Scan: 062949
Type: HCD, FTMS + c NSI d Full ms2 1281.9319@hcd30.00 [120.0000-3944.8535]
Precursor Scan: 062872, m/z: 1281.6017, charge: 3
IIVPLNNRENISDPTSPLR_4_3_1_1_0

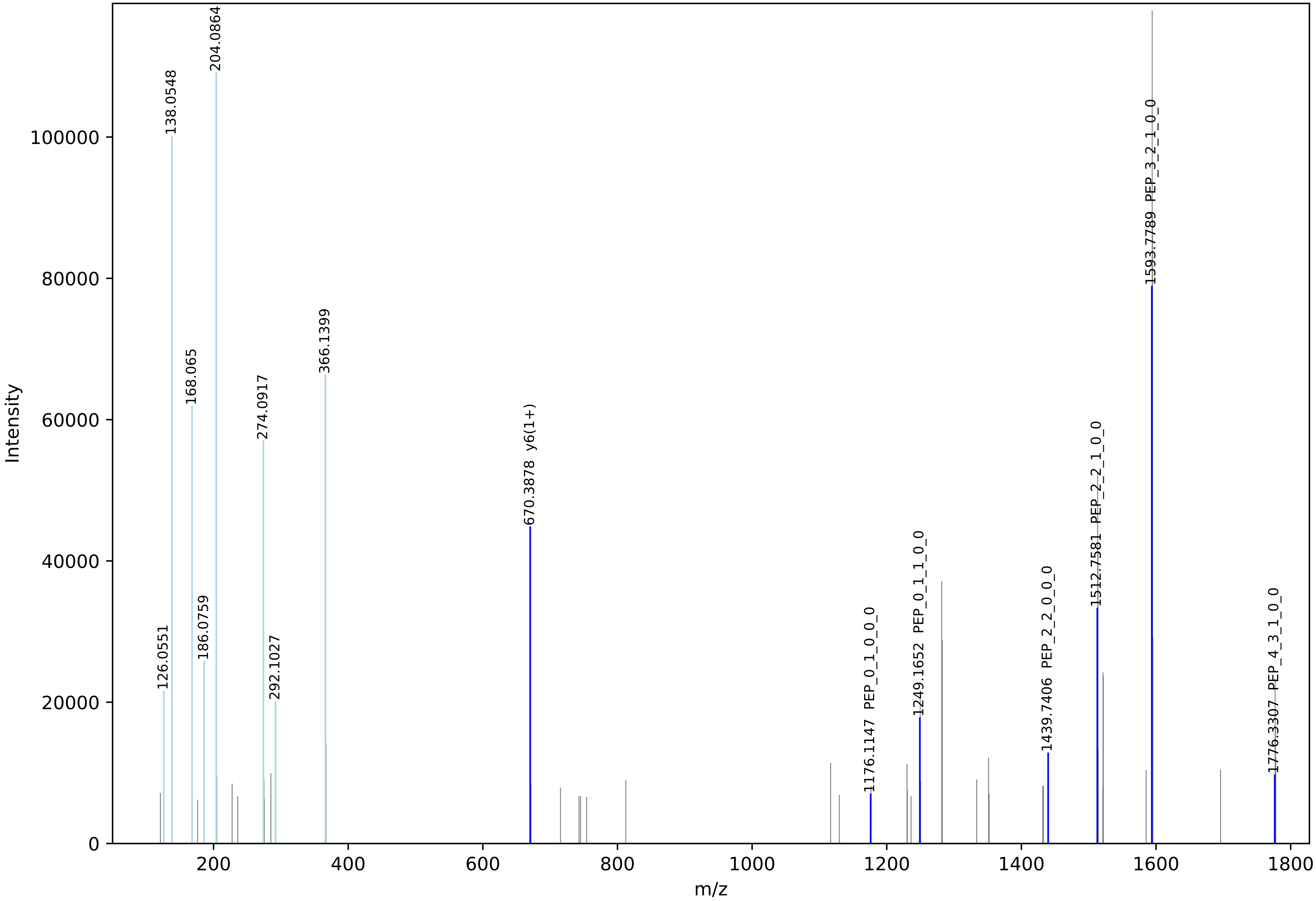

File: 231129_OE_RK_37, RT: 47.567, Scan: 063026
Type: HCD, FTMS + c NSI d Full ms2 1212.5521@hcd30.00 [120.0000-3732.5515]
Precursor Scan: 062945, m/z: 1211.5502, charge: 3
TVLTPATNHMGNVTFTIPANR_6_2_0_0_0

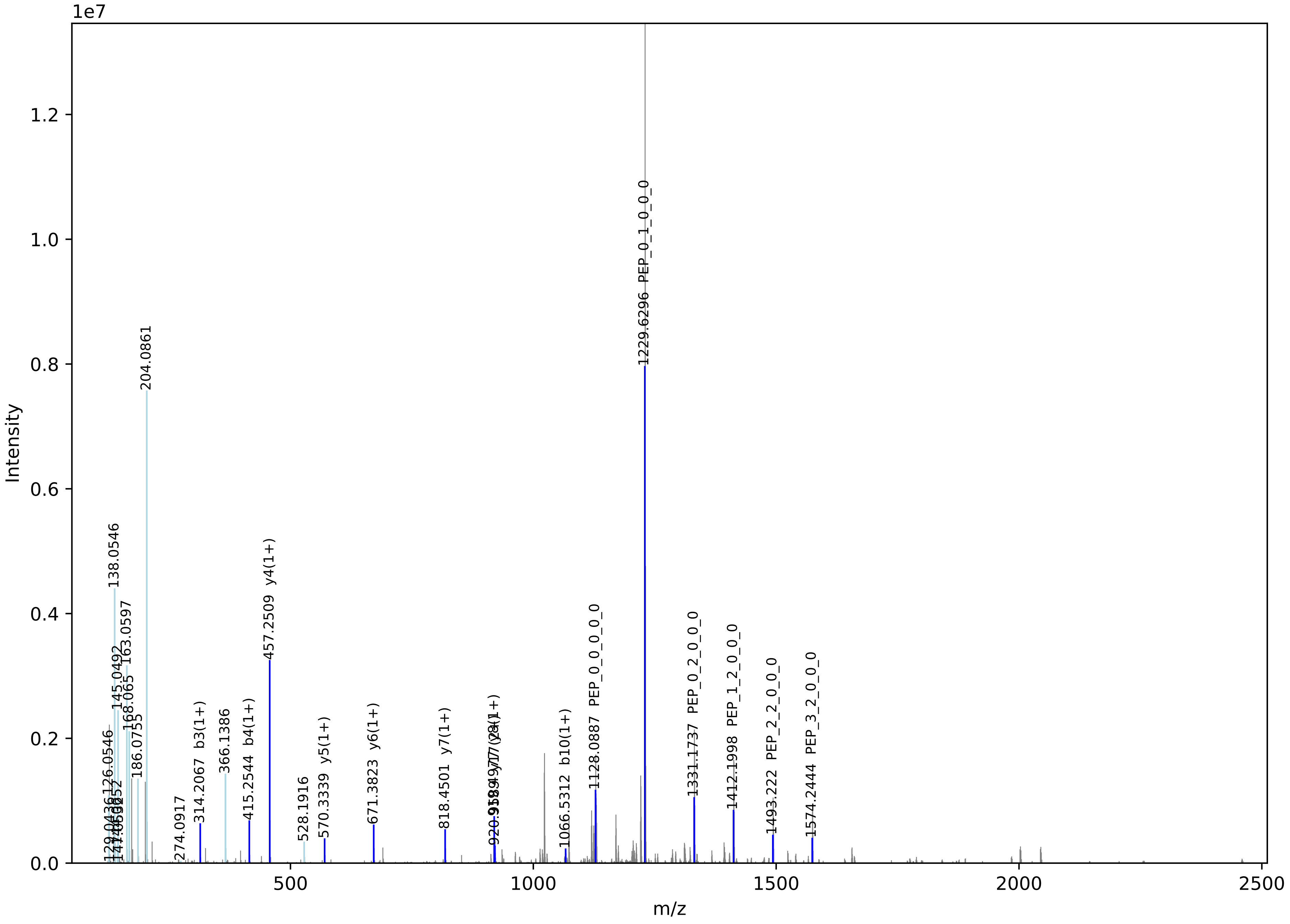

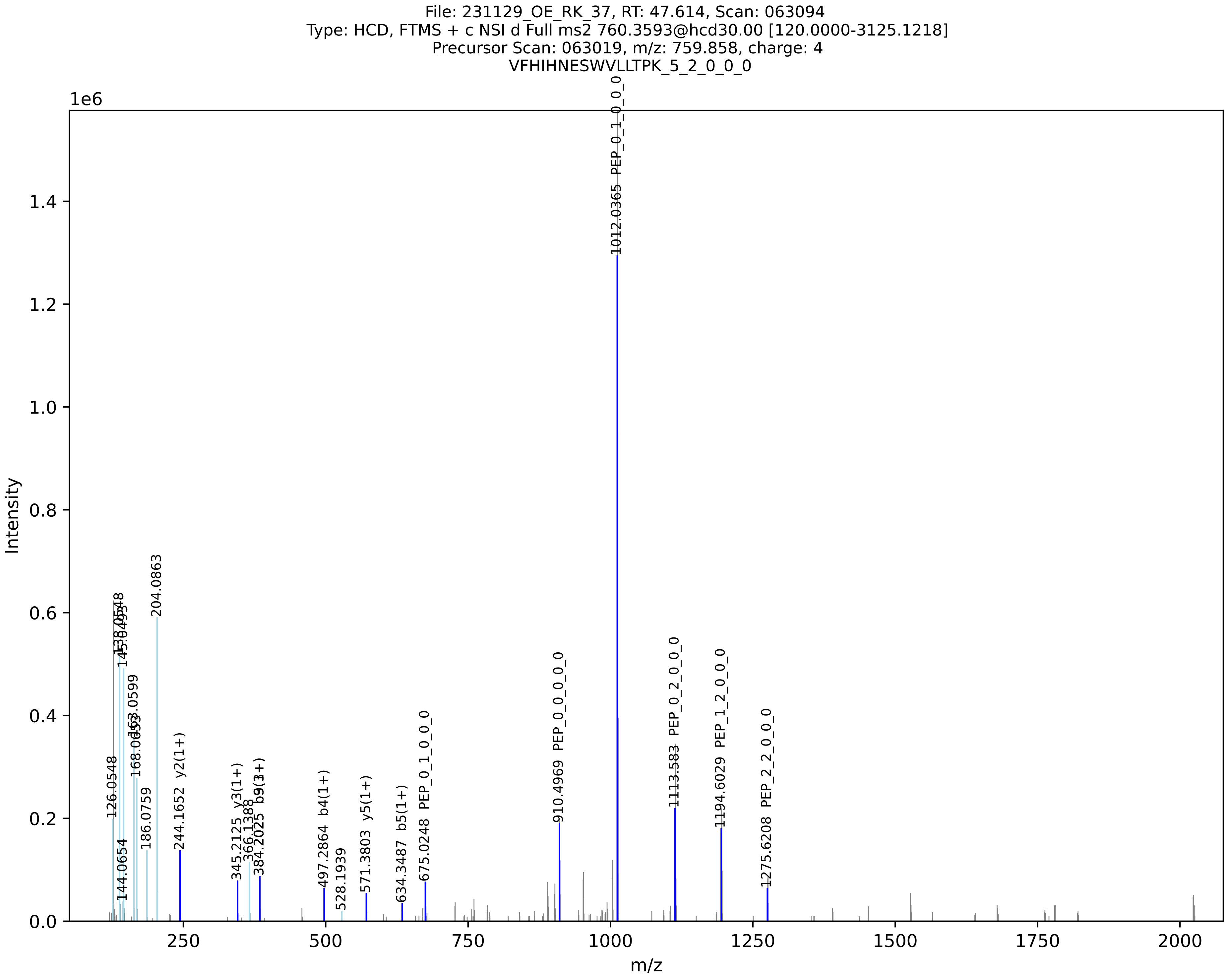

File: 231129_OE_RK_37, RT: 47.614, Scan: 063094
Type: HCD, FTMS + c NSI d Full ms2 760.3593@hcd30.00 [120.0000-3125.1218]
Precursor Scan: 063019, m/z: 759.858, charge: 4
VFHIHNESWVLLTPK_5_2_0_0_0
1e6
Intensity
m/z
1012.0365 PEP_0_1_0_0_0
204.0863
138.0548
145.0493
163.0599
168.0653
126.0548
186.0759
144.0654
244.1652 y2(1+)
345.2125 y3(1+)
366.1388
384.2025 b3(1+)
497.2864 b4(1+)
528.1939
571.3803 y5(1+)
634.3487 b5(1+)
675.0248 PEP_0_1_0_0_0
910.4969 PEP_0_0_0_0_0
1113.583 PEP_0_2_0_0_0
1194.6029 PEP_1_2_0_0_0
1275.6208 PEP_2_2_0_0_0

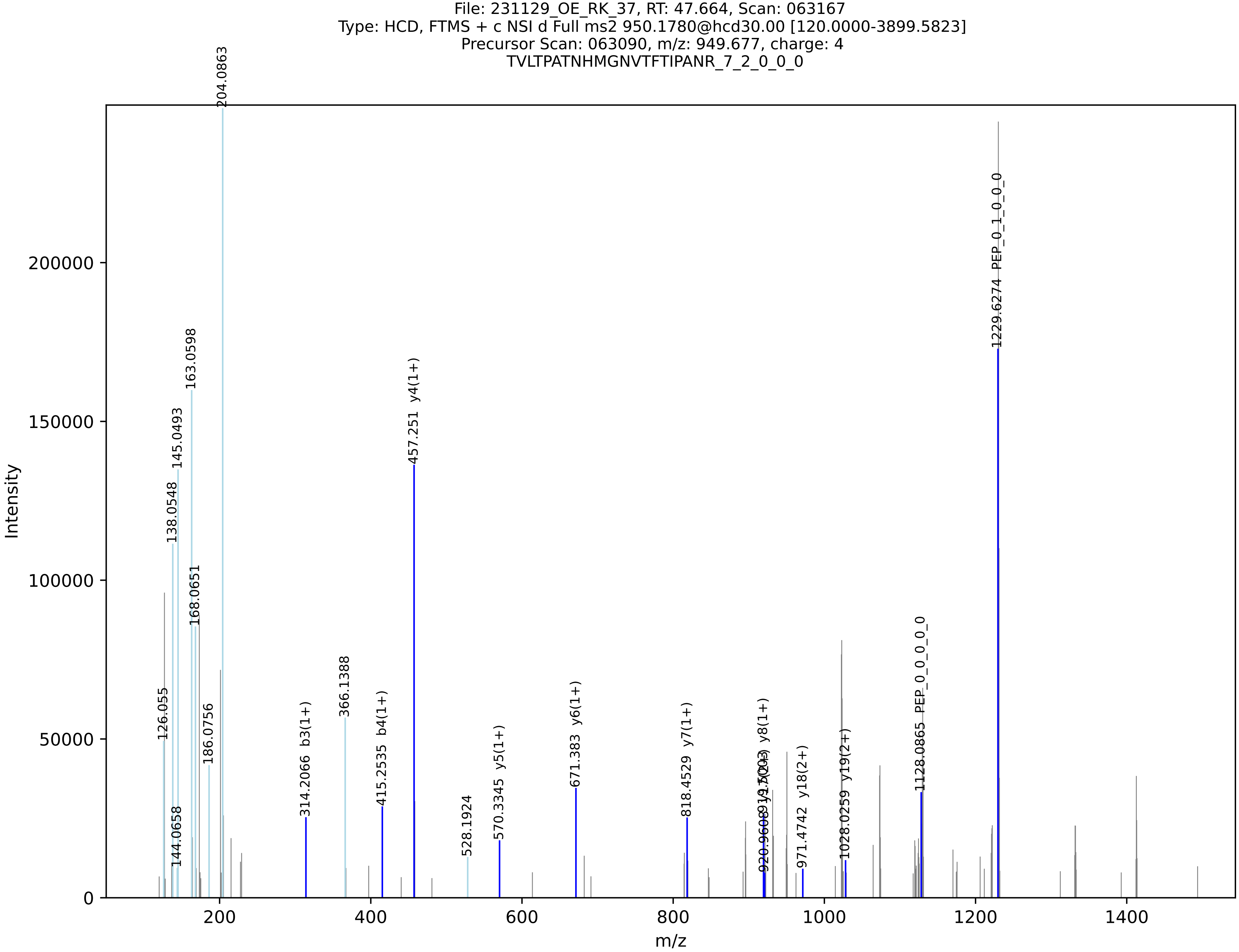
File: 231129_OE_RK_37, RT: 47.664, Scan: 063167
Type: HCD, FTMS + c NSI d Full ms2 950.1780@hcd30.00 [120.0000-3899.5823]
Precursor Scan: 063090, m/z: 949.677, charge: 4
TVLTPATNHMGNVTFTIPANR_7_2_0_0_0
Intensity
m/z
204.0863
163.0598
145.0493
138.0548
168.0651
126.055
144.0658
186.0756
314.2066 b3(1+)
366.1388
415.2535 b4(1+)
457.251 y4(1+)
528.1924
570.3345 y5(1+)
671.383 y6(1+)
818.4529 y7(1+)
y8(1+)
971.4742 y18(2+)
1028.0259 y19(2+)
1128.0865 PEP_0_0_0_0_0
1229.6274 PEP_0_1_0_0_0

File: 231129_OE_RK_37, RT: 47.706, Scan: 063235
Type: HCD, FTMS + c NSI d Full ms2 949.4443@hcd30.00 [120.0000-3896.5889]
Precursor Scan: 063019, m/z: 949.6771, charge: 4
TVLTPATNHMGNVTFTIPANR_7_2_0_0_0

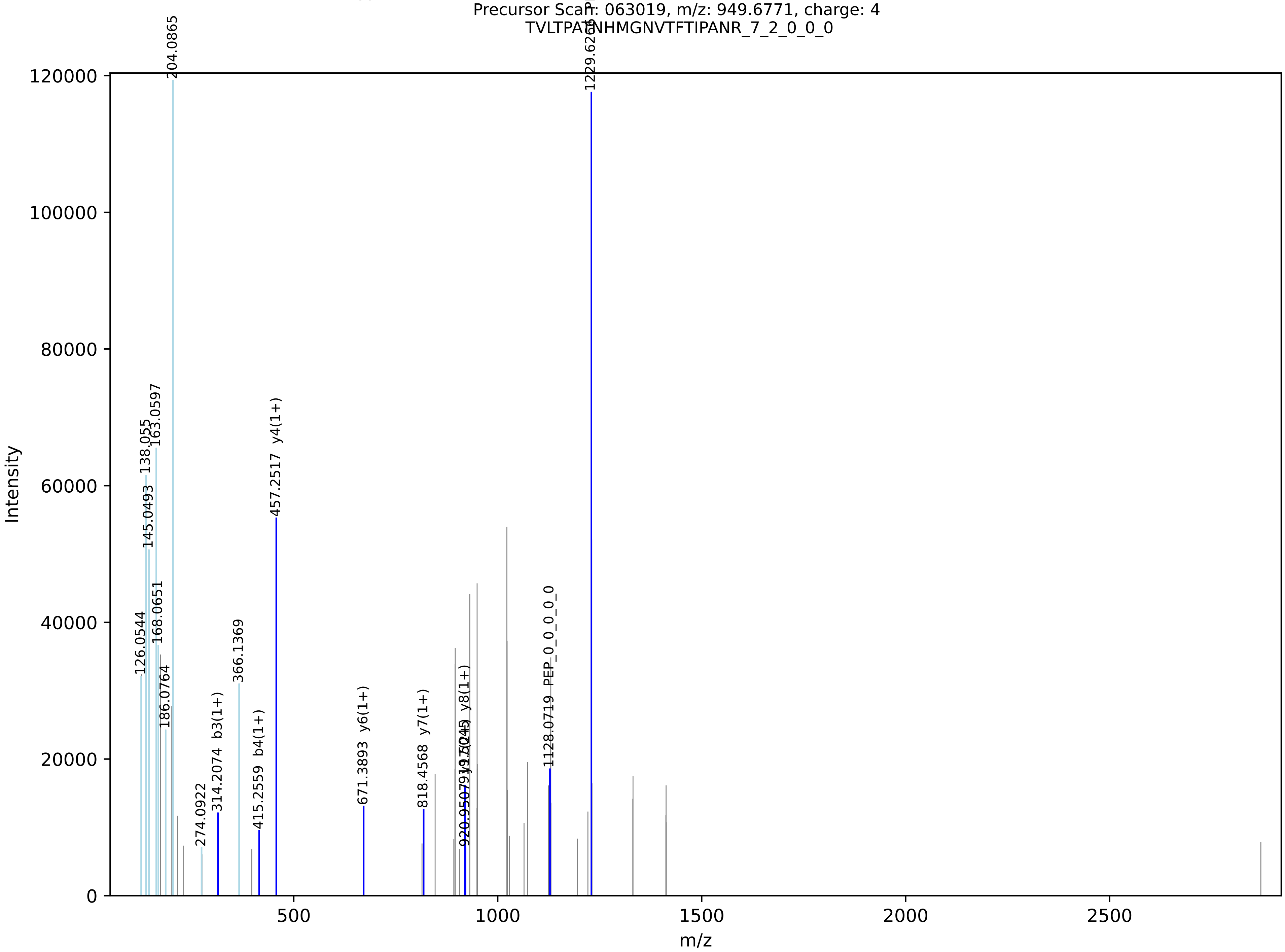

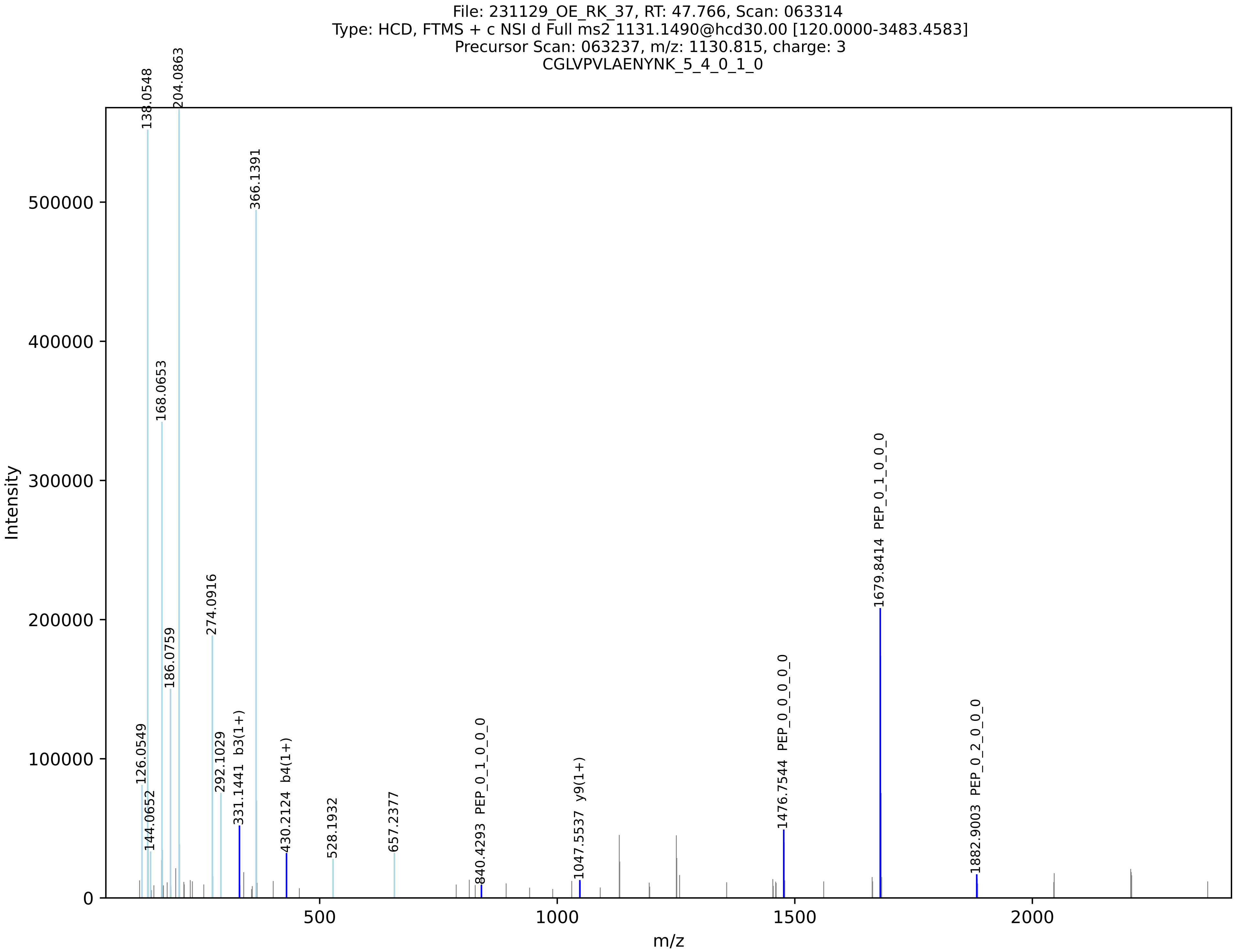
File: 231129_OE_RK_37, RT: 47.766, Scan: 063314
Type: HCD, FTMS + c NSI d Full ms2 1131.1490@hcd30.00 [120.0000-3483.4583]
Precursor Scan: 063237, m/z: 1130.815, charge: 3
CGLVPVLAENYNK_5_4_0_1_0
Intensity
m/z
138.0548
204.0863
366.1391
168.0653
274.0916
186.0759
126.0549
144.0652
292.1029
331.1441 b3(1+)
430.2124 b4(1+)
528.1932
657.2377
840.4293 PEP_0_1_0_0_0
1047.5537 y9(1+)
1476.7544 PEP_0_0_0_0_0
1679.8414 PEP_0_1_0_0_0
1882.9003 PEP_0_2_0_0_0

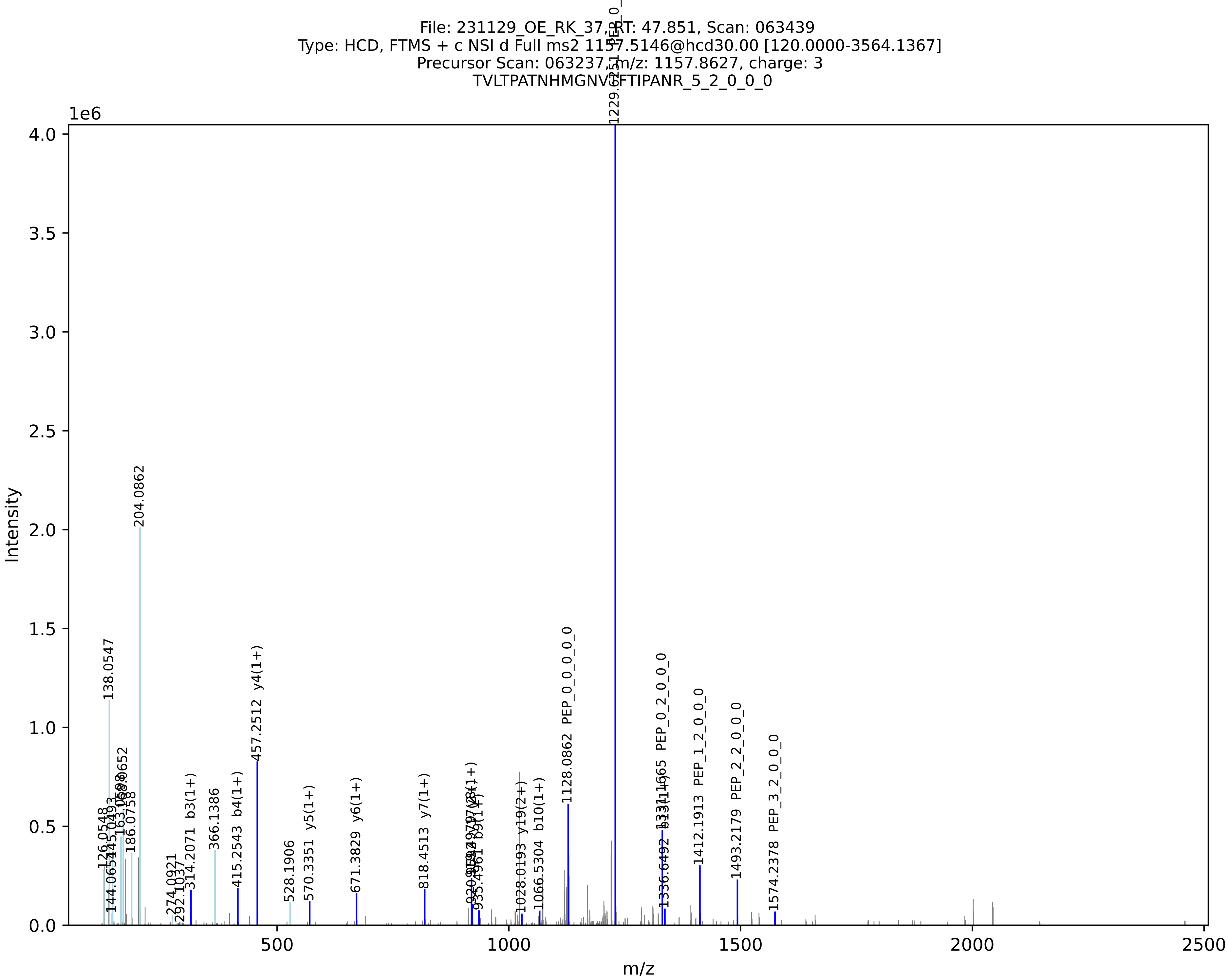
File: 231129_OE_RK_37, RT: 47.851, Scan: 063439
Type: HCD, FTMS + c NSI d Full ms2 1157.5146@hcd30.00 [120.0000-3564.1367]
Precursor Scan: 063237, m/z: 1157.8627, charge: 3
TVLTPATNHMGNVcFTIPANR_5_2_0_0_0
1e6
Intensity
m/z
0.0
0.5
1.0
1.5
2.0
2.5
3.0
3.5
4.0
500
1000
1500
2000
2500
138.0547
204.0862
186.0758
274.0921
314.2071 b3(1+)
366.1386
415.2543 b4(1+)
457.2512 y4(1+)
528.1906
570.3351 y5(1+)
671.3829 y6(1+)
818.4513 y7(1+)
935.4961 b9(1+)
1028.0193 y19(2+)
1066.5304 b10(1+)
1128.0862 PEP_0_0_0_0_0
1331.1665 PEP_0_2_0_0_0
1336.6492 b13(1+)
1412.1913 PEP_1_2_0_0_0
1493.2179 PEP_2_2_0_0_0
1574.2378 PEP_3_2_0_0_0

File: 231129_OE_RK_37, RT: 47.864, Scan: 063453
Type: HCD, FTMS + c NSI d Full ms2 1158.5339@hcd30.00 [120.0000-3567.2561]
Precursor Scan: 063381, m/z: 1157.866, charge: 3
TVLTPATNHMGNVTFTIPANR_5_2_0_0_0

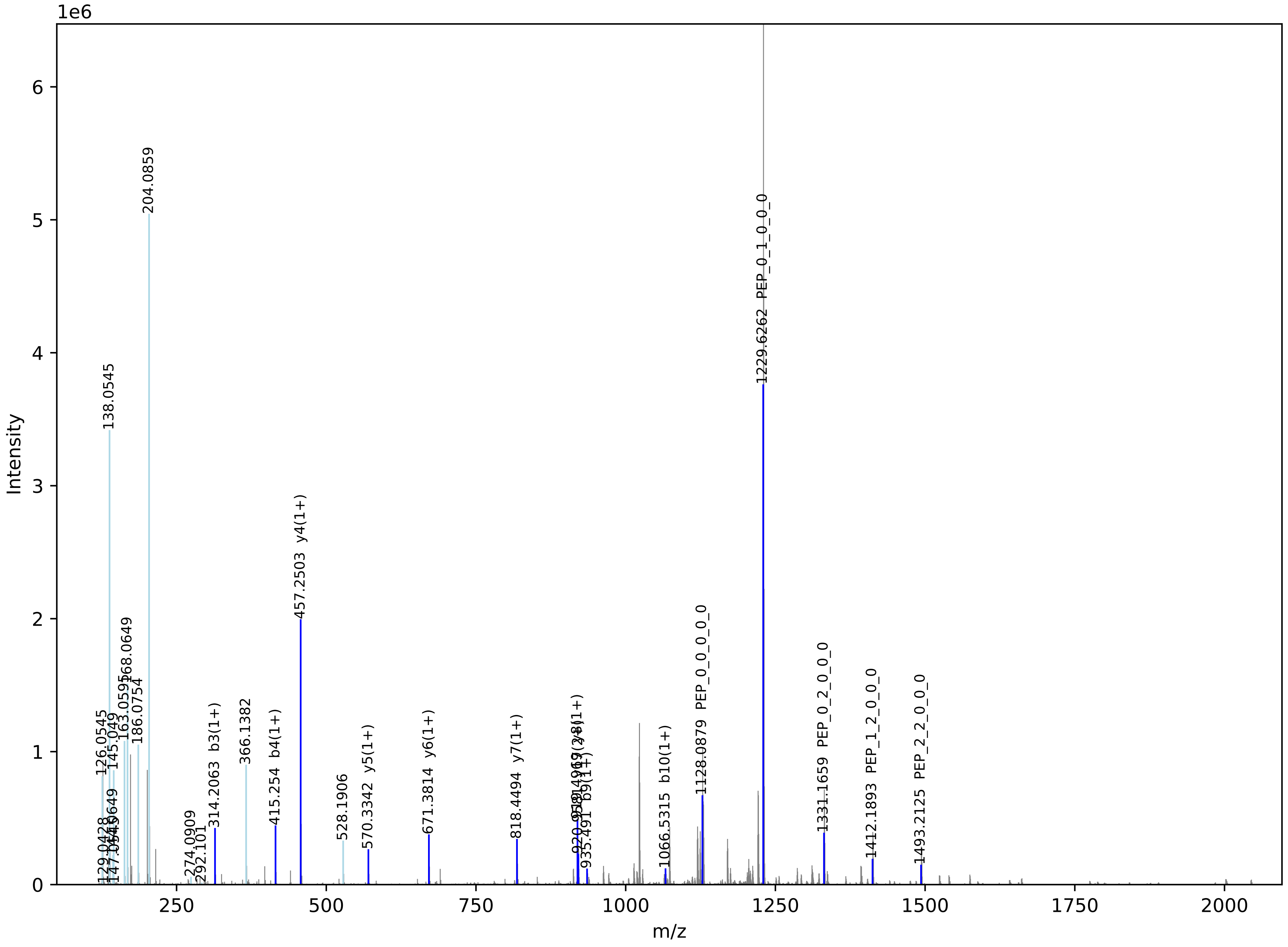

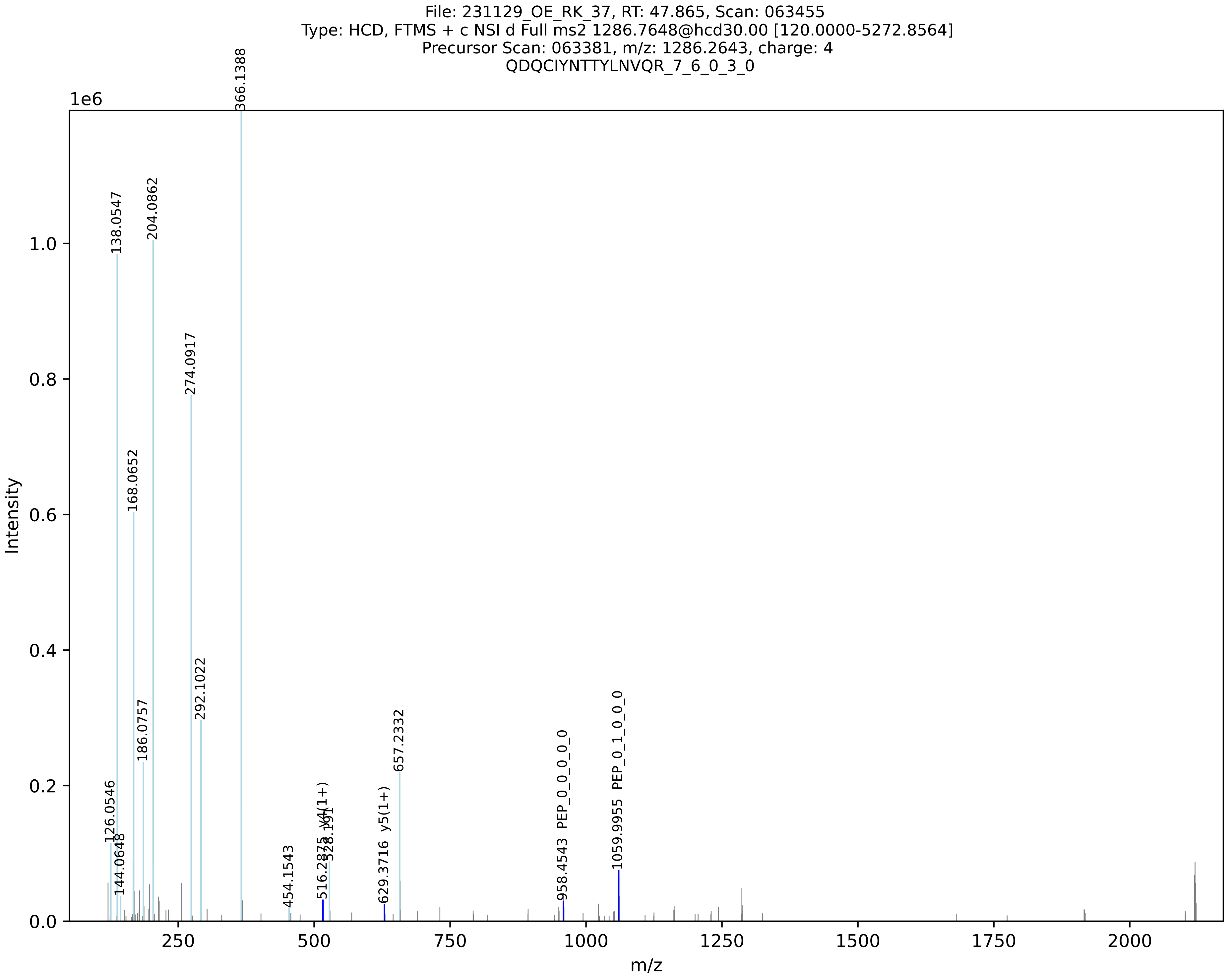
File: 231129_OE_RK_37, RT: 47.865, Scan: 063455
Type: HCD, FTMS + c NSI d Full ms2 1286.7648@hcd30.00 [120.0000-5272.8564]
Precursor Scan: 063381, m/z: 1286.2643, charge: 4
QDQCIYNTTYLNVQR_7_6_0_3_0
1e6
Intensity
m/z
366.1388
138.0547
204.0862
274.0917
168.0652
292.1022
186.0757
126.0546
144.0648
454.1543
516.2875 y4(1+)
528.191
629.3716 y5(1+)
657.2332
958.4543 PEP_0_0_0_0_0
1059.9955 PEP_0_1_0_0_0
0.0
0.2
0.4
0.6
0.8
1.0
250
500
750
1000
1250
1500
1750
2000

File: 231129_OE_RK_37, RT: 47.917, Scan: 063530
Type: HCD, FTMS + c NSI d Full ms2 1221.5499@hcd30.00 [120.0000-3760.0847]
Precursor Scan: 063452, m/z: 1221.217, charge: 3
QDNTTQLVQDDQVKGPLR_4_4_1_0_0

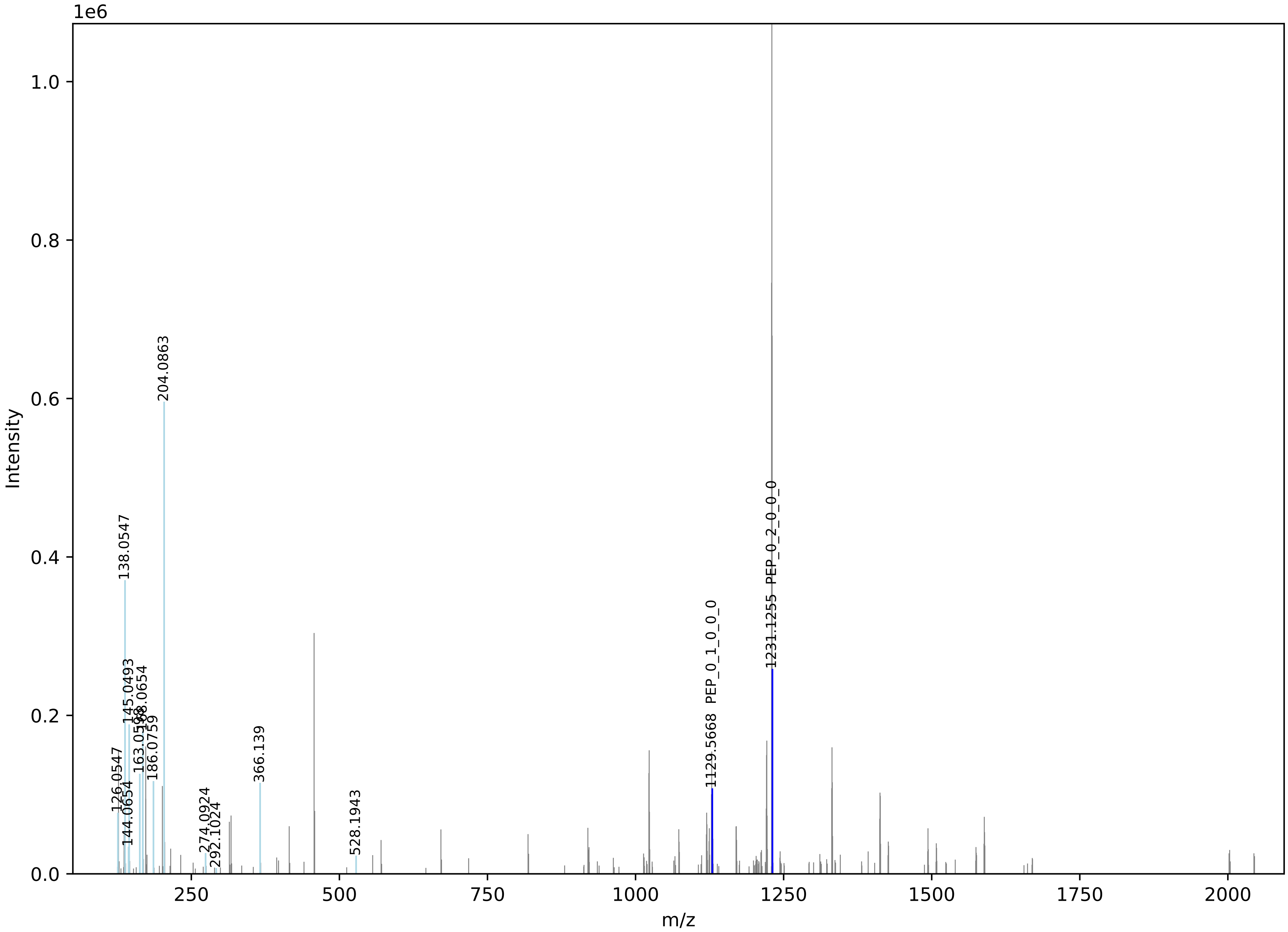

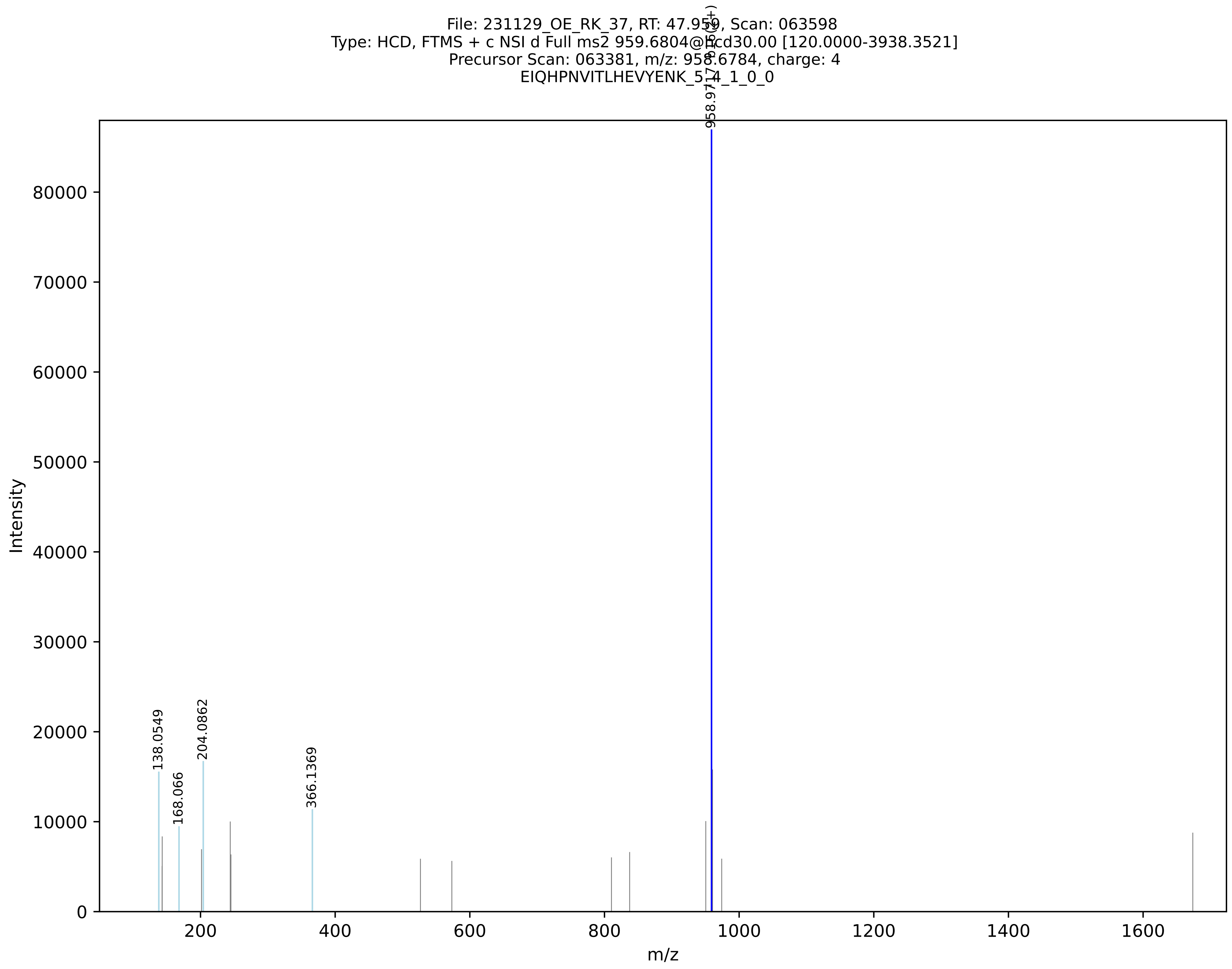
File: 231129_OE_RK_37, RT: 47.959, Scan: 063598
Type: HCD, FTMS + c NSI d Full ms2 959.6804@hcd30.00 [120.0000-3938.3521]
Precursor Scan: 063381, m/z: 958.6784, charge: 4
EIQHPNVITLHEVYENK_5_4_1_0_0
958.9717
138.0549
168.066
204.0862
366.1369
Intensity
m/z
0
10000
20000
30000
40000
50000
60000
70000
80000
200
400
600
800
1000
1200
1400
1600

File: 231129_OE_RK_37, RT: 48.049, Scan: 063722
Type: HCD, FTMS + c NSI d Full ms2 1158.2000@hcd30.00 [120.0000-3566.2341]
Precursor Scan: 063673, m/z: 1157.8659, charge: 3
TVLTPATNHMGNVTFTIPANR_5_2_0_0_0

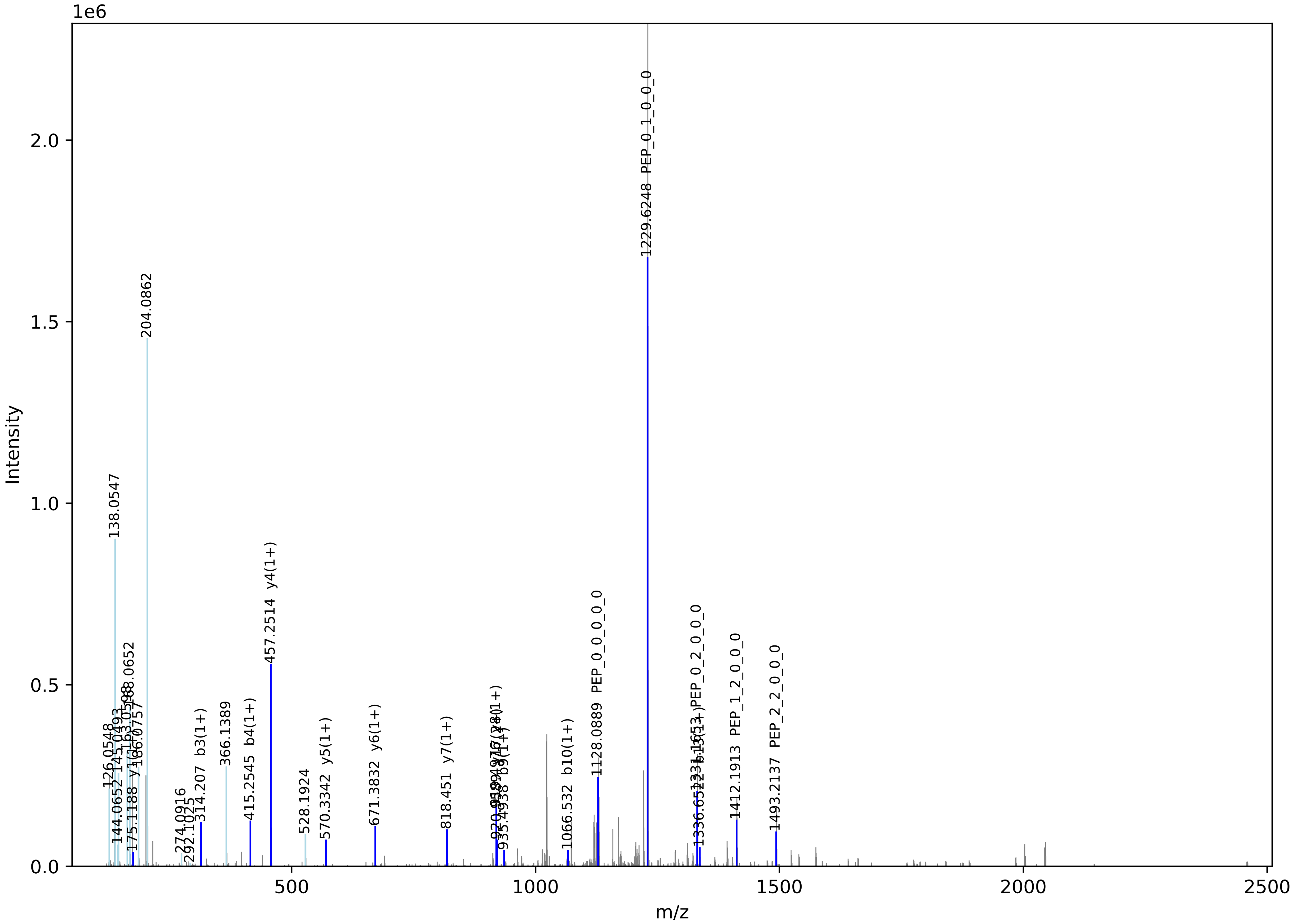

File: 231129_OE_RK_37, RT: 48.098, Scan: 063784
Type: HCD, FTMS + c NSI d Full ms2 1715.6801@hcd30.00 [120.0000-5272.1230]
Precursor Scan: 063747, m/z: 1715.6801, charge: 3
QDQCIYNTTYLNVQR_7_6_2_2_0

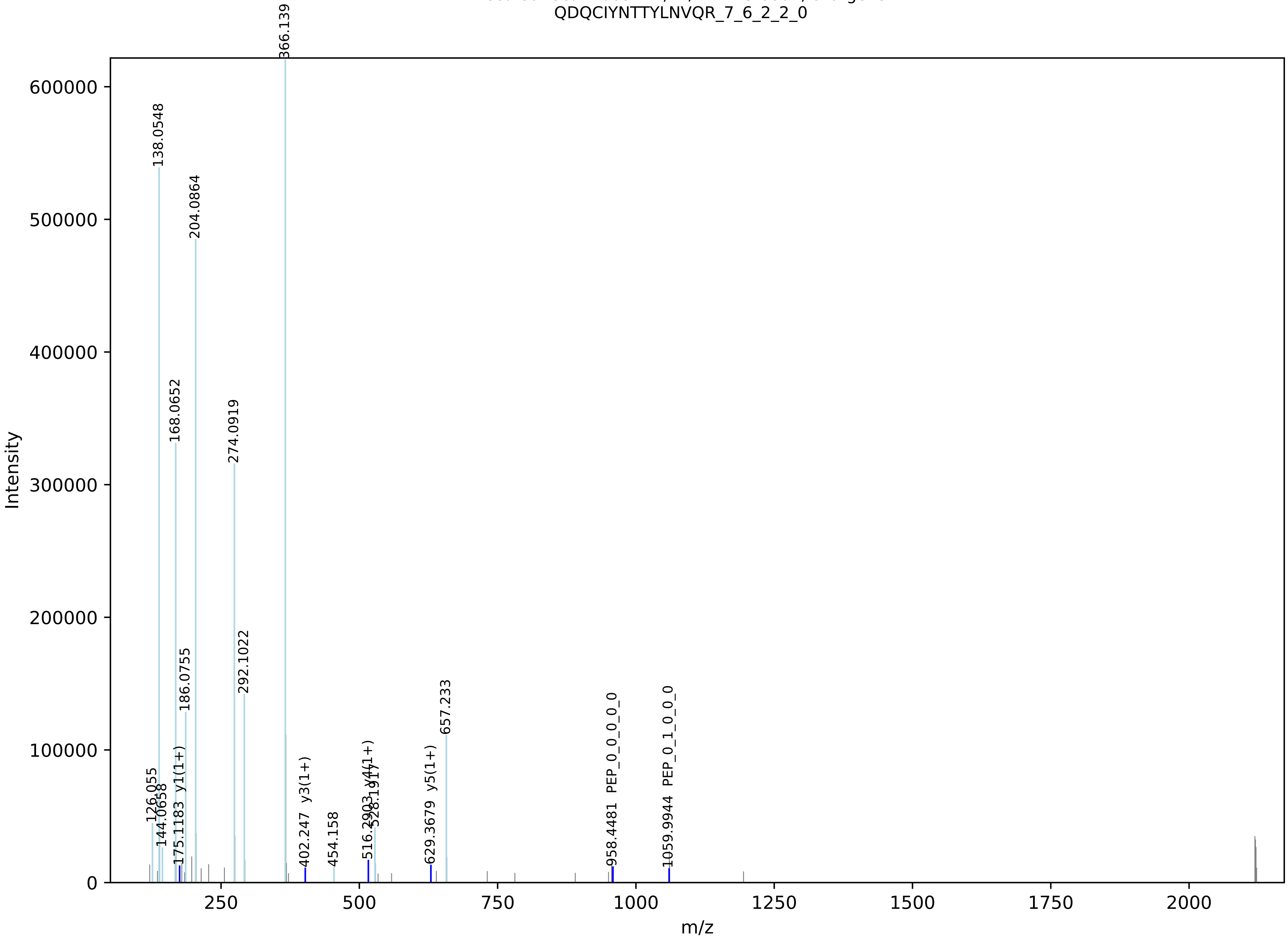

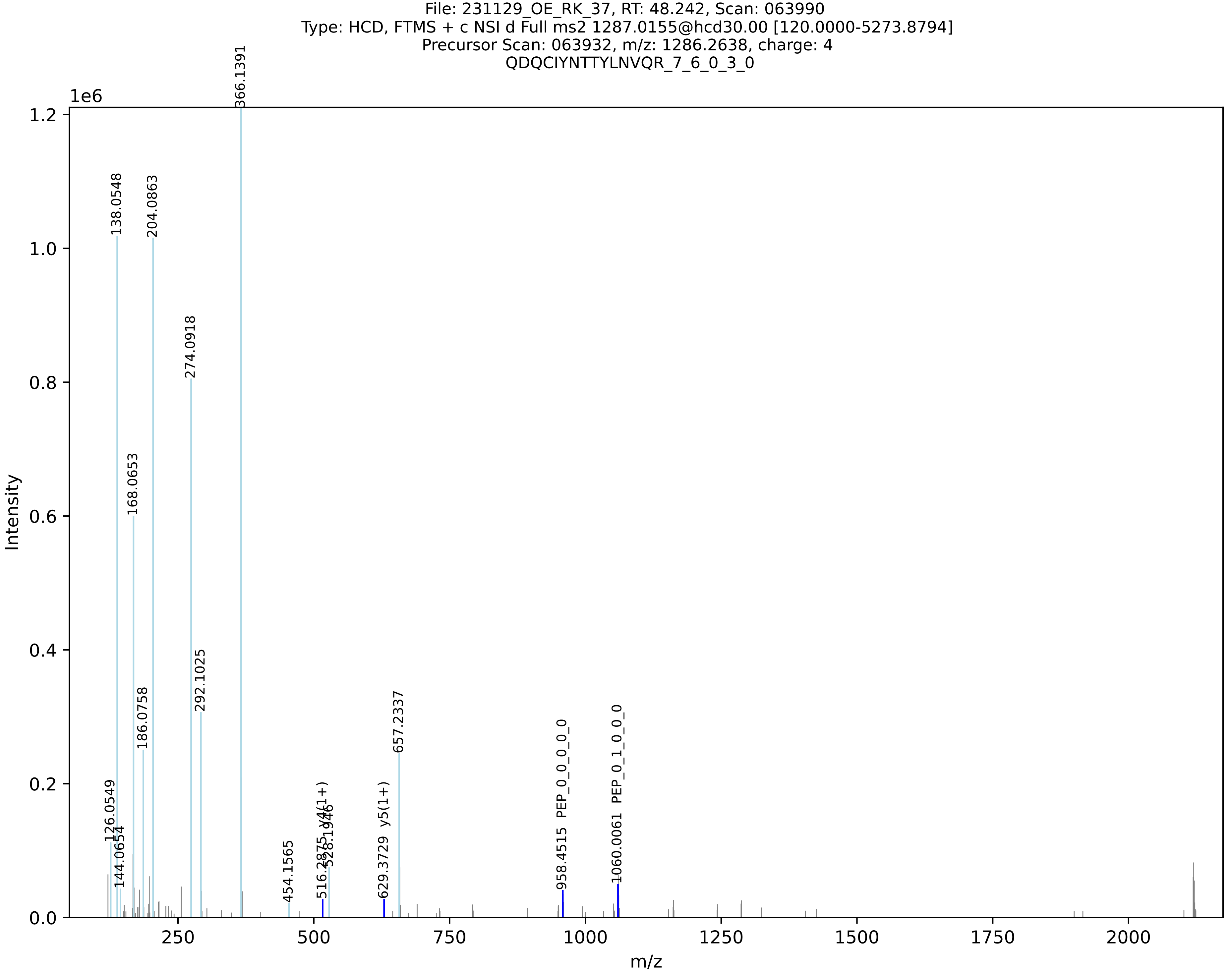

File: 231129_OE_RK_37, RT: 48.242, Scan: 063990
Type: HCD, FTMS + c NSI d Full ms2 1287.0155@hcd30.00 [120.0000-5273.8794]
Precursor Scan: 063932, m/z: 1286.2638, charge: 4
QDQCIYNTTYLNVQR_7_6_0_3_0
1e6
Intensity
m/z
366.1391
138.0548
204.0863
274.0918
168.0653
292.1025
186.0758
126.0549
144.0654
454.1565
516.2875 y4(1+)
528.1946
629.3729 y5(1+)
657.2337
958.4515 PEP_0_0_0_0_0
1060.0061 PEP_0_1_0_0_0

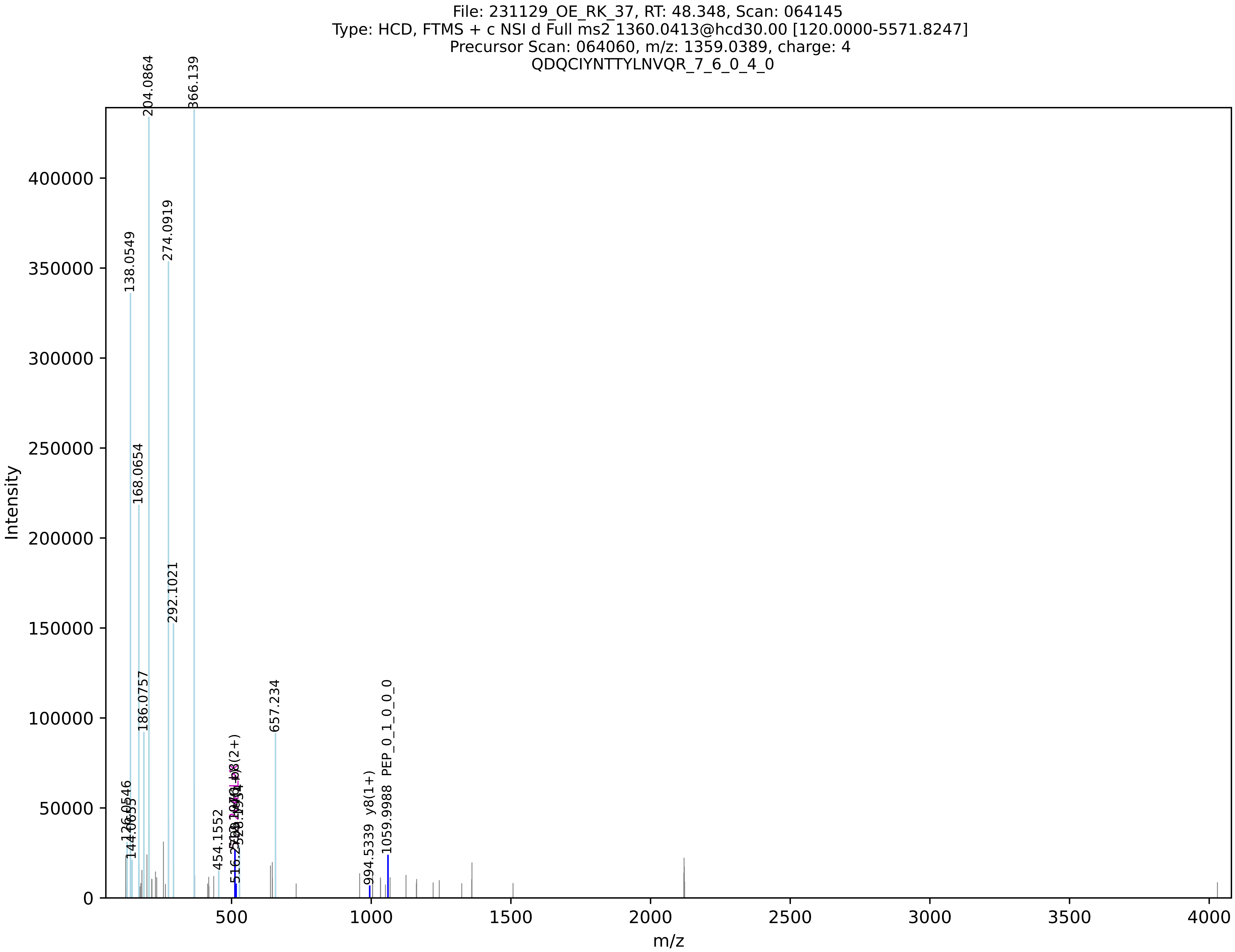
File: 231129_OE_RK_37, RT: 48.348, Scan: 064145
Type: HCD, FTMS + c NSI d Full ms2 1360.0413@hcd30.00 [120.0000-5571.8247]
Precursor Scan: 064060, m/z: 1359.0389, charge: 4
QDQCIYNTTYLNVQR_7_6_0_4_0
Intensity
m/z
0
50000
100000
150000
200000
250000
300000
350000
400000
500
1000
1500
2000
2500
3000
3500
4000
204.0864
366.139
274.0919
138.0549
168.0654
292.1021
186.0757
657.234
126.0546
144.0655
454.1552
994.5339 y8(1+)
1059.9988 PEP_0_1_0_0_0

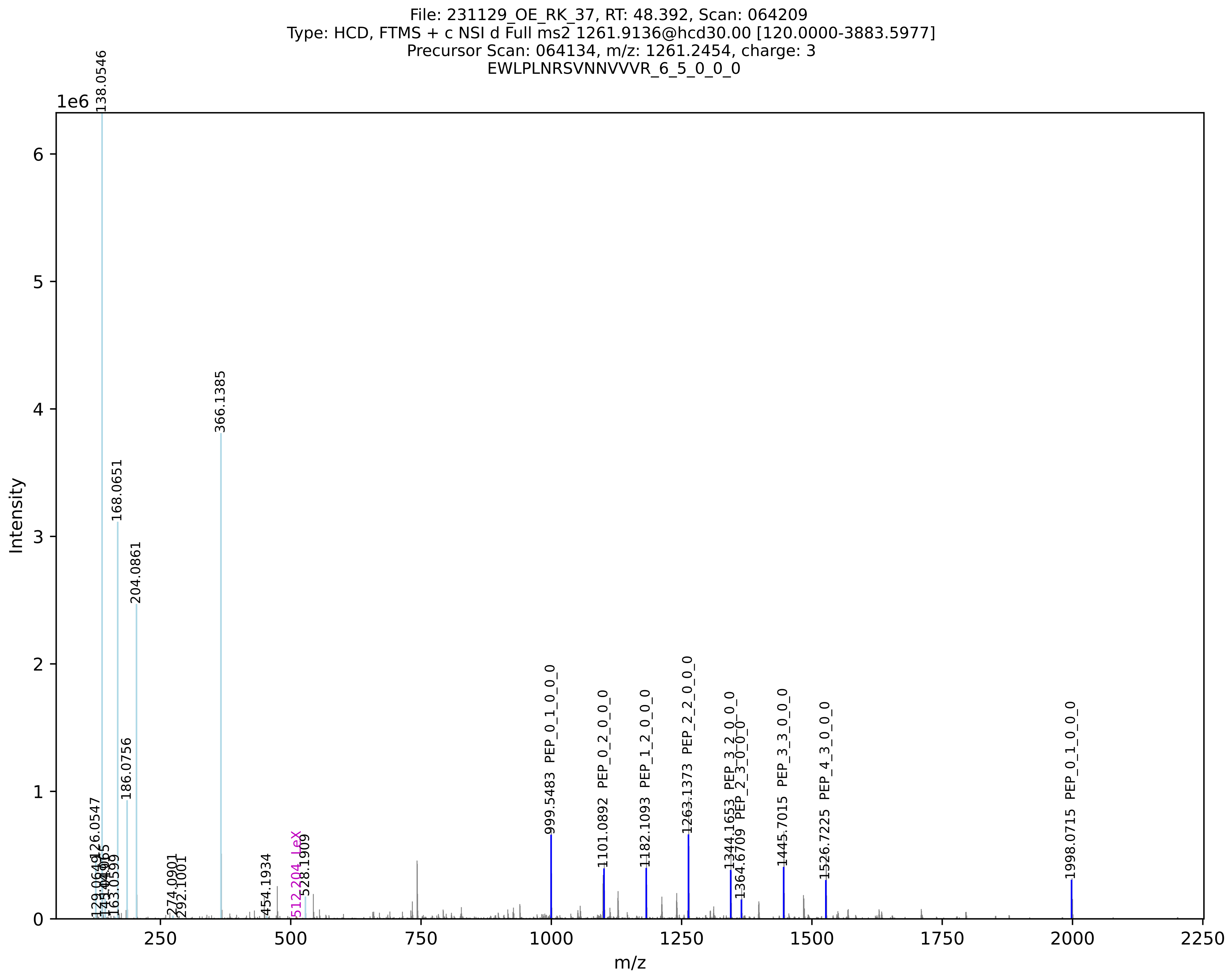
File: 231129_OE_RK_37, RT: 48.392, Scan: 064209
Type: HCD, FTMS + c NSI d Full ms2 1261.9136@hcd30.00 [120.0000-3883.5977]
Precursor Scan: 064134, m/z: 1261.2454, charge: 3
EWLPLNRSVNNVVVR_6_5_0_0_0
1e6
Intensity
m/z
138.0546
366.1385
168.0651
204.0861
186.0756
126.0547
274.0901
292.1001
454.1934
512.204_LeX
528.1909
999.5483 PEP_0_1_0_0_0
1101.0892 PEP_0_2_0_0_0
1182.1093 PEP_1_2_0_0_0
1263.1373 PEP_2_2_0_0_0
1344.1653 PEP_3_2_0_0_0
1364.6709 PEP_2_3_0_0_0
1445.7015 PEP_3_3_0_0_0
1526.7225 PEP_4_3_0_0_0
1998.0715 PEP_0_1_0_0_0

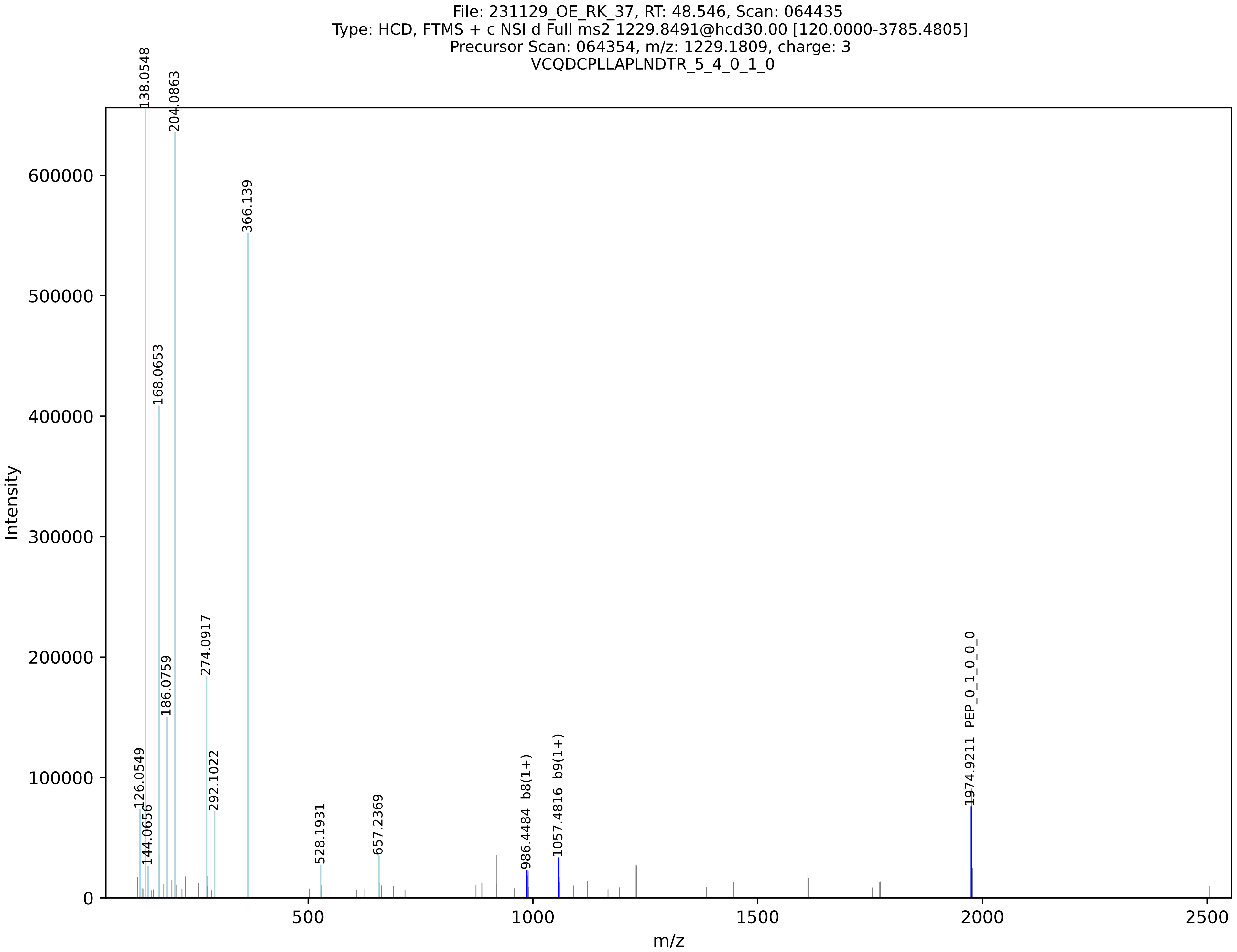
File: 231129_OE_RK_37, RT: 48.546, Scan: 064435
Type: HCD, FTMS + c NSI d Full ms2 1229.8491@hcd30.00 [120.0000-3785.4805]
Precursor Scan: 064354, m/z: 1229.1809, charge: 3
VCQDCPLLAPLNDTR_5_4_0_1_0
Intensity
m/z
138.0548
204.0863
366.139
168.0653
186.0759
274.0917
292.1022
126.0549
144.0656
528.1931
657.2369
986.4484 b8(1+)
1057.4816 b9(1+)
1974.9211 PEP_0_1_0_0_0

File: 231129_OE_RK_37, RT: 48.614, Scan: 064537
Type: HCD, FTMS + c NSI d Full ms2 1501.0106@hcd30.00 [120.0000-4615.2344]
Precursor Scan: 064428, m/z: 1500.684, charge: 3
IIVPLNNRENISDPTSPLR_5_4_1_2_0

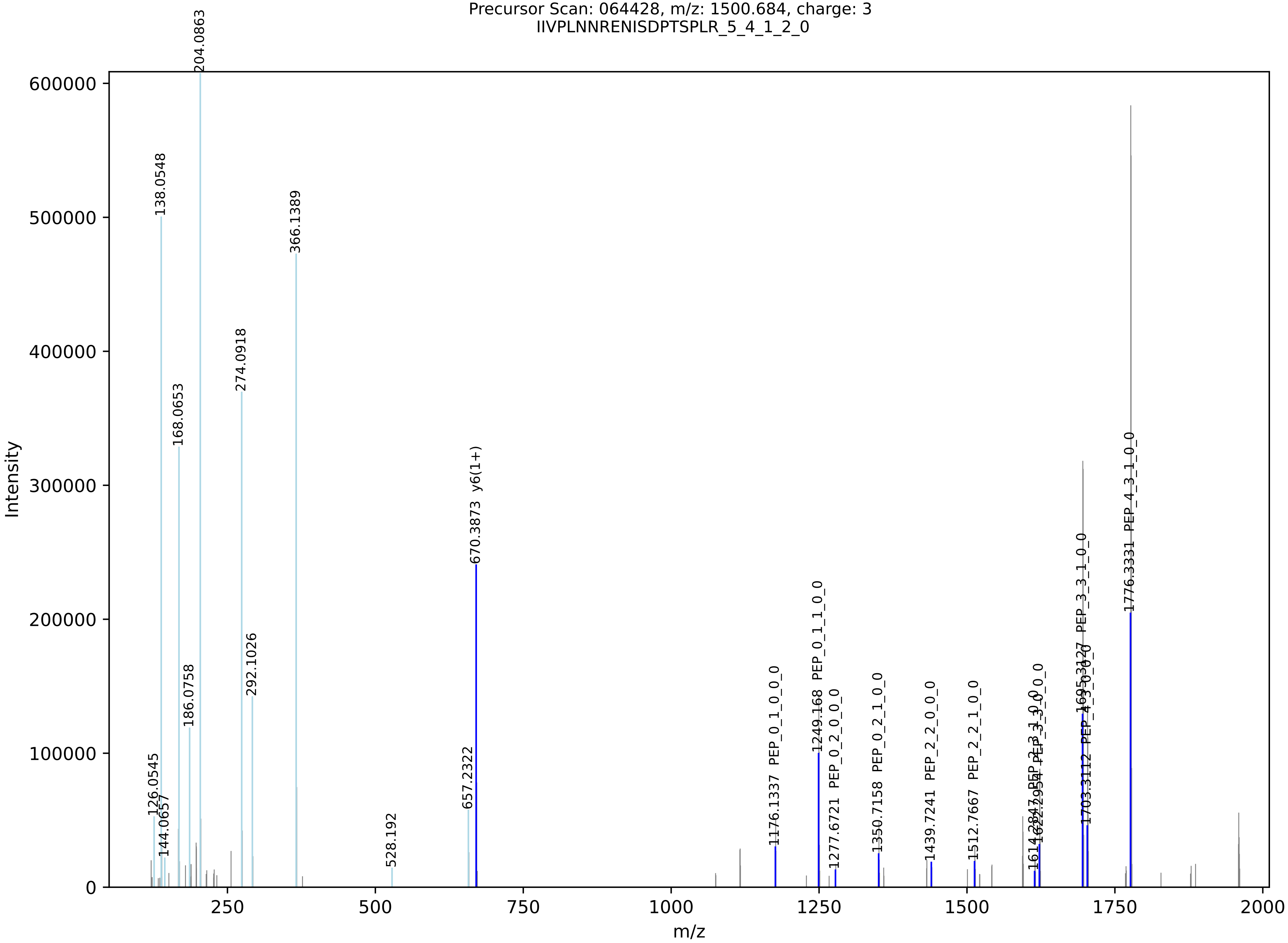

File: 231129_OE_RK_37, RT: 48.65, Scan: 064585
Type: HCD, FTMS + c NSI d Full ms2 1360.0446@hcd30.00 [120.0000-5571.8374]
Precursor Scan: 064502, m/z: 1359.0421, charge: 4
QDQCIYNTTYLNVQR_7_6_0_4_0

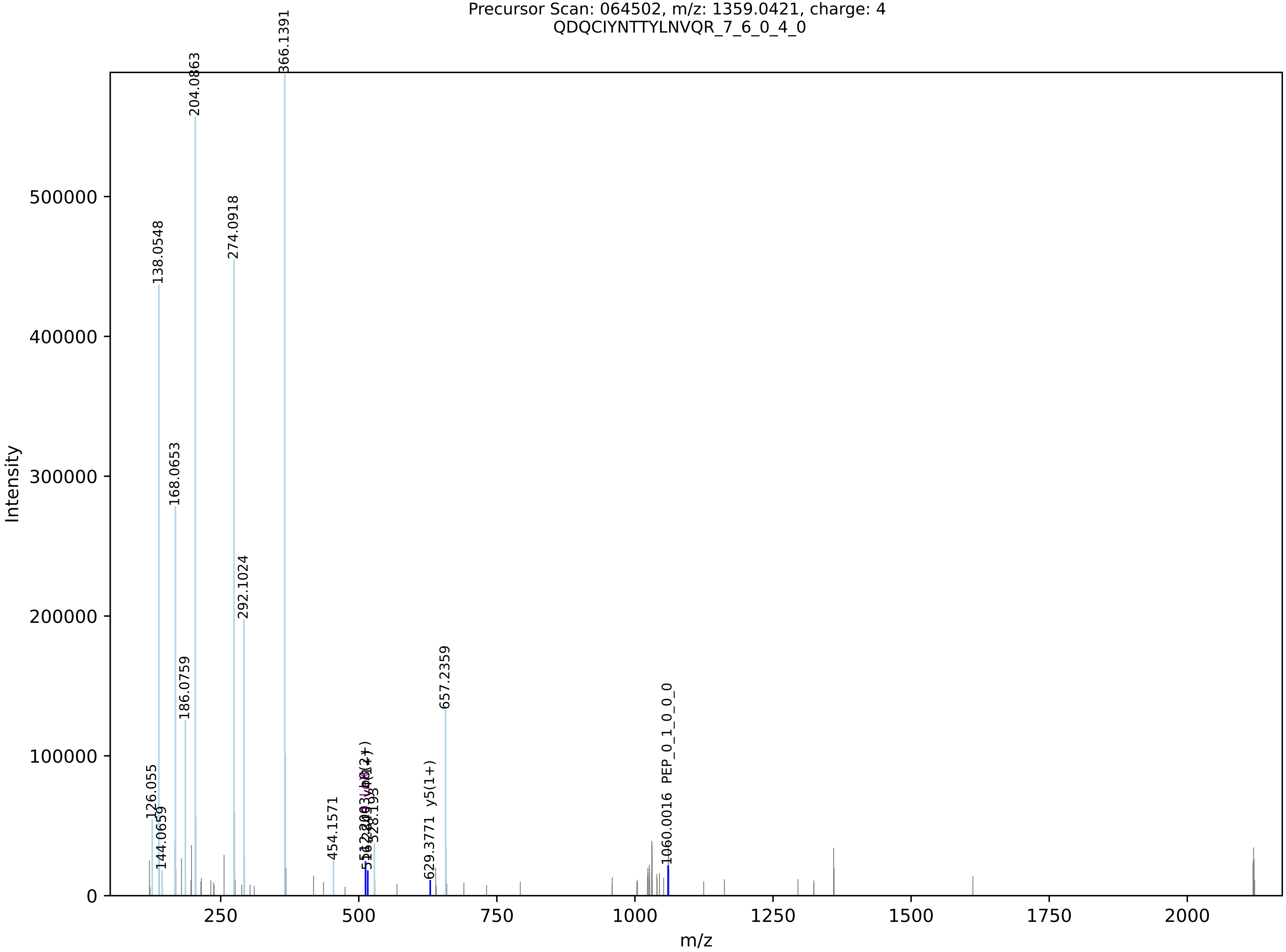

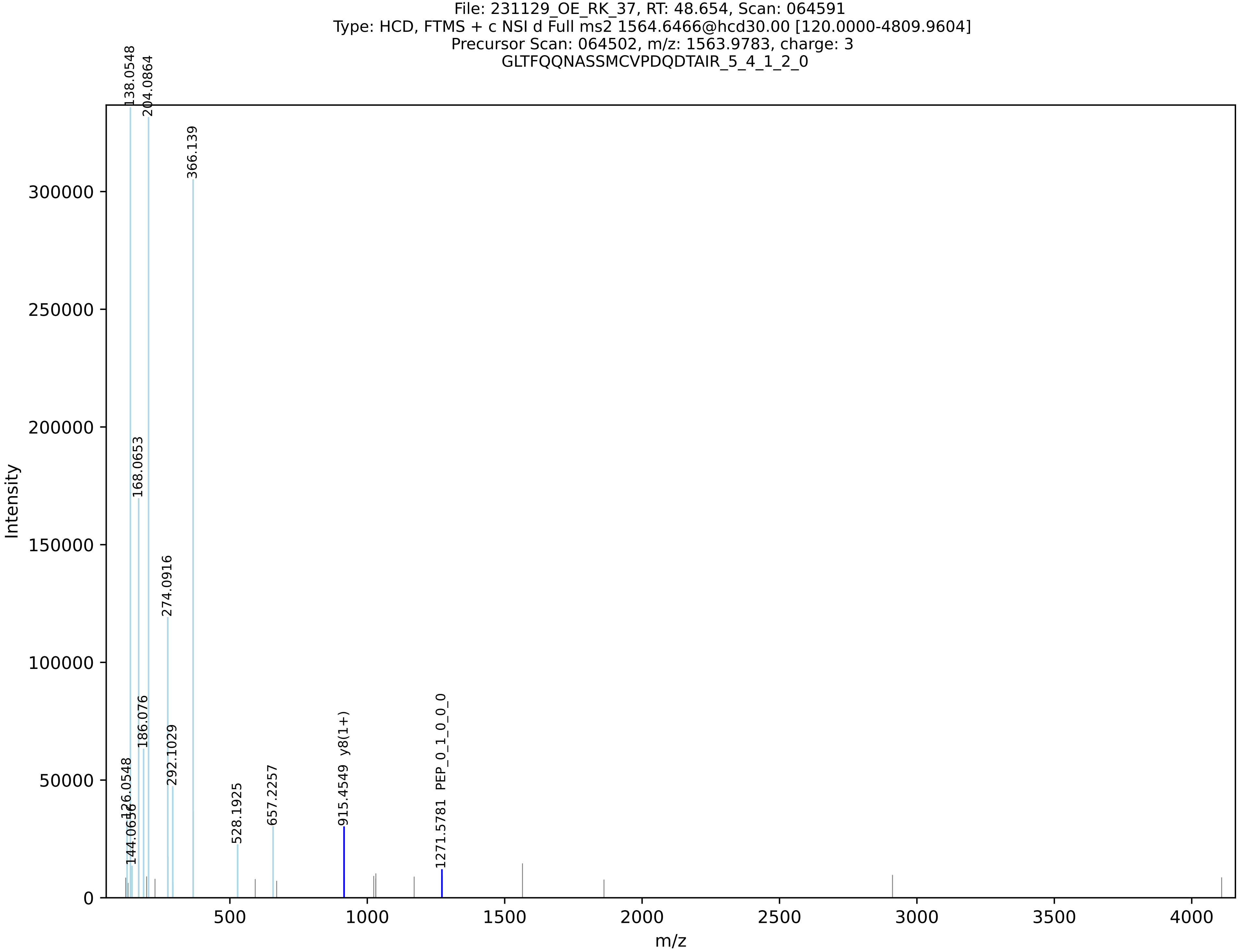

File: 231129_OE_RK_37, RT: 48.654, Scan: 064591
Type: HCD, FTMS + c NSI d Full ms2 1564.6466@hcd30.00 [120.0000-4809.9604]
Precursor Scan: 064502, m/z: 1563.9783, charge: 3
GLTFQQNASSMCVPDQDTAIR_5_4_1_2_0
Intensity
m/z
0
50000
100000
150000
200000
250000
300000
500
1000
1500
2000
2500
3000
3500
4000
138.0548
204.0864
366.139
168.0653
274.0916
186.076
292.1029
126.0548
144.0656
528.1925
657.2257
915.4549 y8(1+)
1271.5781 PEP_0_1_0_0_0

File: 231129_OE_RK_37, RT: 48.66, Scan: 064600
Type: HCD, FTMS + c NSI d Full ms2 1173.7338@hcd30.00 [120.0000-4811.6899]
Precursor Scan: 064502, m/z: 1173.2356, charge: 4
GLTFQQNASSMCVPDQDTAIR_5_4_1_2_0

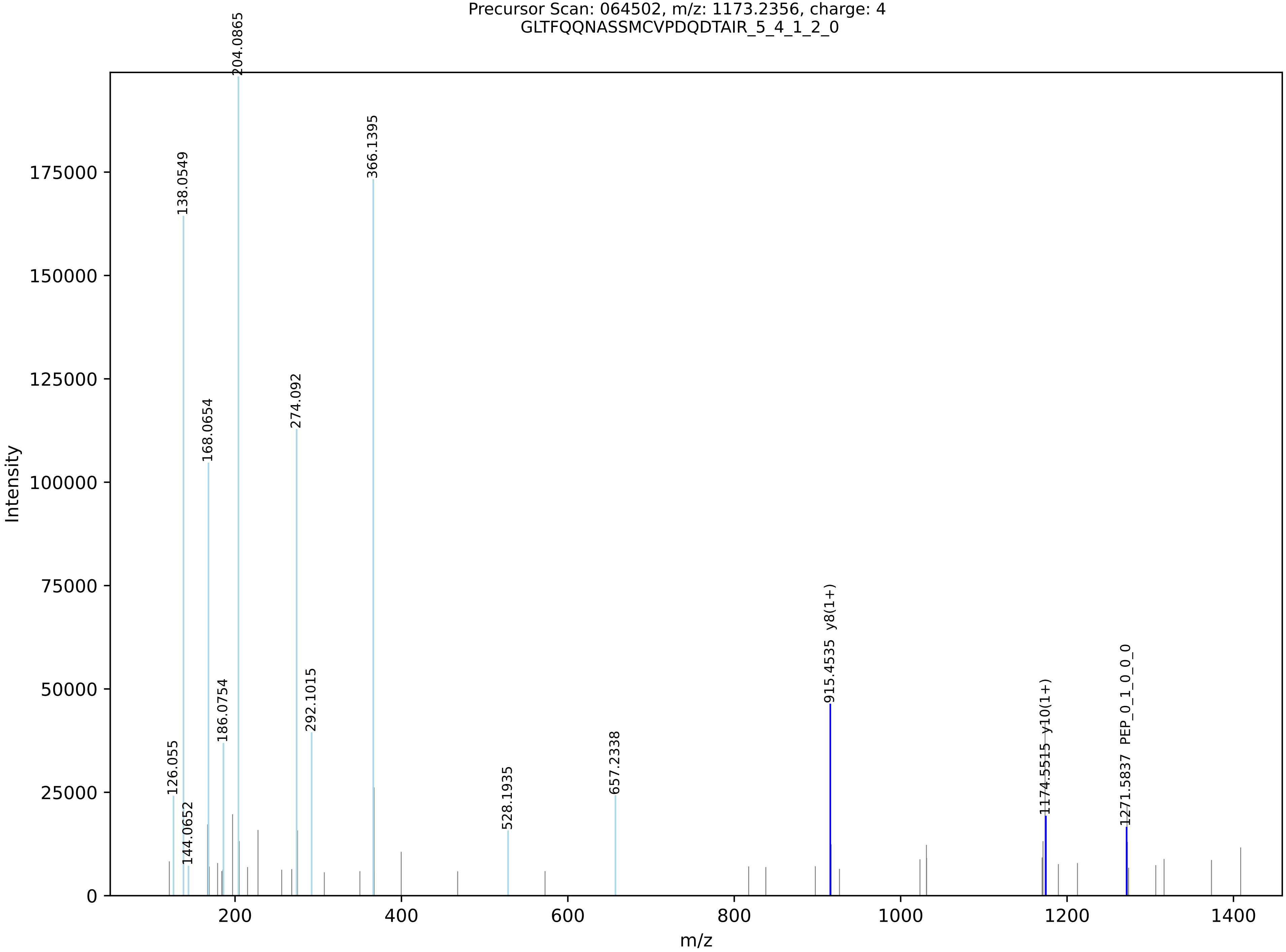

File: 231129_OE_RK_37, RT: 48.748, Scan: 064727
Type: HCD, FTMS + c NSI d Full ms2 1229.8461@hcd30.00 [120.0000-3785.4709]
Precursor Scan: 064647, m/z: 1229.1811, charge: 3
VCQDCPLLAPLNDTR_5_4_0_1_0

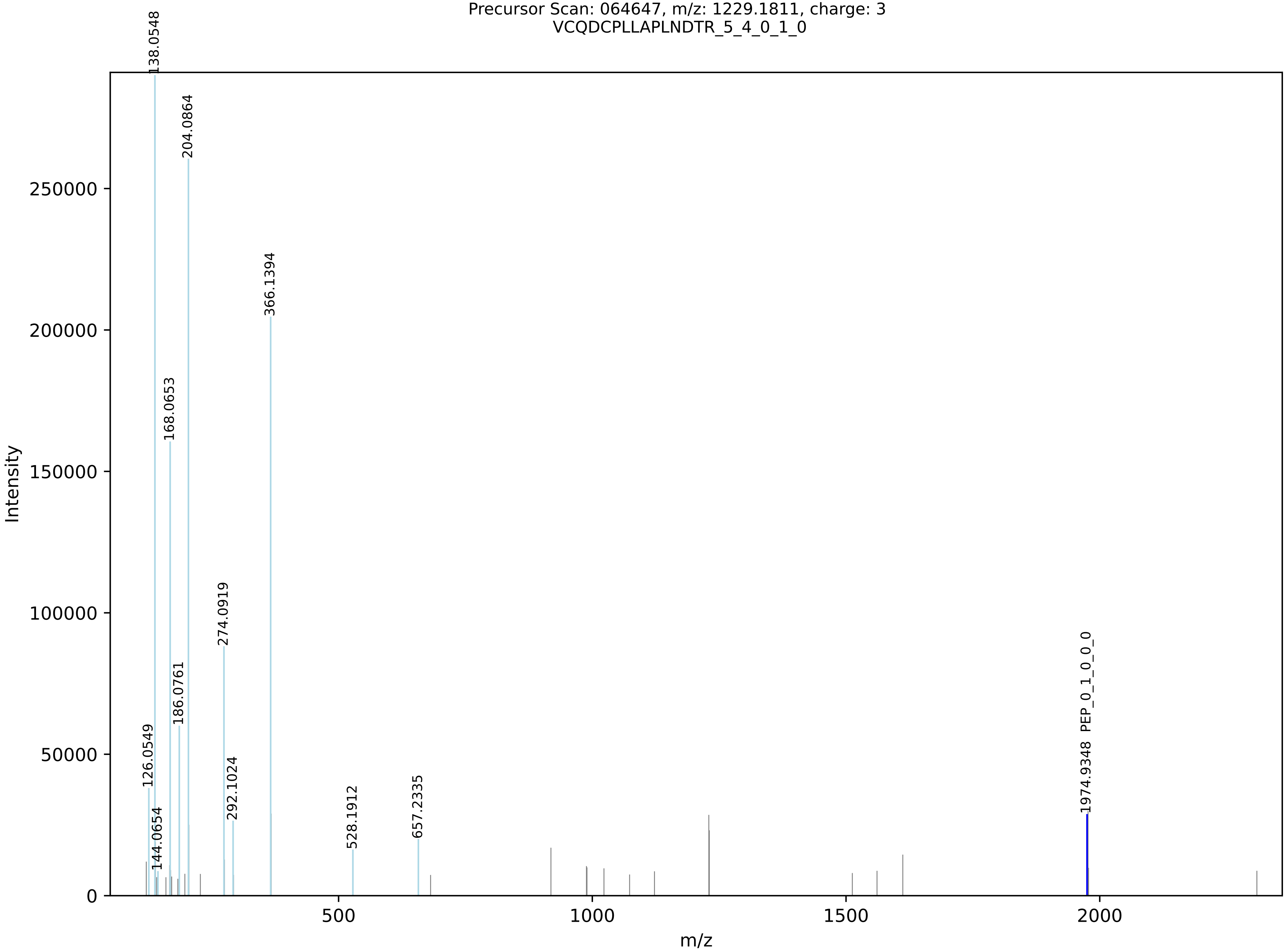

File: 231129_OE_RK_37, RT: 48.75, Scan: 064731
Type: HCD, FTMS + c NSI d Full ms2 1130.2673@hcd30.00 [120.0000-4634.3467]
Precursor Scan: 064647, m/z: 1129.7682, charge: 4
IIVPLNNRENISDPTSPLR_6_4_2_1_0

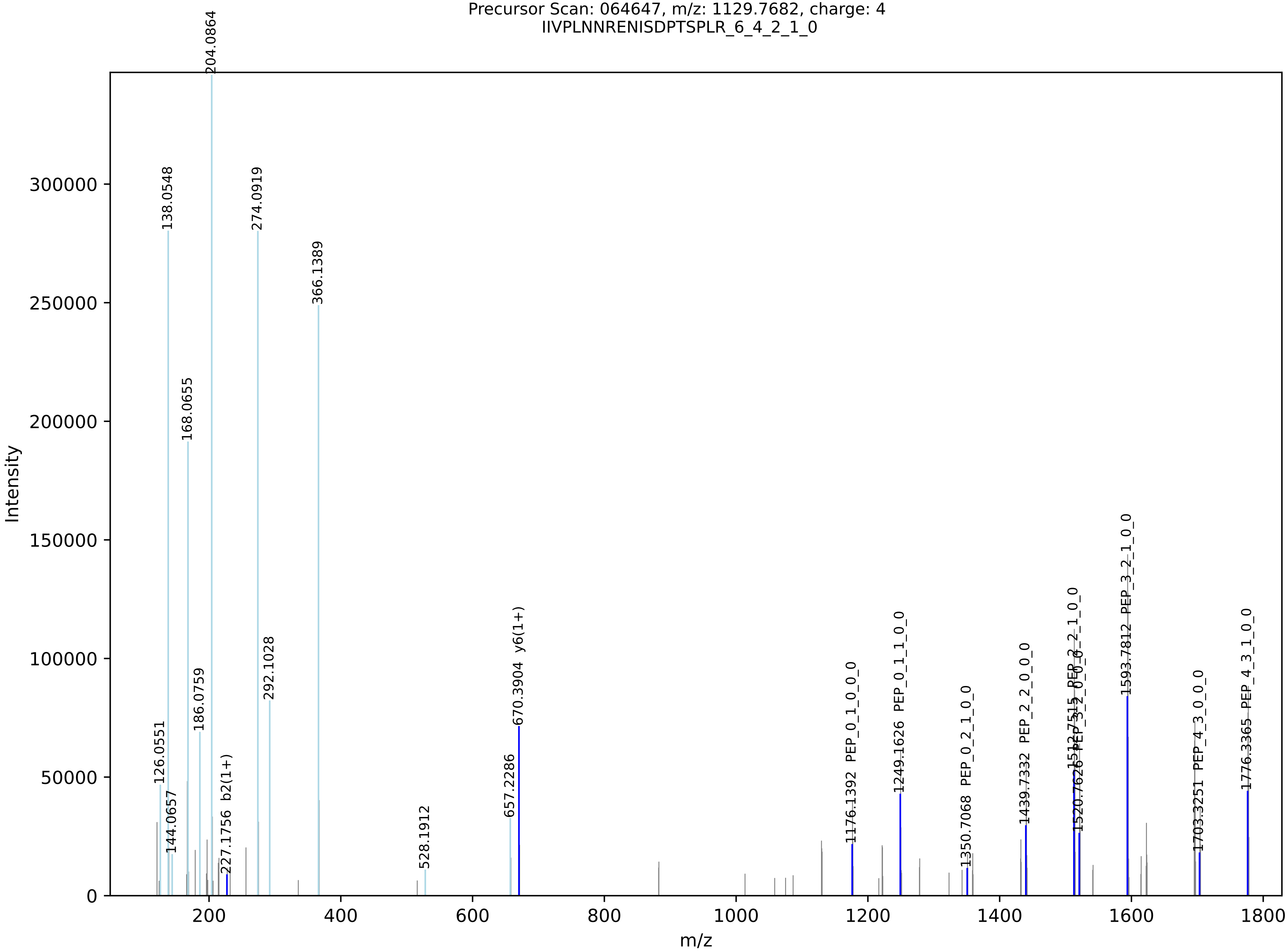

File: 231129_OE_RK_37, RT: 48.798, Scan: 064801
Type: HCD, FTMS + c NSI d Full ms2 1185.5031@hcd30.00 [120.0000-4859.7085]
Precursor Scan: 064720, m/z: 1185.0019, charge: 4
NNVSMPSVASHTRGPEQQVK_6_5_2_1_0

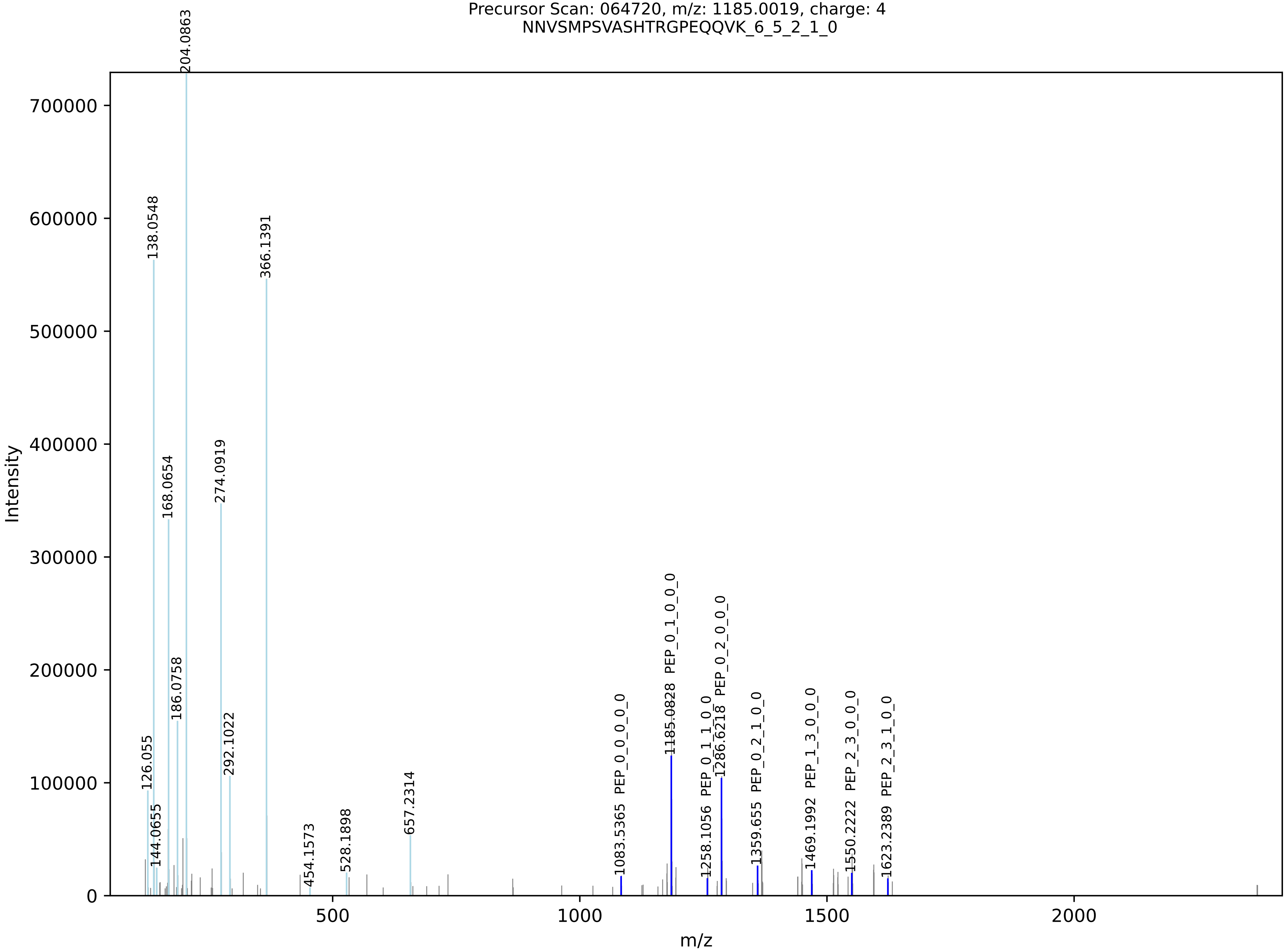

File: 231129_OE_RK_37, RT: 48.901, Scan: 064948
Type: HCD, FTMS + c NSI d Full ms2 1360.0428@hcd30.00 [120.0000-5571.8311]
Precursor Scan: 064868, m/z: 1359.2911, charge: 4
QDQCIYNTTYLNVQR_7_6_2_3_0

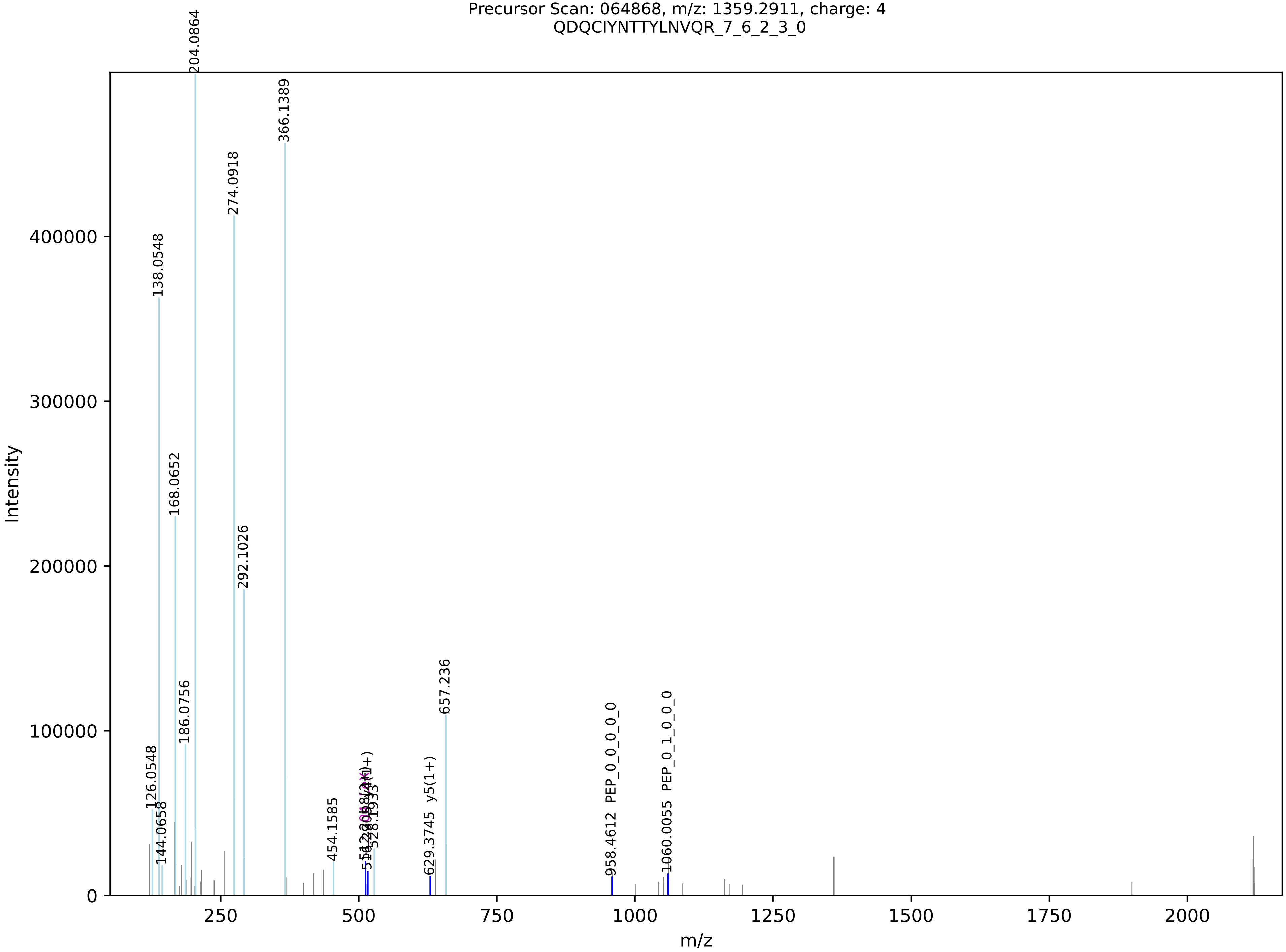

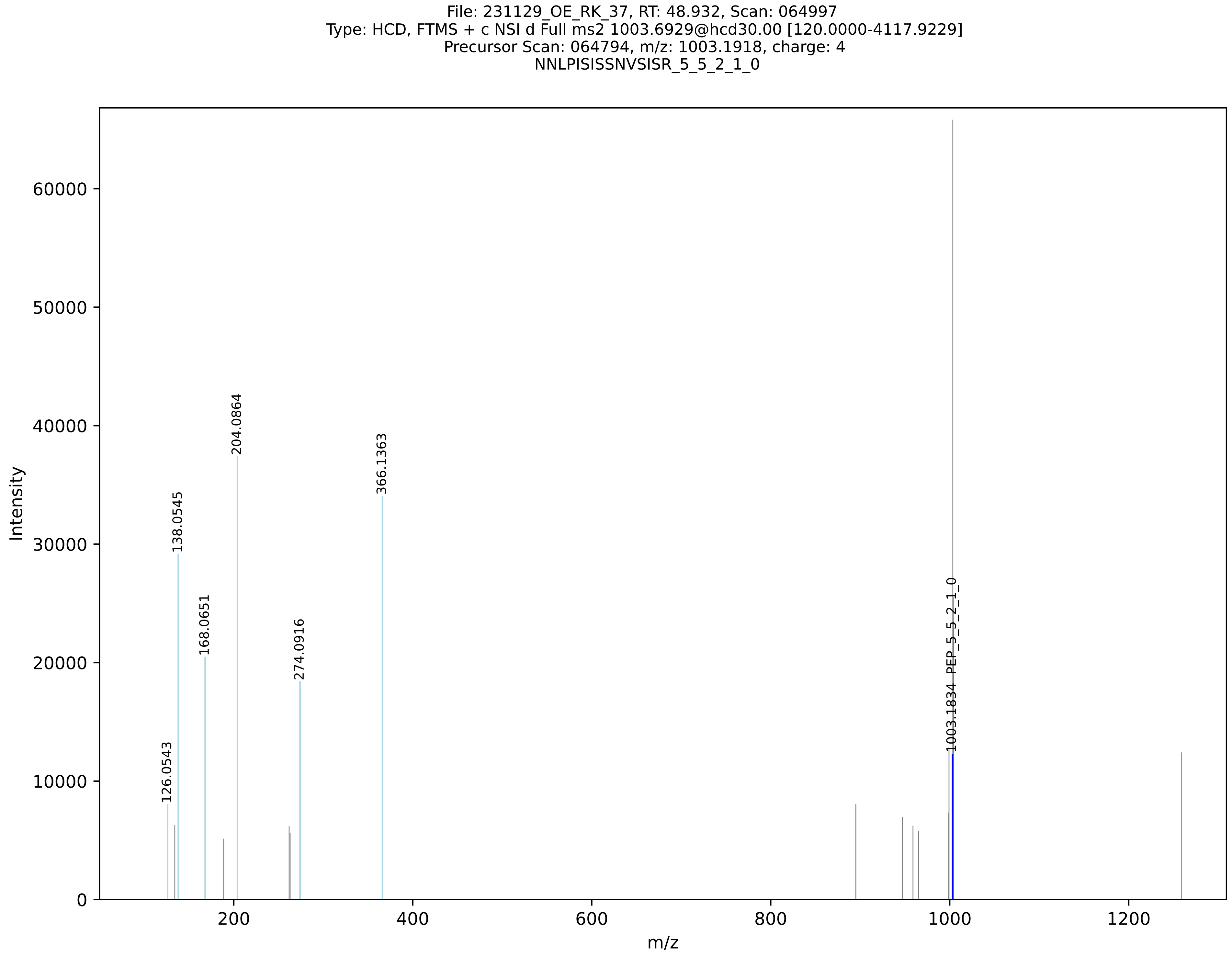
File: 231129_OE_RK_37, RT: 48.932, Scan: 064997
Type: HCD, FTMS + c NSI d Full ms2 1003.6929@hcd30.00 [120.0000-4117.9229]
Precursor Scan: 064794, m/z: 1003.1918, charge: 4
NNLPISISSNVSISR_5_5_2_1_0
Intensity
m/z
126.0543
138.0545
168.0651
204.0864
274.0916
366.1363
1003.1834_PEP_5_5_2_1_0_
0
10000
20000
30000
40000
50000
60000
200
400
600
800
1000
1200

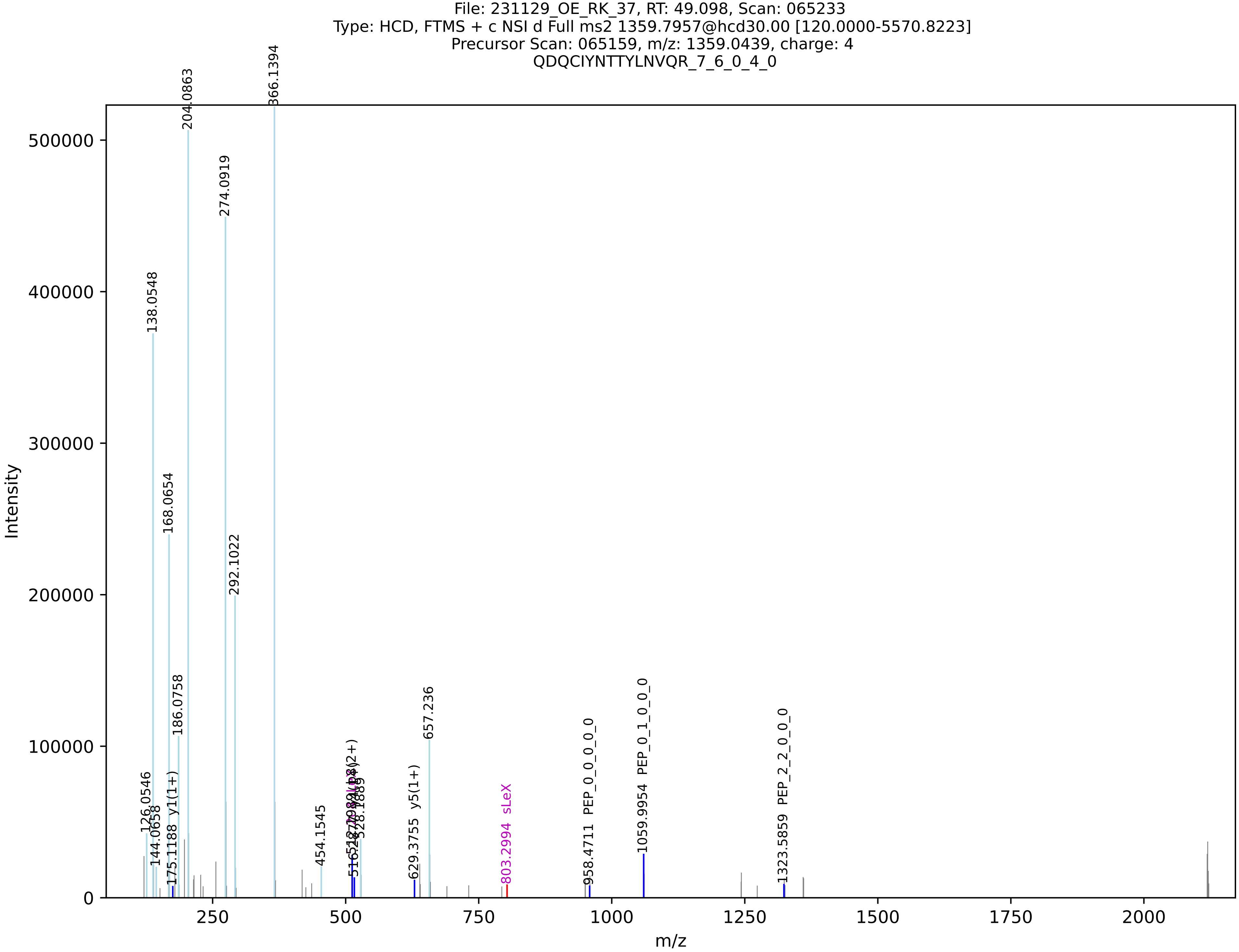
File: 231129_OE_RK_37, RT: 49.098, Scan: 065233
Type: HCD, FTMS + c NSI d Full ms2 1359.7957@hcd30.00 [120.0000-5570.8223]
Precursor Scan: 065159, m/z: 1359.0439, charge: 4
QDQCIYNTTYLNVQR_7_6_0_4_0
Intensity
m/z
366.1394
204.0863
274.0919
138.0548
168.0654
292.1022
186.0758
126.0546
144.0658
175.1188 y1(1+)
454.1545
528.1889
629.3755 y5(1+)
657.236
803.2994 sLeX
958.4711 PEP_0_0_0_0_0
1059.9954 PEP_0_1_0_0_0
1323.5859 PEP_2_2_0_0_0
0
100000
200000
300000
400000
500000
250
500
750
1000
1250
1500
1750
2000

File: 231129_OE_RK_37, RT: 49.1, Scan: 065236
Type: HCD, FTMS + c NSI d Full ms2 1089.4958@hcd30.00 [120.0000-4467.9995]
Precursor Scan: 065159, m/z: 1088.9988, charge: 4
IIVPLNNRENISDPTSPLR_5_4_0_2_0

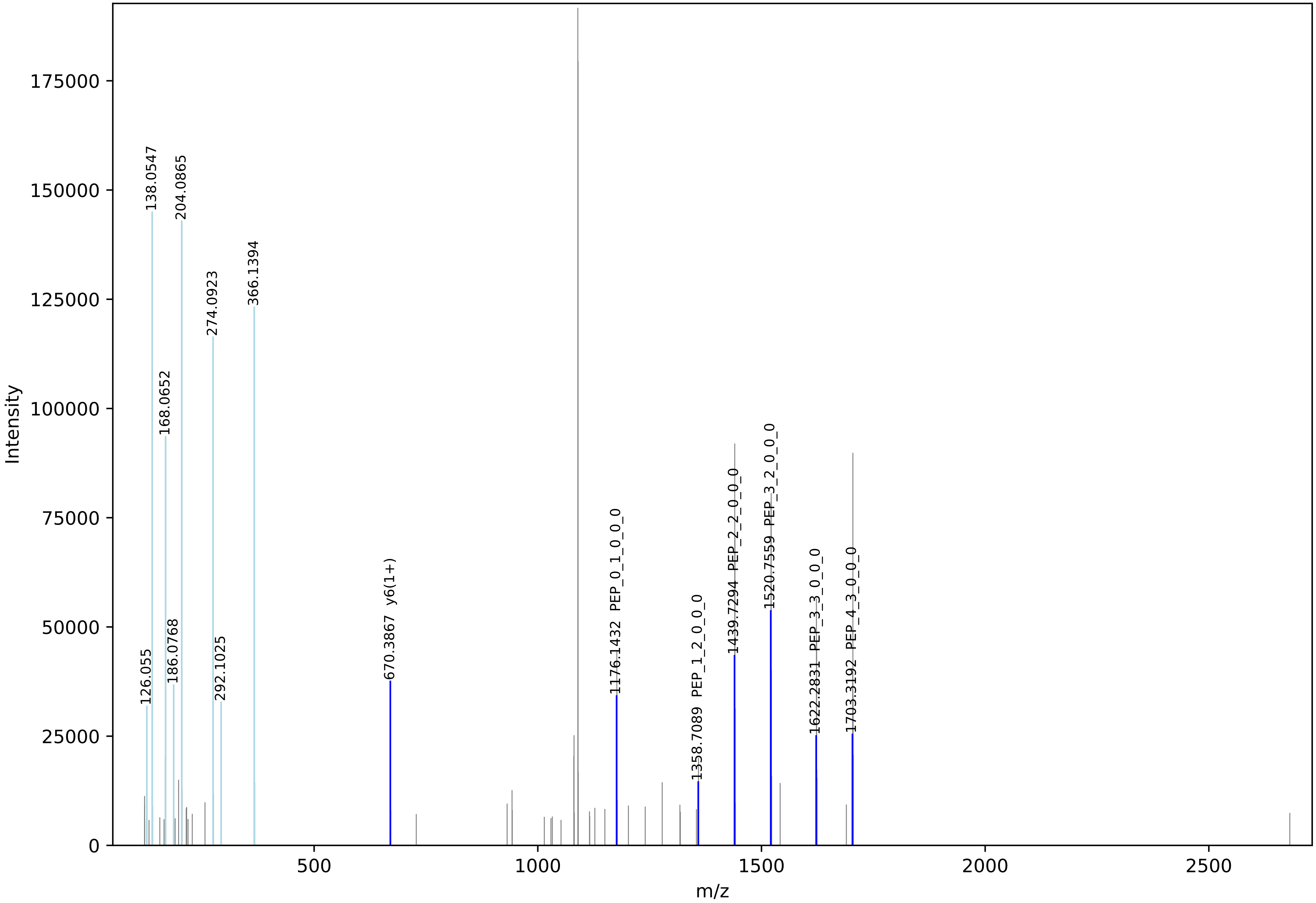

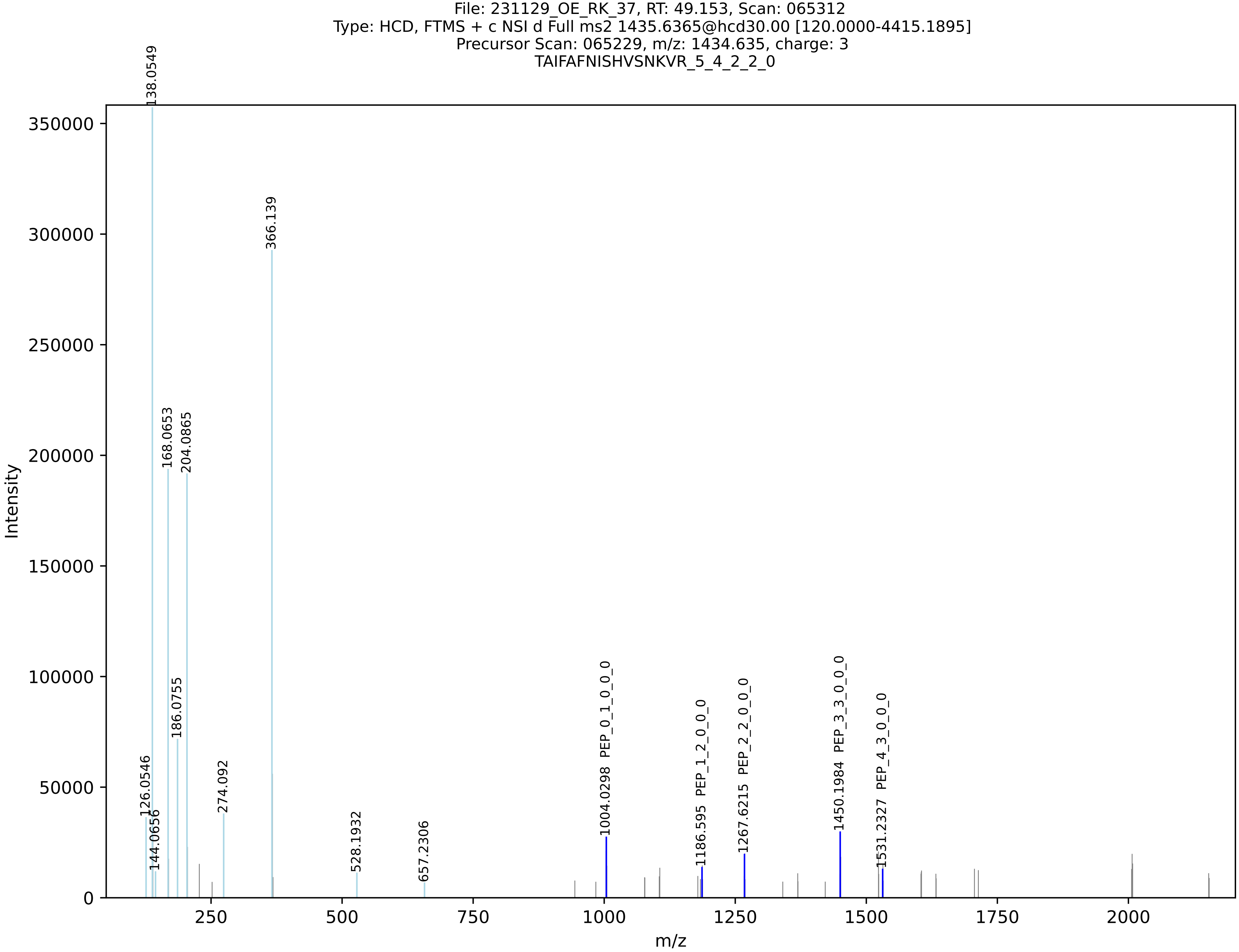
File: 231129_OE_RK_37, RT: 49.153, Scan: 065312
Type: HCD, FTMS + c NSI d Full ms2 1435.6365@hcd30.00 [120.0000-4415.1895]
Precursor Scan: 065229, m/z: 1434.635, charge: 3
TAIFAFNISHVSNKVR_5_4_2_2_0
138.0549
366.139
168.0653
204.0865
186.0755
126.0546
144.0656
274.092
528.1932
657.2306
1004.0298 PEP_0_1_0_0_0
1186.595 PEP_1_2_0_0_0
1267.6215 PEP_2_2_0_0_0
1450.1984 PEP_3_3_0_0_0
1531.2327 PEP_4_3_0_0_0
Intensity
m/z

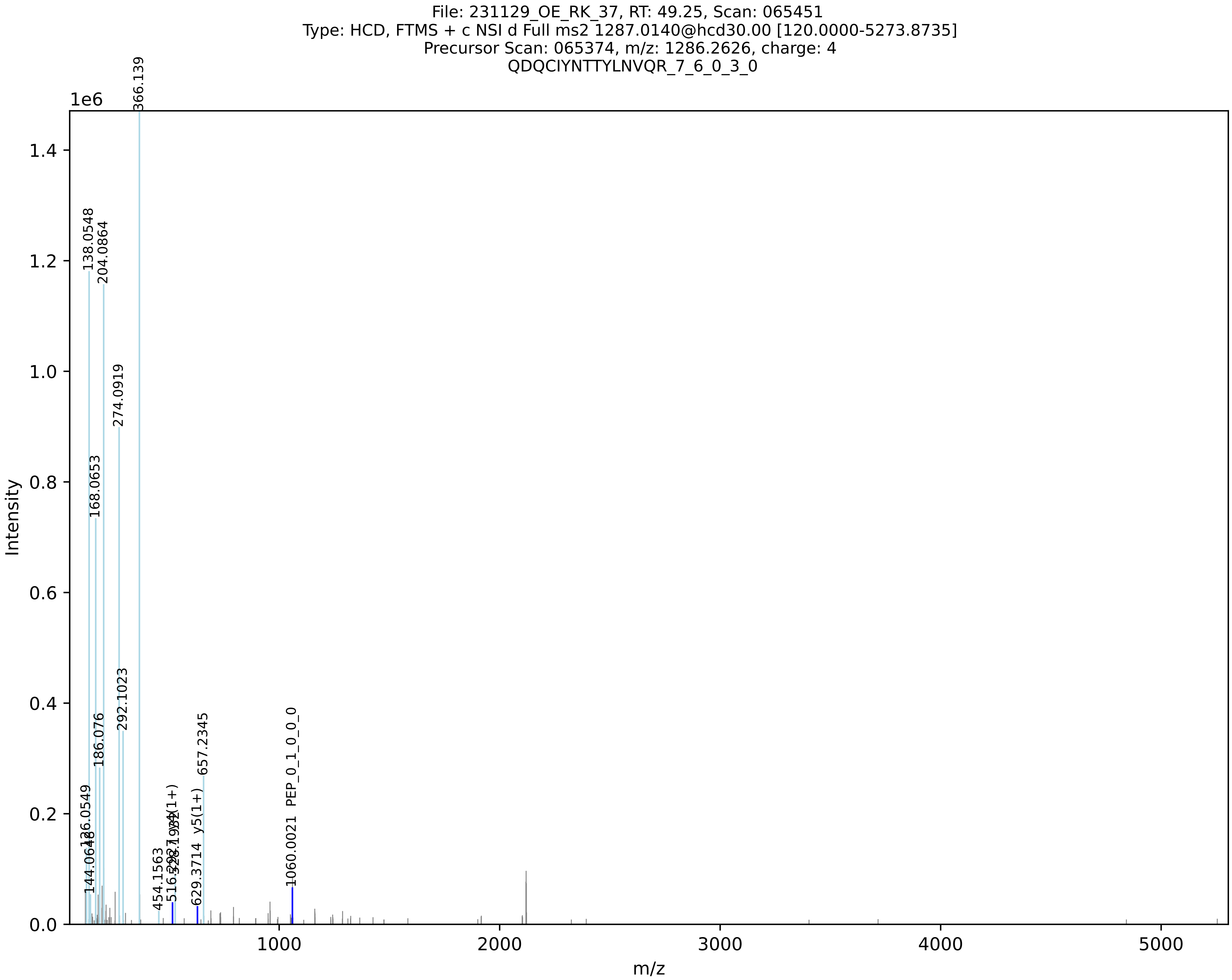
File: 231129_OE_RK_37, RT: 49.25, Scan: 065451
Type: HCD, FTMS + c NSI d Full ms2 1287.0140@hcd30.00 [120.0000-5273.8735]
Precursor Scan: 065374, m/z: 1286.2626, charge: 4
QDQCIYNTTYLNVQR_7_6_0_3_0
1e6
Intensity
m/z
366.139
138.0548
204.0864
274.0919
168.0653
292.1023
186.076
126.0549
144.0648
454.1563
657.2345
y5(1+)
629.3714
1060.0021
PEP_0_1_0_0_0
1.4
1.2
1.0
0.8
0.6
0.4
0.2
0.0
1000
2000
3000
4000
5000

File: 231129_OE_RK_37, RT: 49.302, Scan: 065528
Type: HCD, FTMS + c NSI d Full ms2 1359.7905@hcd30.00 [120.0000-5570.8013]
Precursor Scan: 065447, m/z: 1359.0387, charge: 4
QDQCIYNTTYLNVQR_7_6_0_4_0

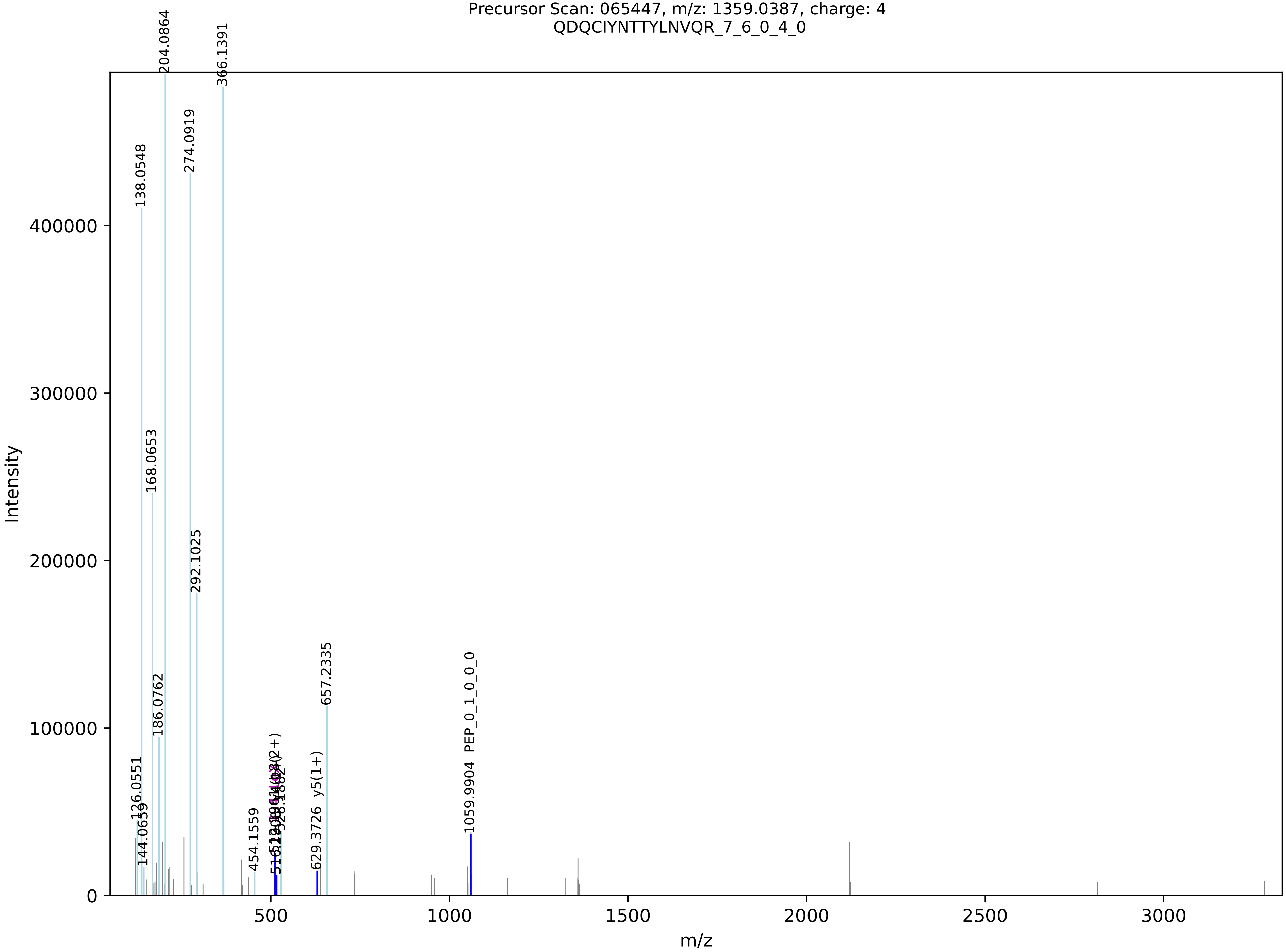

File: 231129_OE_RK_37, RT: 49.501, Scan: 065819
Type: HCD, FTMS + c NSI d Full ms2 1359.7942@hcd30.00 [120.0000-5570.8164]
Precursor Scan: 065739, m/z: 1359.0424, charge: 4
QDQCIYNTTYLNVQR_7_6_0_4_0

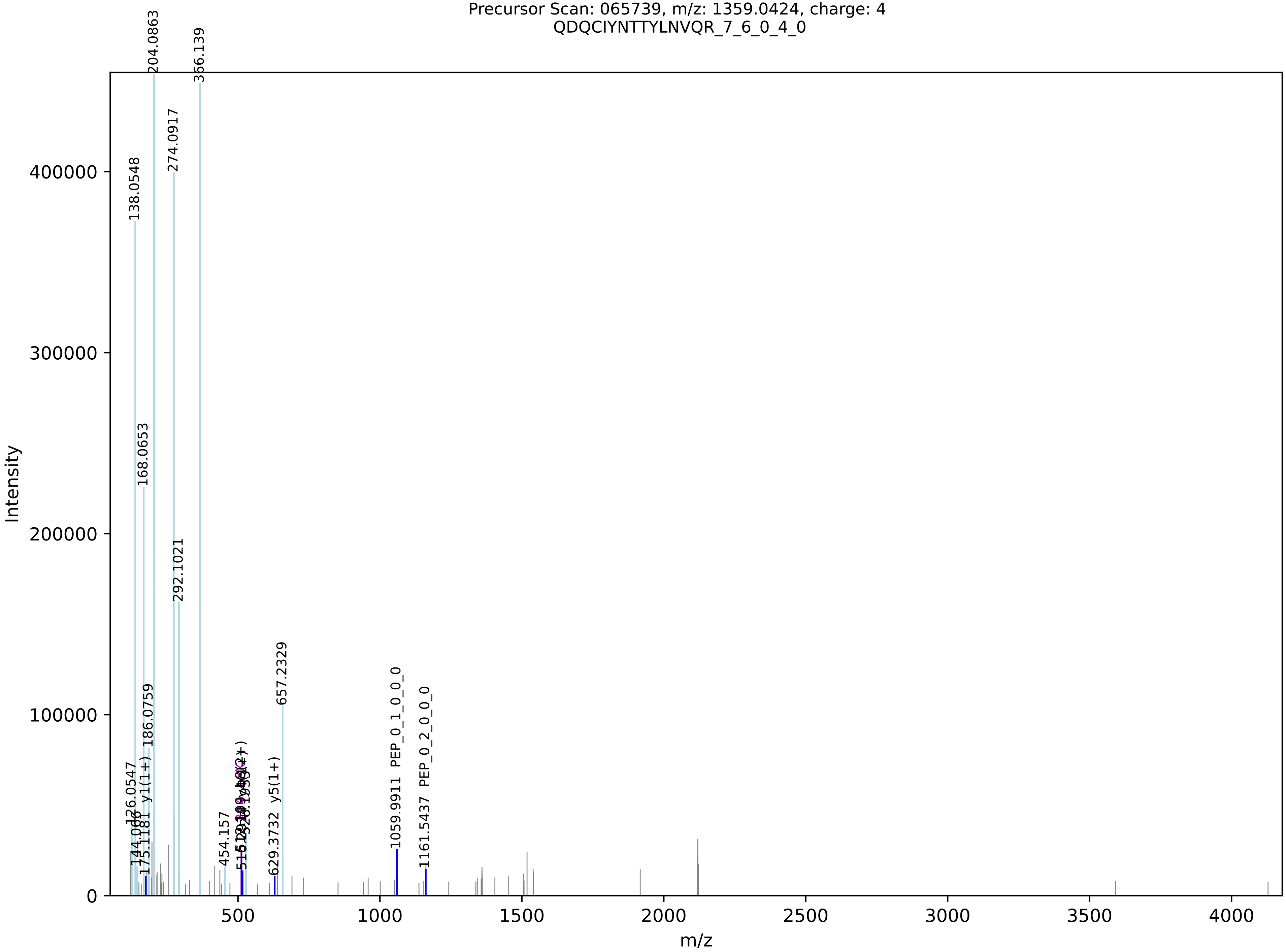

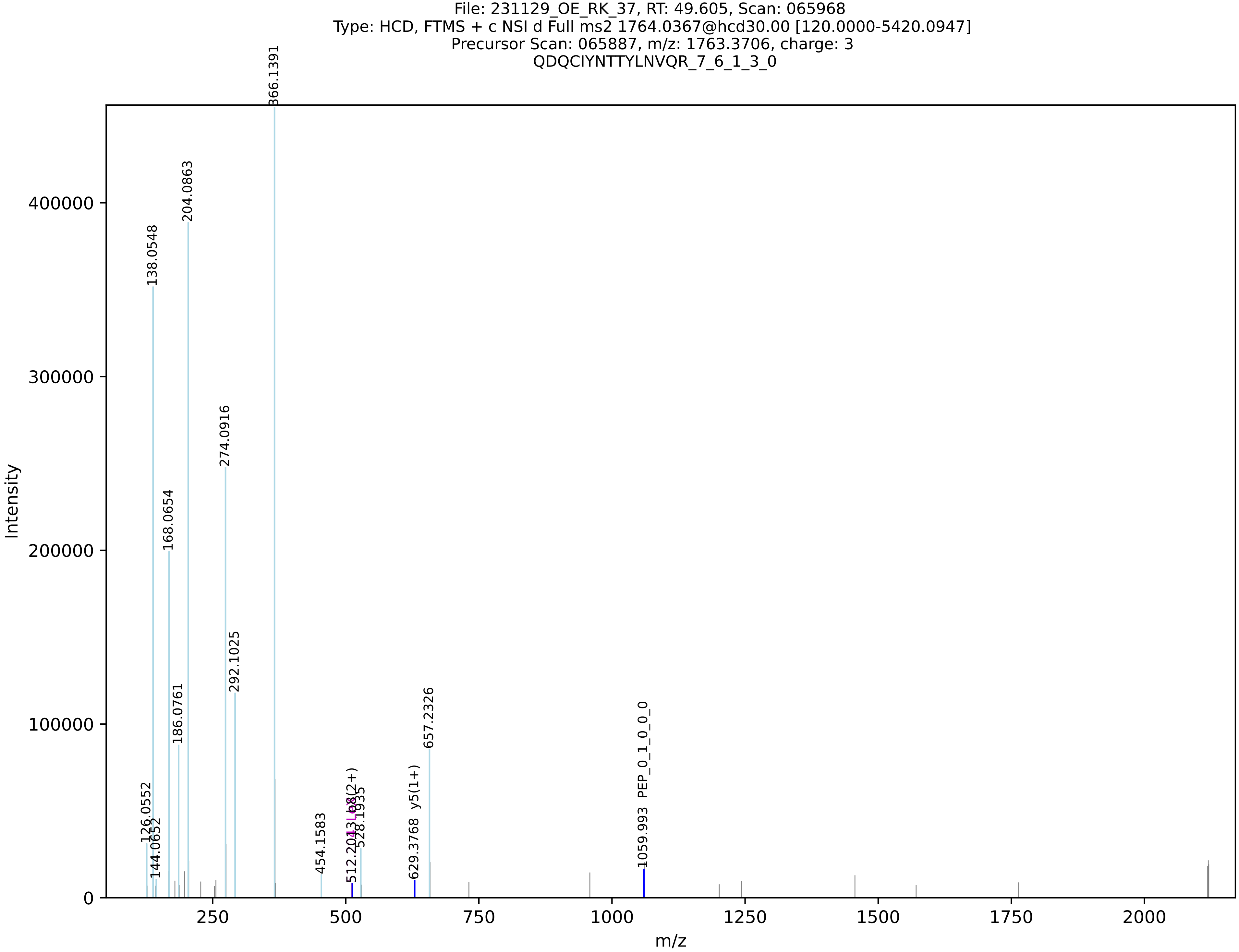
File: 231129_OE_RK_37, RT: 49.605, Scan: 065968
Type: HCD, FTMS + c NSI d Full ms2 1764.0367@hcd30.00 [120.0000-5420.0947]
Precursor Scan: 065887, m/z: 1763.3706, charge: 3
QDQCIYNTTYLNVQR_7_6_1_3_0
Intensity
m/z
366.1391
204.0863
138.0548
274.0916
168.0654
292.1025
186.0761
657.2326
126.0552
144.0652
454.1583
512.2013 b8(2+)
528.1935
629.3768 y5(1+)
1059.993 PEP_0_1_0_0_0

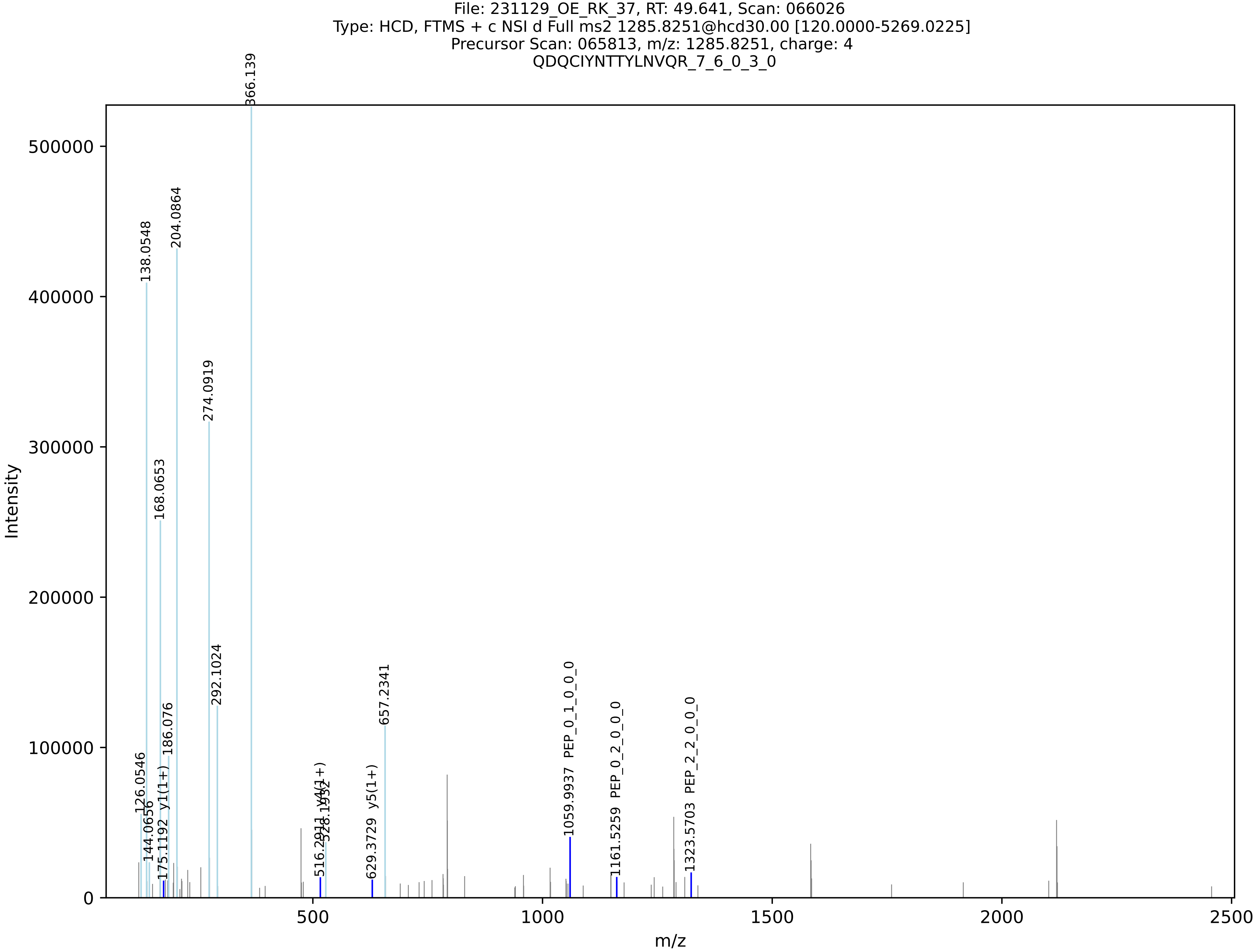
File: 231129_OE_RK_37, RT: 49.641, Scan: 066026
Type: HCD, FTMS + c NSI d Full ms2 1285.8251@hcd30.00 [120.0000-5269.0225]
Precursor Scan: 065813, m/z: 1285.8251, charge: 4
QDQCIYNTTYLNVQR_7_6_0_3_0
366.139
204.0864
138.0548
274.0919
168.0653
292.1024
186.076
126.0546
144.0656
175.1192 y1(1+)
516.2914 y4(1+)
528.1932
629.3729 y5(1+)
657.2341
1059.9937 PEP_0_1_0_0_0
1161.5259 PEP_0_2_0_0_0
1323.5703 PEP_2_2_0_0_0
Intensity
m/z
0
100000
200000
300000
400000
500000
500
1000
1500
2000
2500

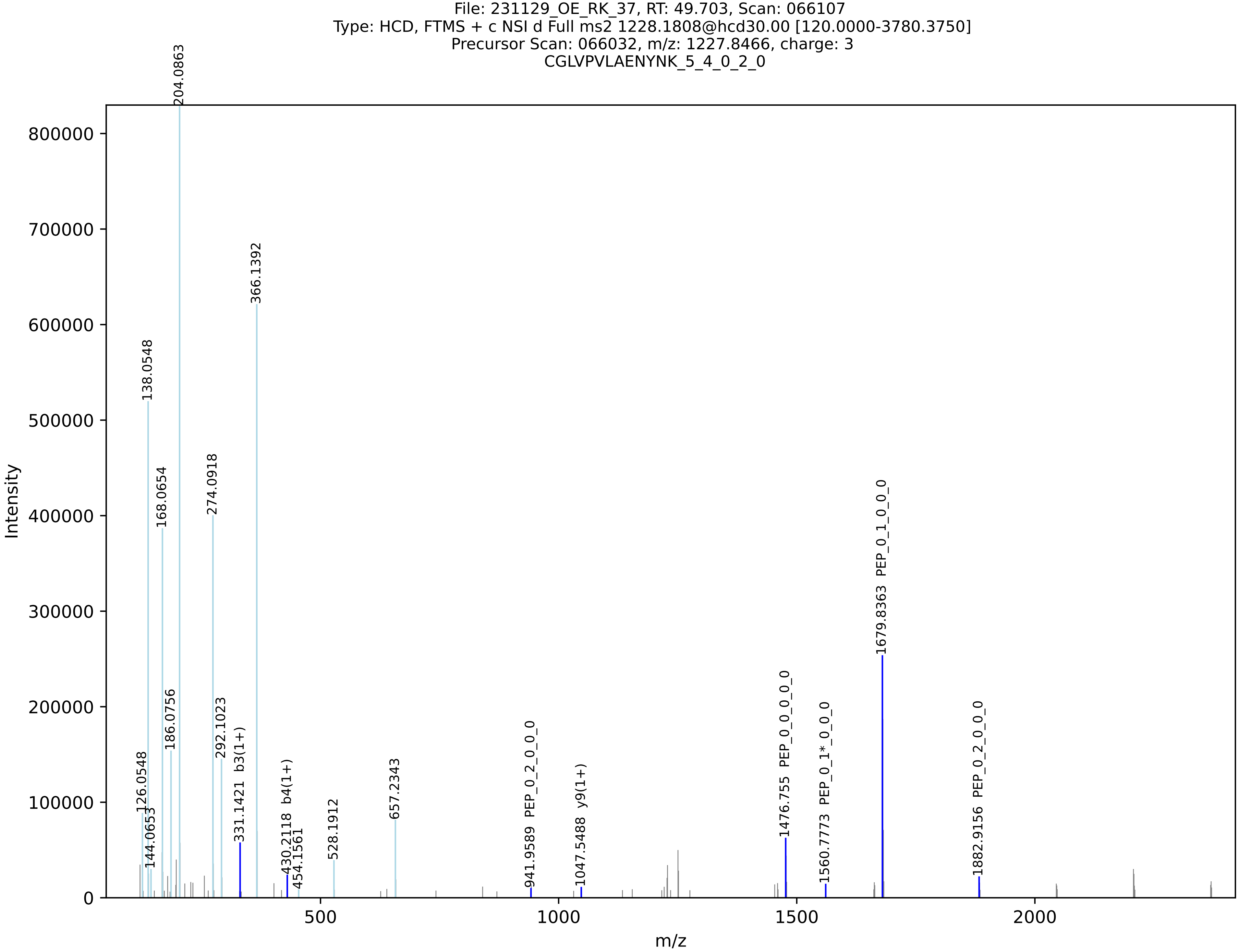
File: 231129_OE_RK_37, RT: 49.703, Scan: 066107
Type: HCD, FTMS + c NSI d Full ms2 1228.1808@hcd30.00 [120.0000-3780.3750]
Precursor Scan: 066032, m/z: 1227.8466, charge: 3
CGLVPVLAENYNK_5_4_0_2_0
Intensity
m/z
204.0863
366.1392
138.0548
168.0654
274.0918
186.0756
292.1023
126.0548
144.0653
331.1421 b3(1+)
430.2118 b4(1+)
454.1561
528.1912
657.2343
941.9589 PEP_0_2_0_0_0
1047.5488 y9(1+)
1476.755 PEP_0_0_0_0_0
1560.7773 PEP_0_1*_0_0_0
1679.8363 PEP_0_1_0_0_0
1882.9156 PEP_0_2_0_0_0

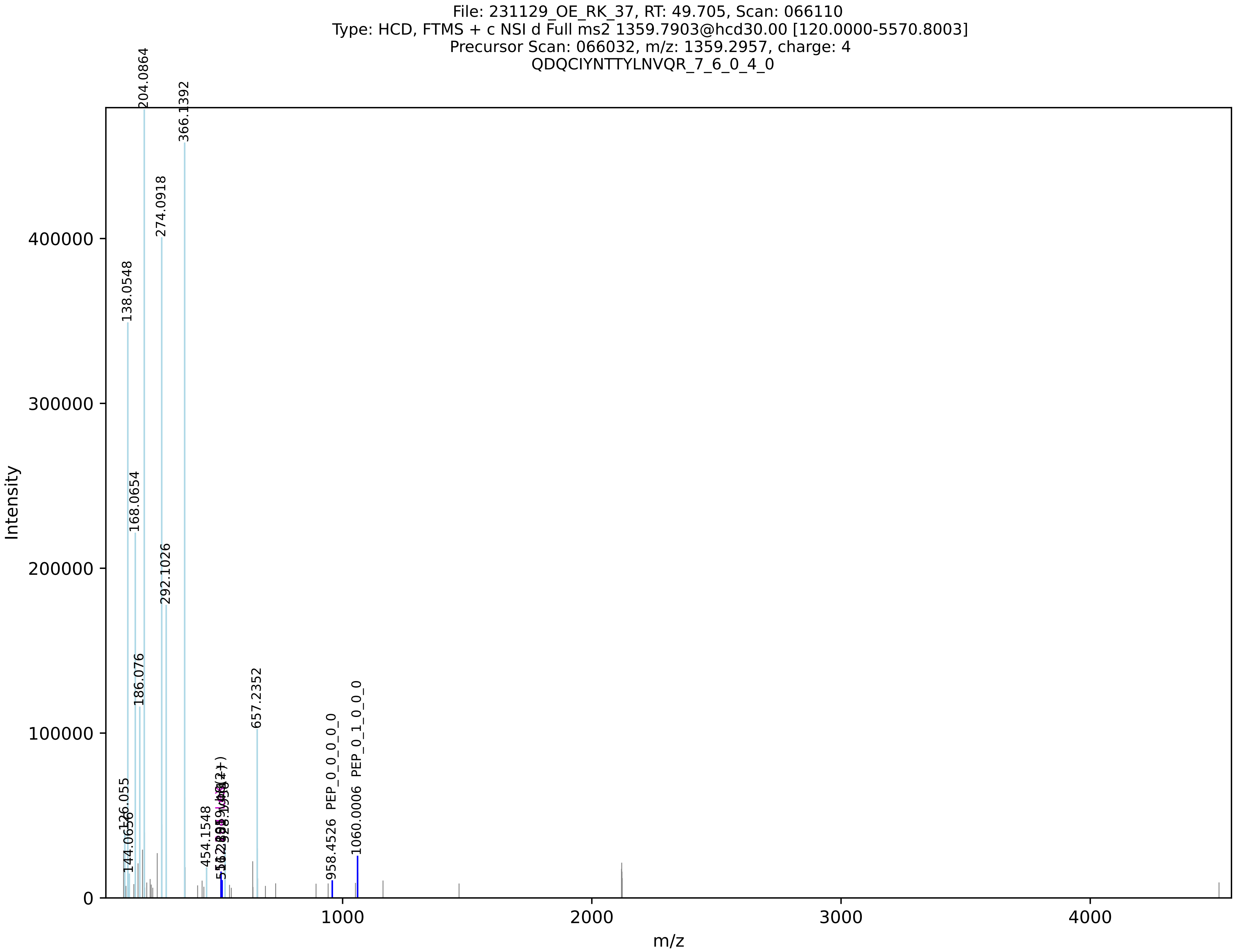
File: 231129_OE_RK_37, RT: 49.705, Scan: 066110
Type: HCD, FTMS + c NSI d Full ms2 1359.7903@hcd30.00 [120.0000-5570.8003]
Precursor Scan: 066032, m/z: 1359.2957, charge: 4
QDQCIYNTTYLNVQR_7_6_0_4_0
Intensity
m/z
204.0864
366.1392
274.0918
138.0548
168.0654
292.1026
186.076
657.2352
126.055
144.0656
454.1548
958.4526 PEP_0_0_0_0_0
1060.0006 PEP_0_1_0_0_0
0
100000
200000
300000
400000
1000
2000
3000
4000

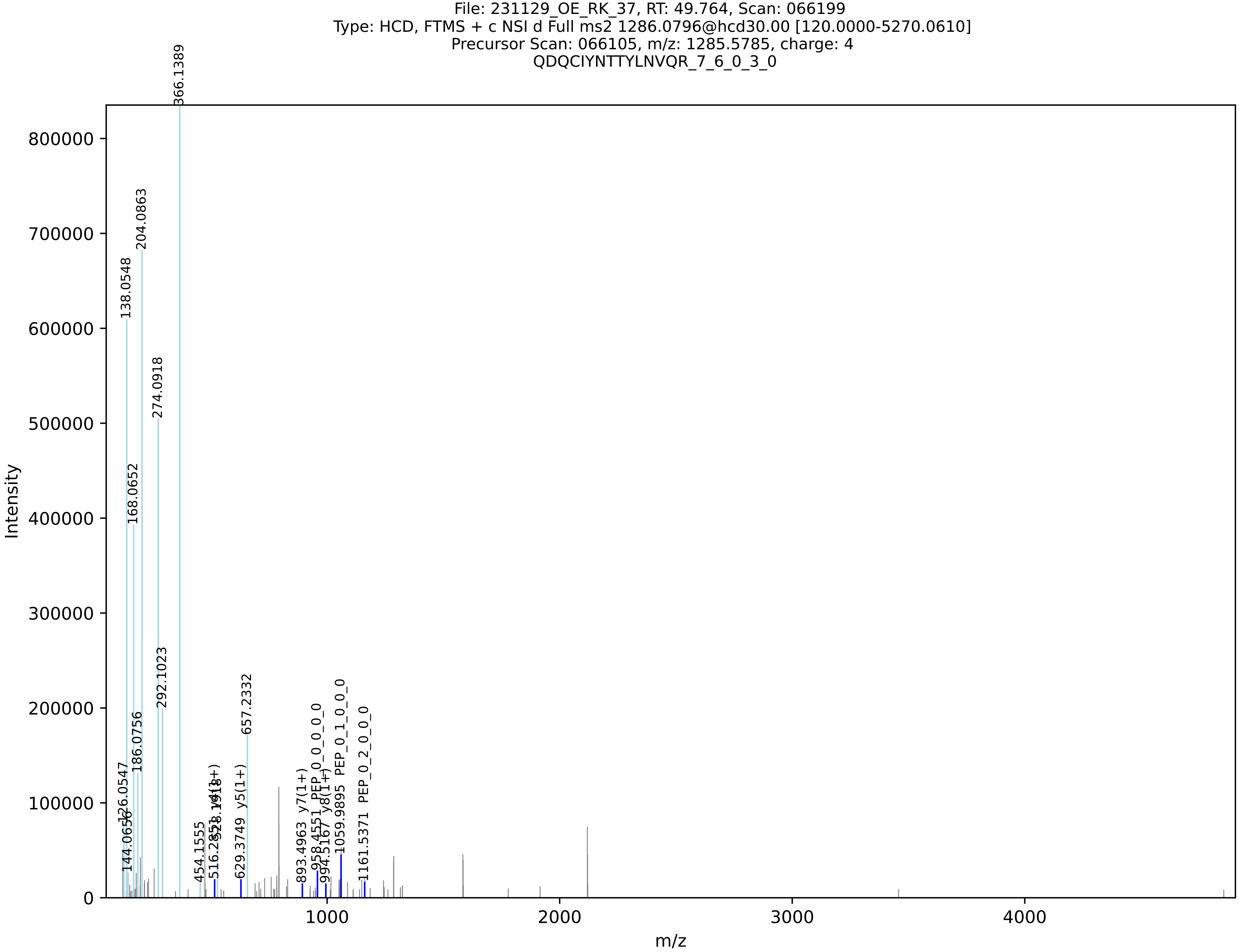

File: 231129_OE_RK_37, RT: 49.764, Scan: 066199
Type: HCD, FTMS + c NSI d Full ms2 1286.0796@hcd30.00 [120.0000-5270.0610]
Precursor Scan: 066105, m/z: 1285.5785, charge: 4
QDQCIYNTTYLNVQR_7_6_0_3_0
Intensity
m/z
0
100000
200000
300000
400000
500000
600000
700000
800000
1000
2000
3000
4000
366.1389
204.0863
138.0548
274.0918
168.0652
292.1023
186.0756
126.0547
144.0656
657.2332
454.1555
629.3749 y5(1+)
893.4963 y7(1+)
958.4551 PEP_0_0_0_0_0
994.5167 y8(1+)
1059.9895 PEP_0_1_0_0_0
1161.5371 PEP_0_2_0_0_0

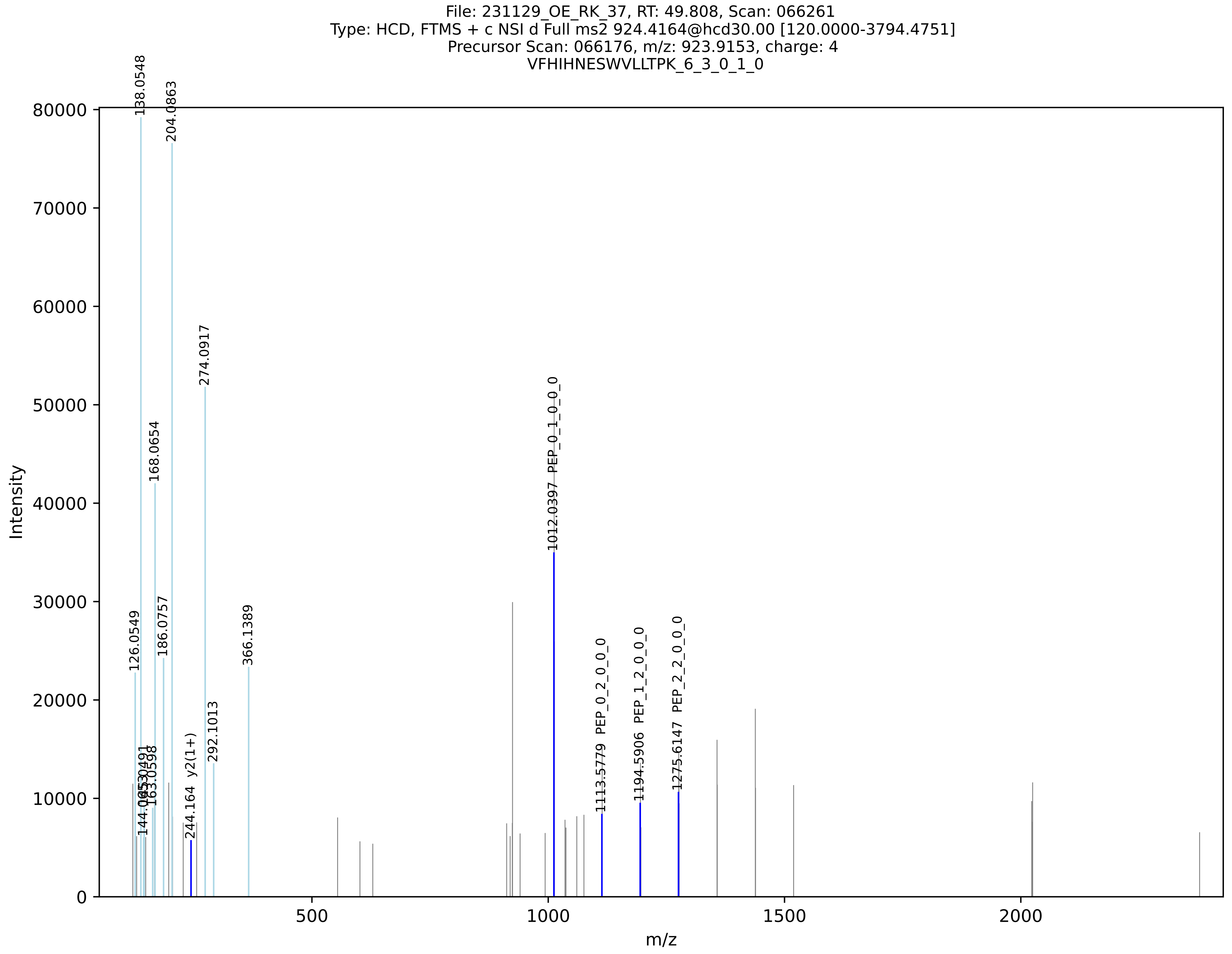
File: 231129_OE_RK_37, RT: 49.808, Scan: 066261
Type: HCD, FTMS + c NSI d Full ms2 924.4164@hcd30.00 [120.0000-3794.4751]
Precursor Scan: 066176, m/z: 923.9153, charge: 4
VFHIHNESWVLLTPK_6_3_0_1_0
138.0548
204.0863
274.0917
168.0654
1012.0397 PEP_0_1_0_0_
126.0549
186.0757
366.1389
1113.5779 PEP_0_2_0_0_0
1194.5906 PEP_1_2_0_0_0
1275.6147 PEP_2_2_0_0_0
292.1013
244.164 y2(1+)
163.0598
Intensity
m/z
0
10000
20000
30000
40000
50000
60000
70000
80000
500
1000
1500
2000

File: 231129_OE_RK_37, RT: 49.854, Scan: 066327
Type: HCD, FTMS + c NSI d Full ms2 1323.2777@hcd30.00 [120.0000-5421.8291]
Precursor Scan: 066250, m/z: 1322.5263, charge: 4
QDQCIYNTTYLNVQR_7_6_1_3_0

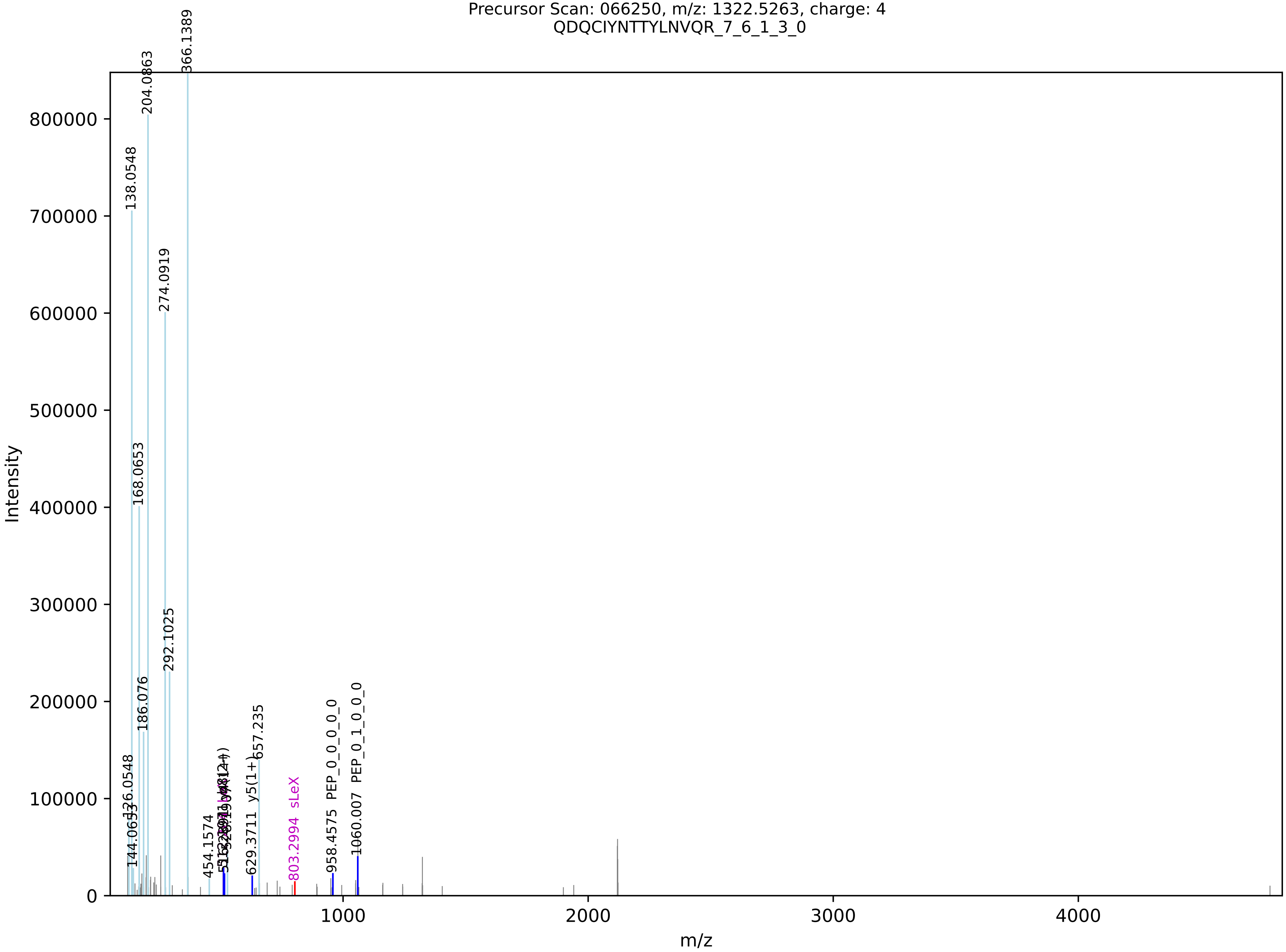

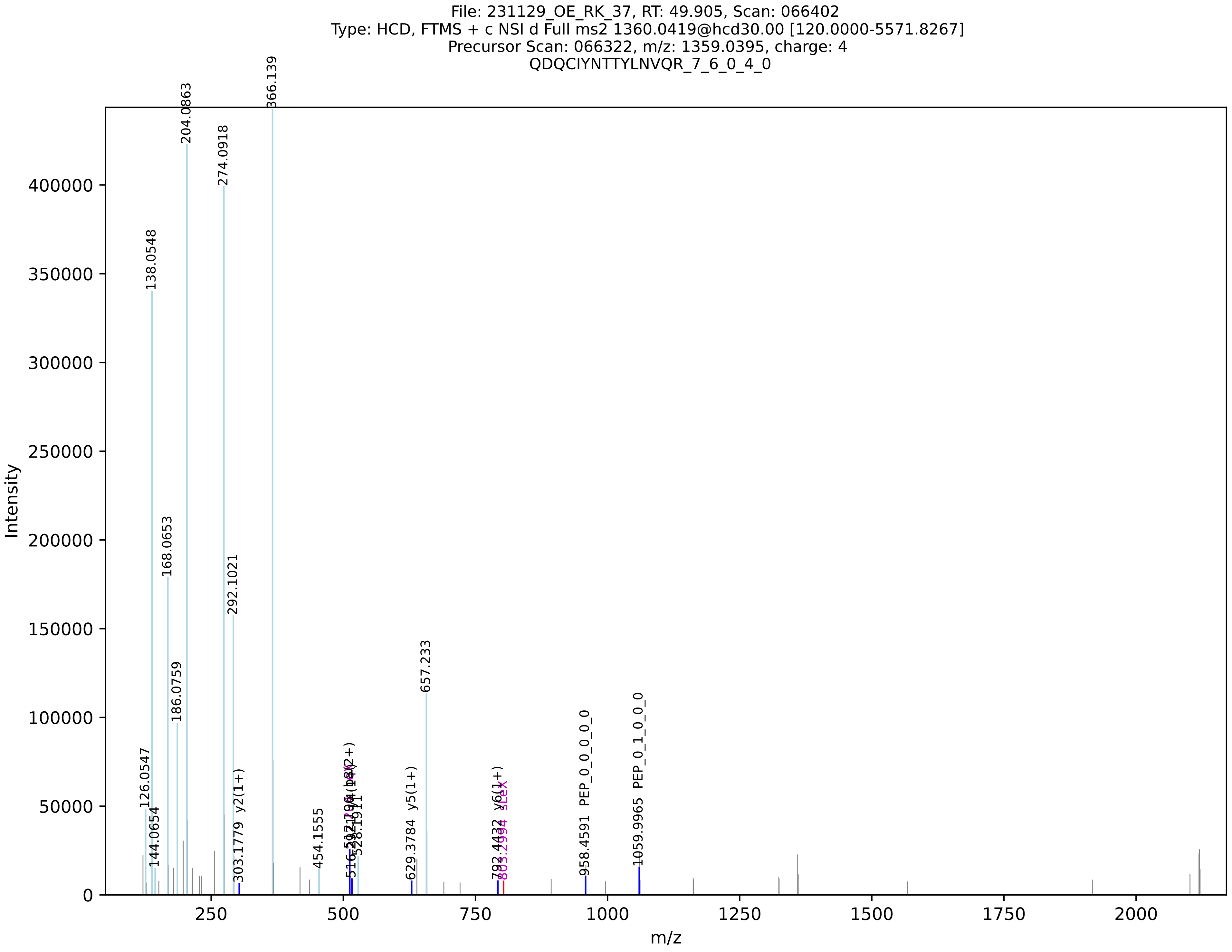
File: 231129_OE_RK_37, RT: 49.905, Scan: 066402
Type: HCD, FTMS + c NSI d Full ms2 1360.0419@hcd30.00 [120.0000-5571.8267]
Precursor Scan: 066322, m/z: 1359.0395, charge: 4
QDQCIYNTTYLNVQR_7_6_0_4_0
Intensity
m/z
366.139
204.0863
274.0918
138.0548
168.0653
292.1021
186.0759
657.233
126.0547
144.0654
303.1779 y2(1+)
454.1555
528.1911
629.3784 y5(1+)
793.4432 y6(1+)
sLeX
958.4591 PEP_0_0_0_0_0
1059.9965 PEP_0_1_0_0_0

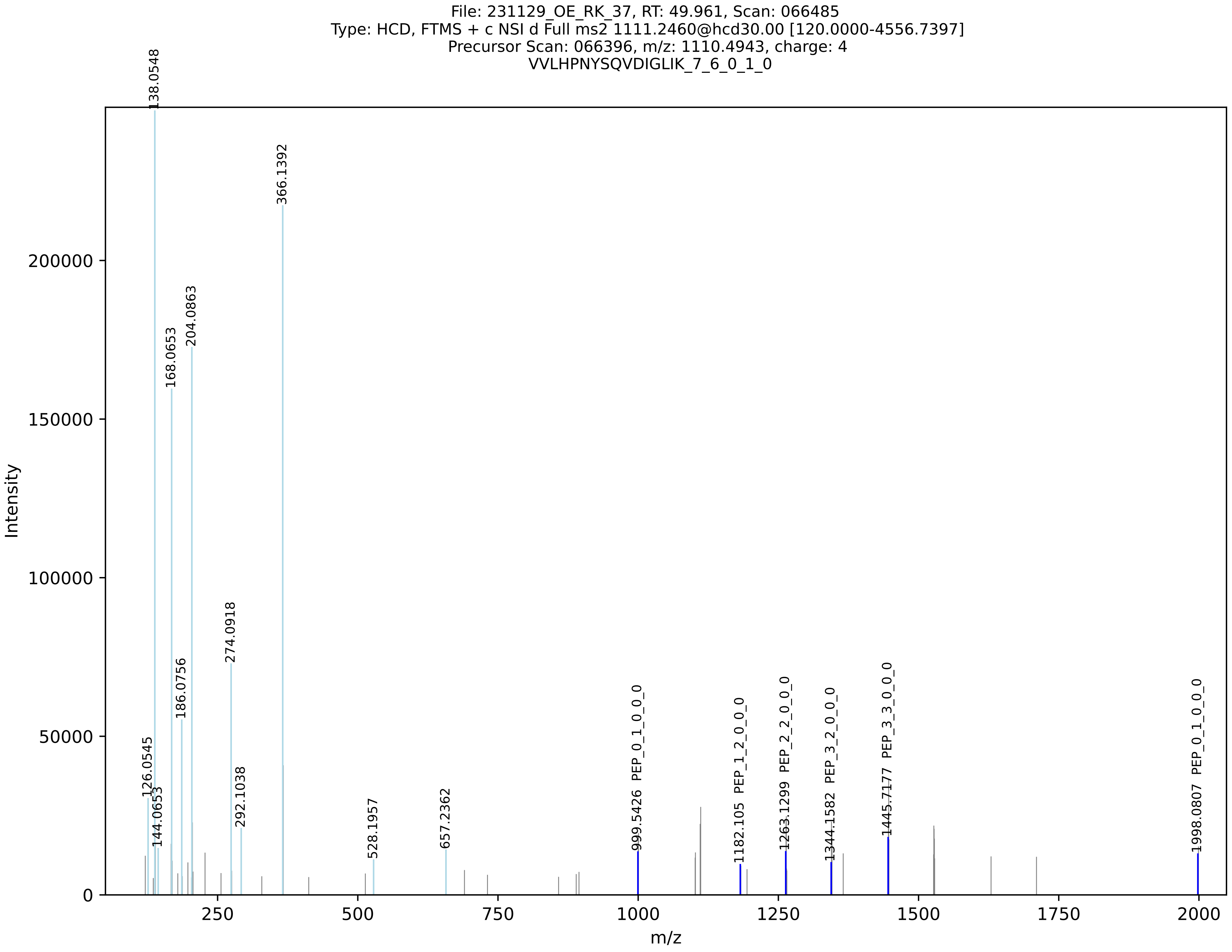
File: 231129_OE_RK_37, RT: 49.961, Scan: 066485
Type: HCD, FTMS + c NSI d Full ms2 1111.2460@hcd30.00 [120.0000-4556.7397]
Precursor Scan: 066396, m/z: 1110.4943, charge: 4
VVLHPNYSQVDIGLIK_7_6_0_1_0
Intensity
m/z
0
50000
100000
150000
200000
250
500
750
1000
1250
1500
1750
2000
138.0548
366.1392
204.0863
168.0653
274.0918
186.0756
126.0545
144.0653
292.1038
528.1957
657.2362
999.5426 PEP_0_1_0_0_0
1182.105 PEP_1_2_0_0_0
1263.1299 PEP_2_2_0_0_0
1344.1582 PEP_3_2_0_0_0
1445.7177 PEP_3_3_0_0_0
1998.0807 PEP_0_1_0_0_0

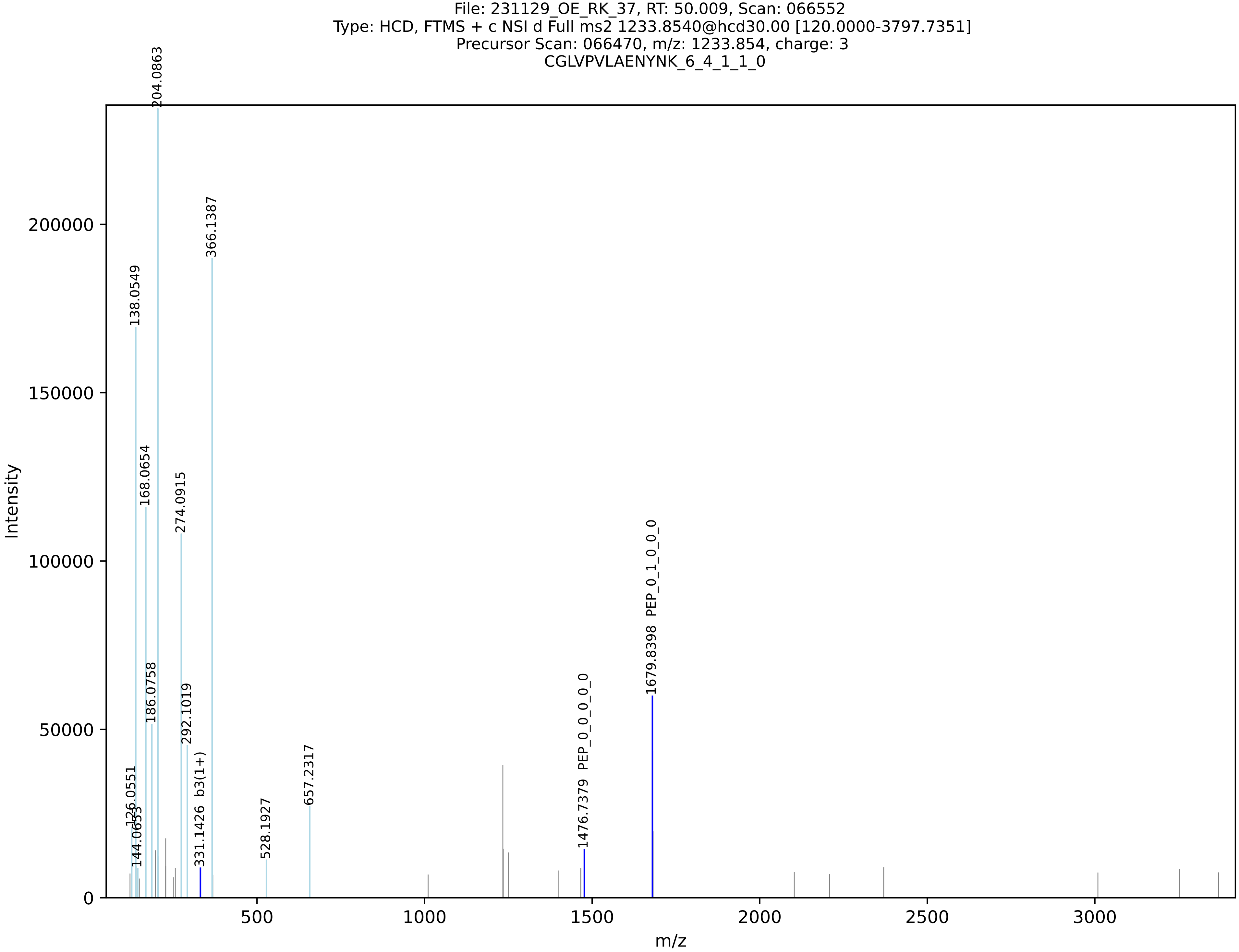
File: 231129_OE_RK_37, RT: 50.009, Scan: 066552
Type: HCD, FTMS + c NSI d Full ms2 1233.8540@hcd30.00 [120.0000-3797.7351]
Precursor Scan: 066470, m/z: 1233.854, charge: 3
CGLVPVLAENYNK_6_4_1_1_0
Intensity
m/z
204.0863
366.1387
138.0549
168.0654
274.0915
186.0758
292.1019
126.0551
144.0655
331.1426 b3(1+)
528.1927
657.2317
1476.7379 PEP_0_0_0_0_0_
1679.8398 PEP_0_1_0_0_0_
0
50000
100000
150000
200000
500
1000
1500
2000
2500
3000

File: 231129_OE_RK_37, RT: 50.035, Scan: 066593
Type: HCD, FTMS + c NSI d Full ms2 1350.2260@hcd30.00 [120.0000-4153.8335]
Precursor Scan: 066396, m/z: 1349.5577, charge: 3
CGLVPVLAENYNK_6_5_0_2_0

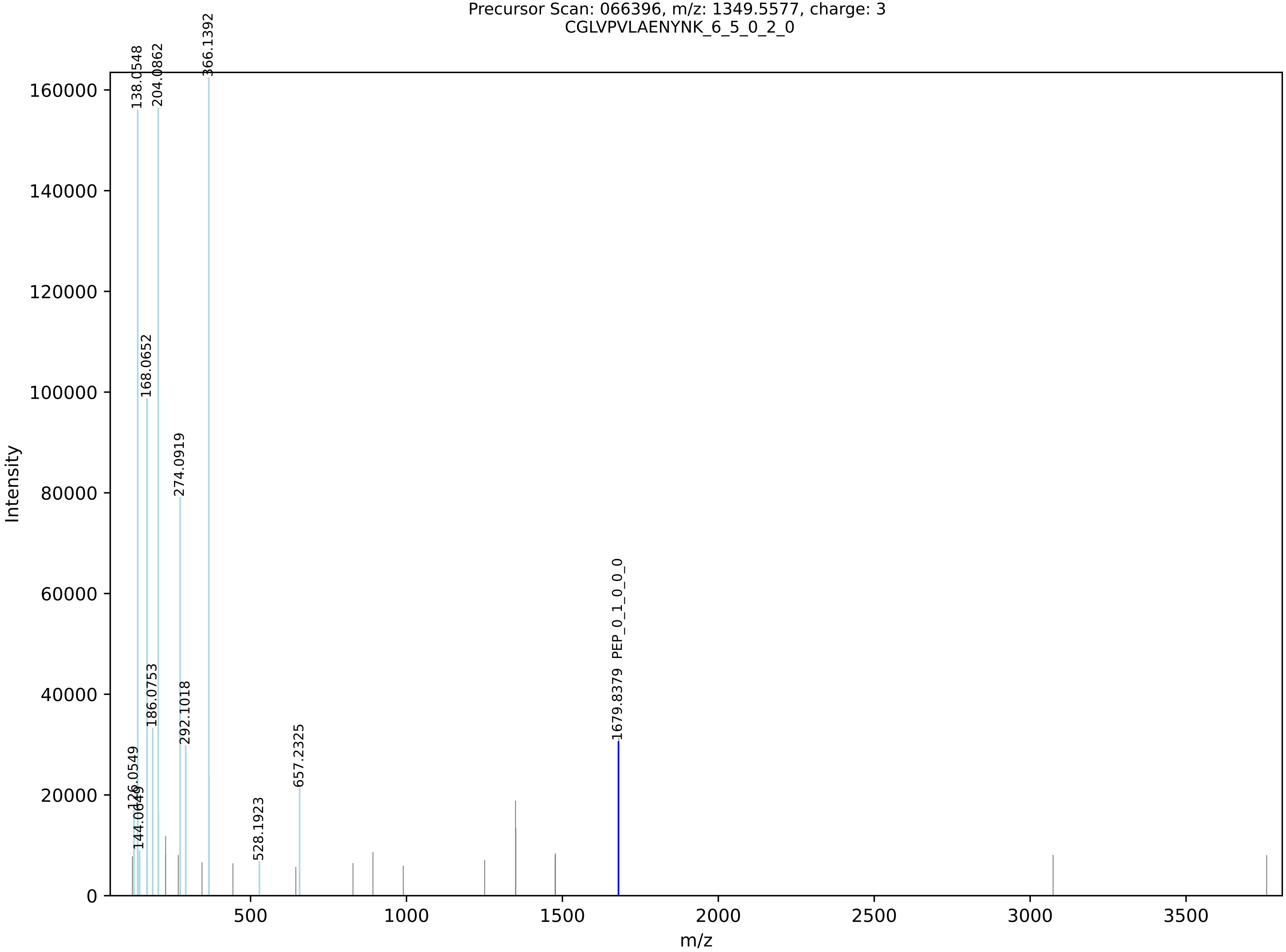

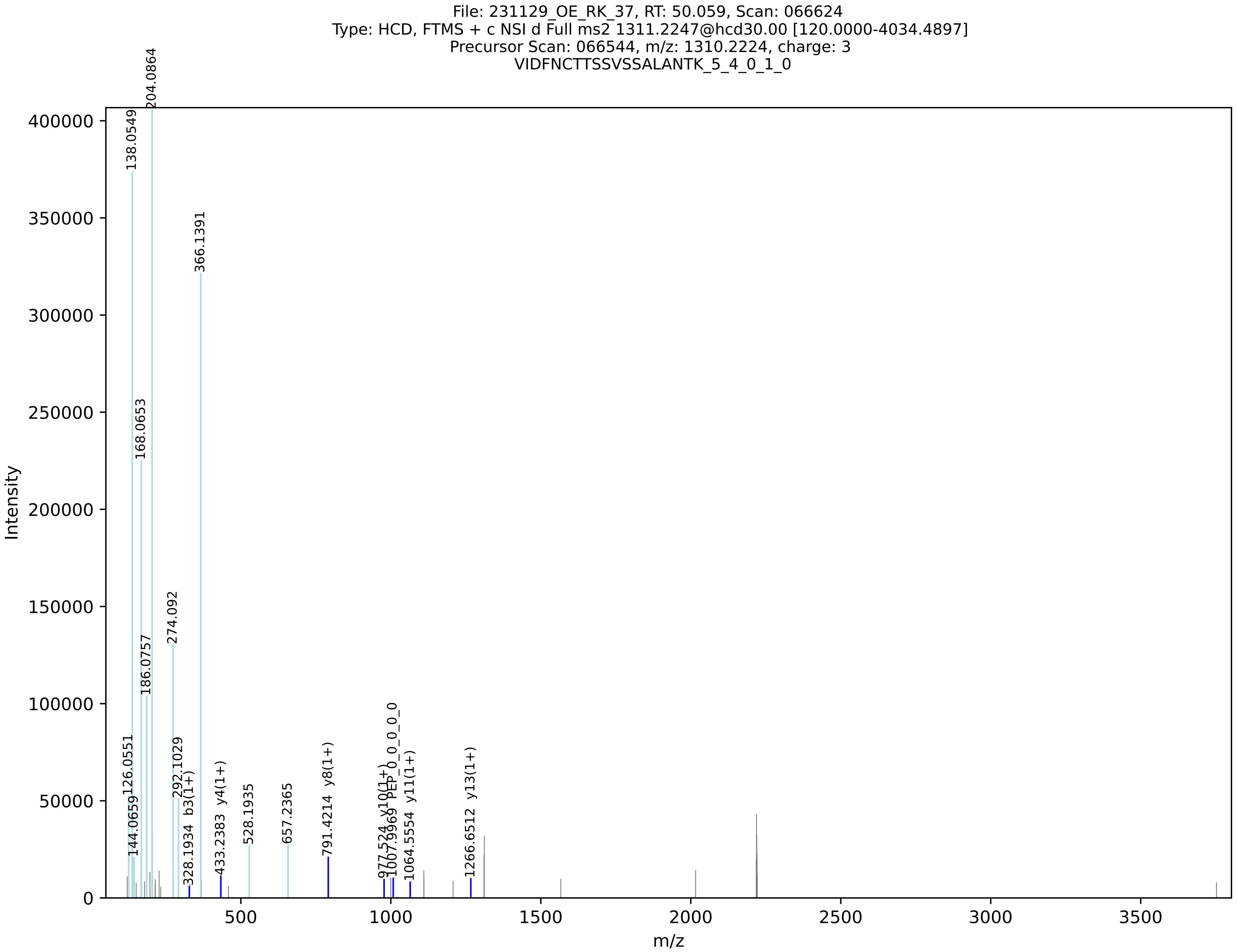
File: 231129_OE_RK_37, RT: 50.059, Scan: 066624
Type: HCD, FTMS + c NSI d Full ms2 1311.2247@hcd30.00 [120.0000-4034.4897]
Precursor Scan: 066544, m/z: 1310.2224, charge: 3
VIDFNCTTSSVSSALANTK_5_4_0_1_0
Intensity
m/z
204.0864
138.0549
366.1391
168.0653
274.092
186.0757
126.0551
292.1029
144.0659
328.1934 b3(1+)
433.2383 y4(1+)
528.1935
657.2365
791.4214 y8(1+)
977.524 y10(1+)
1007.9969 PEP_0_0_0_0_0
1064.5554 y11(1+)
1266.6512 y13(1+)

File: 231129_OE_RK_37, RT: 50.06, Scan: 066626
Type: HCD, FTMS + c NSI d Full ms2 1764.3706@hcd30.00 [120.0000-5421.1162]
Precursor Scan: 066544, m/z: 1763.3683, charge: 3
QDQCIYNTTYLNVQR_7_6_1_3_0

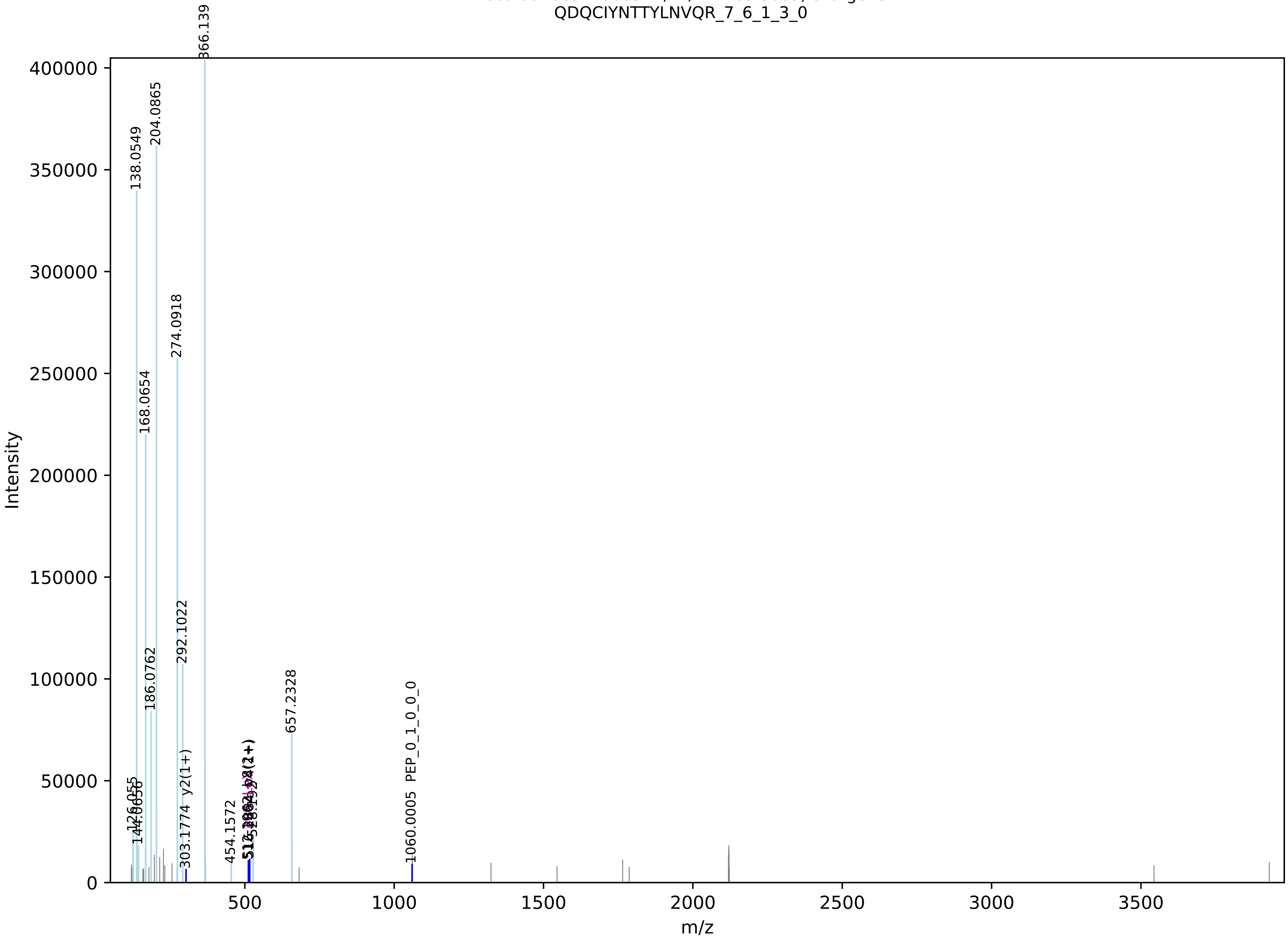

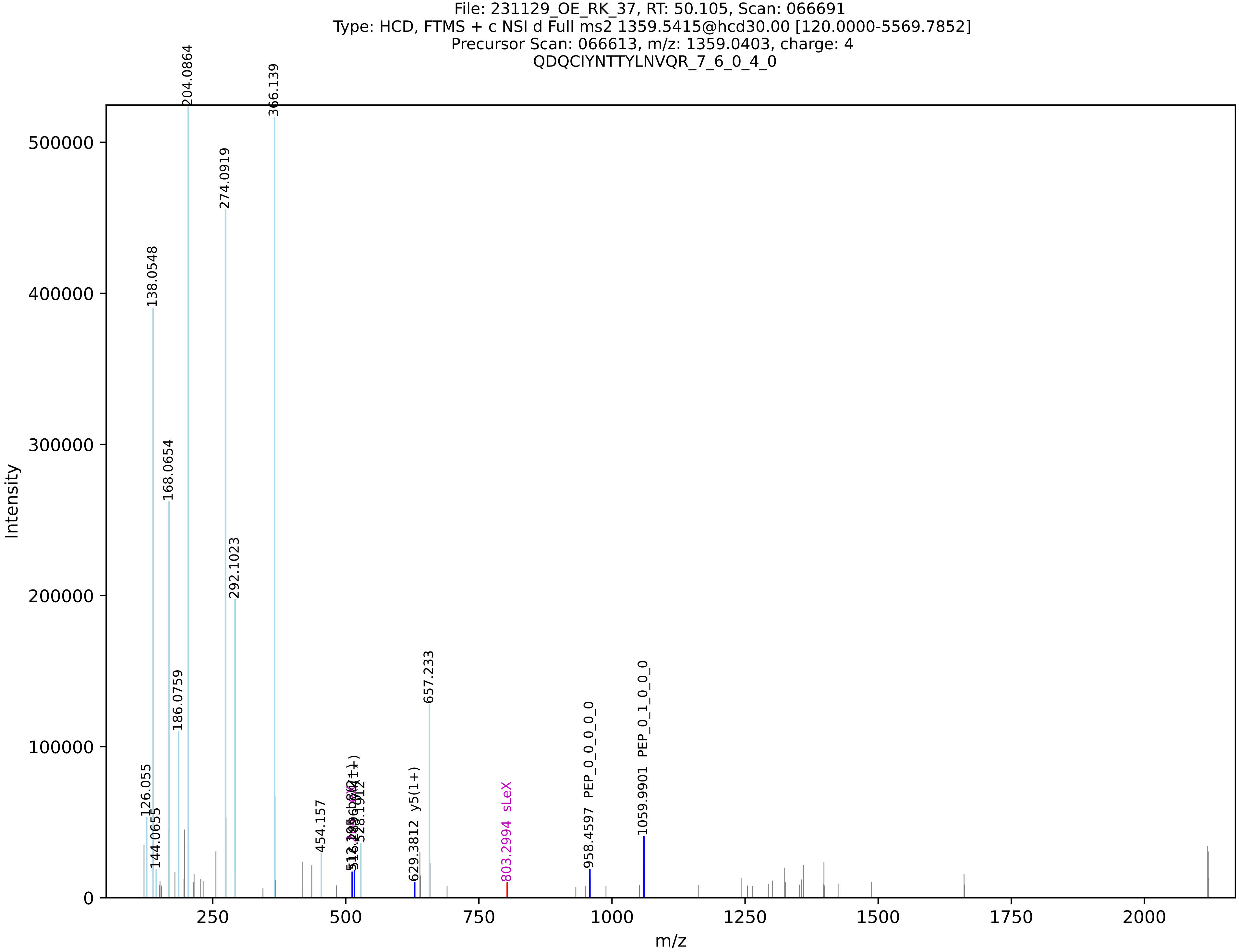
File: 231129_OE_RK_37, RT: 50.105, Scan: 066691
Type: HCD, FTMS + c NSI d Full ms2 1359.5415@hcd30.00 [120.0000-5569.7852]
Precursor Scan: 066613, m/z: 1359.0403, charge: 4
QDQCIYNTTYLNVQR_7_6_0_4_0
204.0864
366.139
274.0919
138.0548
168.0654
292.1023
186.0759
126.055
144.0655
454.157
528.1912
629.3812 y5(1+)
657.233
803.2994 sLeX
958.4597 PEP_0_0_0_0_0
1059.9901 PEP_0_1_0_0_0
Intensity
m/z
0
100000
200000
300000
400000
500000
250
500
750
1000
1250
1500
1750
2000

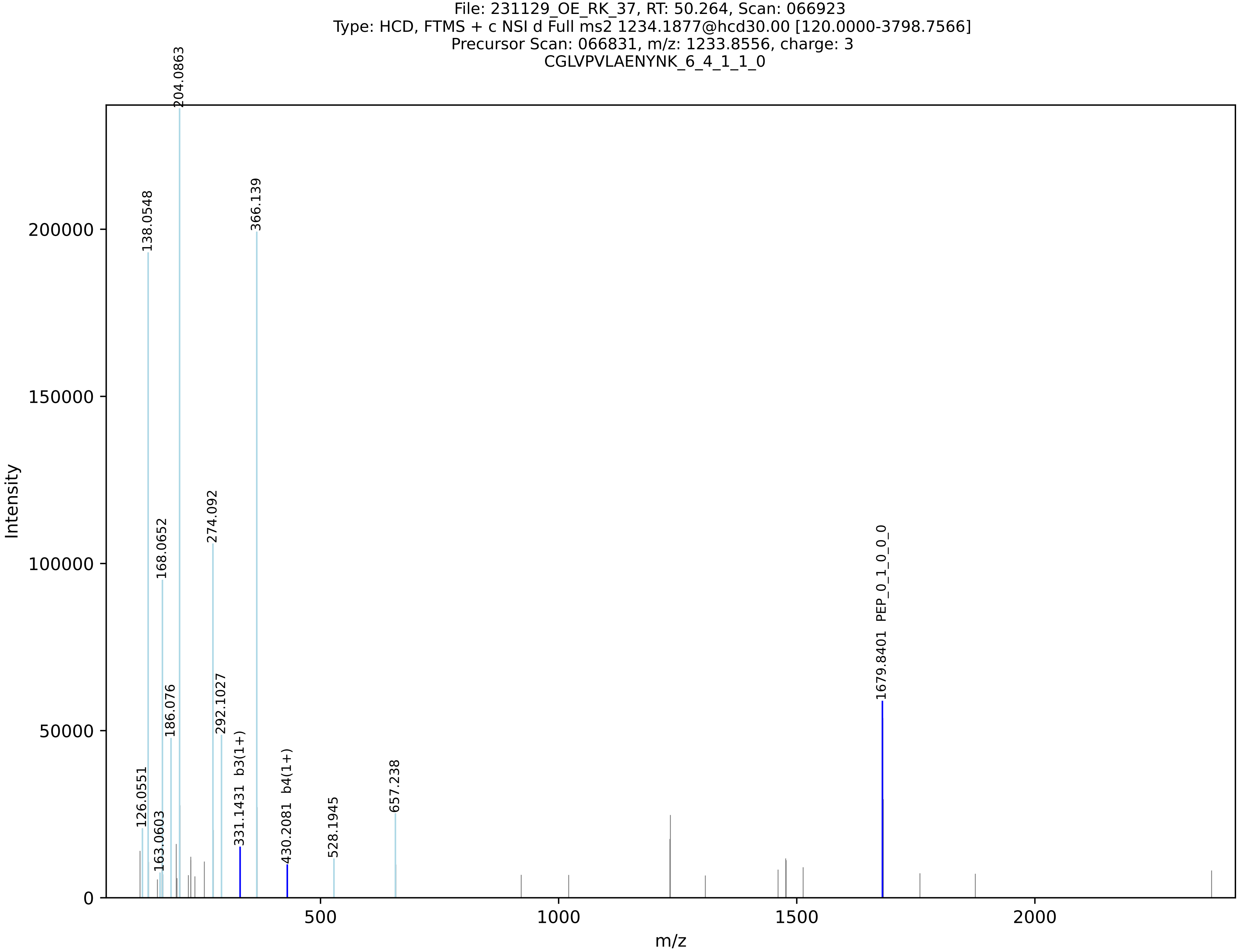
File: 231129_OE_RK_37, RT: 50.264, Scan: 066923
Type: HCD, FTMS + c NSI d Full ms2 1234.1877@hcd30.00 [120.0000-3798.7566]
Precursor Scan: 066831, m/z: 1233.8556, charge: 3
CGLVPVLAENYNK_6_4_1_1_0
Intensity
m/z
204.0863
138.0548
366.139
274.092
168.0652
186.076
292.1027
126.0551
163.0603
331.1431 b3(1+)
430.2081 b4(1+)
528.1945
657.238
1679.8401 PEP_0_1_0_0_0
0
50000
100000
150000
200000
500
1000
1500
2000

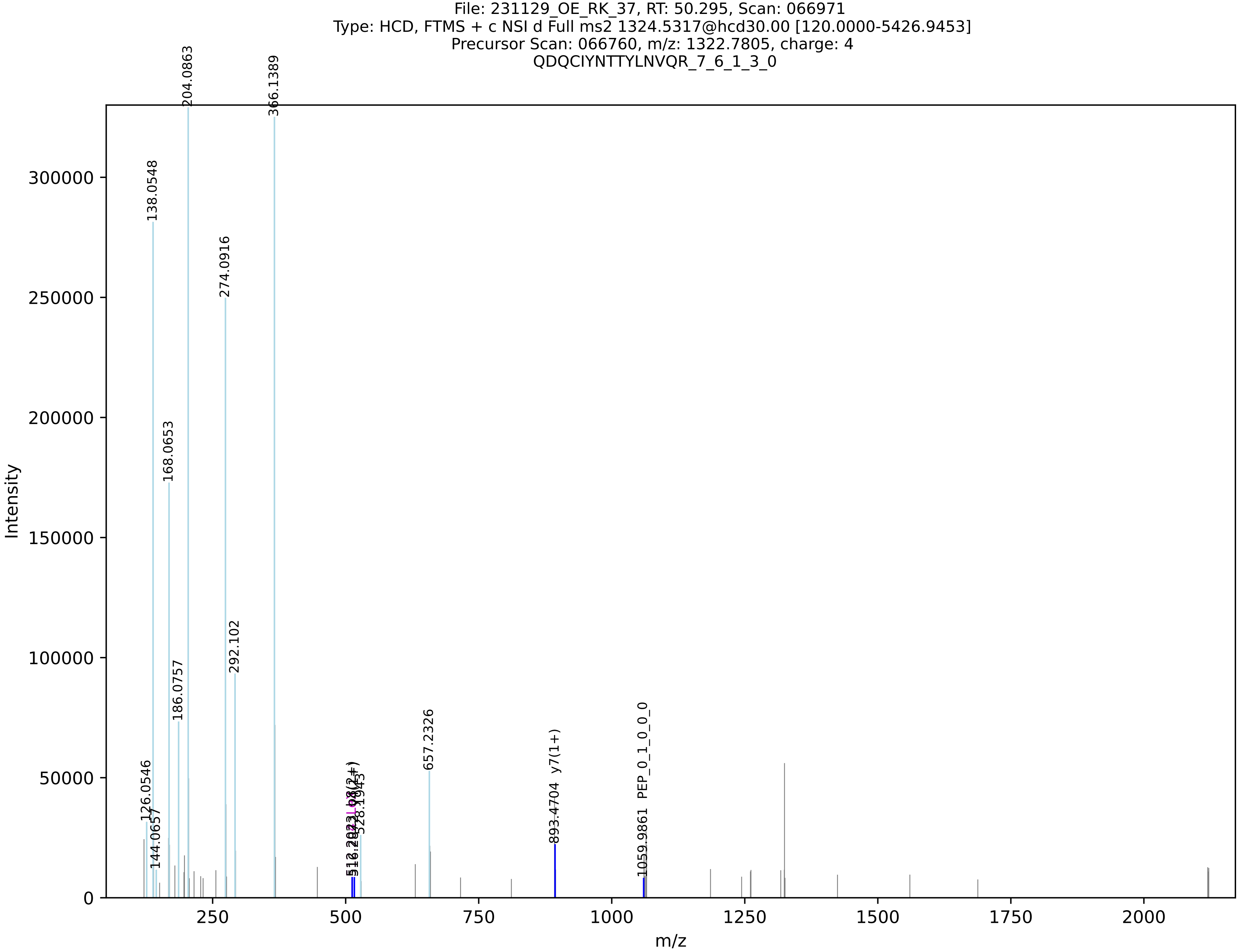
File: 231129_OE_RK_37, RT: 50.295, Scan: 066971
Type: HCD, FTMS + c NSI d Full ms2 1324.5317@hcd30.00 [120.0000-5426.9453]
Precursor Scan: 066760, m/z: 1322.7805, charge: 4
QDQCIYNTTYLNVQR_7_6_1_3_0
Intensity
m/z
204.0863
366.1389
138.0548
274.0916
168.0653
292.102
186.0757
126.0546
144.0657
657.2326
528.1943
893.4704 y7(1+)
1059.9861 PEP_0_1_0_0_0
300000
250000
200000
150000
100000
50000
0
250
500
750
1000
1250
1500
1750
2000

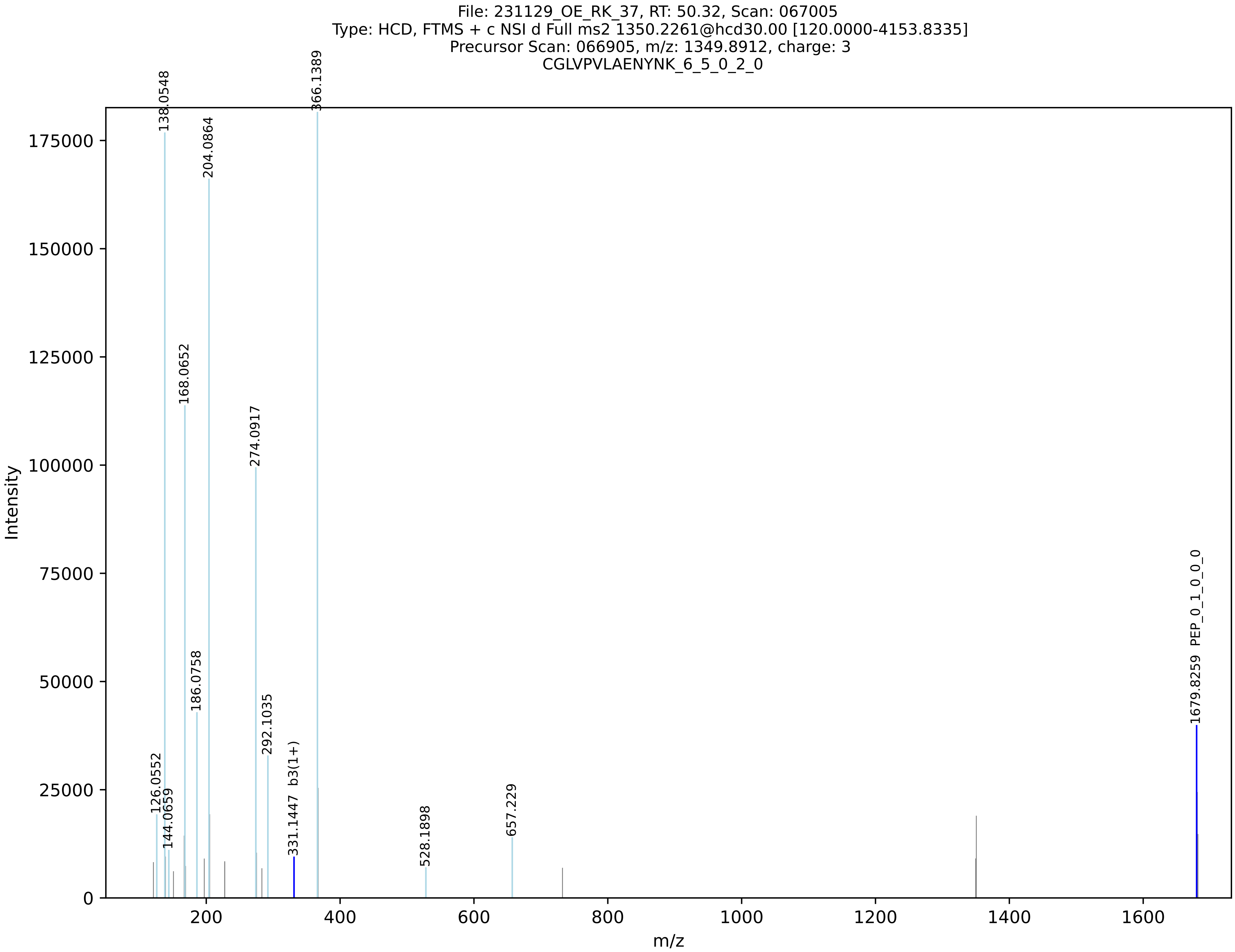
File: 231129_OE_RK_37, RT: 50.32, Scan: 067005
Type: HCD, FTMS + c NSI d Full ms2 1350.2261@hcd30.00 [120.0000-4153.8335]
Precursor Scan: 066905, m/z: 1349.8912, charge: 3
CGLVPVLAENYNK_6_5_0_2_0
Intensity
m/z
138.0548
366.1389
204.0864
168.0652
274.0917
186.0758
292.1035
126.0552
144.0659
331.1447 b3(1+)
528.1898
657.229
1679.8259 PEP_0_1_0_0_0

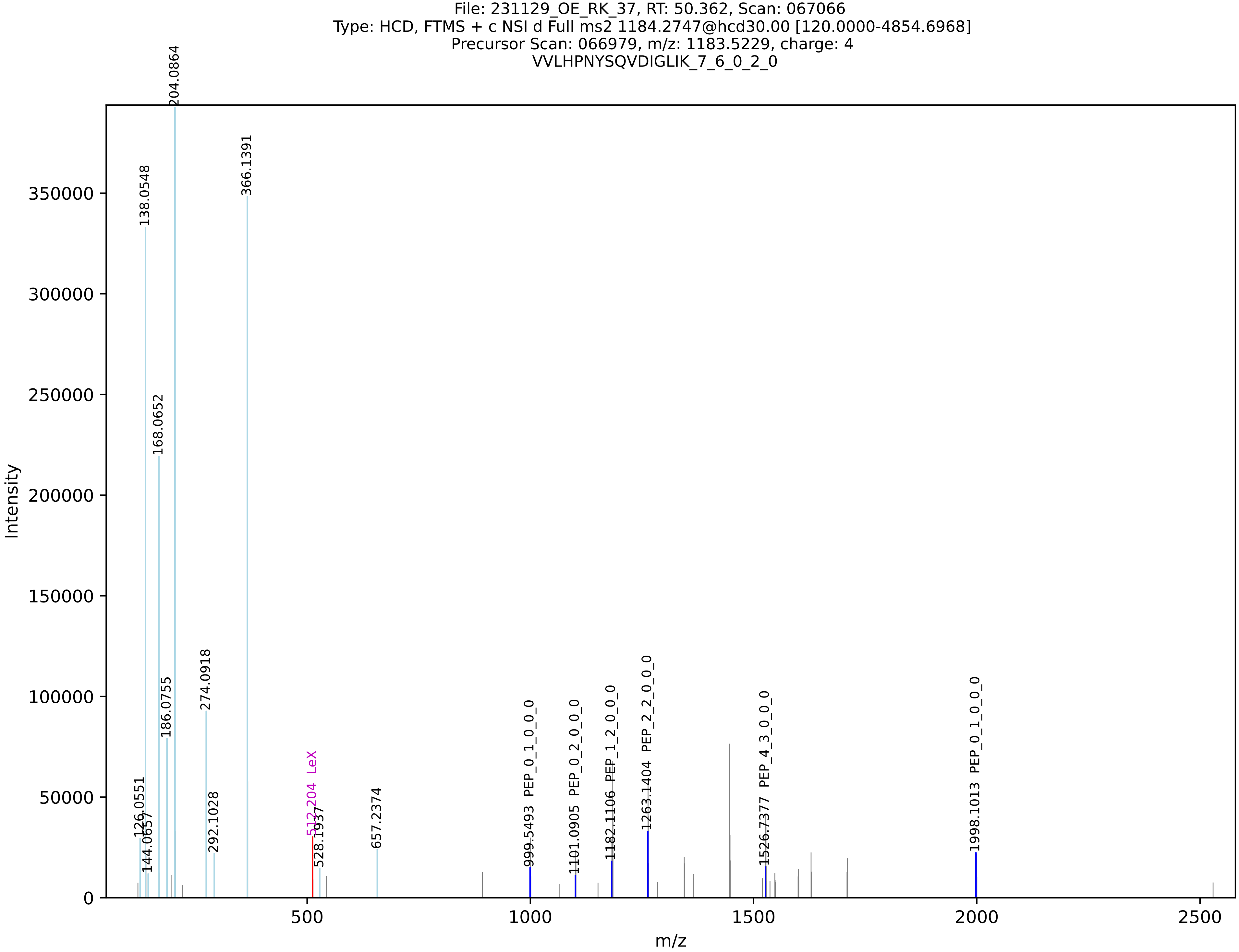

File: 231129_OE_RK_37, RT: 50.362, Scan: 067066
Type: HCD, FTMS + c NSI d Full ms2 1184.2747@hcd30.00 [120.0000-4854.6968]
Precursor Scan: 066979, m/z: 1183.5229, charge: 4
VVLHPNYSQVDIGLIK_7_6_0_2_0
Intensity
m/z
204.0864
366.1391
138.0548
168.0652
274.0918
186.0755
126.0551
144.0657
292.1028
512.204 LeX
528.1937
657.2374
999.5493 PEP_0_1_0_0_0
1101.0905 PEP_0_2_0_0_0
1182.1106 PEP_1_2_0_0_0
1263.1404 PEP_2_2_0_0_0
1526.7377 PEP_4_3_0_0_0
1998.1013 PEP_0_1_0_0_0

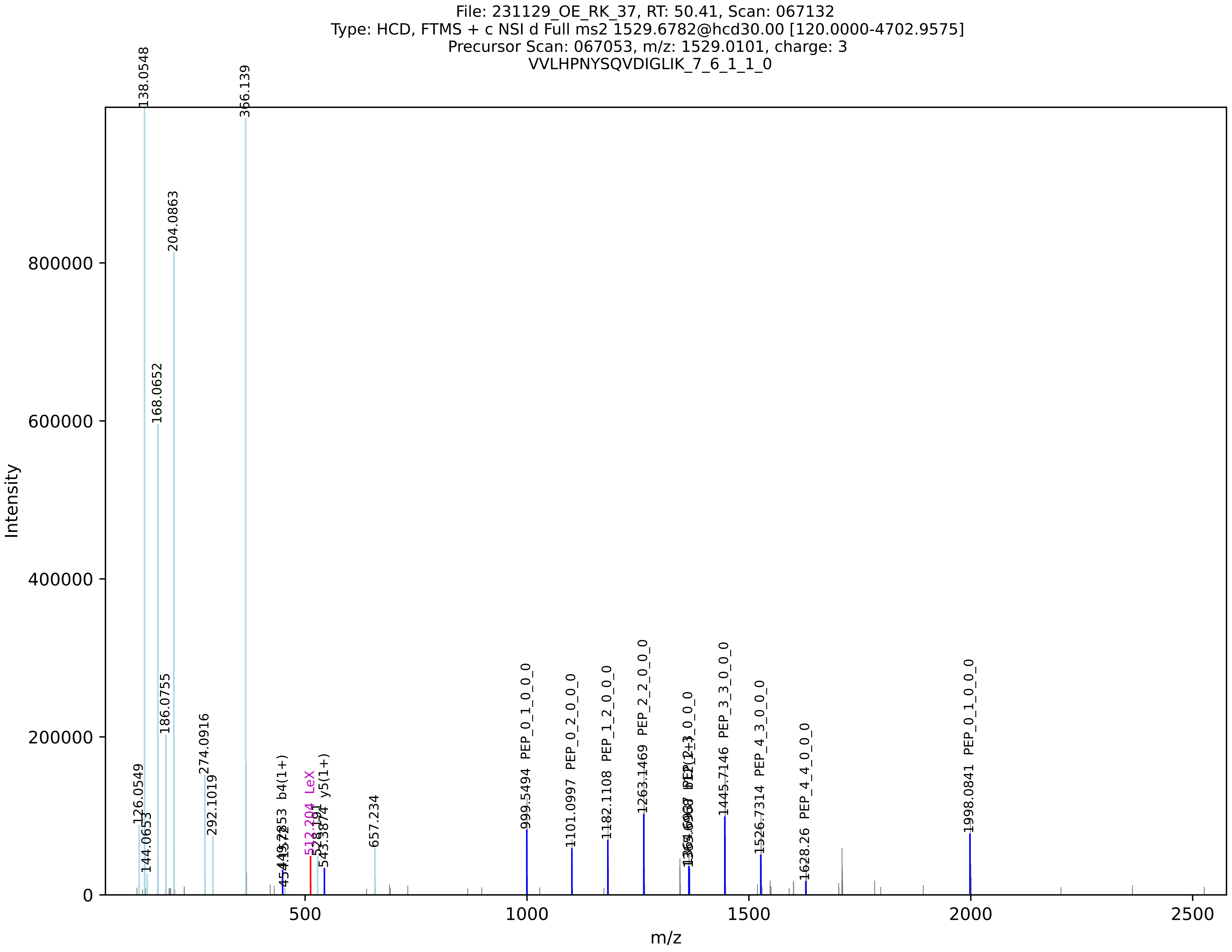
File: 231129_OE_RK_37, RT: 50.41, Scan: 067132
Type: HCD, FTMS + c NSI d Full ms2 1529.6782@hcd30.00 [120.0000-4702.9575]
Precursor Scan: 067053, m/z: 1529.0101, charge: 3
VVLHPNYSQVDIGLIK_7_6_1_1_0
Intensity
m/z
0
200000
400000
600000
800000
500
1000
1500
2000
2500
138.0548
366.139
204.0863
168.0652
186.0755
274.0916
292.1019
126.0549
144.0653
b4(1+)
512.204 LeX
543.3874 y5(1+)
657.234
999.5494 PEP_0_1_0_0_0
1101.0997 PEP_0_2_0_0_0
1182.1108 PEP_1_2_0_0_0
1263.1469 PEP_2_2_0_0_0
1445.7146 PEP_3_3_0_0_0
1526.7314 PEP_4_3_0_0_0
1628.26 PEP_4_4_0_0_0
1998.0841 PEP_0_1_0_0_

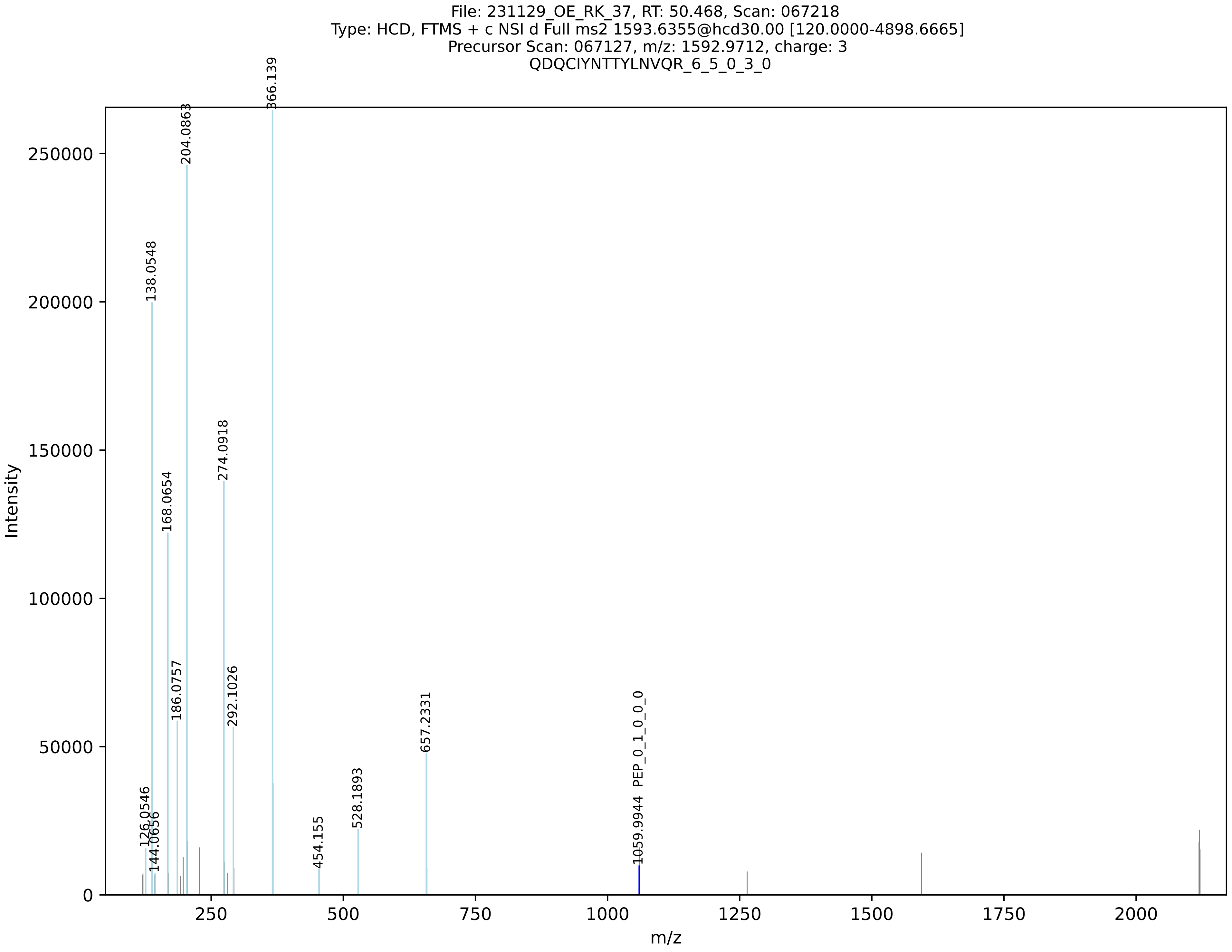

File: 231129_OE_RK_37, RT: 50.468, Scan: 067218
Type: HCD, FTMS + c NSI d Full ms2 1593.6355@hcd30.00 [120.0000-4898.6665]
Precursor Scan: 067127, m/z: 1592.9712, charge: 3
QDQCIYNTTYLNVQR_6_5_0_3_0
Intensity
m/z
366.139
204.0863
138.0548
274.0918
168.0654
186.0757
292.1026
657.2331
PEP_0_1_0_0_0
1059.9944
528.1893
454.155
126.0546
144.0656

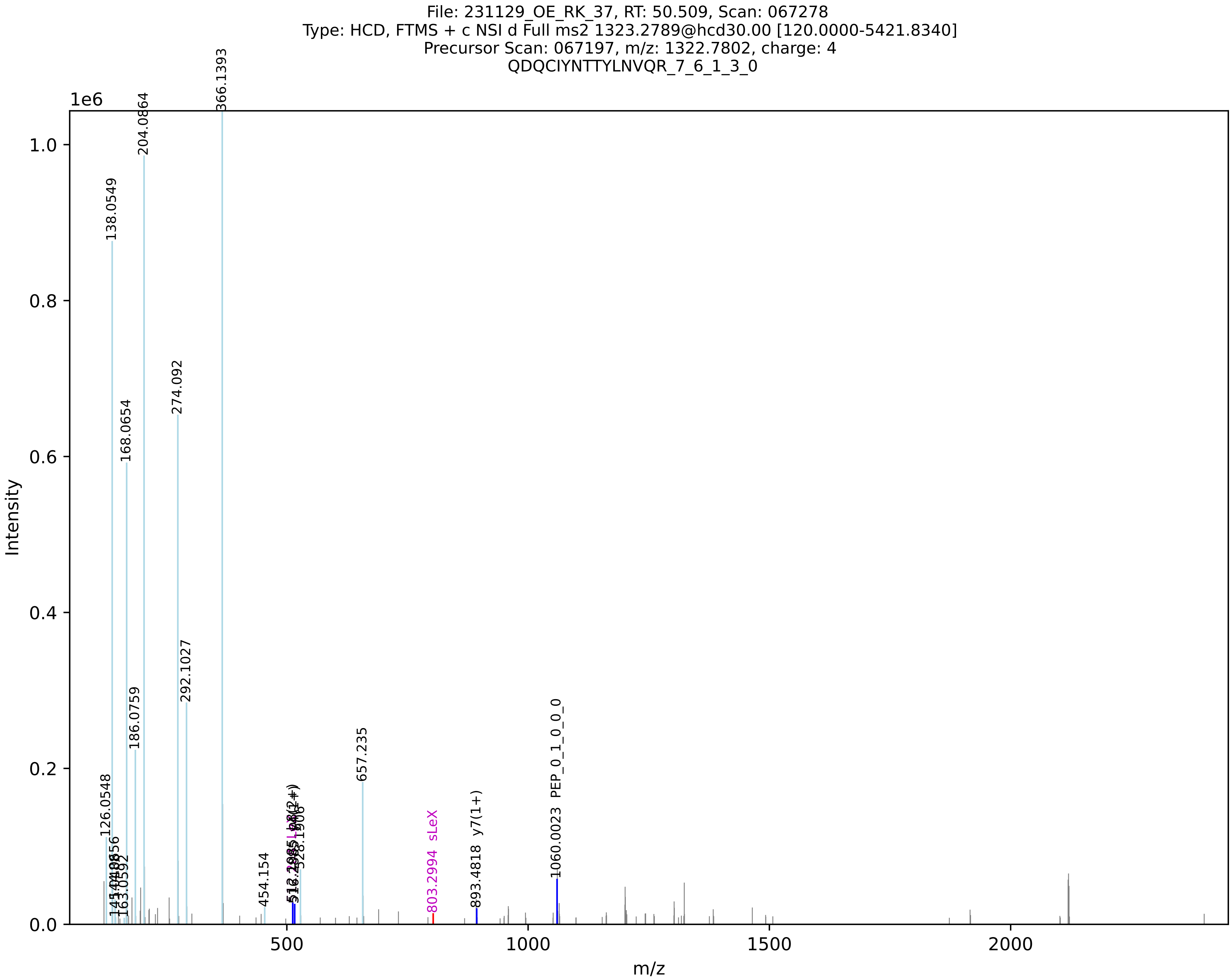

File: 231129_OE_RK_37, RT: 50.509, Scan: 067278
Type: HCD, FTMS + c NSI d Full ms2 1323.2789@hcd30.00 [120.0000-5421.8340]
Precursor Scan: 067197, m/z: 1322.7802, charge: 4
QDQCIYNTTYLNVQR_7_6_1_3_0
1e6
Intensity
m/z
366.1393
204.0864
138.0549
274.092
168.0654
292.1027
186.0759
126.0548
657.235
454.154
803.2994 sLeX
893.4818 y7(1+)
1060.0023 PEP_0_1_0_0_0
163.0592
0.0
0.2
0.4
0.6
0.8
1.0
500
1000
1500
2000

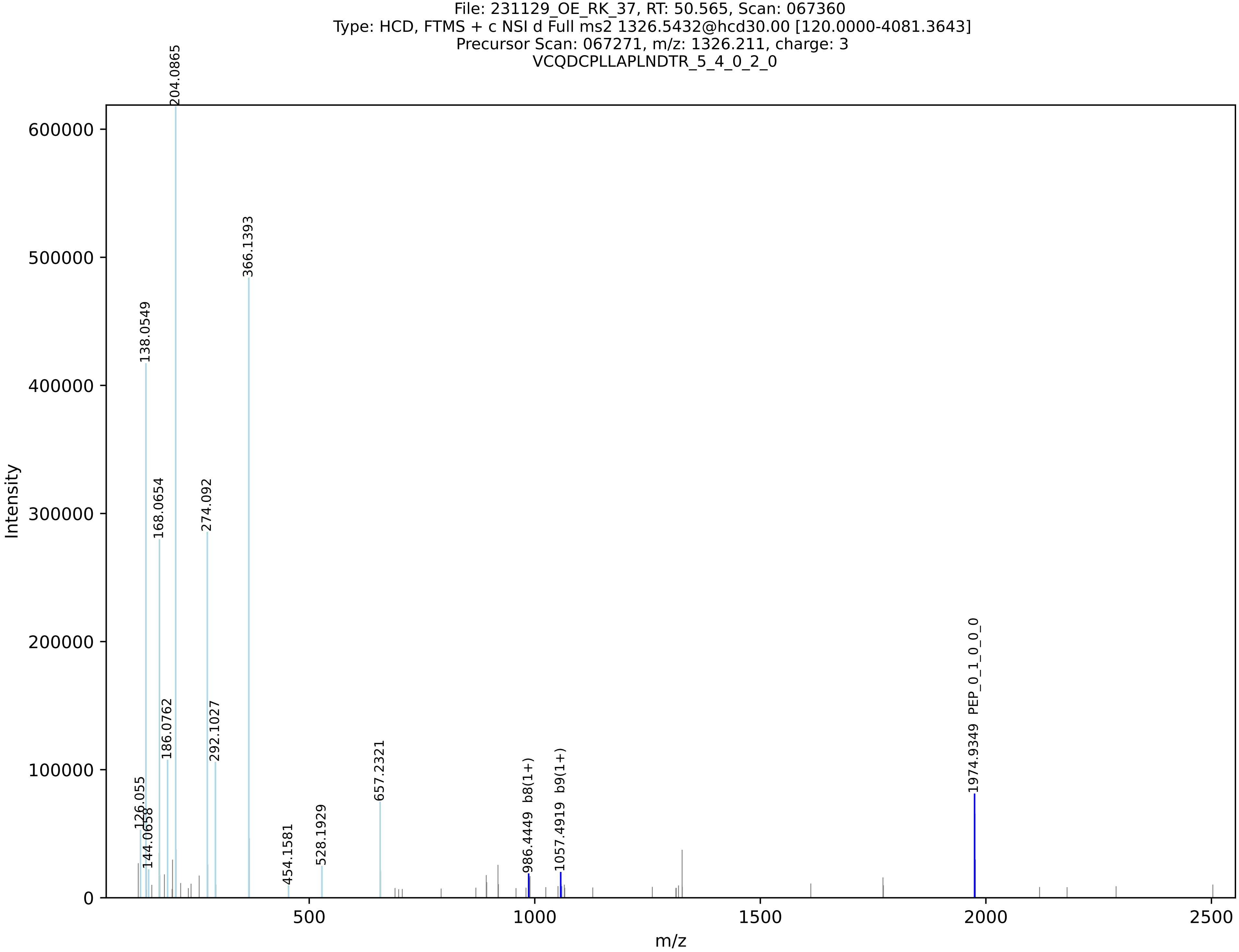

File: 231129_OE_RK_37, RT: 50.565, Scan: 067360
Type: HCD, FTMS + c NSI d Full ms2 1326.5432@hcd30.00 [120.0000-4081.3643]
Precursor Scan: 067271, m/z: 1326.211, charge: 3
VCQDCPLLAPLNDTR_5_4_0_2_0
Intensity
m/z
204.0865
366.1393
138.0549
168.0654
274.092
186.0762
292.1027
126.055
144.0658
454.1581
528.1929
657.2321
986.4449 b8(1+)
1057.4919 b9(1+)
1974.9349 PEP_0_1_0_0_0

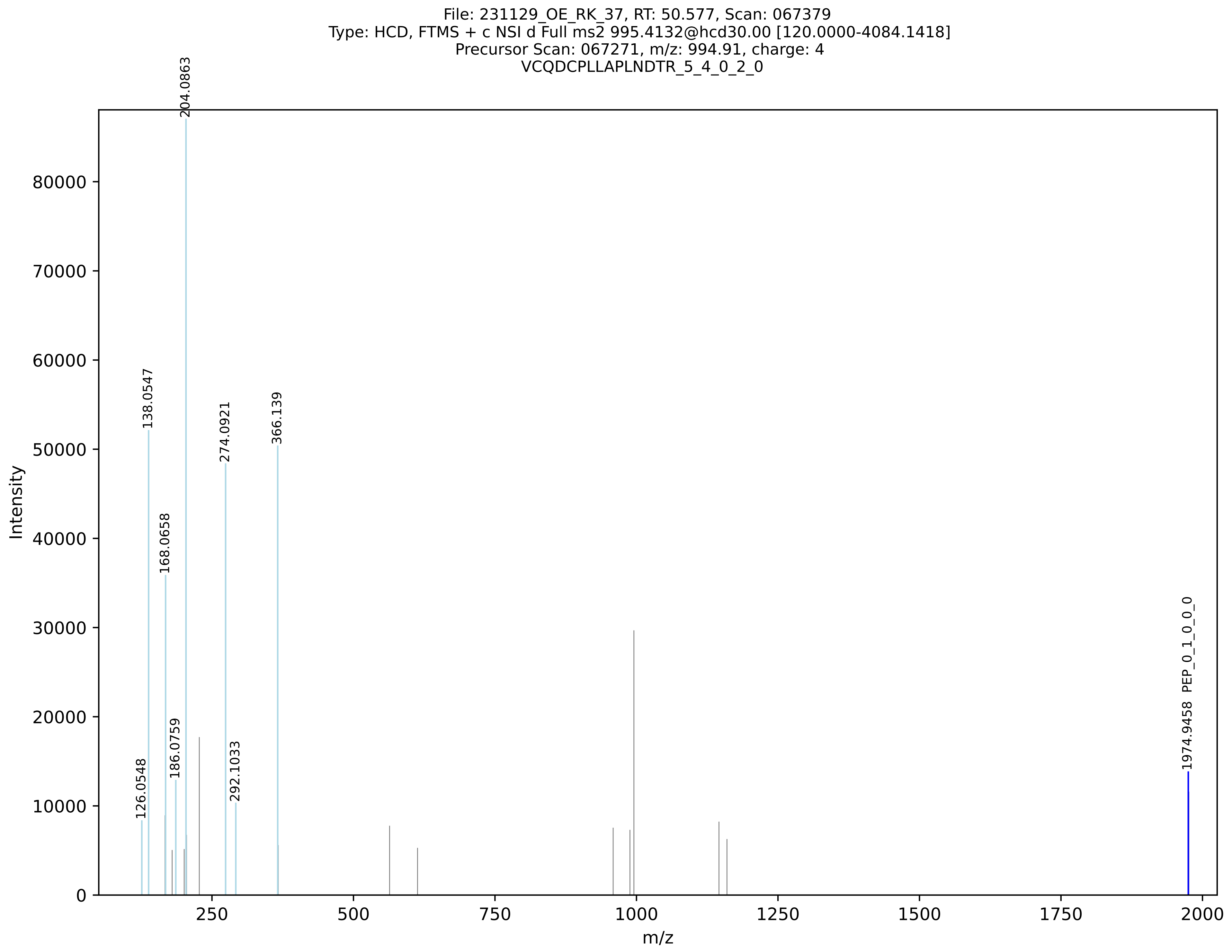
File: 231129_OE_RK_37, RT: 50.577, Scan: 067379
Type: HCD, FTMS + c NSI d Full ms2 995.4132@hcd30.00 [120.0000-4084.1418]
Precursor Scan: 067271, m/z: 994.91, charge: 4
VCQDCPLLAPLNDTR_5_4_0_2_0
204.0863
138.0547
366.139
274.0921
168.0658
PEP_0_1_0_0_0_
1974.9458
186.0759
292.1033
126.0548
Intensity
m/z
0
10000
20000
30000
40000
50000
60000
70000
80000
250
500
750
1000
1250
1500
1750
2000

File: 231129_OE_RK_37, RT: 50.613, Scan: 067427
Type: HCD, FTMS + c NSI d Full ms2 1529.6760@hcd30.00 [120.0000-4702.9507]
Precursor Scan: 067345, m/z: 1529.0097, charge: 3
VVLHPNYSQVDIGLIK_7_6_1_1_0

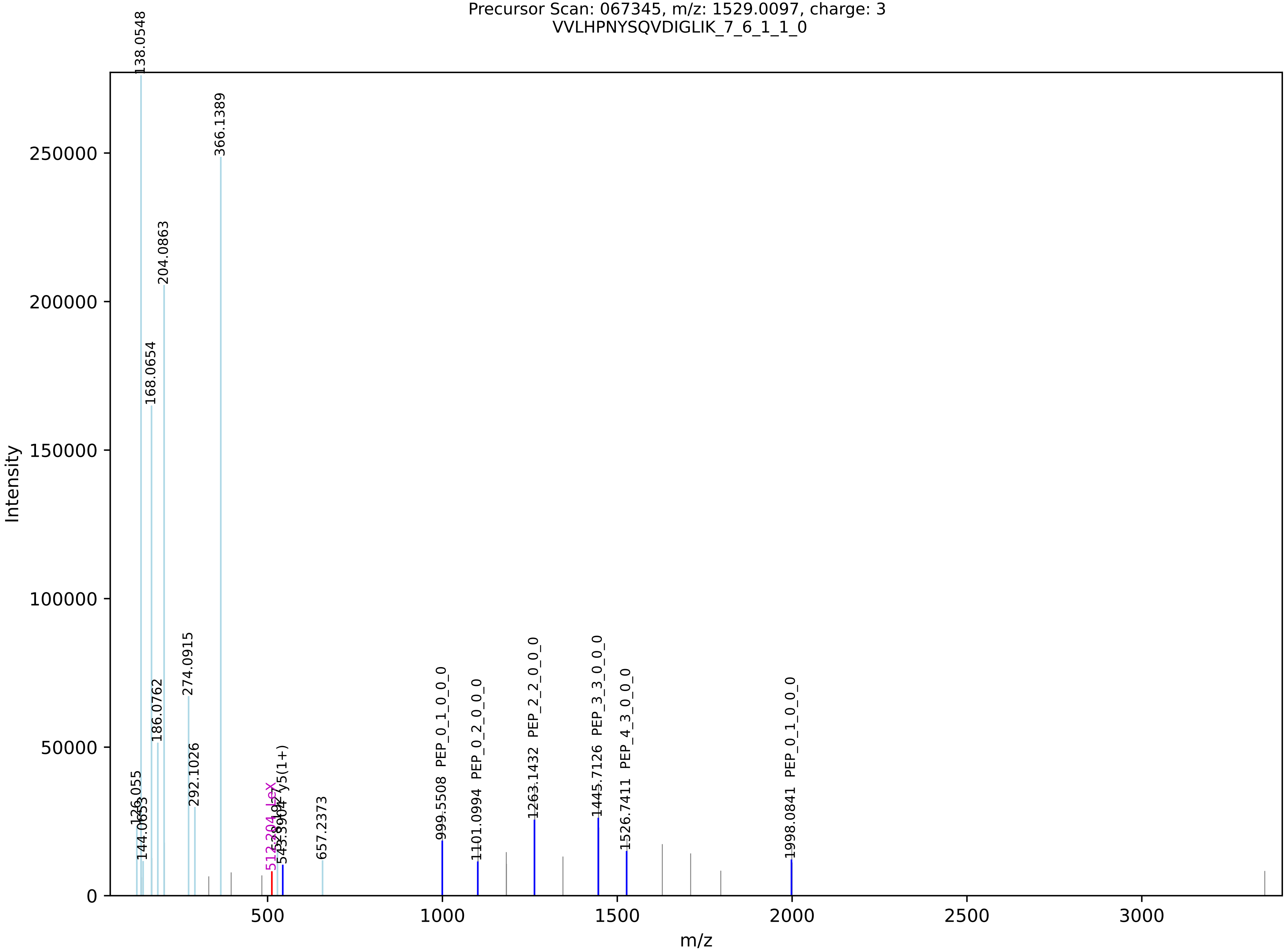

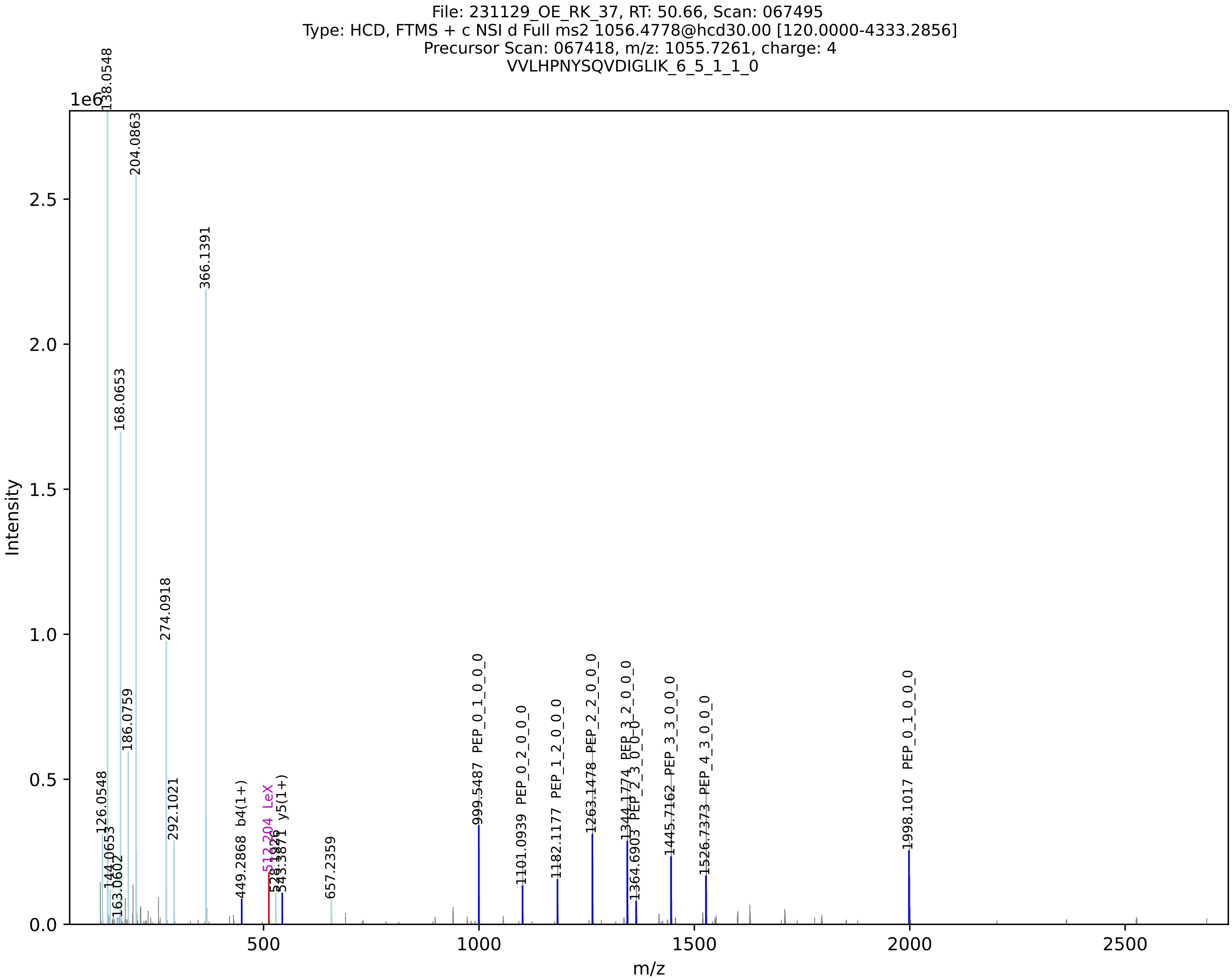
File: 231129_OE_RK_37, RT: 50.66, Scan: 067495
Type: HCD, FTMS + c NSI d Full ms2 1056.4778@hcd30.00 [120.0000-4333.2856]
Precursor Scan: 067418, m/z: 1055.7261, charge: 4
VVLHPNYSQVDIGLIK_6_5_1_1_0
1e6
Intensity
m/z
138.0548
204.0863
366.1391
168.0653
274.0918
186.0759
126.0548
144.0653
163.0602
292.1021
449.2868 b4(1+)
512.204 LeX
657.2359
999.5487 PEP_0_1_0_0_0
1101.0939 PEP_0_2_0_0_0
1182.1177 PEP_1_2_0_0_0
1263.1478 PEP_2_2_0_0_0
1344.1774 PEP_3_2_0_0_0
1364.6903 PEP_2_3_0_0_0
1445.7162 PEP_3_3_0_0_0
1526.7373 PEP_4_3_0_0_0
1998.1017 PEP_0_1_0_0_0

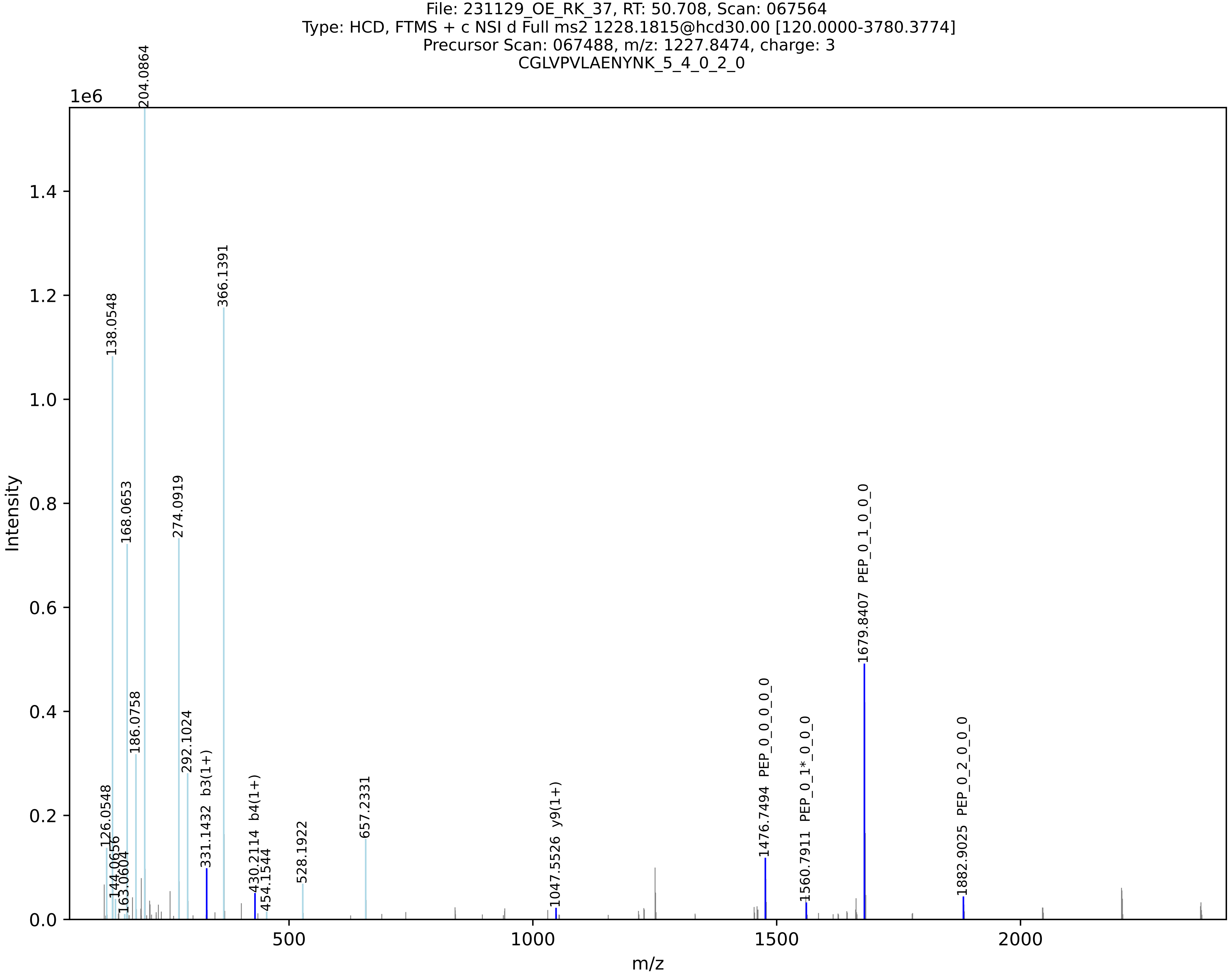

File: 231129_OE_RK_37, RT: 50.708, Scan: 067564
Type: HCD, FTMS + c NSI d Full ms2 1228.1815@hcd30.00 [120.0000-3780.3774]
Precursor Scan: 067488, m/z: 1227.8474, charge: 3
CGLVPVLAENYNK_5_4_0_2_0
1e6
Intensity
m/z
204.0864
366.1391
138.0548
168.0653
274.0919
186.0758
292.1024
331.1432 b3(1+)
430.2114 b4(1+)
454.1544
528.1922
657.2331
126.0548
144.0656
163.0604
1047.5526 y9(1+)
1476.7494 PEP_0_0_0_0_0
1560.7911 PEP_0_1*_0_0_0
1679.8407 PEP_0_1_0_0_0
1882.9025 PEP_0_2_0_0_0

File: 231129_OE_RK_37, RT: 50.712, Scan: 067571
Type: HCD, FTMS + c NSI d Full ms2 1323.2784@hcd30.00 [120.0000-5421.8320]
Precursor Scan: 067488, m/z: 1322.7768, charge: 4
QDQCIYNTTYLNVQR_7_6_1_3_0

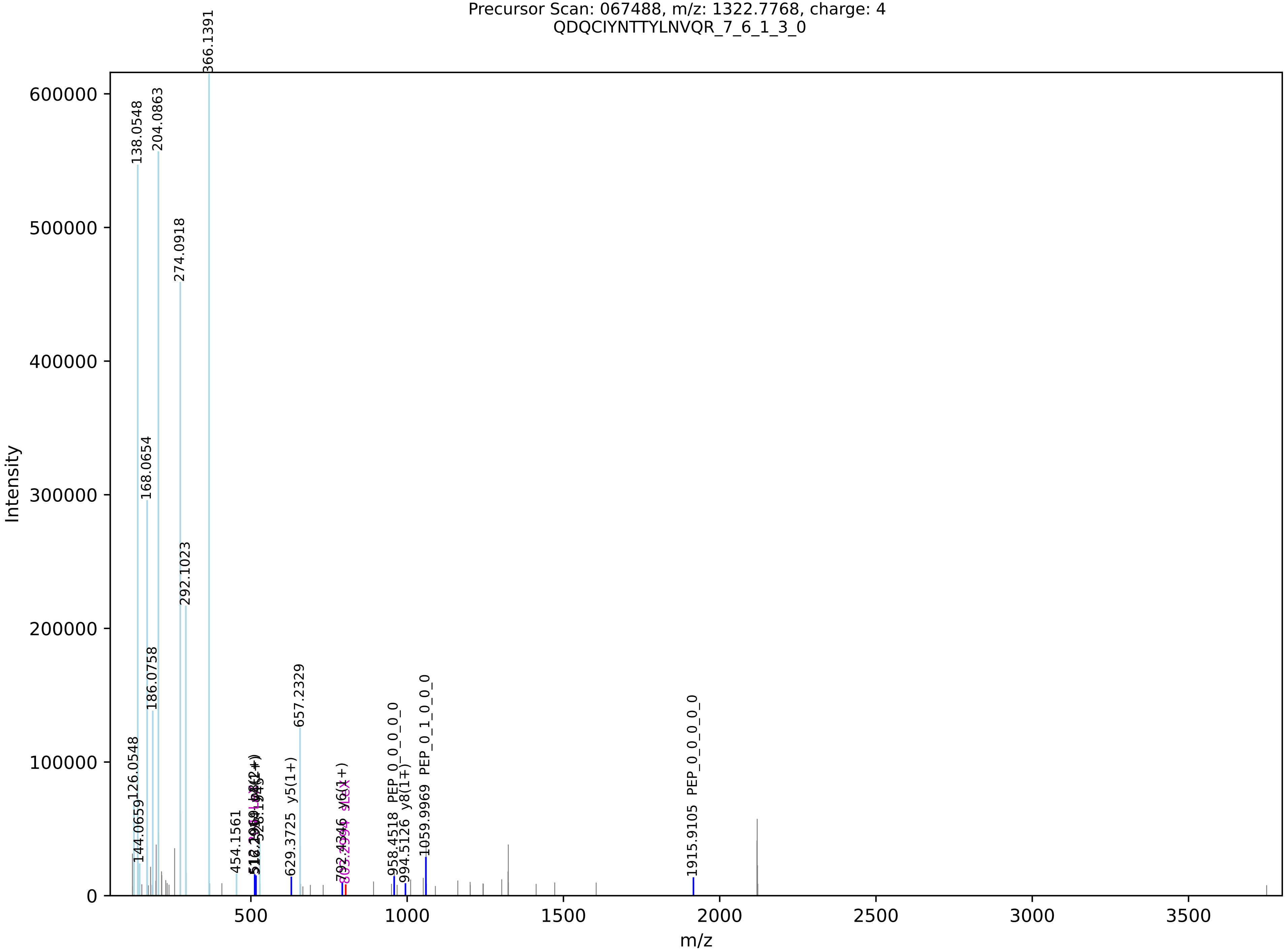

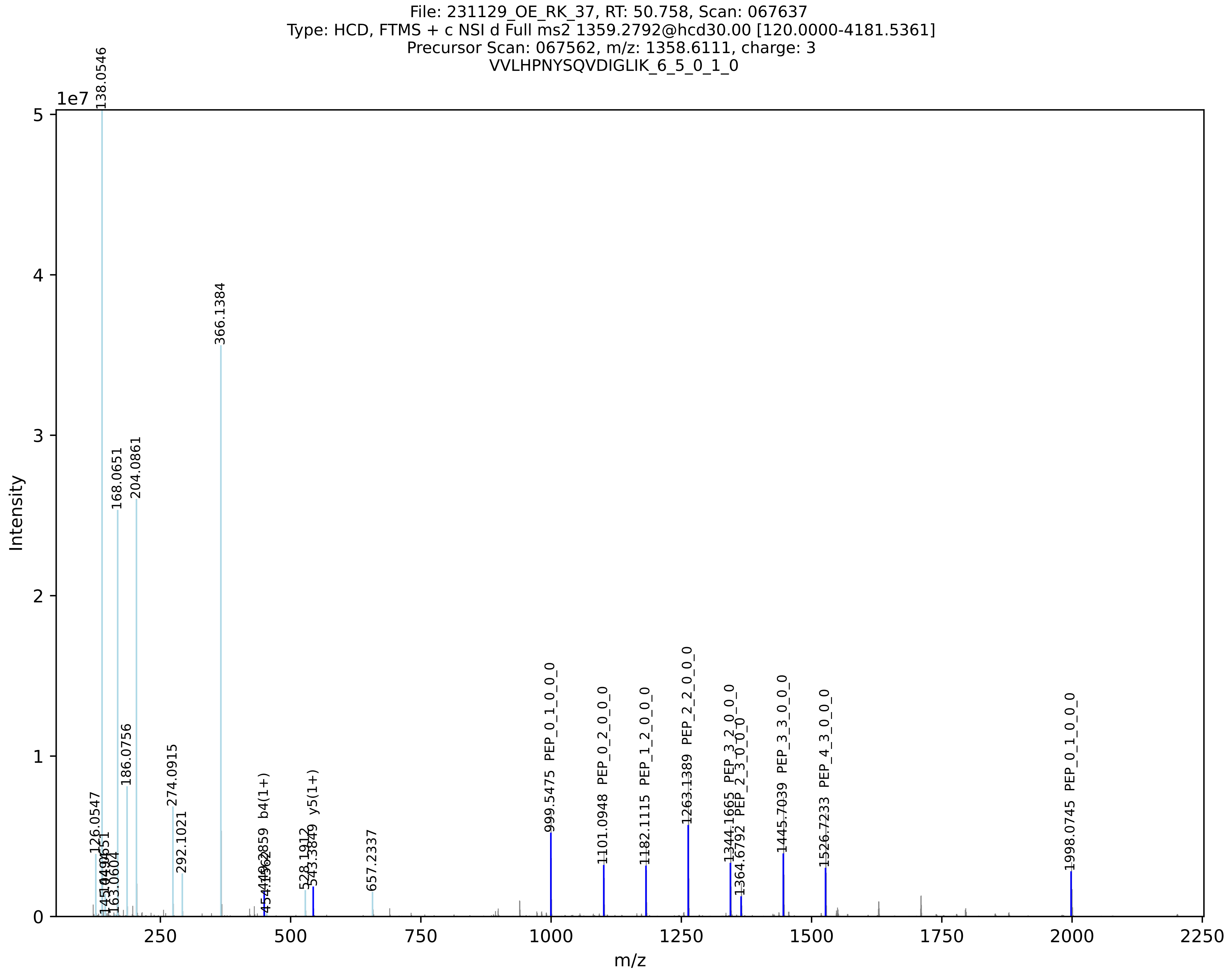
File: 231129_OE_RK_37, RT: 50.758, Scan: 067637
Type: HCD, FTMS + c NSI d Full ms2 1359.2792@hcd30.00 [120.0000-4181.5361]
Precursor Scan: 067562, m/z: 1358.6111, charge: 3
VVLHPNYSQVDIGLIK_6_5_0_1_0
1e7
Intensity
m/z
138.0546
366.1384
168.0651
204.0861
186.0756
274.0915
292.1021
126.0547
163.0604
b4(1+)
y5(1+)
528.1912
543.3849
657.2337
999.5475 PEP_0_1_0_0_0
1101.0948 PEP_0_2_0_0_0
1182.1115 PEP_1_2_0_0_0
1263.1389 PEP_2_2_0_0_0
1344.1665 PEP_3_2_0_0_0
1364.6792 PEP_2_3_0_0_0
1445.7039 PEP_3_3_0_0_0
1526.7233 PEP_4_3_0_0_0
1998.0745 PEP_0_1_0_0_0

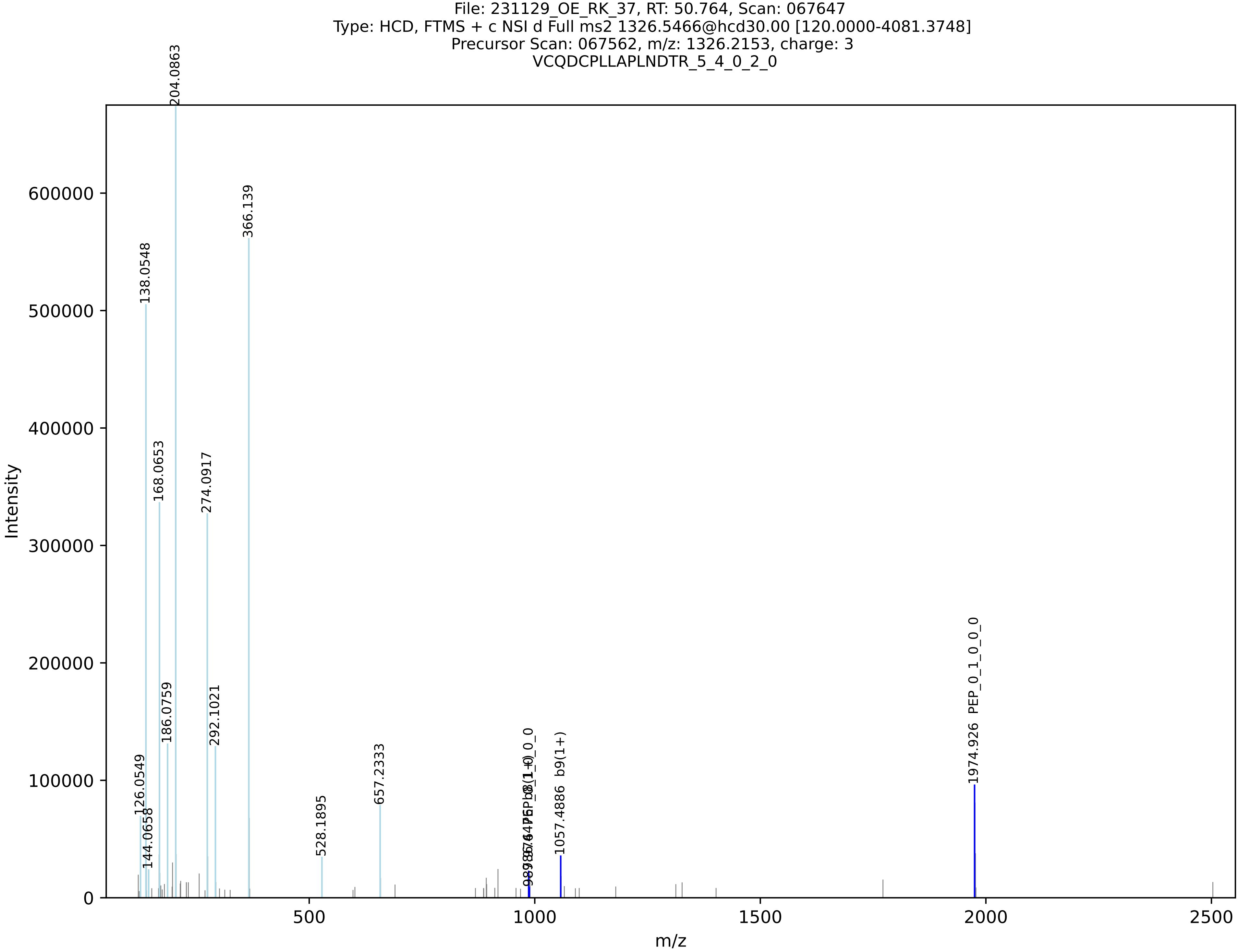
File: 231129_OE_RK_37, RT: 50.764, Scan: 067647
Type: HCD, FTMS + c NSI d Full ms2 1326.5466@hcd30.00 [120.0000-4081.3748]
Precursor Scan: 067562, m/z: 1326.2153, charge: 3
VCQDCPLLAPLNDTR_5_4_0_2_0
Intensity
m/z
204.0863
366.139
138.0548
168.0653
274.0917
186.0759
292.1021
126.0549
144.0658
528.1895
657.2333
1057.4886 b9(1+)
1974.926 PEP_0_1_0_0_0
0
100000
200000
300000
400000
500000
600000
500
1000
1500
2000
2500

File: 231129_OE_RK_37, RT: 50.808, Scan: 067711
Type: HCD, FTMS + c NSI d Full ms2 1359.1128@hcd30.00 [120.0000-4181.0269]
Precursor Scan: 067636, m/z: 1358.4445, charge: 3
VVLHPNYSQVDIGLIK_6_5_0_1_0

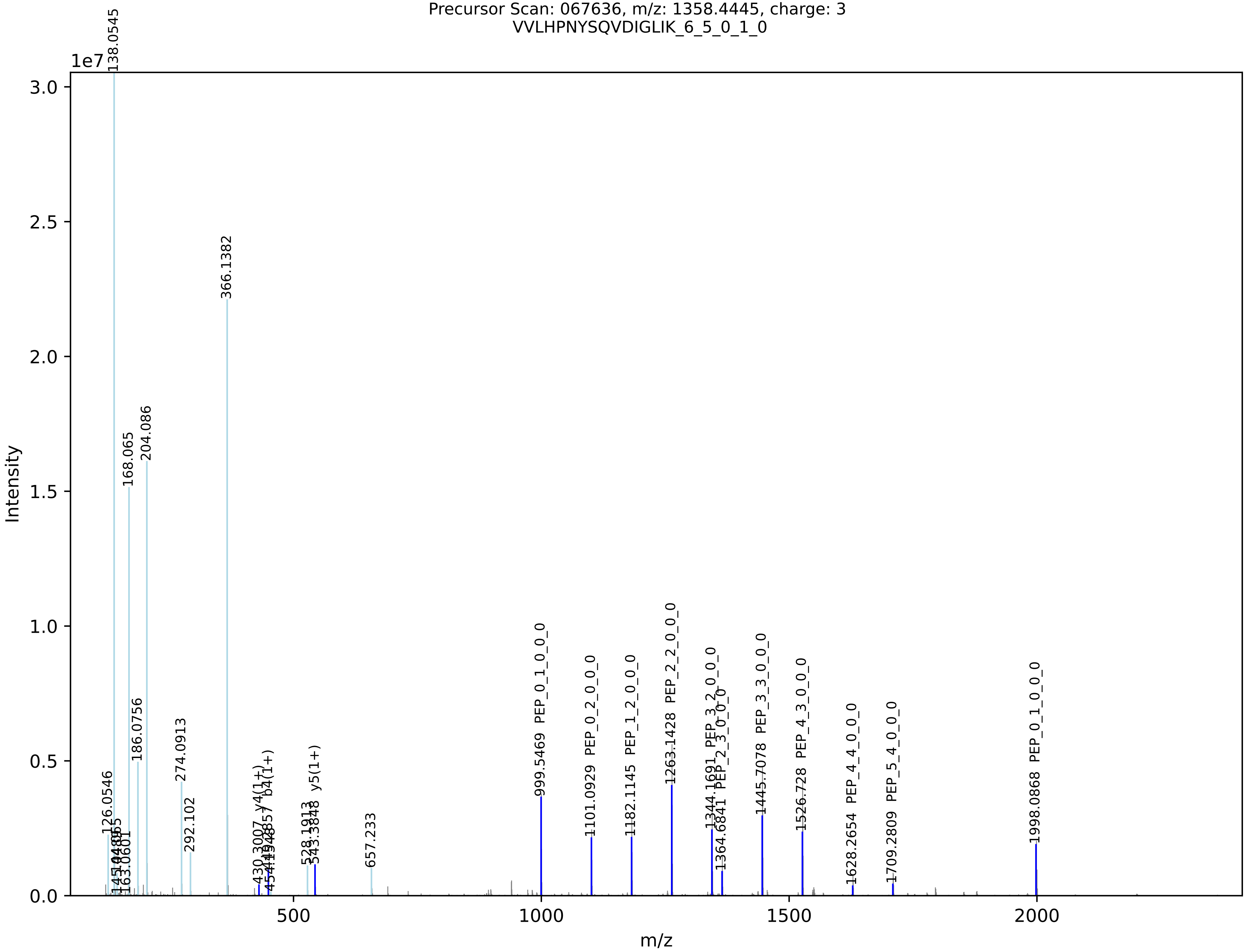

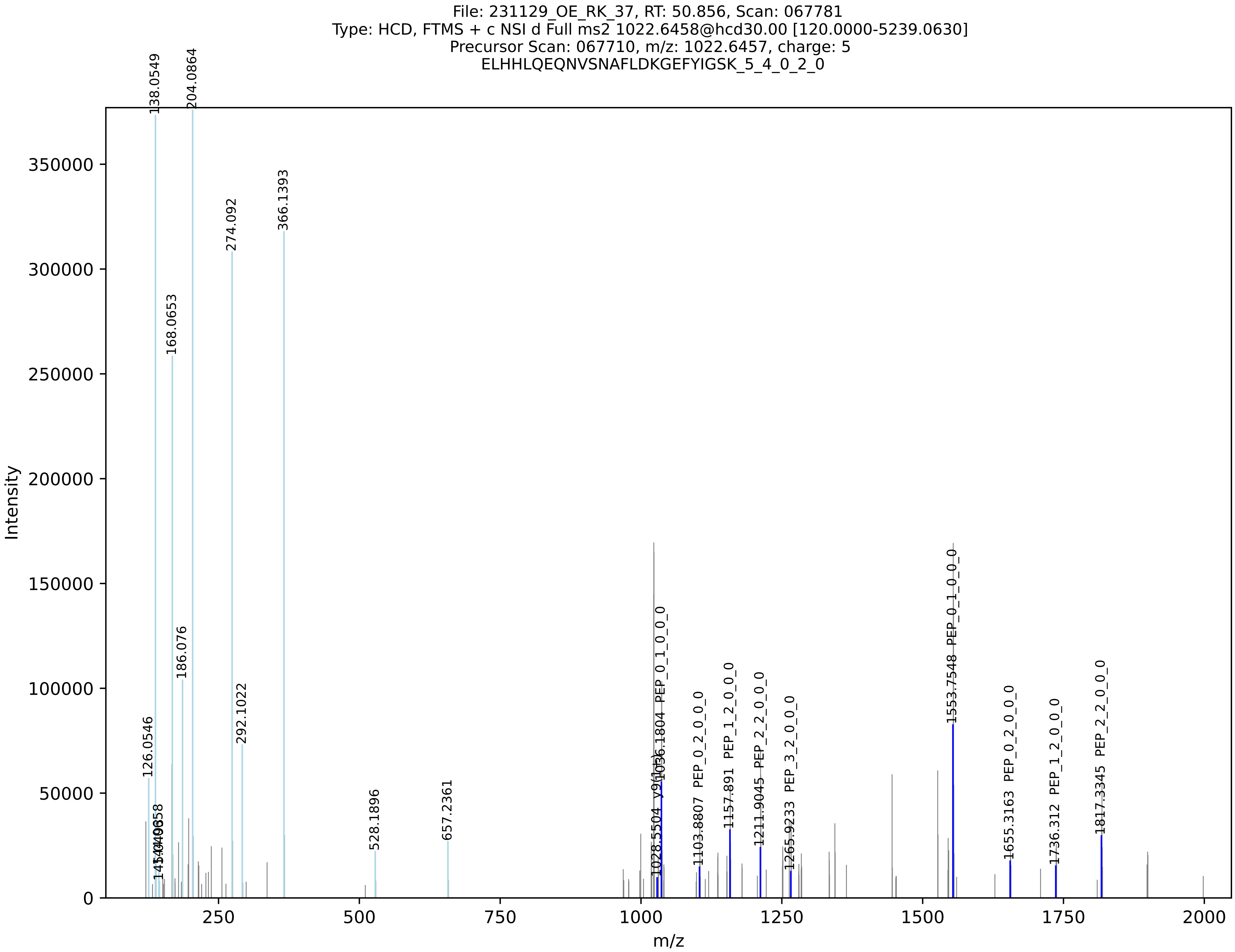

File: 231129_OE_RK_37, RT: 50.856, Scan: 067781
Type: HCD, FTMS + c NSI d Full ms2 1022.6458@hcd30.00 [120.0000-5239.0630]
Precursor Scan: 067710, m/z: 1022.6457, charge: 5
ELHHLQEQNVSNAFLDKGEFYIGSK_5_4_0_2_0
Intensity
m/z
138.0549
204.0864
366.1393
274.092
168.0653
186.076
292.1022
126.0546
528.1896
657.2361
1028.5504_y9(1+)
1036.1804_PEP_0_1_0_0_0
1103.8807_PEP_0_2_0_0_0
1157.891_PEP_1_2_0_0_0
1211.9045_PEP_2_2_0_0_0
1265.9233_PEP_3_2_0_0_0
1553.7548_PEP_0_1_0_0_0
1655.3163_PEP_0_2_0_0_0
1736.312_PEP_1_2_0_0_0
1817.3345_PEP_2_2_0_0_0

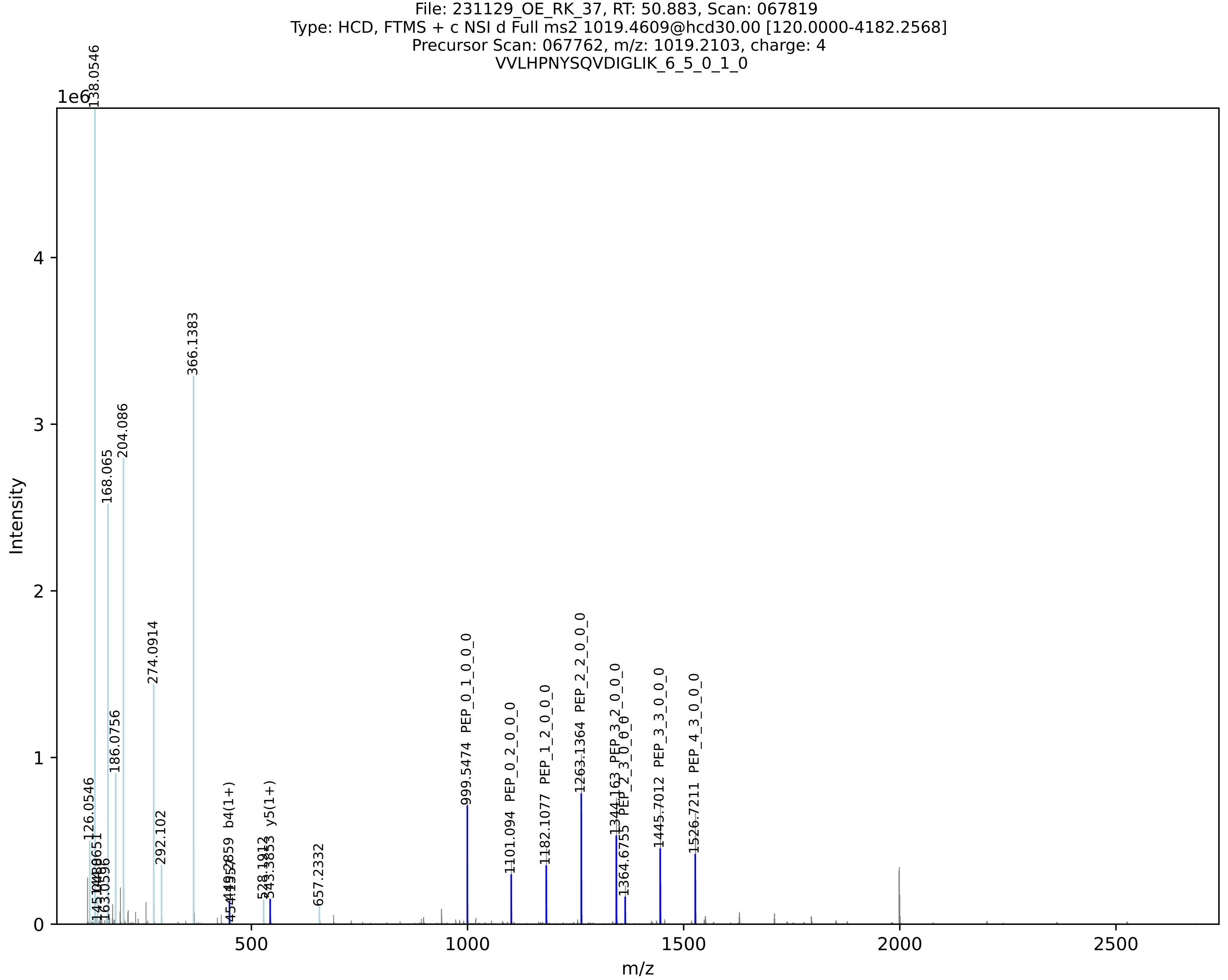

File: 231129_OE_RK_37, RT: 50.883, Scan: 067819
Type: HCD, FTMS + c NSI d Full ms2 1019.4609@hcd30.00 [120.0000-4182.2568]
Precursor Scan: 067762, m/z: 1019.2103, charge: 4
VVLHPNYSQVDIGLIK_6_5_0_1_0
1e6
Intensity
m/z
138.0546
366.1383
204.086
168.065
274.0914
186.0756
126.0546
292.102
b4(1+)
y5(1+)
657.2332
999.5474 PEP_0_1_0_0_0
1101.094 PEP_0_2_0_0_0
1182.1077 PEP_1_2_0_0_0
1263.1364 PEP_2_2_0_0_0
PEP_3_2_0_0_0
PEP_2_3_0_0_0
1364.6755
1445.7012 PEP_3_3_0_0_0
1526.7211 PEP_4_3_0_0_0

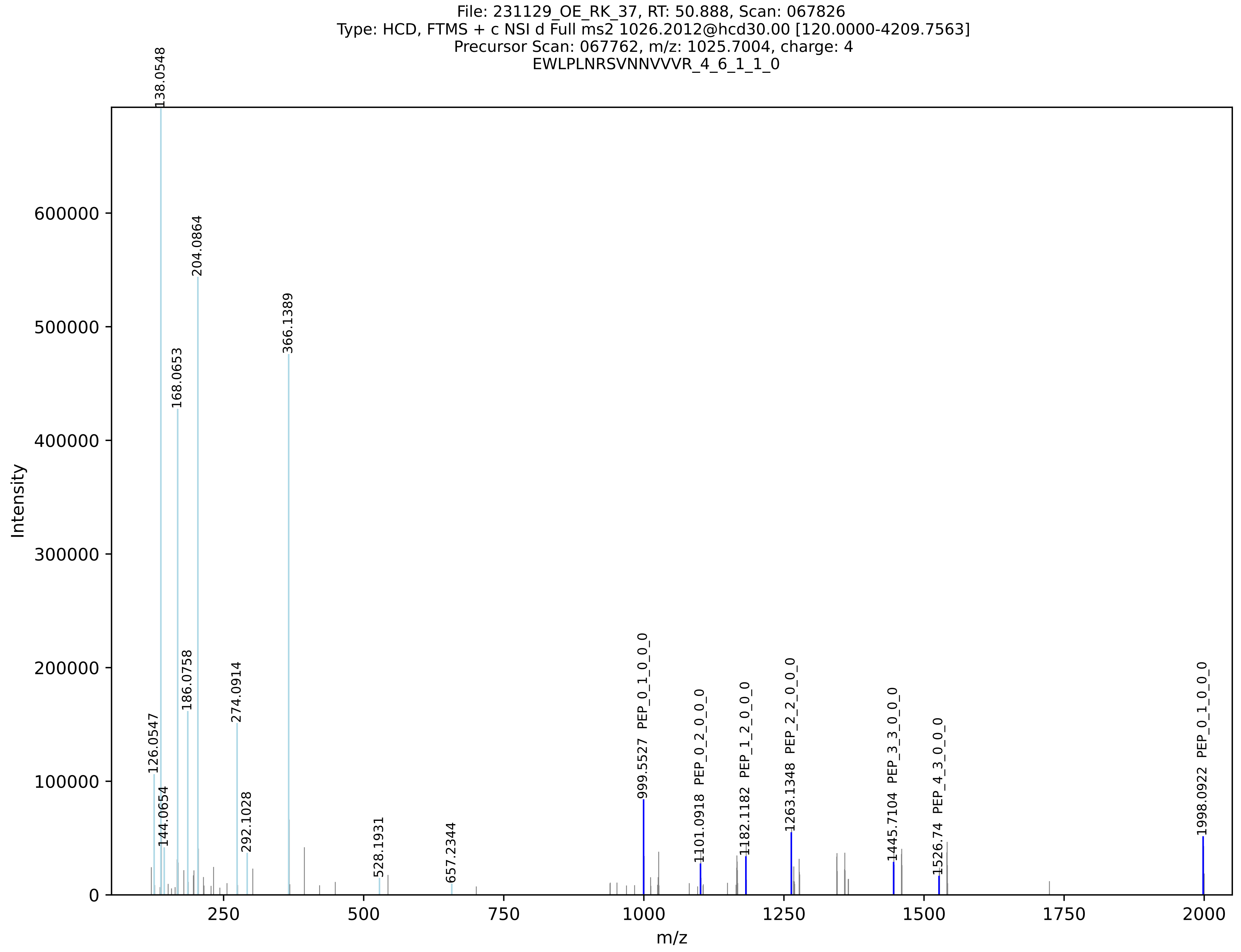
File: 231129_OE_RK_37, RT: 50.888, Scan: 067826
Type: HCD, FTMS + c NSI d Full ms2 1026.2012@hcd30.00 [120.0000-4209.7563]
Precursor Scan: 067762, m/z: 1025.7004, charge: 4
EWLPLNRSVNNVVVR_4_6_1_1_0
Intensity
m/z
138.0548
204.0864
366.1389
168.0653
186.0758
274.0914
126.0547
144.0654
292.1028
528.1931
657.2344
999.5527 PEP_0_1_0_0_
1101.0918 PEP_0_2_0_0_0
1182.1182 PEP_1_2_0_0_0
1263.1348 PEP_2_2_0_0_0
1445.7104 PEP_3_3_0_0_0
1526.74 PEP_4_3_0_0_0
1998.0922 PEP_0_1_0_0_0

File: 231129_OE_RK_37, RT: 50.892, Scan: 067834
Type: HCD, FTMS + c NSI d Full ms2 1532.3330@hcd30.00 [120.0000-4711.0811]
Precursor Scan: 067762, m/z: 1531.6613, charge: 3
SVTLQIYNHSLTLSAR_7_6_1_1_0

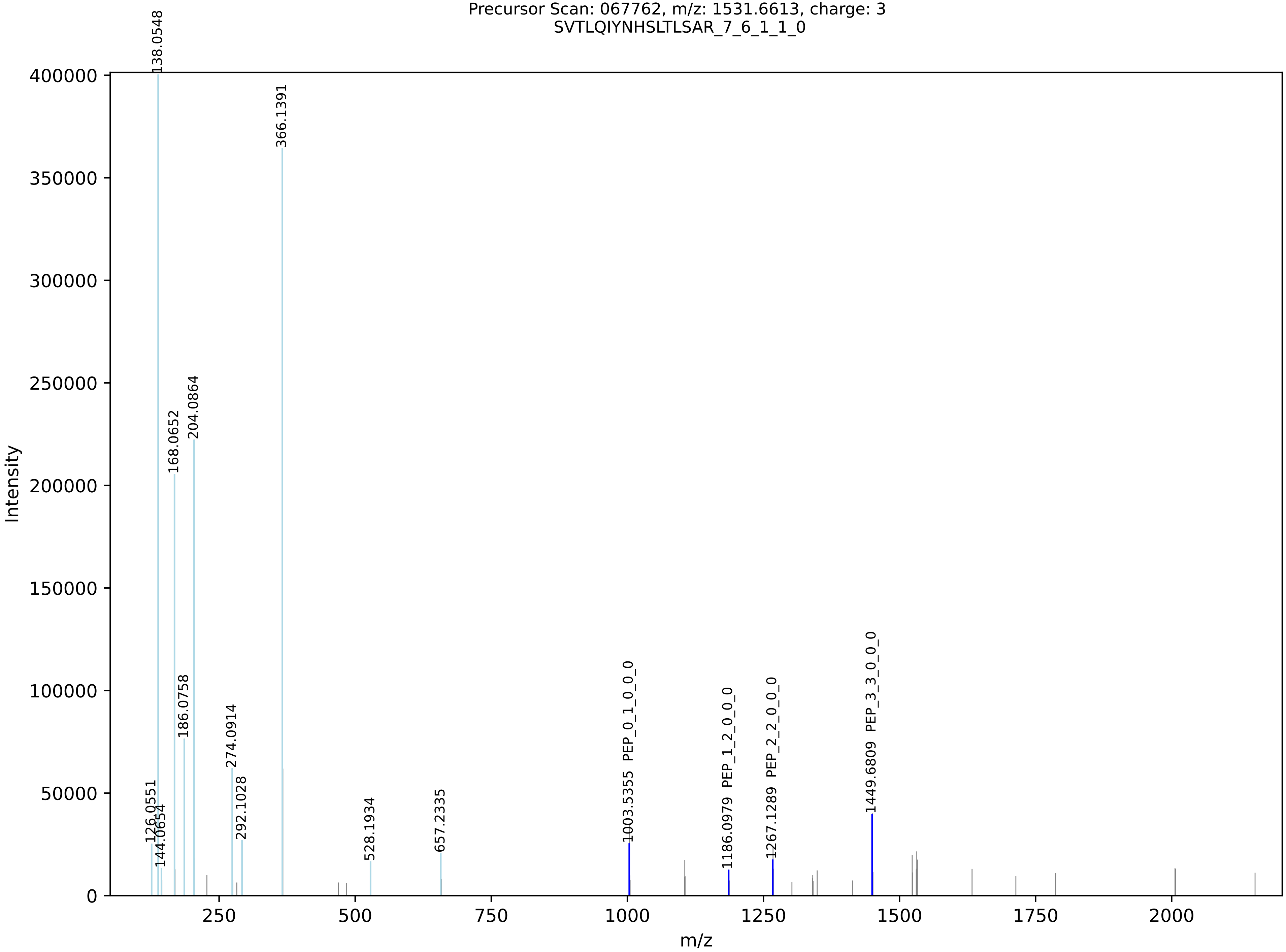

File: 231129_OE_RK_37, RT: 50.935, Scan: 067893
Type: HCD, FTMS + c NSI d Full ms2 1359.2802@hcd30.00 [120.0000-4181.5391]
Precursor Scan: 067818, m/z: 1358.612, charge: 3
VVLHPNYSQVDIGLIK_6_5_0_1_0

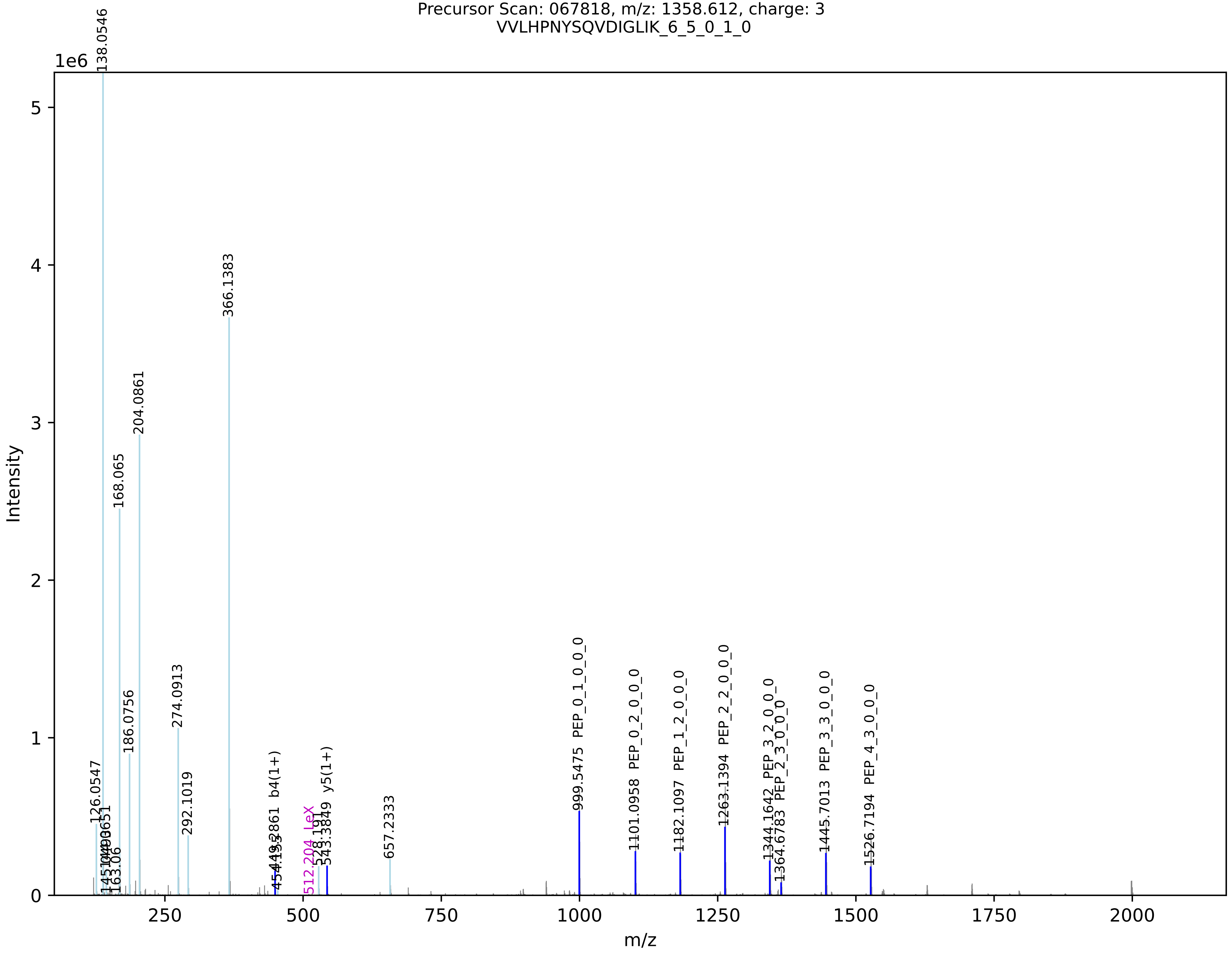

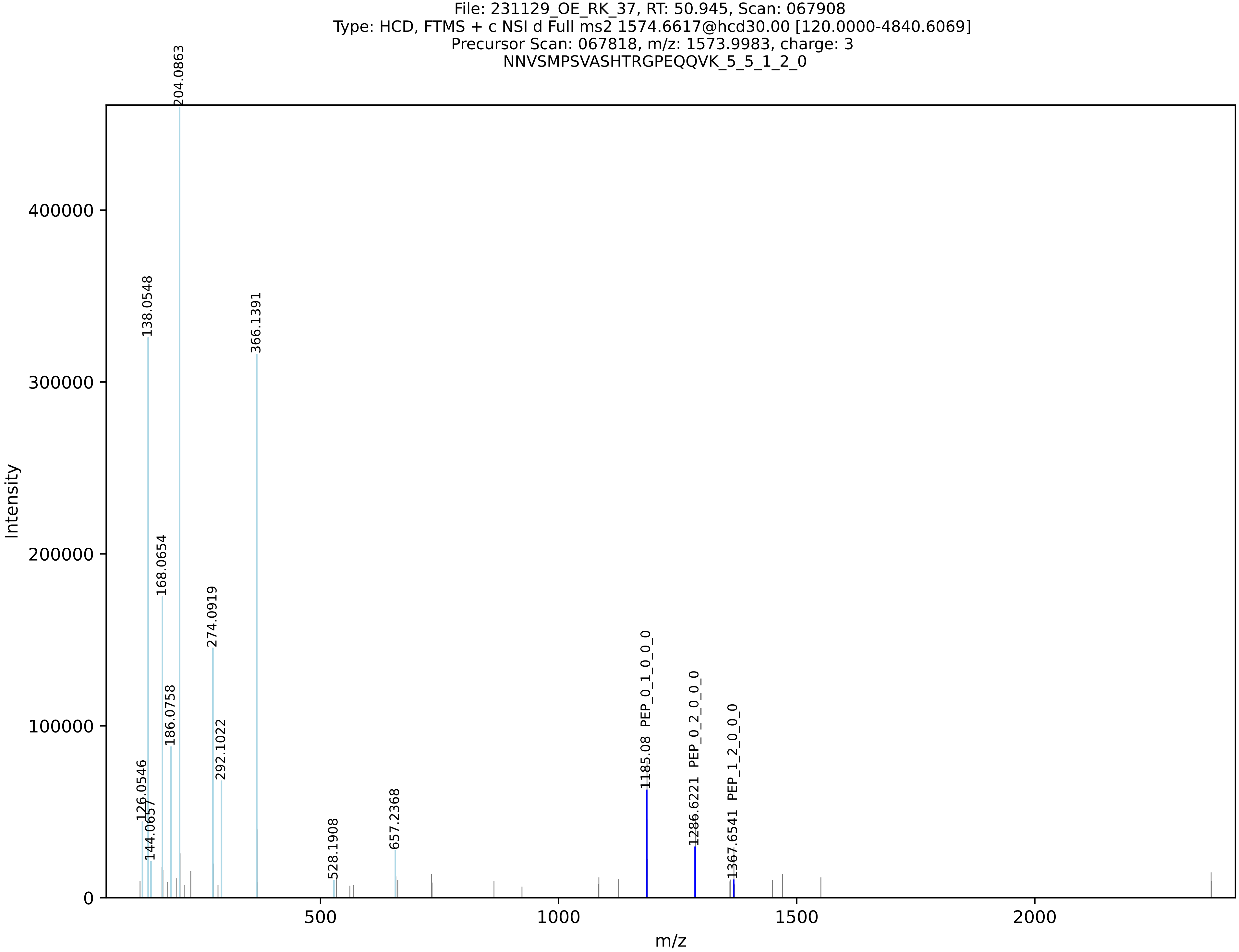

File: 231129_OE_RK_37, RT: 50.945, Scan: 067908
Type: HCD, FTMS + c NSI d Full ms2 1574.6617@hcd30.00 [120.0000-4840.6069]
Precursor Scan: 067818, m/z: 1573.9983, charge: 3
NNVSMPSVASHTRGPEQQVK_5_5_1_2_0
Intensity
m/z
204.0863
138.0548
366.1391
168.0654
274.0919
186.0758
292.1022
126.0546
144.0657
528.1908
657.2368
1185.08 PEP_0_1_0_0_0
1286.6221 PEP_0_2_0_0_0
1367.6541 PEP_1_2_0_0_0
0
100000
200000
300000
400000
500
1000
1500
2000

File: 231129_OE_RK_37, RT: 50.947, Scan: 067911
Type: HCD, FTMS + c NSI d Full ms2 968.6916@hcd30.00 [120.0000-3975.1177]
Precursor Scan: 067818, m/z: 968.6916, charge: 4
VVLHPNYSQVDIGLIK_6_4_0_1_0

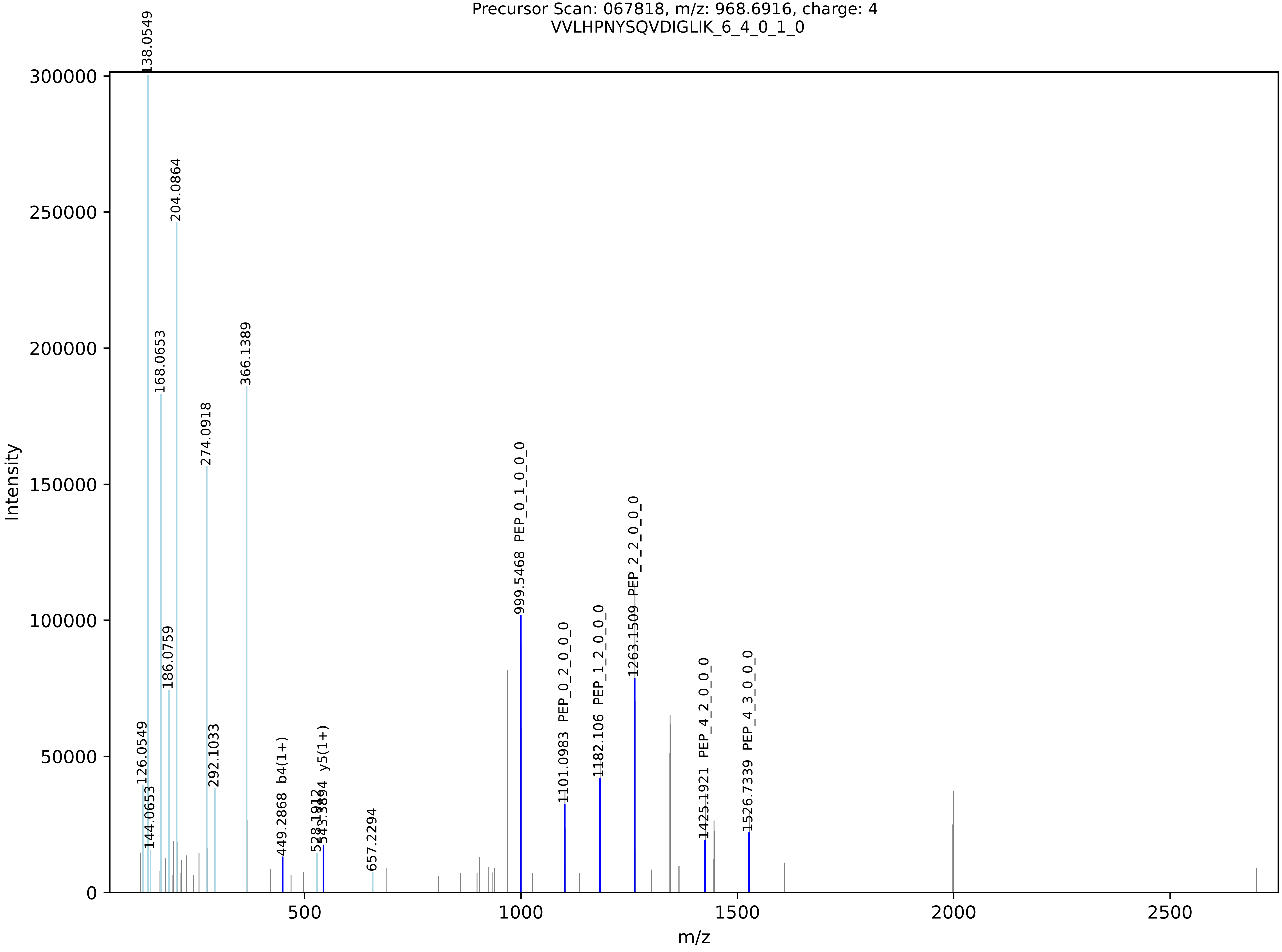

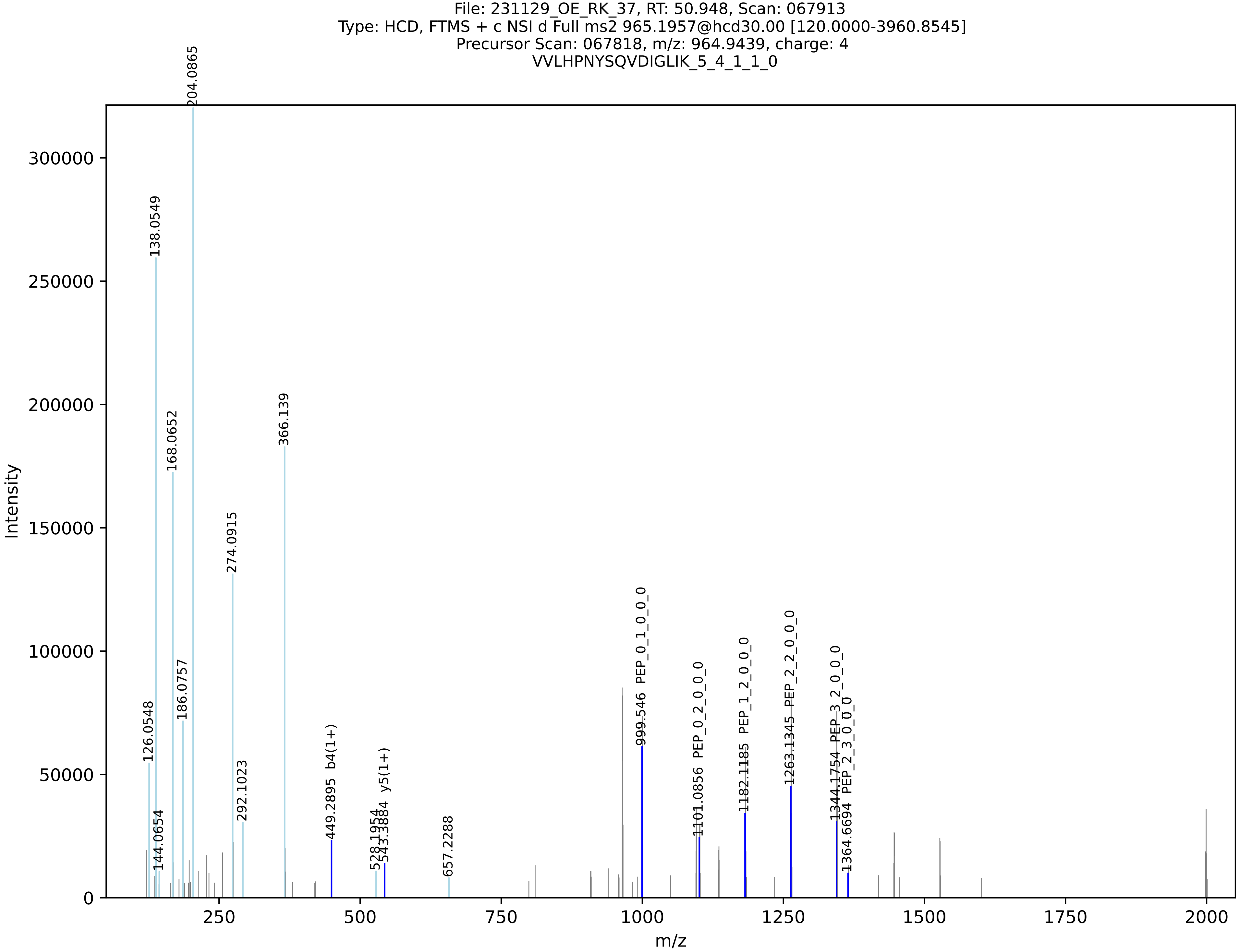
File: 231129_OE_RK_37, RT: 50.948, Scan: 067913
Type: HCD, FTMS + c NSI d Full ms2 965.1957@hcd30.00 [120.0000-3960.8545]
Precursor Scan: 067818, m/z: 964.9439, charge: 4
VVLHPNYSQVDIGLIK_5_4_1_1_0
Intensity
m/z
204.0865
138.0549
168.0652
366.139
274.0915
186.0757
126.0548
292.1023
144.0654
449.2895 b4(1+)
528.1954
543.3884 y5(1+)
657.2288
999.546 PEP_0_1_0_0_0
1101.0856 PEP_0_2_0_0_0
1182.1185 PEP_1_2_0_0_0
1263.1345 PEP_2_2_0_0_0
1344.1754 PEP_3_2_0_0_0
1364.6694 PEP_2_3_0_0_0

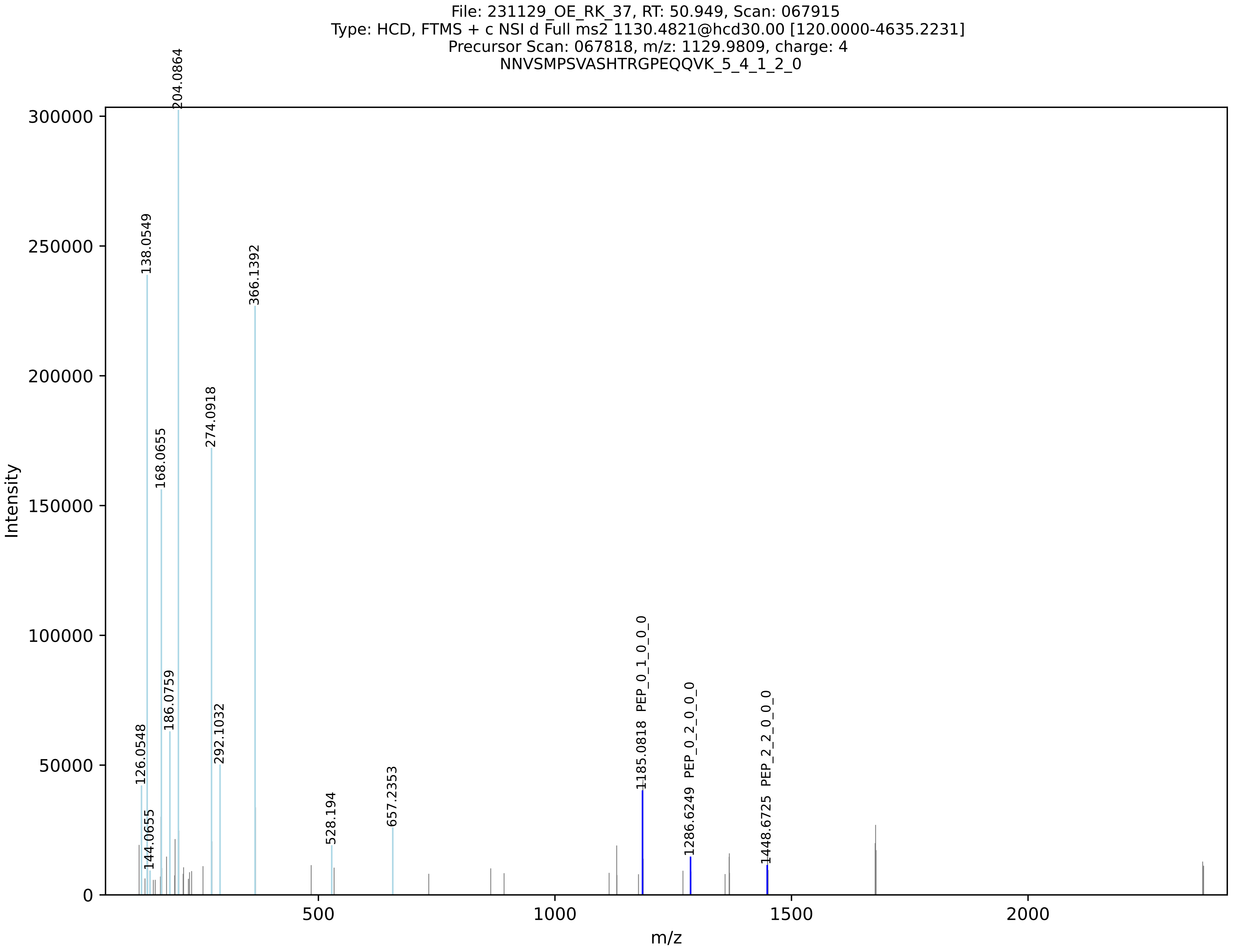

File: 231129_OE_RK_37, RT: 50.949, Scan: 067915
Type: HCD, FTMS + c NSI d Full ms2 1130.4821@hcd30.00 [120.0000-4635.2231]
Precursor Scan: 067818, m/z: 1129.9809, charge: 4
NNVSMPSVASHTRGPEQQVK_5_4_1_2_0
Intensity
m/z
300000
250000
200000
150000
100000
50000
0
500
1000
1500
2000
204.0864
138.0549
366.1392
274.0918
168.0655
186.0759
292.1032
126.0548
144.0655
528.194
657.2353
1185.0818 PEP_0_1_0_0_0
1286.6249 PEP_0_2_0_0_0
1448.6725 PEP_2_2_0_0_0

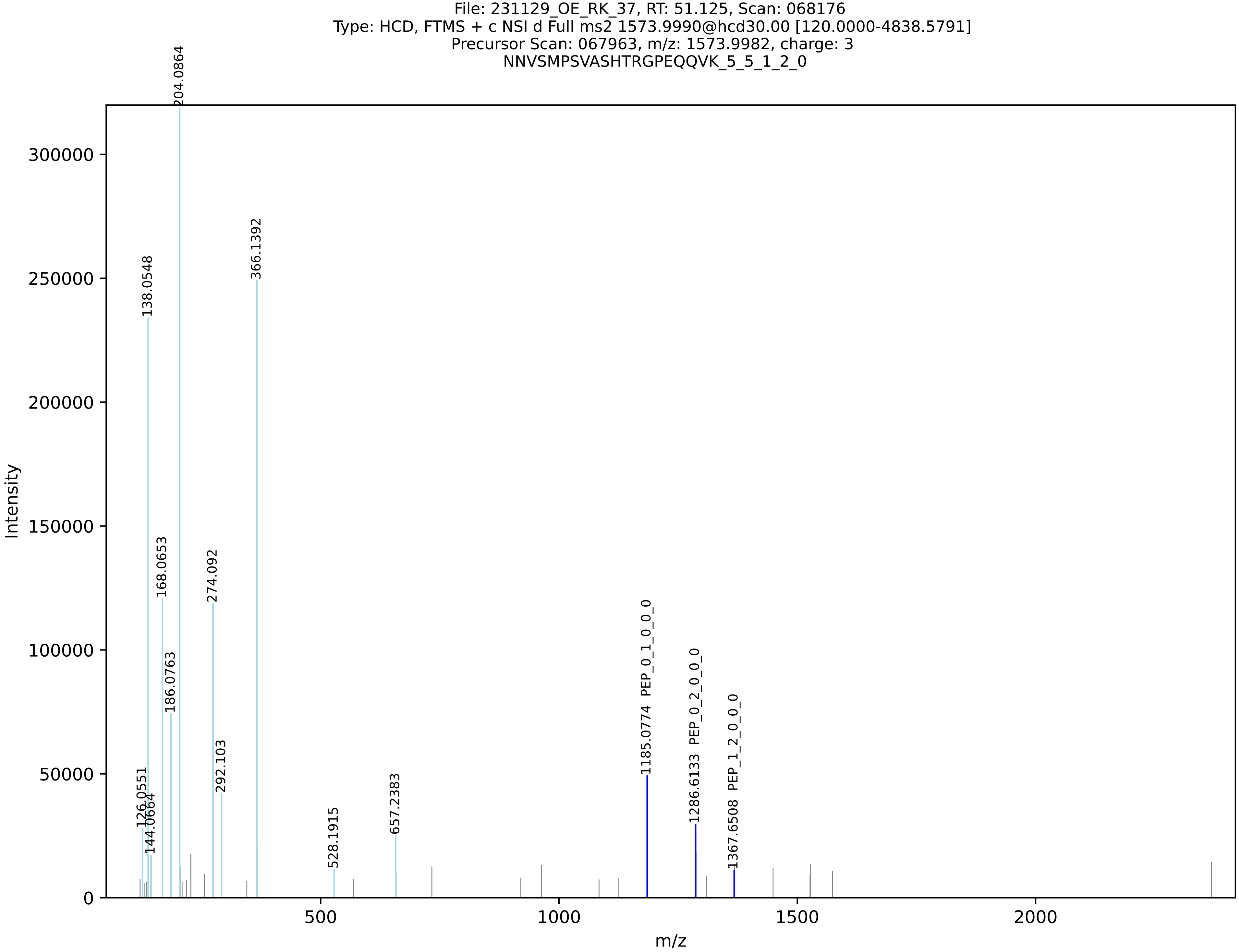

File: 231129_OE_RK_37, RT: 51.125, Scan: 068176
Type: HCD, FTMS + c NSI d Full ms2 1573.9990@hcd30.00 [120.0000-4838.5791]
Precursor Scan: 067963, m/z: 1573.9982, charge: 3
NNVSMPSVASHTRGPEQQVK_5_5_1_2_0
Intensity
m/z
204.0864
366.1392
138.0548
168.0653
274.092
186.0763
292.103
126.0551
144.0664
528.1915
657.2383
1185.0774 PEP_0_1_0_0_0
1286.6133 PEP_0_2_0_0_0
1367.6508 PEP_1_2_0_0_0

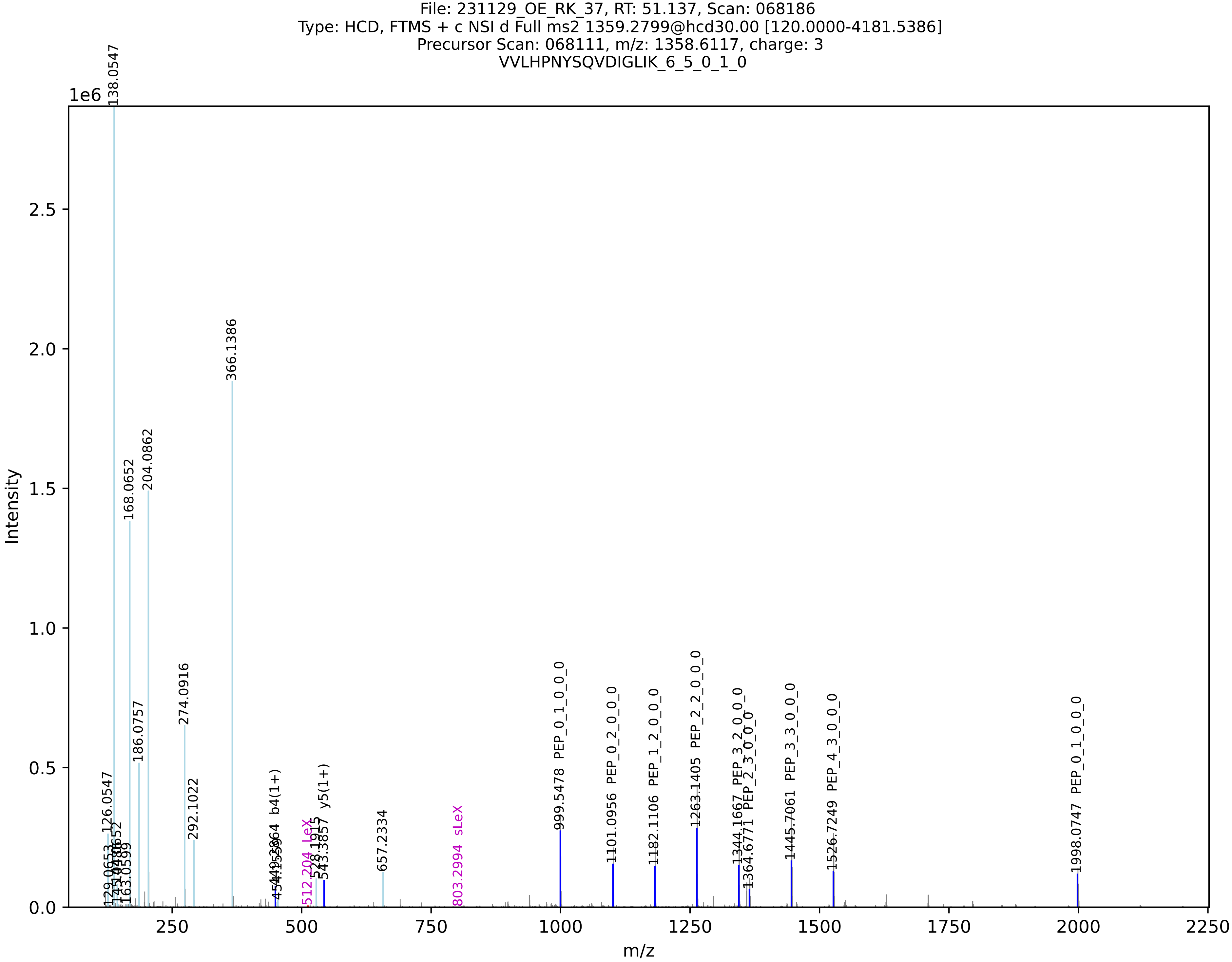
File: 231129_OE_RK_37, RT: 51.137, Scan: 068186
Type: HCD, FTMS + c NSI d Full ms2 1359.2799@hcd30.00 [120.0000-4181.5386]
Precursor Scan: 068111, m/z: 1358.6117, charge: 3
VVLHPNYSQVDIGLIK_6_5_0_1_0
1e6
Intensity
m/z
138.0547
366.1386
204.0862
168.0652
186.0757
274.0916
292.1022
126.0547
129.0653
163.0589
b4(1+)
y5(1+)
512.204 LeX
543.3857
657.2334
803.2994 sLeX
999.5478 PEP_0_1_0_0_0
1101.0956 PEP_0_2_0_0_0
1182.1106 PEP_1_2_0_0_0
1263.1405 PEP_2_2_0_0_0
1344.1667 PEP_3_2_0_0_0
1364.6771 PEP_2_3_0_0_0
1445.7061 PEP_3_3_0_0_0
1526.7249 PEP_4_3_0_0_0
1998.0747 PEP_0_1_0_0_0

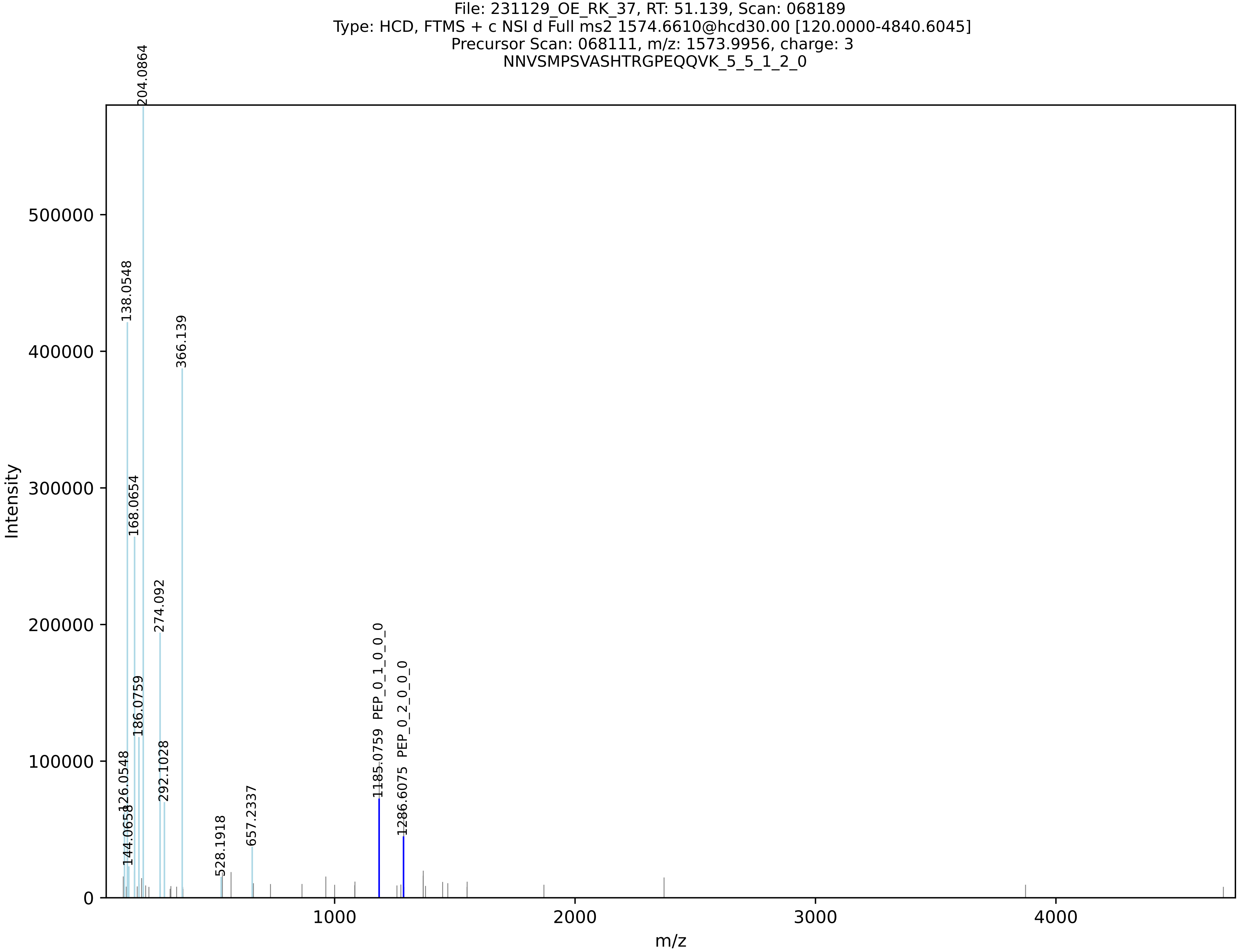
File: 231129_OE_RK_37, RT: 51.139, Scan: 068189
Type: HCD, FTMS + c NSI d Full ms2 1574.6610@hcd30.00 [120.0000-4840.6045]
Precursor Scan: 068111, m/z: 1573.9956, charge: 3
NNVSMPSVASHTRGPEQQVK_5_5_1_2_0
Intensity
m/z
204.0864
138.0548
366.139
168.0654
274.092
186.0759
126.0548
292.1028
144.0658
528.1918
657.2337
1185.0759 PEP_0_1_0_0_0
1286.6075 PEP_0_2_0_0_0
0
100000
200000
300000
400000
500000
1000
2000
3000
4000

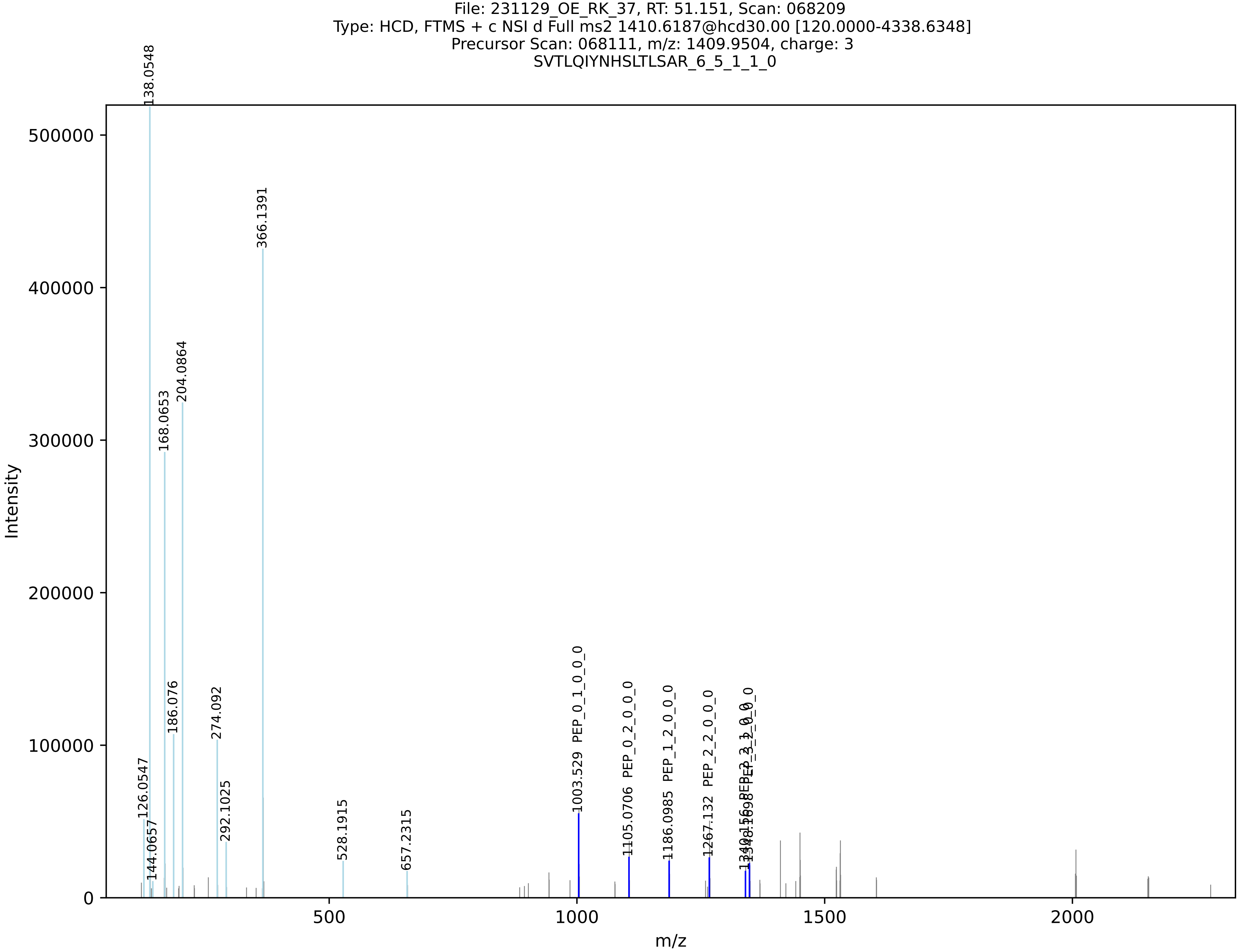

File: 231129_OE_RK_37, RT: 51.151, Scan: 068209
Type: HCD, FTMS + c NSI d Full ms2 1410.6187@hcd30.00 [120.0000-4338.6348]
Precursor Scan: 068111, m/z: 1409.9504, charge: 3
SVTLQIYNHSLTLSAR_6_5_1_1_0
Intensity
m/z
138.0548
366.1391
204.0864
168.0653
186.076
274.092
126.0547
292.1025
144.0657
528.1915
657.2315
1003.529 PEP_0_1_0_0_0
1105.0706 PEP_0_2_0_0_0
1186.0985 PEP_1_2_0_0_0
1267.132 PEP_2_2_0_0_0

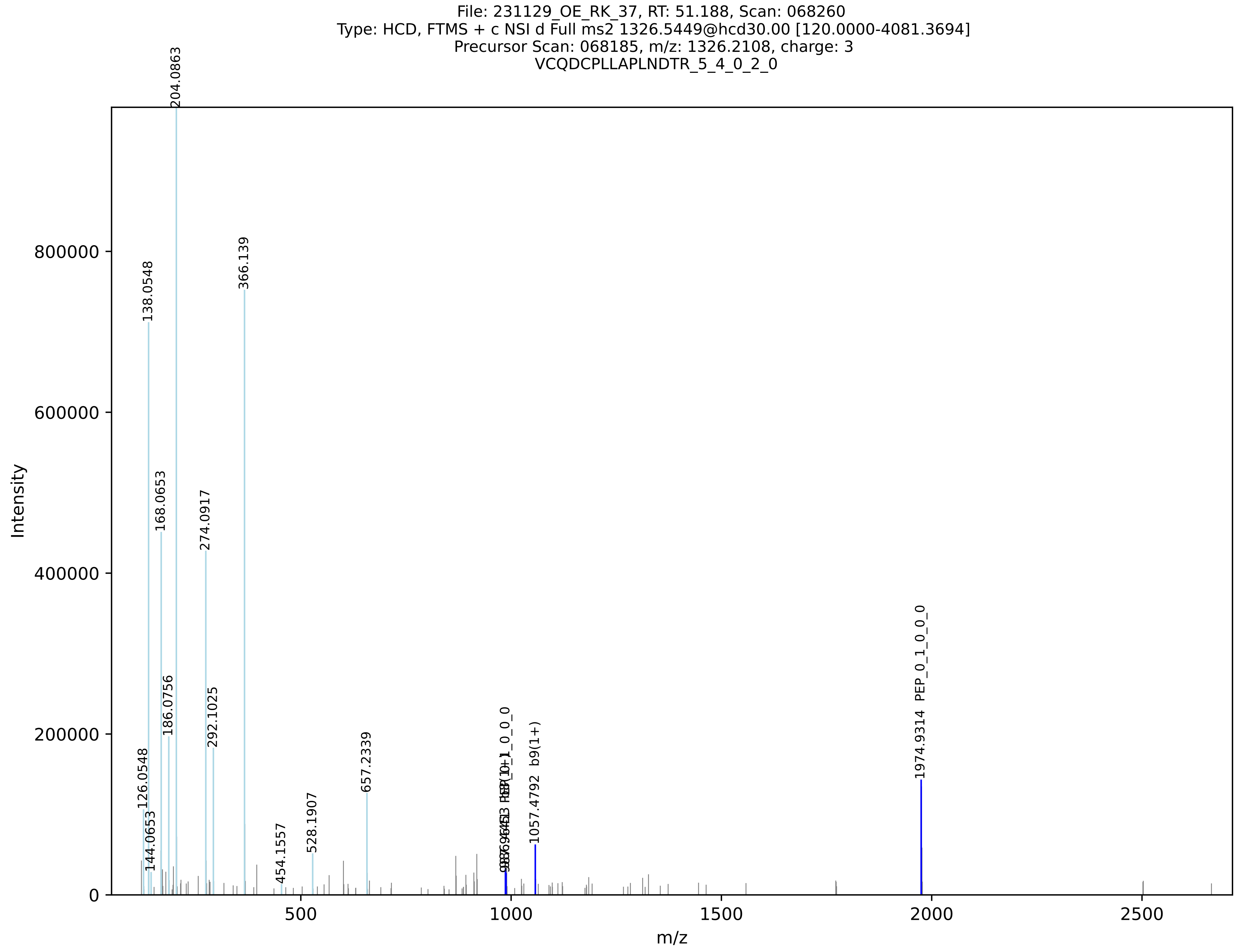

File: 231129_OE_RK_37, RT: 51.188, Scan: 068260
Type: HCD, FTMS + c NSI d Full ms2 1326.5449@hcd30.00 [120.0000-4081.3694]
Precursor Scan: 068185, m/z: 1326.2108, charge: 3
VCQDCPLLAPLNDTR_5_4_0_2_0
Intensity
m/z
204.0863
138.0548
366.139
168.0653
274.0917
186.0756
292.1025
126.0548
144.0653
454.1557
528.1907
657.2339
1057.4792 b9(1+)
1974.9314 PEP_0_1_0_0_
0
200000
400000
600000
800000
500
1000
1500
2000
2500

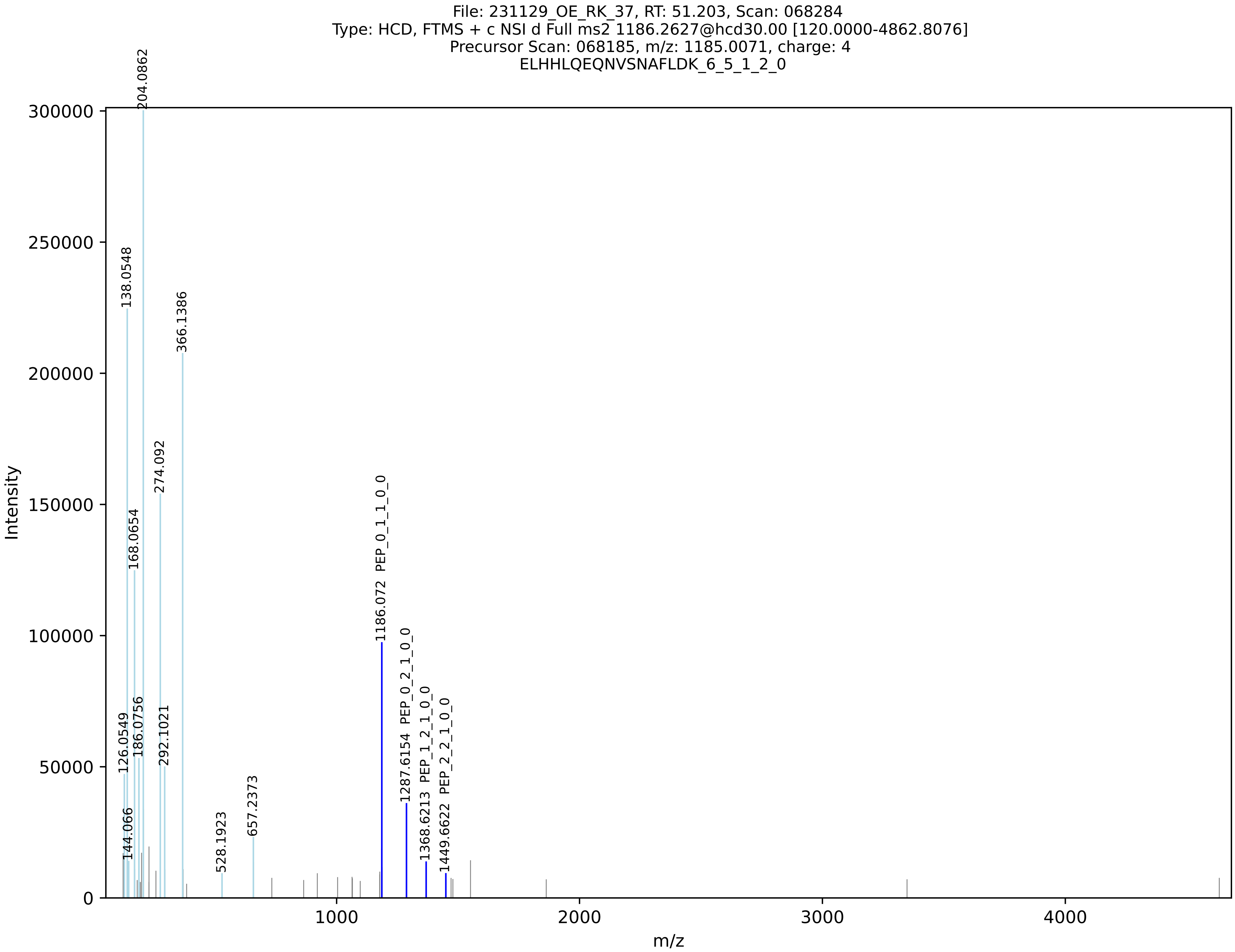
File: 231129_OE_RK_37, RT: 51.203, Scan: 068284
Type: HCD, FTMS + c NSI d Full ms2 1186.2627@hcd30.00 [120.0000-4862.8076]
Precursor Scan: 068185, m/z: 1185.0071, charge: 4
ELHHLQEQNVSNAFLDK_6_5_1_2_0
204.0862
138.0548
366.1386
274.092
168.0654
1186.072 PEP_0_1_1_0_0_
1287.6154 PEP_0_2_1_0_0_
1368.6213 PEP_1_2_1_0_0
1449.6622 PEP_2_2_1_0_0
126.0549
186.0756
292.1021
144.066
528.1923
657.2373
Intensity
m/z
0
50000
100000
150000
200000
250000
300000
1000
2000
3000
4000

File: 231129_OE_RK_37, RT: 51.238, Scan: 068331
Type: HCD, FTMS + c NSI d Full ms2 1237.5688@hcd30.00 [120.0000-3809.1028]
Precursor Scan: 068256, m/z: 1236.9007, charge: 3
VVLHPNYSQVDIGLIK_5_4_0_1_0

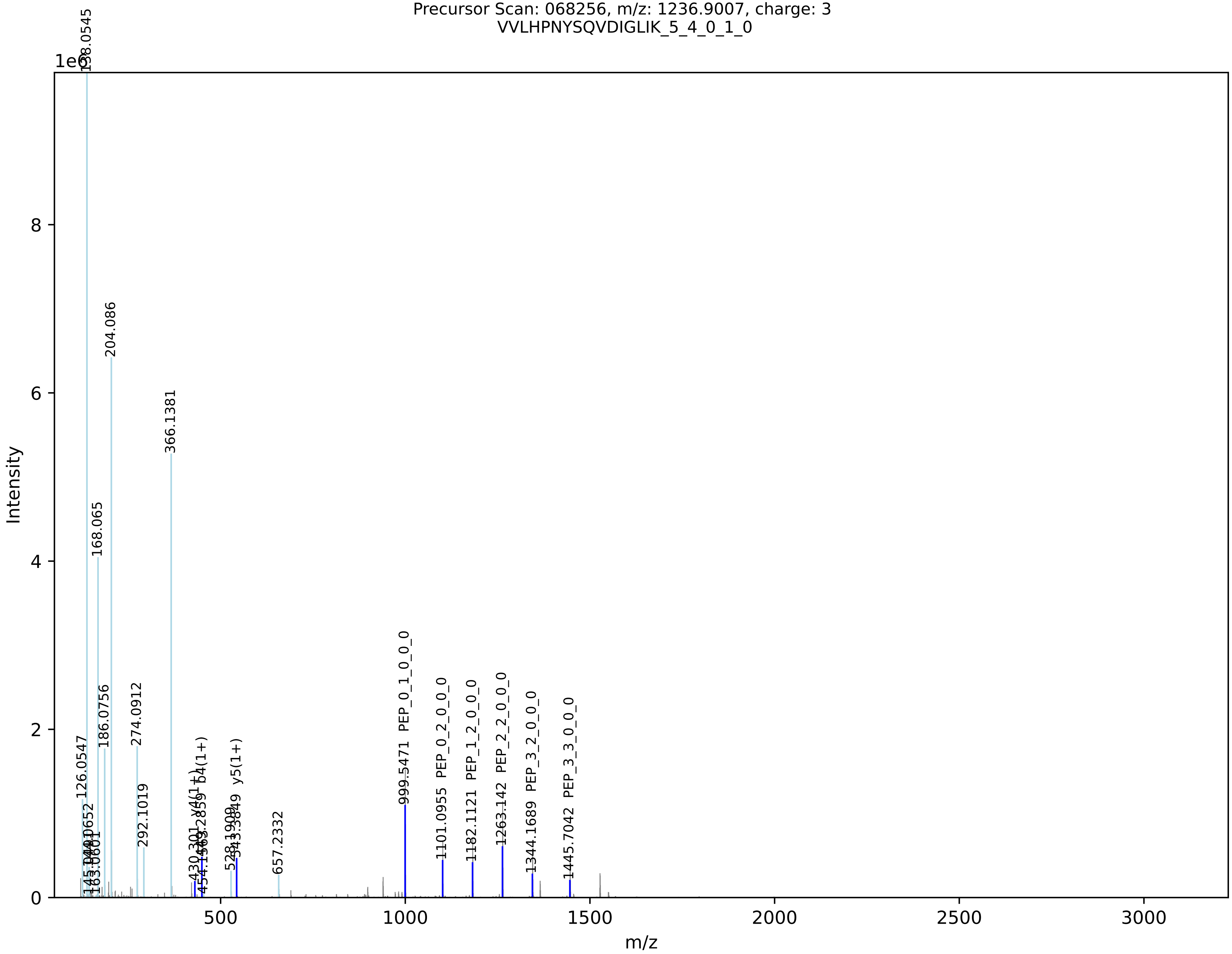

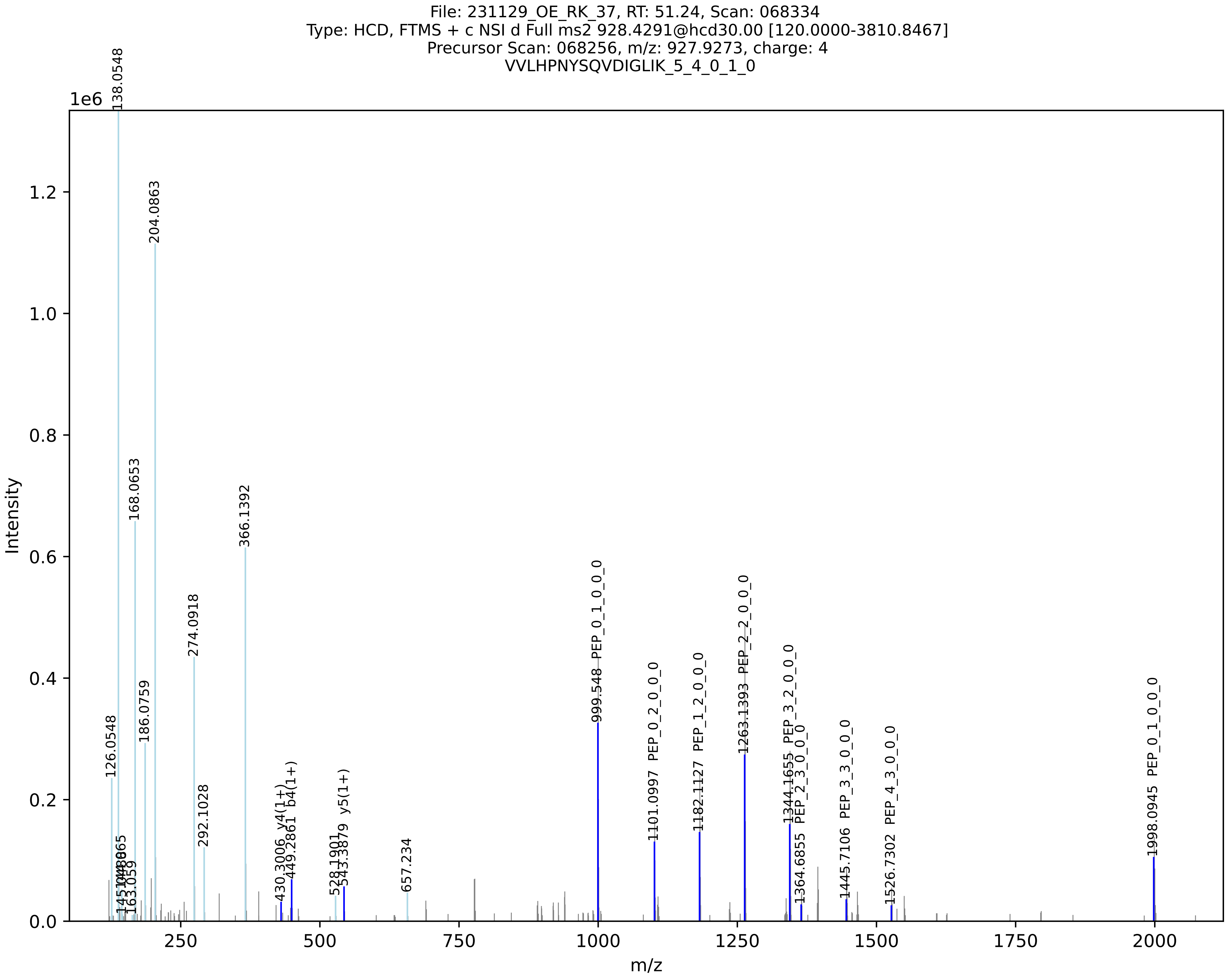
File: 231129_OE_RK_37, RT: 51.24, Scan: 068334
Type: HCD, FTMS + c NSI d Full ms2 928.4291@hcd30.00 [120.0000-3810.8467]
Precursor Scan: 068256, m/z: 927.9273, charge: 4
VVLHPNYSQVDIGLIK_5_4_0_1_0
1e6
Intensity
m/z
138.0548
204.0863
168.0653
366.1392
274.0918
186.0759
126.0548
292.1028
163.059
430.3006 y4(1+)
449.2861 b4(1+)
528.1901
543.3879 y5(1+)
657.234
999.548 PEP_0_1_0_0_0
1101.0997 PEP_0_2_0_0_0
1182.1127 PEP_1_2_0_0_0
1263.1393 PEP_2_2_0_0_0
1344.1655 PEP_3_2_0_0_0
1364.6855 PEP_2_3_0_0_0
1445.7106 PEP_3_3_0_0_0
1526.7302 PEP_4_3_0_0_0
1998.0945 PEP_0_1_0_0_0

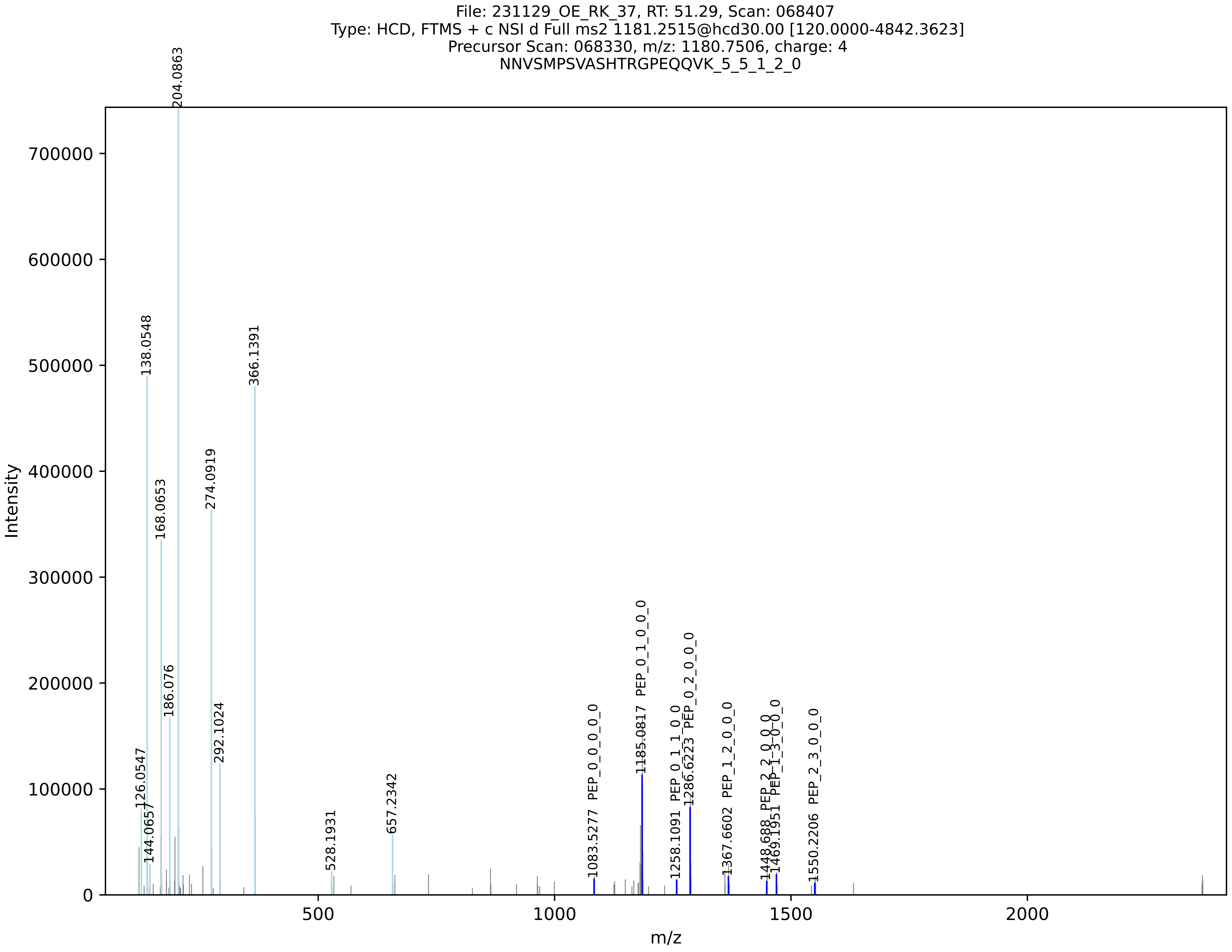

File: 231129_OE_RK_37, RT: 51.29, Scan: 068407
Type: HCD, FTMS + c NSI d Full ms2 1181.2515@hcd30.00 [120.0000-4842.3623]
Precursor Scan: 068330, m/z: 1180.7506, charge: 4
NNVSMPSVASHTRGPEQQVK_5_5_1_2_0
Intensity
m/z
204.0863
138.0548
366.1391
274.0919
168.0653
186.076
292.1024
126.0547
144.0657
528.1931
657.2342
1083.5277 PEP_0_0_0_0_0
1185.0817 PEP_0_1_0_0_0
1258.1091 PEP_0_1_1_0_0
1286.6223 PEP_0_2_0_0_0
1367.6602 PEP_1_2_0_0_0
1448.688 PEP_2_2_0_0_0
1469.1951 PEP_1_3_0_0_0
1550.2206 PEP_2_3_0_0_0

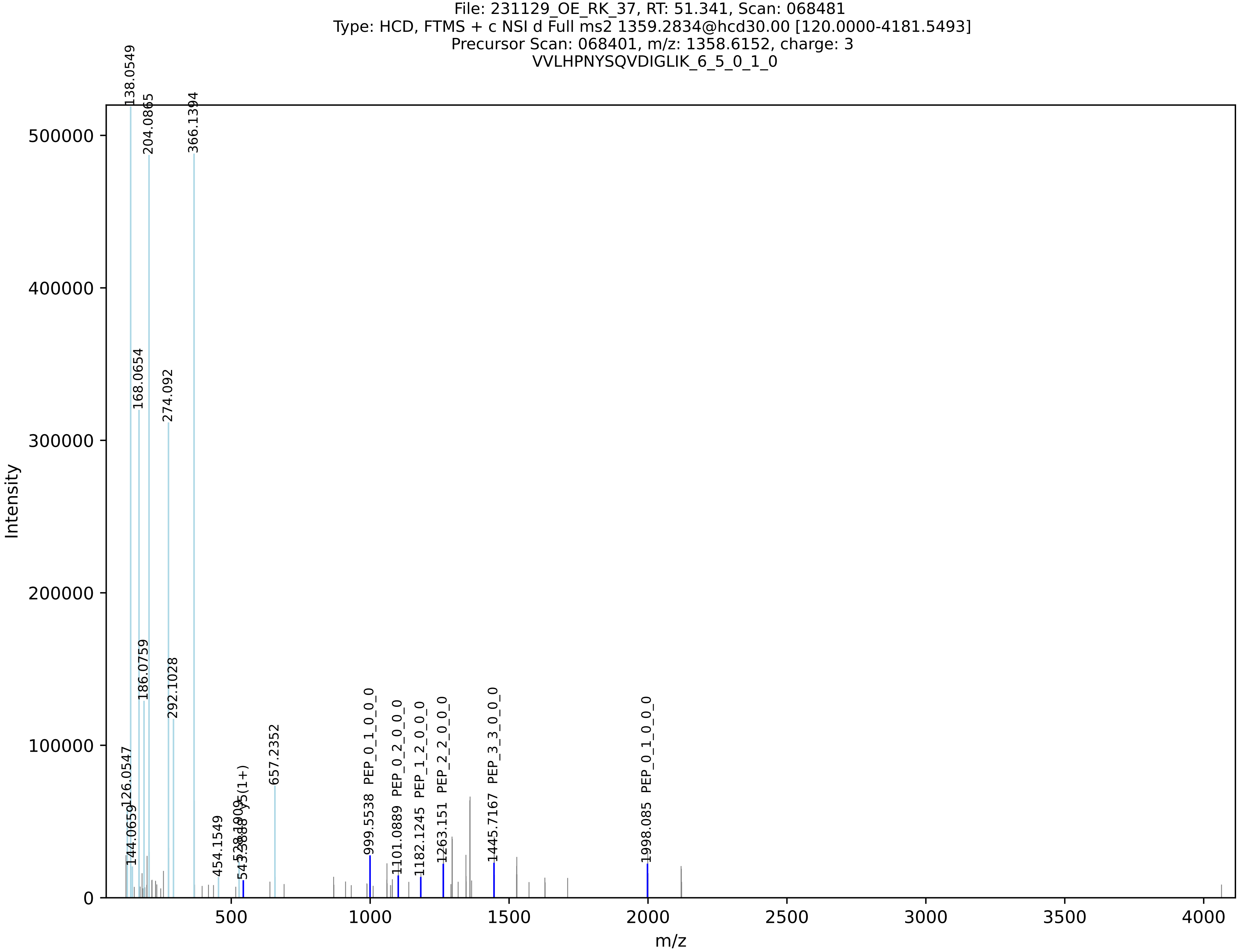
File: 231129_OE_RK_37, RT: 51.341, Scan: 068481
Type: HCD, FTMS + c NSI d Full ms2 1359.2834@hcd30.00 [120.0000-4181.5493]
Precursor Scan: 068401, m/z: 1358.6152, charge: 3
VVLHPNYSQVDIGLIK_6_5_0_1_0
Intensity
m/z
138.0549
204.0865
366.1394
168.0654
274.092
186.0759
292.1028
126.0547
144.0659
454.1549
657.2352
999.5538 PEP_0_1_0_0_
1101.0889 PEP_0_2_0_0_0
1182.1245 PEP_1_2_0_0_0
1263.151 PEP_2_2_0_0_0
1445.7167 PEP_3_3_0_0_0
1998.085 PEP_0_1_0_0_0
y5(1+)

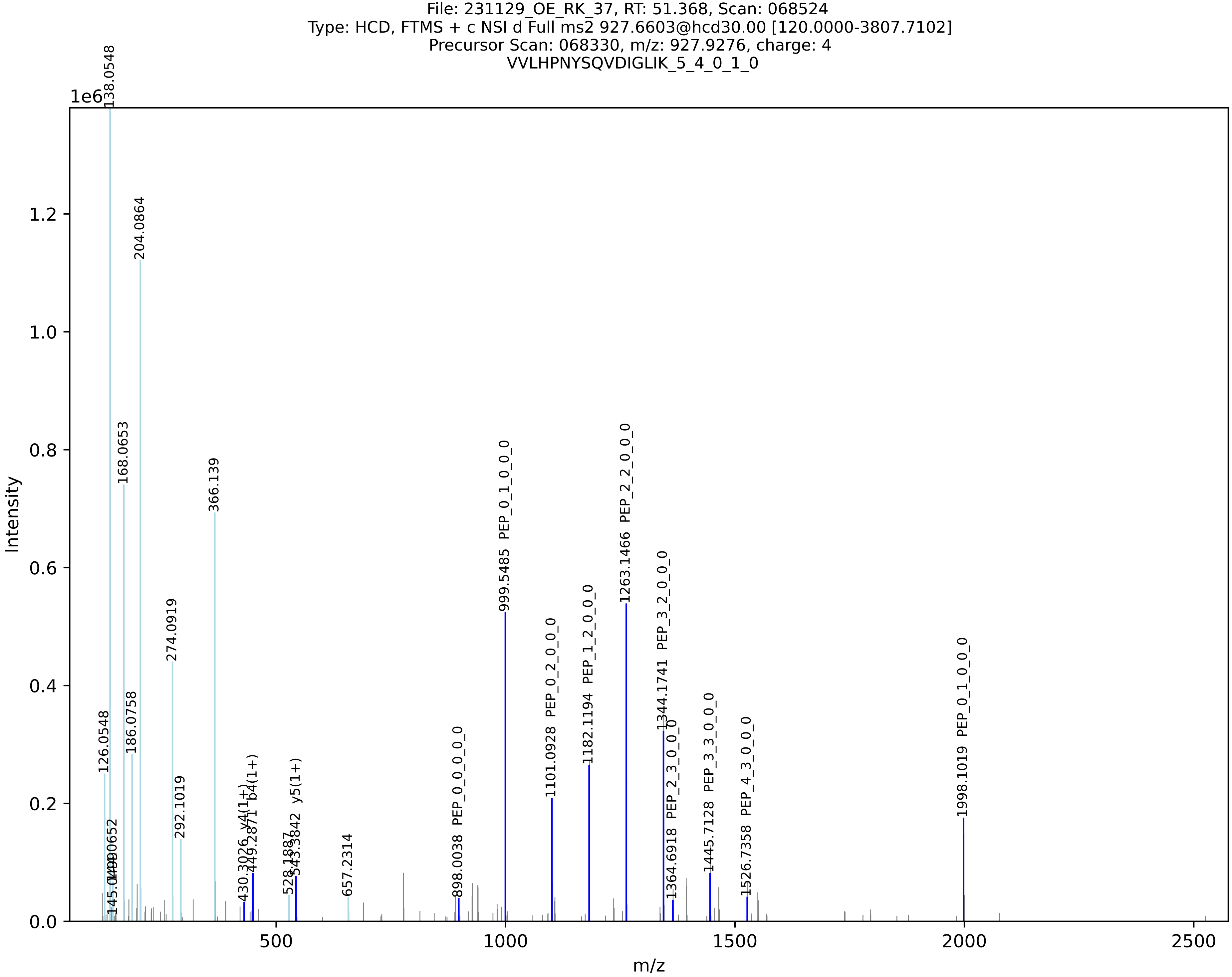
File: 231129_OE_RK_37, RT: 51.368, Scan: 068524
Type: HCD, FTMS + c NSI d Full ms2 927.6603@hcd30.00 [120.0000-3807.7102]
Precursor Scan: 068330, m/z: 927.9276, charge: 4
VVLHPNYSQVDIGLIK_5_4_0_1_0
1e6
Intensity
m/z
138.0548
204.0864
168.0653
366.139
274.0919
126.0548
186.0758
292.1019
430.3026 y4(1+)
449.2871 b4(1+)
528.1887
543.3842 y5(1+)
657.2314
898.0038 PEP_0_0_0_0_0
999.5485 PEP_0_1_0_0_0
1101.0928 PEP_0_2_0_0_0
1182.1194 PEP_1_2_0_0_0
1263.1466 PEP_2_2_0_0_0
1344.1741 PEP_3_2_0_0_0
1364.6918 PEP_2_3_0_0_0
1445.7128 PEP_3_3_0_0_0
1526.7358 PEP_4_3_0_0_0
1998.1019 PEP_0_1_0_0_0

File: 231129_OE_RK_37, RT: 51.392, Scan: 068558
Type: HCD, FTMS + c NSI d Full ms2 1325.8943@hcd30.00 [120.0000-5432.5049]
Precursor Scan: 068475, m/z: 1324.6414, charge: 4
VCQDCPLLAPLNDTR_9_8_1_1_0

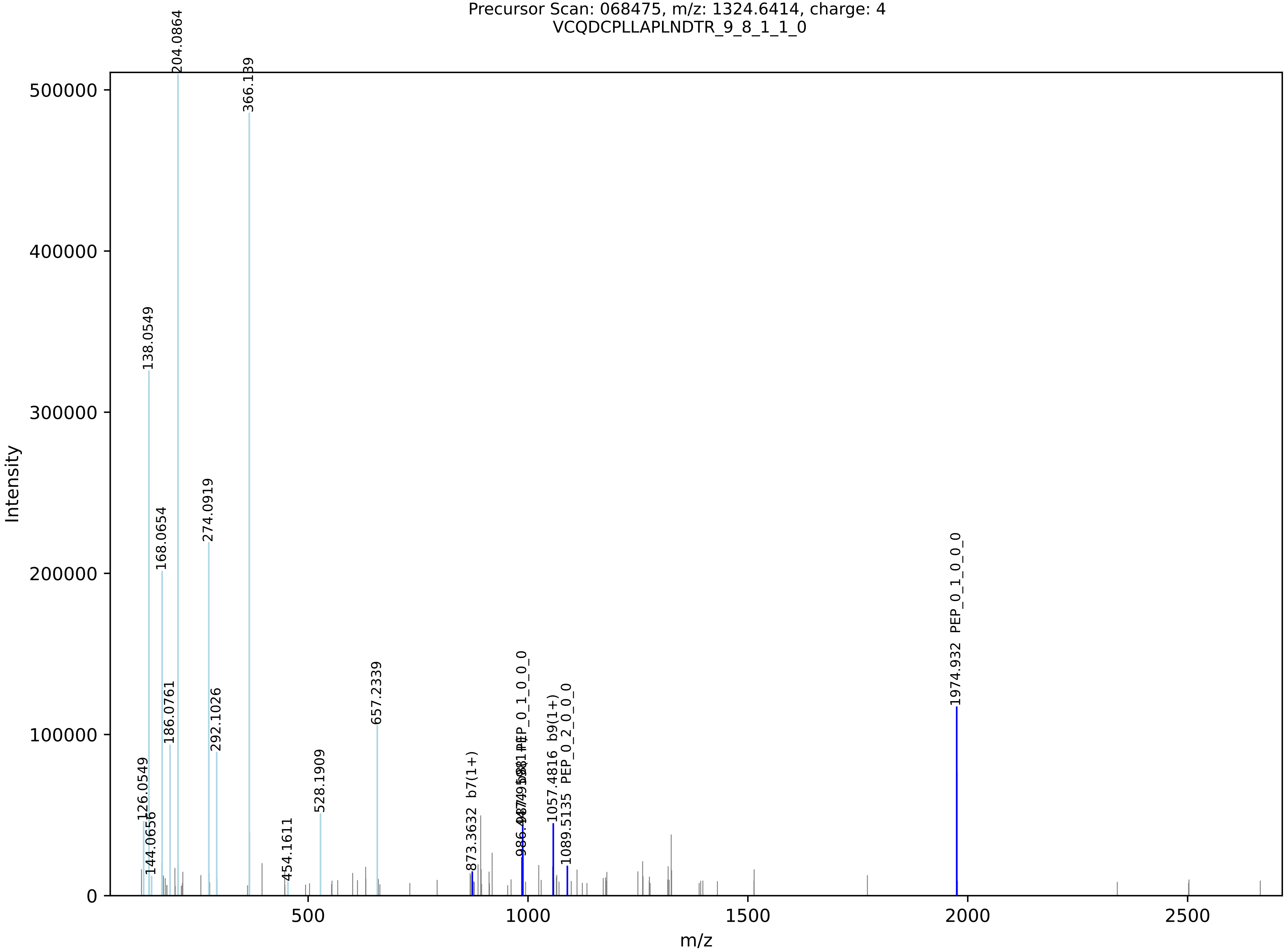

File: 231129_OE_RK_37, RT: 51.428, Scan: 068608
Type: HCD, FTMS + c NSI d Full ms2 1237.5695@hcd30.00 [120.0000-3809.1045]
Precursor Scan: 068533, m/z: 1236.9013, charge: 3
VVLHPNYSQVDIGLIK_5_4_0_1_0

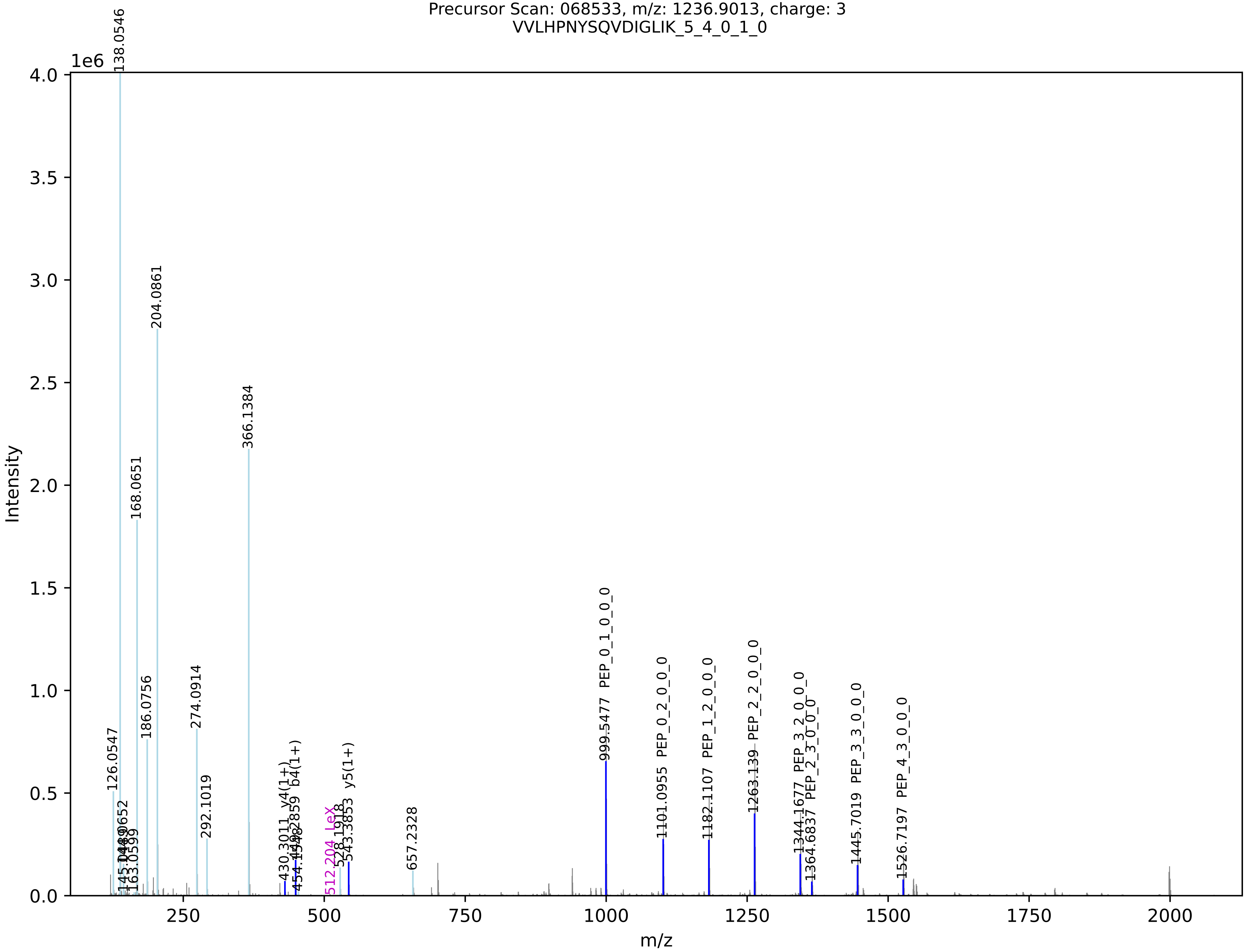

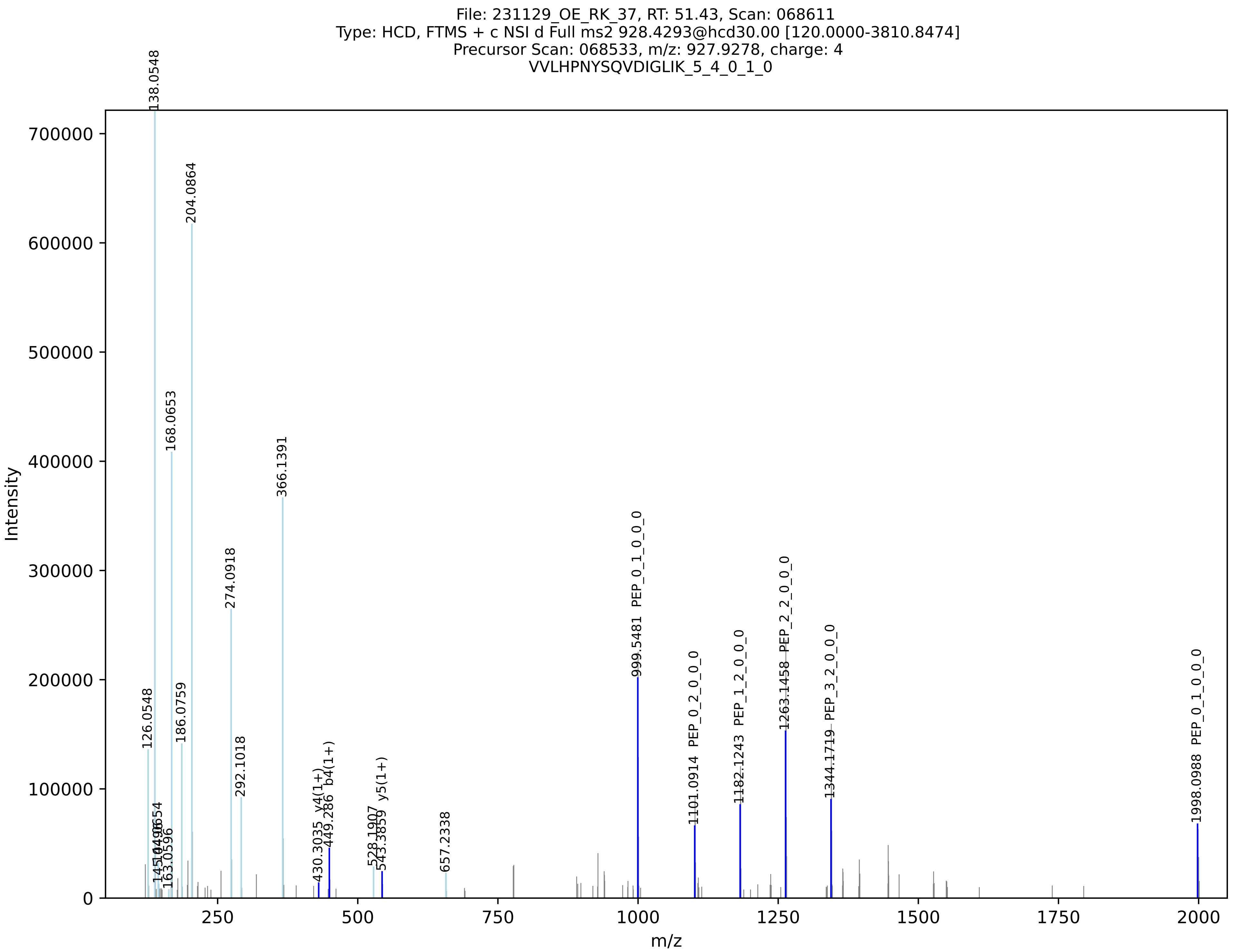

File: 231129_OE_RK_37, RT: 51.43, Scan: 068611
Type: HCD, FTMS + c NSI d Full ms2 928.4293@hcd30.00 [120.0000-3810.8474]
Precursor Scan: 068533, m/z: 927.9278, charge: 4
VVLHPNYSQVDIGLIK_5_4_0_1_0
Intensity
m/z
138.0548
204.0864
168.0653
366.1391
274.0918
292.1018
126.0548
186.0759
163.0596
430.3035 y4(1+)
449.286 b4(1+)
528.1907
543.3859 y5(1+)
657.2338
999.5481 PEP_0_1_0_0_0
1101.0914 PEP_0_2_0_0_0
1182.1243 PEP_1_2_0_0_0
1263.1458 PEP_2_2_0_0_0
1344.1719 PEP_3_2_0_0_0
1998.0988 PEP_0_1_0_0_0

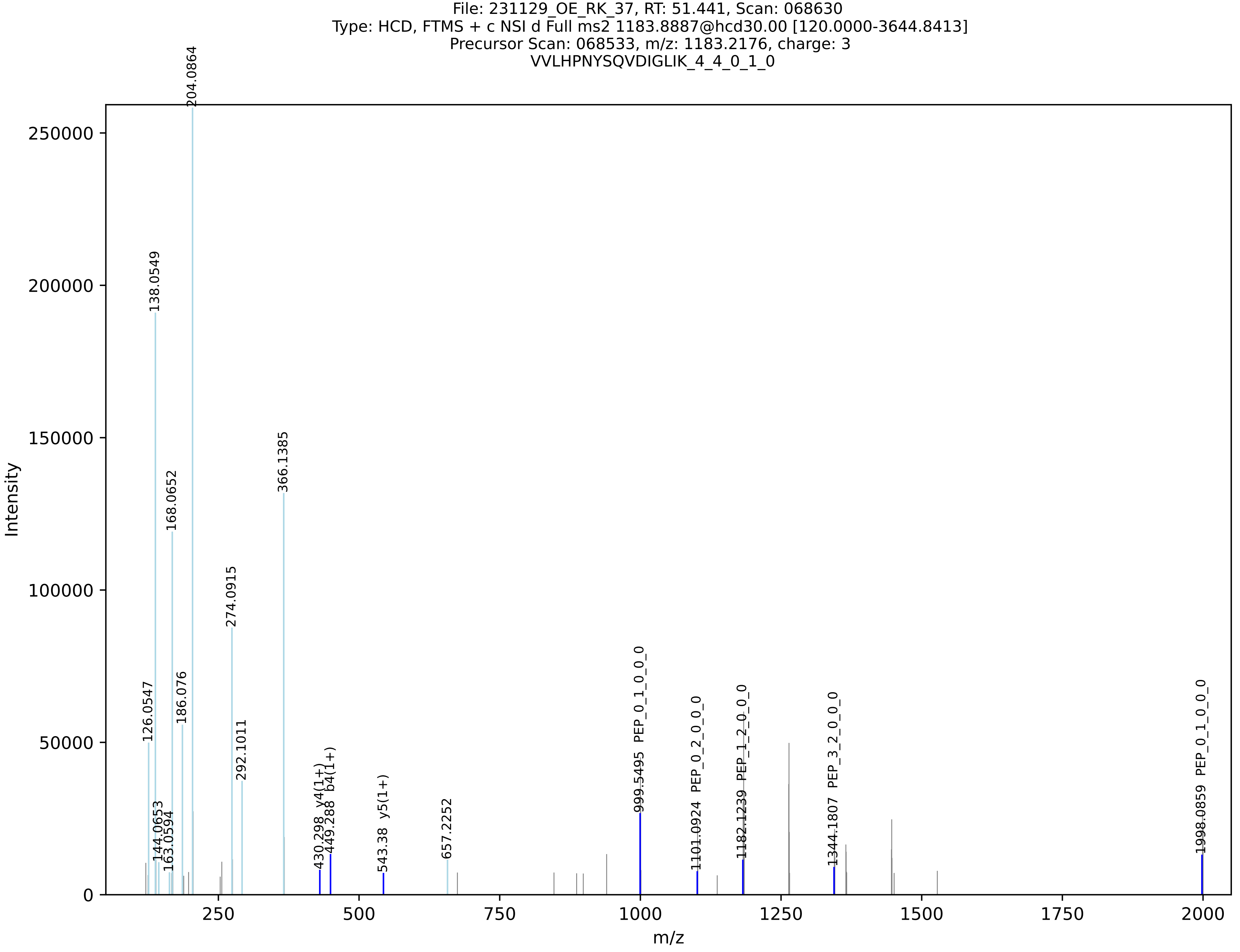
File: 231129_OE_RK_37, RT: 51.441, Scan: 068630
Type: HCD, FTMS + c NSI d Full ms2 1183.8887@hcd30.00 [120.0000-3644.8413]
Precursor Scan: 068533, m/z: 1183.2176, charge: 3
VVLHPNYSQVDIGLIK_4_4_0_1_0
Intensity
m/z
204.0864
138.0549
168.0652
366.1385
274.0915
126.0547
186.076
292.1011
144.0653
163.0594
430.298 y4(1+)
449.288 b4(1+)
543.38 y5(1+)
657.2252
999.5495 PEP_0_1_0_0_0
1101.0924 PEP_0_2_0_0_0
1182.1239 PEP_1_2_0_0_0
1344.1807 PEP_3_2_0_0_0
1998.0859 PEP_0_1_0_0_0

File: 231129_OE_RK_37, RT: 51.449, Scan: 068643
Type: HCD, FTMS + c NSI d Full ms2 1182.4843@hcd30.00 [120.0000-3640.5437]
Precursor Scan: 068533, m/z: 1182.4842, charge: 3
VVLHPNYSQVDIGLIK_4_4_0_1_0

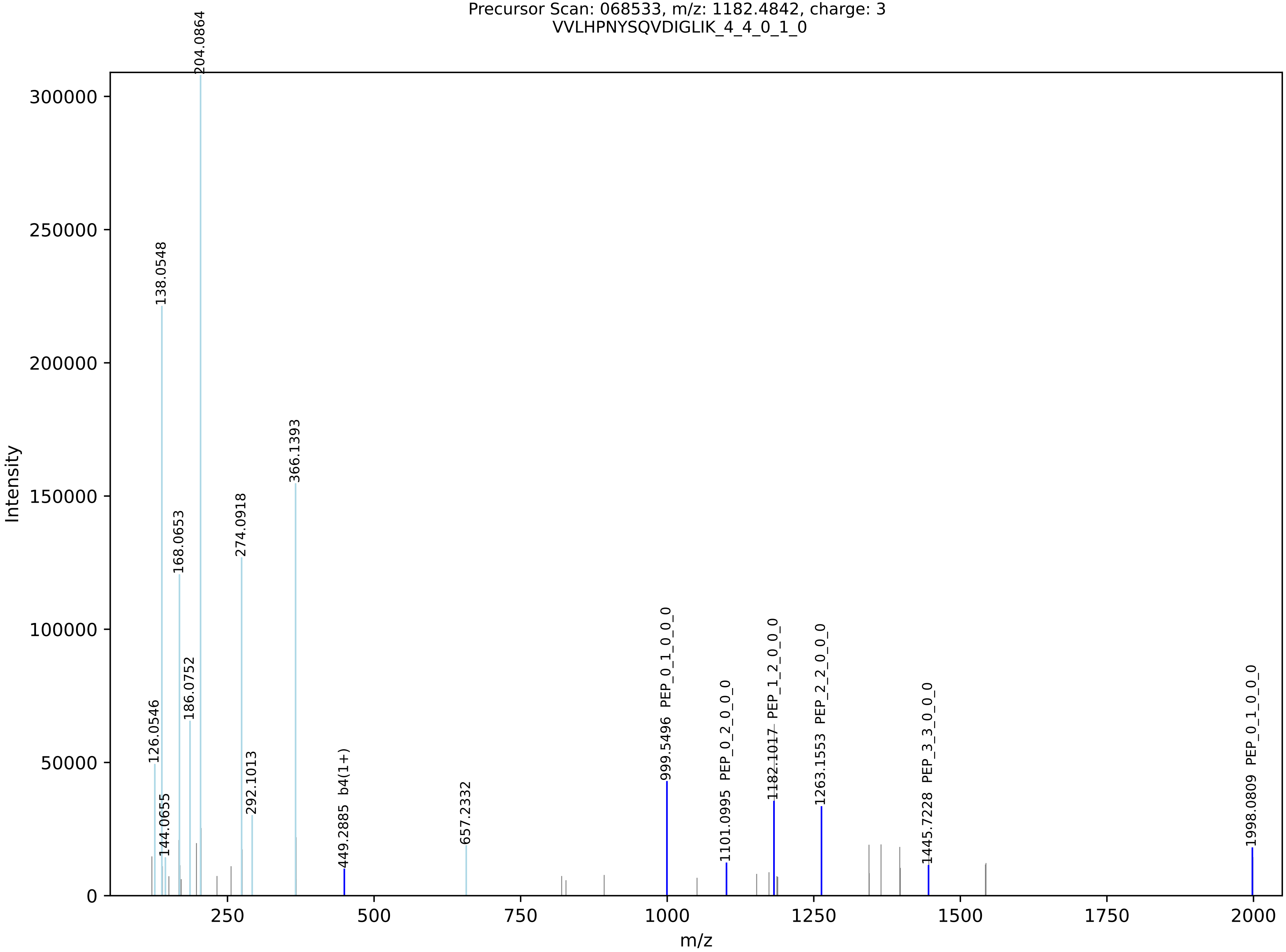

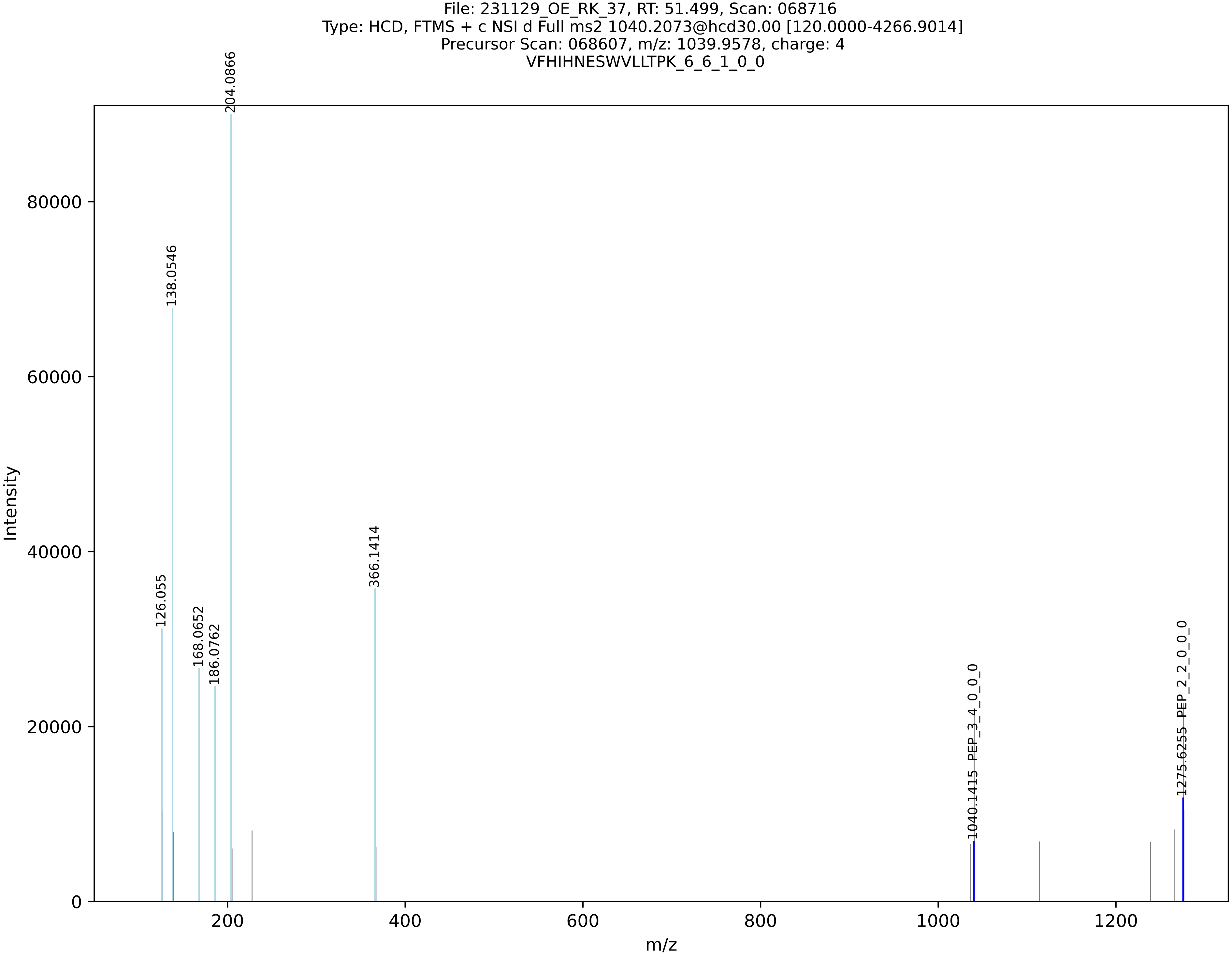

File: 231129_OE_RK_37, RT: 51.499, Scan: 068716
Type: HCD, FTMS + c NSI d Full ms2 1040.2073@hcd30.00 [120.0000-4266.9014]
Precursor Scan: 068607, m/z: 1039.9578, charge: 4
VFHIHNESWVLLTPK_6_6_1_0_0
Intensity
m/z
0
20000
40000
60000
80000
200
400
600
800
1000
1200
204.0866
138.0546
126.055
168.0652
186.0762
366.1414
1040.1415_PEP_3_4_0_0_0
1275.6255_PEP_2_2_0_0_0

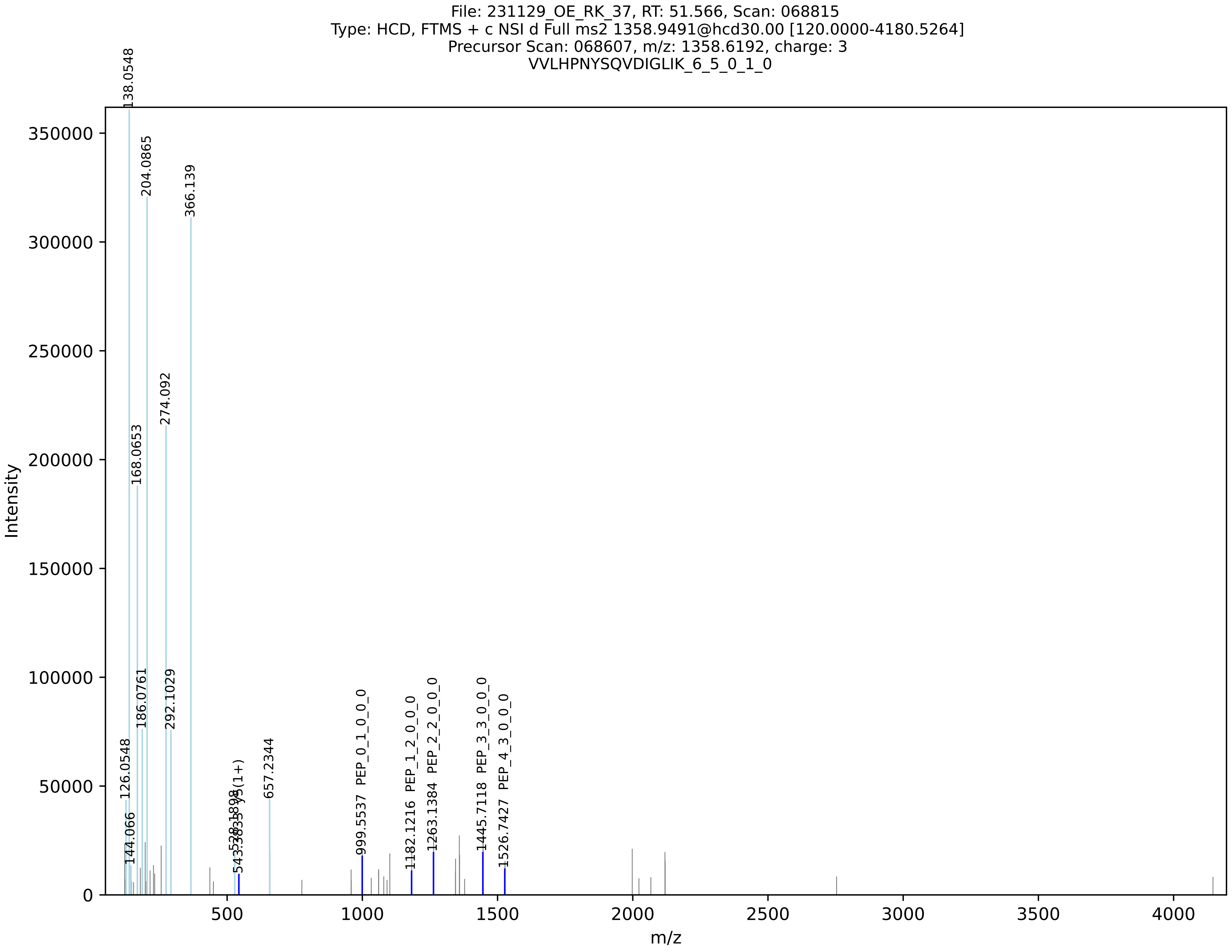

File: 231129_OE_RK_37, RT: 51.566, Scan: 068815
Type: HCD, FTMS + c NSI d Full ms2 1358.9491@hcd30.00 [120.0000-4180.5264]
Precursor Scan: 068607, m/z: 1358.6192, charge: 3
VVLHPNYSQVDIGLIK_6_5_0_1_0
Intensity
m/z
0
50000
100000
150000
200000
250000
300000
350000
500
1000
1500
2000
2500
3000
3500
4000
138.0548
204.0865
366.139
274.092
168.0653
186.0761
292.1029
126.0548
144.066
528.1898
543.3835 y5(1+)
657.2344
999.5537 PEP_0_1_0_0_0
1182.1216 PEP_1_2_0_0_0
1263.1384 PEP_2_2_0_0_0
1445.7118 PEP_3_3_0_0_0
1526.7427 PEP_4_3_0_0_0

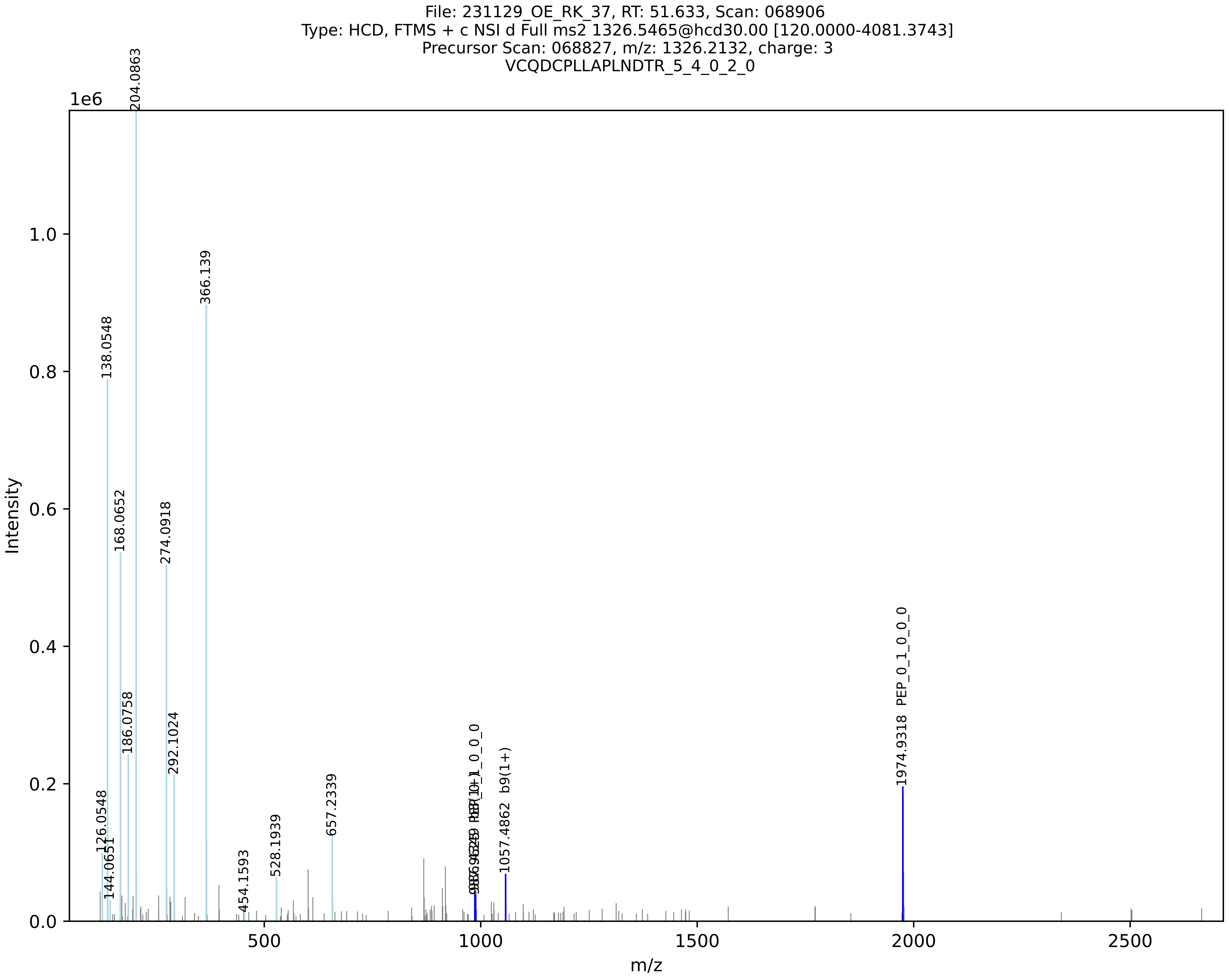
File: 231129_OE_RK_37, RT: 51.633, Scan: 068906
Type: HCD, FTMS + c NSI d Full ms2 1326.5465@hcd30.00 [120.0000-4081.3743]
Precursor Scan: 068827, m/z: 1326.2132, charge: 3
VCQDCPLLAPLNDTR_5_4_0_2_0
1e6
Intensity
m/z
0.0
0.2
0.4
0.6
0.8
1.0
500
1000
1500
2000
2500
204.0863
366.139
138.0548
168.0652
274.0918
186.0758
292.1024
126.0548
144.0651
454.1593
528.1939
657.2339
1057.4862 b9(1+)
1974.9318 PEP_0_1_0_0_0

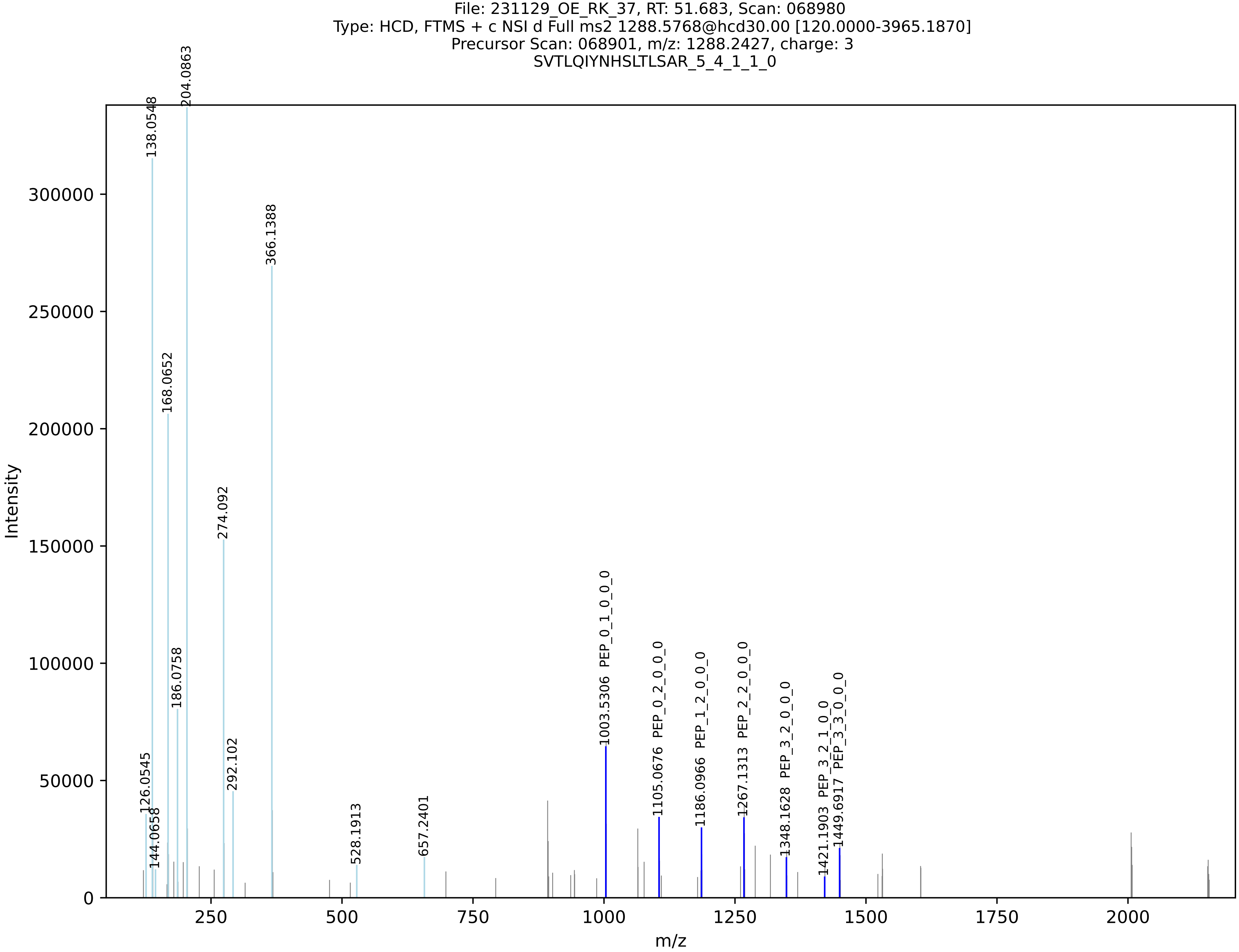
File: 231129_OE_RK_37, RT: 51.683, Scan: 068980
Type: HCD, FTMS + c NSI d Full ms2 1288.5768@hcd30.00 [120.0000-3965.1870]
Precursor Scan: 068901, m/z: 1288.2427, charge: 3
SVTLQIYNHSLTLSAR_5_4_1_1_0
Intensity
m/z
0
50000
100000
150000
200000
250000
300000
250
500
750
1000
1250
1500
1750
2000
126.0545
138.0548
144.0658
168.0652
186.0758
204.0863
274.092
292.102
366.1388
528.1913
657.2401
1003.5306 PEP_0_1_0_0_0
1105.0676 PEP_0_2_0_0_0
1186.0966 PEP_1_2_0_0_0
1267.1313 PEP_2_2_0_0_0
1348.1628 PEP_3_2_0_0_0
1421.1903 PEP_3_2_1_0_0
1449.6917 PEP_3_3_0_0_0

File: 231129_OE_RK_37, RT: 51.687, Scan: 068986
Type: HCD, FTMS + c NSI d Full ms2 1228.5138@hcd30.00 [120.0000-3781.3940]
Precursor Scan: 068901, m/z: 1227.8455, charge: 3
CGLVPVLAENYNK_5_4_0_2_0

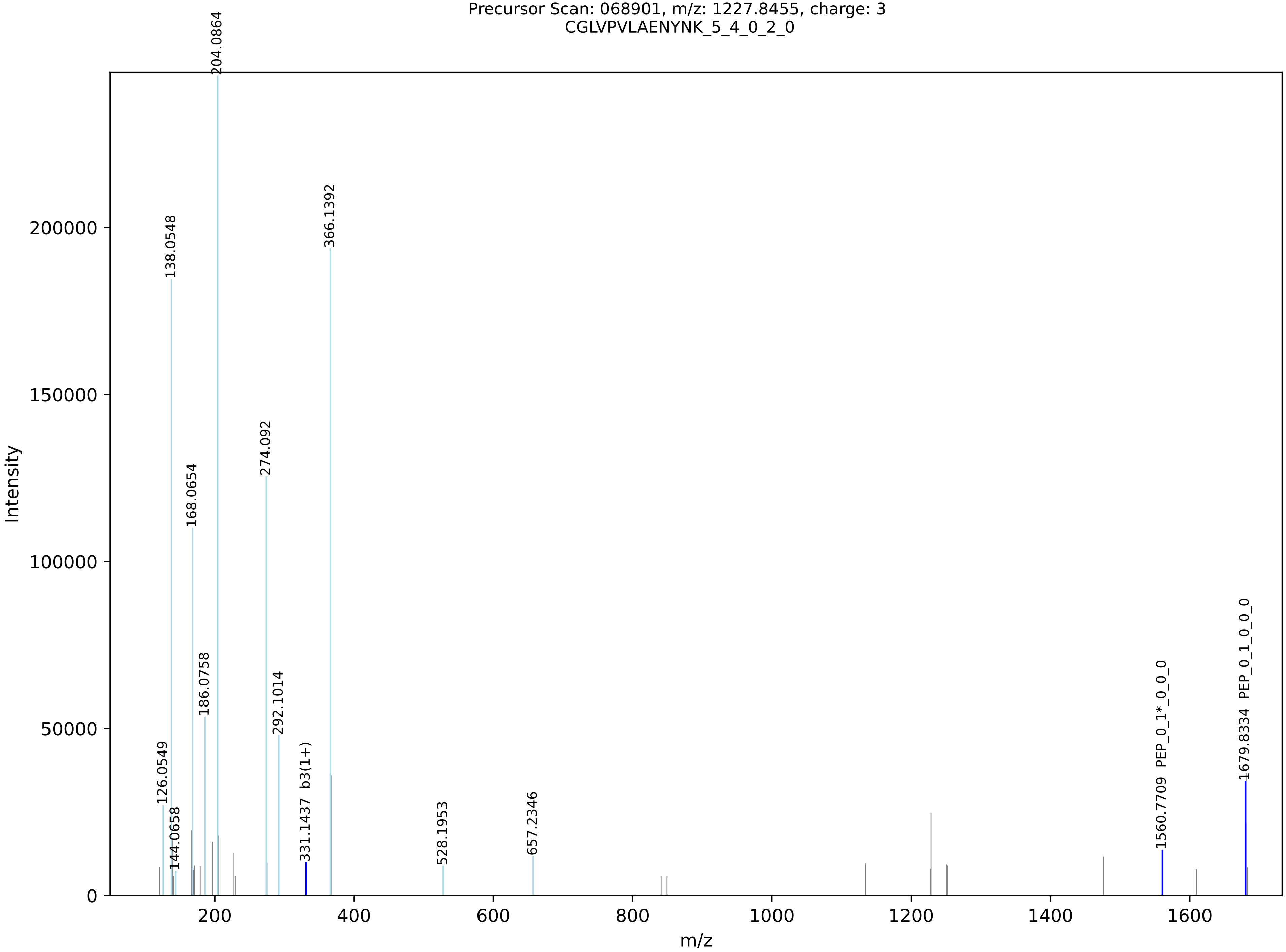

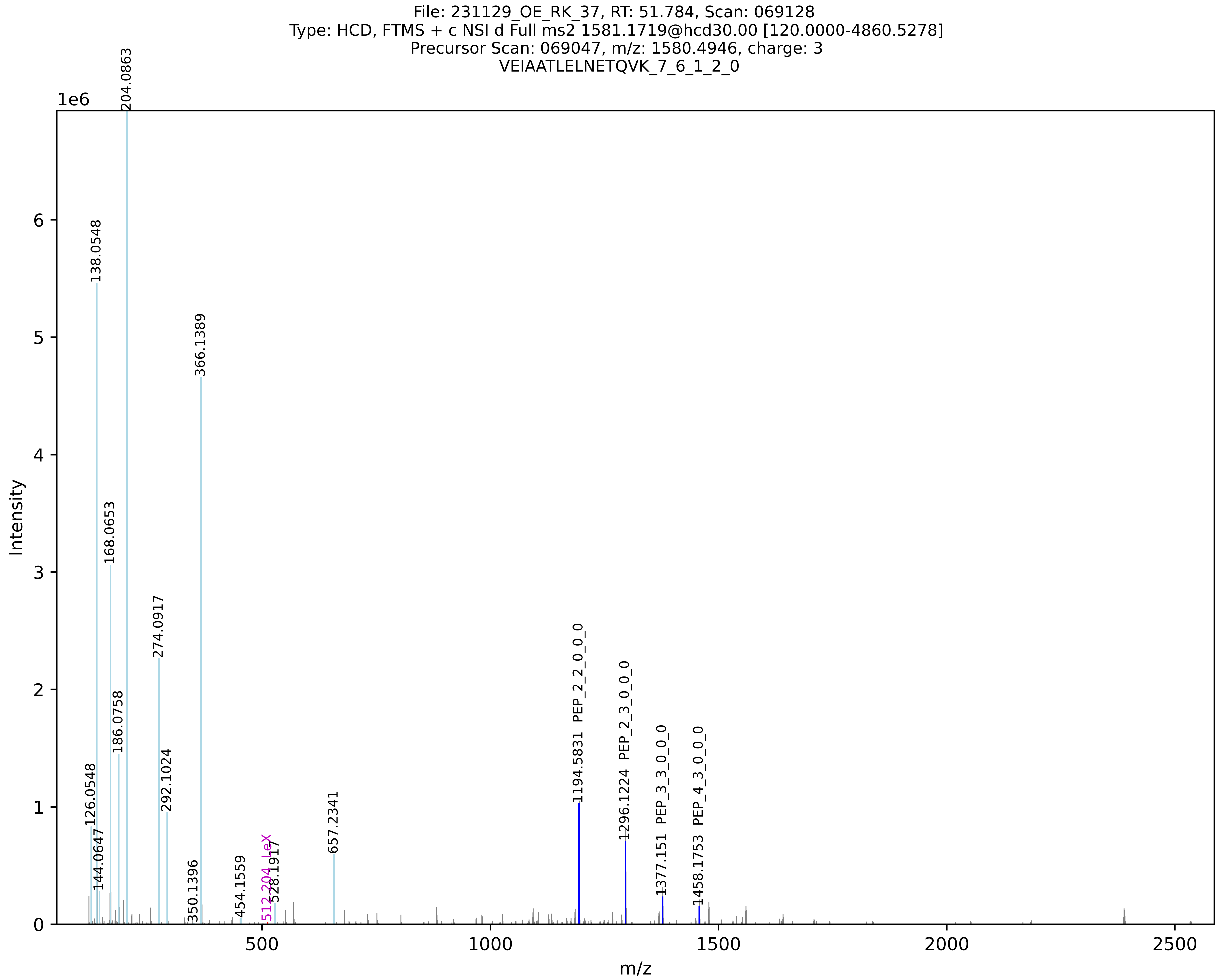
File: 231129_OE_RK_37, RT: 51.784, Scan: 069128
Type: HCD, FTMS + c NSI d Full ms2 1581.1719@hcd30.00 [120.0000-4860.5278]
Precursor Scan: 069047, m/z: 1580.4946, charge: 3
VEIAATLELNETQVK_7_6_1_2_0
1e6
204.0863
138.0548
366.1389
168.0653
274.0917
186.0758
292.1024
126.0548
144.0647
350.1396
454.1559
512.204_LeX
528.1917
657.2341
1194.5831 PEP_2_2_0_0_0
1296.1224 PEP_2_3_0_0_0
1377.151 PEP_3_3_0_0_0
1458.1753 PEP_4_3_0_0_0
Intensity
m/z
0
1
2
3
4
5
6
500
1000
1500
2000
2500

File: 231129_OE_RK_37, RT: 51.978, Scan: 069411
Type: HCD, FTMS + c NSI d Full ms2 1581.1680@hcd30.00 [120.0000-4860.5161]
Precursor Scan: 069343, m/z: 1580.4973, charge: 3
VEIAATLELNETQVK_7_6_1_2_0

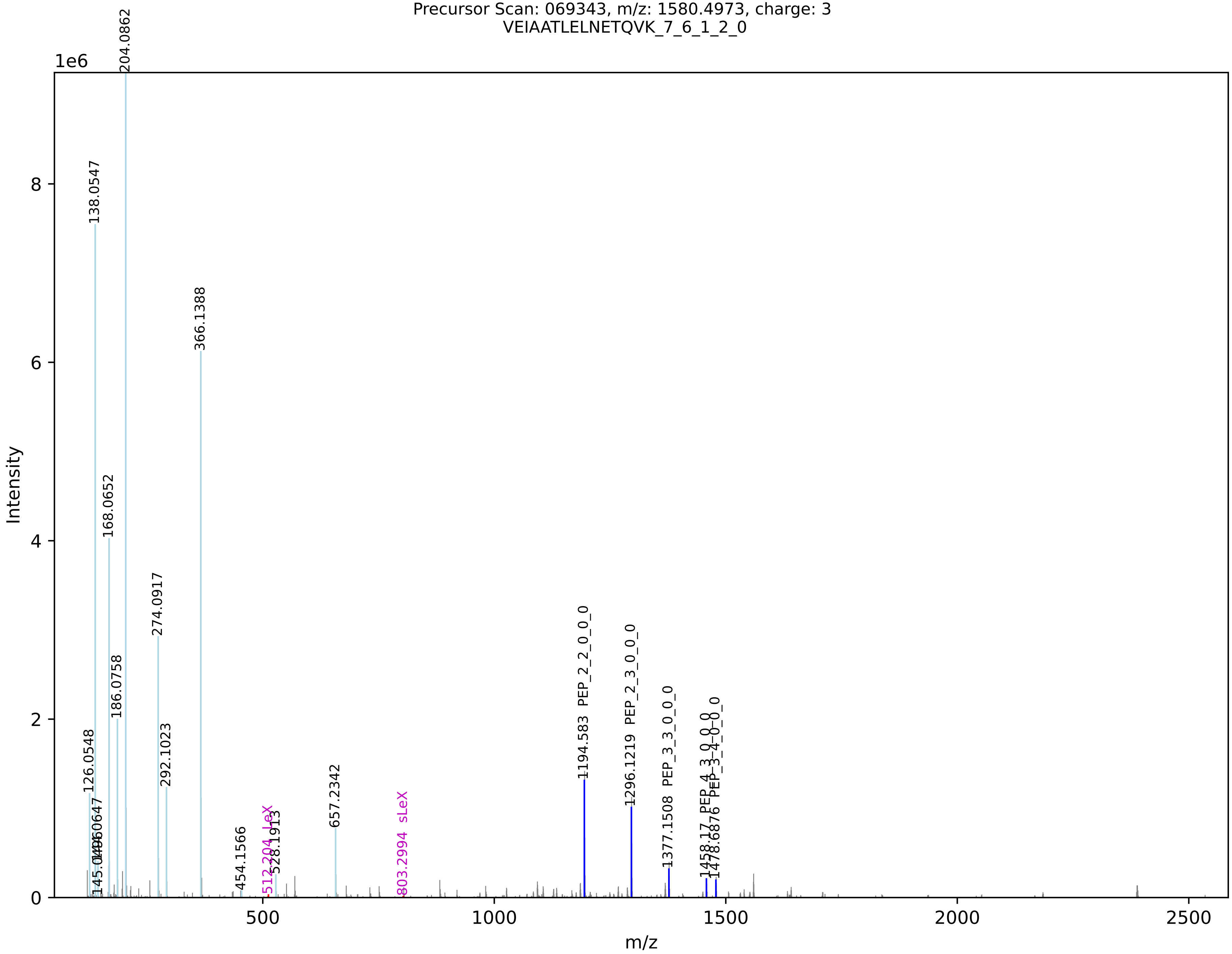

File: 231129_OE_RK_37, RT: 52.072, Scan: 069543
Type: HCD, FTMS + c NSI d Full ms2 1327.2125@hcd30.00 [120.0000-4083.4124]
Precursor Scan: 069505, m/z: 1326.2102, charge: 3
VCQDCPLLAPLNDTR_5_4_0_2_0

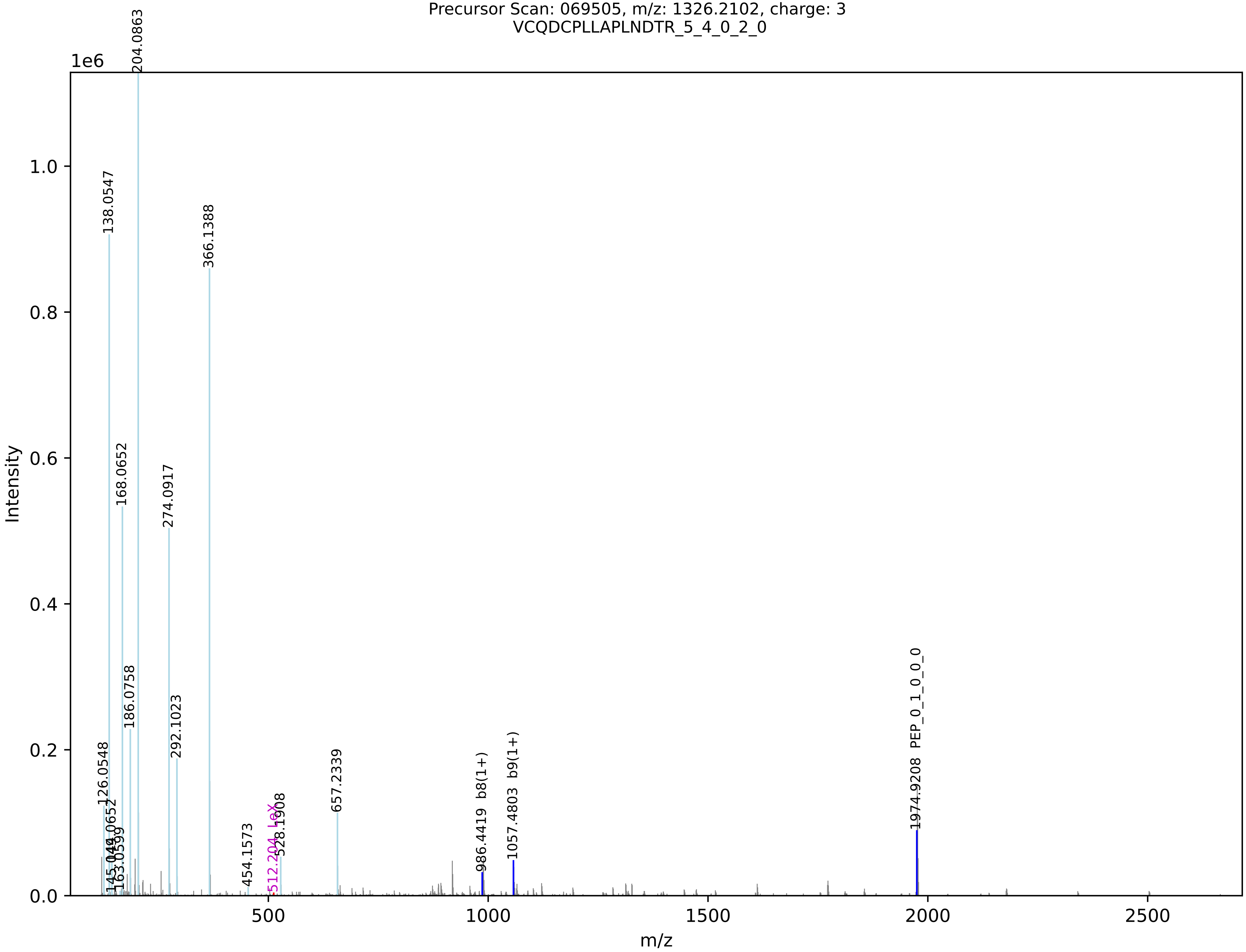

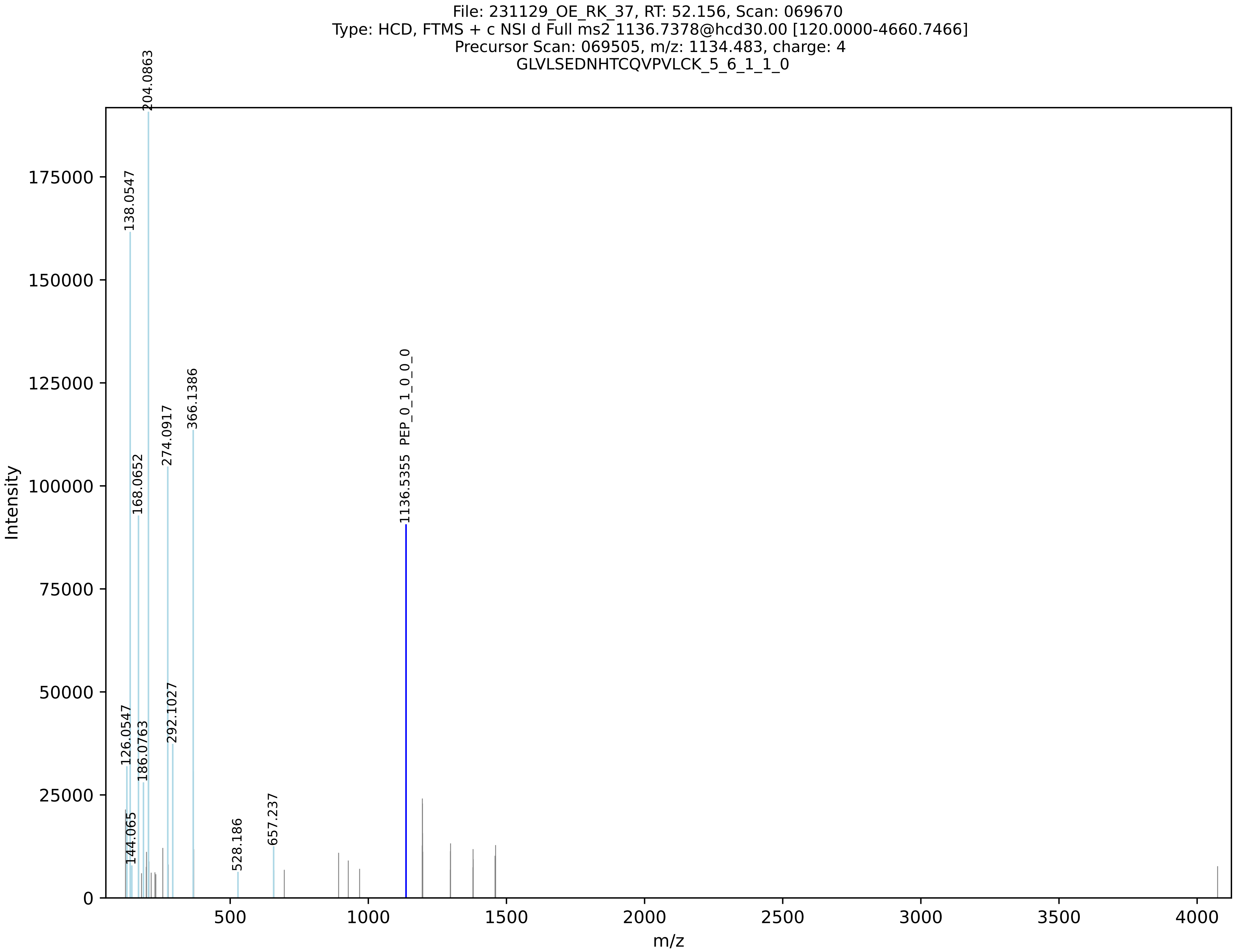
File: 231129_OE_RK_37, RT: 52.156, Scan: 069670
Type: HCD, FTMS + c NSI d Full ms2 1136.7378@hcd30.00 [120.0000-4660.7466]
Precursor Scan: 069505, m/z: 1134.483, charge: 4
GLVLSEDNHTCQVPVLCK_5_6_1_1_0
Intensity
m/z
204.0863
138.0547
168.0652
274.0917
366.1386
1136.5355 PEP_0_1_0_0_0_
126.0547
186.0763
292.1027
144.065
528.186
657.237
0
25000
50000
75000
100000
125000
150000
175000
500
1000
1500
2000
2500
3000
3500
4000

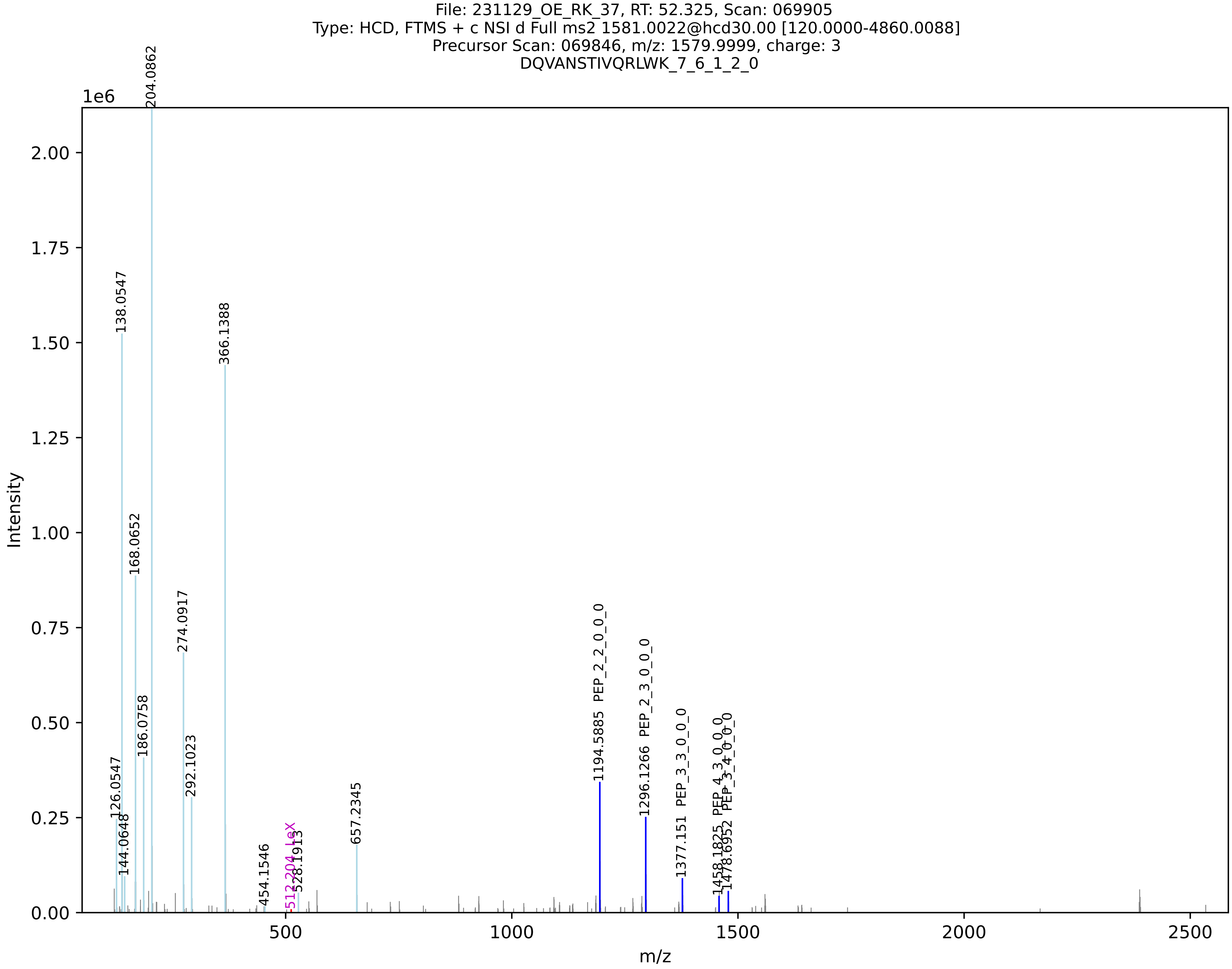
File: 231129_OE_RK_37, RT: 52.325, Scan: 069905
Type: HCD, FTMS + c NSI d Full ms2 1581.0022@hcd30.00 [120.0000-4860.0088]
Precursor Scan: 069846, m/z: 1579.9999, charge: 3
DQVANSTIVQRLWK_7_6_1_2_0
1e6
Intensity
m/z
204.0862
138.0547
366.1388
168.0652
274.0917
186.0758
292.1023
126.0547
144.0648
454.1546
-512.204_LeX
528.1913
657.2345
1194.5885 PEP_2_2_0_0_0
1296.1266 PEP_2_3_0_0_0
1377.151 PEP_3_3_0_0_0
1458.1825 PEP_4_3_0_0_0
1478.6952 PEP_3_4_0_0_0
0.00
0.25
0.50
0.75
1.00
1.25
1.50
1.75
2.00
500
1000
1500
2000
2500

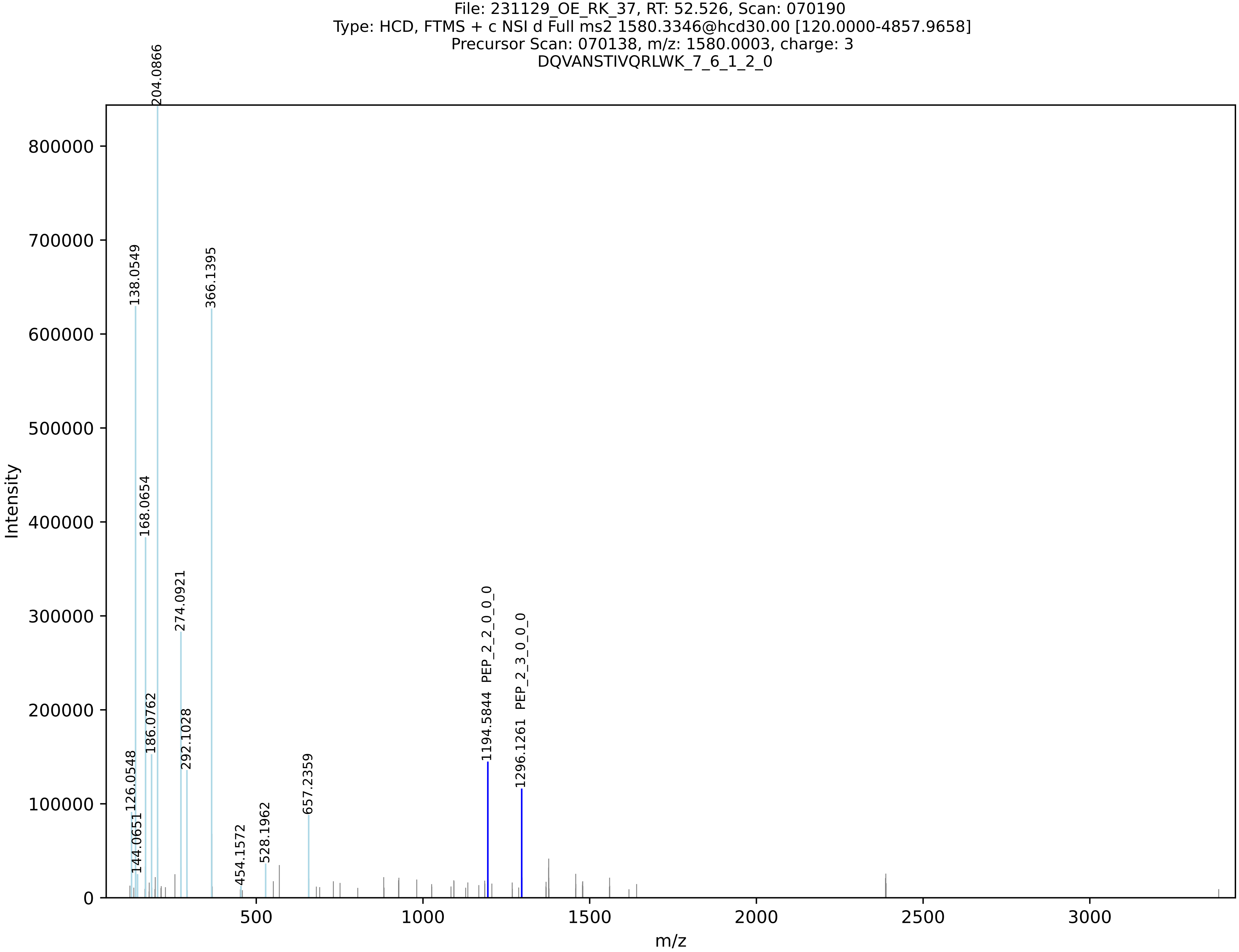
File: 231129_OE_RK_37, RT: 52.526, Scan: 070190
Type: HCD, FTMS + c NSI d Full ms2 1580.3346@hcd30.00 [120.0000-4857.9658]
Precursor Scan: 070138, m/z: 1580.0003, charge: 3
DQVANSTIVQRLWK_7_6_1_2_0
Intensity
m/z
204.0866
138.0549
366.1395
168.0654
274.0921
186.0762
292.1028
126.0548
144.0651
454.1572
528.1962
657.2359
1194.5844 PEP_2_2_0_0_0_
1296.1261 PEP_2_3_0_0_0
0
100000
200000
300000
400000
500000
600000
700000
800000
500
1000
1500
2000
2500
3000

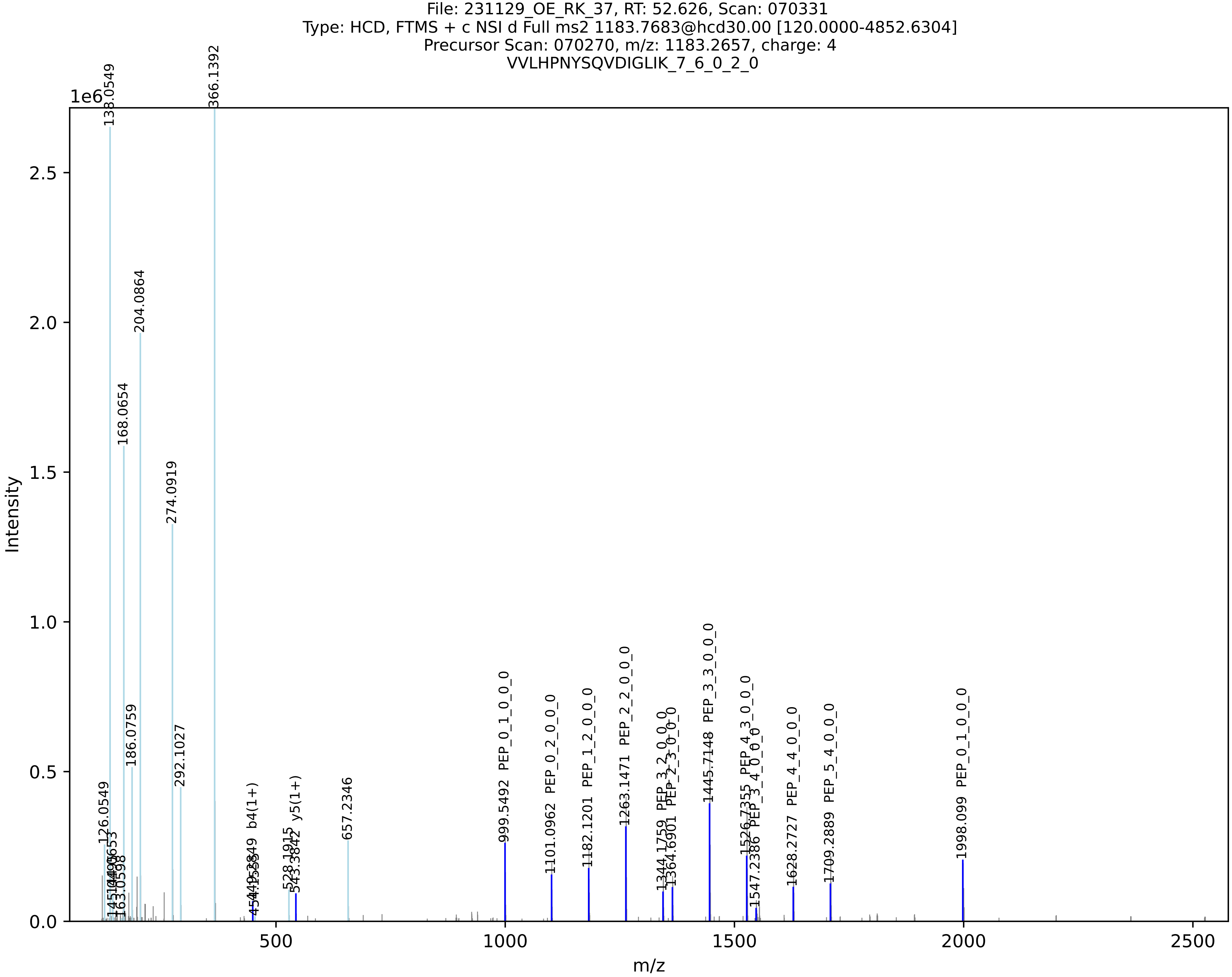
File: 231129_OE_RK_37, RT: 52.626, Scan: 070331
Type: HCD, FTMS + c NSI d Full ms2 1183.7683@hcd30.00 [120.0000-4852.6304]
Precursor Scan: 070270, m/z: 1183.2657, charge: 4
VVLHPNYSQVDIGLIK_7_6_0_2_0
1e6
Intensity
m/z
138.0549
366.1392
204.0864
168.0654
274.0919
186.0759
292.1027
126.0549
163.0598
b4(1+)
528.1915
543.3842 y5(1+)
657.2346
999.5492 PEP_0_1_0_0_0_
1101.0962 PEP_0_2_0_0_0_
1182.1201 PEP_1_2_0_0_0_
1263.1471 PEP_2_2_0_0_0_
1344.1759 PEP_3_2_0_0_0_
1364.6901 PEP_2_3_0_0_0_
1445.7148 PEP_3_3_0_0_0_
1526.7355 PEP_4_3_0_0_0_
1547.2386 PEP_3_4_0_0_0_
1628.2727 PEP_4_4_0_0_0_
1709.2889 PEP_5_4_0_0_0_
1998.099 PEP_0_1_0_0_0_
0.0
0.5
1.0
1.5
2.0
2.5
500
1000
1500
2000
2500

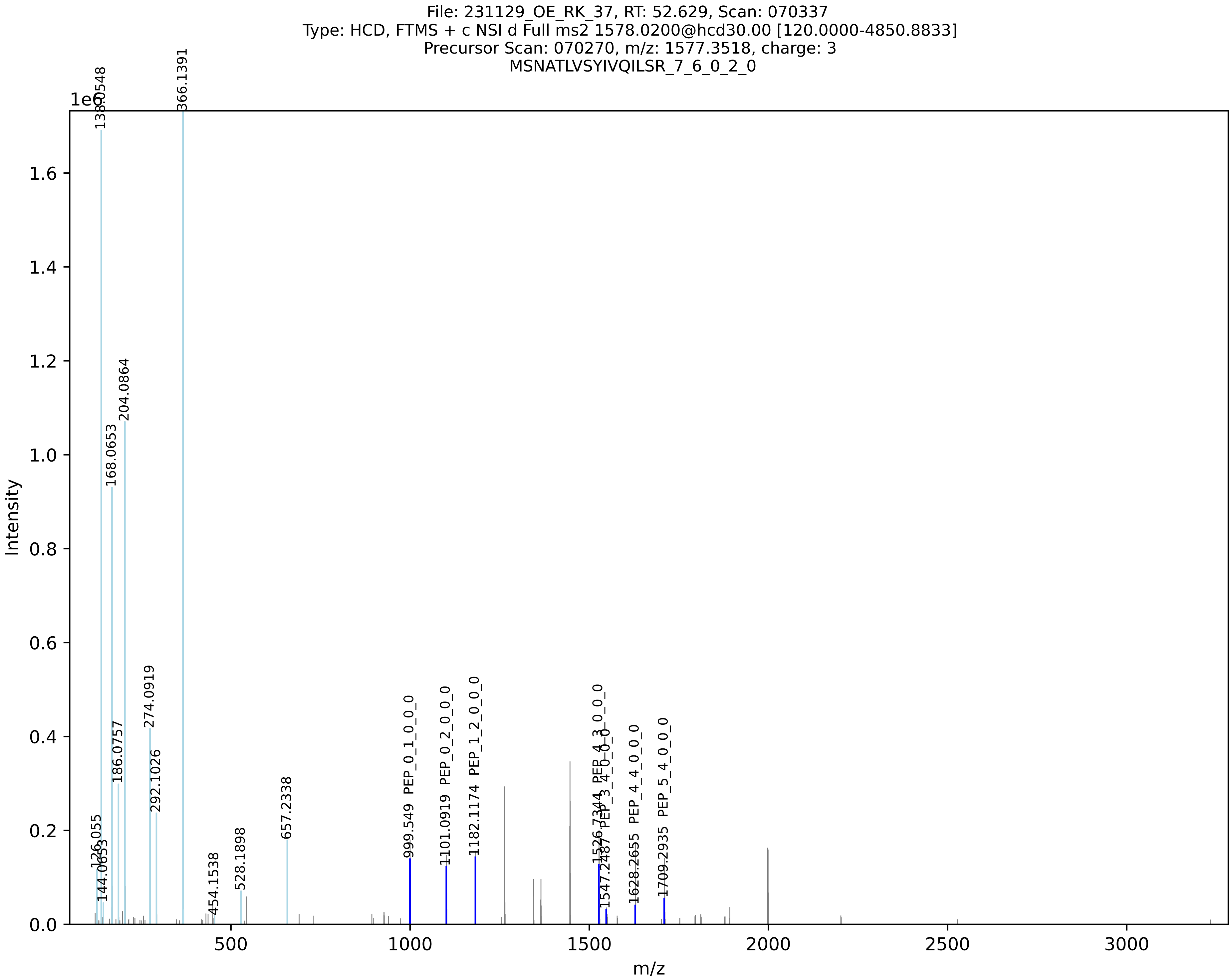
File: 231129_OE_RK_37, RT: 52.629, Scan: 070337
Type: HCD, FTMS + c NSI d Full ms2 1578.0200@hcd30.00 [120.0000-4850.8833]
Precursor Scan: 070270, m/z: 1577.3518, charge: 3
MSNATLVSYIVQILSR_7_6_0_2_0
1e6
366.1391
204.0864
168.0653
274.0919
186.0757
292.1026
126.055
144.0653
454.1538
528.1898
657.2338
999.549 PEP_0_1_0_0_0_
1101.0919 PEP_0_2_0_0_0_
1182.1174 PEP_1_2_0_0_0_
1526.7344 PEP_4_3_0_0_0_
1547.2487 PEP_3_4_0_0_0_
1628.2655 PEP_4_4_0_0_0_
1709.2935 PEP_5_4_0_0_0_
Intensity
m/z
0.0
0.2
0.4
0.6
0.8
1.0
1.2
1.4
1.6
500
1000
1500
2000
2500
3000

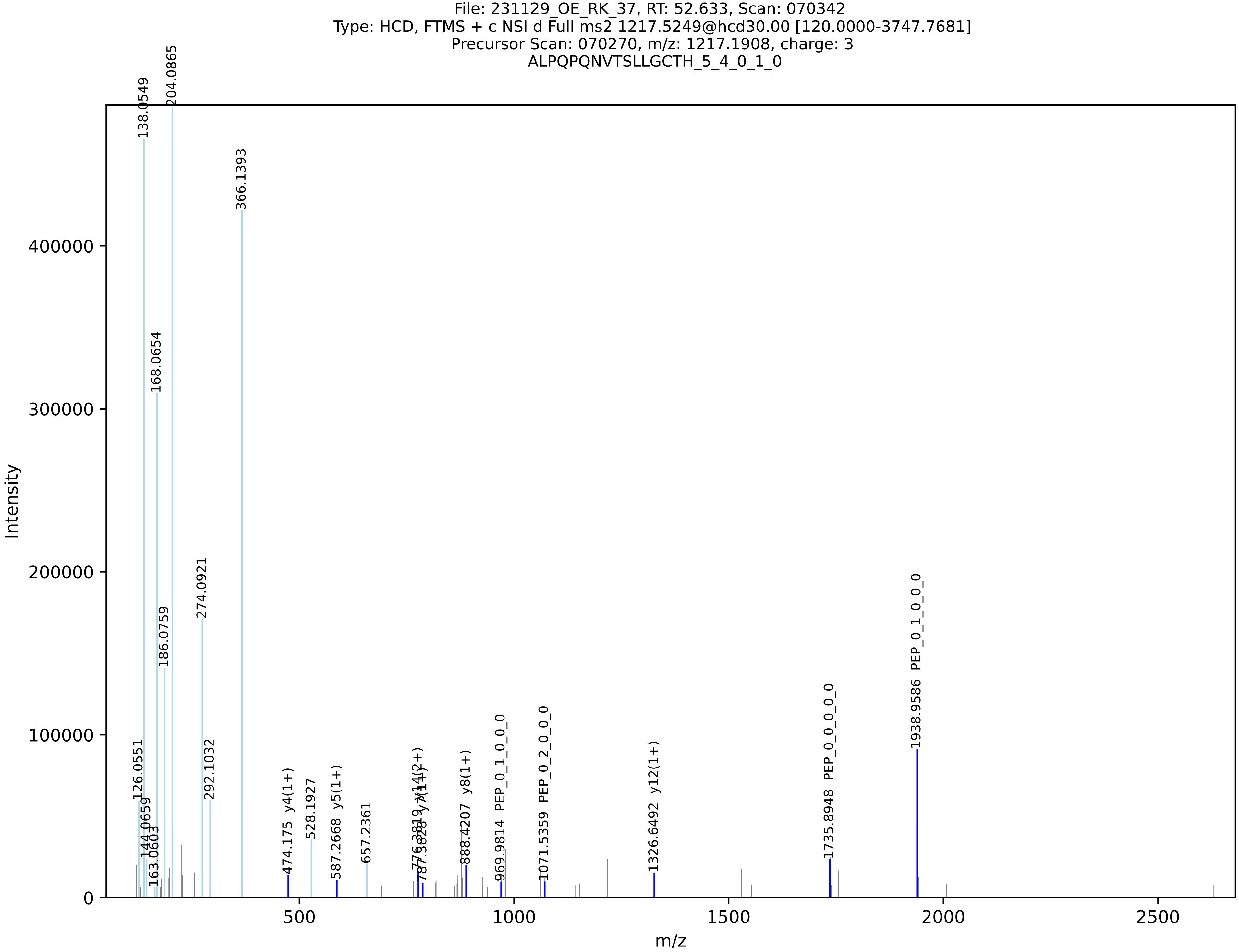
File: 231129_OE_RK_37, RT: 52.633, Scan: 070342
Type: HCD, FTMS + c NSI d Full ms2 1217.5249@hcd30.00 [120.0000-3747.7681]
Precursor Scan: 070270, m/z: 1217.1908, charge: 3
ALPQPQNVTSLLGCTH_5_4_0_1_0
Intensity
m/z
138.0549
204.0865
366.1393
168.0654
186.0759
274.0921
126.0551
292.1032
144.0659
163.0603
474.175 y4(1+)
528.1927
587.2668 y5(1+)
657.2361
776.3819 y14(2+)
787.3828 y7(1+)
888.4207 y8(1+)
969.9814 PEP_0_1_0_0_0
1071.5359 PEP_0_2_0_0_0
1326.6492 y12(1+)
1735.8948 PEP_0_0_0_0_0
1938.9586 PEP_0_1_0_0_0
0
100000
200000
300000
400000
500
1000
1500
2000
2500

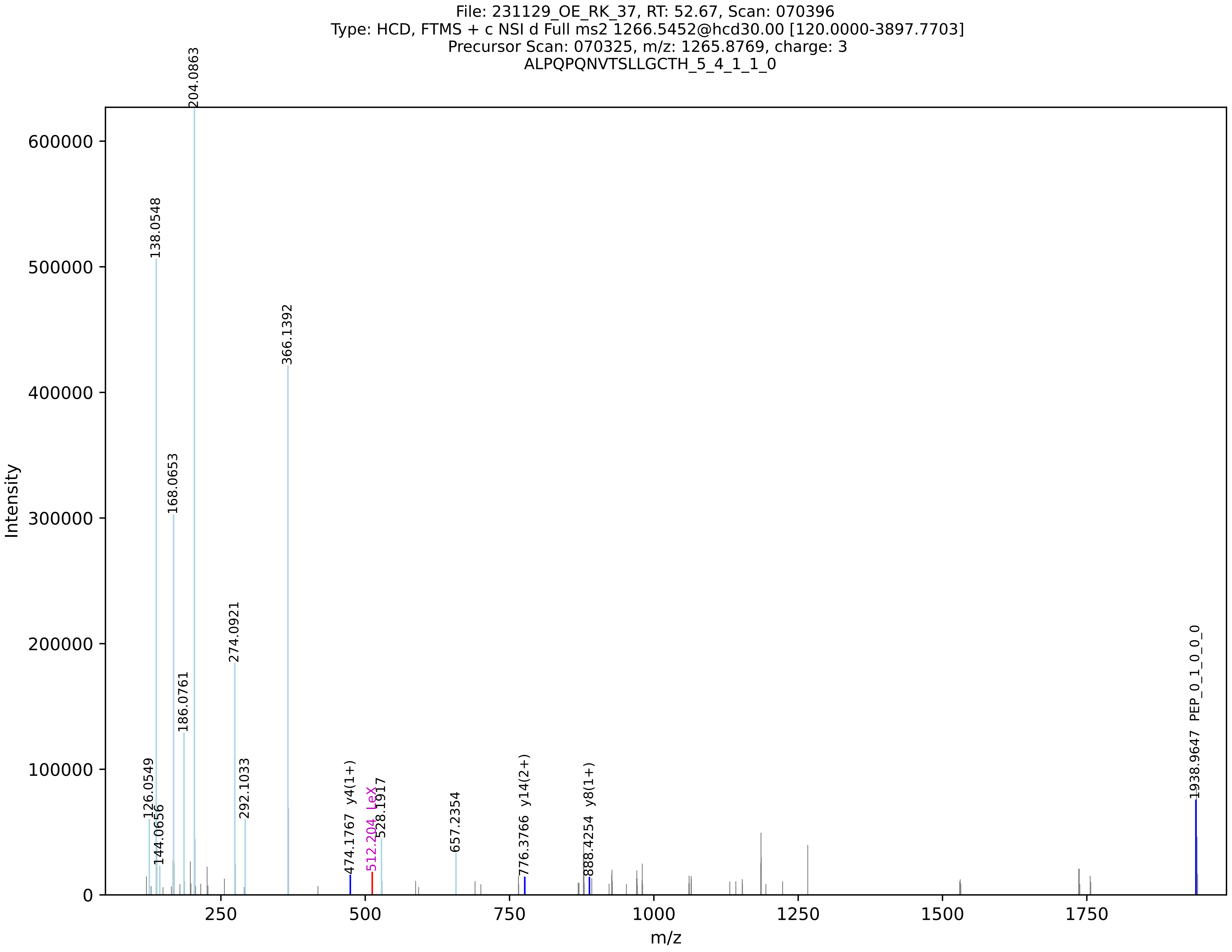

File: 231129_OE_RK_37, RT: 52.67, Scan: 070396
Type: HCD, FTMS + c NSI d Full ms2 1266.5452@hcd30.00 [120.0000-3897.7703]
Precursor Scan: 070325, m/z: 1265.8769, charge: 3
ALPQPQNVTSLLGCTH_5_4_1_1_0
Intensity
m/z
204.0863
138.0548
366.1392
168.0653
274.0921
186.0761
126.0549
144.0656
292.1033
474.1767 y4(1+)
512.204 LeX
528.1917
657.2354
776.3766 y14(2+)
888.4254 y8(1+)
1938.9647 PEP_0_1_0_0_0

File: 231129_OE_RK_37, RT: 52.711, Scan: 070456
Type: HCD, FTMS + c NSI d Full ms2 1220.5336@hcd30.00 [120.0000-5002.6333]
Precursor Scan: 070386, m/z: 1219.7818, charge: 4
VVLHPNYSQVDIGLIK_7_6_1_2_0

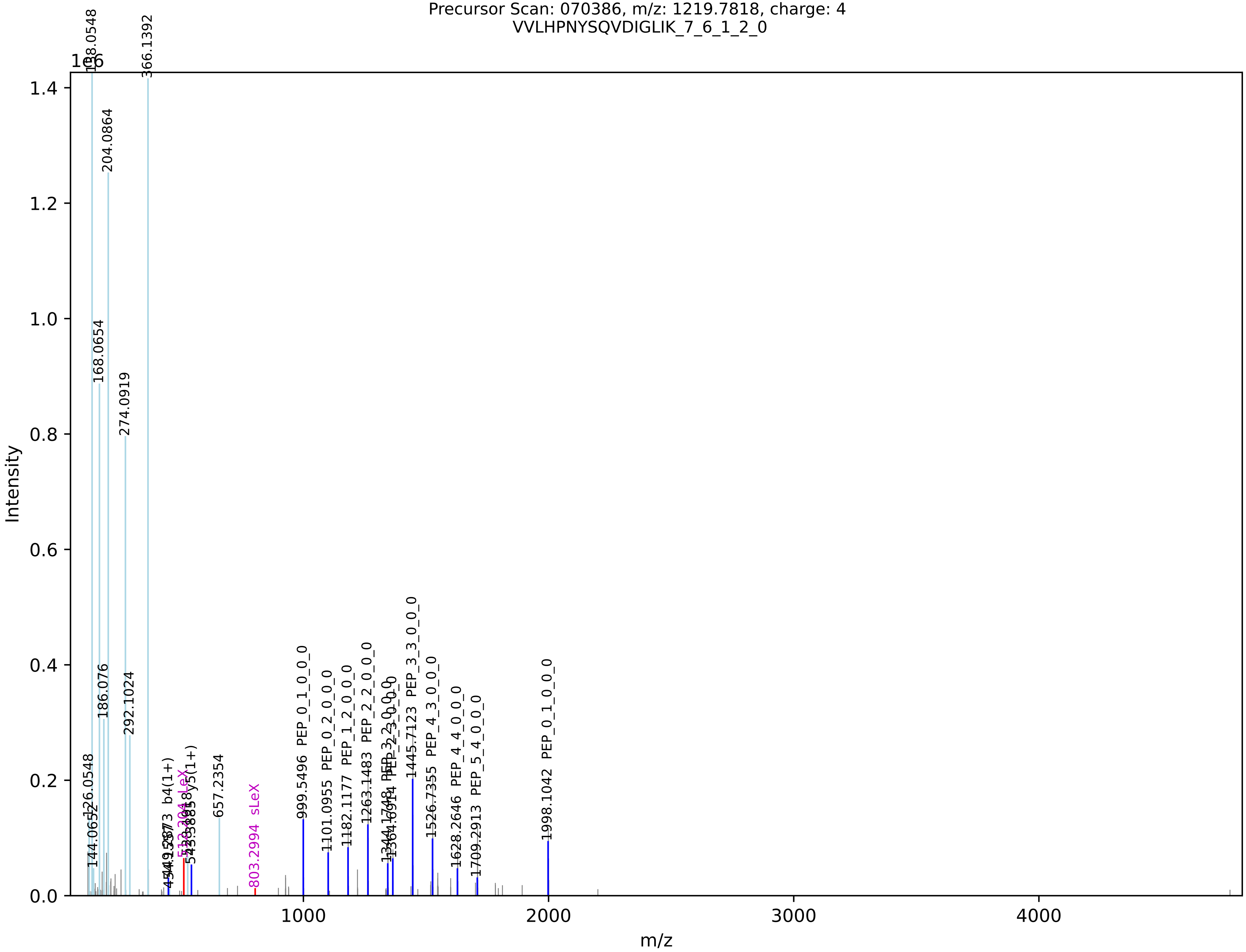

File: 231129_OE_RK_37, RT: 52.804, Scan: 070591
Type: HCD, FTMS + c NSI d Full ms2 1183.7698@hcd30.00 [120.0000-4852.6367]
Precursor Scan: 070527, m/z: 1183.2686, charge: 4
VVLHPNYSQVDIGLIK_7_6_0_2_0

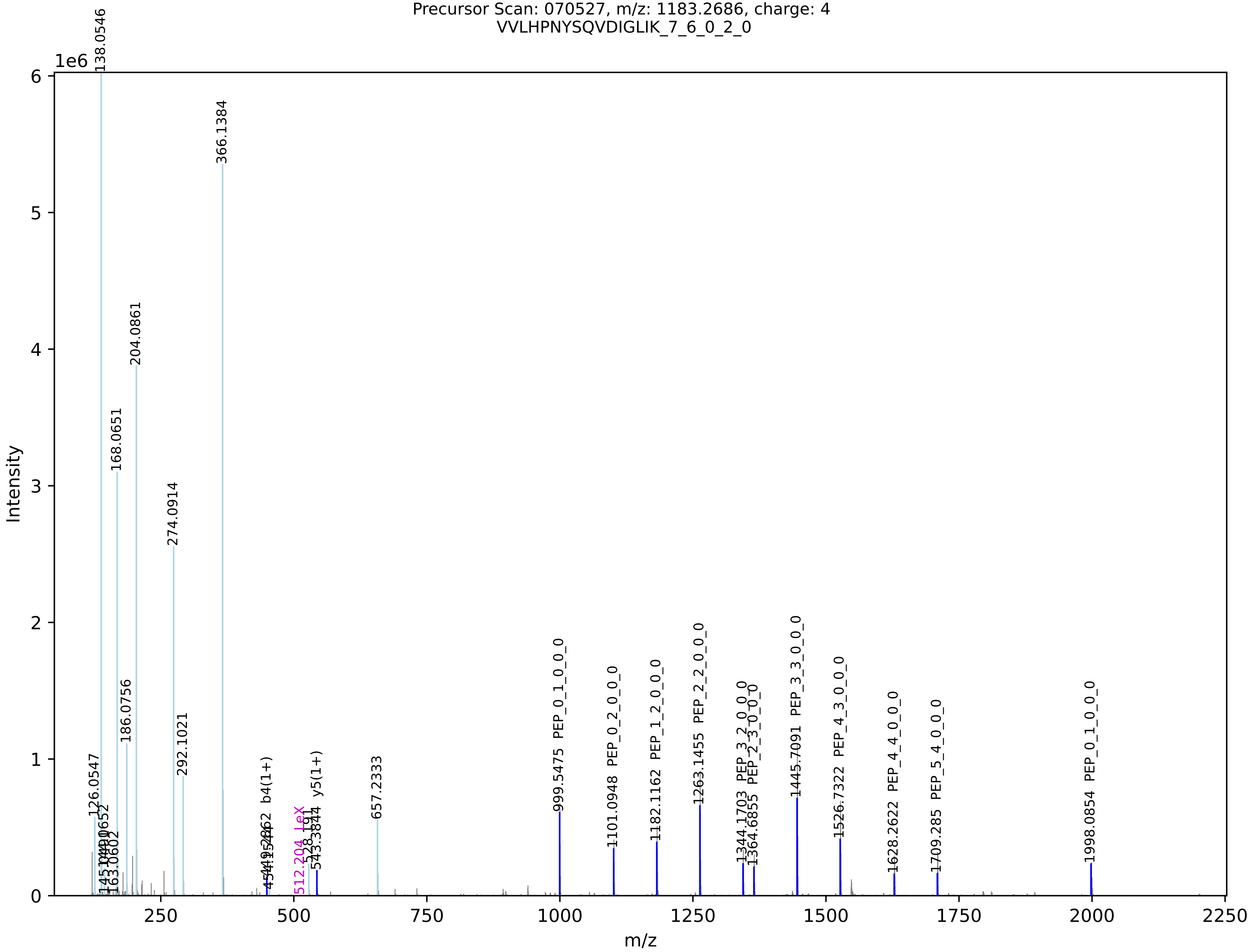

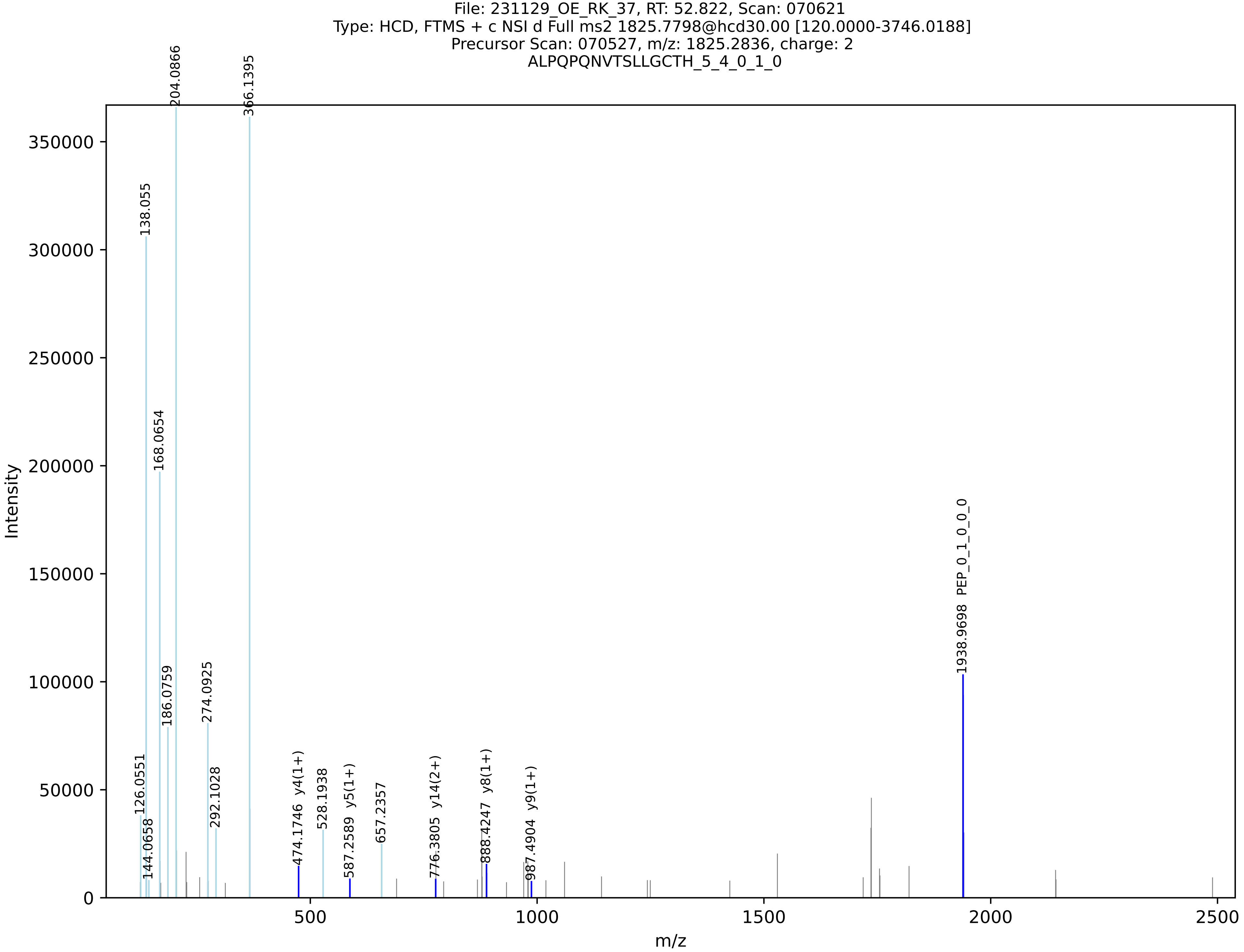

File: 231129_OE_RK_37, RT: 52.822, Scan: 070621
Type: HCD, FTMS + c NSI d Full ms2 1825.7798@hcd30.00 [120.0000-3746.0188]
Precursor Scan: 070527, m/z: 1825.2836, charge: 2
ALPQPQNVTSLLGCTH_5_4_0_1_0
Intensity
m/z
204.0866
366.1395
138.055
168.0654
186.0759
274.0925
126.0551
144.0658
292.1028
474.1746 y4(1+)
528.1938
587.2589 y5(1+)
657.2357
776.3805 y14(2+)
888.4247 y8(1+)
987.4904 y9(1+)
1938.9698 PEP_0_1_0_0_0
0
50000
100000
150000
200000
250000
300000
350000
500
1000
1500
2000
2500

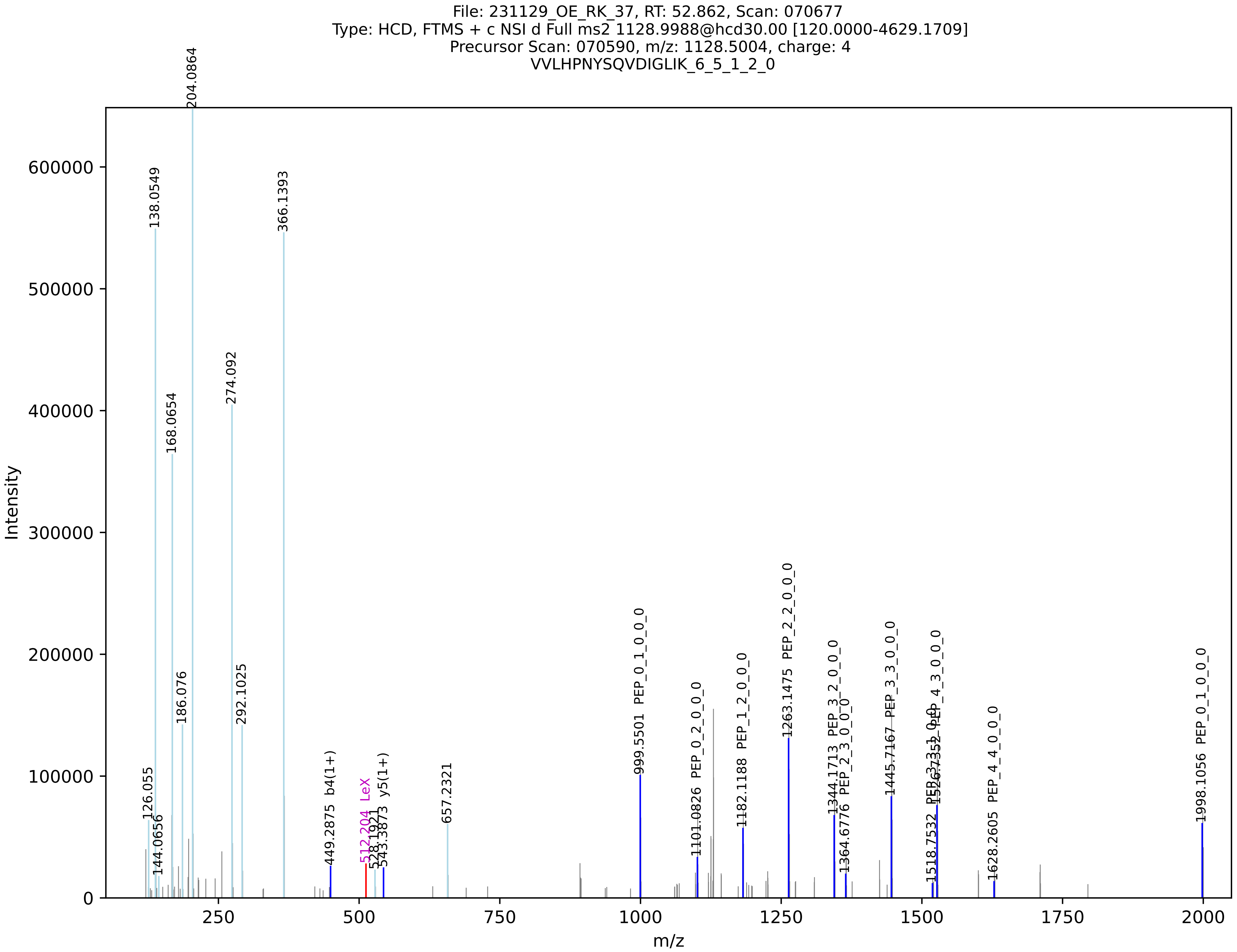
File: 231129_OE_RK_37, RT: 52.862, Scan: 070677
Type: HCD, FTMS + c NSI d Full ms2 1128.9988@hcd30.00 [120.0000-4629.1709]
Precursor Scan: 070590, m/z: 1128.5004, charge: 4
VVLHPNYSQVDIGLIK_6_5_1_2_0
Intensity
m/z
204.0864
138.0549
366.1393
274.092
168.0654
186.076
292.1025
126.055
144.0656
449.2875 b4(1+)
512.204 LeX
528.1921
543.3873 y5(1+)
657.2321
999.5501 PEP_0_1_0_0_0
1101.0826 PEP_0_2_0_0_0
1182.1188 PEP_1_2_0_0_0
1263.1475 PEP_2_2_0_0_0
1344.1713 PEP_3_2_0_0_0
1364.6776 PEP_2_3_0_0_0
1445.7167 PEP_3_3_0_0_0
1518.7532 PEP_3_3_1_0_0
1526.7352 PEP_4_3_0_0_0
1628.2605 PEP_4_4_0_0_0
1998.1056 PEP_0_1_0_0_0

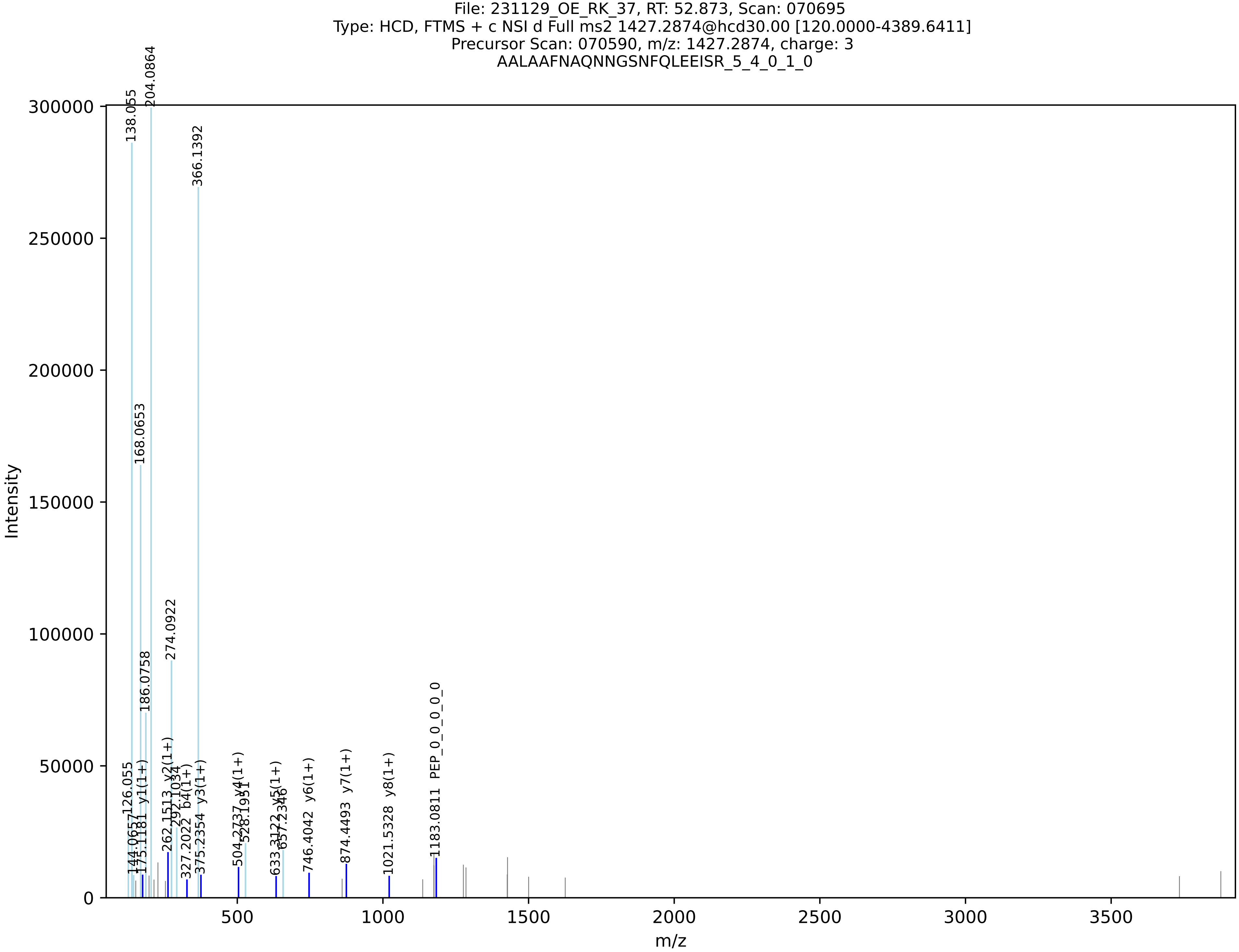

File: 231129_OE_RK_37, RT: 52.873, Scan: 070695
Type: HCD, FTMS + c NSI d Full ms2 1427.2874@hcd30.00 [120.0000-4389.6411]
Precursor Scan: 070590, m/z: 1427.2874, charge: 3
AALAAFNAQNNGSNFQLEEISR_5_4_0_1_0
Intensity
m/z
204.0864
138.055
366.1392
168.0653
274.0922
186.0758
126.055
144.0657
175.1181 y1(1+)
262.1513 y2(1+)
292.1034
327.2022 b4(1+)
375.2354 y3(1+)
504.2737 y4(1+)
528.1951
633.3122 y5(1+)
657.2346
746.4042 y6(1+)
874.4493 y7(1+)
1021.5328 y8(1+)
1183.0811 PEP_0_0_0_0_0

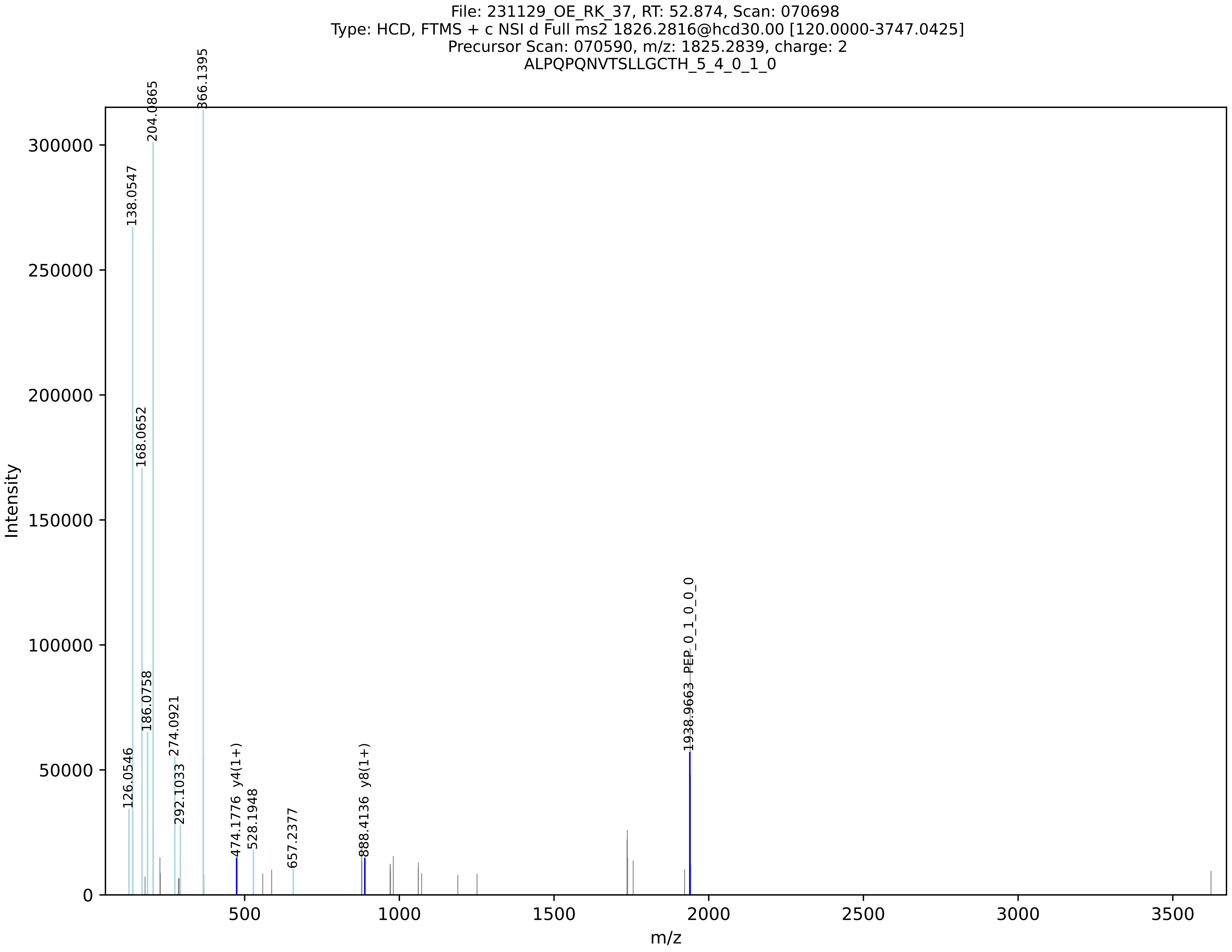
File: 231129_OE_RK_37, RT: 52.874, Scan: 070698
Type: HCD, FTMS + c NSI d Full ms2 1826.2816@hcd30.00 [120.0000-3747.0425]
Precursor Scan: 070590, m/z: 1825.2839, charge: 2
ALPQPQNVTSLLGCTH_5_4_0_1_0
366.1395
204.0865
138.0547
168.0652
186.0758
274.0921
126.0546
292.1033
474.1776_y4(1+)
528.1948
657.2377
888.4136_y8(1+)
1938.9663_PEP_0_1_0_0_0_
Intensity
m/z
0
50000
100000
150000
200000
250000
300000
500
1000
1500
2000
2500
3000
3500

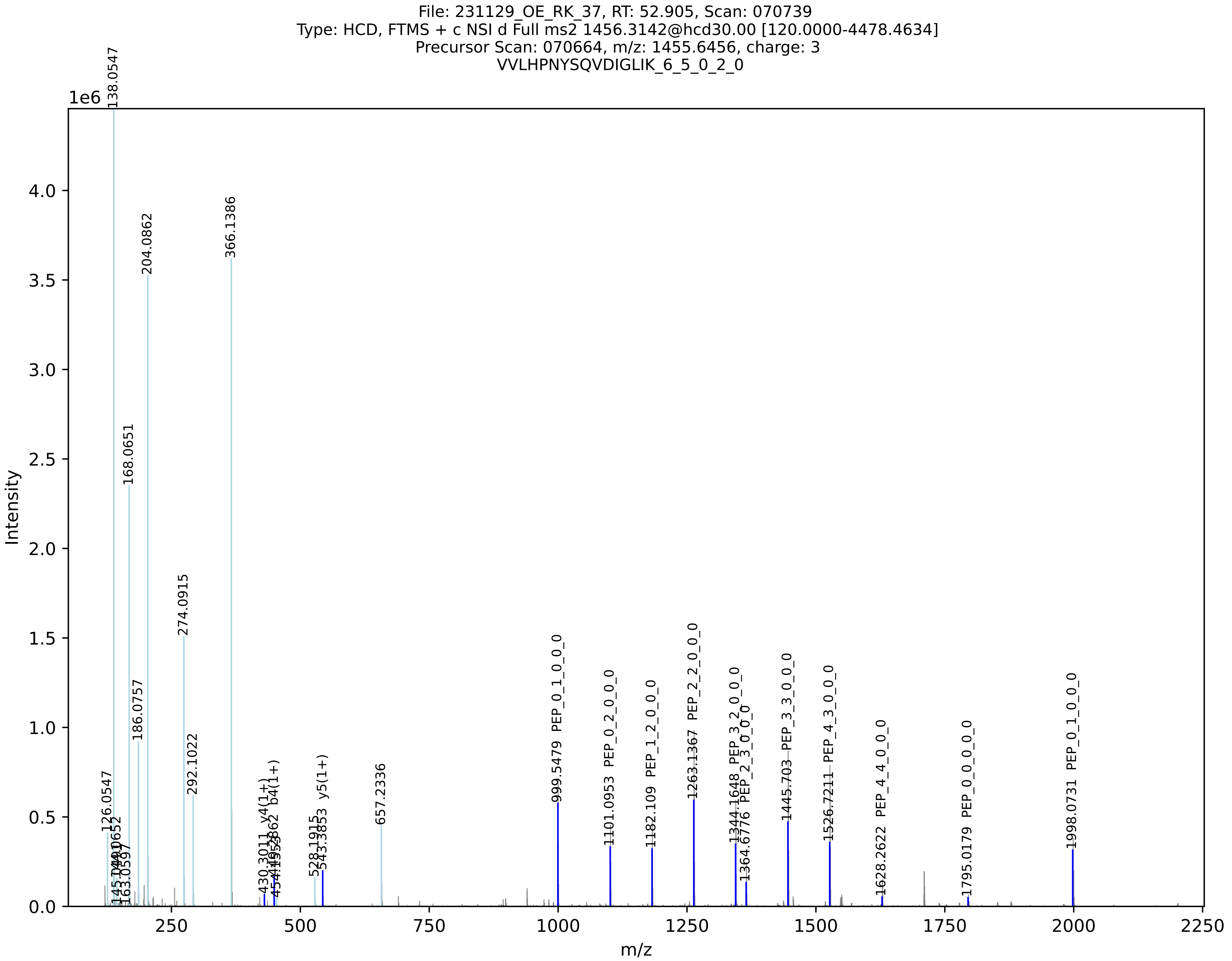
File: 231129_OE_RK_37, RT: 52.905, Scan: 070739
Type: HCD, FTMS + c NSI d Full ms2 1456.3142@hcd30.00 [120.0000-4478.4634]
Precursor Scan: 070664, m/z: 1455.6456, charge: 3
VVLHPNYSQVDIGLIK_6_5_0_2_0
1e6
Intensity
m/z
138.0547
204.0862
366.1386
168.0651
274.0915
186.0757
292.1022
126.0547
657.2336
430.3011 y4(1+)
447.2862 b4(1+)
543.3853 y5(1+)
528.1915
999.5479 PEP_0_1_0_0_0
1101.0953 PEP_0_2_0_0_0
1182.109 PEP_1_2_0_0_0
1263.1367 PEP_2_2_0_0_0
1344.1648 PEP_3_2_0_0_0
1364.6776 PEP_2_3_0_0_0
1445.703 PEP_3_3_0_0_0
1526.7211 PEP_4_3_0_0_0
1628.2622 PEP_4_4_0_0_0
1795.0179 PEP_0_0_0_0_0
1998.0731 PEP_0_1_0_0_0

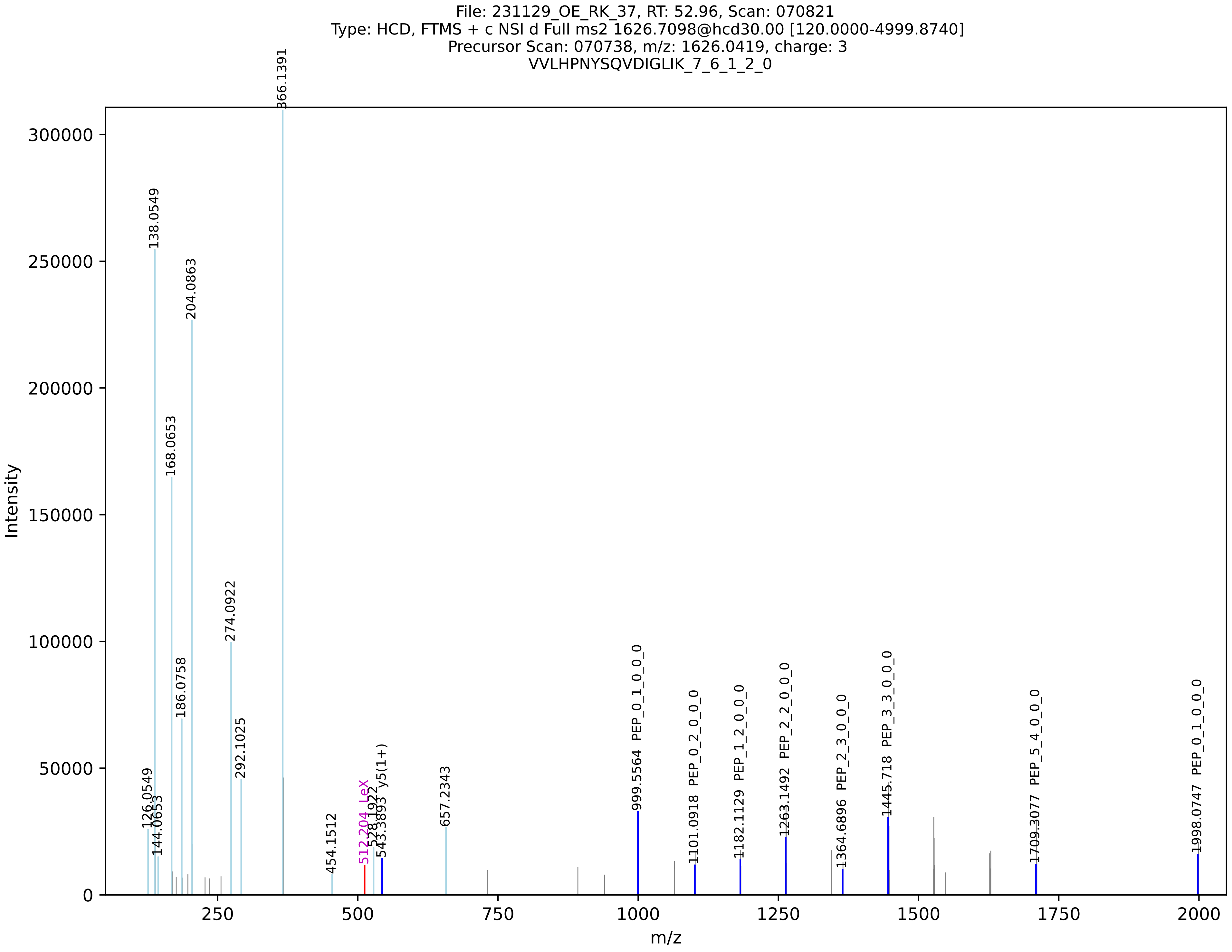
File: 231129_OE_RK_37, RT: 52.96, Scan: 070821
Type: HCD, FTMS + c NSI d Full ms2 1626.7098@hcd30.00 [120.0000-4999.8740]
Precursor Scan: 070738, m/z: 1626.0419, charge: 3
VVLHPNYSQVDIGLIK_7_6_1_2_0
Intensity
m/z
366.1391
138.0549
204.0863
168.0653
274.0922
186.0758
292.1025
126.0549
144.0653
454.1512
512.204_LeX
528.1922
543.3893 y5(1+)
657.2343
999.5564 PEP_0_1_0_0_0
1101.0918 PEP_0_2_0_0_0
1182.1129 PEP_1_2_0_0_0
1263.1492 PEP_2_2_0_0_0
1364.6896 PEP_2_3_0_0_0
1445.718 PEP_3_3_0_0_0
1709.3077 PEP_5_4_0_0_0
1998.0747 PEP_0_1_0_0_0

File: 231129_OE_RK_37, RT: 53.007, Scan: 070889
Type: HCD, FTMS + c NSI d Full ms2 1183.7700@hcd30.00 [120.0000-4852.6377]
Precursor Scan: 070812, m/z: 1183.2687, charge: 4
VVLHPNYSQVDIGLIK_7_6_0_2_0

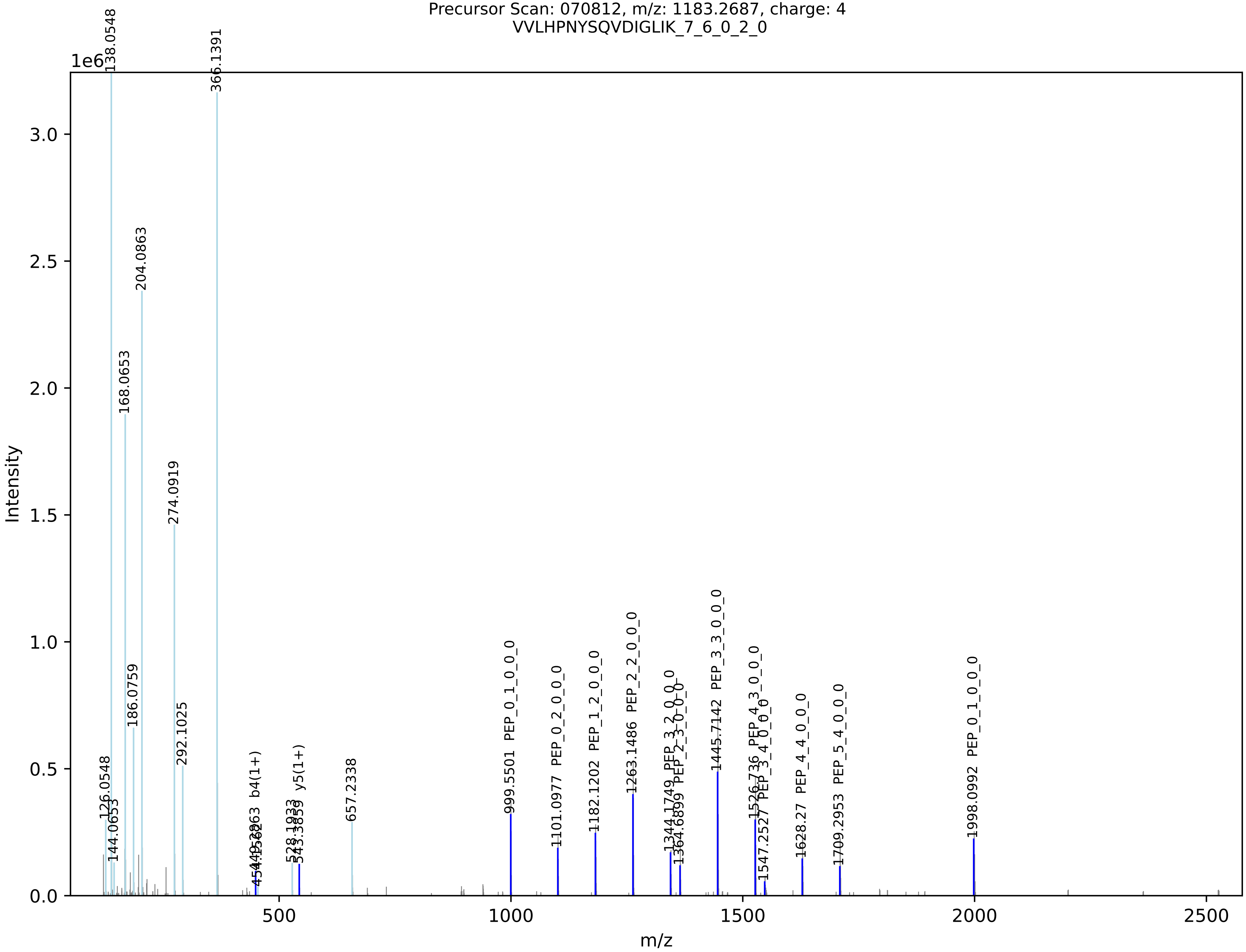

File: 231129_OE_RK_37, RT: 53.008, Scan: 070891
Type: HCD, FTMS + c NSI d Full ms2 1578.0239@hcd30.00 [120.0000-4850.8950]
Precursor Scan: 070812, m/z: 1577.3559, charge: 3
VVLHPNYSQVDIGLIK_7_6_0_2_0

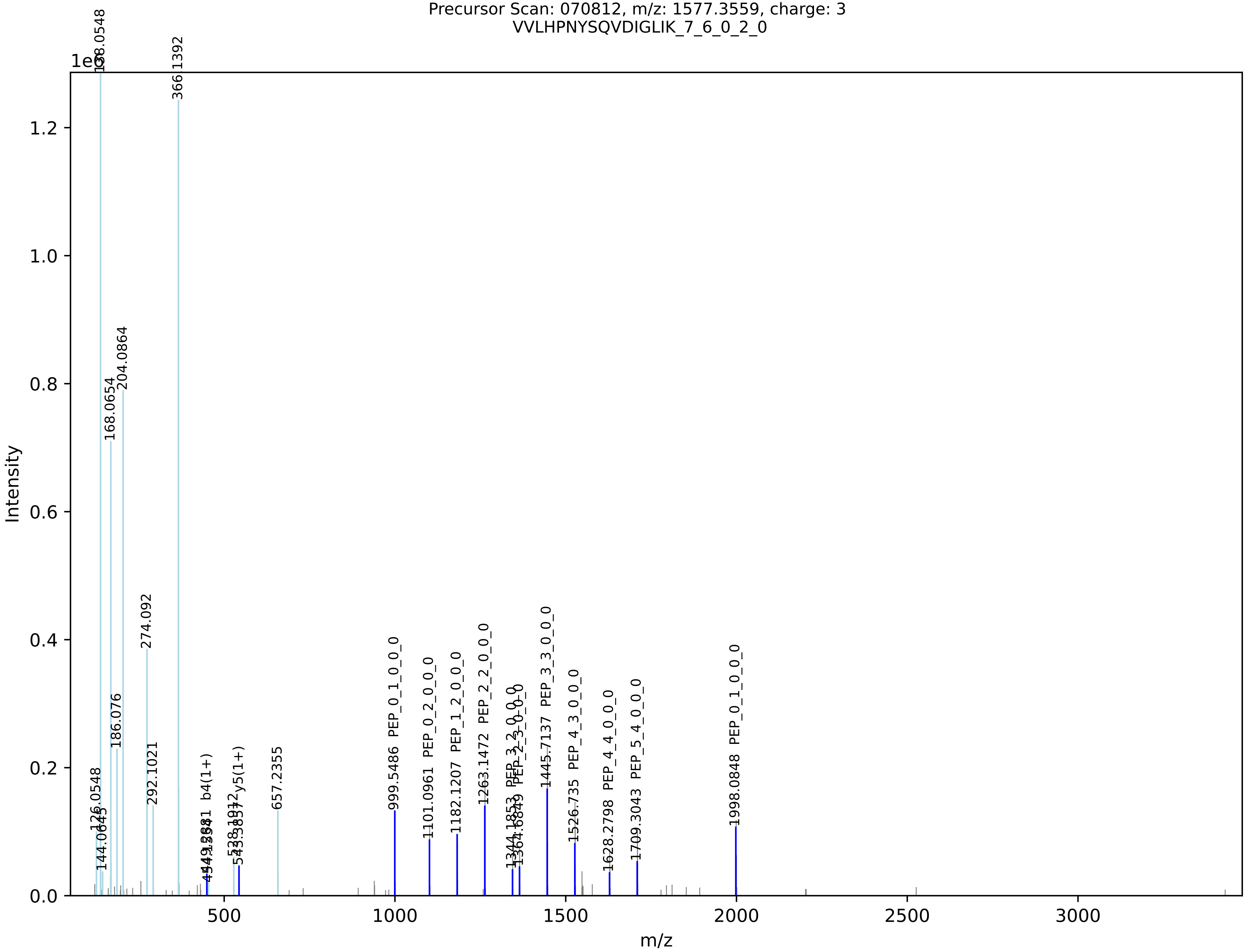

File: 231129_OE_RK_37, RT: 53.014, Scan: 070900
Type: HCD, FTMS + c NSI d Full ms2 1096.7433@hcd30.00 [120.0000-4497.5688]
Precursor Scan: 070812, m/z: 1096.2431, charge: 4
VVLHPNYSQVDIGLIK_7_5_1_1_0

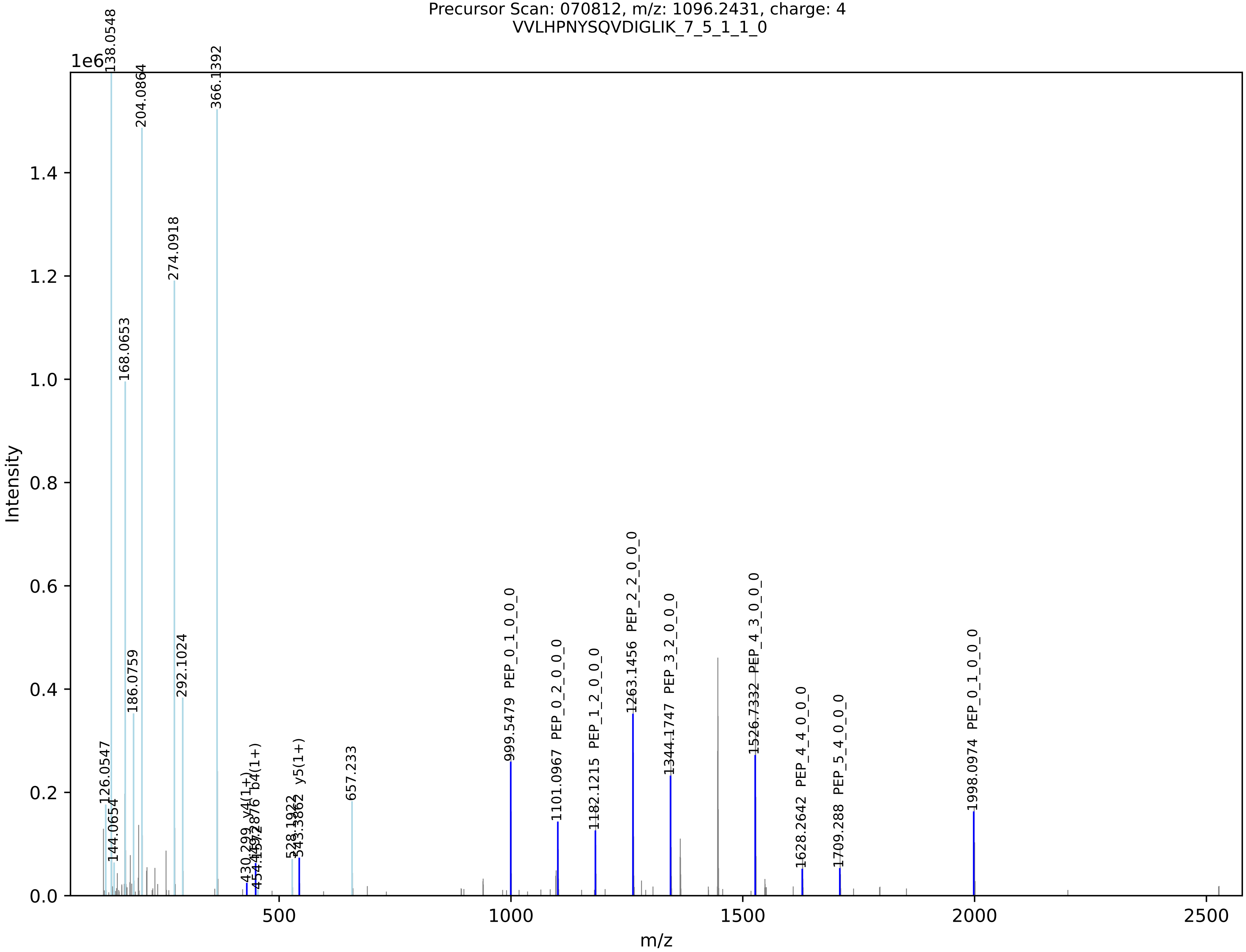

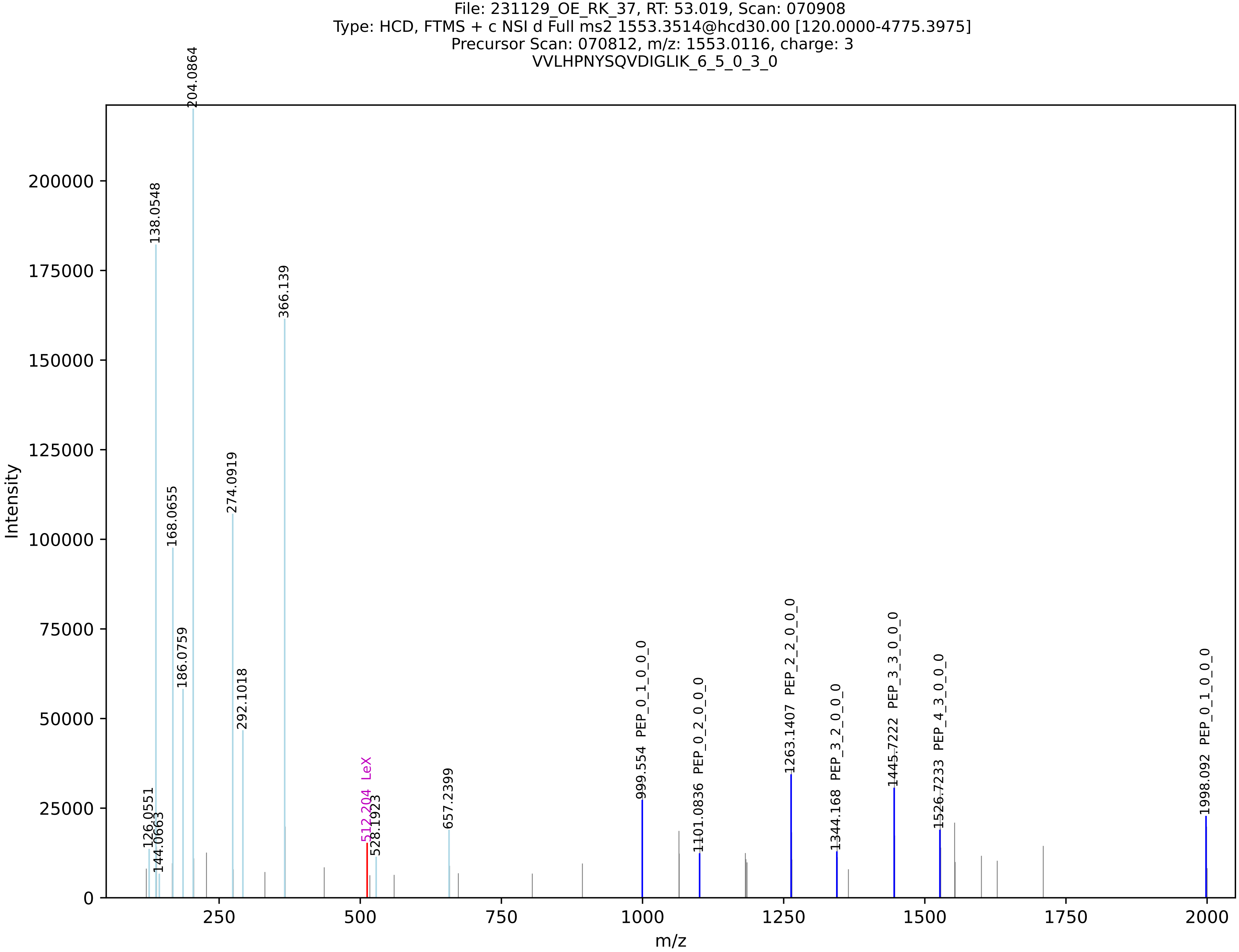

File: 231129_OE_RK_37, RT: 53.019, Scan: 070908
Type: HCD, FTMS + c NSI d Full ms2 1553.3514@hcd30.00 [120.0000-4775.3975]
Precursor Scan: 070812, m/z: 1553.0116, charge: 3
VVLHPNYSQVDIGLIK_6_5_0_3_0
Intensity
m/z
204.0864
138.0548
366.139
274.0919
168.0655
186.0759
292.1018
126.0551
144.0663
512.204 LeX
528.1923
657.2399
999.554 PEP_0_1_0_0_0
1101.0836 PEP_0_2_0_0_0
1263.1407 PEP_2_2_0_0_0
1344.168 PEP_3_2_0_0_0
1445.7222 PEP_3_3_0_0_0
1526.7233 PEP_4_3_0_0_0
1998.092 PEP_0_1_0_0_0

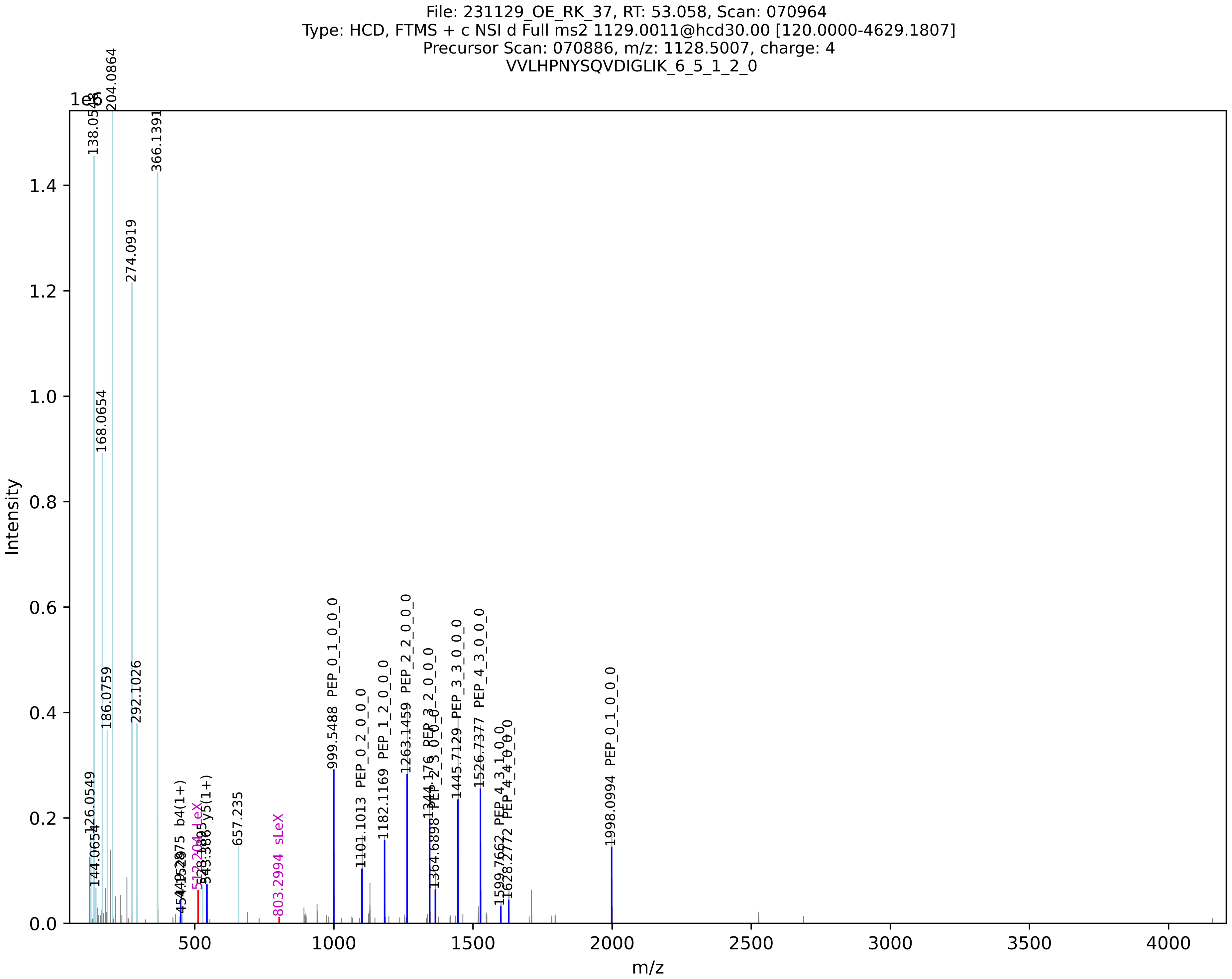

File: 231129_OE_RK_37, RT: 53.058, Scan: 070964
Type: HCD, FTMS + c NSI d Full ms2 1129.0011@hcd30.00 [120.0000-4629.1807]
Precursor Scan: 070886, m/z: 1128.5007, charge: 4
VVLHPNYSQVDIGLIK_6_5_1_2_0
1e6
Intensity
m/z
204.0864
138.0549
366.1391
274.0919
168.0654
186.0759
292.1026
126.0549
144.0654
999.5488 PEP_0_1_0_0_0
1101.1013 PEP_0_2_0_0_0
1182.1169 PEP_1_2_0_0_0
1263.1459 PEP_2_2_0_0_0
1445.7129 PEP_3_3_0_0_0
1526.7377 PEP_4_3_0_0_0
1998.0994 PEP_0_1_0_0_0
657.235
803.2994 sLeX

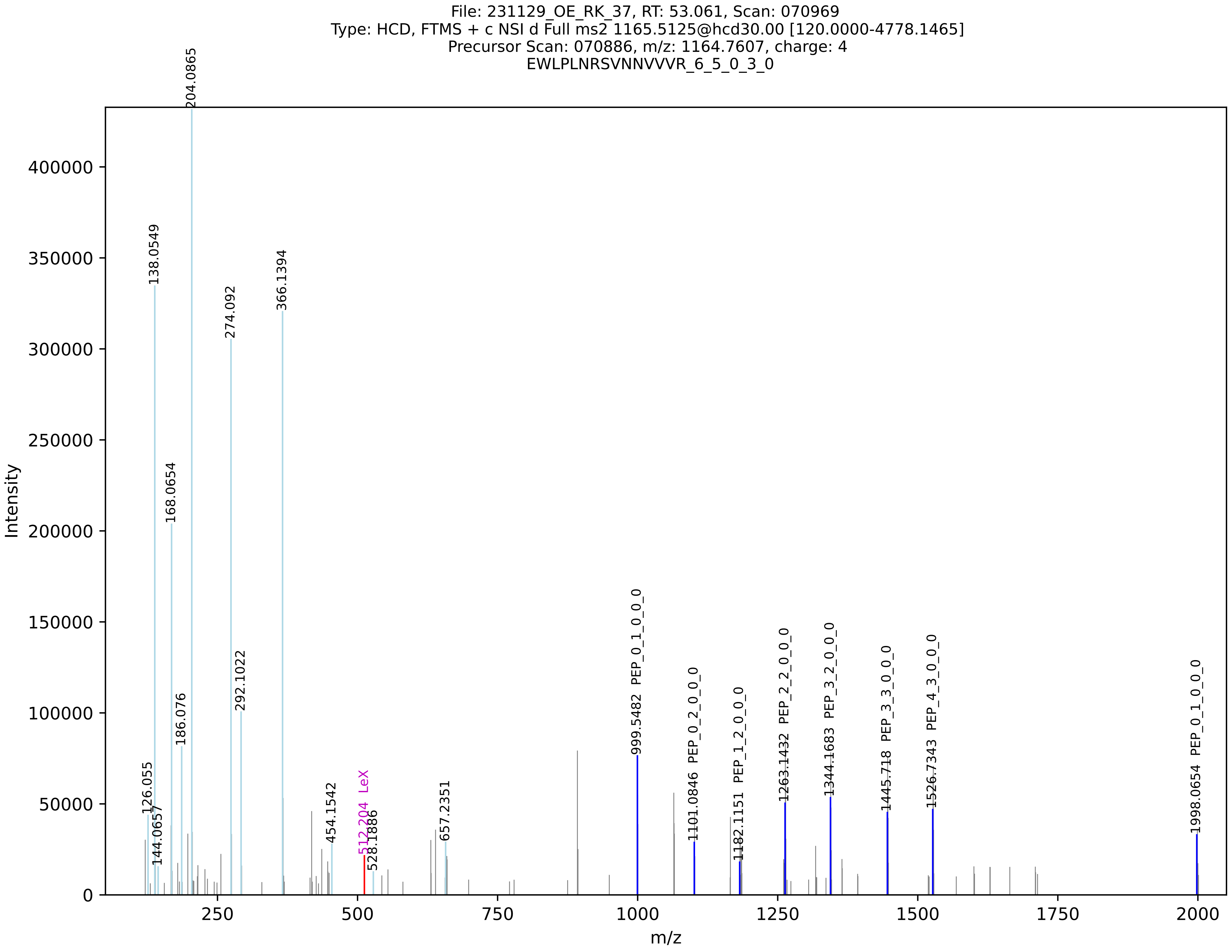
File: 231129_OE_RK_37, RT: 53.061, Scan: 070969
Type: HCD, FTMS + c NSI d Full ms2 1165.5125@hcd30.00 [120.0000-4778.1465]
Precursor Scan: 070886, m/z: 1164.7607, charge: 4
EWLPLNRSVNNVVVR_6_5_0_3_0
Intensity
m/z
204.0865
138.0549
366.1394
274.092
168.0654
292.1022
186.076
126.055
144.0657
454.1542
512.204 LeX
528.1886
657.2351
999.5482 PEP_0_1_0_0_0
1101.0846 PEP_0_2_0_0_0
1182.1151 PEP_1_2_0_0_0
1263.1432 PEP_2_2_0_0_0
1344.1683 PEP_3_2_0_0_0
1445.718 PEP_3_3_0_0_0
1526.7343 PEP_4_3_0_0_0
1998.0654 PEP_0_1_0_0_0

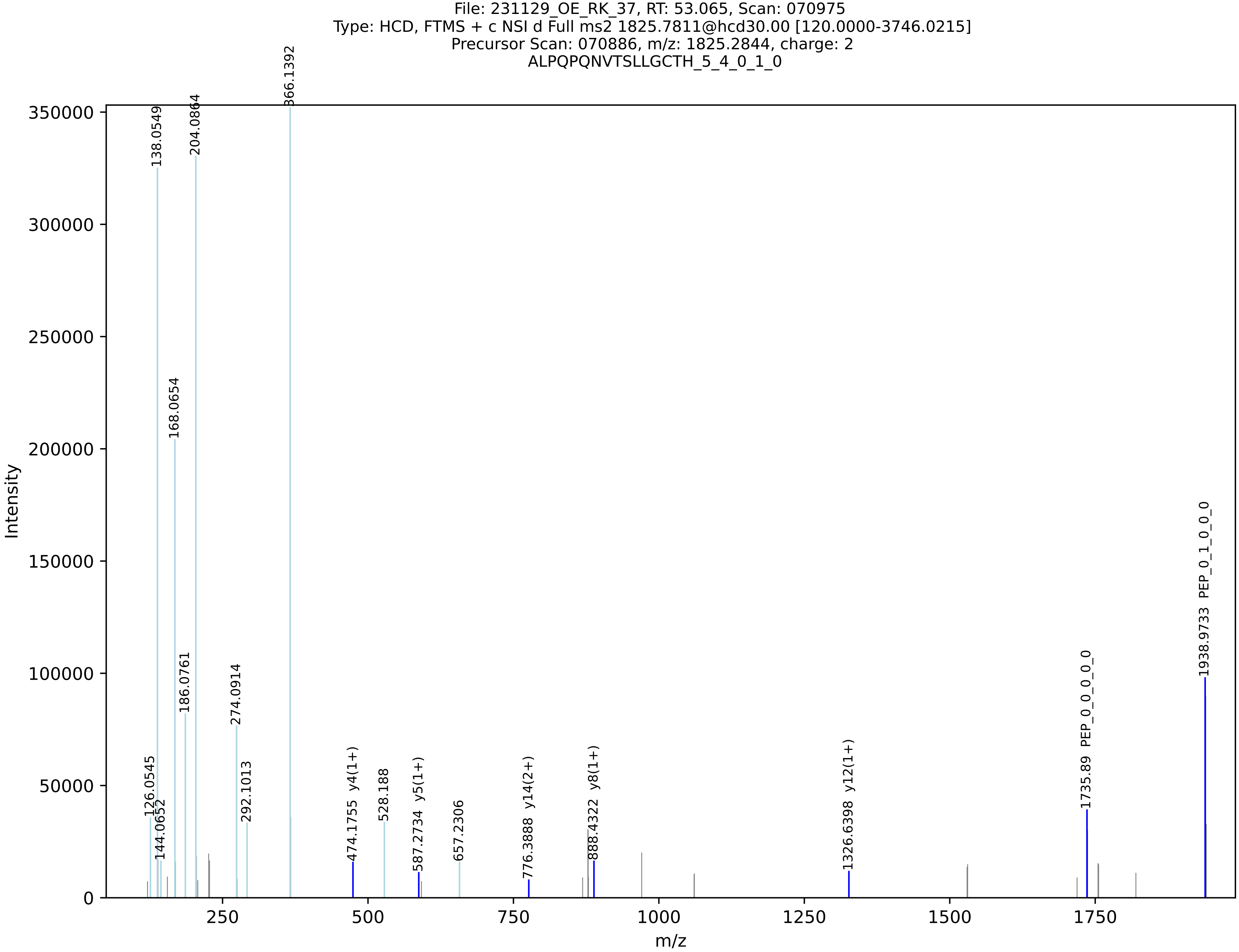
File: 231129_OE_RK_37, RT: 53.065, Scan: 070975
Type: HCD, FTMS + c NSI d Full ms2 1825.7811@hcd30.00 [120.0000-3746.0215]
Precursor Scan: 070886, m/z: 1825.2844, charge: 2
ALPQPQNVTSLLGCTH_5_4_0_1_0
Intensity
m/z
138.0549
204.0864
366.1392
168.0654
186.0761
274.0914
292.1013
126.0545
144.0652
474.1755 y4(1+)
528.188
587.2734 y5(1+)
657.2306
776.3888 y14(2+)
888.4322 y8(1+)
1326.6398 y12(1+)
1735.89 PEP_0_0_0_0_0
1938.9733 PEP_0_1_0_0_0

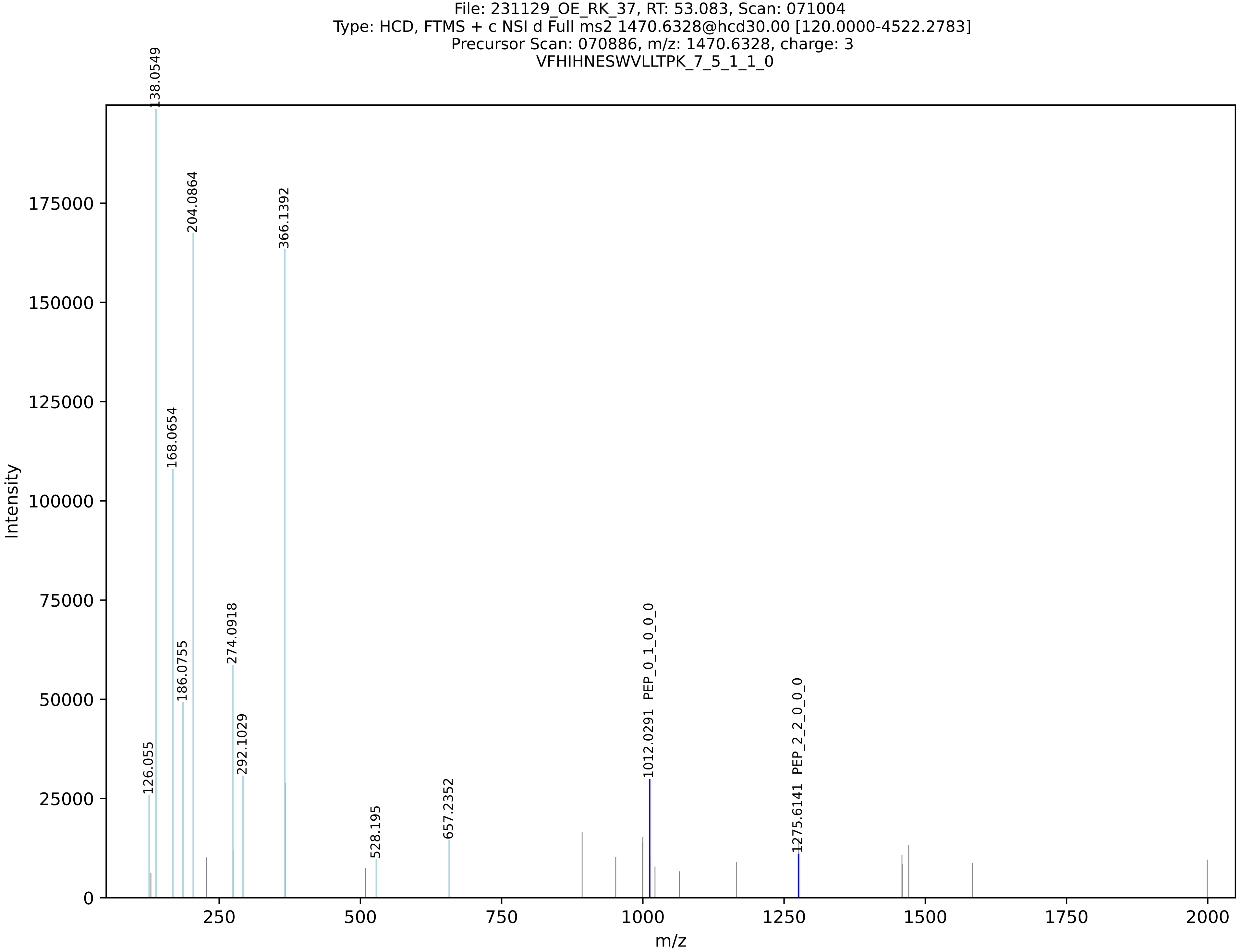
File: 231129_OE_RK_37, RT: 53.083, Scan: 071004
Type: HCD, FTMS + c NSI d Full ms2 1470.6328@hcd30.00 [120.0000-4522.2783]
Precursor Scan: 070886, m/z: 1470.6328, charge: 3
VFHIHNESWVLLTPK_7_5_1_1_0
138.0549
204.0864
366.1392
168.0654
186.0755
274.0918
292.1029
126.055
528.195
657.2352
1012.0291 PEP_0_1_0_0_0
1275.6141 PEP_2_2_0_0_0
Intensity
m/z
0
25000
50000
75000
100000
125000
150000
175000
250
500
750
1000
1250
1500
1750
2000

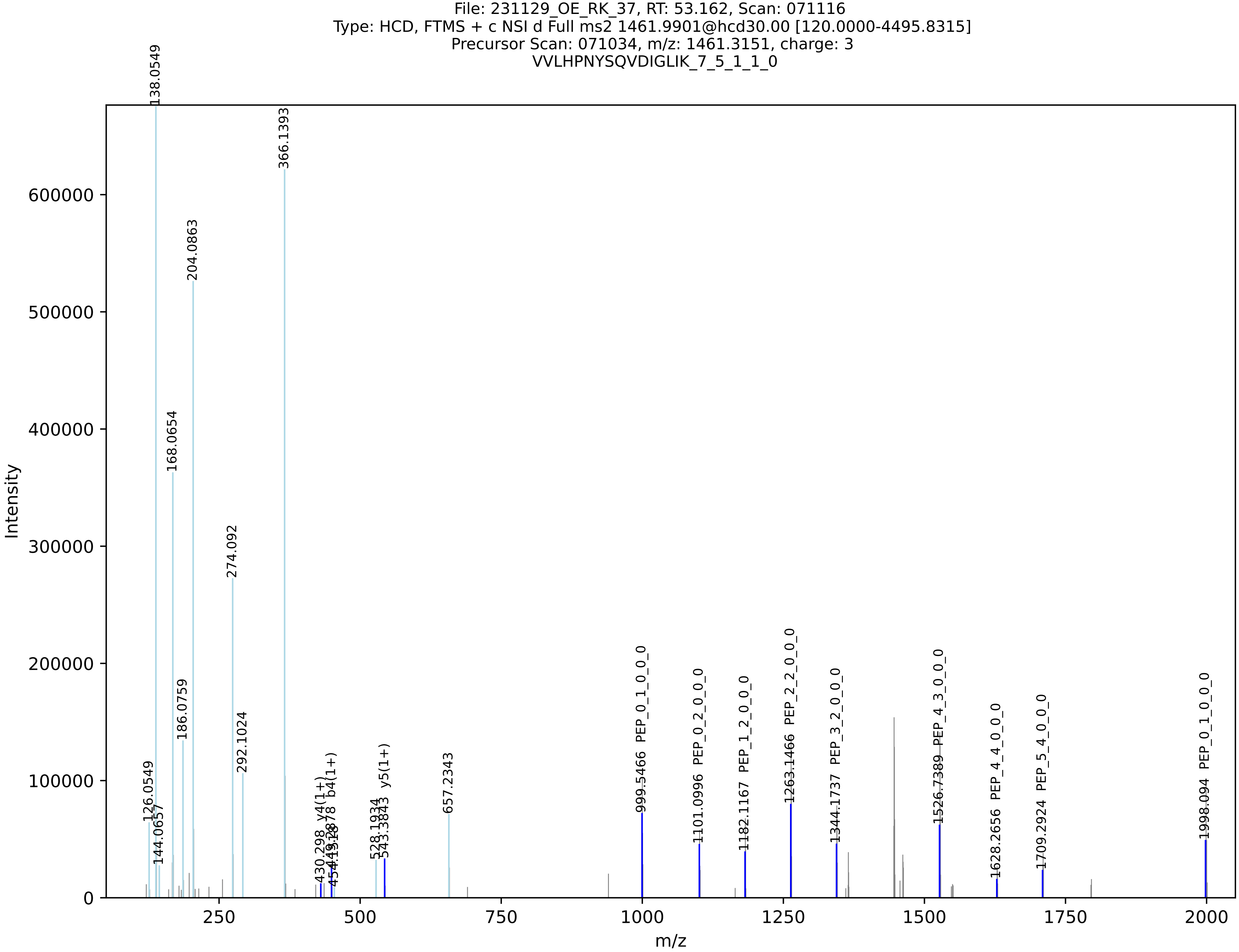
File: 231129_OE_RK_37, RT: 53.162, Scan: 071116
Type: HCD, FTMS + c NSI d Full ms2 1461.9901@hcd30.00 [120.0000-4495.8315]
Precursor Scan: 071034, m/z: 1461.3151, charge: 3
VVLHPNYSQVDIGLIK_7_5_1_1_0
Intensity
m/z
0
100000
200000
300000
400000
500000
600000
250
500
750
1000
1250
1500
1750
2000
138.0549
366.1393
204.0863
168.0654
274.092
186.0759
292.1024
126.0549
144.0657
430.298 y4(1+)
b4(1+)
528.1934
543.3843 y5(1+)
657.2343
999.5466 PEP_0_1_0_0_0
1101.0996 PEP_0_2_0_0_0
1182.1167 PEP_1_2_0_0_0
1263.1466 PEP_2_2_0_0_0
1344.1737 PEP_3_2_0_0_0
1526.7389 PEP_4_3_0_0_0
1628.2656 PEP_4_4_0_0_0
1709.2924 PEP_5_4_0_0_0
1998.094 PEP_0_1_0_0_0

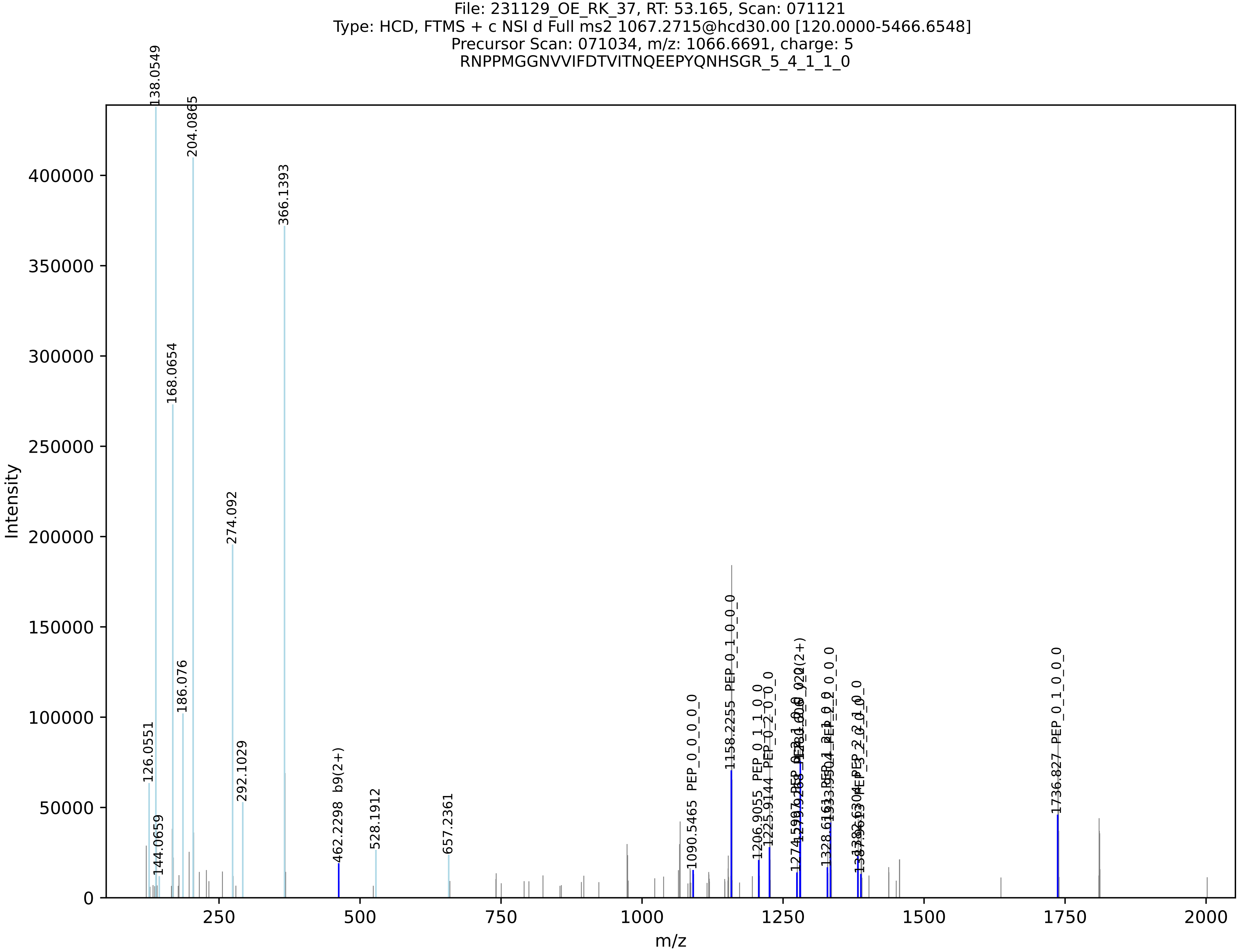
File: 231129_OE_RK_37, RT: 53.165, Scan: 071121
Type: HCD, FTMS + c NSI d Full ms2 1067.2715@hcd30.00 [120.0000-5466.6548]
Precursor Scan: 071034, m/z: 1066.6691, charge: 5
RNPPMGGNVVIFDTVITNQEEPYQNHSGR_5_4_1_1_0
Intensity
m/z
138.0549
204.0865
366.1393
168.0654
274.092
186.076
126.0551
292.1029
144.0659
462.2298 b9(2+)
528.1912
657.2361
1090.5465 PEP_0_0_0_0_0
1158.2255 PEP_0_1_0_0_0
1206.9055 PEP_0_1_1_0_0
1225.9144 PEP_0_2_0_0_0
1736.827 PEP_0_1_0_0_0

File: 231129_OE_RK_37, RT: 53.168, Scan: 071126
Type: HCD, FTMS + c NSI d Full ms2 1456.4751@hcd30.00 [120.0000-4478.9561]
Precursor Scan: 071034, m/z: 1456.1453, charge: 3
VVLHPNYSQVDIGLIK_6_5_0_2_0

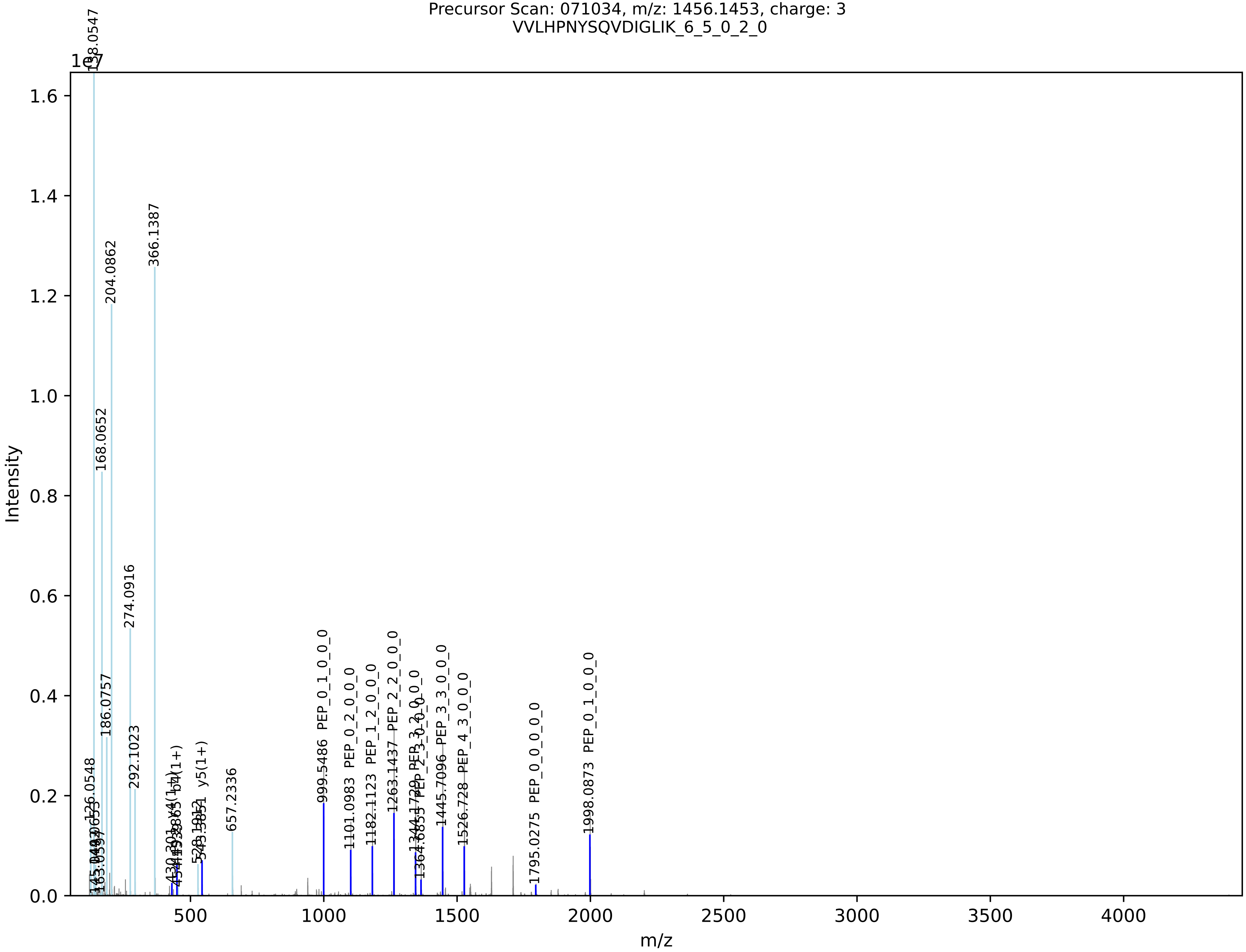

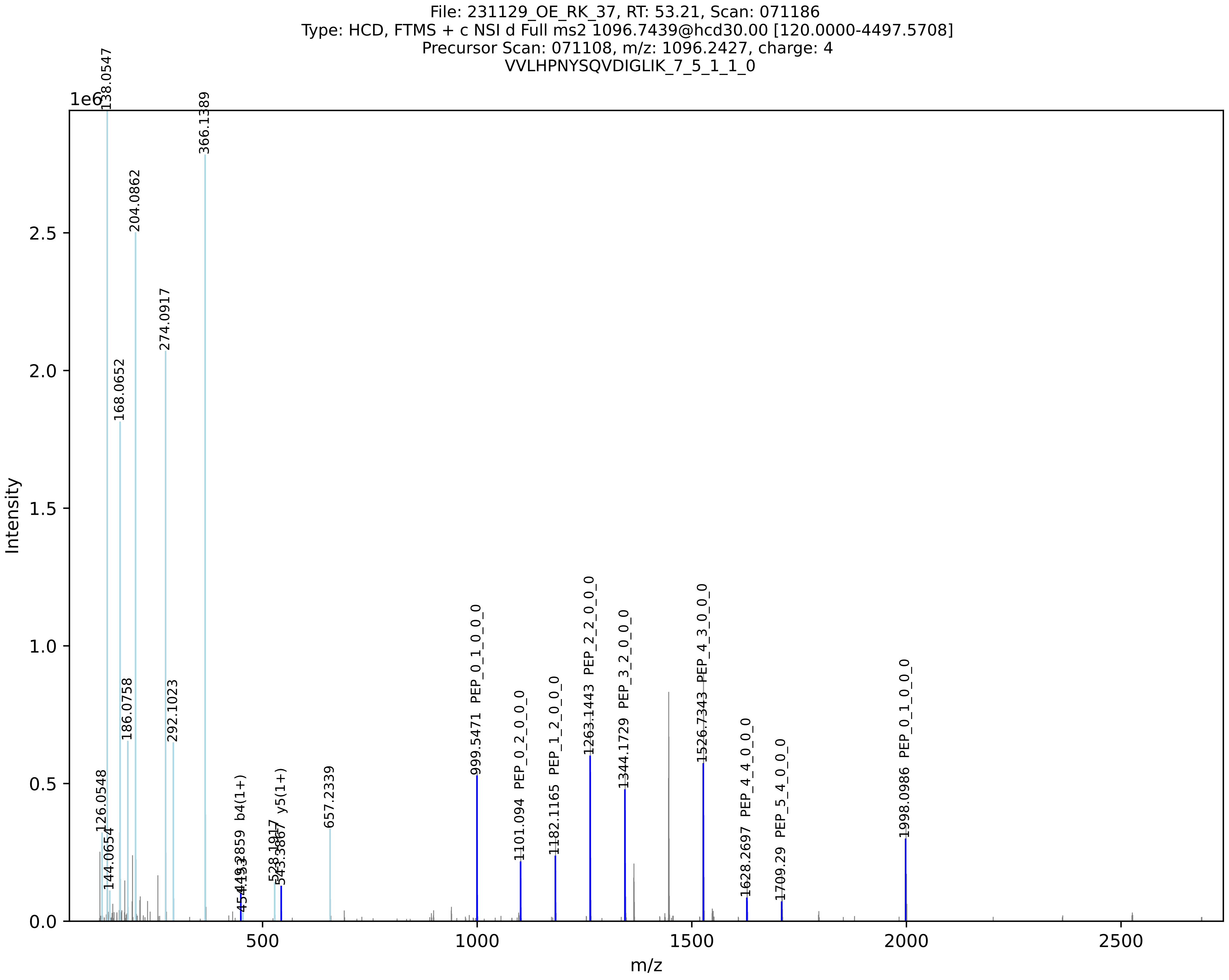
File: 231129_OE_RK_37, RT: 53.21, Scan: 071186
Type: HCD, FTMS + c NSI d Full ms2 1096.7439@hcd30.00 [120.0000-4497.5708]
Precursor Scan: 071108, m/z: 1096.2427, charge: 4
VVLHPNYSQVDIGLIK_7_5_1_1_0
1e6
Intensity
m/z
0.0
0.5
1.0
1.5
2.0
2.5
500
1000
1500
2000
2500
138.0547
366.1389
204.0862
274.0917
168.0652
186.0758
292.1023
126.0548
144.0654
b4(1+)
528.1917
543.3867 y5(1+)
657.2339
999.5471 PEP_0_1_0_0_0
1101.094 PEP_0_2_0_0_0
1182.1165 PEP_1_2_0_0_0
1263.1443 PEP_2_2_0_0_0
1344.1729 PEP_3_2_0_0_0
1526.7343 PEP_4_3_0_0_0
1628.2697 PEP_4_4_0_0_0
1709.29 PEP_5_4_0_0_0
1998.0986 PEP_0_1_0_0_0

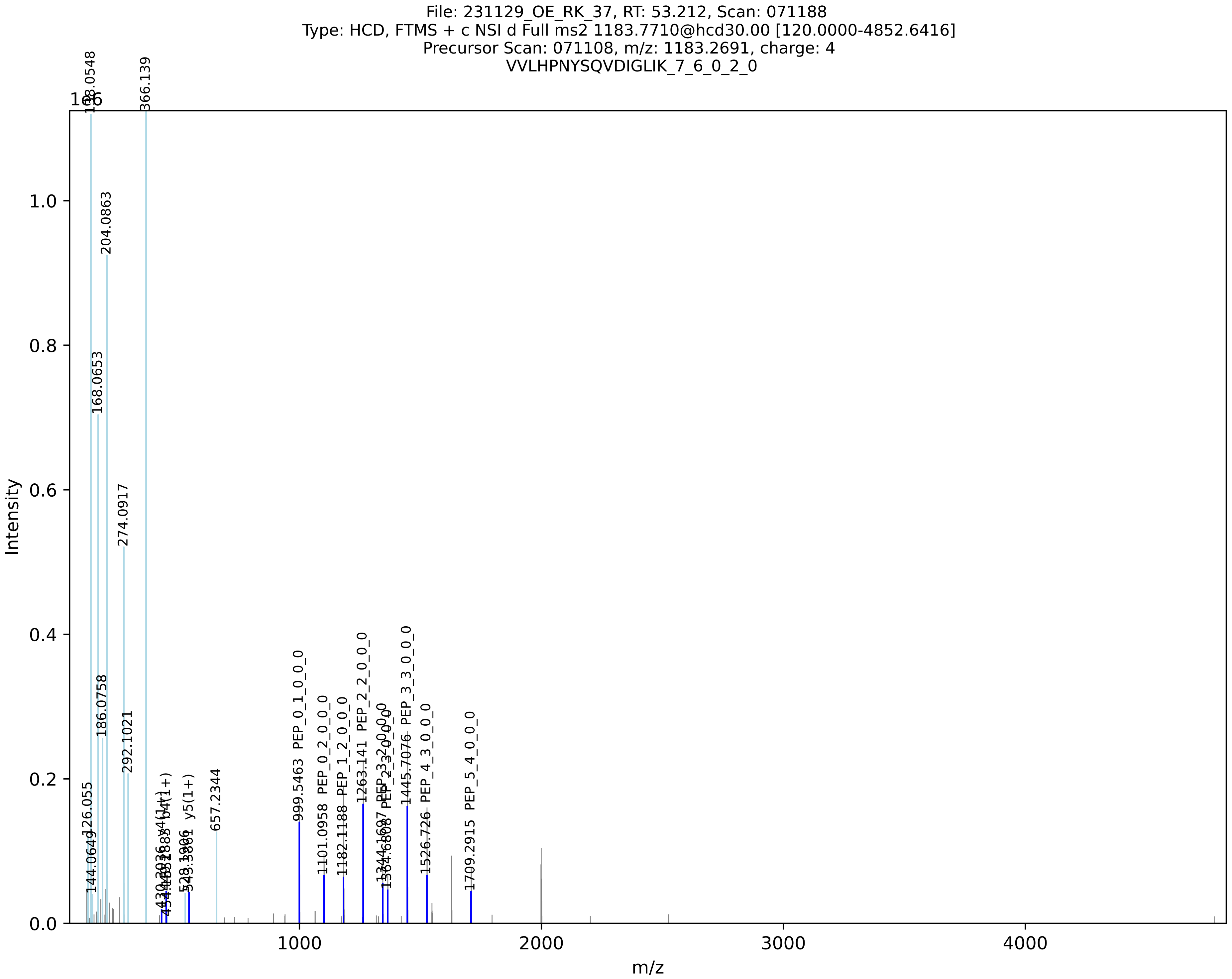

File: 231129_OE_RK_37, RT: 53.212, Scan: 071188
Type: HCD, FTMS + c NSI d Full ms2 1183.7710@hcd30.00 [120.0000-4852.6416]
Precursor Scan: 071108, m/z: 1183.2691, charge: 4
VVLHPNYSQVDIGLIK_7_6_0_2_0
Intensity
m/z
366.139
204.0863
168.0653
274.0917
186.0758
292.1021
126.055
144.0649
657.2344
y5(1+)
999.5463 PEP_0_1_0_0_0
1101.0958 PEP_0_2_0_0_0
1182.1188 PEP_1_2_0_0_0
1263.141 PEP_2_2_0_0_0
1445.7076 PEP_3_3_0_0_0
1526.726 PEP_4_3_0_0_0
1709.2915 PEP_5_4_0_0_0

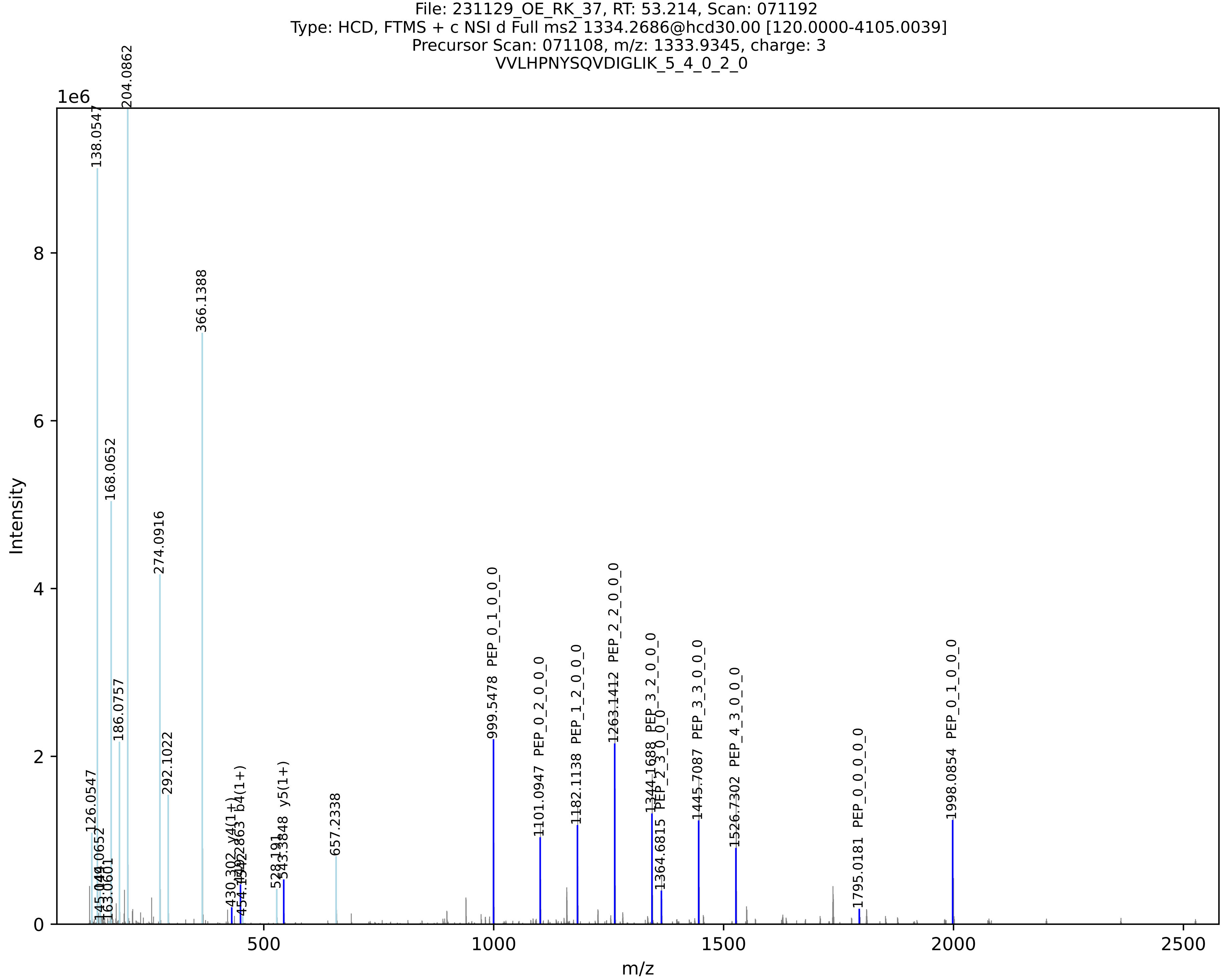
File: 231129_OE_RK_37, RT: 53.214, Scan: 071192
Type: HCD, FTMS + c NSI d Full ms2 1334.2686@hcd30.00 [120.0000-4105.0039]
Precursor Scan: 071108, m/z: 1333.9345, charge: 3
VVLHPNYSQVDIGLIK_5_4_0_2_0
1e6
Intensity
m/z
204.0862
138.0547
366.1388
168.0652
274.0916
186.0757
292.1022
126.0547
657.2338
543.3848 y5(1+)
999.5478 PEP_0_1_0_0_0
1101.0947 PEP_0_2_0_0_0
1182.1138 PEP_1_2_0_0_0
1263.1412 PEP_2_2_0_0_0
1344.1688 PEP_3_2_0_0_0
1364.6815 PEP_2_3_0_0_0
1445.7087 PEP_3_3_0_0_0
1526.7302 PEP_4_3_0_0_0
1795.0181 PEP_0_0_0_0_0
1998.0854 PEP_0_1_0_0_0

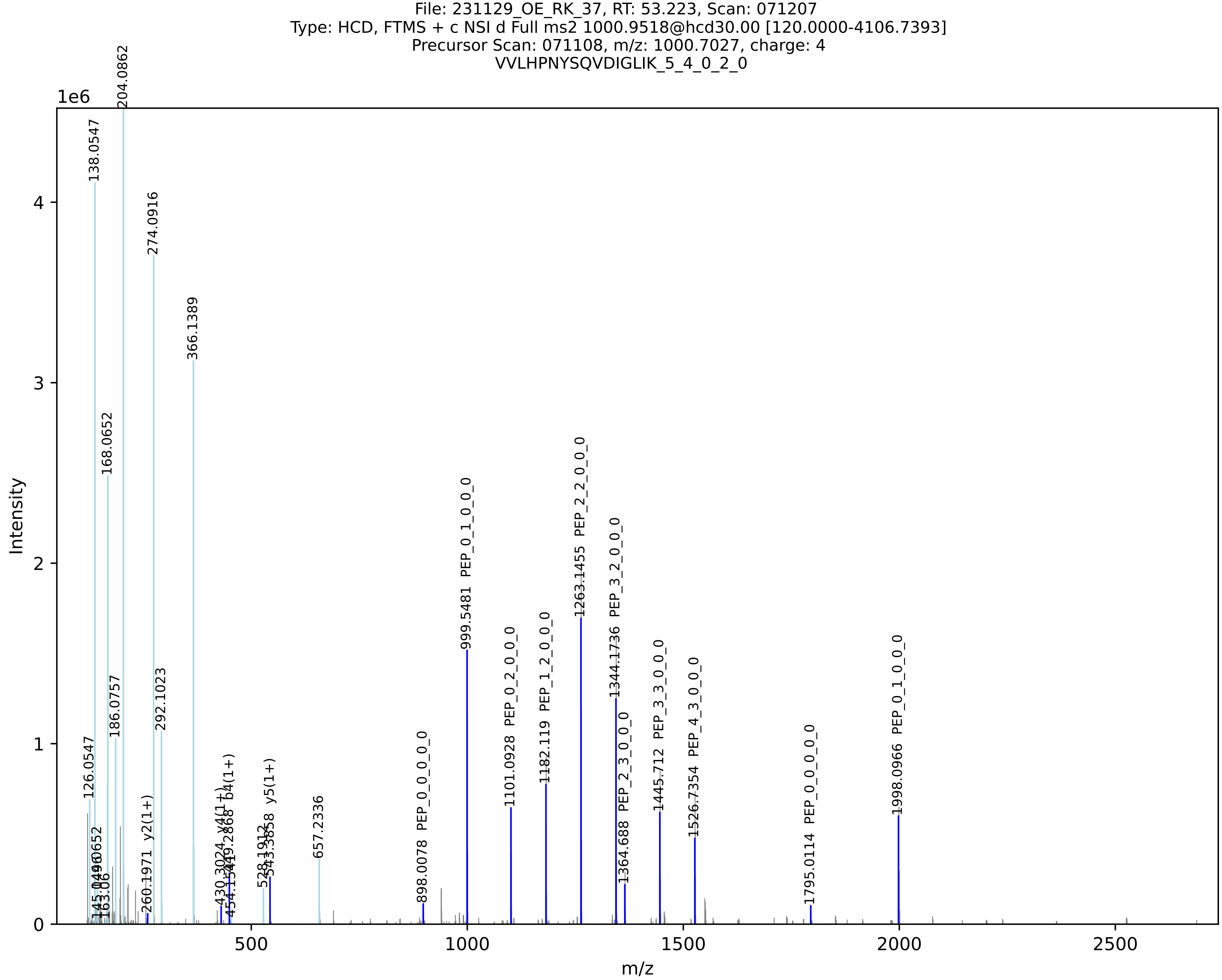
File: 231129_OE_RK_37, RT: 53.223, Scan: 071207
Type: HCD, FTMS + c NSI d Full ms2 1000.9518@hcd30.00 [120.0000-4106.7393]
Precursor Scan: 071108, m/z: 1000.7027, charge: 4
VVLHPNYSQVDIGLIK_5_4_0_2_0
1e6
Intensity
m/z
204.0862
138.0547
274.0916
366.1389
168.0652
186.0757
292.1023
126.0547
260.1971 y2(1+)
657.2336
898.0078 PEP_0_0_0_0_0
999.5481 PEP_0_1_0_0_0
1101.0928 PEP_0_2_0_0_0
1182.119 PEP_1_2_0_0_0
1263.1455 PEP_2_2_0_0_0
1344.1736 PEP_3_2_0_0_0
1364.688 PEP_2_3_0_0_0
1445.712 PEP_3_3_0_0_0
1526.7354 PEP_4_3_0_0_0
1795.0114 PEP_0_0_0_0_0
1998.0966 PEP_0_1_0_0_0
543.3858 y5(1+)
449.2868 b4(1+)
430.3024 y4(1+)
528.1912

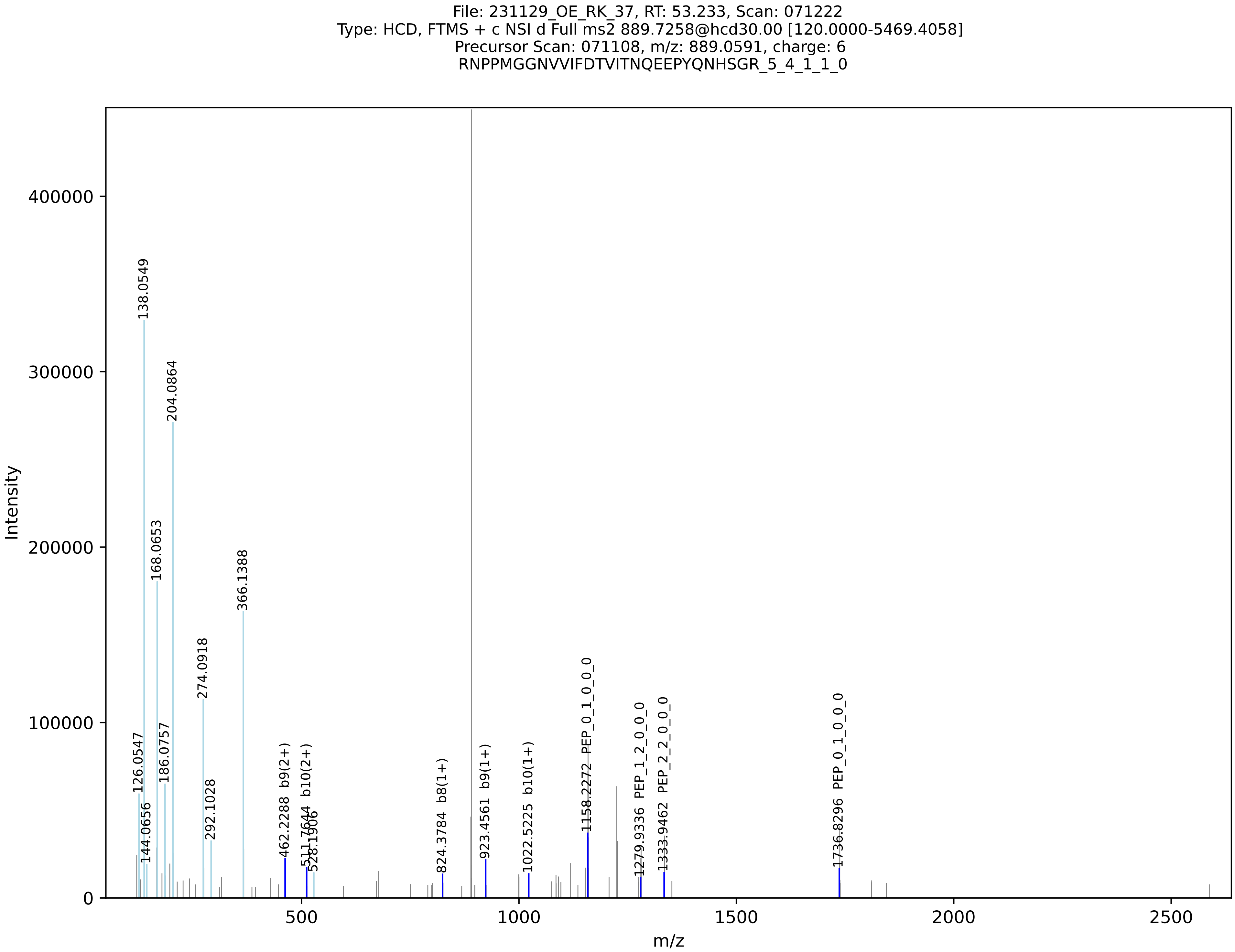
File: 231129_OE_RK_37, RT: 53.233, Scan: 071222
Type: HCD, FTMS + c NSI d Full ms2 889.7258@hcd30.00 [120.0000-5469.4058]
Precursor Scan: 071108, m/z: 889.0591, charge: 6
RNPPMGGNVVIFDTVITNQEEPYQNHSGR_5_4_1_1_0
Intensity
m/z
138.0549
204.0864
168.0653
366.1388
274.0918
126.0547
186.0757
144.0656
292.1028
462.2288 b9(2+)
511.7644 b10(2+)
528.1906
824.3784 b8(1+)
923.4561 b9(1+)
1022.5225 b10(1+)
1158.2272 PEP_0_1_0_0_0
1279.9336 PEP_1_2_0_0_0
1333.9462 PEP_2_2_0_0_0
1736.8296 PEP_0_1_0_0_0

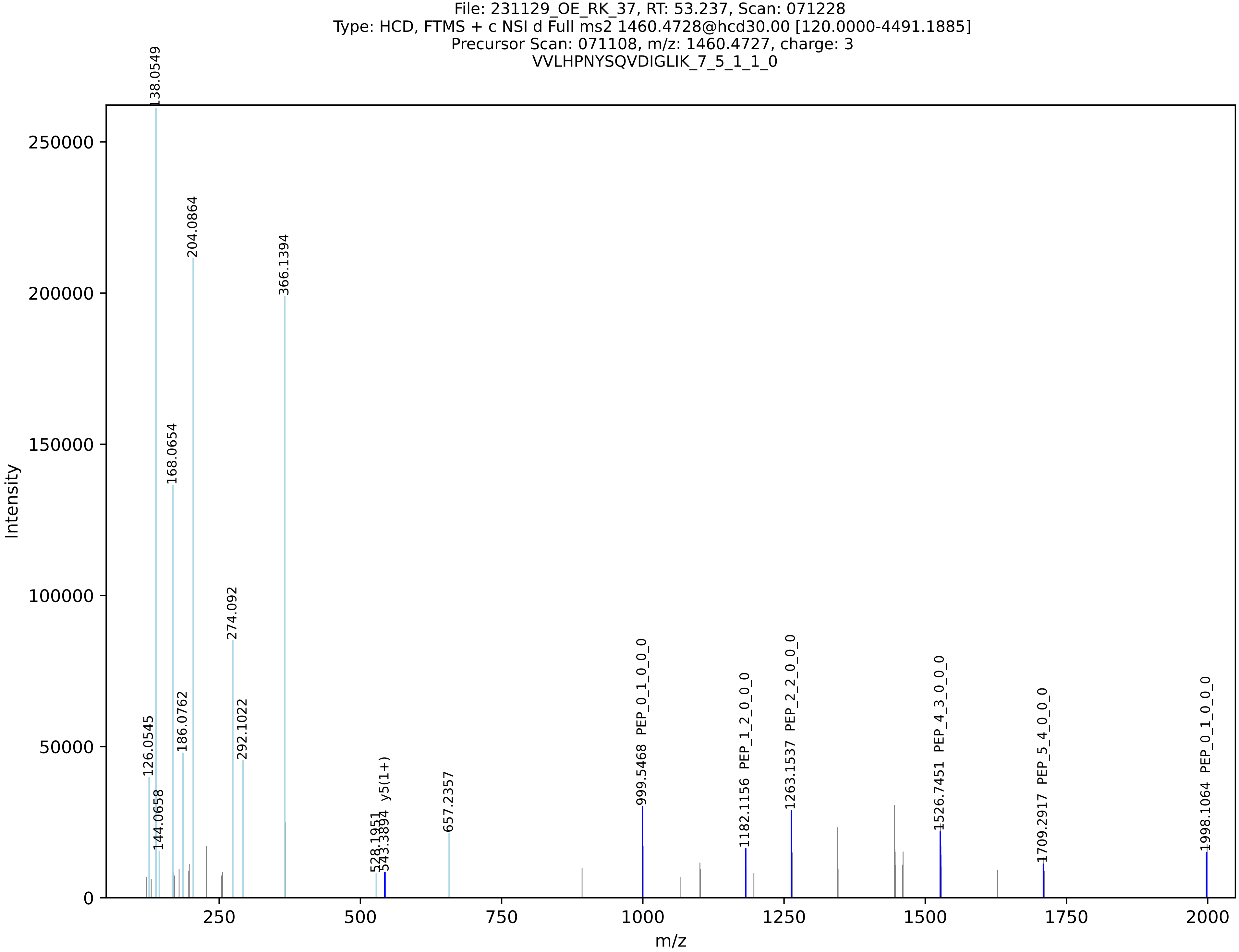
File: 231129_OE_RK_37, RT: 53.237, Scan: 071228
Type: HCD, FTMS + c NSI d Full ms2 1460.4728@hcd30.00 [120.0000-4491.1885]
Precursor Scan: 071108, m/z: 1460.4727, charge: 3
VVLHPNYSQVDIGLIK_7_5_1_1_0
Intensity
m/z
138.0549
204.0864
366.1394
168.0654
274.092
126.0545
186.0762
292.1022
144.0658
528.1951
543.3894 y5(1+)
657.2357
999.5468 PEP_0_1_0_0_0
1182.1156 PEP_1_2_0_0_0
1263.1537 PEP_2_2_0_0_0
1526.7451 PEP_4_3_0_0_0
1709.2917 PEP_5_4_0_0_0
1998.1064 PEP_0_1_0_0_0

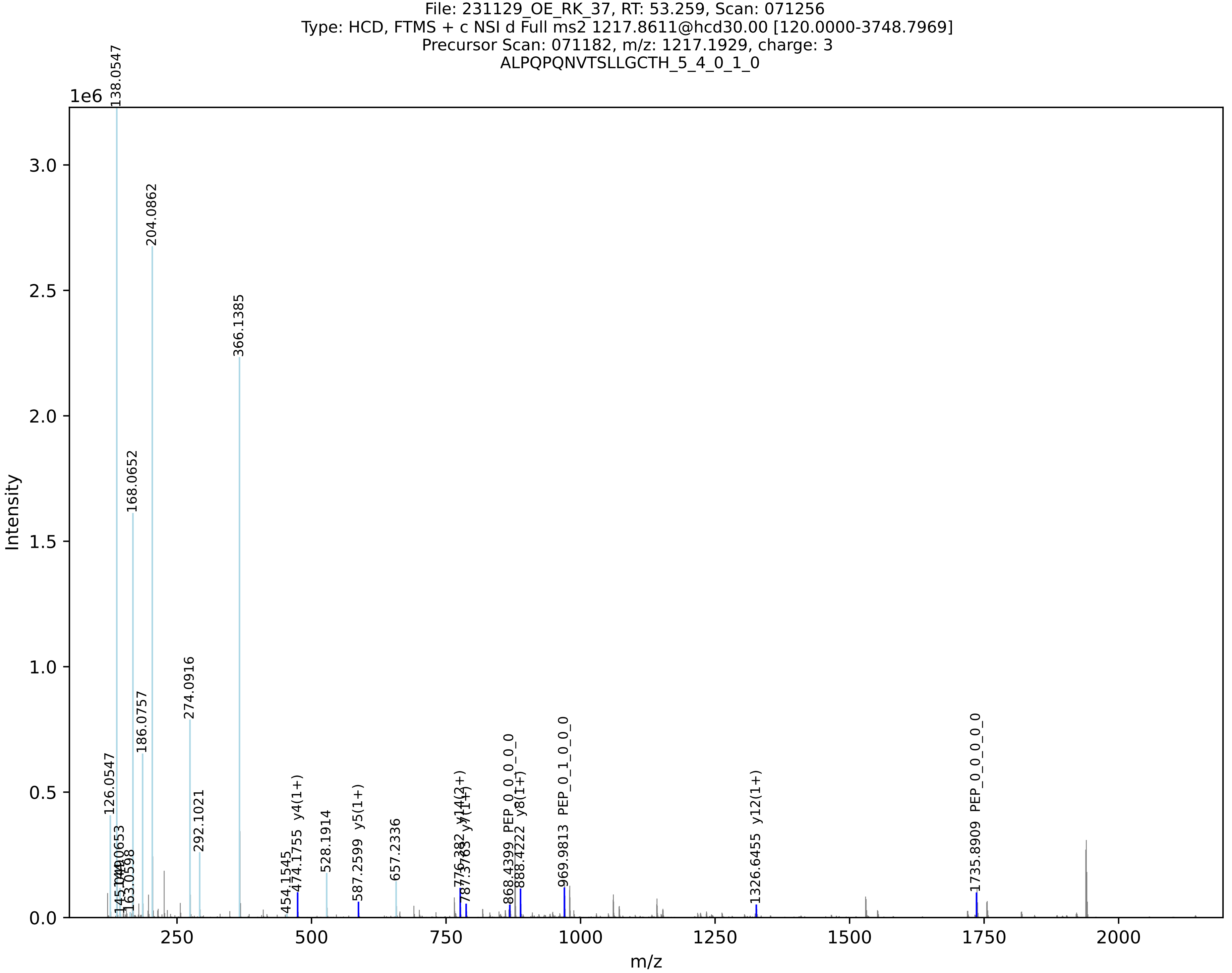

File: 231129_OE_RK_37, RT: 53.259, Scan: 071256
Type: HCD, FTMS + c NSI d Full ms2 1217.8611@hcd30.00 [120.0000-3748.7969]
Precursor Scan: 071182, m/z: 1217.1929, charge: 3
ALPQPQNVTSLLGCTH_5_4_0_1_0
1e6
Intensity
m/z
138.0547
204.0862
366.1385
168.0652
274.0916
186.0757
126.0547
292.1021
528.1914
657.2336
474.1755 y4(1+)
587.2599 y5(1+)
454.1545
776.382 y14(2+)
787.3763 y7(1+)
868.4399 PEP_0_0_0_0_0
888.4222 y8(1+)
969.9813 PEP_0_1_0_0_0
1326.6455 y12(1+)
1735.8909 PEP_0_0_0_0_0

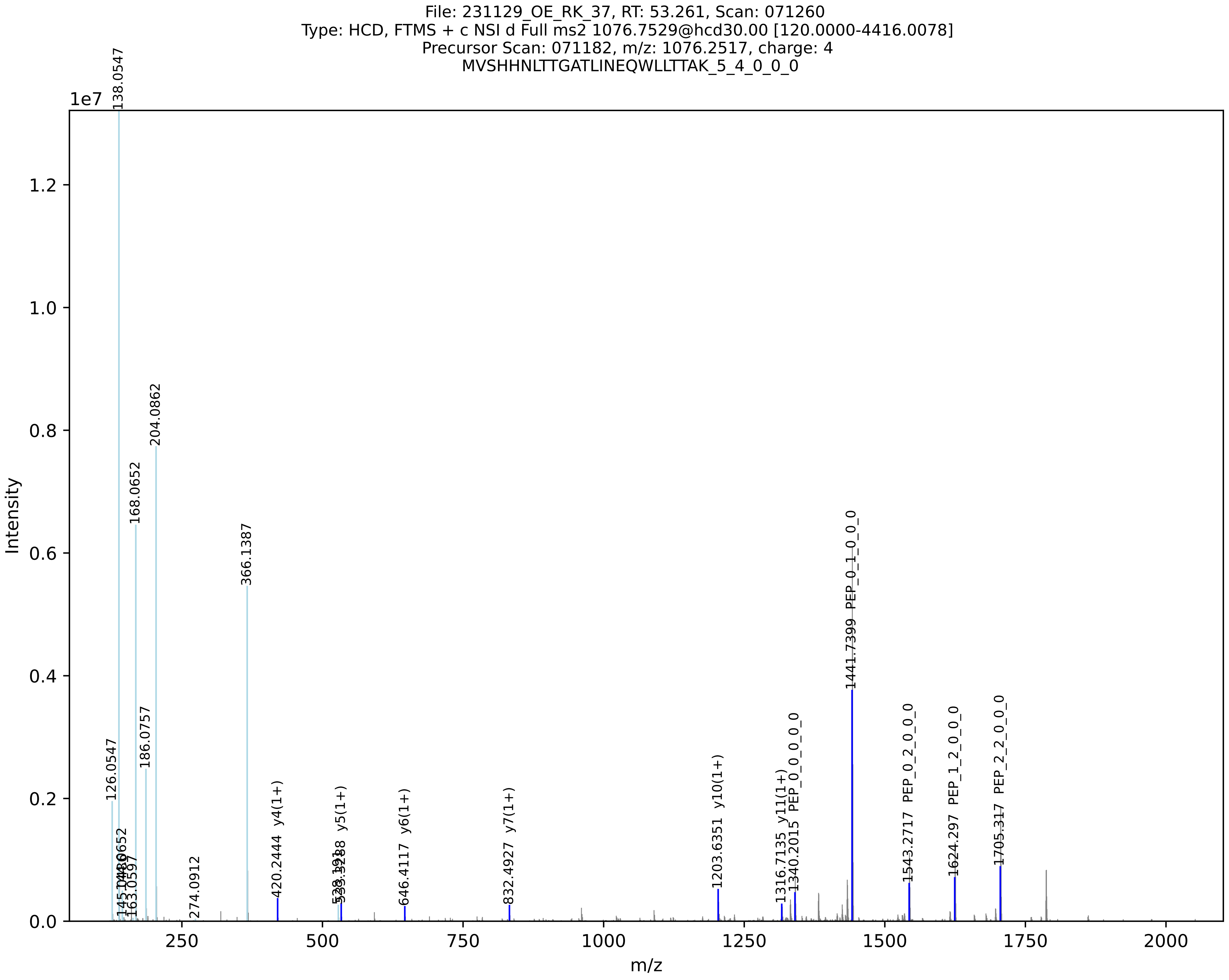
File: 231129_OE_RK_37, RT: 53.261, Scan: 071260
Type: HCD, FTMS + c NSI d Full ms2 1076.7529@hcd30.00 [120.0000-4416.0078]
Precursor Scan: 071182, m/z: 1076.2517, charge: 4
MVSHHNLTTGATLINEQWLLTTAK_5_4_0_0_0
1e7
Intensity
m/z
138.0547
204.0862
168.0652
366.1387
186.0757
126.0547
163.0597
274.0912
420.2444 y4(1+)
533.3288 y5(1+)
646.4117 y6(1+)
832.4927 y7(1+)
1203.6351 y10(1+)
1316.7135 y11(1+)
1340.2015 PEP_0_0_0_0_0
1441.7399 PEP_0_1_0_0_0
1543.2717 PEP_0_2_0_0_0
1624.297 PEP_1_2_0_0_0
1705.317 PEP_2_2_0_0_0
0.0
0.2
0.4
0.6
0.8
1.0
1.2
250
500
750
1000
1250
1500
1750
2000

File: 231129_OE_RK_37, RT: 53.277, Scan: 071285
Type: HCD, FTMS + c NSI d Full ms2 1435.6719@hcd30.00 [120.0000-4415.2983]
Precursor Scan: 071182, m/z: 1434.6666, charge: 3
MVSHHNLTTGATLINEQWLLTTAK_5_4_0_0_0

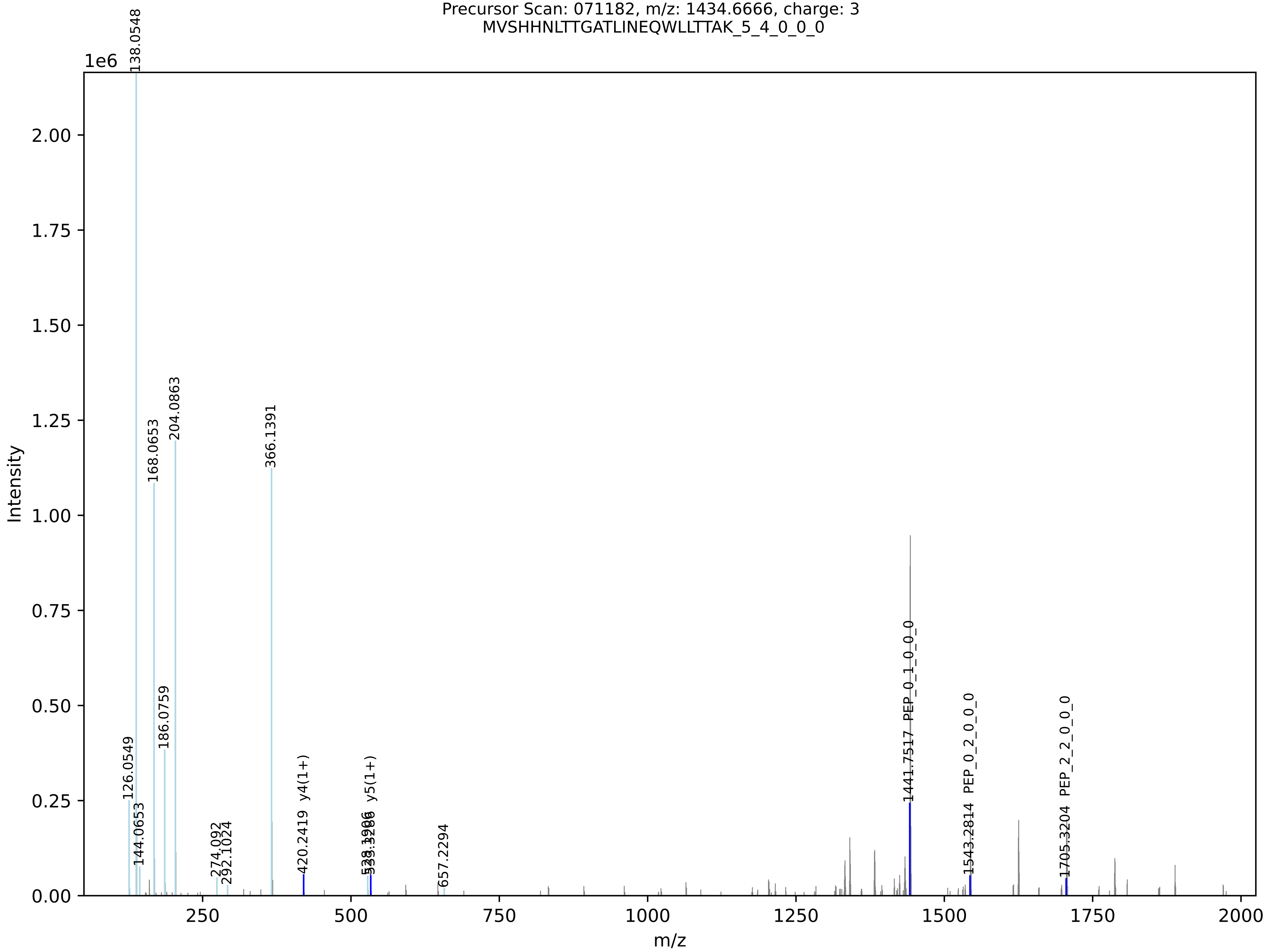

File: 231129_OE_RK_37, RT: 53.332, Scan: 071367
Type: HCD, FTMS + c NSI d Full ms2 1237.2583@hcd30.00 [120.0000-3808.1523]
Precursor Scan: 071255, m/z: 1237.2583, charge: 3
VVLHPNYSQVDIGLIK_5_4_0_1_0

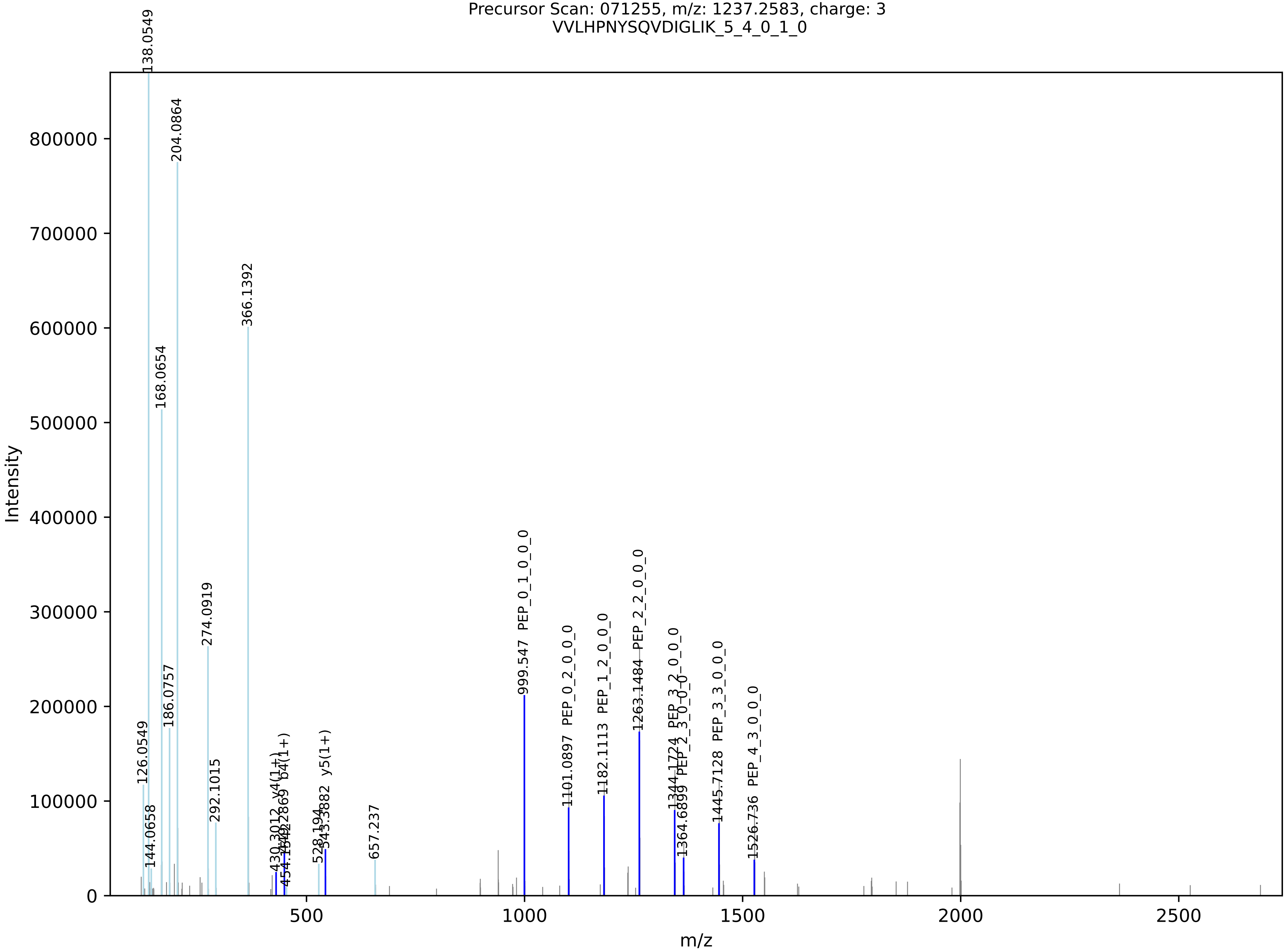

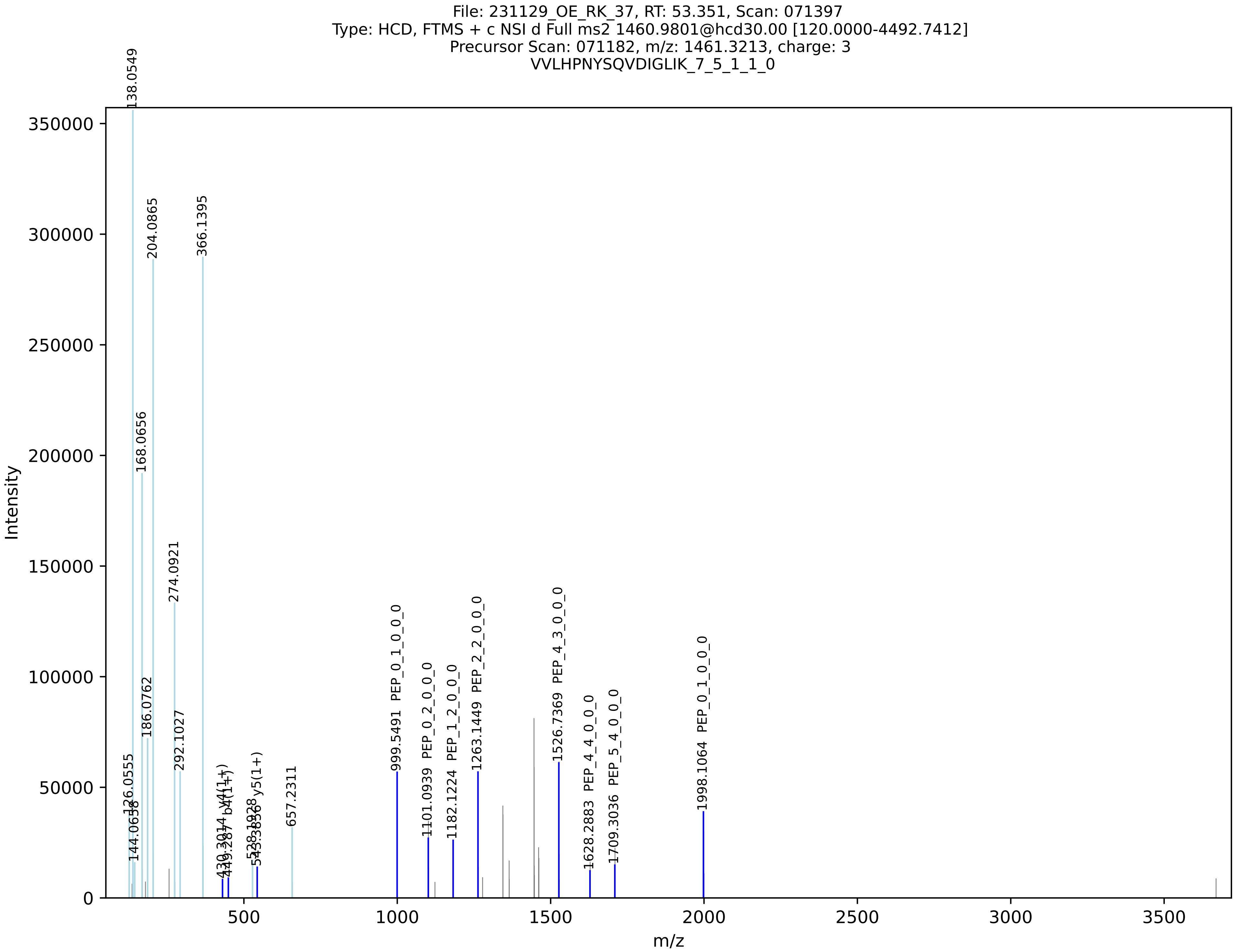

File: 231129_OE_RK_37, RT: 53.351, Scan: 071397
Type: HCD, FTMS + c NSI d Full ms2 1460.9801@hcd30.00 [120.0000-4492.7412]
Precursor Scan: 071182, m/z: 1461.3213, charge: 3
VVLHPNYSQVDIGLIK_7_5_1_1_0
Intensity
m/z
138.0549
204.0865
366.1395
168.0656
274.0921
186.0762
292.1027
126.0555
144.0658
657.2311
999.5491 PEP_0_1_0_0_0
1101.0939 PEP_0_2_0_0_0
1182.1224 PEP_1_2_0_0_0
1263.1449 PEP_2_2_0_0_0
1526.7369 PEP_4_3_0_0_0
1628.2883 PEP_4_4_0_0_0
1709.3036 PEP_5_4_0_0_0
1998.1064 PEP_0_1_0_0_0

File: 231129_OE_RK_37, RT: 53.362, Scan: 071404
Type: HCD, FTMS + c NSI d Full ms2 1334.6012@hcd30.00 [120.0000-4106.0215]
Precursor Scan: 071329, m/z: 1333.9334, charge: 3
VVLHPNYSQVDIGLIK_5_4_0_2_0

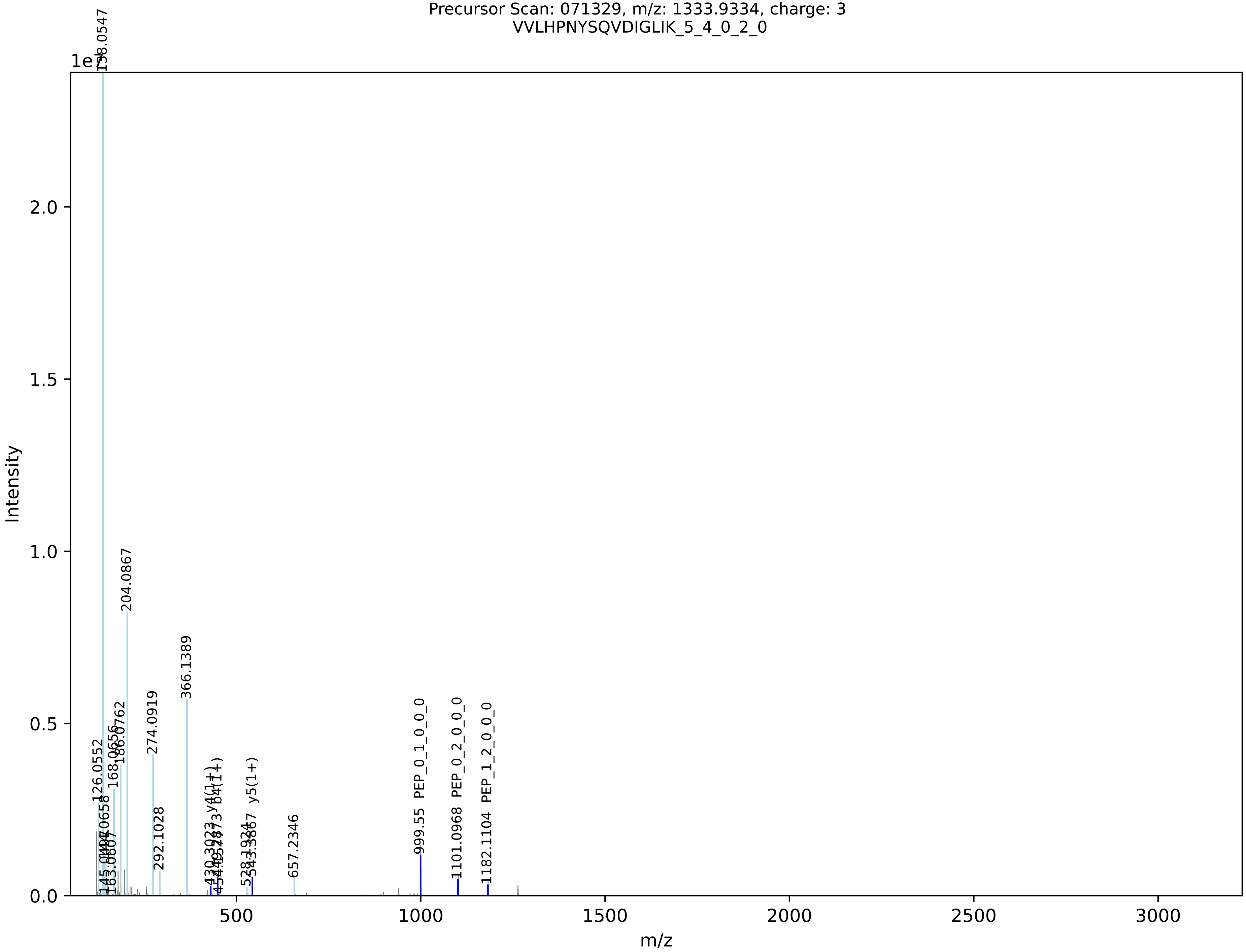

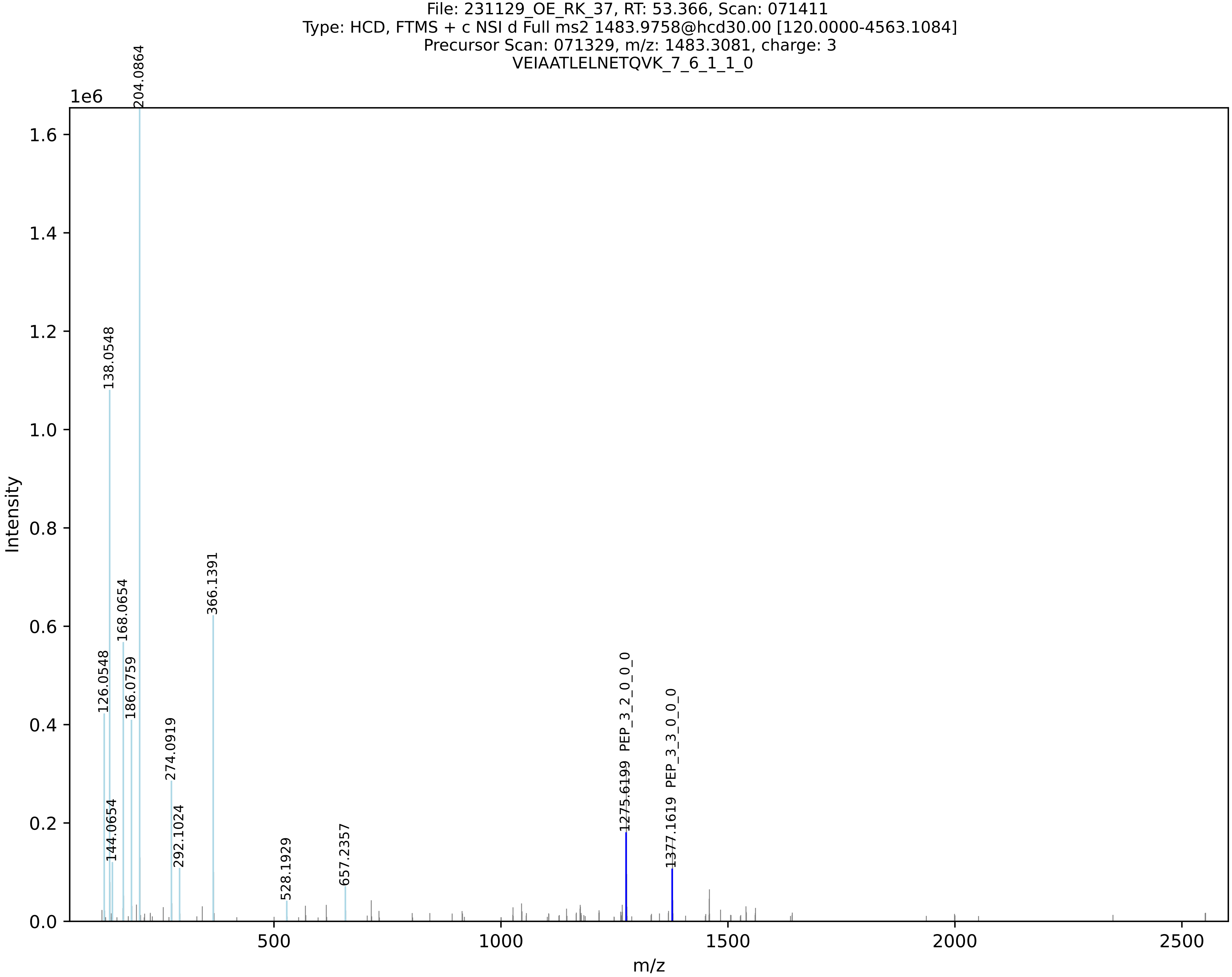
File: 231129_OE_RK_37, RT: 53.366, Scan: 071411
Type: HCD, FTMS + c NSI d Full ms2 1483.9758@hcd30.00 [120.0000-4563.1084]
Precursor Scan: 071329, m/z: 1483.3081, charge: 3
VEIAATLELNETQVK_7_6_1_1_0
1e6
Intensity
m/z
204.0864
138.0548
168.0654
126.0548
186.0759
366.1391
274.0919
144.0654
292.1024
528.1929
657.2357
1275.6199 PEP_3_2_0_0_0
1377.1619 PEP_3_3_0_0_0

File: 231129_OE_RK_37, RT: 53.378, Scan: 071431
Type: HCD, FTMS + c NSI d Full ms2 1005.4551@hcd30.00 [120.0000-4125.1128]
Precursor Scan: 071329, m/z: 1004.9556, charge: 4
VVLHPNYSQVDIGLIK_6_4_1_1_0

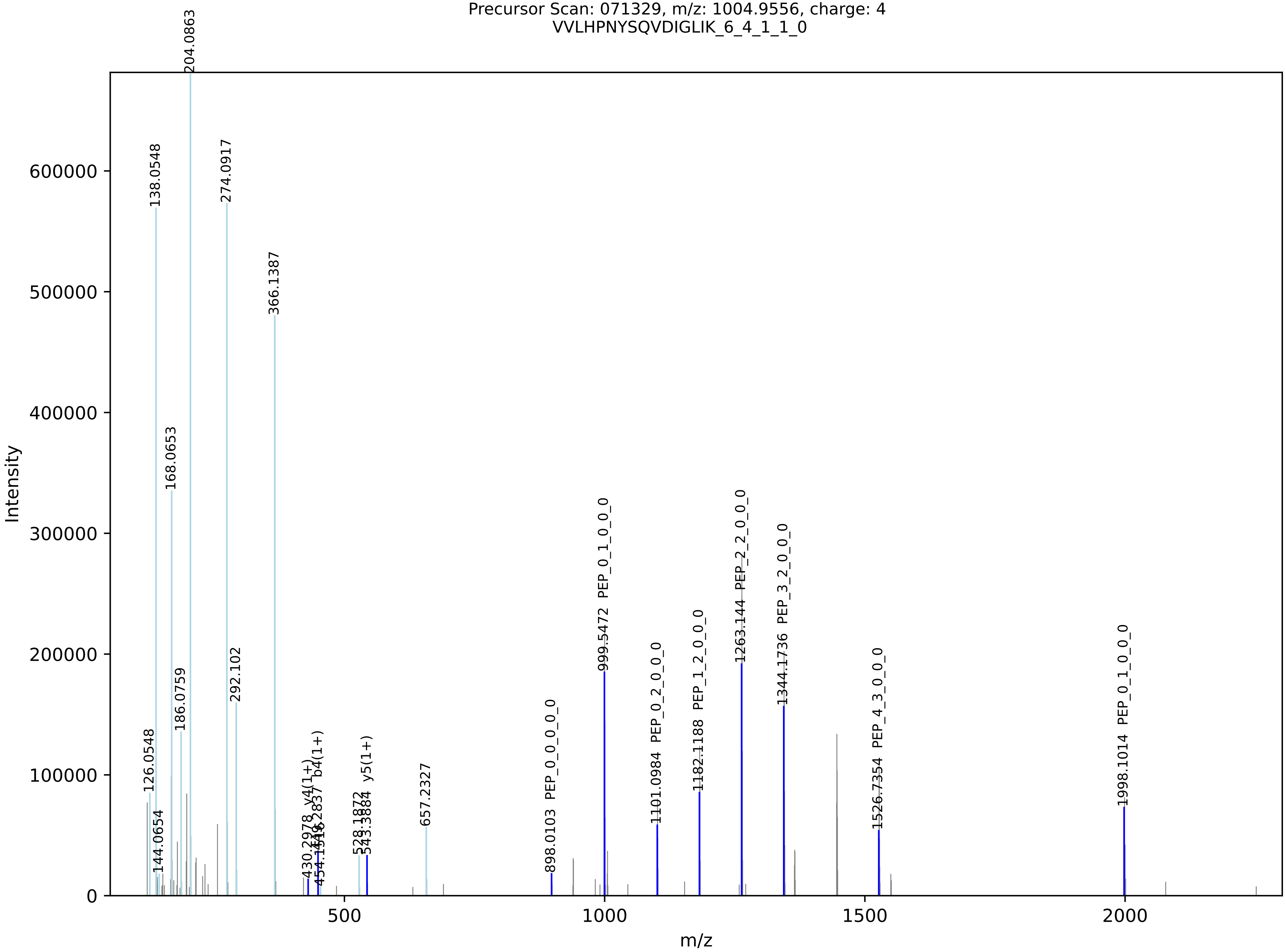

File: 231129_OE_RK_37, RT: 53.41, Scan: 071474
Type: HCD, FTMS + c NSI d Full ms2 1001.2034@hcd30.00 [120.0000-4107.7656]
Precursor Scan: 071403, m/z: 1000.7018, charge: 4
VVLHPNYSQVDIGLIK_5_4_0_2_0

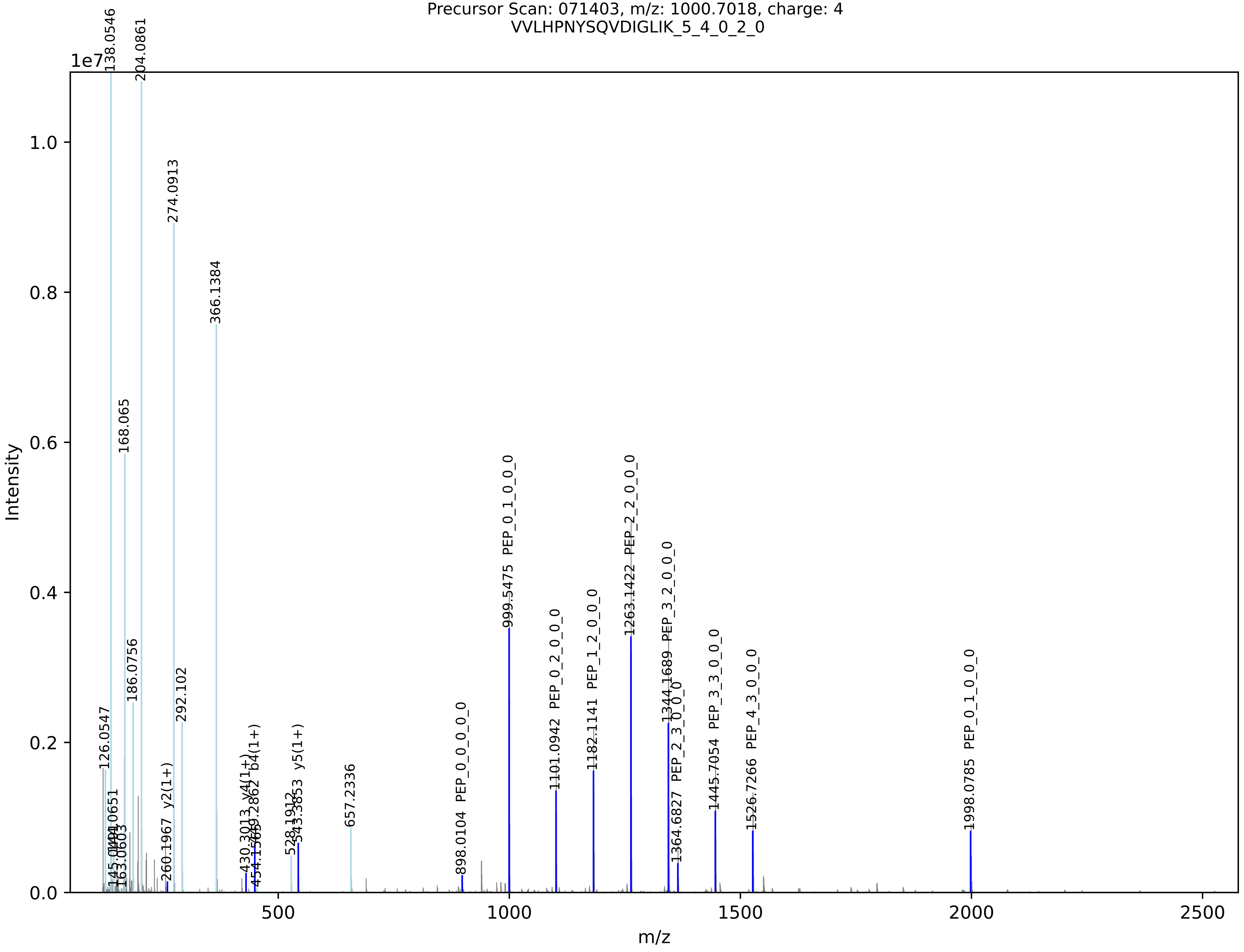

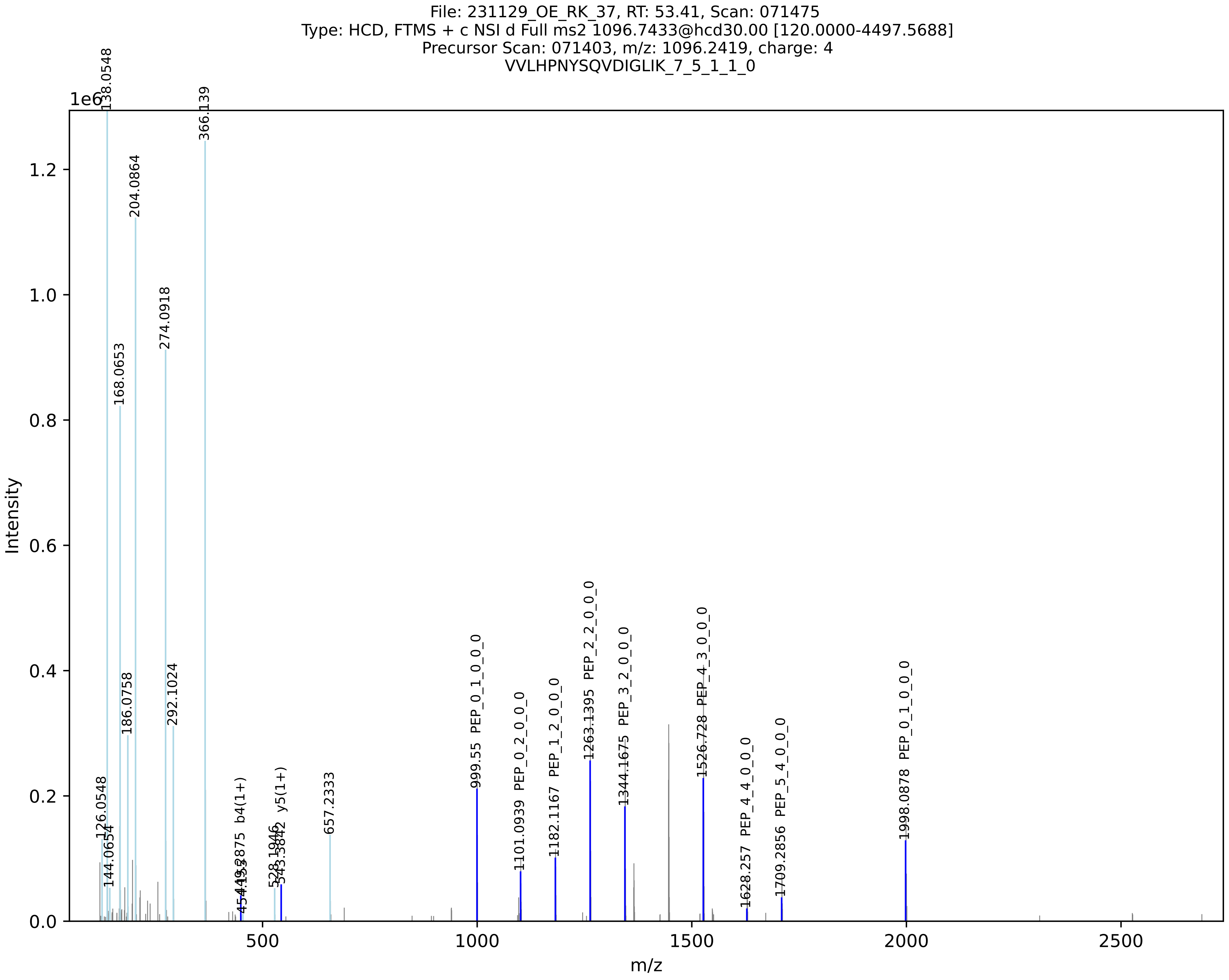
File: 231129_OE_RK_37, RT: 53.41, Scan: 071475
Type: HCD, FTMS + c NSI d Full ms2 1096.7433@hcd30.00 [120.0000-4497.5688]
Precursor Scan: 071403, m/z: 1096.2419, charge: 4
VVLHPNYSQVDIGLIK_7_5_1_1_0
1e6
Intensity
m/z
0.0
0.2
0.4
0.6
0.8
1.0
1.2
500
1000
1500
2000
2500
138.0548
366.139
204.0864
274.0918
168.0653
186.0758
292.1024
126.0548
144.0654
449.2875 b4(1+)
528.1946
543.3842 y5(1+)
657.2333
999.55 PEP_0_1_0_0_0
1101.0939 PEP_0_2_0_0_0
1182.1167 PEP_1_2_0_0_0
1263.1395 PEP_2_2_0_0_0
1344.1675 PEP_3_2_0_0_0
1526.728 PEP_4_3_0_0_0
1628.257 PEP_4_4_0_0_0
1709.2856 PEP_5_4_0_0_0
1998.0878 PEP_0_1_0_0_0

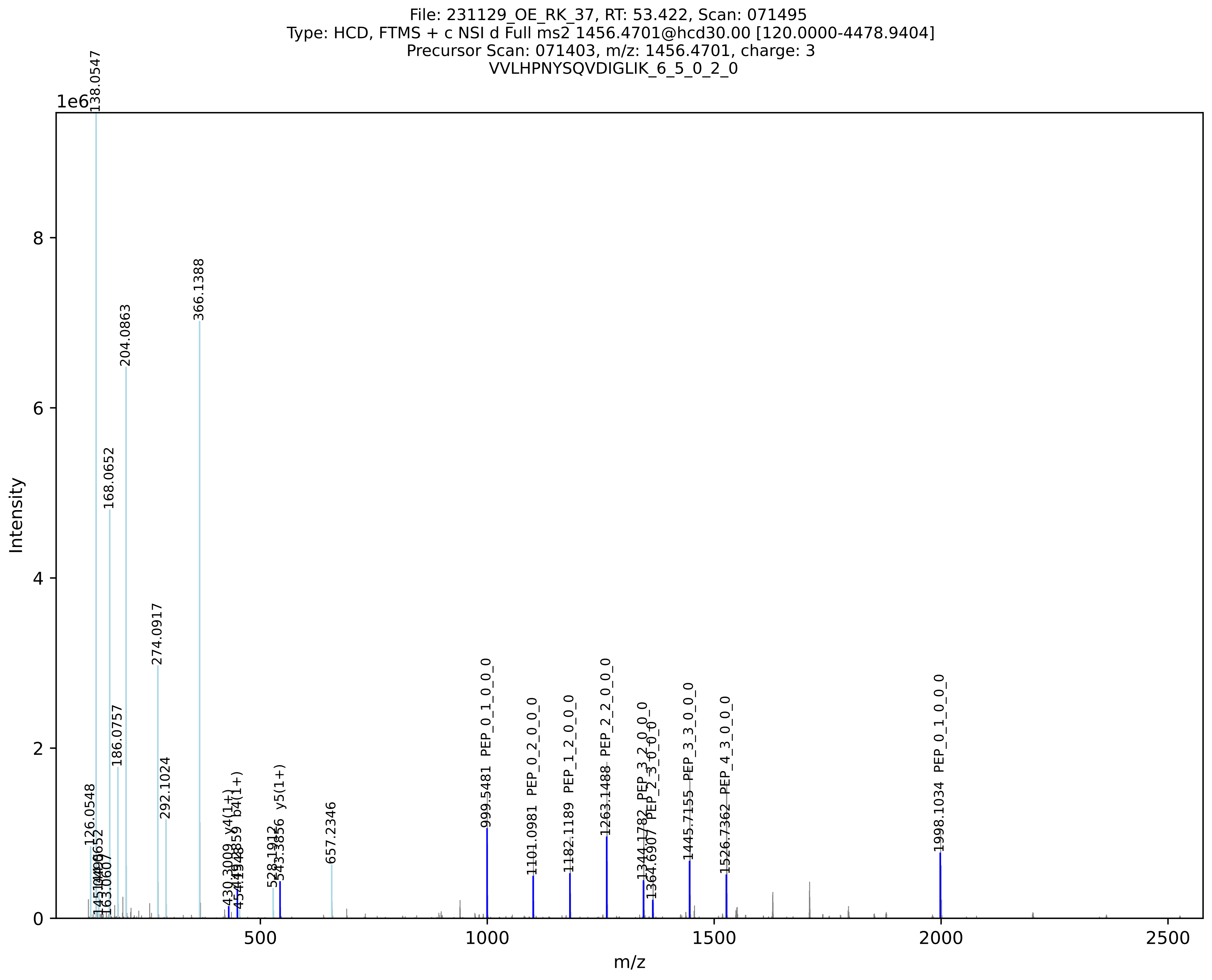
File: 231129_OE_RK_37, RT: 53.422, Scan: 071495
Type: HCD, FTMS + c NSI d Full ms2 1456.4701@hcd30.00 [120.0000-4478.9404]
Precursor Scan: 071403, m/z: 1456.4701, charge: 3
VVLHPNYSQVDIGLIK_6_5_0_2_0
1e6
Intensity
m/z
138.0547
366.1388
204.0863
168.0652
274.0917
186.0757
292.1024
126.0548
163.0607
657.2346
528.1912
543.3856 y5(1+)
430.3009 y4(1+)
999.5481 PEP_0_1_0_0_0
1101.0981 PEP_0_2_0_0_0
1182.1189 PEP_1_2_0_0_0
1263.1488 PEP_2_2_0_0_0
1344.1782 PEP_3_2_0_0_0
1364.6907 PEP_2_3_0_0_0
1445.7155 PEP_3_3_0_0_0
1526.7362 PEP_4_3_0_0_0
1998.1034 PEP_0_1_0_0_0

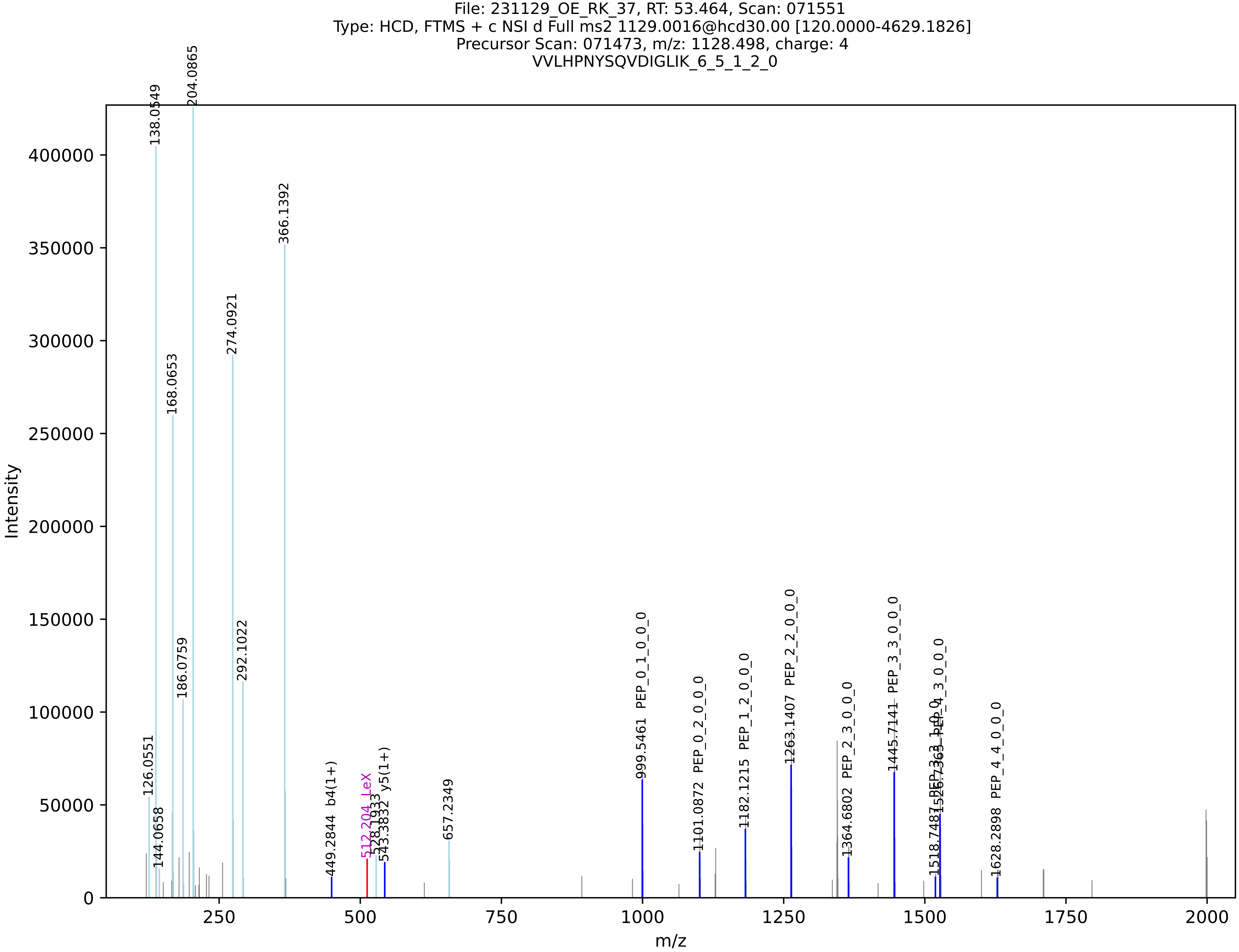
File: 231129_OE_RK_37, RT: 53.464, Scan: 071551
Type: HCD, FTMS + c NSI d Full ms2 1129.0016@hcd30.00 [120.0000-4629.1826]
Precursor Scan: 071473, m/z: 1128.498, charge: 4
VVLHPNYSQVDIGLIK_6_5_1_2_0
Intensity
m/z
204.0865
138.0549
366.1392
274.0921
168.0653
186.0759
292.1022
126.0551
144.0658
449.2844 b4(1+)
512.204_LeX
528.1933
543.3832 y5(1+)
657.2349
999.5461 PEP_0_1_0_0_0
1101.0872 PEP_0_2_0_0_0
1182.1215 PEP_1_2_0_0_0
1263.1407 PEP_2_2_0_0_0
1364.6802 PEP_2_3_0_0_0
1445.7141 PEP_3_3_0_0_0
1518.7487 PEP_3_3_1_0_0
1526.7365 PEP_4_3_0_0_0
1628.2898 PEP_4_4_0_0_0

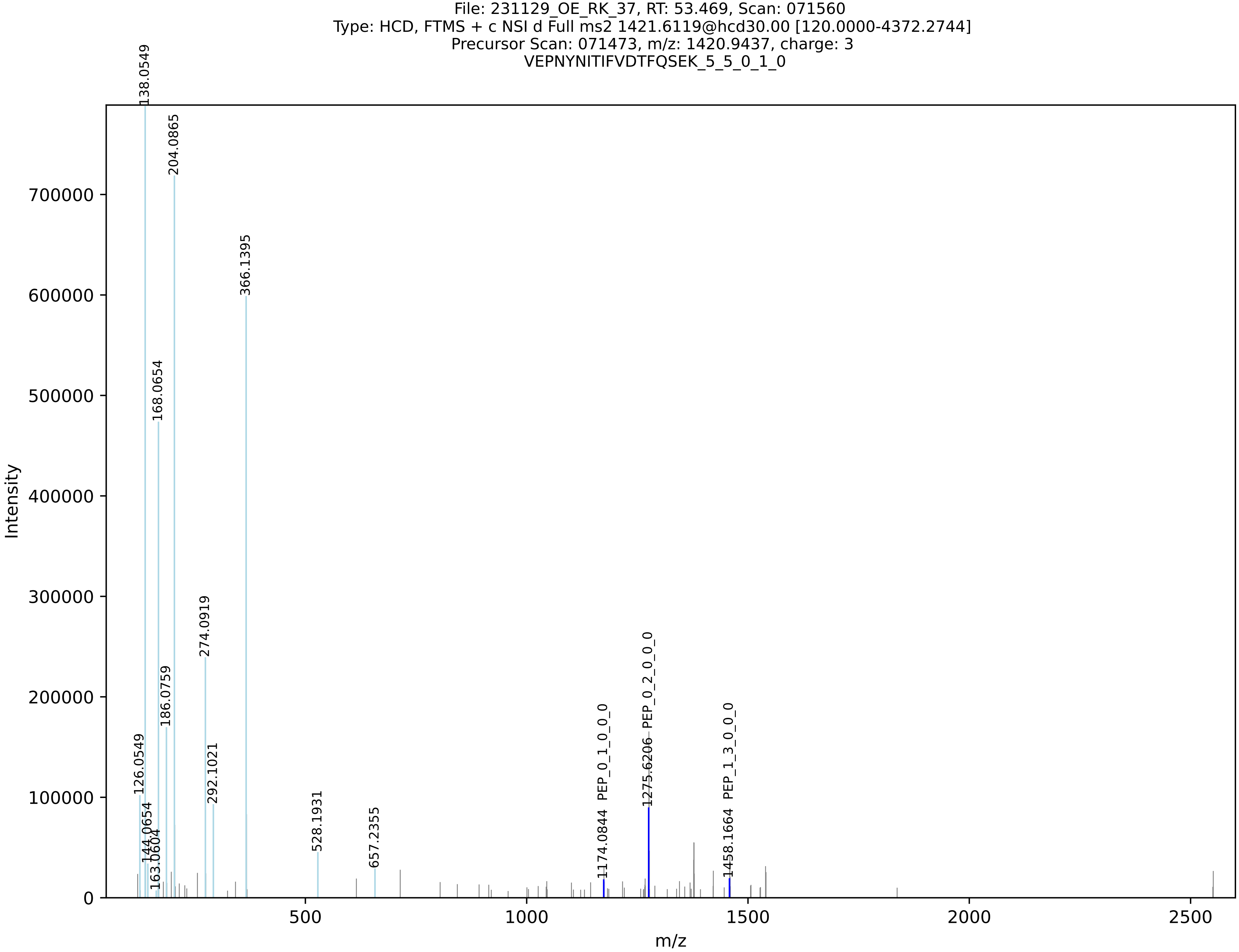
File: 231129_OE_RK_37, RT: 53.469, Scan: 071560
Type: HCD, FTMS + c NSI d Full ms2 1421.6119@hcd30.00 [120.0000-4372.2744]
Precursor Scan: 071473, m/z: 1420.9437, charge: 3
VEPNYNITIFVDTFQSEK_5_5_0_1_0
138.0549
204.0865
366.1395
168.0654
274.0919
186.0759
126.0549
292.1021
144.0654
163.0604
528.1931
657.2355
PEP_0_1_0_0_0
1174.0844
PEP_0_2_0_0_0
1275.6206
PEP_1_3_0_0_0
1458.1664
Intensity
m/z
0
100000
200000
300000
400000
500000
600000
700000
500
1000
1500
2000
2500

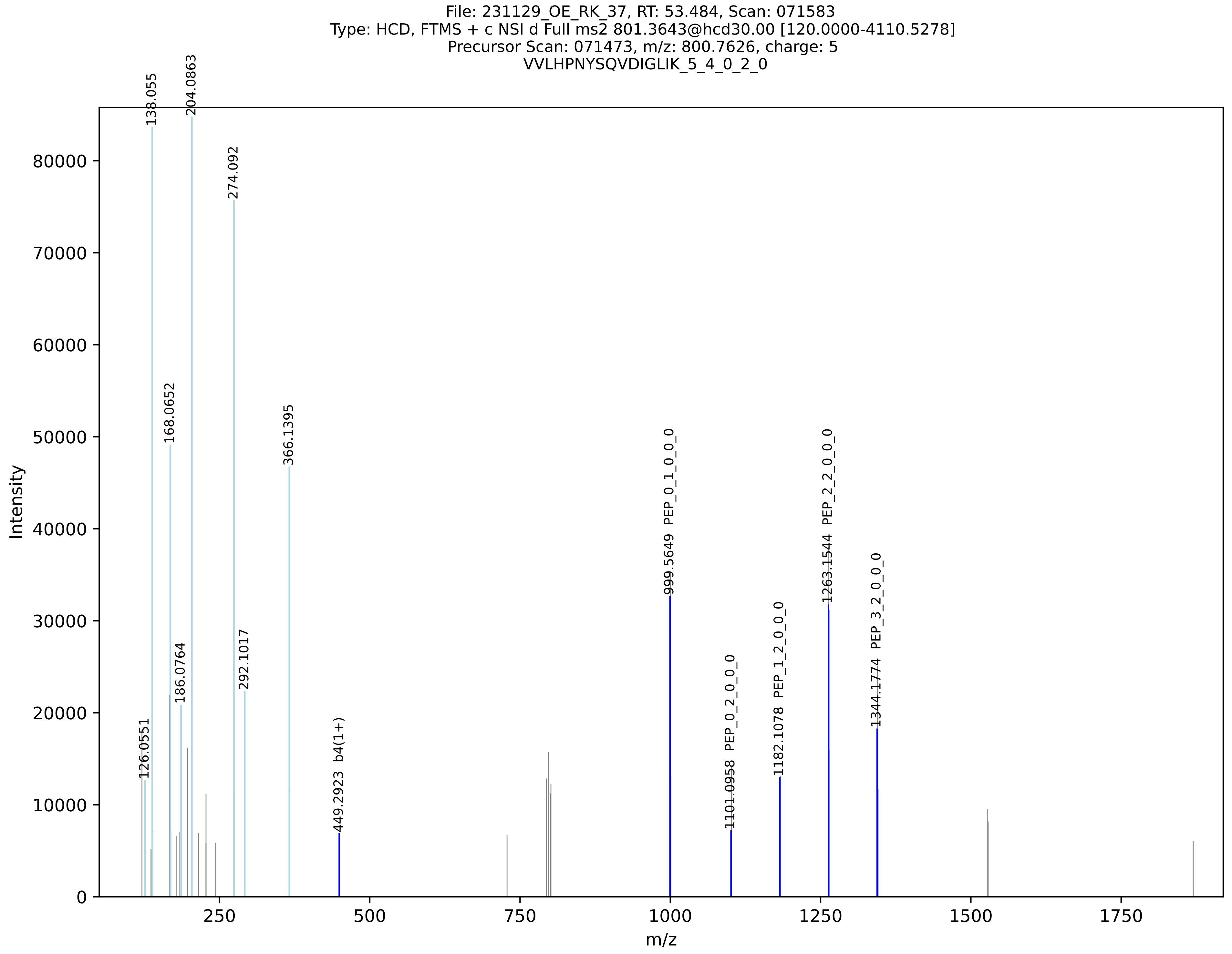
File: 231129_OE_RK_37, RT: 53.484, Scan: 071583
Type: HCD, FTMS + c NSI d Full ms2 801.3643@hcd30.00 [120.0000-4110.5278]
Precursor Scan: 071473, m/z: 800.7626, charge: 5
VVLHPNYSQVDIGLIK_5_4_0_2_0
Intensity
m/z
138.055
204.0863
274.092
168.0652
366.1395
186.0764
292.1017
126.0551
449.2923 b4(1+)
999.5649 PEP_0_1_0_0_0
1101.0958 PEP_0_2_0_0_0
1182.1078 PEP_1_2_0_0_0
1263.1544 PEP_2_2_0_0_0
1344.1774 PEP_3_2_0_0_0

File: 231129_OE_RK_37, RT: 53.511, Scan: 071620
Type: HCD, FTMS + c NSI d Full ms2 1092.4861@hcd30.00 [120.0000-4480.1992]
Precursor Scan: 071547, m/z: 1091.9849, charge: 4
VVLHPNYSQVDIGLIK_6_5_0_2_0

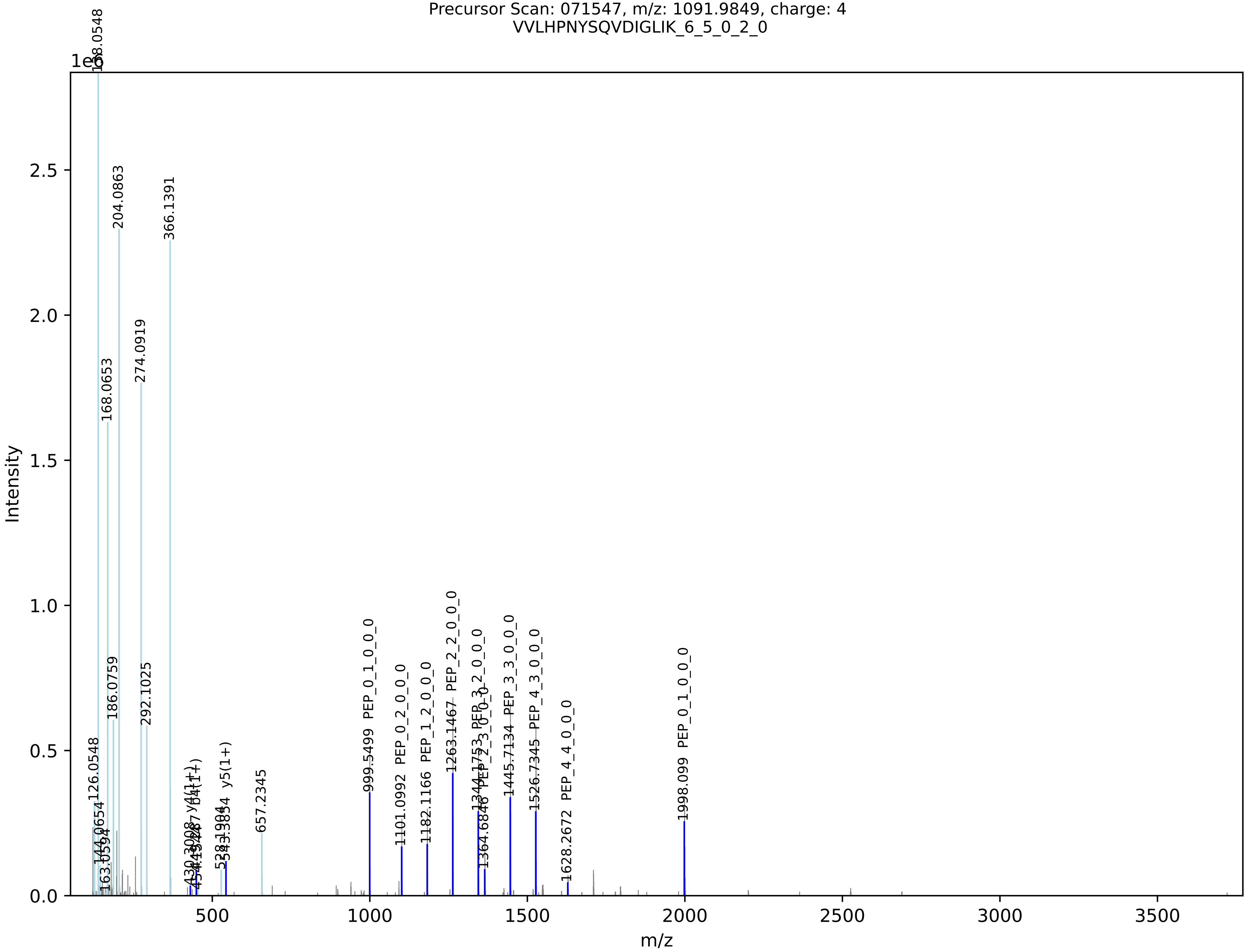

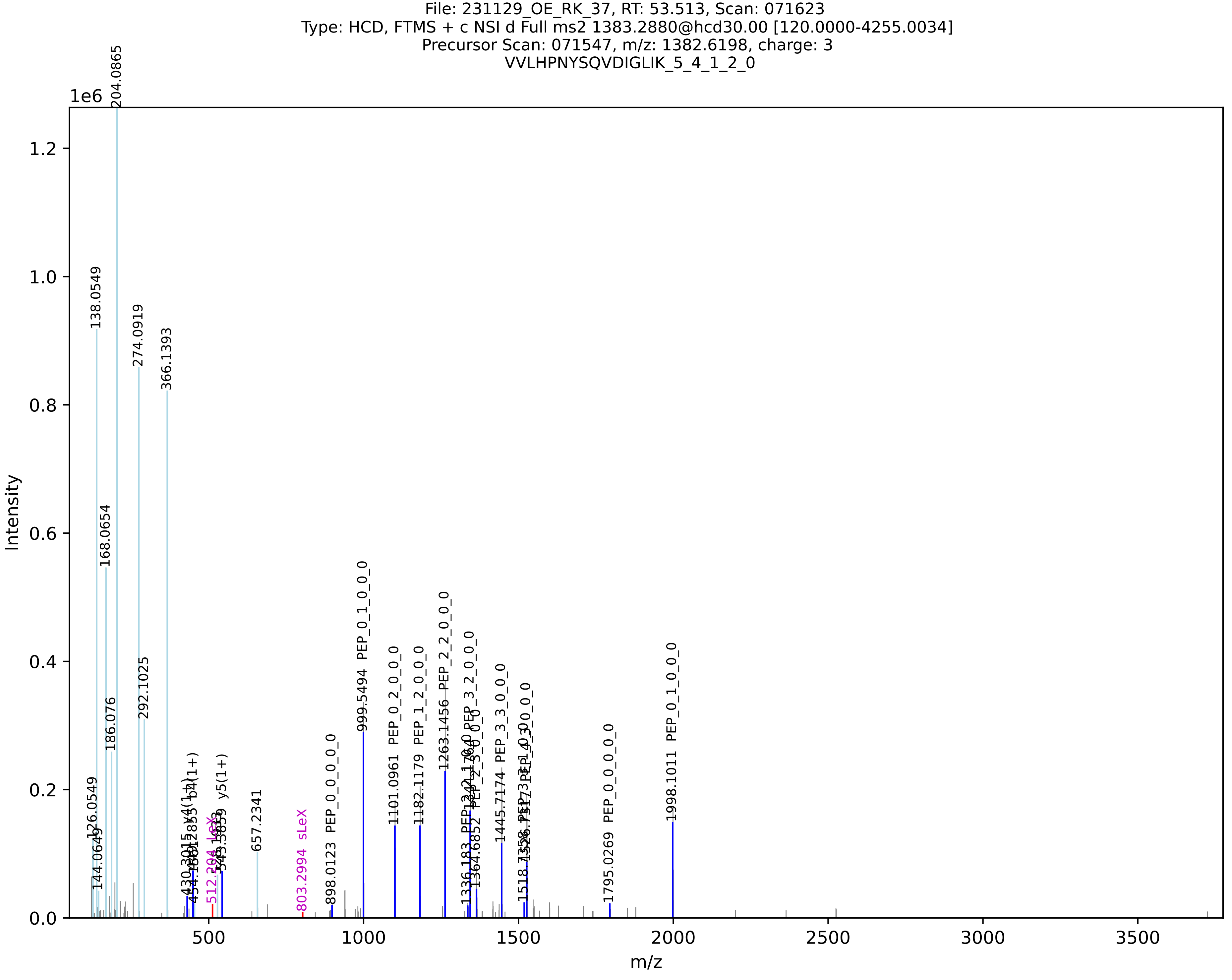
File: 231129_OE_RK_37, RT: 53.513, Scan: 071623
Type: HCD, FTMS + c NSI d Full ms2 1383.2880@hcd30.00 [120.0000-4255.0034]
Precursor Scan: 071547, m/z: 1382.6198, charge: 3
VVLHPNYSQVDIGLIK_5_4_1_2_0
1e6
Intensity
m/z
204.0865
138.0549
274.0919
366.1393
168.0654
292.1025
186.076
126.0549
144.0649
657.2341
803.2994 sLeX
898.0123 PEP_0_0_0_0_0
999.5494 PEP_0_1_0_0_0
1101.0961 PEP_0_2_0_0_0
1182.1179 PEP_1_2_0_0_0
1263.1456 PEP_2_2_0_0_0
1364.6852 PEP_3_2_0_0_0
1445.7174 PEP_3_3_0_0_0
1795.0269 PEP_0_0_0_0_0
1998.1011 PEP_0_1_0_0_0
543.3859 y5(1+)

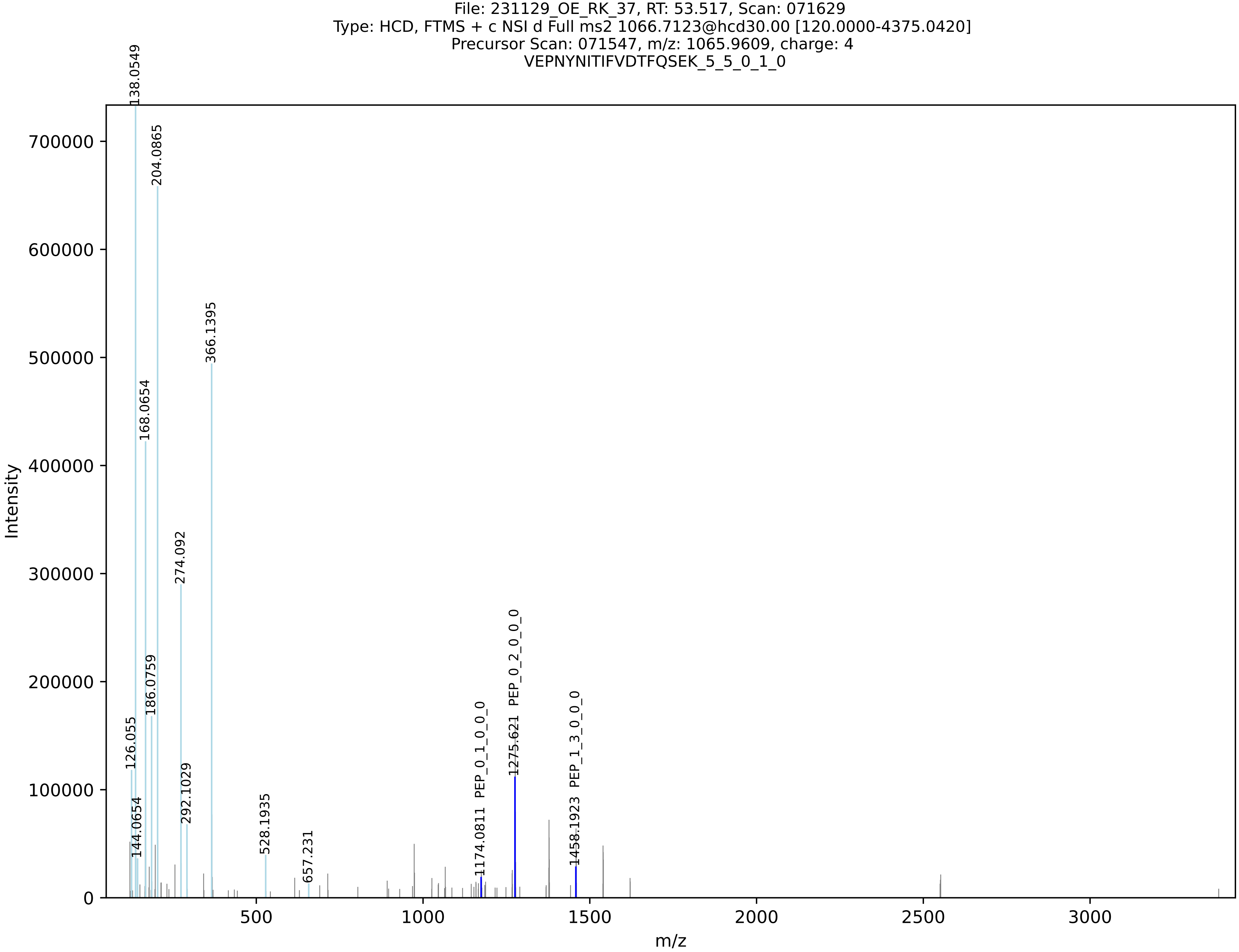
File: 231129_OE_RK_37, RT: 53.517, Scan: 071629
Type: HCD, FTMS + c NSI d Full ms2 1066.7123@hcd30.00 [120.0000-4375.0420]
Precursor Scan: 071547, m/z: 1065.9609, charge: 4
VEPNYNITIFVDTFQSEK_5_5_0_1_0
138.0549
204.0865
366.1395
168.0654
274.092
186.0759
126.055
292.1029
144.0654
528.1935
657.231
1174.0811 PEP_0_1_0_0_0
1275.621 PEP_0_2_0_0_0
1458.1923 PEP_1_3_0_0_0
Intensity
m/z
0
100000
200000
300000
400000
500000
600000
700000
500
1000
1500
2000
2500
3000

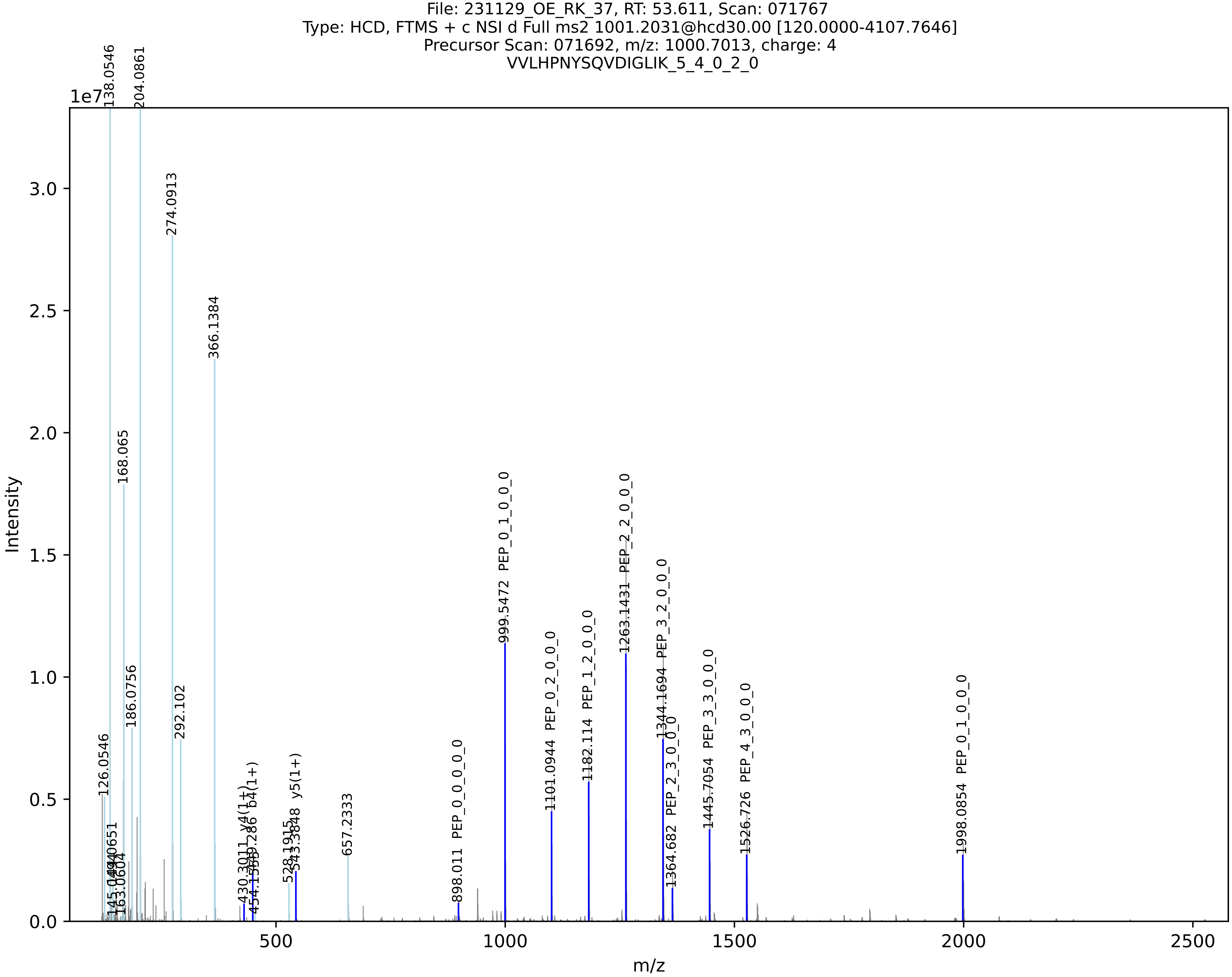
File: 231129_OE_RK_37, RT: 53.611, Scan: 071767
Type: HCD, FTMS + c NSI d Full ms2 1001.2031@hcd30.00 [120.0000-4107.7646]
Precursor Scan: 071692, m/z: 1000.7013, charge: 4
VVLHPNYSQVDIGLIK_5_4_0_2_0
1e7
Intensity
m/z
138.0546
204.0861
274.0913
366.1384
168.065
186.0756
292.102
126.0546
999.5472 PEP_0_1_0_0_0
1263.1431 PEP_2_2_0_0_0
1344.1694 PEP_3_2_0_0_0
1182.114 PEP_1_2_0_0_0
1101.0944 PEP_0_2_0_0_0
1445.7054 PEP_3_3_0_0_0
1526.726 PEP_4_3_0_0_0
1998.0854 PEP_0_1_0_0_0
1364.682 PEP_2_3_0_0_0
898.011 PEP_0_0_0_0_0
657.2333
543.3848 y5(1+)
528.1915
430.3011 y4(1+)
454.1555
449.286 b4(1+)
163.0604
145.0494
144.0651

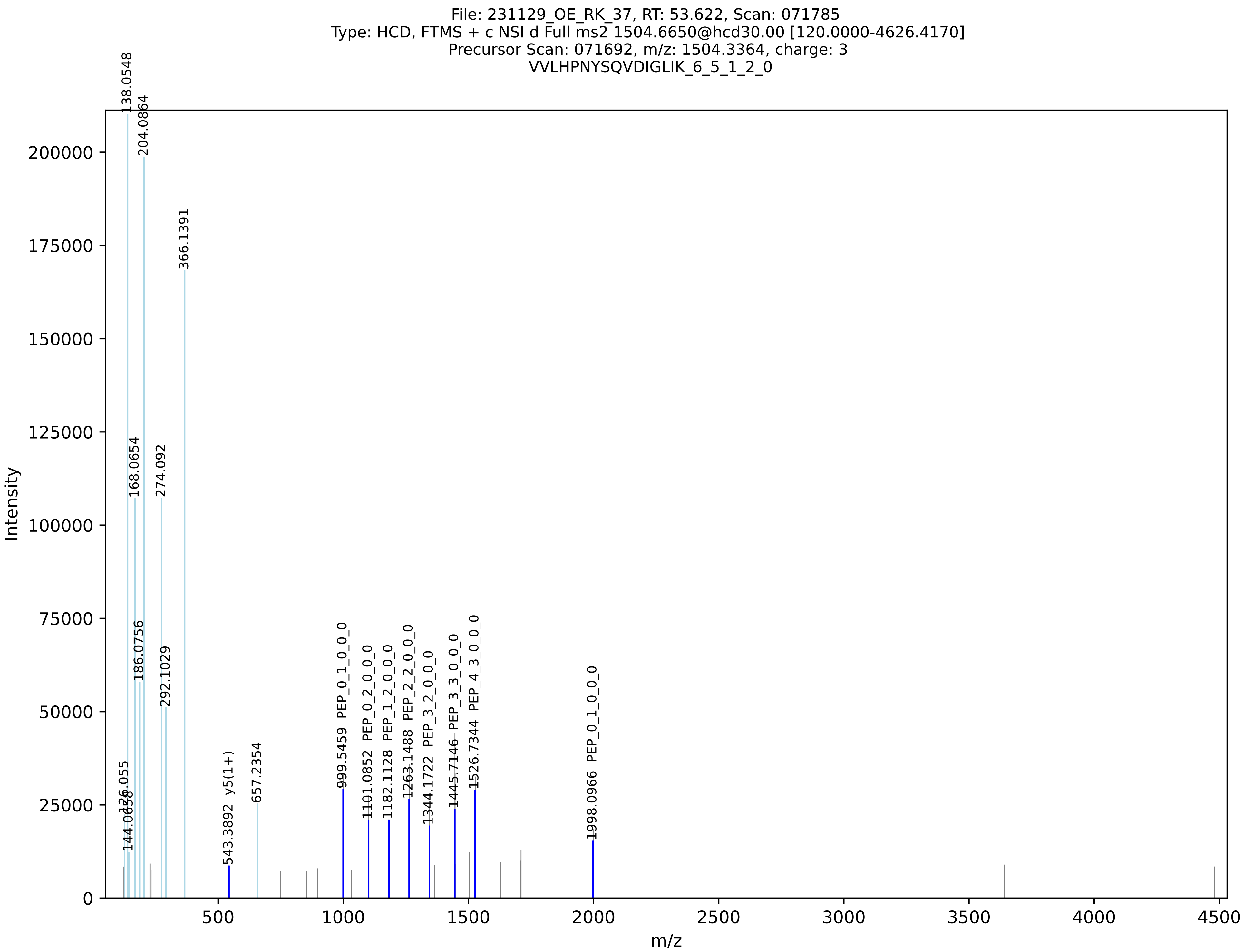

File: 231129_OE_RK_37, RT: 53.622, Scan: 071785
Type: HCD, FTMS + c NSI d Full ms2 1504.6650@hcd30.00 [120.0000-4626.4170]
Precursor Scan: 071692, m/z: 1504.3364, charge: 3
VVLHPNYSQVDIGLIK_6_5_1_2_0
Intensity
m/z
138.0548
204.0864
366.1391
168.0654
274.092
186.0756
292.1029
126.055
144.0658
543.3892 y5(1+)
657.2354
999.5459 PEP_0_1_0_0_0
1101.0852 PEP_0_2_0_0_0
1182.1128 PEP_1_2_0_0_0
1263.1488 PEP_2_2_0_0_0
1344.1722 PEP_3_2_0_0_0
1445.7146 PEP_3_3_0_0_0
1526.7344 PEP_4_3_0_0_0
1998.0966 PEP_0_1_0_0_0

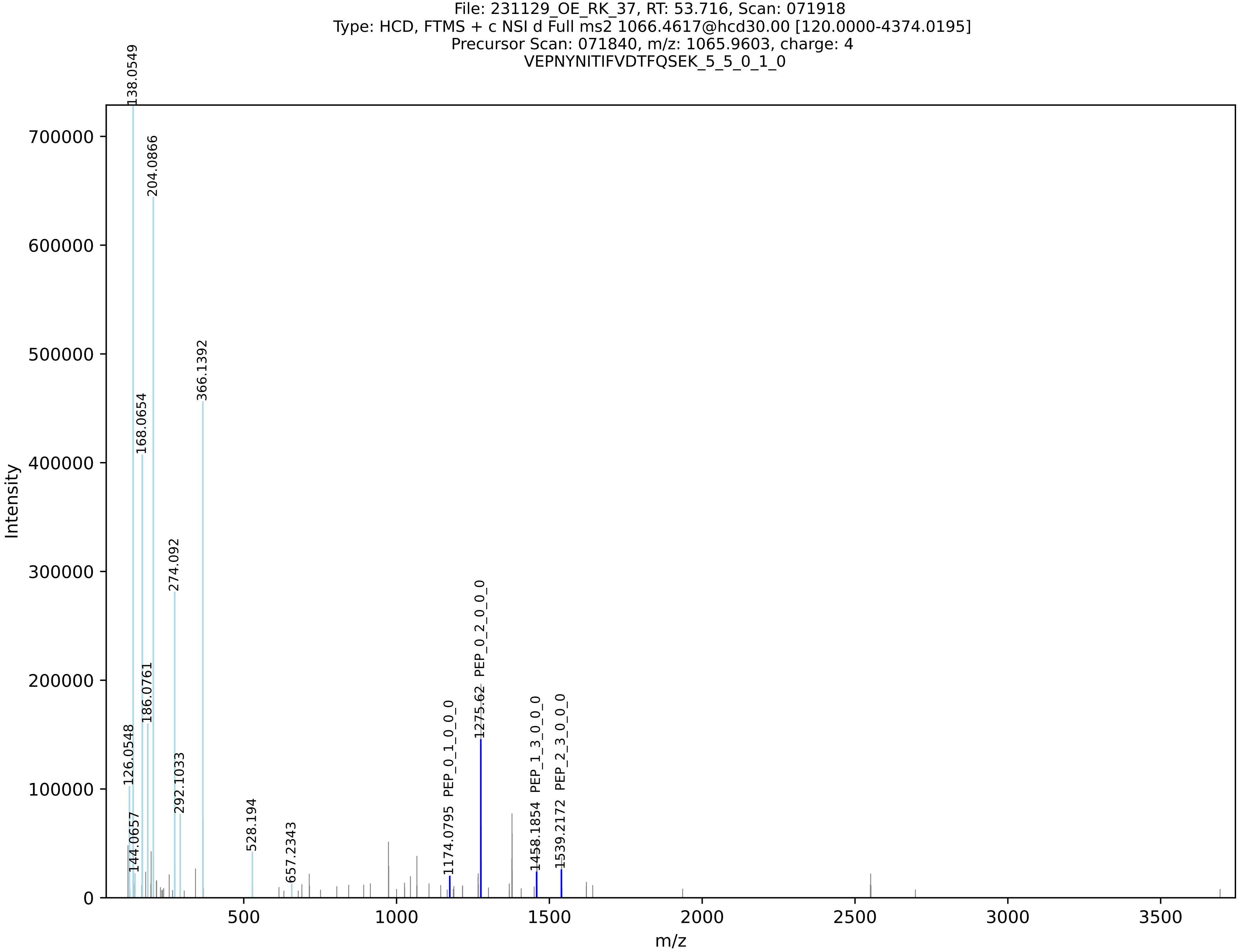
File: 231129_OE_RK_37, RT: 53.716, Scan: 071918
Type: HCD, FTMS + c NSI d Full ms2 1066.4617@hcd30.00 [120.0000-4374.0195]
Precursor Scan: 071840, m/z: 1065.9603, charge: 4
VEPNYNITIFVDTFQSEK_5_5_0_1_0
Intensity
m/z
138.0549
204.0866
366.1392
168.0654
274.092
186.0761
126.0548
292.1033
144.0657
528.194
657.2343
1174.0795
PEP_0_1_0_0_0
1275.62
PEP_0_2_0_0_0
1458.1854
PEP_1_3_0_0_0
1539.2172
PEP_2_3_0_0_0

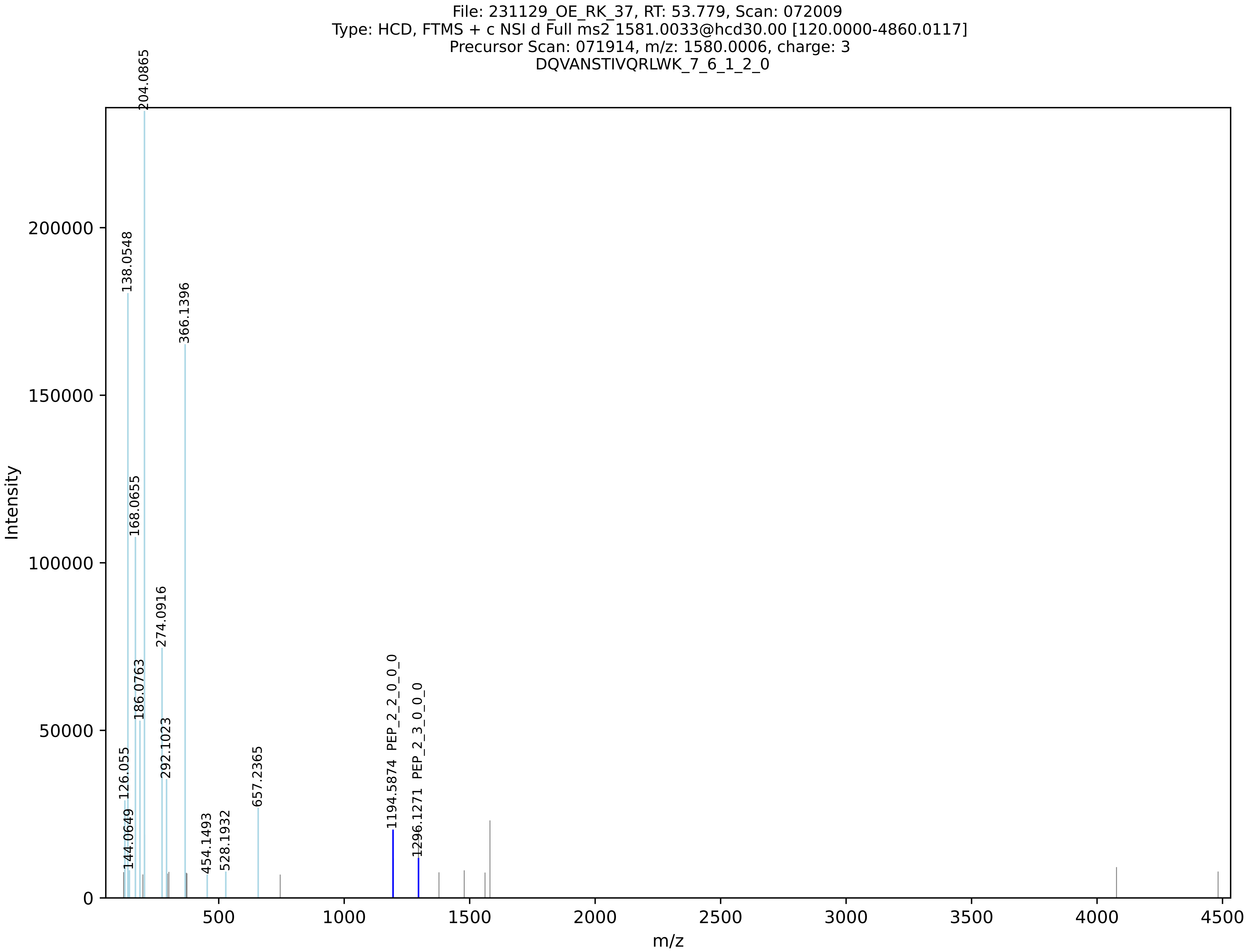
File: 231129_OE_RK_37, RT: 53.779, Scan: 072009
Type: HCD, FTMS + c NSI d Full ms2 1581.0033@hcd30.00 [120.0000-4860.0117]
Precursor Scan: 071914, m/z: 1580.0006, charge: 3
DQVANSTIVQRLWK_7_6_1_2_0
Intensity
m/z
204.0865
138.0548
366.1396
168.0655
274.0916
186.0763
292.1023
126.055
144.0649
454.1493
528.1932
657.2365
1194.5874 PEP_2_2_0_0_0
1296.1271 PEP_2_3_0_0_0
0
50000
100000
150000
200000
500
1000
1500
2000
2500
3000
3500
4000
4500

File: 231129_OE_RK_37, RT: 53.814, Scan: 072057
Type: HCD, FTMS + c NSI d Full ms2 1001.2040@hcd30.00 [120.0000-4107.7686]
Precursor Scan: 071985, m/z: 1000.7025, charge: 4
VVLHPNYSQVDIGLIK_5_4_0_2_0

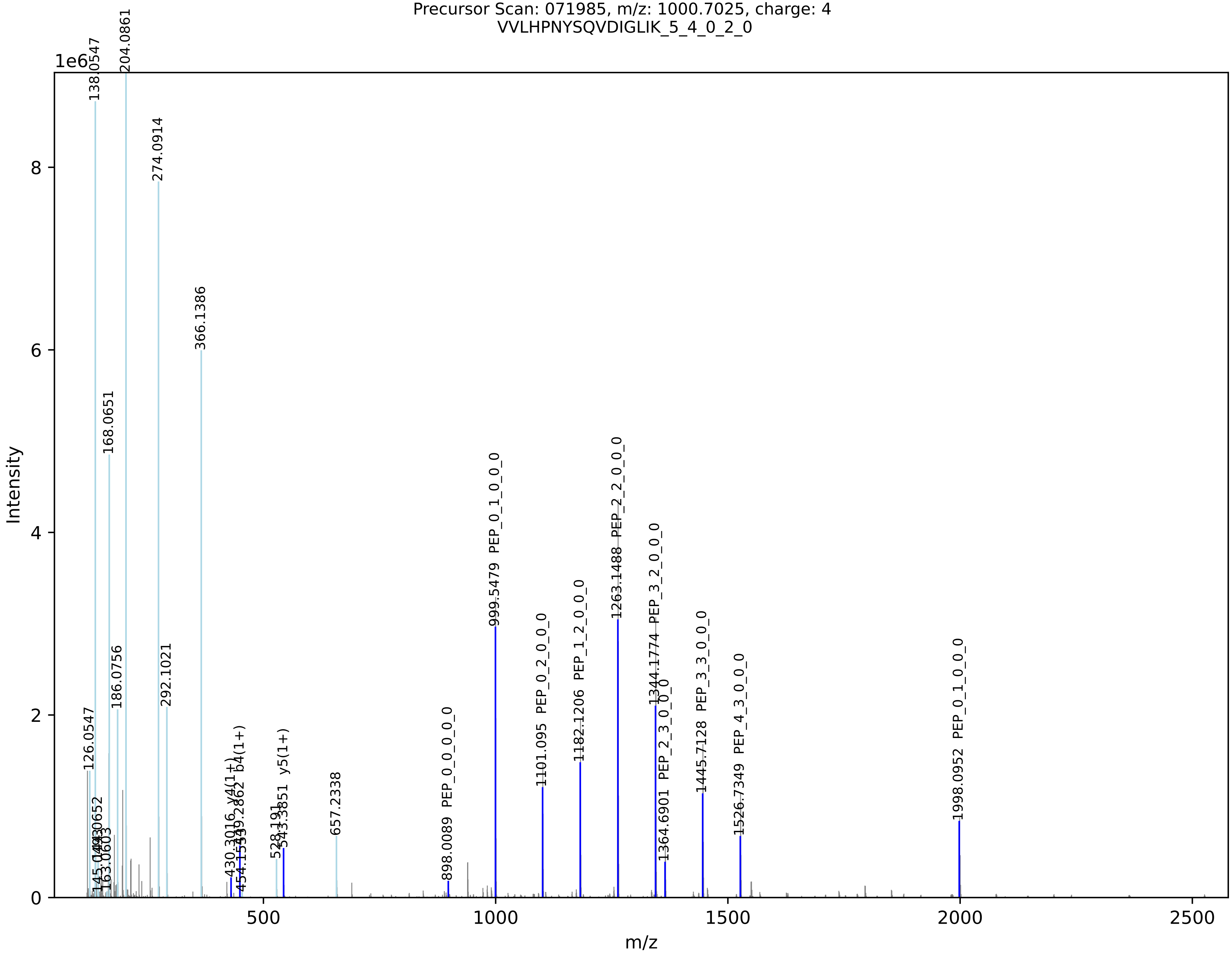

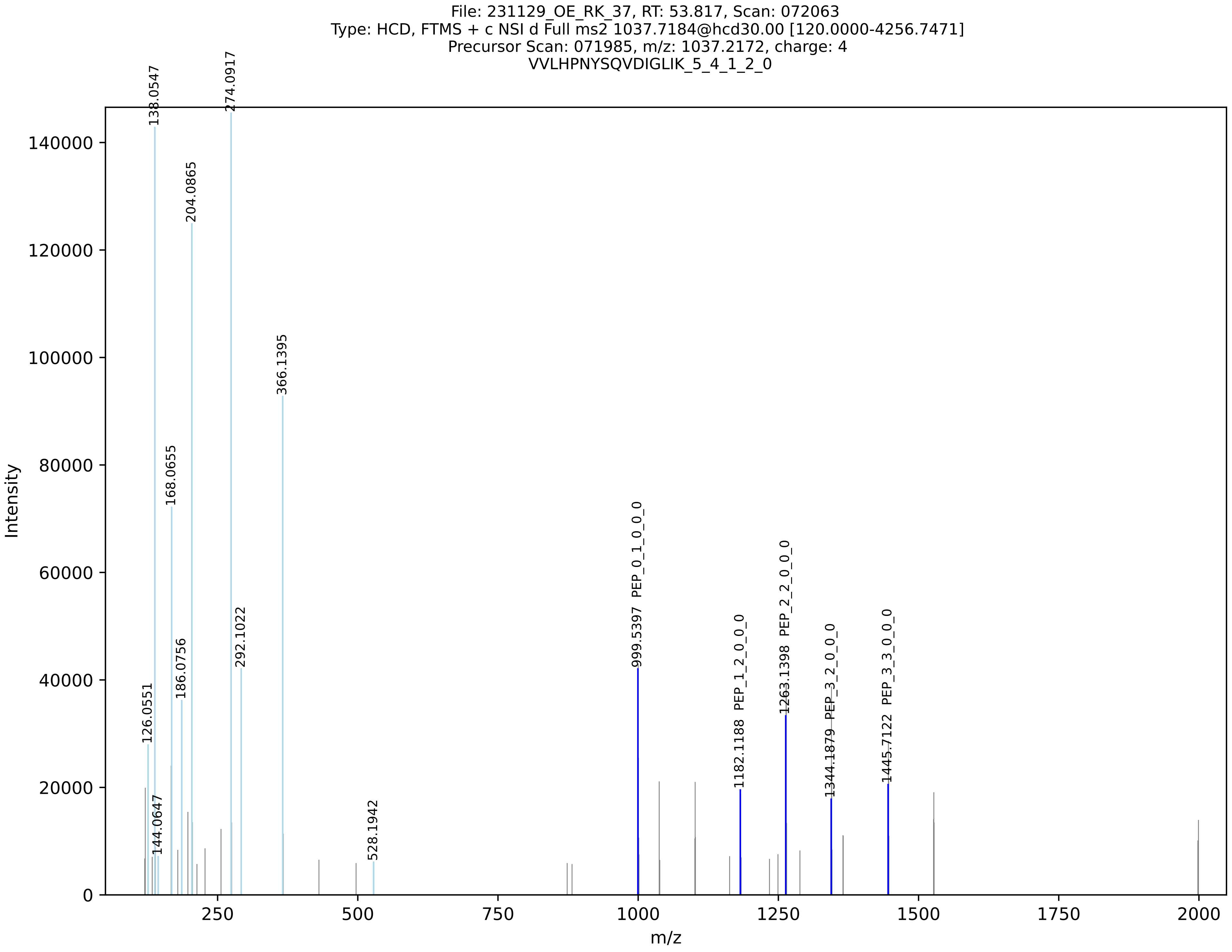
File: 231129_OE_RK_37, RT: 53.817, Scan: 072063
Type: HCD, FTMS + c NSI d Full ms2 1037.7184@hcd30.00 [120.0000-4256.7471]
Precursor Scan: 071985, m/z: 1037.2172, charge: 4
VVLHPNYSQVDIGLIK_5_4_1_2_0
Intensity
m/z
138.0547
274.0917
204.0865
366.1395
168.0655
292.1022
186.0756
126.0551
144.0647
528.1942
999.5397 PEP_0_1_0_0_0
1182.1188 PEP_1_2_0_0_0
1263.1398 PEP_2_2_0_0_0
1344.1879 PEP_3_2_0_0_0
1445.7122 PEP_3_3_0_0_0

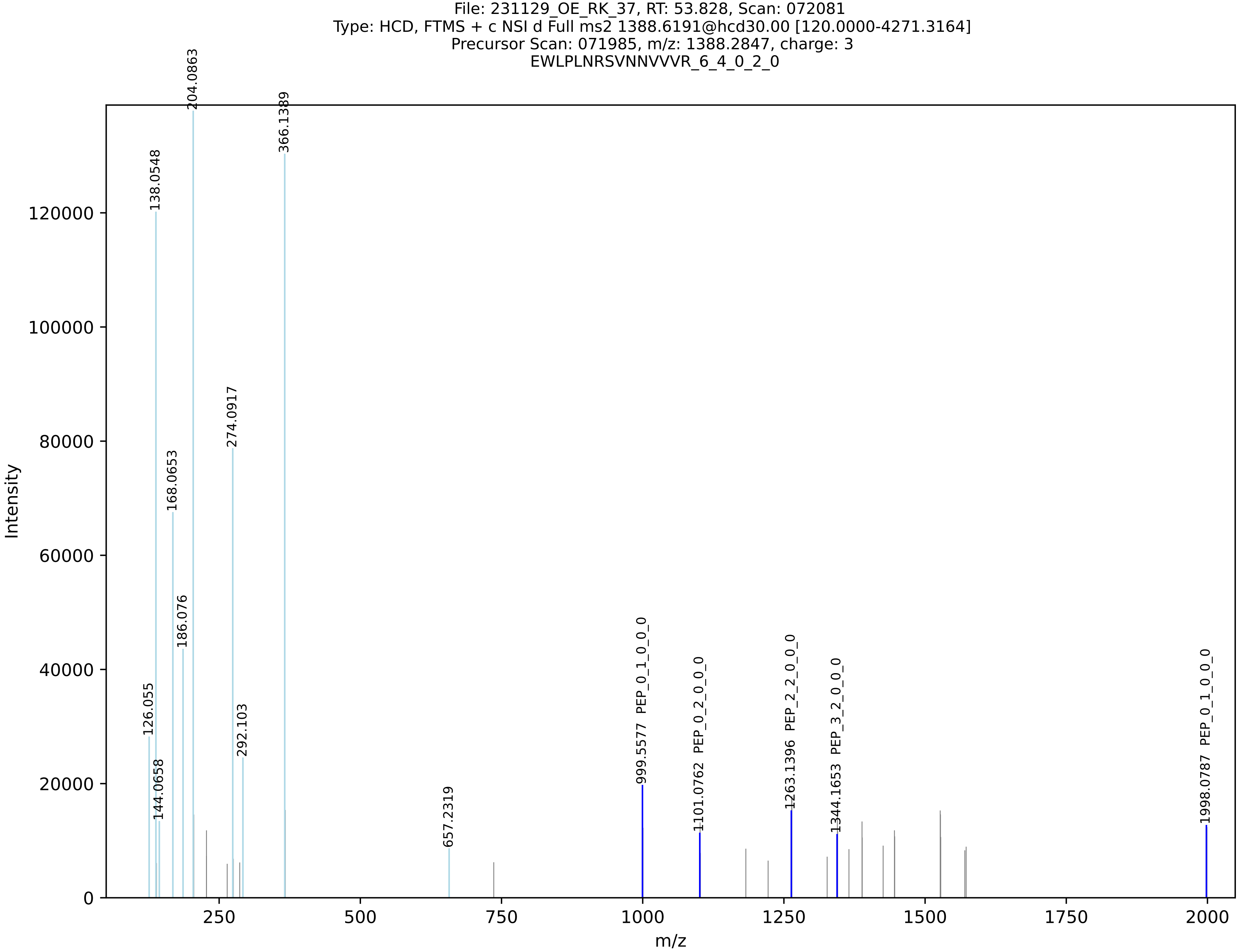

File: 231129_OE_RK_37, RT: 53.828, Scan: 072081
Type: HCD, FTMS + c NSI d Full ms2 1388.6191@hcd30.00 [120.0000-4271.3164]
Precursor Scan: 071985, m/z: 1388.2847, charge: 3
EWLPLNRSVNNVVVR_6_4_0_2_0
Intensity
m/z
204.0863
366.1389
138.0548
274.0917
168.0653
186.076
126.055
292.103
144.0658
657.2319
999.5577 PEP_0_1_0_0_0
1101.0762 PEP_0_2_0_0_0
1263.1396 PEP_2_2_0_0_0
1344.1653 PEP_3_2_0_0_0
1998.0787 PEP_0_1_0_0_0

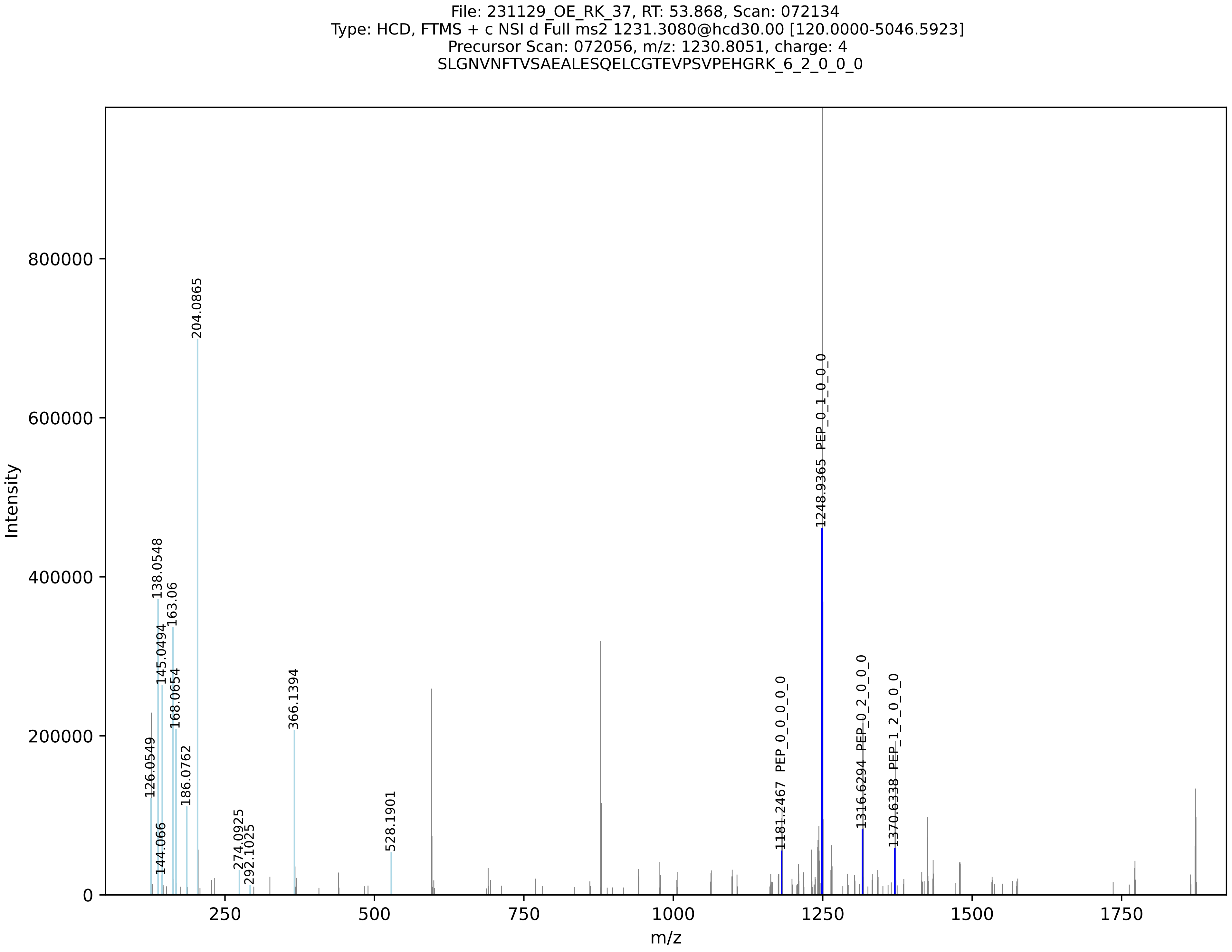
File: 231129_OE_RK_37, RT: 53.868, Scan: 072134
Type: HCD, FTMS + c NSI d Full ms2 1231.3080@hcd30.00 [120.0000-5046.5923]
Precursor Scan: 072056, m/z: 1230.8051, charge: 4
SLGNVNFTVSAEALESQELCGTEVPSVPEHGRK_6_2_0_0_0
Intensity
m/z
0
200000
400000
600000
800000
250
500
750
1000
1250
1500
1750
204.0865
138.0548
163.06
145.0494
168.0654
126.0549
186.0762
144.066
274.0925
292.1025
366.1394
528.1901
1181.2467 PEP_0_0_0_0_0
1248.9365 PEP_0_1_0_0_0
1316.6294 PEP_0_2_0_0_0
1370.6338 PEP_1_2_0_0_0

File: 231129_OE_RK_37, RT: 53.919, Scan: 072208
Type: HCD, FTMS + c NSI d Full ms2 1190.7965@hcd30.00 [120.0000-4881.3057]
Precursor Scan: 072130, m/z: 1190.2953, charge: 4
SLGNVNFTVSAEALESQELCGTEVPSVPEHGRK_5_2_0_0_0

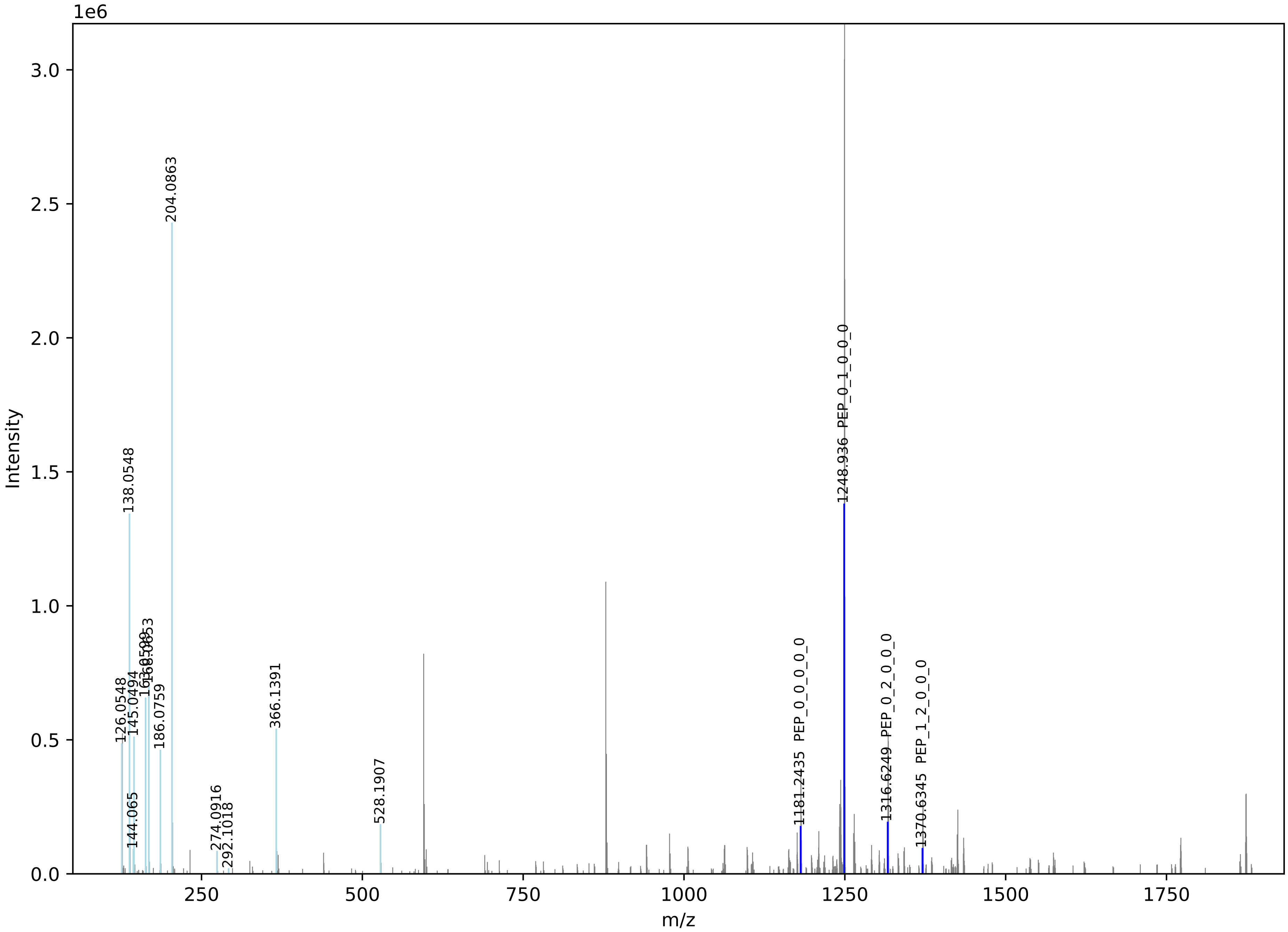

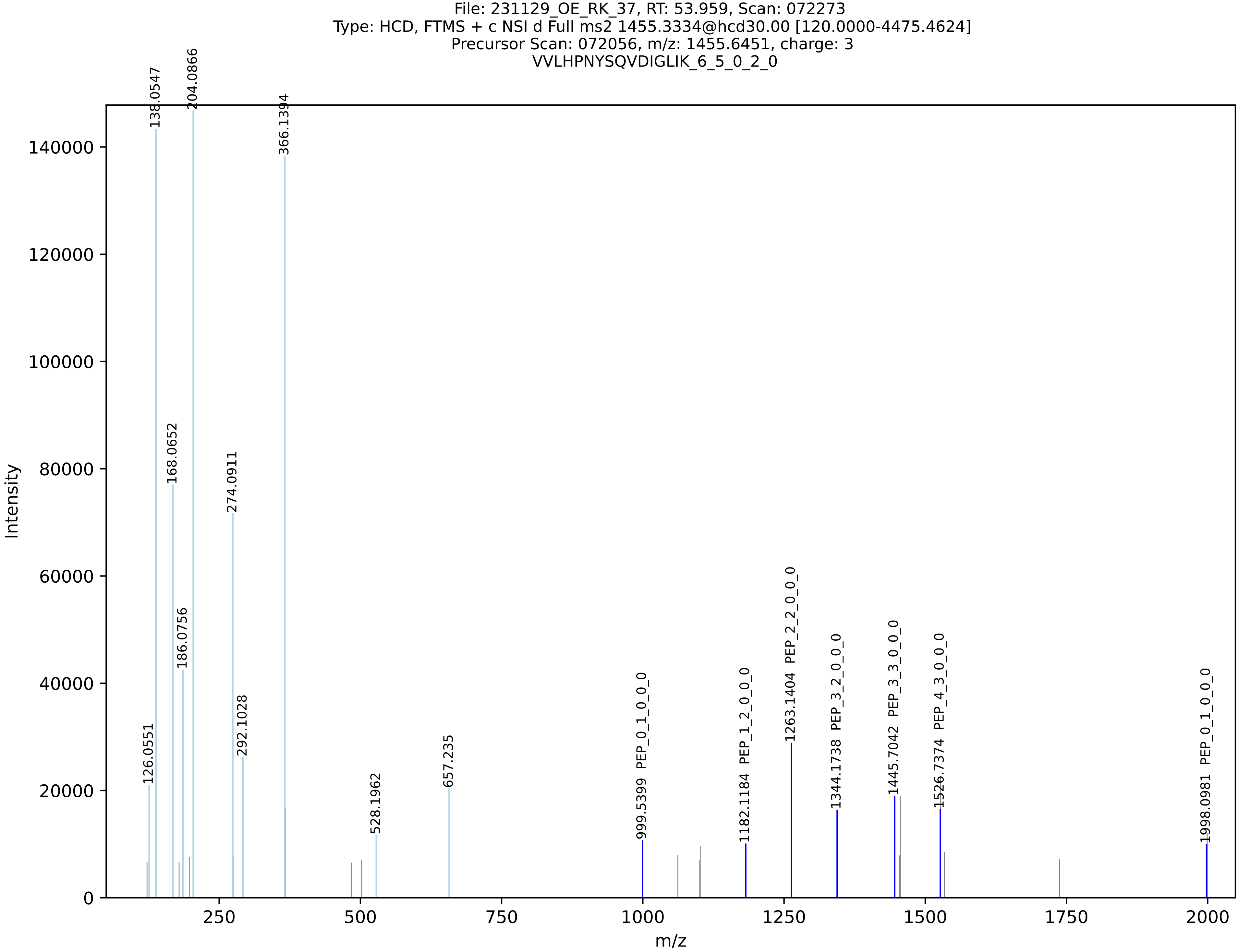

File: 231129_OE_RK_37, RT: 53.959, Scan: 072273
Type: HCD, FTMS + c NSI d Full ms2 1455.3334@hcd30.00 [120.0000-4475.4624]
Precursor Scan: 072056, m/z: 1455.6451, charge: 3
VVLHPNYSQVDIGLIK_6_5_0_2_0
Intensity
m/z
138.0547
204.0866
366.1394
168.0652
274.0911
186.0756
292.1028
126.0551
528.1962
657.235
999.5399 PEP_0_1_0_0_0
1182.1184 PEP_1_2_0_0_0
1263.1404 PEP_2_2_0_0_0
1344.1738 PEP_3_2_0_0_0
1445.7042 PEP_3_3_0_0_0
1526.7374 PEP_4_3_0_0_0
1998.0981 PEP_0_1_0_0_0

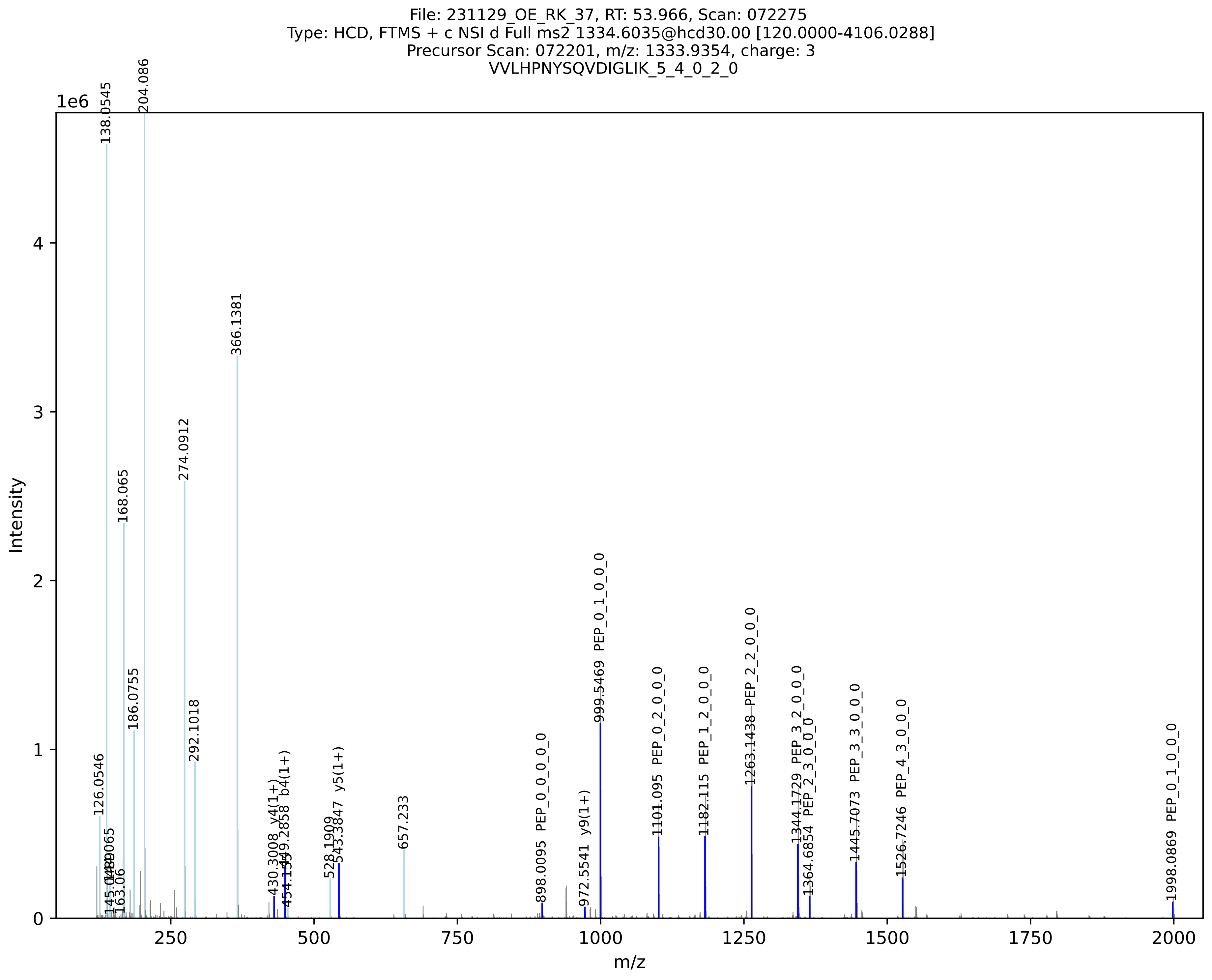

File: 231129_OE_RK_37, RT: 53.966, Scan: 072275
Type: HCD, FTMS + c NSI d Full ms2 1334.6035@hcd30.00 [120.0000-4106.0288]
Precursor Scan: 072201, m/z: 1333.9354, charge: 3
VVLHPNYSQVDIGLIK_5_4_0_2_0
1e6
Intensity
m/z
204.086
138.0545
366.1381
274.0912
168.065
186.0755
292.1018
126.0546
657.233
430.3008 y4(1+)
449.2858 b4(1+)
543.3847 y5(1+)
528.1909
898.0095 PEP_0_0_0_0_0
972.5541 y9(1+)
999.5469 PEP_0_1_0_0_0
1101.095 PEP_0_2_0_0_0
1182.115 PEP_1_2_0_0_0
1263.1438 PEP_2_2_0_0_0
1344.1729 PEP_3_2_0_0_0
1364.6854 PEP_2_3_0_0_0
1445.7073 PEP_3_3_0_0_0
1526.7246 PEP_4_3_0_0_0
1998.0869 PEP_0_1_0_0_0

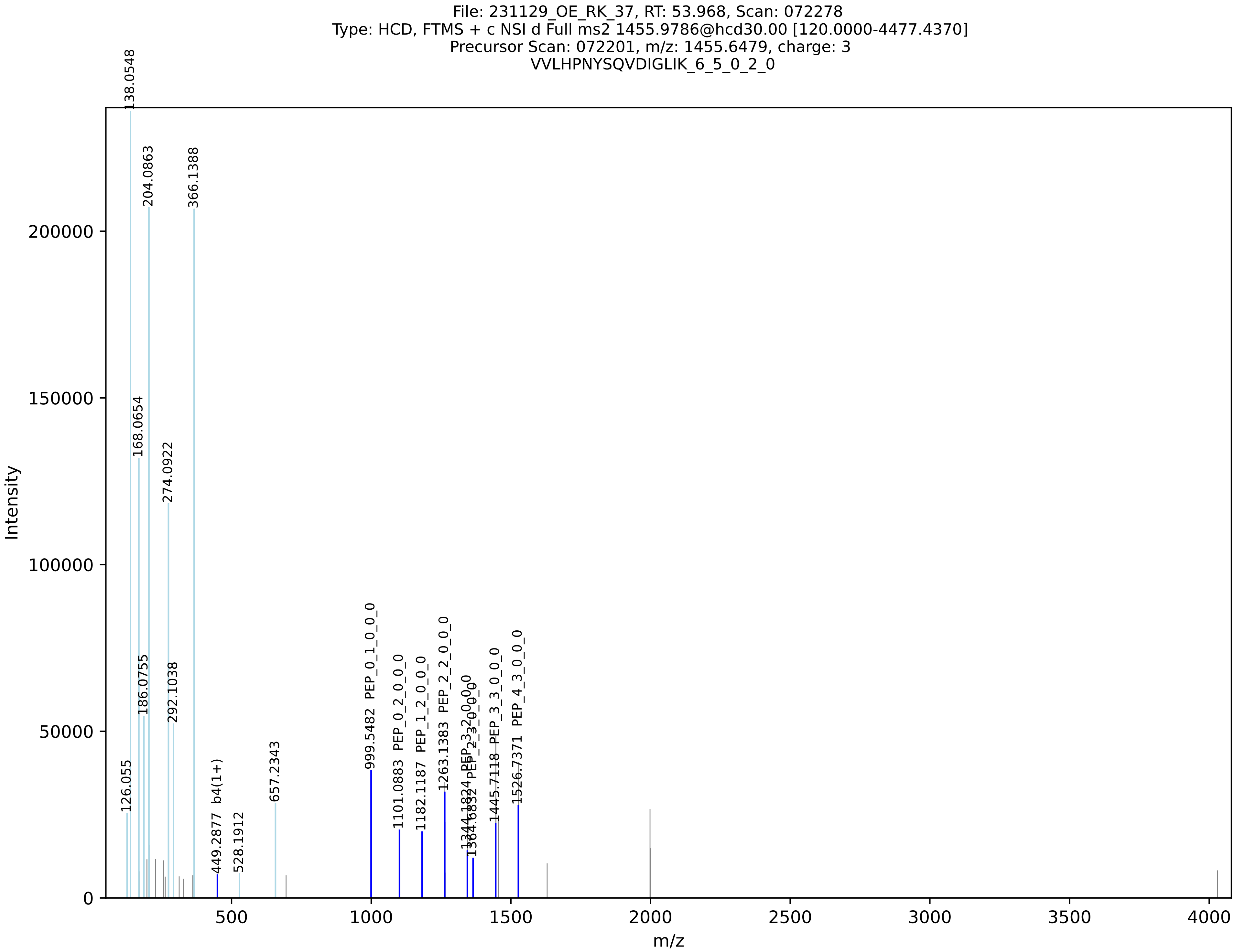
File: 231129_OE_RK_37, RT: 53.968, Scan: 072278
Type: HCD, FTMS + c NSI d Full ms2 1455.9786@hcd30.00 [120.0000-4477.4370]
Precursor Scan: 072201, m/z: 1455.6479, charge: 3
VVLHPNYSQVDIGLIK_6_5_0_2_0
138.0548
204.0863
366.1388
168.0654
274.0922
186.0755
292.1038
126.055
449.2877 b4(1+)
528.1912
657.2343
999.5482 PEP_0_1_0_0_0
1101.0883 PEP_0_2_0_0_0
1182.1187 PEP_1_2_0_0_0
1263.1383 PEP_2_2_0_0_0
1445.7118 PEP_3_3_0_0_0
1526.7371 PEP_4_3_0_0_0
Intensity
m/z
0
50000
100000
150000
200000
500
1000
1500
2000
2500
3000
3500
4000

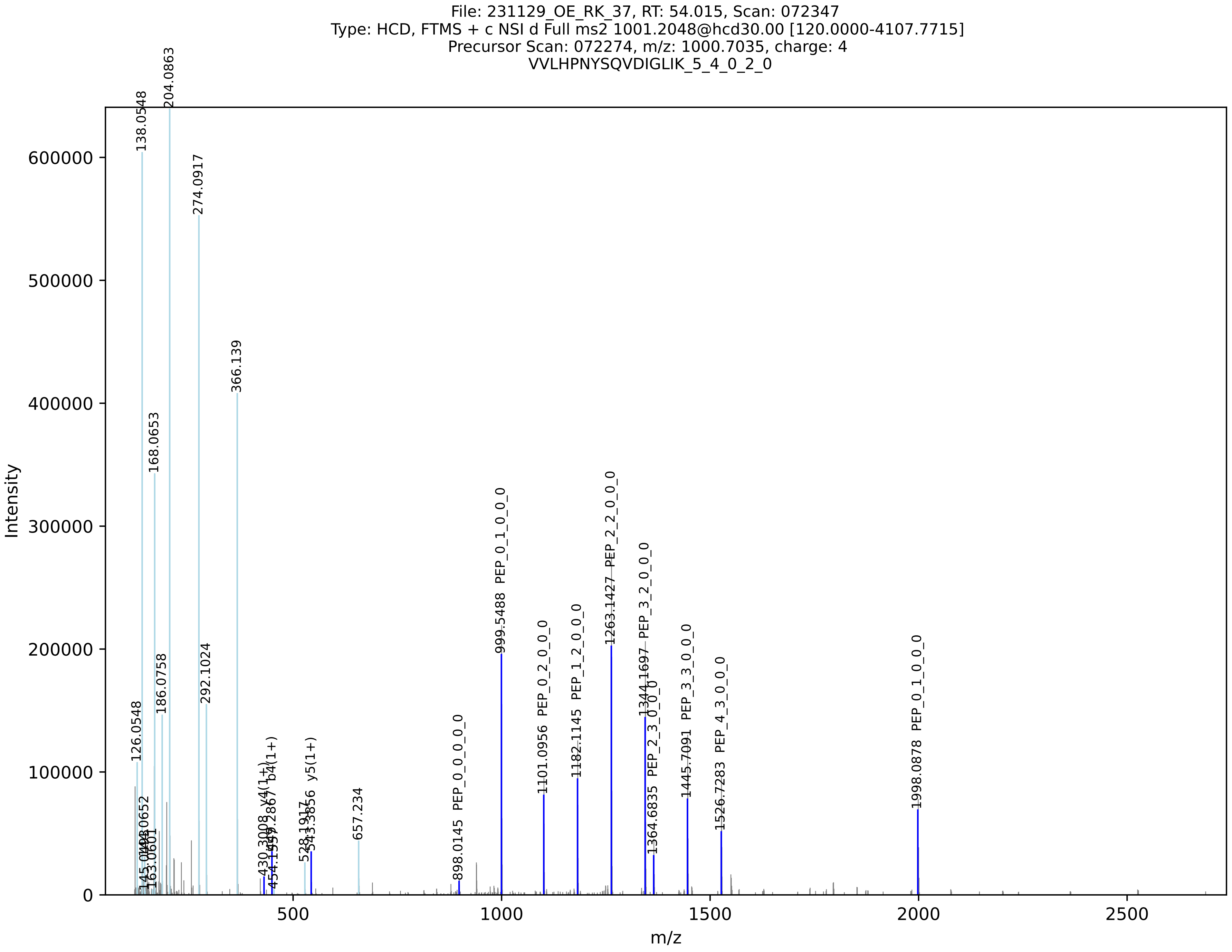
File: 231129_OE_RK_37, RT: 54.015, Scan: 072347
Type: HCD, FTMS + c NSI d Full ms2 1001.2048@hcd30.00 [120.0000-4107.7715]
Precursor Scan: 072274, m/z: 1000.7035, charge: 4
VVLHPNYSQVDIGLIK_5_4_0_2_0
Intensity
m/z
0
100000
200000
300000
400000
500000
600000
500
1000
1500
2000
2500
204.0863
138.0548
274.0917
366.139
168.0653
186.0758
292.1024
126.0548
657.234
898.0145 PEP_0_0_0_0_0
999.5488 PEP_0_1_0_0_0
1101.0956 PEP_0_2_0_0_0
1182.1145 PEP_1_2_0_0_0
1263.1427 PEP_2_2_0_0_0
1344.1697 PEP_3_2_0_0_0
1364.6835 PEP_2_3_0_0_0
1445.7091 PEP_3_3_0_0_0
1526.7283 PEP_4_3_0_0_0
1998.0878 PEP_0_1_0_0_0
430.3008 y4(1+)
b4(1+)
543.3856 y5(1+)
528.1917

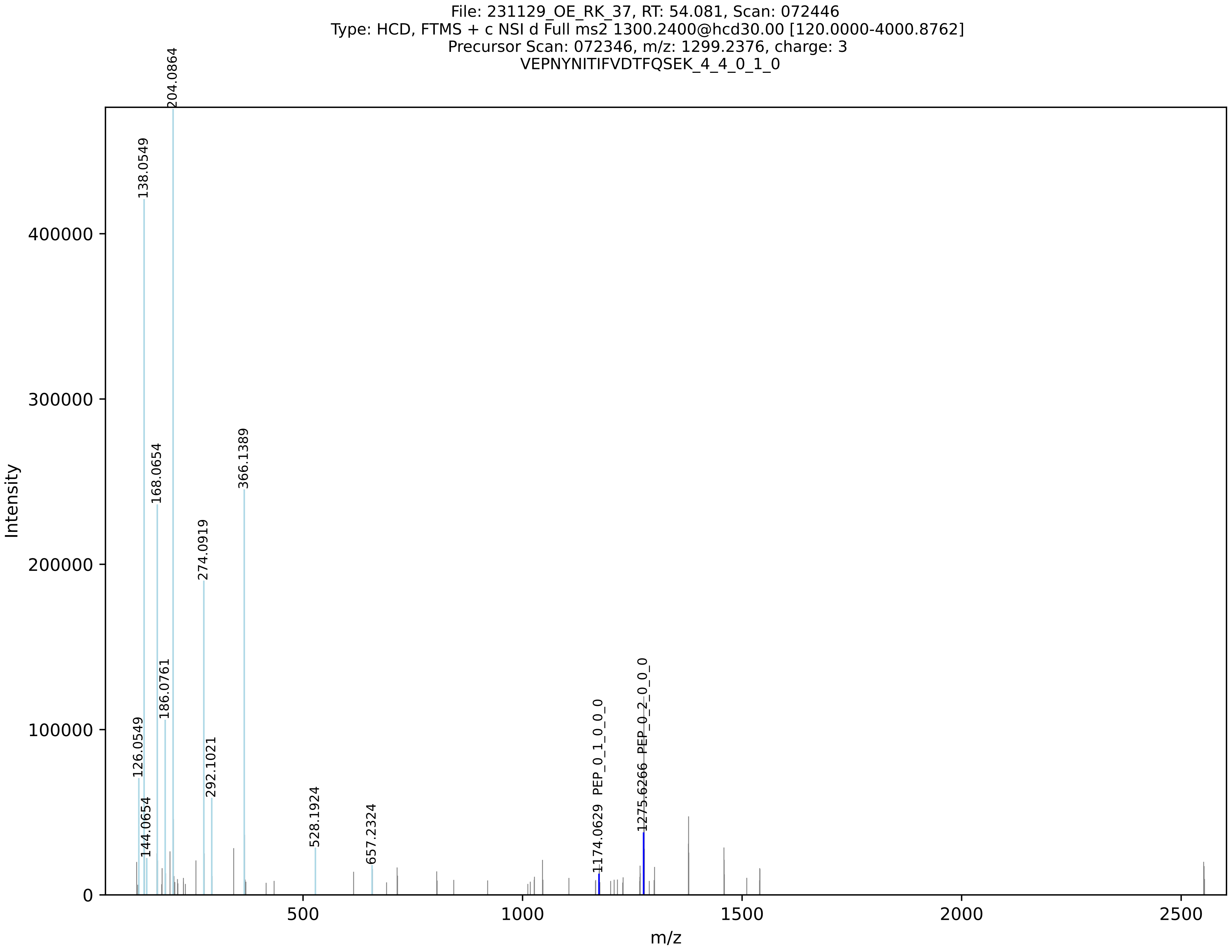

File: 231129_OE_RK_37, RT: 54.081, Scan: 072446
Type: HCD, FTMS + c NSI d Full ms2 1300.2400@hcd30.00 [120.0000-4000.8762]
Precursor Scan: 072346, m/z: 1299.2376, charge: 3
VEPNYNITIFVDTFQSEK_4_4_0_1_0
Intensity
m/z
204.0864
138.0549
168.0654
366.1389
274.0919
186.0761
126.0549
292.1021
144.0654
528.1924
657.2324
1174.0629 PEP_0_1_0_0_0
1275.6266 PEP_0_2_0_0_0

File: 231129_OE_RK_37, RT: 54.217, Scan: 072639
Type: HCD, FTMS + c NSI d Full ms2 1501.2742@hcd30.00 [120.0000-4616.0410]
Precursor Scan: 072566, m/z: 1500.6059, charge: 3
SPYYNVSDEISFHCYDGYTLR_5_4_0_1_0

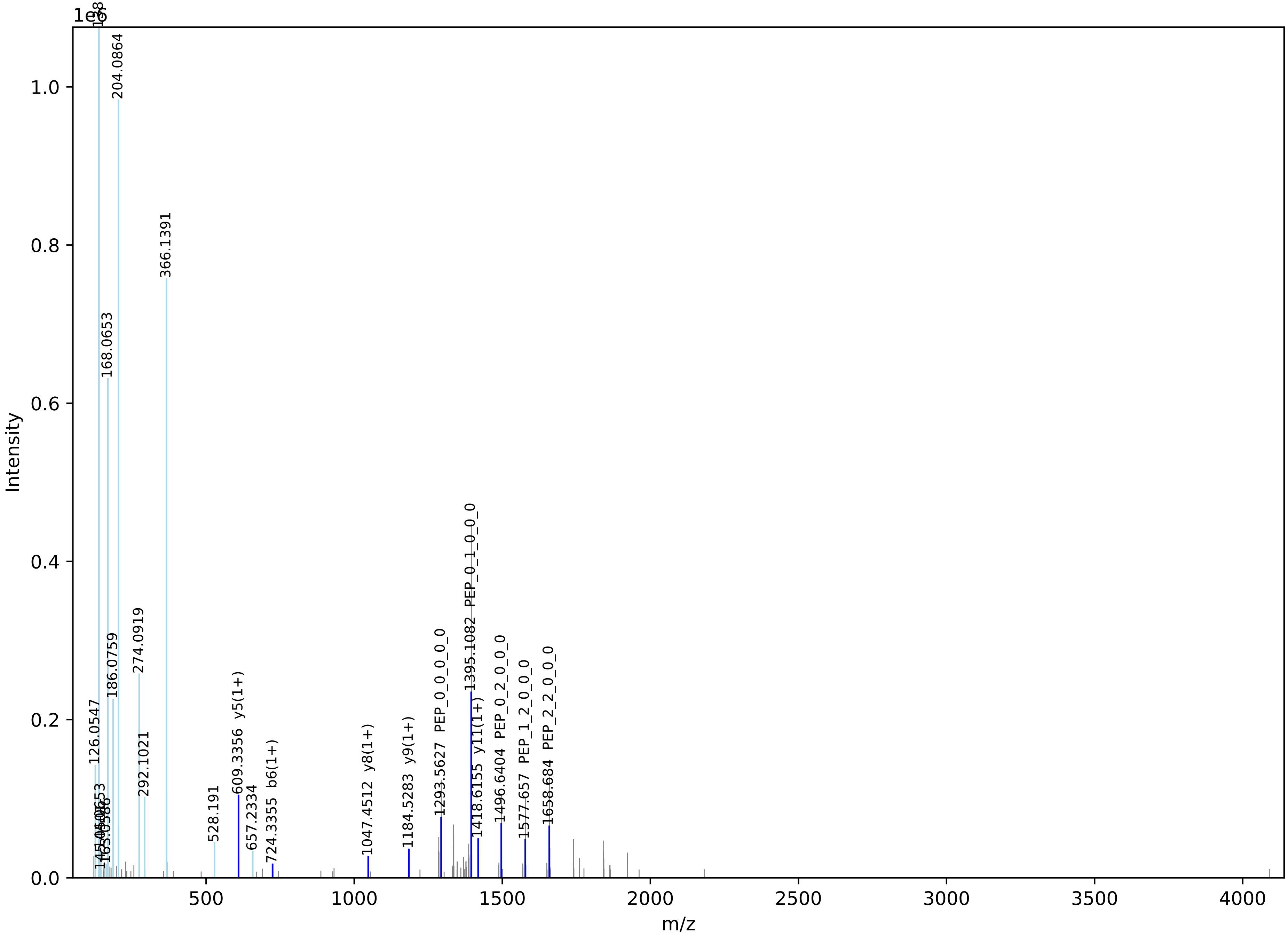

File: 231129_OE_RK_37, RT: 54.374, Scan: 072867
Type: HCD, FTMS + c NSI d Full ms2 1226.3013@hcd30.00 [120.0000-5026.1650]
Precursor Scan: 072785, m/z: 1225.8001, charge: 4
NTTSYVLRGLEPGQEYNVLLTAEK_5_4_0_2_0

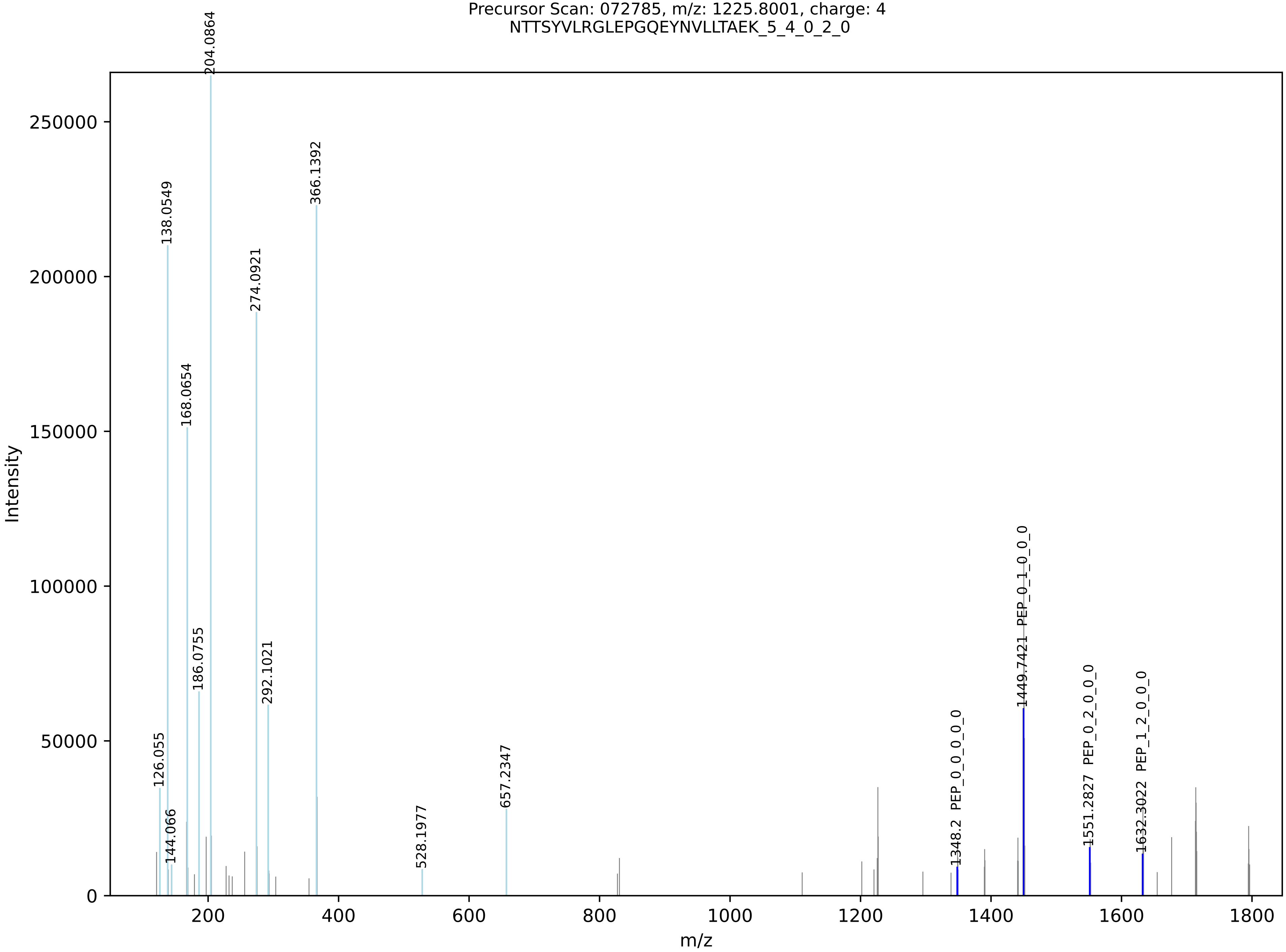

File: 231129_OE_RK_37, RT: 54.421, Scan: 072932
Type: HCD, FTMS + c NSI d Full ms2 1501.2758@hcd30.00 [120.0000-4616.0459]
Precursor Scan: 072856, m/z: 1500.6074, charge: 3
SPYYNVSDEISFHCYDGYTLR_5_4_0_1_0

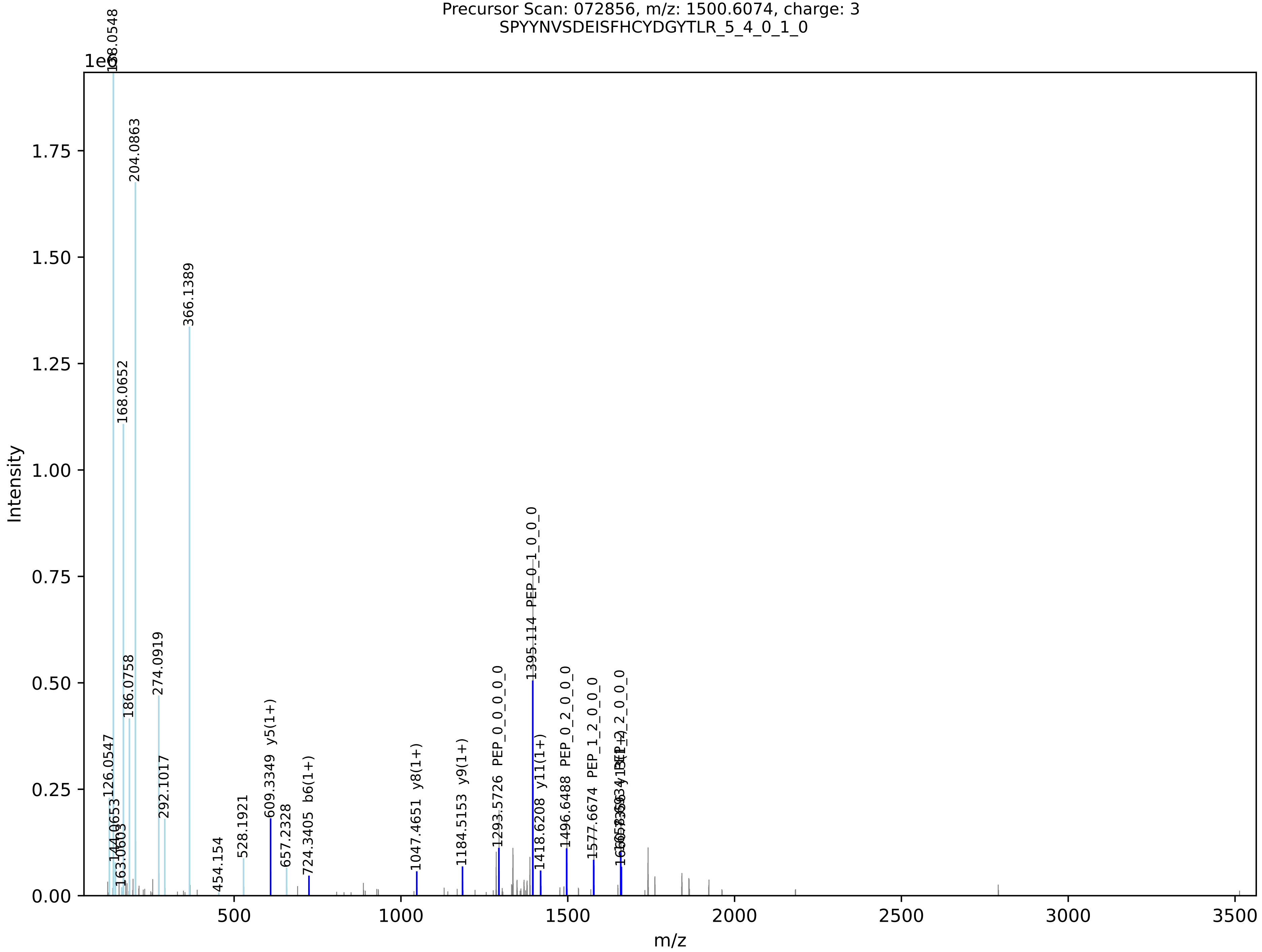

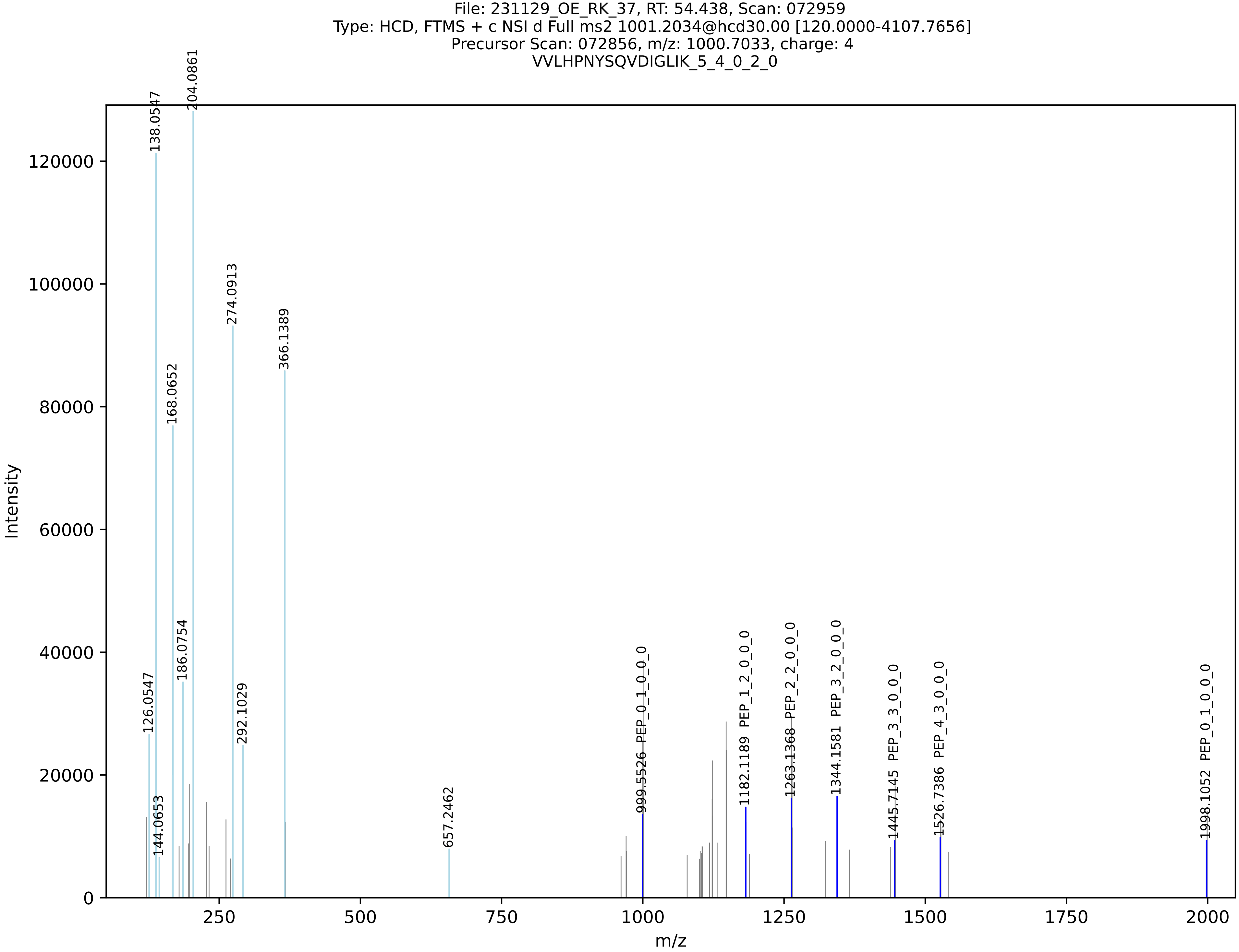
File: 231129_OE_RK_37, RT: 54.438, Scan: 072959
Type: HCD, FTMS + c NSI d Full ms2 1001.2034@hcd30.00 [120.0000-4107.7656]
Precursor Scan: 072856, m/z: 1000.7033, charge: 4
VVLHPNYSQVDIGLIK_5_4_0_2_0
Intensity
m/z
138.0547
204.0861
274.0913
366.1389
168.0652
186.0754
126.0547
292.1029
144.0653
657.2462
999.5526 PEP_0_1_0_0_0
1182.1189 PEP_1_2_0_0_0
1263.1368 PEP_2_2_0_0_0
1344.1581 PEP_3_2_0_0_0
1445.7145 PEP_3_3_0_0_0
1526.7386 PEP_4_3_0_0_0
1998.1052 PEP_0_1_0_0_0

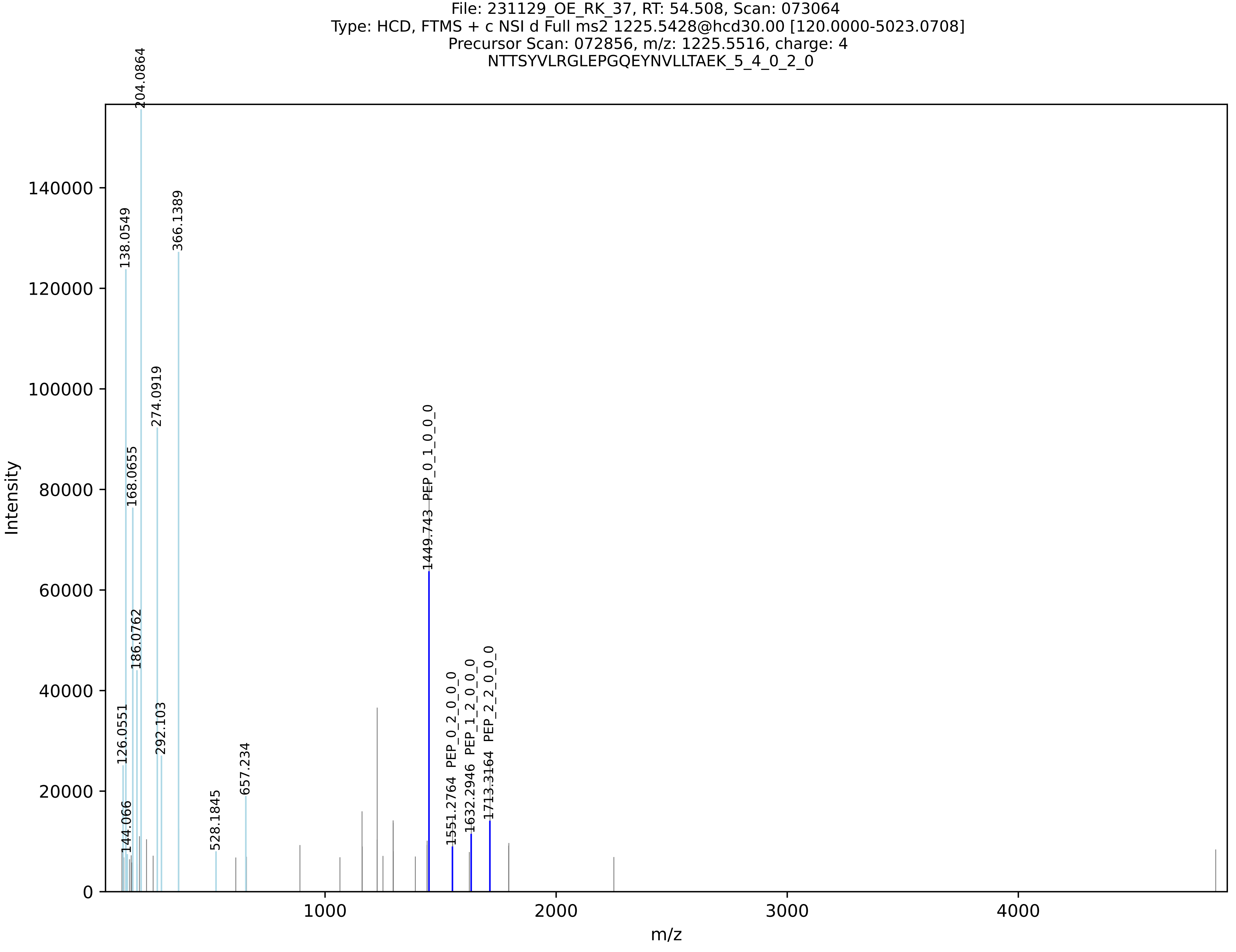

File: 231129_OE_RK_37, RT: 54.508, Scan: 073064
Type: HCD, FTMS + c NSI d Full ms2 1225.5428@hcd30.00 [120.0000-5023.0708]
Precursor Scan: 072856, m/z: 1225.5516, charge: 4
NTTSYVLRGLEPGQEYNVLLTAEK_5_4_0_2_0
Intensity
m/z
204.0864
366.1389
138.0549
274.0919
168.0655
186.0762
126.0551
292.103
144.066
528.1845
657.234
1449.743 PEP_0_1_0_0_0
1551.2764 PEP_0_2_0_0_0
1632.2946 PEP_1_2_0_0_0
1713.3164 PEP_2_2_0_0_0
0
20000
40000
60000
80000
100000
120000
140000
1000
2000
3000
4000

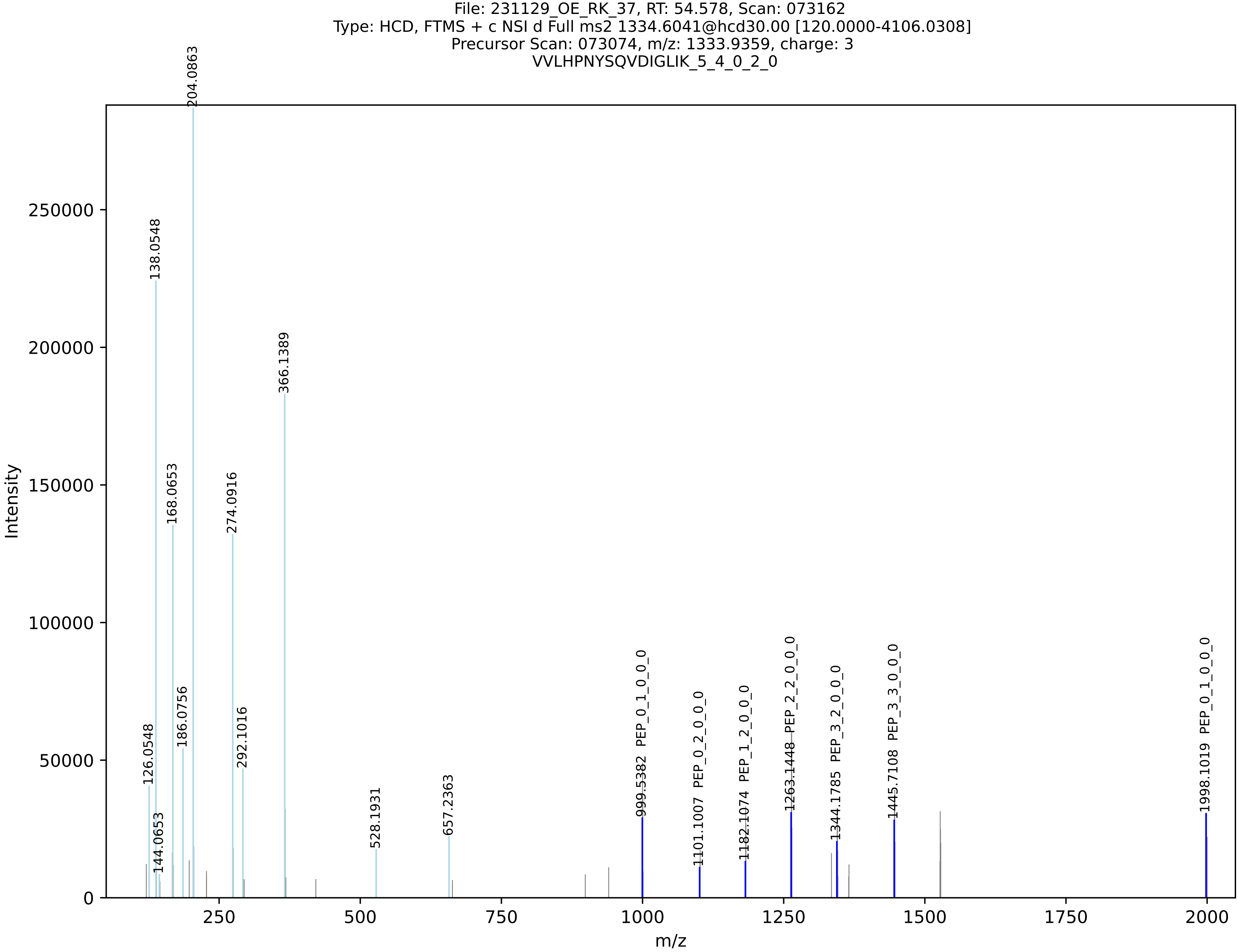
File: 231129_OE_RK_37, RT: 54.578, Scan: 073162
Type: HCD, FTMS + c NSI d Full ms2 1334.6041@hcd30.00 [120.0000-4106.0308]
Precursor Scan: 073074, m/z: 1333.9359, charge: 3
VVLHPNYSQVDIGLIK_5_4_0_2_0
Intensity
m/z
204.0863
138.0548
366.1389
168.0653
274.0916
186.0756
292.1016
126.0548
144.0653
528.1931
657.2363
999.5382 PEP_0_1_0_0_0
1101.1007 PEP_0_2_0_0_0
1182.1074 PEP_1_2_0_0_0
1263.1448 PEP_2_2_0_0_0
1344.1785 PEP_3_2_0_0_0
1445.7108 PEP_3_3_0_0_0
1998.1019 PEP_0_1_0_0_0

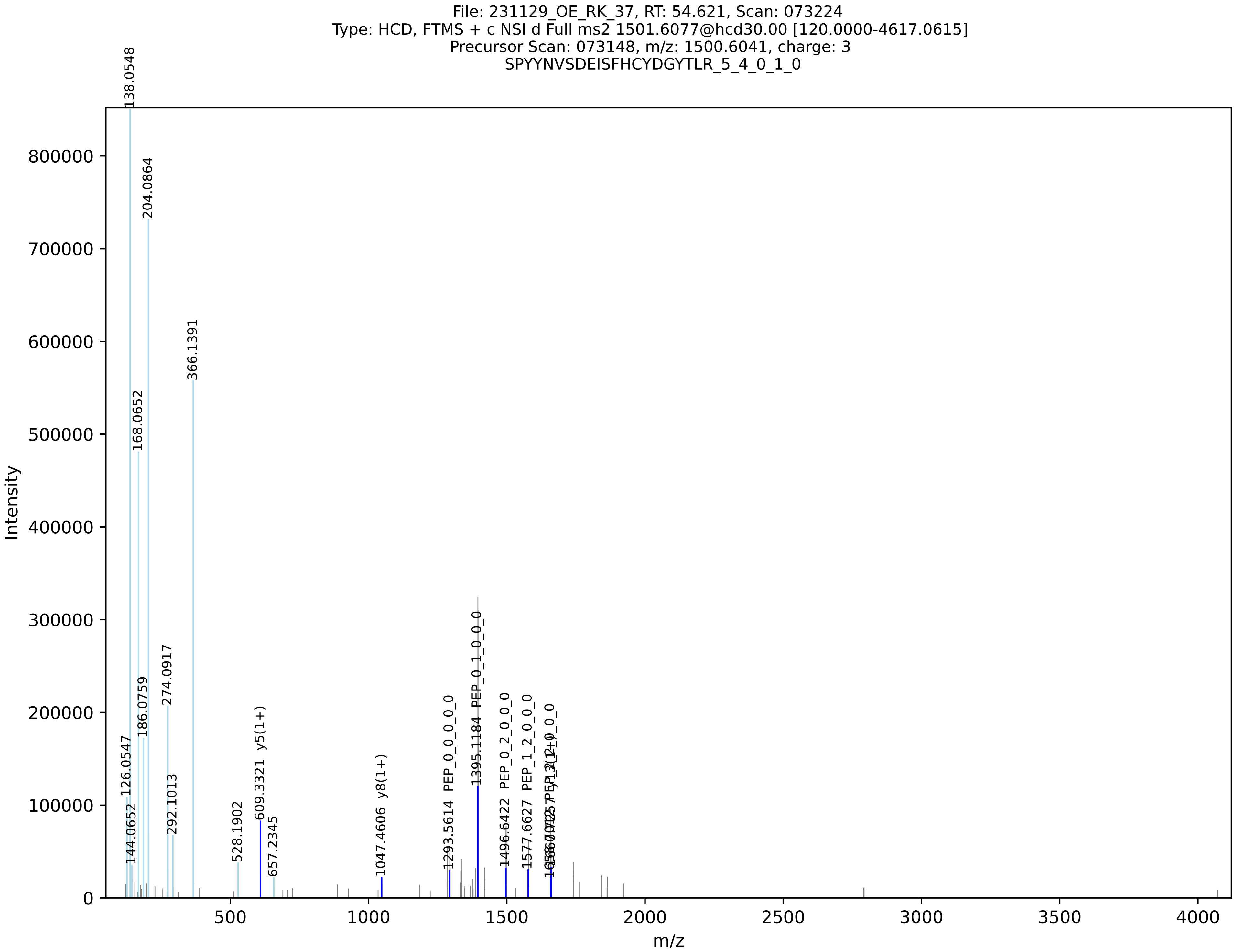
File: 231129_OE_RK_37, RT: 54.621, Scan: 073224
Type: HCD, FTMS + c NSI d Full ms2 1501.6077@hcd30.00 [120.0000-4617.0615]
Precursor Scan: 073148, m/z: 1500.6041, charge: 3
SPYYNVSDEISFHCYDGYTLR_5_4_0_1_0
Intensity
m/z
138.0548
204.0864
366.1391
168.0652
274.0917
186.0759
126.0547
144.0652
292.1013
528.1902
609.3321 y5(1+)
657.2345
1047.4606 y8(1+)
1293.5614 PEP_0_0_0_0_0
1395.1184 PEP_0_1_0_0_0
1496.6422 PEP_0_2_0_0_0
1577.6627 PEP_1_2_0_0_0
0
100000
200000
300000
400000
500000
600000
700000
800000
500
1000
1500
2000
2500
3000
3500
4000

File: 231129_OE_RK_37, RT: 54.624, Scan: 073229
Type: HCD, FTMS + c NSI d Full ms2 1126.2048@hcd30.00 [120.0000-4617.7715]
Precursor Scan: 073148, m/z: 1125.7049, charge: 4
SPYYNVSDEISFHCYDGYTLR_5_4_0_1_0

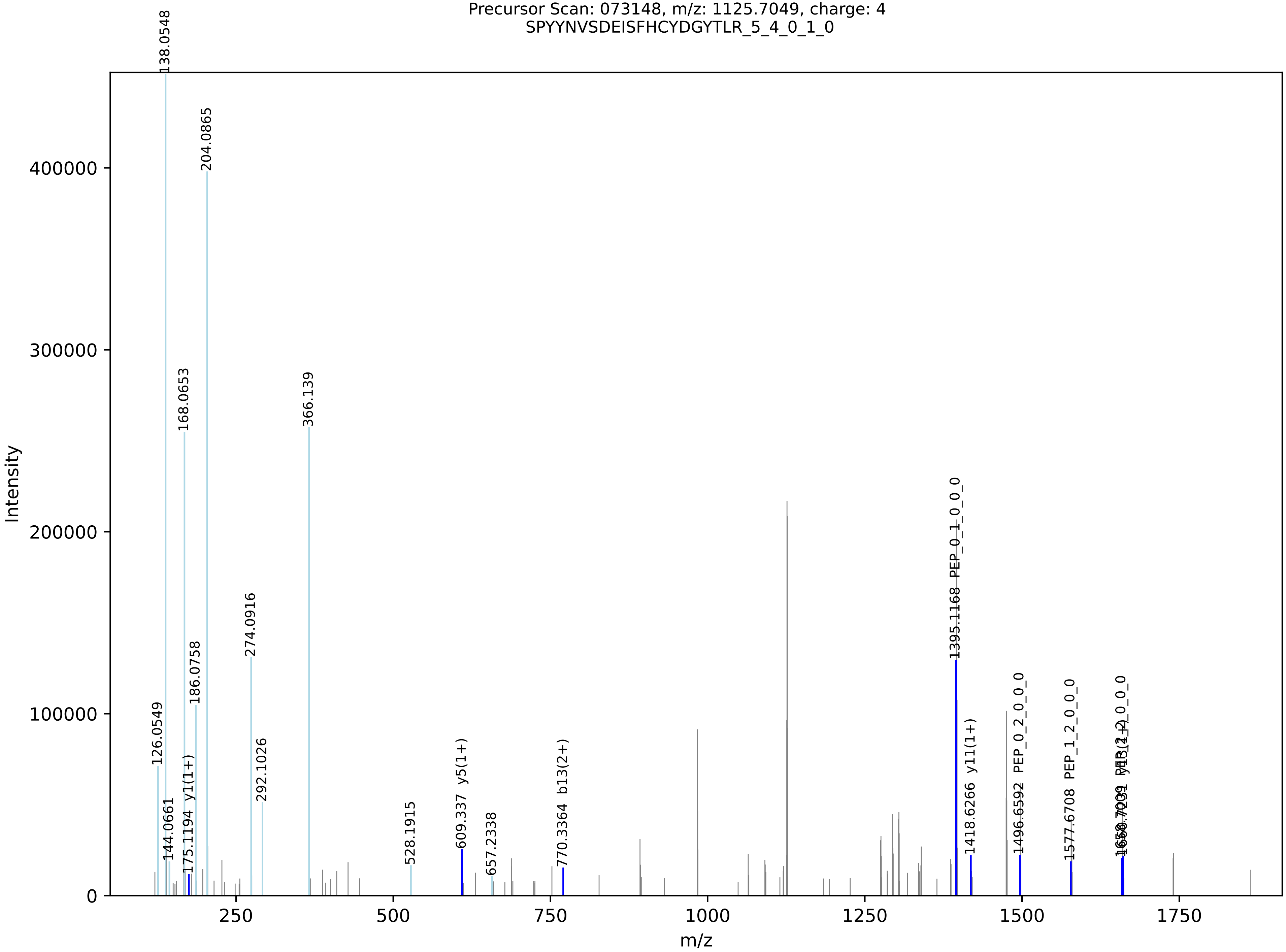

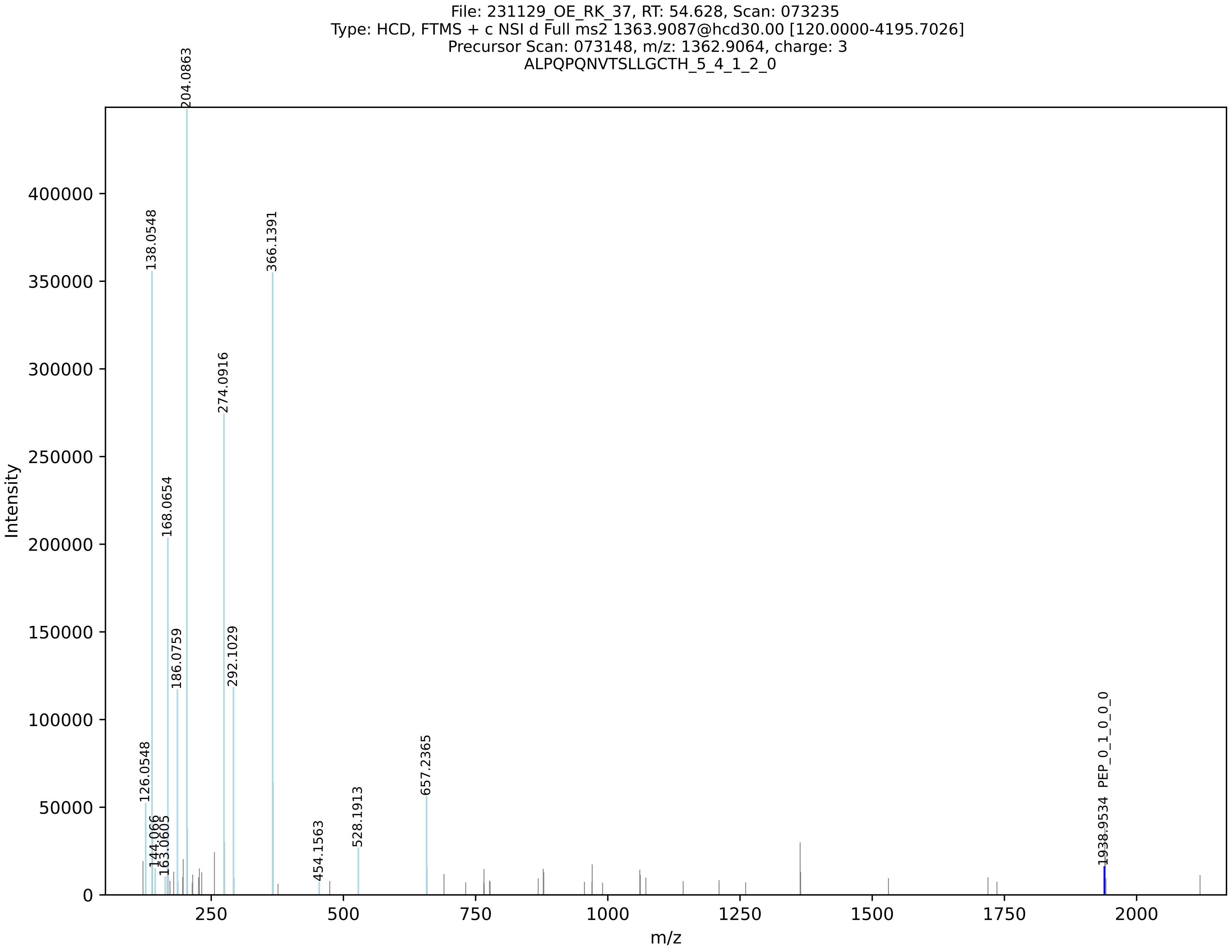
File: 231129_OE_RK_37, RT: 54.628, Scan: 073235
Type: HCD, FTMS + c NSI d Full ms2 1363.9087@hcd30.00 [120.0000-4195.7026]
Precursor Scan: 073148, m/z: 1362.9064, charge: 3
ALPQPQNVTSLLGCTH_5_4_1_2_0
204.0863
138.0548
366.1391
274.0916
168.0654
186.0759
292.1029
126.0548
144.066
163.0605
454.1563
528.1913
657.2365
1938.9534
PEP_0_1_0_0_0
Intensity
m/z
0
50000
100000
150000
200000
250000
300000
350000
400000
250
500
750
1000
1250
1500
1750
2000

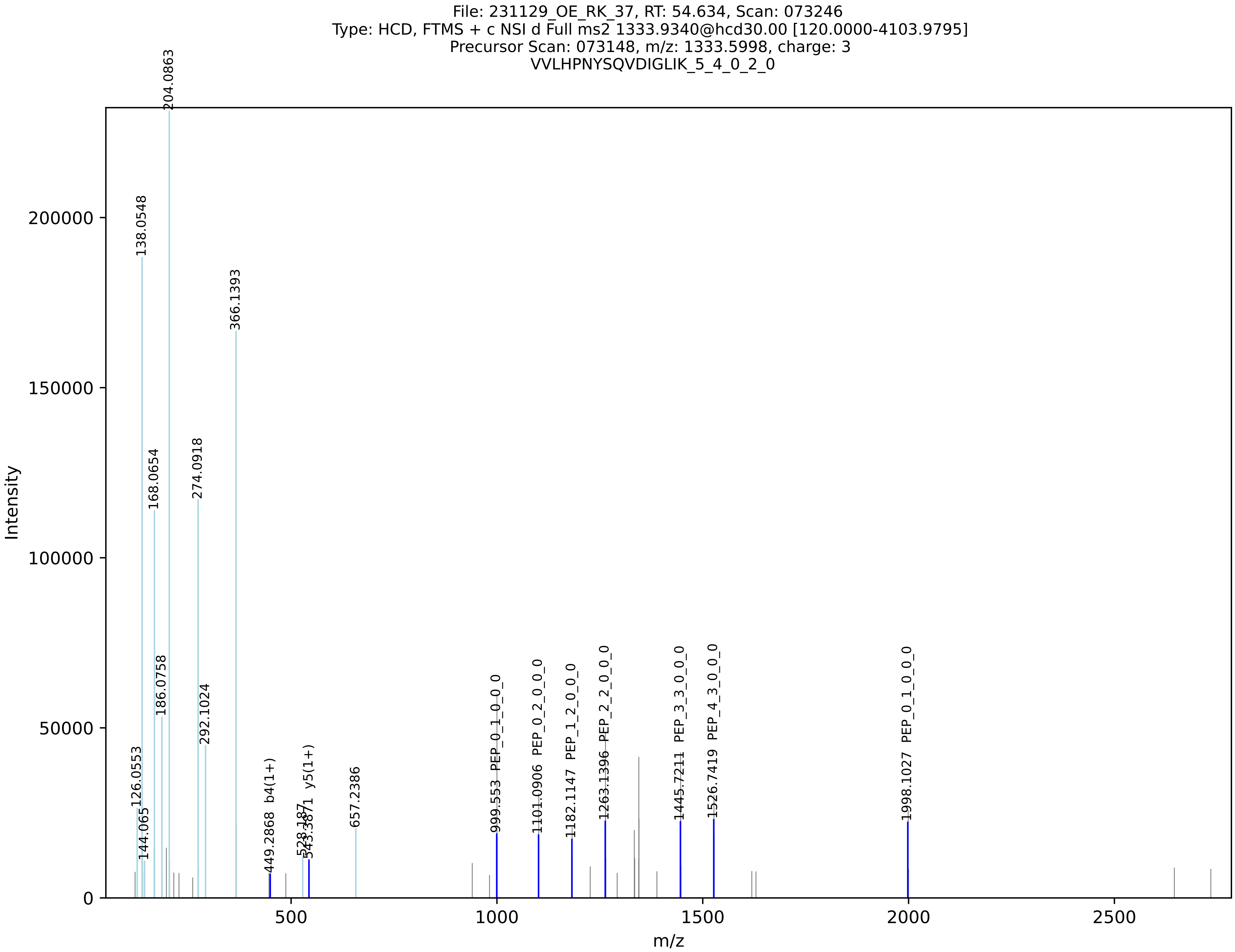
File: 231129_OE_RK_37, RT: 54.634, Scan: 073246
Type: HCD, FTMS + c NSI d Full ms2 1333.9340@hcd30.00 [120.0000-4103.9795]
Precursor Scan: 073148, m/z: 1333.5998, charge: 3
VVLHPNYSQVDIGLIK_5_4_0_2_0
Intensity
m/z
204.0863
138.0548
366.1393
168.0654
274.0918
186.0758
292.1024
126.0553
144.065
449.2868 b4(1+)
528.187
543.3871 y5(1+)
657.2386
999.553 PEP_0_1_0_0_0
1101.0906 PEP_0_2_0_0_0
1182.1147 PEP_1_2_0_0_0
1263.1396 PEP_2_2_0_0_0
1445.7211 PEP_3_3_0_0_0
1526.7419 PEP_4_3_0_0_0
1998.1027 PEP_0_1_0_0_0
0
50000
100000
150000
200000
500
1000
1500
2000
2500

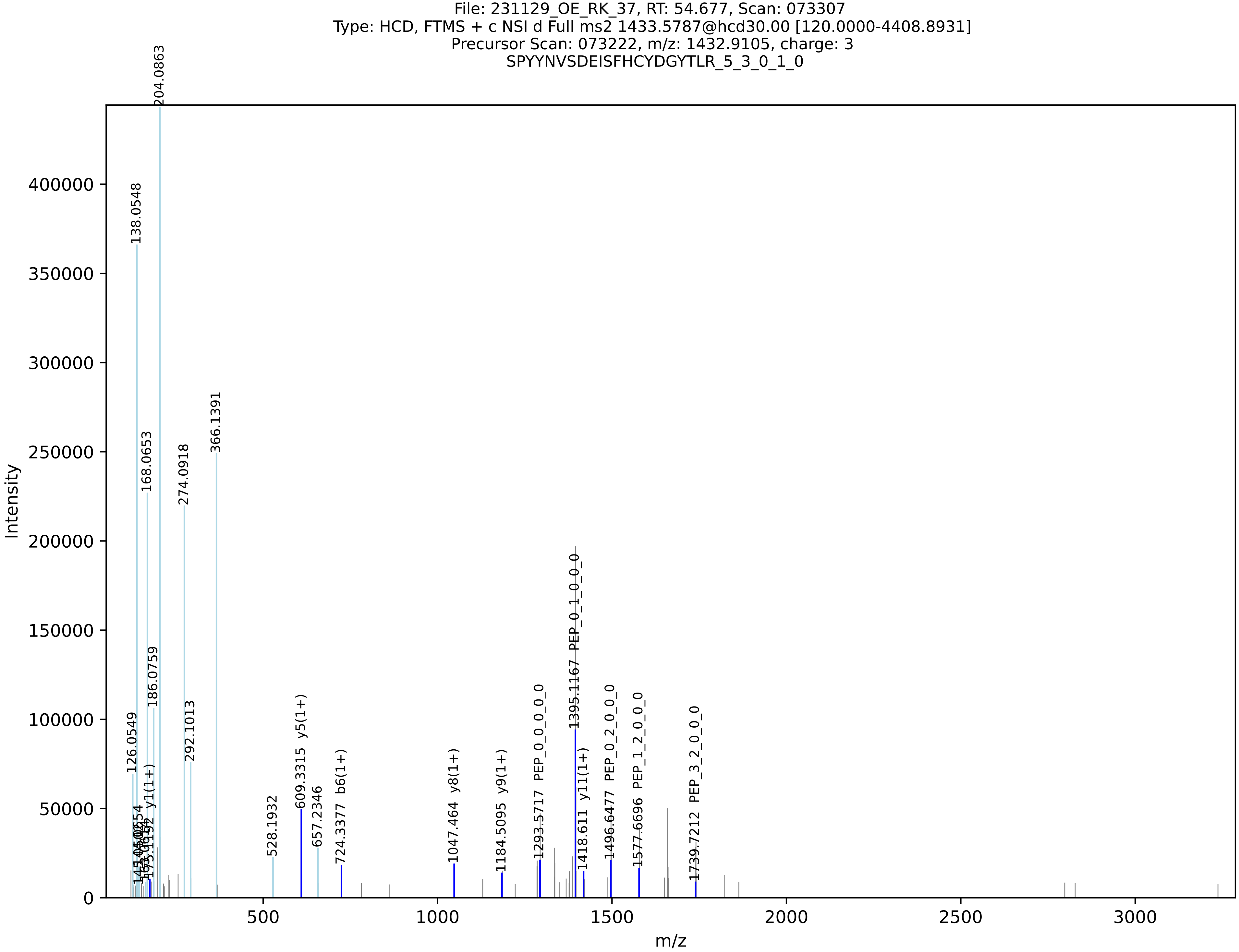
File: 231129_OE_RK_37, RT: 54.677, Scan: 073307
Type: HCD, FTMS + c NSI d Full ms2 1433.5787@hcd30.00 [120.0000-4408.8931]
Precursor Scan: 073222, m/z: 1432.9105, charge: 3
SPYYNVSDEISFHCYDGYTLR_5_3_0_1_0
Intensity
m/z
204.0863
138.0548
168.0653
274.0918
366.1391
186.0759
292.1013
126.0549
y1(1+)
528.1932
609.3315 y5(1+)
657.2346
724.3377 b6(1+)
1047.464 y8(1+)
1184.5095 y9(1+)
1293.5717 PEP_0_0_0_0_0
1395.1167 PEP_0_1_0_0_0
1418.611 y11(1+)
1496.6477 PEP_0_2_0_0_0
1577.6696 PEP_1_2_0_0_0
1739.7212 PEP_3_2_0_0_0

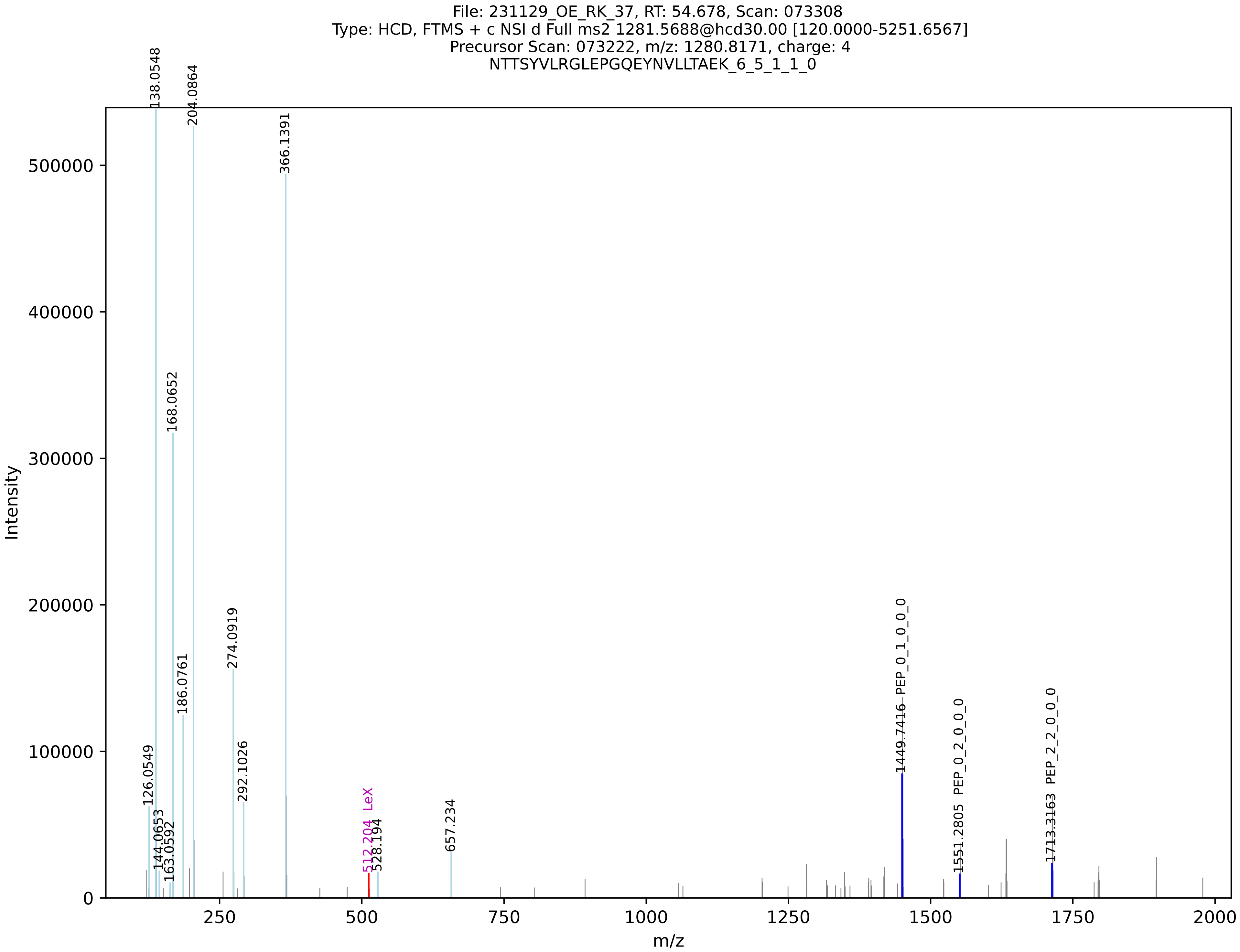
File: 231129_OE_RK_37, RT: 54.678, Scan: 073308
Type: HCD, FTMS + c NSI d Full ms2 1281.5688@hcd30.00 [120.0000-5251.6567]
Precursor Scan: 073222, m/z: 1280.8171, charge: 4
NTTSYVLRGLEPGQEYNVLLTAEK_6_5_1_1_0
138.0548
204.0864
366.1391
168.0652
186.0761
274.0919
292.1026
126.0549
144.0653
163.0592
512.204 LeX
528.194
657.234
1449.7416 PEP_0_1_0_0_0
1551.2805 PEP_0_2_0_0_0
1713.3163 PEP_2_2_0_0_0
Intensity
m/z
0
100000
200000
300000
400000
500000
250
500
750
1000
1250
1500
1750
2000

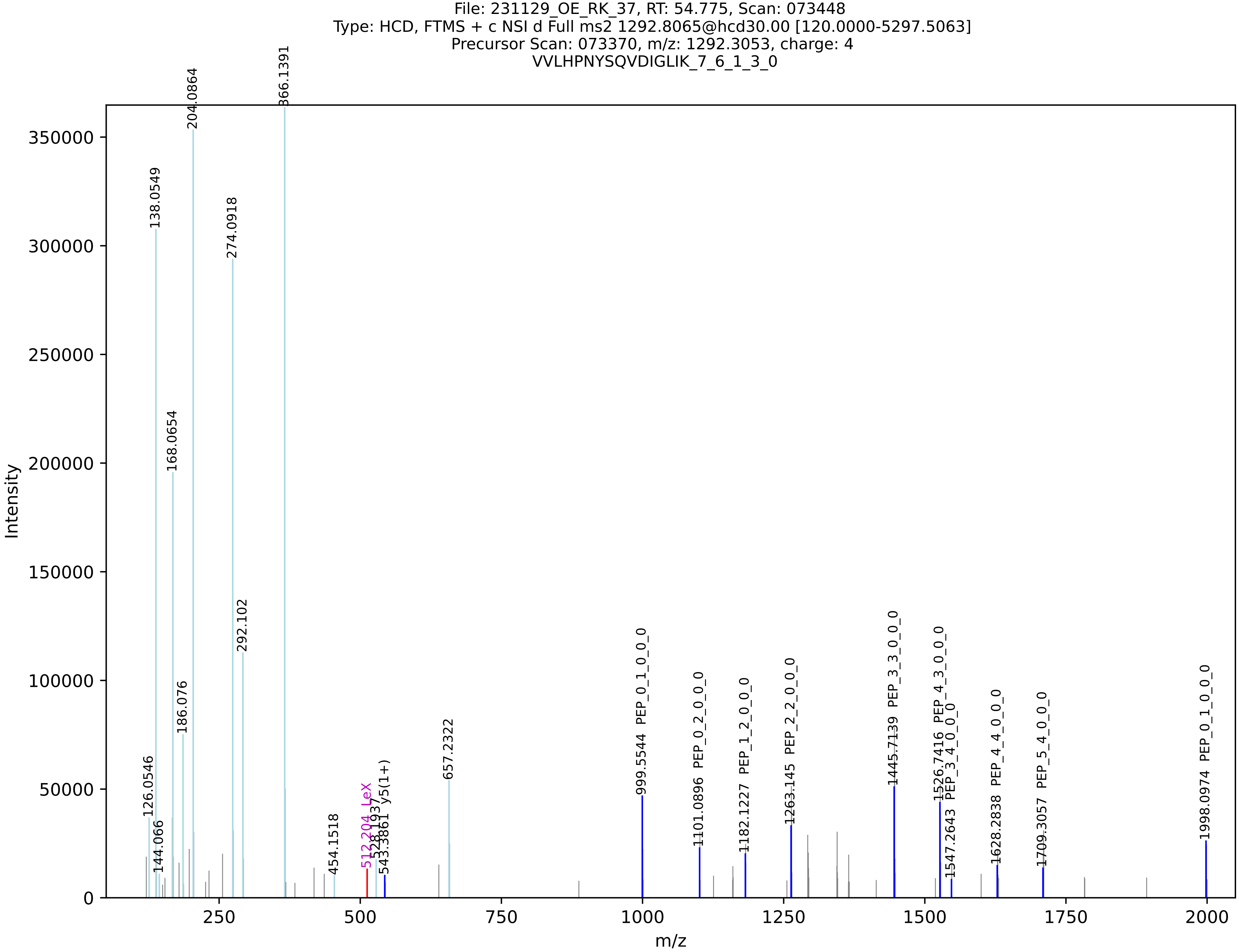

File: 231129_OE_RK_37, RT: 54.775, Scan: 073448
Type: HCD, FTMS + c NSI d Full ms2 1292.8065@hcd30.00 [120.0000-5297.5063]
Precursor Scan: 073370, m/z: 1292.3053, charge: 4
VVLHPNYSQVDIGLIK_7_6_1_3_0
Intensity
m/z
366.1391
204.0864
138.0549
274.0918
168.0654
292.102
186.076
126.0546
144.066
454.1518
512.204 LeX
528.1937
543.3861 y5(1+)
657.2322
999.5544 PEP_0_1_0_0_0
1101.0896 PEP_0_2_0_0_0
1182.1227 PEP_1_2_0_0_0
1263.145 PEP_2_2_0_0_0
1445.7139 PEP_3_3_0_0_0
1526.7416 PEP_4_3_0_0_0
1547.2643 PEP_3_4_0_0_0
1628.2838 PEP_4_4_0_0_0
1709.3057 PEP_5_4_0_0_0
1998.0974 PEP_0_1_0_0_0

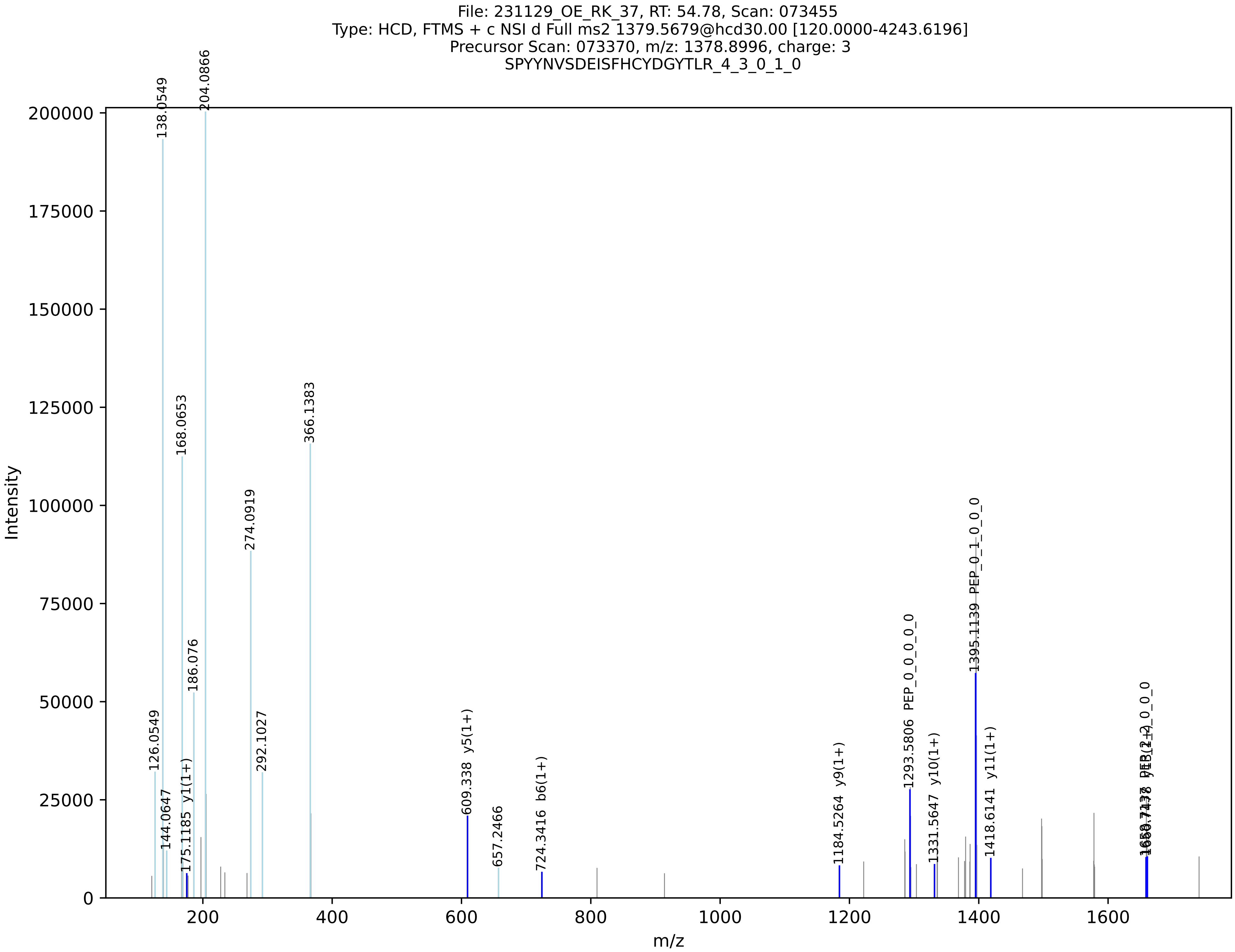

File: 231129_OE_RK_37, RT: 54.78, Scan: 073455
Type: HCD, FTMS + c NSI d Full ms2 1379.5679@hcd30.00 [120.0000-4243.6196]
Precursor Scan: 073370, m/z: 1378.8996, charge: 3
SPYYNVSDEISFHCYDGYTLR_4_3_0_1_0
Intensity
m/z
138.0549
204.0866
168.0653
366.1383
274.0919
186.076
126.0549
292.1027
144.0647
175.1185 y1(1+)
609.338 y5(1+)
657.2466
724.3416 b6(1+)
1184.5264 y9(1+)
1293.5806 PEP_0_0_0_0_
1331.5647 y10(1+)
1395.1139 PEP_0_1_0_0_0
1418.6141 y11(1+)

File: 231129_OE_RK_37, RT: 54.824, Scan: 073516
Type: HCD, FTMS + c NSI d Full ms2 1244.8075@hcd30.00 [120.0000-5101.6704]
Precursor Scan: 073441, m/z: 1244.3063, charge: 4
NTTSYVLRGLEPGQEYNVLLTAEK_6_5_0_1_0

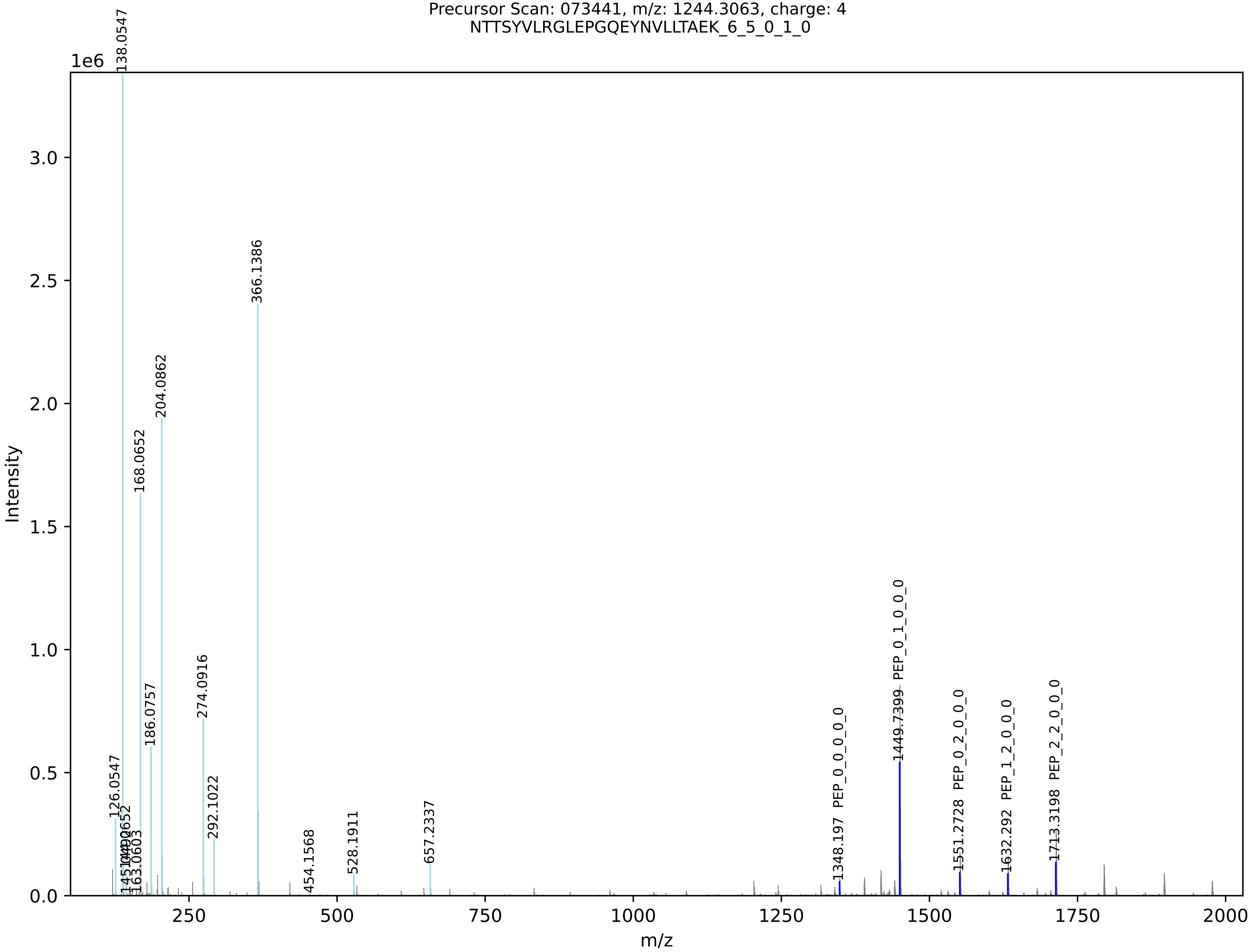

File: 231129_OE_RK_37, RT: 54.827, Scan: 073521
Type: HCD, FTMS + c NSI d Full ms2 1334.5970@hcd30.00 [120.0000-4106.0088]
Precursor Scan: 073441, m/z: 1334.2649, charge: 3
VVLHPNYSQVDIGLIK_5_4_0_2_0

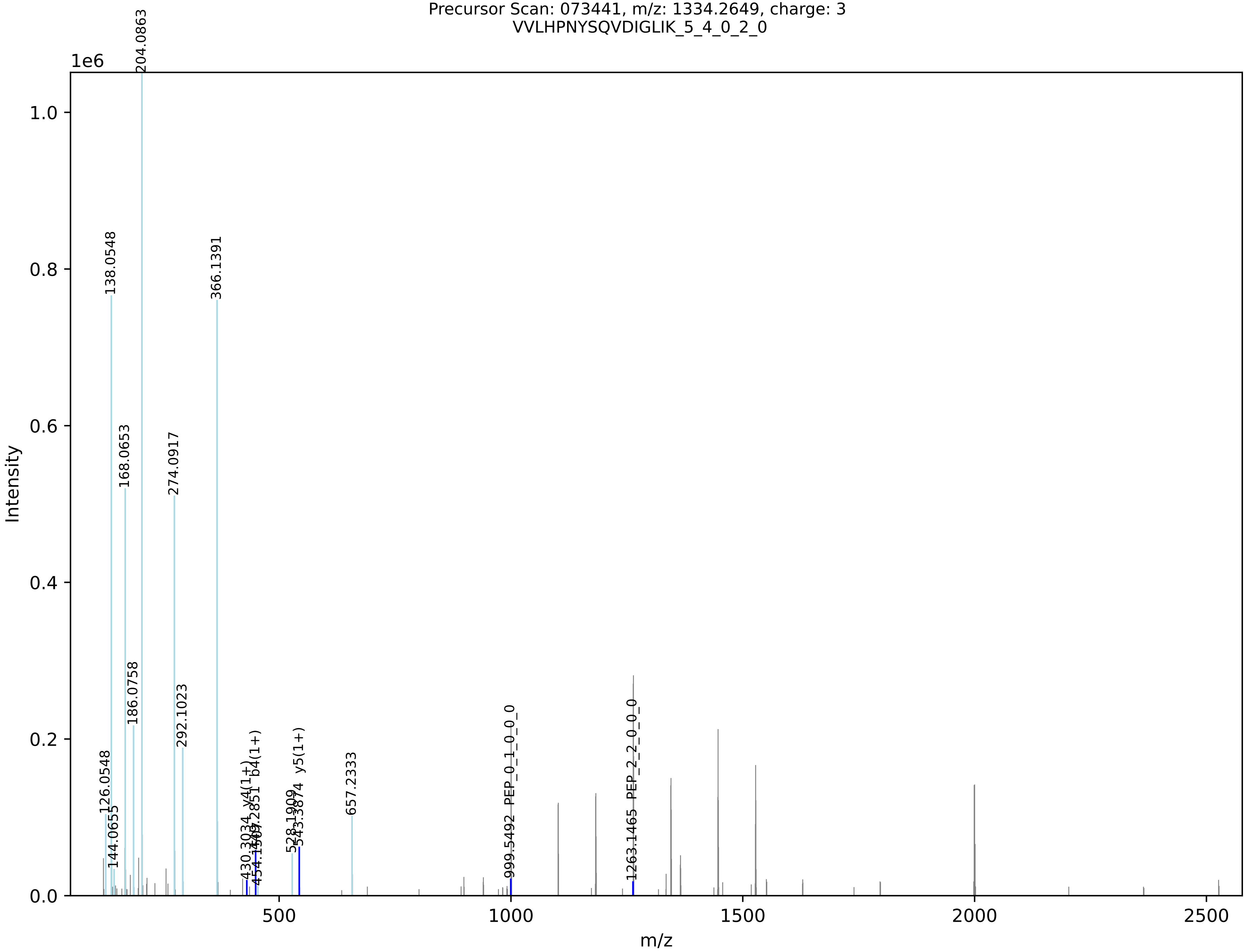

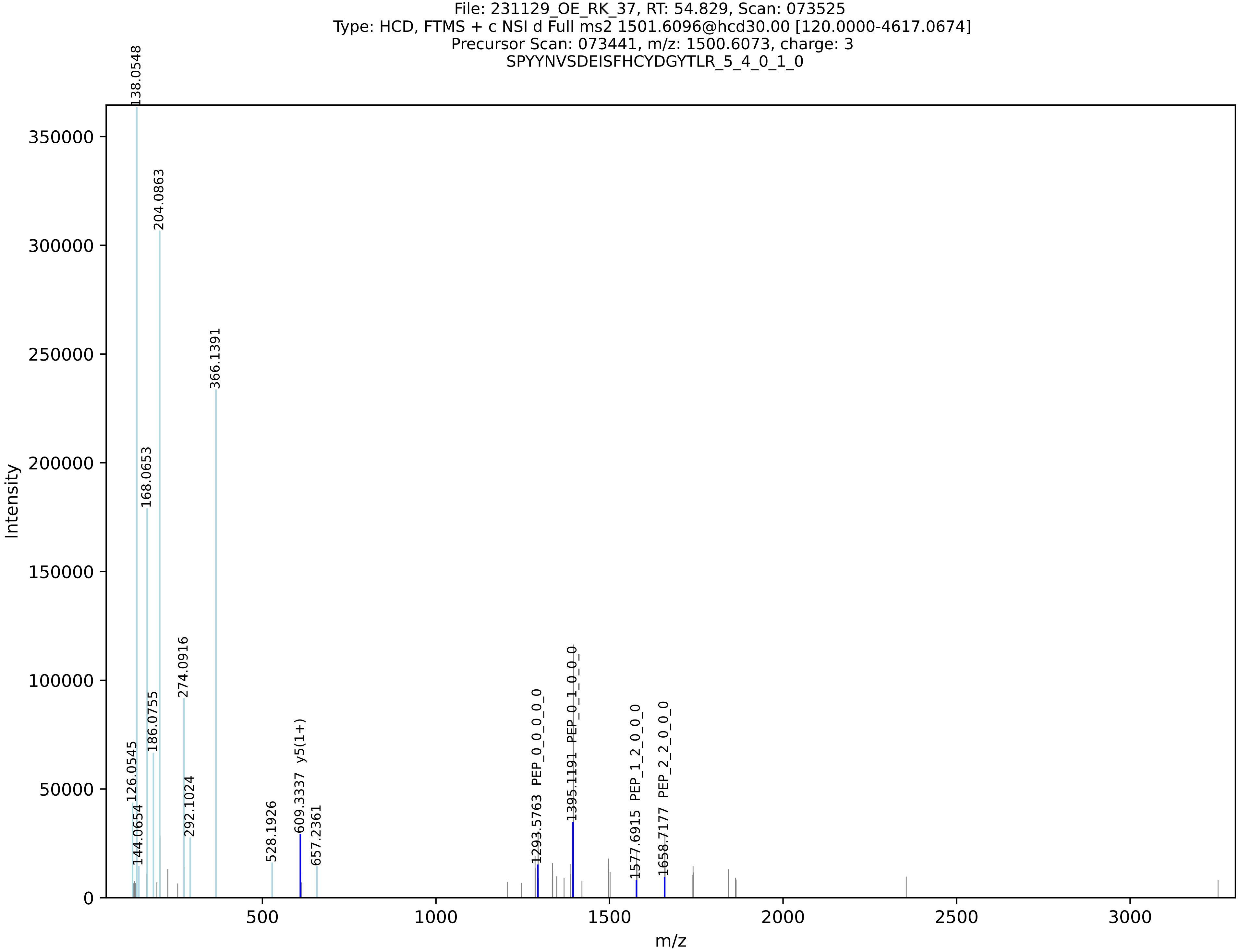

File: 231129_OE_RK_37, RT: 54.829, Scan: 073525
Type: HCD, FTMS + c NSI d Full ms2 1501.6096@hcd30.00 [120.0000-4617.0674]
Precursor Scan: 073441, m/z: 1500.6073, charge: 3
SPYYNVSDEISFHCYDGYTLR_5_4_0_1_0
Intensity
m/z
138.0548
204.0863
366.1391
168.0653
274.0916
186.0755
126.0545
144.0654
292.1024
528.1926
609.3337 y5(1+)
657.2361
1293.5763 PEP_0_0_0_0_0
1395.1191 PEP_0_1_0_0_0
1577.6915 PEP_1_2_0_0_0
1658.7177 PEP_2_2_0_0_0

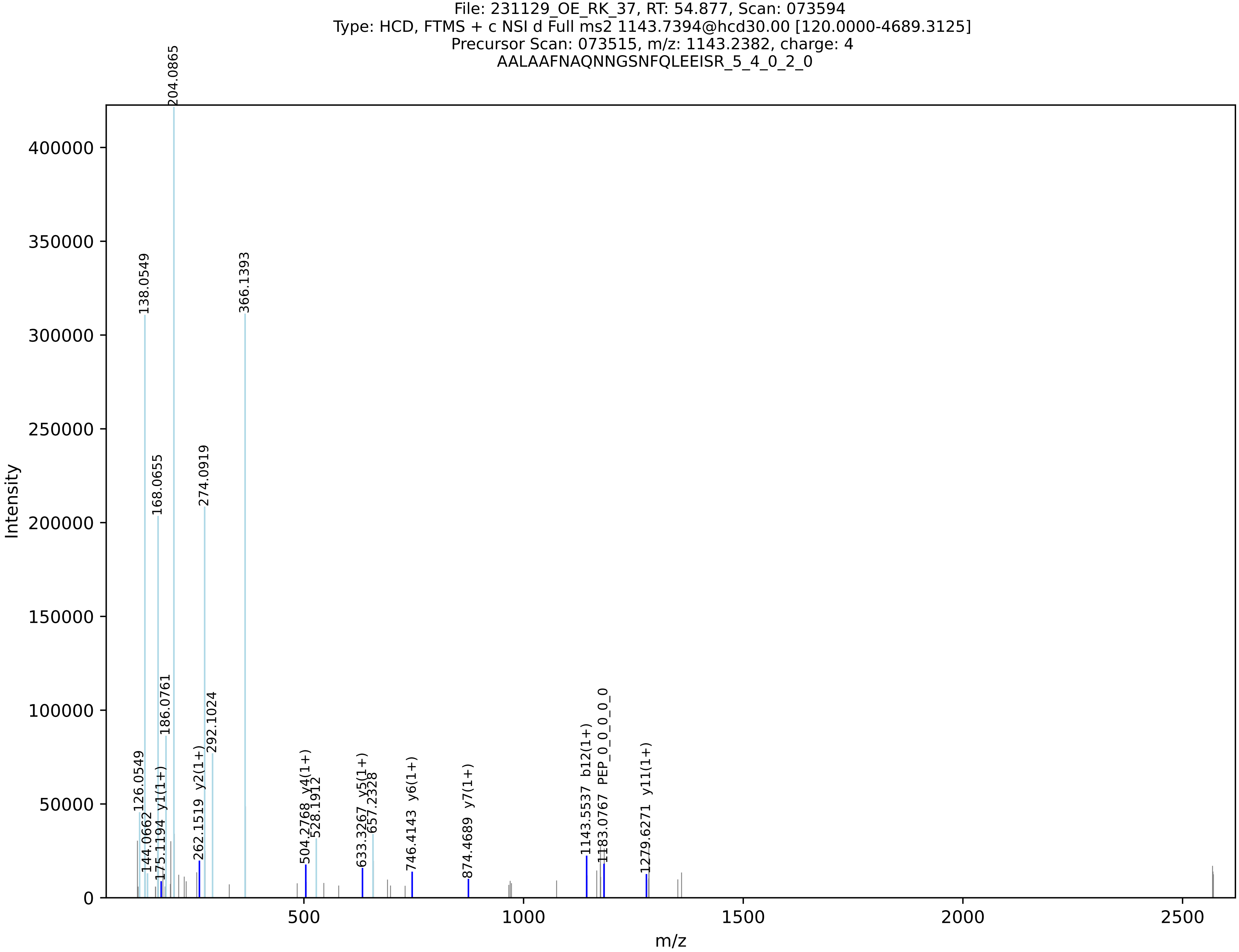

File: 231129_OE_RK_37, RT: 54.877, Scan: 073594
Type: HCD, FTMS + c NSI d Full ms2 1143.7394@hcd30.00 [120.0000-4689.3125]
Precursor Scan: 073515, m/z: 1143.2382, charge: 4
AALAAFNAQNNGSNFQLEEISR_5_4_0_2_0
Intensity
m/z
204.0865
138.0549
366.1393
168.0655
274.0919
186.0761
292.1024
126.0549
144.0662
175.1194 y1(1+)
262.1519 y2(1+)
504.2768 y4(1+)
528.1912
633.3267 y5(1+)
657.2328
746.4143 y6(1+)
874.4689 y7(1+)
1143.5537 b12(1+)
1183.0767 PEP_0_0_0_0_0
1279.6271 y11(1+)

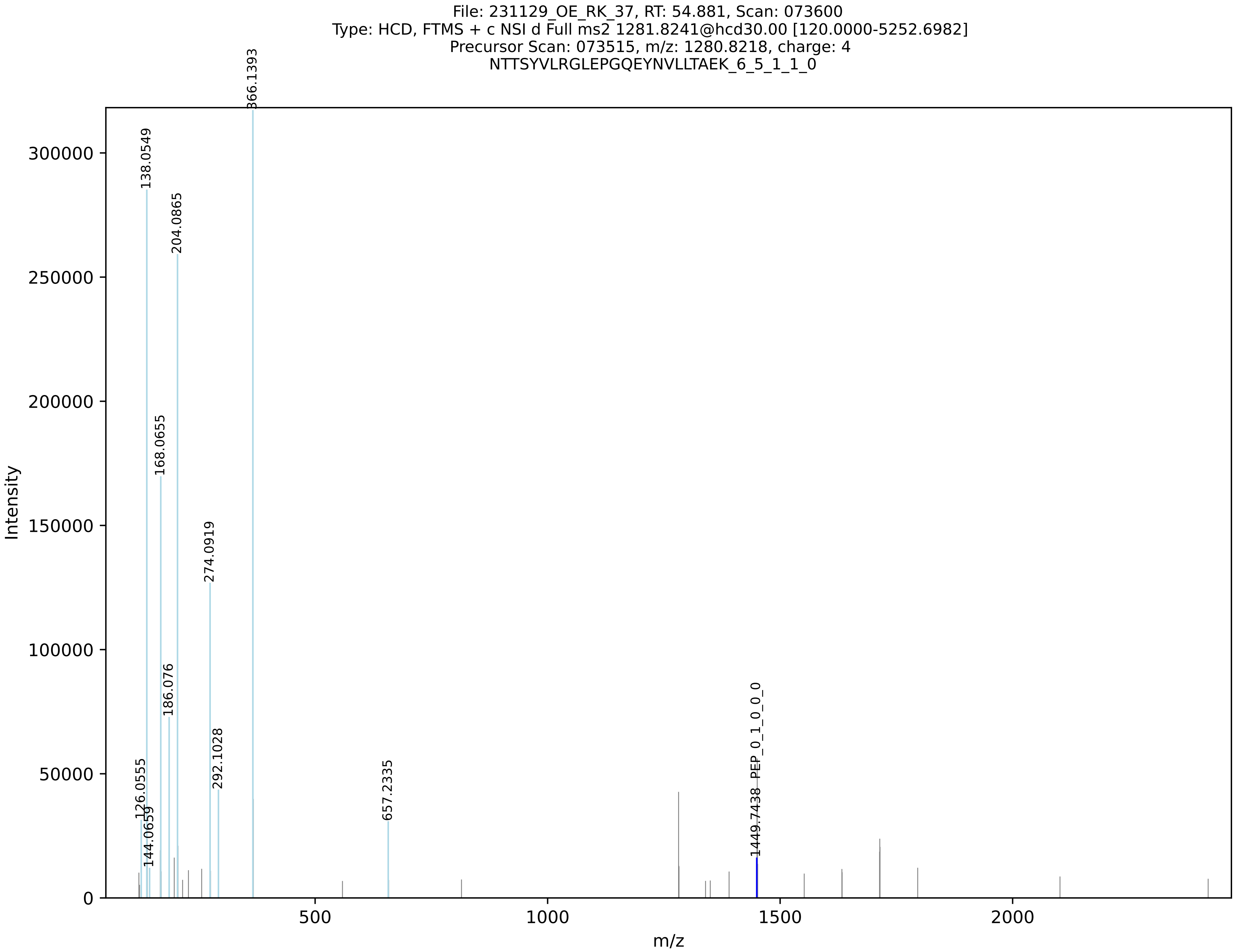

File: 231129_OE_RK_37, RT: 54.881, Scan: 073600
Type: HCD, FTMS + c NSI d Full ms2 1281.8241@hcd30.00 [120.0000-5252.6982]
Precursor Scan: 073515, m/z: 1280.8218, charge: 4
NTTTSYVLRGLEPGQEYNVLLTAEK_6_5_1_1_0
366.1393
138.0549
204.0865
168.0655
274.0919
186.076
292.1028
126.0555
144.0659
657.2335
1449.7438_PEP_0_1_0_0_0
Intensity
m/z

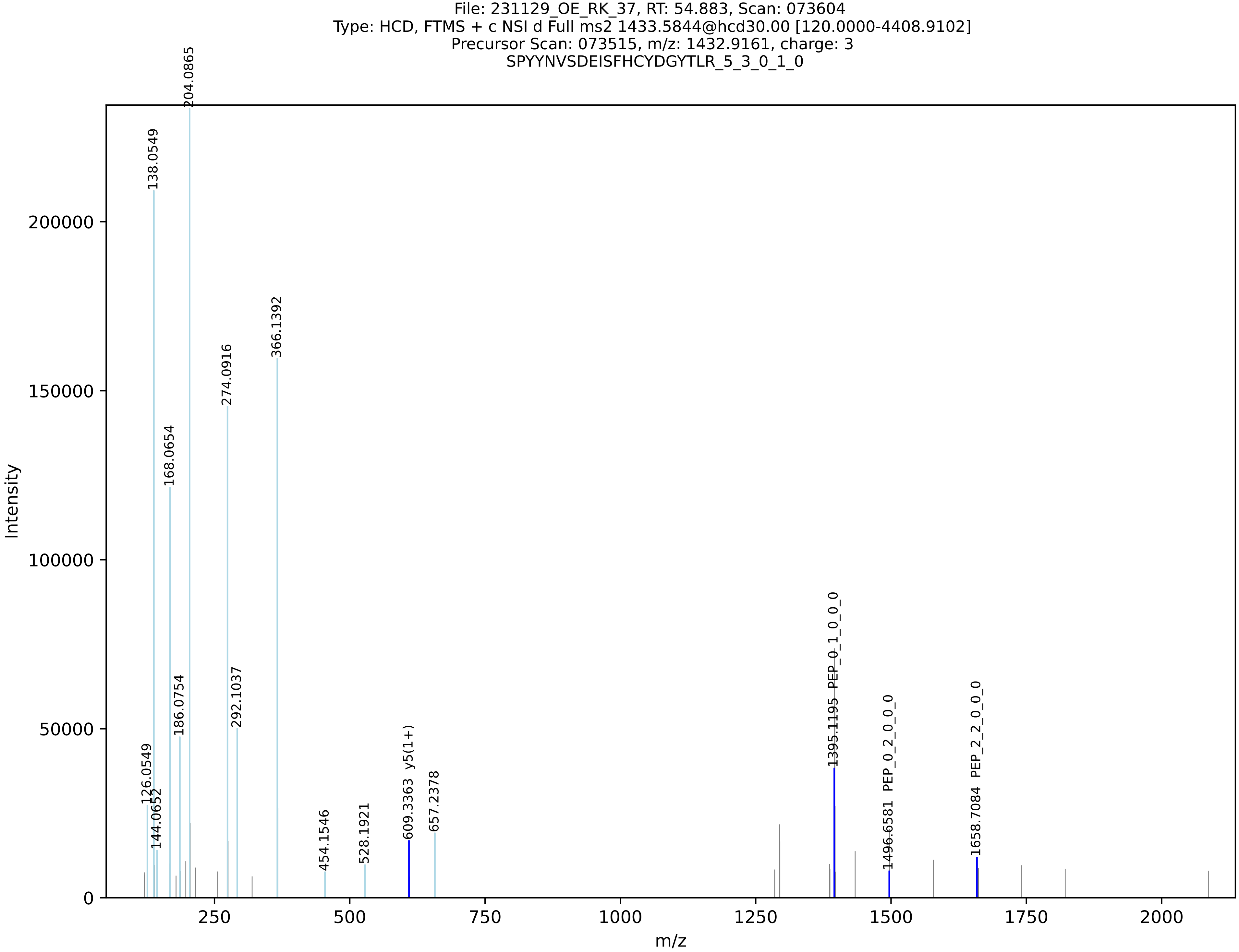
File: 231129_OE_RK_37, RT: 54.883, Scan: 073604
Type: HCD, FTMS + c NSI d Full ms2 1433.5844@hcd30.00 [120.0000-4408.9102]
Precursor Scan: 073515, m/z: 1432.9161, charge: 3
SPYYNVSDEISFHCYDGYTLR_5_3_0_1_0
Intensity
m/z
204.0865
138.0549
366.1392
274.0916
168.0654
292.1037
186.0754
126.0549
144.0652
454.1546
528.1921
609.3363 y5(1+)
657.2378
1395.1195 PEP_0_1_0_0_0
1496.6581 PEP_0_2_0_0_0
1658.7084 PEP_2_2_0_0_0

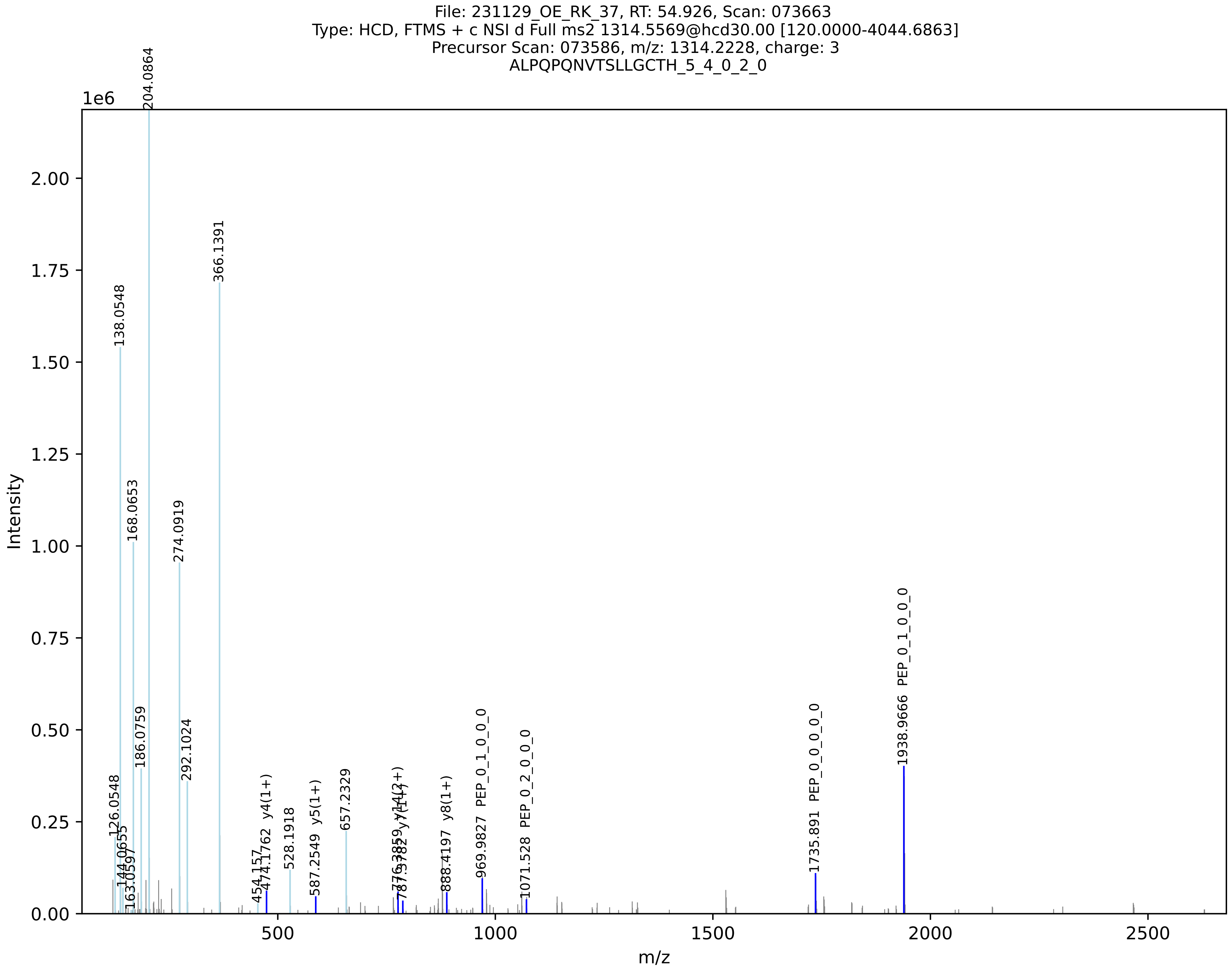
File: 231129_OE_RK_37, RT: 54.926, Scan: 073663
Type: HCD, FTMS + c NSI d Full ms2 1314.5569@hcd30.00 [120.0000-4044.6863]
Precursor Scan: 073586, m/z: 1314.2228, charge: 3
ALPQPQNVTSLLGCTH_5_4_0_2_0
1e6
Intensity
m/z
204.0864
366.1391
138.0548
168.0653
274.0919
186.0759
292.1024
126.0548
144.0655
163.0597
454.157
474.1762 y4(1+)
528.1918
587.2549 y5(1+)
657.2329
776.3859 y14(2+)
787.3782 y7(1+)
888.4197 y8(1+)
969.9827 PEP_0_1_0_0_0
1071.528 PEP_0_2_0_0_0
1735.891 PEP_0_0_0_0_0
1938.9666 PEP_0_1_0_0_0
0.00
0.25
0.50
0.75
1.00
1.25
1.50
1.75
2.00
500
1000
1500
2000
2500

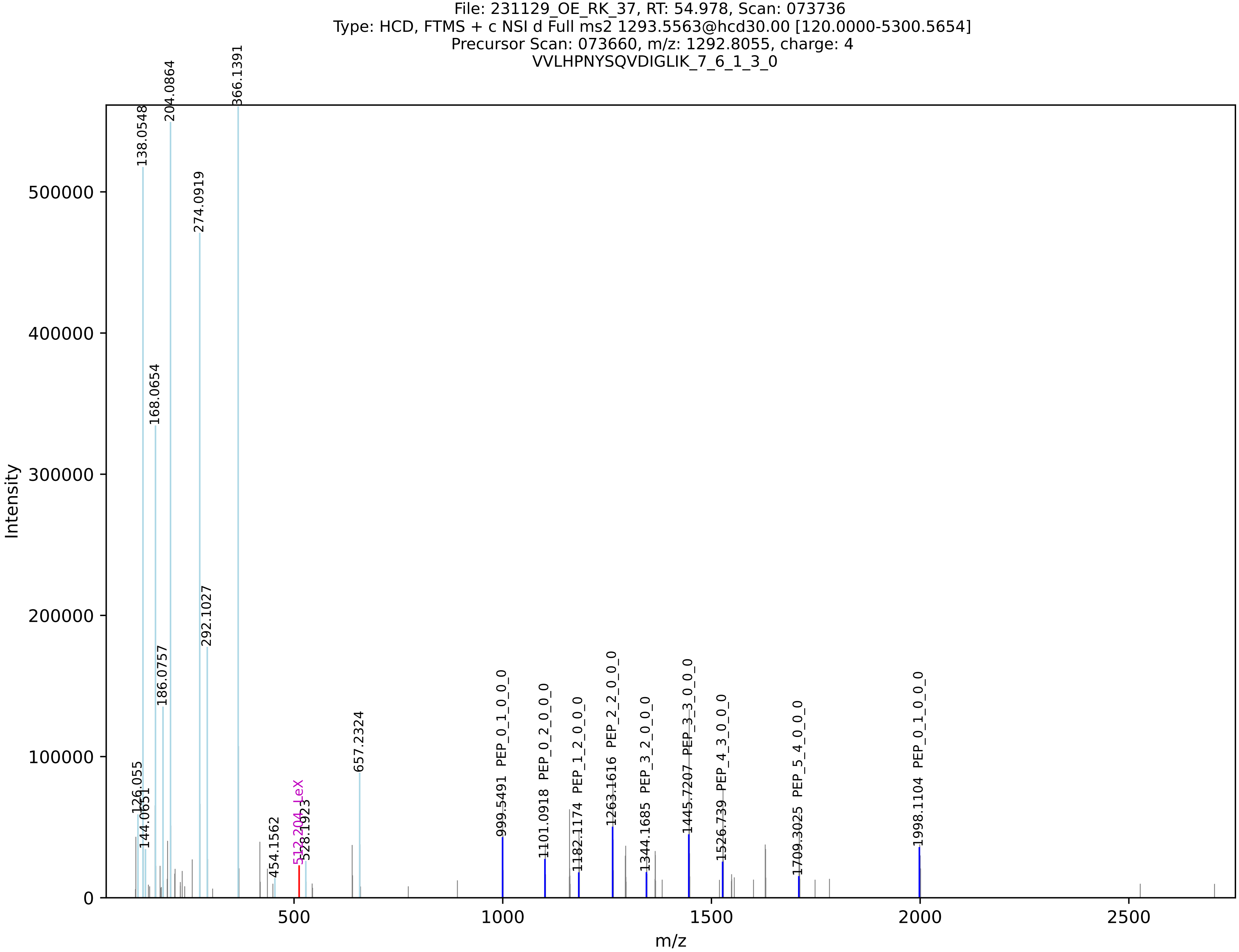

File: 231129_OE_RK_37, RT: 54.978, Scan: 073736
Type: HCD, FTMS + c NSI d Full ms2 1293.5563@hcd30.00 [120.0000-5300.5654]
Precursor Scan: 073660, m/z: 1292.8055, charge: 4
VVLHPNYSQVDIGLIK_7_6_1_3_0
Intensity
m/z
366.1391
204.0864
138.0548
274.0919
168.0654
292.1027
186.0757
126.055
144.0651
454.1562
512.204_LeX
528.1923
657.2324
999.5491 PEP_0_1_0_0_0
1101.0918 PEP_0_2_0_0_0
1182.1174 PEP_1_2_0_0_0
1263.1616 PEP_2_2_0_0_0
1344.1685 PEP_3_2_0_0_0
1445.7207 PEP_3_3_0_0_0
1526.739 PEP_4_3_0_0_0
1709.3025 PEP_5_4_0_0_0
1998.1104 PEP_0_1_0_0_0

File: 231129_OE_RK_37, RT: 54.981, Scan: 073740
Type: HCD, FTMS + c NSI d Full ms2 1320.5670@hcd30.00 [120.0000-4063.0771]
Precursor Scan: 073660, m/z: 1319.8988, charge: 3
ALPQPQNVTSLLGCTH_6_4_1_1_0

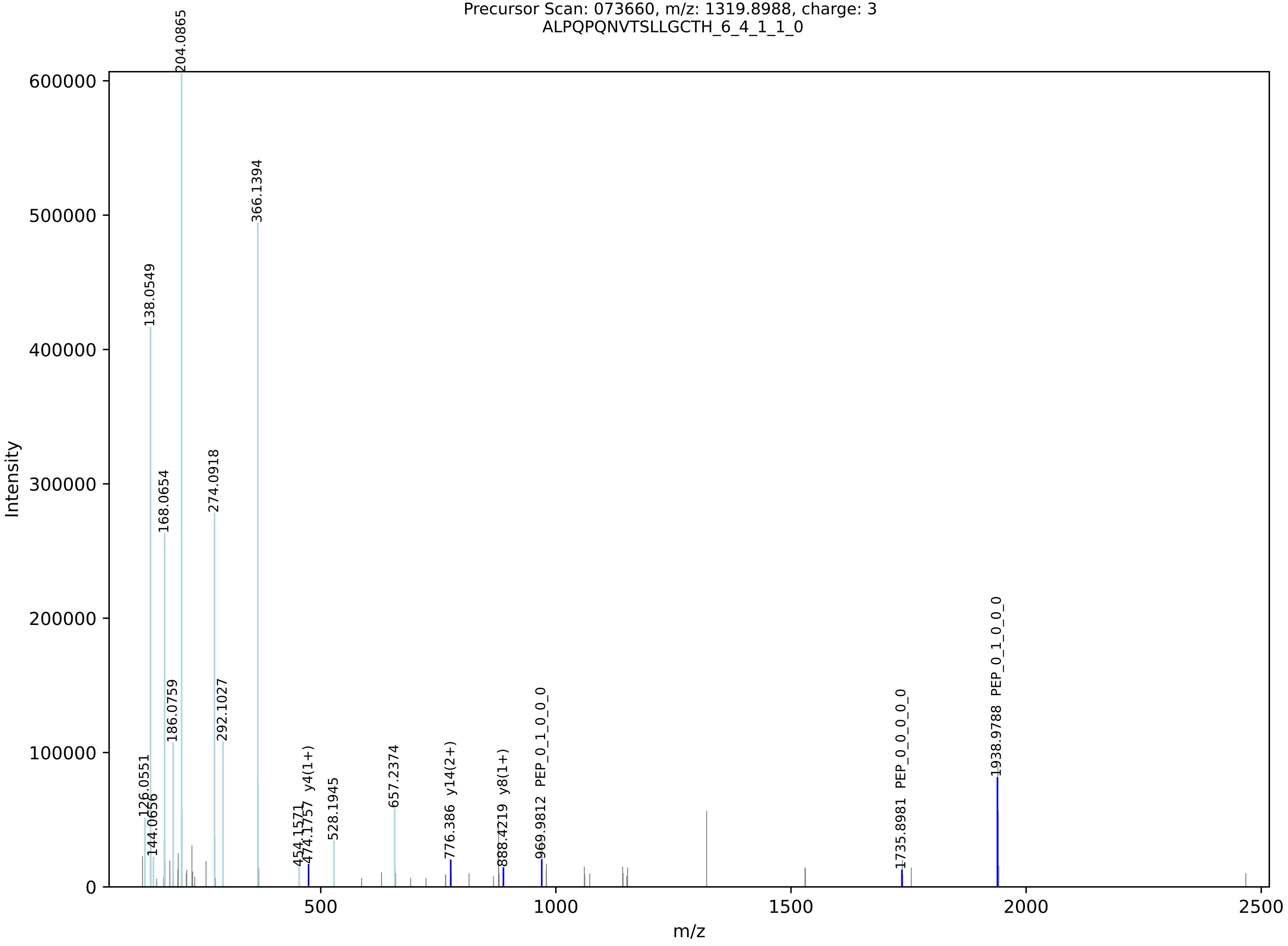

File: 231129_OE_RK_37, RT: 55.011, Scan: 073788
Type: HCD, FTMS + c NSI d Full ms2 1261.8312@hcd30.00 [120.0000-5171.1274]
Precursor Scan: 073586, m/z: 1260.8289, charge: 4
NPVTGNYVKMSLQLYLVDNR_6_5_1_2_0

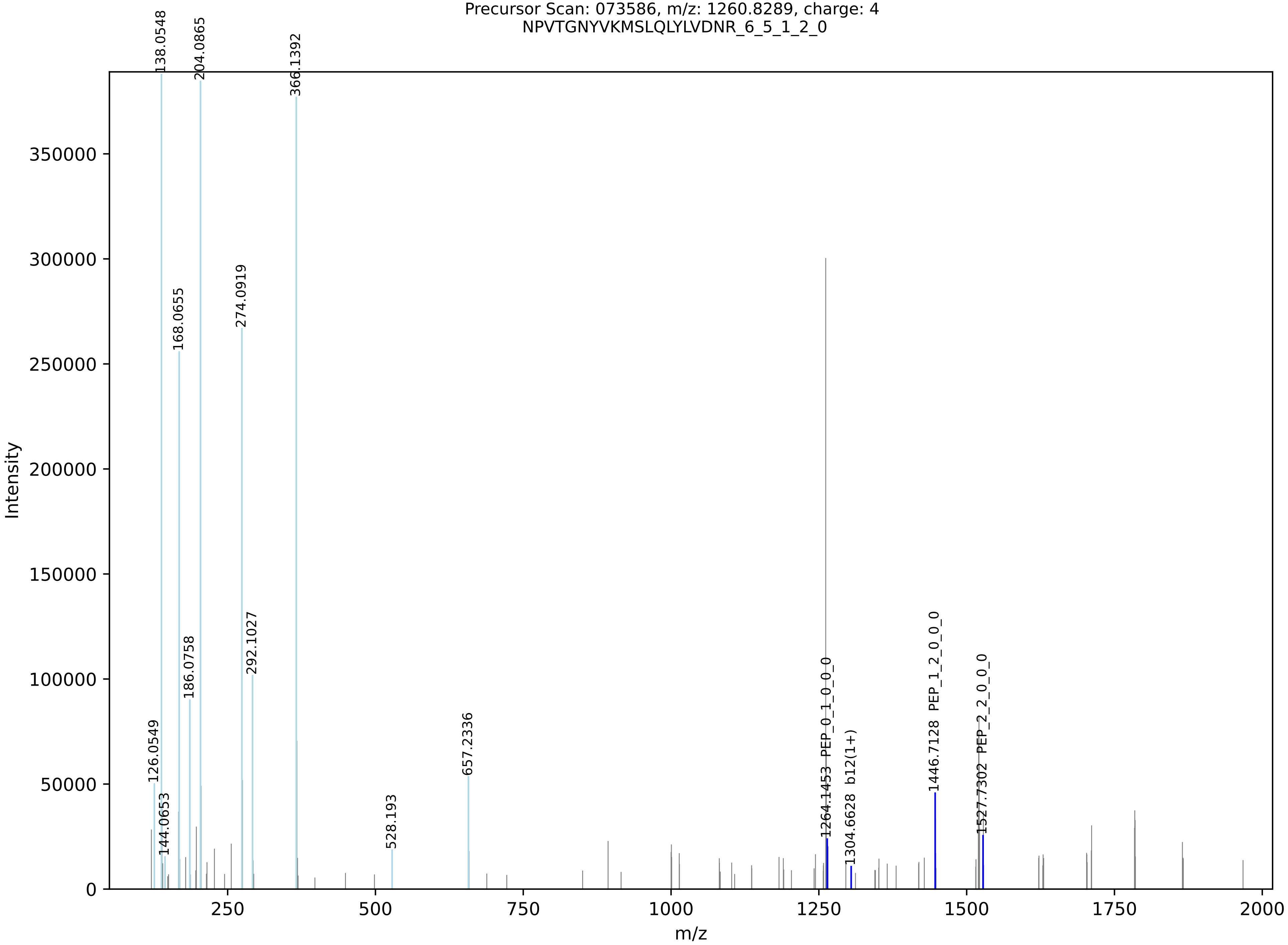

File: 231129_OE_RK_37, RT: 55.014, Scan: 073793
Type: HCD, FTMS + c NSI d Full ms2 1000.9493@hcd30.00 [120.0000-4106.7290]
Precursor Scan: 073586, m/z: 1000.9485, charge: 4
VVLHPNYSQVDIGLIK_5_4_0_2_0

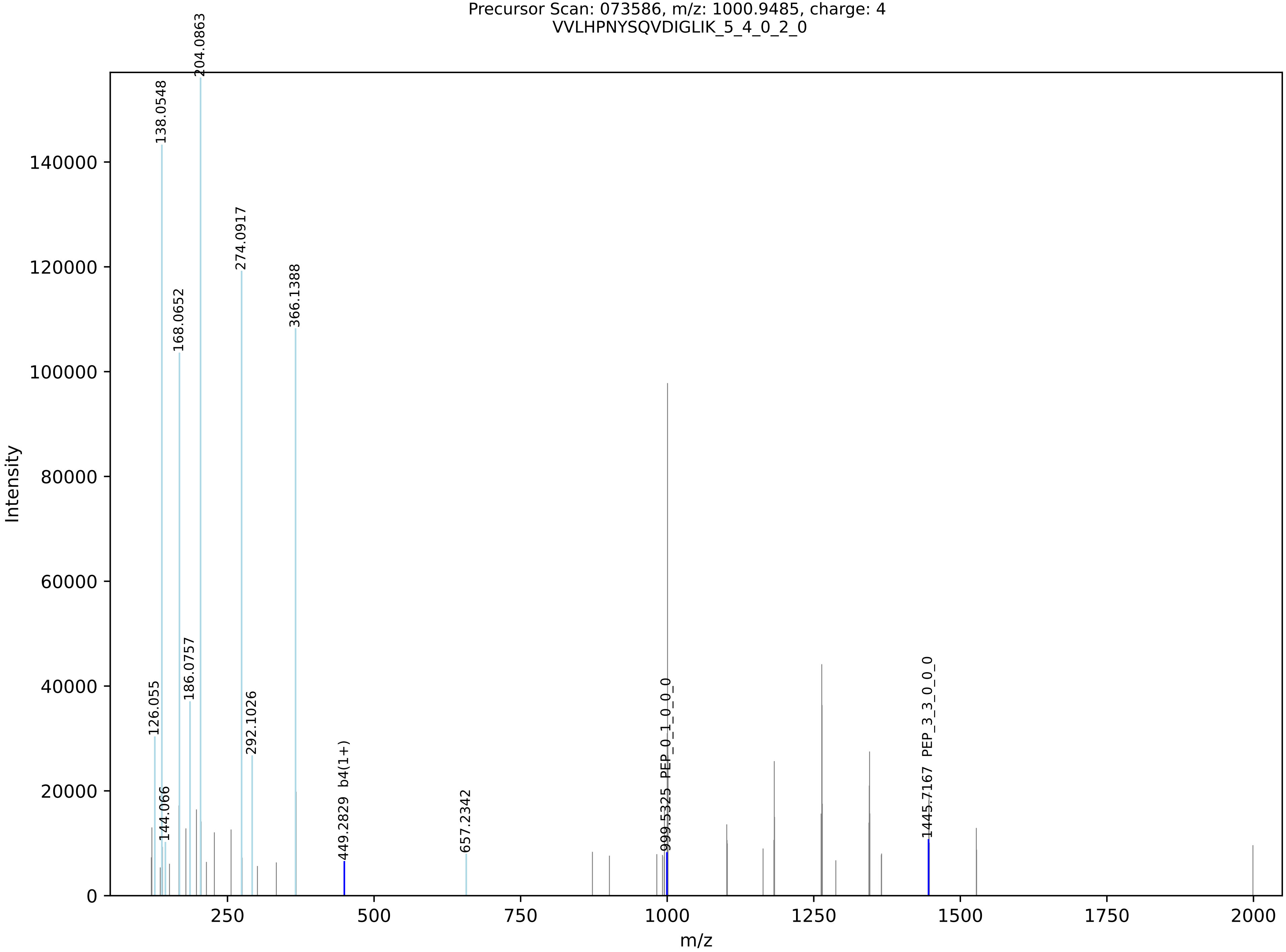

File: 231129_OE_RK_37, RT: 55.025, Scan: 073804
Type: HCD, FTMS + c NSI d Full ms2 1256.5417@hcd30.00 [120.0000-5149.5464]
Precursor Scan: 073730, m/z: 1256.0405, charge: 4
NHPNITFFVYYVSNFTWPIK_5_5_2_2_0

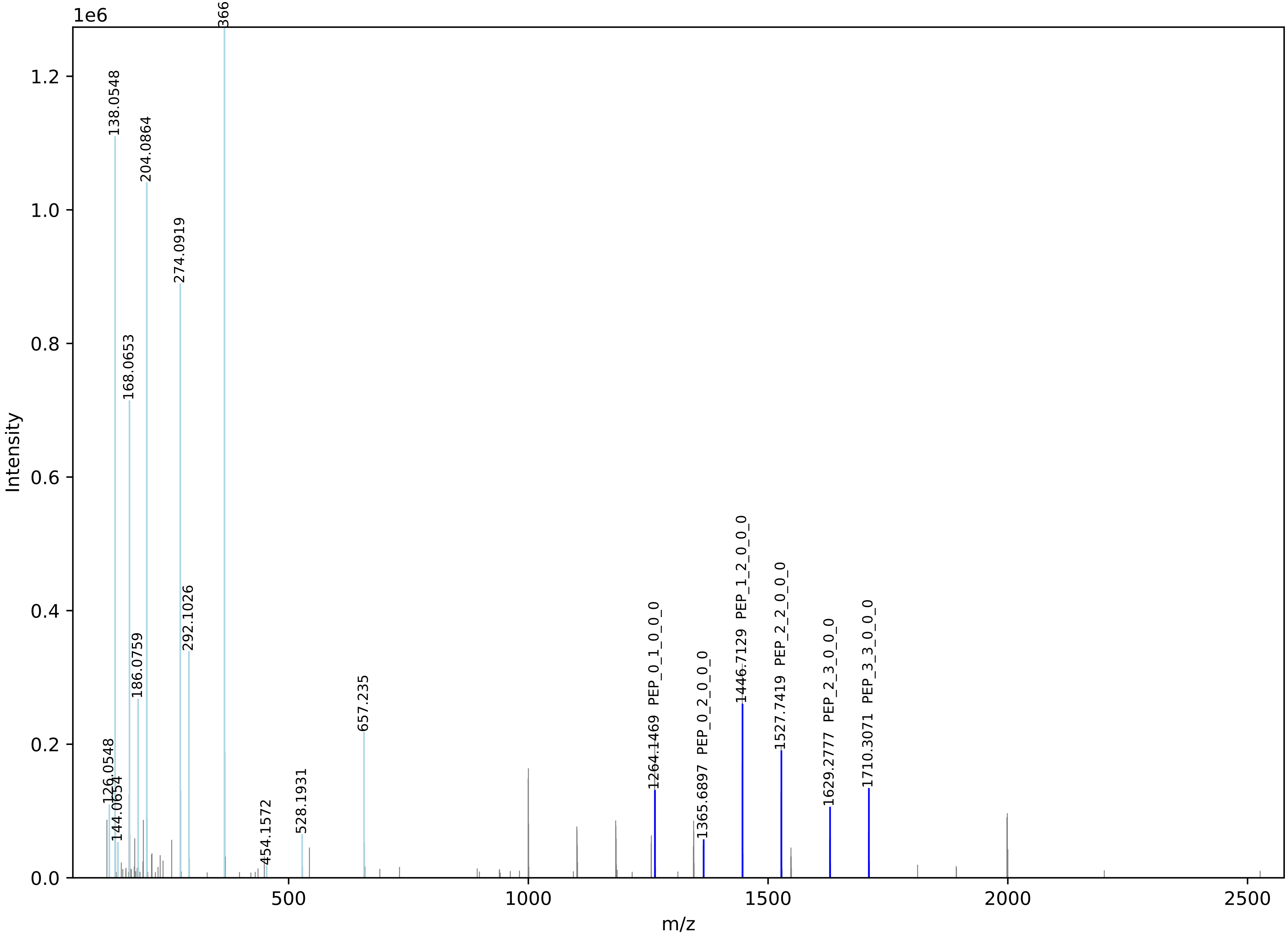

File: 231129_OE_RK_37, RT: 55.032, Scan: 073816
Type: HCD, FTMS + c NSI d Full ms2 1674.7175@hcd30.00 [120.0000-5146.7773]
Precursor Scan: 073730, m/z: 1674.385, charge: 3
VVLHPNYSQVDIGLIK_7_6_0_3_0

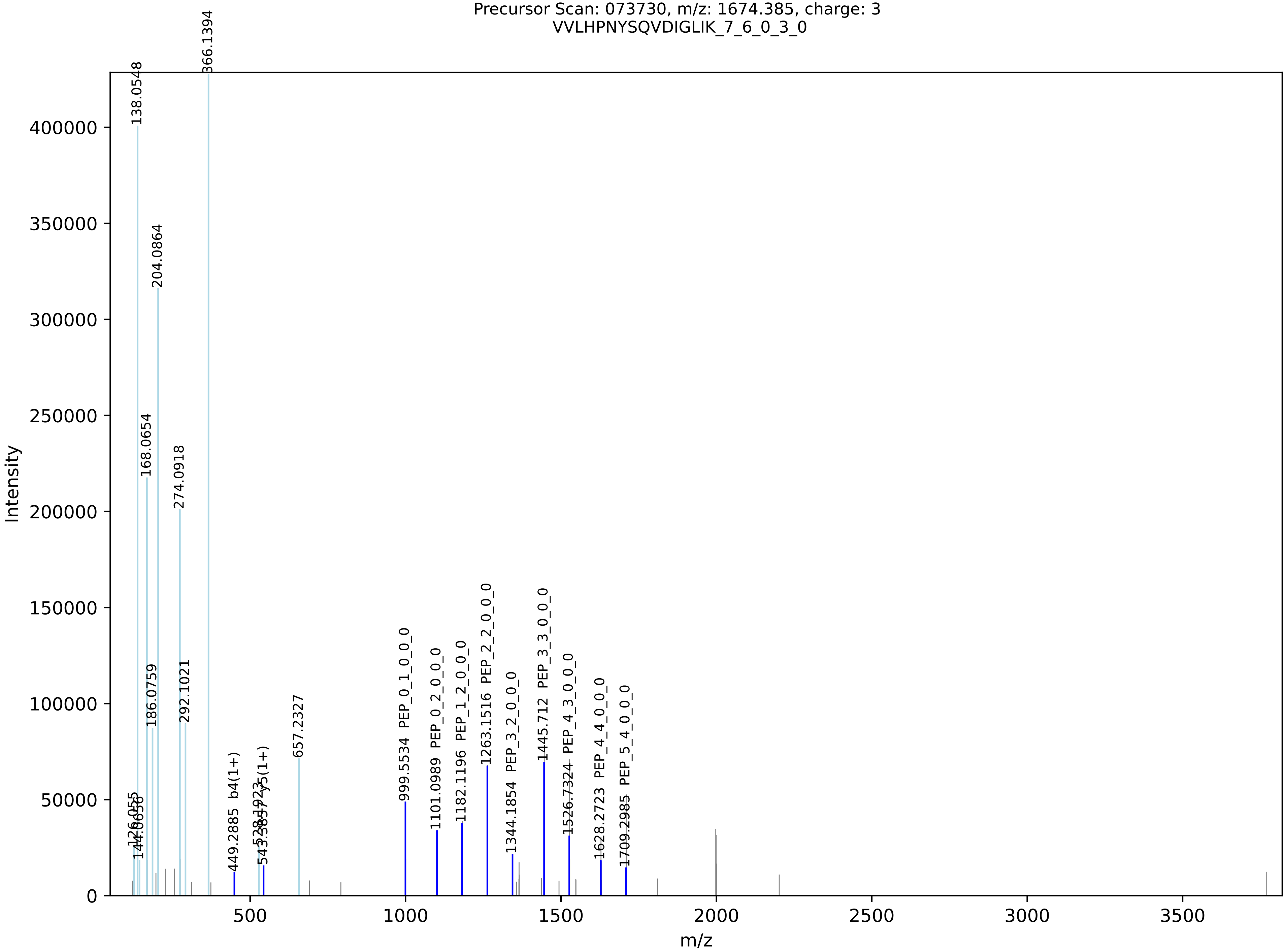

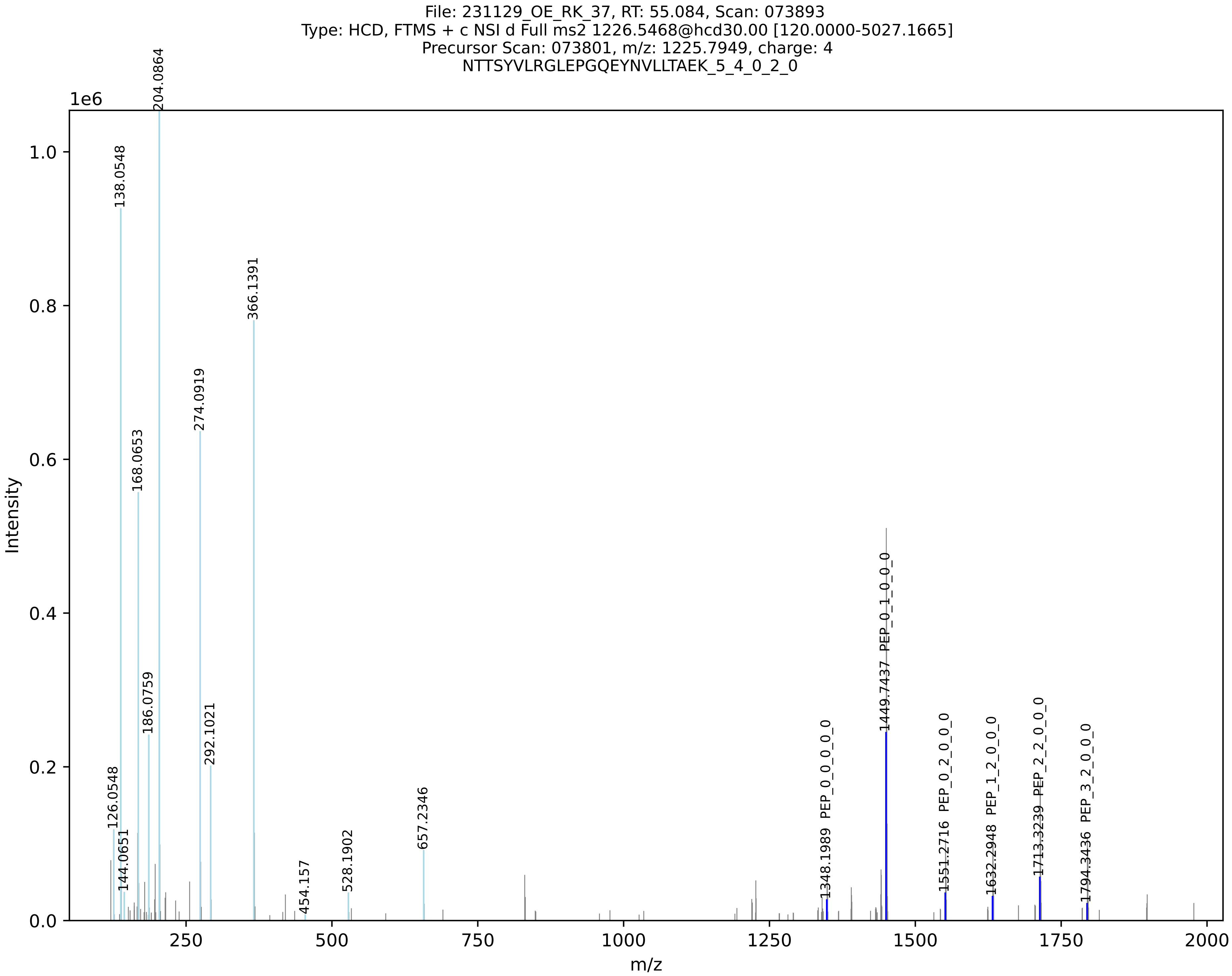
File: 231129_OE_RK_37, RT: 55.084, Scan: 073893
Type: HCD, FTMS + c NSI d Full ms2 1226.5468@hcd30.00 [120.0000-5027.1665]
Precursor Scan: 073801, m/z: 1225.7949, charge: 4
NTTSYVLRGLEPGQEYNVLLTAEK_5_4_0_2_0
1e6
Intensity
m/z
204.0864
138.0548
366.1391
274.0919
168.0653
186.0759
292.1021
126.0548
144.0651
454.157
528.1902
657.2346
1348.1989 PEP_0_0_0_0_0_
1449.7437 PEP_0_1_0_0_0_
1551.2716 PEP_0_2_0_0_0_
1632.2948 PEP_1_2_0_0_0_
1713.3239 PEP_2_2_0_0_0_
1794.3436 PEP_3_2_0_0_0_

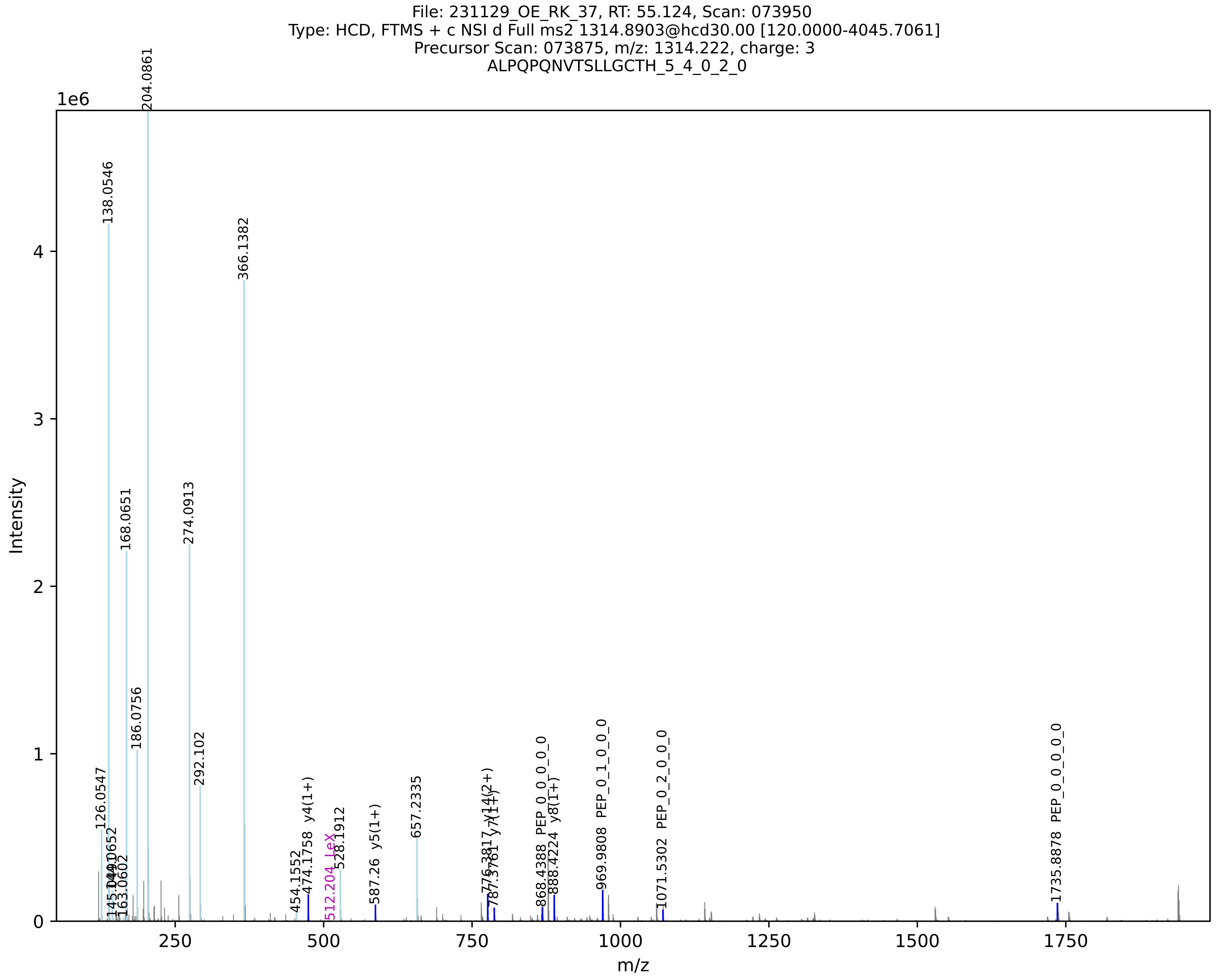

File: 231129_OE_RK_37, RT: 55.124, Scan: 073950
Type: HCD, FTMS + c NSI d Full ms2 1314.8903@hcd30.00 [120.0000-4045.7061]
Precursor Scan: 073875, m/z: 1314.222, charge: 3
ALPQPQNVTSLLGCTH_5_4_0_2_0
1e6
Intensity
m/z
204.0861
138.0546
366.1382
168.0651
274.0913
186.0756
292.102
126.0547
163.0602
454.1552
474.1758 y4(1+)
512.204_LeX
528.1912
587.26 y5(1+)
657.2335
776.3817 y14(2+)
787.3761 y7(1+)
868.4388 PEP_0_0_0_0_0
888.4224 y8(1+)
969.9808 PEP_0_1_0_0_0
1071.5302 PEP_0_2_0_0_0
1735.8878 PEP_0_0_0_0_0

File: 231129_OE_RK_37, RT: 55.131, Scan: 073961
Type: HCD, FTMS + c NSI d Full ms2 1201.7753@hcd30.00 [120.0000-4926.0991]
Precursor Scan: 073875, m/z: 1201.2729, charge: 4
VVLHPNYSQVDIGLIK_6_5_1_3_0

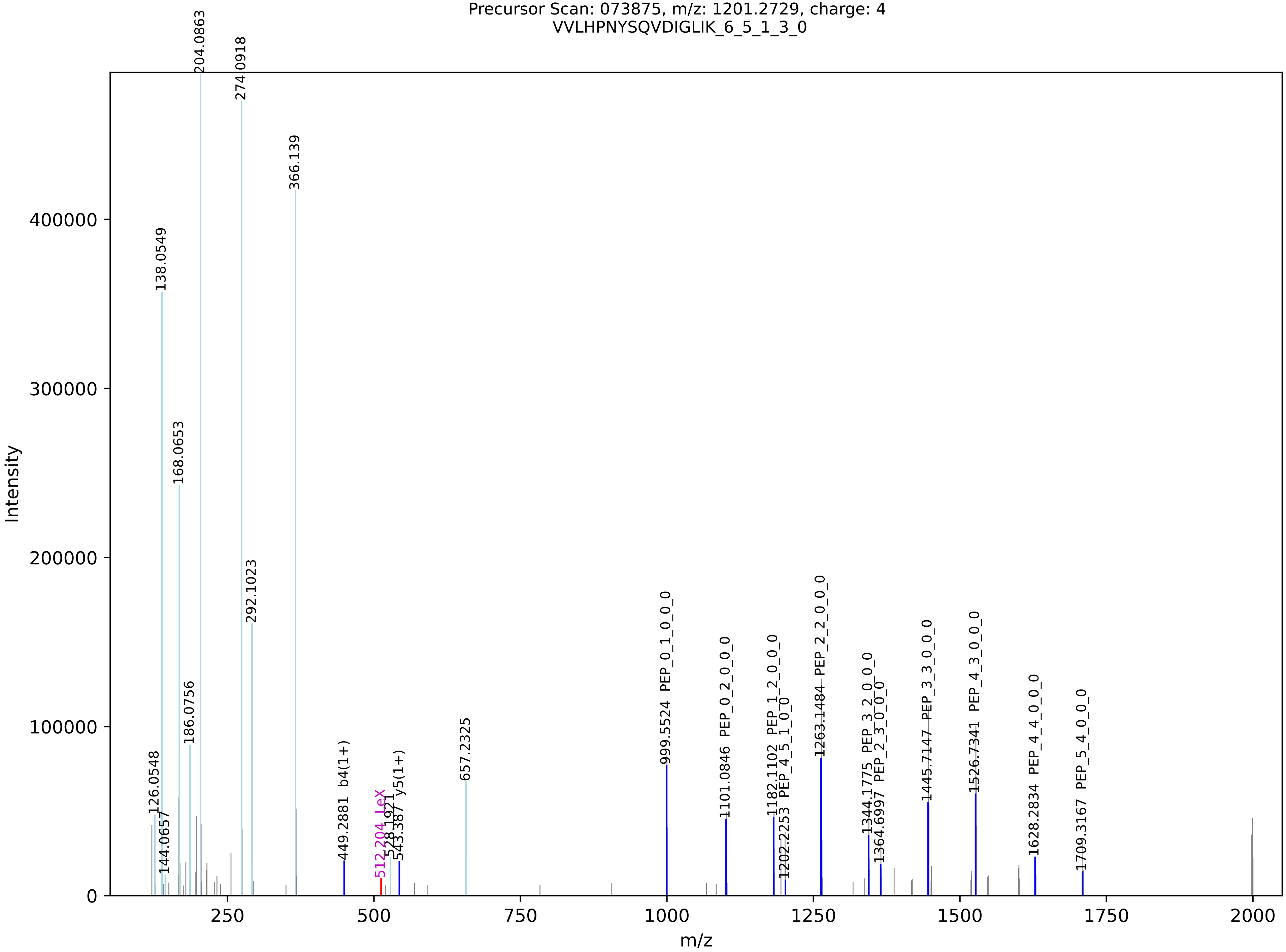

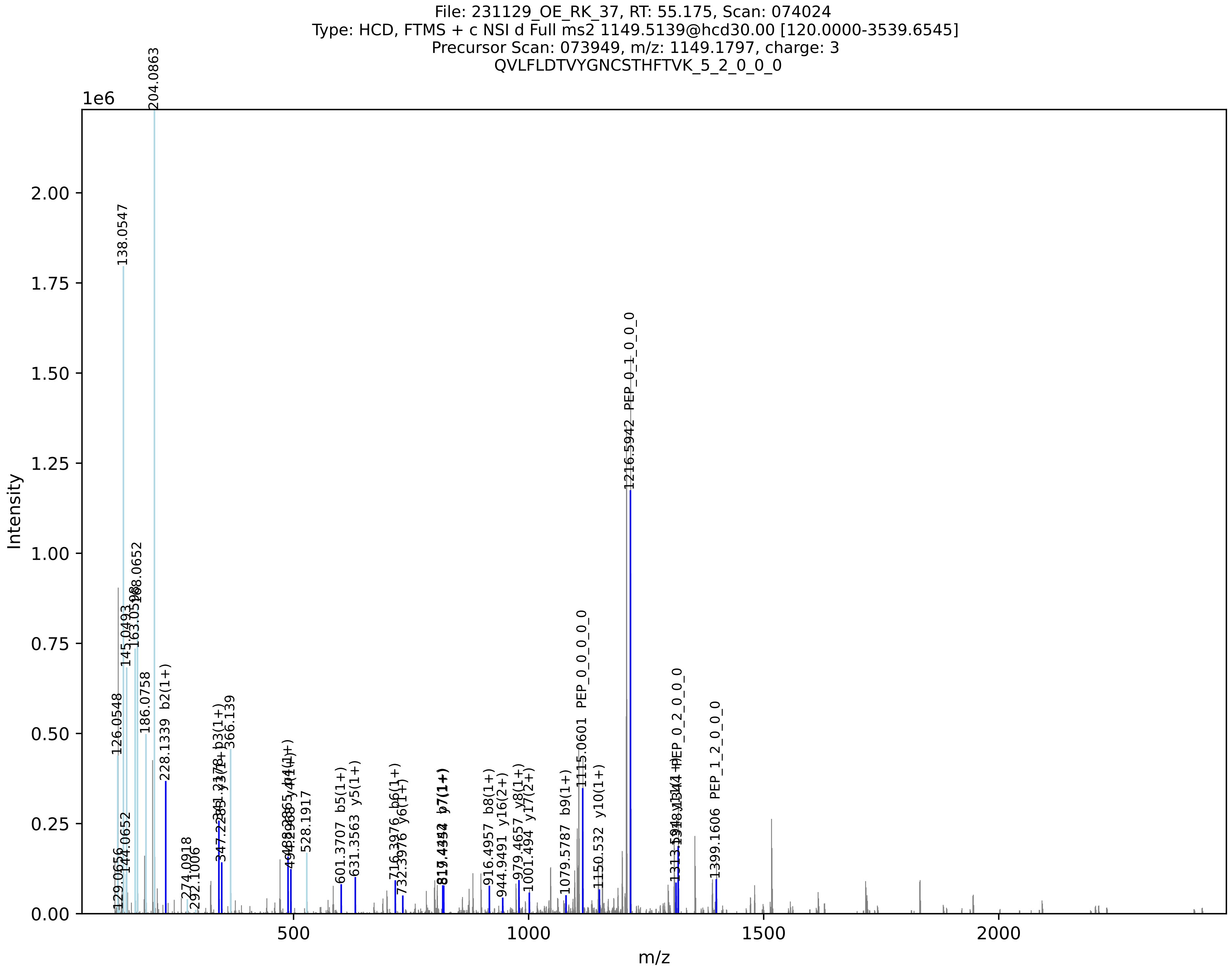

File: 231129_OE_RK_37, RT: 55.175, Scan: 074024
Type: HCD, FTMS + c NSI d Full ms2 1149.5139@hcd30.00 [120.0000-3539.6545]
Precursor Scan: 073949, m/z: 1149.1797, charge: 3
QVLFLDTVYGNCSTHFTVK_5_2_0_0_0
1e6
Intensity
m/z
0.00
0.25
0.50
0.75
1.00
1.25
1.50
1.75
2.00
500
1000
1500
2000
204.0863
138.0547
168.0652
145.0493
126.0548
186.0758
228.1339 b2(1+)
366.139
528.1917
144.0652
129.0656
274.0918
292.1006
347.2263 y3(1+)
601.3707 b5(1+)
631.3563 y5(1+)
732.3976 y6(1+)
916.4957 b8(1+)
944.9491 y16(2+)
979.4657 y8(1+)
1001.494 y17(2+)
1079.5787 b9(1+)
1115.0601 PEP_0_0_0_0_0
1150.532 y10(1+)
1216.5942 PEP_0_1_0_0_0
PEP_0_2_0_0_0
1399.1606 PEP_1_2_0_0_0

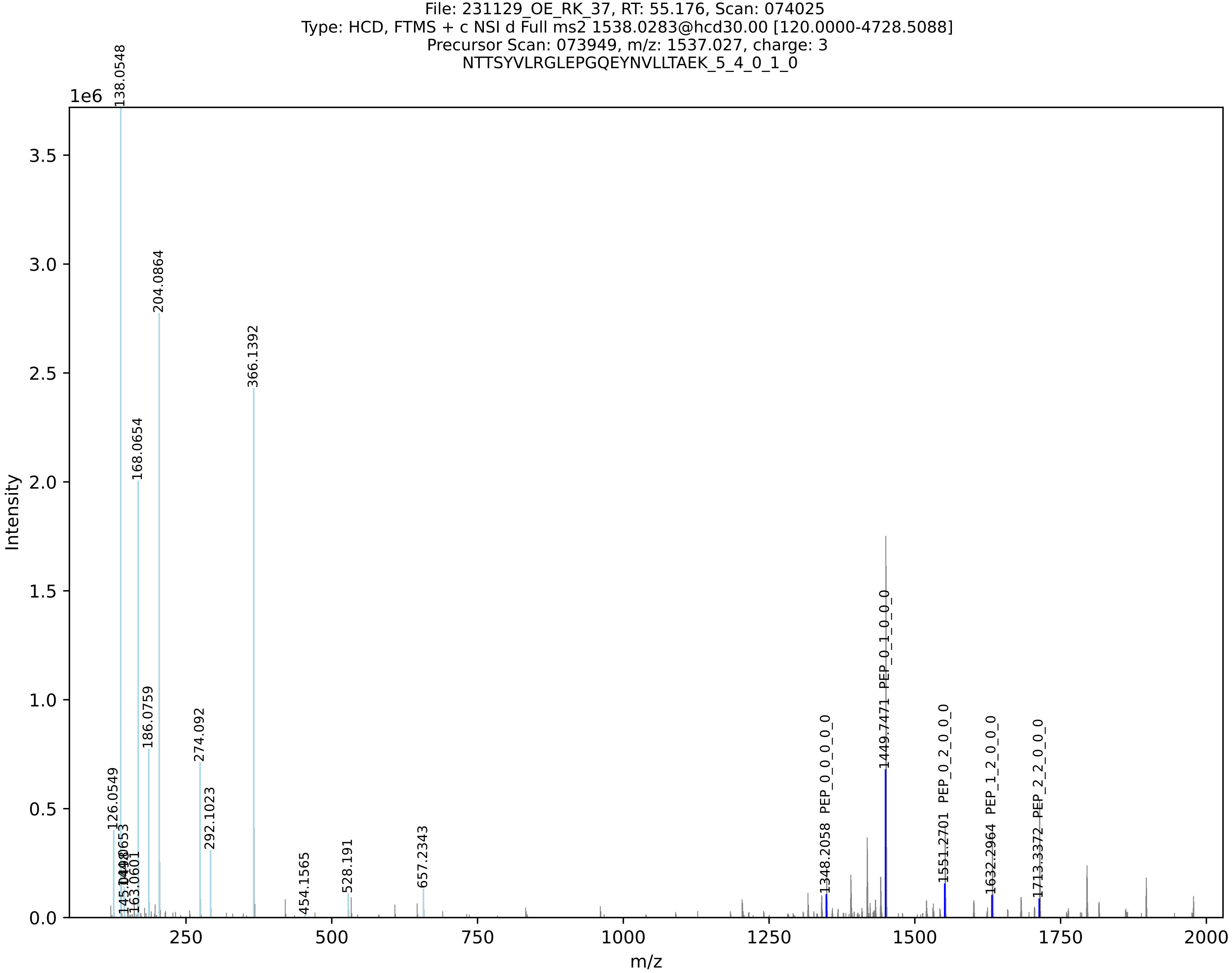
File: 231129_OE_RK_37, RT: 55.176, Scan: 074025
Type: HCD, FTMS + c NSI d Full ms2 1538.0283@hcd30.00 [120.0000-4728.5088]
Precursor Scan: 073949, m/z: 1537.027, charge: 3
NTTSYVLRGLEPGQEYNVLLTAEK_5_4_0_1_0
1e6
Intensity
m/z
138.0548
204.0864
366.1392
168.0654
186.0759
274.092
292.1023
126.0549
163.0601
454.1565
528.191
657.2343
1348.2058 PEP_0_0_0_0_0
1449.7471 PEP_0_1_0_0_0
1551.2701 PEP_0_2_0_0_0
1632.2964 PEP_1_2_0_0_0
1713.3372 PEP_2_2_0_0_0

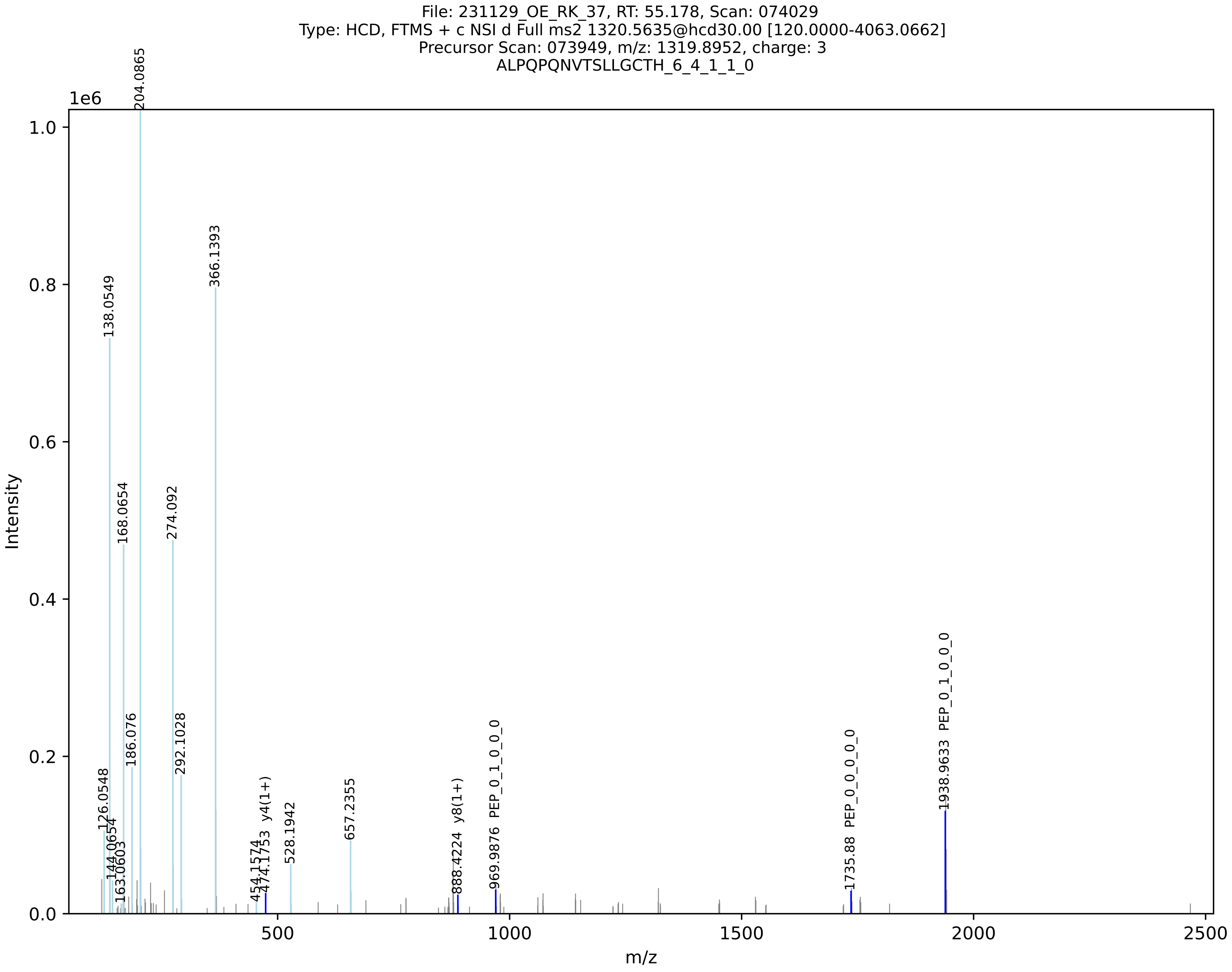
File: 231129_OE_RK_37, RT: 55.178, Scan: 074029
Type: HCD, FTMS + c NSI d Full ms2 1320.5635@hcd30.00 [120.0000-4063.0662]
Precursor Scan: 073949, m/z: 1319.8952, charge: 3
ALPQPQNVTSLLGCTH_6_4_1_1_0
1e6
Intensity
m/z
204.0865
366.1393
138.0549
168.0654
274.092
186.076
292.1028
126.0548
144.0654
163.0603
454.1574
474.1753 y4(1+)
528.1942
657.2355
888.4224 y8(1+)
969.9876 PEP_0_1_0_0_0_
1735.88 PEP_0_0_0_0_0_
1938.9633 PEP_0_1_0_0_0_

File: 231129_OE_RK_37, RT: 55.181, Scan: 074034
Type: HCD, FTMS + c NSI d Full ms2 1601.6948@hcd30.00 [120.0000-4923.3281]
Precursor Scan: 073949, m/z: 1601.3628, charge: 3
VVLHPNYSQVDIGLIK_6_5_1_3_0

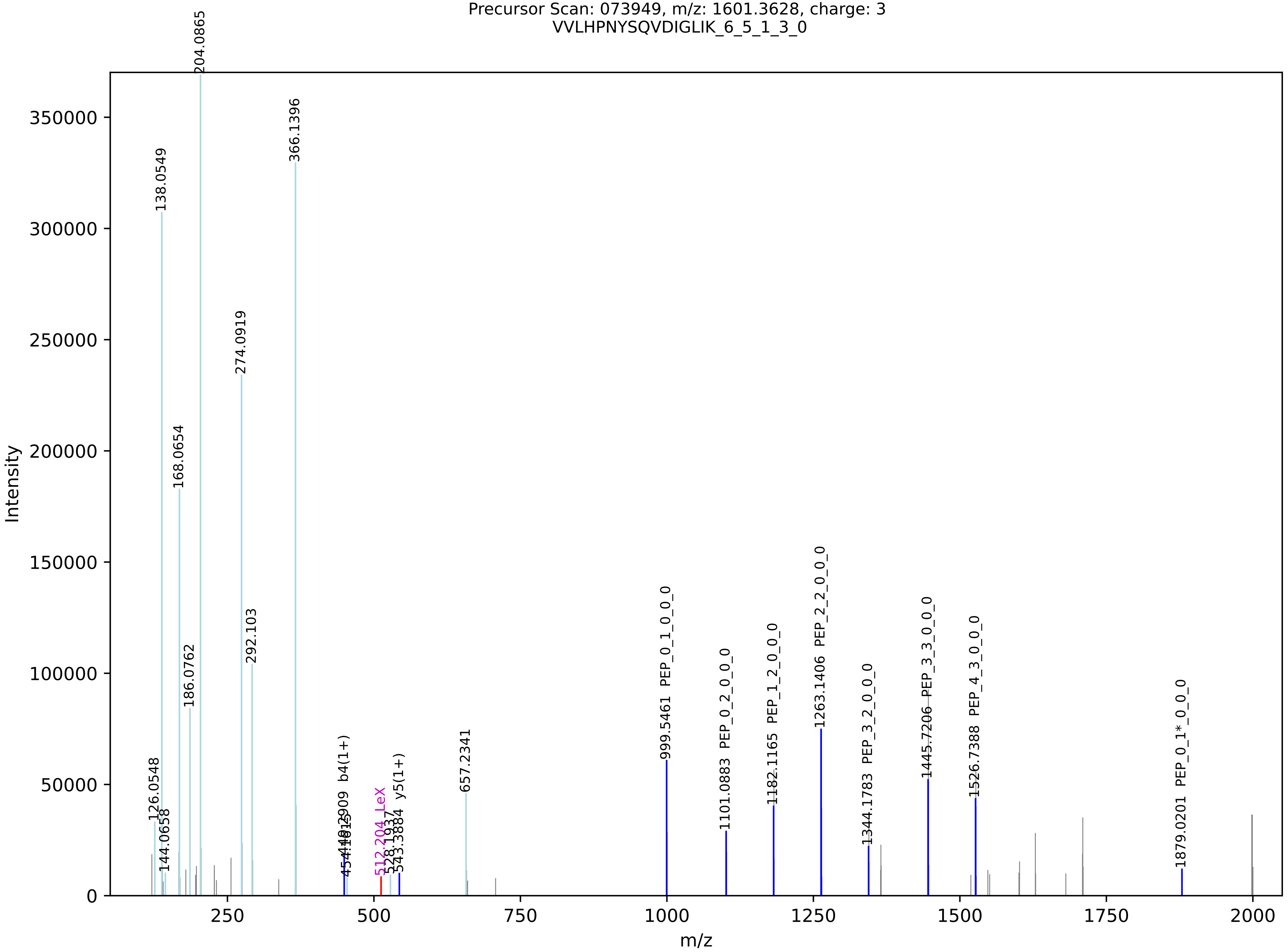

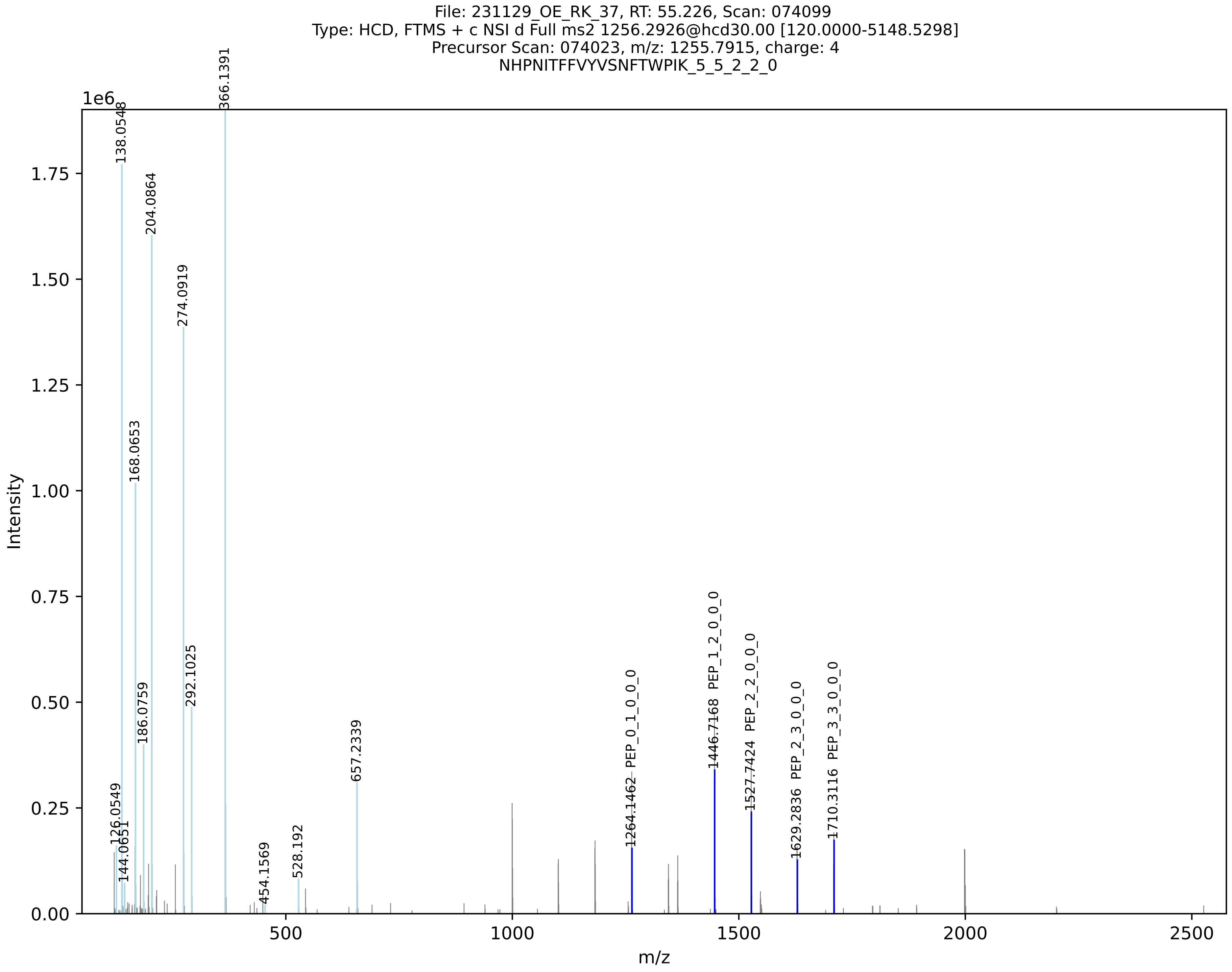

File: 231129_OE_RK_37, RT: 55.226, Scan: 074099
Type: HCD, FTMS + c NSI d Full ms2 1256.2926@hcd30.00 [120.0000-5148.5298]
Precursor Scan: 074023, m/z: 1255.7915, charge: 4
NHPNITFFVYYVSNFTWPIK_5_5_2_2_0
1e6
Intensity
m/z
366.1391
138.0548
204.0864
274.0919
168.0653
292.1025
186.0759
126.0549
144.0651
454.1569
528.192
657.2339
1264.1462 PEP_0_1_0_0_0
1446.7168 PEP_1_2_0_0_0
1527.7424 PEP_2_2_0_0_0
1629.2836 PEP_2_3_0_0_0
1710.3116 PEP_3_3_0_0_0

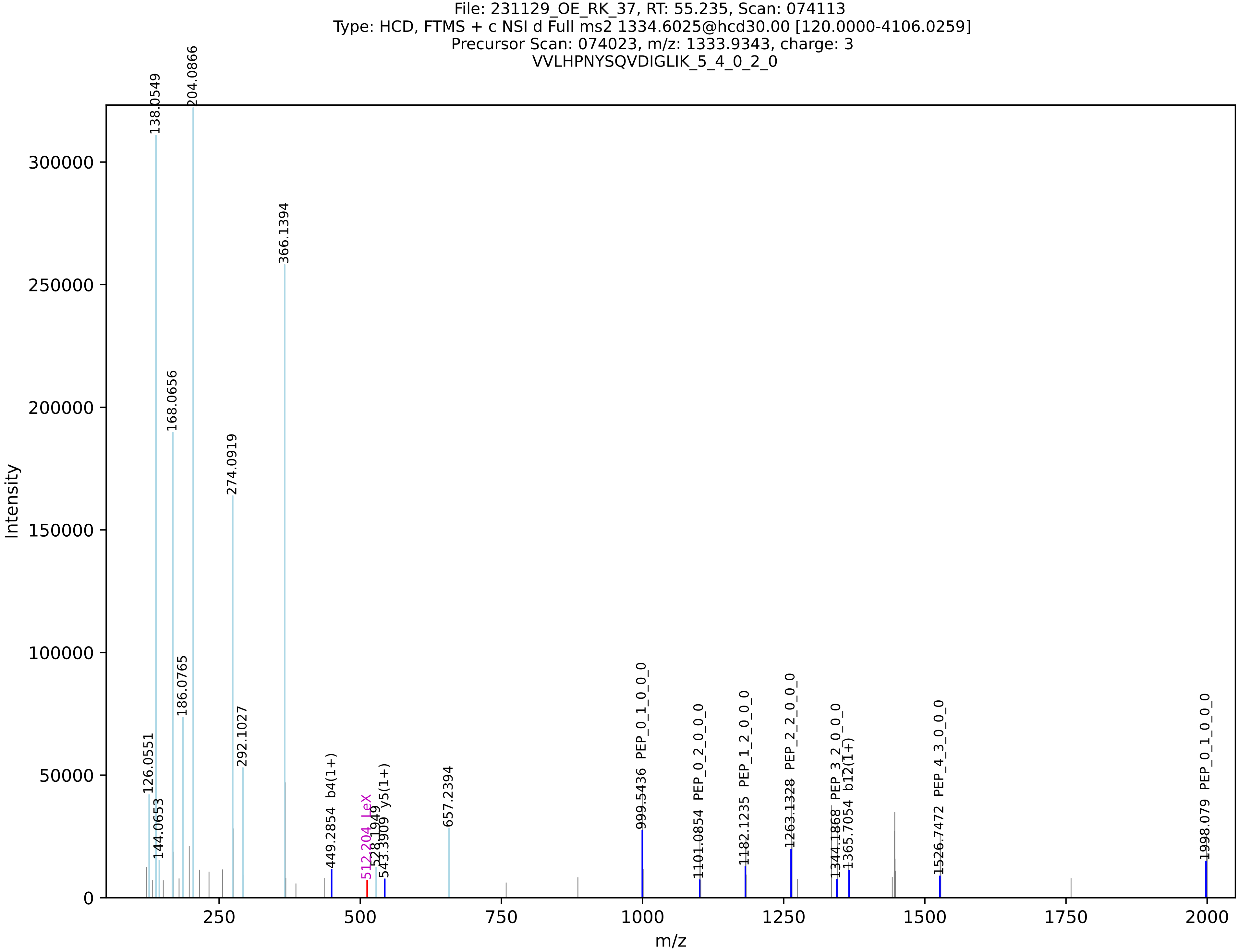
File: 231129_OE_RK_37, RT: 55.235, Scan: 074113
Type: HCD, FTMS + c NSI d Full ms2 1334.6025@hcd30.00 [120.0000-4106.0259]
Precursor Scan: 074023, m/z: 1333.9343, charge: 3
VVLHPNYSQVDIGLIK_5_4_0_2_0
Intensity
m/z
138.0549
204.0866
366.1394
168.0656
274.0919
186.0765
292.1027
126.0551
144.0653
449.2854 b4(1+)
512.204_LeX
528.1949
543.3909 y5(1+)
657.2394
999.5436 PEP_0_1_0_0_0
1101.0854 PEP_0_2_0_0_0
1182.1235 PEP_1_2_0_0_0
1263.1328 PEP_2_2_0_0_0
1344.1868 PEP_3_2_0_0_0
1365.7054 b12(1+)
1526.7472 PEP_4_3_0_0_0
1998.079 PEP_0_1_0_0_0

File: 231129_OE_RK_37, RT: 55.26, Scan: 074154
Type: HCD, FTMS + c NSI d Full ms2 987.1740@hcd30.00 [120.0000-4050.5256]
Precursor Scan: 073949, m/z: 985.9189, charge: 4
ALPQPQNVTSLLGCTH_5_4_0_2_0

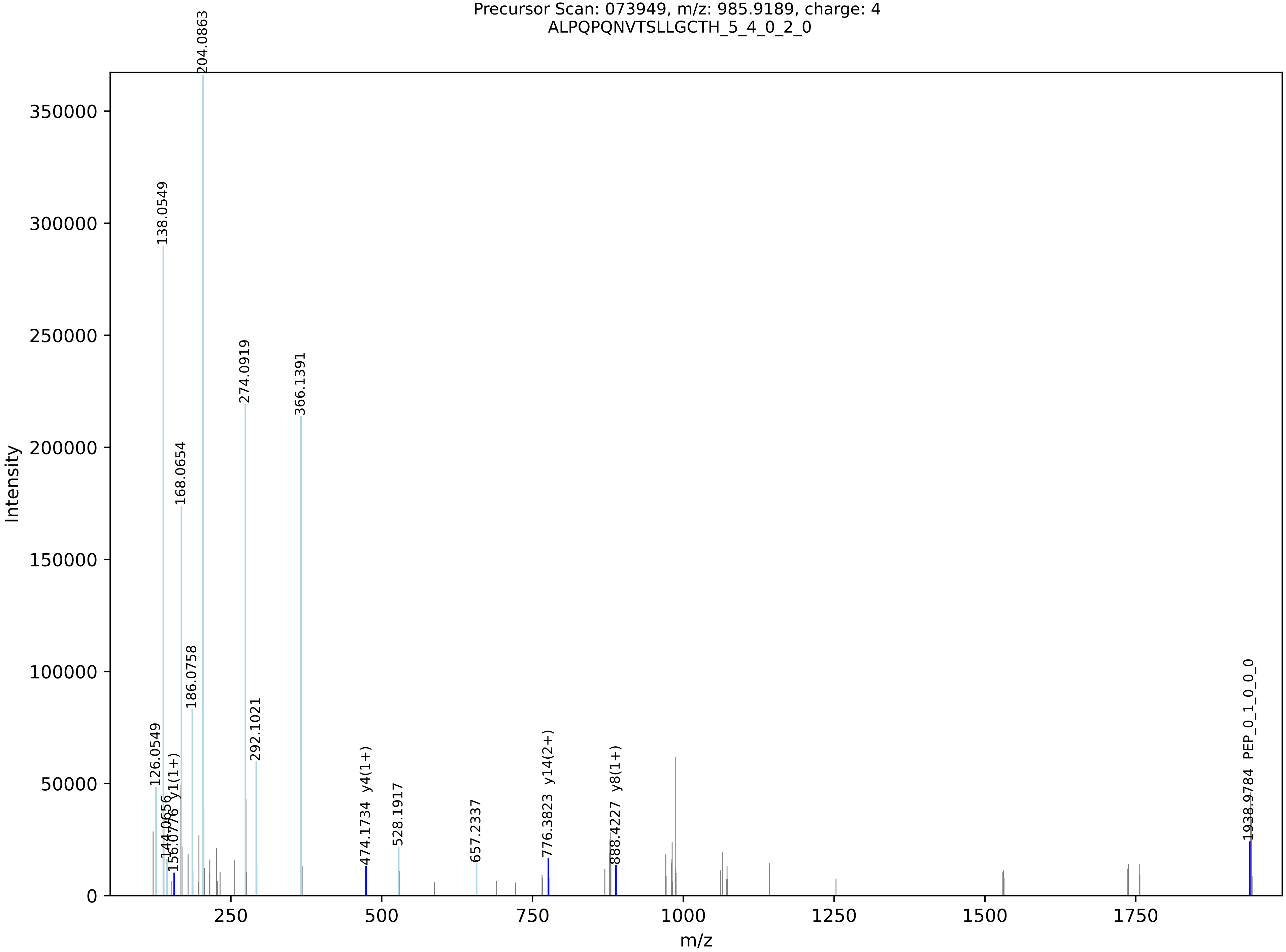

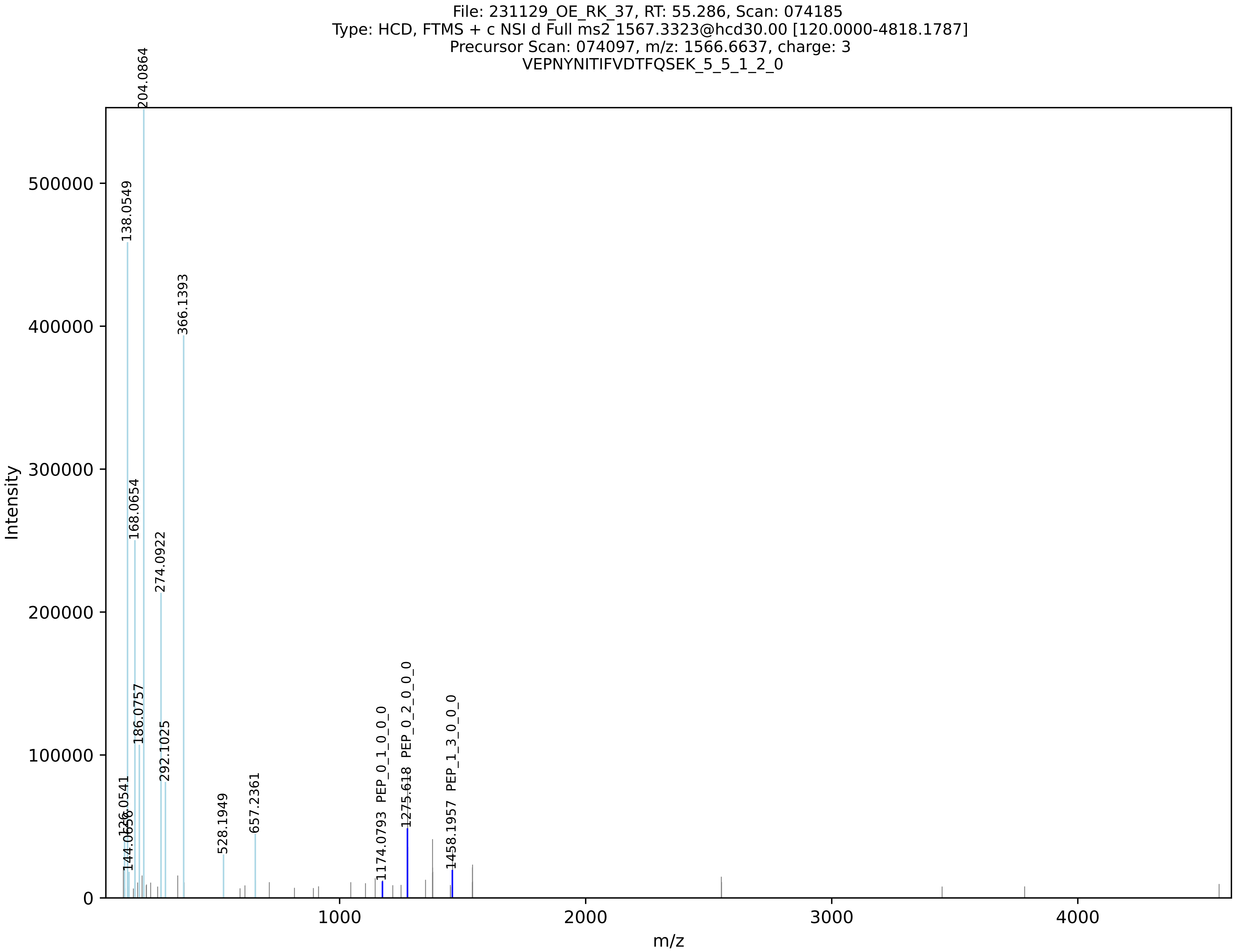
File: 231129_OE_RK_37, RT: 55.286, Scan: 074185
Type: HCD, FTMS + c NSI d Full ms2 1567.3323@hcd30.00 [120.0000-4818.1787]
Precursor Scan: 074097, m/z: 1566.6637, charge: 3
VEPNYNITIFVDTFQSEK_5_5_1_2_0
Intensity
m/z
204.0864
138.0549
366.1393
168.0654
274.0922
186.0757
292.1025
126.0541
144.0656
528.1949
657.2361
1174.0793 PEP_0_1_0_0_0
1275.618 PEP_0_2_0_0_0
1458.1957 PEP_1_3_0_0_0
500000
400000
300000
200000
100000
0
1000
2000
3000
4000

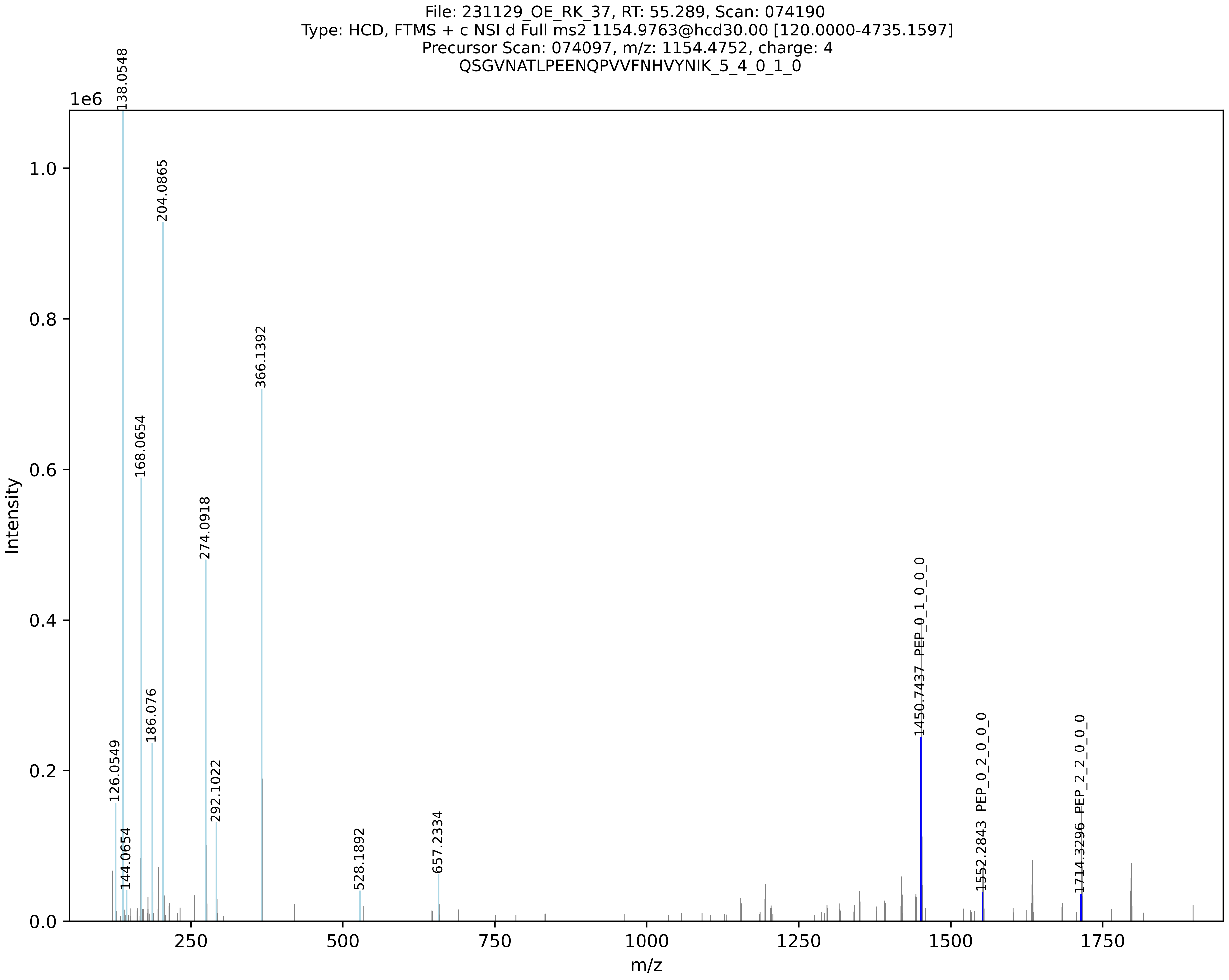
File: 231129_OE_RK_37, RT: 55.289, Scan: 074190
Type: HCD, FTMS + c NSI d Full ms2 1154.9763@hcd30.00 [120.0000-4735.1597]
Precursor Scan: 074097, m/z: 1154.4752, charge: 4
QSGVNATLPEENQPVVFNHVYNIK_5_4_0_1_0
1e6
138.0548
204.0865
366.1392
168.0654
274.0918
186.076
126.0549
292.1022
144.0654
528.1892
657.2334
1450.7437_PEP_0_1_0_0_0_
1552.2843_PEP_0_2_0_0_0_
1714.3296_PEP_2_2_0_0_0_
Intensity
m/z
0.0
0.2
0.4
0.6
0.8
1.0
250
500
750
1000
1250
1500
1750

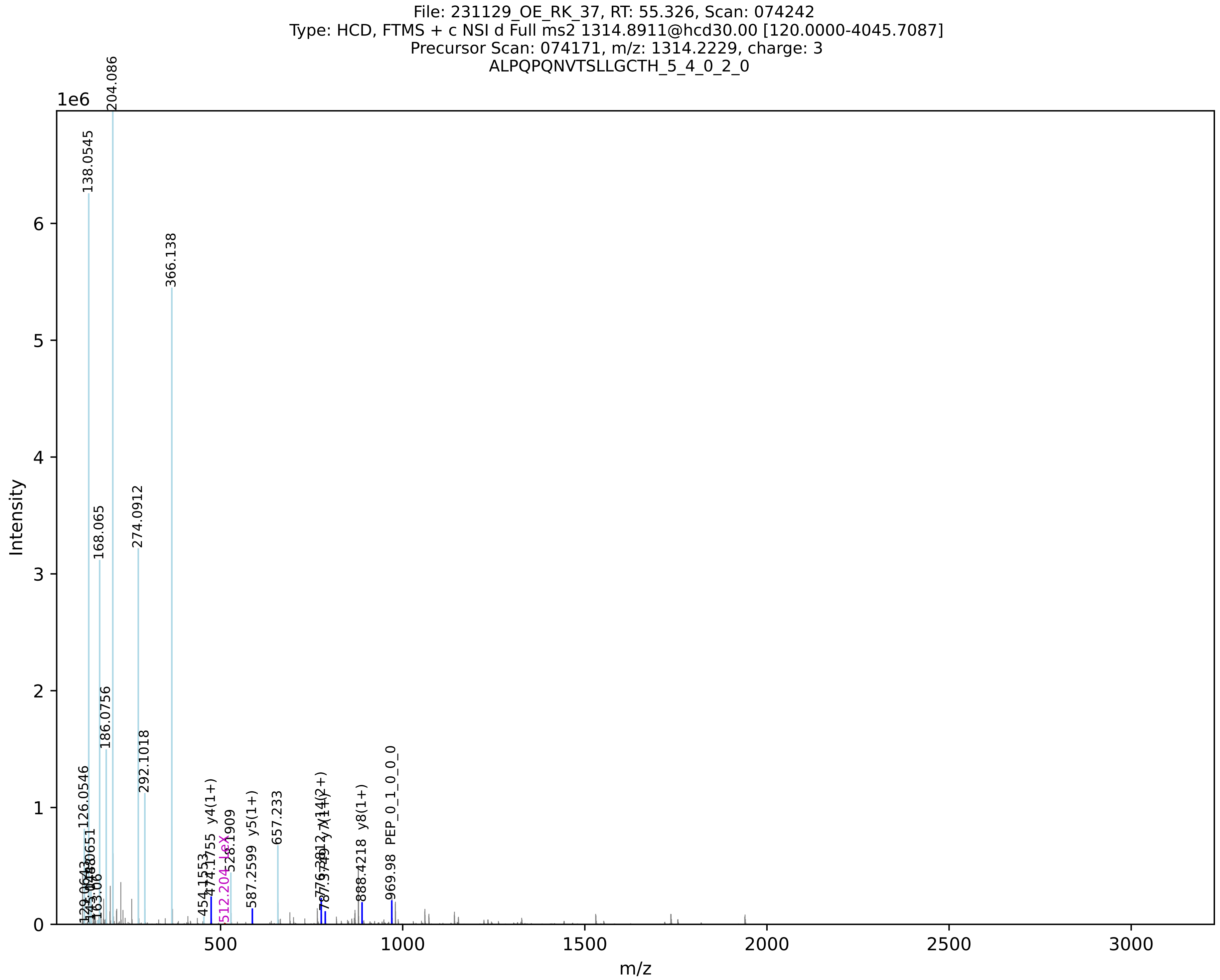
File: 231129_OE_RK_37, RT: 55.326, Scan: 074242
Type: HCD, FTMS + c NSI d Full ms2 1314.8911@hcd30.00 [120.0000-4045.7087]
Precursor Scan: 074171, m/z: 1314.2229, charge: 3
ALPQPQNVTSLLGCTH_5_4_0_2_0
1e6
Intensity
m/z
204.086
138.0545
366.138
168.065
274.0912
186.0756
292.1018
126.0546
y4(1+)
y5(1+)
657.233
y8(1+)
PEP_0_1_0_0_0
587.2599
888.4218
969.98
528.1909
454.1553
474.1755
512.204 LeX
0
1
2
3
4
5
6
500
1000
1500
2000
2500
3000

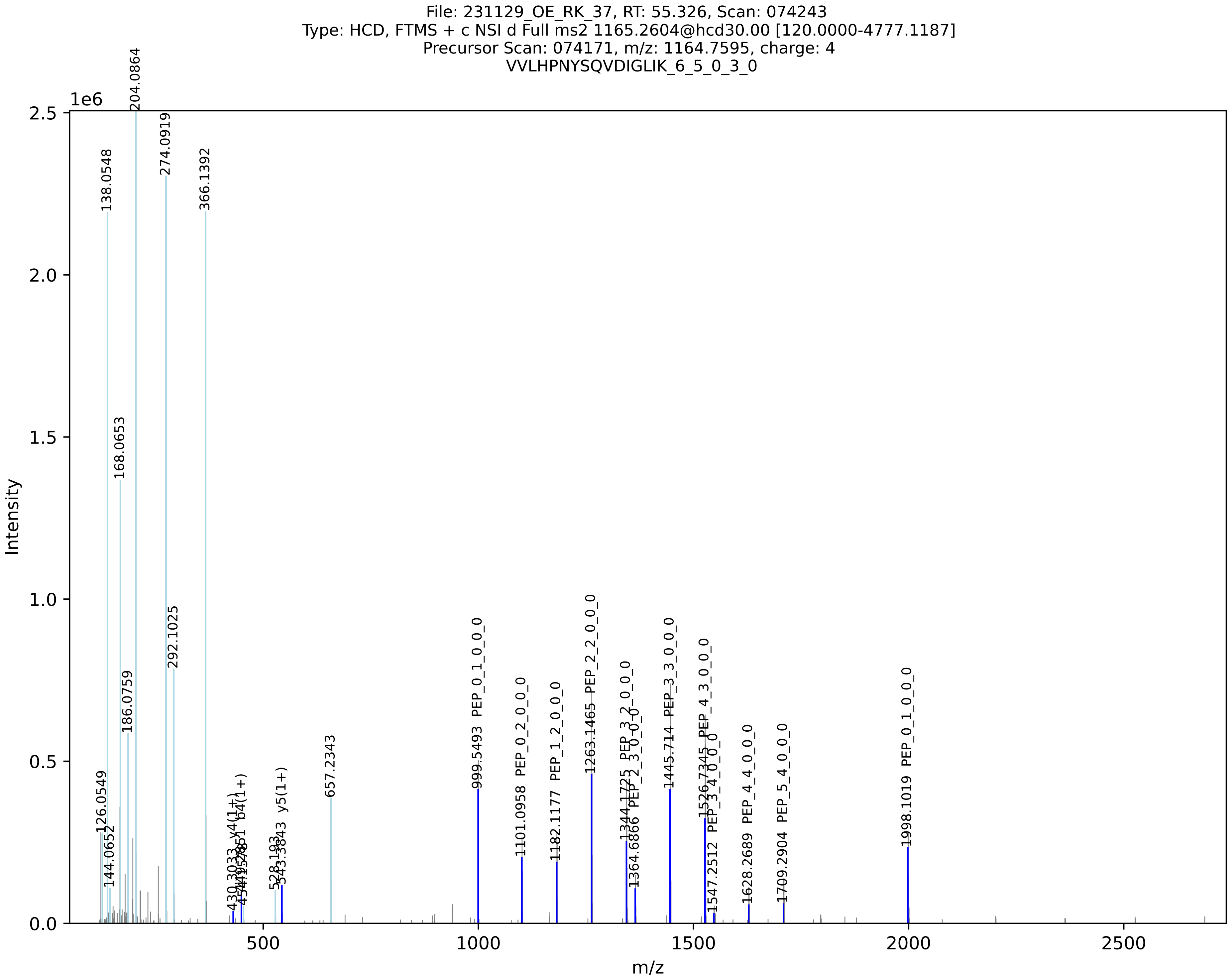

File: 231129_OE_RK_37, RT: 55.326, Scan: 074243
Type: HCD, FTMS + c NSI d Full ms2 1165.2604@hcd30.00 [120.0000-4777.1187]
Precursor Scan: 074171, m/z: 1164.7595, charge: 4
VVLHPNYSQVDIGLIK_6_5_0_3_0
1e6
Intensity
m/z
204.0864
138.0548
274.0919
366.1392
168.0653
292.1025
186.0759
126.0549
144.0652
528.193
543.3843 y5(1+)
657.2343
999.5493 PEP_0_1_0_0_0
1101.0958 PEP_0_2_0_0_0
1182.1177 PEP_1_2_0_0_0
1263.1465 PEP_2_2_0_0_0
1344.1725 PEP_3_2_0_0_0
1364.6866 PEP_2_3_0_0_0
1445.714 PEP_3_3_0_0_0
1526.7345 PEP_4_3_0_0_0
1547.2512 PEP_3_4_0_0_0
1628.2689 PEP_4_4_0_0_0
1709.2904 PEP_5_4_0_0_0
1998.1019 PEP_0_1_0_0_0

File: 231129_OE_RK_37, RT: 55.329, Scan: 074248
Type: HCD, FTMS + c NSI d Full ms2 1092.2870@hcd30.00 [120.0000-5594.2339]
Precursor Scan: 074171, m/z: 1091.8866, charge: 5
SLGNVNFTVSAEALESQELCGTEVPSVPEHGRK_5_4_0_1_0

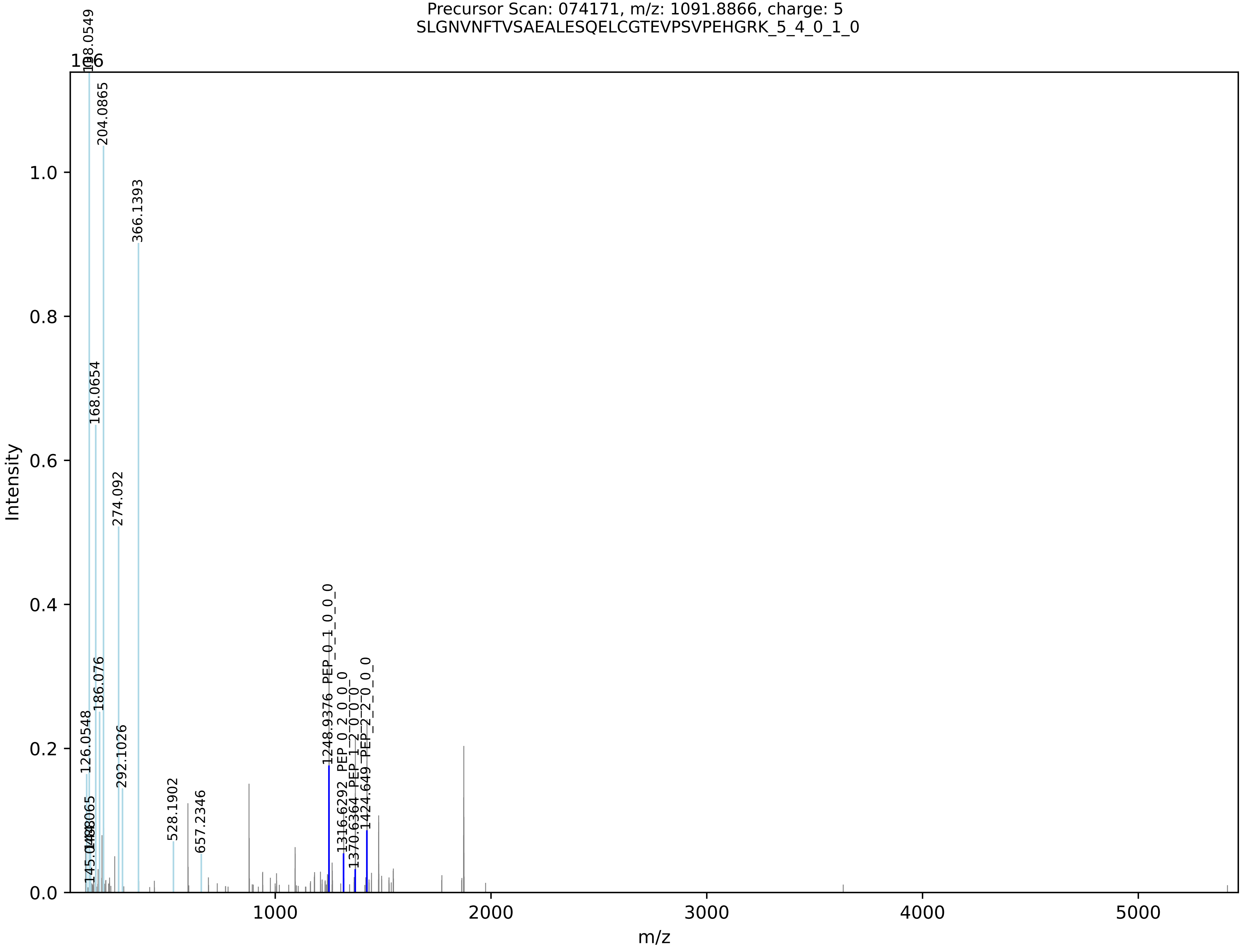

File: 231129_OE_RK_37, RT: 55.33, Scan: 074249
Type: HCD, FTMS + c NSI d Full ms2 986.1695@hcd30.00 [120.0000-4046.4275]
Precursor Scan: 074171, m/z: 985.919, charge: 4
ALPQPQNVTSLLGCTH_5_4_0_2_0

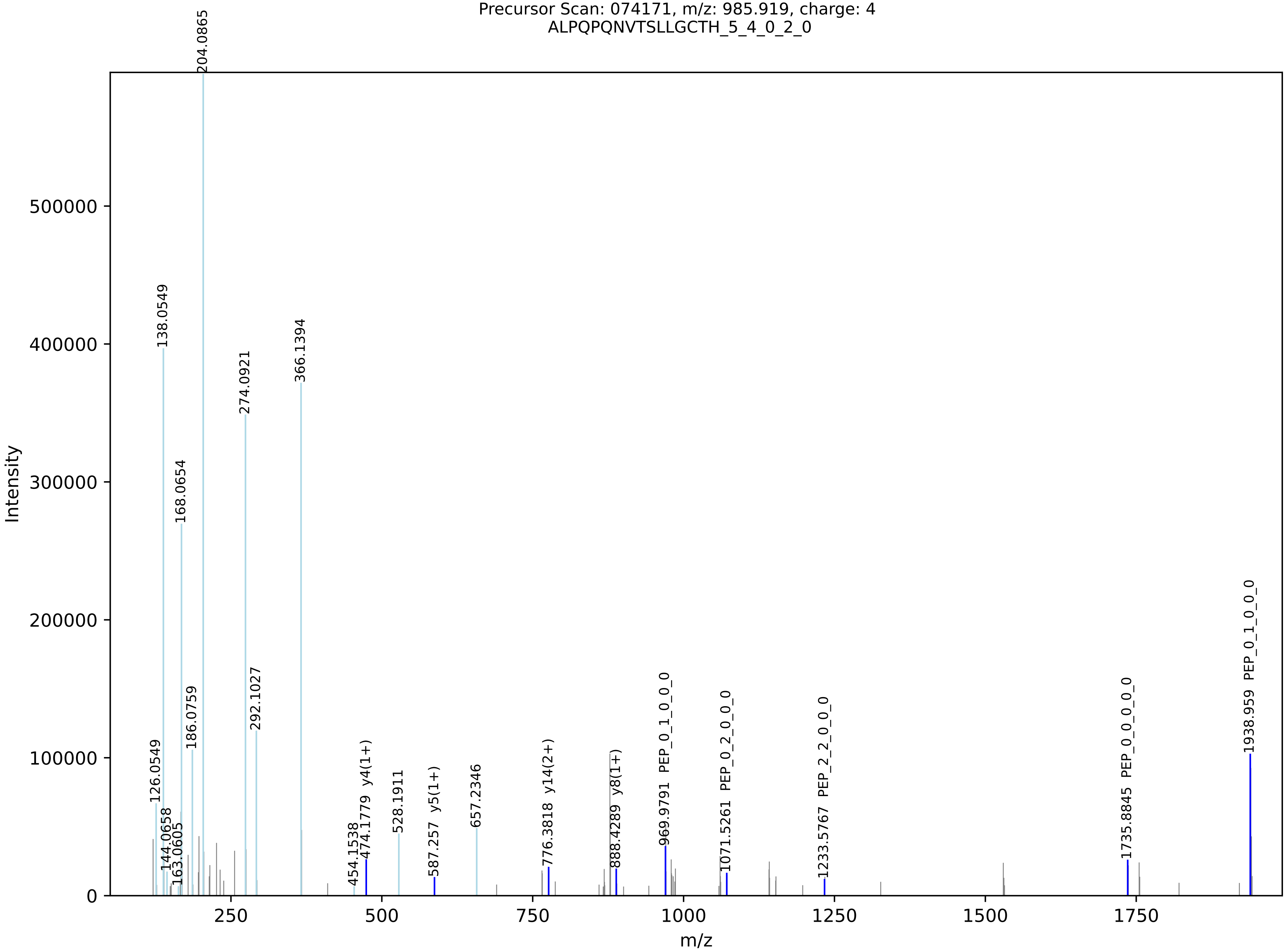

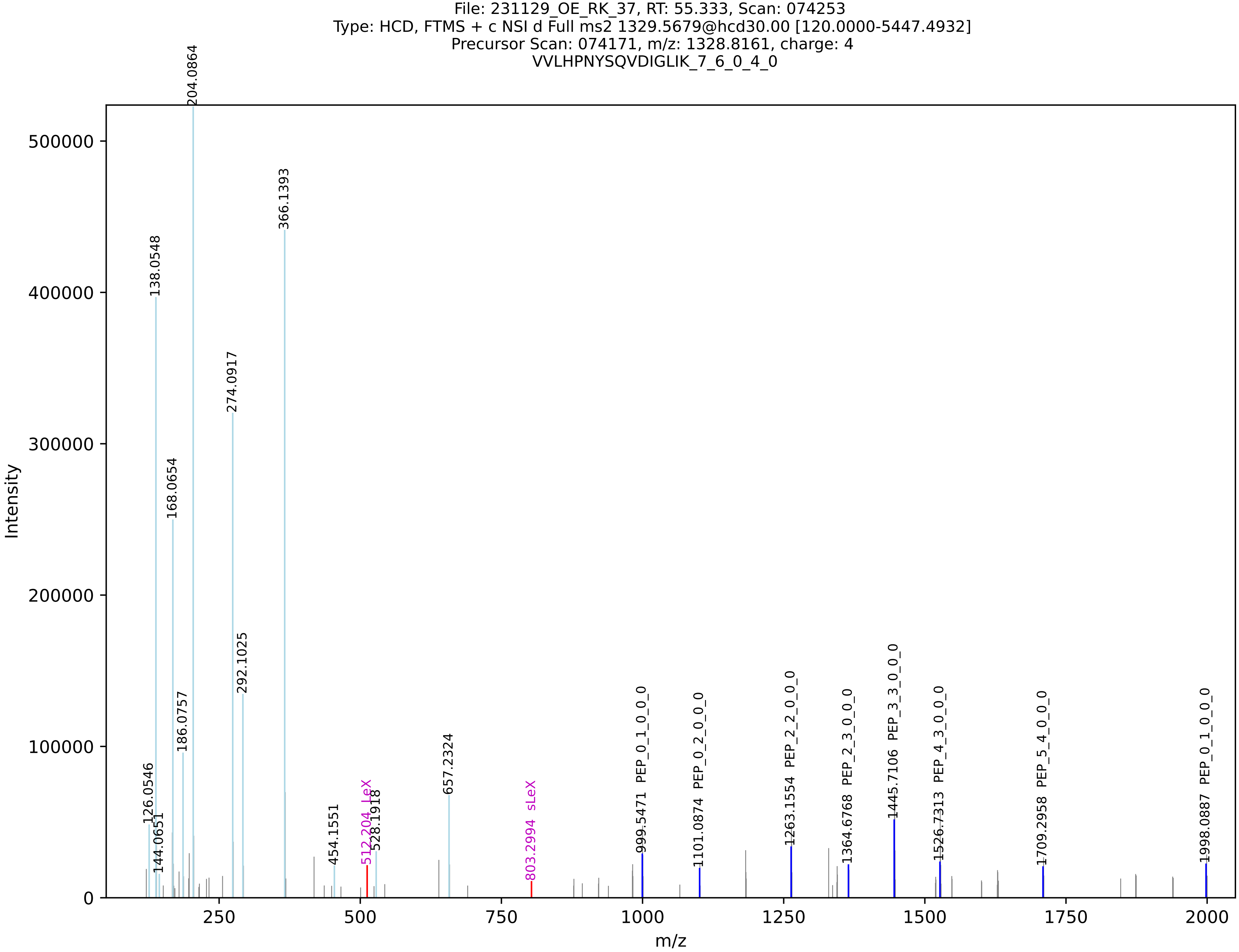
File: 231129_OE_RK_37, RT: 55.333, Scan: 074253
Type: HCD, FTMS + c NSI d Full ms2 1329.5679@hcd30.00 [120.0000-5447.4932]
Precursor Scan: 074171, m/z: 1328.8161, charge: 4
VVLHPNYSQVDIGLIK_7_6_0_4_0
Intensity
m/z
204.0864
366.1393
138.0548
274.0917
168.0654
292.1025
186.0757
126.0546
144.0651
454.1551
512.204 LeX
528.1918
657.2324
803.2994 sLeX
999.5471 PEP_0_1_0_0_0
1101.0874 PEP_0_2_0_0_0
1263.1554 PEP_2_2_0_0_0
1364.6768 PEP_2_3_0_0_0
1445.7106 PEP_3_3_0_0_0
1526.7313 PEP_4_3_0_0_0
1709.2958 PEP_5_4_0_0_0
1998.0887 PEP_0_1_0_0_0

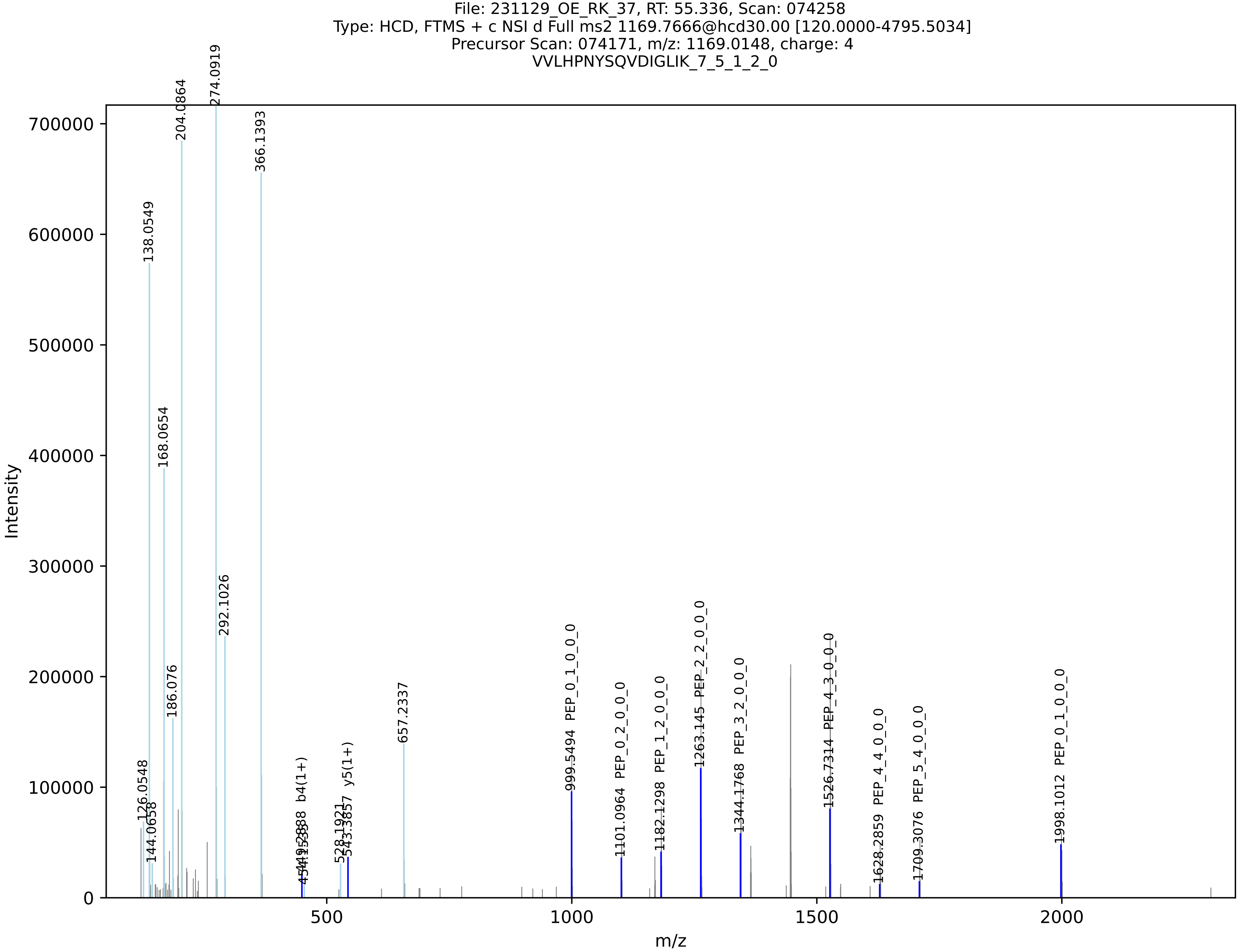

File: 231129_OE_RK_37, RT: 55.336, Scan: 074258
Type: HCD, FTMS + c NSI d Full ms2 1169.7666@hcd30.00 [120.0000-4795.5034]
Precursor Scan: 074171, m/z: 1169.0148, charge: 4
VVLHPNYSQVDIGLIK_7_5_1_2_0
Intensity
m/z
700000
600000
500000
400000
300000
200000
100000
0
500
1000
1500
2000
274.0919
204.0864
366.1393
138.0549
168.0654
292.1026
186.076
126.0548
144.0658
449.2388 b4(1+)
543.3857 y5(1+)
528.1921
657.2337
999.5494 PEP_0_1_0_0_0
1101.0964 PEP_0_2_0_0_0
1182.1298 PEP_1_2_0_0_0
1263.145 PEP_2_2_0_0_0
1344.1768 PEP_3_2_0_0_0
1526.7314 PEP_4_3_0_0_0
1628.2859 PEP_4_4_0_0_0
1709.3076 PEP_5_4_0_0_0
1998.1012 PEP_0_1_0_0_0

File: 231129_OE_RK_37, RT: 55.376, Scan: 074316
Type: HCD, FTMS + c NSI d Full ms2 1153.5239@hcd30.00 [120.0000-4729.2334]
Precursor Scan: 074241, m/z: 1153.0227, charge: 4
NTTSYVLRGLEPGQEYNVLLTAEK_5_4_0_1_0

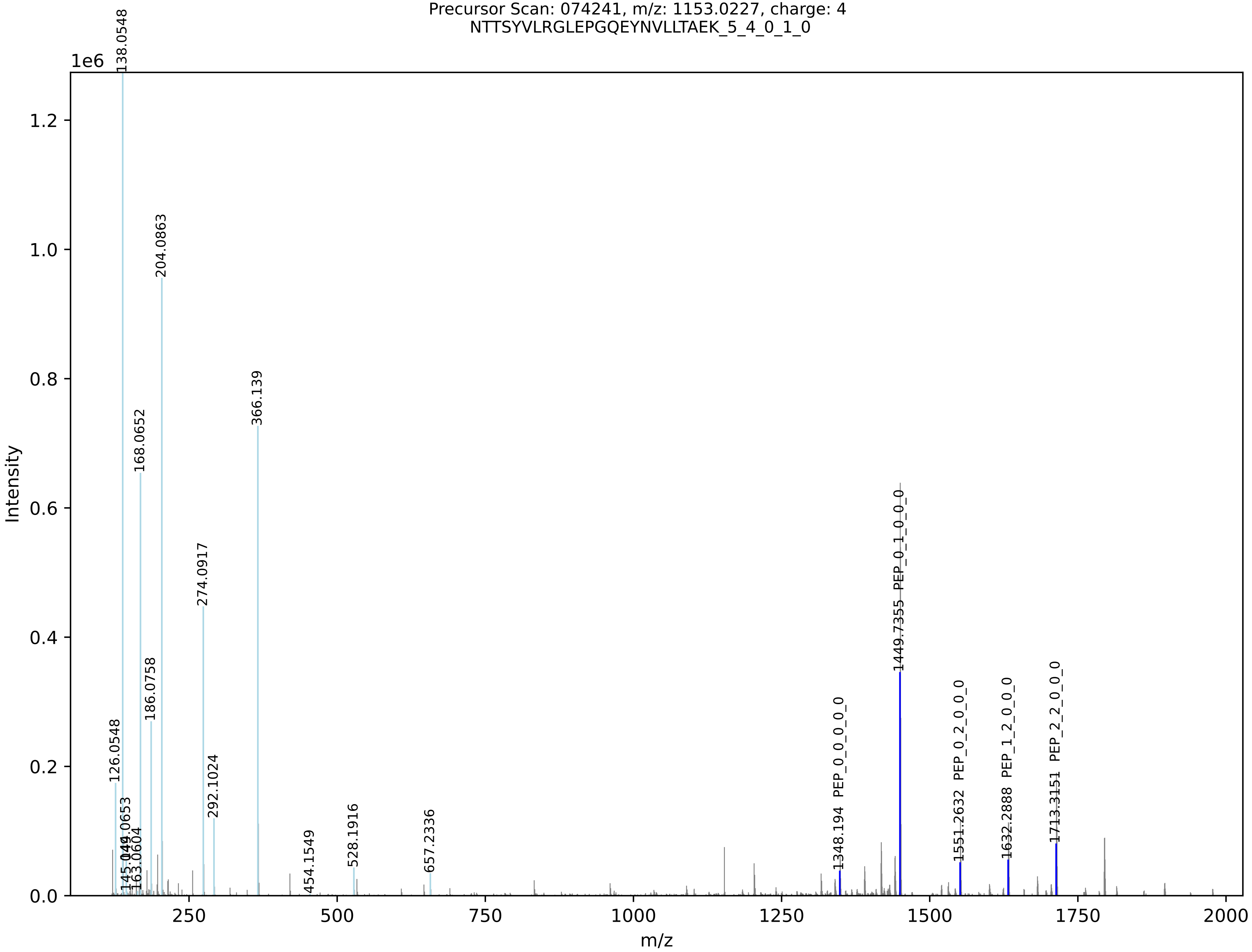

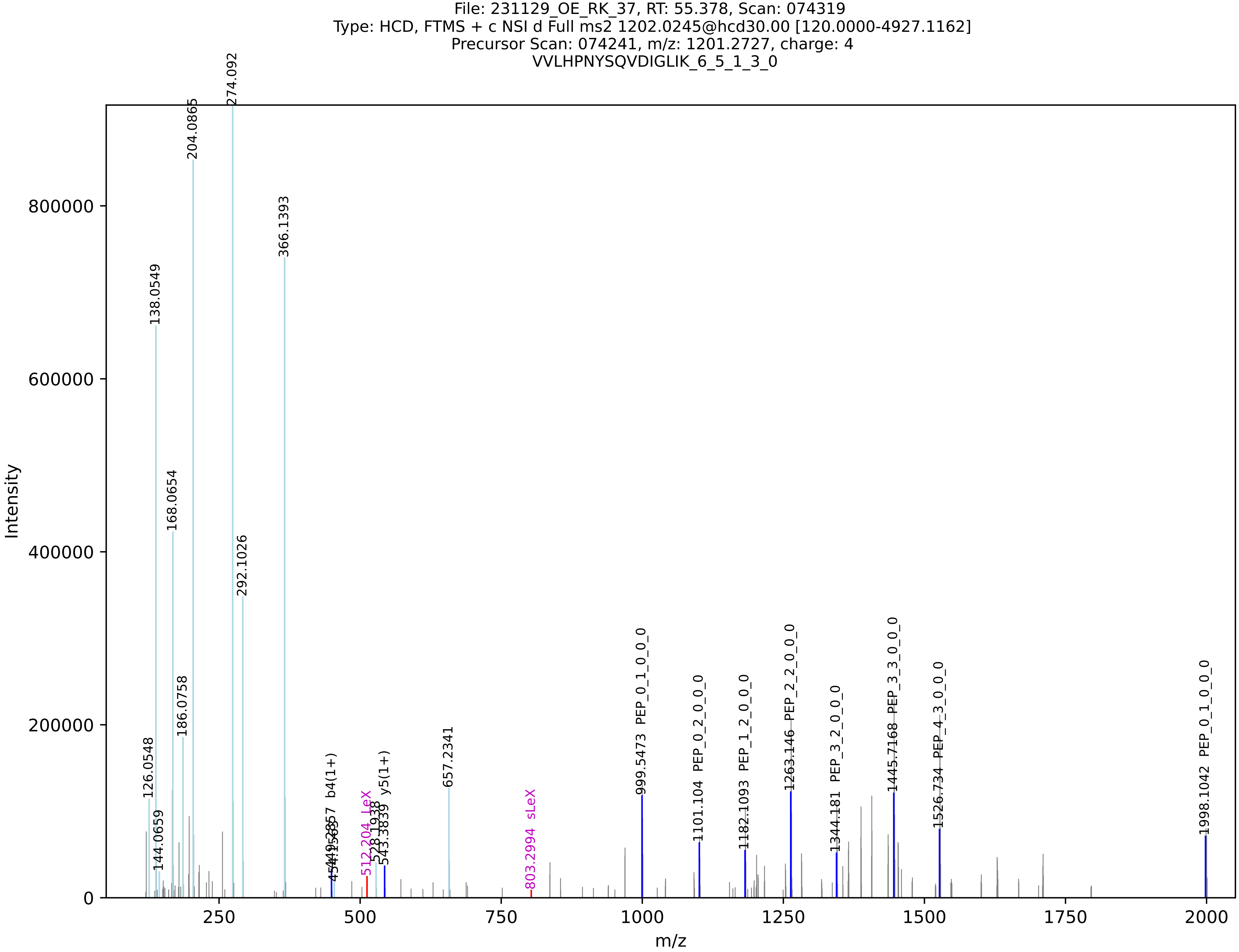
File: 231129_OE_RK_37, RT: 55.378, Scan: 074319
Type: HCD, FTMS + c NSI d Full ms2 1202.0245@hcd30.00 [120.0000-4927.1162]
Precursor Scan: 074241, m/z: 1201.2727, charge: 4
VVLHPNYSQVDIGLIK_6_5_1_3_0
Intensity
m/z
0
200000
400000
600000
800000
250
500
750
1000
1250
1500
1750
2000
274.092
204.0865
366.1393
138.0549
168.0654
292.1026
186.0758
126.0548
144.0659
b4(1+)
512.204 LeX
528.1938
543.3839 y5(1+)
657.2341
803.2994 sLeX
999.5473 PEP_0_1_0_0_0
1101.104 PEP_0_2_0_0_0
1182.1093 PEP_1_2_0_0_0
1263.146 PEP_2_2_0_0_0
1344.181 PEP_3_2_0_0_0
1445.7168 PEP_3_3_0_0_
1526.734 PEP_4_3_0_0_0
1998.1042 PEP_0_1_0_0_

File: 231129_OE_RK_37, RT: 55.383, Scan: 074326
Type: HCD, FTMS + c NSI d Full ms2 1602.0287@hcd30.00 [120.0000-4924.3496]
Precursor Scan: 074241, m/z: 1601.3612, charge: 3
VVLHPNYSQVDIGLIK_6_5_1_3_0

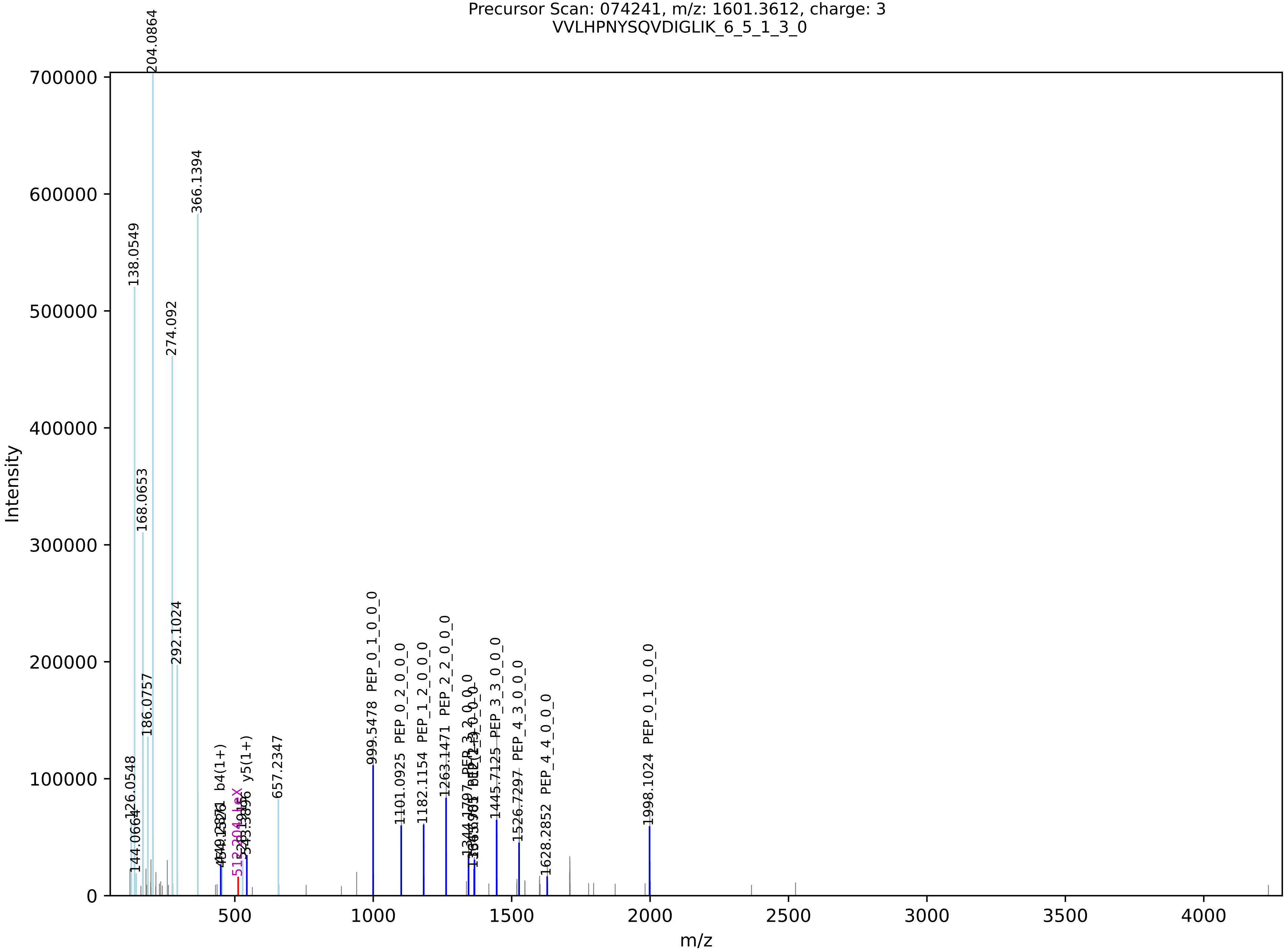

File: 231129_OE_RK_37, RT: 55.44, Scan: 074408
Type: HCD, FTMS + c NSI d Full ms2 1654.0709@hcd30.00 [120.0000-5083.5991]
Precursor Scan: 074315, m/z: 1653.4075, charge: 3
MVSHHNLTTGATLINEQWLLTTAK_6_5_0_1_0

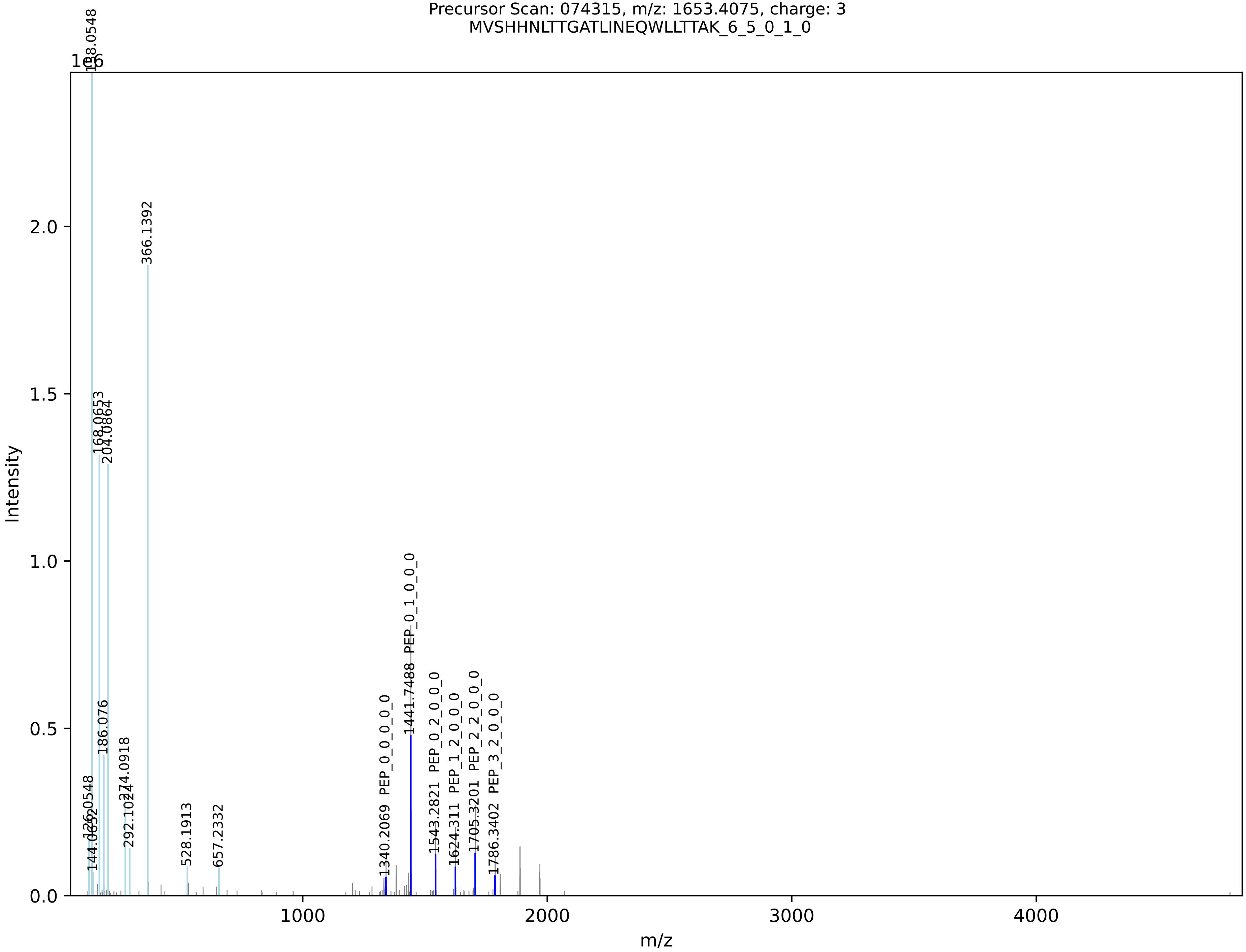

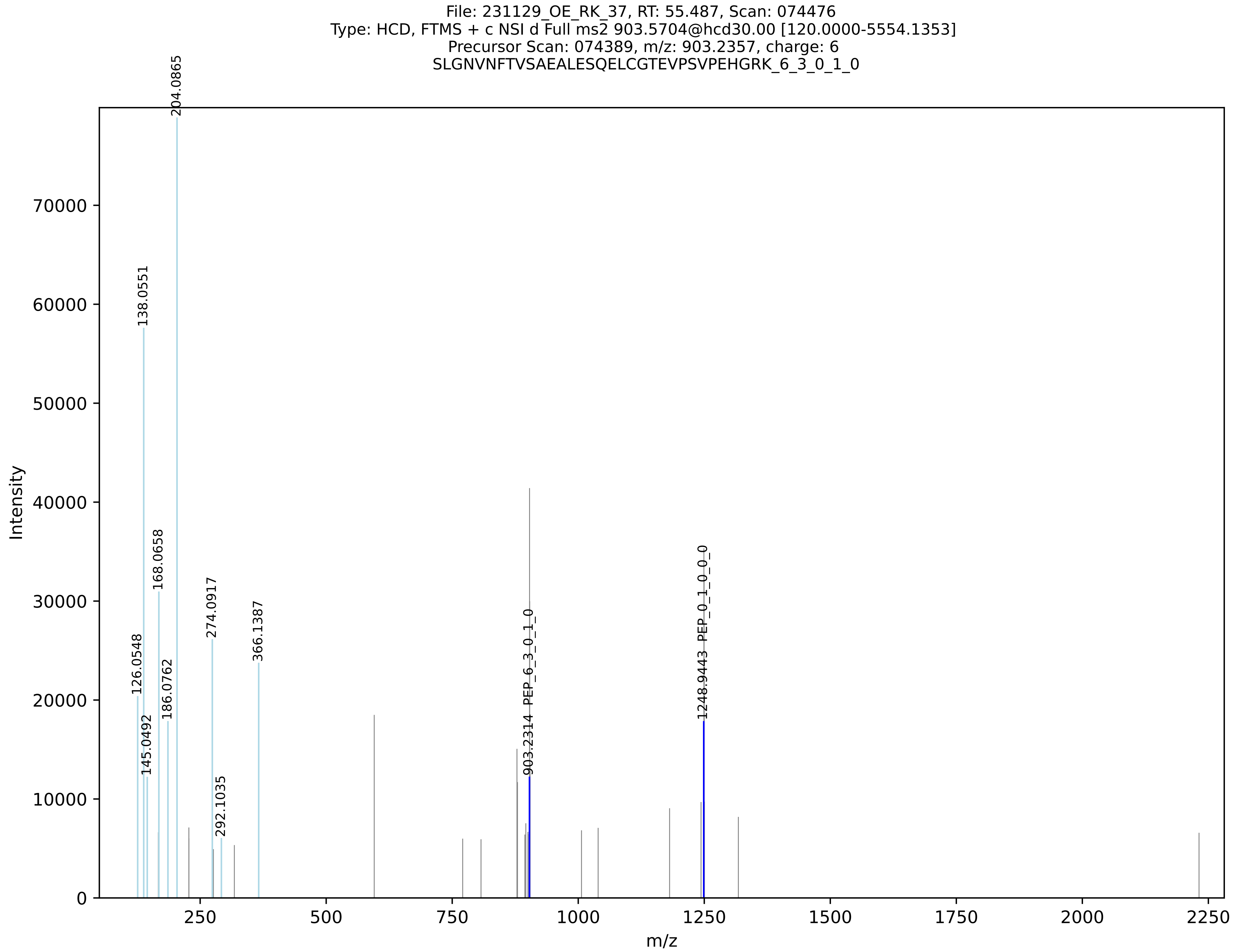

File: 231129_OE_RK_37, RT: 55.487, Scan: 074476
Type: HCD, FTMS + c NSI d Full ms2 903.5704@hcd30.00 [120.0000-5554.1353]
Precursor Scan: 074389, m/z: 903.2357, charge: 6
SLGNVNFTVSAEALESQELCGTEVPSVPEHGRK_6_3_0_1_0
204.0865
138.0551
168.0658
126.0548
145.0492
186.0762
274.0917
292.1035
366.1387
903.2314_PEP_6_3_0_1_0
1248.9443_PEP_0_1_0_0_0
Intensity
m/z

File: 231129_OE_RK_37, RT: 55.53, Scan: 074535
Type: HCD, FTMS + c NSI d Full ms2 1314.8909@hcd30.00 [120.0000-4045.7080]
Precursor Scan: 074460, m/z: 1314.2227, charge: 3
ALPQPQNVTSLLGCTH_5_4_0_2_0

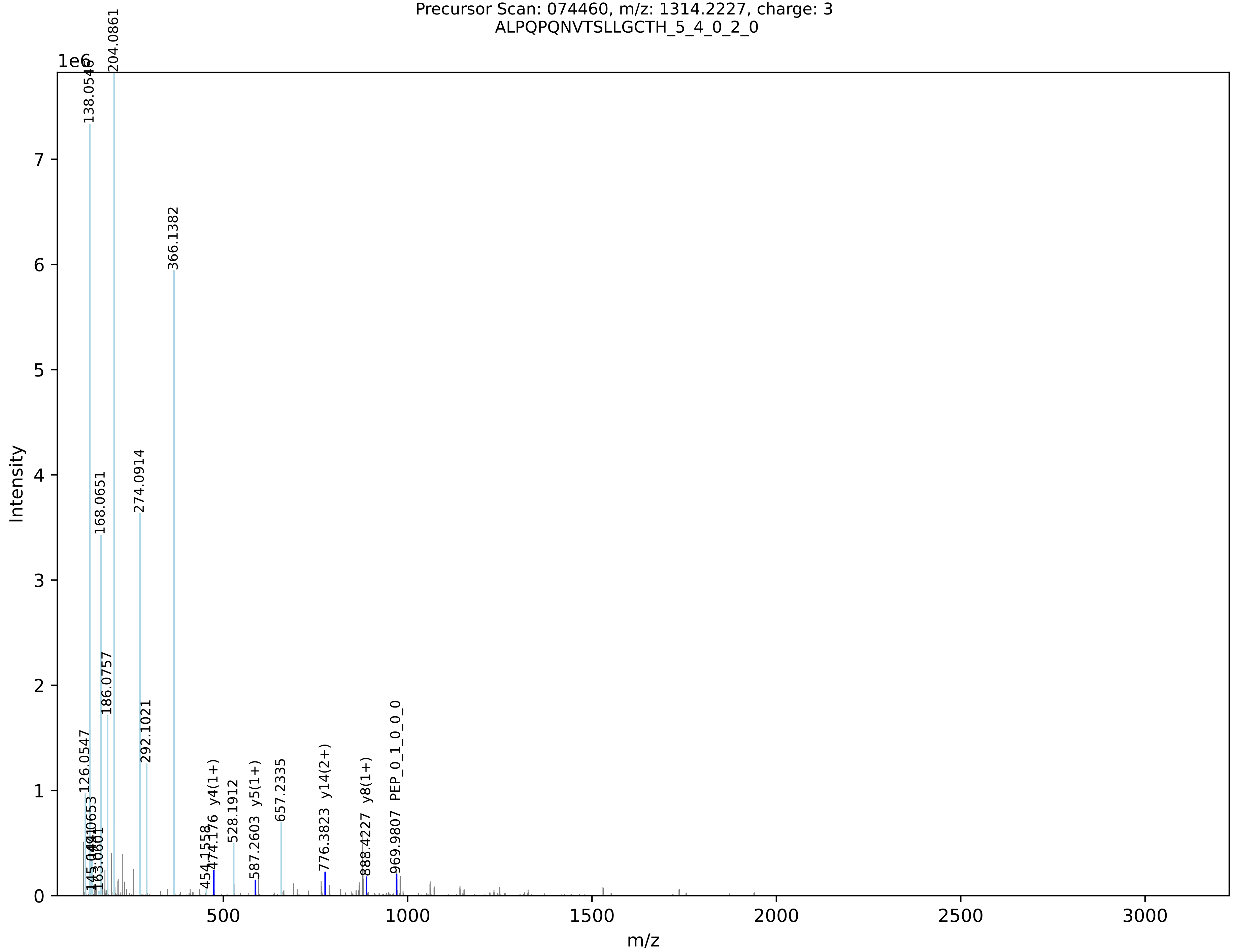

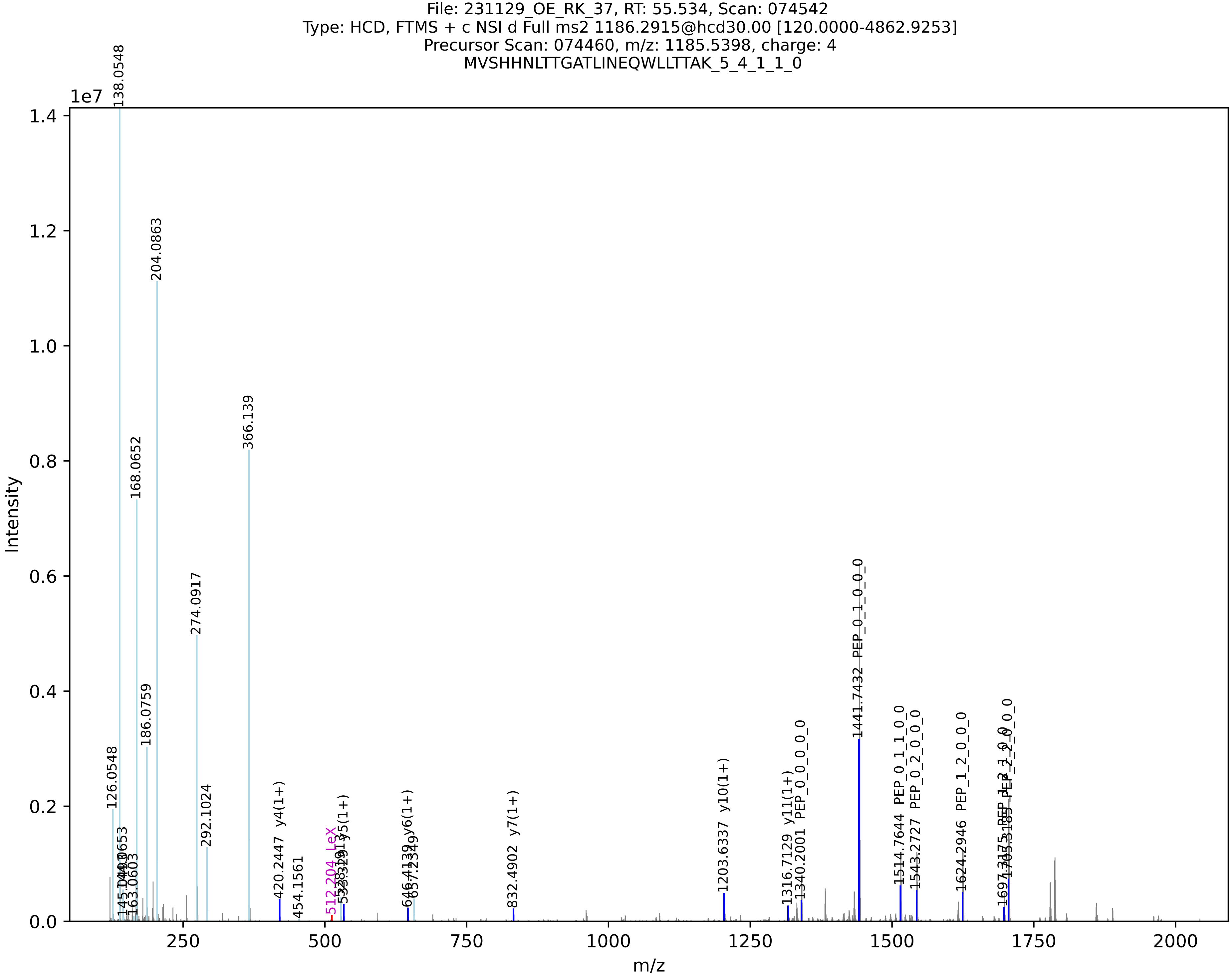

File: 231129_OE_RK_37, RT: 55.534, Scan: 074542
Type: HCD, FTMS + c NSI d Full ms2 1186.2915@hcd30.00 [120.0000-4862.9253]
Precursor Scan: 074460, m/z: 1185.5398, charge: 4
MVSHHNLTTGATLINEQWLLTTAK_5_4_1_1_0
1e7
Intensity
m/z
138.0548
204.0863
168.0652
366.139
274.0917
186.0759
126.0548
292.1024
163.0603
420.2447 y4(1+)
454.1561
512.204 LeX
y5(1+)
y6(1+)
657.2349
832.4902 y7(1+)
1203.6337 y10(1+)
1316.7129 y11(1+)
1340.2001 PEP_0_0_0_0_0
1441.7432 PEP_0_1_0_0_0
1514.7644 PEP_0_1_1_0_0
1543.2727 PEP_0_2_0_0_0
1624.2946 PEP_1_2_0_0_0

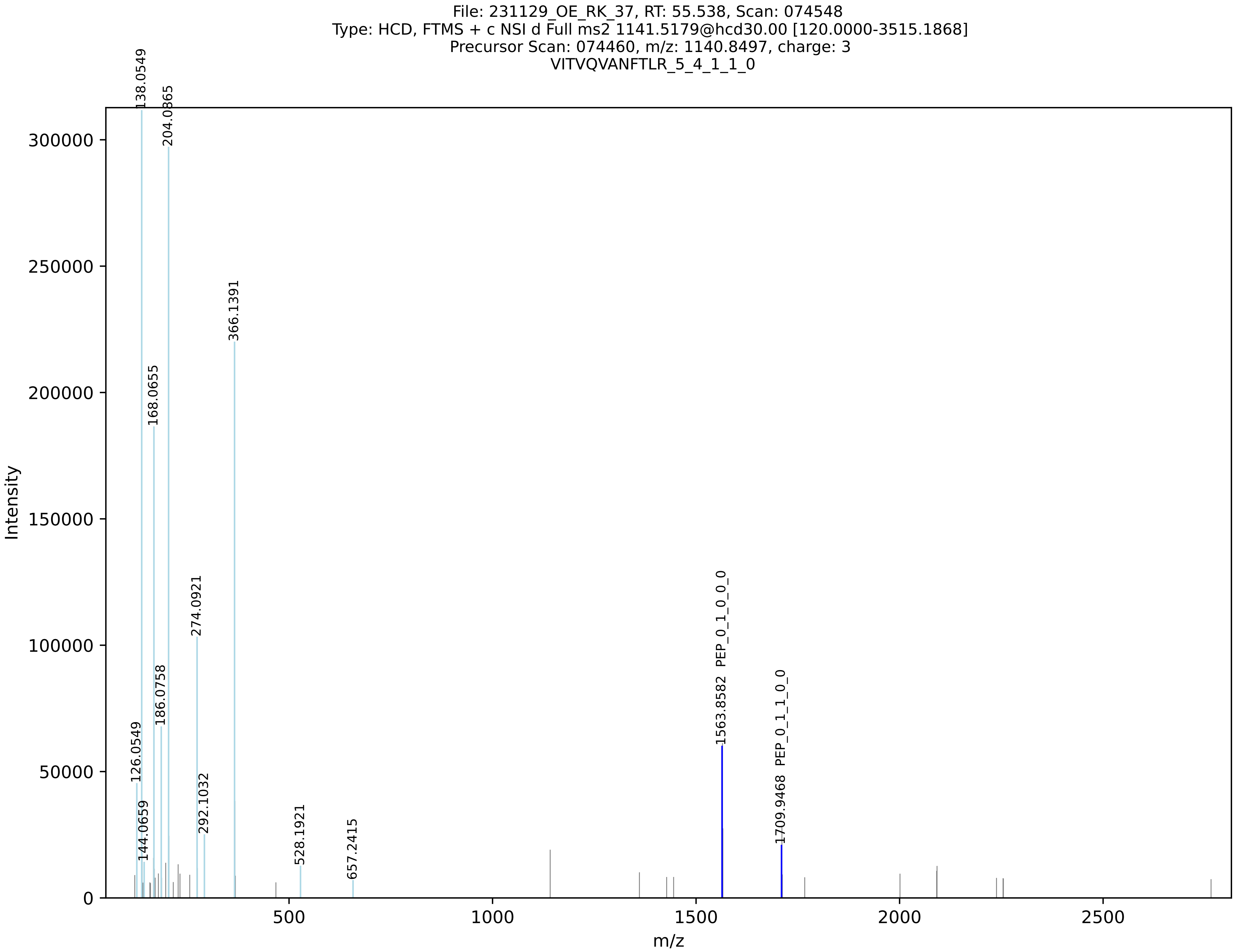

File: 231129_OE_RK_37, RT: 55.538, Scan: 074548
Type: HCD, FTMS + c NSI d Full ms2 1141.5179@hcd30.00 [120.0000-3515.1868]
Precursor Scan: 074460, m/z: 1140.8497, charge: 3
VITVQVANFTLR_5_4_1_1_0
138.0549
204.0865
168.0655
366.1391
274.0921
186.0758
126.0549
144.0659
292.1032
528.1921
657.2415
1563.8582 PEP_0_1_0_0_0
1709.9468 PEP_0_1_1_0_0
Intensity
m/z
0
50000
100000
150000
200000
250000
300000
500
1000
1500
2000
2500

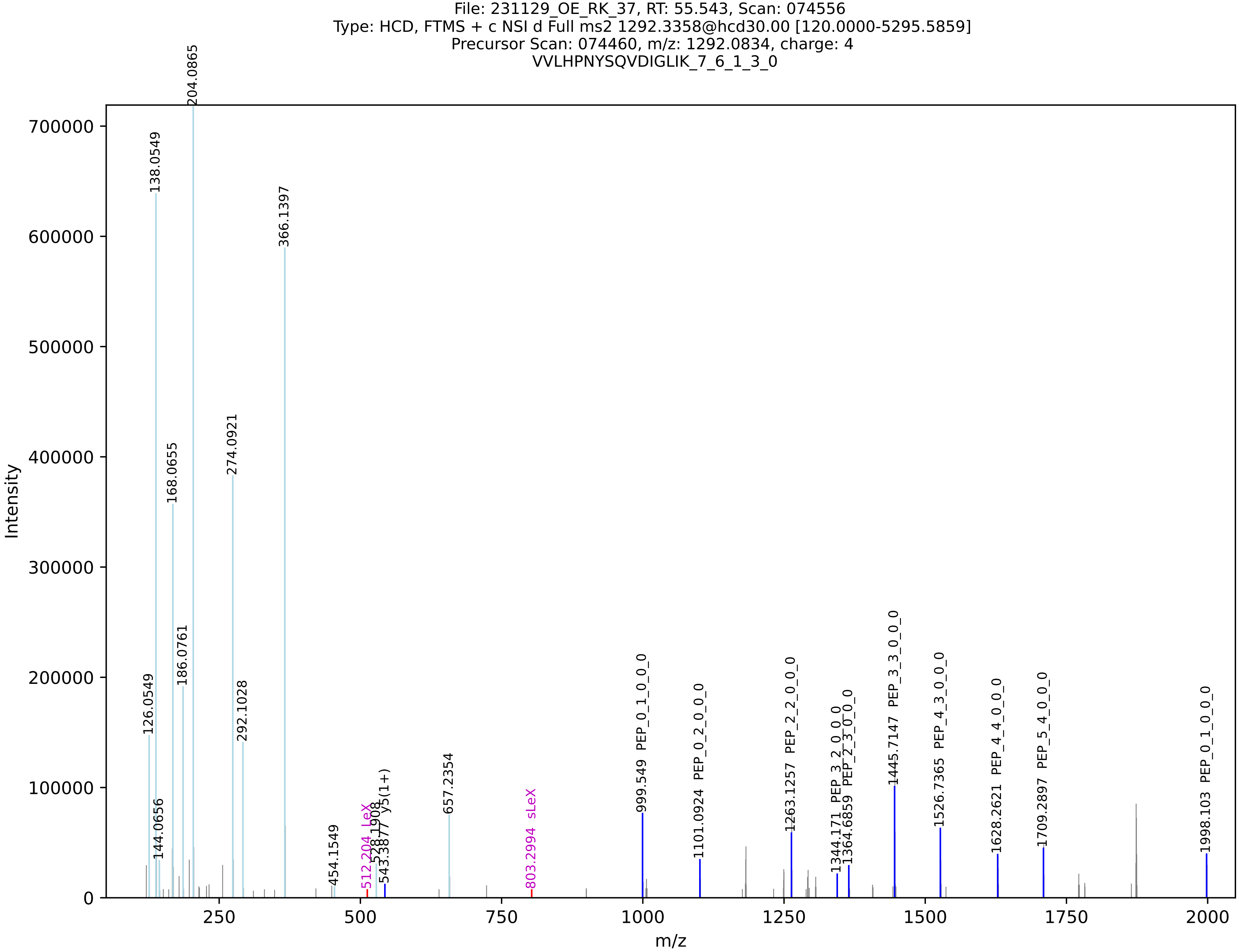
File: 231129_OE_RK_37, RT: 55.543, Scan: 074556
Type: HCD, FTMS + c NSI d Full ms2 1292.3358@hcd30.00 [120.0000-5295.5859]
Precursor Scan: 074460, m/z: 1292.0834, charge: 4
VVLHPNYSQVDIGLIK_7_6_1_3_0
Intensity
m/z
204.0865
138.0549
366.1397
274.0921
168.0655
186.0761
126.0549
292.1028
144.0656
454.1549
512.204 LeX
528.1908
543.3877 y5(1+)
657.2354
803.2994 sLeX
999.549 PEP_0_1_0_0_0
1101.0924 PEP_0_2_0_0_0
1263.1257 PEP_2_2_0_0_0
1344.171 PEP_3_2_0_0_0
1364.6859 PEP_2_3_0_0_0
1445.7147 PEP_3_3_0_0_0
1526.7365 PEP_4_3_0_0_0
1628.2621 PEP_4_4_0_0_0
1709.2897 PEP_5_4_0_0_0
1998.103 PEP_0_1_0_0_0

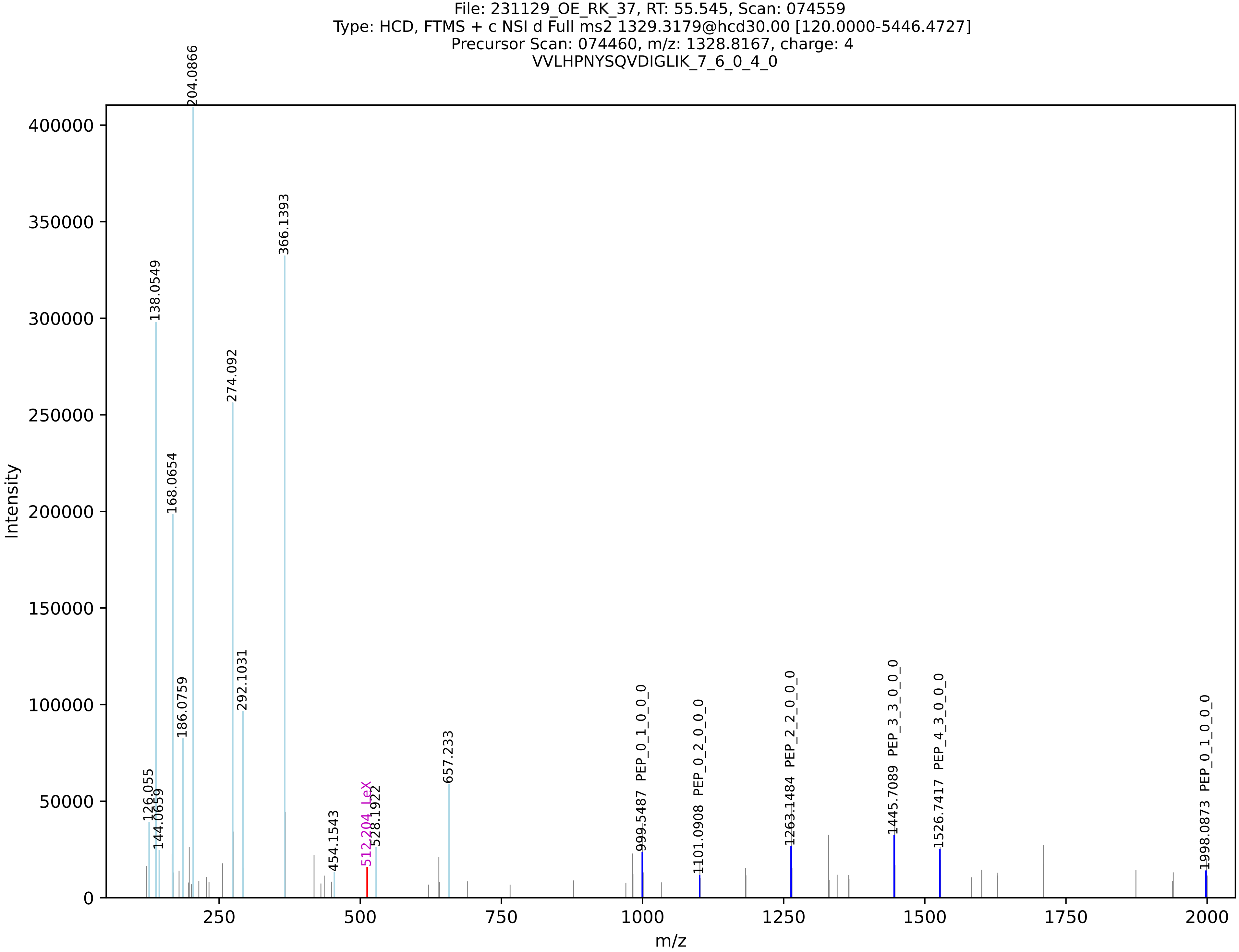
File: 231129_OE_RK_37, RT: 55.545, Scan: 074559
Type: HCD, FTMS + c NSI d Full ms2 1329.3179@hcd30.00 [120.0000-5446.4727]
Precursor Scan: 074460, m/z: 1328.8167, charge: 4
VVLHPNYSQVDIGLIK_7_6_0_4_0
Intensity
m/z
204.0866
366.1393
138.0549
274.092
168.0654
292.1031
186.0759
126.055
144.0659
454.1543
512.204_LeX
528.1922
657.233
999.5487 PEP_0_1_0_0_0
1101.0908 PEP_0_2_0_0_0
1263.1484 PEP_2_2_0_0_0
1445.7089 PEP_3_3_0_0_0
1526.7417 PEP_4_3_0_0_0
1998.0873 PEP_0_1_0_0_0

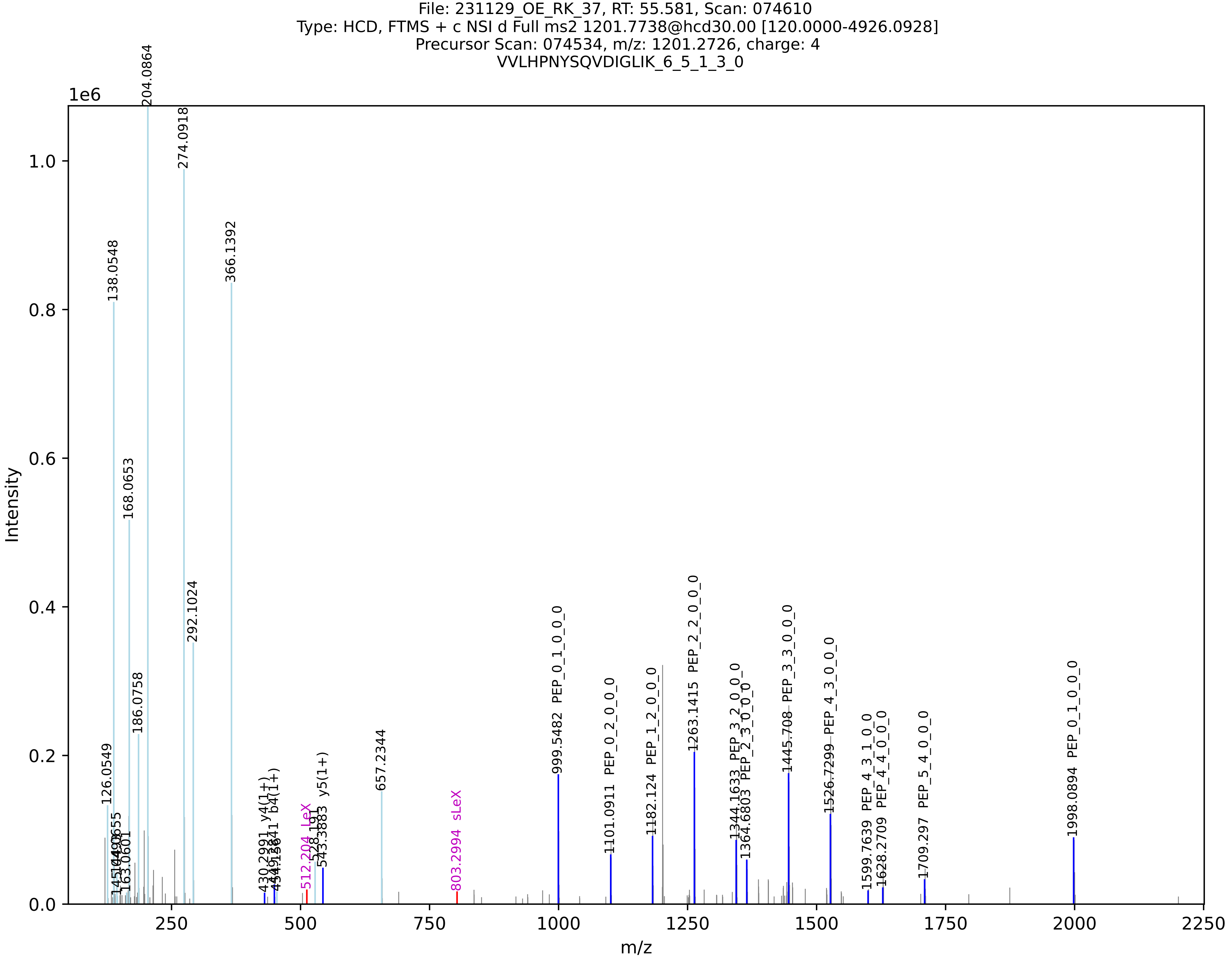
File: 231129_OE_RK_37, RT: 55.581, Scan: 074610
Type: HCD, FTMS + c NSI d Full ms2 1201.7738@hcd30.00 [120.0000-4926.0928]
Precursor Scan: 074534, m/z: 1201.2726, charge: 4
VVLHPNYSQVDIGLIK_6_5_1_3_0
1e6
Intensity
m/z
204.0864
274.0918
366.1392
138.0548
168.0653
292.1024
186.0758
126.0549
163.0601
657.2344
430.2991 y4(1+)
543.3883 y5(1+)
512.204 LeX
803.2994 sLeX
999.5482 PEP_0_1_0_0_0
1101.0911 PEP_0_2_0_0_0
1182.124 PEP_1_2_0_0_0
1263.1415 PEP_2_2_0_0_0
1344.1633 PEP_3_2_0_0_0
1364.6803 PEP_2_3_0_0_0
1445.708 PEP_3_3_0_0_0
1526.7299 PEP_4_3_0_0_0
1599.7639 PEP_4_3_1_0_0
1628.2709 PEP_4_4_0_0_0
1709.297 PEP_5_4_0_0_0
1998.0894 PEP_0_1_0_0_0

File: 231129_OE_RK_37, RT: 55.582, Scan: 074612
Type: HCD, FTMS + c NSI d Full ms2 1538.0306@hcd30.00 [120.0000-4728.5161]
Precursor Scan: 074534, m/z: 1537.0278, charge: 3
NTTSYVLRGLEPGQEYNVLLTAEK_5_4_0_1_0

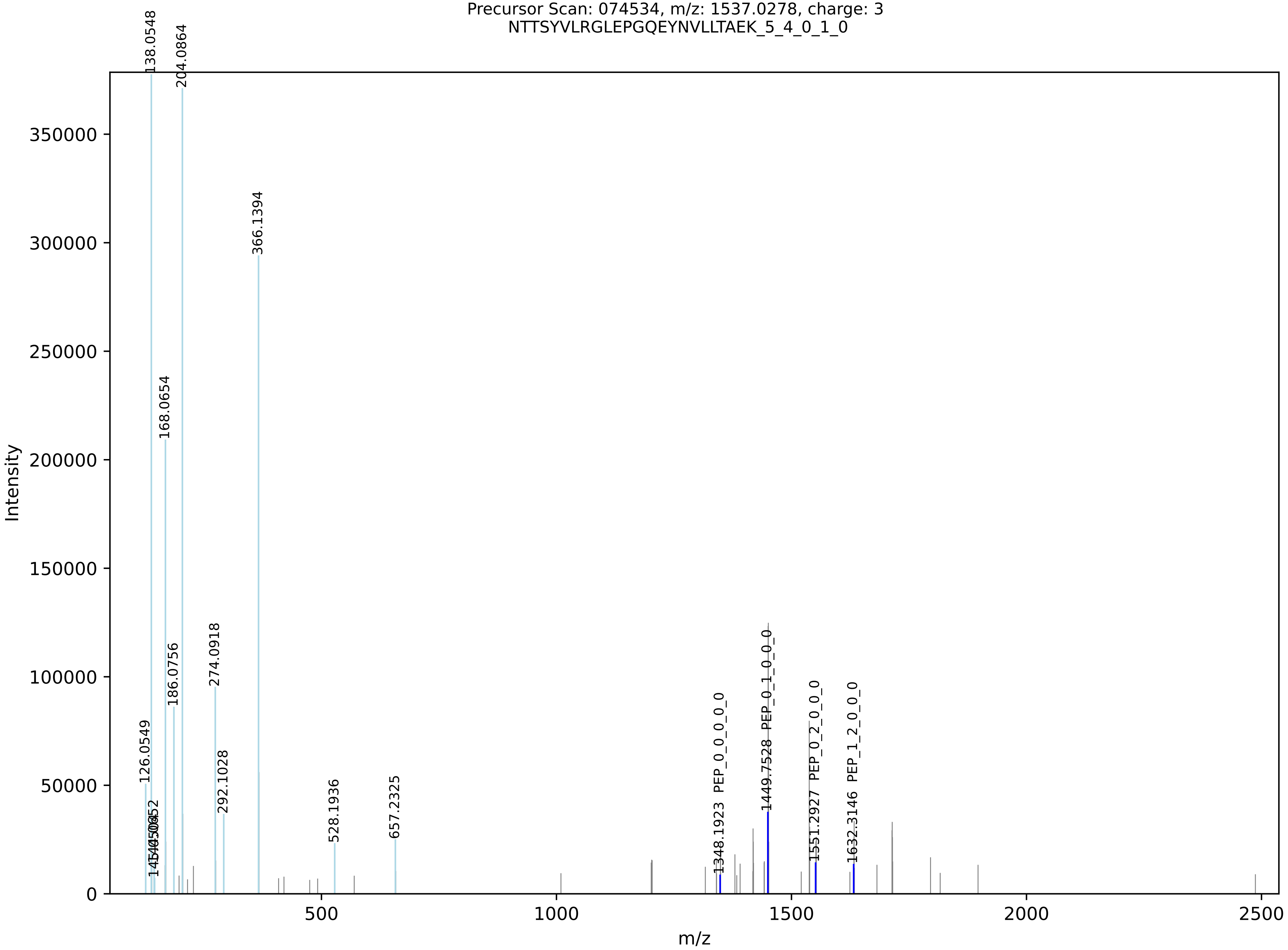

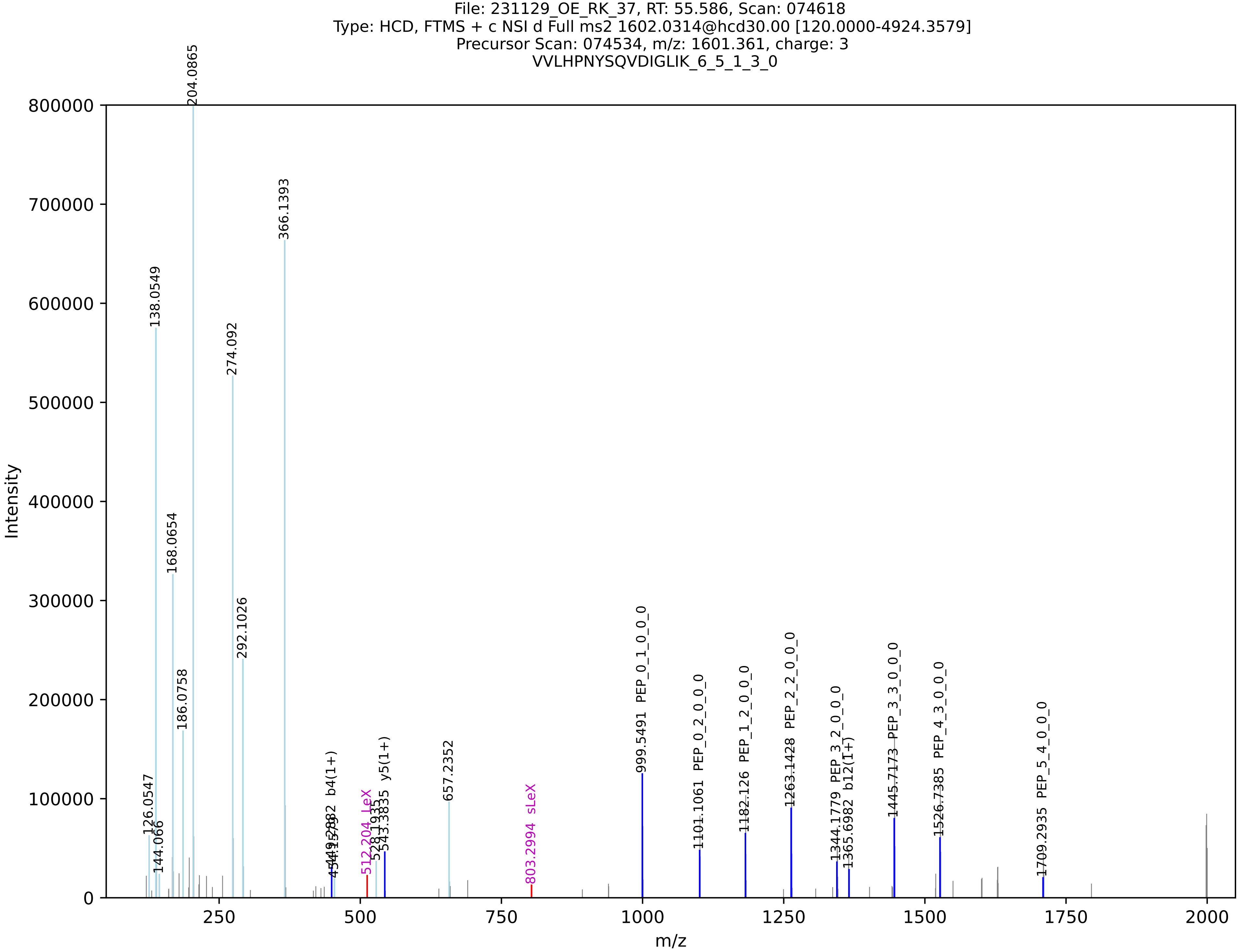
File: 231129_OE_RK_37, RT: 55.586, Scan: 074618
Type: HCD, FTMS + c NSI d Full ms2 1602.0314@hcd30.00 [120.0000-4924.3579]
Precursor Scan: 074534, m/z: 1601.361, charge: 3
VVLHPNYSQVDIGLIK_6_5_1_3_0
Intensity
m/z
204.0865
366.1393
138.0549
274.092
168.0654
292.1026
186.0758
126.0547
144.066
512.204 LeX
528.1935
543.3835 y5(1+)
657.2352
803.2994 sLeX
999.5491 PEP_0_1_0_0_0
1101.1061 PEP_0_2_0_0_0
1182.126 PEP_1_2_0_0_0
1263.1428 PEP_2_2_0_0_0
1344.1779 PEP_3_2_0_0_0
1365.6982 b12(1+)
1445.7173 PEP_3_3_0_0_0
1526.7385 PEP_4_3_0_0_0
1709.2935 PEP_5_4_0_0_0

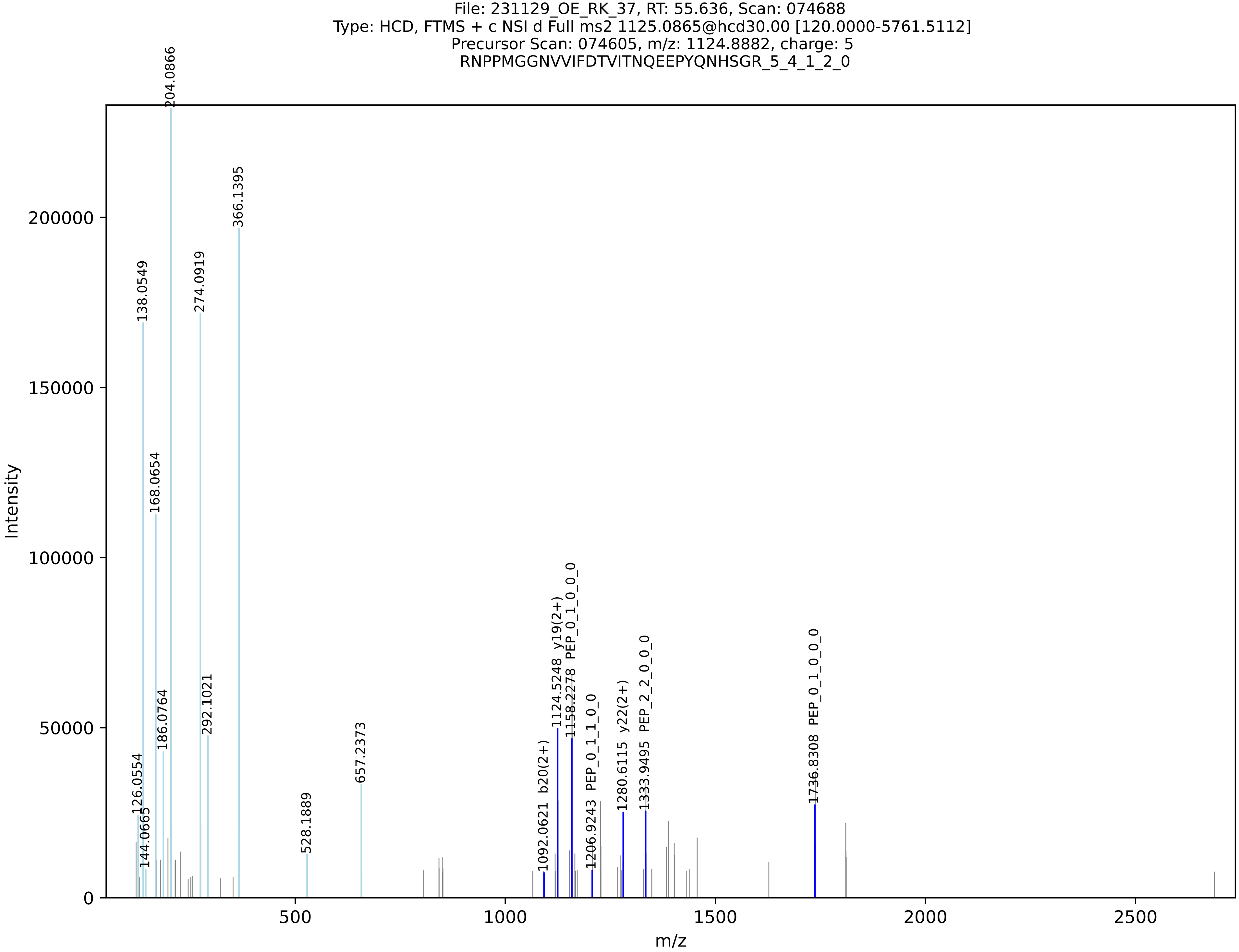
File: 231129_OE_RK_37, RT: 55.636, Scan: 074688
Type: HCD, FTMS + c NSI d Full ms2 1125.0865@hcd30.00 [120.0000-5761.5112]
Precursor Scan: 074605, m/z: 1124.8882, charge: 5
RNPPMGGNVVIFDTVITNQEEPYQNHSGR_5_4_1_2_0
Intensity
m/z
204.0866
366.1395
138.0549
274.0919
168.0654
186.0764
292.1021
126.0554
144.0665
528.1889
657.2373
1092.0621 b20(2+)
1124.5248 y19(2+)
1158.2278 PEP_0_1_0_0_0
1206.9243 PEP_0_1_1_0_0
1280.6115 y22(2+)
1333.9495 PEP_2_2_0_0_0
1736.8308 PEP_0_1_0_0_0

File: 231129_OE_RK_37, RT: 55.639, Scan: 074693
Type: HCD, FTMS + c NSI d Full ms2 1117.7230@hcd30.00 [120.0000-4583.1655]
Precursor Scan: 074605, m/z: 1117.2218, charge: 4
NVSCPQLEVPVCPSGFQLSCK_5_4_1_1_0

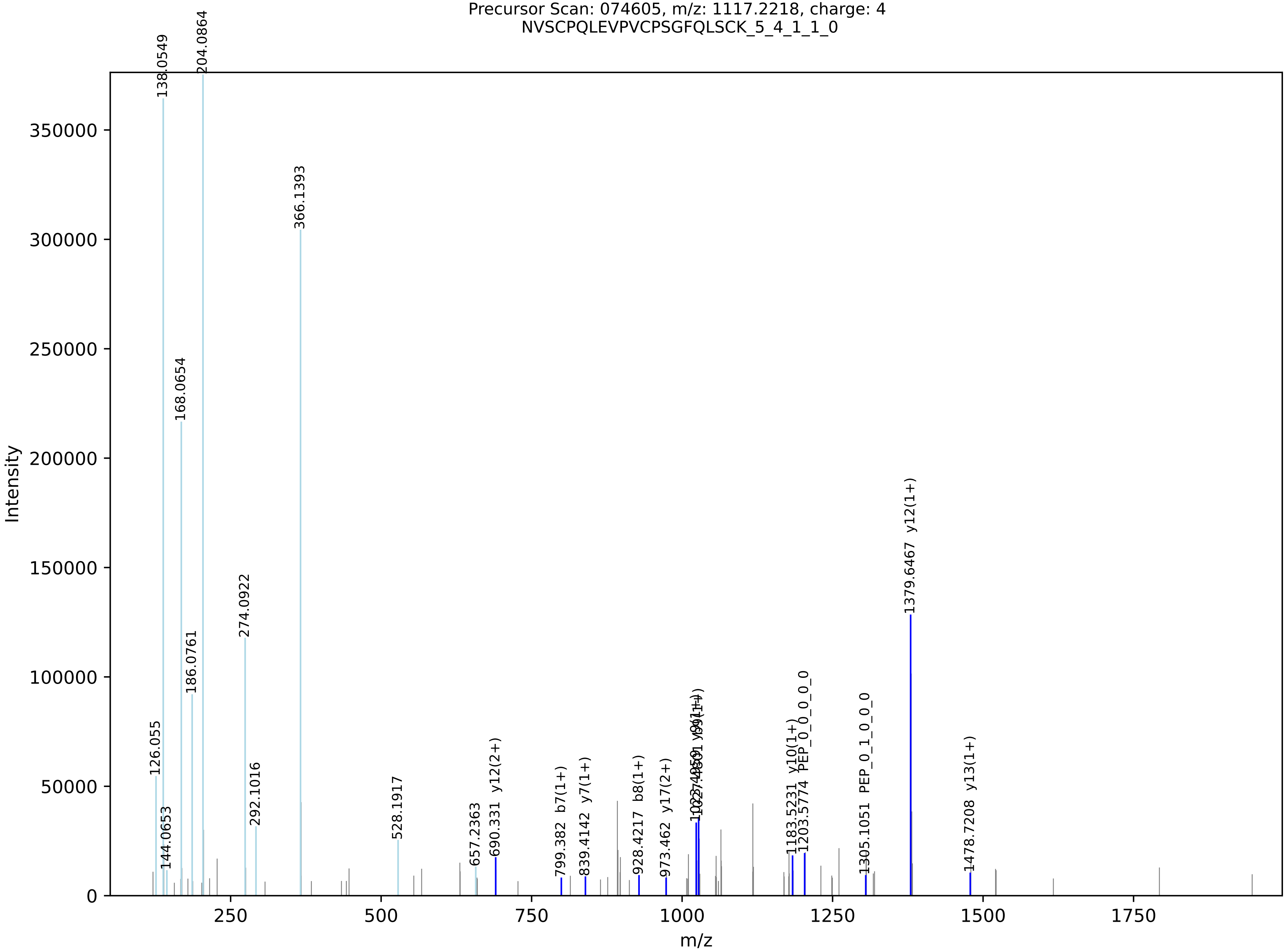

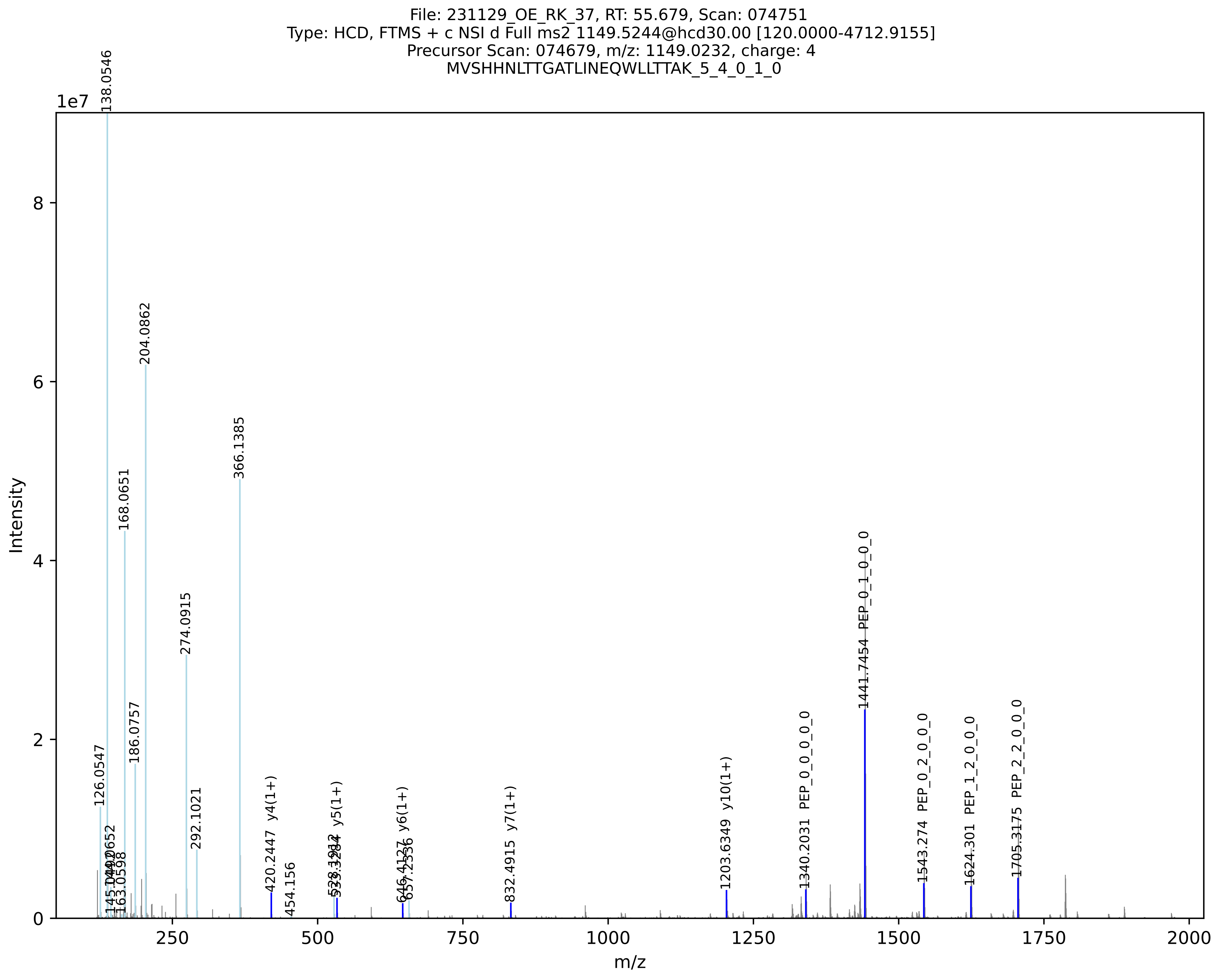

File: 231129_OE_RK_37, RT: 55.679, Scan: 074751
Type: HCD, FTMS + c NSI d Full ms2 1149.5244@hcd30.00 [120.0000-4712.9155]
Precursor Scan: 074679, m/z: 1149.0232, charge: 4
MVSHHNLTTGATLINEQWLLTTAK_5_4_0_1_0
1e7
Intensity
m/z
138.0546
204.0862
366.1385
168.0651
274.0915
186.0757
126.0547
292.1021
163.0598
420.2447 y4(1+)
454.156
y5(1+)
646.4127 y6(1+)
657.2536
832.4915 y7(1+)
1203.6349 y10(1+)
1340.2031 PEP_0_0_0_0_0
1441.7454 PEP_0_1_0_0_0
1543.274 PEP_0_2_0_0_0
1624.301 PEP_1_2_0_0_0
1705.3175 PEP_2_2_0_0_0

File: 231129_OE_RK_37, RT: 55.731, Scan: 074826
Type: HCD, FTMS + c NSI d Full ms2 1314.8895@hcd30.00 [120.0000-4045.7039]
Precursor Scan: 074750, m/z: 1314.2212, charge: 3
ALPQPQNVTSLLGCTH_5_4_0_2_0

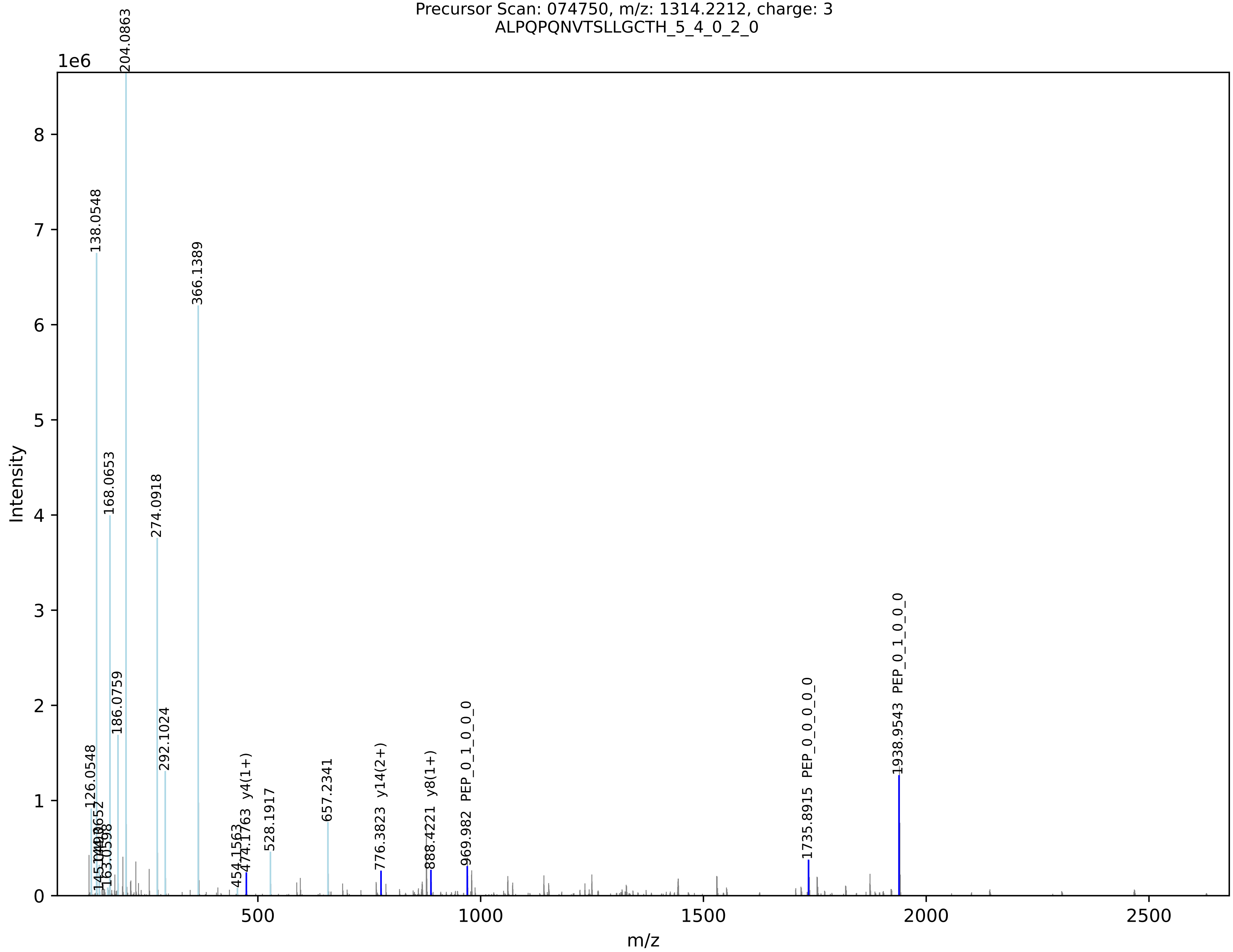

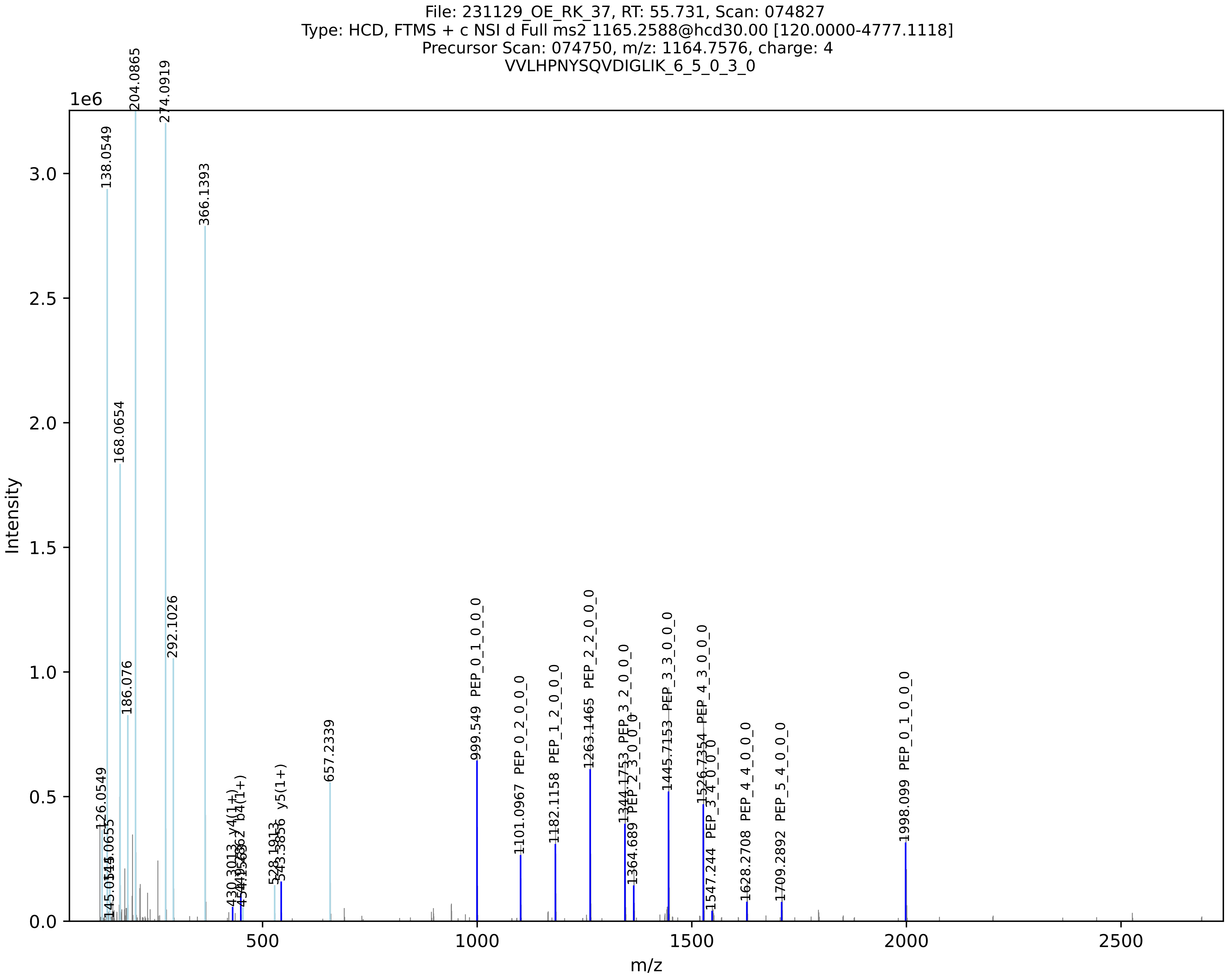
File: 231129_OE_RK_37, RT: 55.731, Scan: 074827
Type: HCD, FTMS + c NSI d Full ms2 1165.2588@hcd30.00 [120.0000-4777.1118]
Precursor Scan: 074750, m/z: 1164.7576, charge: 4
VVLHPNYSQVDIGLIK_6_5_0_3_0
1e6
Intensity
m/z
204.0865
274.0919
138.0549
366.1393
168.0654
292.1026
186.076
126.0549
657.2339
528.1913
543.3856 y5(1+)
999.549 PEP_0_1_0_0_0
1101.0967 PEP_0_2_0_0_0
1182.1158 PEP_1_2_0_0_0
1263.1465 PEP_2_2_0_0_0
1344.1753 PEP_3_2_0_0_0
1364.689 PEP_2_3_0_0_0
1445.7153 PEP_3_3_0_0_0
1526.7354 PEP_4_3_0_0_0
1547.244 PEP_3_4_0_0_0
1628.2708 PEP_4_4_0_0_0
1709.2892 PEP_5_4_0_0_0
1998.099 PEP_0_1_0_0_0

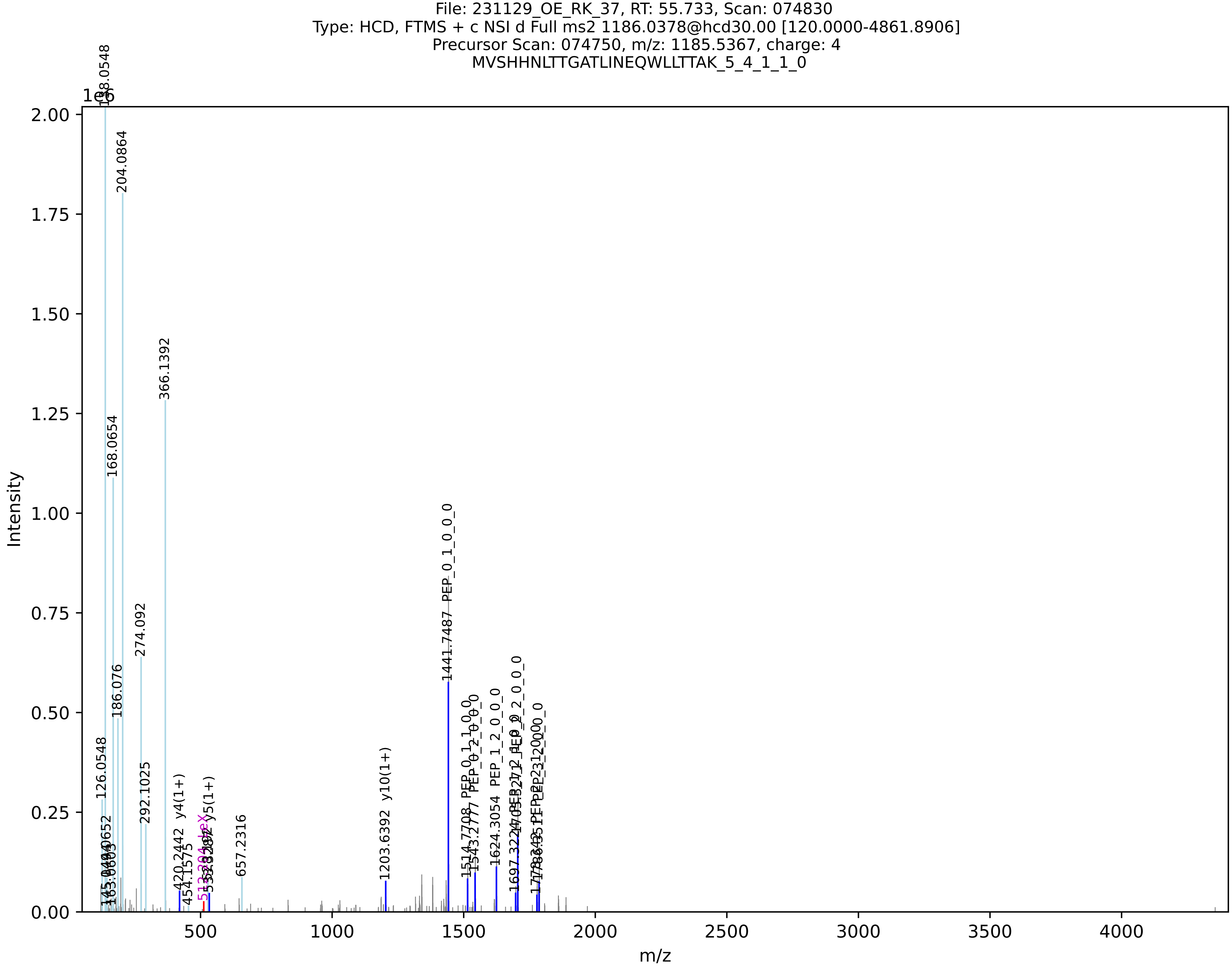
File: 231129_OE_RK_37, RT: 55.733, Scan: 074830
Type: HCD, FTMS + c NSI d Full ms2 1186.0378@hcd30.00 [120.0000-4861.8906]
Precursor Scan: 074750, m/z: 1185.5367, charge: 4
MVSHHNLTTGATLINEQWLLTTAK_5_4_1_1_0
1e6
Intensity
m/z
204.0864
366.1392
168.0654
274.092
186.076
126.0548
292.1025
420.2442 y4(1+)
454.1575
657.2316
1203.6392 y10(1+)
1441.7487 PEP_0_1_0_0_0
1624.3054 PEP_1_2_0_0_0
0.00
0.25
0.50
0.75
1.00
1.25
1.50
1.75
2.00
500
1000
1500
2000
2500
3000
3500
4000

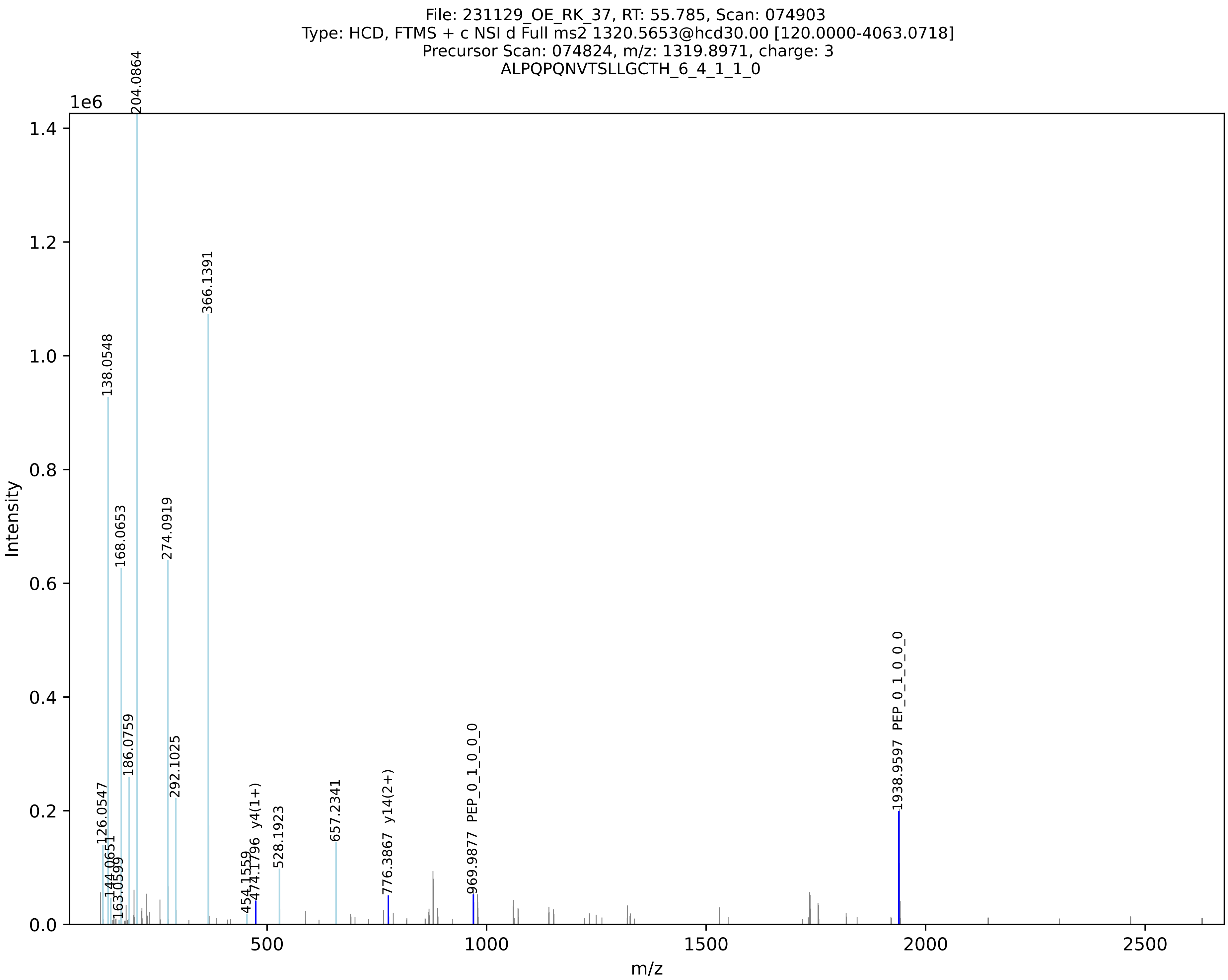
File: 231129_OE_RK_37, RT: 55.785, Scan: 074903
Type: HCD, FTMS + c NSI d Full ms2 1320.5653@hcd30.00 [120.0000-4063.0718]
Precursor Scan: 074824, m/z: 1319.8971, charge: 3
ALPQPQNVTSLLGCTH_6_4_1_1_0
1e6
Intensity
m/z
204.0864
366.1391
138.0548
168.0653
274.0919
186.0759
292.1025
126.0547
144.0651
163.0599
454.1559
474.1796 y4(1+)
528.1923
657.2341
776.3867 y14(2+)
969.9877 PEP_0_1_0_0_0
1938.9597 PEP_0_1_0_0_0
0.0
0.2
0.4
0.6
0.8
1.0
1.2
1.4
500
1000
1500
2000
2500

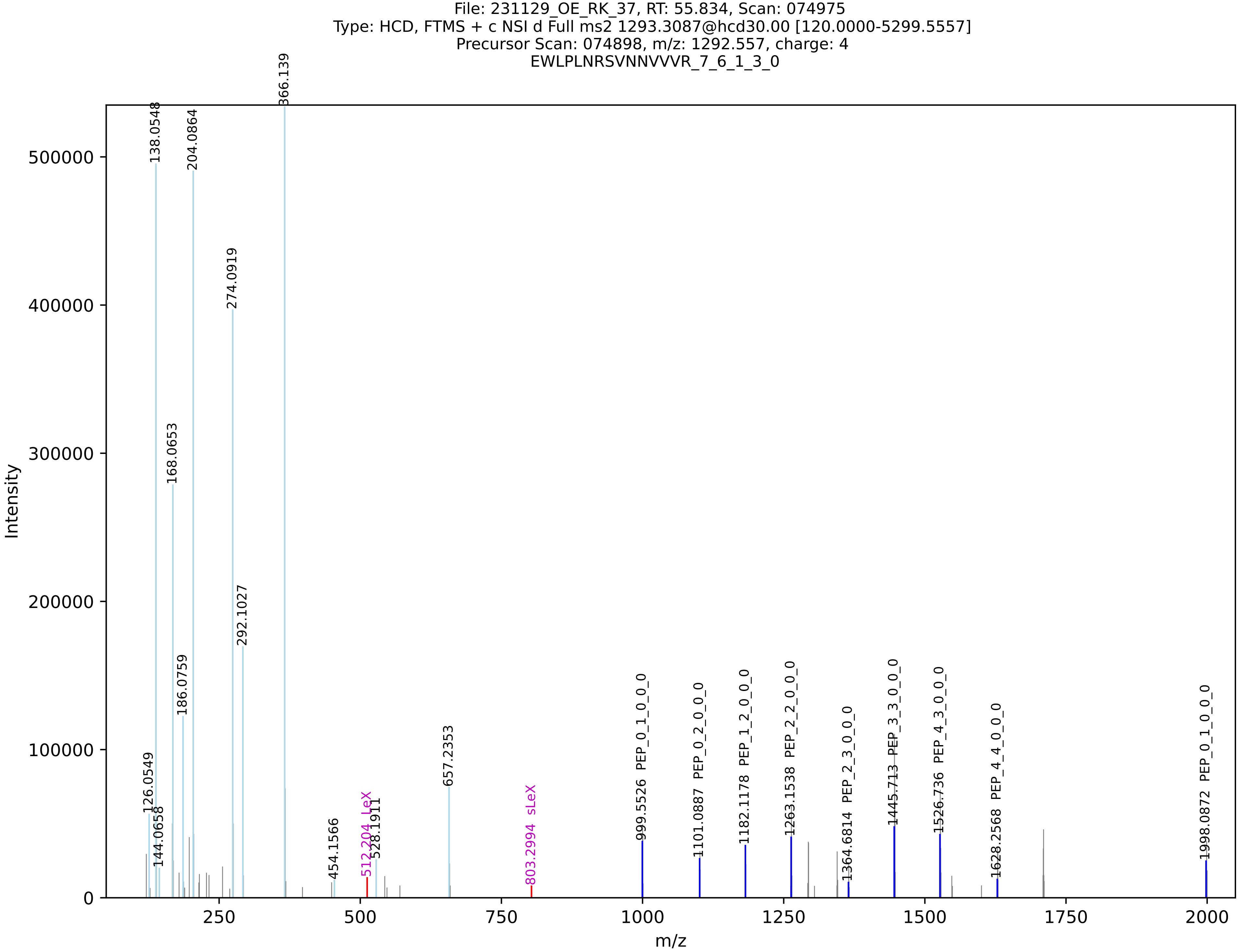

File: 231129_OE_RK_37, RT: 55.834, Scan: 074975
Type: HCD, FTMS + c NSI d Full ms2 1293.3087@hcd30.00 [120.0000-5299.5557]
Precursor Scan: 074898, m/z: 1292.557, charge: 4
EWLPLNRSVNNVVVR_7_6_1_3_0
Intensity
m/z
366.139
138.0548
204.0864
274.0919
168.0653
292.1027
186.0759
126.0549
144.0658
454.1566
512.204 LeX
528.1911
657.2353
803.2994 sLeX
999.5526 PEP_0_1_0_0_0
1101.0887 PEP_0_2_0_0_0
1182.1178 PEP_1_2_0_0_0
1263.1538 PEP_2_2_0_0_0
1364.6814 PEP_2_3_0_0_0
1445.713 PEP_3_3_0_0_0
1526.736 PEP_4_3_0_0_0
1628.2568 PEP_4_4_0_0_0
1998.0872 PEP_0_1_0_0_0

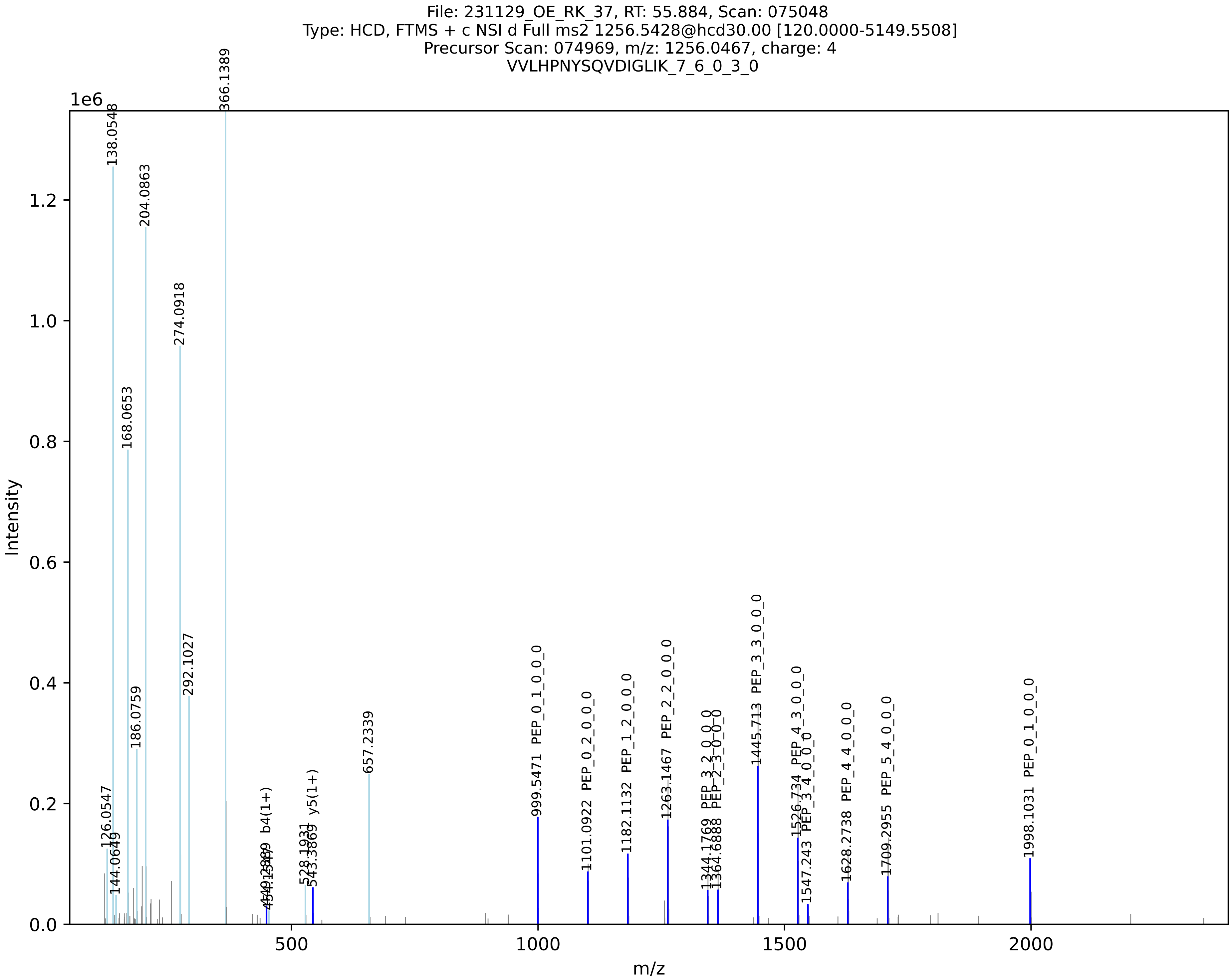
File: 231129_OE_RK_37, RT: 55.884, Scan: 075048
Type: HCD, FTMS + c NSI d Full ms2 1256.5428@hcd30.00 [120.0000-5149.5508]
Precursor Scan: 074969, m/z: 1256.0467, charge: 4
VVLHPNYSQVDIGLIK_7_6_0_3_0
1e6
Intensity
m/z
366.1389
138.0548
204.0863
274.0918
168.0653
292.1027
186.0759
126.0547
144.0649
b4(1+)
528.1931
543.3869 y5(1+)
657.2339
999.5471 PEP_0_1_0_0_0
1101.0922 PEP_0_2_0_0_0
1182.1132 PEP_1_2_0_0_0
1263.1467 PEP_2_2_0_0_0
1344.1769 PEP_3_2_0_0_0
1364.6888 PEP_2_3_0_0_0
1445.713 PEP_3_3_0_0_0
1526.734 PEP_4_3_0_0_0
1547.243 PEP_3_4_0_0_0
1628.2738 PEP_4_4_0_0_0
1709.2955 PEP_5_4_0_0_0
1998.1031 PEP_0_1_0_0_0

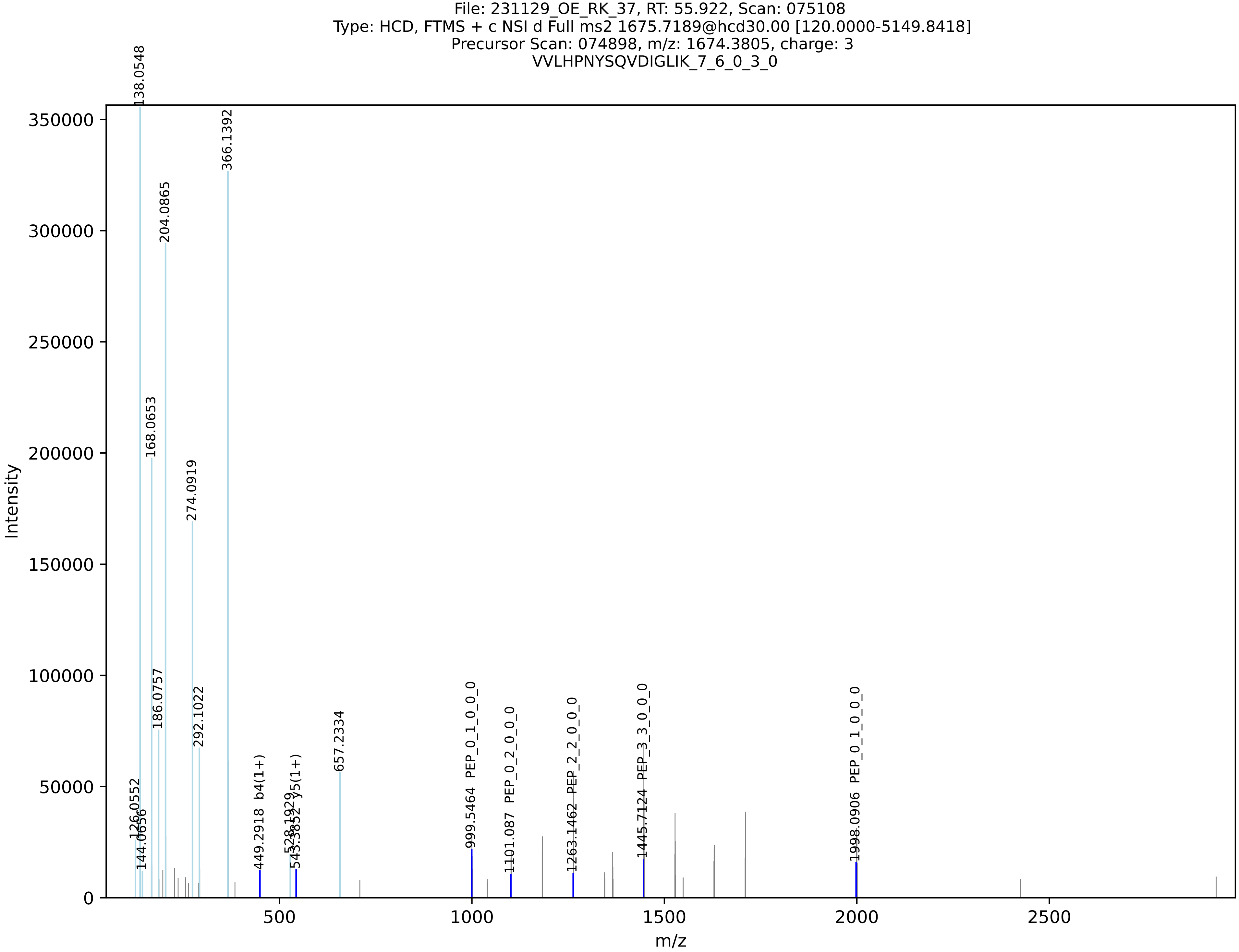
File: 231129_OE_RK_37, RT: 55.922, Scan: 075108
Type: HCD, FTMS + c NSI d Full ms2 1675.7189@hcd30.00 [120.0000-5149.8418]
Precursor Scan: 074898, m/z: 1674.3805, charge: 3
VVLHPNYSQVDIGLIK_7_6_0_3_0
Intensity
m/z
138.0548
366.1392
204.0865
168.0653
274.0919
186.0757
292.1022
126.0552
144.0656
449.2918 b4(1+)
528.1929
543.3852 y5(1+)
657.2334
999.5464 PEP_0_1_0_0_0
1101.087 PEP_0_2_0_0_0
1263.1462 PEP_2_2_0_0_0
1445.7124 PEP_3_3_0_0_0
1998.0906 PEP_0_1_0_0_0
0
50000
100000
150000
200000
250000
300000
350000
500
1000
1500
2000
2500

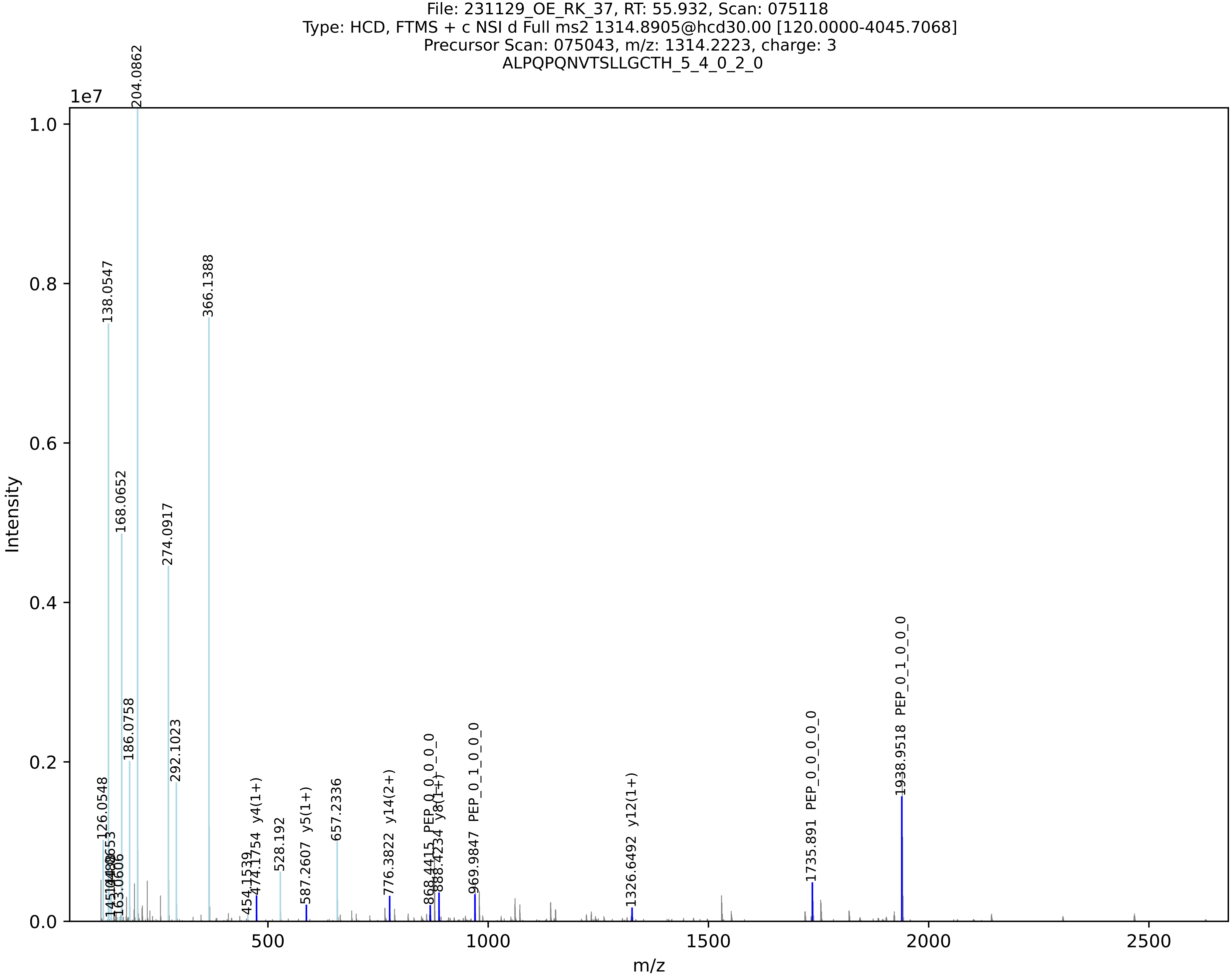
File: 231129_OE_RK_37, RT: 55.932, Scan: 075118
Type: HCD, FTMS + c NSI d Full ms2 1314.8905@hcd30.00 [120.0000-4045.7068]
Precursor Scan: 075043, m/z: 1314.2223, charge: 3
ALPQPQNVTSLLGCTH_5_4_0_2_0
1e7
Intensity
m/z
204.0862
138.0547
366.1388
168.0652
274.0917
186.0758
292.1023
126.0548
474.1754 y4(1+)
528.192
587.2607 y5(1+)
657.2336
776.3822 y14(2+)
868.4415 PEP_0_0_0_0_0
888.4234 y8(1+)
969.9847 PEP_0_1_0_0_0
1326.6492 y12(1+)
1735.891 PEP_0_0_0_0_0
1938.9518 PEP_0_1_0_0_0
454.1539

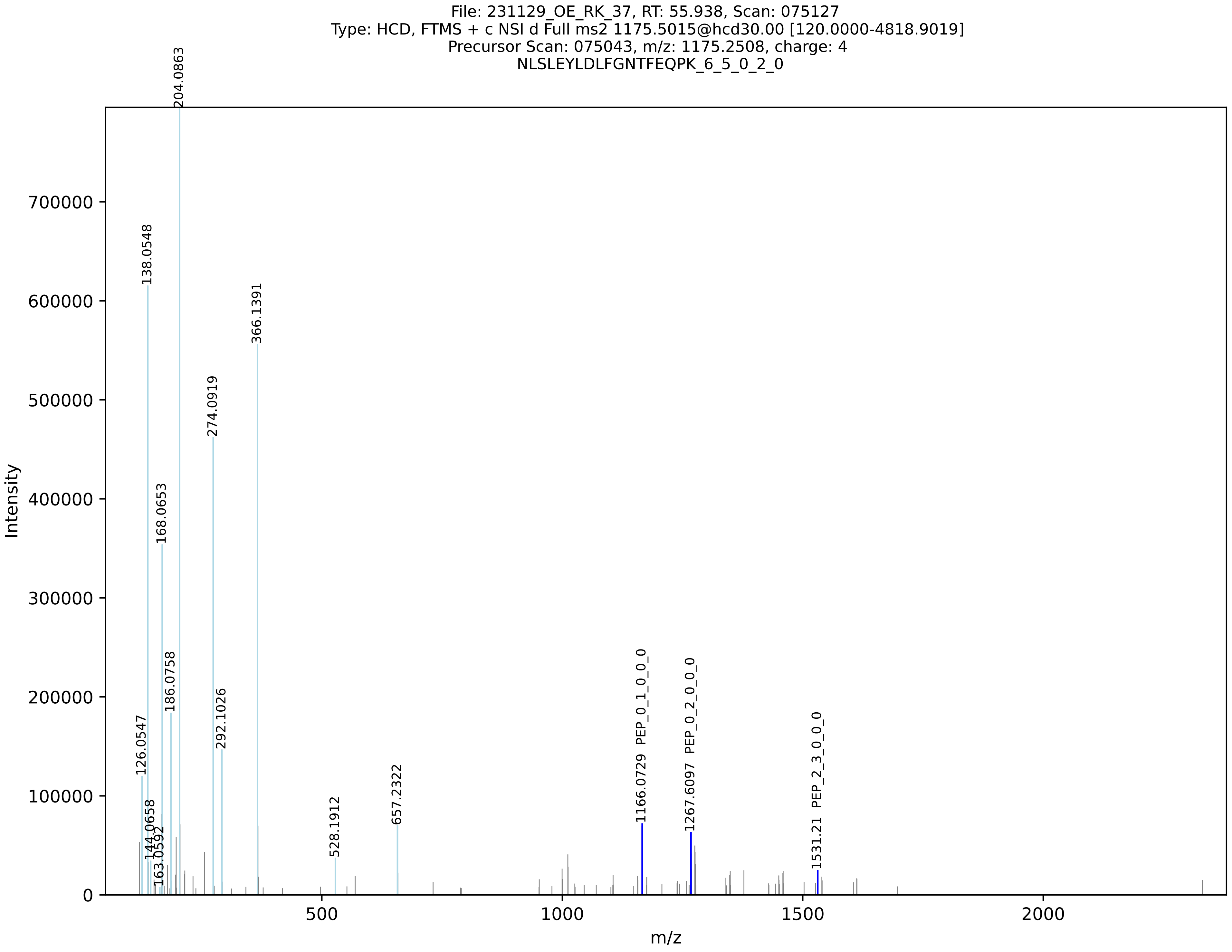

File: 231129_OE_RK_37, RT: 55.938, Scan: 075127
Type: HCD, FTMS + c NSI d Full ms2 1175.5015@hcd30.00 [120.0000-4818.9019]
Precursor Scan: 075043, m/z: 1175.2508, charge: 4
NLSLEYLDLFGNTFEQPK_6_5_0_2_0
Intensity
m/z
204.0863
138.0548
366.1391
274.0919
168.0653
186.0758
292.1026
126.0547
144.0658
163.0592
528.1912
657.2322
1166.0729 PEP_0_1_0_0_0
1267.6097 PEP_0_2_0_0_0
1531.21 PEP_2_3_0_0_0
0
100000
200000
300000
400000
500000
600000
700000
500
1000
1500
2000

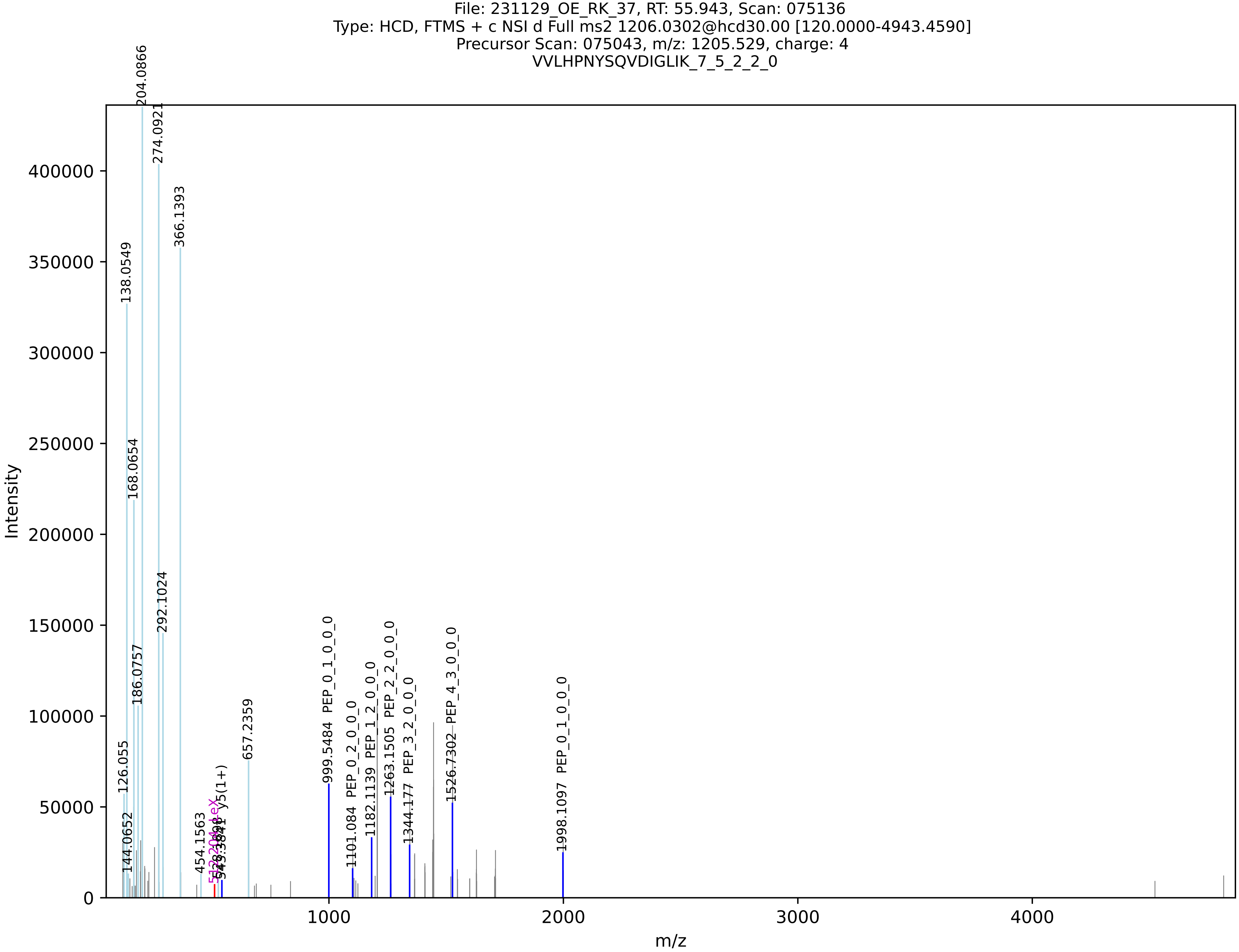
File: 231129_OE_RK_37, RT: 55.943, Scan: 075136
Type: HCD, FTMS + c NSI d Full ms2 1206.0302@hcd30.00 [120.0000-4943.4590]
Precursor Scan: 075043, m/z: 1205.529, charge: 4
VVLHPNYSQVDIGLIK_7_5_2_2_0
Intensity
m/z
204.0866
274.0921
366.1393
138.0549
168.0654
292.1024
186.0757
126.055
144.0652
454.1563
y5(1+)
657.2359
999.5484 PEP_0_1_0_0_0_
1101.084 PEP_0_2_0_0_0_
1182.1139 PEP_1_2_0_0_0_
1263.1505 PEP_2_2_0_0_0_
1344.177 PEP_3_2_0_0_0_
1526.7302 PEP_4_3_0_0_0_
1998.1097 PEP_0_1_0_0_0_
0
50000
100000
150000
200000
250000
300000
350000
400000
1000
2000
3000
4000

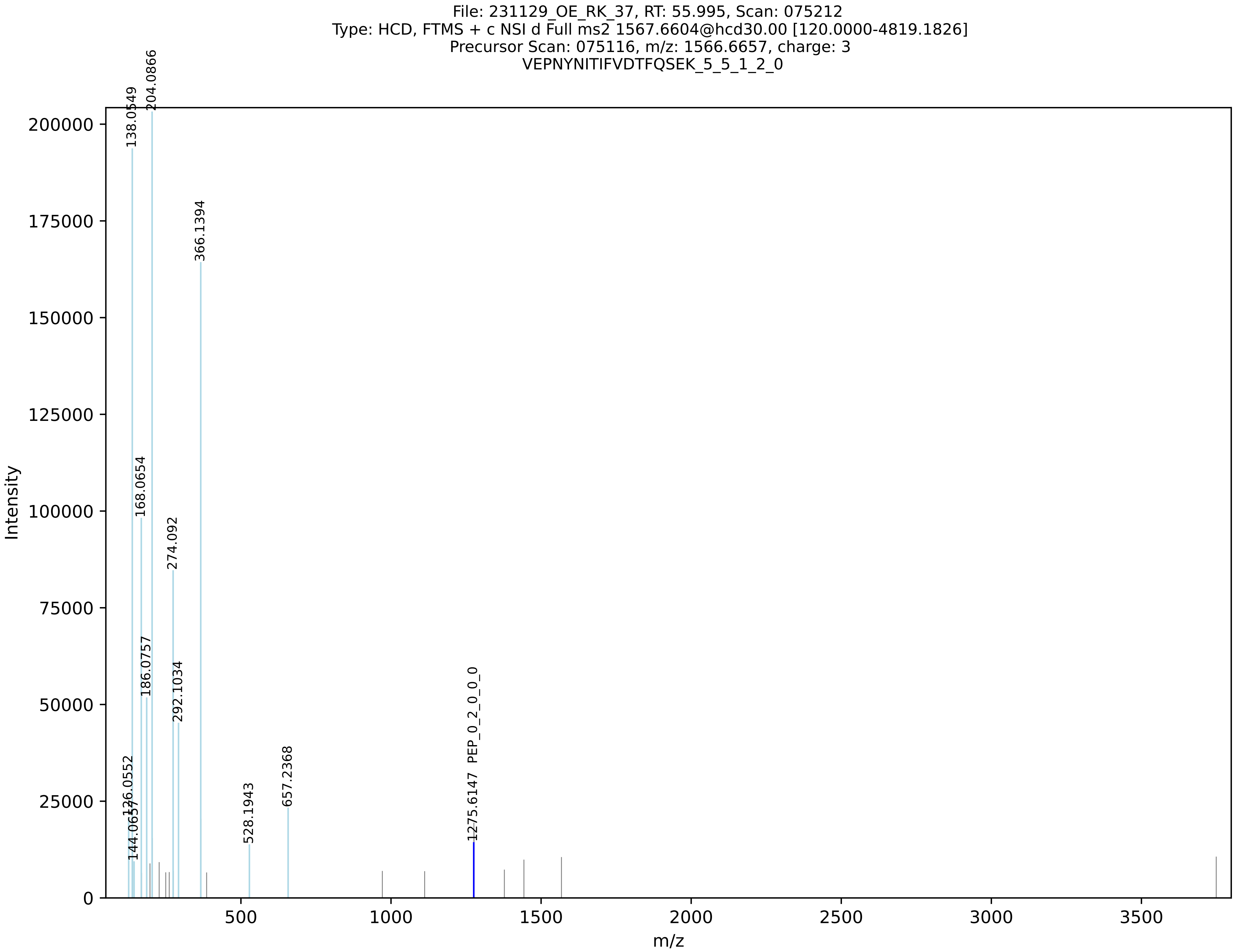
File: 231129_OE_RK_37, RT: 55.995, Scan: 075212
Type: HCD, FTMS + c NSI d Full ms2 1567.6604@hcd30.00 [120.0000-4819.1826]
Precursor Scan: 075116, m/z: 1566.6657, charge: 3
VEPNYNITIFVDTFQSEK_5_5_1_2_0
Intensity
m/z
138.0549
204.0866
366.1394
168.0654
274.092
186.0757
292.1034
126.0552
144.0657
528.1943
657.2368
1275.6147 PEP_0_2_0_0_0_
0
25000
50000
75000
100000
125000
150000
175000
200000
500
1000
1500
2000
2500
3000
3500

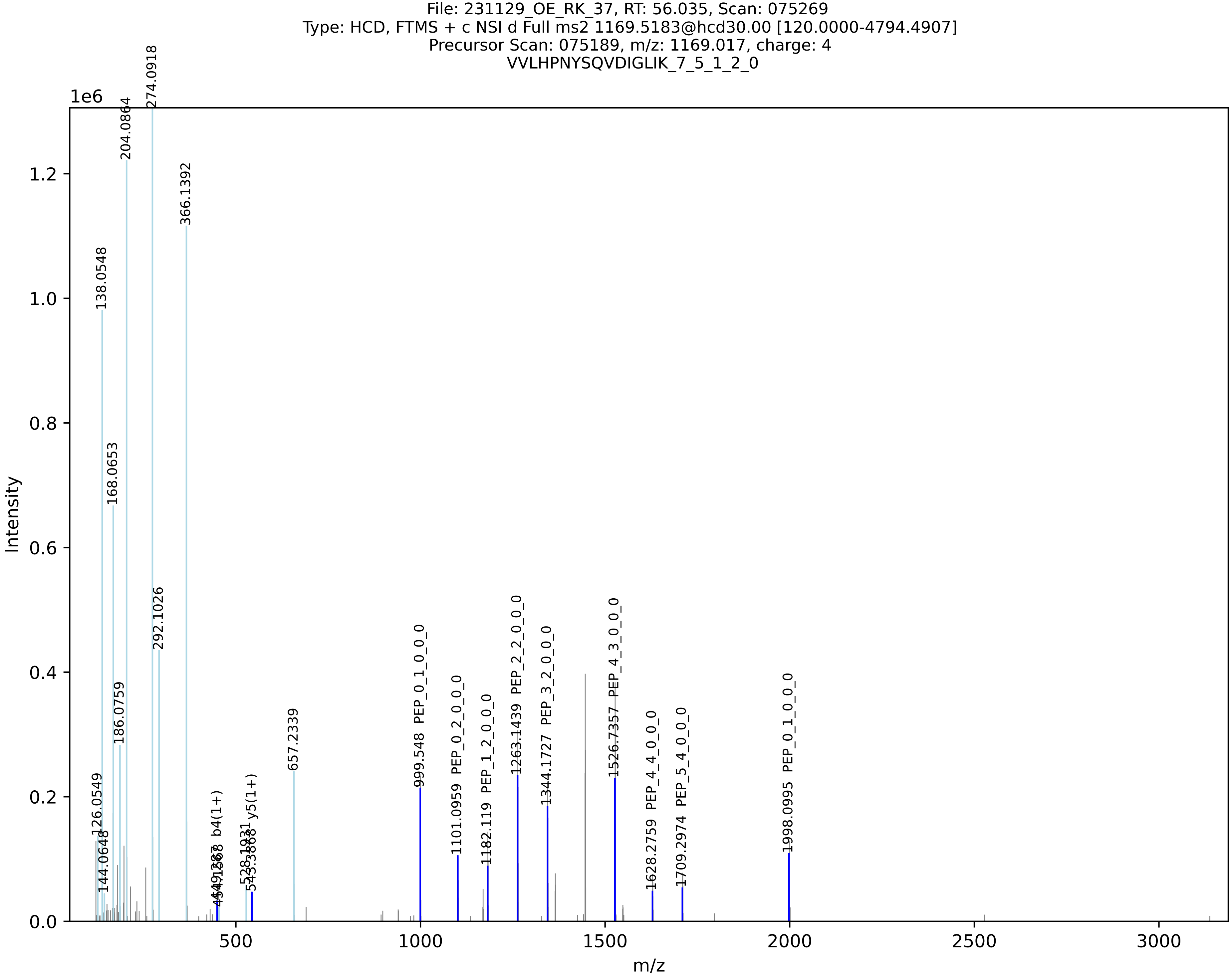
File: 231129_OE_RK_37, RT: 56.035, Scan: 075269
Type: HCD, FTMS + c NSI d Full ms2 1169.5183@hcd30.00 [120.0000-4794.4907]
Precursor Scan: 075189, m/z: 1169.017, charge: 4
VVLHPNYSQVDIGLIK_7_5_1_2_0
1e6
Intensity
m/z
0.0
0.2
0.4
0.6
0.8
1.0
1.2
500
1000
1500
2000
2500
3000
274.0918
204.0864
366.1392
138.0548
168.0653
292.1026
186.0759
126.0549
144.0648
b4(1+)
y5(1+)
657.2339
999.548 PEP_0_1_0_0_0
1101.0959 PEP_0_2_0_0_0
1182.119 PEP_1_2_0_0_0
1263.1439 PEP_2_2_0_0_0
1344.1727 PEP_3_2_0_0_0
1526.7357 PEP_4_3_0_0_0
1628.2759 PEP_4_4_0_0_0
1709.2974 PEP_5_4_0_0_0
1998.0995 PEP_0_1_0_0_0

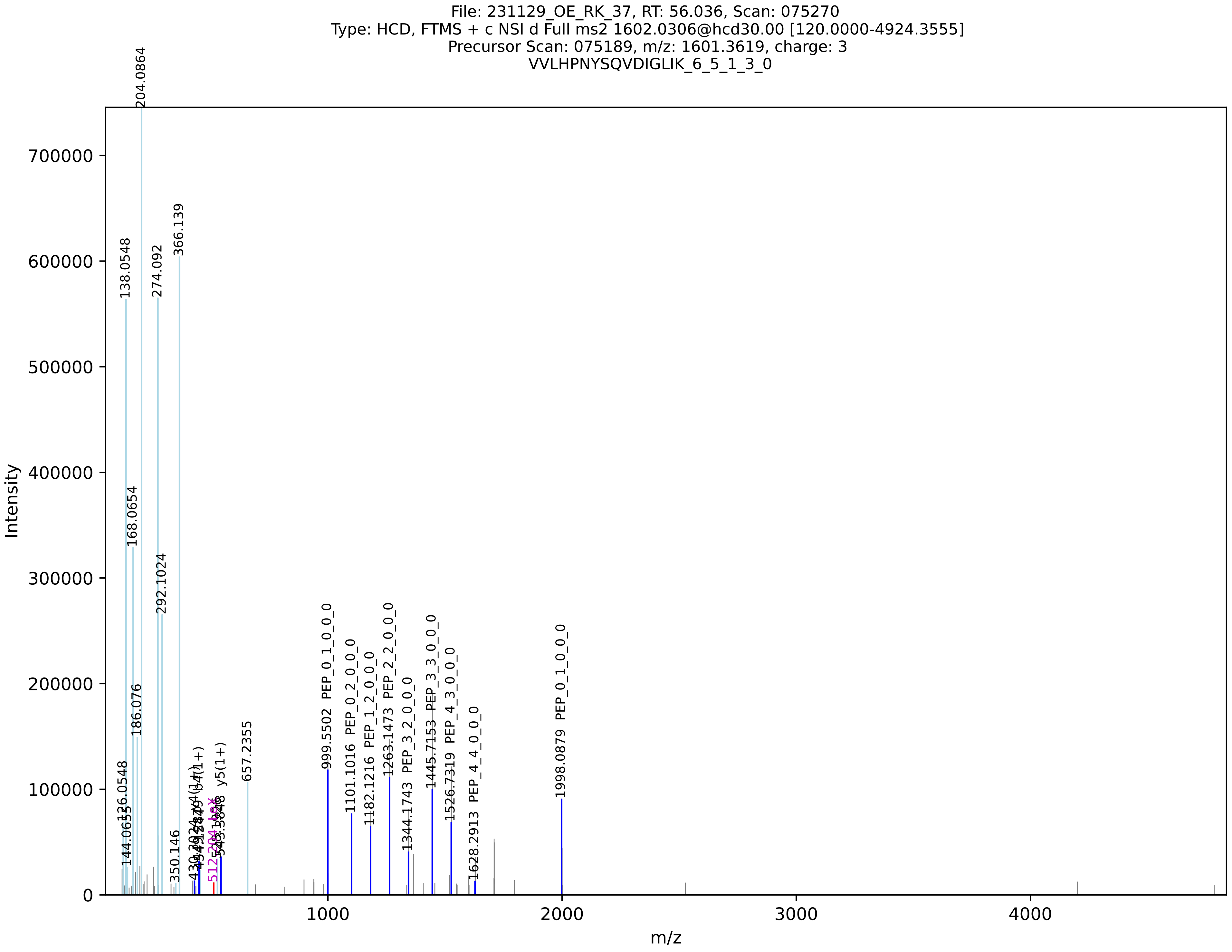

File: 231129_OE_RK_37, RT: 56.036, Scan: 075270
Type: HCD, FTMS + c NSI d Full ms2 1602.0306@hcd30.00 [120.0000-4924.3555]
Precursor Scan: 075189, m/z: 1601.3619, charge: 3
VVLHPNYSQVDIGLIK_6_5_1_3_0
Intensity
m/z
204.0864
366.139
138.0548
274.092
168.0654
292.1024
186.076
126.0548
144.0655
350.146
657.2355
999.5502 PEP_0_1_0_0_0
1101.1016 PEP_0_2_0_0_0
1182.1216 PEP_1_2_0_0_0
1263.1473 PEP_2_2_0_0_0
1344.1743 PEP_3_2_0_0_0
1445.7153 PEP_3_3_0_0_0
1526.7319 PEP_4_3_0_0_0
1628.2913 PEP_4_4_0_0_0
1998.0879 PEP_0_1_0_0_0
y5(1+)

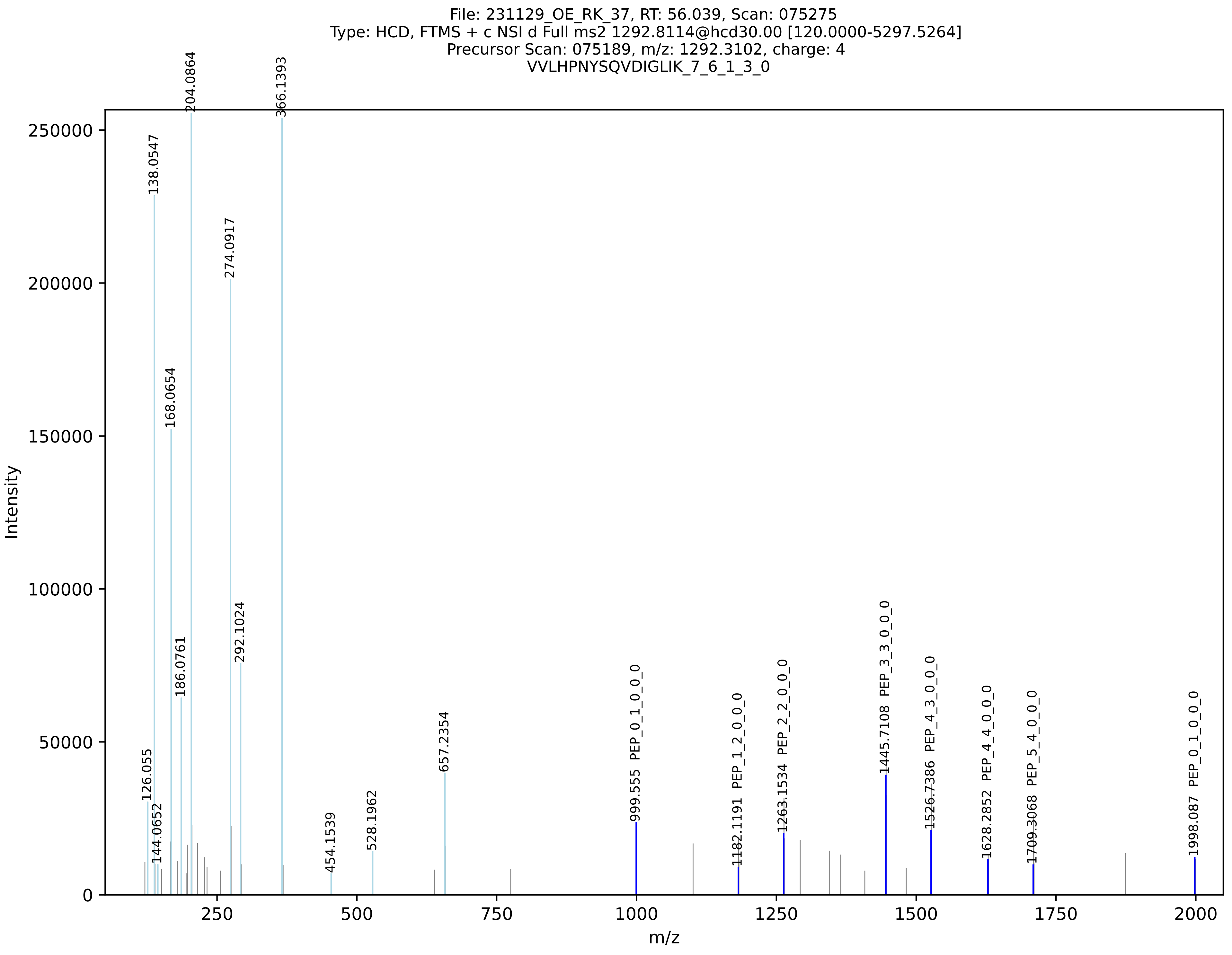
File: 231129_OE_RK_37, RT: 56.039, Scan: 075275
Type: HCD, FTMS + c NSI d Full ms2 1292.8114@hcd30.00 [120.0000-5297.5264]
Precursor Scan: 075189, m/z: 1292.3102, charge: 4
VVLHPNYSQVDIGLIK_7_6_1_3_0
Intensity
m/z
204.0864
366.1393
138.0547
274.0917
168.0654
186.0761
292.1024
126.055
144.0652
454.1539
528.1962
657.2354
999.555 PEP_0_1_0_0_0
1182.1191 PEP_1_2_0_0_0
1263.1534 PEP_2_2_0_0_0
1445.7108 PEP_3_3_0_0_0
1526.7386 PEP_4_3_0_0_0
1628.2852 PEP_4_4_0_0_0
1709.3068 PEP_5_4_0_0_0
1998.087 PEP_0_1_0_0_0

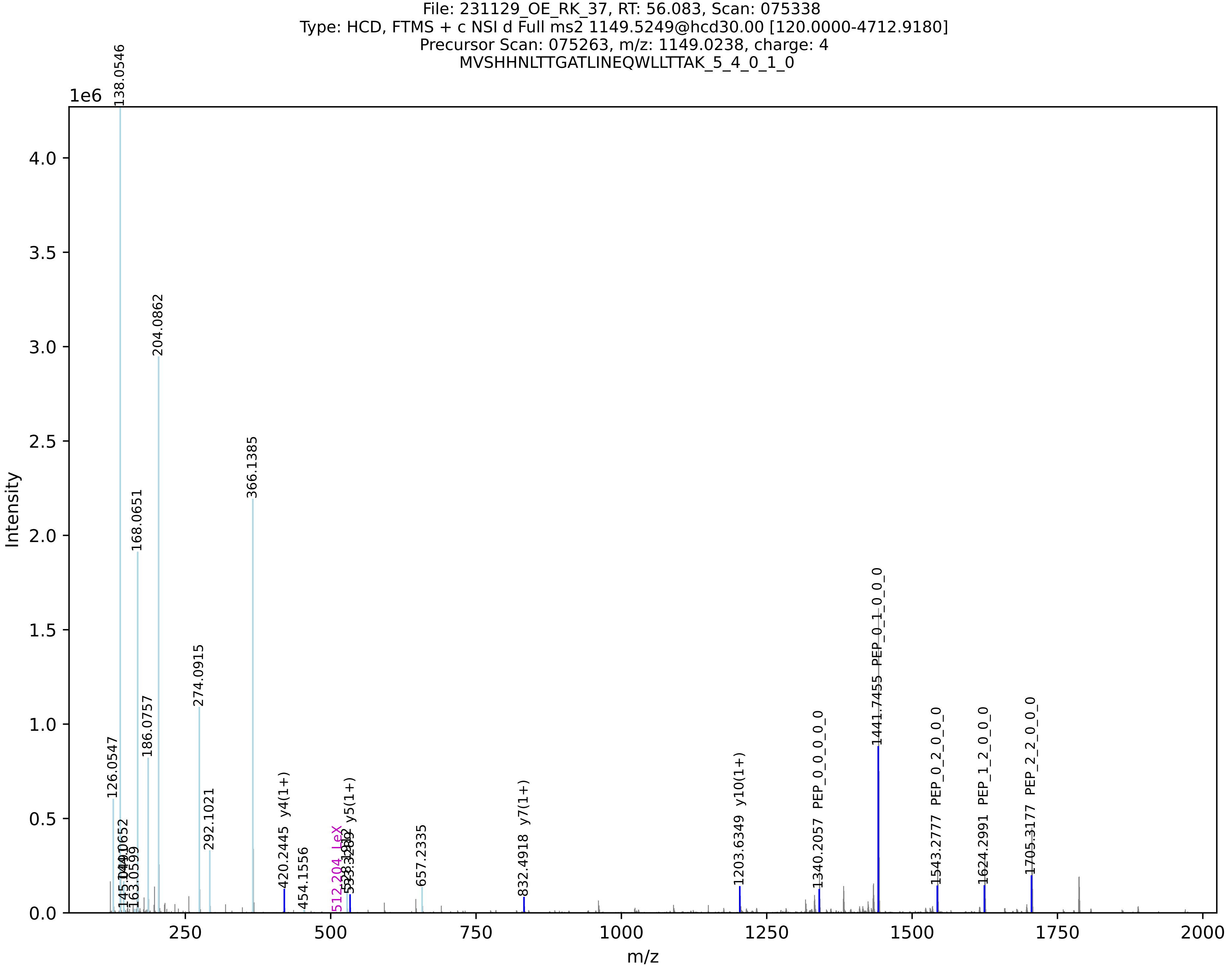
File: 231129_OE_RK_37, RT: 56.083, Scan: 075338
Type: HCD, FTMS + c NSI d Full ms2 1149.5249@hcd30.00 [120.0000-4712.9180]
Precursor Scan: 075263, m/z: 1149.0238, charge: 4
MVSHHNLTTGATLINEQWLLTTAK_5_4_0_1_0
1e6
Intensity
m/z
138.0546
204.0862
366.1385
168.0651
274.0915
186.0757
126.0547
292.1021
420.2445 y4(1+)
454.1556
512.204_LeX
y5(1+)
657.2335
832.4918 y7(1+)
1203.6349 y10(1+)
1340.2057 PEP_0_0_0_0_0
1441.7455 PEP_0_1_0_0_0
1543.2777 PEP_0_2_0_0_0
1624.2991 PEP_1_2_0_0_0
1705.3177 PEP_2_2_0_0_0

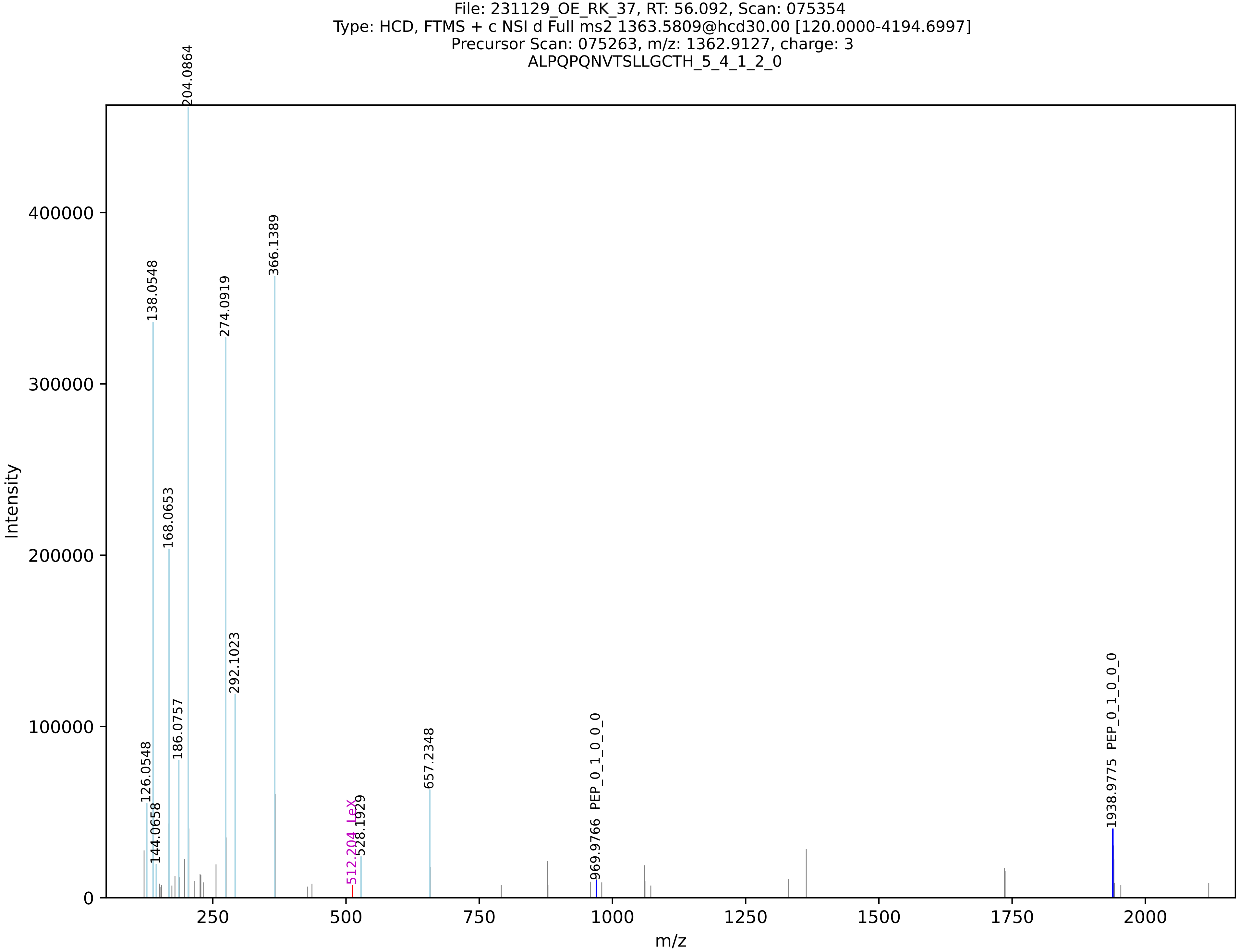
File: 231129_OE_RK_37, RT: 56.092, Scan: 075354
Type: HCD, FTMS + c NSI d Full ms2 1363.5809@hcd30.00 [120.0000-4194.6997]
Precursor Scan: 075263, m/z: 1362.9127, charge: 3
ALPQPQNVTSLLGCTH_5_4_1_2_0
Intensity
m/z
204.0864
366.1389
138.0548
274.0919
168.0653
292.1023
186.0757
126.0548
144.0658
512.204_LeX
528.1929
657.2348
969.9766 PEP_0_1_0_0_0
1938.9775 PEP_0_1_0_0_0
0
100000
200000
300000
400000
250
500
750
1000
1250
1500
1750
2000

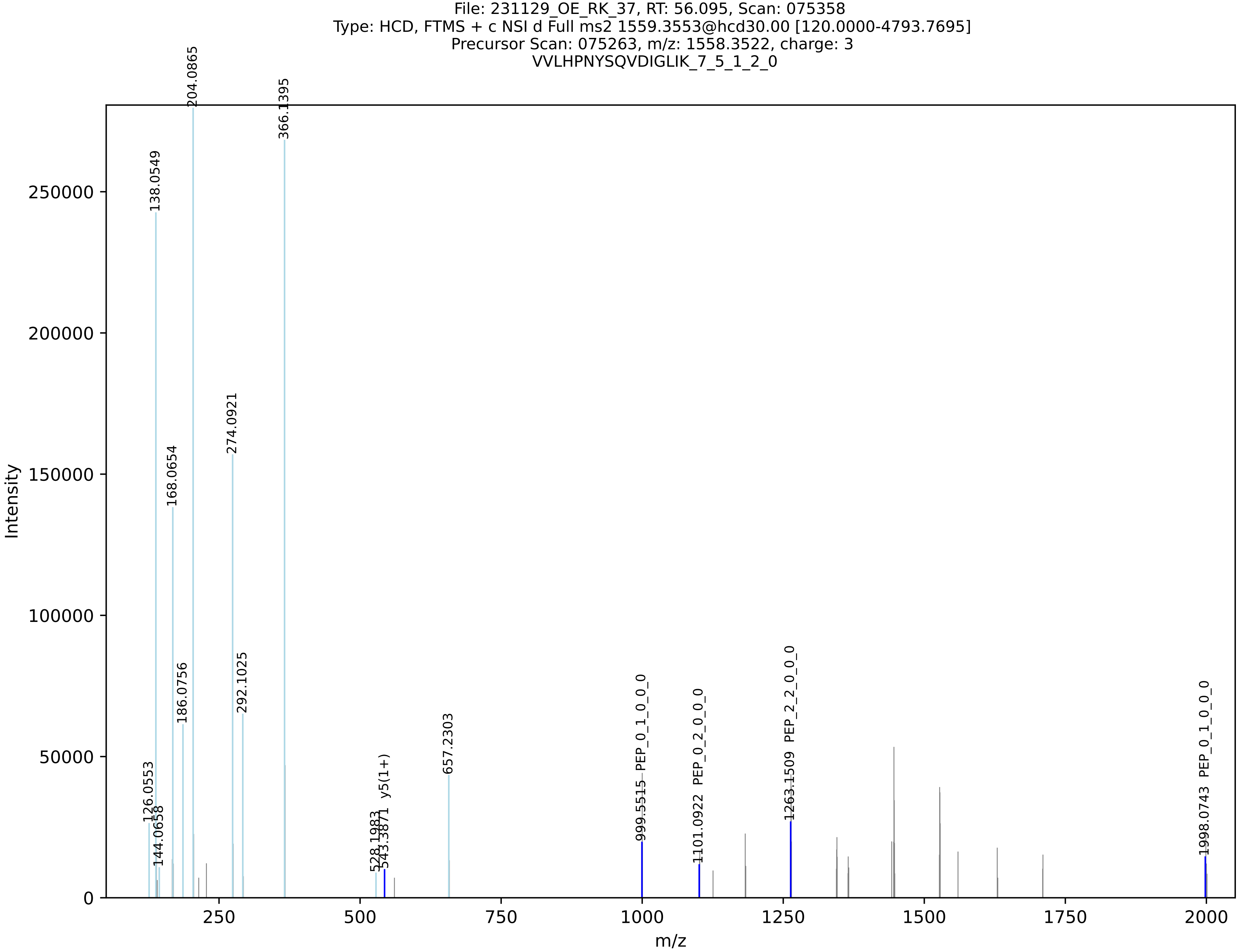

File: 231129_OE_RK_37, RT: 56.095, Scan: 075358
Type: HCD, FTMS + c NSI d Full ms2 1559.3553@hcd30.00 [120.0000-4793.7695]
Precursor Scan: 075263, m/z: 1558.3522, charge: 3
VVLHPNYSQVDIGLIK_7_5_1_2_0
Intensity
m/z
204.0865
366.1395
138.0549
274.0921
168.0654
292.1025
186.0756
126.0553
144.0658
528.1983
543.3871 y5(1+)
657.2303
999.5515 PEP_0_1_0_0_0
1101.0922 PEP_0_2_0_0_0
1263.1509 PEP_2_2_0_0_0
1998.0743 PEP_0_1_0_0_0

File: 231129_OE_RK_37, RT: 56.133, Scan: 075412
Type: HCD, FTMS + c NSI d Full ms2 1314.8922@hcd30.00 [120.0000-4045.7124]
Precursor Scan: 075337, m/z: 1314.224, charge: 3
ALPQPQNVTSLLGCTH_5_4_0_2_0

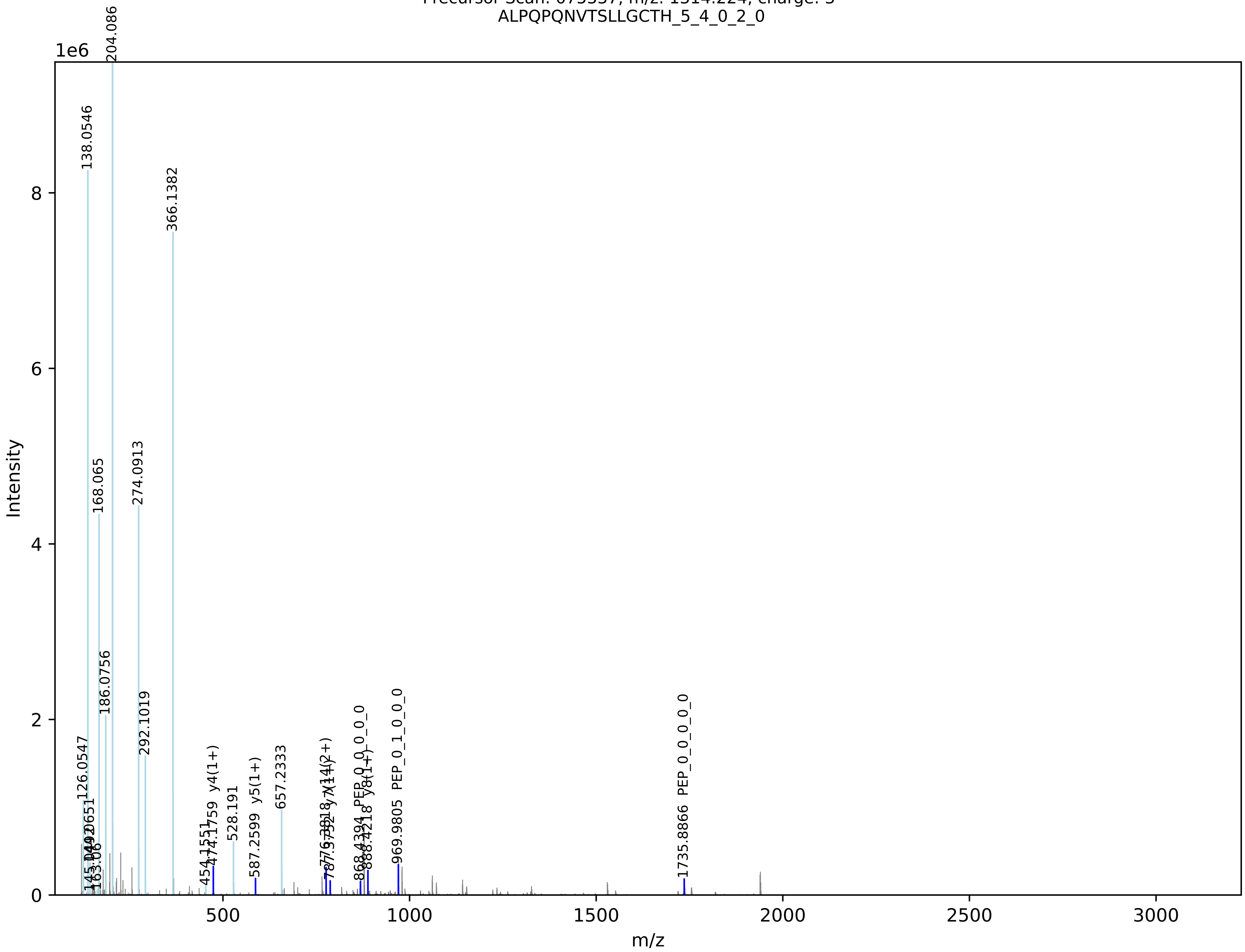

File: 231129_OE_RK_37, RT: 56.134, Scan: 075413
Type: HCD, FTMS + c NSI d Full ms2 1165.5120@hcd30.00 [120.0000-4778.1450]
Precursor Scan: 075337, m/z: 1164.7602, charge: 4
VVLHPNYSQVDIGLIK_6_5_0_3_0

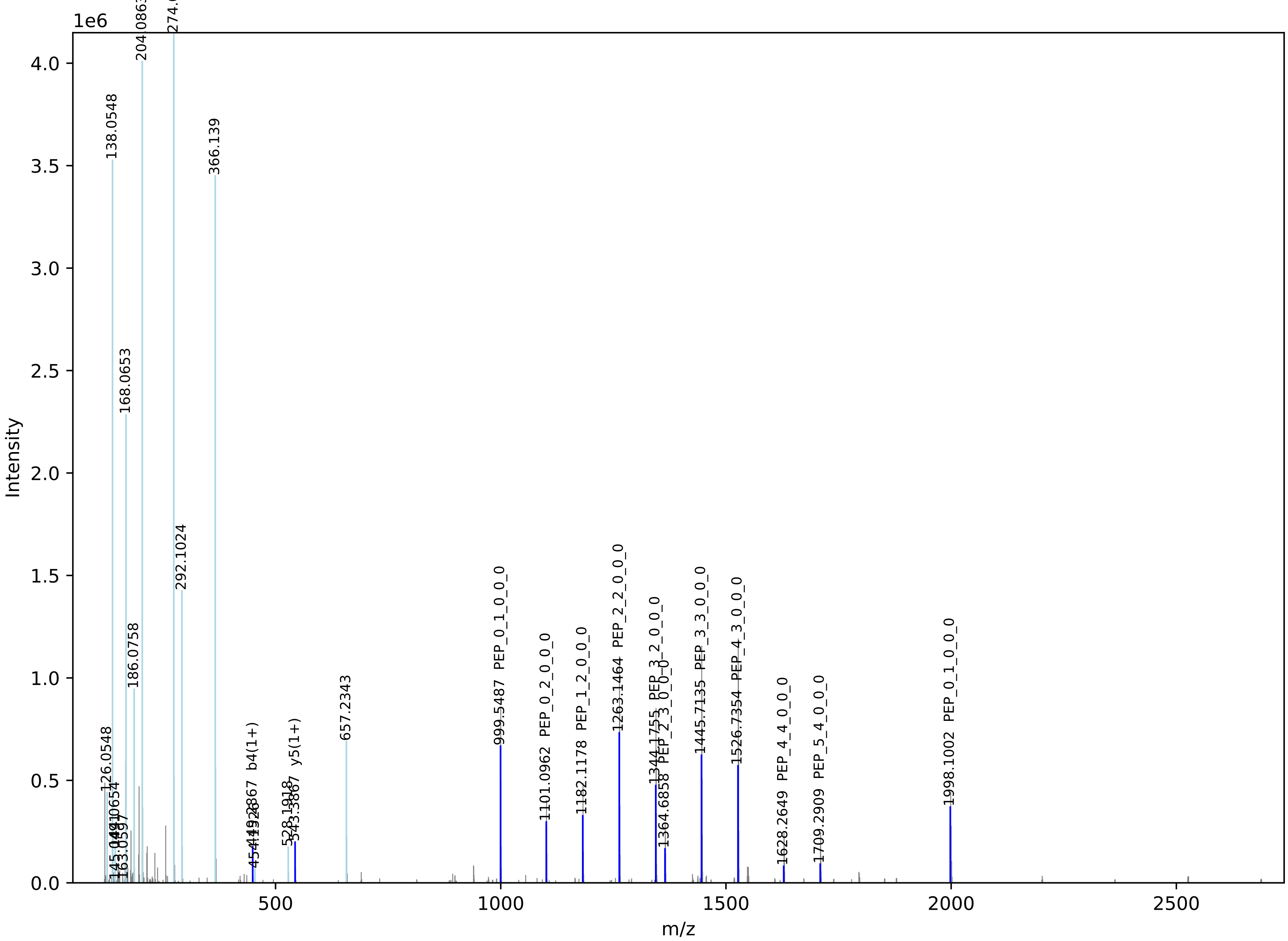

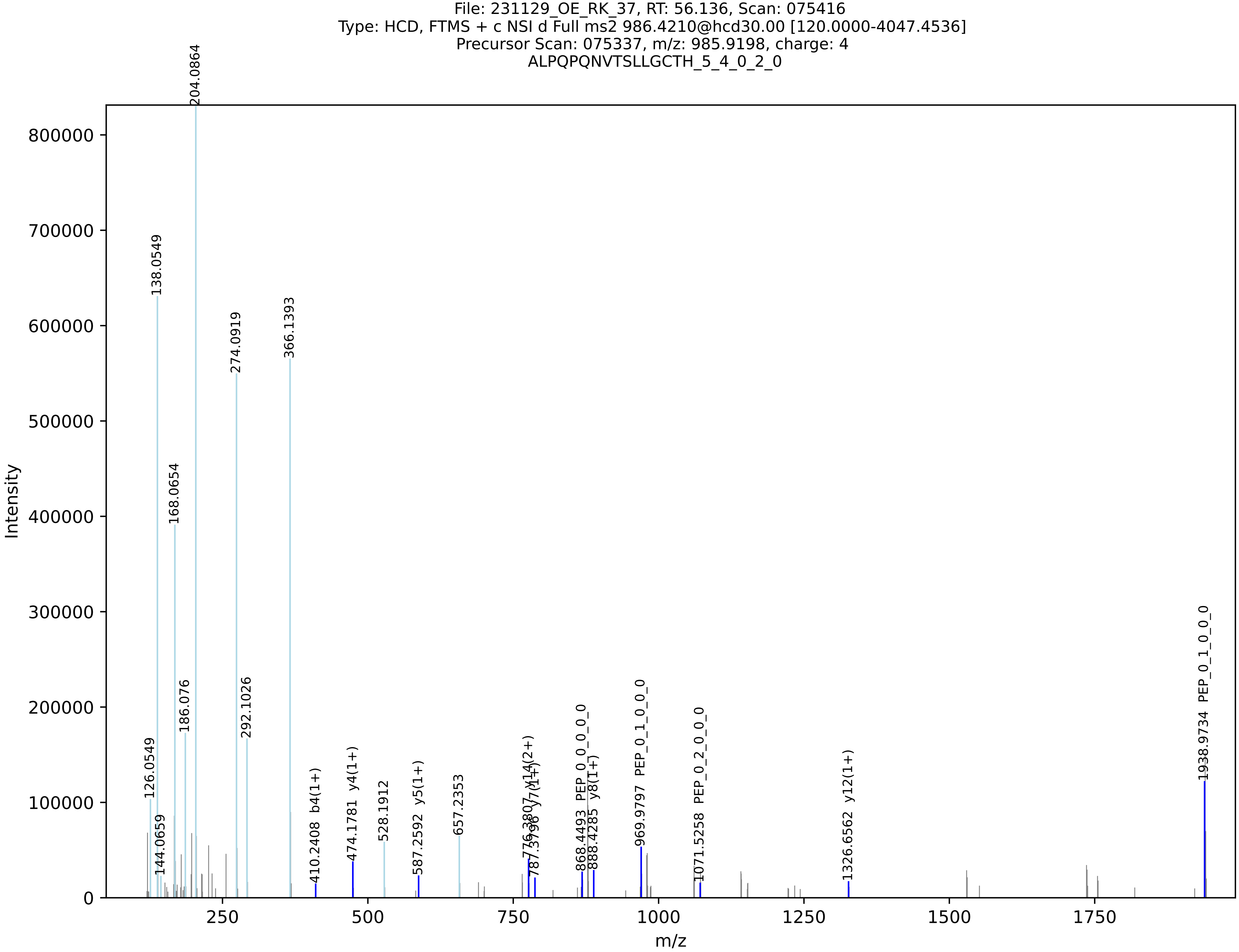
File: 231129_OE_RK_37, RT: 56.136, Scan: 075416
Type: HCD, FTMS + c NSI d Full ms2 986.4210@hcd30.00 [120.0000-4047.4536]
Precursor Scan: 075337, m/z: 985.9198, charge: 4
ALPQPQNVTSLLGCTH_5_4_0_2_0
Intensity
m/z
204.0864
138.0549
274.0919
366.1393
168.0654
186.076
292.1026
126.0549
144.0659
410.2408 b4(1+)
474.1781 y4(1+)
528.1912
587.2592 y5(1+)
657.2353
776.3807 y14(2+)
787.3796 y7(1+)
868.4493 PEP_0_0_0_0_0
888.4285 y8(1+)
969.9797 PEP_0_1_0_0_0
1071.5258 PEP_0_2_0_0_0
1326.6562 y12(1+)
1938.9734 PEP_0_1_0_0_0

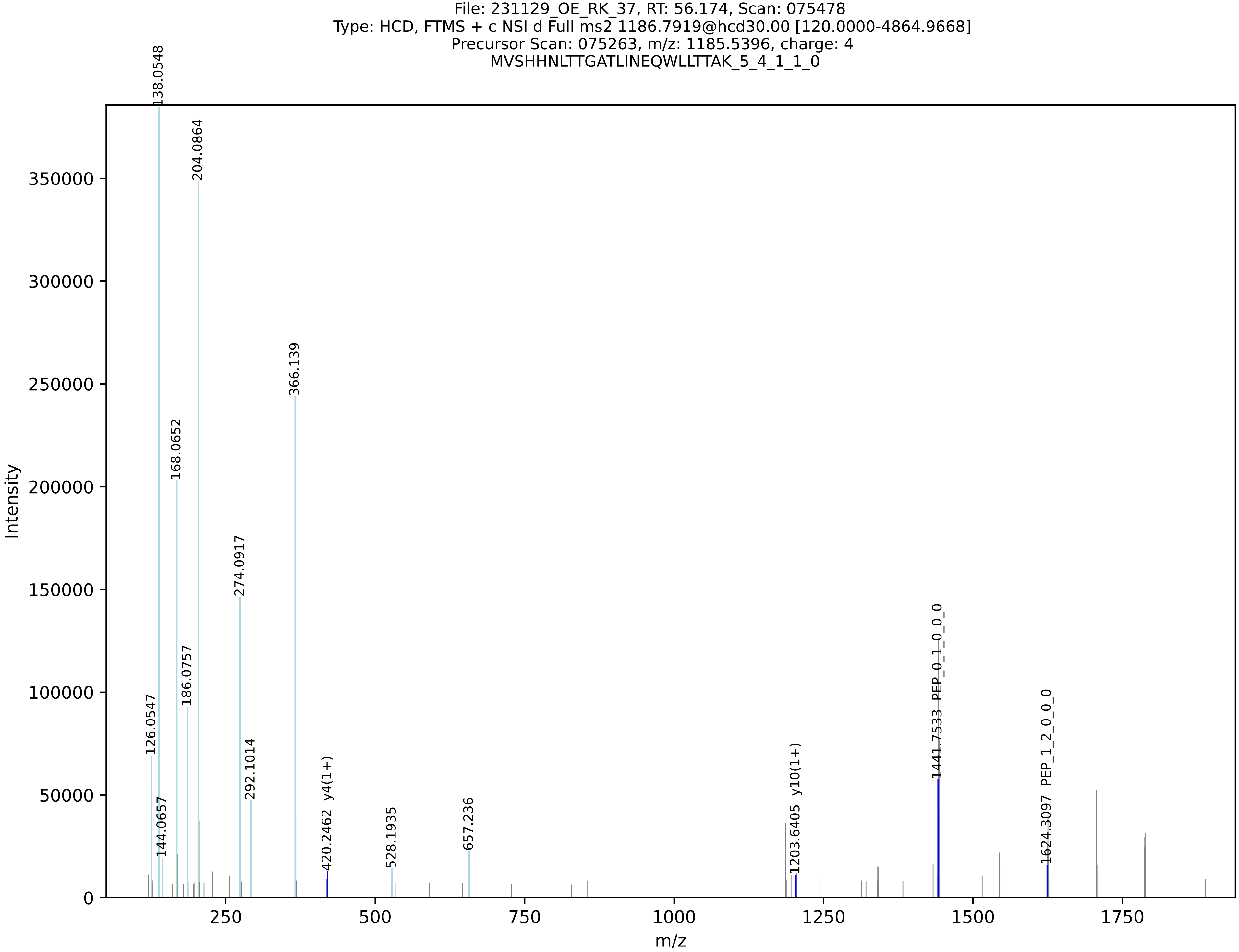
File: 231129_OE_RK_37, RT: 56.174, Scan: 075478
Type: HCD, FTMS + c NSI d Full ms2 1186.7919@hcd30.00 [120.0000-4864.9668]
Precursor Scan: 075263, m/z: 1185.5396, charge: 4
MVSHHNLTTGATLINEQWLLTTAK_5_4_1_1_0
138.0548
204.0864
366.139
168.0652
274.0917
186.0757
126.0547
292.1014
144.0657
420.2462 y4(1+)
528.1935
657.236
1203.6405 y10(1+)
1441.7533 PEP_0_1_0_0_0_
1624.3097 PEP_1_2_0_0_0_
Intensity
m/z
0
50000
100000
150000
200000
250000
300000
350000
250
500
750
1000
1250
1500
1750

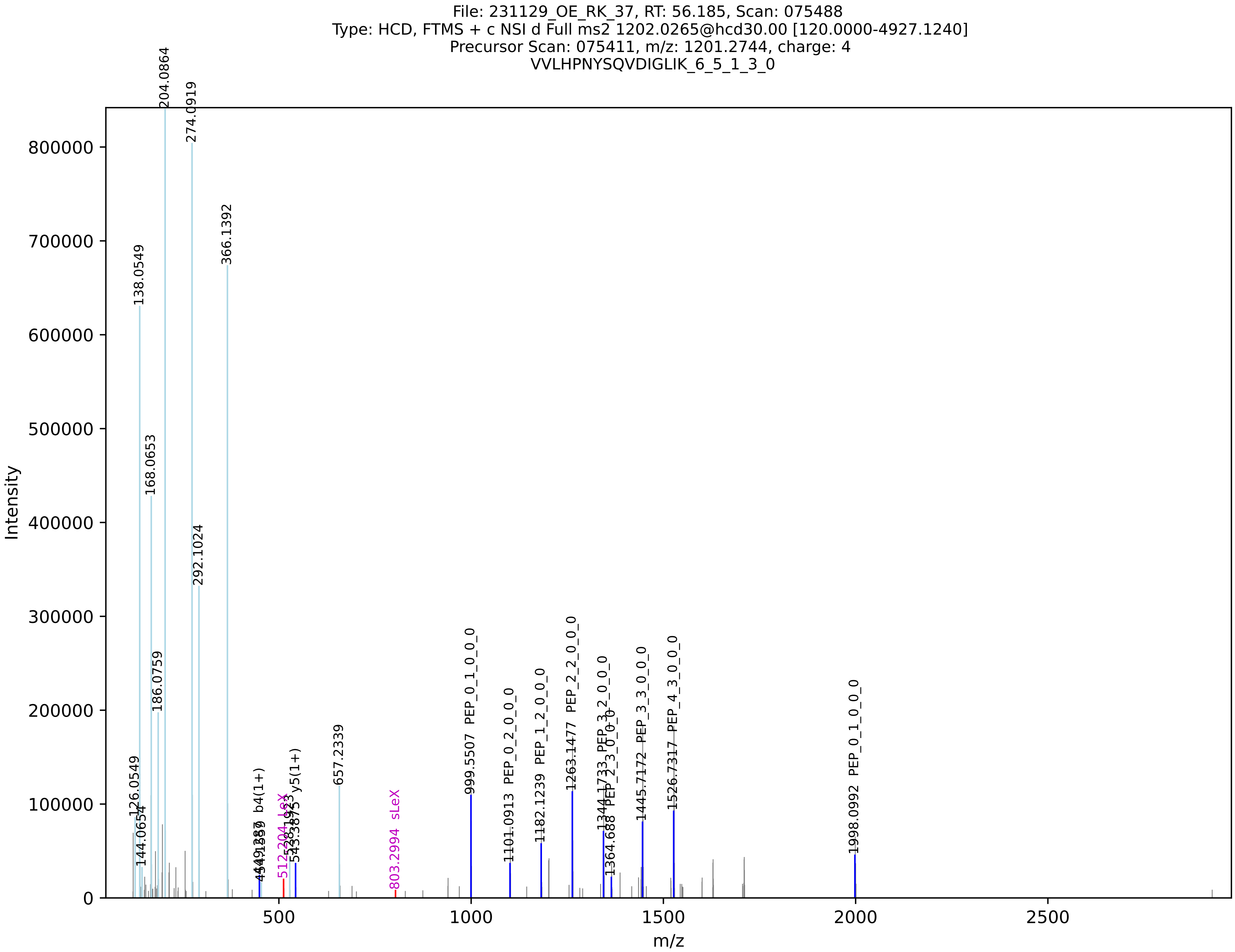
File: 231129_OE_RK_37, RT: 56.185, Scan: 075488
Type: HCD, FTMS + c NSI d Full ms2 1202.0265@hcd30.00 [120.0000-4927.1240]
Precursor Scan: 075411, m/z: 1201.2744, charge: 4
VVLHPNYSQVDIGLIK_6_5_1_3_0
Intensity
m/z
204.0864
274.0919
366.1392
138.0549
168.0653
292.1024
186.0759
126.0549
144.0654
b4(1+)
y5(1+)
543.3875
512.204 LeX
657.2339
803.2994 sLeX
999.5507 PEP_0_1_0_0_0
1101.0913 PEP_0_2_0_0_0
1182.1239 PEP_1_2_0_0_0
1263.1477 PEP_2_2_0_0_0
1344.1733 PEP_3_2_0_0_0
1364.688 PEP_2_3_0_0_0
1445.7172 PEP_3_3_0_0_0
1526.7317 PEP_4_3_0_0_0
1998.0992 PEP_0_1_0_0_0

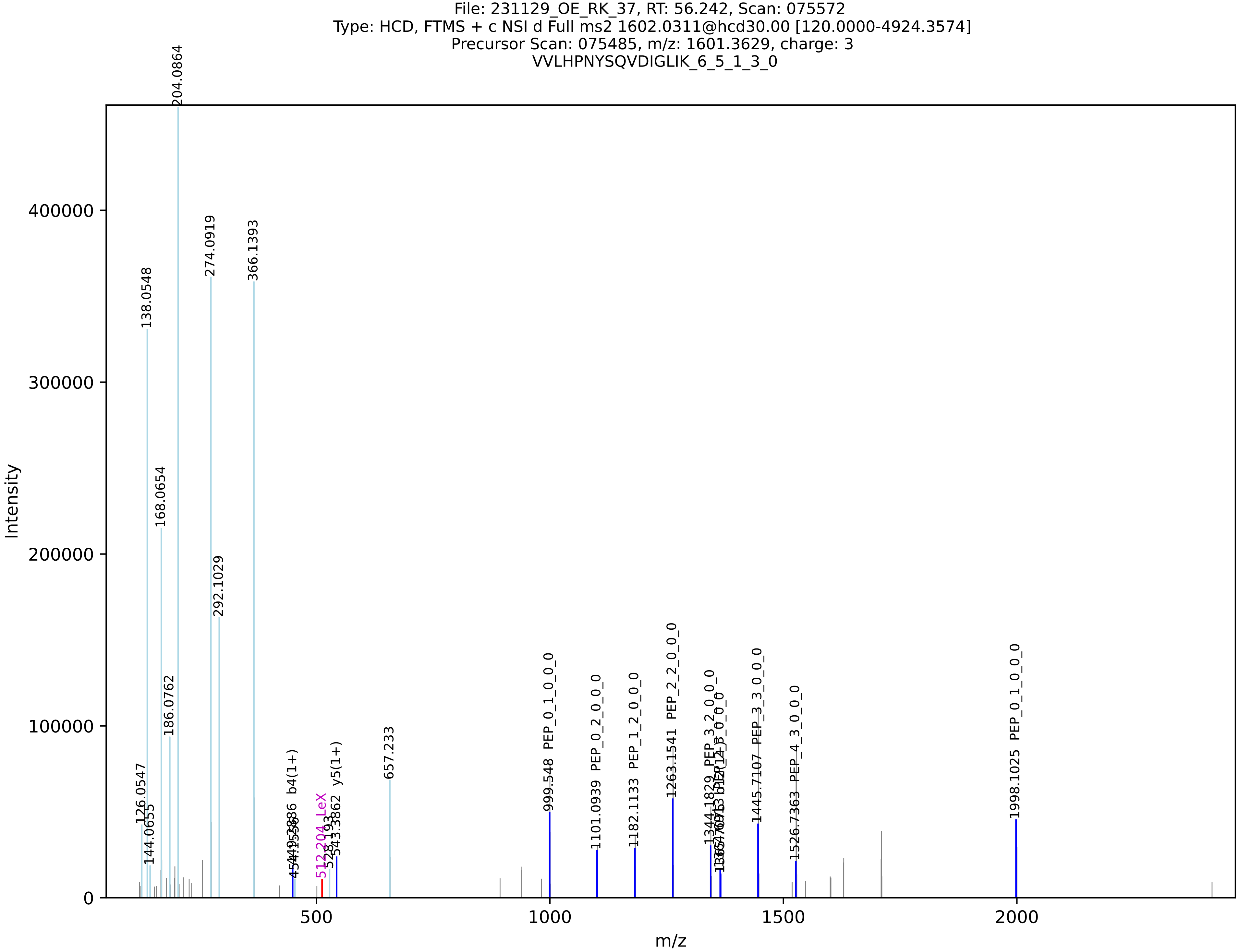
File: 231129_OE_RK_37, RT: 56.242, Scan: 075572
Type: HCD, FTMS + c NSI d Full ms2 1602.0311@hcd30.00 [120.0000-4924.3574]
Precursor Scan: 075485, m/z: 1601.3629, charge: 3
VVLHPNYSQVDIGLIK_6_5_1_3_0
Intensity
m/z
204.0864
274.0919
366.1393
138.0548
168.0654
292.1029
186.0762
126.0547
144.0655
b4(1+)
512.204_LeX
543.3862 y5(1+)
657.233
999.548 PEP_0_1_0_0_0
1101.0939 PEP_0_2_0_0_0
1182.1133 PEP_1_2_0_0_0
1263.1541 PEP_2_2_0_0_0
1445.7107 PEP_3_3_0_0_0
1526.7363 PEP_4_3_0_0_0
1998.1025 PEP_0_1_0_0_0

File: 231129_OE_RK_37, RT: 56.285, Scan: 075634
Type: HCD, FTMS + c NSI d Full ms2 1149.5247@hcd30.00 [120.0000-4712.9170]
Precursor Scan: 075559, m/z: 1149.0235, charge: 4
MVSHHNLTTGATLINEQWLLTTAK_5_4_0_1_0

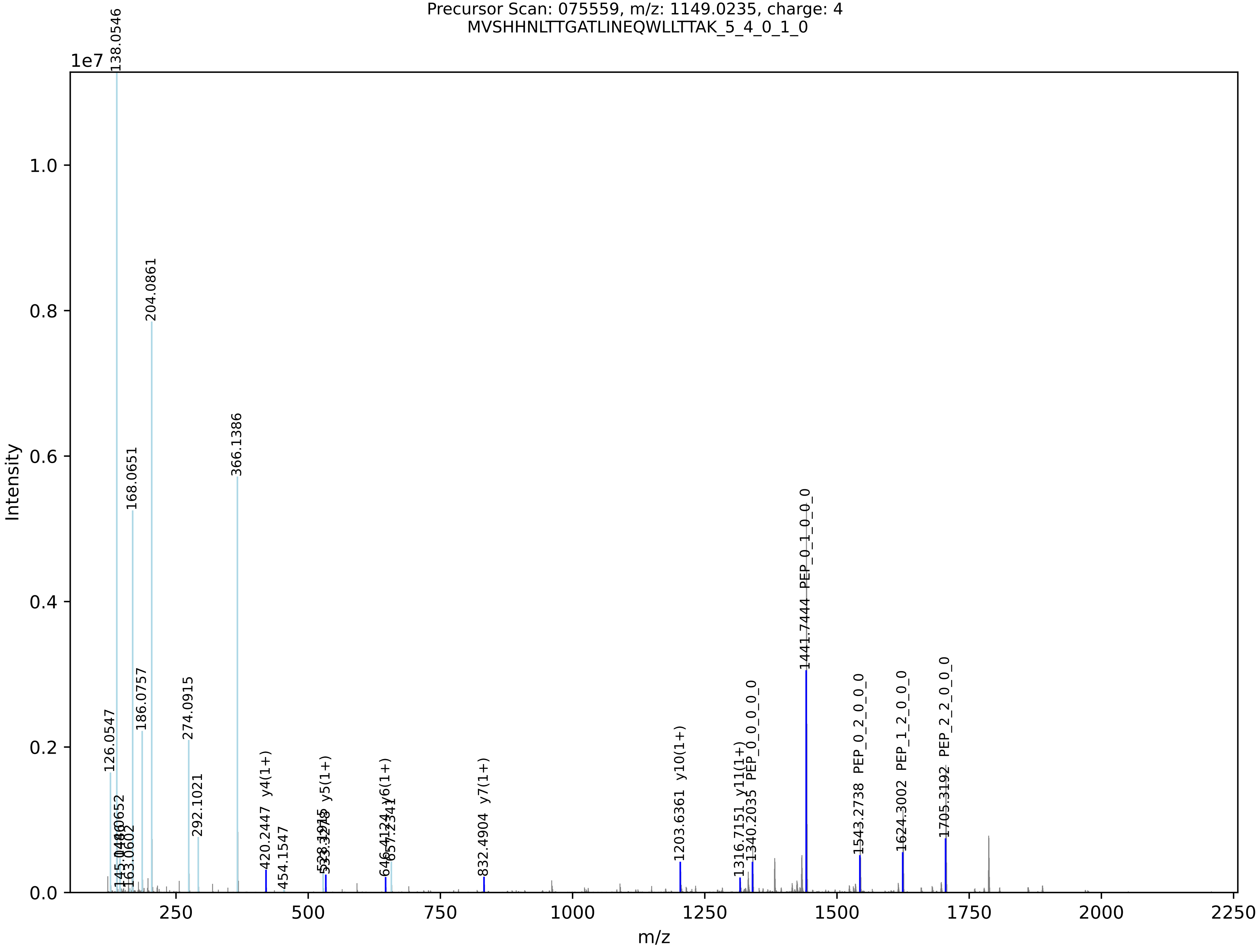

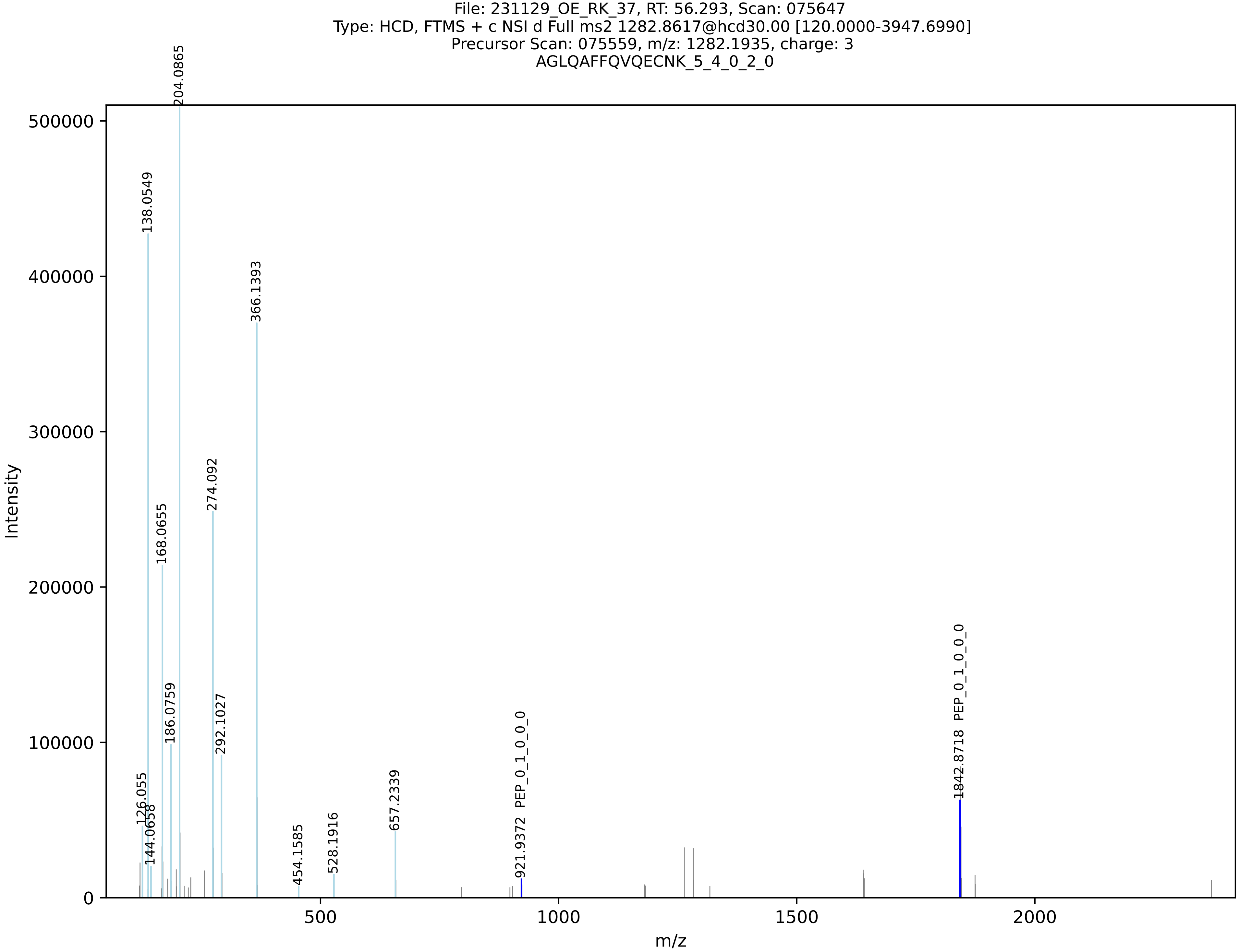

File: 231129_OE_RK_37, RT: 56.293, Scan: 075647
Type: HCD, FTMS + c NSI d Full ms2 1282.8617@hcd30.00 [120.0000-3947.6990]
Precursor Scan: 075559, m/z: 1282.1935, charge: 3
AGLQAFFQVQECNK_5_4_0_2_0
Intensity
m/z
204.0865
138.0549
366.1393
274.092
168.0655
186.0759
292.1027
126.055
144.0658
454.1585
528.1916
657.2339
921.9372 PEP_0_1_0_0_0
1842.8718 PEP_0_1_0_0_0

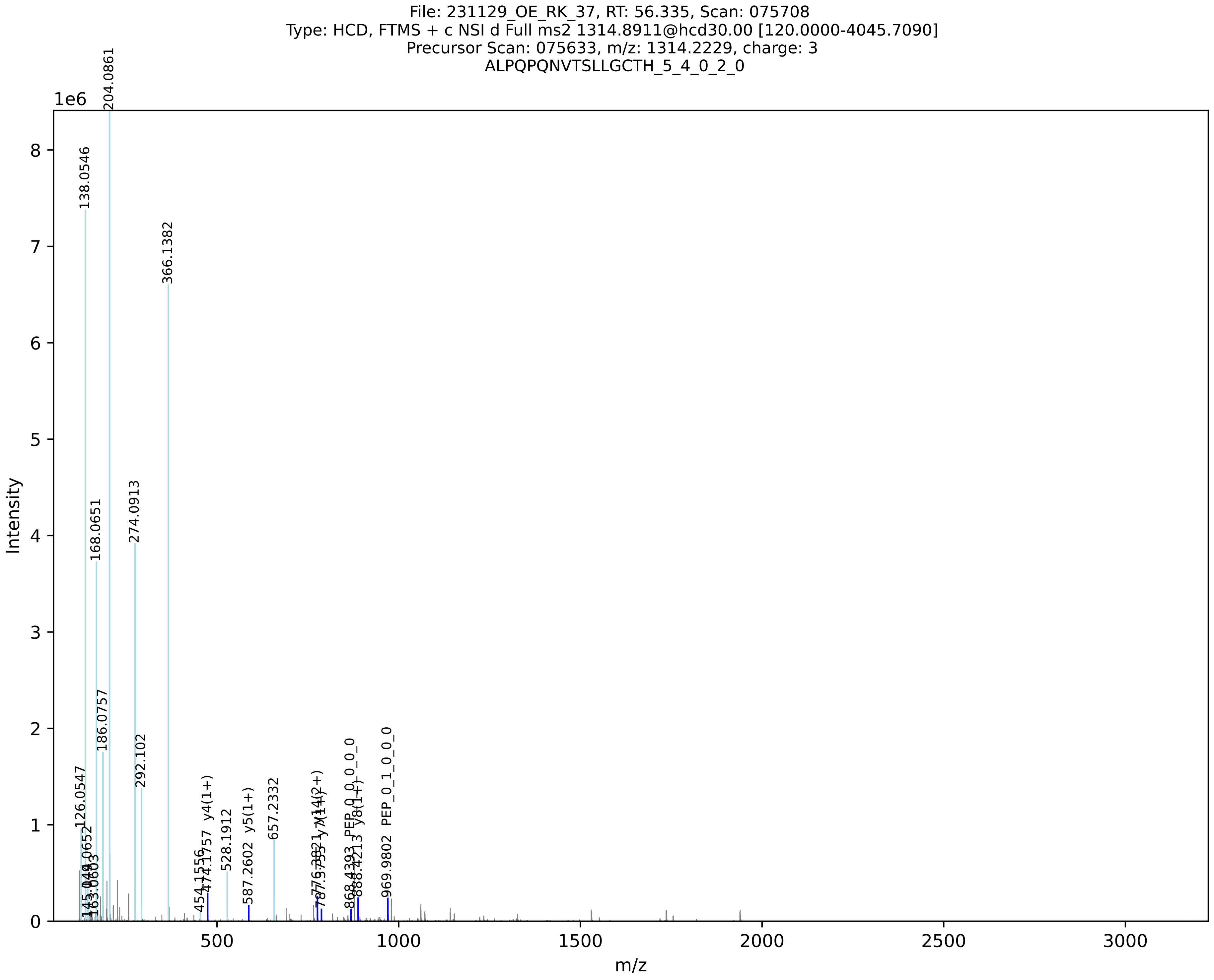

File: 231129_OE_RK_37, RT: 56.335, Scan: 075708
Type: HCD, FTMS + c NSI d Full ms2 1314.8911@hcd30.00 [120.0000-4045.7090]
Precursor Scan: 075633, m/z: 1314.2229, charge: 3
ALPQPQNVTSLLGCTH_5_4_0_2_0
1e6
Intensity
m/z
204.0861
138.0546
366.1382
274.0913
168.0651
186.0757
292.102
126.0547
474.1757 y4(1+)
528.1912
587.2602 y5(1+)
657.2332
969.9802 PEP_0_1_0_0_0_
868.4393 PEP_0_0_0_0_0_
888.4213 y8(1+)
454.1556

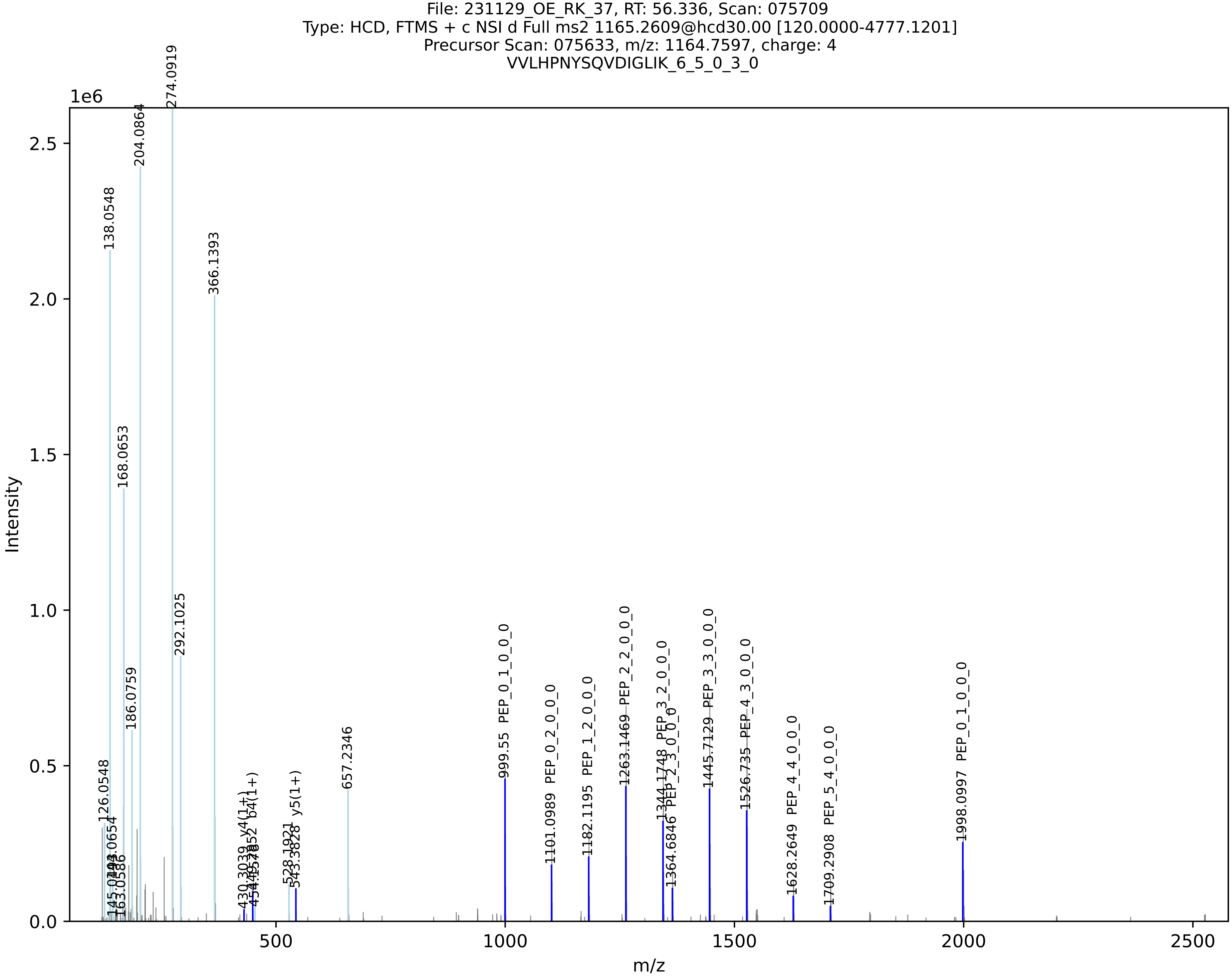
File: 231129_OE_RK_37, RT: 56.336, Scan: 075709
Type: HCD, FTMS + c NSI d Full ms2 1165.2609@hcd30.00 [120.0000-4777.1201]
Precursor Scan: 075633, m/z: 1164.7597, charge: 4
VVLHPNYSQVDIGLIK_6_5_0_3_0
1e6
Intensity
m/z
274.0919
204.0864
138.0548
366.1393
168.0653
292.1025
186.0759
126.0548
163.0586
430.3039 y4(1+)
b4(1+)
528.1921
543.3828 y5(1+)
657.2346
999.55 PEP_0_1_0_0_0
1101.0989 PEP_0_2_0_0_0
1182.1195 PEP_1_2_0_0_0
1263.1469 PEP_2_2_0_0_0
1344.1748 PEP_3_2_0_0_0
1364.6846 PEP_2_3_0_0_0
1445.7129 PEP_3_3_0_0_0
1526.735 PEP_4_3_0_0_0
1628.2649 PEP_4_4_0_0_0
1709.2908 PEP_5_4_0_0_0
1998.0997 PEP_0_1_0_0_0
0.0
0.5
1.0
1.5
2.0
2.5
500
1000
1500
2000
2500

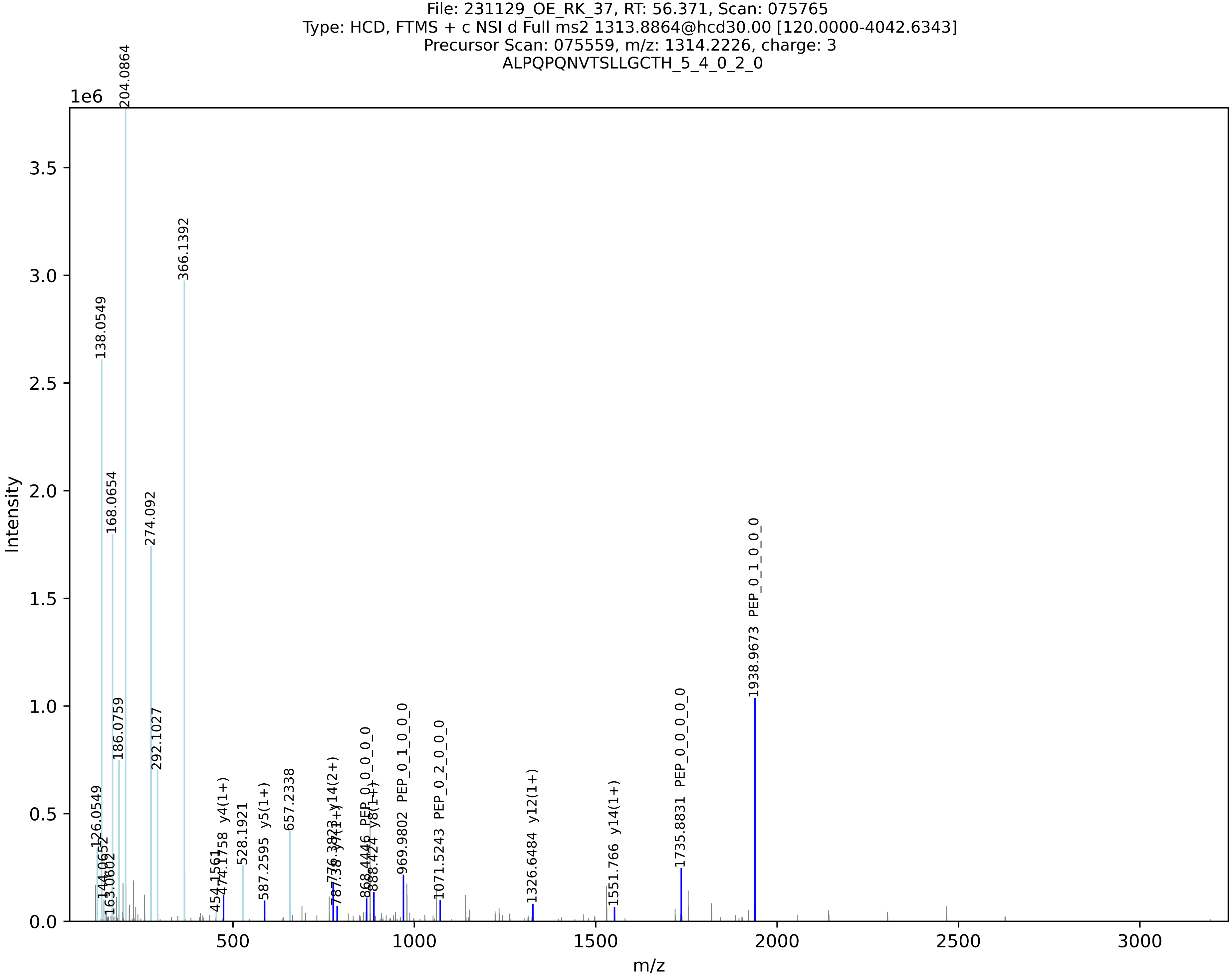

File: 231129_OE_RK_37, RT: 56.371, Scan: 075765
Type: HCD, FTMS + c NSI d Full ms2 1313.8864@hcd30.00 [120.0000-4042.6343]
Precursor Scan: 075559, m/z: 1314.2226, charge: 3
ALPQPQNVTSLLGCTH_5_4_0_2_0
1e6
Intensity
m/z
204.0864
366.1392
138.0549
168.0654
274.092
186.0759
292.1027
126.0549
144.0652
163.0602
454.1561
474.1758 y4(1+)
528.1921
587.2595 y5(1+)
657.2338
776.3823 y14(2+)
787.38 y7(1+)
868.4446 PEP_0_0_0_0_0
888.424 y8(1+)
969.9802 PEP_0_1_0_0_0
1071.5243 PEP_0_2_0_0_0
1326.6484 y12(1+)
1551.766 y14(1+)
1735.8831 PEP_0_0_0_0_0
1938.9673 PEP_0_1_0_0_0

File: 231129_OE_RK_37, RT: 56.374, Scan: 075771
Type: HCD, FTMS + c NSI d Full ms2 1171.2837@hcd30.00 [120.0000-4801.6934]
Precursor Scan: 075559, m/z: 1169.2666, charge: 4
VVLHPNYSQVDIGLIK_7_5_1_2_0

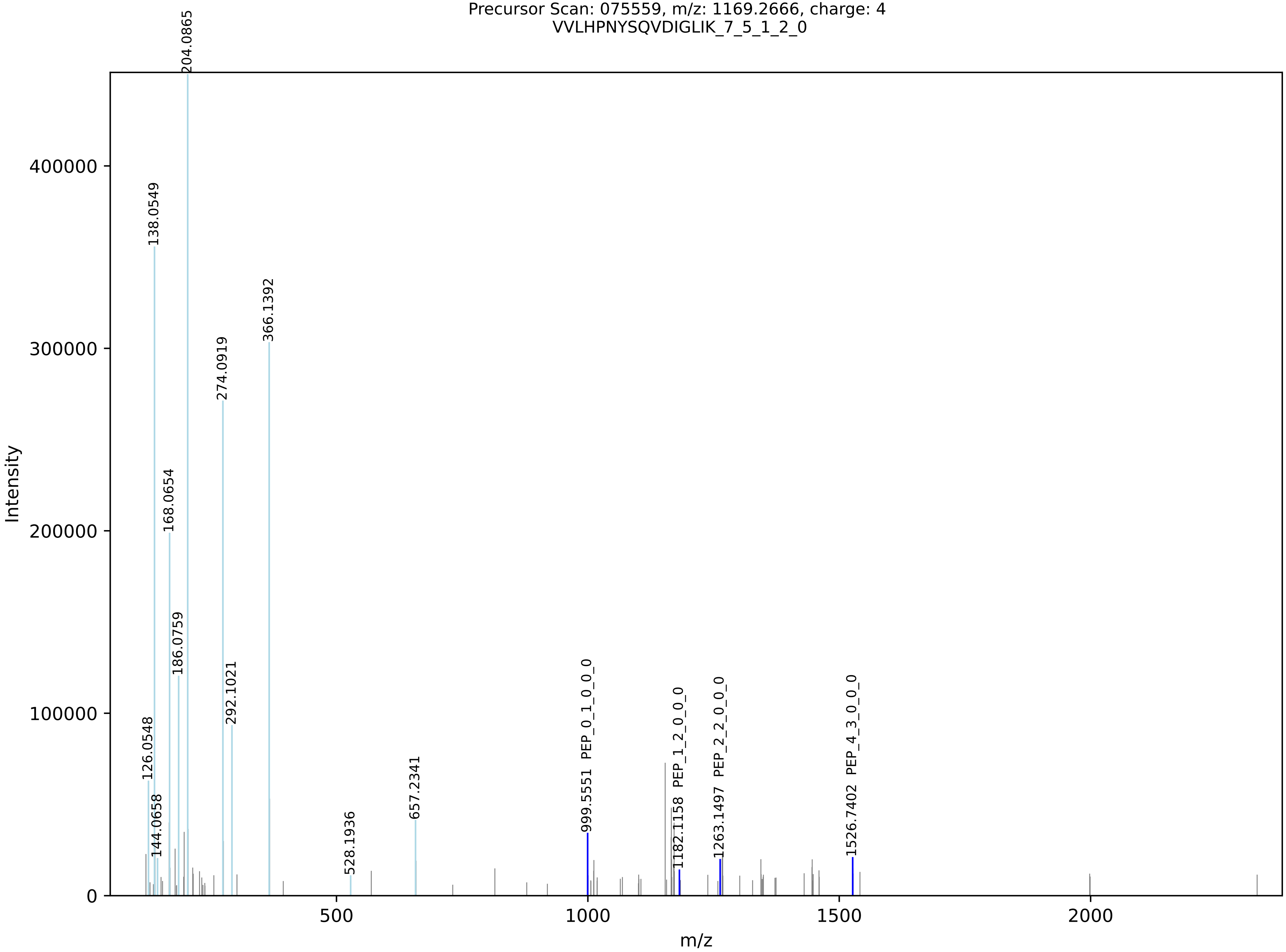

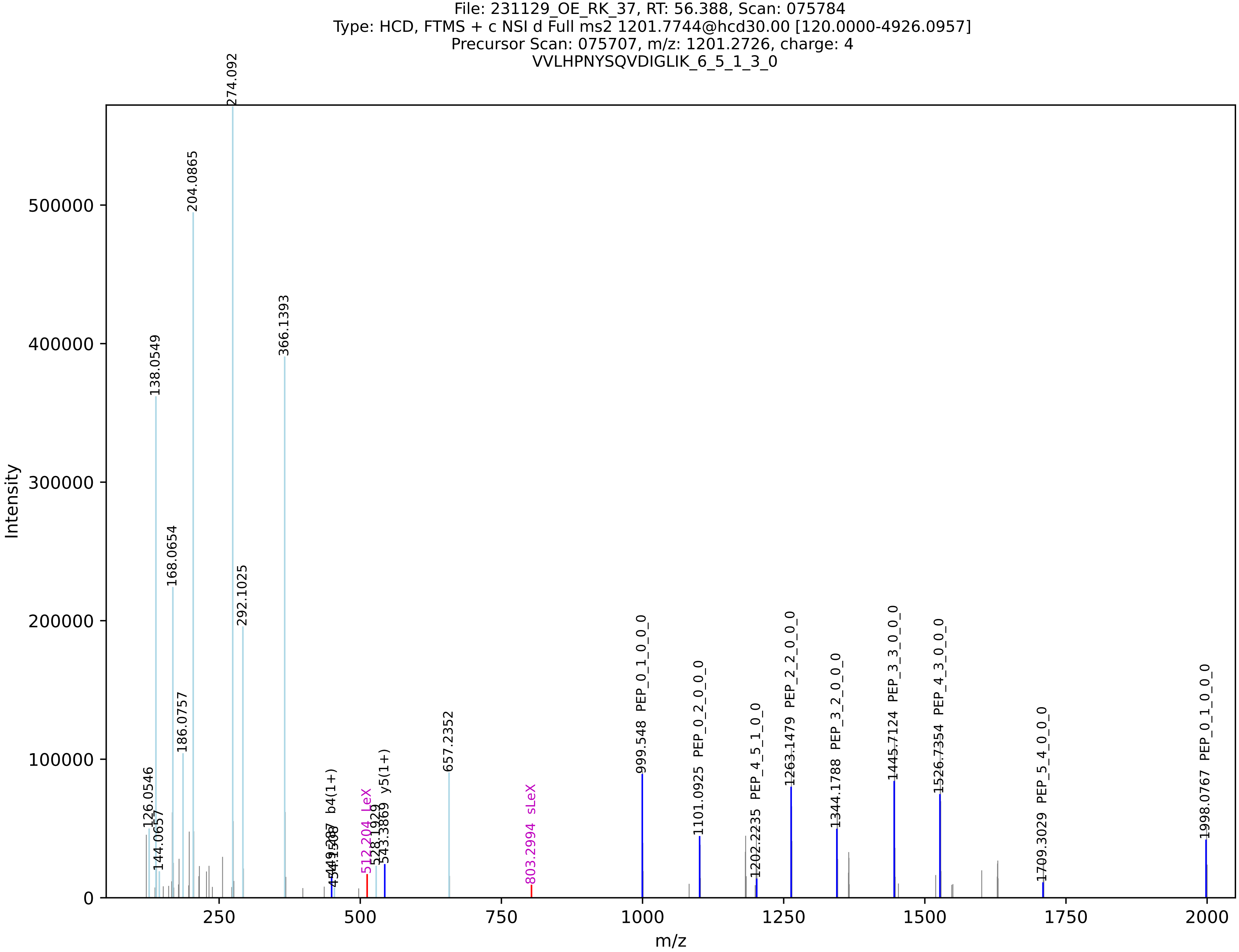
File: 231129_OE_RK_37, RT: 56.388, Scan: 075784
Type: HCD, FTMS + c NSI d Full ms2 1201.7744@hcd30.00 [120.0000-4926.0957]
Precursor Scan: 075707, m/z: 1201.2726, charge: 4
VVLHPNYSQVDIGLIK_6_5_1_3_0
Intensity
m/z
0
100000
200000
300000
400000
500000
250
500
750
1000
1250
1500
1750
2000
274.092
204.0865
366.1393
138.0549
168.0654
292.1025
186.0757
126.0546
144.0657
b4(1+)
512.204 LeX
y5(1+)
543.3869
657.2352
803.2994 sLeX
999.548 PEP_0_1_0_0_0
1101.0925 PEP_0_2_0_0_0
1202.2235 PEP_4_5_1_0_0
1263.1479 PEP_2_2_0_0_0
1344.1788 PEP_3_2_0_0_0
1445.7124 PEP_3_3_0_0_0
1526.7354 PEP_4_3_0_0_0
1709.3029 PEP_5_4_0_0_0
1998.0767 PEP_0_1_0_0_0

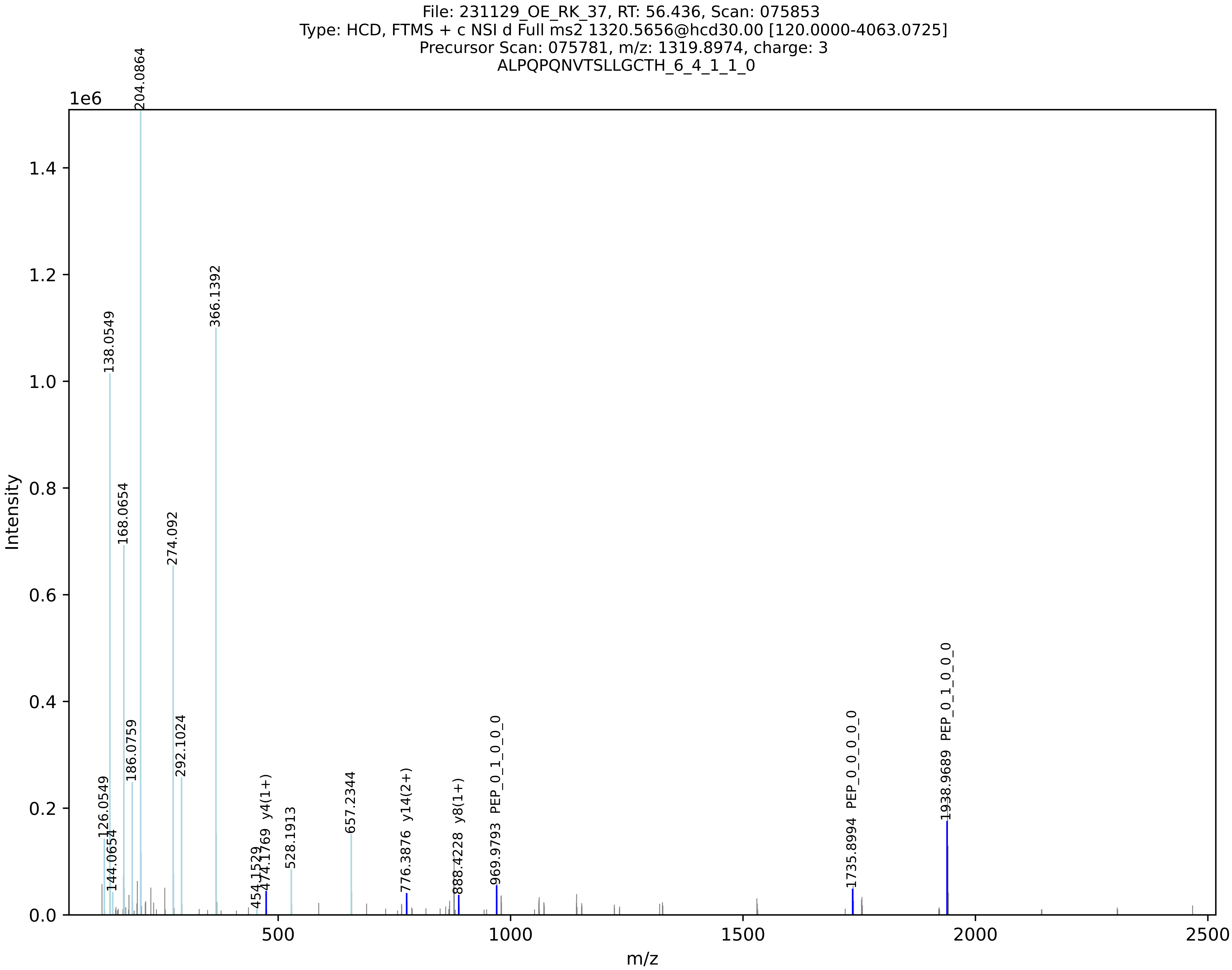
File: 231129_OE_RK_37, RT: 56.436, Scan: 075853
Type: HCD, FTMS + c NSI d Full ms2 1320.5656@hcd30.00 [120.0000-4063.0725]
Precursor Scan: 075781, m/z: 1319.8974, charge: 3
ALPQPQNVTSLLGCTH_6_4_1_1_0
1e6
Intensity
m/z
204.0864
366.1392
138.0549
168.0654
274.092
186.0759
292.1024
126.0549
144.0654
454.1529
474.1769 y4(1+)
528.1913
657.2344
776.3876 y14(2+)
888.4228 y8(1+)
969.9793 PEP_0_1_0_0_0
1735.8994 PEP_0_0_0_0_0
1938.9689 PEP_0_1_0_0_0

File: 231129_OE_RK_37, RT: 56.438, Scan: 075856
Type: HCD, FTMS + c NSI d Full ms2 1169.2670@hcd30.00 [120.0000-4793.4653]
Precursor Scan: 075781, m/z: 1169.0164, charge: 4
VVLHPNYSQVDIGLIK_7_5_1_2_0

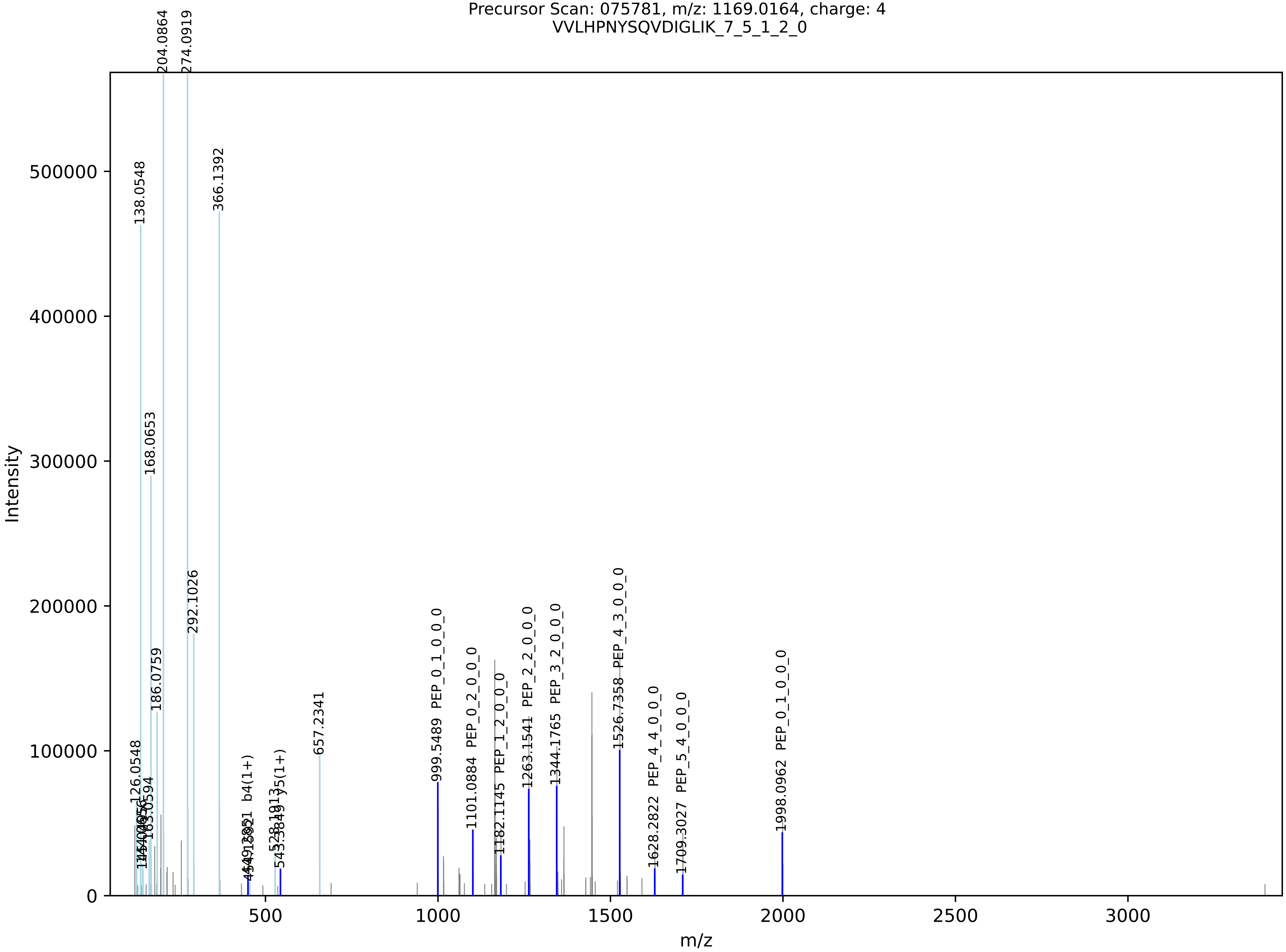

File: 231129_OE_RK_37, RT: 56.443, Scan: 075864
Type: HCD, FTMS + c NSI d Full ms2 1601.6963@hcd30.00 [120.0000-4923.3325]
Precursor Scan: 075781, m/z: 1601.3612, charge: 3
VVLHPNYSQVDIGLIK_6_5_1_3_0

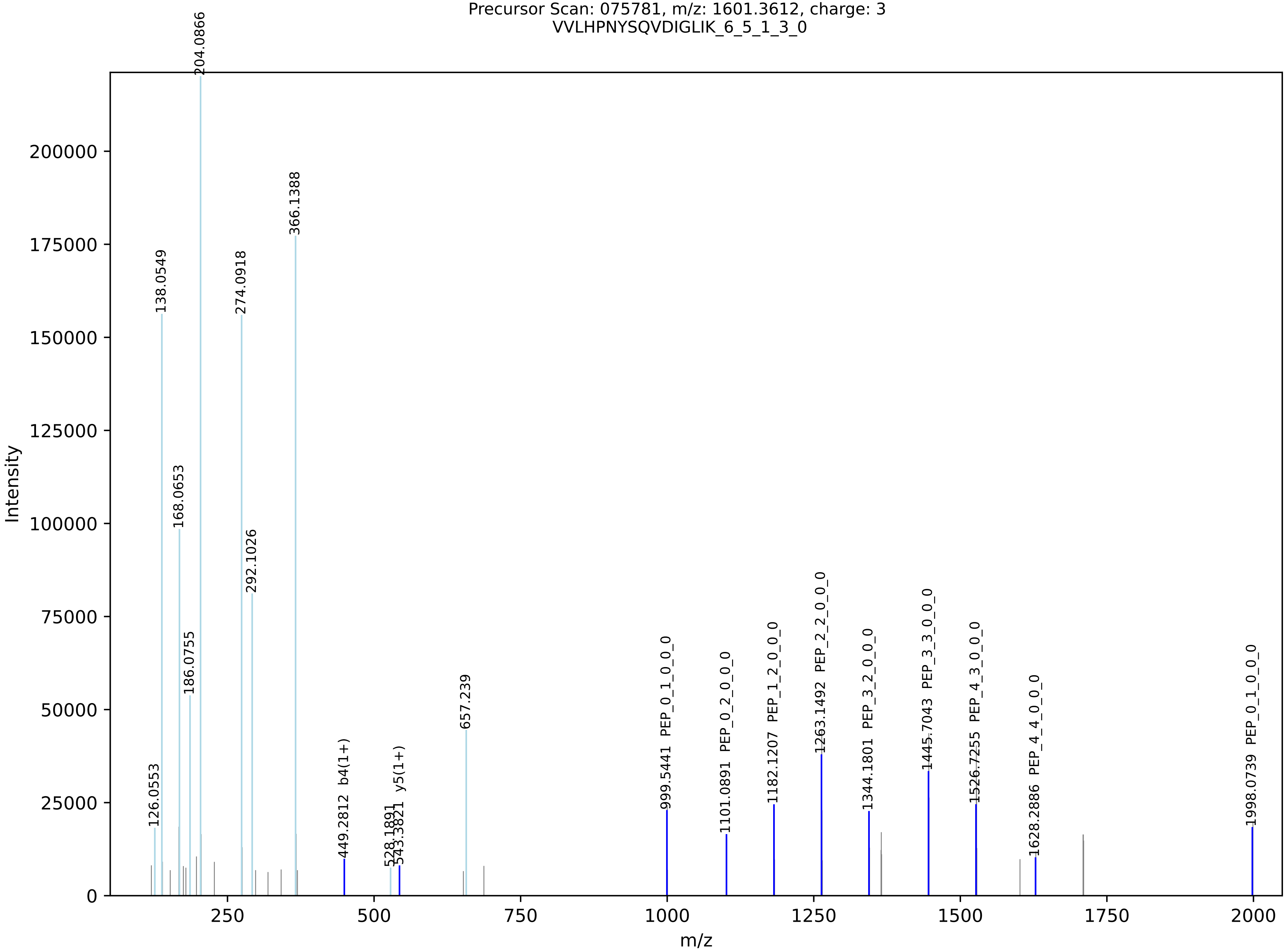

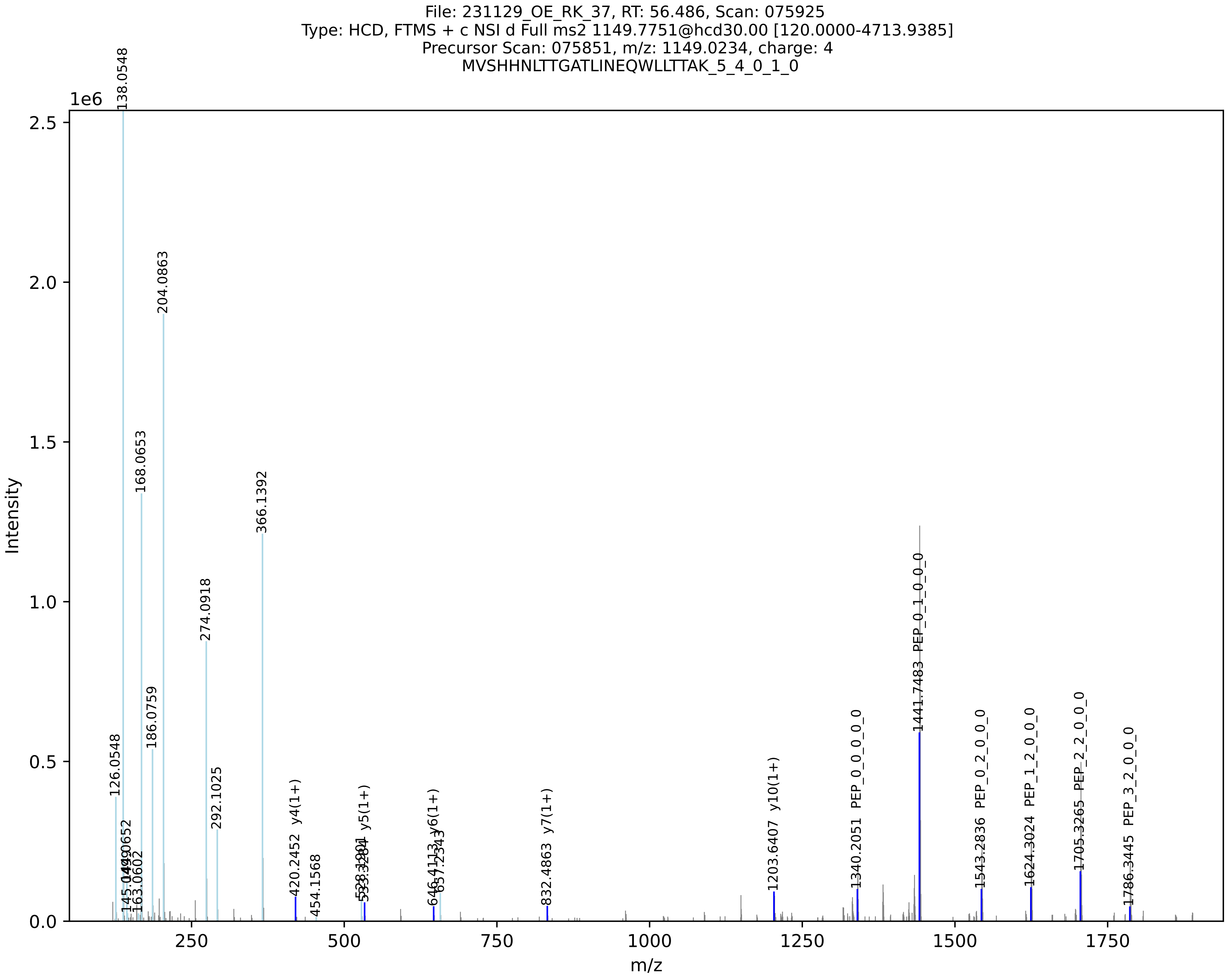
File: 231129_OE_RK_37, RT: 56.486, Scan: 075925
Type: HCD, FTMS + c NSI d Full ms2 1149.7751@hcd30.00 [120.0000-4713.9385]
Precursor Scan: 075851, m/z: 1149.0234, charge: 4
MVSHHNLTTGATLINEQWLLTTAK_5_4_0_1_0
1e6
2.5
2.0
1.5
1.0
0.5
0.0
Intensity
250
500
750
1000
1250
1500
1750
m/z
138.0548
204.0863
168.0653
366.1392
274.0918
186.0759
126.0548
292.1025
163.0602
420.2452 y4(1+)
454.1568
y5(1+)
y6(1+)
657.2343
832.4863 y7(1+)
1203.6407 y10(1+)
1340.2051 PEP_0_0_0_0_0
1441.7483 PEP_0_1_0_0_0
1543.2836 PEP_0_2_0_0_0
1624.3024 PEP_1_2_0_0_0
1705.3265 PEP_2_2_0_0_0
1786.3445 PEP_3_2_0_0_0

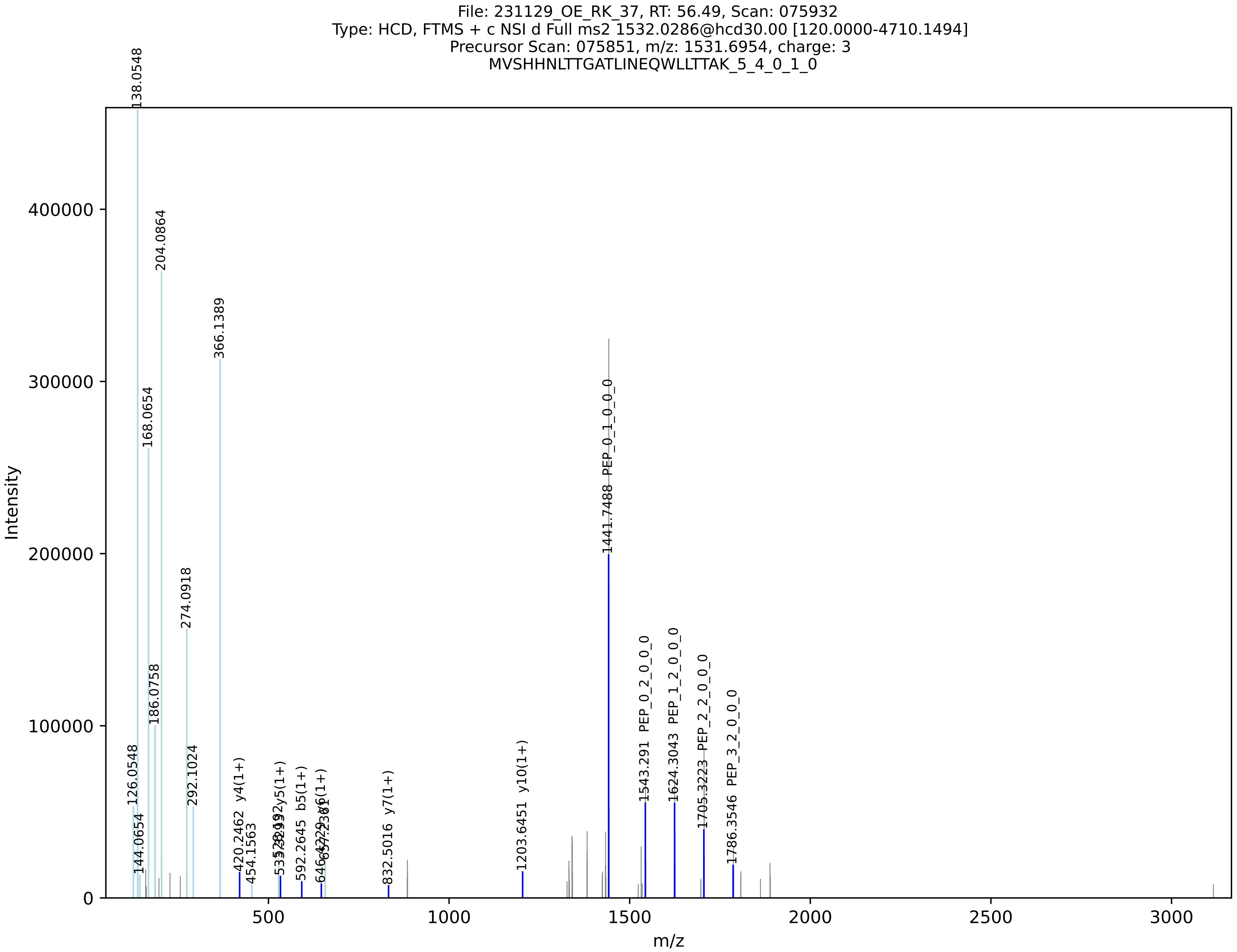
File: 231129_OE_RK_37, RT: 56.49, Scan: 075932
Type: HCD, FTMS + c NSI d Full ms2 1532.0286@hcd30.00 [120.0000-4710.1494]
Precursor Scan: 075851, m/z: 1531.6954, charge: 3
MVSHHNLTTGATLINEQWLLTTAK_5_4_0_1_0
Intensity
m/z
138.0548
204.0864
366.1389
168.0654
274.0918
186.0758
126.0548
292.1024
144.0654
420.2462 y4(1+)
454.1563
592.2645 b5(1+)
832.5016 y7(1+)
1203.6451 y10(1+)
1441.7488 PEP_0_1_0_0_0
1543.291 PEP_0_2_0_0_0
1624.3043 PEP_1_2_0_0_0
1705.3223 PEP_2_2_0_0_0
1786.3546 PEP_3_2_0_0_0

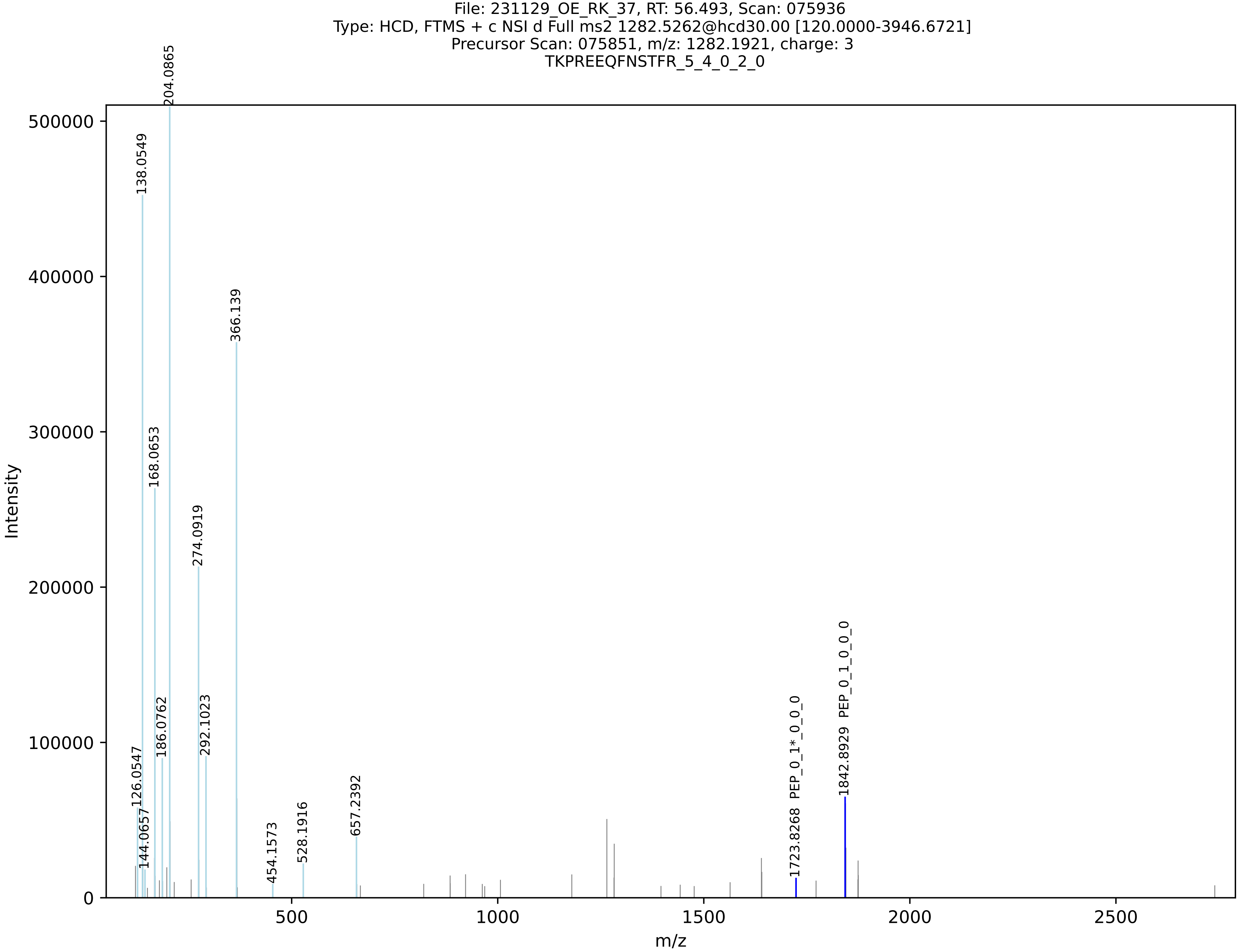
File: 231129_OE_RK_37, RT: 56.493, Scan: 075936
Type: HCD, FTMS + c NSI d Full ms2 1282.5262@hcd30.00 [120.0000-3946.6721]
Precursor Scan: 075851, m/z: 1282.1921, charge: 3
TKPREEQFNSTFR_5_4_0_2_0
Intensity
m/z
204.0865
138.0549
366.139
168.0653
274.0919
186.0762
292.1023
126.0547
144.0657
454.1573
528.1916
657.2392
1723.8268 PEP_0_1*_0_0_0
1842.8929 PEP_0_1_0_0_0

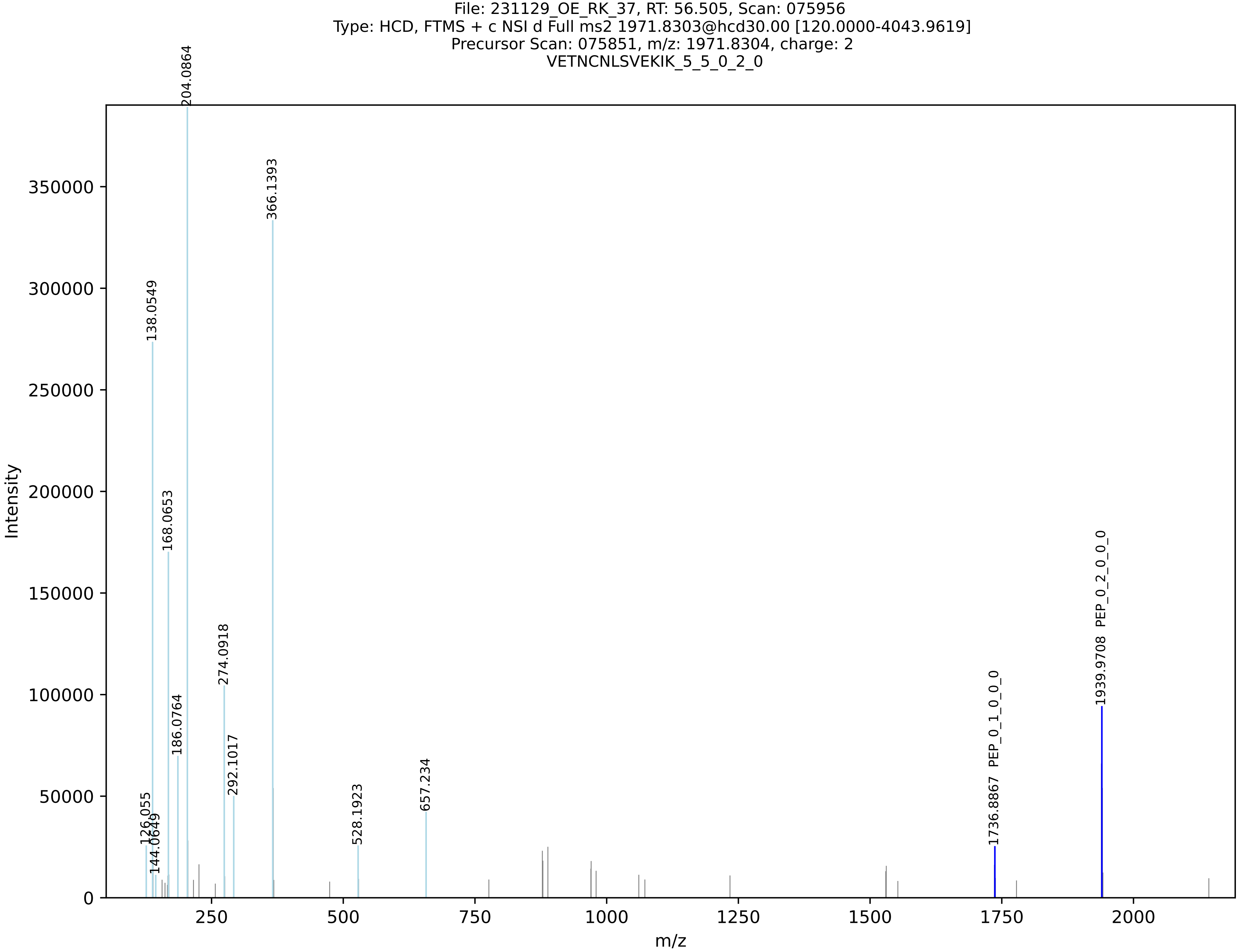

File: 231129_OE_RK_37, RT: 56.505, Scan: 075956
Type: HCD, FTMS + c NSI d Full ms2 1971.8303@hcd30.00 [120.0000-4043.9619]
Precursor Scan: 075851, m/z: 1971.8304, charge: 2
VETNCNLSVEKIK_5_5_0_2_0
Intensity
m/z
204.0864
366.1393
138.0549
168.0653
274.0918
186.0764
292.1017
126.055
144.0649
528.1923
657.234
1736.8867 PEP_0_1_0_0_0
1939.9708 PEP_0_2_0_0_0

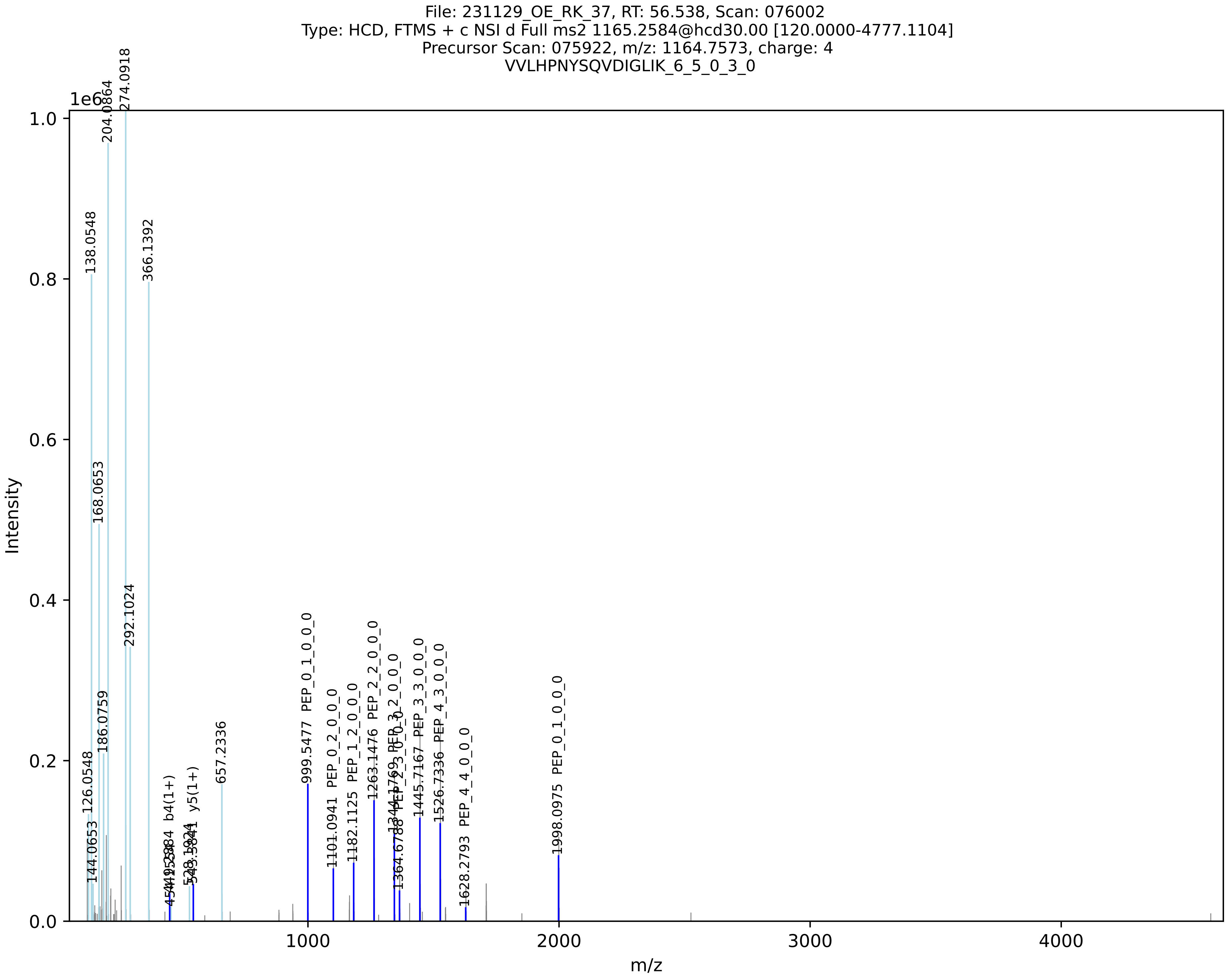
File: 231129_OE_RK_37, RT: 56.538, Scan: 076002
Type: HCD, FTMS + c NSI d Full ms2 1165.2584@hcd30.00 [120.0000-4777.1104]
Precursor Scan: 075922, m/z: 1164.7573, charge: 4
VVLHPNYSQVDIGLIK_6_5_0_3_0
1e6
Intensity
m/z
274.0918
204.0864
138.0548
366.1392
168.0653
292.1024
186.0759
126.0548
144.0653
b4(1+)
y5(1+)
657.2336
999.5477 PEP_0_1_0_0_0
1101.0941 PEP_0_2_0_0_0
1182.1125 PEP_1_2_0_0_0
1263.1476 PEP_2_2_0_0_0
PEP_3_2_0_0_0
1445.7167 PEP_3_3_0_0_0
1526.7336 PEP_4_3_0_0_0
1628.2793 PEP_4_4_0_0_0
1998.0975 PEP_0_1_0_0_0

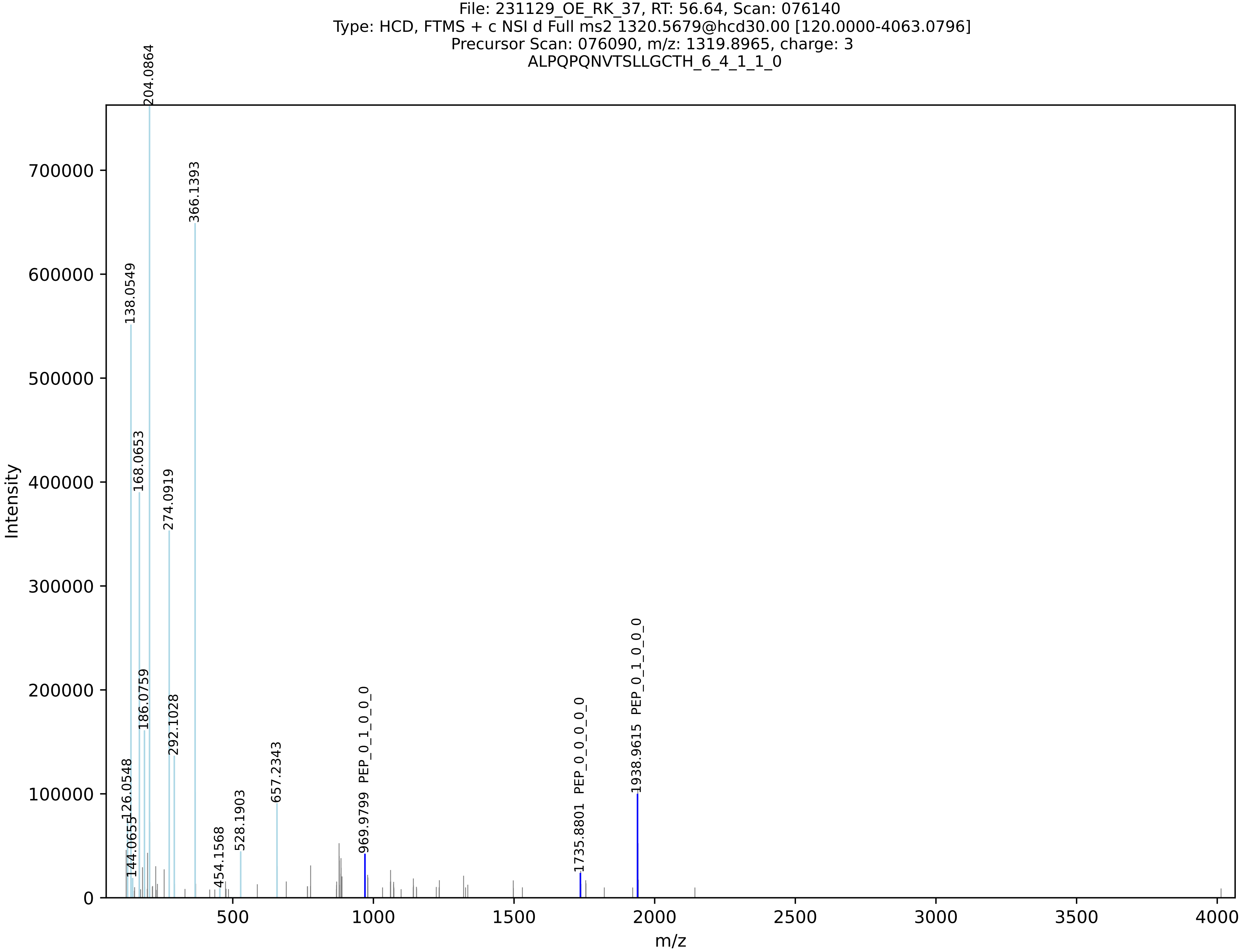
File: 231129_OE_RK_37, RT: 56.64, Scan: 076140
Type: HCD, FTMS + c NSI d Full ms2 1320.5679@hcd30.00 [120.0000-4063.0796]
Precursor Scan: 076090, m/z: 1319.8965, charge: 3
ALPQPQNVTSLLGCTH_6_4_1_1_0
Intensity
m/z
204.0864
366.1393
138.0549
168.0653
274.0919
186.0759
292.1028
126.0548
144.0655
454.1568
528.1903
657.2343
969.9799 PEP_0_1_0_0_0_
1735.8801 PEP_0_0_0_0_0_
1938.9615 PEP_0_1_0_0_0_

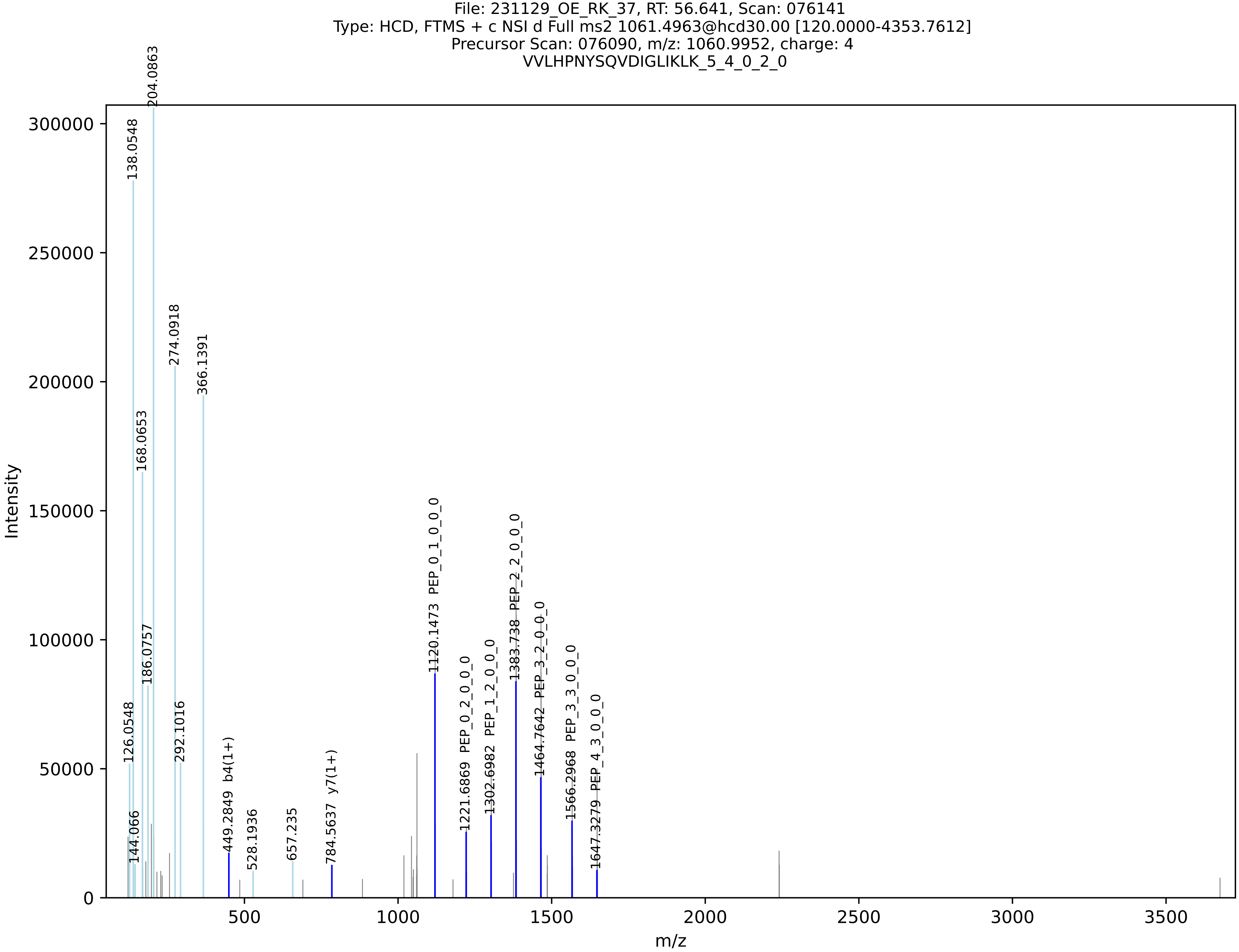
File: 231129_OE_RK_37, RT: 56.641, Scan: 076141
Type: HCD, FTMS + c NSI d Full ms2 1061.4963@hcd30.00 [120.0000-4353.7612]
Precursor Scan: 076090, m/z: 1060.9952, charge: 4
VVLHPNYSQVDIGLIKLK_5_4_0_2_0
Intensity
m/z
204.0863
138.0548
168.0653
186.0757
126.0548
144.066
274.0918
292.1016
366.1391
449.2849 b4(1+)
528.1936
657.235
784.5637 y7(1+)
1120.1473 PEP_0_1_0_0_0
1221.6869 PEP_0_2_0_0_0
1302.6982 PEP_1_2_0_0_0
1383.738 PEP_2_2_0_0_0
1464.7642 PEP_3_2_0_0_0
1566.2968 PEP_3_3_0_0_0
1647.3279 PEP_4_3_0_0_0

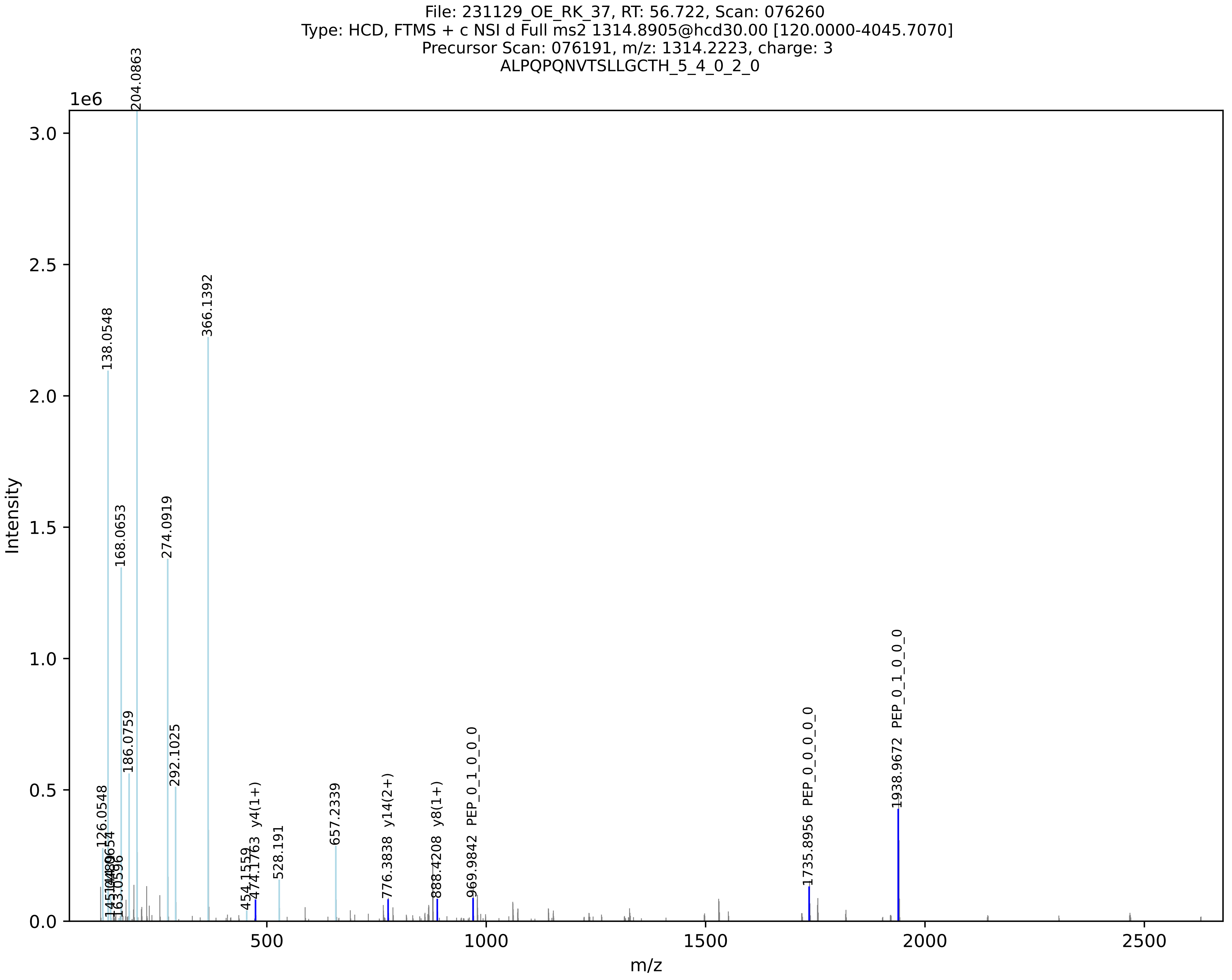
File: 231129_OE_RK_37, RT: 56.722, Scan: 076260
Type: HCD, FTMS + c NSI d Full ms2 1314.8905@hcd30.00 [120.0000-4045.7070]
Precursor Scan: 076191, m/z: 1314.2223, charge: 3
ALPQPQNVTSLLGCTH_5_4_0_2_0
1e6
Intensity
m/z
204.0863
366.1392
138.0548
168.0653
274.0919
186.0759
292.1025
126.0548
657.2339
528.191
454.1559
474.1763 y4(1+)
776.3838 y14(2+)
888.4208 y8(1+)
969.9842 PEP_0_1_0_0_0_
1735.8956 PEP_0_0_0_0_0
1938.9672 PEP_0_1_0_0_0_

File: 231129_OE_RK_37, RT: 56.726, Scan: 076266
Type: HCD, FTMS + c NSI d Full ms2 1165.2599@hcd30.00 [120.0000-4777.1167]
Precursor Scan: 076191, m/z: 1164.7588, charge: 4
VVLHPNYSQVDIGLIK_6_5_0_3_0

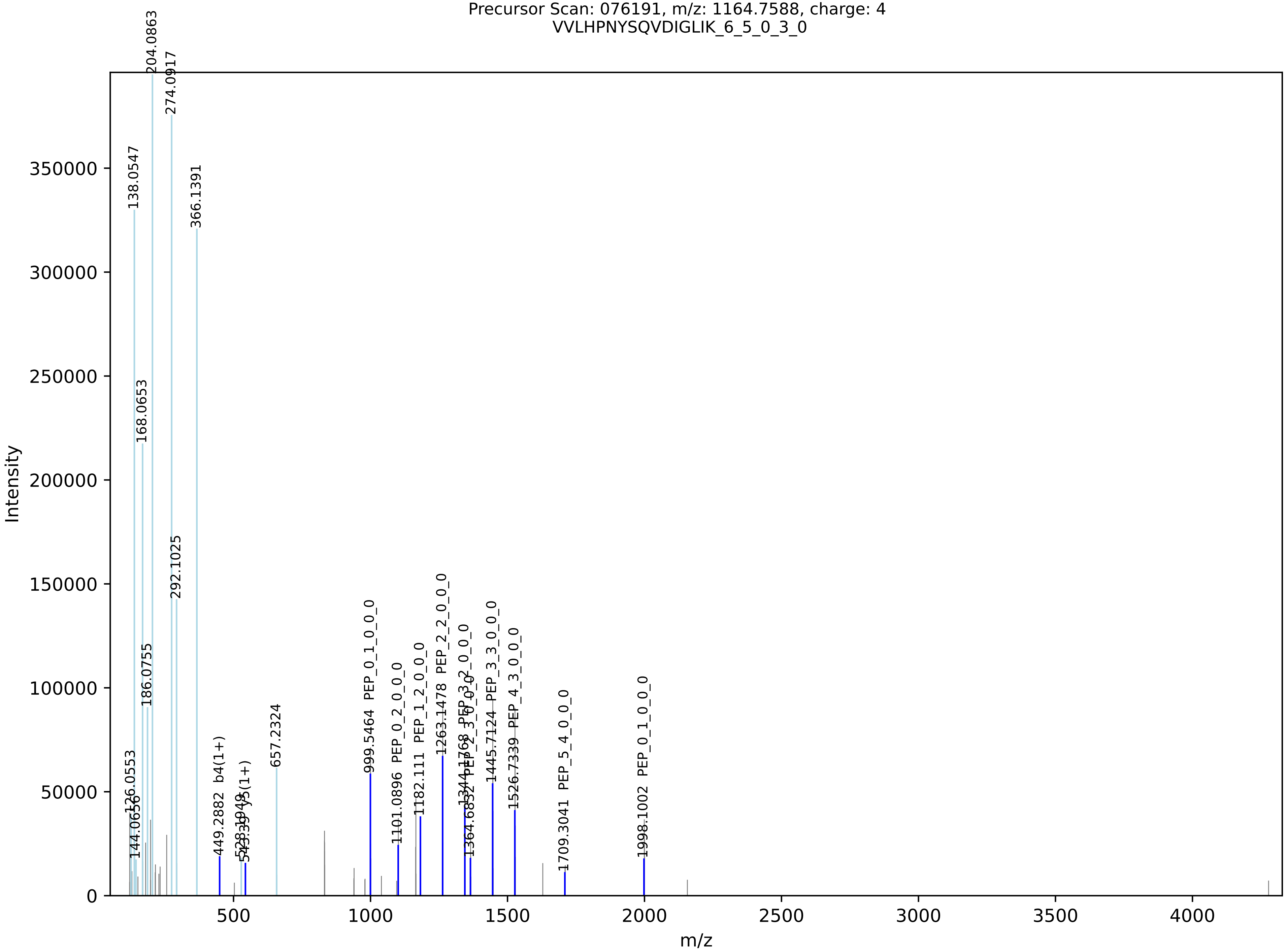

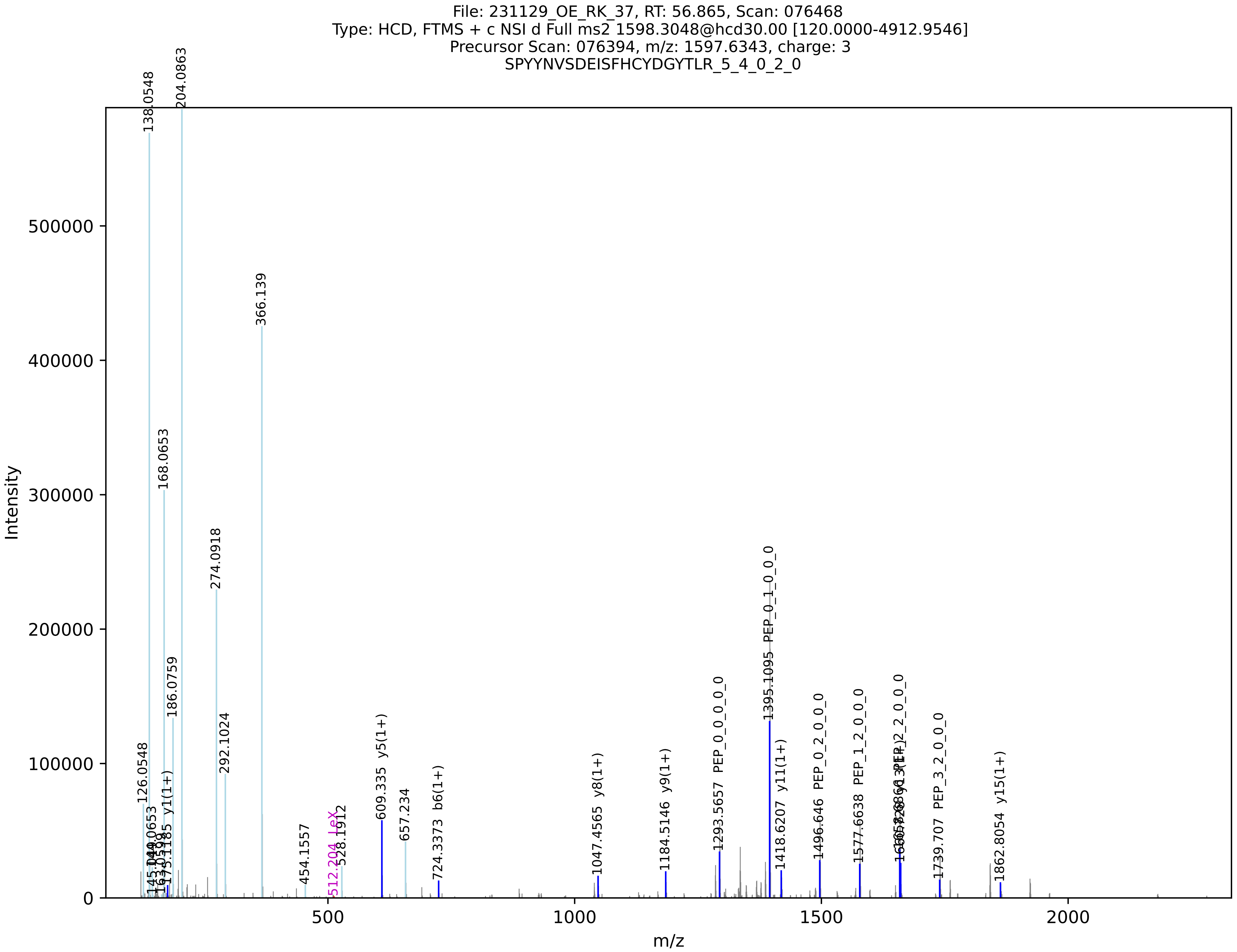
File: 231129_OE_RK_37, RT: 56.865, Scan: 076468
Type: HCD, FTMS + c NSI d Full ms2 1598.3048@hcd30.00 [120.0000-4912.9546]
Precursor Scan: 076394, m/z: 1597.6343, charge: 3
SPYYNVSDEISFHCYDGYTLR_5_4_0_2_0
Intensity
m/z
138.0548
204.0863
366.139
168.0653
274.0918
186.0759
292.1024
126.0548
175.1185 y1(1+)
454.1557
512.204 LeX
528.1912
609.335 y5(1+)
657.234
724.3373 b6(1+)
1047.4565 y8(1+)
1184.5146 y9(1+)
1293.5657 PEP_0_0_0_0_0
1395.1095 PEP_0_1_0_0_0
1418.6207 y11(1+)
1496.646 PEP_0_2_0_0_0
1577.6638 PEP_1_2_0_0_0
PEP_2_2_0_0_0
1739.707 PEP_3_2_0_0_0
1862.8054 y15(1+)

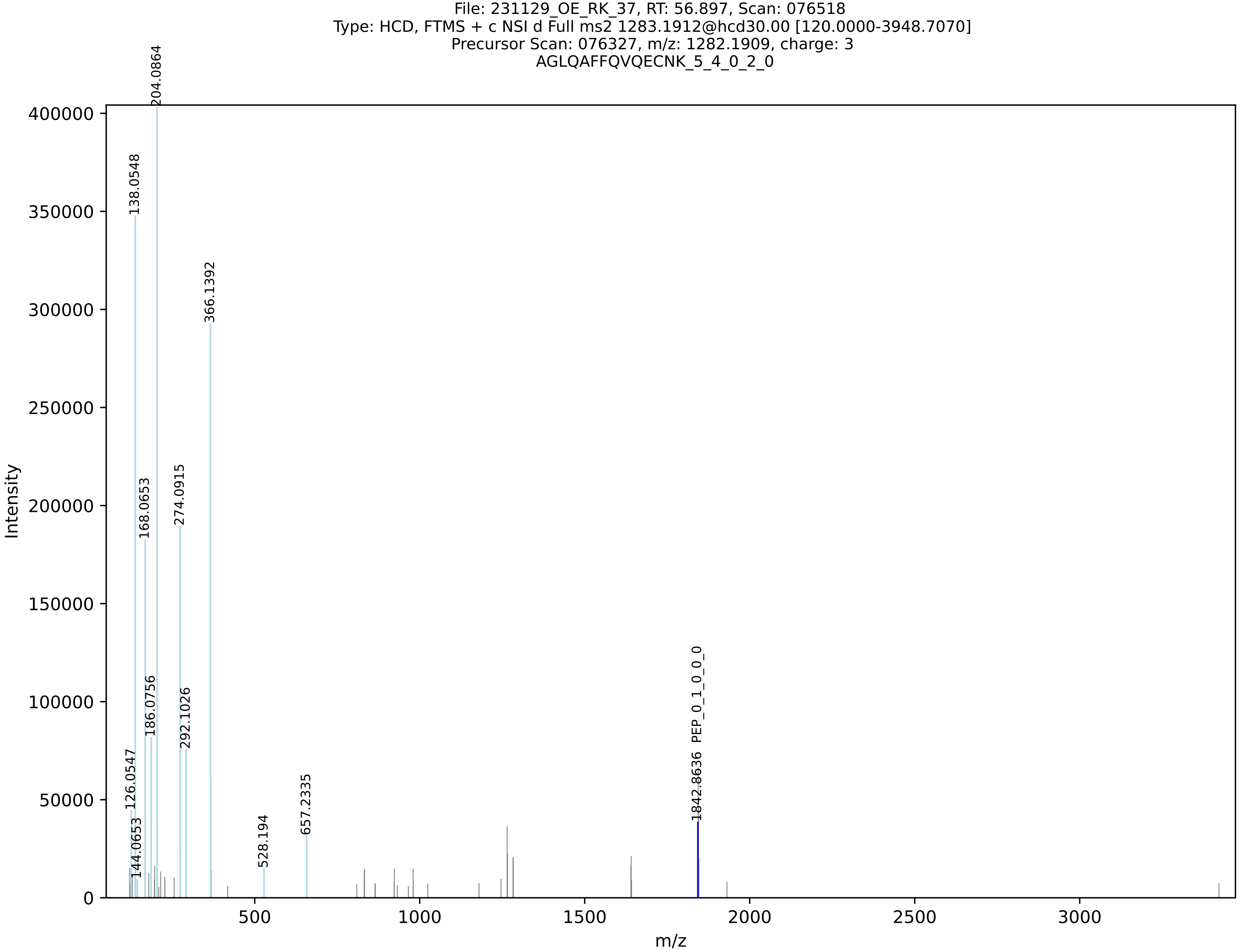

File: 231129_OE_RK_37, RT: 56.897, Scan: 076518
Type: HCD, FTMS + c NSI d Full ms2 1283.1912@hcd30.00 [120.0000-3948.7070]
Precursor Scan: 076327, m/z: 1282.1909, charge: 3
AGLQAFFQVQECNK_5_4_0_2_0
Intensity
m/z
204.0864
138.0548
366.1392
274.0915
168.0653
186.0756
292.1026
126.0547
144.0653
528.194
657.2335
1842.8636 PEP_0_1_0_0_0
0
50000
100000
150000
200000
250000
300000
350000
400000
500
1000
1500
2000
2500
3000

File: 231129_OE_RK_37, RT: 57.061, Scan: 076752
Type: HCD, FTMS + c NSI d Full ms2 1149.5208@hcd30.00 [120.0000-4712.9004]
Precursor Scan: 076676, m/z: 1149.0196, charge: 4
MVSHHNLTTGATLINEQWLLTTAK_5_4_0_1_0

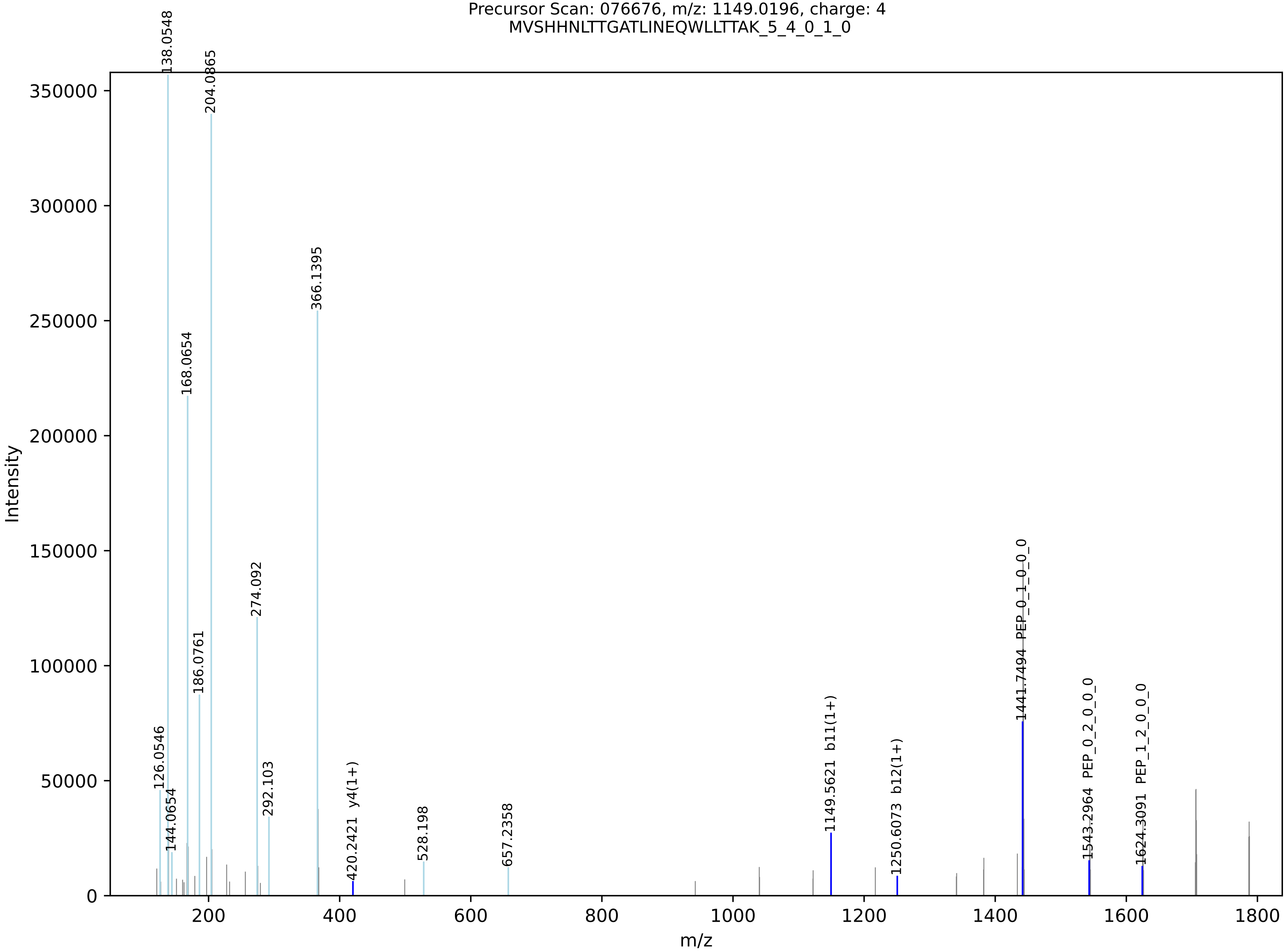

File: 231129_OE_RK_37, RT: 57.062, Scan: 076754
Type: HCD, FTMS + c NSI d Full ms2 1217.7457@hcd30.00 [120.0000-4991.2583]
Precursor Scan: 076676, m/z: 1217.2445, charge: 4
QQQHLFGSNVTDCSGNFCLFR_5_4_1_2_0

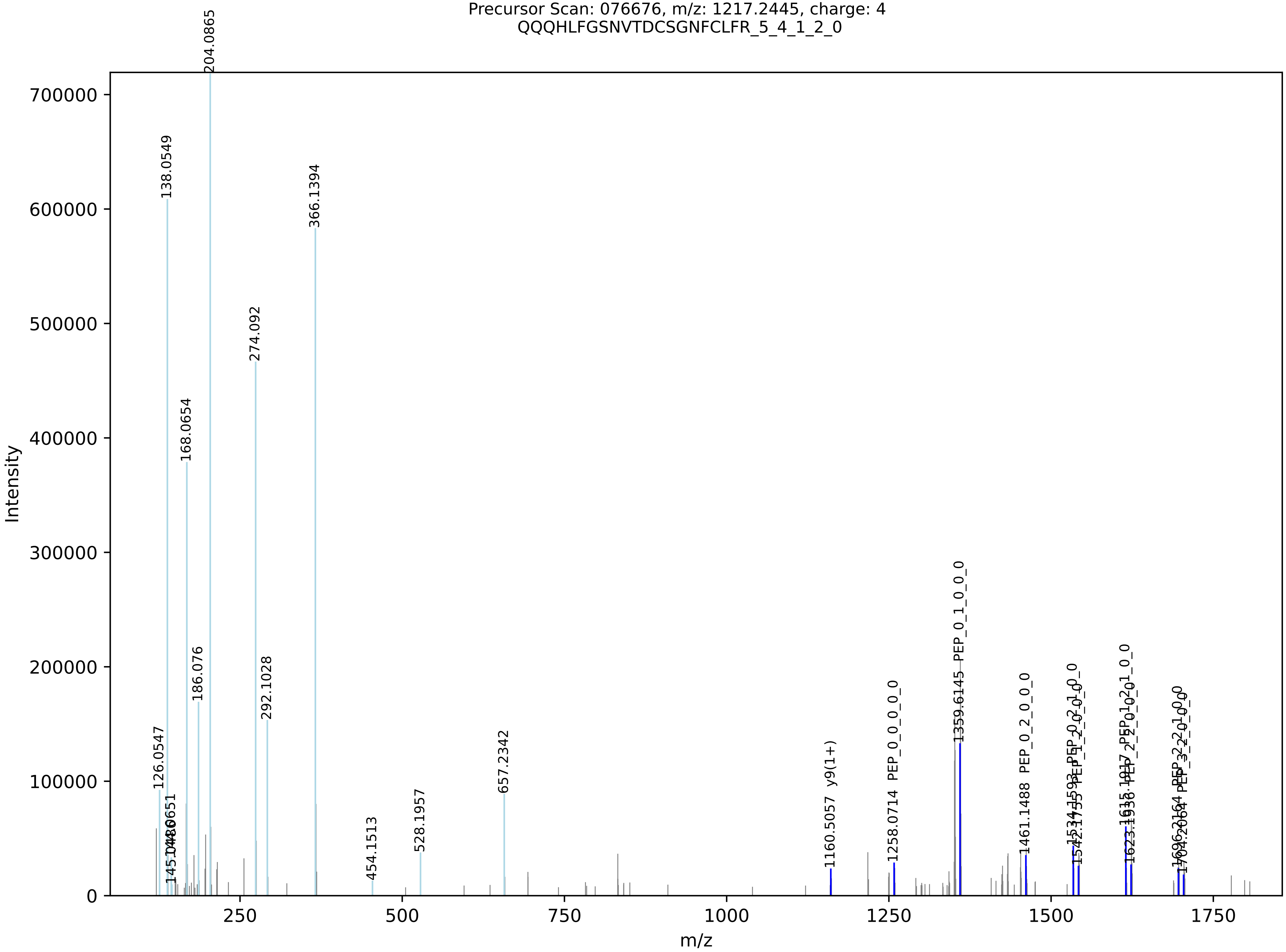

File: 231129_OE_RK_37, RT: 57.066, Scan: 076759
Type: HCD, FTMS + c NSI d Full ms2 910.2399@hcd30.00 [120.0000-5594.9526]
Precursor Scan: 076676, m/z: 910.0725, charge: 6
SLGNVNFTVSAEALESQELCGTEVPSVPEHGRK_5_4_0_1_0

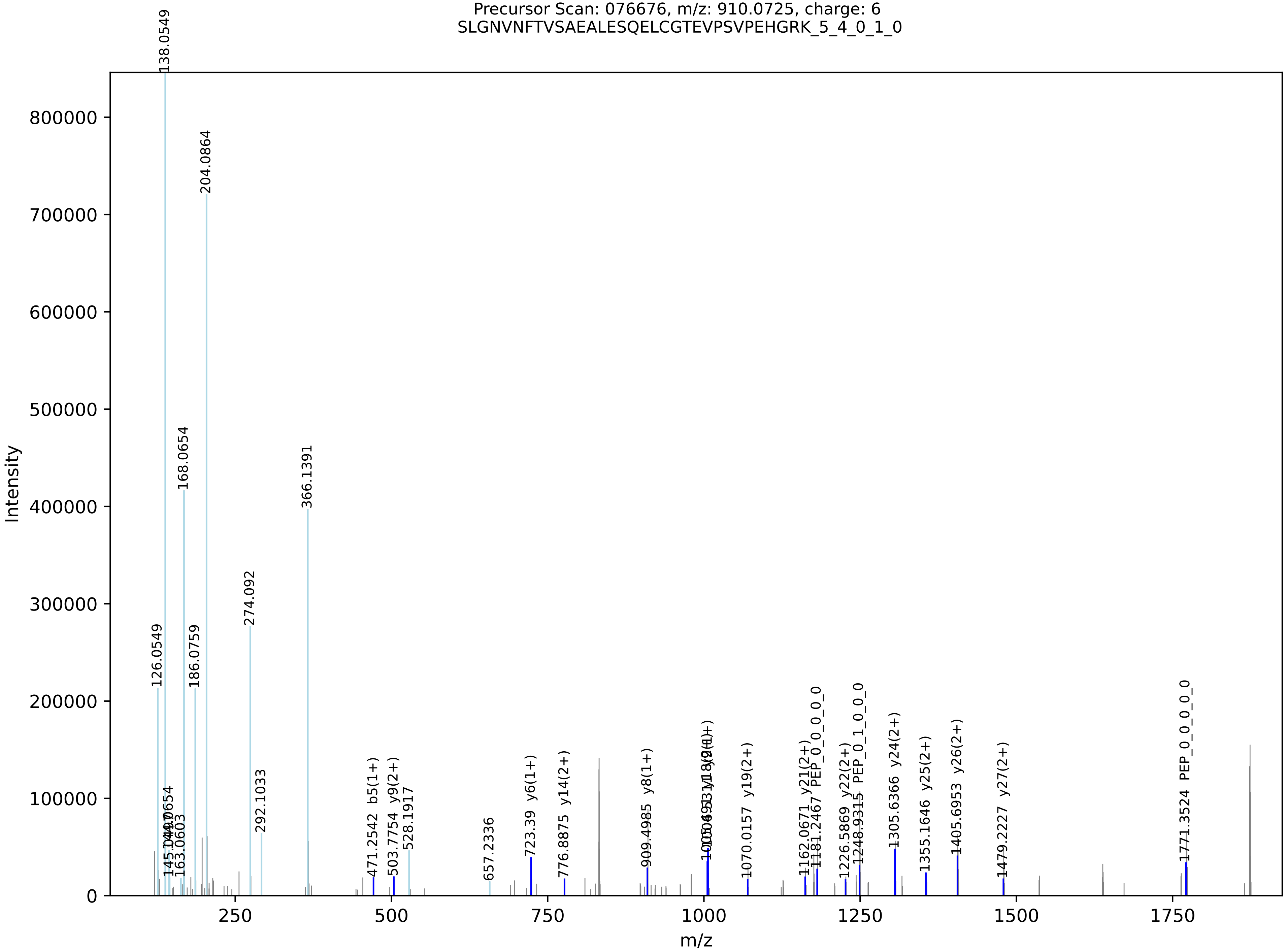

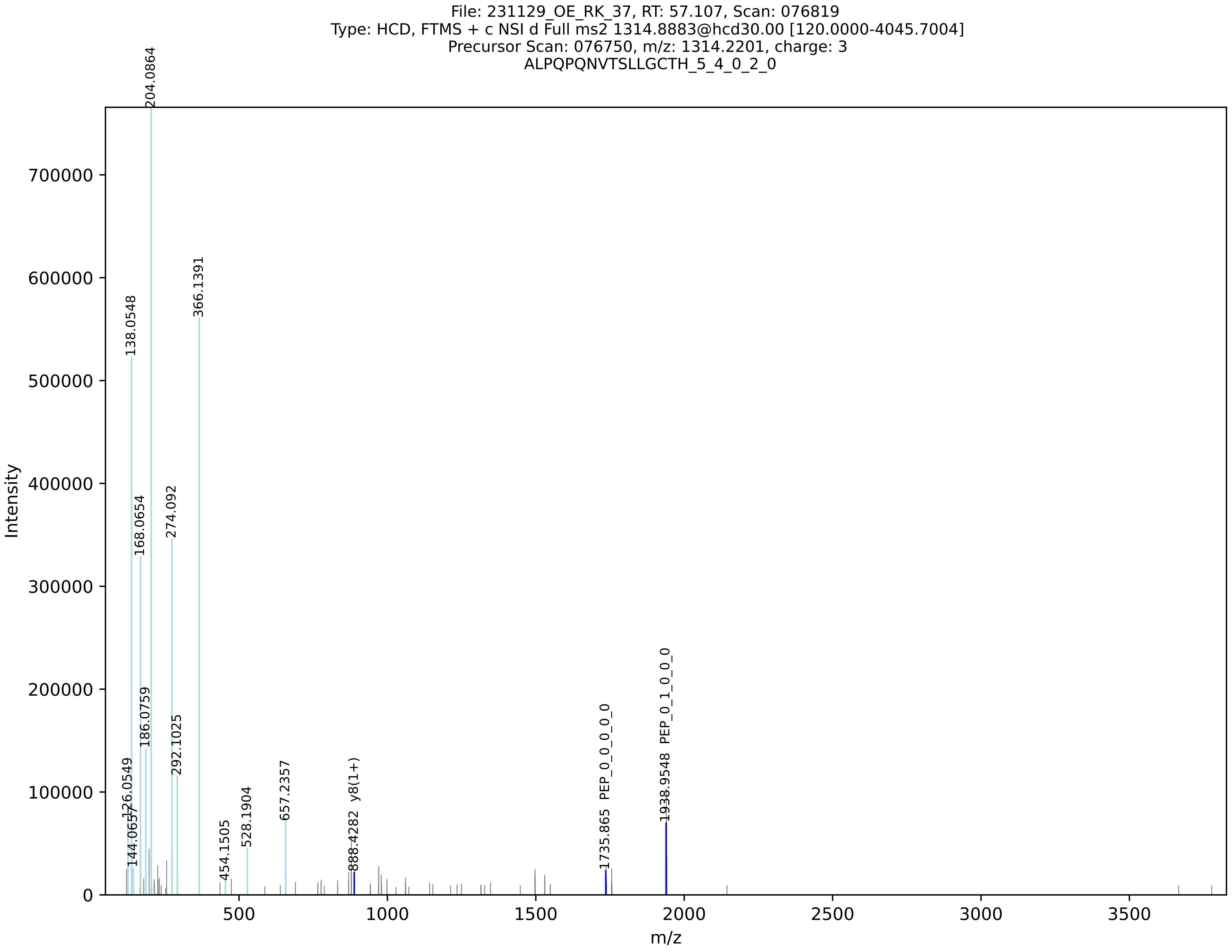

File: 231129_OE_RK_37, RT: 57.107, Scan: 076819
Type: HCD, FTMS + c NSI d Full ms2 1314.8883@hcd30.00 [120.0000-4045.7004]
Precursor Scan: 076750, m/z: 1314.2201, charge: 3
ALPQPQNVTSLLGCTH_5_4_0_2_0
Intensity
m/z
204.0864
366.1391
138.0548
168.0654
274.092
186.0759
292.1025
126.0549
144.0657
454.1505
528.1904
657.2357
888.4282 y8(1+)
1735.865 PEP_0_0_0_0_0
1938.9548 PEP_0_1_0_0_0

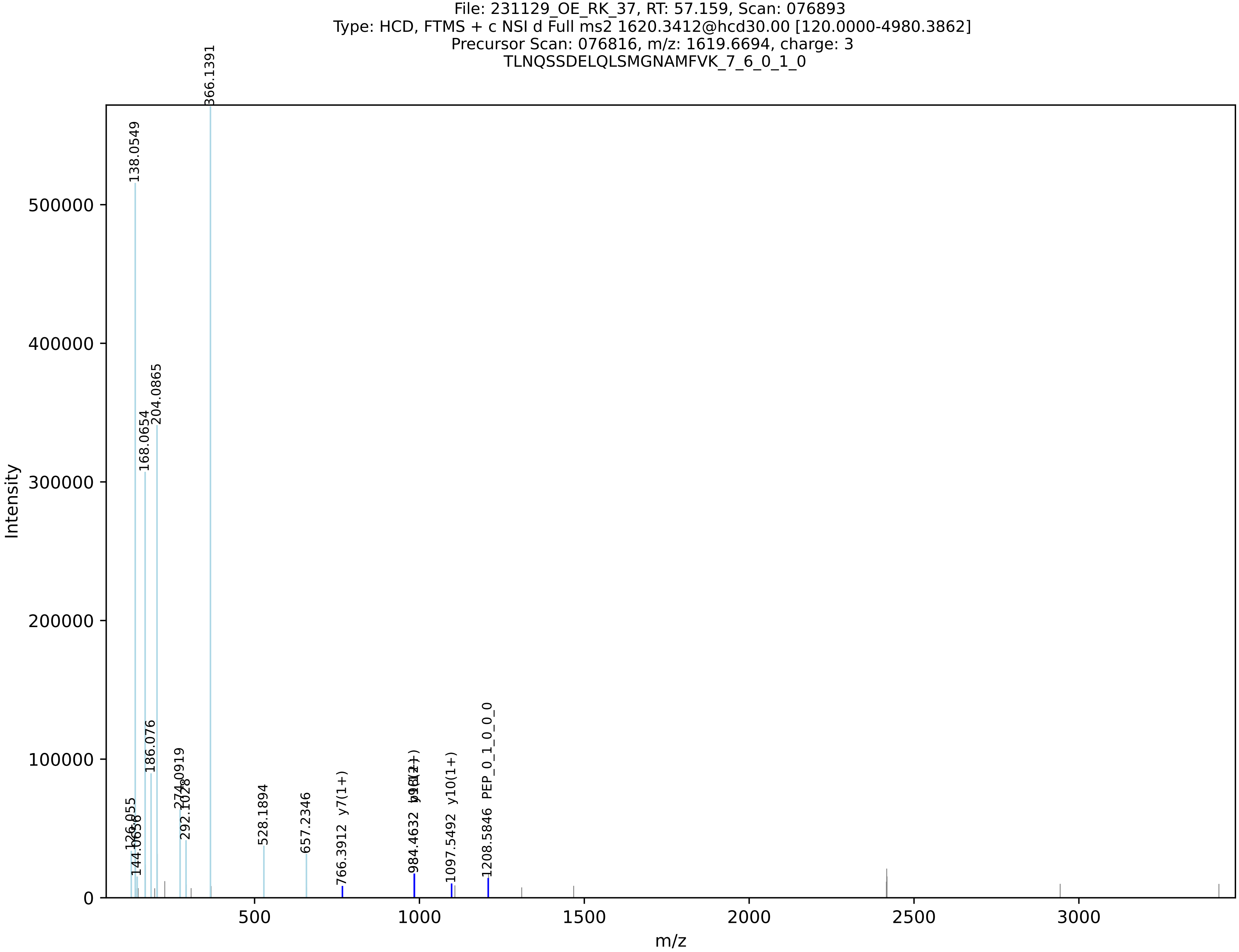
File: 231129_OE_RK_37, RT: 57.159, Scan: 076893
Type: HCD, FTMS + c NSI d Full ms2 1620.3412@hcd30.00 [120.0000-4980.3862]
Precursor Scan: 076816, m/z: 1619.6694, charge: 3
TLNQSSDELQLSMGNAMFVK_7_6_0_1_0
366.1391
138.0549
204.0865
168.0654
186.076
274.0919
292.1028
126.055
144.0658
528.1894
657.2346
766.3912 y7(1+)
984.4632
1097.5492 y10(1+)
1208.5846 PEP_0_1_0_0_0
Intensity
m/z
0
100000
200000
300000
400000
500000
500
1000
1500
2000
2500
3000

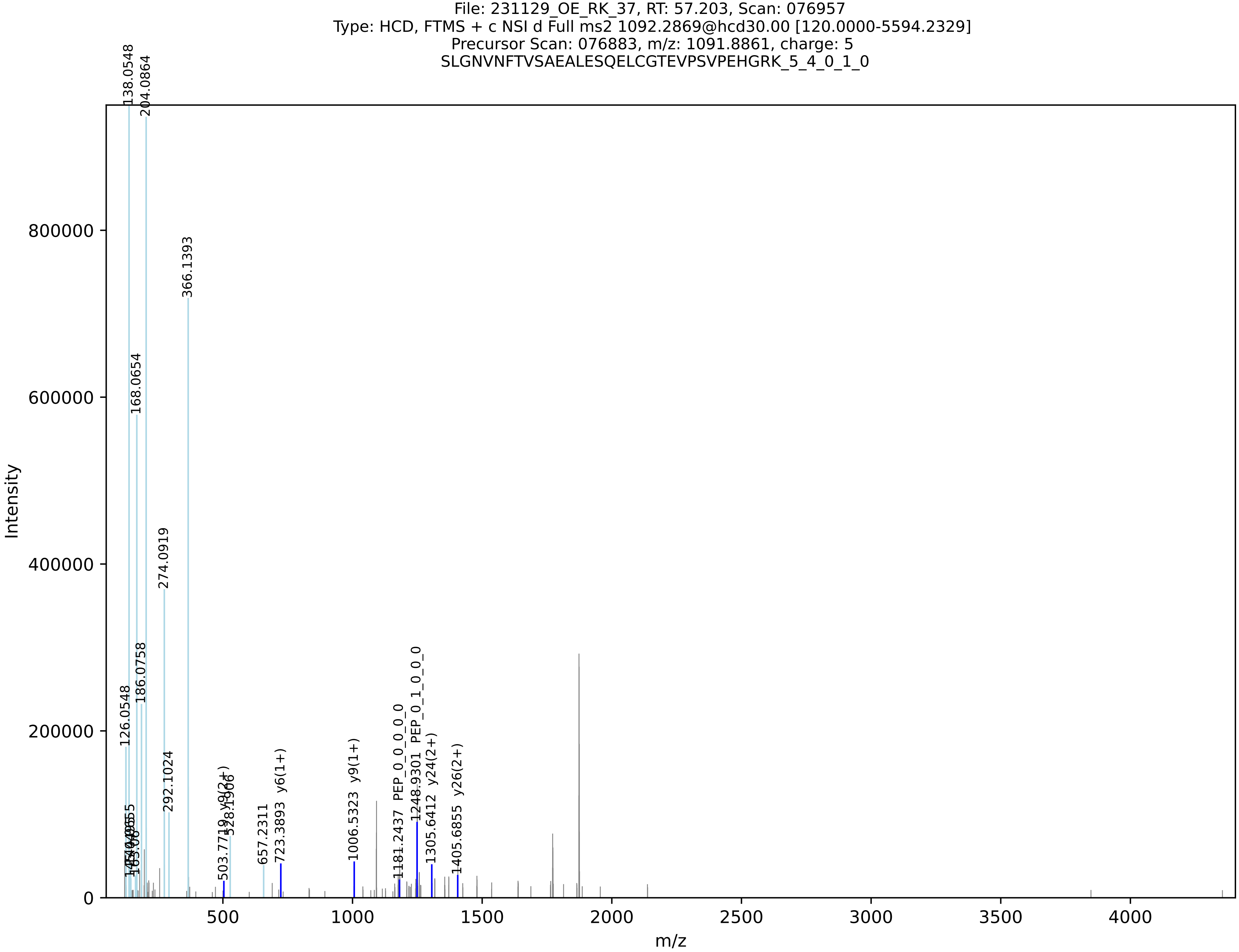
File: 231129_OE_RK_37, RT: 57.203, Scan: 076957
Type: HCD, FTMS + c NSI d Full ms2 1092.2869@hcd30.00 [120.0000-5594.2329]
Precursor Scan: 076883, m/z: 1091.8861, charge: 5
SLGNVNFTVSAEALESQELCGTEVPSVPEHGRK_5_4_0_1_0
138.0548
204.0864
366.1393
168.0654
274.0919
186.0758
126.0548
292.1024
503.7719 y9(2+)
528.1906
657.2311
723.3893 y6(1+)
1006.5323 y9(1+)
1181.2437 PEP_0_0_0_0_0
1248.9301 PEP_0_1_0_0_0
1305.6412 y24(2+)
1405.6855 y26(2+)
Intensity
m/z
0
200000
400000
600000
800000
500
1000
1500
2000
2500
3000
3500
4000

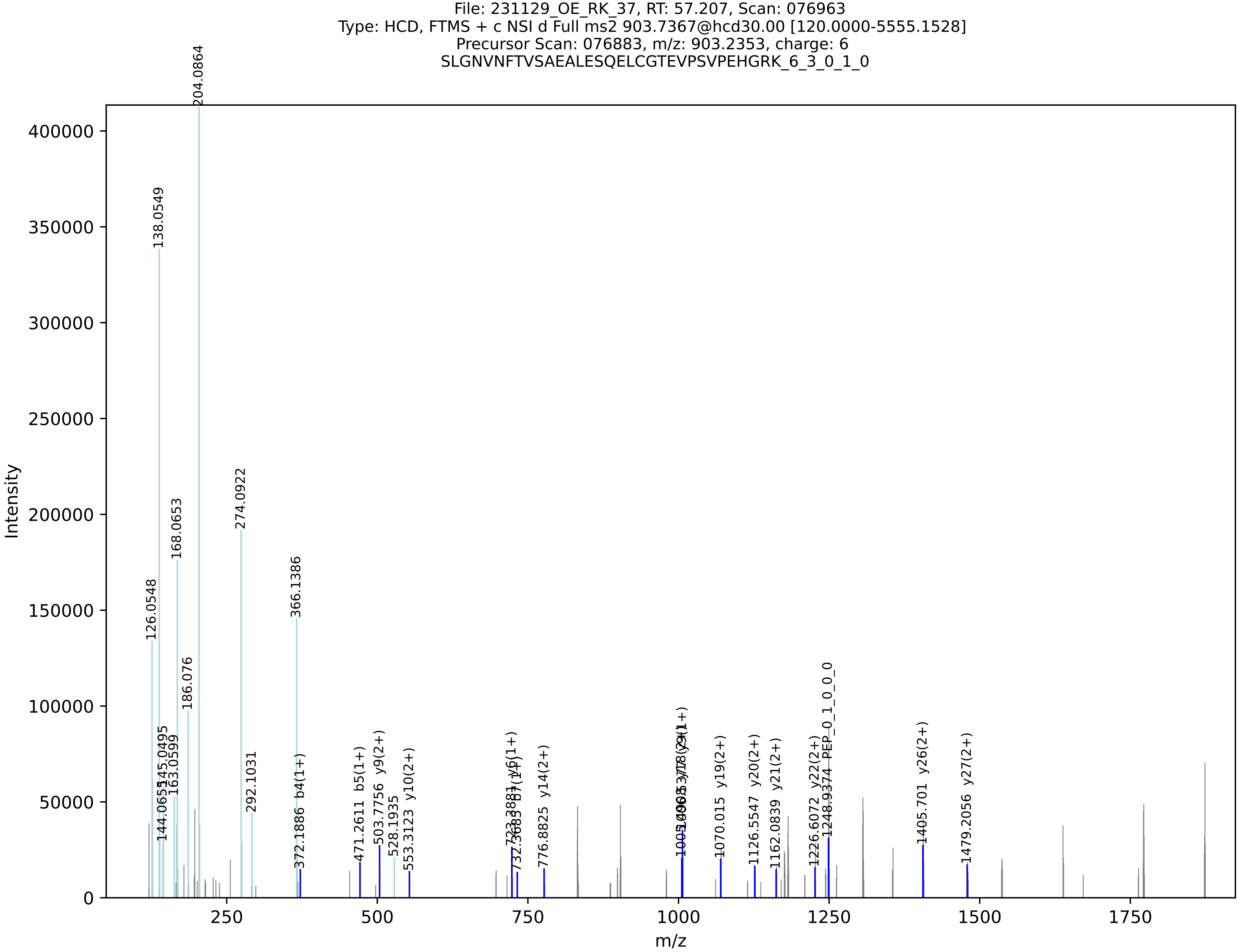
File: 231129_OE_RK_37, RT: 57.207, Scan: 076963
Type: HCD, FTMS + c NSI d Full ms2 903.7367@hcd30.00 [120.0000-5555.1528]
Precursor Scan: 076883, m/z: 903.2353, charge: 6
SLGNVNFTVSAEALESQELCGTEVPSVPEHGRK_6_3_0_1_0
Intensity
m/z
204.0864
138.0549
274.0922
168.0653
126.0548
366.1386
186.076
292.1031
144.0655
145.0495
163.0599
372.1886 b4(1+)
471.2611 b5(1+)
503.7756 y9(2+)
528.1935
553.3123 y10(2+)
723.3881 y6(1+)
776.8825 y14(2+)
1070.015 y19(2+)
1126.5547 y20(2+)
1162.0839 y21(2+)
1226.6072 y22(2+)
1248.9374 PEP_0_1_0_0_0
1405.701 y26(2+)
1479.2056 y27(2+)

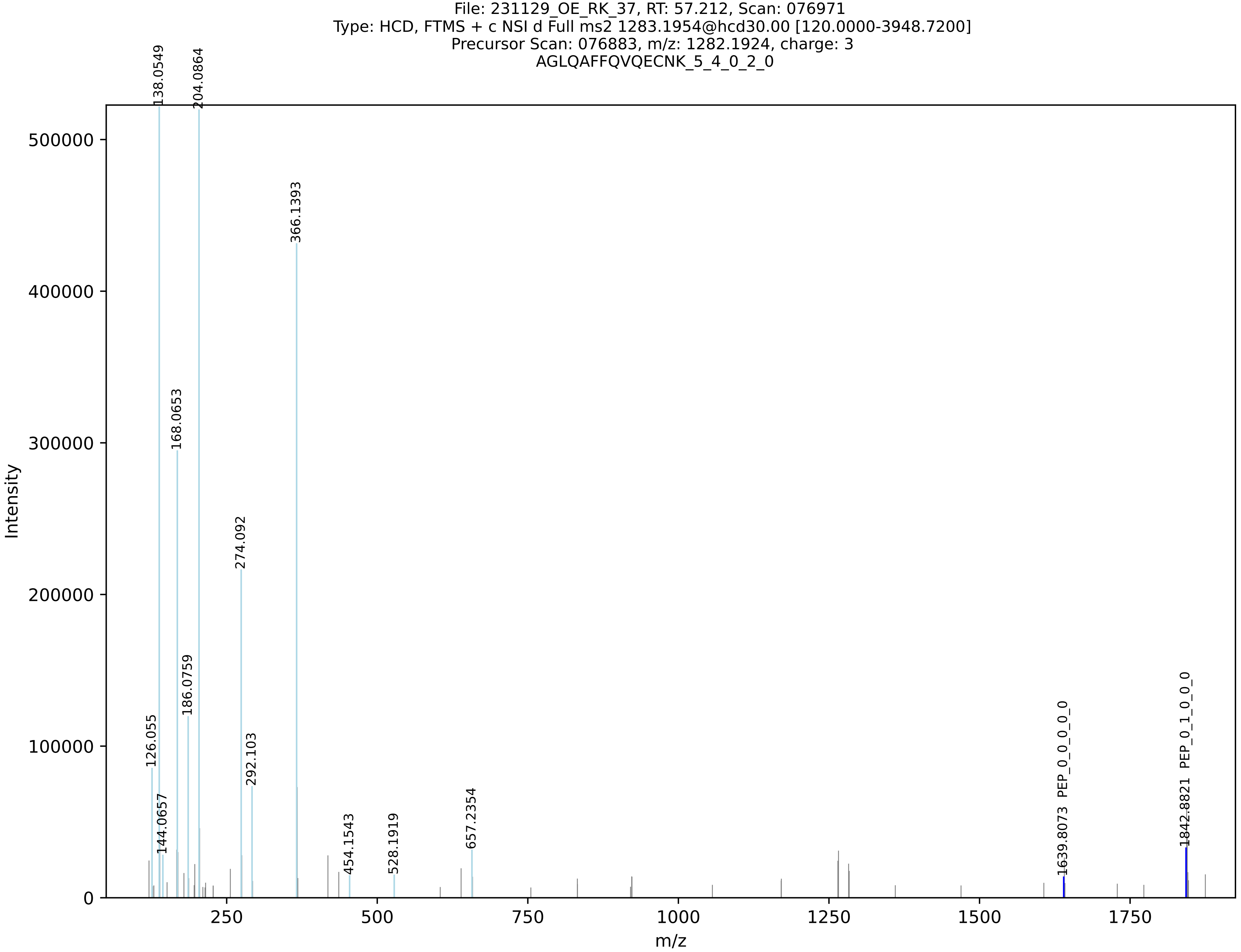

File: 231129_OE_RK_37, RT: 57.212, Scan: 076971
Type: HCD, FTMS + c NSI d Full ms2 1283.1954@hcd30.00 [120.0000-3948.7200]
Precursor Scan: 076883, m/z: 1282.1924, charge: 3
AGLQAFFQVQECNK_5_4_0_2_0
138.0549
204.0864
366.1393
168.0653
274.092
186.0759
126.055
292.103
144.0657
454.1543
528.1919
657.2354
1639.8073 PEP_0_0_0_0_0
1842.8821 PEP_0_1_0_0_0
Intensity
m/z
500000
400000
300000
200000
100000
0
250
500
750
1000
1250
1500
1750

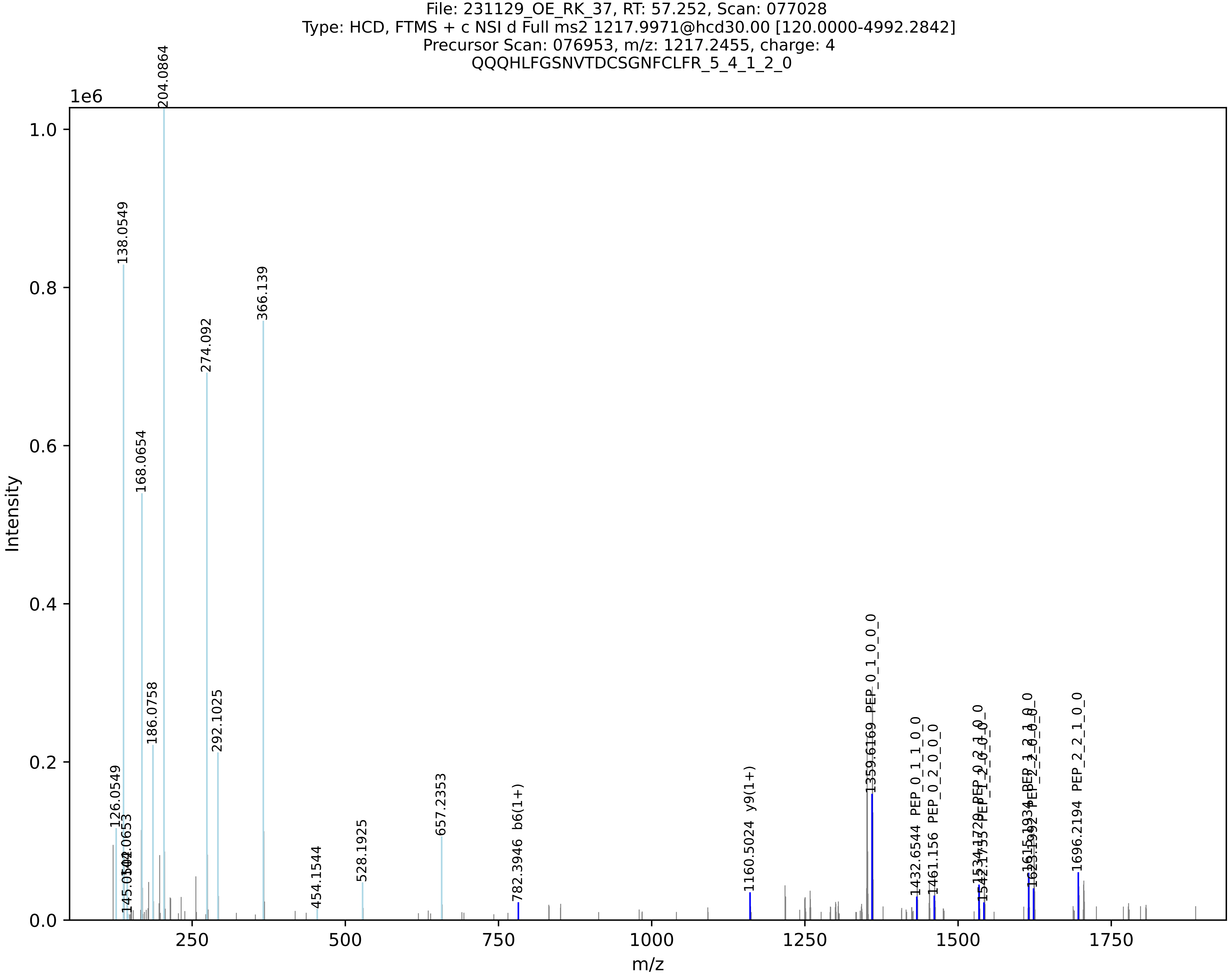
File: 231129_OE_RK_37, RT: 57.252, Scan: 077028
Type: HCD, FTMS + c NSI d Full ms2 1217.9971@hcd30.00 [120.0000-4992.2842]
Precursor Scan: 076953, m/z: 1217.2455, charge: 4
QQQHLFGSNVTDCSGNFCLFR_5_4_1_2_0
1e6
Intensity
m/z
204.0864
138.0549
366.139
274.092
168.0654
186.0758
292.1025
126.0549
657.2353
528.1925
454.1544
782.3946 b6(1+)
1160.5024 y9(1+)
1359.6169 PEP_0_1_0_0_0
1432.6544 PEP_0_1_1_0_0
1461.156 PEP_0_2_0_0_0
1696.2194 PEP_2_2_1_0_0

File: 231129_OE_RK_37, RT: 57.256, Scan: 077034
Type: HCD, FTMS + c NSI d Full ms2 1199.2321@hcd30.00 [120.0000-4915.7231]
Precursor Scan: 076953, m/z: 1198.4805, charge: 4
SPYYNVSDEISFHCYDGYTLR_5_4_0_2_0

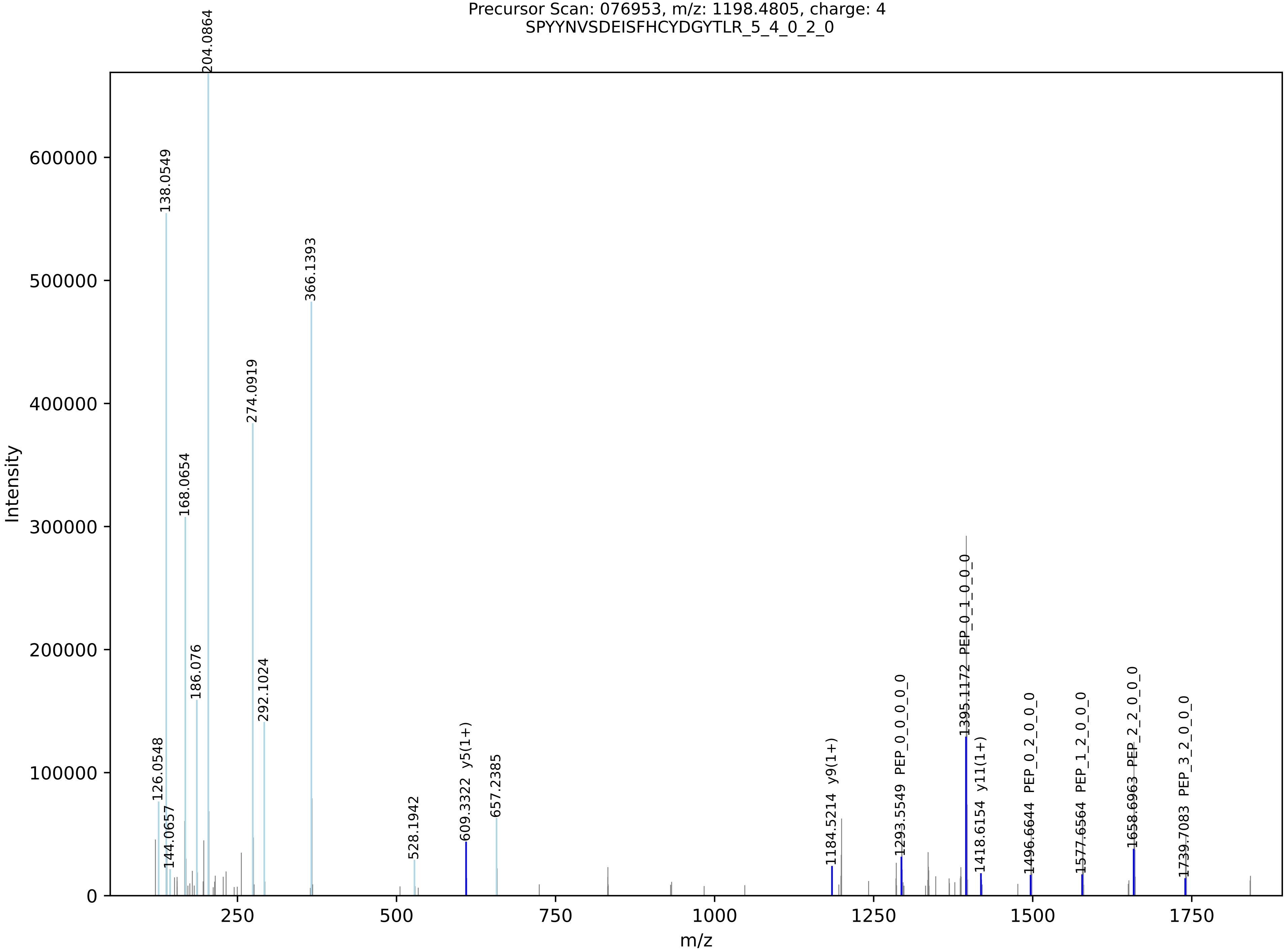

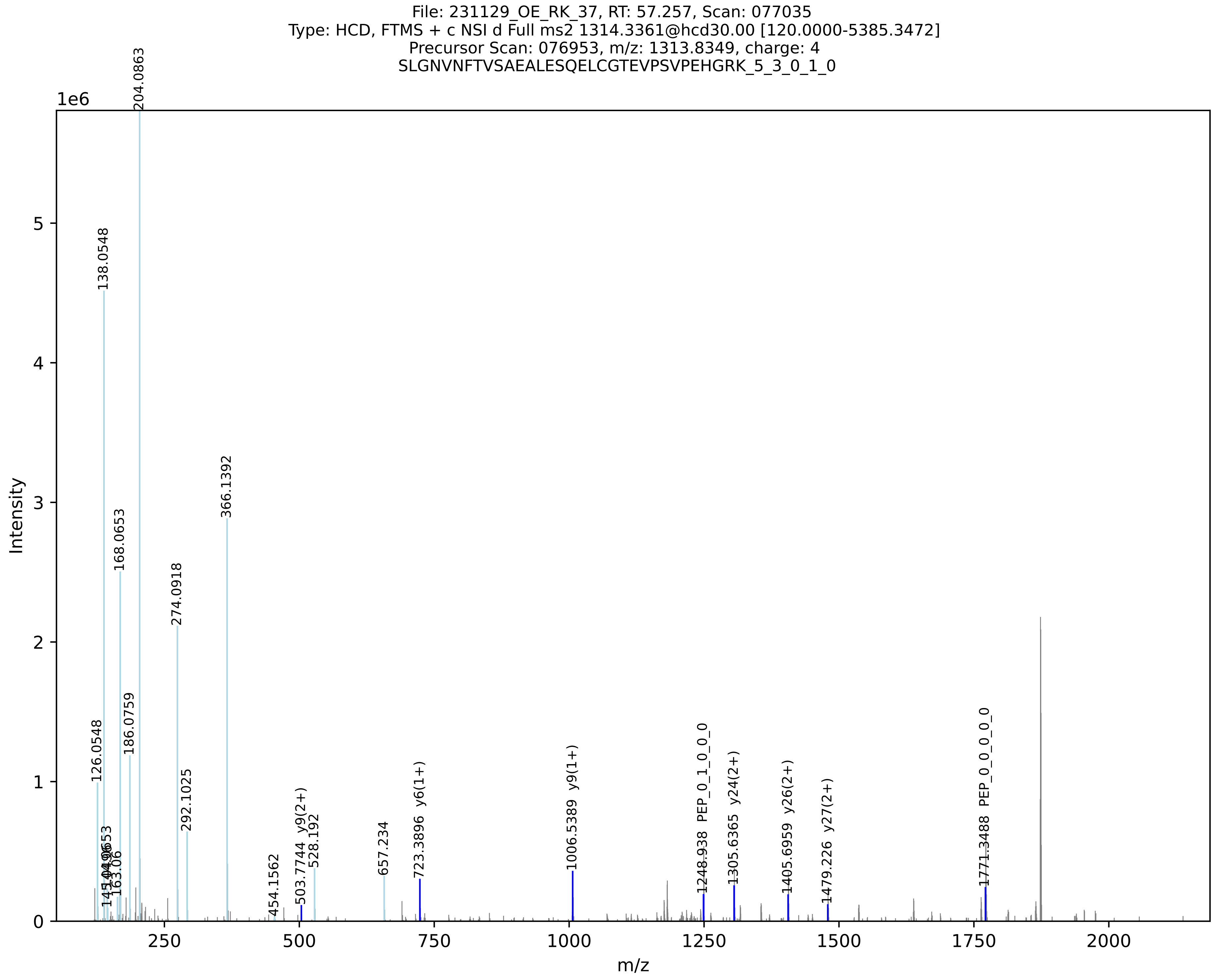
File: 231129_OE_RK_37, RT: 57.257, Scan: 077035
Type: HCD, FTMS + c NSI d Full ms2 1314.3361@hcd30.00 [120.0000-5385.3472]
Precursor Scan: 076953, m/z: 1313.8349, charge: 4
SLGNVNFTVSAEALESQELCGTEVPSVPEHGRK_5_3_0_1_0
1e6
Intensity
m/z
204.0863
138.0548
168.0653
366.1392
274.0918
186.0759
126.0548
292.1025
163.06
454.1562
503.7744 y9(2+)
528.192
657.234
723.3896 y6(1+)
1006.5389 y9(1+)
1248.938 PEP_0_1_0_0_0
1305.6365 y24(2+)
1405.6959 y26(2+)
1479.226 y27(2+)
1771.3488 PEP_0_0_0_0_0

File: 231129_OE_RK_37, RT: 57.26, Scan: 077041
Type: HCD, FTMS + c NSI d Full ms2 1054.0660@hcd30.00 [120.0000-5399.3066]
Precursor Scan: 076953, m/z: 1053.8652, charge: 5
NTTSYVLRGLEPGQEYNVLLTAEK_6_5_0_2_0

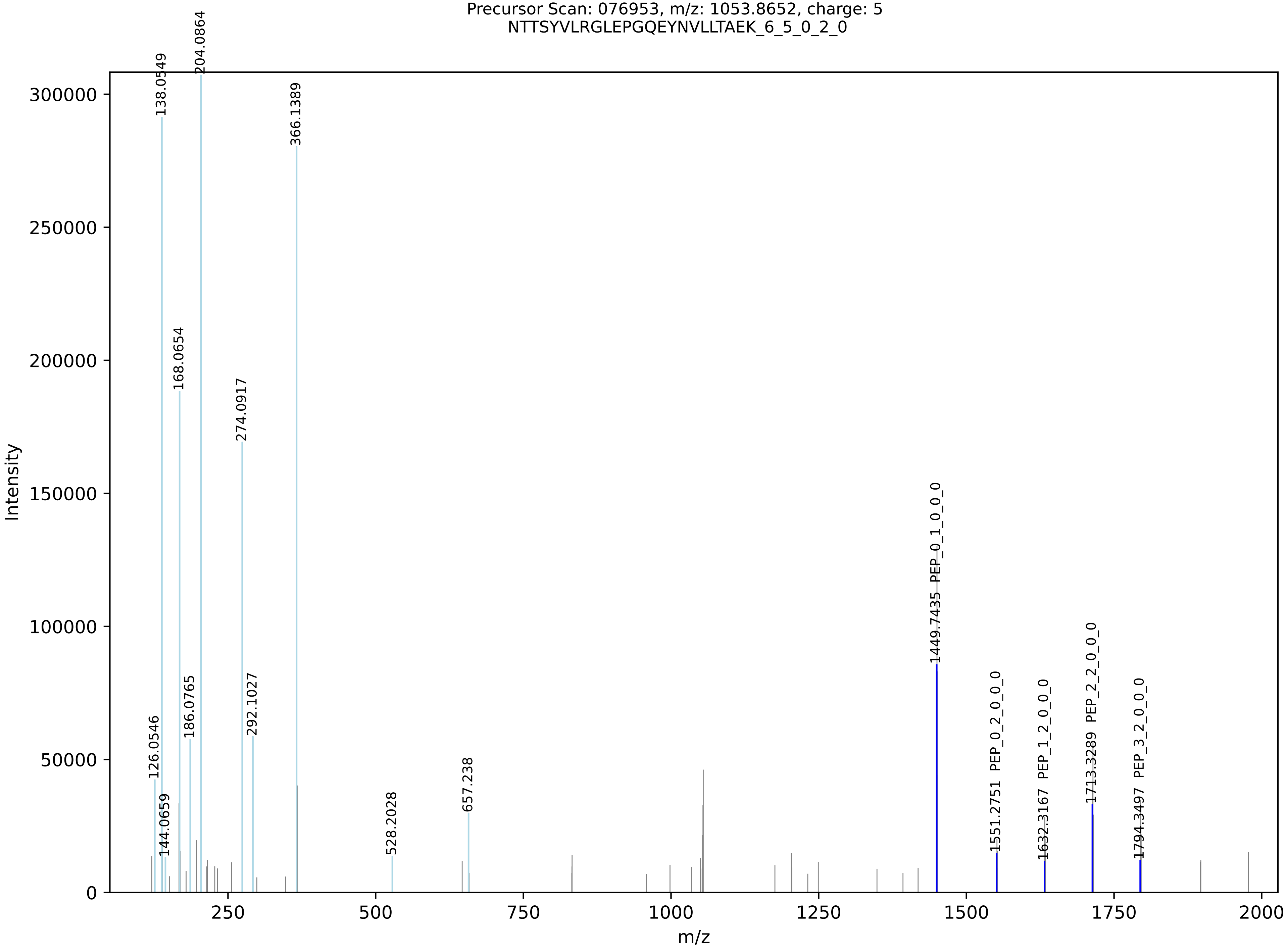

File: 231129_OE_RK_37, RT: 57.27, Scan: 077057
Type: HCD, FTMS + c NSI d Full ms2 1051.8719@hcd30.00 [120.0000-5388.1172]
Precursor Scan: 076953, m/z: 1051.2694, charge: 5
SLGNVNFTVSAEALESQELCGTEVPSVPEHGRK_5_3_0_1_0

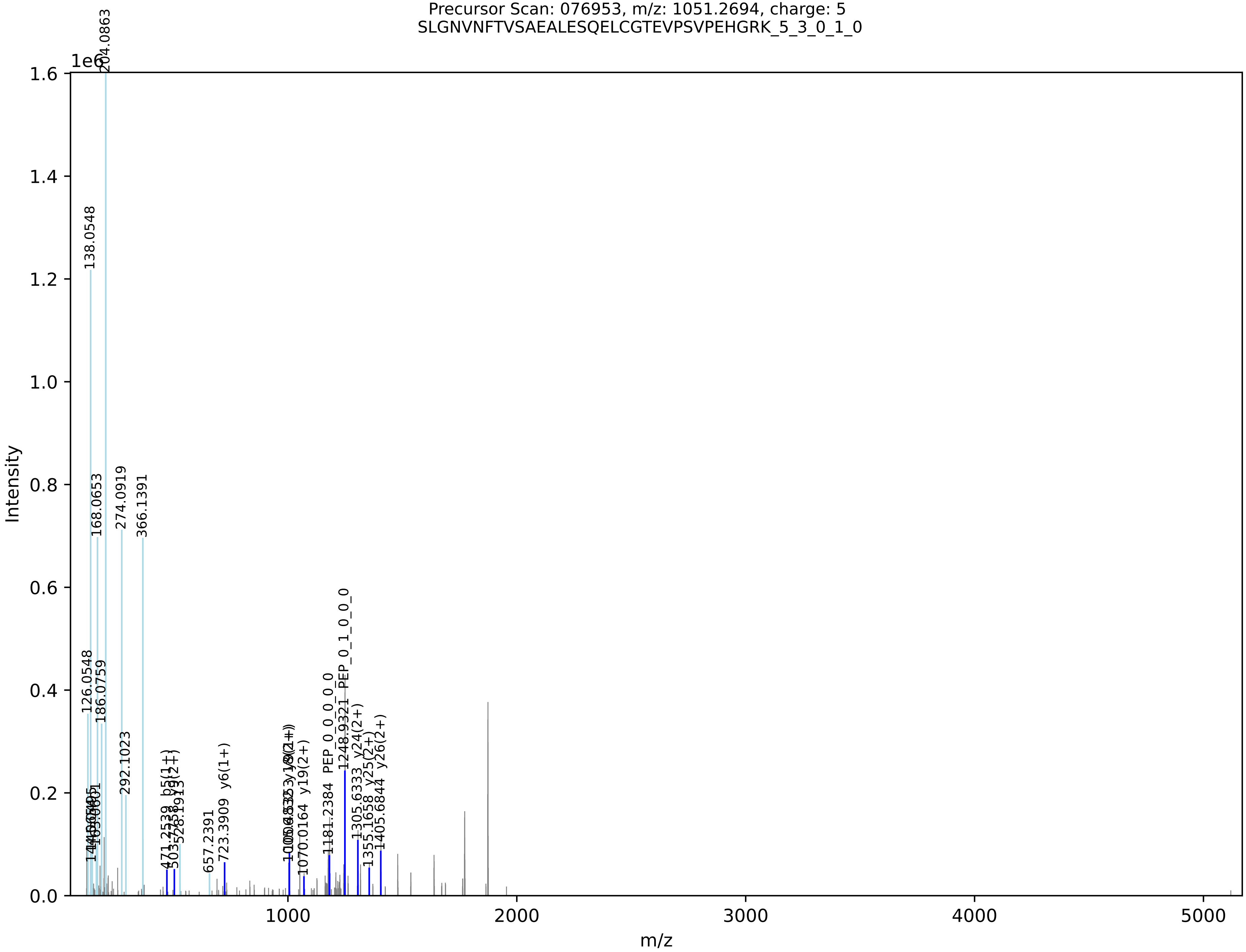

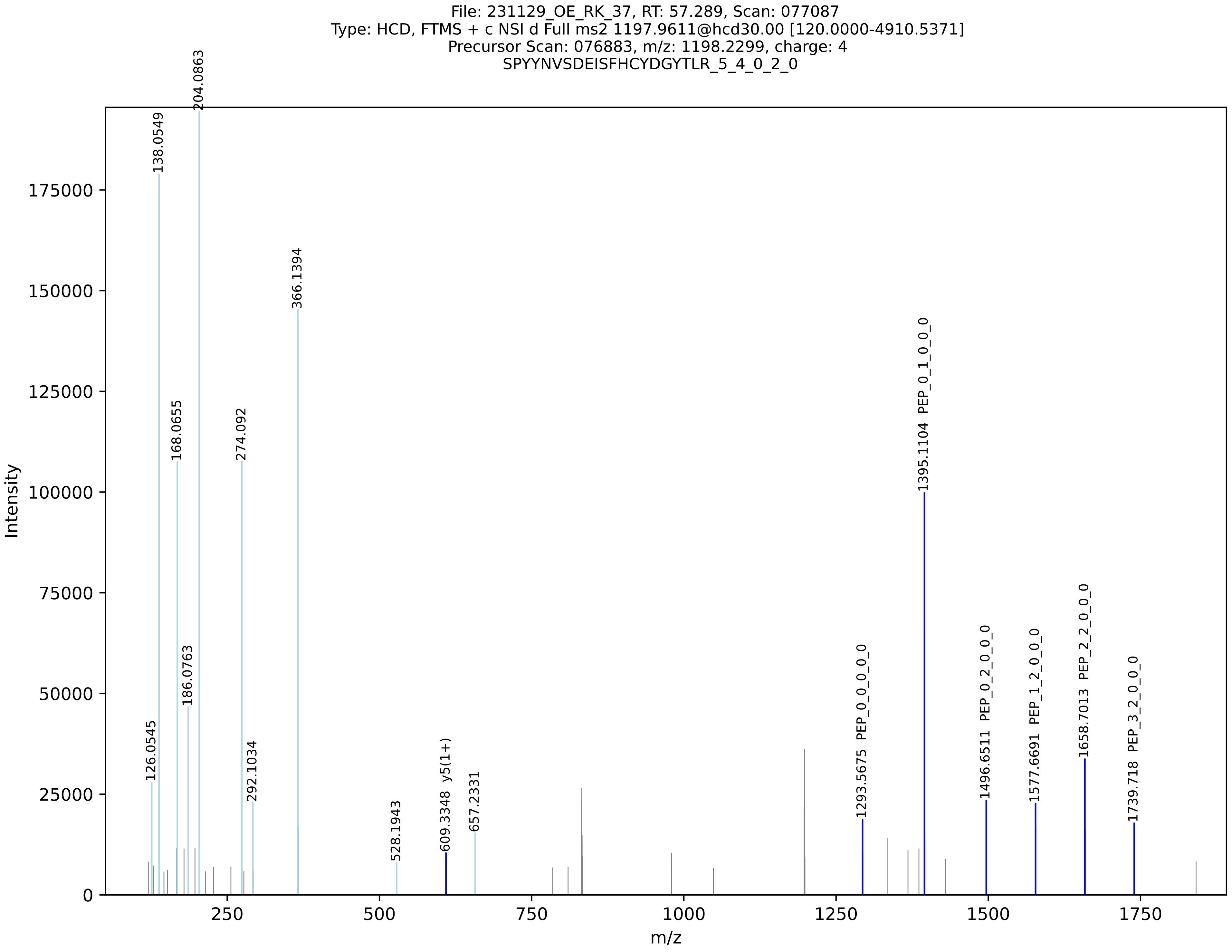
File: 231129_OE_RK_37, RT: 57.289, Scan: 077087
Type: HCD, FTMS + c NSI d Full ms2 1197.9611@hcd30.00 [120.0000-4910.5371]
Precursor Scan: 076883, m/z: 1198.2299, charge: 4
SPYYNVSDEISFHCYDGYTLR_5_4_0_2_0
Intensity
m/z
204.0863
138.0549
168.0655
186.0763
126.0545
274.092
292.1034
366.1394
528.1943
609.3348 y5(1+)
657.2331
1293.5675 PEP_0_0_0_0_0
1395.1104 PEP_0_1_0_0_0
1496.6511 PEP_0_2_0_0_0
1577.6691 PEP_1_2_0_0_0
1658.7013 PEP_2_2_0_0_0
1739.718 PEP_3_2_0_0_0

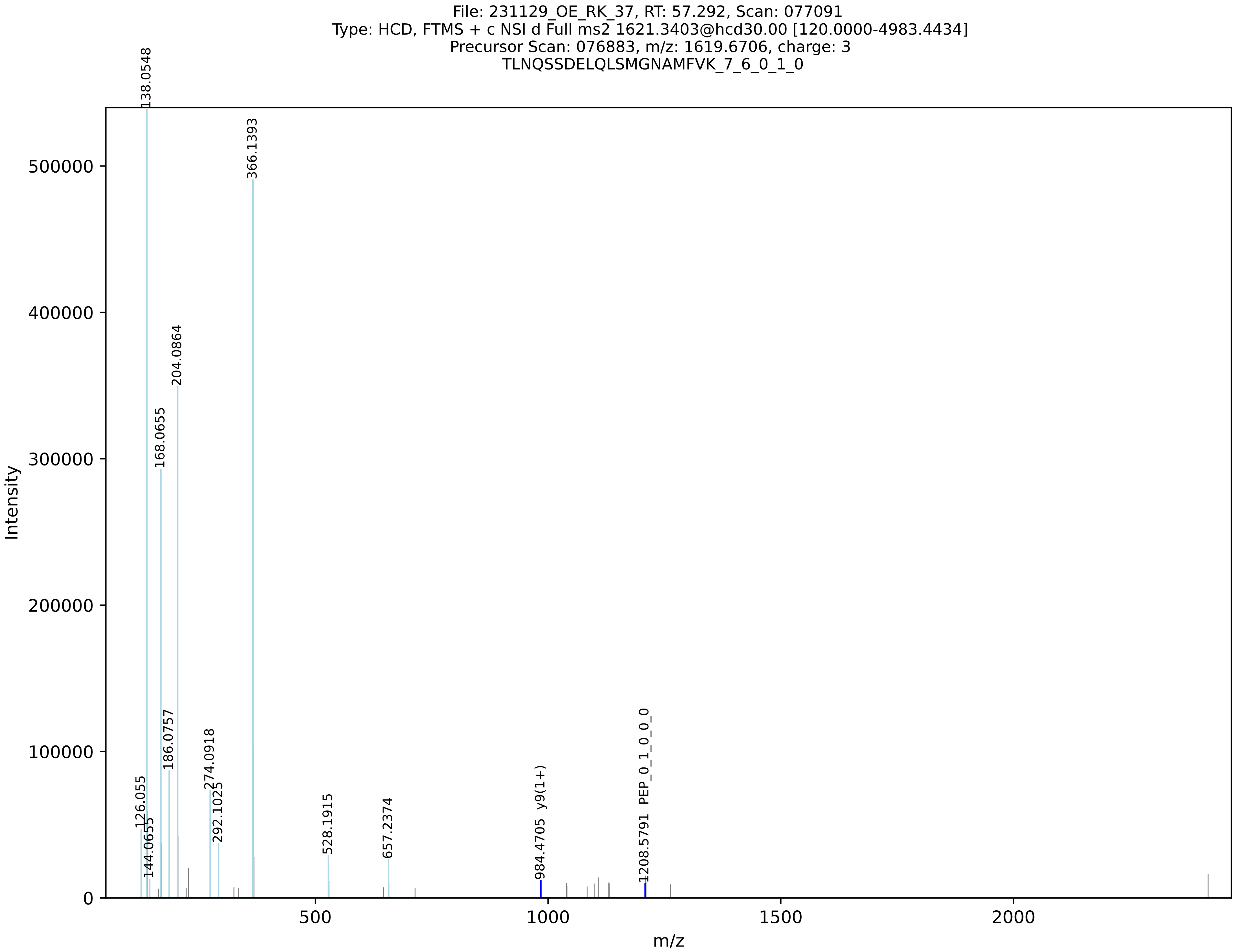
File: 231129_OE_RK_37, RT: 57.292, Scan: 077091
Type: HCD, FTMS + c NSI d Full ms2 1621.3403@hcd30.00 [120.0000-4983.4434]
Precursor Scan: 076883, m/z: 1619.6706, charge: 3
TLNQSSDELQLSMGNAMFVK_7_6_0_1_0
138.0548
366.1393
204.0864
168.0655
186.0757
274.0918
126.055
292.1025
144.0655
528.1915
657.2374
984.4705 y9(1+)
1208.5791 PEP_0_1_0_0_0
Intensity
m/z
0
100000
200000
300000
400000
500000
500
1000
1500
2000

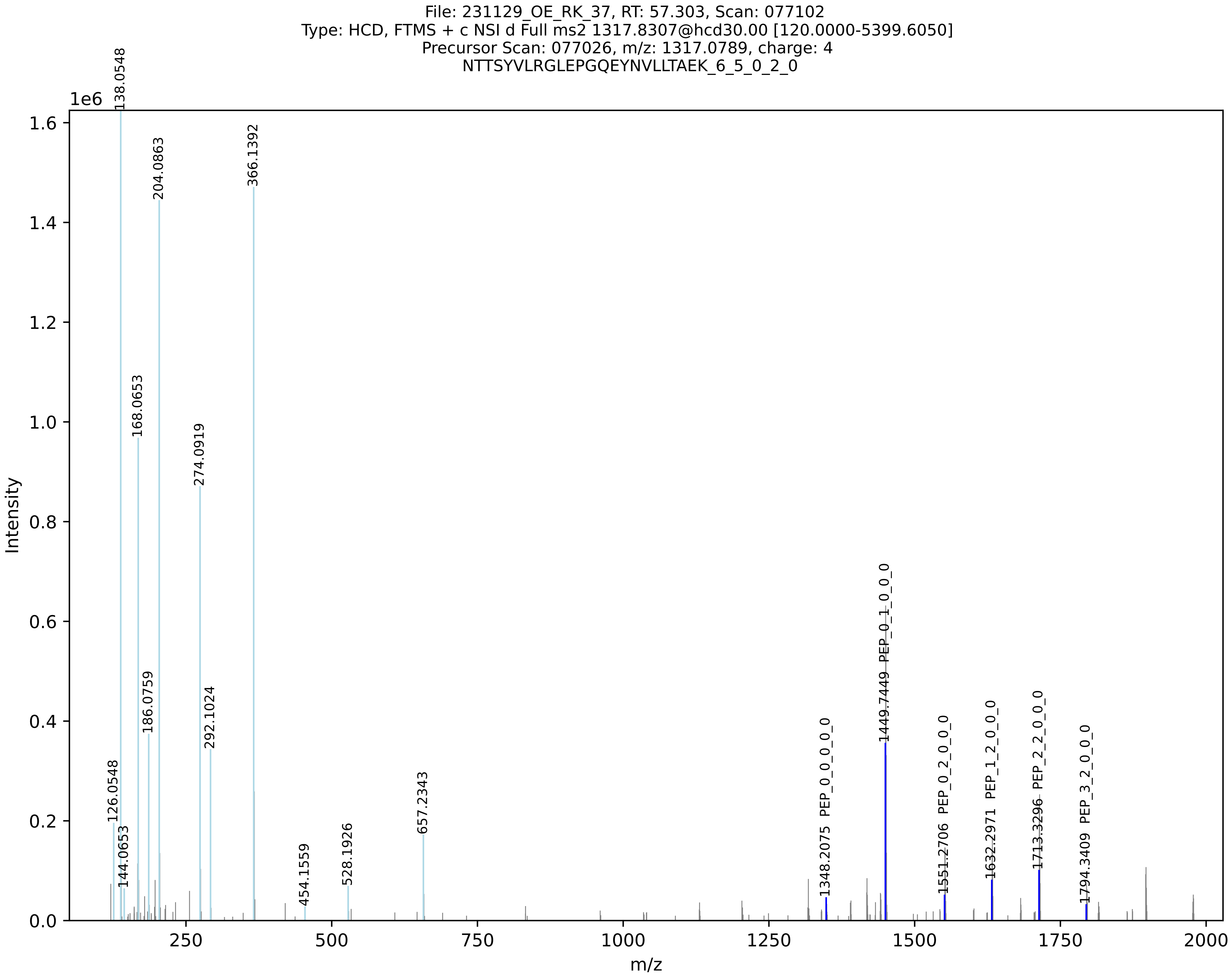
File: 231129_OE_RK_37, RT: 57.303, Scan: 077102
Type: HCD, FTMS + c NSI d Full ms2 1317.8307@hcd30.00 [120.0000-5399.6050]
Precursor Scan: 077026, m/z: 1317.0789, charge: 4
NTTSYVLRGLEPGQEYNVLLTAEK_6_5_0_2_0
1e6
Intensity
m/z
138.0548
204.0863
366.1392
168.0653
274.0919
186.0759
292.1024
126.0548
144.0653
454.1559
528.1926
657.2343
1348.2075 PEP_0_0_0_0_0
1449.7449 PEP_0_1_0_0_0
1551.2706 PEP_0_2_0_0_0
1632.2971 PEP_1_2_0_0_0
1713.3296 PEP_2_2_0_0_0
1794.3409 PEP_3_2_0_0_0

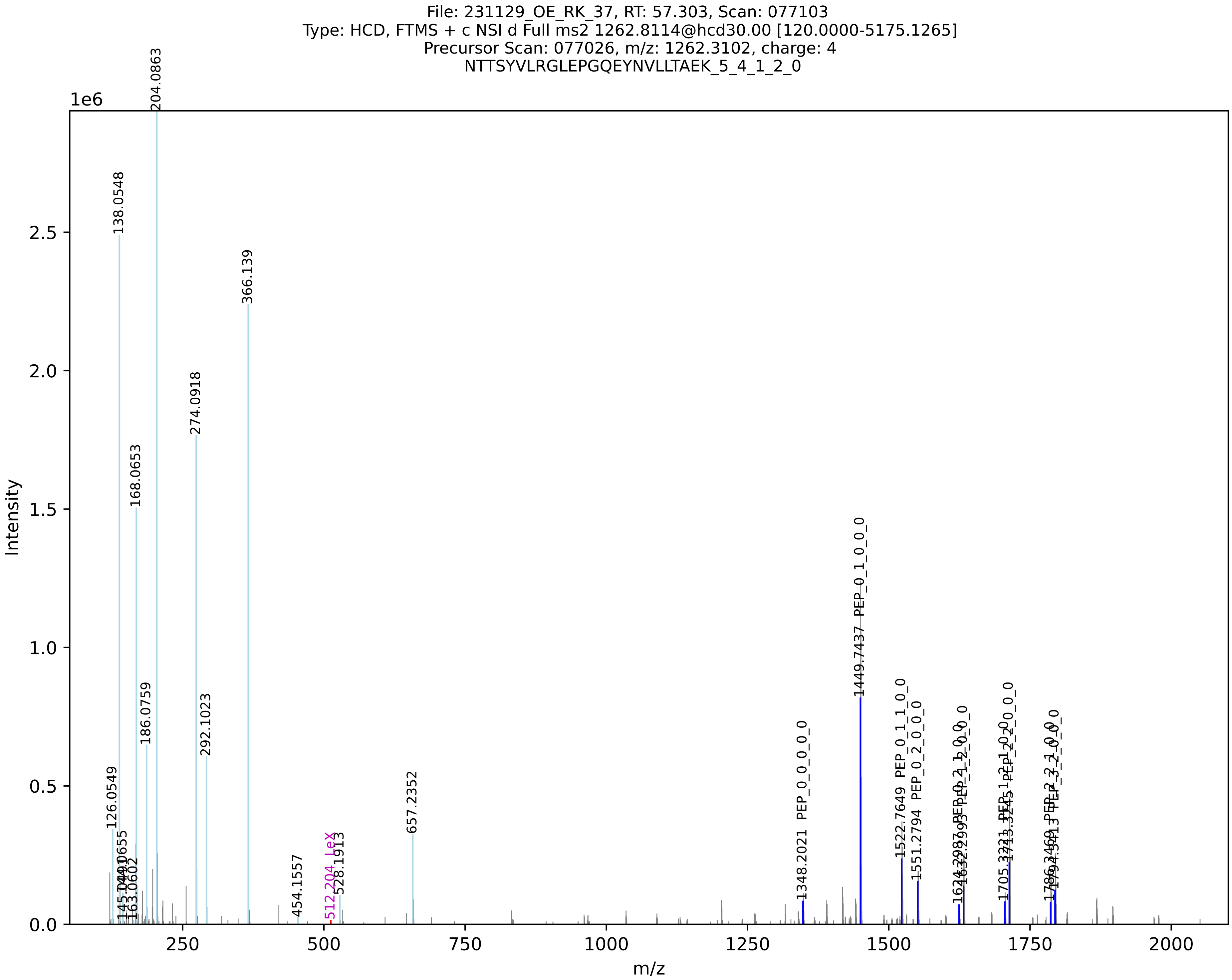
File: 231129_OE_RK_37, RT: 57.303, Scan: 077103
Type: HCD, FTMS + c NSI d Full ms2 1262.8114@hcd30.00 [120.0000-5175.1265]
Precursor Scan: 077026, m/z: 1262.3102, charge: 4
NTTSYVLRGLEPGQEYNVLLTAEK_5_4_1_2_0
1e6
Intensity
m/z
204.0863
138.0548
366.139
274.0918
168.0653
186.0759
292.1023
126.0549
163.0602
454.1557
512.204_LeX
528.1913
657.2352
1348.2021 PEP_0_0_0_0_0
1449.7437 PEP_0_1_0_0_0
1522.7649 PEP_0_1_1_0_0
1551.2794 PEP_0_2_0_0_0
1705.3221
1786.3469
0.0
0.5
1.0
1.5
2.0
2.5
250
500
750
1000
1250
1500
1750
2000

File: 231129_OE_RK_37, RT: 57.305, Scan: 077105
Type: HCD, FTMS + c NSI d Full ms2 1405.1155@hcd30.00 [120.0000-5755.7271]
Precursor Scan: 077026, m/z: 1404.8845, charge: 4
MVSHHNLTTGATLINEQWLLTTAK_7_6_0_2_0

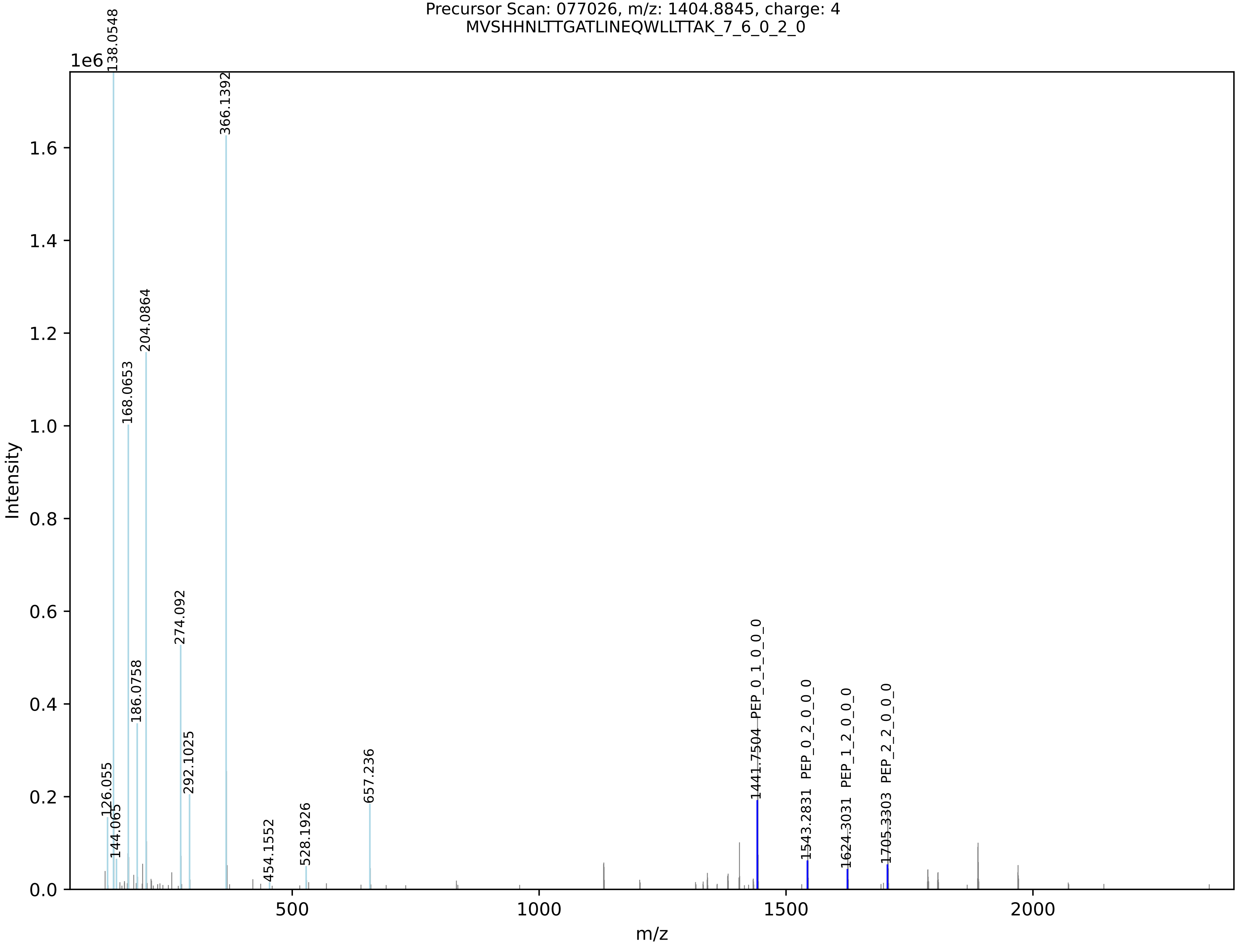

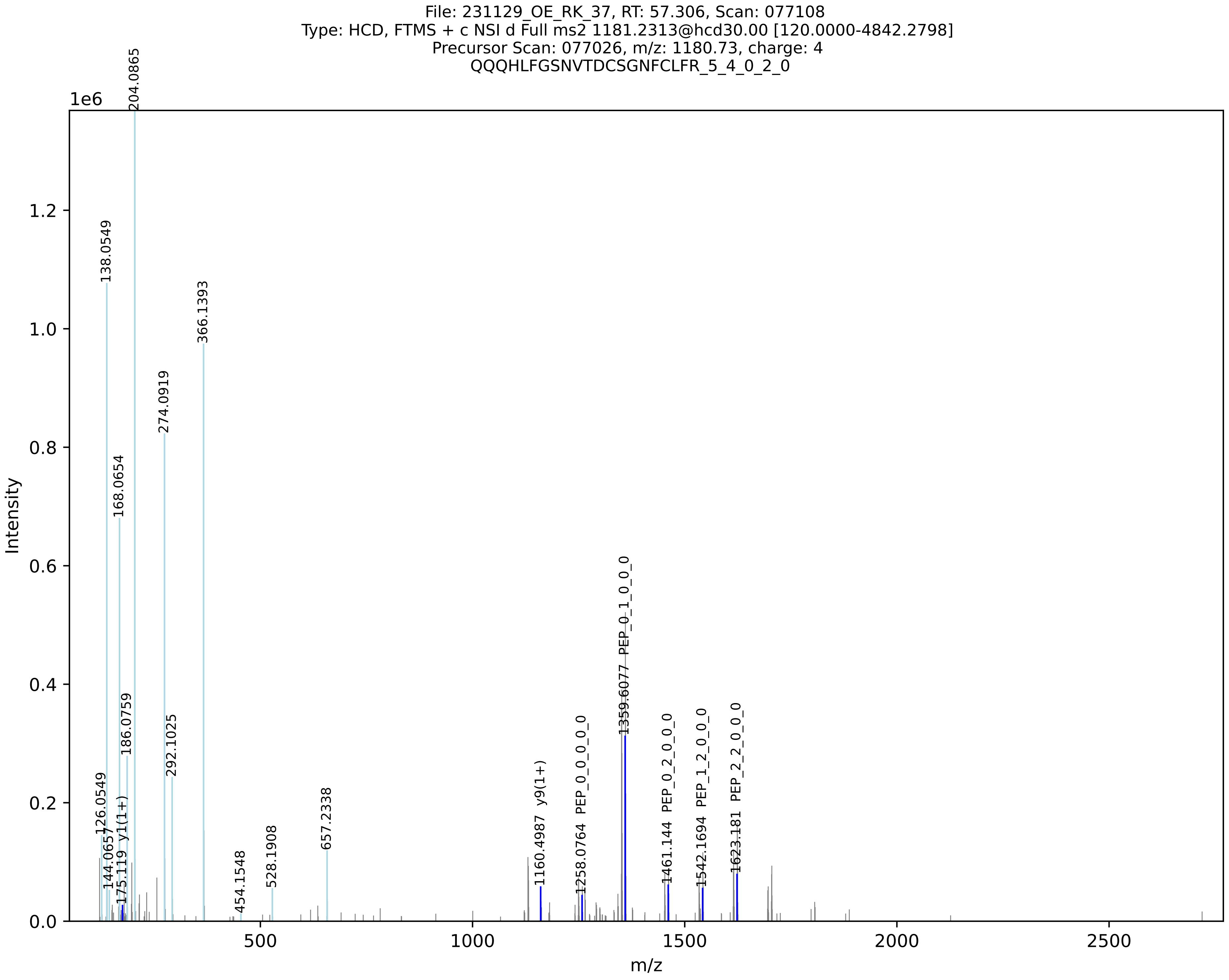

File: 231129_OE_RK_37, RT: 57.306, Scan: 077108
Type: HCD, FTMS + c NSI d Full ms2 1181.2313@hcd30.00 [120.0000-4842.2798]
Precursor Scan: 077026, m/z: 1180.73, charge: 4
QQQHLFGSNVTDCSGNFCLFR_5_4_0_2_0
1e6
Intensity
m/z
204.0865
138.0549
366.1393
274.0919
168.0654
186.0759
292.1025
126.0549
144.0657
175.119 y1(1+)
454.1548
528.1908
657.2338
1160.4987 y9(1+)
1258.0764 PEP_0_0_0_0_0
1359.6077 PEP_0_1_0_0_0
1461.144 PEP_0_2_0_0_0
1542.1694 PEP_1_2_0_0_0
1623.181 PEP_2_2_0_0_0

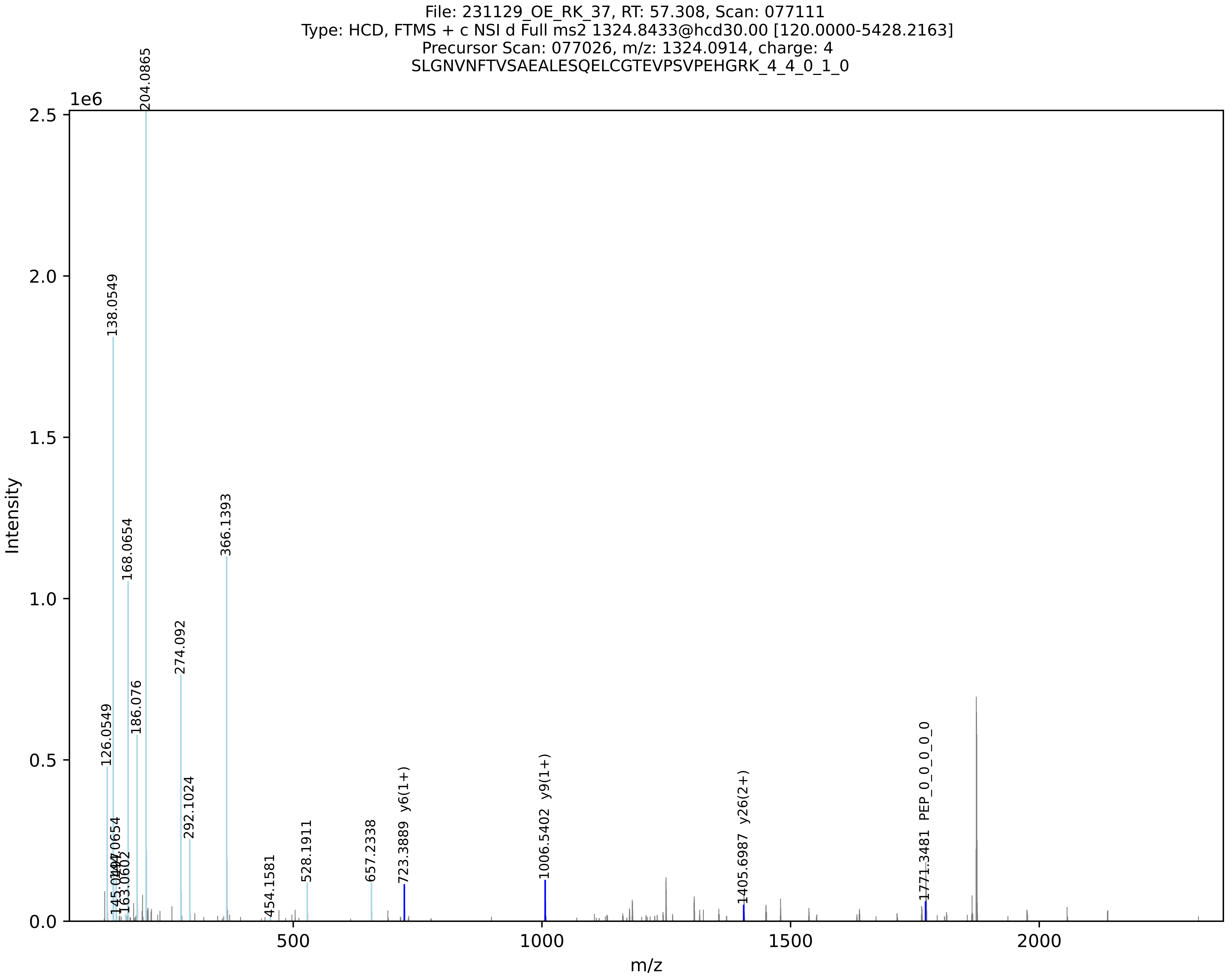

File: 231129_OE_RK_37, RT: 57.308, Scan: 077111
Type: HCD, FTMS + c NSI d Full ms2 1324.8433@hcd30.00 [120.0000-5428.2163]
Precursor Scan: 077026, m/z: 1324.0914, charge: 4
SLGNVNFTVSAEALESQELCGTEVPSVPEHGRK_4_4_0_1_0
1e6
Intensity
m/z
204.0865
138.0549
168.0654
366.1393
274.092
186.076
126.0549
292.1024
145.0494
147.0654
163.0602
454.1581
528.1911
657.2338
723.3889 y6(1+)
1006.5402 y9(1+)
1405.6987 y26(2+)
1771.3481 PEP_0_0_0_0_0

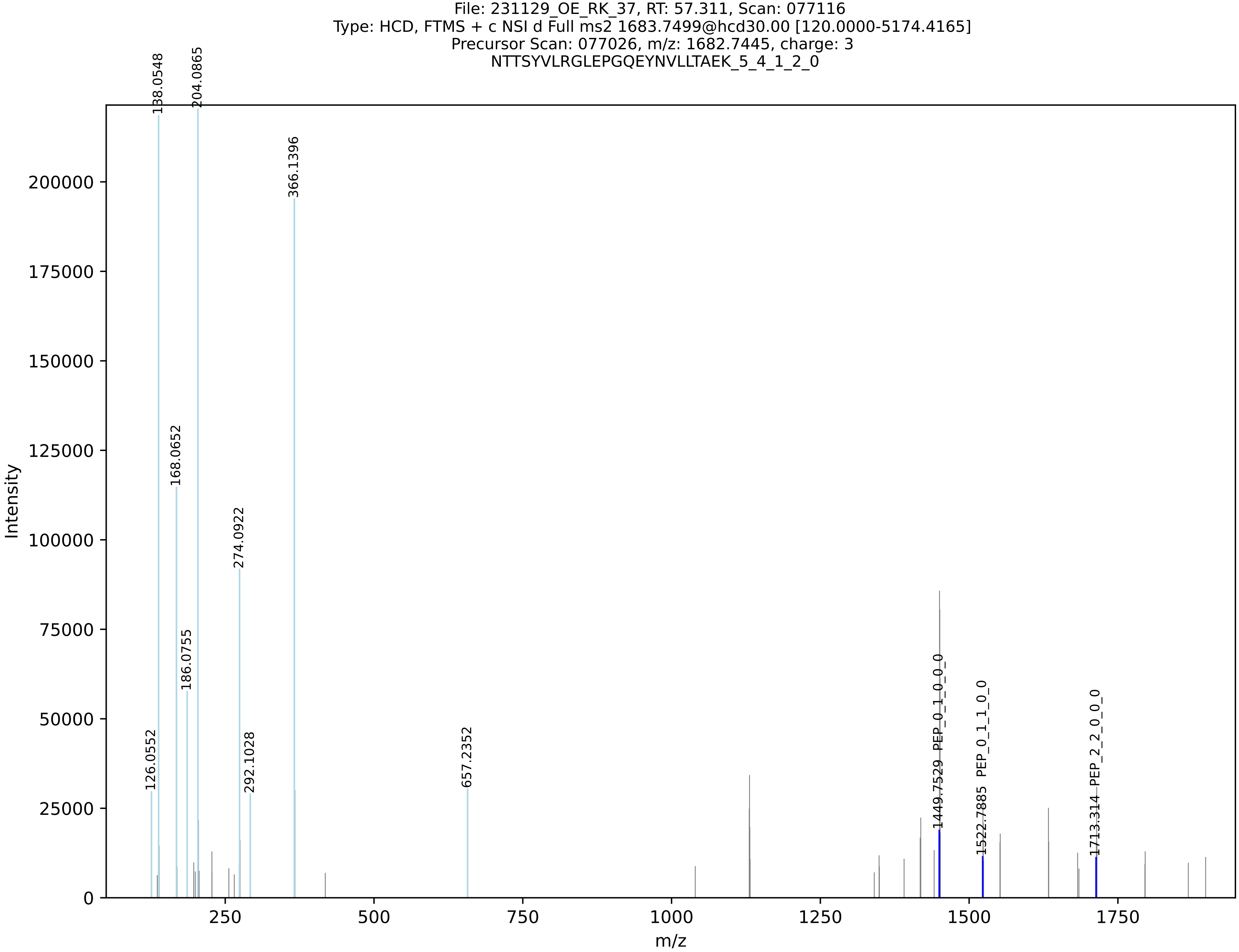

File: 231129_OE_RK_37, RT: 57.311, Scan: 077116
Type: HCD, FTMS + c NSI d Full ms2 1683.7499@hcd30.00 [120.0000-5174.4165]
Precursor Scan: 077026, m/z: 1682.7445, charge: 3
NTTSYVLRGLEPGQEYNVLLTAEK_5_4_1_2_0
138.0548
204.0865
366.1396
168.0652
274.0922
186.0755
126.0552
292.1028
657.2352
1449.7529_PEP_0_1_0_0_0_
1522.7885_PEP_0_1_1_0_0
1713.314_PEP_2_2_0_0_0_
Intensity
m/z
0
25000
50000
75000
100000
125000
150000
175000
200000
250
500
750
1000
1250
1500
1750

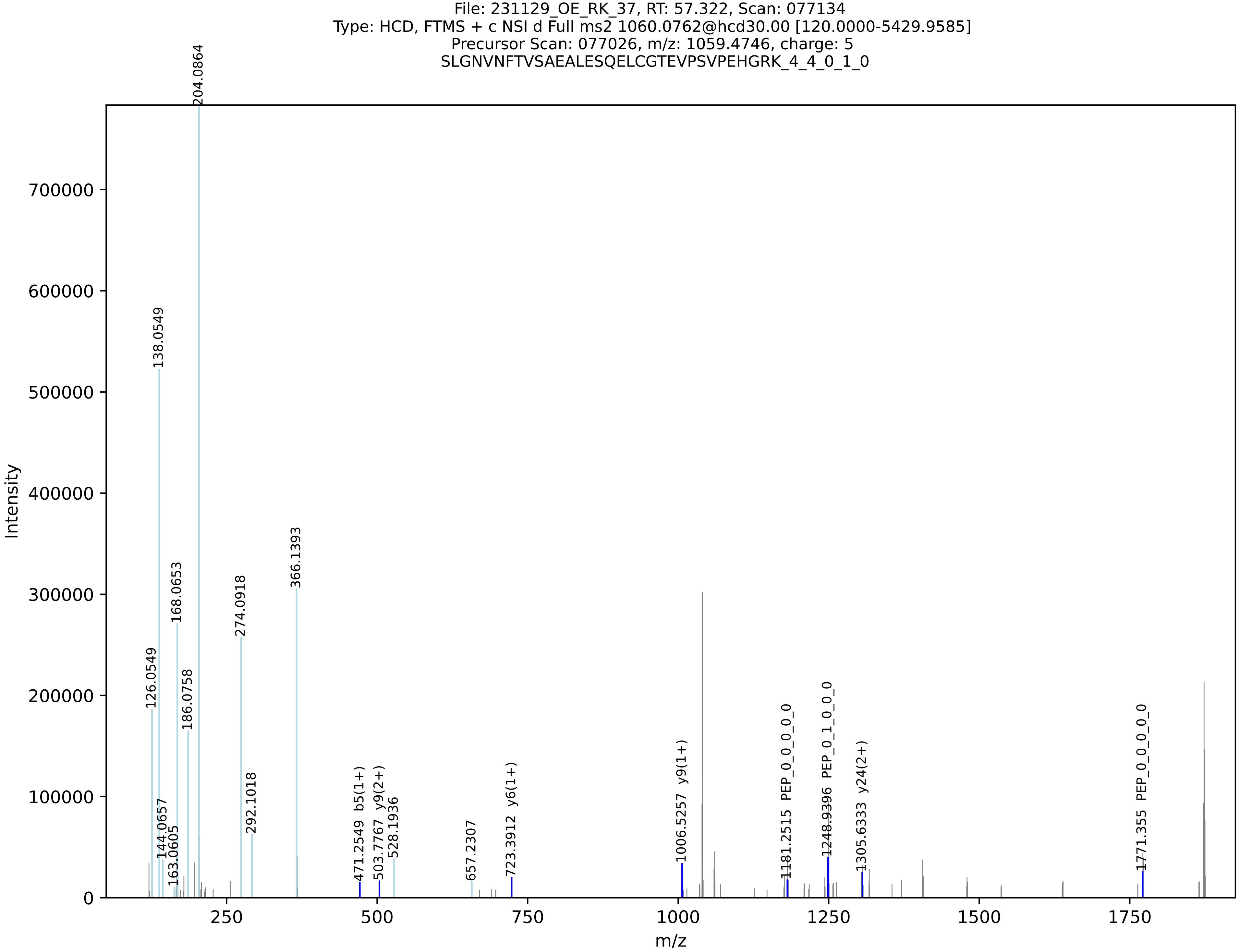

File: 231129_OE_RK_37, RT: 57.322, Scan: 077134
Type: HCD, FTMS + c NSI d Full ms2 1060.0762@hcd30.00 [120.0000-5429.9585]
Precursor Scan: 077026, m/z: 1059.4746, charge: 5
SLGNVNFTVSAEALESQELCGTEVPSVPEHGRK_4_4_0_1_0
Intensity
m/z
0
100000
200000
300000
400000
500000
600000
700000
250
500
750
1000
1250
1500
1750
204.0864
138.0549
126.0549
168.0653
186.0758
144.0657
163.0605
274.0918
292.1018
366.1393
471.2549 b5(1+)
503.7767 y9(2+)
528.1936
657.2307
723.3912 y6(1+)
1006.5257 y9(1+)
1181.2515 PEP_0_0_0_0_0
1248.9396 PEP_0_1_0_0_0
1305.6333 y24(2+)
1771.355 PEP_0_0_0_0_0

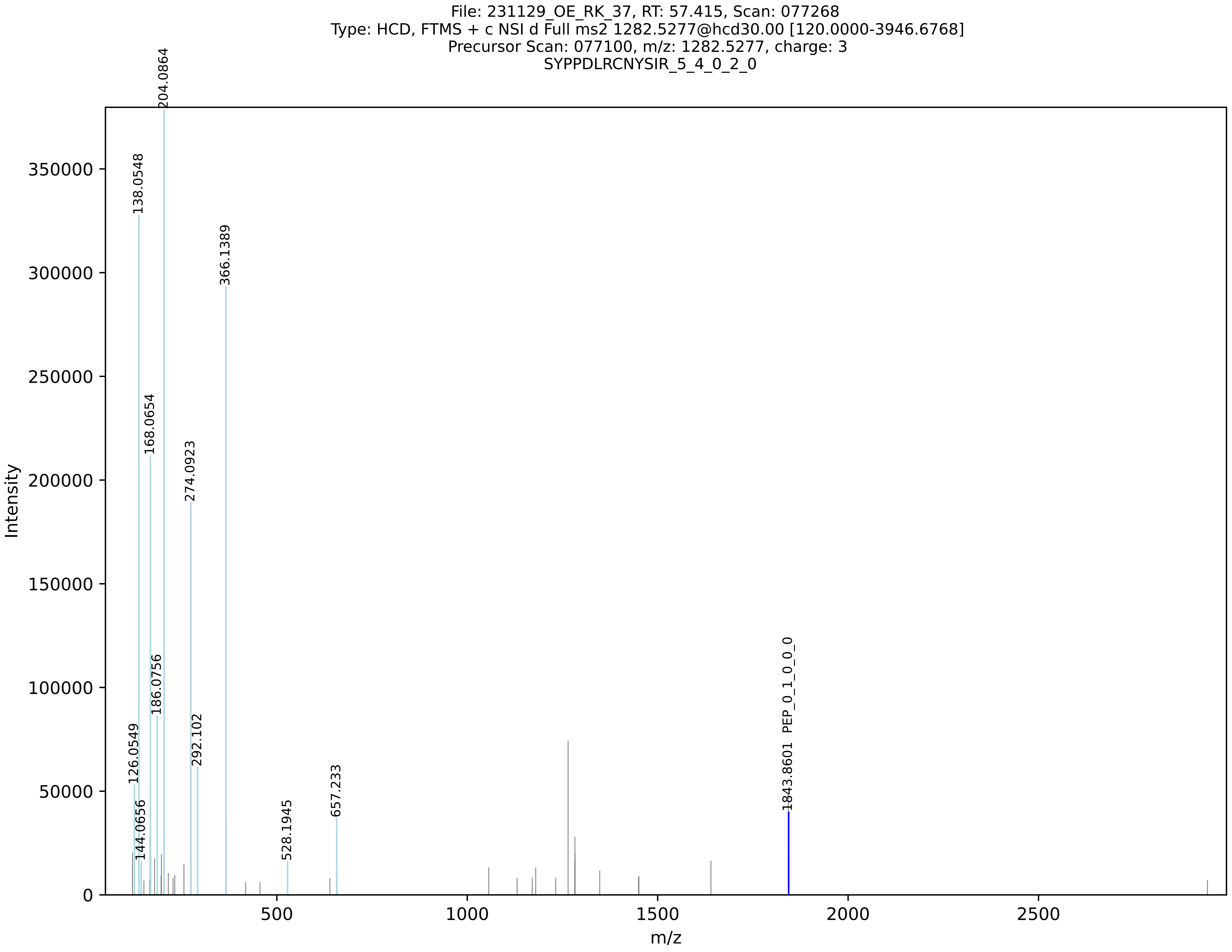
File: 231129_OE_RK_37, RT: 57.415, Scan: 077268
Type: HCD, FTMS + c NSI d Full ms2 1282.5277@hcd30.00 [120.0000-3946.6768]
Precursor Scan: 077100, m/z: 1282.5277, charge: 3
SYPPDLRCNYSIR_5_4_0_2_0
204.0864
138.0548
366.1389
168.0654
274.0923
186.0756
292.102
126.0549
144.0656
528.1945
657.233
PEP_0_1_0_0_0
1843.8601
Intensity
m/z
0
50000
100000
150000
200000
250000
300000
350000
500
1000
1500
2000
2500

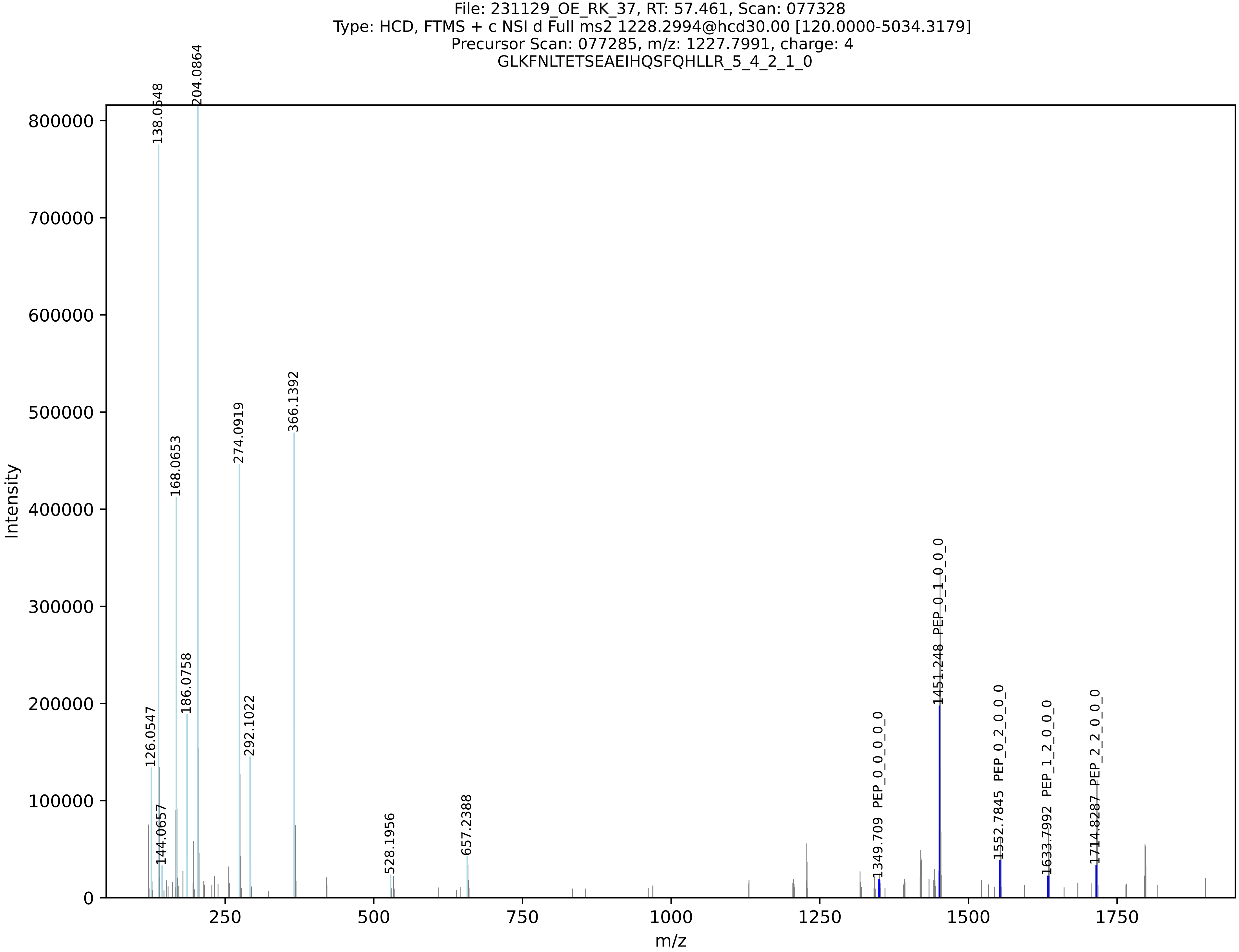

File: 231129_OE_RK_37, RT: 57.461, Scan: 077328
Type: HCD, FTMS + c NSI d Full ms2 1228.2994@hcd30.00 [120.0000-5034.3179]
Precursor Scan: 077285, m/z: 1227.7991, charge: 4
GLKFNLTETSEAEIHQSFQHLLR_5_4_2_1_0
Intensity
m/z
204.0864
138.0548
168.0653
186.0758
126.0547
144.0657
274.0919
292.1022
366.1392
528.1956
657.2388
1349.709 PEP_0_0_0_0_0
1451.248 PEP_0_1_0_0_0
1552.7845 PEP_0_2_0_0_0
1633.7992 PEP_1_2_0_0_0
1714.8287 PEP_2_2_0_0_0

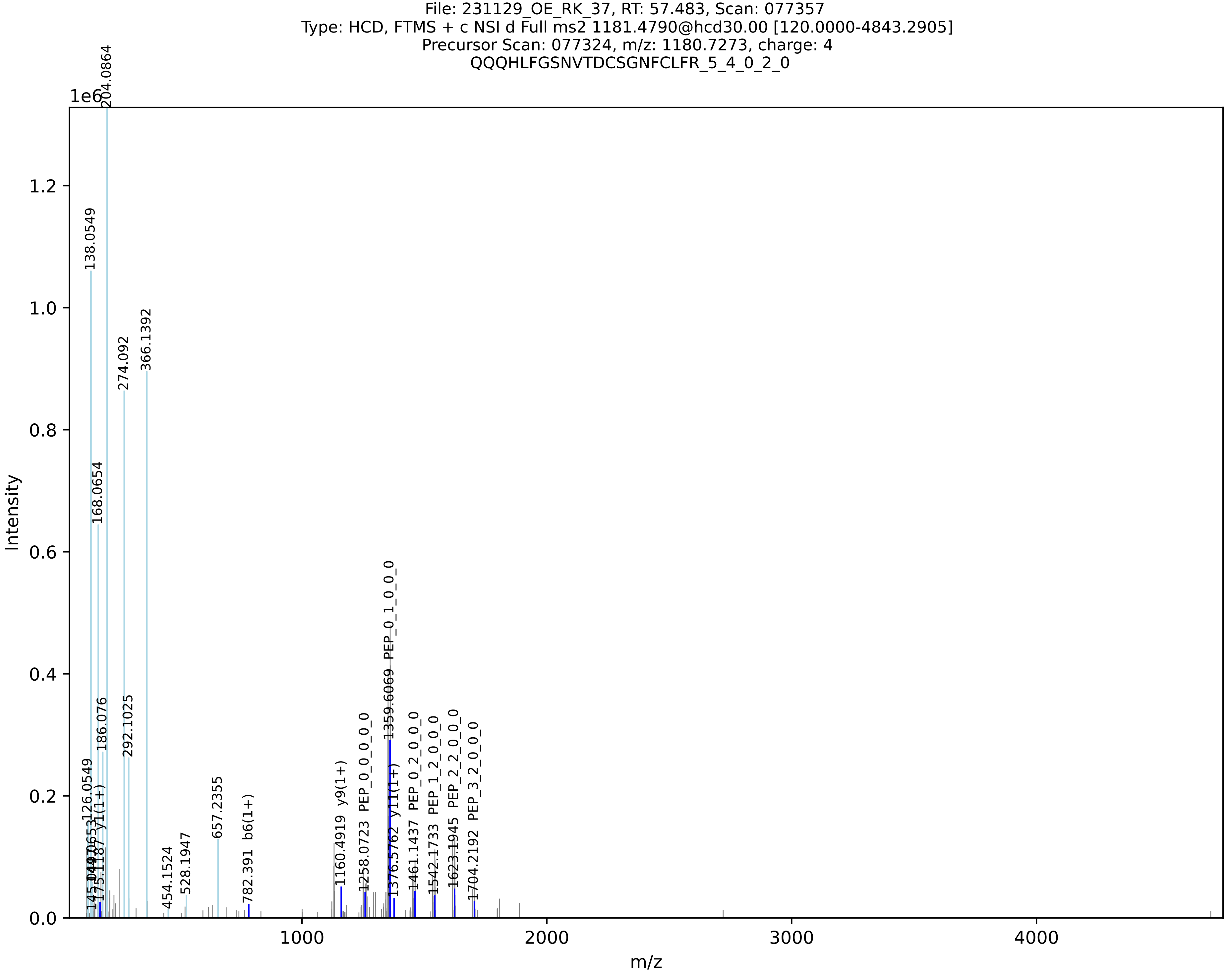
File: 231129_OE_RK_37, RT: 57.483, Scan: 077357
Type: HCD, FTMS + c NSI d Full ms2 1181.4790@hcd30.00 [120.0000-4843.2905]
Precursor Scan: 077324, m/z: 1180.7273, charge: 4
QQQHLFGSNVTDCSGNFCLFR_5_4_0_2_0
1e6
Intensity
m/z
204.0864
138.0549
366.1392
274.092
168.0654
186.076
292.1025
126.0549
175.1187 y1(1+)
454.1524
528.1947
657.2355
782.391 b6(1+)
1160.4919 y9(1+)
1258.0723 PEP_0_0_0_0_0
1359.6069 PEP_0_1_0_0_0
1376.5762 y11(1+)
1461.1437 PEP_0_2_0_0_0
1542.1733 PEP_1_2_0_0_0
1623.1945 PEP_2_2_0_0_0
1704.2192 PEP_3_2_0_0_0

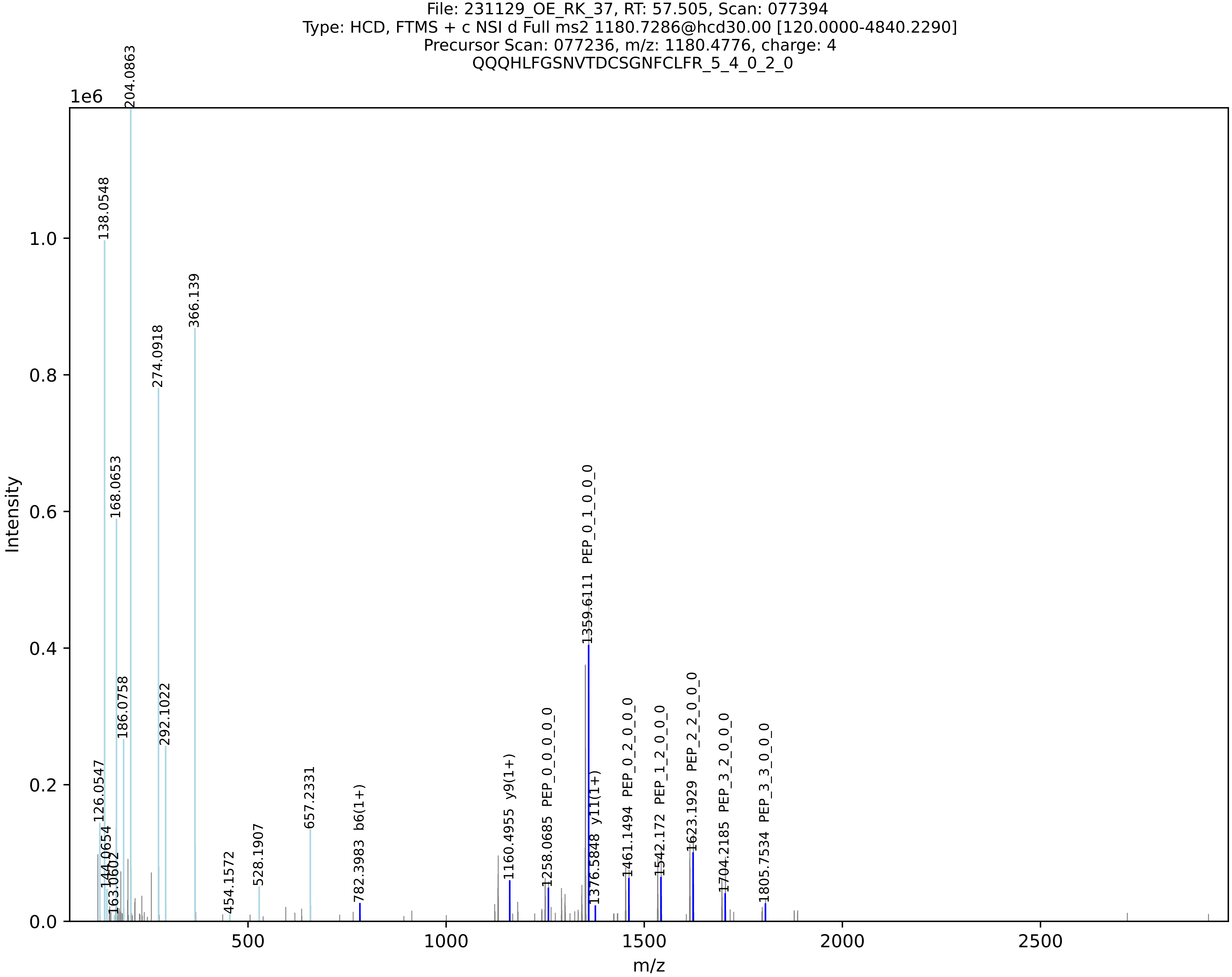
File: 231129_OE_RK_37, RT: 57.505, Scan: 077394
Type: HCD, FTMS + c NSI d Full ms2 1180.7286@hcd30.00 [120.0000-4840.2290]
Precursor Scan: 077236, m/z: 1180.4776, charge: 4
QQQHLFGSNVTDCSGNFCLFR_5_4_0_2_0
1e6
Intensity
m/z
204.0863
138.0548
366.139
274.0918
168.0653
186.0758
292.1022
126.0547
144.0654
163.0602
454.1572
528.1907
657.2331
782.3983 b6(1+)
1160.4955 y9(1+)
1258.0685 PEP_0_0_0_0_0
1359.6111 PEP_0_1_0_0_0
1376.5848 y11(1+)
1461.1494 PEP_0_2_0_0_0
1542.172 PEP_1_2_0_0_0
1623.1929 PEP_2_2_0_0_0
1704.2185 PEP_3_2_0_0_0
1805.7534 PEP_3_3_0_0_0

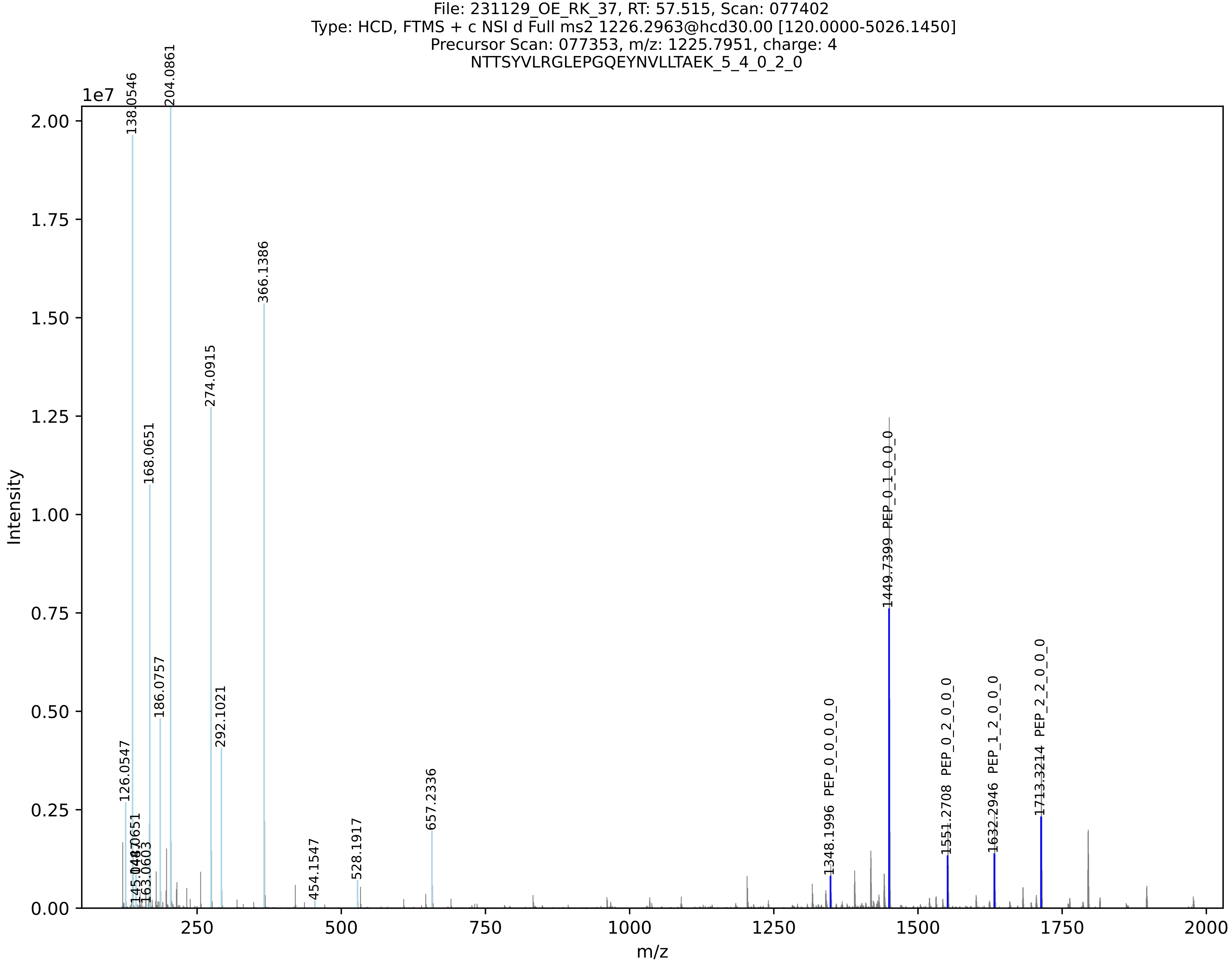
File: 231129_OE_RK_37, RT: 57.515, Scan: 077402
Type: HCD, FTMS + c NSI d Full ms2 1226.2963@hcd30.00 [120.0000-5026.1450]
Precursor Scan: 077353, m/z: 1225.7951, charge: 4
NTTSYVLRGLEPGQEYNVLLTAEK_5_4_0_2_0
1e7
Intensity
m/z
138.0546
204.0861
366.1386
274.0915
168.0651
186.0757
292.1021
126.0547
163.0603
454.1547
528.1917
657.2336
1348.1996 PEP_0_0_0_0_0
1449.7399 PEP_0_1_0_0_0
1551.2708 PEP_0_2_0_0_0
1632.2946 PEP_1_2_0_0_0
1713.3214 PEP_2_2_0_0_0

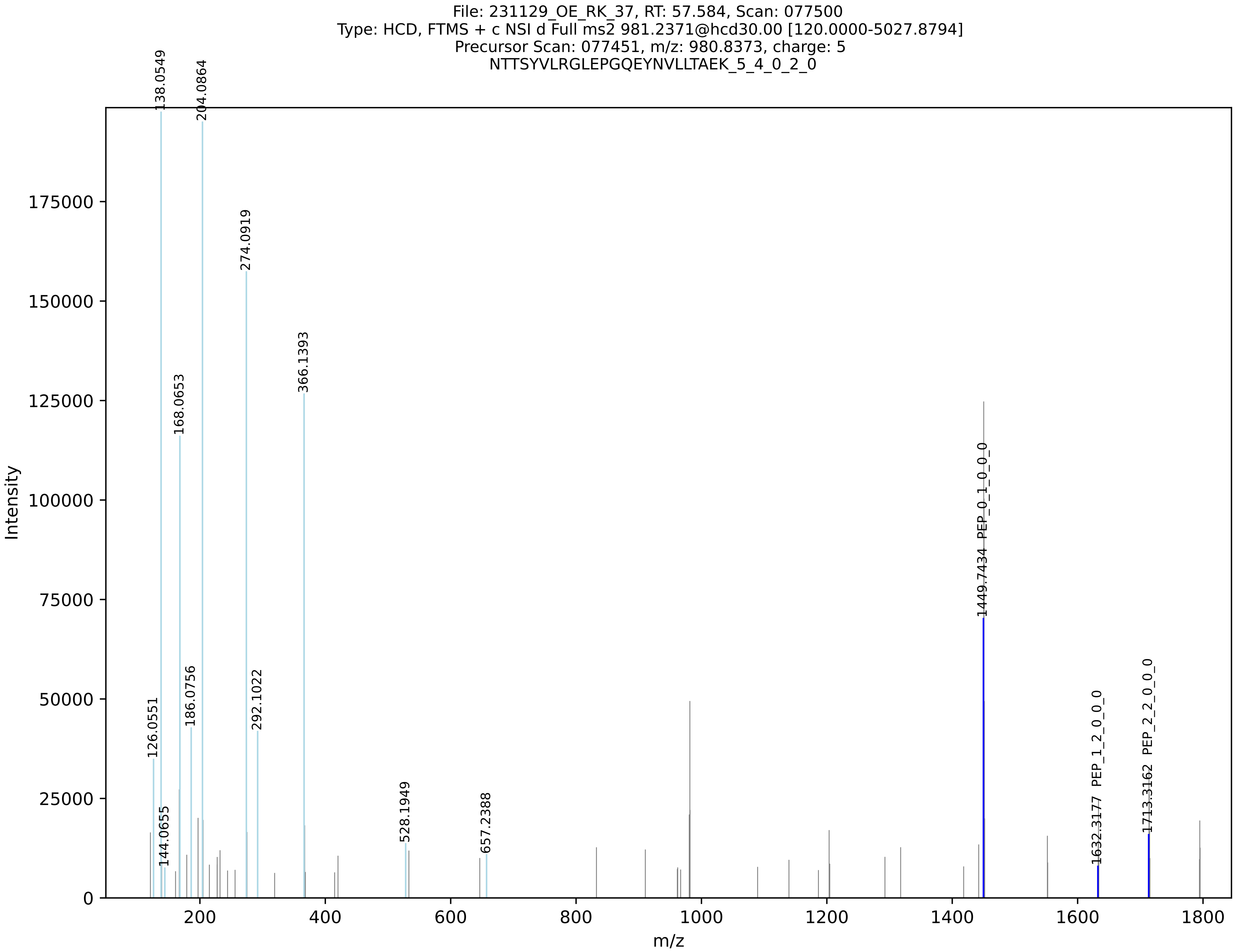

File: 231129_OE_RK_37, RT: 57.584, Scan: 077500
Type: HCD, FTMS + c NSI d Full ms2 981.2371@hcd30.00 [120.0000-5027.8794]
Precursor Scan: 077451, m/z: 980.8373, charge: 5
NTTSYVLRGLEPGQEYNVLLTAEK_5_4_0_2_0
Intensity
m/z
138.0549
204.0864
274.0919
366.1393
168.0653
186.0756
292.1022
126.0551
144.0655
528.1949
657.2388
1449.7434 PEP_0_1_0_0_0
1632.3177 PEP_1_2_0_0_0
1713.3162 PEP_2_2_0_0_0

File: 231129_OE_RK_37, RT: 57.619, Scan: 077551
Type: HCD, FTMS + c NSI d Full ms2 1597.9680@hcd30.00 [120.0000-4911.9238]
Precursor Scan: 077494, m/z: 1597.6346, charge: 3
SPYYNVSDEISFHCYDGYTLR_5_4_0_2_0

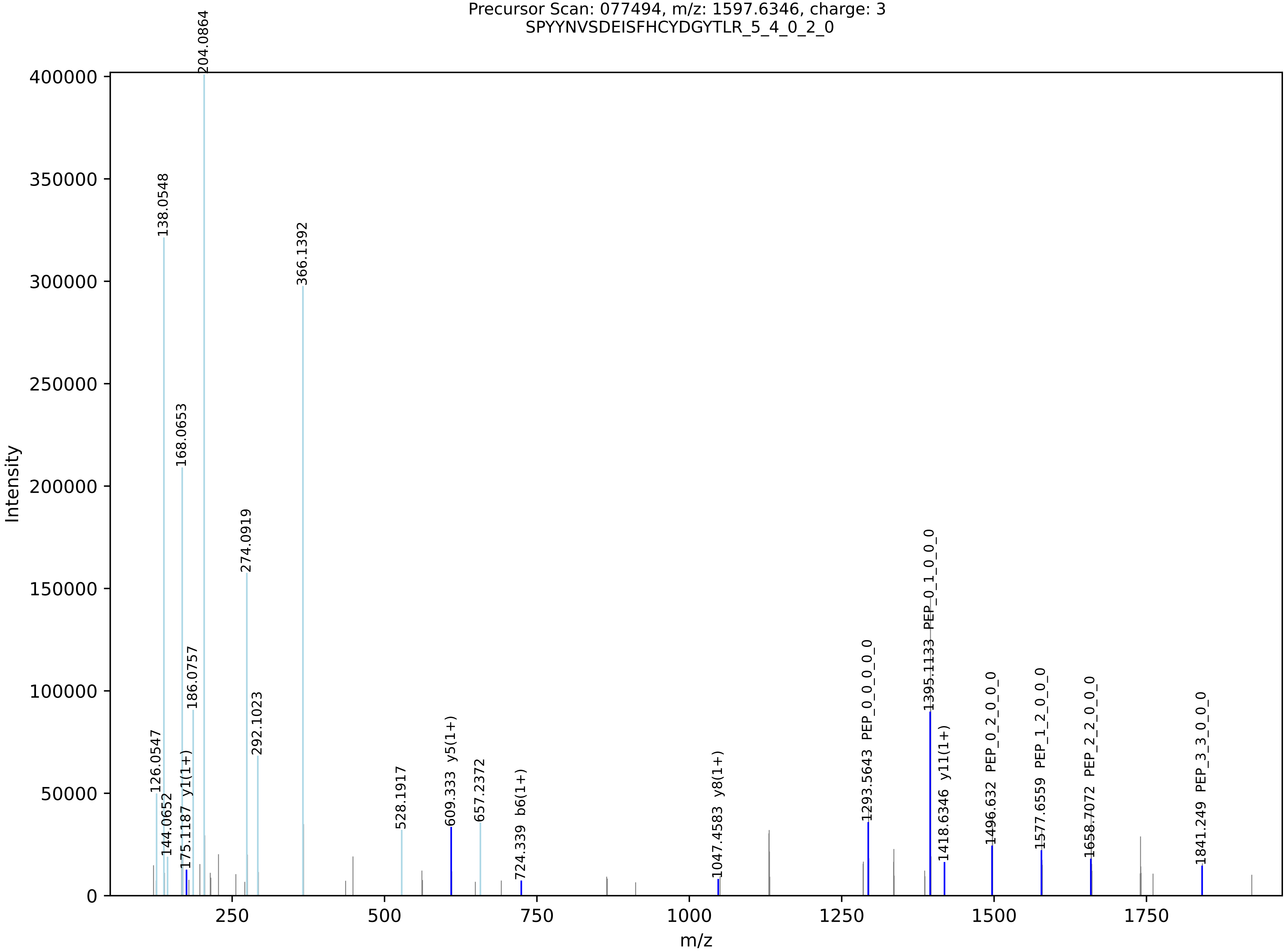

File: 231129_OE_RK_37, RT: 57.643, Scan: 077588
Type: HCD, FTMS + c NSI d Full ms2 1018.6394@hcd30.00 [120.0000-5218.6309]
Precursor Scan: 077451, m/z: 1018.859, charge: 5
SLGNVNFTVSAEALESQELCGTEVPSVPEHGRK_4_3_0_1_0

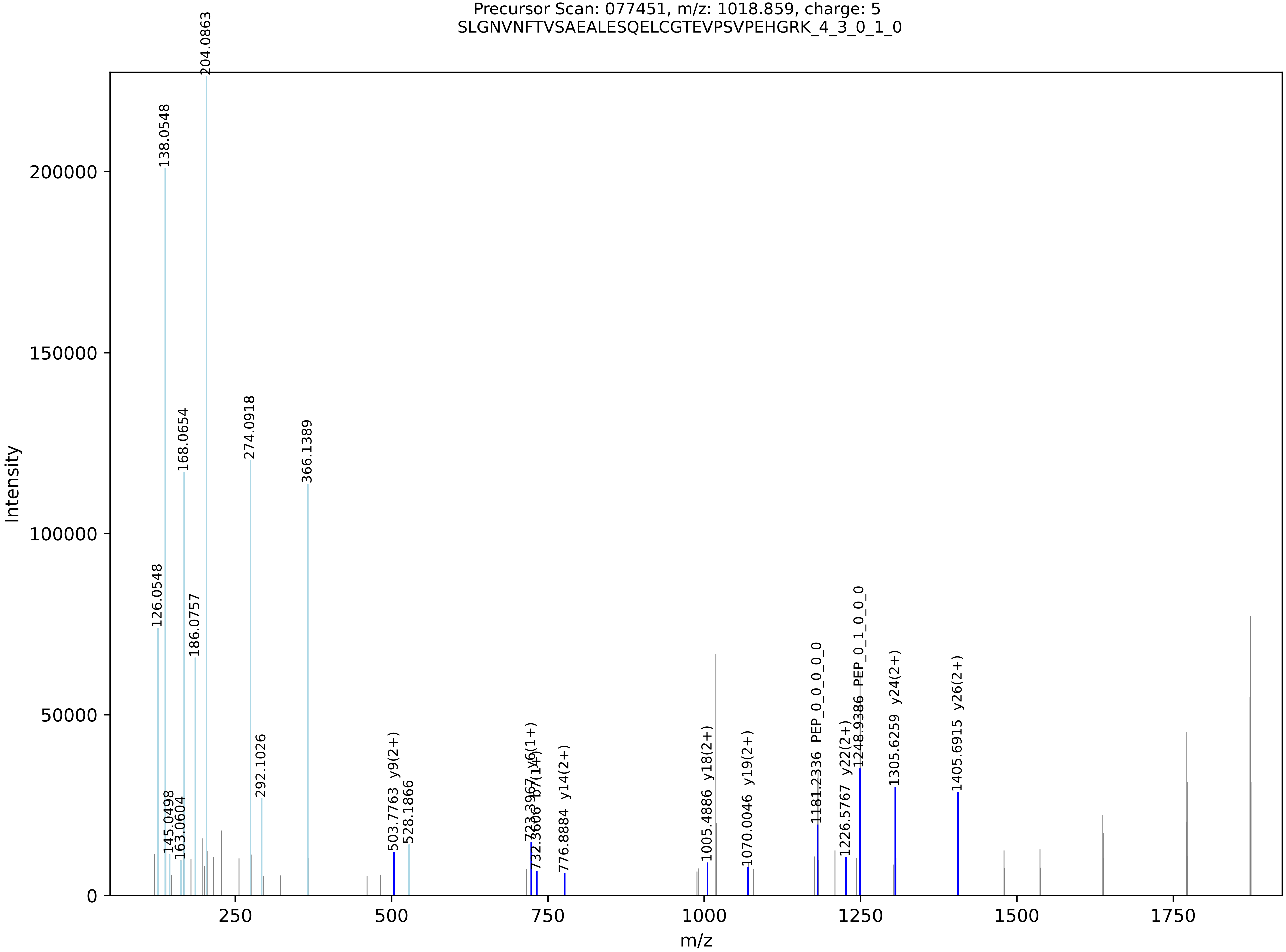

File: 231129_OE_RK_37, RT: 57.644, Scan: 077590
Type: HCD, FTMS + c NSI d Full ms2 980.8375@hcd30.00 [120.0000-5025.8408]
Precursor Scan: 077401, m/z: 980.8374, charge: 5
NTTSYVLRGLEPGQEYNVLLTAEK_5_4_0_2_0

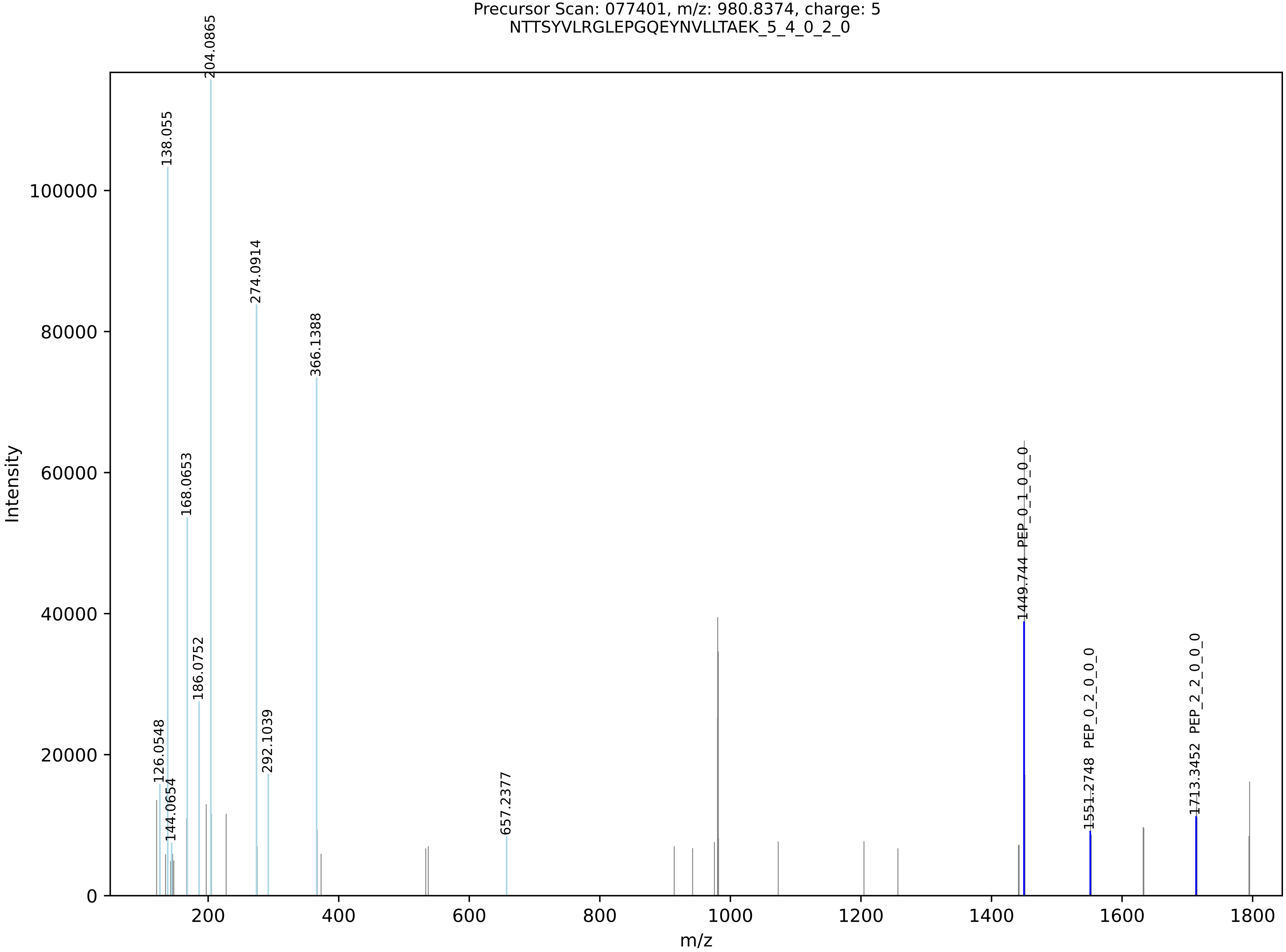

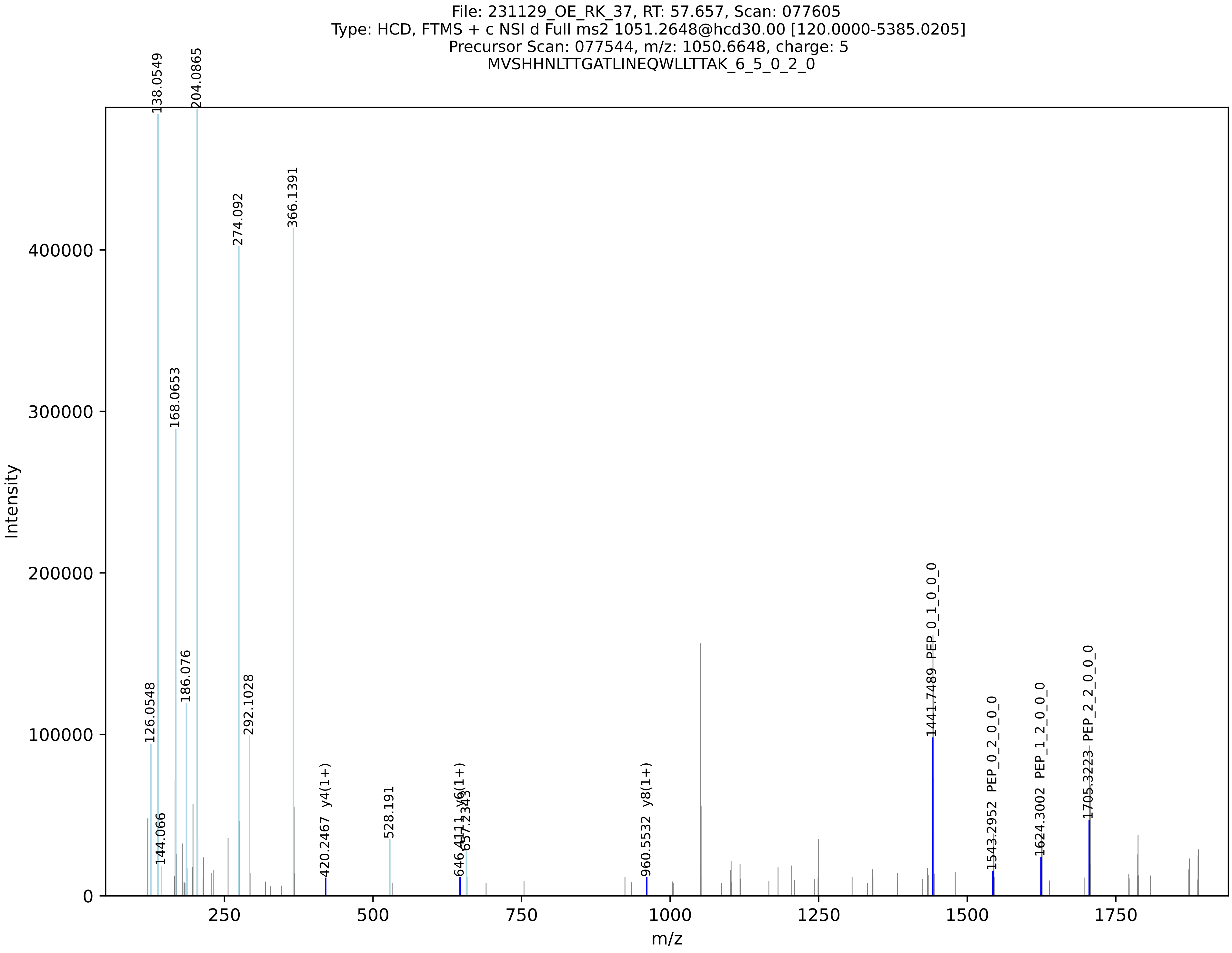

File: 231129_OE_RK_37, RT: 57.657, Scan: 077605
Type: HCD, FTMS + c NSI d Full ms2 1051.2648@hcd30.00 [120.0000-5385.0205]
Precursor Scan: 077544, m/z: 1050.6648, charge: 5
MVSHHNLTTGATLINEQWLLTTAK_6_5_0_2_0
138.0549
204.0865
366.1391
274.092
168.0653
186.076
126.0548
292.1028
144.066
420.2467 y4(1+)
528.191
646.4111 y6(1+)
657.2343
960.5532 y8(1+)
1441.7489 PEP_0_1_0_0_0
1543.2952 PEP_0_2_0_0_0
1624.3002 PEP_1_2_0_0_0
1705.3223 PEP_2_2_0_0_0
Intensity
m/z
0
100000
200000
300000
400000
250
500
750
1000
1250
1500
1750

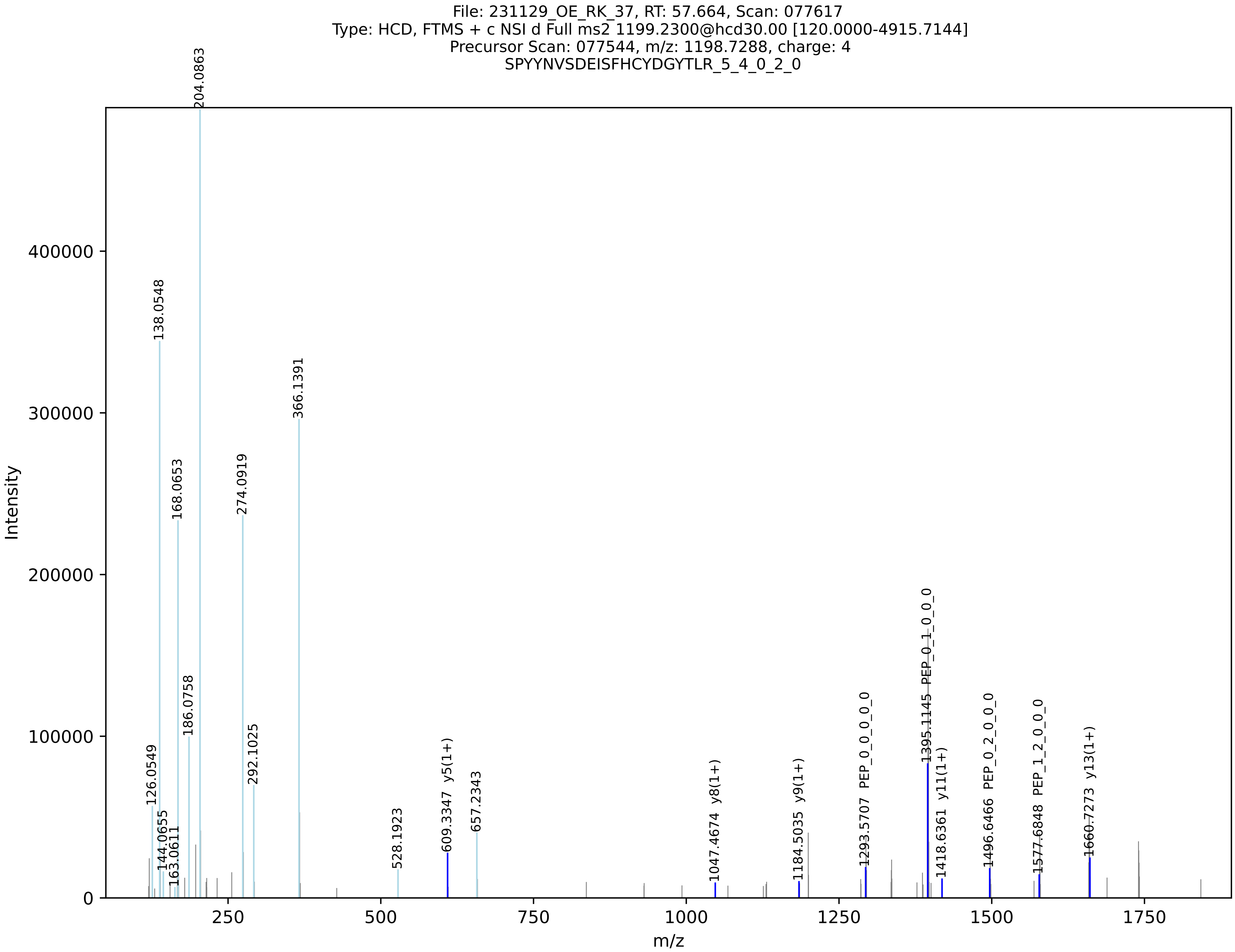
File: 231129_OE_RK_37, RT: 57.664, Scan: 077617
Type: HCD, FTMS + c NSI d Full ms2 1199.2300@hcd30.00 [120.0000-4915.7144]
Precursor Scan: 077544, m/z: 1198.7288, charge: 4
SPYYNVSDEISFHCYDGYTLR_5_4_0_2_0
Intensity
m/z
0
100000
200000
300000
400000
250
500
750
1000
1250
1500
1750
204.0863
138.0548
366.1391
168.0653
274.0919
186.0758
292.1025
126.0549
144.0655
163.0611
528.1923
609.3347 y5(1+)
657.2343
1047.4674 y8(1+)
1184.5035 y9(1+)
1293.5707 PEP_0_0_0_0_0
1395.1145 PEP_0_1_0_0_0
1418.6361 y11(1+)
1496.6466 PEP_0_2_0_0_0
1577.6848 PEP_1_2_0_0_0
1660.7273 y13(1+)

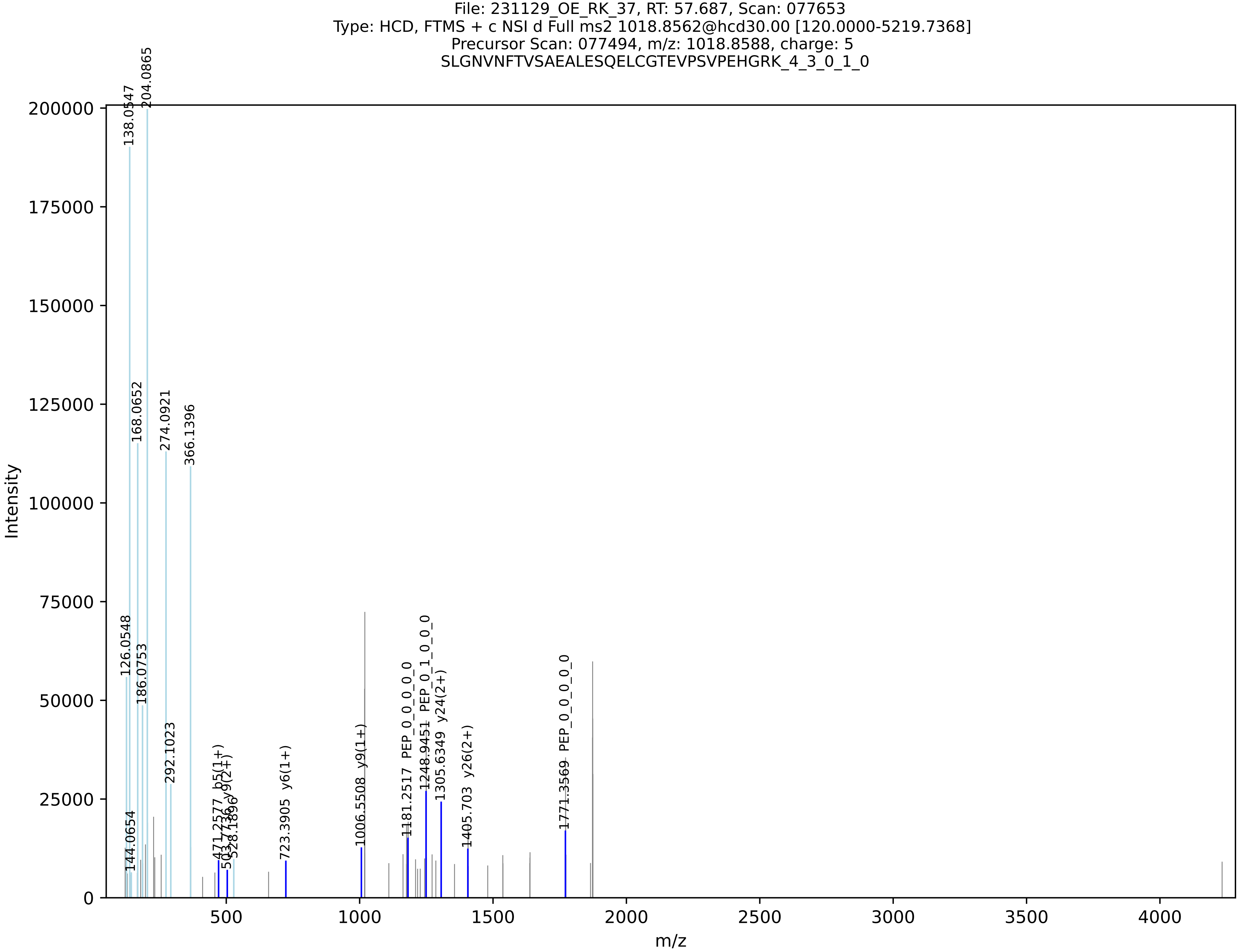
File: 231129_OE_RK_37, RT: 57.687, Scan: 077653
Type: HCD, FTMS + c NSI d Full ms2 1018.8562@hcd30.00 [120.0000-5219.7368]
Precursor Scan: 077494, m/z: 1018.8588, charge: 5
SLGNVNFTVSAEALESQELCGTEVPSVPEHGRK_4_3_0_1_0
Intensity
m/z
0
25000
50000
75000
100000
125000
150000
175000
200000
500
1000
1500
2000
2500
3000
3500
4000
138.0547
204.0865
168.0652
274.0921
366.1396
126.0548
186.0753
292.1023
144.0654
471.2577 b5(1+)
y9(2+)
528.1896
723.3905 y6(1+)
1006.5508 y9(1+)
1181.2517 PEP_0_0_0_0_0
1248.9451 PEP_0_1_0_0_0
1305.6349 y24(2+)
1405.703 y26(2+)
1771.3569 PEP_0_0_0_0_0

File: 231129_OE_RK_37, RT: 57.702, Scan: 077670
Type: HCD, FTMS + c NSI d Full ms2 1226.5464@hcd30.00 [120.0000-5027.1655]
Precursor Scan: 077599, m/z: 1225.7947, charge: 4
NTTTSYVLRGLEPGQEYNVLLTAEK_5_4_0_2_0

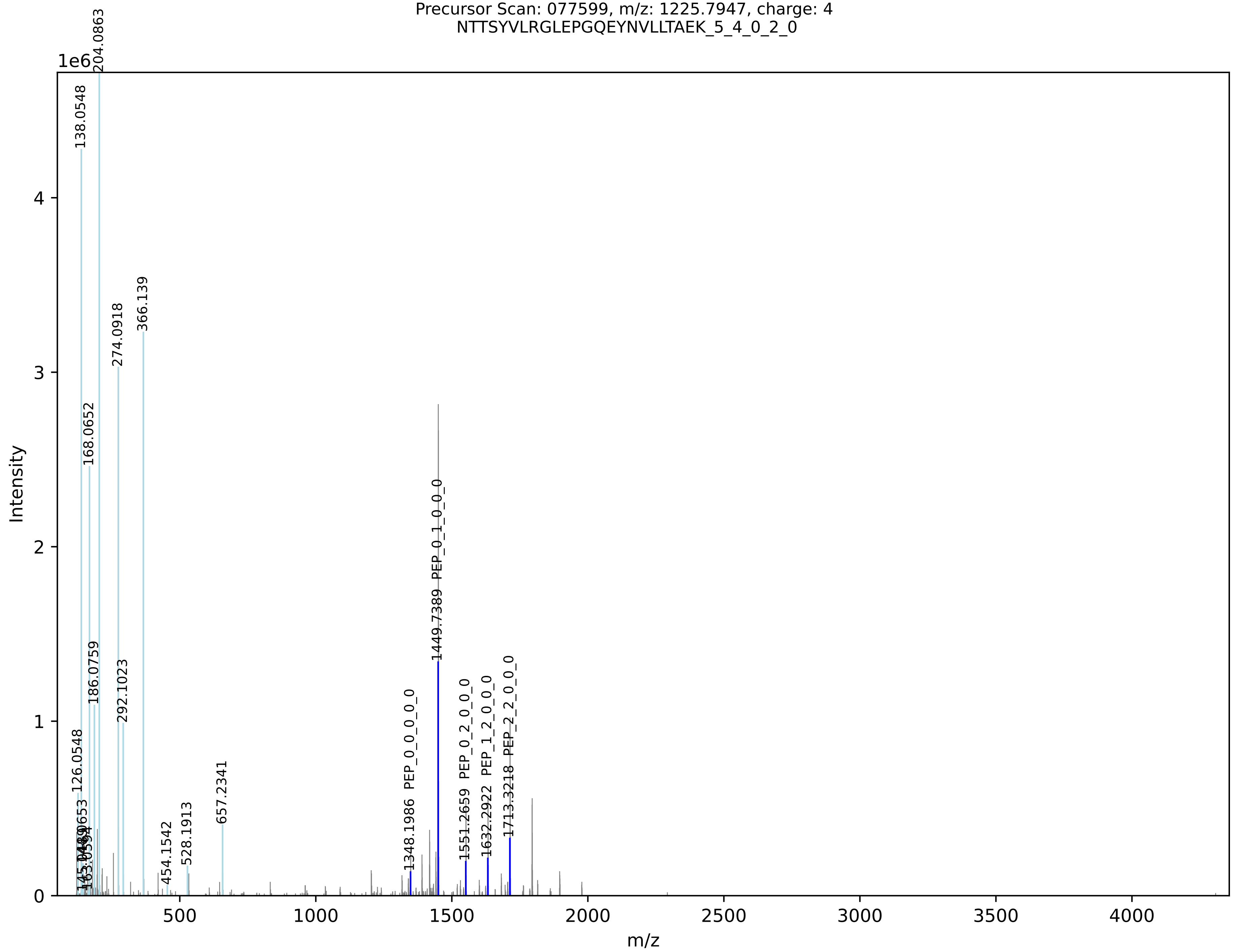

File: 231129_OE_RK_37, RT: 57.703, Scan: 077672
Type: HCD, FTMS + c NSI d Full ms2 1181.2297@hcd30.00 [120.0000-4842.2734]
Precursor Scan: 077599, m/z: 1180.7286, charge: 4
QQQHLFGSNVTDCSGNFCLFR_5_4_0_2_0

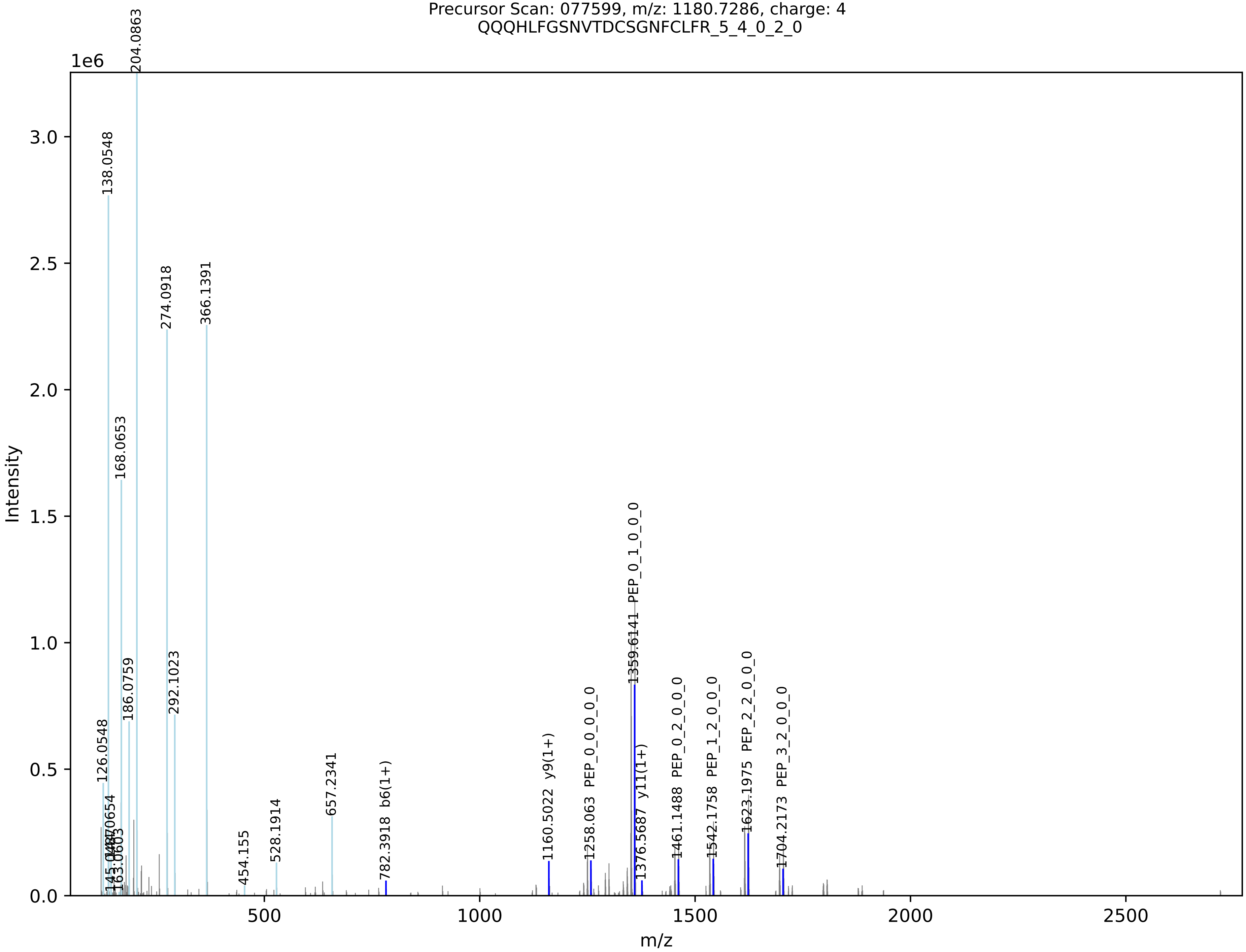

File: 231129_OE_RK_37, RT: 57.752, Scan: 077743
Type: HCD, FTMS + c NSI d Full ms2 1799.4557@hcd30.00 [120.0000-5528.4761]
Precursor Scan: 077668, m/z: 1799.4556, charge: 3
MLFVEPILEVSSLPTTNSTTNSATK_6_5_1_2_0

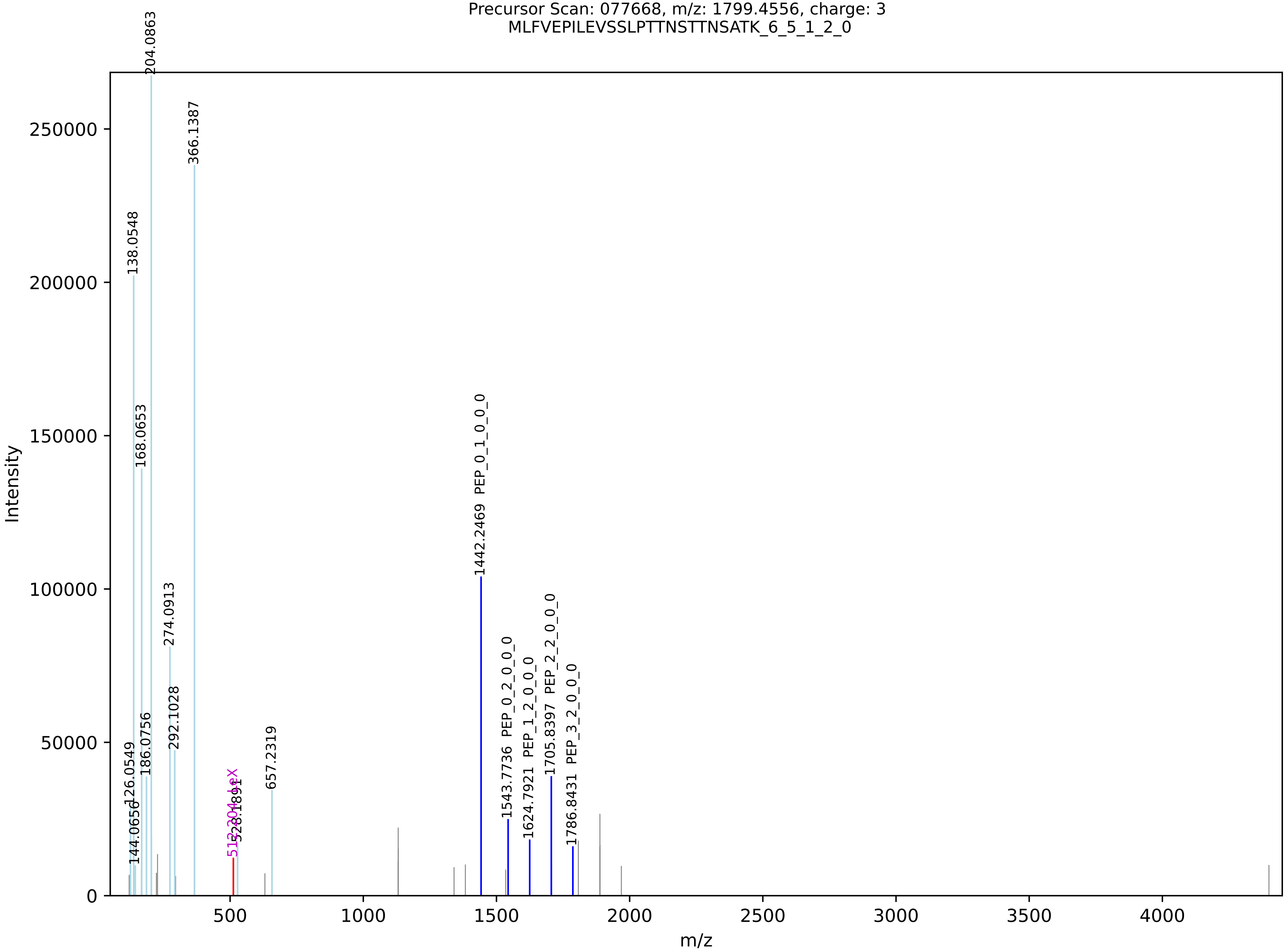

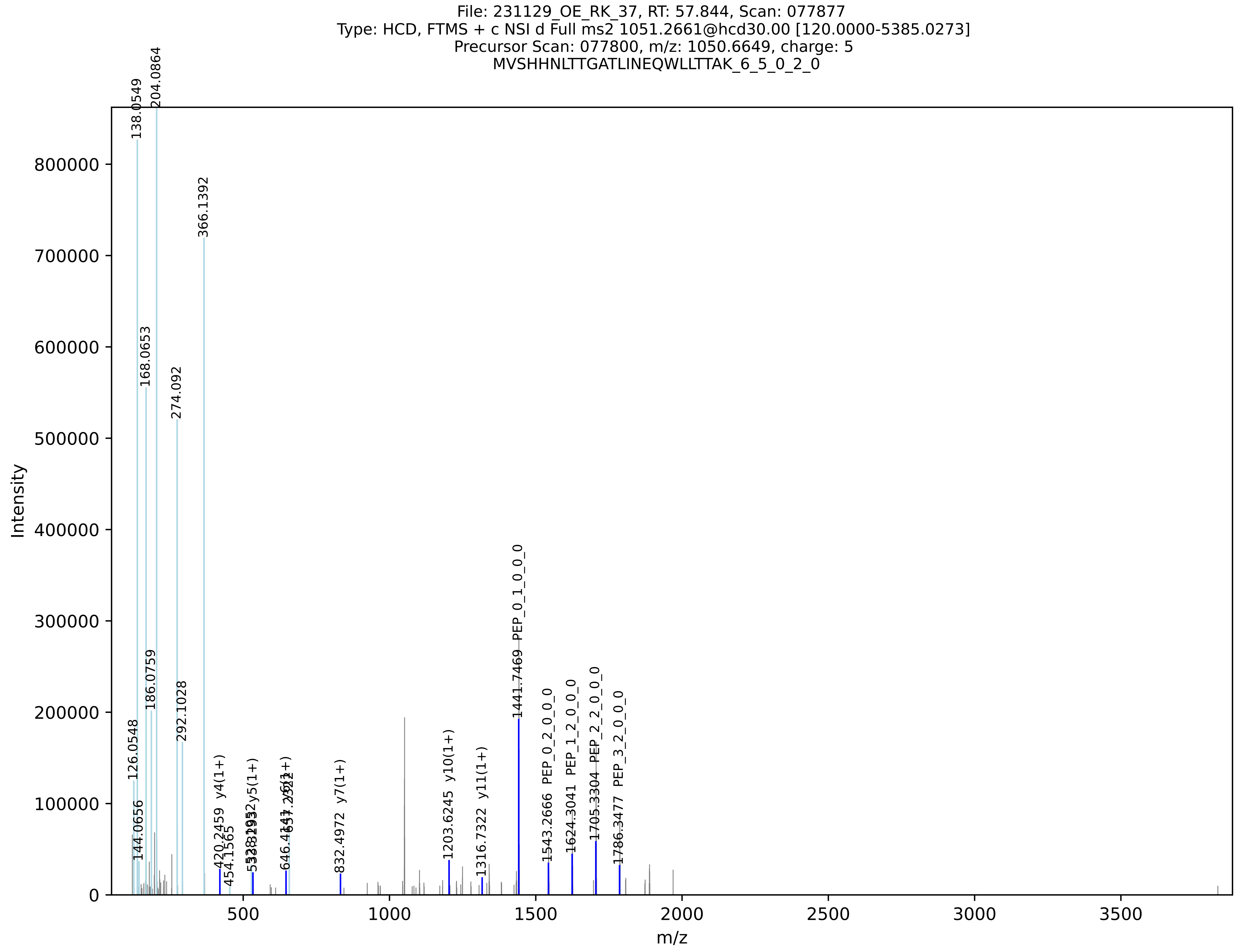
File: 231129_OE_RK_37, RT: 57.844, Scan: 077877
Type: HCD, FTMS + c NSI d Full ms2 1051.2661@hcd30.00 [120.0000-5385.0273]
Precursor Scan: 077800, m/z: 1050.6649, charge: 5
MVSHHNLTTGATLINEQWLLTTAK_6_5_0_2_0
Intensity
m/z
138.0549
204.0864
366.1392
168.0653
274.092
186.0759
292.1028
126.0548
144.0656
420.2459 y4(1+)
454.1565
y5(1+)
y6(1+)
832.4972 y7(1+)
1203.6245 y10(1+)
1316.7322 y11(1+)
1441.7469 PEP_0_1_0_0_0
1543.2666 PEP_0_2_0_0_0
1624.3041 PEP_1_2_0_0_0
1705.3304 PEP_2_2_0_0_0
1786.3477 PEP_3_2_0_0_0

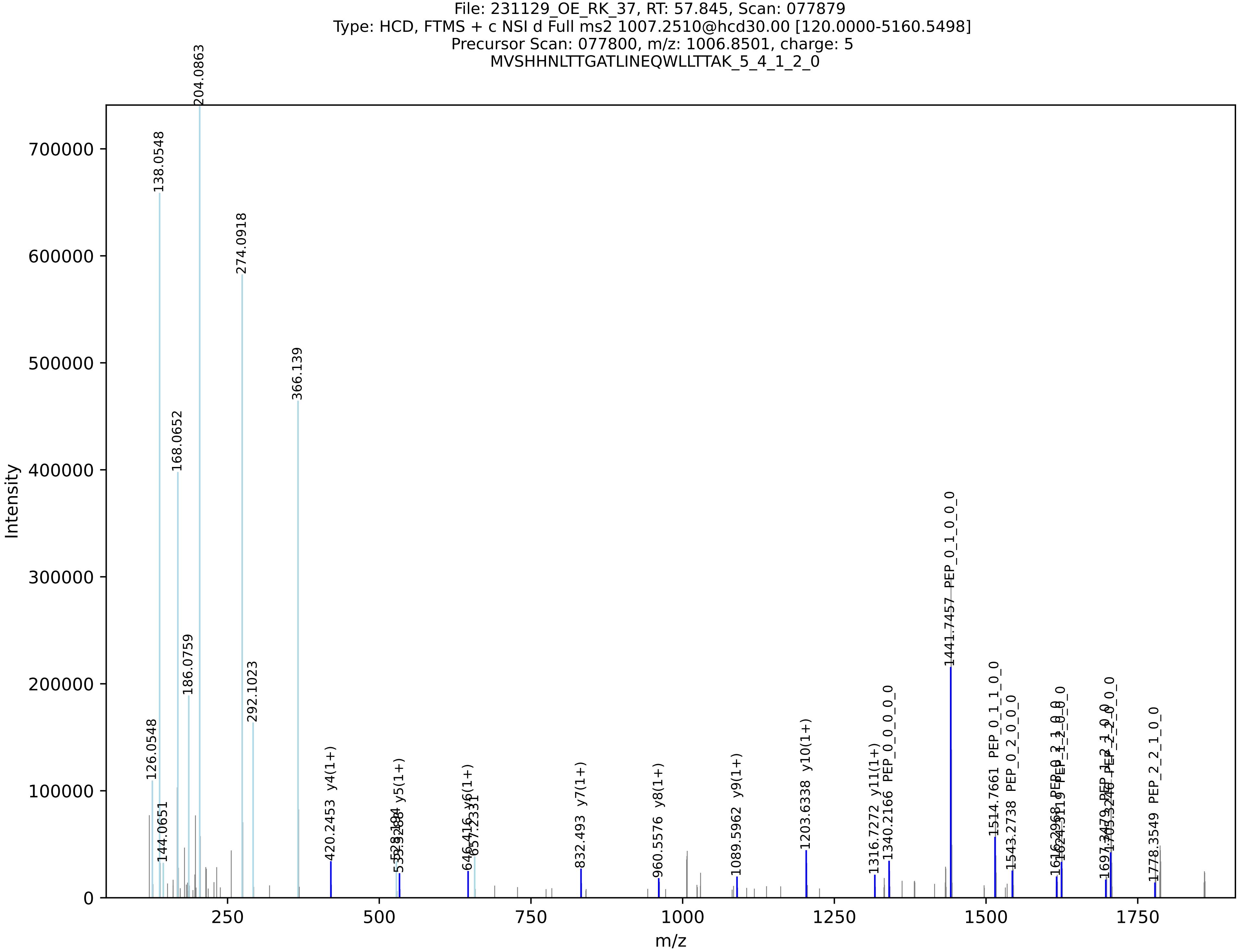

File: 231129_OE_RK_37, RT: 57.845, Scan: 077879
Type: HCD, FTMS + c NSI d Full ms2 1007.2510@hcd30.00 [120.0000-5160.5498]
Precursor Scan: 077800, m/z: 1006.8501, charge: 5
MVSHHNLTTGATLINEQWLLTTAK_5_4_1_2_0
Intensity
m/z
204.0863
138.0548
274.0918
366.139
168.0652
186.0759
292.1023
126.0548
144.0651
420.2453 y4(1+)
535.3208 y5(1+)
646.416 y6(1+)
657.2331
832.493 y7(1+)
960.5576 y8(1+)
1089.5962 y9(1+)
1203.6338 y10(1+)
1316.7272 y11(1+)
1340.2166 PEP_0_0_0_0_0
1441.7457 PEP_0_1_0_0_0
1514.7661 PEP_0_1_1_0_0
1543.2738 PEP_0_2_0_0_0
1778.3549 PEP_2_2_1_0_0

File: 231129_OE_RK_37, RT: 57.893, Scan: 077949
Type: HCD, FTMS + c NSI d Full ms2 1181.2311@hcd30.00 [120.0000-4842.2788]
Precursor Scan: 077874, m/z: 1180.73, charge: 4
QQQHLFGSNVTDCSGNFCLFR_5_4_0_2_0

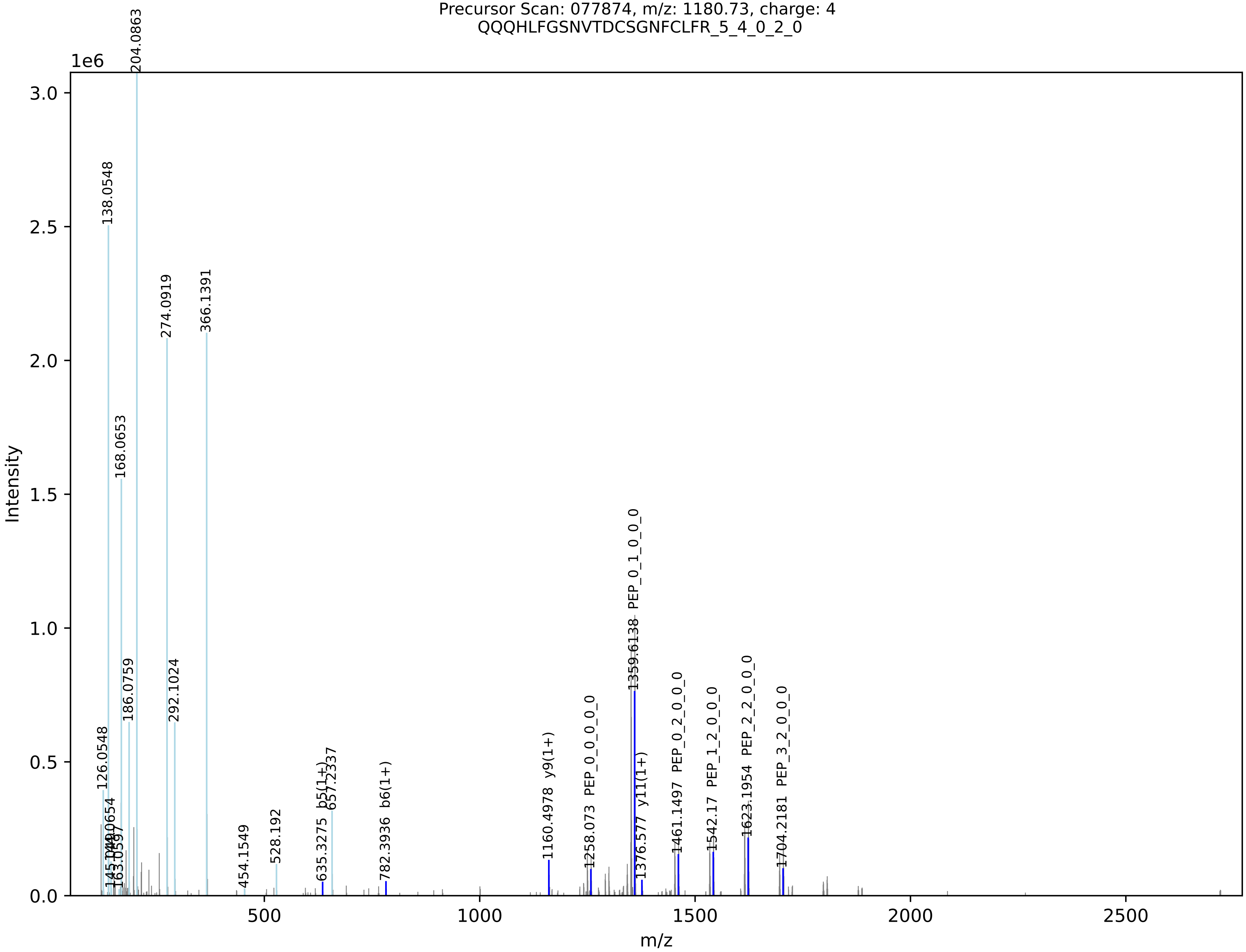

File: 231129_OE_RK_37, RT: 57.894, Scan: 077950
Type: HCD, FTMS + c NSI d Full ms2 1226.2970@hcd30.00 [120.0000-5026.1475]
Precursor Scan: 077874, m/z: 1225.7958, charge: 4
NTTSYVLRGLEPGQEYNVLLTAEK_5_4_0_2_0

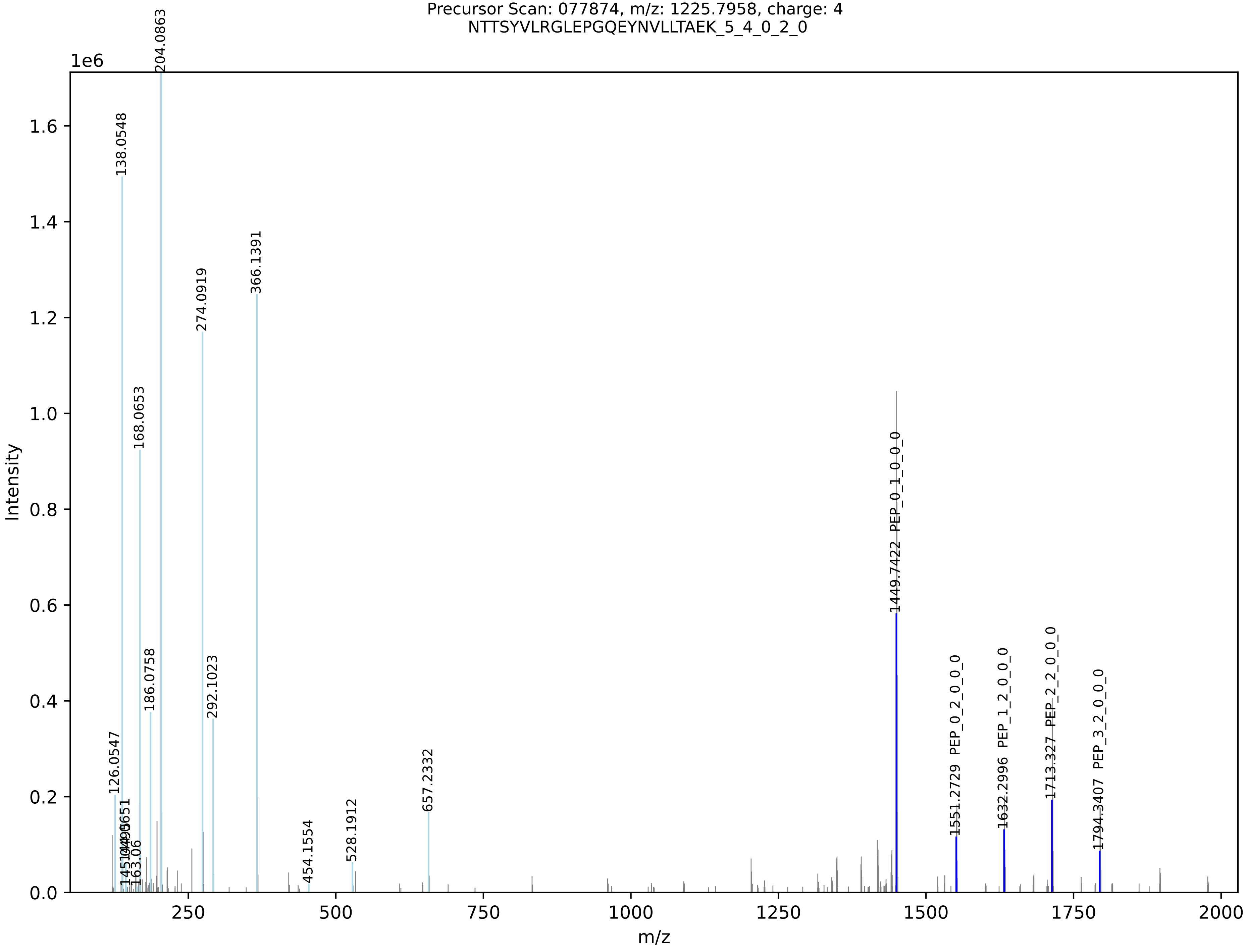

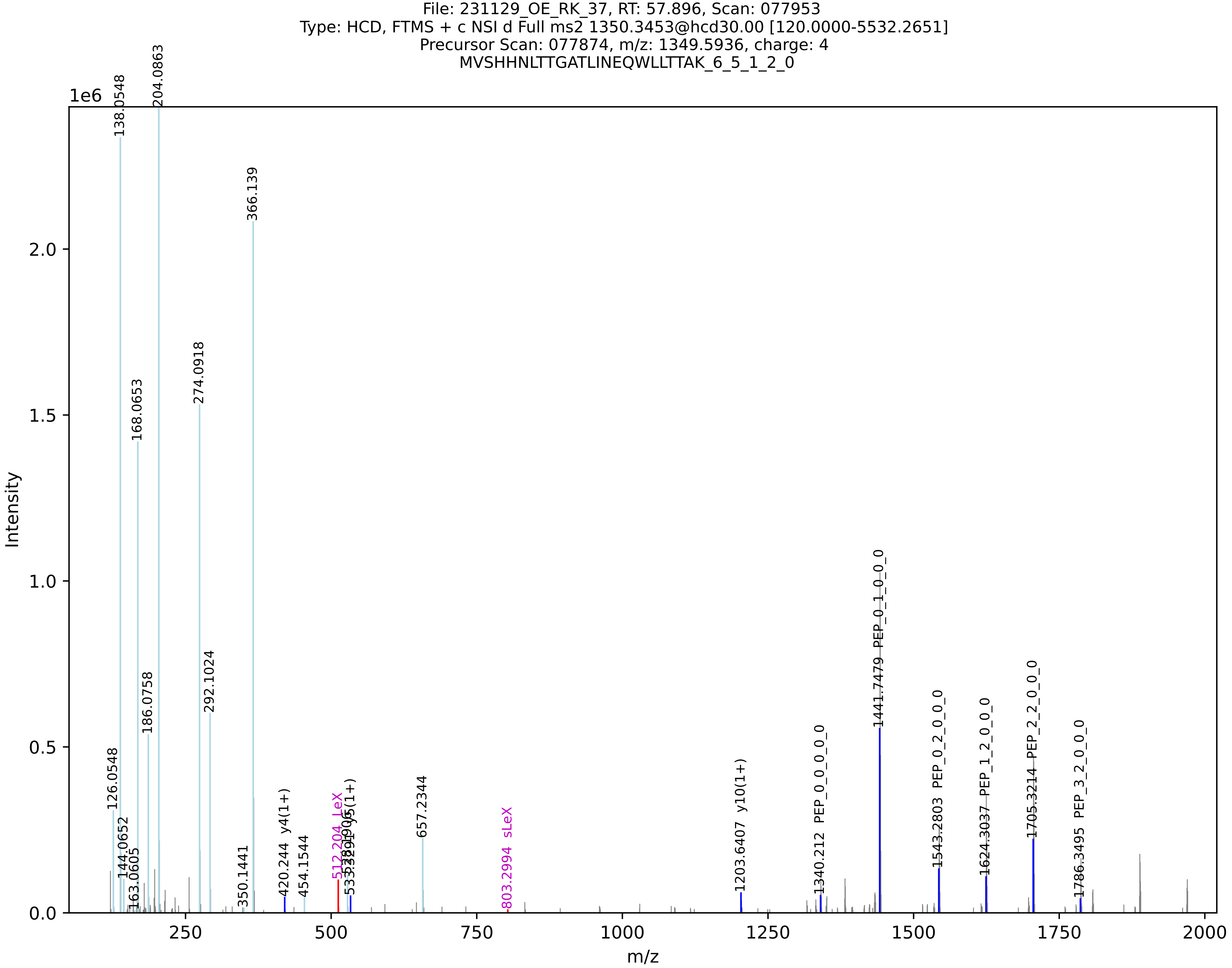
File: 231129_OE_RK_37, RT: 57.896, Scan: 077953
Type: HCD, FTMS + c NSI d Full ms2 1350.3453@hcd30.00 [120.0000-5532.2651]
Precursor Scan: 077874, m/z: 1349.5936, charge: 4
MVSHHNLTTGATLINEQWLLTTAK_6_5_1_2_0
1e6
Intensity
m/z
138.0548
204.0863
366.139
274.0918
168.0653
292.1024
186.0758
126.0548
144.0652
163.0605
350.1441
420.244 y4(1+)
454.1544
512.204 LeX
657.2344
803.2994 sLeX
1203.6407 y10(1+)
1340.212 PEP_0_0_0_0_0
1441.7479 PEP_0_1_0_0_0
1543.2803 PEP_0_2_0_0_0
1624.3037 PEP_1_2_0_0_0
1705.3214 PEP_2_2_0_0_0
1786.3495 PEP_3_2_0_0_0

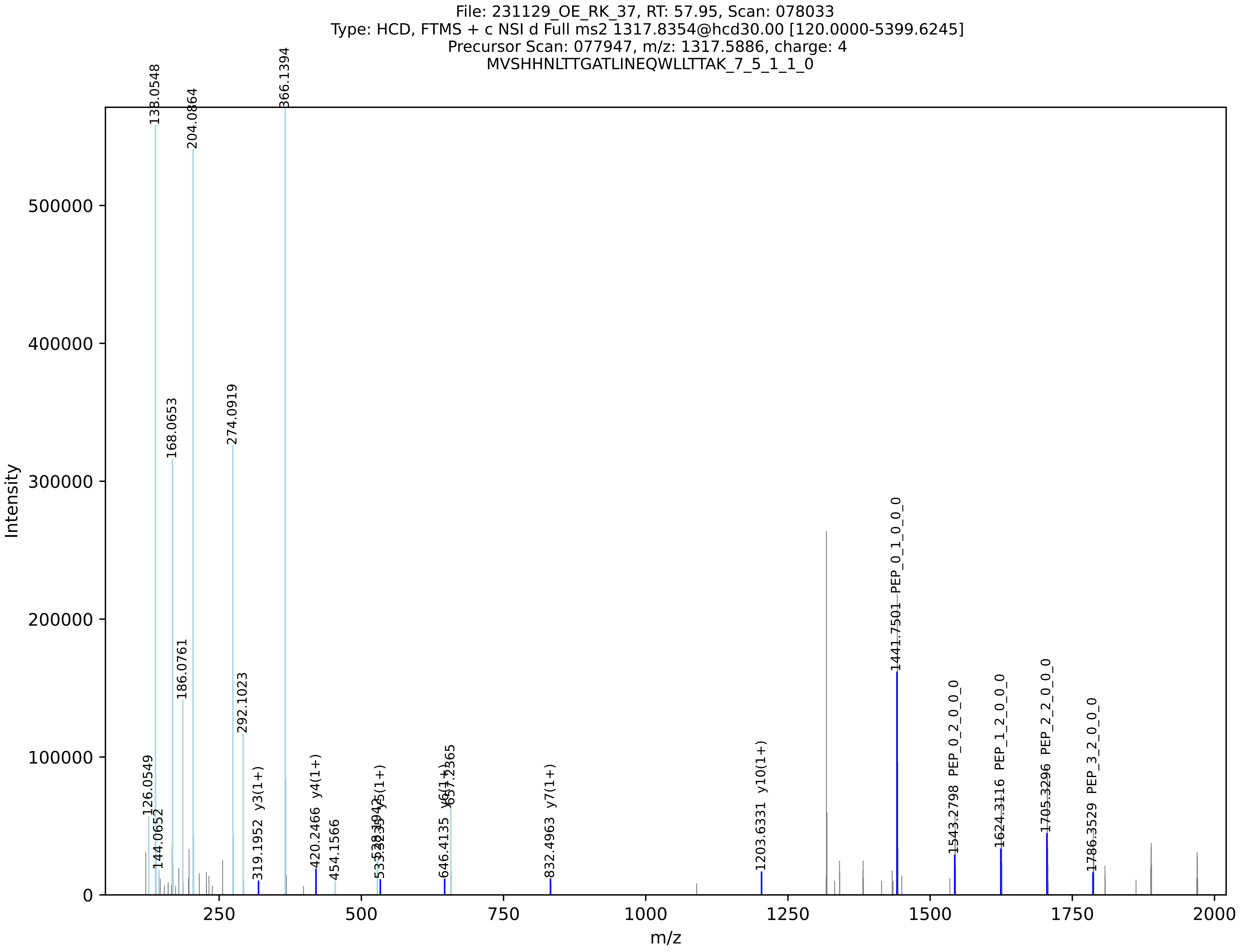

File: 231129_OE_RK_37, RT: 57.95, Scan: 078033
Type: HCD, FTMS + c NSI d Full ms2 1317.8354@hcd30.00 [120.0000-5399.6245]
Precursor Scan: 077947, m/z: 1317.5886, charge: 4
MVSHHNLTTGATLINEQWLLTTAK_7_5_1_1_0
Intensity
m/z
138.0548
366.1394
204.0864
168.0653
274.0919
186.0761
292.1023
126.0549
144.0652
319.1952 y3(1+)
420.2466 y4(1+)
454.1566
533.3235 y5(1+)
646.4135 y6(1+)
657.2365
832.4963 y7(1+)
1203.6331 y10(1+)
1441.7501 PEP_0_1_0_0_0
1543.2798 PEP_0_2_0_0_0
1624.3116 PEP_1_2_0_0_0
1705.3296 PEP_2_2_0_0_0
1786.3529 PEP_3_2_0_0_0

File: 231129_OE_RK_37, RT: 57.993, Scan: 078096
Type: HCD, FTMS + c NSI d Full ms2 1222.5482@hcd30.00 [120.0000-5010.8525]
Precursor Scan: 078021, m/z: 1221.7964, charge: 4
MVSHHNLTTGATLINEQWLLTTAK_5_4_0_2_0

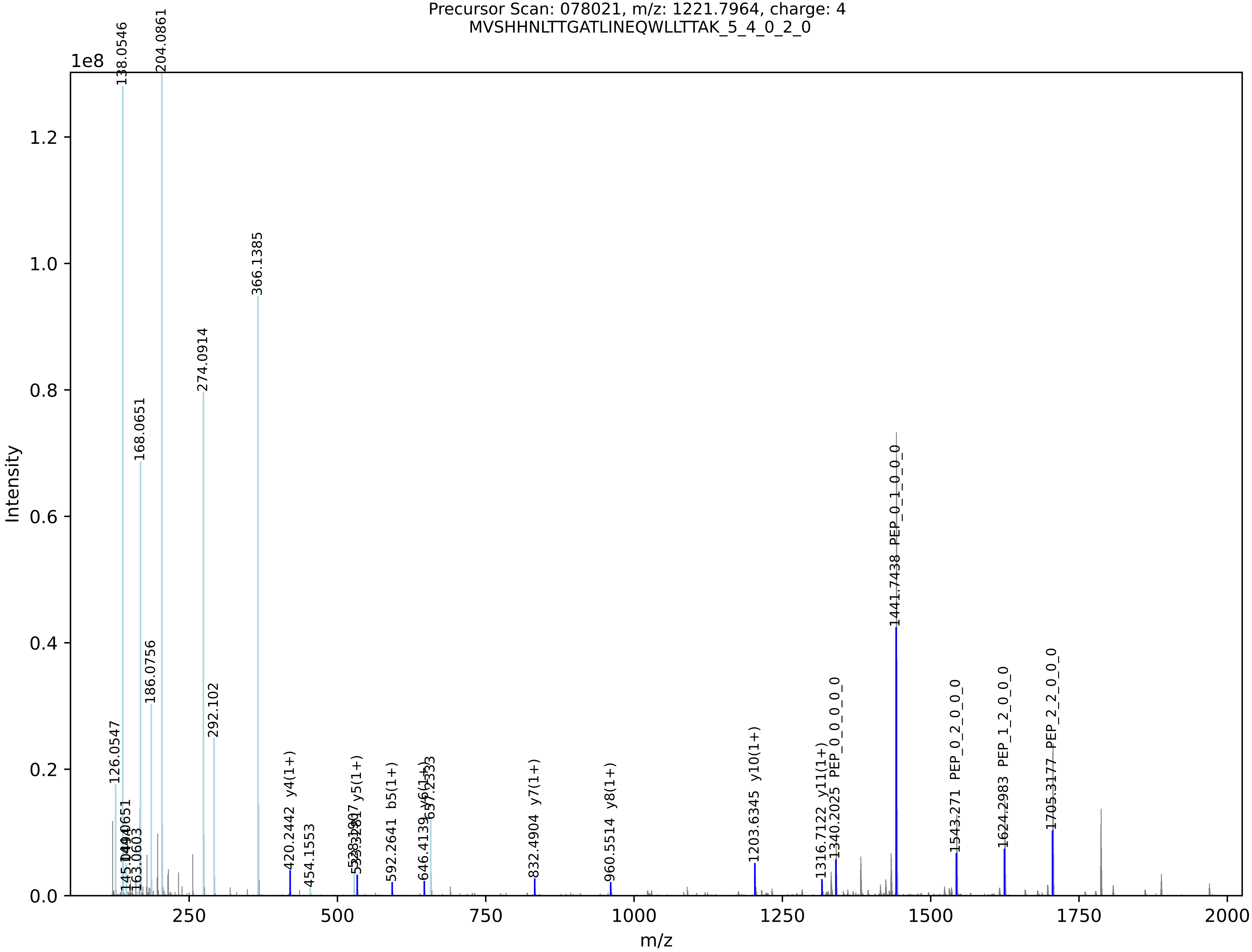

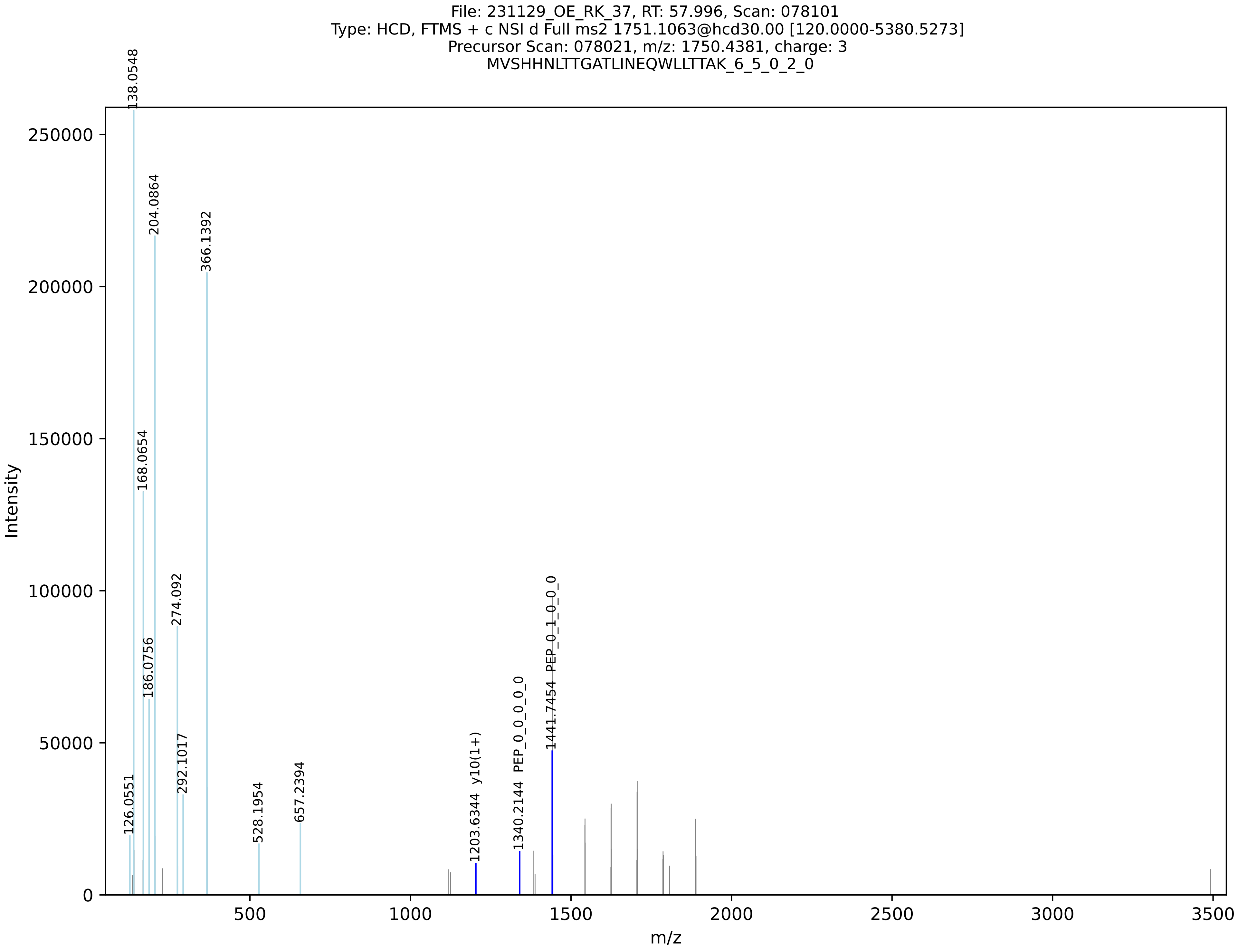
File: 231129_OE_RK_37, RT: 57.996, Scan: 078101
Type: HCD, FTMS + c NSI d Full ms2 1751.1063@hcd30.00 [120.0000-5380.5273]
Precursor Scan: 078021, m/z: 1750.4381, charge: 3
MVSHHNLTTGATLINEQWLLTTAK_6_5_0_2_0
Intensity
m/z
138.0548
204.0864
366.1392
168.0654
274.092
186.0756
292.1017
126.0551
528.1954
657.2394
1203.6344 y10(1+)
1340.2144 PEP_0_0_0_0_0_
1441.7454 PEP_0_1_0_0_0_

File: 231129_OE_RK_37, RT: 58.15, Scan: 078325
Type: HCD, FTMS + c NSI d Full ms2 1376.9160@hcd30.00 [120.0000-4235.5054]
Precursor Scan: 078242, m/z: 1376.2478, charge: 3
TLNQSSDELQLSMGNAMFVK_5_4_0_1_0

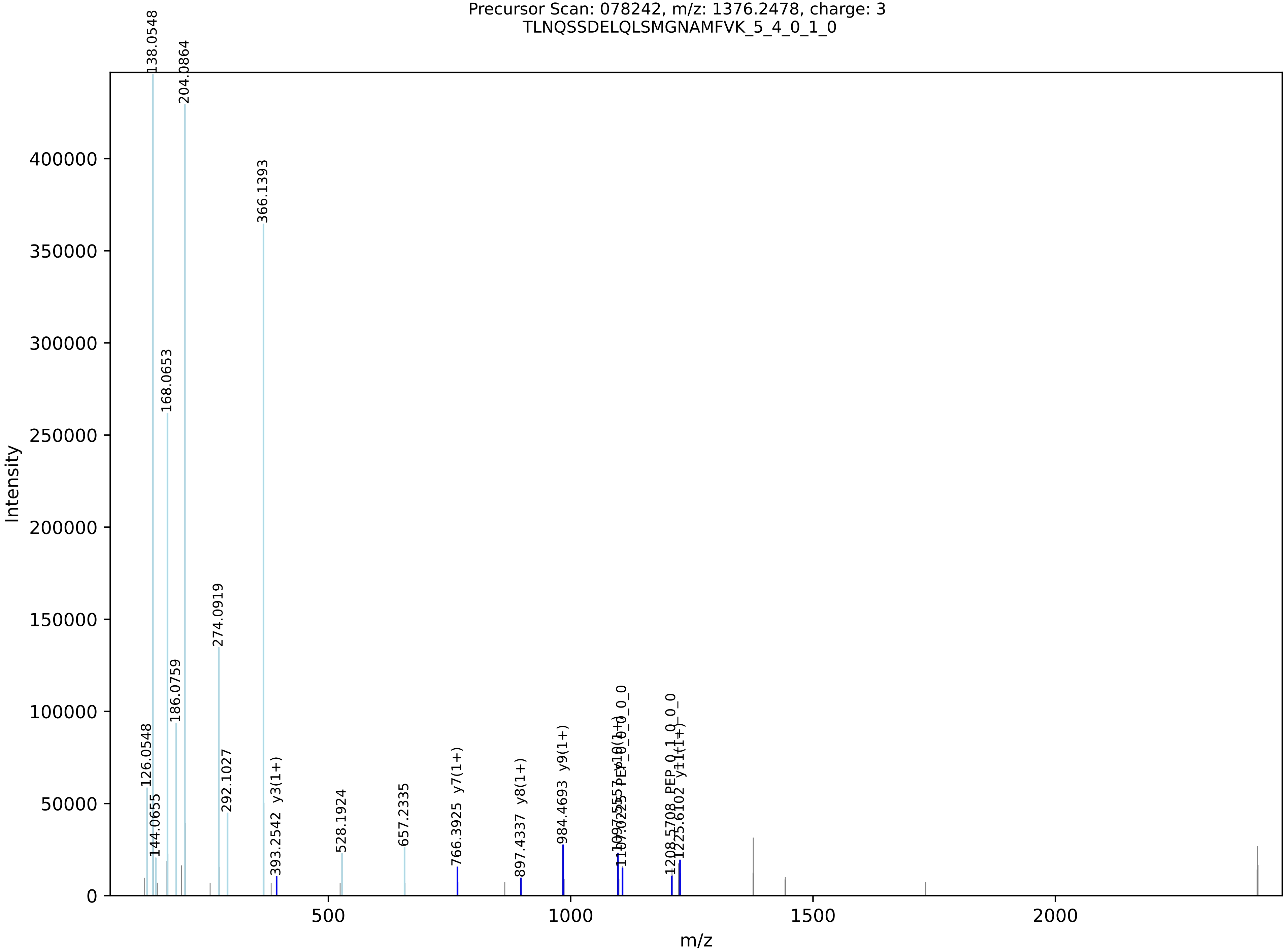

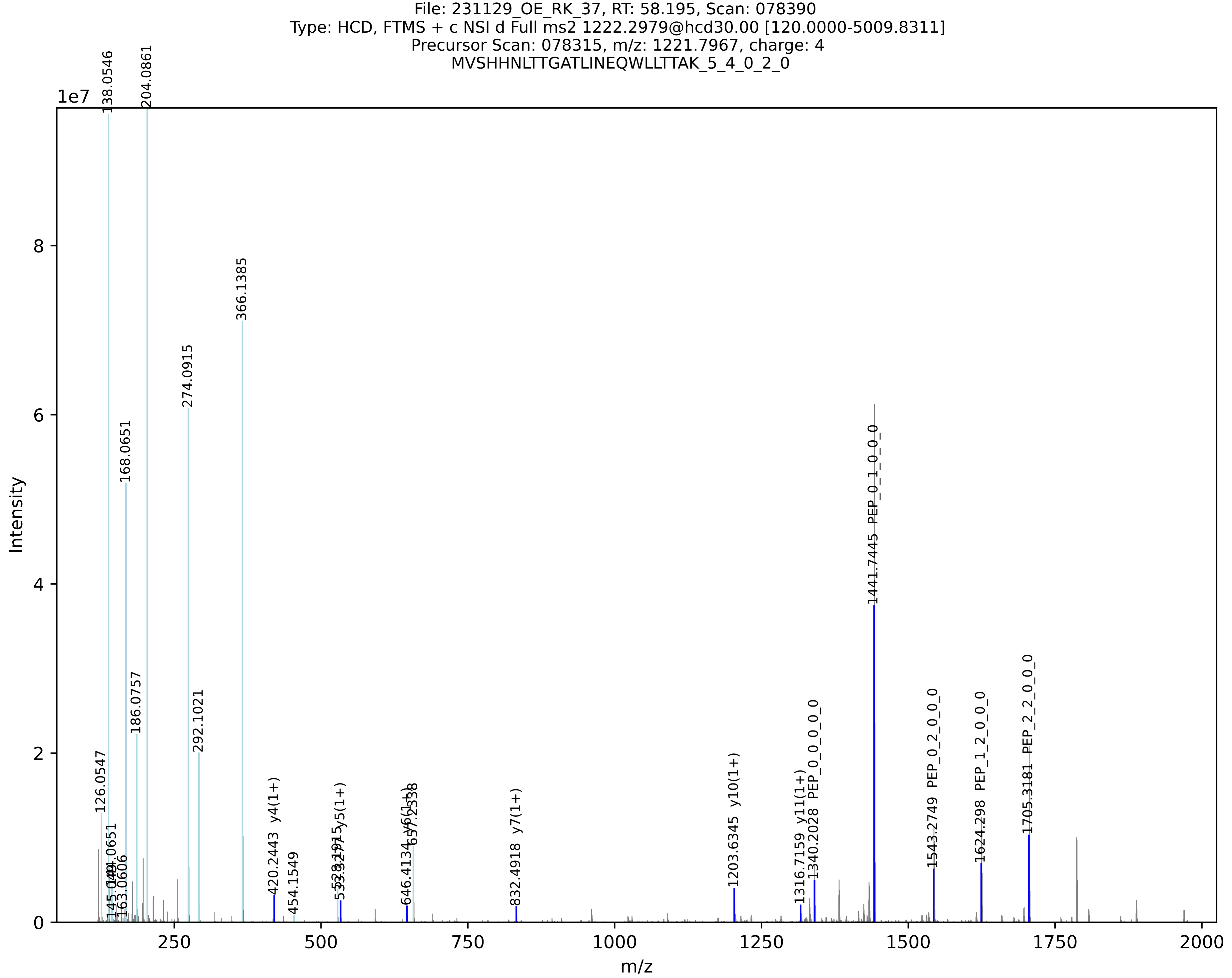
File: 231129_OE_RK_37, RT: 58.195, Scan: 078390
Type: HCD, FTMS + c NSI d Full ms2 1222.2979@hcd30.00 [120.0000-5009.8311]
Precursor Scan: 078315, m/z: 1221.7967, charge: 4
MVSHHNLTTGATLINEQWLLTTAK_5_4_0_2_0
1e7
Intensity
m/z
138.0546
204.0861
366.1385
274.0915
168.0651
186.0757
292.1021
126.0547
163.0606
420.2443 y4(1+)
454.1549
533.3177 y5(1+)
646.4134 y6(1+)
657.2338
832.4918 y7(1+)
1203.6345 y10(1+)
1316.7159 y11(1+)
1340.2028 PEP_0_0_0_0_0
1441.7445 PEP_0_1_0_0_0
1543.2749 PEP_0_2_0_0_0
1624.298 PEP_1_2_0_0_0
1705.3181 PEP_2_2_0_0_0

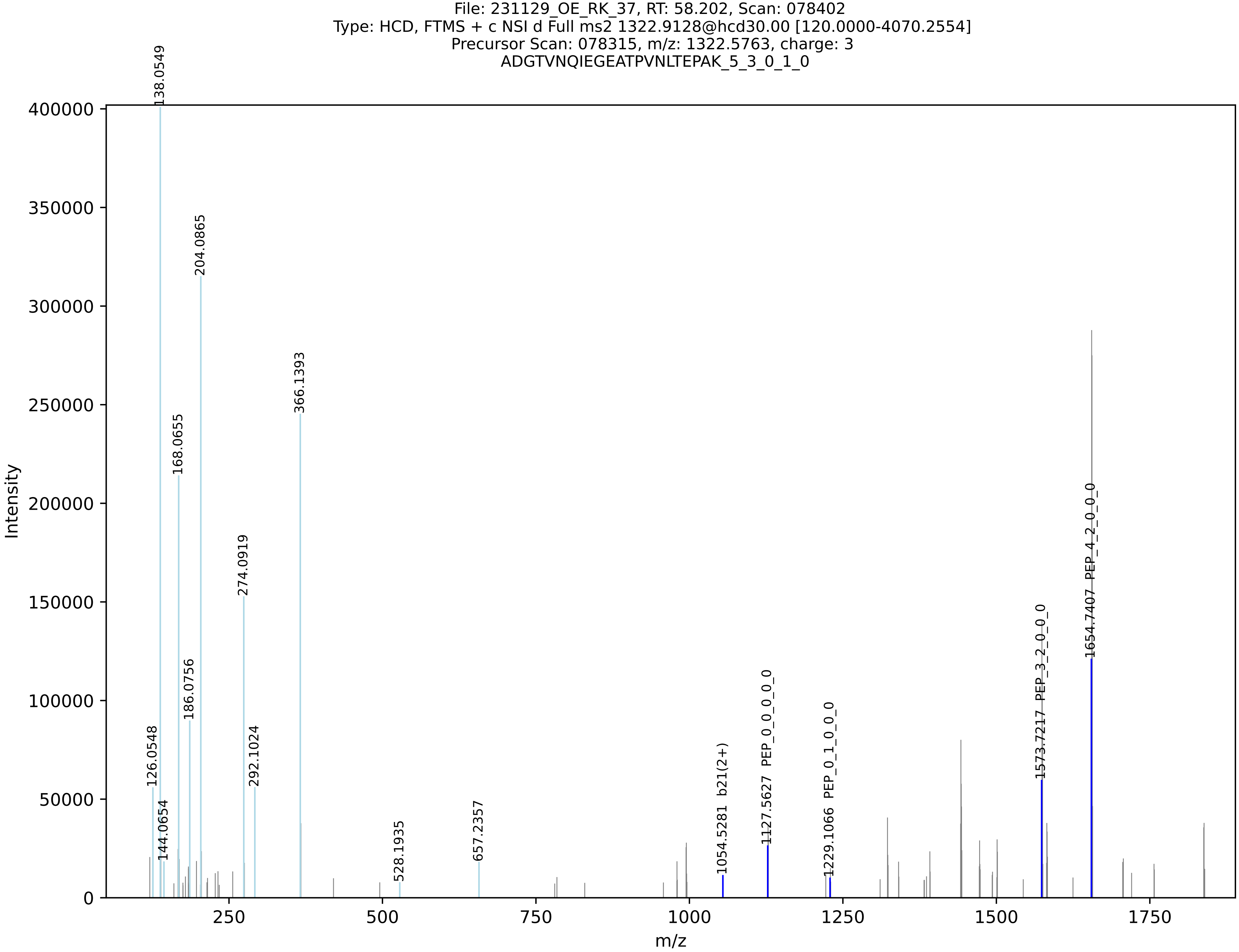
File: 231129_OE_RK_37, RT: 58.202, Scan: 078402
Type: HCD, FTMS + c NSI d Full ms2 1322.9128@hcd30.00 [120.0000-4070.2554]
Precursor Scan: 078315, m/z: 1322.5763, charge: 3
ADGTVNQIEGEATPVNLTEPAK_5_3_0_1_0
Intensity
m/z
138.0549
204.0865
366.1393
168.0655
274.0919
186.0756
126.0548
292.1024
144.0654
528.1935
657.2357
1054.5281 b21(2+)
1127.5627 PEP_0_0_0_0_0
1229.1066 PEP_0_1_0_0_0
1573.7217 PEP_3_2_0_0_0
1654.7407 PEP_4_2_0_0_0

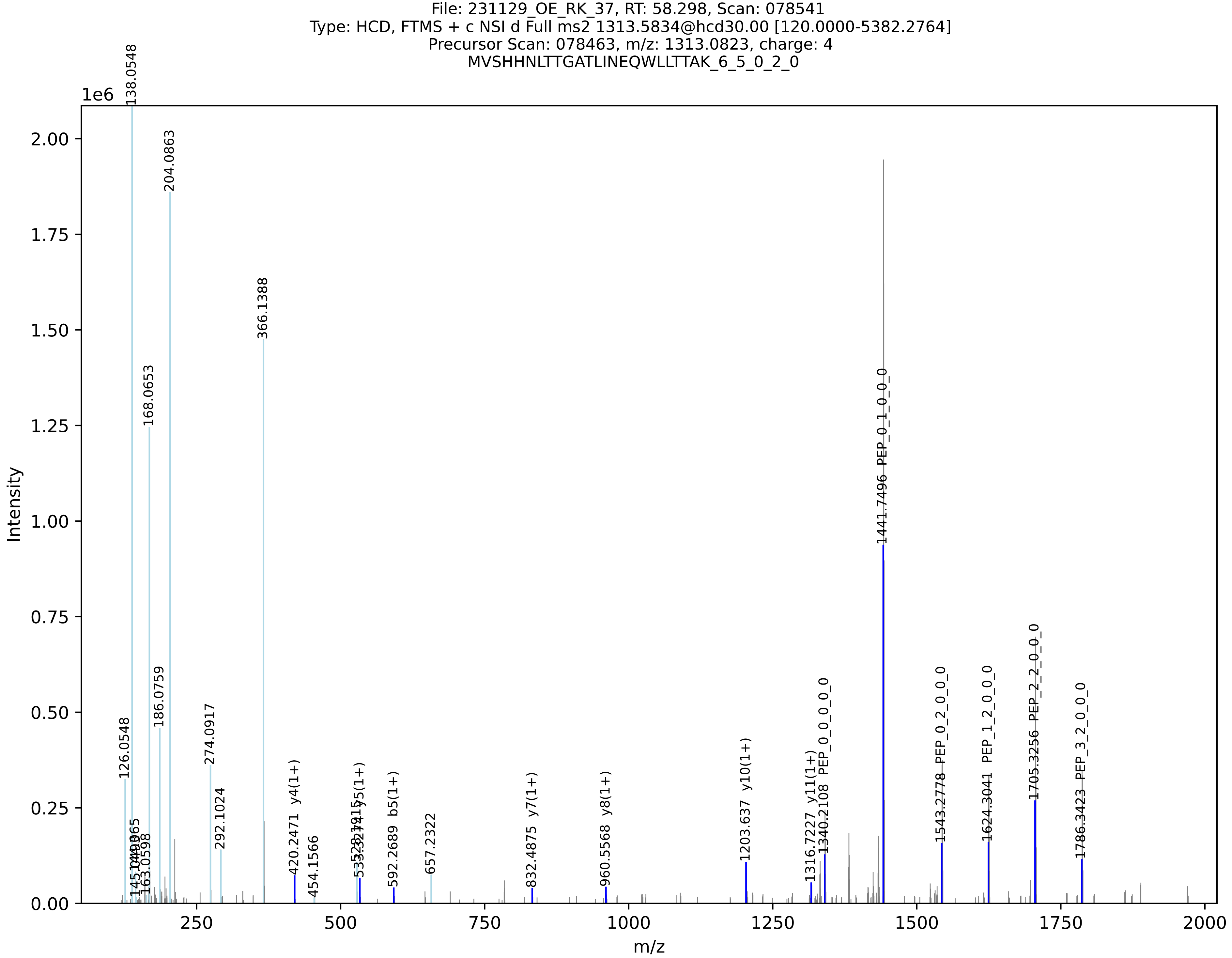
File: 231129_OE_RK_37, RT: 58.298, Scan: 078541
Type: HCD, FTMS + c NSI d Full ms2 1313.5834@hcd30.00 [120.0000-5382.2764]
Precursor Scan: 078463, m/z: 1313.0823, charge: 4
MVSHHNLTTGATLINEQWLLTTAK_6_5_0_2_0
1e6
Intensity
m/z
138.0548
204.0863
366.1388
168.0653
186.0759
126.0548
274.0917
292.1024
163.0598
420.2471 y4(1+)
454.1566
592.2689 b5(1+)
657.2322
832.4875 y7(1+)
960.5568 y8(1+)
1203.637 y10(1+)
1316.7227 y11(1+)
1340.2108 PEP_0_0_0_0_0
1441.7496 PEP_0_1_0_0_0
1543.2778 PEP_0_2_0_0_0
1624.3041 PEP_1_2_0_0_0
1705.3256 PEP_2_2_0_0_0
1786.3423 PEP_3_2_0_0_0

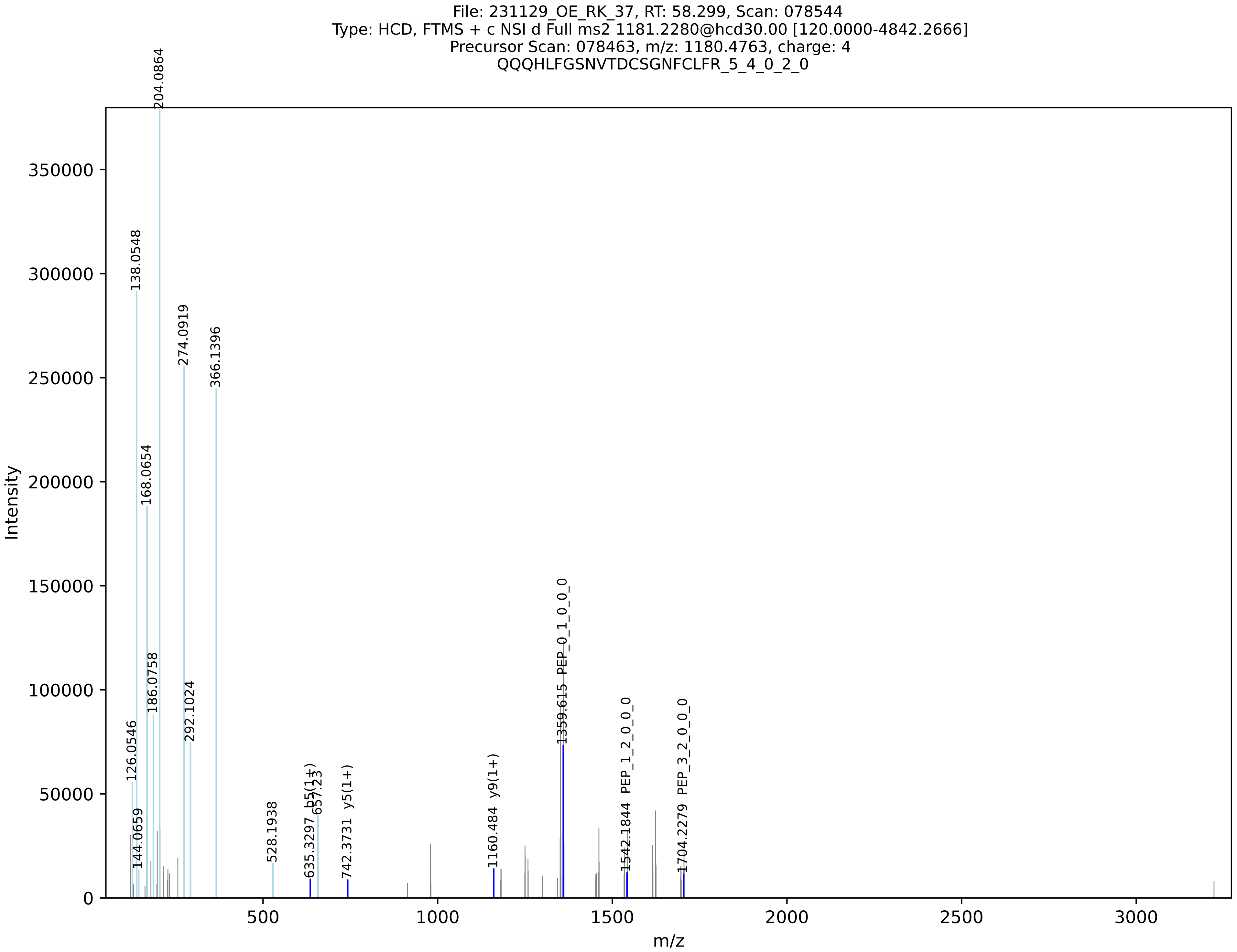

File: 231129_OE_RK_37, RT: 58.299, Scan: 078544
Type: HCD, FTMS + c NSI d Full ms2 1181.2280@hcd30.00 [120.0000-4842.2666]
Precursor Scan: 078463, m/z: 1180.4763, charge: 4
QQQHLFGSNVTDCSGNFCLFR_5_4_0_2_0
Intensity
m/z
204.0864
138.0548
274.0919
366.1396
168.0654
186.0758
292.1024
126.0546
144.0659
528.1938
635.3297 b5(1+)
657.23
742.3731 y5(1+)
1160.484 y9(1+)
1359.615 PEP_0_1_0_0_0
1542.1844 PEP_1_2_0_0_0
1704.2279 PEP_3_2_0_0_0

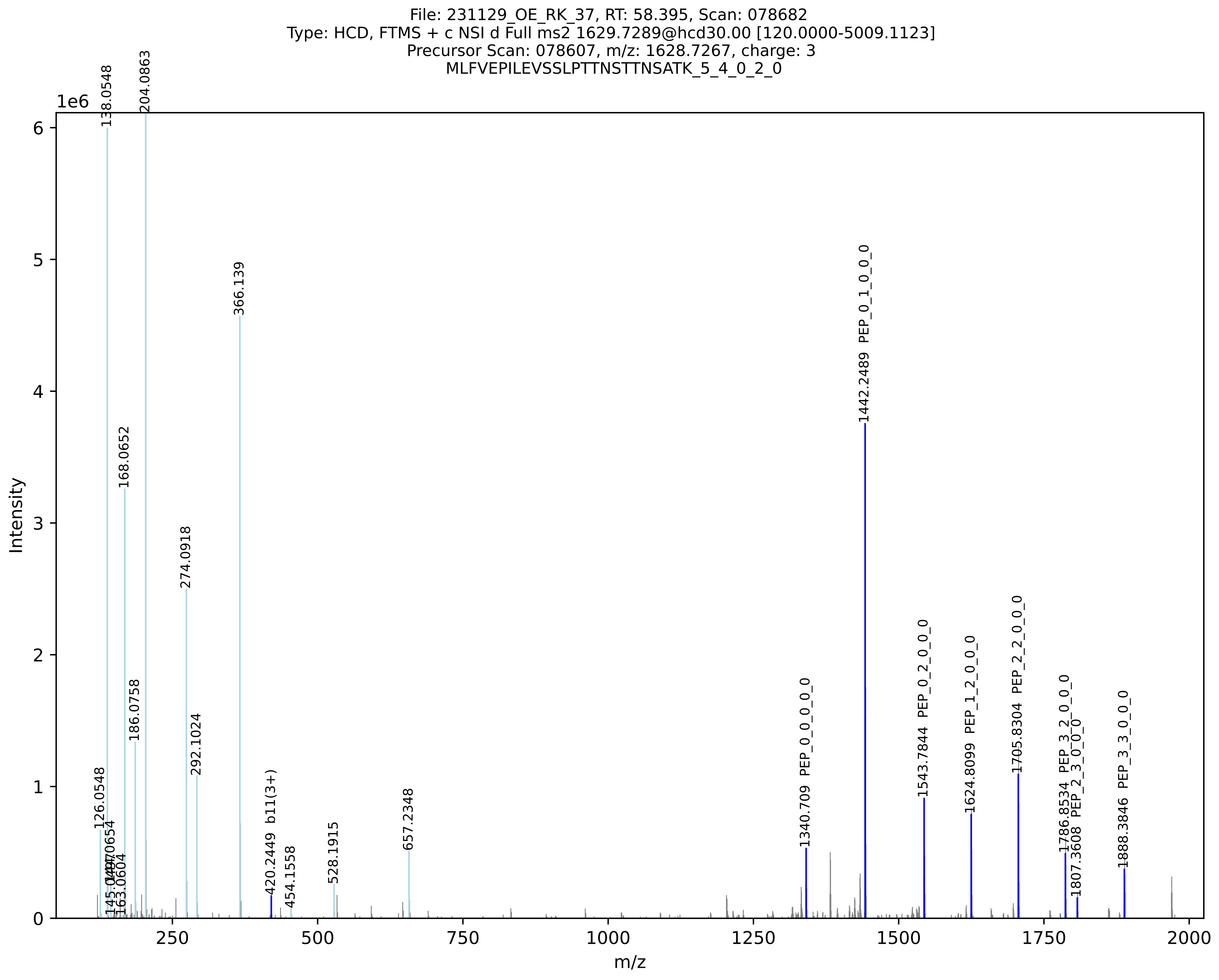

File: 231129_OE_RK_37, RT: 58.395, Scan: 078682
Type: HCD, FTMS + c NSI d Full ms2 1629.7289@hcd30.00 [120.0000-5009.1123]
Precursor Scan: 078607, m/z: 1628.7267, charge: 3
MLFVEPILEVSSLPTTNSTTNSATK_5_4_0_2_0
1e6
Intensity
m/z
138.0548
204.0863
366.139
168.0652
274.0918
186.0758
292.1024
126.0548
163.0604
420.2449 b11(3+)
454.1558
528.1915
657.2348
1340.709 PEP_0_0_0_0_0
1442.2489 PEP_0_1_0_0_0
1543.7844 PEP_0_2_0_0_0
1624.8099 PEP_1_2_0_0_0
1705.8304 PEP_2_2_0_0_0
1786.8534 PEP_3_2_0_0_0
1807.3608 PEP_2_3_0_0_0
1888.3846 PEP_3_3_0_0_0

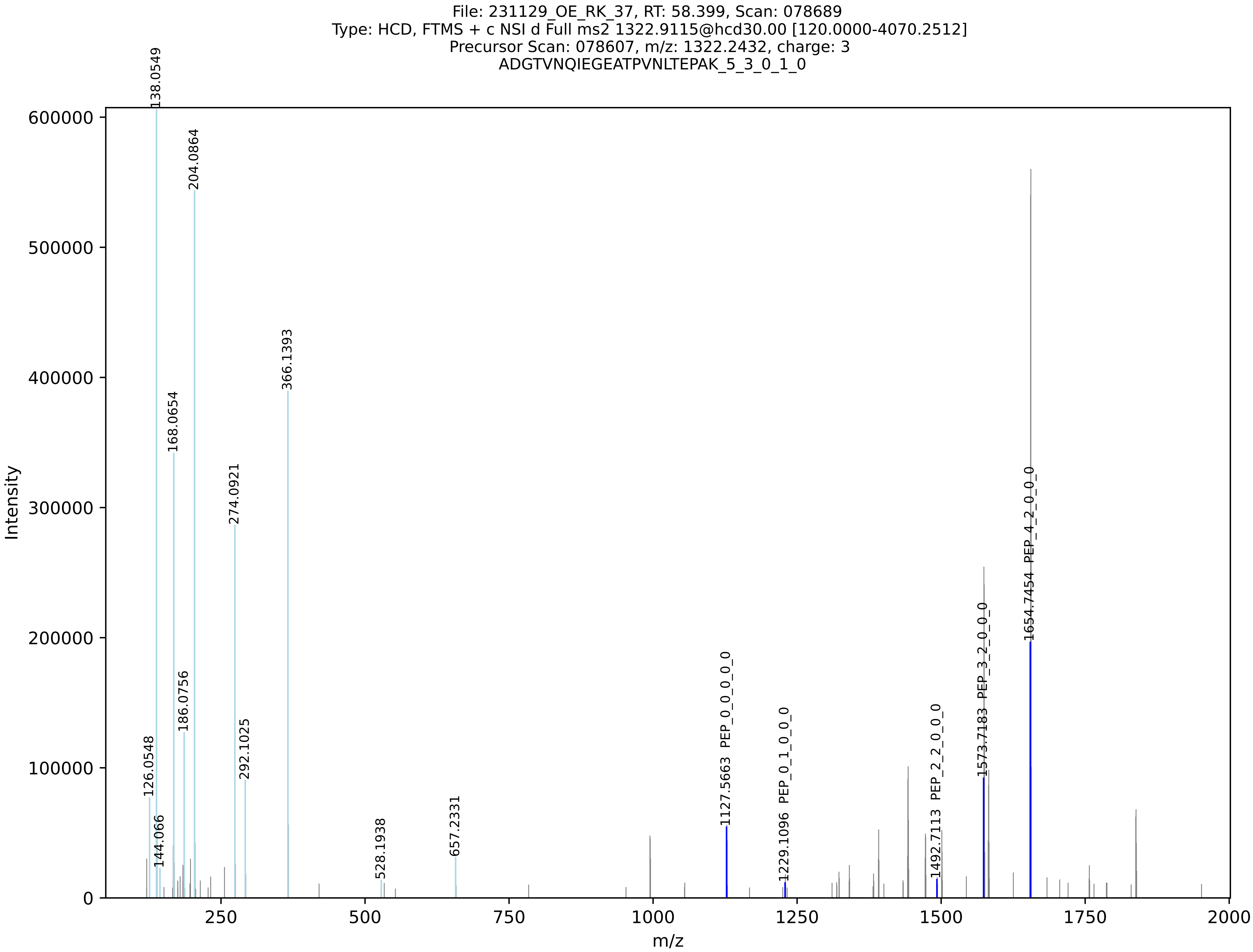
File: 231129_OE_RK_37, RT: 58.399, Scan: 078689
Type: HCD, FTMS + c NSI d Full ms2 1322.9115@hcd30.00 [120.0000-4070.2512]
Precursor Scan: 078607, m/z: 1322.2432, charge: 3
ADGTVNQIEGEATPVNLTEPAK_5_3_0_1_0
Intensity
m/z
138.0549
204.0864
366.1393
168.0654
274.0921
186.0756
292.1025
126.0548
144.066
528.1938
657.2331
1127.5663 PEP_0_0_0_0_0
1229.1096 PEP_0_1_0_0_0
1492.7113 PEP_2_2_0_0_0
1573.7183 PEP_3_2_0_0_0
1654.7454 PEP_4_2_0_0_0

File: 231129_OE_RK_37, RT: 58.427, Scan: 078734
Type: HCD, FTMS + c NSI d Full ms2 1631.7275@hcd30.00 [120.0000-5015.2280]
Precursor Scan: 078534, m/z: 1628.7262, charge: 3
MLFVEPILEVSSLPTTNSTTNSATK_5_4_0_2_0

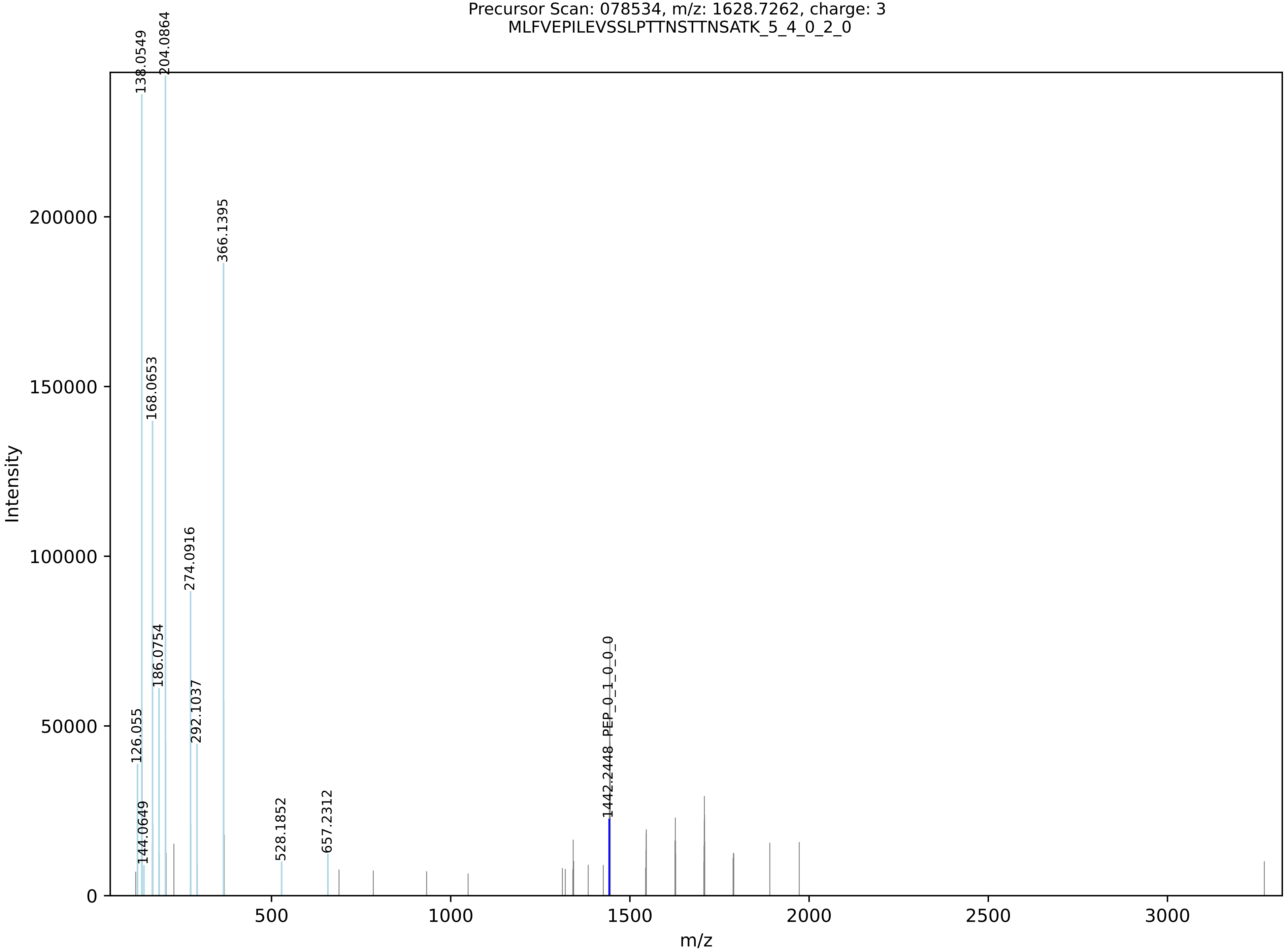

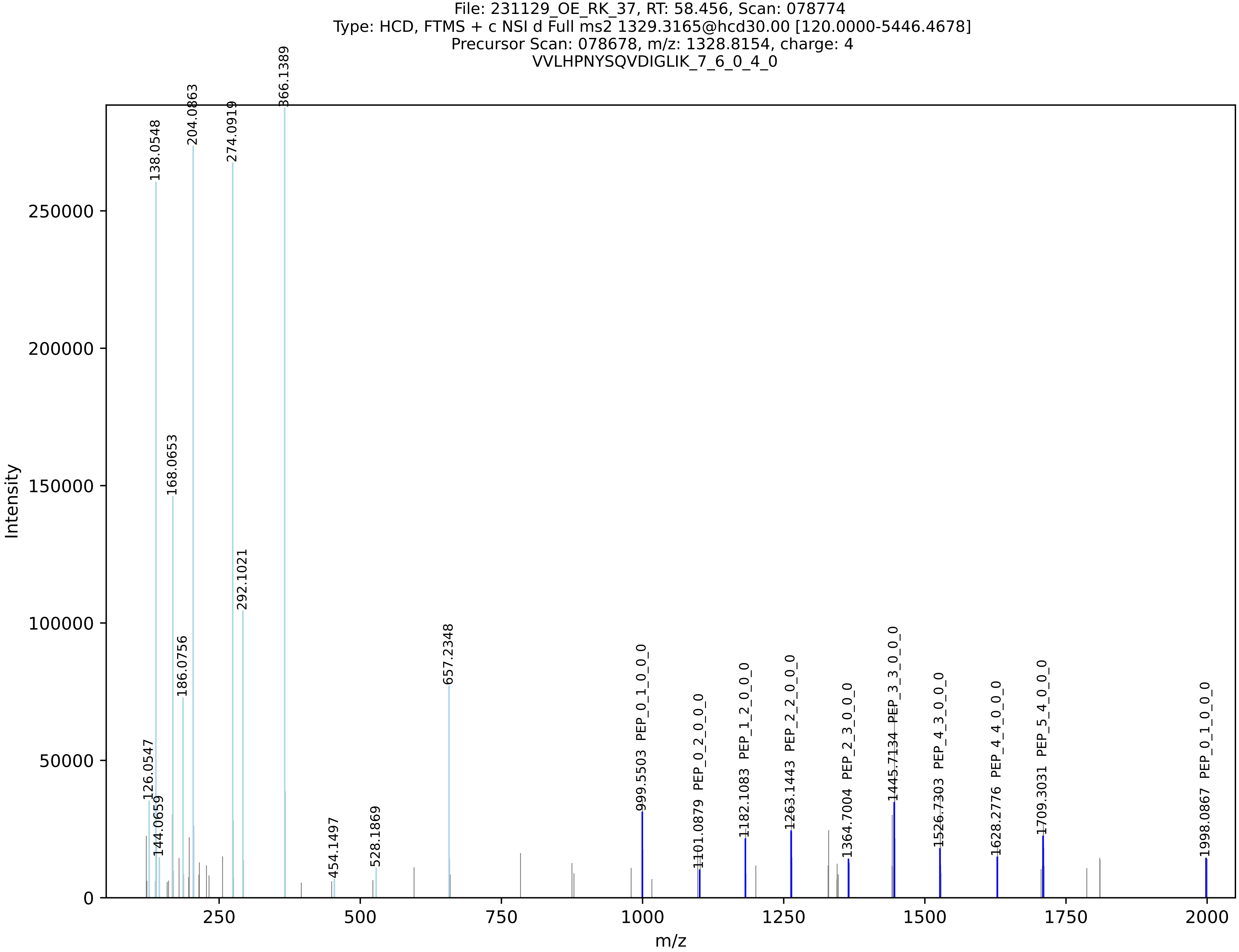

File: 231129_OE_RK_37, RT: 58.456, Scan: 078774
Type: HCD, FTMS + c NSI d Full ms2 1329.3165@hcd30.00 [120.0000-5446.4678]
Precursor Scan: 078678, m/z: 1328.8154, charge: 4
VVLHPNYSQVDIGLIK_7_6_0_4_0
Intensity
m/z
366.1389
204.0863
274.0919
138.0548
168.0653
292.1021
186.0756
126.0547
144.0659
657.2348
454.1497
528.1869
999.5503 PEP_0_1_0_0_0
1101.0879 PEP_0_2_0_0_0
1182.1083 PEP_1_2_0_0_0
1263.1443 PEP_2_2_0_0_0
1364.7004 PEP_2_3_0_0_0
1445.7134 PEP_3_3_0_0_0
1526.7303 PEP_4_3_0_0_0
1628.2776 PEP_4_4_0_0_0
1709.3031 PEP_5_4_0_0_0
1998.0867 PEP_0_1_0_0_0

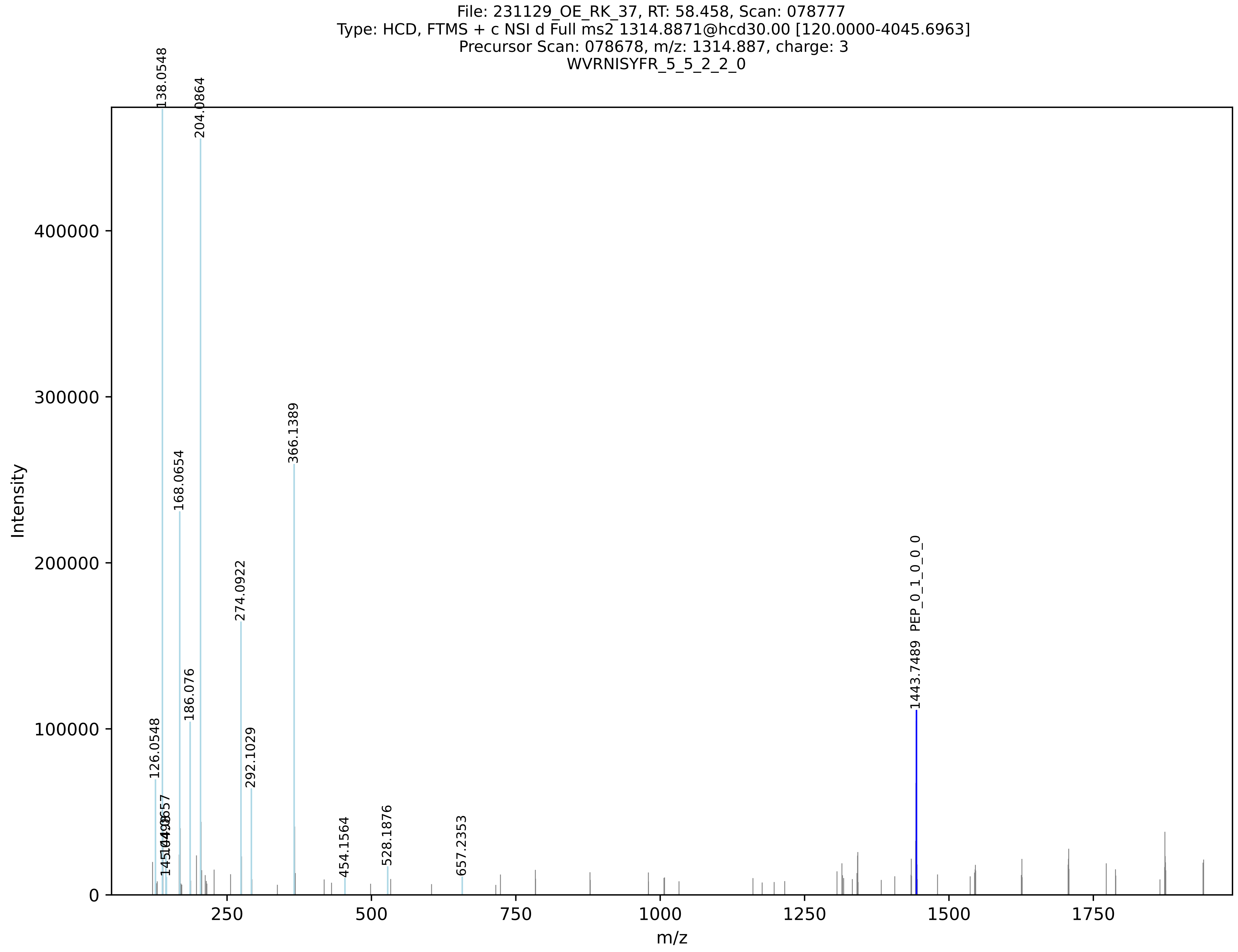

File: 231129_OE_RK_37, RT: 58.458, Scan: 078777
Type: HCD, FTMS + c NSI d Full ms2 1314.8871@hcd30.00 [120.0000-4045.6963]
Precursor Scan: 078678, m/z: 1314.887, charge: 3
WVRNISYFR_5_5_2_2_0
138.0548
204.0864
366.1389
168.0654
274.0922
186.076
126.0548
292.1029
454.1564
528.1876
657.2353
1443.7489 PEP_0_1_0_0_0
Intensity
0
100000
200000
300000
400000
250
500
750
1000
1250
1500
1750
m/z

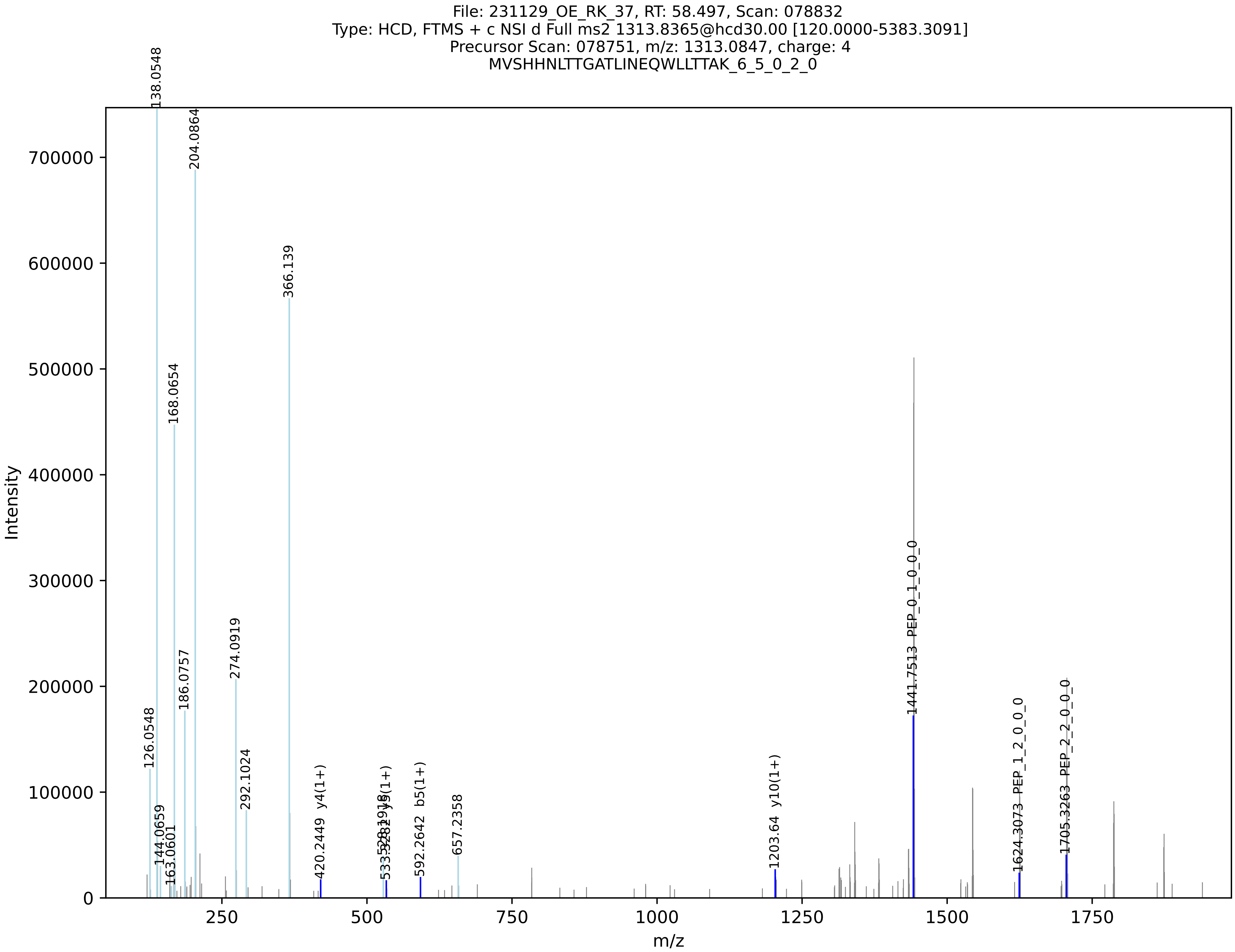
File: 231129_OE_RK_37, RT: 58.497, Scan: 078832
Type: HCD, FTMS + c NSI d Full ms2 1313.8365@hcd30.00 [120.0000-5383.3091]
Precursor Scan: 078751, m/z: 1313.0847, charge: 4
MVSHHNLTTGATLINEQWLLTTAK_6_5_0_2_0
Intensity
m/z
138.0548
204.0864
366.139
168.0654
186.0757
274.0919
126.0548
292.1024
144.0659
163.0601
420.2449 y4(1+)
533.3282 y5(1+)
592.2642 b5(1+)
657.2358
1203.64 y10(1+)
1441.7513_PEP_0_1_0_0_0_
1624.3073_PEP_1_2_0_0_0
1705.3263_PEP_2_2_0_0_0_

File: 231129_OE_RK_37, RT: 58.595, Scan: 078976
Type: HCD, FTMS + c NSI d Full ms2 1629.7311@hcd30.00 [120.0000-5009.1191]
Precursor Scan: 078898, m/z: 1628.7288, charge: 3
MLFVEPILEVSSLPTTNSTTNSATK_5_4_0_2_0

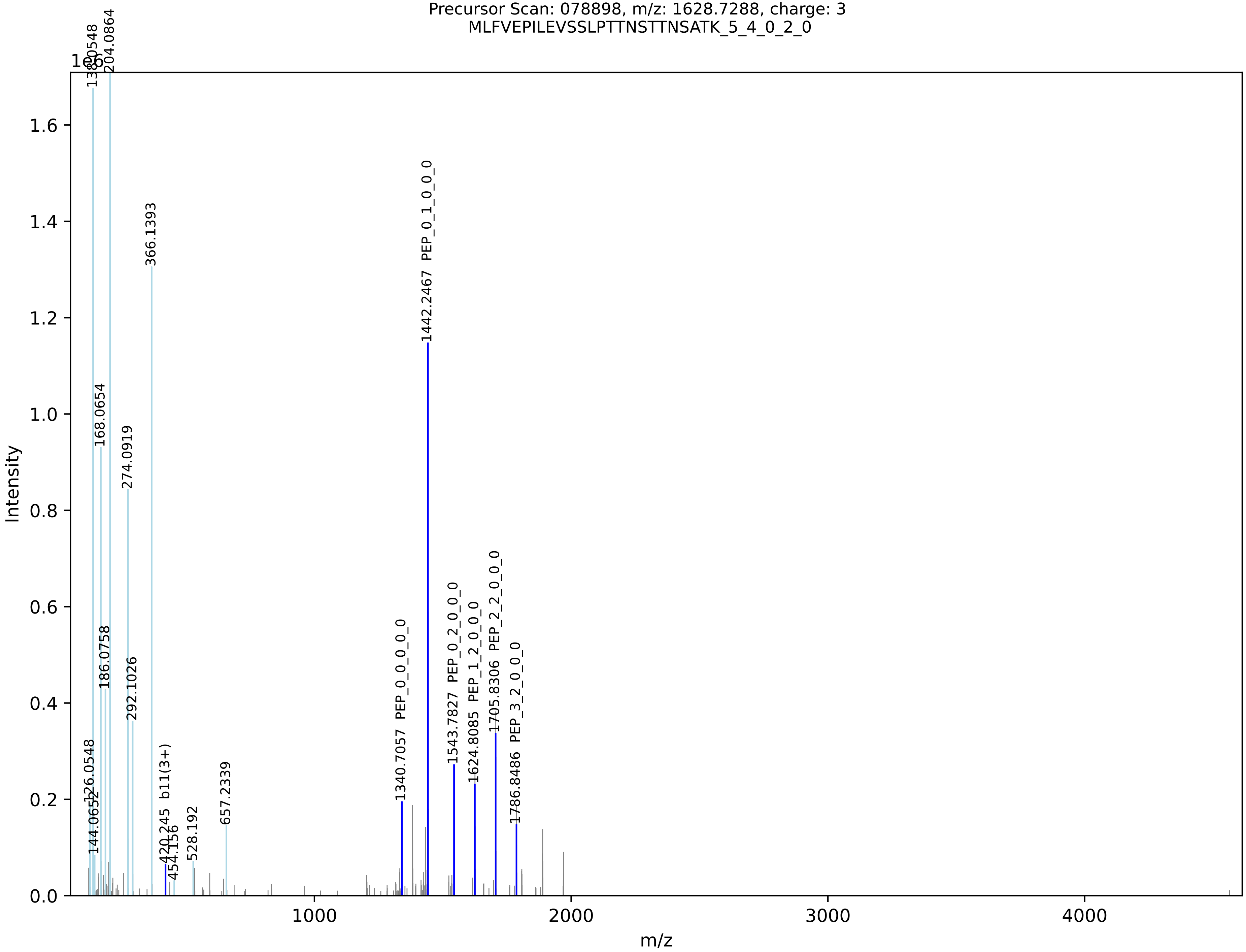

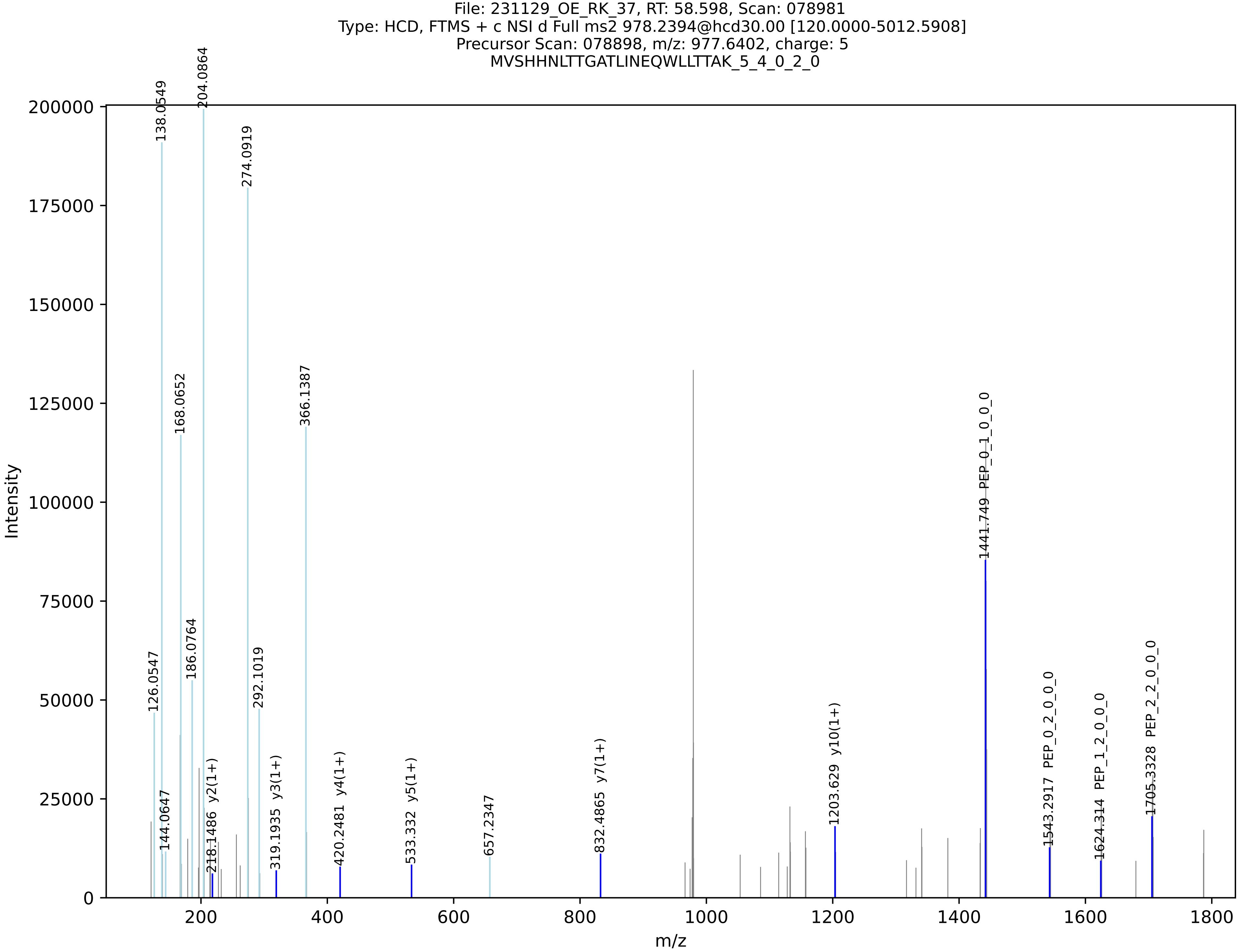
File: 231129_OE_RK_37, RT: 58.598, Scan: 078981
Type: HCD, FTMS + c NSI d Full ms2 978.2394@hcd30.00 [120.0000-5012.5908]
Precursor Scan: 078898, m/z: 977.6402, charge: 5
MVSHHNLTTGATLINEQWLLTTAK_5_4_0_2_0
Intensity
m/z
138.0549
204.0864
274.0919
168.0652
366.1387
126.0547
186.0764
292.1019
144.0647
218.1486 y2(1+)
319.1935 y3(1+)
420.2481 y4(1+)
533.332 y5(1+)
657.2347
832.4865 y7(1+)
1203.629 y10(1+)
1441.749 PEP_0_1_0_0_0
1543.2917 PEP_0_2_0_0_0
1624.314 PEP_1_2_0_0_0
1705.3328 PEP_2_2_0_0_0

File: 231129_OE_RK_37, RT: 58.599, Scan: 078982
Type: HCD, FTMS + c NSI d Full ms2 1322.5769@hcd30.00 [120.0000-4069.2275]
Precursor Scan: 078898, m/z: 1322.2425, charge: 3
ADGTVNQIEGEATPVNLTEPAK_5_3_0_1_0

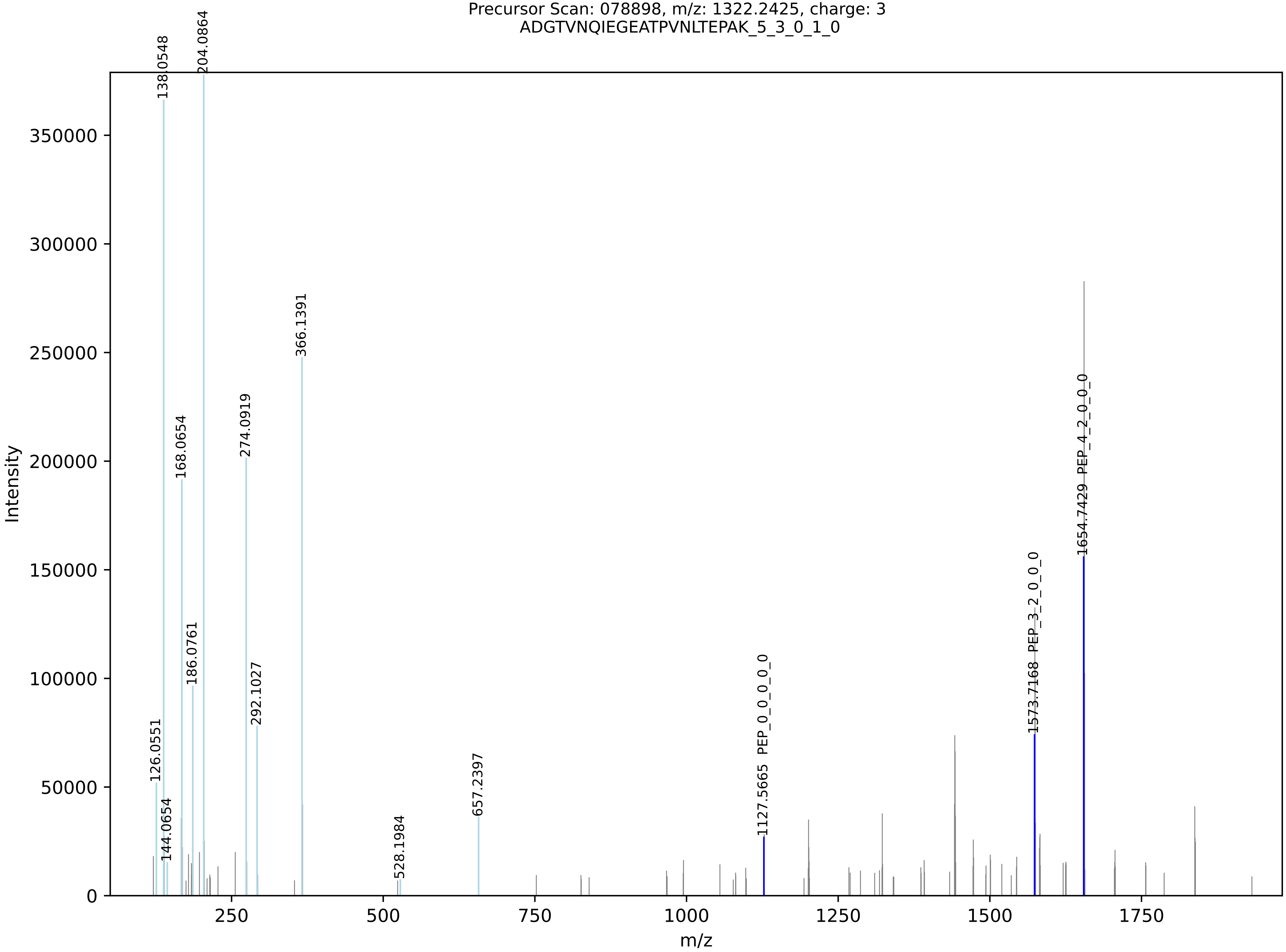

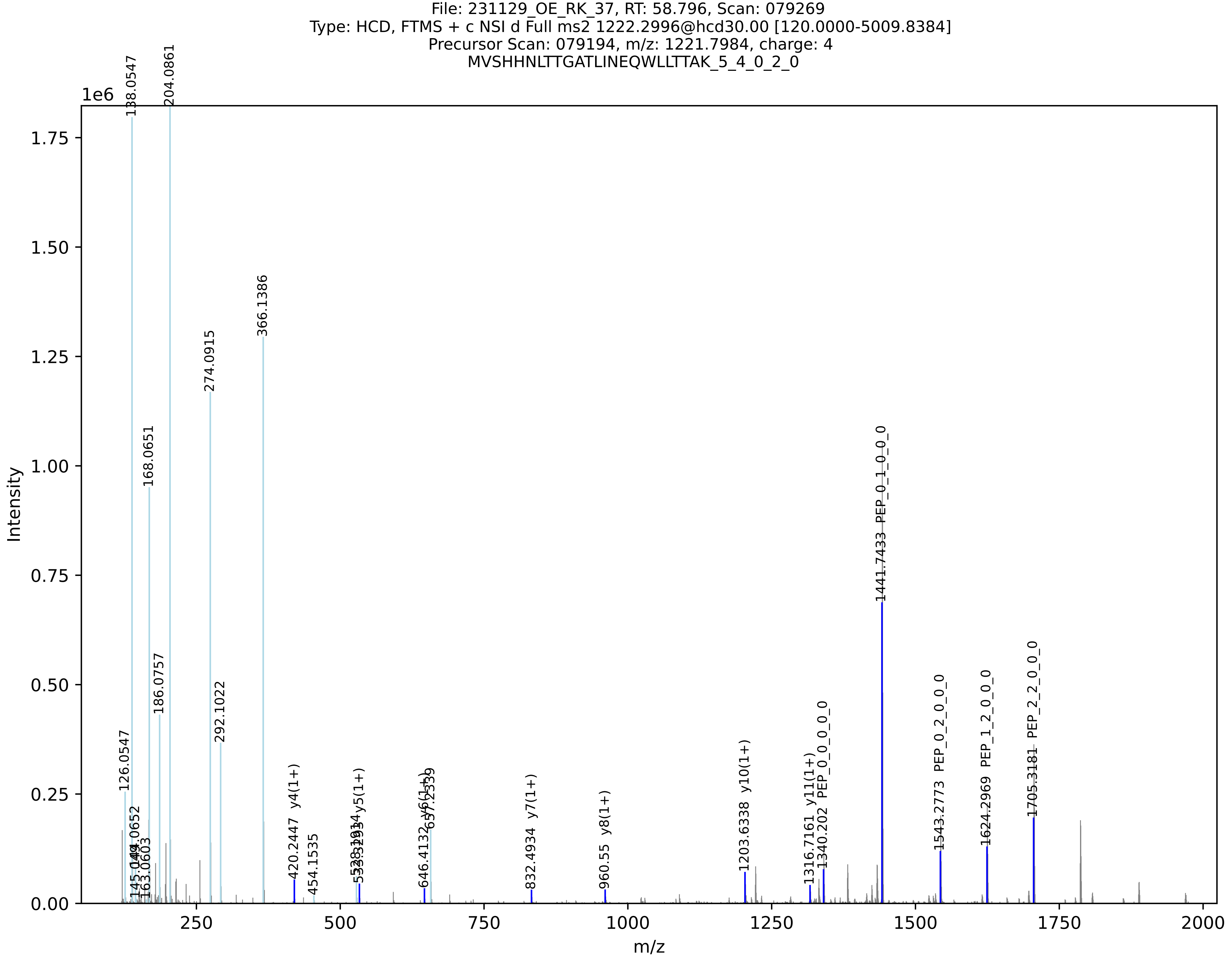
File: 231129_OE_RK_37, RT: 58.796, Scan: 079269
Type: HCD, FTMS + c NSI d Full ms2 1222.2996@hcd30.00 [120.0000-5009.8384]
Precursor Scan: 079194, m/z: 1221.7984, charge: 4
MVSHHNLTTGATLINEQWLLTTAK_5_4_0_2_0
1e6
Intensity
m/z
138.0547
204.0861
366.1386
274.0915
168.0651
186.0757
292.1022
126.0547
163.0603
420.2447 y4(1+)
454.1535
y5(1+)
646.4132 y6(1+)
657.2339
832.4934 y7(1+)
960.55 y8(1+)
1203.6338 y10(1+)
1316.7161 y11(1+)
1340.202 PEP_0_0_0_0_0
1441.7433 PEP_0_1_0_0_0
1543.2773 PEP_0_2_0_0_0
1624.2969 PEP_1_2_0_0_0
1705.3181 PEP_2_2_0_0_0

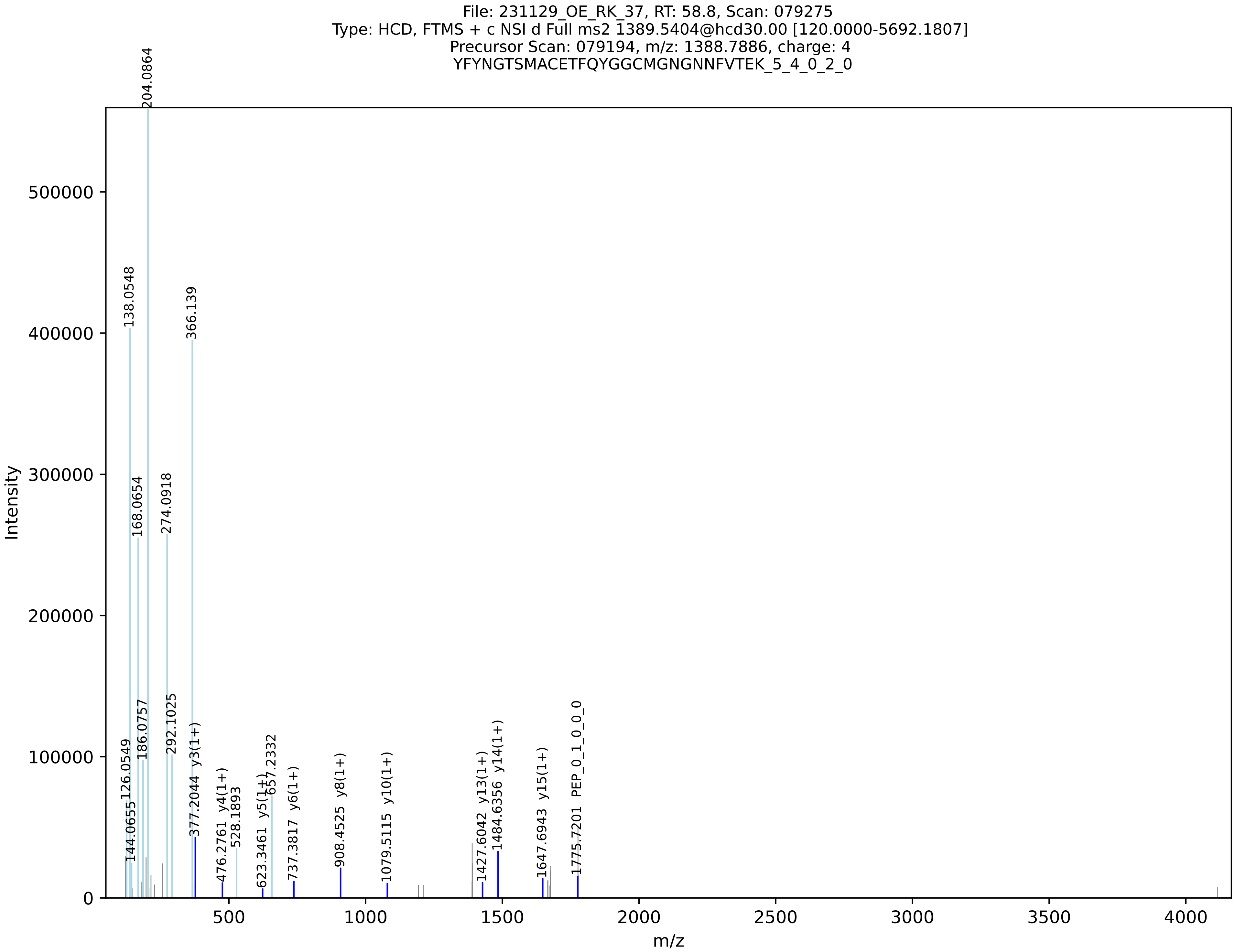

File: 231129_OE_RK_37, RT: 58.8, Scan: 079275
Type: HCD, FTMS + c NSI d Full ms2 1389.5404@hcd30.00 [120.0000-5692.1807]
Precursor Scan: 079194, m/z: 1388.7886, charge: 4
YFYNGTSMACETFQYGGCMGNGNNFVTEK_5_4_0_2_0
Intensity
m/z
204.0864
138.0548
366.139
168.0654
274.0918
186.0757
292.1025
126.0549
144.0655
377.2044 y3(1+)
476.2761 y4(1+)
528.1893
623.3461 y5(1+)
657.2332
737.3817 y6(1+)
908.4525 y8(1+)
1079.5115 y10(1+)
1427.6042 y13(1+)
1484.6356 y14(1+)
1647.6943 y15(1+)
1775.7201 PEP_0_1_0_0_0

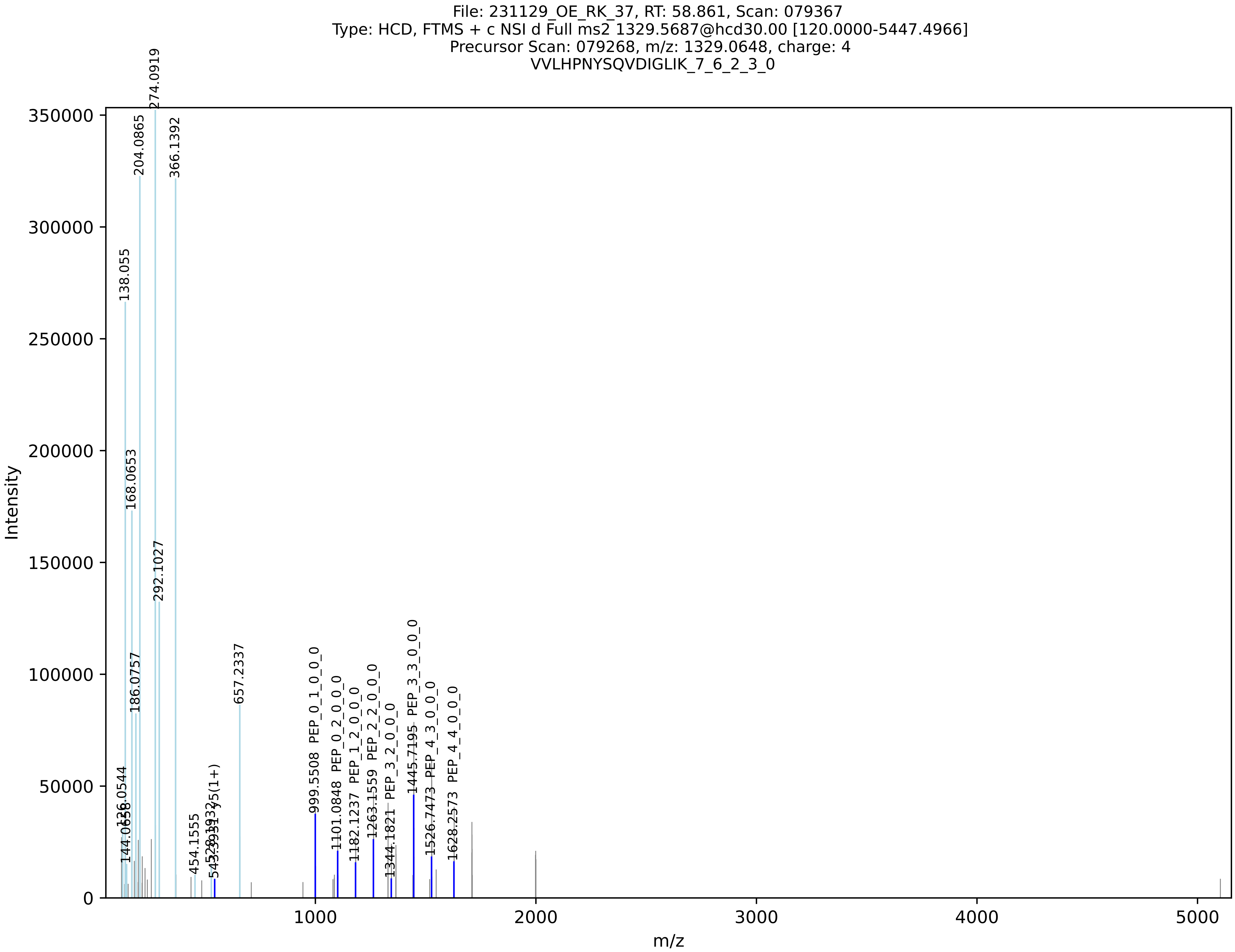

File: 231129_OE_RK_37, RT: 58.861, Scan: 079367
Type: HCD, FTMS + c NSI d Full ms2 1329.5687@hcd30.00 [120.0000-5447.4966]
Precursor Scan: 079268, m/z: 1329.0648, charge: 4
VVLHPNYSQVDIGLIK_7_6_2_3_0
Intensity
m/z
274.0919
204.0865
366.1392
138.055
168.0653
292.1027
186.0757
657.2337
126.0544
144.0658
454.1555
528.1932
543.3351 y5(1+)
999.5508 PEP_0_1_0_0_0
1101.0848 PEP_0_2_0_0_0
1182.1237 PEP_1_2_0_0_0
1263.1559 PEP_2_2_0_0_0
1344.1821 PEP_3_2_0_0_0
1445.7195 PEP_3_3_0_0_0
1526.7473 PEP_4_3_0_0_0
1628.2573 PEP_4_4_0_0_0

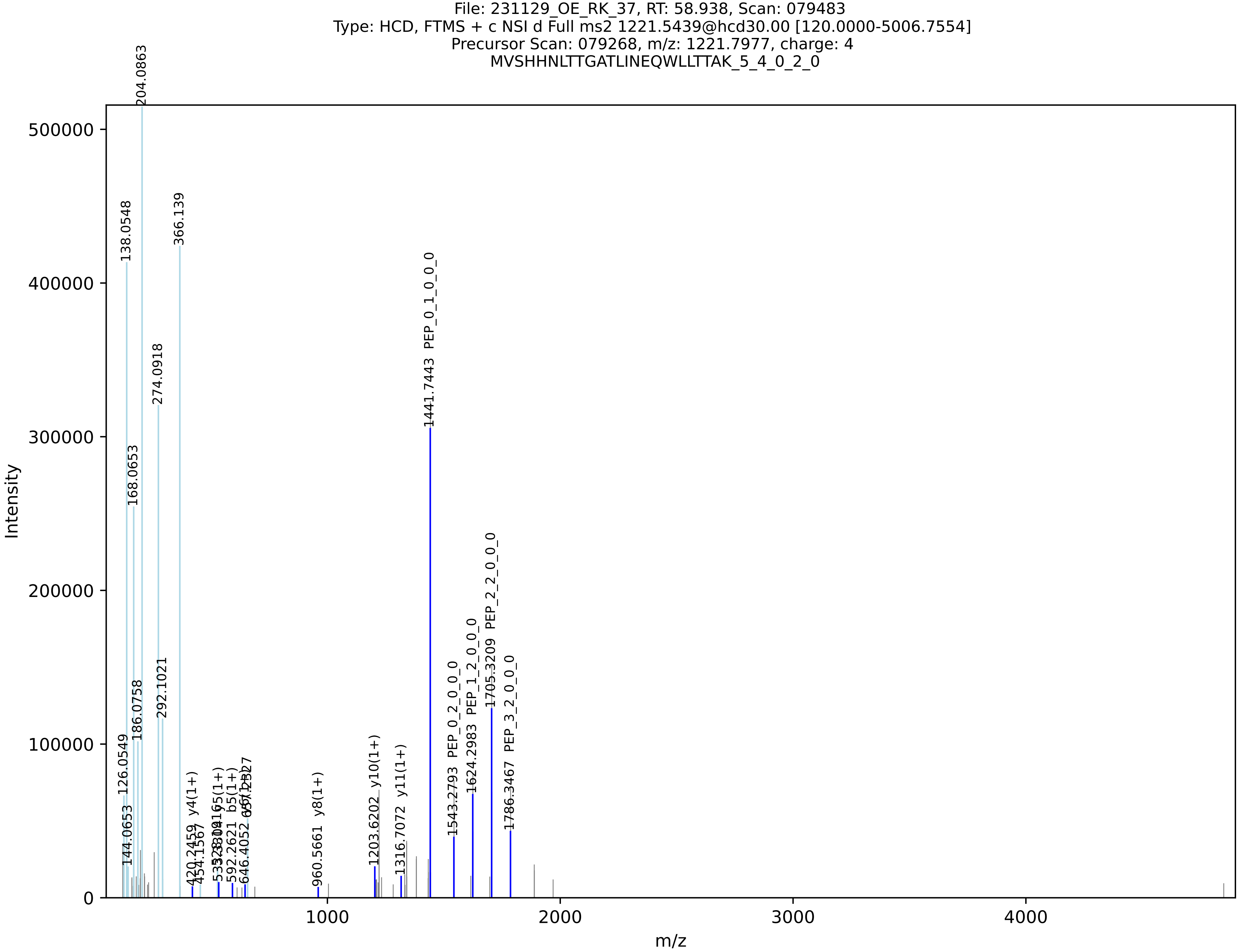
File: 231129_OE_RK_37, RT: 58.938, Scan: 079483
Type: HCD, FTMS + c NSI d Full ms2 1221.5439@hcd30.00 [120.0000-5006.7554]
Precursor Scan: 079268, m/z: 1221.7977, charge: 4
MVSHHNLTTGATLINEQWLLTTAK_5_4_0_2_0
Intensity
m/z
204.0863
138.0548
366.139
274.0918
168.0653
186.0758
292.1021
126.0549
144.0653
420.2459 y4(1+)
454.1567
592.2621 b5(1+)
646.4052
960.5661 y8(1+)
1203.6202 y10(1+)
1316.7072 y11(1+)
1441.7443 PEP_0_1_0_0_0
1543.2793 PEP_0_2_0_0_0
1624.2983 PEP_1_2_0_0_0
1705.3209 PEP_2_2_0_0_0
1786.3467 PEP_3_2_0_0_0

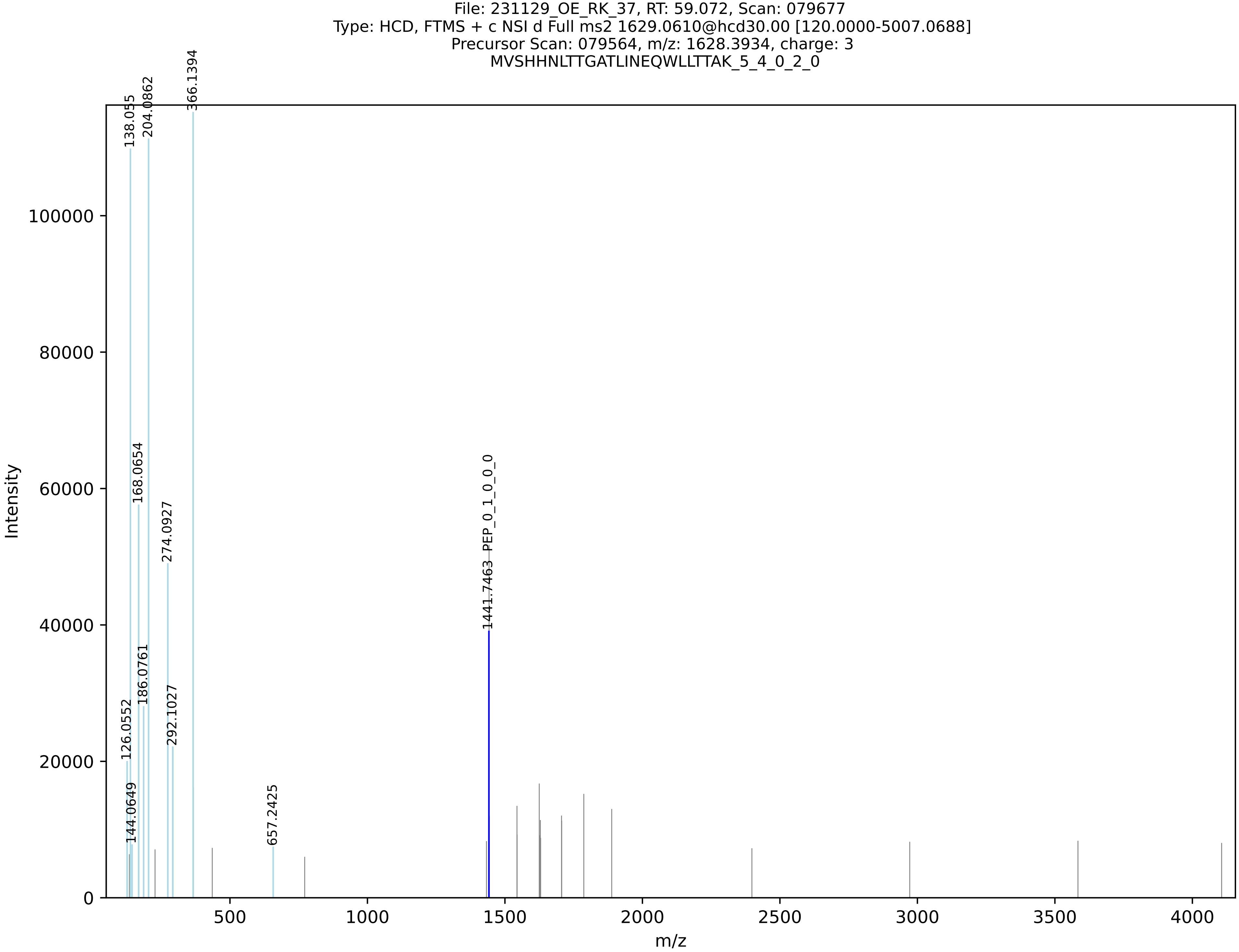
File: 231129_OE_RK_37, RT: 59.072, Scan: 079677
Type: HCD, FTMS + c NSI d Full ms2 1629.0610@hcd30.00 [120.0000-5007.0688]
Precursor Scan: 079564, m/z: 1628.3934, charge: 3
MVSHHNLTTGATLINEQWLLTTAK_5_4_0_2_0
Intensity
m/z
0
20000
40000
60000
80000
100000
500
1000
1500
2000
2500
3000
3500
4000
138.055
204.0862
366.1394
168.0654
274.0927
186.0761
126.0552
292.1027
144.0649
657.2425
1441.7463_PEP_0_1_0_0_0_

File: 231129_OE_RK_37, RT: 59.202, Scan: 079863
Type: HCD, FTMS + c NSI d Full ms2 1222.2996@hcd30.00 [120.0000-5009.8384]
Precursor Scan: 079786, m/z: 1221.5478, charge: 4
MVSHHNLTTGATLINEQWLLTTAK_5_4_0_2_0

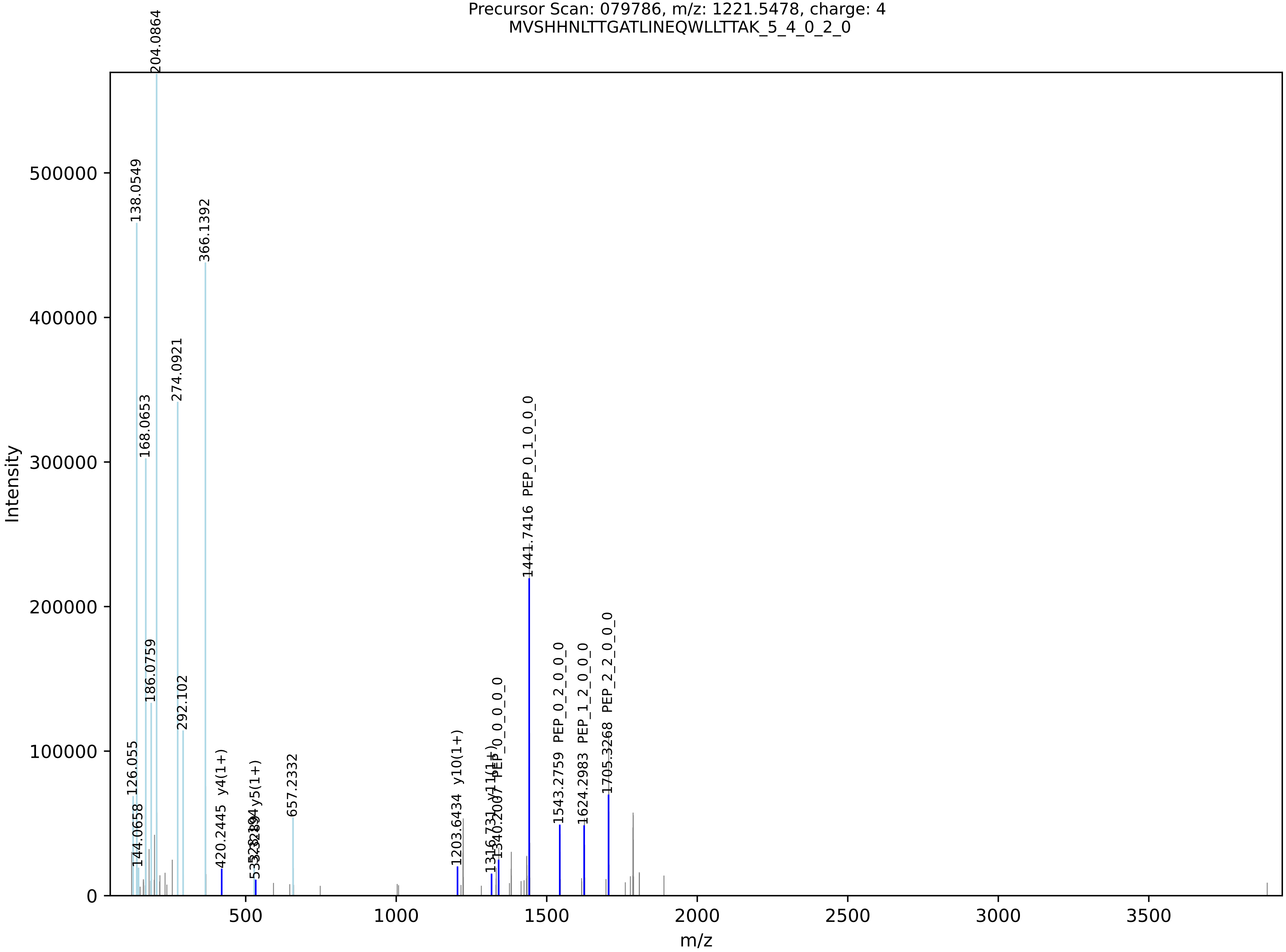

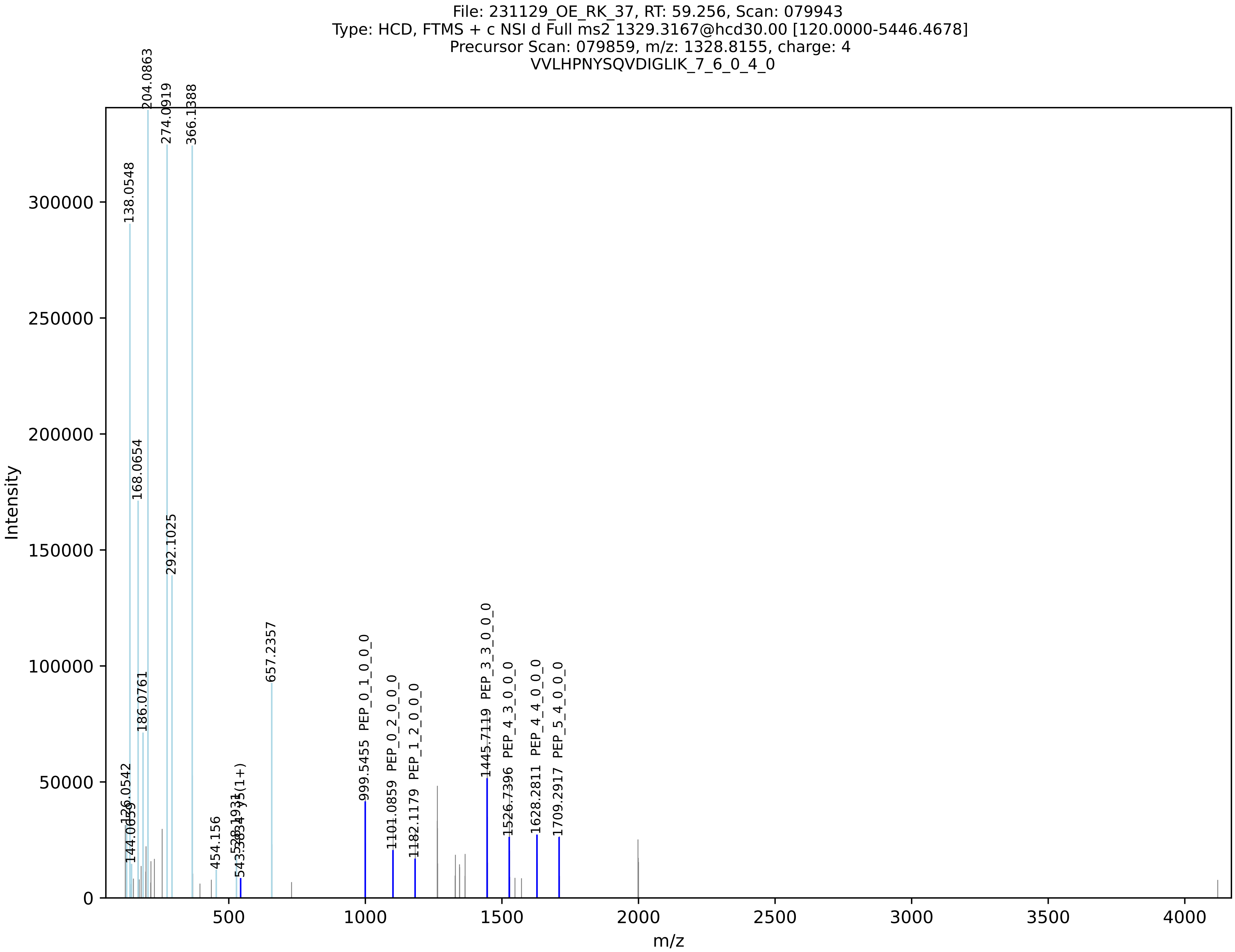

File: 231129_OE_RK_37, RT: 59.256, Scan: 079943
Type: HCD, FTMS + c NSI d Full ms2 1329.3167@hcd30.00 [120.0000-5446.4678]
Precursor Scan: 079859, m/z: 1328.8155, charge: 4
VVLHPNYSQVDIGLIK_7_6_0_4_0
Intensity
m/z
204.0863
274.0919
366.1388
138.0548
168.0654
292.1025
186.0761
126.0542
144.0659
454.156
528.1931
543.3034 y5(1+)
657.2357
999.5455 PEP_0_1_0_0_0
1101.0859 PEP_0_2_0_0_0
1182.1179 PEP_1_2_0_0_0
1445.7119 PEP_3_3_0_0_0
1526.7396 PEP_4_3_0_0_0
1628.2811 PEP_4_4_0_0_0
1709.2917 PEP_5_4_0_0_0

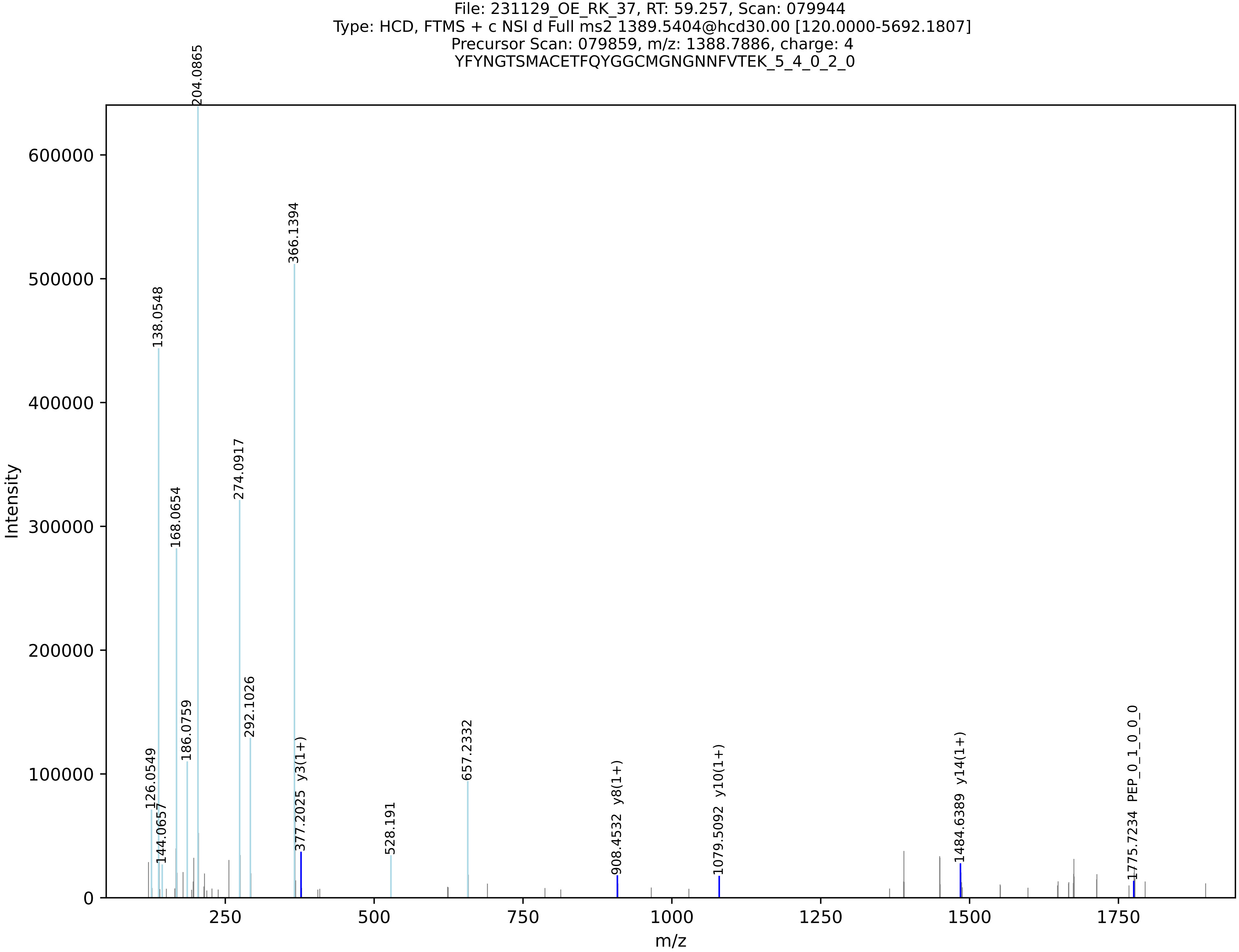

File: 231129_OE_RK_37, RT: 59.257, Scan: 079944
Type: HCD, FTMS + c NSI d Full ms2 1389.5404@hcd30.00 [120.0000-5692.1807]
Precursor Scan: 079859, m/z: 1388.7886, charge: 4
YFYNGTSMACETFQYGGCMGNGNNFVTEK_5_4_0_2_0
Intensity
m/z
204.0865
366.1394
138.0548
274.0917
168.0654
292.1026
186.0759
126.0549
144.0657
377.2025 y3(1+)
528.191
657.2332
908.4532 y8(1+)
1079.5092 y10(1+)
1484.6389 y14(1+)
1775.7234 PEP_0_1_0_0_0
0
100000
200000
300000
400000
500000
600000
250
500
750
1000
1250
1500
1750

File: 231129_OE_RK_37, RT: 59.314, Scan: 080030
Type: HCD, FTMS + c NSI d Full ms2 1390.1016@hcd30.00 [120.0000-5694.4707]
Precursor Scan: 079932, m/z: 1389.6004, charge: 4
NTTSYVLRGLEPGQEYNVLLTAEK_6_5_2_2_0

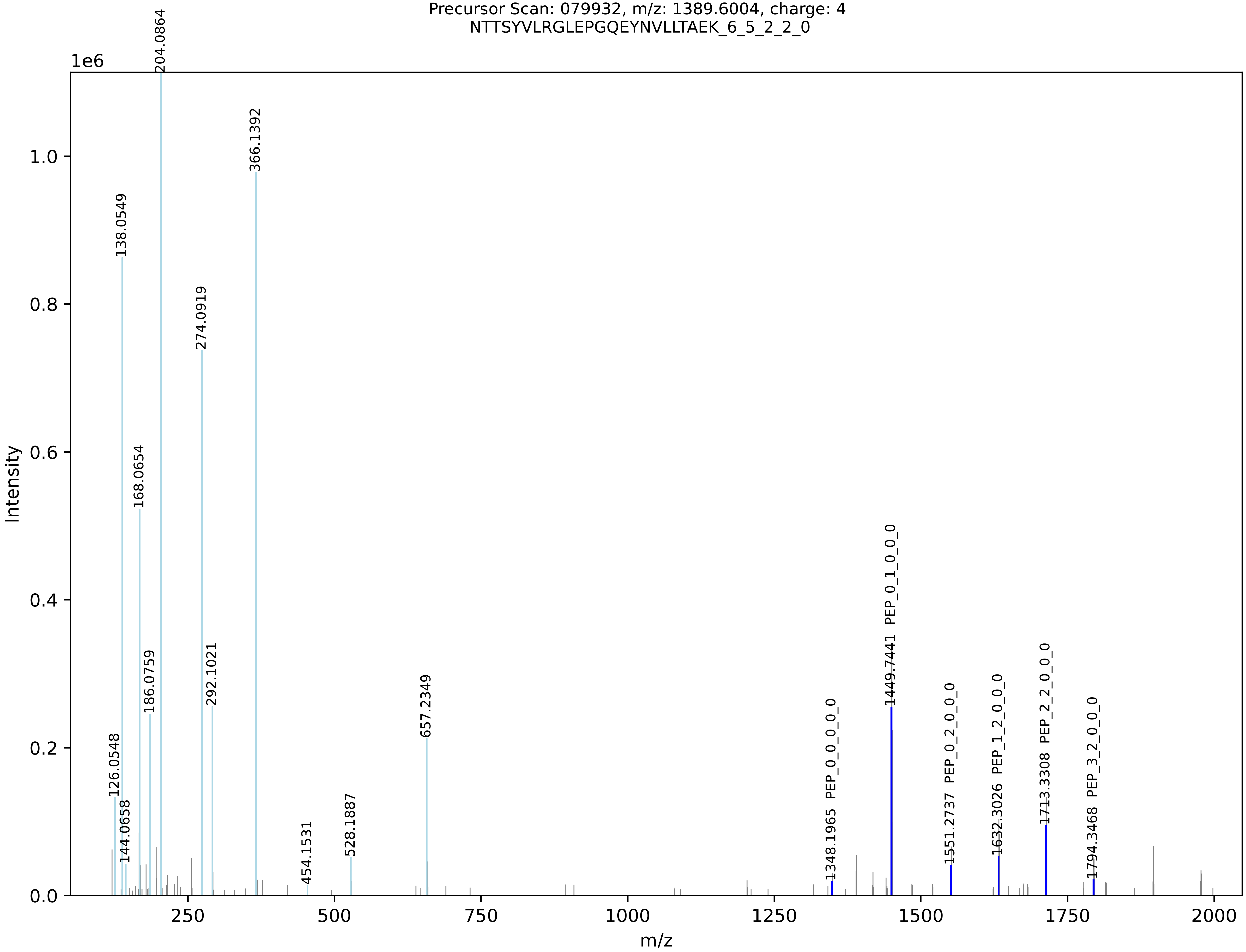

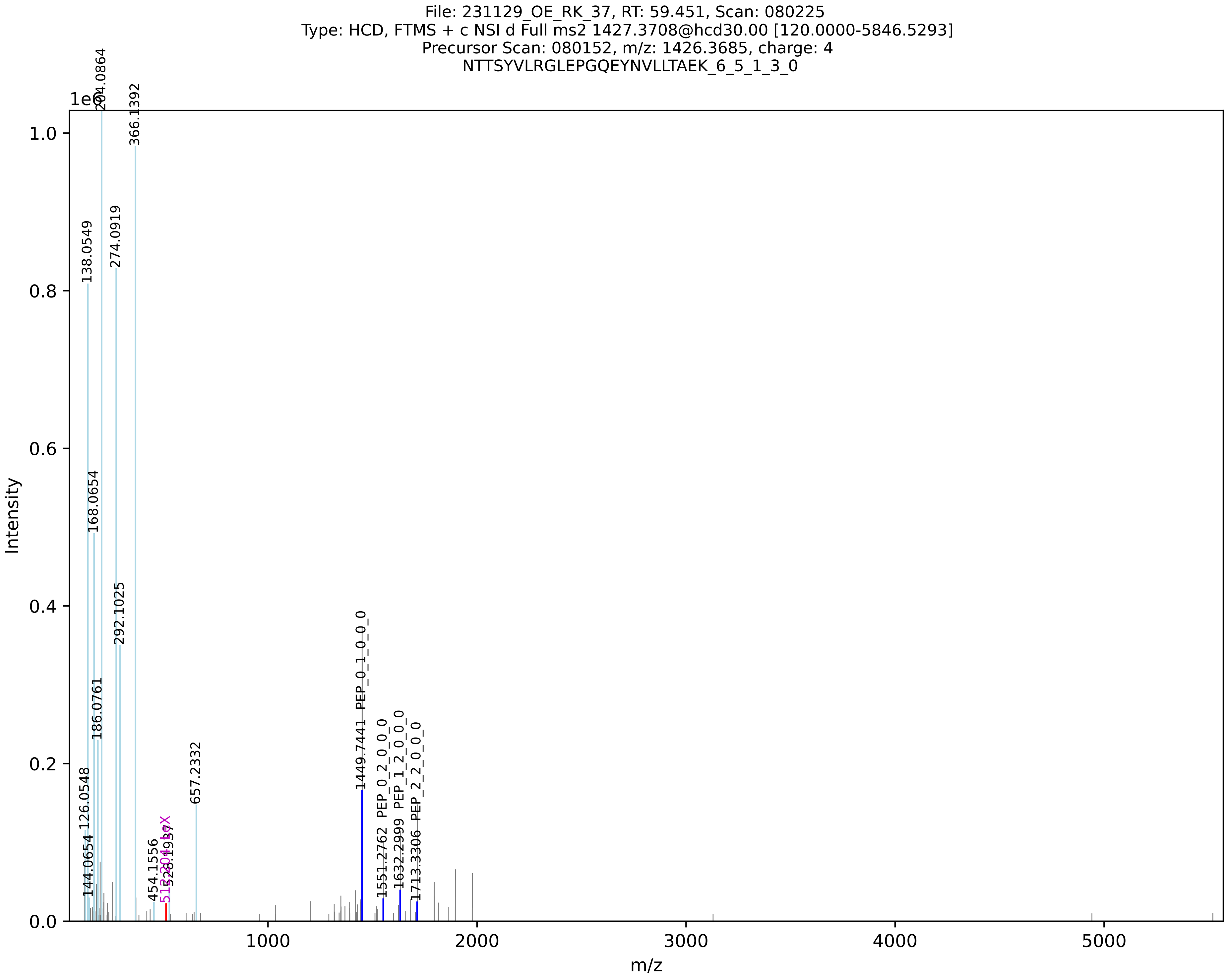
File: 231129_OE_RK_37, RT: 59.451, Scan: 080225
Type: HCD, FTMS + c NSI d Full ms2 1427.3708@hcd30.00 [120.0000-5846.5293]
Precursor Scan: 080152, m/z: 1426.3685, charge: 4
NTTSYVLRGLEPGQEYNVLLTAEK_6_5_1_3_0
1e6
Intensity
m/z
204.0864
366.1392
138.0549
274.0919
168.0654
292.1025
186.0761
126.0548
144.0654
454.1556
513.2204_beX
528.1937
657.2332
1449.7441_PEP_0_1_0_0_0
1551.2762_PEP_0_2_0_0_0
1632.2999_PEP_1_2_0_0_0
1713.3306_PEP_2_2_0_0_0

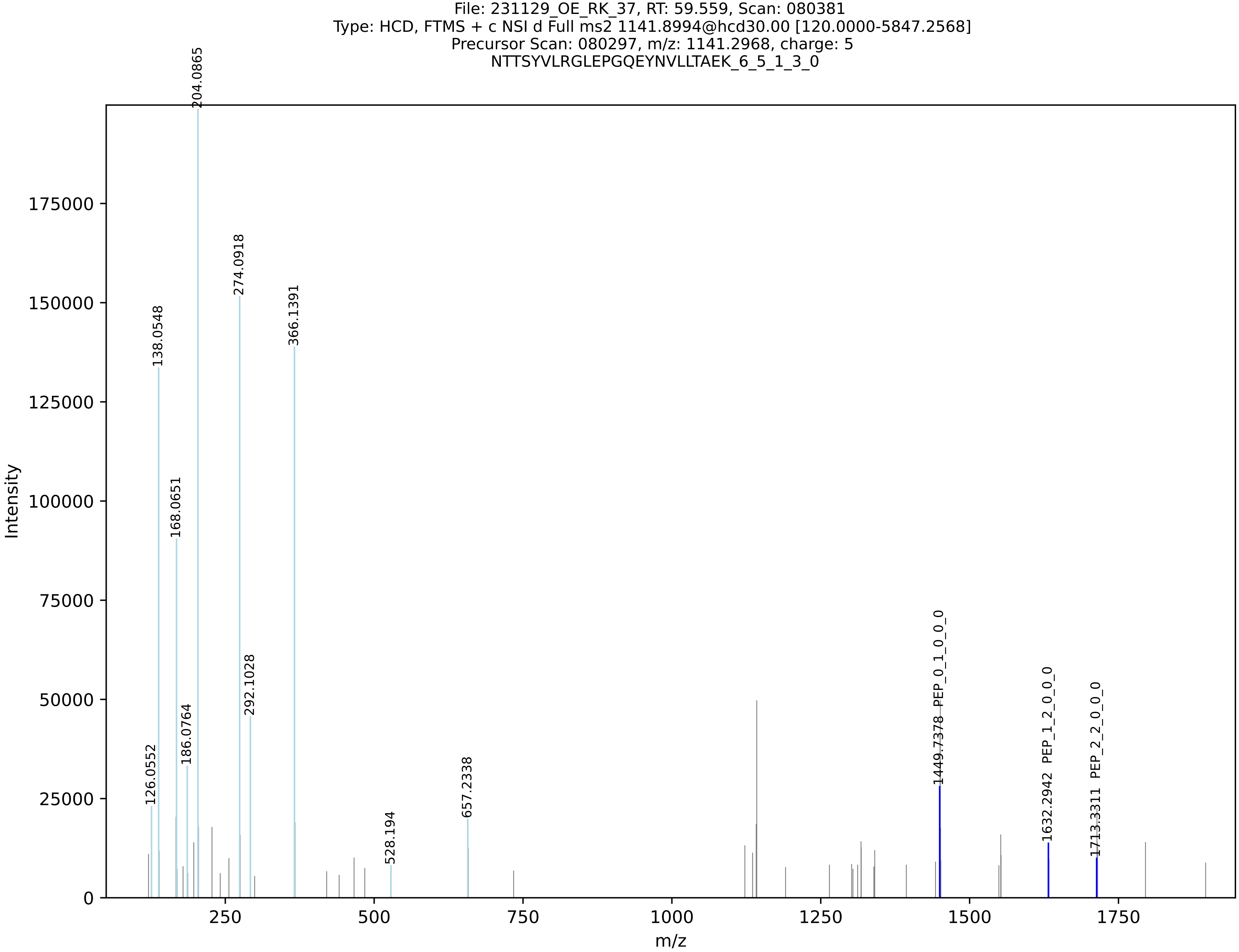

File: 231129_OE_RK_37, RT: 59.559, Scan: 080381
Type: HCD, FTMS + c NSI d Full ms2 1141.8994@hcd30.00 [120.0000-5847.2568]
Precursor Scan: 080297, m/z: 1141.2968, charge: 5
NTTSYVLRGLEPGQEYNVLLTAEK_6_5_1_3_0
Intensity
m/z
204.0865
274.0918
366.1391
138.0548
168.0651
292.1028
186.0764
126.0552
657.2338
528.194
1449.7378 PEP_0_1_0_0_0
1632.2942 PEP_1_2_0_0_0
1713.3311 PEP_2_2_0_0_0

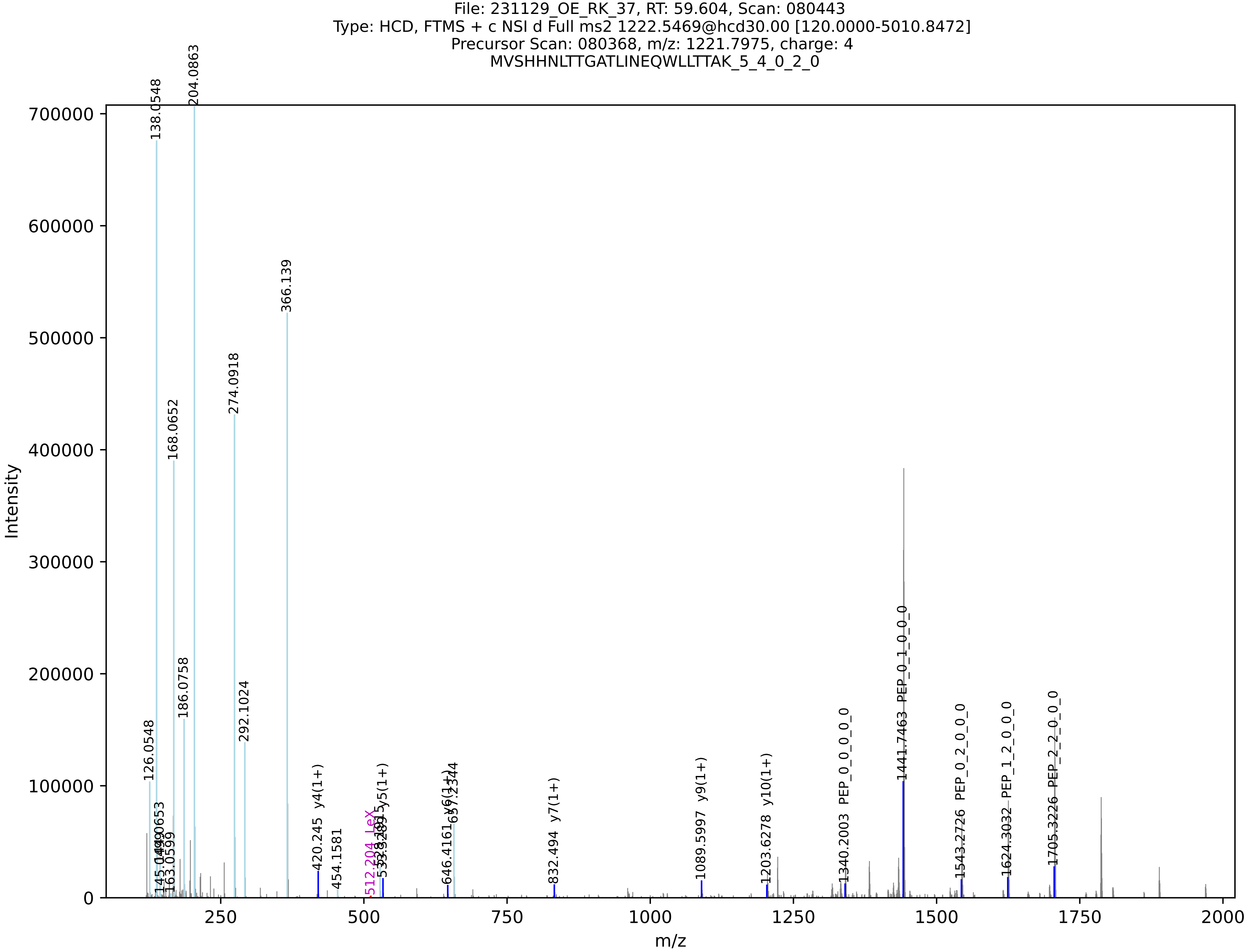

File: 231129_OE_RK_37, RT: 59.604, Scan: 080443
Type: HCD, FTMS + c NSI d Full ms2 1222.5469@hcd30.00 [120.0000-5010.8472]
Precursor Scan: 080368, m/z: 1221.7975, charge: 4
MVSHHNLTTGATLINEQWLLTTAK_5_4_0_2_0
Intensity
m/z
0
100000
200000
300000
400000
500000
600000
700000
250
500
750
1000
1250
1500
1750
2000
204.0863
138.0548
366.139
274.0918
168.0652
292.1024
186.0758
126.0548
163.0599
420.245 y4(1+)
454.1581
512.204 LeX
y5(1+)
646.4161 y6(1+)
657.2344
832.494 y7(1+)
1089.5997 y9(1+)
1203.6278 y10(1+)
1340.2003 PEP_0_0_0_0_0_
1441.7463 PEP_0_1_0_0_0_
1543.2726 PEP_0_2_0_0_0_
1624.3032 PEP_1_2_0_0_0_
1705.3226 PEP_2_2_0_0_0_

File: 231129_OE_RK_37, RT: 59.611, Scan: 080454
Type: HCD, FTMS + c NSI d Full ms2 1459.8875@hcd30.00 [120.0000-5979.1968]
Precursor Scan: 080368, m/z: 1458.885, charge: 4
MVSHHNLTTGATLINEQWLLTTAK_6_5_2_3_0

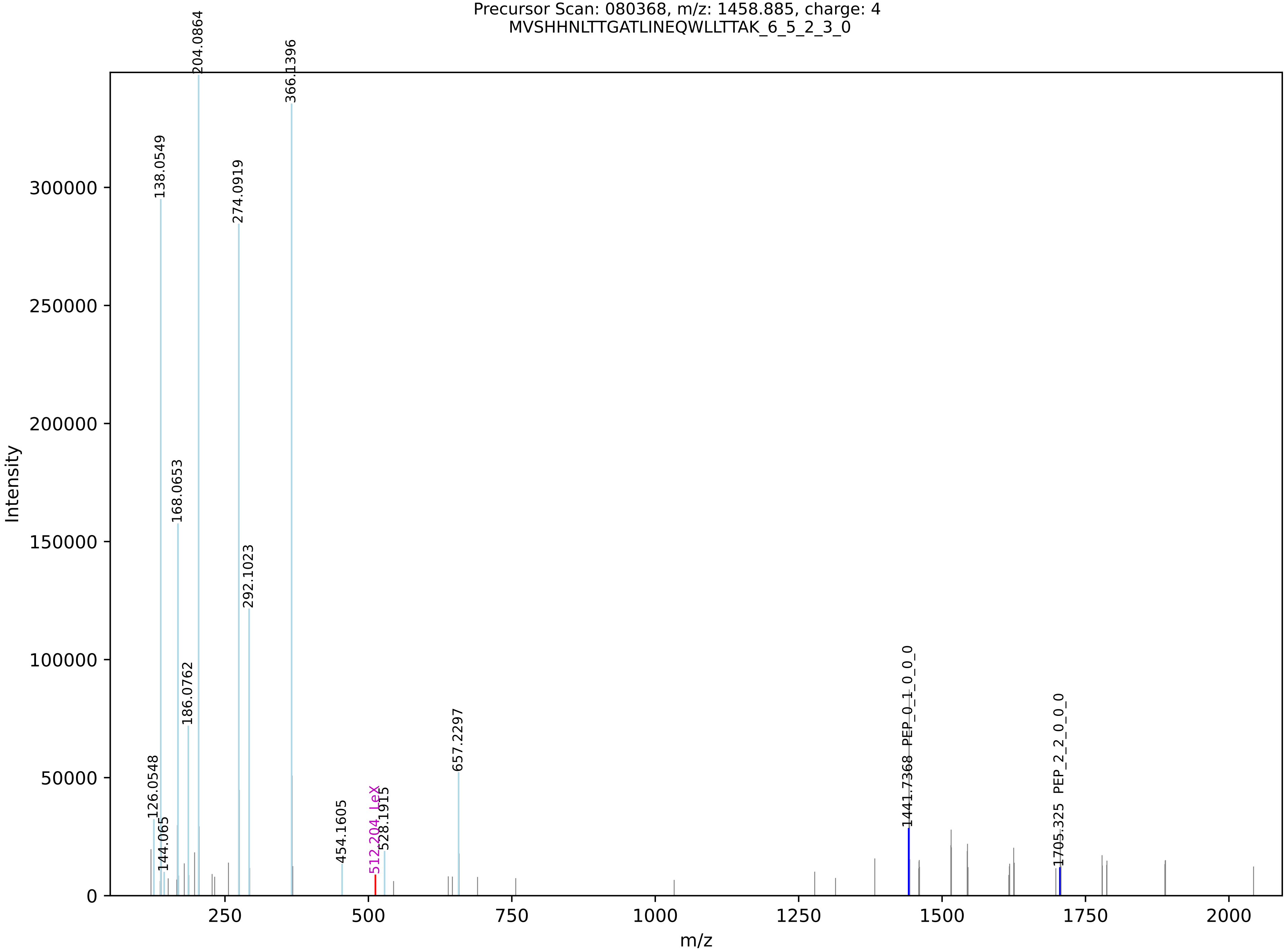

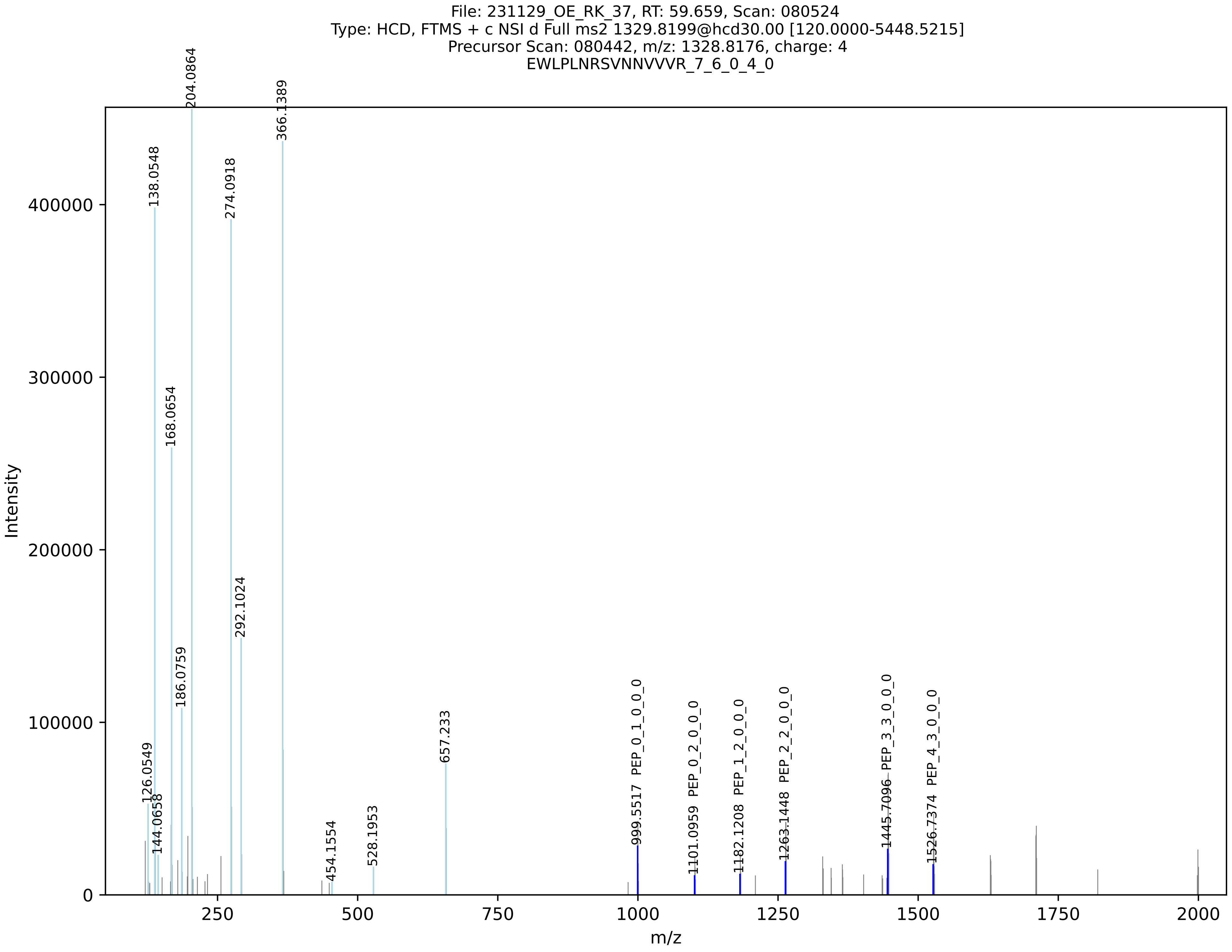
File: 231129_OE_RK_37, RT: 59.659, Scan: 080524
Type: HCD, FTMS + c NSI d Full ms2 1329.8199@hcd30.00 [120.0000-5448.5215]
Precursor Scan: 080442, m/z: 1328.8176, charge: 4
EWLPLNRSVNNVVVR_7_6_0_4_0
Intensity
m/z
0
100000
200000
300000
400000
250
500
750
1000
1250
1500
1750
2000
204.0864
366.1389
138.0548
274.0918
168.0654
292.1024
186.0759
126.0549
144.0658
454.1554
528.1953
657.233
999.5517 PEP_0_1_0_0_0
1101.0959 PEP_0_2_0_0_0
1182.1208 PEP_1_2_0_0_0
1263.1448 PEP_2_2_0_0_0
1445.7096 PEP_3_3_0_0_0
1526.7374 PEP_4_3_0_0_0

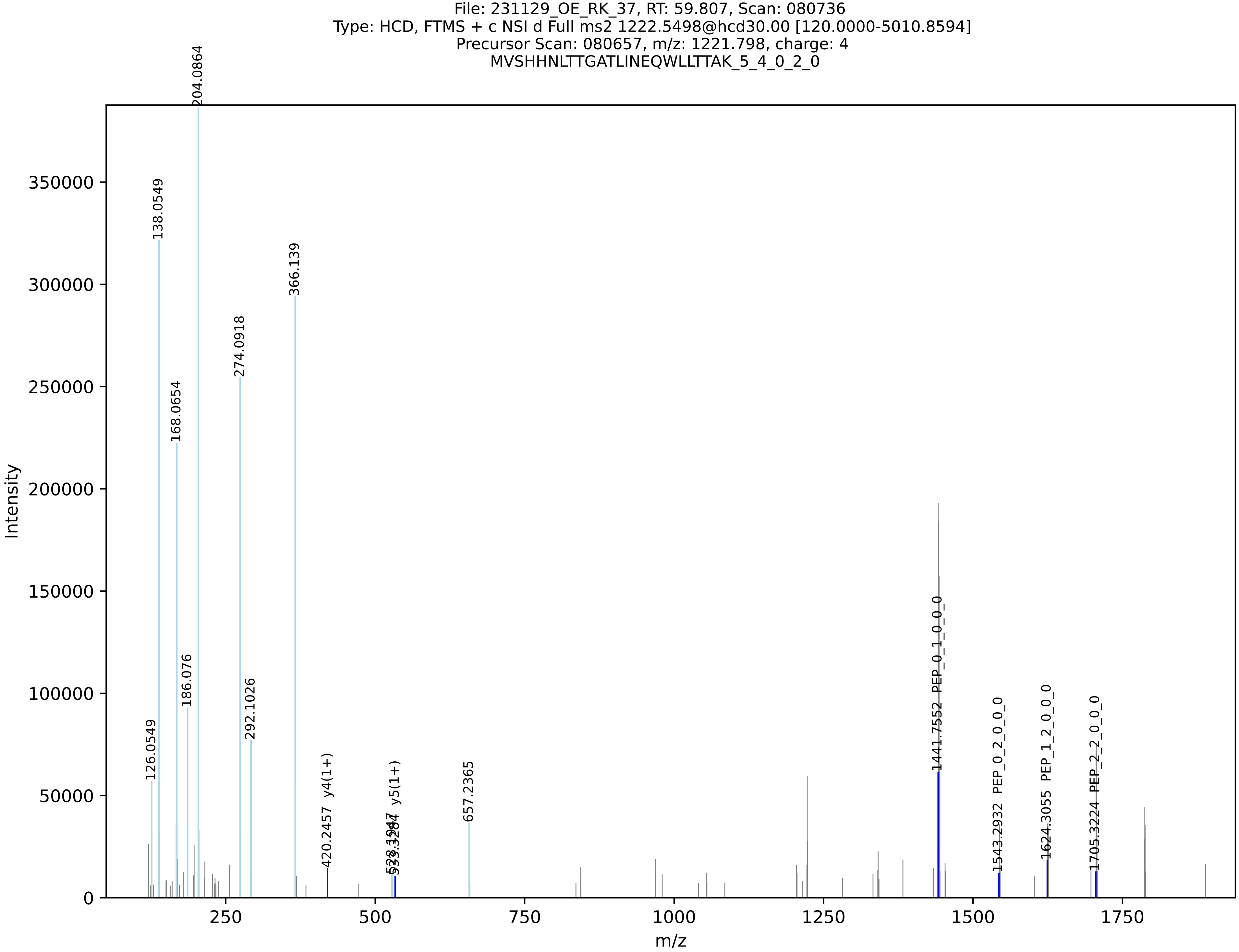
File: 231129_OE_RK_37, RT: 59.807, Scan: 080736
Type: HCD, FTMS + c NSI d Full ms2 1222.5498@hcd30.00 [120.0000-5010.8594]
Precursor Scan: 080657, m/z: 1221.798, charge: 4
MVSHHNLTTGATLINEQWLLTTAK_5_4_0_2_0
Intensity
m/z
204.0864
138.0549
366.139
274.0918
168.0654
186.076
292.1026
126.0549
420.2457 y4(1+)
y5(1+)
657.2365
1441.7552 PEP_0_1_0_0_0_
1543.2932 PEP_0_2_0_0_0
1624.3055 PEP_1_2_0_0_0
1705.3224 PEP_2_2_0_0_0

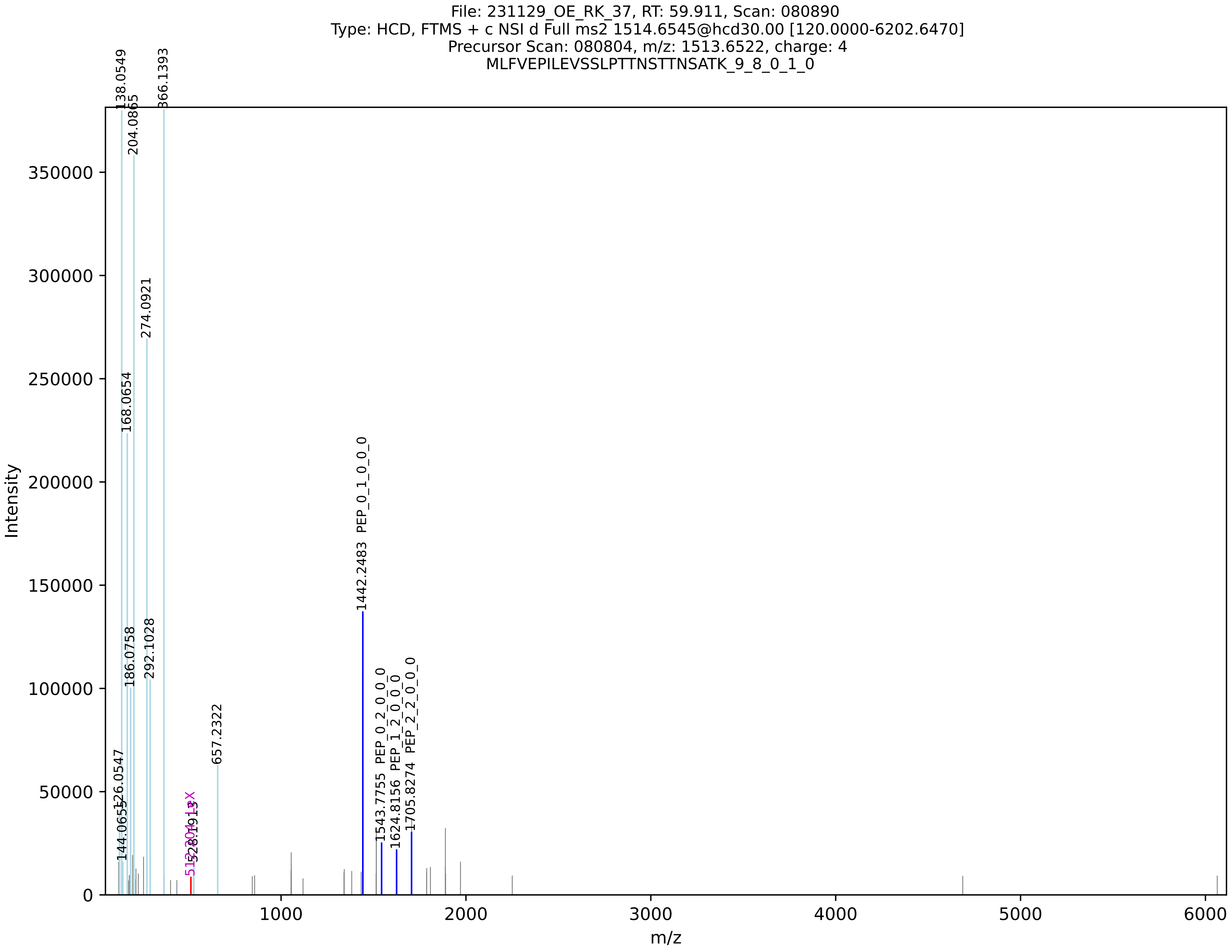
File: 231129_OE_RK_37, RT: 59.911, Scan: 080890
Type: HCD, FTMS + c NSI d Full ms2 1514.6545@hcd30.00 [120.0000-6202.6470]
Precursor Scan: 080804, m/z: 1513.6522, charge: 4
MLFVEPILEVSSLPTTNSTTNSATK_9_8_0_1_0
Intensity
m/z
0
50000
100000
150000
200000
250000
300000
350000
1000
2000
3000
4000
5000
6000
138.0549
204.0865
366.1393
274.0921
168.0654
186.0758
292.1028
126.0547
144.0655
512.2304 LeX
528.1913
657.2322
1442.2483 PEP_0_1_0_0_0
1543.7755 PEP_0_2_0_0_0
1624.8156 PEP_1_2_0_0_0
1705.8274 PEP_2_2_0_0_0

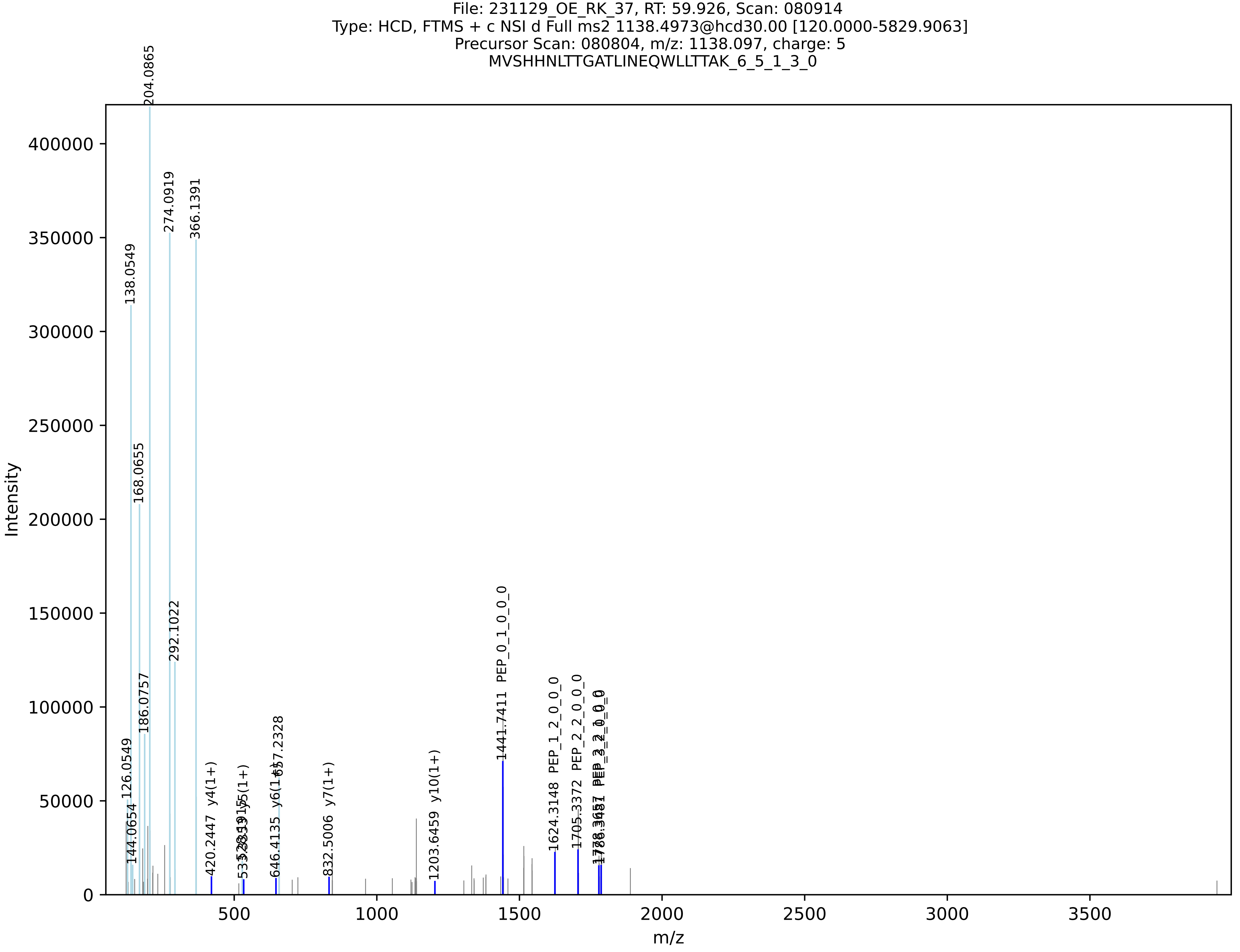

File: 231129_OE_RK_37, RT: 59.926, Scan: 080914
Type: HCD, FTMS + c NSI d Full ms2 1138.4973@hcd30.00 [120.0000-5829.9063]
Precursor Scan: 080804, m/z: 1138.097, charge: 5
MVSHHNLTTGATLINEQWLLTTAK_6_5_1_3_0
Intensity
m/z
204.0865
274.0919
366.1391
138.0549
168.0655
292.1022
186.0757
126.0549
144.0654
420.2447 y4(1+)
646.4135 y6(1+)
657.2328
832.5006 y7(1+)
1203.6459 y10(1+)
1441.7411 PEP_0_1_0_0_0
1624.3148 PEP_1_2_0_0_0
1705.3372 PEP_2_2_0_0_0

File: 231129_OE_RK_37, RT: 60.01, Scan: 081032
Type: HCD, FTMS + c NSI d Full ms2 1459.6345@hcd30.00 [120.0000-5978.1650]
Precursor Scan: 080952, m/z: 1459.1327, charge: 4
MLFVEPILEVSSLPTTNSTTNSATK_6_5_2_3_0

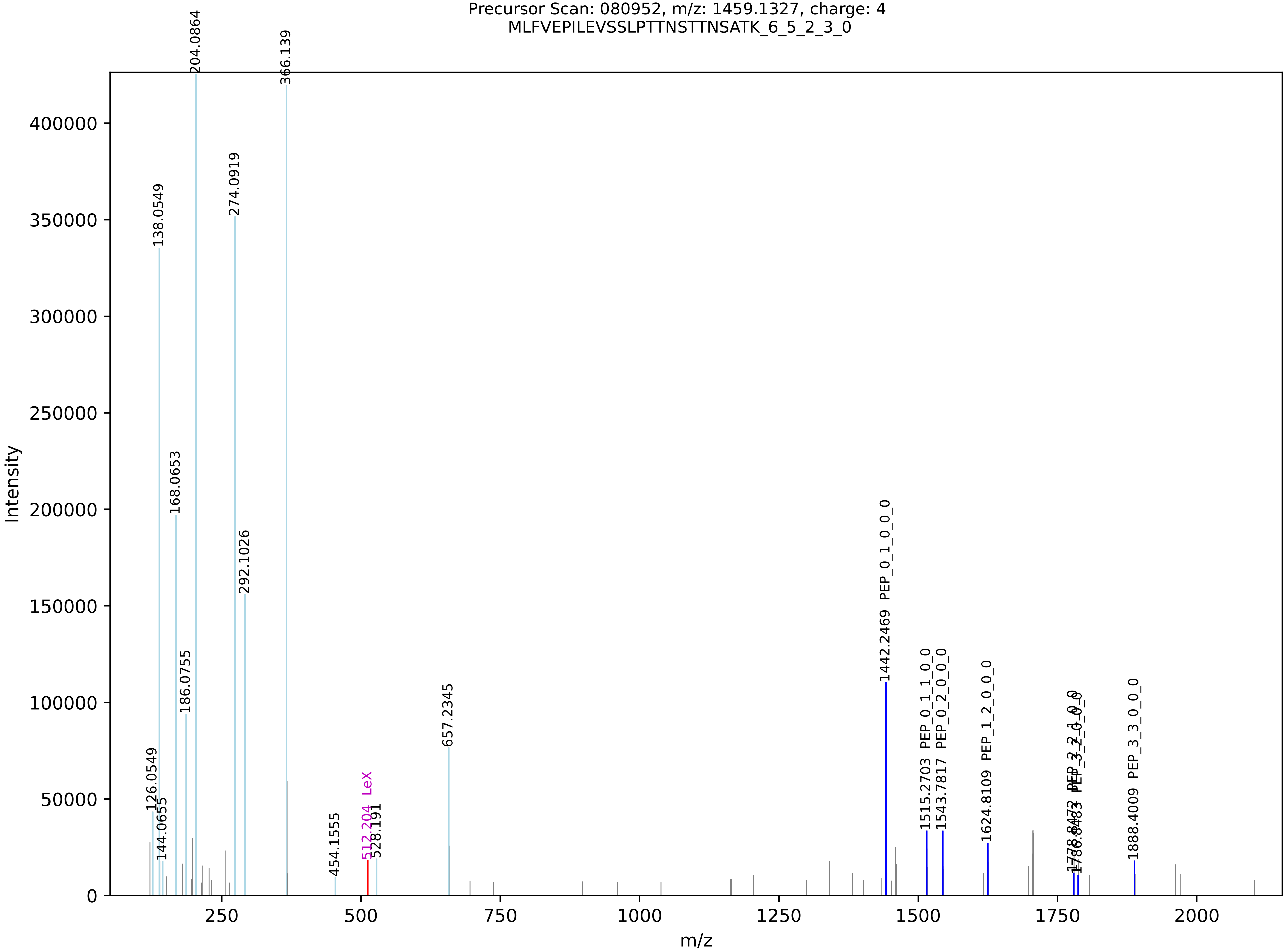

File: 231129_OE_RK_37, RT: 60.062, Scan: 081106
Type: HCD, FTMS + c NSI d Full ms2 1322.8279@hcd30.00 [120.0000-5419.9937]
Precursor Scan: 081022, m/z: 1322.3267, charge: 4
SLGNVNFTVSAEALESQELCGTEVPSVPEHGR_6_3_0_1_0

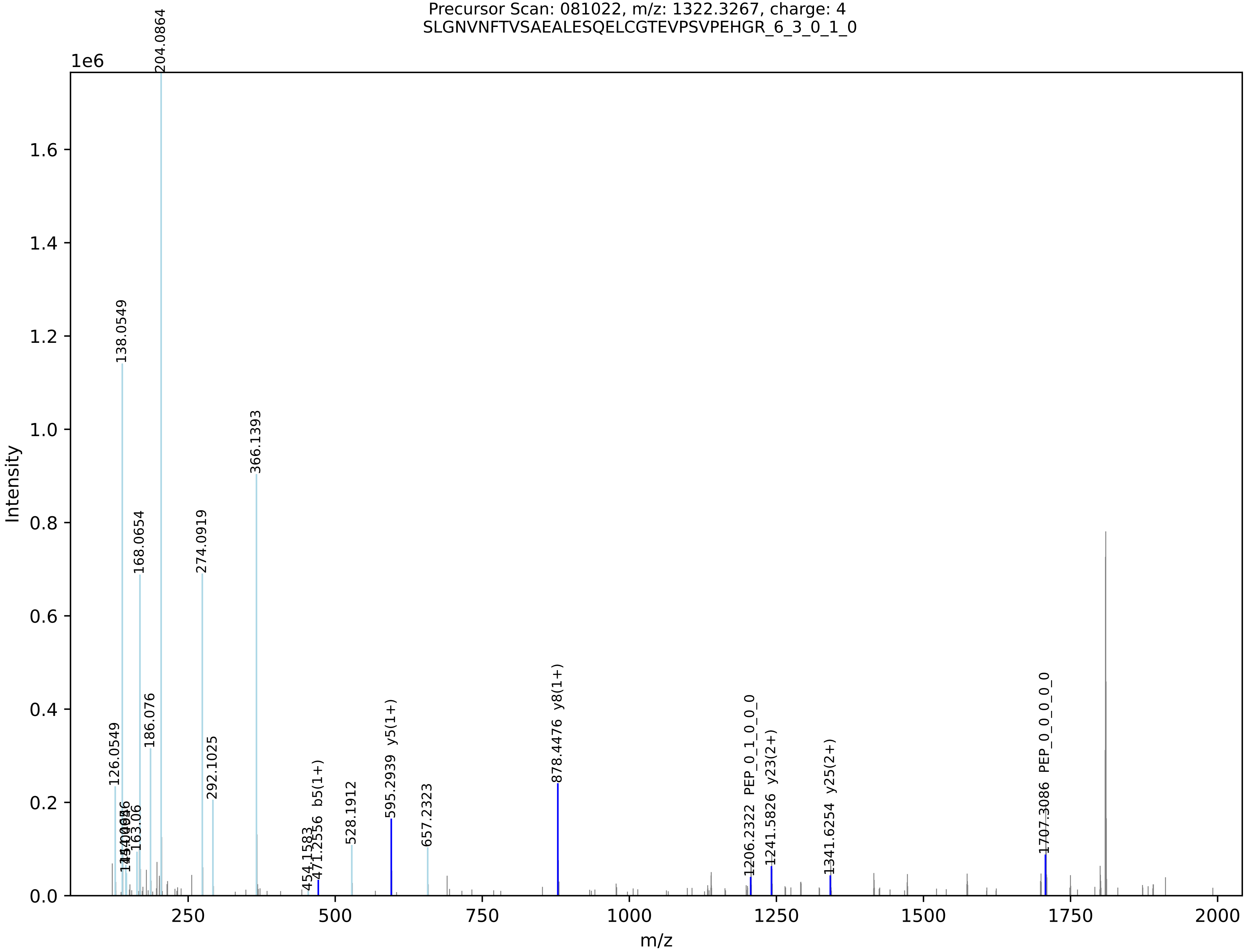

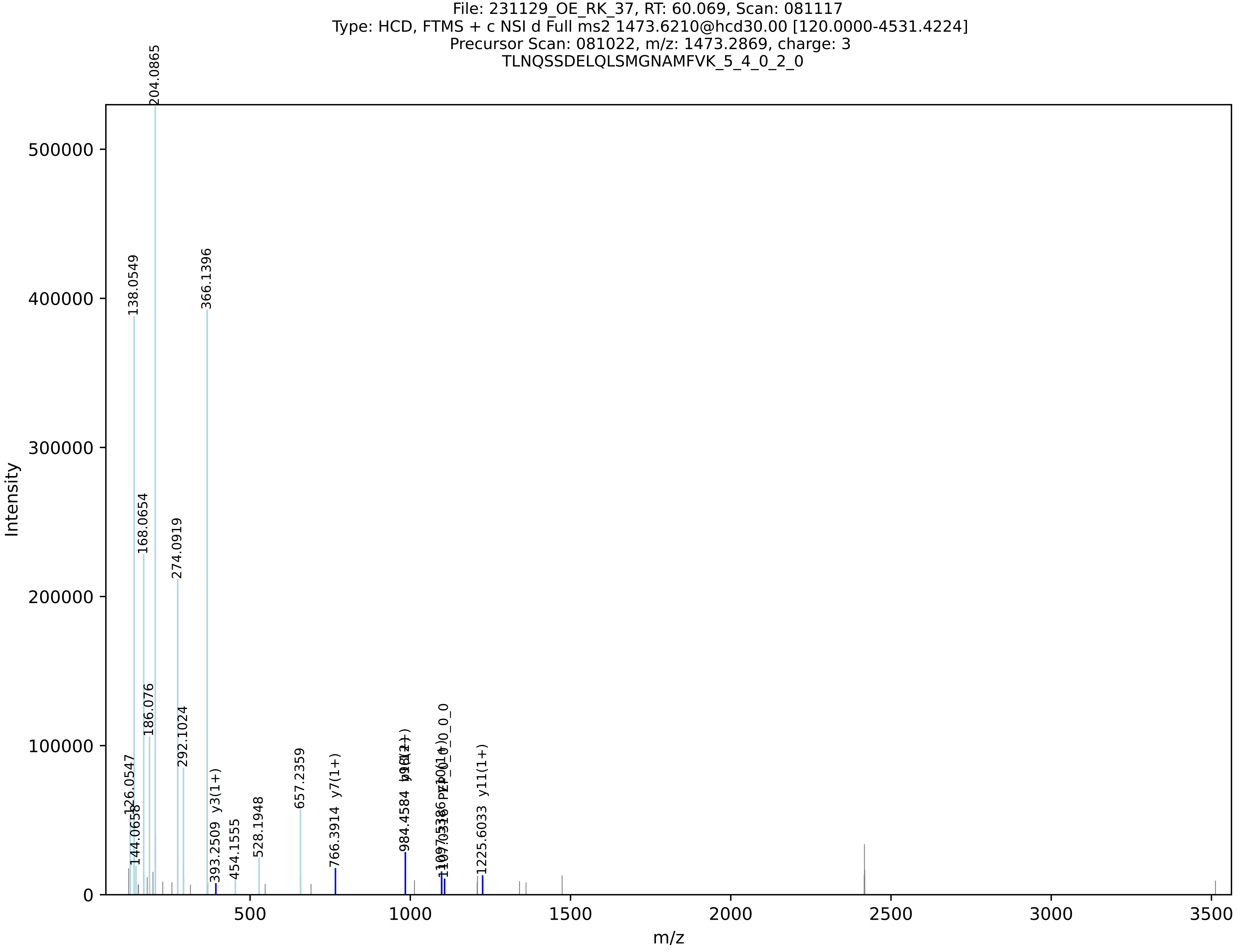
File: 231129_OE_RK_37, RT: 60.069, Scan: 081117
Type: HCD, FTMS + c NSI d Full ms2 1473.6210@hcd30.00 [120.0000-4531.4224]
Precursor Scan: 081022, m/z: 1473.2869, charge: 3
TLNQSSDELQLSMGNAMFVK_5_4_0_2_0
Intensity
m/z
204.0865
138.0549
366.1396
168.0654
274.0919
186.076
292.1024
126.0547
144.0658
393.2509 y3(1+)
454.1555
528.1948
657.2359
766.3914 y7(1+)
984.4584
1225.6033 y11(1+)

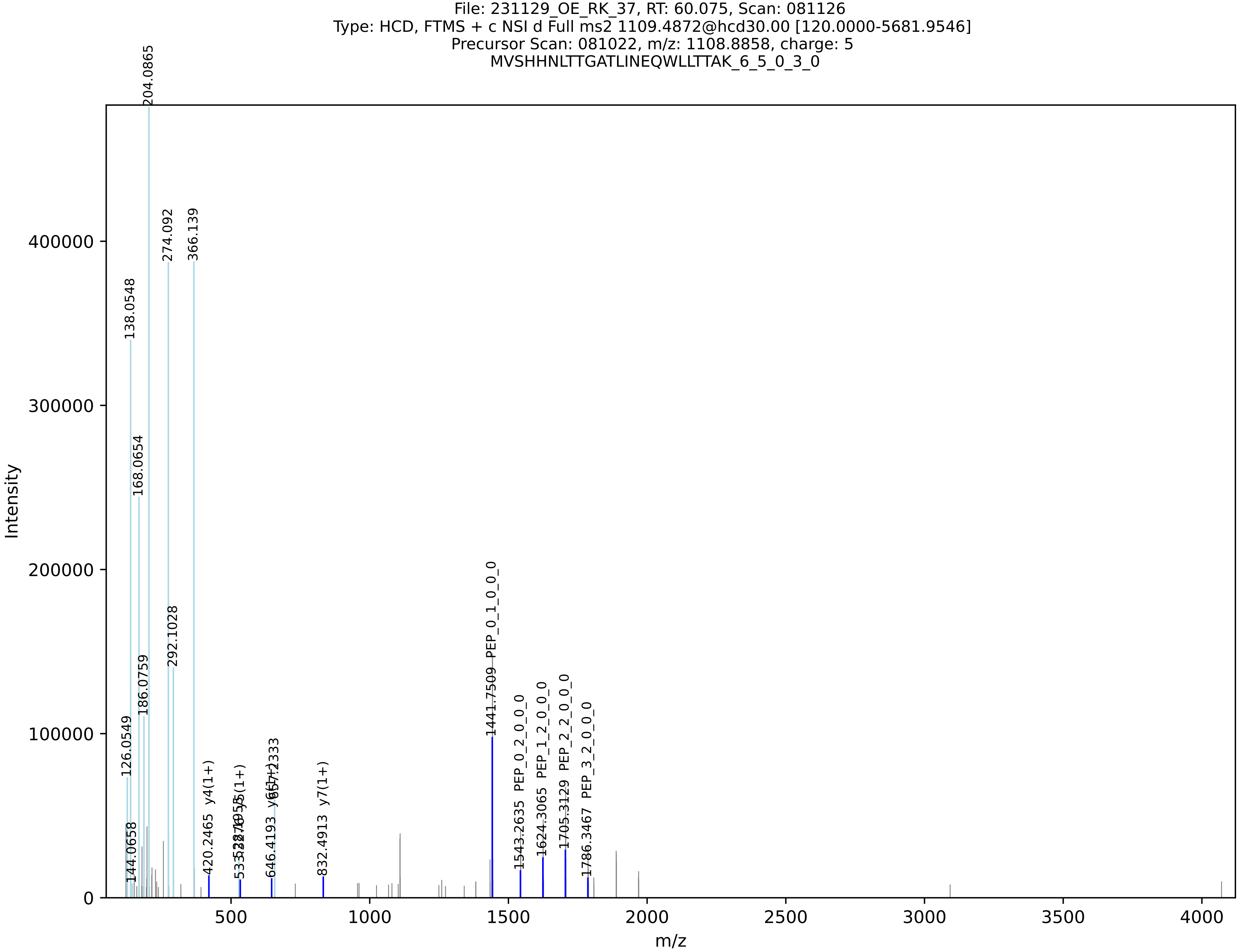
File: 231129_OE_RK_37, RT: 60.075, Scan: 081126
Type: HCD, FTMS + c NSI d Full ms2 1109.4872@hcd30.00 [120.0000-5681.9546]
Precursor Scan: 081022, m/z: 1108.8858, charge: 5
MVSHHNLTTGATLINEQWLLTTAK_6_5_0_3_0
Intensity
m/z
204.0865
274.092
366.139
138.0548
168.0654
292.1028
186.0759
126.0549
144.0658
420.2465 y4(1+)
646.4193 y6(1+)
832.4913 y7(1+)
1441.7509 PEP_0_1_0_0_0
1543.2635 PEP_0_2_0_0_0
1624.3065 PEP_1_2_0_0_0
1705.3129 PEP_2_2_0_0_0
1786.3467 PEP_3_2_0_0_0

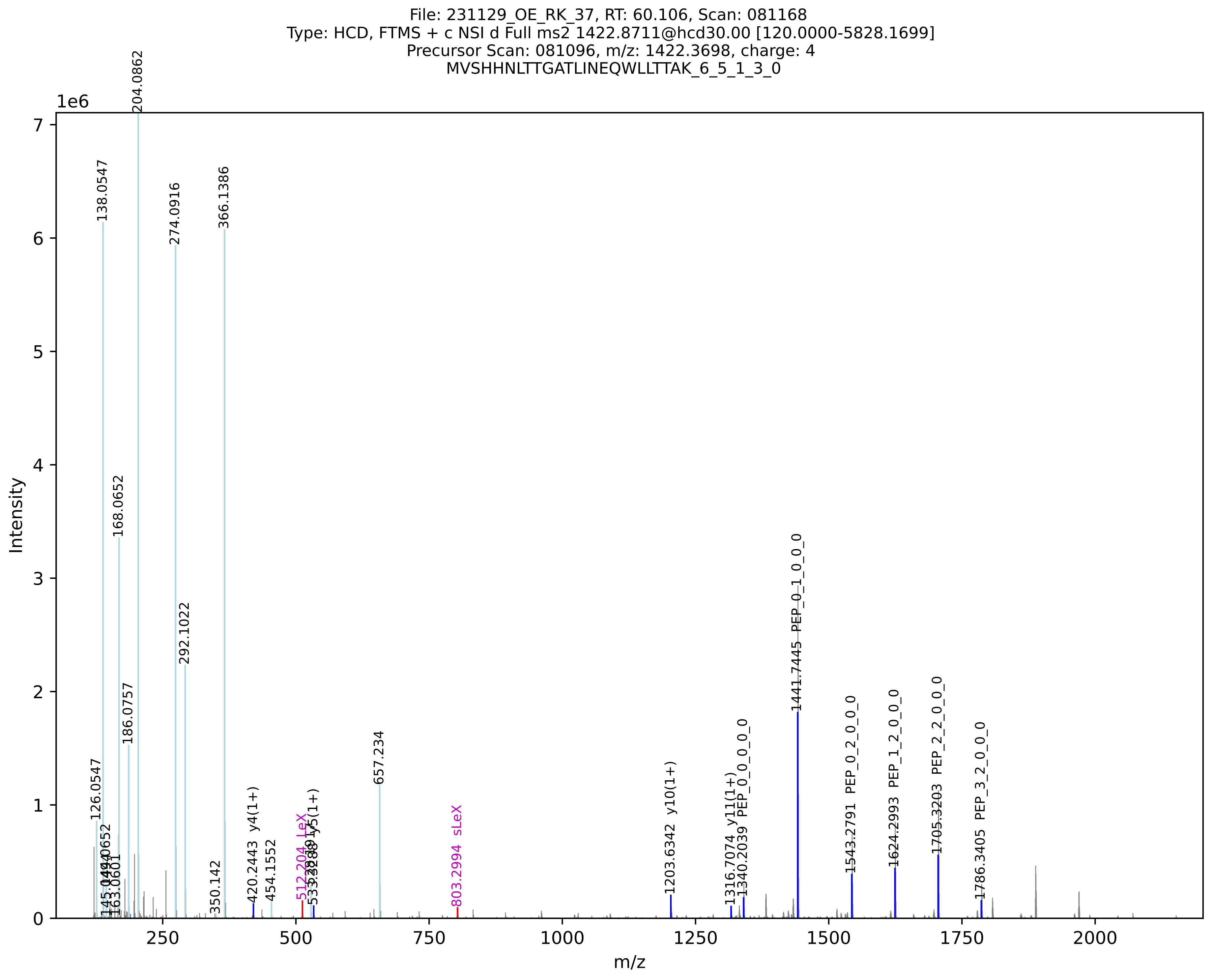

File: 231129_OE_RK_37, RT: 60.106, Scan: 081168
Type: HCD, FTMS + c NSI d Full ms2 1422.8711@hcd30.00 [120.0000-5828.1699]
Precursor Scan: 081096, m/z: 1422.3698, charge: 4
MVSHHNLTTGATLINEQWLLTTAK_6_5_1_3_0
1e6
Intensity
m/z
204.0862
138.0547
274.0916
366.1386
168.0652
292.1022
186.0757
126.0547
657.234
350.142
420.2443 y4(1+)
454.1552
512.204 LeX
y5(1+)
803.2994 sLeX
1203.6342 y10(1+)
1316.7074 y11(1+)
1340.2039 PEP_0_0_0_0_0
1441.7445 PEP_0_1_0_0_0
1543.2791 PEP_0_2_0_0_0
1624.2993 PEP_1_2_0_0_0
1705.3203 PEP_2_2_0_0_0
1786.3405 PEP_3_2_0_0_0

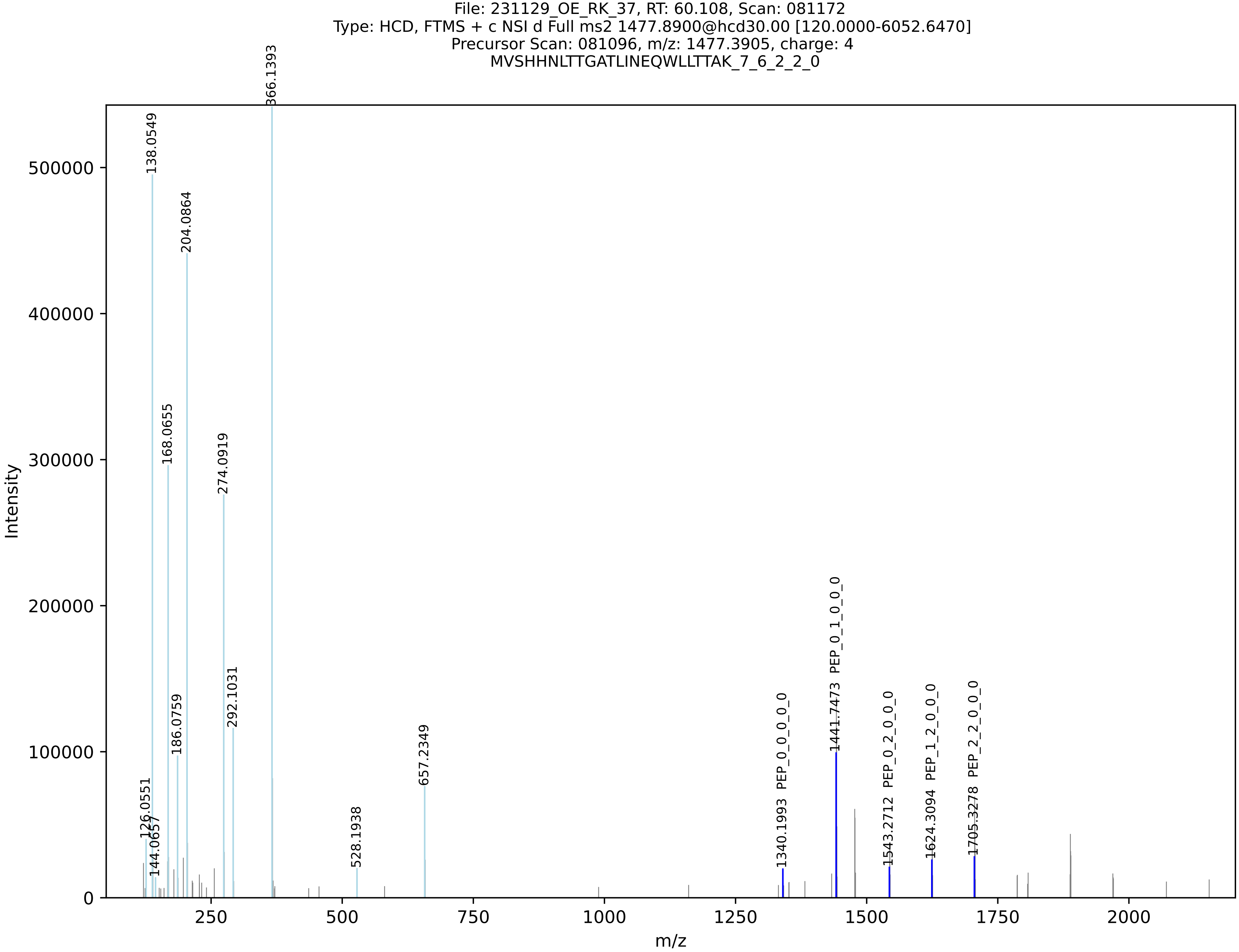

File: 231129_OE_RK_37, RT: 60.108, Scan: 081172
Type: HCD, FTMS + c NSI d Full ms2 1477.8900@hcd30.00 [120.0000-6052.6470]
Precursor Scan: 081096, m/z: 1477.3905, charge: 4
MVSHHNLTTGATLINEQWLLTTAK_7_6_2_2_0
Intensity
m/z
366.1393
138.0549
204.0864
168.0655
274.0919
292.1031
186.0759
126.0551
144.0657
528.1938
657.2349
1340.1993 PEP_0_0_0_0_0
1441.7473 PEP_0_1_0_0_0
1543.2712 PEP_0_2_0_0_0
1624.3094 PEP_1_2_0_0_0
1705.3278 PEP_2_2_0_0_0

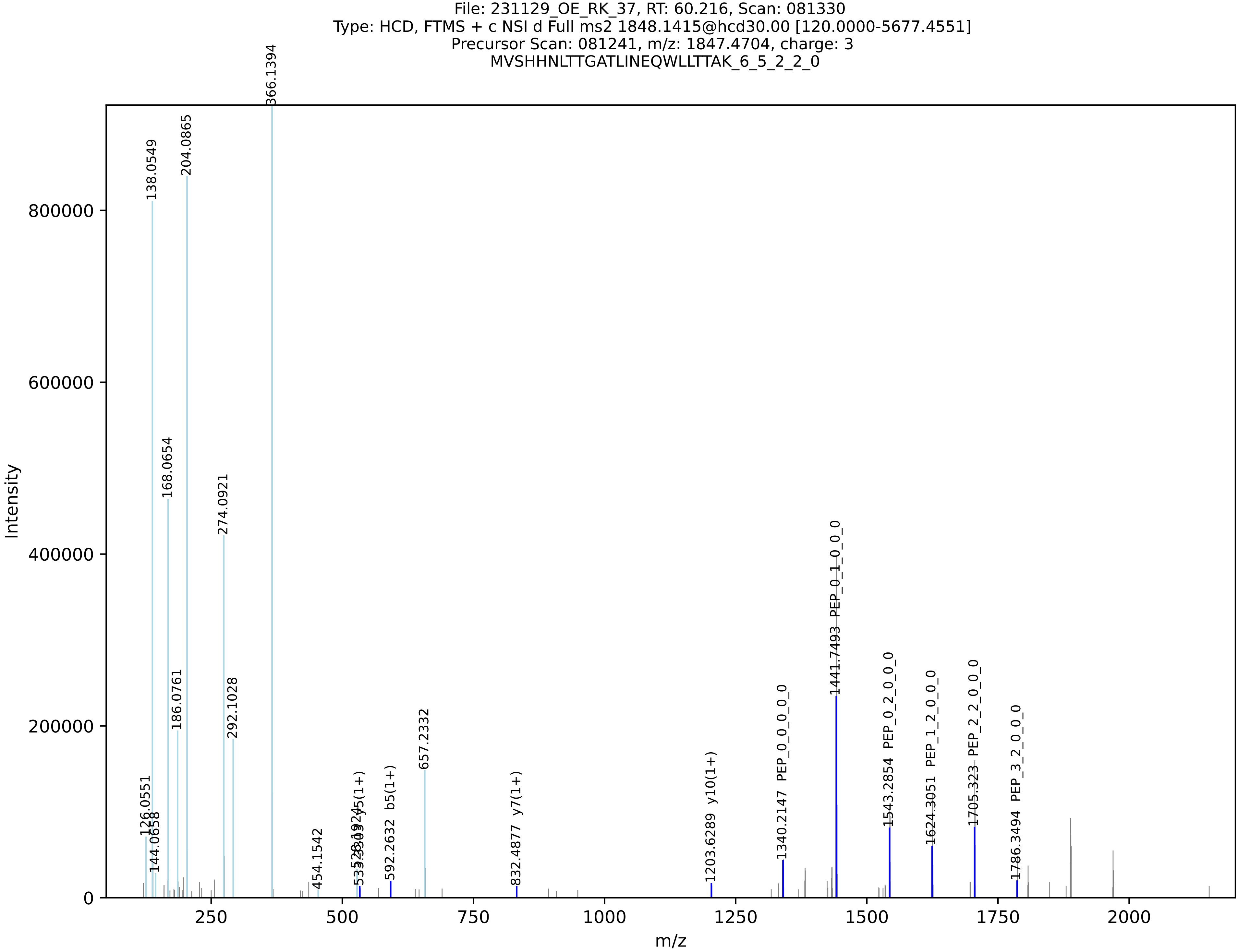
File: 231129_OE_RK_37, RT: 60.216, Scan: 081330
Type: HCD, FTMS + c NSI d Full ms2 1848.1415@hcd30.00 [120.0000-5677.4551]
Precursor Scan: 081241, m/z: 1847.4704, charge: 3
MVSHHNLTTGATLINEQWLLTTAK_6_5_2_2_0
Intensity
m/z
366.1394
204.0865
138.0549
168.0654
274.0921
186.0761
292.1028
126.0551
144.0658
454.1542
592.2632 b5(1+)
657.2332
832.4877 y7(1+)
1203.6289 y10(1+)
1340.2147 PEP_0_0_0_0_0
1441.7493 PEP_0_1_0_0_0
1543.2854 PEP_0_2_0_0_0
1624.3051 PEP_1_2_0_0_0
1705.323 PEP_2_2_0_0_0
1786.3494 PEP_3_2_0_0_0
0
200000
400000
600000
800000
250
500
750
1000
1250
1500
1750
2000

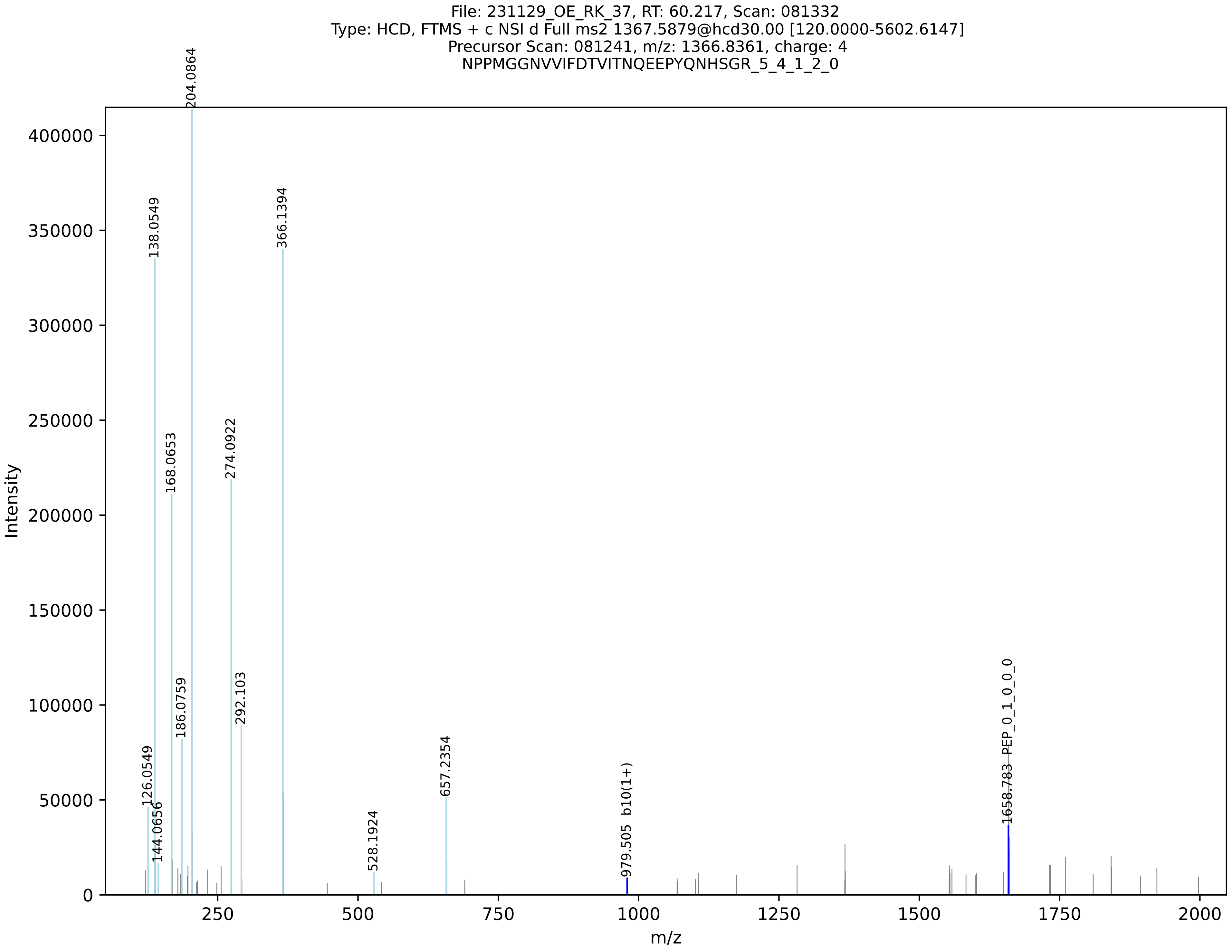
File: 231129_OE_RK_37, RT: 60.217, Scan: 081332
Type: HCD, FTMS + c NSI d Full ms2 1367.5879@hcd30.00 [120.0000-5602.6147]
Precursor Scan: 081241, m/z: 1366.8361, charge: 4
NPPMGGNVVIFDTVITNQEEPYQNHSGR_5_4_1_2_0
Intensity
m/z
204.0864
138.0549
366.1394
168.0653
274.0922
186.0759
292.103
126.0549
144.0656
528.1924
657.2354
979.505_b10(1+)
1658.783_PEP_0_1_0_0_0

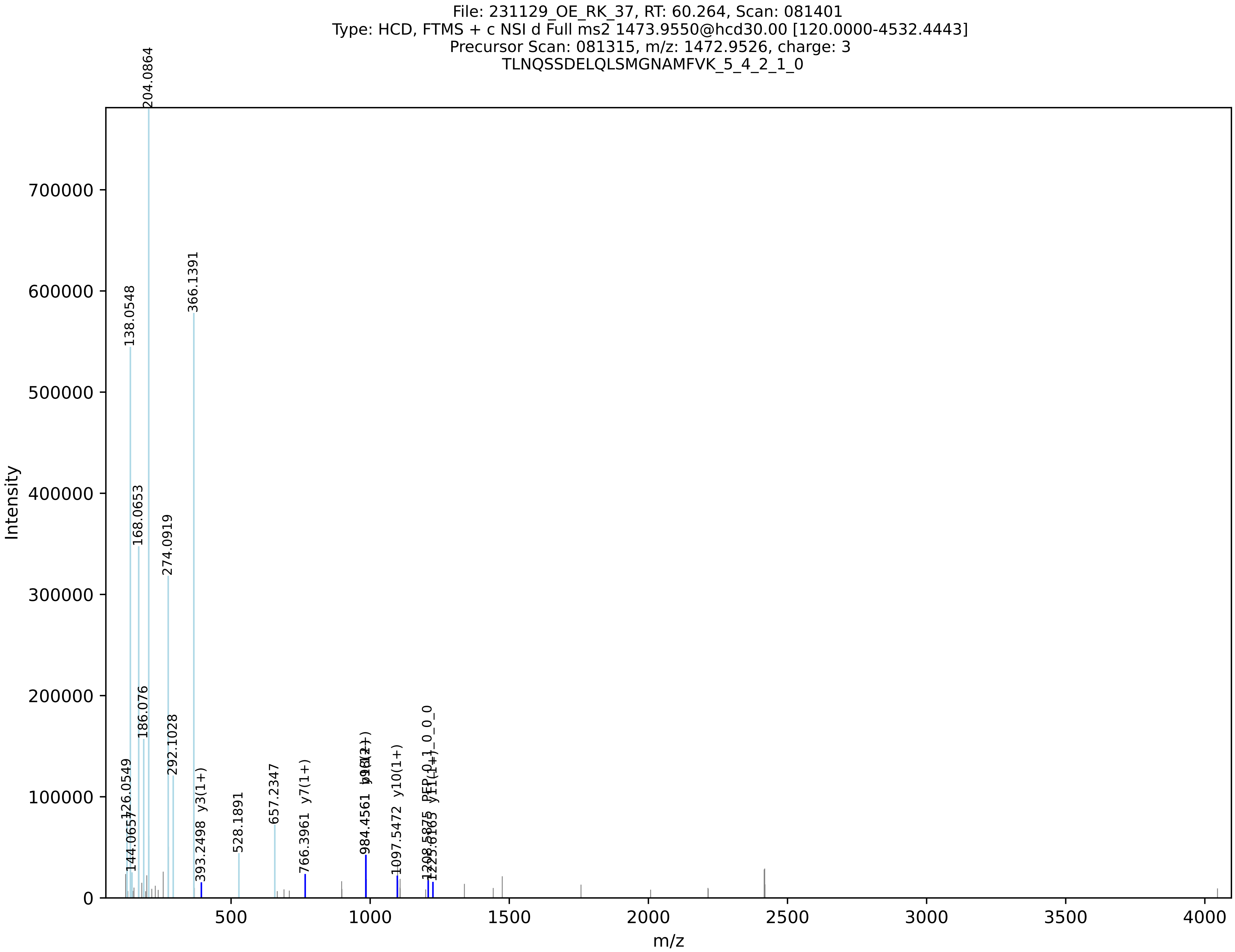
File: 231129_OE_RK_37, RT: 60.264, Scan: 081401
Type: HCD, FTMS + c NSI d Full ms2 1473.9550@hcd30.00 [120.0000-4532.4443]
Precursor Scan: 081315, m/z: 1472.9526, charge: 3
TLNQSSDELQLSMGNAMFVK_5_4_2_1_0
Intensity
m/z
204.0864
366.1391
138.0548
168.0653
274.0919
186.076
292.1028
126.0549
144.0657
393.2498 y3(1+)
528.1891
657.2347
766.3961 y7(1+)
984.4561 y9(1+)
1097.5472 y10(1+)
1208.5875 PEP_0_1_0_0_0_
1225.6165 y11(1+)

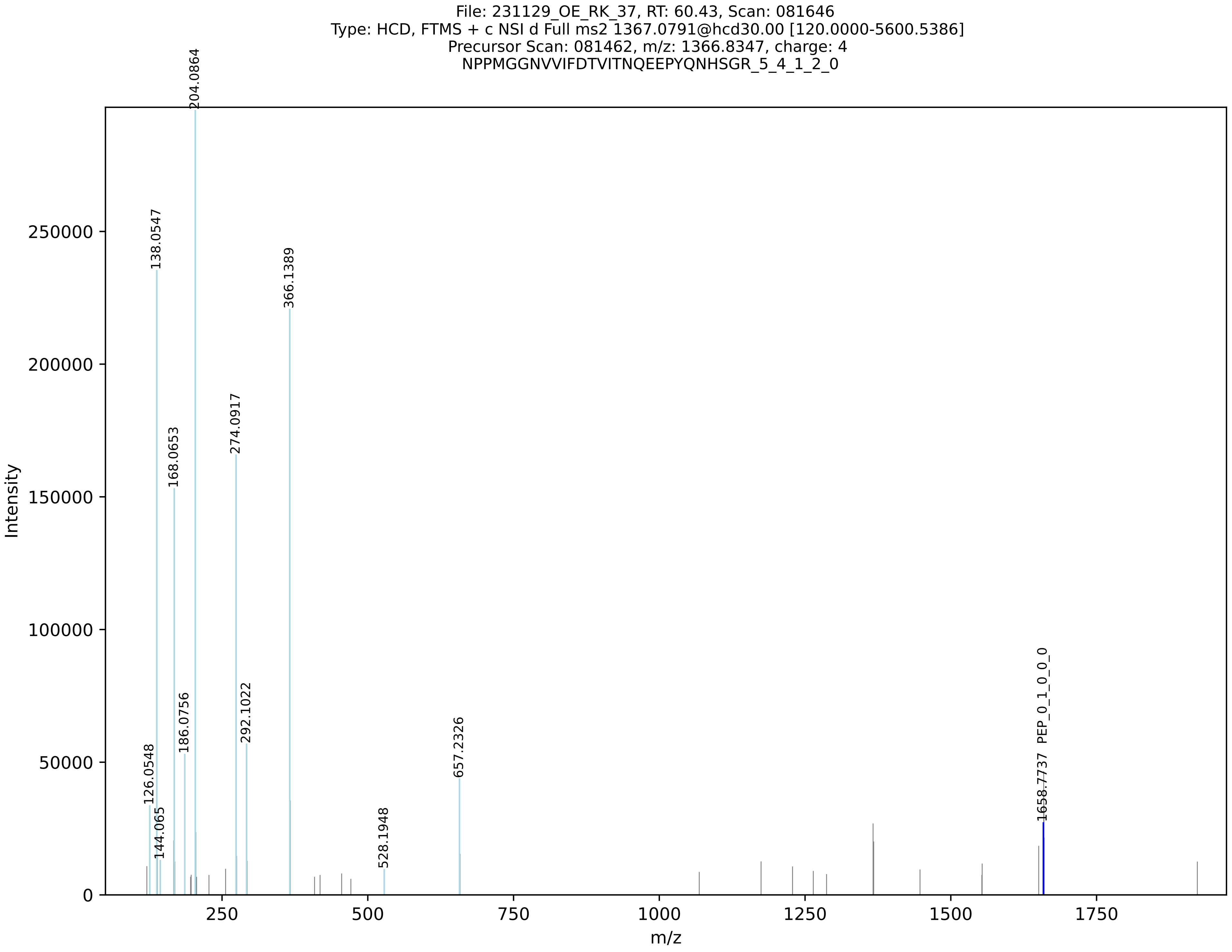

File: 231129_OE_RK_37, RT: 60.43, Scan: 081646
Type: HCD, FTMS + c NSI d Full ms2 1367.0791@hcd30.00 [120.0000-5600.5386]
Precursor Scan: 081462, m/z: 1366.8347, charge: 4
NPPMGGNVVIFDTVITNQEEPYQNHSGR_5_4_1_2_0
204.0864
138.0547
366.1389
274.0917
168.0653
186.0756
292.1022
126.0548
144.065
528.1948
657.2326
PEP_0_1_0_0_0
1658.7737
Intensity
m/z
0
50000
100000
150000
200000
250000
250
500
750
1000
1250
1500
1750

File: 231129_OE_RK_37, RT: 60.456, Scan: 081681
Type: HCD, FTMS + c NSI d Full ms2 1109.2837@hcd30.00 [120.0000-5680.9165]
Precursor Scan: 081610, m/z: 1108.8832, charge: 5
MVSHHNLTTGATLINEQWLLTTAK_6_5_0_3_0

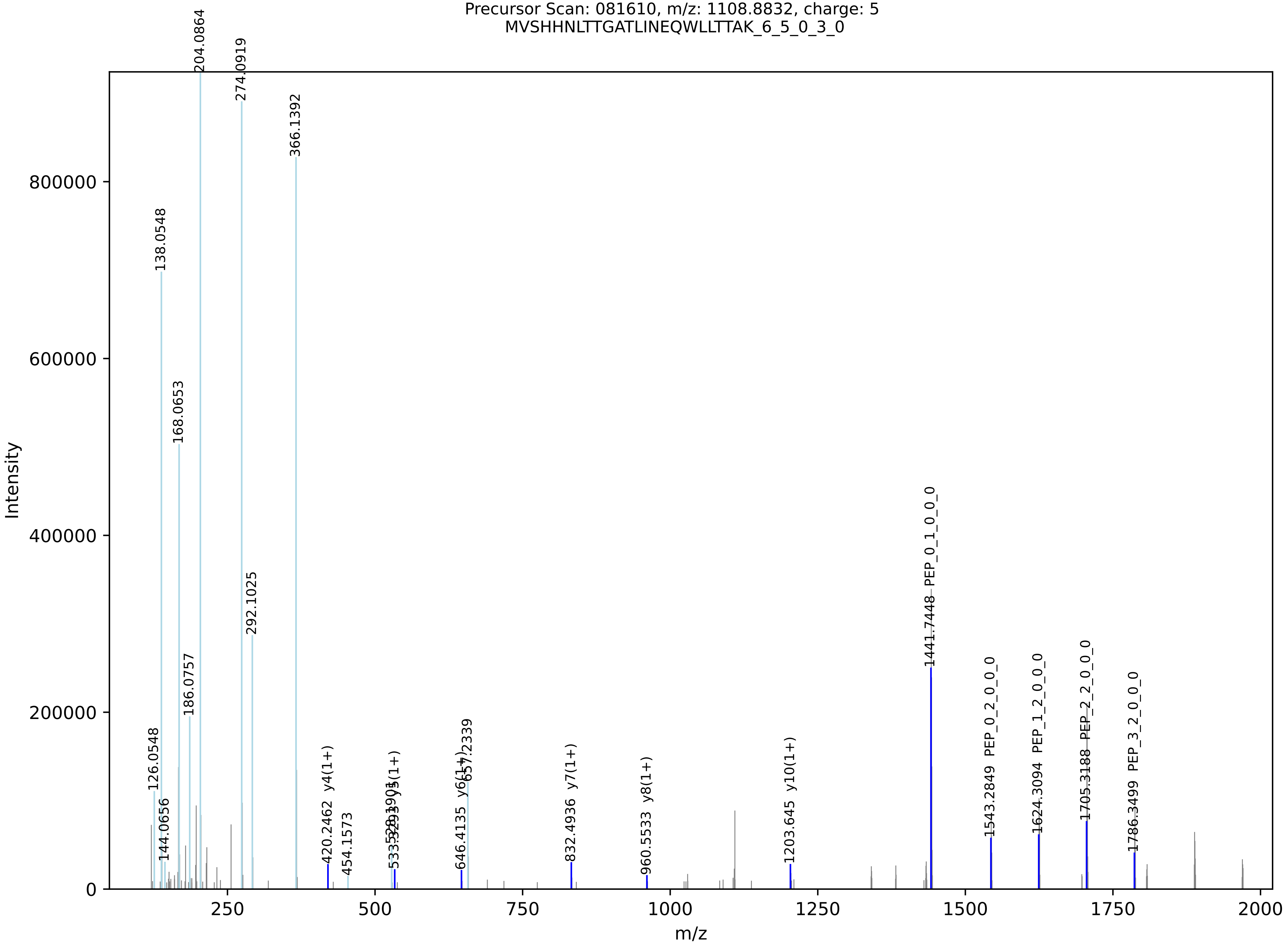

File: 231129_OE_RK_37, RT: 60.503, Scan: 081749
Type: HCD, FTMS + c NSI d Full ms2 1422.8687@hcd30.00 [120.0000-5828.1602]
Precursor Scan: 081677, m/z: 1422.1169, charge: 4
LPVDVVVNASNEDLKHYGGLAAALSK_6_5_1_3_0

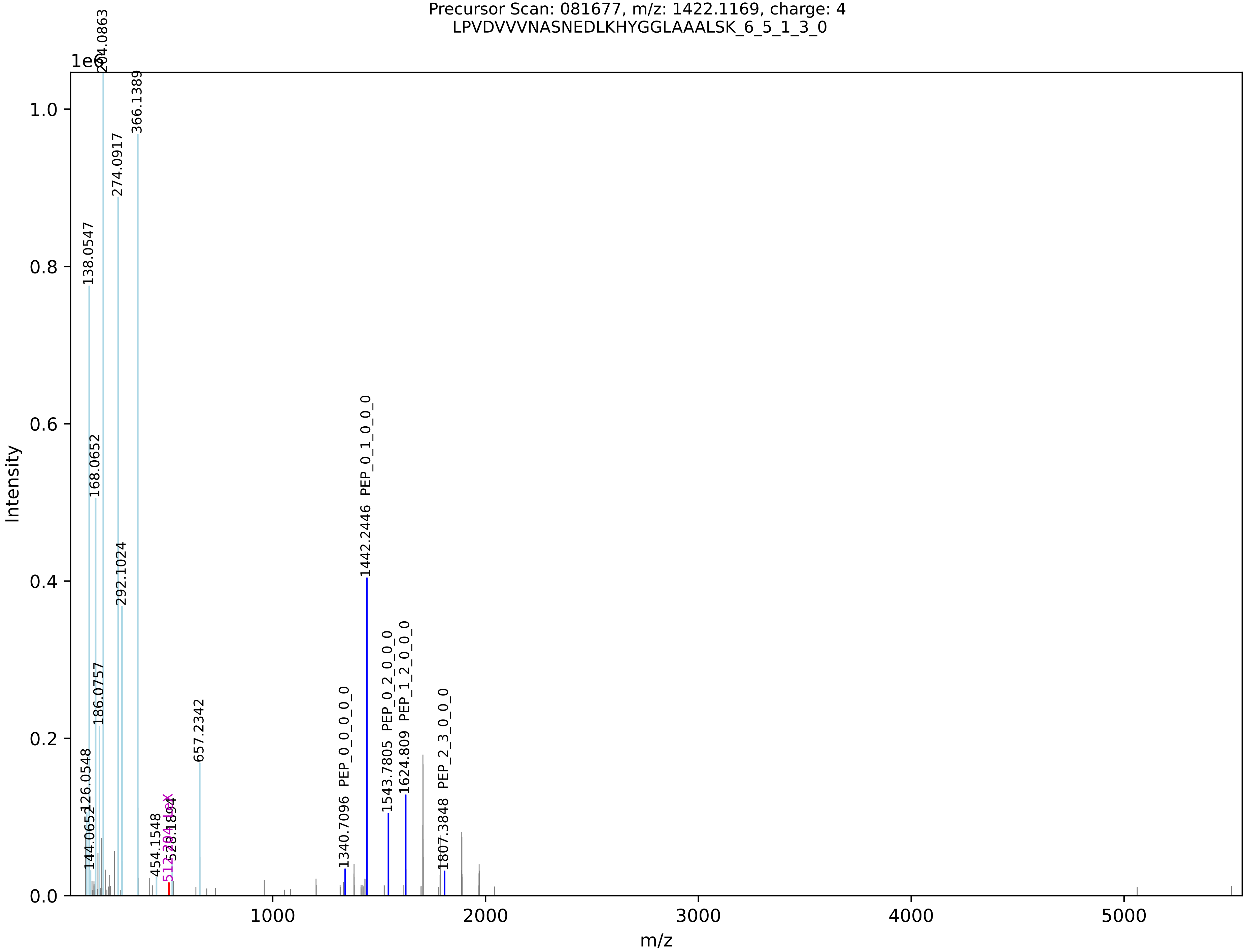

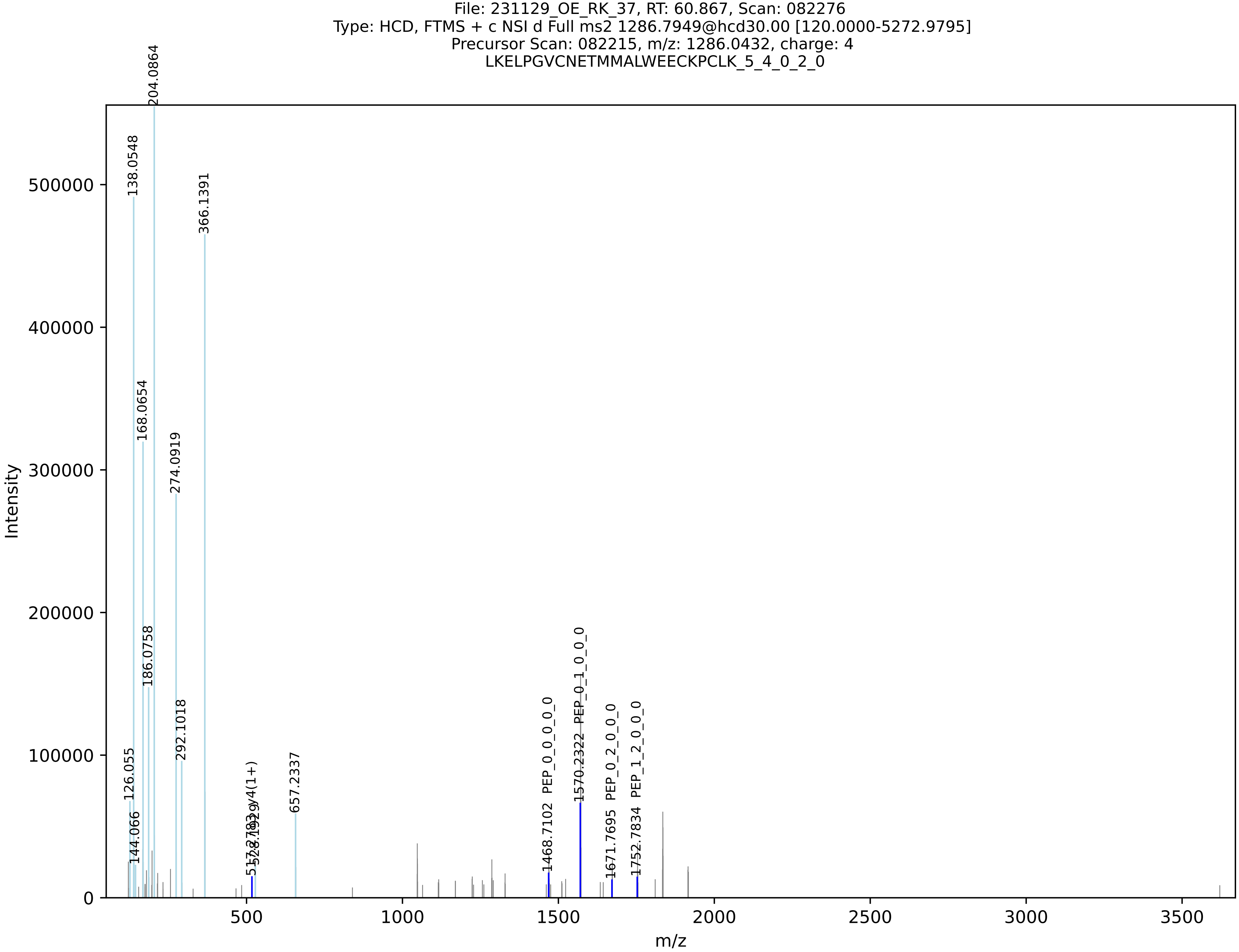
File: 231129_OE_RK_37, RT: 60.867, Scan: 082276
Type: HCD, FTMS + c NSI d Full ms2 1286.7949@hcd30.00 [120.0000-5272.9795]
Precursor Scan: 082215, m/z: 1286.0432, charge: 4
LKELPGVCNETMMALWEECKPCLK_5_4_0_2_0
204.0864
138.0548
366.1391
168.0654
274.0919
186.0758
292.1018
126.055
144.066
517.2783 y4(1+)
528.1929
657.2337
1468.7102 PEP_0_0_0_0_0
1570.2322 PEP_0_1_0_0_0
1671.7695 PEP_0_2_0_0_0
1752.7834 PEP_1_2_0_0_0
Intensity
m/z
0
100000
200000
300000
400000
500000
500
1000
1500
2000
2500
3000
3500

File: 231129_OE_RK_37, RT: 61.017, Scan: 082492
Type: HCD, FTMS + c NSI d Full ms2 1105.7158@hcd30.00 [120.0000-4534.1763]
Precursor Scan: 082410, m/z: 1105.2132, charge: 4
TLNQSSDELQLSMGNAMFVK_5_4_0_2_0

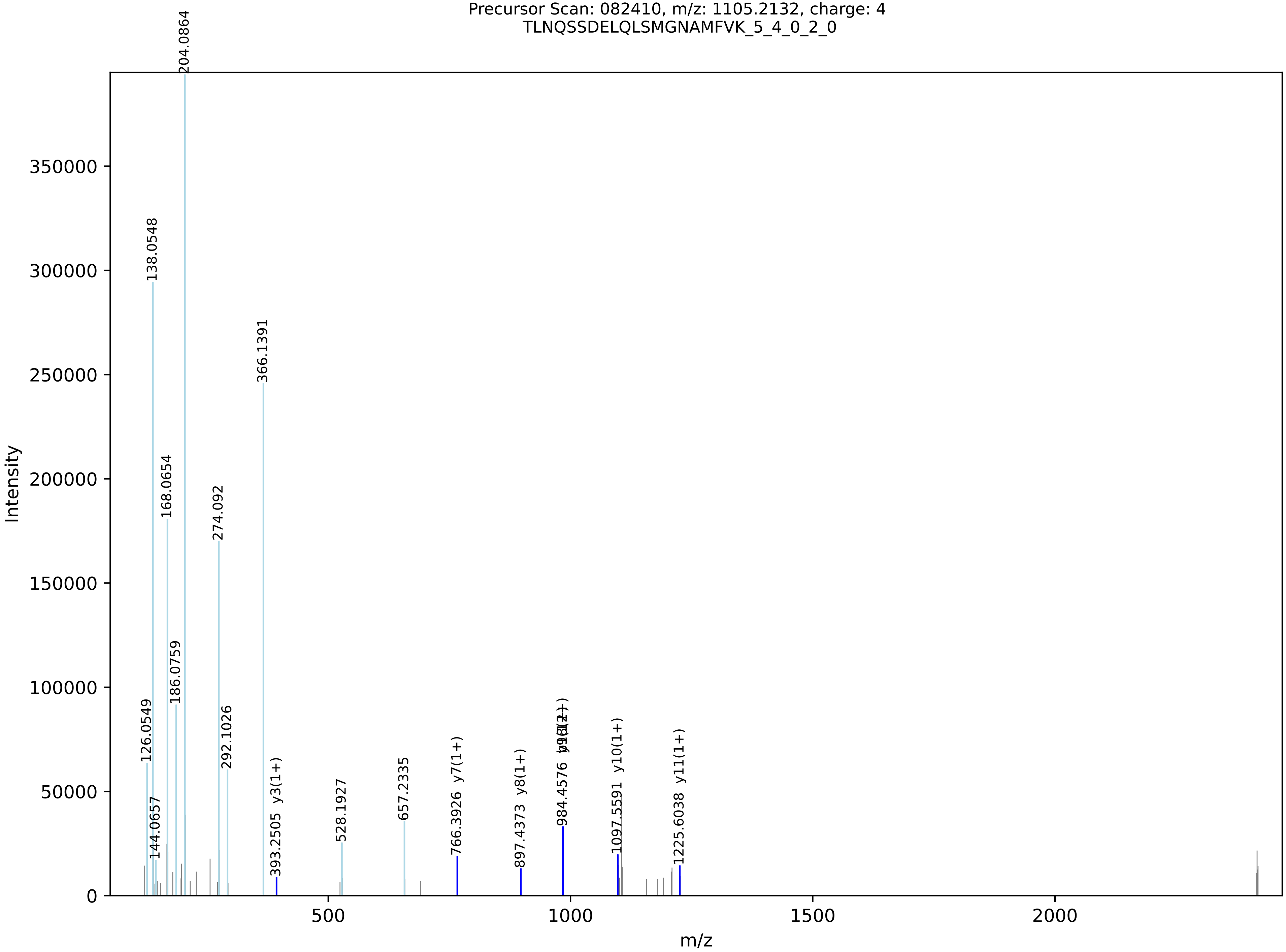

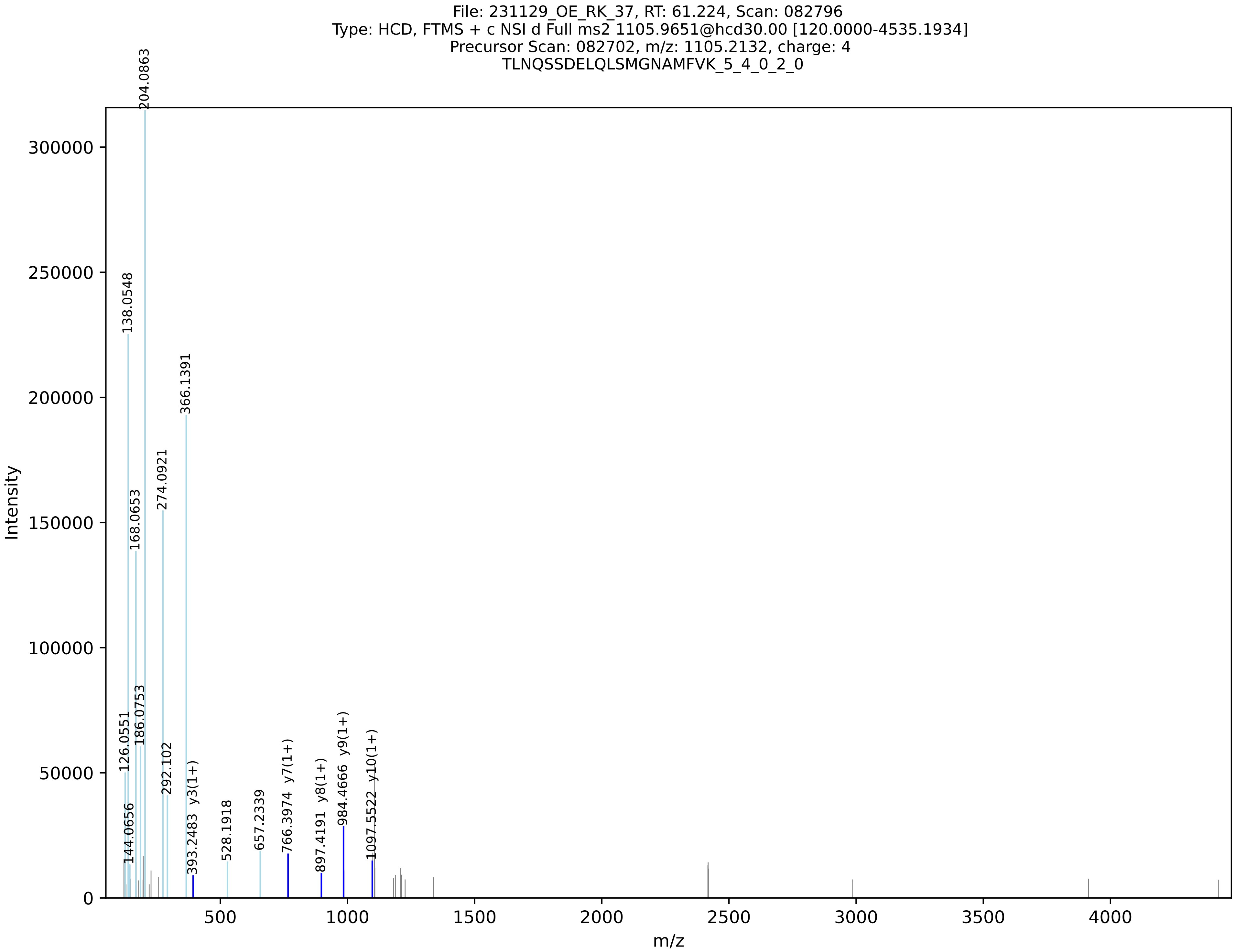

File: 231129_OE_RK_37, RT: 61.224, Scan: 082796
Type: HCD, FTMS + c NSI d Full ms2 1105.9651@hcd30.00 [120.0000-4535.1934]
Precursor Scan: 082702, m/z: 1105.2132, charge: 4
TLNQSSDELQLSMGNAMFVK_5_4_0_2_0
Intensity
m/z
204.0863
138.0548
366.1391
274.0921
168.0653
186.0753
126.0551
292.102
144.0656
393.2483 y3(1+)
528.1918
657.2339
766.3974 y7(1+)
897.4191 y8(1+)
984.4666 y9(1+)
1097.5522 y10(1+)

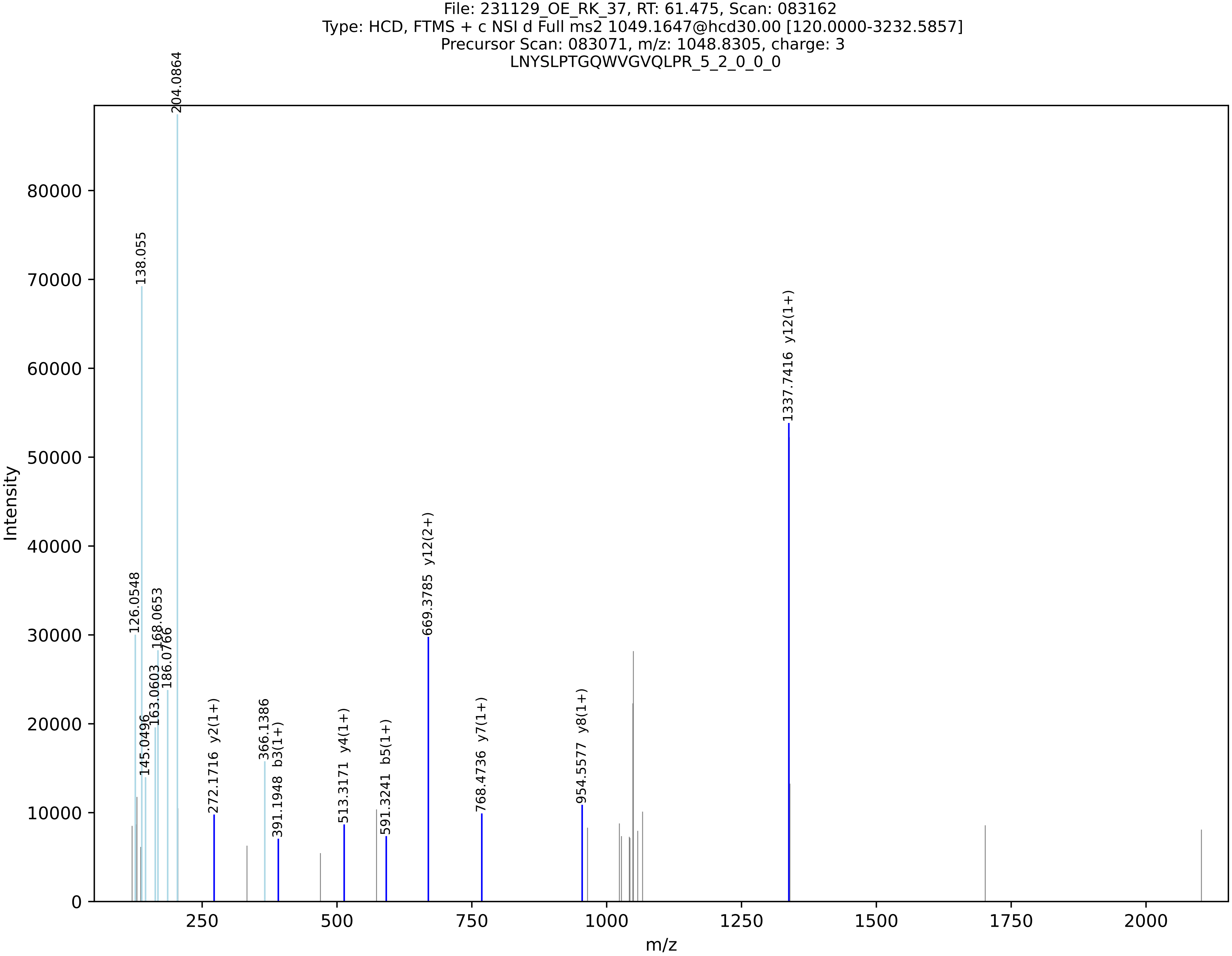
File: 231129_OE_RK_37, RT: 61.475, Scan: 083162
Type: HCD, FTMS + c NSI d Full ms2 1049.1647@hcd30.00 [120.0000-3232.5857]
Precursor Scan: 083071, m/z: 1048.8305, charge: 3
LNYSLPTGQWVGVQLPR_5_2_0_0_0
Intensity
m/z
204.0864
138.055
126.0548
168.0653
186.0766
163.0603
145.0496
272.1716 y2(1+)
366.1386
391.1948 b3(1+)
513.3171 y4(1+)
591.3241 b5(1+)
669.3785 y12(2+)
768.4736 y7(1+)
954.5577 y8(1+)
1337.7416 y12(1+)
0
10000
20000
30000
40000
50000
60000
70000
80000
250
500
750
1000
1250
1500
1750
2000

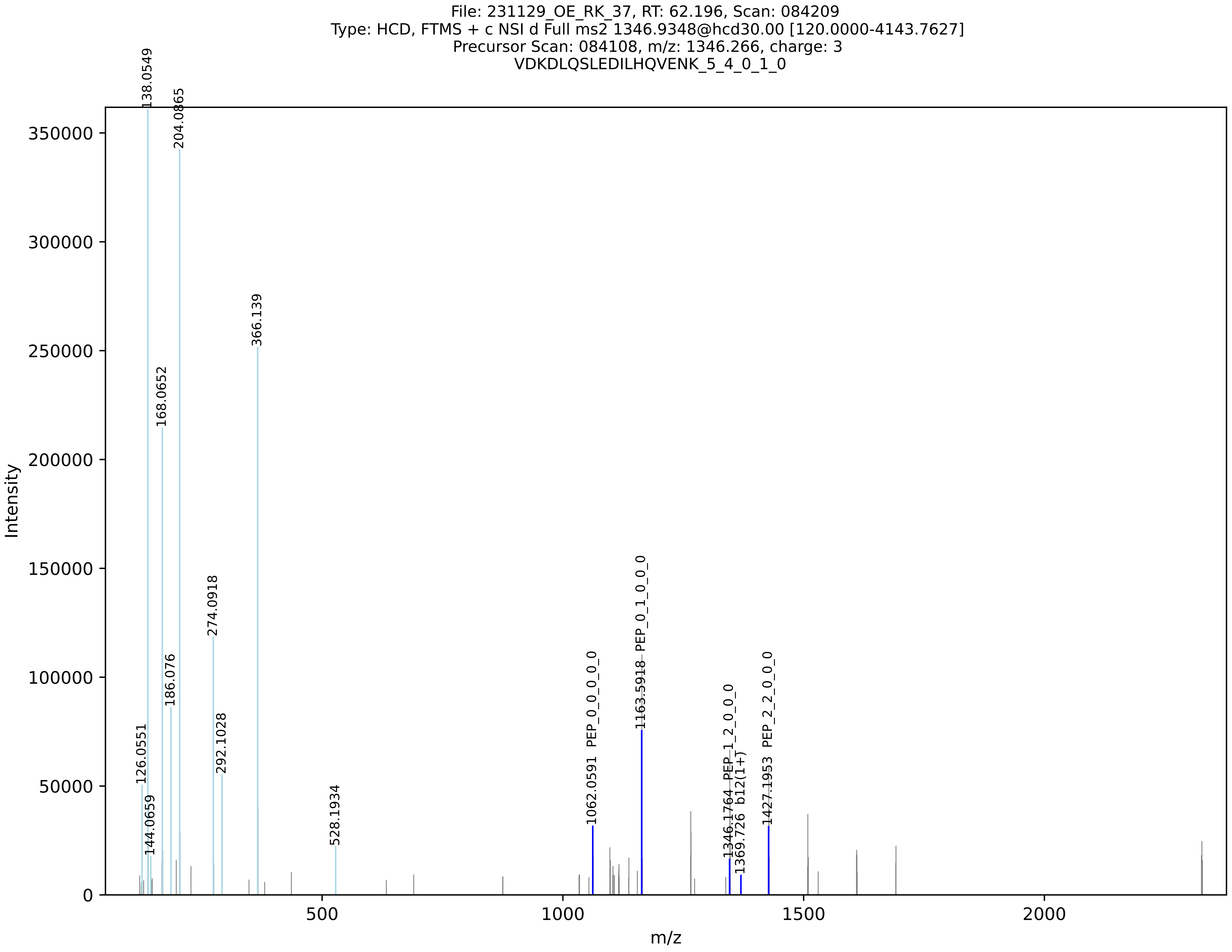

File: 231129_OE_RK_37, RT: 62.196, Scan: 084209
Type: HCD, FTMS + c NSI d Full ms2 1346.9348@hcd30.00 [120.0000-4143.7627]
Precursor Scan: 084108, m/z: 1346.266, charge: 3
VDKDLQSLEDILHQVENK_5_4_0_1_0
138.0549
204.0865
366.139
168.0652
274.0918
186.076
126.0551
292.1028
144.0659
528.1934
1062.0591 PEP_0_0_0_0_0_
1163.5918 PEP_0_1_0_0_0_
1346.1764 PEP_1_2_0_0_0_
1369.726 b12(1+)
1427.1953 PEP_2_2_0_0_0_
Intensity
m/z

File: 231129_OE_RK_37, RT: 62.342, Scan: 084421
Type: HCD, FTMS + c NSI d Full ms2 982.1385@hcd30.00 [120.0000-6034.9717]
Precursor Scan: 084330, m/z: 981.6384, charge: 6
QKVEGSSSHLVTFTVLPLEIGLHNINFSLETWFGK_4_5_2_0_0

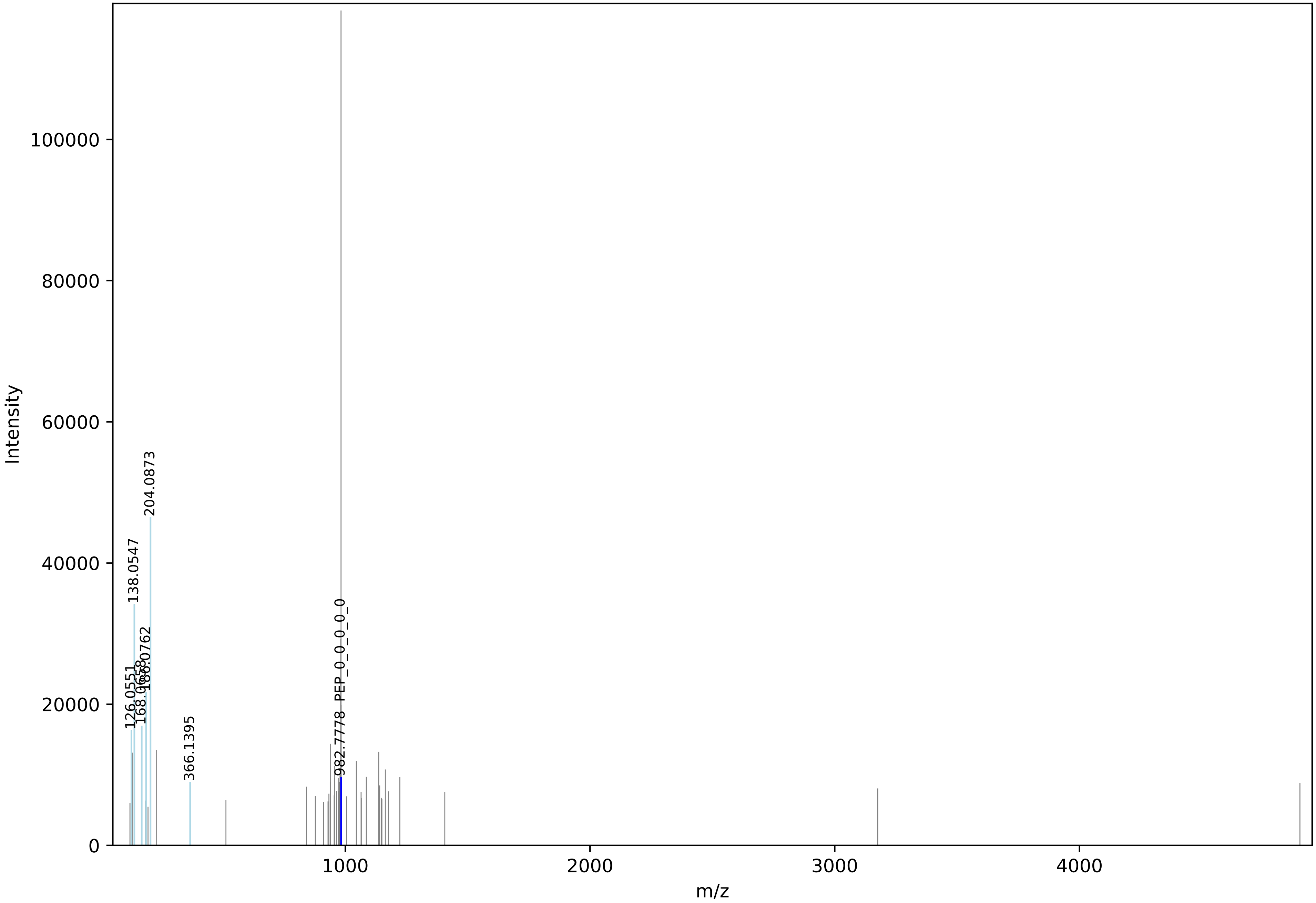

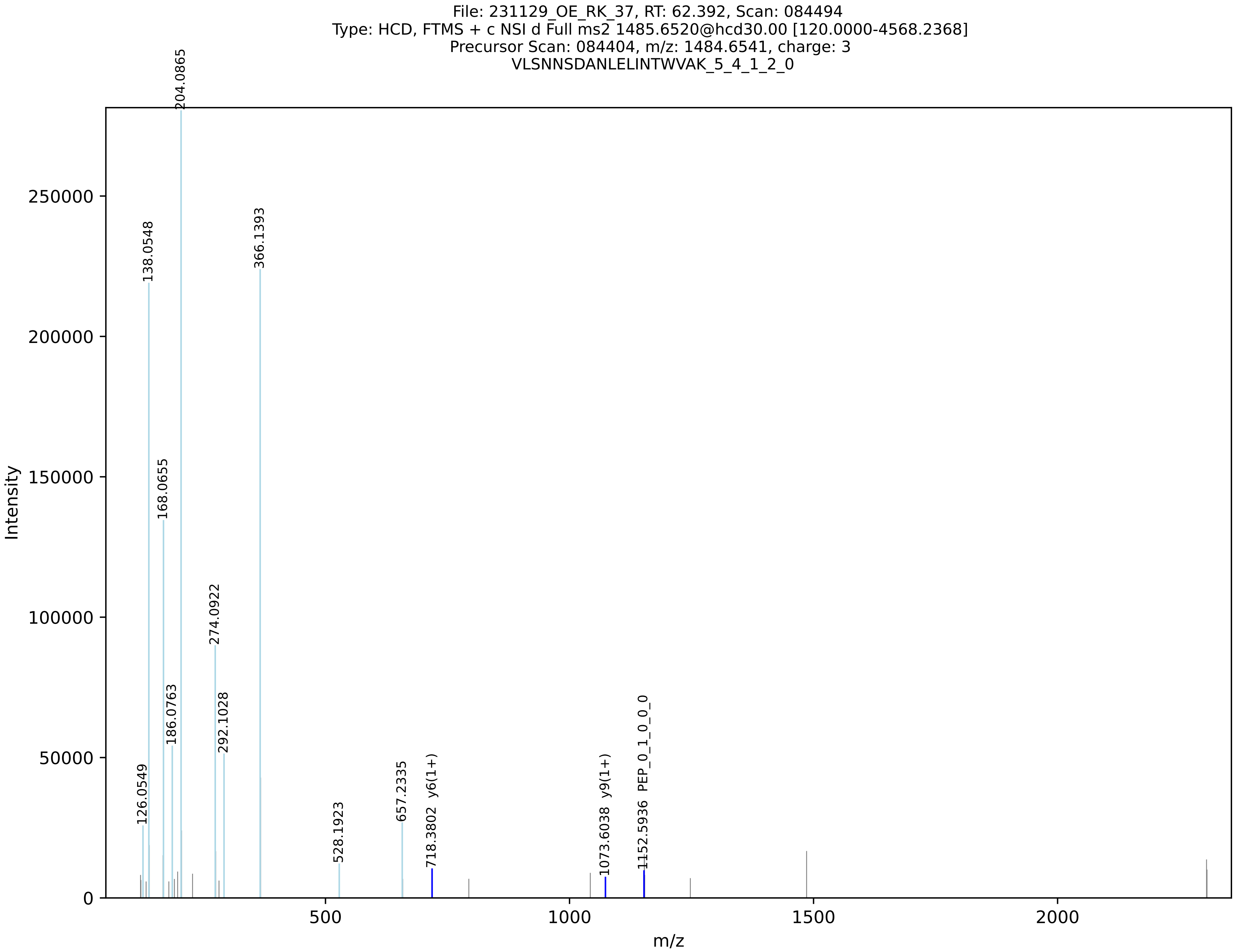
File: 231129_OE_RK_37, RT: 62.392, Scan: 084494
Type: HCD, FTMS + c NSI d Full ms2 1485.6520@hcd30.00 [120.0000-4568.2368]
Precursor Scan: 084404, m/z: 1484.6541, charge: 3
VLSNNSDANLELINTWVAK_5_4_1_2_0
Intensity
m/z
0
50000
100000
150000
200000
250000
500
1000
1500
2000
204.0865
138.0548
366.1393
168.0655
274.0922
186.0763
292.1028
126.0549
528.1923
657.2335
718.3802 y6(1+)
1073.6038 y9(1+)
1152.5936 PEP_0_1_0_0_0

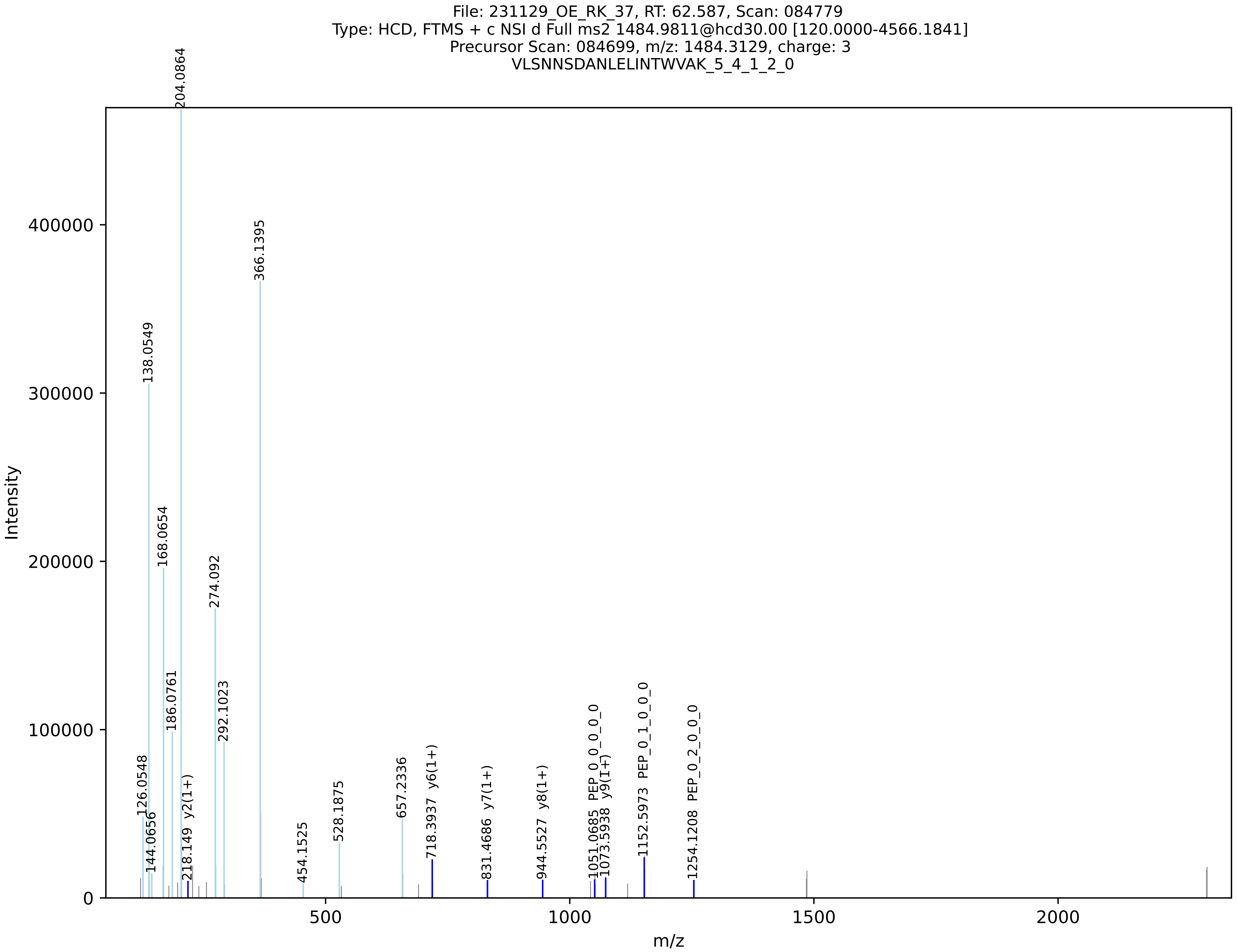
File: 231129_OE_RK_37, RT: 62.587, Scan: 084779
Type: HCD, FTMS + c NSI d Full ms2 1484.9811@hcd30.00 [120.0000-4566.1841]
Precursor Scan: 084699, m/z: 1484.3129, charge: 3
VLSNNSDANLELINTWVAK_5_4_1_2_0
Intensity
m/z
0
100000
200000
300000
400000
500
1000
1500
2000
204.0864
366.1395
138.0549
168.0654
274.092
186.0761
292.1023
126.0548
144.0656
218.149 y2(1+)
454.1525
528.1875
657.2336
718.3937 y6(1+)
831.4686 y7(1+)
944.5527 y8(1+)
1051.0685 PEP_0_0_0_0_
1073.5938 y9(1+)
1152.5973 PEP_0_1_0_0_0_
1254.1208 PEP_0_2_0_0_

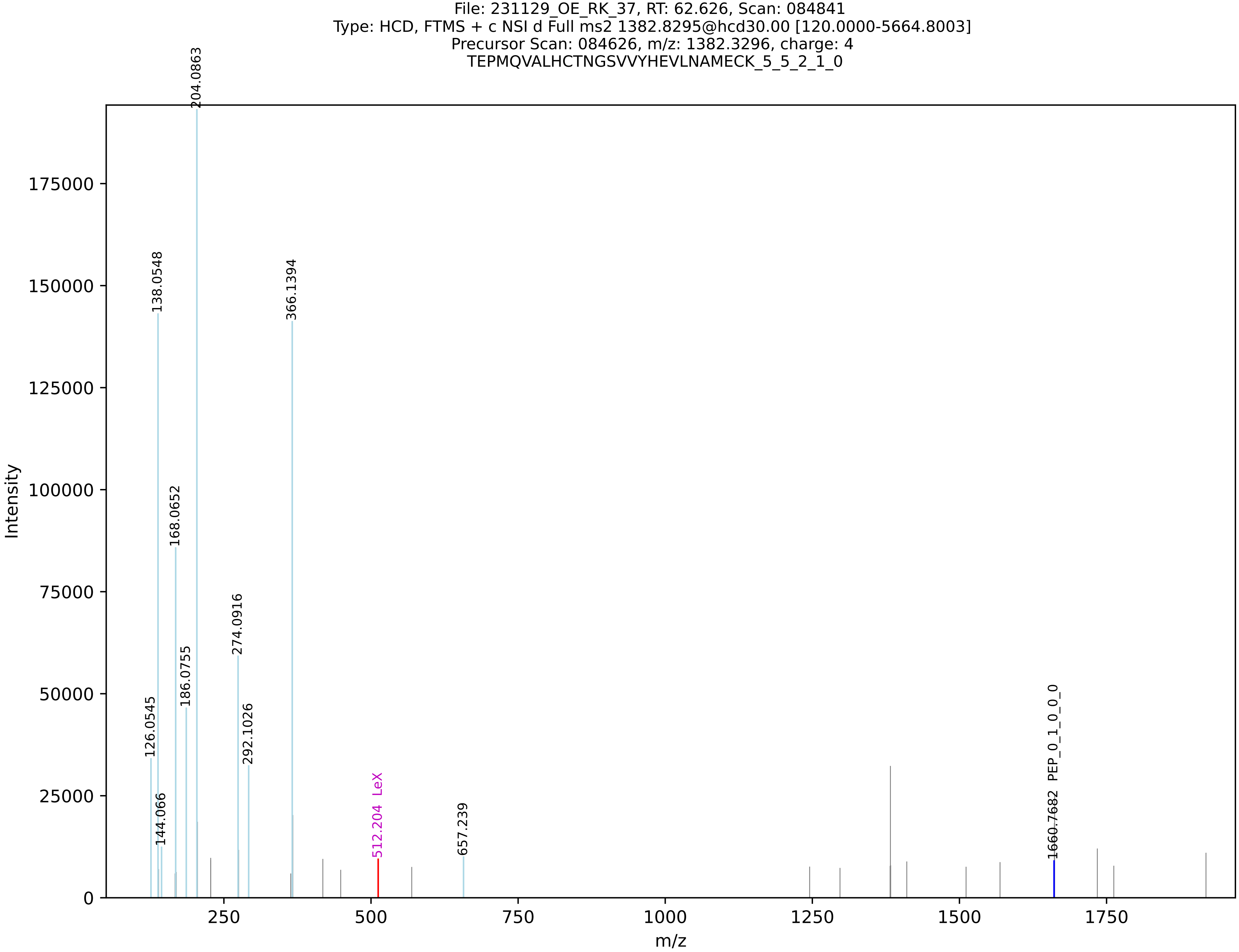
File: 231129_OE_RK_37, RT: 62.626, Scan: 084841
Type: HCD, FTMS + c NSI d Full ms2 1382.8295@hcd30.00 [120.0000-5664.8003]
Precursor Scan: 084626, m/z: 1382.3296, charge: 4
TEPMQVALHCTNGSVVYHEVLNAMECK_5_5_2_1_0
Intensity
m/z
204.0863
138.0548
366.1394
168.0652
274.0916
186.0755
126.0545
292.1026
144.066
512.204 LeX
657.239
PEP_0_1_0_0_0
1660.7682

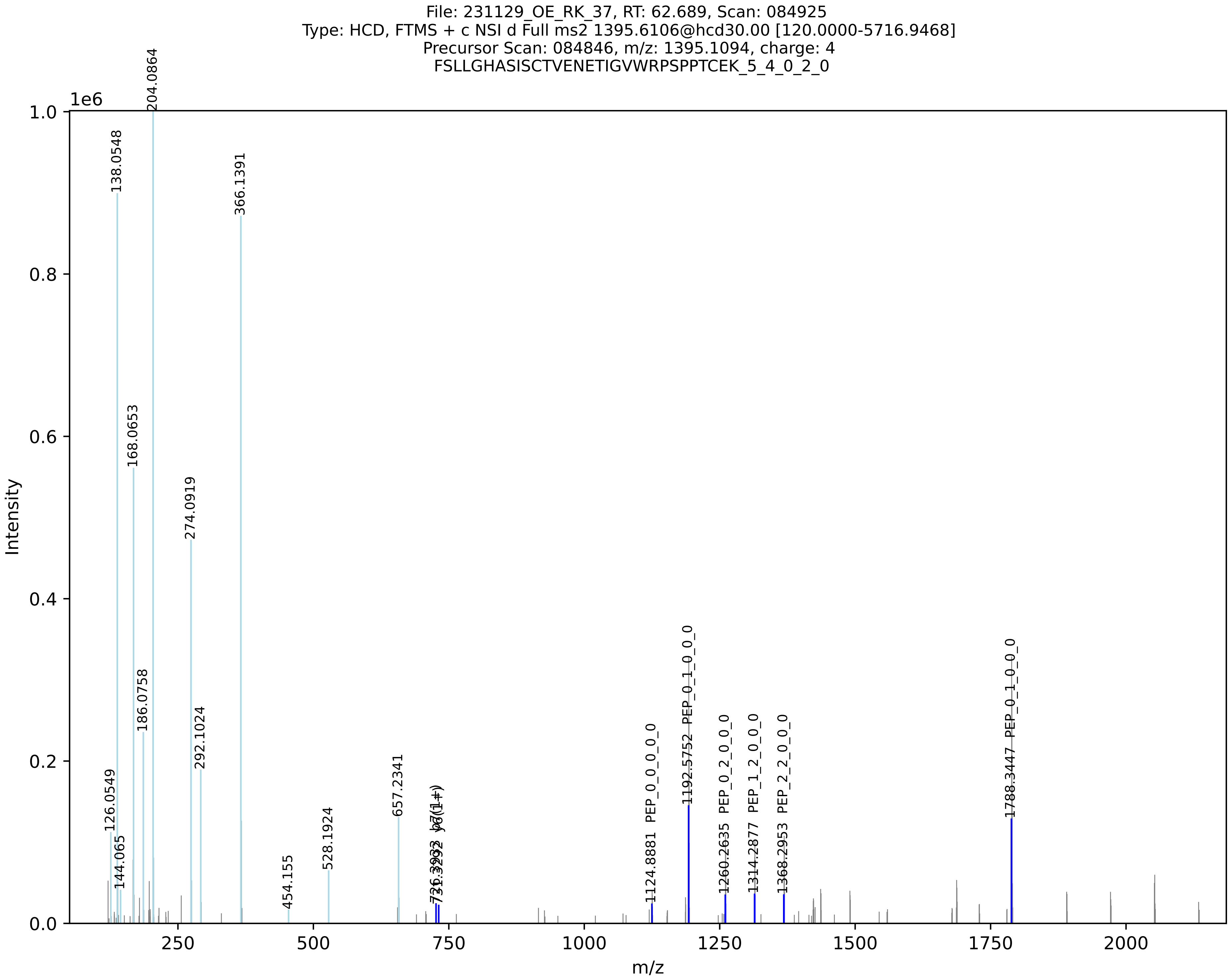
File: 231129_OE_RK_37, RT: 62.689, Scan: 084925
Type: HCD, FTMS + c NSI d Full ms2 1395.6106@hcd30.00 [120.0000-5716.9468]
Precursor Scan: 084846, m/z: 1395.1094, charge: 4
FSLLGHASISCTVENETIGVWRPSPPTCEK_5_4_0_2_0
1e6
Intensity
m/z
204.0864
138.0548
366.1391
168.0653
274.0919
186.0758
292.1024
126.0549
144.065
454.155
528.1924
657.2341
726.3963 b7(1+)
731.3292 y6(1+)
1124.8881 PEP_0_0_0_0_0
1192.5752 PEP_0_1_0_0_0
1260.2635 PEP_0_2_0_0_0
1314.2877 PEP_1_2_0_0_0
1368.2953 PEP_2_2_0_0_0
1788.3447 PEP_0_1_0_0_0

File: 231129_OE_RK_37, RT: 62.735, Scan: 084992
Type: HCD, FTMS + c NSI d Full ms2 1295.7994@hcd30.00 [120.0000-5309.7178]
Precursor Scan: 084917, m/z: 1295.0477, charge: 4
TEPMQVALHCTNGSVVYHEVLNAMECK_5_4_1_1_0

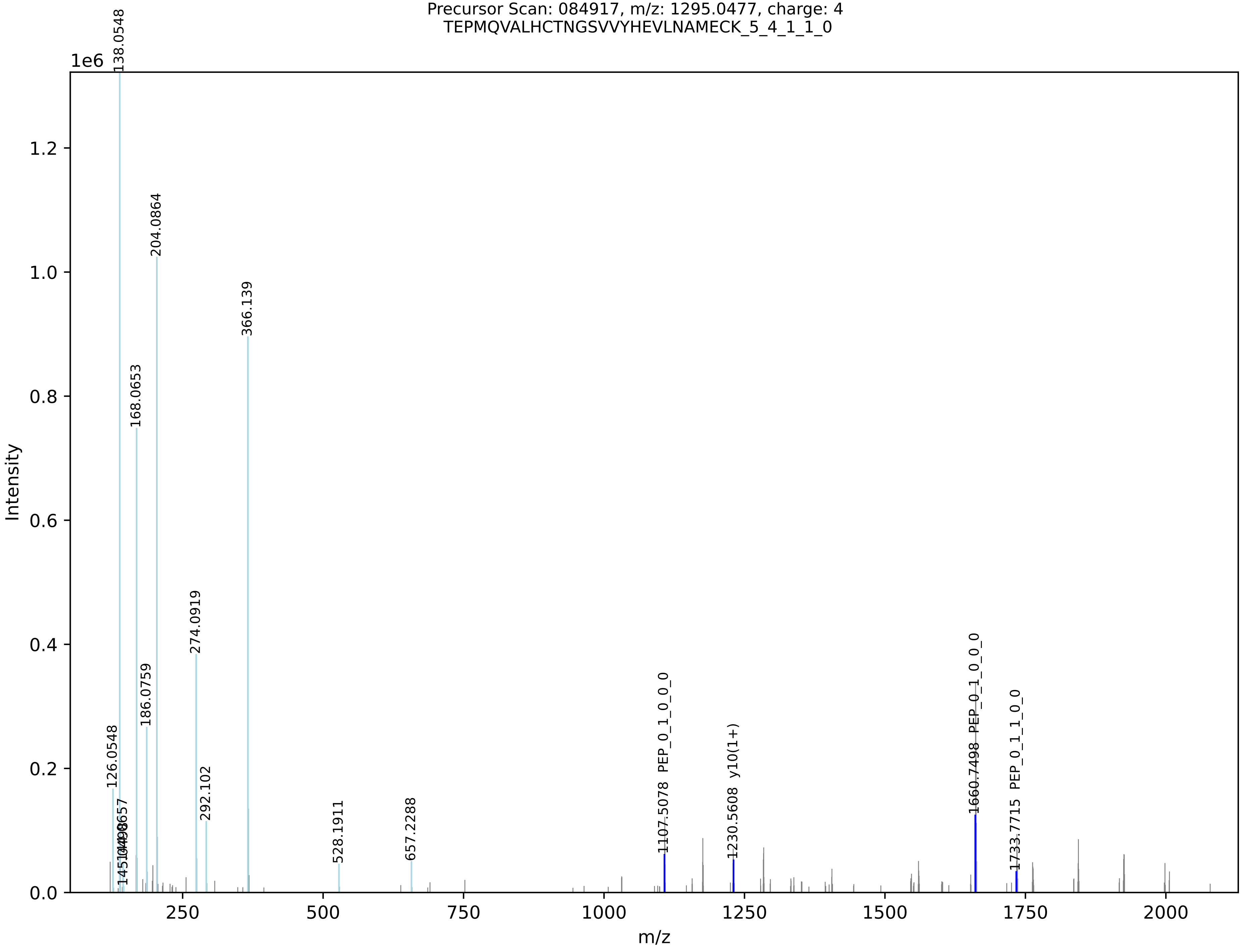

File: 231129_OE_RK_37, RT: 62.75, Scan: 085017
Type: HCD, FTMS + c NSI d Full ms2 1116.8910@hcd30.00 [120.0000-5719.7144]
Precursor Scan: 084917, m/z: 1116.2888, charge: 5
FSLLGHASISCTVENETIGVWRPSPPTCEK_5_4_0_2_0

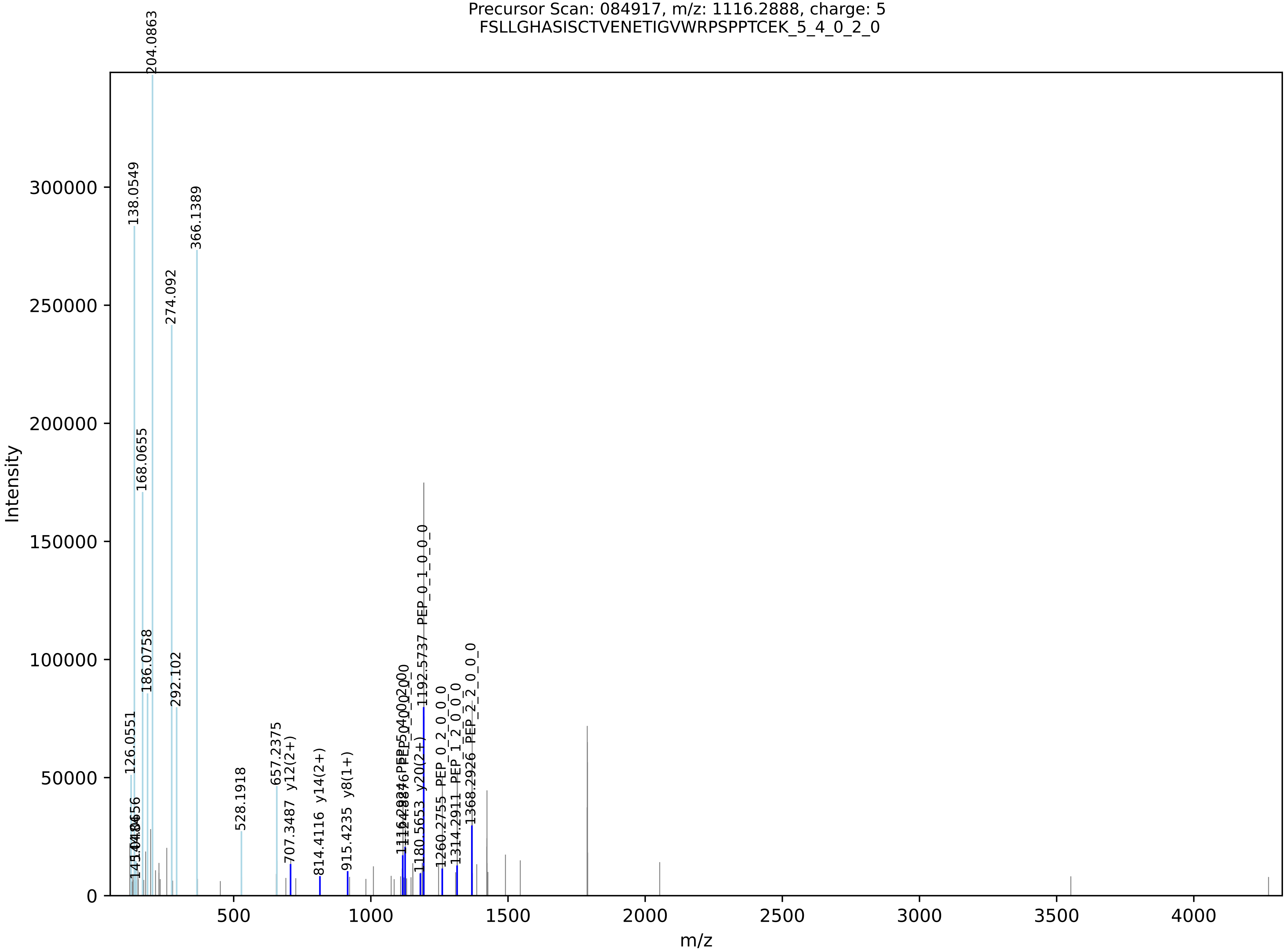

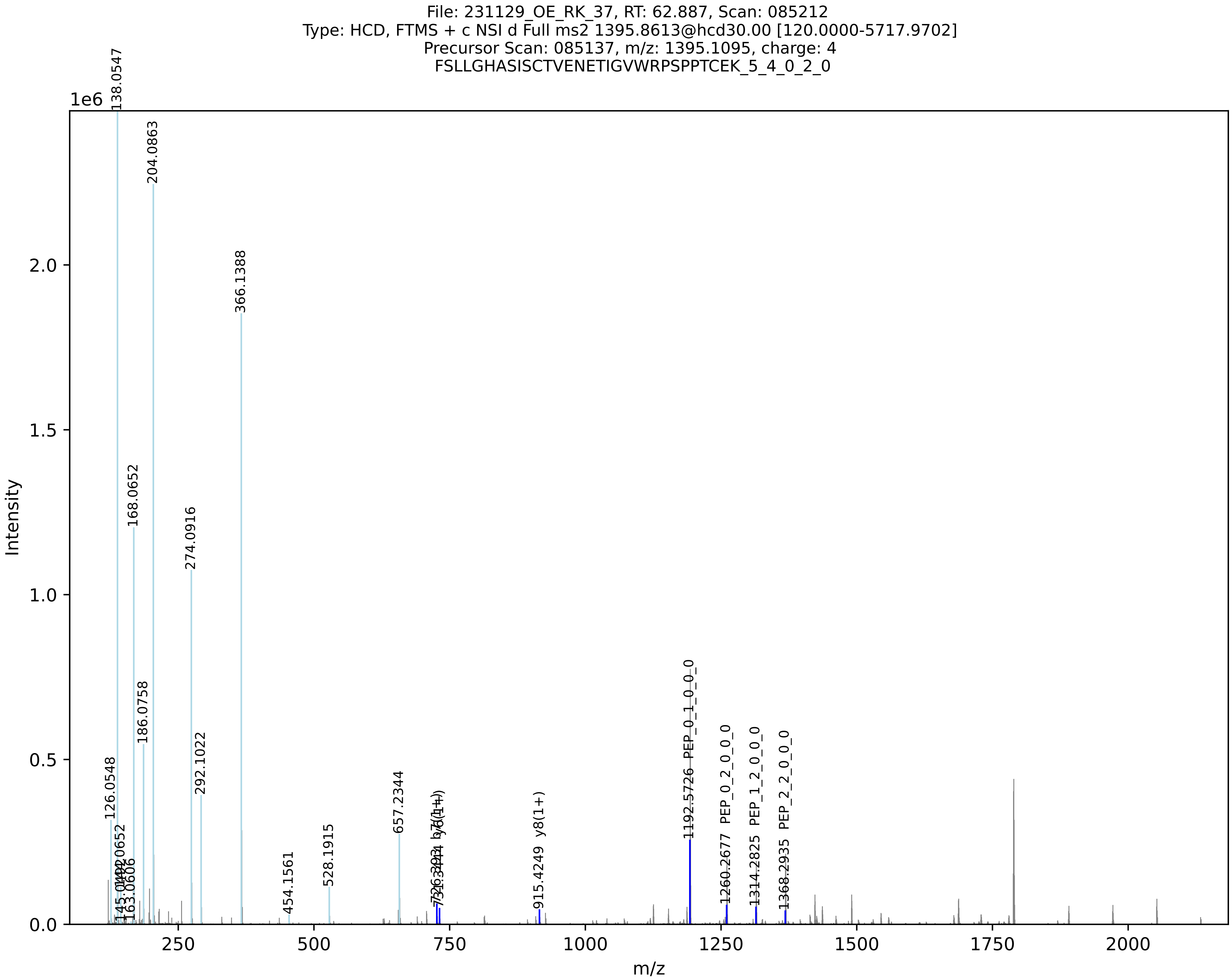

File: 231129_OE_RK_37, RT: 62.887, Scan: 085212
Type: HCD, FTMS + c NSI d Full ms2 1395.8613@hcd30.00 [120.0000-5717.9702]
Precursor Scan: 085137, m/z: 1395.1095, charge: 4
FSLLGHASISCTVENETIGVWRPSPPTCEK_5_4_0_2_0
1e6
Intensity
m/z
138.0547
204.0863
366.1388
168.0652
274.0916
186.0758
292.1022
126.0548
454.1561
528.1915
657.2344
915.4249 y8(1+)
1192.5726 PEP_0_1_0_0_0
1260.2677 PEP_0_2_0_0_0
1314.2825 PEP_1_2_0_0_0
1368.2935 PEP_2_2_0_0_0

File: 231129_OE_RK_37, RT: 62.989, Scan: 085358
Type: HCD, FTMS + c NSI d Full ms2 1485.3195@hcd30.00 [120.0000-4567.2197]
Precursor Scan: 085282, m/z: 1484.6505, charge: 3
VLSNNSDANLELINTWVAK_5_4_1_2_0

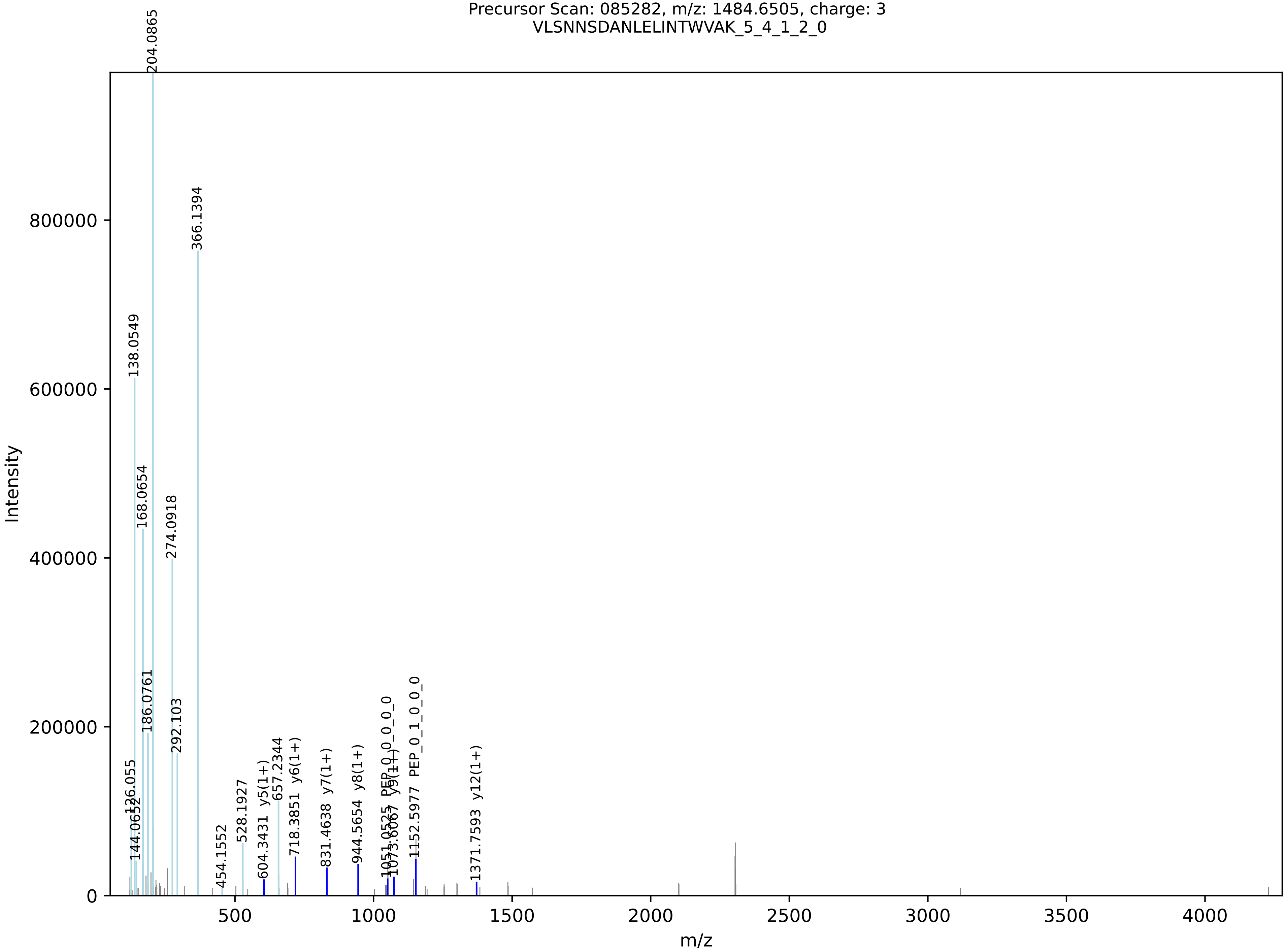

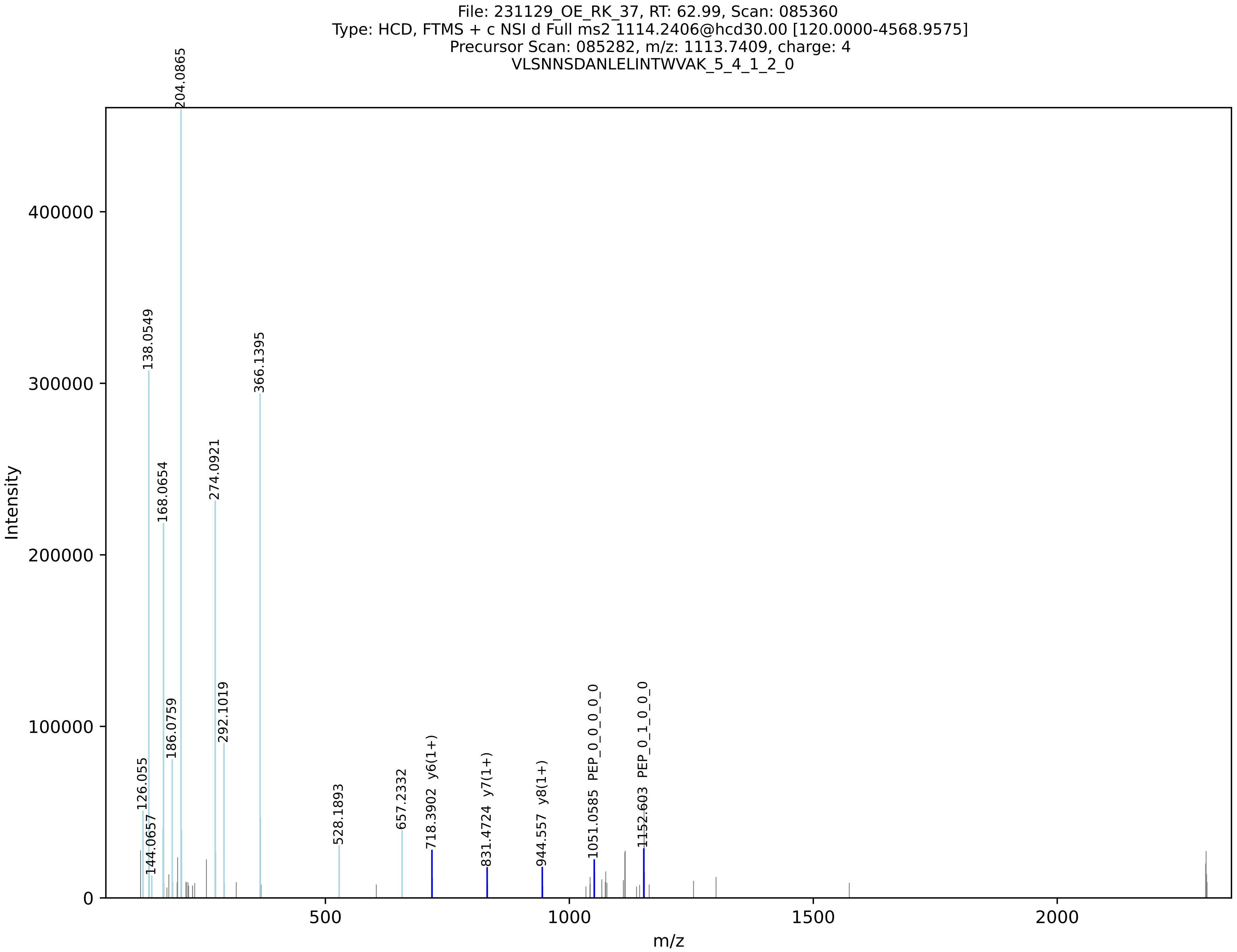
File: 231129_OE_RK_37, RT: 62.99, Scan: 085360
Type: HCD, FTMS + c NSI d Full ms2 1114.2406@hcd30.00 [120.0000-4568.9575]
Precursor Scan: 085282, m/z: 1113.7409, charge: 4
VLSNNSDANLELINTWVAK_5_4_1_2_0
204.0865
138.0549
366.1395
274.0921
168.0654
186.0759
292.1019
126.055
144.0657
528.1893
657.2332
718.3902 y6(1+)
831.4724 y7(1+)
944.557 y8(1+)
1051.0585 PEP_0_0_0_0_0
1152.603 PEP_0_1_0_0_0
Intensity
0
100000
200000
300000
400000
500
1000
1500
2000
m/z

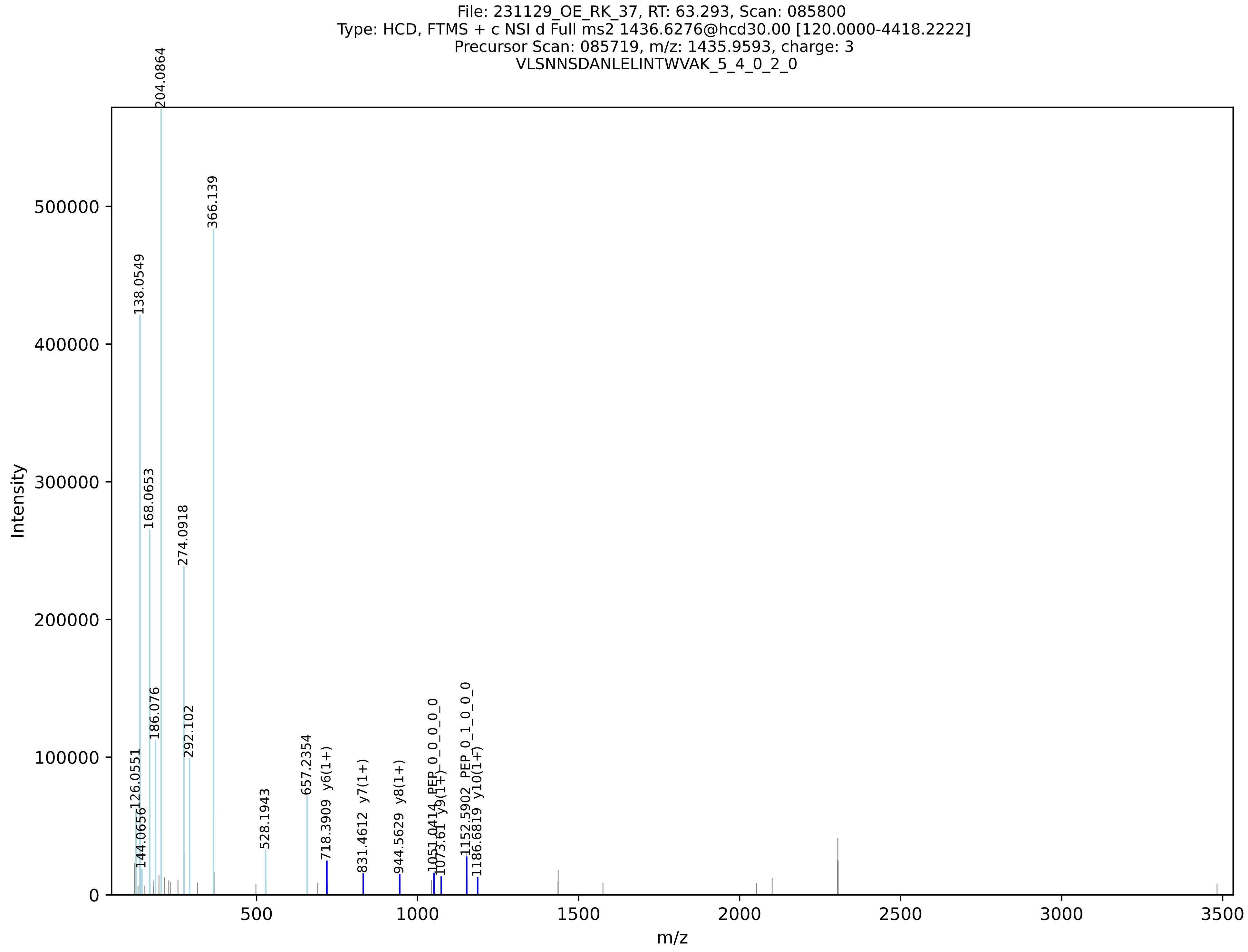
File: 231129_OE_RK_37, RT: 63.293, Scan: 085800
Type: HCD, FTMS + c NSI d Full ms2 1436.6276@hcd30.00 [120.0000-4418.2222]
Precursor Scan: 085719, m/z: 1435.9593, charge: 3
VLSNNSDANLELINTWVAK_5_4_0_2_0
Intensity
m/z
204.0864
366.139
138.0549
168.0653
274.0918
186.076
292.102
126.0551
144.0656
528.1943
657.2354
718.3909 y6(1+)
831.4612 y7(1+)
944.5629 y8(1+)
1051.0414 PEP_0_0_0_0_0
1073.61 y9(1+)
1152.5902 PEP_0_1_0_0_0
1186.6819 y10(1+)
0
100000
200000
300000
400000
500000
500
1000
1500
2000
2500
3000
3500

File: 231129_OE_RK_37, RT: 63.391, Scan: 085943
Type: HCD, FTMS + c NSI d Full ms2 1485.3162@hcd30.00 [120.0000-4567.2095]
Precursor Scan: 085866, m/z: 1484.6467, charge: 3
VLSNNSDANLELINTWVAK_5_4_1_2_0

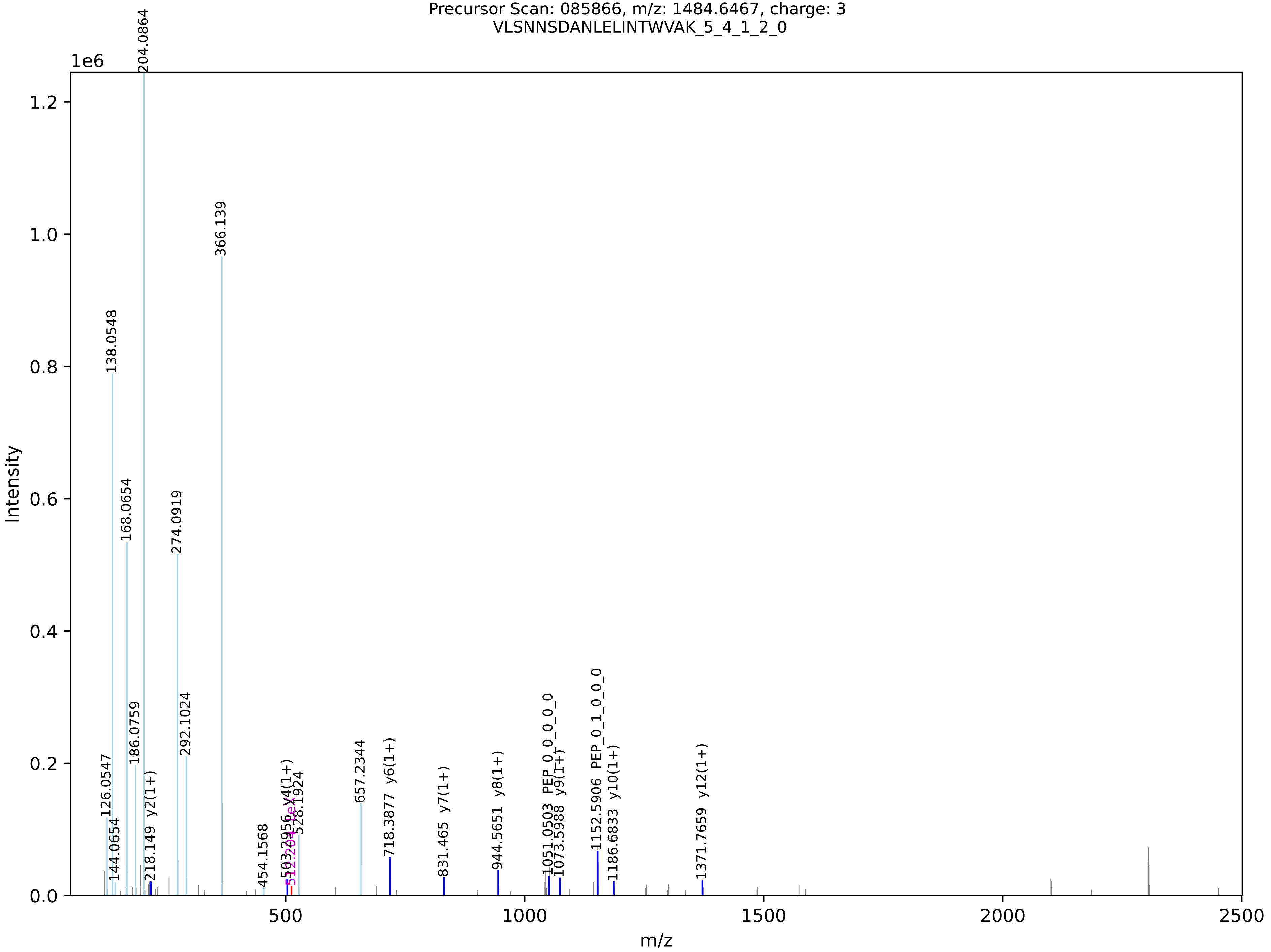

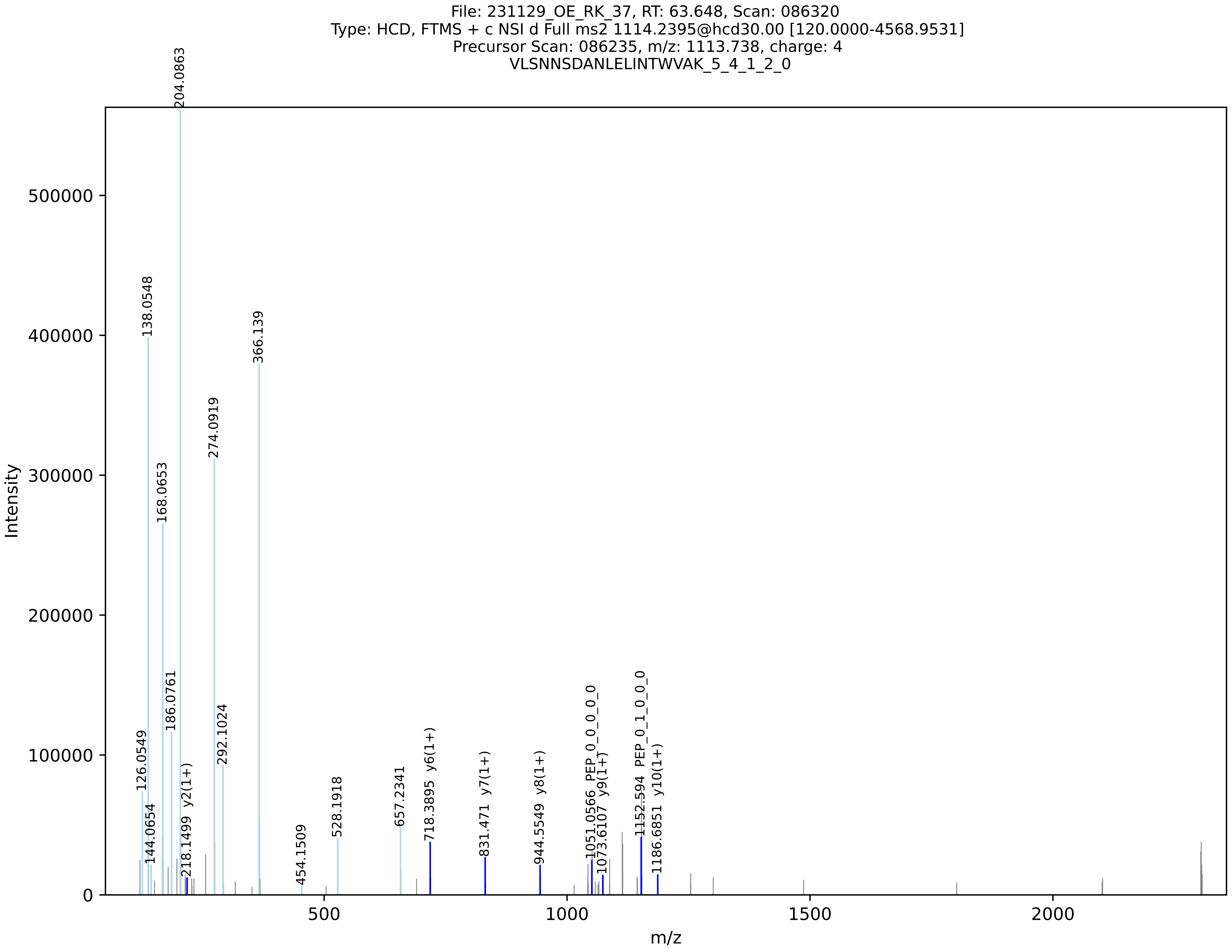
File: 231129_OE_RK_37, RT: 63.648, Scan: 086320
Type: HCD, FTMS + c NSI d Full ms2 1114.2395@hcd30.00 [120.0000-4568.9531]
Precursor Scan: 086235, m/z: 1113.738, charge: 4
VLSNNSDANLELINTWVAK_5_4_1_2_0
Intensity
m/z
204.0863
138.0548
366.139
274.0919
168.0653
186.0761
292.1024
126.0549
144.0654
218.1499 y2(1+)
454.1509
528.1918
657.2341
718.3895 y6(1+)
831.471 y7(1+)
944.5549 y8(1+)
1051.0566 PEP_0_0_0_0_0
1073.6107 y9(1+)
1152.594 PEP_0_1_0_0_0
1186.6851 y10(1+)
0
100000
200000
300000
400000
500000
500
1000
1500
2000

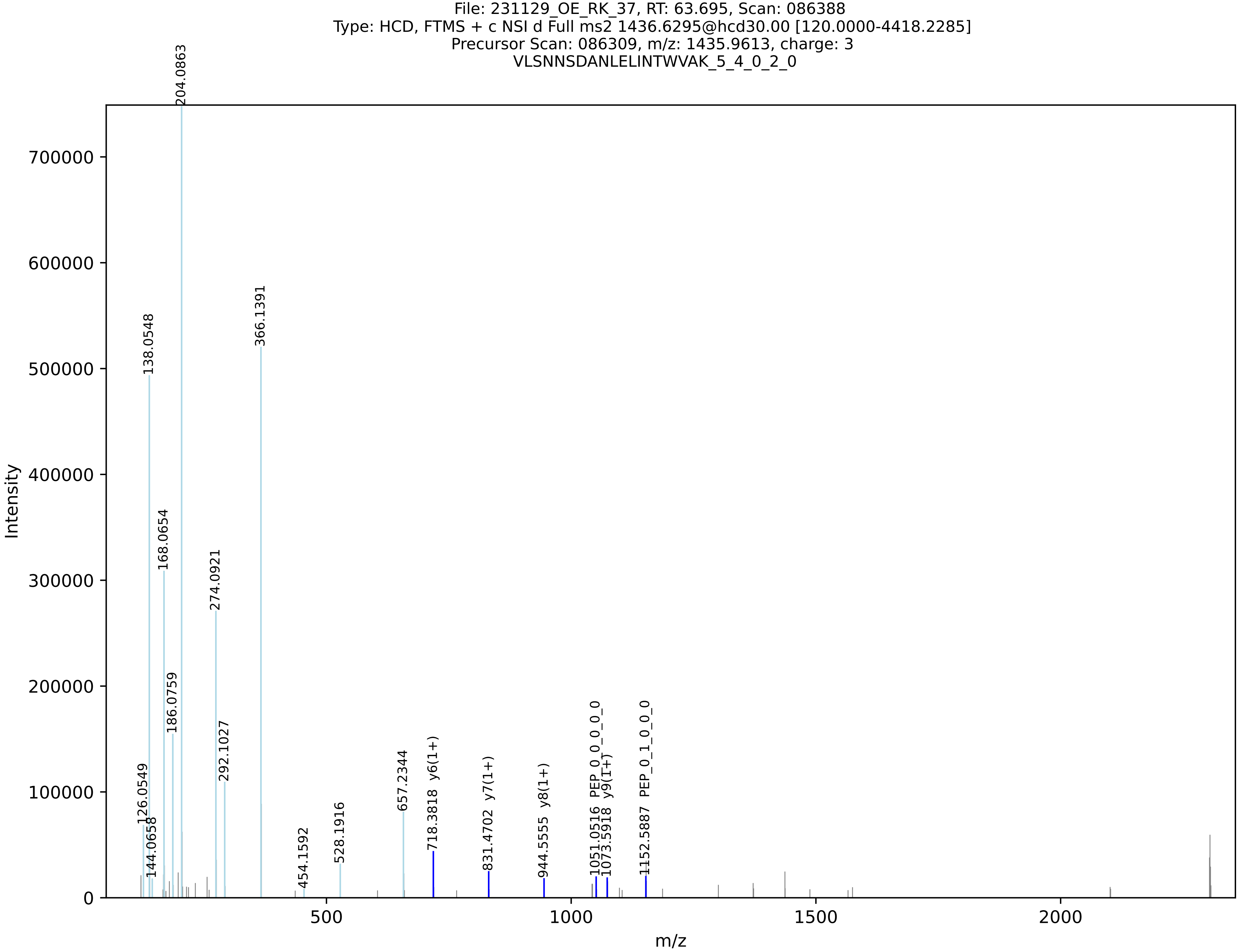
File: 231129_OE_RK_37, RT: 63.695, Scan: 086388
Type: HCD, FTMS + c NSI d Full ms2 1436.6295@hcd30.00 [120.0000-4418.2285]
Precursor Scan: 086309, m/z: 1435.9613, charge: 3
VLSNNSDANLELINTWVAK_5_4_0_2_0
Intensity
m/z
204.0863
138.0548
366.1391
168.0654
274.0921
186.0759
292.1027
126.0549
144.0658
454.1592
528.1916
657.2344
718.3818 y6(1+)
831.4702 y7(1+)
944.5555 y8(1+)
1051.0516 PEP_0_0_0_0_0_
1073.5918 y9(1+)
1152.5887 PEP_0_1_0_0_0_
0
100000
200000
300000
400000
500000
600000
700000
500
1000
1500
2000

File: 231129_OE_RK_37, RT: 63.706, Scan: 086406
Type: HCD, FTMS + c NSI d Full ms2 1105.3145@hcd30.00 [120.0000-5660.6738]
Precursor Scan: 086309, m/z: 1104.7131, charge: 5
VPHSRNSITLTNLTPGTEYVVSIVALNGR_5_5_0_2_0

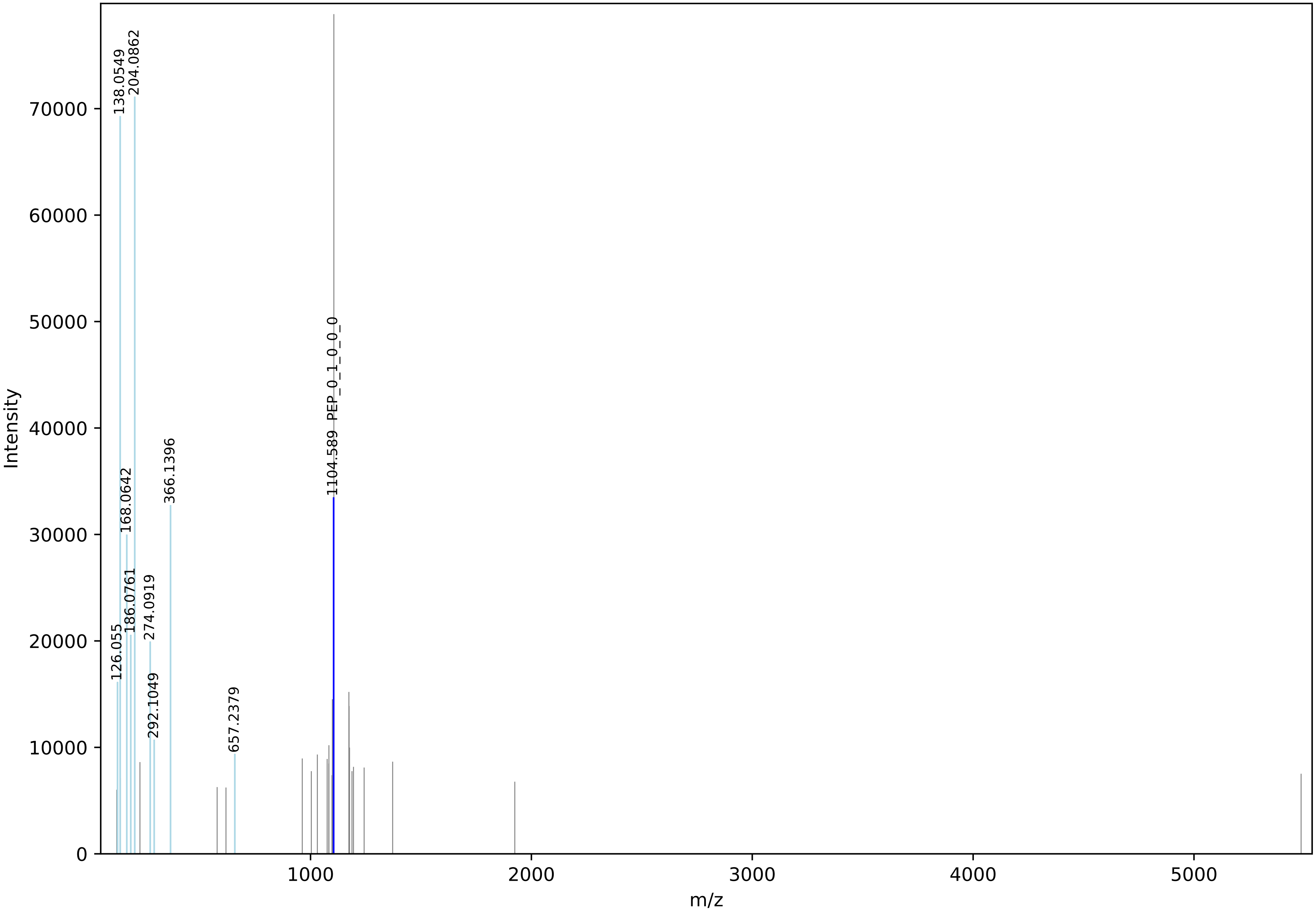

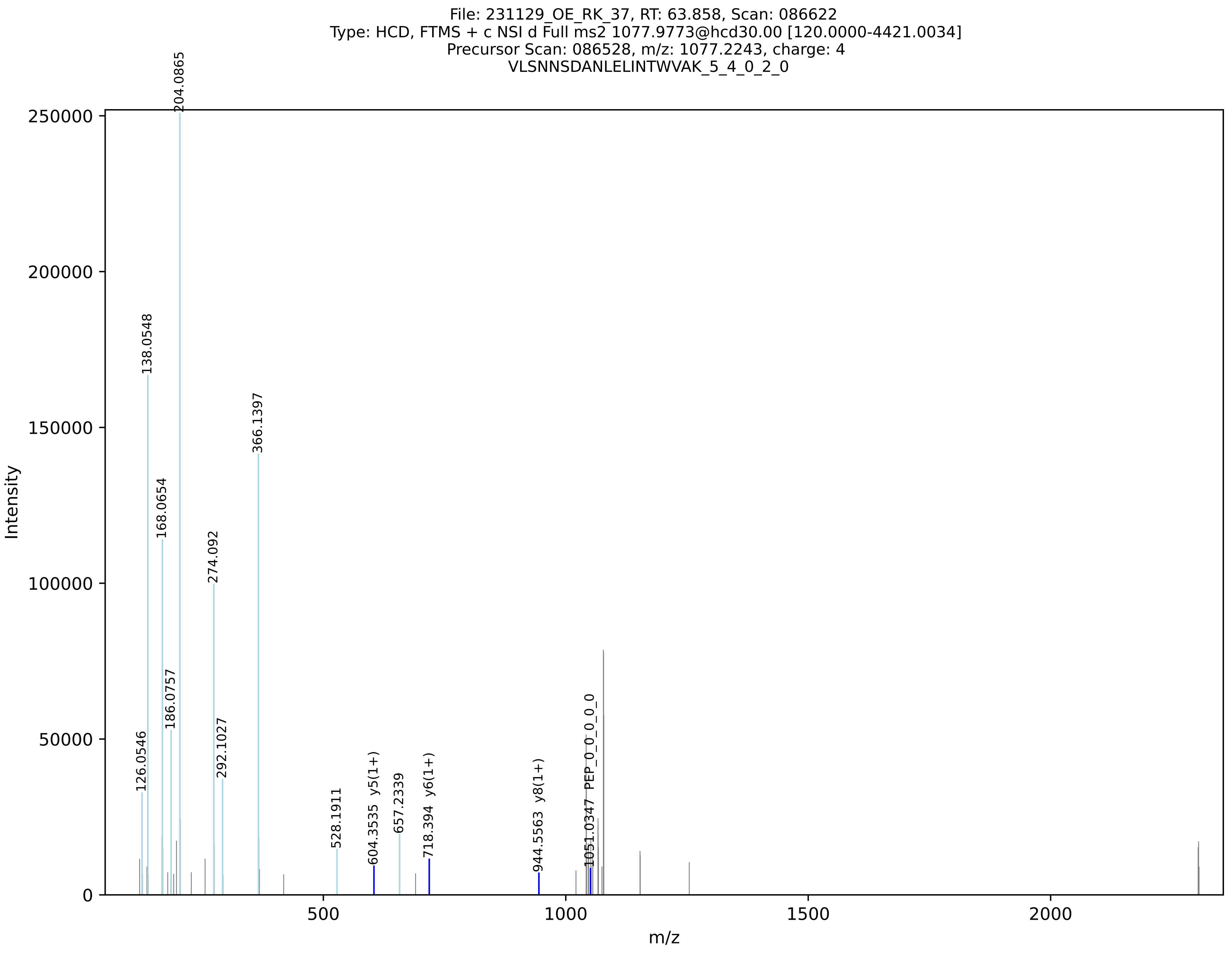

File: 231129_OE_RK_37, RT: 63.858, Scan: 086622
Type: HCD, FTMS + c NSI d Full ms2 1077.9773@hcd30.00 [120.0000-4421.0034]
Precursor Scan: 086528, m/z: 1077.2243, charge: 4
VLSNNSDANLELINTWVAK_5_4_0_2_0
204.0865
138.0548
366.1397
168.0654
274.092
186.0757
292.1027
126.0546
528.1911
604.3535 y5(1+)
657.2339
718.394 y6(1+)
944.5563 y8(1+)
1051.0347 PEP_0_0_0_0_0
Intensity
250000
200000
150000
100000
50000
0
500
1000
1500
2000
m/z

File: 231129_OE_RK_37, RT: 63.989, Scan: 086818
Type: HCD, FTMS + c NSI d Full ms2 1484.3011@hcd30.00 [120.0000-4564.1035]
Precursor Scan: 086602, m/z: 1484.3132, charge: 3
VLSNNSDANLELINTWVAK_5_4_1_2_0

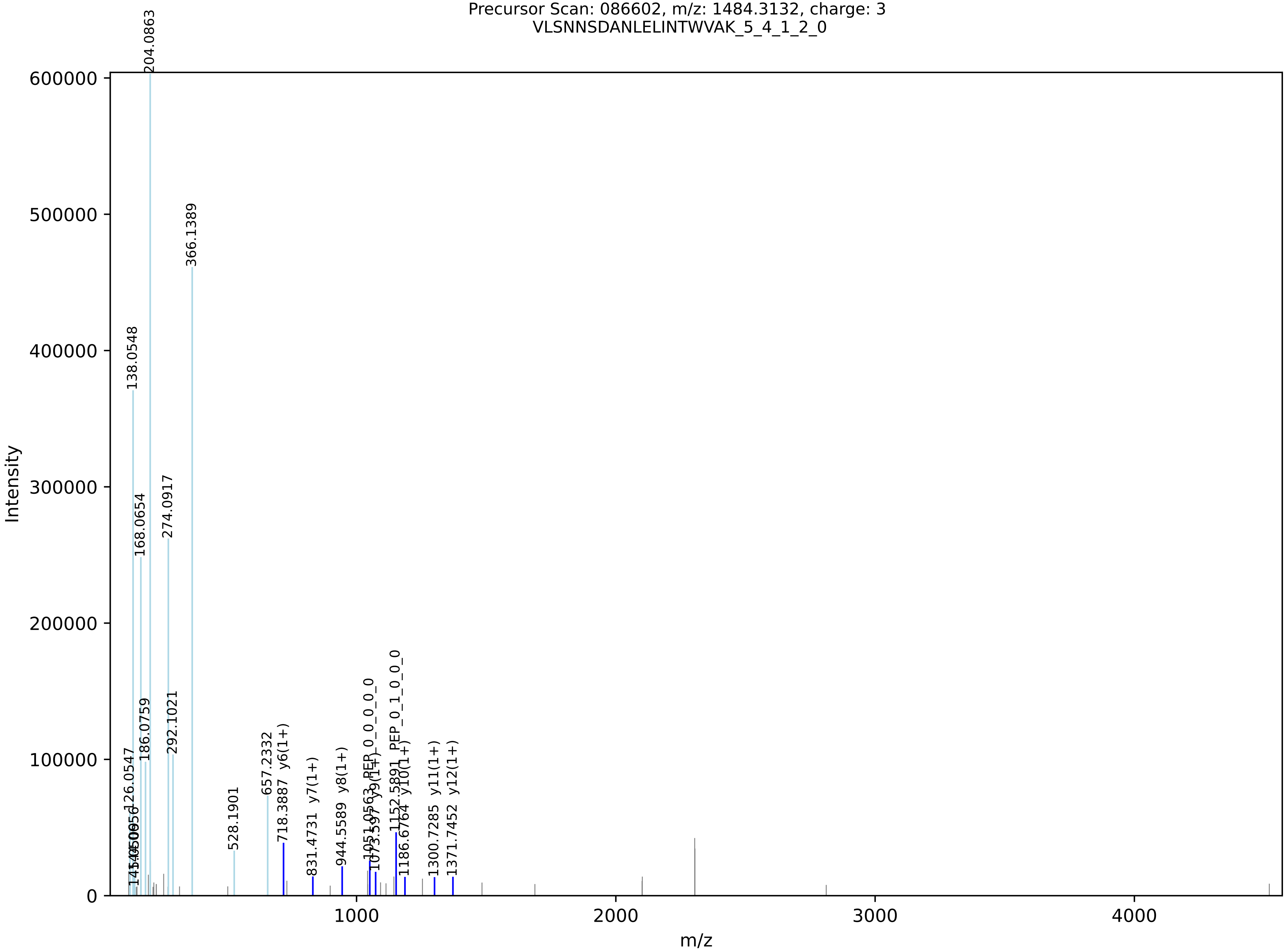

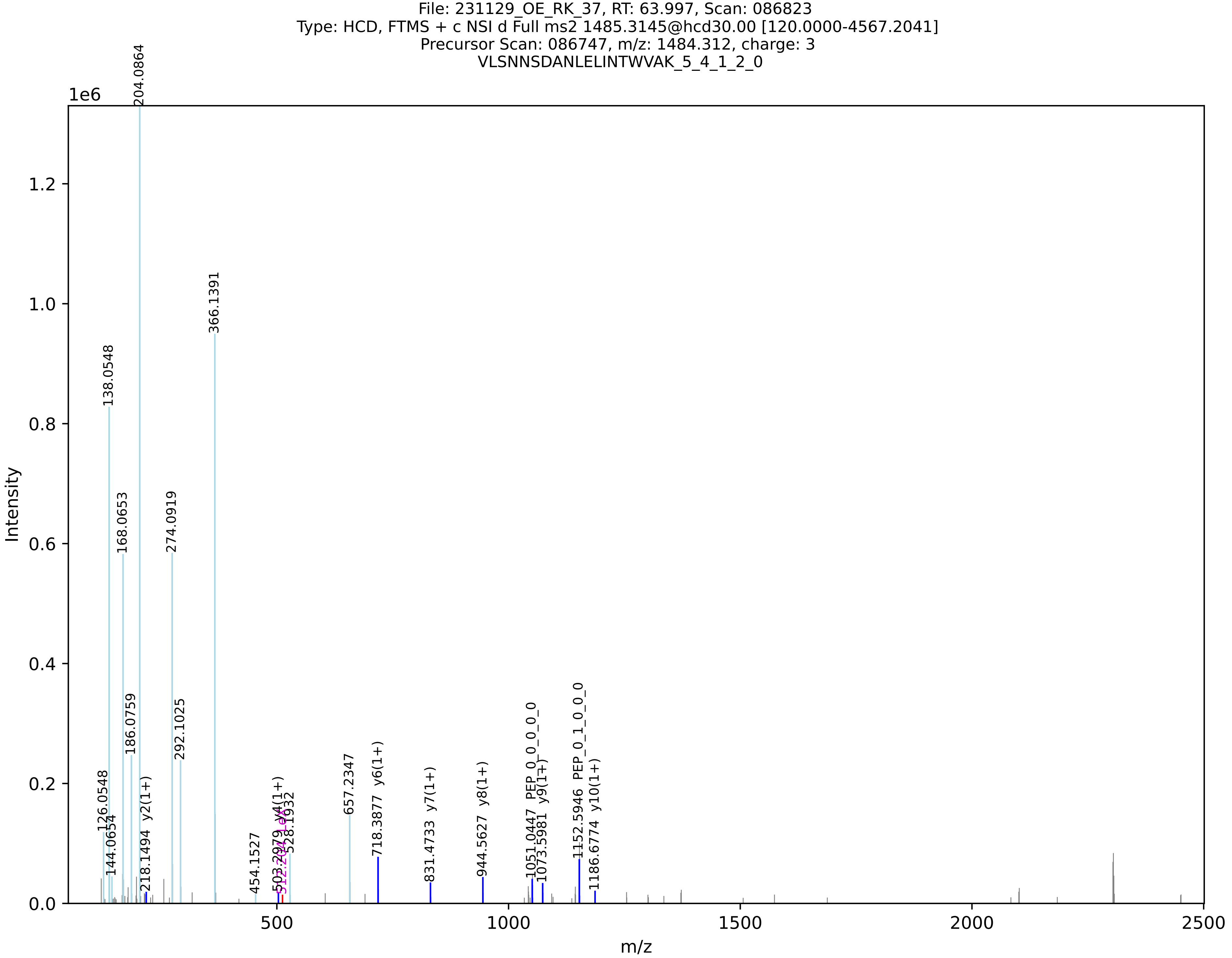

File: 231129_OE_RK_37, RT: 63.997, Scan: 086823
Type: HCD, FTMS + c NSI d Full ms2 1485.3145@hcd30.00 [120.0000-4567.2041]
Precursor Scan: 086747, m/z: 1484.312, charge: 3
VLSNNSDANLELINTWVAK_5_4_1_2_0
1e6
Intensity
m/z
204.0864
366.1391
138.0548
168.0653
274.0919
186.0759
292.1025
126.0548
144.0654
218.1494 y2(1+)
454.1527
503.2979 y4(1+)
528.1932
657.2347
718.3877 y6(1+)
831.4733 y7(1+)
944.5627 y8(1+)
1051.0447 PEP_0_0_0_0_0
1073.5981 y9(1+)
1152.5946 PEP_0_1_0_0_0
1186.6774 y10(1+)

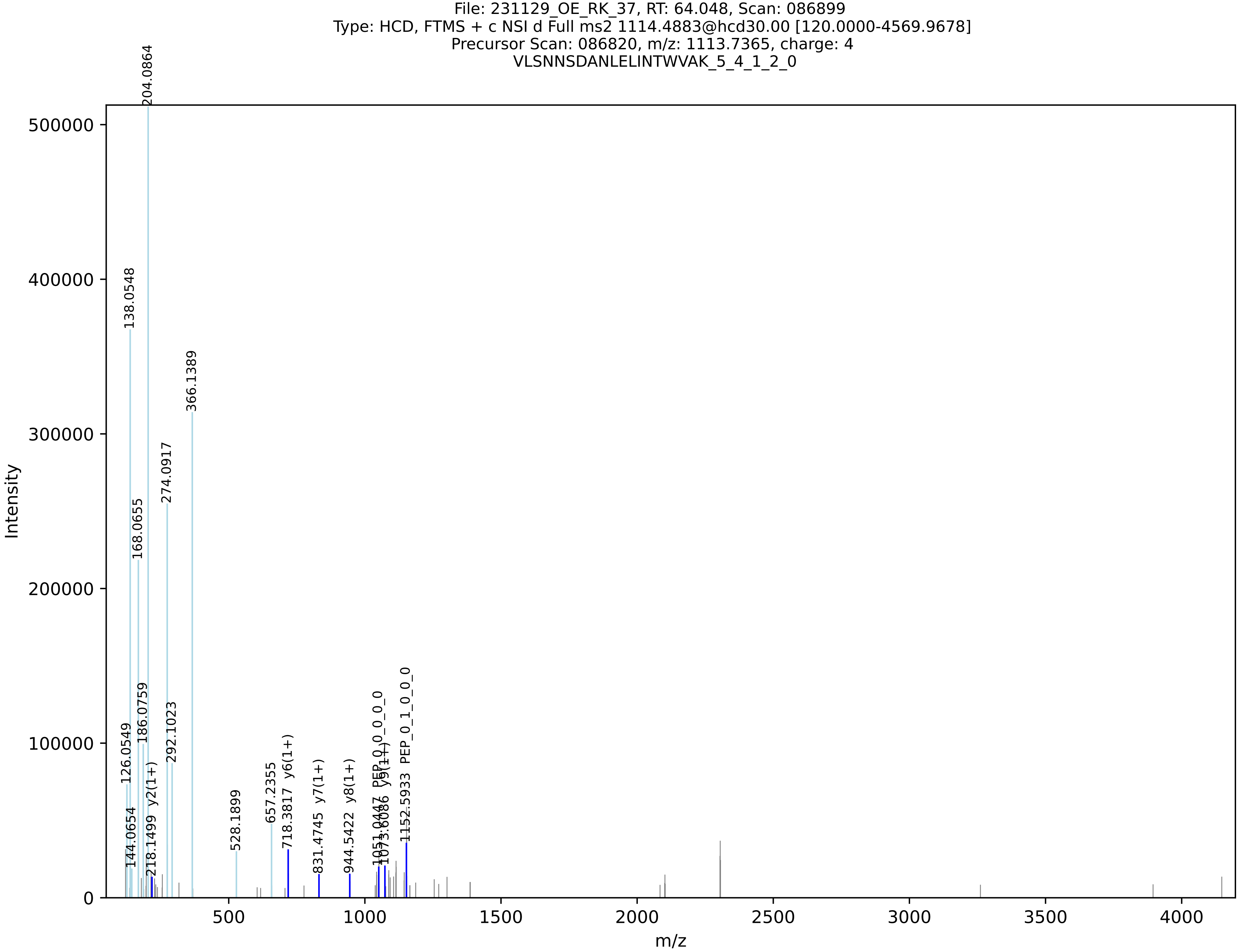
File: 231129_OE_RK_37, RT: 64.048, Scan: 086899
Type: HCD, FTMS + c NSI d Full ms2 1114.4883@hcd30.00 [120.0000-4569.9678]
Precursor Scan: 086820, m/z: 1113.7365, charge: 4
VLSNNSDANLELINTWVAK_5_4_1_2_0
Intensity
m/z
204.0864
138.0548
366.1389
274.0917
168.0655
186.0759
292.1023
126.0549
144.0654
218.1499 y2(1+)
528.1899
657.2355
718.3817 y6(1+)
831.4745 y7(1+)
944.5422 y8(1+)
1051.0447 PEP_0_0_0_0_0
1073.6086 y9(1+)
1152.5933 PEP_0_1_0_0_0
0
100000
200000
300000
400000
500000
500
1000
1500
2000
2500
3000
3500
4000

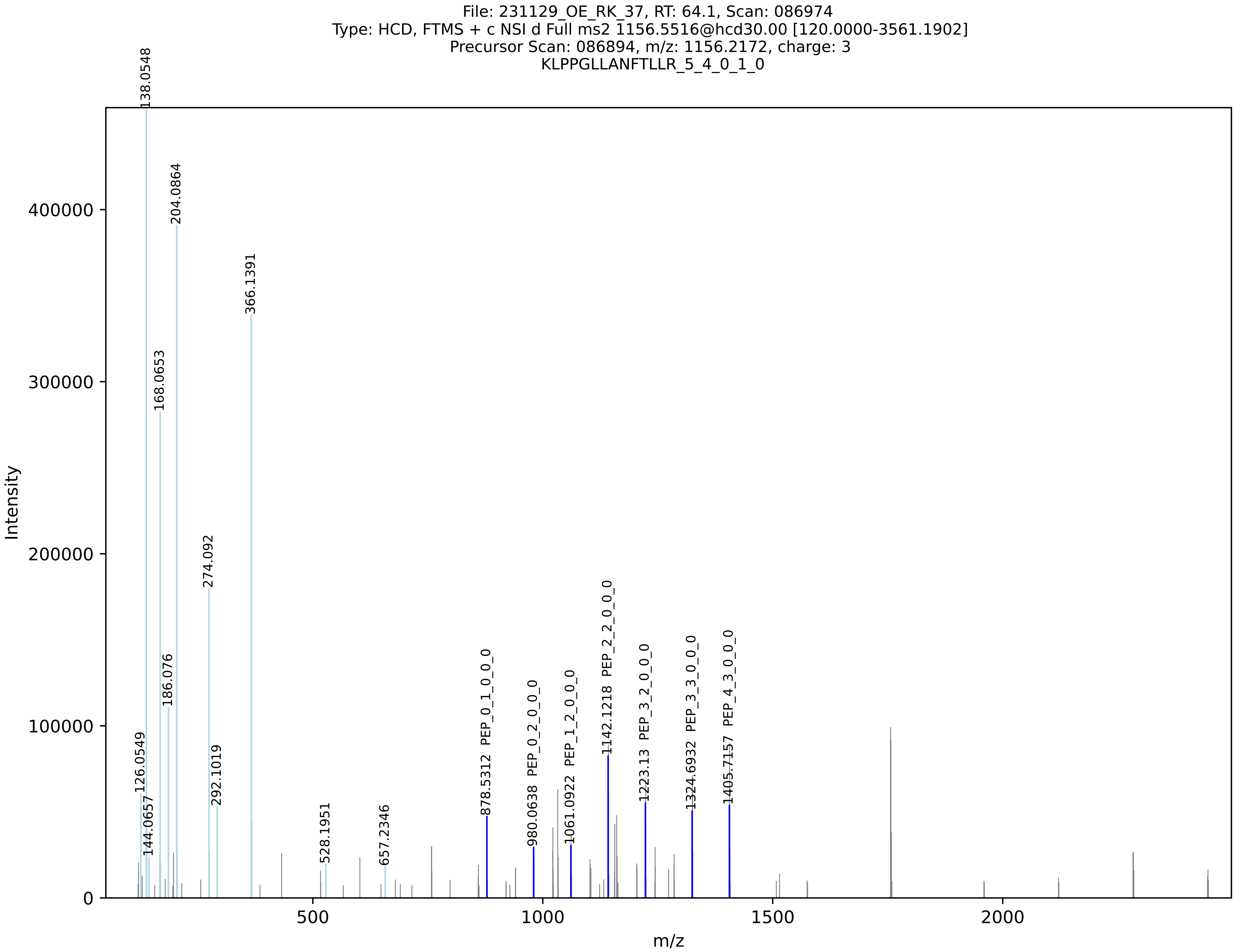

File: 231129_OE_RK_37, RT: 64.1, Scan: 086974
Type: HCD, FTMS + c NSI d Full ms2 1156.5516@hcd30.00 [120.0000-3561.1902]
Precursor Scan: 086894, m/z: 1156.2172, charge: 3
KLPPGLLANFTLLR_5_4_0_1_0
Intensity
m/z
138.0548
204.0864
366.1391
168.0653
274.092
186.076
126.0549
144.0657
292.1019
528.1951
657.2346
878.5312 PEP_0_1_0_0_0
980.0638 PEP_0_2_0_0_0
1061.0922 PEP_1_2_0_0_0
1142.1218 PEP_2_2_0_0_0
1223.13 PEP_3_2_0_0_0
1324.6932 PEP_3_3_0_0_0
1405.7157 PEP_4_3_0_0_0

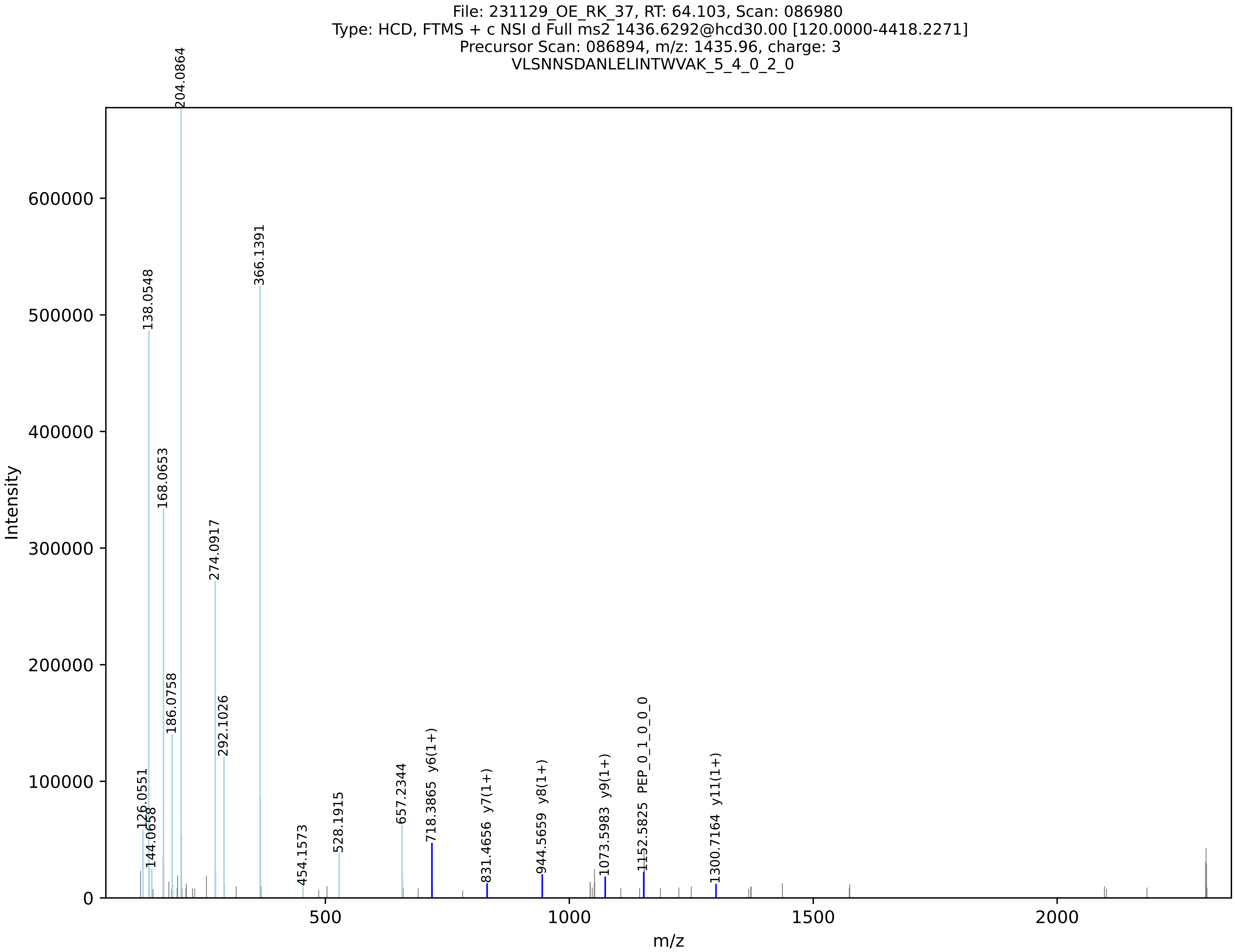
File: 231129_OE_RK_37, RT: 64.103, Scan: 086980
Type: HCD, FTMS + c NSI d Full ms2 1436.6292@hcd30.00 [120.0000-4418.2271]
Precursor Scan: 086894, m/z: 1435.96, charge: 3
VLSNNSDANLELINTWVAK_5_4_0_2_0
Intensity
m/z
204.0864
366.1391
138.0548
168.0653
274.0917
186.0758
292.1026
126.0551
144.0658
454.1573
528.1915
657.2344
718.3865 y6(1+)
831.4656 y7(1+)
944.5659 y8(1+)
1073.5983 y9(1+)
1152.5825 PEP_0_1_0_0_0
1300.7164 y11(1+)

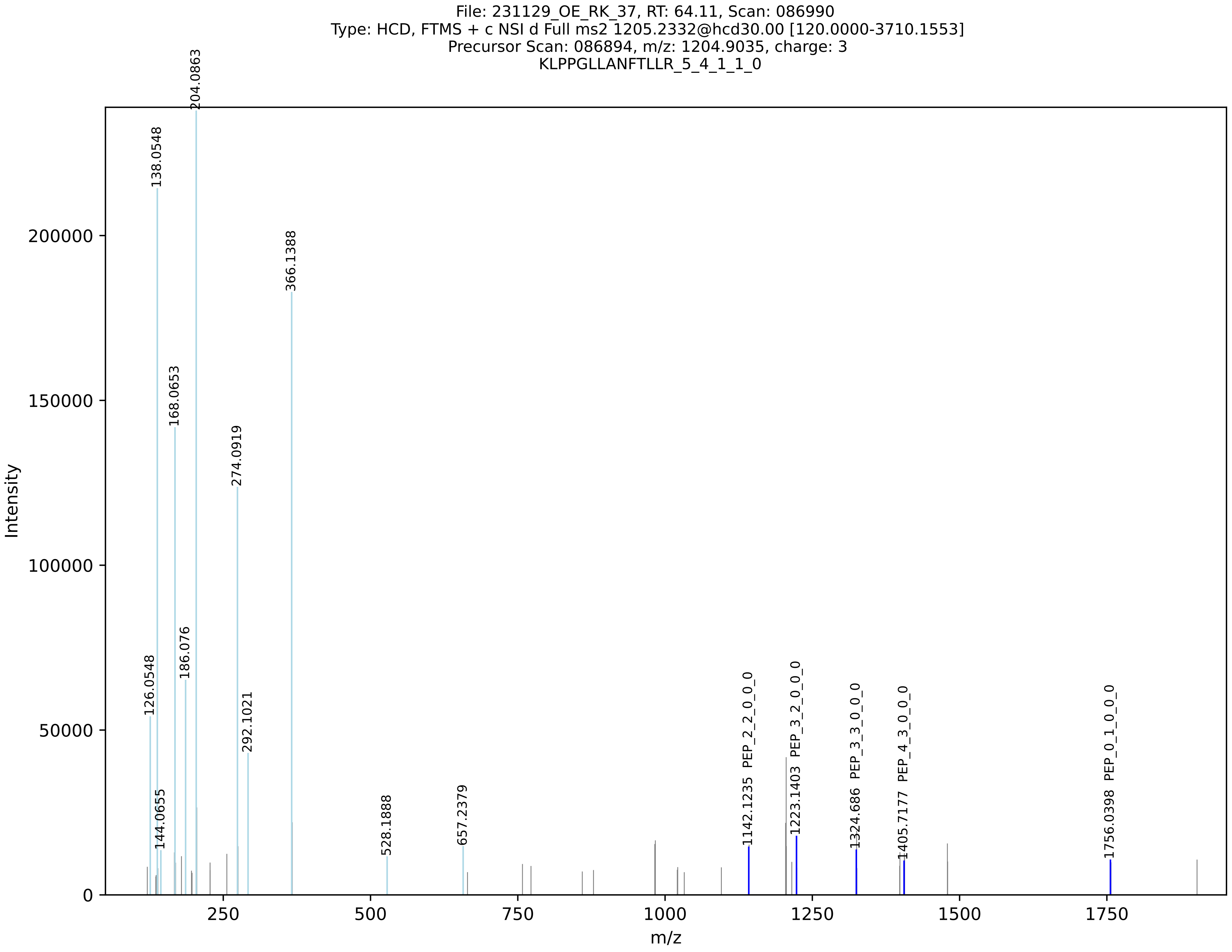
File: 231129_OE_RK_37, RT: 64.11, Scan: 086990
Type: HCD, FTMS + c NSI d Full ms2 1205.2332@hcd30.00 [120.0000-3710.1553]
Precursor Scan: 086894, m/z: 1204.9035, charge: 3
KLPPGLLANFTLLR_5_4_1_1_0
Intensity
m/z
204.0863
138.0548
366.1388
168.0653
274.0919
186.076
126.0548
292.1021
144.0655
528.1888
657.2379
1142.1235 PEP_2_2_0_0_0
1223.1403 PEP_3_2_0_0_0
1324.686 PEP_3_3_0_0_0
1405.7177 PEP_4_3_0_0_0
1756.0398 PEP_0_1_0_0_0
0
50000
100000
150000
200000
250
500
750
1000
1250
1500
1750

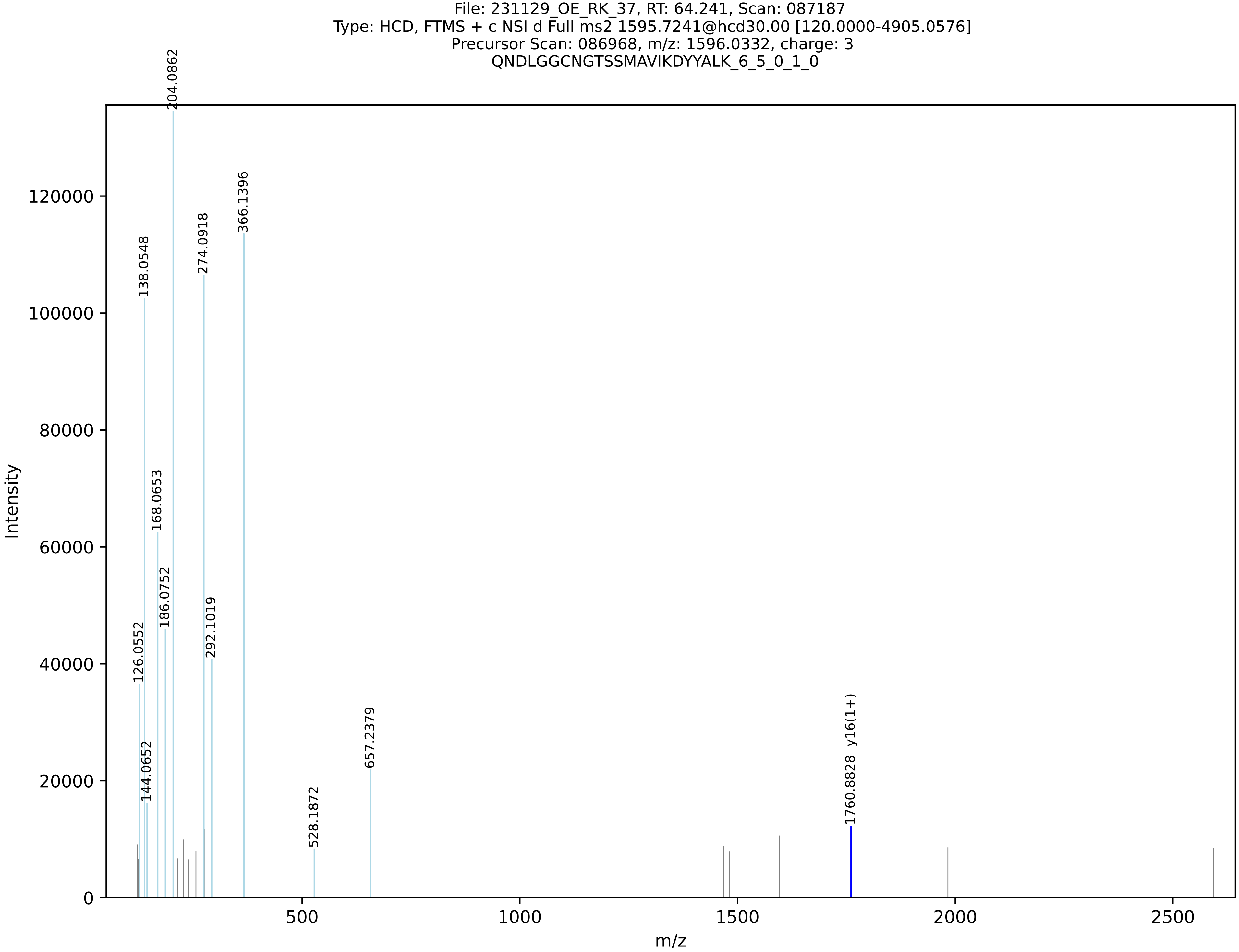
File: 231129_OE_RK_37, RT: 64.241, Scan: 087187
Type: HCD, FTMS + c NSI d Full ms2 1595.7241@hcd30.00 [120.0000-4905.0576]
Precursor Scan: 086968, m/z: 1596.0332, charge: 3
QNDLGGCNGTSSMAVIKDYYALK_6_5_0_1_0
204.0862
366.1396
274.0918
138.0548
168.0653
186.0752
292.1019
126.0552
144.0652
657.2379
528.1872
1760.8828 y16(1+)
Intensity
m/z
0
20000
40000
60000
80000
100000
120000
500
1000
1500
2000
2500

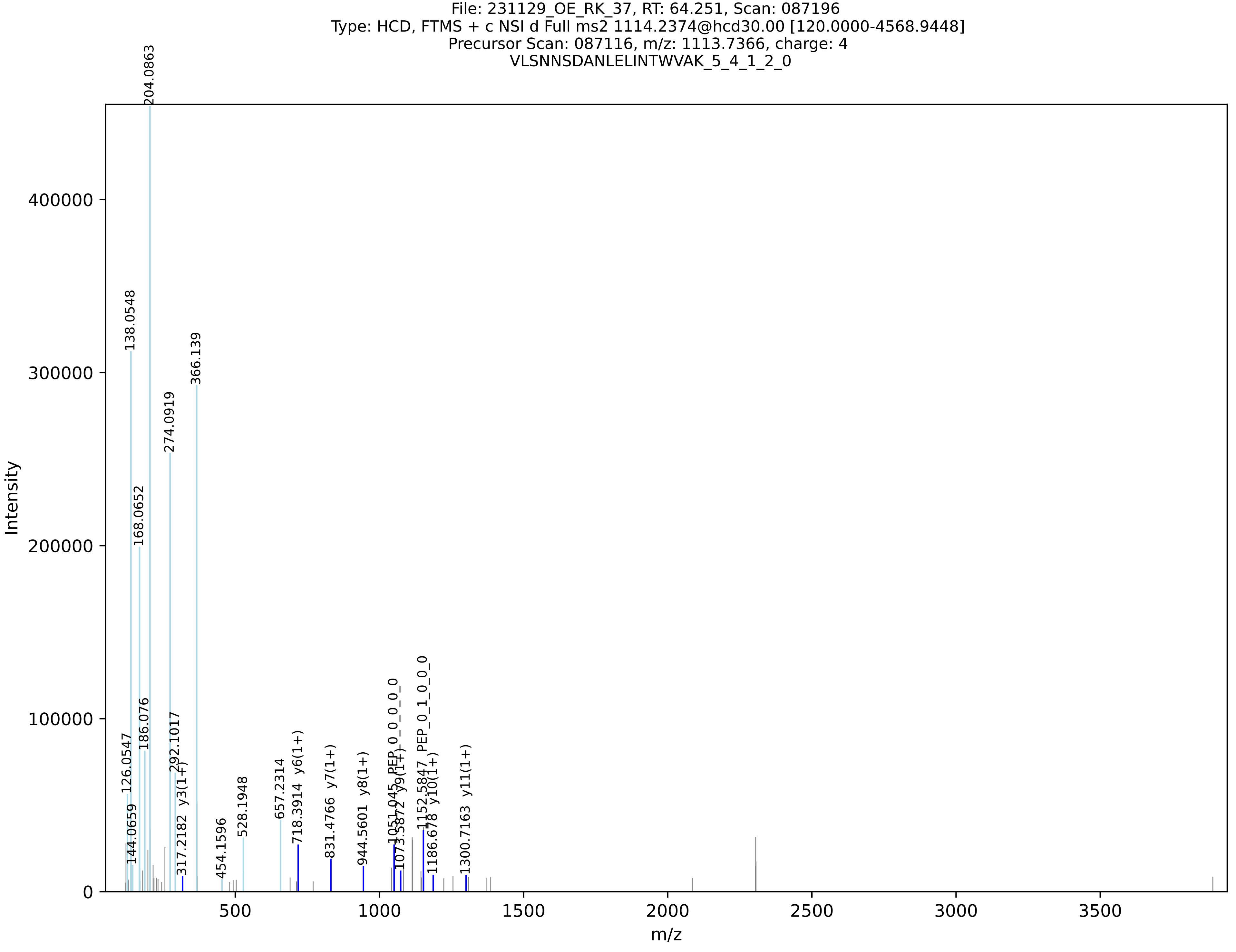
File: 231129_OE_RK_37, RT: 64.251, Scan: 087196
Type: HCD, FTMS + c NSI d Full ms2 1114.2374@hcd30.00 [120.0000-4568.9448]
Precursor Scan: 087116, m/z: 1113.7366, charge: 4
VLSNNSDANLELINTWVAK_5_4_1_2_0
Intensity
m/z
204.0863
138.0548
366.139
274.0919
168.0652
186.076
126.0547
144.0659
292.1017
317.2182 y3(1+)
454.1596
528.1948
657.2314
718.3914 y6(1+)
831.4766 y7(1+)
944.5601 y8(1+)
1051.045_PEP_0_0_0_0_0
1073.5872 y9(1+)
1152.5847_PEP_0_1_0_0_0
1186.678 y10(1+)
1300.7163 y11(1+)
0
100000
200000
300000
400000
500
1000
1500
2000
2500
3000
3500

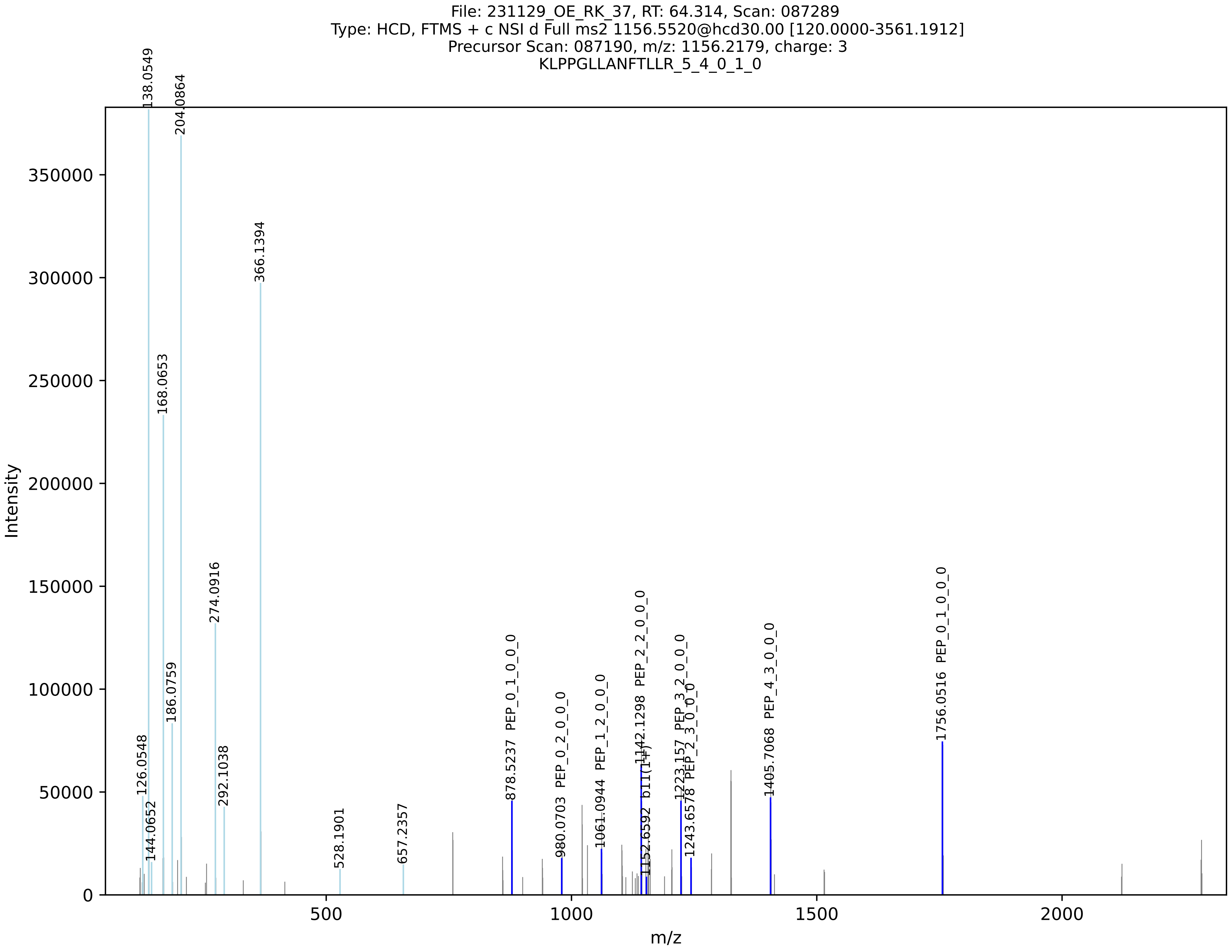

File: 231129_OE_RK_37, RT: 64.314, Scan: 087289
Type: HCD, FTMS + c NSI d Full ms2 1156.5520@hcd30.00 [120.0000-3561.1912]
Precursor Scan: 087190, m/z: 1156.2179, charge: 3
KLPPGLLANFTLLR_5_4_0_1_0
Intensity
m/z
0
50000
100000
150000
200000
250000
300000
350000
500
1000
1500
2000
138.0549
204.0864
366.1394
168.0653
274.0916
186.0759
126.0548
144.0652
292.1038
528.1901
657.2357
878.5237 PEP_0_1_0_0_0
980.0703 PEP_0_2_0_0_0
1061.0944 PEP_1_2_0_0_0
1142.1298 PEP_2_2_0_0_0
1152.6592 b11(1+)
1223.157 PEP_3_2_0_0_0
1243.6578 PEP_2_3_0_0_0
1405.7068 PEP_4_3_0_0_0
1756.0516 PEP_0_1_0_0_0

File: 231129_OE_RK_37, RT: 64.717, Scan: 087876
Type: HCD, FTMS + c NSI d Full ms2 1224.1949@hcd30.00 [120.0000-3768.1787]
Precursor Scan: 087812, m/z: 1223.5268, charge: 3
YLGNATAIFFLPDEGK_5_4_0_1_0

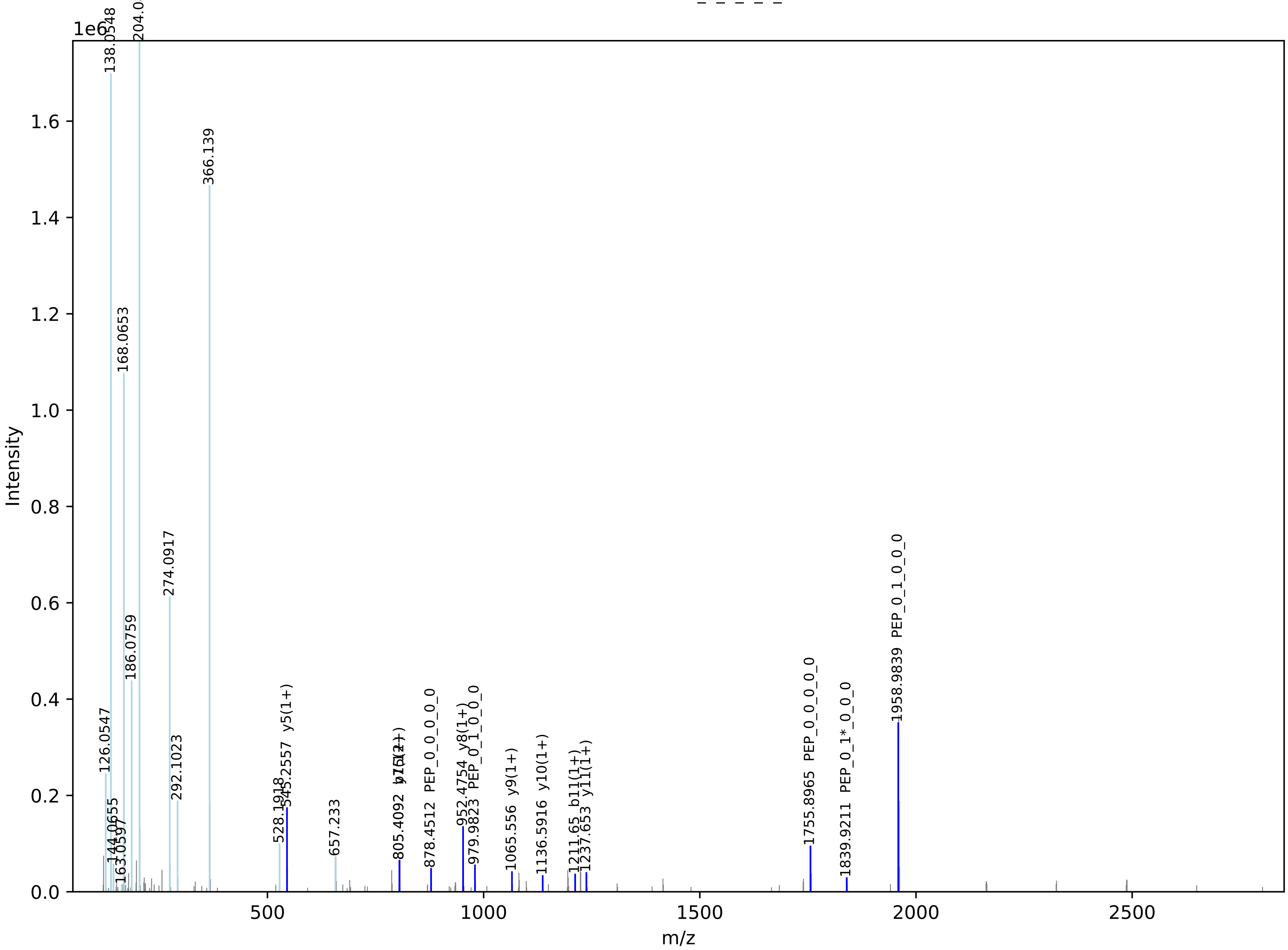

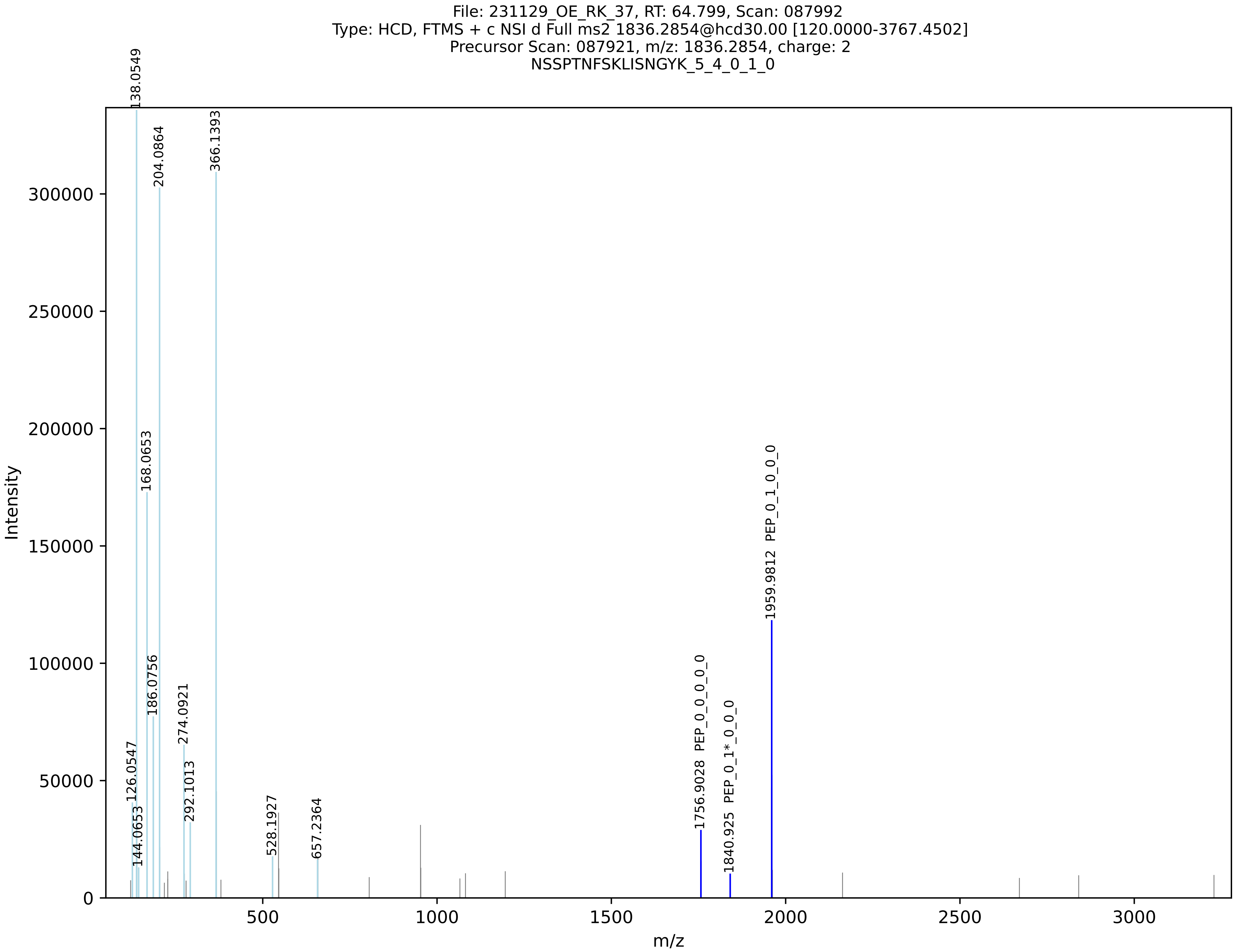

File: 231129_OE_RK_37, RT: 64.799, Scan: 087992
Type: HCD, FTMS + c NSI d Full ms2 1836.2854@hcd30.00 [120.0000-3767.4502]
Precursor Scan: 087921, m/z: 1836.2854, charge: 2
NSSPTNFSKLISNGYK_5_4_0_1_0
Intensity
m/z
138.0549
204.0864
366.1393
168.0653
186.0756
274.0921
126.0547
292.1013
144.0653
528.1927
657.2364
1756.9028 PEP_0_0_0_0_0
1840.925 PEP_0_1*_0_0_0
1959.9812 PEP_0_1_0_0_0
0
50000
100000
150000
200000
250000
300000
500
1000
1500
2000
2500
3000

File: 231129_OE_RK_37, RT: 64.81, Scan: 088010
Type: HCD, FTMS + c NSI d Full ms2 1224.1266@hcd30.00 [120.0000-3767.9695]
Precursor Scan: 087921, m/z: 1224.4906, charge: 3
YLGNATAIFFLPDEGK_5_4_0_1_0

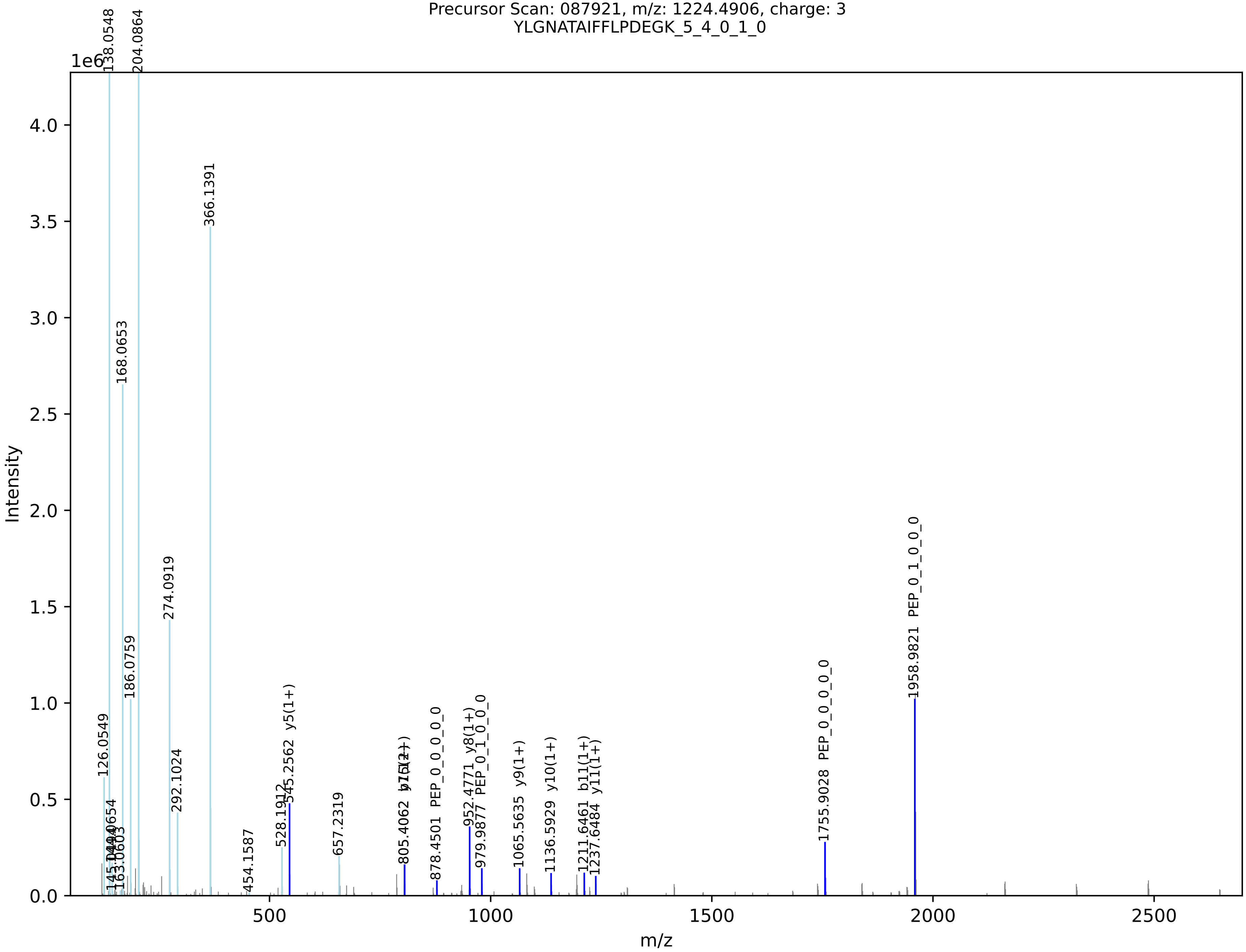

File: 231129_OE_RK_37, RT: 64.856, Scan: 088077
Type: HCD, FTMS + c NSI d Full ms2 1444.5989@hcd30.00 [120.0000-5916.8193]
Precursor Scan: 087976, m/z: 1444.3488, charge: 4
EIRPGGNYTITCVGNTPHLR_7_6_2_3_0

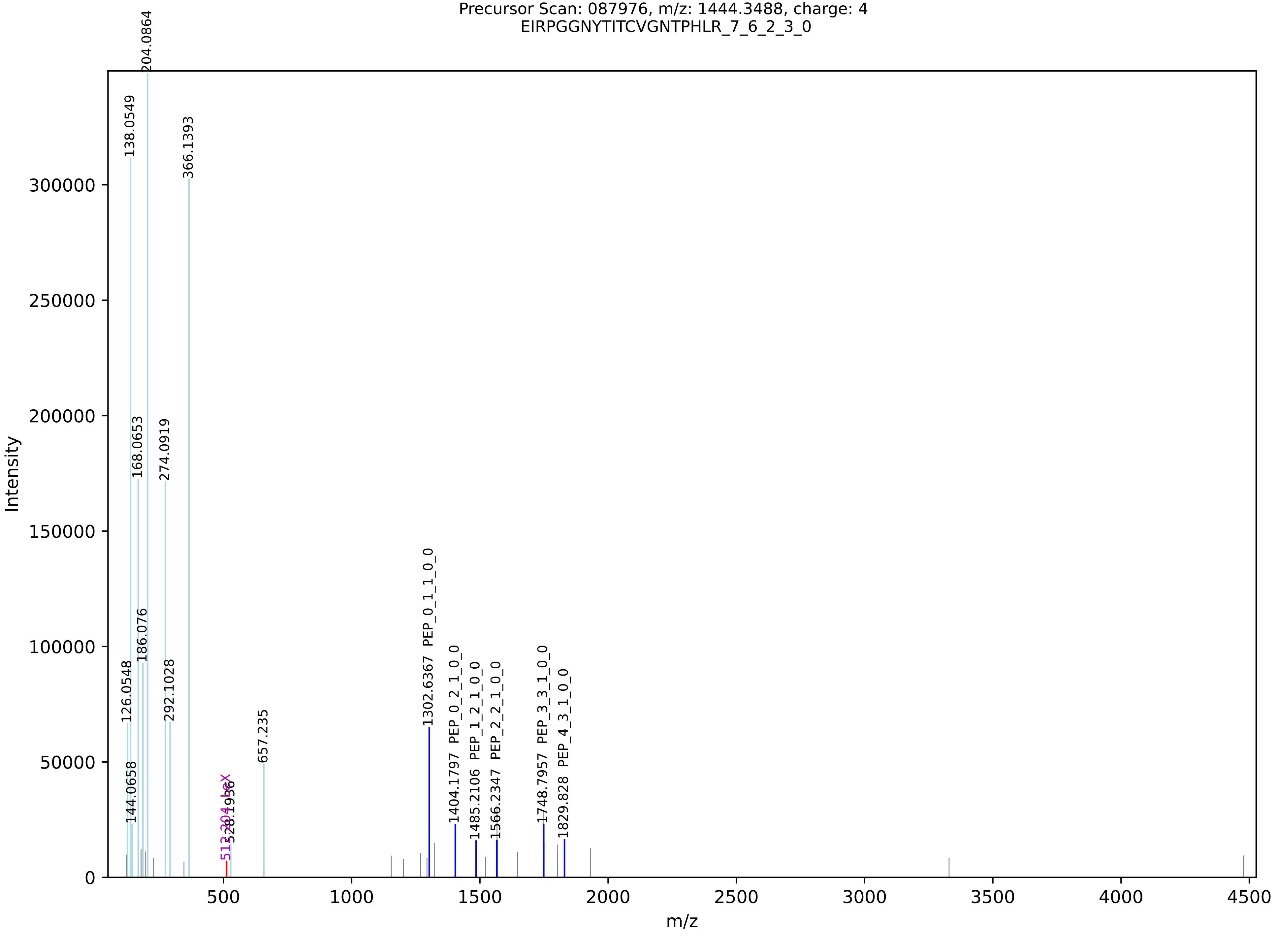

File: 231129_OE_RK_37, RT: 65.036, Scan: 088338
Type: HCD, FTMS + c NSI d Full ms2 1836.2903@hcd30.00 [120.0000-3767.4602]
Precursor Scan: 088269, m/z: 1835.2891, charge: 2
YLGNATAIFFLPDEGK_5_4_0_1_0

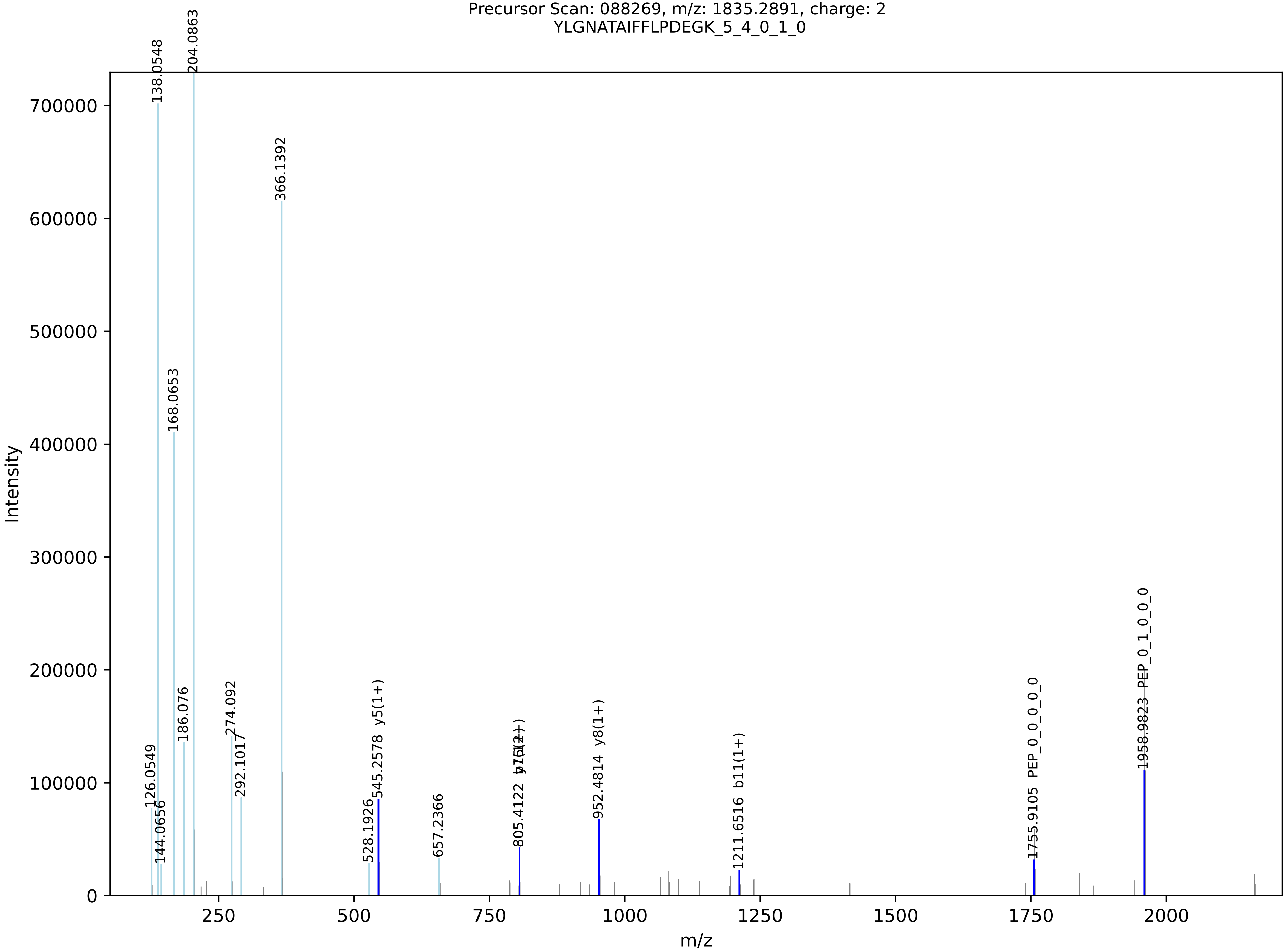

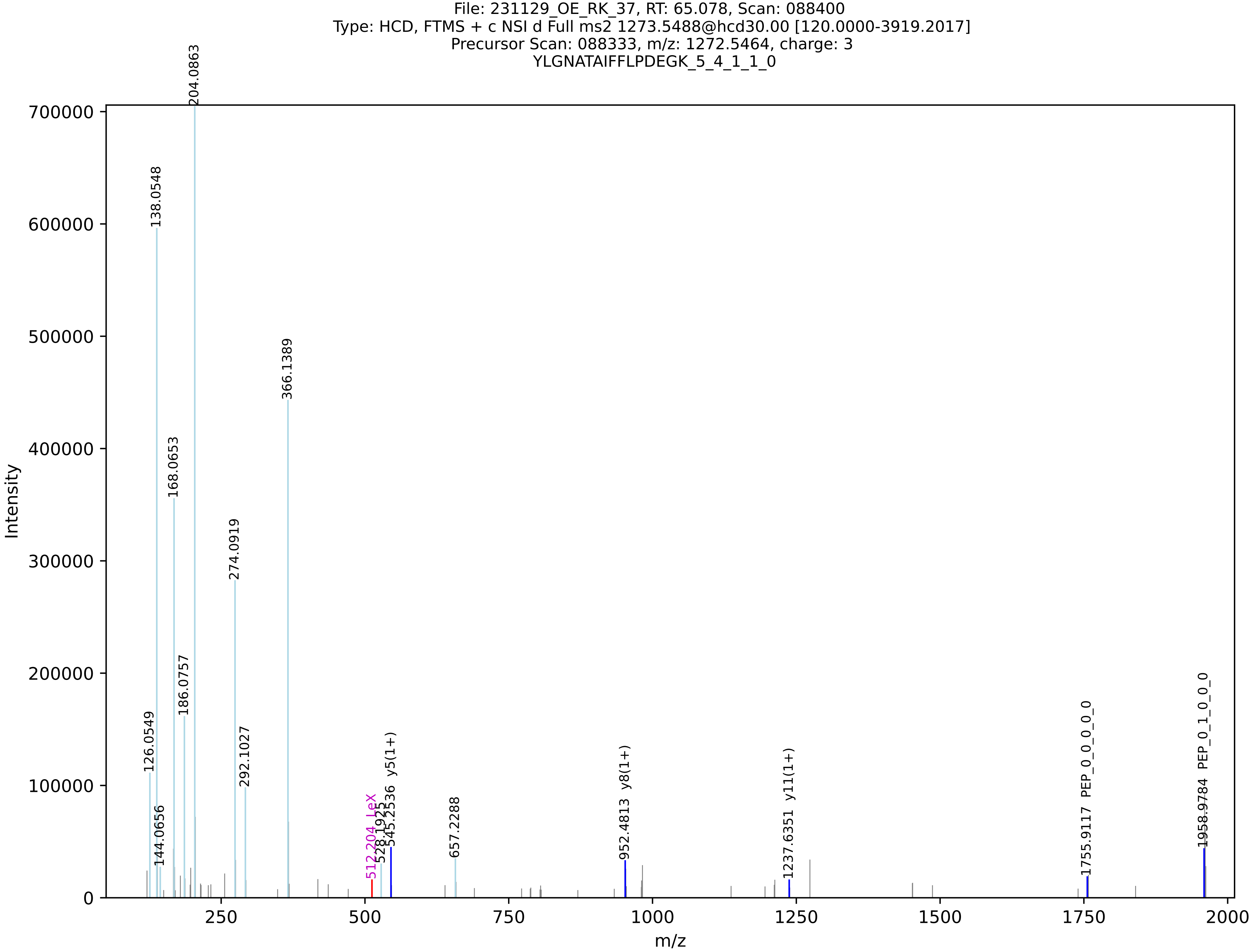

File: 231129_OE_RK_37, RT: 65.078, Scan: 088400
Type: HCD, FTMS + c NSI d Full ms2 1273.5488@hcd30.00 [120.0000-3919.2017]
Precursor Scan: 088333, m/z: 1272.5464, charge: 3
YLGNATAIFFLPDEGK_5_4_1_1_0
Intensity
m/z
204.0863
138.0548
168.0653
366.1389
274.0919
186.0757
126.0549
292.1027
144.0656
512.204 LeX
528.1925
545.2536 y5(1+)
657.2288
952.4813 y8(1+)
1237.6351 y11(1+)
1755.9117 PEP_0_0_0_0_0
1958.9784 PEP_0_1_0_0_0

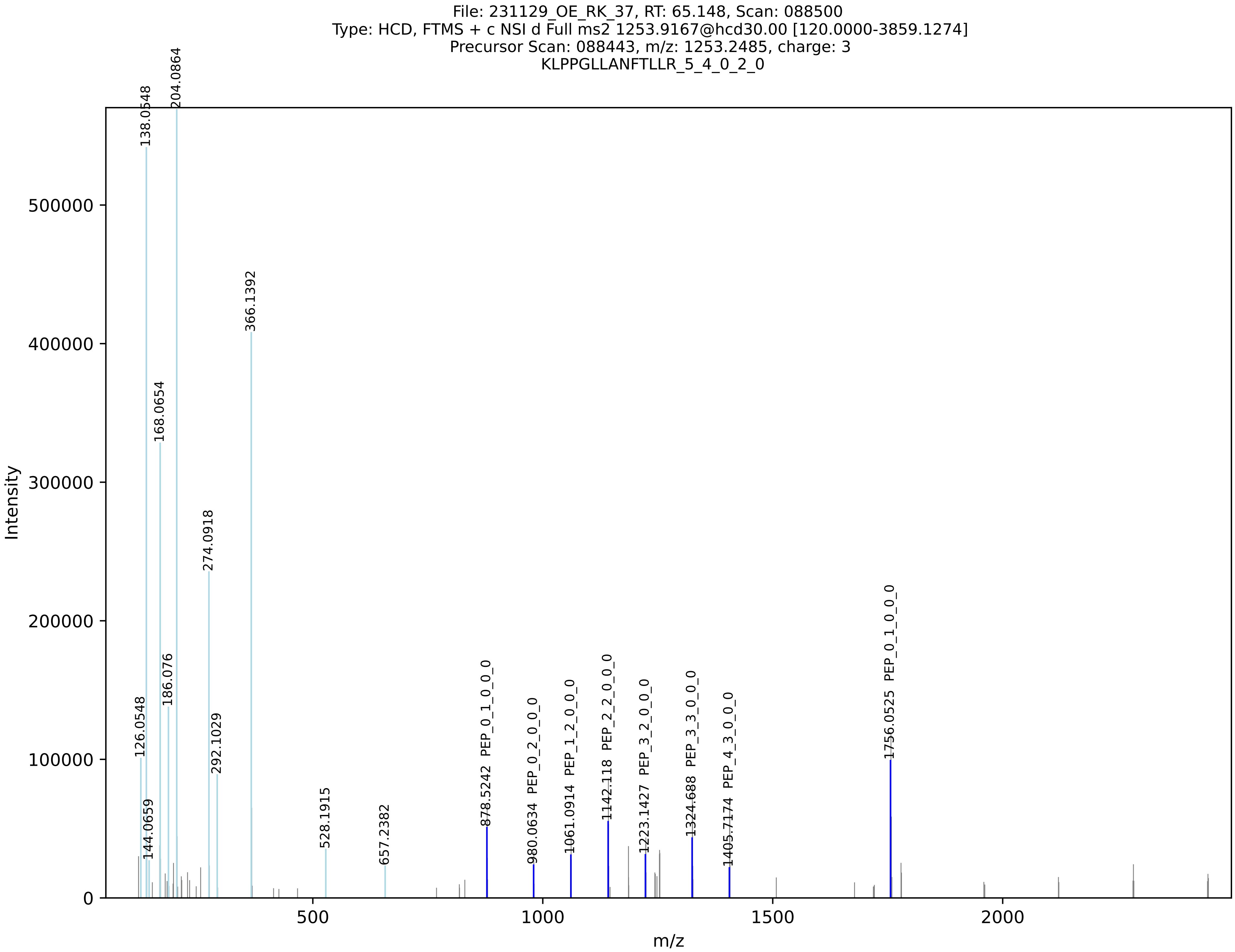

File: 231129_OE_RK_37, RT: 65.148, Scan: 088500
Type: HCD, FTMS + c NSI d Full ms2 1253.9167@hcd30.00 [120.0000-3859.1274]
Precursor Scan: 088443, m/z: 1253.2485, charge: 3
KLPPGLLANFTLLR_5_4_0_2_0
Intensity
m/z
0
100000
200000
300000
400000
500000
500
1000
1500
2000
138.0548
204.0864
366.1392
168.0654
274.0918
186.076
126.0548
292.1029
144.0659
528.1915
657.2382
878.5242 PEP_0_1_0_0_0
980.0634 PEP_0_2_0_0_0
1061.0914 PEP_1_2_0_0_0
1142.118 PEP_2_2_0_0_0
1223.1427 PEP_3_2_0_0_0
1324.688 PEP_3_3_0_0_0
1405.7174 PEP_4_3_0_0_0
1756.0525 PEP_0_1_0_0_0

File: 231129_OE_RK_37, RT: 65.234, Scan: 088624
Type: HCD, FTMS + c NSI d Full ms2 1245.2805@hcd30.00 [120.0000-5103.6006]
Precursor Scan: 088547, m/z: 1245.03, charge: 4
NISDGFDGIPDNVDAALALPAHSYSGR_5_4_0_2_0

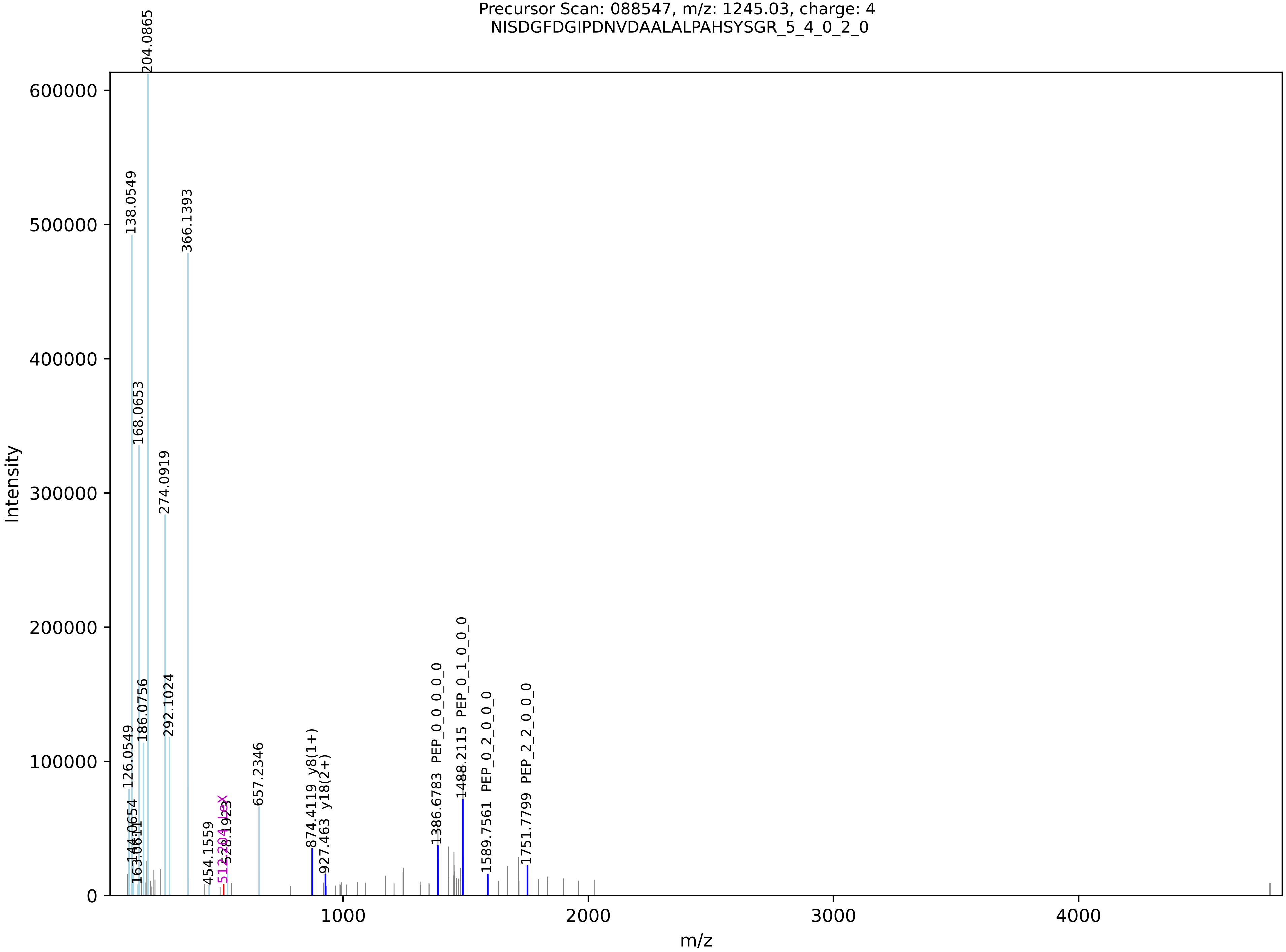

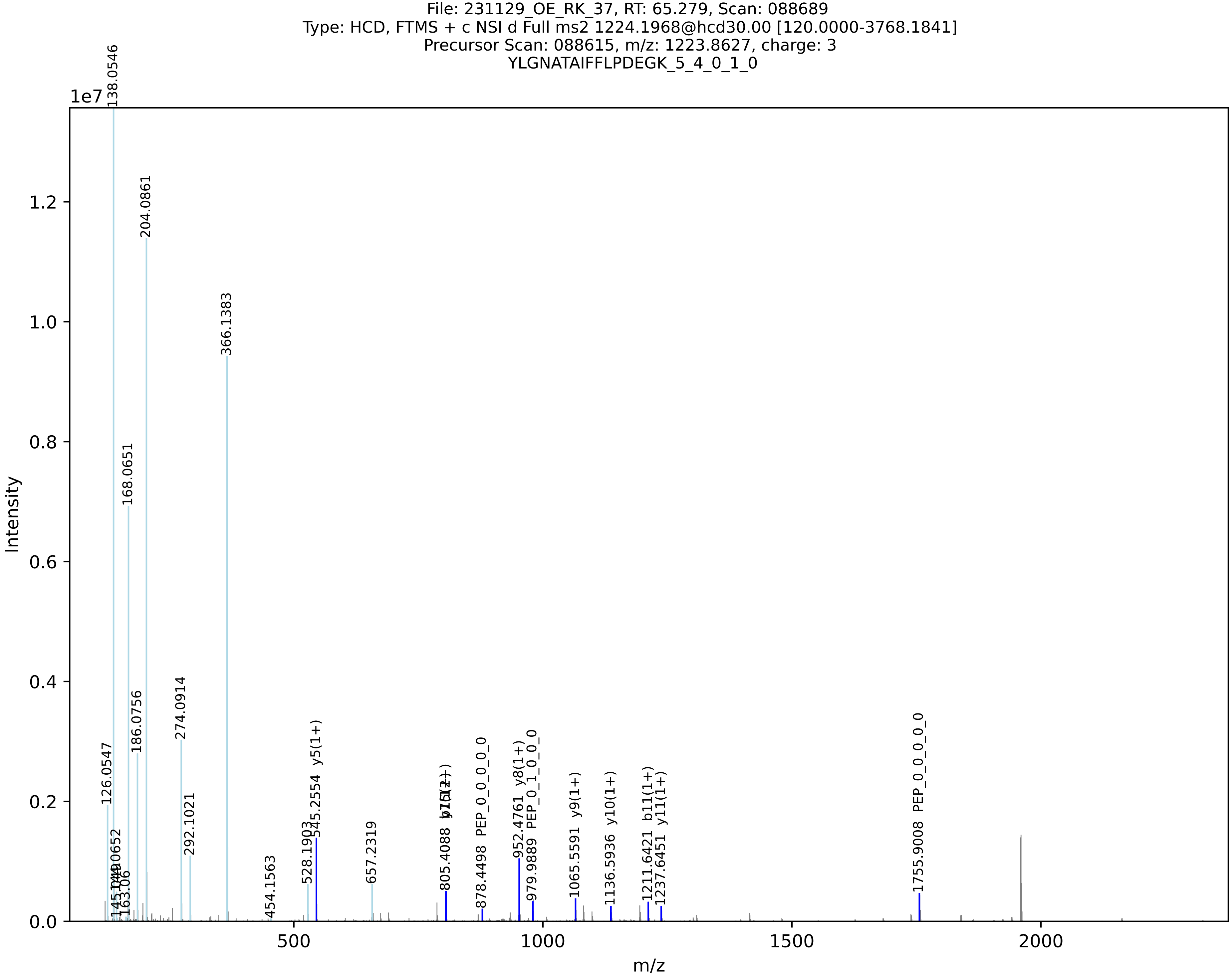

File: 231129_OE_RK_37, RT: 65.279, Scan: 088689
Type: HCD, FTMS + c NSI d Full ms2 1224.1968@hcd30.00 [120.0000-3768.1841]
Precursor Scan: 088615, m/z: 1223.8627, charge: 3
YLGNATAIFFLPDEGK_5_4_0_1_0
1e7
Intensity
m/z
138.0546
204.0861
366.1383
168.0651
186.0756
274.0914
126.0547
292.1021
454.1563
528.1903
545.2554 y5(1+)
657.2319
805.4088 y7(1+)
878.4498 PEP_0_0_0_0_0
952.4761 y8(1+)
979.9889 PEP_0_1_0_0_0
1065.5591 y9(1+)
1136.5936 y10(1+)
1211.6421 b11(1+)
1237.6451 y11(1+)
1755.9008 PEP_0_0_0_0_0

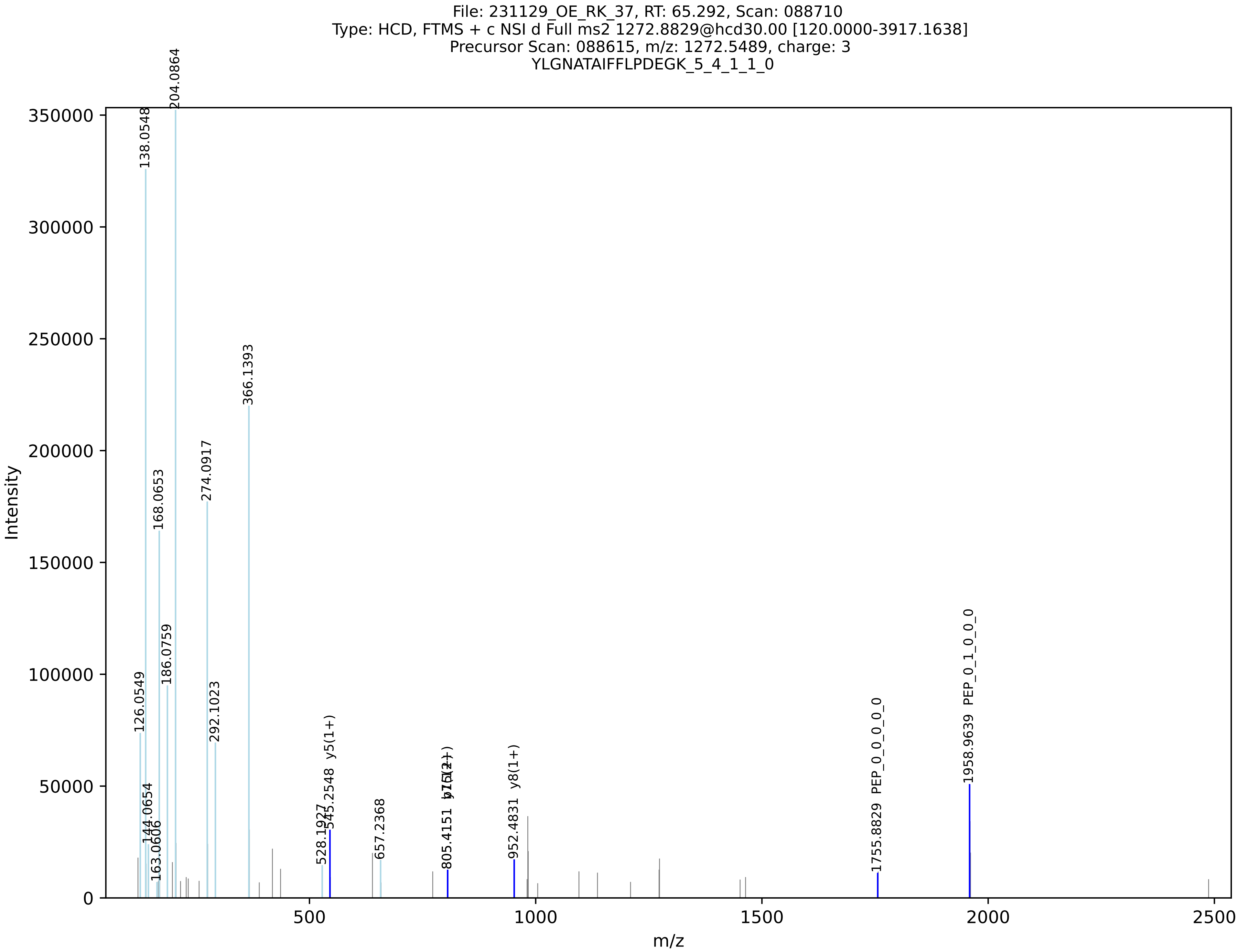

File: 231129_OE_RK_37, RT: 65.292, Scan: 088710
Type: HCD, FTMS + c NSI d Full ms2 1272.8829@hcd30.00 [120.0000-3917.1638]
Precursor Scan: 088615, m/z: 1272.5489, charge: 3
YLGNATAIFFLPDEGK_5_4_1_1_0
Intensity
m/z
204.0864
138.0548
366.1393
274.0917
168.0653
186.0759
126.0549
292.1023
144.0654
163.0606
528.1927
545.2548 y5(1+)
657.2368
805.4151
952.4831 y8(1+)
1755.8829 PEP_0_0_0_0_0
1958.9639 PEP_0_1_0_0_0

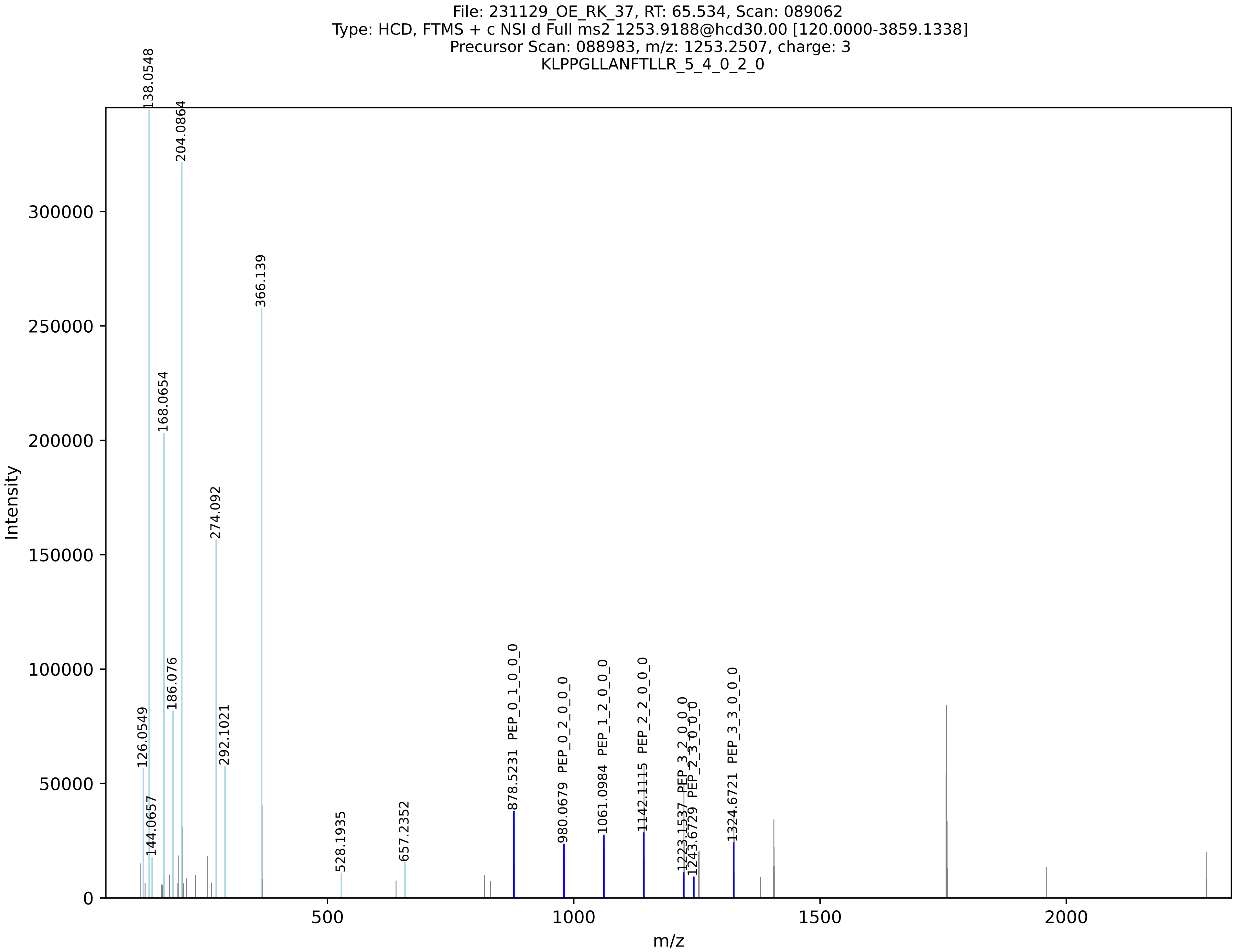

File: 231129_OE_RK_37, RT: 65.534, Scan: 089062
Type: HCD, FTMS + c NSI d Full ms2 1253.9188@hcd30.00 [120.0000-3859.1338]
Precursor Scan: 088983, m/z: 1253.2507, charge: 3
KLPPGLLANFTLLR_5_4_0_2_0
Intensity
m/z
138.0548
204.0864
366.139
168.0654
274.092
186.076
126.0549
292.1021
144.0657
528.1935
657.2352
878.5231 PEP_0_1_0_0_
980.0679 PEP_0_2_0_0_0
1061.0984 PEP_1_2_0_0_0
1142.1115 PEP_2_2_0_0_0
1223.1537 PEP_3_2_0_0_0
1243.6729 PEP_2_3_0_0_0
1324.6721 PEP_3_3_0_0_0
300000
250000
200000
150000
100000
50000
0
500
1000
1500
2000

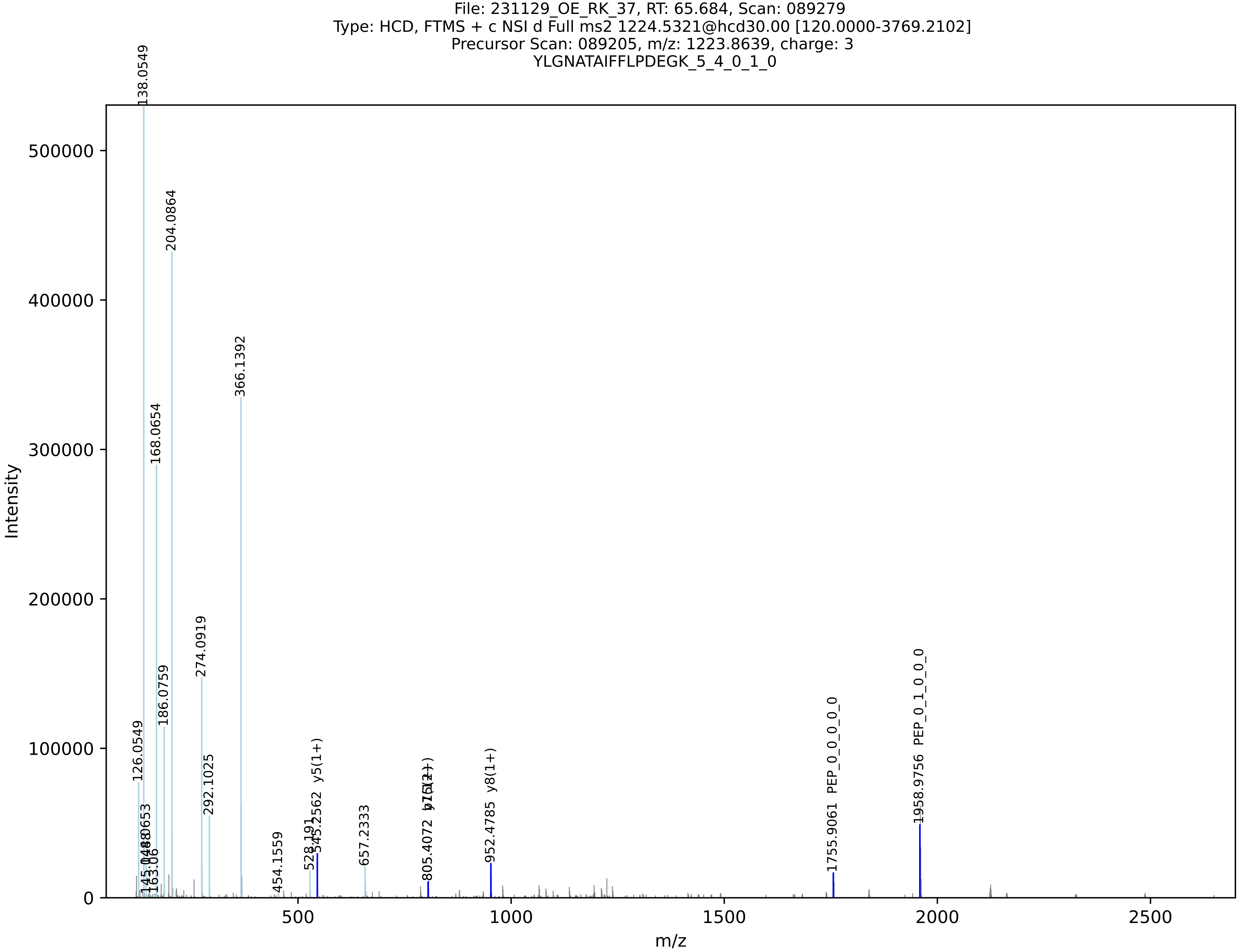
File: 231129_OE_RK_37, RT: 65.684, Scan: 089279
Type: HCD, FTMS + c NSI d Full ms2 1224.5321@hcd30.00 [120.0000-3769.2102]
Precursor Scan: 089205, m/z: 1223.8639, charge: 3
YLGNATAIFFLPDEGK_5_4_0_1_0
Intensity
m/z
138.0549
204.0864
366.1392
168.0654
274.0919
186.0759
126.0549
292.1025
454.1559
528.191
545.2562 y5(1+)
657.2333
805.4072
952.4785 y8(1+)
1755.9061 PEP_0_0_0_0_0
1958.9756 PEP_0_1_0_0_0
0
100000
200000
300000
400000
500000
500
1000
1500
2000
2500

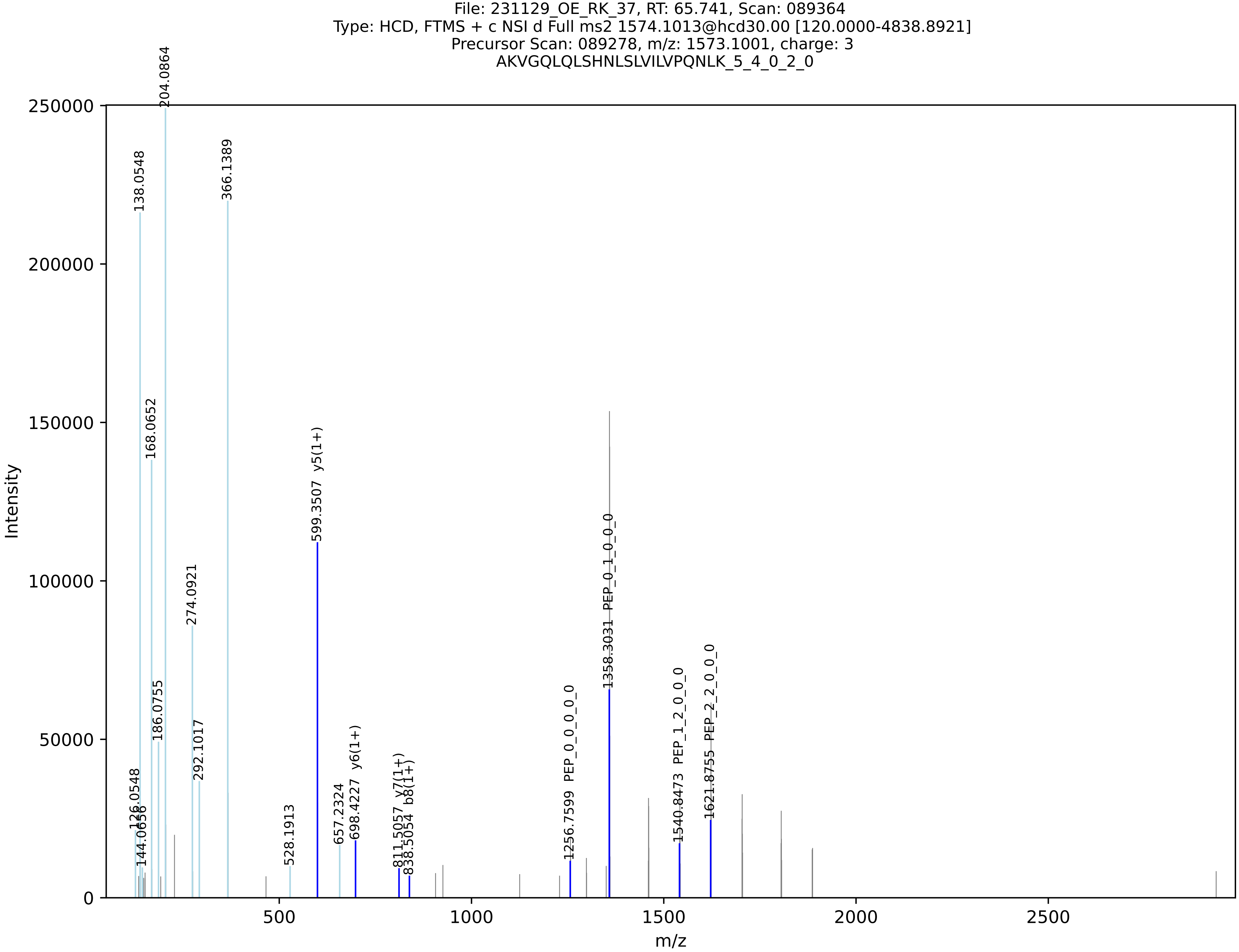

File: 231129_OE_RK_37, RT: 65.741, Scan: 089364
Type: HCD, FTMS + c NSI d Full ms2 1574.1013@hcd30.00 [120.0000-4838.8921]
Precursor Scan: 089278, m/z: 1573.1001, charge: 3
AKVGQLQLSHNLSLVILVPQNLK_5_4_0_2_0
Intensity
m/z
204.0864
138.0548
366.1389
168.0652
599.3507 y5(1+)
274.0921
186.0755
292.1017
126.0548
144.0656
528.1913
657.2324
698.4227 y6(1+)
811.5057 y7(1+)
838.5054 b8(1+)
1256.7599 PEP_0_0_0_0_0
1358.3031 PEP_0_1_0_0_0
1540.8473 PEP_1_2_0_0_0
1621.8755 PEP_2_2_0_0_0

File: 231129_OE_RK_37, RT: 65.744, Scan: 089369
Type: HCD, FTMS + c NSI d Full ms2 1217.0927@hcd30.00 [120.0000-4988.5938]
Precursor Scan: 089278, m/z: 1216.5915, charge: 4
AKVGQLQLSHNLSLVILVPQNLK_5_4_1_2_0

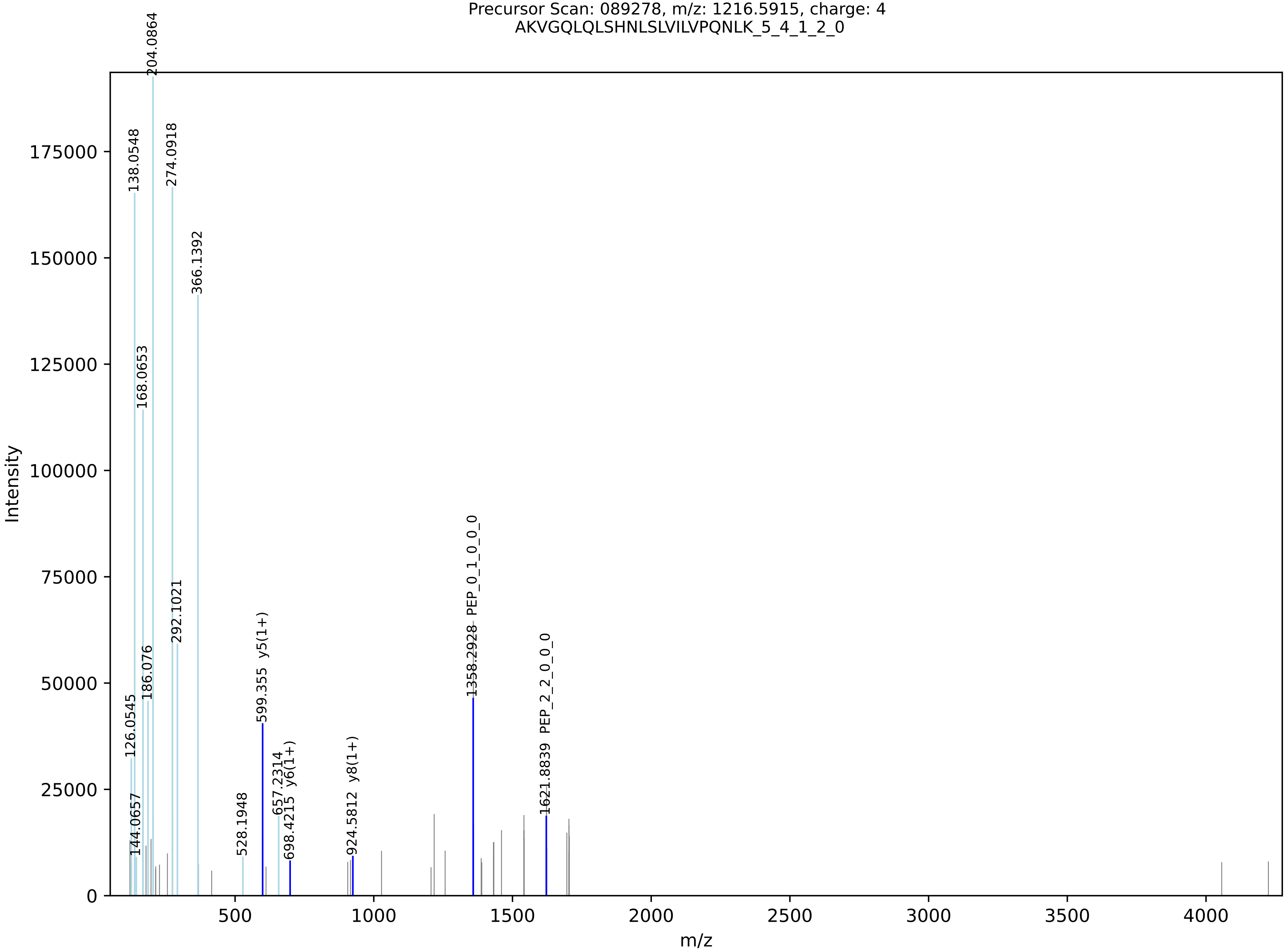

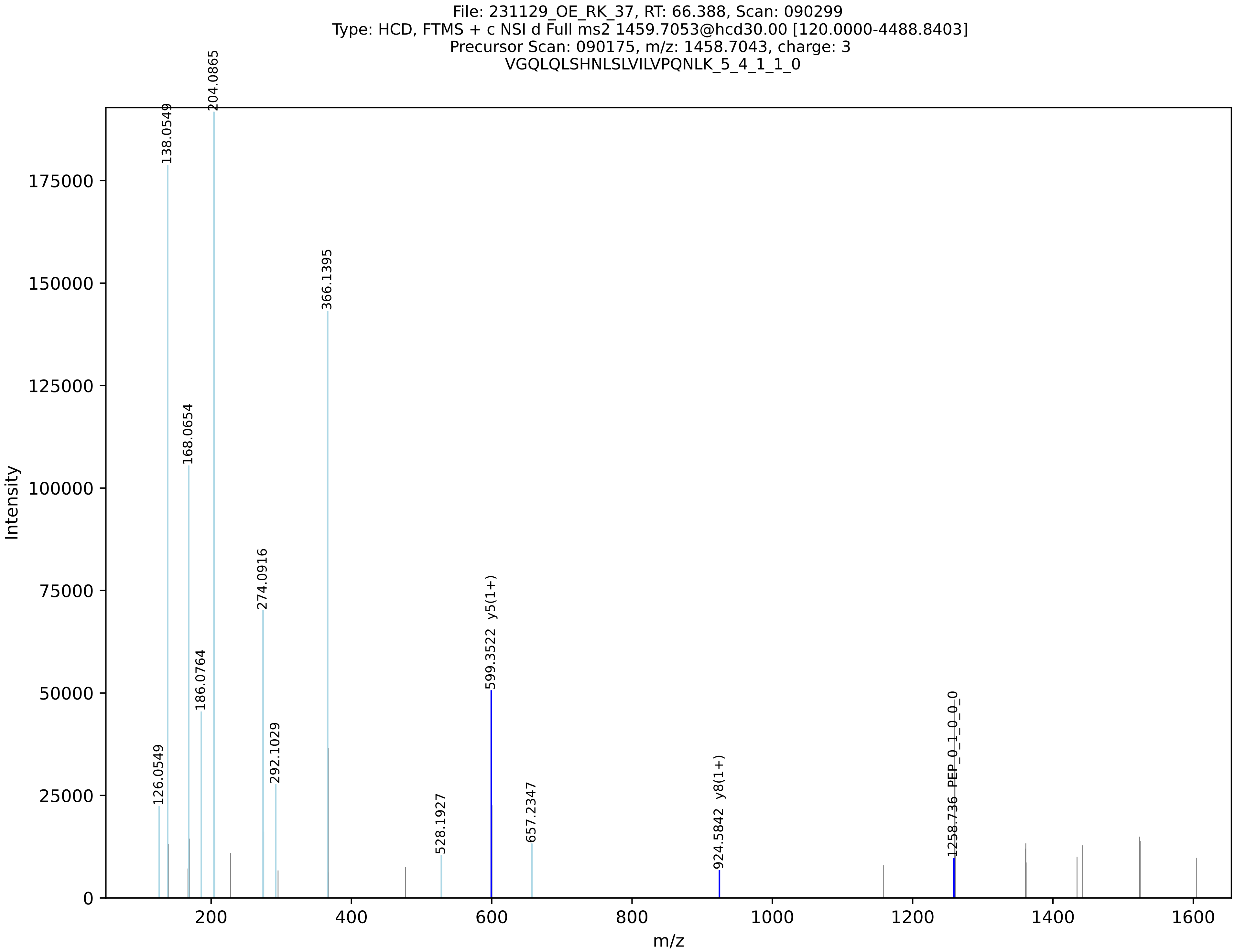
File: 231129_OE_RK_37, RT: 66.388, Scan: 090299
Type: HCD, FTMS + c NSI d Full ms2 1459.7053@hcd30.00 [120.0000-4488.8403]
Precursor Scan: 090175, m/z: 1458.7043, charge: 3
VGQLQLSHNLSLVILVPQNLK_5_4_1_1_0
Intensity
m/z
204.0865
138.0549
366.1395
168.0654
274.0916
599.3522 y5(1+)
186.0764
292.1029
126.0549
528.1927
657.2347
924.5842 y8(1+)
1258.736 PEP_0_1_0_0_0

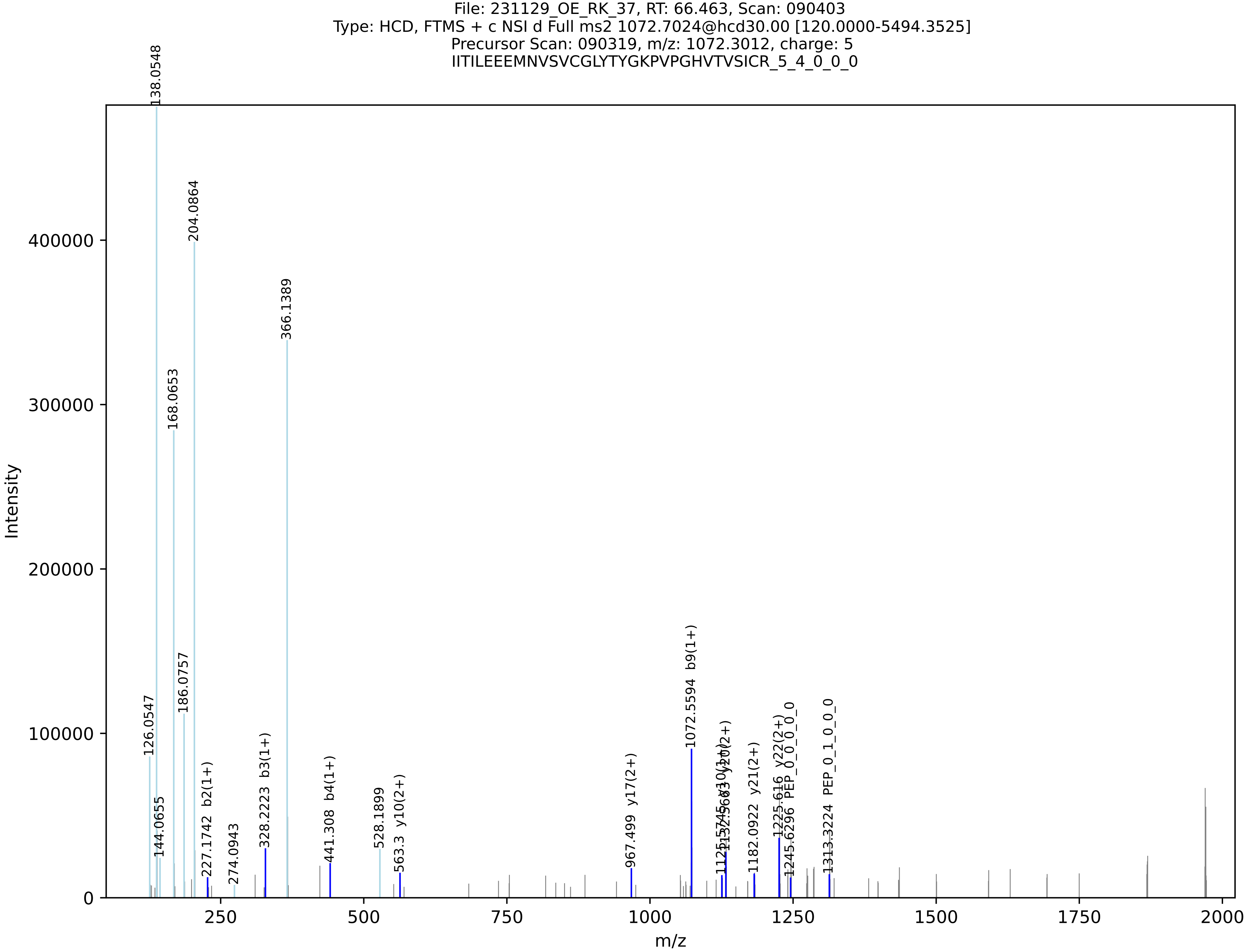
File: 231129_OE_RK_37, RT: 66.463, Scan: 090403
Type: HCD, FTMS + c NSI d Full ms2 1072.7024@hcd30.00 [120.0000-5494.3525]
Precursor Scan: 090319, m/z: 1072.3012, charge: 5
IITILEEEMNVSVCGLYTYGKPVPGHVTVSICR_5_4_0_0_0
Intensity
m/z
138.0548
204.0864
366.1389
168.0653
186.0757
126.0547
144.0655
227.1742 b2(1+)
274.0943
328.2223 b3(1+)
441.308 b4(1+)
528.1899
563.3 y10(2+)
967.499 y17(2+)
1072.5594 b9(1+)
1182.0922 y21(2+)
1225.616 y22(2+)
1245.6296 PEP_0_0_0_0_0
1313.3224 PEP_0_1_0_0_0

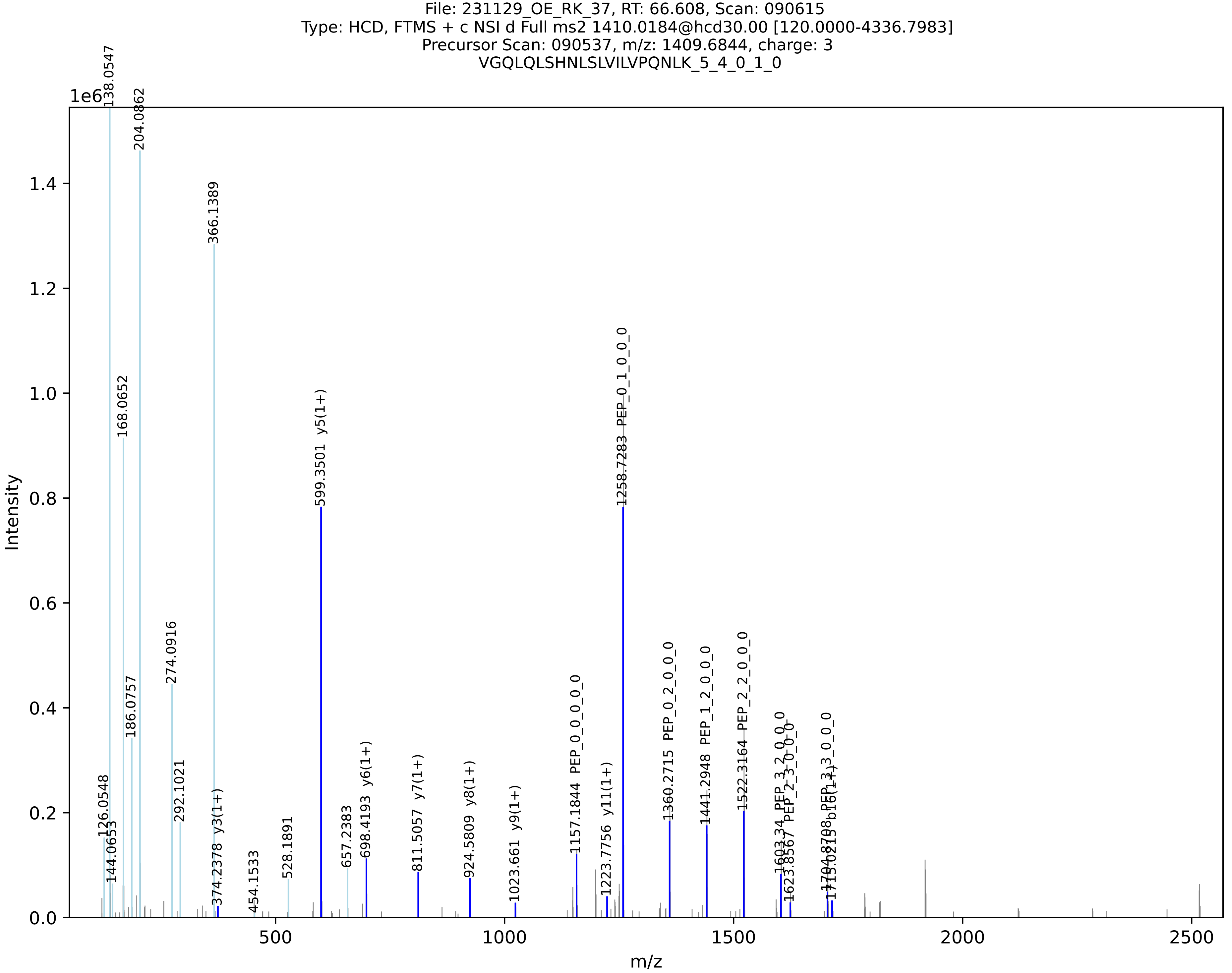
File: 231129_OE_RK_37, RT: 66.608, Scan: 090615
Type: HCD, FTMS + c NSI d Full ms2 1410.0184@hcd30.00 [120.0000-4336.7983]
Precursor Scan: 090537, m/z: 1409.6844, charge: 3
VGQLQLSHNLSLVILVPQNLK_5_4_0_1_0
1e6
Intensity
m/z
138.0547
204.0862
366.1389
168.0652
186.0757
274.0916
292.1021
126.0548
144.0653
374.2378 y3(1+)
454.1533
528.1891
599.3501 y5(1+)
657.2383
698.4193 y6(1+)
811.5057 y7(1+)
924.5809 y8(1+)
1023.661 y9(1+)
1157.1844 PEP_0_0_0_0_0
1223.7756 y11(1+)
1258.7283 PEP_0_1_0_0_0
1360.2715 PEP_0_2_0_0_0
1441.2948 PEP_1_2_0_0_0
1522.3164 PEP_2_2_0_0_0
1603.34 PEP_3_2_0_0_0
1623.8567 PEP_2_3_0_0_0
1704.8708 PEP_3_3_0_0_0
1715.0215 b16(1+)

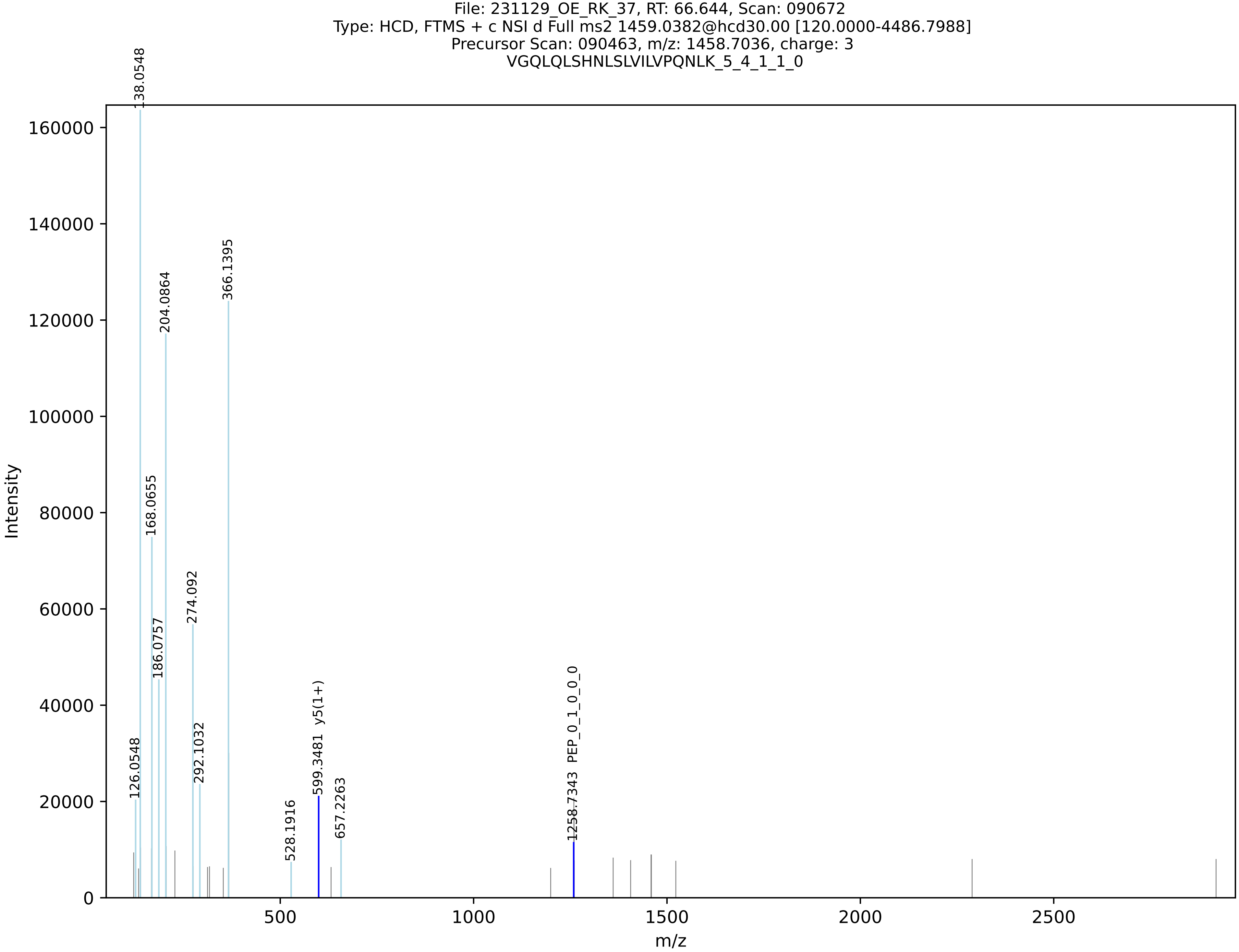

File: 231129_OE_RK_37, RT: 66.644, Scan: 090672
Type: HCD, FTMS + c NSI d Full ms2 1459.0382@hcd30.00 [120.0000-4486.7988]
Precursor Scan: 090463, m/z: 1458.7036, charge: 3
VGQLQLSHNLSLVILVPQNLK_5_4_1_1_0
138.0548
366.1395
204.0864
168.0655
274.092
186.0757
599.3481 y5(1+)
1258.7343 PEP_0_1_0_0_0
126.0548
292.1032
657.2263
528.1916
Intensity
m/z
0
20000
40000
60000
80000
100000
120000
140000
160000
500
1000
1500
2000
2500

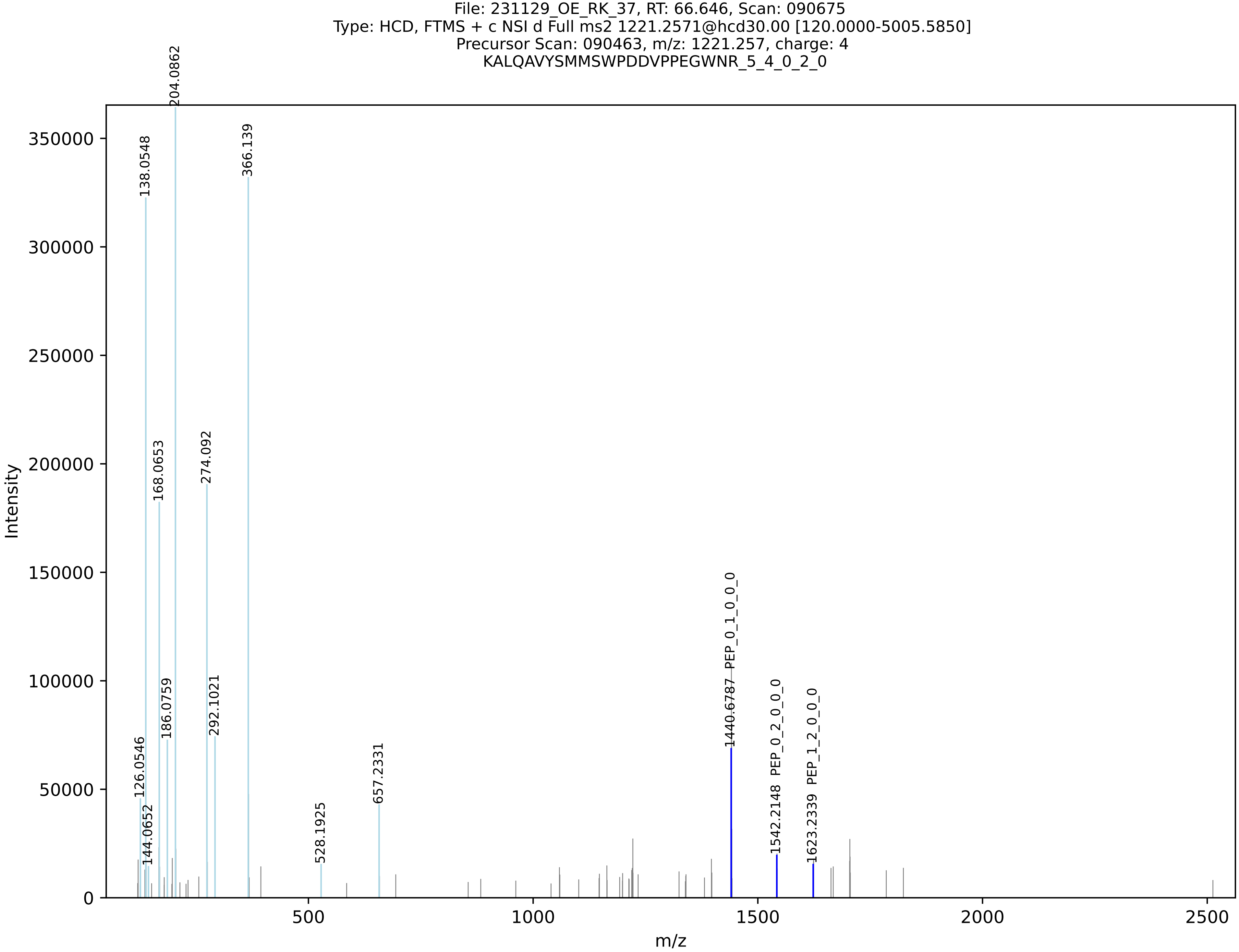
File: 231129_OE_RK_37, RT: 66.646, Scan: 090675
Type: HCD, FTMS + c NSI d Full ms2 1221.2571@hcd30.00 [120.0000-5005.5850]
Precursor Scan: 090463, m/z: 1221.257, charge: 4
KALQAVYSMMSWPDDVPPEGWNR_5_4_0_2_0
Intensity
m/z
204.0862
138.0548
366.139
168.0653
274.092
186.0759
292.1021
126.0546
144.0652
528.1925
657.2331
1440.6787 PEP_0_1_0_0_0
1542.2148 PEP_0_2_0_0_0
1623.2339 PEP_1_2_0_0_0

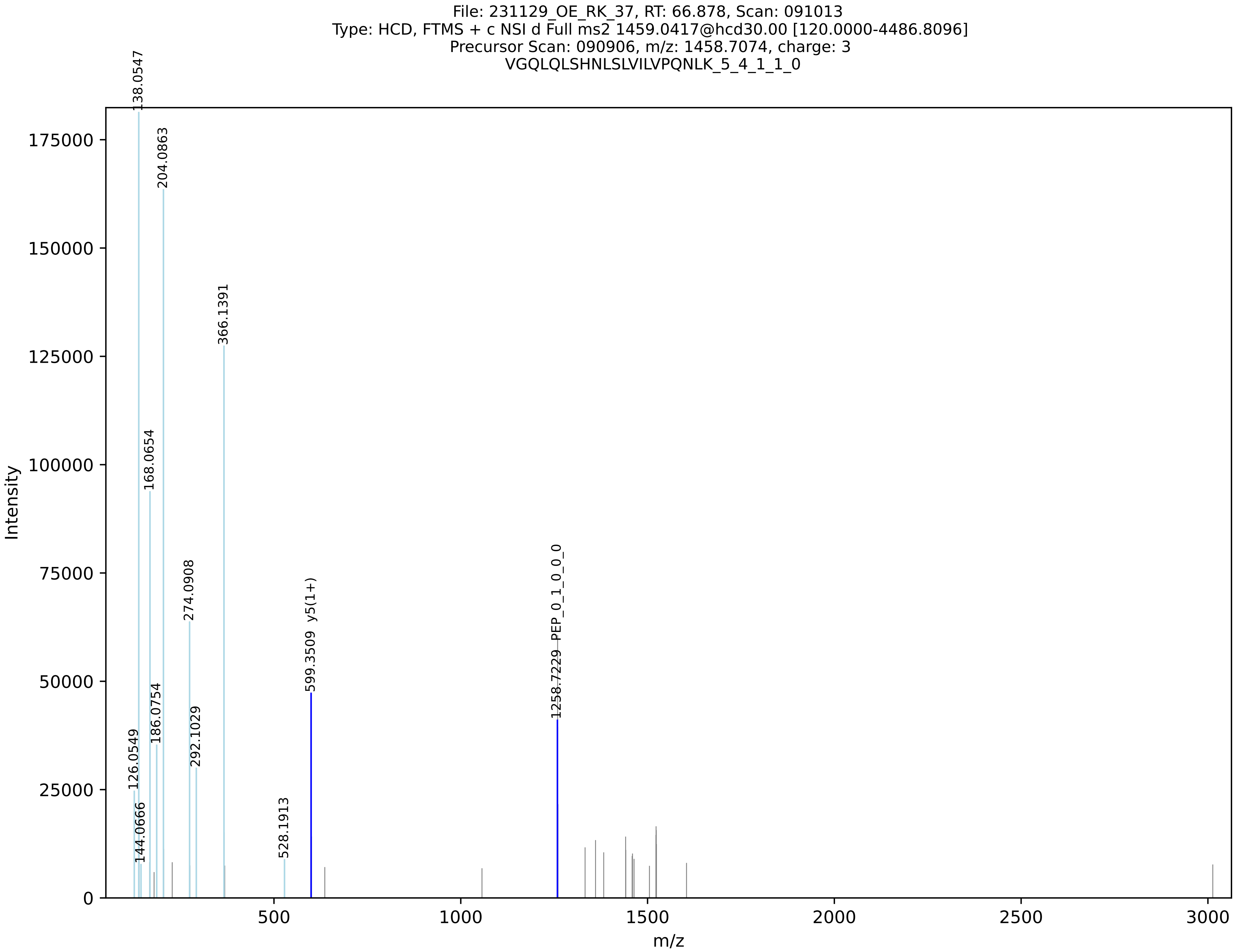

File: 231129_OE_RK_37, RT: 66.878, Scan: 091013
Type: HCD, FTMS + c NSI d Full ms2 1459.0417@hcd30.00 [120.0000-4486.8096]
Precursor Scan: 090906, m/z: 1458.7074, charge: 3
VGQLQLSHNLSLVILVPQNLK_5_4_1_1_0
138.0547
204.0863
366.1391
168.0654
274.0908
599.3509 y5(1+)
1258.7229 PEP_0_1_0_0_0
186.0754
292.1029
126.0549
144.0666
528.1913
Intensity
m/z
175000
150000
125000
100000
75000
50000
25000
0
500
1000
1500
2000
2500
3000

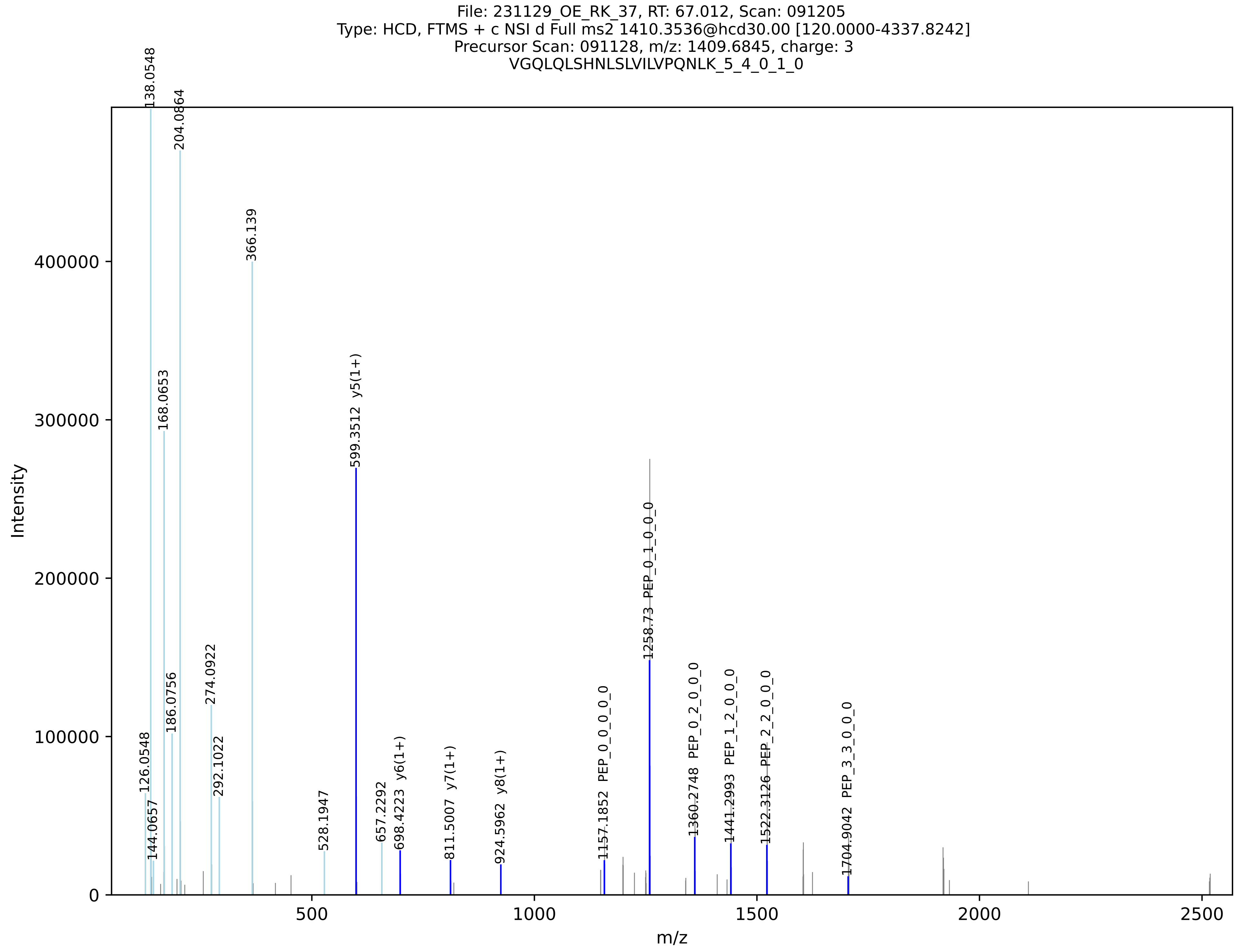
File: 231129_OE_RK_37, RT: 67.012, Scan: 091205
Type: HCD, FTMS + c NSI d Full ms2 1410.3536@hcd30.00 [120.0000-4337.8242]
Precursor Scan: 091128, m/z: 1409.6845, charge: 3
VGQLQLSHNLSLVILVPQNLK_5_4_0_1_0
Intensity
m/z
138.0548
204.0864
366.139
168.0653
599.3512 y5(1+)
1258.73 PEP_0_1_0_0_
274.0922
186.0756
126.0548
292.1022
144.0657
528.1947
657.2292
698.4223 y6(1+)
811.5007 y7(1+)
924.5962 y8(1+)
1157.1852 PEP_0_0_0_0_0
1360.2748 PEP_0_2_0_0_0
1441.2993 PEP_1_2_0_0_0
1522.3126 PEP_2_2_0_0_0
1704.9042 PEP_3_3_0_0_0

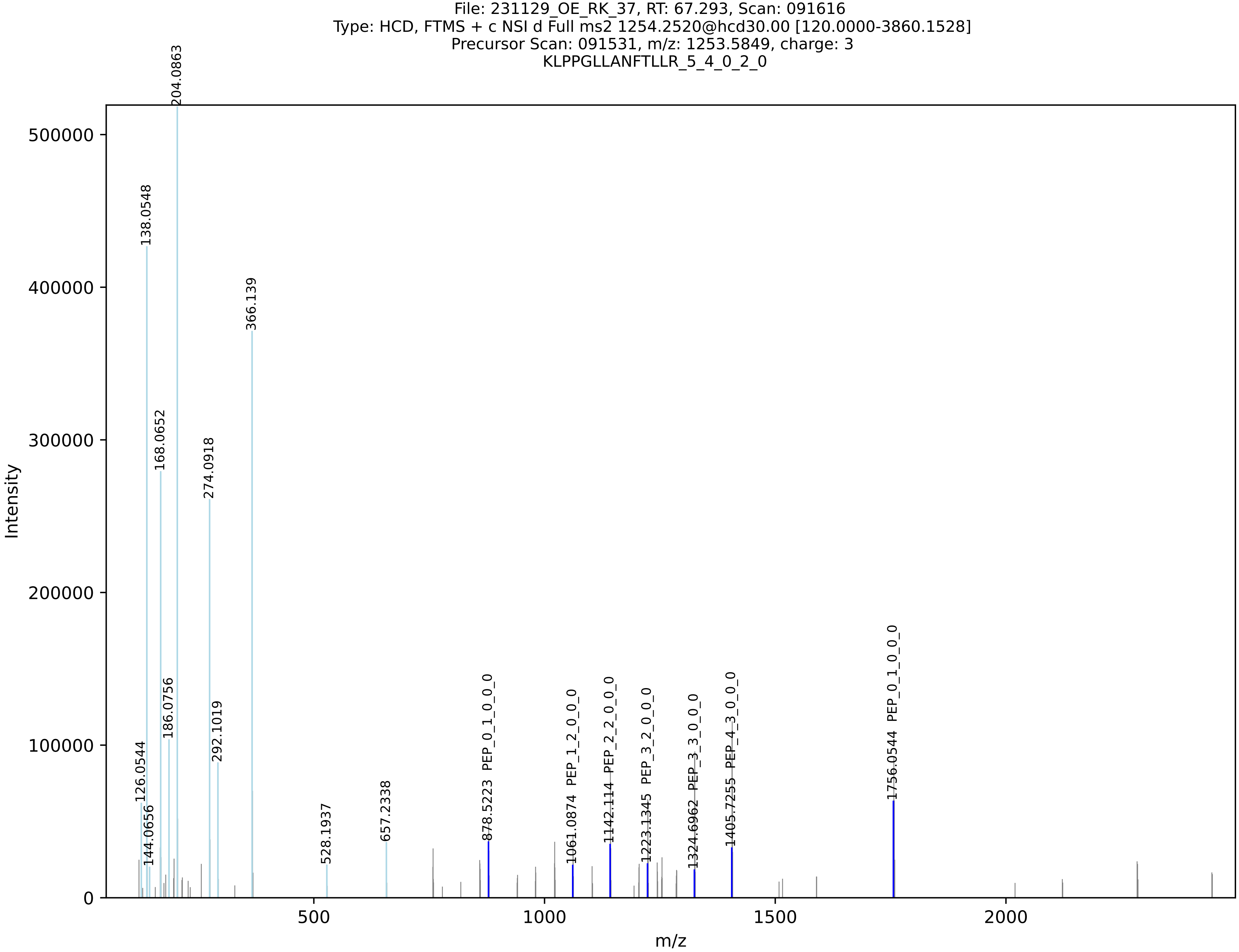
File: 231129_OE_RK_37, RT: 67.293, Scan: 091616
Type: HCD, FTMS + c NSI d Full ms2 1254.2520@hcd30.00 [120.0000-3860.1528]
Precursor Scan: 091531, m/z: 1253.5849, charge: 3
KLPPGLLANFTLLR_5_4_0_2_0
Intensity
m/z
204.0863
138.0548
366.139
168.0652
274.0918
186.0756
292.1019
126.0544
144.0656
528.1937
657.2338
878.5223 PEP_0_1_0_0_0
1061.0874 PEP_1_2_0_0_0
1142.114 PEP_2_2_0_0_0
1223.1345 PEP_3_2_0_0_0
1324.6962 PEP_3_3_0_0_0
1405.7255 PEP_4_3_0_0_0
1756.0544 PEP_0_1_0_0_0
0
100000
200000
300000
400000
500000
500
1000
1500
2000

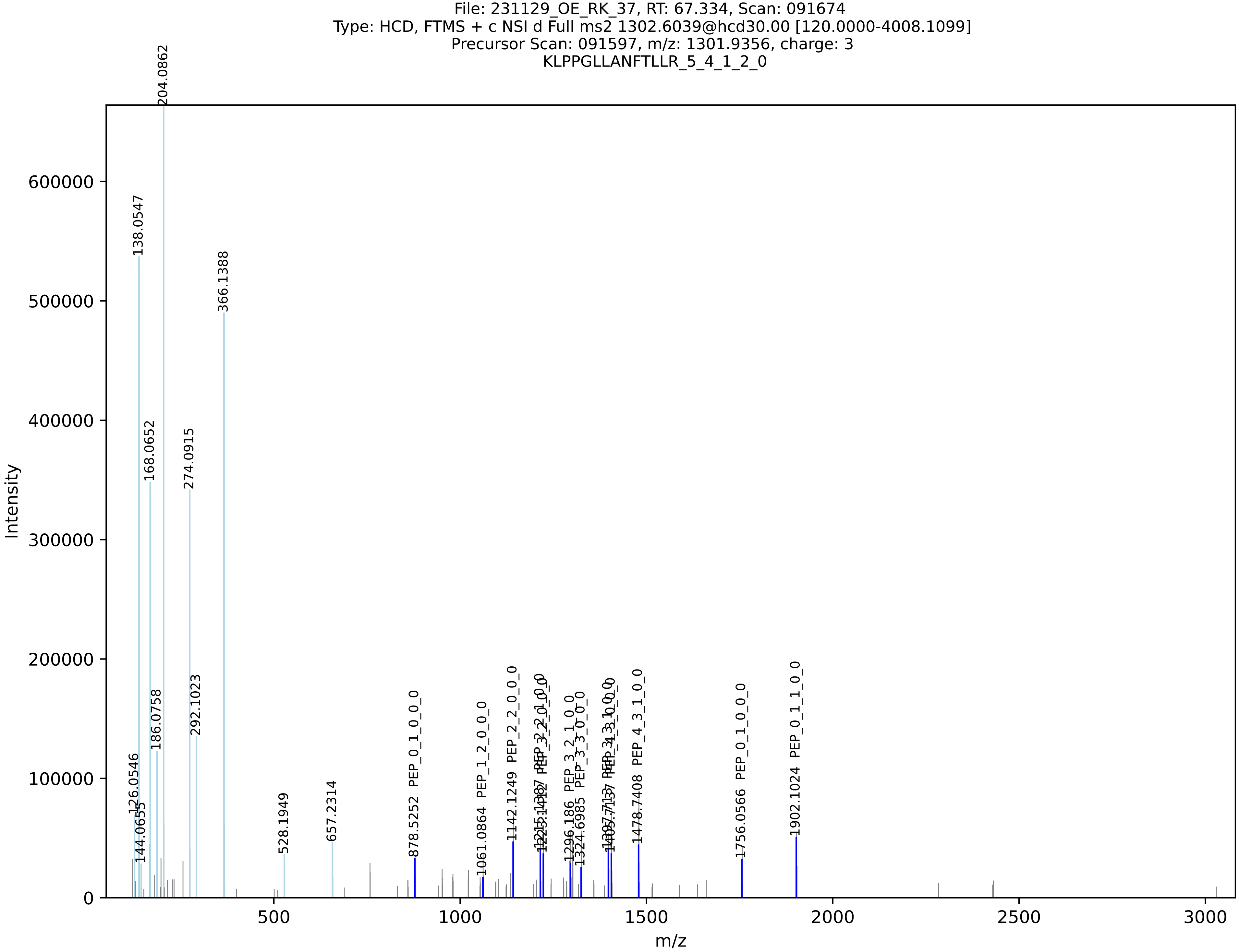
File: 231129_OE_RK_37, RT: 67.334, Scan: 091674
Type: HCD, FTMS + c NSI d Full ms2 1302.6039@hcd30.00 [120.0000-4008.1099]
Precursor Scan: 091597, m/z: 1301.9356, charge: 3
KLPPGLLANFTLLR_5_4_1_2_0
Intensity
m/z
204.0862
138.0547
366.1388
168.0652
274.0915
186.0758
292.1023
126.0546
144.0655
528.1949
657.2314
878.5252 PEP_0_1_0_0_0
1061.0864 PEP_1_2_0_0_0
1142.1249 PEP_2_2_0_0_0
1478.7408 PEP_4_3_1_0_0
1756.0566 PEP_0_1_0_0_0
1902.1024 PEP_0_1_1_0_0

File: 231129_OE_RK_37, RT: 67.389, Scan: 091754
Type: HCD, FTMS + c NSI d Full ms2 1370.5847@hcd30.00 [120.0000-4216.1313]
Precursor Scan: 091670, m/z: 1369.582, charge: 3
YLGNATAIFFLPDEGK_5_4_1_2_0

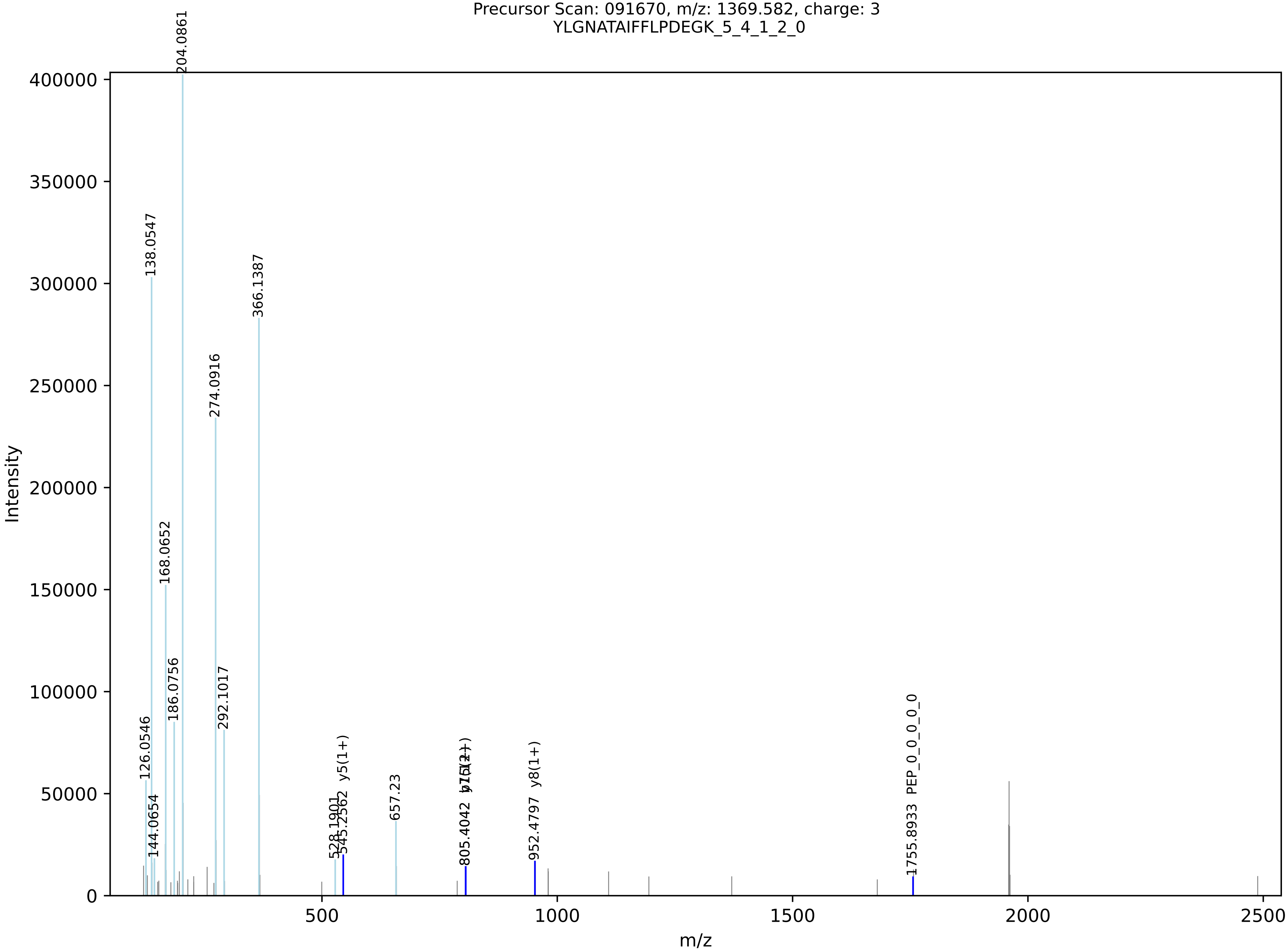

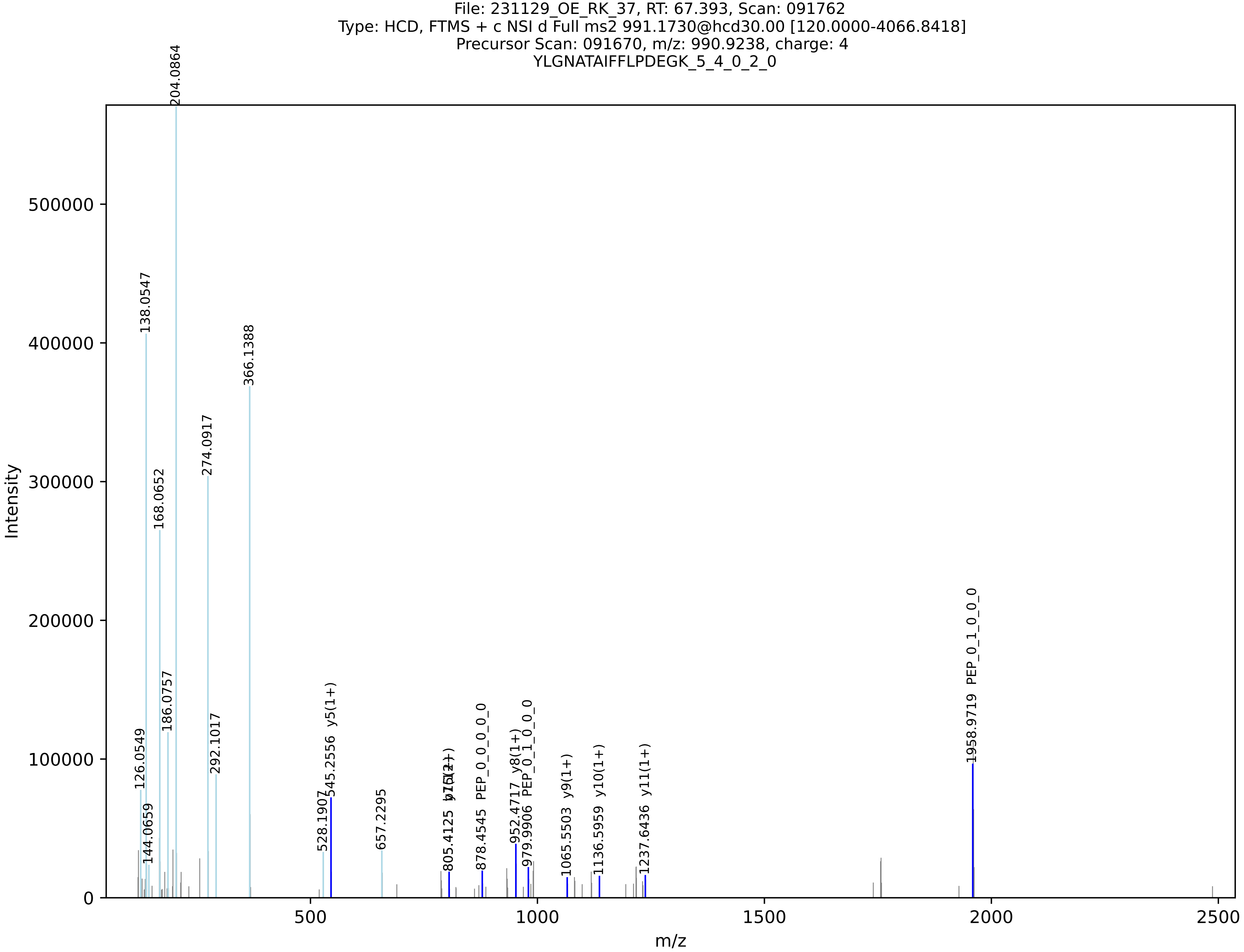
File: 231129_OE_RK_37, RT: 67.393, Scan: 091762
Type: HCD, FTMS + c NSI d Full ms2 991.1730@hcd30.00 [120.0000-4066.8418]
Precursor Scan: 091670, m/z: 990.9238, charge: 4
YLGNATAIFFLPDEGK_5_4_0_2_0
Intensity
m/z
204.0864
138.0547
366.1388
274.0917
168.0652
186.0757
292.1017
126.0549
144.0659
528.1907
545.2556 y5(1+)
657.2295
805.4125
878.4545 PEP_0_0_0_0_0
952.4717 y8(1+)
979.9906 PEP_0_1_0_0_0
1065.5503 y9(1+)
1136.5959 y10(1+)
1237.6436 y11(1+)
1958.9719 PEP_0_1_0_0_0
0
100000
200000
300000
400000
500000
500
1000
1500
2000
2500

File: 231129_OE_RK_37, RT: 67.417, Scan: 091799
Type: HCD, FTMS + c NSI d Full ms2 1981.3381@hcd30.00 [120.0000-4063.3579]
Precursor Scan: 091670, m/z: 1981.3382, charge: 2
YLGNATAIFFLPDEGK_5_4_0_2_0

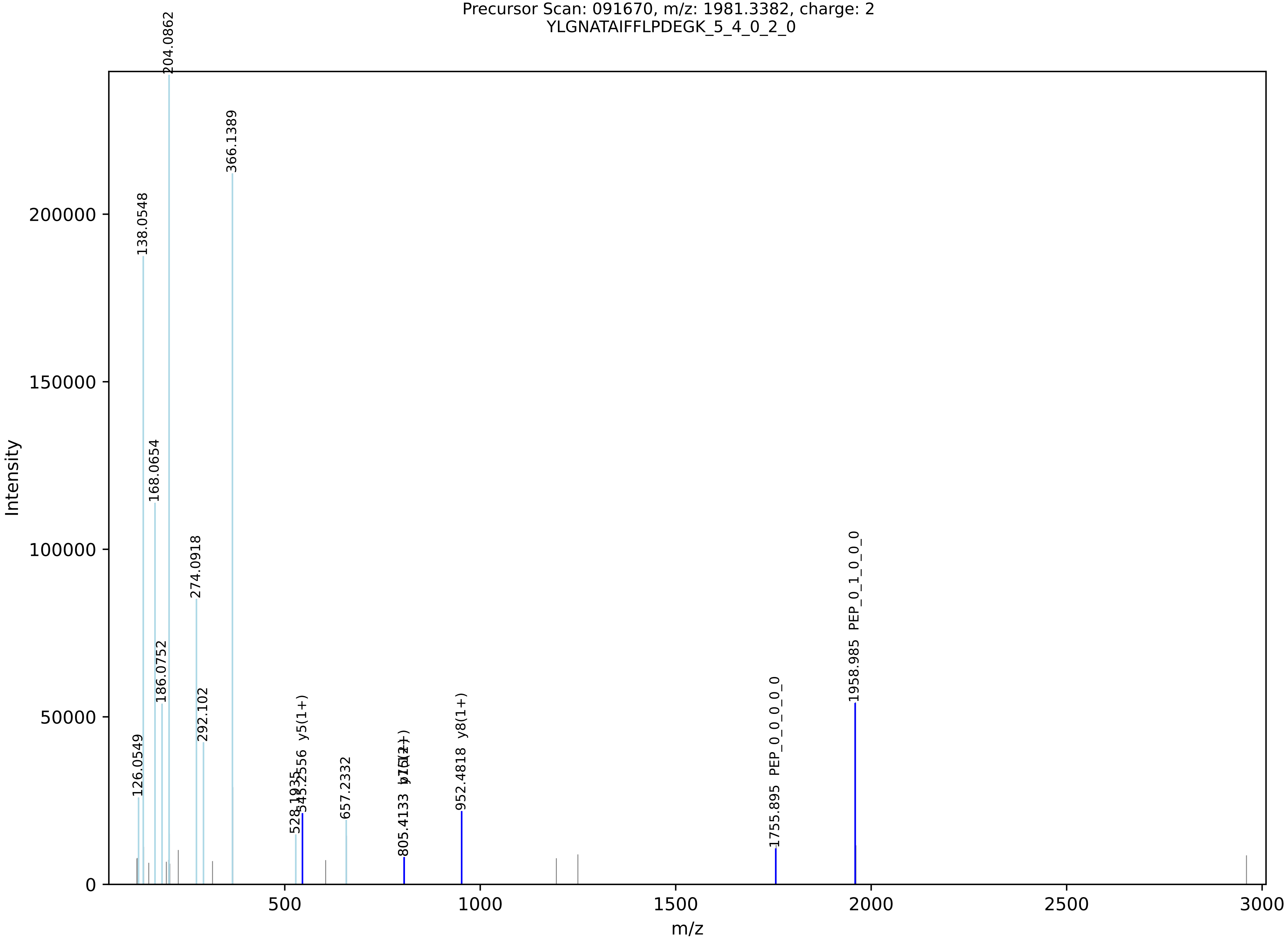

File: 231129_OE_RK_37, RT: 67.445, Scan: 091835
Type: HCD, FTMS + c NSI d Full ms2 917.0964@hcd30.00 [120.0000-5636.9136]
Precursor Scan: 091744, m/z: 916.7622, charge: 6
LQDSQNNLQINVSELQTLQSEHDTLLERHNK_3_6_1_0_0

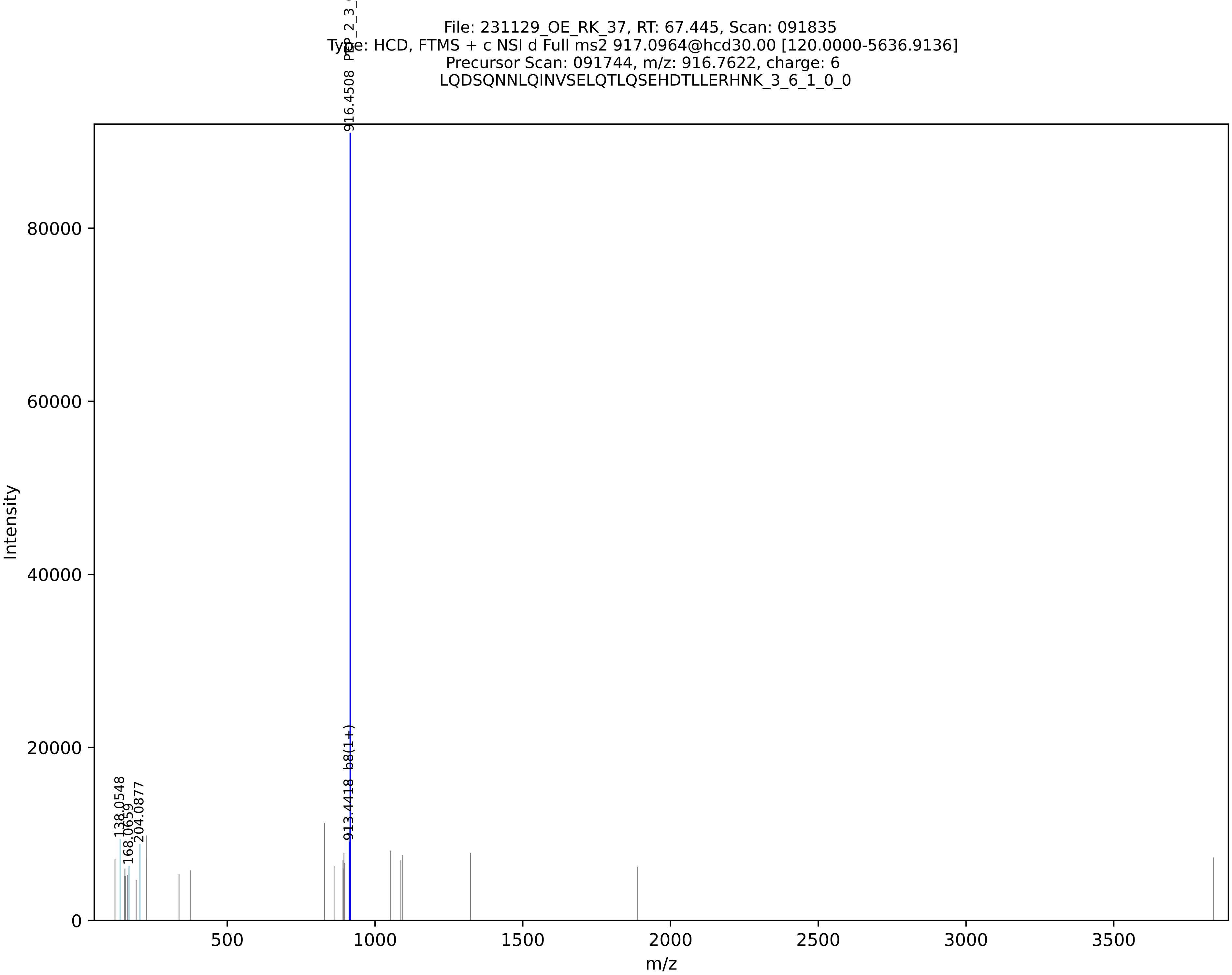

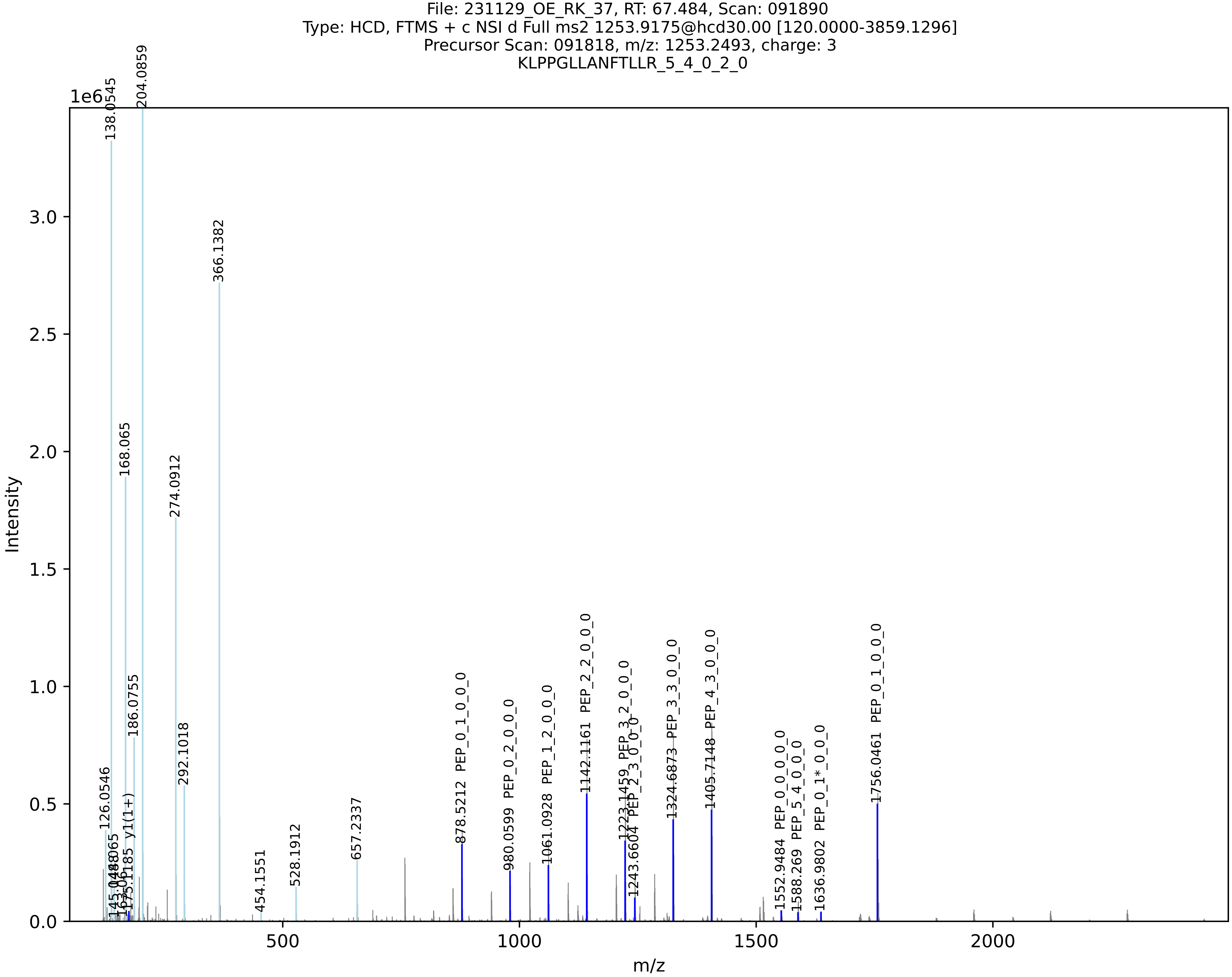

File: 231129_OE_RK_37, RT: 67.484, Scan: 091890
Type: HCD, FTMS + c NSI d Full ms2 1253.9175@hcd30.00 [120.0000-3859.1296]
Precursor Scan: 091818, m/z: 1253.2493, charge: 3
KLPPGLLANFTLLR_5_4_0_2_0
1e6
Intensity
m/z
138.0545
204.0859
366.1382
168.065
274.0912
186.0755
292.1018
126.0546
175.1185 y1(1+)
454.1551
528.1912
657.2337
878.5212 PEP_0_1_0_0_0
980.0599 PEP_0_2_0_0_0
1061.0928 PEP_1_2_0_0_0
1142.1161 PEP_2_2_0_0_0
1223.1459 PEP_3_2_0_0_0
1243.6604 PEP_2_3_0_0_0
1324.6873 PEP_3_3_0_0_0
1405.7148 PEP_4_3_0_0_0
1552.9484 PEP_0_0_0_0_0
1588.269 PEP_5_4_0_0_0
1636.9802 PEP_0_1*_0_0_0
1756.0461 PEP_0_1_0_0_0

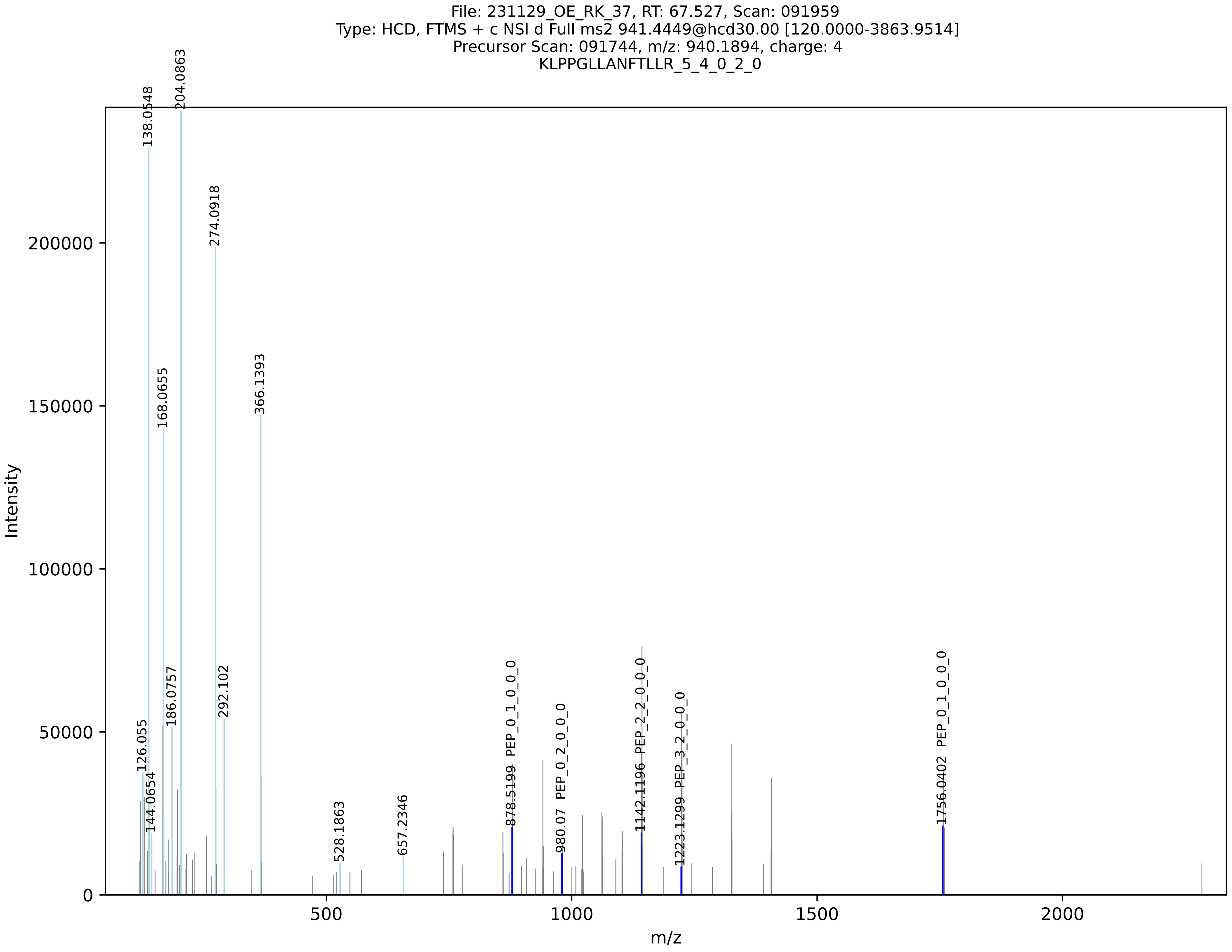

File: 231129_OE_RK_37, RT: 67.527, Scan: 091959
Type: HCD, FTMS + c NSI d Full ms2 941.4449@hcd30.00 [120.0000-3863.9514]
Precursor Scan: 091744, m/z: 940.1894, charge: 4
KLPPGLLANFTLLR_5_4_0_2_0
Intensity
m/z
0
50000
100000
150000
200000
500
1000
1500
2000
204.0863
138.0548
274.0918
168.0655
366.1393
186.0757
292.102
126.055
144.0654
528.1863
657.2346
878.5199 PEP_0_1_0_0_0
980.07 PEP_0_2_0_0_0
1142.1196 PEP_2_2_0_0_0
1223.1299 PEP_3_2_0_0_0
1756.0402 PEP_0_1_0_0_0

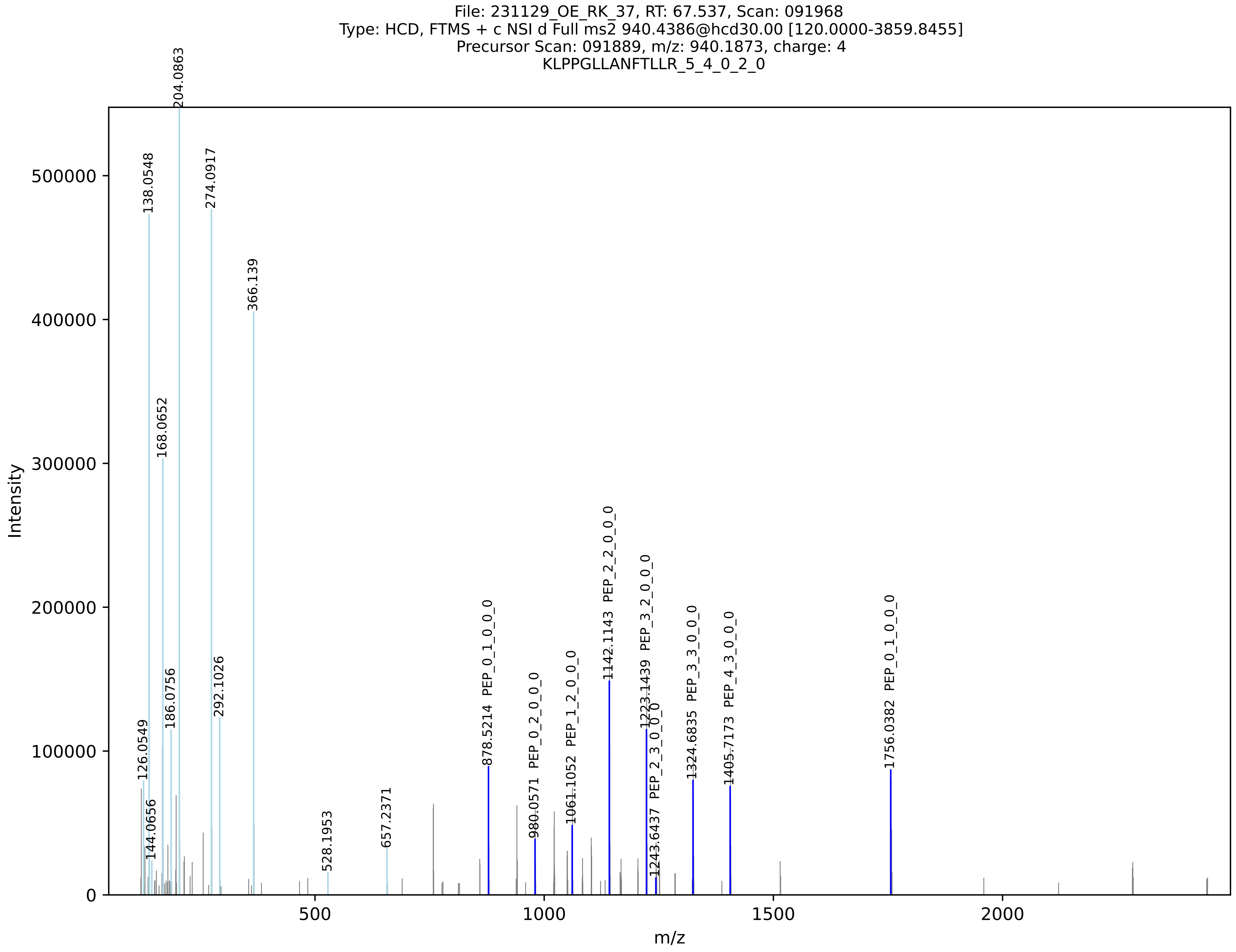

File: 231129_OE_RK_37, RT: 67.537, Scan: 091968
Type: HCD, FTMS + c NSI d Full ms2 940.4386@hcd30.00 [120.0000-3859.8455]
Precursor Scan: 091889, m/z: 940.1873, charge: 4
KLPPGLLANFTLLR_5_4_0_2_0
Intensity
m/z
204.0863
138.0548
274.0917
366.139
168.0652
186.0756
292.1026
126.0549
144.0656
528.1953
657.2371
878.5214 PEP_0_1_0_0_0
980.0571 PEP_0_2_0_0_0
1061.1052 PEP_1_2_0_0_0
1142.1143 PEP_2_2_0_0_0
1223.1439 PEP_3_2_0_0_0
1243.6437 PEP_2_3_0_0_0
1324.6835 PEP_3_3_0_0_0
1405.7173 PEP_4_3_0_0_0
1756.0382 PEP_0_1_0_0_0

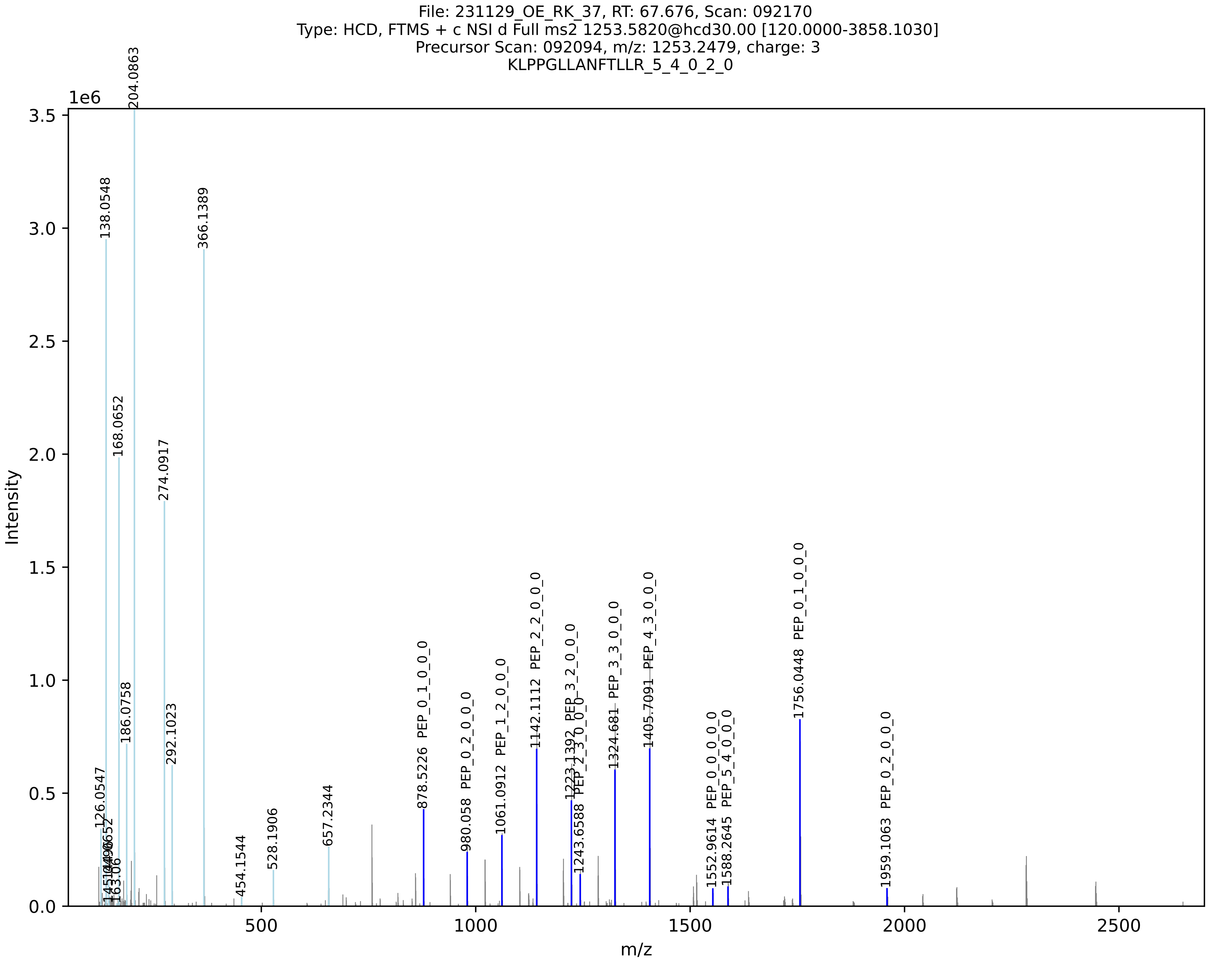
File: 231129_OE_RK_37, RT: 67.676, Scan: 092170
Type: HCD, FTMS + c NSI d Full ms2 1253.5820@hcd30.00 [120.0000-3858.1030]
Precursor Scan: 092094, m/z: 1253.2479, charge: 3
KLPPGLLANFTLLR_5_4_0_2_0
1e6
Intensity
m/z
204.0863
138.0548
366.1389
168.0652
274.0917
186.0758
292.1023
126.0547
454.1544
528.1906
657.2344
878.5226 PEP_0_1_0_0_0
980.058 PEP_0_2_0_0_0
1061.0912 PEP_1_2_0_0_0
1142.1112 PEP_2_2_0_0_0
1223.1392 PEP_3_2_0_0_0
1243.6588 PEP_2_3_0_0_0
1324.681 PEP_3_3_0_0_0
1405.7091 PEP_4_3_0_0_0
1552.9614 PEP_0_0_0_0_0
1588.2645 PEP_5_4_0_0_0
1756.0448 PEP_0_1_0_0_0
1959.1063 PEP_0_2_0_0_0

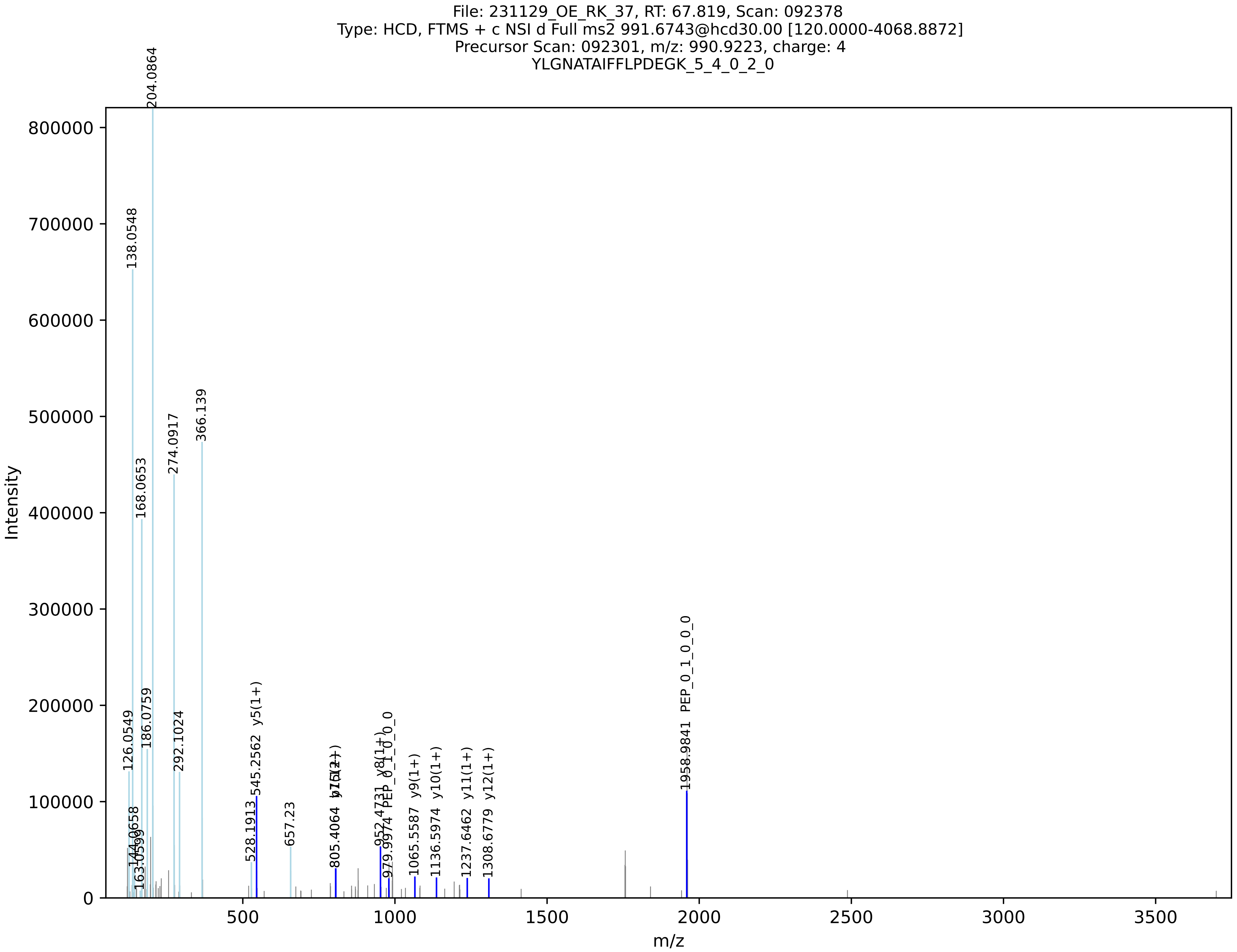

File: 231129_OE_RK_37, RT: 67.819, Scan: 092378
Type: HCD, FTMS + c NSI d Full ms2 991.6743@hcd30.00 [120.0000-4068.8872]
Precursor Scan: 092301, m/z: 990.9223, charge: 4
YLGNATAIFFLPDEGK_5_4_0_2_0
Intensity
m/z
204.0864
138.0548
168.0653
366.139
274.0917
126.0549
186.0759
292.1024
144.0658
163.0599
528.1913
545.2562 y5(1+)
657.23
805.4064
952.4731 y8(1+)
979.9974 PEP_0_1_0_0_0
1065.5587 y9(1+)
1136.5974 y10(1+)
1237.6462 y11(1+)
1308.6779 y12(1+)
1958.9841 PEP_0_1_0_0_0

File: 231129_OE_RK_37, RT: 67.867, Scan: 092448
Type: HCD, FTMS + c NSI d Full ms2 1253.9170@hcd30.00 [120.0000-3859.1282]
Precursor Scan: 092375, m/z: 1253.2488, charge: 3
KLPPGLLANFTLLR_5_4_0_2_0

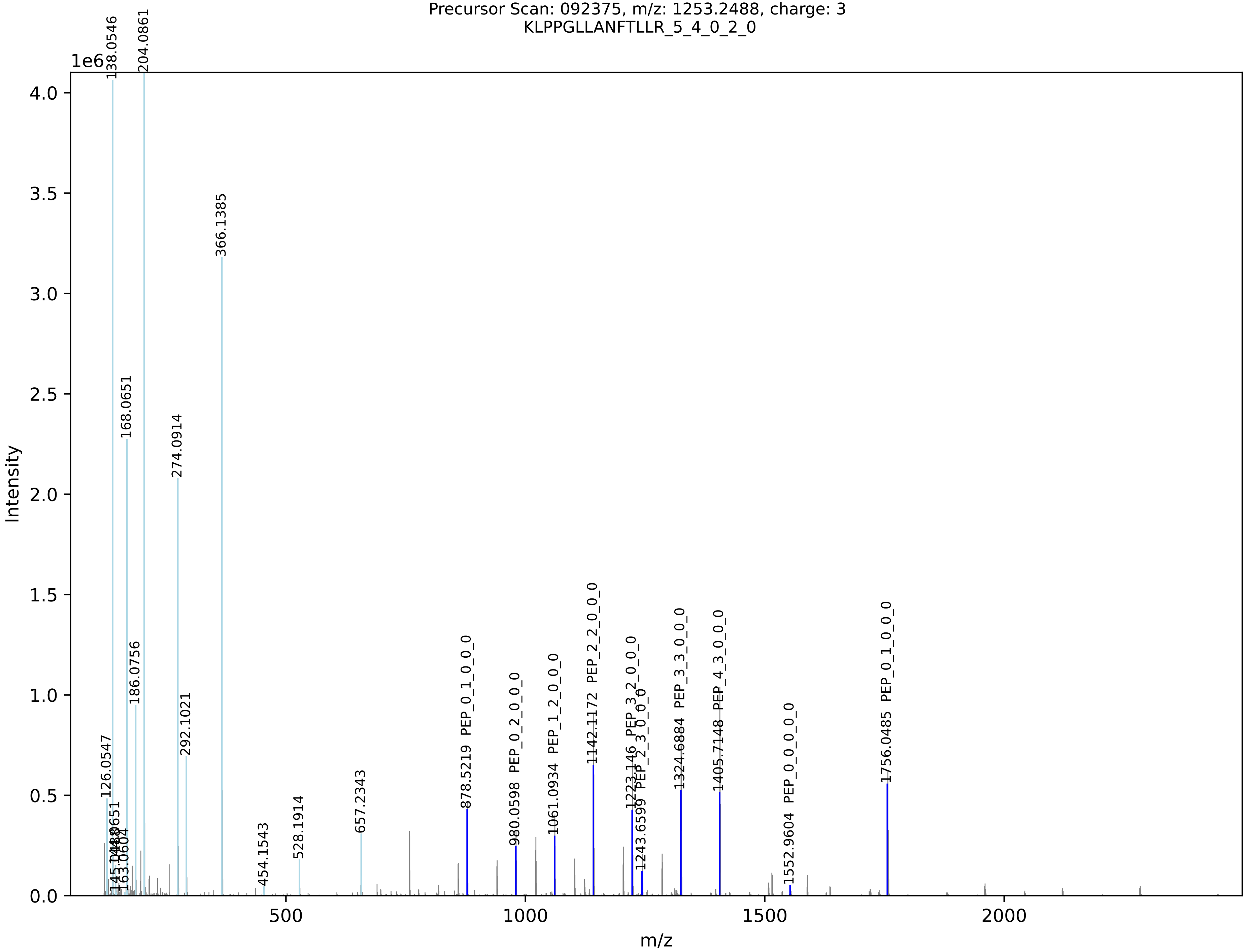

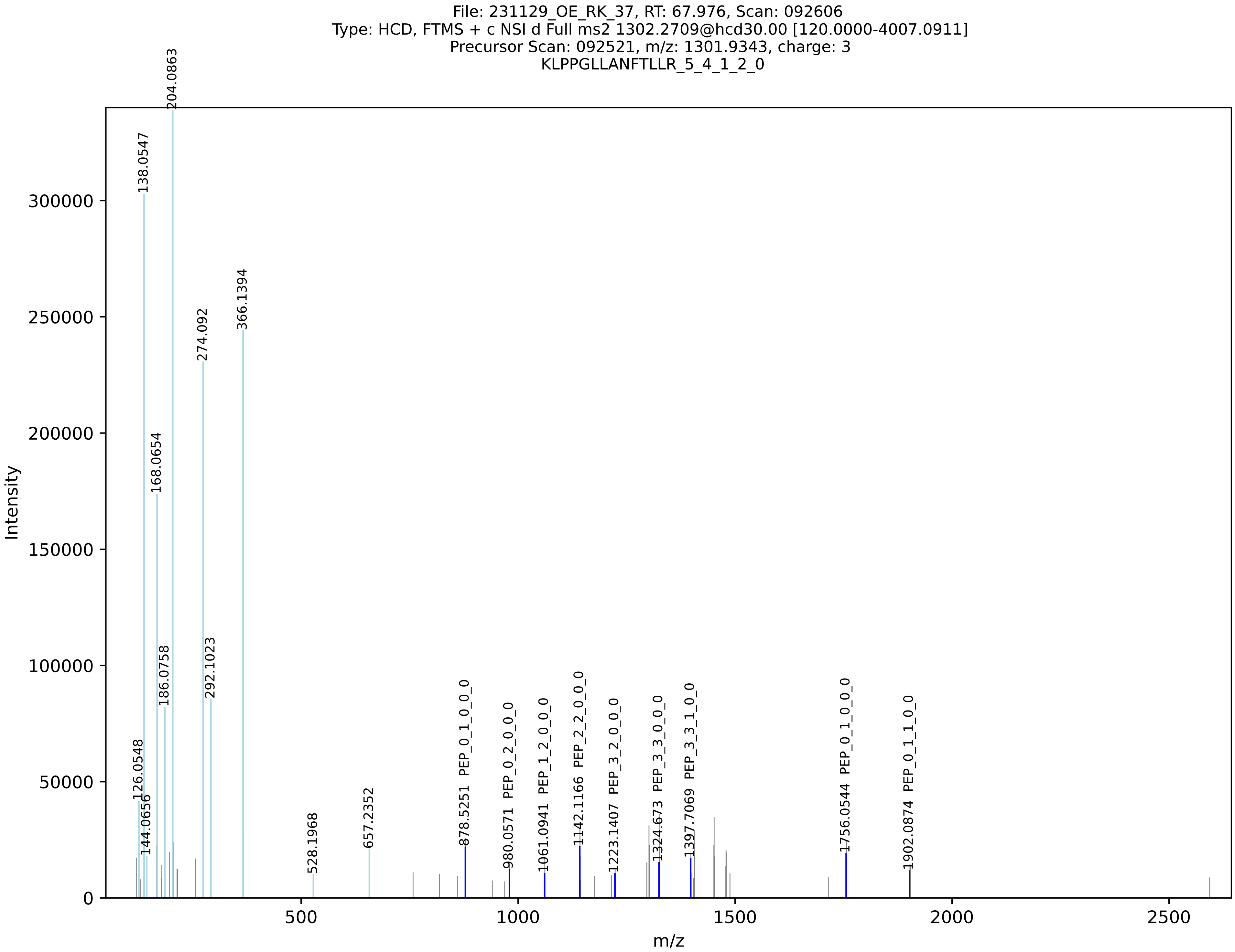
File: 231129_OE_RK_37, RT: 67.976, Scan: 092606
Type: HCD, FTMS + c NSI d Full ms2 1302.2709@hcd30.00 [120.0000-4007.0911]
Precursor Scan: 092521, m/z: 1301.9343, charge: 3
KLPPGLLANFTLLR_5_4_1_2_0
Intensity
m/z
204.0863
138.0547
366.1394
274.092
168.0654
186.0758
292.1023
126.0548
144.0656
528.1968
657.2352
878.5251 PEP_0_1_0_0_0
980.0571 PEP_0_2_0_0_0
1061.0941 PEP_1_2_0_0_0
1142.1166 PEP_2_2_0_0_0
1223.1407 PEP_3_2_0_0_0
1324.673 PEP_3_3_0_0_0
1397.7069 PEP_3_3_1_0_0
1756.0544 PEP_0_1_0_0_0
1902.0874 PEP_0_1_1_0_0
0
50000
100000
150000
200000
250000
300000
500
1000
1500
2000
2500

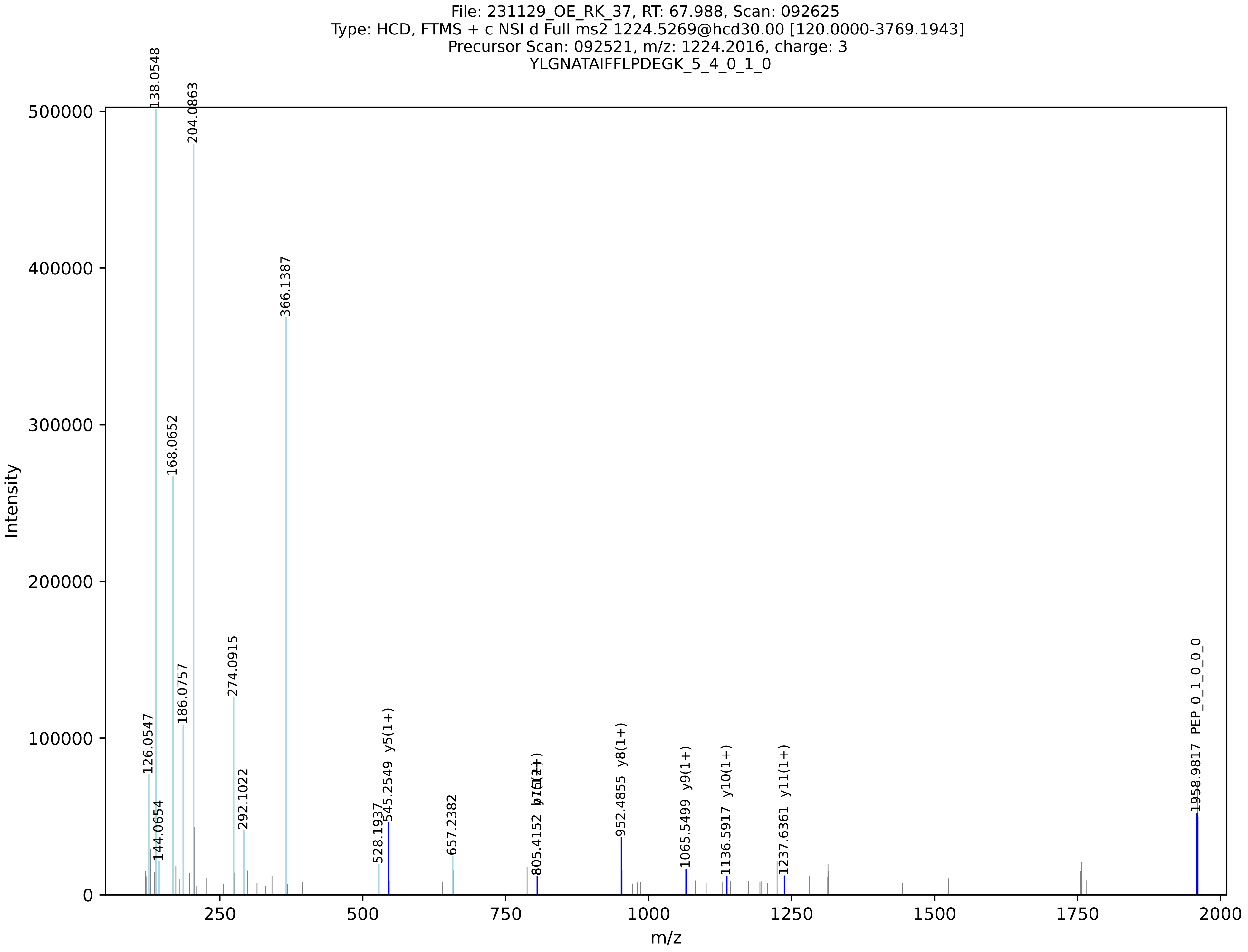

File: 231129_OE_RK_37, RT: 67.988, Scan: 092625
Type: HCD, FTMS + c NSI d Full ms2 1224.5269@hcd30.00 [120.0000-3769.1943]
Precursor Scan: 092521, m/z: 1224.2016, charge: 3
YLGNATAIFFLPDEGK_5_4_0_1_0
Intensity
m/z
138.0548
204.0863
366.1387
168.0652
186.0757
274.0915
126.0547
292.1022
144.0654
528.1937
545.2549 y5(1+)
657.2382
805.4152 y7(1+)
952.4855 y8(1+)
1065.5499 y9(1+)
1136.5917 y10(1+)
1237.6361 y11(1+)
1958.9817 PEP_0_1_0_0_0

File: 231129_OE_RK_37, RT: 68.07, Scan: 092741
Type: HCD, FTMS + c NSI d Full ms2 1253.9172@hcd30.00 [120.0000-3859.1287]
Precursor Scan: 092666, m/z: 1253.249, charge: 3
KLPPGLLANFTLLR_5_4_0_2_0

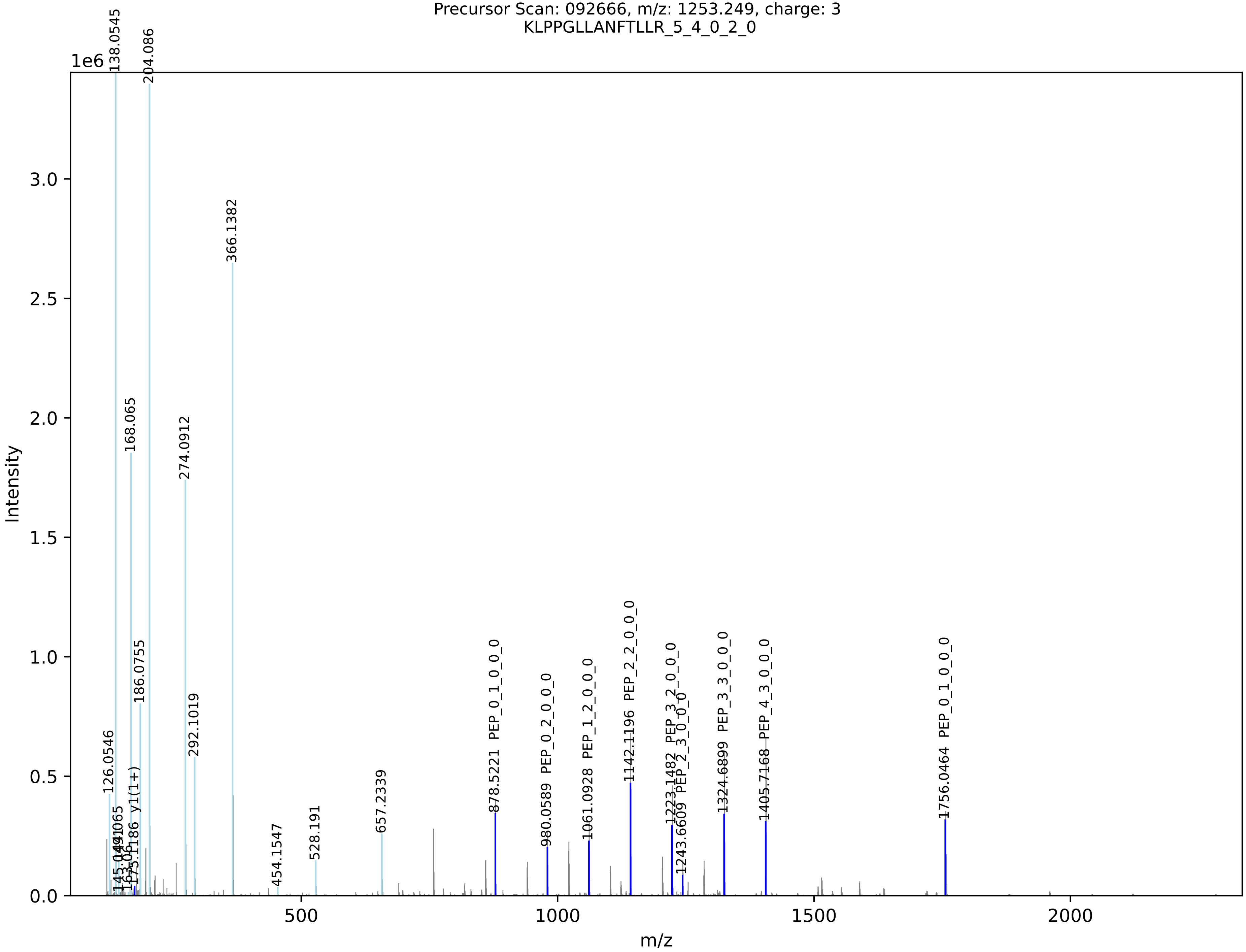

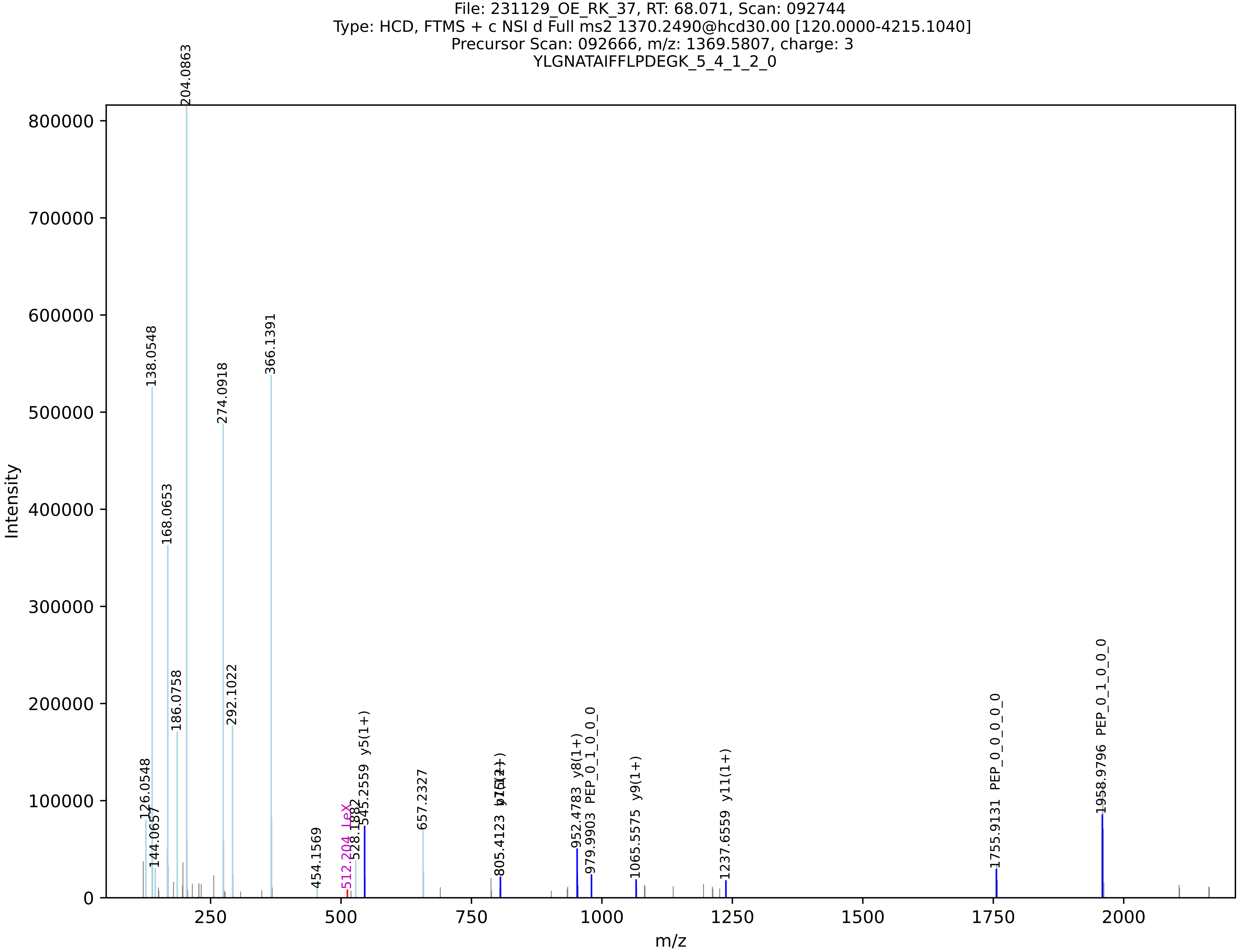

File: 231129_OE_RK_37, RT: 68.071, Scan: 092744
Type: HCD, FTMS + c NSI d Full ms2 1370.2490@hcd30.00 [120.0000-4215.1040]
Precursor Scan: 092666, m/z: 1369.5807, charge: 3
YLGNATAIFFLPDEGK_5_4_1_2_0
Intensity
m/z
204.0863
138.0548
366.1391
274.0918
168.0653
186.0758
292.1022
126.0548
144.0657
454.1569
512.204 LeX
528.1882
545.2559 y5(1+)
657.2327
805.4123 y7(1+)
952.4783 y8(1+)
979.9903 PEP_0_1_0_0_0
1065.5575 y9(1+)
1237.6559 y11(1+)
1755.9131 PEP_0_0_0_0_0
1958.9796 PEP_0_1_0_0_0

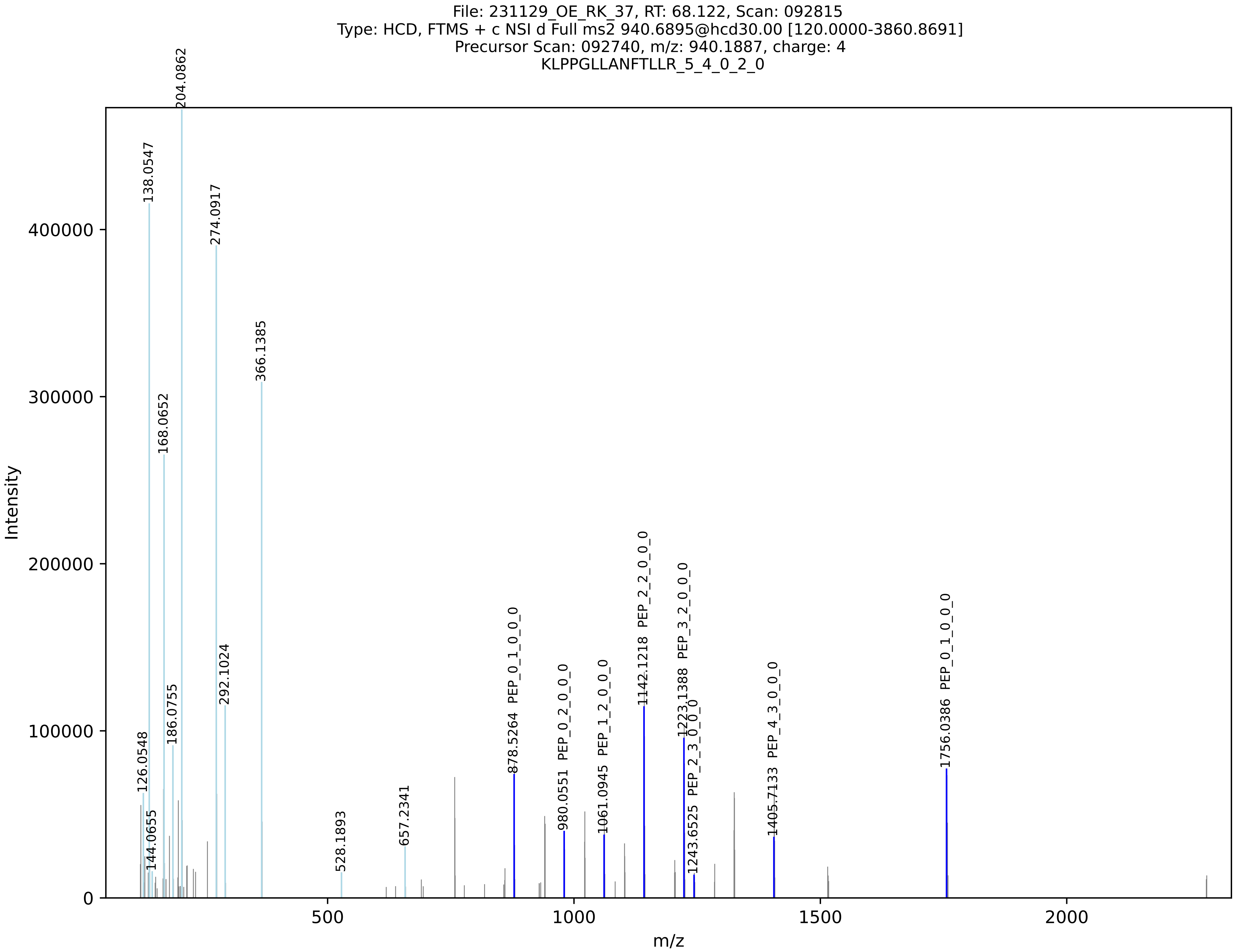
File: 231129_OE_RK_37, RT: 68.122, Scan: 092815
Type: HCD, FTMS + c NSI d Full ms2 940.6895@hcd30.00 [120.0000-3860.8691]
Precursor Scan: 092740, m/z: 940.1887, charge: 4
KLPPGLLANFTLLR_5_4_0_2_0
Intensity
m/z
204.0862
138.0547
274.0917
366.1385
168.0652
186.0755
292.1024
126.0548
144.0655
528.1893
657.2341
878.5264 PEP_0_1_0_0_0
980.0551 PEP_0_2_0_0_0
1061.0945 PEP_1_2_0_0_0
1142.1218 PEP_2_2_0_0_0
1223.1388 PEP_3_2_0_0_0
1243.6525 PEP_2_3_0_0_0
1405.7133 PEP_4_3_0_0_0
1756.0386 PEP_0_1_0_0_0

File: 231129_OE_RK_37, RT: 68.223, Scan: 092964
Type: HCD, FTMS + c NSI d Full ms2 1198.3002@hcd30.00 [120.0000-4911.9209]
Precursor Scan: 092884, m/z: 1197.799, charge: 4
EEQYNSTYRVVSVLTVLHQDWLNGK_4_5_1_0_0

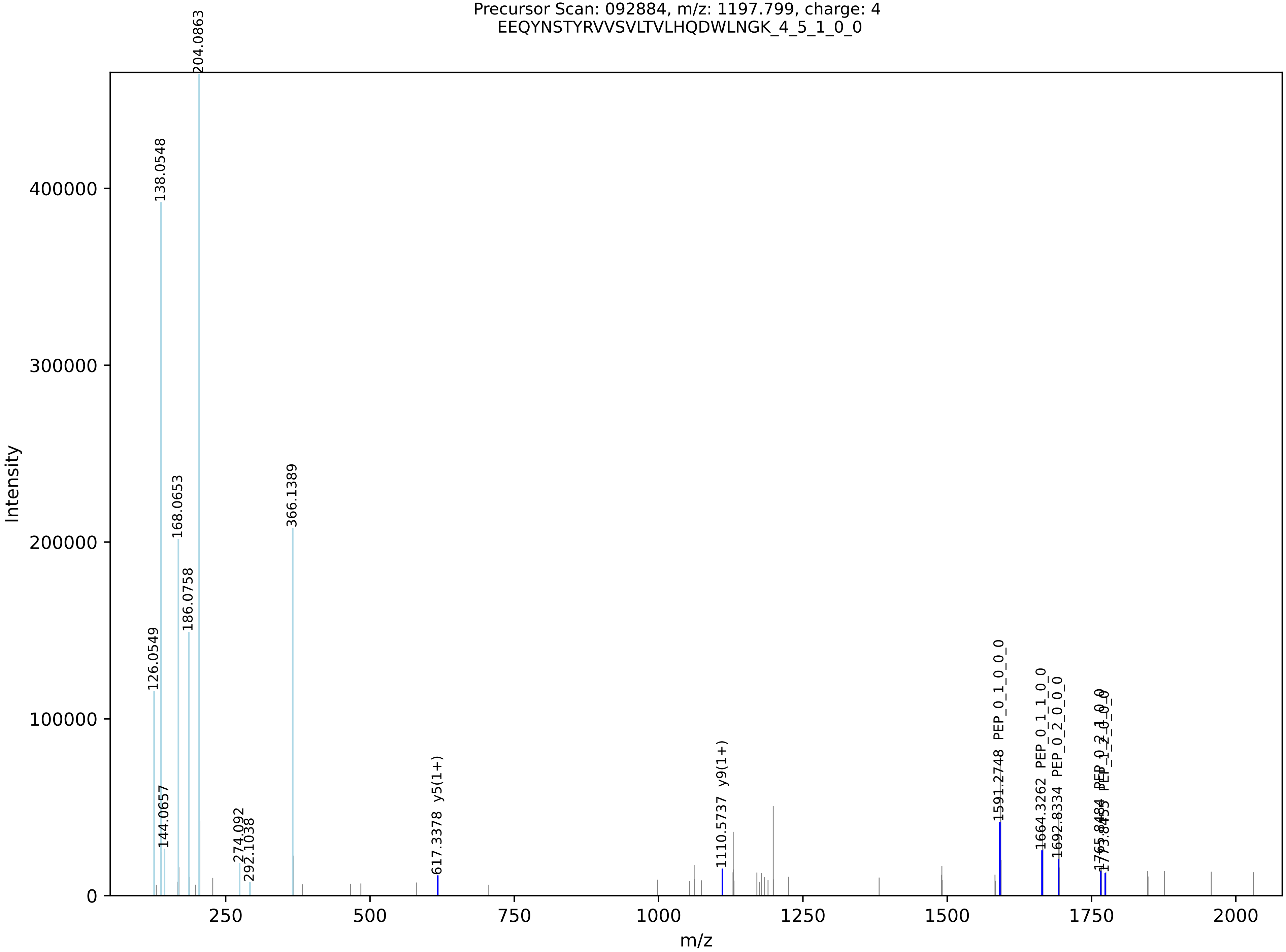

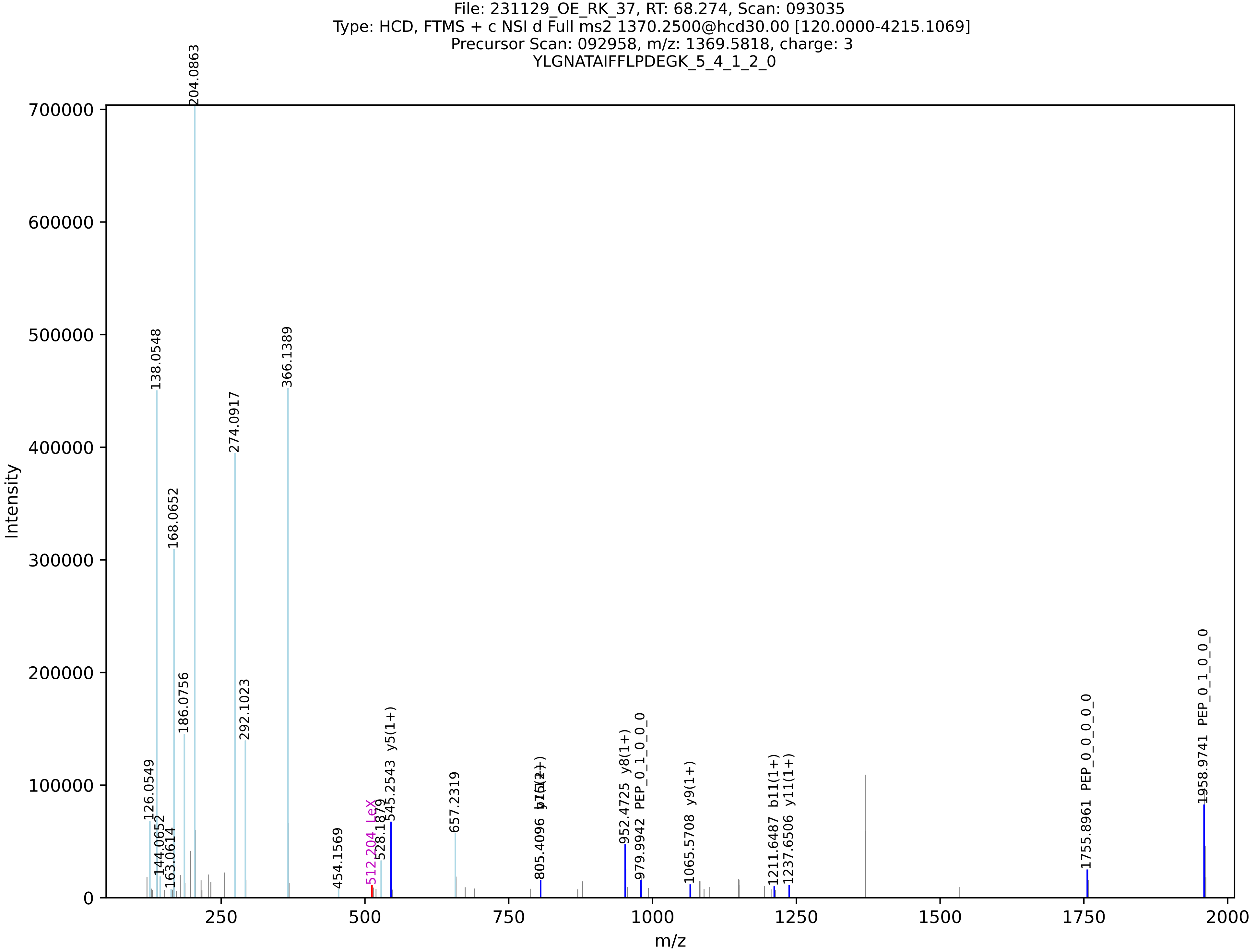
File: 231129_OE_RK_37, RT: 68.274, Scan: 093035
Type: HCD, FTMS + c NSI d Full ms2 1370.2500@hcd30.00 [120.0000-4215.1069]
Precursor Scan: 092958, m/z: 1369.5818, charge: 3
YLGNATAIFFLPDEGK_5_4_1_2_0
Intensity
m/z
204.0863
138.0548
366.1389
274.0917
168.0652
186.0756
292.1023
126.0549
144.0652
163.0614
454.1569
512.204_LeX
528.1879
545.2543 y5(1+)
657.2319
805.4096 b7(1+)
952.4725 y8(1+)
979.9942 PEP_0_1_0_0_0
1065.5708 y9(1+)
1211.6487 b11(1+)
1237.6506 y11(1+)
1755.8961 PEP_0_0_0_0_0
1958.9741 PEP_0_1_0_0_0

File: 231129_OE_RK_37, RT: 68.378, Scan: 093185
Type: HCD, FTMS + c NSI d Full ms2 940.6883@hcd30.00 [120.0000-3860.8643]
Precursor Scan: 093103, m/z: 940.1874, charge: 4
KLPPGLLANFTLLR_5_4_0_2_0

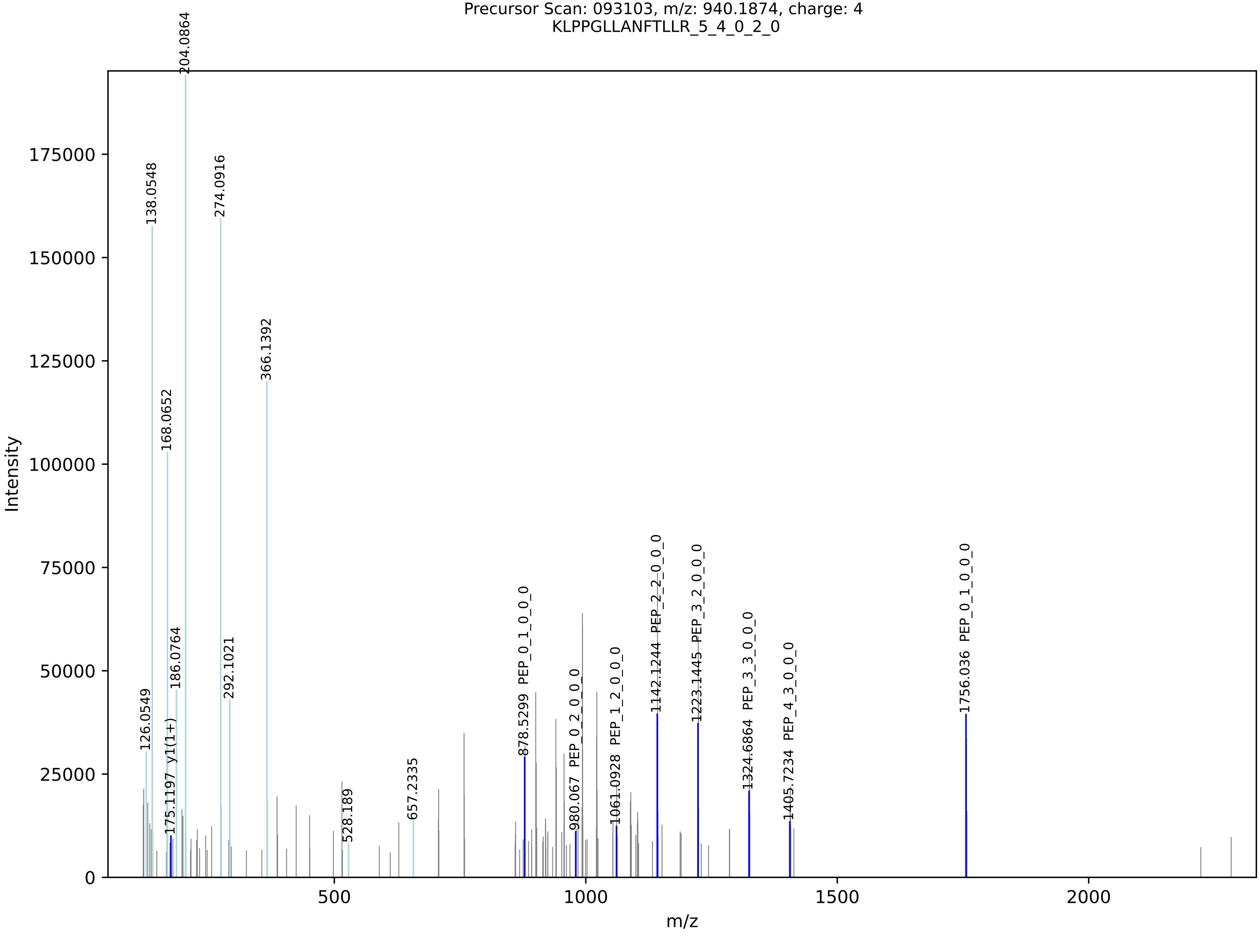

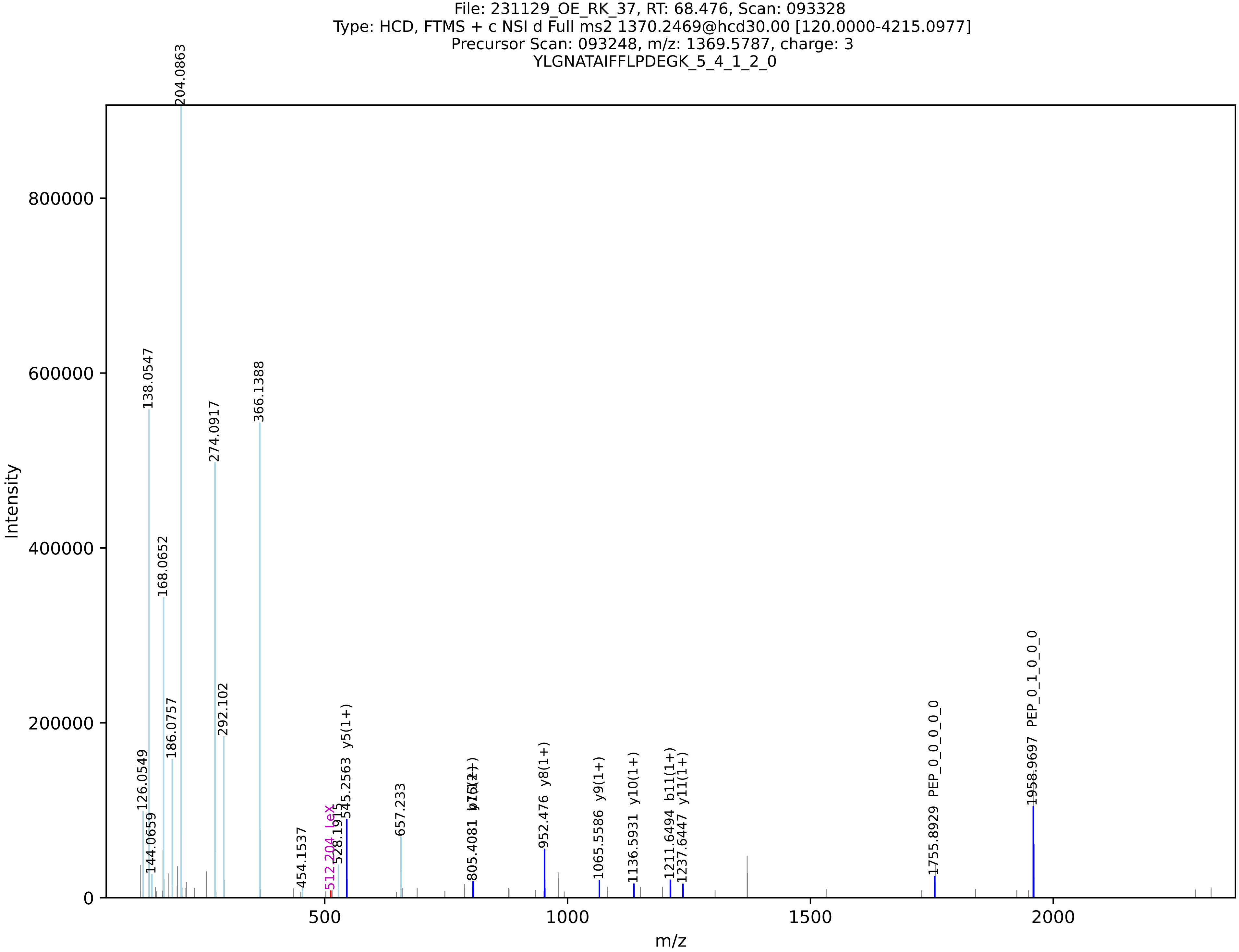
File: 231129_OE_RK_37, RT: 68.476, Scan: 093328
Type: HCD, FTMS + c NSI d Full ms2 1370.2469@hcd30.00 [120.0000-4215.0977]
Precursor Scan: 093248, m/z: 1369.5787, charge: 3
YLGNATAIFFLPDEGK_5_4_1_2_0
Intensity
m/z
204.0863
138.0547
366.1388
274.0917
168.0652
292.102
186.0757
126.0549
144.0659
454.1537
512.204_LeX
528.1915
545.2563 y5(1+)
657.233
805.4081
952.476 y8(1+)
1065.5586 y9(1+)
1136.5931 y10(1+)
1211.6494 b11(1+)
1237.6447 y11(1+)
1755.8929 PEP_0_0_0_0_0
1958.9697 PEP_0_1_0_0_0

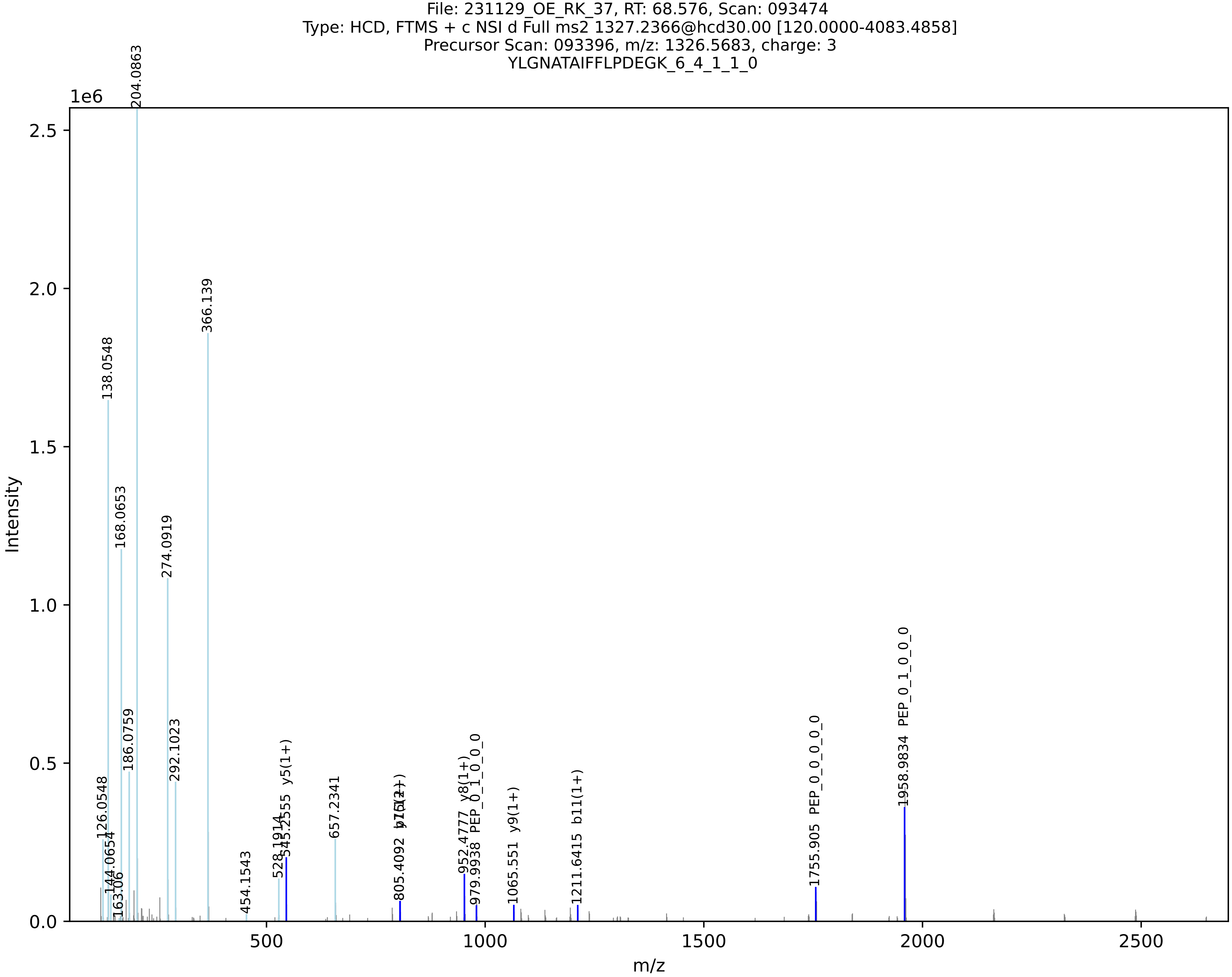
File: 231129_OE_RK_37, RT: 68.576, Scan: 093474
Type: HCD, FTMS + c NSI d Full ms2 1327.2366@hcd30.00 [120.0000-4083.4858]
Precursor Scan: 093396, m/z: 1326.5683, charge: 3
YLGNATAIFFLPDEGK_6_4_1_1_0
1e6
Intensity
m/z
204.0863
366.139
138.0548
168.0653
274.0919
186.0759
292.1023
126.0548
144.0654
163.06
454.1543
528.1914
545.2555 y5(1+)
657.2341
805.4092 y7(1+)
952.4777 y8(1+)
979.9938 PEP_0_1_0_0_0
1065.551 y9(1+)
1211.6415 b11(1+)
1755.905 PEP_0_0_0_0_0
1958.9834 PEP_0_1_0_0_0

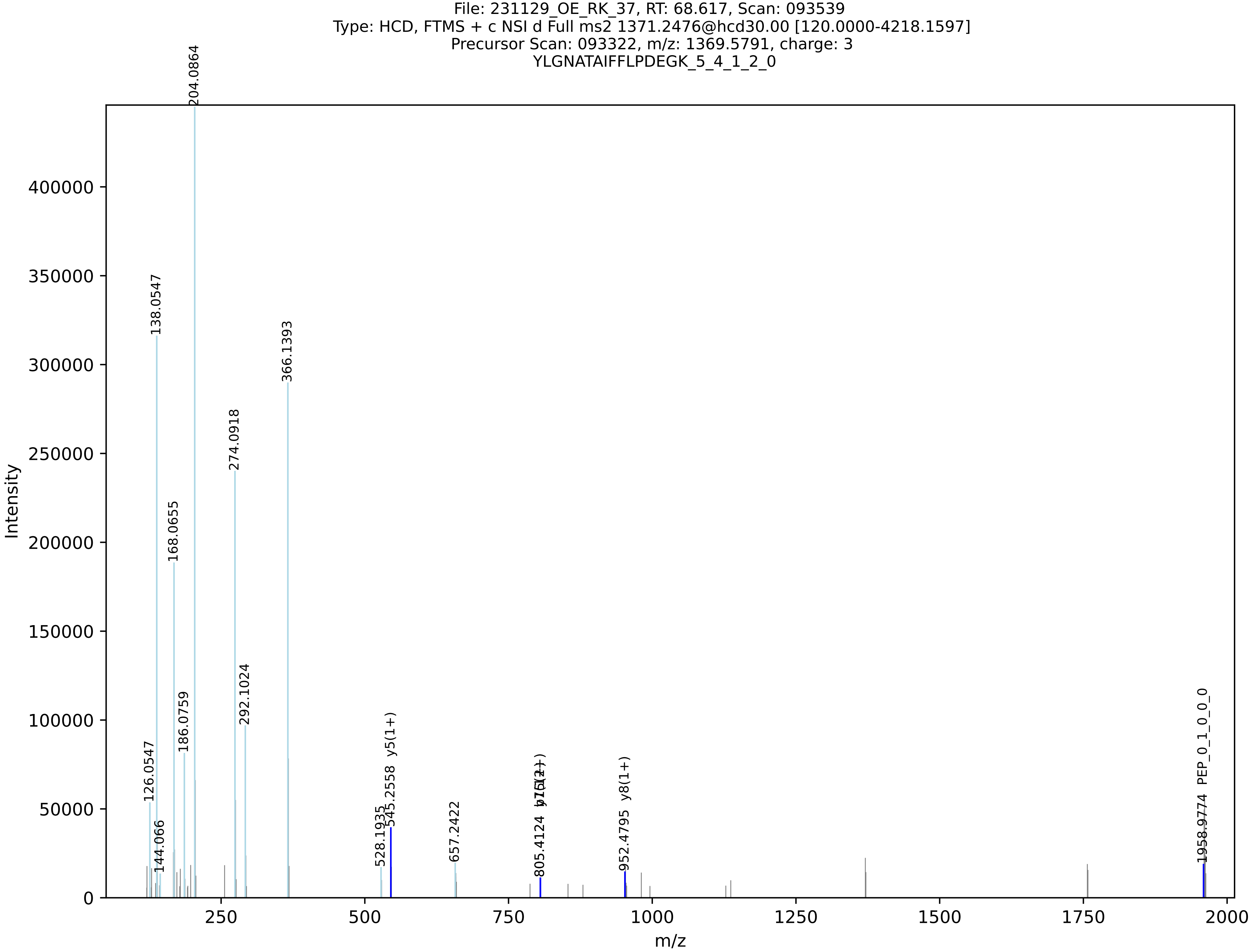
File: 231129_OE_RK_37, RT: 68.617, Scan: 093539
Type: HCD, FTMS + c NSI d Full ms2 1371.2476@hcd30.00 [120.0000-4218.1597]
Precursor Scan: 093322, m/z: 1369.5791, charge: 3
YLGNATAIFFLPDEGK_5_4_1_2_0
Intensity
m/z
204.0864
138.0547
366.1393
274.0918
168.0655
292.1024
186.0759
126.0547
144.066
528.1935
545.2558 y5(1+)
657.2422
805.4124
952.4795 y8(1+)
1958.9774 PEP_0_1_0_0_0

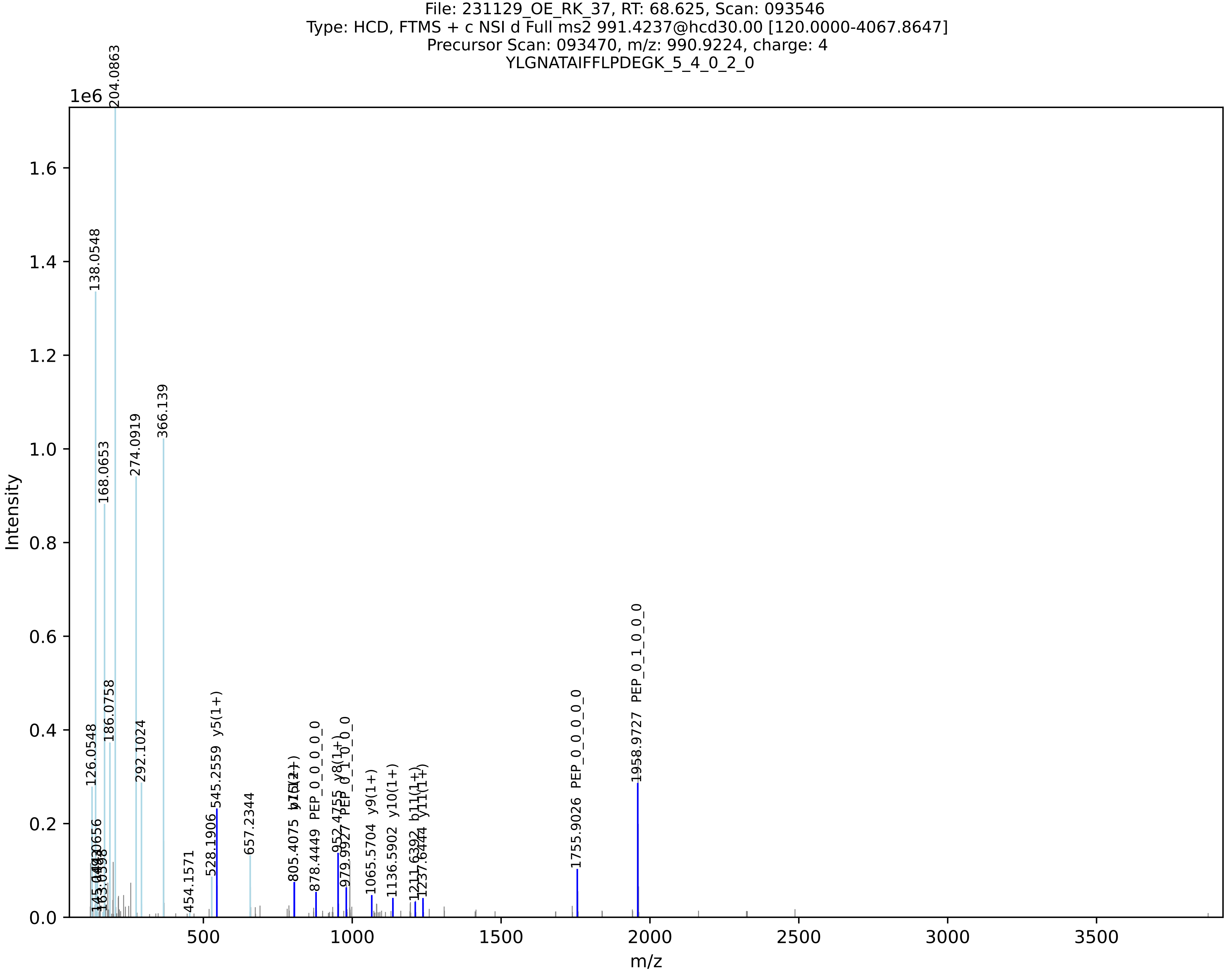

File: 231129_OE_RK_37, RT: 68.625, Scan: 093546
Type: HCD, FTMS + c NSI d Full ms2 991.4237@hcd30.00 [120.0000-4067.8647]
Precursor Scan: 093470, m/z: 990.9224, charge: 4
YLGNATAIFFLPDEGK_5_4_0_2_0
1e6
Intensity
m/z
204.0863
138.0548
168.0653
274.0919
366.139
186.0758
126.0548
292.1024
545.2559 y5(1+)
528.1906
657.2344
454.1571
805.4075
878.4449 PEP_0_0_0_0_0_
952.4755 y8(1+)
979.9927
1065.5704 y9(1+)
1136.5902 y10(1+)
1211.6392 b11(1+)
1237.6444 y11(1+)
1755.9026 PEP_0_0_0_0_0_
1958.9727 PEP_0_1_0_0_0_

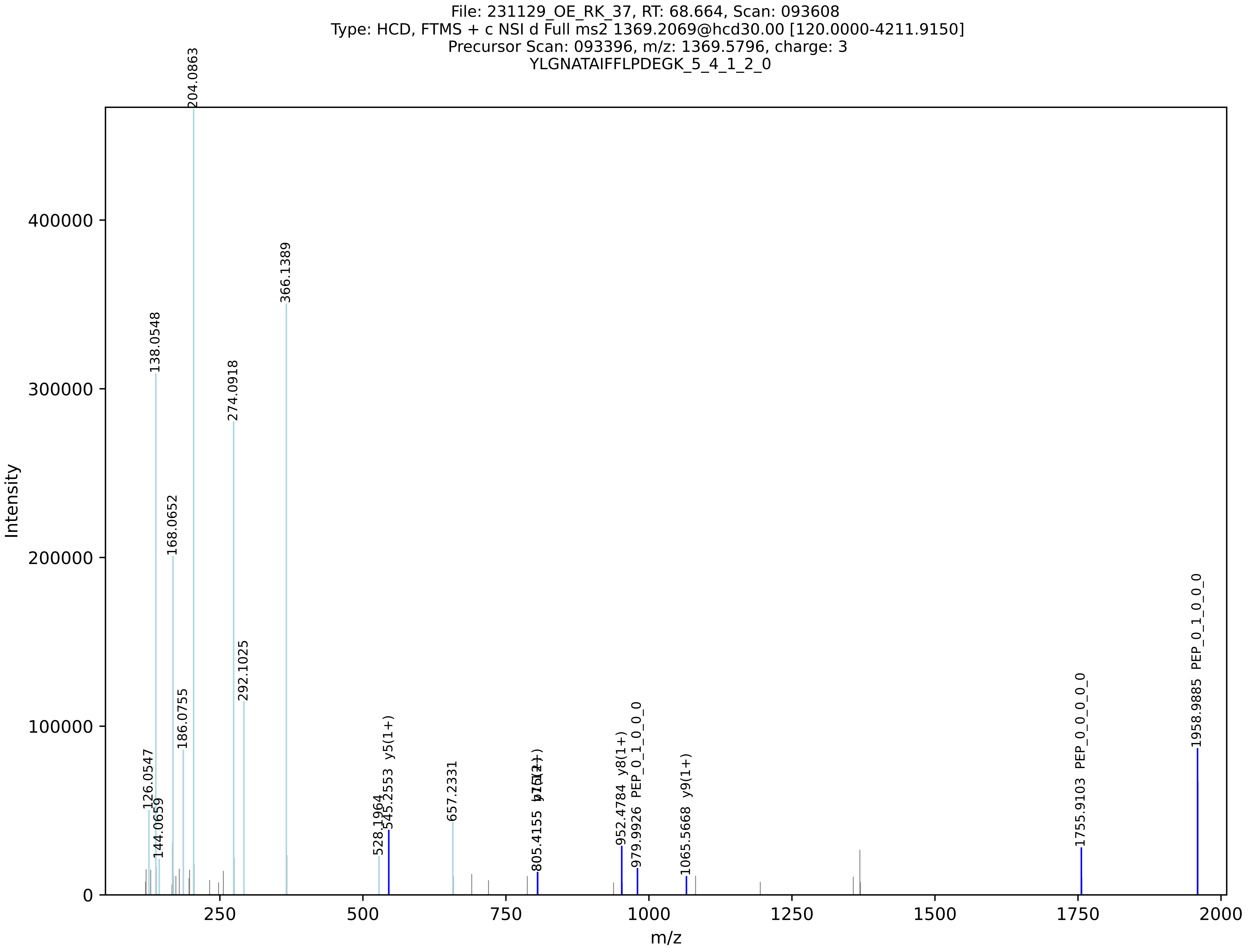
File: 231129_OE_RK_37, RT: 68.664, Scan: 093608
Type: HCD, FTMS + c NSI d Full ms2 1369.2069@hcd30.00 [120.0000-4211.9150]
Precursor Scan: 093396, m/z: 1369.5796, charge: 3
YLGNATAIFFLPDEGK_5_4_1_2_0
Intensity
m/z
204.0863
366.1389
138.0548
274.0918
168.0652
292.1025
186.0755
126.0547
144.0659
528.1964
545.2553 y5(1+)
657.2331
805.4155
952.4784 y8(1+)
979.9926 PEP_0_1_0_0_0
1065.5668 y9(1+)
1755.9103 PEP_0_0_0_0_0
1958.9885 PEP_0_1_0_0_0

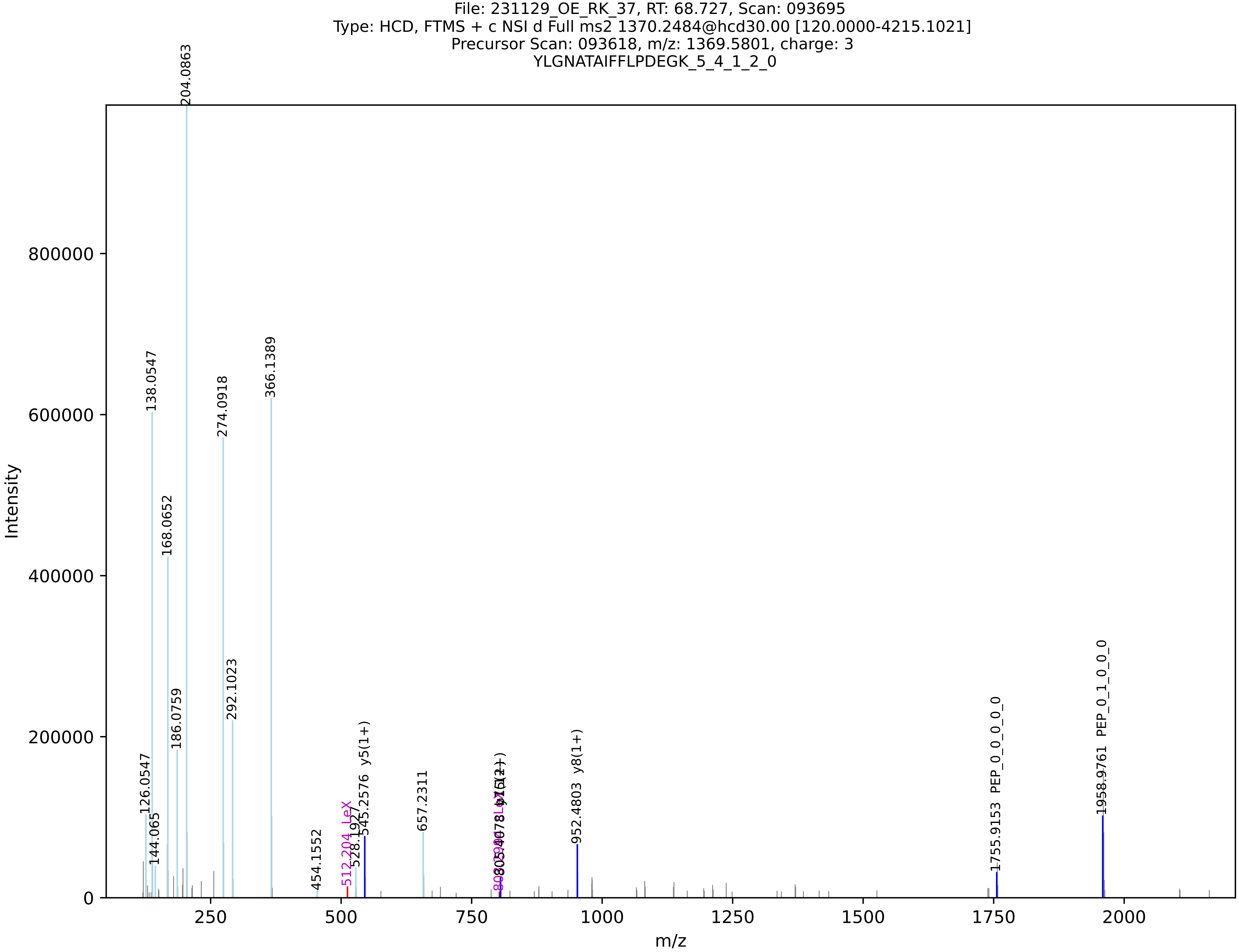
File: 231129_OE_RK_37, RT: 68.727, Scan: 093695
Type: HCD, FTMS + c NSI d Full ms2 1370.2484@hcd30.00 [120.0000-4215.1021]
Precursor Scan: 093618, m/z: 1369.5801, charge: 3
YLGNATAIFFLPDEGK_5_4_1_2_0
Intensity
m/z
204.0863
138.0547
366.1389
274.0918
168.0652
292.1023
186.0759
126.0547
144.065
454.1552
512.204 LeX
528.1927
545.2576 y5(1+)
657.2311
952.4803 y8(1+)
1755.9153 PEP_0_0_0_0_0
1958.9761 PEP_0_1_0_0_0
0
200000
400000
600000
800000
250
500
750
1000
1250
1500
1750
2000

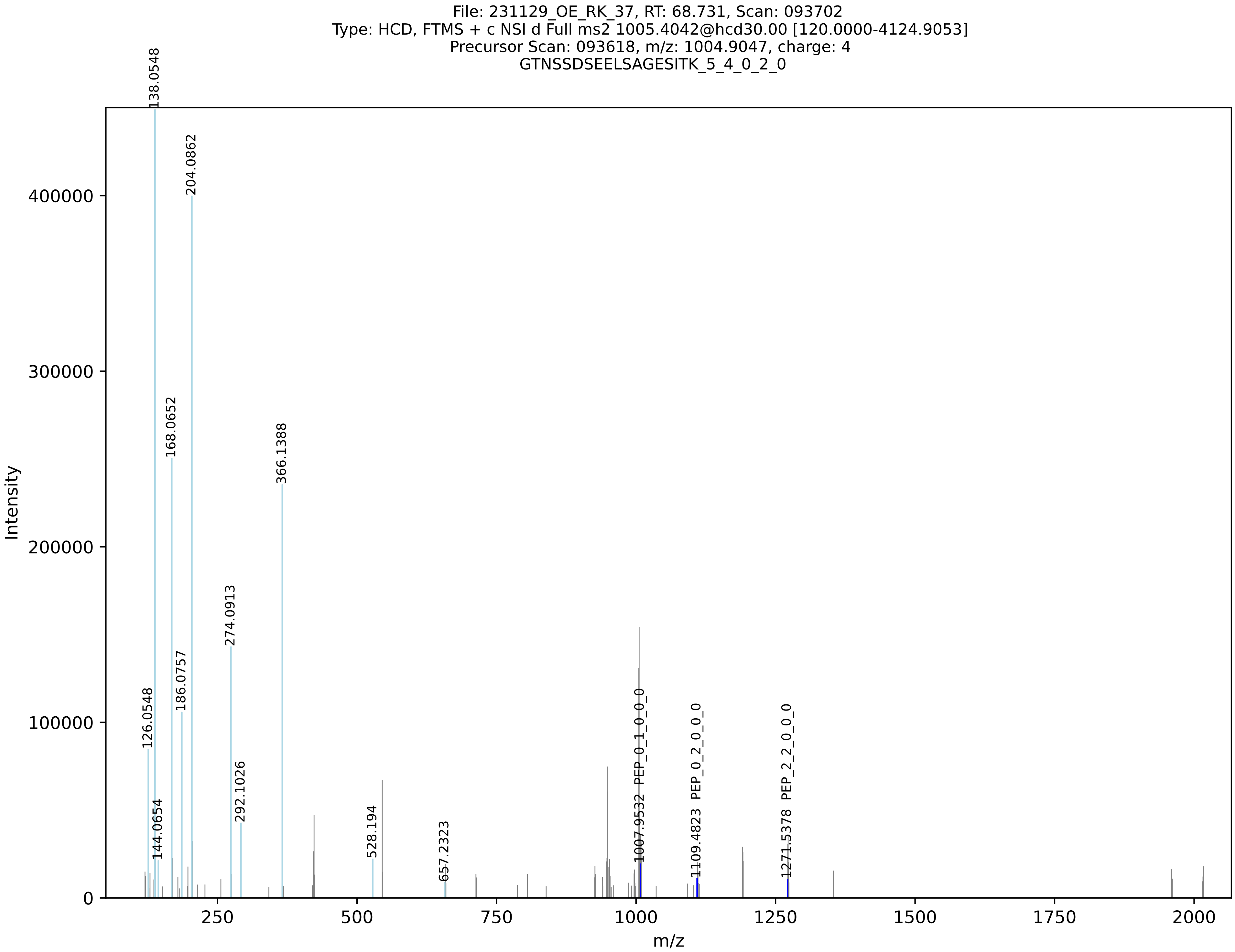
File: 231129_OE_RK_37, RT: 68.731, Scan: 093702
Type: HCD, FTMS + c NSI d Full ms2 1005.4042@hcd30.00 [120.0000-4124.9053]
Precursor Scan: 093618, m/z: 1004.9047, charge: 4
GTNSSDSEELSAGESITK_5_4_0_2_0
Intensity
m/z
138.0548
204.0862
168.0652
366.1388
274.0913
186.0757
126.0548
292.1026
144.0654
528.194
657.2323
1007.9532 PEP_0_1_0_0_0
1109.4823 PEP_0_2_0_0_0
1271.5378 PEP_2_2_0_0_0
0
100000
200000
300000
400000
250
500
750
1000
1250
1500
1750
2000

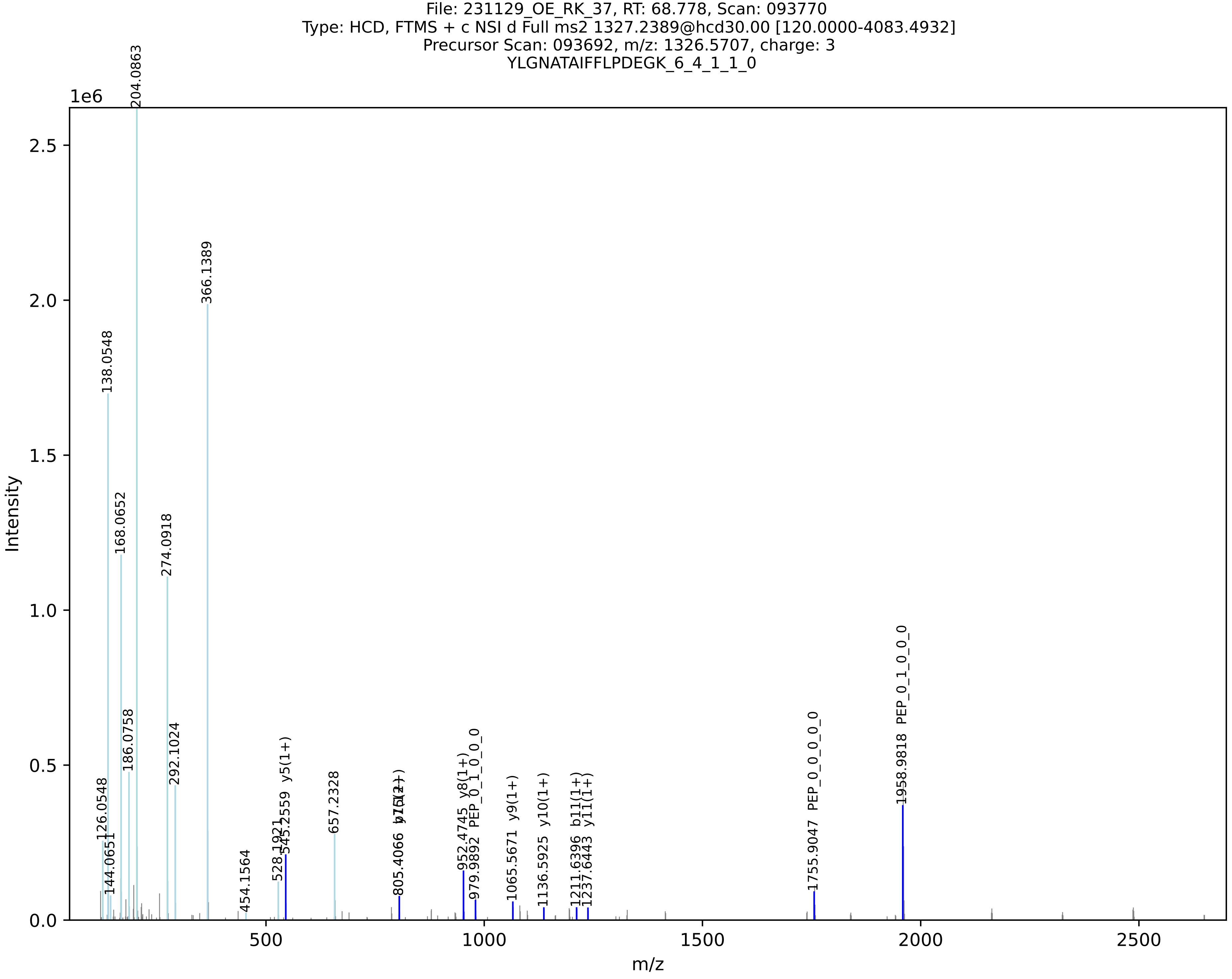

File: 231129_OE_RK_37, RT: 68.778, Scan: 093770
Type: HCD, FTMS + c NSI d Full ms2 1327.2389@hcd30.00 [120.0000-4083.4932]
Precursor Scan: 093692, m/z: 1326.5707, charge: 3
YLGNATAIFFLPDEGK_6_4_1_1_0
1e6
Intensity
m/z
0.0
0.5
1.0
1.5
2.0
2.5
500
1000
1500
2000
2500
204.0863
366.1389
138.0548
168.0652
274.0918
186.0758
292.1024
126.0548
144.0651
454.1564
528.1921
545.2559 y5(1+)
657.2328
805.4066
952.4745 y8(1+)
979.9892 PEP_0_1_0_0_0_
1065.5671 y9(1+)
1136.5925 y10(1+)
1211.6396 b11(1+)
1237.6443 y11(1+)
1755.9047 PEP_0_0_0_0_0_
1958.9818 PEP_0_1_0_0_0_

File: 231129_OE_RK_37, RT: 68.829, Scan: 093844
Type: HCD, FTMS + c NSI d Full ms2 991.1741@hcd30.00 [120.0000-4066.8462]
Precursor Scan: 093766, m/z: 990.9229, charge: 4
YLGNATAIFFLPDEGK_5_4_0_2_0

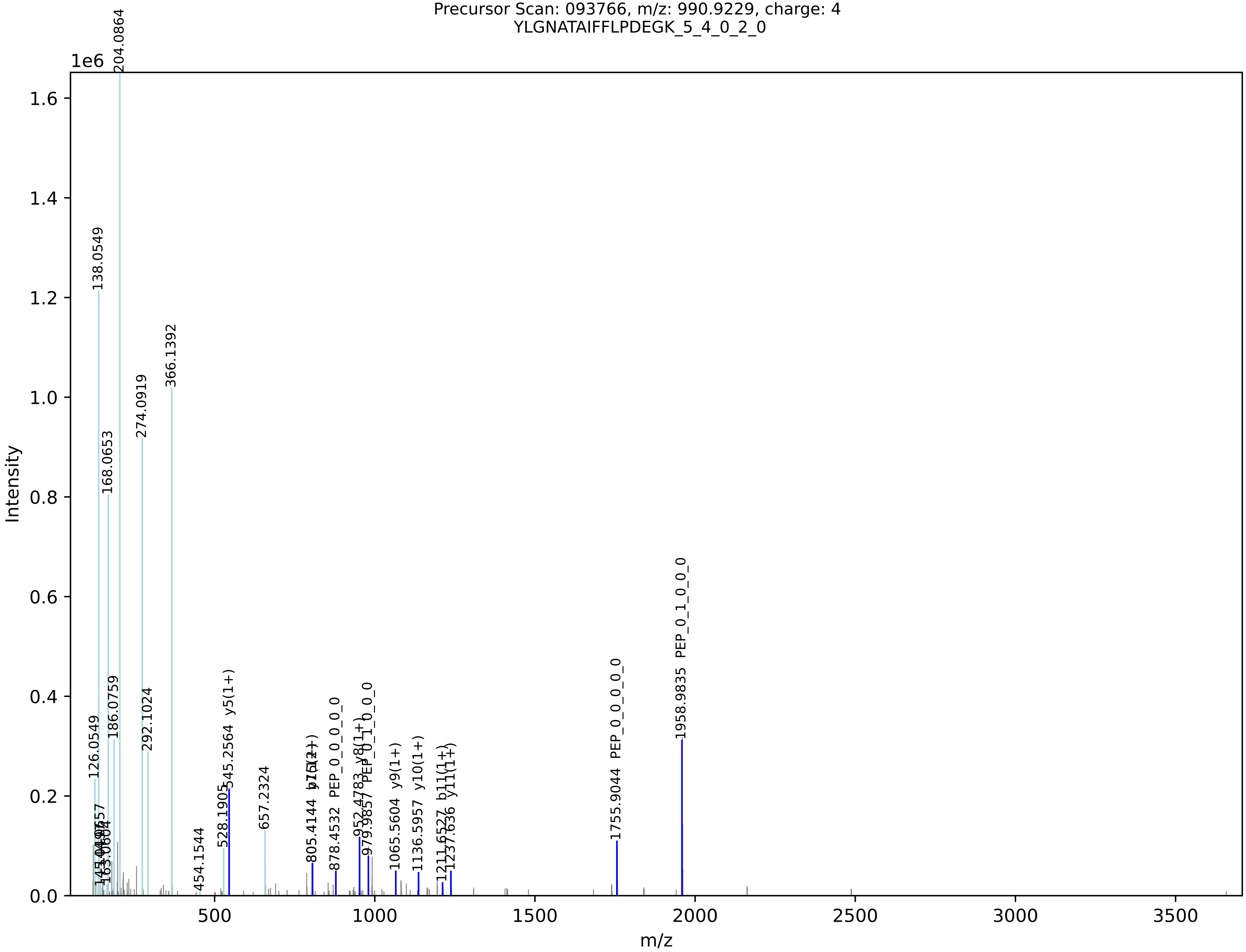

File: 231129_OE_RK_37, RT: 68.931, Scan: 093991
Type: HCD, FTMS + c NSI d Full ms2 1369.9139@hcd30.00 [120.0000-4214.0786]
Precursor Scan: 093913, m/z: 1369.5798, charge: 3
YLGNATAIFFLPDEGK_5_4_1_2_0

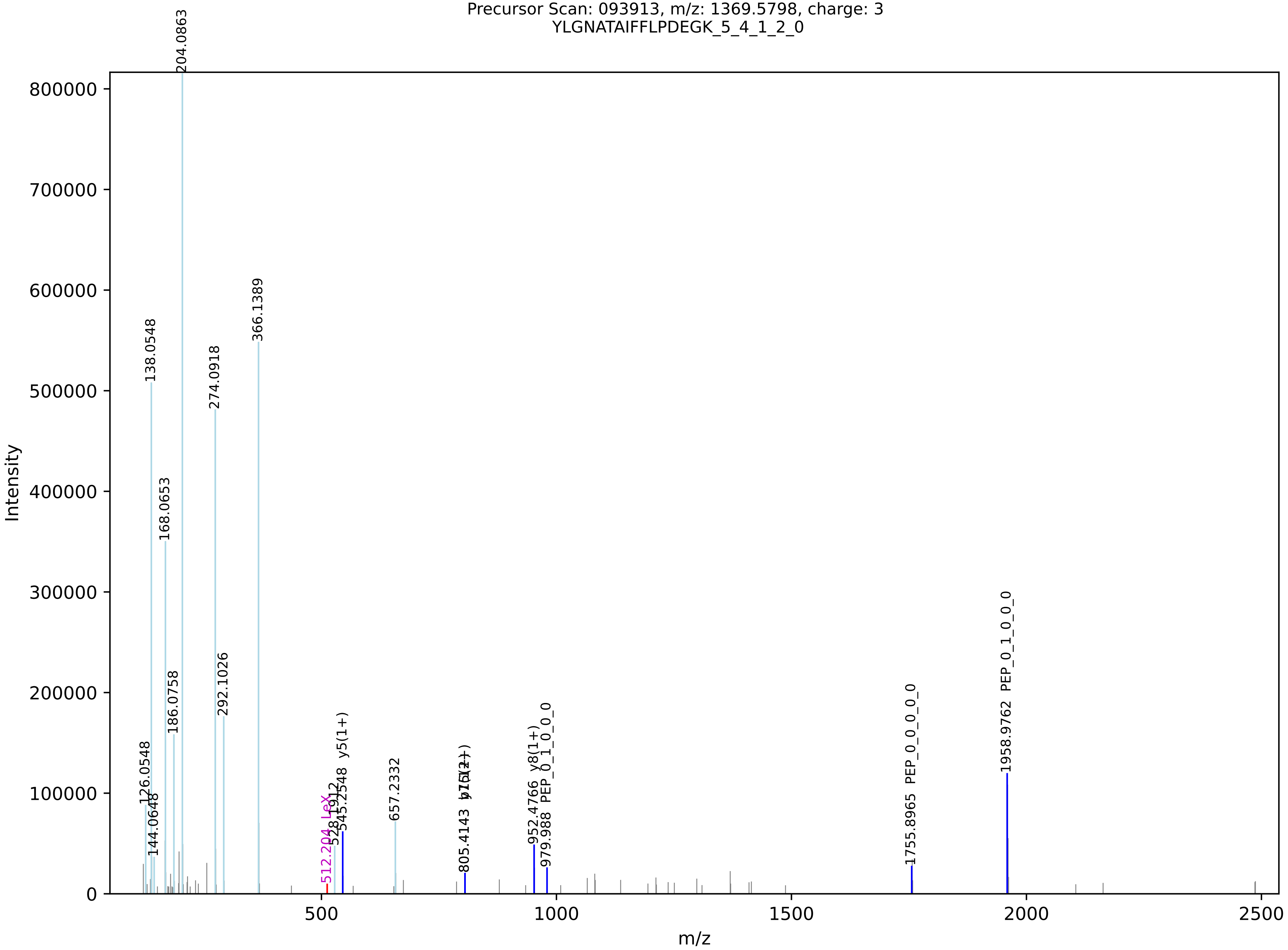

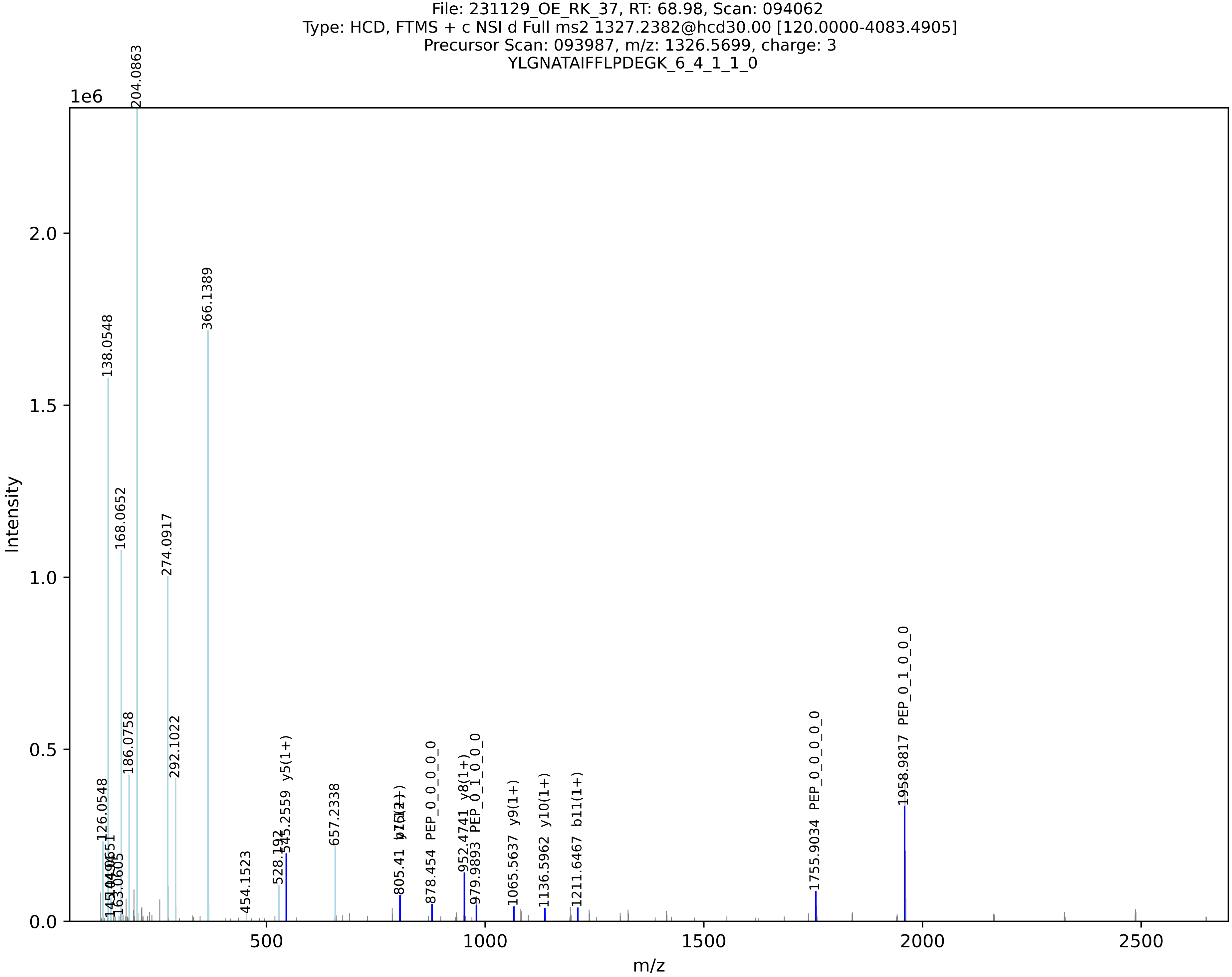

File: 231129_OE_RK_37, RT: 68.98, Scan: 094062
Type: HCD, FTMS + c NSI d Full ms2 1327.2382@hcd30.00 [120.0000-4083.4905]
Precursor Scan: 093987, m/z: 1326.5699, charge: 3
YLGNATAIFFLPDEGK_6_4_1_1_0
1e6
Intensity
m/z
204.0863
138.0548
366.1389
168.0652
274.0917
186.0758
292.1022
126.0548
545.2559 y5(1+)
657.2338
454.1523
528.192
805.41
878.454 PEP_0_0_0_0_0_
952.4741 y8(1+)
979.9893 PEP_0_1_0_0_0_
1065.5637 y9(1+)
1136.5962 y10(1+)
1211.6467 b11(1+)
1755.9034 PEP_0_0_0_0_0_
1958.9817 PEP_0_1_0_0_0_

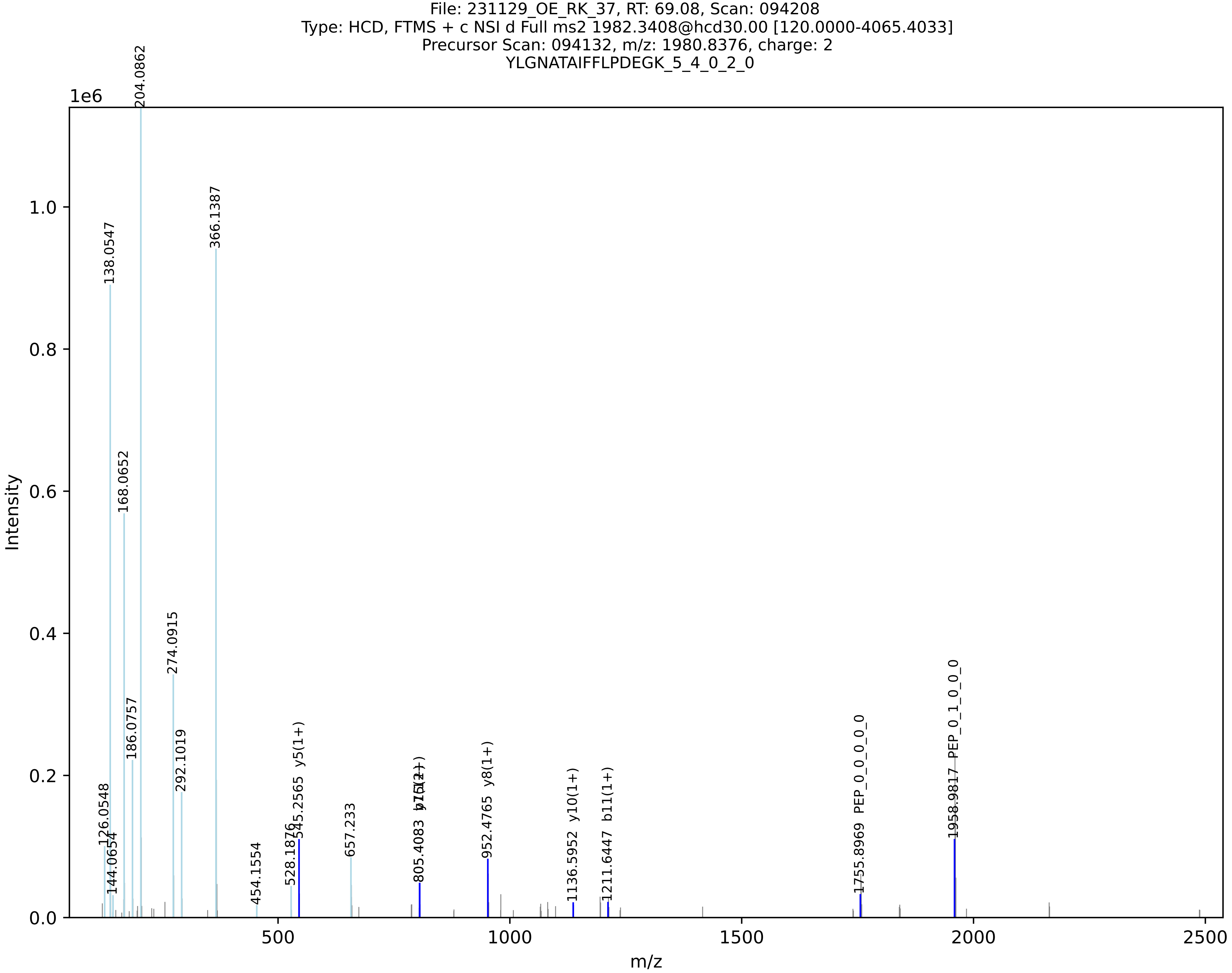
File: 231129_OE_RK_37, RT: 69.08, Scan: 094208
Type: HCD, FTMS + c NSI d Full ms2 1982.3408@hcd30.00 [120.0000-4065.4033]
Precursor Scan: 094132, m/z: 1980.8376, charge: 2
YLGNATAIFFLPDEGK_5_4_0_2_0
1e6
Intensity
m/z
204.0862
366.1387
138.0547
168.0652
274.0915
186.0757
292.1019
126.0548
144.0654
454.1554
528.1876
545.2565 y5(1+)
657.233
805.4083 b7(1+)
952.4765 y8(1+)
1136.5952 y10(1+)
1211.6447 b11(1+)
1755.8969 PEP_0_0_0_0_0
1958.9817 PEP_0_1_0_0_0
0.0
0.2
0.4
0.6
0.8
1.0
500
1000
1500
2000
2500

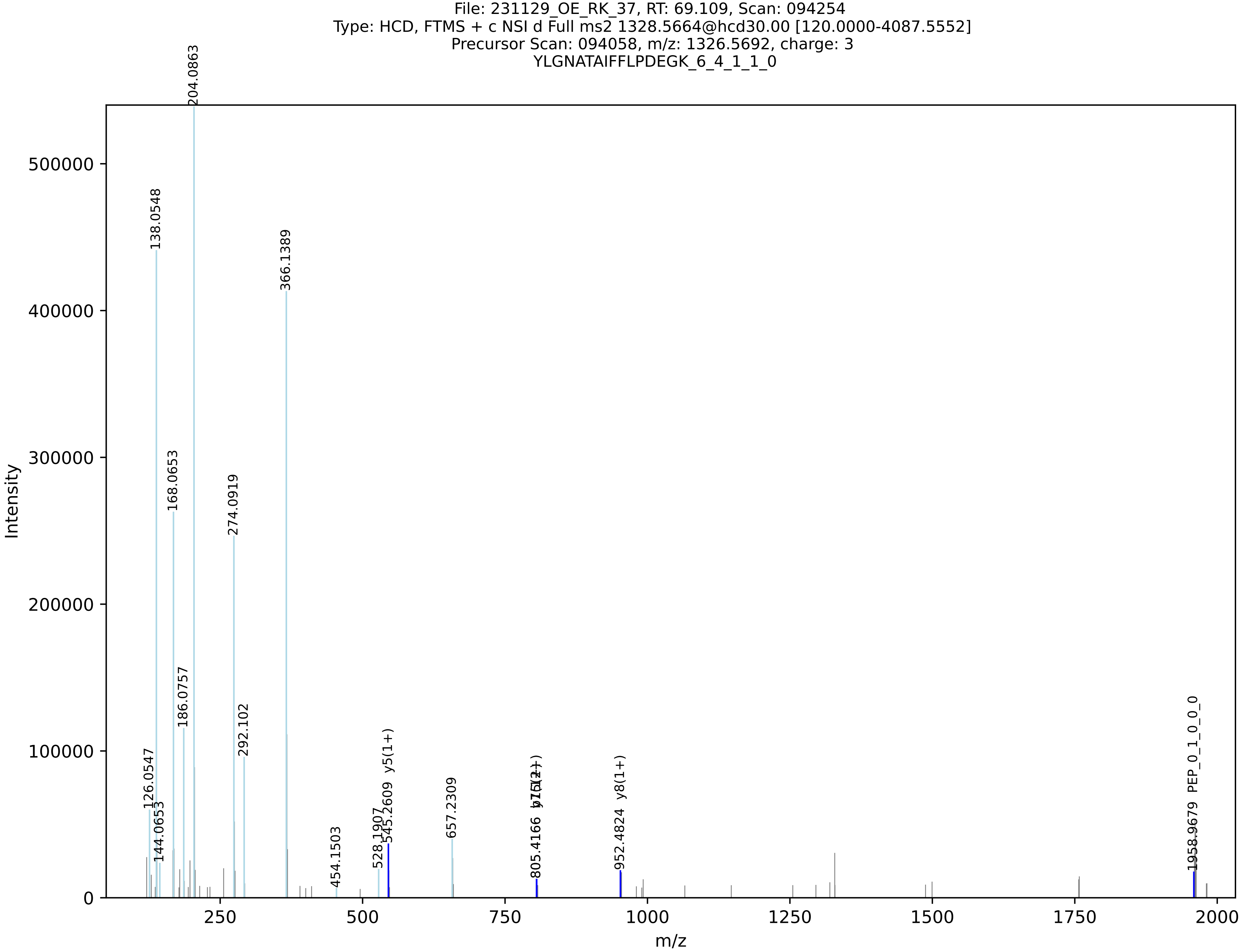
File: 231129_OE_RK_37, RT: 69.109, Scan: 094254
Type: HCD, FTMS + c NSI d Full ms2 1328.5664@hcd30.00 [120.0000-4087.5552]
Precursor Scan: 094058, m/z: 1326.5692, charge: 3
YLGNATAIFFLPDEGK_6_4_1_1_0
Intensity
m/z
204.0863
138.0548
366.1389
168.0653
274.0919
186.0757
292.102
126.0547
144.0653
454.1503
528.1907
545.2609 y5(1+)
657.2309
805.4166
952.4824 y8(1+)
1958.9679 PEP_0_1_0_0_0

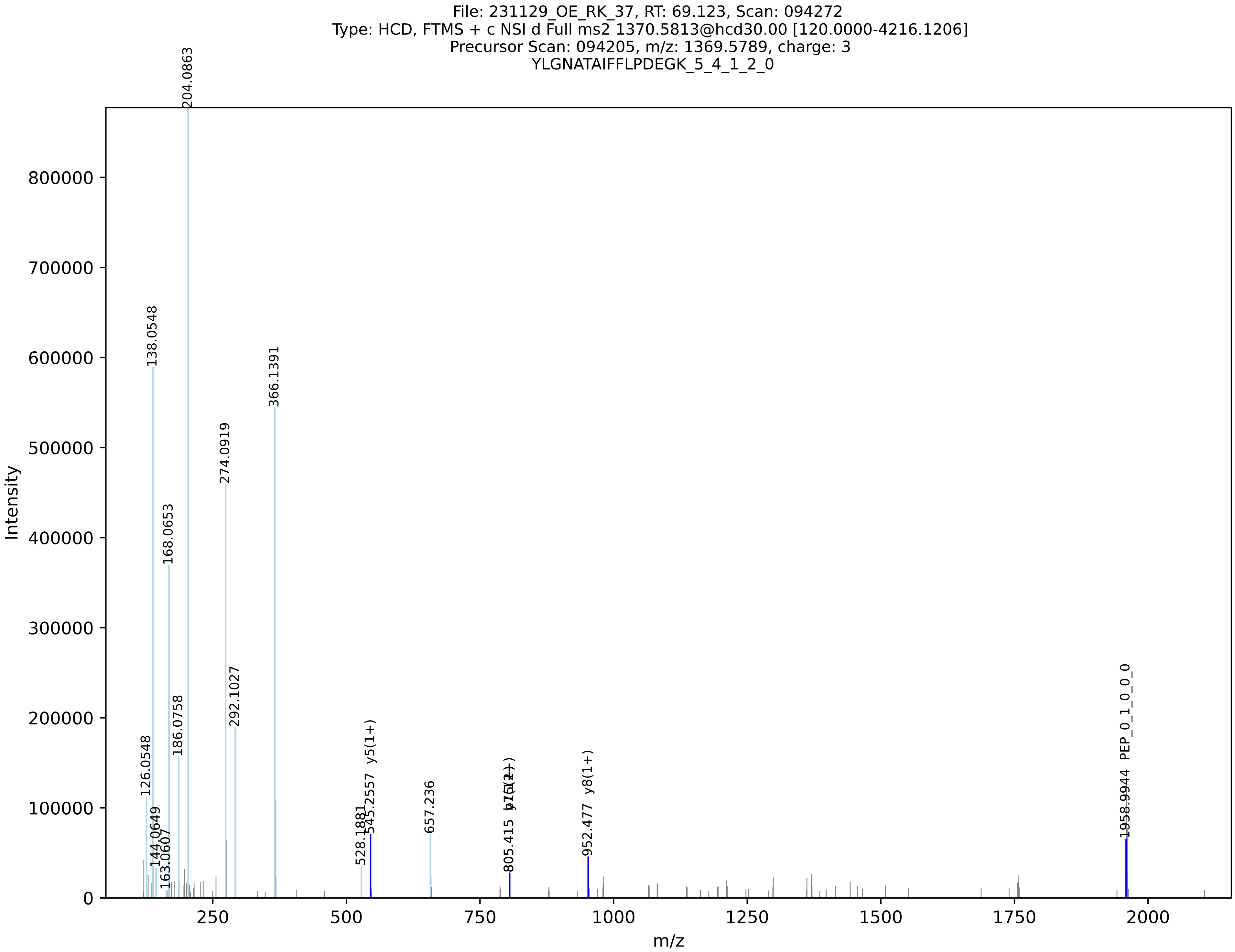
File: 231129_OE_RK_37, RT: 69.123, Scan: 094272
Type: HCD, FTMS + c NSI d Full ms2 1370.5813@hcd30.00 [120.0000-4216.1206]
Precursor Scan: 094205, m/z: 1369.5789, charge: 3
YLGNATAIFFLPDEGK_5_4_1_2_0
Intensity
m/z
204.0863
138.0548
366.1391
274.0919
168.0653
292.1027
186.0758
126.0548
144.0649
163.0607
528.1881
545.2557 y5(1+)
657.236
805.415
952.477 y8(1+)
1958.9944 PEP_0_1_0_0_0
0
100000
200000
300000
400000
500000
600000
700000
800000
250
500
750
1000
1250
1500
1750
2000

File: 231129_OE_RK_37, RT: 69.269, Scan: 094485
Type: HCD, FTMS + c NSI d Full ms2 1981.8435@hcd30.00 [120.0000-4064.3887]
Precursor Scan: 094406, m/z: 1980.837, charge: 2
YLGNATAIFFLPDEGK_5_4_0_2_0

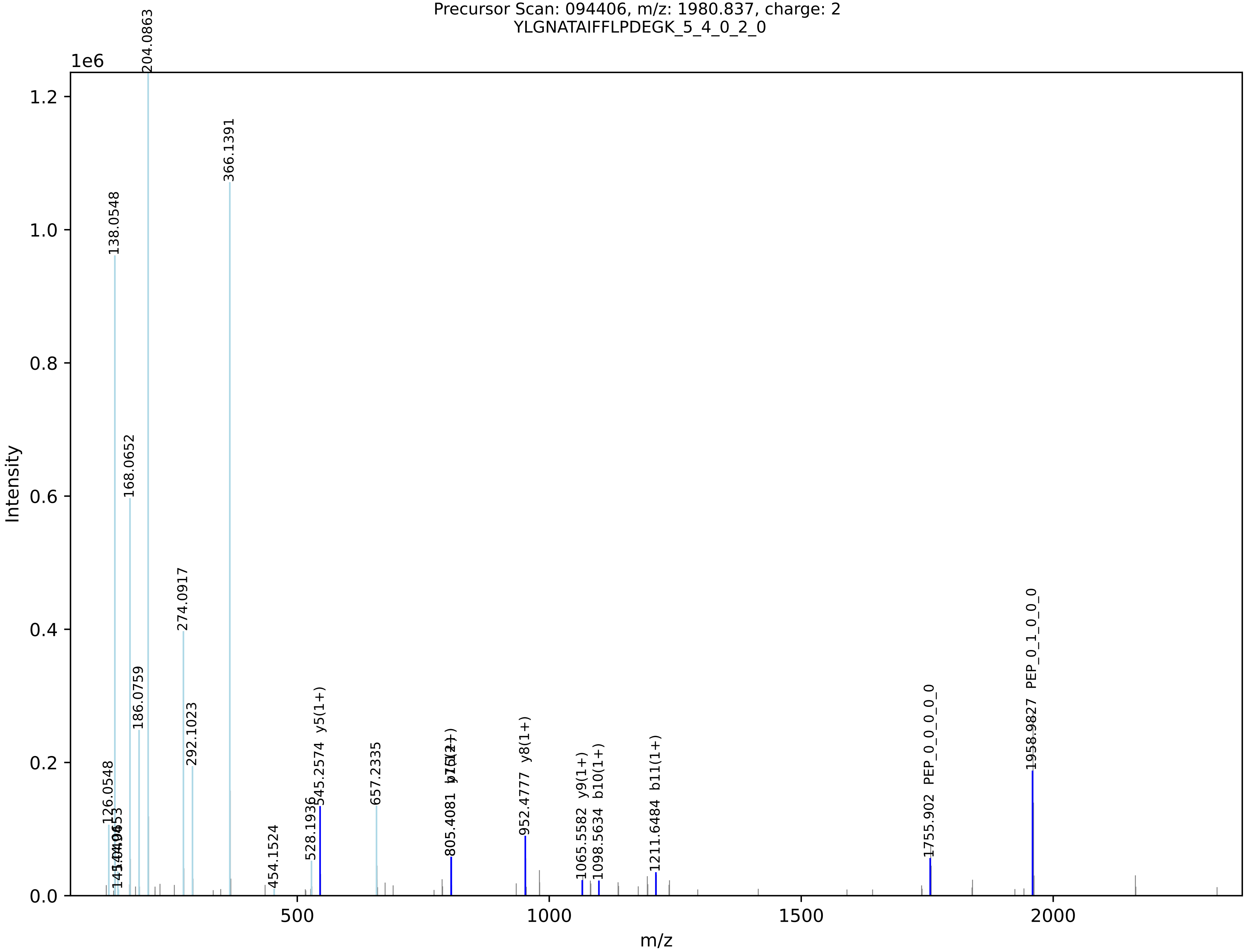

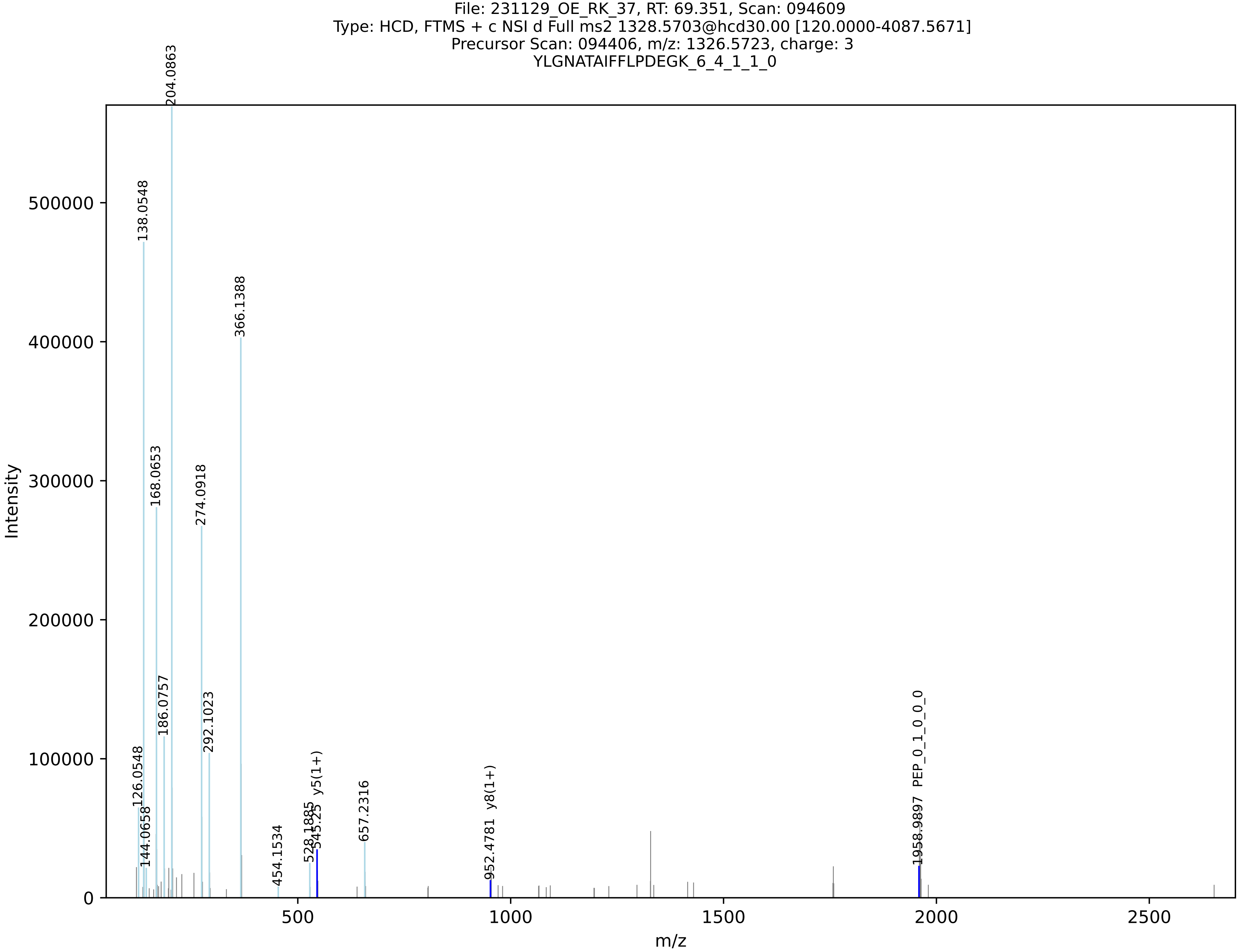
File: 231129_OE_RK_37, RT: 69.351, Scan: 094609
Type: HCD, FTMS + c NSI d Full ms2 1328.5703@hcd30.00 [120.0000-4087.5671]
Precursor Scan: 094406, m/z: 1326.5723, charge: 3
YLGNATAIFFLPDEGK_6_4_1_1_0
Intensity
m/z
204.0863
138.0548
366.1388
168.0653
274.0918
186.0757
292.1023
126.0548
144.0658
454.1534
528.1885
545.25 y5(1+)
657.2316
952.4781 y8(1+)
1958.9897 PEP_0_1_0_0_0_
0
100000
200000
300000
400000
500000
500
1000
1500
2000
2500

File: 231129_OE_RK_37, RT: 69.367, Scan: 094629
Type: HCD, FTMS + c NSI d Full ms2 1327.2369@hcd30.00 [120.0000-4083.4868]
Precursor Scan: 094551, m/z: 1326.5696, charge: 3
YLGNATAIFFLPDEGK_6_4_1_1_0

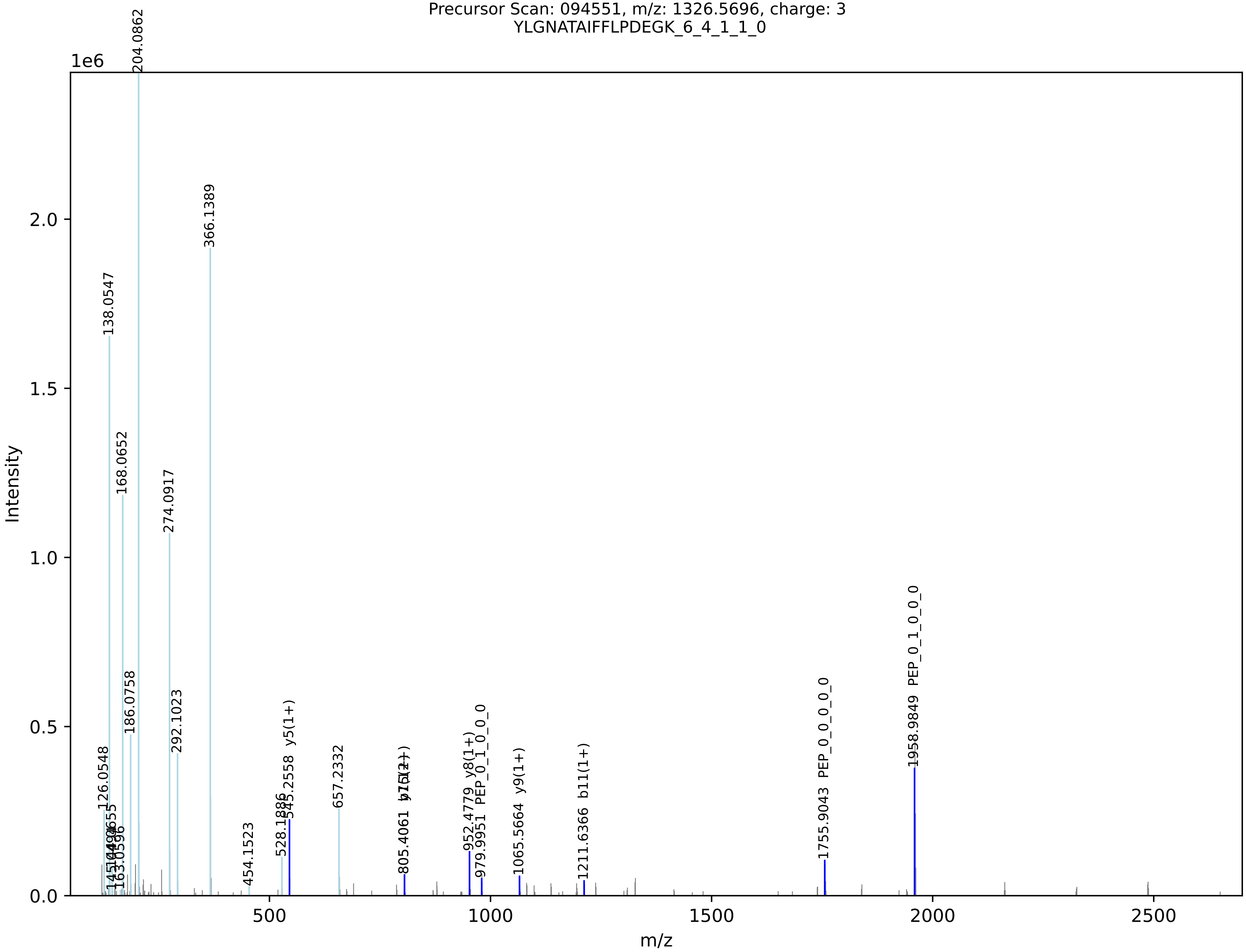

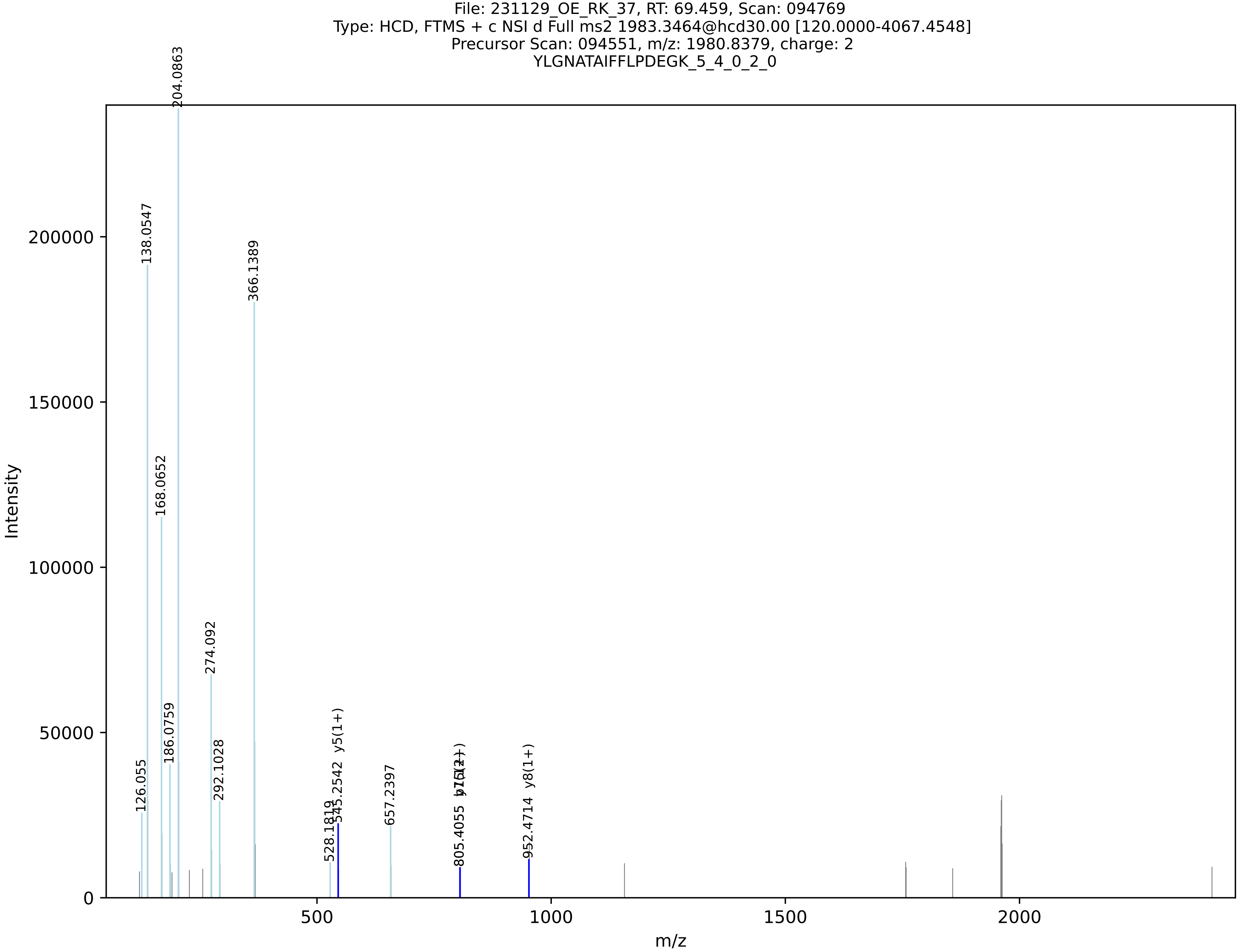
File: 231129_OE_RK_37, RT: 69.459, Scan: 094769
Type: HCD, FTMS + c NSI d Full ms2 1983.3464@hcd30.00 [120.0000-4067.4548]
Precursor Scan: 094551, m/z: 1980.8379, charge: 2
YLGNATAIFFLPDEGK_5_4_0_2_0
Intensity
m/z
0
50000
100000
150000
200000
500
1000
1500
2000
204.0863
138.0547
366.1389
168.0652
274.092
186.0759
126.055
292.1028
528.1819
545.2542 y5(1+)
657.2397
805.4055
952.4714 y8(1+)

File: 231129_OE_RK_37, RT: 69.466, Scan: 094773
Type: HCD, FTMS + c NSI d Full ms2 1130.7910@hcd30.00 [120.0000-4636.4834]
Precursor Scan: 094698, m/z: 1130.2898, charge: 4
VGQLQLSHNLSLVILVPQNLK_5_4_0_2_0

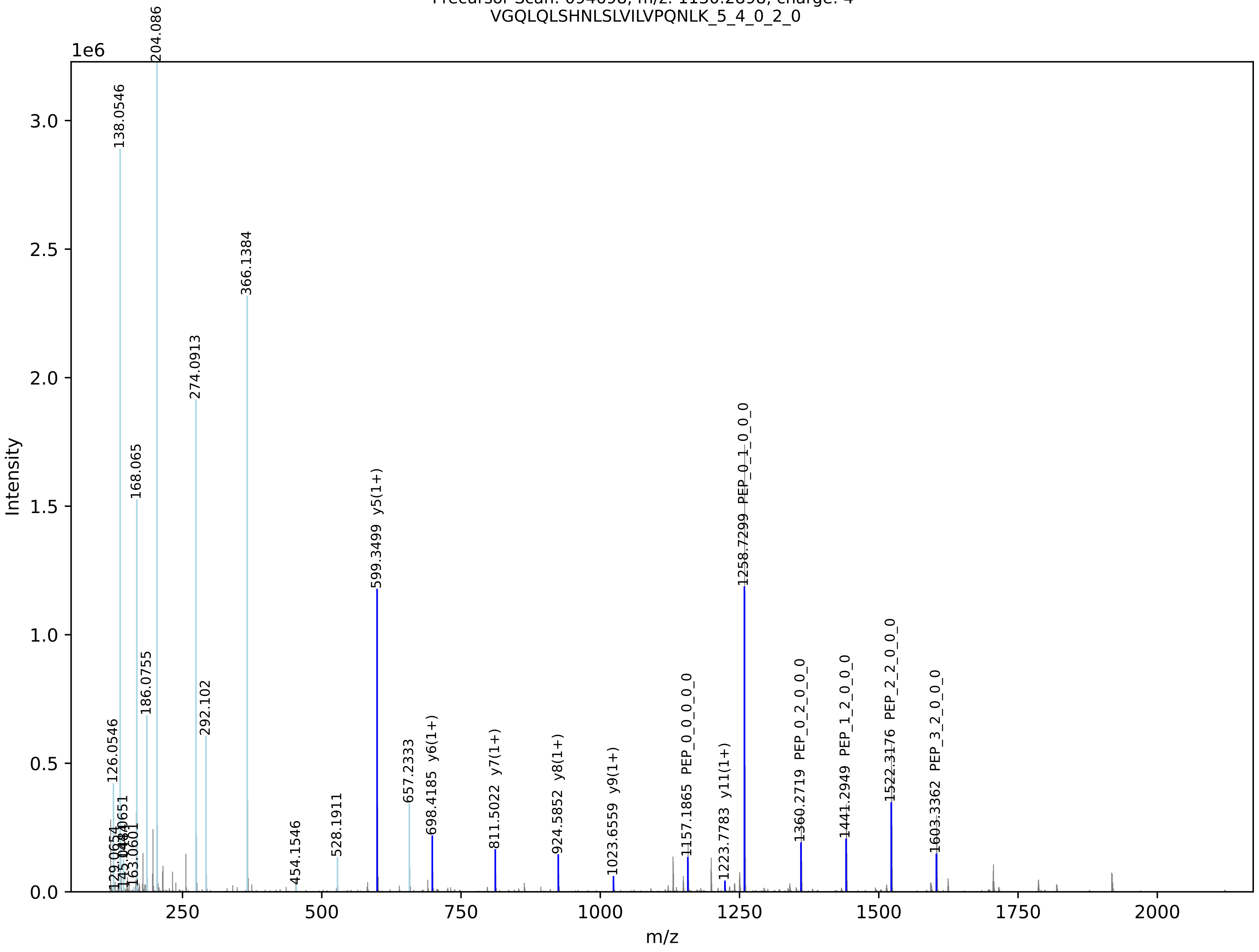

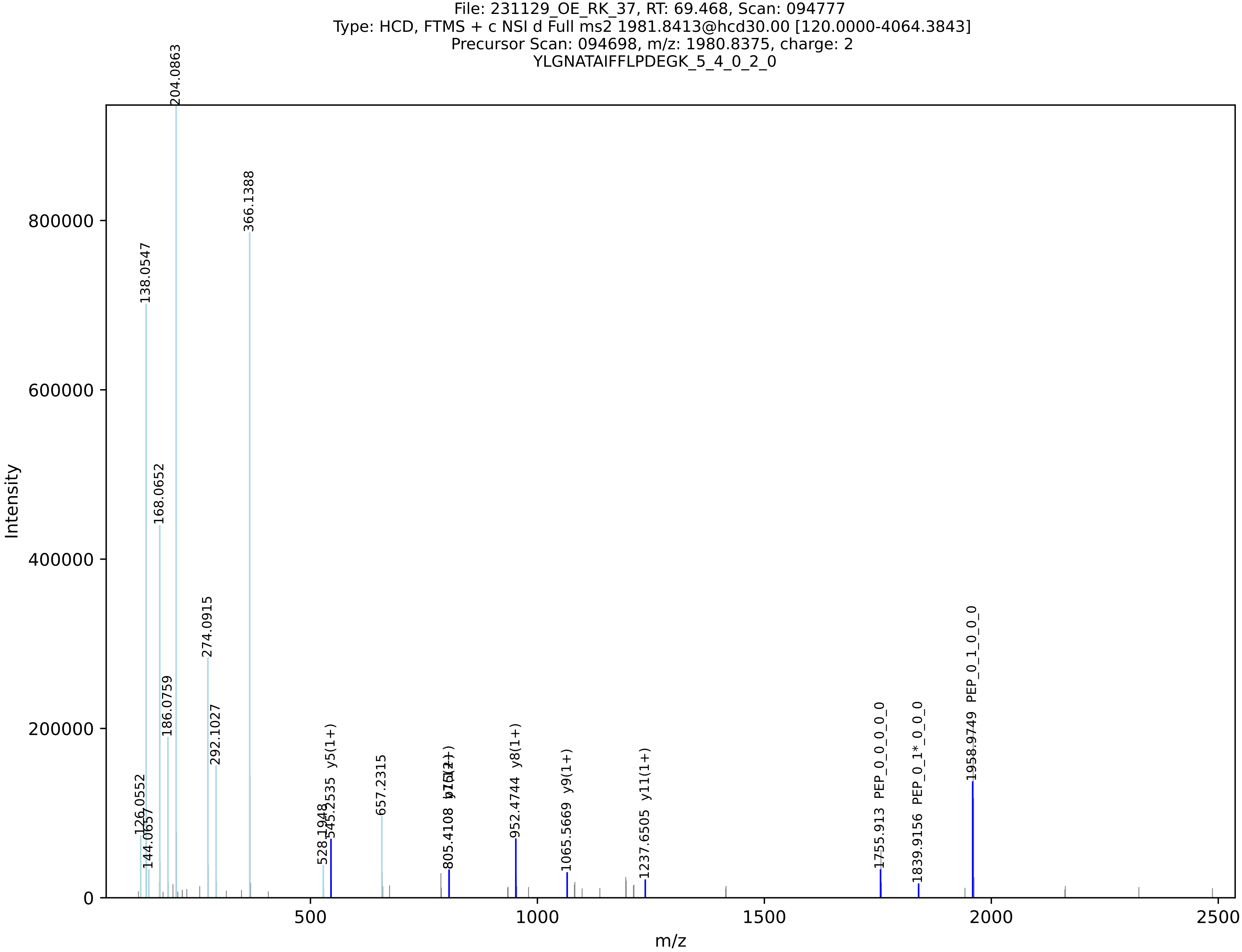
File: 231129_OE_RK_37, RT: 69.468, Scan: 094777
Type: HCD, FTMS + c NSI d Full ms2 1981.8413@hcd30.00 [120.0000-4064.3843]
Precursor Scan: 094698, m/z: 1980.8375, charge: 2
YLGNATAIFFLPDEGK_5_4_0_2_0
Intensity
m/z
204.0863
366.1388
138.0547
168.0652
274.0915
186.0759
292.1027
126.0552
144.0657
528.1948
545.2535 y5(1+)
657.2315
805.4108
952.4744 y8(1+)
1065.5669 y9(1+)
1237.6505 y11(1+)
1755.913 PEP_0_0_0_0_0
1839.9156 PEP_0_1*_0_0_0
1958.9749 PEP_0_1_0_0_0
0
200000
400000
600000
800000
500
1000
1500
2000
2500

File: 231129_OE_RK_37, RT: 69.503, Scan: 094833
Type: HCD, FTMS + c NSI d Full ms2 1982.8385@hcd30.00 [120.0000-4066.4185]
Precursor Scan: 094624, m/z: 1980.8373, charge: 2
YLGNATAIFFLPDEGK_5_4_0_2_0

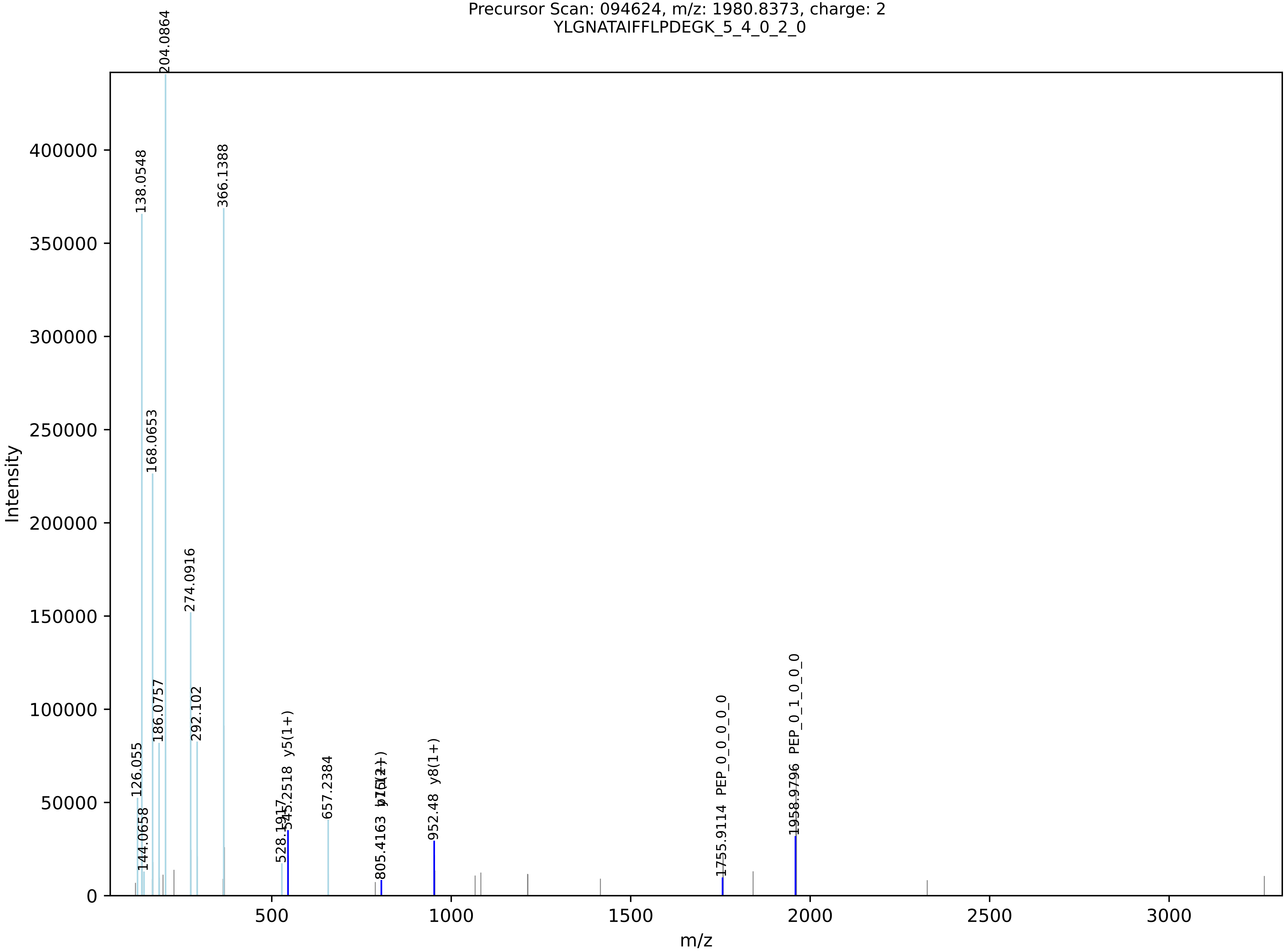

File: 231129_OE_RK_37, RT: 69.532, Scan: 094870
Type: HCD, FTMS + c NSI d Full ms2 1257.1705@hcd30.00 [120.0000-3869.0837]
Precursor Scan: 094772, m/z: 1257.1705, charge: 3
AYATITNNCTK_6_4_1_2_0

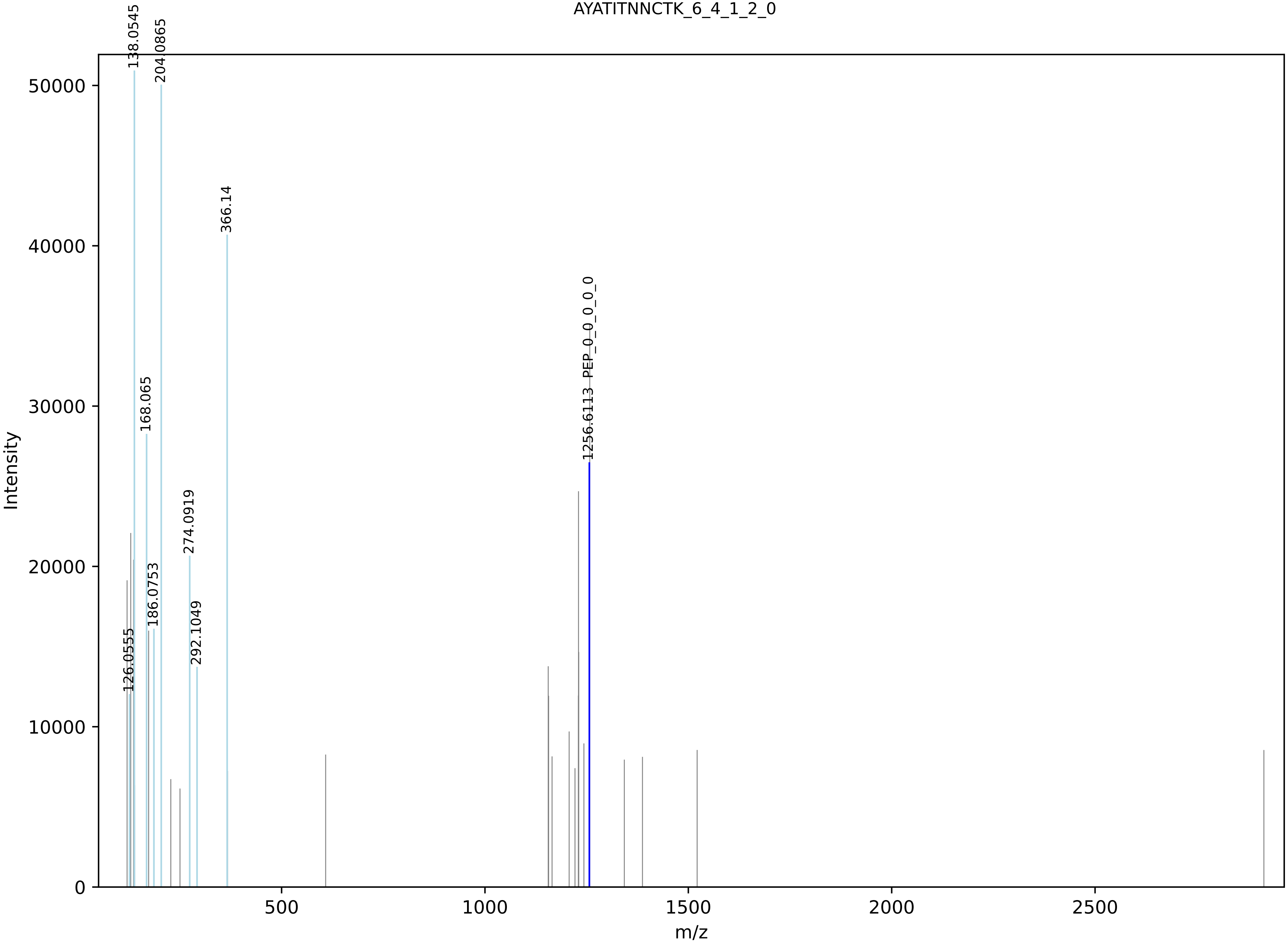

File: 231129_OE_RK_37, RT: 69.57, Scan: 094924
Type: HCD, FTMS + c NSI d Full ms2 1327.2384@hcd30.00 [120.0000-4083.4912]
Precursor Scan: 094846, m/z: 1326.5701, charge: 3
YLGNATAIFFLPDEGK_6_4_1_1_0

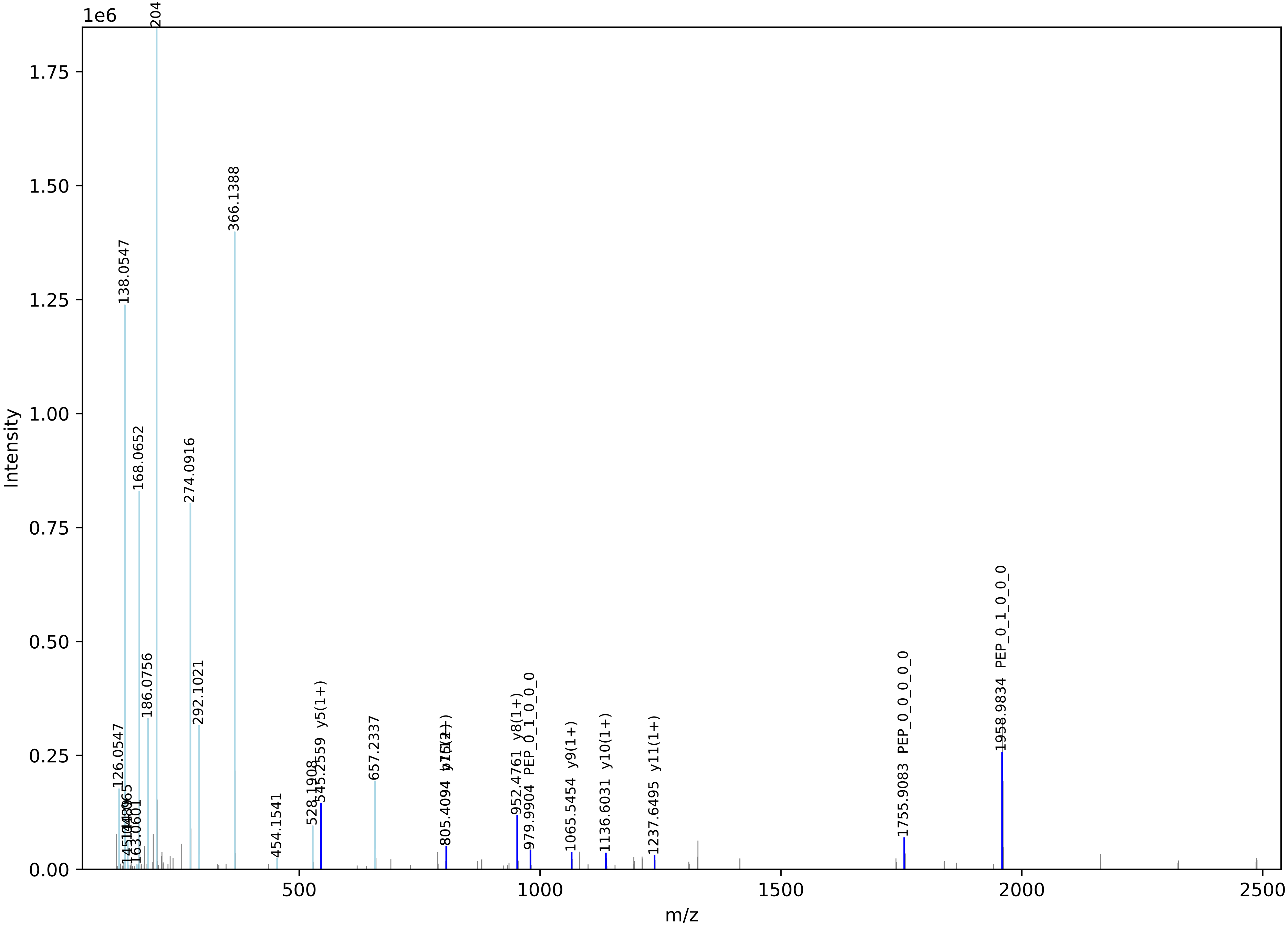

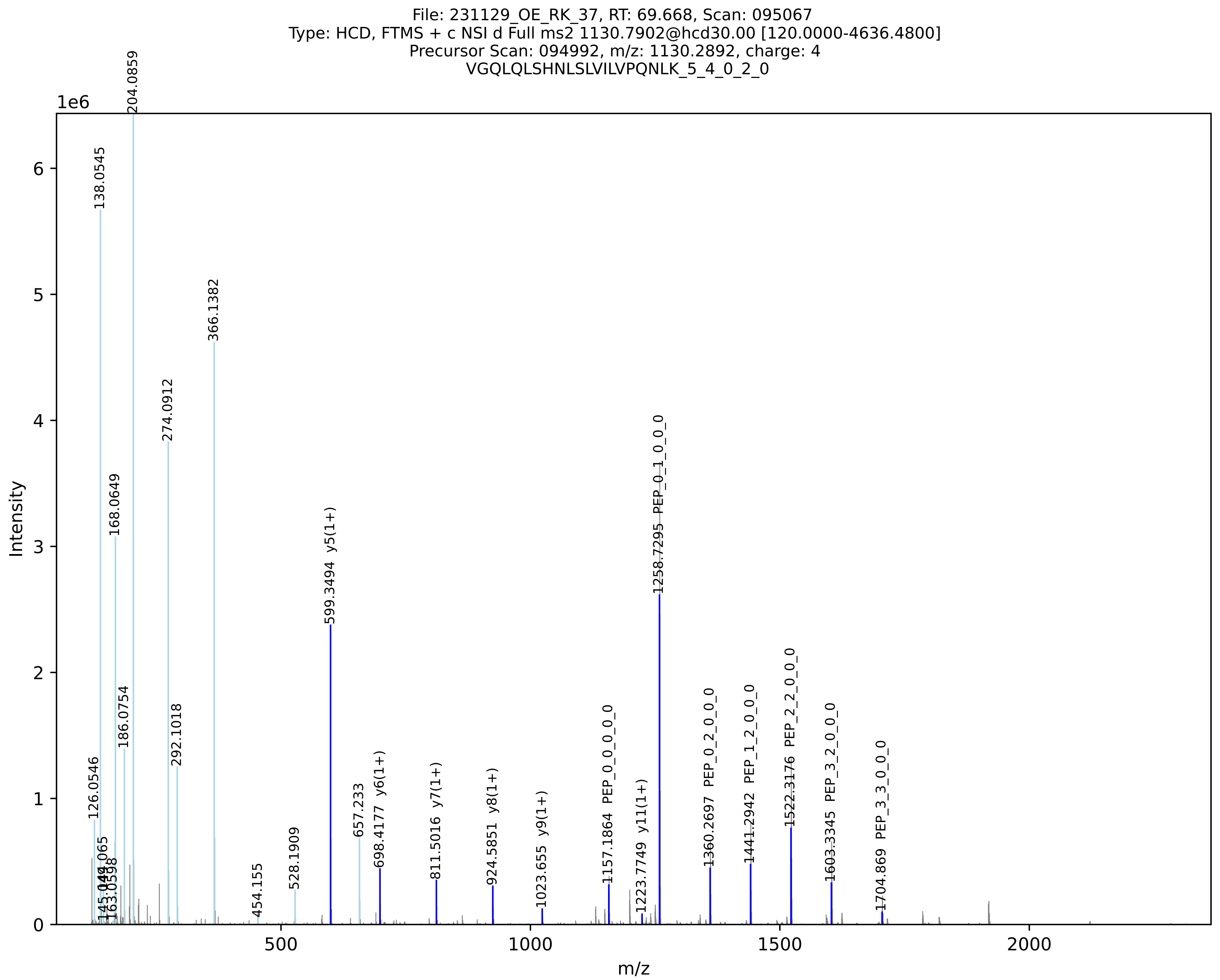

File: 231129_OE_RK_37, RT: 69.668, Scan: 095067
Type: HCD, FTMS + c NSI d Full ms2 1130.7902@hcd30.00 [120.0000-4636.4800]
Precursor Scan: 094992, m/z: 1130.2892, charge: 4
VGQLQLSHNLSLVILVPQNLK_5_4_0_2_0
1e6
Intensity
m/z
204.0859
138.0545
366.1382
274.0912
168.0649
186.0754
292.1018
126.0546
599.3494 y5(1+)
657.233
698.4177 y6(1+)
811.5016 y7(1+)
924.5851 y8(1+)
1023.655 y9(1+)
1157.1864 PEP_0_0_0_0_
1223.7749 y11(1+)
1258.7295 PEP_0_1_0_0_
1360.2697 PEP_0_2_0_0_
1441.2942 PEP_1_2_0_0_
1522.3176 PEP_2_2_0_0_
1603.3345 PEP_3_2_0_0_
1704.869 PEP_3_3_0_0_
454.155
528.1909

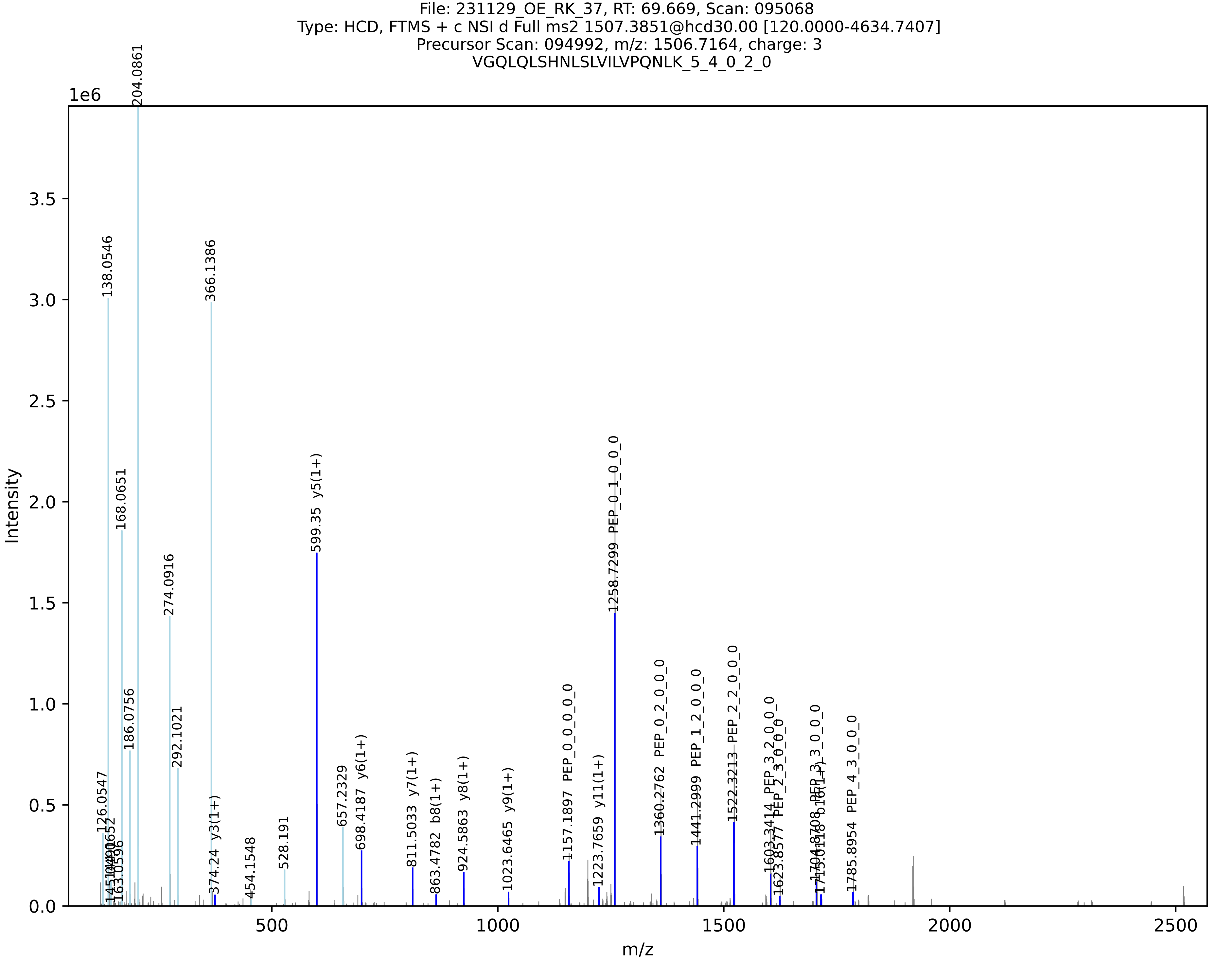

File: 231129_OE_RK_37, RT: 69.669, Scan: 095068
Type: HCD, FTMS + c NSI d Full ms2 1507.3851@hcd30.00 [120.0000-4634.7407]
Precursor Scan: 094992, m/z: 1506.7164, charge: 3
VGQLQLSHNLSLVILVPQNLK_5_4_0_2_0
1e6
Intensity
m/z
204.0861
138.0546
366.1386
168.0651
274.0916
186.0756
292.1021
126.0547
163.0596
374.24 y3(1+)
454.1548
528.191
599.35 y5(1+)
657.2329
698.4187 y6(1+)
811.5033 y7(1+)
863.4782 b8(1+)
924.5863 y8(1+)
1023.6465 y9(1+)
1157.1897 PEP_0_0_0_0_0
1223.7659 y11(1+)
1258.7299 PEP_0_1_0_0_0
1360.2762 PEP_0_2_0_0_0
1441.2999 PEP_1_2_0_0_0
1522.3213 PEP_2_2_0_0_0
1603.3414 PEP_3_2_0_0_0
1623.8577 PEP_2_3_0_0_0
1704.8708 PEP_3_3_0_0_0
1785.8954 PEP_4_3_0_0_0

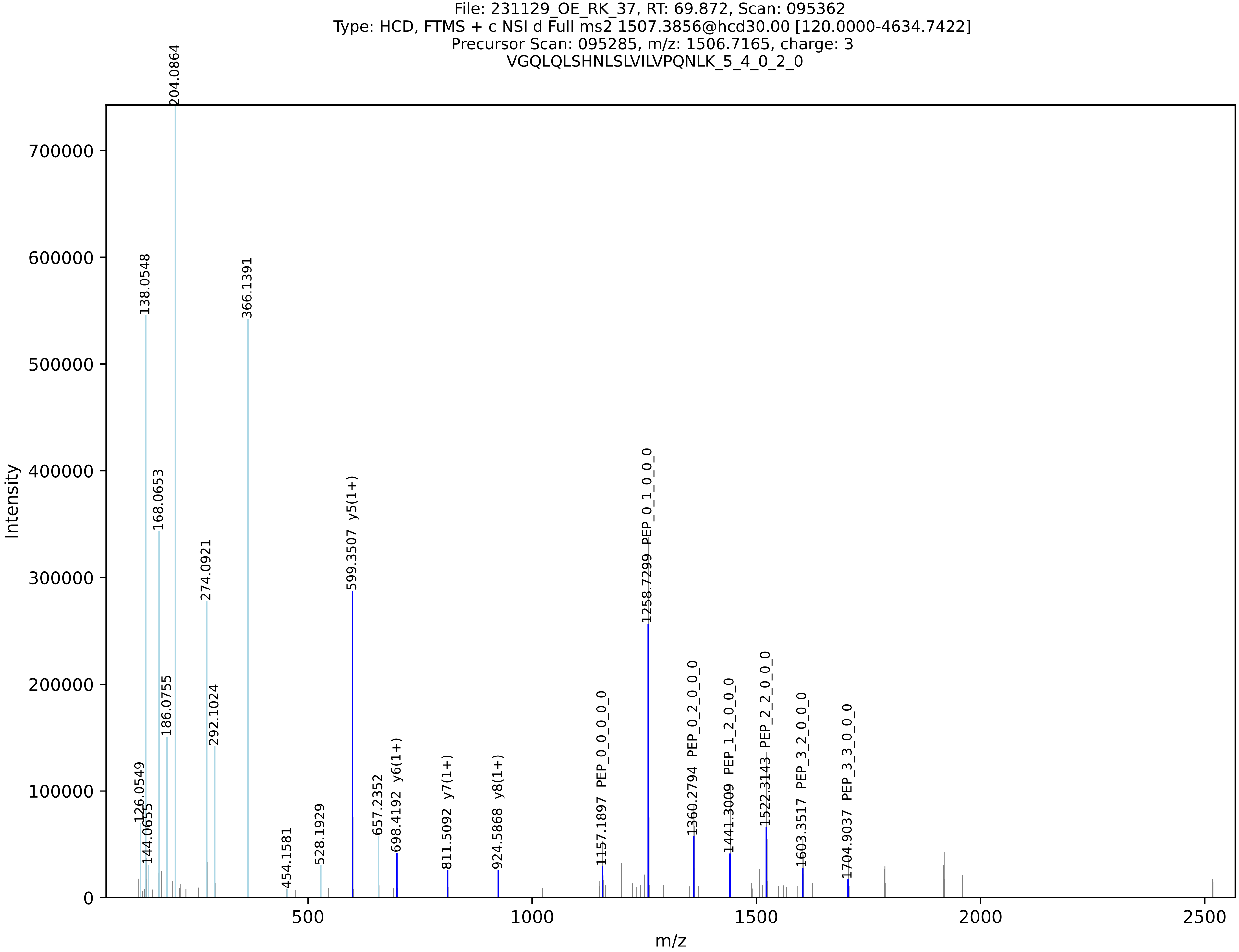
File: 231129_OE_RK_37, RT: 69.872, Scan: 095362
Type: HCD, FTMS + c NSI d Full ms2 1507.3856@hcd30.00 [120.0000-4634.7422]
Precursor Scan: 095285, m/z: 1506.7165, charge: 3
VGQLQLSHNLSLVILVPQNLK_5_4_0_2_0
Intensity
m/z
204.0864
138.0548
366.1391
168.0653
274.0921
186.0755
292.1024
126.0549
144.0655
454.1581
528.1929
599.3507 y5(1+)
657.2352
698.4192 y6(1+)
811.5092 y7(1+)
924.5868 y8(1+)
1157.1897 PEP_0_0_0_0_0
1258.7299 PEP_0_1_0_0_0
1360.2794 PEP_0_2_0_0_0
1441.3009 PEP_1_2_0_0_0
1522.3143 PEP_2_2_0_0_0
1603.3517 PEP_3_2_0_0_0
1704.9037 PEP_3_3_0_0_0

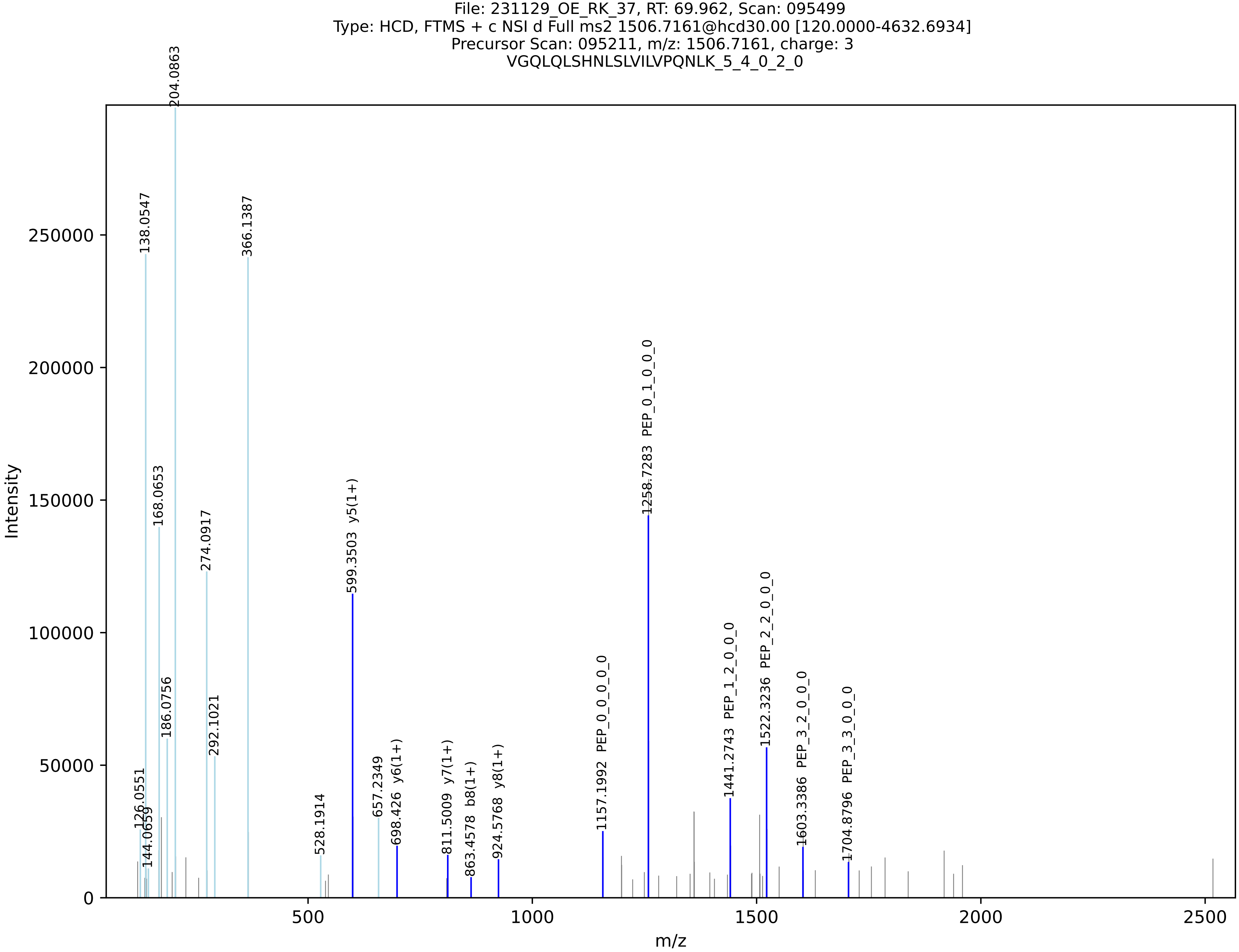
File: 231129_OE_RK_37, RT: 69.962, Scan: 095499
Type: HCD, FTMS + c NSI d Full ms2 1506.7161@hcd30.00 [120.0000-4632.6934]
Precursor Scan: 095211, m/z: 1506.7161, charge: 3
VGQLQLSHNLSLVILVPQNLK_5_4_0_2_0
Intensity
m/z
204.0863
138.0547
366.1387
168.0653
274.0917
186.0756
292.1021
126.0551
144.0659
528.1914
599.3503 y5(1+)
657.2349
698.426 y6(1+)
811.5009 y7(1+)
863.4578 b8(1+)
924.5768 y8(1+)
1157.1992 PEP_0_0_0_0_0
1258.7283 PEP_0_1_0_0_0
1441.2743 PEP_1_2_0_0_0
1522.3236 PEP_2_2_0_0_0
1603.3386 PEP_3_2_0_0_0
1704.8796 PEP_3_3_0_0_0
0
50000
100000
150000
200000
250000
500
1000
1500
2000
2500

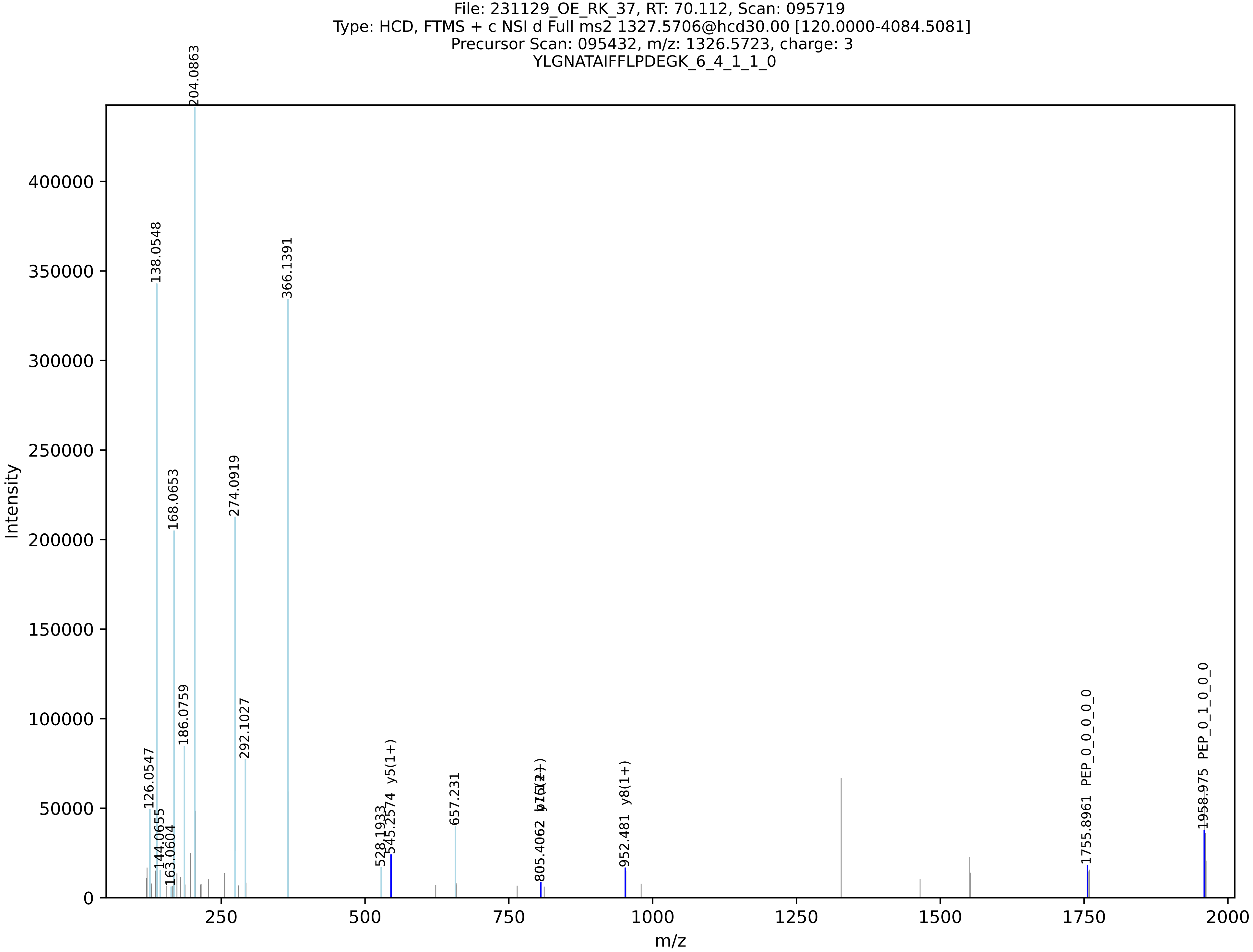

File: 231129_OE_RK_37, RT: 70.112, Scan: 095719
Type: HCD, FTMS + c NSI d Full ms2 1327.5706@hcd30.00 [120.0000-4084.5081]
Precursor Scan: 095432, m/z: 1326.5723, charge: 3
YLGNATAIFFLPDEGK_6_4_1_1_0
Intensity
m/z
204.0863
138.0548
366.1391
274.0919
168.0653
186.0759
292.1027
126.0547
144.0655
163.0604
528.1933
545.2574 y5(1+)
657.231
805.4062
952.481 y8(1+)
1755.8961 PEP_0_0_0_0_0
1958.975 PEP_0_1_0_0_0

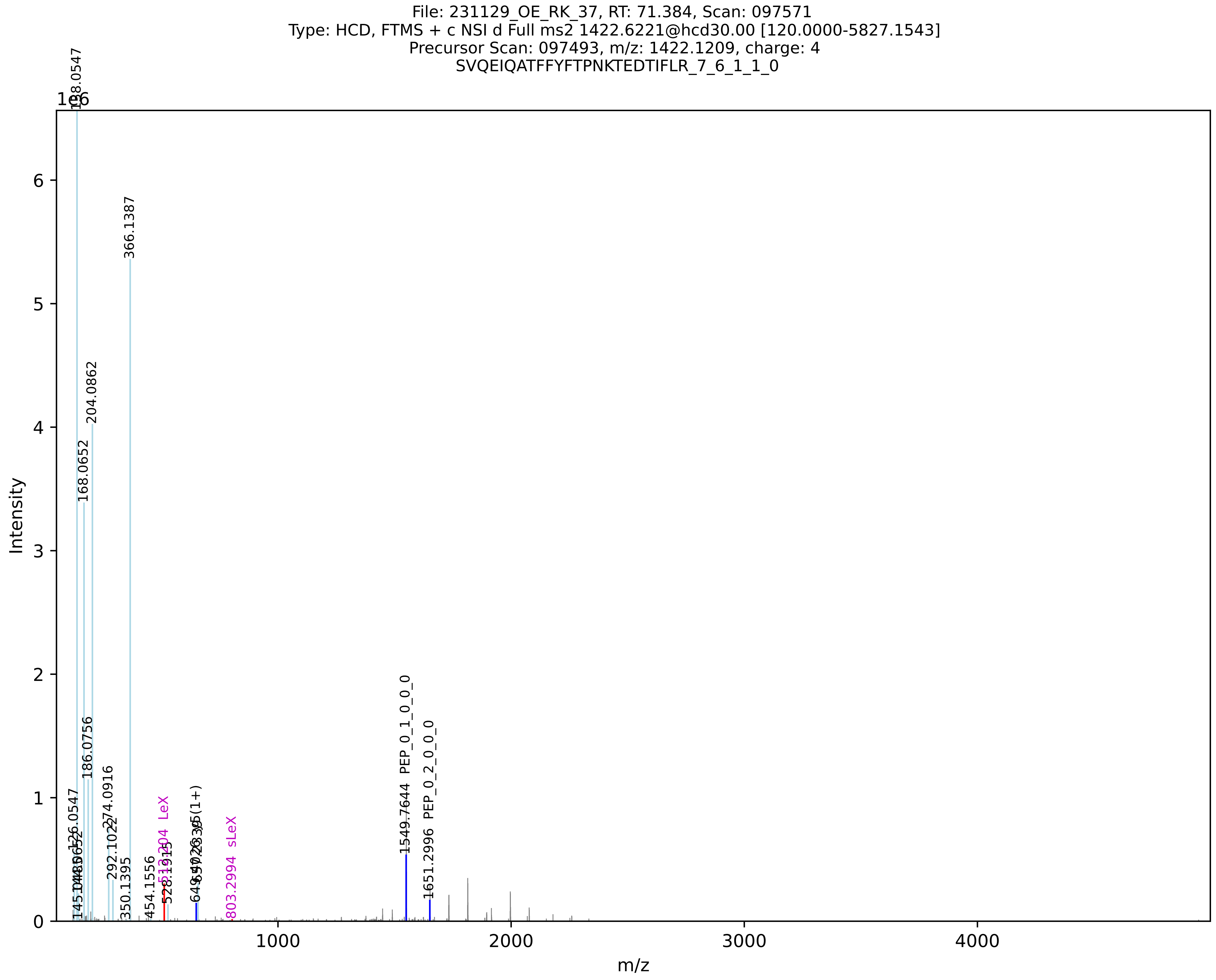
File: 231129_OE_RK_37, RT: 71.384, Scan: 097571
Type: HCD, FTMS + c NSI d Full ms2 1422.6221@hcd30.00 [120.0000-5827.1543]
Precursor Scan: 097493, m/z: 1422.1209, charge: 4
SVQEIQATFFYFTPNKTEDTIFLR_7_6_1_1_0
1e6
Intensity
m/z
366.1387
204.0862
168.0652
186.0756
274.0916
292.1022
350.1395
454.1556
528.1915
LeX
sLeX
803.2994
1549.7644 PEP_0_1_0_0_0
1651.2996 PEP_0_2_0_0_0
0
1
2
3
4
5
6
1000
2000
3000
4000

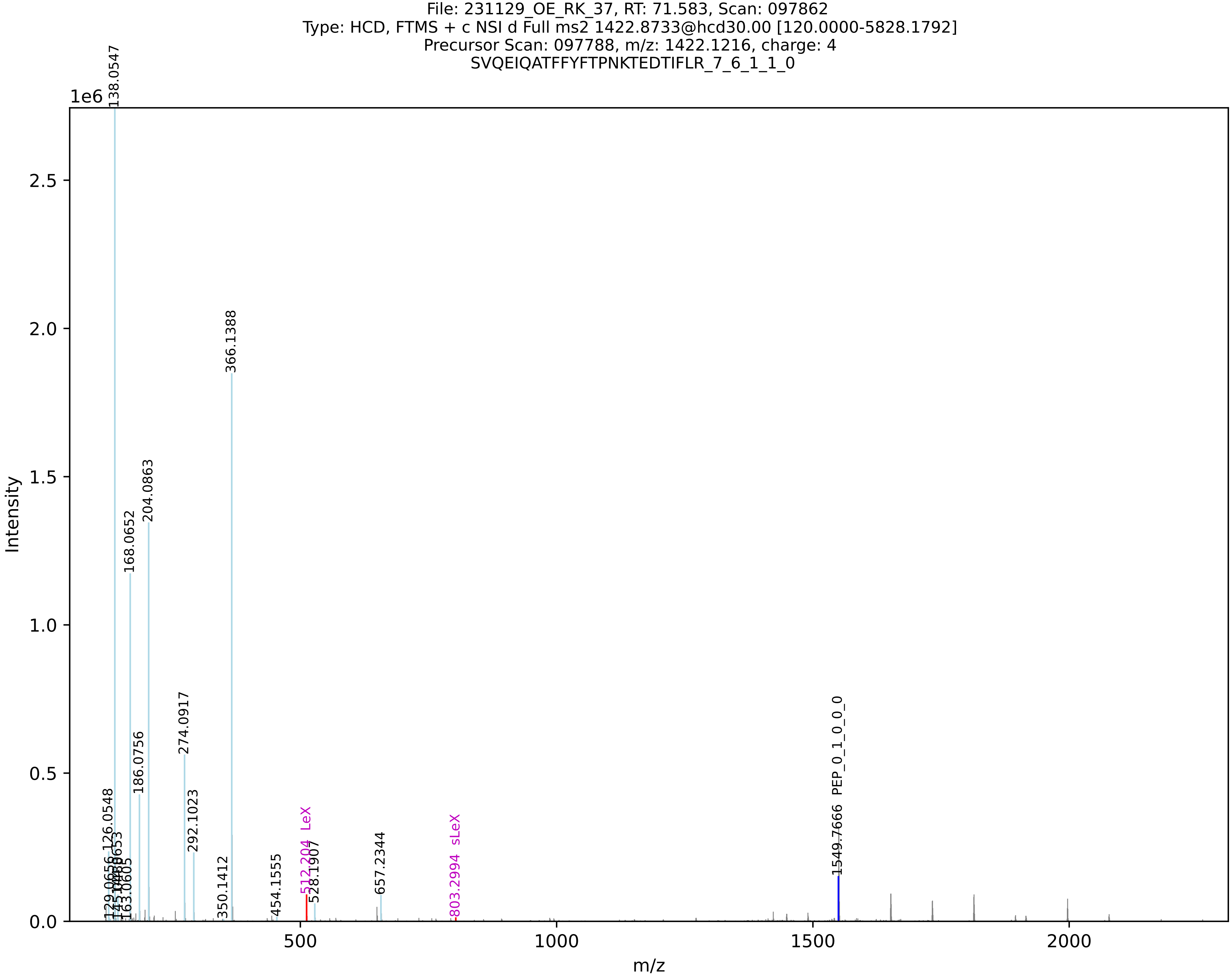
File: 231129_OE_RK_37, RT: 71.583, Scan: 097862
Type: HCD, FTMS + c NSI d Full ms2 1422.8733@hcd30.00 [120.0000-5828.1792]
Precursor Scan: 097788, m/z: 1422.1216, charge: 4
SVQEIQATFFYFTPNKTEDTIFLR_7_6_1_1_0
1e6
Intensity
m/z
138.0547
366.1388
204.0863
168.0652
274.0917
186.0756
126.0548
292.1023
129.0656
163.0605
350.1412
454.1555
512.204 LeX
528.1907
657.2344
803.2994 sLeX
1549.7666 PEP_0_1_0_0_0

File: 231129_OE_RK_37, RT: 71.685, Scan: 098007
Type: HCD, FTMS + c NSI d Full ms2 1386.3584@hcd30.00 [120.0000-5679.1982]
Precursor Scan: 097935, m/z: 1385.6067, charge: 4
AWTPVTYTVTRQNATVQGLIQGK_6_5_1_3_0

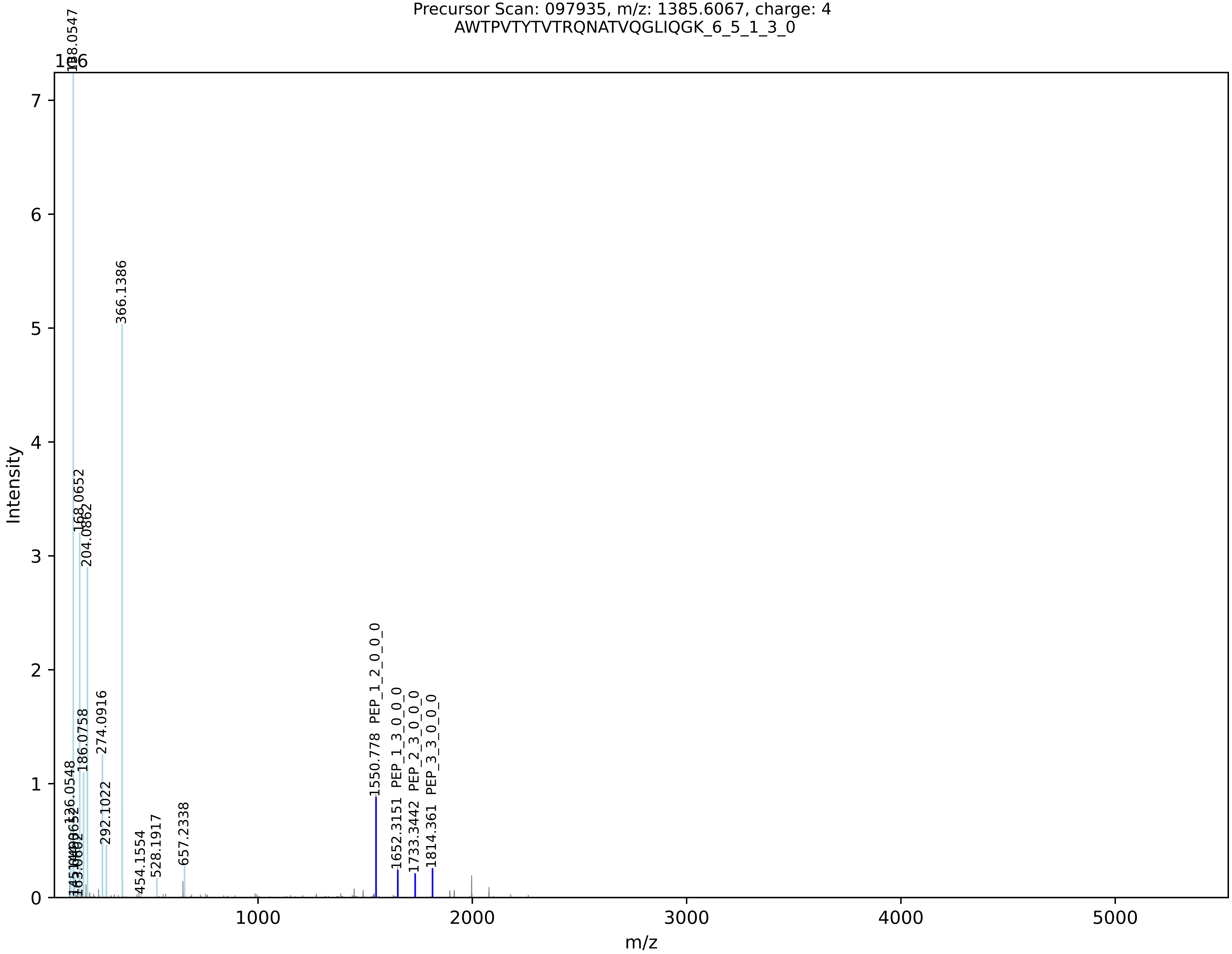

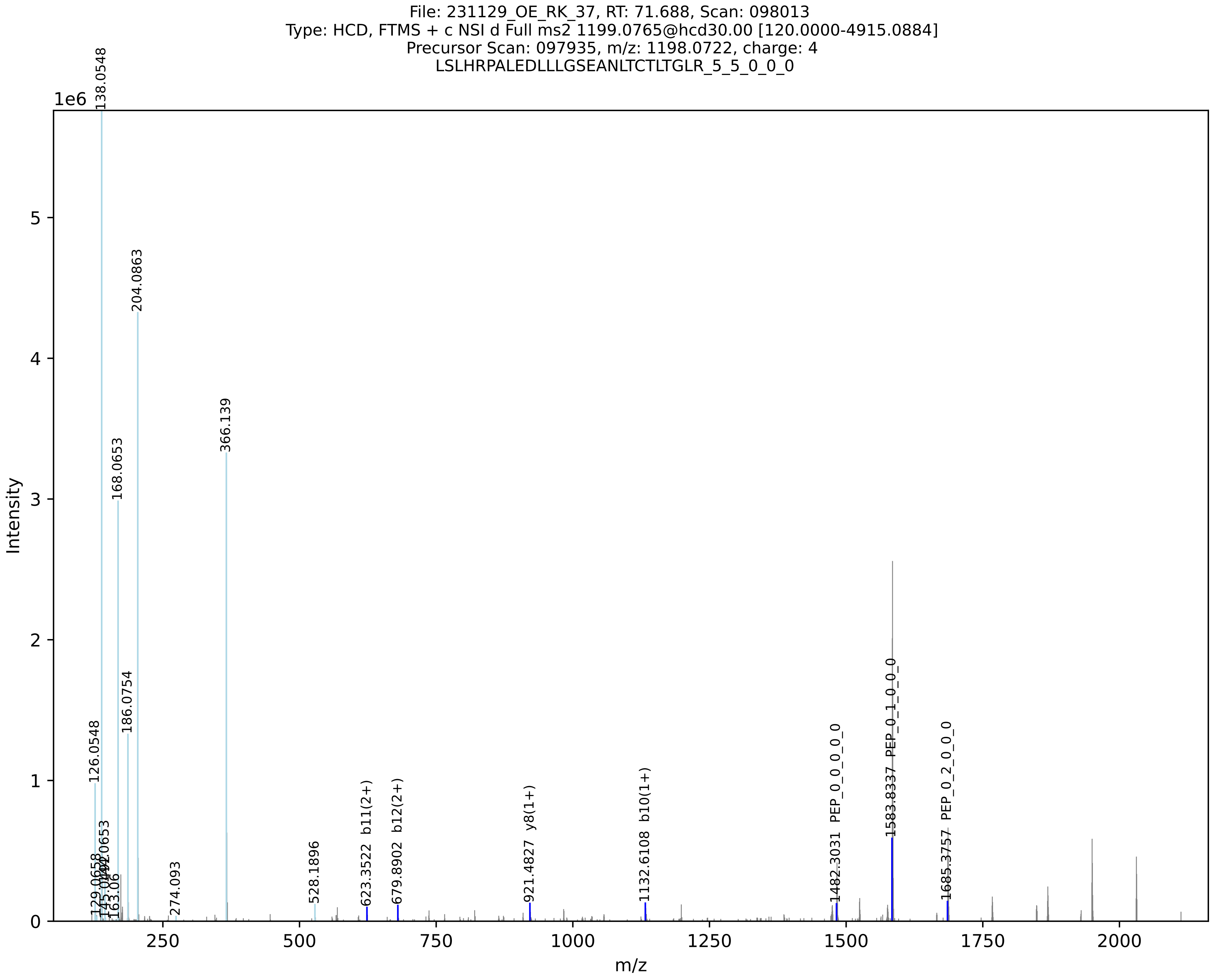
File: 231129_OE_RK_37, RT: 71.688, Scan: 098013
Type: HCD, FTMS + c NSI d Full ms2 1199.0765@hcd30.00 [120.0000-4915.0884]
Precursor Scan: 097935, m/z: 1198.0722, charge: 4
LSLHRPALEDLLLGSEANLTCTLTGLR_5_5_0_0_0
1e6
Intensity
m/z
138.0548
204.0863
366.139
168.0653
186.0754
126.0548
274.093
528.1896
623.3522 b11(2+)
679.8902 b12(2+)
921.4827 y8(1+)
1132.6108 b10(1+)
1482.3031 PEP_0_0_0_0_0
1583.8337 PEP_0_1_0_0_0
1685.3757 PEP_0_2_0_0_0

File: 231129_OE_RK_37, RT: 71.787, Scan: 098155
Type: HCD, FTMS + c NSI d Full ms2 1208.3159@hcd30.00 [120.0000-4952.7852]
Precursor Scan: 098080, m/z: 1207.8148, charge: 4
LSLHRPALEDLLLGSEANLTCTLTGLR_9_2_0_0_0

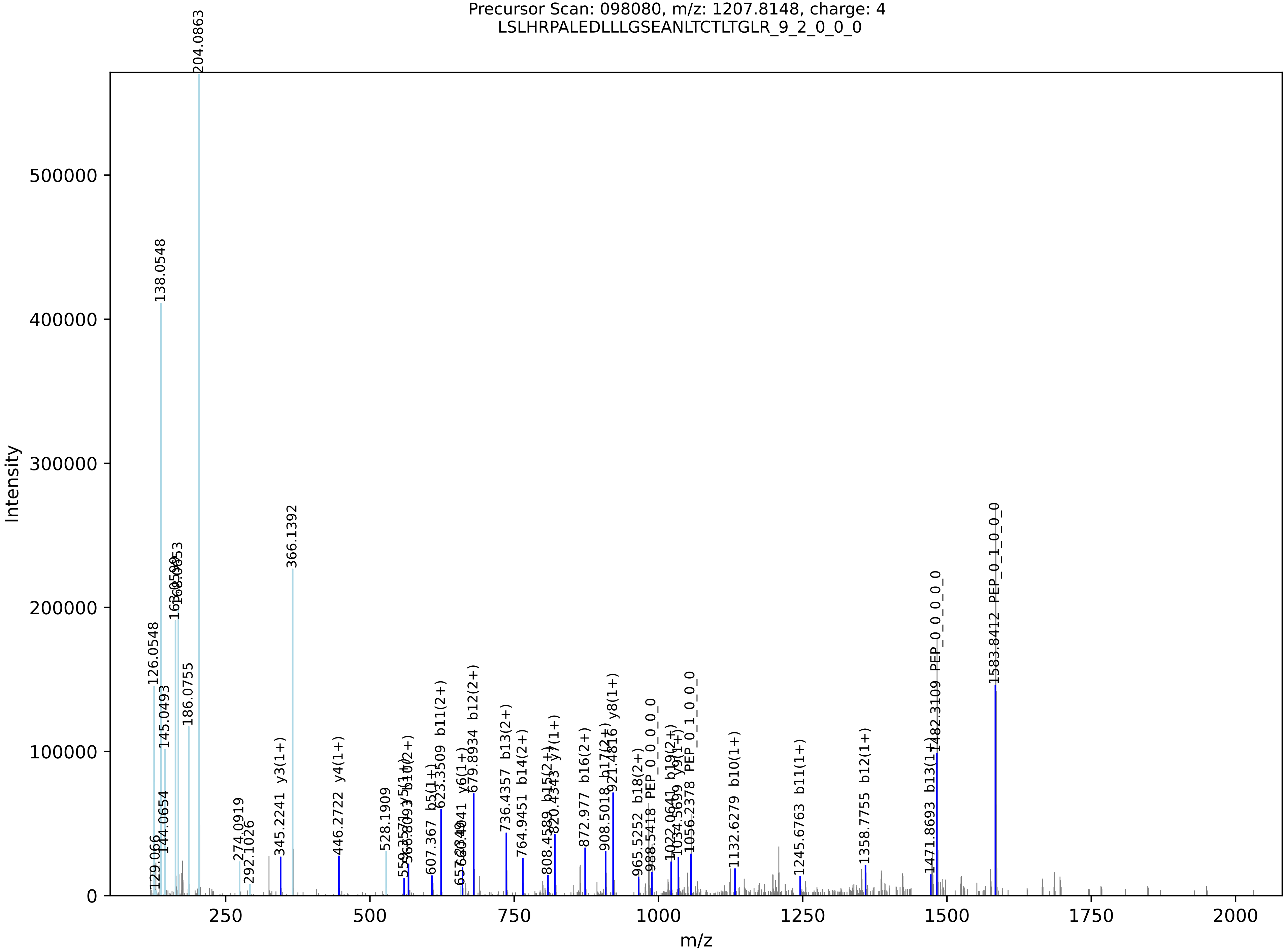

File: 231129_OE_RK_37, RT: 71.788, Scan: 098157
Type: HCD, FTMS + c NSI d Full ms2 1543.7457@hcd30.00 [120.0000-4746.0039]
Precursor Scan: 098080, m/z: 1543.0748, charge: 3
LSLHRPALEDLLLGSEANLTCTLTGLR_4_5_0_0_0

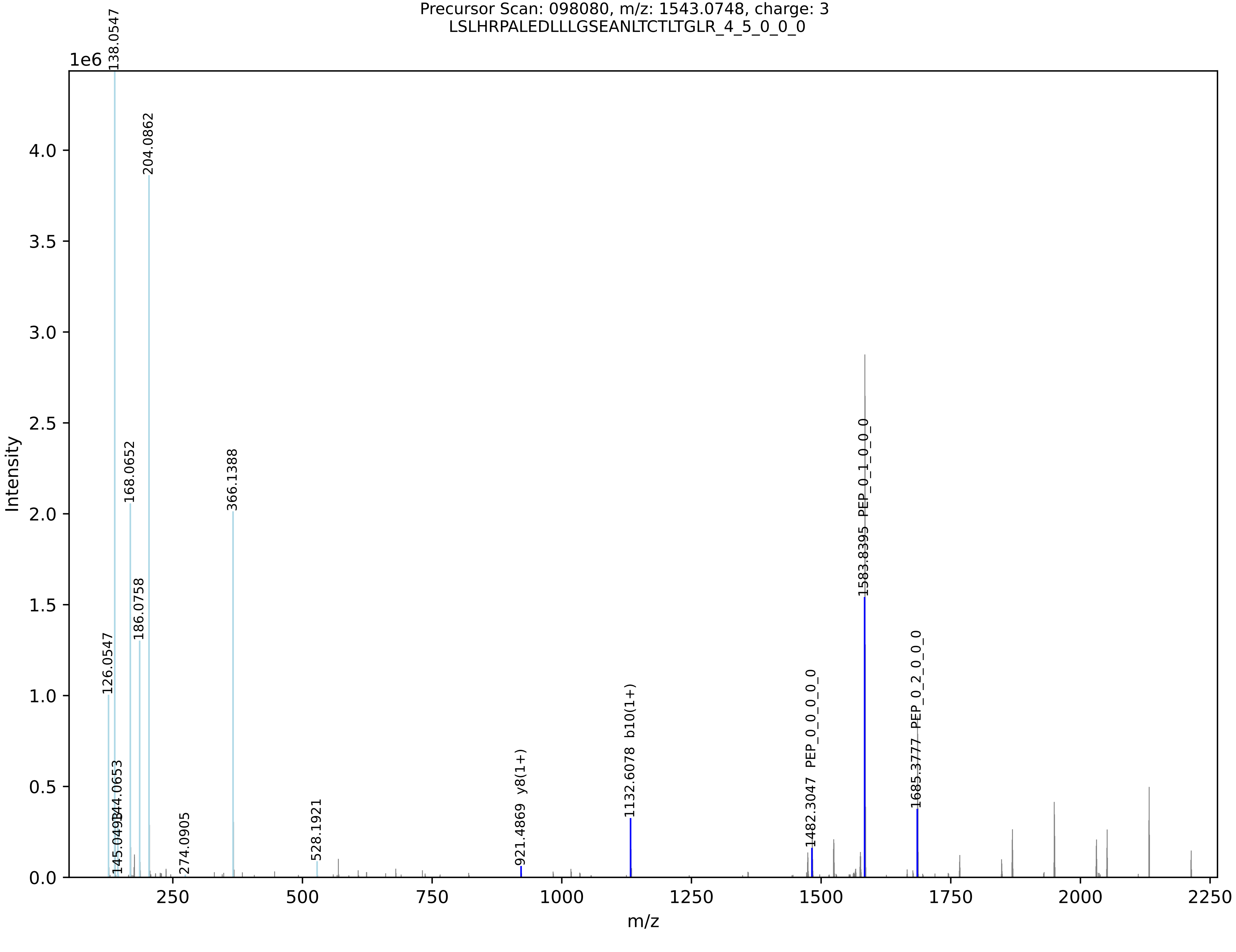

File: 231129_OE_RK_37, RT: 71.806, Scan: 098186
Type: HCD, FTMS + c NSI d Full ms2 1556.4004@hcd30.00 [120.0000-4784.7271]
Precursor Scan: 098080, m/z: 1556.0654, charge: 3
LSLHRPALEDLLLGSEANLTCTLTGLR_8_2_0_0_0

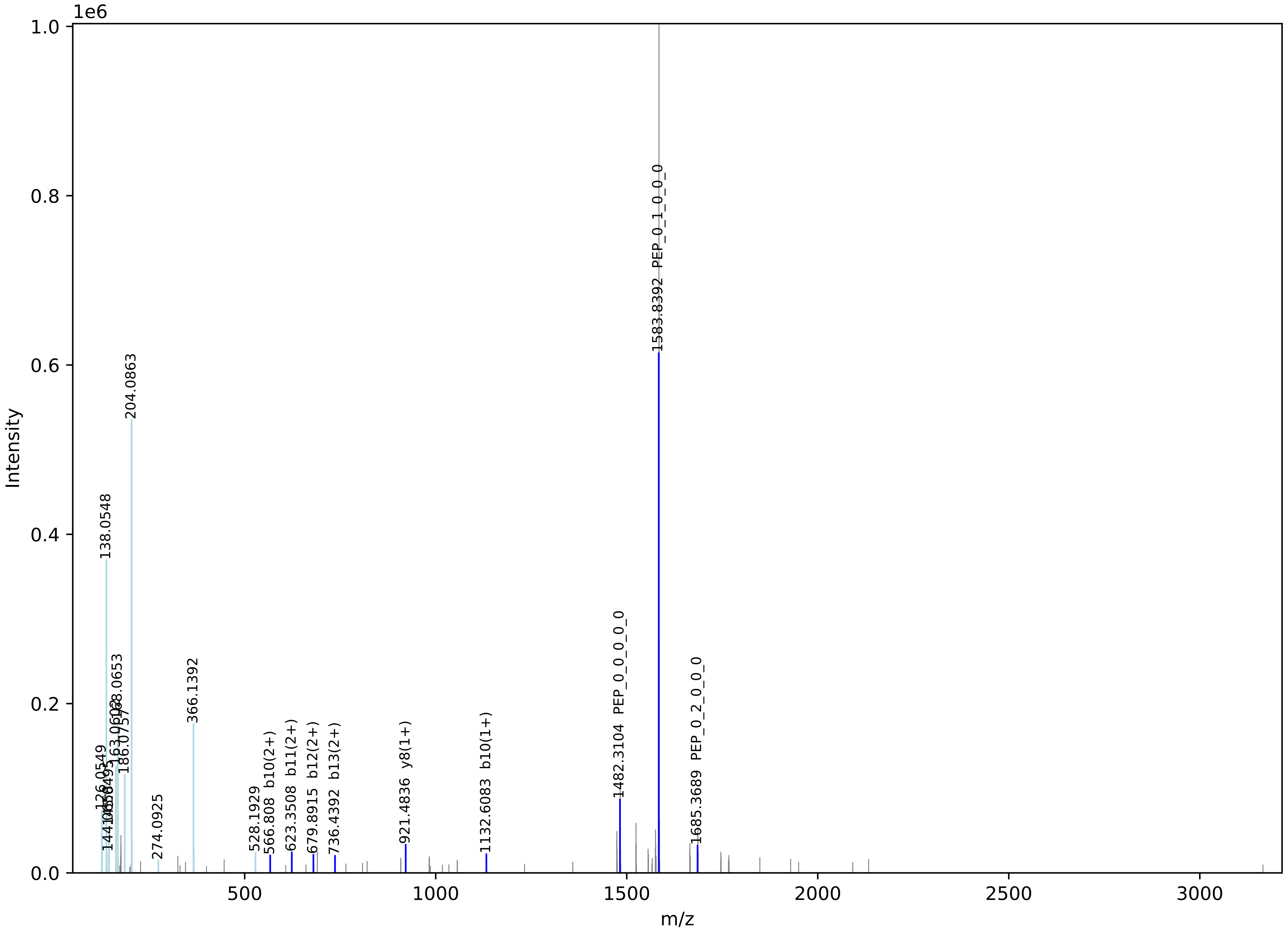

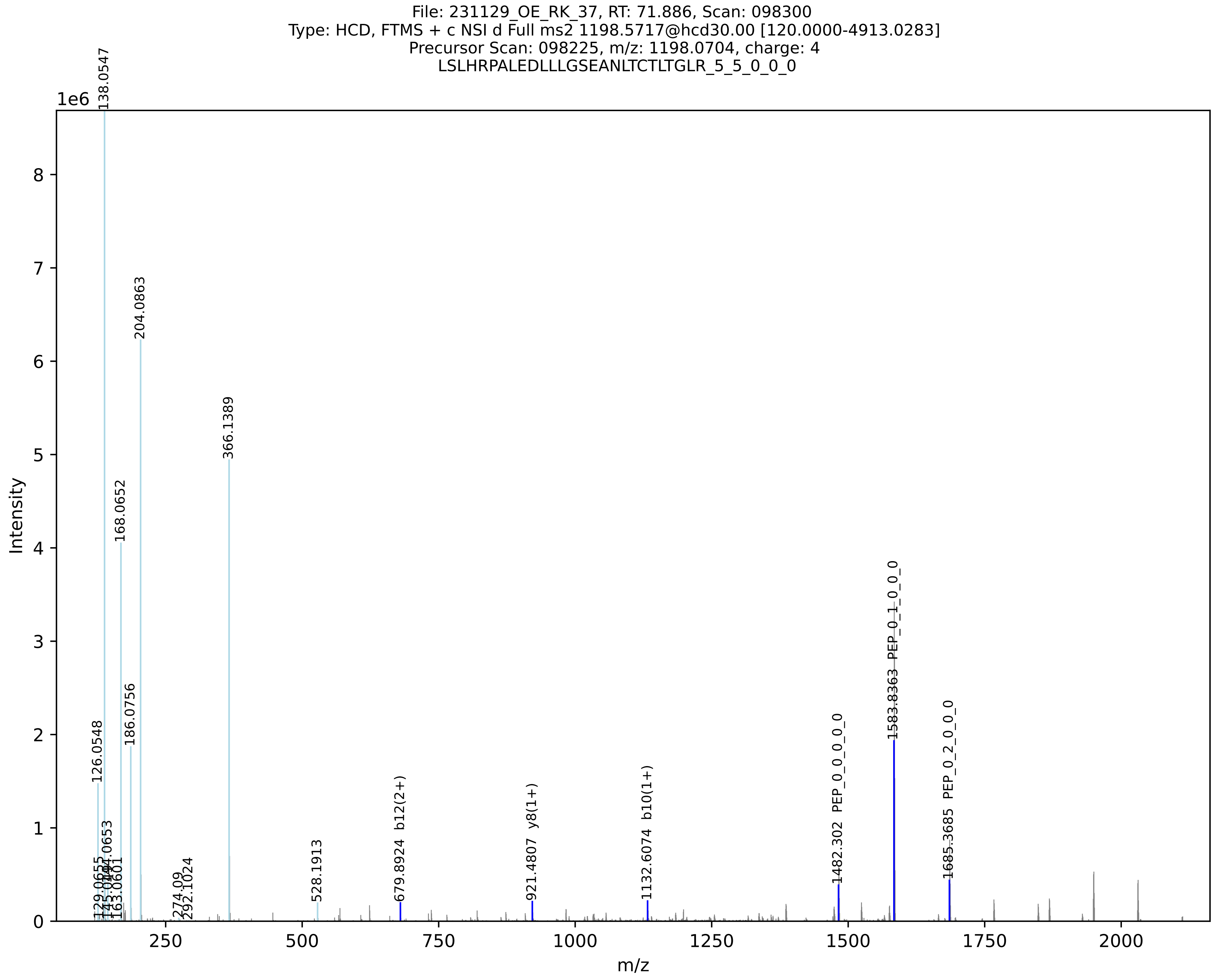
File: 231129_OE_RK_37, RT: 71.886, Scan: 098300
Type: HCD, FTMS + c NSI d Full ms2 1198.5717@hcd30.00 [120.0000-4913.0283]
Precursor Scan: 098225, m/z: 1198.0704, charge: 4
LSLHRPALEDLLLGSEANLTCTLTGLR_5_5_0_0_0
1e6
Intensity
m/z
138.0547
204.0863
366.1389
168.0652
186.0756
126.0548
528.1913
679.8924 b12(2+)
921.4807 y8(1+)
1132.6074 b10(1+)
1482.302 PEP_0_0_0_0_0
1583.8363 PEP_0_1_0_0_0
1685.3685 PEP_0_2_0_0_0
274.09
292.1024
163.0601

File: 231129_OE_RK_37, RT: 72.048, Scan: 098539
Type: HCD, FTMS + c NSI d Full ms2 1097.7859@hcd30.00 [120.0000-4501.8223]
Precursor Scan: 098446, m/z: 1096.7823, charge: 4
LSLHRPALEDLLLGSEANLTCTLTGLR_5_3_0_0_0

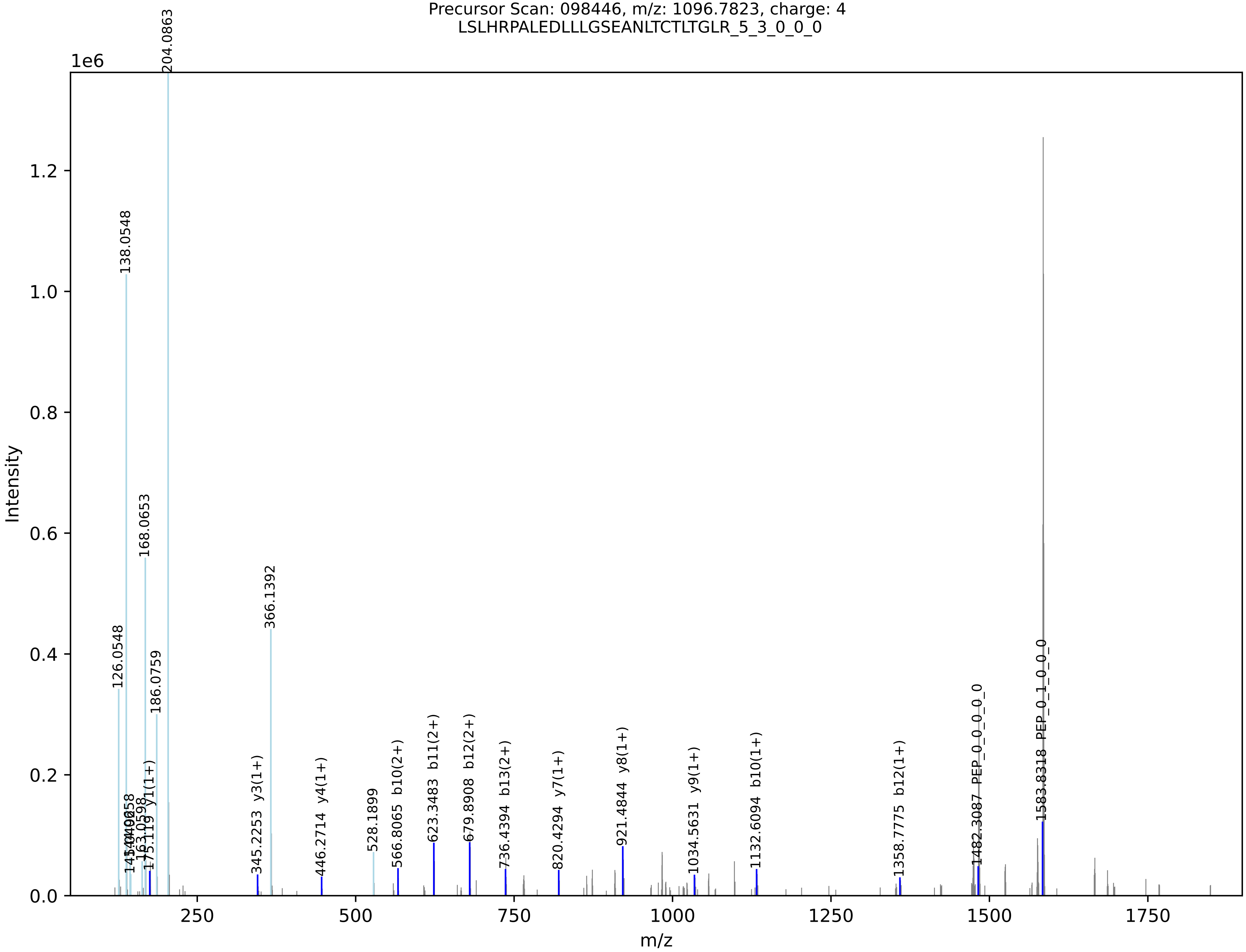

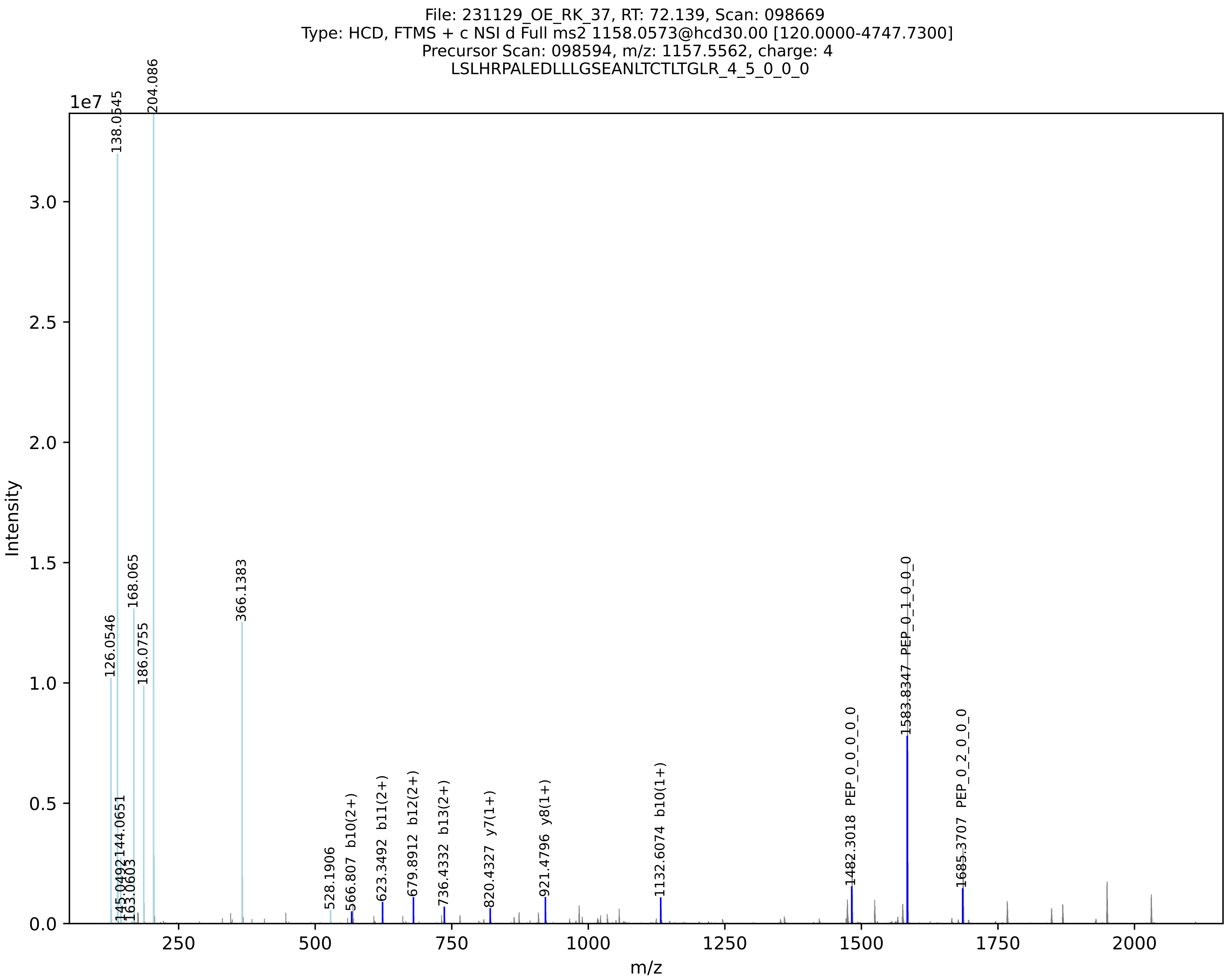

File: 231129_OE_RK_37, RT: 72.139, Scan: 098669
Type: HCD, FTMS + c NSI d Full ms2 1158.0573@hcd30.00 [120.0000-4747.7300]
Precursor Scan: 098594, m/z: 1157.5562, charge: 4
LSLHRPALEDLLLGSEANLTCTLTGLR_4_5_0_0_0
1e7
Intensity
m/z
204.086
138.0545
168.065
366.1383
126.0546
186.0755
144.0651
145.0492
163.0603
528.1906
566.807 b10(2+)
623.3492 b11(2+)
679.8912 b12(2+)
736.4332 b13(2+)
820.4327 y7(1+)
921.4796 y8(1+)
1132.6074 b10(1+)
1482.3018 PEP_0_0_0_0_0
1583.8347 PEP_0_1_0_0_0
1685.3707 PEP_0_2_0_0_0

File: 231129_OE_RK_37, RT: 72.19, Scan: 098744
Type: HCD, FTMS + c NSI d Full ms2 1543.7416@hcd30.00 [120.0000-4745.9912]
Precursor Scan: 098668, m/z: 1543.0733, charge: 3
LSLHRPALEDLLLGSEANLTCTLTGLR_4_5_0_0_0

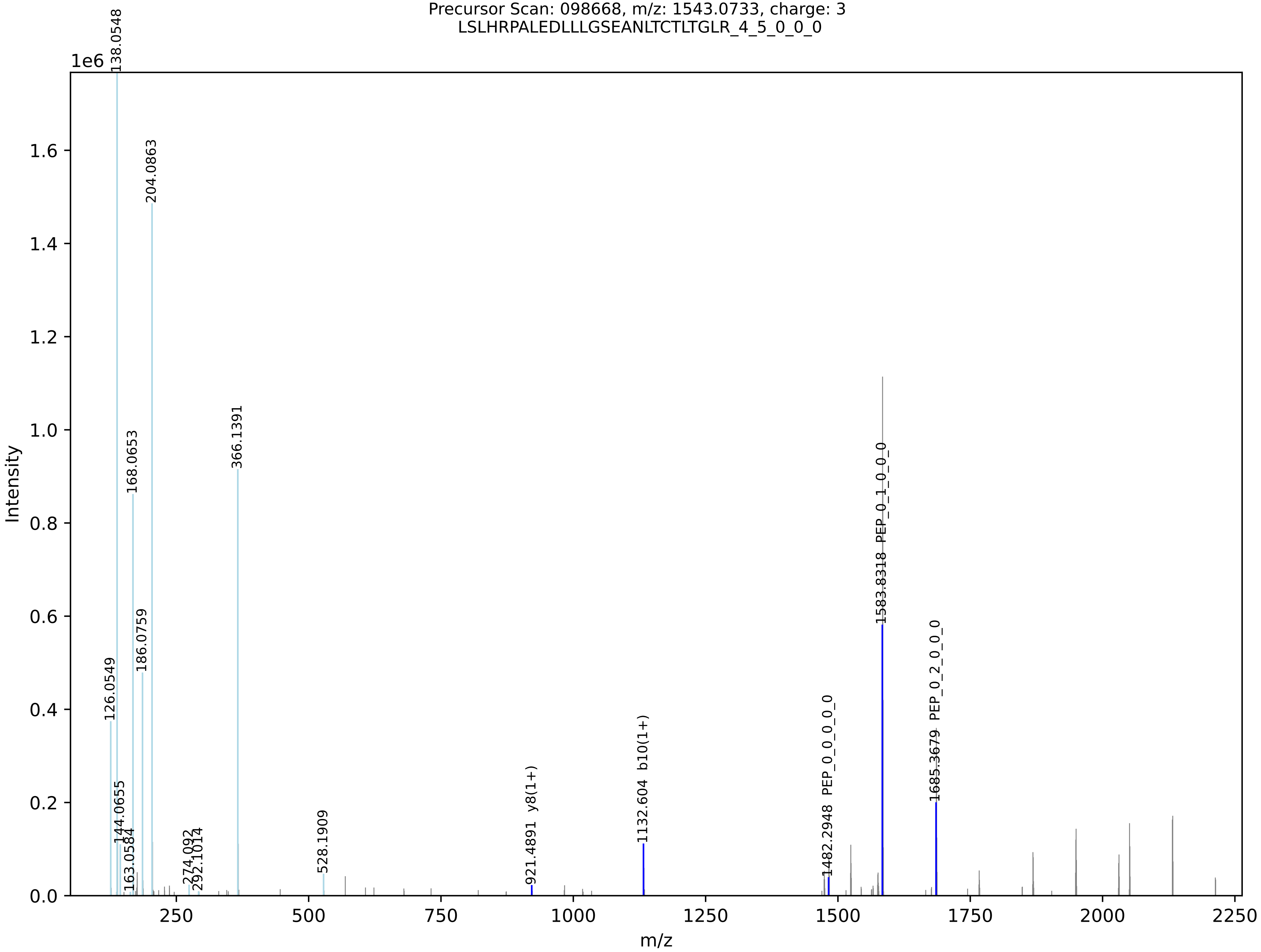

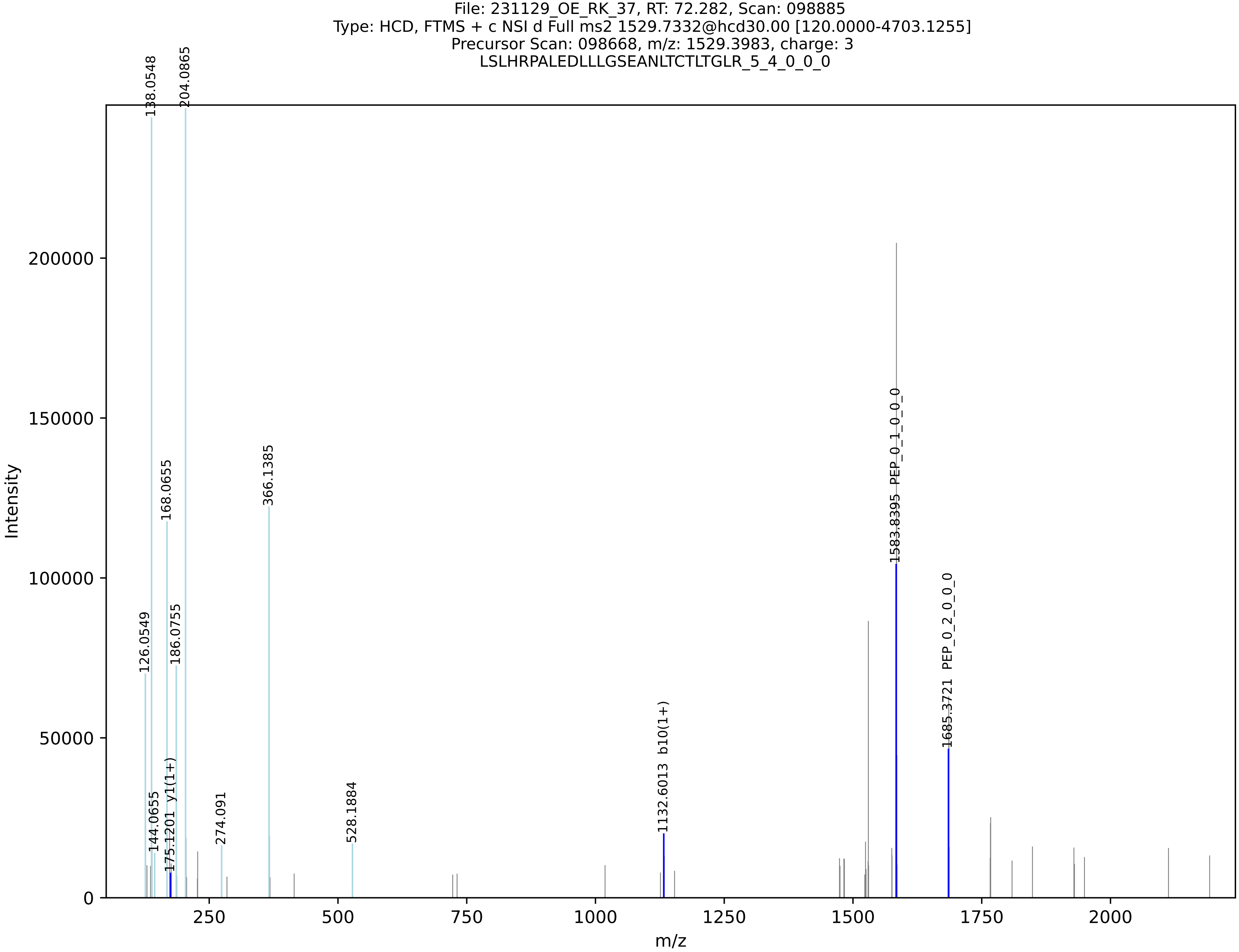

File: 231129_OE_RK_37, RT: 72.282, Scan: 098885
Type: HCD, FTMS + c NSI d Full ms2 1529.7332@hcd30.00 [120.0000-4703.1255]
Precursor Scan: 098668, m/z: 1529.3983, charge: 3
LSLHRPALEDLLLGSEANLTCTLTGLR_5_4_0_0_0
Intensity
m/z
138.0548
204.0865
168.0655
366.1385
126.0549
186.0755
144.0655
175.1201 y1(1+)
274.091
528.1884
1132.6013 b10(1+)
1583.8395 PEP_0_1_0_0_0
1685.3721 PEP_0_2_0_0_0

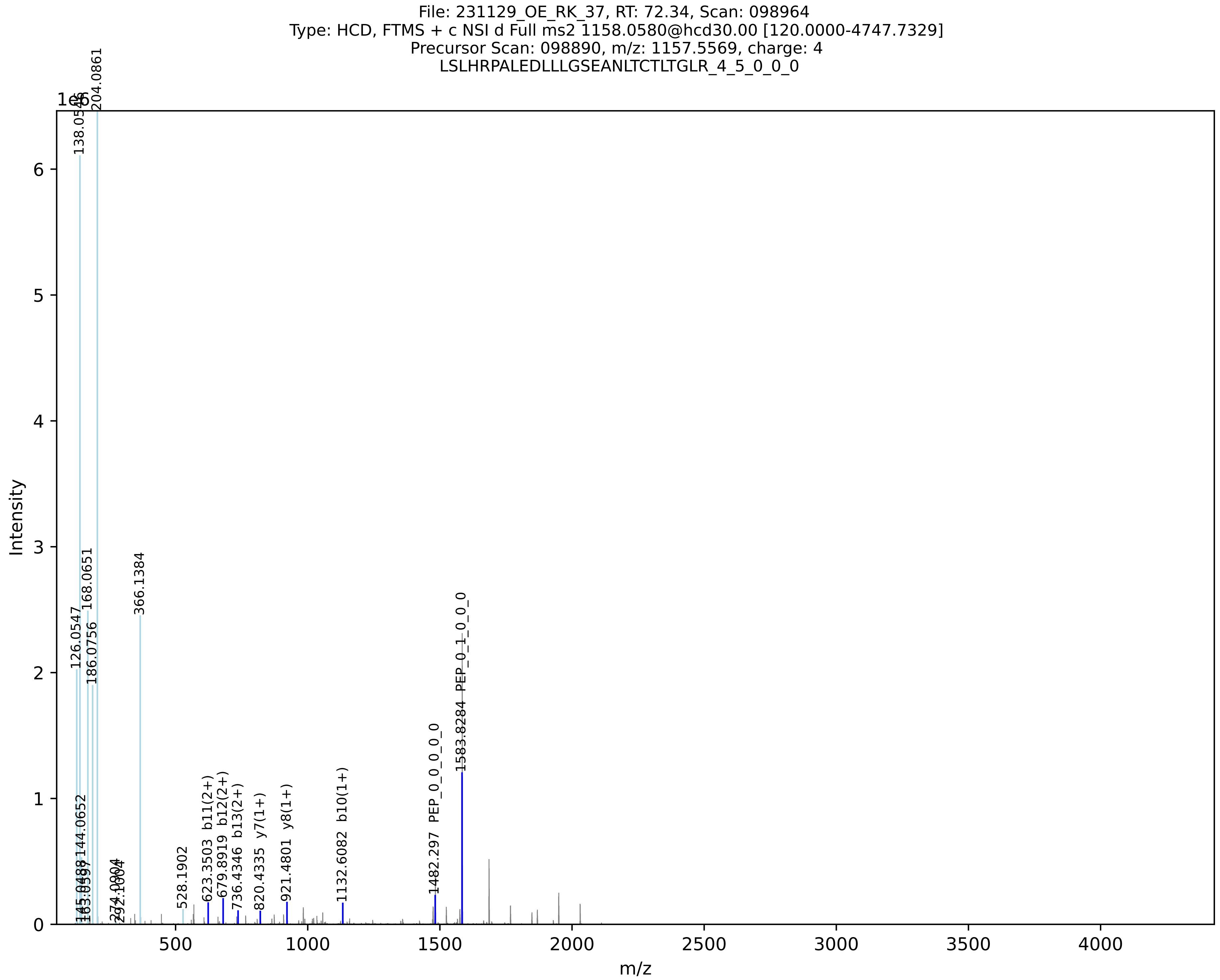

File: 231129_OE_RK_37, RT: 72.34, Scan: 098964
Type: HCD, FTMS + c NSI d Full ms2 1158.0580@hcd30.00 [120.0000-4747.7329]
Precursor Scan: 098890, m/z: 1157.5569, charge: 4
LSLHRPALEDLLLGSEANLTCTLTGLR_4_5_0_0_0
1e6
Intensity
m/z
204.0861
138.0546
168.0651
366.1384
126.0547
186.0756
144.0652
528.1902
623.3503 b11(2+)
679.8919 b12(2+)
736.4346 b13(2+)
820.4335 y7(1+)
921.4801 y8(1+)
1132.6082 b10(1+)
1482.297 PEP_0_0_0_0_0
1583.8284 PEP_0_1_0_0_0

File: 231129_OE_RK_37, RT: 72.586, Scan: 099330
Type: HCD, FTMS + c NSI d Full ms2 1148.3053@hcd30.00 [120.0000-4707.9414]
Precursor Scan: 099110, m/z: 1147.3021, charge: 4
LSLHRPALEDLLLGSEANLTCTLTGLR_5_4_0_0_0

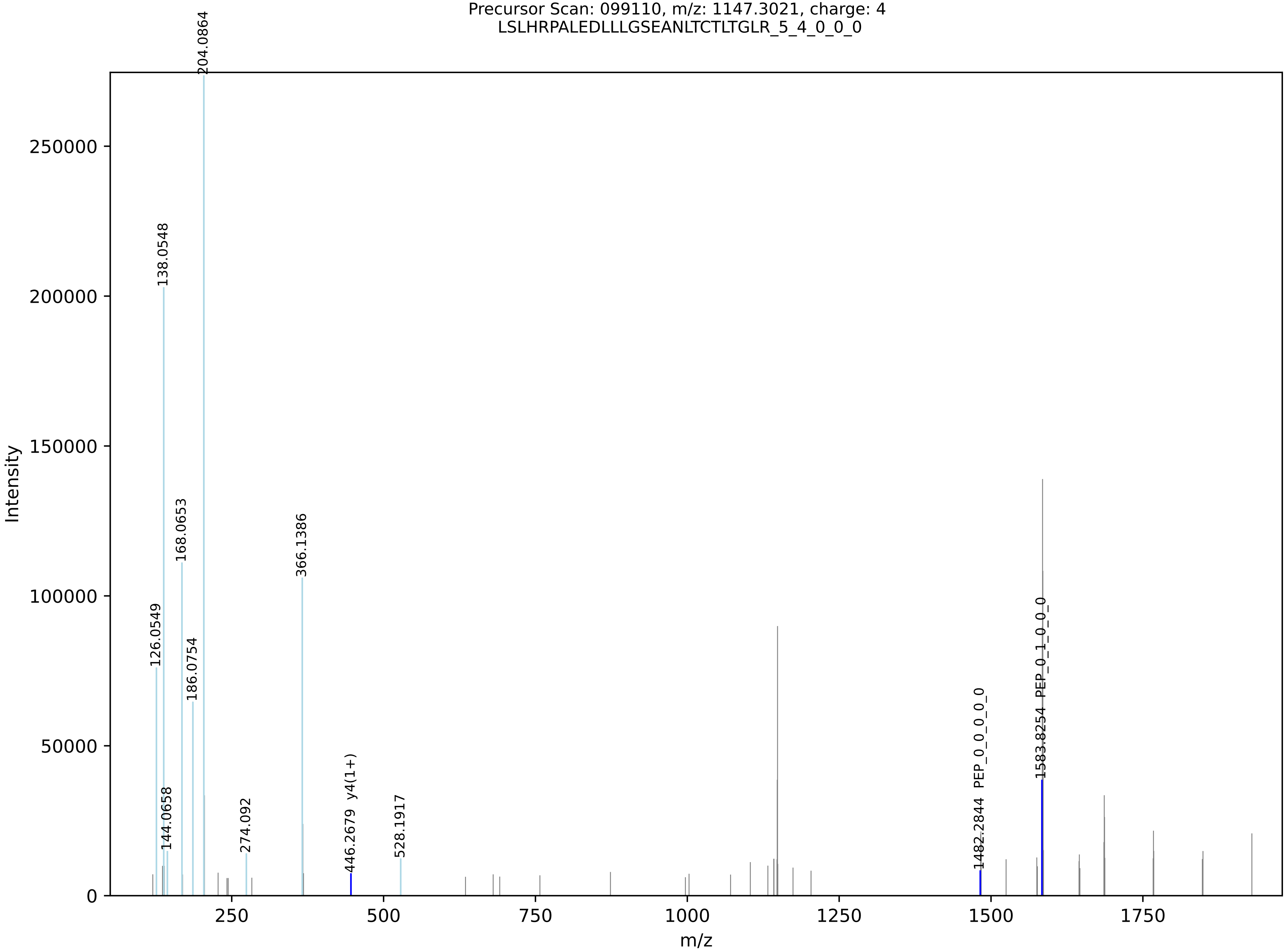

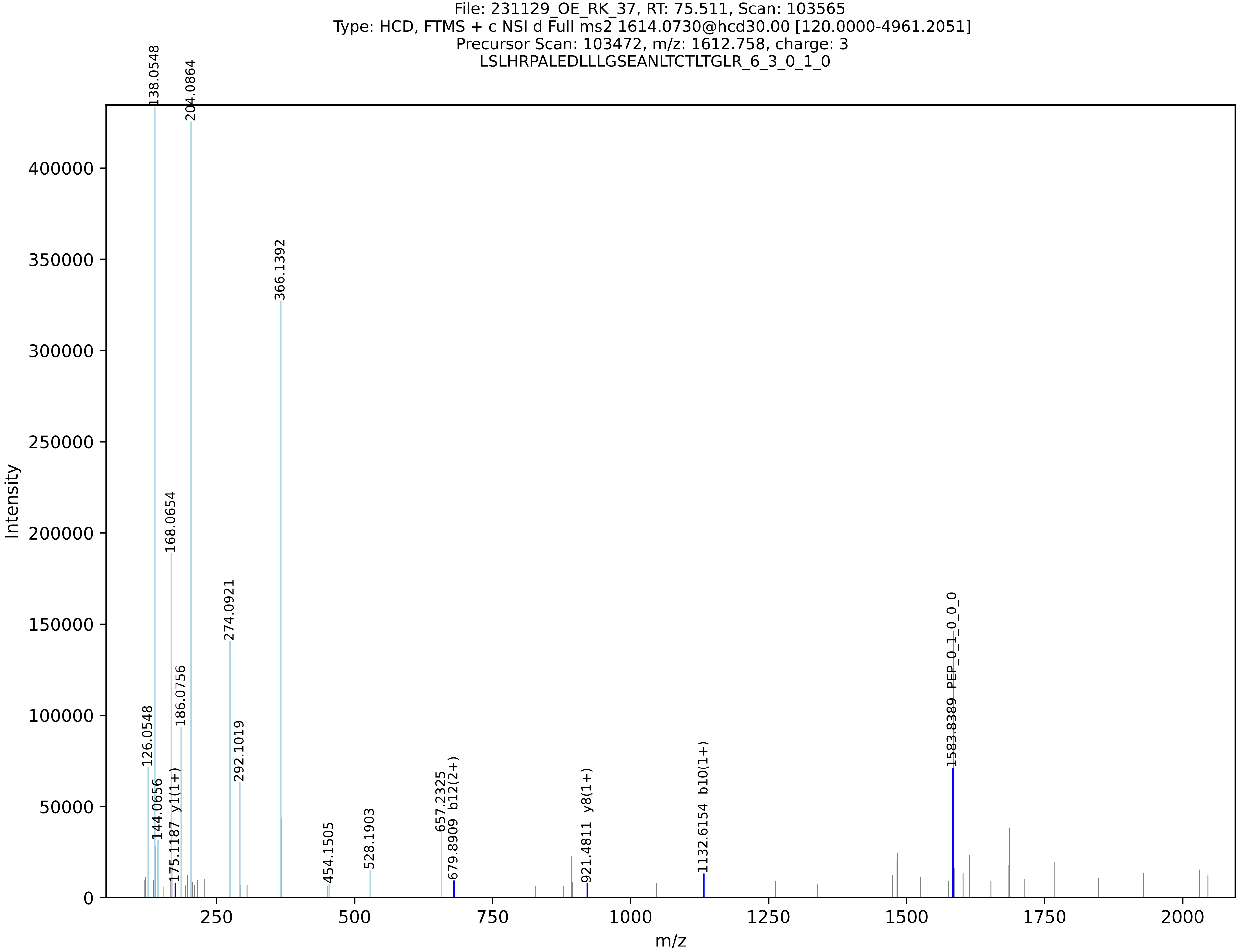
File: 231129_OE_RK_37, RT: 75.511, Scan: 103565
Type: HCD, FTMS + c NSI d Full ms2 1614.0730@hcd30.00 [120.0000-4961.2051]
Precursor Scan: 103472, m/z: 1612.758, charge: 3
LSLHRPALEDLLLGSEANLTCTLTGLR_6_3_0_1_0
Intensity
m/z
138.0548
204.0864
366.1392
168.0654
274.0921
186.0756
126.0548
292.1019
144.0656
175.1187 y1(1+)
454.1505
528.1903
657.2325
679.8909 b12(2+)
921.4811 y8(1+)
1132.6154 b10(1+)
1583.8389 PEP_0_1_0_0_0
0
50000
100000
150000
200000
250000
300000
350000
400000
250
500
750
1000
1250
1500
1750
2000

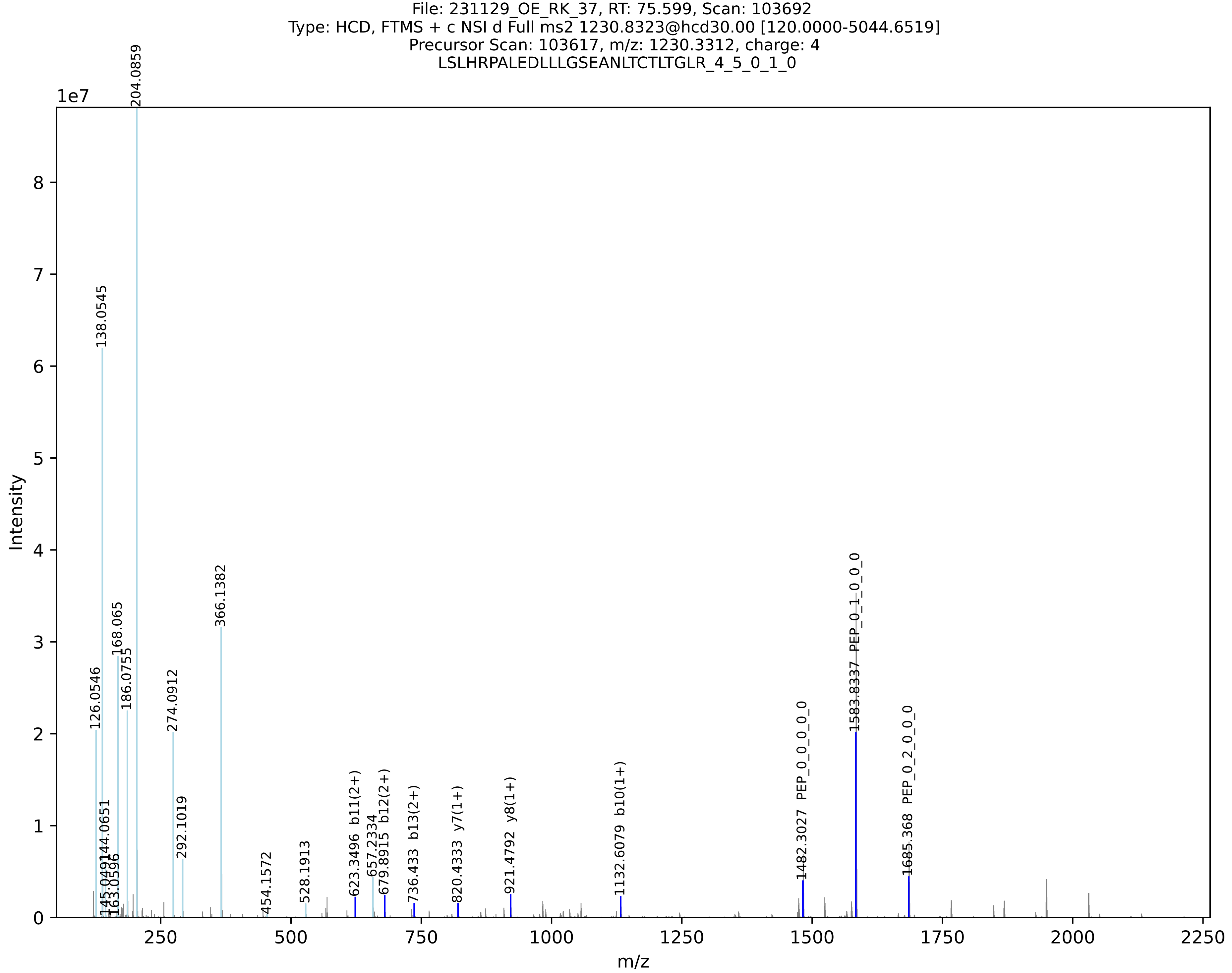
File: 231129_OE_RK_37, RT: 75.599, Scan: 103692
Type: HCD, FTMS + c NSI d Full ms2 1230.8323@hcd30.00 [120.0000-5044.6519]
Precursor Scan: 103617, m/z: 1230.3312, charge: 4
LSLHRPALEDLLLGSEANLTCTLTGLR_4_5_0_1_0
1e7
Intensity
m/z
204.0859
138.0545
366.1382
168.065
186.0755
126.0546
274.0912
292.1019
144.0651
145.0491
163.0596
454.1572
528.1913
623.3496 b11(2+)
657.2334
679.8915 b12(2+)
736.433 b13(2+)
820.4333 y7(1+)
921.4792 y8(1+)
1132.6079 b10(1+)
1482.3027 PEP_0_0_0_0_0
1583.8337 PEP_0_1_0_0_0
1685.368 PEP_0_2_0_0_0

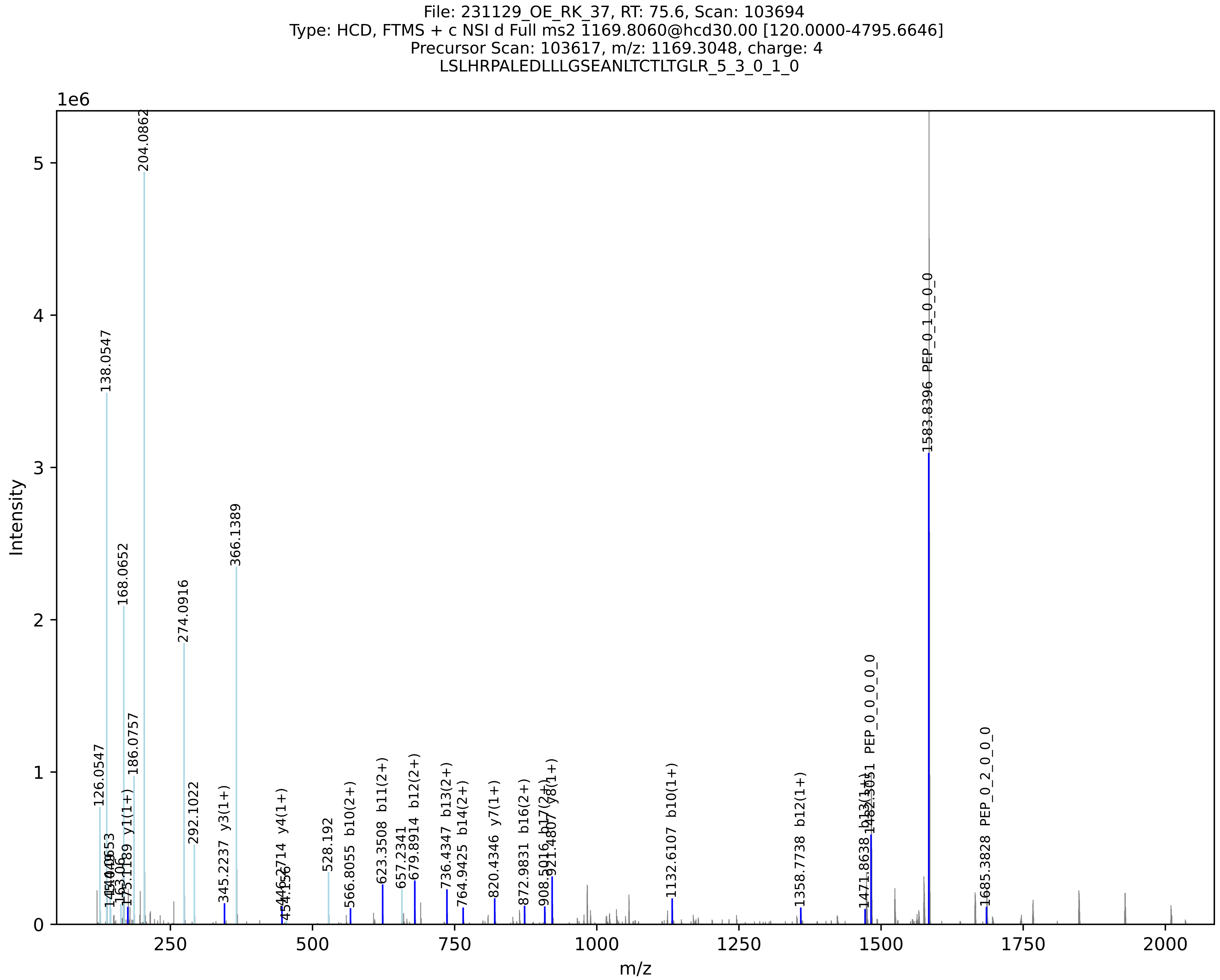

File: 231129_OE_RK_37, RT: 75.6, Scan: 103694
Type: HCD, FTMS + c NSI d Full ms2 1169.8060@hcd30.00 [120.0000-4795.6646]
Precursor Scan: 103617, m/z: 1169.3048, charge: 4
LSLHRPALEDLLLGSEANLTCTLTGLR_5_3_0_1_0
1e6
Intensity
m/z
204.0862
138.0547
168.0652
274.0916
366.1389
186.0757
126.0547
292.1022
175.1189 y1(1+)
345.2237 y3(1+)
446.2714 y4(1+)
528.192
566.8055 b10(2+)
623.3508 b11(2+)
657.2341
679.8914 b12(2+)
736.4347 b13(2+)
764.9425 b14(2+)
820.4346 y7(1+)
872.9831 b16(2+)
1132.6107 b10(1+)
1358.7738 b12(1+)
1471.8638 b13(1+)
1583.8396 PEP_0_1_0_0_0
1685.3828 PEP_0_2_0_0_0
PEP_0_0_0_0_0

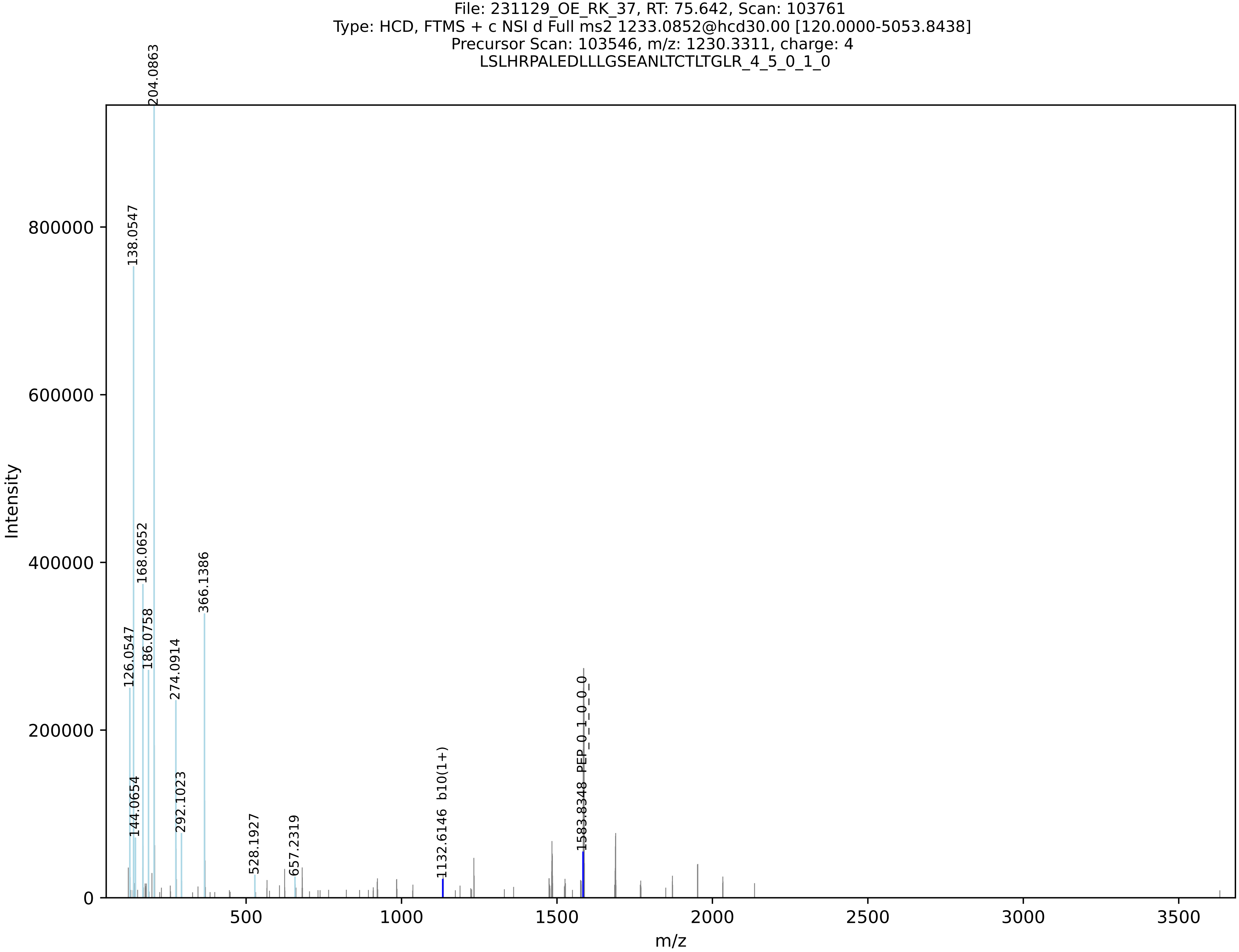

File: 231129_OE_RK_37, RT: 75.642, Scan: 103761
Type: HCD, FTMS + c NSI d Full ms2 1233.0852@hcd30.00 [120.0000-5053.8438]
Precursor Scan: 103546, m/z: 1230.3311, charge: 4
LSLHRPALEDLLLGSEANLTCTLTGLR_4_5_0_1_0
Intensity
m/z
204.0863
138.0547
168.0652
366.1386
126.0547
186.0758
274.0914
144.0654
292.1023
528.1927
657.2319
1132.6146 b10(1+)
1583.8348 PEP_0_1_0_0_0
0
200000
400000
600000
800000
500
1000
1500
2000
2500
3000
3500

File: 231129_OE_RK_37, RT: 75.702, Scan: 103843
Type: HCD, FTMS + c NSI d Full ms2 1220.5734@hcd30.00 [120.0000-5002.7954]
Precursor Scan: 103765, m/z: 1220.0722, charge: 4
LSLHRPALEDLLLGSEANLTCTLTGLR_5_4_0_1_0

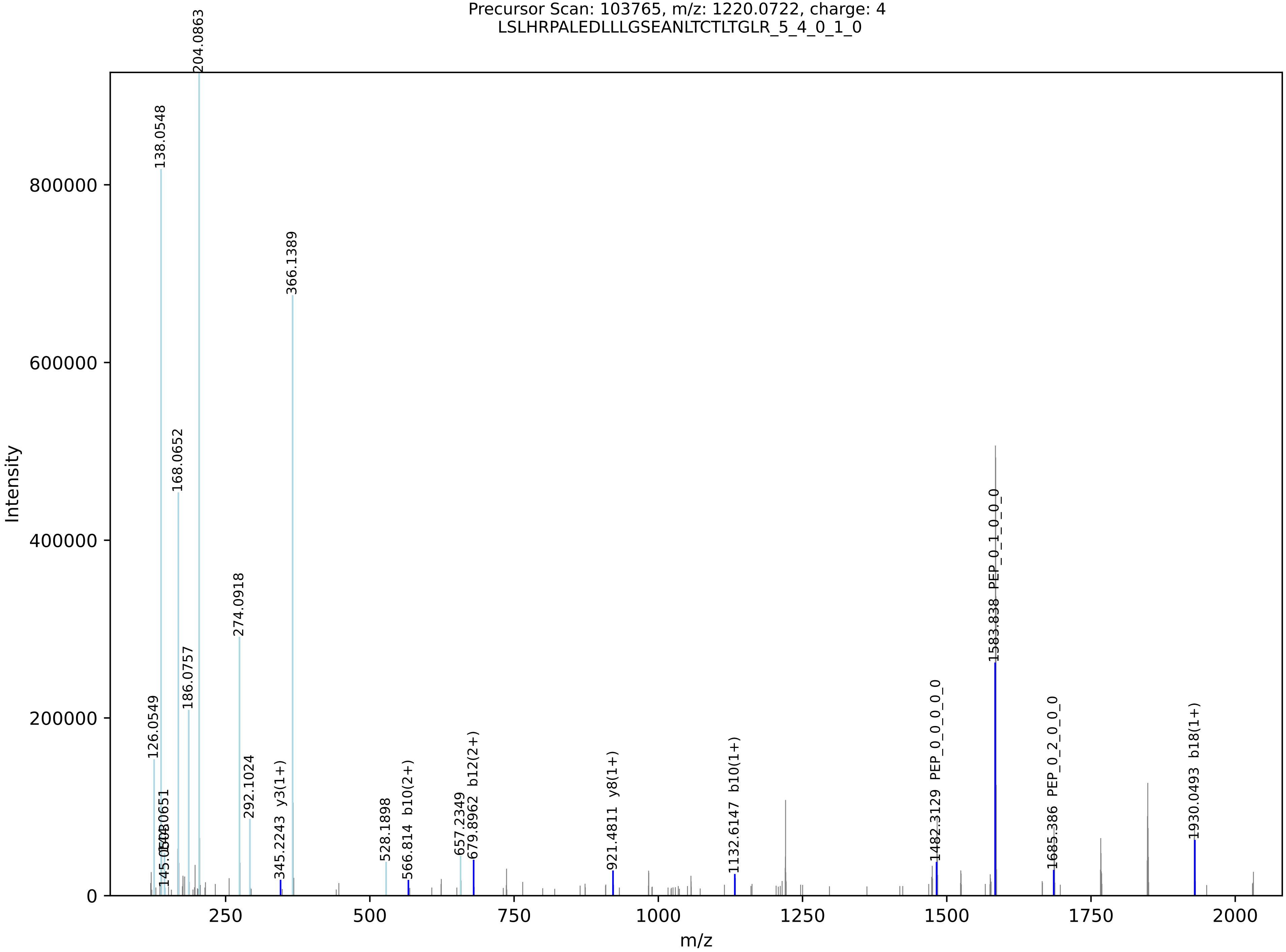

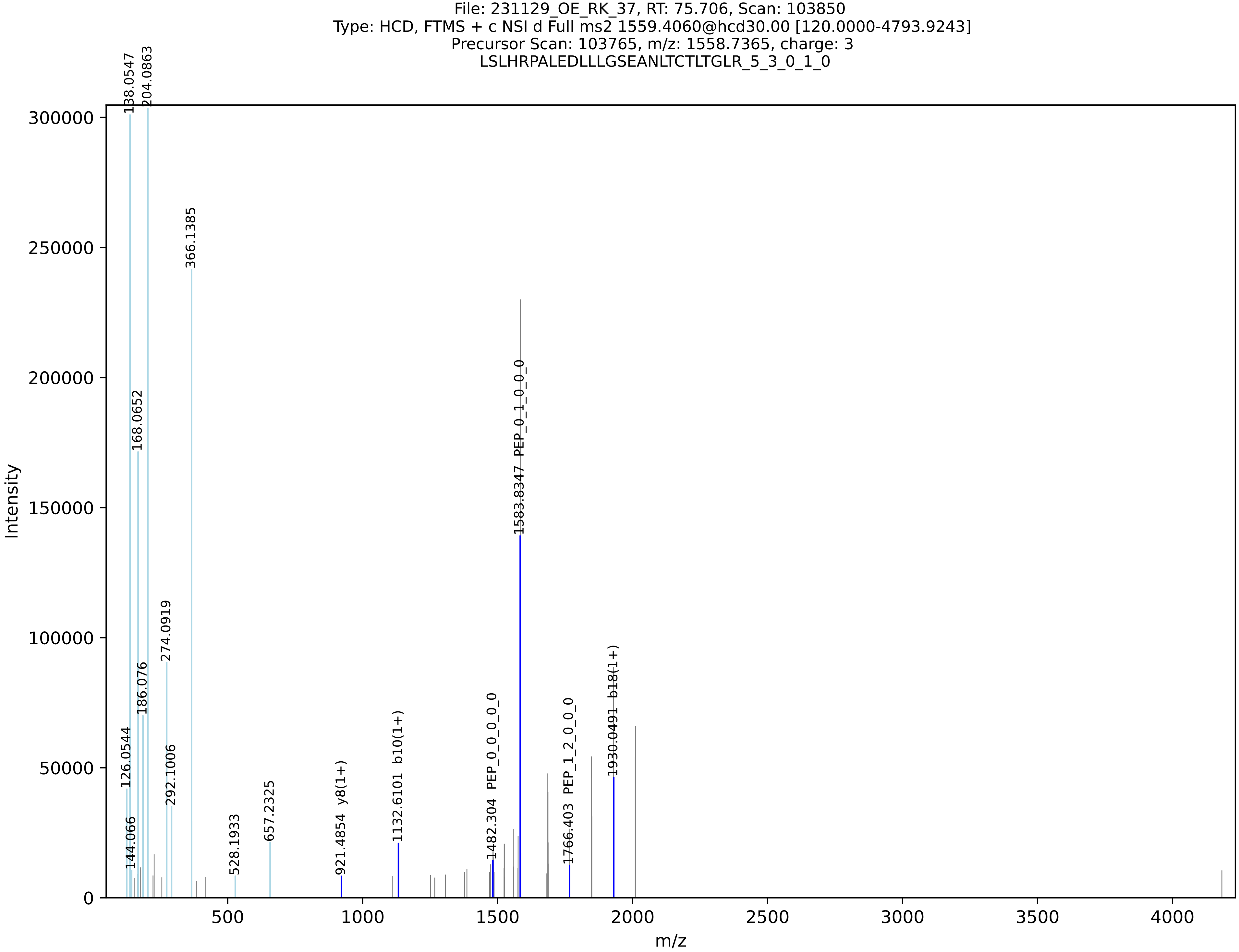

File: 231129_OE_RK_37, RT: 75.706, Scan: 103850
Type: HCD, FTMS + c NSI d Full ms2 1559.4060@hcd30.00 [120.0000-4793.9243]
Precursor Scan: 103765, m/z: 1558.7365, charge: 3
LSLHRPALEDLLLGSEANLTCTLTGLR_5_3_0_1_0
Intensity
m/z
138.0547
204.0863
366.1385
168.0652
274.0919
186.076
126.0544
292.1006
144.066
528.1933
657.2325
921.4854 y8(1+)
1132.6101 b10(1+)
1482.304 PEP_0_0_0_0_0
1583.8347 PEP_0_1_0_0_0
1766.403 PEP_1_2_0_0_0
1930.0491 b18(1+)

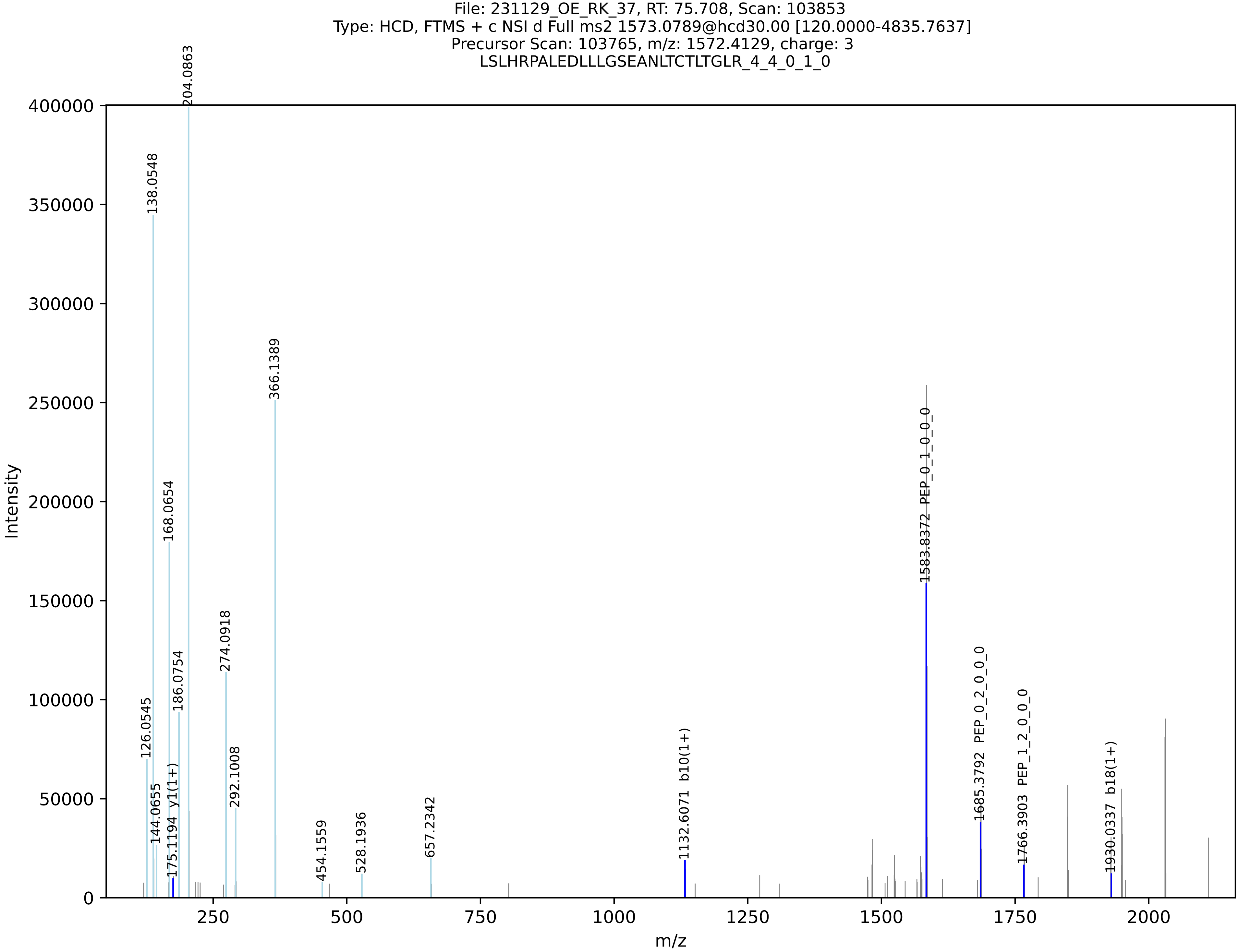

File: 231129_OE_RK_37, RT: 75.708, Scan: 103853
Type: HCD, FTMS + c NSI d Full ms2 1573.0789@hcd30.00 [120.0000-4835.7637]
Precursor Scan: 103765, m/z: 1572.4129, charge: 3
LSLHRPALEDLLLGSEANLTCTLTGLR_4_4_0_1_0
Intensity
m/z
204.0863
138.0548
366.1389
168.0654
274.0918
186.0754
126.0545
292.1008
144.0655
175.1194 y1(1+)
454.1559
528.1936
657.2342
1132.6071 b10(1+)
1583.8372 PEP_0_1_0_0_0
1685.3792 PEP_0_2_0_0_0
1766.3903 PEP_1_2_0_0_0
1930.0337 b18(1+)

File: 231129_OE_RK_37, RT: 75.752, Scan: 103916
Type: HCD, FTMS + c NSI d Full ms2 1129.2919@hcd30.00 [120.0000-4630.3667]
Precursor Scan: 103838, m/z: 1128.7907, charge: 4
LSLHRPALEDLLLGSEANLTCTLTGLR_4_3_0_1_0

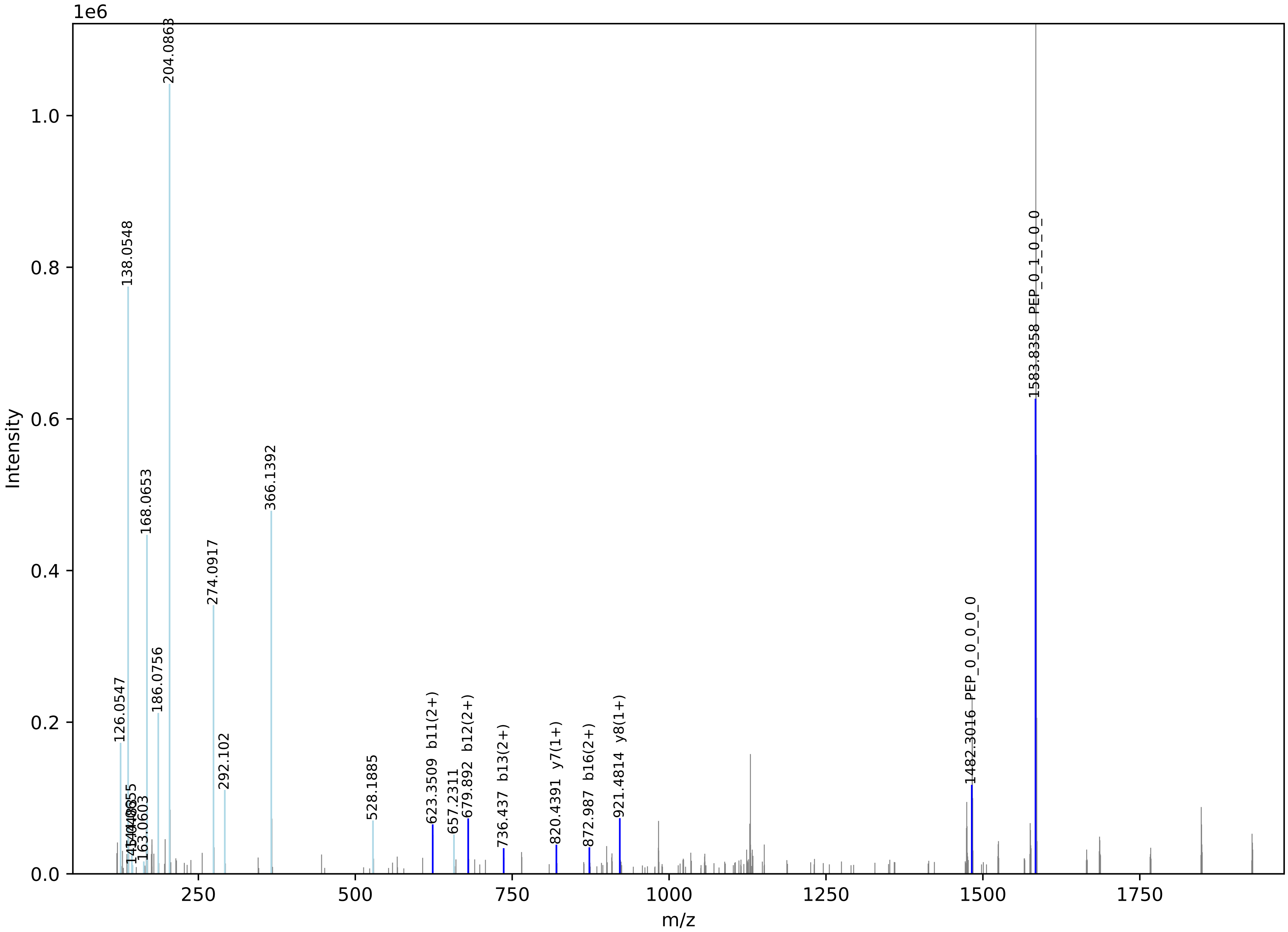

File: 231129_OE_RK_37, RT: 75.802, Scan: 103989
Type: HCD, FTMS + c NSI d Full ms2 1640.7740@hcd30.00 [120.0000-5042.9106]
Precursor Scan: 103912, m/z: 1640.1055, charge: 3
LSLHRPALEDLLLGSEANLTCTLTGLR_4_5_0_1_0

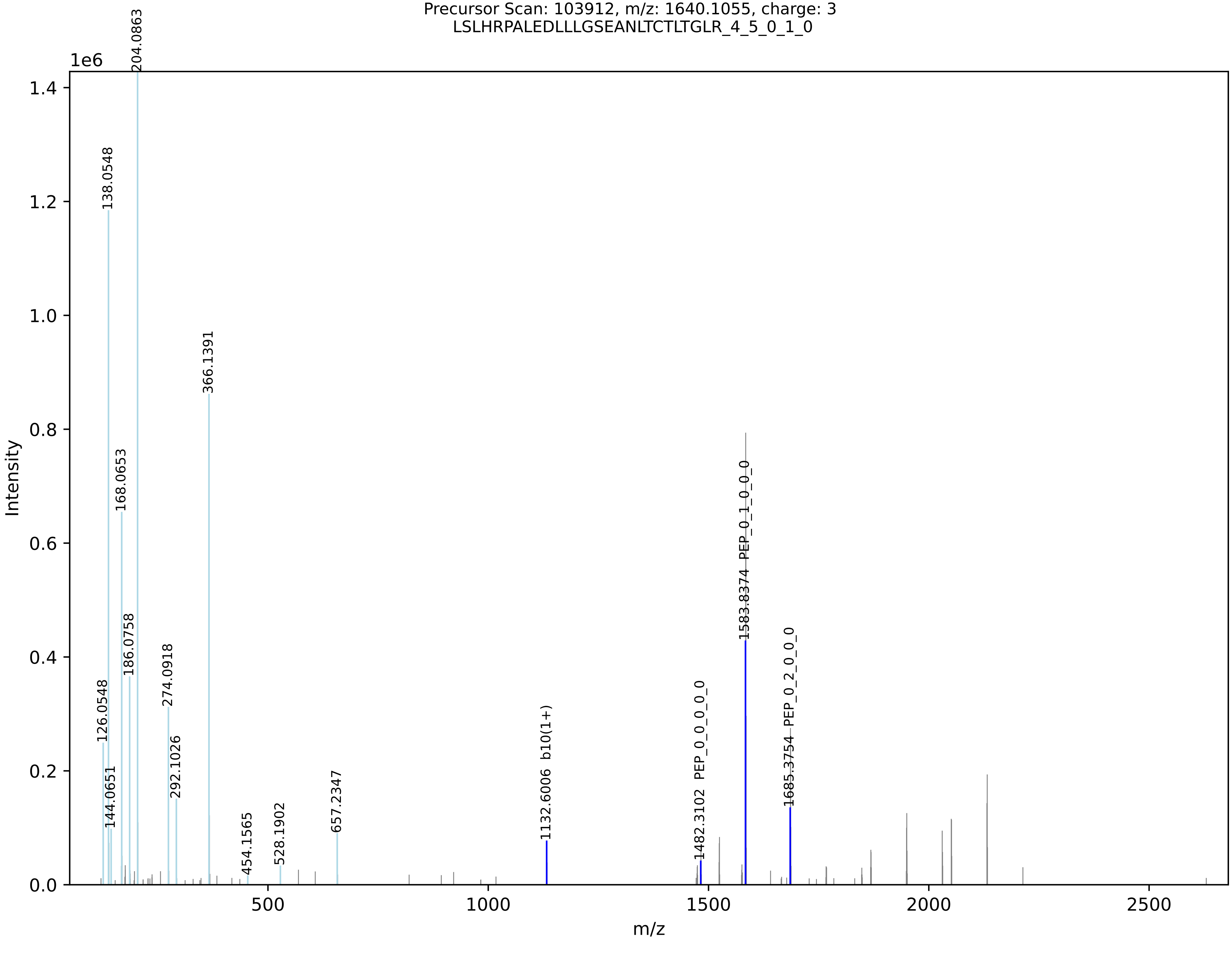

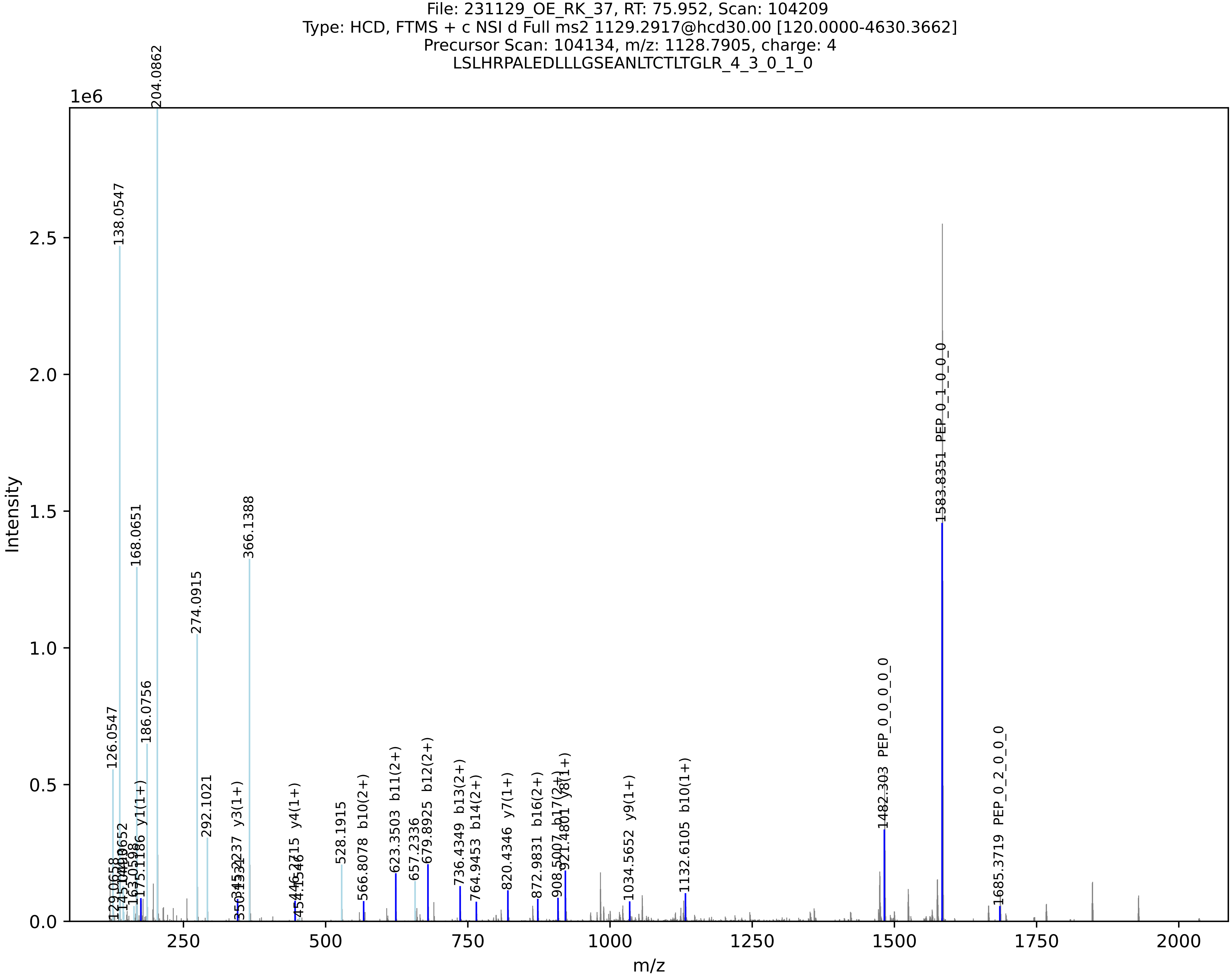
File: 231129_OE_RK_37, RT: 75.952, Scan: 104209
Type: HCD, FTMS + c NSI d Full ms2 1129.2917@hcd30.00 [120.0000-4630.3662]
Precursor Scan: 104134, m/z: 1128.7905, charge: 4
LSLHRPALEDLLLGSEANLTCTLTGLR_4_3_0_1_0
1e6
Intensity
m/z
204.0862
138.0547
168.0651
366.1388
274.0915
186.0756
126.0547
292.1021
175.1186 y1(1+)
345.2237 y3(1+)
446.2715 y4(1+)
454.1546
528.1915
566.8078 b10(2+)
623.3503 b11(2+)
657.2336
679.8925 b12(2+)
736.4349 b13(2+)
764.9453 b14(2+)
820.4346 y7(1+)
872.9831 b16(2+)
908.5007 b17(2+)
921.4801 y8(1+)
1034.5652 y9(1+)
1132.6105 b10(1+)
1482.303 PEP_0_0_0_0_0
1583.8351 PEP_0_1_0_0_0
1685.3719 PEP_0_2_0_0_0

File: 231129_OE_RK_37, RT: 76.046, Scan: 104352
Type: HCD, FTMS + c NSI d Full ms2 1232.0851@hcd30.00 [120.0000-5049.7632]
Precursor Scan: 104134, m/z: 1230.3296, charge: 4
LSLHRPALEDLLLGSEANLTCTLTGLR_4_5_0_1_0

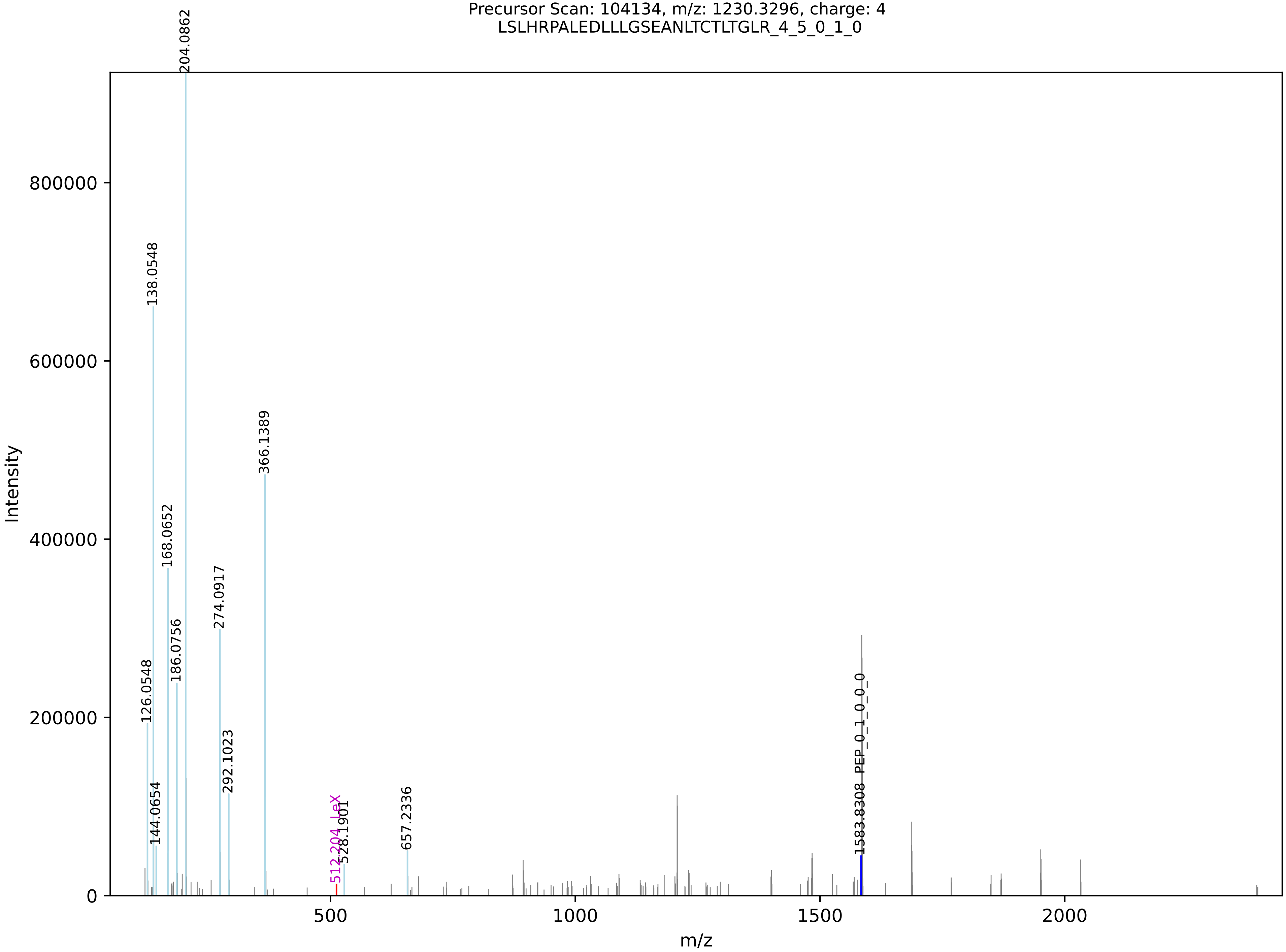

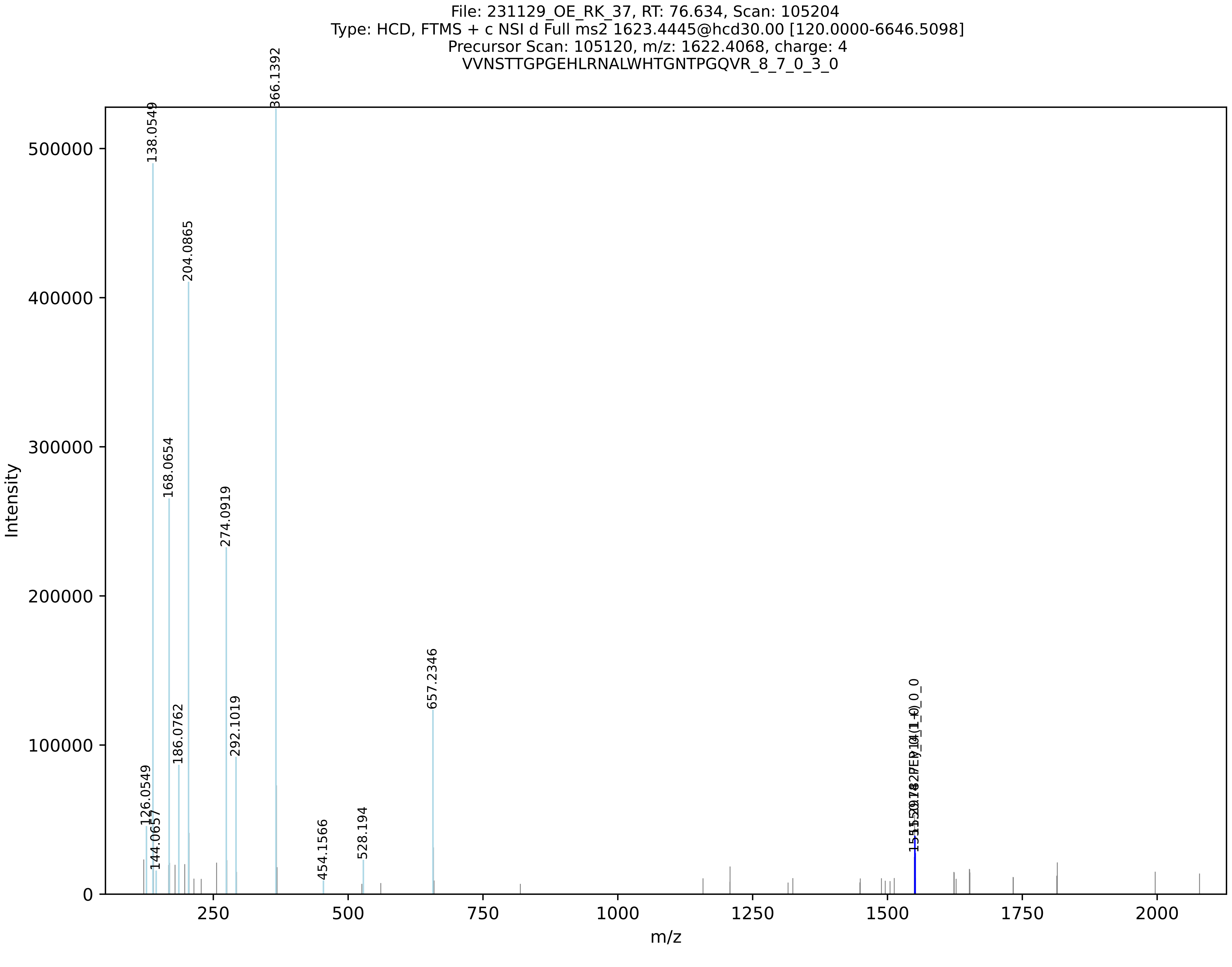
File: 231129_OE_RK_37, RT: 76.634, Scan: 105204
Type: HCD, FTMS + c NSI d Full ms2 1623.4445@hcd30.00 [120.0000-6646.5098]
Precursor Scan: 105120, m/z: 1622.4068, charge: 4
VVNSTTGPGEHLRNALWHTGNTPGQVR_8_7_0_3_0
366.1392
138.0549
204.0865
168.0654
274.0919
657.2346
186.0762
292.1019
126.0549
144.0657
454.1566
528.194
Intensity
m/z
0
100000
200000
300000
400000
500000
250
500
750
1000
1250
1500
1750
2000

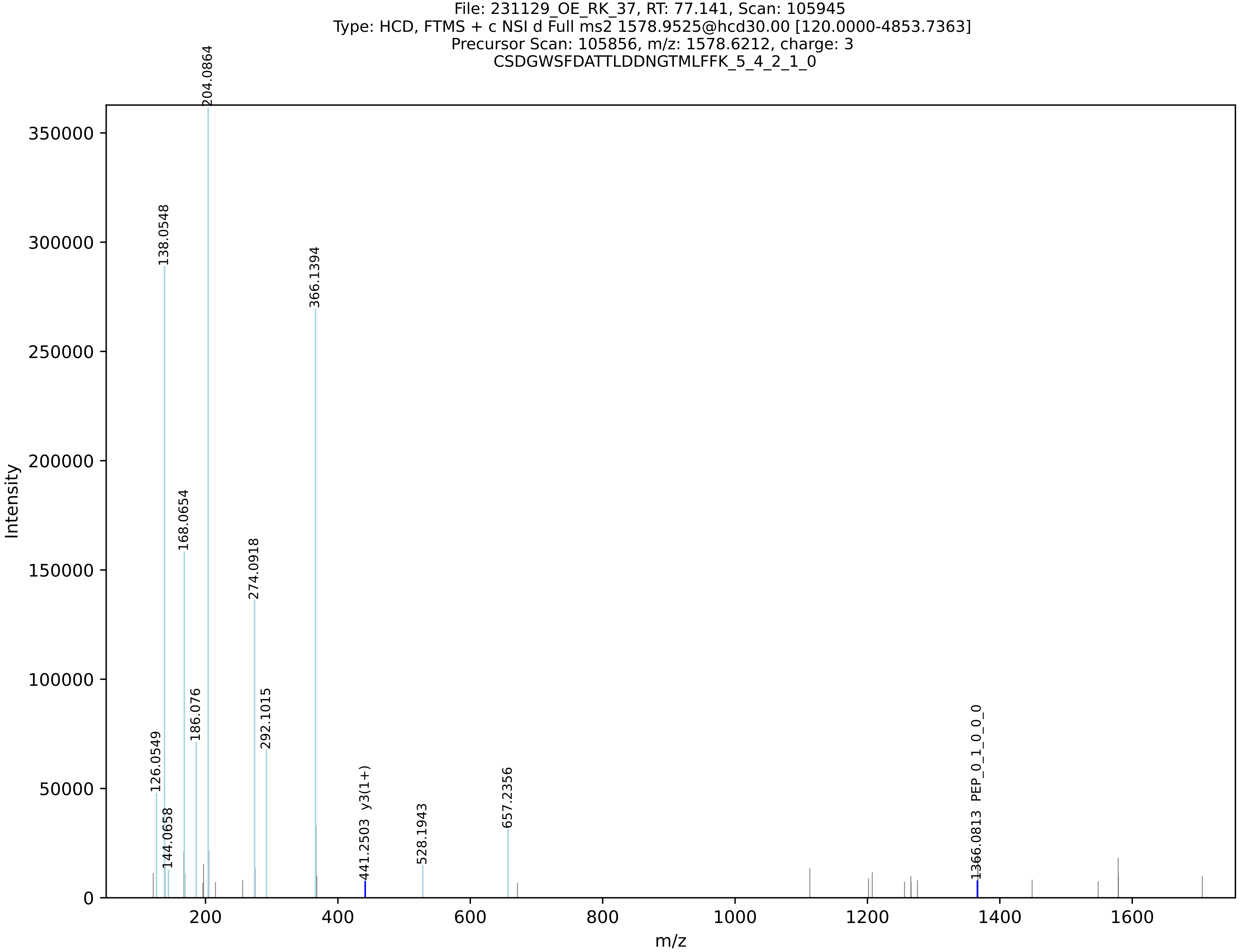

File: 231129_OE_RK_37, RT: 77.141, Scan: 105945
Type: HCD, FTMS + c NSI d Full ms2 1578.9525@hcd30.00 [120.0000-4853.7363]
Precursor Scan: 105856, m/z: 1578.6212, charge: 3
CSDGWSFDATTLDDNGTMLFFK_5_4_2_1_0
Intensity
m/z
204.0864
138.0548
366.1394
168.0654
274.0918
186.076
292.1015
126.0549
144.0658
441.2503 y3(1+)
528.1943
657.2356
1366.0813 PEP_0_1_0_0_0_

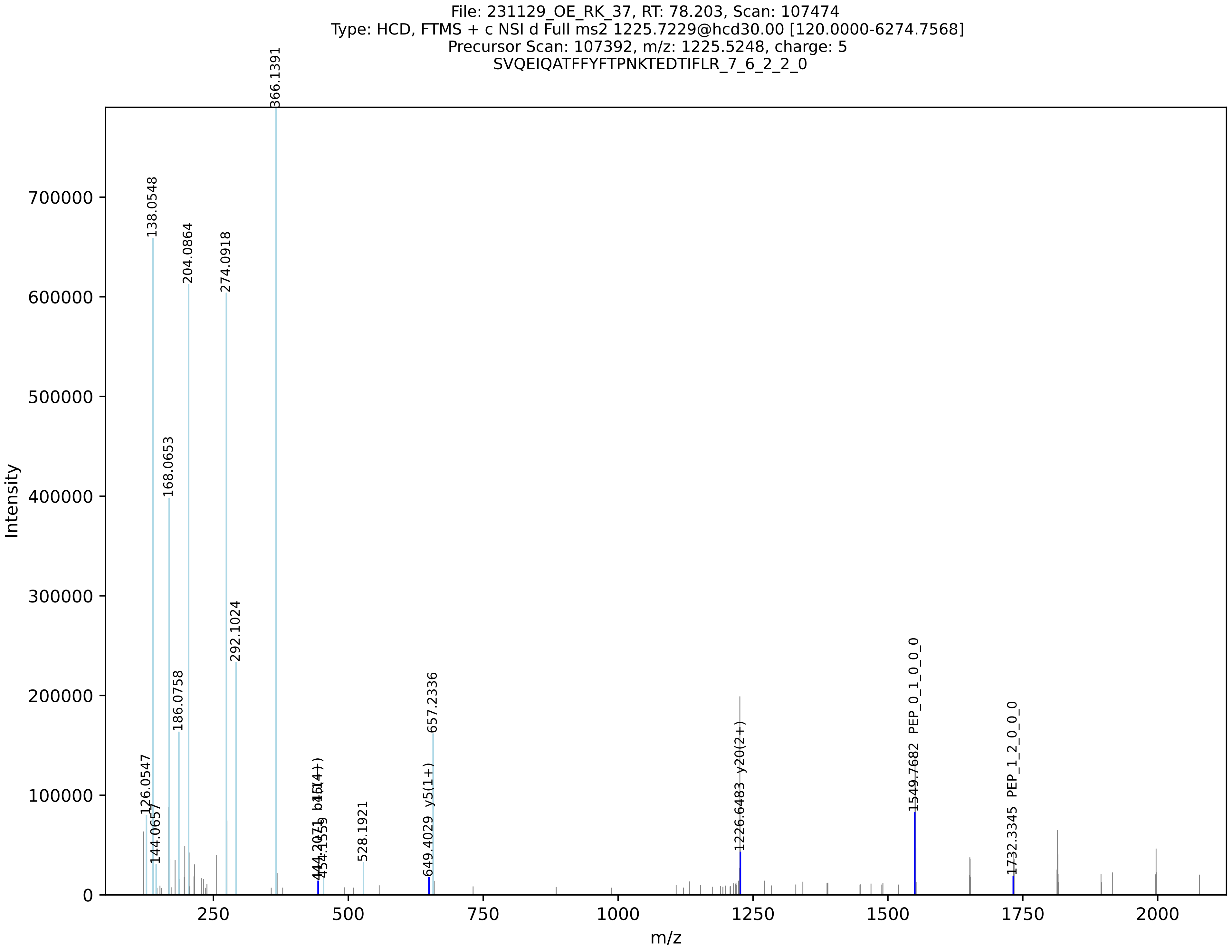
File: 231129_OE_RK_37, RT: 78.203, Scan: 107474
Type: HCD, FTMS + c NSI d Full ms2 1225.7229@hcd30.00 [120.0000-6274.7568]
Precursor Scan: 107392, m/z: 1225.5248, charge: 5
SVQEIQATFFYFTPNKTEDTIFLR_7_6_2_2_0
Intensity
m/z
366.1391
138.0548
204.0864
274.0918
168.0653
292.1024
186.0758
657.2336
126.0547
144.0657
528.1921
454.1559
649.4029 y5(1+)
1226.6483 y20(2+)
1549.7682 PEP_0_1_0_0_0
1732.3345 PEP_1_2_0_0_0

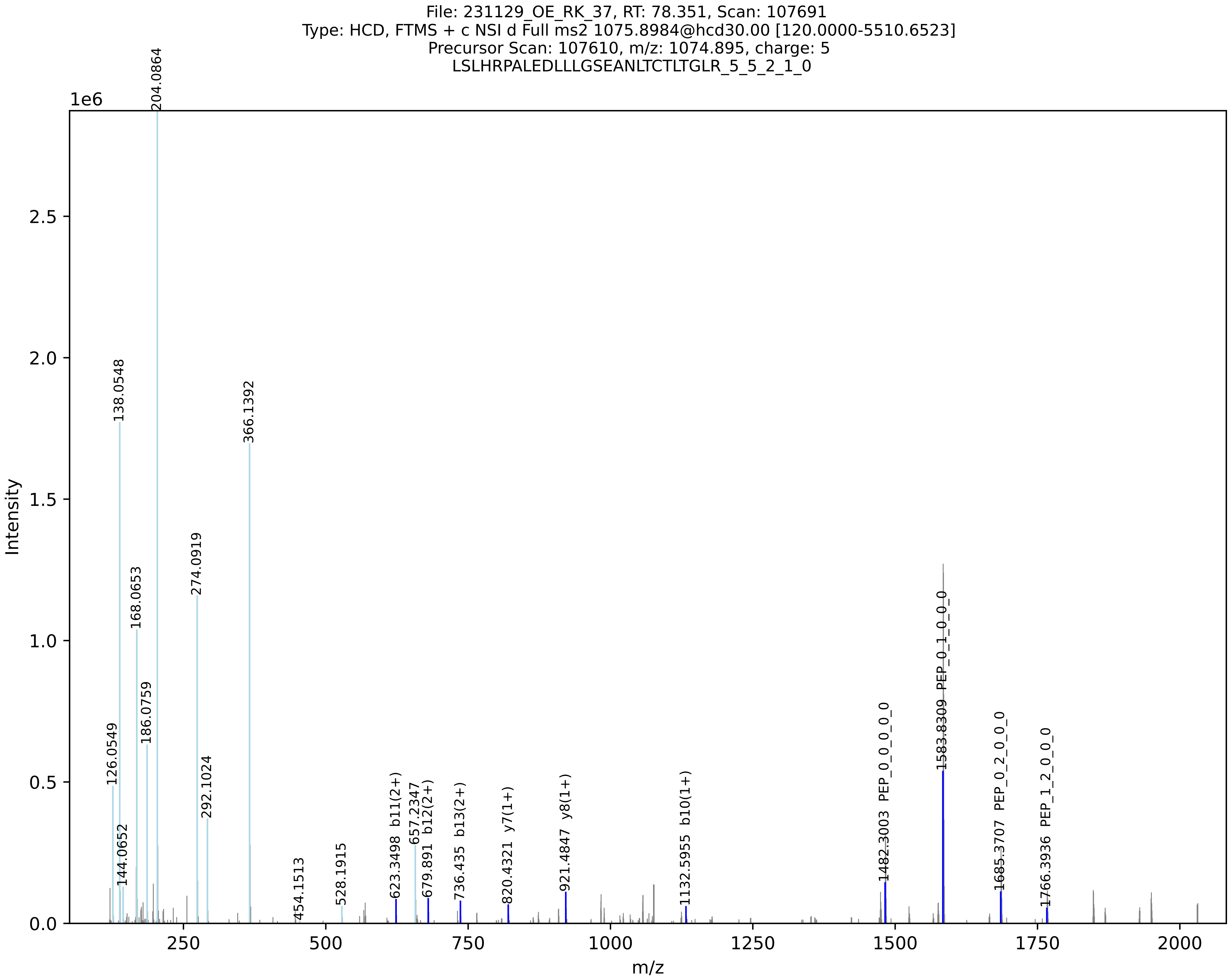

File: 231129_OE_RK_37, RT: 78.351, Scan: 107691
Type: HCD, FTMS + c NSI d Full ms2 1075.8984@hcd30.00 [120.0000-5510.6523]
Precursor Scan: 107610, m/z: 1074.895, charge: 5
LSLHRPALEDLLLGSEANLTCTLTGLR_5_5_2_1_0
1e6
Intensity
m/z
204.0864
138.0548
366.1392
274.0919
168.0653
186.0759
126.0549
292.1024
144.0652
454.1513
528.1915
623.3498 b11(2+)
657.2347
679.891 b12(2+)
736.435 b13(2+)
820.4321 y7(1+)
921.4847 y8(1+)
1132.5955 b10(1+)
1482.3003 PEP_0_0_0_0_0
1583.8309 PEP_0_1_0_0_0
1685.3707 PEP_0_2_0_0_0
1766.3936 PEP_1_2_0_0_0

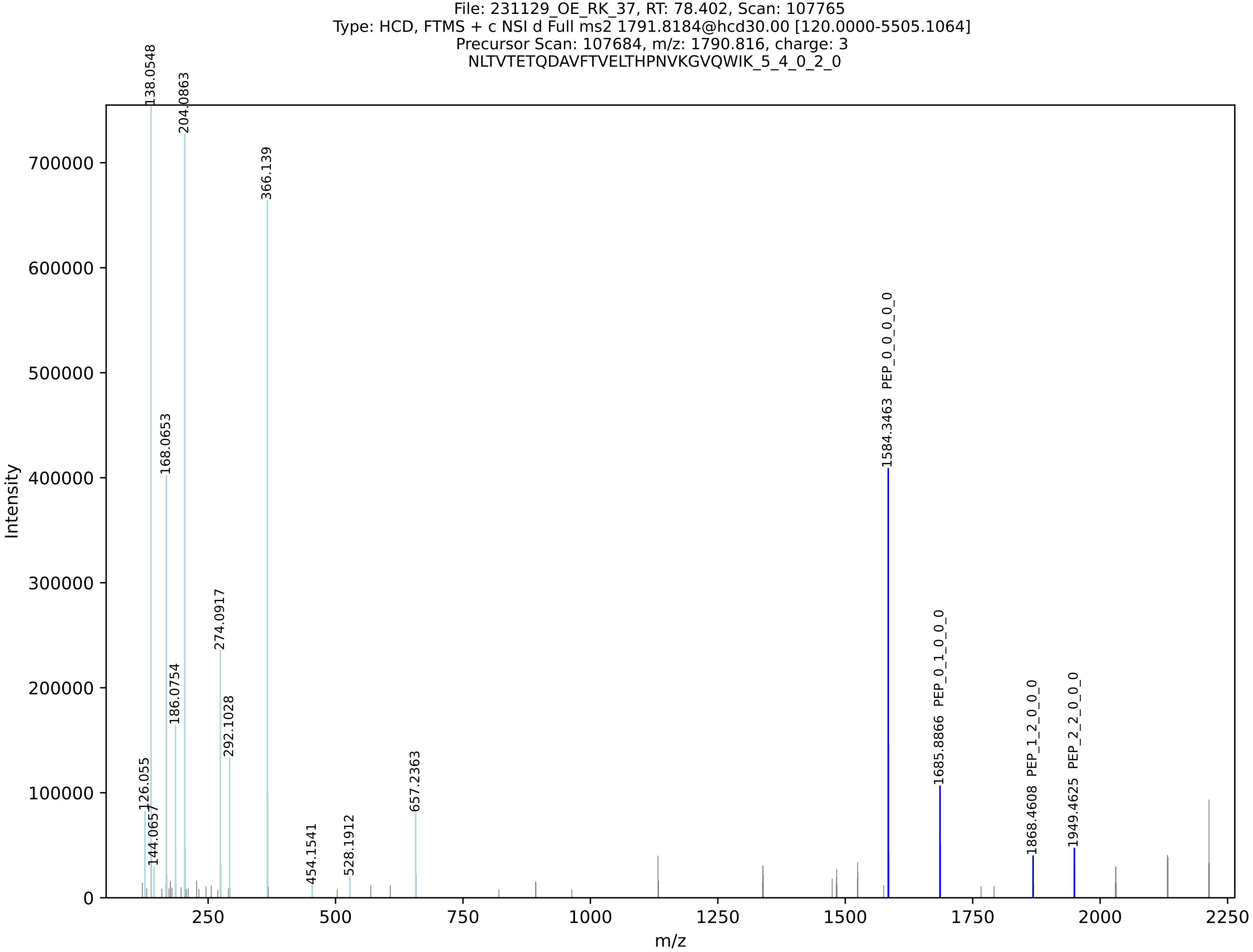

File: 231129_OE_RK_37, RT: 78.402, Scan: 107765
Type: HCD, FTMS + c NSI d Full ms2 1791.8184@hcd30.00 [120.0000-5505.1064]
Precursor Scan: 107684, m/z: 1790.816, charge: 3
NLTVTETQDAVFTVELTHPNVKGVQWIK_5_4_0_2_0
Intensity
m/z
138.0548
204.0863
366.139
168.0653
186.0754
274.0917
292.1028
126.055
144.0657
454.1541
528.1912
657.2363
1584.3463 PEP_0_0_0_0_0
1685.8866 PEP_0_1_0_0_0
1868.4608 PEP_1_2_0_0_0
1949.4625 PEP_2_2_0_0_0

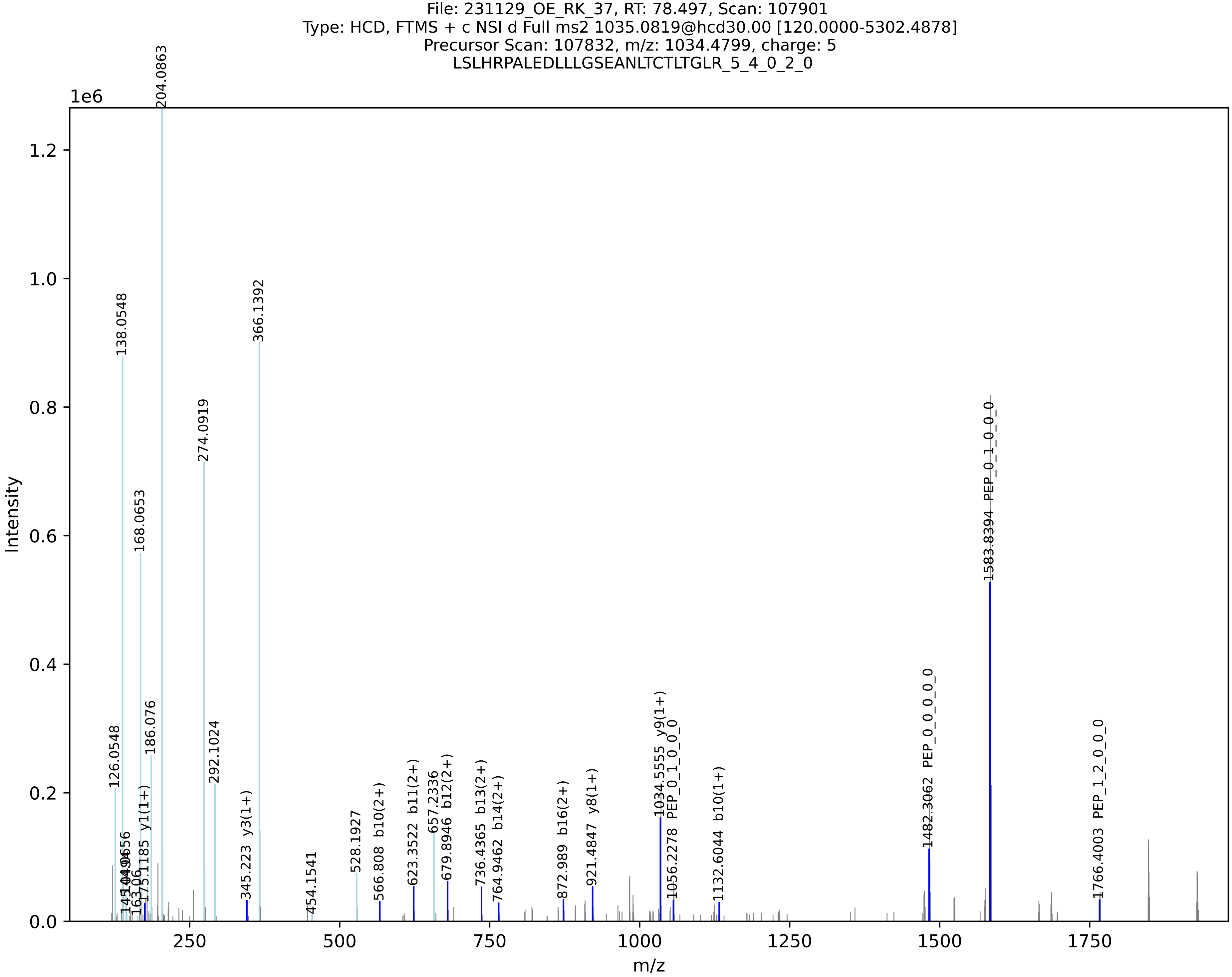
File: 231129_OE_RK_37, RT: 78.497, Scan: 107901
Type: HCD, FTMS + c NSI d Full ms2 1035.0819@hcd30.00 [120.0000-5302.4878]
Precursor Scan: 107832, m/z: 1034.4799, charge: 5
LSLHRPALEDLLLGSEANLTCTLTGLR_5_4_0_2_0
1e6
Intensity
m/z
204.0863
138.0548
366.1392
274.0919
168.0653
126.0548
186.076
292.1024
175.1185 y1(1+)
345.223 y3(1+)
454.1541
528.1927
566.808 b10(2+)
623.3522 b11(2+)
657.2336
679.8946 b12(2+)
736.4365 b13(2+)
764.9462 b14(2+)
872.989 b16(2+)
921.4847 y8(1+)
1034.5555 y9(1+)
1056.2278 PEP_0_1_0_0_0
1132.6044 b10(1+)
1482.3062 PEP_0_0_0_0_0
1583.8394 PEP_0_1_0_0_0
1766.4003 PEP_1_2_0_0_0
0.0
0.2
0.4
0.6
0.8
1.0
1.2
250
500
750
1000
1250
1500
1750

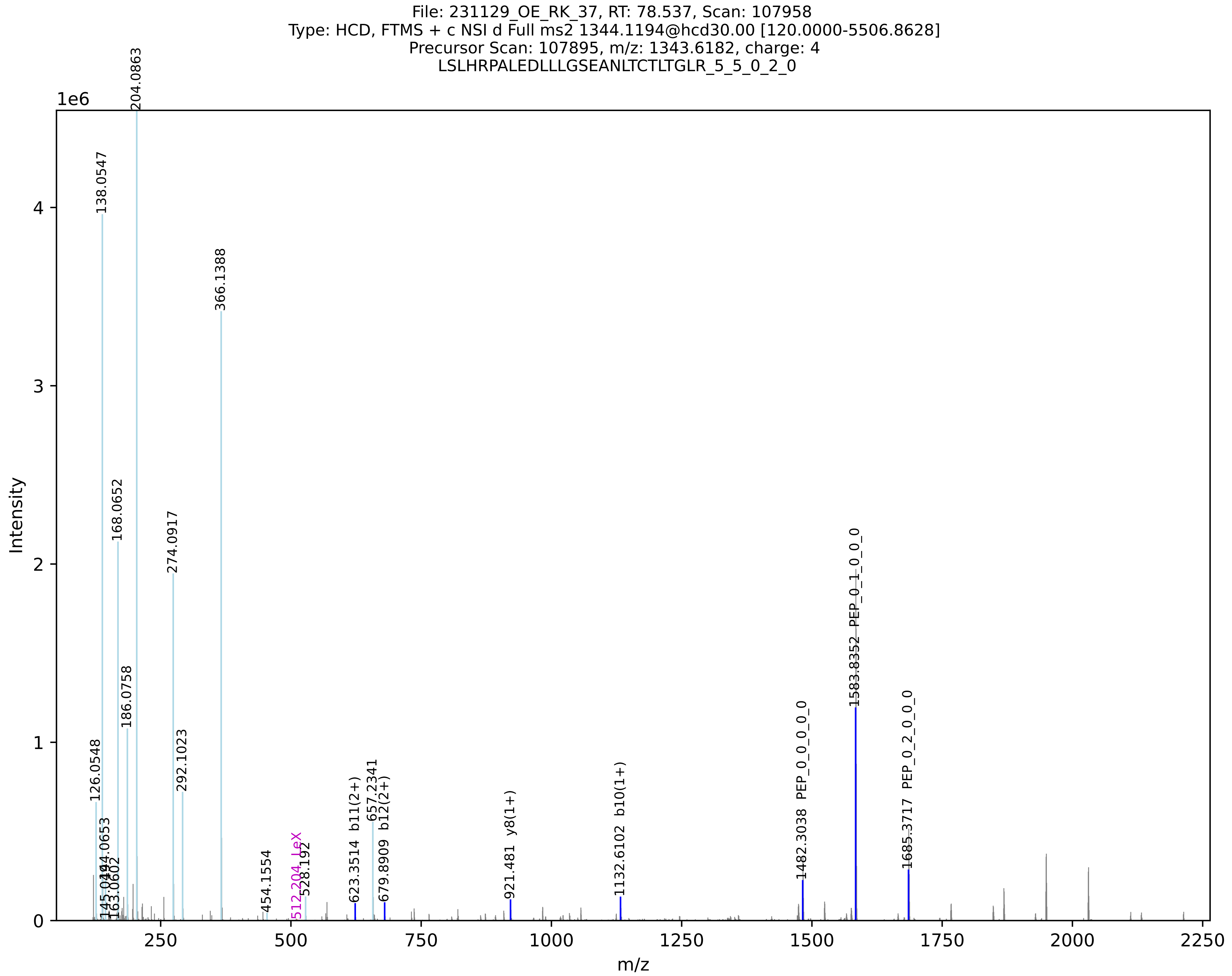
File: 231129_OE_RK_37, RT: 78.537, Scan: 107958
Type: HCD, FTMS + c NSI d Full ms2 1344.1194@hcd30.00 [120.0000-5506.8628]
Precursor Scan: 107895, m/z: 1343.6182, charge: 4
LSLHRPALEDLLLGSEANLTCTLTGLR_5_5_0_2_0
1e6
Intensity
m/z
204.0863
138.0547
366.1388
168.0652
274.0917
186.0758
292.1023
126.0548
144.0653
163.0602
454.1554
512.204_LeX
528.192
623.3514 b11(2+)
657.2341
679.8909 b12(2+)
921.481 y8(1+)
1132.6102 b10(1+)
1482.3038 PEP_0_0_0_0_0
1583.8352 PEP_0_1_0_0_0
1685.3717 PEP_0_2_0_0_0

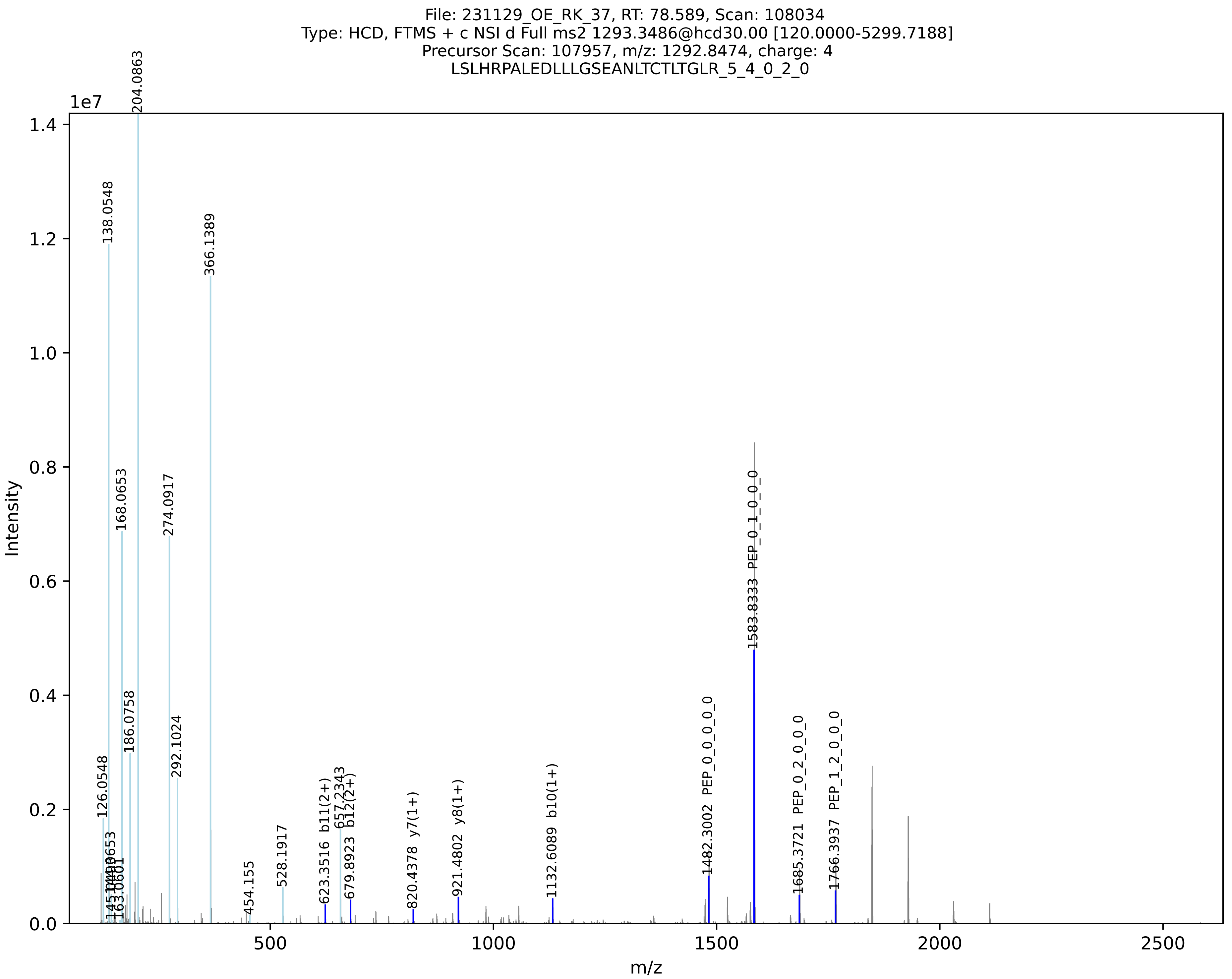
File: 231129_OE_RK_37, RT: 78.589, Scan: 108034
Type: HCD, FTMS + c NSI d Full ms2 1293.3486@hcd30.00 [120.0000-5299.7188]
Precursor Scan: 107957, m/z: 1292.8474, charge: 4
LSLHRPALEDLLLGSEANLTCTLTGLR_5_4_0_2_0
1e7
Intensity
m/z
204.0863
138.0548
366.1389
168.0653
274.0917
186.0758
292.1024
126.0548
163.0601
454.155
528.1917
623.3516 b11(2+)
657.2343
679.8923 b12(2+)
820.4378 y7(1+)
921.4802 y8(1+)
1132.6089 b10(1+)
1482.3002 PEP_0_0_0_0_0
1583.8333 PEP_0_1_0_0_0
1685.3721 PEP_0_2_0_0_0
1766.3937 PEP_1_2_0_0_0

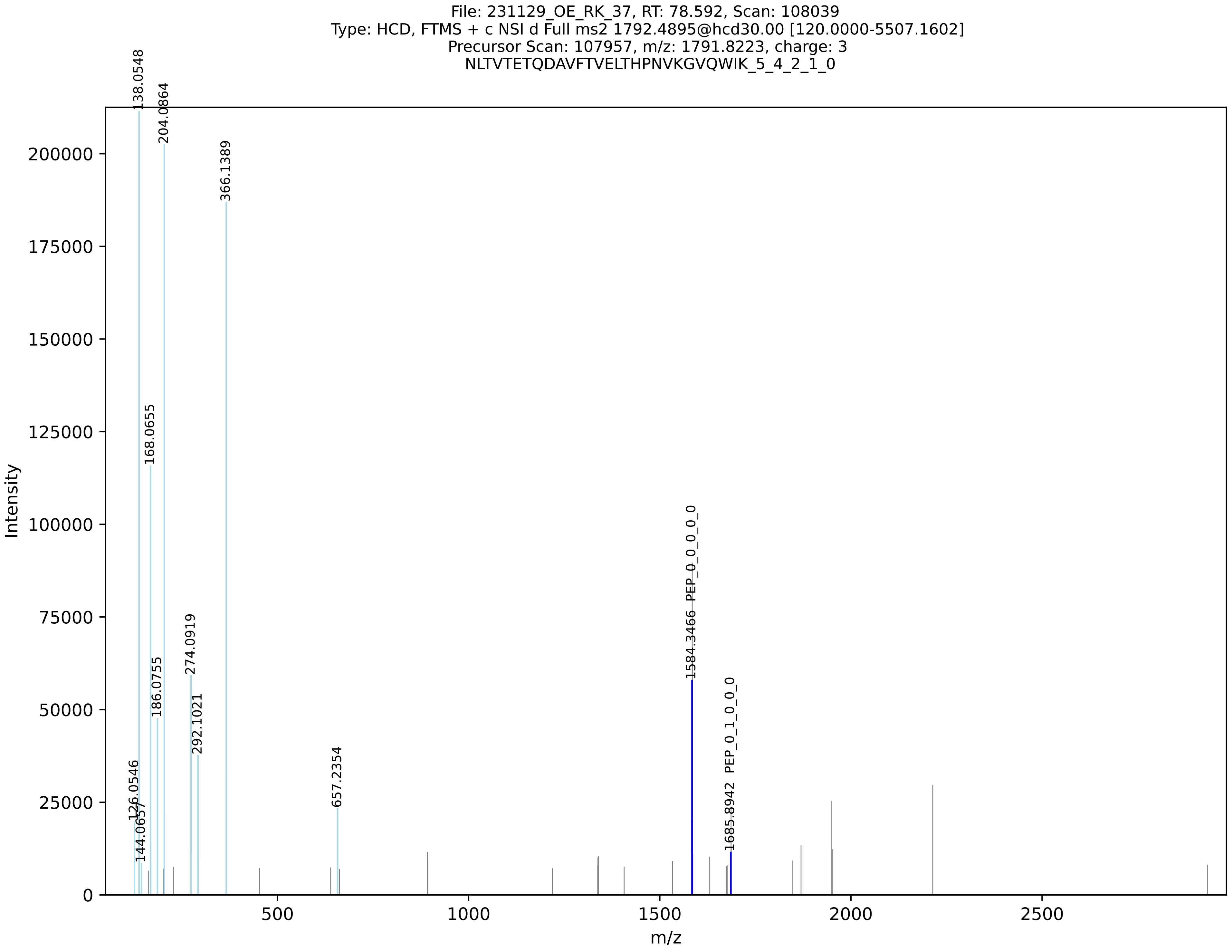

File: 231129_OE_RK_37, RT: 78.592, Scan: 108039
Type: HCD, FTMS + c NSI d Full ms2 1792.4895@hcd30.00 [120.0000-5507.1602]
Precursor Scan: 107957, m/z: 1791.8223, charge: 3
NLTVTETQDAVFTVELTHPNVKGVQWIK_5_4_2_1_0
138.0548
204.0864
366.1389
168.0655
186.0755
274.0919
292.1021
126.0546
144.0657
657.2354
1584.3466 PEP_0_0_0_0_0
1685.8942 PEP_0_1_0_0_0
Intensity
m/z
0
25000
50000
75000
100000
125000
150000
175000
200000
500
1000
1500
2000
2500

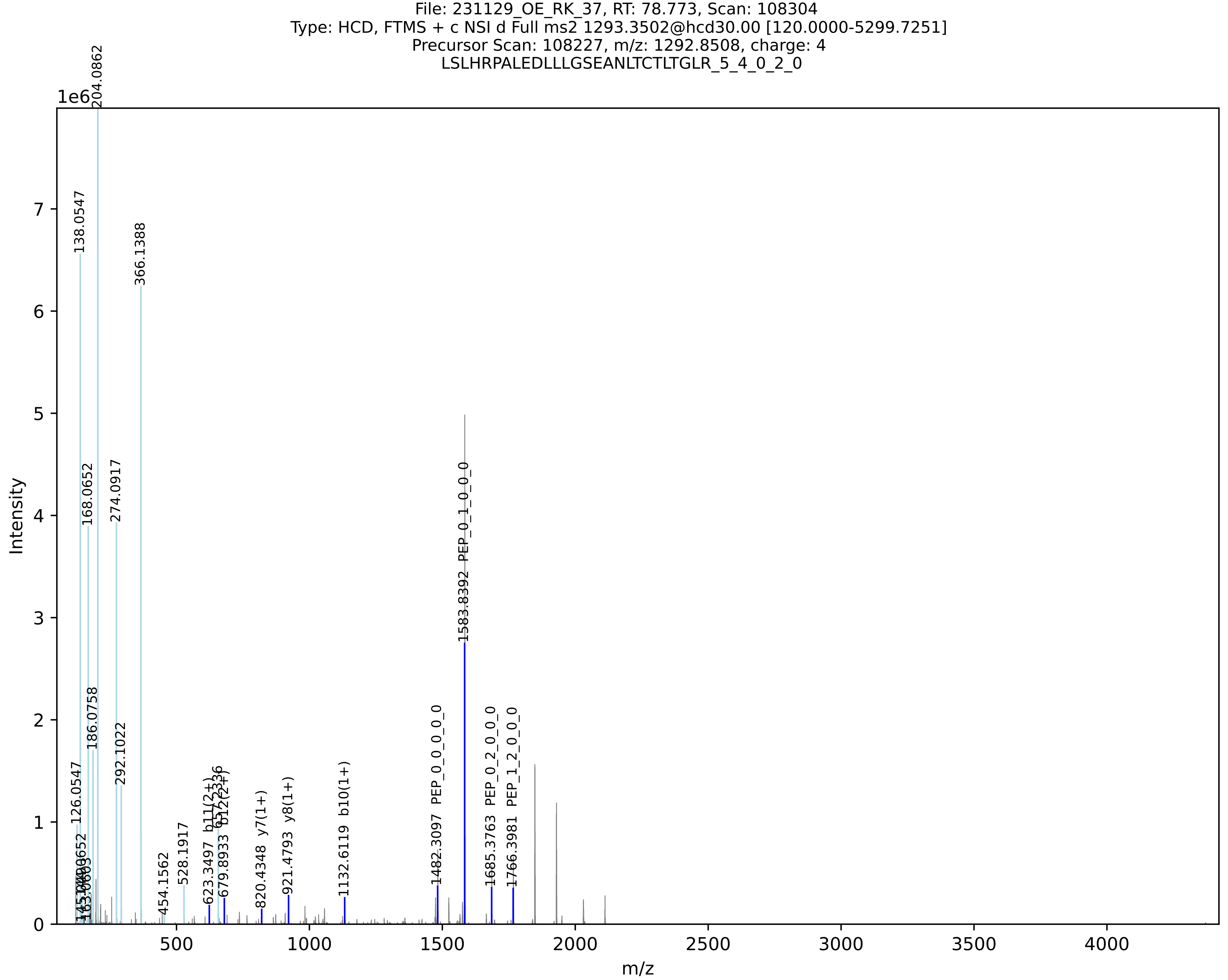
File: 231129_OE_RK_37, RT: 78.773, Scan: 108304
Type: HCD, FTMS + c NSI d Full ms2 1293.3502@hcd30.00 [120.0000-5299.7251]
Precursor Scan: 108227, m/z: 1292.8508, charge: 4
LSLHRPALEDLLLGSEANLTCTLTGLR_5_4_0_2_0
1e6
Intensity
m/z
204.0862
138.0547
366.1388
168.0652
274.0917
186.0758
292.1022
126.0547
454.1562
528.1917
623.3497 b11(2+)
679.8933 b12(2+)
820.4348 y7(1+)
921.4793 y8(1+)
1132.6119 b10(1+)
1482.3097 PEP_0_0_0_0_0
1583.8392 PEP_0_1_0_0_0
1685.3763 PEP_0_2_0_0_0
1766.3981 PEP_1_2_0_0_0

File: 231129_OE_RK_37, RT: 78.875, Scan: 108452
Type: HCD, FTMS + c NSI d Full ms2 1035.0815@hcd30.00 [120.0000-5302.4858]
Precursor Scan: 108374, m/z: 1034.4808, charge: 5
LSLHRPALEDLLLGSEANLTCTLTGLR_5_4_0_2_0

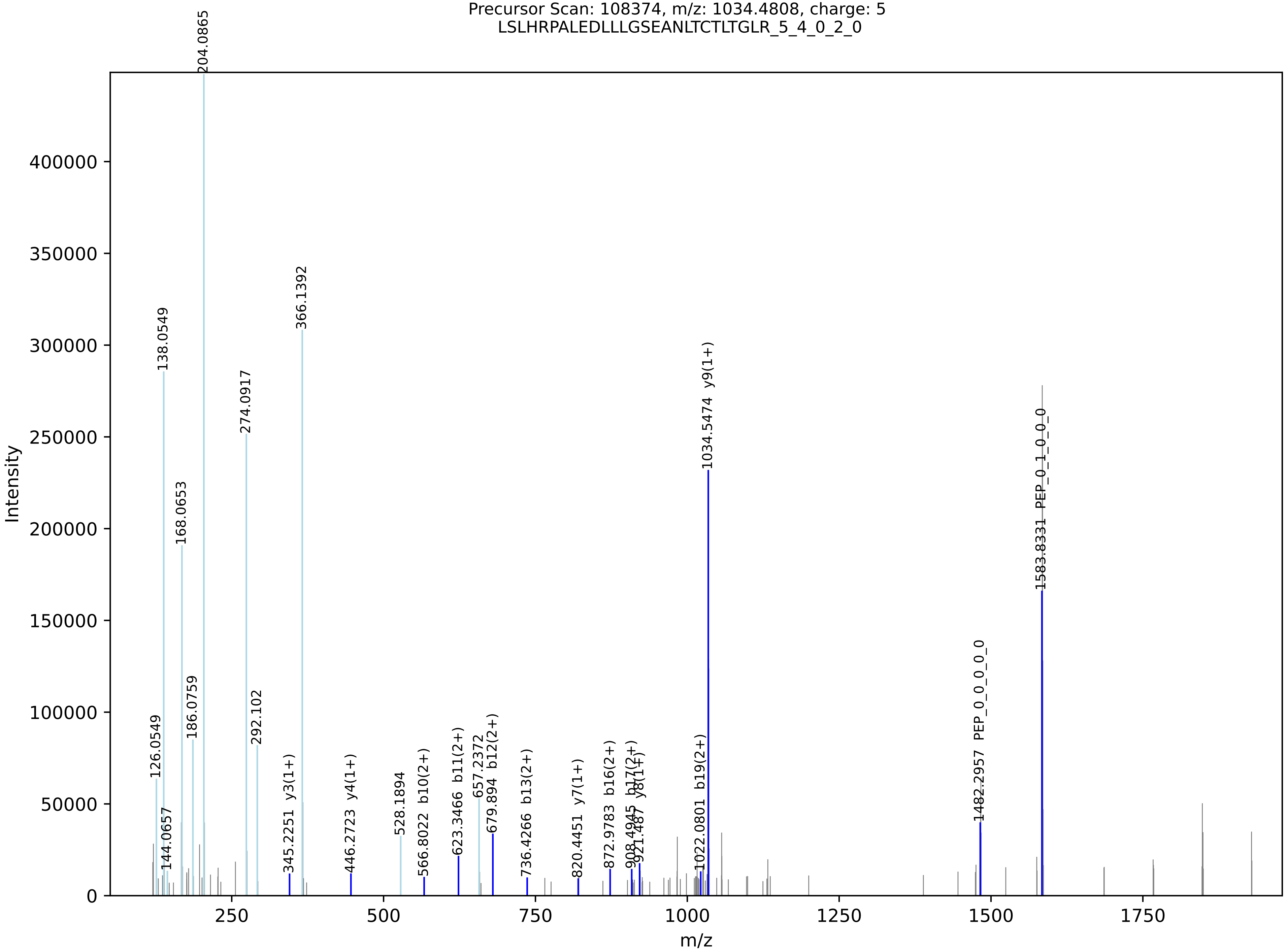

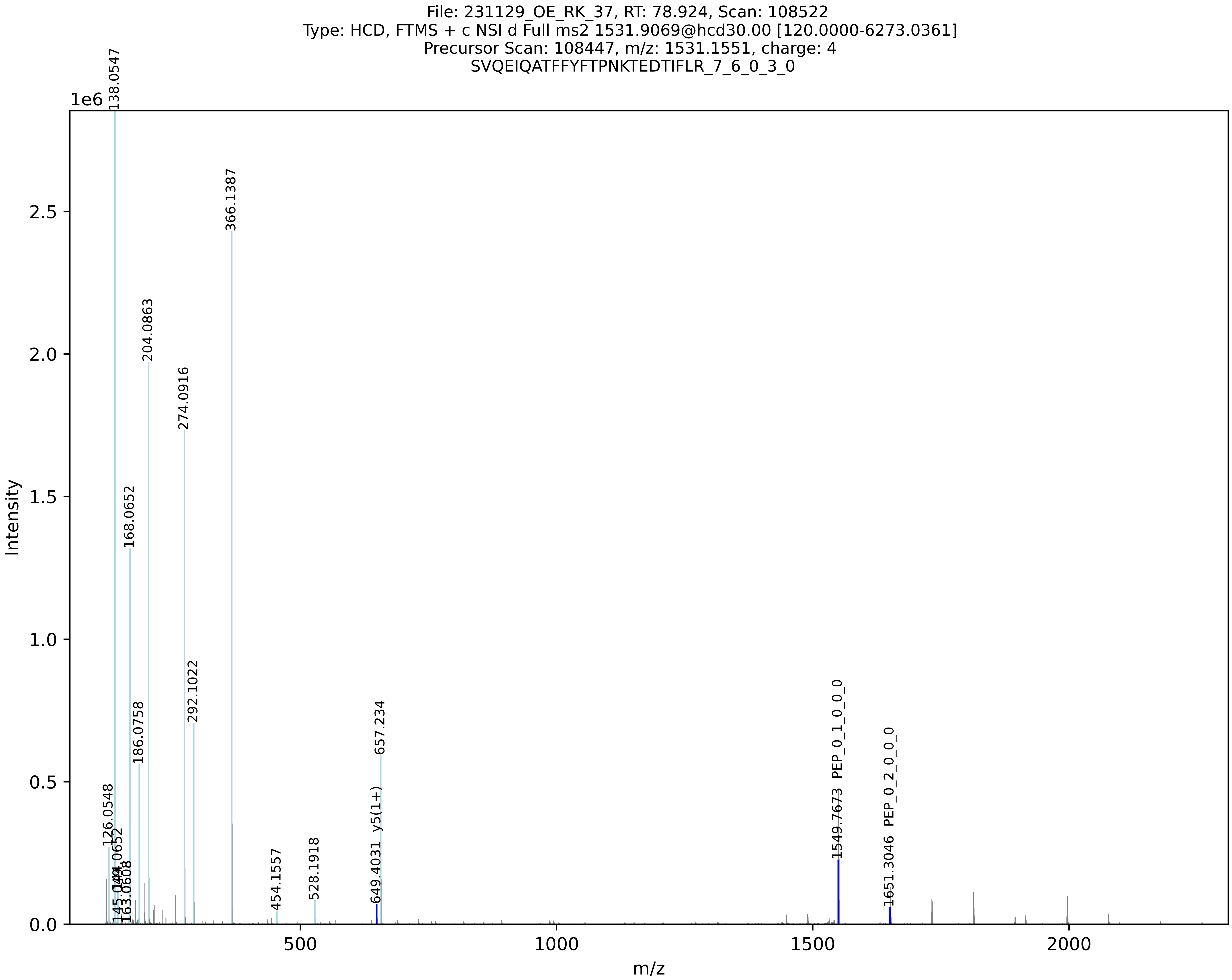

File: 231129_OE_RK_37, RT: 78.924, Scan: 108522
Type: HCD, FTMS + c NSI d Full ms2 1531.9069@hcd30.00 [120.0000-6273.0361]
Precursor Scan: 108447, m/z: 1531.1551, charge: 4
SVQEIQATFFYFTPNKTEDTIFLR_7_6_0_3_0
1e6
Intensity
m/z
138.0547
366.1387
204.0863
274.0916
168.0652
292.1022
186.0758
126.0548
163.0608
454.1557
528.1918
649.4031 y5(1+)
657.234
1549.7673 PEP_0_1_0_0_0
1651.3046 PEP_0_2_0_0_0

File: 231129_OE_RK_37, RT: 78.976, Scan: 108598
Type: HCD, FTMS + c NSI d Full ms2 1293.6017@hcd30.00 [120.0000-5300.7510]
Precursor Scan: 108521, m/z: 1292.8499, charge: 4
LSLHRPALEDLLLGSEANLTCTLTGLR_5_4_0_2_0

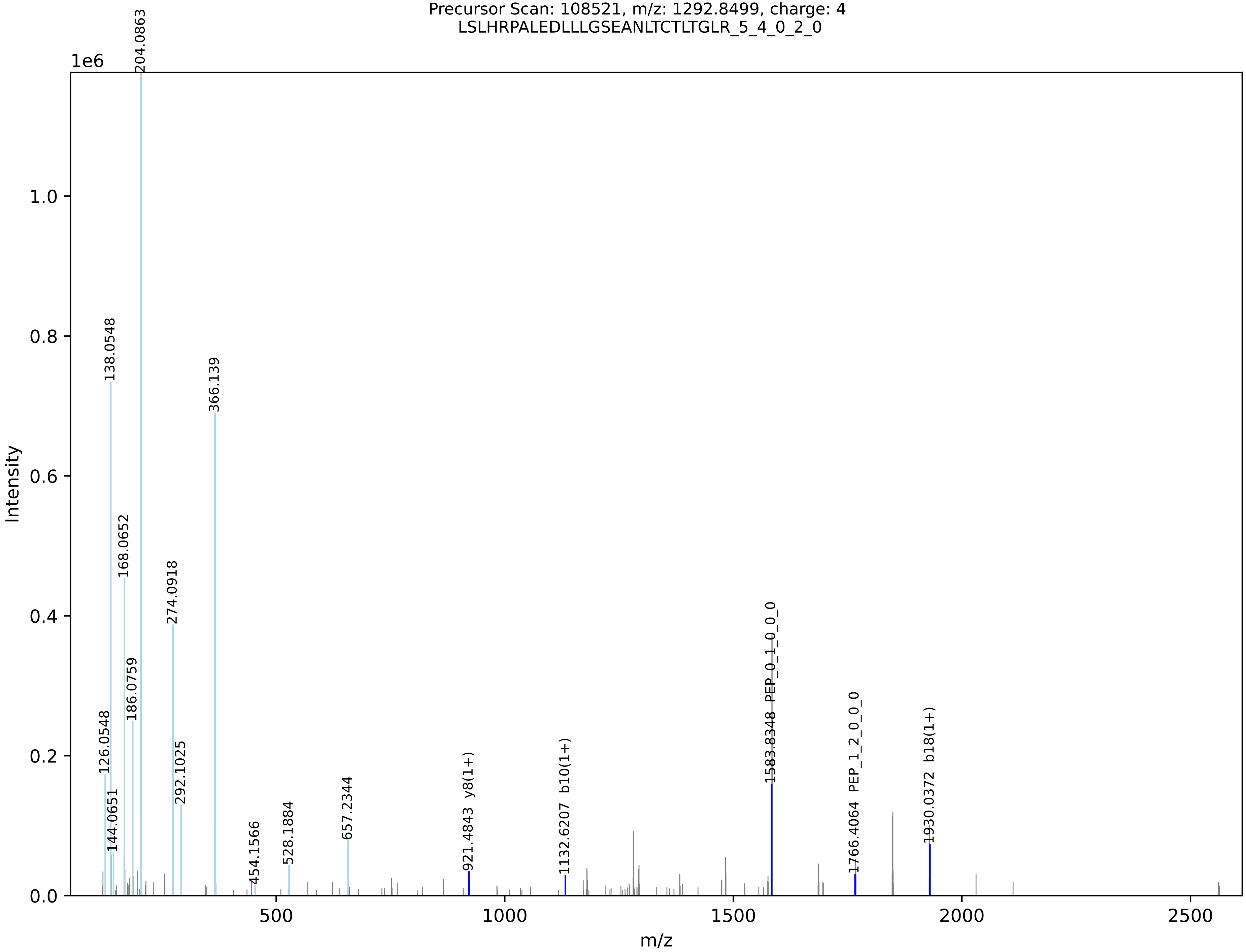

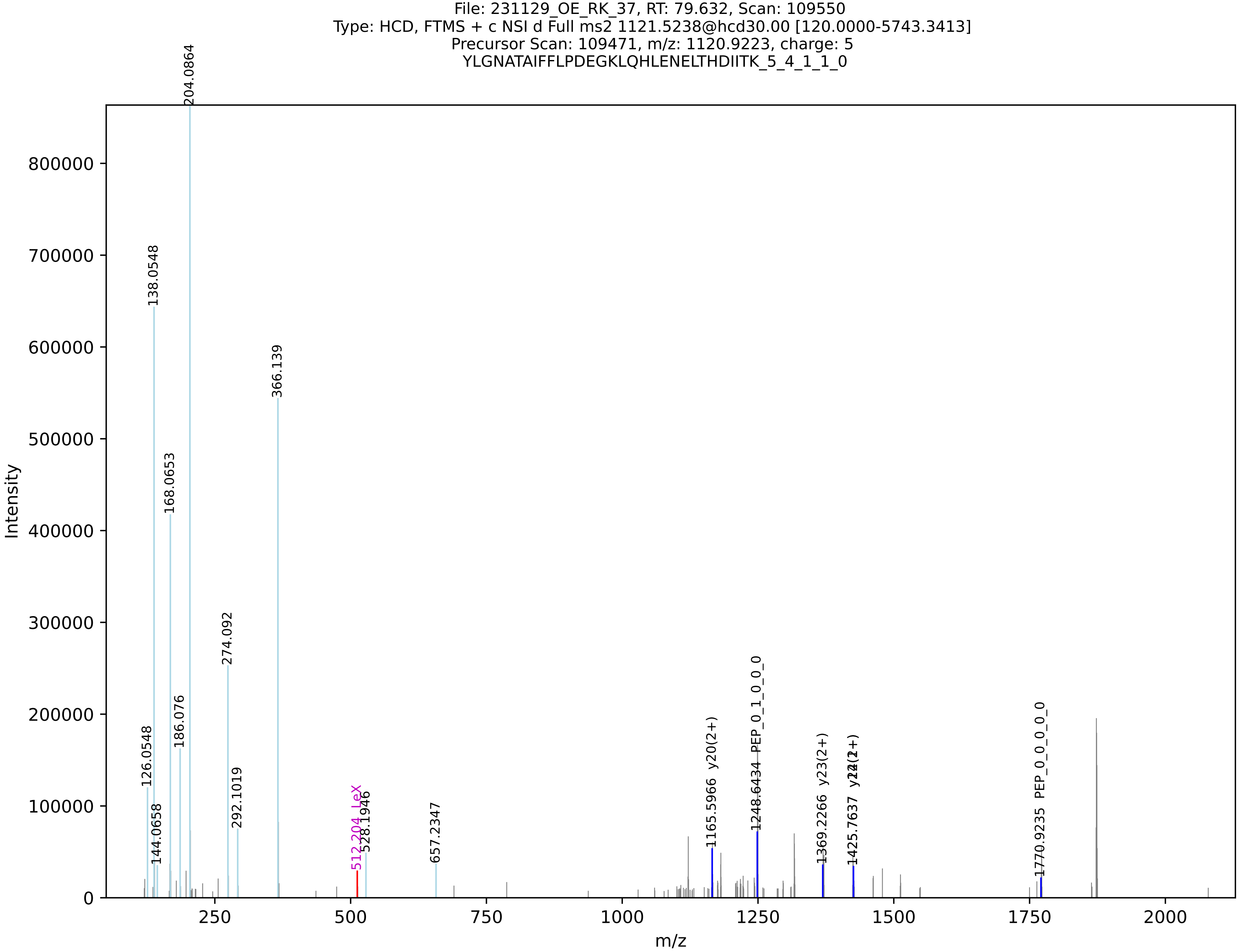

File: 231129_OE_RK_37, RT: 79.632, Scan: 109550
Type: HCD, FTMS + c NSI d Full ms2 1121.5238@hcd30.00 [120.0000-5743.3413]
Precursor Scan: 109471, m/z: 1120.9223, charge: 5
YLGNATAIFFLPDEGKLQHLENELTHDIITK_5_4_1_1_0
Intensity
m/z
0
100000
200000
300000
400000
500000
600000
700000
800000
250
500
750
1000
1250
1500
1750
2000
204.0864
138.0548
366.139
168.0653
274.092
186.076
126.0548
292.1019
144.0658
512.204_LeX
528.1946
657.2347
1165.5966 y20(2+)
1248.6434 PEP_0_1_0_0_0
1369.2266 y23(2+)
1425.7637
1770.9235 PEP_0_0_0_0_0

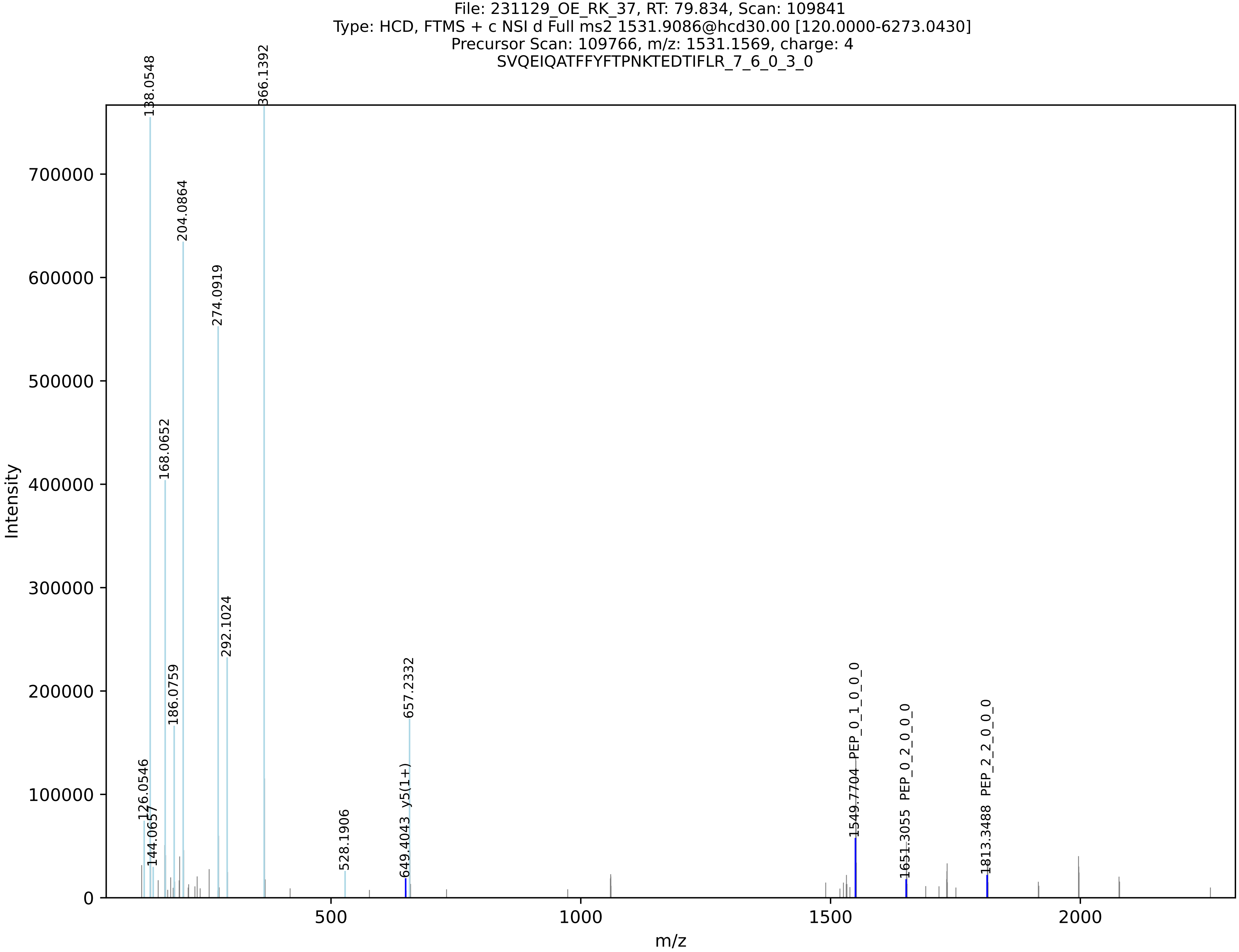
File: 231129_OE_RK_37, RT: 79.834, Scan: 109841
Type: HCD, FTMS + c NSI d Full ms2 1531.9086@hcd30.00 [120.0000-6273.0430]
Precursor Scan: 109766, m/z: 1531.1569, charge: 4
SVQEIQATFFYFTPNKTEDTIFLR_7_6_0_3_0
Intensity
m/z
138.0548
366.1392
204.0864
274.0919
168.0652
292.1024
186.0759
126.0546
144.0657
657.2332
y5(1+)
649.4043
528.1906
1549.7704 PEP_0_1_0_0_0
1651.3055 PEP_0_2_0_0_0
1813.3488 PEP_2_2_0_0_0

File: 231129_OE_RK_37, RT: 81.348, Scan: 112033
Type: HCD, FTMS + c NSI d Full ms2 1604.9314@hcd30.00 [120.0000-6570.9761]
Precursor Scan: 111954, m/z: 1604.1801, charge: 4
SVQEIQATFFYFTPNKTEDTIFLR_7_6_2_3_0

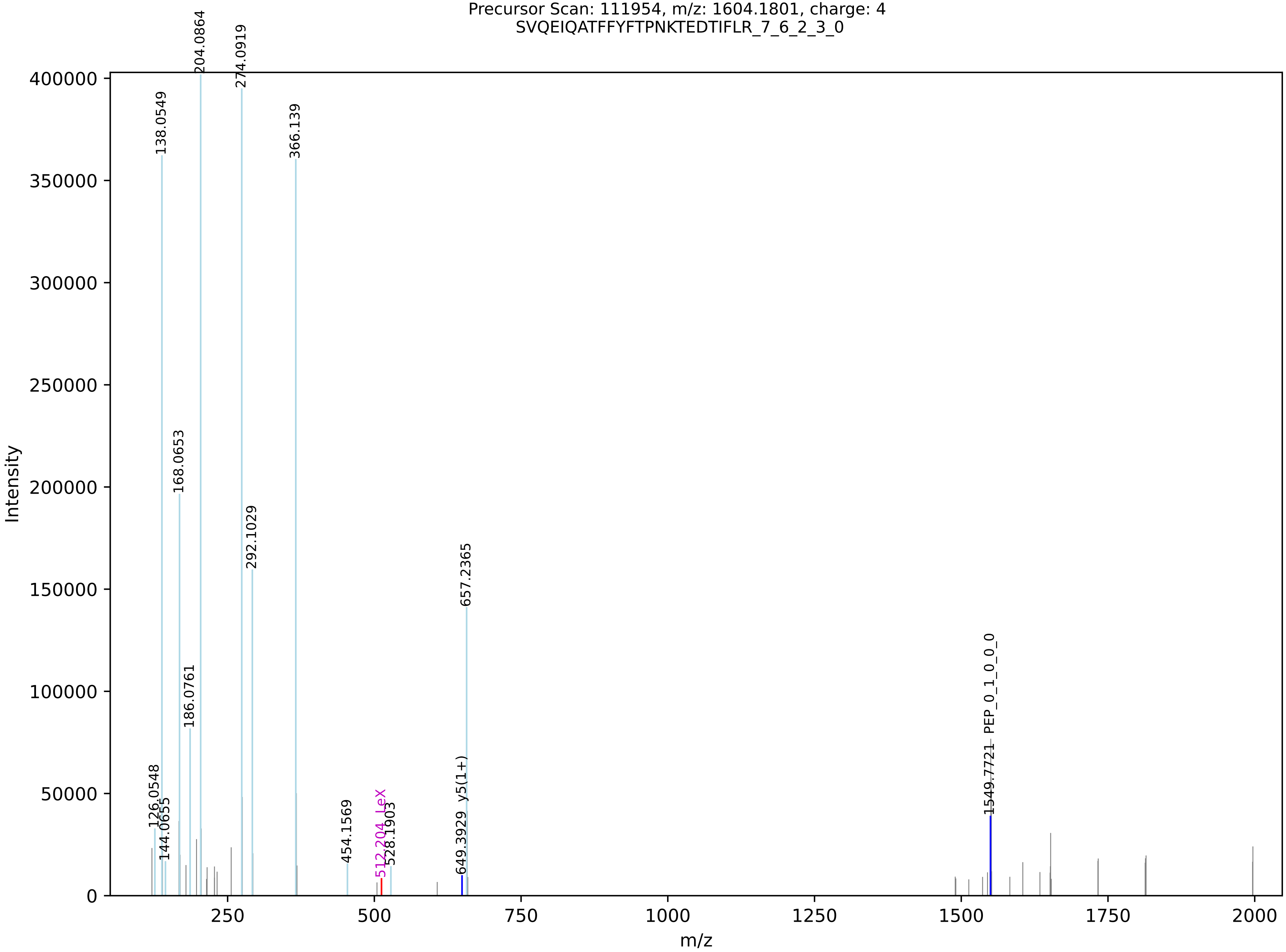

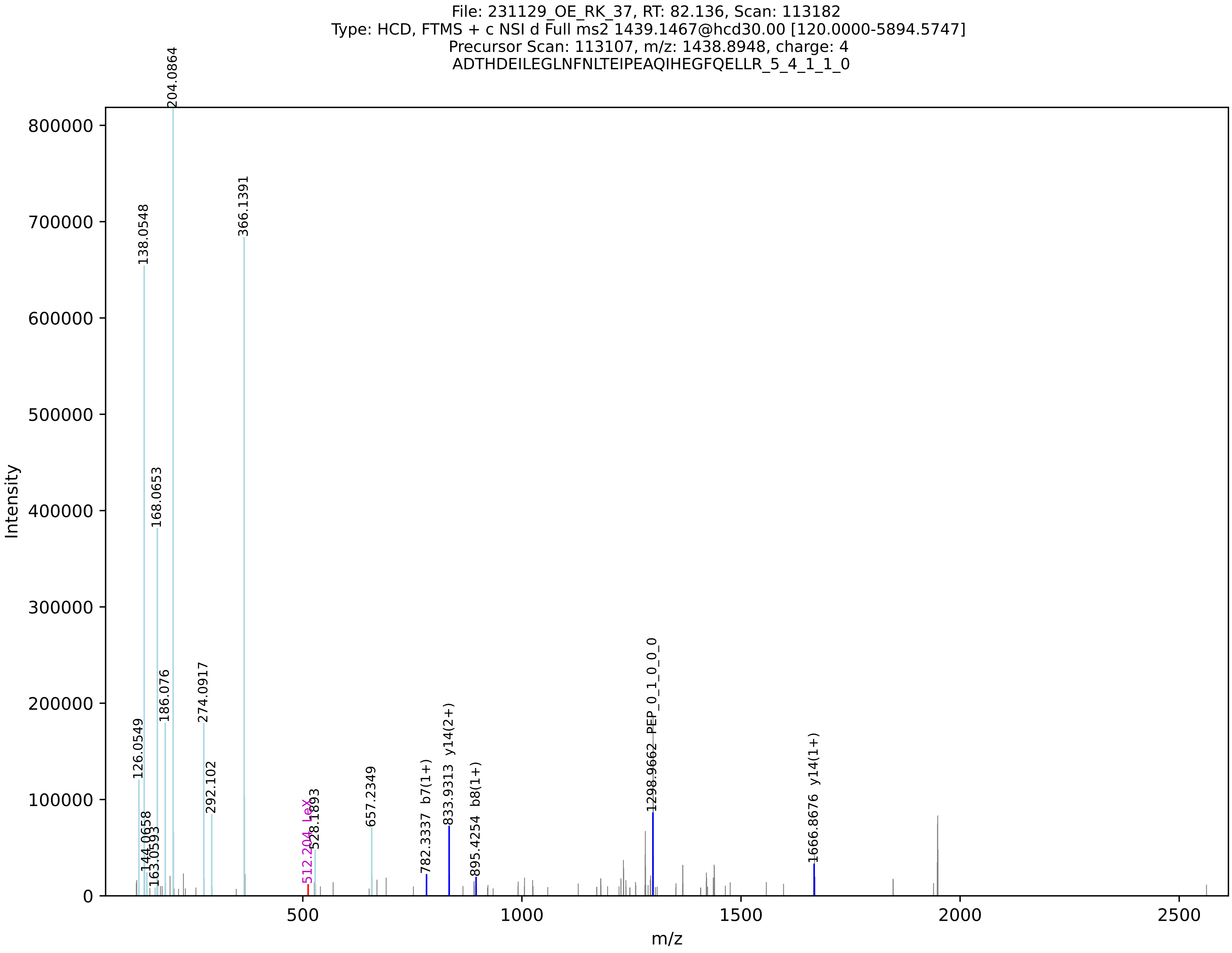

File: 231129_OE_RK_37, RT: 82.136, Scan: 113182
Type: HCD, FTMS + c NSI d Full ms2 1439.1467@hcd30.00 [120.0000-5894.5747]
Precursor Scan: 113107, m/z: 1438.8948, charge: 4
ADTHDEILEGLNFNLTEIPEAQIHEGFQELLR_5_4_1_1_0
Intensity
m/z
204.0864
138.0548
366.1391
168.0653
186.076
274.0917
126.0549
292.102
144.0658
163.0593
512.204_LeX
528.1893
657.2349
782.3337 b7(1+)
833.9313 y14(2+)
895.4254 b8(1+)
1298.9662 PEP_0_1_0_0_0
1666.8676 y14(1+)

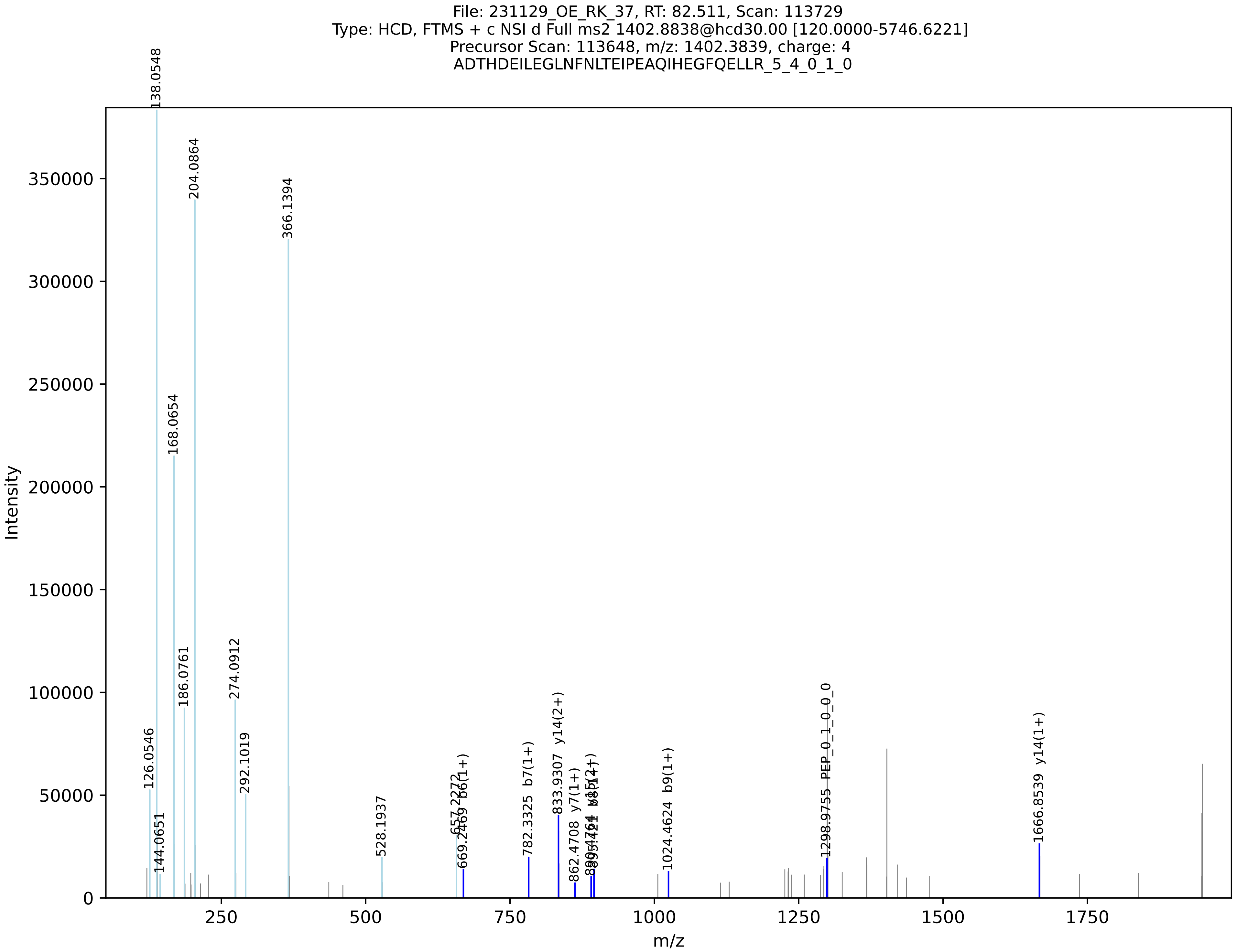
File: 231129_OE_RK_37, RT: 82.511, Scan: 113729
Type: HCD, FTMS + c NSI d Full ms2 1402.8838@hcd30.00 [120.0000-5746.6221]
Precursor Scan: 113648, m/z: 1402.3839, charge: 4
ADTHDEILEGLNFNLTEIPEAQIHEGFQELLR_5_4_0_1_0
Intensity
m/z
138.0548
204.0864
366.1394
168.0654
186.0761
274.0912
126.0546
292.1019
144.0651
528.1937
657.2272
669.2469 b6(1+)
782.3325 b7(1+)
833.9307 y14(2+)
862.4708 y7(1+)
1024.4624 b9(1+)
1298.9755 PEP_0_1_0_0_0
1666.8539 y14(1+)
0
50000
100000
150000
200000
250000
300000
350000
250
500
750
1000
1250
1500
1750

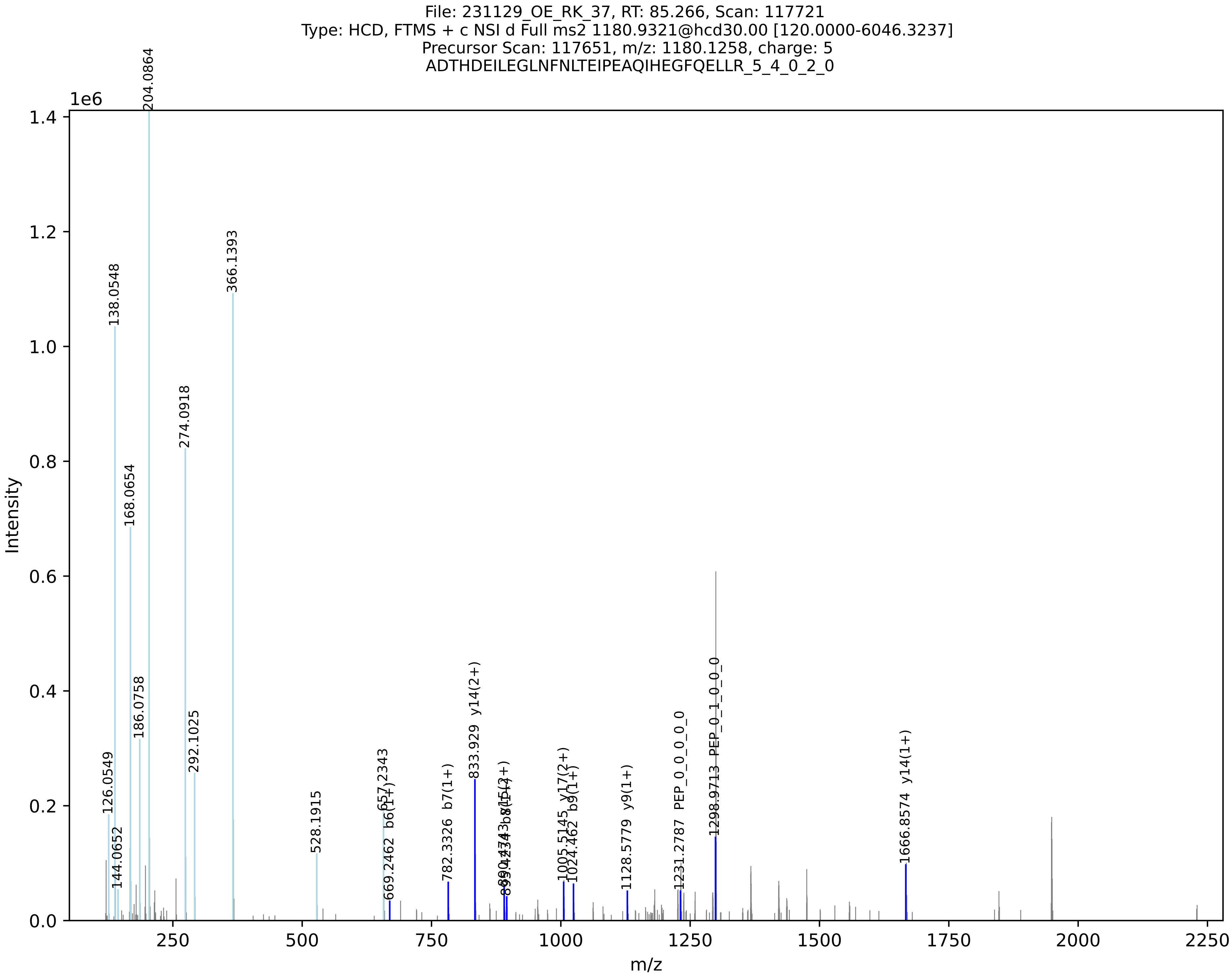

File: 231129_OE_RK_37, RT: 85.266, Scan: 117721
Type: HCD, FTMS + c NSI d Full ms2 1180.9321@hcd30.00 [120.0000-6046.3237]
Precursor Scan: 117651, m/z: 1180.1258, charge: 5
ADTHDEILEGLNFNLTEIPEAQIHEGFQELLR_5_4_0_2_0
1e6
Intensity
m/z
204.0864
138.0548
366.1393
274.0918
168.0654
186.0758
292.1025
126.0549
144.0652
528.1915
657.2343
669.2462 b6(1+)
782.3326 b7(1+)
833.929 y14(2+)
1005.5145 y17(2+)
1024.462 b9(1+)
1128.5779 y9(1+)
1231.2787 PEP_0_0_0_0_0
1298.9713 PEP_0_1_0_0_0
1666.8574 y14(1+)

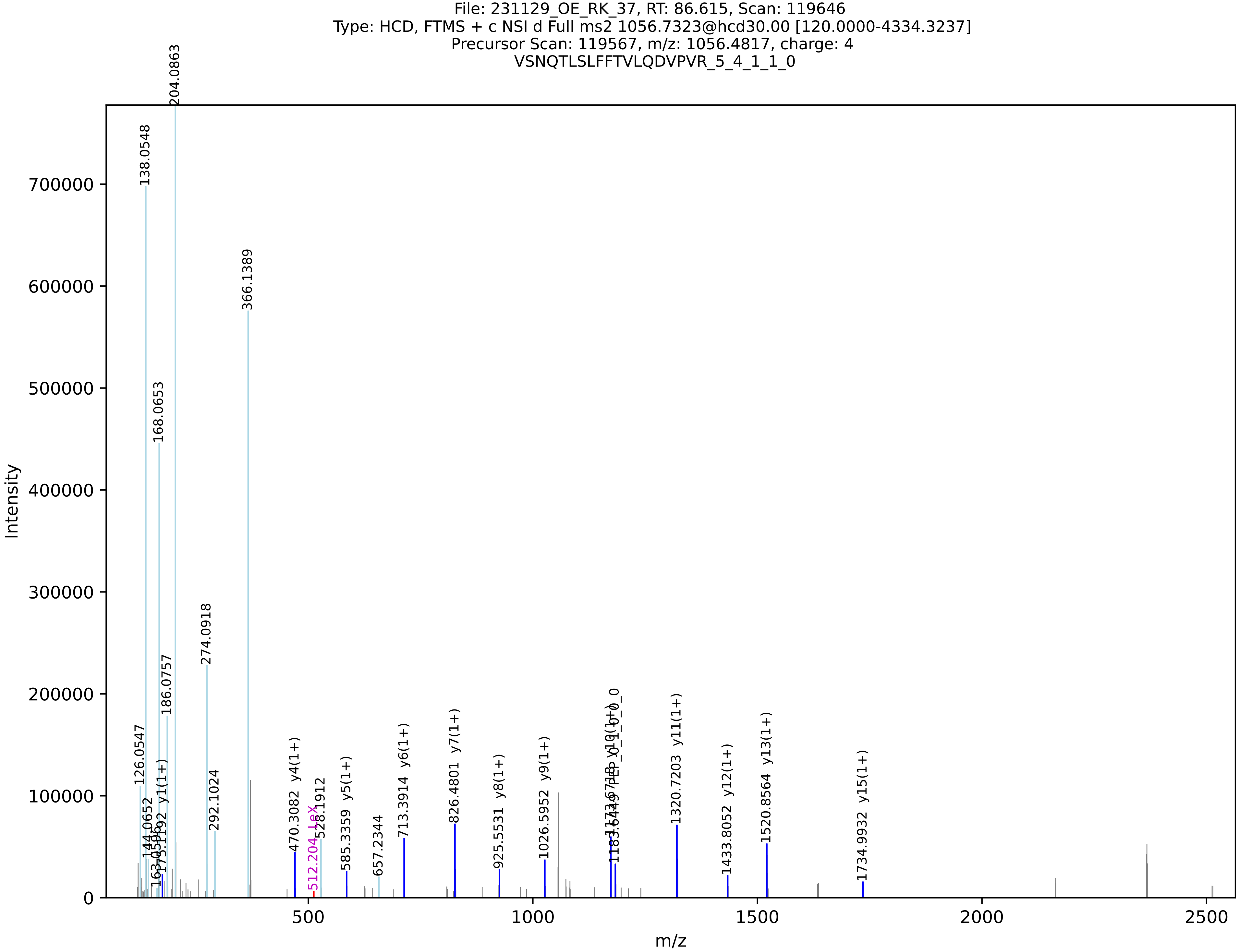
File: 231129_OE_RK_37, RT: 86.615, Scan: 119646
Type: HCD, FTMS + c NSI d Full ms2 1056.7323@hcd30.00 [120.0000-4334.3237]
Precursor Scan: 119567, m/z: 1056.4817, charge: 4
VSNQTLSLFFTVLQDVPVR_5_4_1_1_0
Intensity
m/z
204.0863
138.0548
366.1389
168.0653
274.0918
186.0757
126.0547
292.1024
144.0652
175.1192 y1(1+)
470.3082 y4(1+)
512.204_LeX
528.1912
585.3359 y5(1+)
657.2344
713.3914 y6(1+)
826.4801 y7(1+)
925.5531 y8(1+)
1026.5952 y9(1+)
1320.7203 y11(1+)
1433.8052 y12(1+)
1520.8564 y13(1+)
1734.9932 y15(1+)

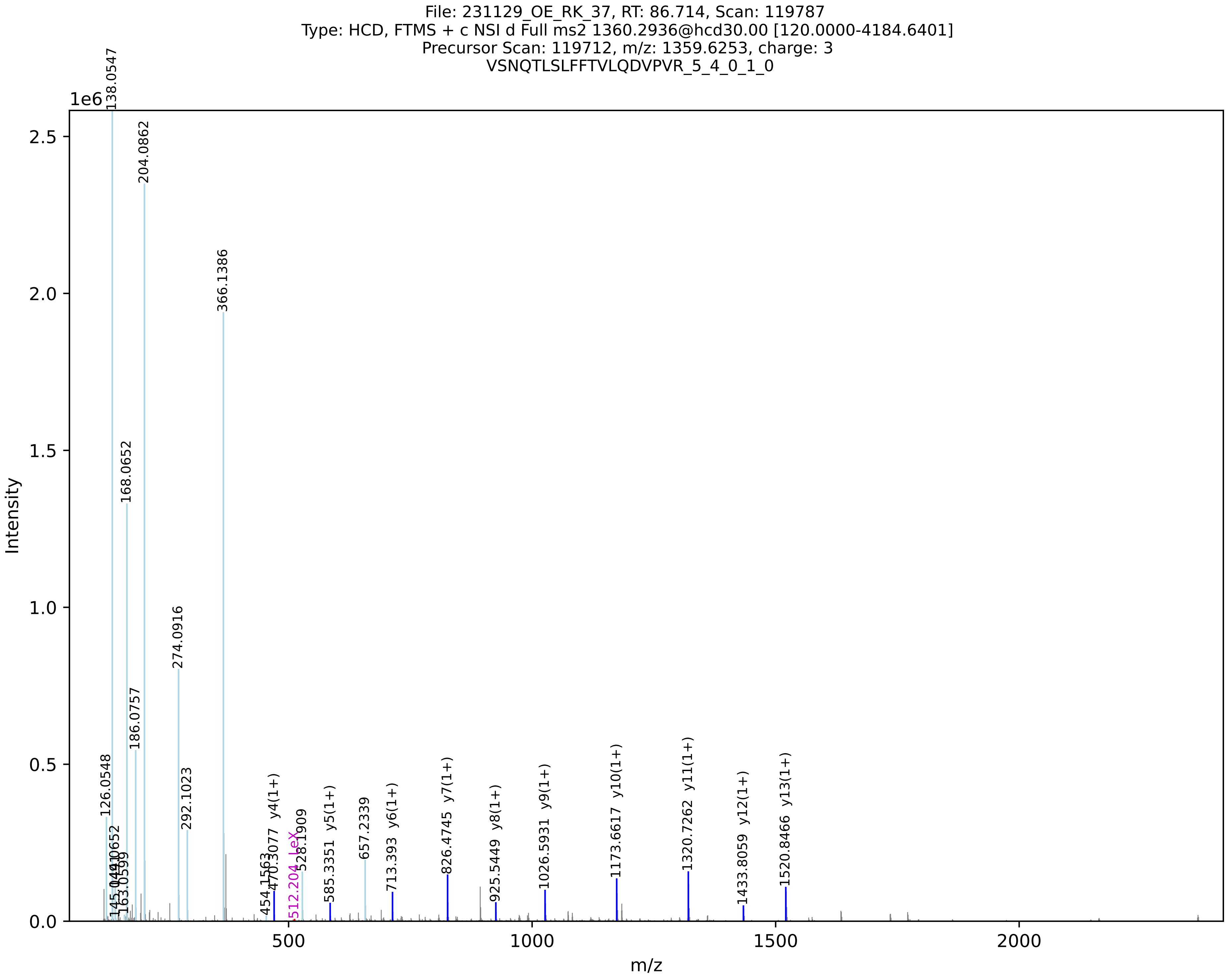

File: 231129_OE_RK_37, RT: 86.714, Scan: 119787
Type: HCD, FTMS + c NSI d Full ms2 1360.2936@hcd30.00 [120.0000-4184.6401]
Precursor Scan: 119712, m/z: 1359.6253, charge: 3
VSNQTLSLFFTVLQDVPVR_5_4_0_1_0
1e6
Intensity
m/z
138.0547
204.0862
366.1386
168.0652
274.0916
186.0757
126.0548
292.1023
163.0599
454.1563
470.3077 y4(1+)
512.204 LeX
528.1909
585.3351 y5(1+)
657.2339
713.393 y6(1+)
826.4745 y7(1+)
925.5449 y8(1+)
1026.5931 y9(1+)
1173.6617 y10(1+)
1320.7262 y11(1+)
1433.8059 y12(1+)
1520.8466 y13(1+)

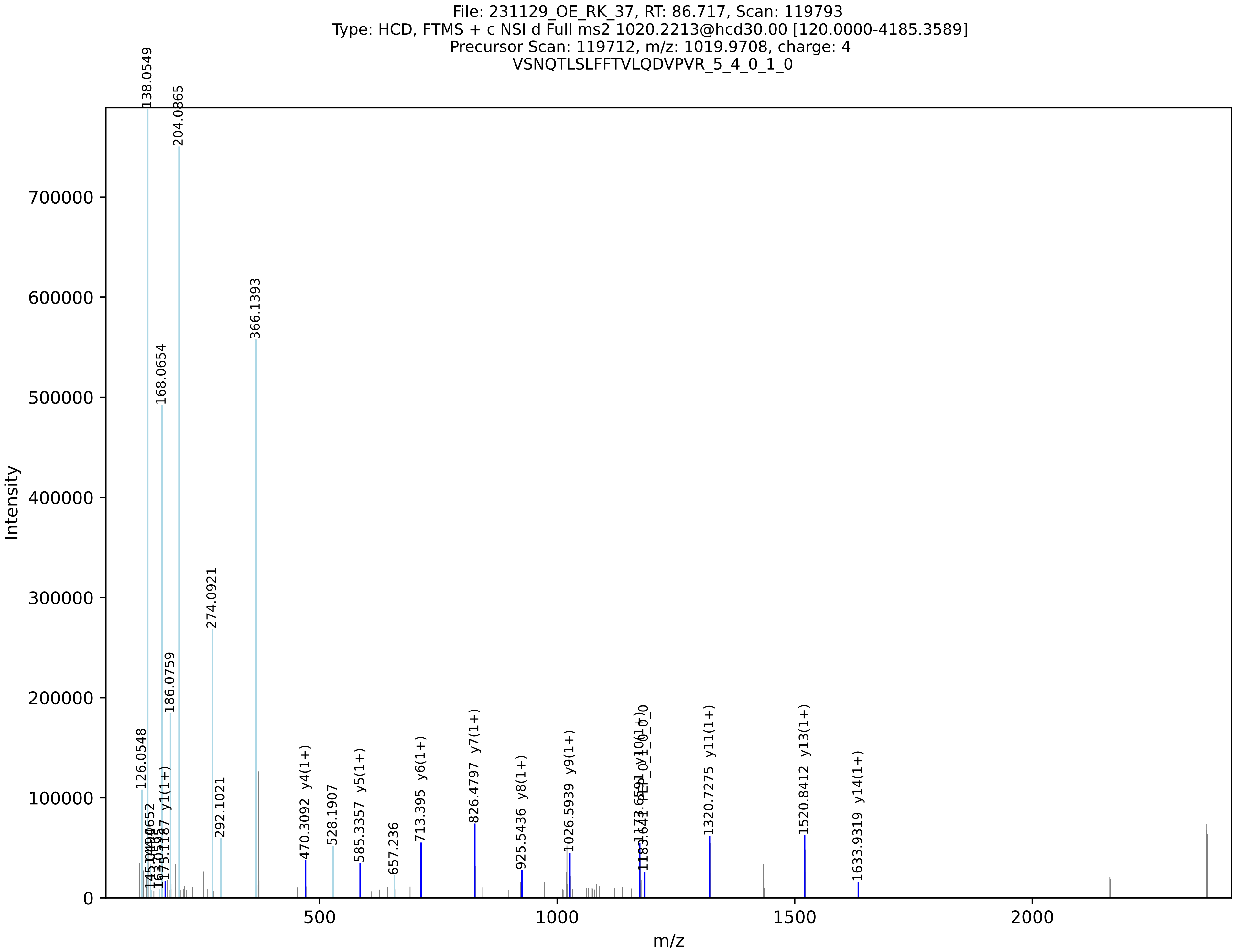
File: 231129_OE_RK_37, RT: 86.717, Scan: 119793
Type: HCD, FTMS + c NSI d Full ms2 1020.2213@hcd30.00 [120.0000-4185.3589]
Precursor Scan: 119712, m/z: 1019.9708, charge: 4
VSNQTLSLFFTVLQDVPVR_5_4_0_1_0
Intensity
m/z
138.0549
204.0865
366.1393
168.0654
274.0921
186.0759
126.0548
292.1021
175.1187 y1(1+)
470.3092 y4(1+)
528.1907
585.3357 y5(1+)
657.236
713.395 y6(1+)
826.4797 y7(1+)
925.5436 y8(1+)
1026.5939 y9(1+)
1320.7275 y11(1+)
1520.8412 y13(1+)
1633.9319 y14(1+)

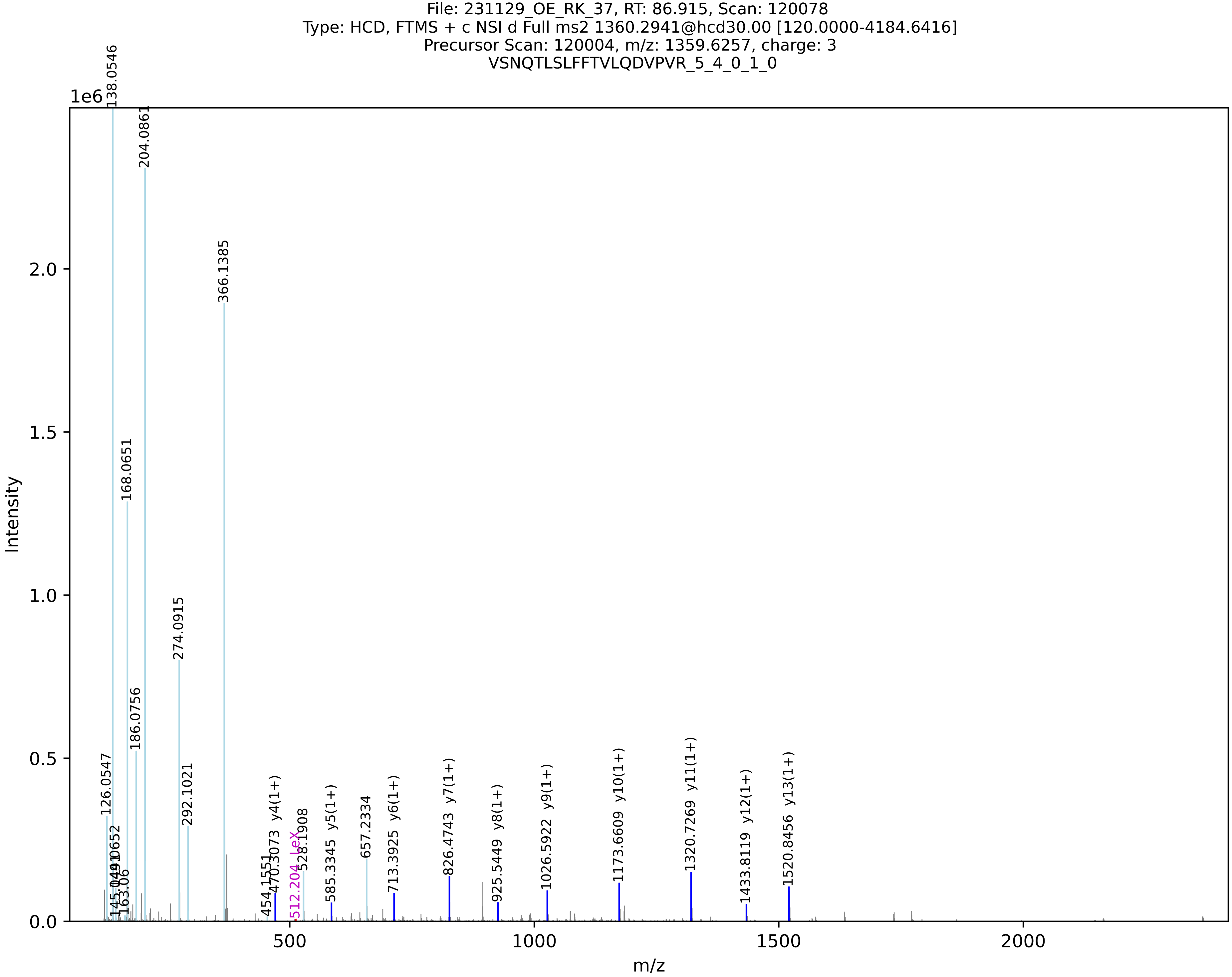
File: 231129_OE_RK_37, RT: 86.915, Scan: 120078
Type: HCD, FTMS + c NSI d Full ms2 1360.2941@hcd30.00 [120.0000-4184.6416]
Precursor Scan: 120004, m/z: 1359.6257, charge: 3
VSNQTLSLFFTVLQDVPVR_5_4_0_1_0
1e6
Intensity
m/z
138.0546
204.0861
366.1385
168.0651
274.0915
186.0756
126.0547
292.1021
470.3073 y4(1+)
512.204 LeX
528.1908
585.3345 y5(1+)
657.2334
713.3925 y6(1+)
826.4743 y7(1+)
925.5449 y8(1+)
1026.5922 y9(1+)
1173.6609 y10(1+)
1320.7269 y11(1+)
1433.8119 y12(1+)
1520.8456 y13(1+)
454.1551
163.06

File: 231129_OE_RK_37, RT: 88.135, Scan: 121846
Type: HCD, FTMS + c NSI d Full ms2 1639.7128@hcd30.00 [120.0000-6712.8838]
Precursor Scan: 121777, m/z: 1638.961, charge: 4
ADTHDEILEGLNFNLTEIPEAQIHEGFQELLR_6_5_0_3_0

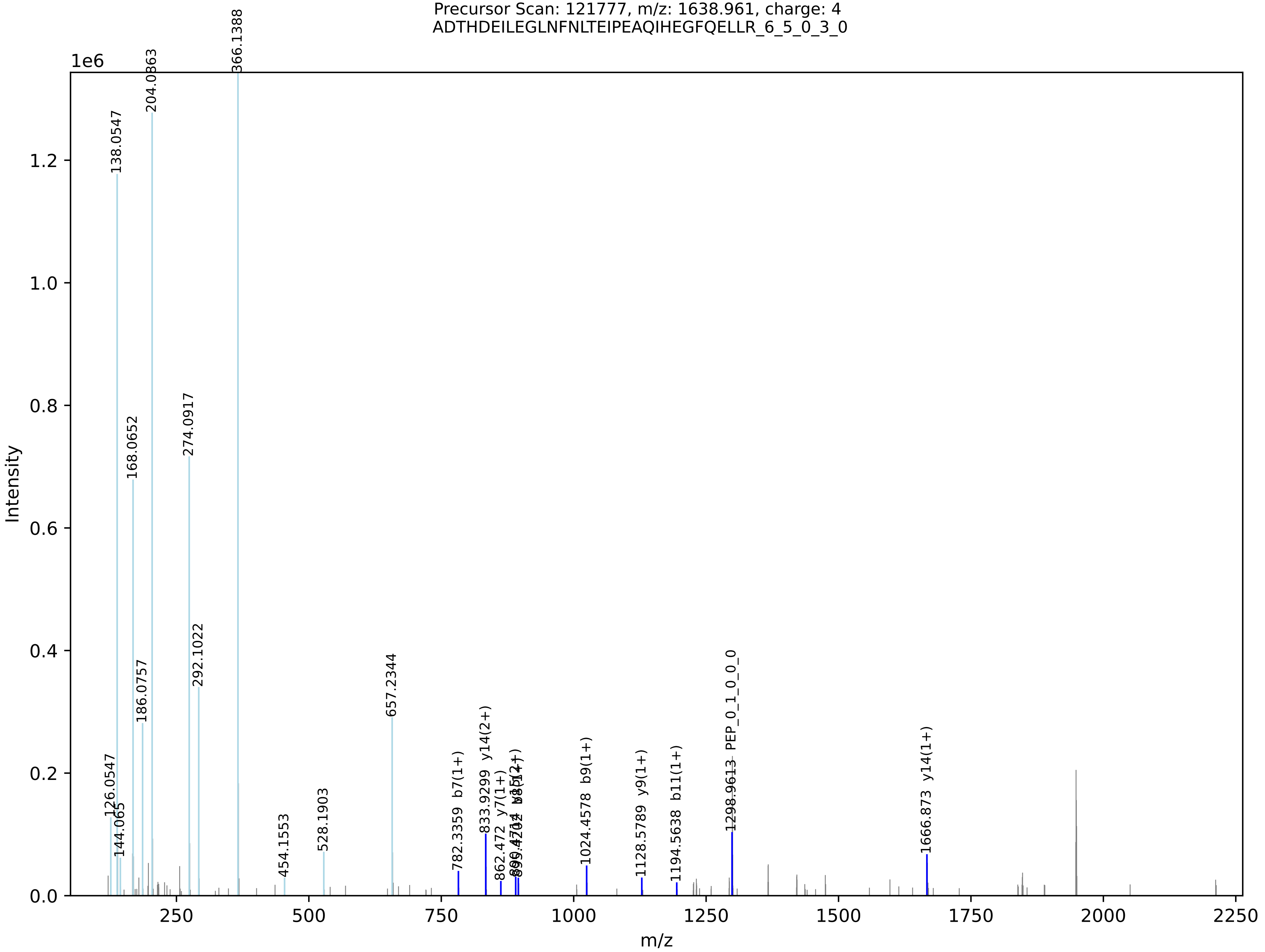

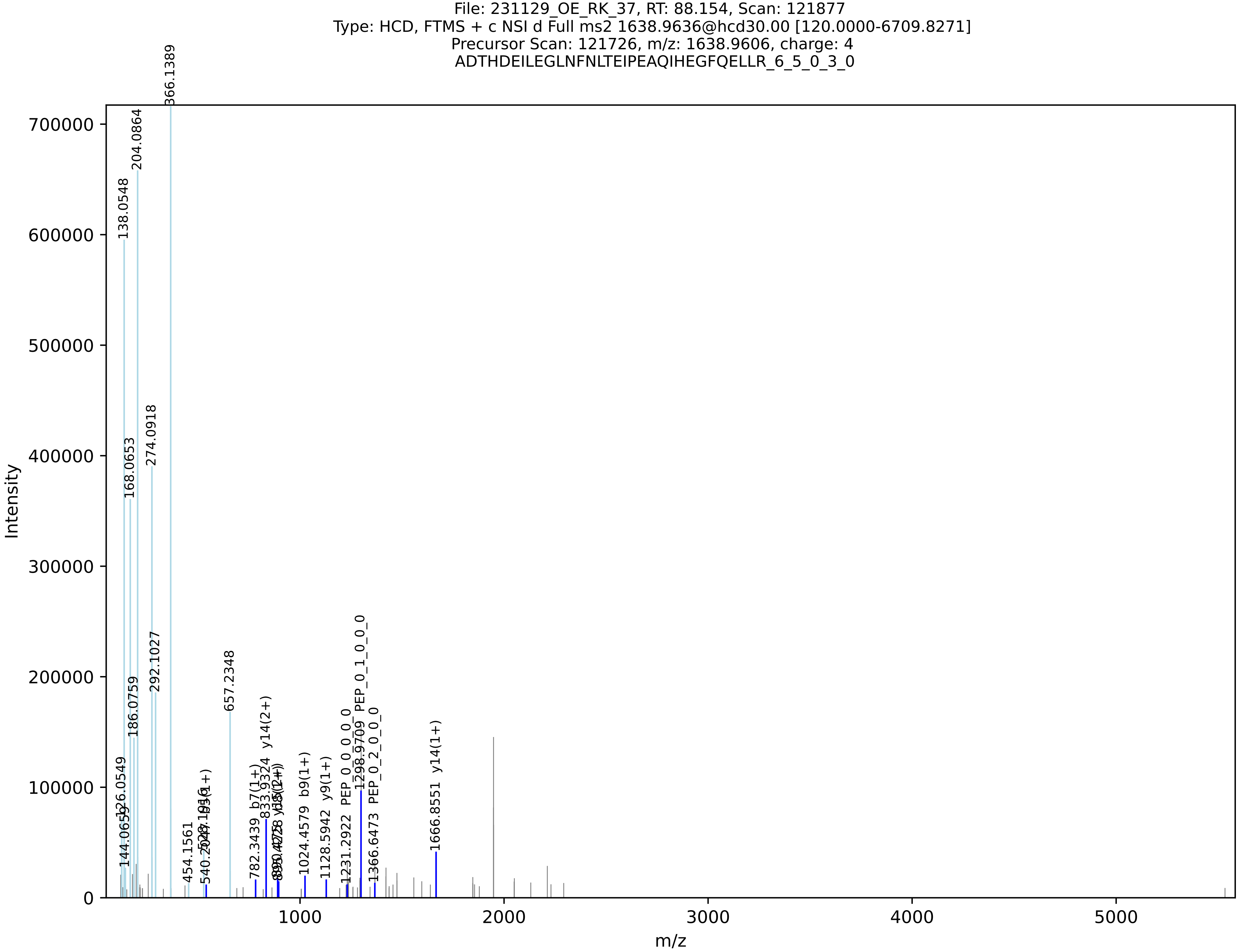
File: 231129_OE_RK_37, RT: 88.154, Scan: 121877
Type: HCD, FTMS + c NSI d Full ms2 1638.9636@hcd30.00 [120.0000-6709.8271]
Precursor Scan: 121726, m/z: 1638.9606, charge: 4
ADTHDEILEGLNFNLTEIPEAQIHEGFQELLR_6_5_0_3_0
366.1389
204.0864
138.0548
274.0918
168.0653
292.1027
186.0759
657.2348
126.0549
144.0659
454.1561
540.2047 b5(1+)
782.3439 b7(1+)
833.9324 y14(2+)
1024.4579 b9(1+)
1128.5942 y9(1+)
1231.2922 PEP_0_0_0_0_0
1298.9709 PEP_0_1_0_0_0
1366.6473 PEP_0_2_0_0_0
1666.8551 y14(1+)
Intensity
m/z

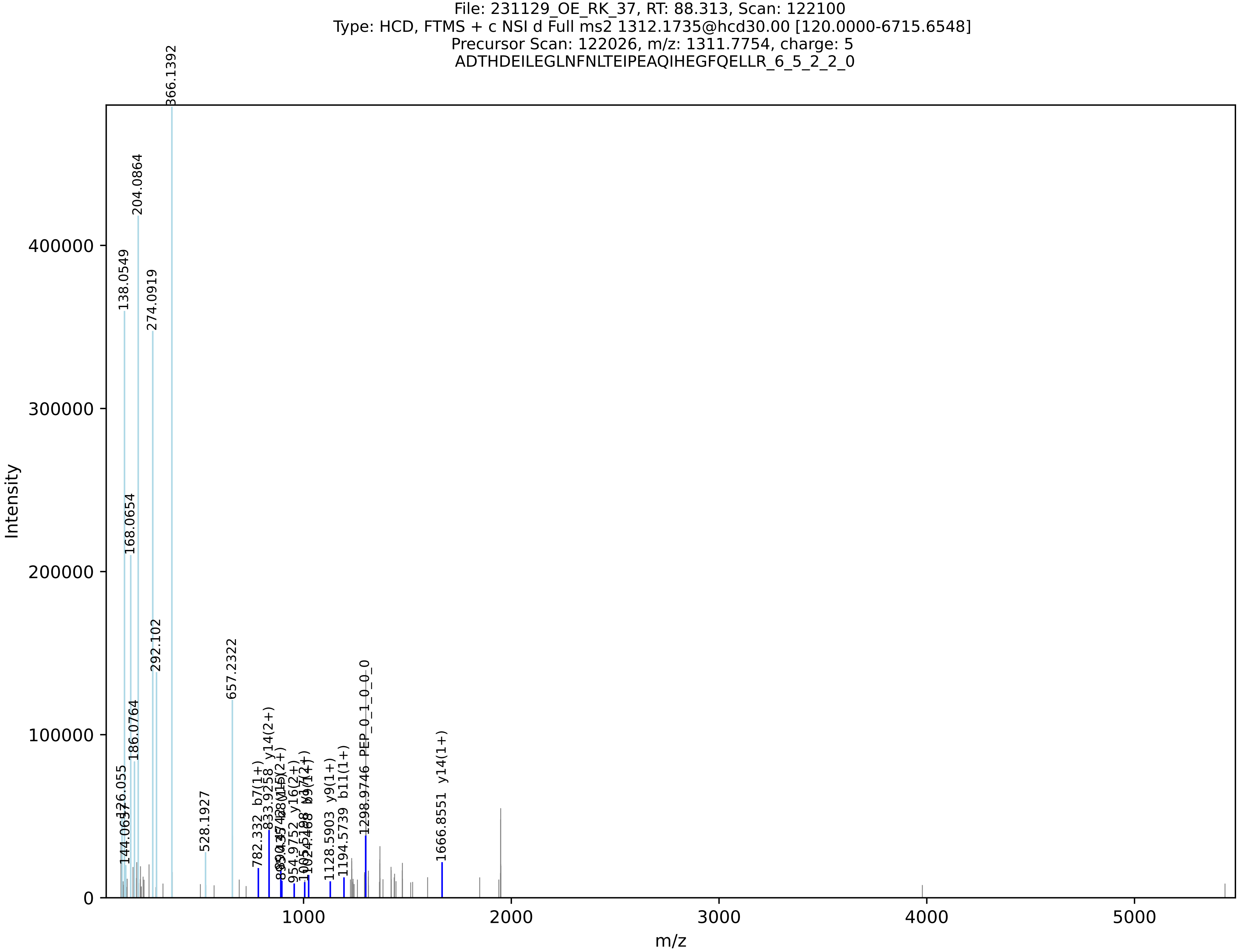
File: 231129_OE_RK_37, RT: 88.313, Scan: 122100
Type: HCD, FTMS + c NSI d Full ms2 1312.1735@hcd30.00 [120.0000-6715.6548]
Precursor Scan: 122026, m/z: 1311.7754, charge: 5
ADTHDEILEGLNFNLTEIPEAQIHEGFQELLR_6_5_2_2_0
Intensity
m/z
366.1392
204.0864
138.0549
274.0919
168.0654
292.102
186.0764
657.2322
528.1927
782.332 b7(1+)
833.9258 y14(2+)
1298.9746 PEP_0_1_0_0_0
1128.5903 y9(1+)
1194.5739 b11(1+)
1666.8551 y14(1+)
954.9752 y16(2+)

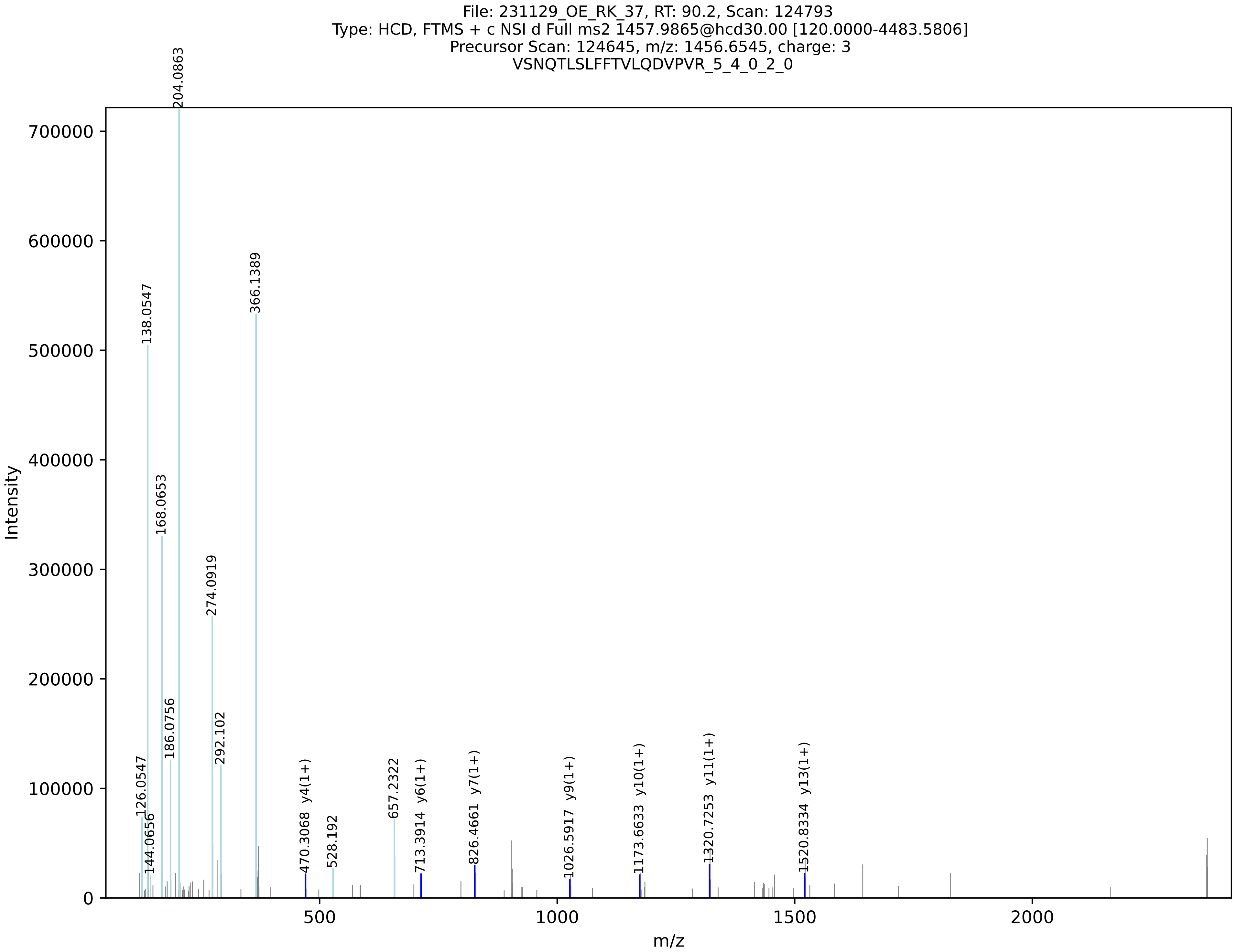
File: 231129_OE_RK_37, RT: 90.2, Scan: 124793
Type: HCD, FTMS + c NSI d Full ms2 1457.9865@hcd30.00 [120.0000-4483.5806]
Precursor Scan: 124645, m/z: 1456.6545, charge: 3
VSNQTLSLFFTVLQDVPVR_5_4_0_2_0
Intensity
m/z
204.0863
138.0547
366.1389
168.0653
274.0919
186.0756
292.102
126.0547
144.0656
470.3068 y4(1+)
528.192
657.2322
713.3914 y6(1+)
826.4661 y7(1+)
1026.5917 y9(1+)
1173.6633 y10(1+)
1320.7253 y11(1+)
1520.8334 y13(1+)

File: 231129_OE_RK_37, RT: 95.857, Scan: 132852
Type: HCD, FTMS + c NSI d Full ms2 1349.5936@hcd30.00 [120.0000-4151.8984]
Precursor Scan: 132766, m/z: 1349.5936, charge: 3
GVTHLNISGLKMPR_5_7_0_1_0

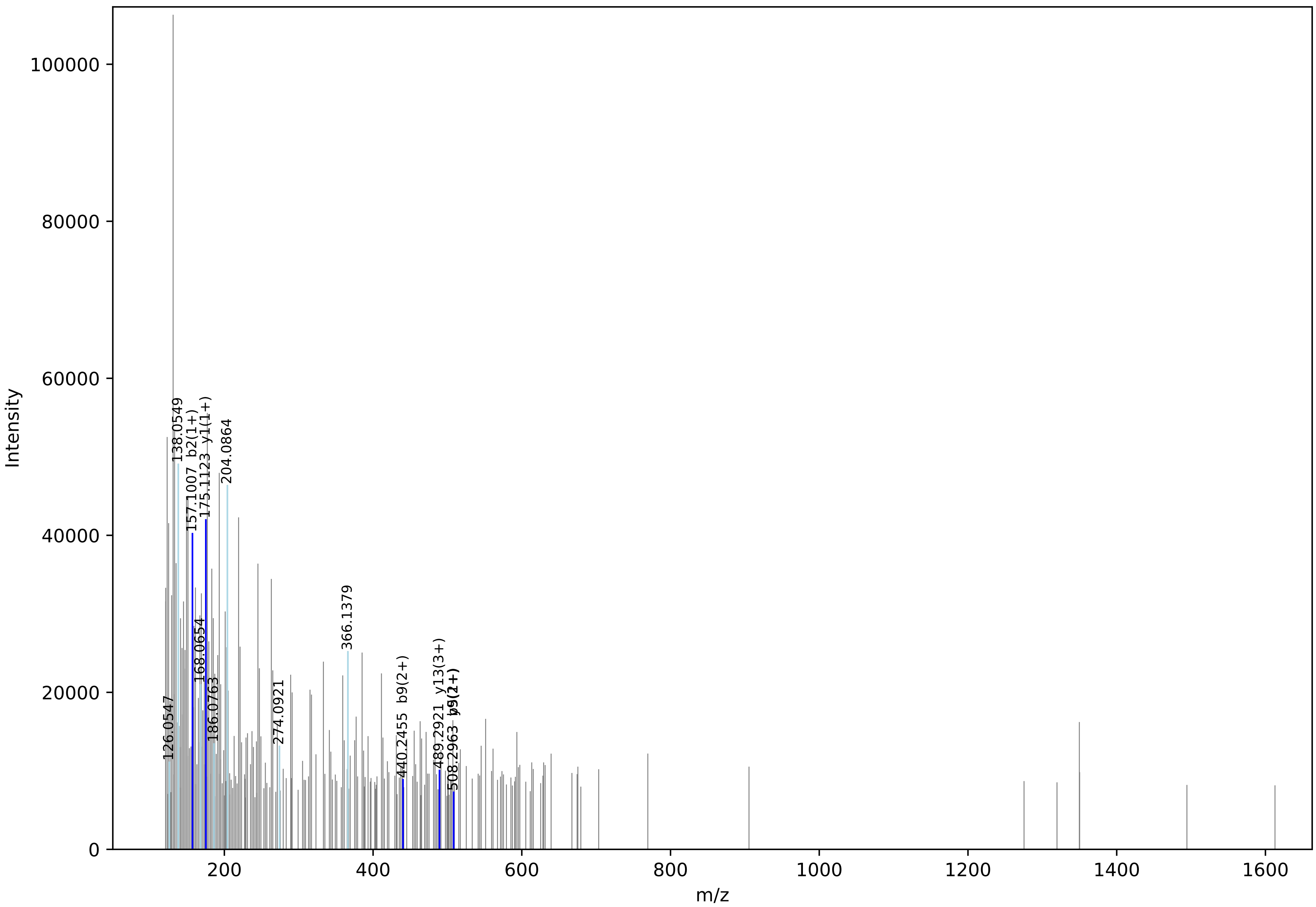

File: 231129_OE_RK_37, RT: 103.376, Scan: 141729
Type: HCD, FTMS + c NSI d Full ms2 1321.2239@hcd30.00 [120.0000-4065.0872]
Precursor Scan: 141723, m/z: 1321.2261, charge: 3
YLGNATAIFFLPDEGK_5_4_0_2_0

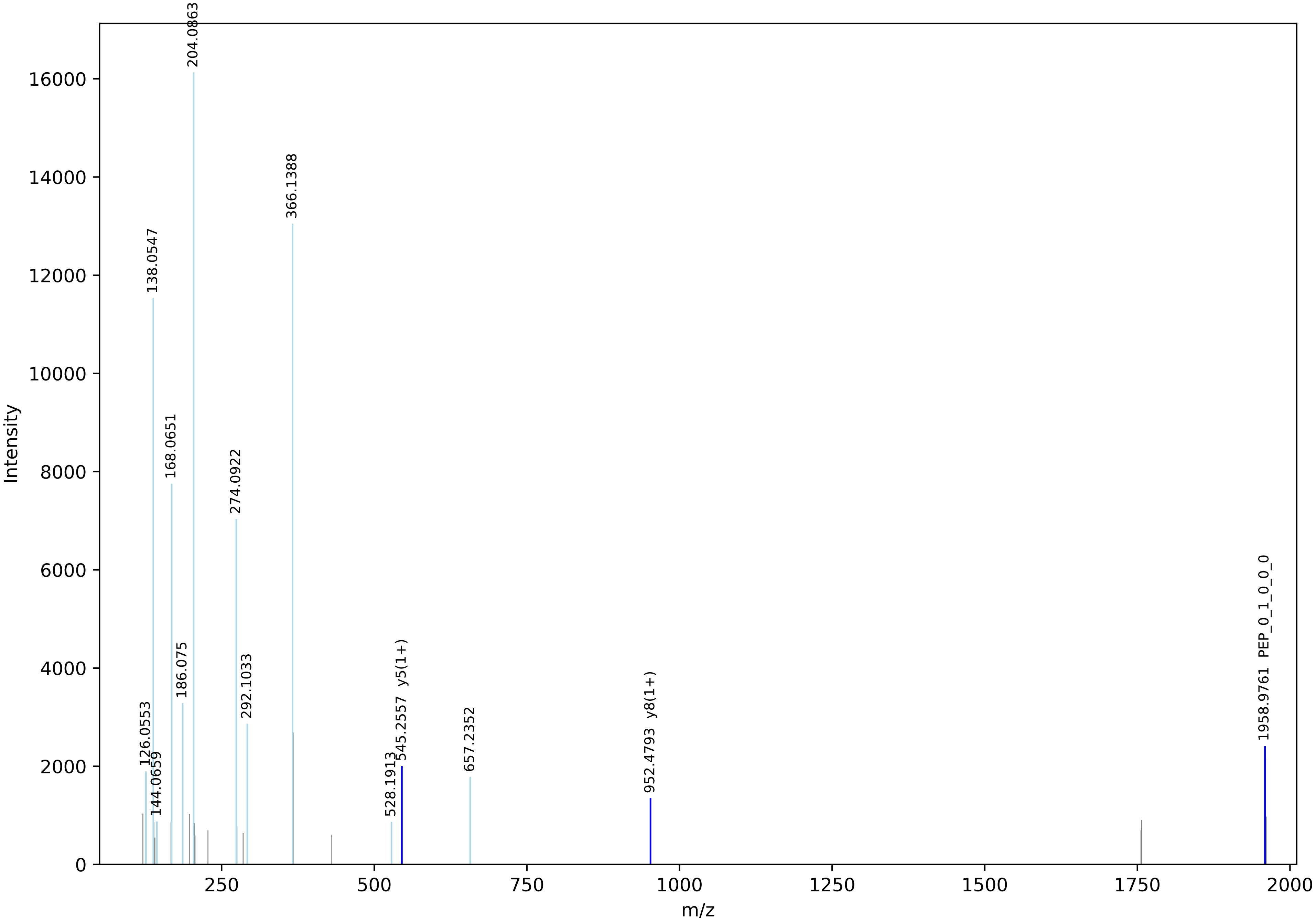

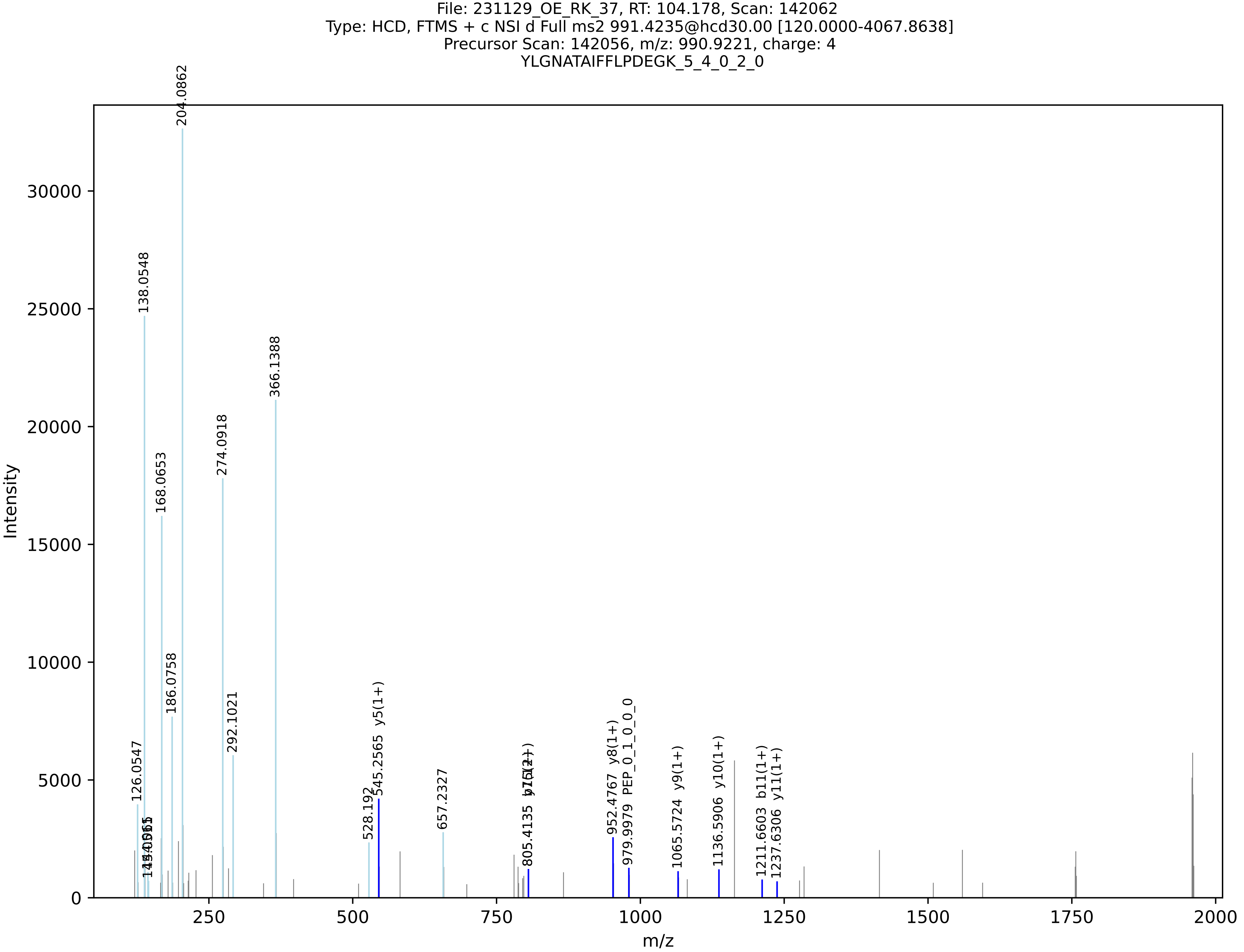
File: 231129_OE_RK_37, RT: 104.178, Scan: 142062
Type: HCD, FTMS + c NSI d Full ms2 991.4235@hcd30.00 [120.0000-4067.8638]
Precursor Scan: 142056, m/z: 990.9221, charge: 4
YLGNATAIFFLPDEGK_5_4_0_2_0
Intensity
m/z
204.0862
138.0548
366.1388
274.0918
168.0653
186.0758
292.1021
126.0547
545.2565 y5(1+)
528.192
657.2327
805.4135
952.4767 y8(1+)
979.9979 PEP_0_1_0_0_0
1065.5724 y9(1+)
1136.5906 y10(1+)
1211.6603 b11(1+)
1237.6306 y11(1+)
0
5000
10000
15000
20000
25000
30000
250
500
750
1000
1250
1500
1750
2000

File: 231129_OE_RK_37, RT: 104.387, Scan: 142221
Type: HCD, FTMS + c NSI d Full ms2 1035.0814@hcd30.00 [120.0000-5302.4854]
Precursor Scan: 142214, m/z: 1034.8802, charge: 5
LSLHRPALEDLLLGSEANLTCTLTGLR_5_4_0_2_0

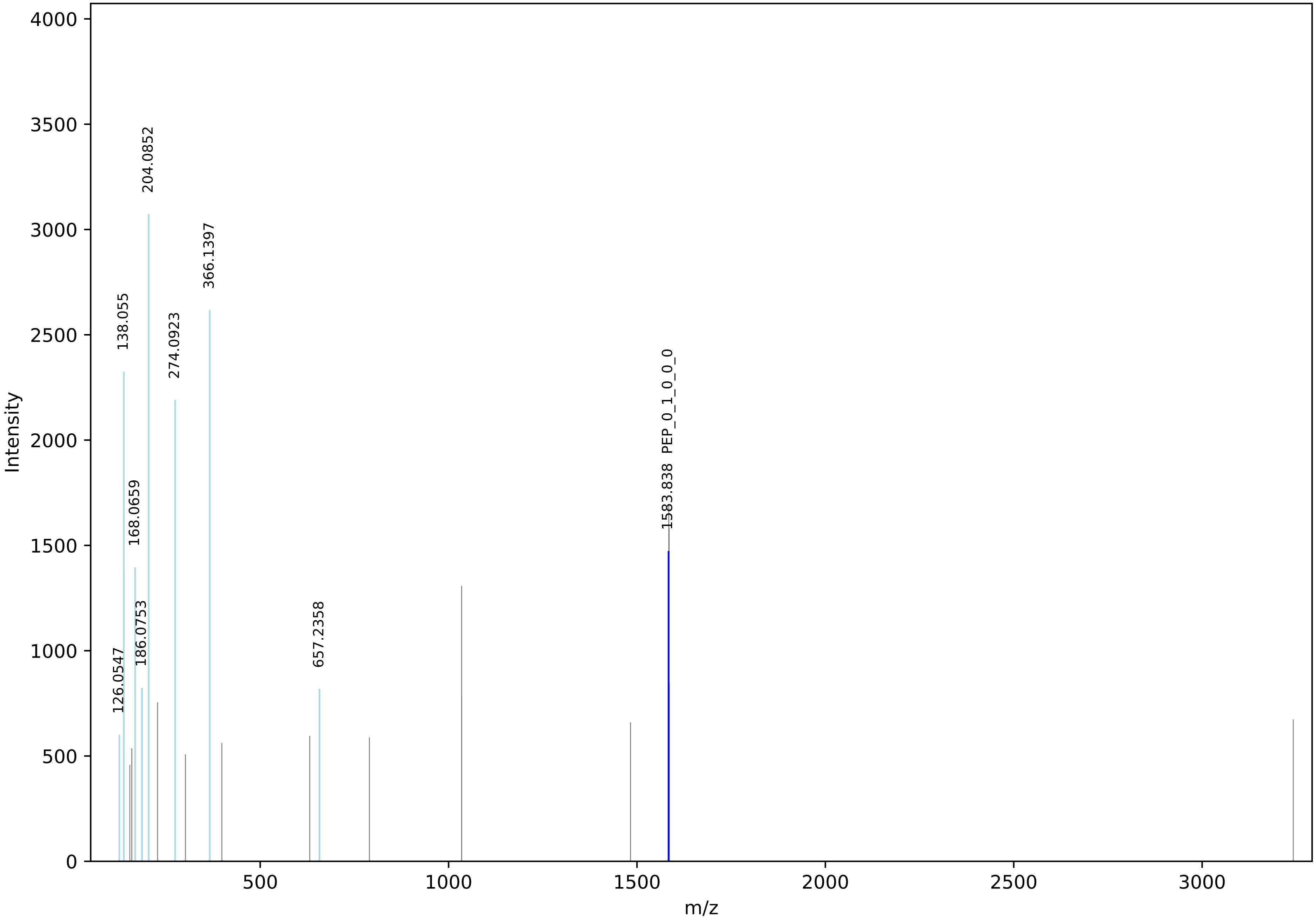

File: 231129_OE_RK_37, RT: 104.654, Scan: 142342
Type: HCD, FTMS + c NSI d Full ms2 1220.5758@hcd30.00 [120.0000-5002.8052]
Precursor Scan: 142337, m/z: 1220.0746, charge: 4
LSLHRPALEDLLLGSEANLTCTLTGLR_5_4_0_1_0

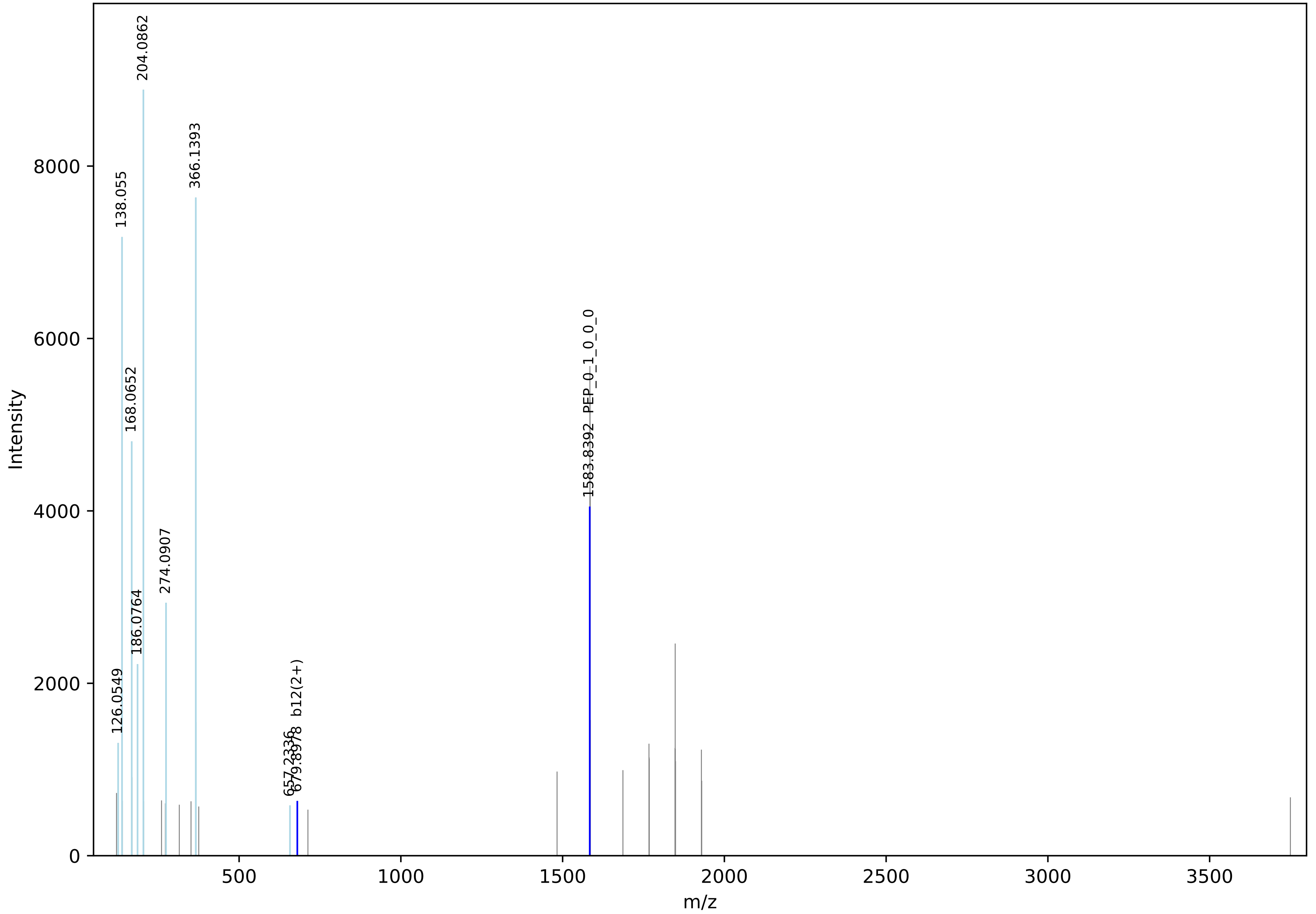

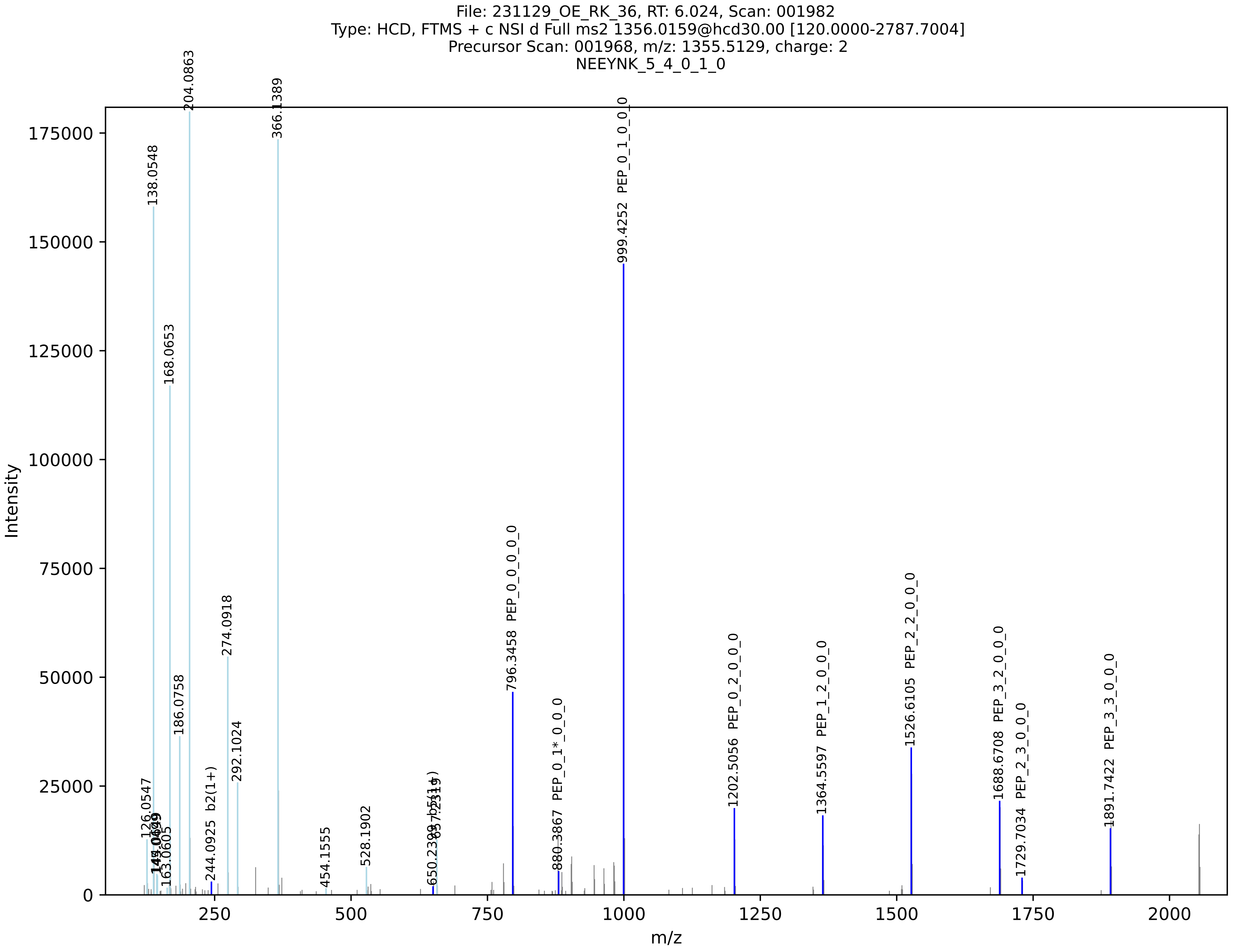
File: 231129_OE_RK_36, RT: 6.024, Scan: 001982
Type: HCD, FTMS + c NSI d Full ms2 1356.0159@hcd30.00 [120.0000-2787.7004]
Precursor Scan: 001968, m/z: 1355.5129, charge: 2
NEEYNK_5_4_0_1_0
Intensity
m/z
204.0863
366.1389
138.0548
168.0653
186.0758
274.0918
292.1024
244.0925 b2(1+)
126.0547
163.0605
454.1555
528.1902
999.4252 PEP_0_1_0_0_0
796.3458 PEP_0_0_0_0_0
880.3867 PEP_0_1*_0_0_0
1202.5056 PEP_0_2_0_0_0
1364.5597 PEP_1_2_0_0_0
1526.6105 PEP_2_2_0_0_0
1688.6708 PEP_3_2_0_0_0
1729.7034 PEP_2_3_0_0_0
1891.7422 PEP_3_3_0_0_0

File: 231129_OE_RK_36, RT: 6.199, Scan: 002167
Type: HCD, FTMS + c NSI d Full ms2 904.3468@hcd30.00 [120.0000-2789.4434]
Precursor Scan: 002152, m/z: 904.0126, charge: 3
NEEYNK_5_4_0_1_0

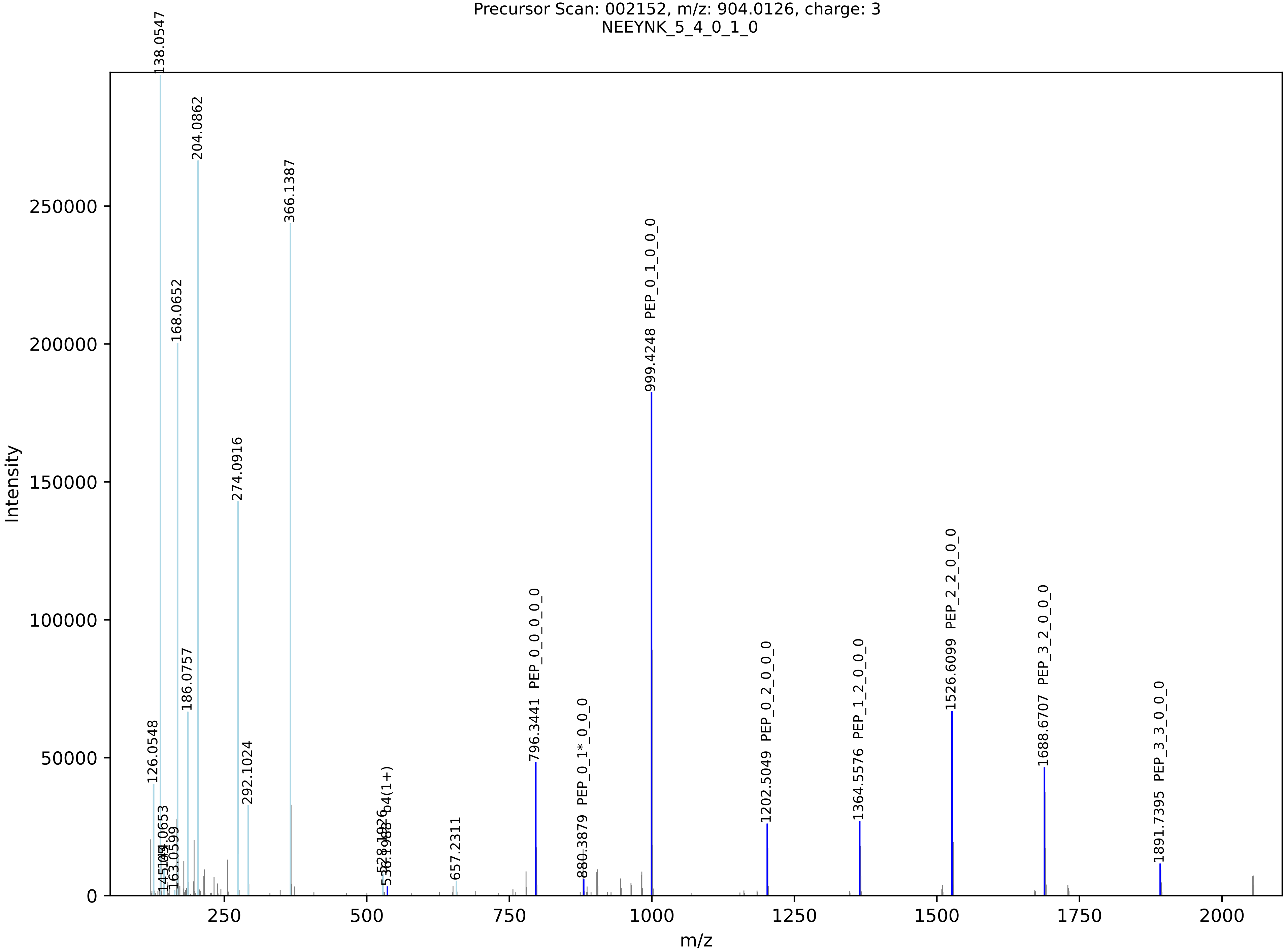

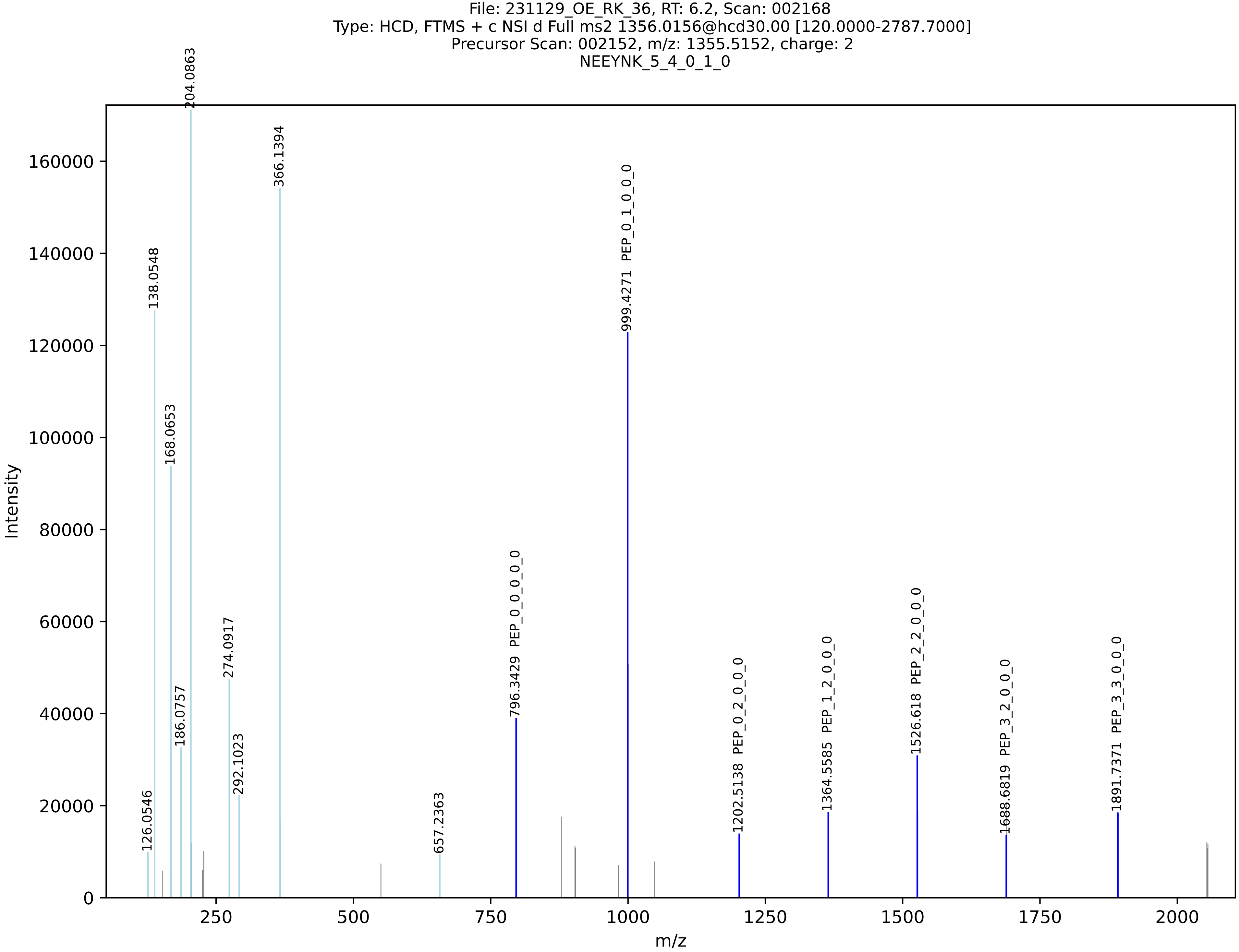

File: 231129_OE_RK_36, RT: 6.2, Scan: 002168
Type: HCD, FTMS + c NSI d Full ms2 1356.0156@hcd30.00 [120.0000-2787.7000]
Precursor Scan: 002152, m/z: 1355.5152, charge: 2
NEEYNK_5_4_0_1_0
Intensity
m/z
204.0863
366.1394
138.0548
168.0653
186.0757
274.0917
292.1023
126.0546
657.2363
796.3429 PEP_0_0_0_0_0
999.4271 PEP_0_1_0_0_0
1202.5138 PEP_0_2_0_0_0
1364.5585 PEP_1_2_0_0_0
1526.618 PEP_2_2_0_0_0
1688.6819 PEP_3_2_0_0_0
1891.7371 PEP_3_3_0_0_0

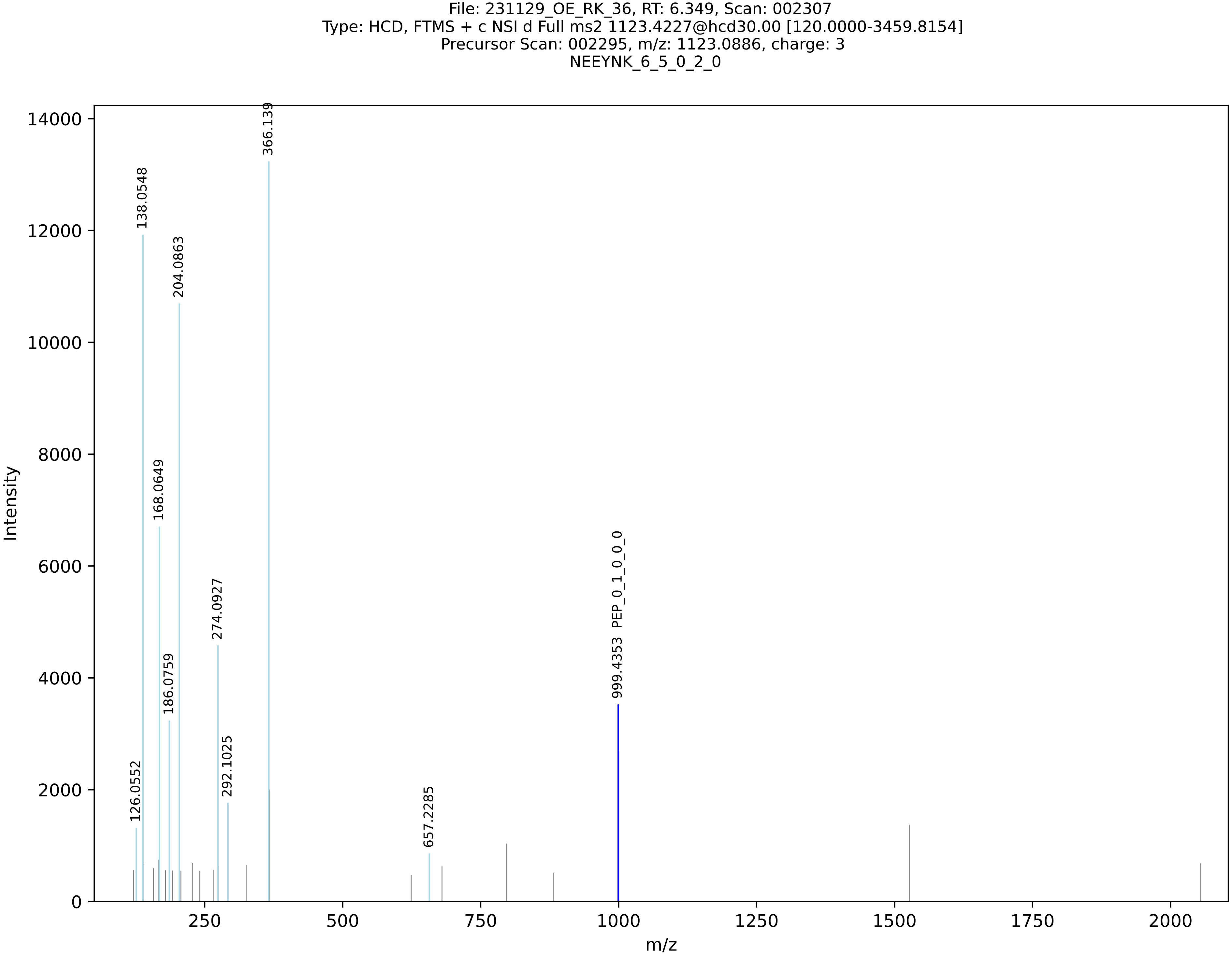
File: 231129_OE_RK_36, RT: 6.349, Scan: 002307
Type: HCD, FTMS + c NSI d Full ms2 1123.4227@hcd30.00 [120.0000-3459.8154]
Precursor Scan: 002295, m/z: 1123.0886, charge: 3
NEEYNK_6_5_0_2_0
Intensity
m/z
366.139
138.0548
204.0863
168.0649
274.0927
186.0759
292.1025
126.0552
657.2285
999.4353 PEP_0_1_0_0_0
0
2000
4000
6000
8000
10000
12000
14000
250
500
750
1000
1250
1500
1750
2000

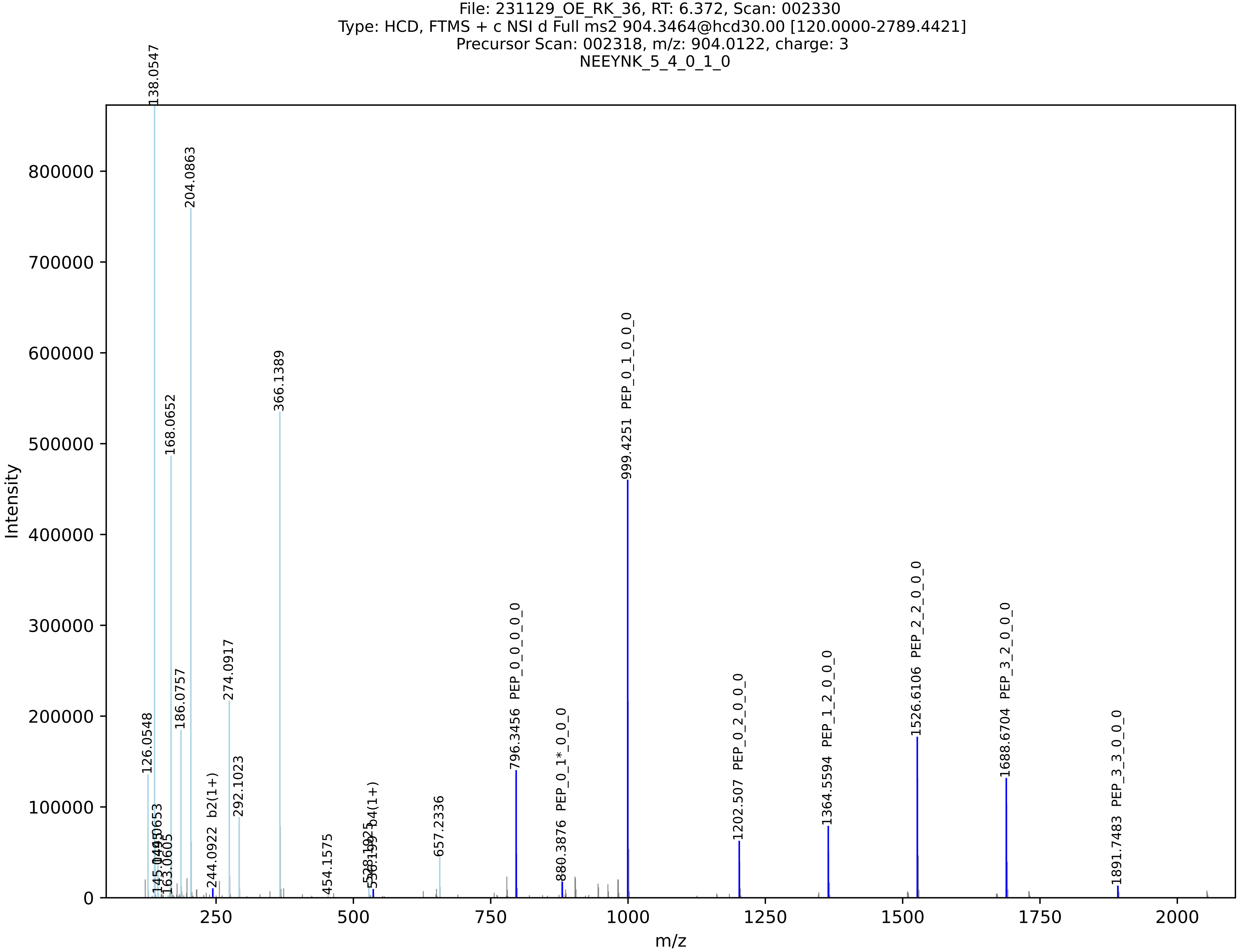

File: 231129_OE_RK_36, RT: 6.372, Scan: 002330
Type: HCD, FTMS + c NSI d Full ms2 904.3464@hcd30.00 [120.0000-2789.4421]
Precursor Scan: 002318, m/z: 904.0122, charge: 3
NEEYNK_5_4_0_1_0
Intensity
m/z
0
100000
200000
300000
400000
500000
600000
700000
800000
250
500
750
1000
1250
1500
1750
2000
138.0547
204.0863
168.0652
366.1389
999.4251 PEP_0_1_0_0_0
186.0757
274.0917
126.0548
292.1023
244.0922 b2(1+)
163.0605
454.1575
657.2336
796.3456 PEP_0_0_0_0_0
880.3876 PEP_0_1*_0_0_0
1202.507 PEP_0_2_0_0_0
1364.5594 PEP_1_2_0_0_0
1526.6106 PEP_2_2_0_0_0
1688.6704 PEP_3_2_0_0_0
1891.7483 PEP_3_3_0_0_0

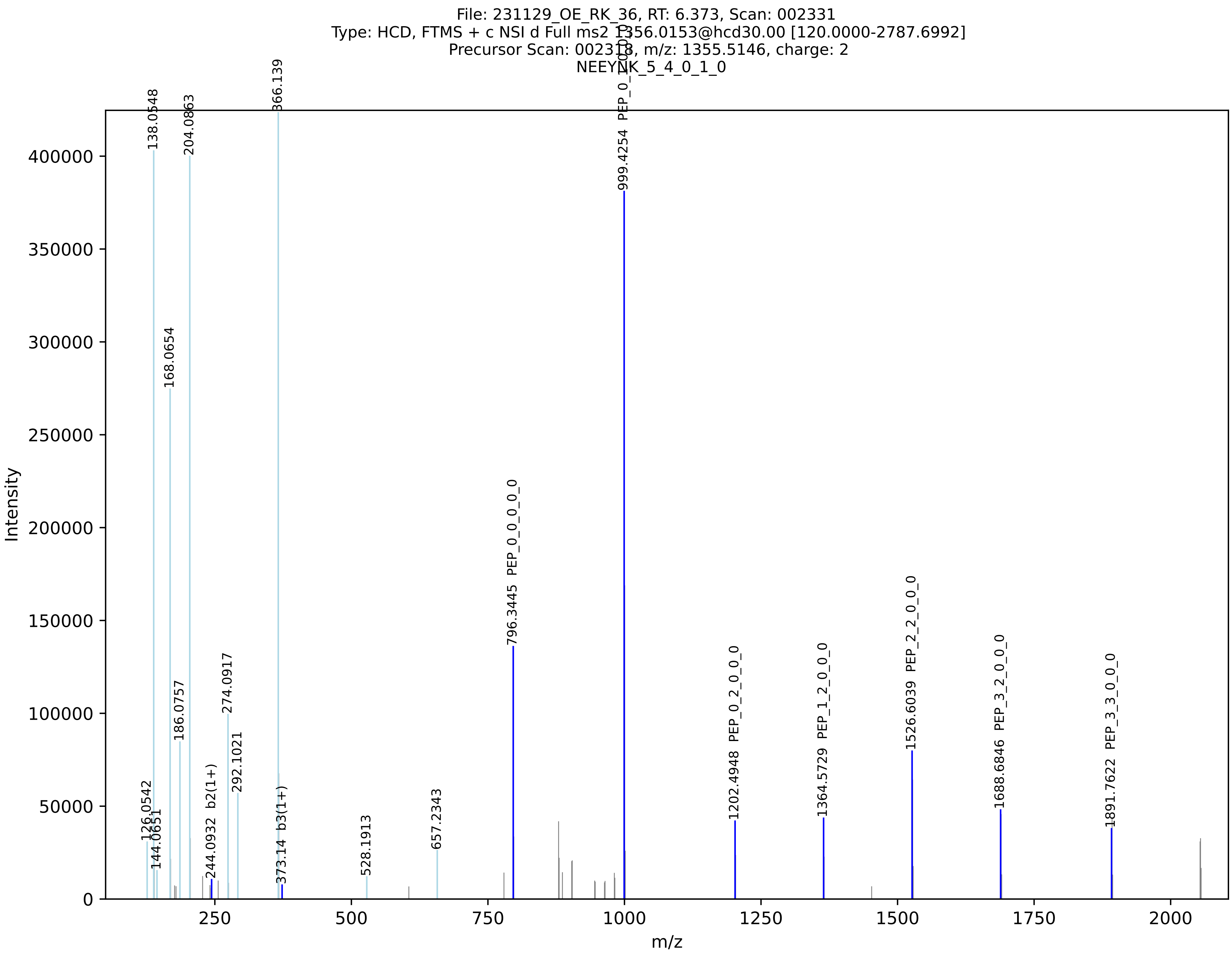
File: 231129_OE_RK_36, RT: 6.373, Scan: 002331
Type: HCD, FTMS + c NSI d Full ms2 1356.0153@hcd30.00 [120.0000-2787.6992]
Precursor Scan: 002318, m/z: 1355.5146, charge: 2
NEEYNK_5_4_0_1_0
Intensity
m/z
138.0548
204.0863
366.139
168.0654
186.0757
274.0917
292.1021
126.0542
144.0651
244.0932 b2(1+)
373.14 b3(1+)
528.1913
657.2343
796.3445 PEP_0_0_0_0_0
999.4254 PEP_0_1_0_0_0
1202.4948 PEP_0_2_0_0_0
1364.5729 PEP_1_2_0_0_0
1526.6039 PEP_2_2_0_0_0
1688.6846 PEP_3_2_0_0_0
1891.7622 PEP_3_3_0_0_0

File: 231129_OE_RK_36, RT: 6.439, Scan: 002394
Type: HCD, FTMS + c NSI d Full ms2 1428.5435@hcd30.00 [120.0000-2935.6565]
Precursor Scan: 002383, m/z: 1428.5434, charge: 2
NEEYNK_5_4_1_1_0

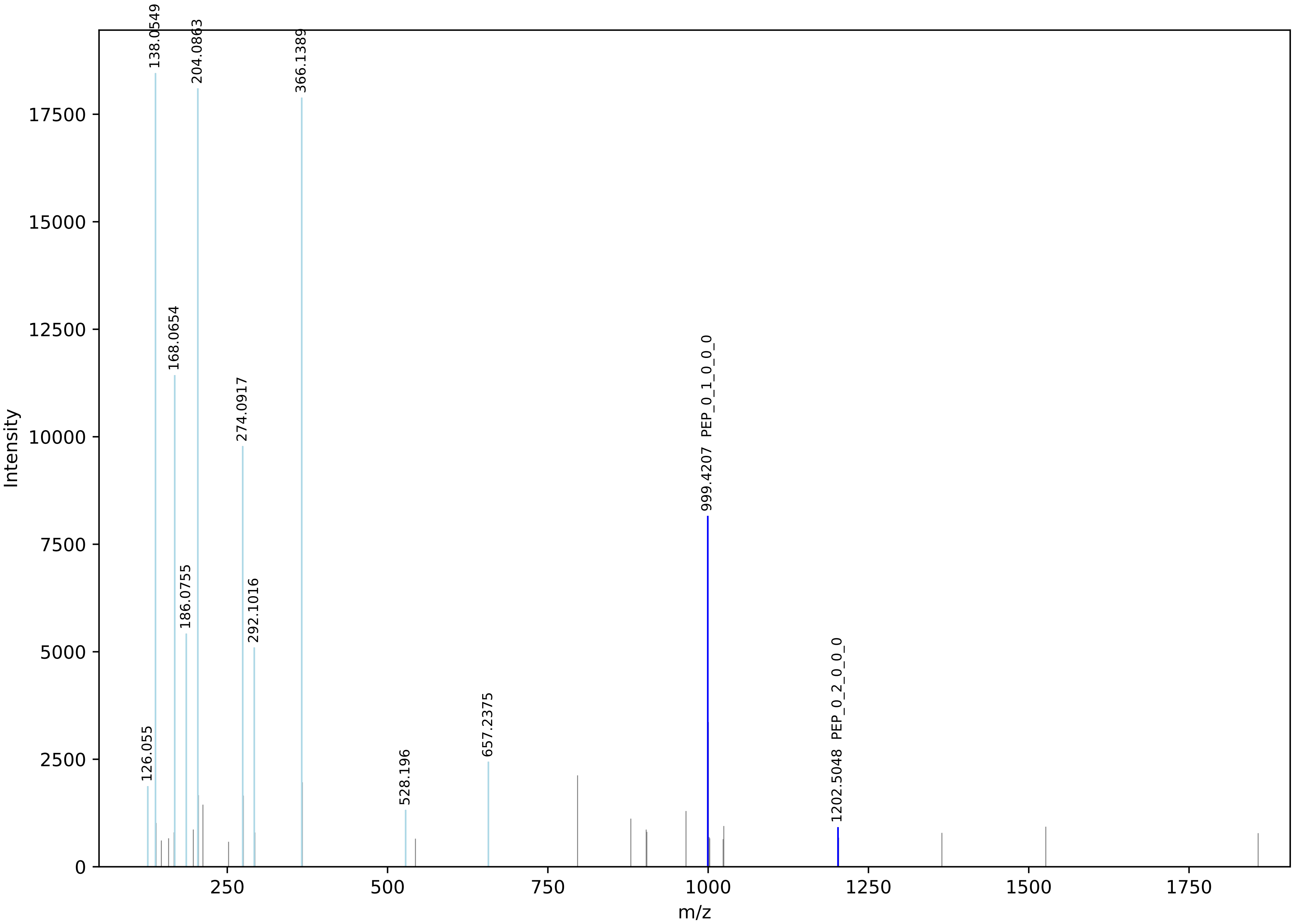

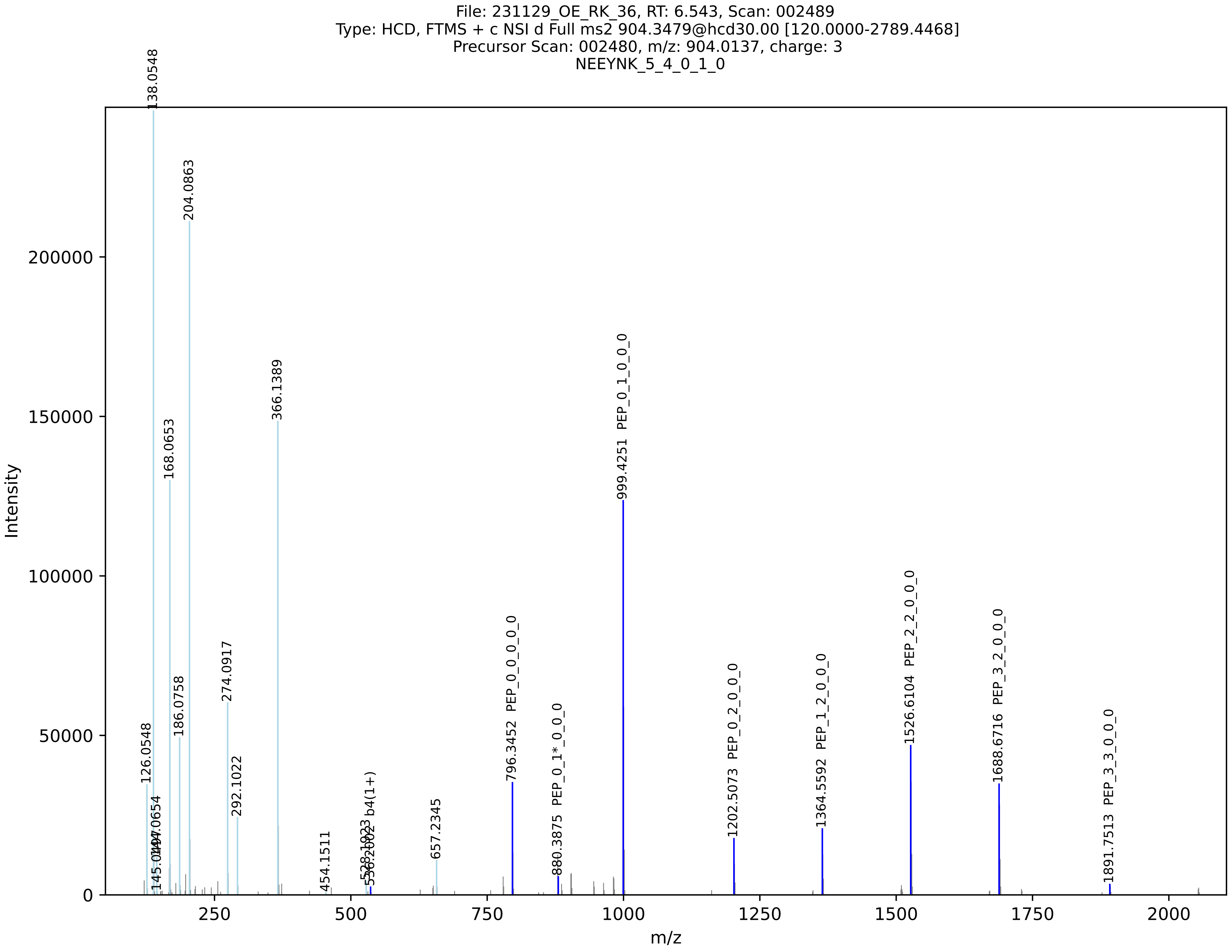
File: 231129_OE_RK_36, RT: 6.543, Scan: 002489
Type: HCD, FTMS + c NSI d Full ms2 904.3479@hcd30.00 [120.0000-2789.4468]
Precursor Scan: 002480, m/z: 904.0137, charge: 3
NEEYNK_5_4_0_1_0
Intensity
m/z
138.0548
204.0863
366.1389
168.0653
274.0917
186.0758
126.0548
292.1022
147.0654
145.0
454.1511
528.1923
536.2002 b4(1+)
657.2345
796.3452 PEP_0_0_0_0_0
880.3875 PEP_0_1*_0_0_0
999.4251 PEP_0_1_0_0_0
1202.5073 PEP_0_2_0_0_0
1364.5592 PEP_1_2_0_0_0
1526.6104 PEP_2_2_0_0_0
1688.6716 PEP_3_2_0_0_0
1891.7513 PEP_3_3_0_0_0
0
50000
100000
150000
200000
250
500
750
1000
1250
1500
1750
2000

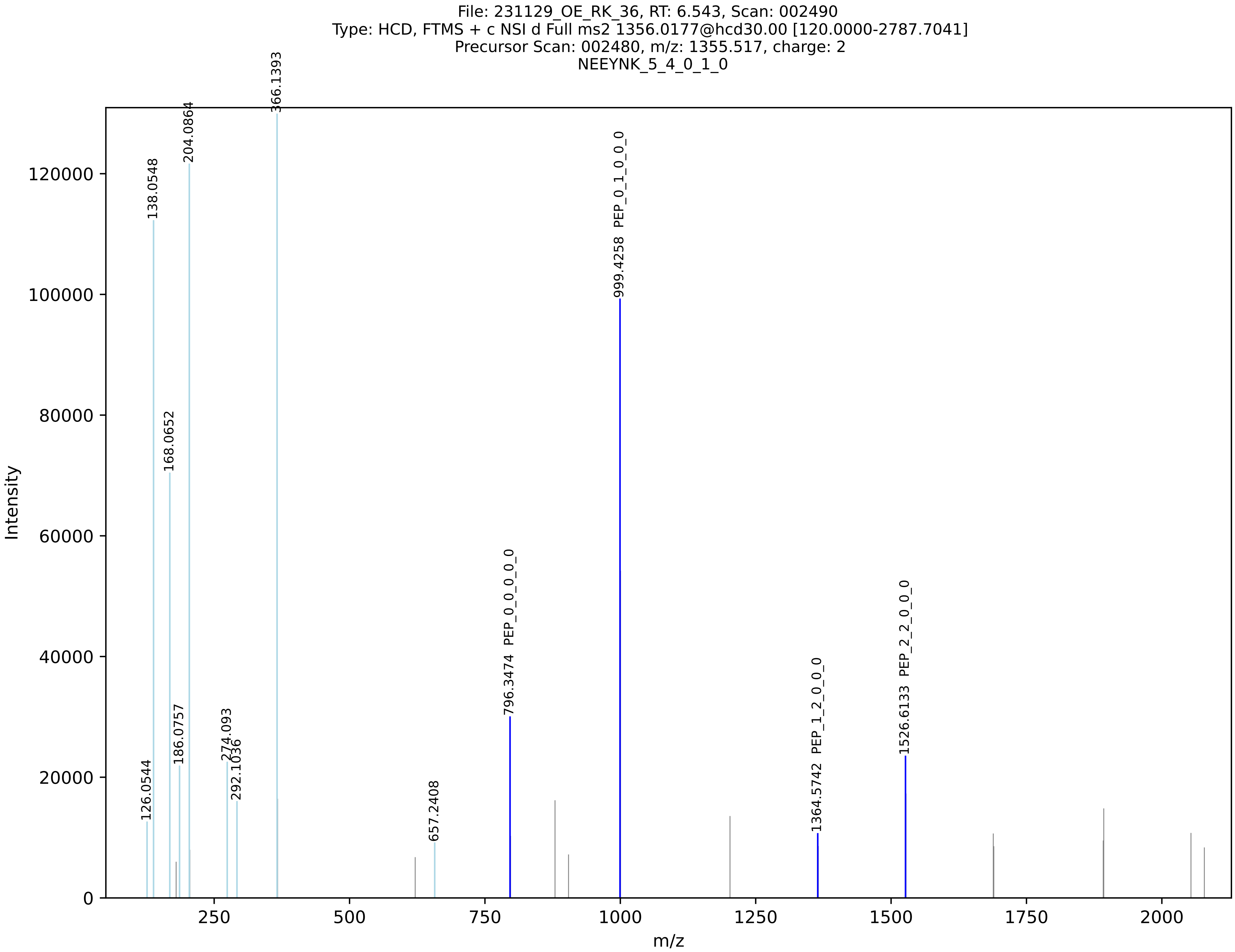
File: 231129_OE_RK_36, RT: 6.543, Scan: 002490
Type: HCD, FTMS + c NSI d Full ms2 1356.0177@hcd30.00 [120.0000-2787.7041]
Precursor Scan: 002480, m/z: 1355.517, charge: 2
NEEYNK_5_4_0_1_0
366.1393
204.0864
138.0548
168.0652
186.0757
126.0544
274.093
292.1036
657.2408
796.3474 PEP_0_0_0_0_0_
999.4258 PEP_0_1_0_0_0_
1364.5742 PEP_1_2_0_0_0_
1526.6133 PEP_2_2_0_0_0_
Intensity
m/z
0
20000
40000
60000
80000
100000
120000
250
500
750
1000
1250
1500
1750
2000

File: 231129_OE_RK_36, RT: 6.561, Scan: 002506
Type: HCD, FTMS + c NSI d Full ms2 1026.0587@hcd30.00 [120.0000-3161.8816]
Precursor Scan: 002496, m/z: 1025.7246, charge: 3
NEEYNK_6_5_0_1_0

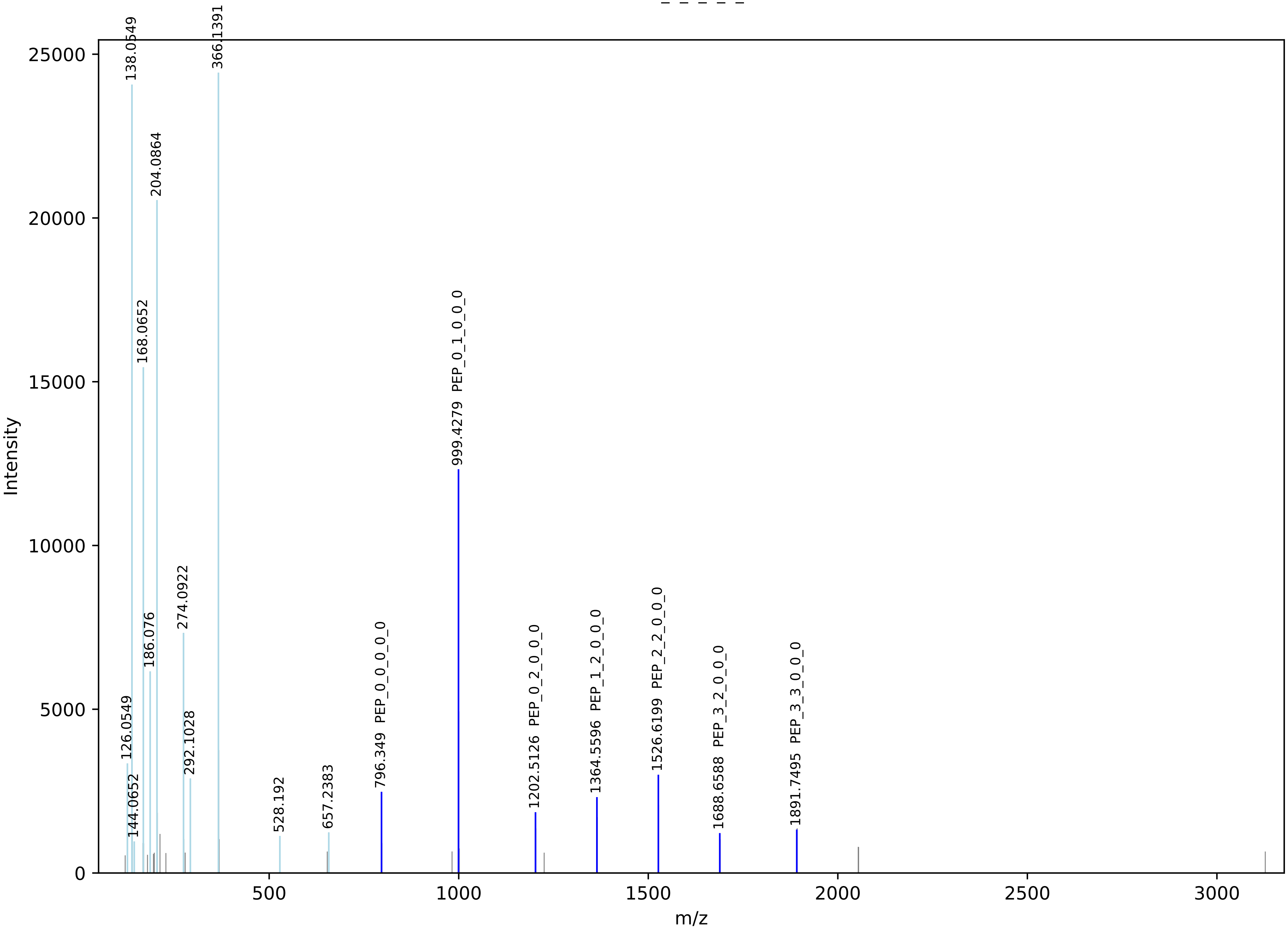

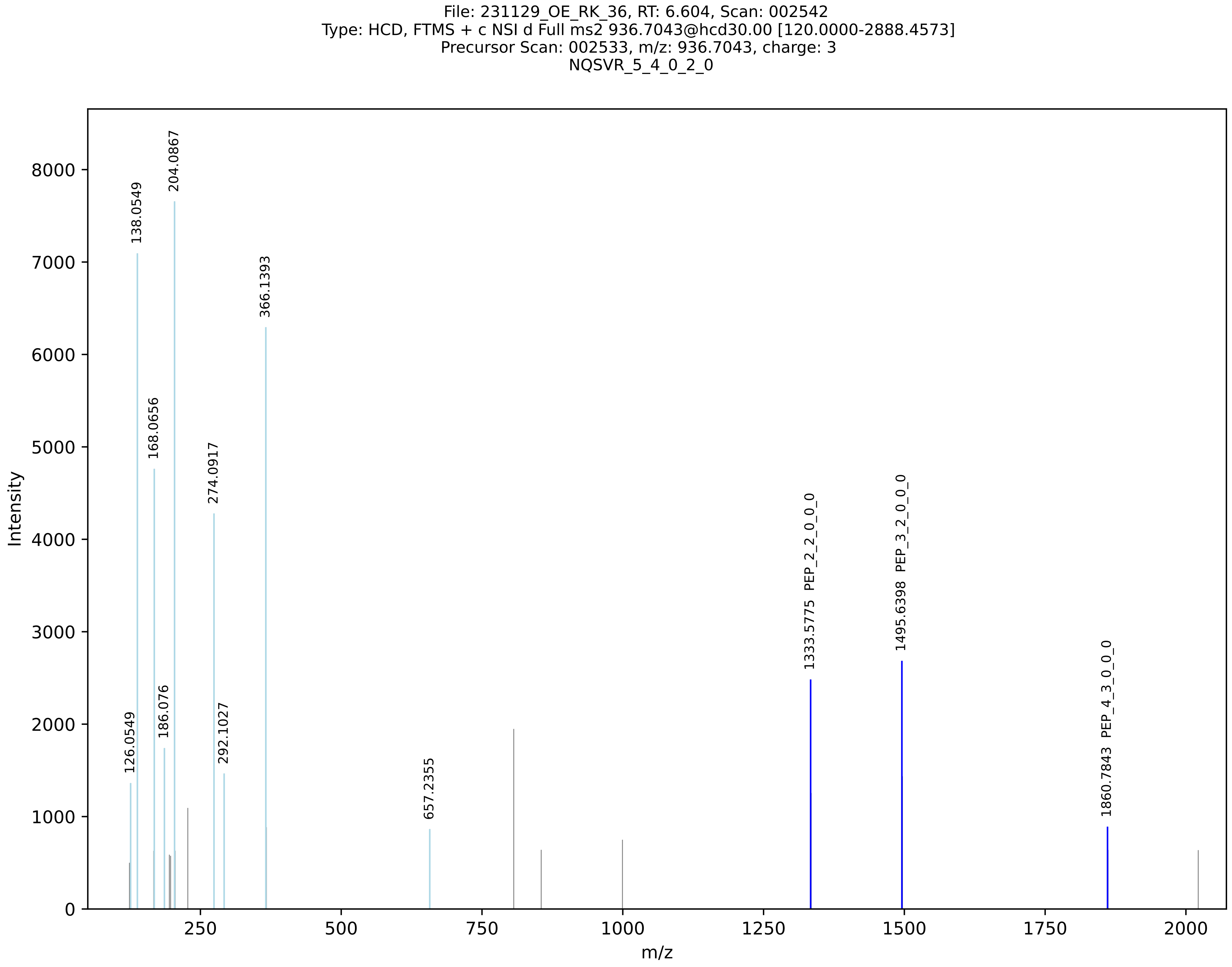

File: 231129_OE_RK_36, RT: 6.604, Scan: 002542
Type: HCD, FTMS + c NSI d Full ms2 936.7043@hcd30.00 [120.0000-2888.4573]
Precursor Scan: 002533, m/z: 936.7043, charge: 3
NQSVR_5_4_0_2_0
Intensity
m/z
138.0549
204.0867
366.1393
168.0656
274.0917
126.0549
186.076
292.1027
657.2355
1333.5775 PEP_2_2_0_0_0
1495.6398 PEP_3_2_0_0_0
1860.7843 PEP_4_3_0_0_0

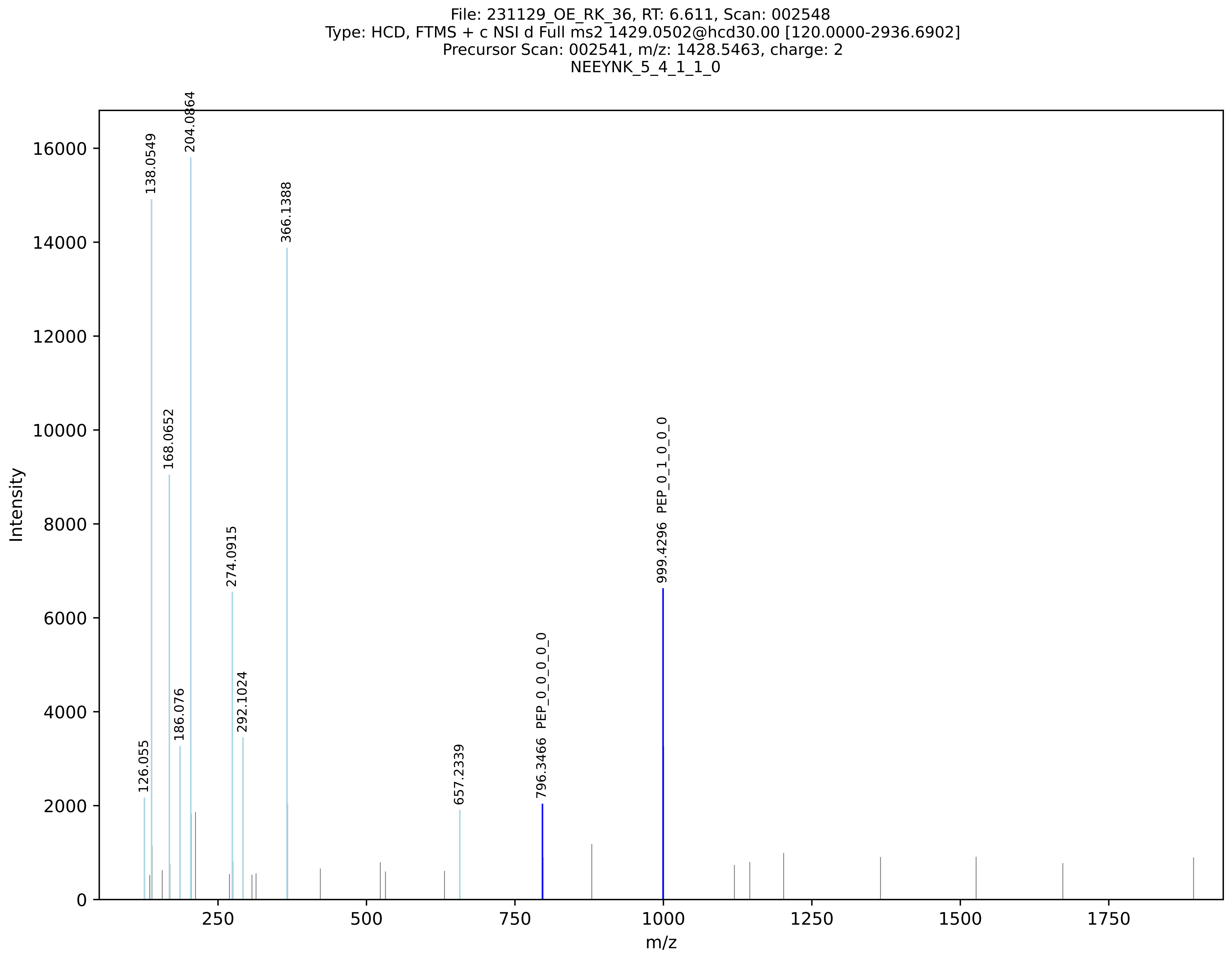
File: 231129_OE_RK_36, RT: 6.611, Scan: 002548
Type: HCD, FTMS + c NSI d Full ms2 1429.0502@hcd30.00 [120.0000-2936.6902]
Precursor Scan: 002541, m/z: 1428.5463, charge: 2
NEEYNK_5_4_1_1_0
Intensity
m/z
138.0549
204.0864
366.1388
168.0652
274.0915
292.1024
186.076
126.055
657.2339
796.3466 PEP_0_0_0_0_0
999.4296 PEP_0_1_0_0_0
0
2000
4000
6000
8000
10000
12000
14000
16000
250
500
750
1000
1250
1500
1750

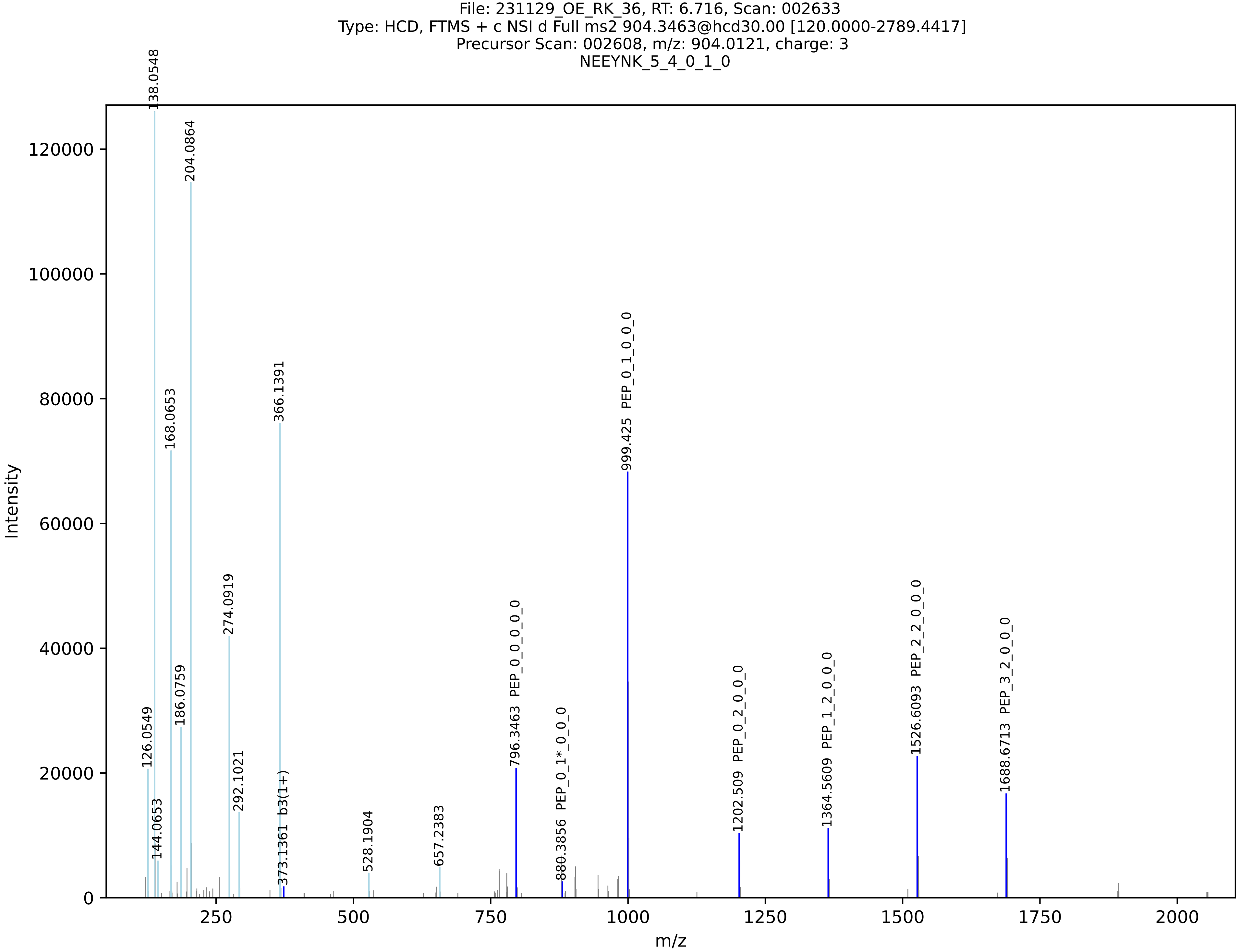
File: 231129_OE_RK_36, RT: 6.716, Scan: 002633
Type: HCD, FTMS + c NSI d Full ms2 904.3463@hcd30.00 [120.0000-2789.4417]
Precursor Scan: 002608, m/z: 904.0121, charge: 3
NEEYNK_5_4_0_1_0
138.0548
204.0864
168.0653
366.1391
999.425 PEP_0_1_0_0_
274.0919
186.0759
126.0549
144.0653
292.1021
373.1361 b3(1+)
528.1904
657.2383
796.3463 PEP_0_0_0_0_0_
880.3856 PEP_0_1*_0_0_0
1202.509 PEP_0_2_0_0_0
1364.5609 PEP_1_2_0_0_0
1526.6093 PEP_2_2_0_0_0
1688.6713 PEP_3_2_0_0_0
Intensity
m/z
0
20000
40000
60000
80000
100000
120000
250
500
750
1000
1250
1500
1750
2000

File: 231129_OE_RK_36, RT: 6.796, Scan: 002709
Type: HCD, FTMS + c NSI d Full ms2 1171.7738@hcd30.00 [120.0000-3607.7698]
Precursor Scan: 002702, m/z: 1171.4397, charge: 3
NEEYNK_6_5_1_2_0

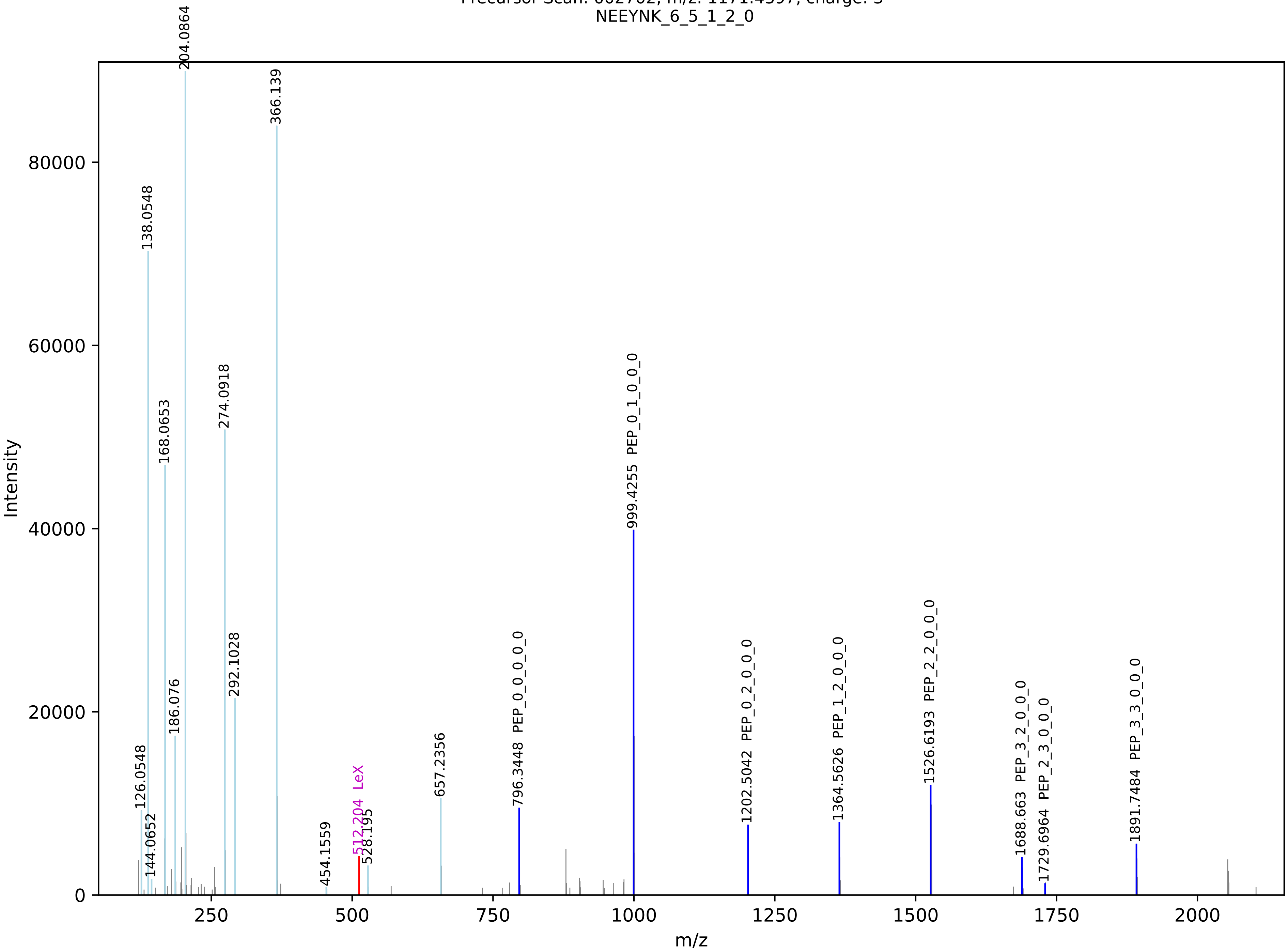

File: 231129_OE_RK_36, RT: 6.834, Scan: 002746
Type: HCD, FTMS + c NSI d Full ms2 1501.5619@hcd30.00 [120.0000-3084.6143]
Precursor Scan: 002738, m/z: 1501.0609, charge: 2
NEEYNK_5_4_0_2_0

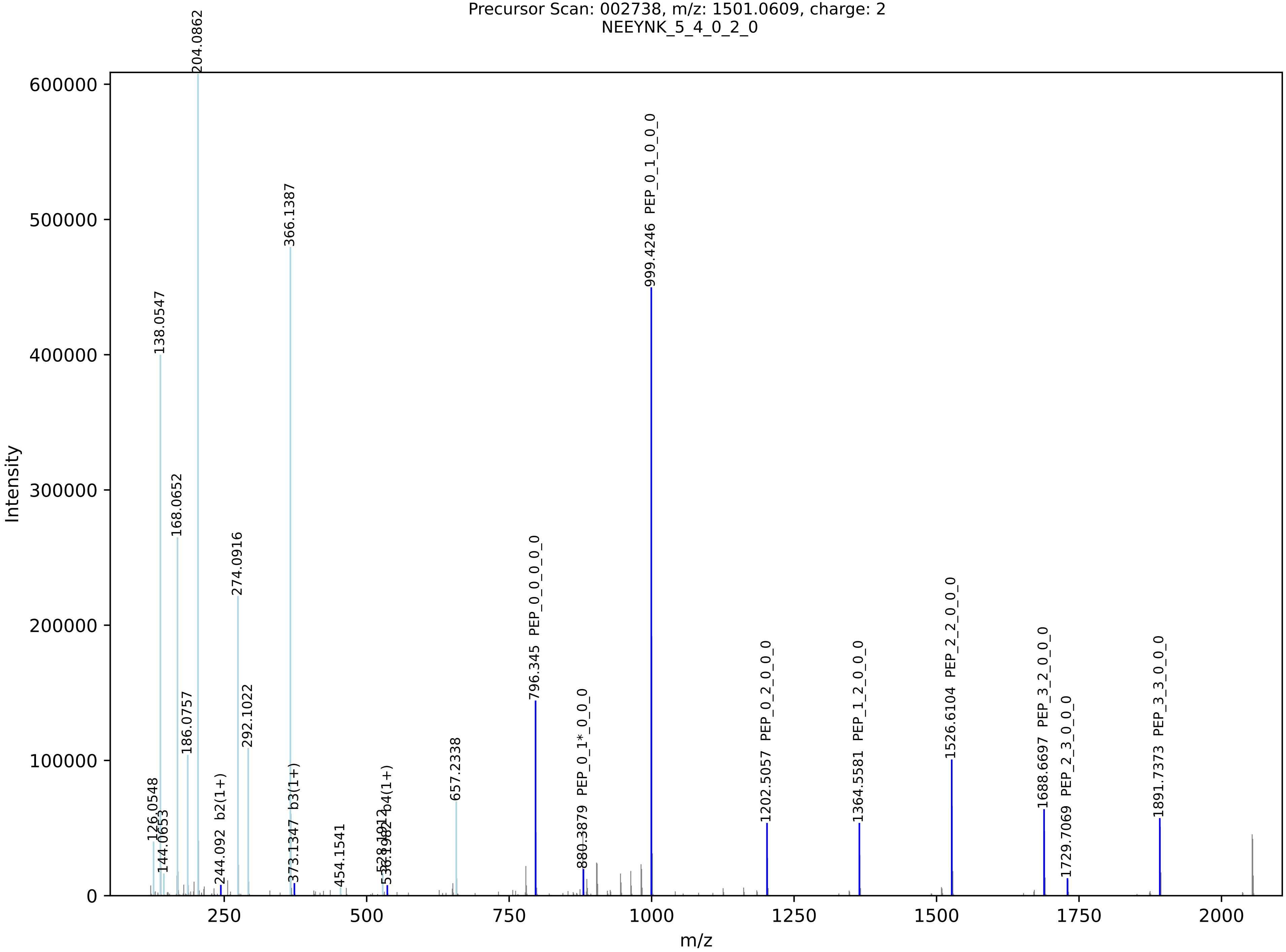

File: 231129_OE_RK_36, RT: 6.861, Scan: 002775
Type: HCD, FTMS + c NSI d Full ms2 1050.0261@hcd30.00 [120.0000-3235.2217]
Precursor Scan: 002758, m/z: 1050.0261, charge: 3
NEEYNK_5_4_1_2_0

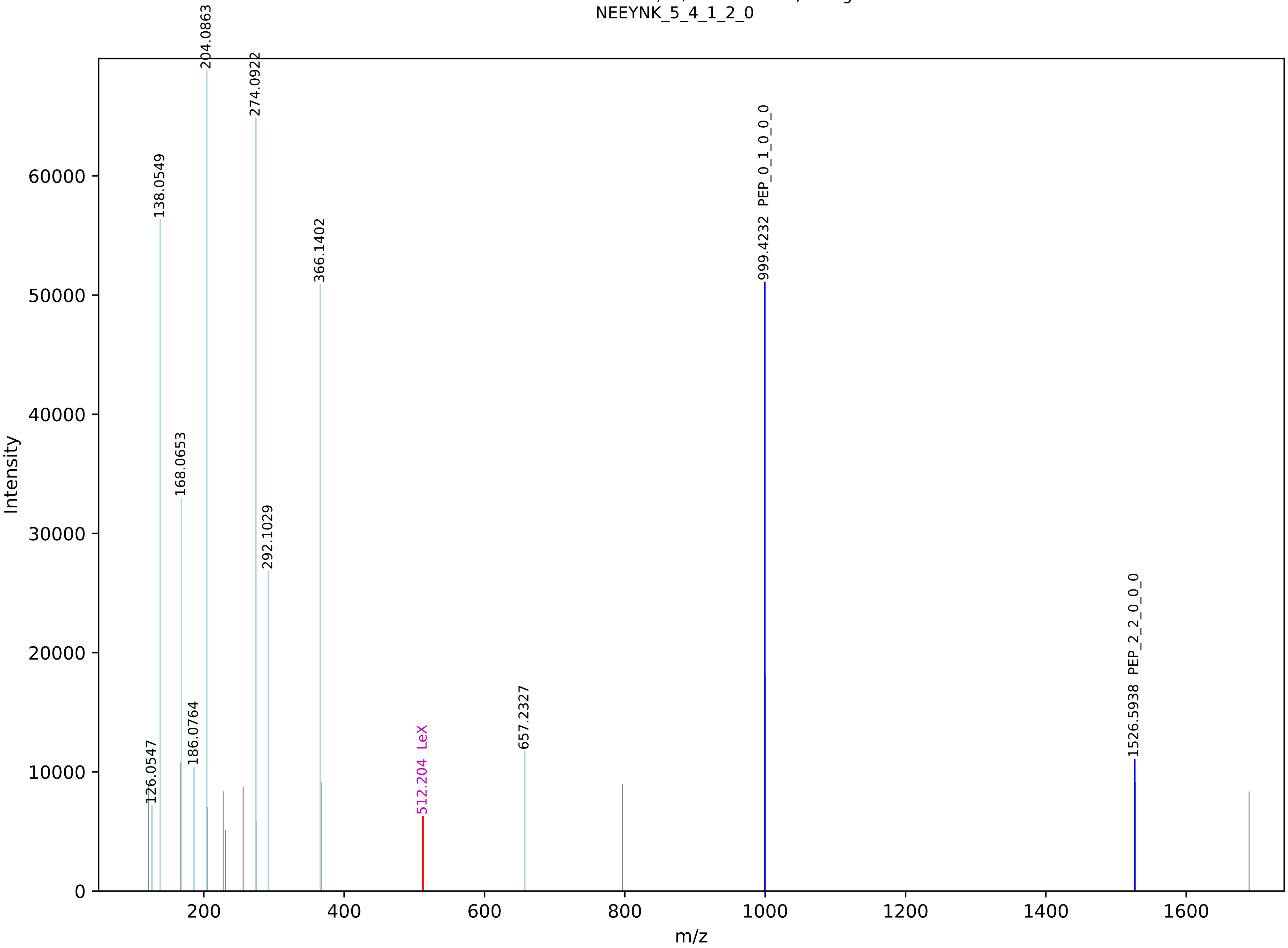

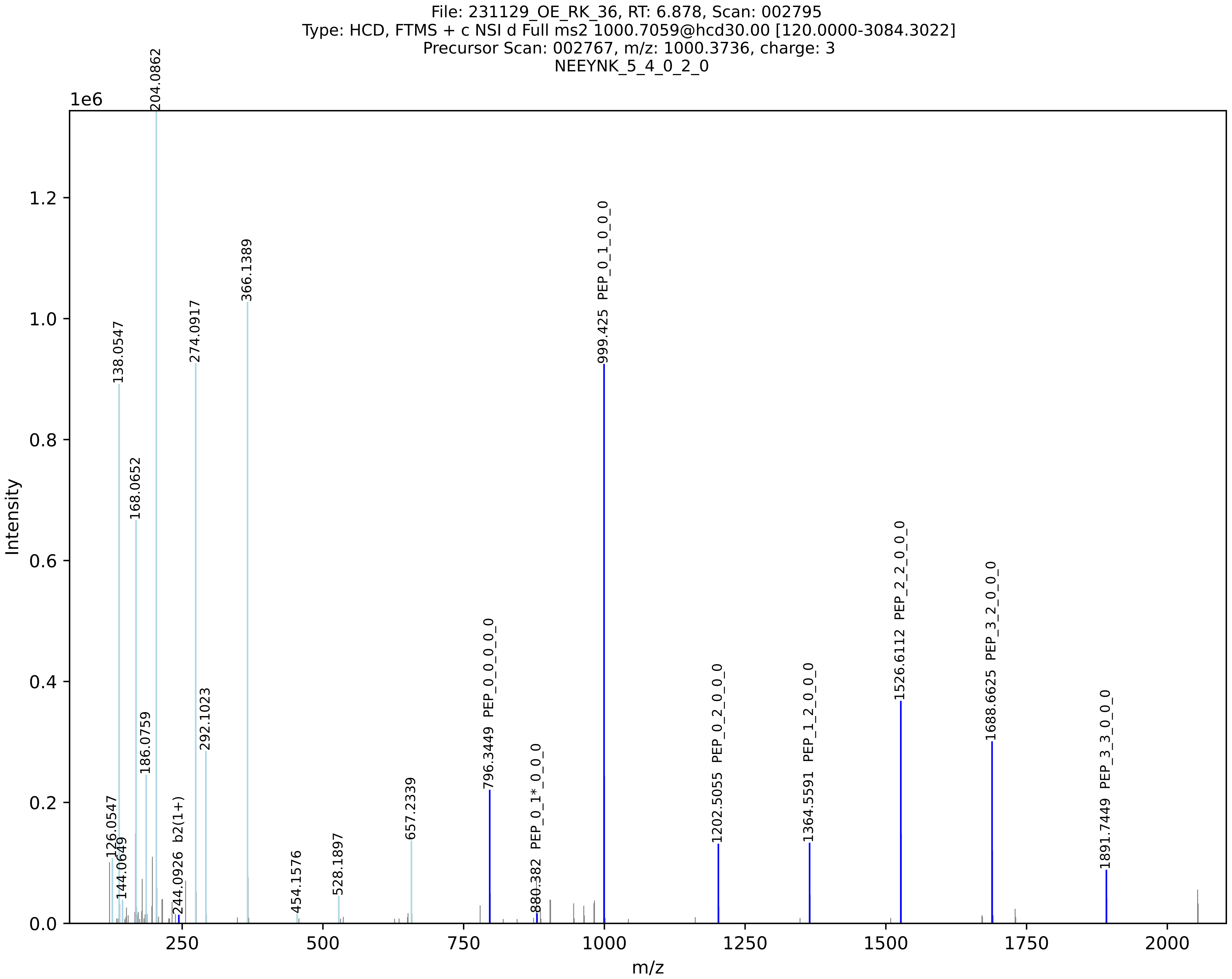
File: 231129_OE_RK_36, RT: 6.878, Scan: 002795
Type: HCD, FTMS + c NSI d Full ms2 1000.7059@hcd30.00 [120.0000-3084.3022]
Precursor Scan: 002767, m/z: 1000.3736, charge: 3
NEEYNK_5_4_0_2_0
1e6
Intensity
m/z
204.0862
138.0547
168.0652
186.0759
126.0547
144.0649
244.0926 b2(1+)
274.0917
292.1023
366.1389
454.1576
528.1897
657.2339
796.3449 PEP_0_0_0_0_0
880.382 PEP_0_1*_0_0_0
999.425 PEP_0_1_0_0_0
1202.5055 PEP_0_2_0_0_0
1364.5591 PEP_1_2_0_0_0
1526.6112 PEP_2_2_0_0_0
1688.6625 PEP_3_2_0_0_0
1891.7449 PEP_3_3_0_0_0

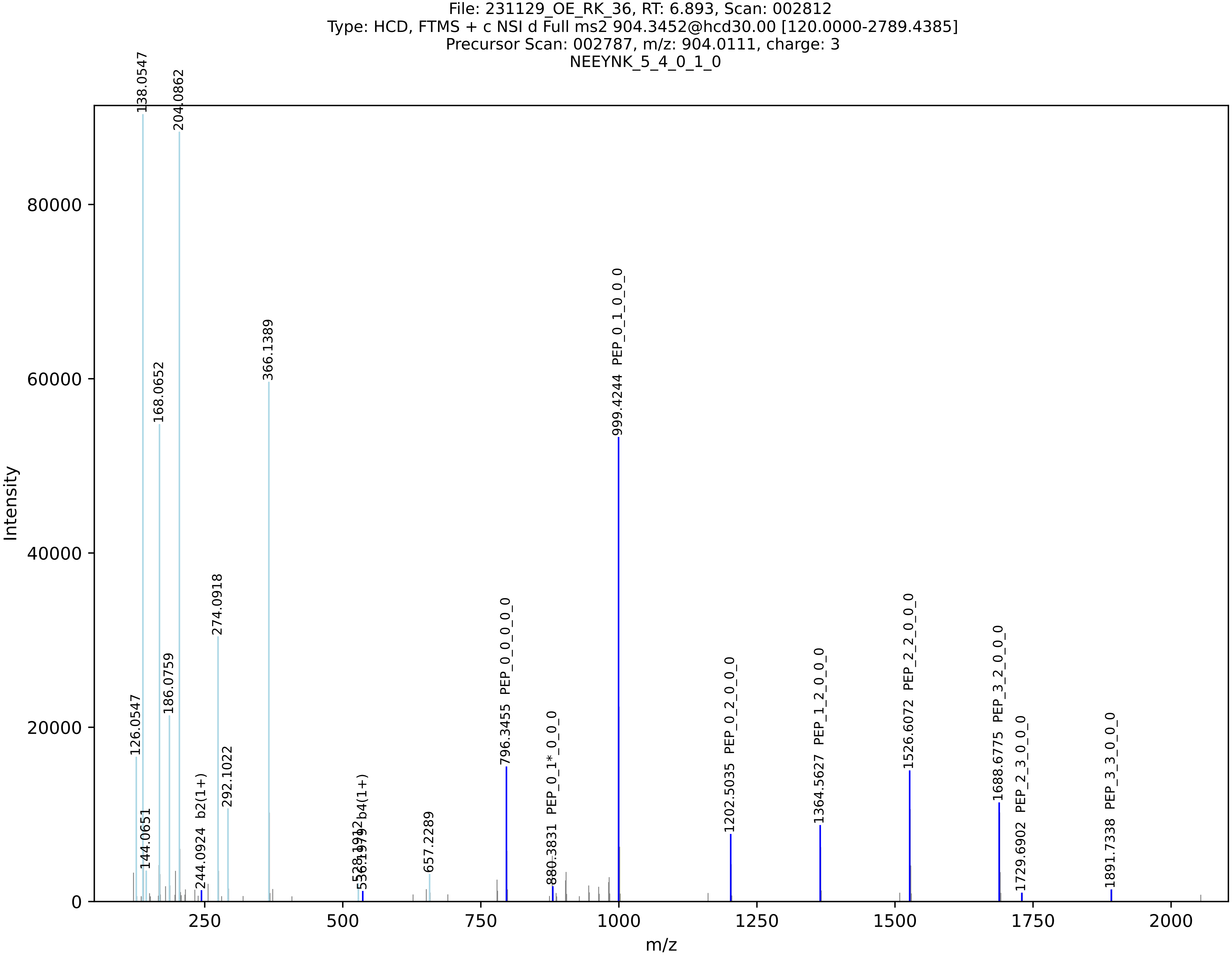
File: 231129_OE_RK_36, RT: 6.893, Scan: 002812
Type: HCD, FTMS + c NSI d Full ms2 904.3452@hcd30.00 [120.0000-2789.4385]
Precursor Scan: 002787, m/z: 904.0111, charge: 3
NEEYNK_5_4_0_1_0
138.0547
204.0862
168.0652
366.1389
999.4244 PEP_0_1_0_0_0
274.0918
796.3455 PEP_0_0_0_0_0
186.0759
126.0547
292.1022
144.0651
244.0924 b2(1+)
528.1912
536.1979 b4(1+)
657.2289
880.3831 PEP_0_1*_0_0_0
1202.5035 PEP_0_2_0_0_0
1364.5627 PEP_1_2_0_0_0
1526.6072 PEP_2_2_0_0_0
1688.6775 PEP_3_2_0_0_0
1729.6902 PEP_2_3_0_0_0
1891.7338 PEP_3_3_0_0_0
Intensity
0
20000
40000
60000
80000
250
500
750
1000
1250
1500
1750
2000
m/z

File: 231129_OE_RK_36, RT: 6.895, Scan: 002816
Type: HCD, FTMS + c NSI d Full ms2 1356.0148@hcd30.00 [120.0000-2787.6982]
Precursor Scan: 002787, m/z: 1355.513, charge: 2
NEEYNK_5_4_0_1_0

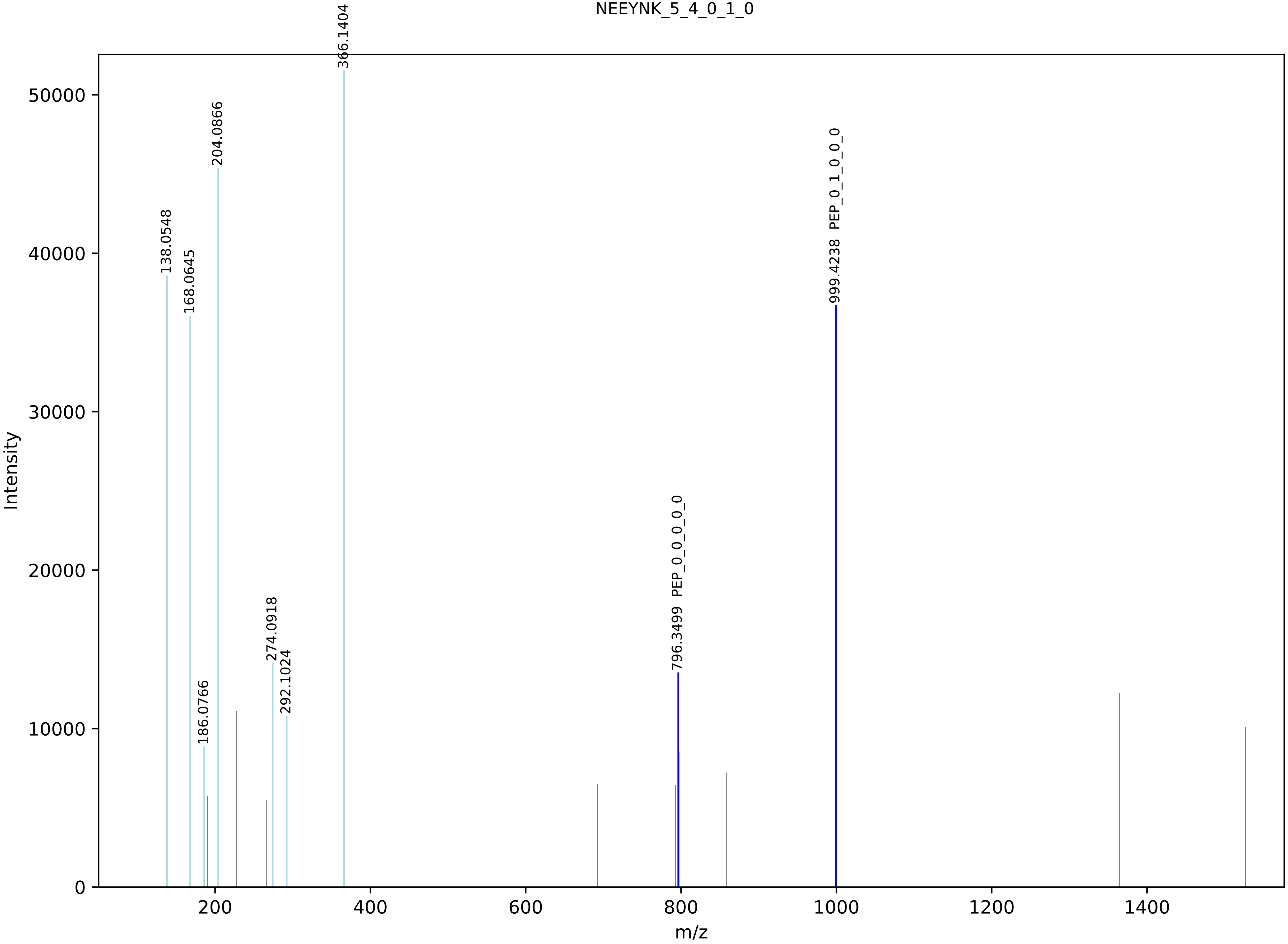

File: 231129_OE_RK_36, RT: 6.911, Scan: 002835
Type: HCD, FTMS + c NSI d Full ms2 1123.4216@hcd30.00 [120.0000-3459.8123]
Precursor Scan: 002811, m/z: 1122.7538, charge: 3
NEEYNK_6_5_0_2_0

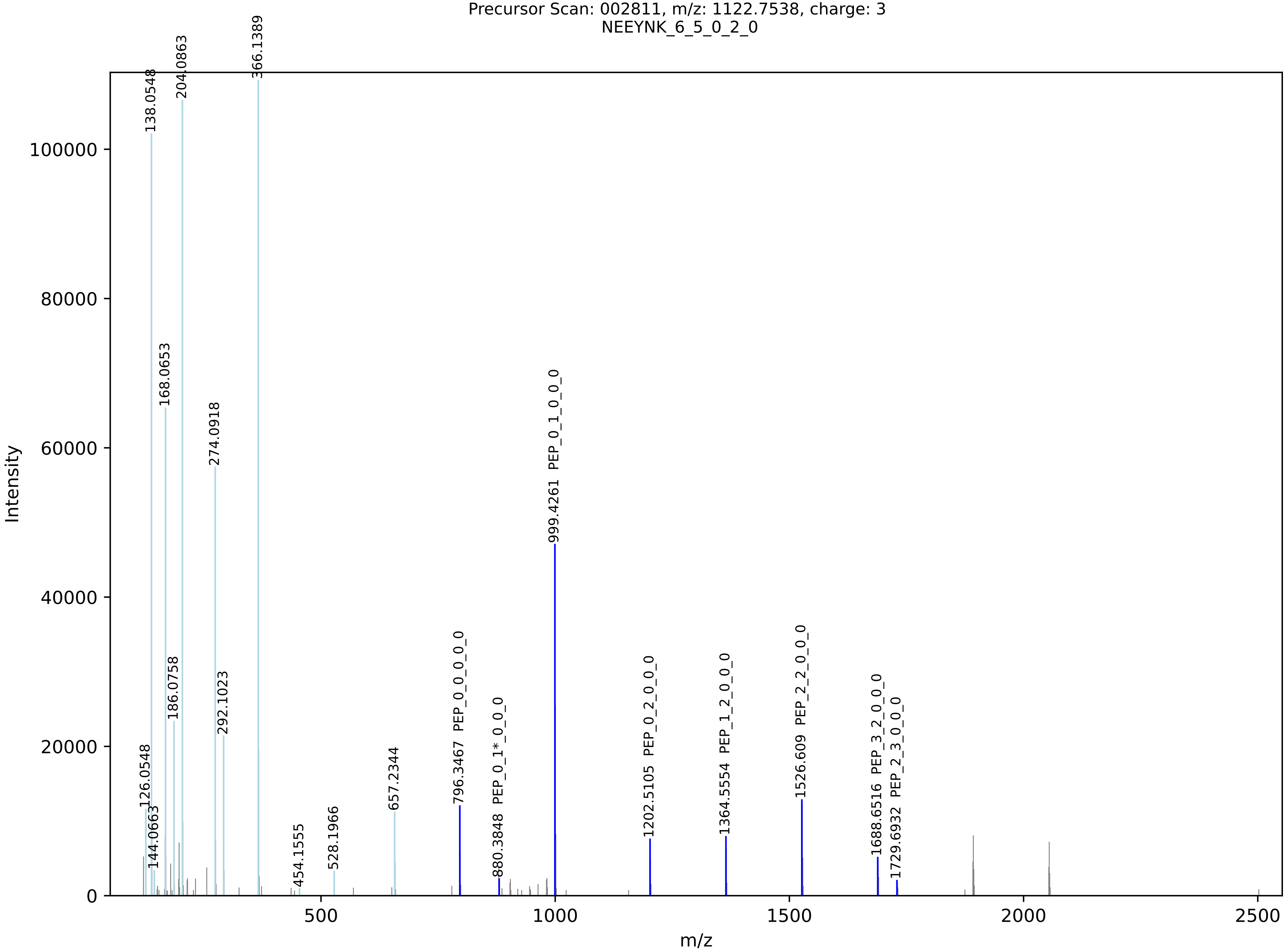

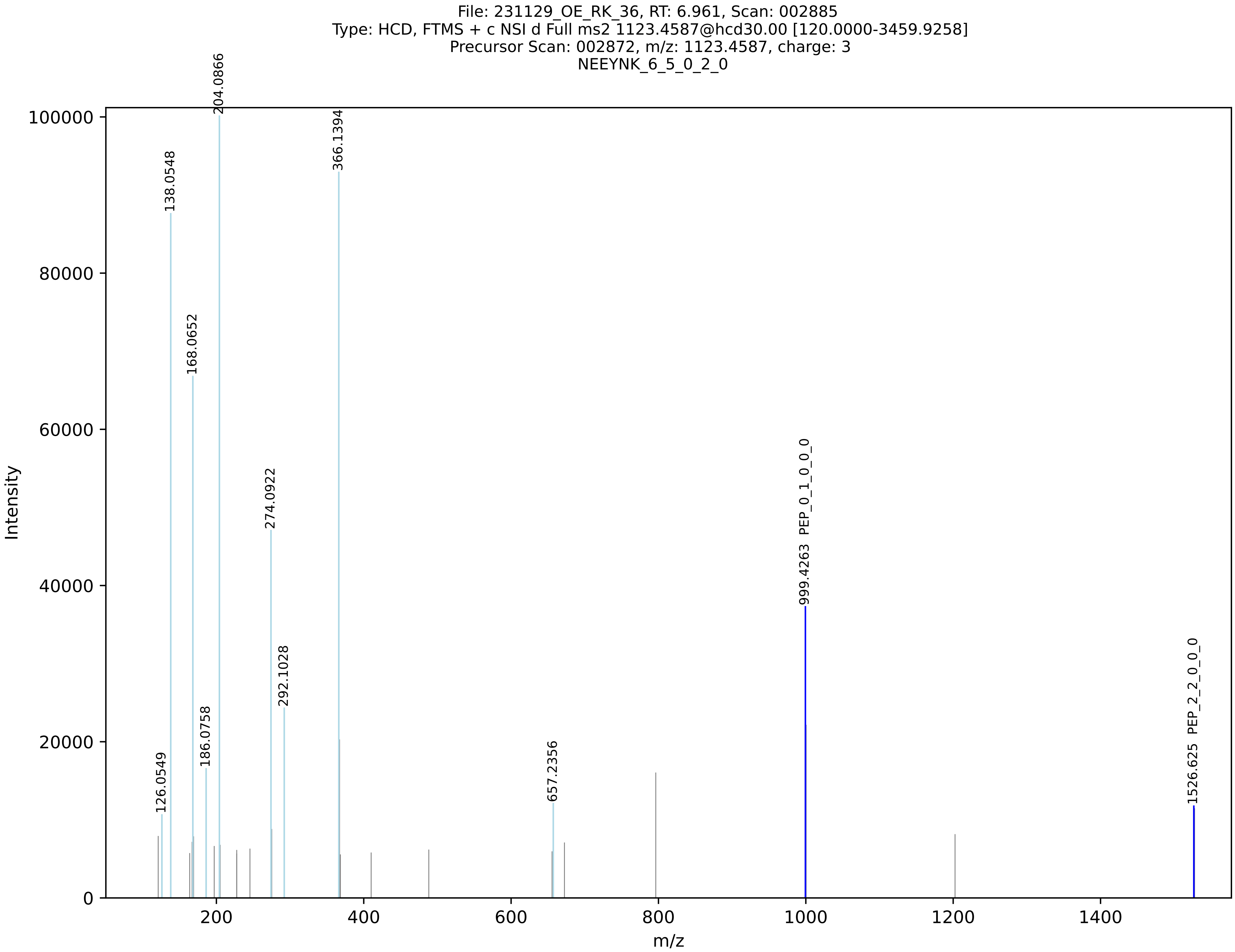
File: 231129_OE_RK_36, RT: 6.961, Scan: 002885
Type: HCD, FTMS + c NSI d Full ms2 1123.4587@hcd30.00 [120.0000-3459.9258]
Precursor Scan: 002872, m/z: 1123.4587, charge: 3
NEEYNK_6_5_0_2_0
Intensity
m/z
0
20000
40000
60000
80000
100000
200
400
600
800
1000
1200
1400
126.0549
138.0548
168.0652
186.0758
204.0866
274.0922
292.1028
366.1394
657.2356
999.4263 PEP_0_1_0_0_0
1526.625 PEP_2_2_0_0_0

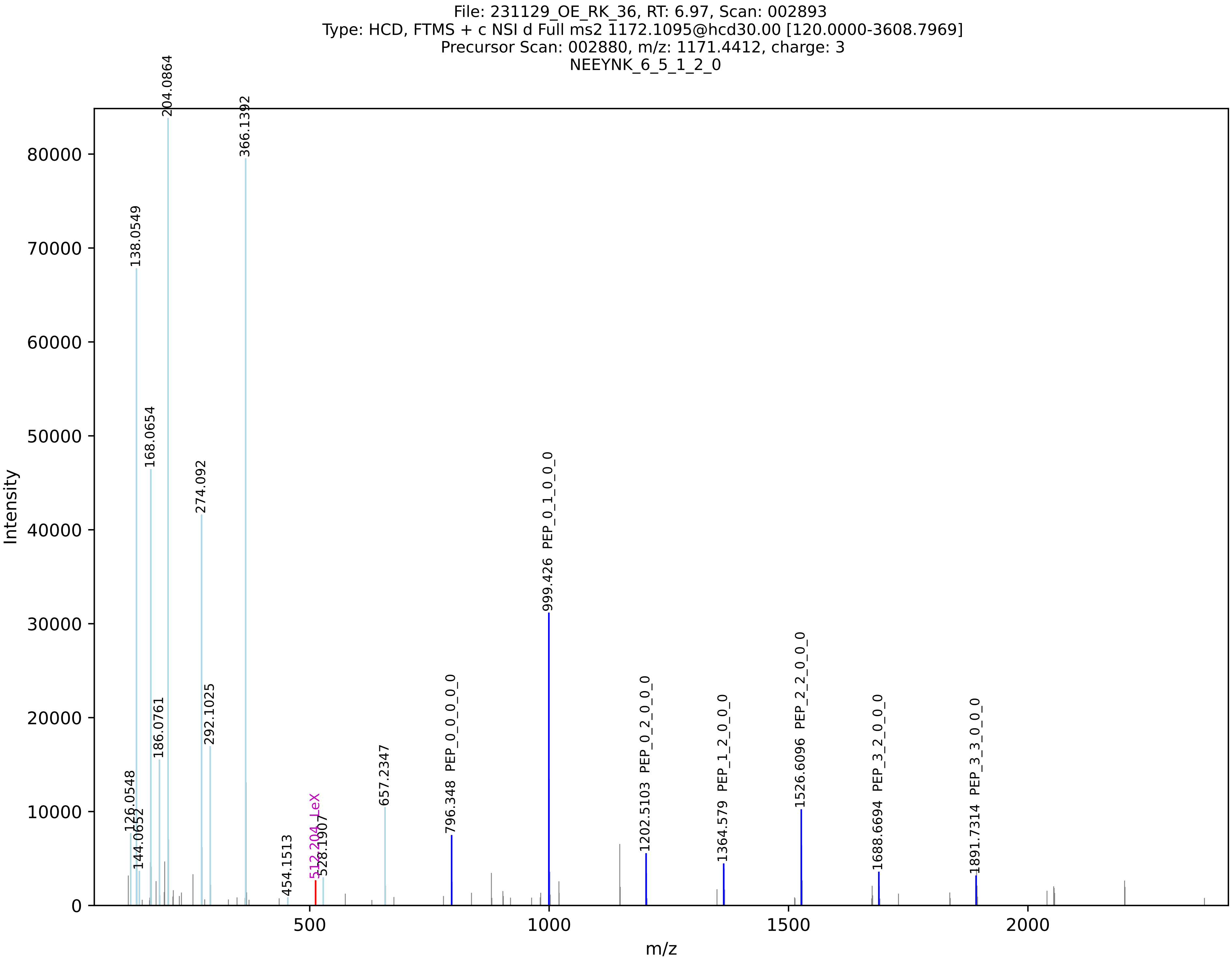
File: 231129_OE_RK_36, RT: 6.97, Scan: 002893
Type: HCD, FTMS + c NSI d Full ms2 1172.1095@hcd30.00 [120.0000-3608.7969]
Precursor Scan: 002880, m/z: 1171.4412, charge: 3
NEEYNK_6_5_1_2_0
Intensity
m/z
204.0864
366.1392
138.0549
168.0654
274.092
186.0761
292.1025
126.0548
144.0652
454.1513
512.204 LeX
528.1907
657.2347
796.348 PEP_0_0_0_0_0
999.426 PEP_0_1_0_0_0
1202.5103 PEP_0_2_0_0_0
1364.579 PEP_1_2_0_0_0
1526.6096 PEP_2_2_0_0_0
1688.6694 PEP_3_2_0_0_0
1891.7314 PEP_3_3_0_0_0

File: 231129_OE_RK_36, RT: 6.98, Scan: 002903
Type: HCD, FTMS + c NSI d Full ms2 953.7089@hcd30.00 [120.0000-2940.4912]
Precursor Scan: 002892, m/z: 953.3743, charge: 3
HNSTGCLR_5_4_0_1_0

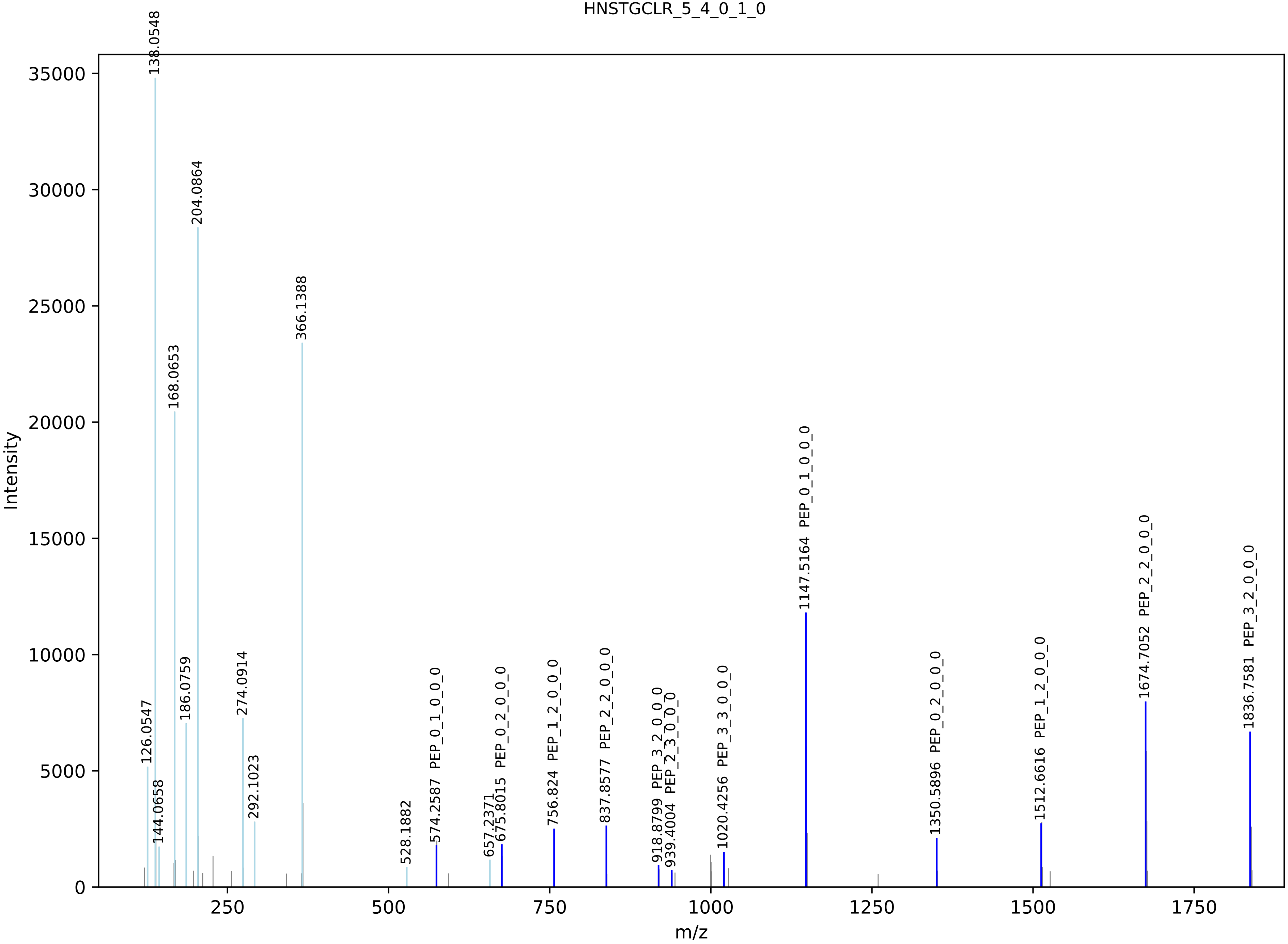

File: 231129_OE_RK_36, RT: 7.009, Scan: 002932
Type: HCD, FTMS + c NSI d Full ms2 1501.5631@hcd30.00 [120.0000-3084.6167]
Precursor Scan: 002915, m/z: 1501.0617, charge: 2
NEEYNK_5_4_0_2_0

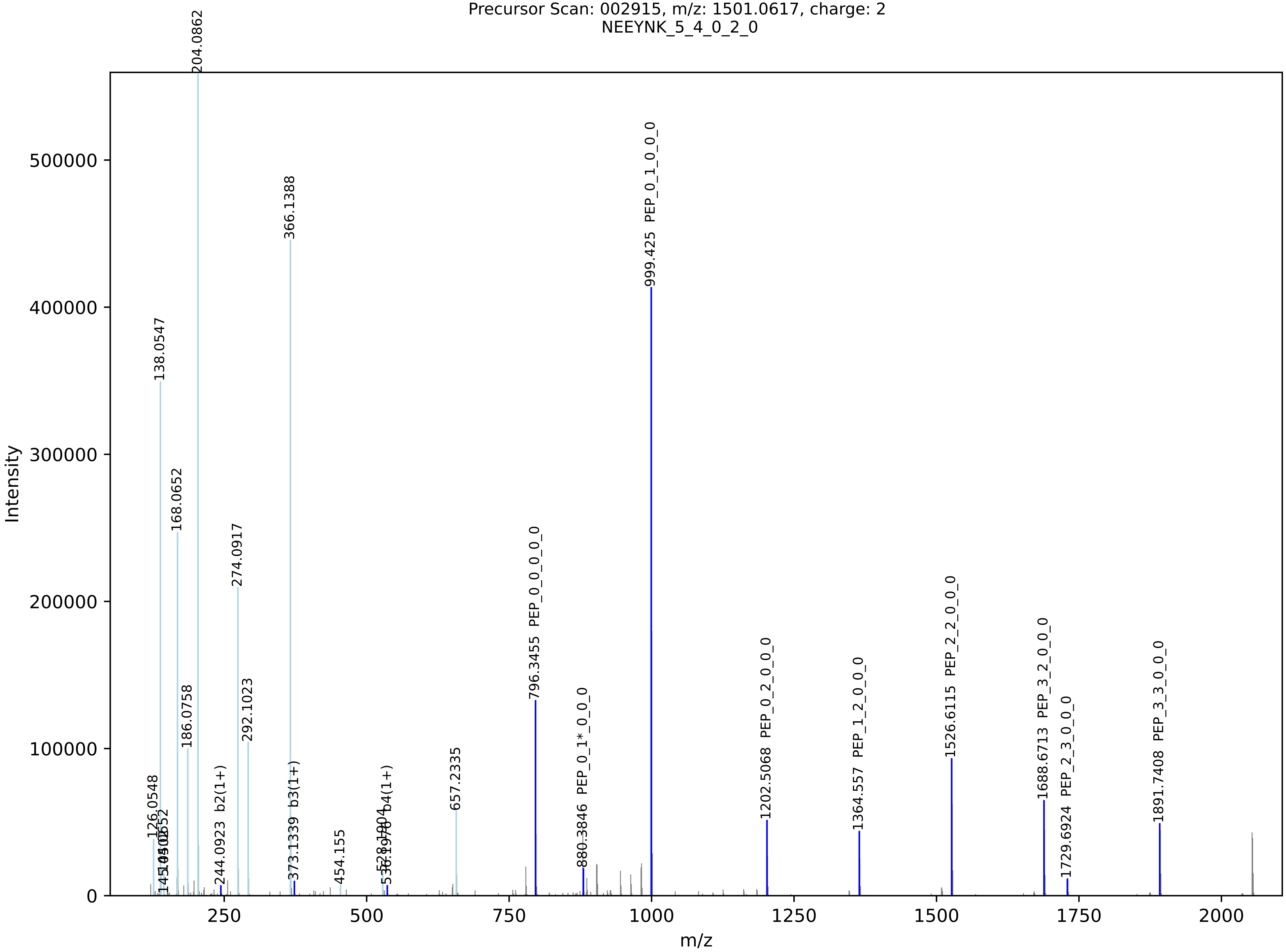

File: 231129_OE_RK_36, RT: 7.01, Scan: 002934
Type: HCD, FTMS + c NSI d Full ms2 1007.0523@hcd30.00 [120.0000-3103.7219]
Precursor Scan: 002915, m/z: 1006.7182, charge: 3
NEEYNK_6_4_1_1_0

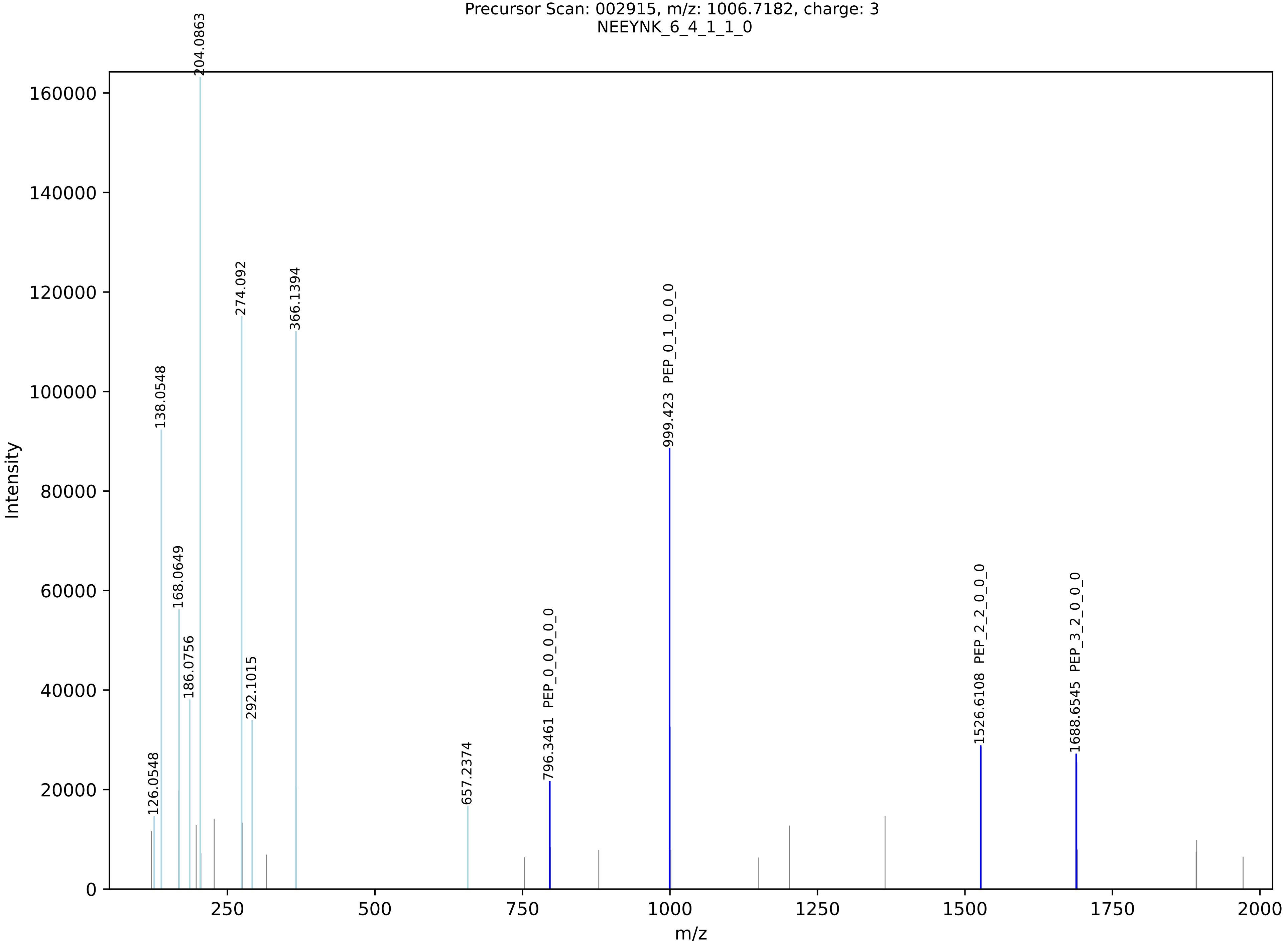

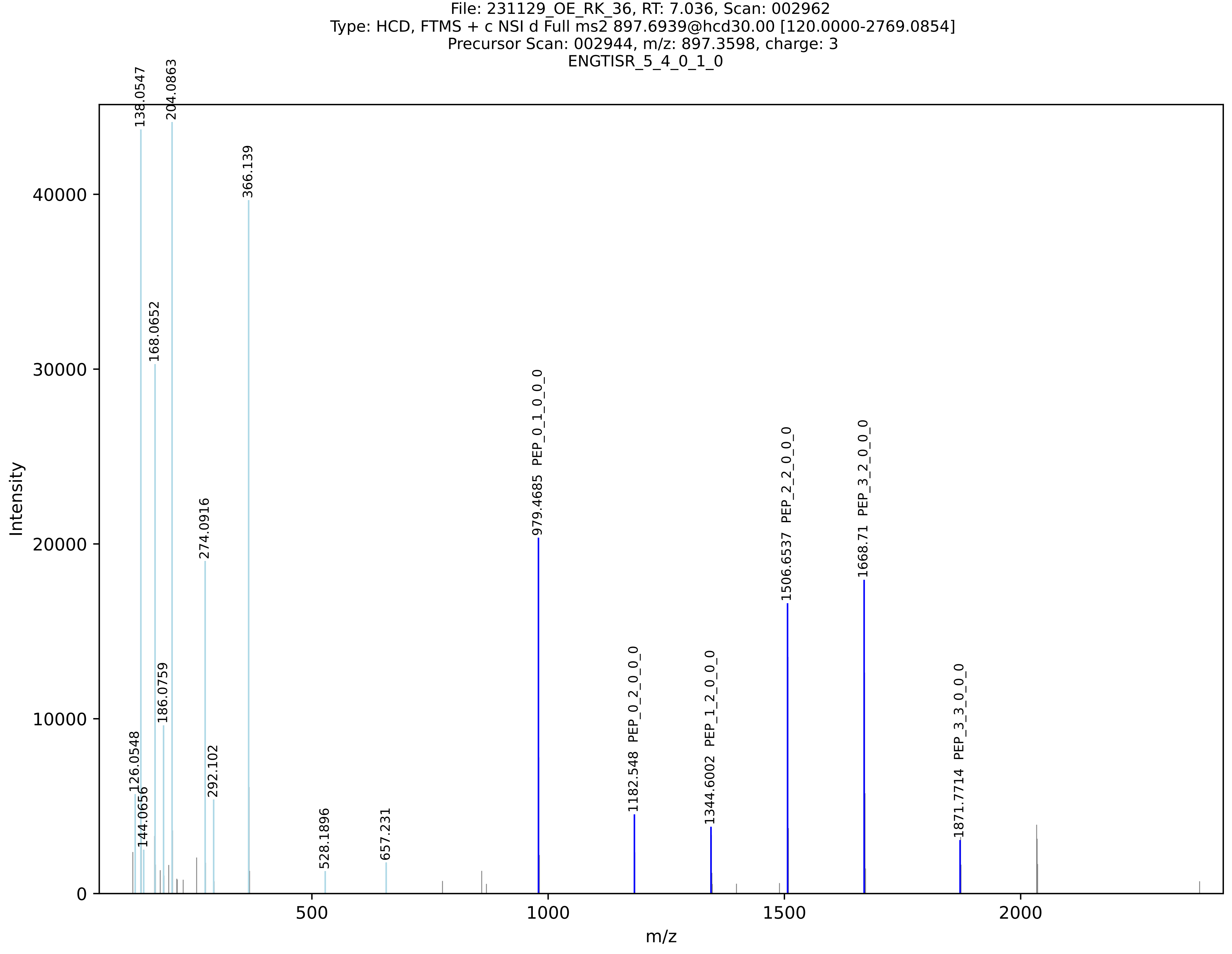
File: 231129_OE_RK_36, RT: 7.036, Scan: 002962
Type: HCD, FTMS + c NSI d Full ms2 897.6939@hcd30.00 [120.0000-2769.0854]
Precursor Scan: 002944, m/z: 897.3598, charge: 3
ENGTISR_5_4_0_1_0
Intensity
m/z
138.0547
204.0863
366.139
168.0652
274.0916
186.0759
126.0548
144.0656
292.102
528.1896
657.231
979.4685 PEP_0_1_0_0_0
1182.548 PEP_0_2_0_0_0
1344.6002 PEP_1_2_0_0_0
1506.6537 PEP_2_2_0_0_0
1668.71 PEP_3_2_0_0_0
1871.7714 PEP_3_3_0_0_0
0
10000
20000
30000
40000
500
1000
1500
2000

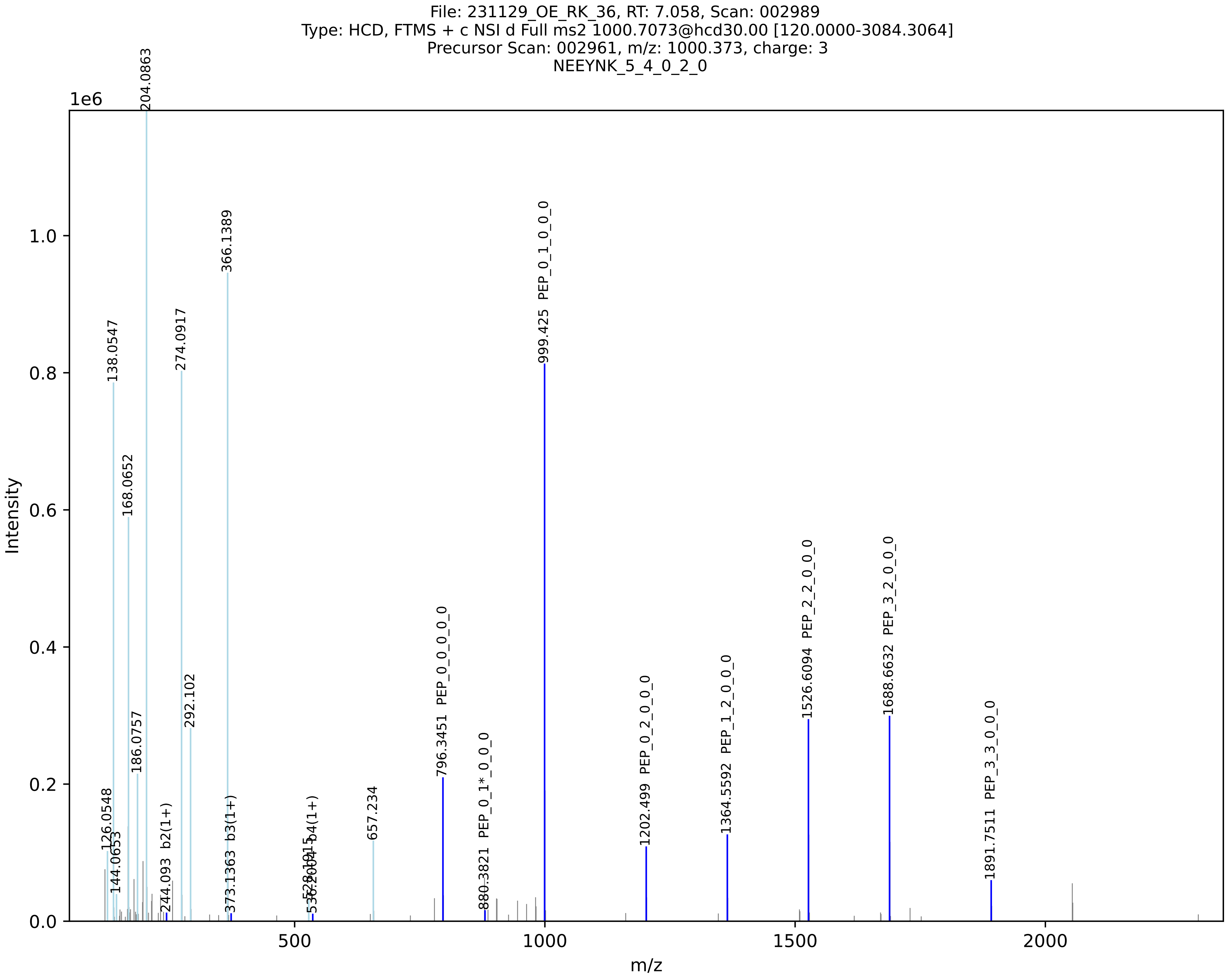
File: 231129_OE_RK_36, RT: 7.058, Scan: 002989
Type: HCD, FTMS + c NSI d Full ms2 1000.7073@hcd30.00 [120.0000-3084.3064]
Precursor Scan: 002961, m/z: 1000.373, charge: 3
NEEYNK_5_4_0_2_0
1e6
Intensity
m/z
204.0863
366.1389
274.0917
138.0547
168.0652
292.102
186.0757
126.0548
144.0653
244.093 b2(1+)
373.1363 b3(1+)
528.1915
530.2004 b4(1+)
657.234
796.3451 PEP_0_0_0_0_0
880.3821 PEP_0_1*_0_0_0
999.425 PEP_0_1_0_0_0
1202.499 PEP_0_2_0_0_0
1364.5592 PEP_1_2_0_0_0
1526.6094 PEP_2_2_0_0_0
1688.6632 PEP_3_2_0_0_0
1891.7511 PEP_3_3_0_0_0

File: 231129_OE_RK_36, RT: 7.153, Scan: 003104
Type: HCD, FTMS + c NSI d Full ms2 1051.0322@hcd30.00 [120.0000-3238.3005]
Precursor Scan: 003068, m/z: 1050.6929, charge: 3
NEEYNK_5_4_1_2_0

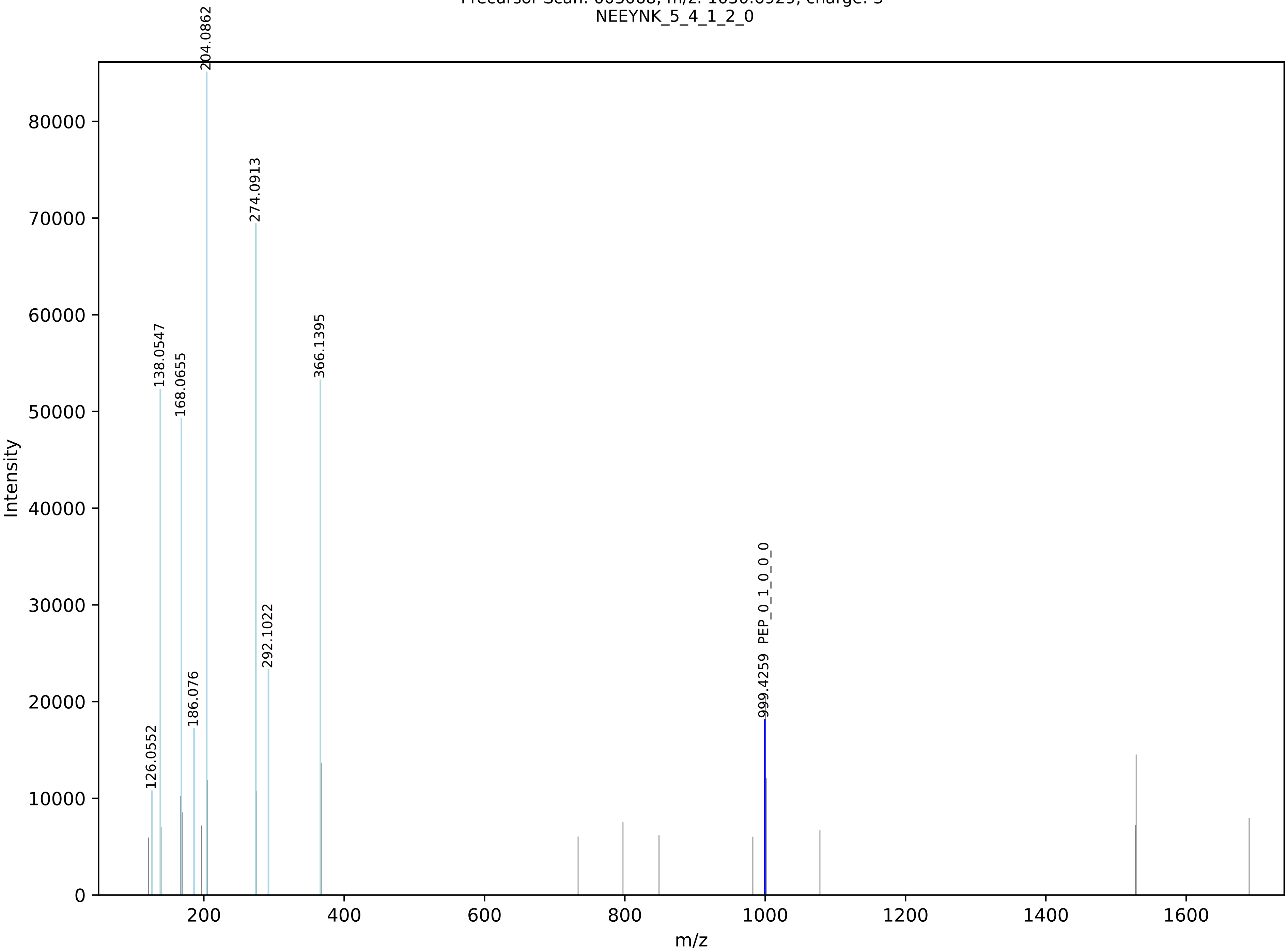

File: 231129_OE_RK_36, RT: 7.162, Scan: 003112
Type: HCD, FTMS + c NSI d Full ms2 953.7095@hcd30.00 [120.0000-2940.4929]
Precursor Scan: 003092, m/z: 953.3753, charge: 3
HNSTGCLR_5_4_0_1_0

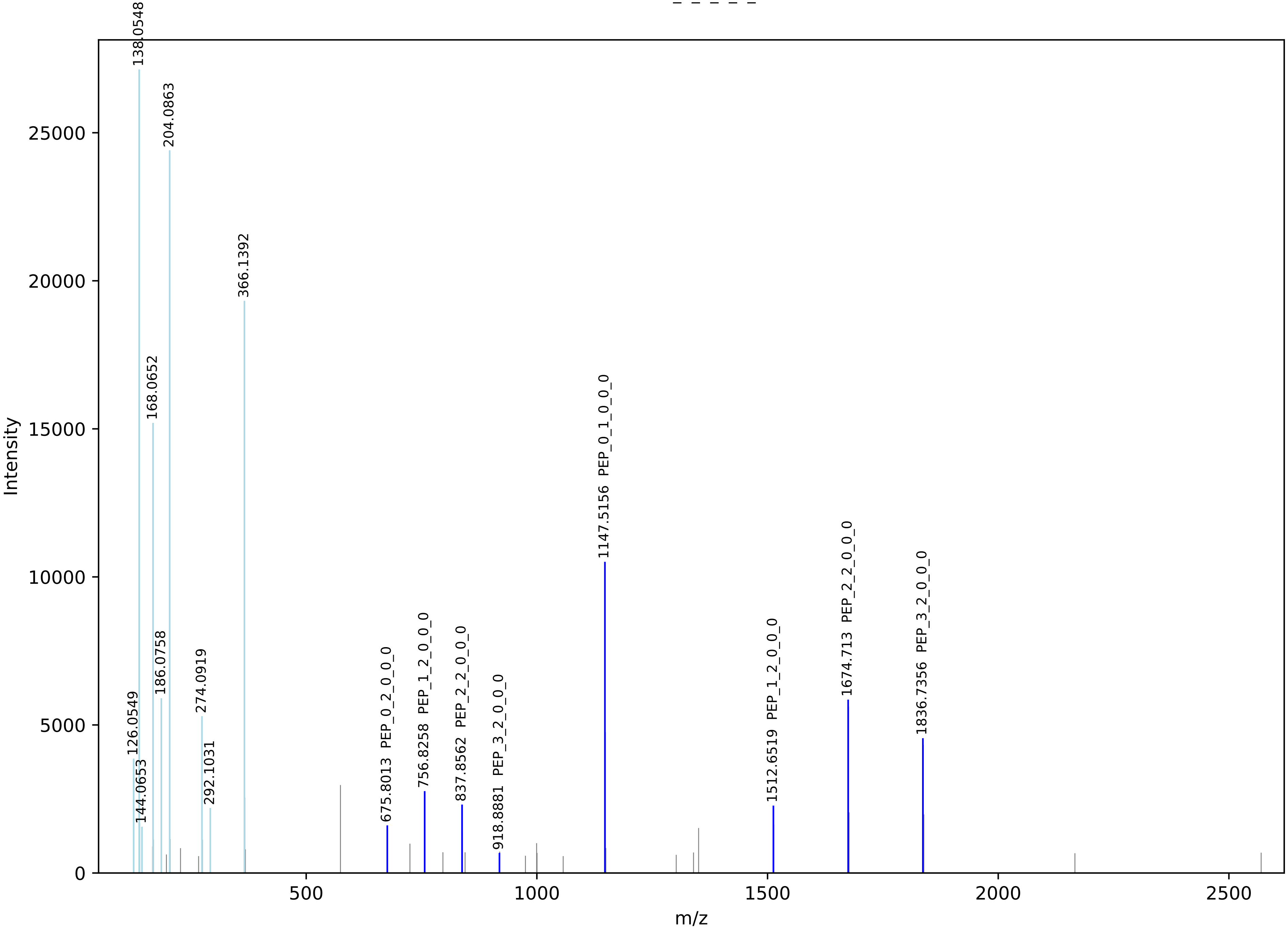

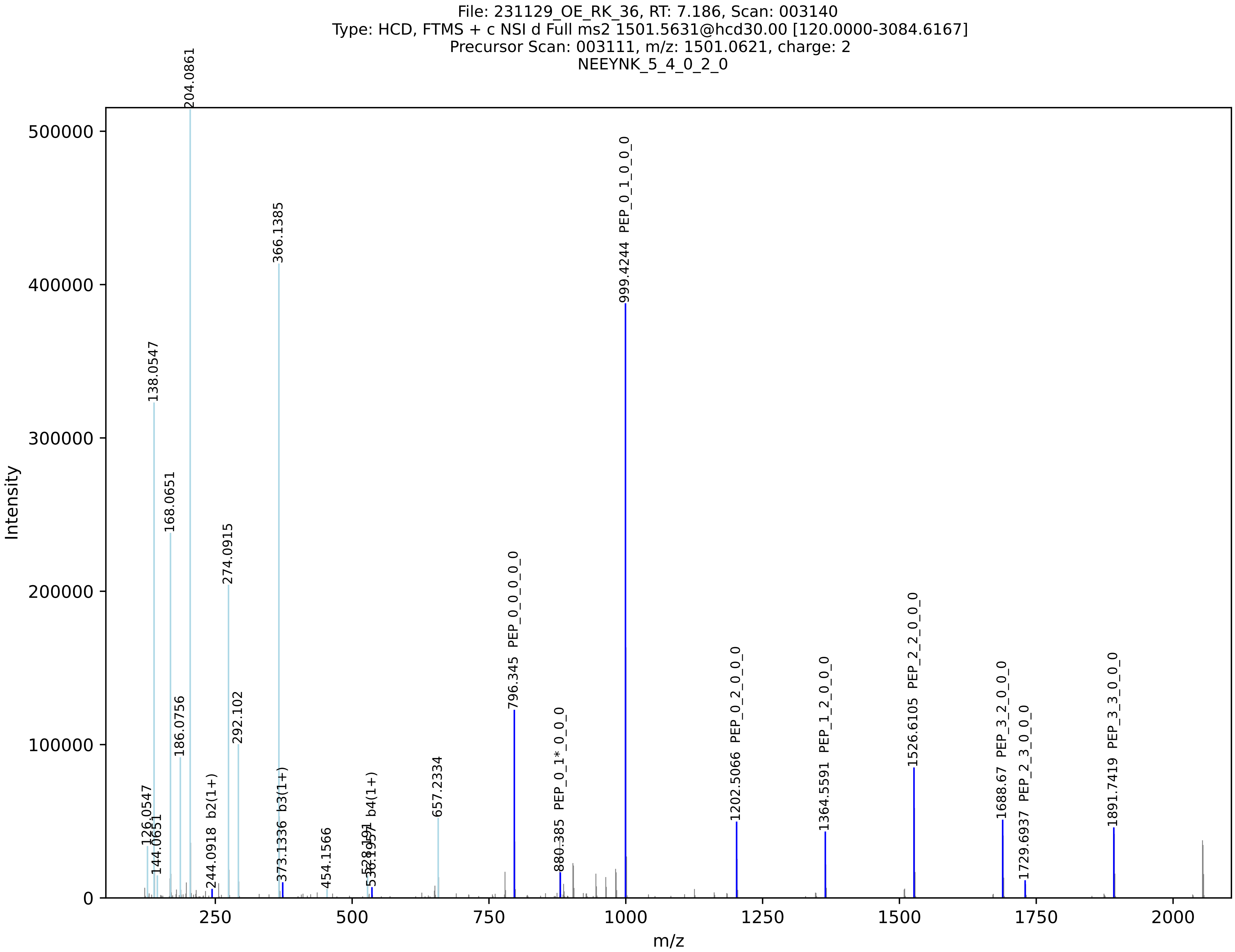
File: 231129_OE_RK_36, RT: 7.186, Scan: 003140
Type: HCD, FTMS + c NSI d Full ms2 1501.5631@hcd30.00 [120.0000-3084.6167]
Precursor Scan: 003111, m/z: 1501.0621, charge: 2
NEEYNK_5_4_0_2_0
Intensity
m/z
204.0861
366.1385
999.4244 PEP_0_1_0_0_0
138.0547
168.0651
274.0915
796.345 PEP_0_0_0_0_0
1526.6105 PEP_2_2_0_0_0
186.0756
292.102
880.385 PEP_0_1*_0_0_0
1202.5066 PEP_0_2_0_0_0
1364.5591 PEP_1_2_0_0_0
1688.67 PEP_3_2_0_0_0
1729.6937 PEP_2_3_0_0_0
1891.7419 PEP_3_3_0_0_0
126.0547
144.0651
244.0918 b2(1+)
373.1336 b3(1+)
454.1566
528.191
536.1957 b4(1+)
657.2334

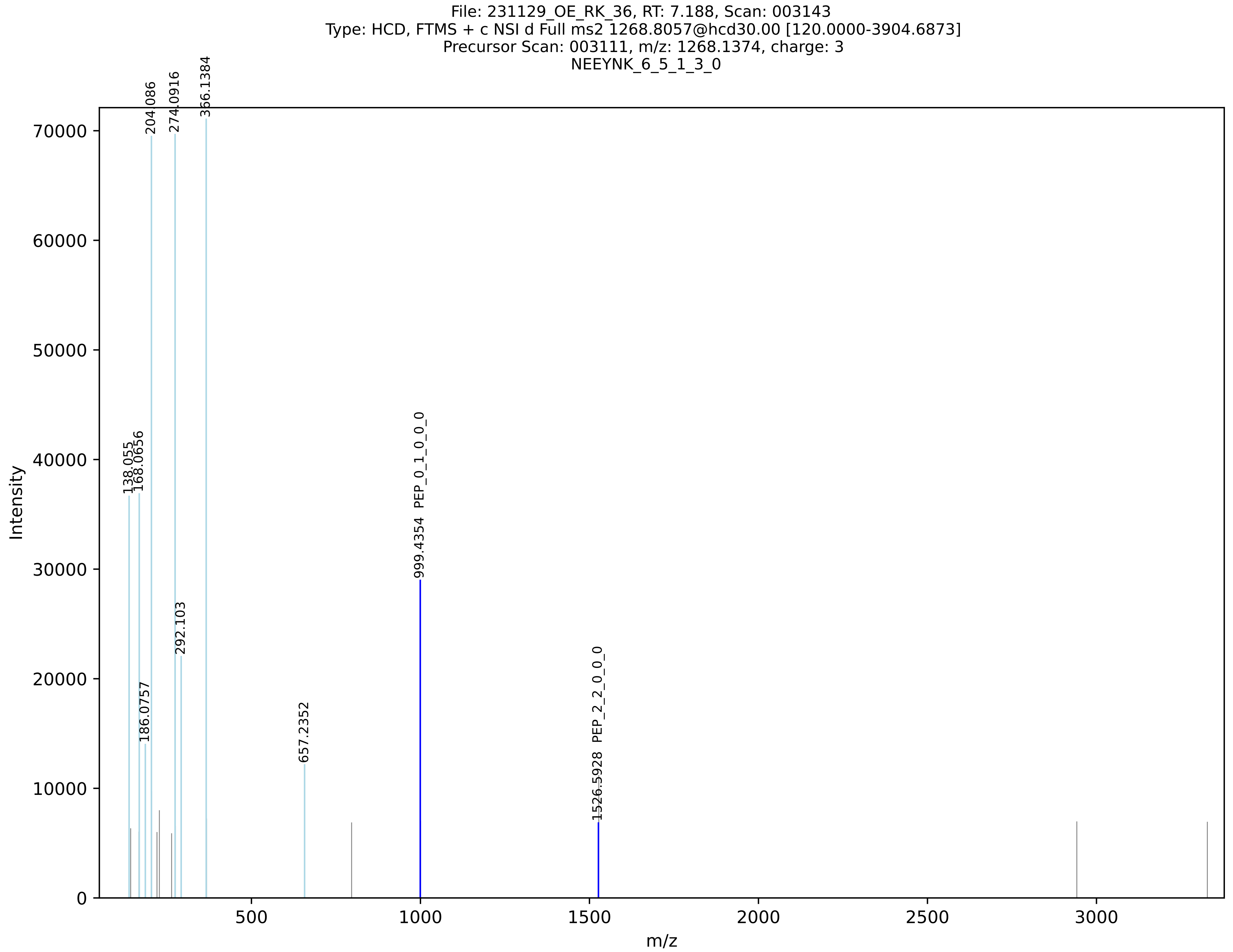

File: 231129_OE_RK_36, RT: 7.188, Scan: 003143
Type: HCD, FTMS + c NSI d Full ms2 1268.8057@hcd30.00 [120.0000-3904.6873]
Precursor Scan: 003111, m/z: 1268.1374, charge: 3
NEEYNK_6_5_1_3_0
204.086
274.0916
366.1384
138.055
168.0656
186.0757
292.103
657.2352
999.4354 PEP_0_1_0_0_0
1526.5928 PEP_2_2_0_0_0
Intensity
m/z
0
10000
20000
30000
40000
50000
60000
70000
500
1000
1500
2000
2500
3000

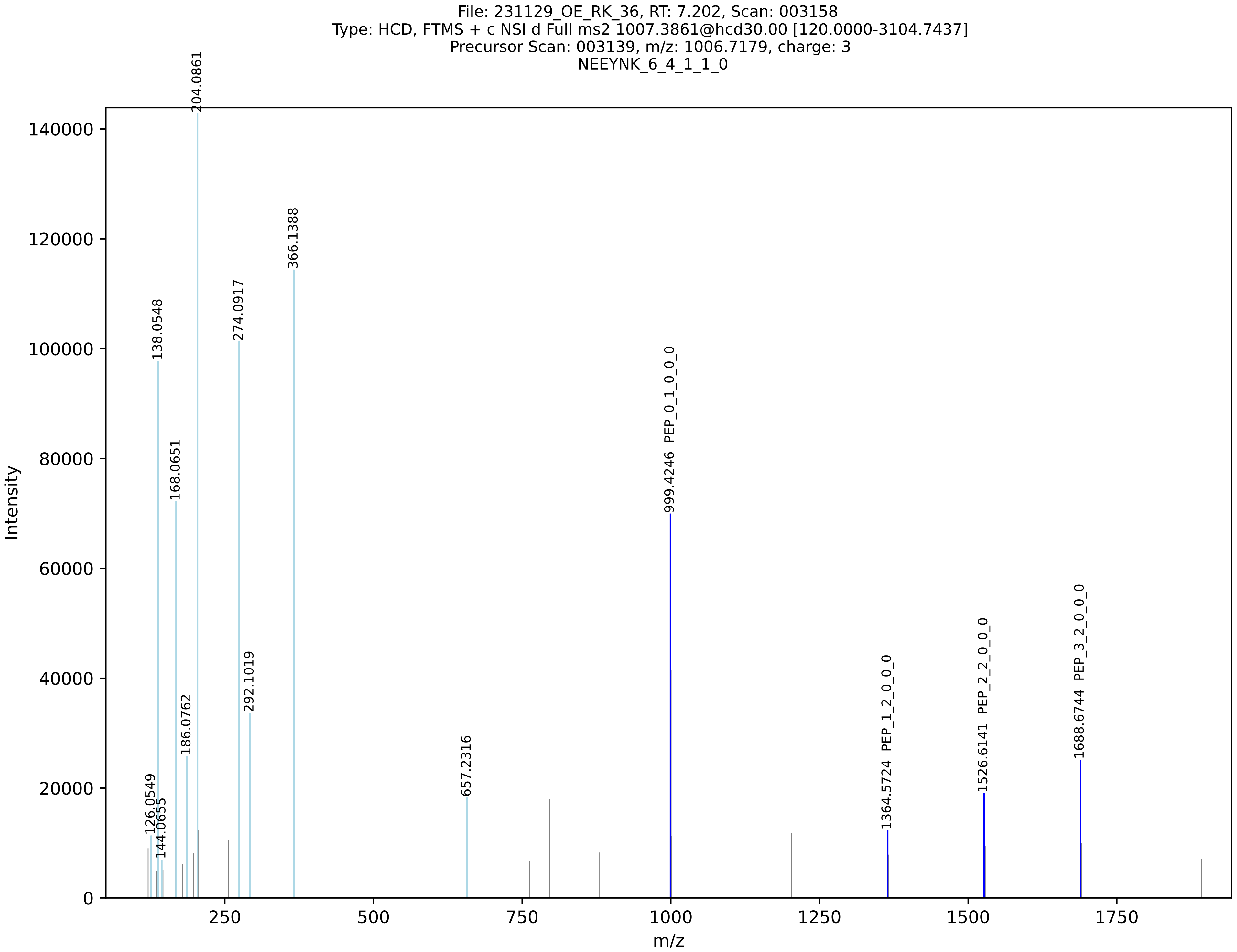
File: 231129_OE_RK_36, RT: 7.202, Scan: 003158
Type: HCD, FTMS + c NSI d Full ms2 1007.3861@hcd30.00 [120.0000-3104.7437]
Precursor Scan: 003139, m/z: 1006.7179, charge: 3
NEEYNK_6_4_1_1_0
Intensity
m/z
204.0861
366.1388
274.0917
138.0548
168.0651
292.1019
186.0762
126.0549
144.0655
657.2316
999.4246 PEP_0_1_0_0_0
1364.5724 PEP_1_2_0_0_0
1526.6141 PEP_2_2_0_0_0
1688.6744 PEP_3_2_0_0_0

File: 231129_OE_RK_36, RT: 7.227, Scan: 003191
Type: HCD, FTMS + c NSI d Full ms2 897.6938@hcd30.00 [120.0000-2769.0850]
Precursor Scan: 003156, m/z: 897.3594, charge: 3
ENGTISR_5_4_0_1_0

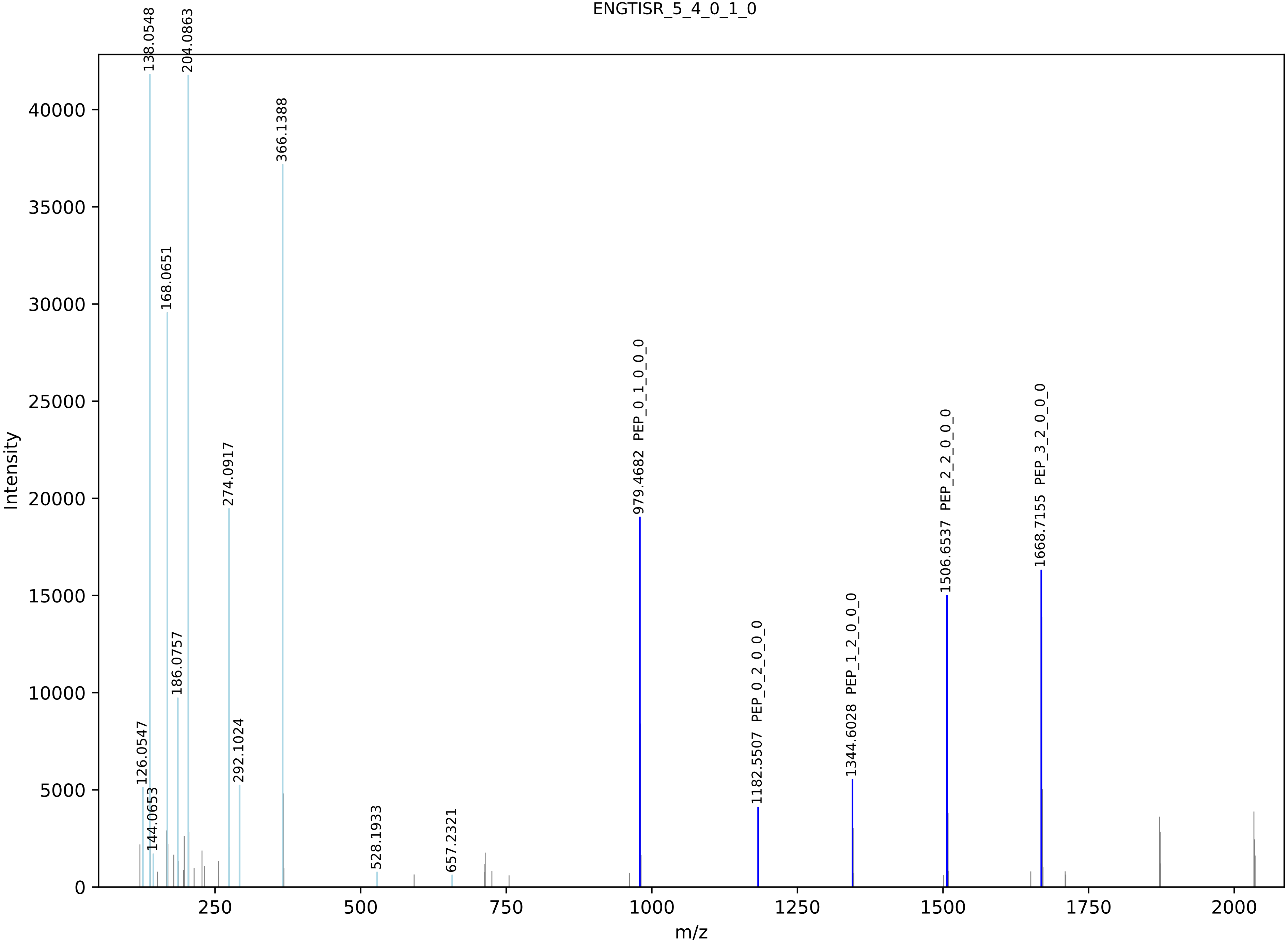

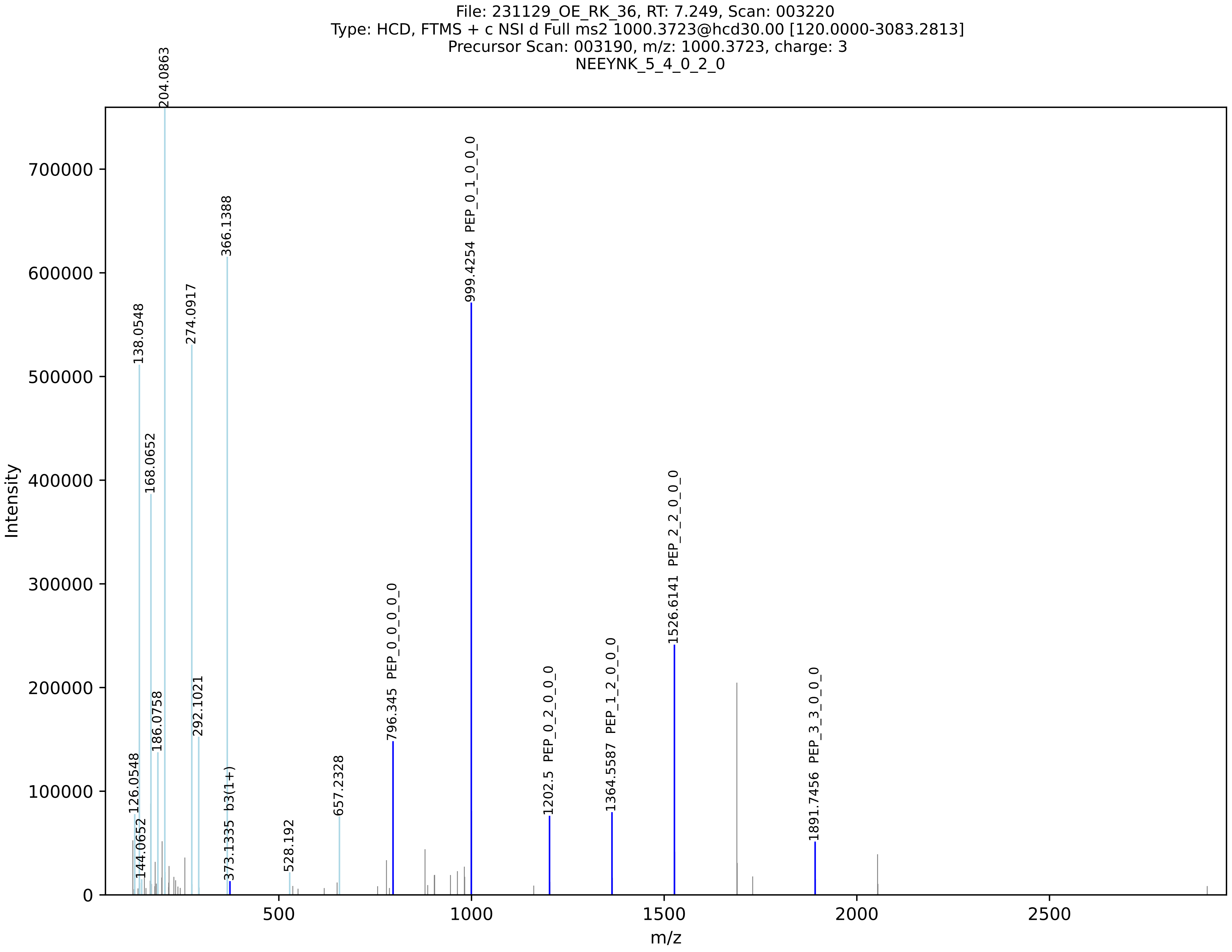
File: 231129_OE_RK_36, RT: 7.249, Scan: 003220
Type: HCD, FTMS + c NSI d Full ms2 1000.3723@hcd30.00 [120.0000-3083.2813]
Precursor Scan: 003190, m/z: 1000.3723, charge: 3
NEEYNK_5_4_0_2_0
Intensity
m/z
204.0863
366.1388
274.0917
138.0548
168.0652
186.0758
292.1021
126.0548
144.0652
373.1335 b3(1+)
528.192
657.2328
796.345 PEP_0_0_0_0_0
999.4254 PEP_0_1_0_0_0
1202.5 PEP_0_2_0_0_0
1364.5587 PEP_1_2_0_0_0
1526.6141 PEP_2_2_0_0_0
1891.7456 PEP_3_3_0_0_0

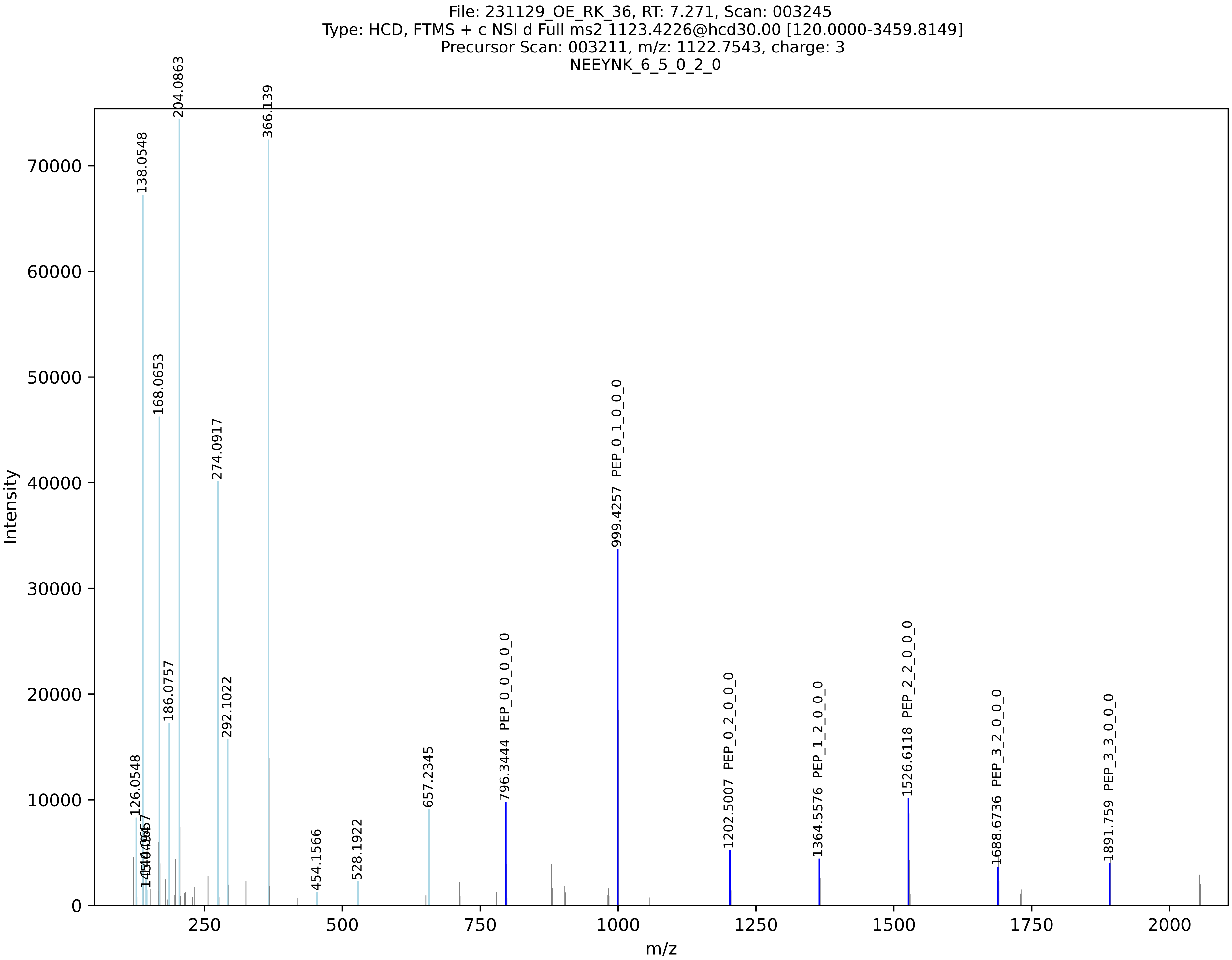
File: 231129_OE_RK_36, RT: 7.271, Scan: 003245
Type: HCD, FTMS + c NSI d Full ms2 1123.4226@hcd30.00 [120.0000-3459.8149]
Precursor Scan: 003211, m/z: 1122.7543, charge: 3
NEEYNK_6_5_0_2_0
Intensity
m/z
0
10000
20000
30000
40000
50000
60000
70000
250
500
750
1000
1250
1500
1750
2000
204.0863
366.139
138.0548
168.0653
274.0917
186.0757
292.1022
126.0548
454.1566
528.1922
657.2345
796.3444 PEP_0_0_0_0_0
999.4257 PEP_0_1_0_0_0
1202.5007 PEP_0_2_0_0_0
1364.5576 PEP_1_2_0_0_0
1526.6118 PEP_2_2_0_0_0
1688.6736 PEP_3_2_0_0_0
1891.759 PEP_3_3_0_0_0

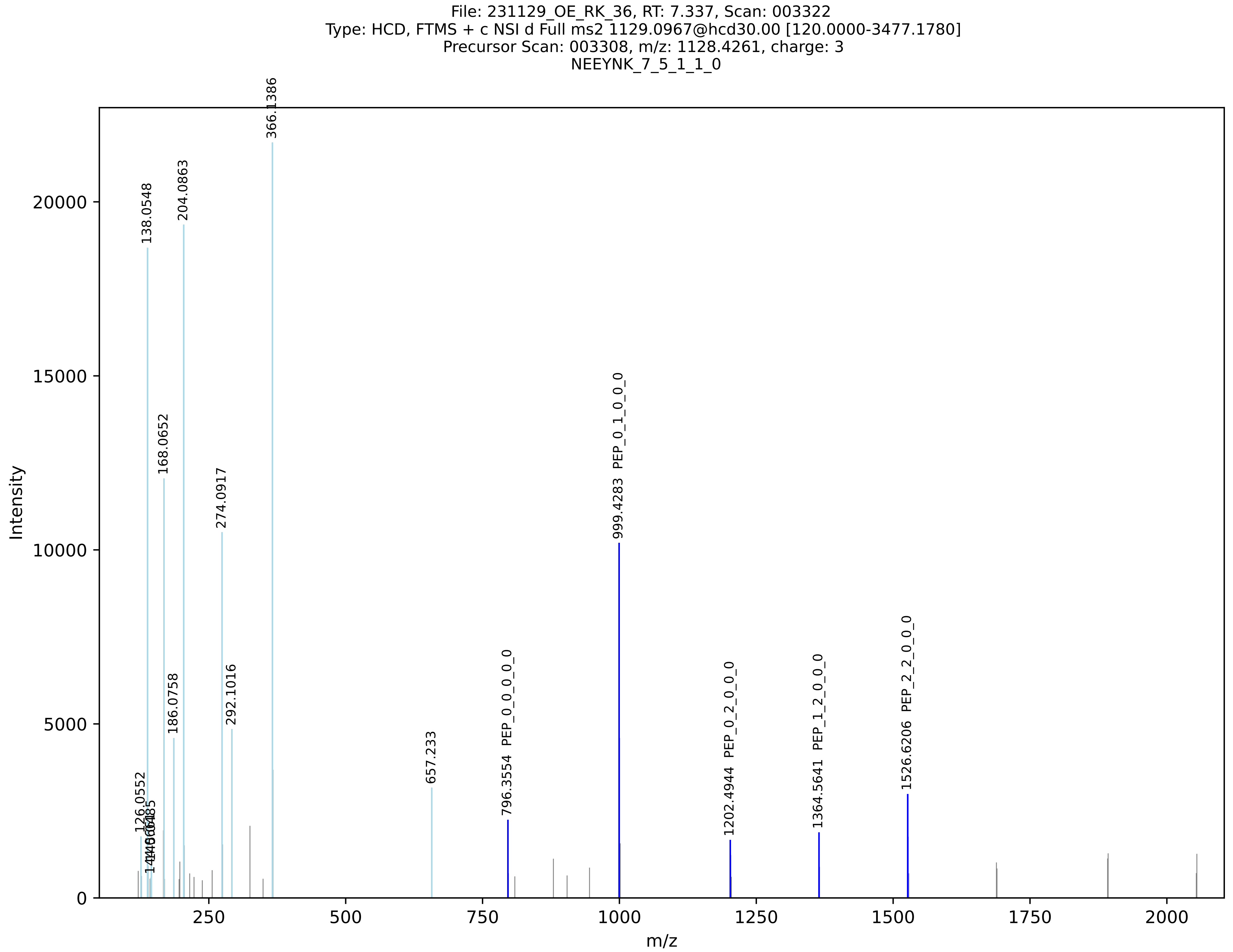
File: 231129_OE_RK_36, RT: 7.337, Scan: 003322
Type: HCD, FTMS + c NSI d Full ms2 1129.0967@hcd30.00 [120.0000-3477.1780]
Precursor Scan: 003308, m/z: 1128.4261, charge: 3
NEEYNK_7_5_1_1_0
Intensity
m/z
366.1386
138.0548
204.0863
168.0652
274.0917
186.0758
292.1016
126.0552
657.233
796.3554 PEP_0_0_0_0_0
999.4283 PEP_0_1_0_0_0
1202.4944 PEP_0_2_0_0_0
1364.5641 PEP_1_2_0_0_0
1526.6206 PEP_2_2_0_0_0

File: 231129_OE_RK_36, RT: 7.355, Scan: 003341
Type: HCD, FTMS + c NSI d Full ms2 1501.5635@hcd30.00 [120.0000-3084.6177]
Precursor Scan: 003321, m/z: 1501.0625, charge: 2
NEEYNK_5_4_0_2_0

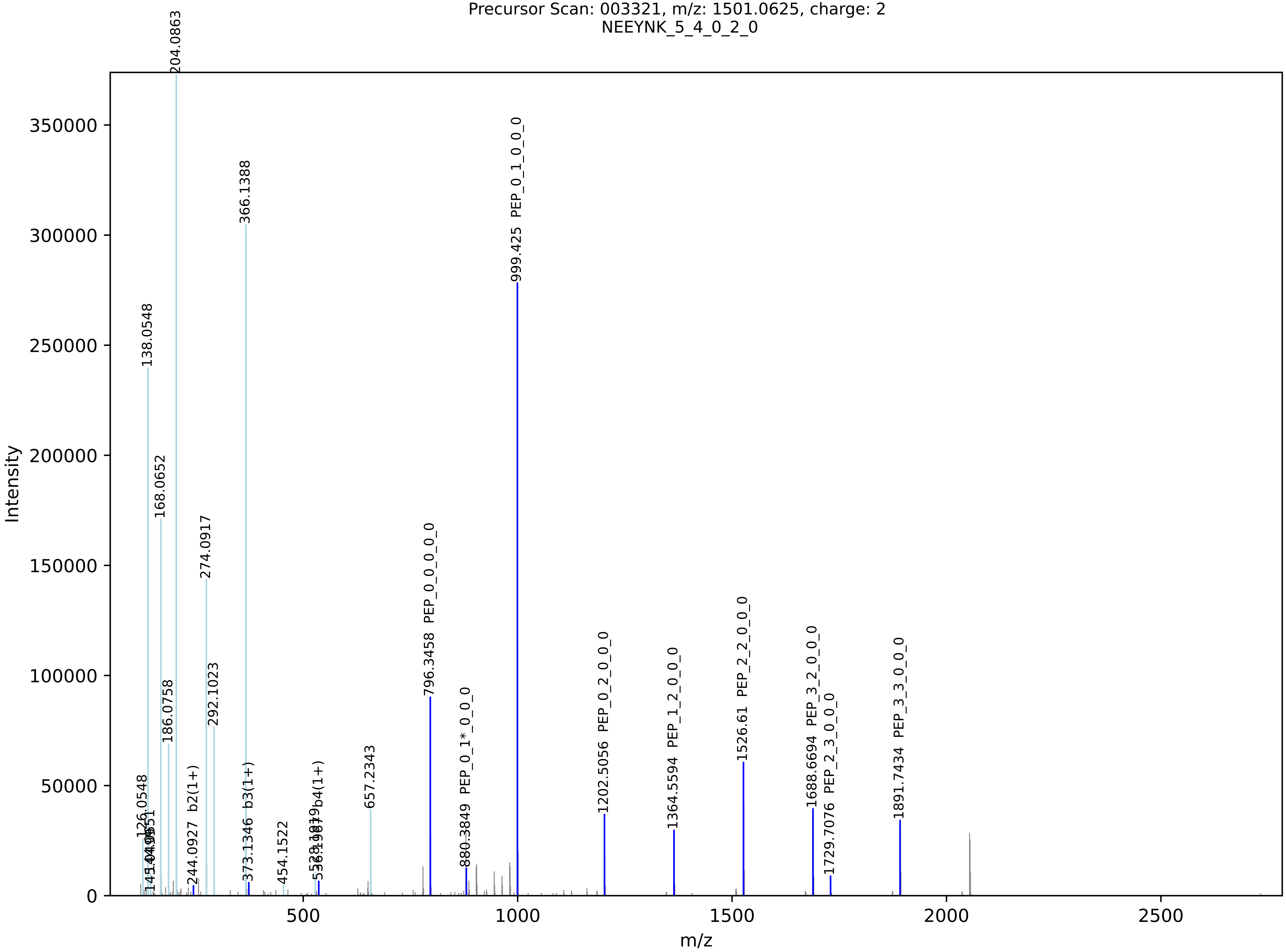

File: 231129_OE_RK_36, RT: 7.369, Scan: 003356
Type: HCD, FTMS + c NSI d Full ms2 1050.0637@hcd30.00 [120.0000-3235.3372]
Precursor Scan: 003340, m/z: 1049.7297, charge: 3
NEEYNK_5_4_1_2_0

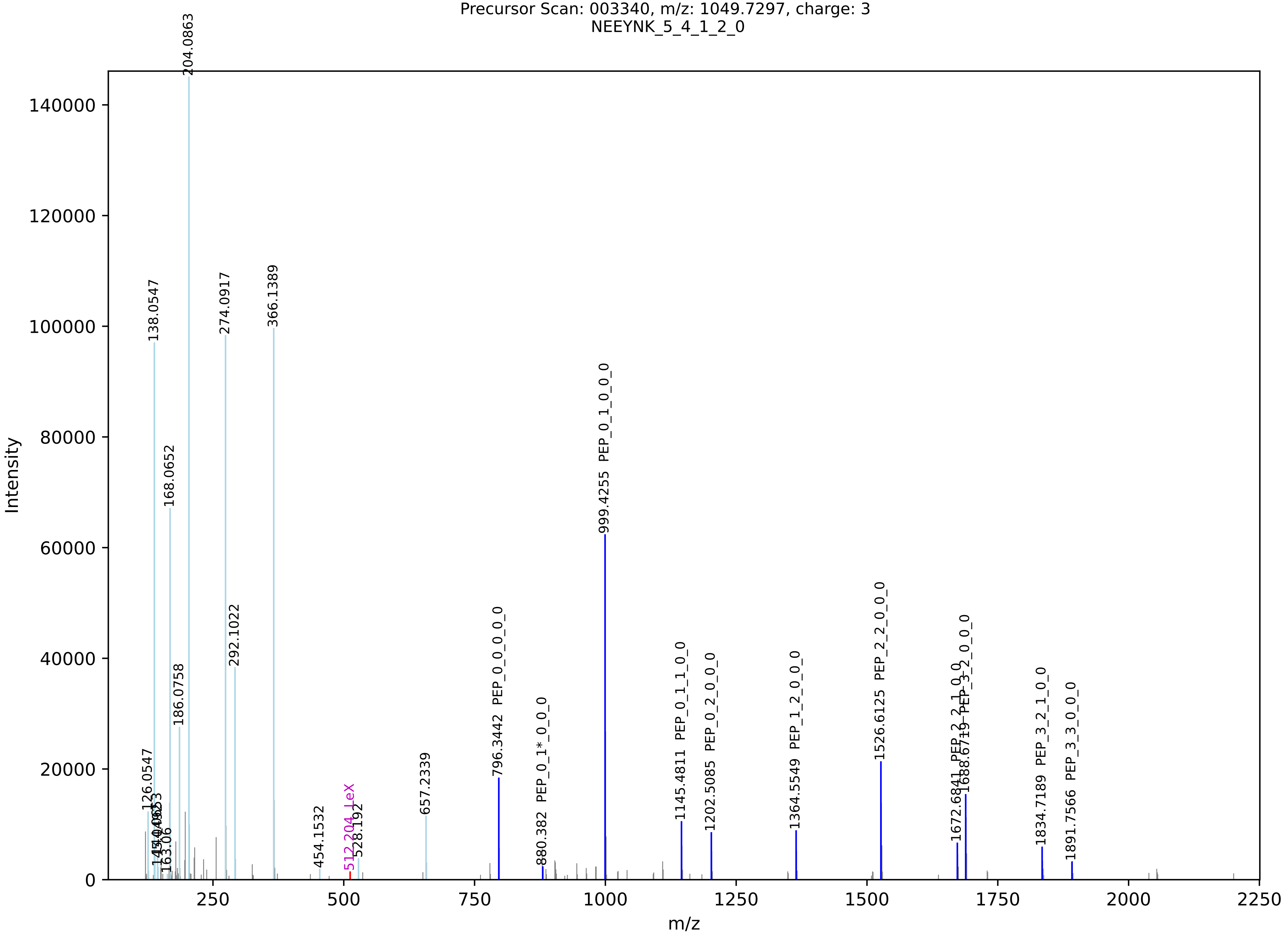

File: 231129_OE_RK_36, RT: 7.369, Scan: 003357
Type: HCD, FTMS + c NSI d Full ms2 1269.1406@hcd30.00 [120.0000-3905.7122]
Precursor Scan: 003340, m/z: 1268.4724, charge: 3
NEEYNK_6_5_1_3_0

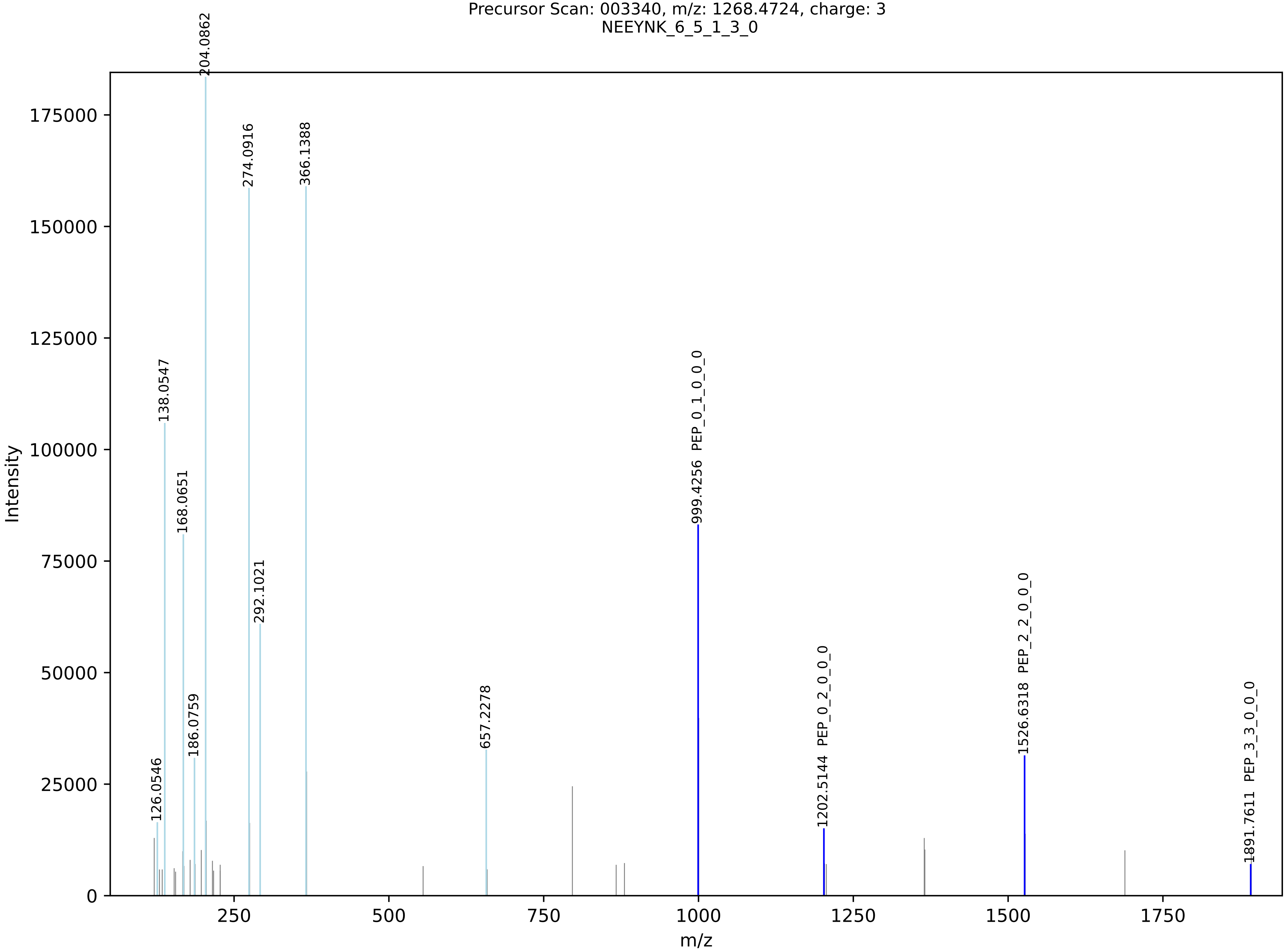

File: 231129_OE_RK_36, RT: 7.387, Scan: 003378
Type: HCD, FTMS + c NSI d Full ms2 1001.3786@hcd30.00 [120.0000-3086.3606]
Precursor Scan: 003355, m/z: 1001.0445, charge: 3
NEEYNK_5_4_0_2_0

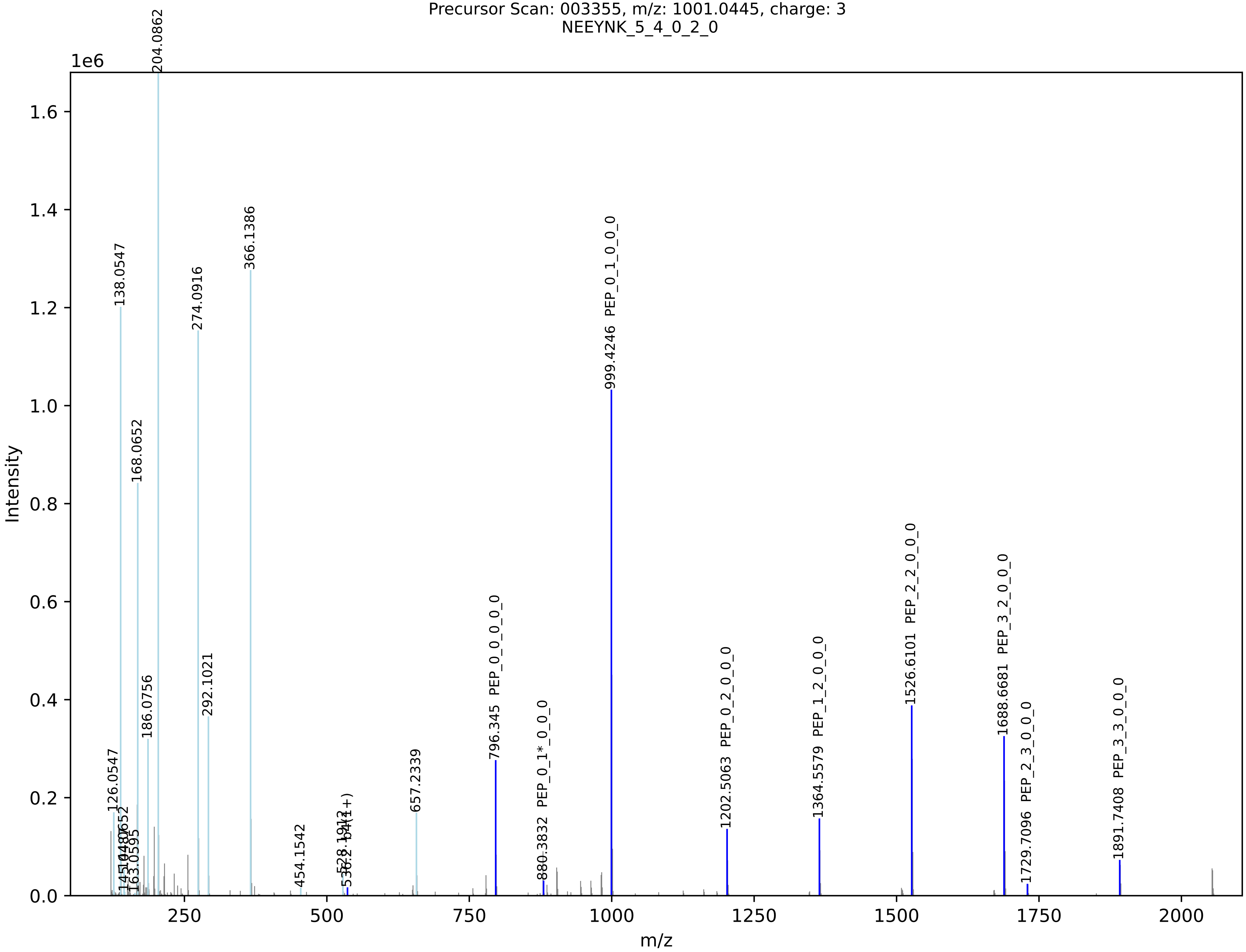

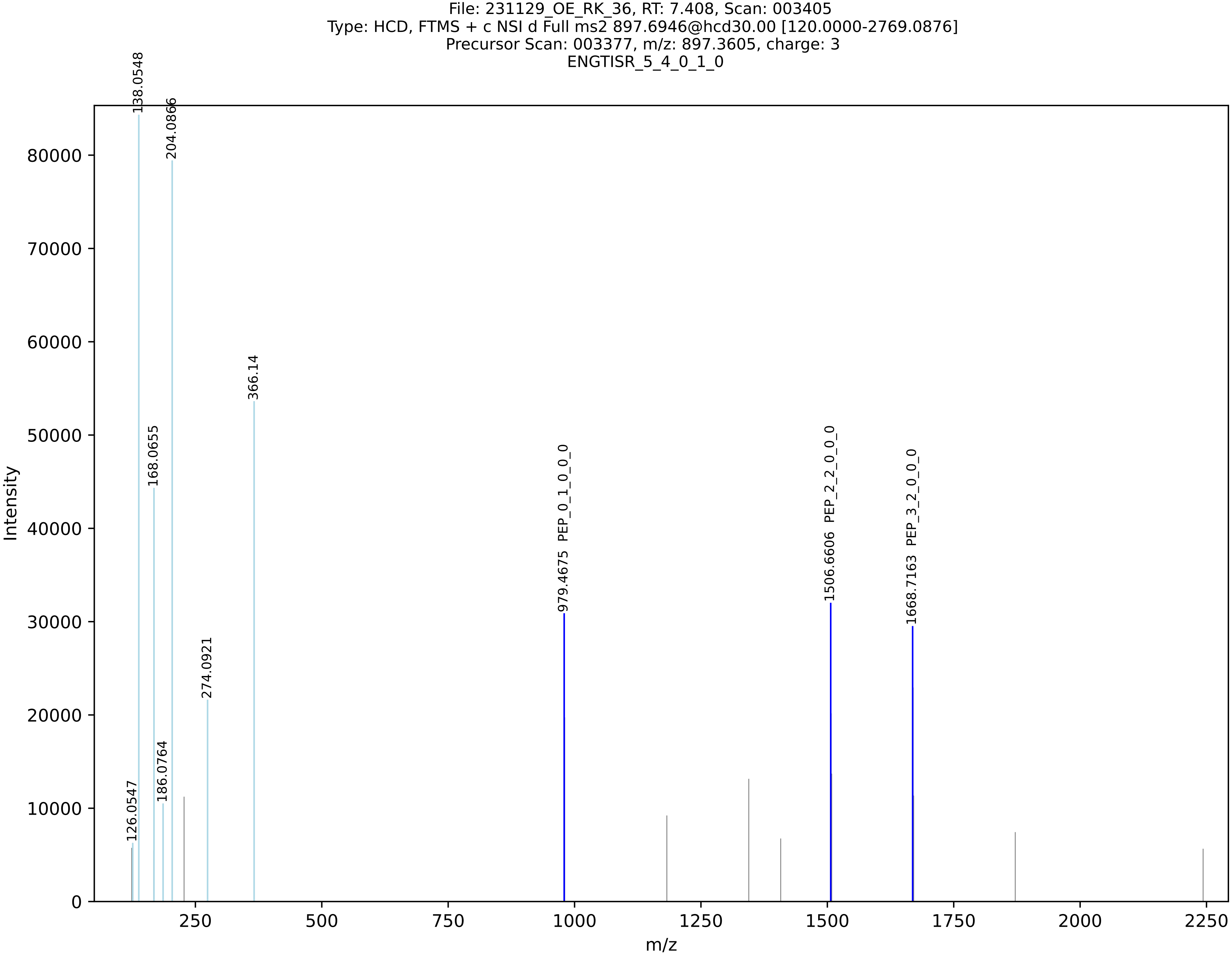
File: 231129_OE_RK_36, RT: 7.408, Scan: 003405
Type: HCD, FTMS + c NSI d Full ms2 897.6946@hcd30.00 [120.0000-2769.0876]
Precursor Scan: 003377, m/z: 897.3605, charge: 3
ENGTISR_5_4_0_1_0
138.0548
204.0866
366.14
168.0655
979.4675 PEP_0_1_0_0_0
1506.6606 PEP_2_2_0_0_0
1668.7163 PEP_3_2_0_0_0
274.0921
186.0764
126.0547
Intensity
m/z
0
10000
20000
30000
40000
50000
60000
70000
80000
250
500
750
1000
1250
1500
1750
2000
2250

File: 231129_OE_RK_36, RT: 7.42, Scan: 003424
Type: HCD, FTMS + c NSI d Full ms2 1050.6968@hcd30.00 [120.0000-3237.2742]
Precursor Scan: 003377, m/z: 1050.6968, charge: 3
NEEYNK_5_4_1_2_0

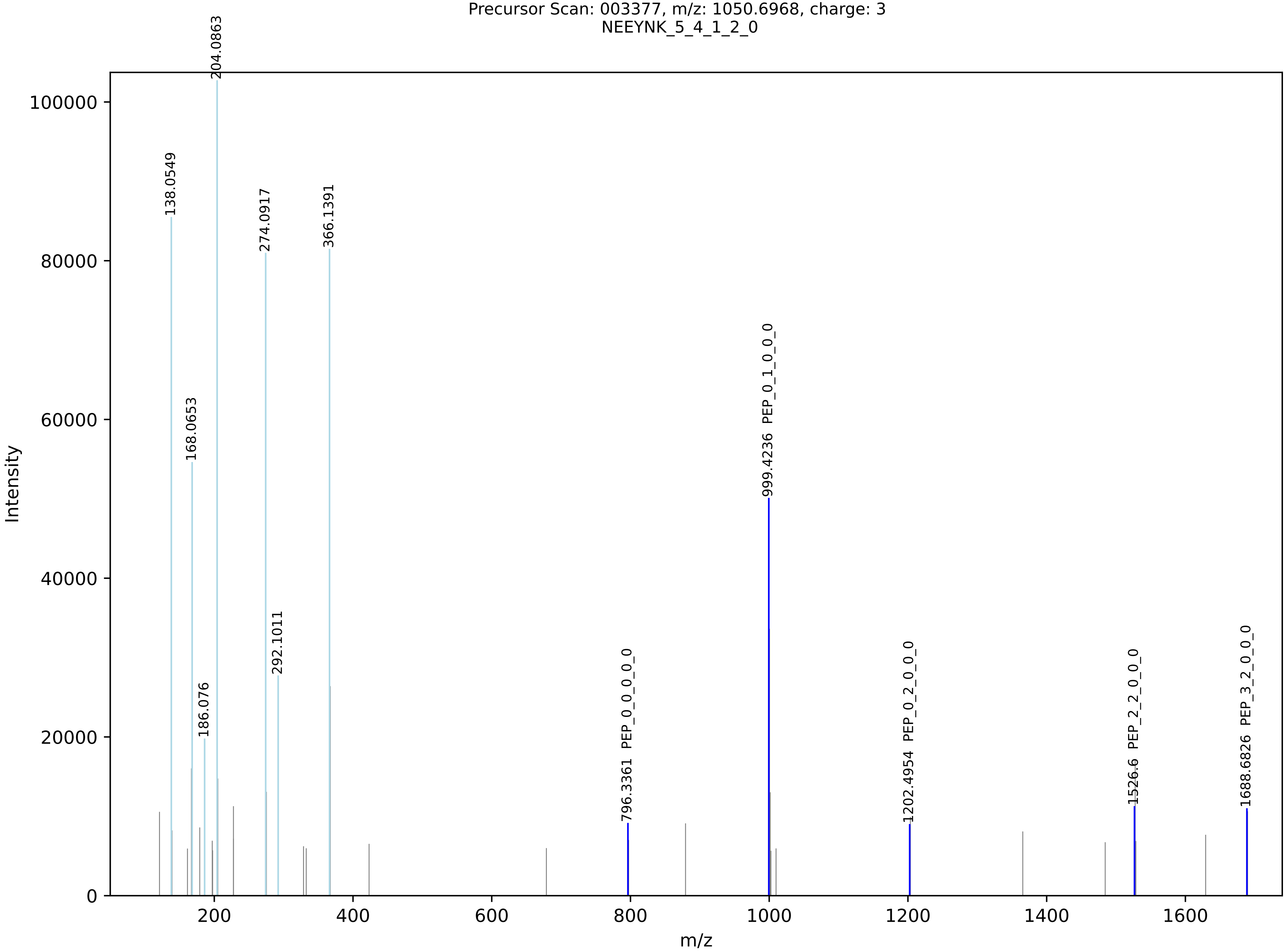

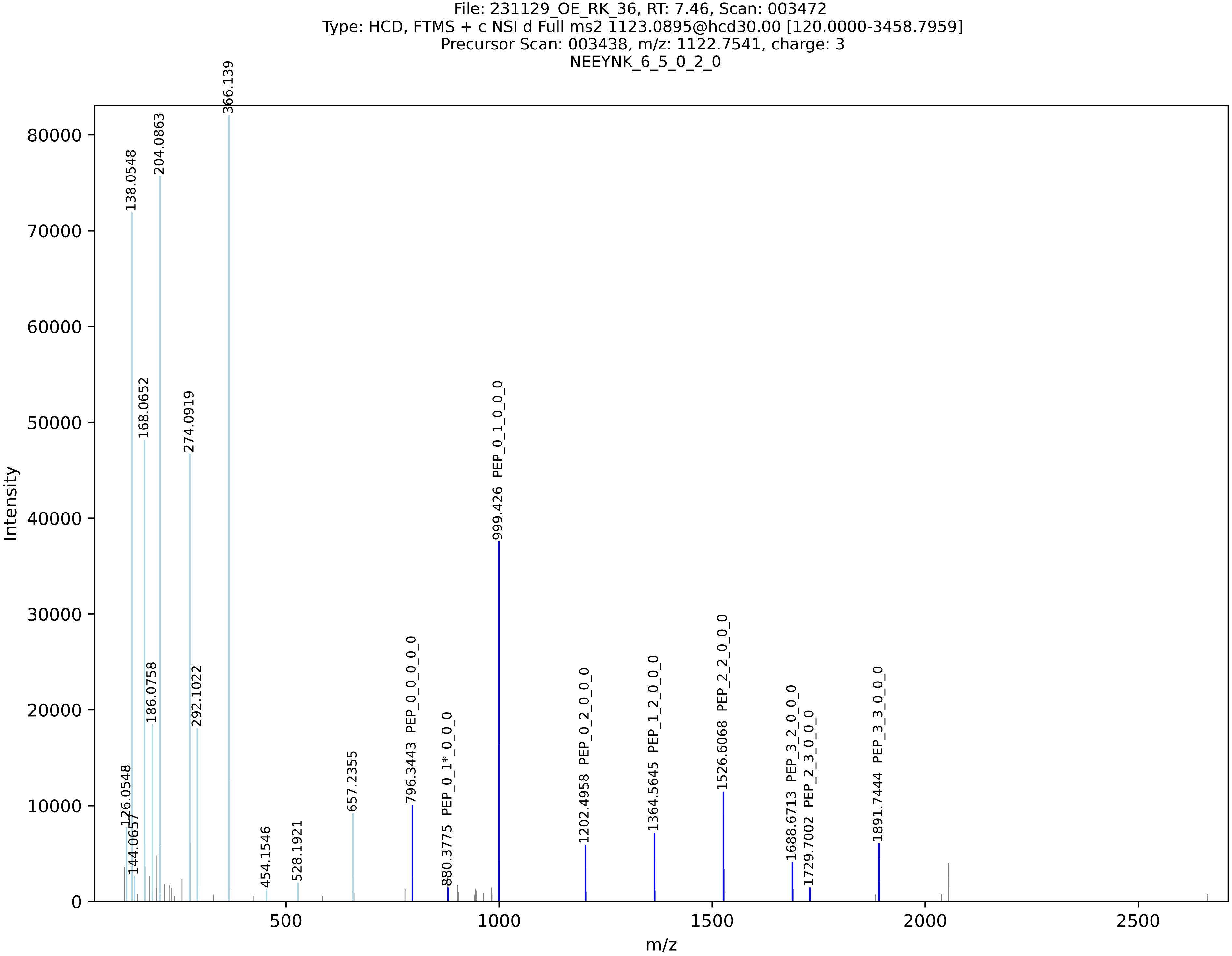

File: 231129_OE_RK_36, RT: 7.46, Scan: 003472
Type: HCD, FTMS + c NSI d Full ms2 1123.0895@hcd30.00 [120.0000-3458.7959]
Precursor Scan: 003438, m/z: 1122.7541, charge: 3
NEEYNK_6_5_0_2_0
Intensity
m/z
366.139
204.0863
138.0548
168.0652
274.0919
186.0758
292.1022
126.0548
144.0657
454.1546
528.1921
657.2355
796.3443 PEP_0_0_0_0_0
880.3775 PEP_0_1*_0_0_0
999.426 PEP_0_1_0_0_0
1202.4958 PEP_0_2_0_0_0
1364.5645 PEP_1_2_0_0_0
1526.6068 PEP_2_2_0_0_0
1688.6713 PEP_3_2_0_0_0
1729.7002 PEP_2_3_0_0_0
1891.7444 PEP_3_3_0_0_0

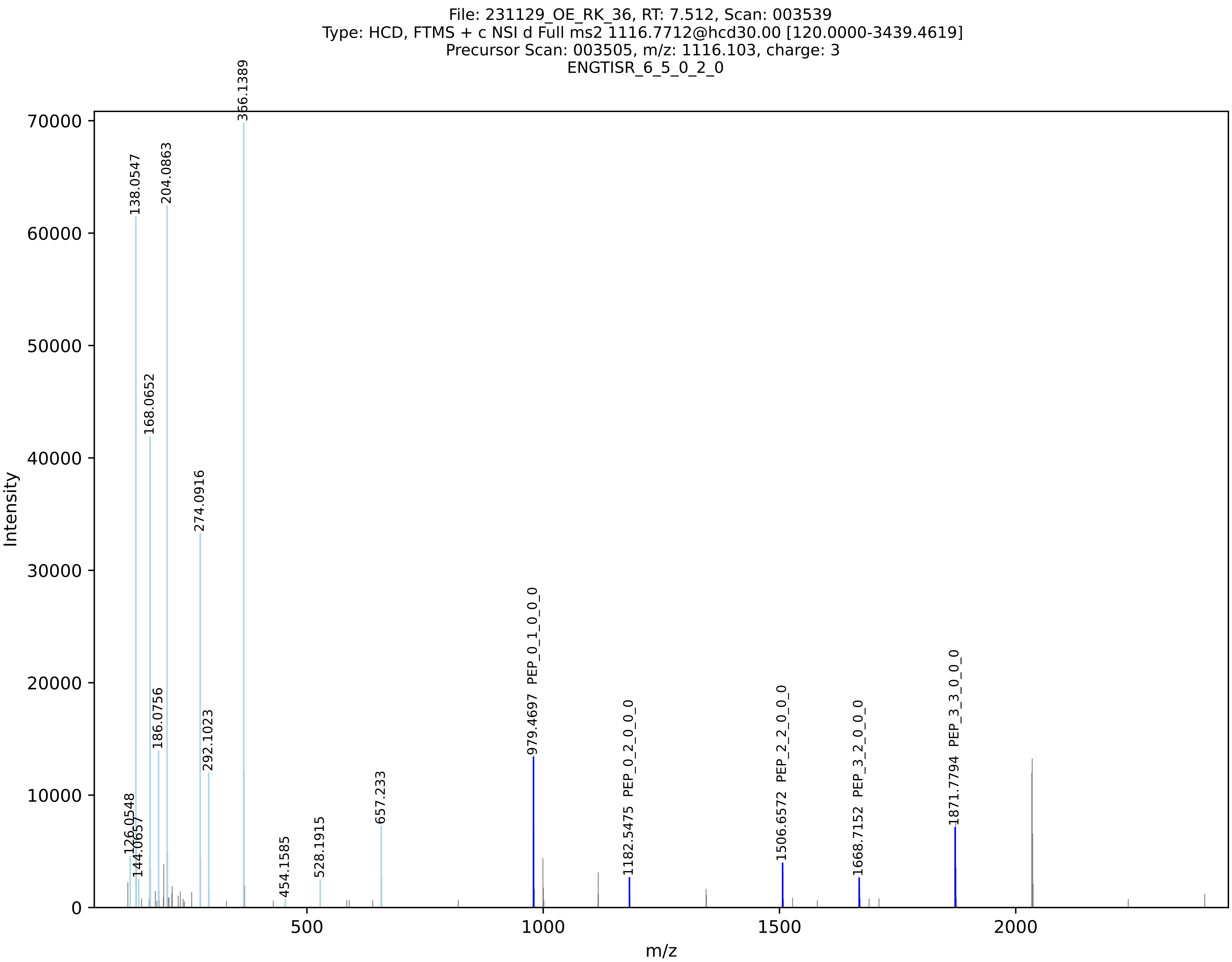
File: 231129_OE_RK_36, RT: 7.512, Scan: 003539
Type: HCD, FTMS + c NSI d Full ms2 1116.7712@hcd30.00 [120.0000-3439.4619]
Precursor Scan: 003505, m/z: 1116.103, charge: 3
ENGTISR_6_5_0_2_0
Intensity
m/z
366.1389
138.0547
204.0863
168.0652
274.0916
186.0756
292.1023
126.0548
144.0657
454.1585
528.1915
657.233
979.4697 PEP_0_1_0_0_0
1182.5475 PEP_0_2_0_0_0
1506.6572 PEP_2_2_0_0_0
1668.7152 PEP_3_2_0_0_0
1871.7794 PEP_3_3_0_0_0

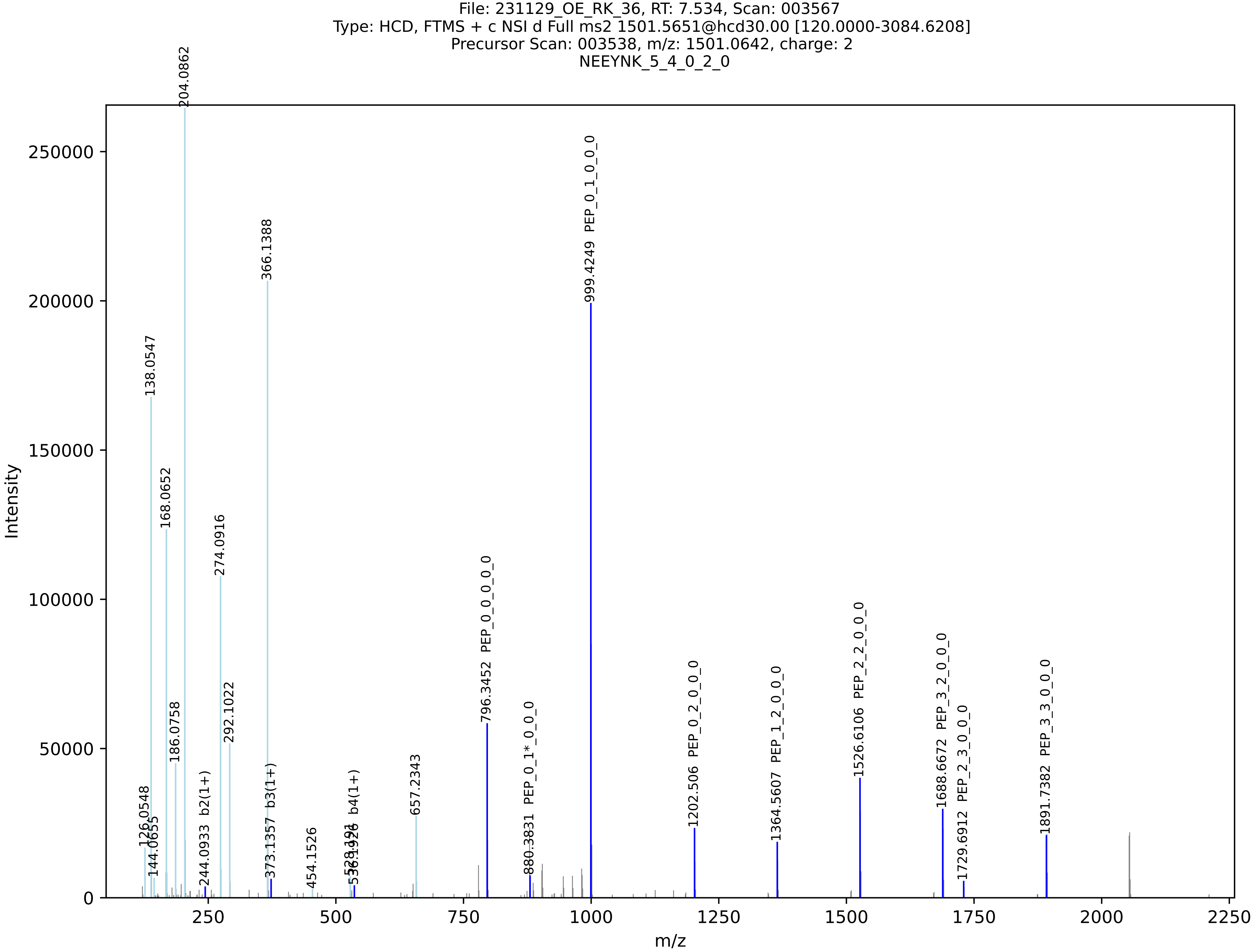
File: 231129_OE_RK_36, RT: 7.534, Scan: 003567
Type: HCD, FTMS + c NSI d Full ms2 1501.5651@hcd30.00 [120.0000-3084.6208]
Precursor Scan: 003538, m/z: 1501.0642, charge: 2
NEEYNK_5_4_0_2_0
Intensity
m/z
204.0862
366.1388
999.4249 PEP_0_1_0_0_0
138.0547
168.0652
274.0916
796.3452 PEP_0_0_0_0_0
292.1022
186.0758
126.0548
144.0655
244.0933 b2(1+)
373.1357 b3(1+)
454.1526
b4(1+)
657.2343
880.3831 PEP_0_1*_0_0_0
1202.506 PEP_0_2_0_0_0
1364.5607 PEP_1_2_0_0_0
1526.6106 PEP_2_2_0_0_0
1688.6672 PEP_3_2_0_0_0
1729.6912 PEP_2_3_0_0_0
1891.7382 PEP_3_3_0_0_0
0
50000
100000
150000
200000
250000
250
500
750
1000
1250
1500
1750
2000
2250

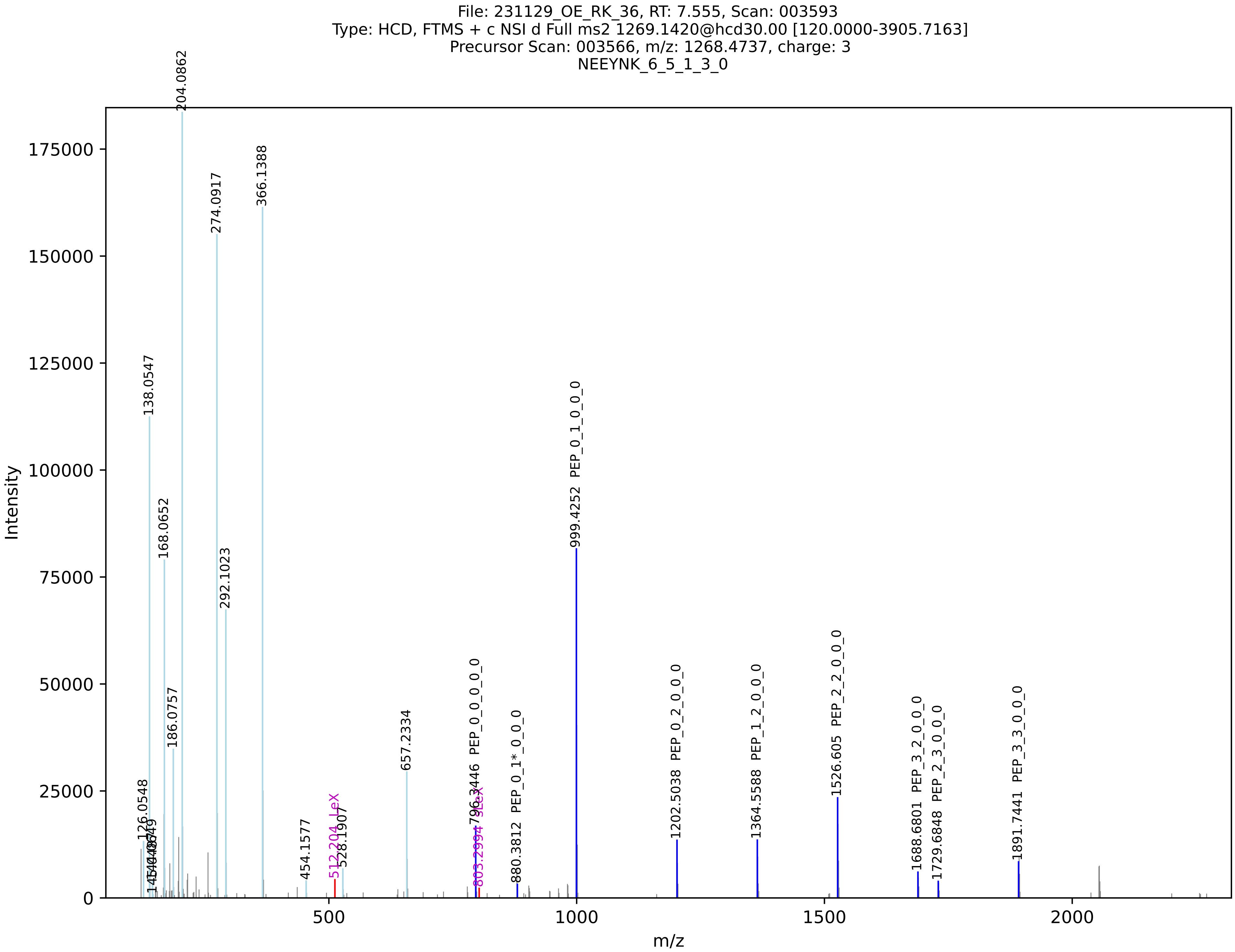
File: 231129_OE_RK_36, RT: 7.555, Scan: 003593
Type: HCD, FTMS + c NSI d Full ms2 1269.1420@hcd30.00 [120.0000-3905.7163]
Precursor Scan: 003566, m/z: 1268.4737, charge: 3
NEEYNK_6_5_1_3_0
Intensity
m/z
204.0862
274.0917
366.1388
138.0547
168.0652
292.1023
186.0757
126.0548
454.1577
512.204_LeX
528.1907
657.2334
796.3446 PEP_0_0_0_0_0
803.2994 sLeX
880.3812 PEP_0_1*_0_0_0
999.4252 PEP_0_1_0_0_0
1202.5038 PEP_0_2_0_0_0
1364.5588 PEP_1_2_0_0_0
1526.605 PEP_2_2_0_0_0
1688.6801 PEP_3_2_0_0_0
1729.6848 PEP_2_3_0_0_0
1891.7441 PEP_3_3_0_0_0
0
25000
50000
75000
100000
125000
150000
175000
500
1000
1500
2000

File: 231129_OE_RK_36, RT: 7.572, Scan: 003612
Type: HCD, FTMS + c NSI d Full ms2 1001.3790@hcd30.00 [120.0000-3086.3616]
Precursor Scan: 003592, m/z: 1001.0449, charge: 3
NEEYNK_5_4_0_2_0

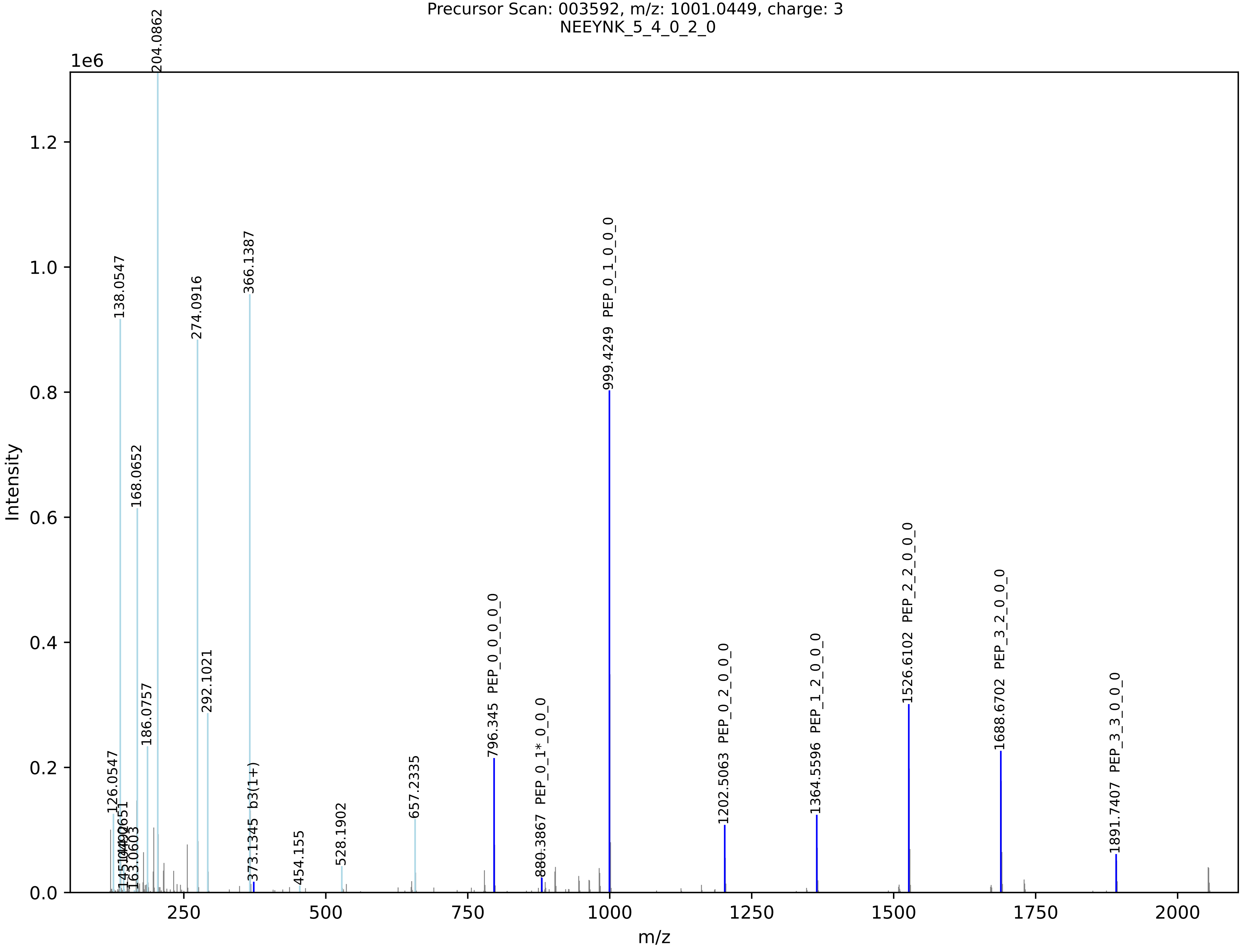

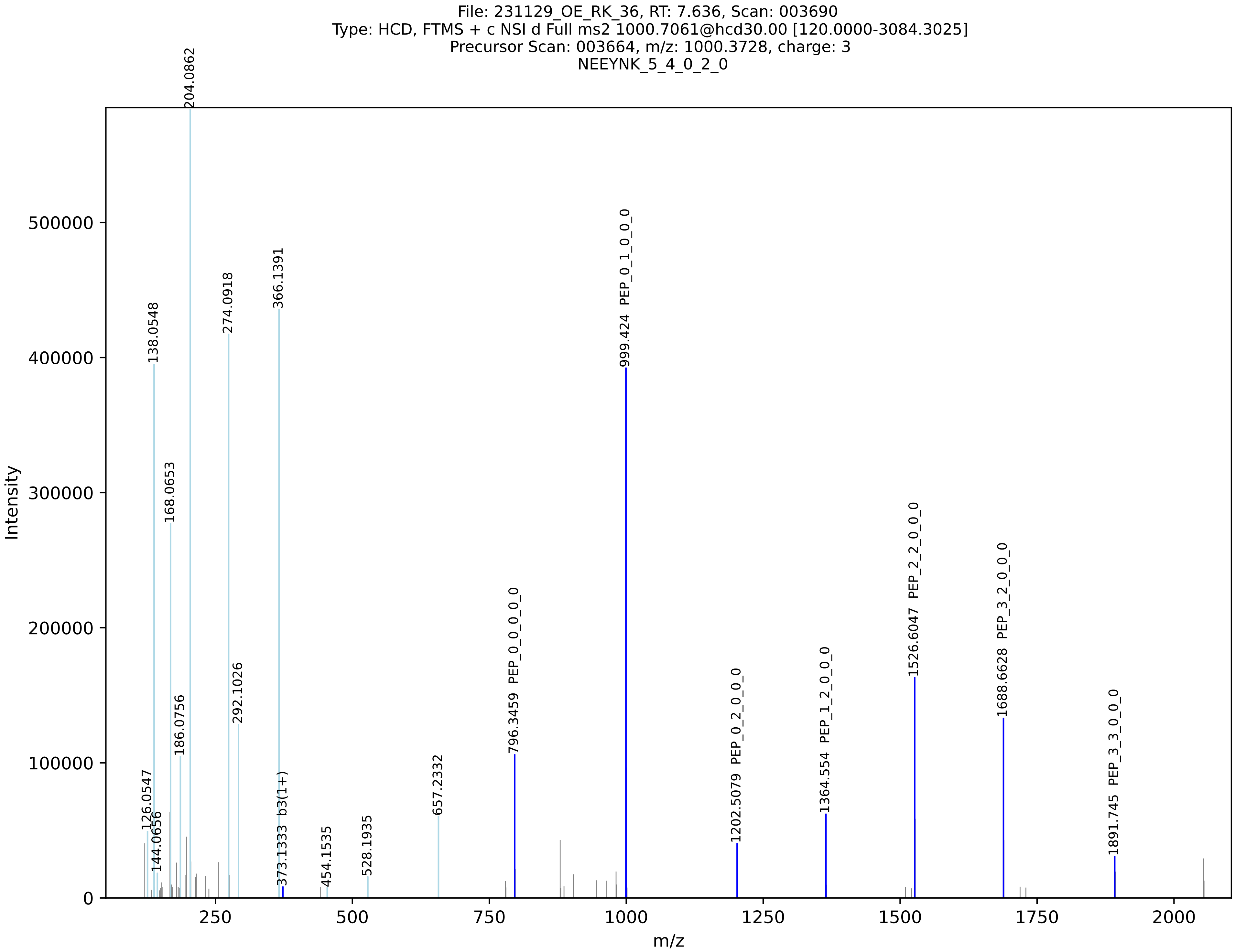
File: 231129_OE_RK_36, RT: 7.636, Scan: 003690
Type: HCD, FTMS + c NSI d Full ms2 1000.7061@hcd30.00 [120.0000-3084.3025]
Precursor Scan: 003664, m/z: 1000.3728, charge: 3
NEEYNK_5_4_0_2_0
Intensity
m/z
204.0862
138.0548
274.0918
366.1391
168.0653
186.0756
292.1026
126.0547
144.0656
373.1333 b3(1+)
454.1535
528.1935
657.2332
796.3459 PEP_0_0_0_0_0
999.424 PEP_0_1_0_0_0
1202.5079 PEP_0_2_0_0_0
1364.554 PEP_1_2_0_0_0
1526.6047 PEP_2_2_0_0_0
1688.6628 PEP_3_2_0_0_0
1891.745 PEP_3_3_0_0_0

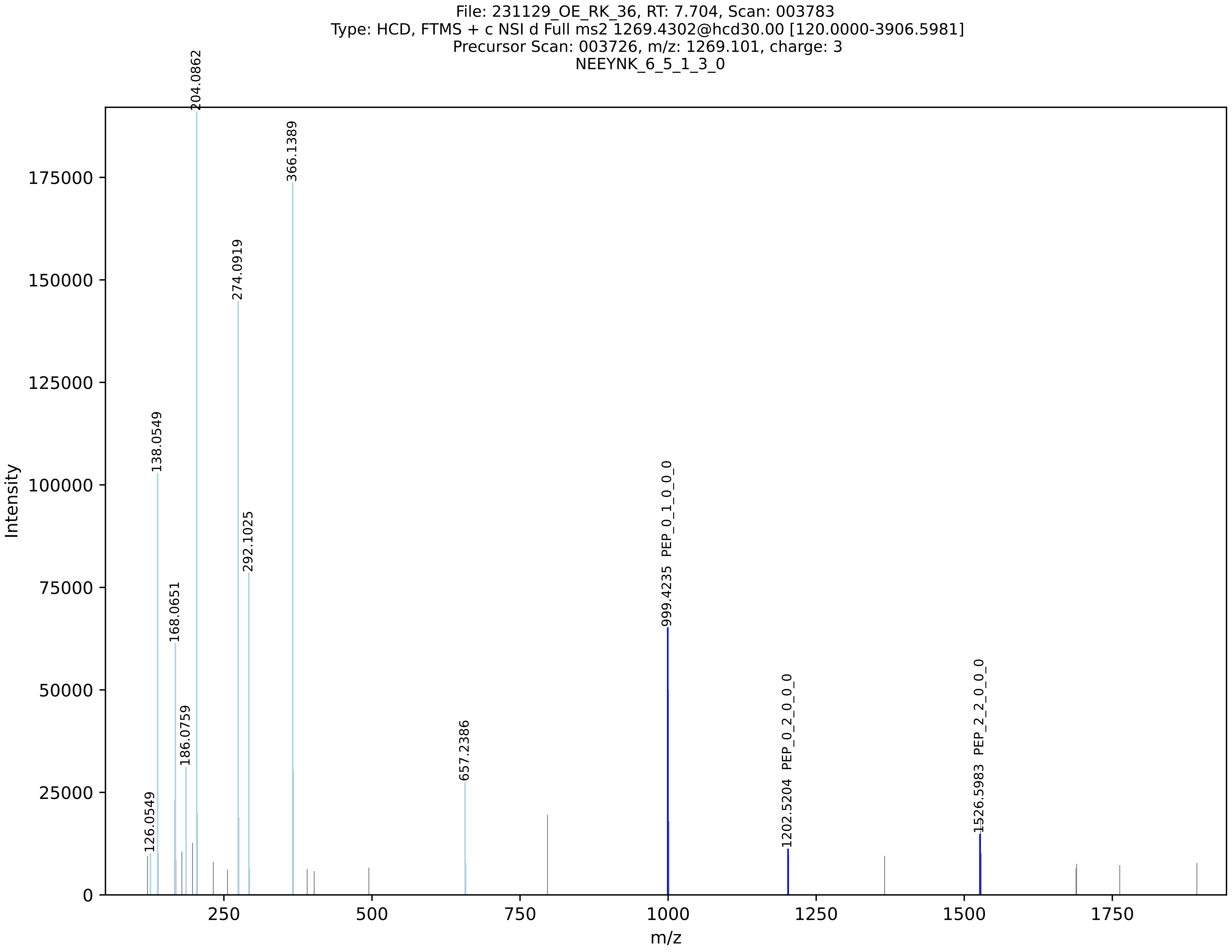
File: 231129_OE_RK_36, RT: 7.704, Scan: 003783
Type: HCD, FTMS + c NSI d Full ms2 1269.4302@hcd30.00 [120.0000-3906.5981]
Precursor Scan: 003726, m/z: 1269.101, charge: 3
NEEYNK_6_5_1_3_0
204.0862
366.1389
274.0919
138.0549
292.1025
168.0651
186.0759
126.0549
657.2386
999.4235 PEP_0_1_0_0_0
1202.5204 PEP_0_2_0_0_0
1526.5983 PEP_2_2_0_0_0
Intensity
m/z
0
25000
50000
75000
100000
125000
150000
175000
250
500
750
1000
1250
1500
1750

File: 231129_OE_RK_36, RT: 7.718, Scan: 003798
Type: HCD, FTMS + c NSI d Full ms2 1501.5645@hcd30.00 [120.0000-3084.6194]
Precursor Scan: 003763, m/z: 1501.0626, charge: 2
NEEYNK_5_4_0_2_0

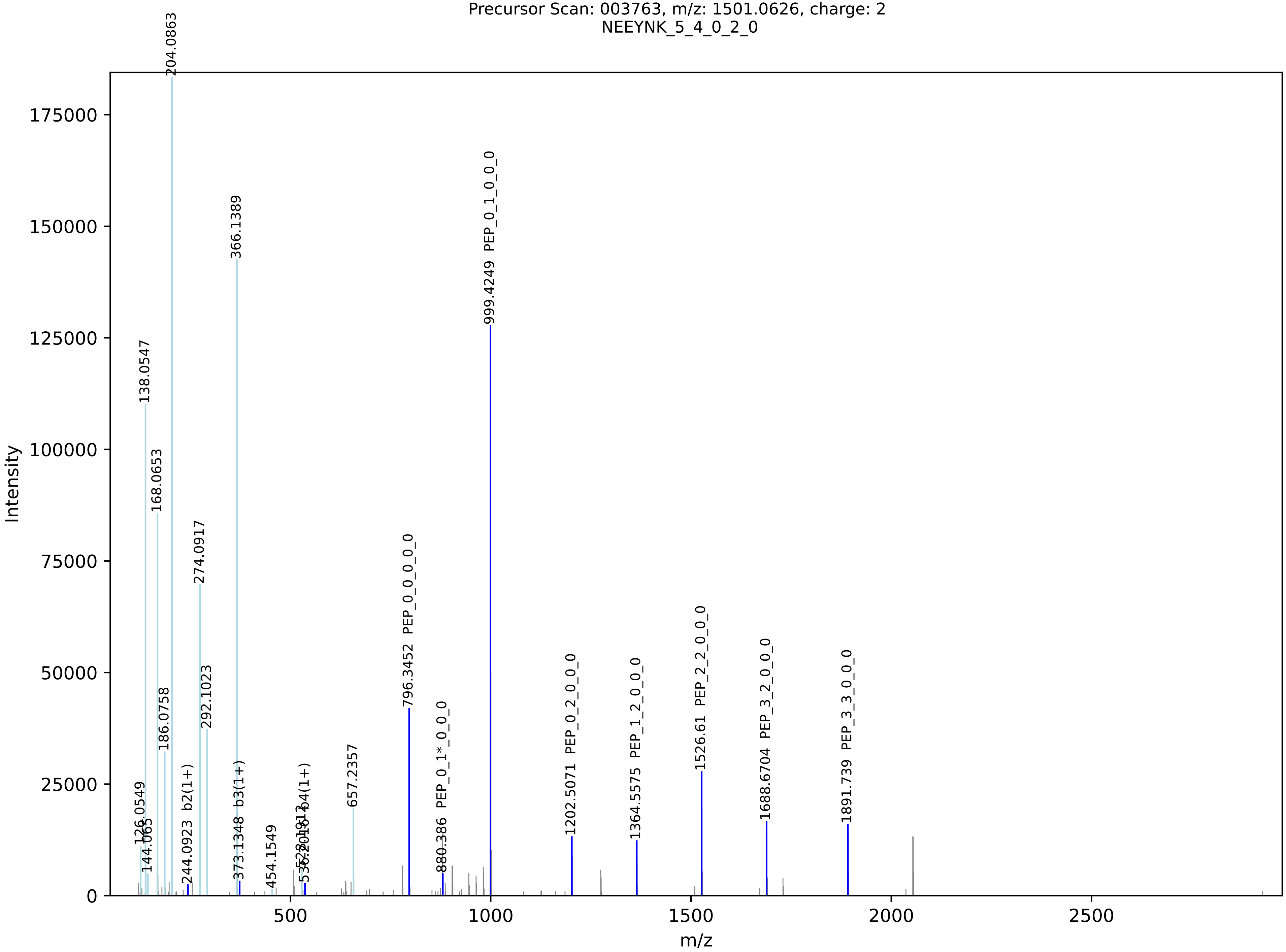

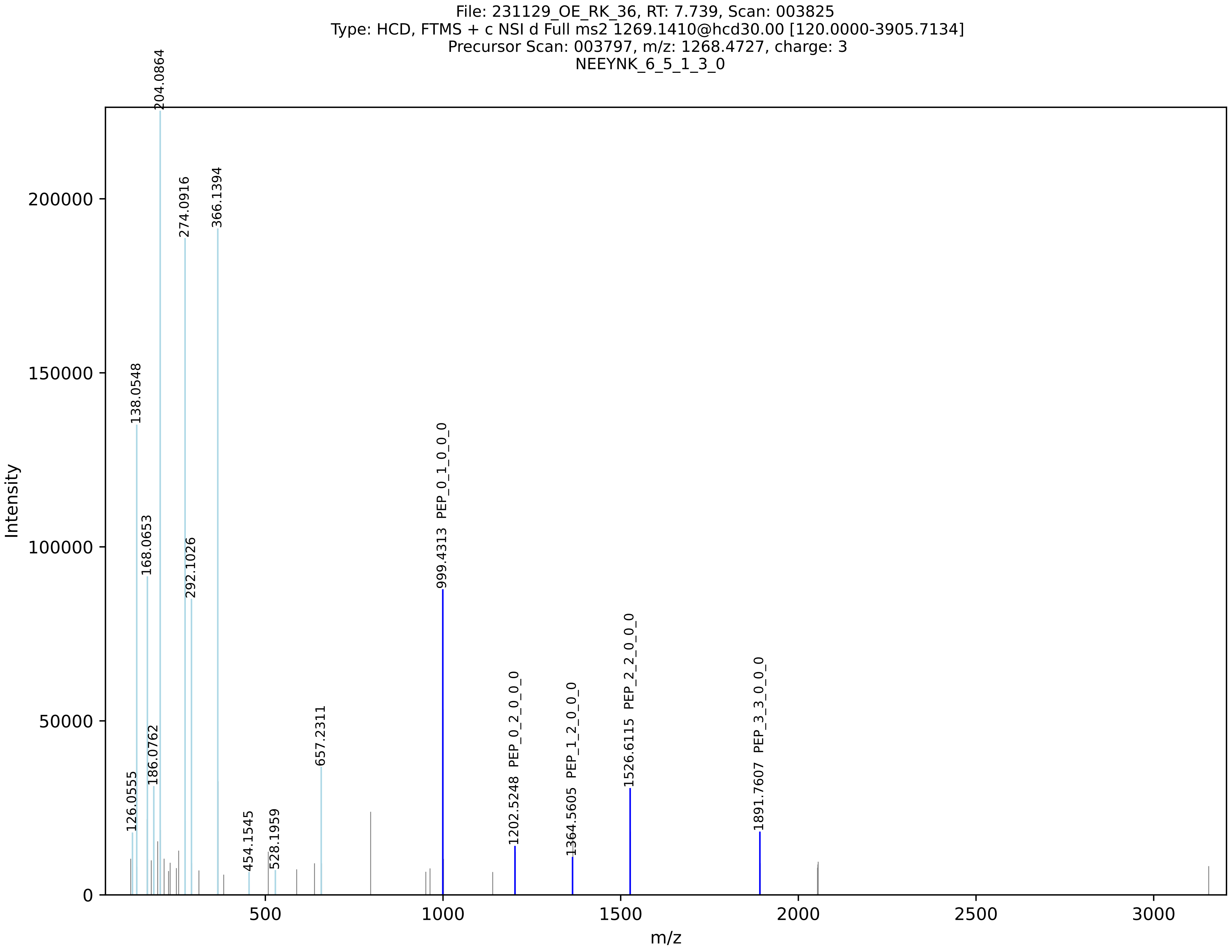

File: 231129_OE_RK_36, RT: 7.739, Scan: 003825
Type: HCD, FTMS + c NSI d Full ms2 1269.1410@hcd30.00 [120.0000-3905.7134]
Precursor Scan: 003797, m/z: 1268.4727, charge: 3
NEEYNK_6_5_1_3_0
Intensity
m/z
0
50000
100000
150000
200000
500
1000
1500
2000
2500
3000
204.0864
274.0916
366.1394
138.0548
168.0653
292.1026
186.0762
126.0555
454.1545
528.1959
657.2311
999.4313 PEP_0_1_0_0_0
1202.5248 PEP_0_2_0_0_0
1364.5605 PEP_1_2_0_0_0
1526.6115 PEP_2_2_0_0_0
1891.7607 PEP_3_3_0_0_0

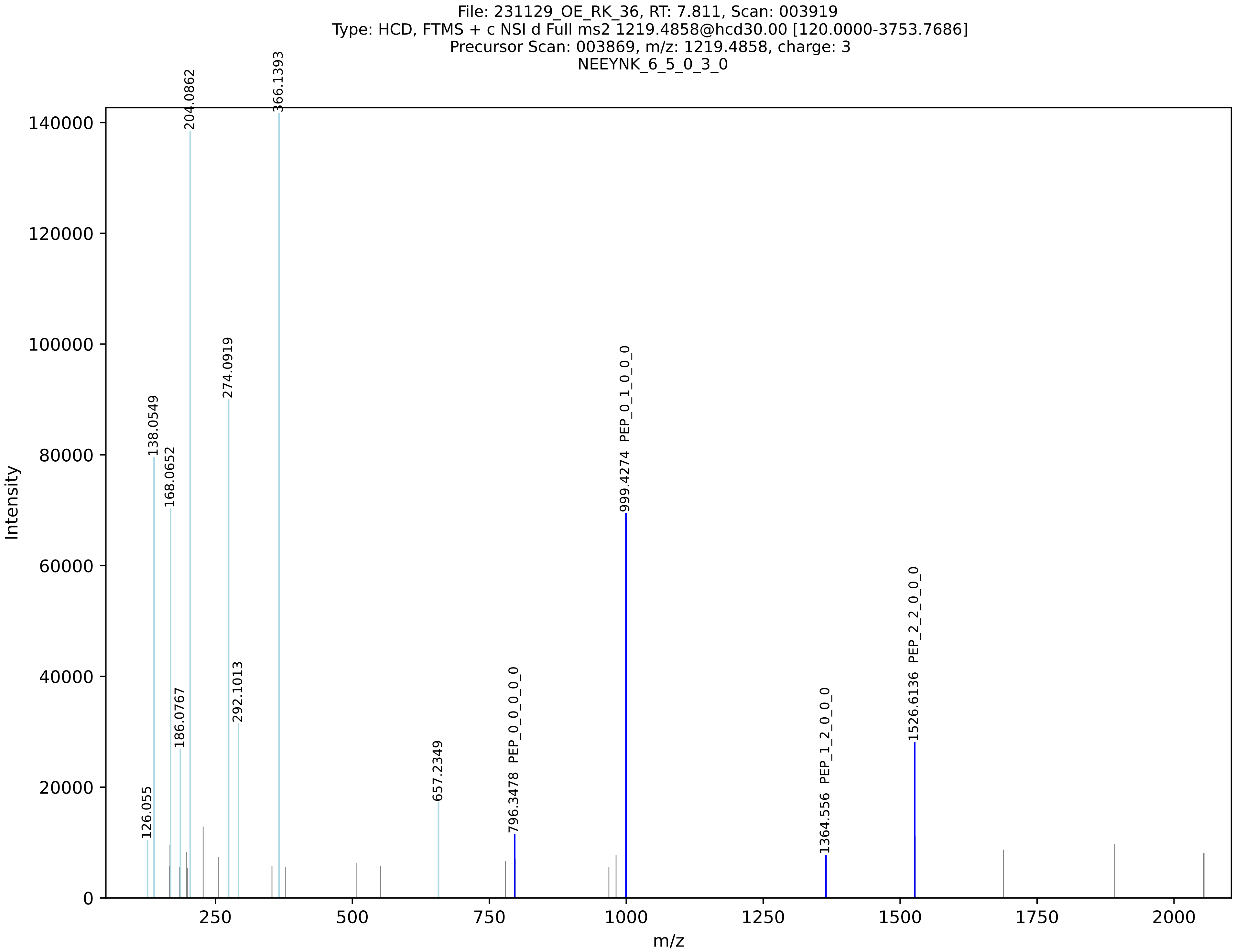
File: 231129_OE_RK_36, RT: 7.811, Scan: 003919
Type: HCD, FTMS + c NSI d Full ms2 1219.4858@hcd30.00 [120.0000-3753.7686]
Precursor Scan: 003869, m/z: 1219.4858, charge: 3
NEEYNK_6_5_0_3_0
Intensity
m/z
126.055
138.0549
168.0652
186.0767
204.0862
274.0919
292.1013
366.1393
657.2349
796.3478 PEP_0_0_0_0_0
999.4274 PEP_0_1_0_0_0
1364.556 PEP_1_2_0_0_0
1526.6136 PEP_2_2_0_0_0

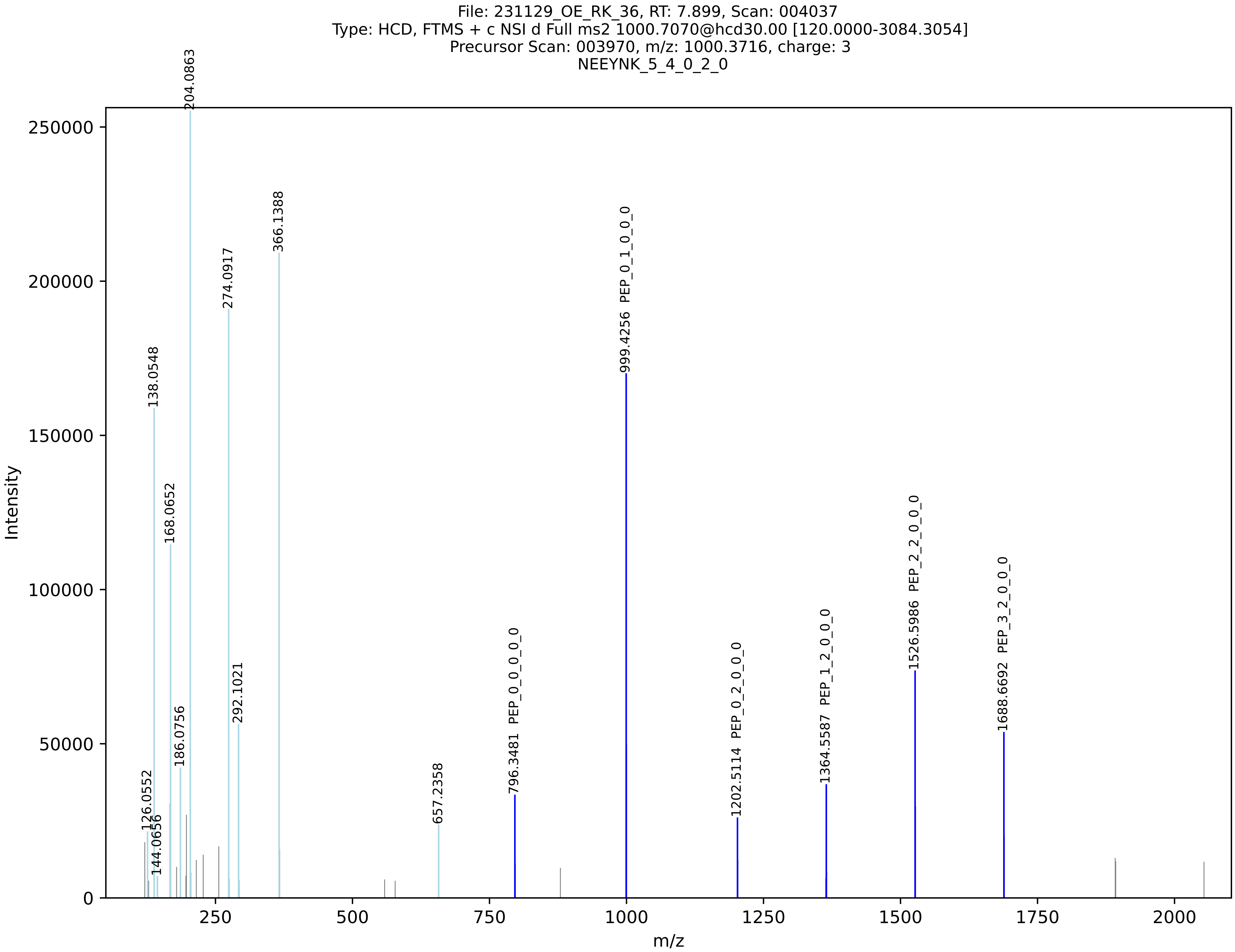
File: 231129_OE_RK_36, RT: 7.899, Scan: 004037
Type: HCD, FTMS + c NSI d Full ms2 1000.7070@hcd30.00 [120.0000-3084.3054]
Precursor Scan: 003970, m/z: 1000.3716, charge: 3
NEEYNK_5_4_0_2_0
Intensity
m/z
204.0863
366.1388
274.0917
138.0548
168.0652
186.0756
292.1021
126.0552
144.0656
657.2358
796.3481 PEP_0_0_0_0_0
999.4256 PEP_0_1_0_0_0
1202.5114 PEP_0_2_0_0_0
1364.5587 PEP_1_2_0_0_0
1526.5986 PEP_2_2_0_0_0
1688.6692 PEP_3_2_0_0_0

File: 231129_OE_RK_36, RT: 7.919, Scan: 004062
Type: HCD, FTMS + c NSI d Full ms2 1268.8060@hcd30.00 [120.0000-3904.6885]
Precursor Scan: 004013, m/z: 1268.4719, charge: 3
NEEYNK_6_5_1_3_0

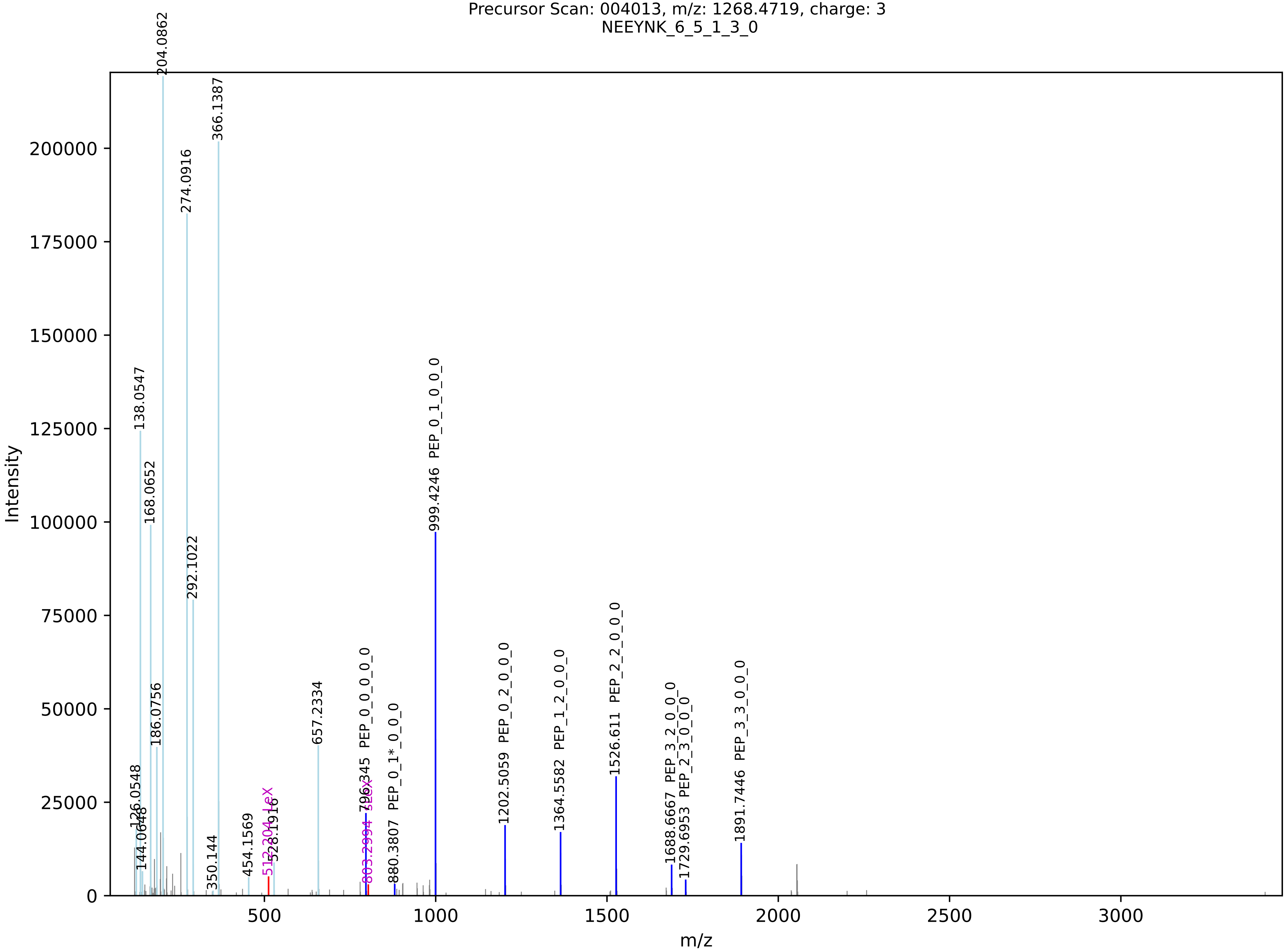

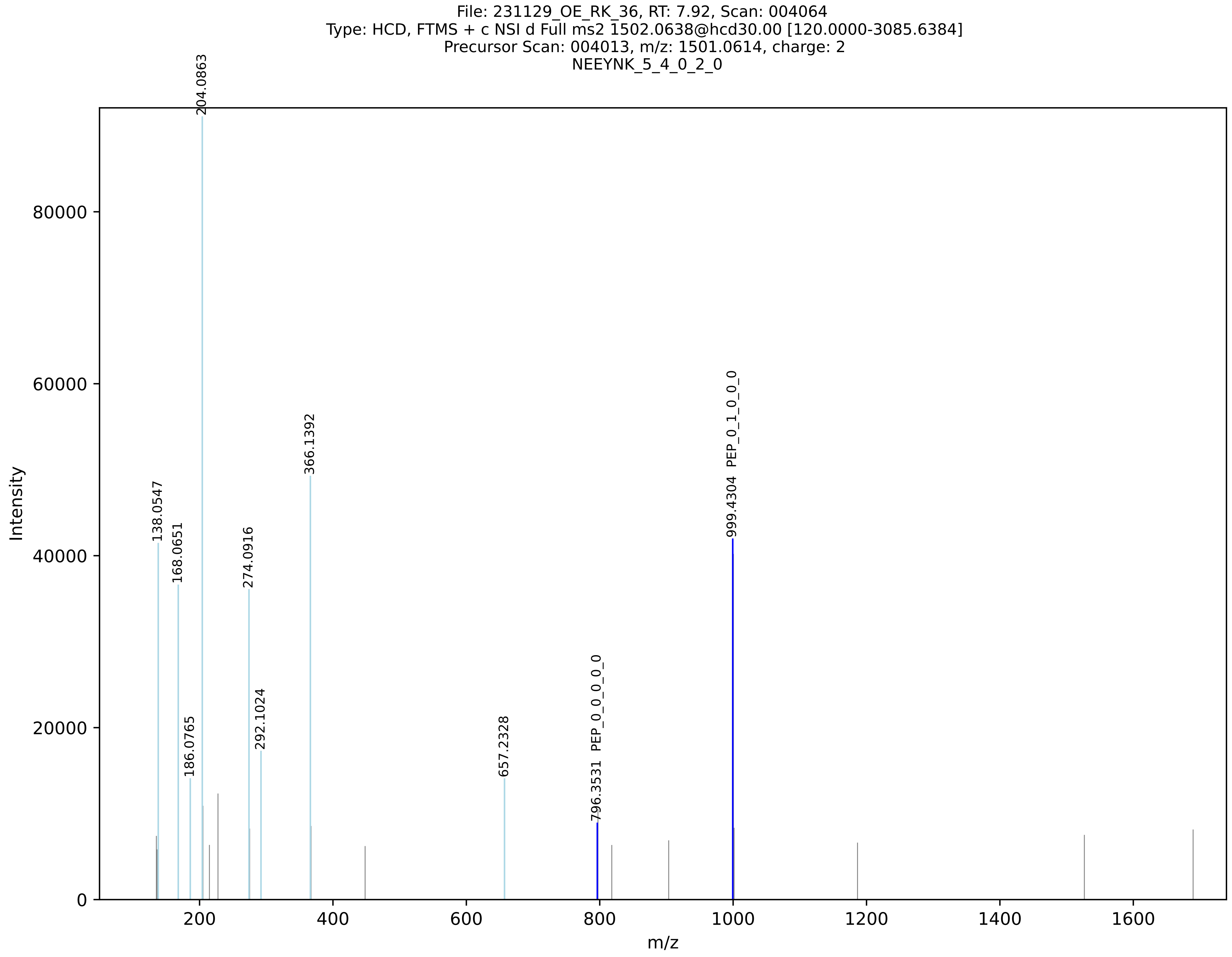
File: 231129_OE_RK_36, RT: 7.92, Scan: 004064
Type: HCD, FTMS + c NSI d Full ms2 1502.0638@hcd30.00 [120.0000-3085.6384]
Precursor Scan: 004013, m/z: 1501.0614, charge: 2
NEEYNK_5_4_0_2_0
Intensity
m/z
0
20000
40000
60000
80000
200
400
600
800
1000
1200
1400
1600
204.0863
138.0547
168.0651
186.0765
274.0916
292.1024
366.1392
657.2328
796.3531 PEP_0_0_0_0_0
999.4304 PEP_0_1_0_0_0

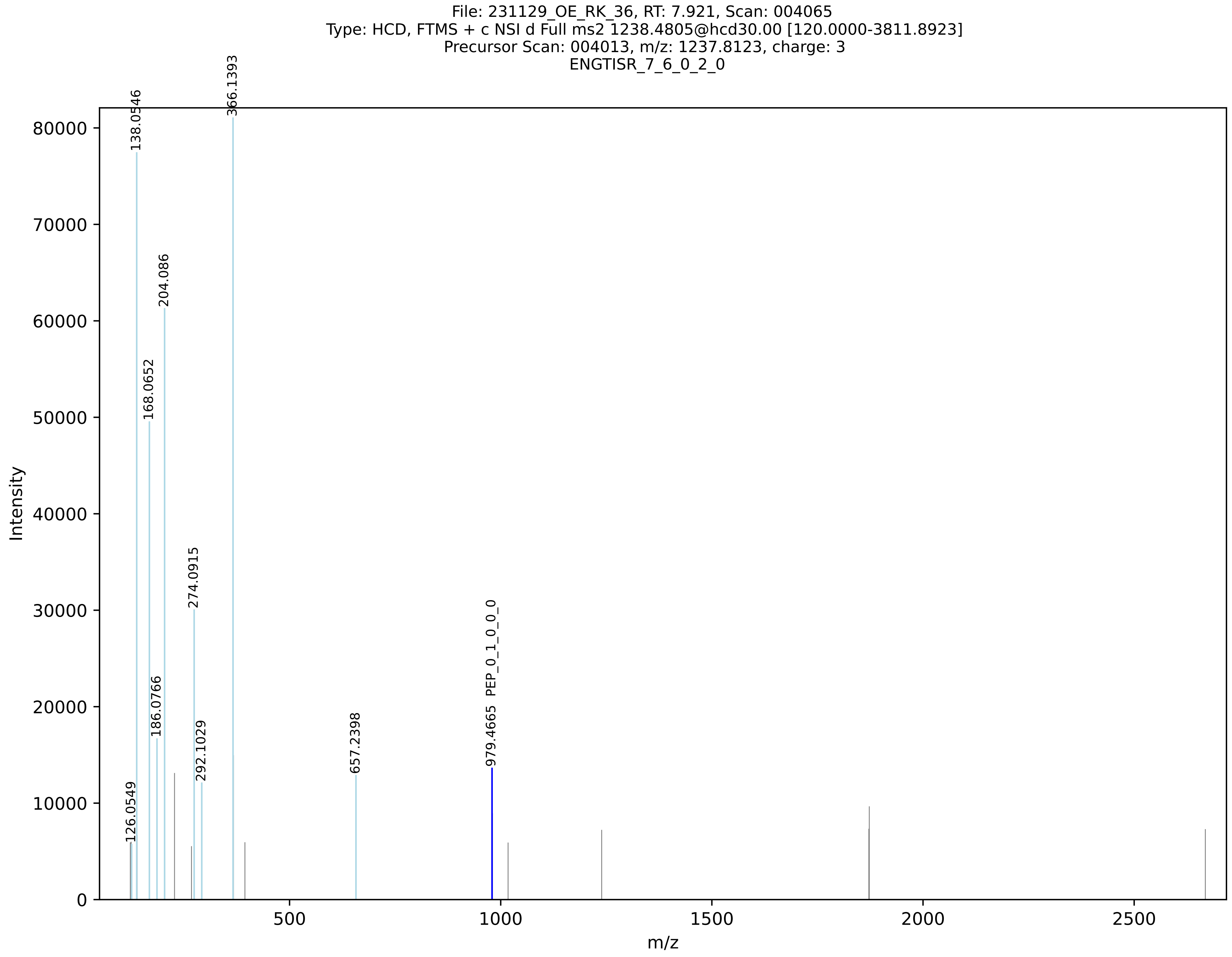
File: 231129_OE_RK_36, RT: 7.921, Scan: 004065
Type: HCD, FTMS + c NSI d Full ms2 1238.4805@hcd30.00 [120.0000-3811.8923]
Precursor Scan: 004013, m/z: 1237.8123, charge: 3
ENGTISR_7_6_0_2_0
Intensity
m/z
366.1393
138.0546
204.086
168.0652
274.0915
186.0766
292.1029
126.0549
657.2398
979.4665 PEP_0_1_0_0_0
80000
70000
60000
50000
40000
30000
20000
10000
0
500
1000
1500
2000
2500

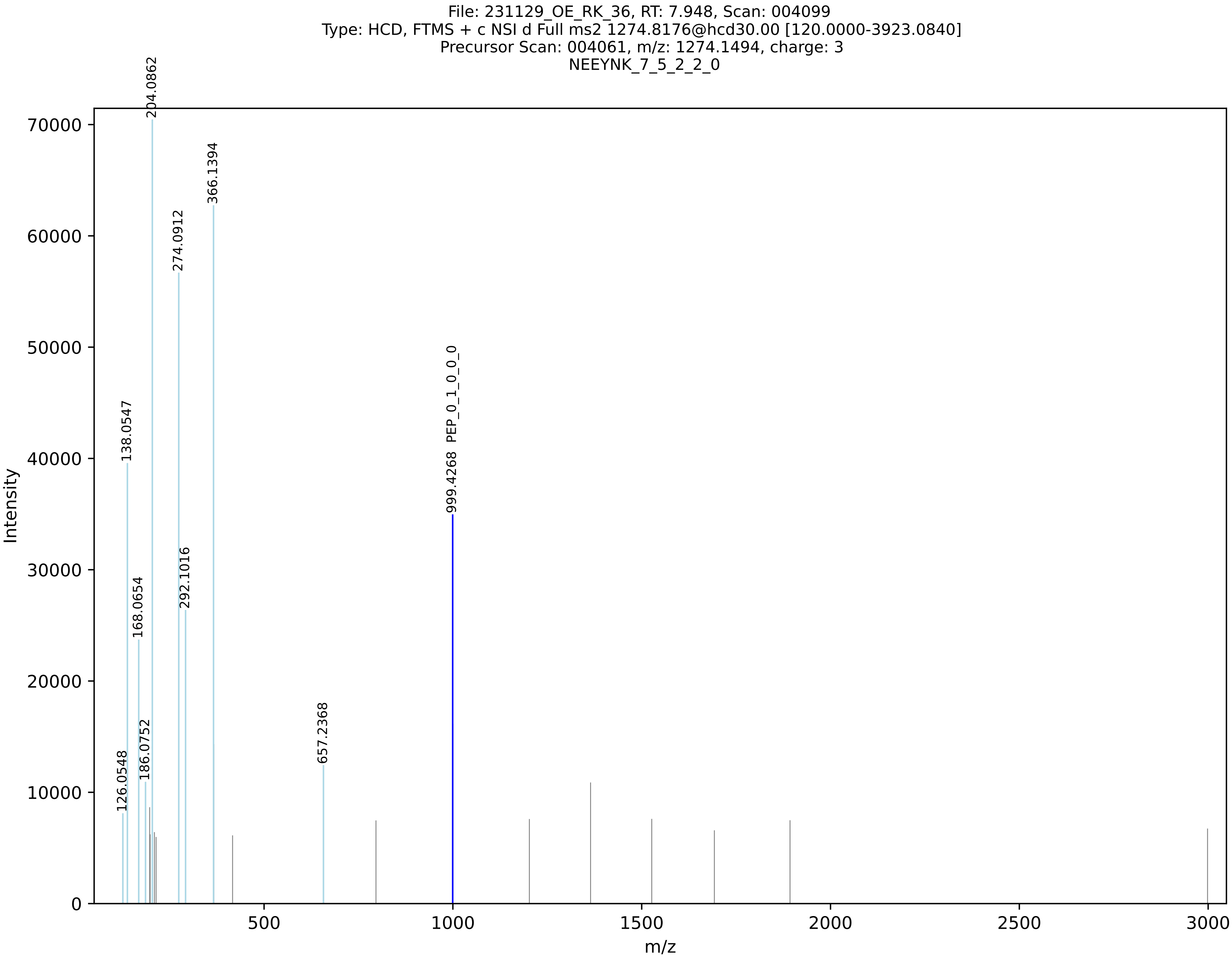
File: 231129_OE_RK_36, RT: 7.948, Scan: 004099
Type: HCD, FTMS + c NSI d Full ms2 1274.8176@hcd30.00 [120.0000-3923.0840]
Precursor Scan: 004061, m/z: 1274.1494, charge: 3
NEEYNK_7_5_2_2_0
Intensity
m/z
204.0862
366.1394
274.0912
999.4268 PEP_0_1_0_0_0_
138.0547
292.1016
168.0654
186.0752
126.0548
657.2368
0
10000
20000
30000
40000
50000
60000
70000
500
1000
1500
2000
2500
3000

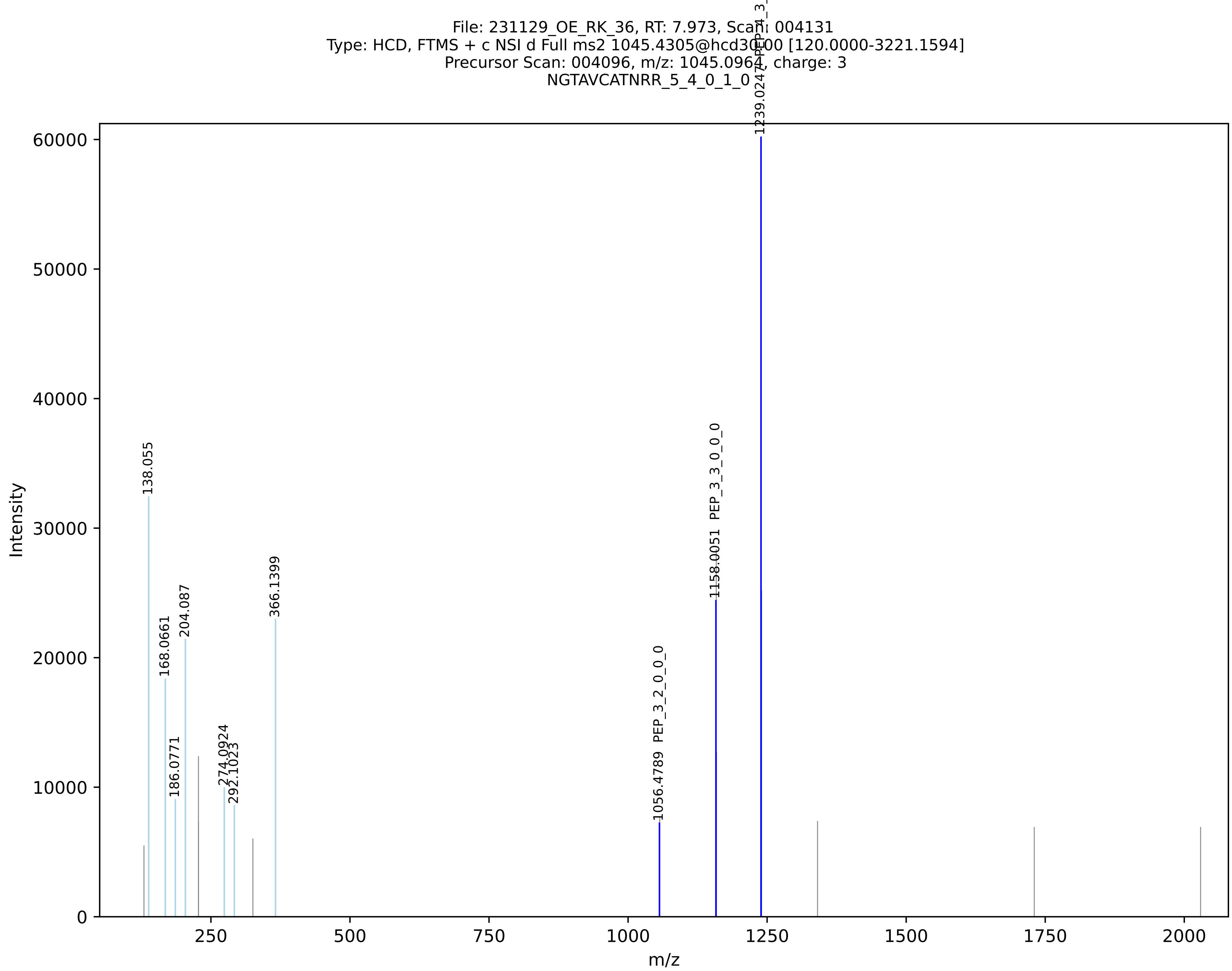

File: 231129_OE_RK_36, RT: 7.973, Scan: 004131
Type: HCD, FTMS + c NSI d Full ms2 1045.4305@hcd30.00 [120.0000-3221.1594]
Precursor Scan: 004096, m/z: 1045.0964, charge: 3
NGTAVCATNRR_5_4_0_1_0
1239.024
138.055
168.0661
186.0771
204.087
274.0924
292.1023
366.1399
1056.4789 PEP_3_2_0_0_0
1158.0051 PEP_3_3_0_0_0
Intensity
m/z
0
10000
20000
30000
40000
50000
60000
250
500
750
1000
1250
1500
1750
2000

File: 231129_OE_RK_36, RT: 7.982, Scan: 004145
Type: HCD, FTMS + c NSI d Full ms2 994.7958@hcd30.00 [120.0000-3066.2173]
Precursor Scan: 004096, m/z: 994.7958, charge: 3
ENGTISR_5_4_0_2_0

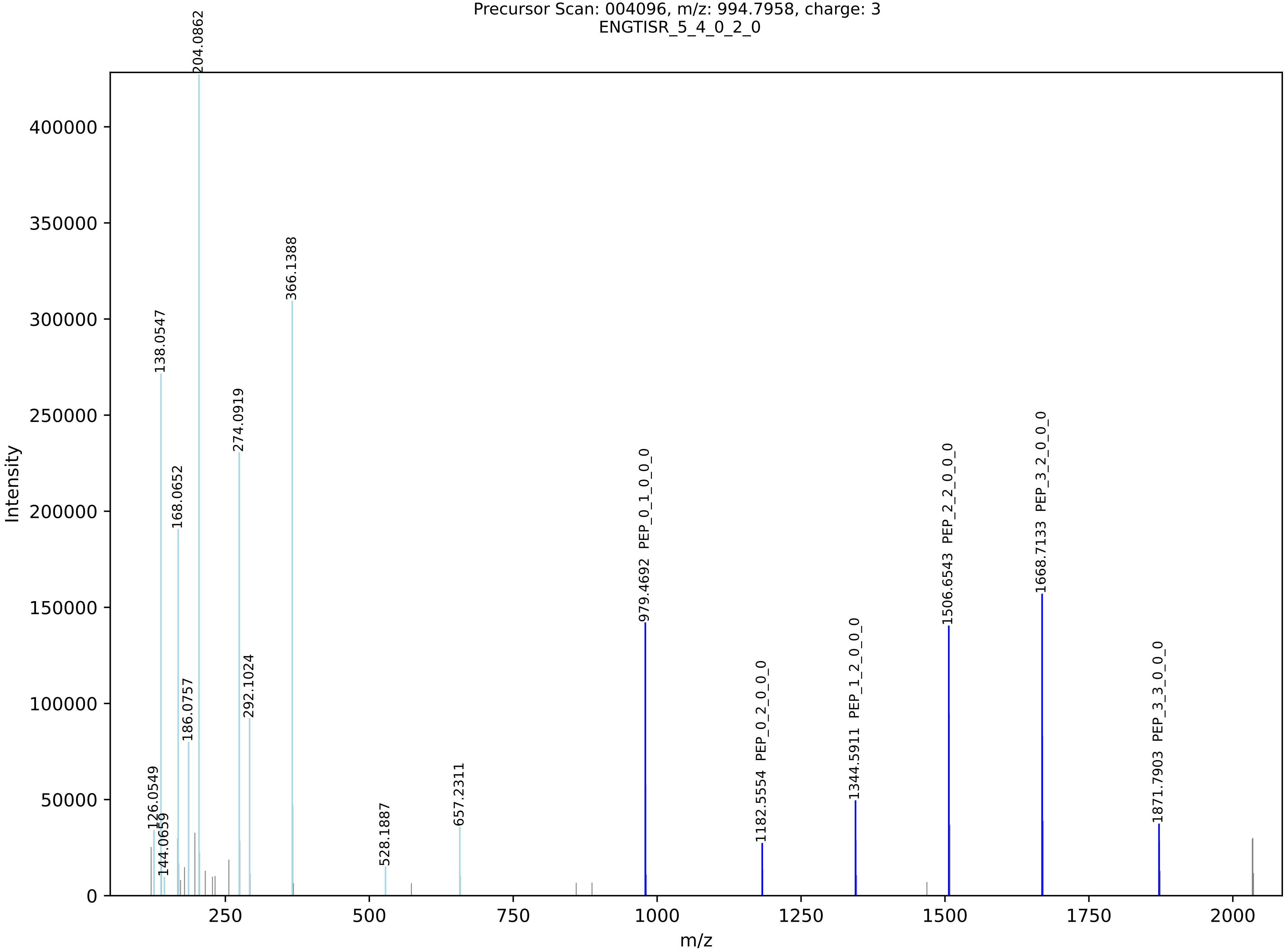

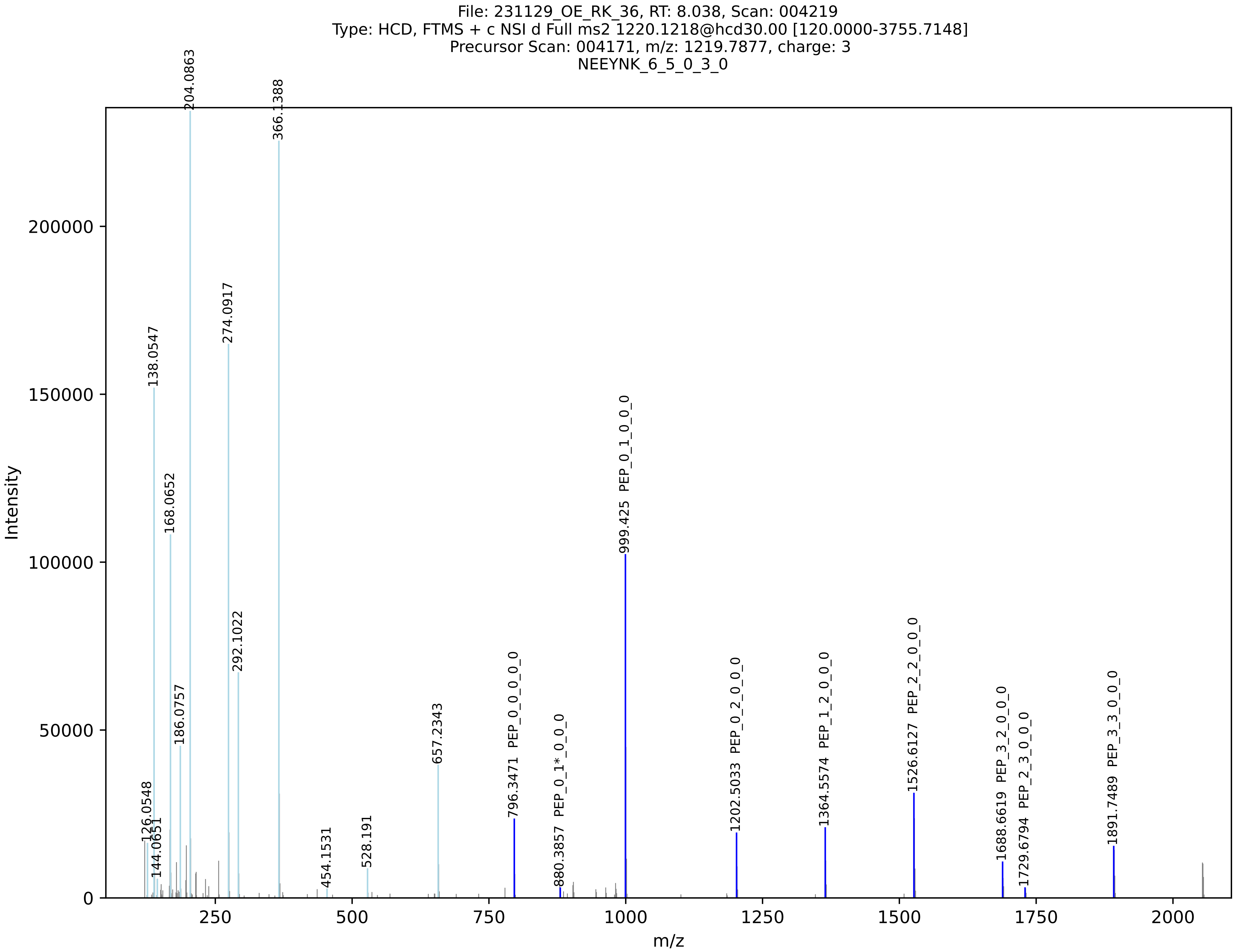

File: 231129_OE_RK_36, RT: 8.038, Scan: 004219
Type: HCD, FTMS + c NSI d Full ms2 1220.1218@hcd30.00 [120.0000-3755.7148]
Precursor Scan: 004171, m/z: 1219.7877, charge: 3
NEEYNK_6_5_0_3_0
Intensity
m/z
204.0863
366.1388
274.0917
138.0547
168.0652
292.1022
186.0757
126.0548
144.0651
454.1531
528.191
657.2343
796.3471 PEP_0_0_0_0_0
880.3857 PEP_0_1*_0_0_0
999.425 PEP_0_1_0_0_0
1202.5033 PEP_0_2_0_0_0
1364.5574 PEP_1_2_0_0_0
1526.6127 PEP_2_2_0_0_0
1688.6619 PEP_3_2_0_0_0
1729.6794 PEP_2_3_0_0_0
1891.7489 PEP_3_3_0_0_0

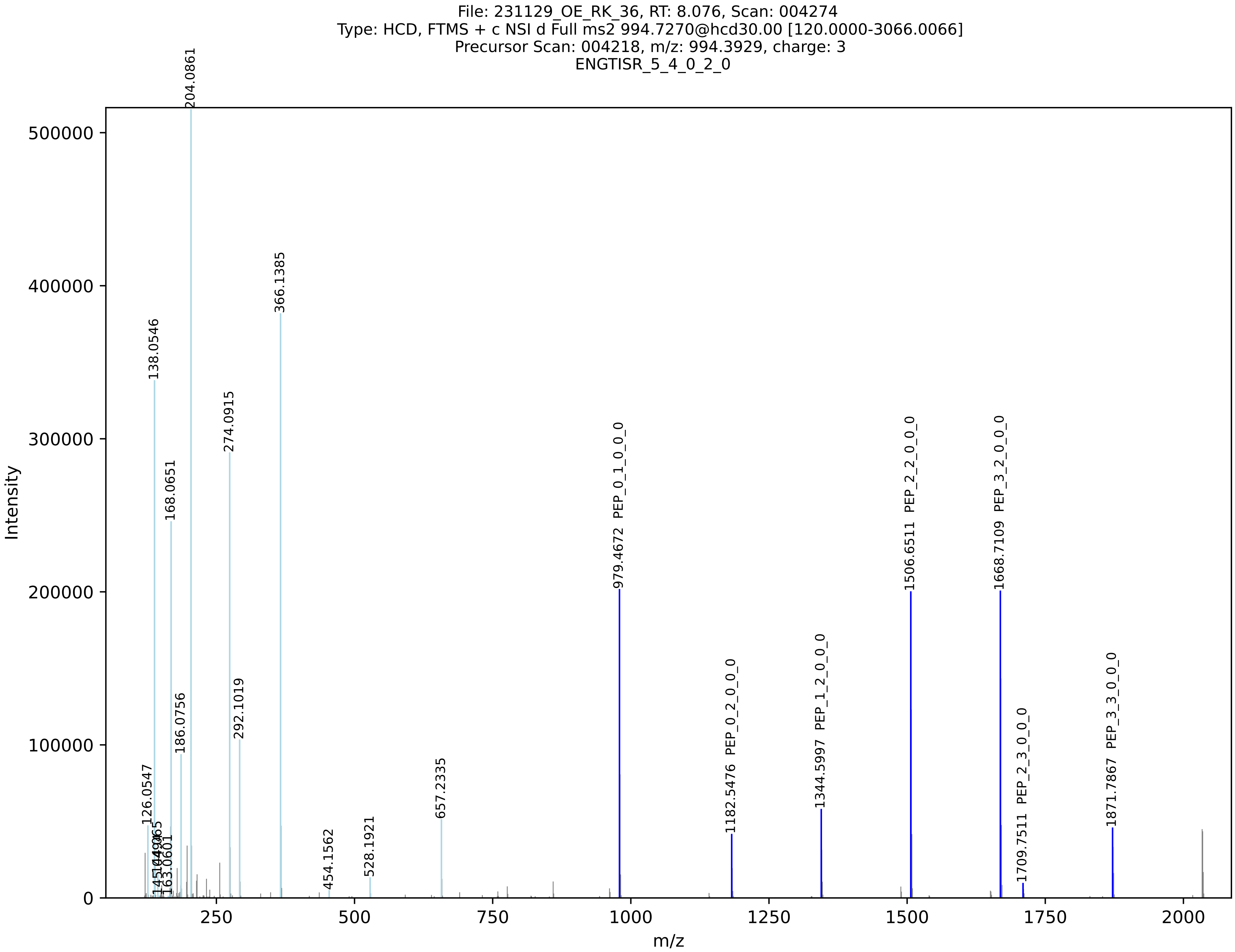
File: 231129_OE_RK_36, RT: 8.076, Scan: 004274
Type: HCD, FTMS + c NSI d Full ms2 994.7270@hcd30.00 [120.0000-3066.0066]
Precursor Scan: 004218, m/z: 994.3929, charge: 3
ENGTISR_5_4_0_2_0
Intensity
m/z
0
100000
200000
300000
400000
500000
250
500
750
1000
1250
1500
1750
2000
126.0547
138.0546
163.0601
168.0651
186.0756
204.0861
274.0915
292.1019
366.1385
454.1562
528.1921
657.2335
979.4672 PEP_0_1_0_0_0
1182.5476 PEP_0_2_0_0_0
1344.5997 PEP_1_2_0_0_0
1506.6511 PEP_2_2_0_0_0
1668.7109 PEP_3_2_0_0_0
1709.7511 PEP_2_3_0_0_0
1871.7867 PEP_3_3_0_0_0

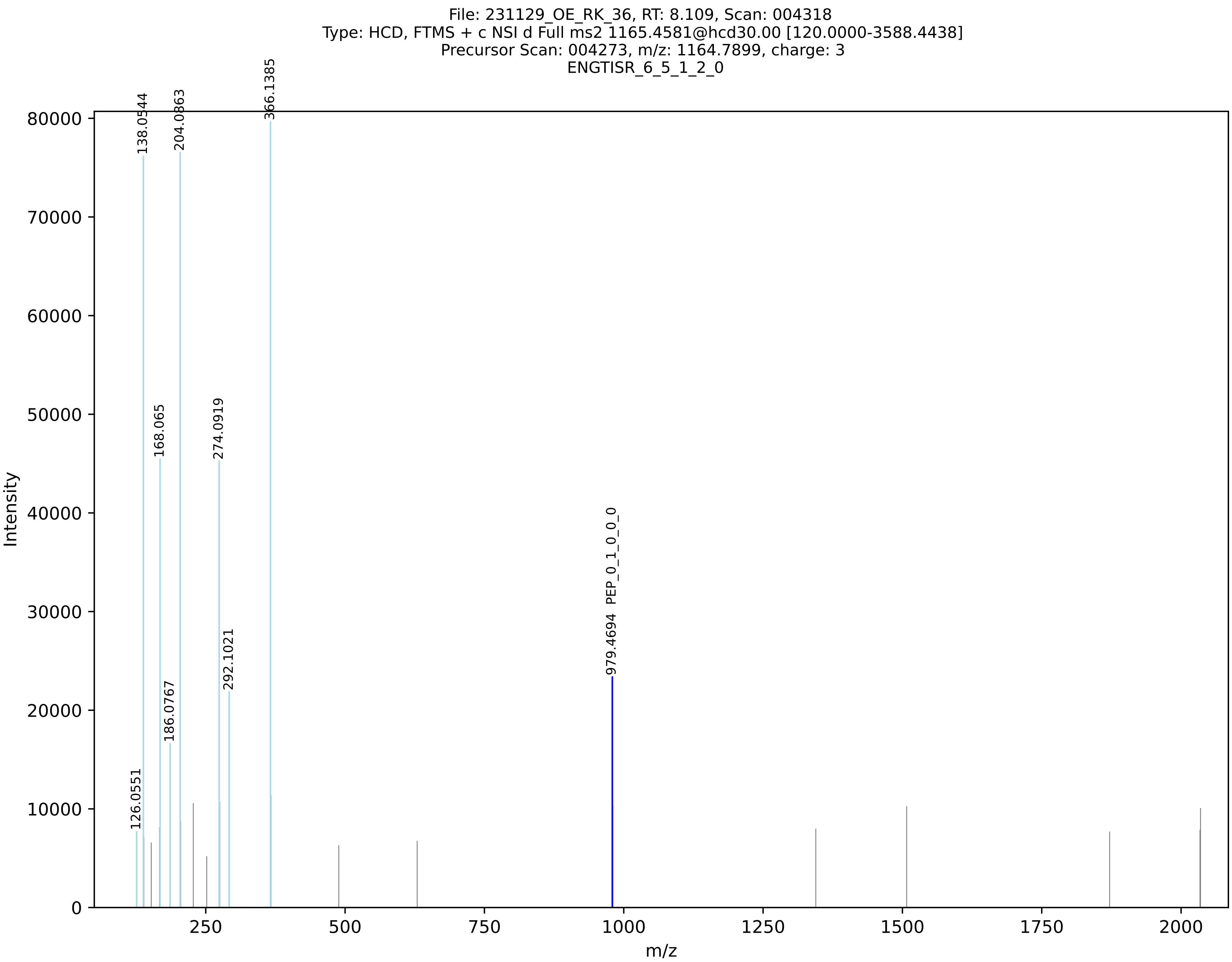
File: 231129_OE_RK_36, RT: 8.109, Scan: 004318
Type: HCD, FTMS + c NSI d Full ms2 1165.4581@hcd30.00 [120.0000-3588.4438]
Precursor Scan: 004273, m/z: 1164.7899, charge: 3
ENGTISR_6_5_1_2_0
138.0544
204.0863
366.1385
168.065
274.0919
292.1021
186.0767
126.0551
979.4694 PEP_0_1_0_0_0
Intensity
m/z
0
10000
20000
30000
40000
50000
60000
70000
80000
250
500
750
1000
1250
1500
1750
2000

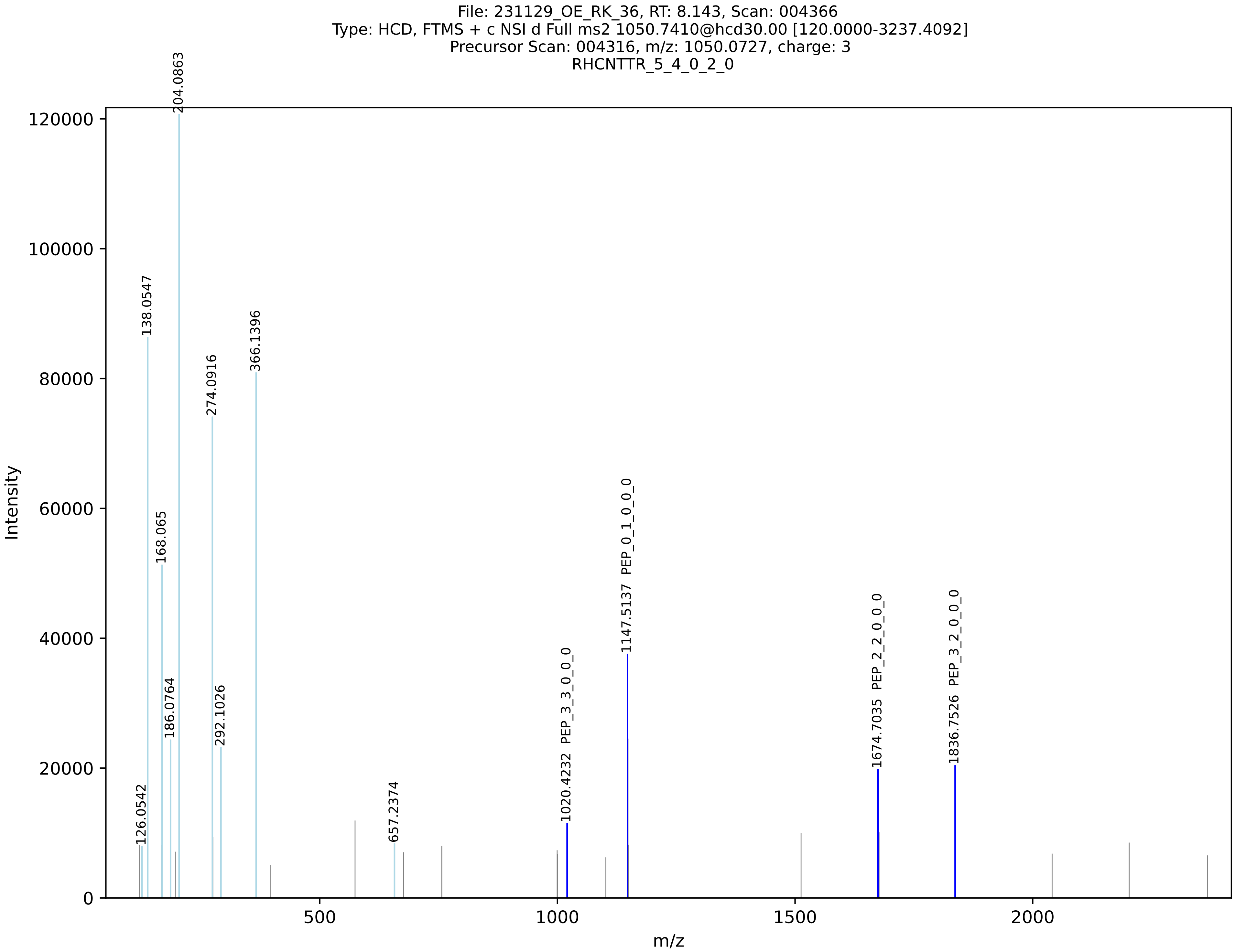
File: 231129_OE_RK_36, RT: 8.143, Scan: 004366
Type: HCD, FTMS + c NSI d Full ms2 1050.7410@hcd30.00 [120.0000-3237.4092]
Precursor Scan: 004316, m/z: 1050.0727, charge: 3
RHCNTTR_5_4_0_2_0
Intensity
m/z
0
20000
40000
60000
80000
100000
120000
500
1000
1500
2000
204.0863
138.0547
274.0916
366.1396
168.065
186.0764
292.1026
126.0542
657.2374
1020.4232 PEP_3_3_0_0_0
1147.5137 PEP_0_1_0_0_0
1674.7035 PEP_2_2_0_0_0
1836.7526 PEP_3_2_0_0_0

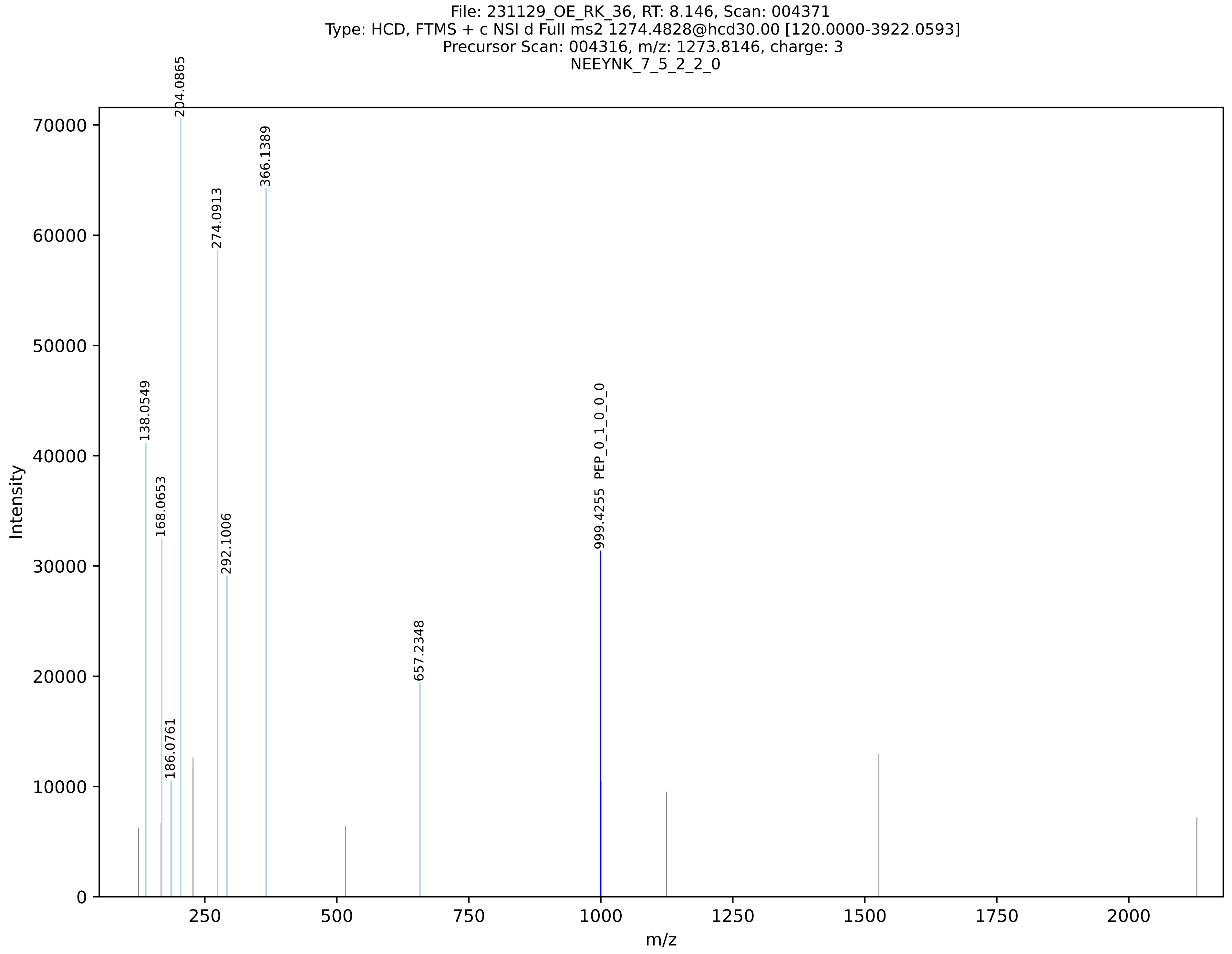
File: 231129_OE_RK_36, RT: 8.146, Scan: 004371
Type: HCD, FTMS + c NSI d Full ms2 1274.4828@hcd30.00 [120.0000-3922.0593]
Precursor Scan: 004316, m/z: 1273.8146, charge: 3
NEEYNK_7_5_2_2_0
Intensity
m/z
204.0865
366.1389
274.0913
138.0549
168.0653
292.1006
186.0761
657.2348
999.4255 PEP_0_1_0_0_0
0
10000
20000
30000
40000
50000
60000
70000
250
500
750
1000
1250
1500
1750
2000

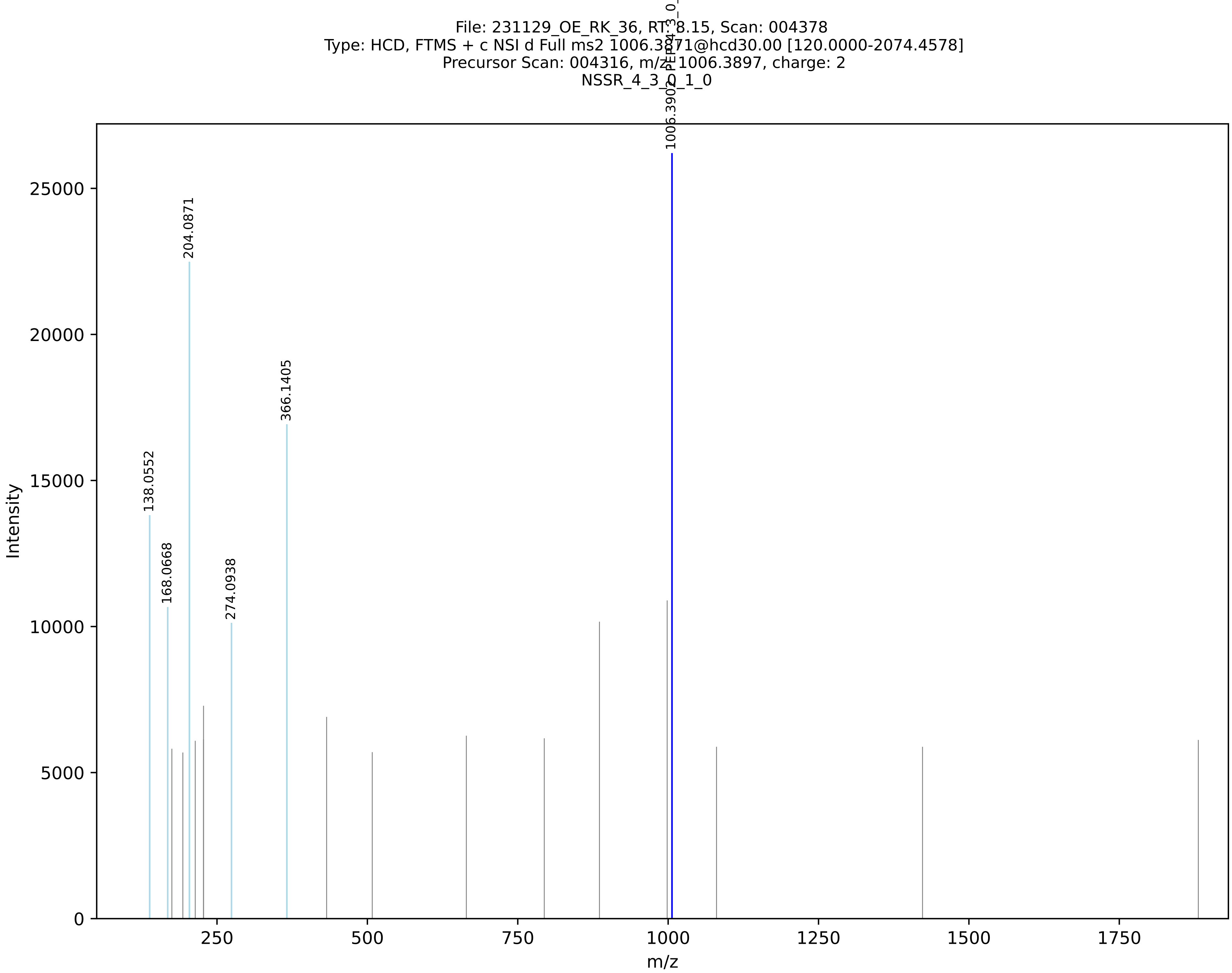
File: 231129_OE_RK_36, RT: 8.15, Scan: 004378
Type: HCD, FTMS + c NSI d Full ms2 1006.3871@hcd30.00 [120.0000-2074.4578]
Precursor Scan: 004316, m/z: 1006.3897, charge: 2
NSSR_4_3_0_1_0
1006.3902_PEP4_3_0_
204.0871
366.1405
138.0552
168.0668
274.0938
Intensity
m/z
0
5000
10000
15000
20000
25000
250
500
750
1000
1250
1500
1750

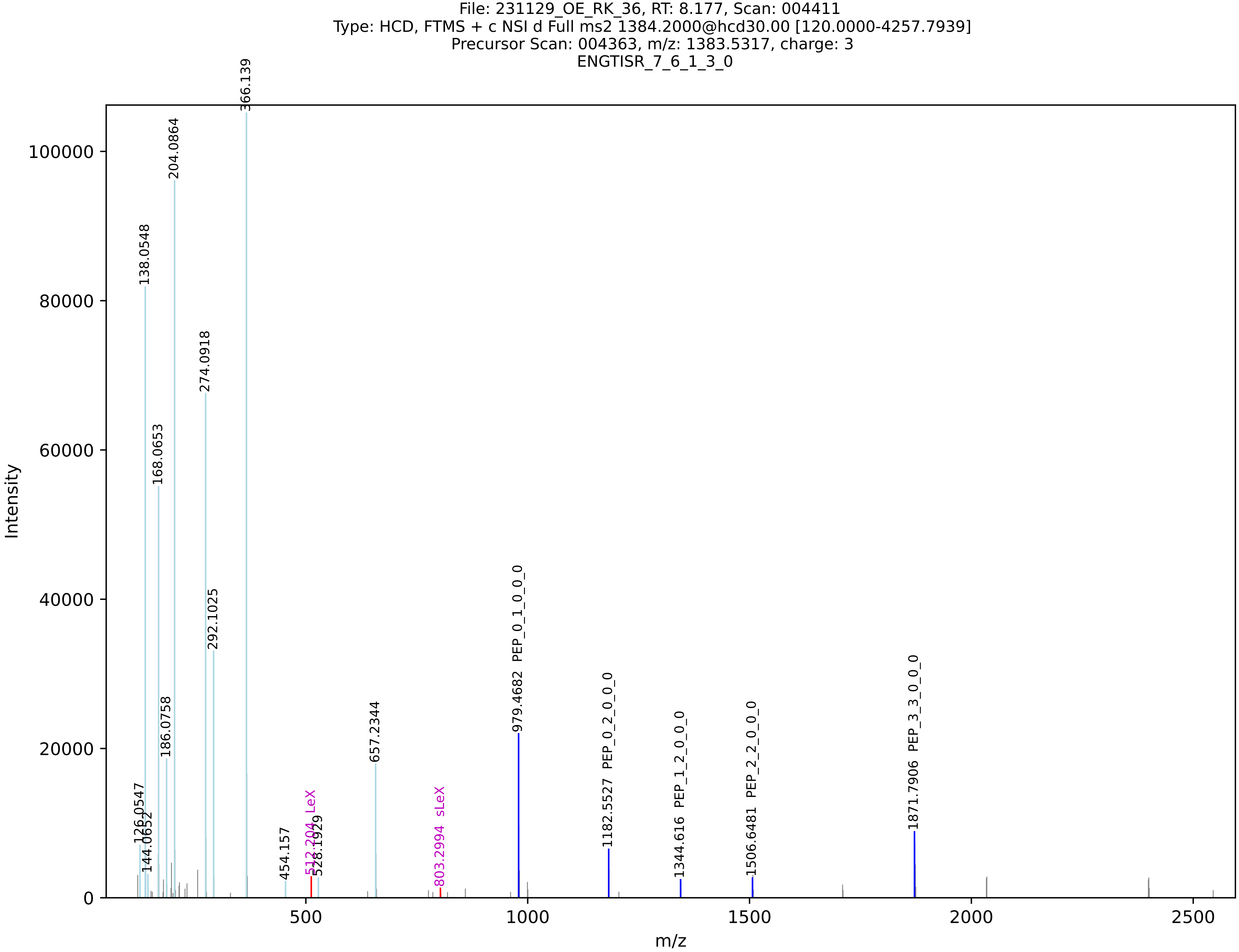
File: 231129_OE_RK_36, RT: 8.177, Scan: 004411
Type: HCD, FTMS + c NSI d Full ms2 1384.2000@hcd30.00 [120.0000-4257.7939]
Precursor Scan: 004363, m/z: 1383.5317, charge: 3
ENGTISR_7_6_1_3_0
Intensity
m/z
126.0547
138.0548
144.0652
168.0653
186.0758
204.0864
274.0918
292.1025
366.139
454.157
512.204 LeX
528.1929
657.2344
803.2994 sLeX
979.4682 PEP_0_1_0_0_0
1182.5527 PEP_0_2_0_0_0
1344.616 PEP_1_2_0_0_0
1506.6481 PEP_2_2_0_0_0
1871.7906 PEP_3_3_0_0_0

File: 231129_OE_RK_36, RT: 8.223, Scan: 004471
Type: HCD, FTMS + c NSI d Full ms2 1220.4568@hcd30.00 [120.0000-3756.7397]
Precursor Scan: 004442, m/z: 1219.7888, charge: 3
NEEYNK_6_5_0_3_0

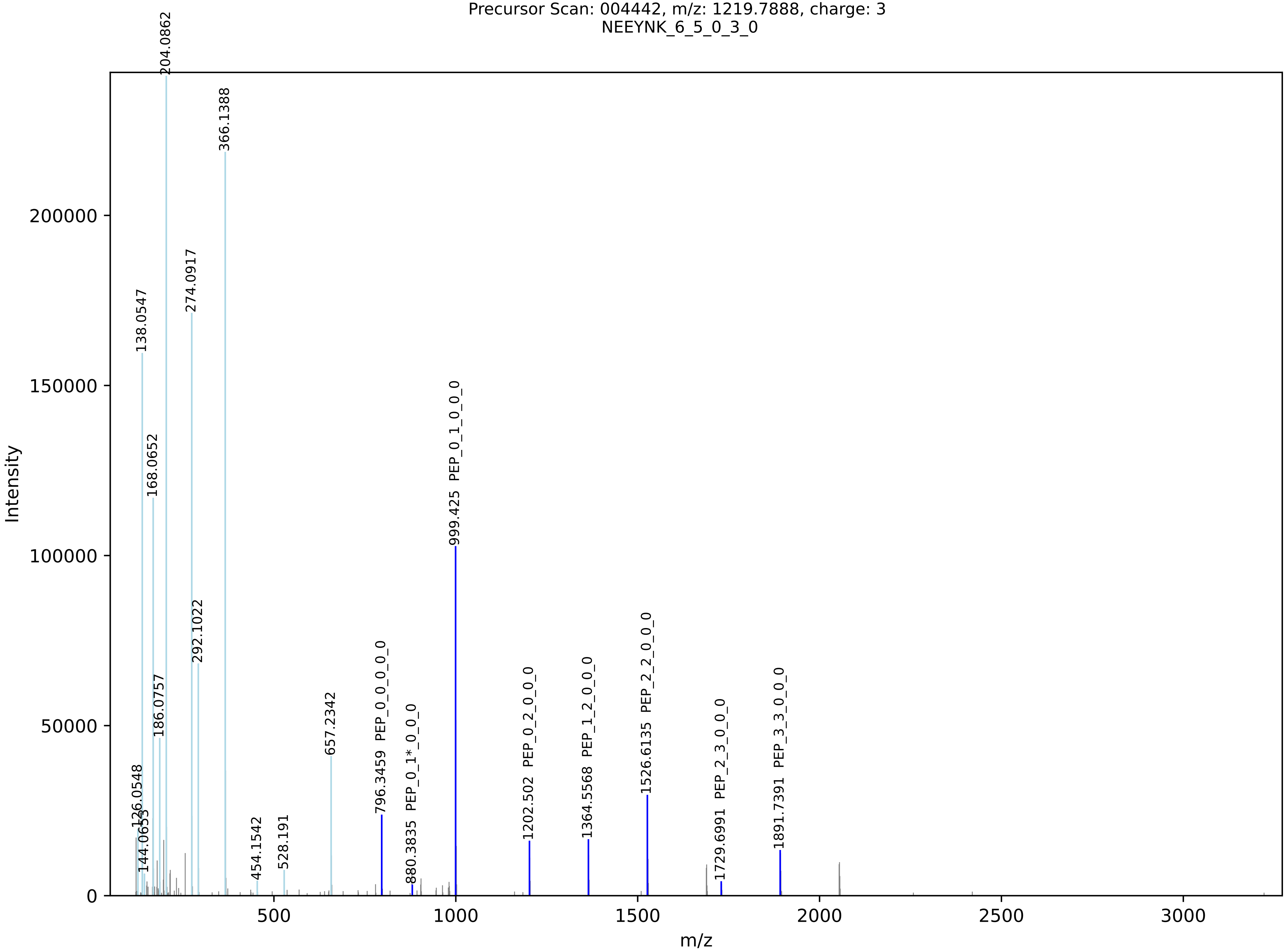

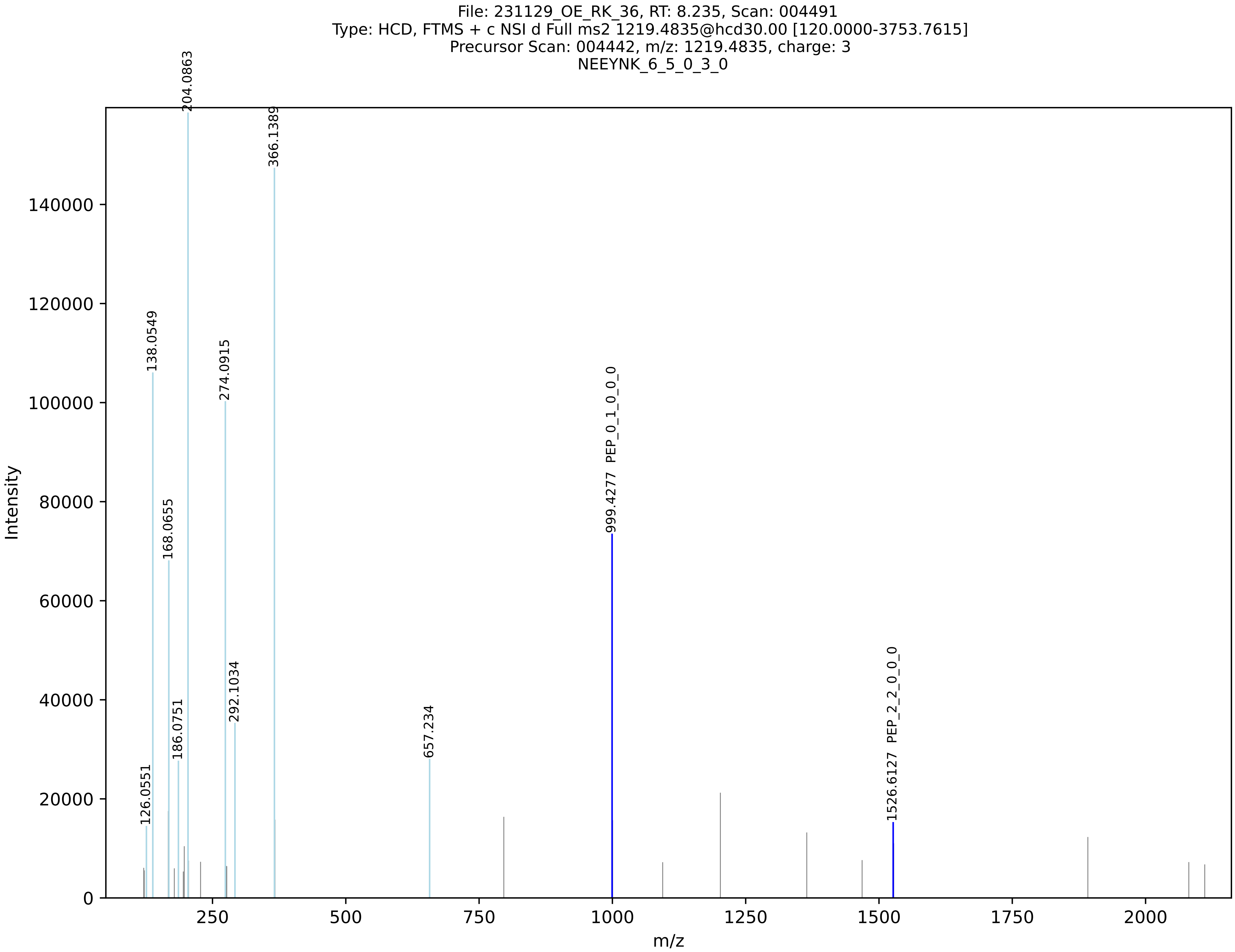
File: 231129_OE_RK_36, RT: 8.235, Scan: 004491
Type: HCD, FTMS + c NSI d Full ms2 1219.4835@hcd30.00 [120.0000-3753.7615]
Precursor Scan: 004442, m/z: 1219.4835, charge: 3
NEEYNK_6_5_0_3_0
Intensity
m/z
204.0863
366.1389
138.0549
274.0915
168.0655
999.4277 PEP_0_1_0_0_0
292.1034
186.0751
657.234
126.0551
1526.6127 PEP_2_2_0_0_0
0
20000
40000
60000
80000
100000
120000
140000
250
500
750
1000
1250
1500
1750
2000

File: 231129_OE_RK_36, RT: 8.256, Scan: 004517
Type: HCD, FTMS + c NSI d Full ms2 994.7275@hcd30.00 [120.0000-3066.0081]
Precursor Scan: 004470, m/z: 994.3933, charge: 3
ENGTISR_5_4_0_2_0

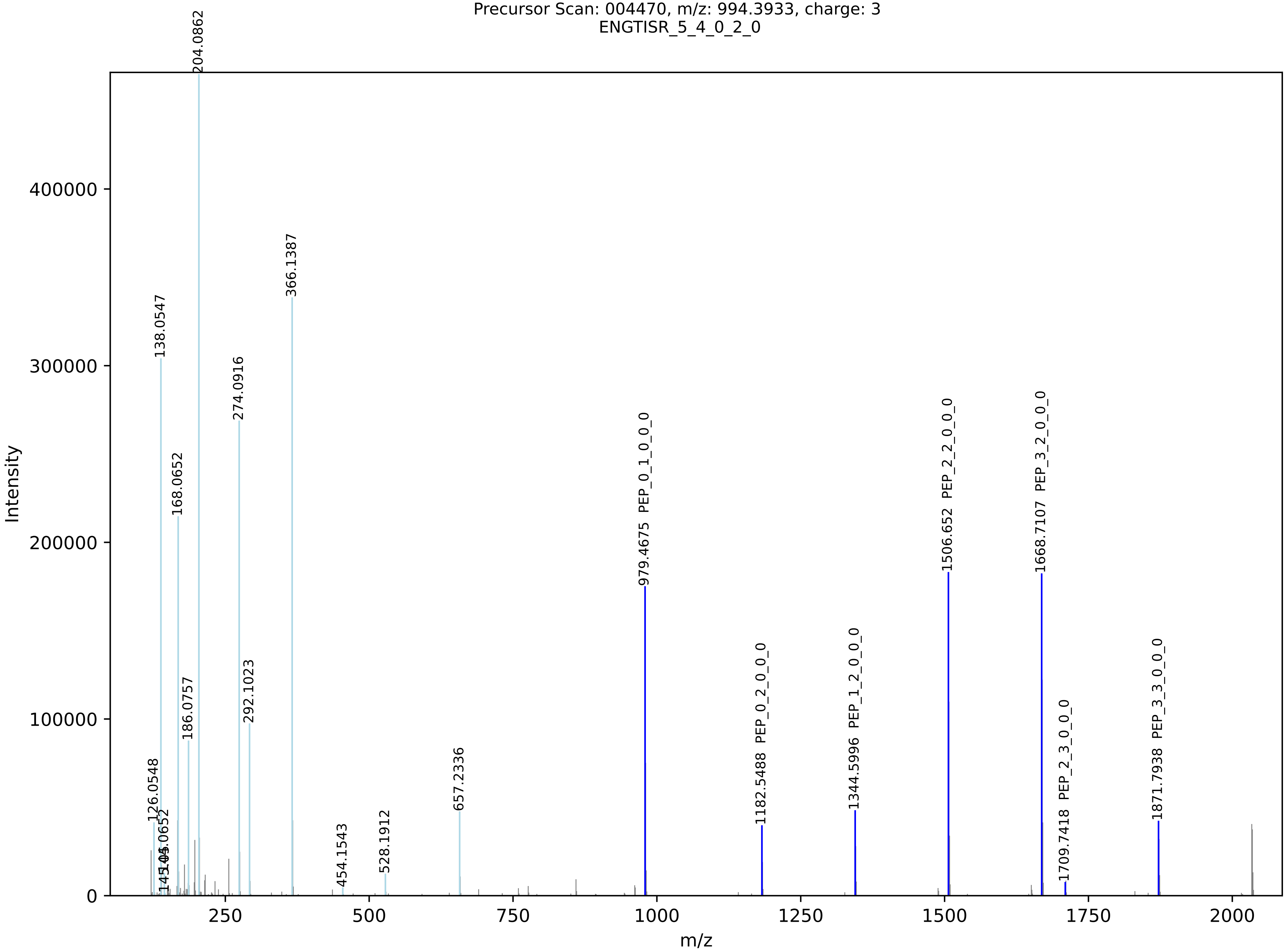

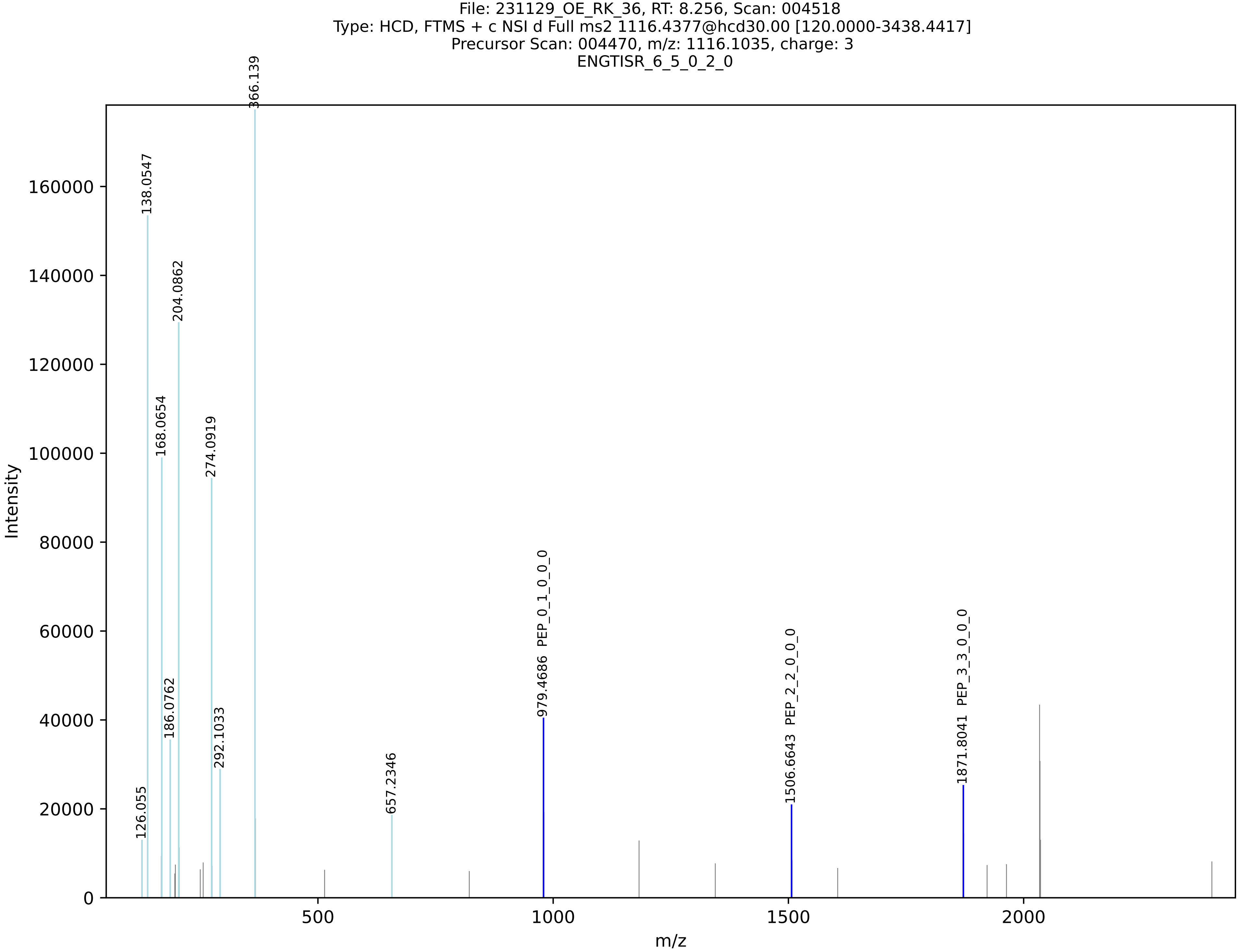
File: 231129_OE_RK_36, RT: 8.256, Scan: 004518
Type: HCD, FTMS + c NSI d Full ms2 1116.4377@hcd30.00 [120.0000-3438.4417]
Precursor Scan: 004470, m/z: 1116.1035, charge: 3
ENGTISR_6_5_0_2_0
366.139
138.0547
204.0862
168.0654
274.0919
186.0762
292.1033
126.055
657.2346
979.4686 PEP_0_1_0_0_0
1506.6643 PEP_2_2_0_0_0
1871.8041 PEP_3_3_0_0_0
Intensity
m/z
0
20000
40000
60000
80000
100000
120000
140000
160000
500
1000
1500
2000

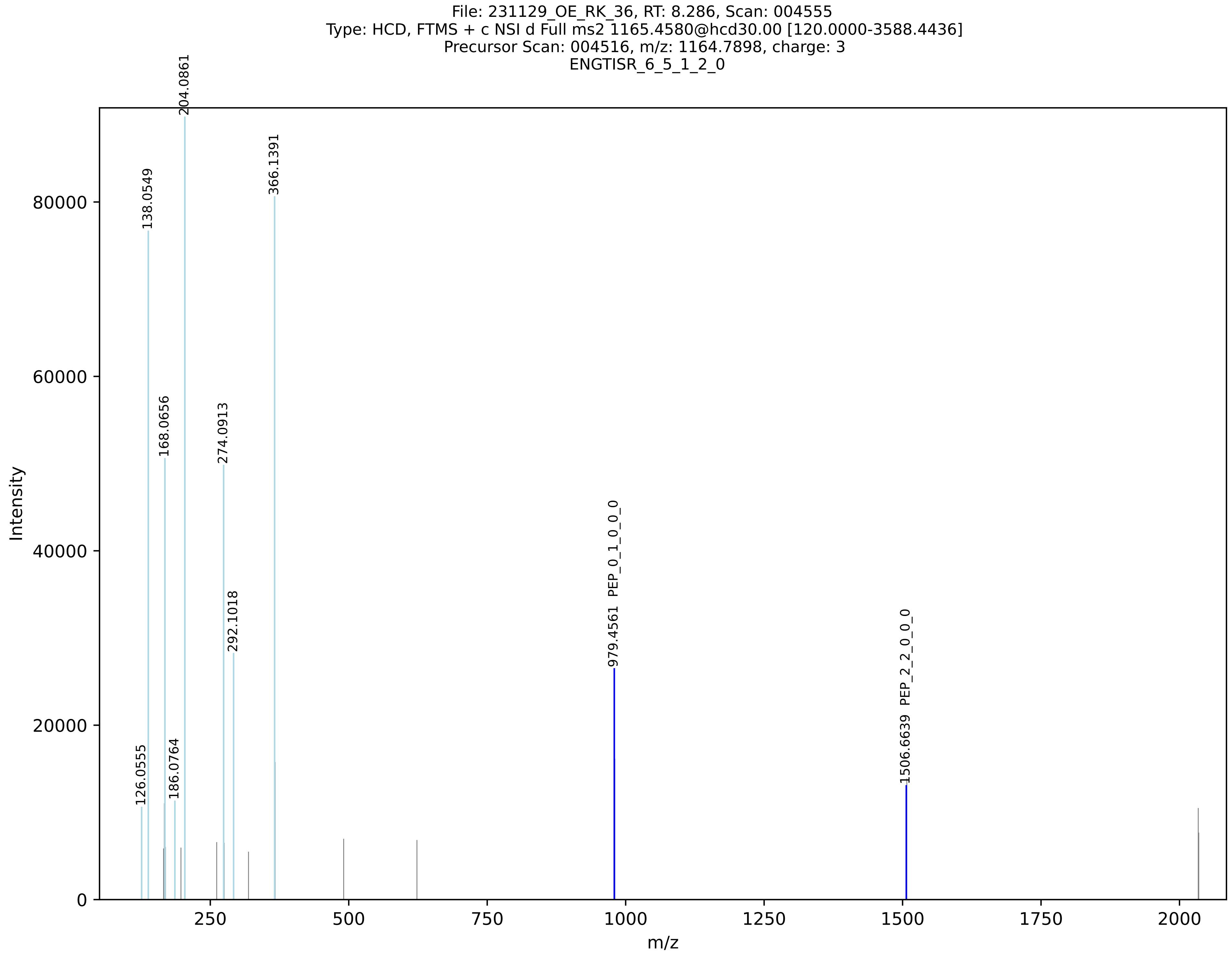

File: 231129_OE_RK_36, RT: 8.286, Scan: 004555
Type: HCD, FTMS + c NSI d Full ms2 1165.4580@hcd30.00 [120.0000-3588.4436]
Precursor Scan: 004516, m/z: 1164.7898, charge: 3
ENGTISR_6_5_1_2_0
Intensity
m/z
204.0861
366.1391
138.0549
168.0656
274.0913
292.1018
126.0555
186.0764
979.4561 PEP_0_1_0_0_0
1506.6639 PEP_2_2_0_0_0
0
20000
40000
60000
80000
250
500
750
1000
1250
1500
1750
2000

File: 231129_OE_RK_36, RT: 8.316, Scan: 004597
Type: HCD, FTMS + c NSI d Full ms2 1043.7030@hcd30.00 [120.0000-3215.8733]
Precursor Scan: 004553, m/z: 1043.7031, charge: 3
ENGTISR_5_4_1_2_0

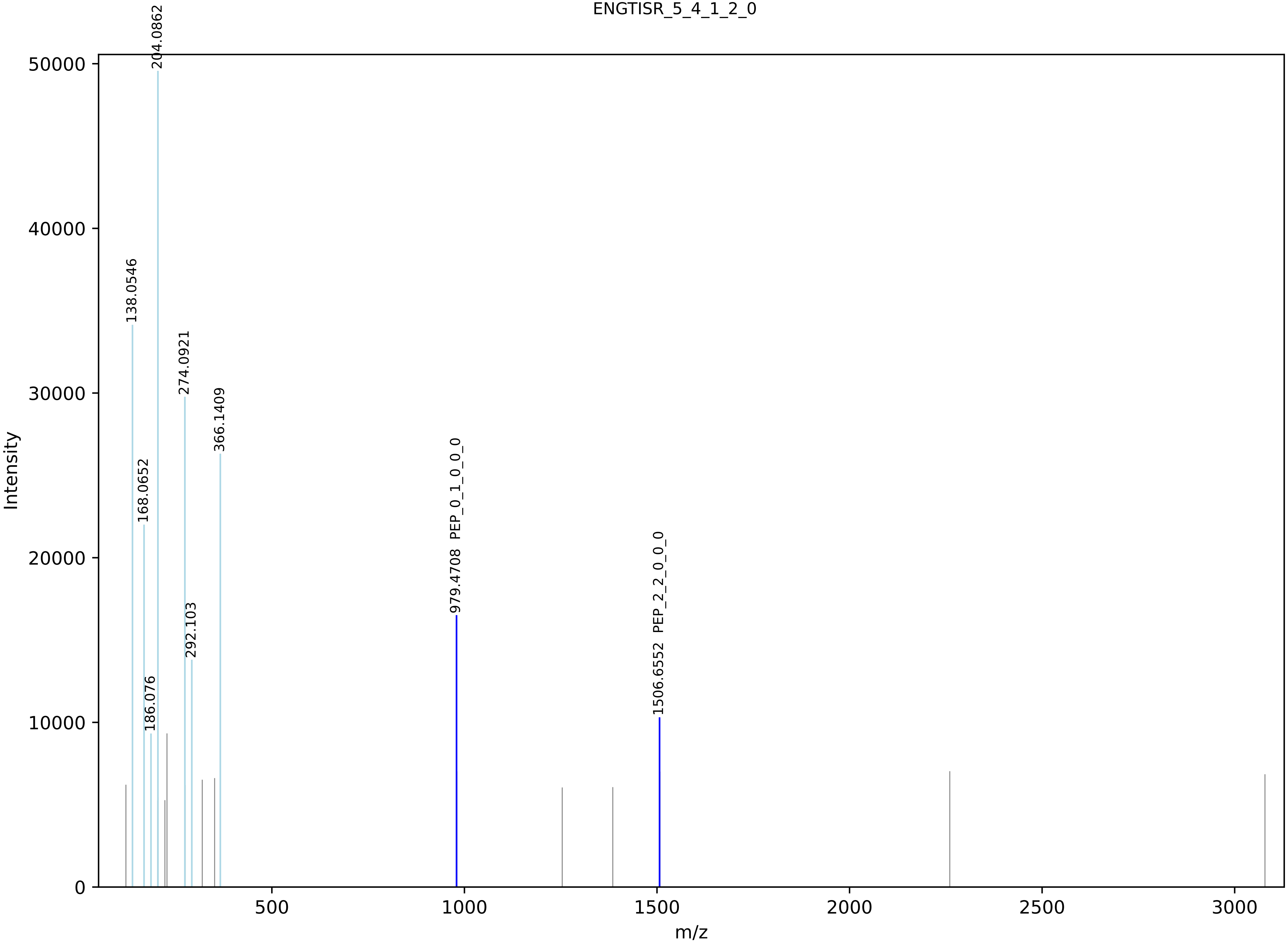

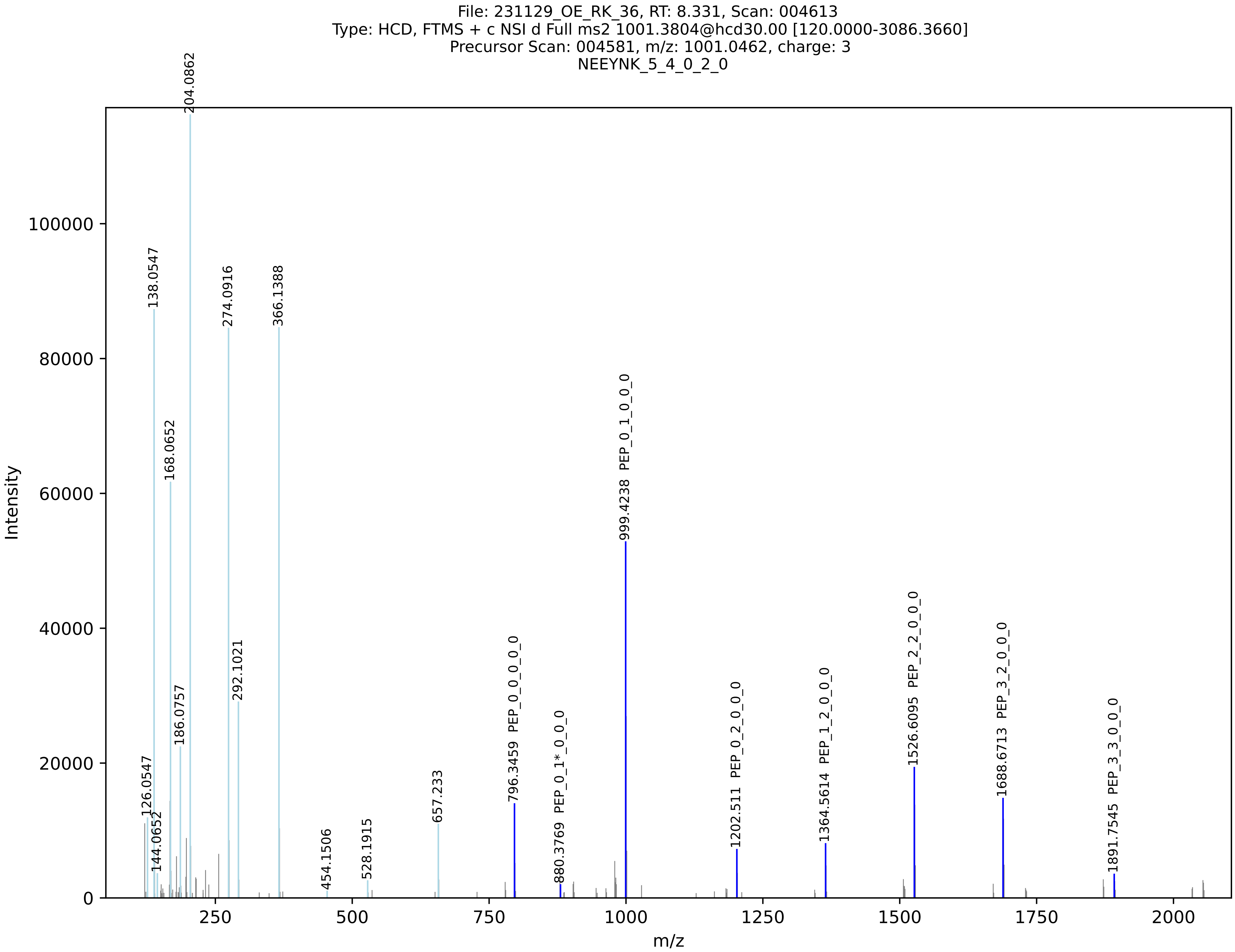

File: 231129_OE_RK_36, RT: 8.331, Scan: 004613
Type: HCD, FTMS + c NSI d Full ms2 1001.3804@hcd30.00 [120.0000-3086.3660]
Precursor Scan: 004581, m/z: 1001.0462, charge: 3
NEEYNK_5_4_0_2_0
Intensity
m/z
204.0862
138.0547
274.0916
366.1388
168.0652
186.0757
292.1021
126.0547
144.0652
454.1506
528.1915
657.233
796.3459 PEP_0_0_0_0_0
880.3769 PEP_0_1*_0_0_0
999.4238 PEP_0_1_0_0_0
1202.511 PEP_0_2_0_0_0
1364.5614 PEP_1_2_0_0_0
1526.6095 PEP_2_2_0_0_0
1688.6713 PEP_3_2_0_0_0
1891.7545 PEP_3_3_0_0_0

File: 231129_OE_RK_36, RT: 8.393, Scan: 004691
Type: HCD, FTMS + c NSI d Full ms2 1220.4563@hcd30.00 [120.0000-3756.7385]
Precursor Scan: 004666, m/z: 1219.7882, charge: 3
NEEYNK_6_5_0_3_0

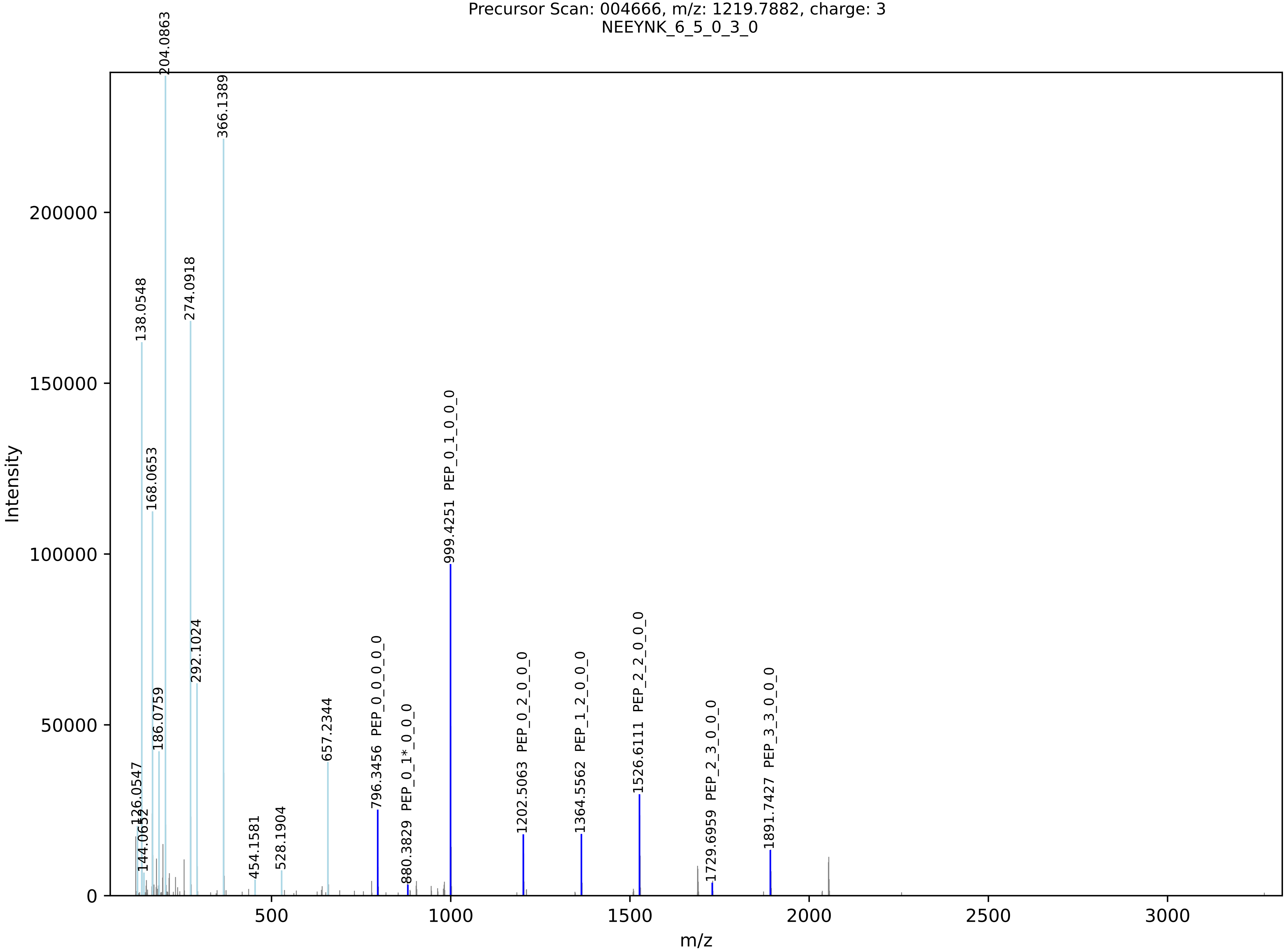

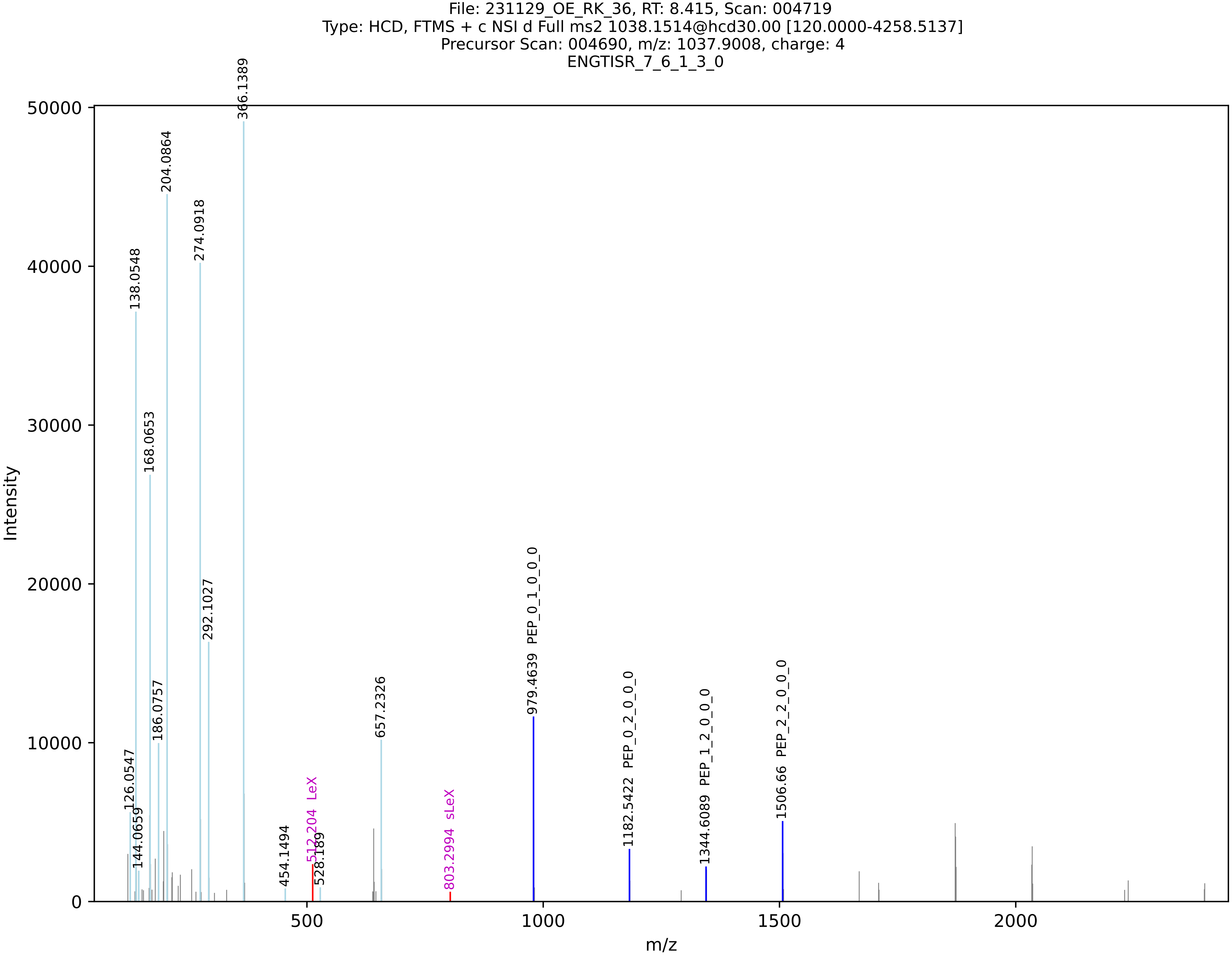
File: 231129_OE_RK_36, RT: 8.415, Scan: 004719
Type: HCD, FTMS + c NSI d Full ms2 1038.1514@hcd30.00 [120.0000-4258.5137]
Precursor Scan: 004690, m/z: 1037.9008, charge: 4
ENGTISR_7_6_1_3_0
Intensity
m/z
0
10000
20000
30000
40000
50000
500
1000
1500
2000
366.1389
204.0864
274.0918
138.0548
168.0653
292.1027
186.0757
126.0547
144.0659
454.1494
512.204 LeX
528.189
657.2326
803.2994 sLeX
979.4639 PEP_0_1_0_0_0
1182.5422 PEP_0_2_0_0_0
1344.6089 PEP_1_2_0_0_0
1506.66 PEP_2_2_0_0_0

File: 231129_OE_RK_36, RT: 8.437, Scan: 004745
Type: HCD, FTMS + c NSI d Full ms2 994.7263@hcd30.00 [120.0000-3066.0044]
Precursor Scan: 004718, m/z: 994.3931, charge: 3
ENGTISR_5_4_0_2_0

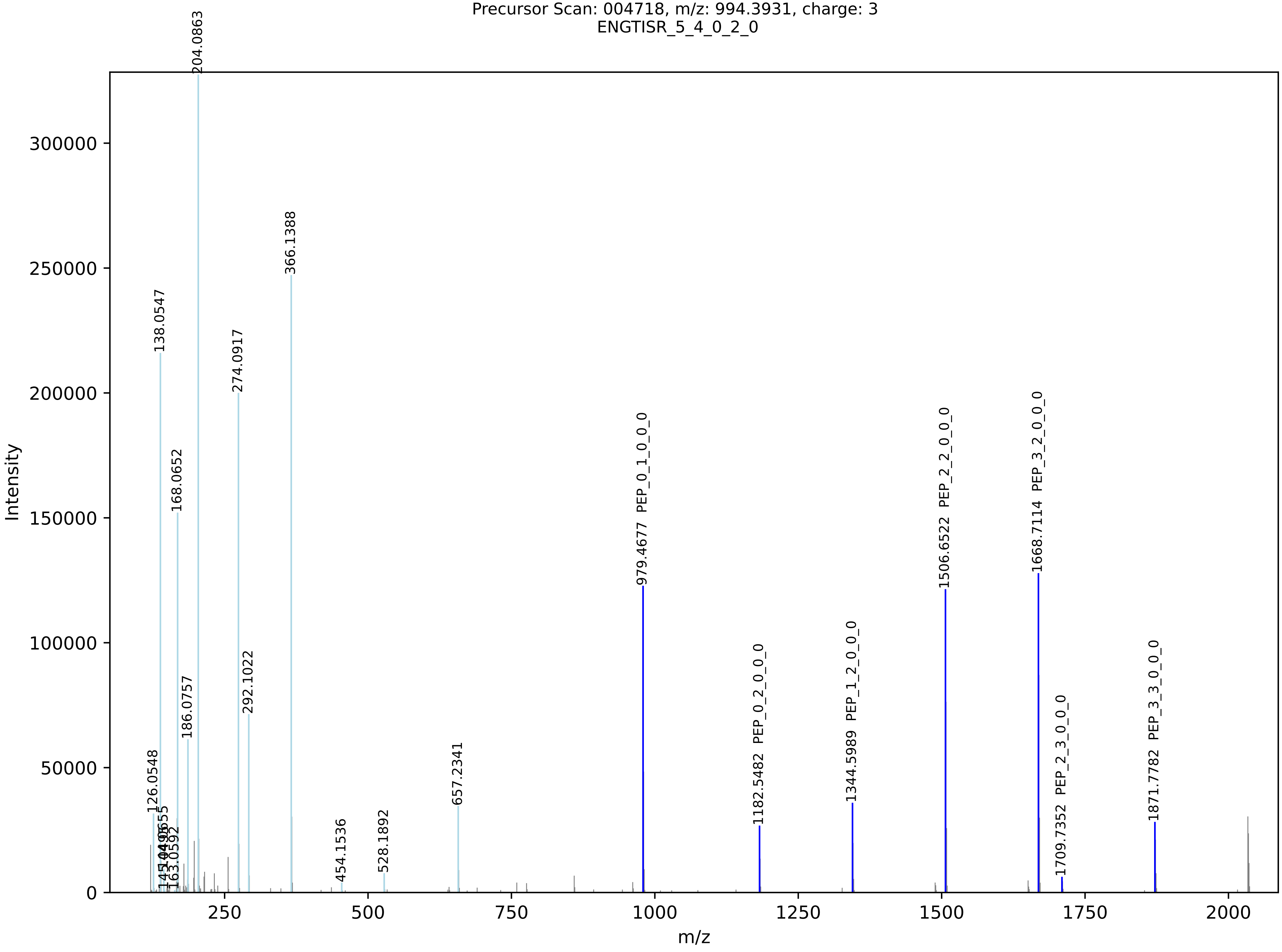

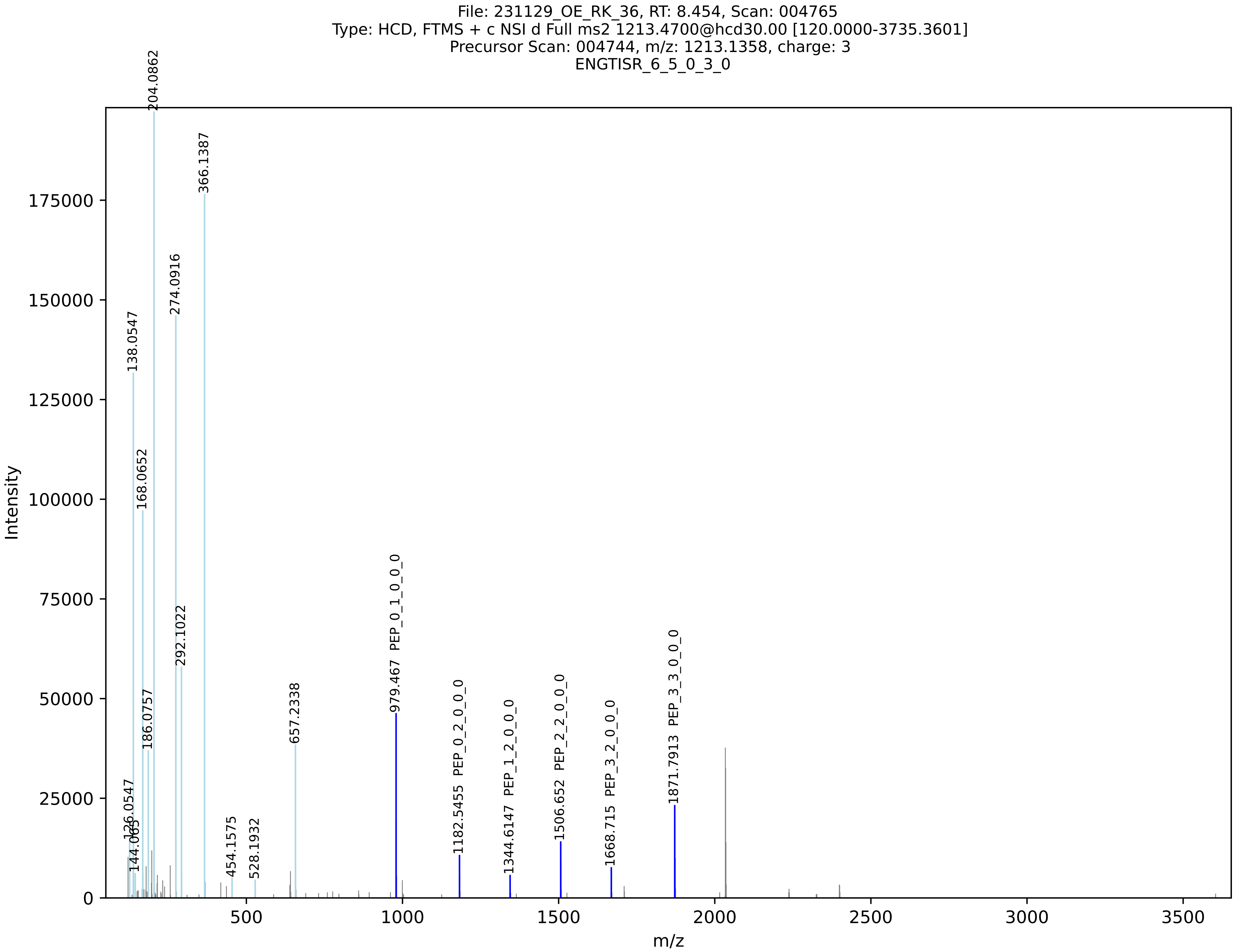
File: 231129_OE_RK_36, RT: 8.454, Scan: 004765
Type: HCD, FTMS + c NSI d Full ms2 1213.4700@hcd30.00 [120.0000-3735.3601]
Precursor Scan: 004744, m/z: 1213.1358, charge: 3
ENGTISR_6_5_0_3_0
Intensity
m/z
204.0862
366.1387
274.0916
138.0547
168.0652
292.1022
186.0757
126.0547
144.065
454.1575
528.1932
657.2338
979.467 PEP_0_1_0_0_
1182.5455 PEP_0_2_0_0_0
1344.6147 PEP_1_2_0_0_0
1506.652 PEP_2_2_0_0_0
1668.715 PEP_3_2_0_0_0
1871.7913 PEP_3_3_0_0_0

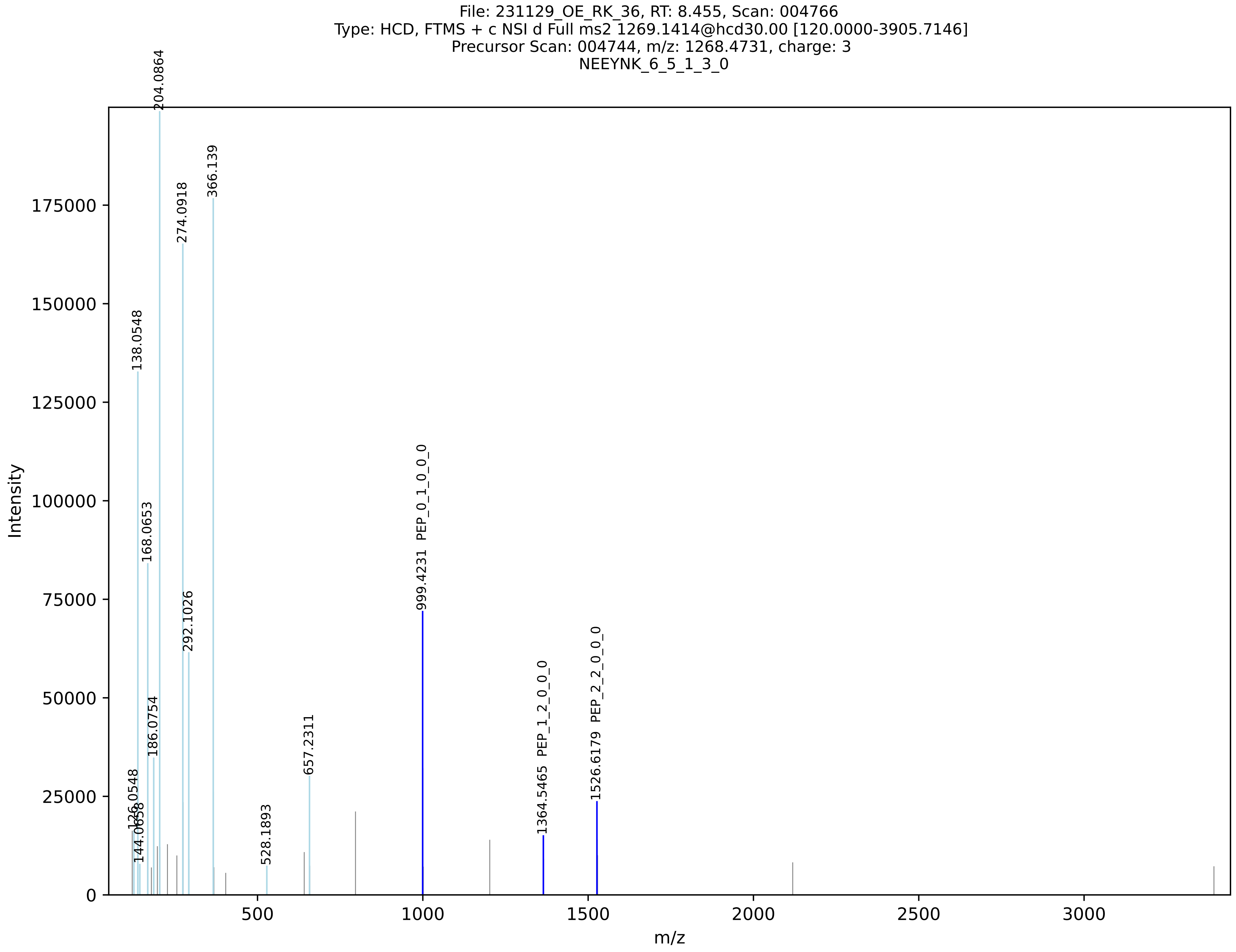
File: 231129_OE_RK_36, RT: 8.455, Scan: 004766
Type: HCD, FTMS + c NSI d Full ms2 1269.1414@hcd30.00 [120.0000-3905.7146]
Precursor Scan: 004744, m/z: 1268.4731, charge: 3
NEEYNK_6_5_1_3_0
Intensity
m/z
0
25000
50000
75000
100000
125000
150000
175000
500
1000
1500
2000
2500
3000
204.0864
366.139
274.0918
138.0548
168.0653
292.1026
186.0754
126.0548
144.0658
528.1893
657.2311
999.4231 PEP_0_1_0_0_0
1364.5465 PEP_1_2_0_0_0
1526.6179 PEP_2_2_0_0_0

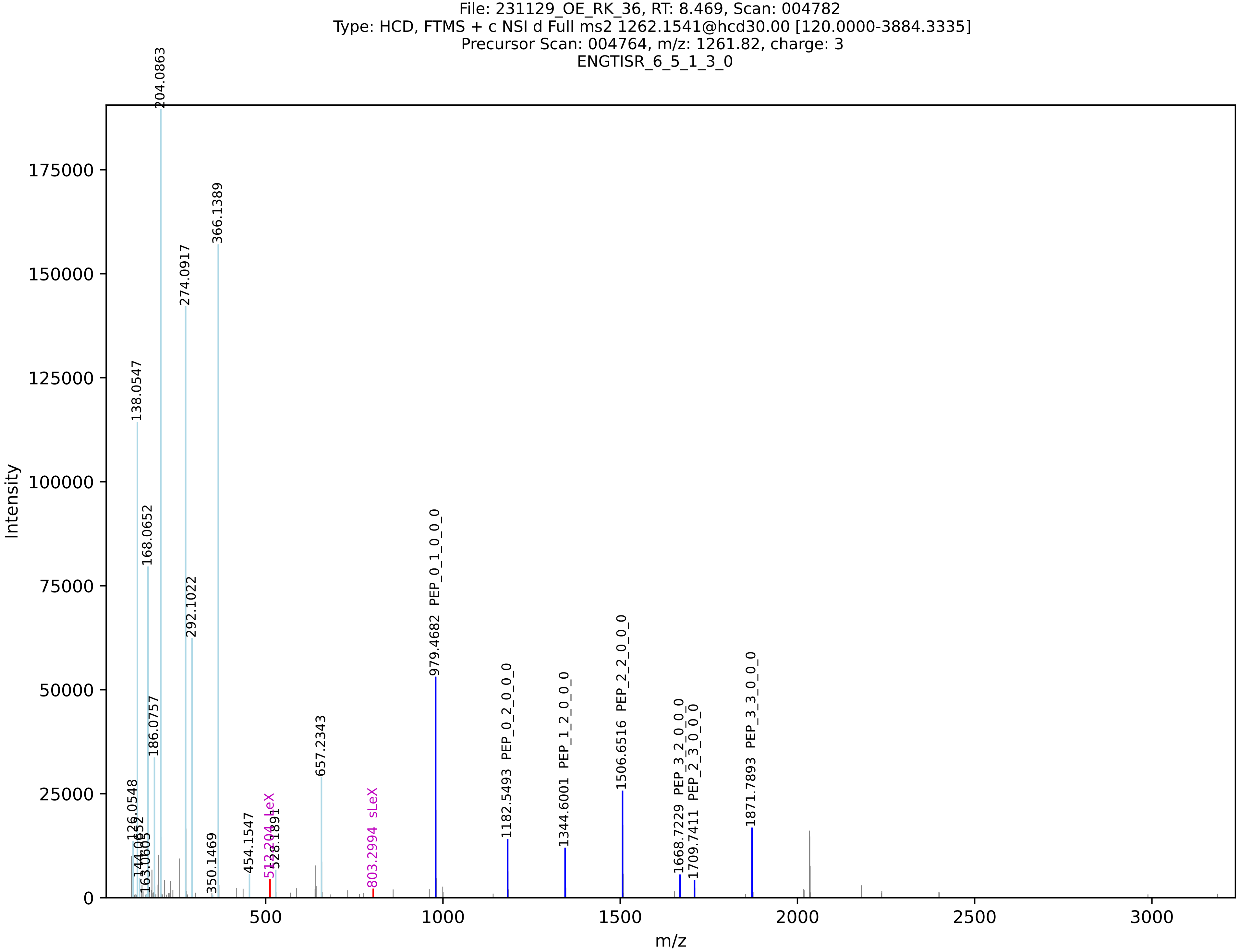
File: 231129_OE_RK_36, RT: 8.469, Scan: 004782
Type: HCD, FTMS + c NSI d Full ms2 1262.1541@hcd30.00 [120.0000-3884.3335]
Precursor Scan: 004764, m/z: 1261.82, charge: 3
ENGTISR_6_5_1_3_0
Intensity
m/z
204.0863
366.1389
274.0917
138.0547
168.0652
292.1022
186.0757
126.0548
350.1469
454.1547
657.2343
803.2994 sLeX
979.4682 PEP_0_1_0_0_0
1182.5493 PEP_0_2_0_0_0
1344.6001 PEP_1_2_0_0_0
1506.6516 PEP_2_2_0_0_0
1668.7229 PEP_3_2_0_0_0
1709.7411 PEP_2_3_0_0_0
1871.7893 PEP_3_3_0_0_0
0
25000
50000
75000
100000
125000
150000
175000
500
1000
1500
2000
2500
3000

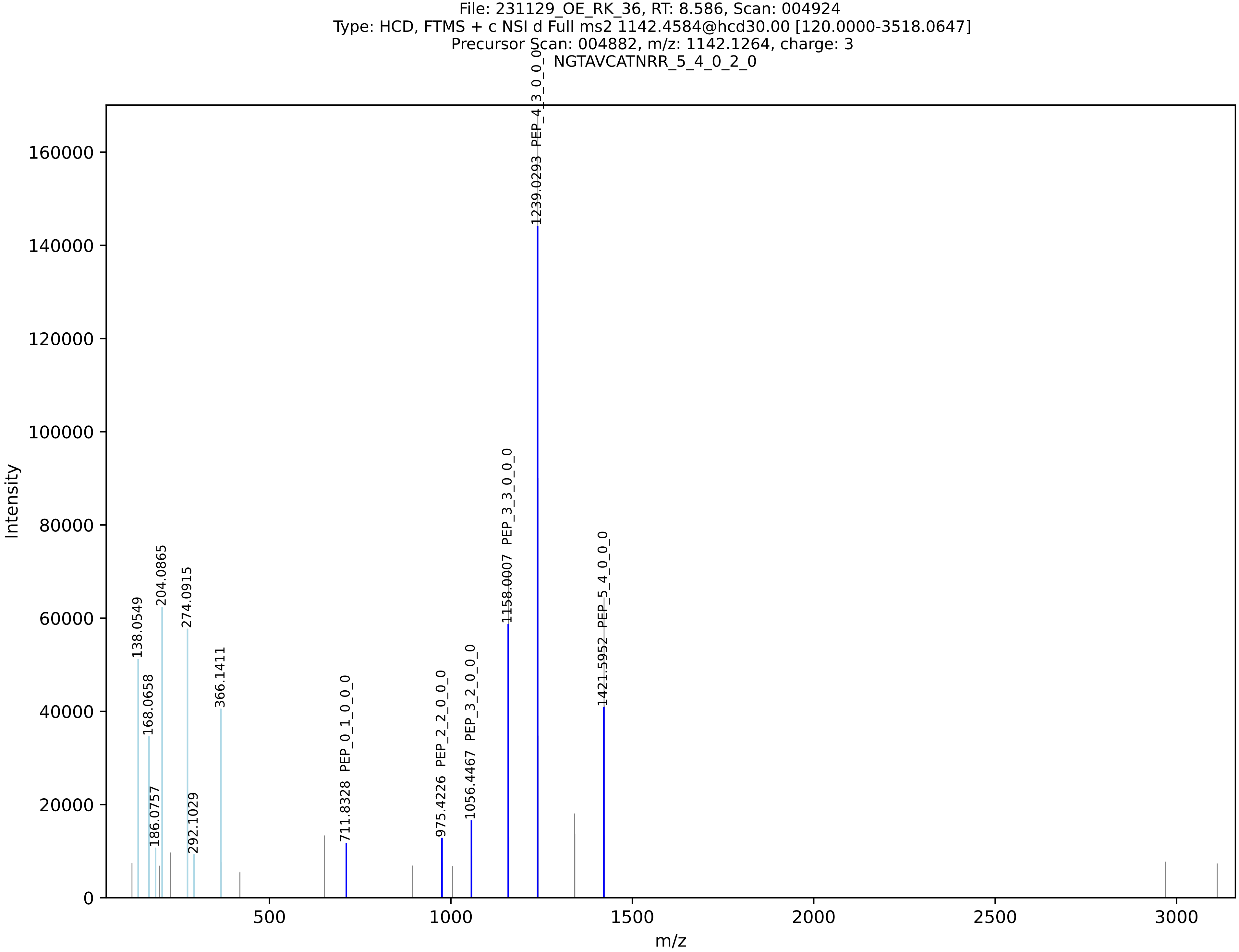

File: 231129_OE_RK_36, RT: 8.586, Scan: 004924
Type: HCD, FTMS + c NSI d Full ms2 1142.4584@hcd30.00 [120.0000-3518.0647]
Precursor Scan: 004882, m/z: 1142.1264, charge: 3
NGTAVCATNRR_5_4_0_2_0
Intensity
m/z
138.0549
168.0658
186.0757
204.0865
274.0915
292.1029
366.1411
711.8328 PEP_0_1_0_0_0
975.4226 PEP_2_2_0_0_0
1056.4467 PEP_3_2_0_0_0
1158.0007 PEP_3_3_0_0_0
1239.0293 PEP_4_3_0_0_0
1421.5952 PEP_5_4_0_0_0

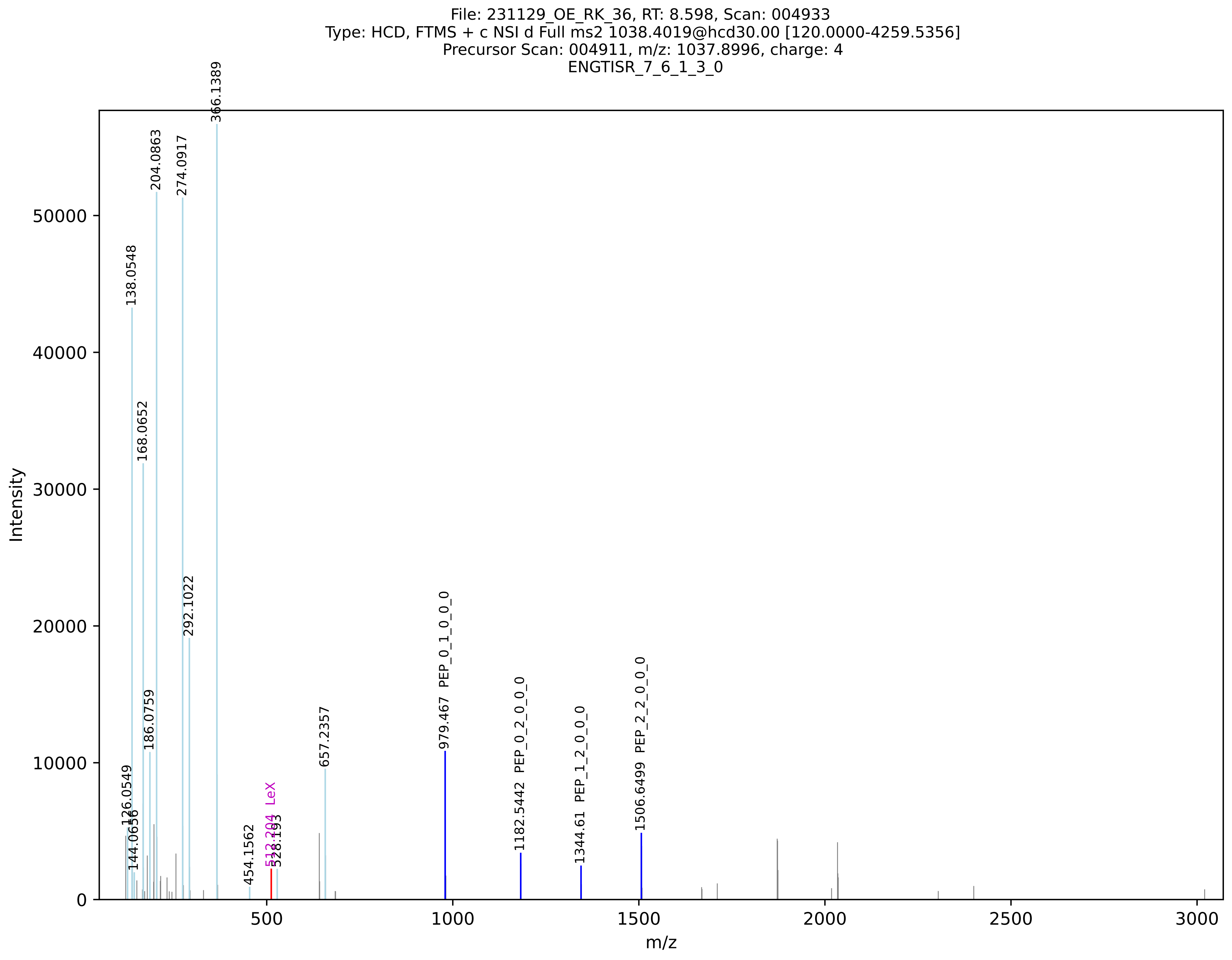
File: 231129_OE_RK_36, RT: 8.598, Scan: 004933
Type: HCD, FTMS + c NSI d Full ms2 1038.4019@hcd30.00 [120.0000-4259.5356]
Precursor Scan: 004911, m/z: 1037.8996, charge: 4
ENGTISR_7_6_1_3_0
Intensity
m/z
366.1389
204.0863
274.0917
138.0548
168.0652
292.1022
186.0759
126.0549
144.0656
454.1562
512.204 LeX
528.193
657.2357
979.467 PEP_0_1_0_0_0
1182.5442 PEP_0_2_0_0_0
1344.61 PEP_1_2_0_0_0
1506.6499 PEP_2_2_0_0_0
0
10000
20000
30000
40000
50000
500
1000
1500
2000
2500
3000

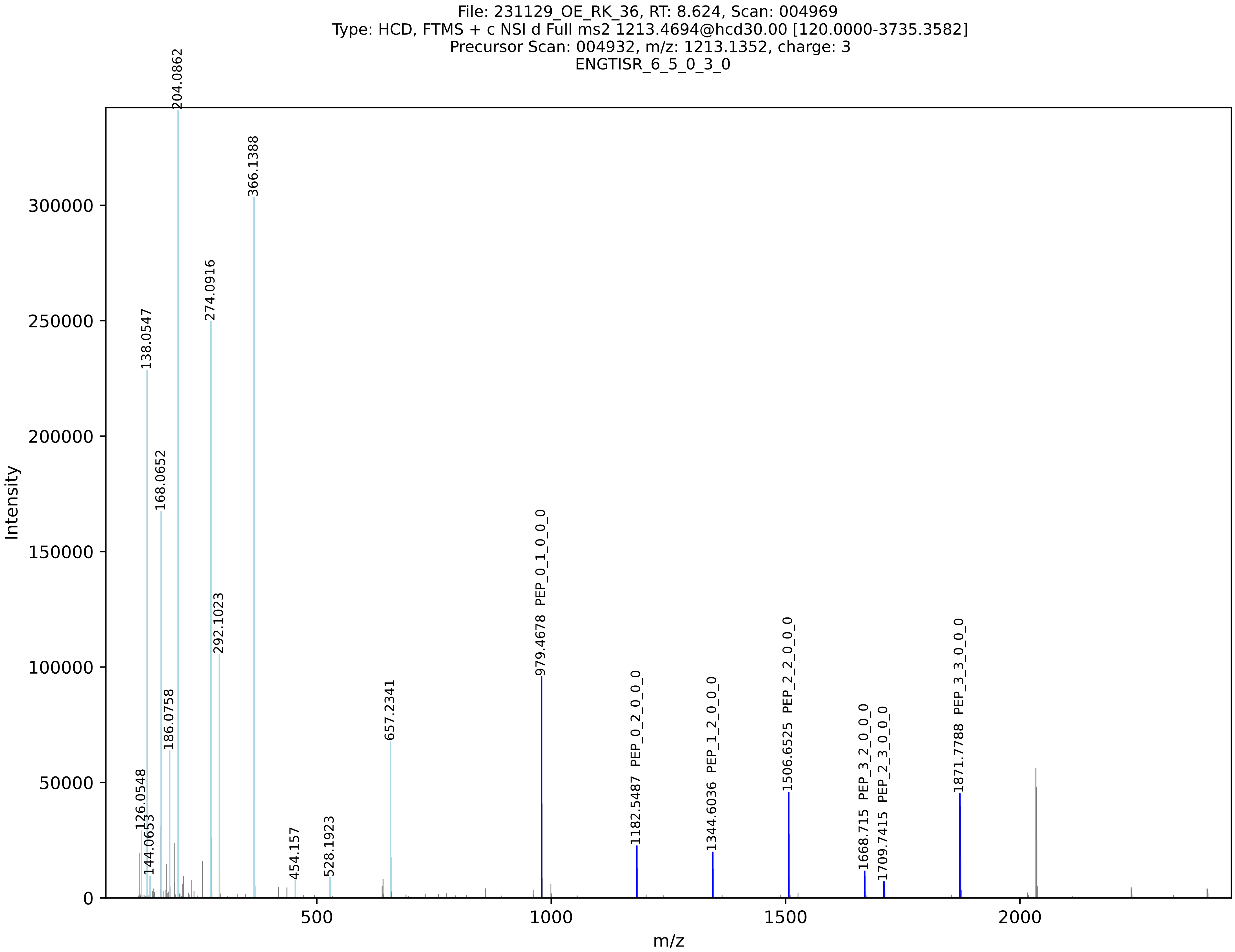
File: 231129_OE_RK_36, RT: 8.624, Scan: 004969
Type: HCD, FTMS + c NSI d Full ms2 1213.4694@hcd30.00 [120.0000-3735.3582]
Precursor Scan: 004932, m/z: 1213.1352, charge: 3
ENGTISR_6_5_0_3_0
Intensity
m/z
204.0862
366.1388
274.0916
138.0547
168.0652
292.1023
186.0758
126.0548
144.0653
454.157
528.1923
657.2341
979.4678 PEP_0_1_0_0_0_
1182.5487 PEP_0_2_0_0_0_
1344.6036 PEP_1_2_0_0_0_
1506.6525 PEP_2_2_0_0_0_
1668.715 PEP_3_2_0_0_0_
1709.7415 PEP_2_3_0_0_0_
1871.7788 PEP_3_3_0_0_0_

File: 231129_OE_RK_36, RT: 8.625, Scan: 004970
Type: HCD, FTMS + c NSI d Full ms2 994.7264@hcd30.00 [120.0000-3066.0049]
Precursor Scan: 004932, m/z: 994.3923, charge: 3
ENGTISR_5_4_0_2_0

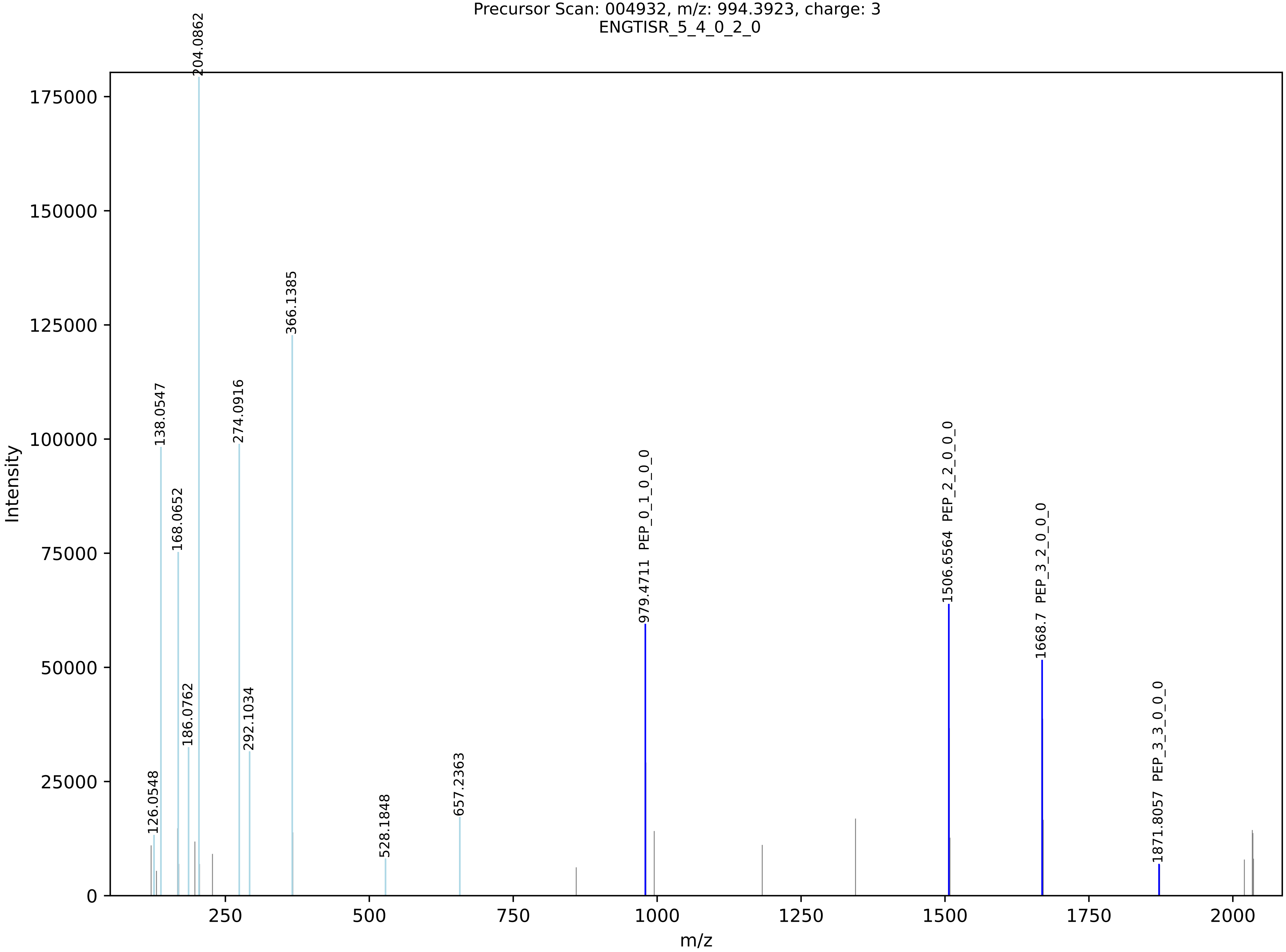

File: 231129_OE_RK_36, RT: 8.625, Scan: 004971
Type: HCD, FTMS + c NSI d Full ms2 1269.1412@hcd30.00 [120.0000-3905.7141]
Precursor Scan: 004932, m/z: 1268.473, charge: 3
NEEYNK_6_5_1_3_0

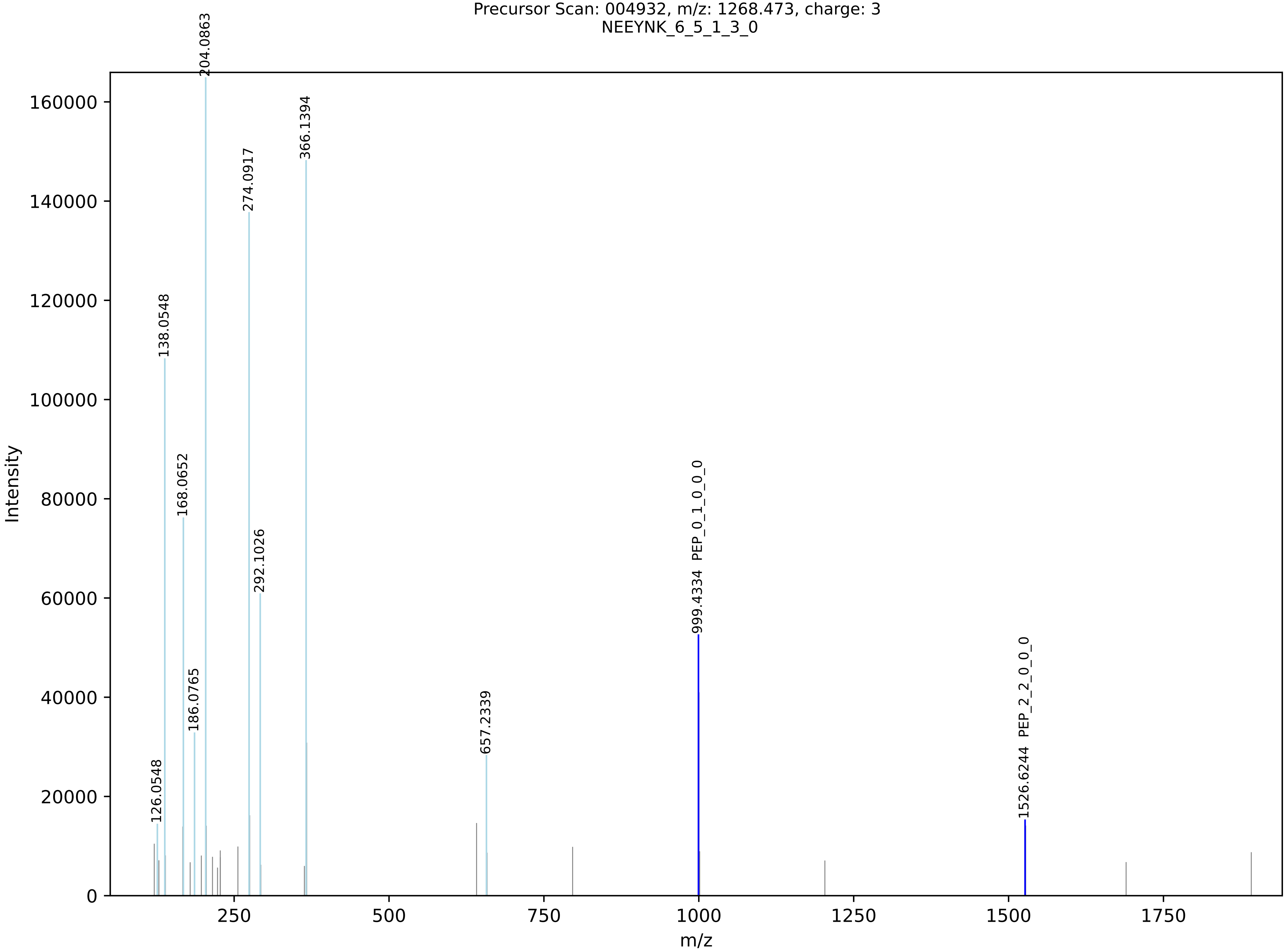

File: 231129_OE_RK_36, RT: 8.652, Scan: 005006
Type: HCD, FTMS + c NSI d Full ms2 1262.4886@hcd30.00 [120.0000-3885.3572]
Precursor Scan: 004968, m/z: 1261.8204, charge: 3
ENGTISR_6_5_1_3_0

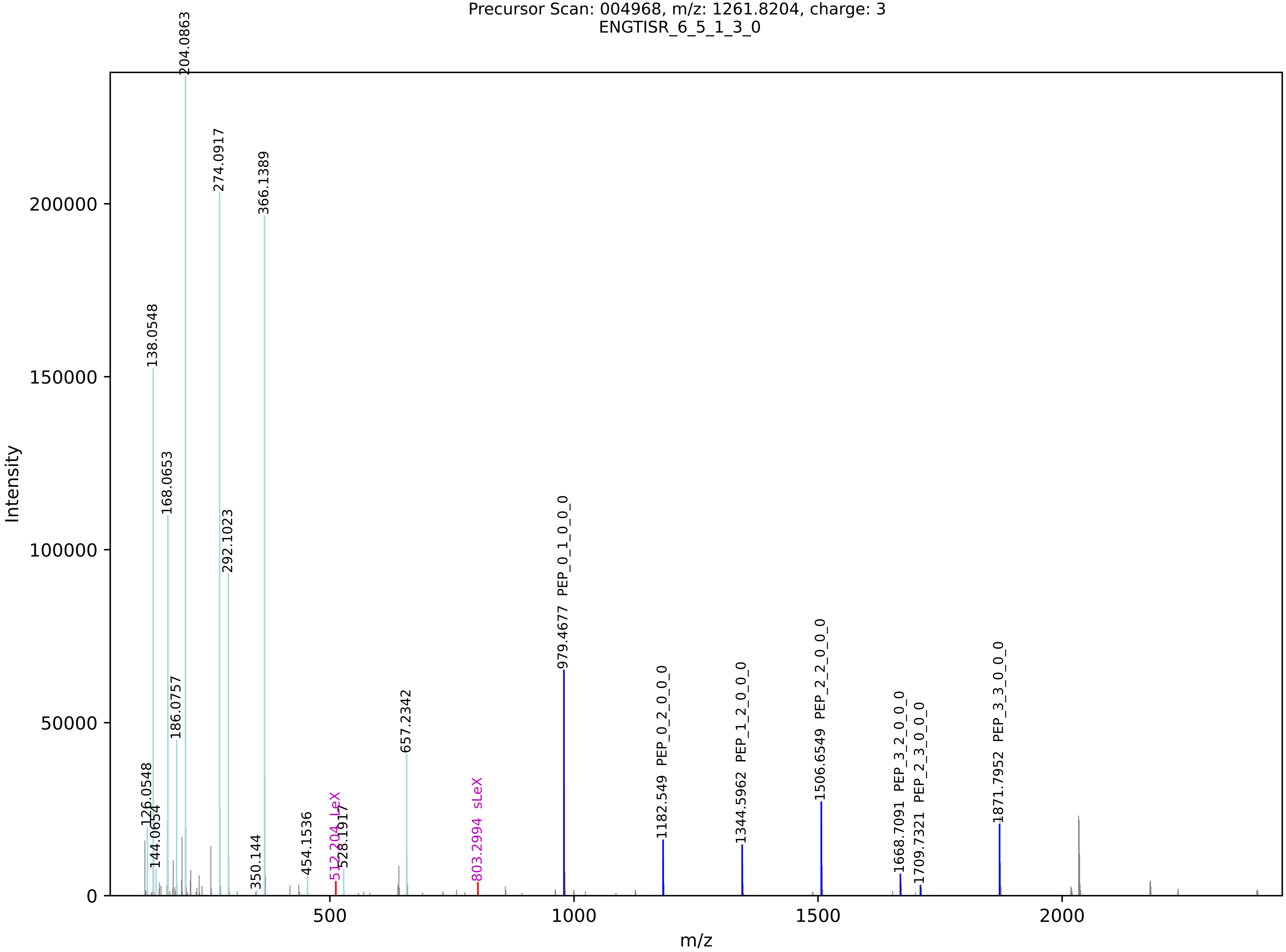

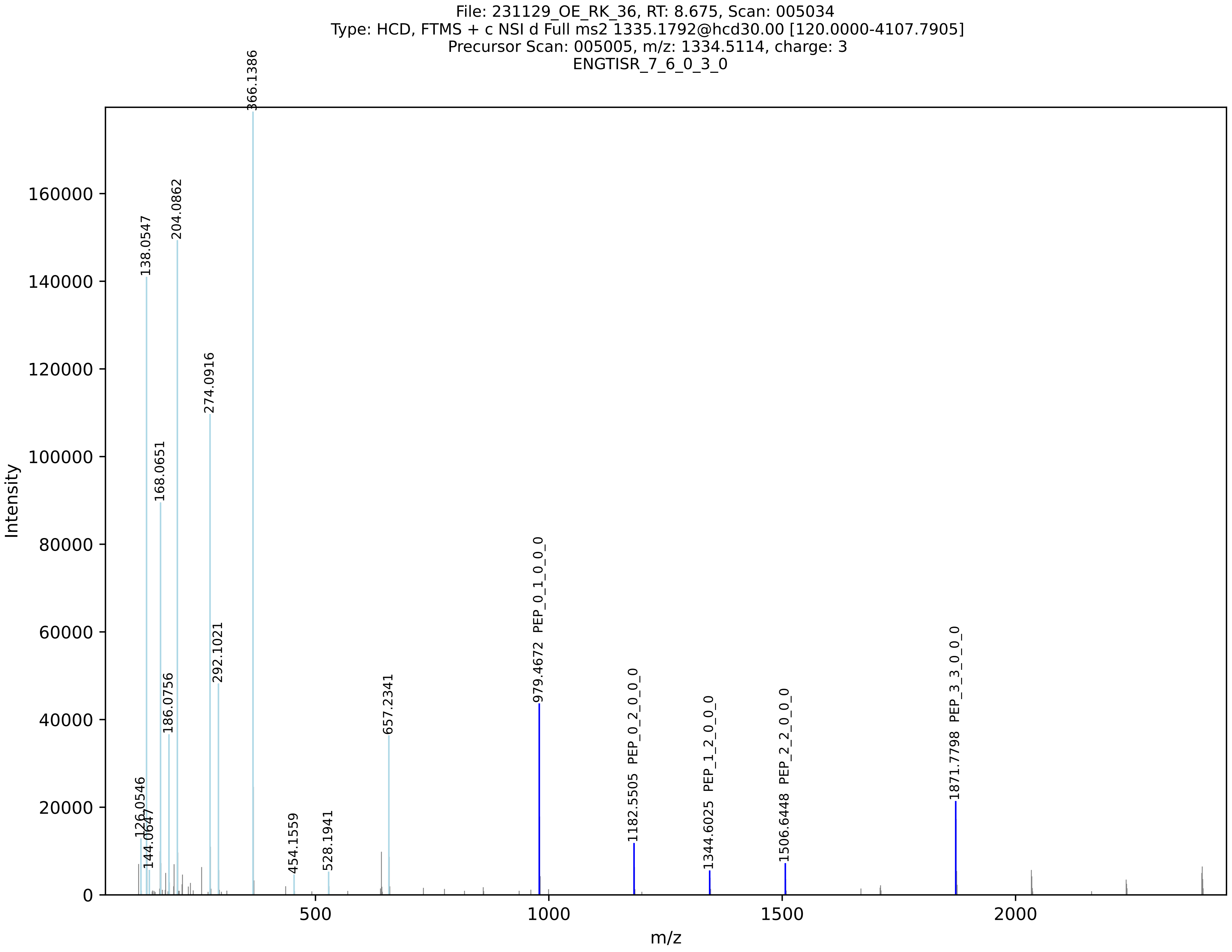
File: 231129_OE_RK_36, RT: 8.675, Scan: 005034
Type: HCD, FTMS + c NSI d Full ms2 1335.1792@hcd30.00 [120.0000-4107.7905]
Precursor Scan: 005005, m/z: 1334.5114, charge: 3
ENGTISR_7_6_0_3_0
Intensity
m/z
366.1386
204.0862
138.0547
274.0916
168.0651
292.1021
186.0756
126.0546
144.0647
454.1559
528.1941
657.2341
979.4672 PEP_0_1_0_0_0
1182.5505 PEP_0_2_0_0_0
1344.6025 PEP_1_2_0_0_0
1506.6448 PEP_2_2_0_0_0
1871.7798 PEP_3_3_0_0_0
0
20000
40000
60000
80000
100000
120000
140000
160000
500
1000
1500
2000

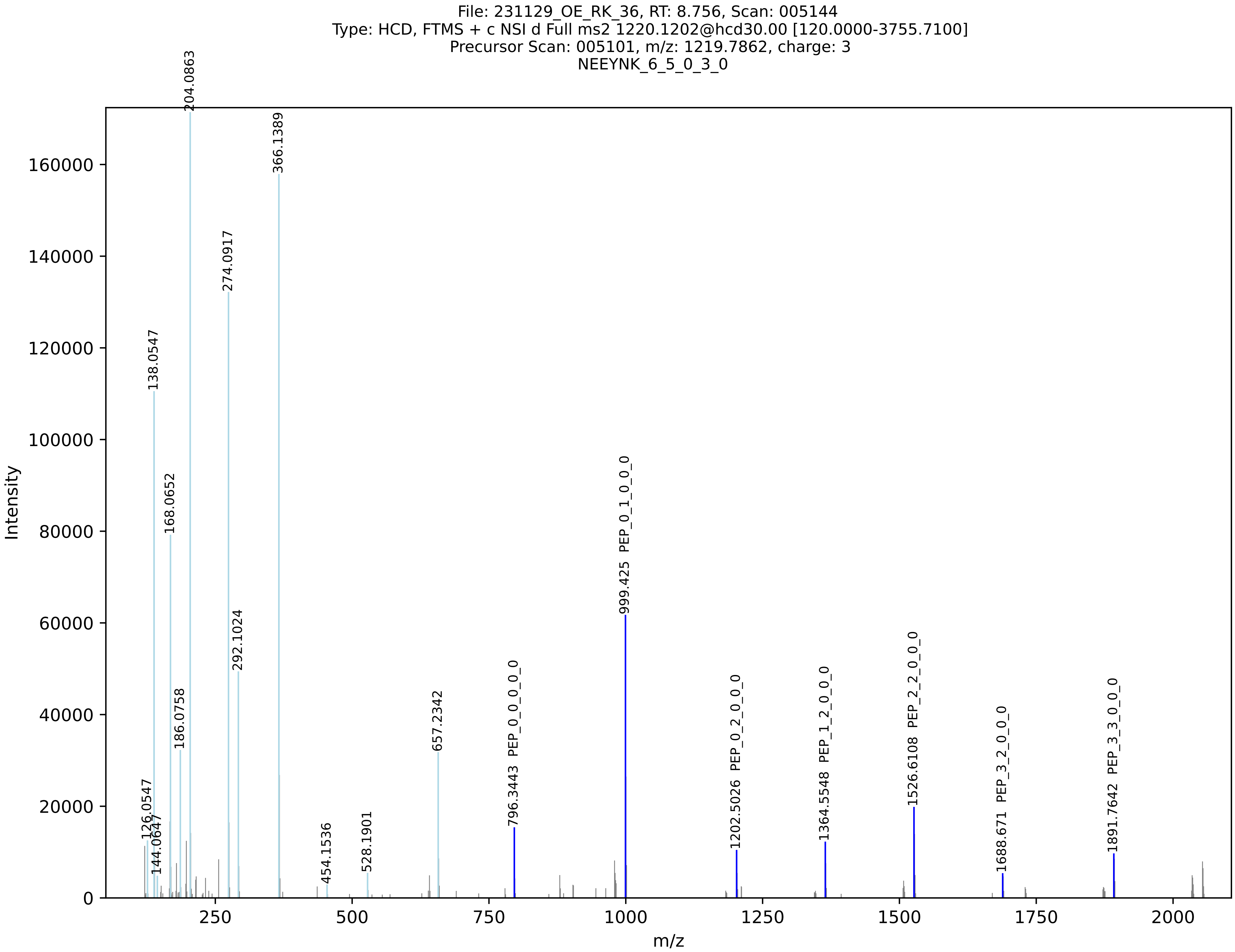

File: 231129_OE_RK_36, RT: 8.756, Scan: 005144
Type: HCD, FTMS + c NSI d Full ms2 1220.1202@hcd30.00 [120.0000-3755.7100]
Precursor Scan: 005101, m/z: 1219.7862, charge: 3
NEEYNK_6_5_0_3_0
Intensity
m/z
204.0863
366.1389
274.0917
138.0547
168.0652
292.1024
186.0758
126.0547
144.0647
454.1536
528.1901
657.2342
796.3443 PEP_0_0_0_0_0
999.425 PEP_0_1_0_0_0
1202.5026 PEP_0_2_0_0_0
1364.5548 PEP_1_2_0_0_0
1526.6108 PEP_2_2_0_0_0
1688.671 PEP_3_2_0_0_0
1891.7642 PEP_3_3_0_0_0

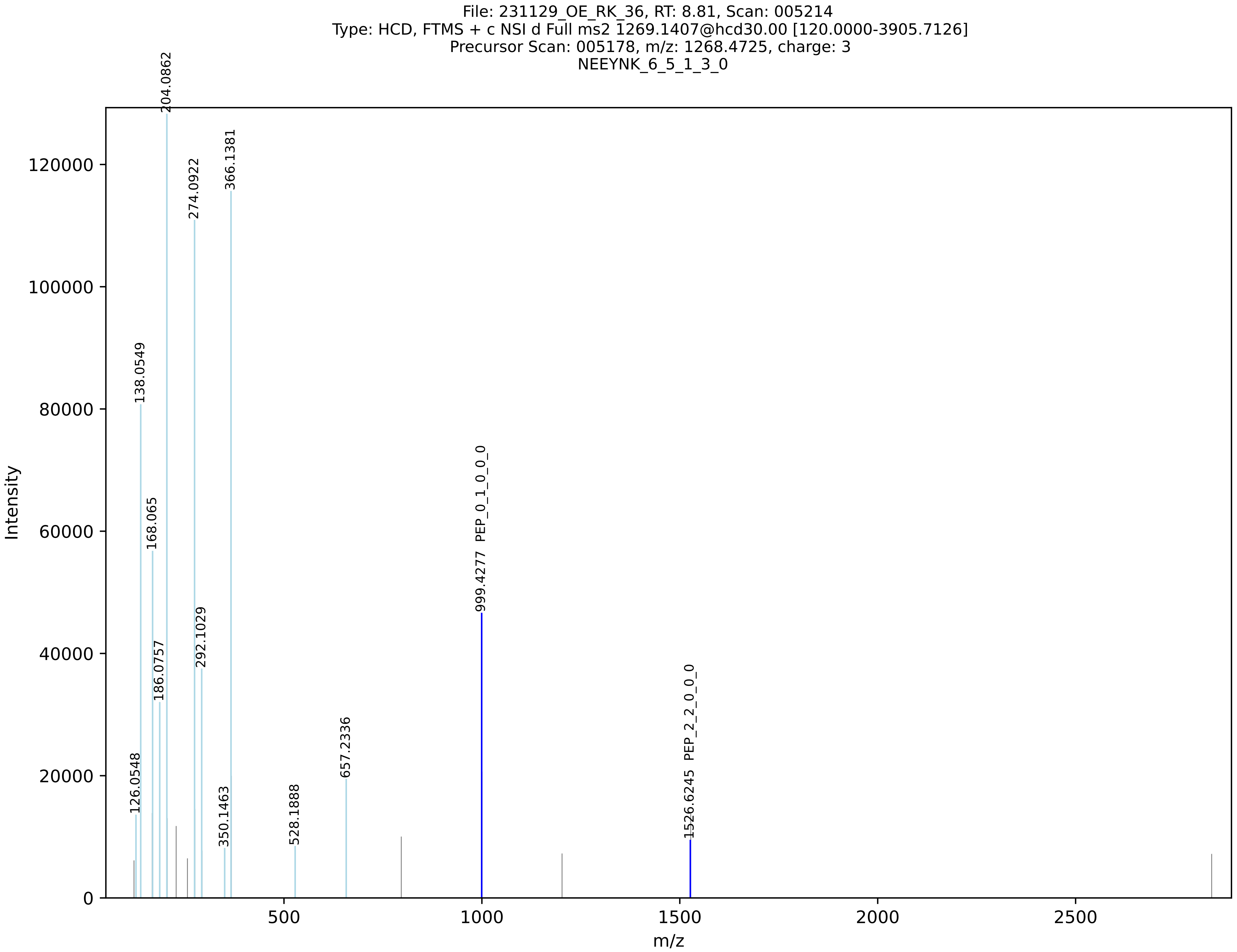

File: 231129_OE_RK_36, RT: 8.81, Scan: 005214
Type: HCD, FTMS + c NSI d Full ms2 1269.1407@hcd30.00 [120.0000-3905.7126]
Precursor Scan: 005178, m/z: 1268.4725, charge: 3
NEEYNK_6_5_1_3_0
Intensity
m/z
204.0862
366.1381
274.0922
138.0549
168.065
186.0757
292.1029
126.0548
350.1463
528.1888
657.2336
999.4277 PEP_0_1_0_0_0
1526.6245 PEP_2_2_0_0_0

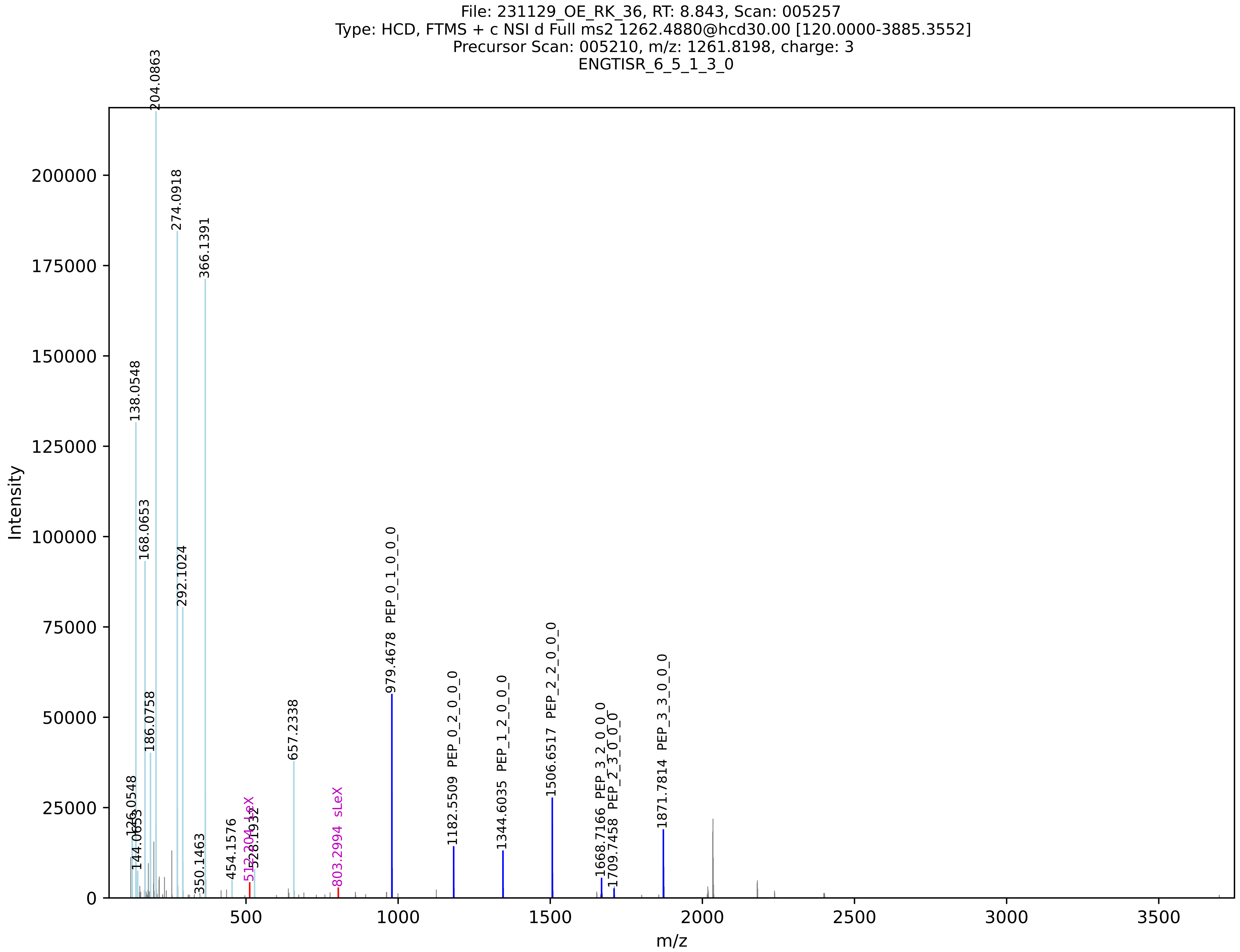
File: 231129_OE_RK_36, RT: 8.843, Scan: 005257
Type: HCD, FTMS + c NSI d Full ms2 1262.4880@hcd30.00 [120.0000-3885.3552]
Precursor Scan: 005210, m/z: 1261.8198, charge: 3
ENGTISR_6_5_1_3_0
Intensity
m/z
204.0863
274.0918
366.1391
138.0548
168.0653
292.1024
186.0758
126.0548
144.0653
350.1463
454.1576
512.204 LeX
528.1932
657.2338
803.2994 sLeX
979.4678 PEP_0_1_0_0_0
1182.5509 PEP_0_2_0_0_0
1344.6035 PEP_1_2_0_0_0
1506.6517 PEP_2_2_0_0_0
1668.7166 PEP_3_2_0_0_0
1709.7458 PEP_2_3_0_0_0
1871.7814 PEP_3_3_0_0_0

File: 231129_OE_RK_36, RT: 8.844, Scan: 005259
Type: HCD, FTMS + c NSI d Full ms2 1219.4779@hcd30.00 [120.0000-3753.7444]
Precursor Scan: 005210, m/z: 1218.8095, charge: 3
NEEYNK_6_5_0_3_0

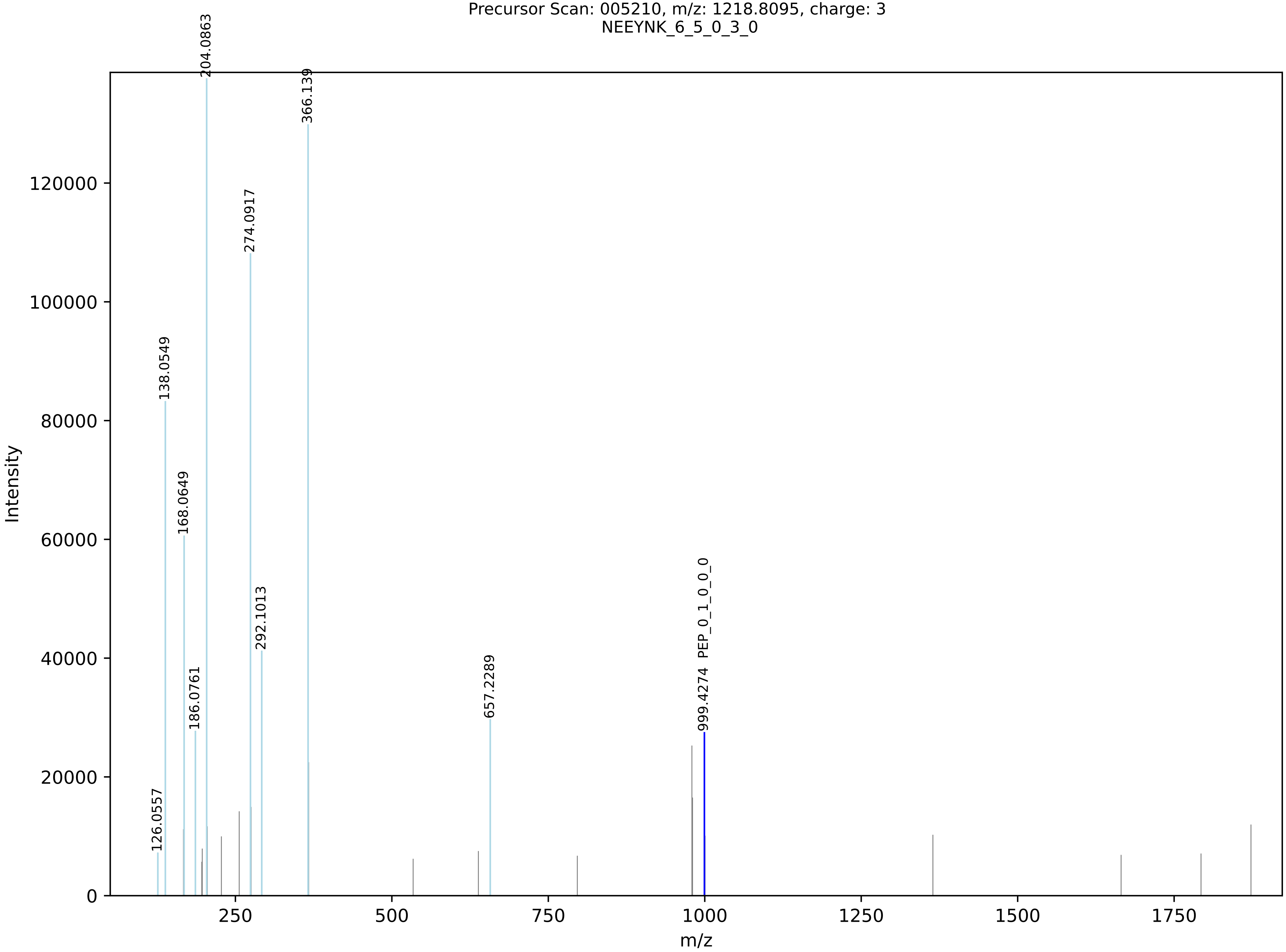

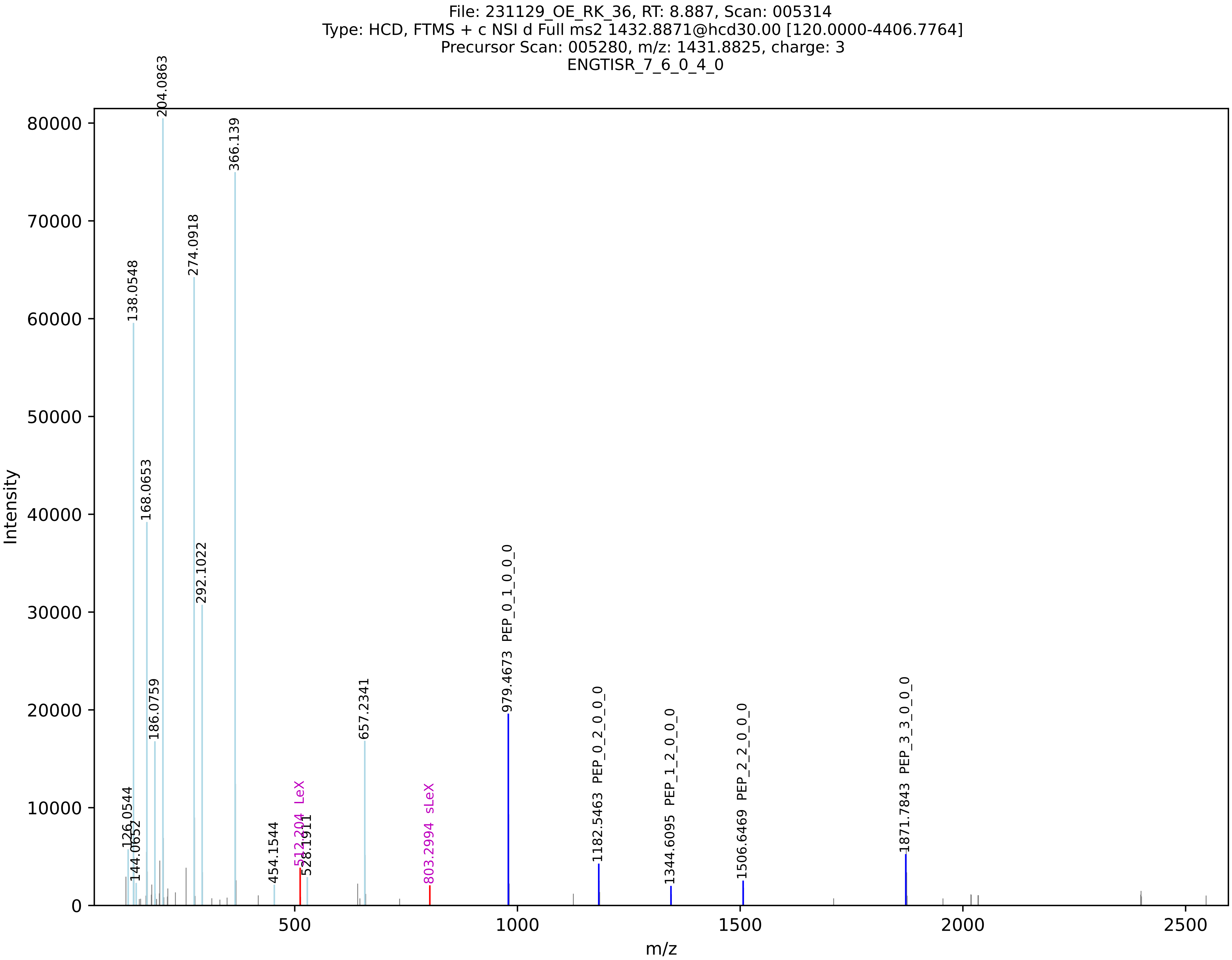
File: 231129_OE_RK_36, RT: 8.887, Scan: 005314
Type: HCD, FTMS + c NSI d Full ms2 1432.8871@hcd30.00 [120.0000-4406.7764]
Precursor Scan: 005280, m/z: 1431.8825, charge: 3
ENGTISR_7_6_0_4_0
Intensity
m/z
204.0863
366.139
274.0918
138.0548
168.0653
292.1022
186.0759
126.0544
144.0652
454.1544
512.204 LeX
528.1911
657.2341
803.2994 sLeX
979.4673 PEP_0_1_0_0_0
1182.5463 PEP_0_2_0_0_0
1344.6095 PEP_1_2_0_0_0
1506.6469 PEP_2_2_0_0_0
1871.7843 PEP_3_3_0_0_0

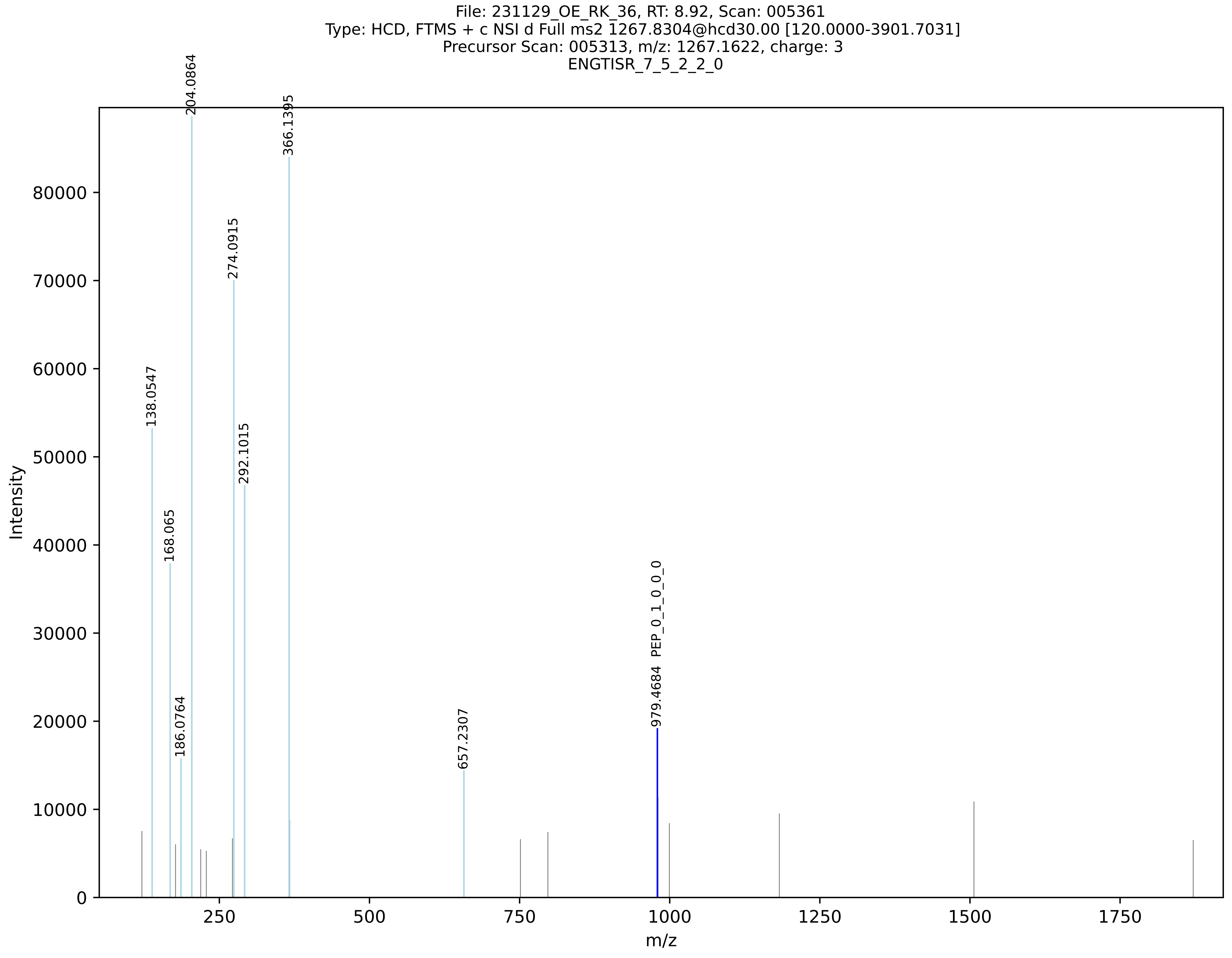
File: 231129_OE_RK_36, RT: 8.92, Scan: 005361
Type: HCD, FTMS + c NSI d Full ms2 1267.8304@hcd30.00 [120.0000-3901.7031]
Precursor Scan: 005313, m/z: 1267.1622, charge: 3
ENGTISR_7_5_2_2_0
204.0864
366.1395
274.0915
138.0547
292.1015
168.065
PEP_0_1_0_0_0
979.4684
186.0764
657.2307
Intensity
m/z
0
10000
20000
30000
40000
50000
60000
70000
80000
250
500
750
1000
1250
1500
1750

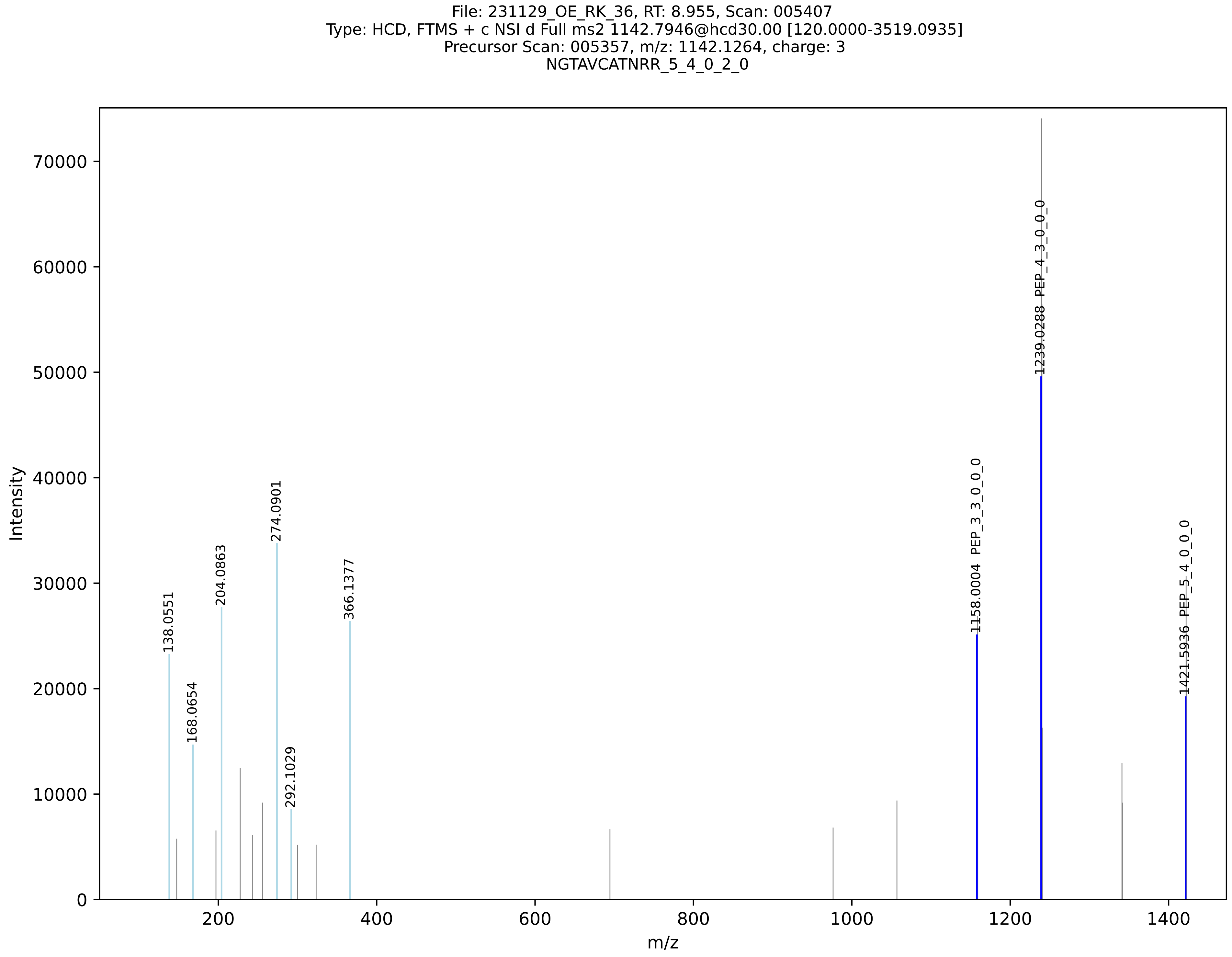
File: 231129_OE_RK_36, RT: 8.955, Scan: 005407
Type: HCD, FTMS + c NSI d Full ms2 1142.7946@hcd30.00 [120.0000-3519.0935]
Precursor Scan: 005357, m/z: 1142.1264, charge: 3
NGTAVCATNRR_5_4_0_2_0
Intensity
m/z
138.0551
168.0654
204.0863
274.0901
292.1029
366.1377
1158.0004 PEP_3_3_0_0_0
1239.0288 PEP_4_3_0_0_0
1421.5936 PEP_5_4_0_0_0
0
10000
20000
30000
40000
50000
60000
70000
200
400
600
800
1000
1200
1400

File: 231129_OE_RK_36, RT: 8.977, Scan: 005435
Type: HCD, FTMS + c NSI d Full ms2 1038.4030@hcd30.00 [120.0000-4259.5400]
Precursor Scan: 005405, m/z: 1037.9017, charge: 4
ENGTISR_7_6_1_3_0

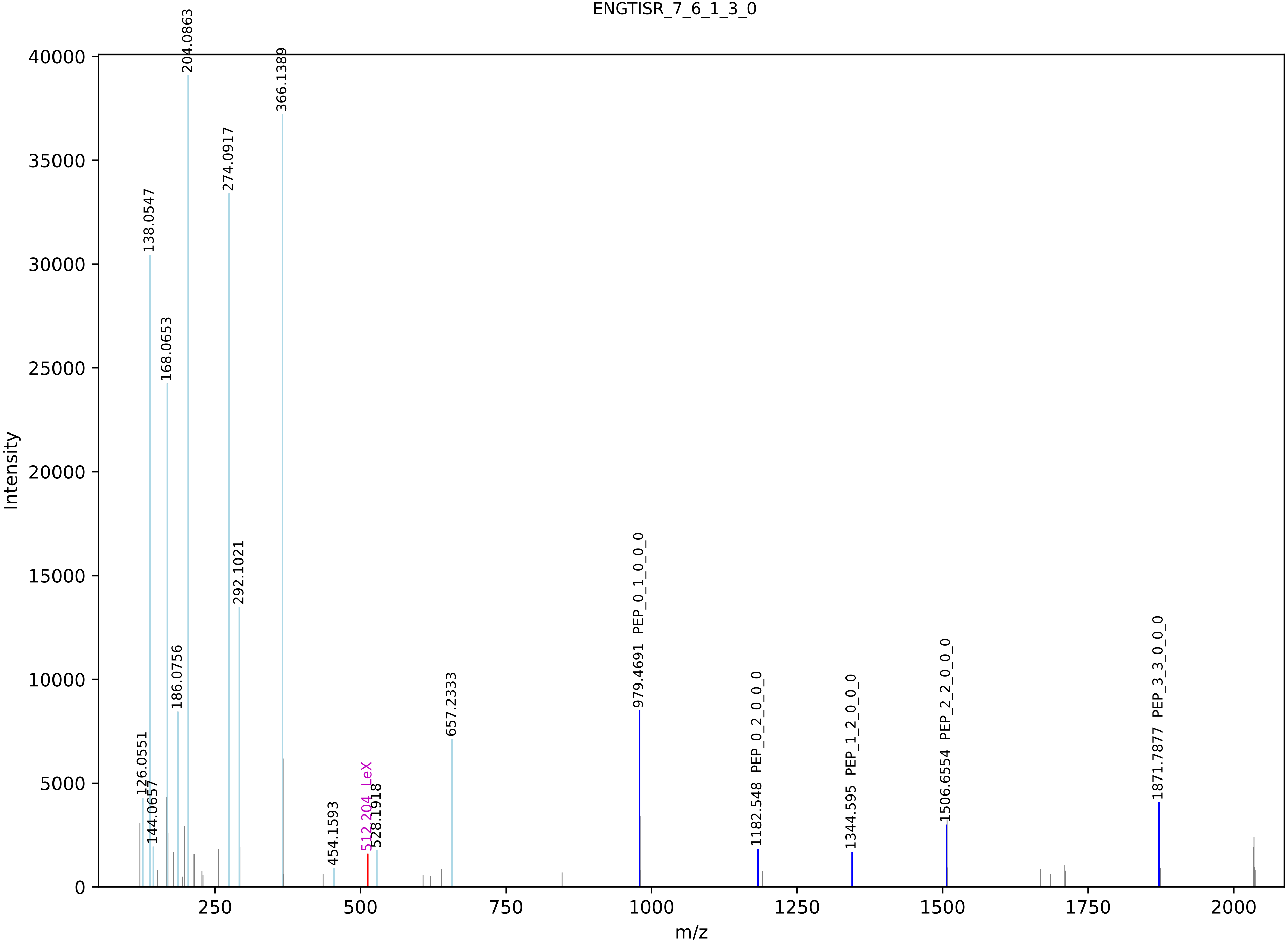

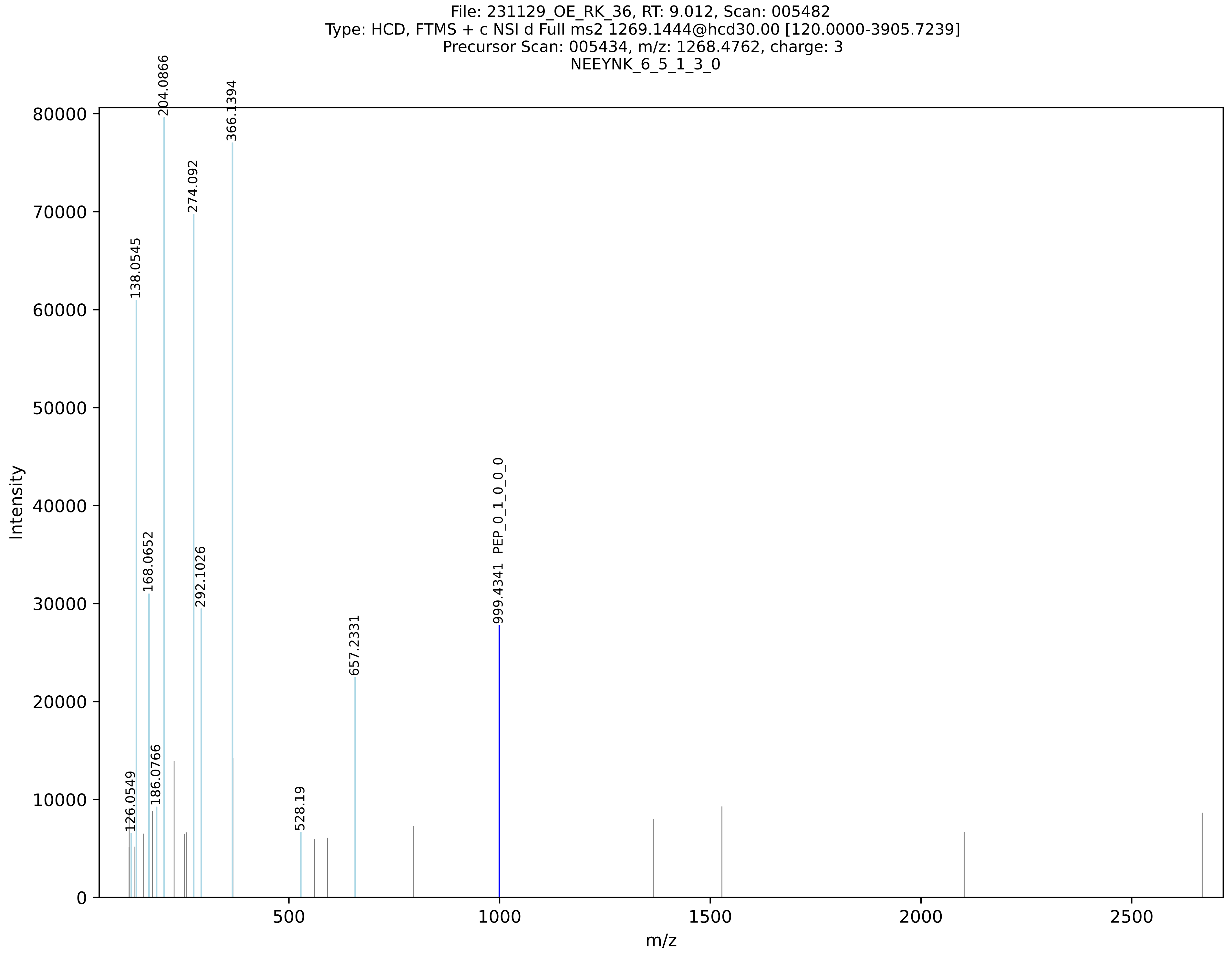
File: 231129_OE_RK_36, RT: 9.012, Scan: 005482
Type: HCD, FTMS + c NSI d Full ms2 1269.1444@hcd30.00 [120.0000-3905.7239]
Precursor Scan: 005434, m/z: 1268.4762, charge: 3
NEEYNK_6_5_1_3_0
Intensity
m/z
204.0866
366.1394
274.092
138.0545
168.0652
292.1026
657.2331
999.4341 PEP_0_1_0_0_0
126.0549
186.0766
528.19
0
10000
20000
30000
40000
50000
60000
70000
80000
500
1000
1500
2000
2500

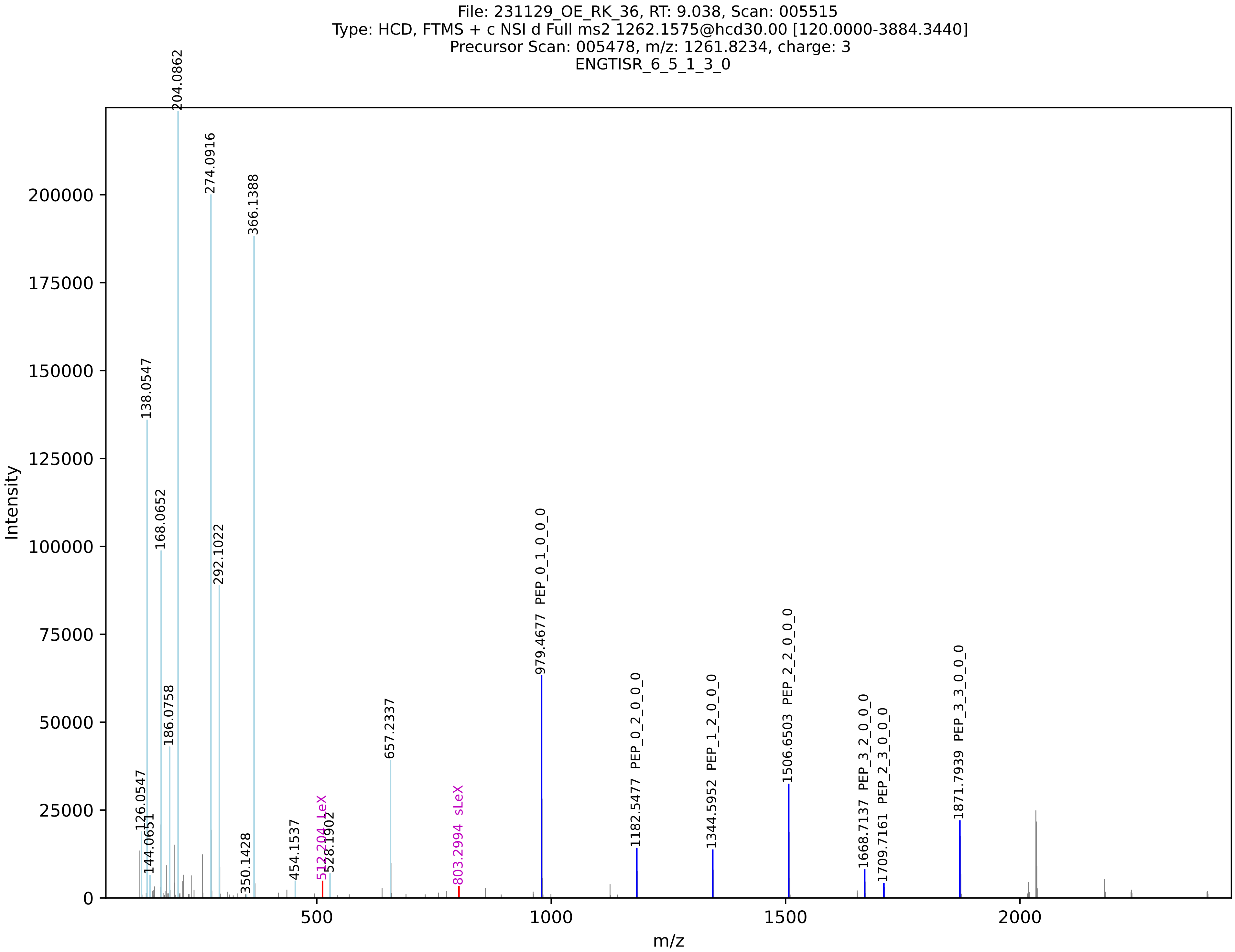
File: 231129_OE_RK_36, RT: 9.038, Scan: 005515
Type: HCD, FTMS + c NSI d Full ms2 1262.1575@hcd30.00 [120.0000-3884.3440]
Precursor Scan: 005478, m/z: 1261.8234, charge: 3
ENGTISR_6_5_1_3_0
Intensity
m/z
204.0862
274.0916
366.1388
138.0547
168.0652
292.1022
186.0758
126.0547
144.0651
350.1428
454.1537
512.204 LeX
528.1902
657.2337
803.2994 sLeX
979.4677 PEP_0_1_0_0_0
1182.5477 PEP_0_2_0_0_0
1344.5952 PEP_1_2_0_0_0
1506.6503 PEP_2_2_0_0_0
1668.7137 PEP_3_2_0_0_0
1709.7161 PEP_2_3_0_0_0
1871.7939 PEP_3_3_0_0_0

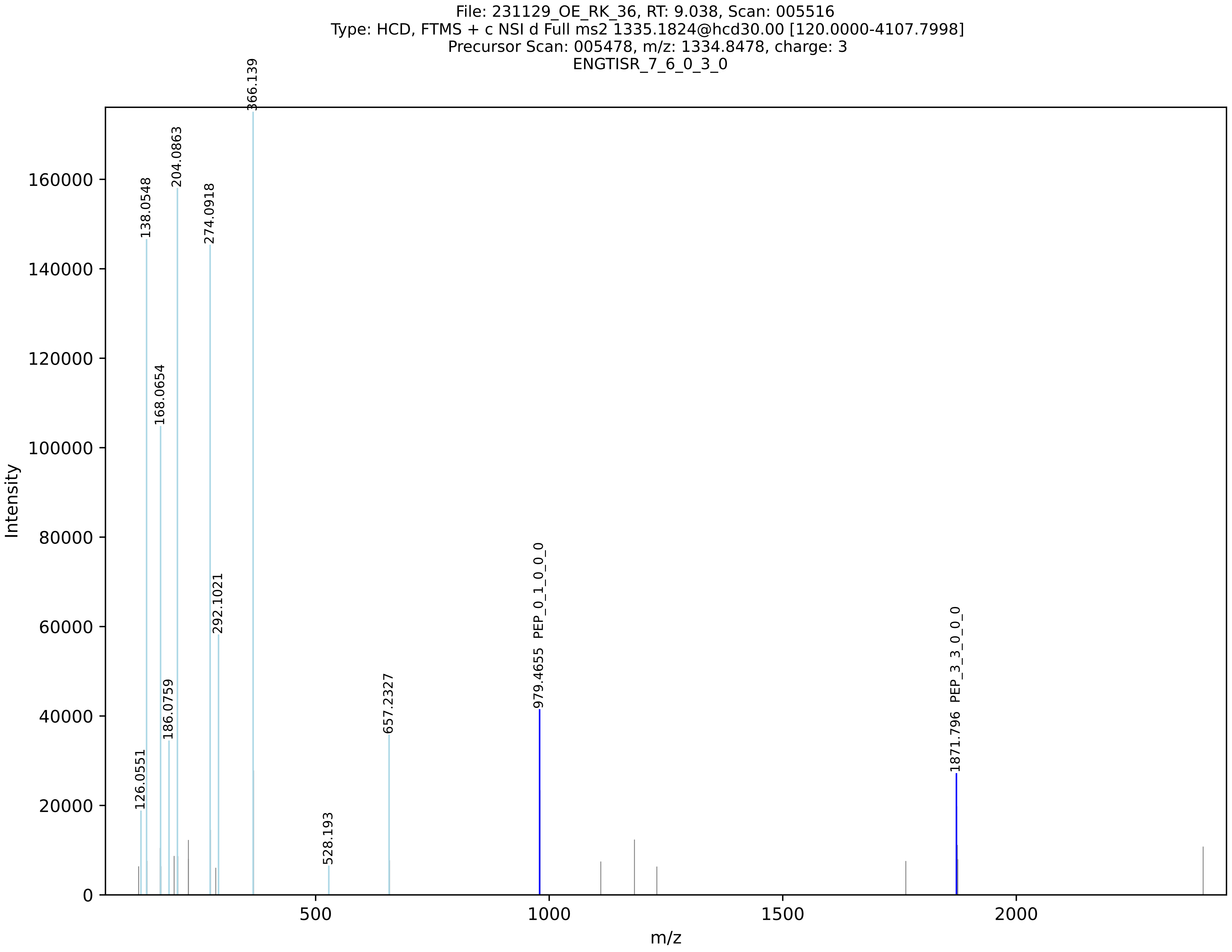

File: 231129_OE_RK_36, RT: 9.038, Scan: 005516
Type: HCD, FTMS + c NSI d Full ms2 1335.1824@hcd30.00 [120.0000-4107.7998]
Precursor Scan: 005478, m/z: 1334.8478, charge: 3
ENGTISR_7_6_0_3_0
Intensity
m/z
366.139
204.0863
138.0548
274.0918
168.0654
292.1021
186.0759
126.0551
528.193
657.2327
979.4655 PEP_0_1_0_0_0
1871.796 PEP_3_3_0_0_0
0
20000
40000
60000
80000
100000
120000
140000
160000
500
1000
1500
2000

File: 231129_OE_RK_36, RT: 9.053, Scan: 005539
Type: HCD, FTMS + c NSI d Full ms2 1212.8064@hcd30.00 [120.0000-3733.3293]
Precursor Scan: 005478, m/z: 1212.8063, charge: 3
ENGTISR_6_5_0_3_0

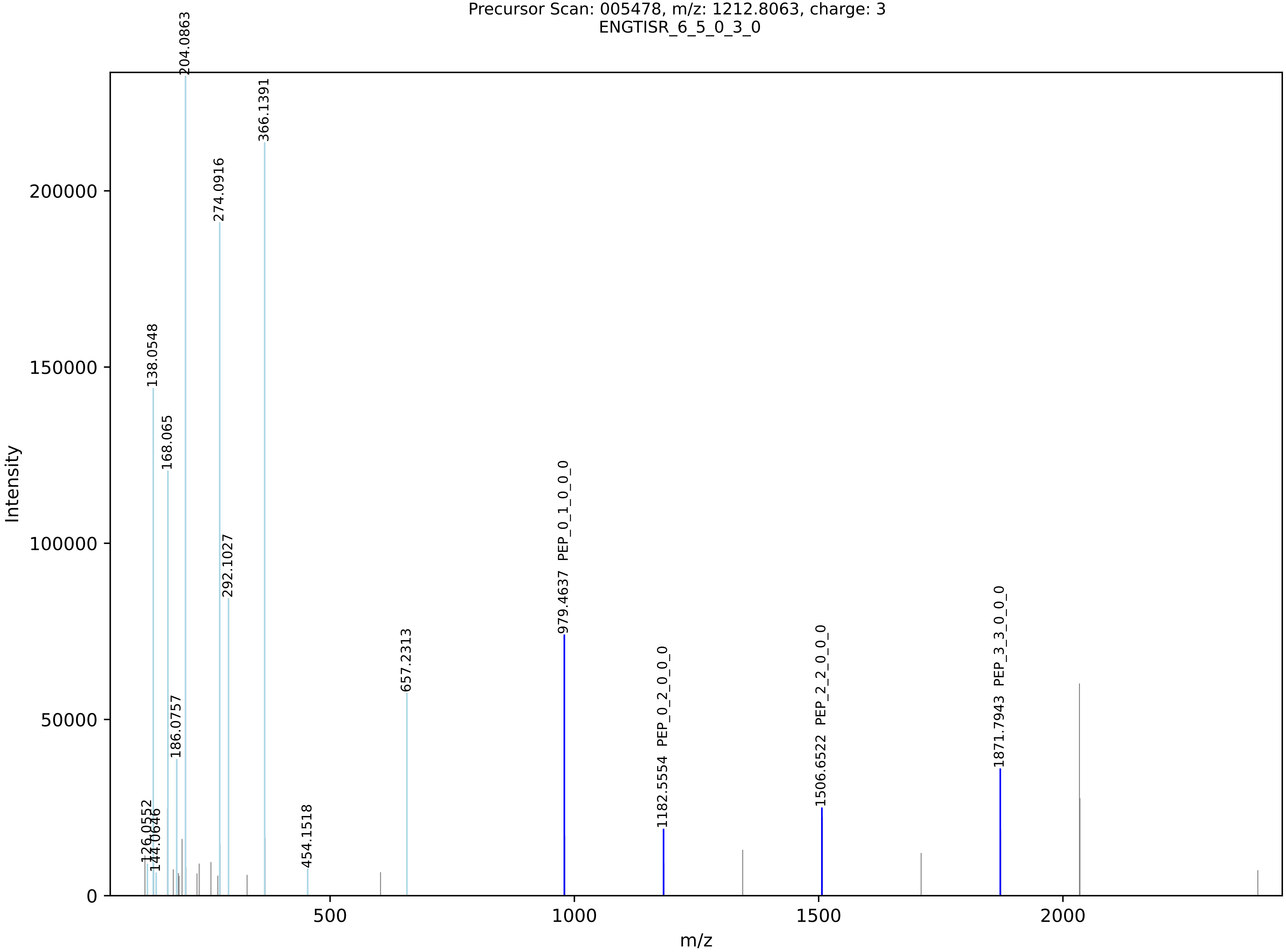

File: 231129_OE_RK_36, RT: 9.09, Scan: 005583
Type: HCD, FTMS + c NSI d Full ms2 1383.8691@hcd30.00 [120.0000-4256.7813]
Precursor Scan: 005549, m/z: 1383.5351, charge: 3
ENGTISR_7_6_1_3_0

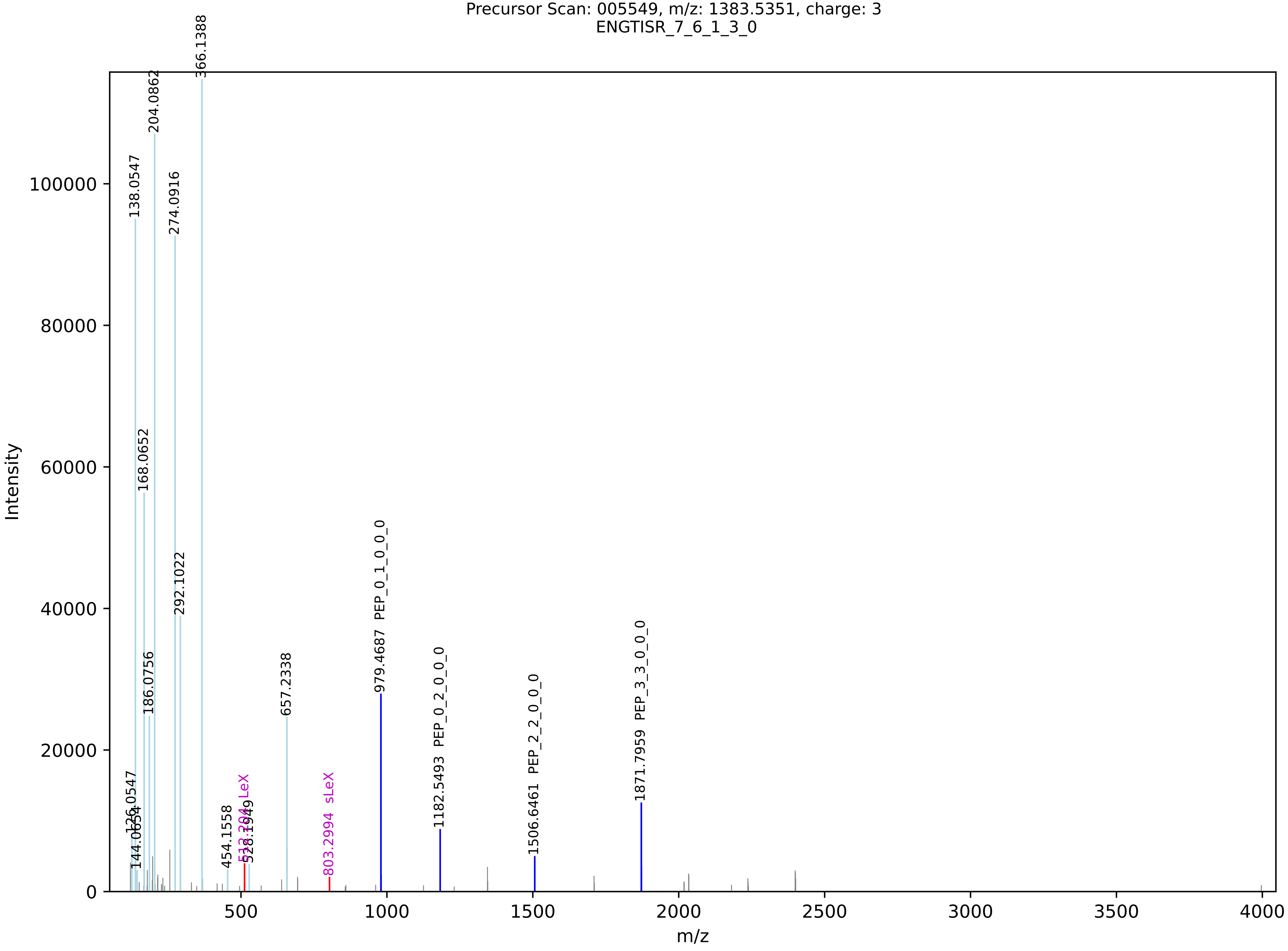

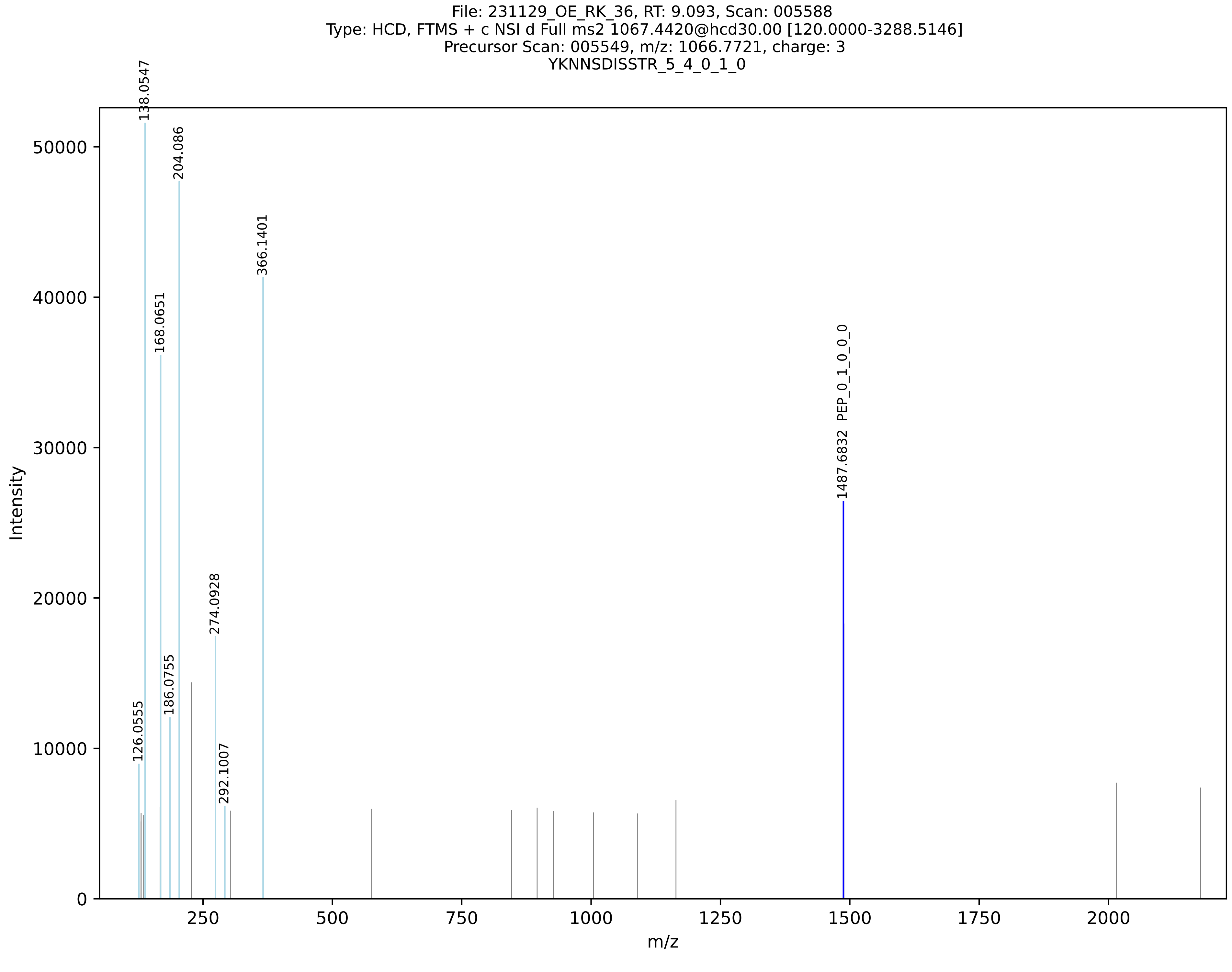
File: 231129_OE_RK_36, RT: 9.093, Scan: 005588
Type: HCD, FTMS + c NSI d Full ms2 1067.4420@hcd30.00 [120.0000-3288.5146]
Precursor Scan: 005549, m/z: 1066.7721, charge: 3
YKNNSDISSTR_5_4_0_1_0
138.0547
204.086
366.1401
168.0651
1487.6832_PEP_0_1_0_0_0
274.0928
186.0755
126.0555
292.1007
Intensity
m/z
0
10000
20000
30000
40000
50000
250
500
750
1000
1250
1500
1750
2000

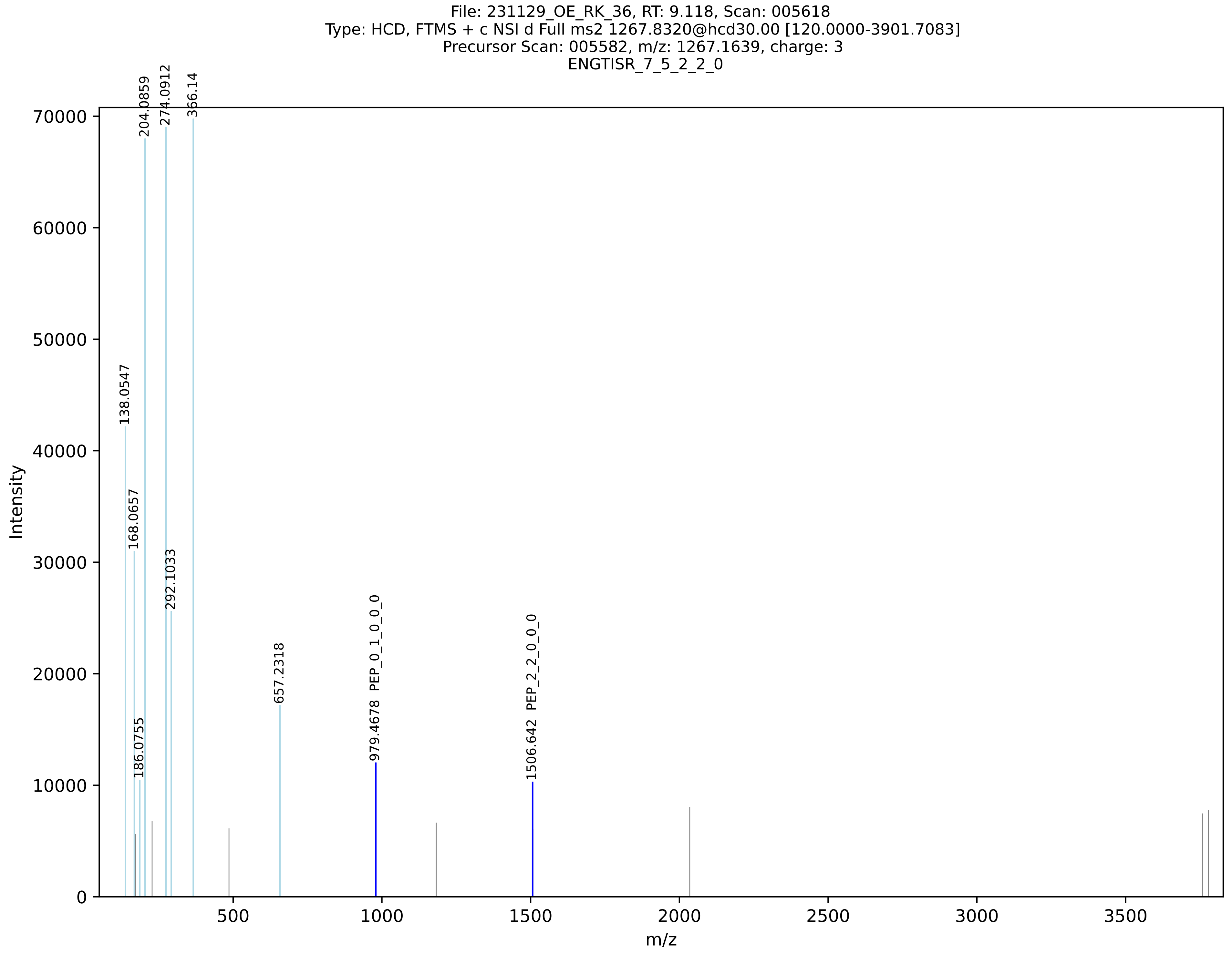
File: 231129_OE_RK_36, RT: 9.118, Scan: 005618
Type: HCD, FTMS + c NSI d Full ms2 1267.8320@hcd30.00 [120.0000-3901.7083]
Precursor Scan: 005582, m/z: 1267.1639, charge: 3
ENGTISR_7_5_2_2_0
204.0859
274.0912
366.14
138.0547
168.0657
292.1033
186.0755
657.2318
979.4678 PEP_0_1_0_0_0_
1506.642 PEP_2_2_0_0_0
Intensity
m/z
70000
60000
50000
40000
30000
20000
10000
0
500
1000
1500
2000
2500
3000
3500

File: 231129_OE_RK_36, RT: 9.199, Scan: 005725
Type: HCD, FTMS + c NSI d Full ms2 1213.4691@hcd30.00 [120.0000-3735.3577]
Precursor Scan: 005691, m/z: 1213.135, charge: 3
ENGTISR_6_5_0_3_0

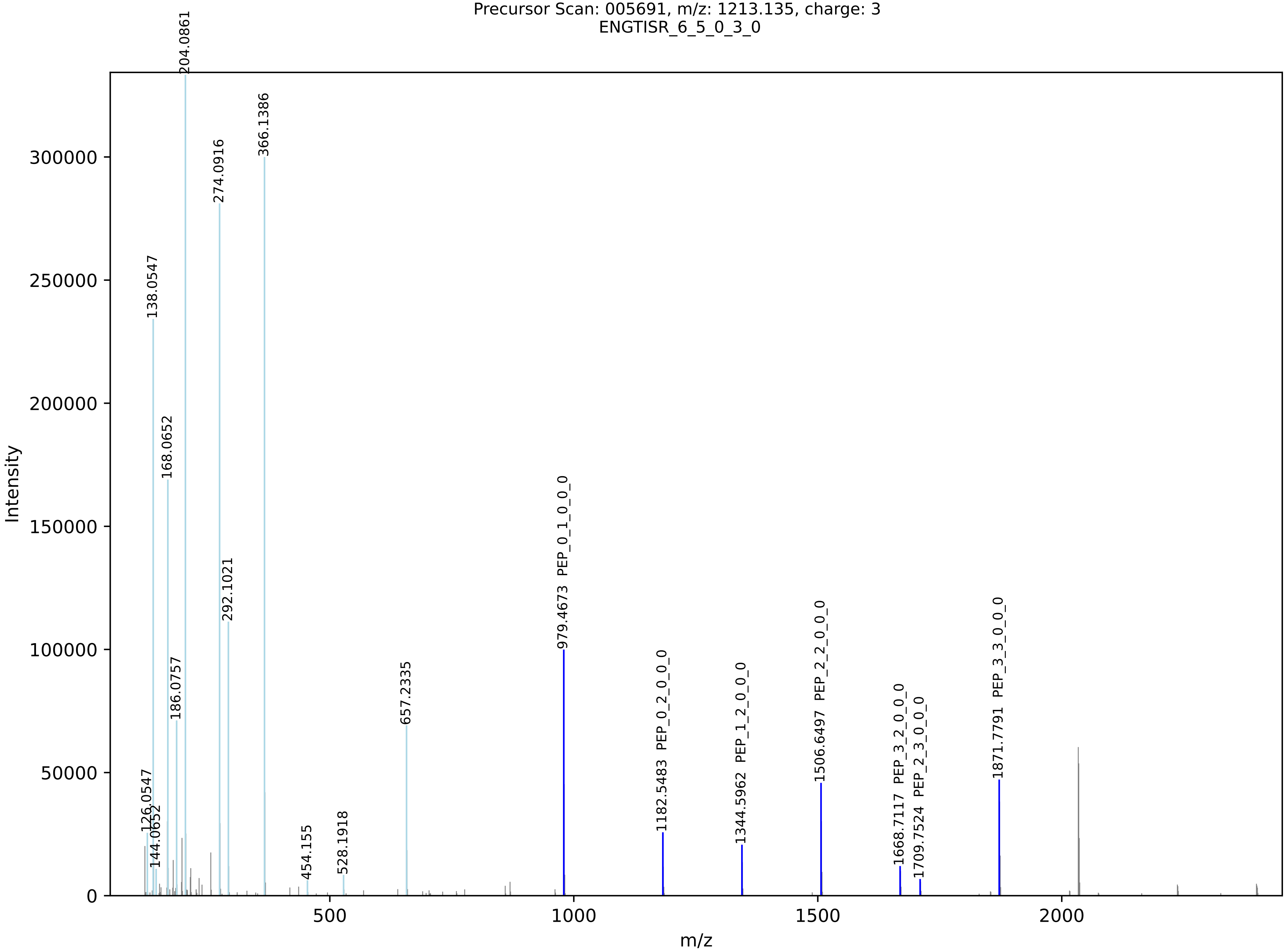

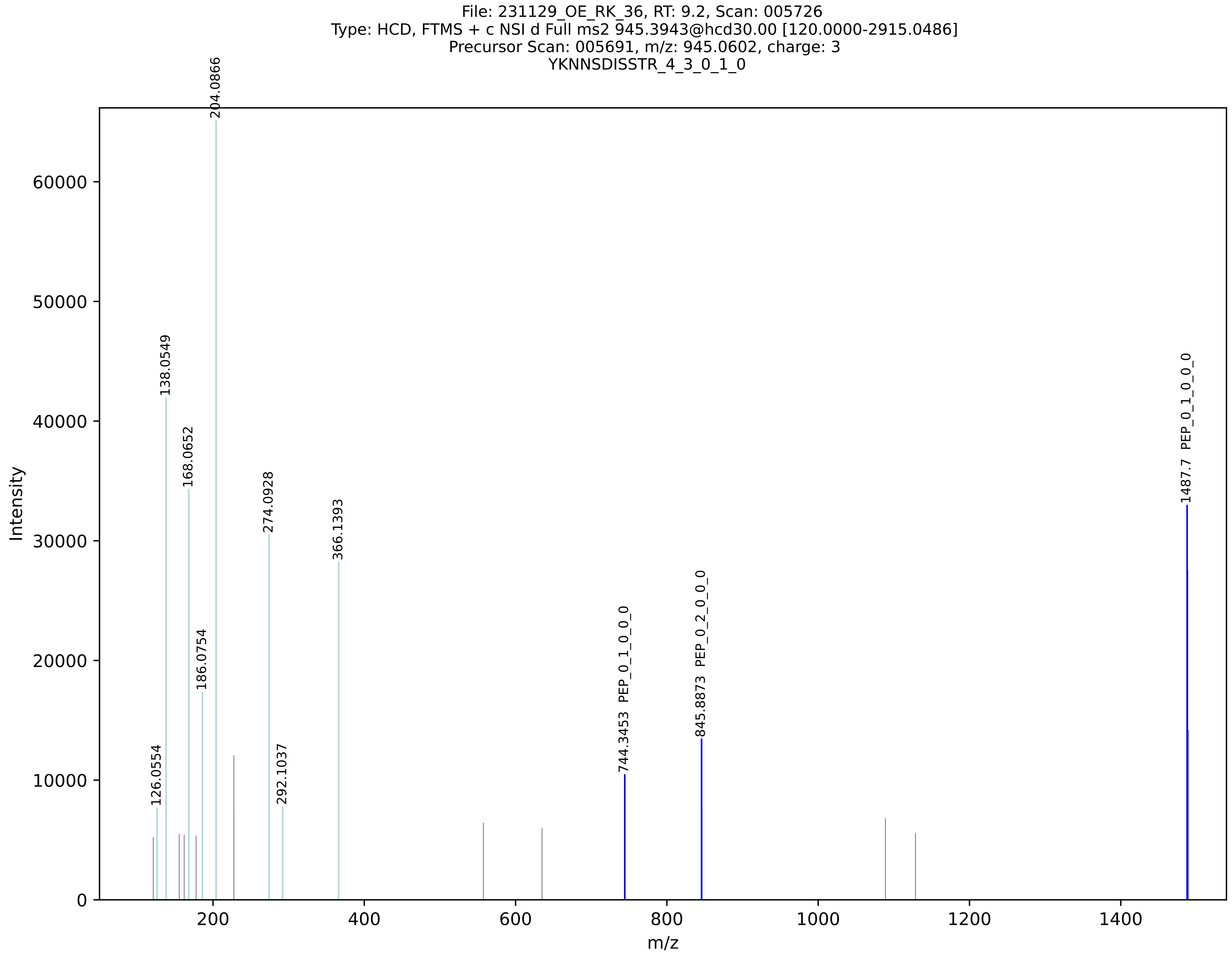
File: 231129_OE_RK_36, RT: 9.2, Scan: 005726
Type: HCD, FTMS + c NSI d Full ms2 945.3943@hcd30.00 [120.0000-2915.0486]
Precursor Scan: 005691, m/z: 945.0602, charge: 3
YKNNSDISSTR_4_3_0_1_0
Intensity
m/z
126.0554
138.0549
168.0652
186.0754
204.0866
274.0928
292.1037
366.1393
744.3453 PEP_0_1_0_0_0
845.8873 PEP_0_2_0_0_0
1487.7 PEP_0_1_0_0_0

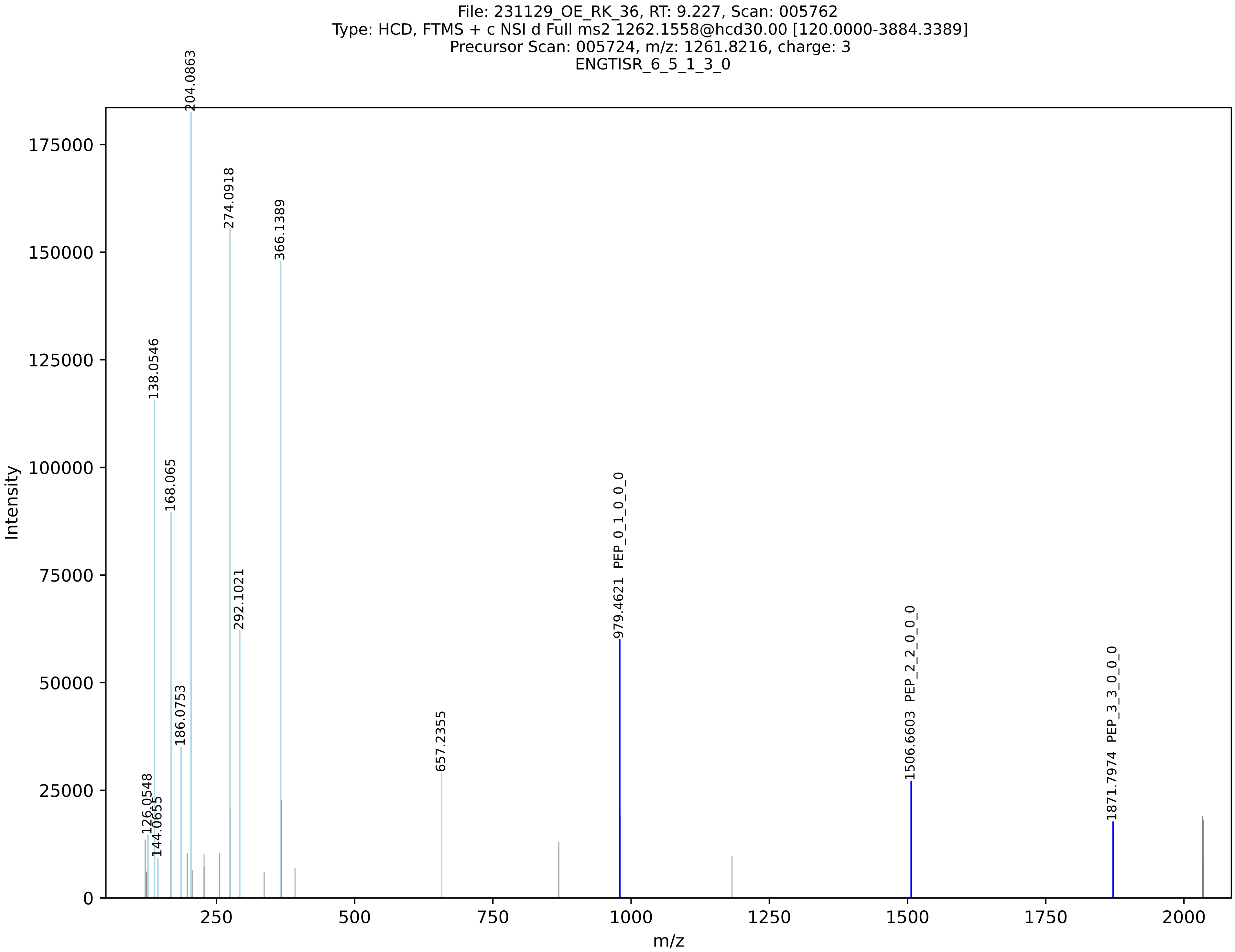
File: 231129_OE_RK_36, RT: 9.227, Scan: 005762
Type: HCD, FTMS + c NSI d Full ms2 1262.1558@hcd30.00 [120.0000-3884.3389]
Precursor Scan: 005724, m/z: 1261.8216, charge: 3
ENGTISR_6_5_1_3_0
Intensity
m/z
204.0863
274.0918
366.1389
138.0546
168.065
292.1021
186.0753
126.0548
144.0655
657.2355
979.4621 PEP_0_1_0_0_0_
1506.6603 PEP_2_2_0_0_0_
1871.7974 PEP_3_3_0_0_0_
0
25000
50000
75000
100000
125000
150000
175000
250
500
750
1000
1250
1500
1750
2000

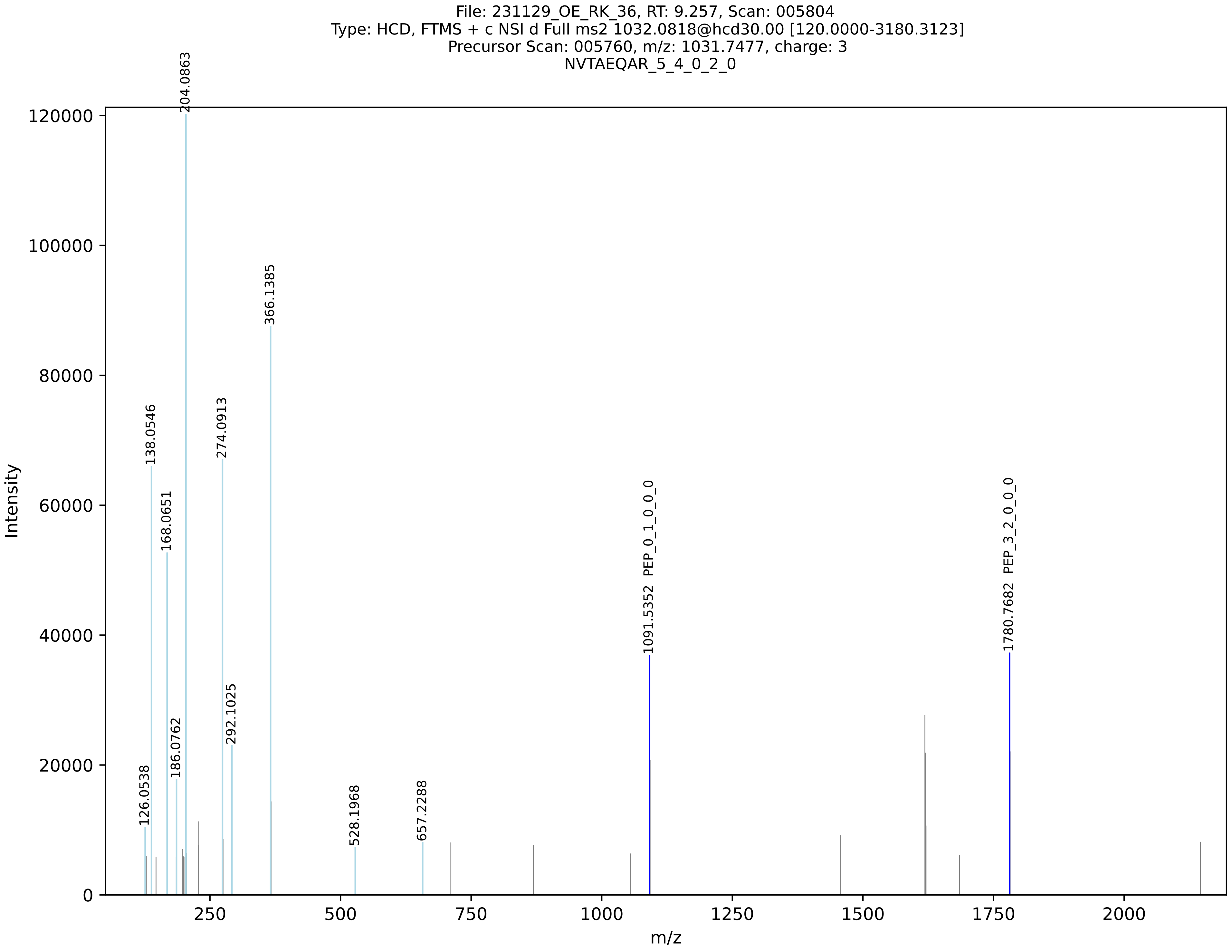
File: 231129_OE_RK_36, RT: 9.257, Scan: 005804
Type: HCD, FTMS + c NSI d Full ms2 1032.0818@hcd30.00 [120.0000-3180.3123]
Precursor Scan: 005760, m/z: 1031.7477, charge: 3
NVTAEQAR_5_4_0_2_0
Intensity
m/z
204.0863
366.1385
138.0546
274.0913
168.0651
186.0762
292.1025
126.0538
528.1968
657.2288
1091.5352 PEP_0_1_0_0_0
1780.7682 PEP_3_2_0_0_0
120000
100000
80000
60000
40000
20000
0
250
500
750
1000
1250
1500
1750
2000

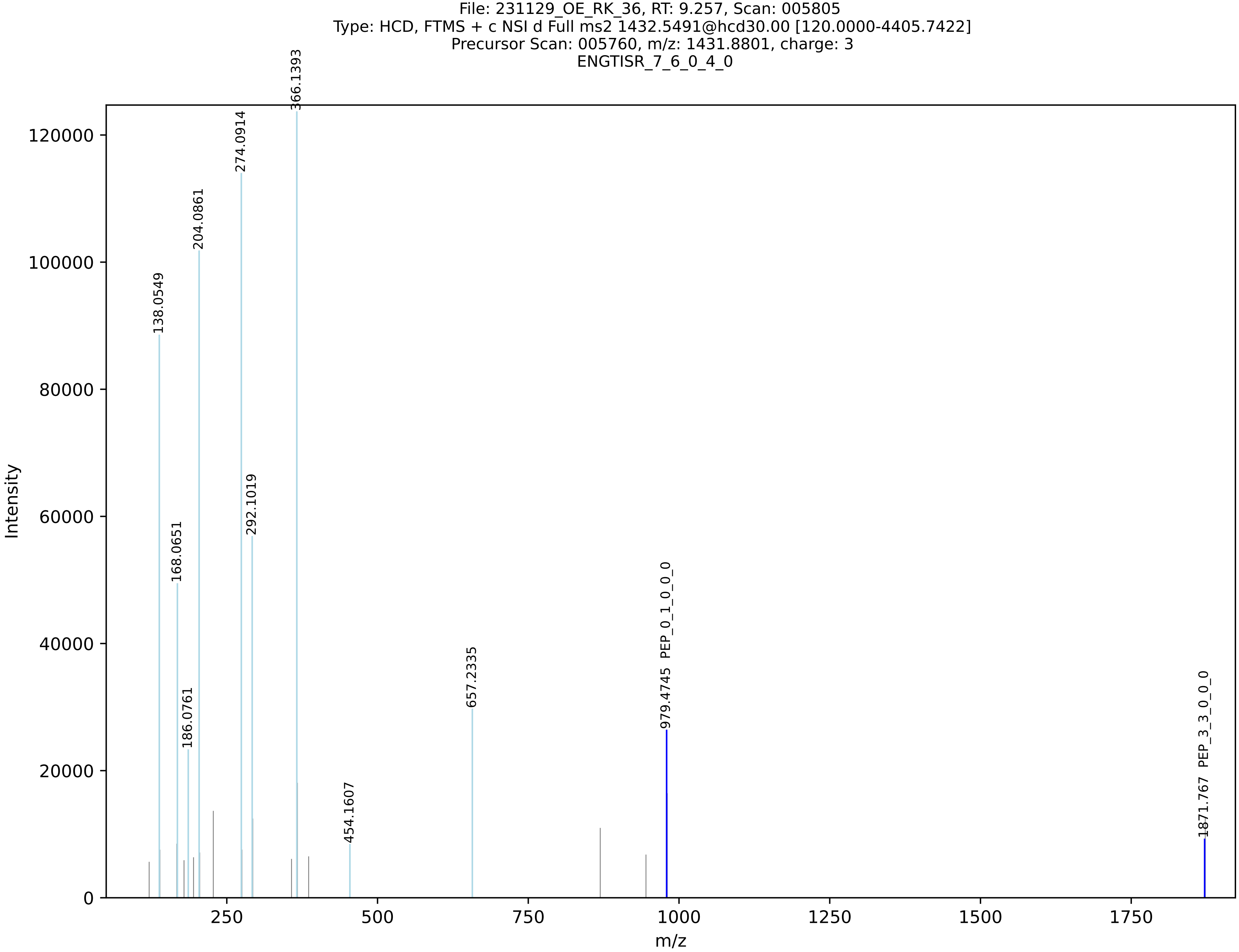
File: 231129_OE_RK_36, RT: 9.257, Scan: 005805
Type: HCD, FTMS + c NSI d Full ms2 1432.5491@hcd30.00 [120.0000-4405.7422]
Precursor Scan: 005760, m/z: 1431.8801, charge: 3
ENGTISR_7_6_0_4_0
Intensity
m/z
366.1393
274.0914
204.0861
138.0549
168.0651
292.1019
186.0761
454.1607
657.2335
979.4745 PEP_0_1_0_0_0
1871.767 PEP_3_3_0_0_0
0
20000
40000
60000
80000
100000
120000
250
500
750
1000
1250
1500
1750

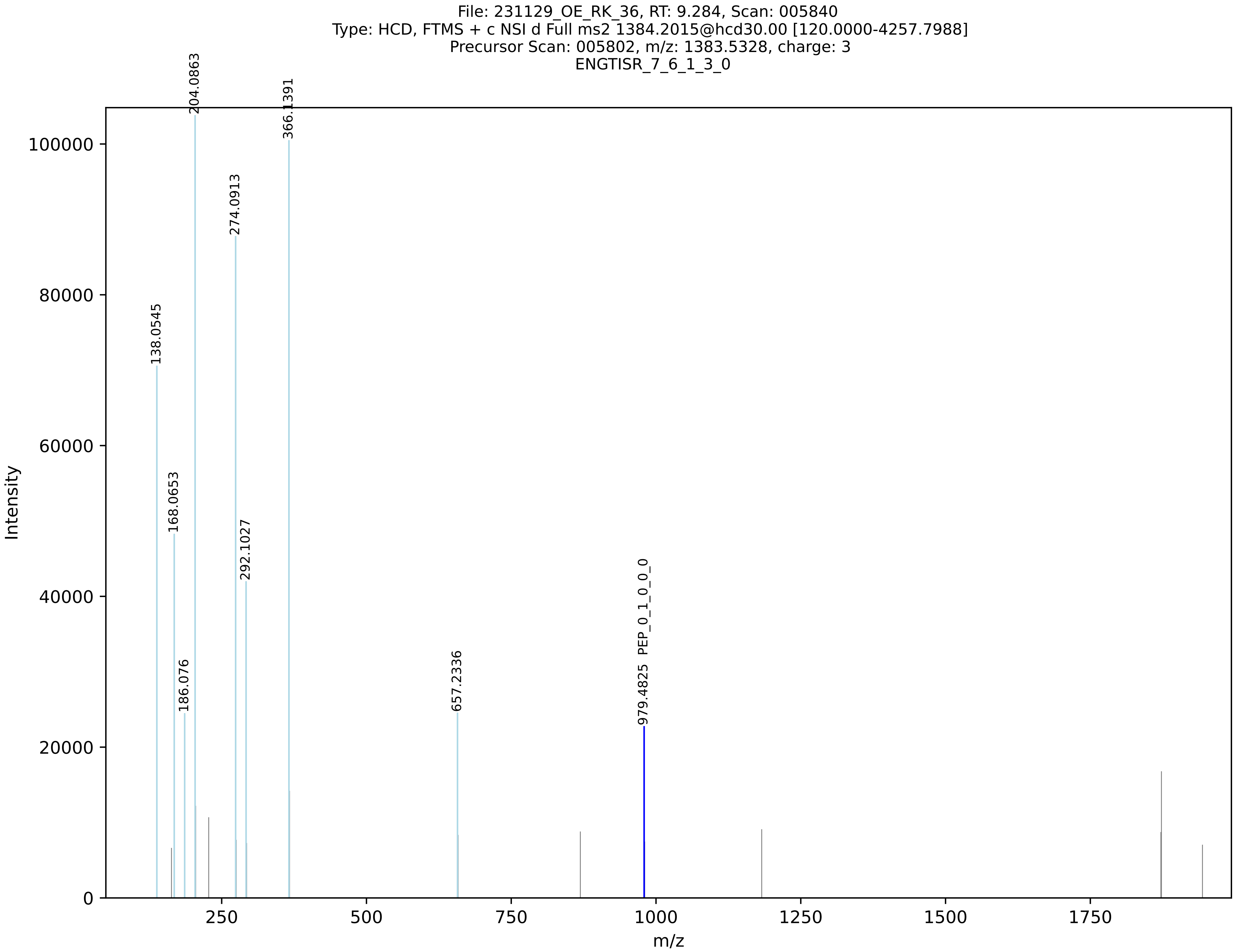

File: 231129_OE_RK_36, RT: 9.284, Scan: 005840
Type: HCD, FTMS + c NSI d Full ms2 1384.2015@hcd30.00 [120.0000-4257.7988]
Precursor Scan: 005802, m/z: 1383.5328, charge: 3
ENGTISR_7_6_1_3_0
204.0863
366.1391
274.0913
138.0545
168.0653
292.1027
186.076
657.2336
979.4825 PEP_0_1_0_0_0
Intensity
m/z
0
20000
40000
60000
80000
100000
250
500
750
1000
1250
1500
1750

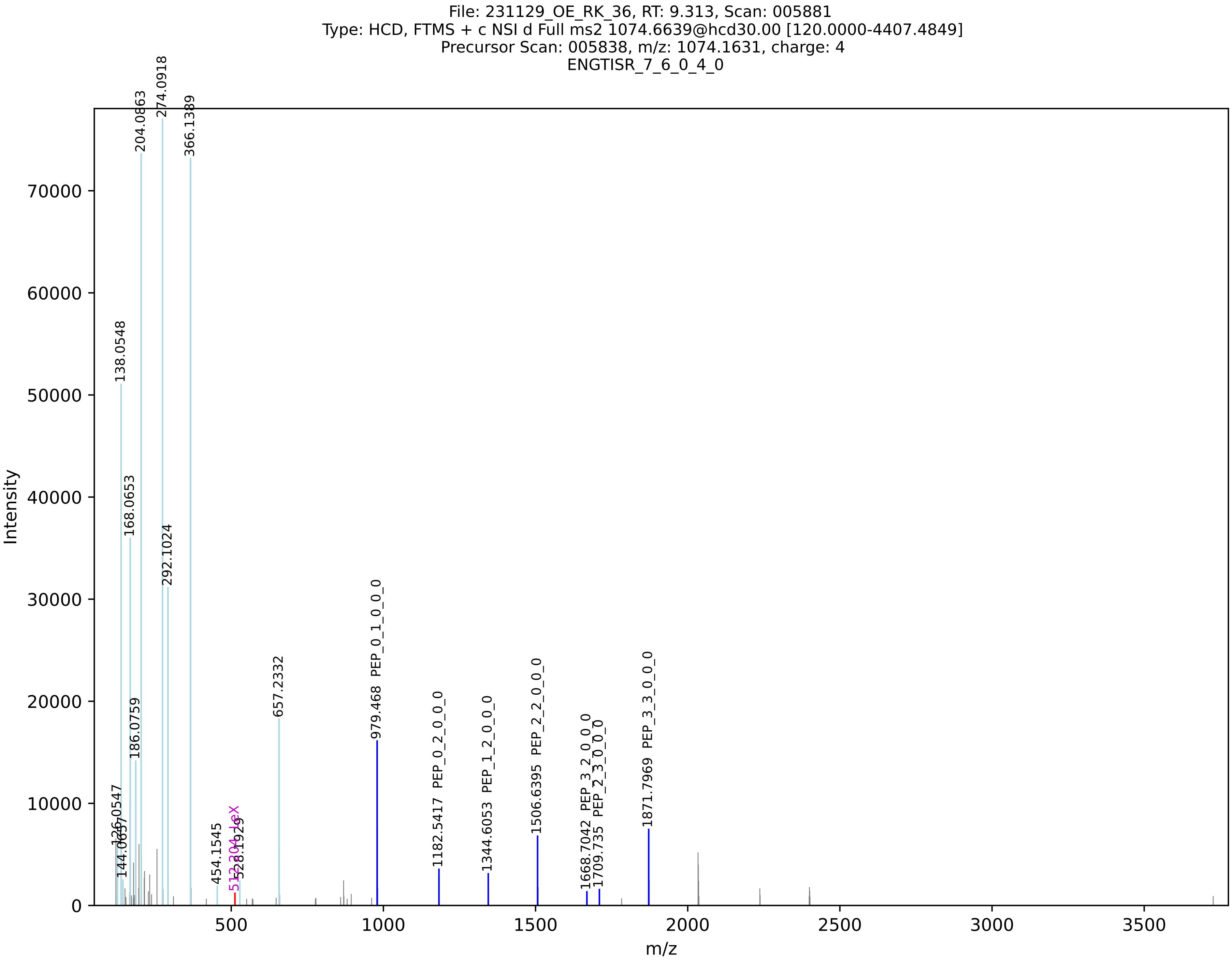
File: 231129_OE_RK_36, RT: 9.313, Scan: 005881
Type: HCD, FTMS + c NSI d Full ms2 1074.6639@hcd30.00 [120.0000-4407.4849]
Precursor Scan: 005838, m/z: 1074.1631, charge: 4
ENGTISR_7_6_0_4_0
Intensity
m/z
126.0547
138.0548
144.0657
168.0653
186.0759
204.0863
274.0918
292.1024
366.1389
454.1545
512.204_LeX
528.1929
657.2332
979.468 PEP_0_1_0_0_0
1182.5417 PEP_0_2_0_0_0
1344.6053 PEP_1_2_0_0_0
1506.6395 PEP_2_2_0_0_0
1668.7042 PEP_3_2_0_0_0
1709.735 PEP_2_3_0_0_0
1871.7969 PEP_3_3_0_0_0

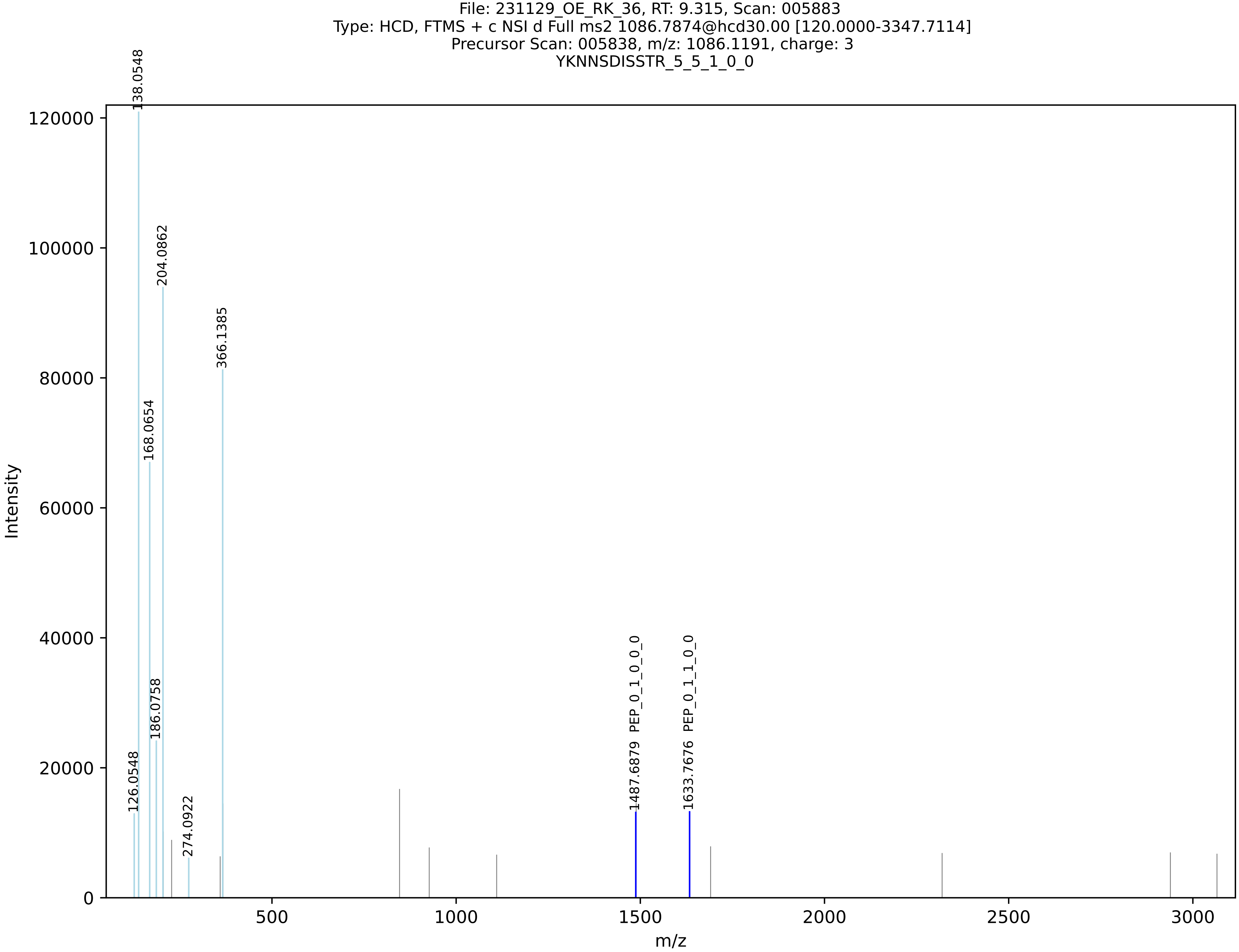
File: 231129_OE_RK_36, RT: 9.315, Scan: 005883
Type: HCD, FTMS + c NSI d Full ms2 1086.7874@hcd30.00 [120.0000-3347.7114]
Precursor Scan: 005838, m/z: 1086.1191, charge: 3
YKNNSDISSTR_5_5_1_0_0
Intensity
m/z
138.0548
204.0862
366.1385
168.0654
186.0758
126.0548
274.0922
1487.6879 PEP_0_1_0_0_0
1633.7676 PEP_0_1_1_0_0
0
20000
40000
60000
80000
100000
120000
500
1000
1500
2000
2500
3000

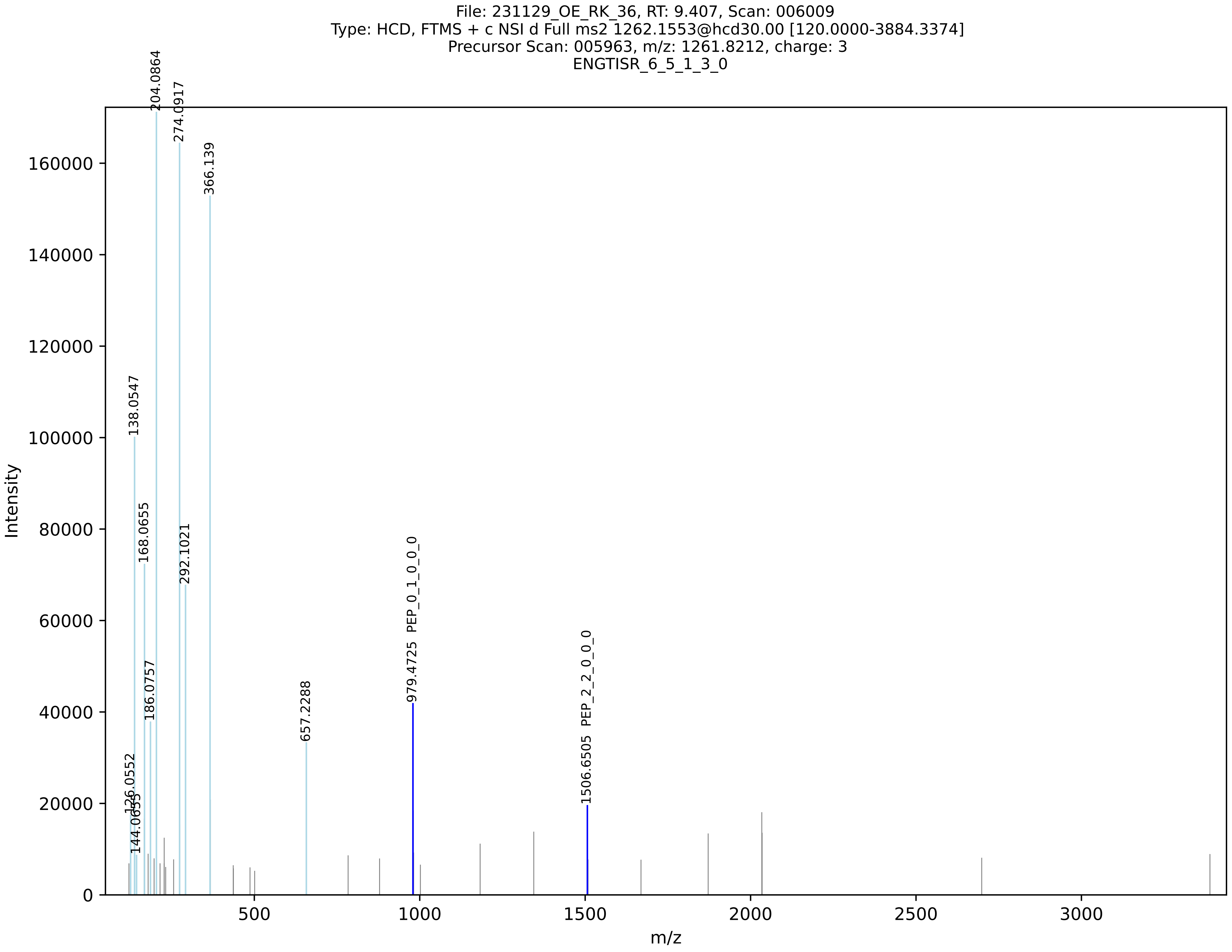
File: 231129_OE_RK_36, RT: 9.407, Scan: 006009
Type: HCD, FTMS + c NSI d Full ms2 1262.1553@hcd30.00 [120.0000-3884.3374]
Precursor Scan: 005963, m/z: 1261.8212, charge: 3
ENGTISR_6_5_1_3_0
204.0864
274.0917
366.139
138.0547
168.0655
292.1021
186.0757
126.0552
144.0655
657.2288
979.4725 PEP_0_1_0_0_
1506.6505 PEP_2_2_0_0_
Intensity
0
20000
40000
60000
80000
100000
120000
140000
160000
500
1000
1500
2000
2500
3000
m/z

File: 231129_OE_RK_36, RT: 9.408, Scan: 006010
Type: HCD, FTMS + c NSI d Full ms2 1481.2332@hcd30.00 [120.0000-4554.7158]
Precursor Scan: 005963, m/z: 1480.2303, charge: 3
ENGTISR_7_6_1_4_0

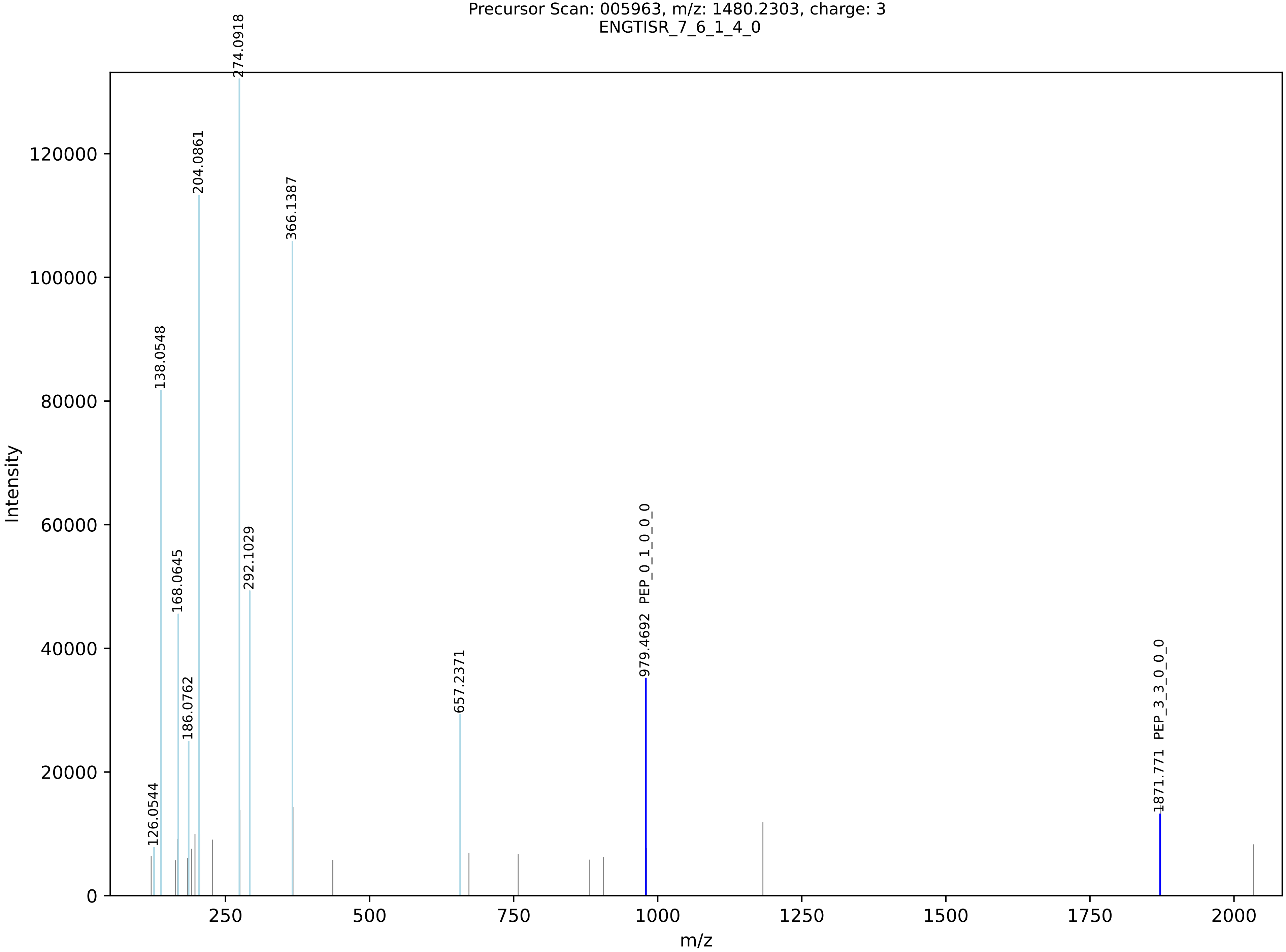

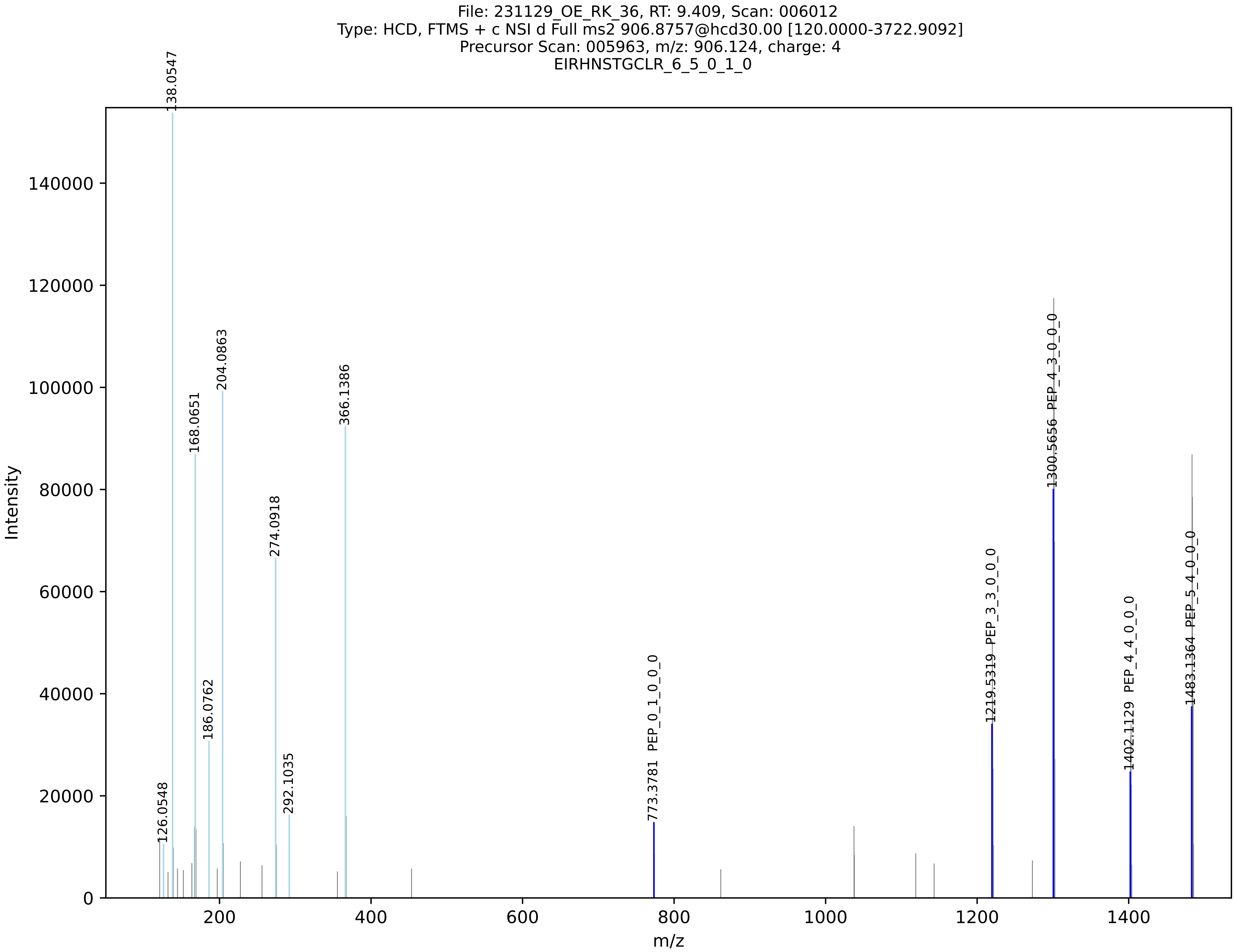
File: 231129_OE_RK_36, RT: 9.409, Scan: 006012
Type: HCD, FTMS + c NSI d Full ms2 906.8757@hcd30.00 [120.0000-3722.9092]
Precursor Scan: 005963, m/z: 906.124, charge: 4
EIRHNSTGCLR_6_5_0_1_0
Intensity
m/z
138.0547
168.0651
204.0863
186.0762
274.0918
292.1035
366.1386
126.0548
773.3781 PEP_0_1_0_0_0
1219.5319 PEP_3_3_0_0_0
1300.5656 PEP_4_3_0_0_0
1402.1129 PEP_4_4_0_0_0
1483.1364 PEP_5_4_0_0_0

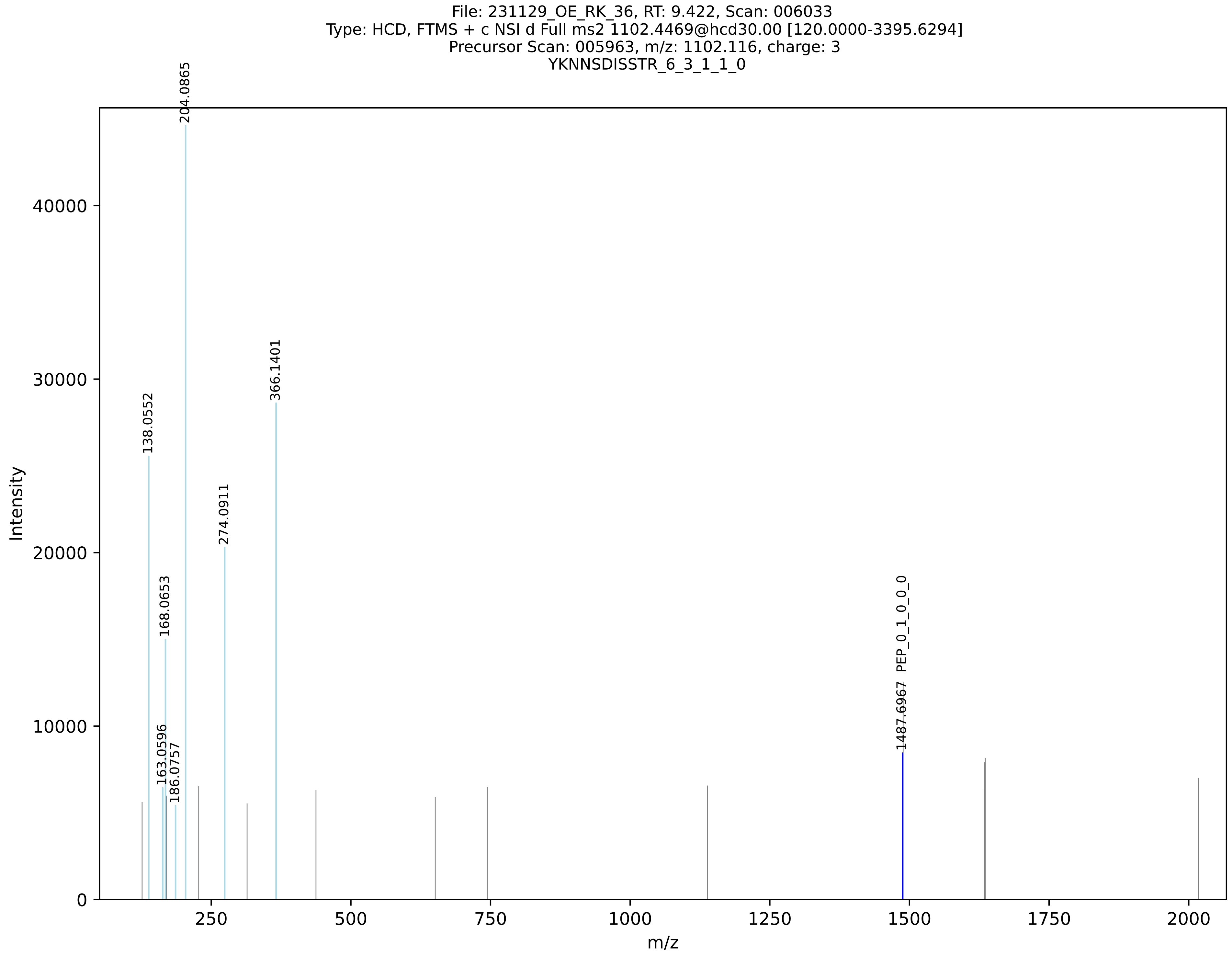
File: 231129_OE_RK_36, RT: 9.422, Scan: 006033
Type: HCD, FTMS + c NSI d Full ms2 1102.4469@hcd30.00 [120.0000-3395.6294]
Precursor Scan: 005963, m/z: 1102.116, charge: 3
YKNNSDISSTR_6_3_1_1_0
204.0865
138.0552
366.1401
274.0911
168.0653
163.0596
186.0757
1487.6967 PEP_0_1_0_0_0
Intensity
m/z
0
10000
20000
30000
40000
250
500
750
1000
1250
1500
1750
2000

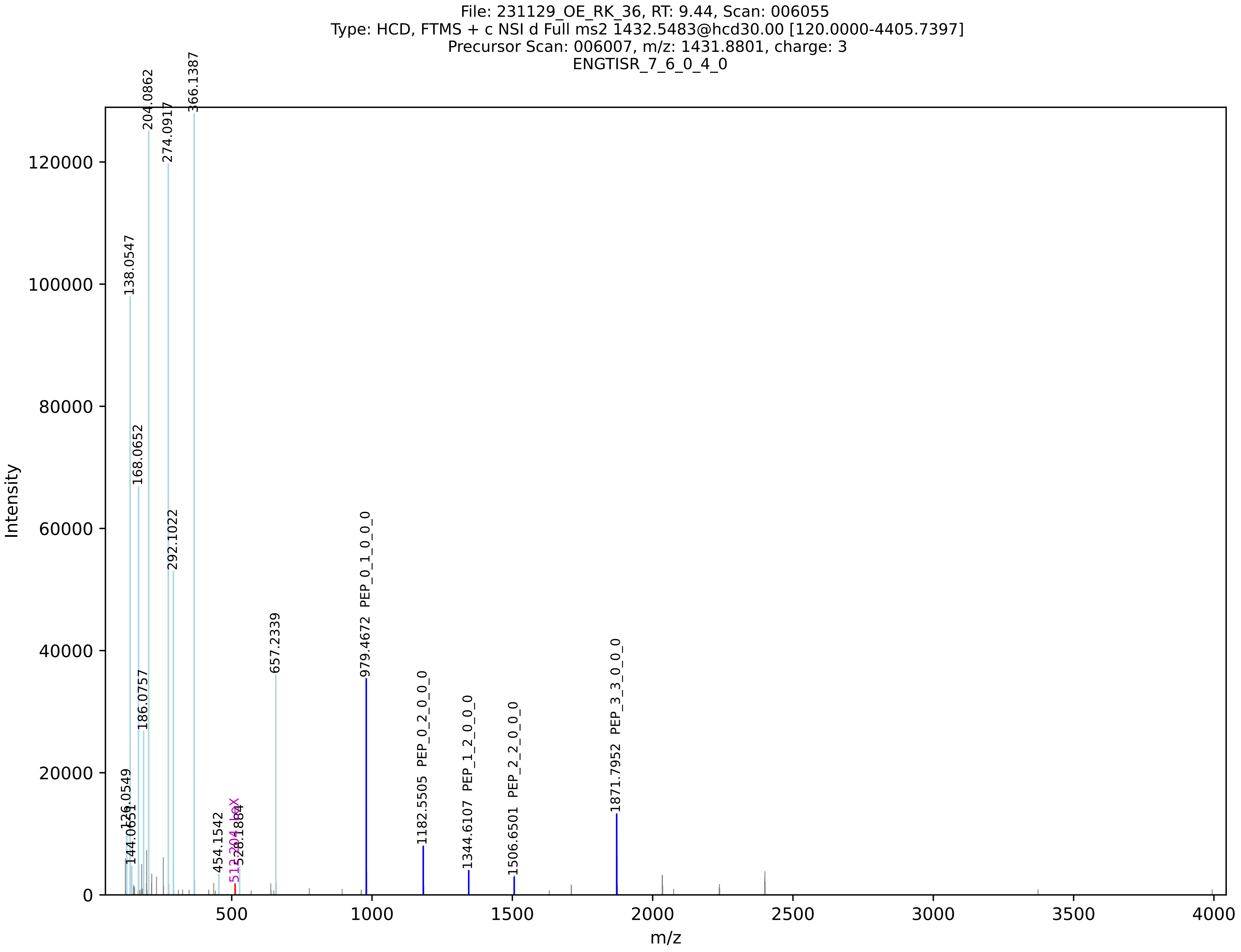
File: 231129_OE_RK_36, RT: 9.44, Scan: 006055
Type: HCD, FTMS + c NSI d Full ms2 1432.5483@hcd30.00 [120.0000-4405.7397]
Precursor Scan: 006007, m/z: 1431.8801, charge: 3
ENGTISR_7_6_0_4_0
Intensity
m/z
366.1387
204.0862
274.0917
138.0547
168.0652
292.1022
186.0757
126.0549
144.0651
454.1542
512.204 LeX
528.1884
657.2339
979.4672 PEP_0_1_0_0_0
1182.5505 PEP_0_2_0_0_0
1344.6107 PEP_1_2_0_0_0
1506.6501 PEP_2_2_0_0_0
1871.7952 PEP_3_3_0_0_0

File: 231129_OE_RK_36, RT: 9.506, Scan: 006148
Type: HCD, FTMS + c NSI d Full ms2 994.0820@hcd30.00 [120.0000-3064.0330]
Precursor Scan: 006103, m/z: 993.7479, charge: 3
YKNNSDISSTR_4_3_1_1_0

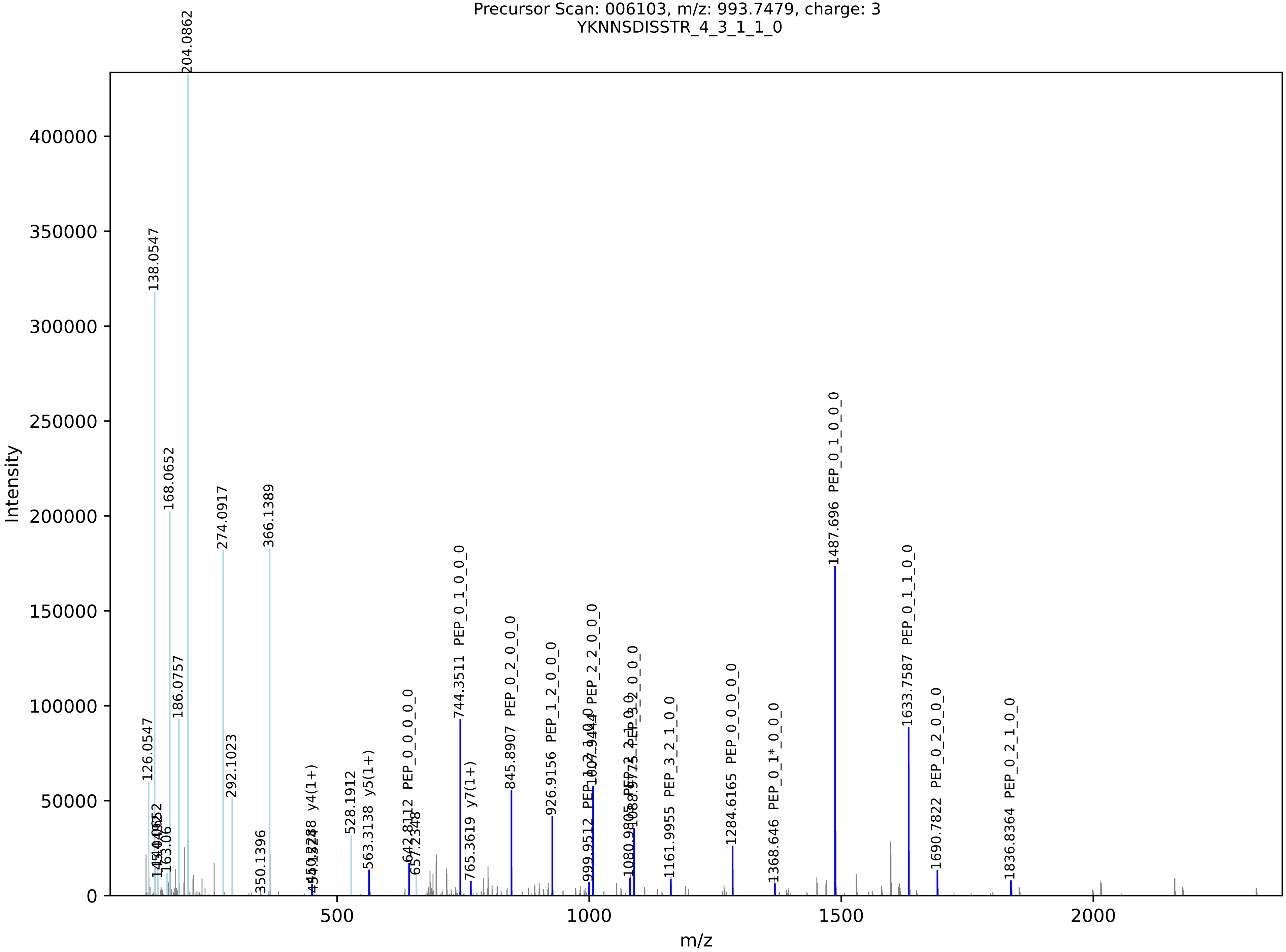

File: 231129_OE_RK_36, RT: 9.607, Scan: 006287
Type: HCD, FTMS + c NSI d Full ms2 1111.1763@hcd30.00 [120.0000-4556.4551]
Precursor Scan: 006228, m/z: 1110.675, charge: 4
ENGTISR_7_6_1_4_0

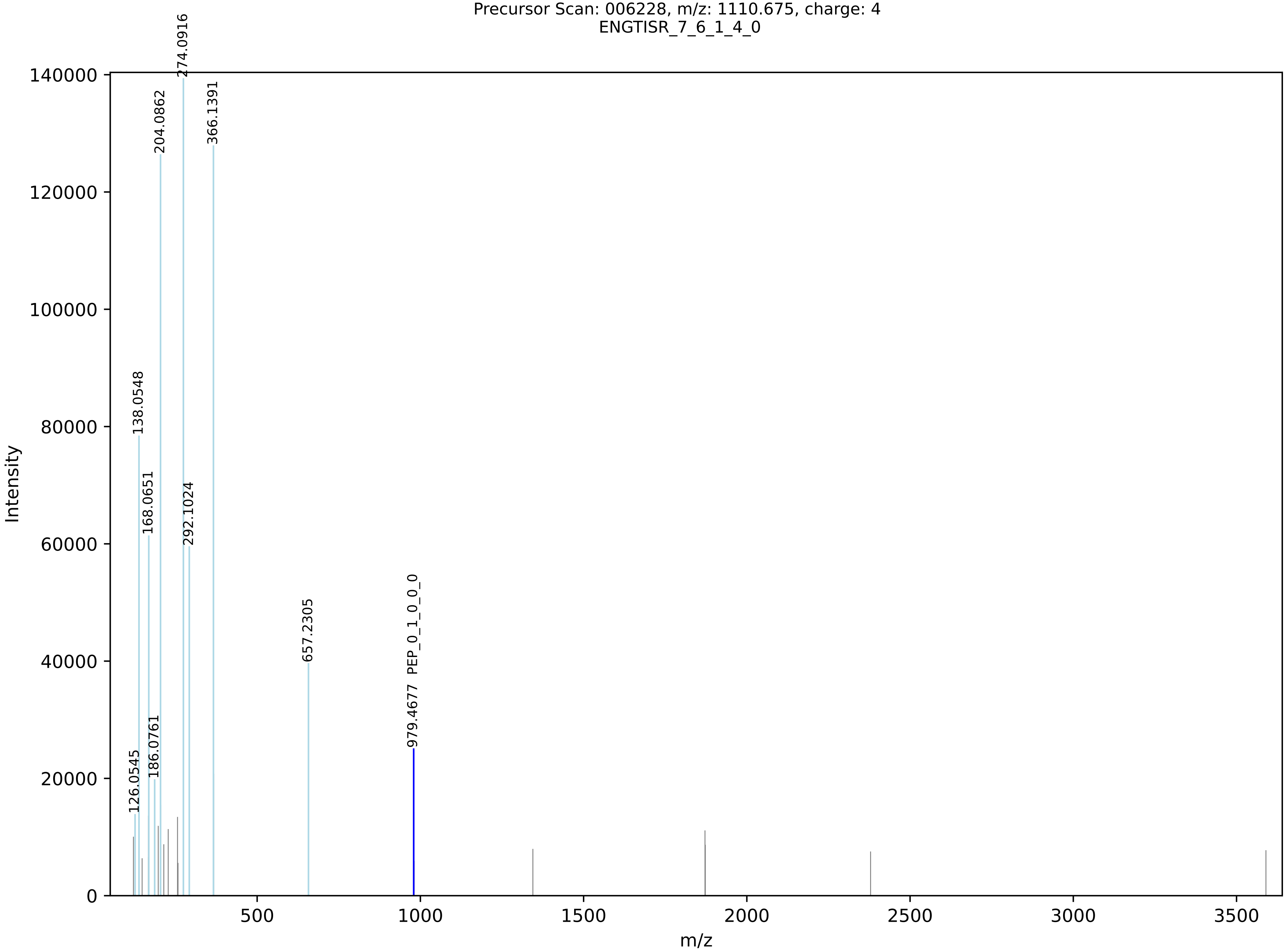

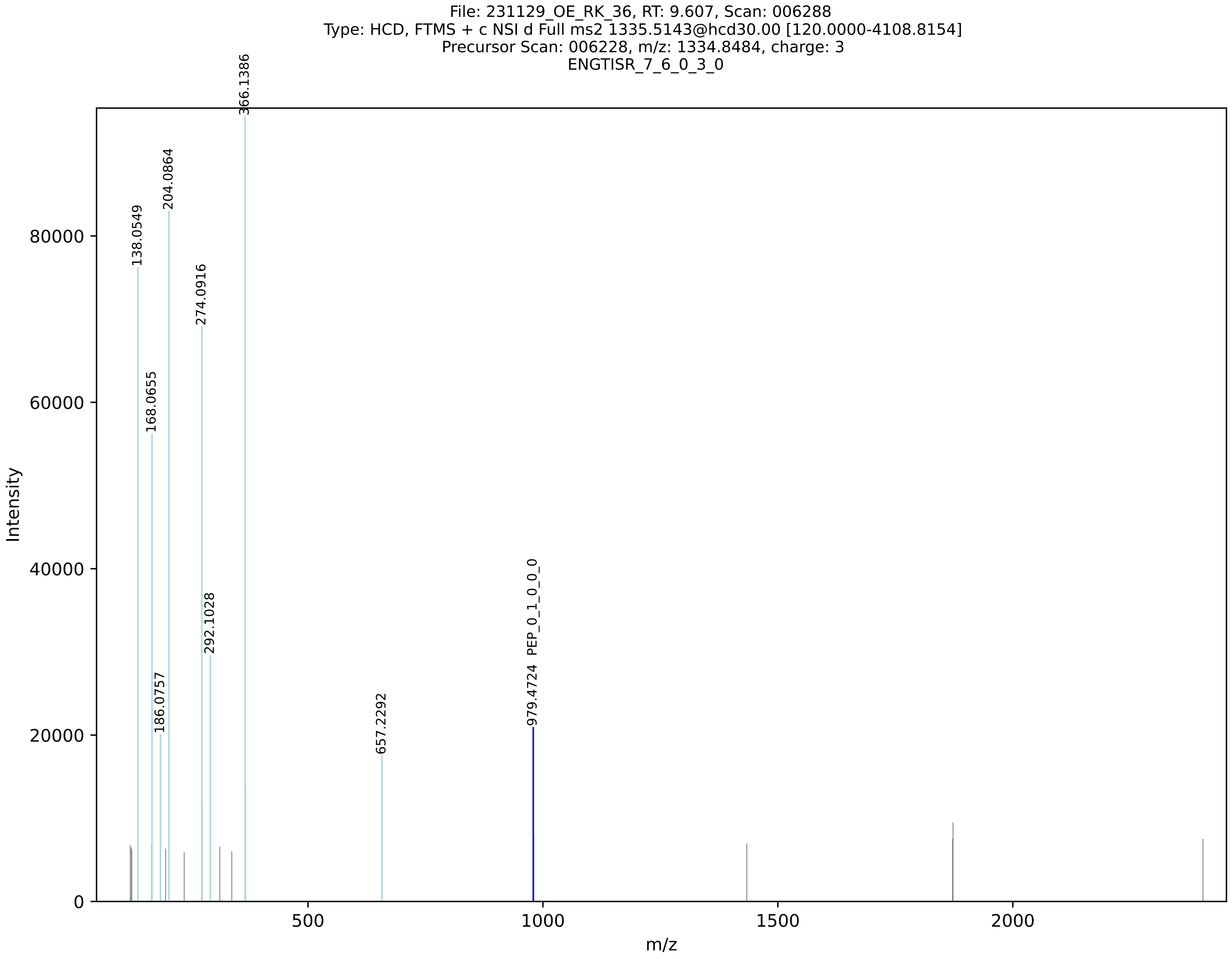
File: 231129_OE_RK_36, RT: 9.607, Scan: 006288
Type: HCD, FTMS + c NSI d Full ms2 1335.5143@hcd30.00 [120.0000-4108.8154]
Precursor Scan: 006228, m/z: 1334.8484, charge: 3
ENGTISR_7_6_0_3_0
Intensity
m/z
366.1386
204.0864
138.0549
274.0916
168.0655
292.1028
186.0757
657.2292
979.4724 PEP_0_1_0_0_0
0
20000
40000
60000
80000
500
1000
1500
2000

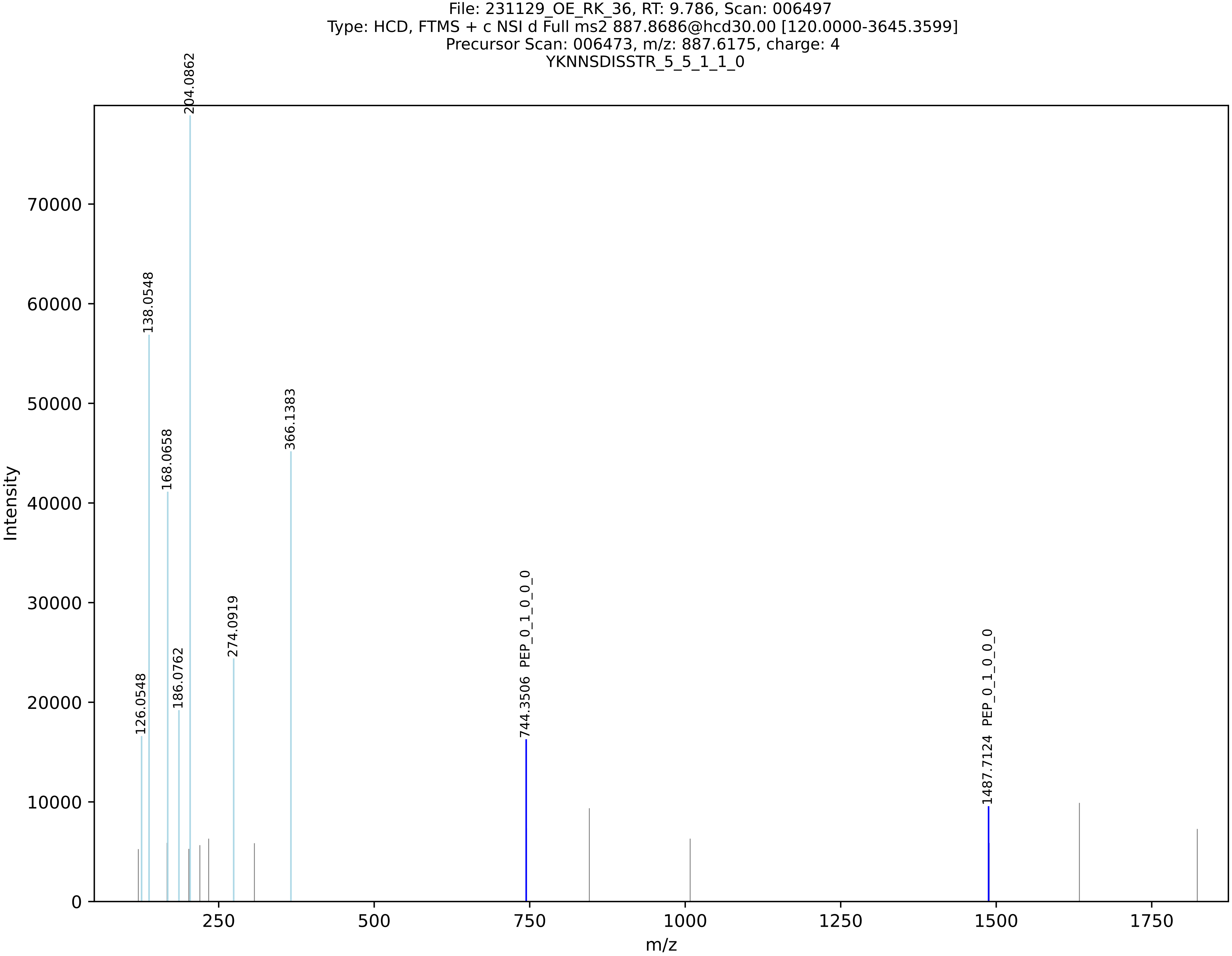

File: 231129_OE_RK_36, RT: 9.786, Scan: 006497
Type: HCD, FTMS + c NSI d Full ms2 887.8686@hcd30.00 [120.0000-3645.3599]
Precursor Scan: 006473, m/z: 887.6175, charge: 4
YKNNSDISSTR_5_5_1_1_0
204.0862
138.0548
168.0658
366.1383
274.0919
186.0762
126.0548
744.3506 PEP_0_1_0_0_0
1487.7124 PEP_0_1_0_0_0
Intensity
m/z
0
10000
20000
30000
40000
50000
60000
70000
250
500
750
1000
1250
1500
1750

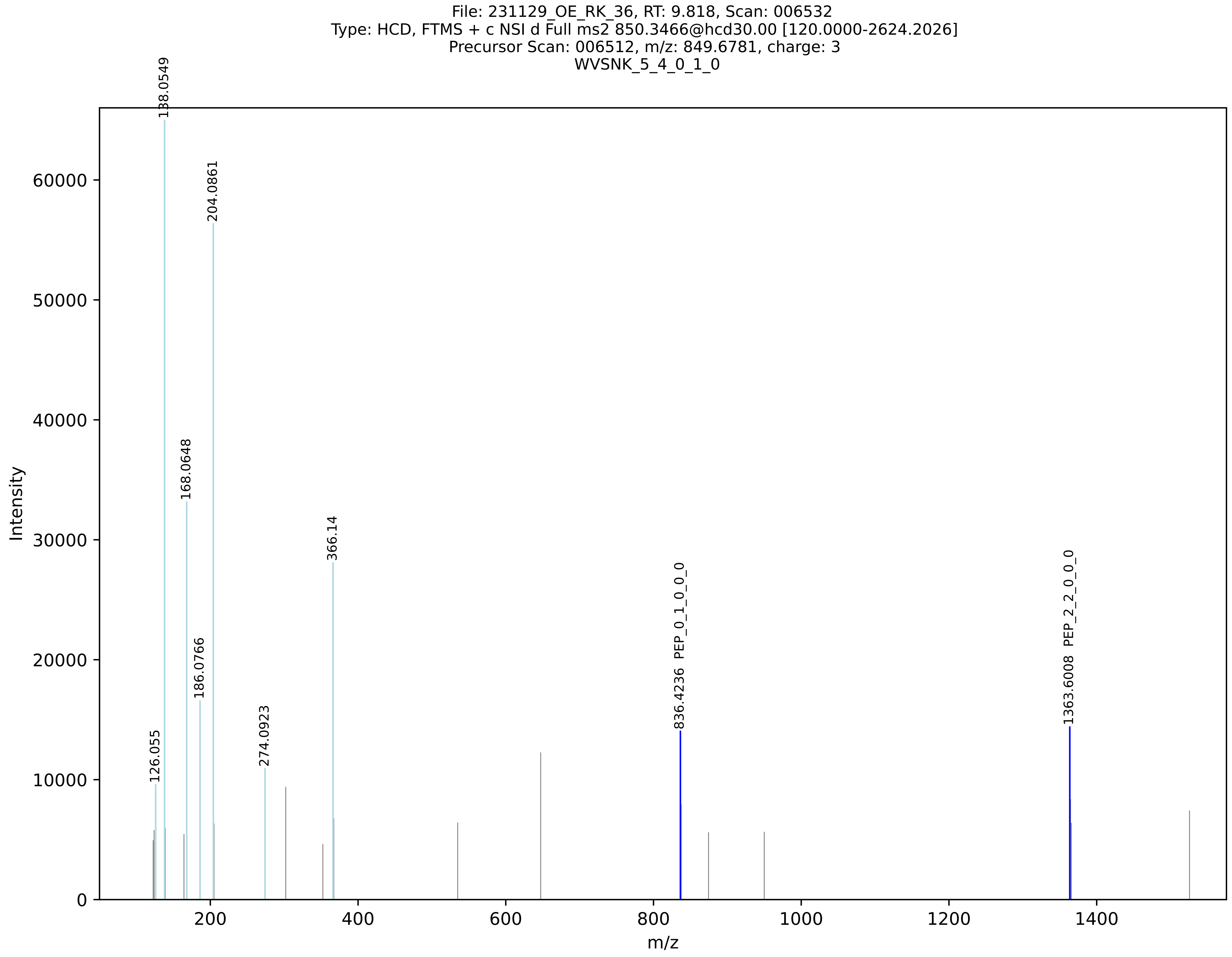
File: 231129_OE_RK_36, RT: 9.818, Scan: 006532
Type: HCD, FTMS + c NSI d Full ms2 850.3466@hcd30.00 [120.0000-2624.2026]
Precursor Scan: 006512, m/z: 849.6781, charge: 3
WVSNK_5_4_0_1_0
138.0549
204.0861
168.0648
186.0766
126.055
274.0923
366.14
836.4236 PEP_0_1_0_0_0
1363.6008 PEP_2_2_0_0_0
Intensity
m/z
0
10000
20000
30000
40000
50000
60000
200
400
600
800
1000
1200
1400

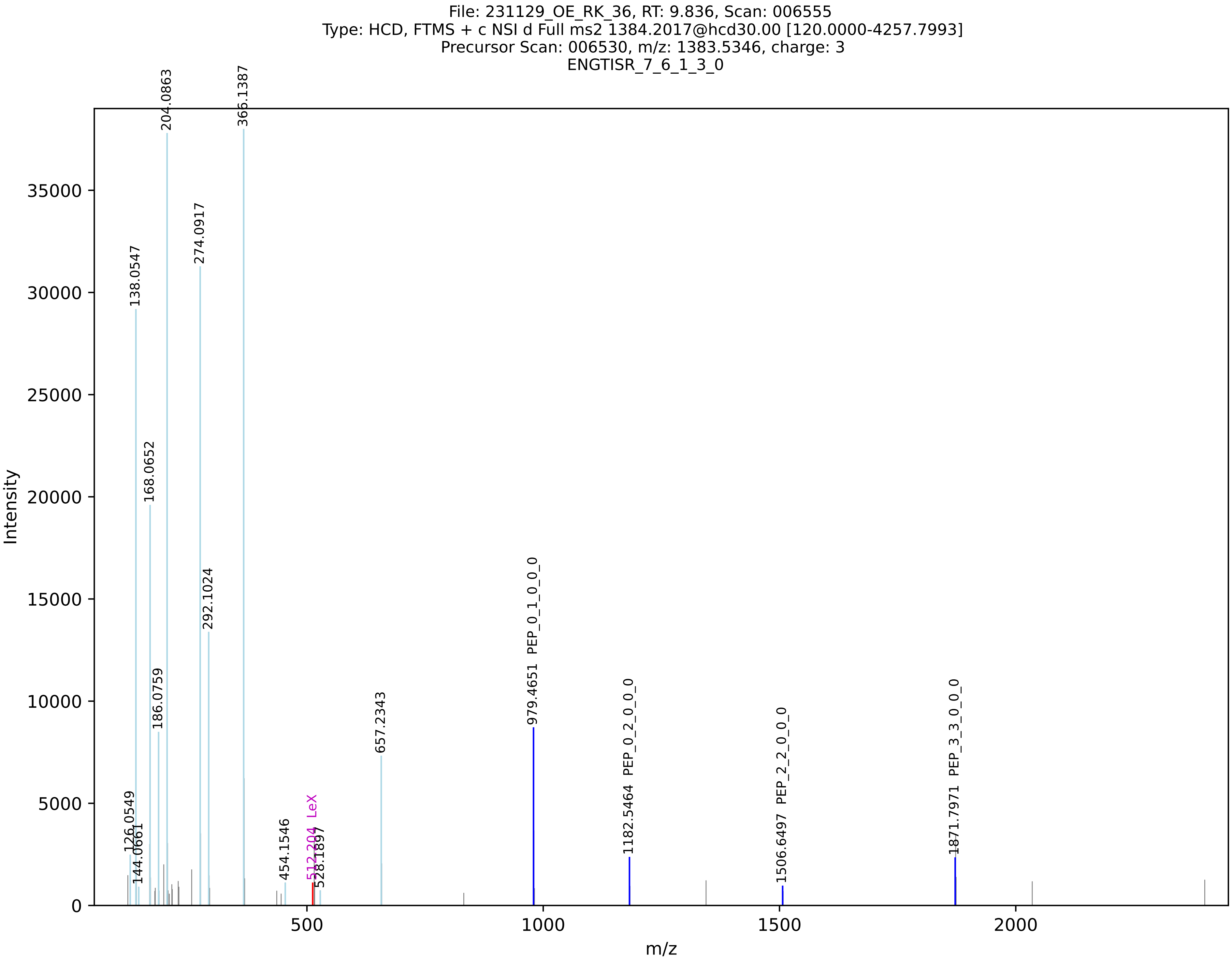
File: 231129_OE_RK_36, RT: 9.836, Scan: 006555
Type: HCD, FTMS + c NSI d Full ms2 1384.2017@hcd30.00 [120.0000-4257.7993]
Precursor Scan: 006530, m/z: 1383.5346, charge: 3
ENGTISR_7_6_1_3_0
Intensity
m/z
204.0863
366.1387
274.0917
138.0547
168.0652
292.1024
186.0759
657.2343
979.4651 PEP_0_1_0_0_0
1182.5464 PEP_0_2_0_0_0
1506.6497 PEP_2_2_0_0_0
1871.7971 PEP_3_3_0_0_0
126.0549
144.0661
454.1546
512.204 LeX
528.1897

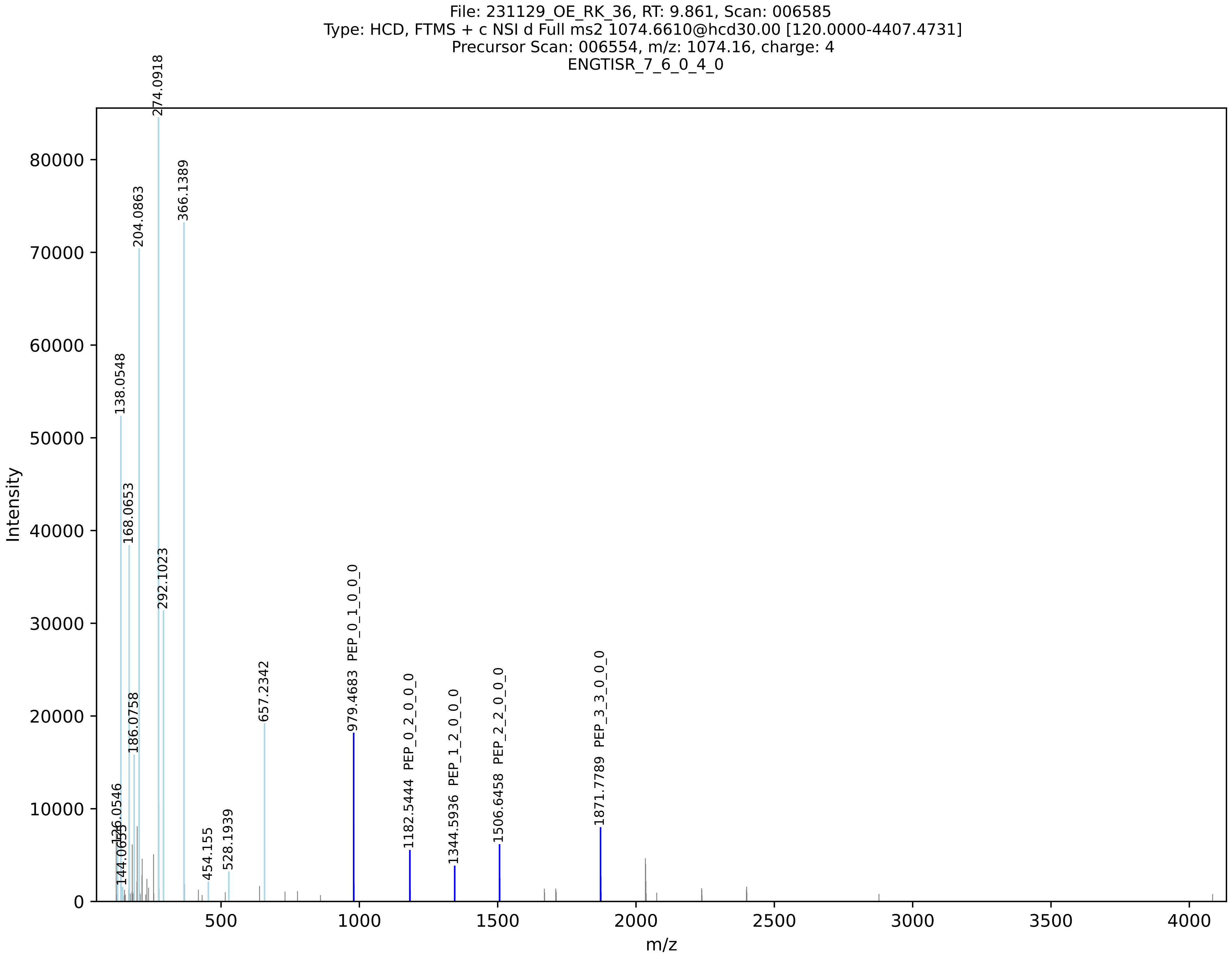

File: 231129_OE_RK_36, RT: 9.861, Scan: 006585
Type: HCD, FTMS + c NSI d Full ms2 1074.6610@hcd30.00 [120.0000-4407.4731]
Precursor Scan: 006554, m/z: 1074.16, charge: 4
ENGTISR_7_6_0_4_0
274.0918
366.1389
204.0863
138.0548
168.0653
292.1023
657.2342
979.4683 PEP_0_1_0_0_0
186.0758
1182.5444 PEP_0_2_0_0_0
1344.5936 PEP_1_2_0_0_0
1506.6458 PEP_2_2_0_0_0
1871.7789 PEP_3_3_0_0_0
126.0546
144.0655
454.155
528.1939
Intensity
m/z

File: 231129_OE_RK_36, RT: 9.97, Scan: 006730
Type: HCD, FTMS + c NSI d Full ms2 1480.9020@hcd30.00 [120.0000-4553.7017]
Precursor Scan: 006702, m/z: 1480.5672, charge: 3
ENGTISR_7_6_1_4_0

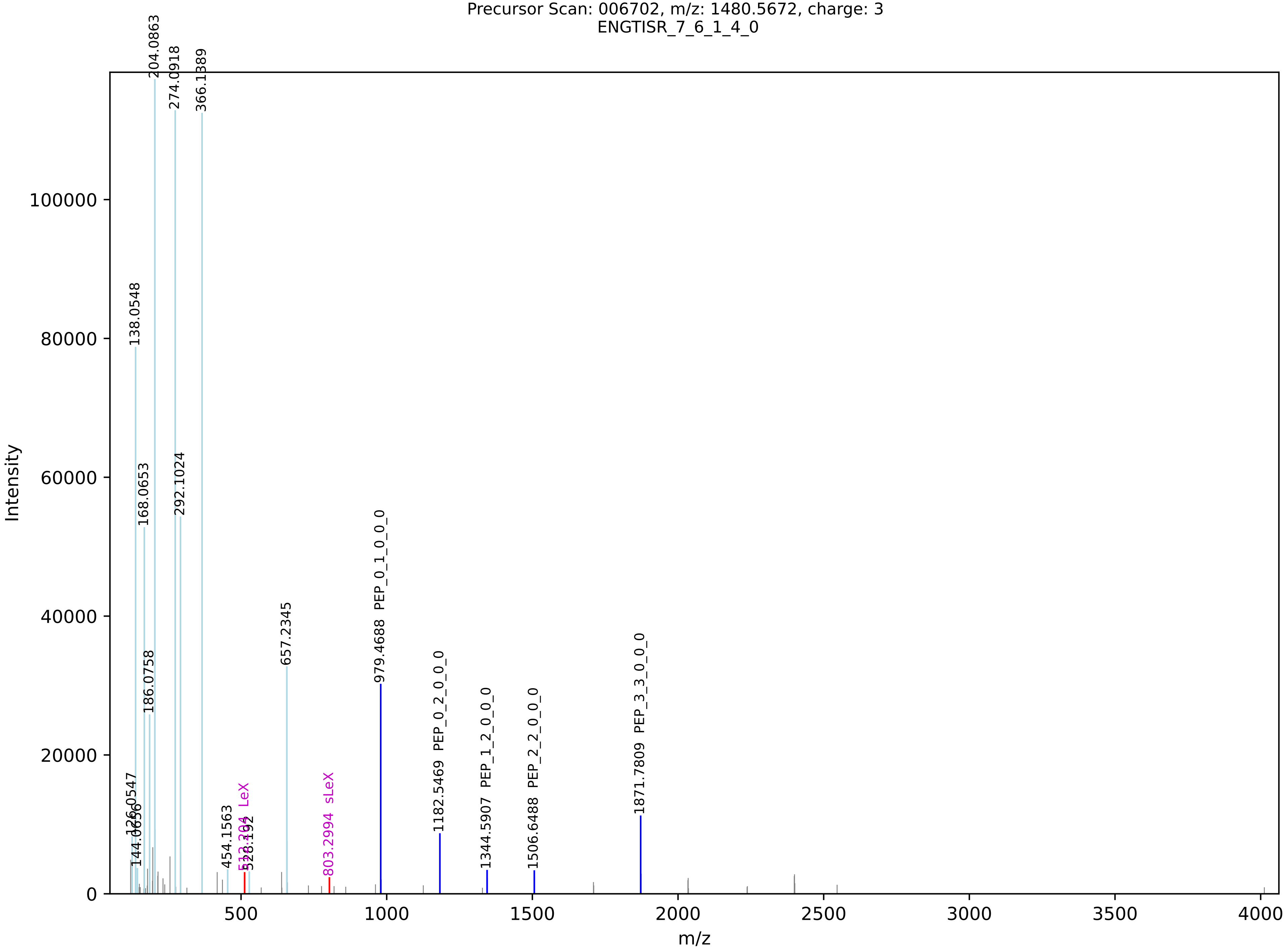

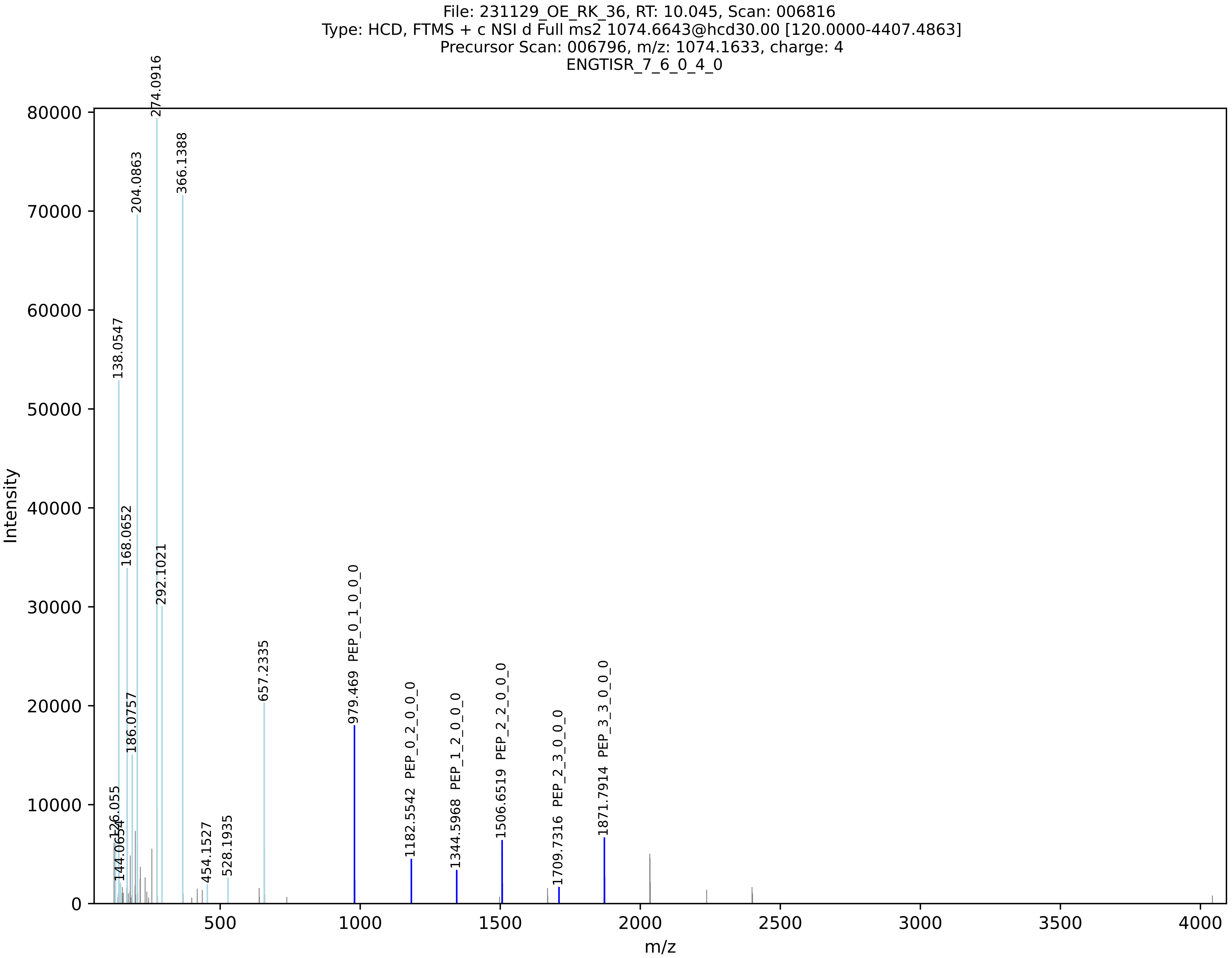
File: 231129_OE_RK_36, RT: 10.045, Scan: 006816
Type: HCD, FTMS + c NSI d Full ms2 1074.6643@hcd30.00 [120.0000-4407.4863]
Precursor Scan: 006796, m/z: 1074.1633, charge: 4
ENGTISR_7_6_0_4_0
Intensity
m/z
0
10000
20000
30000
40000
50000
60000
70000
80000
500
1000
1500
2000
2500
3000
3500
4000
274.0916
366.1388
204.0863
138.0547
168.0652
292.1021
186.0757
126.055
144.0654
454.1527
528.1935
657.2335
979.469 PEP_0_1_0_0_0
1182.5542 PEP_0_2_0_0_0
1344.5968 PEP_1_2_0_0_0
1506.6519 PEP_2_2_0_0_0
1709.7316 PEP_2_3_0_0_0
1871.7914 PEP_3_3_0_0_0

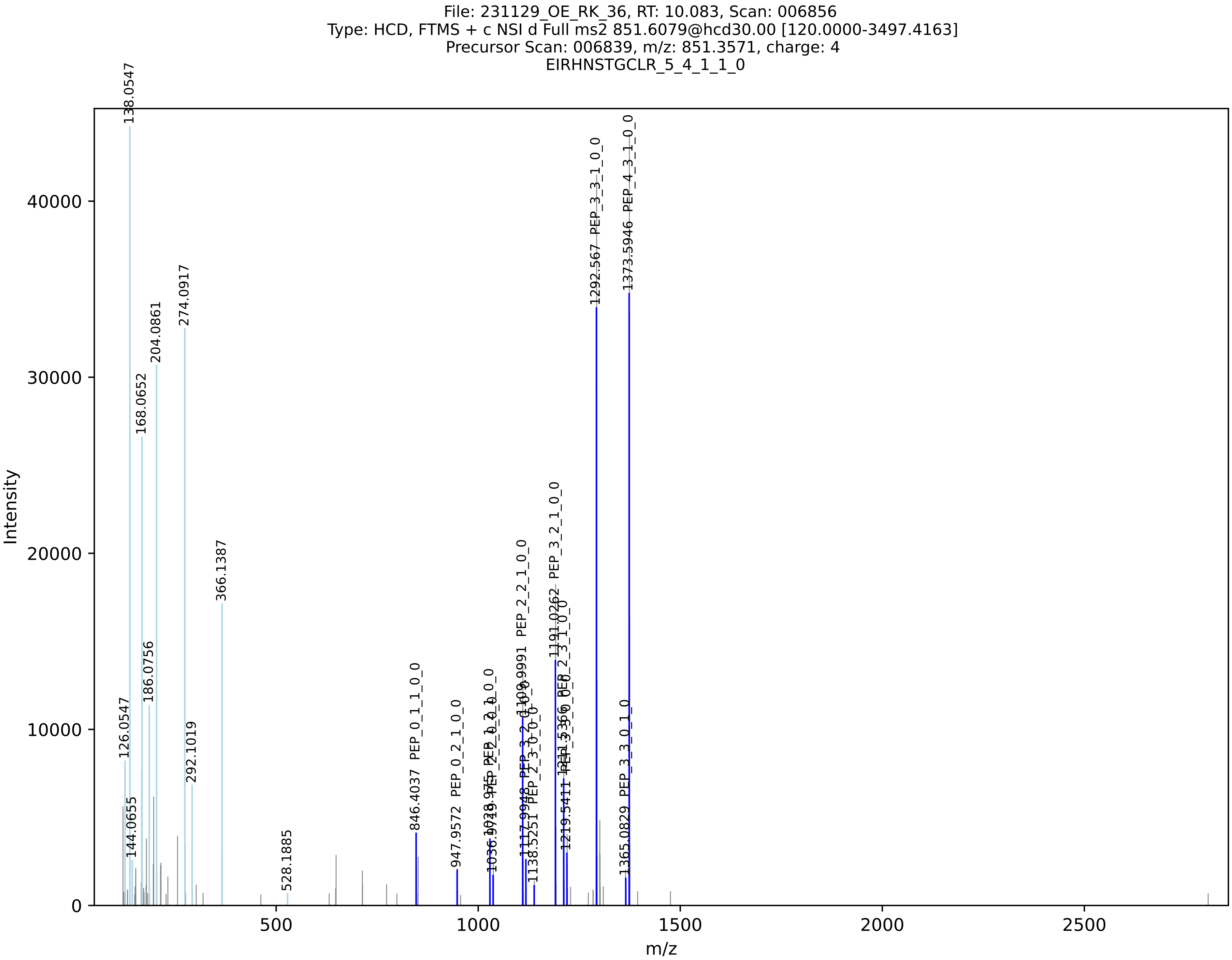
File: 231129_OE_RK_36, RT: 10.083, Scan: 006856
Type: HCD, FTMS + c NSI d Full ms2 851.6079@hcd30.00 [120.0000-3497.4163]
Precursor Scan: 006839, m/z: 851.3571, charge: 4
EIRHNSTGCLR_5_4_1_1_0
Intensity
m/z
0
10000
20000
30000
40000
500
1000
1500
2000
2500
138.0547
274.0917
204.0861
168.0652
366.1387
186.0756
126.0547
292.1019
144.0655
528.1885
846.4037 PEP_0_1_1_0_0
947.9572 PEP_0_2_1_0_0
1292.567 PEP_3_3_1_0_0
1373.5946 PEP_4_3_1_0_0
1191.0262 PEP_3_2_1_0_0
1109.9991 PEP_2_2_1_0_0
1365.0829 PEP_3_3_0_1_0
1138.5251
1219.5411
1117.9948
1036.9719
1028.975

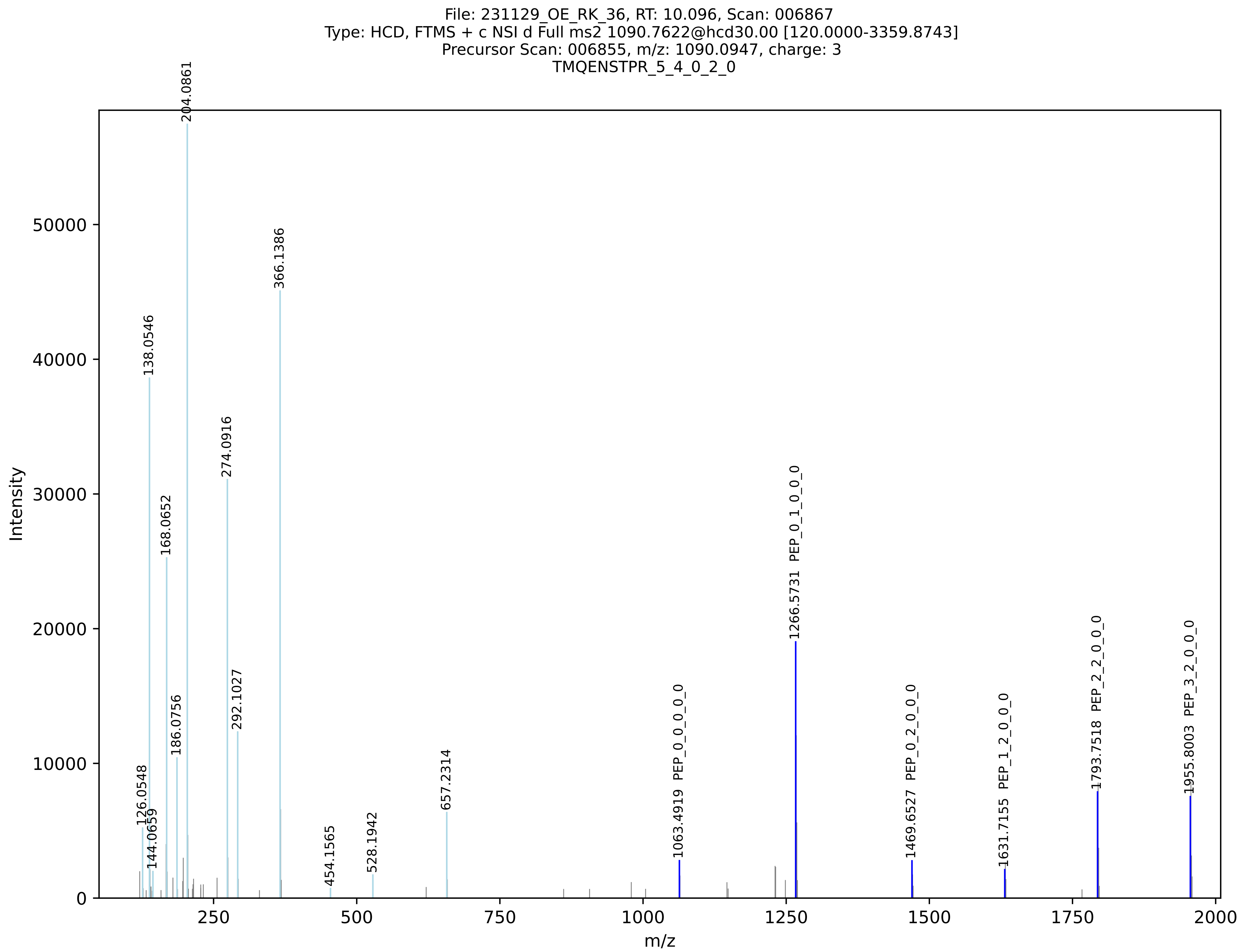

File: 231129_OE_RK_36, RT: 10.096, Scan: 006867
Type: HCD, FTMS + c NSI d Full ms2 1090.7622@hcd30.00 [120.0000-3359.8743]
Precursor Scan: 006855, m/z: 1090.0947, charge: 3
TMQENSTPR_5_4_0_2_0
Intensity
m/z
204.0861
366.1386
138.0546
274.0916
168.0652
292.1027
186.0756
126.0548
144.0659
454.1565
528.1942
657.2314
1063.4919 PEP_0_0_0_0_0
1266.5731 PEP_0_1_0_0_0
1469.6527 PEP_0_2_0_0_0
1631.7155 PEP_1_2_0_0_0
1793.7518 PEP_2_2_0_0_0
1955.8003 PEP_3_2_0_0_0

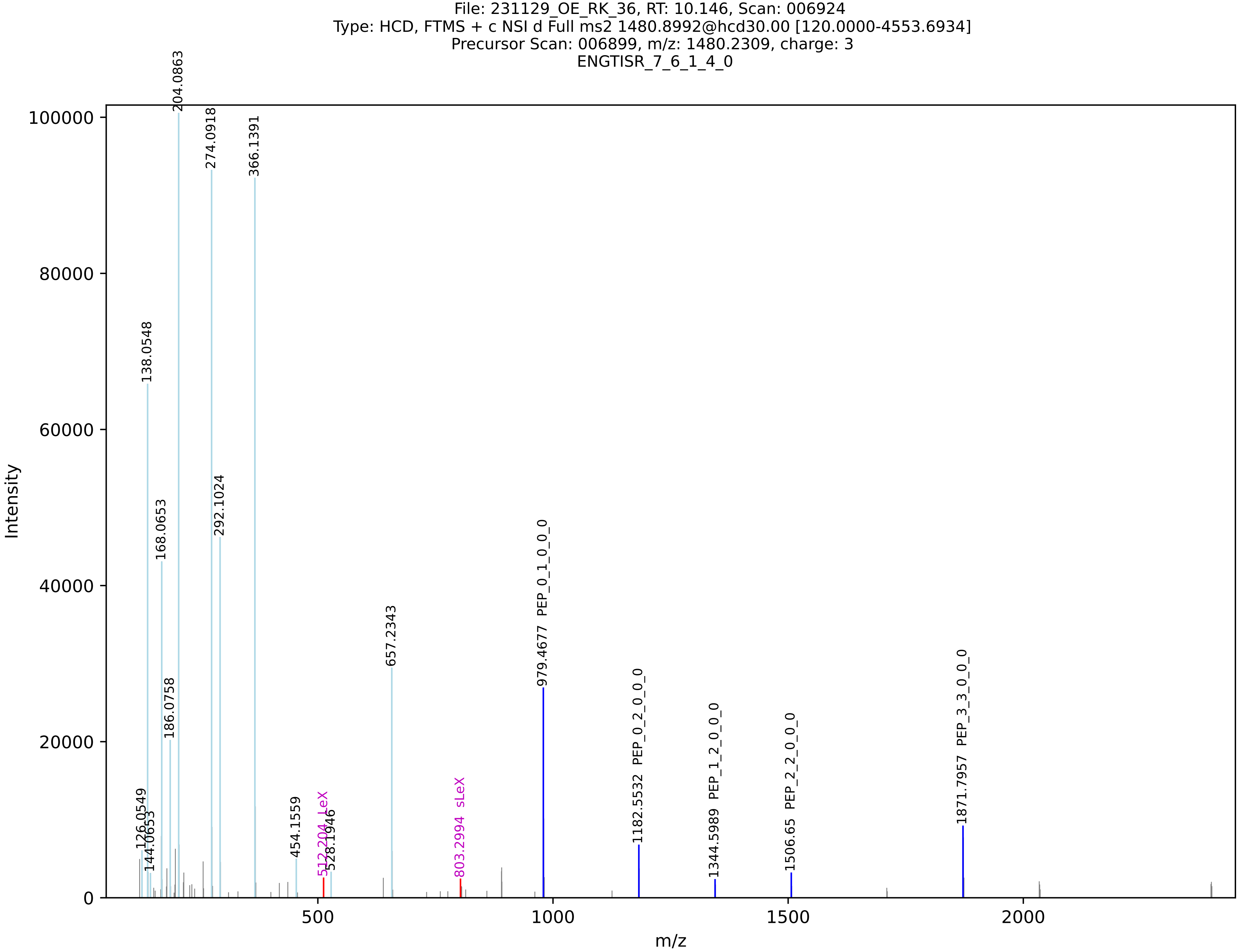

File: 231129_OE_RK_36, RT: 10.146, Scan: 006924
Type: HCD, FTMS + c NSI d Full ms2 1480.8992@hcd30.00 [120.0000-4553.6934]
Precursor Scan: 006899, m/z: 1480.2309, charge: 3
ENGTISR_7_6_1_4_0
Intensity
m/z
204.0863
274.0918
366.1391
138.0548
168.0653
292.1024
186.0758
126.0549
144.0653
454.1559
512.204 LeX
528.1946
657.2343
803.2994 sLeX
979.4677 PEP_0_1_0_0_0
1182.5532 PEP_0_2_0_0_0
1344.5989 PEP_1_2_0_0_0
1506.65 PEP_2_2_0_0_0
1871.7957 PEP_3_3_0_0_0

File: 231129_OE_RK_36, RT: 10.17, Scan: 006956
Type: HCD, FTMS + c NSI d Full ms2 1432.2123@hcd30.00 [120.0000-4404.7114]
Precursor Scan: 006923, m/z: 1431.8803, charge: 3
ENGTISR_7_6_0_4_0

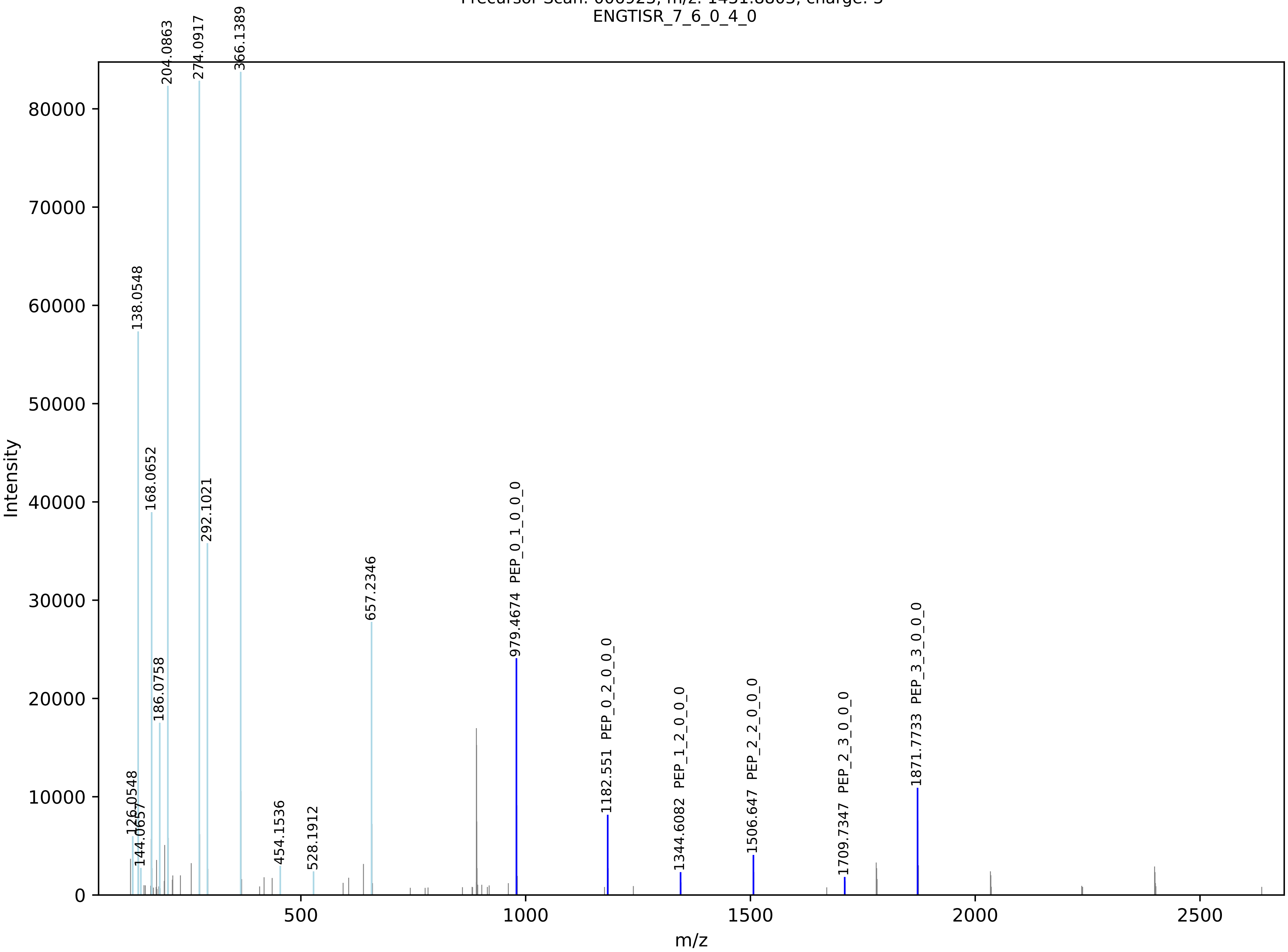

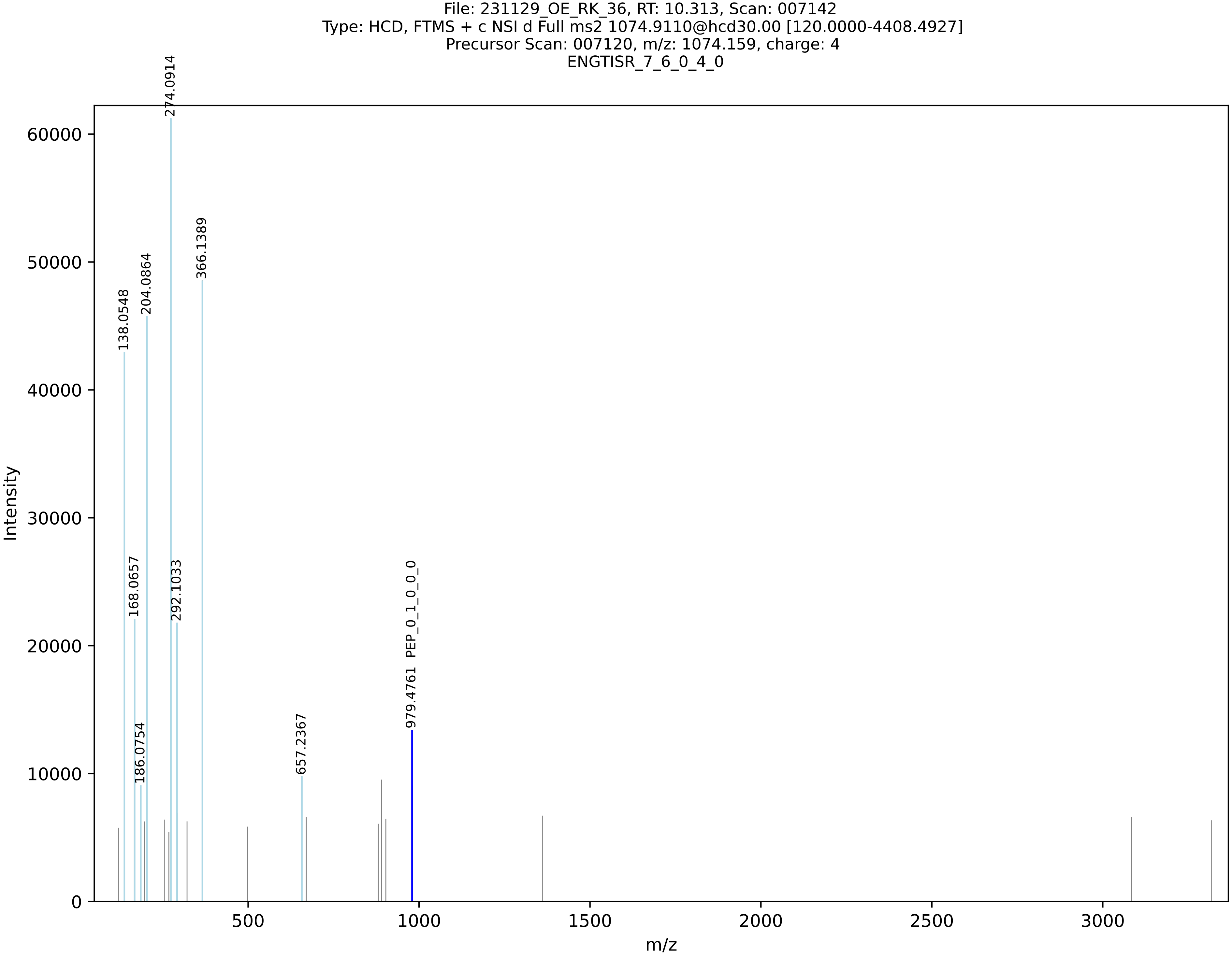
File: 231129_OE_RK_36, RT: 10.313, Scan: 007142
Type: HCD, FTMS + c NSI d Full ms2 1074.9110@hcd30.00 [120.0000-4408.4927]
Precursor Scan: 007120, m/z: 1074.159, charge: 4
ENGTISR_7_6_0_4_0
274.0914
366.1389
204.0864
138.0548
168.0657
292.1033
PEP_0_1_0_0_0
979.4761
186.0754
657.2367
Intensity
m/z
0
10000
20000
30000
40000
50000
60000
500
1000
1500
2000
2500
3000

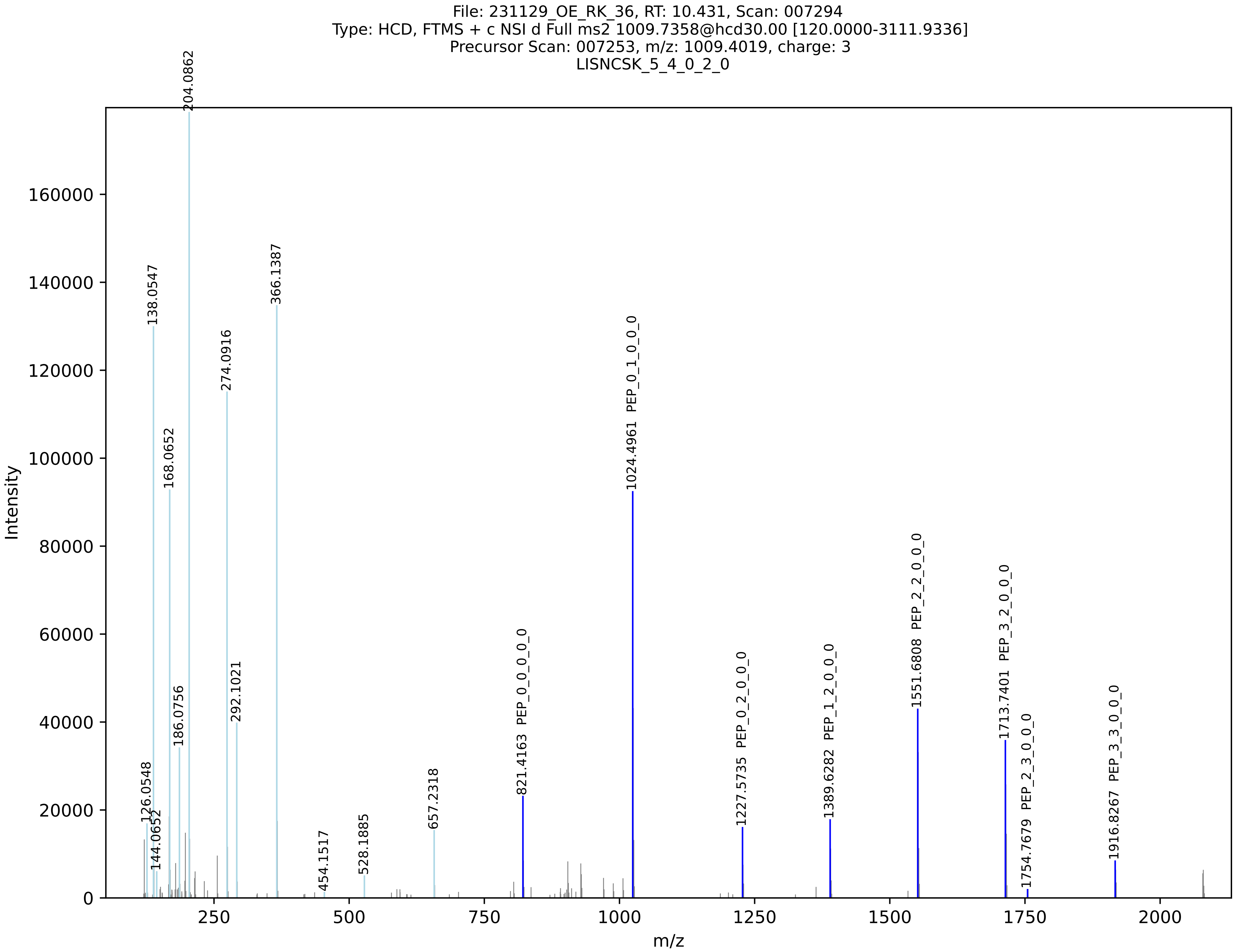

File: 231129_OE_RK_36, RT: 10.431, Scan: 007294
Type: HCD, FTMS + c NSI d Full ms2 1009.7358@hcd30.00 [120.0000-3111.9336]
Precursor Scan: 007253, m/z: 1009.4019, charge: 3
LISNCSK_5_4_0_2_0
Intensity
m/z
204.0862
138.0547
366.1387
274.0916
168.0652
186.0756
292.1021
126.0548
144.0652
454.1517
528.1885
657.2318
821.4163 PEP_0_0_0_0_0
1024.4961 PEP_0_1_0_0_0
1227.5735 PEP_0_2_0_0_0
1389.6282 PEP_1_2_0_0_0
1551.6808 PEP_2_2_0_0_0
1713.7401 PEP_3_2_0_0_0
1754.7679 PEP_2_3_0_0_0
1916.8267 PEP_3_3_0_0_0

File: 231129_OE_RK_36, RT: 10.523, Scan: 007422
Type: HCD, FTMS + c NSI d Full ms2 1480.9004@hcd30.00 [120.0000-4553.6973]
Precursor Scan: 007371, m/z: 1480.5661, charge: 3
ENGTISR_7_6_1_4_0

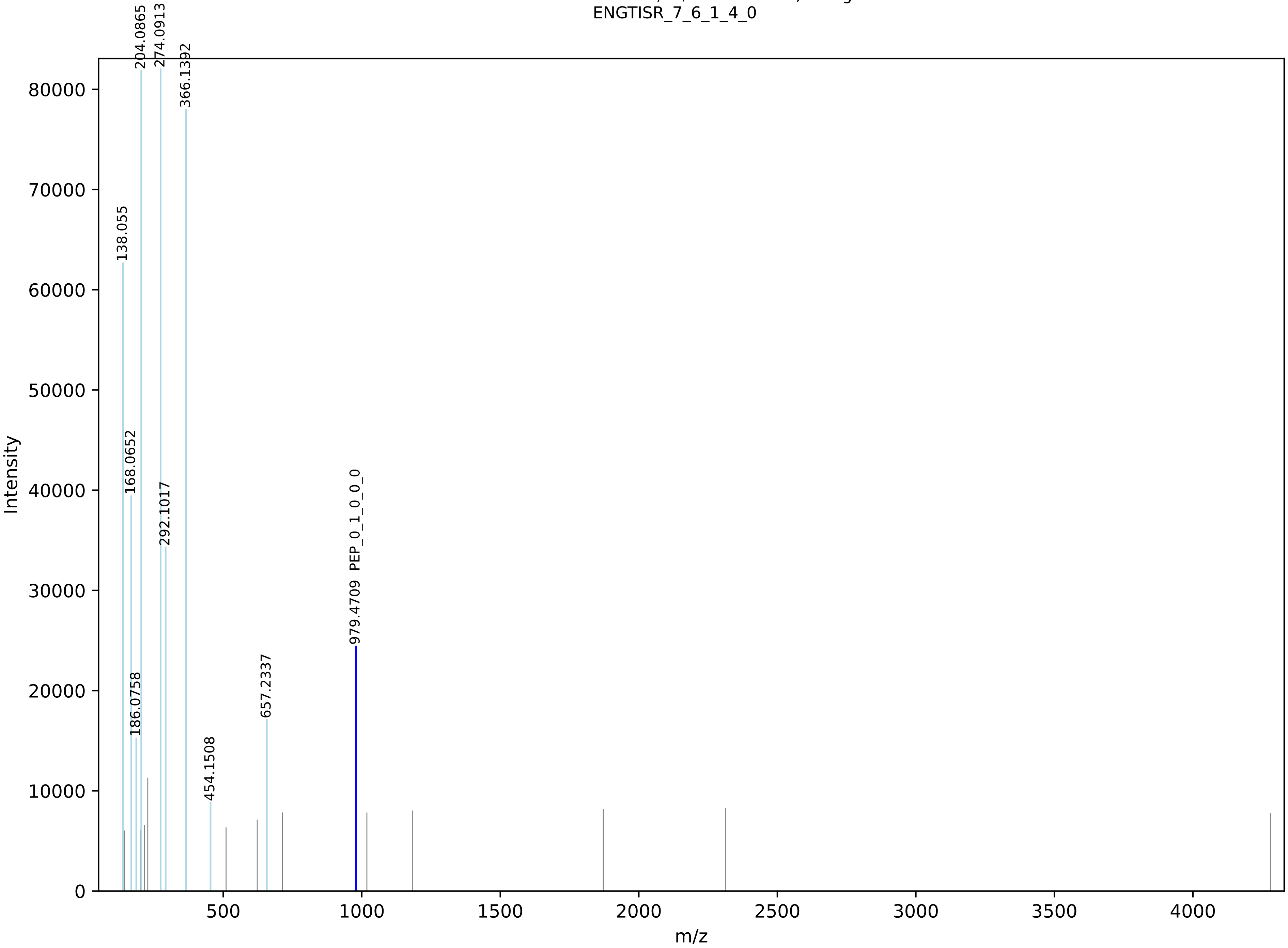

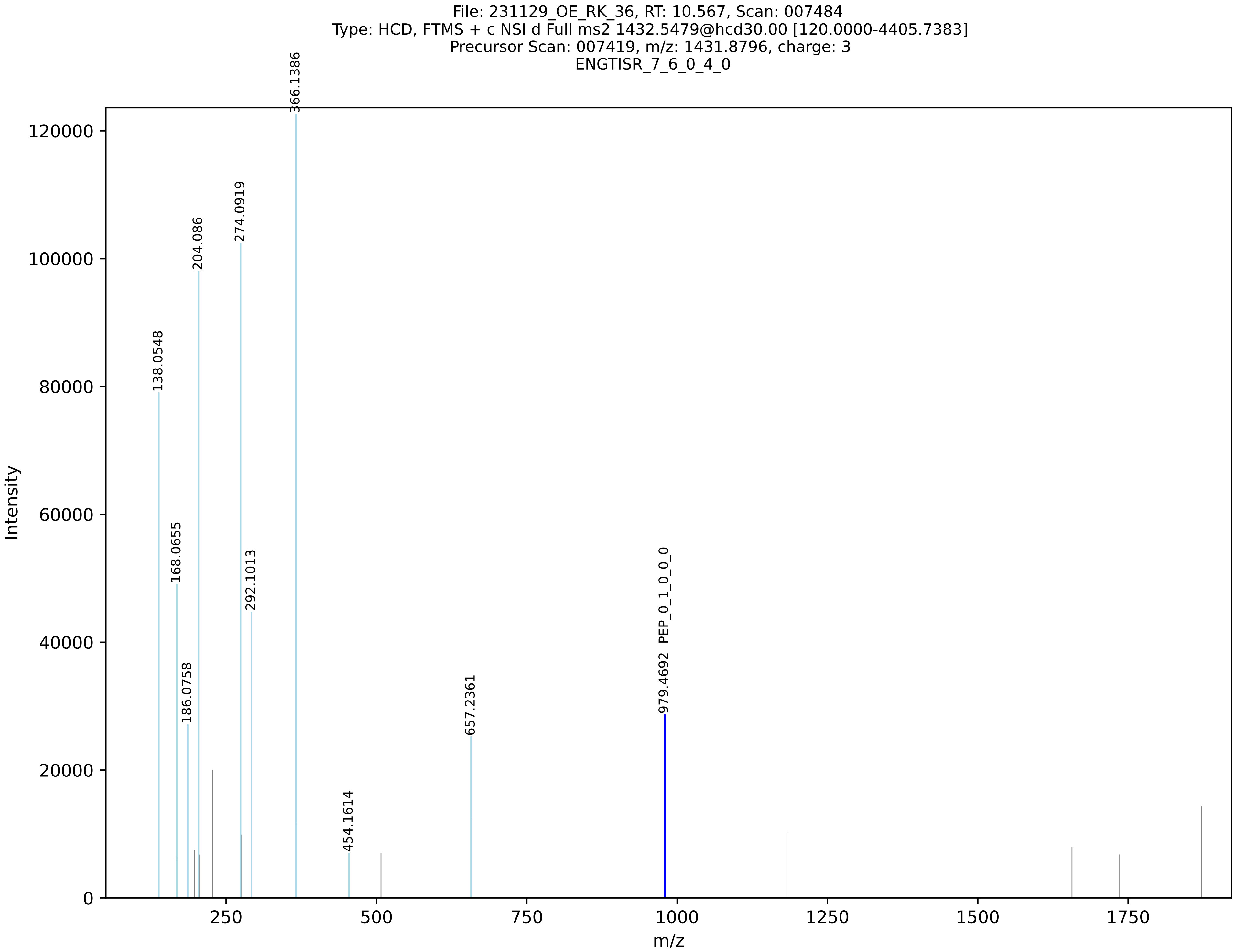

File: 231129_OE_RK_36, RT: 10.567, Scan: 007484
Type: HCD, FTMS + c NSI d Full ms2 1432.5479@hcd30.00 [120.0000-4405.7383]
Precursor Scan: 007419, m/z: 1431.8796, charge: 3
ENGTISR_7_6_0_4_0
366.1386
274.0919
204.086
138.0548
168.0655
292.1013
186.0758
454.1614
657.2361
979.4692 PEP_0_1_0_0_0
Intensity
m/z
0
20000
40000
60000
80000
100000
120000
250
500
750
1000
1250
1500
1750

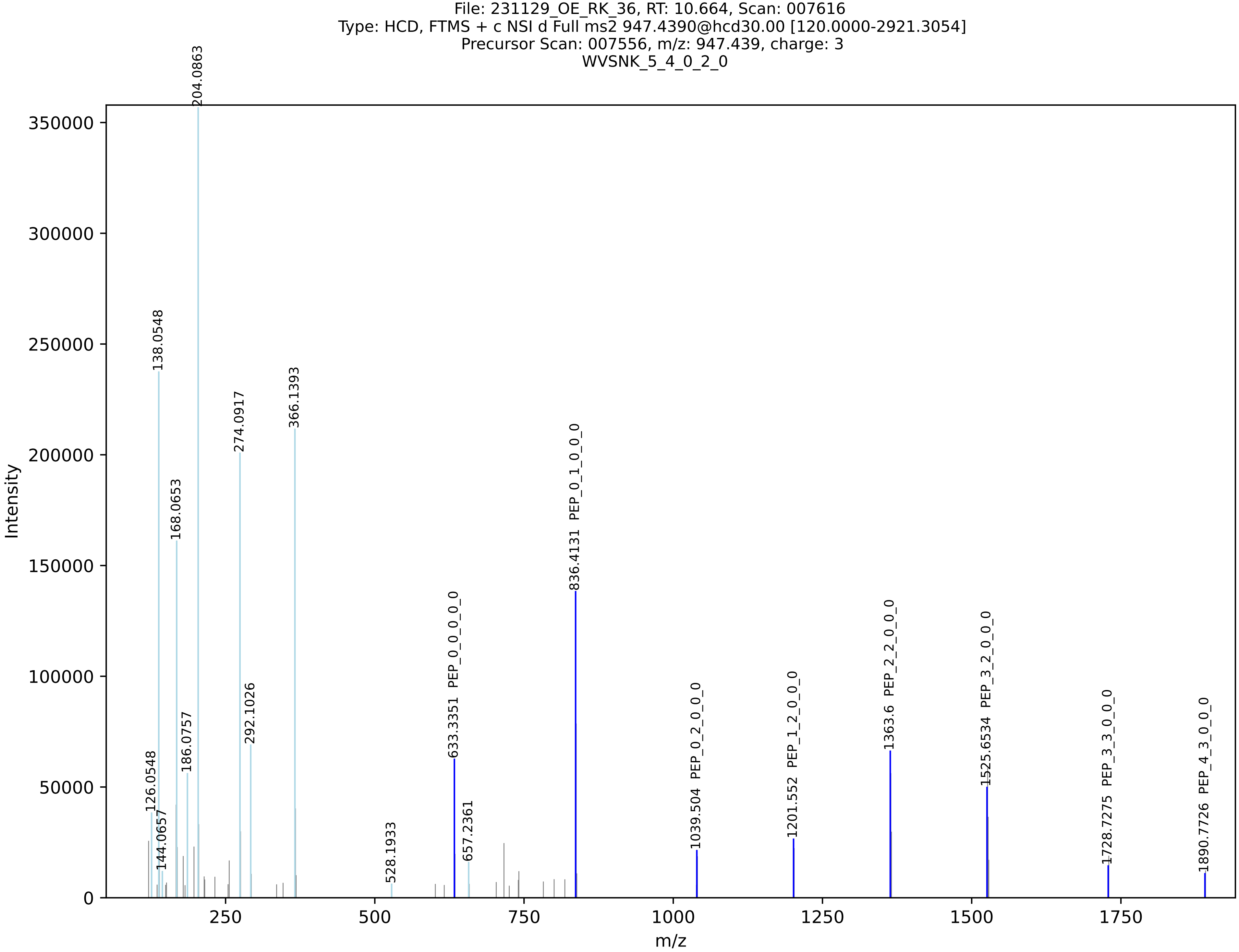

File: 231129_OE_RK_36, RT: 10.664, Scan: 007616
Type: HCD, FTMS + c NSI d Full ms2 947.4390@hcd30.00 [120.0000-2921.3054]
Precursor Scan: 007556, m/z: 947.439, charge: 3
WVSNK_5_4_0_2_0
Intensity
m/z
204.0863
138.0548
168.0653
274.0917
366.1393
292.1026
186.0757
126.0548
144.0657
528.1933
633.3351 PEP_0_0_0_0_0
657.2361
836.4131 PEP_0_1_0_0_0
1039.504 PEP_0_2_0_0_0
1201.552 PEP_1_2_0_0_0
1363.6 PEP_2_2_0_0_0
1525.6534 PEP_3_2_0_0_0
1728.7275 PEP_3_3_0_0_0
1890.7726 PEP_4_3_0_0_0

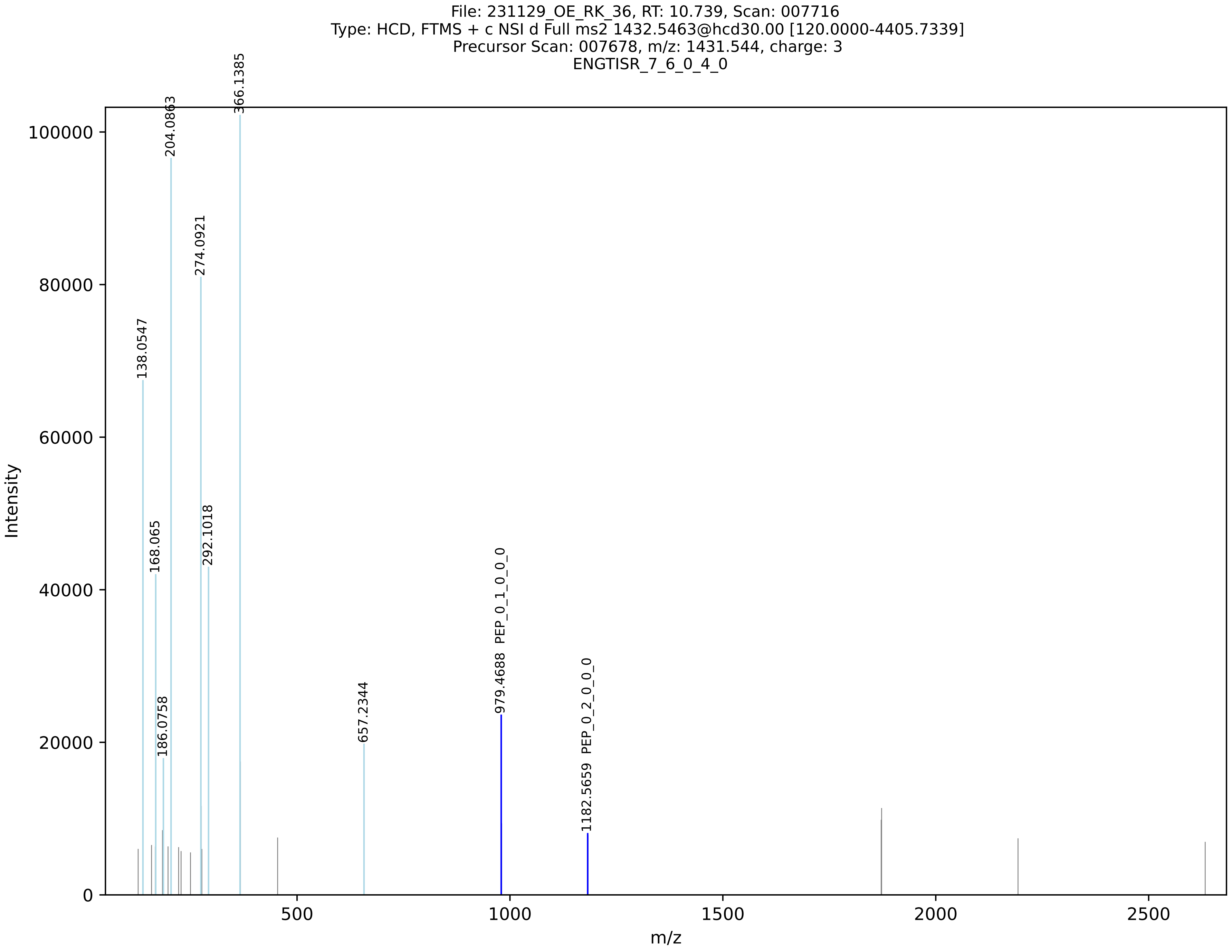

File: 231129_OE_RK_36, RT: 10.739, Scan: 007716
Type: HCD, FTMS + c NSI d Full ms2 1432.5463@hcd30.00 [120.0000-4405.7339]
Precursor Scan: 007678, m/z: 1431.544, charge: 3
ENGTISR_7_6_0_4_0
Intensity
m/z
366.1385
204.0863
274.0921
138.0547
168.065
292.1018
186.0758
657.2344
979.4688 PEP_0_1_0_0_0
1182.5659 PEP_0_2_0_0_0
0
20000
40000
60000
80000
100000
500
1000
1500
2000
2500

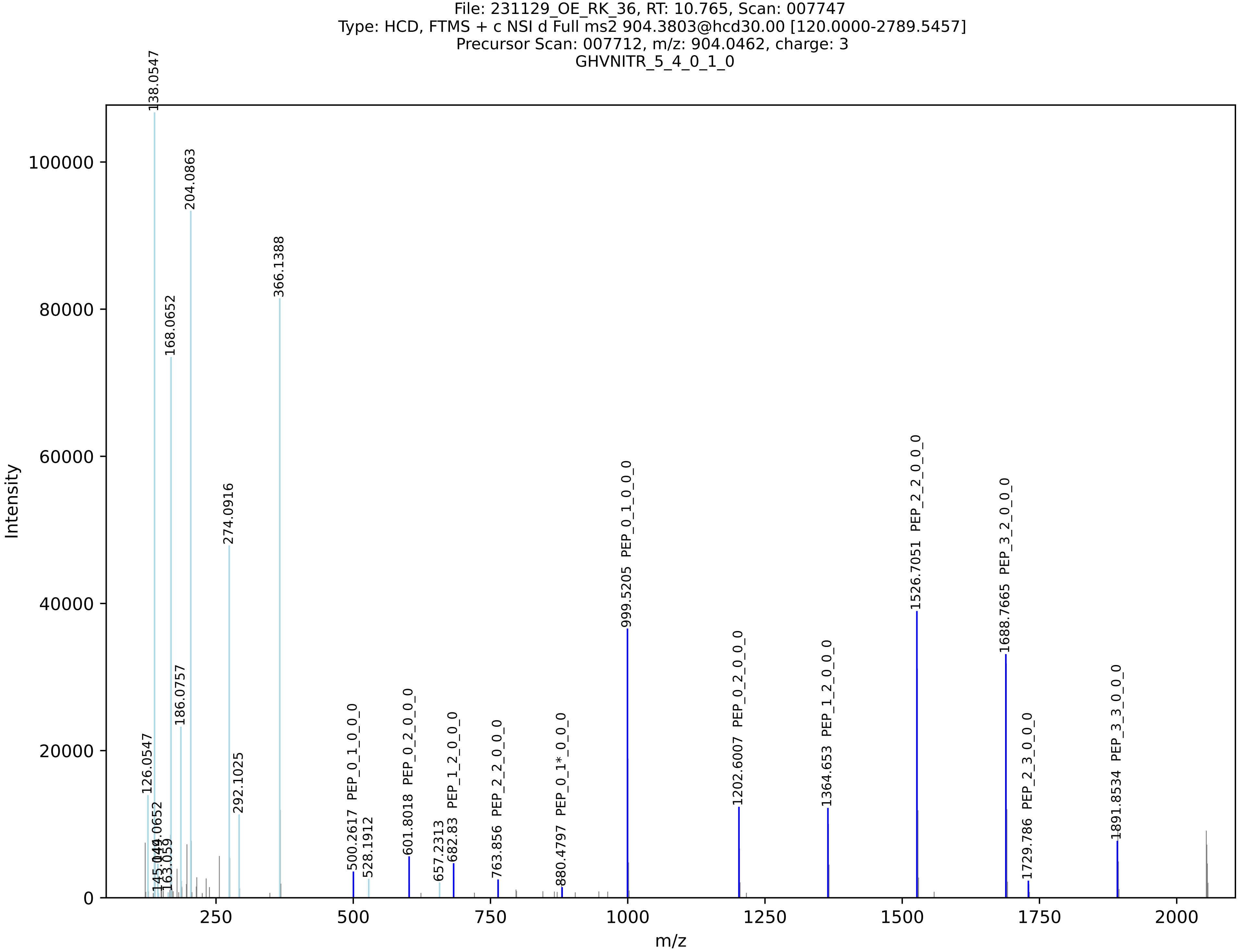
File: 231129_OE_RK_36, RT: 10.765, Scan: 007747
Type: HCD, FTMS + c NSI d Full ms2 904.3803@hcd30.00 [120.0000-2789.5457]
Precursor Scan: 007712, m/z: 904.0462, charge: 3
GHVNITR_5_4_0_1_0
Intensity
m/z
0
20000
40000
60000
80000
100000
250
500
750
1000
1250
1500
1750
2000
138.0547
204.0863
366.1388
168.0652
274.0916
186.0757
126.0547
292.1025
500.2617 PEP_0_1_0_0_0
528.1912
601.8018 PEP_0_2_0_0_0
657.2313
682.83 PEP_1_2_0_0_0
763.856 PEP_2_2_0_0_0
880.4797 PEP_0_1*_0_0_0
999.5205 PEP_0_1_0_0_0
1202.6007 PEP_0_2_0_0_0
1364.653 PEP_1_2_0_0_0
1526.7051 PEP_2_2_0_0_0
1688.7665 PEP_3_2_0_0_0
1729.786 PEP_2_3_0_0_0
1891.8534 PEP_3_3_0_0_0

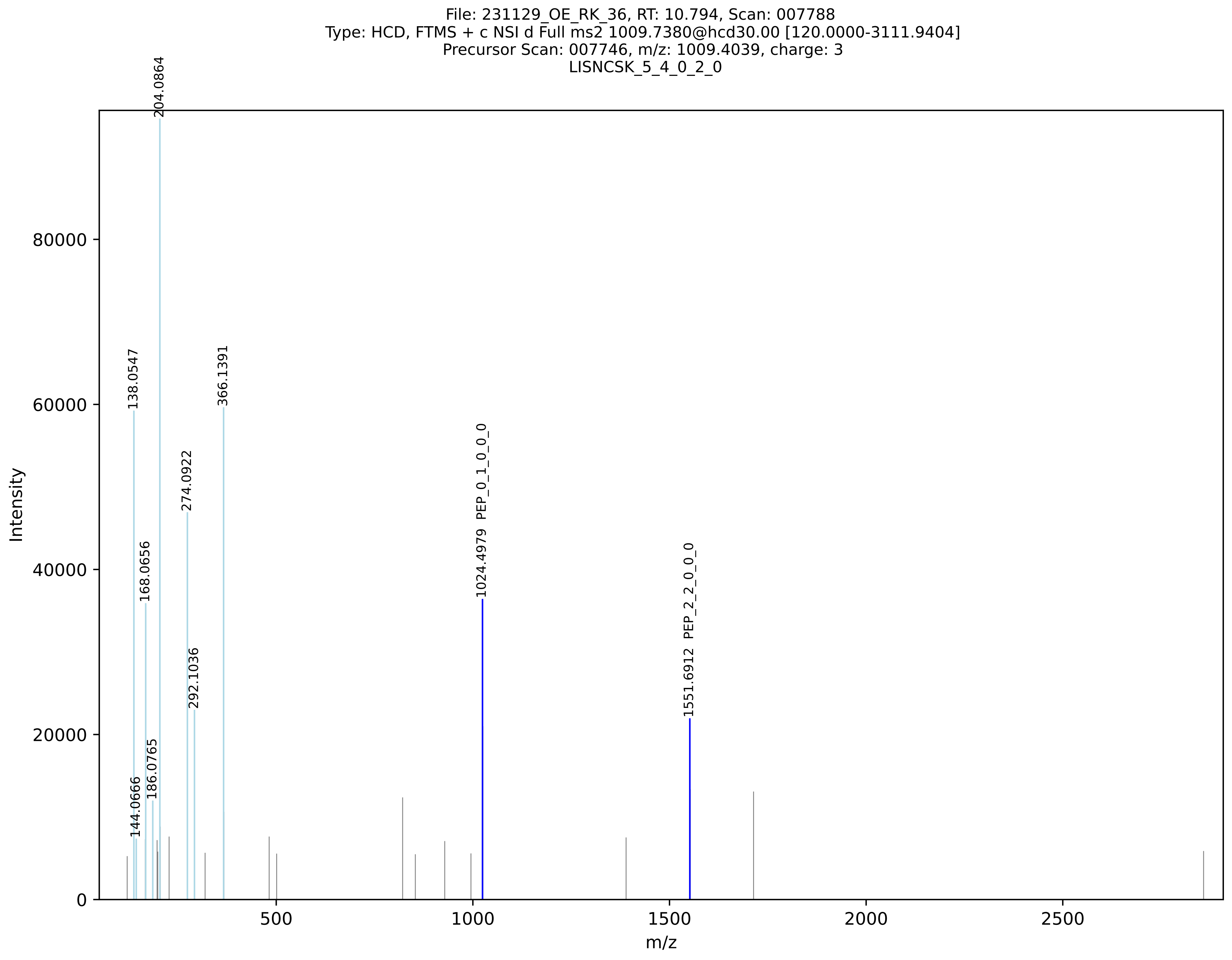
File: 231129_OE_RK_36, RT: 10.794, Scan: 007788
Type: HCD, FTMS + c NSI d Full ms2 1009.7380@hcd30.00 [120.0000-3111.9404]
Precursor Scan: 007746, m/z: 1009.4039, charge: 3
LISNCSK_5_4_0_2_0
204.0864
138.0547
366.1391
274.0922
168.0656
292.1036
186.0765
144.0666
1024.4979 PEP_0_1_0_0_0
1551.6912 PEP_2_2_0_0_0
Intensity
m/z
0
20000
40000
60000
80000
500
1000
1500
2000
2500

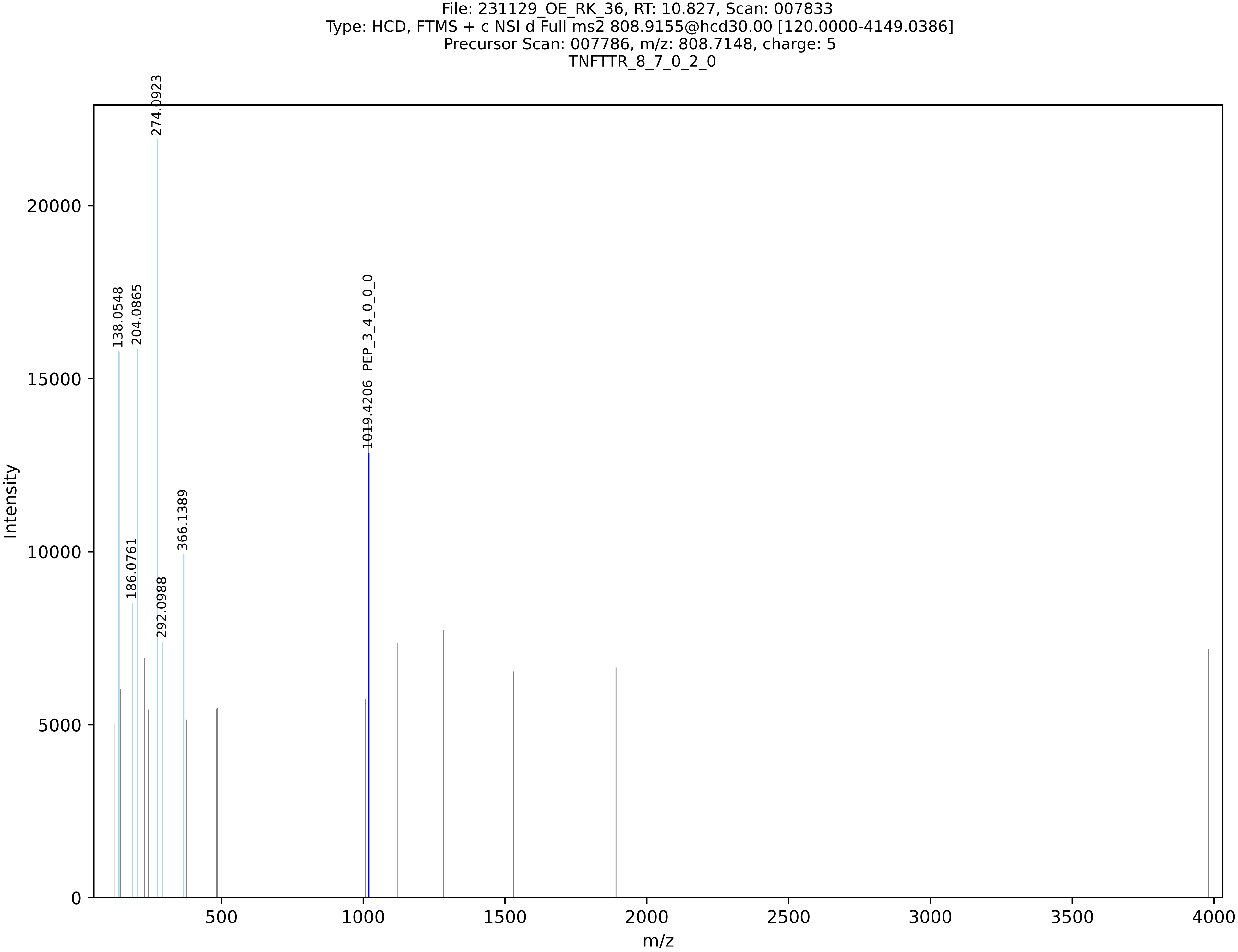

File: 231129_OE_RK_36, RT: 10.827, Scan: 007833
Type: HCD, FTMS + c NSI d Full ms2 808.9155@hcd30.00 [120.0000-4149.0386]
Precursor Scan: 007786, m/z: 808.7148, charge: 5
TNFTTR_8_7_0_2_0
274.0923
138.0548
204.0865
1019.4206 PEP_3_4_0_0_0
366.1389
186.0761
292.0988
Intensity
m/z
0
5000
10000
15000
20000
500
1000
1500
2000
2500
3000
3500
4000

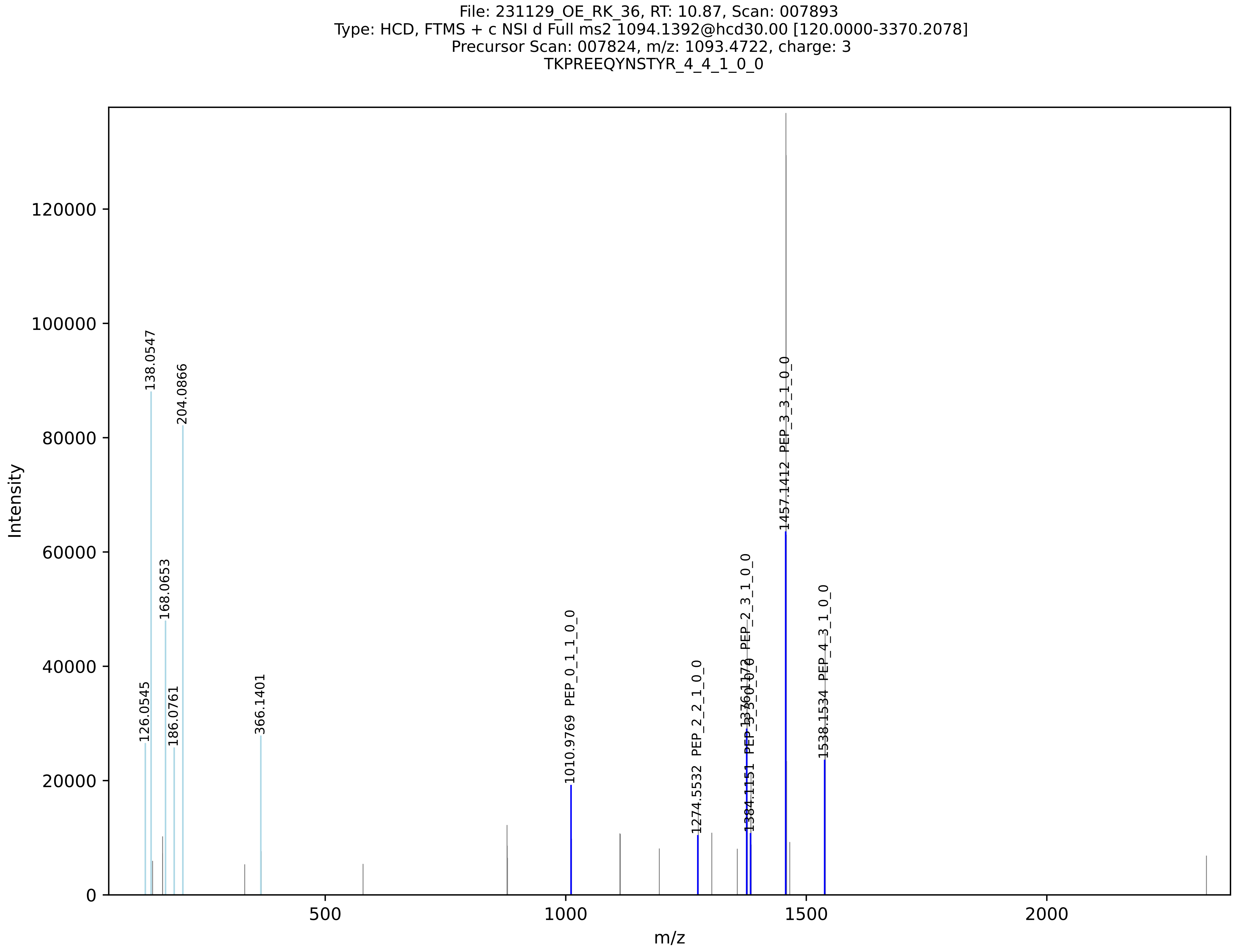
File: 231129_OE_RK_36, RT: 10.87, Scan: 007893
Type: HCD, FTMS + c NSI d Full ms2 1094.1392@hcd30.00 [120.0000-3370.2078]
Precursor Scan: 007824, m/z: 1093.4722, charge: 3
TKPREEQYNSTYR_4_4_1_0_0
Intensity
m/z
0
20000
40000
60000
80000
100000
120000
500
1000
1500
2000
126.0545
138.0547
168.0653
186.0761
204.0866
366.1401
1010.9769 PEP_0_1_1_0_0
1274.5532 PEP_2_2_1_0_0
1376.1072 PEP_2_3_1_0_0
1384.1151 PEP_3_3_0_0_0
1457.1412 PEP_3_3_1_0_0
1538.1534 PEP_4_3_1_0_0

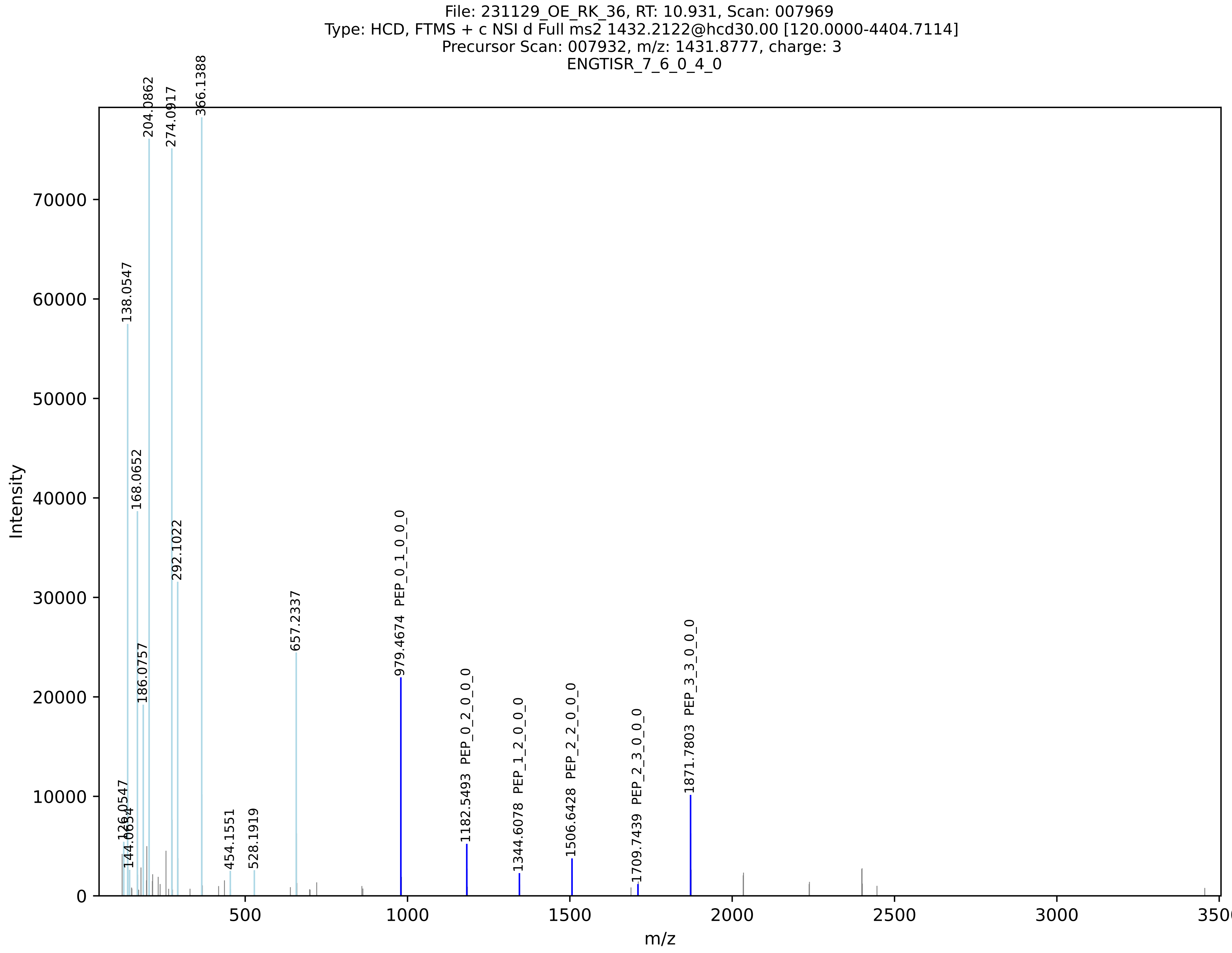
File: 231129_OE_RK_36, RT: 10.931, Scan: 007969
Type: HCD, FTMS + c NSI d Full ms2 1432.2122@hcd30.00 [120.0000-4404.7114]
Precursor Scan: 007932, m/z: 1431.8777, charge: 3
ENGTISR_7_6_0_4_0
366.1388
204.0862
274.0917
138.0547
168.0652
292.1022
186.0757
657.2337
979.4674 PEP_0_1_0_0_0
1182.5493 PEP_0_2_0_0_0
1344.6078 PEP_1_2_0_0_0
1506.6428 PEP_2_2_0_0_0
1709.7439 PEP_2_3_0_0_0
1871.7803 PEP_3_3_0_0_0
126.0547
144.0654
454.1551
528.1919
Intensity
m/z
0
10000
20000
30000
40000
50000
60000
70000
500
1000
1500
2000
2500
3000
3500

File: 231129_OE_RK_36, RT: 10.985, Scan: 008041
Type: HCD, FTMS + c NSI d Full ms2 919.3781@hcd30.00 [120.0000-3773.9187]
Precursor Scan: 007996, m/z: 919.1275, charge: 4
NAHGEEKENLTAR_5_4_0_2_0

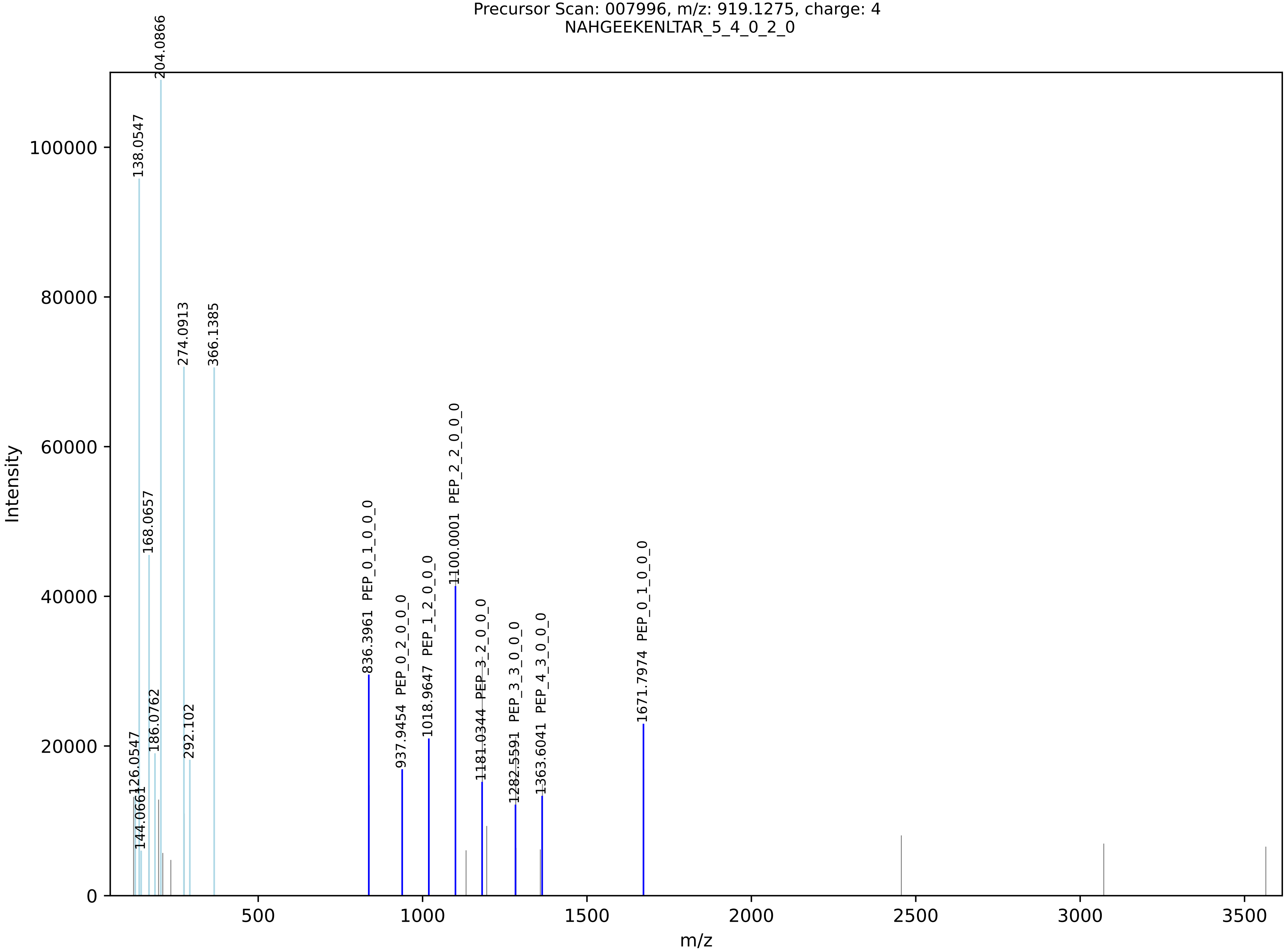

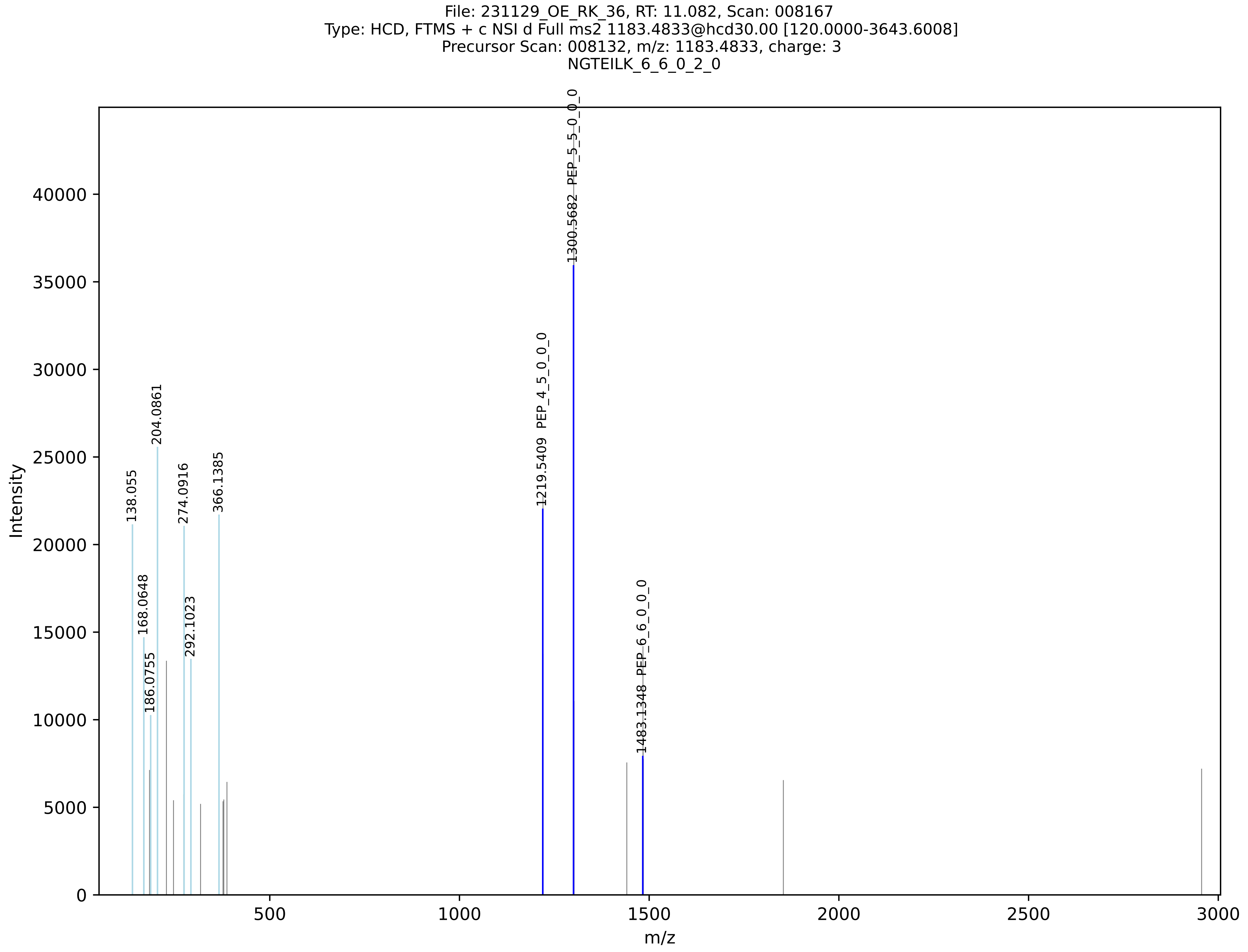
File: 231129_OE_RK_36, RT: 11.082, Scan: 008167
Type: HCD, FTMS + c NSI d Full ms2 1183.4833@hcd30.00 [120.0000-3643.6008]
Precursor Scan: 008132, m/z: 1183.4833, charge: 3
NGTEILK_6_6_0_2_0
Intensity
m/z
138.055
168.0648
186.0755
204.0861
274.0916
292.1023
366.1385
1219.5409_PEP_4_5_0_0_0_
1300.5682_PEP_5_5_0_0_0_
1483.1348_PEP_6_6_0_0_0_

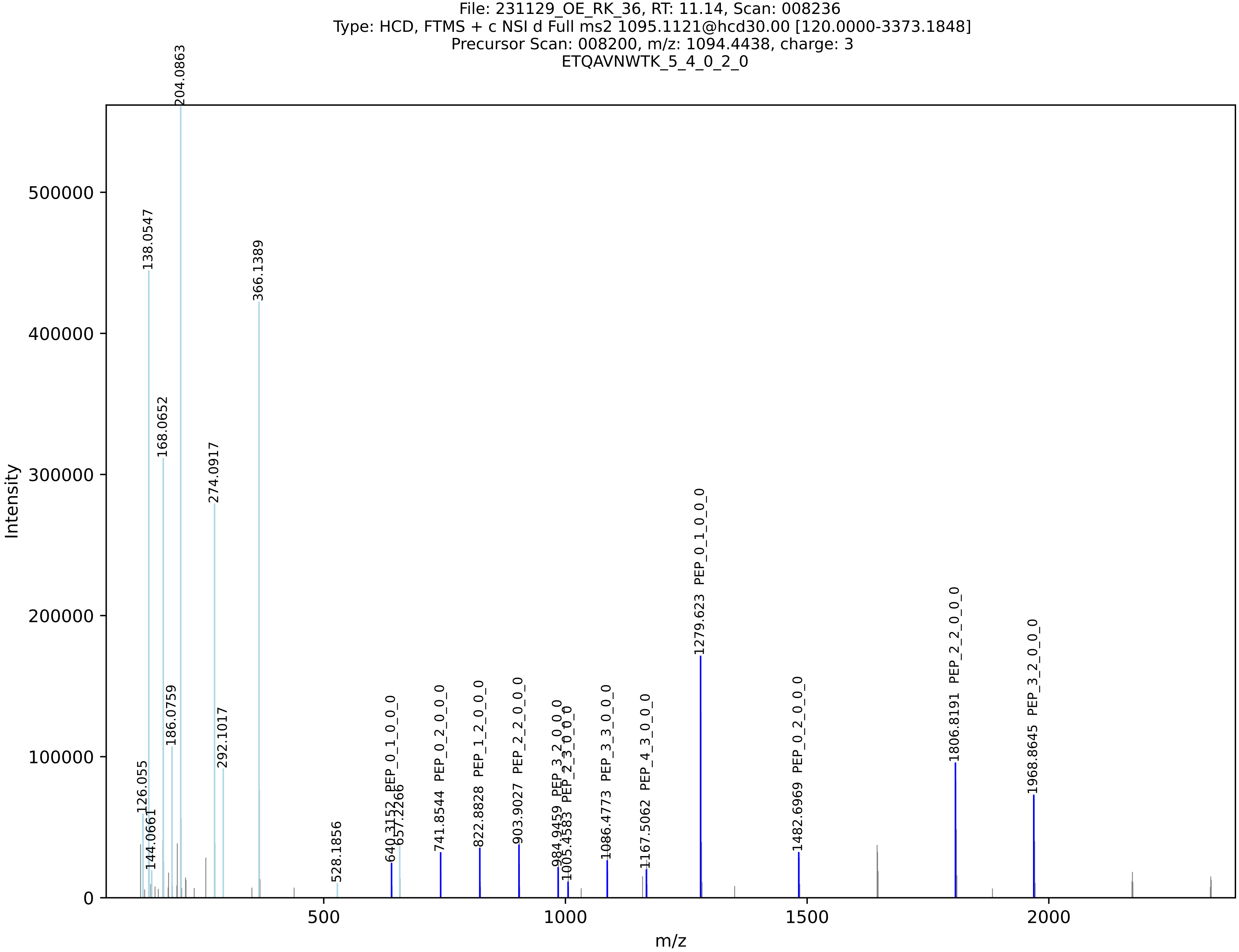
File: 231129_OE_RK_36, RT: 11.14, Scan: 008236
Type: HCD, FTMS + c NSI d Full ms2 1095.1121@hcd30.00 [120.0000-3373.1848]
Precursor Scan: 008200, m/z: 1094.4438, charge: 3
ETQAVNWTK_5_4_0_2_0
Intensity
m/z
204.0863
138.0547
366.1389
168.0652
274.0917
186.0759
292.1017
126.055
144.0661
528.1856
640.3152 PEP_0_1_0_0_
657.2266
741.8544 PEP_0_2_0_0_0
822.8828 PEP_1_2_0_0_0
903.9027 PEP_2_2_0_0_0
984.9459 PEP_3_2_0_0_0
1005.4583 PEP_2_3_0_0_0
1086.4773 PEP_3_3_0_0_0
1167.5062 PEP_4_3_0_0_0
1279.623 PEP_0_1_0_0_0
1482.6969 PEP_0_2_0_0_0
1806.8191 PEP_2_2_0_0_0
1968.8645 PEP_3_2_0_0_0

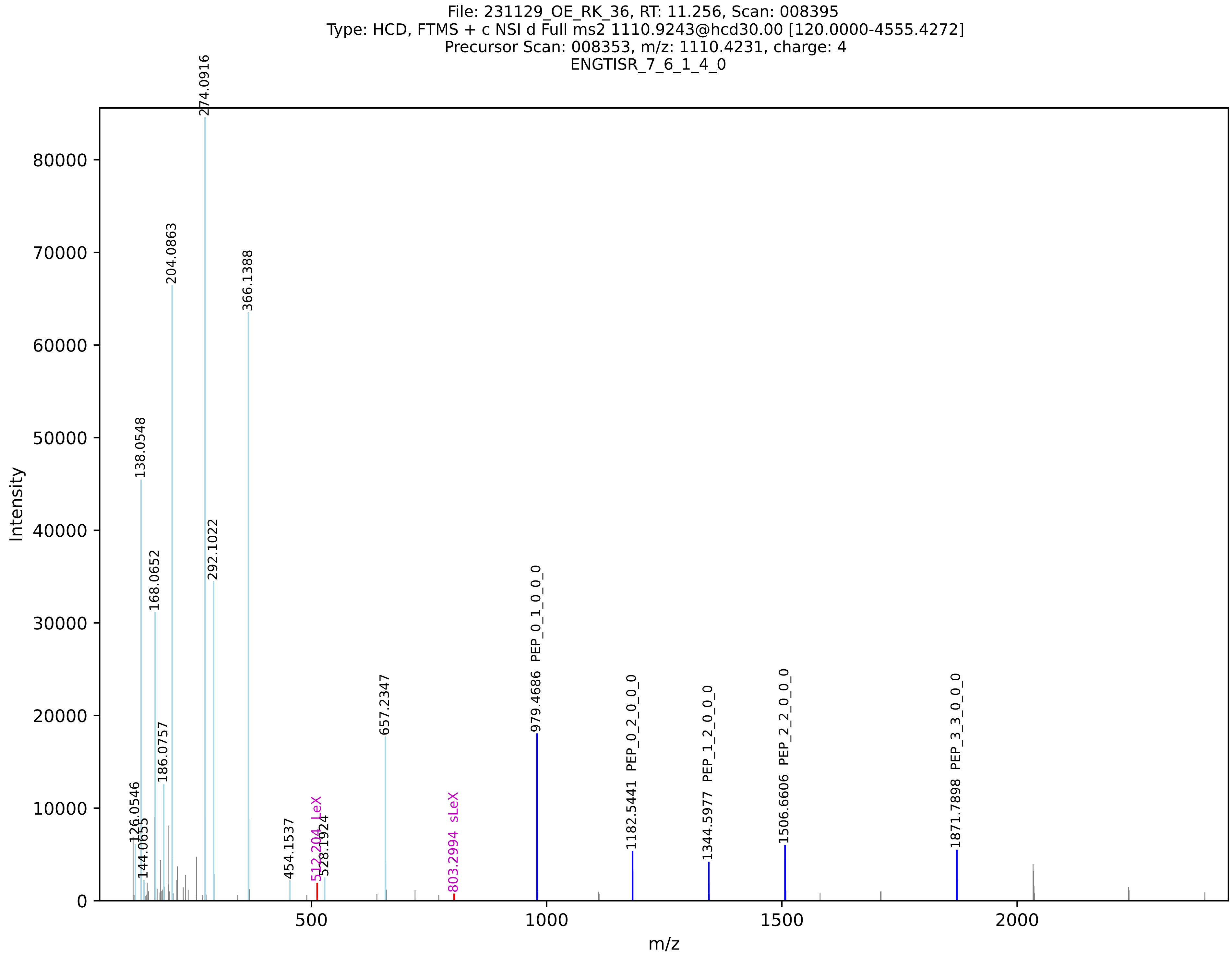

File: 231129_OE_RK_36, RT: 11.256, Scan: 008395
Type: HCD, FTMS + c NSI d Full ms2 1110.9243@hcd30.00 [120.0000-4555.4272]
Precursor Scan: 008353, m/z: 1110.4231, charge: 4
ENGTISR_7_6_1_4_0
Intensity
m/z
274.0916
204.0863
366.1388
138.0548
292.1022
168.0652
186.0757
126.0546
144.0655
454.1537
512.204 LeX
528.1924
657.2347
803.2994 sLeX
979.4686 PEP_0_1_0_0_0
1182.5441 PEP_0_2_0_0_0
1344.5977 PEP_1_2_0_0_0
1506.6606 PEP_2_2_0_0_0
1871.7898 PEP_3_3_0_0_0

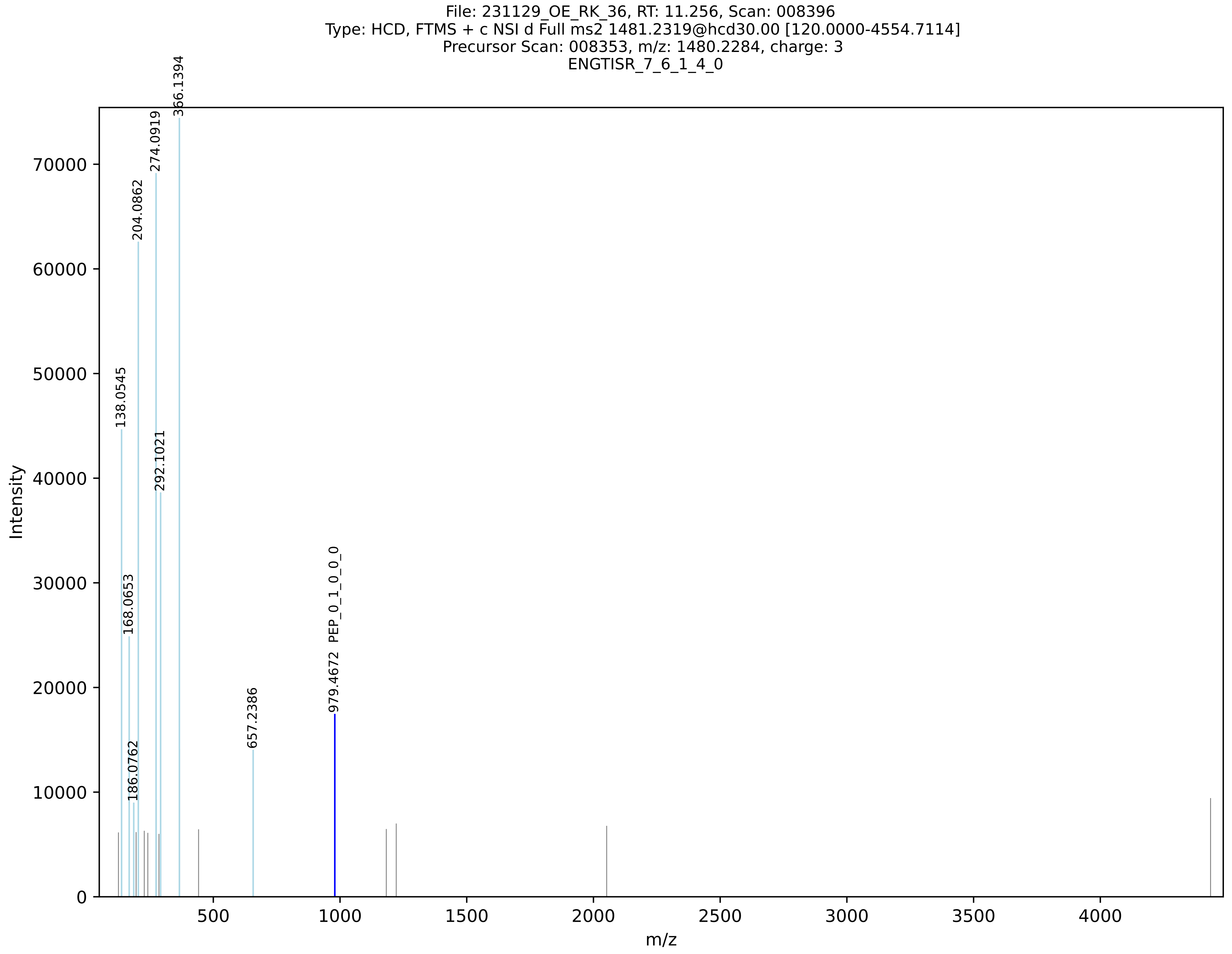
File: 231129_OE_RK_36, RT: 11.256, Scan: 008396
Type: HCD, FTMS + c NSI d Full ms2 1481.2319@hcd30.00 [120.0000-4554.7114]
Precursor Scan: 008353, m/z: 1480.2284, charge: 3
ENGTISR_7_6_1_4_0
366.1394
274.0919
204.0862
138.0545
292.1021
168.0653
186.0762
657.2386
979.4672 PEP_0_1_0_0_0
Intensity
m/z
0
10000
20000
30000
40000
50000
60000
70000
500
1000
1500
2000
2500
3000
3500
4000

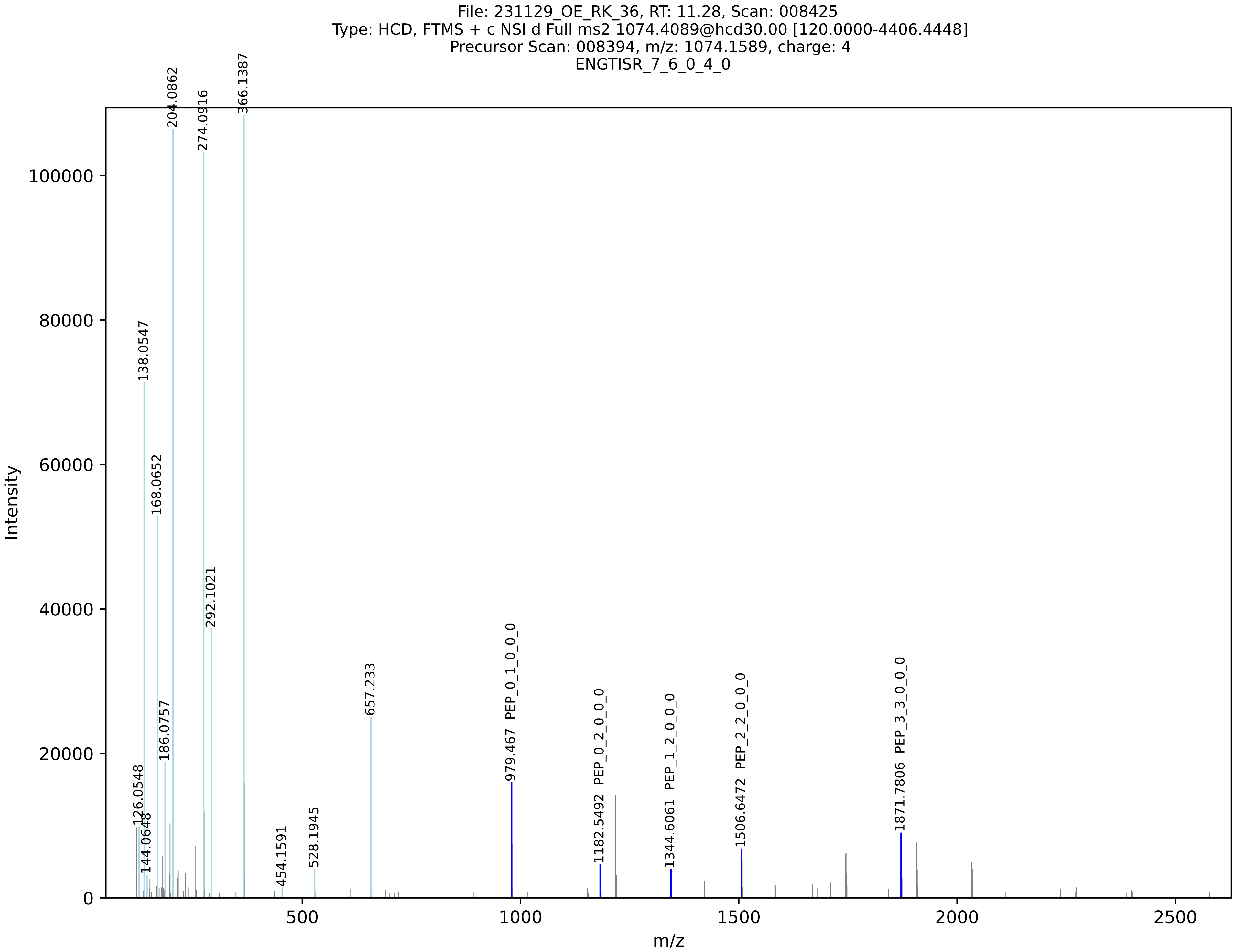
File: 231129_OE_RK_36, RT: 11.28, Scan: 008425
Type: HCD, FTMS + c NSI d Full ms2 1074.4089@hcd30.00 [120.0000-4406.4448]
Precursor Scan: 008394, m/z: 1074.1589, charge: 4
ENGTISR_7_6_0_4_0
Intensity
m/z
126.0548
144.0648
138.0547
168.0652
186.0757
204.0862
274.0916
292.1021
366.1387
454.1591
528.1945
657.233
979.467 PEP_0_1_0_0_0
1182.5492 PEP_0_2_0_0_0
1344.6061 PEP_1_2_0_0_0
1506.6472 PEP_2_2_0_0_0
1871.7806 PEP_3_3_0_0_0

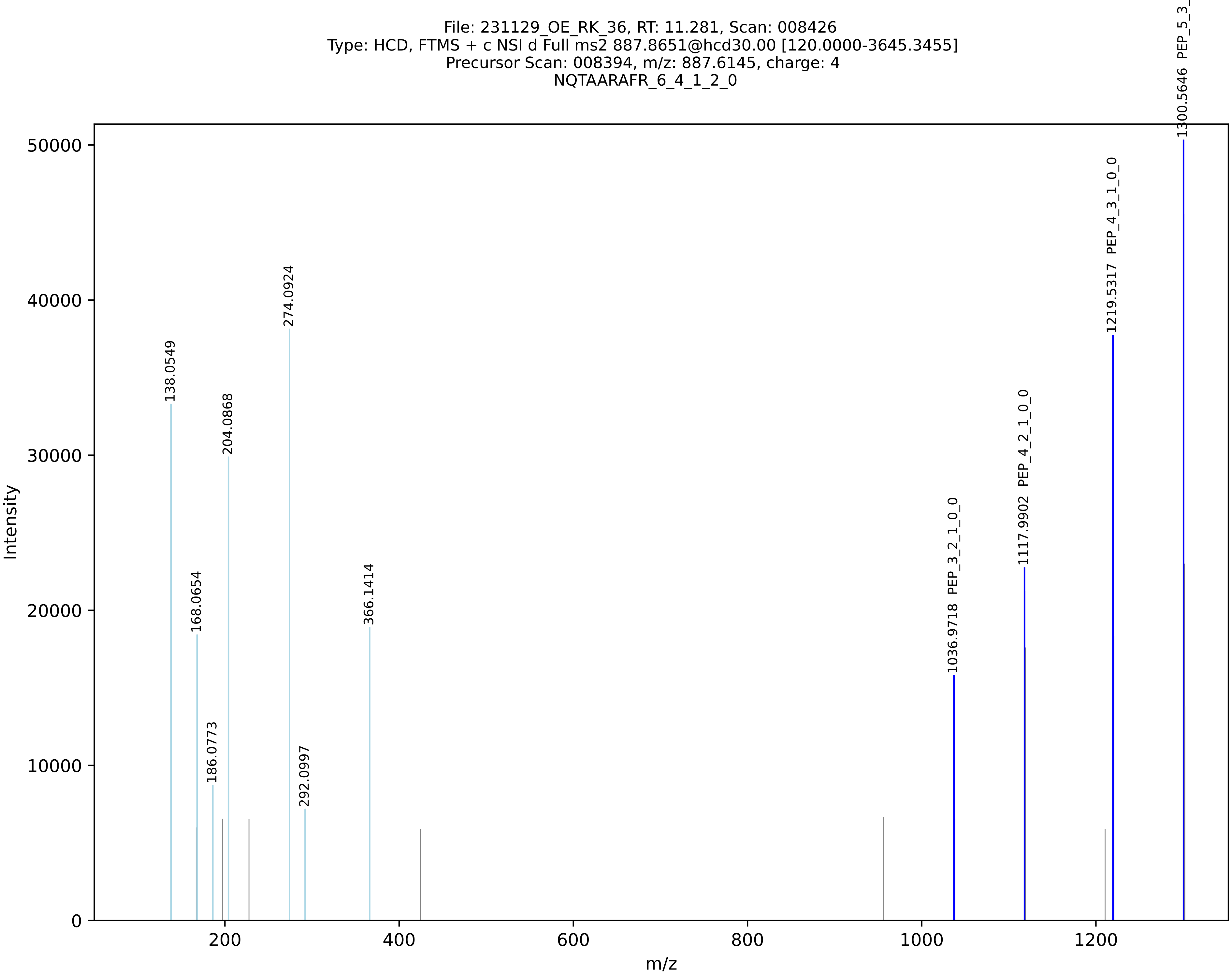
File: 231129_OE_RK_36, RT: 11.281, Scan: 008426
Type: HCD, FTMS + c NSI d Full ms2 887.8651@hcd30.00 [120.0000-3645.3455]
Precursor Scan: 008394, m/z: 887.6145, charge: 4
NQTAARAFR_6_4_1_2_0
138.0549
168.0654
186.0773
204.0868
274.0924
292.0997
366.1414
1036.9718 PEP_3_2_1_0_0
1117.9902 PEP_4_2_1_0_0
1219.5317 PEP_4_3_1_0_0
1300.5646 PEP_5_3_
Intensity
m/z
0
10000
20000
30000
40000
50000
200
400
600
800
1000
1200

File: 231129_OE_RK_36, RT: 11.339, Scan: 008506
Type: HCD, FTMS + c NSI d Full ms2 1094.7781@hcd30.00 [120.0000-3372.1628]
Precursor Scan: 008465, m/z: 1094.444, charge: 3
WVSNKTEGR_5_4_0_2_0

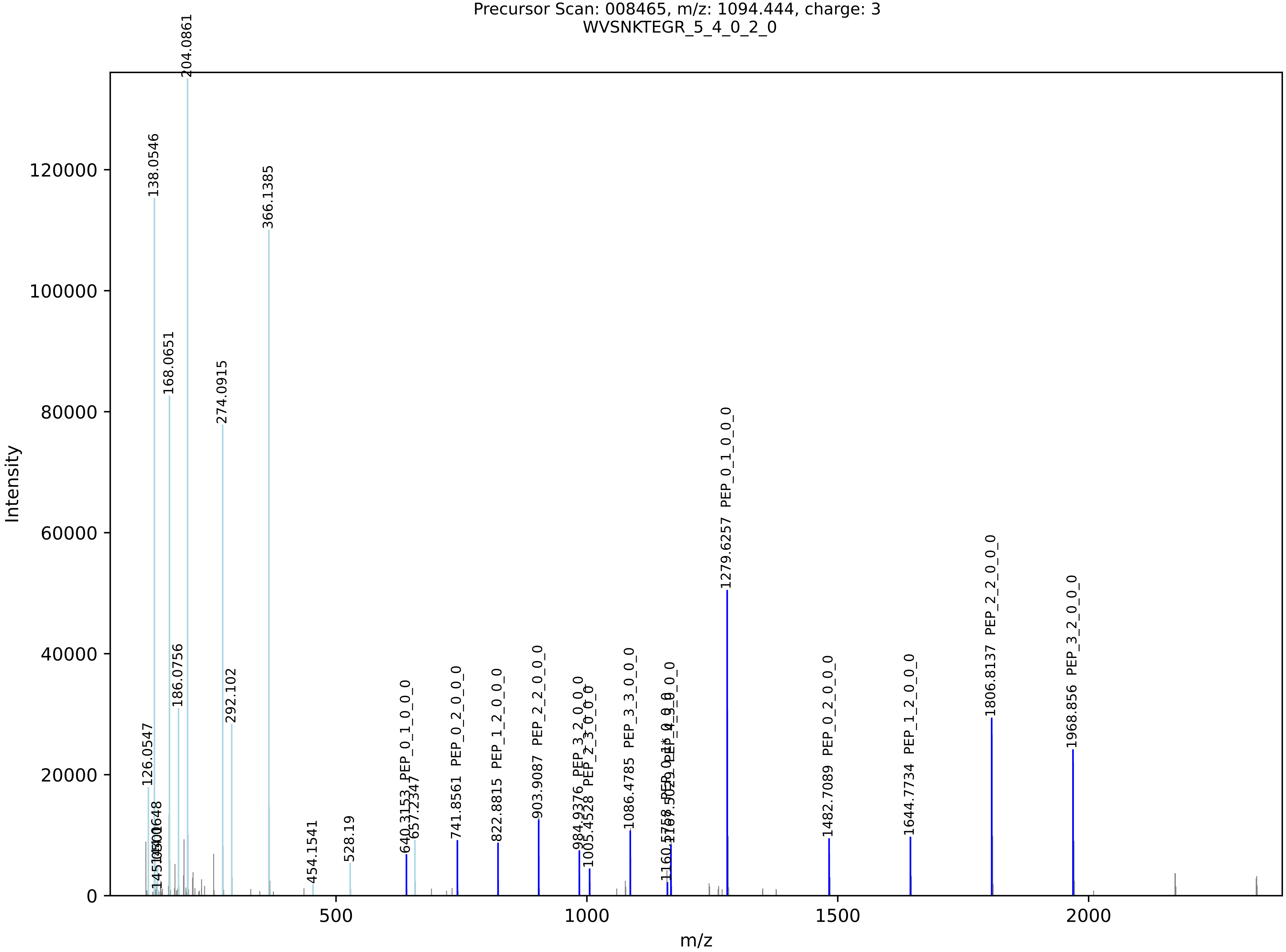

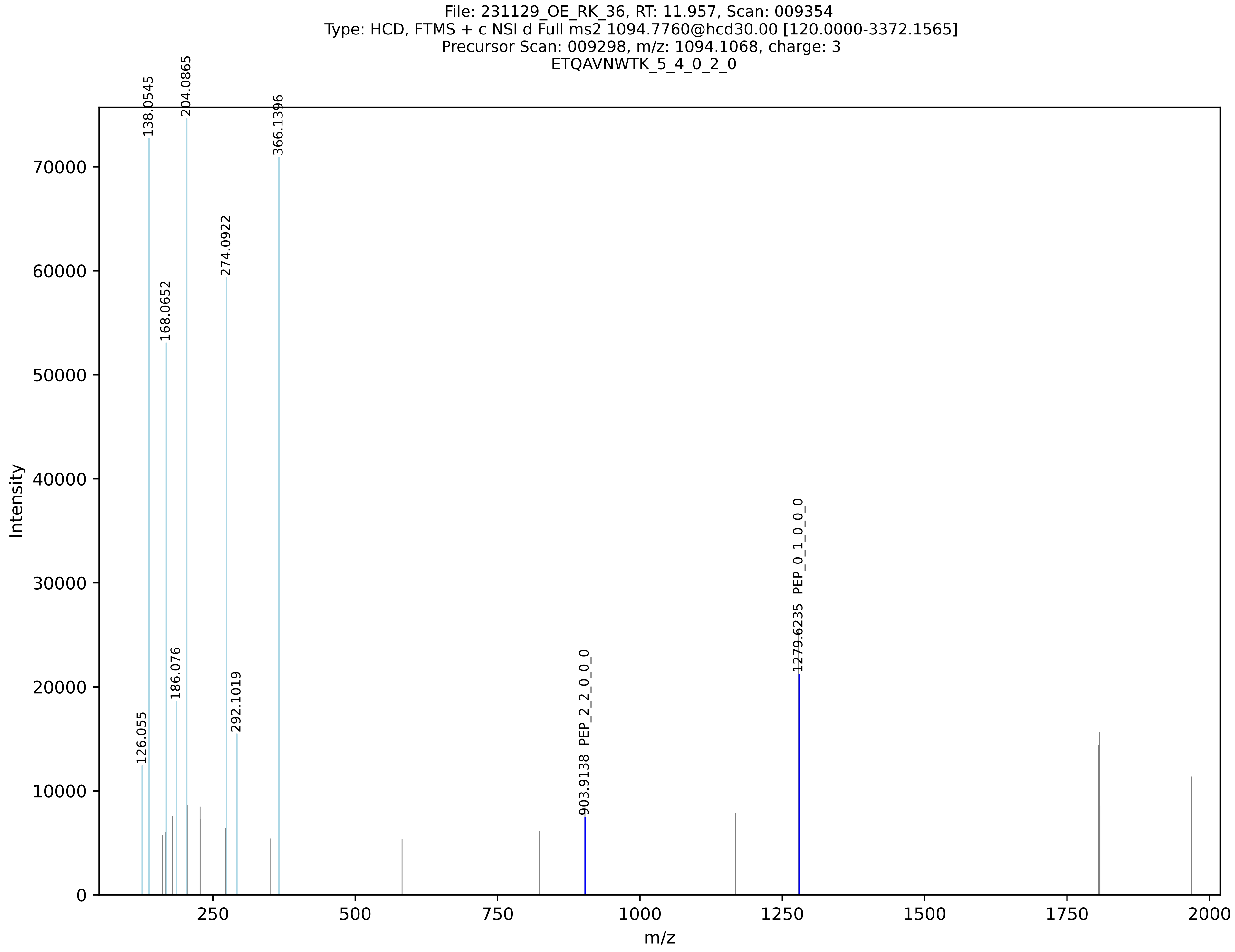
File: 231129_OE_RK_36, RT: 11.957, Scan: 009354
Type: HCD, FTMS + c NSI d Full ms2 1094.7760@hcd30.00 [120.0000-3372.1565]
Precursor Scan: 009298, m/z: 1094.1068, charge: 3
ETQAVNWTK_5_4_0_2_0
138.0545
204.0865
366.1396
274.0922
168.0652
186.076
292.1019
126.055
903.9138 PEP_2_2_0_0_0
1279.6235 PEP_0_1_0_0_0
Intensity
m/z
0
10000
20000
30000
40000
50000
60000
70000
250
500
750
1000
1250
1500
1750
2000

File: 231129_OE_RK_36, RT: 12.382, Scan: 009855
Type: HCD, FTMS + c NSI d Full ms2 1191.1658@hcd30.00 [120.0000-3667.1094]
Precursor Scan: 009797, m/z: 1190.4976, charge: 3
IKLLNNSDER_6_4_2_1_0

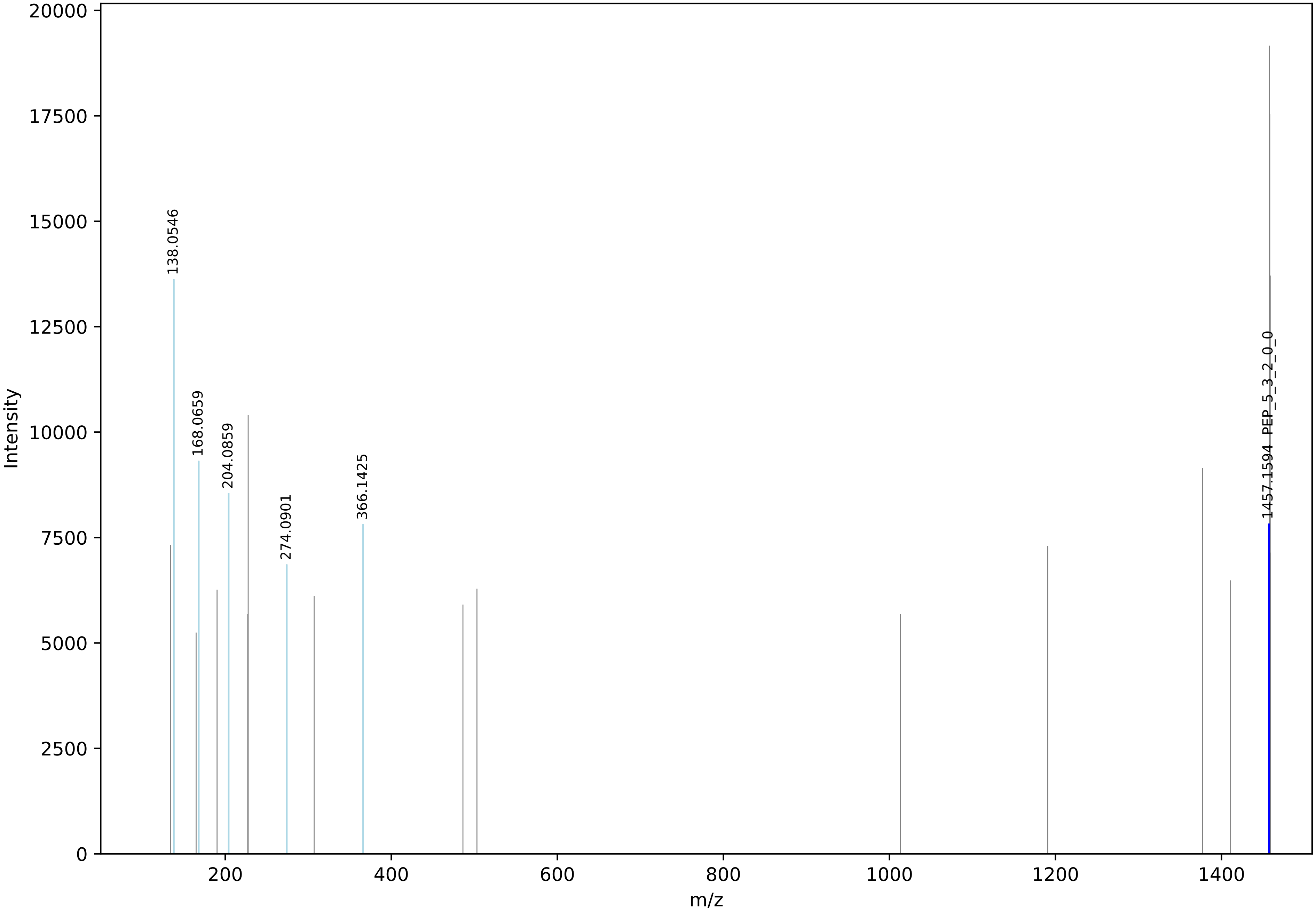

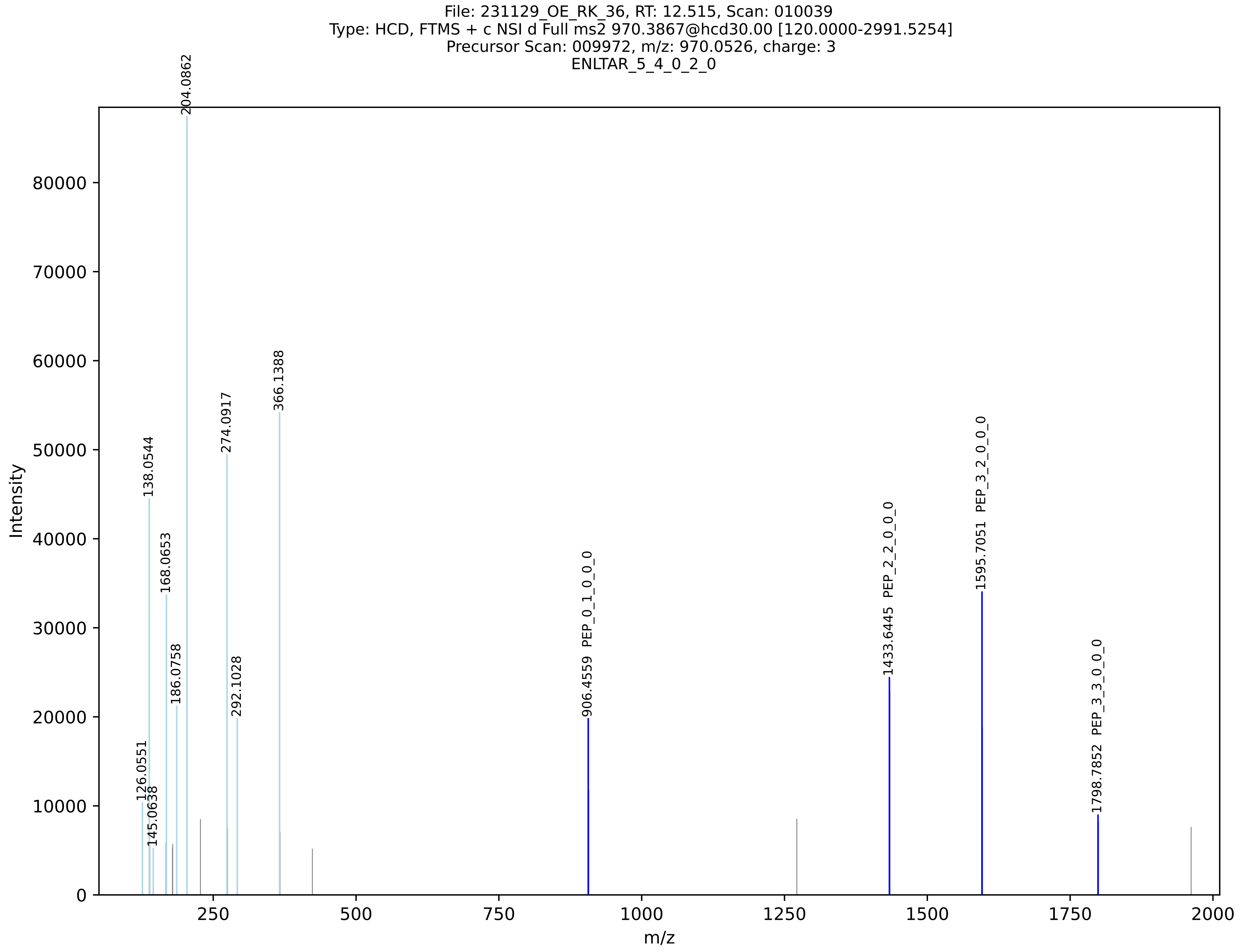
File: 231129_OE_RK_36, RT: 12.515, Scan: 010039
Type: HCD, FTMS + c NSI d Full ms2 970.3867@hcd30.00 [120.0000-2991.5254]
Precursor Scan: 009972, m/z: 970.0526, charge: 3
ENLTAR_5_4_0_2_0
204.0862
366.1388
274.0917
138.0544
168.0653
186.0758
292.1028
126.0551
145.0638
906.4559 PEP_0_1_0_0_0
1433.6445 PEP_2_2_0_0_0
1595.7051 PEP_3_2_0_0_0
1798.7852 PEP_3_3_0_0_0
Intensity
m/z
0
10000
20000
30000
40000
50000
60000
70000
80000
250
500
750
1000
1250
1500
1750
2000

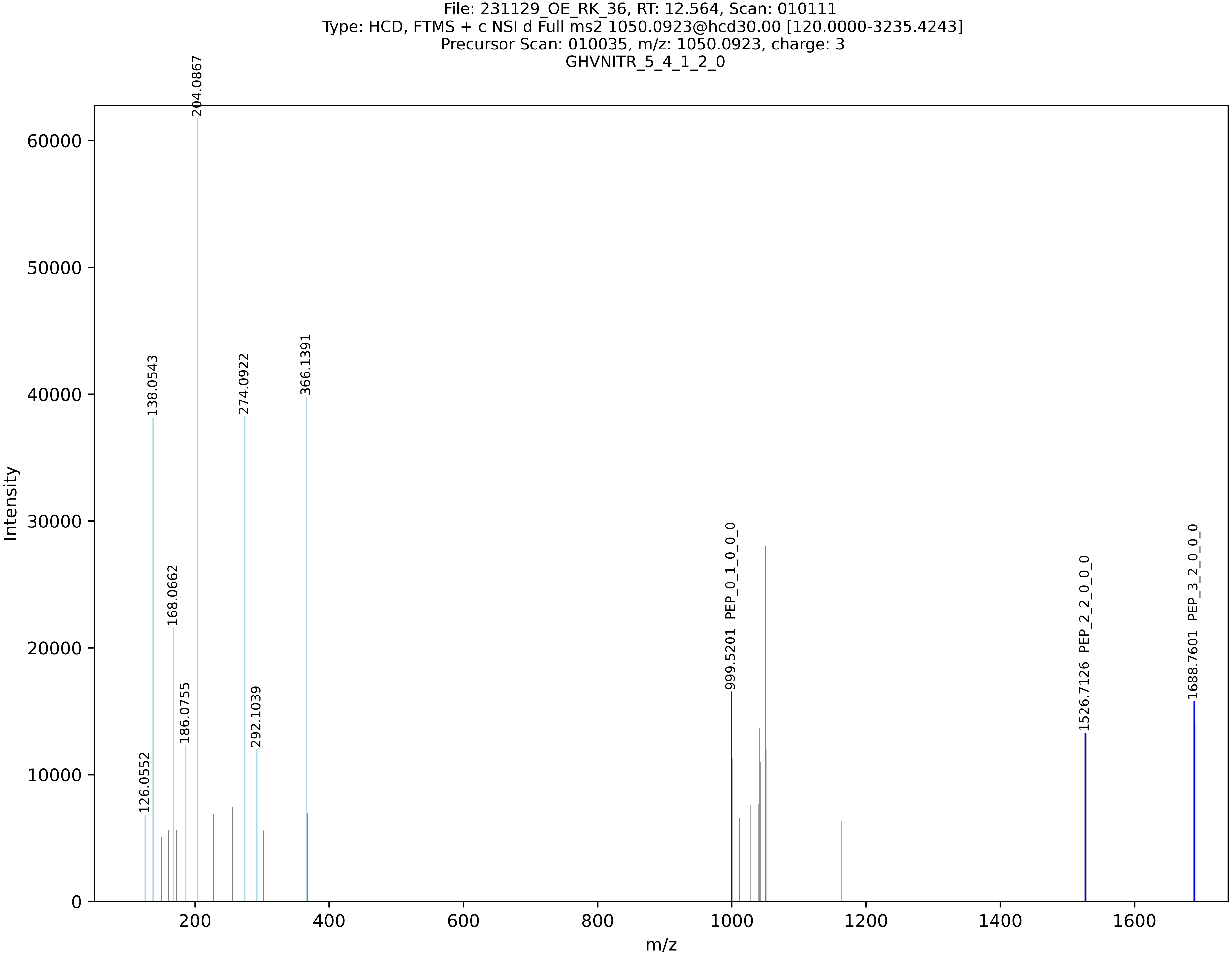

File: 231129_OE_RK_36, RT: 12.564, Scan: 010111
Type: HCD, FTMS + c NSI d Full ms2 1050.0923@hcd30.00 [120.0000-3235.4243]
Precursor Scan: 010035, m/z: 1050.0923, charge: 3
GHVNITR_5_4_1_2_0
Intensity
m/z
204.0867
138.0543
274.0922
366.1391
168.0662
186.0755
292.1039
126.0552
999.5201 PEP_0_1_0_0_0
1526.7126 PEP_2_2_0_0_0
1688.7601 PEP_3_2_0_0_0

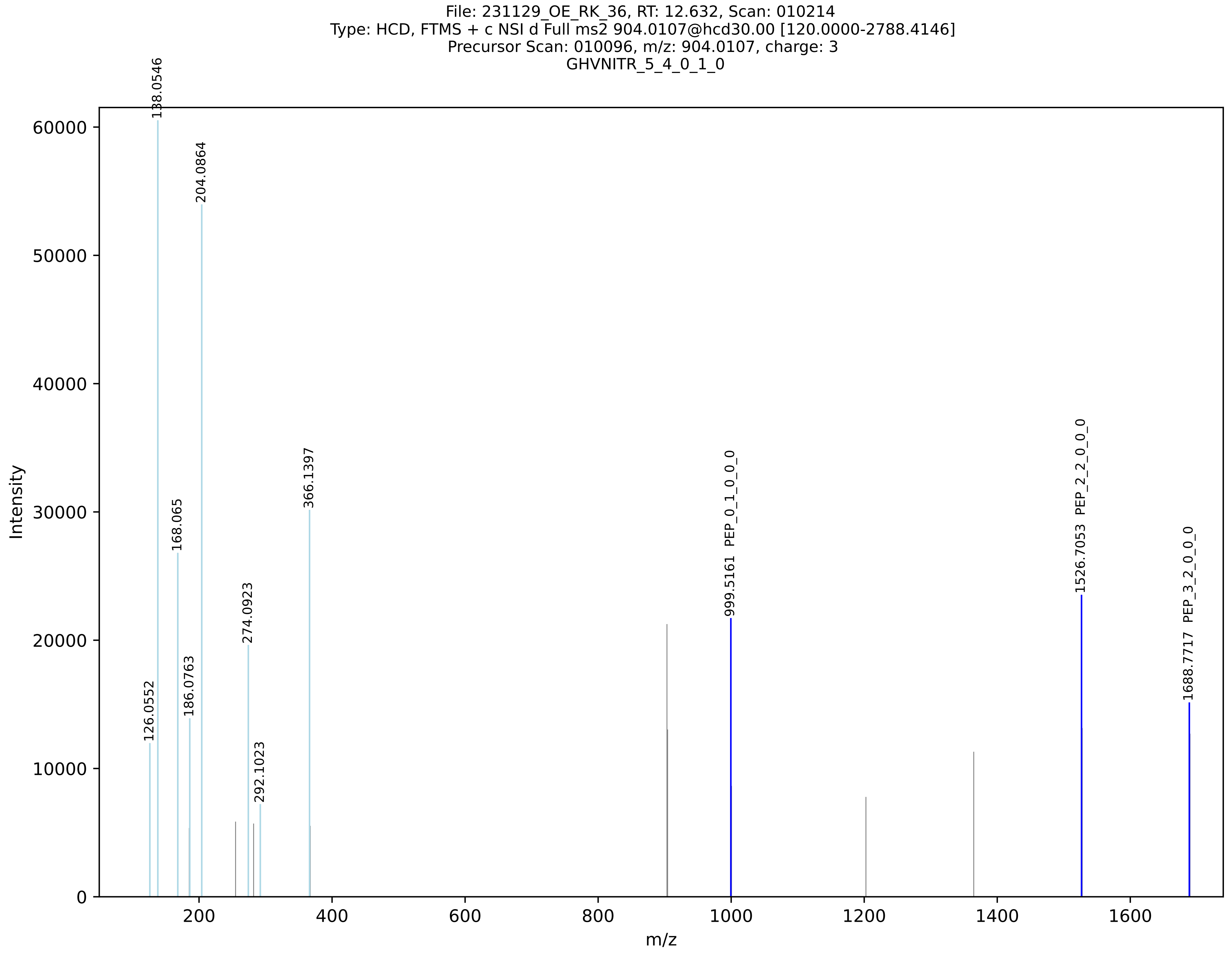

File: 231129_OE_RK_36, RT: 12.632, Scan: 010214
Type: HCD, FTMS + c NSI d Full ms2 904.0107@hcd30.00 [120.0000-2788.4146]
Precursor Scan: 010096, m/z: 904.0107, charge: 3
GHVNITR_5_4_0_1_0
Intensity
m/z
138.0546
204.0864
168.065
366.1397
274.0923
186.0763
126.0552
292.1023
999.5161 PEP_0_1_0_0_0
1526.7053 PEP_2_2_0_0_0
1688.7717 PEP_3_2_0_0_0

File: 231129_OE_RK_36, RT: 12.726, Scan: 010343
Type: HCD, FTMS + c NSI d Full ms2 970.3868@hcd30.00 [120.0000-2991.5259]
Precursor Scan: 010280, m/z: 970.0527, charge: 3
ENLTAR_5_4_0_2_0

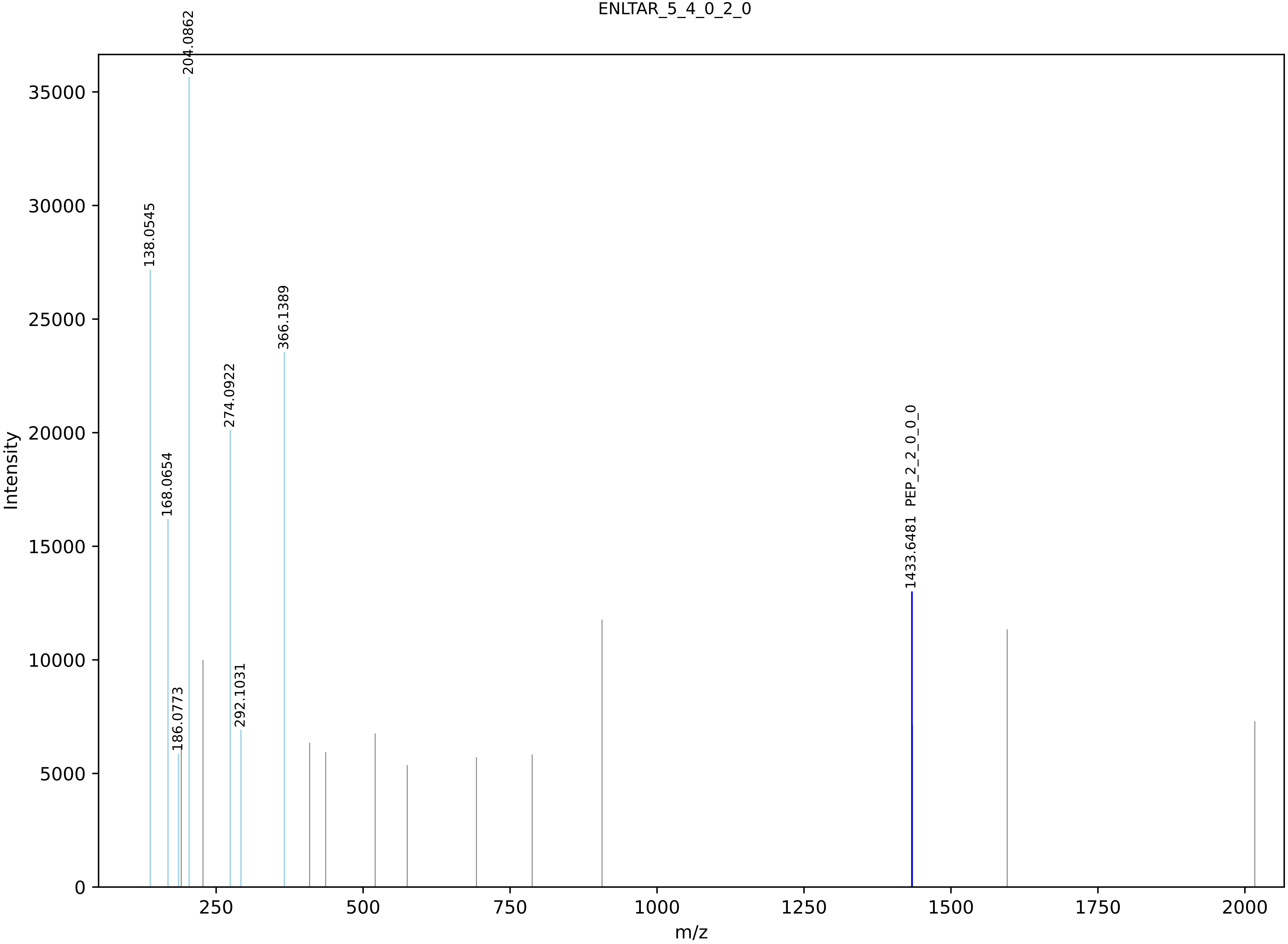

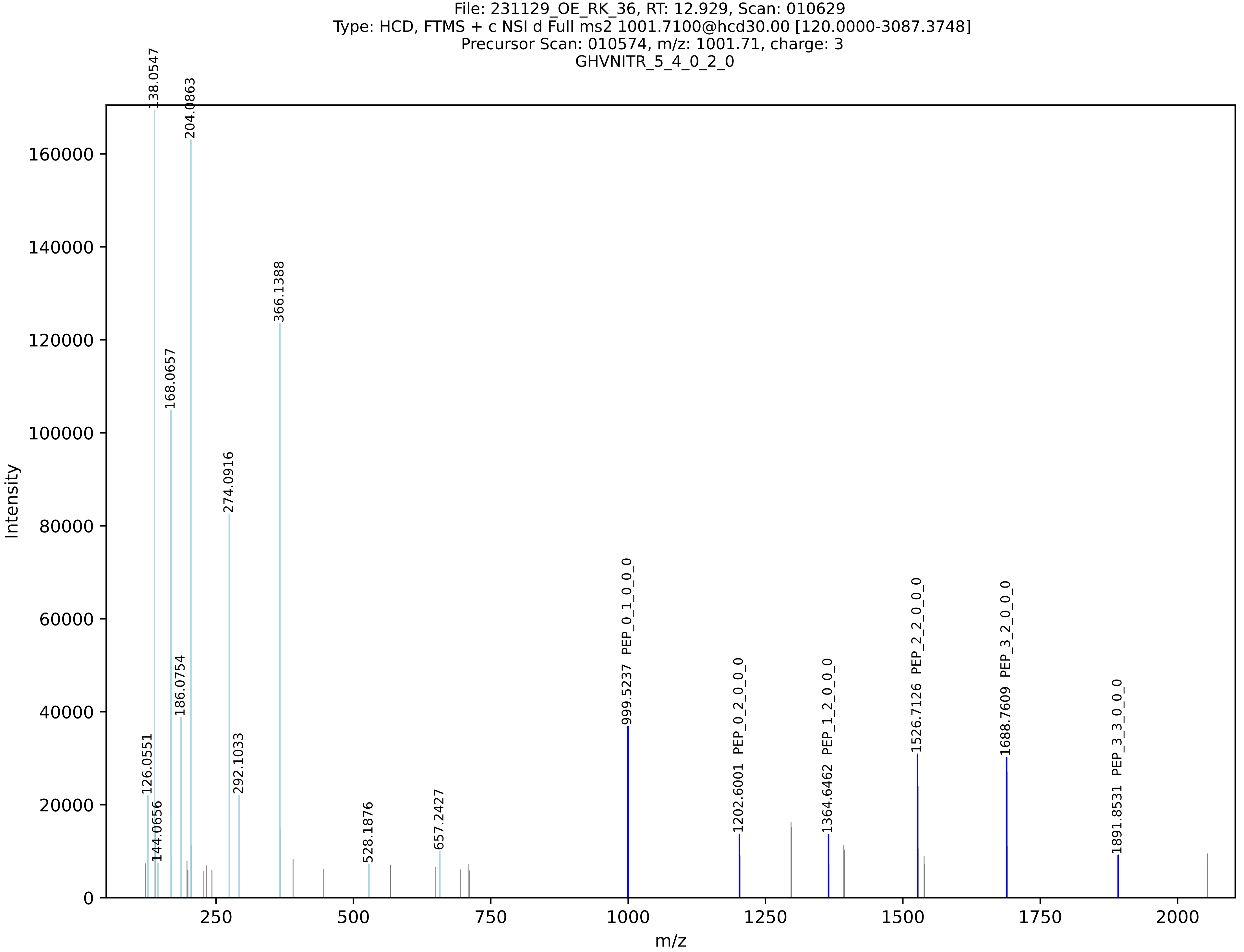

File: 231129_OE_RK_36, RT: 12.929, Scan: 010629
Type: HCD, FTMS + c NSI d Full ms2 1001.7100@hcd30.00 [120.0000-3087.3748]
Precursor Scan: 010574, m/z: 1001.71, charge: 3
GHVNITR_5_4_0_2_0
Intensity
m/z
138.0547
204.0863
366.1388
168.0657
274.0916
186.0754
126.0551
292.1033
144.0656
528.1876
657.2427
999.5237 PEP_0_1_0_0_0
1202.6001 PEP_0_2_0_0_0
1364.6462 PEP_1_2_0_0_0
1526.7126 PEP_2_2_0_0_0
1688.7609 PEP_3_2_0_0_0
1891.8531 PEP_3_3_0_0_0

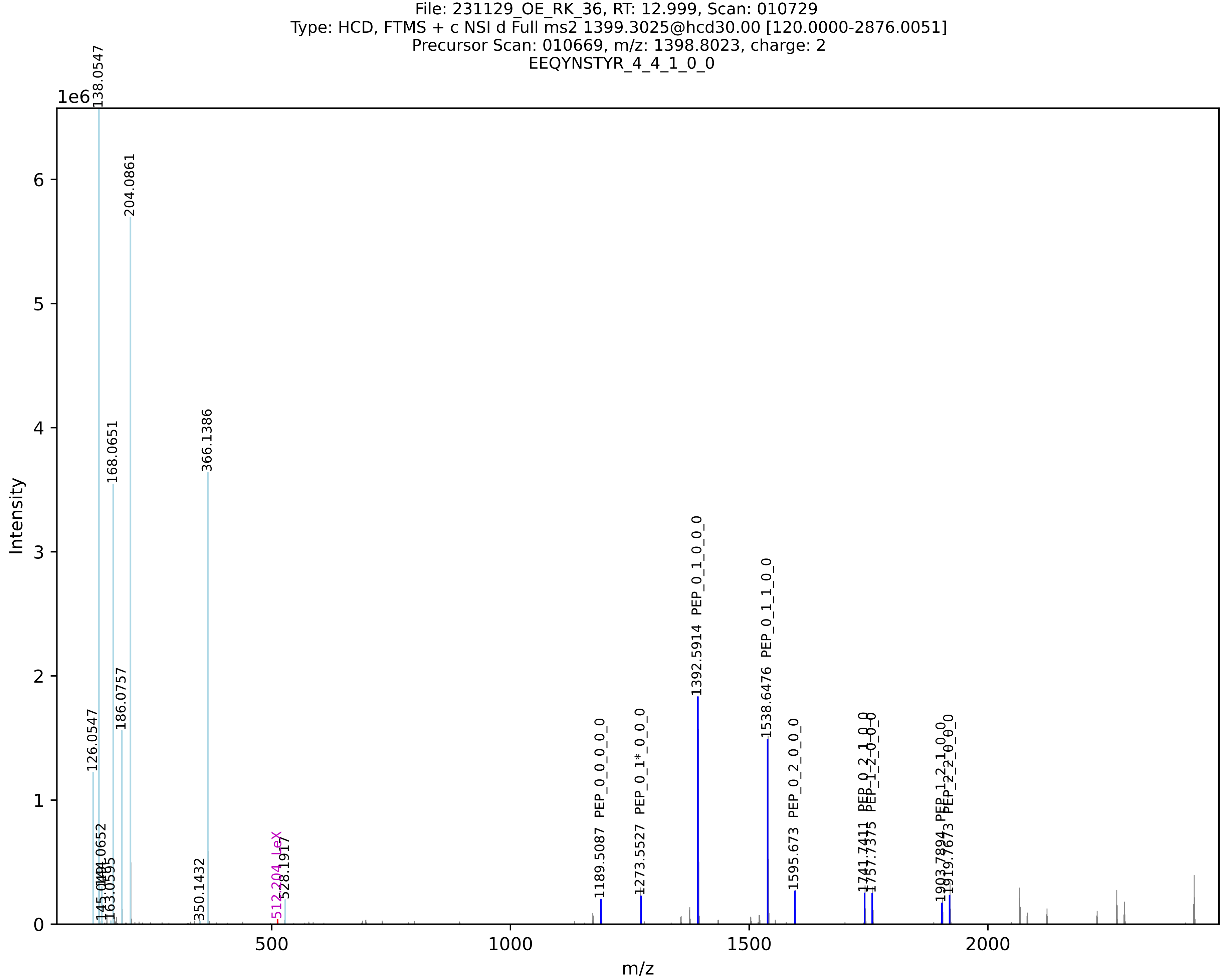
File: 231129_OE_RK_36, RT: 12.999, Scan: 010729
Type: HCD, FTMS + c NSI d Full ms2 1399.3025@hcd30.00 [120.0000-2876.0051]
Precursor Scan: 010669, m/z: 1398.8023, charge: 2
EEQYNSTYR_4_4_1_0_0
1e6
Intensity
m/z
138.0547
204.0861
366.1386
168.0651
186.0757
126.0547
350.1432
512.204_LeX
528.1917
163.0595
1189.5087 PEP_0_0_0_0_0
1273.5527 PEP_0_1*_0_0_0
1392.5914 PEP_0_1_0_0_0
1538.6476 PEP_0_1_1_0_0
1595.673 PEP_0_2_0_0_0
1741.7411 PEP_0_2_1_0_0
1757.7375 PEP_1_2_0_0_0
1903.7894 PEP_1_2_1_0_0
1919.7673 PEP_2_2_0_0_0

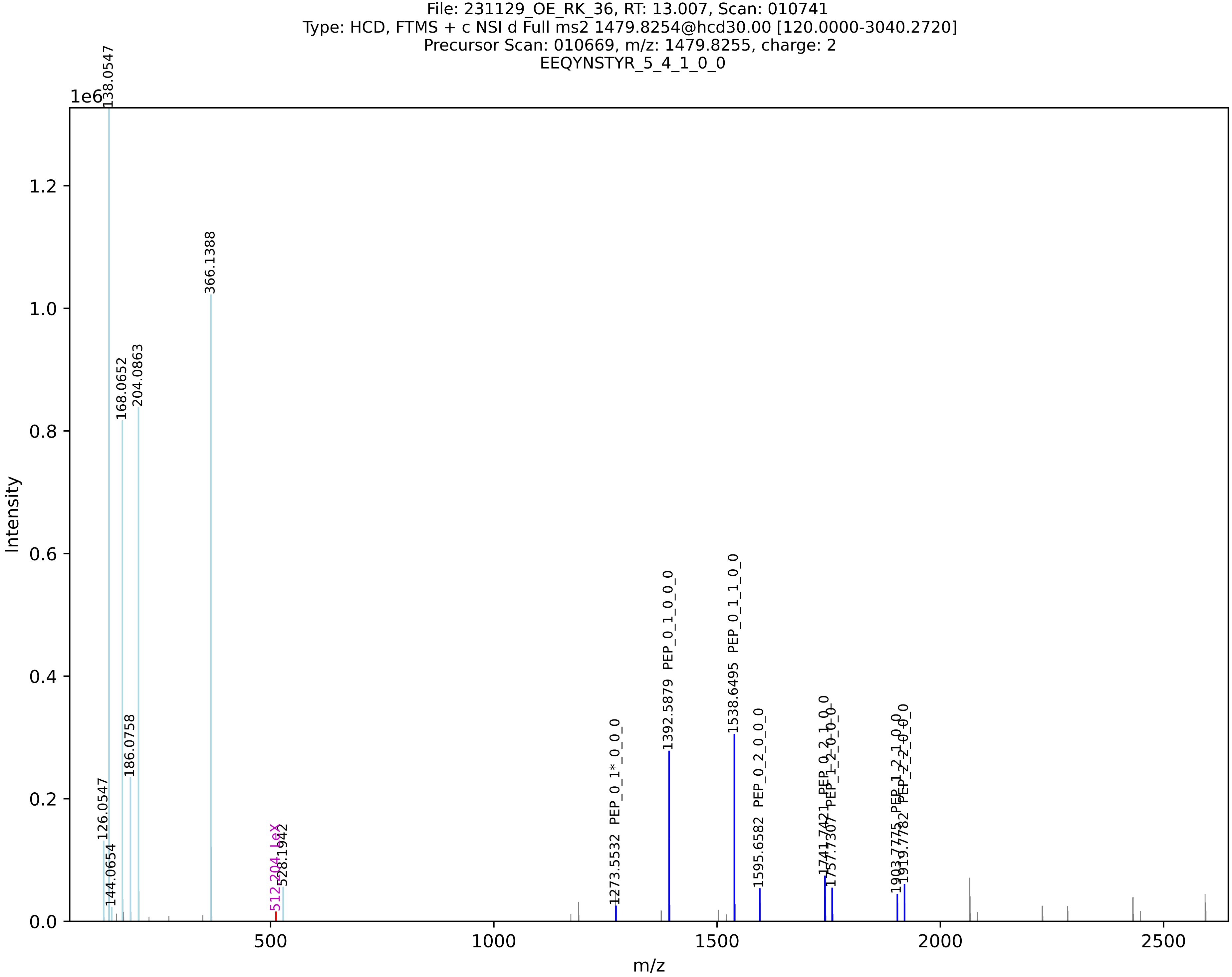
File: 231129_OE_RK_36, RT: 13.007, Scan: 010741
Type: HCD, FTMS + c NSI d Full ms2 1479.8254@hcd30.00 [120.0000-3040.2720]
Precursor Scan: 010669, m/z: 1479.8255, charge: 2
EEQYNSTYR_5_4_1_0_0
1e6
138.0547
366.1388
168.0652
204.0863
186.0758
126.0547
144.0654
512.204 LeX
528.1942
1273.5532 PEP_0_1*_0_0_0
1392.5879 PEP_0_1_0_0_0
1538.6495 PEP_0_1_1_0_0
1595.6582 PEP_0_2_0_0_0
1741.7421 PEP_0_2_1_0_0
1757.7307 PEP_1_2_0_0_0
1903.7775 PEP_1_2_1_0_0
1919.7782 PEP_2_2_0_0_0
Intensity
m/z
0.0
0.2
0.4
0.6
0.8
1.0
1.2
500
1000
1500
2000
2500

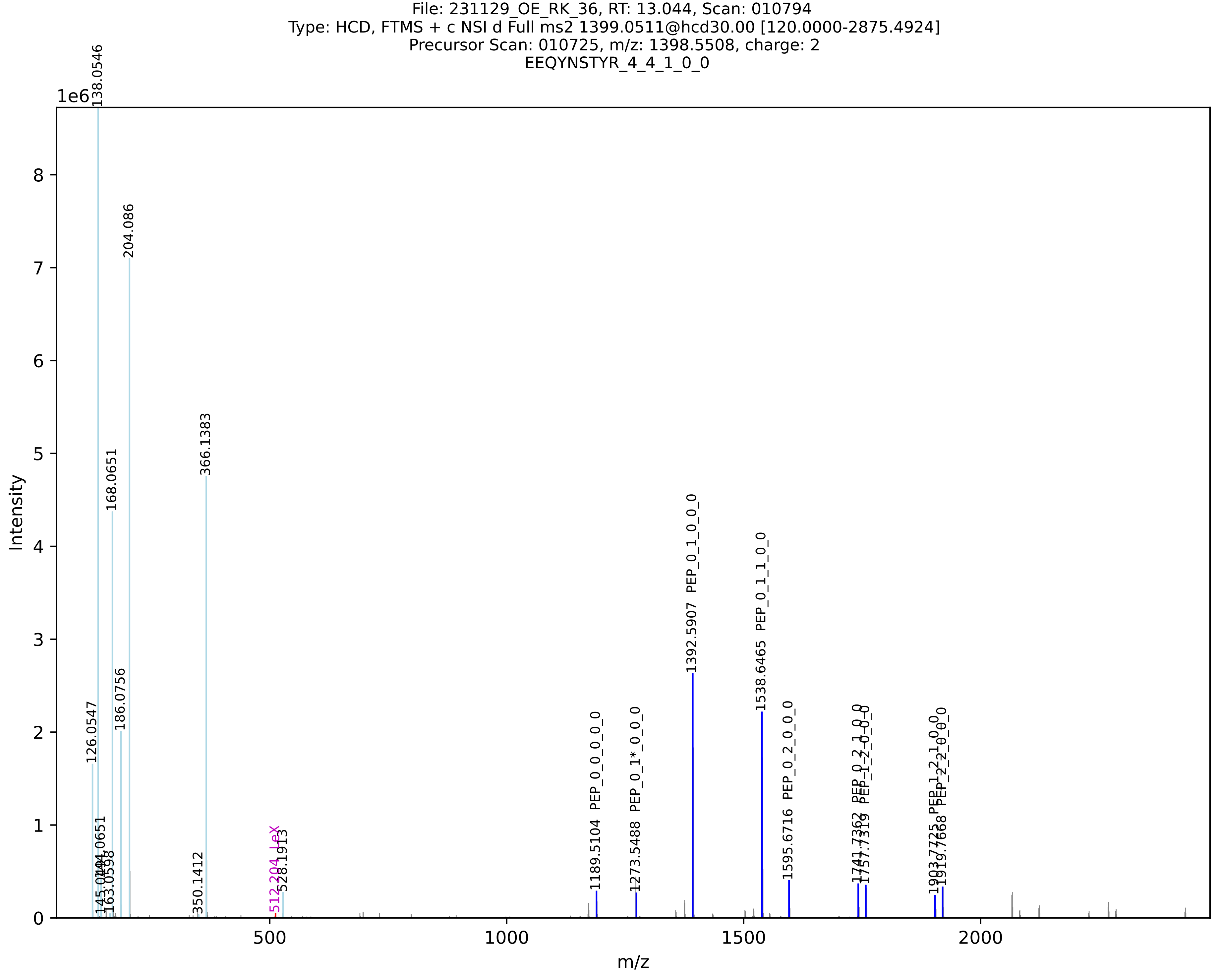

File: 231129_OE_RK_36, RT: 13.044, Scan: 010794
Type: HCD, FTMS + c NSI d Full ms2 1399.0511@hcd30.00 [120.0000-2875.4924]
Precursor Scan: 010725, m/z: 1398.5508, charge: 2
EEQYNSTYR_4_4_1_0_0
1e6
Intensity
m/z
138.0546
204.086
366.1383
168.0651
186.0756
126.0547
144.0651
163.0598
350.1412
512.204_LeX
528.1913
1189.5104 PEP_0_0_0_0_0
1273.5488 PEP_0_1*_0_0_0
1392.5907 PEP_0_1_0_0_0
1538.6465 PEP_0_1_1_0_0
1595.6716 PEP_0_2_0_0_0
1741.7362 PEP_0_2_1_0_0
1757.7319 PEP_1_2_0_0_0
1903.7725 PEP_1_2_1_0_0
1919.7668 PEP_2_2_0_0_0

File: 231129_OE_RK_36, RT: 13.045, Scan: 010795
Type: HCD, FTMS + c NSI d Full ms2 987.0541@hcd30.00 [120.0000-3042.5276]
Precursor Scan: 010725, m/z: 986.7201, charge: 3
EEQYNSTYR_5_4_1_0_0

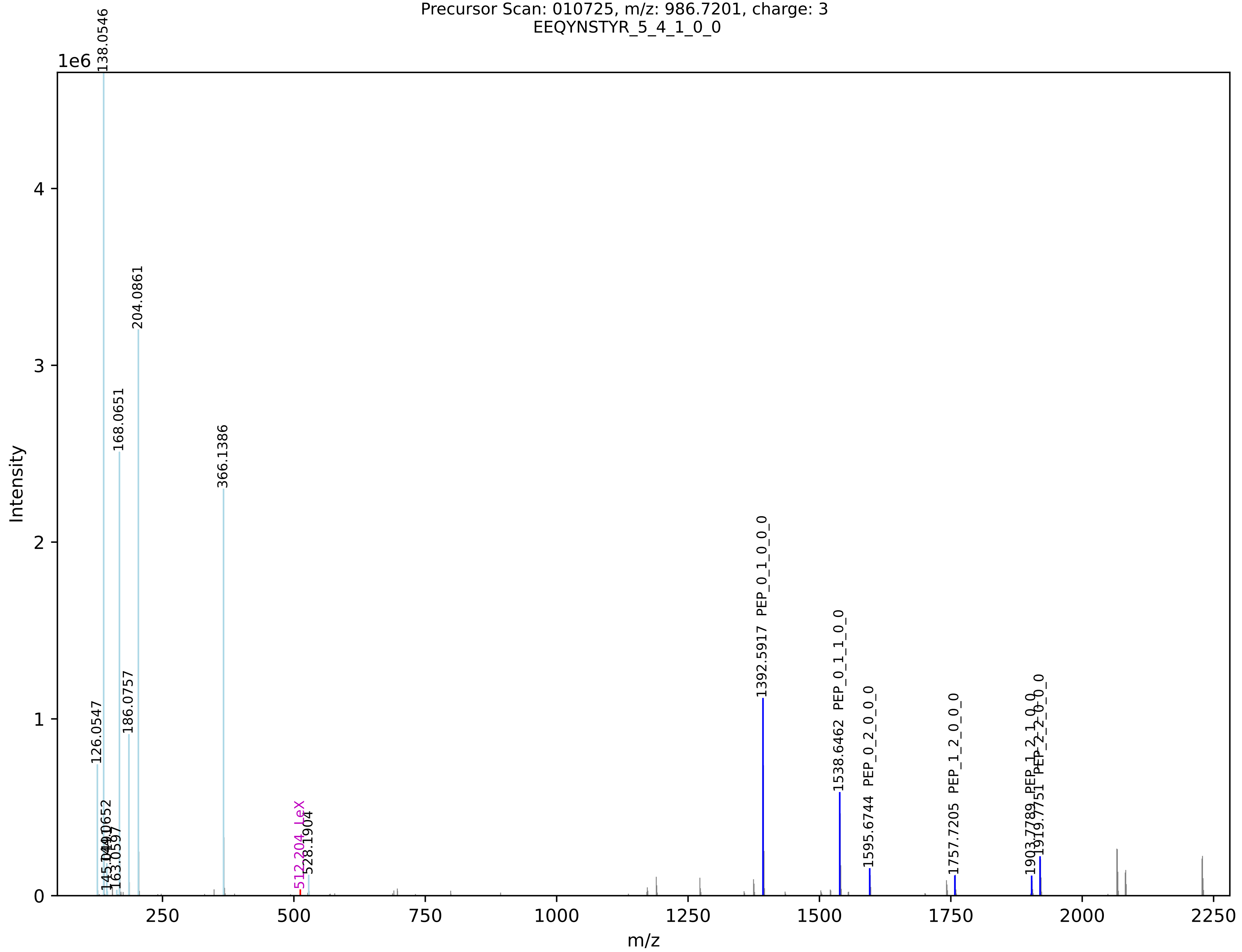

File: 231129_OE_RK_36, RT: 13.266, Scan: 011104
Type: HCD, FTMS + c NSI d Full ms2 1326.0205@hcd30.00 [120.0000-2726.5098]
Precursor Scan: 011026, m/z: 1325.5228, charge: 2
EEQYNSTYR_4_4_0_0_0

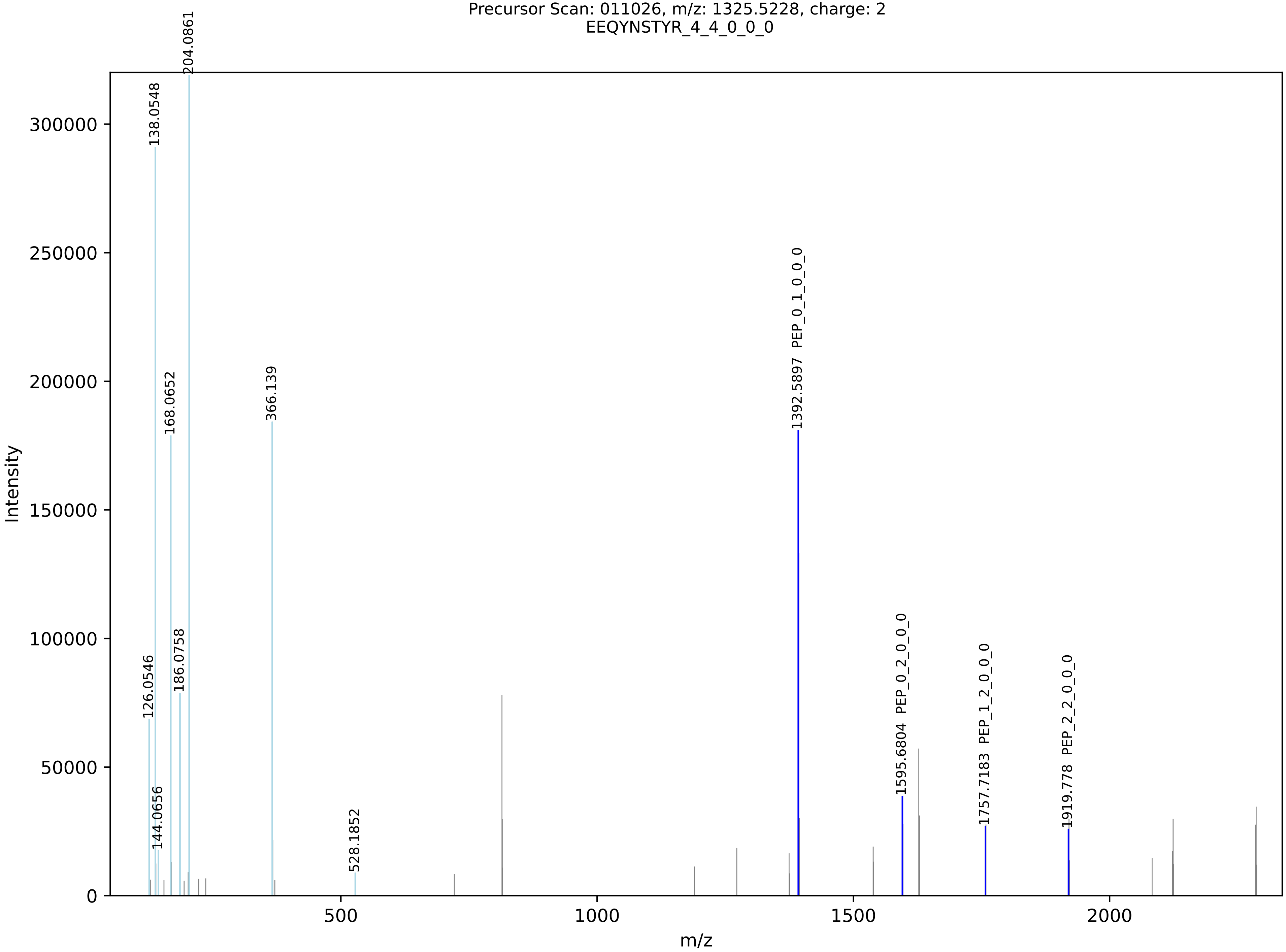

File: 231129_OE_RK_36, RT: 13.324, Scan: 011184
Type: HCD, FTMS + c NSI d Full ms2 933.0372@hcd30.00 [120.0000-2877.2358]
Precursor Scan: 011144, m/z: 932.703, charge: 3
EEQYNSTYR_4_4_1_0_0

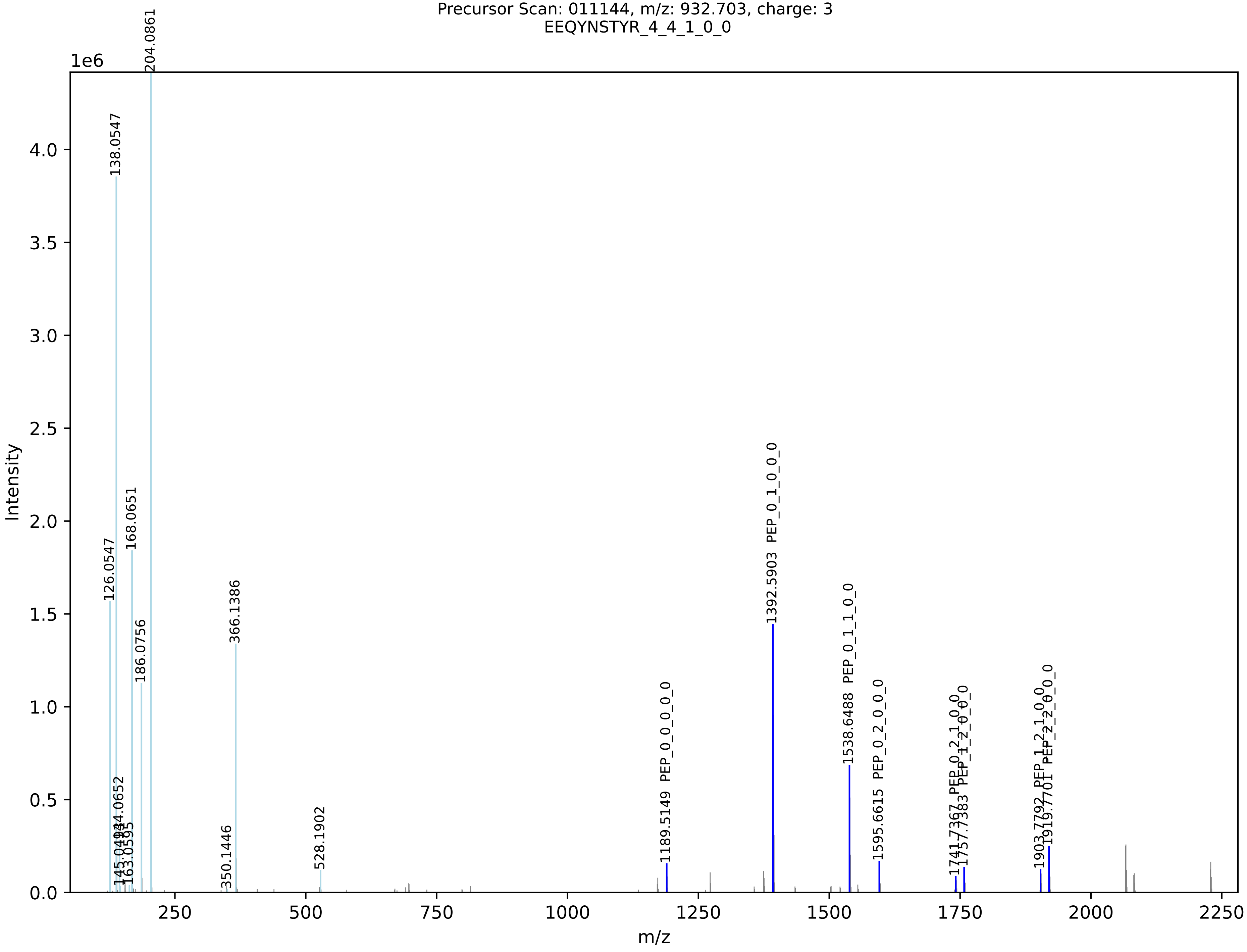

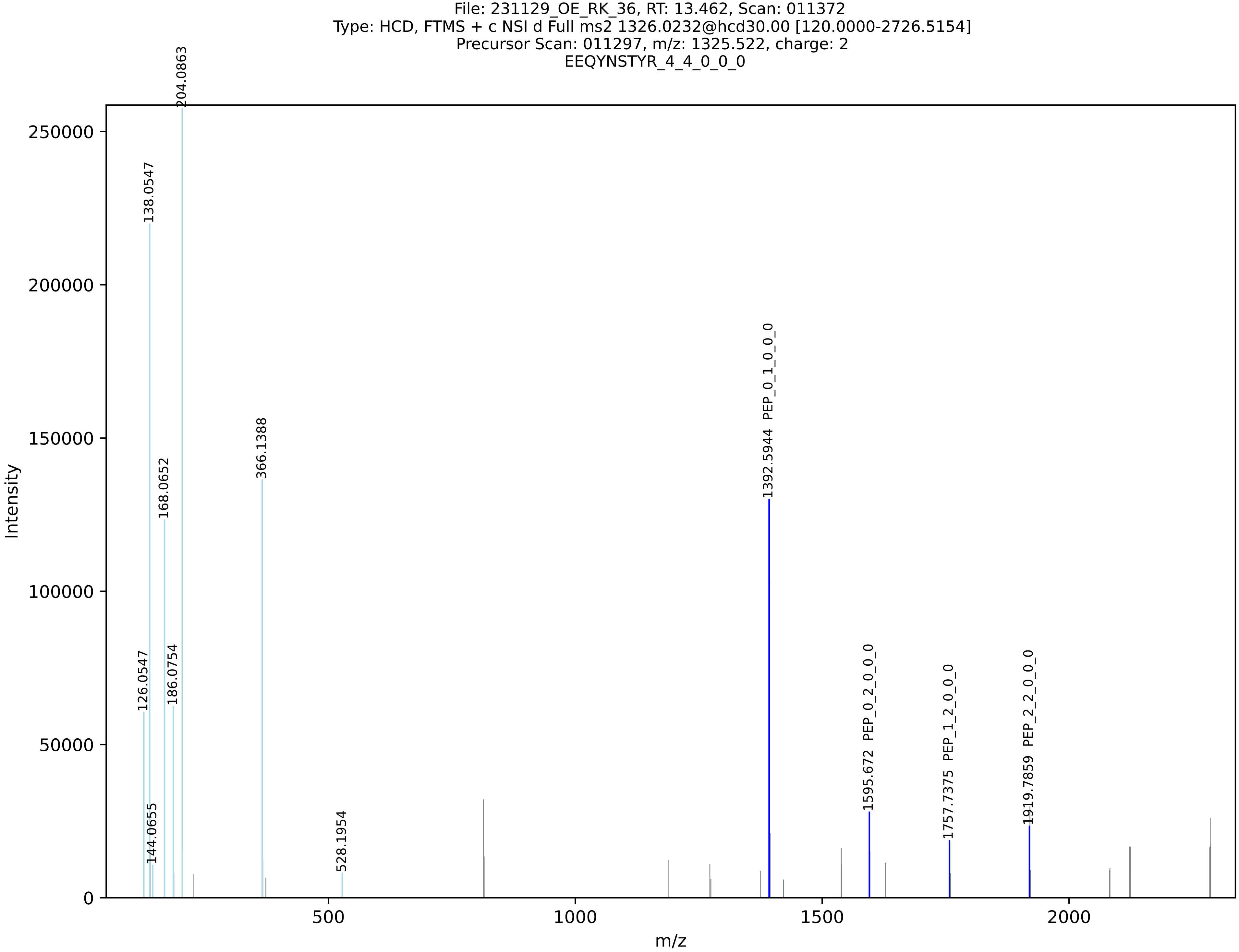
File: 231129_OE_RK_36, RT: 13.462, Scan: 011372
Type: HCD, FTMS + c NSI d Full ms2 1326.0232@hcd30.00 [120.0000-2726.5154]
Precursor Scan: 011297, m/z: 1325.522, charge: 2
EEQYNSTYR_4_4_0_0_0
Intensity
m/z
204.0863
138.0547
168.0652
366.1388
126.0547
186.0754
144.0655
528.1954
1392.5944 PEP_0_1_0_0_0
1595.672 PEP_0_2_0_0_0
1757.7375 PEP_1_2_0_0_0
1919.7859 PEP_2_2_0_0_0
0
50000
100000
150000
200000
250000
500
1000
1500
2000

File: 231129_OE_RK_36, RT: 13.501, Scan: 011428
Type: HCD, FTMS + c NSI d Full ms2 933.0372@hcd30.00 [120.0000-2877.2356]
Precursor Scan: 011368, m/z: 932.7027, charge: 3
EEQYNSTYR_4_4_1_0_0

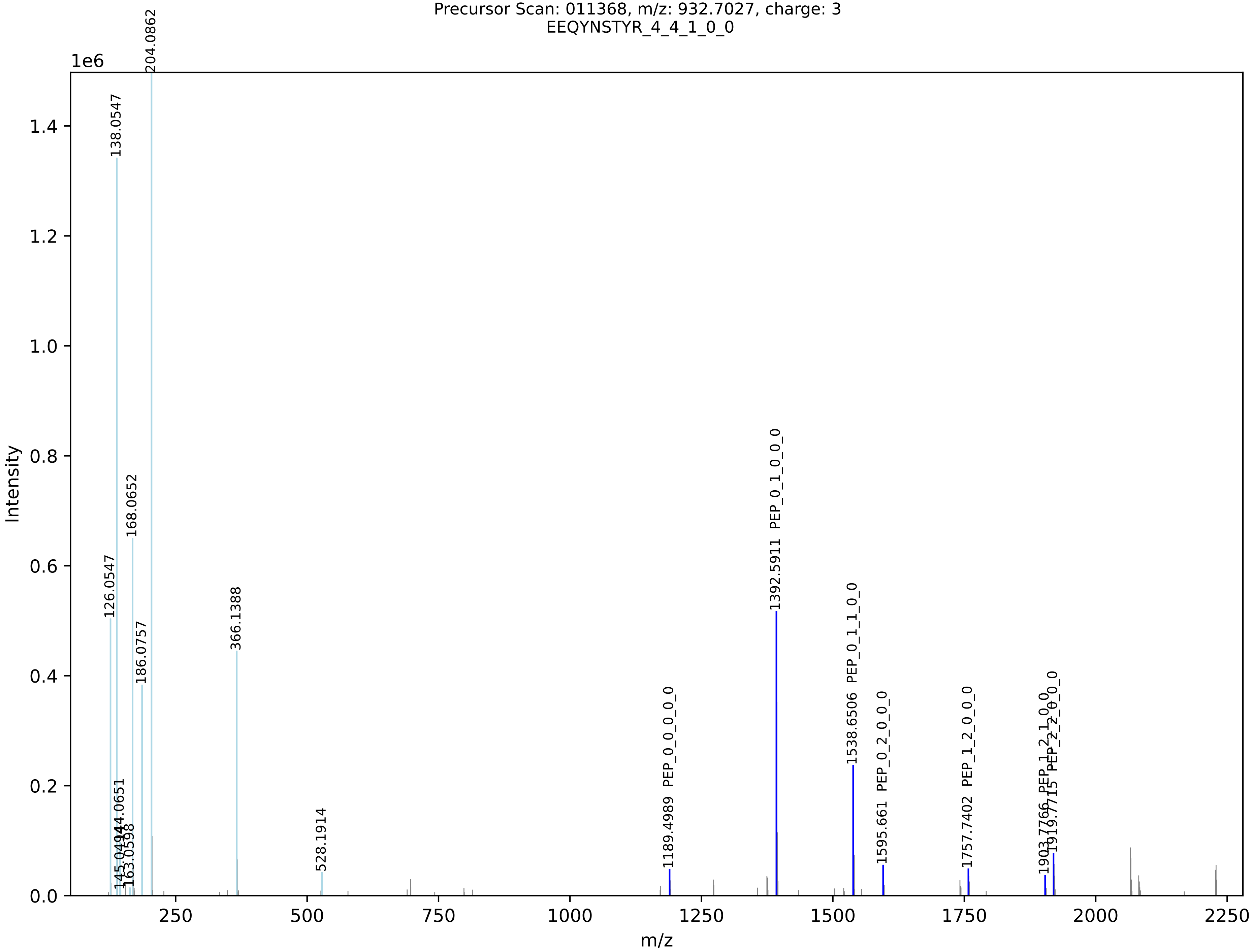

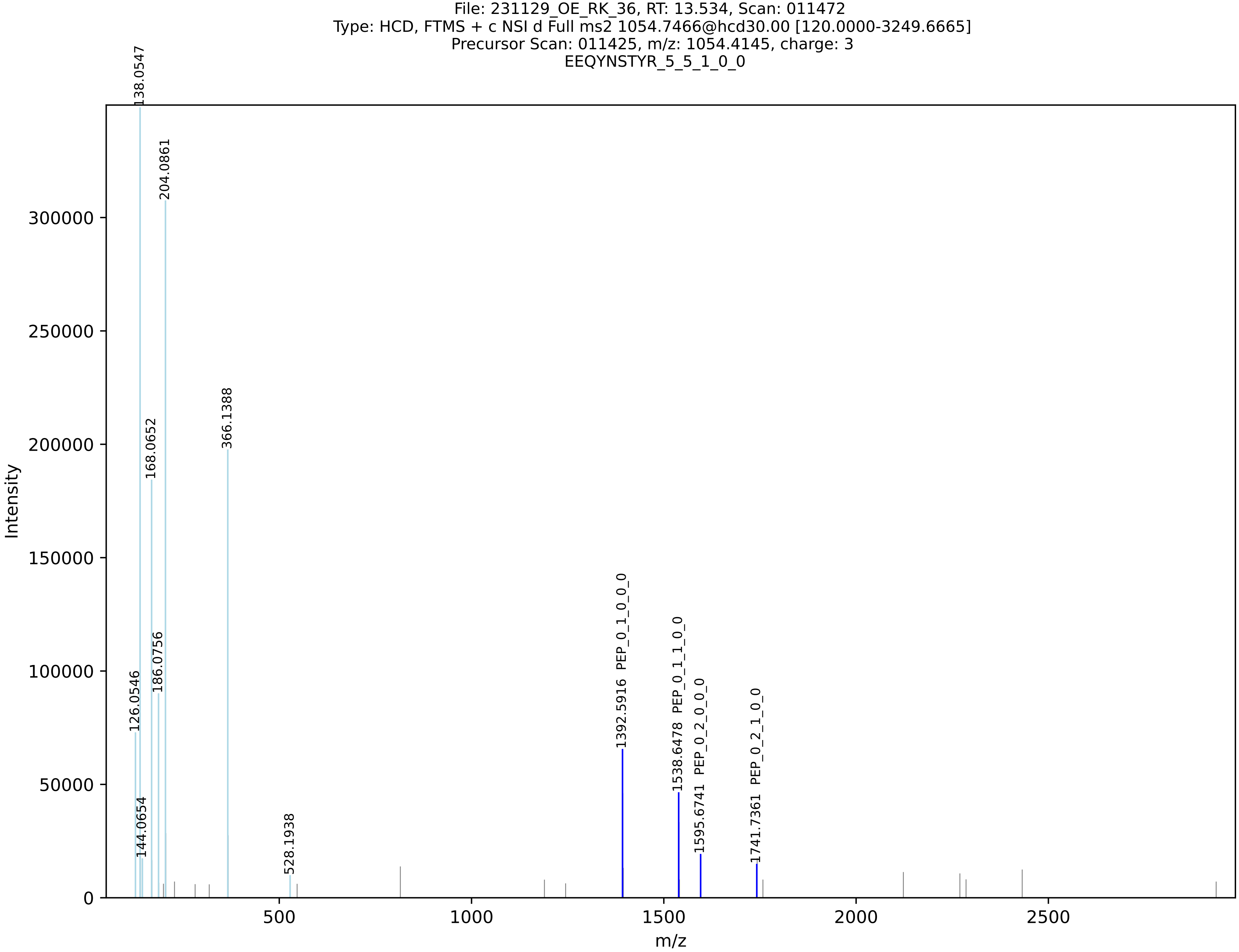

File: 231129_OE_RK_36, RT: 13.534, Scan: 011472
Type: HCD, FTMS + c NSI d Full ms2 1054.7466@hcd30.00 [120.0000-3249.6665]
Precursor Scan: 011425, m/z: 1054.4145, charge: 3
EEQYNSTYR_5_5_1_0_0
138.0547
204.0861
366.1388
168.0652
186.0756
126.0546
144.0654
528.1938
1392.5916 PEP_0_1_0_0_0
1538.6478 PEP_0_1_1_0_0
1595.6741 PEP_0_2_0_0_0
1741.7361 PEP_0_2_1_0_0
Intensity
m/z
0
50000
100000
150000
200000
250000
300000
500
1000
1500
2000
2500

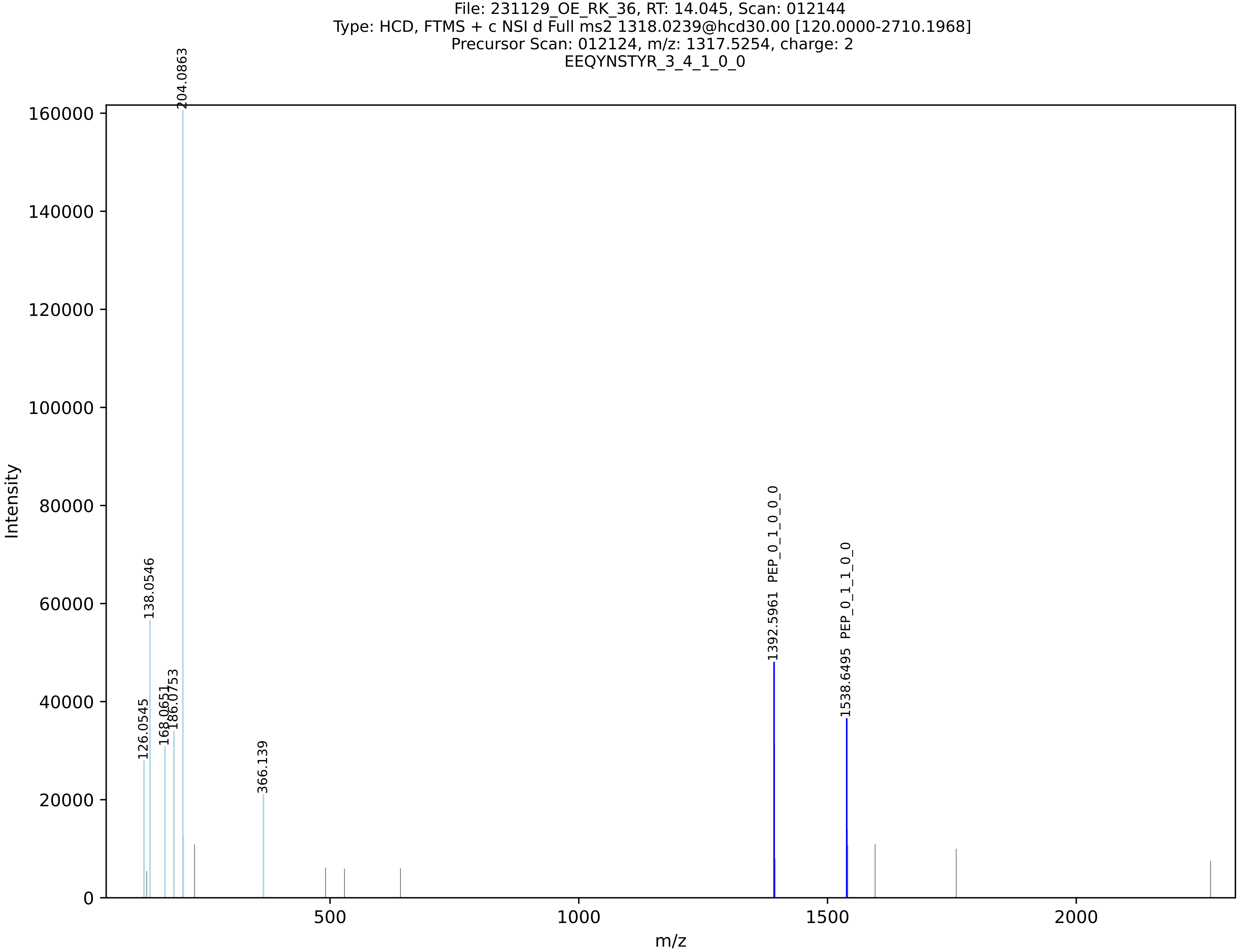
File: 231129_OE_RK_36, RT: 14.045, Scan: 012144
Type: HCD, FTMS + c NSI d Full ms2 1318.0239@hcd30.00 [120.0000-2710.1968]
Precursor Scan: 012124, m/z: 1317.5254, charge: 2
EEQYNSTYR_3_4_1_0_0
Intensity
m/z
204.0863
138.0546
126.0545
168.0651
186.0753
366.139
1392.5961 PEP_0_1_0_0_0
1538.6495 PEP_0_1_1_0_0
0
20000
40000
60000
80000
100000
120000
140000
160000
500
1000
1500
2000

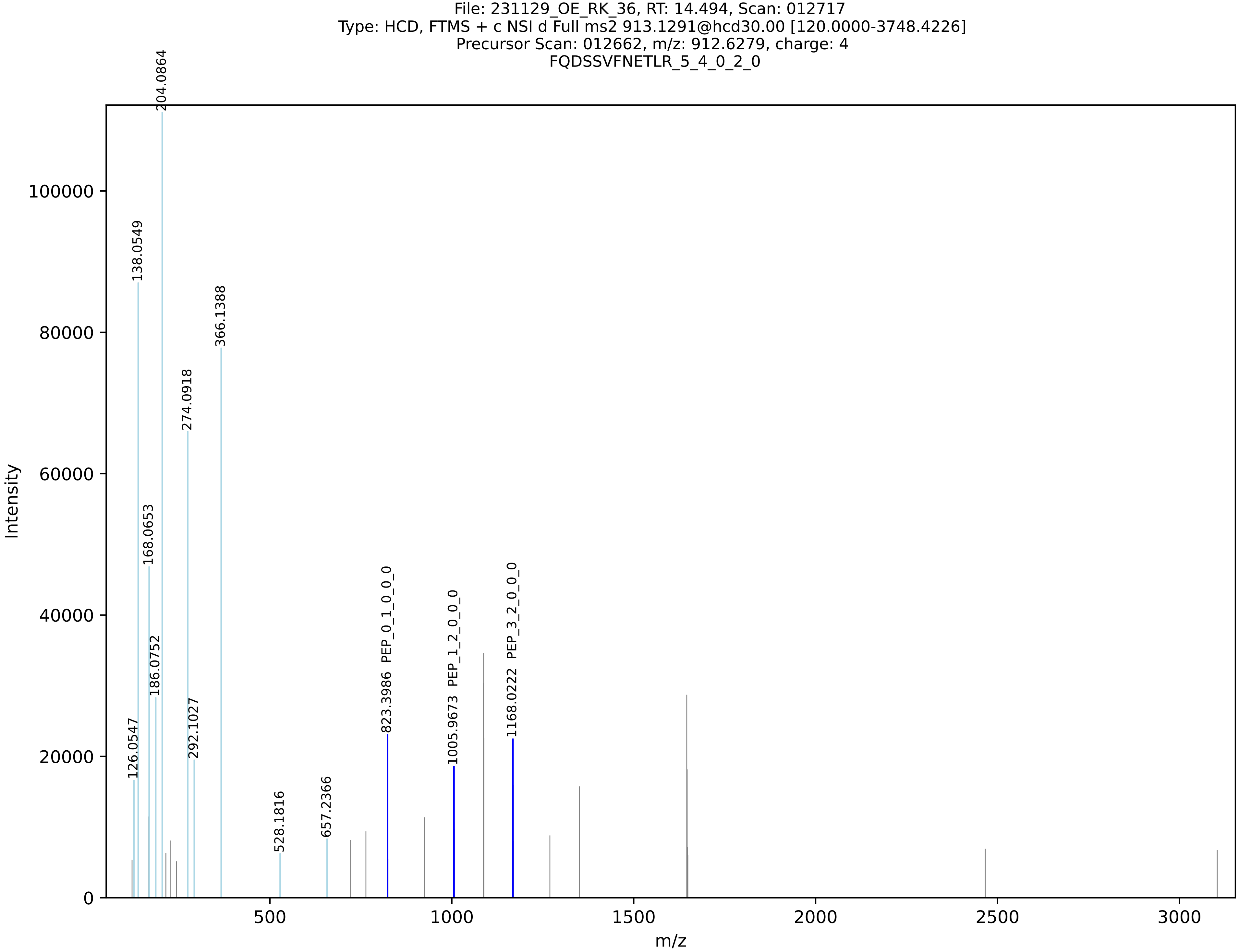
File: 231129_OE_RK_36, RT: 14.494, Scan: 012717
Type: HCD, FTMS + c NSI d Full ms2 913.1291@hcd30.00 [120.0000-3748.4226]
Precursor Scan: 012662, m/z: 912.6279, charge: 4
FQDSSVFNETLR_5_4_0_2_0
204.0864
138.0549
366.1388
274.0918
168.0653
186.0752
292.1027
126.0547
528.1816
657.2366
823.3986 PEP_0_1_0_0_0
1005.9673 PEP_1_2_0_0_0
1168.0222 PEP_3_2_0_0_0
Intensity
m/z
0
20000
40000
60000
80000
100000
500
1000
1500
2000
2500
3000

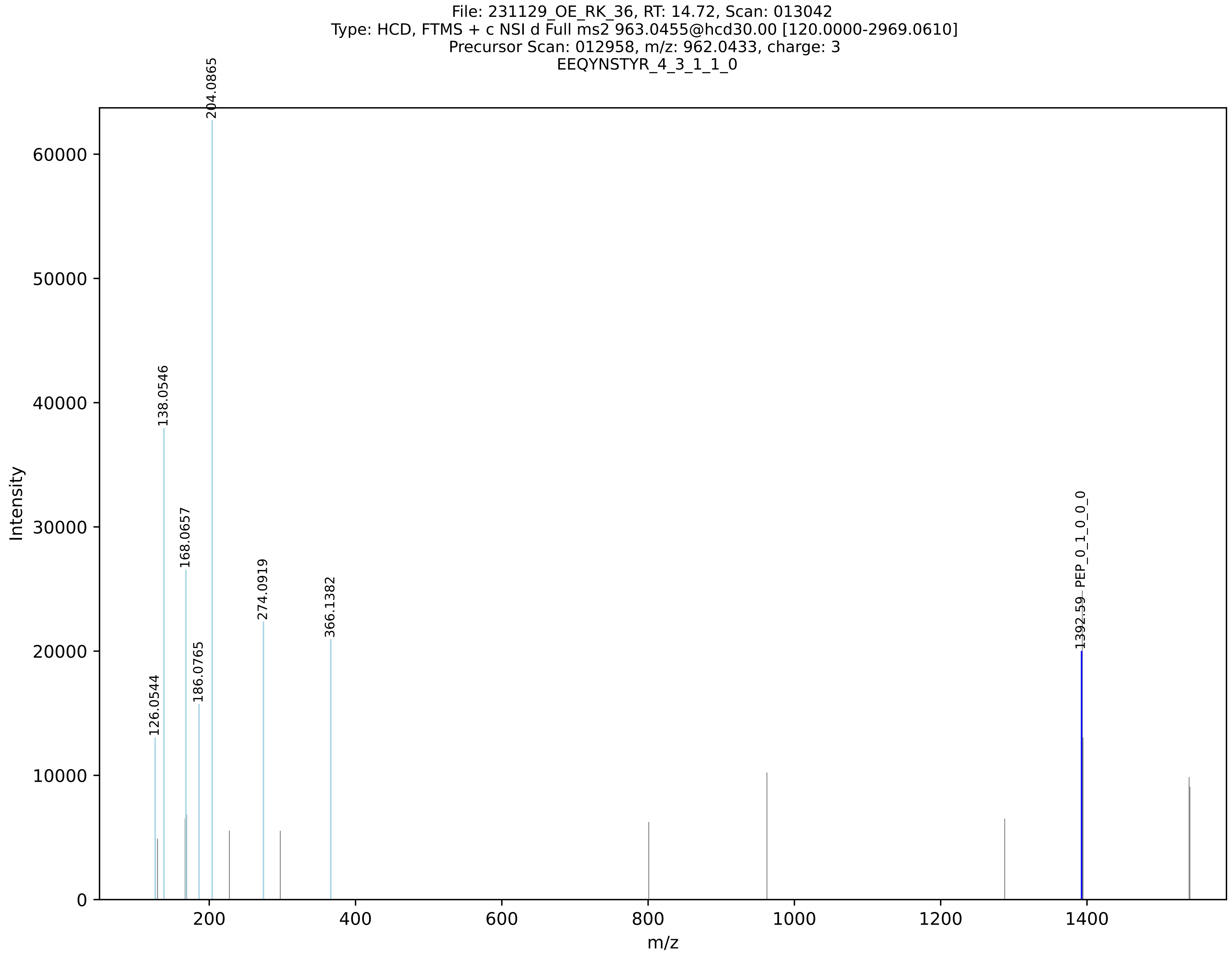
File: 231129_OE_RK_36, RT: 14.72, Scan: 013042
Type: HCD, FTMS + c NSI d Full ms2 963.0455@hcd30.00 [120.0000-2969.0610]
Precursor Scan: 012958, m/z: 962.0433, charge: 3
EEQYNSTYR_4_3_1_1_0
204.0865
138.0546
168.0657
186.0765
126.0544
274.0919
366.1382
1392.59_PEP_0_1_0_0_0_
Intensity
0
10000
20000
30000
40000
50000
60000
200
400
600
800
1000
1200
1400
m/z

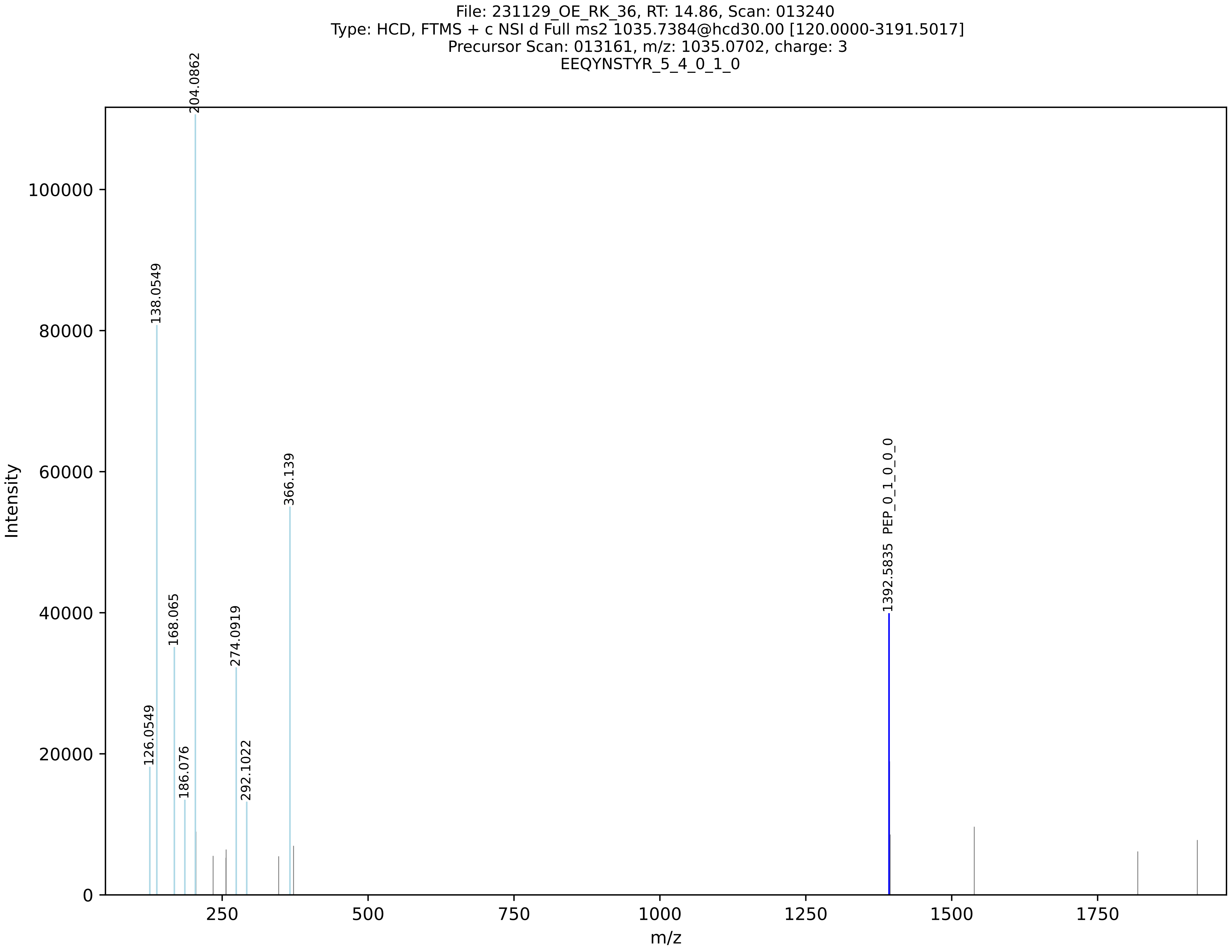
File: 231129_OE_RK_36, RT: 14.86, Scan: 013240
Type: HCD, FTMS + c NSI d Full ms2 1035.7384@hcd30.00 [120.0000-3191.5017]
Precursor Scan: 013161, m/z: 1035.0702, charge: 3
EEQYNSTYR_5_4_0_1_0
204.0862
138.0549
366.139
1392.5835 PEP_0_1_0_0_0
168.065
274.0919
126.0549
186.076
292.1022
Intensity
m/z
0
20000
40000
60000
80000
100000
250
500
750
1000
1250
1500
1750

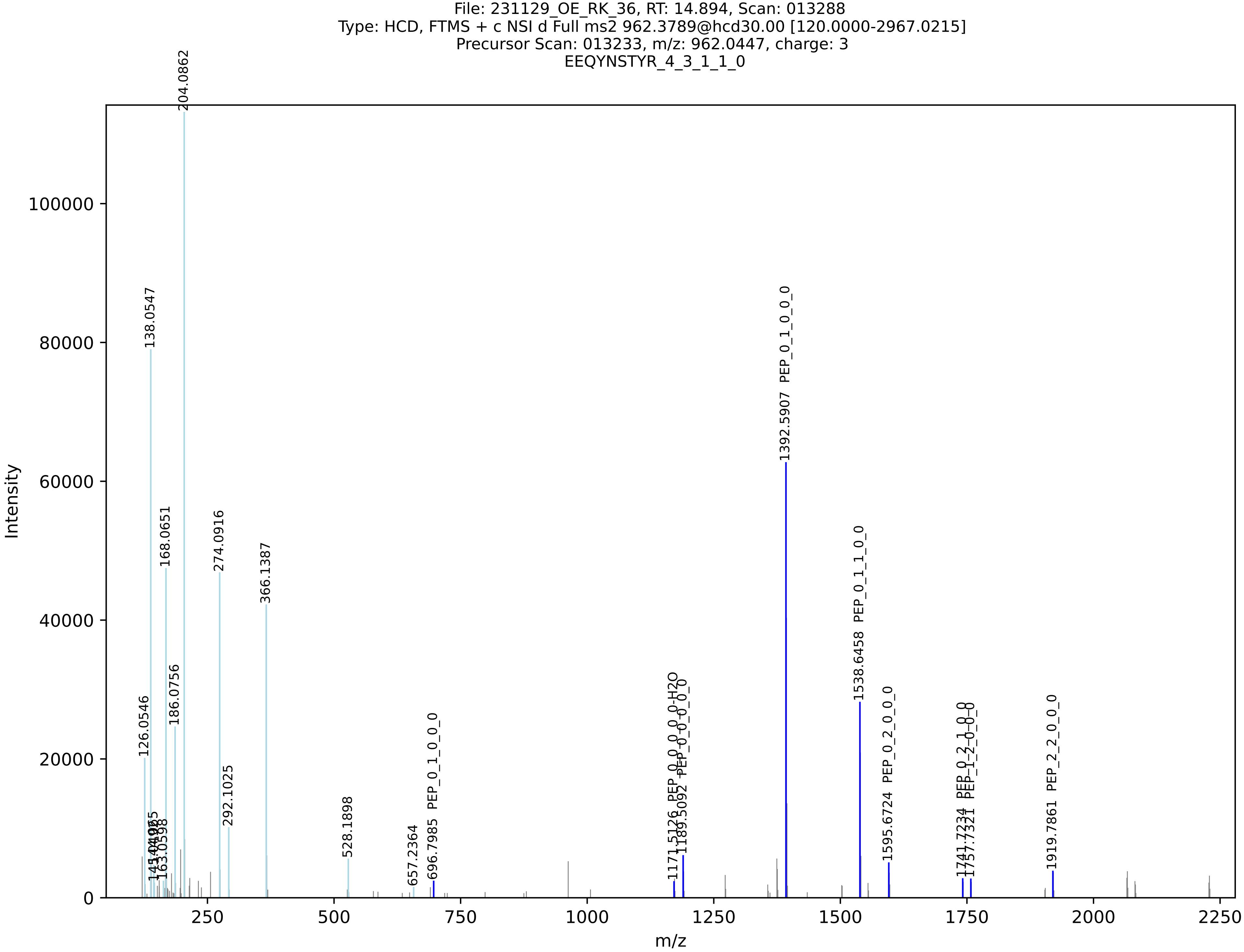
File: 231129_OE_RK_36, RT: 14.894, Scan: 013288
Type: HCD, FTMS + c NSI d Full ms2 962.3789@hcd30.00 [120.0000-2967.0215]
Precursor Scan: 013233, m/z: 962.0447, charge: 3
EEQYNSTYR_4_3_1_1_0
Intensity
m/z
204.0862
138.0547
168.0651
274.0916
366.1387
186.0756
126.0546
292.1025
528.1898
657.2364
696.7985 PEP_0_1_0_0_
1171.5126 PEP_0_0_0_0-H2O
1189.5092 PEP_0_0_0_0_
1392.5907 PEP_0_1_0_0_
1538.6458 PEP_0_1_1_0_
1595.6724 PEP_0_2_0_0_
1741.7234 PEP_0_2_1_0_
1757.7321 PEP_1_2_0_0_
1919.7861 PEP_2_2_0_0_
163.0598

File: 231129_OE_RK_36, RT: 14.946, Scan: 013362
Type: HCD, FTMS + c NSI d Full ms2 1084.0889@hcd30.00 [120.0000-3339.4541]
Precursor Scan: 013287, m/z: 1083.7548, charge: 3
EEQYNSTYR_5_4_1_1_0

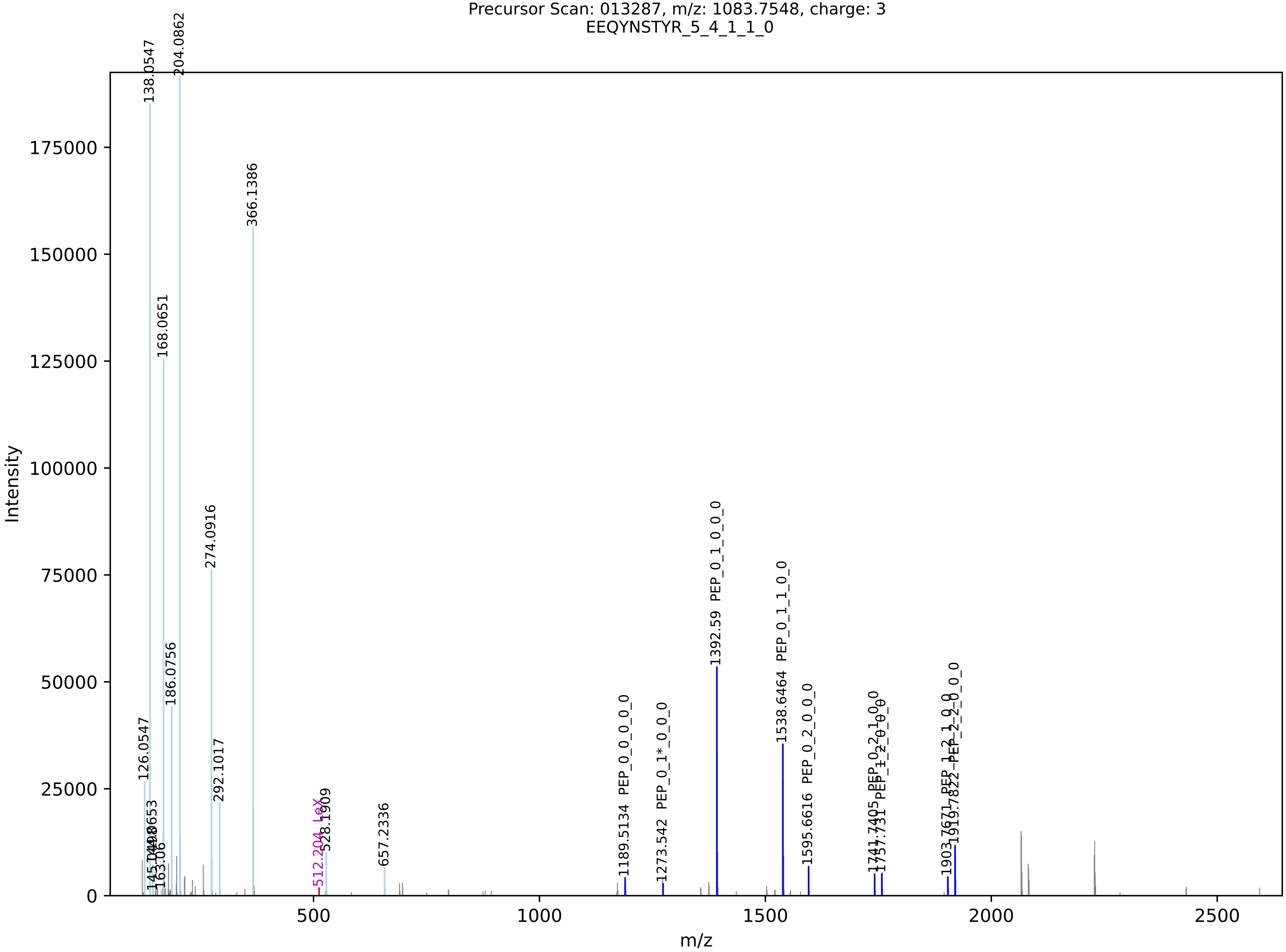

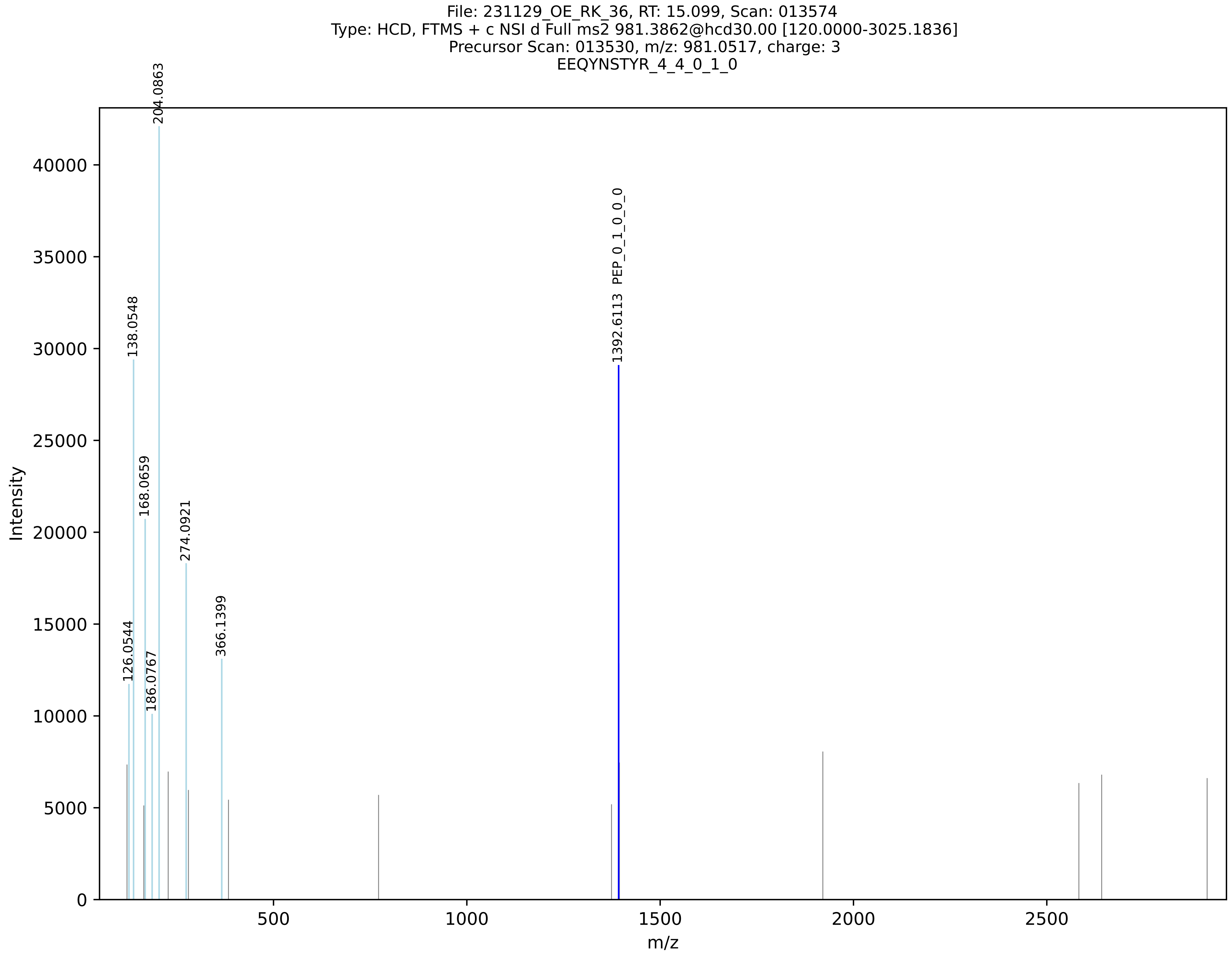
File: 231129_OE_RK_36, RT: 15.099, Scan: 013574
Type: HCD, FTMS + c NSI d Full ms2 981.3862@hcd30.00 [120.0000-3025.1836]
Precursor Scan: 013530, m/z: 981.0517, charge: 3
EEQYNSTYR_4_4_0_1_0
204.0863
138.0548
168.0659
274.0921
366.1399
126.0544
186.0767
1392.6113 PEP_0_1_0_0_0
Intensity
m/z
0
5000
10000
15000
20000
25000
30000
35000
40000
500
1000
1500
2000
2500

File: 231129_OE_RK_36, RT: 15.513, Scan: 014159
Type: HCD, FTMS + c NSI d Full ms2 1051.7444@hcd30.00 [120.0000-3240.4797]
Precursor Scan: 014097, m/z: 1051.4102, charge: 3
EDALNETR_5_4_0_2_0

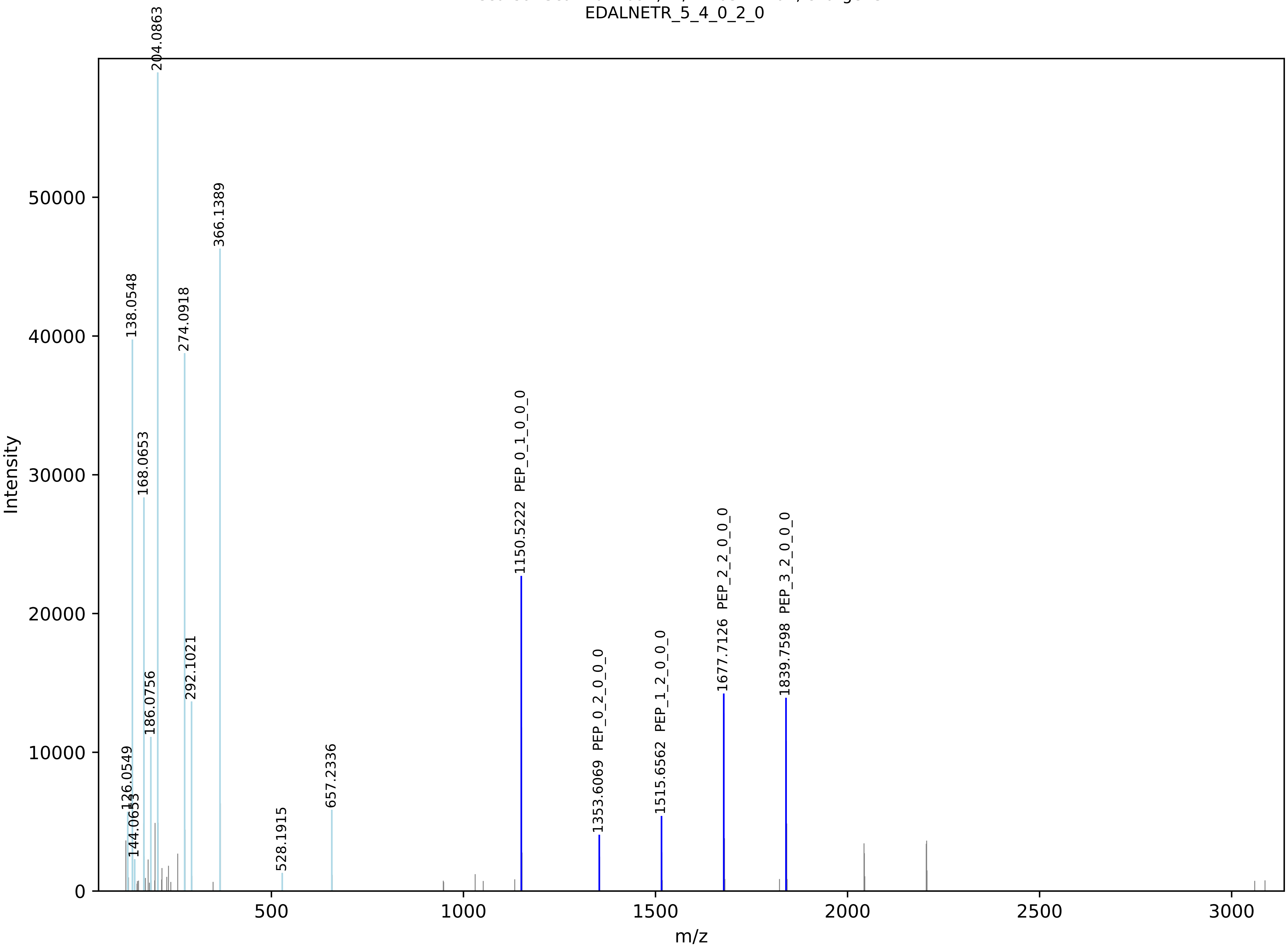

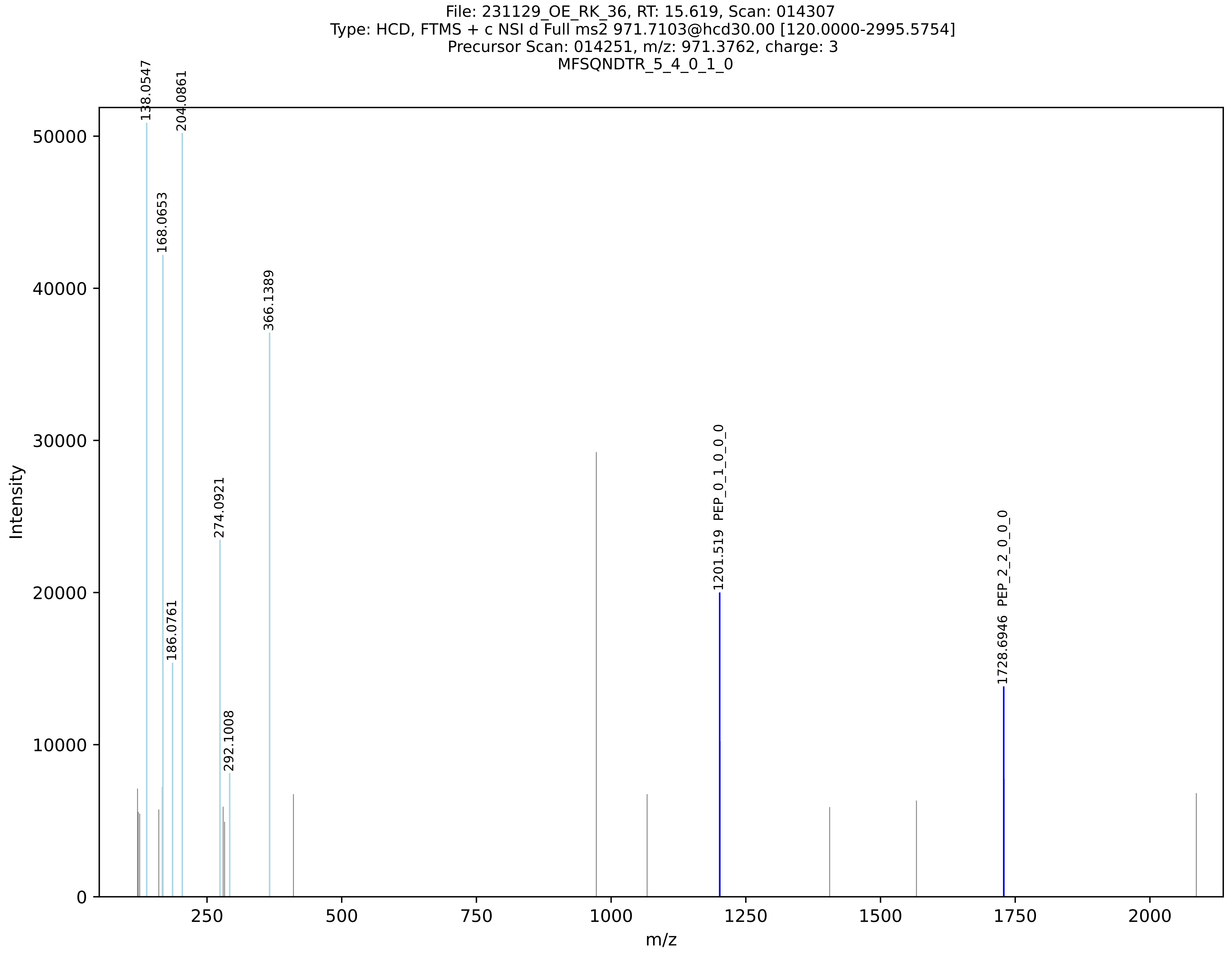
File: 231129_OE_RK_36, RT: 15.619, Scan: 014307
Type: HCD, FTMS + c NSI d Full ms2 971.7103@hcd30.00 [120.0000-2995.5754]
Precursor Scan: 014251, m/z: 971.3762, charge: 3
MFSQNDTR_5_4_0_1_0
138.0547
204.0861
168.0653
366.1389
274.0921
186.0761
292.1008
1201.519 PEP_0_1_0_0_0
1728.6946 PEP_2_2_0_0_0
Intensity
m/z
0
10000
20000
30000
40000
50000
250
500
750
1000
1250
1500
1750
2000

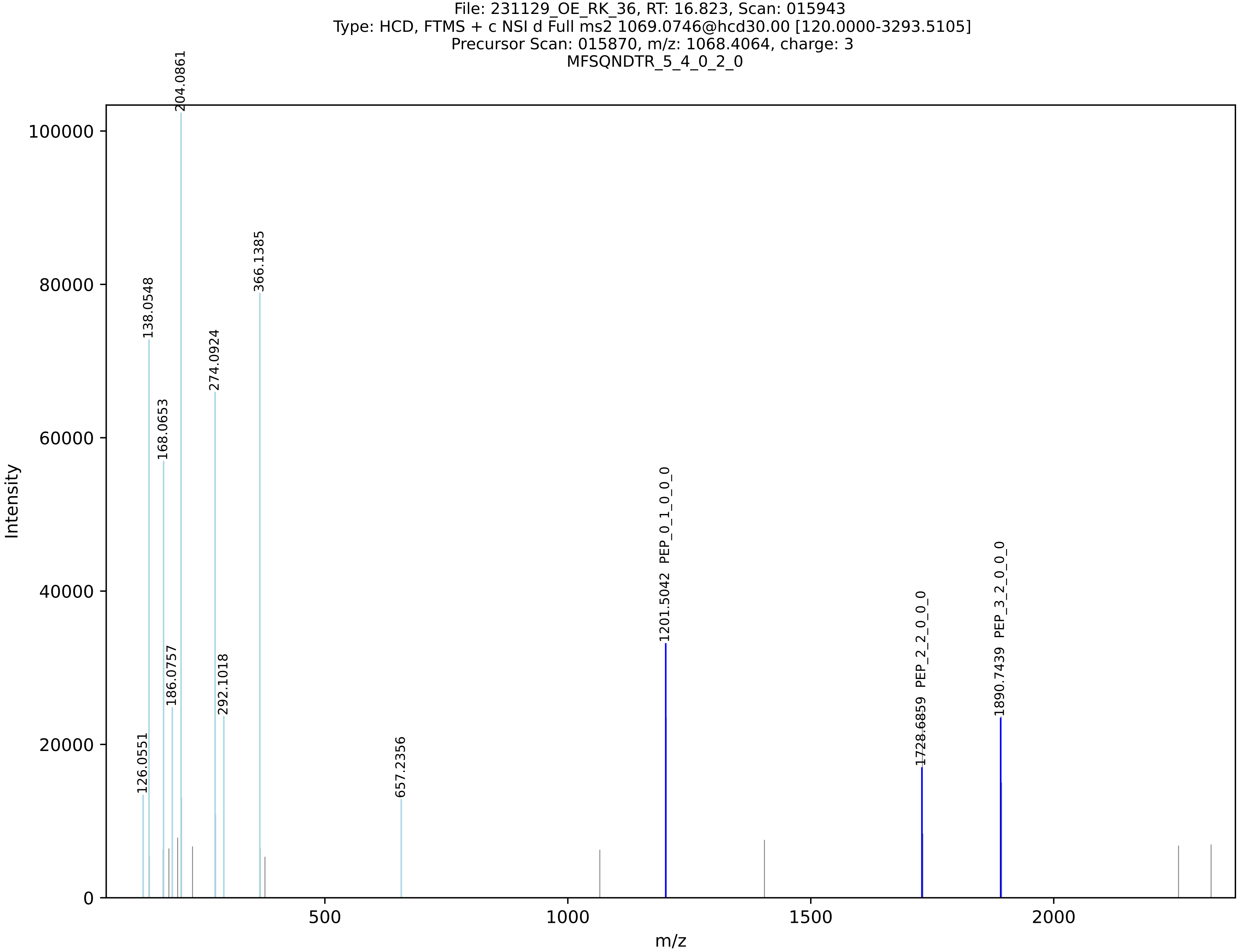

File: 231129_OE_RK_36, RT: 16.823, Scan: 015943
Type: HCD, FTMS + c NSI d Full ms2 1069.0746@hcd30.00 [120.0000-3293.5105]
Precursor Scan: 015870, m/z: 1068.4064, charge: 3
MFSQNDTR_5_4_0_2_0
Intensity
m/z
204.0861
366.1385
138.0548
274.0924
168.0653
186.0757
292.1018
126.0551
657.2356
1201.5042 PEP_0_1_0_0_0
1728.6859 PEP_2_2_0_0_0
1890.7439 PEP_3_2_0_0_0

File: 231129_OE_RK_36, RT: 17.01, Scan: 016208
Type: HCD, FTMS + c NSI d Full ms2 1068.7411@hcd30.00 [120.0000-3292.4895]
Precursor Scan: 016148, m/z: 1068.407, charge: 3
MFSQNDTR_5_4_0_2_0

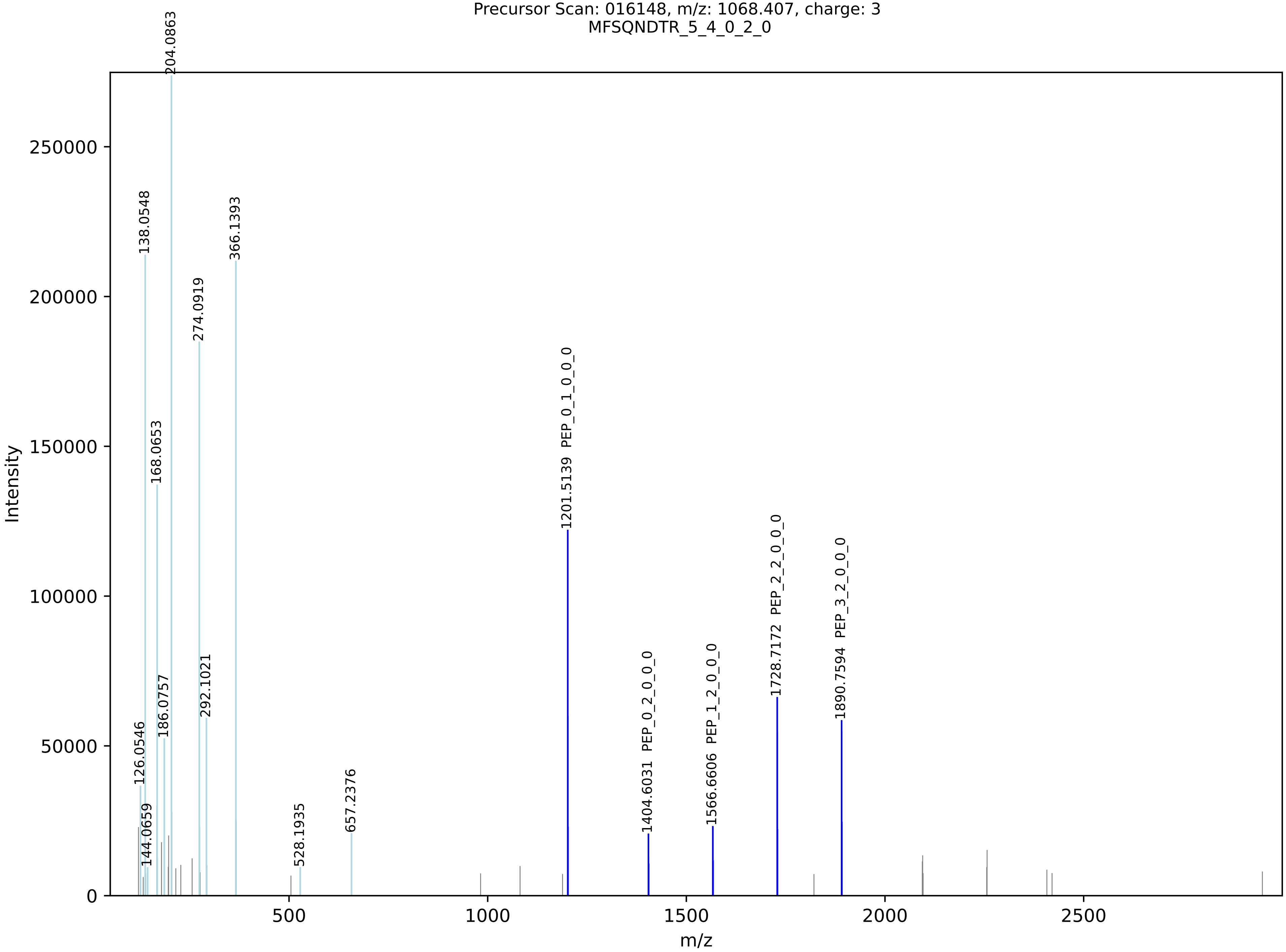

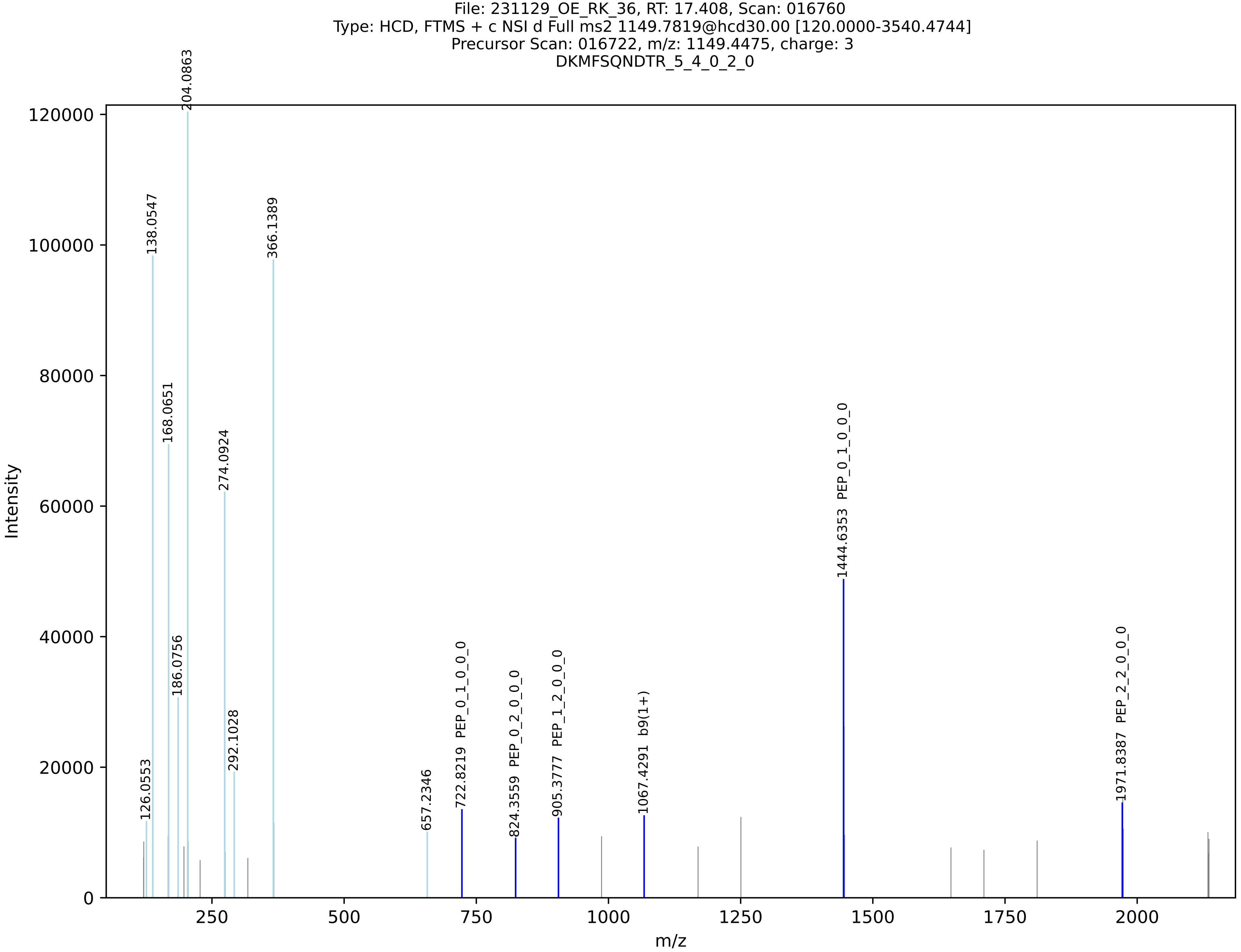

File: 231129_OE_RK_36, RT: 17.408, Scan: 016760
Type: HCD, FTMS + c NSI d Full ms2 1149.7819@hcd30.00 [120.0000-3540.4744]
Precursor Scan: 016722, m/z: 1149.4475, charge: 3
DKMFSQNDTR_5_4_0_2_0
Intensity
m/z
204.0863
138.0547
366.1389
168.0651
274.0924
186.0756
292.1028
126.0553
657.2346
722.8219 PEP_0_1_0_0_0
824.3559 PEP_0_2_0_0_0
905.3777 PEP_1_2_0_0_0
1067.4291 b9(1+)
1444.6353 PEP_0_1_0_0_0
1971.8387 PEP_2_2_0_0_0

File: 231129_OE_RK_36, RT: 17.584, Scan: 017009
Type: HCD, FTMS + c NSI d Full ms2 1149.7831@hcd30.00 [120.0000-3540.4783]
Precursor Scan: 016954, m/z: 1149.4489, charge: 3
DKMFSQNDTR_5_4_0_2_0

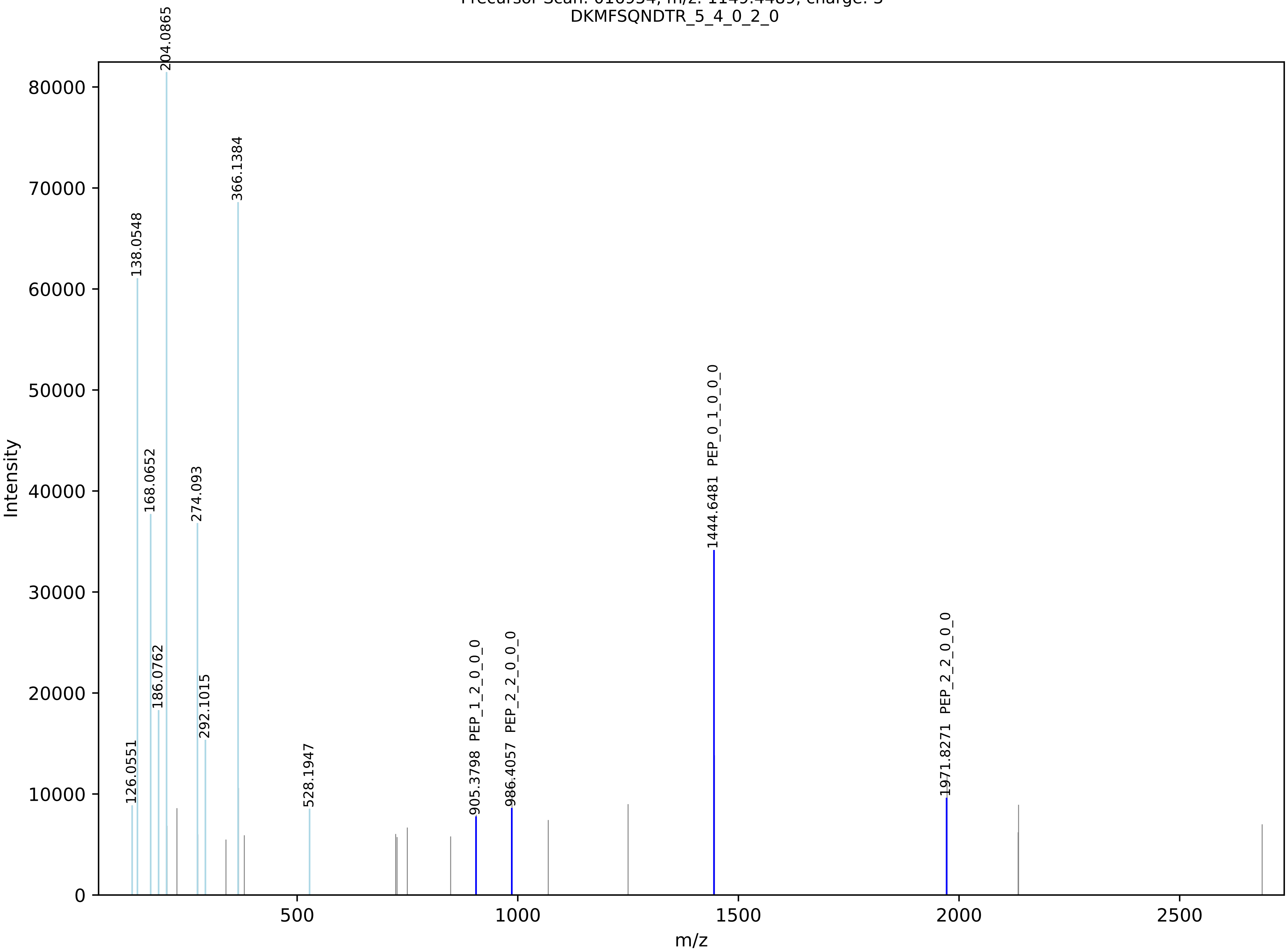

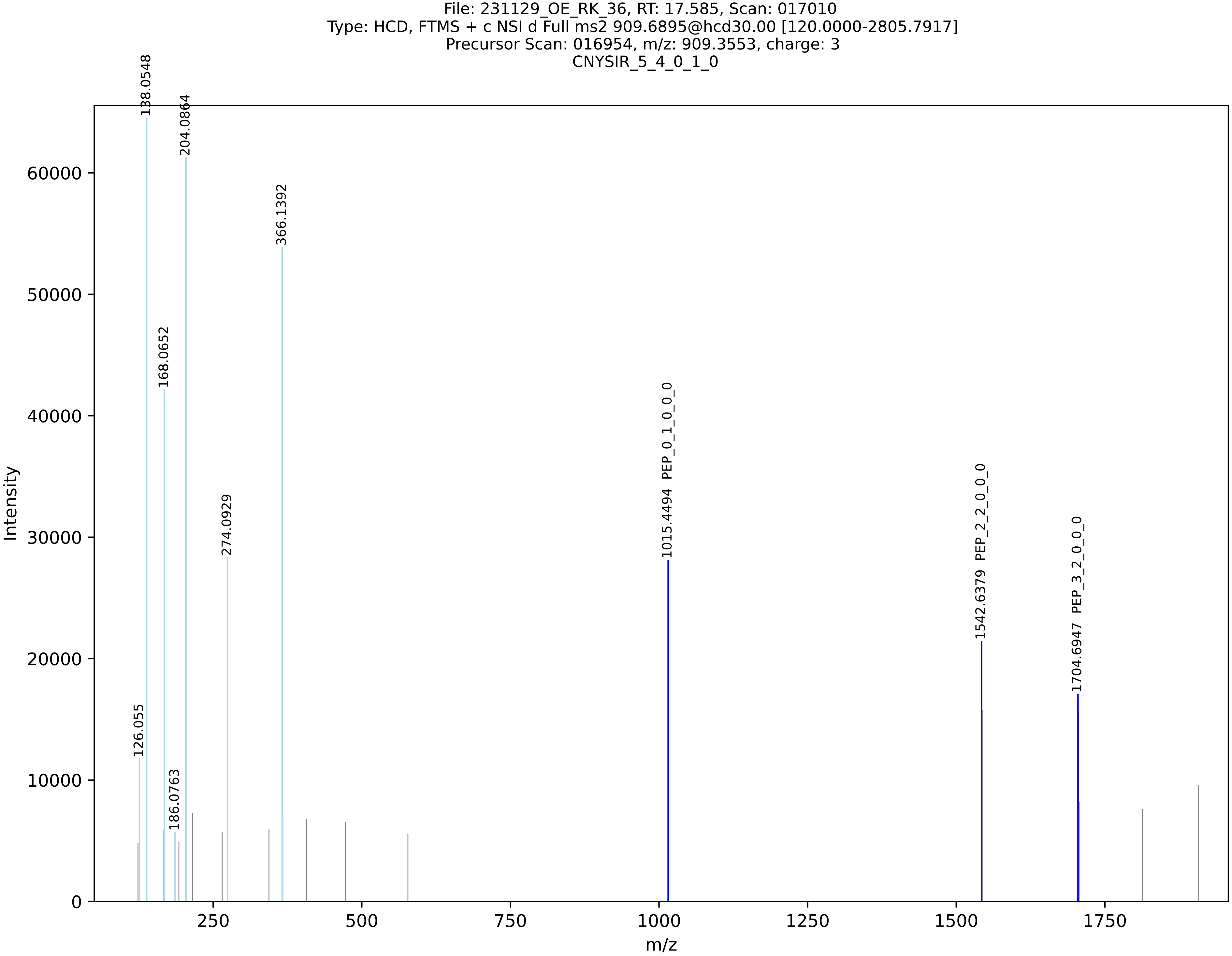
File: 231129_OE_RK_36, RT: 17.585, Scan: 017010
Type: HCD, FTMS + c NSI d Full ms2 909.6895@hcd30.00 [120.0000-2805.7917]
Precursor Scan: 016954, m/z: 909.3553, charge: 3
CNYSIR_5_4_0_1_0
138.0548
204.0864
366.1392
168.0652
274.0929
126.055
186.0763
1015.4494 PEP_0_1_0_0_0
1542.6379 PEP_2_2_0_0_0
1704.6947 PEP_3_2_0_0_0
Intensity
0
10000
20000
30000
40000
50000
60000
250
500
750
1000
1250
1500
1750
m/z

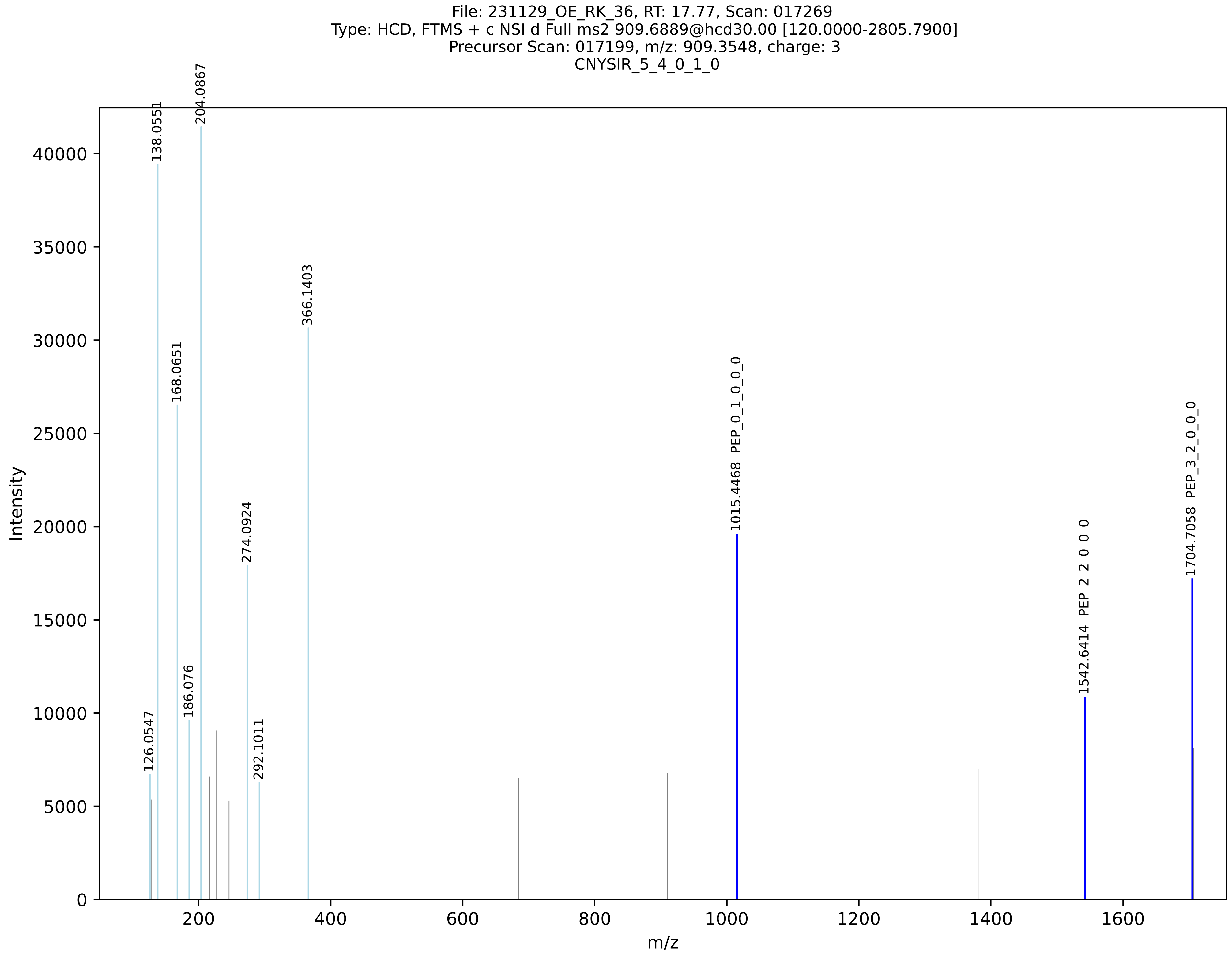
File: 231129_OE_RK_36, RT: 17.77, Scan: 017269
Type: HCD, FTMS + c NSI d Full ms2 909.6889@hcd30.00 [120.0000-2805.7900]
Precursor Scan: 017199, m/z: 909.3548, charge: 3
CNYSIR_5_4_0_1_0
Intensity
m/z
126.0547
138.0551
168.0651
186.076
204.0867
274.0924
292.1011
366.1403
1015.4468 PEP_0_1_0_0_0
1542.6414 PEP_2_2_0_0_0
1704.7058 PEP_3_2_0_0_0
0
5000
10000
15000
20000
25000
30000
35000
40000
200
400
600
800
1000
1200
1400
1600

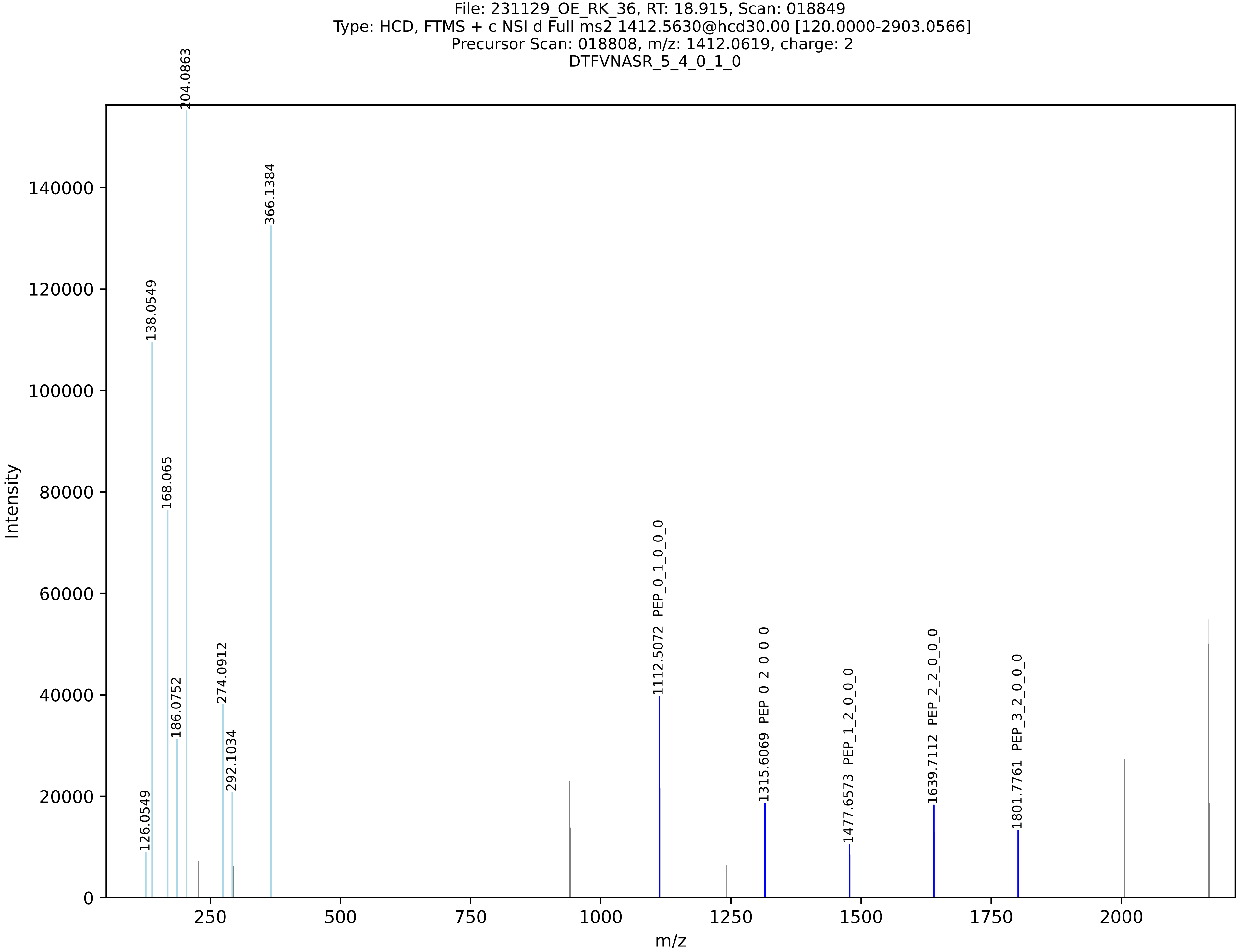
File: 231129_OE_RK_36, RT: 18.915, Scan: 018849
Type: HCD, FTMS + c NSI d Full ms2 1412.5630@hcd30.00 [120.0000-2903.0566]
Precursor Scan: 018808, m/z: 1412.0619, charge: 2
DTFVNASR_5_4_0_1_0
Intensity
m/z
204.0863
366.1384
138.0549
168.065
186.0752
274.0912
292.1034
126.0549
1112.5072 PEP_0_1_0_0_0
1315.6069 PEP_0_2_0_0_0
1477.6573 PEP_1_2_0_0_0
1639.7112 PEP_2_2_0_0_0
1801.7761 PEP_3_2_0_0_0
0
20000
40000
60000
80000
100000
120000
140000
250
500
750
1000
1250
1500
1750
2000

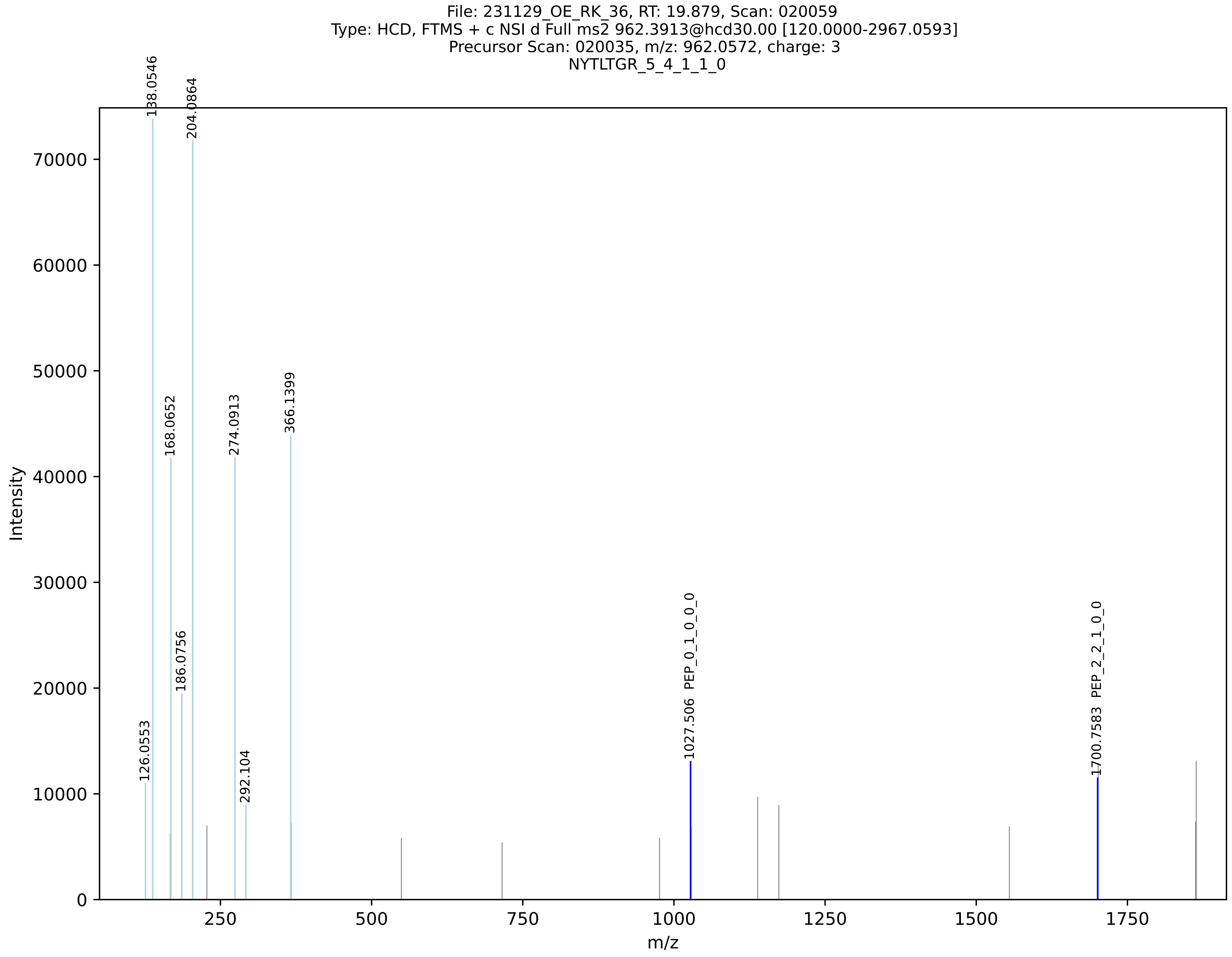

File: 231129_OE_RK_36, RT: 19.879, Scan: 020059
Type: HCD, FTMS + c NSI d Full ms2 962.3913@hcd30.00 [120.0000-2967.0593]
Precursor Scan: 020035, m/z: 962.0572, charge: 3
NYTLTGR_5_4_1_1_0
138.0546
204.0864
168.0652
274.0913
366.1399
186.0756
126.0553
292.104
1027.506 PEP_0_1_0_0_0
1700.7583 PEP_2_2_1_0_0
Intensity
m/z
0
10000
20000
30000
40000
50000
60000
70000
250
500
750
1000
1250
1500
1750

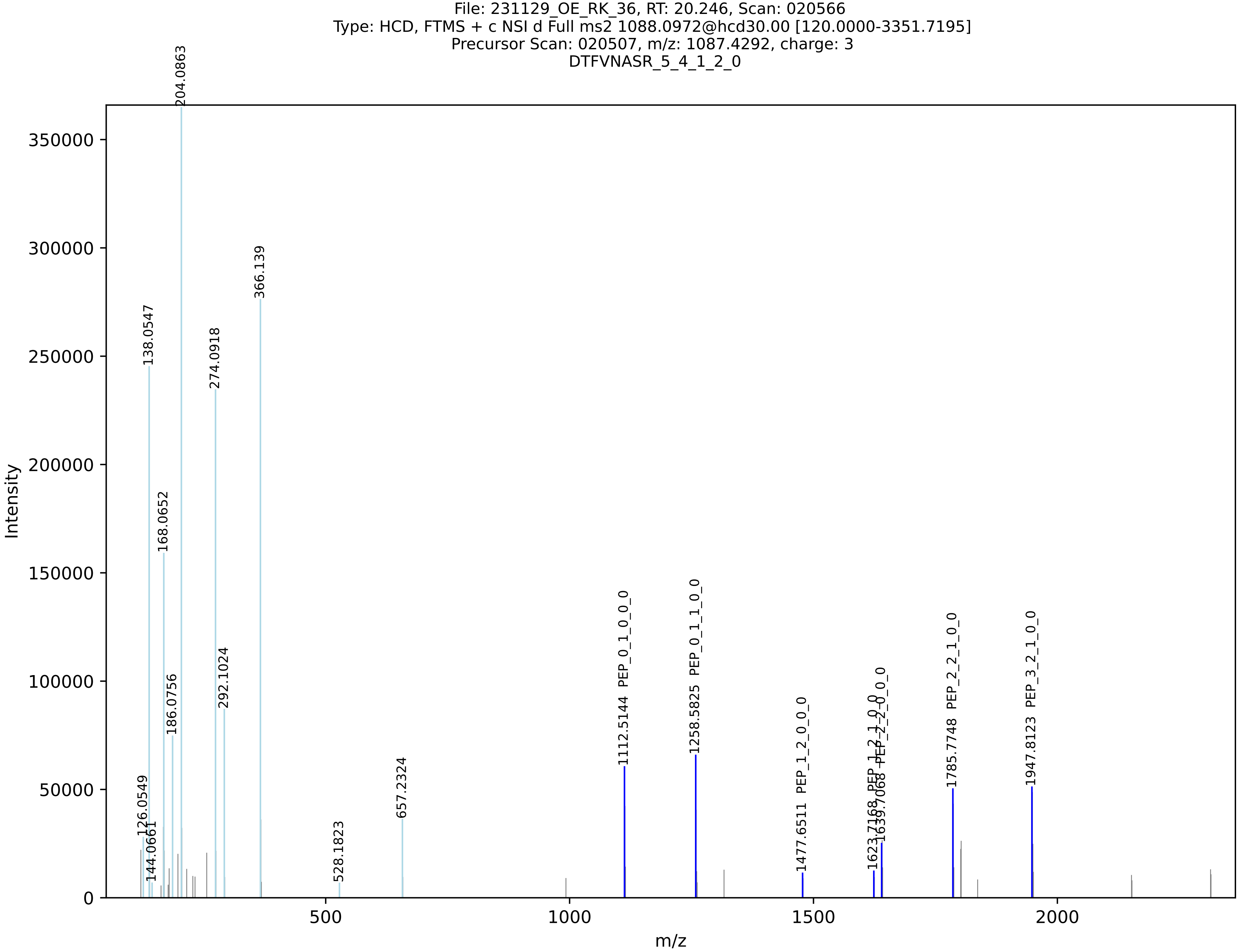
File: 231129_OE_RK_36, RT: 20.246, Scan: 020566
Type: HCD, FTMS + c NSI d Full ms2 1088.0972@hcd30.00 [120.0000-3351.7195]
Precursor Scan: 020507, m/z: 1087.4292, charge: 3
DTFVNASR_5_4_1_2_0
Intensity
m/z
204.0863
366.139
138.0547
274.0918
168.0652
292.1024
186.0756
126.0549
144.0661
528.1823
657.2324
1112.5144 PEP_0_1_0_0_0
1258.5825 PEP_0_1_1_0_0
1477.6511 PEP_1_2_0_0_0
1623.7168 PEP_1_2_1_0_0
1639.7068 PEP_2_2_0_0_0
1785.7748 PEP_2_2_1_0_0
1947.8123 PEP_3_2_1_0_0
0
50000
100000
150000
200000
250000
300000
350000
500
1000
1500
2000

File: 231129_OE_RK_36, RT: 20.247, Scan: 020567
Type: HCD, FTMS + c NSI d Full ms2 1227.8214@hcd30.00 [120.0000-3779.2754]
Precursor Scan: 020507, m/z: 1227.1531, charge: 3
YAEDKFNETTEK_5_4_0_2_0

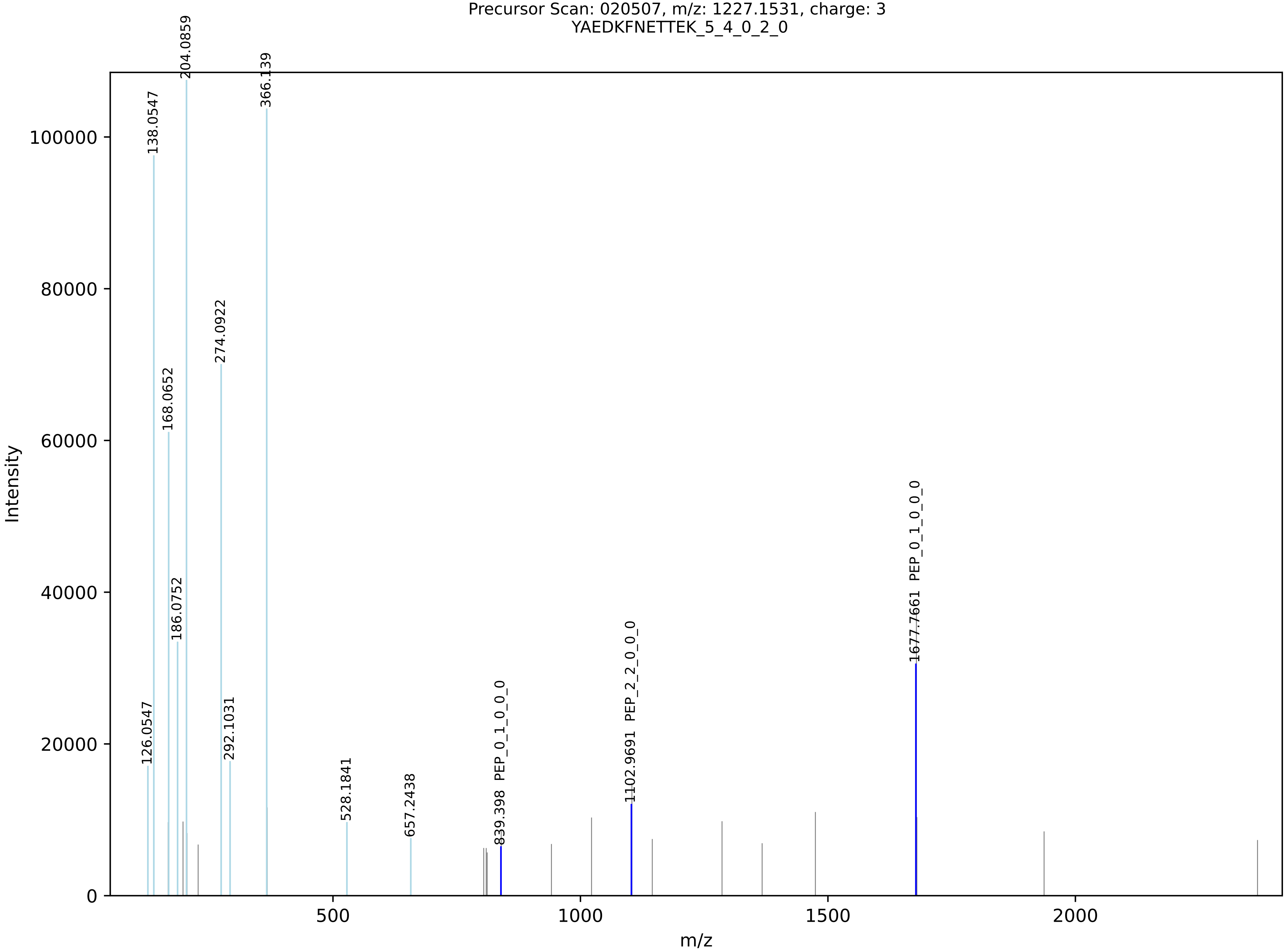

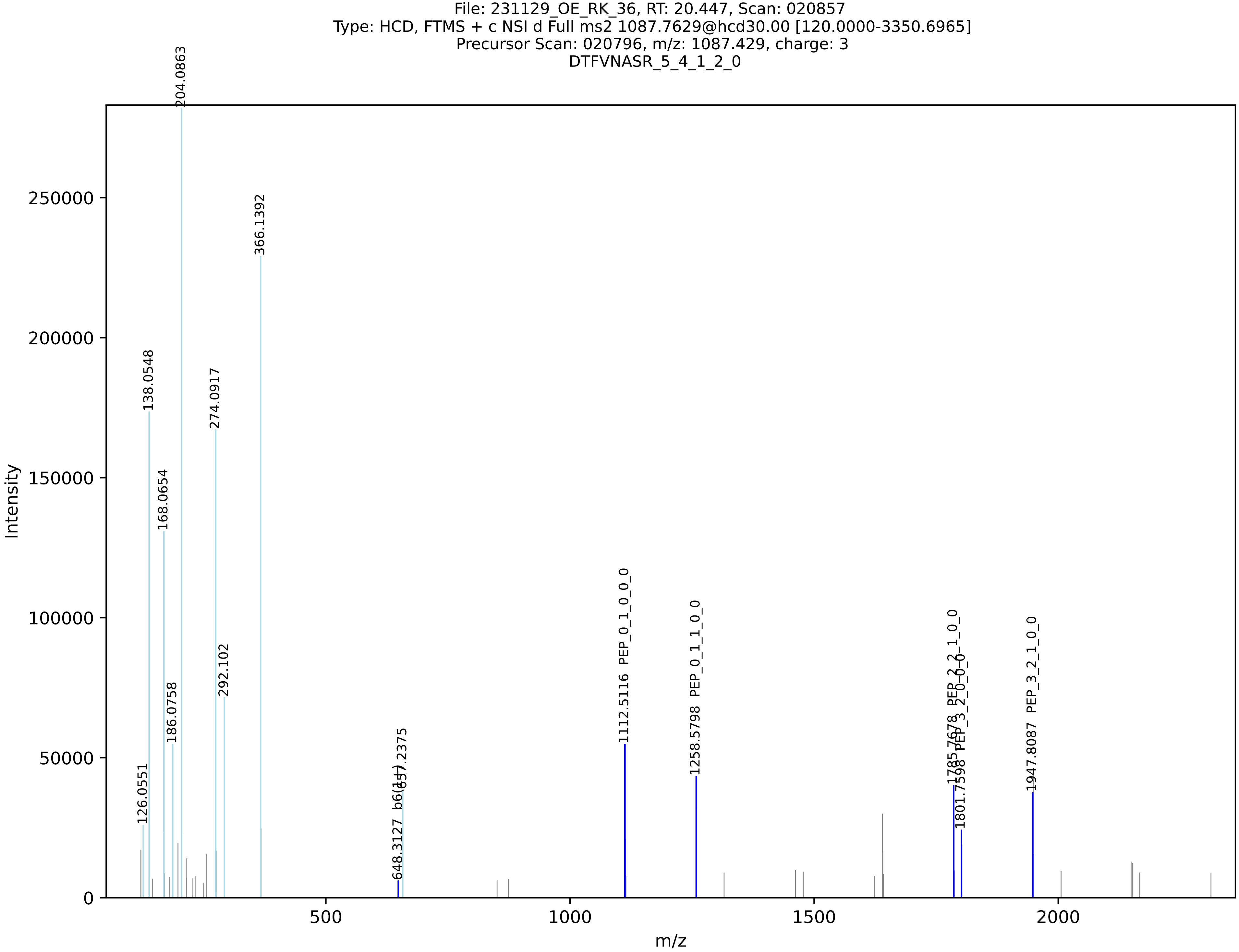
File: 231129_OE_RK_36, RT: 20.447, Scan: 020857
Type: HCD, FTMS + c NSI d Full ms2 1087.7629@hcd30.00 [120.0000-3350.6965]
Precursor Scan: 020796, m/z: 1087.429, charge: 3
DTFVNASR_5_4_1_2_0
204.0863
366.1392
138.0548
274.0917
168.0654
292.102
186.0758
126.0551
648.3127 b6(1+)
657.2375
1112.5116 PEP_0_1_0_0_0
1258.5798 PEP_0_1_1_0_0
1785.7678 PEP_2_2_1_0_0
1801.7598 PEP_3_2_0_0_0
1947.8087 PEP_3_2_1_0_0
Intensity
0
50000
100000
150000
200000
250000
500
1000
1500
2000
m/z

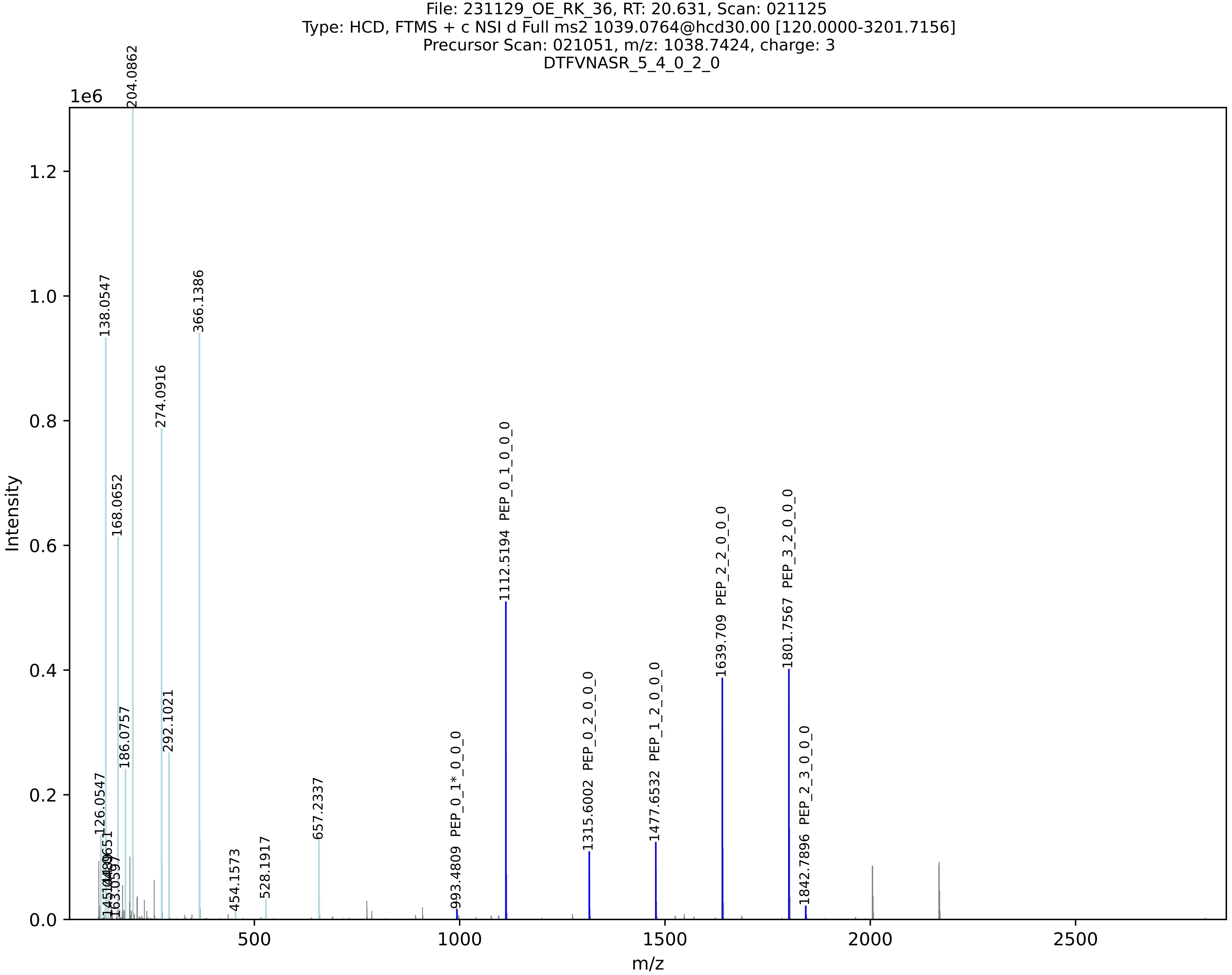
File: 231129_OE_RK_36, RT: 20.631, Scan: 021125
Type: HCD, FTMS + c NSI d Full ms2 1039.0764@hcd30.00 [120.0000-3201.7156]
Precursor Scan: 021051, m/z: 1038.7424, charge: 3
DTFVNASR_5_4_0_2_0
1e6
Intensity
m/z
204.0862
138.0547
366.1386
274.0916
168.0652
186.0757
292.1021
126.0547
163.0597
454.1573
528.1917
657.2337
993.4809 PEP_0_1*_0_0_0
1112.5194 PEP_0_1_0_0_0
1315.6002 PEP_0_2_0_0_0
1477.6532 PEP_1_2_0_0_0
1639.709 PEP_2_2_0_0_0
1801.7567 PEP_3_2_0_0_0
1842.7896 PEP_2_3_0_0_0

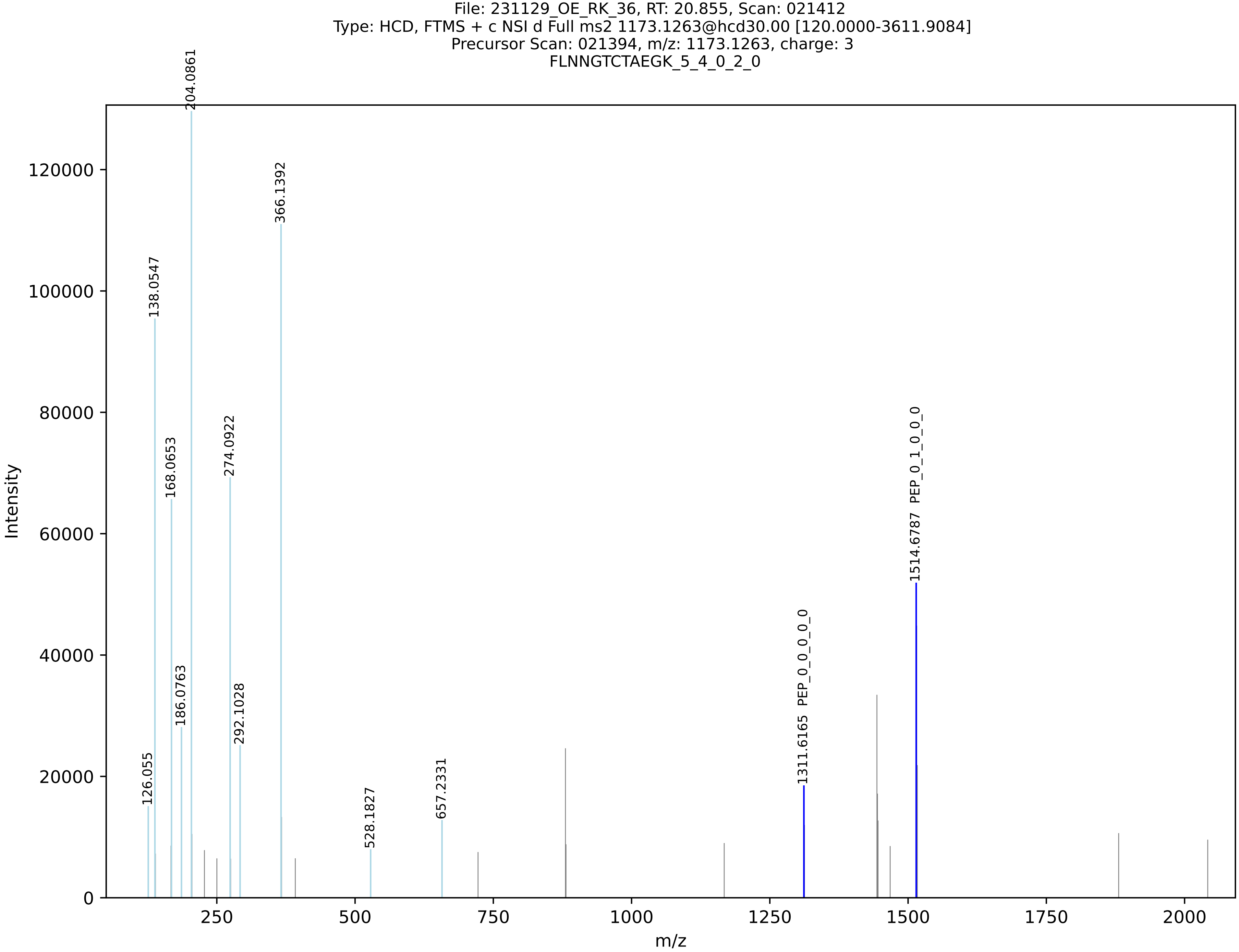
File: 231129_OE_RK_36, RT: 20.855, Scan: 021412
Type: HCD, FTMS + c NSI d Full ms2 1173.1263@hcd30.00 [120.0000-3611.9084]
Precursor Scan: 021394, m/z: 1173.1263, charge: 3
FLNNGTCTAEGK_5_4_0_2_0
Intensity
m/z
204.0861
366.1392
138.0547
168.0653
274.0922
186.0763
292.1028
126.055
528.1827
657.2331
1311.6165 PEP_0_0_0_0_0
1514.6787 PEP_0_1_0_0_0
0
20000
40000
60000
80000
100000
120000
250
500
750
1000
1250
1500
1750
2000

File: 231129_OE_RK_36, RT: 21.541, Scan: 022281
Type: HCD, FTMS + c NSI d Full ms2 1039.0773@hcd30.00 [120.0000-3201.7185]
Precursor Scan: 022205, m/z: 1038.7432, charge: 3
DTFVNASR_5_4_0_2_0

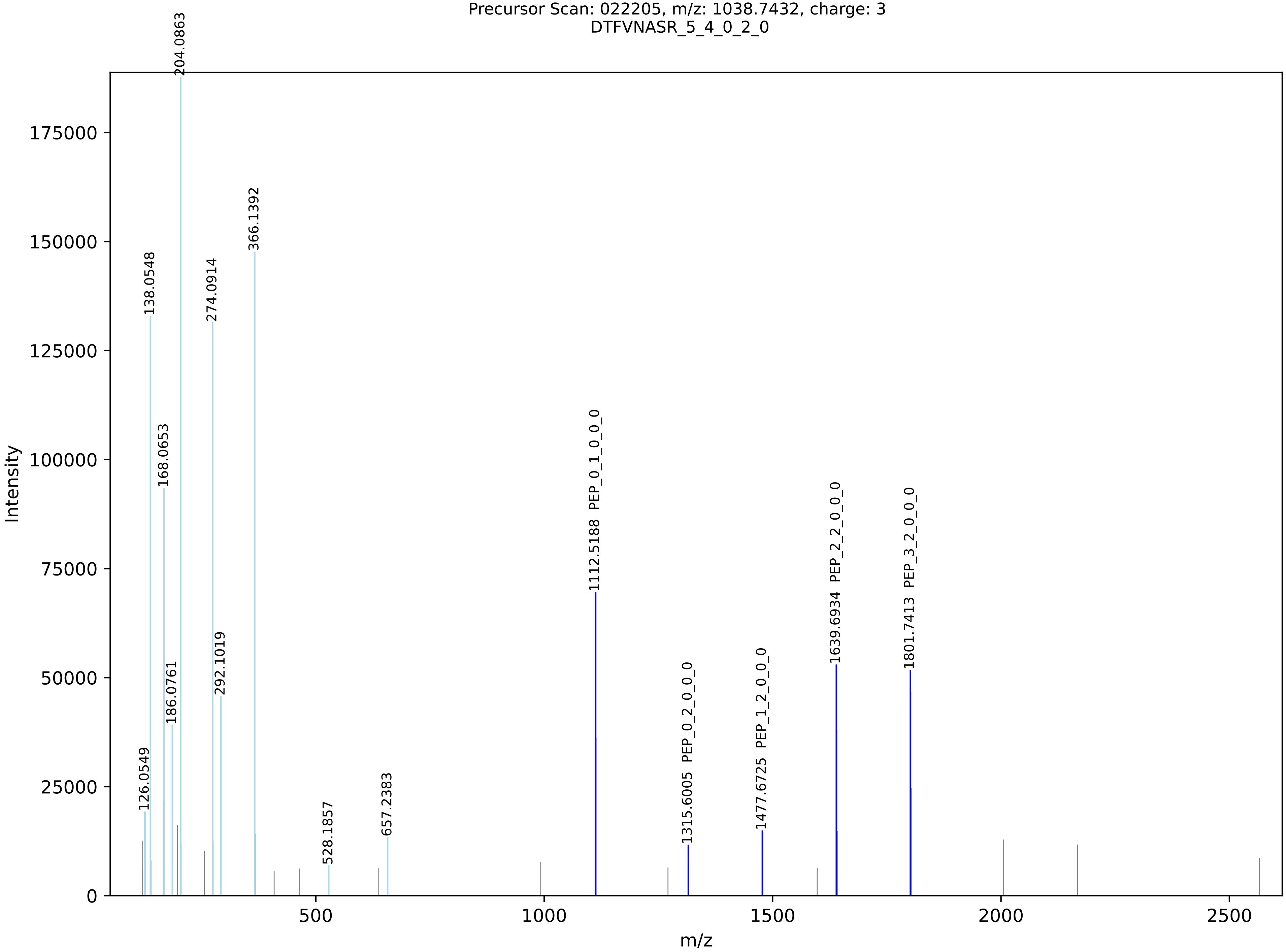

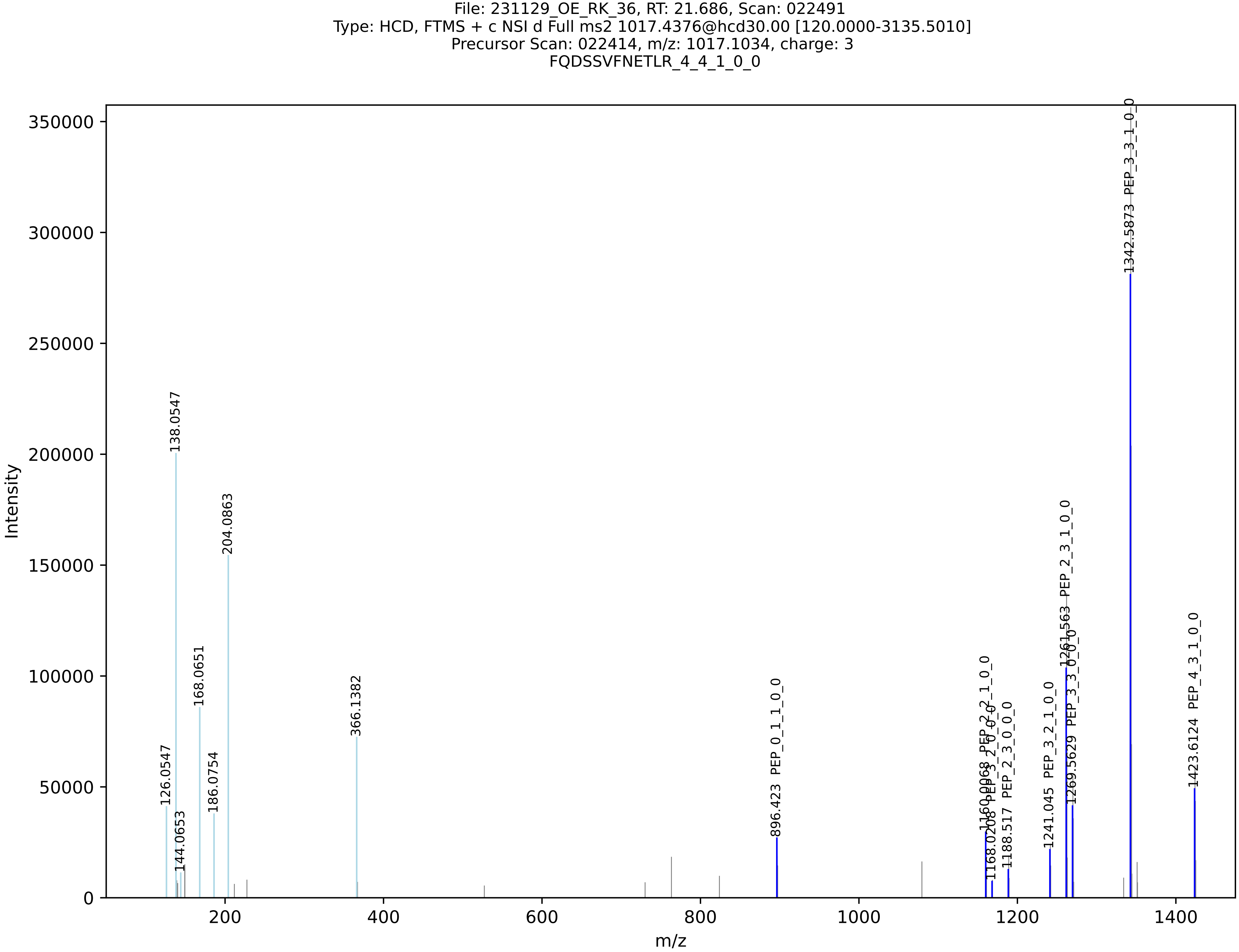

File: 231129_OE_RK_36, RT: 21.686, Scan: 022491
Type: HCD, FTMS + c NSI d Full ms2 1017.4376@hcd30.00 [120.0000-3135.5010]
Precursor Scan: 022414, m/z: 1017.1034, charge: 3
FQDSSVFNETLR_4_4_1_0_0
Intensity
m/z
138.0547
204.0863
168.0651
366.1382
126.0547
186.0754
144.0653
896.423 PEP_0_1_1_0_0
1160.0063 PEP_2_2_1_0_0
1168.0208 PEP_3_2_0_0
1188.517 PEP_2_3_0_0_0
1241.045 PEP_3_2_1_0_0
1261.563 PEP_2_3_1_0_0
1269.5629 PEP_3_3_0_0
1342.5873 PEP_3_3_1_0_0
1423.6124 PEP_4_3_1_0_0

File: 231129_OE_RK_36, RT: 22.296, Scan: 023365
Type: HCD, FTMS + c NSI d Full ms2 1294.8661@hcd30.00 [120.0000-3984.4321]
Precursor Scan: 023279, m/z: 1294.5319, charge: 3
VVNCSDPGFVENAIR_5_4_0_2_0

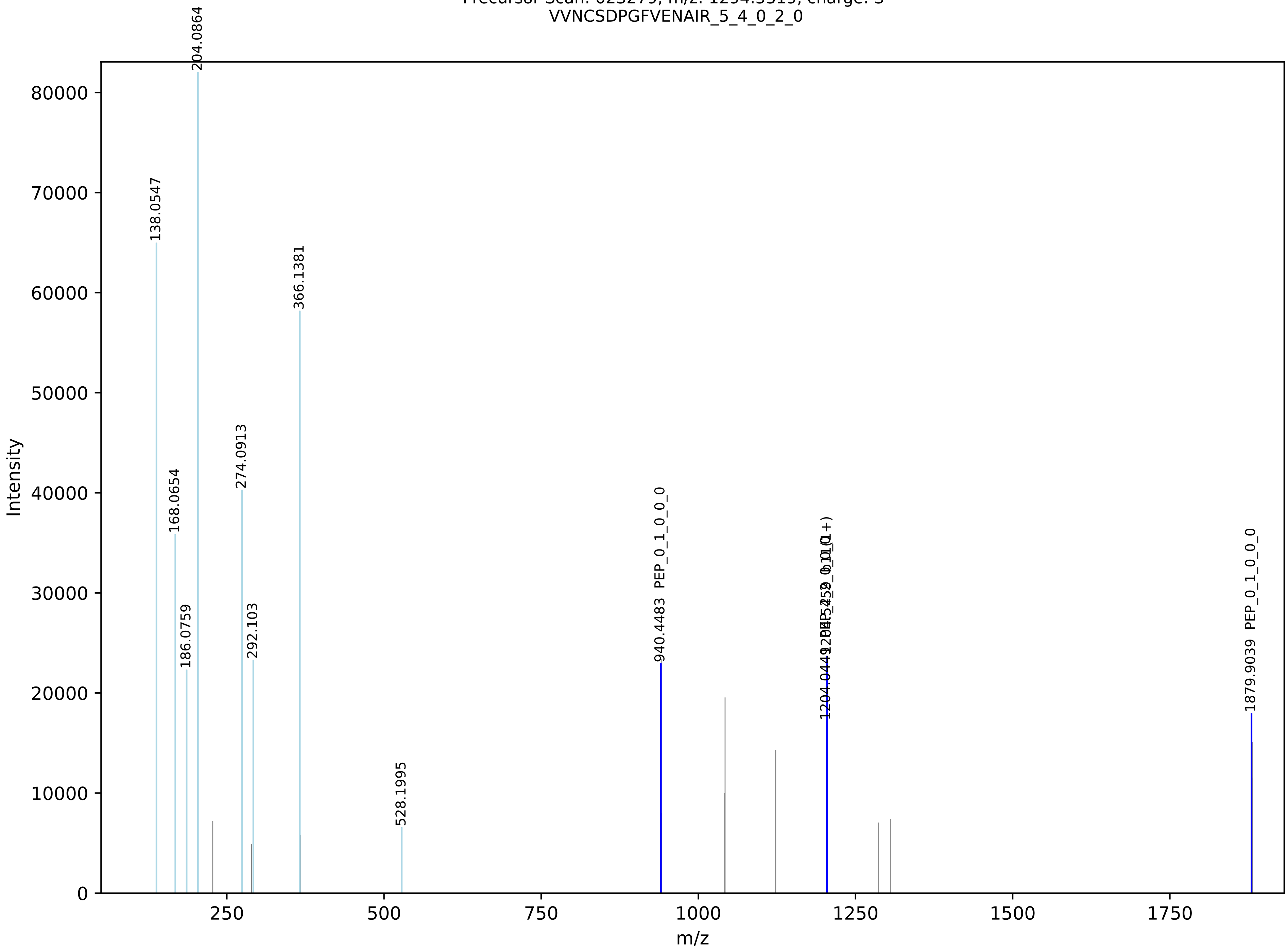

File: 231129_OE_RK_36, RT: 22.297, Scan: 023367
Type: HCD, FTMS + c NSI d Full ms2 971.6521@hcd30.00 [120.0000-3987.1965]
Precursor Scan: 023279, m/z: 971.1508, charge: 4
VVNCSDPGFVENAIR_5_4_0_2_0

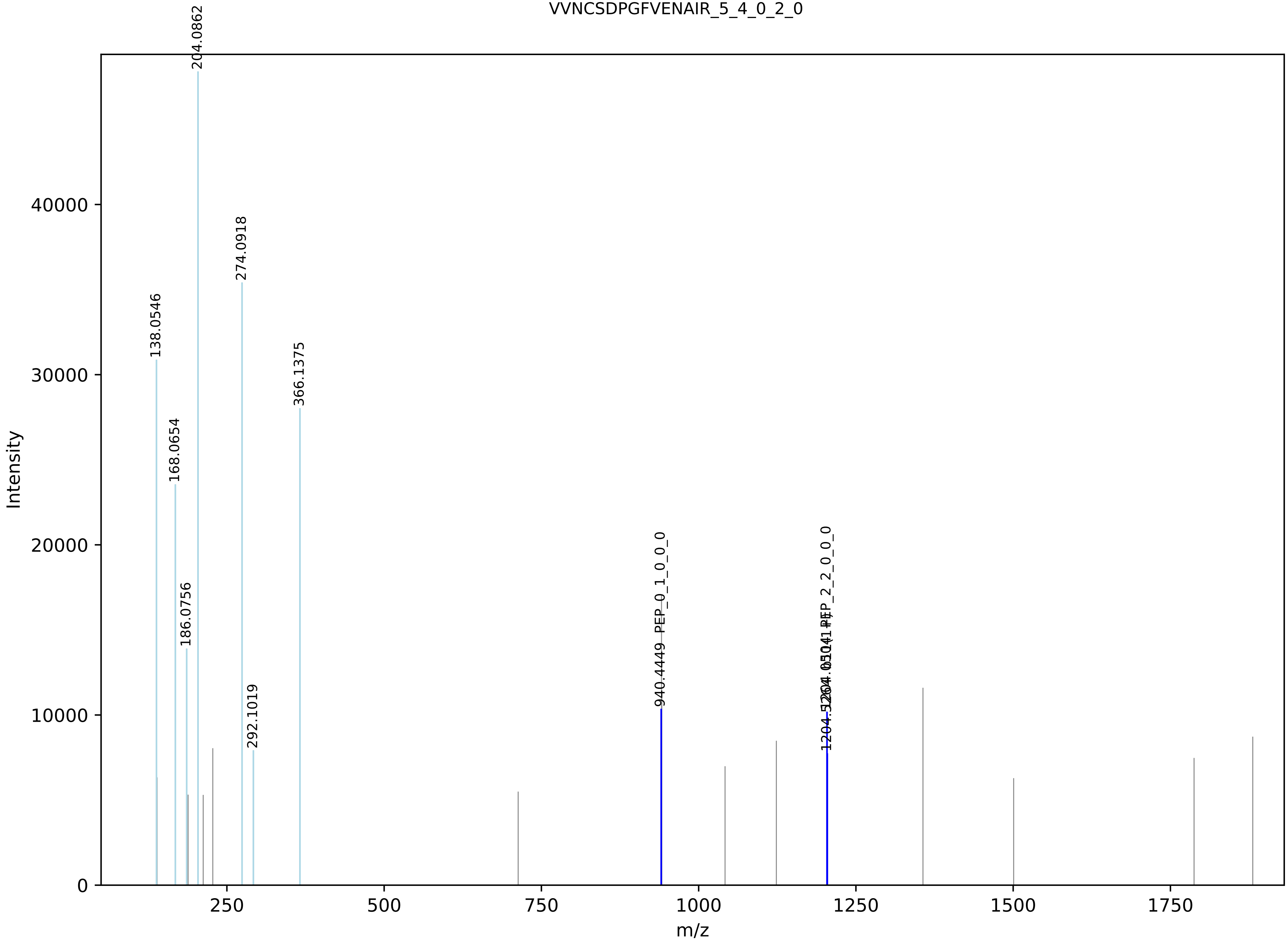

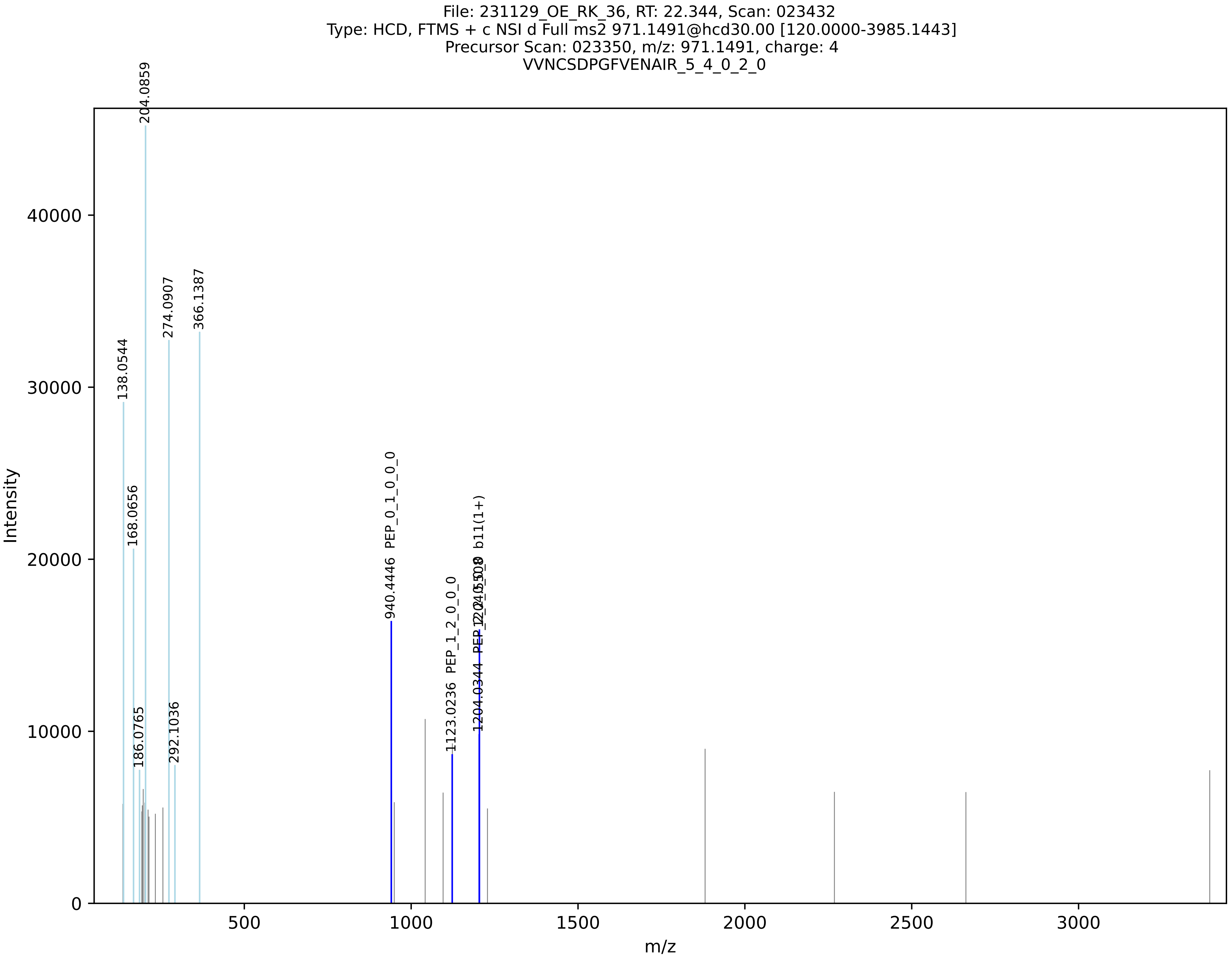
File: 231129_OE_RK_36, RT: 22.344, Scan: 023432
Type: HCD, FTMS + c NSI d Full ms2 971.1491@hcd30.00 [120.0000-3985.1443]
Precursor Scan: 023350, m/z: 971.1491, charge: 4
VVNCSDPGFVENAIR_5_4_0_2_0
Intensity
m/z
204.0859
138.0544
274.0907
366.1387
168.0656
186.0765
292.1036
940.4446 PEP_0_1_0_0_0
1123.0236 PEP_1_2_0_0_0
1204.0344 PEP_1_2_0_1_0
1204.5508 b11(1+)
0
10000
20000
30000
40000
500
1000
1500
2000
2500
3000

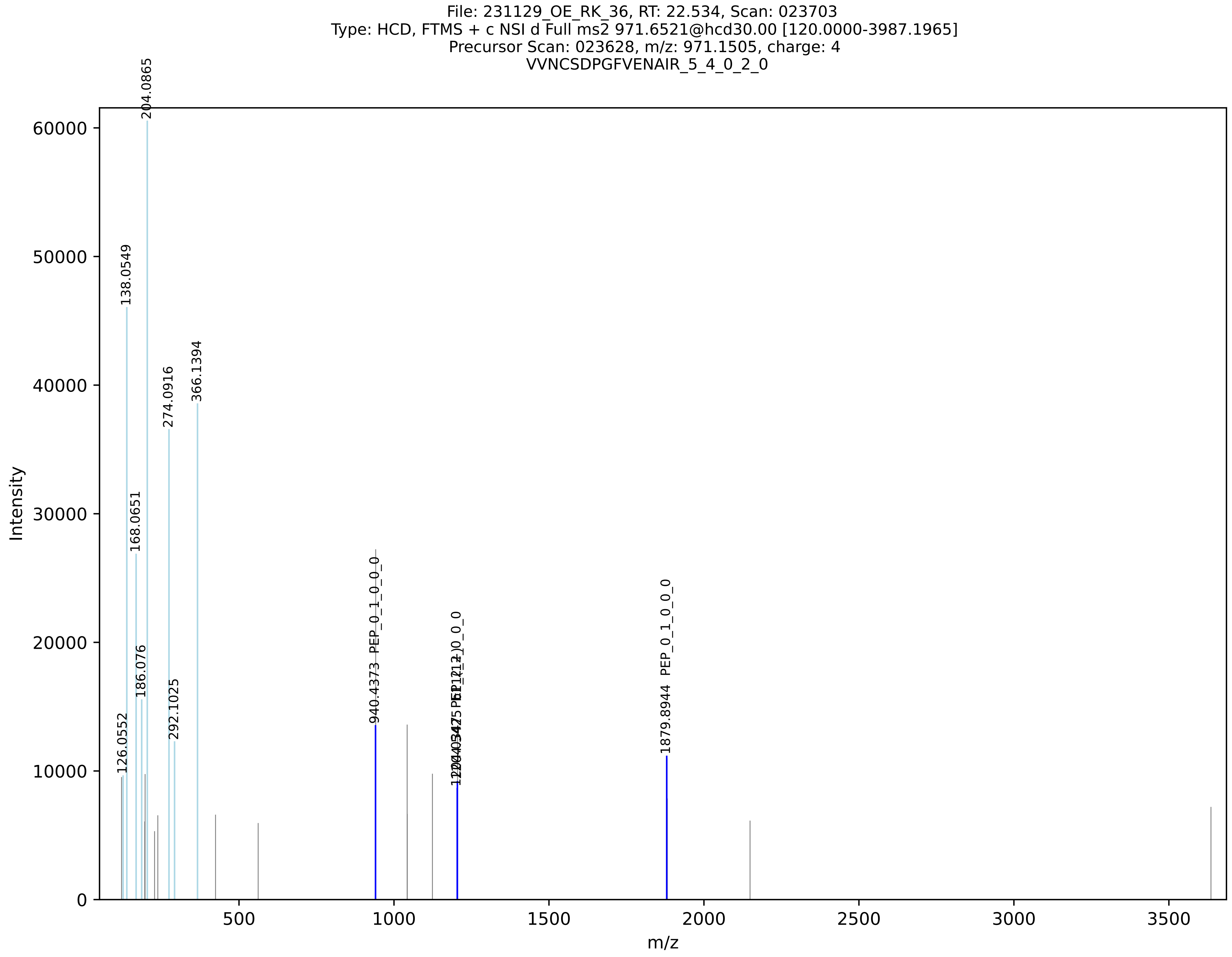

File: 231129_OE_RK_36, RT: 22.534, Scan: 023703
Type: HCD, FTMS + c NSI d Full ms2 971.6521@hcd30.00 [120.0000-3987.1965]
Precursor Scan: 023628, m/z: 971.1505, charge: 4
VVNCSDPGFVENAIR_5_4_0_2_0
204.0865
138.0549
366.1394
274.0916
168.0651
186.076
292.1025
126.0552
940.4373 PEP_0_1_0_0_0_
1879.8944 PEP_0_1_0_0_0
Intensity
m/z
0
10000
20000
30000
40000
50000
60000
500
1000
1500
2000
2500
3000
3500

File: 231129_OE_RK_36, RT: 22.686, Scan: 023922
Type: HCD, FTMS + c NSI d Full ms2 1294.8647@hcd30.00 [120.0000-3984.4282]
Precursor Scan: 023845, m/z: 1294.5306, charge: 3
VVNCSDPGFVENAIR_5_4_0_2_0

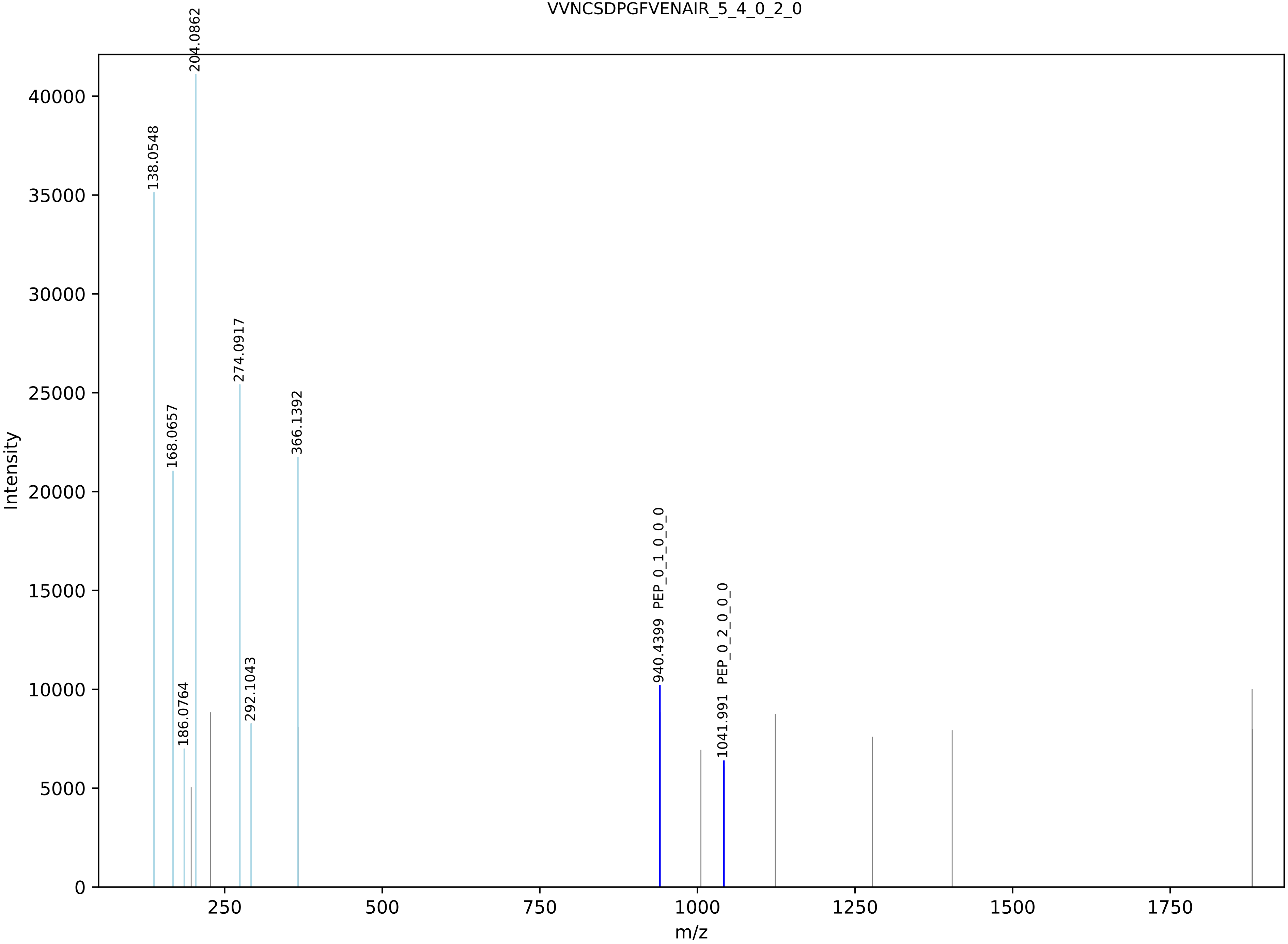

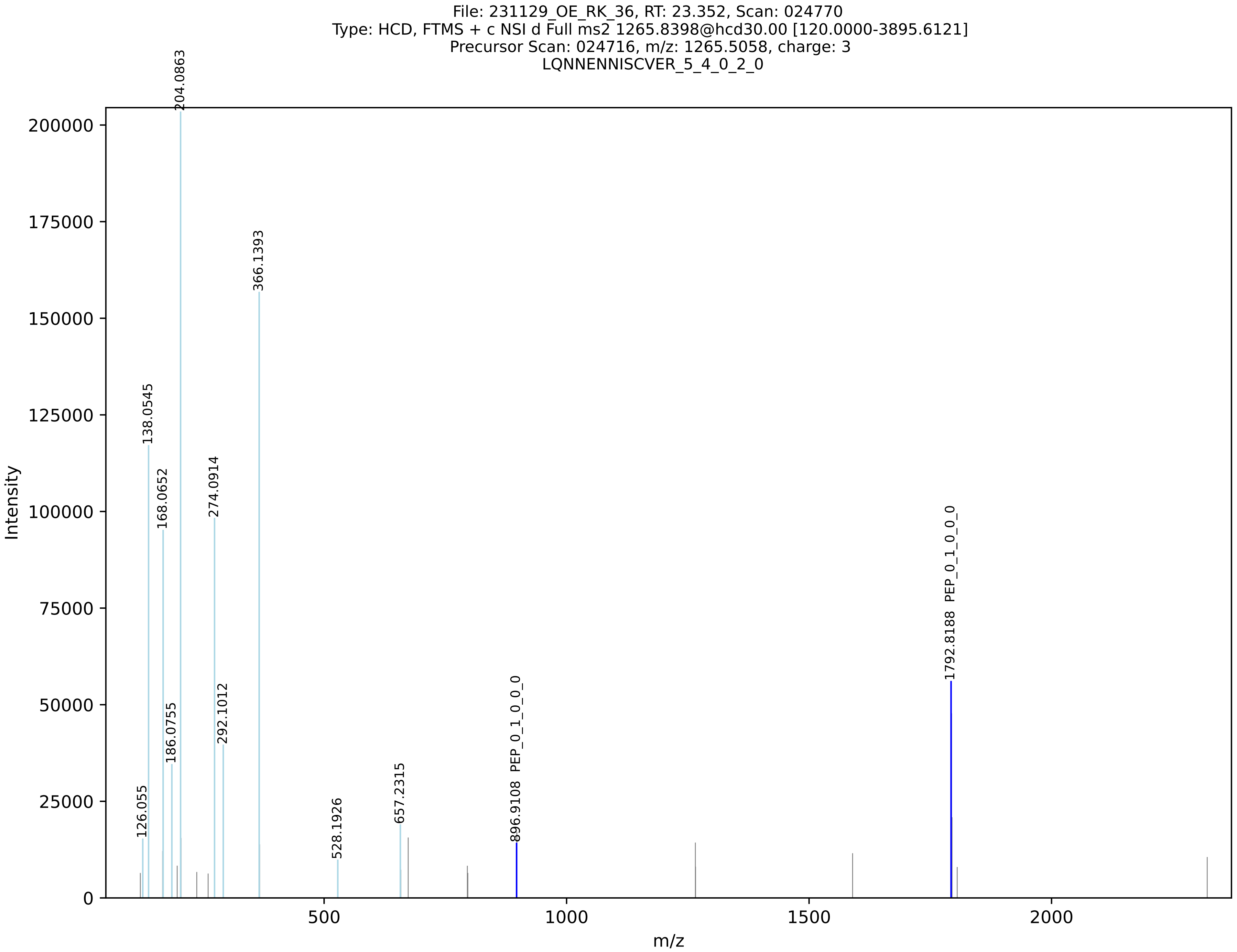
File: 231129_OE_RK_36, RT: 23.352, Scan: 024770
Type: HCD, FTMS + c NSI d Full ms2 1265.8398@hcd30.00 [120.0000-3895.6121]
Precursor Scan: 024716, m/z: 1265.5058, charge: 3
LQNNENNISCVER_5_4_0_2_0
Intensity
m/z
204.0863
366.1393
138.0545
168.0652
274.0914
292.1012
186.0755
126.055
528.1926
657.2315
896.9108 PEP_0_1_0_0_0
1792.8188 PEP_0_1_0_0_0
0
25000
50000
75000
100000
125000
150000
175000
200000
500
1000
1500
2000

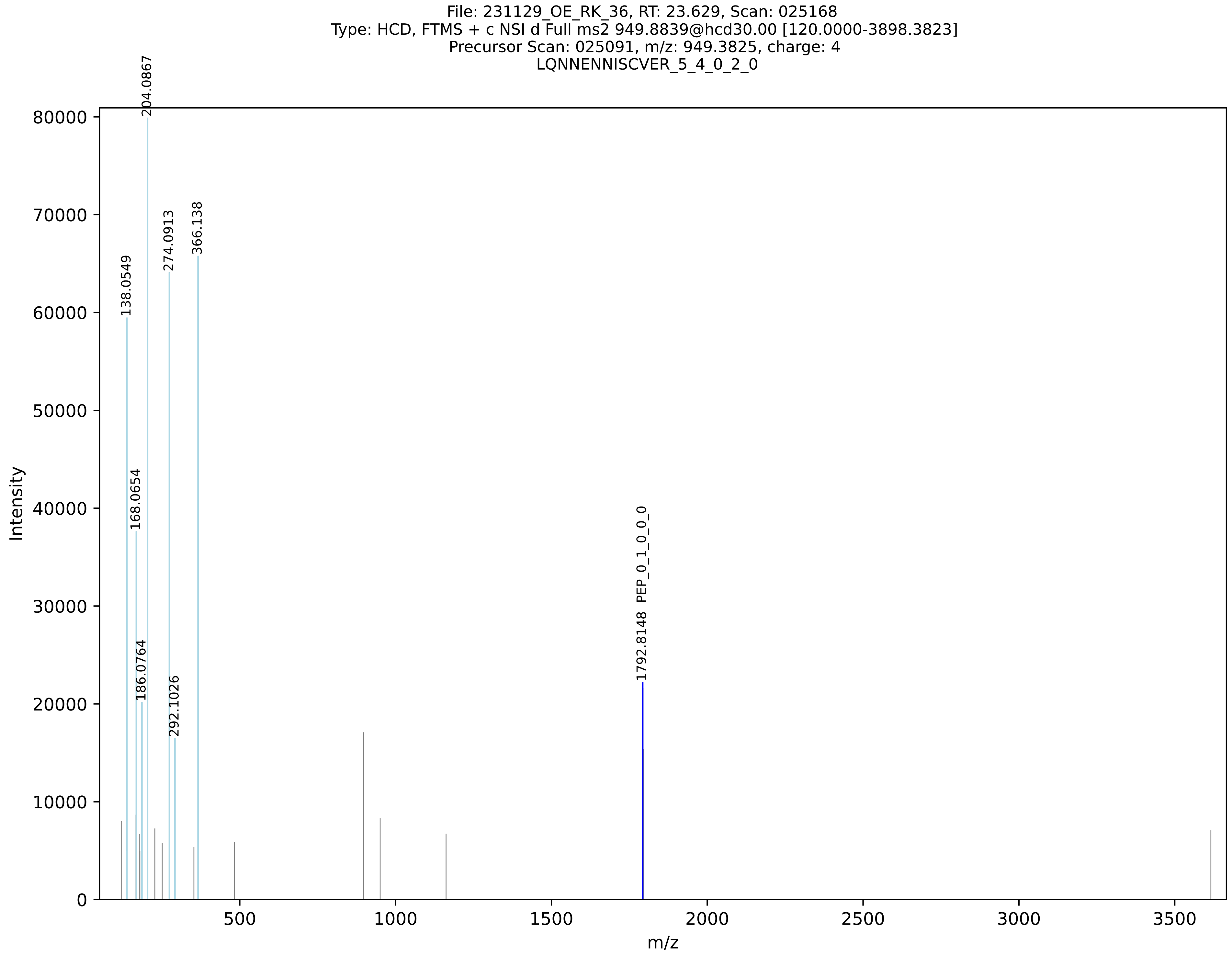

File: 231129_OE_RK_36, RT: 23.629, Scan: 025168
Type: HCD, FTMS + c NSI d Full ms2 949.8839@hcd30.00 [120.0000-3898.3823]
Precursor Scan: 025091, m/z: 949.3825, charge: 4
LQNNENNISCVER_5_4_0_2_0
204.0867
138.0549
274.0913
366.138
168.0654
186.0764
292.1026
1792.8148 PEP_0_1_0_0_0_
Intensity
m/z
80000
70000
60000
50000
40000
30000
20000
10000
0
500
1000
1500
2000
2500
3000
3500

File: 231129_OE_RK_36, RT: 23.728, Scan: 025312
Type: HCD, FTMS + c NSI d Full ms2 1265.8411@hcd30.00 [120.0000-3895.6157]
Precursor Scan: 025235, m/z: 1265.5069, charge: 3
LQNNENNISCVER_5_4_0_2_0

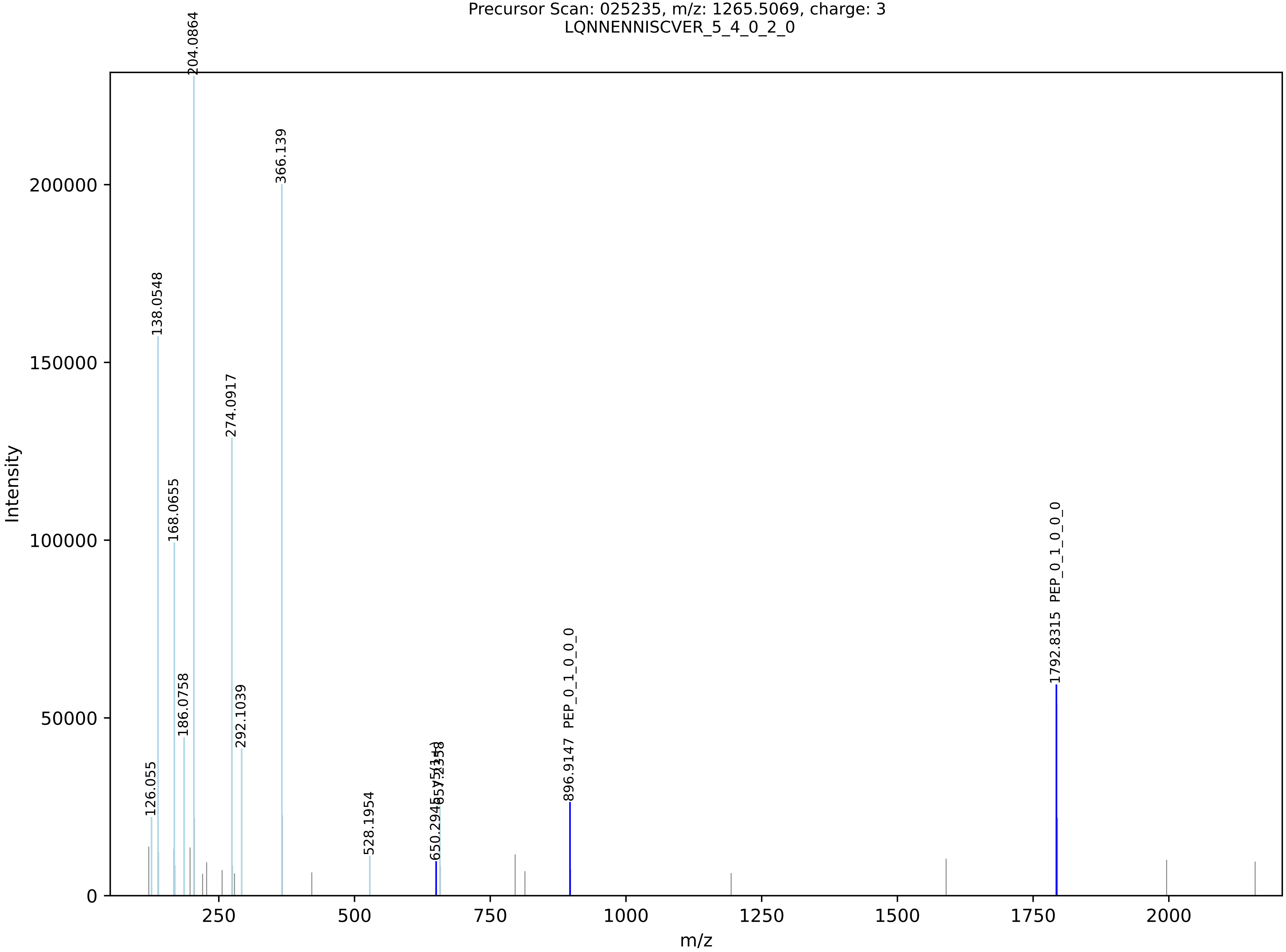

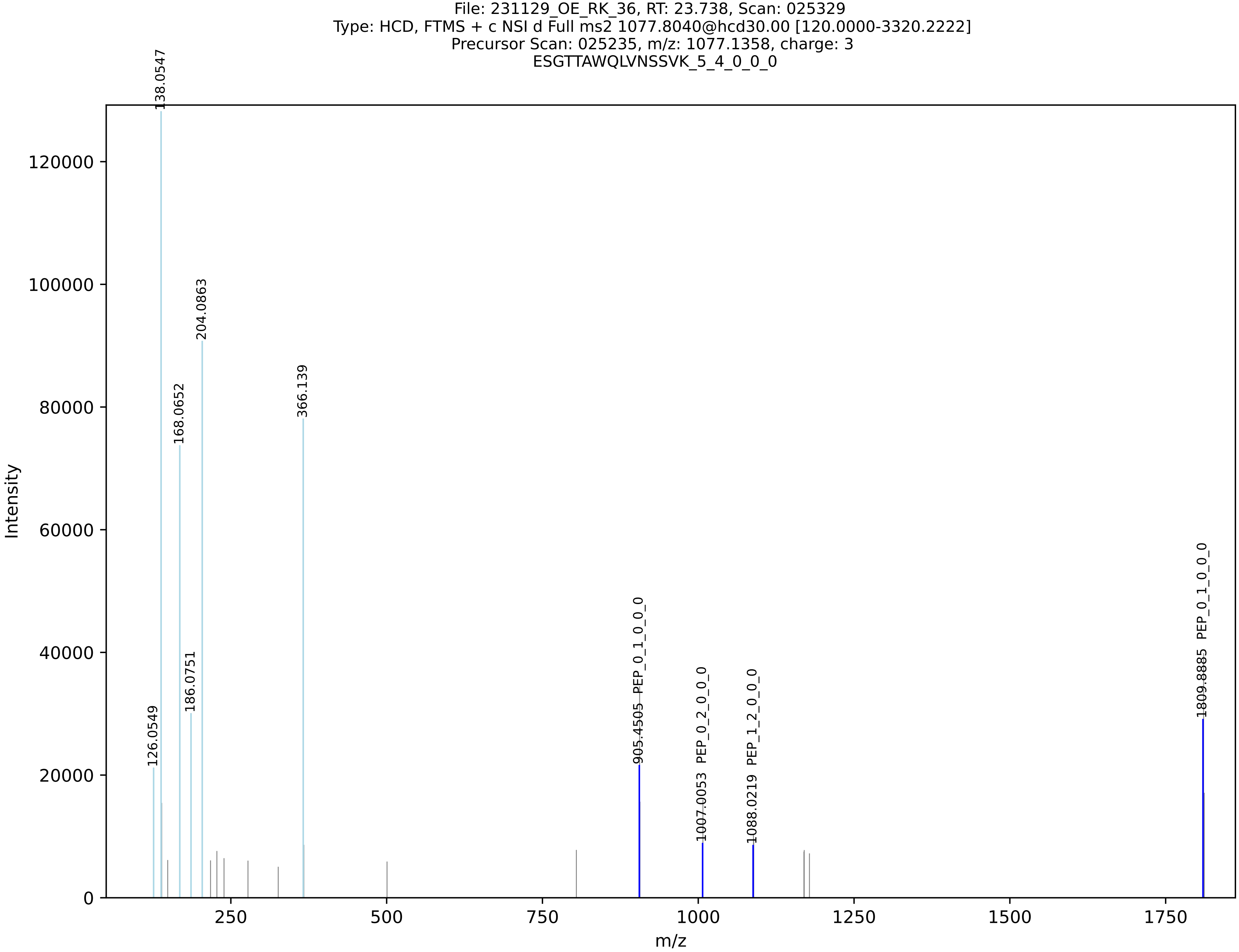
File: 231129_OE_RK_36, RT: 23.738, Scan: 025329
Type: HCD, FTMS + c NSI d Full ms2 1077.8040@hcd30.00 [120.0000-3320.2222]
Precursor Scan: 025235, m/z: 1077.1358, charge: 3
ESGTTAWQLVNSSVK_5_4_0_0_0
Intensity
m/z
138.0547
204.0863
168.0652
366.139
186.0751
126.0549
905.4505 PEP_0_1_0_0_0
1007.0053 PEP_0_2_0_0_0
1088.0219 PEP_1_2_0_0_0
1809.8885 PEP_0_1_0_0_0
0
20000
40000
60000
80000
100000
120000
250
500
750
1000
1250
1500
1750

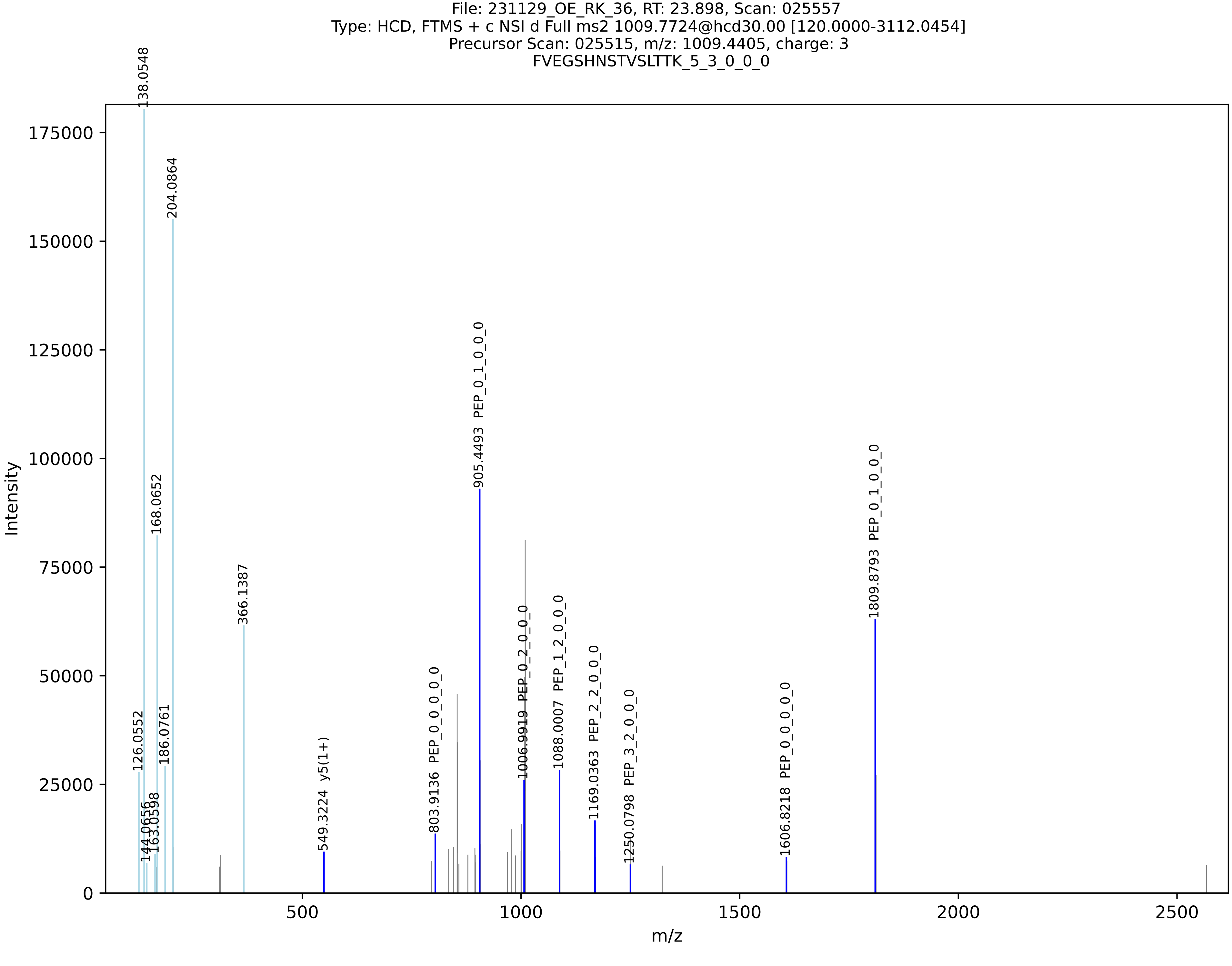

File: 231129_OE_RK_36, RT: 23.898, Scan: 025557
Type: HCD, FTMS + c NSI d Full ms2 1009.7724@hcd30.00 [120.0000-3112.0454]
Precursor Scan: 025515, m/z: 1009.4405, charge: 3
FVEGSHNSTVSLTTK_5_3_0_0_0
Intensity
m/z
138.0548
204.0864
168.0652
366.1387
126.0552
186.0761
144.0656
163.0598
549.3224 y5(1+)
803.9136 PEP_0_0_0_0_0
905.4493 PEP_0_1_0_0_0
1006.9919 PEP_0_2_0_0_0
1088.0007 PEP_1_2_0_0_0
1169.0363 PEP_2_2_0_0_0
1250.0798 PEP_3_2_0_0_0
1606.8218 PEP_0_0_0_0_0
1809.8793 PEP_0_1_0_0_0

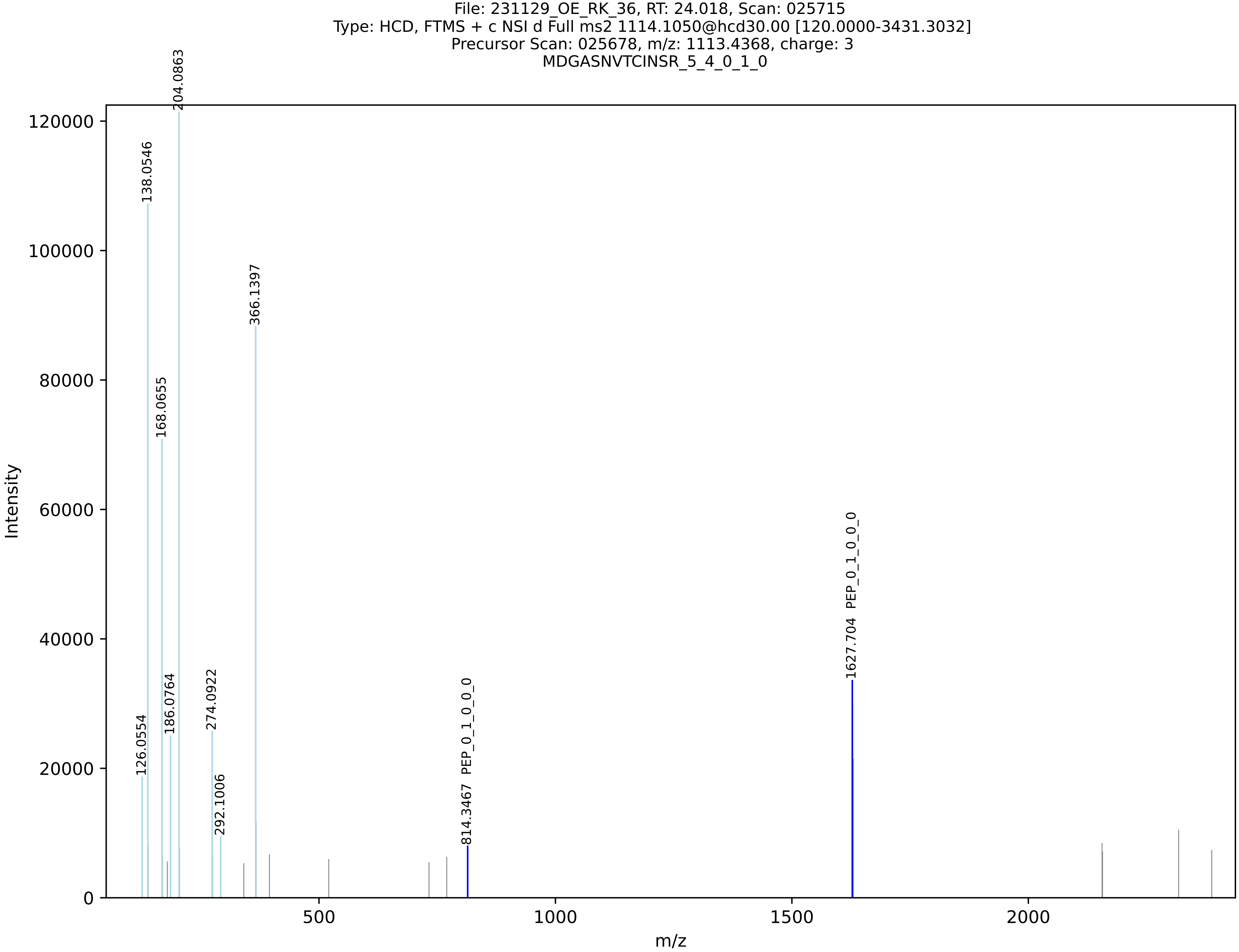
File: 231129_OE_RK_36, RT: 24.018, Scan: 025715
Type: HCD, FTMS + c NSI d Full ms2 1114.1050@hcd30.00 [120.0000-3431.3032]
Precursor Scan: 025678, m/z: 1113.4368, charge: 3
MDGASNVTCINSR_5_4_0_1_0
Intensity
m/z
204.0863
138.0546
168.0655
366.1397
186.0764
274.0922
126.0554
292.1006
814.3467 PEP_0_1_0_0_0
1627.704 PEP_0_1_0_0_0
0
20000
40000
60000
80000
100000
120000
500
1000
1500
2000

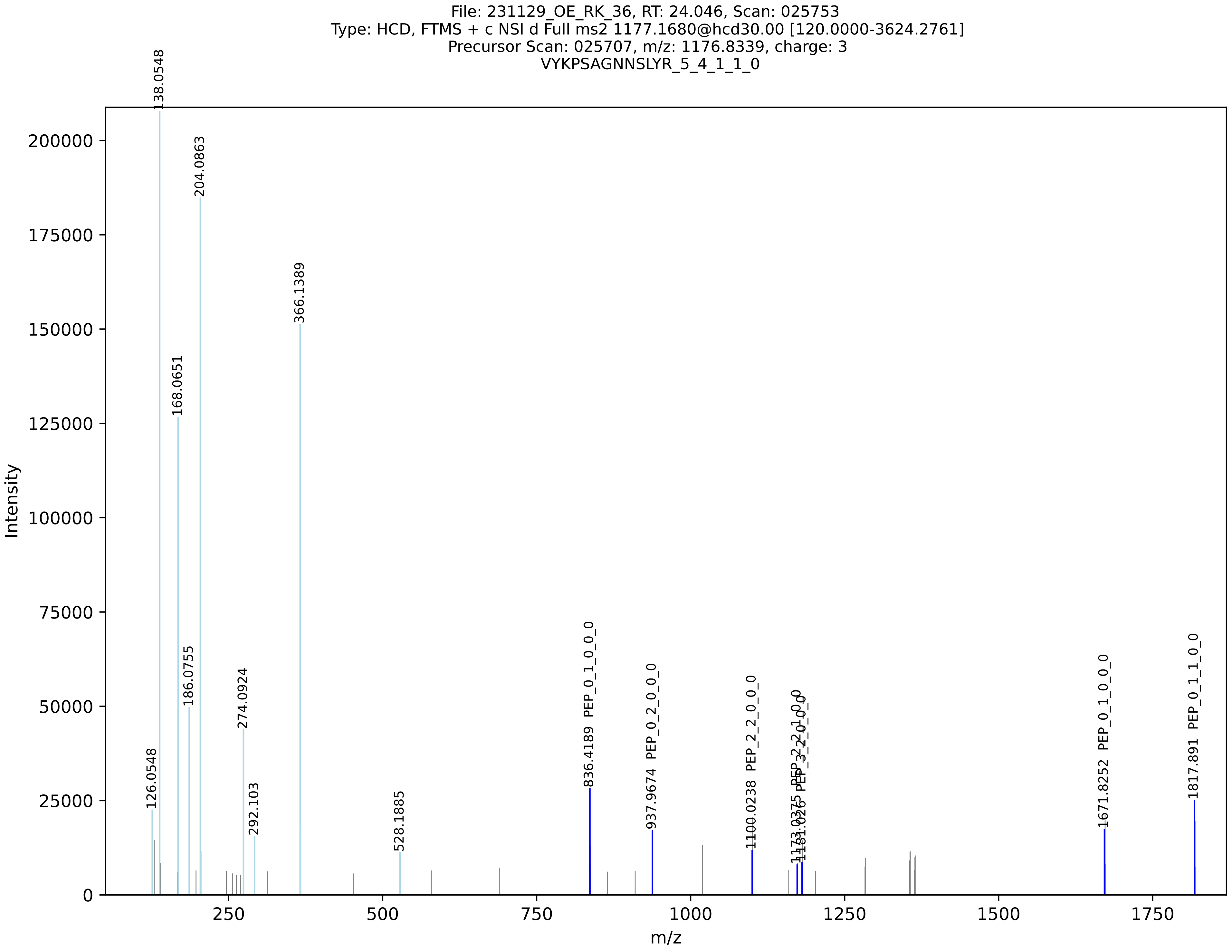
File: 231129_OE_RK_36, RT: 24.046, Scan: 025753
Type: HCD, FTMS + c NSI d Full ms2 1177.1680@hcd30.00 [120.0000-3624.2761]
Precursor Scan: 025707, m/z: 1176.8339, charge: 3
VYKPSAGNNSLYR_5_4_1_1_0
Intensity
m/z
0
25000
50000
75000
100000
125000
150000
175000
200000
250
500
750
1000
1250
1500
1750
138.0548
204.0863
366.1389
168.0651
186.0755
274.0924
126.0548
292.103
528.1885
836.4189 PEP_0_1_0_0_0
937.9674 PEP_0_2_0_0_0
1100.0238 PEP_2_2_0_0_0
1173.0375 PEP_2_2_1_0_0
1181.026 PEP_3_2_0_0_0
1671.8252 PEP_0_1_0_0_0
1817.891 PEP_0_1_1_0_0

File: 231129_OE_RK_36, RT: 24.094, Scan: 025813
Type: HCD, FTMS + c NSI d Full ms2 1128.4816@hcd30.00 [120.0000-3475.2957]
Precursor Scan: 025779, m/z: 1128.1475, charge: 3
VYKPSAGNNSLYR_5_4_0_1_0

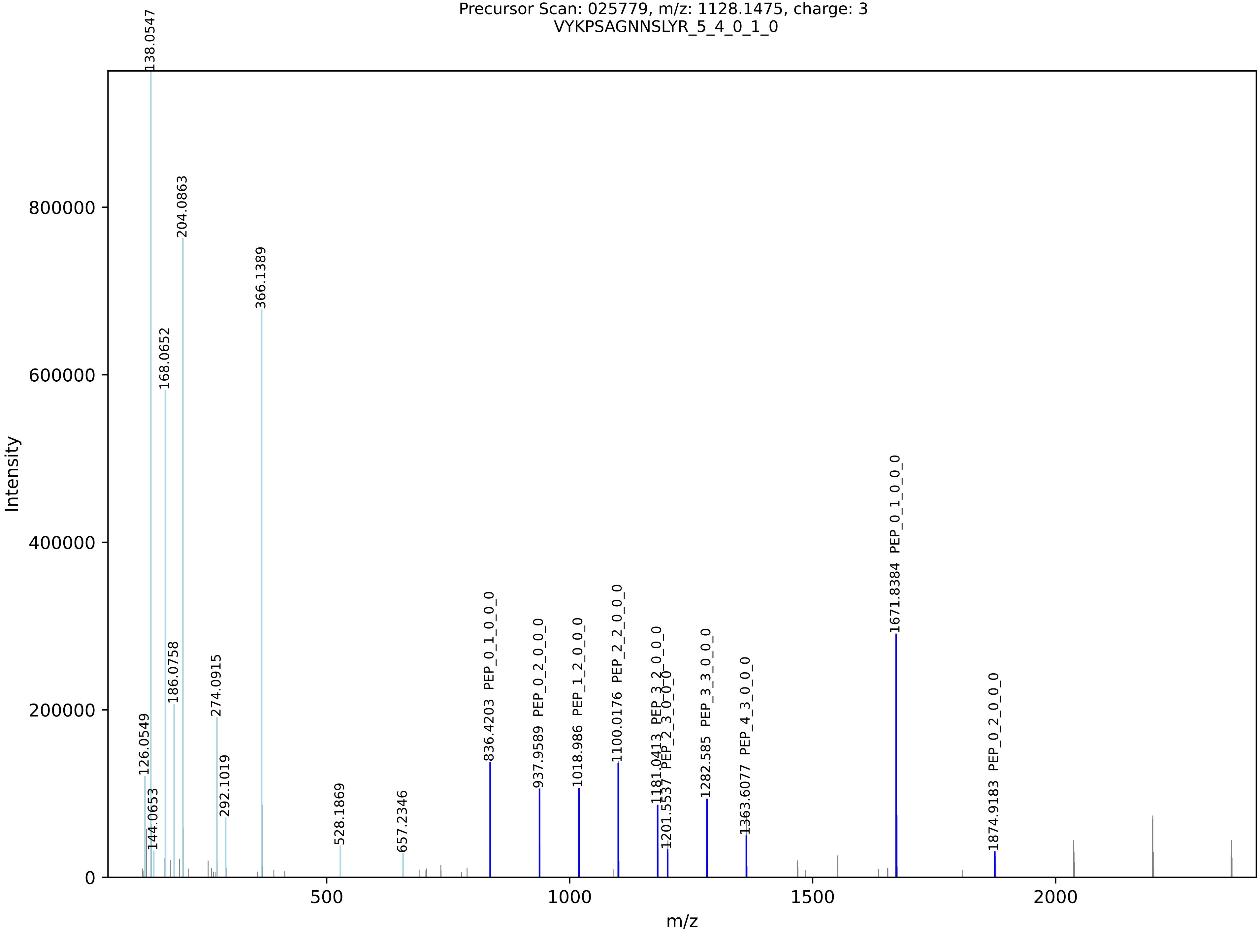

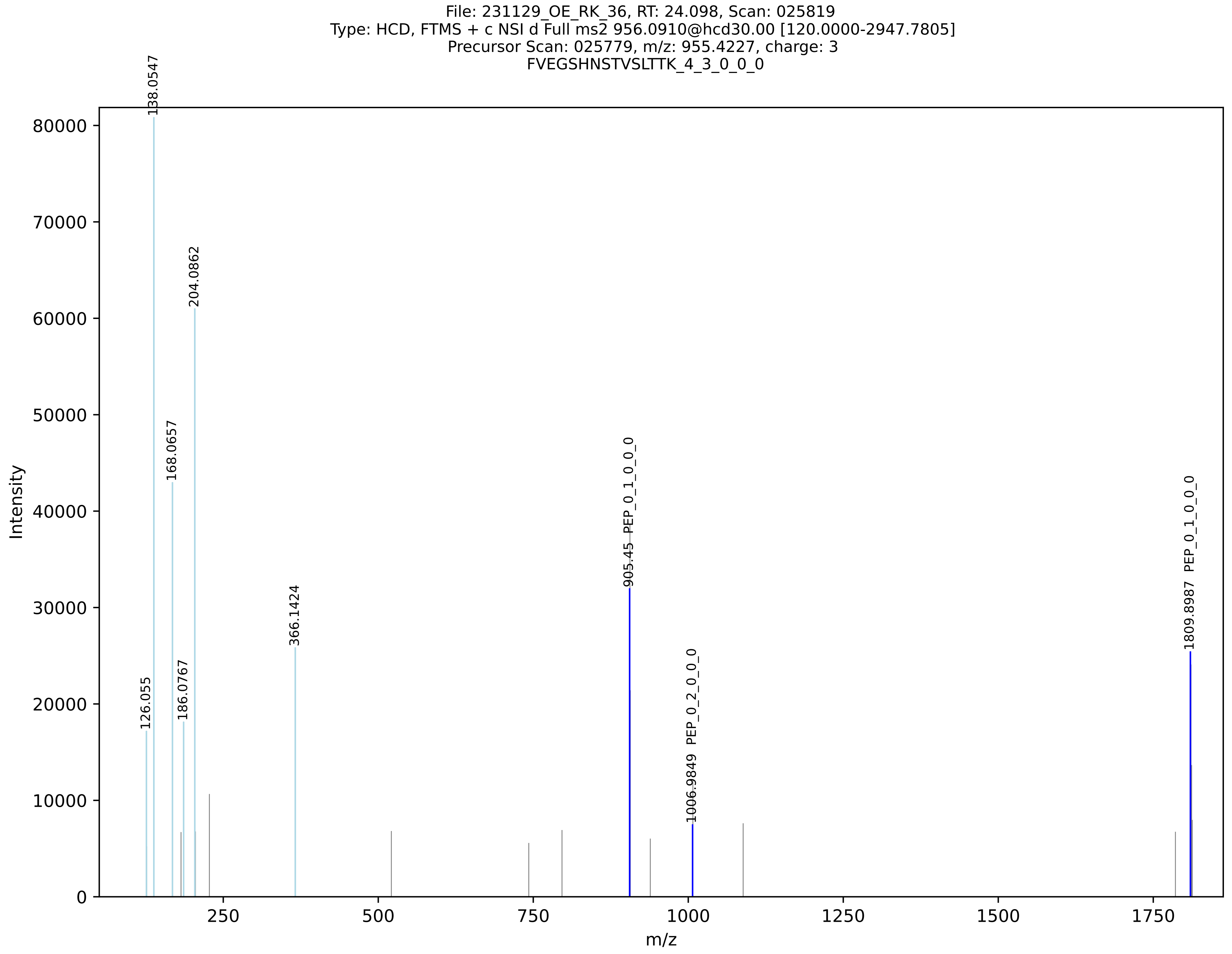

File: 231129_OE_RK_36, RT: 24.098, Scan: 025819
Type: HCD, FTMS + c NSI d Full ms2 956.0910@hcd30.00 [120.0000-2947.7805]
Precursor Scan: 025779, m/z: 955.4227, charge: 3
FVEGSHNSTVSLTTK_4_3_0_0_0
138.0547
204.0862
168.0657
126.055
186.0767
366.1424
905.45 PEP_0_1_0_0_0
1006.9849 PEP_0_2_0_0_0
1809.8987 PEP_0_1_0_0_0
Intensity
m/z
0
10000
20000
30000
40000
50000
60000
70000
80000
250
500
750
1000
1250
1500
1750

File: 231129_OE_RK_36, RT: 24.431, Scan: 026294
Type: HCD, FTMS + c NSI d Full ms2 1134.4512@hcd30.00 [120.0000-3493.5625]
Precursor Scan: 026212, m/z: 1134.1167, charge: 3
DIENFNSTQK_5_4_0_2_0

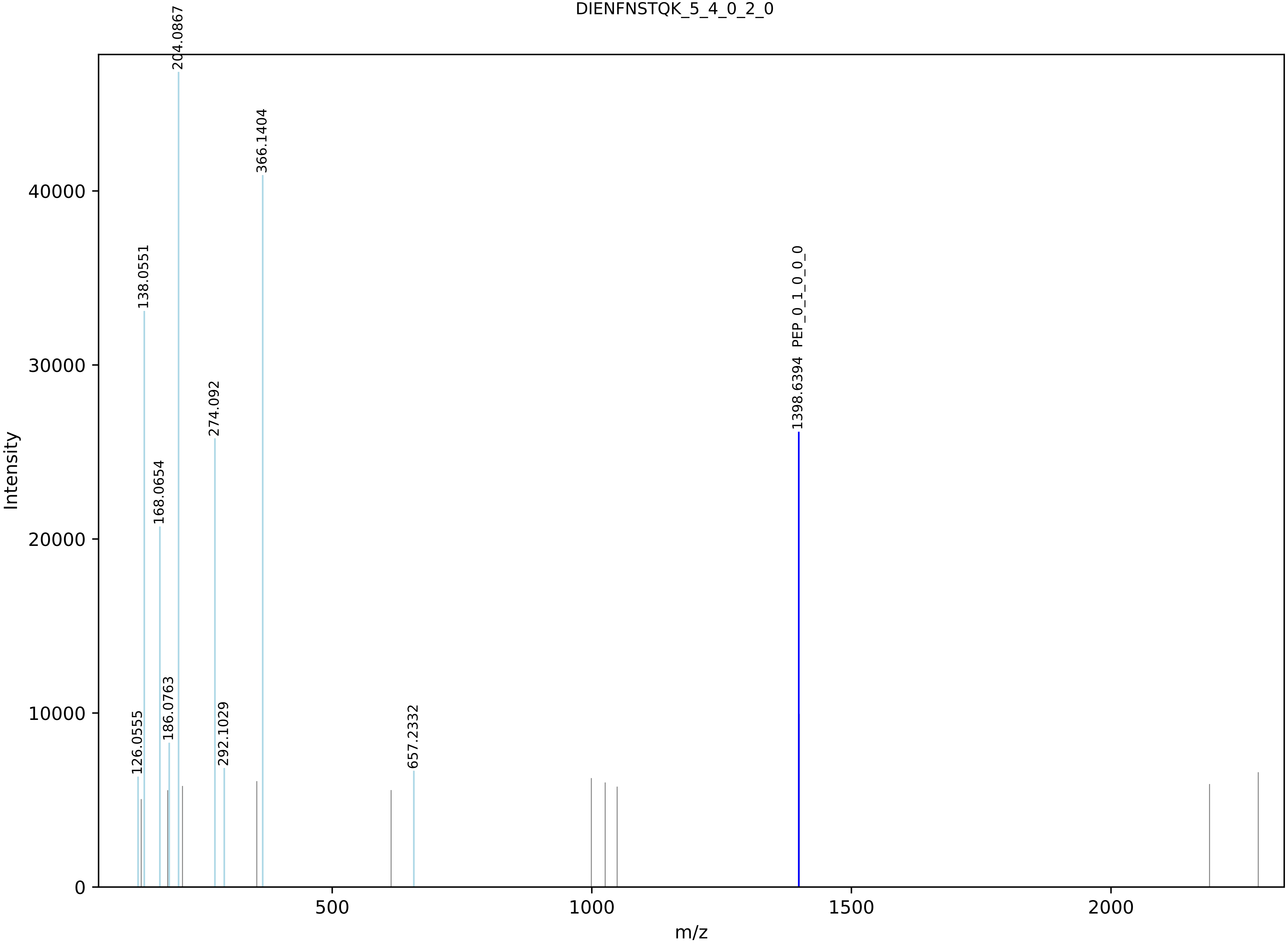

File: 231129_OE_RK_36, RT: 24.476, Scan: 026360
Type: HCD, FTMS + c NSI d Full ms2 945.1271@hcd30.00 [120.0000-3878.9749]
Precursor Scan: 026285, m/z: 944.8762, charge: 4
LTNGSRYPNCAYR_5_4_0_2_0

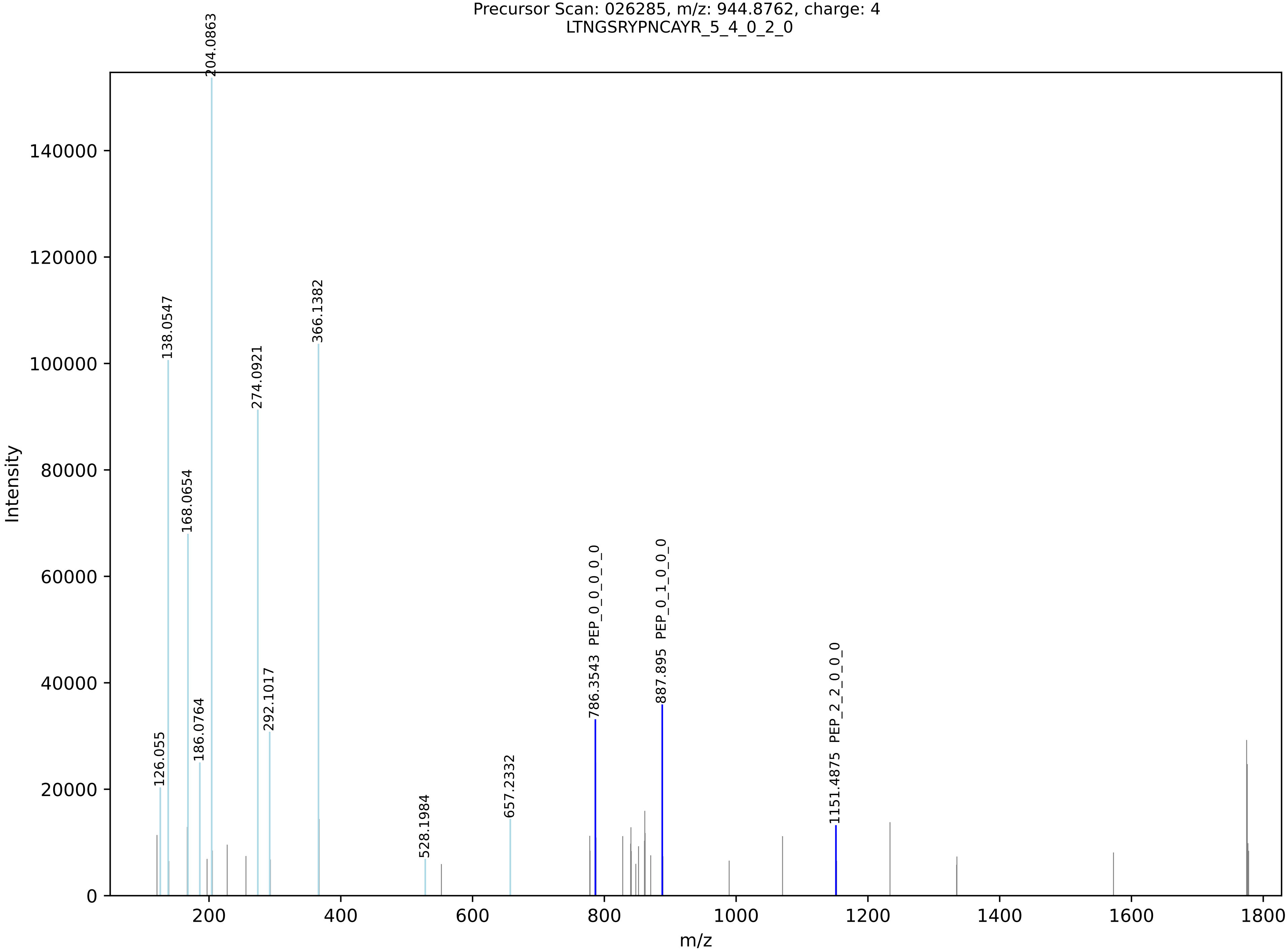

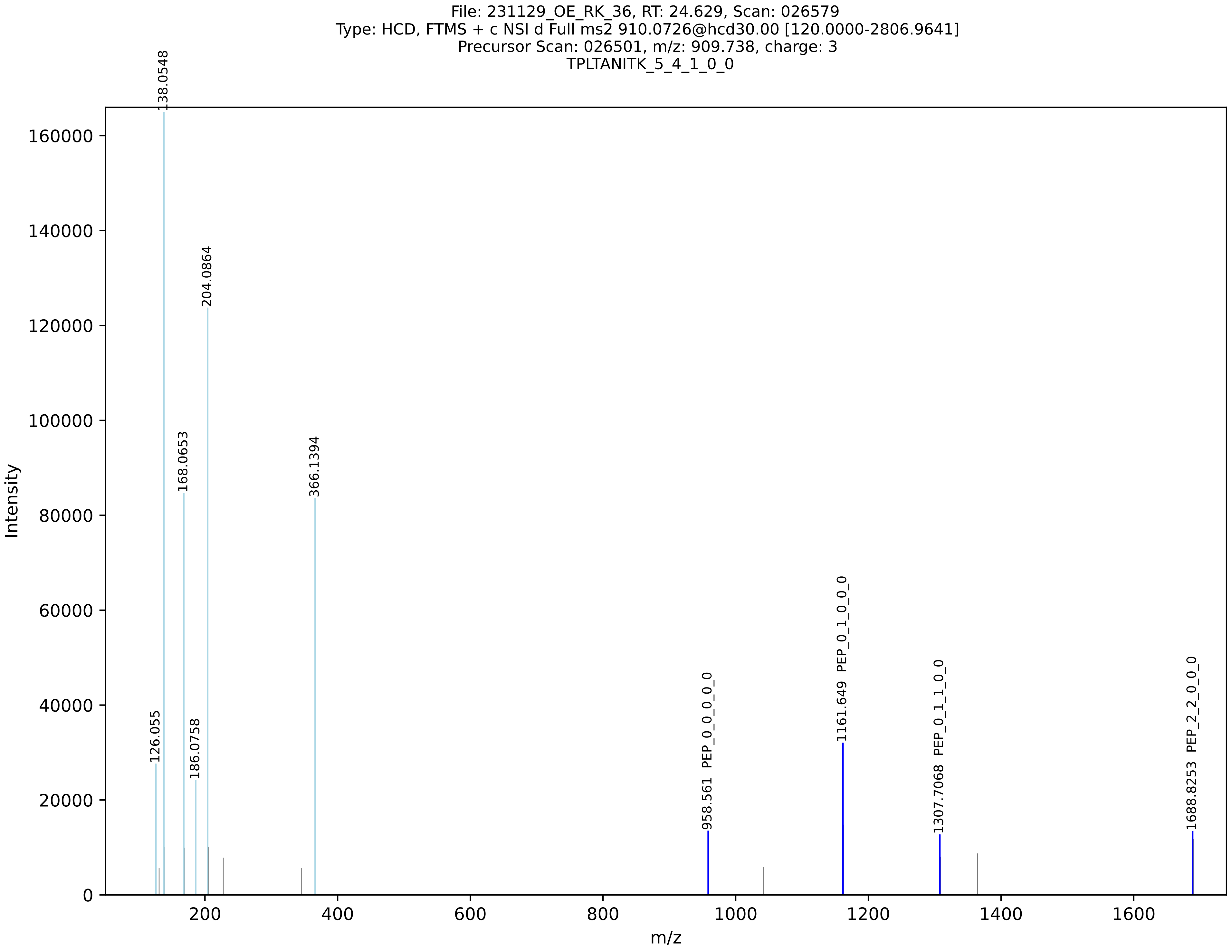
File: 231129_OE_RK_36, RT: 24.629, Scan: 026579
Type: HCD, FTMS + c NSI d Full ms2 910.0726@hcd30.00 [120.0000-2806.9641]
Precursor Scan: 026501, m/z: 909.738, charge: 3
TPLTANITK_5_4_1_0_0
Intensity
m/z
138.0548
204.0864
168.0653
366.1394
126.055
186.0758
958.561 PEP_0_0_0_0_0
1161.649 PEP_0_1_0_0_0
1307.7068 PEP_0_1_1_0_0
1688.8253 PEP_2_2_0_0_0
0
20000
40000
60000
80000
100000
120000
140000
160000
200
400
600
800
1000
1200
1400
1600

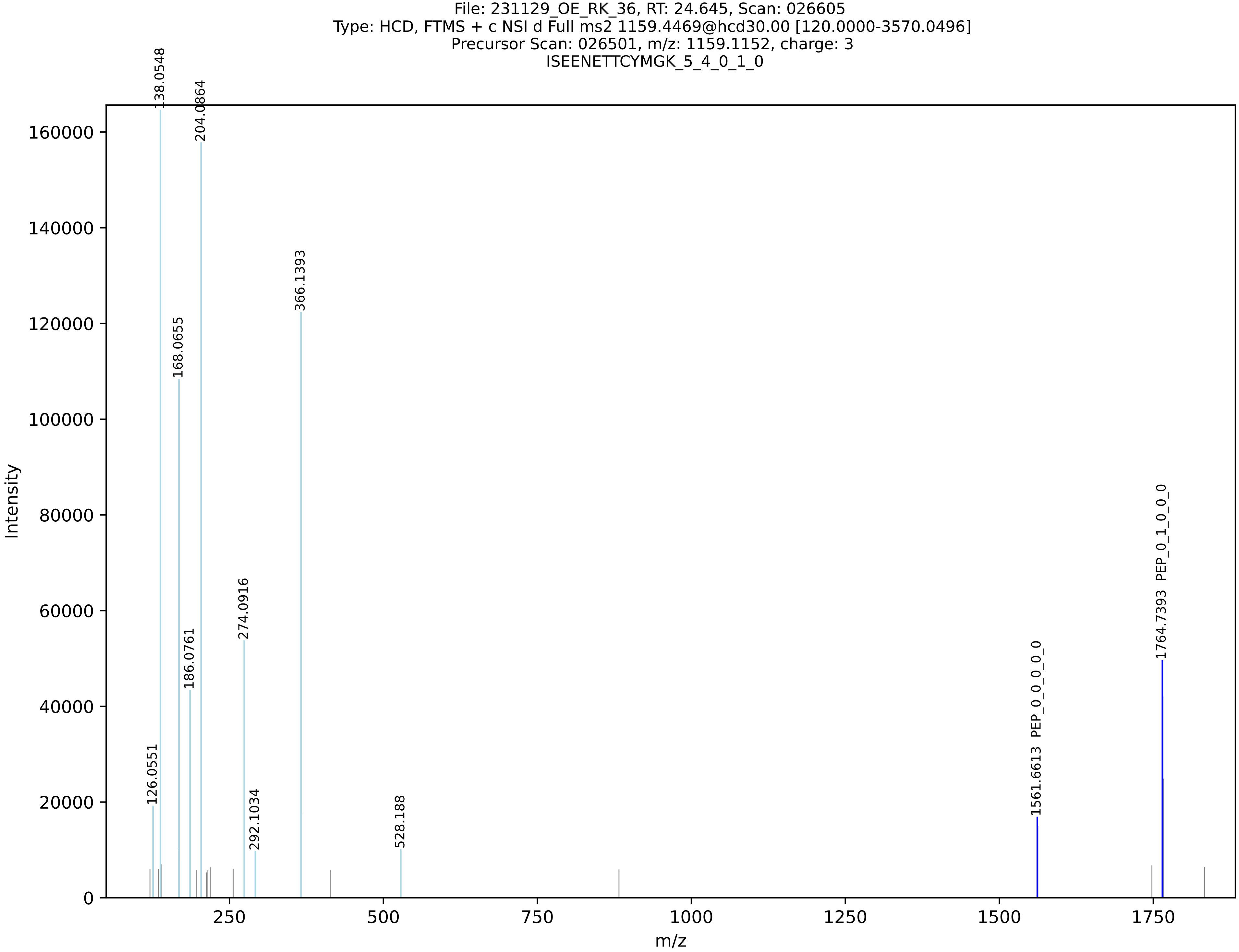
File: 231129_OE_RK_36, RT: 24.645, Scan: 026605
Type: HCD, FTMS + c NSI d Full ms2 1159.4469@hcd30.00 [120.0000-3570.0496]
Precursor Scan: 026501, m/z: 1159.1152, charge: 3
ISEENETTCYMGK_5_4_0_1_0
Intensity
m/z
138.0548
204.0864
366.1393
168.0655
274.0916
186.0761
126.0551
292.1034
528.188
1561.6613 PEP_0_0_0_0_0
1764.7393 PEP_0_1_0_0_0

File: 231129_OE_RK_36, RT: 24.827, Scan: 026868
Type: HCD, FTMS + c NSI d Full ms2 1159.4487@hcd30.00 [120.0000-3570.0552]
Precursor Scan: 026793, m/z: 1159.1146, charge: 3
ISEENETTCYMGK_5_4_0_1_0

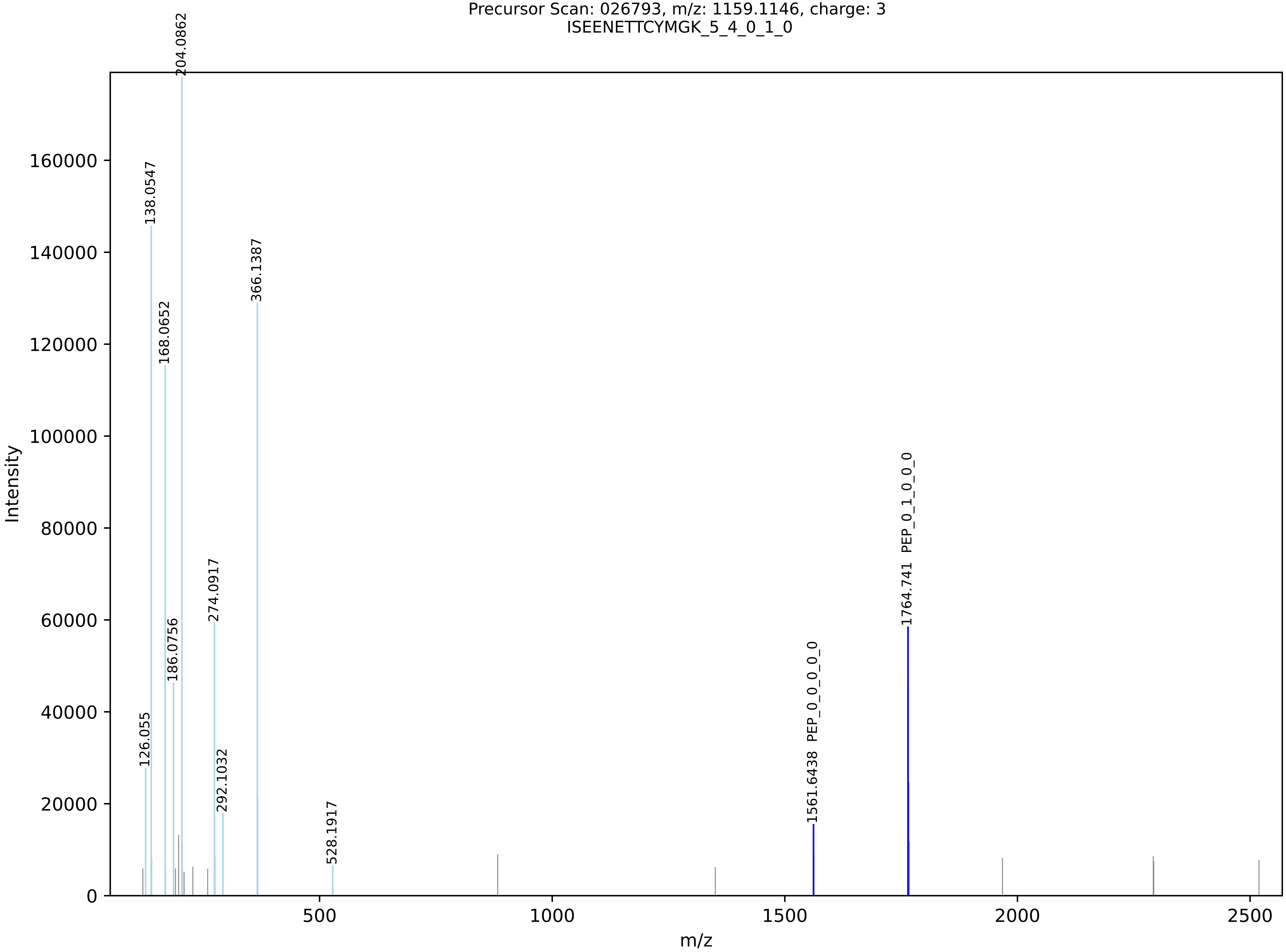

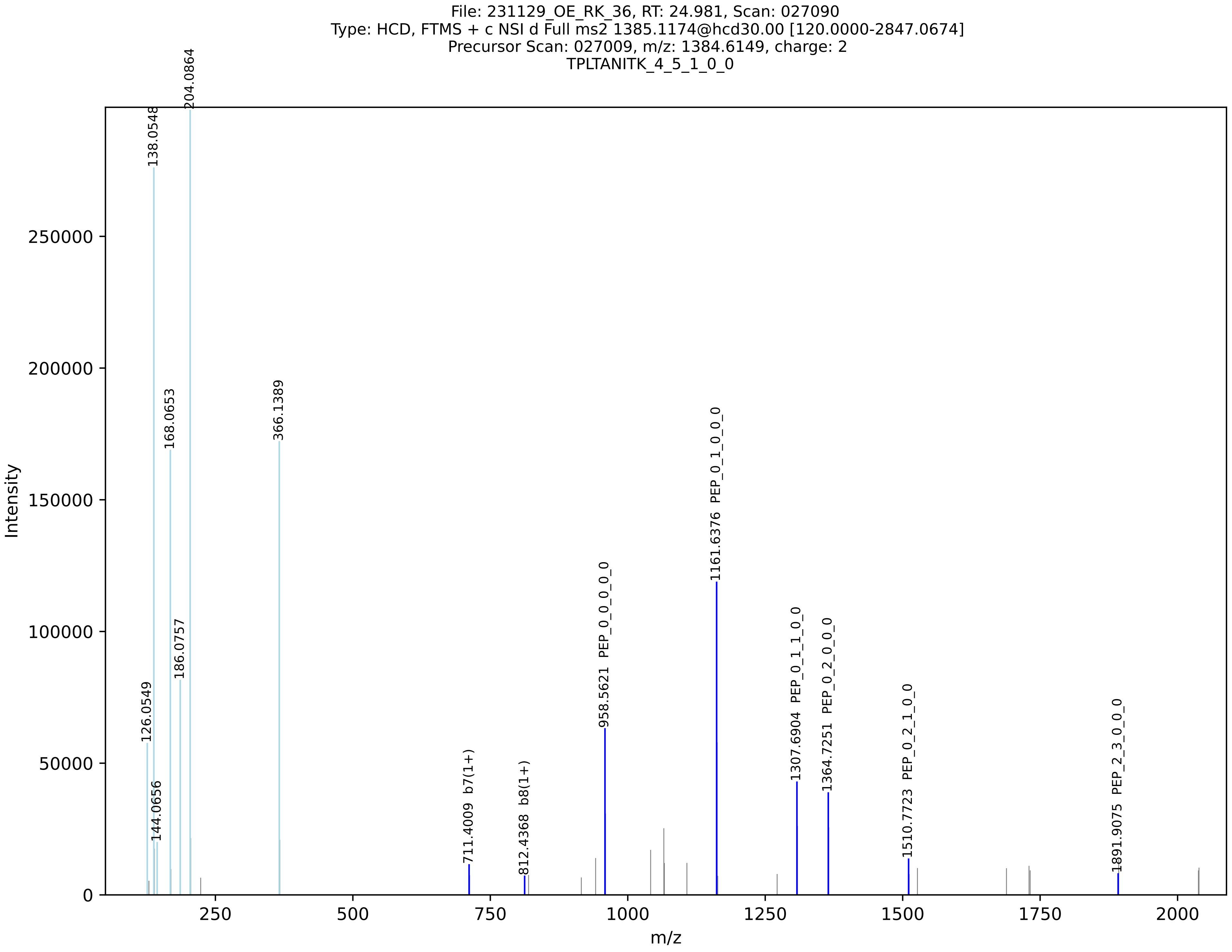

File: 231129_OE_RK_36, RT: 24.981, Scan: 027090
Type: HCD, FTMS + c NSI d Full ms2 1385.1174@hcd30.00 [120.0000-2847.0674]
Precursor Scan: 027009, m/z: 1384.6149, charge: 2
TPLTANITK_4_5_1_0_0
Intensity
m/z
204.0864
138.0548
168.0653
366.1389
186.0757
126.0549
144.0656
711.4009 b7(1+)
812.4368 b8(1+)
958.5621 PEP_0_0_0_0_0
1161.6376 PEP_0_1_0_0_0
1307.6904 PEP_0_1_1_0_0
1364.7251 PEP_0_2_0_0_0
1510.7723 PEP_0_2_1_0_0
1891.9075 PEP_2_3_0_0_0
0
50000
100000
150000
200000
250000
250
500
750
1000
1250
1500
1750
2000

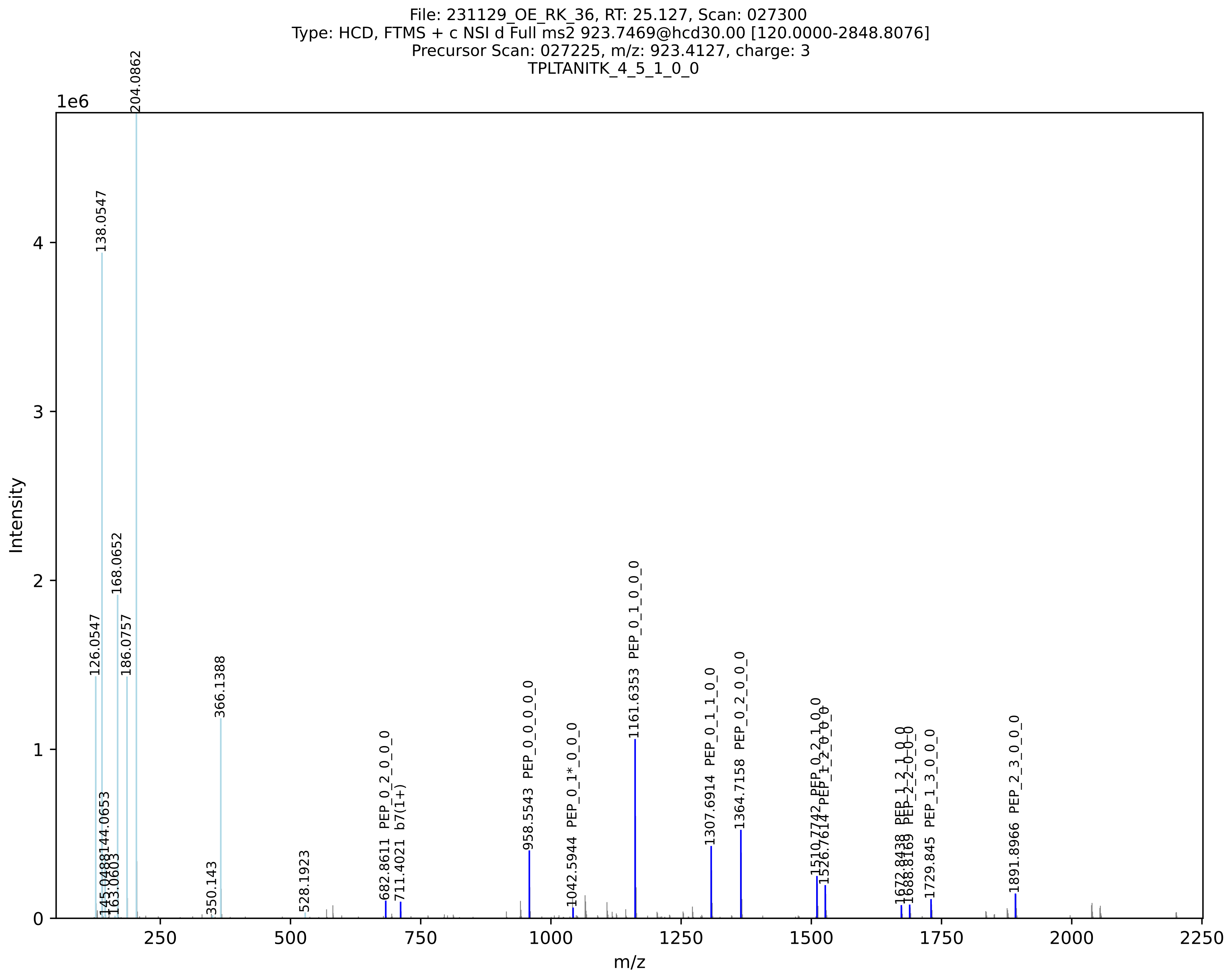
File: 231129_OE_RK_36, RT: 25.127, Scan: 027300
Type: HCD, FTMS + c NSI d Full ms2 923.7469@hcd30.00 [120.0000-2848.8076]
Precursor Scan: 027225, m/z: 923.4127, charge: 3
TPLTANITK_4_5_1_0_0
1e6
Intensity
m/z
204.0862
138.0547
168.0652
126.0547
186.0757
366.1388
144.0653
145.0488
163.0603
350.143
528.1923
682.8611 PEP_0_2_0_0_0
711.4021 b7(1+)
958.5543 PEP_0_0_0_0_0
1042.5944 PEP_0_1*_0_0_0
1161.6353 PEP_0_1_0_0_0
1307.6914 PEP_0_1_1_0_0
1364.7158 PEP_0_2_0_0_0
1510.7742 PEP_0_2_1_0_0
1526.7614 PEP_1_2_0_0_0
1672.8438 PEP_1_2_1_0_0
1688.8169 PEP_2_2_0_0_0
1729.845 PEP_1_3_0_0_0
1891.8966 PEP_2_3_0_0_0

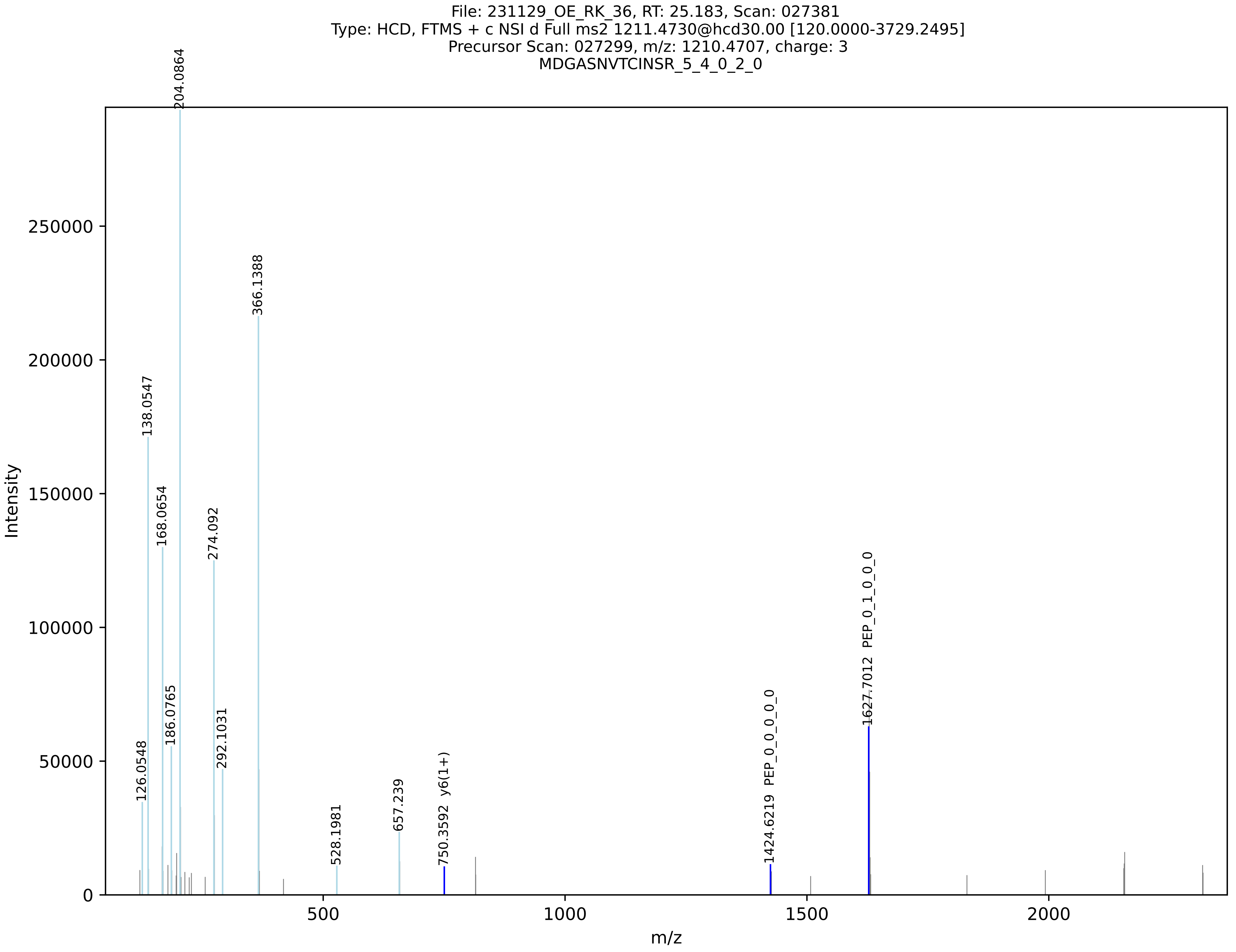

File: 231129_OE_RK_36, RT: 25.183, Scan: 027381
Type: HCD, FTMS + c NSI d Full ms2 1211.4730@hcd30.00 [120.0000-3729.2495]
Precursor Scan: 027299, m/z: 1210.4707, charge: 3
MDGASNVTCINSR_5_4_0_2_0
Intensity
m/z
204.0864
366.1388
138.0547
168.0654
274.092
186.0765
292.1031
126.0548
528.1981
657.239
750.3592 y6(1+)
1424.6219 PEP_0_0_0_0_0
1627.7012 PEP_0_1_0_0_0

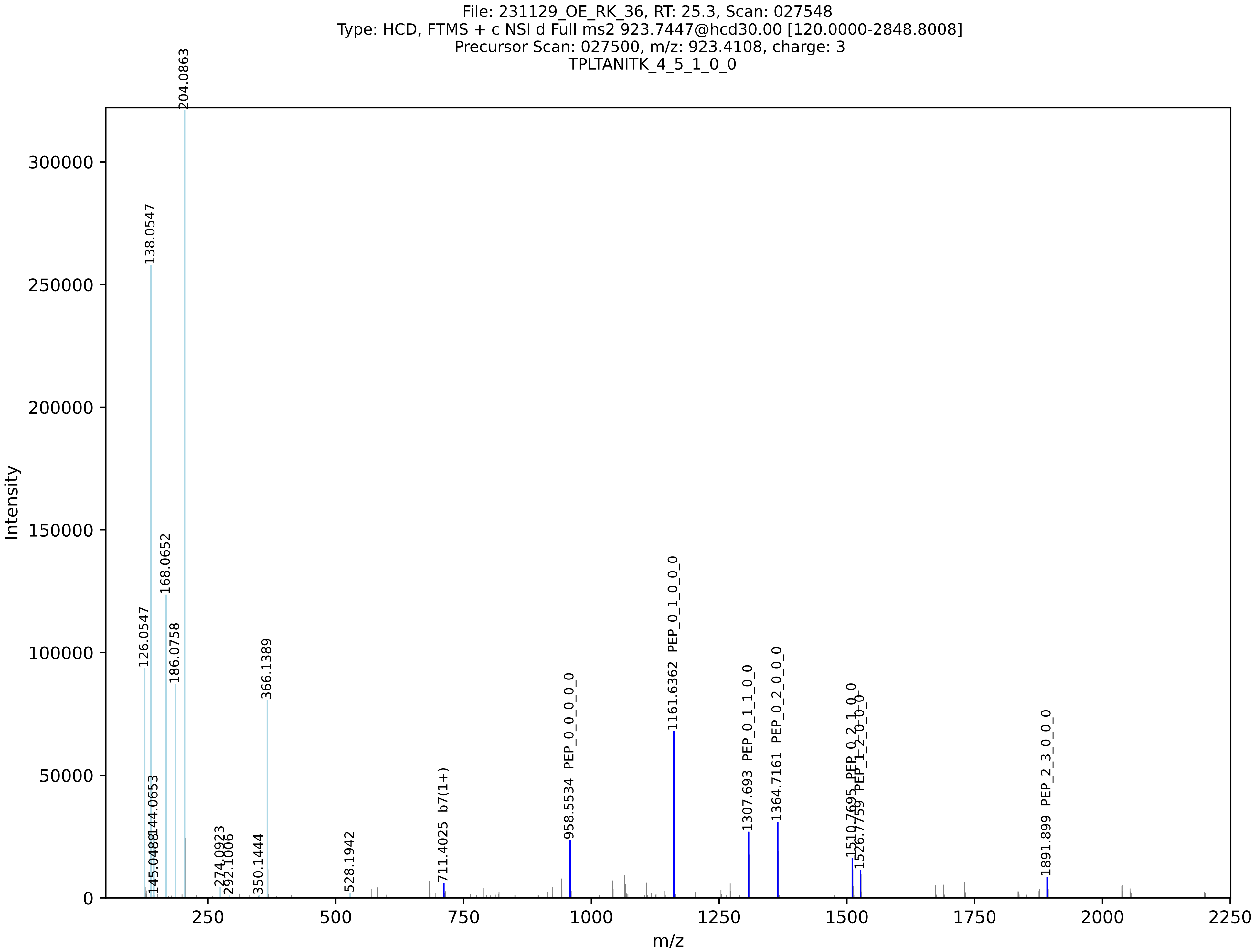
File: 231129_OE_RK_36, RT: 25.3, Scan: 027548
Type: HCD, FTMS + c NSI d Full ms2 923.7447@hcd30.00 [120.0000-2848.8008]
Precursor Scan: 027500, m/z: 923.4108, charge: 3
TPLTANITK_4_5_1_0_0
204.0863
138.0547
168.0652
126.0547
186.0758
366.1389
144.0653
145.0488
274.0923
292.1006
350.1444
528.1942
711.4025 b7(1+)
958.5534 PEP_0_0_0_0_0
1161.6362 PEP_0_1_0_0_0
1307.693 PEP_0_1_1_0_0
1364.7161 PEP_0_2_0_0_0
1510.7695 PEP_0_2_1_0_0
1526.7759 PEP_1_2_0_0_0
1891.899 PEP_2_3_0_0_0
Intensity
m/z
0
50000
100000
150000
200000
250000
300000
250
500
750
1000
1250
1500
1750
2000
2250

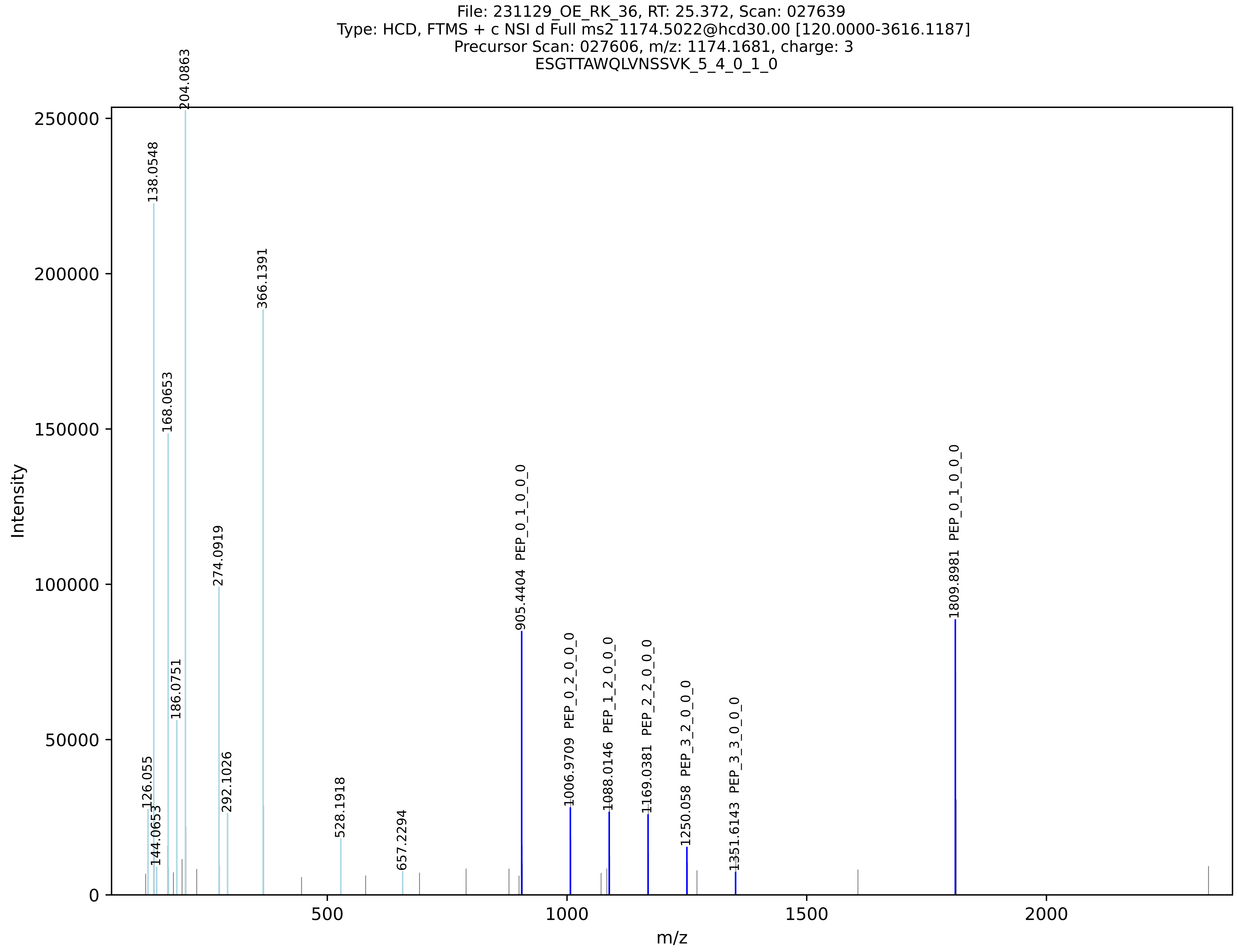
File: 231129_OE_RK_36, RT: 25.372, Scan: 027639
Type: HCD, FTMS + c NSI d Full ms2 1174.5022@hcd30.00 [120.0000-3616.1187]
Precursor Scan: 027606, m/z: 1174.1681, charge: 3
ESGTTAWQLVNSSVK_5_4_0_1_0
Intensity
m/z
0
50000
100000
150000
200000
250000
500
1000
1500
2000
204.0863
138.0548
366.1391
168.0653
274.0919
186.0751
126.055
144.0653
292.1026
528.1918
657.2294
905.4404 PEP_0_1_0_0_0
1006.9709 PEP_0_2_0_0_0
1088.0146 PEP_1_2_0_0_0
1169.0381 PEP_2_2_0_0_0
1250.058 PEP_3_2_0_0_0
1351.6143 PEP_3_3_0_0_0
1809.8981 PEP_0_1_0_0_0

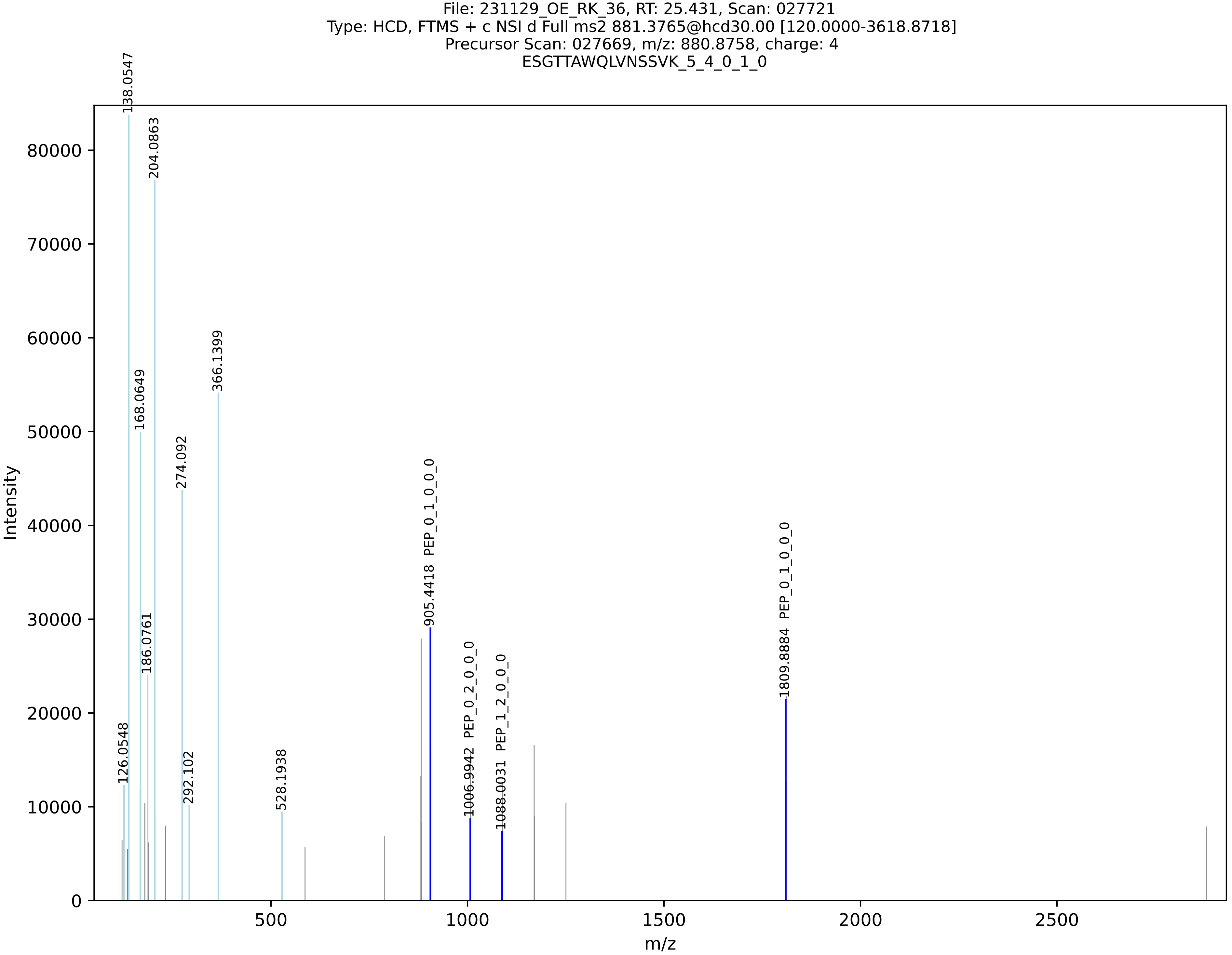
File: 231129_OE_RK_36, RT: 25.431, Scan: 027721
Type: HCD, FTMS + c NSI d Full ms2 881.3765@hcd30.00 [120.0000-3618.8718]
Precursor Scan: 027669, m/z: 880.8758, charge: 4
ESGTTAWQLVNSSVK_5_4_0_1_0
Intensity
m/z
138.0547
204.0863
366.1399
168.0649
274.092
905.4418 PEP_0_1_0_0_0
1809.8884 PEP_0_1_0_0_0
186.0761
1006.9942 PEP_0_2_0_0_0
1088.0031 PEP_1_2_0_0_0
126.0548
292.102
528.1938

File: 231129_OE_RK_36, RT: 25.576, Scan: 027922
Type: HCD, FTMS + c NSI d Full ms2 1210.8049@hcd30.00 [120.0000-3727.2051]
Precursor Scan: 027852, m/z: 1210.4708, charge: 3
MDGASNVTCINSR_5_4_0_2_0

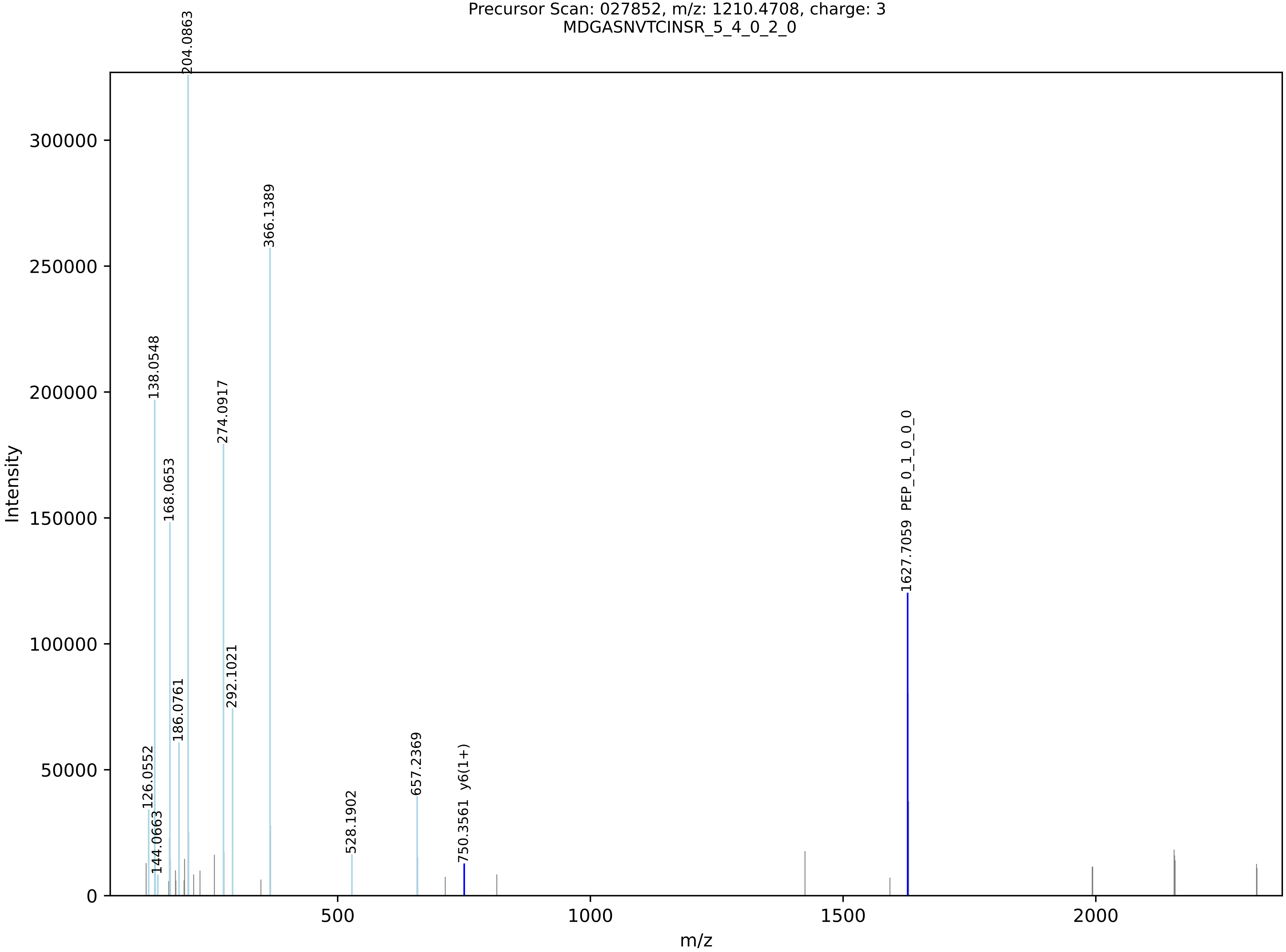

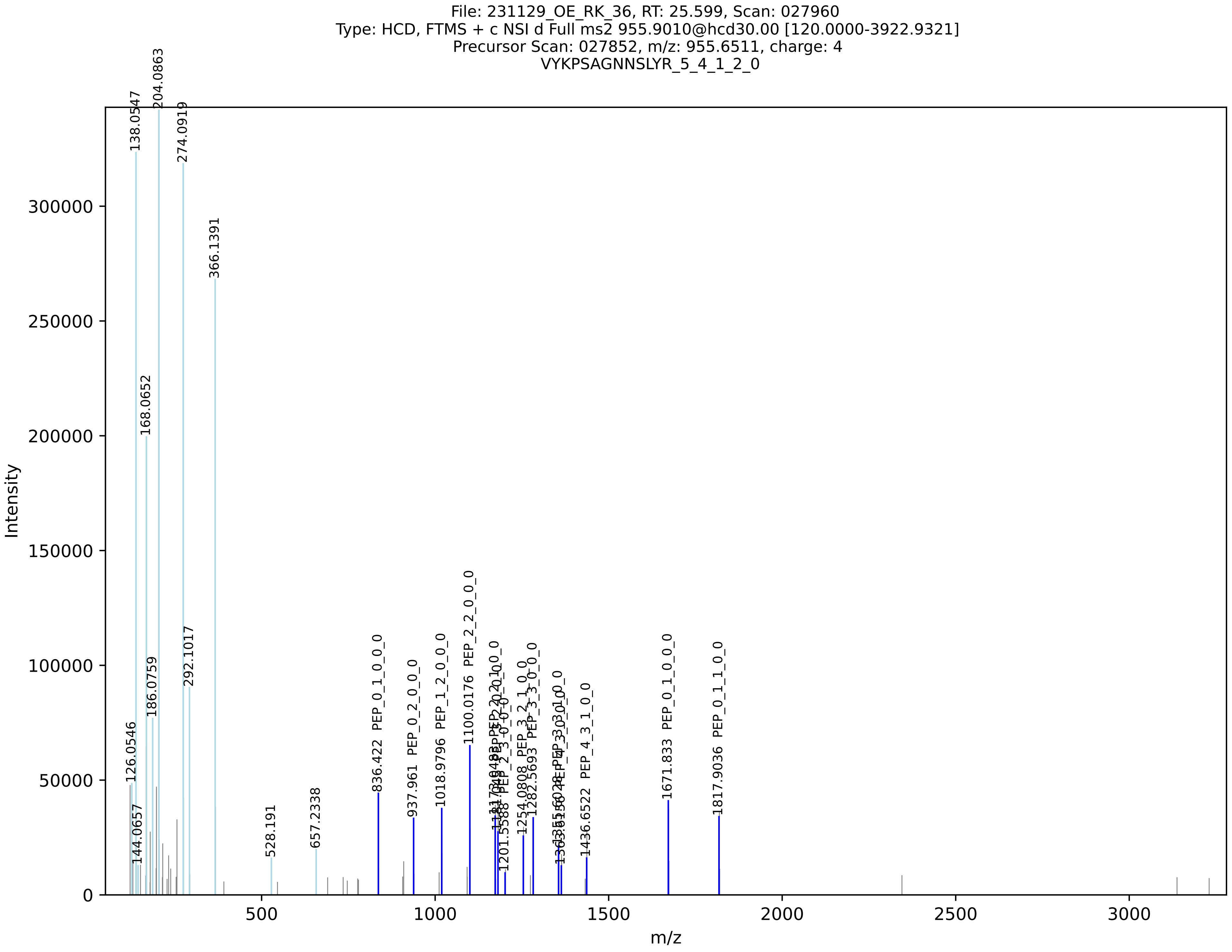
File: 231129_OE_RK_36, RT: 25.599, Scan: 027960
Type: HCD, FTMS + c NSI d Full ms2 955.9010@hcd30.00 [120.0000-3922.9321]
Precursor Scan: 027852, m/z: 955.6511, charge: 4
VYKPSAGNNSLYR_5_4_1_2_0
Intensity
m/z
138.0547
204.0863
274.0919
366.1391
168.0652
292.1017
186.0759
126.0546
144.0657
528.191
657.2338
836.422 PEP_0_1_0_0_0
937.961 PEP_0_2_0_0_0
1018.9796 PEP_1_2_0_0_0
1100.0176 PEP_2_2_0_0_0
1254.0808 PEP_3_2_1_0_0
1282.5693 PEP_3_3_0_0_0
1436.6522 PEP_4_3_1_0_0
1671.833 PEP_0_1_0_0_0
1817.9036 PEP_0_1_1_0_0

File: 231129_OE_RK_36, RT: 25.625, Scan: 027993
Type: HCD, FTMS + c NSI d Full ms2 1107.1416@hcd30.00 [120.0000-3409.9951]
Precursor Scan: 027919, m/z: 1106.4733, charge: 3
FVEGSHNSTVSLTTK_5_3_0_1_0

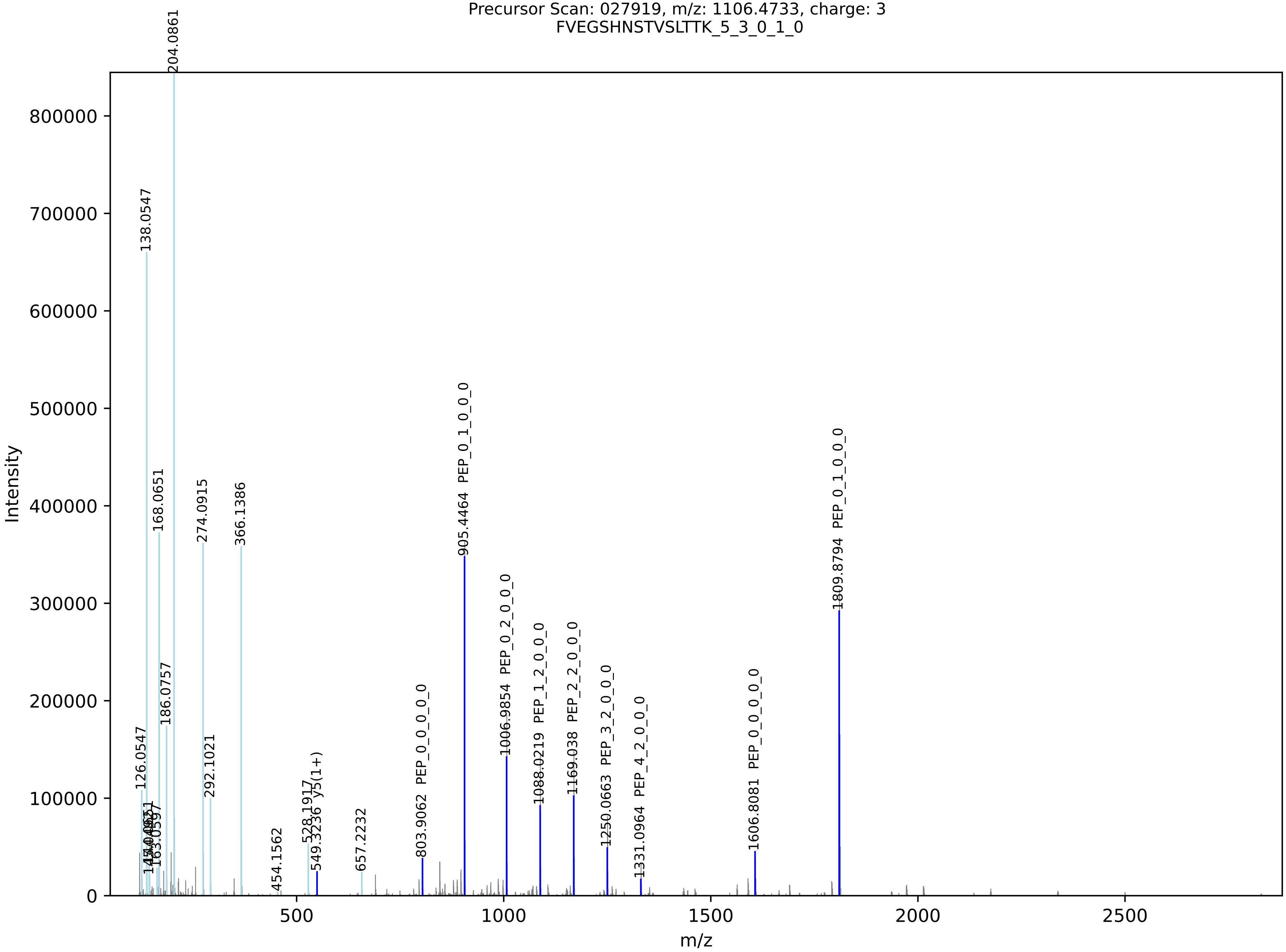

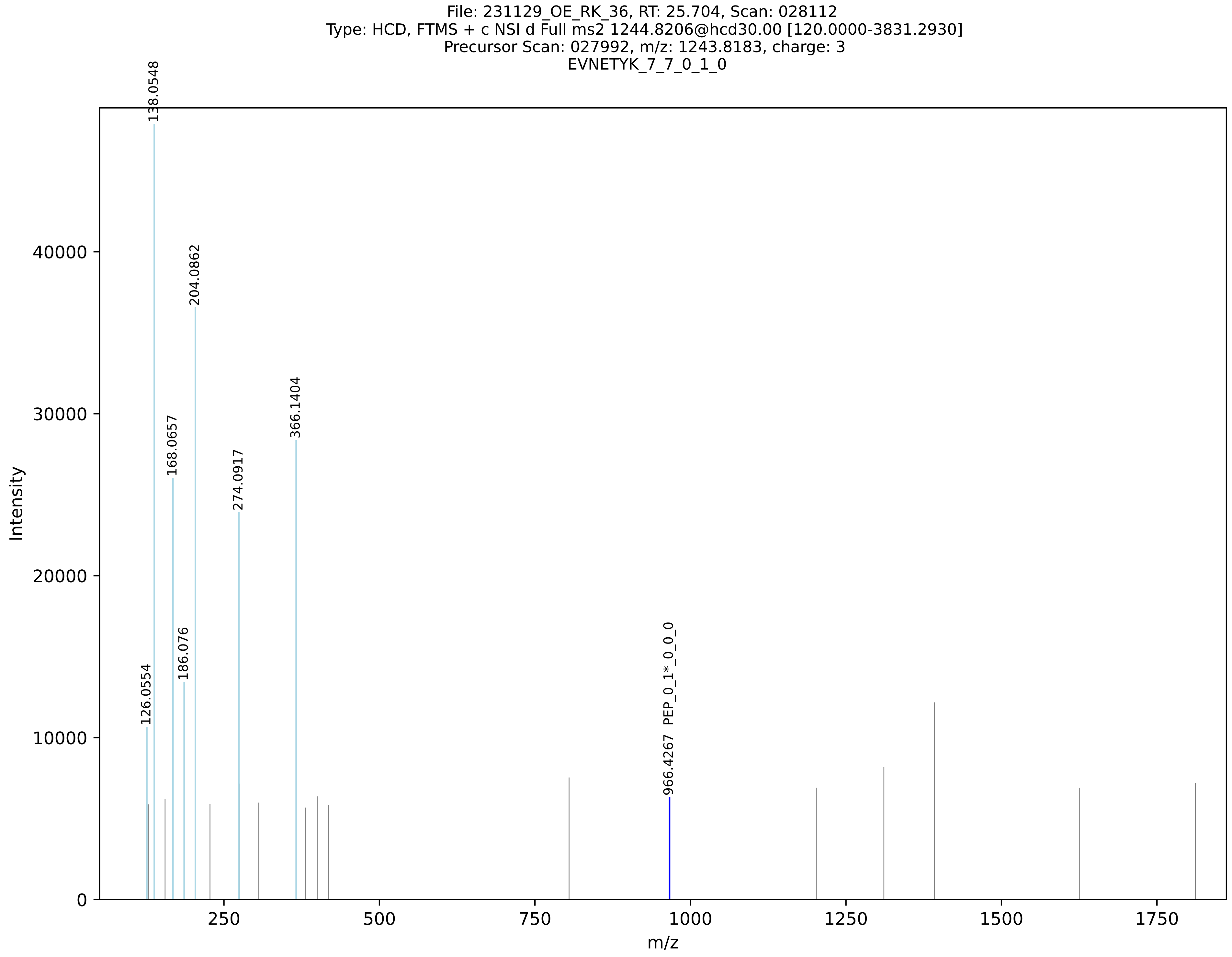

File: 231129_OE_RK_36, RT: 25.704, Scan: 028112
Type: HCD, FTMS + c NSI d Full ms2 1244.8206@hcd30.00 [120.0000-3831.2930]
Precursor Scan: 027992, m/z: 1243.8183, charge: 3
EVNETYK_7_7_0_1_0
138.0548
204.0862
366.1404
168.0657
274.0917
186.076
126.0554
966.4267 PEP_0_1*_0_0_0
Intensity
40000
30000
20000
10000
0
250
500
750
1000
1250
1500
1750
m/z

File: 231129_OE_RK_36, RT: 25.776, Scan: 028210
Type: HCD, FTMS + c NSI d Full ms2 1225.5144@hcd30.00 [120.0000-3772.2161]
Precursor Scan: 028138, m/z: 1225.1804, charge: 3
VYKPSAGNNSLYR_5_4_0_2_0

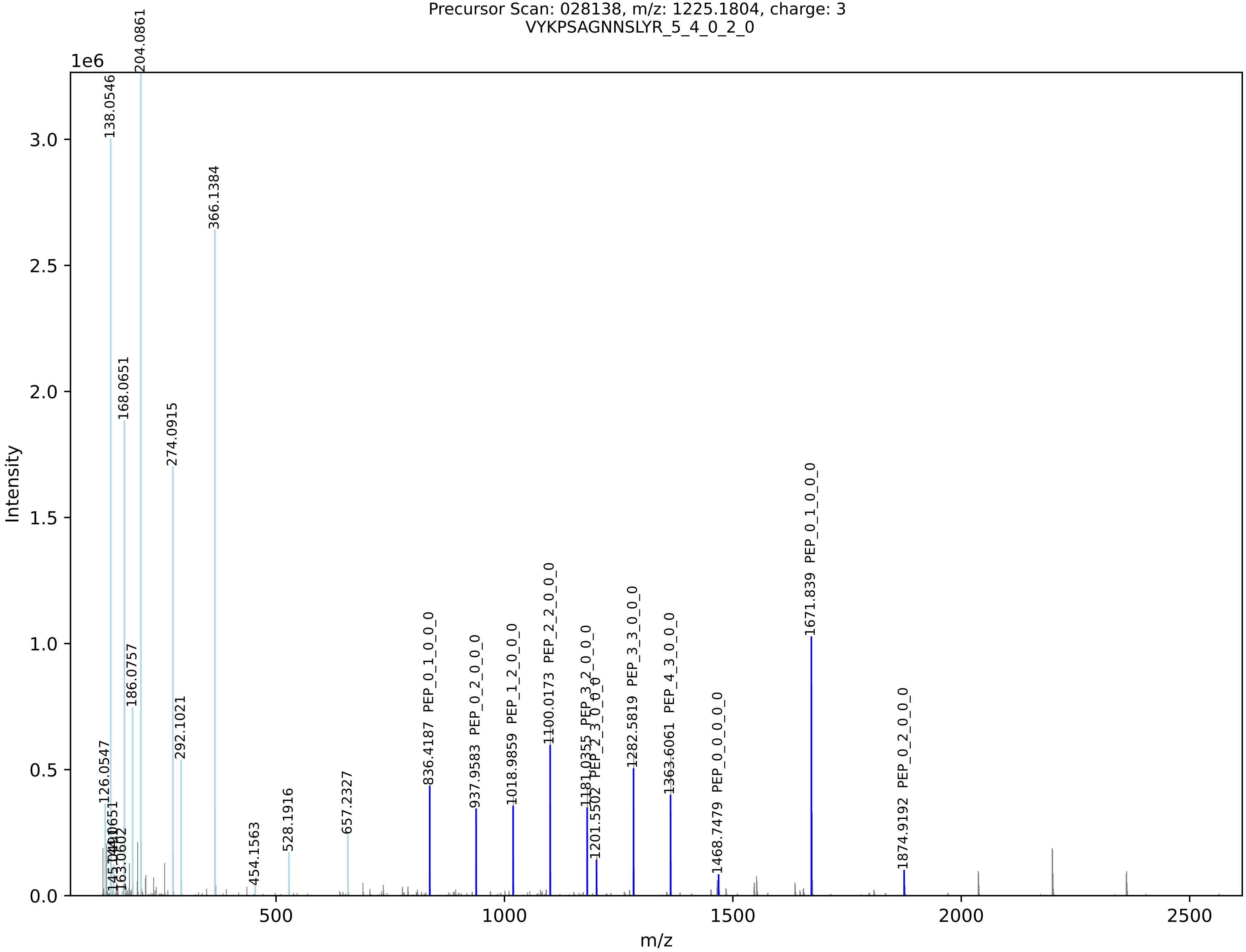

File: 231129_OE_RK_36, RT: 25.779, Scan: 028216
Type: HCD, FTMS + c NSI d Full ms2 1211.1396@hcd30.00 [120.0000-3728.2292]
Precursor Scan: 028138, m/z: 1210.4714, charge: 3
MDGASNVTCINSR_5_4_0_2_0

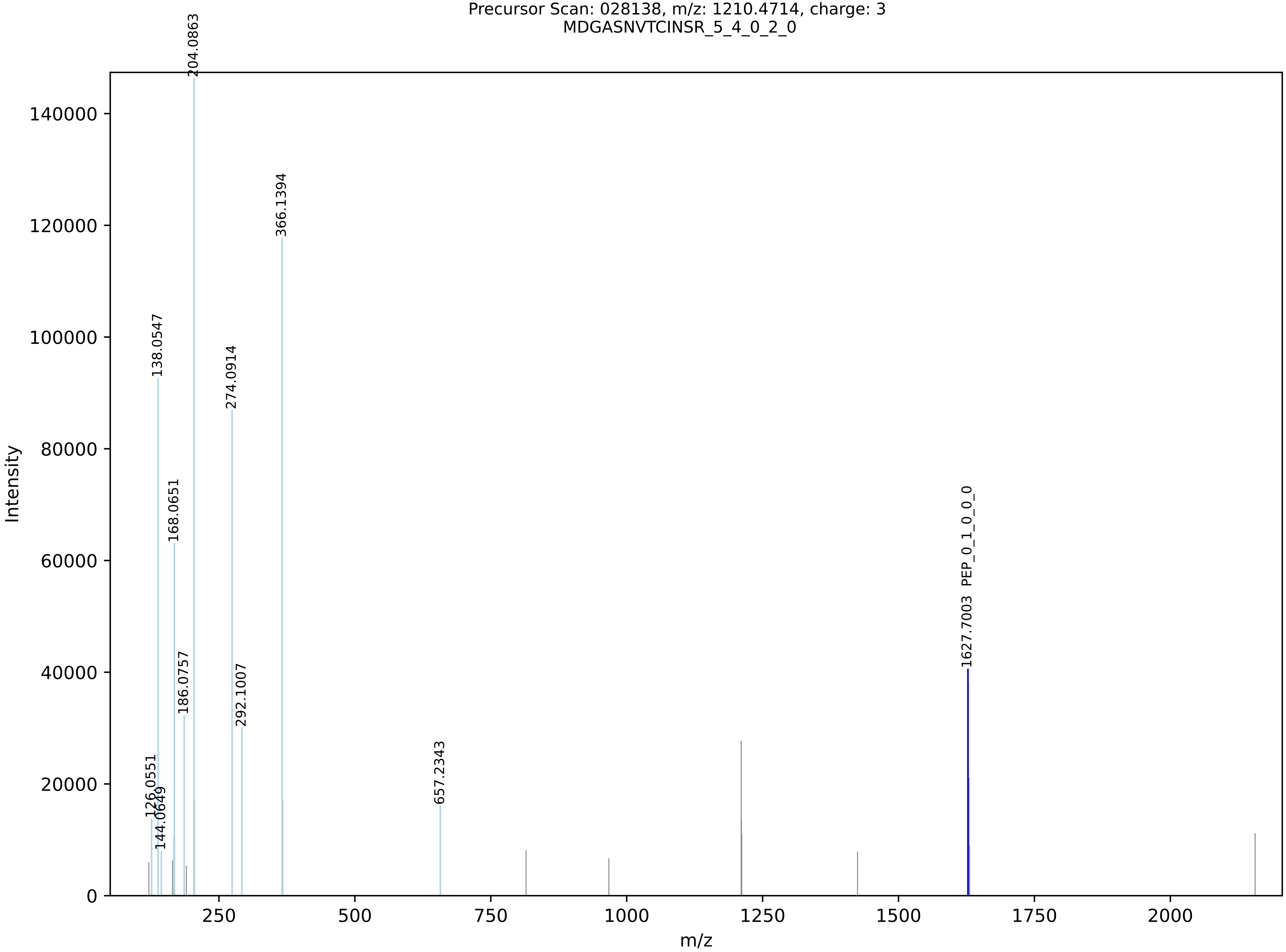

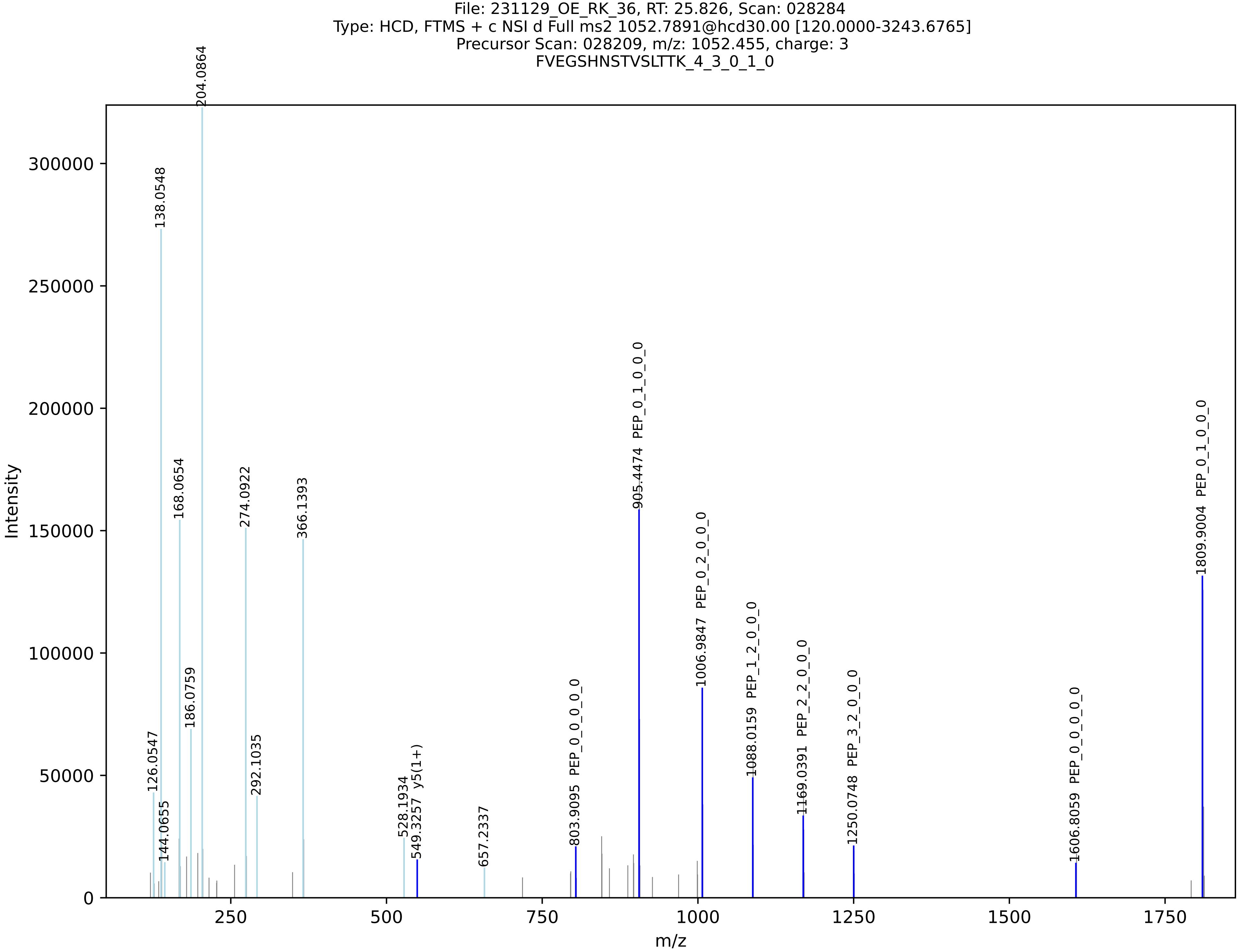
File: 231129_OE_RK_36, RT: 25.826, Scan: 028284
Type: HCD, FTMS + c NSI d Full ms2 1052.7891@hcd30.00 [120.0000-3243.6765]
Precursor Scan: 028209, m/z: 1052.455, charge: 3
FVEGSHNSTVSLTTK_4_3_0_1_0
Intensity
m/z
204.0864
138.0548
168.0654
274.0922
366.1393
186.0759
126.0547
292.1035
144.0655
528.1934
549.3257 y5(1+)
657.2337
803.9095 PEP_0_0_0_0_0_
905.4474 PEP_0_1_0_0_0_
1006.9847 PEP_0_2_0_0_0_
1088.0159 PEP_1_2_0_0_0_
1169.0391 PEP_2_2_0_0_0_
1250.0748 PEP_3_2_0_0_0_
1606.8059 PEP_0_0_0_0_0_
1809.9004 PEP_0_1_0_0_0_

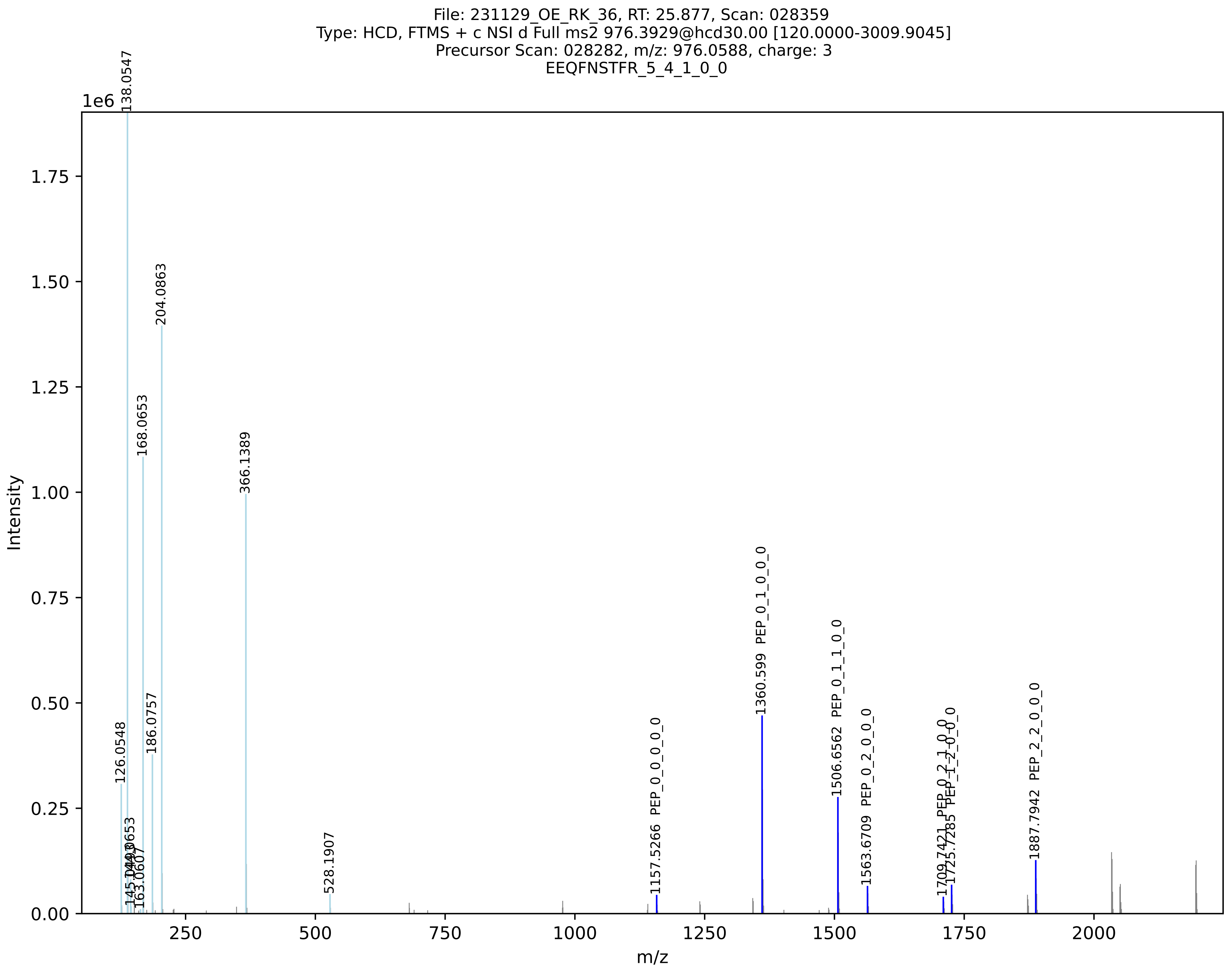
File: 231129_OE_RK_36, RT: 25.877, Scan: 028359
Type: HCD, FTMS + c NSI d Full ms2 976.3929@hcd30.00 [120.0000-3009.9045]
Precursor Scan: 028282, m/z: 976.0588, charge: 3
EEQFNSTFR_5_4_1_0_0
1e6
Intensity
m/z
138.0547
204.0863
168.0653
366.1389
126.0548
186.0757
163.0607
528.1907
1157.5266 PEP_0_0_0_0_0
1360.599 PEP_0_1_0_0_0
1506.6562 PEP_0_1_1_0_0
1563.6709 PEP_0_2_0_0_0
1709.7421 PEP_0_2_1_0_0
1725.7285 PEP_1_2_0_0_0
1887.7942 PEP_2_2_0_0_0

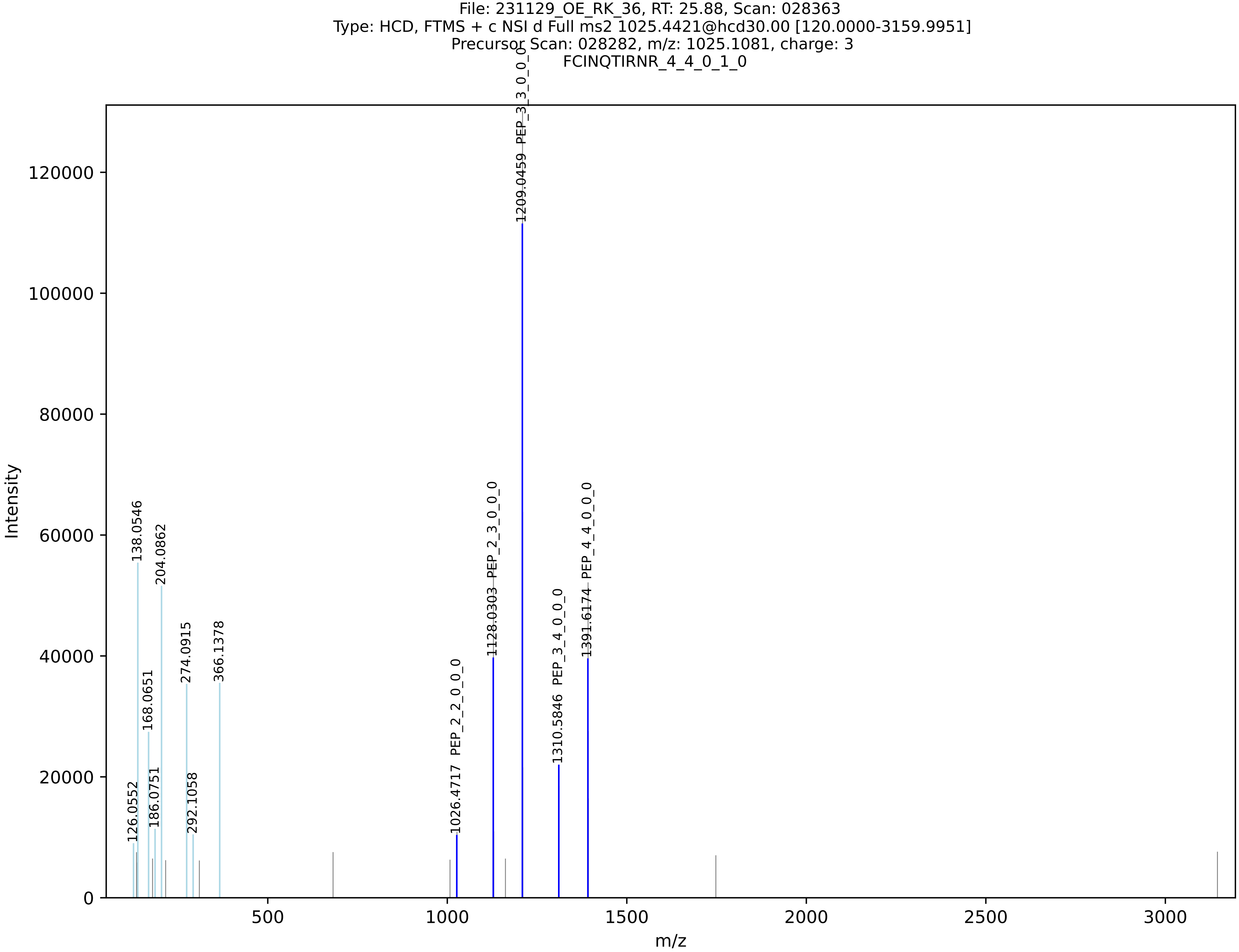
File: 231129_OE_RK_36, RT: 25.88, Scan: 028363
Type: HCD, FTMS + c NSI d Full ms2 1025.4421@hcd30.00 [120.0000-3159.9951]
Precursor Scan: 028282, m/z: 1025.1081, charge: 3
FCINQTIRNR_4_4_0_1_0
Intensity
m/z
0
20000
40000
60000
80000
100000
120000
500
1000
1500
2000
2500
3000
126.0552
138.0546
168.0651
186.0751
204.0862
274.0915
292.1058
366.1378
1026.4717 PEP_2_2_0_0_0
1128.0303 PEP_2_3_0_0_0
1209.0459 PEP_3_3_0_0_0
1310.5846 PEP_3_4_0_0_0
1391.6174 PEP_4_4_0_0_0

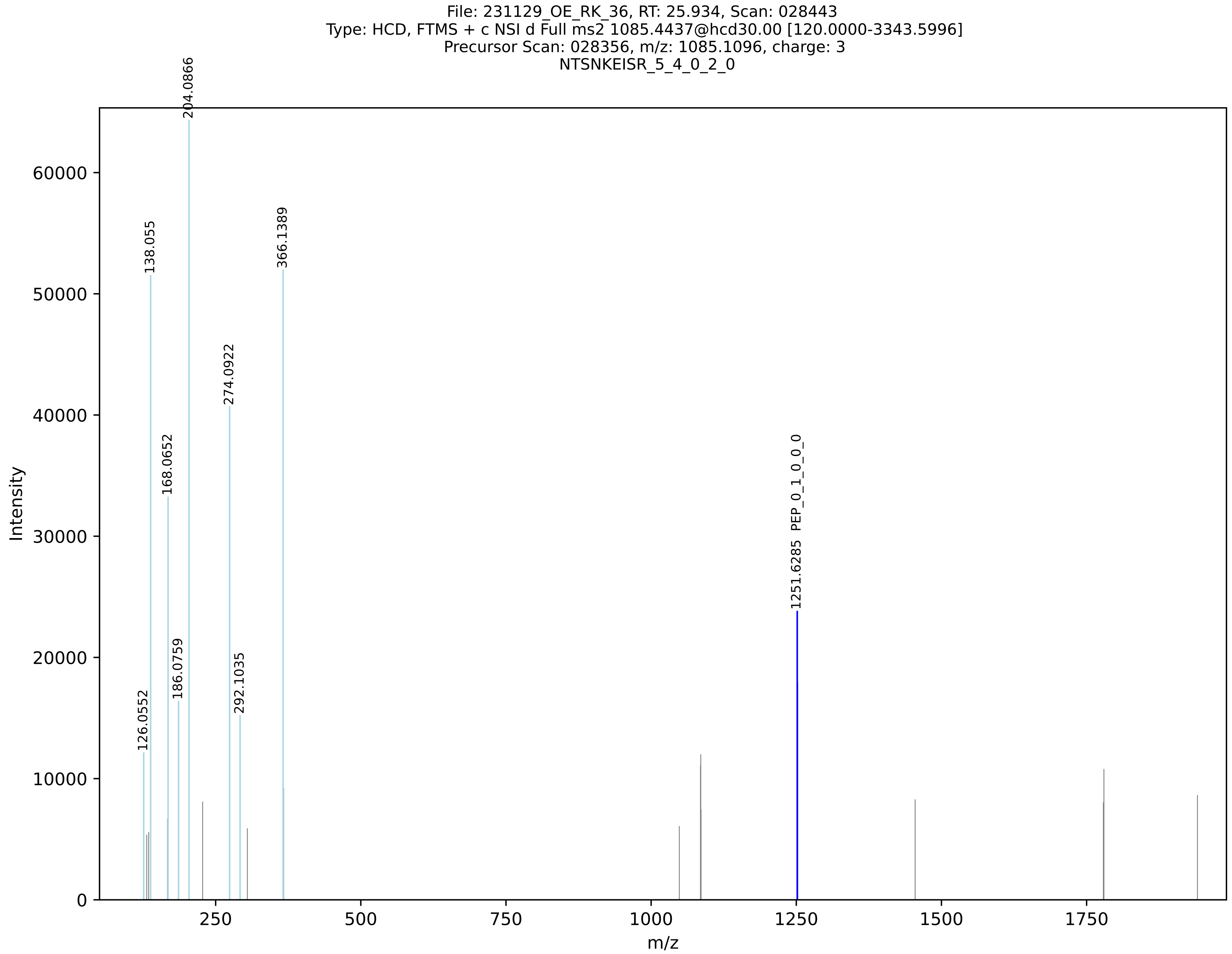
File: 231129_OE_RK_36, RT: 25.934, Scan: 028443
Type: HCD, FTMS + c NSI d Full ms2 1085.4437@hcd30.00 [120.0000-3343.5996]
Precursor Scan: 028356, m/z: 1085.1096, charge: 3
NTSNKEISR_5_4_0_2_0
204.0866
138.055
366.1389
274.0922
168.0652
1251.6285 PEP_0_1_0_0_0
186.0759
292.1035
126.0552
Intensity
m/z
0
10000
20000
30000
40000
50000
60000
250
500
750
1000
1250
1500
1750

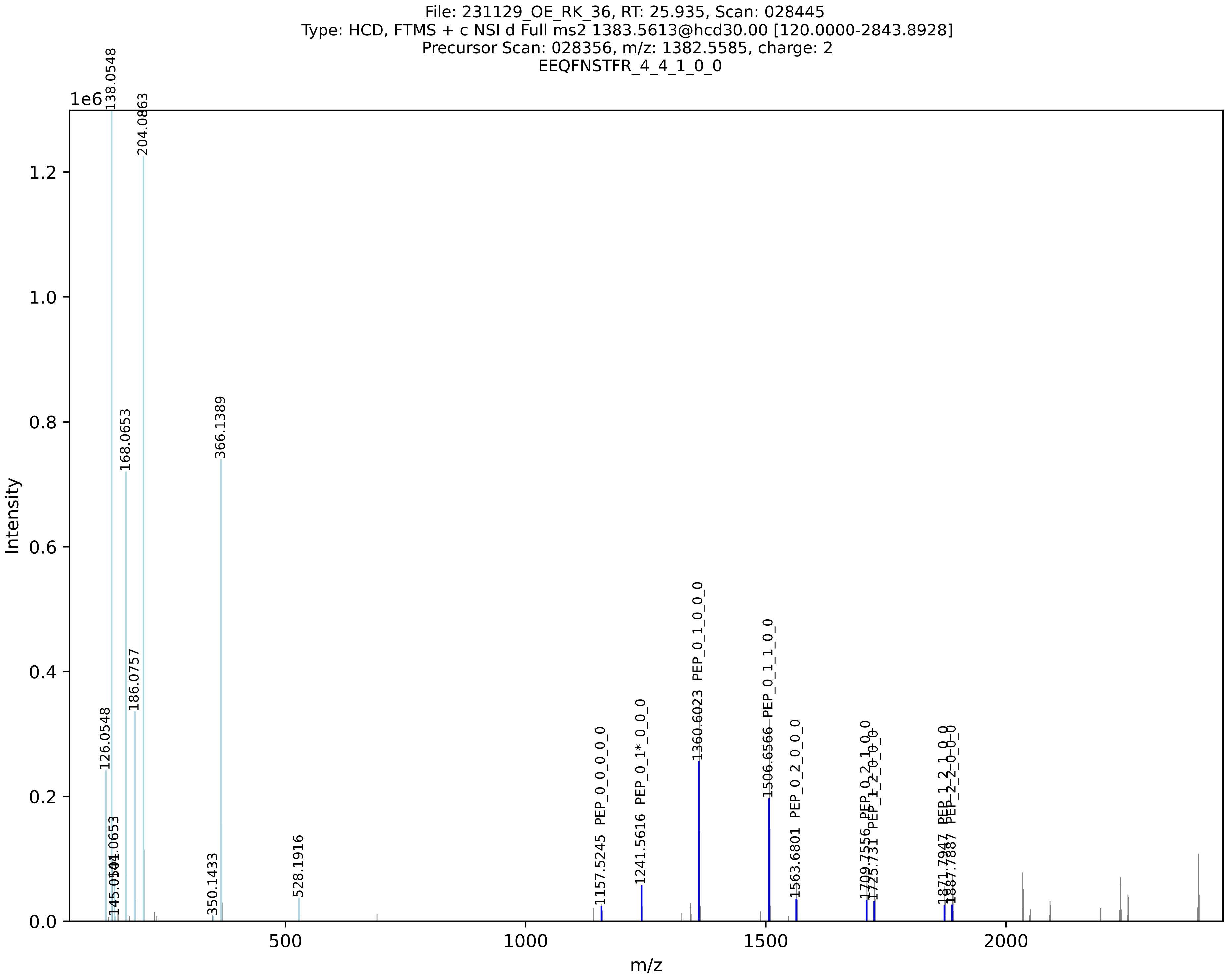
File: 231129_OE_RK_36, RT: 25.935, Scan: 028445
Type: HCD, FTMS + c NSI d Full ms2 1383.5613@hcd30.00 [120.0000-2843.8928]
Precursor Scan: 028356, m/z: 1382.5585, charge: 2
EEQFNSTFR_4_4_1_0_0
1e6
Intensity
m/z
138.0548
204.0863
168.0653
366.1389
186.0757
126.0548
145.0504
350.1433
528.1916
1157.5245 PEP_0_0_0_0_0
1241.5616 PEP_0_1*_0_0_0
1360.6023 PEP_0_1_0_0_0
1506.6566 PEP_0_1_1_0_0
1563.6801 PEP_0_2_0_0_0
1709.7556 PEP_0_2_1_0_0
1725.731 PEP_1_2_0_0_0
1871.7947 PEP_1_2_1_0_0
1887.7887 PEP_2_2_0_0_0

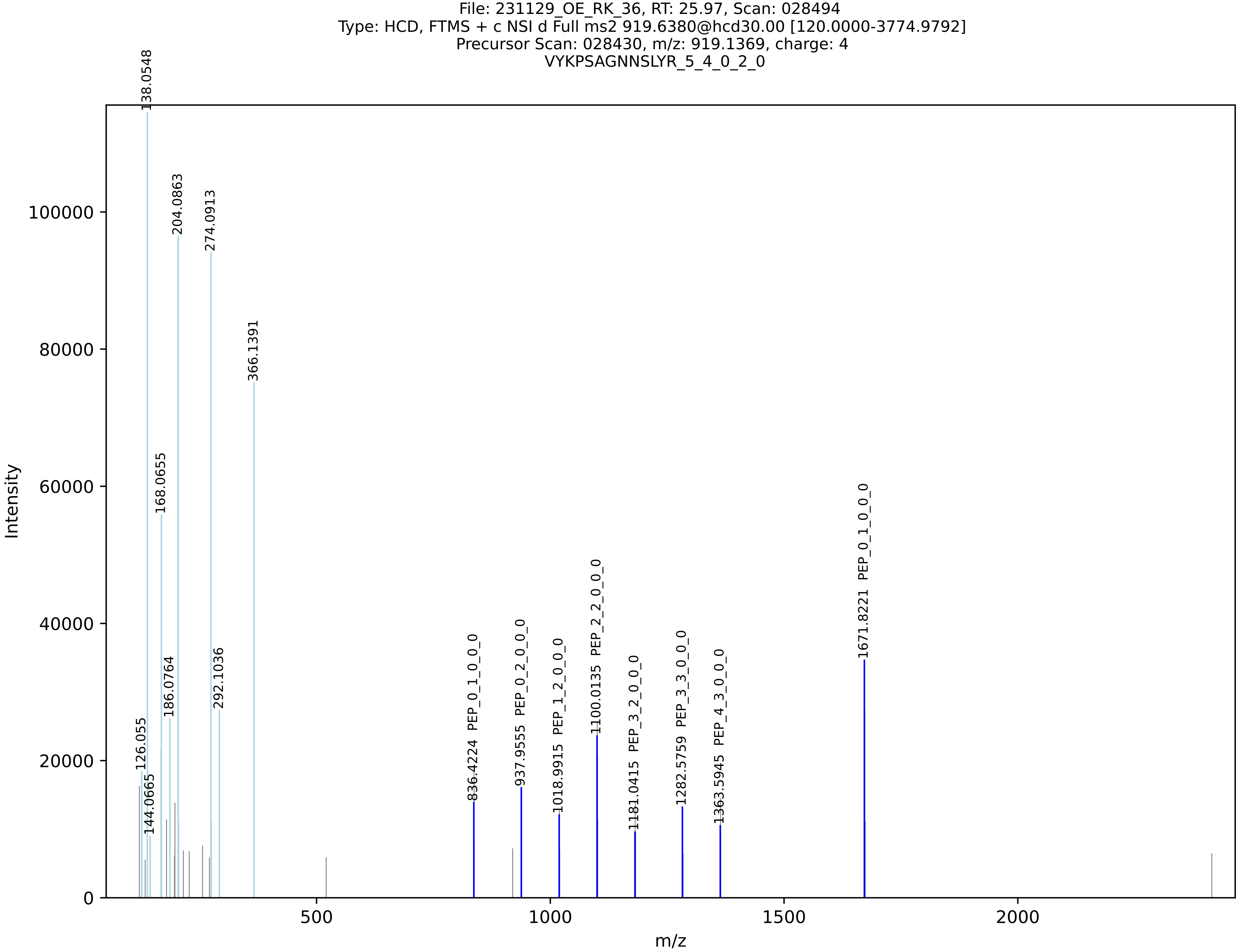
File: 231129_OE_RK_36, RT: 25.97, Scan: 028494
Type: HCD, FTMS + c NSI d Full ms2 919.6380@hcd30.00 [120.0000-3774.9792]
Precursor Scan: 028430, m/z: 919.1369, charge: 4
VYKPSAGNNSLYR_5_4_0_2_0
138.0548
204.0863
274.0913
366.1391
168.0655
186.0764
292.1036
126.055
144.0665
836.4224 PEP_0_1_0_0_0
937.9555 PEP_0_2_0_0_0
1018.9915 PEP_1_2_0_0_0
1100.0135 PEP_2_2_0_0_0
1181.0415 PEP_3_2_0_0_0
1282.5759 PEP_3_3_0_0_0
1363.5945 PEP_4_3_0_0_0
1671.8221 PEP_0_1_0_0_0
Intensity
m/z
0
20000
40000
60000
80000
100000
500
1000
1500
2000

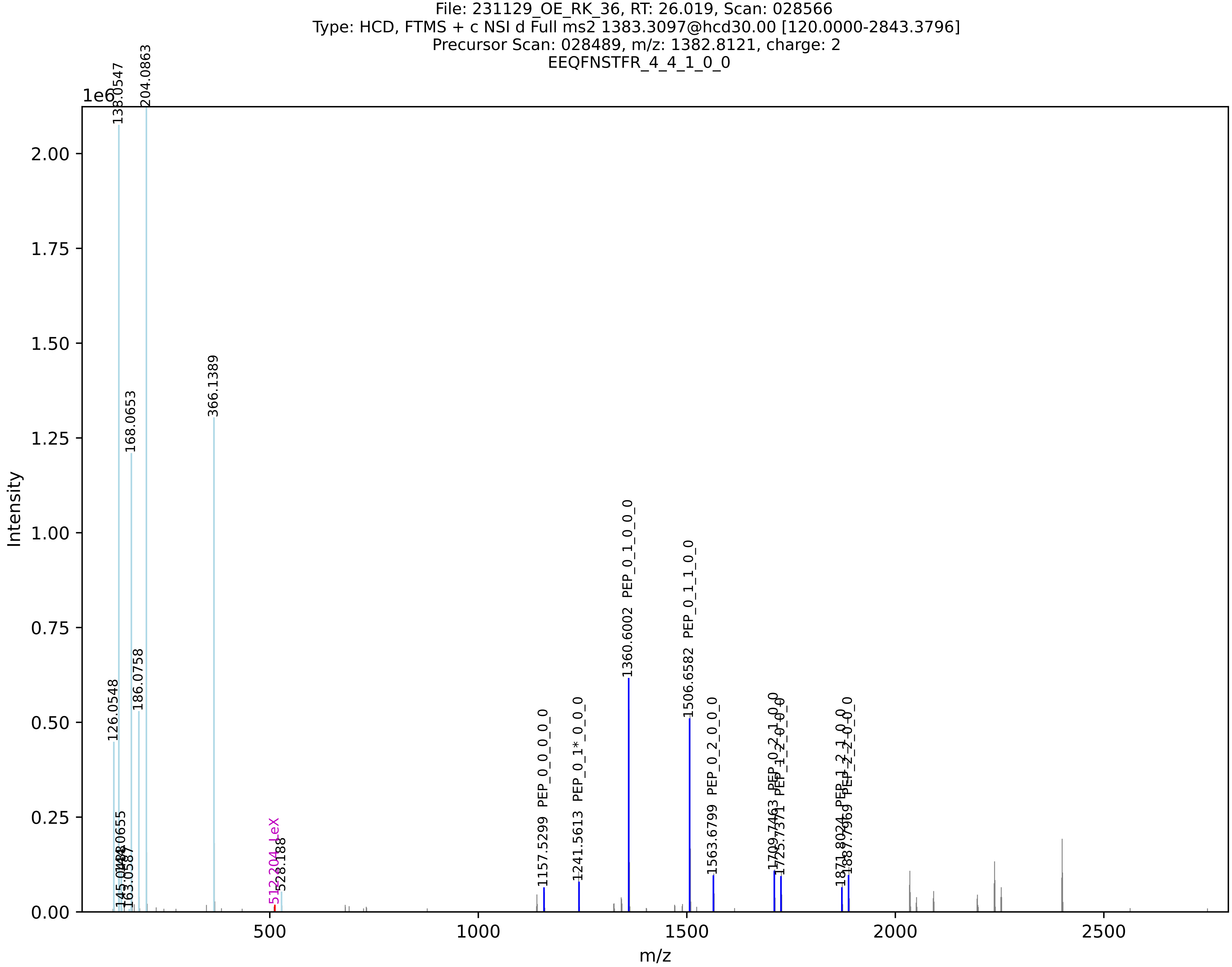
File: 231129_OE_RK_36, RT: 26.019, Scan: 028566
Type: HCD, FTMS + c NSI d Full ms2 1383.3097@hcd30.00 [120.0000-2843.3796]
Precursor Scan: 028489, m/z: 1382.8121, charge: 2
EEQFNSTFR_4_4_1_0_0
1e6
Intensity
m/z
138.0547
204.0863
366.1389
168.0653
186.0758
126.0548
512.204_LeX
528.188
1157.5299 PEP_0_0_0_0_0
1241.5613 PEP_0_1*_0_0_0
1360.6002 PEP_0_1_0_0_0
1506.6582 PEP_0_1_1_0_0
1563.6799 PEP_0_2_0_0_0
1709.7463 PEP_0_2_1_0_0
1725.7371 PEP_1_2_0_0_0
1871.8024 PEP_1_2_1_0_0
1887.7969 PEP_2_2_0_0_0

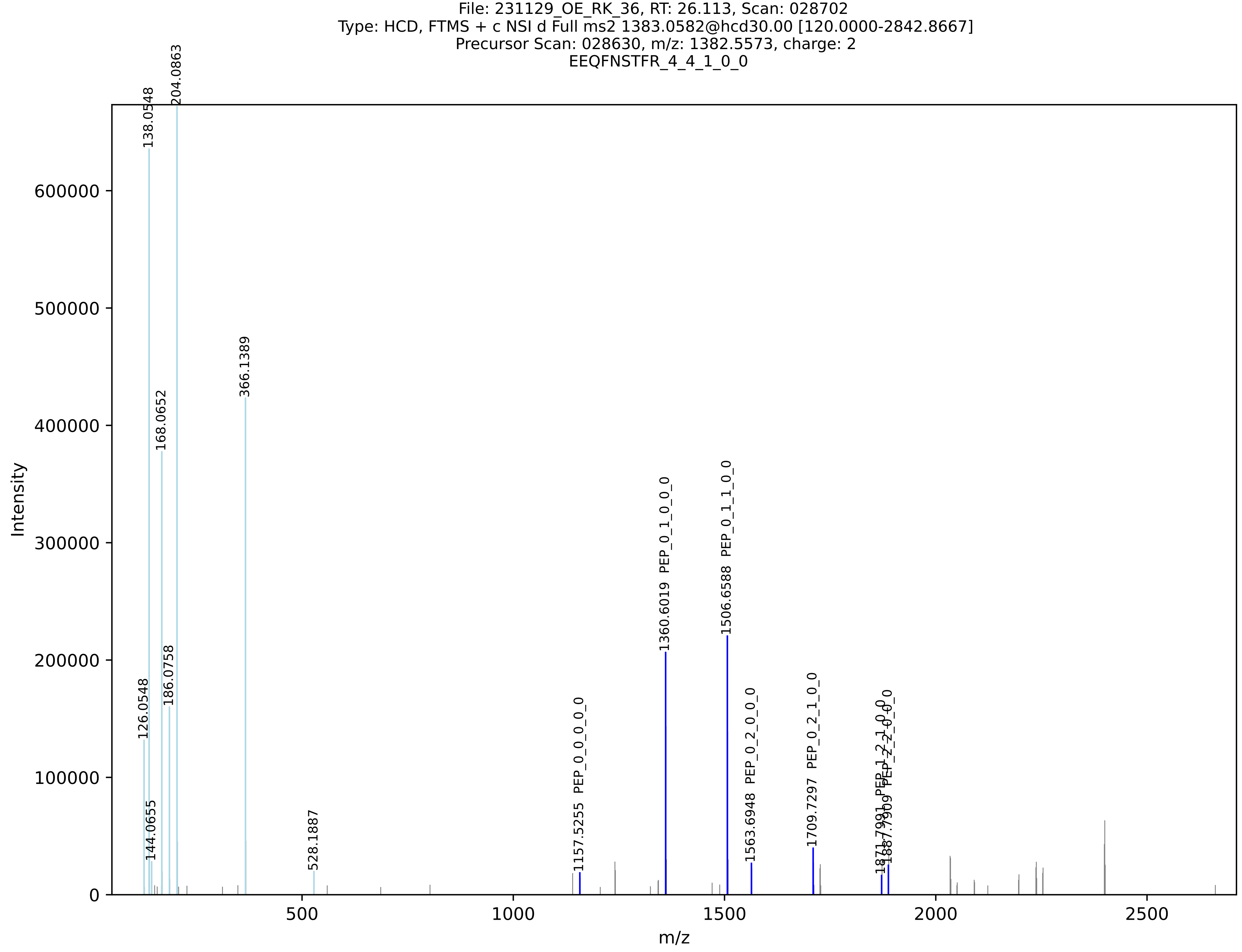

File: 231129_OE_RK_36, RT: 26.113, Scan: 028702
Type: HCD, FTMS + c NSI d Full ms2 1383.0582@hcd30.00 [120.0000-2842.8667]
Precursor Scan: 028630, m/z: 1382.5573, charge: 2
EEQFNSTFR_4_4_1_0_0
Intensity
m/z
0
100000
200000
300000
400000
500000
600000
500
1000
1500
2000
2500
204.0863
138.0548
366.1389
168.0652
186.0758
126.0548
144.0655
528.1887
1157.5255 PEP_0_0_0_0_0
1360.6019 PEP_0_1_0_0_0
1506.6588 PEP_0_1_1_0_0
1563.6948 PEP_0_2_0_0_0
1709.7297 PEP_0_2_1_0_0
1871.7991 PEP_1_2_1_0_0
1887.7909 PEP_2_2_0_0_0

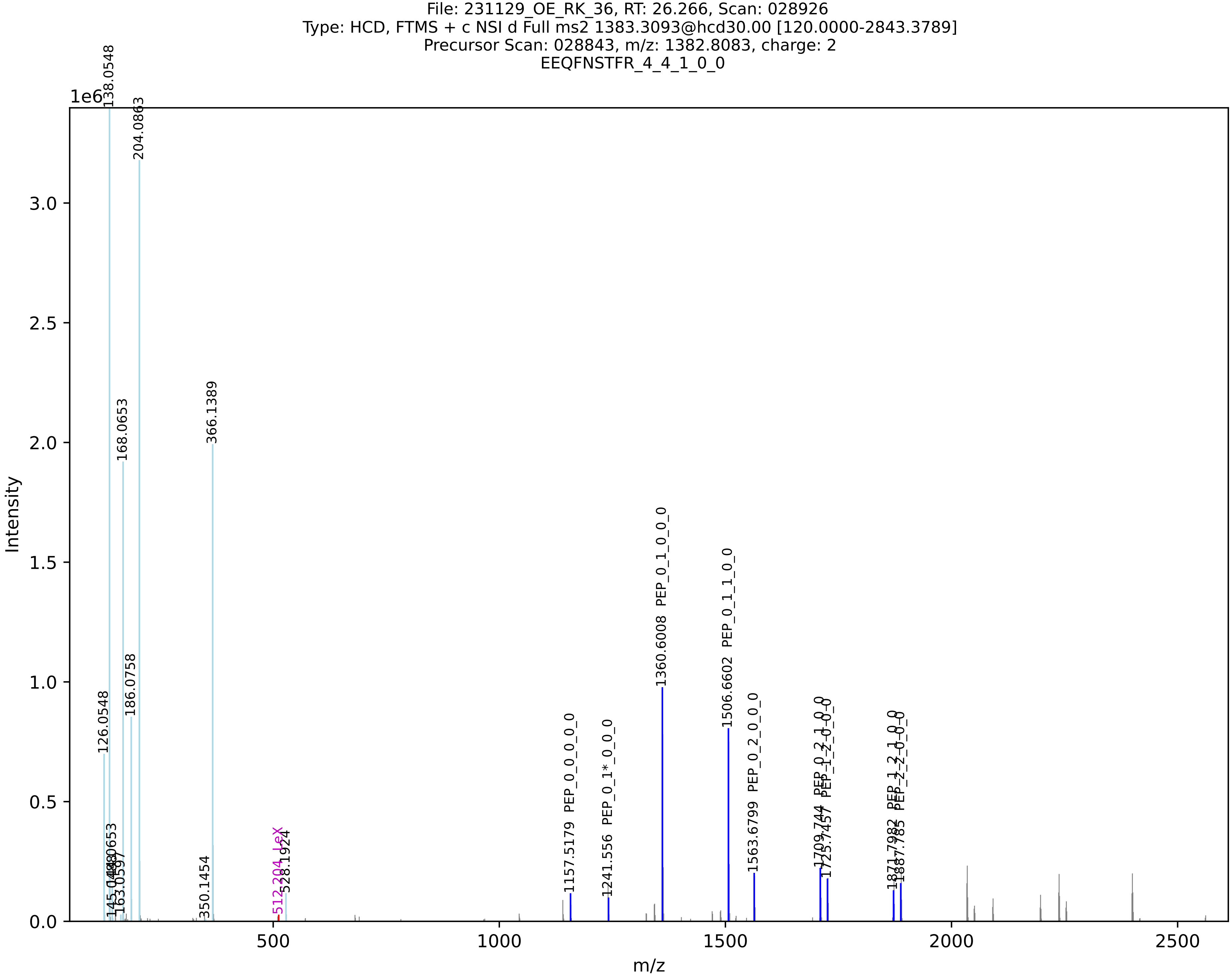
File: 231129_OE_RK_36, RT: 26.266, Scan: 028926
Type: HCD, FTMS + c NSI d Full ms2 1383.3093@hcd30.00 [120.0000-2843.3789]
Precursor Scan: 028843, m/z: 1382.8083, charge: 2
EEQFNSTFR_4_4_1_0_0
1e6
138.0548
204.0863
168.0653
366.1389
186.0758
126.0548
350.1454
512.204 LeX
528.1924
1157.5179 PEP_0_0_0_0_0
1241.556 PEP_0_1*_0_0_0
1360.6008 PEP_0_1_0_0_0
1506.6602 PEP_0_1_1_0_0
1563.6799 PEP_0_2_0_0_0
1709.744 PEP_0_2_1_0_0
1725.7457 PEP_1_2_0_0_0
1871.7982 PEP_1_2_1_0_0
1887.785 PEP_2_2_0_0_0
Intensity
m/z

File: 231129_OE_RK_36, RT: 26.286, Scan: 028958
Type: HCD, FTMS + c NSI d Full ms2 1281.5179@hcd30.00 [120.0000-2635.7246]
Precursor Scan: 028843, m/z: 1281.0157, charge: 2
YQHNESHSR_4_3_1_0_0

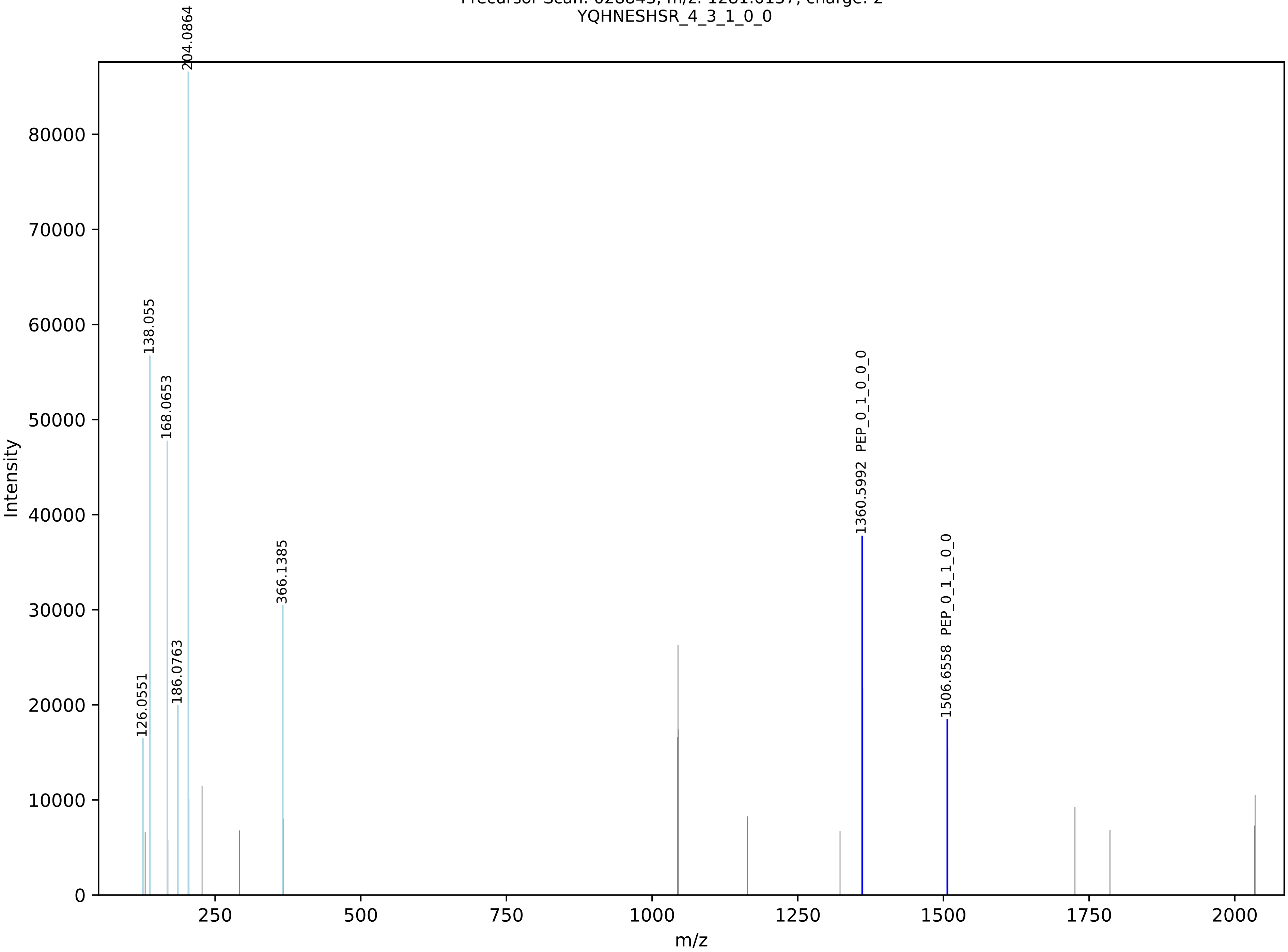

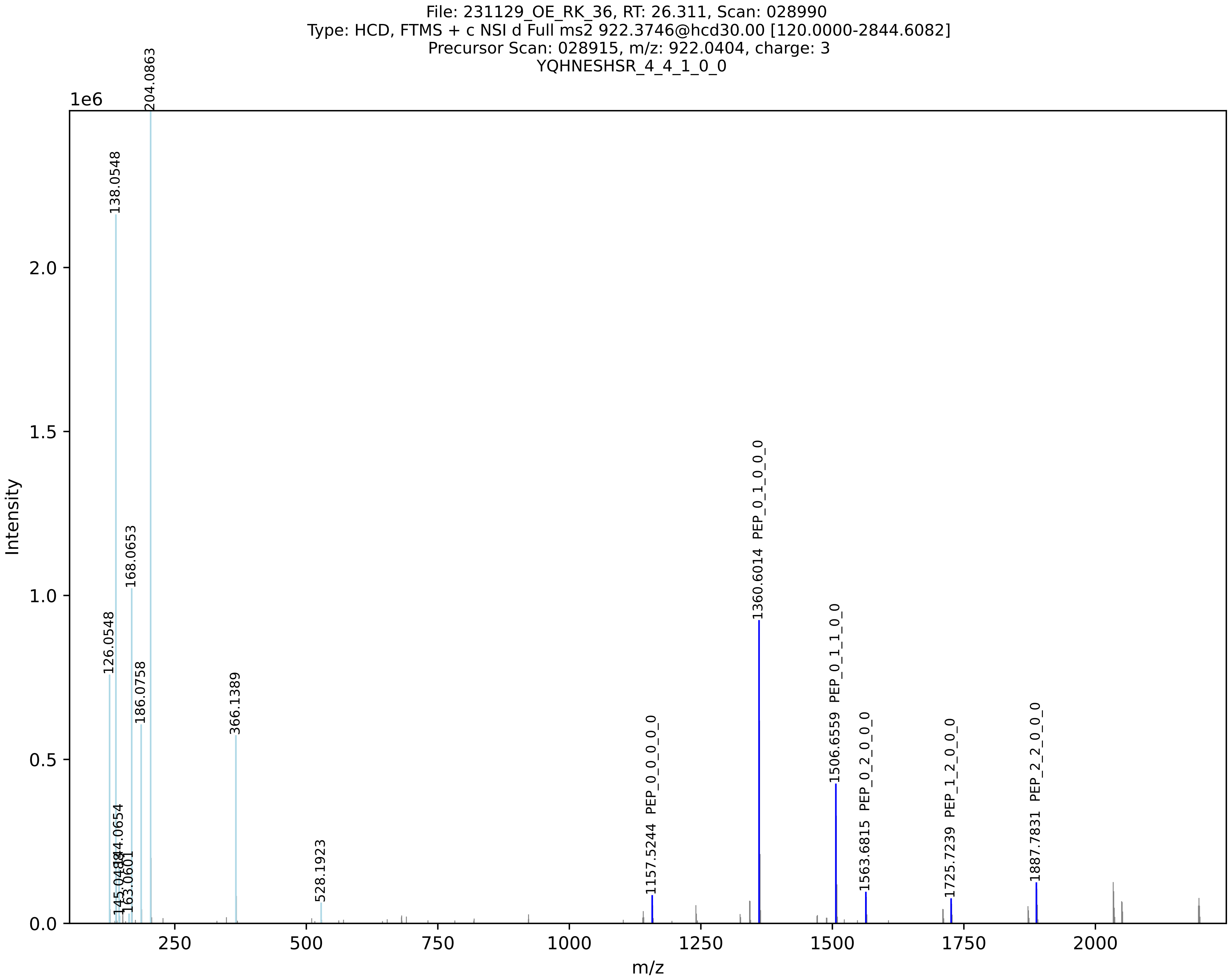
File: 231129_OE_RK_36, RT: 26.311, Scan: 028990
Type: HCD, FTMS + c NSI d Full ms2 922.3746@hcd30.00 [120.0000-2844.6082]
Precursor Scan: 028915, m/z: 922.0404, charge: 3
YQHNESHSR_4_4_1_0_0
1e6
Intensity
m/z
204.0863
138.0548
168.0653
126.0548
186.0758
366.1389
144.0654
145.0498
163.0601
528.1923
1157.5244 PEP_0_0_0_0_0
1360.6014 PEP_0_1_0_0_0
1506.6559 PEP_0_1_1_0_0
1563.6815 PEP_0_2_0_0_0
1725.7239 PEP_1_2_0_0_0
1887.7831 PEP_2_2_0_0_0

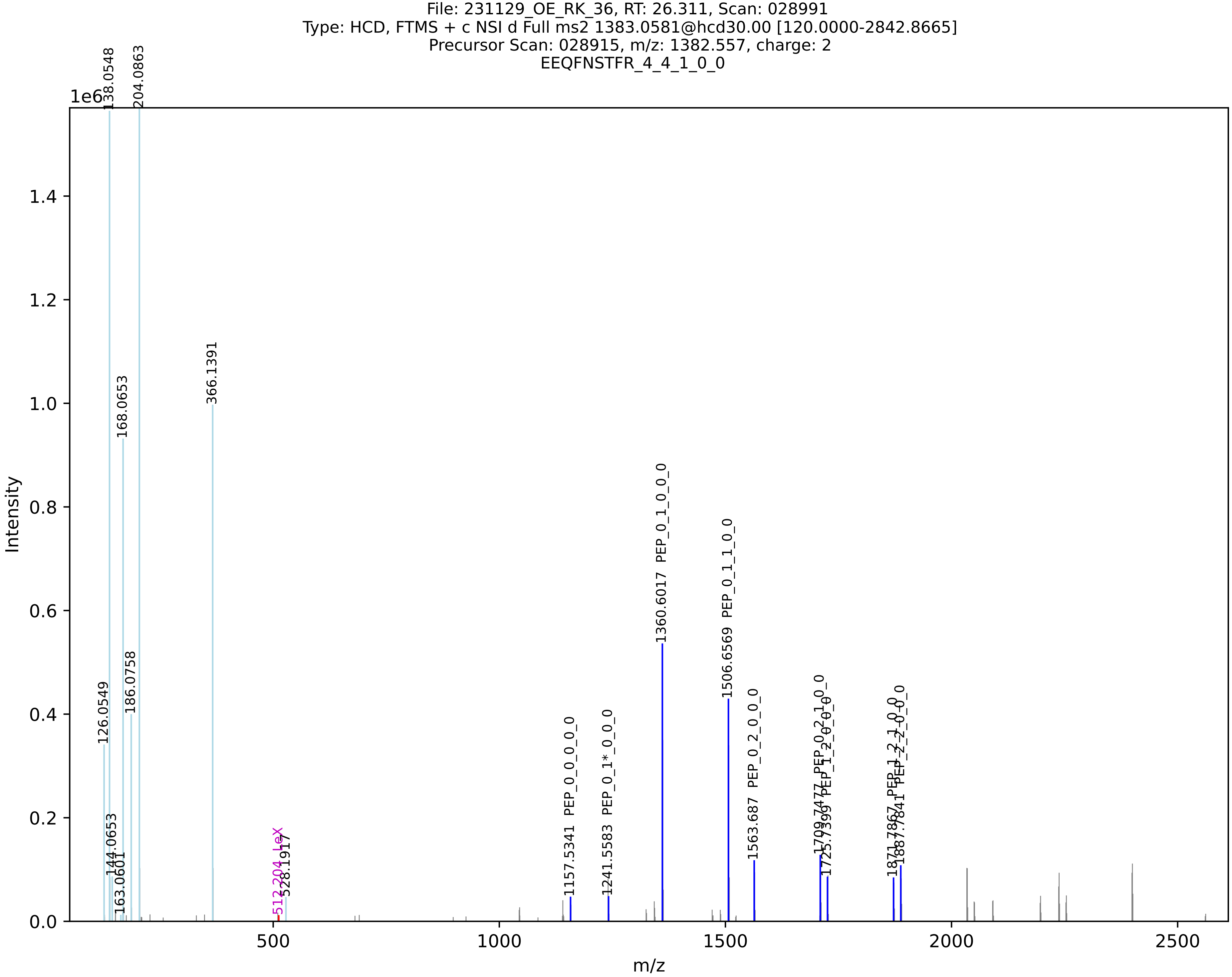

File: 231129_OE_RK_36, RT: 26.311, Scan: 028991
Type: HCD, FTMS + c NSI d Full ms2 1383.0581@hcd30.00 [120.0000-2842.8665]
Precursor Scan: 028915, m/z: 1382.557, charge: 2
EEQFNSTFR_4_4_1_0_0
1e6
Intensity
m/z
138.0548
204.0863
366.1391
168.0653
186.0758
126.0549
144.0653
163.0601
512.204 LeX
528.1917
1157.5341 PEP_0_0_0_0_0
1241.5583 PEP_0_1*_0_0_0
1360.6017 PEP_0_1_0_0_0
1506.6569 PEP_0_1_1_0_0
1563.687 PEP_0_2_0_0_0
1709.7477 PEP_0_2_1_0_0
1725.7399 PEP_1_2_0_0_0
1871.7867 PEP_1_2_1_0_0
1887.7841 PEP_2_2_0_0_0

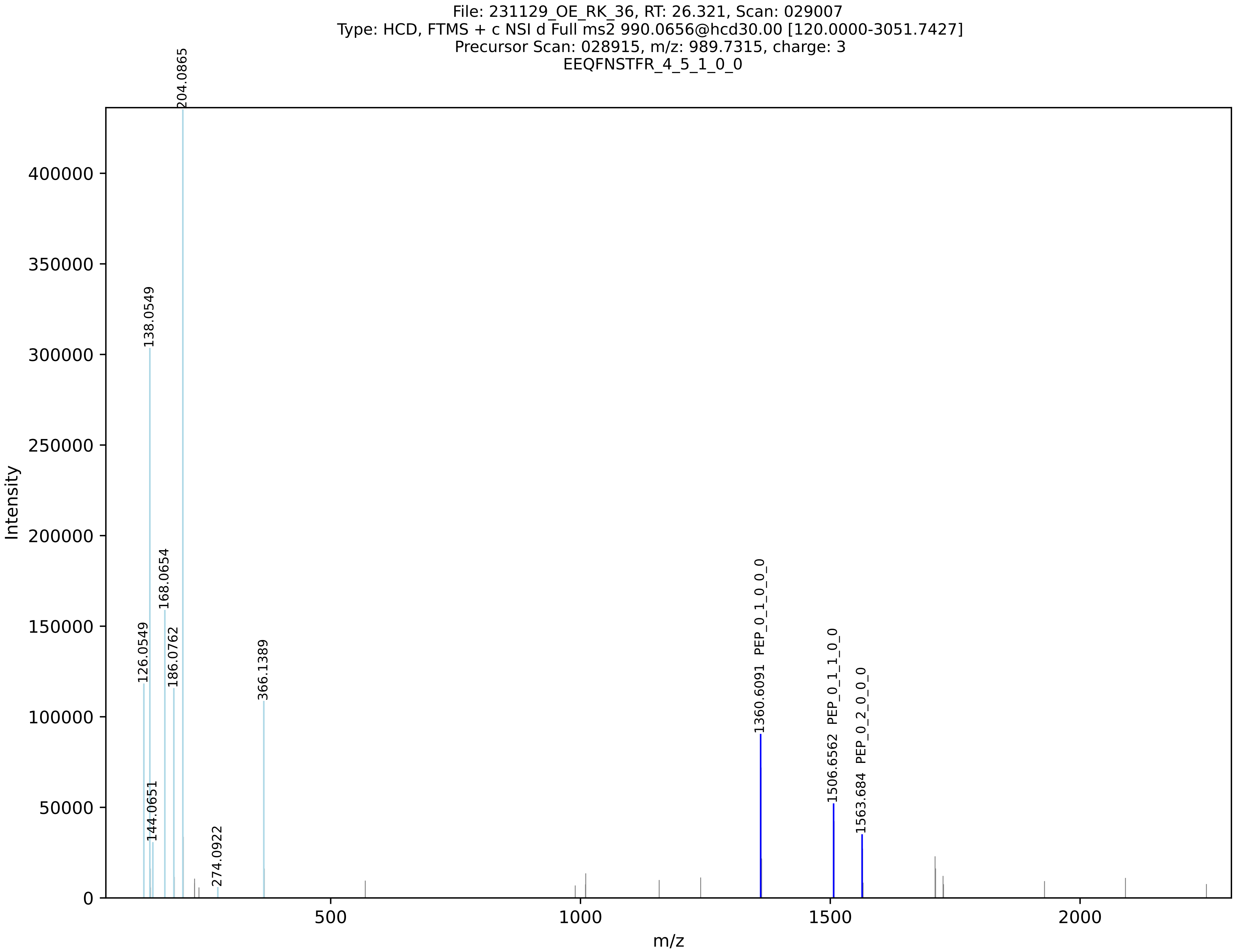
File: 231129_OE_RK_36, RT: 26.321, Scan: 029007
Type: HCD, FTMS + c NSI d Full ms2 990.0656@hcd30.00 [120.0000-3051.7427]
Precursor Scan: 028915, m/z: 989.7315, charge: 3
EEQFNSTFR_4_5_1_0_0
Intensity
m/z
204.0865
138.0549
168.0654
126.0549
186.0762
366.1389
144.0651
274.0922
1360.6091 PEP_0_1_0_0_0
1506.6562 PEP_0_1_1_0_0
1563.684 PEP_0_2_0_0_0

File: 231129_OE_RK_36, RT: 26.461, Scan: 029204
Type: HCD, FTMS + c NSI d Full ms2 1074.4604@hcd30.00 [120.0000-3309.9910]
Precursor Scan: 029158, m/z: 1074.4604, charge: 3
TPLTANITK_5_5_1_1_0

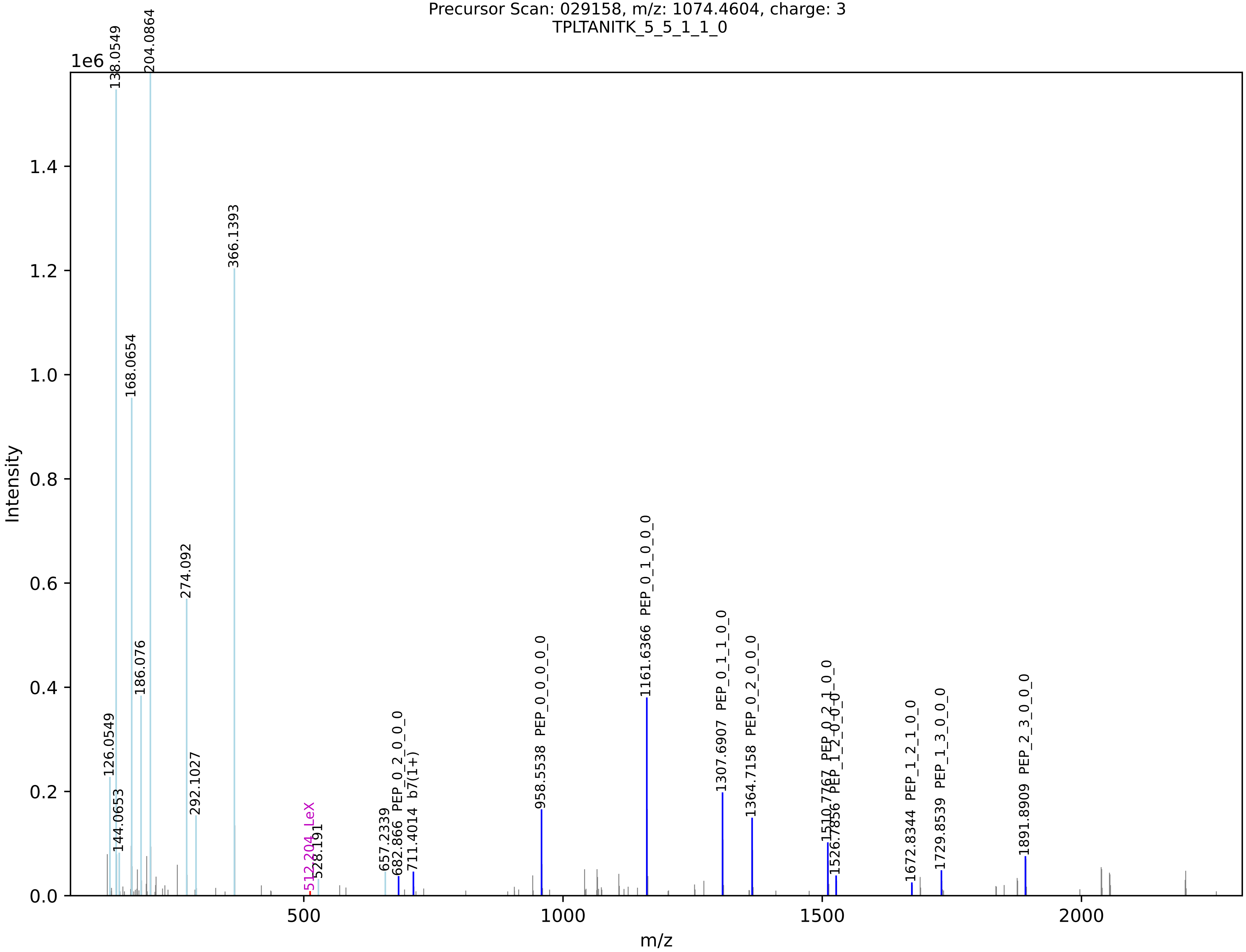

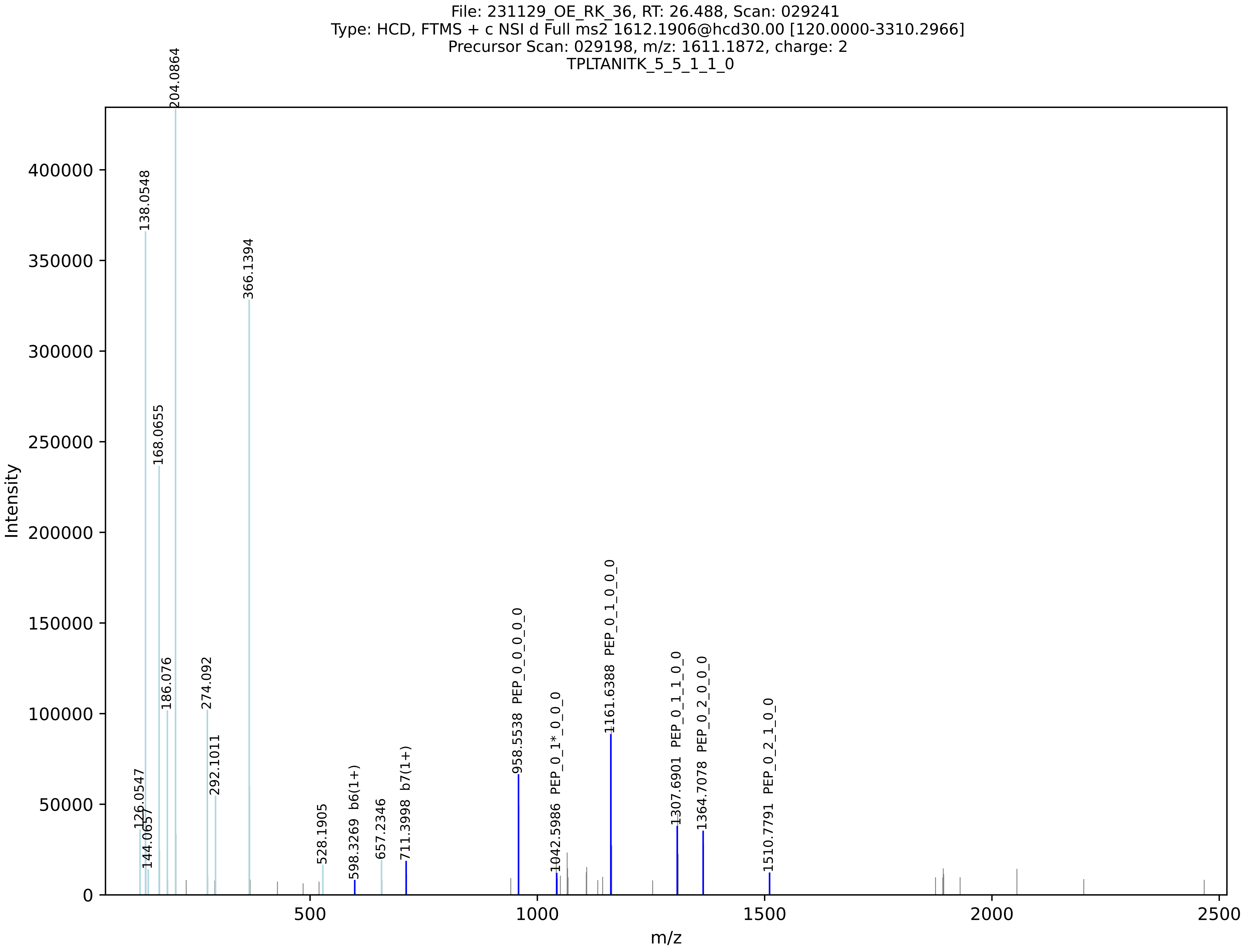

File: 231129_OE_RK_36, RT: 26.488, Scan: 029241
Type: HCD, FTMS + c NSI d Full ms2 1612.1906@hcd30.00 [120.0000-3310.2966]
Precursor Scan: 029198, m/z: 1611.1872, charge: 2
TPLTANITK_5_5_1_1_0
Intensity
m/z
0
50000
100000
150000
200000
250000
300000
350000
400000
500
1000
1500
2000
2500
204.0864
138.0548
366.1394
168.0655
186.076
274.092
292.1011
126.0547
144.0657
528.1905
598.3269 b6(1+)
657.2346
711.3998 b7(1+)
958.5538 PEP_0_0_0_0_
1042.5986 PEP_0_1*_0_0_
1161.6388 PEP_0_1_0_0_
1307.6901 PEP_0_1_1_0_
1364.7078 PEP_0_2_0_0_
1510.7791 PEP_0_2_1_0_

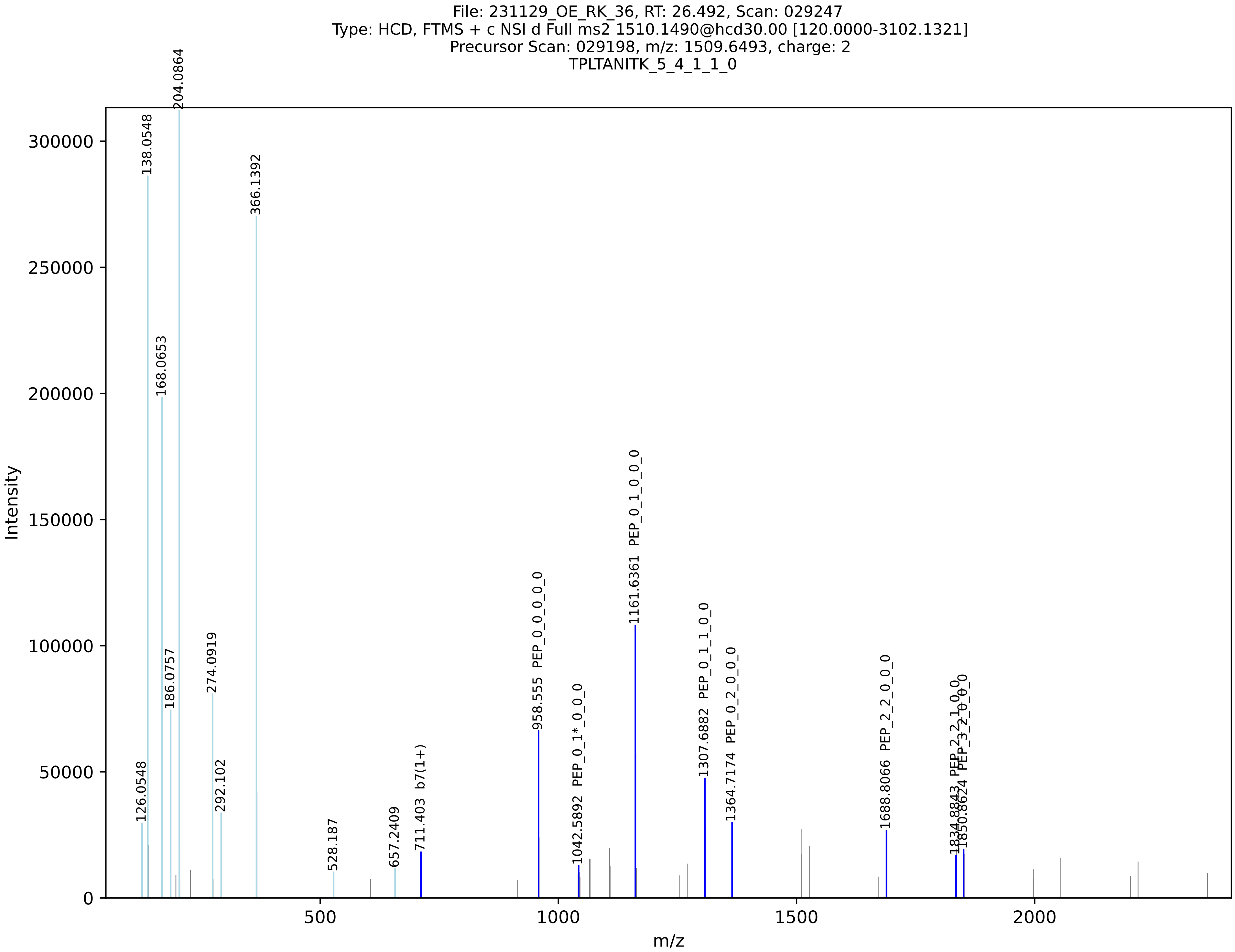

File: 231129_OE_RK_36, RT: 26.492, Scan: 029247
Type: HCD, FTMS + c NSI d Full ms2 1510.1490@hcd30.00 [120.0000-3102.1321]
Precursor Scan: 029198, m/z: 1509.6493, charge: 2
TPLTANITK_5_4_1_1_0
Intensity
m/z
204.0864
138.0548
366.1392
168.0653
186.0757
274.0919
126.0548
292.102
528.187
657.2409
711.403 b7(1+)
958.555 PEP_0_0_0_0_0_
1042.5892 PEP_0_1*_0_0_0_
1161.6361 PEP_0_1_0_0_0_
1307.6882 PEP_0_1_1_0_0_
1364.7174 PEP_0_2_0_0_0_
1688.8066 PEP_2_2_0_0_0_
1834.8843 PEP_2_2_1_0_0_
1850.8624 PEP_3_2_0_0_0_

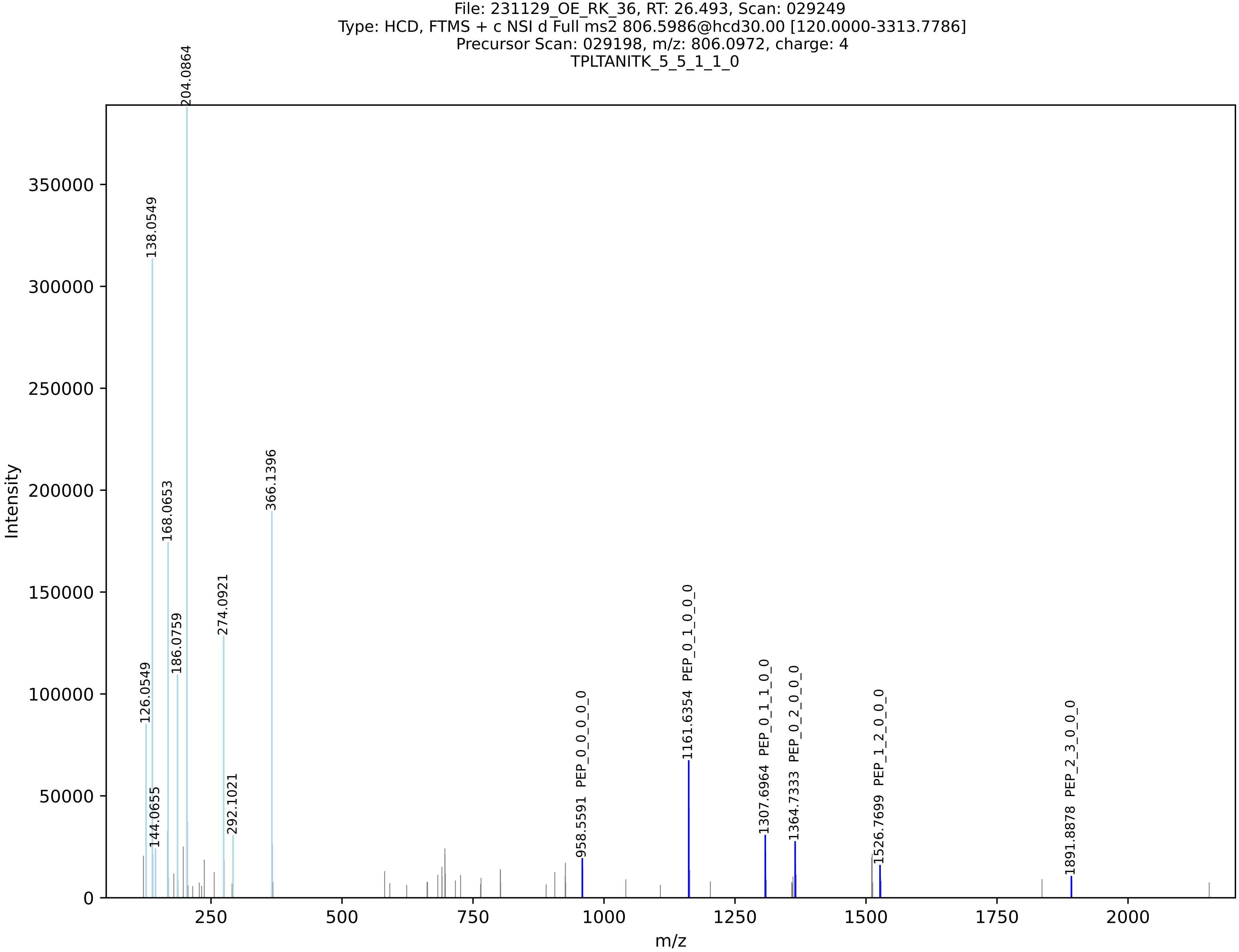

File: 231129_OE_RK_36, RT: 26.493, Scan: 029249
Type: HCD, FTMS + c NSI d Full ms2 806.5986@hcd30.00 [120.0000-3313.7786]
Precursor Scan: 029198, m/z: 806.0972, charge: 4
TPLTANITK_5_5_1_1_0
Intensity
m/z
204.0864
138.0549
168.0653
366.1396
274.0921
186.0759
126.0549
144.0655
292.1021
958.5591 PEP_0_0_0_0_0
1161.6354 PEP_0_1_0_0_0
1307.6964 PEP_0_1_1_0_0
1364.7333 PEP_0_2_0_0_0
1526.7699 PEP_1_2_0_0_0
1891.8878 PEP_2_3_0_0_0

File: 231129_OE_RK_36, RT: 26.6, Scan: 029401
Type: HCD, FTMS + c NSI d Full ms2 1026.1084@hcd30.00 [120.0000-3162.0337]
Precursor Scan: 029330, m/z: 1026.1084, charge: 3
TPLTANITK_5_5_0_1_0

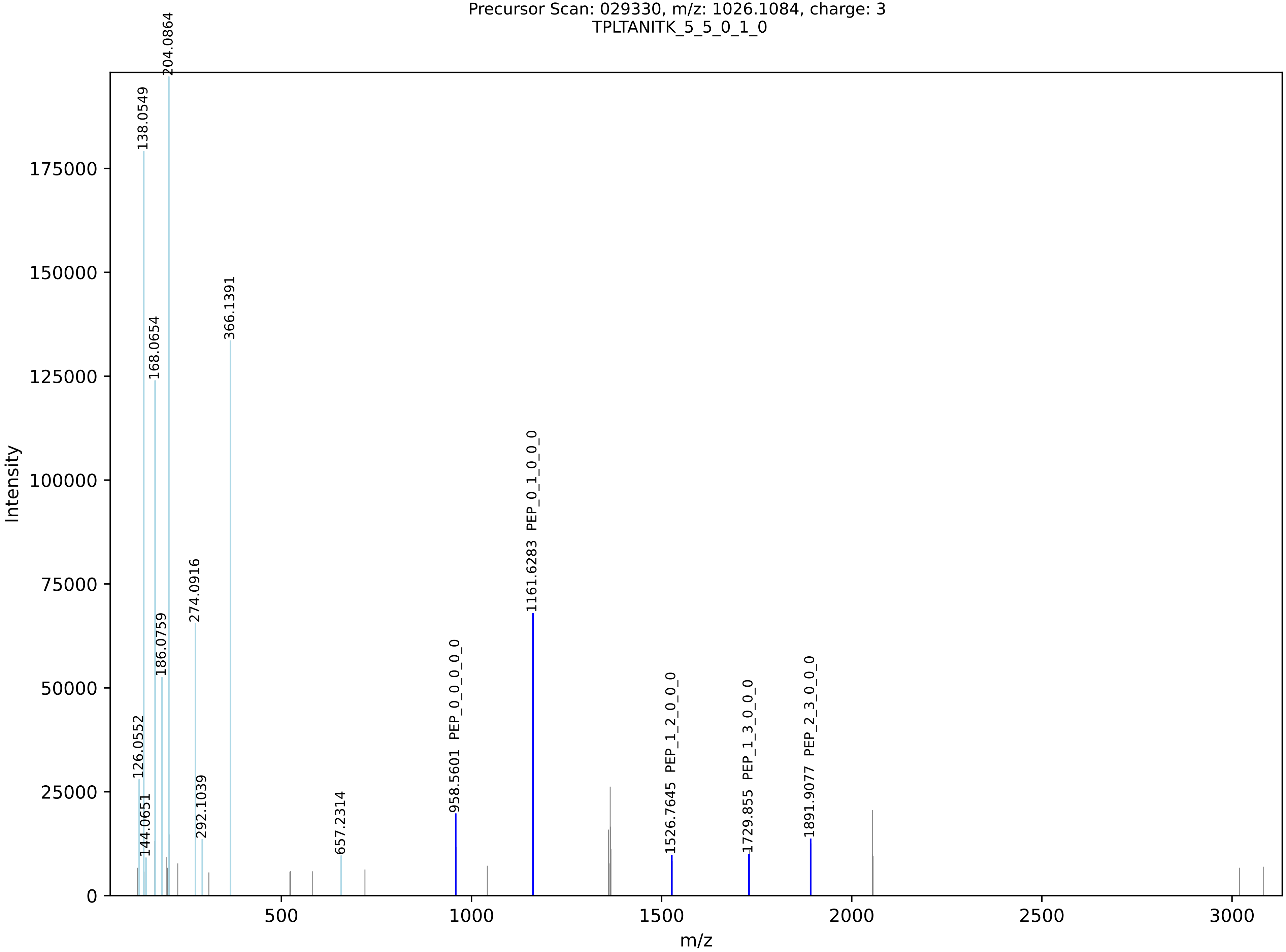

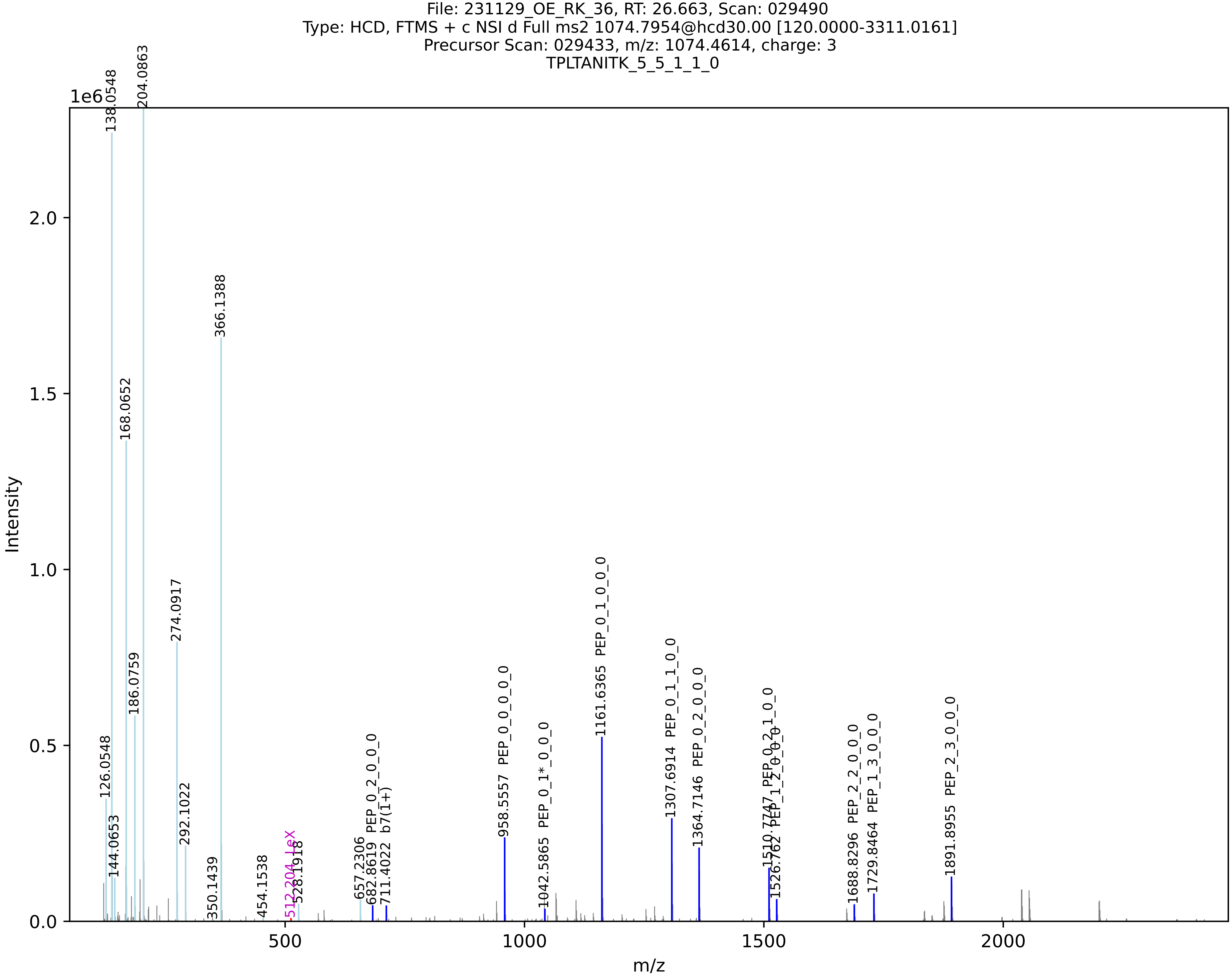
File: 231129_OE_RK_36, RT: 26.663, Scan: 029490
Type: HCD, FTMS + c NSI d Full ms2 1074.7954@hcd30.00 [120.0000-3311.0161]
Precursor Scan: 029433, m/z: 1074.4614, charge: 3
TPLTANITK_5_5_1_1_0
1e6
Intensity
m/z
0.0
0.5
1.0
1.5
2.0
500
1000
1500
2000
126.0548
138.0548
144.0653
168.0652
186.0759
204.0863
274.0917
292.1022
350.1439
366.1388
454.1538
512.204_LeX
528.1918
657.2306
682.8619 PEP_0_2_0_0_0
711.4022 b7(1+)
958.5557 PEP_0_0_0_0_0
1042.5865 PEP_0_1*_0_0_0
1161.6365 PEP_0_1_0_0_0
1307.6914 PEP_0_1_1_0_0
1364.7146 PEP_0_2_0_0_0
1510.7747 PEP_0_2_1_0_0
1526.762 PEP_1_2_0_0_0
1688.8296 PEP_2_2_0_0_0
1729.8464 PEP_1_3_0_0_0
1891.8955 PEP_2_3_0_0_0

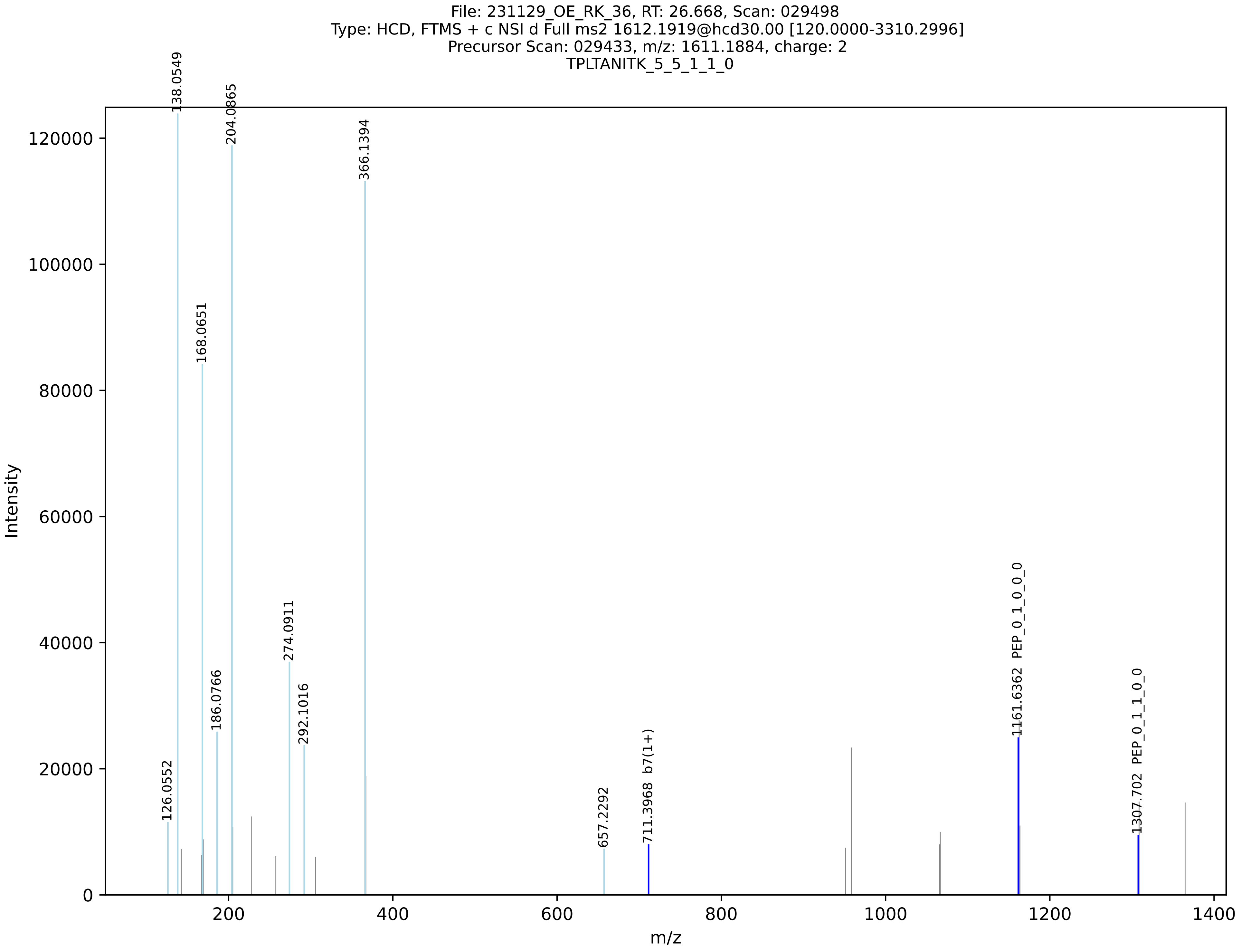
File: 231129_OE_RK_36, RT: 26.668, Scan: 029498
Type: HCD, FTMS + c NSI d Full ms2 1612.1919@hcd30.00 [120.0000-3310.2996]
Precursor Scan: 029433, m/z: 1611.1884, charge: 2
TPLTANITK_5_5_1_1_0
138.0549
204.0865
366.1394
168.0651
186.0766
274.0911
292.1016
126.0552
657.2292
711.3968 b7(1+)
1161.6362 PEP_0_1_0_0_0
1307.702 PEP_0_1_1_0_0
Intensity
m/z
0
20000
40000
60000
80000
100000
120000
200
400
600
800
1000
1200
1400

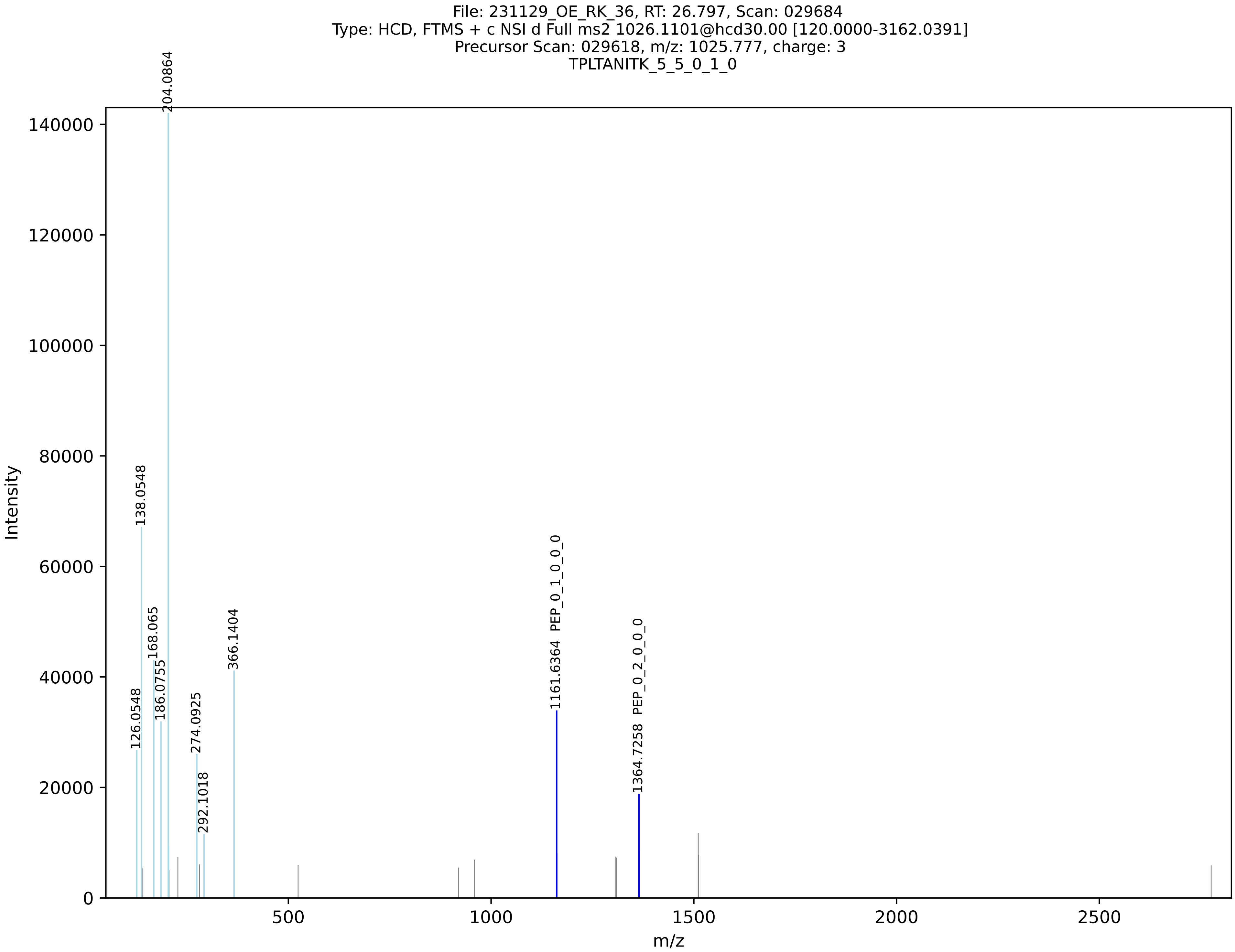
File: 231129_OE_RK_36, RT: 26.797, Scan: 029684
Type: HCD, FTMS + c NSI d Full ms2 1026.1101@hcd30.00 [120.0000-3162.0391]
Precursor Scan: 029618, m/z: 1025.777, charge: 3
TPLTANITK_5_5_0_1_0
Intensity
m/z
204.0864
138.0548
168.065
186.0755
126.0548
366.1404
274.0925
292.1018
1161.6364 PEP_0_1_0_0_0
1364.7258 PEP_0_2_0_0_0
0
20000
40000
60000
80000
100000
120000
140000
500
1000
1500
2000
2500

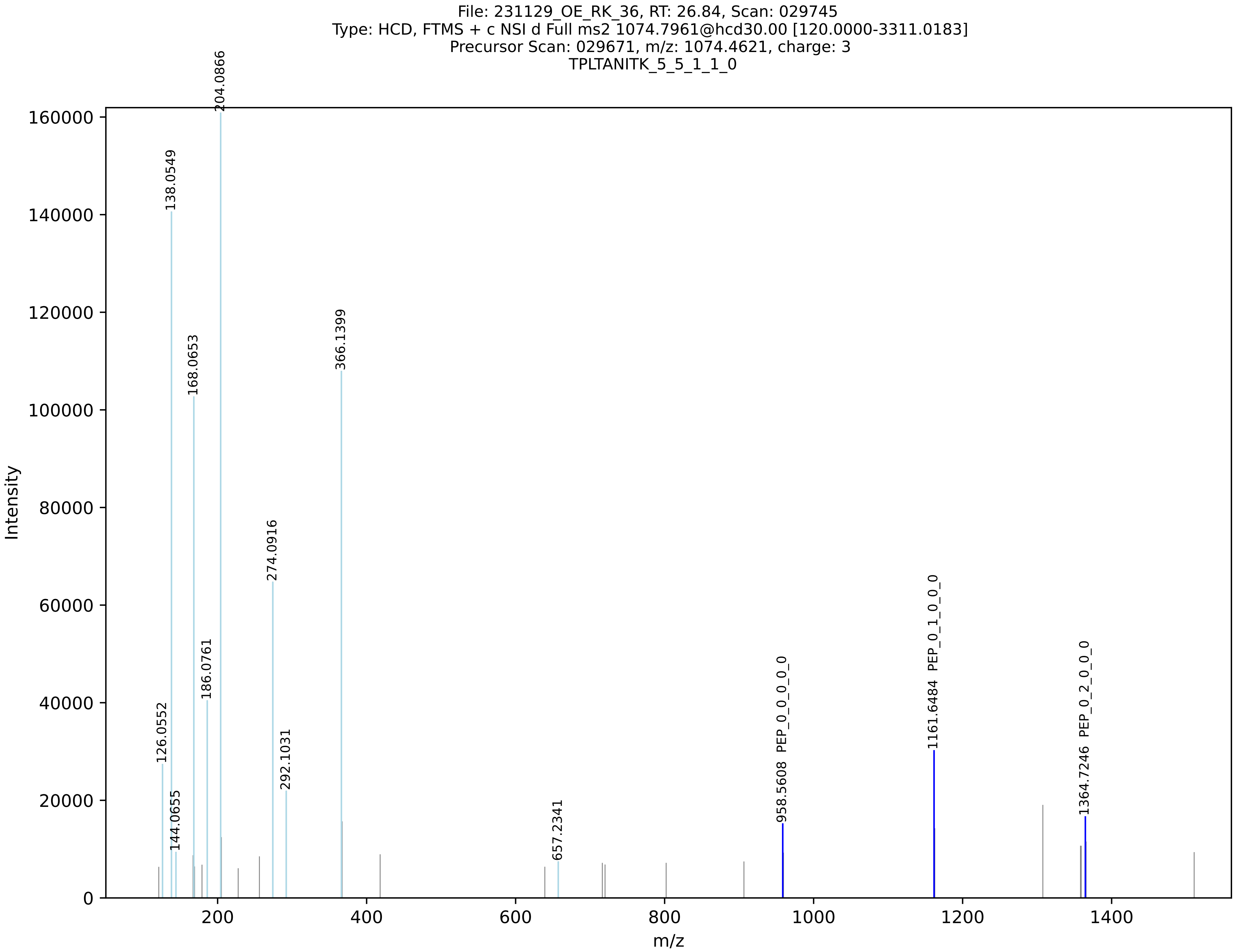

File: 231129_OE_RK_36, RT: 26.84, Scan: 029745
Type: HCD, FTMS + c NSI d Full ms2 1074.7961@hcd30.00 [120.0000-3311.0183]
Precursor Scan: 029671, m/z: 1074.4621, charge: 3
TPLTANITK_5_5_1_1_0
Intensity
m/z
0
20000
40000
60000
80000
100000
120000
140000
160000
200
400
600
800
1000
1200
1400
126.0552
138.0549
144.0655
168.0653
186.0761
204.0866
274.0916
292.1031
366.1399
657.2341
958.5608 PEP_0_0_0_0_0
1161.6484 PEP_0_1_0_0_0
1364.7246 PEP_0_2_0_0_0

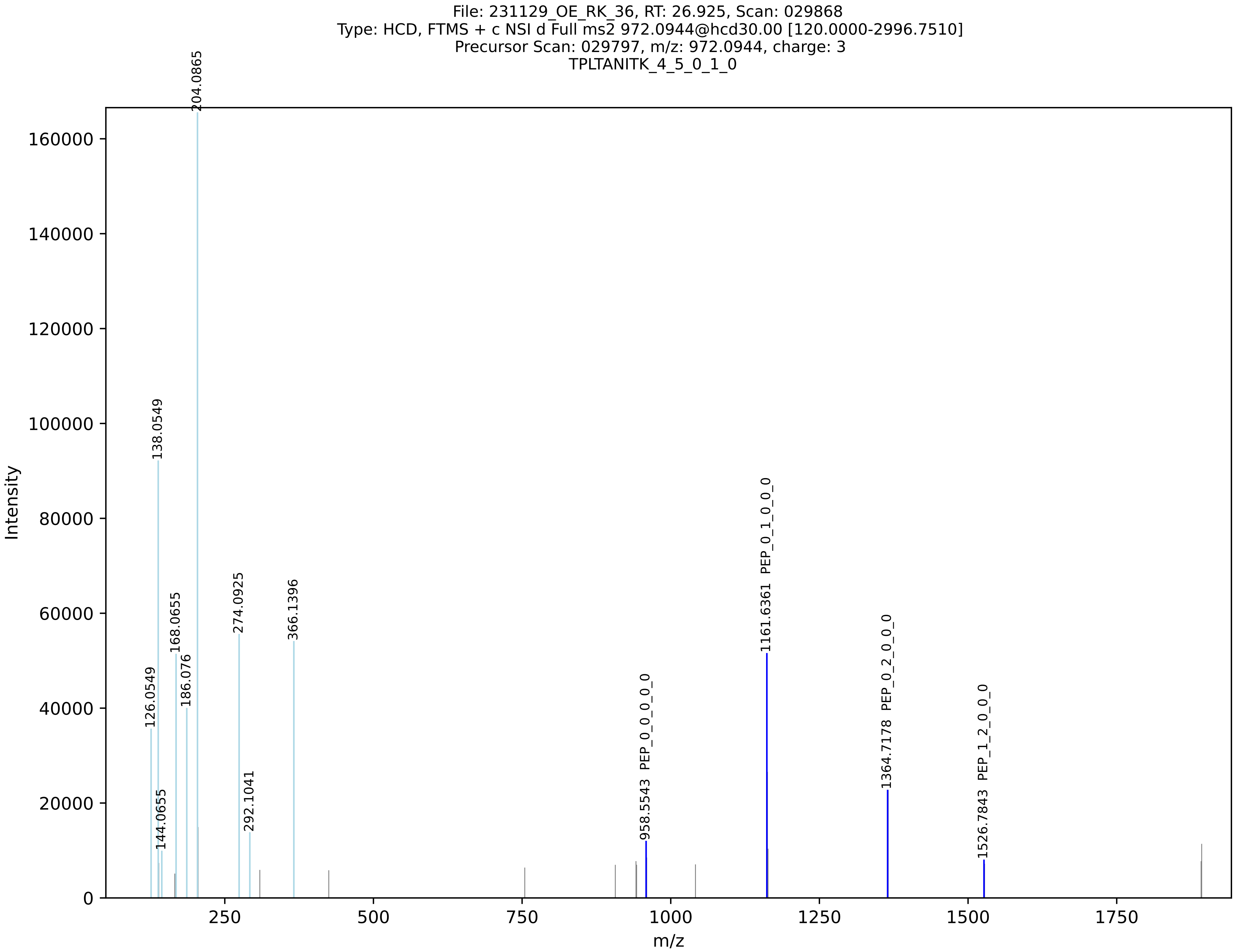

File: 231129_OE_RK_36, RT: 26.925, Scan: 029868
Type: HCD, FTMS + c NSI d Full ms2 972.0944@hcd30.00 [120.0000-2996.7510]
Precursor Scan: 029797, m/z: 972.0944, charge: 3
TPLTANITK_4_5_0_1_0
Intensity
m/z
204.0865
138.0549
168.0655
186.076
126.0549
144.0655
274.0925
292.1041
366.1396
958.5543 PEP_0_0_0_0_0
1161.6361 PEP_0_1_0_0_0
1364.7178 PEP_0_2_0_0_0
1526.7843 PEP_1_2_0_0_0

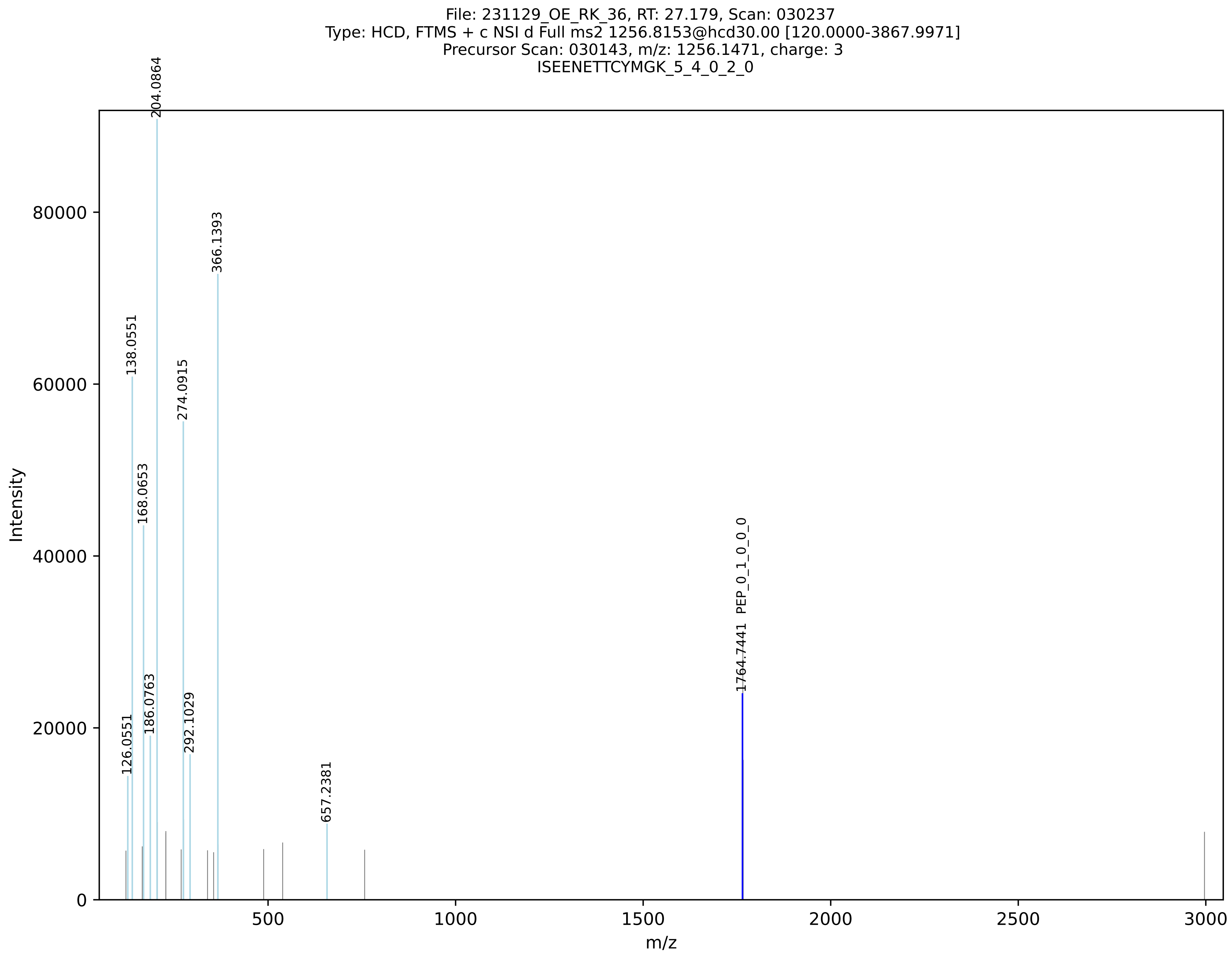

File: 231129_OE_RK_36, RT: 27.179, Scan: 030237
Type: HCD, FTMS + c NSI d Full ms2 1256.8153@hcd30.00 [120.0000-3867.9971]
Precursor Scan: 030143, m/z: 1256.1471, charge: 3
ISEENETTCYMGK_5_4_0_2_0
204.0864
366.1393
138.0551
274.0915
168.0653
186.0763
126.0551
292.1029
657.2381
1764.7441 PEP_0_1_0_0_0
Intensity
m/z
0
20000
40000
60000
80000
500
1000
1500
2000
2500
3000

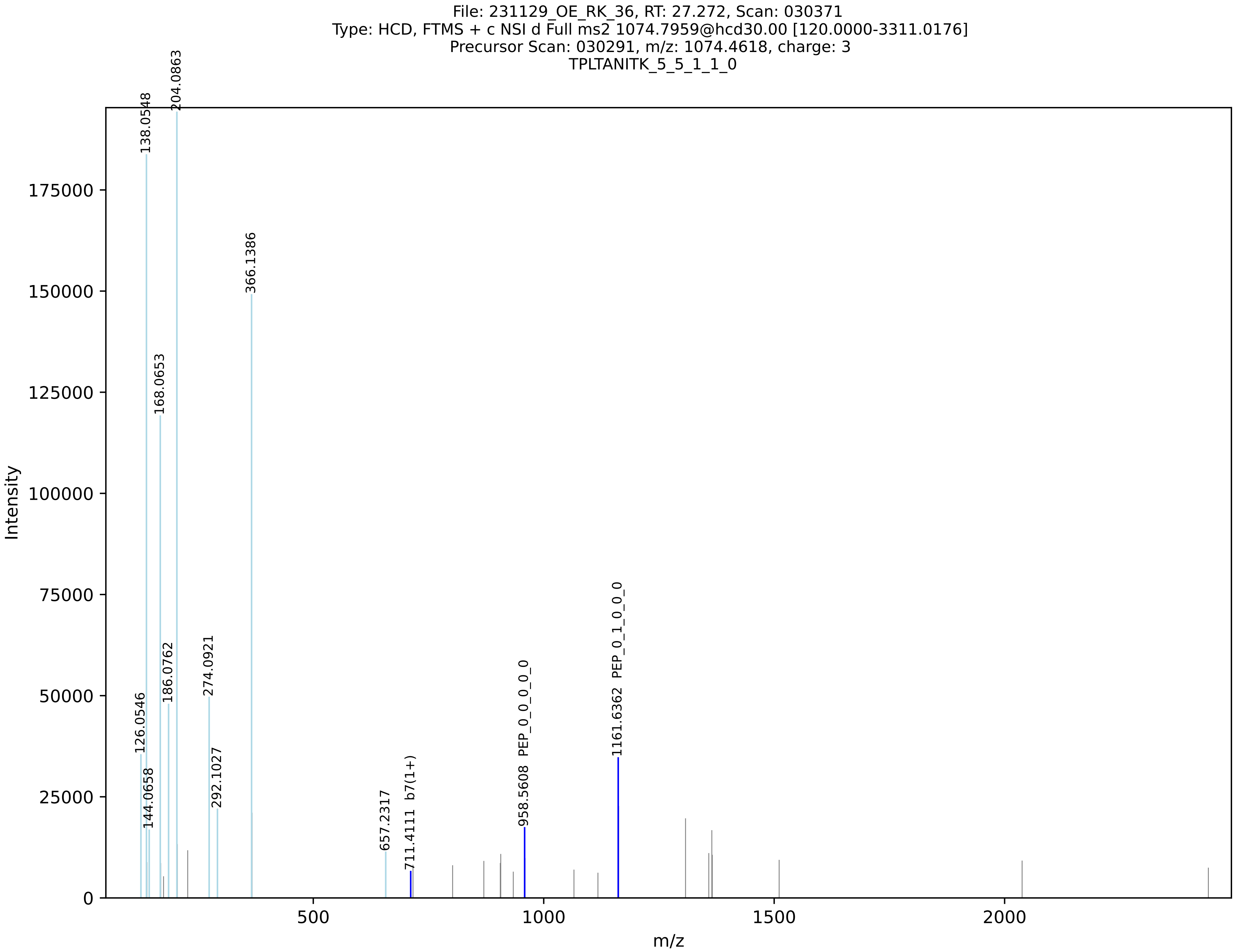

File: 231129_OE_RK_36, RT: 27.272, Scan: 030371
Type: HCD, FTMS + c NSI d Full ms2 1074.7959@hcd30.00 [120.0000-3311.0176]
Precursor Scan: 030291, m/z: 1074.4618, charge: 3
TPLTANITK_5_5_1_1_0
Intensity
m/z
204.0863
138.0548
366.1386
168.0653
186.0762
274.0921
126.0546
292.1027
144.0658
657.2317
711.4111 b7(1+)
958.5608 PEP_0_0_0_0_0
1161.6362 PEP_0_1_0_0_0

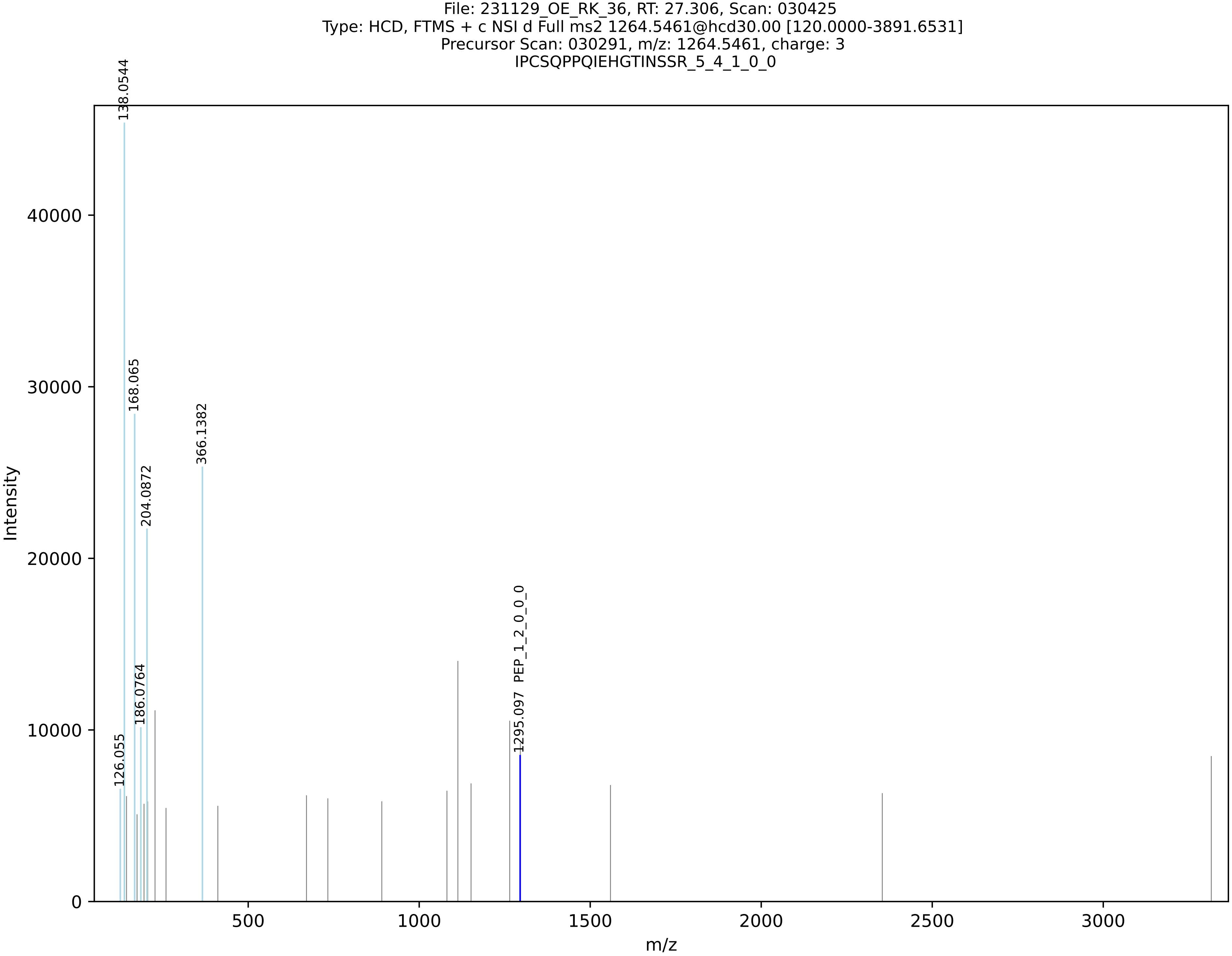
File: 231129_OE_RK_36, RT: 27.306, Scan: 030425
Type: HCD, FTMS + c NSI d Full ms2 1264.5461@hcd30.00 [120.0000-3891.6531]
Precursor Scan: 030291, m/z: 1264.5461, charge: 3
IPCSQPPQIEHGTINSSR_5_4_1_0_0
138.0544
168.065
366.1382
204.0872
186.0764
126.055
1295.097 PEP_1_2_0_0_0
Intensity
m/z
0
10000
20000
30000
40000
500
1000
1500
2000
2500
3000

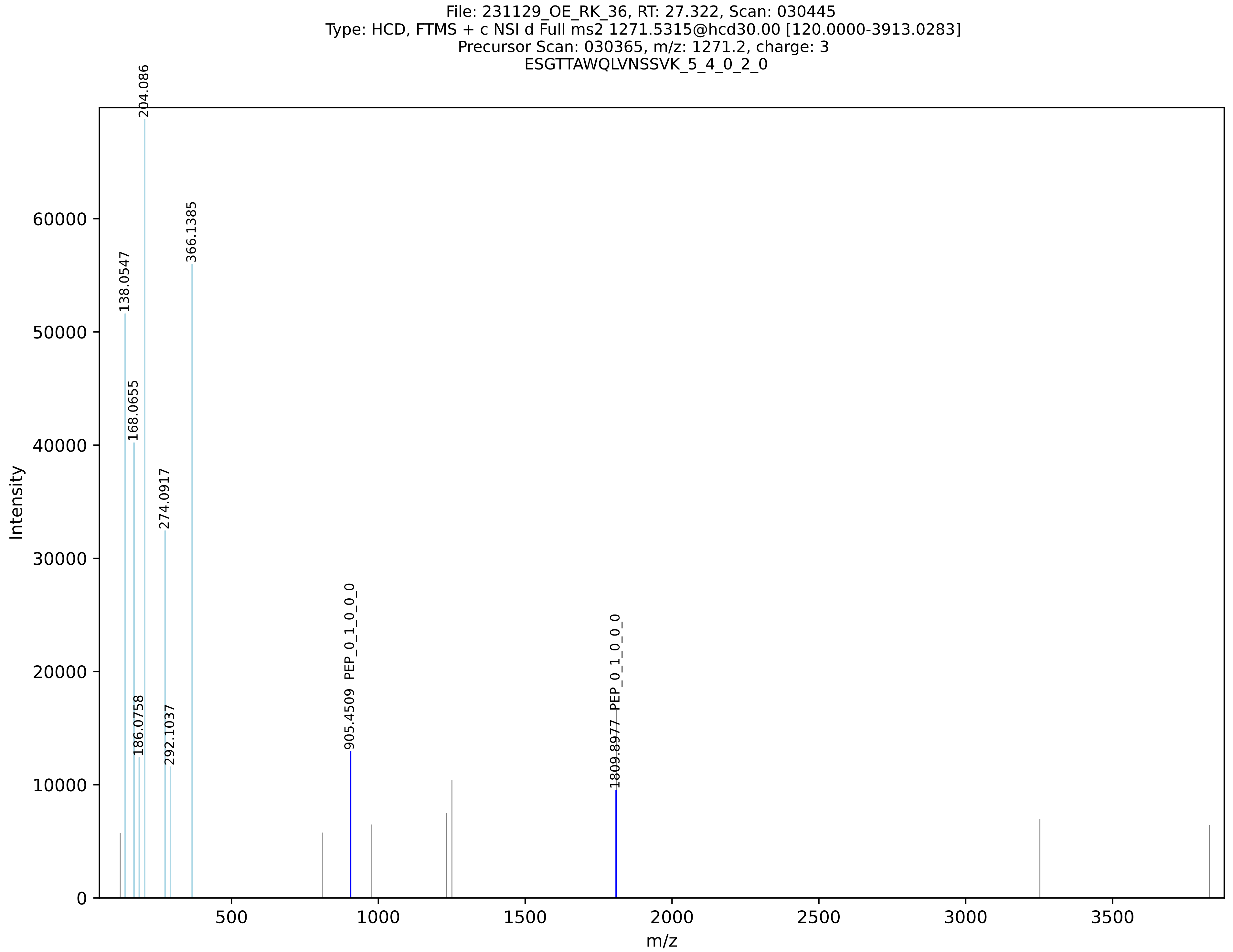

File: 231129_OE_RK_36, RT: 27.322, Scan: 030445
Type: HCD, FTMS + c NSI d Full ms2 1271.5315@hcd30.00 [120.0000-3913.0283]
Precursor Scan: 030365, m/z: 1271.2, charge: 3
ESGTTAWQLVNSSVK_5_4_0_2_0
Intensity
m/z
204.086
366.1385
138.0547
168.0655
274.0917
186.0758
292.1037
905.4509 PEP_0_1_0_0_0
1809.8977 PEP_0_1_0_0_0
0
10000
20000
30000
40000
50000
60000
500
1000
1500
2000
2500
3000
3500

File: 231129_OE_RK_36, RT: 27.53, Scan: 030750
Type: HCD, FTMS + c NSI d Full ms2 1268.5450@hcd30.00 [120.0000-2609.2600]
Precursor Scan: 030657, m/z: 1268.0448, charge: 2
IDSTGNVTNELR_5_2_0_0_0

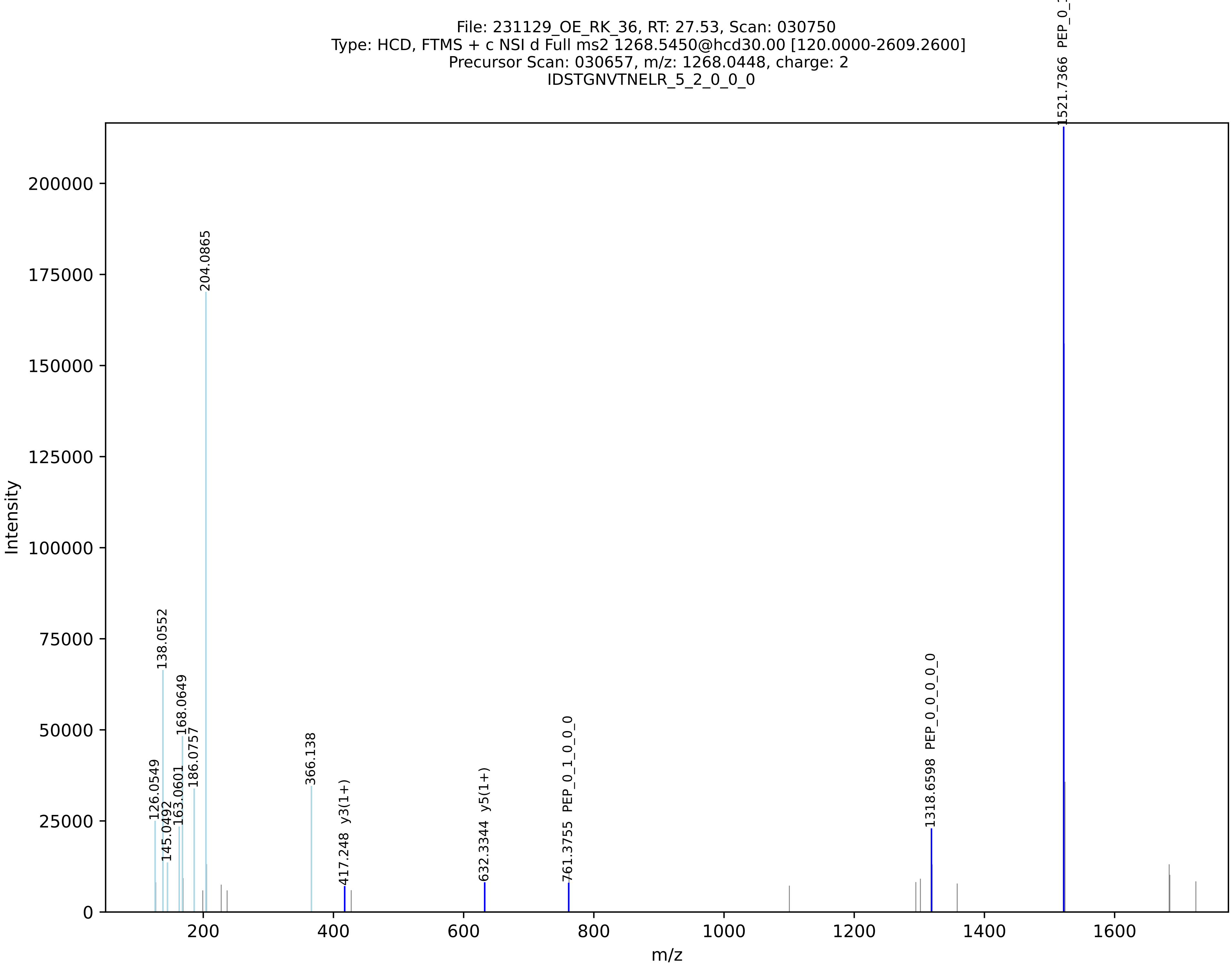

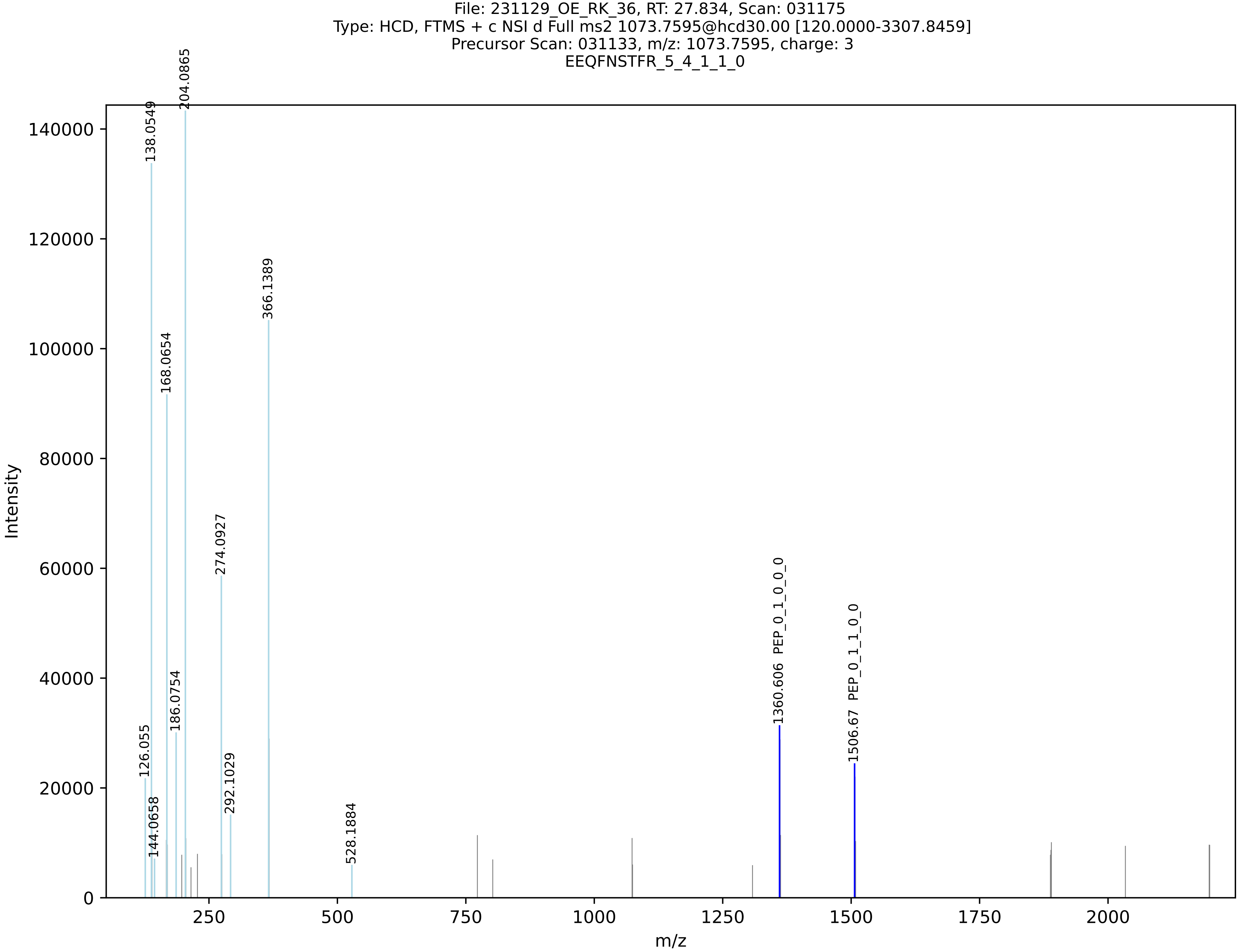
File: 231129_OE_RK_36, RT: 27.834, Scan: 031175
Type: HCD, FTMS + c NSI d Full ms2 1073.7595@hcd30.00 [120.0000-3307.8459]
Precursor Scan: 031133, m/z: 1073.7595, charge: 3
EEQFNSTFR_5_4_1_1_0
Intensity
m/z
204.0865
138.0549
366.1389
168.0654
274.0927
186.0754
126.055
292.1029
144.0658
528.1884
1360.606 PEP_0_1_0_0_0
1506.67 PEP_0_1_1_0_0
0
20000
40000
60000
80000
100000
120000
140000
250
500
750
1000
1250
1500
1750
2000

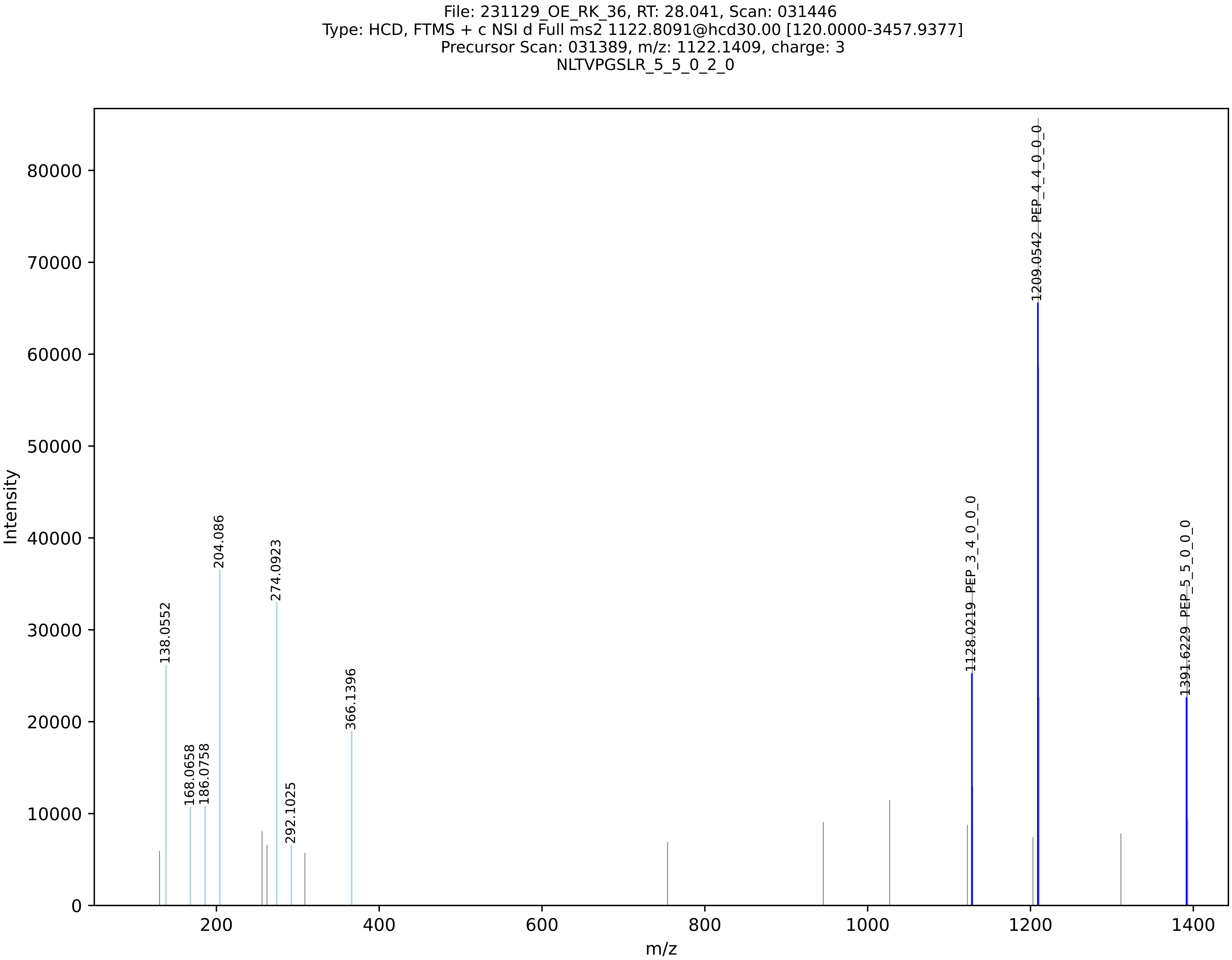
File: 231129_OE_RK_36, RT: 28.041, Scan: 031446
Type: HCD, FTMS + c NSI d Full ms2 1122.8091@hcd30.00 [120.0000-3457.9377]
Precursor Scan: 031389, m/z: 1122.1409, charge: 3
NLTVPGSLR_5_5_0_2_0
Intensity
m/z
138.0552
168.0658
186.0758
204.086
274.0923
292.1025
366.1396
1128.0219_PEP_3_4_0_0_0
1209.0542_PEP_4_4_0_0_0
1391.6229_PEP_5_5_0_0_0

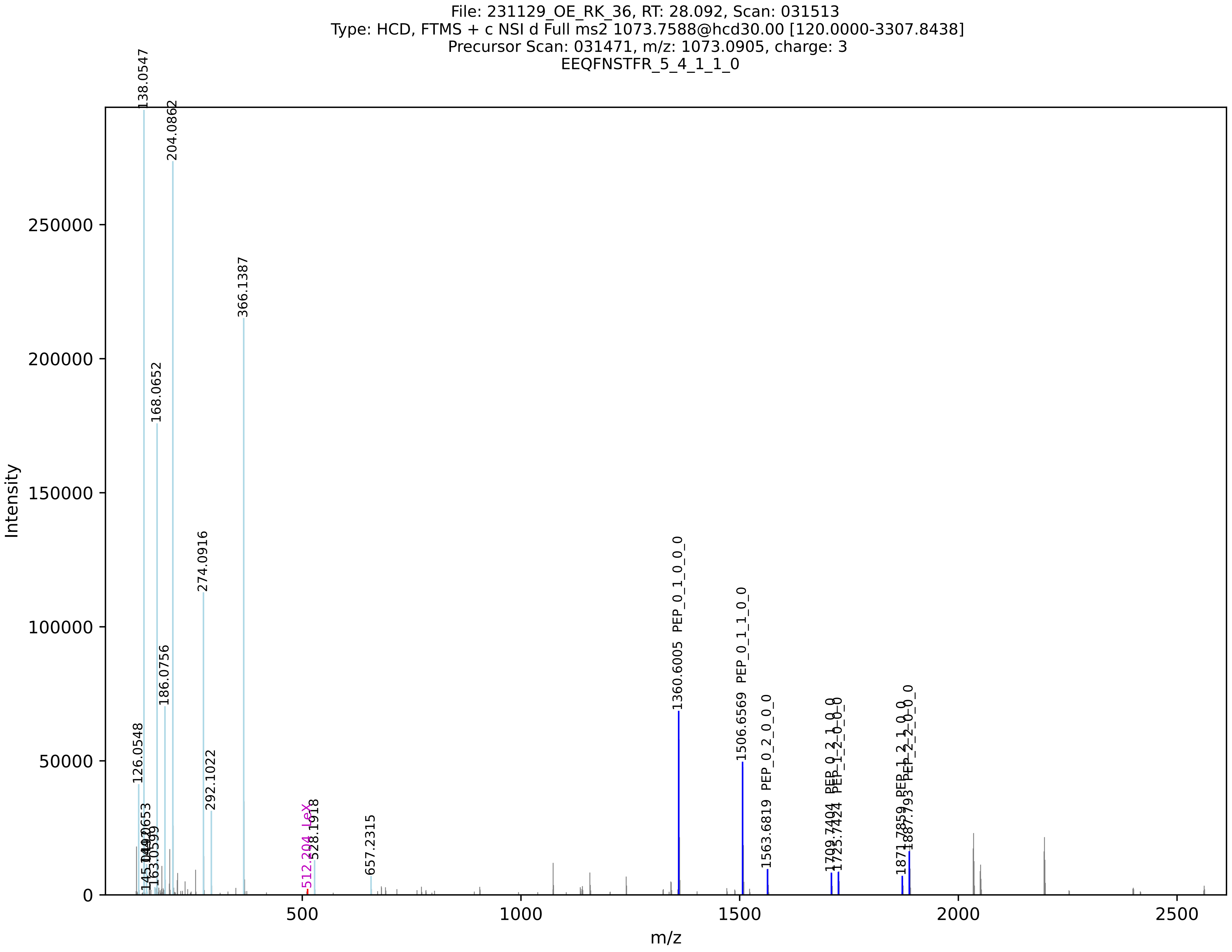
File: 231129_OE_RK_36, RT: 28.092, Scan: 031513
Type: HCD, FTMS + c NSI d Full ms2 1073.7588@hcd30.00 [120.0000-3307.8438]
Precursor Scan: 031471, m/z: 1073.0905, charge: 3
EEQFNSTFR_5_4_1_1_0
Intensity
m/z
138.0547
204.0862
366.1387
168.0652
274.0916
186.0756
126.0548
292.1022
163.0599
512.204 LeX
528.1918
657.2315
1360.6005 PEP_0_1_0_0_0
1506.6569 PEP_0_1_1_0_0
1563.6819 PEP_0_2_0_0_0
1709.7404 PEP_0_2_1_0_0
1725.7424 PEP_1_2_0_0_0
1871.7859 PEP_1_2_1_0_0
1887.793 PEP_2_2_0_0_0
0
50000
100000
150000
200000
250000
500
1000
1500
2000
2500

File: 231129_OE_RK_36, RT: 28.14, Scan: 031584
Type: HCD, FTMS + c NSI d Full ms2 1122.1385@hcd30.00 [120.0000-3455.8860]
Precursor Scan: 031471, m/z: 1121.8057, charge: 3
NNTRGPGLK_5_5_2_1_0

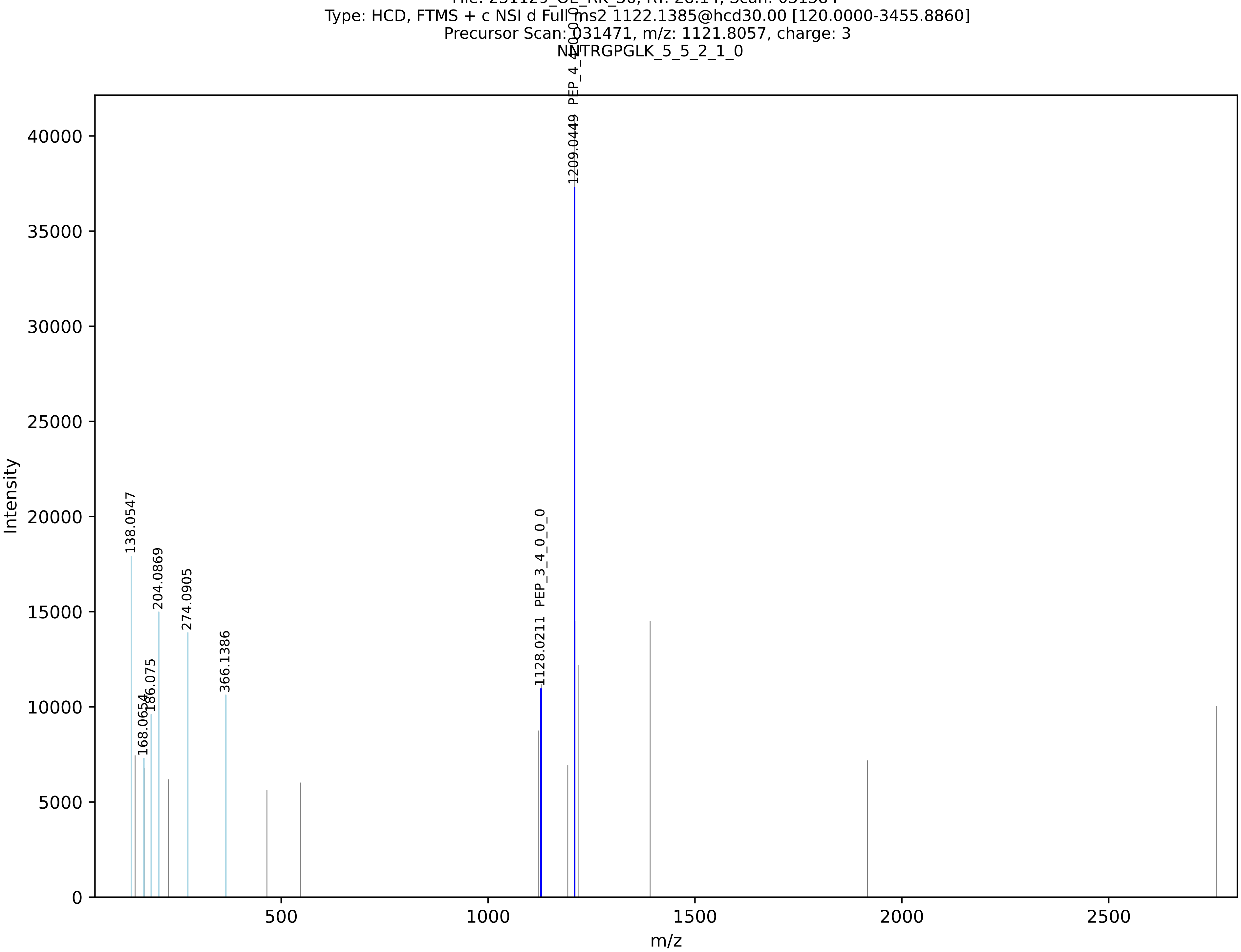

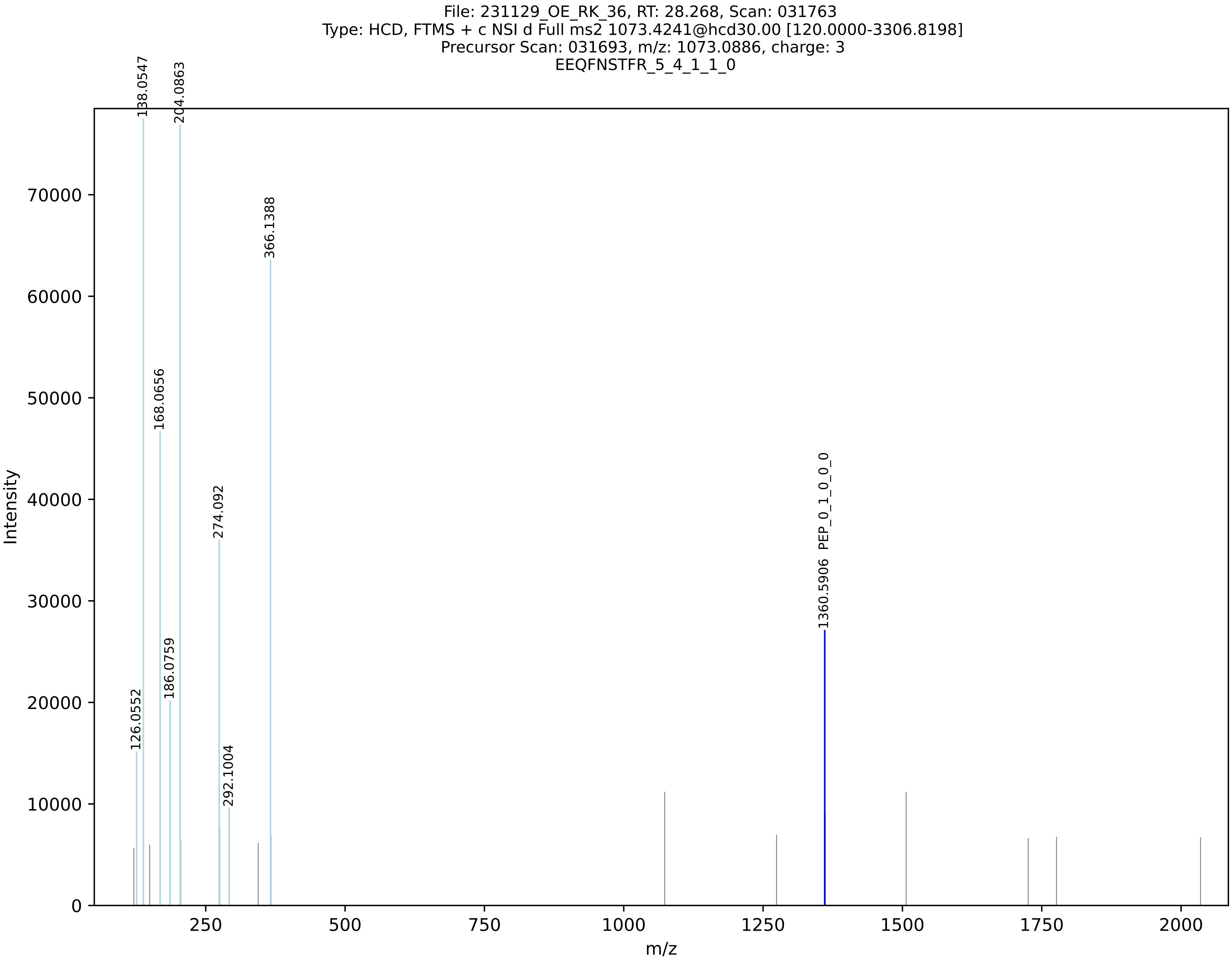

File: 231129_OE_RK_36, RT: 28.268, Scan: 031763
Type: HCD, FTMS + c NSI d Full ms2 1073.4241@hcd30.00 [120.0000-3306.8198]
Precursor Scan: 031693, m/z: 1073.0886, charge: 3
EEQFNSTFR_5_4_1_1_0
138.0547
204.0863
366.1388
168.0656
274.092
186.0759
126.0552
292.1004
1360.5906 PEP_0_1_0_0_0
Intensity
m/z
0
10000
20000
30000
40000
50000
60000
70000
250
500
750
1000
1250
1500
1750
2000

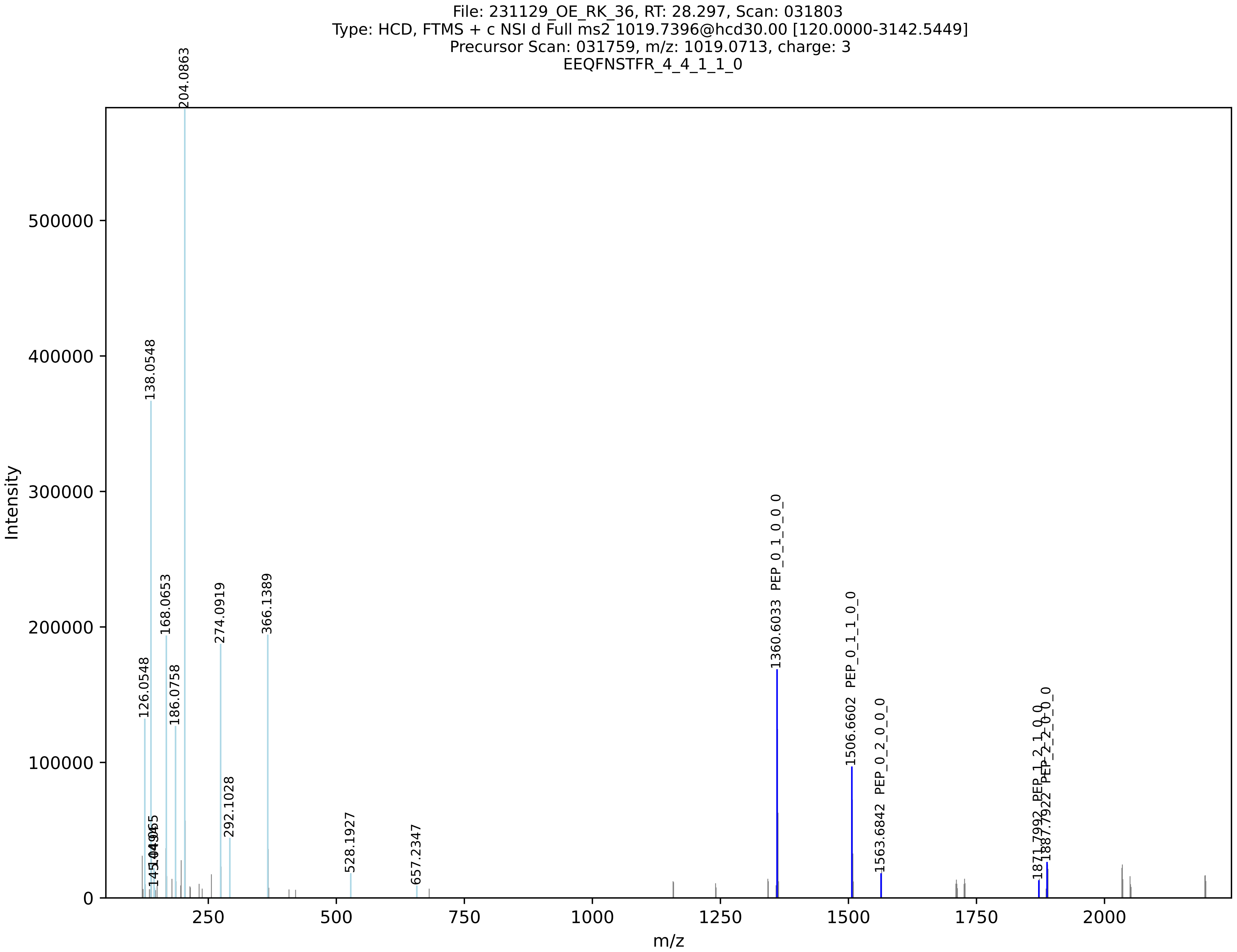

File: 231129_OE_RK_36, RT: 28.297, Scan: 031803
Type: HCD, FTMS + c NSI d Full ms2 1019.7396@hcd30.00 [120.0000-3142.5449]
Precursor Scan: 031759, m/z: 1019.0713, charge: 3
EEQFNSTFR_4_4_1_1_0
Intensity
m/z
204.0863
138.0548
126.0548
168.0653
186.0758
274.0919
292.1028
366.1389
528.1927
657.2347
1360.6033 PEP_0_1_0_0_0
1506.6602 PEP_0_1_1_0_0
1563.6842 PEP_0_2_0_0_0
1871.7992 PEP_1_2_1_0_0
1887.7922 PEP_2_2_0_0_0

File: 231129_OE_RK_36, RT: 28.33, Scan: 031847
Type: HCD, FTMS + c NSI d Full ms2 1528.6056@hcd30.00 [120.0000-3139.7832]
Precursor Scan: 031800, m/z: 1528.1041, charge: 2
EEQFNSTFR_4_4_1_1_0

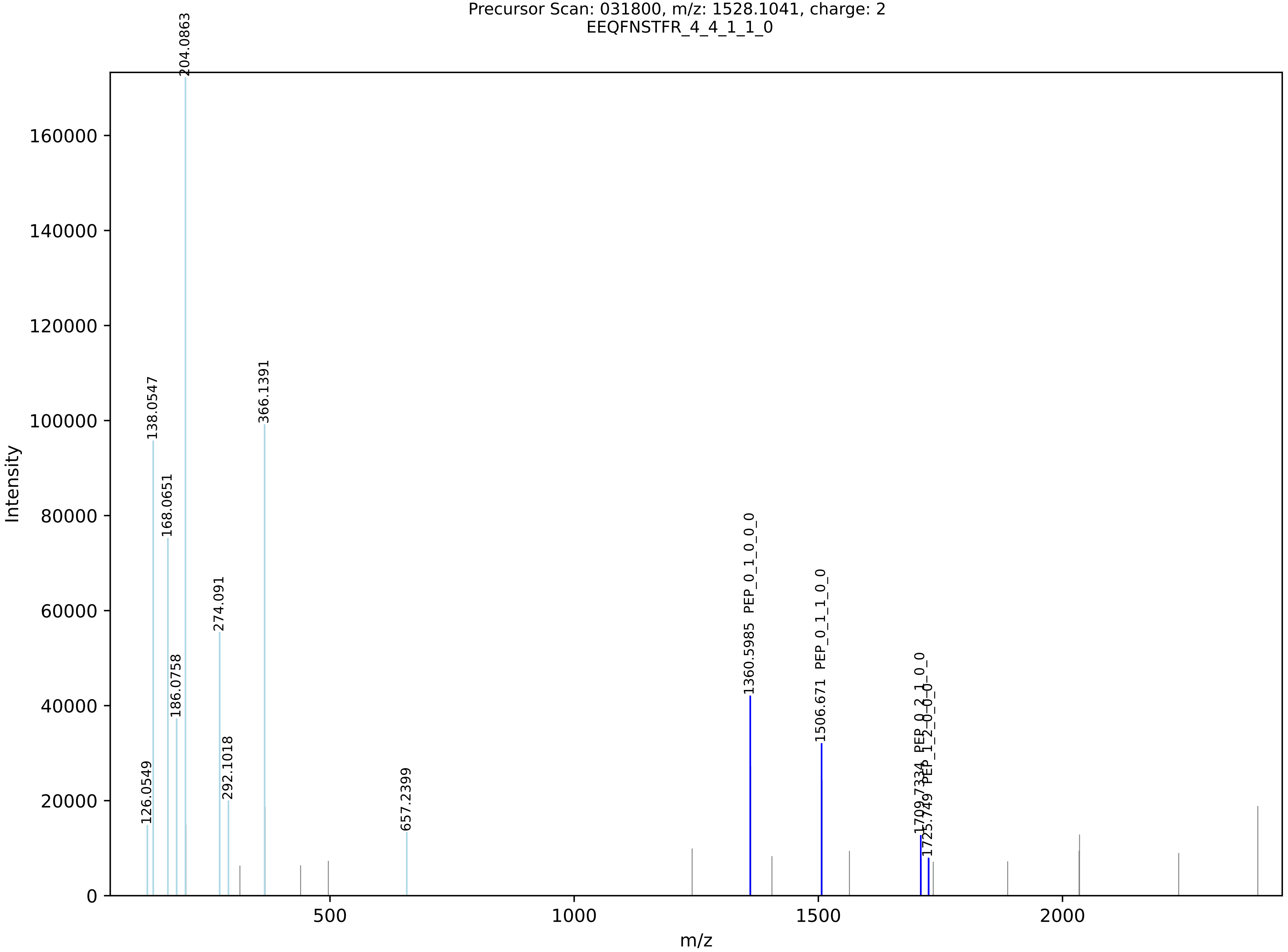

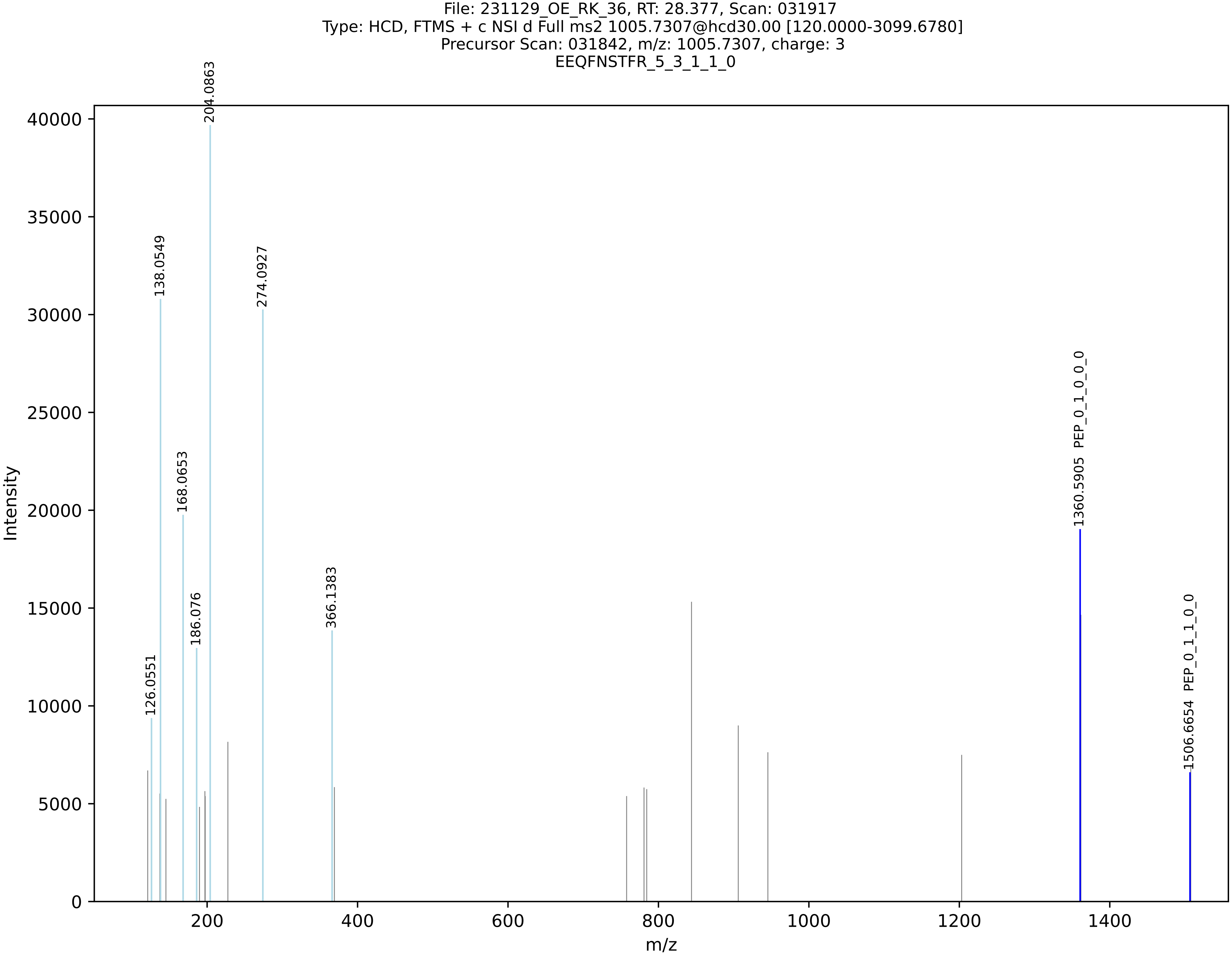
File: 231129_OE_RK_36, RT: 28.377, Scan: 031917
Type: HCD, FTMS + c NSI d Full ms2 1005.7307@hcd30.00 [120.0000-3099.6780]
Precursor Scan: 031842, m/z: 1005.7307, charge: 3
EEQFNSTFR_5_3_1_1_0
Intensity
m/z
204.0863
138.0549
274.0927
168.0653
186.076
126.0551
366.1383
1360.5905 PEP_0_1_0_0_0
1506.6654 PEP_0_1_1_0_0
0
5000
10000
15000
20000
25000
30000
35000
40000
200
400
600
800
1000
1200
1400

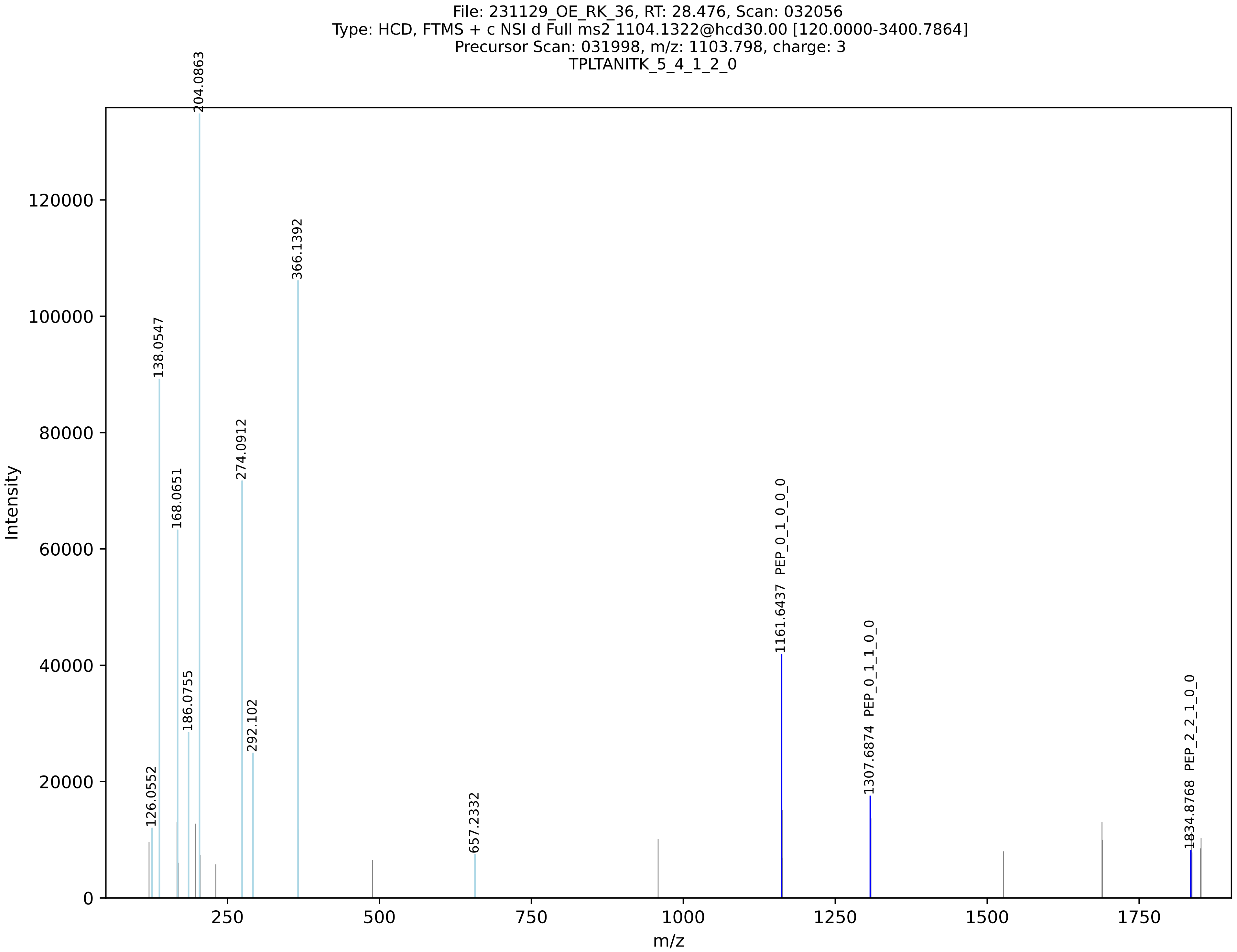
File: 231129_OE_RK_36, RT: 28.476, Scan: 032056
Type: HCD, FTMS + c NSI d Full ms2 1104.1322@hcd30.00 [120.0000-3400.7864]
Precursor Scan: 031998, m/z: 1103.798, charge: 3
TPLTANITK_5_4_1_2_0
Intensity
m/z
204.0863
366.1392
138.0547
274.0912
168.0651
186.0755
292.102
126.0552
657.2332
1161.6437 PEP_0_1_0_0_0
1307.6874 PEP_0_1_1_0_0
1834.8768 PEP_2_2_1_0_0
0
20000
40000
60000
80000
100000
120000
250
500
750
1000
1250
1500
1750

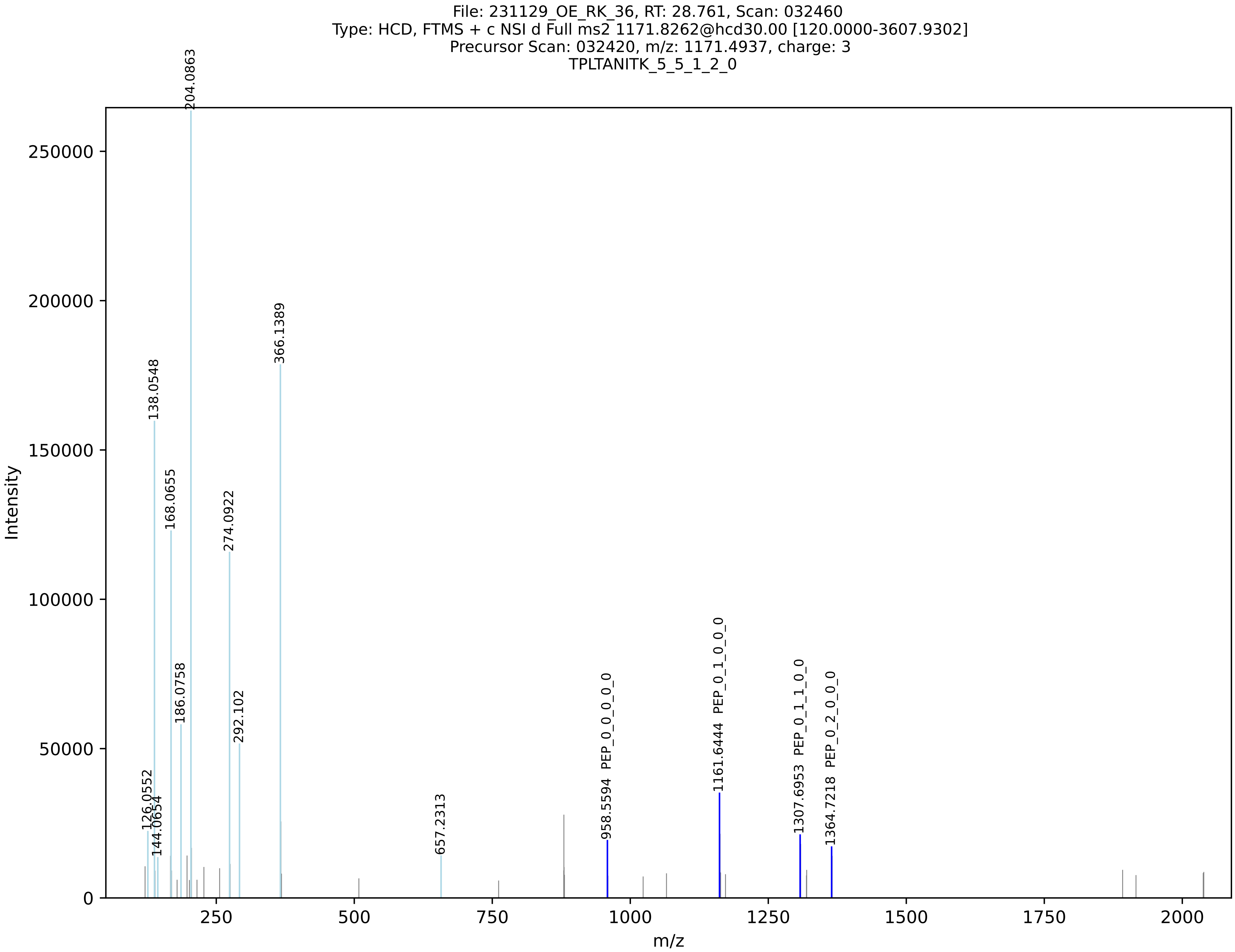
File: 231129_OE_RK_36, RT: 28.761, Scan: 032460
Type: HCD, FTMS + c NSI d Full ms2 1171.8262@hcd30.00 [120.0000-3607.9302]
Precursor Scan: 032420, m/z: 1171.4937, charge: 3
TPLTANITK_5_5_1_2_0
Intensity
m/z
204.0863
366.1389
138.0548
168.0655
274.0922
186.0758
292.102
126.0552
144.0654
657.2313
958.5594 PEP_0_0_0_0_0
1161.6444 PEP_0_1_0_0_0
1307.6953 PEP_0_1_1_0_0
1364.7218 PEP_0_2_0_0_0
0
50000
100000
150000
200000
250000
250
500
750
1000
1250
1500
1750
2000

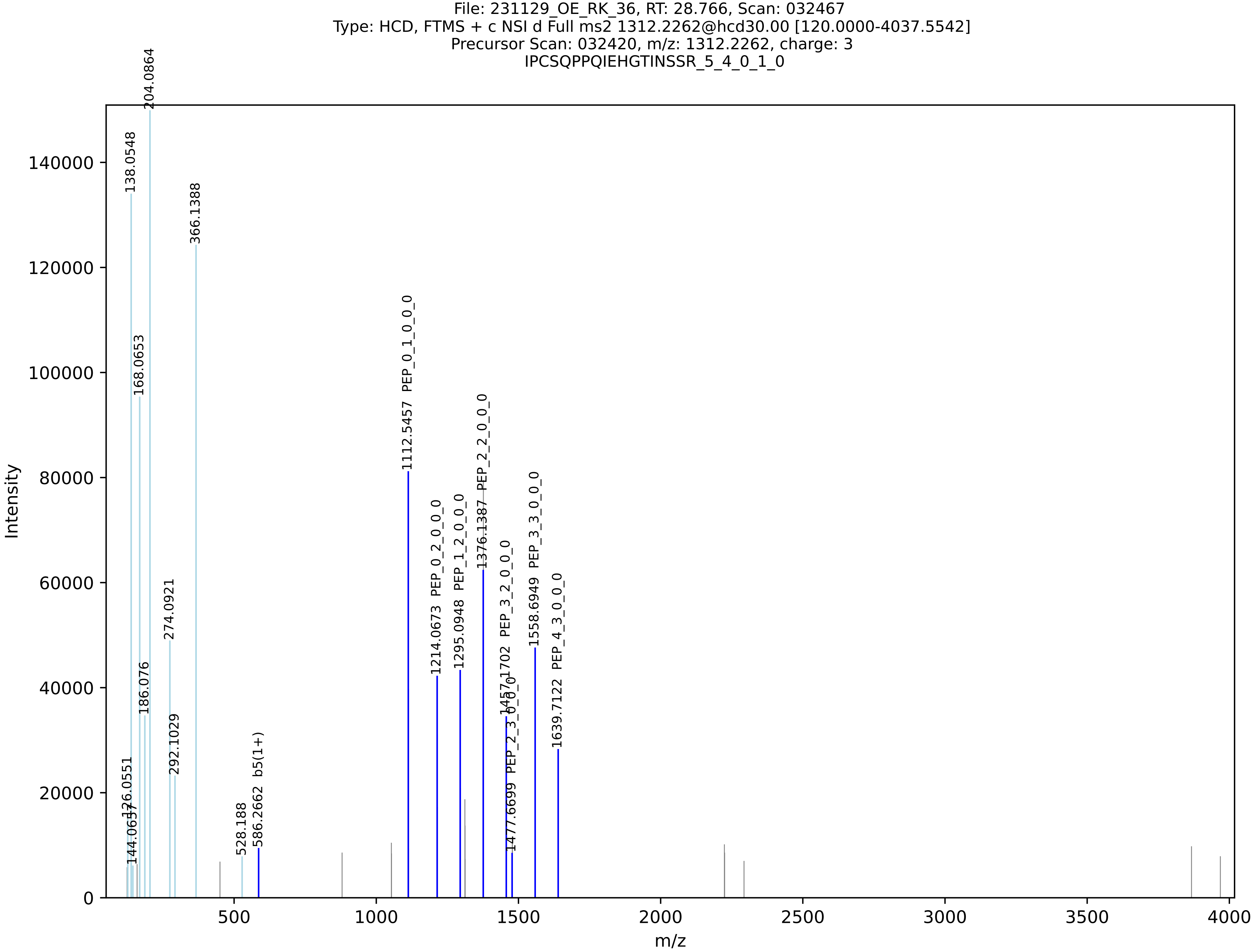
File: 231129_OE_RK_36, RT: 28.766, Scan: 032467
Type: HCD, FTMS + c NSI d Full ms2 1312.2262@hcd30.00 [120.0000-4037.5542]
Precursor Scan: 032420, m/z: 1312.2262, charge: 3
IPCSQPPQIEHGTINSSR_5_4_0_1_0
Intensity
m/z
204.0864
138.0548
366.1388
168.0653
274.0921
186.076
292.1029
126.0551
144.0657
528.188
586.2662 b5(1+)
1112.5457 PEP_0_1_0_0_0
1214.0673 PEP_0_2_0_0_0
1295.0948 PEP_1_2_0_0_0
1376.1387 PEP_2_2_0_0_0
1457.1702 PEP_3_2_0_0_0
1477.6699 PEP_2_3_0_0_0
1558.6949 PEP_3_3_0_0_0
1639.7122 PEP_4_3_0_0_0

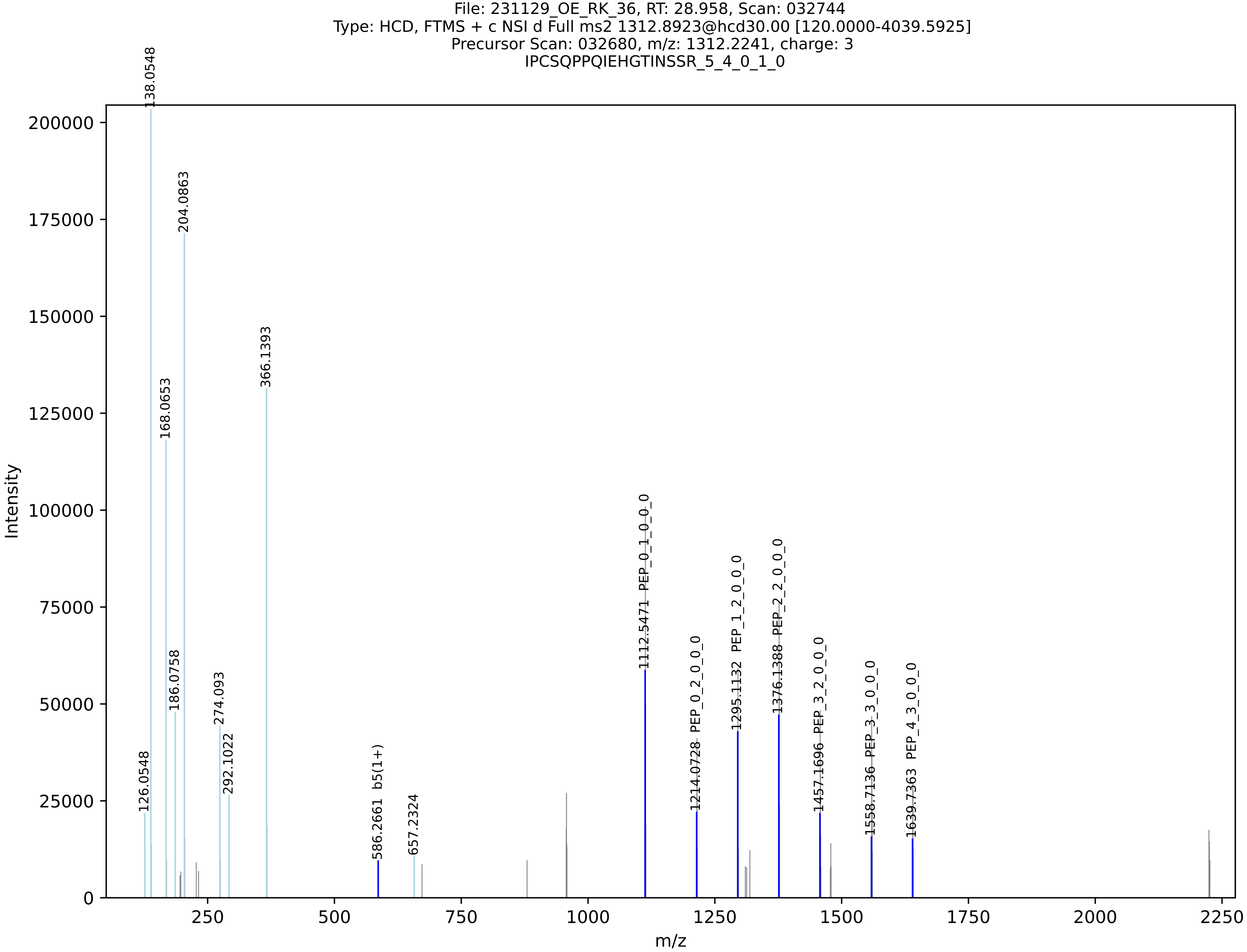
File: 231129_OE_RK_36, RT: 28.958, Scan: 032744
Type: HCD, FTMS + c NSI d Full ms2 1312.8923@hcd30.00 [120.0000-4039.5925]
Precursor Scan: 032680, m/z: 1312.2241, charge: 3
IPCSQPPQIEHGTINSSR_5_4_0_1_0
Intensity
m/z
138.0548
204.0863
168.0653
366.1393
186.0758
274.093
292.1022
126.0548
586.2661 b5(1+)
657.2324
1112.5471 PEP_0_1_0_0_0
1214.0728 PEP_0_2_0_0_0
1295.1132 PEP_1_2_0_0_0
1376.1388 PEP_2_2_0_0_0
1457.1696 PEP_3_2_0_0_0
1558.7136 PEP_3_3_0_0_0
1639.7363 PEP_4_3_0_0_0

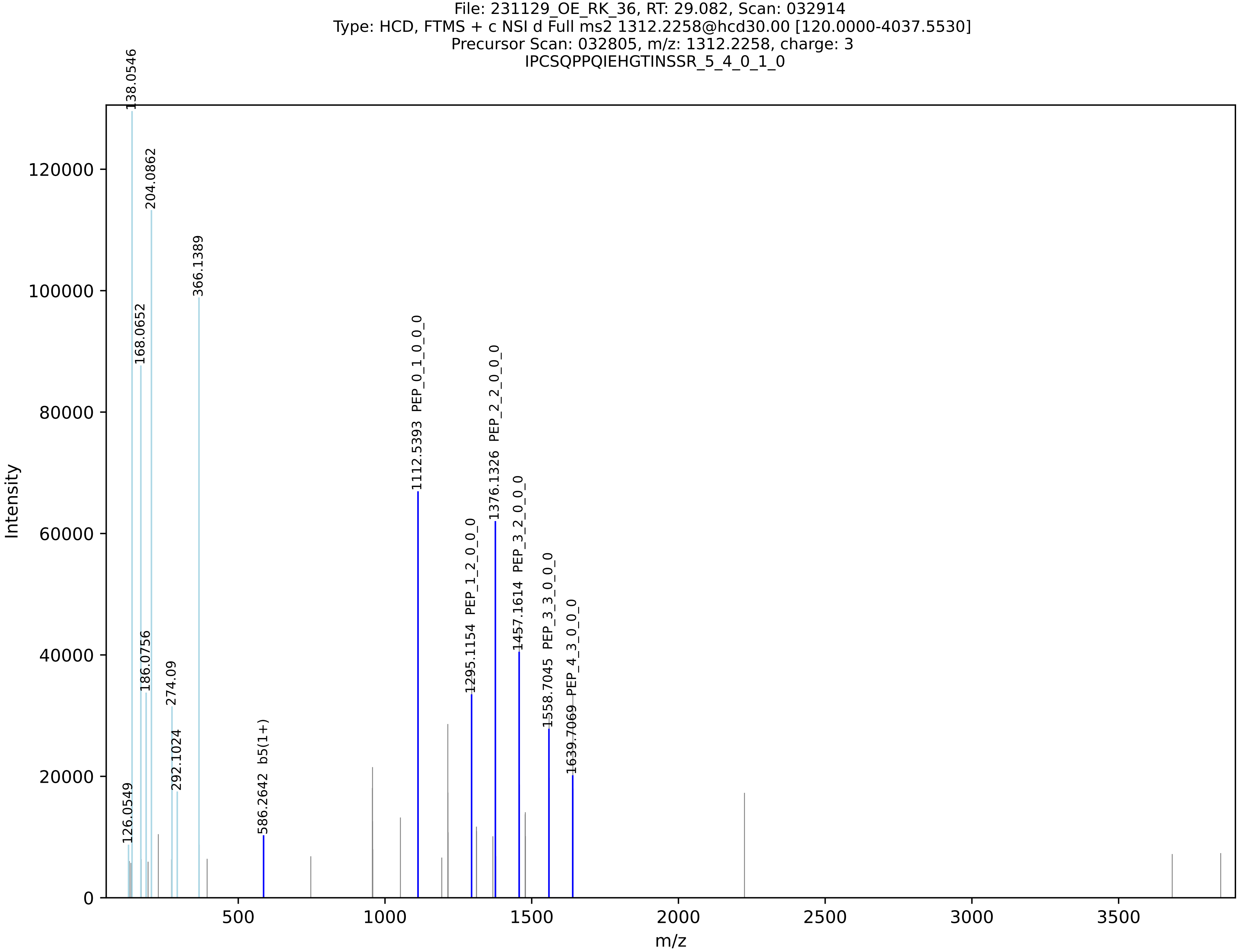
File: 231129_OE_RK_36, RT: 29.082, Scan: 032914
Type: HCD, FTMS + c NSI d Full ms2 1312.2258@hcd30.00 [120.0000-4037.5530]
Precursor Scan: 032805, m/z: 1312.2258, charge: 3
IPCSQPPQIEHGTINSSR_5_4_0_1_0
138.0546
204.0862
366.1389
168.0652
186.0756
274.09
292.1024
126.0549
586.2642 b5(1+)
1112.5393 PEP_0_1_0_0_0_
1295.1154 PEP_1_2_0_0_0_
1376.1326 PEP_2_2_0_0_0_
1457.1614 PEP_3_2_0_0_0_
1558.7045 PEP_3_3_0_0_0_
1639.7069 PEP_4_3_0_0_0_
Intensity
m/z
0
20000
40000
60000
80000
100000
120000
500
1000
1500
2000
2500
3000
3500

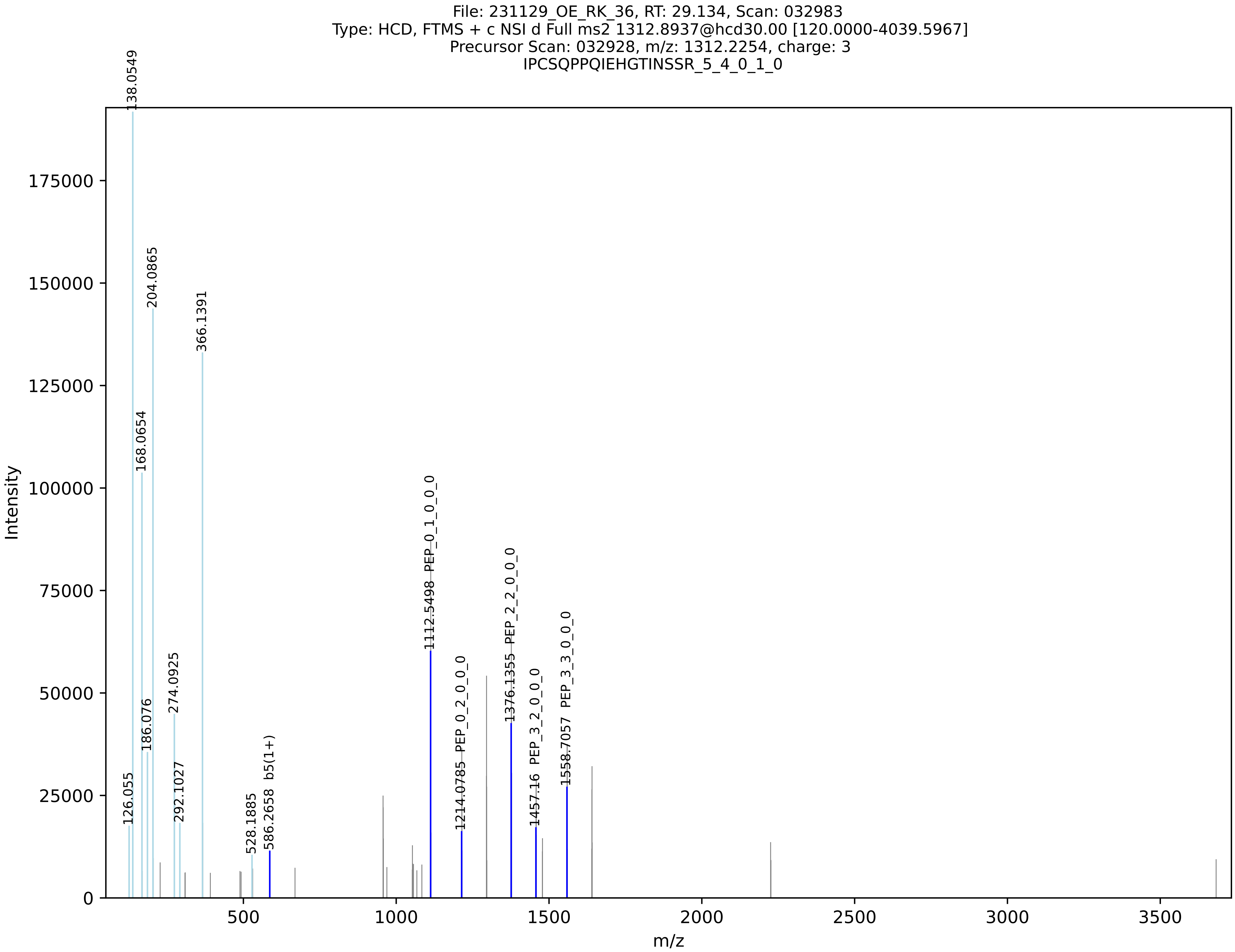

File: 231129_OE_RK_36, RT: 29.134, Scan: 032983
Type: HCD, FTMS + c NSI d Full ms2 1312.8937@hcd30.00 [120.0000-4039.5967]
Precursor Scan: 032928, m/z: 1312.2254, charge: 3
IPCSQPPQIEHGTINSSR_5_4_0_1_0
Intensity
m/z
138.0549
204.0865
366.1391
168.0654
274.0925
186.076
292.1027
126.055
528.1885
586.2658 b5(1+)
1112.5498 PEP_0_1_0_0_0
1214.0785 PEP_0_2_0_0_0
1376.1355 PEP_2_2_0_0_0
1457.16 PEP_3_2_0_0_0
1558.7057 PEP_3_3_0_0_0
0
25000
50000
75000
100000
125000
150000
175000
500
1000
1500
2000
2500
3000
3500

File: 231129_OE_RK_36, RT: 29.196, Scan: 033074
Type: HCD, FTMS + c NSI d Full ms2 1024.7491@hcd30.00 [120.0000-3157.8743]
Precursor Scan: 032928, m/z: 1024.0815, charge: 3
GVNFNVSK_5_4_0_2_0

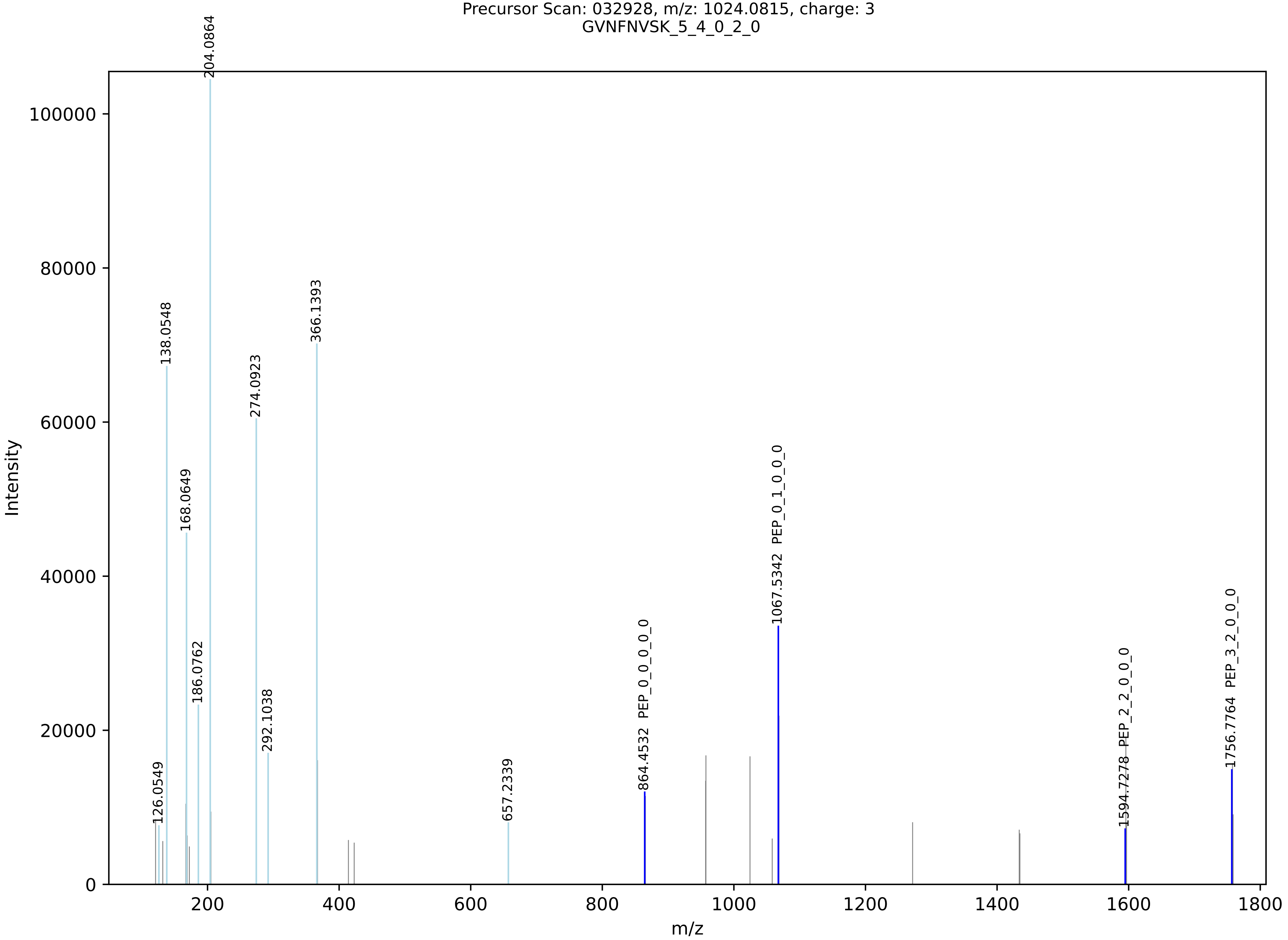

File: 231129_OE_RK_36, RT: 29.296, Scan: 033214
Type: HCD, FTMS + c NSI d Full ms2 1025.0853@hcd30.00 [120.0000-3158.9031]
Precursor Scan: 033079, m/z: 1023.7473, charge: 3
GVNFNVSK_5_4_0_2_0

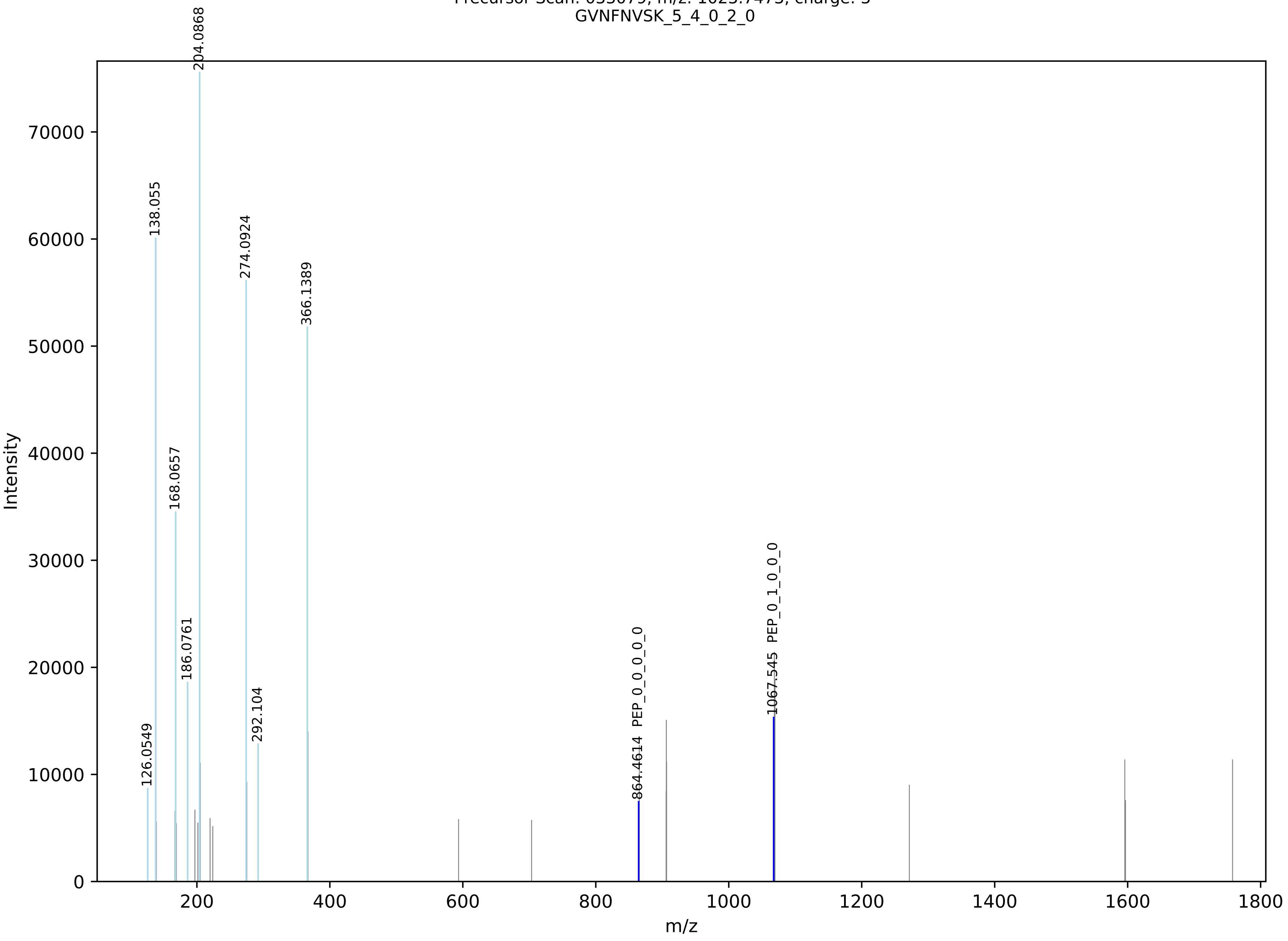

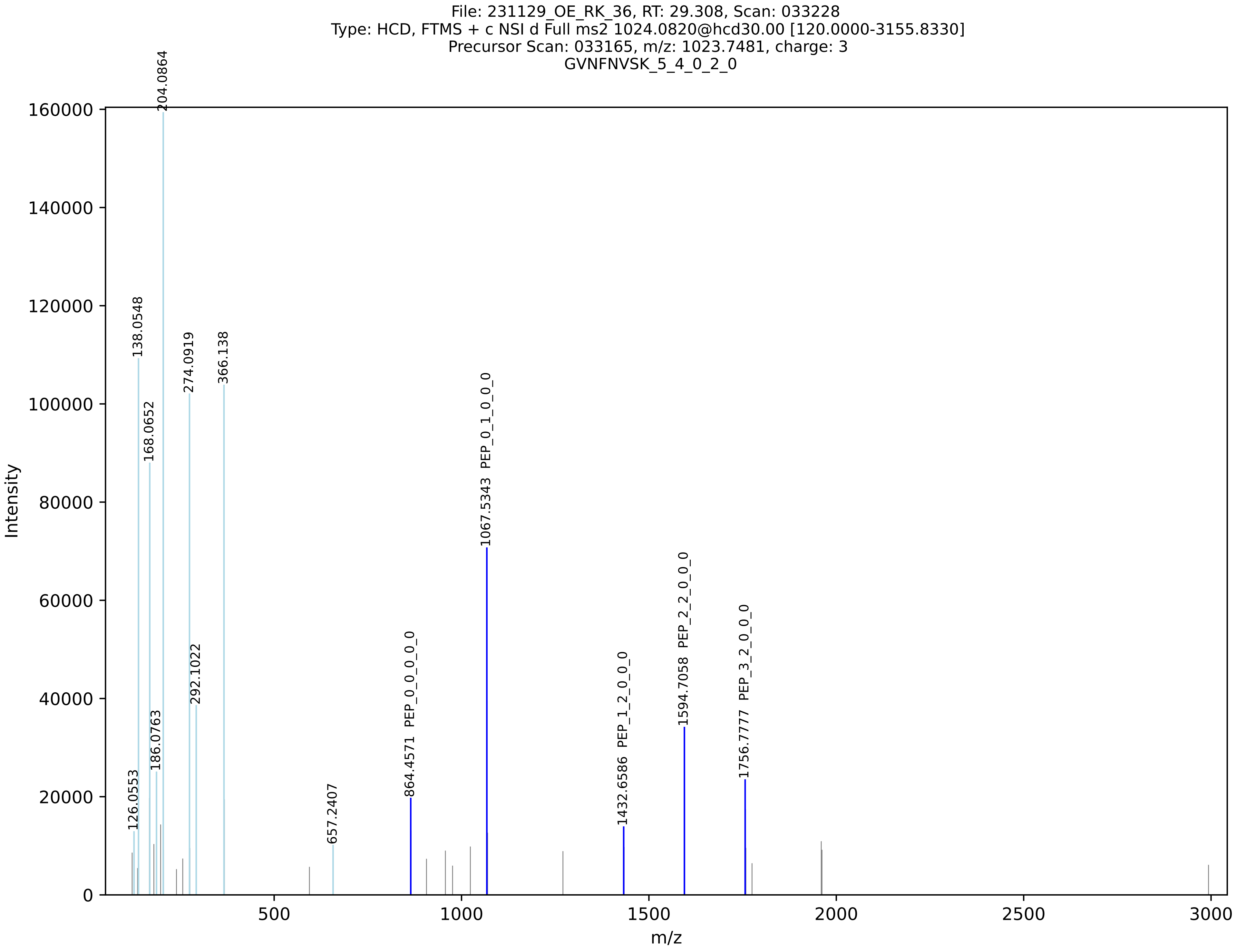

File: 231129_OE_RK_36, RT: 29.308, Scan: 033228
Type: HCD, FTMS + c NSI d Full ms2 1024.0820@hcd30.00 [120.0000-3155.8330]
Precursor Scan: 033165, m/z: 1023.7481, charge: 3
GVNFNVSK_5_4_0_2_0
Intensity
m/z
204.0864
138.0548
168.0652
274.0919
366.138
292.1022
186.0763
126.0553
657.2407
864.4571 PEP_0_0_0_0_0
1067.5343 PEP_0_1_0_0_0
1432.6586 PEP_1_2_0_0_0
1594.7058 PEP_2_2_0_0_0
1756.7777 PEP_3_2_0_0_0

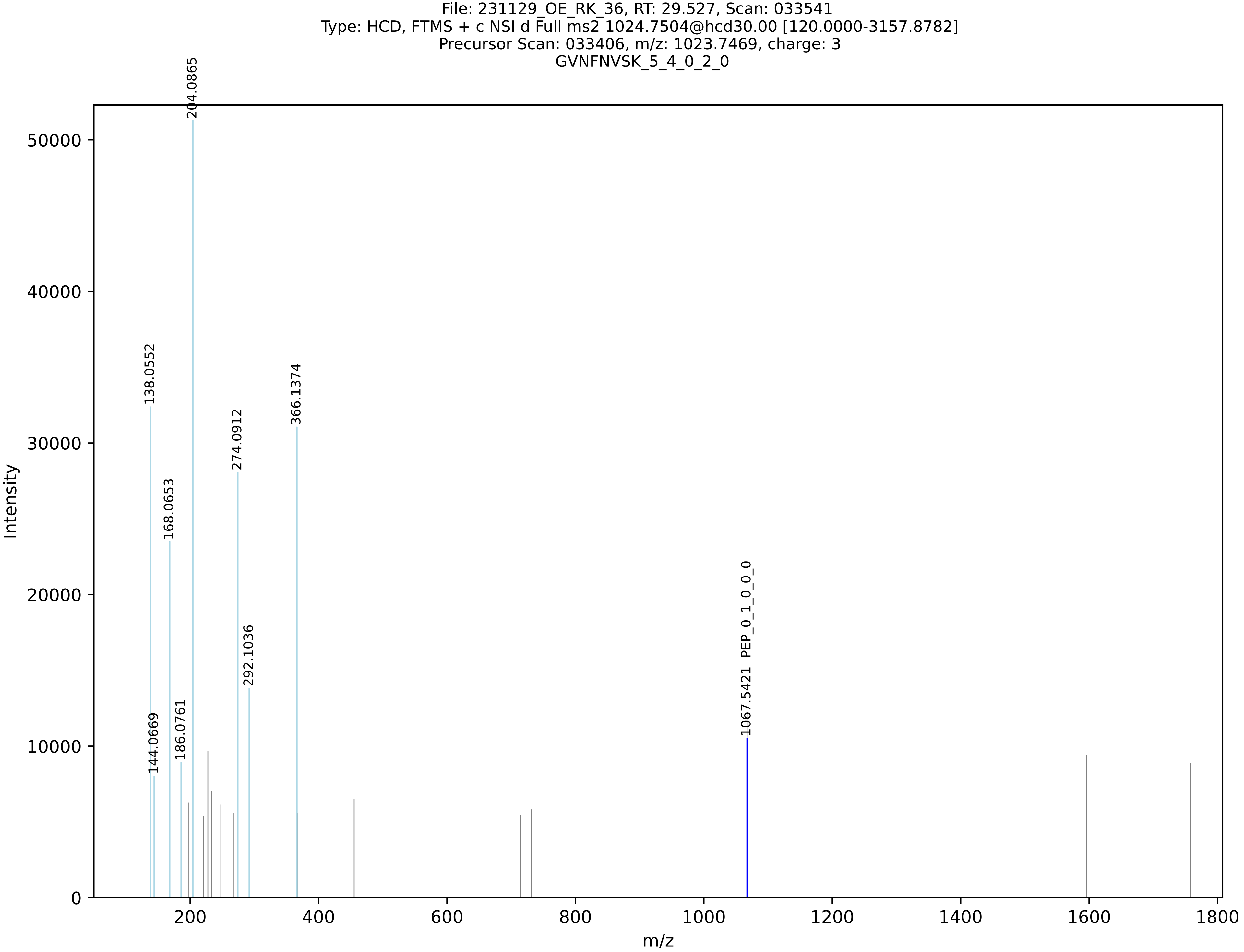
File: 231129_OE_RK_36, RT: 29.527, Scan: 033541
Type: HCD, FTMS + c NSI d Full ms2 1024.7504@hcd30.00 [120.0000-3157.8782]
Precursor Scan: 033406, m/z: 1023.7469, charge: 3
GVNFNVSK_5_4_0_2_0
204.0865
138.0552
366.1374
274.0912
168.0653
292.1036
186.0761
144.0669
1067.5421 PEP_0_1_0_0_0_
Intensity
m/z
0
10000
20000
30000
40000
50000
200
400
600
800
1000
1200
1400
1600
1800

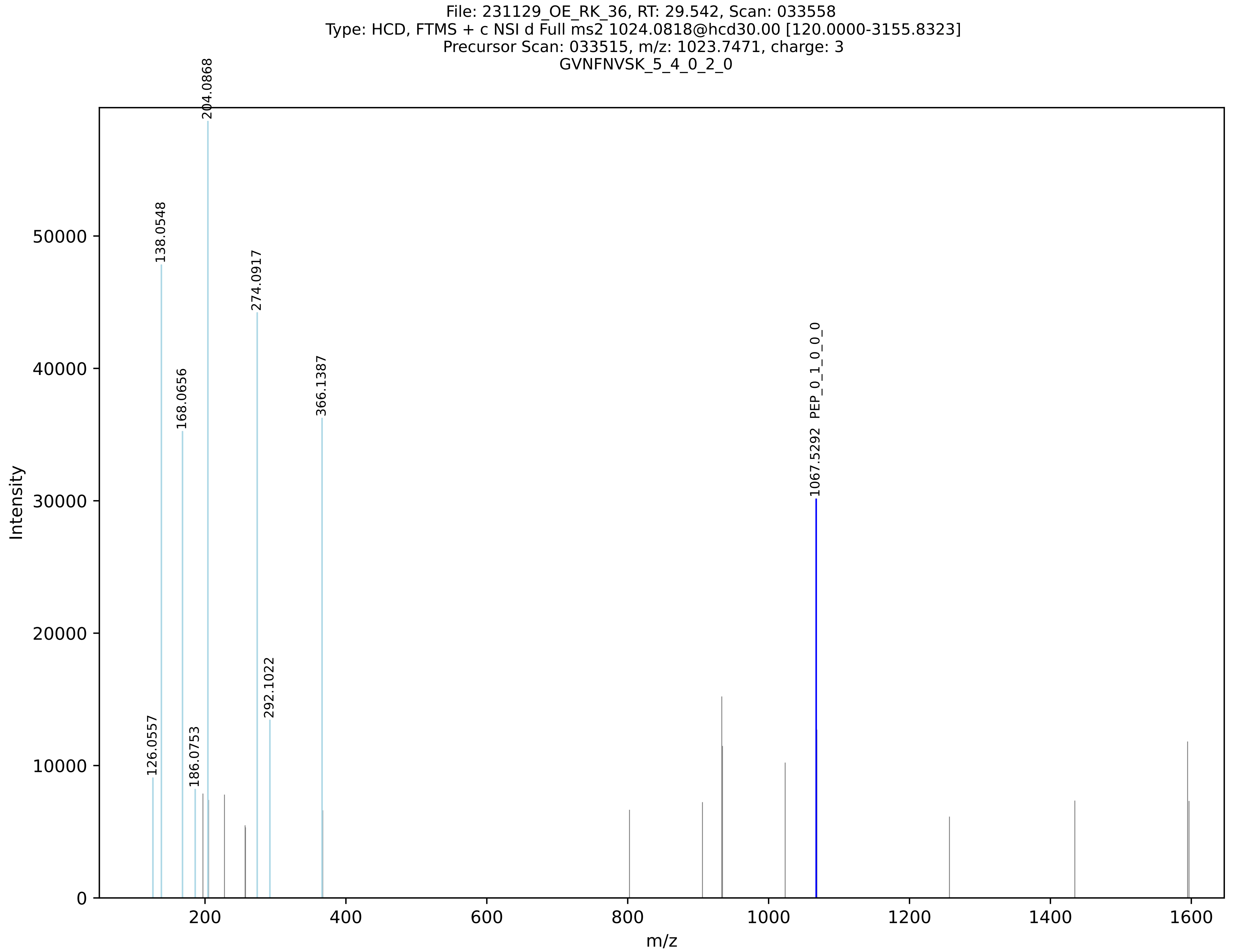
File: 231129_OE_RK_36, RT: 29.542, Scan: 033558
Type: HCD, FTMS + c NSI d Full ms2 1024.0818@hcd30.00 [120.0000-3155.8323]
Precursor Scan: 033515, m/z: 1023.7471, charge: 3
GVNFNVSK_5_4_0_2_0
204.0868
138.0548
274.0917
168.0656
366.1387
1067.5292 PEP_0_1_0_0_0
292.1022
126.0557
186.0753
Intensity
m/z
0
10000
20000
30000
40000
50000
200
400
600
800
1000
1200
1400
1600

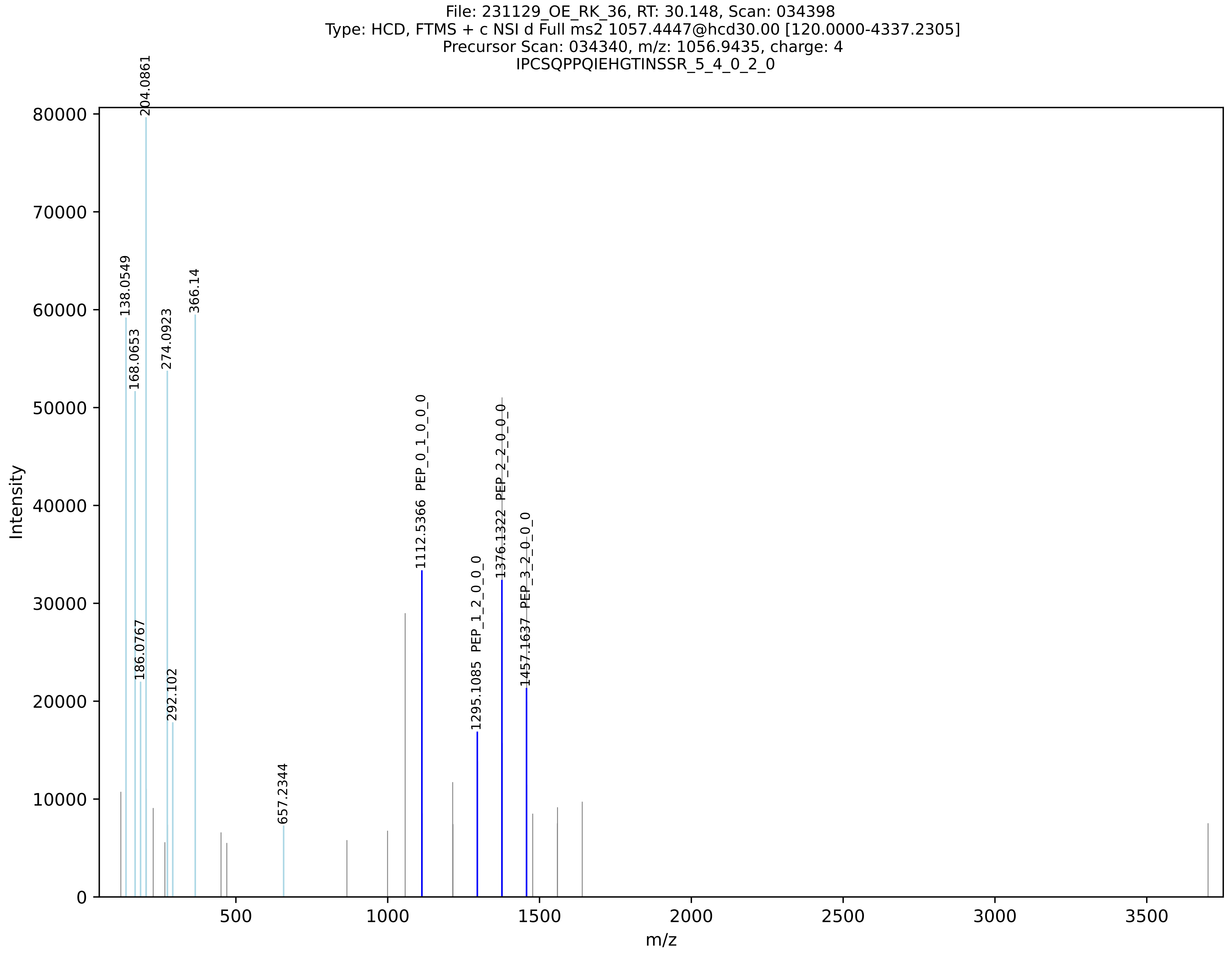
File: 231129_OE_RK_36, RT: 30.148, Scan: 034398
Type: HCD, FTMS + c NSI d Full ms2 1057.4447@hcd30.00 [120.0000-4337.2305]
Precursor Scan: 034340, m/z: 1056.9435, charge: 4
IPCSQPPQIEHGTINSSR_5_4_0_2_0
Intensity
m/z
204.0861
138.0549
168.0653
274.0923
366.14
186.0767
292.102
657.2344
1112.5366 PEP_0_1_0_0_0
1295.1085 PEP_1_2_0_0_0
1376.1322 PEP_2_2_0_0_0
1457.1637 PEP_3_2_0_0_0

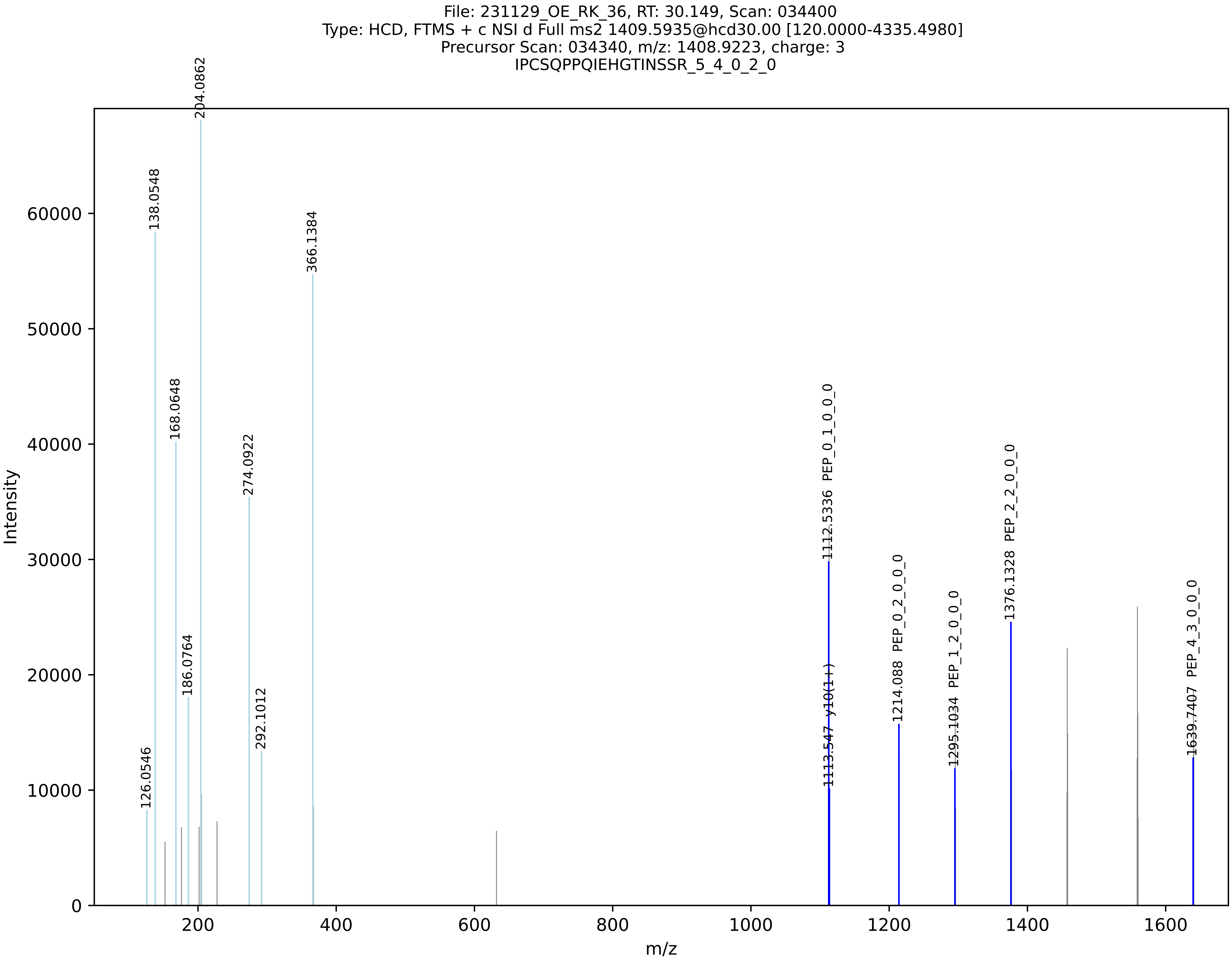
File: 231129_OE_RK_36, RT: 30.149, Scan: 034400
Type: HCD, FTMS + c NSI d Full ms2 1409.5935@hcd30.00 [120.0000-4335.4980]
Precursor Scan: 034340, m/z: 1408.9223, charge: 3
IPCSQPPQIEHGTINSSR_5_4_0_2_0
204.0862
138.0548
366.1384
168.0648
274.0922
186.0764
292.1012
126.0546
1112.5336 PEP_0_1_0_0_0
1113.547 y10(1+)
1214.088 PEP_0_2_0_0_0
1295.1034 PEP_1_2_0_0_0
1376.1328 PEP_2_2_0_0_0
1639.7407 PEP_4_3_0_0_0
Intensity
m/z
0
10000
20000
30000
40000
50000
60000
200
400
600
800
1000
1200
1400
1600

File: 231129_OE_RK_36, RT: 30.325, Scan: 034633
Type: HCD, FTMS + c NSI d Full ms2 1409.9255@hcd30.00 [120.0000-4336.5142]
Precursor Scan: 034612, m/z: 1409.257, charge: 3
IPCSQPPQIEHGTINSSR_5_4_0_2_0

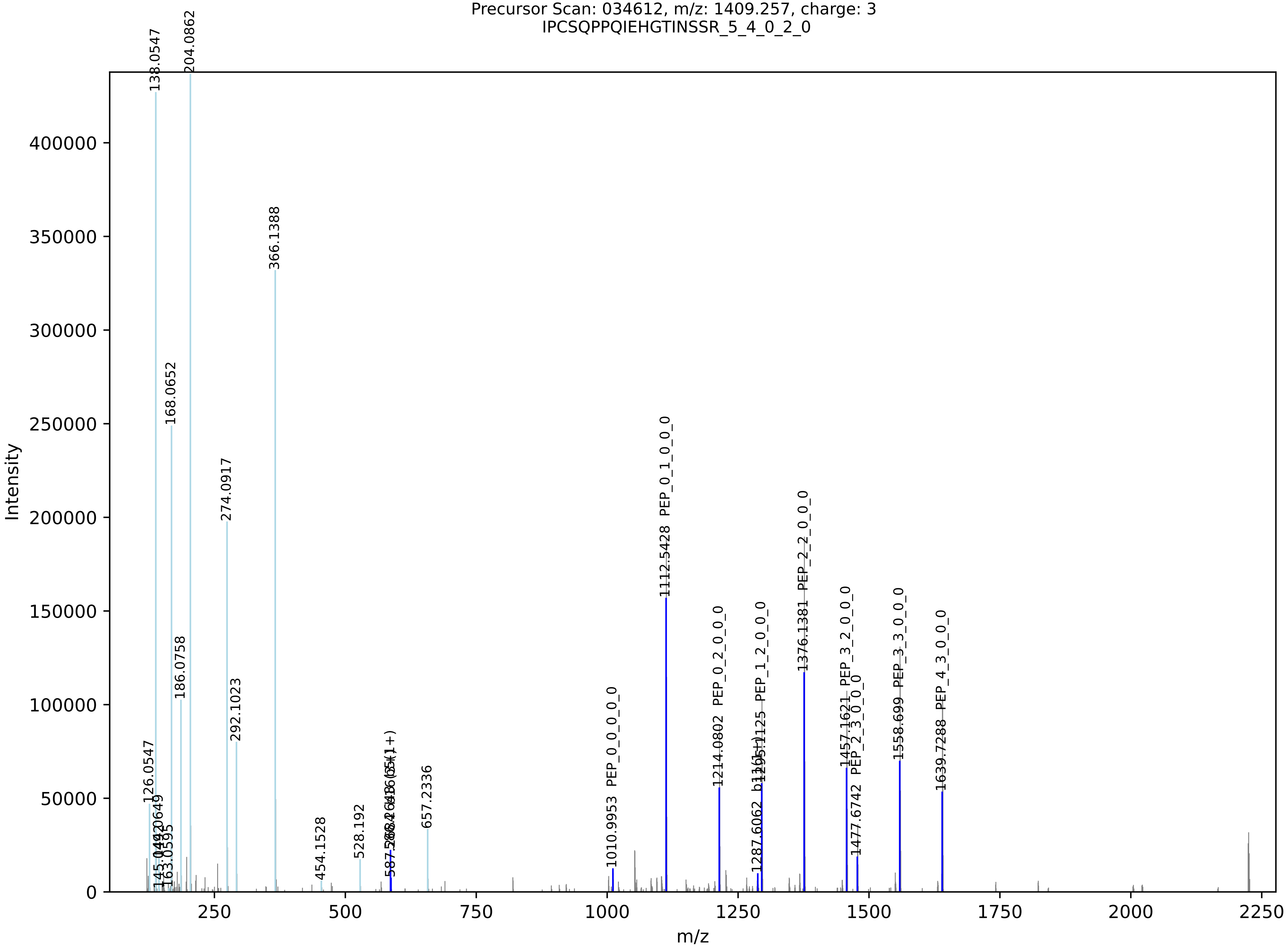

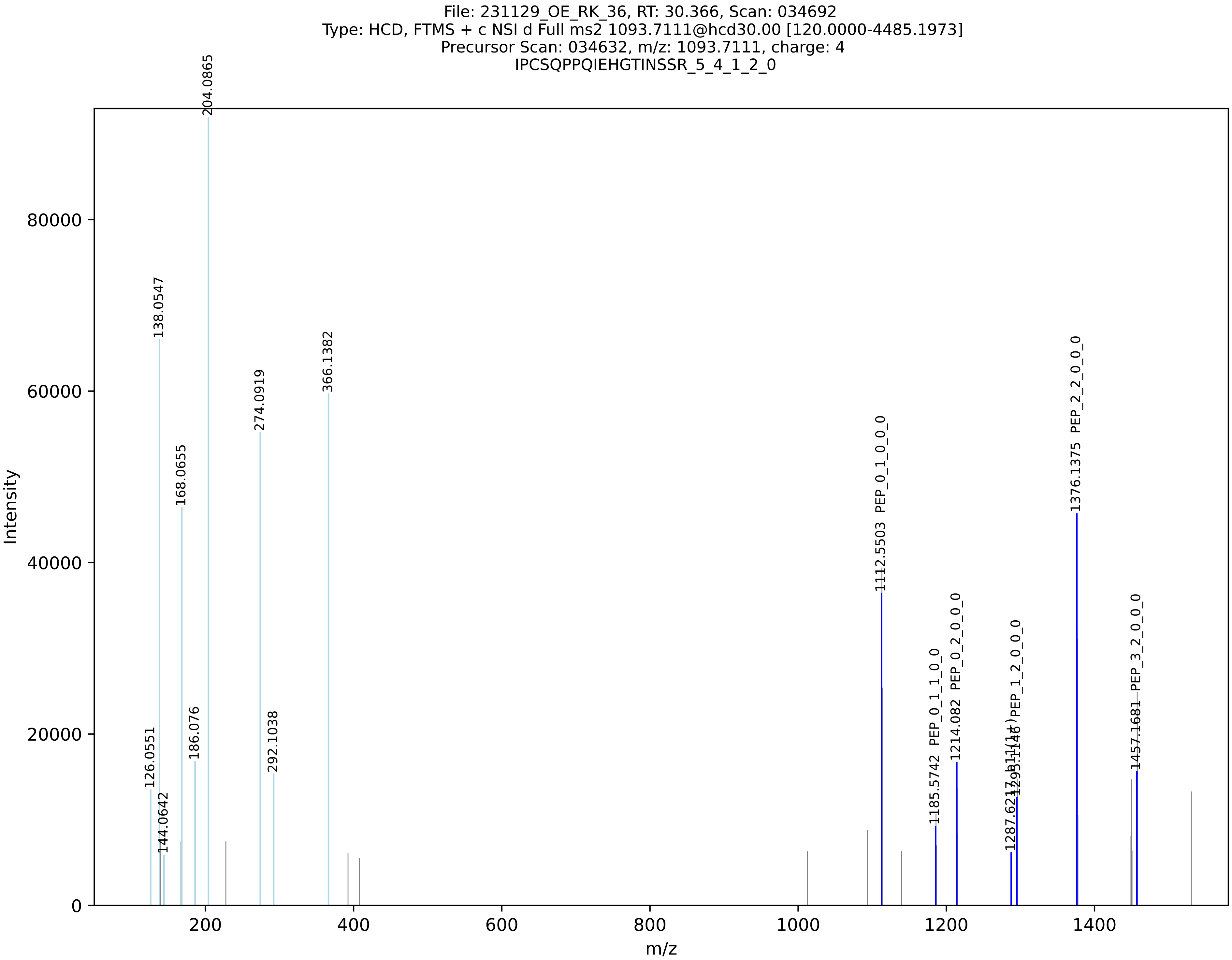

File: 231129_OE_RK_36, RT: 30.366, Scan: 034692
Type: HCD, FTMS + c NSI d Full ms2 1093.7111@hcd30.00 [120.0000-4485.1973]
Precursor Scan: 034632, m/z: 1093.7111, charge: 4
IPCSQPPQIEHGTINSSR_5_4_1_2_0
Intensity
m/z
204.0865
138.0547
168.0655
274.0919
366.1382
126.0551
144.0642
186.076
292.1038
1112.5503 PEP_0_1_0_0_
1185.5742 PEP_0_1_1_0_0
1214.082 PEP_0_2_0_0_0
1287.6217 b11(1+)
1295.1146 PEP_1_2_0_0_0
1376.1375 PEP_2_2_0_0_
1457.1681 PEP_3_2_0_0_0

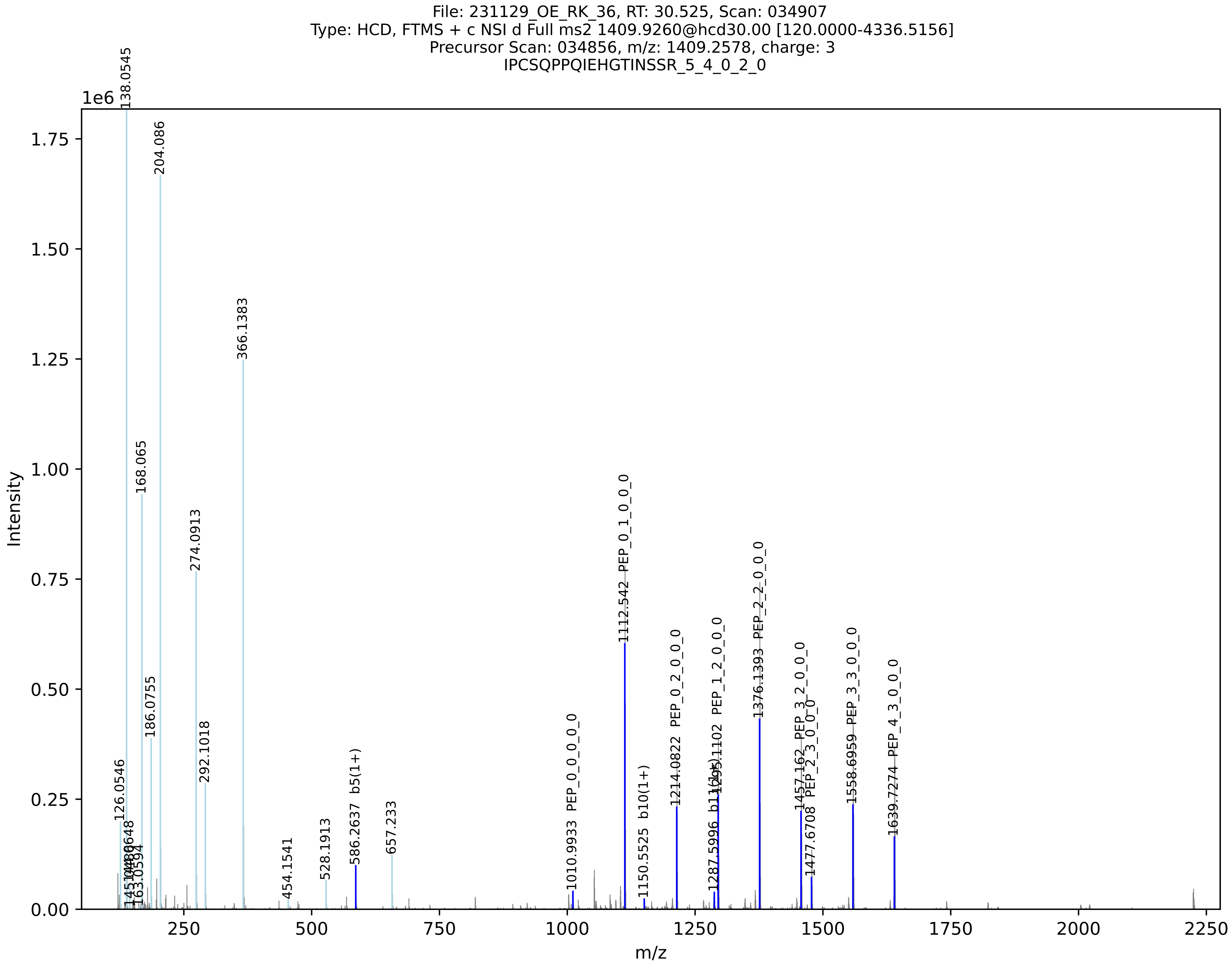

File: 231129_OE_RK_36, RT: 30.525, Scan: 034907
Type: HCD, FTMS + c NSI d Full ms2 1409.9260@hcd30.00 [120.0000-4336.5156]
Precursor Scan: 034856, m/z: 1409.2578, charge: 3
IPCSQPPQIEHGTINSSR_5_4_0_2_0
1e6
Intensity
m/z
138.0545
204.086
366.1383
168.065
274.0913
186.0755
292.1018
126.0546
163.0594
454.1541
528.1913
586.2637 b5(1+)
657.233
1010.9933 PEP_0_0_0_0_0_
1112.542 PEP_0_1_0_0_0_
1150.5525 b10(1+)
1214.0822 PEP_0_2_0_0_0_
1287.5996 b11(1+)
1295.1102 PEP_1_2_0_0_0_
1376.1393 PEP_2_2_0_0_0_
1457.162 PEP_3_2_0_0_0_
1477.6708 PEP_2_3_0_0_0_
1558.6959 PEP_3_3_0_0_0_
1639.7274 PEP_4_3_0_0_0_

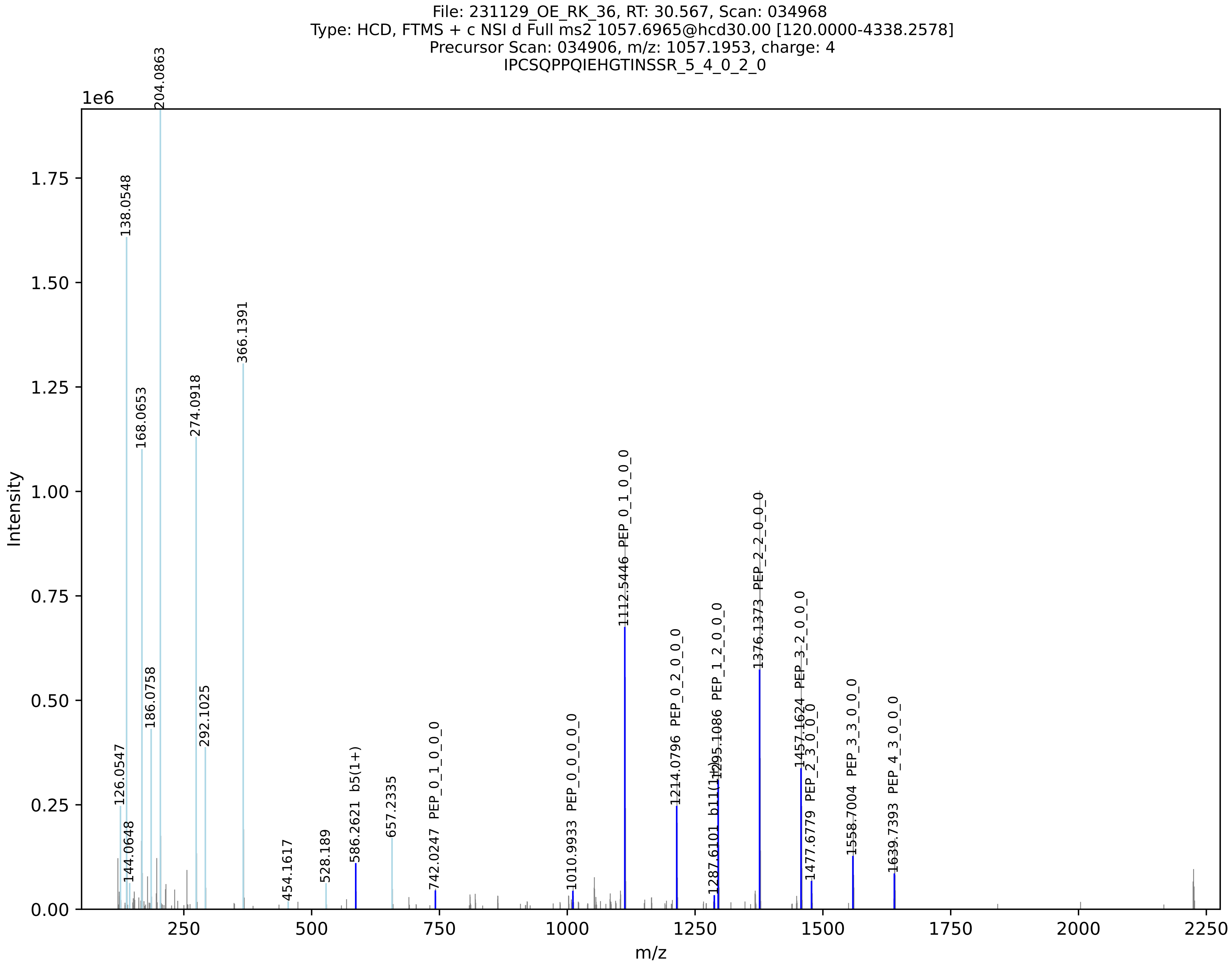

File: 231129_OE_RK_36, RT: 30.567, Scan: 034968
Type: HCD, FTMS + c NSI d Full ms2 1057.6965@hcd30.00 [120.0000-4338.2578]
Precursor Scan: 034906, m/z: 1057.1953, charge: 4
IPCSQPPQIEHGTINSSR_5_4_0_2_0
1e6
Intensity
m/z
204.0863
138.0548
168.0653
274.0918
366.1391
186.0758
292.1025
126.0547
144.0648
454.1617
528.189
586.2621 b5(1+)
657.2335
742.0247 PEP_0_1_0_0_0
1010.9933 PEP_0_0_0_0_0
1112.5446 PEP_0_1_0_0_0
1214.0796 PEP_0_2_0_0_0
1287.6101 b11(1+)
1295.1086 PEP_1_2_0_0_0
1376.1373 PEP_2_2_0_0_0
1457.1624 PEP_3_2_0_0_0
1477.6779 PEP_2_3_0_0_0
1558.7004 PEP_3_3_0_0_0
1639.7393 PEP_4_3_0_0_0

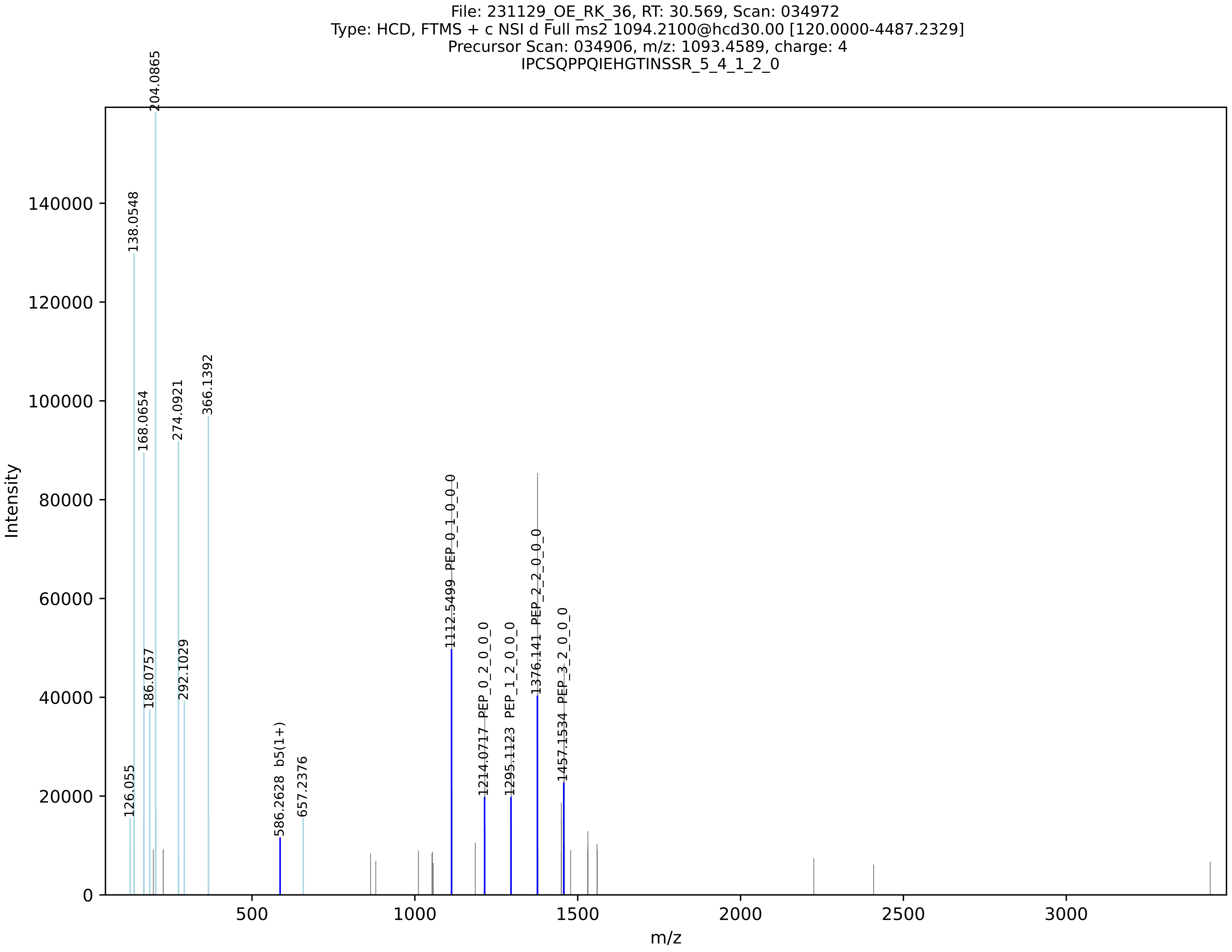

File: 231129_OE_RK_36, RT: 30.569, Scan: 034972
Type: HCD, FTMS + c NSI d Full ms2 1094.2100@hcd30.00 [120.0000-4487.2329]
Precursor Scan: 034906, m/z: 1093.4589, charge: 4
IPCSQPPQIEHGTINSSR_5_4_1_2_0
204.0865
138.0548
168.0654
274.0921
366.1392
186.0757
292.1029
126.055
586.2628 b5(1+)
657.2376
1112.5499 PEP_0_1_0_0_0
1214.0717 PEP_0_2_0_0_0
1295.1123 PEP_1_2_0_0_0
1376.141 PEP_2_2_0_0_0
1457.1534 PEP_3_2_0_0_0
Intensity
m/z
0
20000
40000
60000
80000
100000
120000
140000
500
1000
1500
2000
2500
3000

File: 231129_OE_RK_36, RT: 30.701, Scan: 035162
Type: HCD, FTMS + c NSI d Full ms2 1409.9257@hcd30.00 [120.0000-4336.5146]
Precursor Scan: 035087, m/z: 1409.2573, charge: 3
IPCSQPPQIEHGTINSSR_5_4_0_2_0

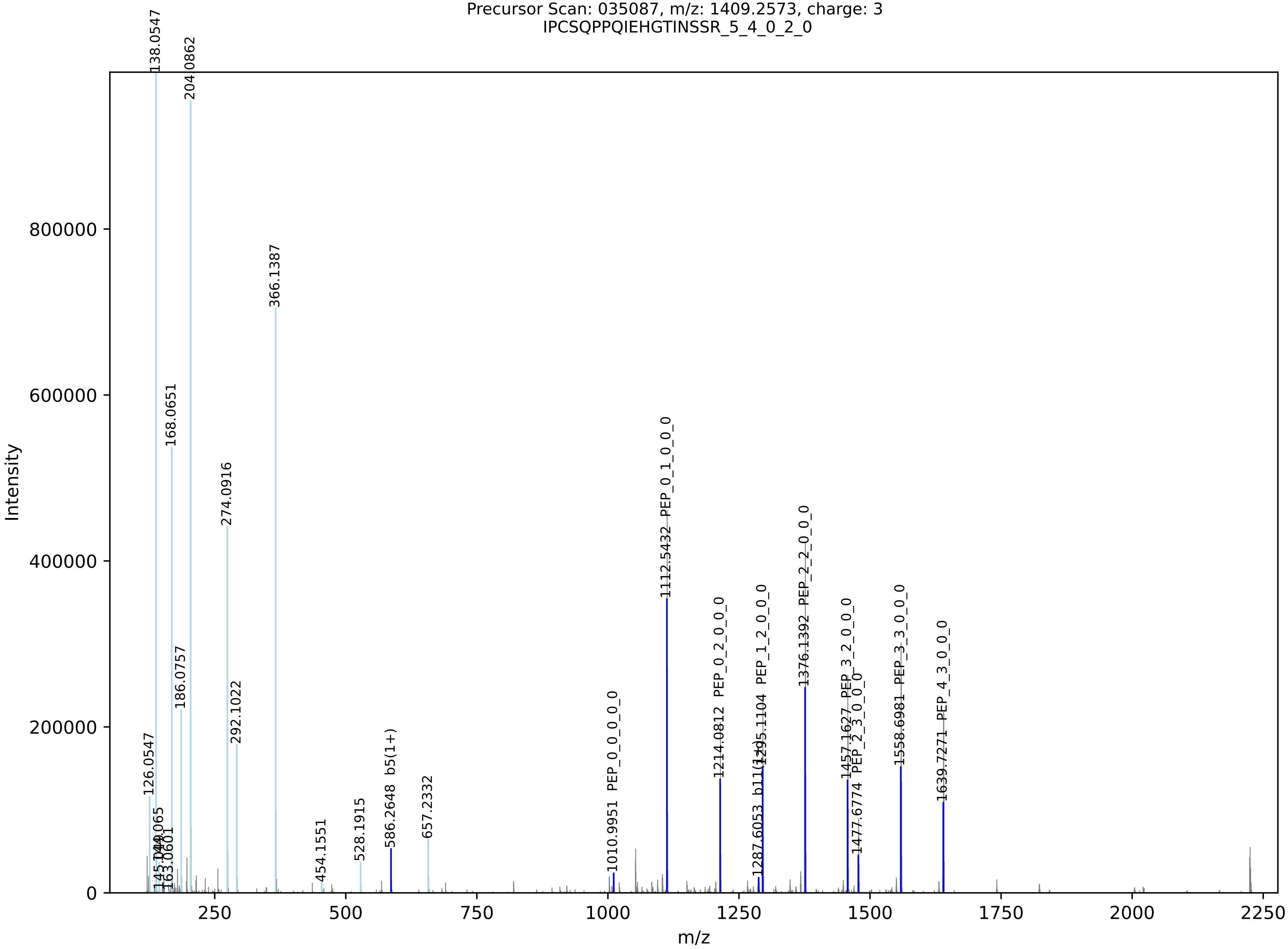

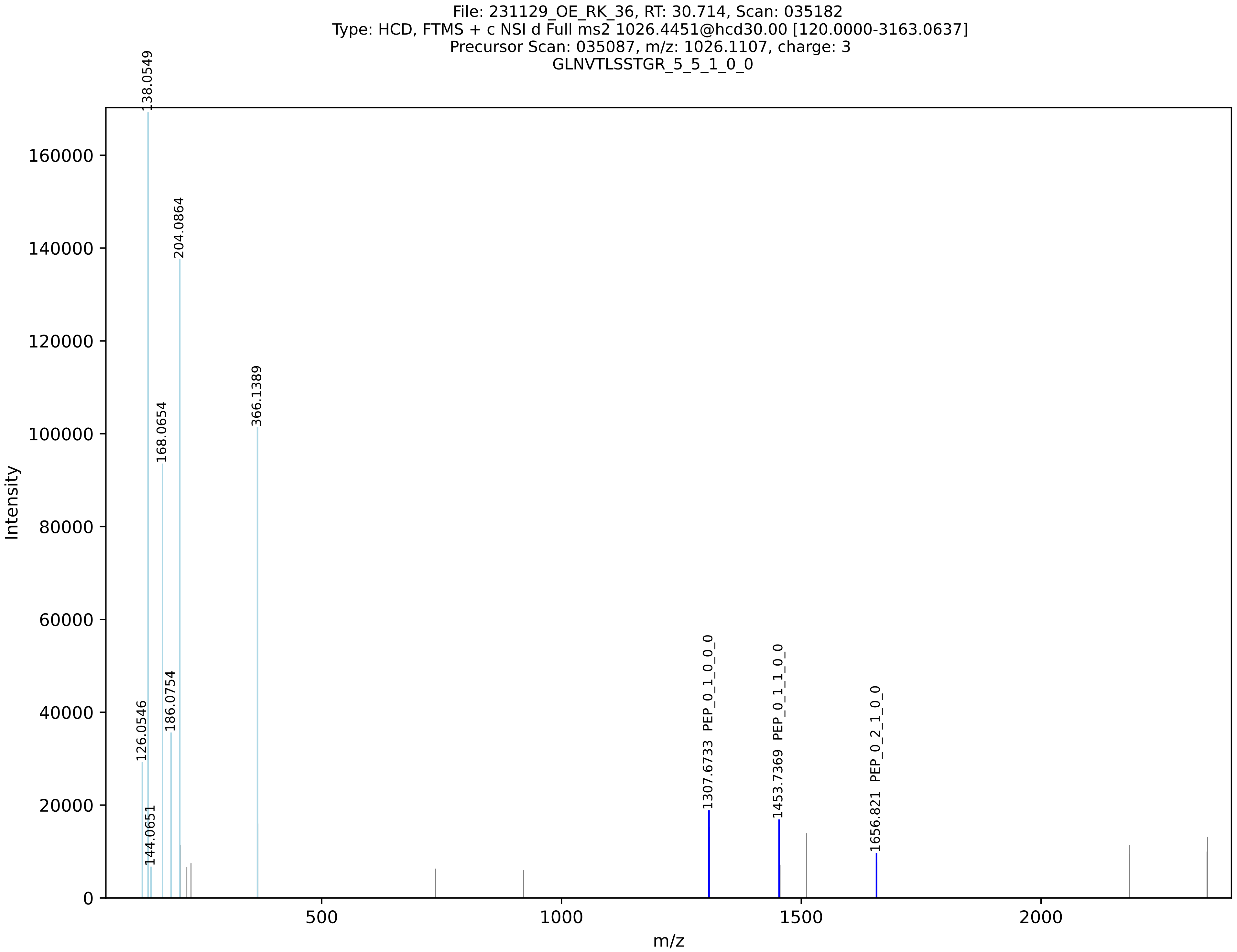
File: 231129_OE_RK_36, RT: 30.714, Scan: 035182
Type: HCD, FTMS + c NSI d Full ms2 1026.4451@hcd30.00 [120.0000-3163.0637]
Precursor Scan: 035087, m/z: 1026.1107, charge: 3
GLNVTLSSTGR_5_5_1_0_0
138.0549
204.0864
366.1389
168.0654
186.0754
126.0546
144.0651
1307.6733 PEP_0_1_0_0_0
1453.7369 PEP_0_1_1_0_0
1656.821 PEP_0_2_1_0_0
Intensity
160000
140000
120000
100000
80000
60000
40000
20000
0
500
1000
1500
2000
m/z

File: 231129_OE_RK_36, RT: 30.72, Scan: 035193
Type: HCD, FTMS + c NSI d Full ms2 1157.8169@hcd30.00 [120.0000-3565.0618]
Precursor Scan: 035087, m/z: 1157.4822, charge: 3
ERTVNNQSQR_7_4_2_0_0

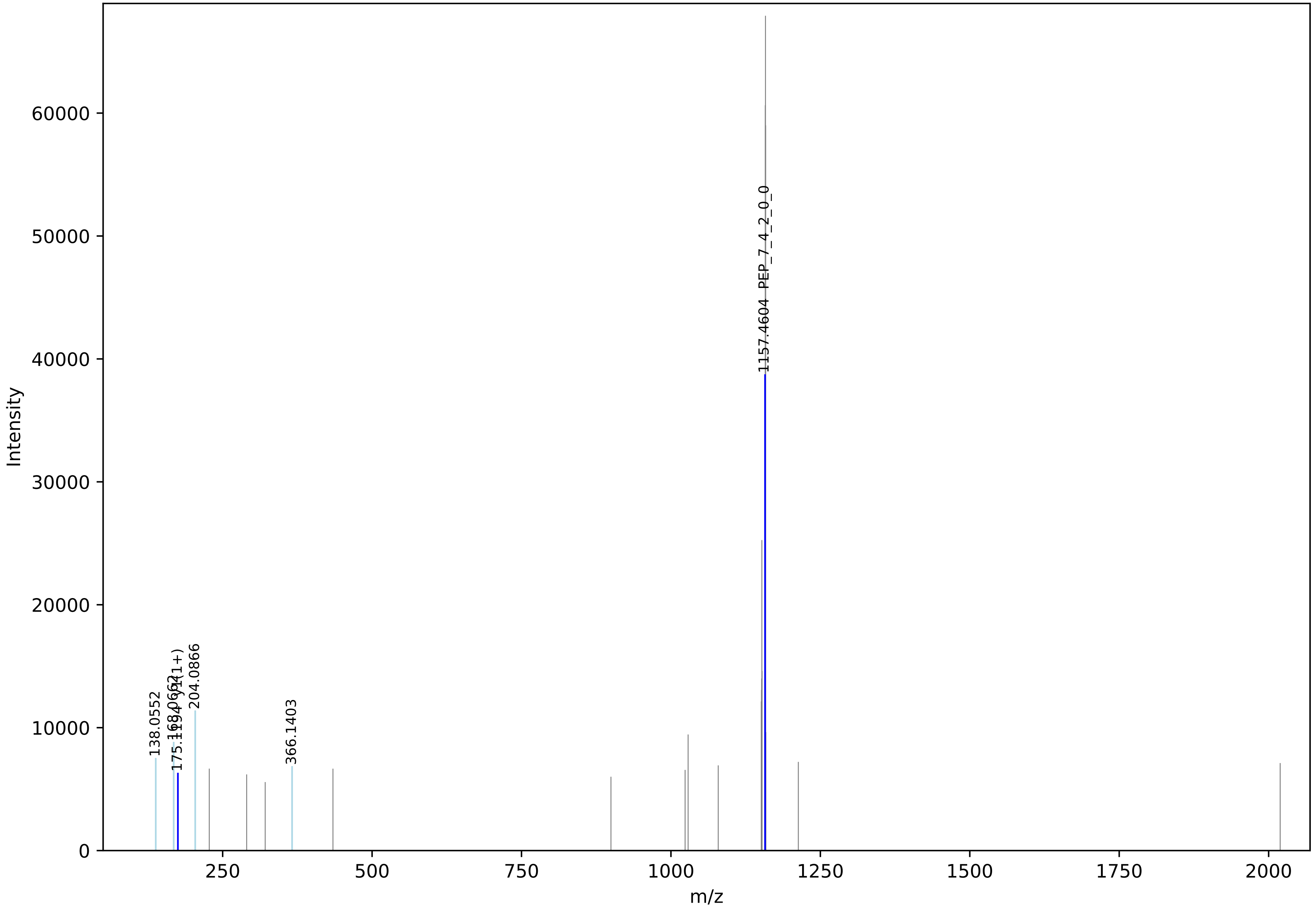

File: 231129_OE_RK_36, RT: 30.751, Scan: 035235
Type: HCD, FTMS + c NSI d Full ms2 1057.6963@hcd30.00 [120.0000-4338.2568]
Precursor Scan: 035161, m/z: 1057.1953, charge: 4
IPCSQPPQIEHGTINSSR_5_4_0_2_0

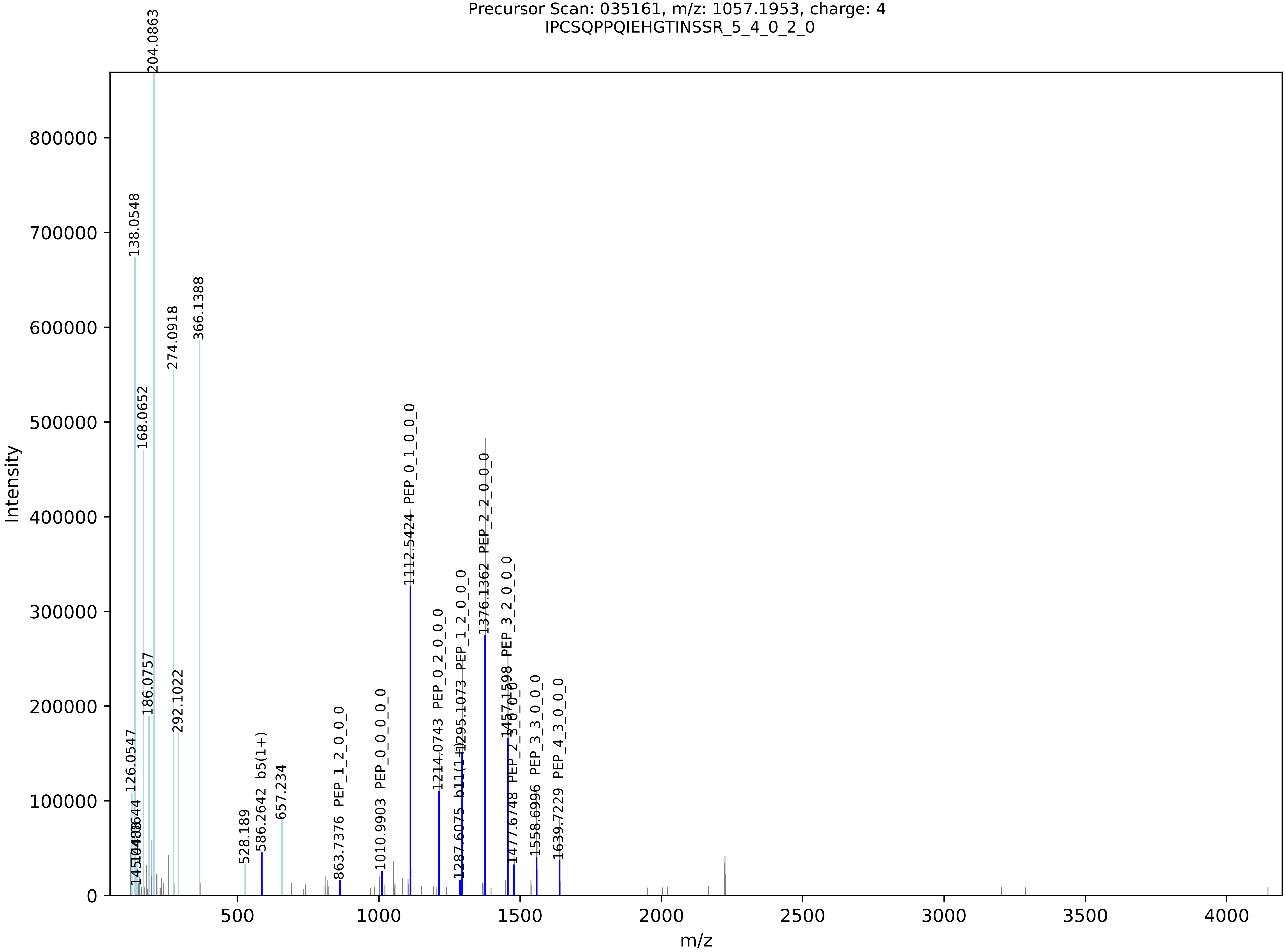

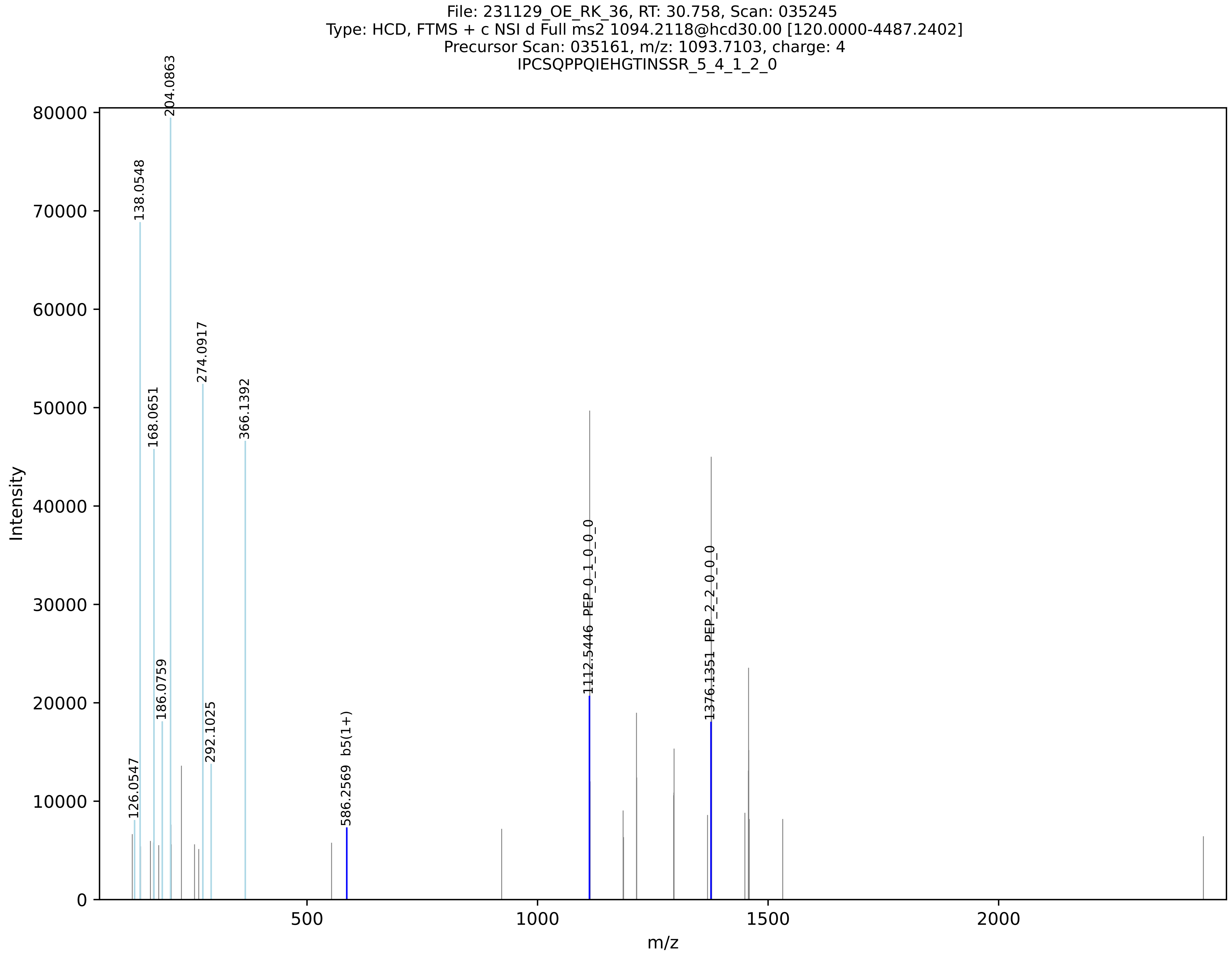
File: 231129_OE_RK_36, RT: 30.758, Scan: 035245
Type: HCD, FTMS + c NSI d Full ms2 1094.2118@hcd30.00 [120.0000-4487.2402]
Precursor Scan: 035161, m/z: 1093.7103, charge: 4
IPCSQPPQIEHGTINSSR_5_4_1_2_0
Intensity
m/z
204.0863
138.0548
274.0917
366.1392
168.0651
186.0759
292.1025
126.0547
586.2569 b5(1+)
1112.5446 PEP_0_1_0_0_0
1376.1351 PEP_2_2_0_0_0
0
10000
20000
30000
40000
50000
60000
70000
80000
500
1000
1500
2000

File: 231129_OE_RK_36, RT: 30.783, Scan: 035286
Type: HCD, FTMS + c NSI d Full ms2 1061.4504@hcd30.00 [120.0000-4353.5742]
Precursor Scan: 035087, m/z: 1061.4505, charge: 4
IPCSQPPQIEHGTINSSR_6_4_1_1_0

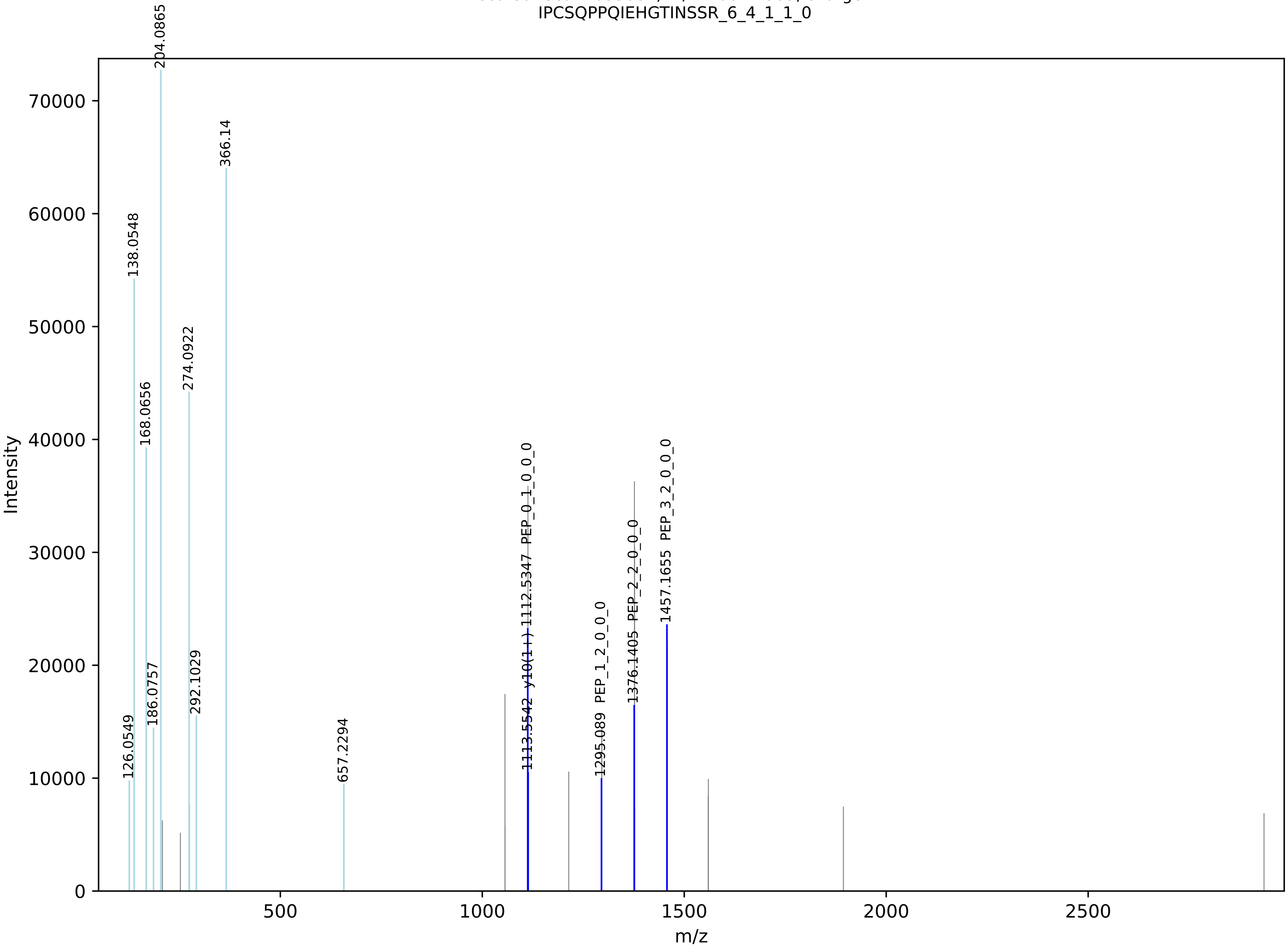

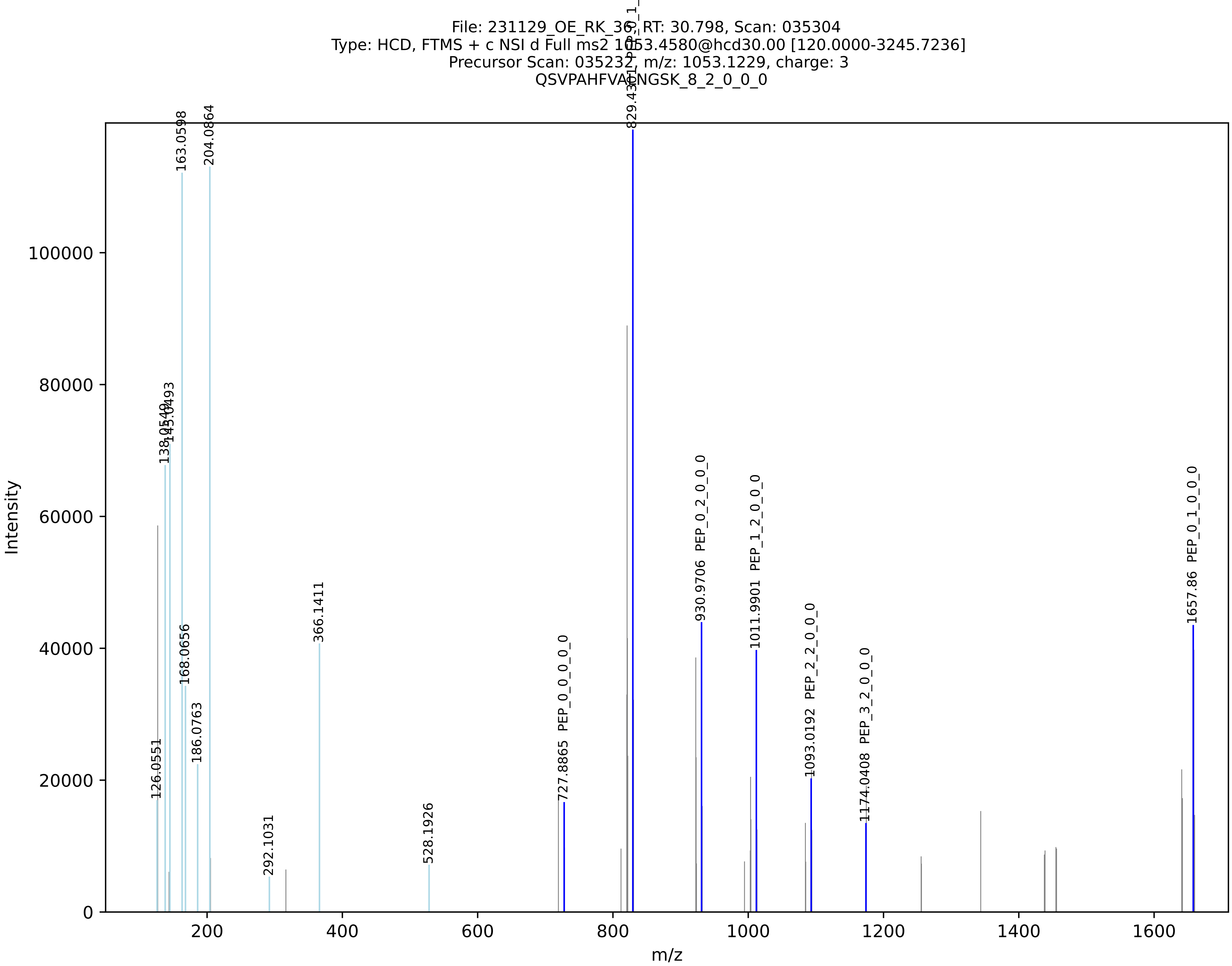
File: 231129_OE_RK_36, RT: 30.798, Scan: 035304
Type: HCD, FTMS + c NSI d Full ms2 1053.4580@hcd30.00 [120.0000-3245.7236]
Precursor Scan: 035232, m/z: 1053.1229, charge: 3
QSVPAHFVACNGSK_8_2_0_0_0
Intensity
m/z
126.0551
143.0493
163.0598
168.0656
186.0763
204.0864
292.1031
366.1411
528.1926
727.8865 PEP_0_0_0_0_0
930.9706 PEP_0_2_0_0_0
1011.9901 PEP_1_2_0_0_0
1093.0192 PEP_2_2_0_0_0
1174.0408 PEP_3_2_0_0_0
1657.86 PEP_0_1_0_0_0

File: 231129_OE_RK_36, RT: 30.936, Scan: 035501
Type: HCD, FTMS + c NSI d Full ms2 1057.6970@hcd30.00 [120.0000-4338.2598]
Precursor Scan: 035427, m/z: 1057.1958, charge: 4
IPCSQPPQIEHGTINSSR_5_4_0_2_0

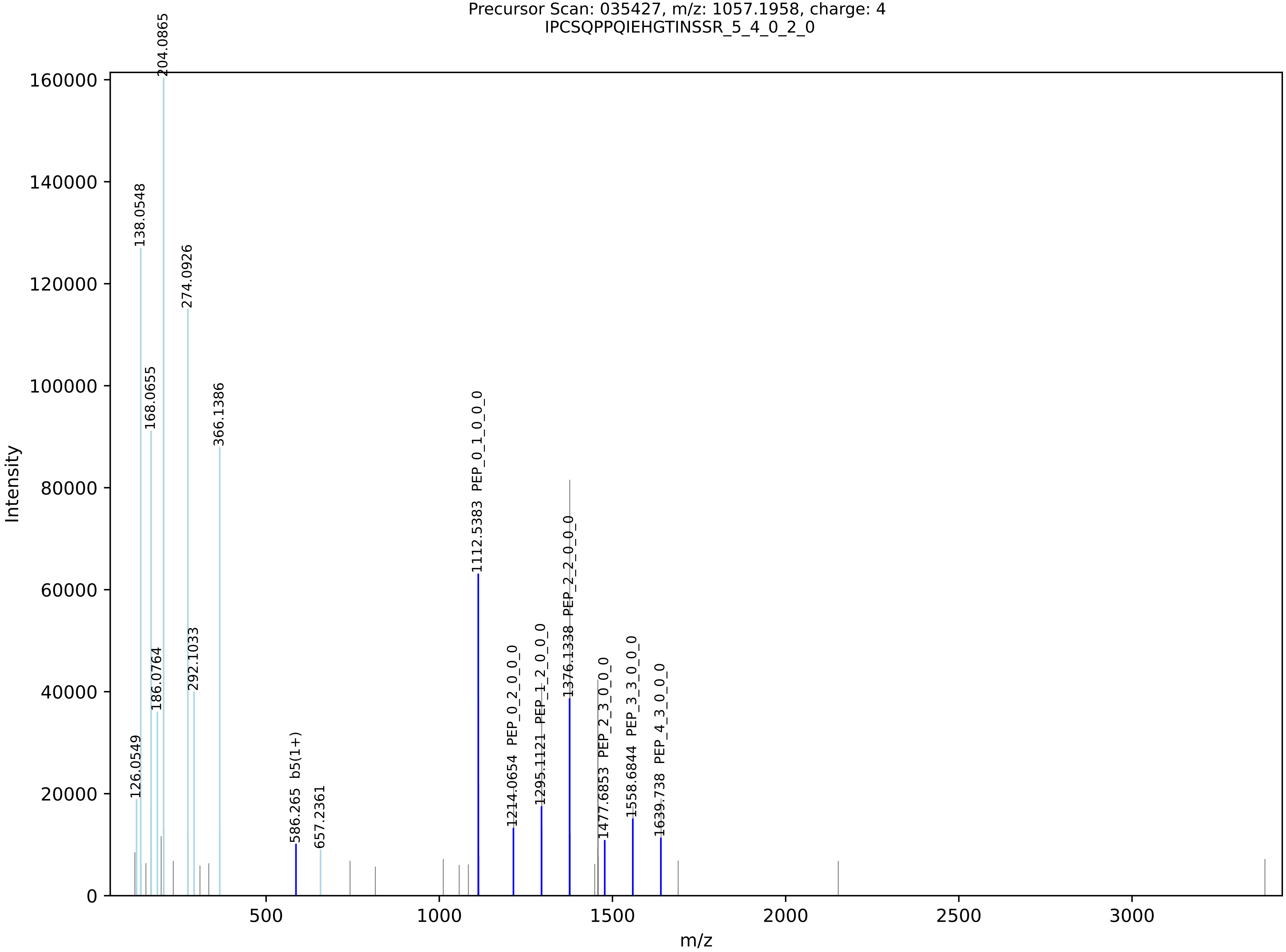

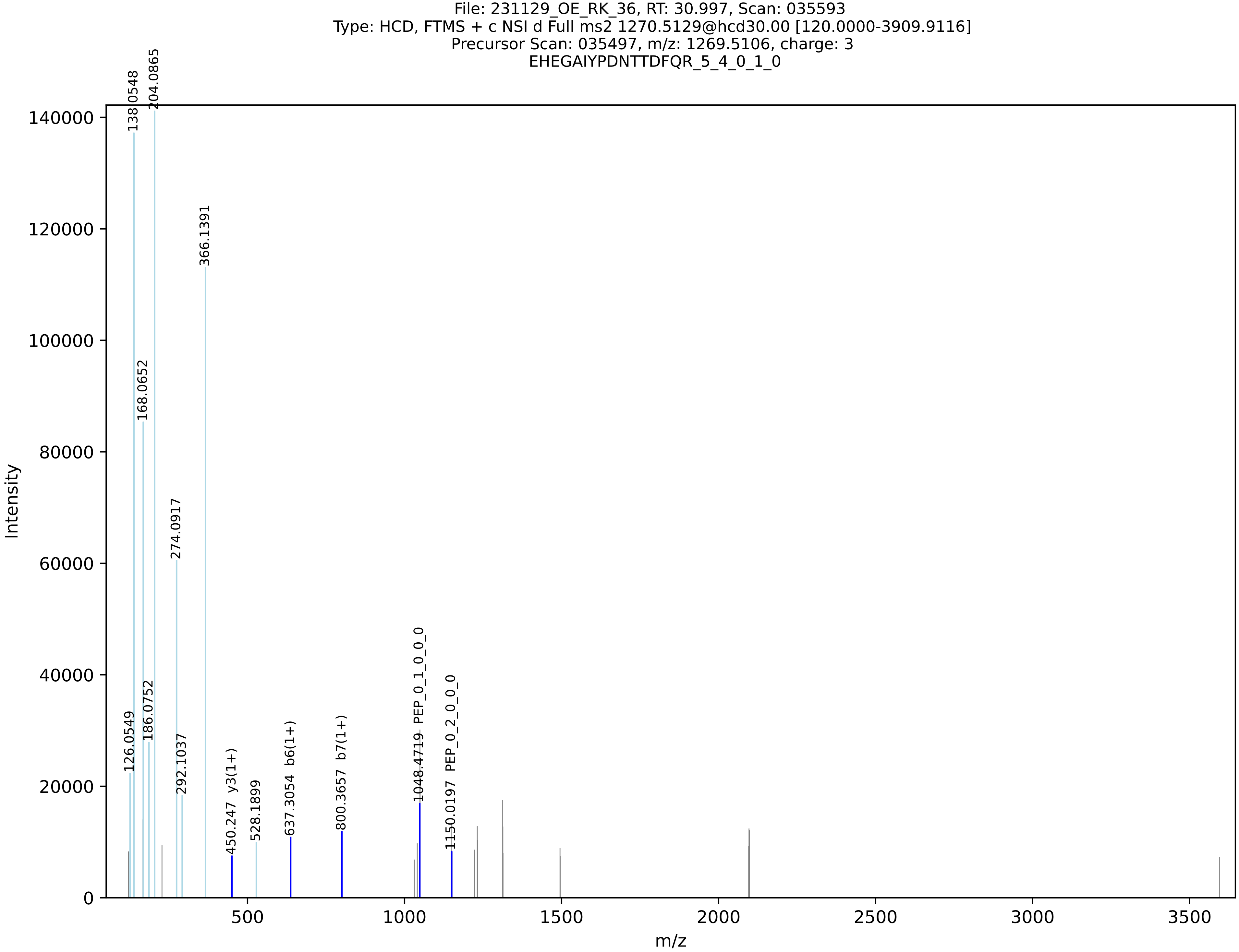
File: 231129_OE_RK_36, RT: 30.997, Scan: 035593
Type: HCD, FTMS + c NSI d Full ms2 1270.5129@hcd30.00 [120.0000-3909.9116]
Precursor Scan: 035497, m/z: 1269.5106, charge: 3
EHEGAIYPDNTTDFQR_5_4_0_1_0
138.0548
204.0865
366.1391
168.0652
274.0917
126.0549
186.0752
292.1037
450.247 y3(1+)
528.1899
637.3054 b6(1+)
800.3657 b7(1+)
1048.4719 PEP_0_1_0_0_0
1150.0197 PEP_0_2_0_0_0
Intensity
m/z
0
20000
40000
60000
80000
100000
120000
140000
500
1000
1500
2000
2500
3000
3500

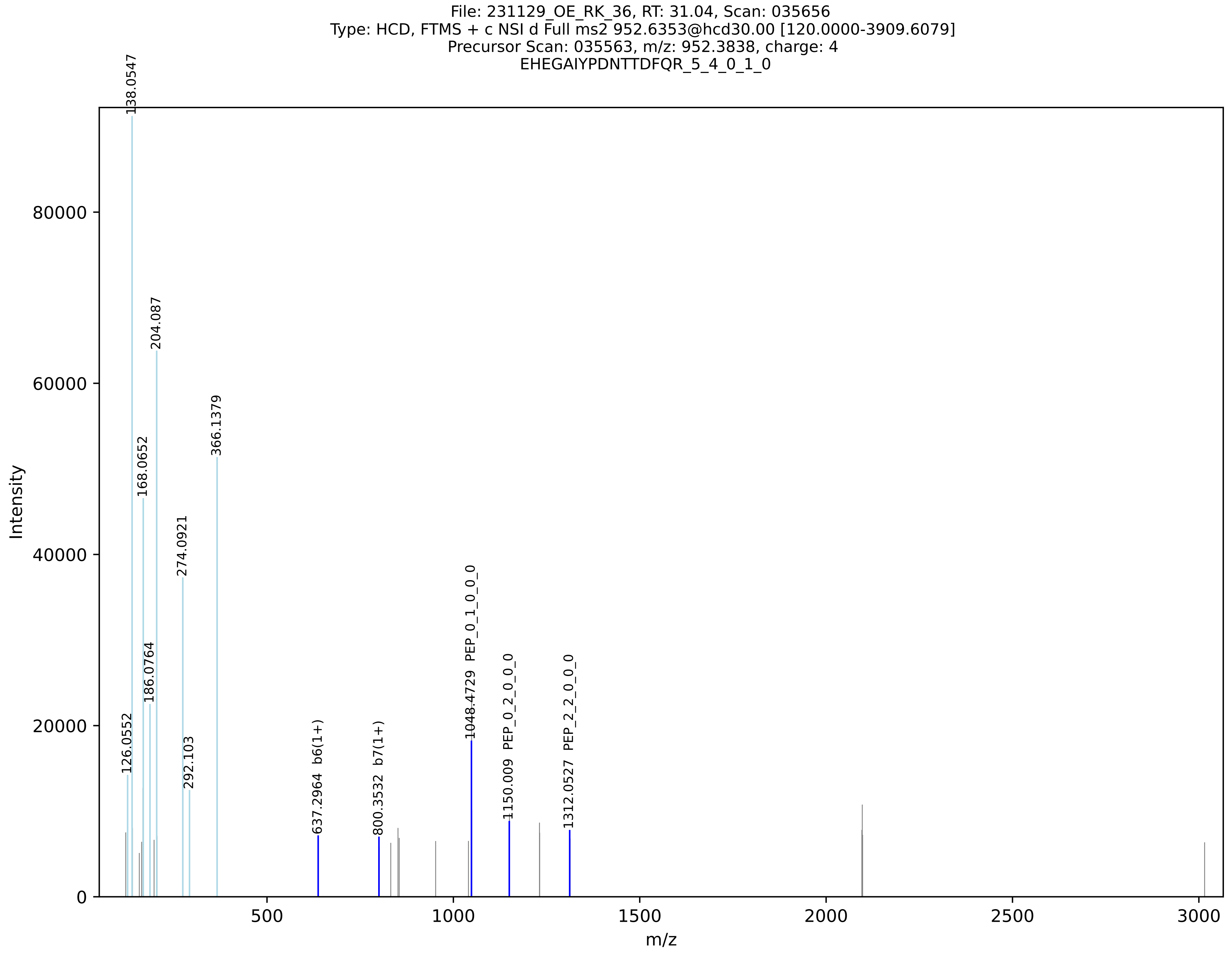

File: 231129_OE_RK_36, RT: 31.04, Scan: 035656
Type: HCD, FTMS + c NSI d Full ms2 952.6353@hcd30.00 [120.0000-3909.6079]
Precursor Scan: 035563, m/z: 952.3838, charge: 4
EHEGAIYPDNTTDFQR_5_4_0_1_0
Intensity
m/z
138.0547
204.087
366.1379
168.0652
274.0921
186.0764
126.0552
292.103
637.2964 b6(1+)
800.3532 b7(1+)
1048.4729 PEP_0_1_0_0_0_
1150.009 PEP_0_2_0_0_0_
1312.0527 PEP_2_2_0_0_0_

File: 231129_OE_RK_36, RT: 31.07, Scan: 035697
Type: HCD, FTMS + c NSI d Full ms2 999.7744@hcd30.00 [120.0000-3081.4514]
Precursor Scan: 035629, m/z: 999.1061, charge: 3
QSVPAHFVALNGSK_7_2_0_0_0

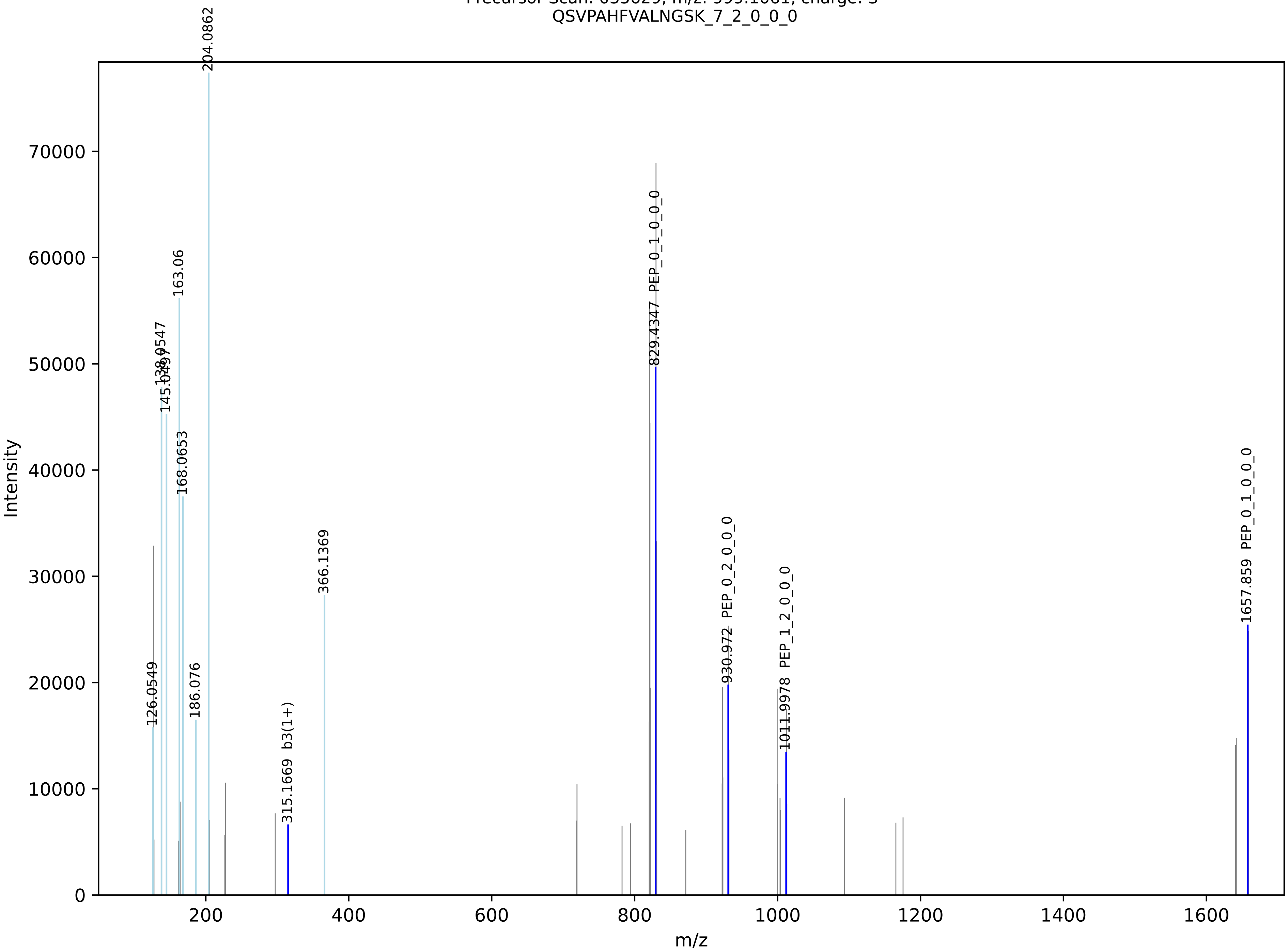

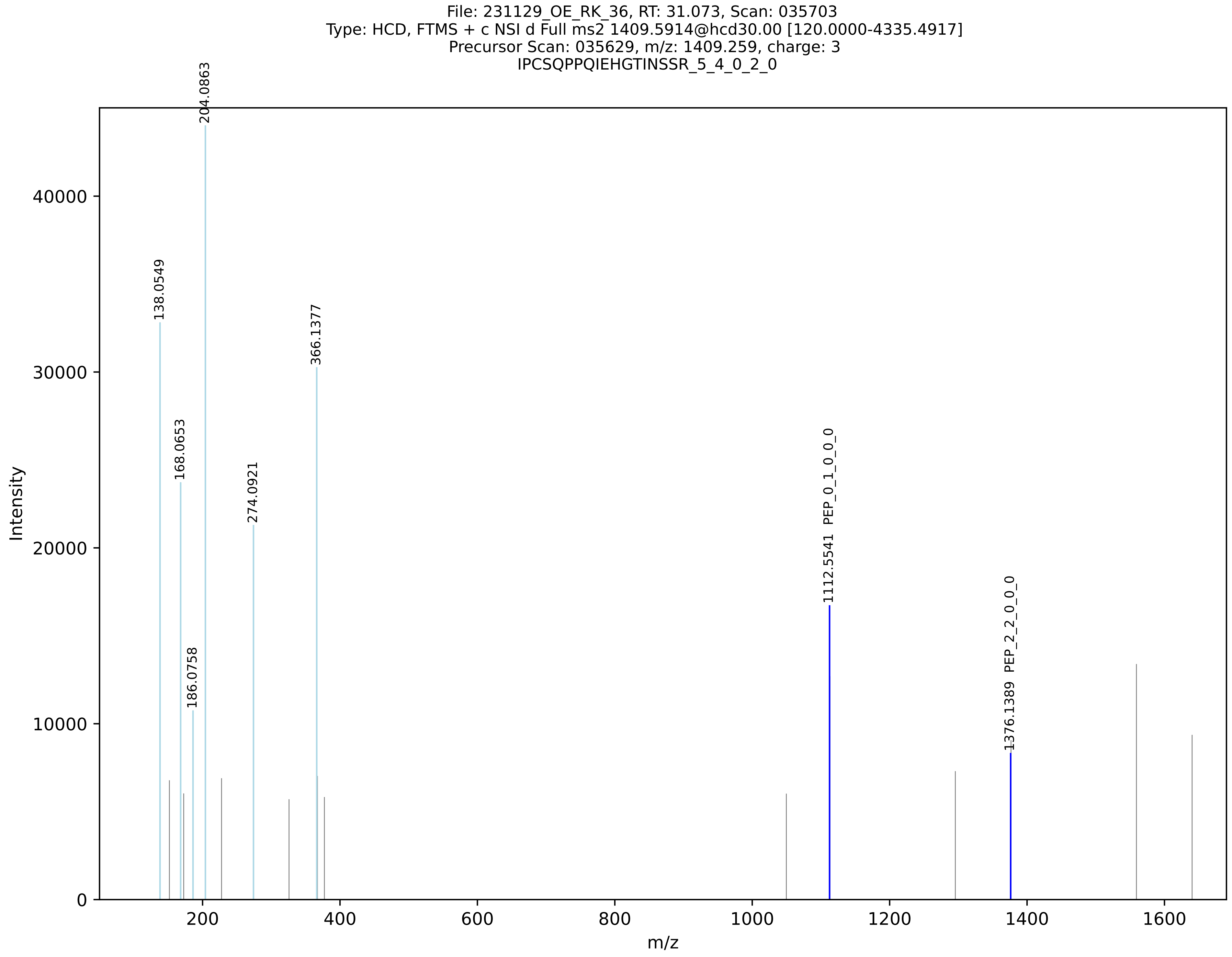

File: 231129_OE_RK_36, RT: 31.073, Scan: 035703
Type: HCD, FTMS + c NSI d Full ms2 1409.5914@hcd30.00 [120.0000-4335.4917]
Precursor Scan: 035629, m/z: 1409.259, charge: 3
IPCSQPPQIEHGTINSSR_5_4_0_2_0
204.0863
138.0549
366.1377
168.0653
274.0921
186.0758
1112.5541 PEP_0_1_0_0_0
1376.1389 PEP_2_2_0_0_0
Intensity
m/z
0
10000
20000
30000
40000
200
400
600
800
1000
1200
1400
1600

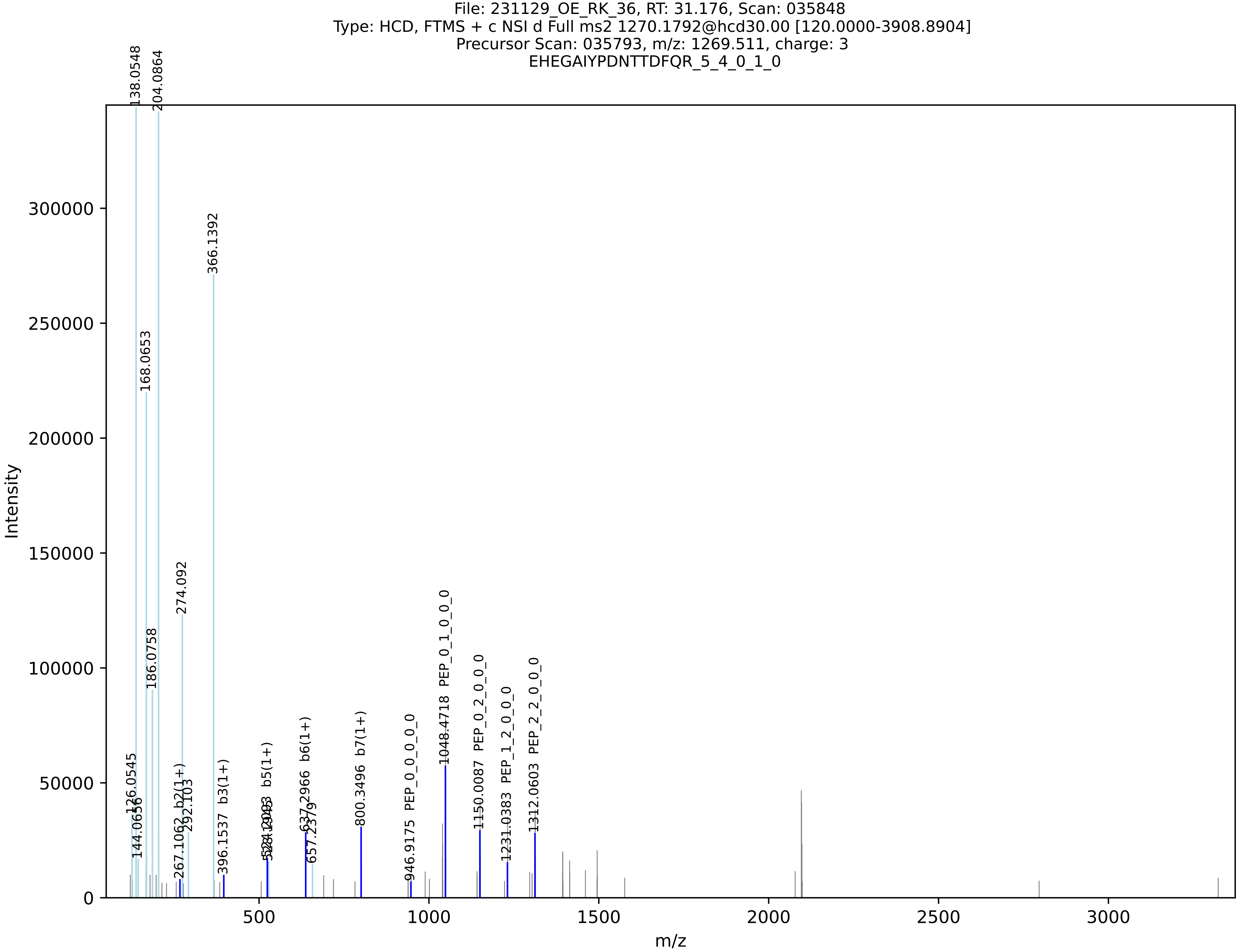
File: 231129_OE_RK_36, RT: 31.176, Scan: 035848
Type: HCD, FTMS + c NSI d Full ms2 1270.1792@hcd30.00 [120.0000-3908.8904]
Precursor Scan: 035793, m/z: 1269.511, charge: 3
EHEGAIYPDNTTDFQR_5_4_0_1_0
Intensity
m/z
138.0548
204.0864
366.1392
168.0653
274.092
186.0758
126.0545
144.0656
267.1062 b2(1+)
292.103
396.1537 b3(1+)
b5(1+)
637.2966 b6(1+)
657.2379
800.3496 b7(1+)
946.9175 PEP_0_0_0_0_0
1048.4718 PEP_0_1_0_0_0
1150.0087 PEP_0_2_0_0_0
1231.0383 PEP_1_2_0_0_0
1312.0603 PEP_2_2_0_0_0

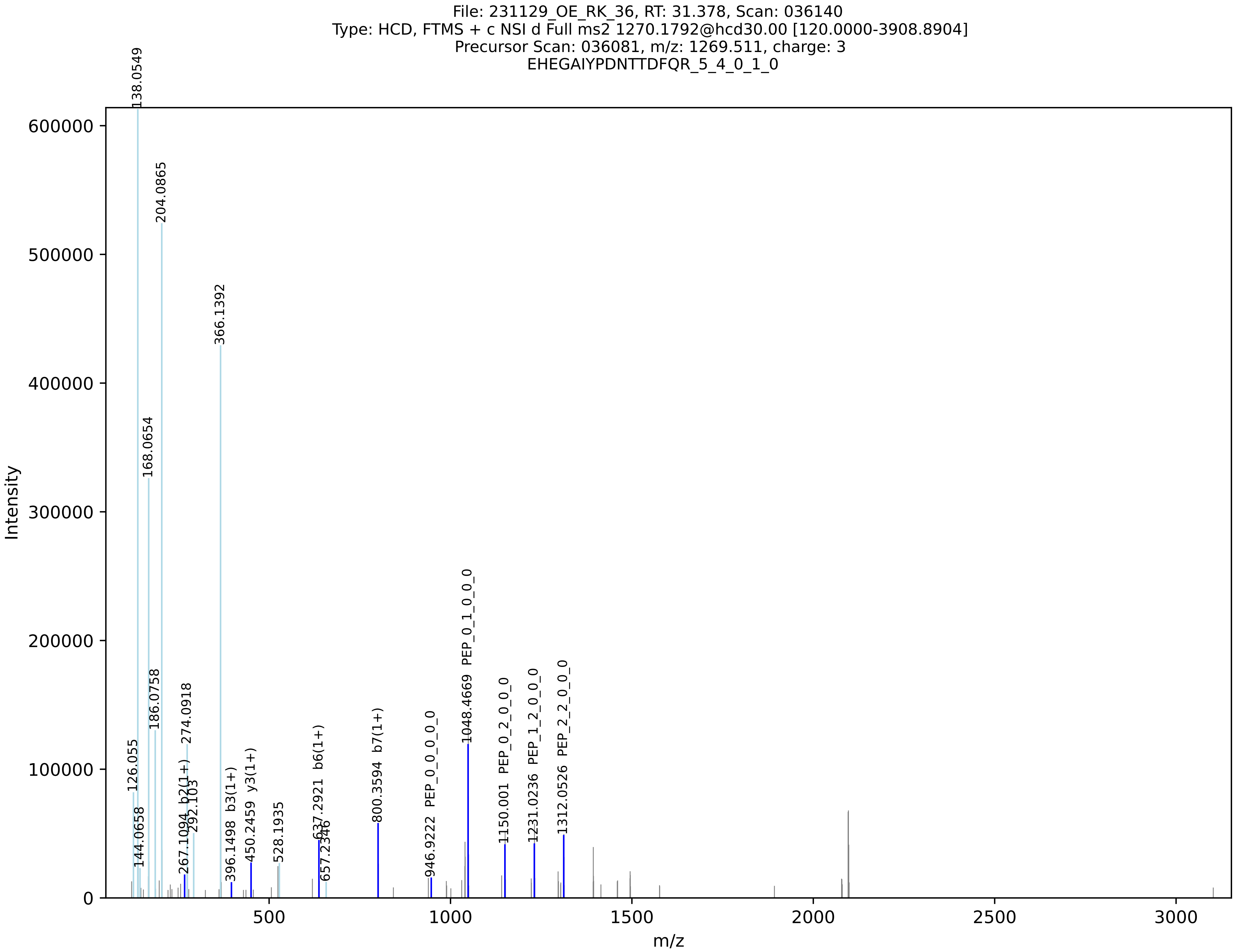

File: 231129_OE_RK_36, RT: 31.378, Scan: 036140
Type: HCD, FTMS + c NSI d Full ms2 1270.1792@hcd30.00 [120.0000-3908.8904]
Precursor Scan: 036081, m/z: 1269.511, charge: 3
EHEGAIYPDNTTDFQR_5_4_0_1_0
138.0549
204.0865
366.1392
168.0654
186.0758
274.0918
126.055
144.0658
267.1094 b2(1+)
292.103
396.1498 b3(1+)
450.2459 y3(1+)
528.1935
637.2921 b6(1+)
657.2346
800.3594 b7(1+)
946.9222 PEP_0_0_0_0_0
1048.4669 PEP_0_1_0_0_0
1150.001 PEP_0_2_0_0_0
1231.0236 PEP_1_2_0_0_0
1312.0526 PEP_2_2_0_0_0
Intensity
m/z

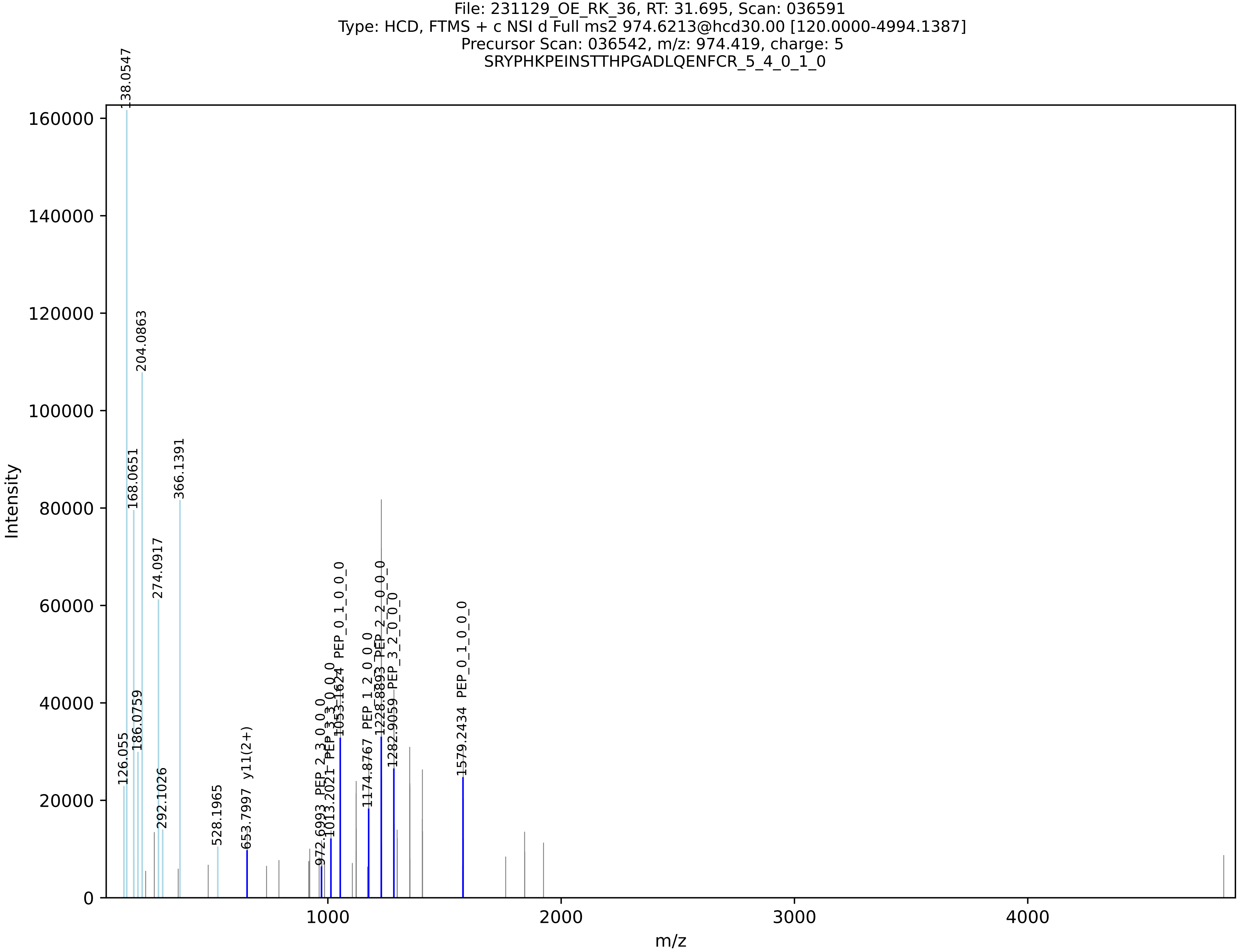
File: 231129_OE_RK_36, RT: 31.695, Scan: 036591
Type: HCD, FTMS + c NSI d Full ms2 974.6213@hcd30.00 [120.0000-4994.1387]
Precursor Scan: 036542, m/z: 974.419, charge: 5
SRYPHKPEINSTTHPGADLQENFCR_5_4_0_1_0
138.0547
204.0863
168.0651
366.1391
274.0917
186.0759
126.055
292.1026
528.1965
653.7997 y11(2+)
972.6993 PEP_2_3_0_0_0
1013.2021 PEP_3_3_0_0_0
1053.1624 PEP_0_1_0_0_0
1174.8767 PEP_1_2_0_0_0
1228.8893 PEP_2_2_0_0_0
1282.9059 PEP_3_2_0_0_0
1579.2434 PEP_0_1_0_0_0
Intensity
m/z
0
20000
40000
60000
80000
100000
120000
140000
160000
1000
2000
3000
4000

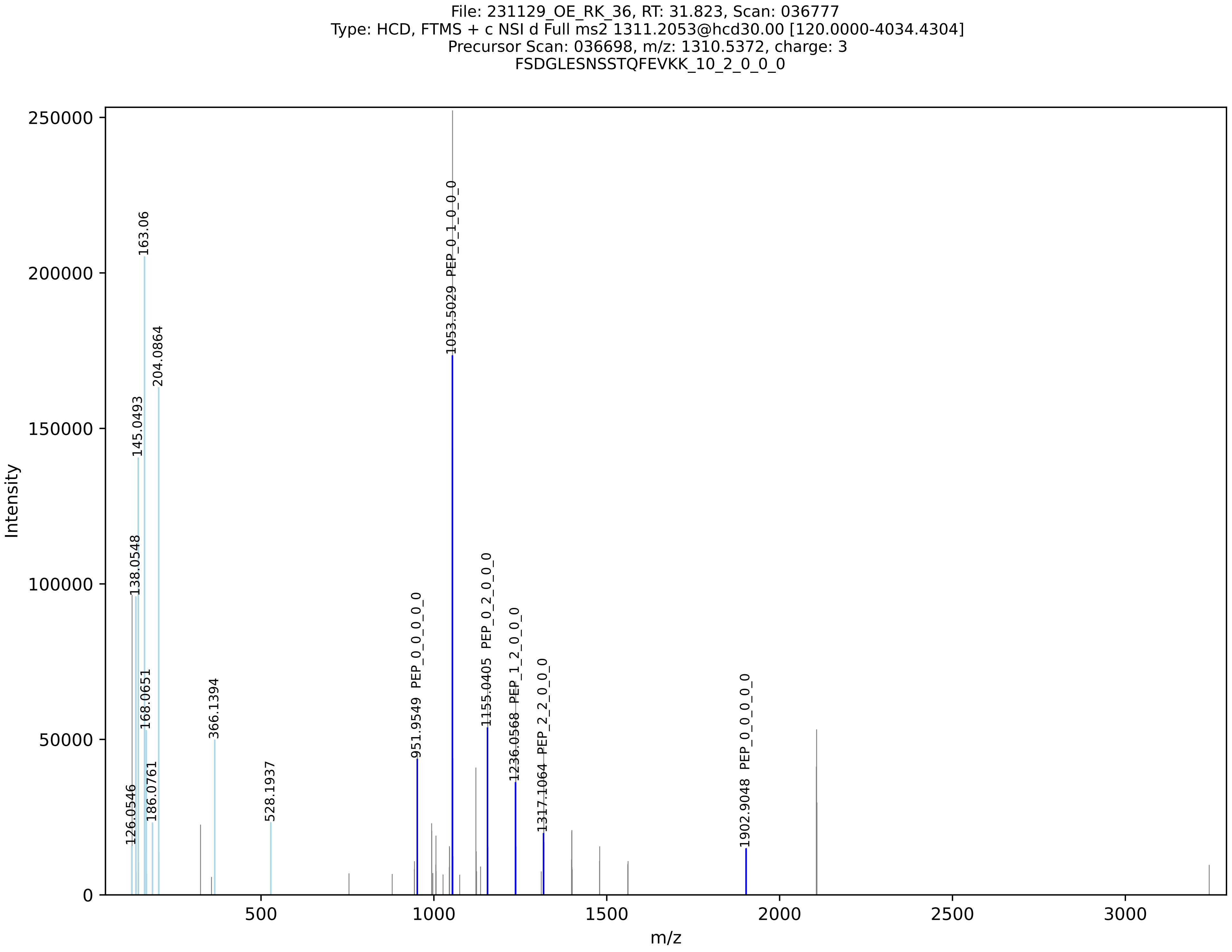
File: 231129_OE_RK_36, RT: 31.823, Scan: 036777
Type: HCD, FTMS + c NSI d Full ms2 1311.2053@hcd30.00 [120.0000-4034.4304]
Precursor Scan: 036698, m/z: 1310.5372, charge: 3
FSDGLESNSSTQFEVKK_10_2_0_0_0
Intensity
m/z
163.06
204.0864
145.0493
138.0548
168.0651
186.0761
126.0546
366.1394
528.1937
951.9549 PEP_0_0_0_0_0
1053.5029 PEP_0_1_0_0_0
1155.0405 PEP_0_2_0_0_0
1236.0568 PEP_1_2_0_0_0
1317.1064 PEP_2_2_0_0_0
1902.9048 PEP_0_0_0_0_0

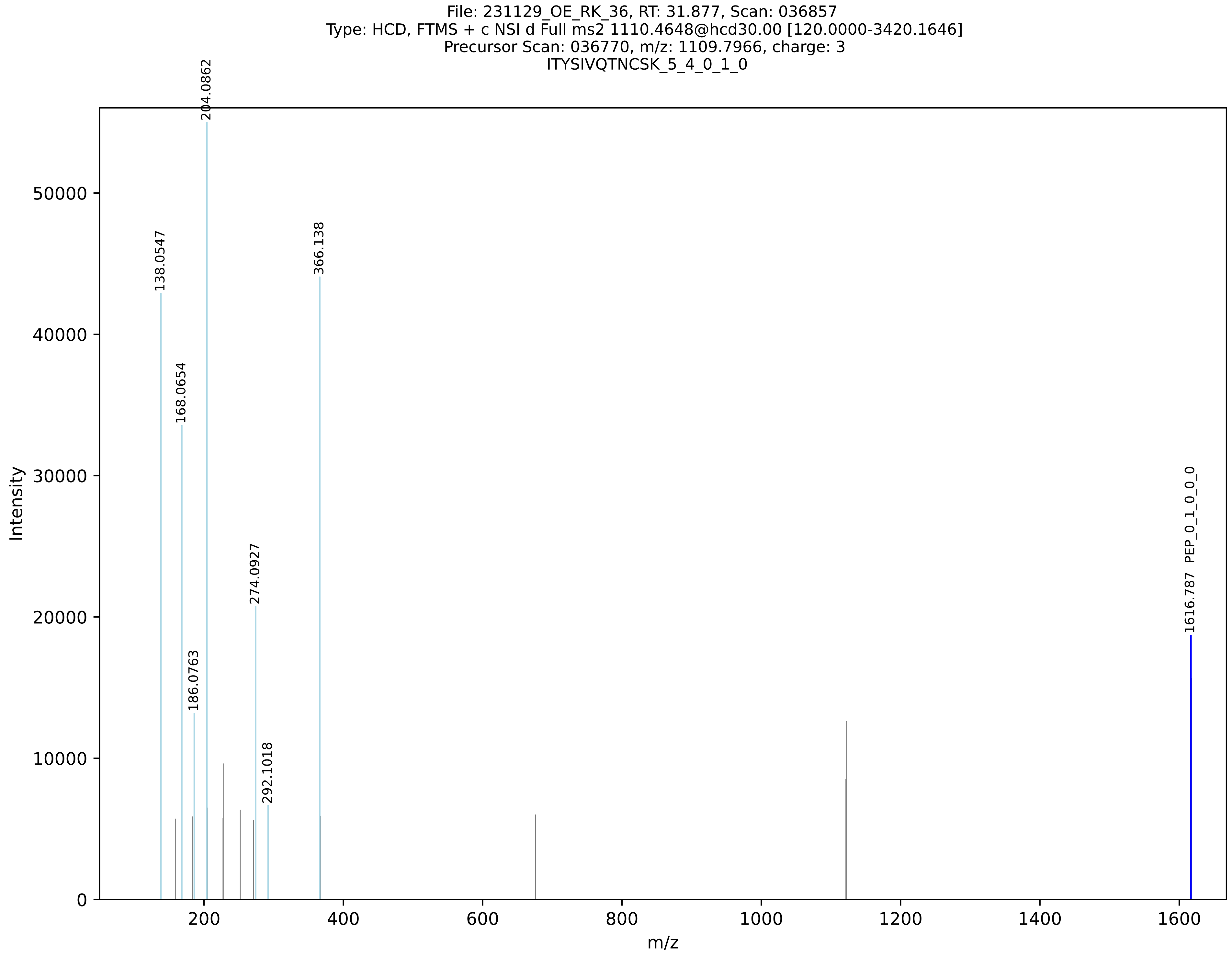

File: 231129_OE_RK_36, RT: 31.877, Scan: 036857
Type: HCD, FTMS + c NSI d Full ms2 1110.4648@hcd30.00 [120.0000-3420.1646]
Precursor Scan: 036770, m/z: 1109.7966, charge: 3
ITYSIVQTNCSK_5_4_0_1_0
204.0862
138.0547
366.138
168.0654
274.0927
186.0763
292.1018
1616.787 PEP_0_1_0_0_0
Intensity
m/z
0
10000
20000
30000
40000
50000
200
400
600
800
1000
1200
1400
1600

File: 231129_OE_RK_36, RT: 31.912, Scan: 036908
Type: HCD, FTMS + c NSI d Full ms2 1203.1700@hcd30.00 [120.0000-3703.8423]
Precursor Scan: 036832, m/z: 1202.8369, charge: 3
FSDGLESNSSTQFEVKK_8_2_0_0_0

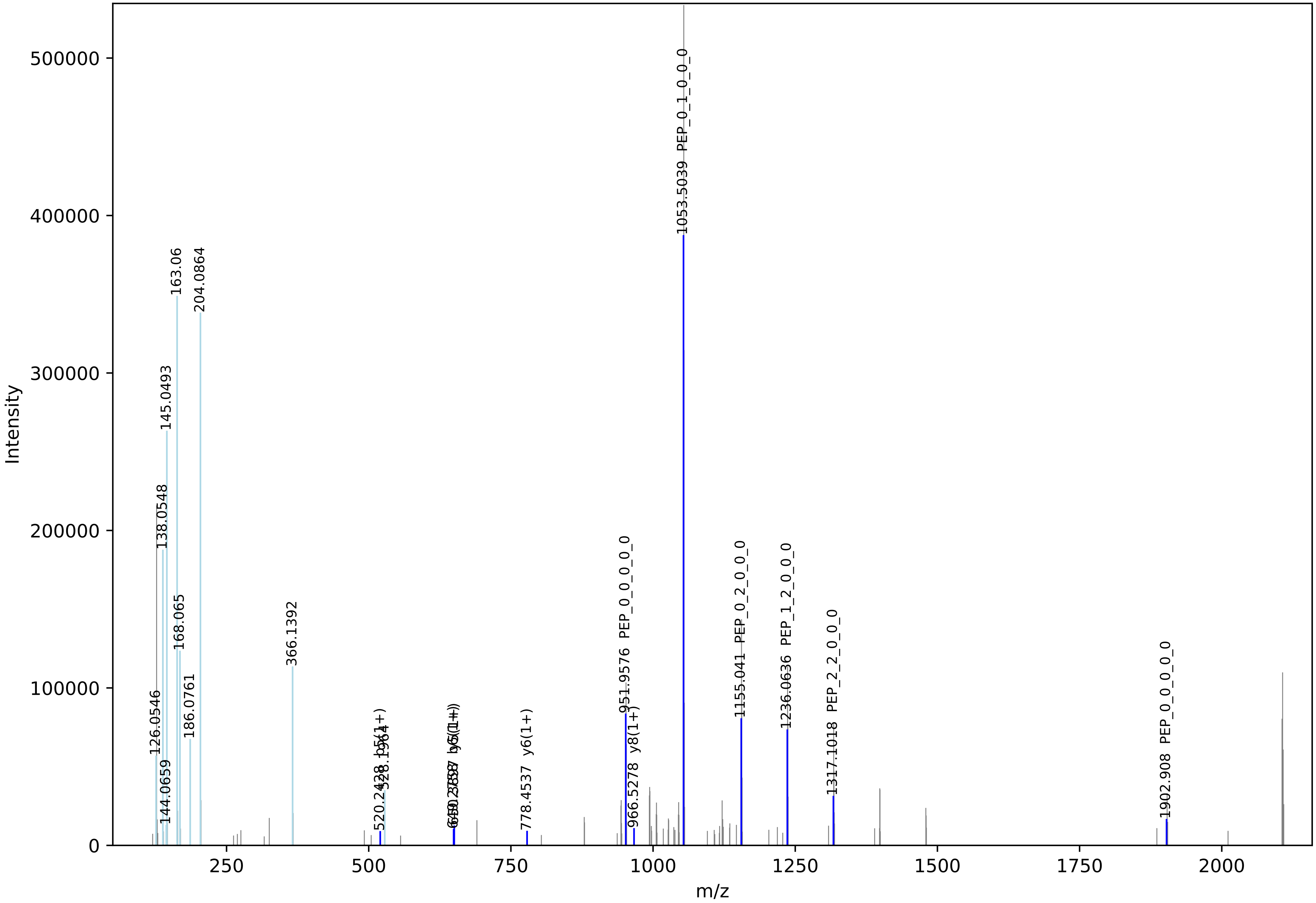

File: 231129_OE_RK_36, RT: 31.975, Scan: 036995
Type: HCD, FTMS + c NSI d Full ms2 902.6289@hcd30.00 [120.0000-3705.5818]
Precursor Scan: 036940, m/z: 902.1279, charge: 4
FSDGLESNSSTQFEVKK_8_2_0_0_0

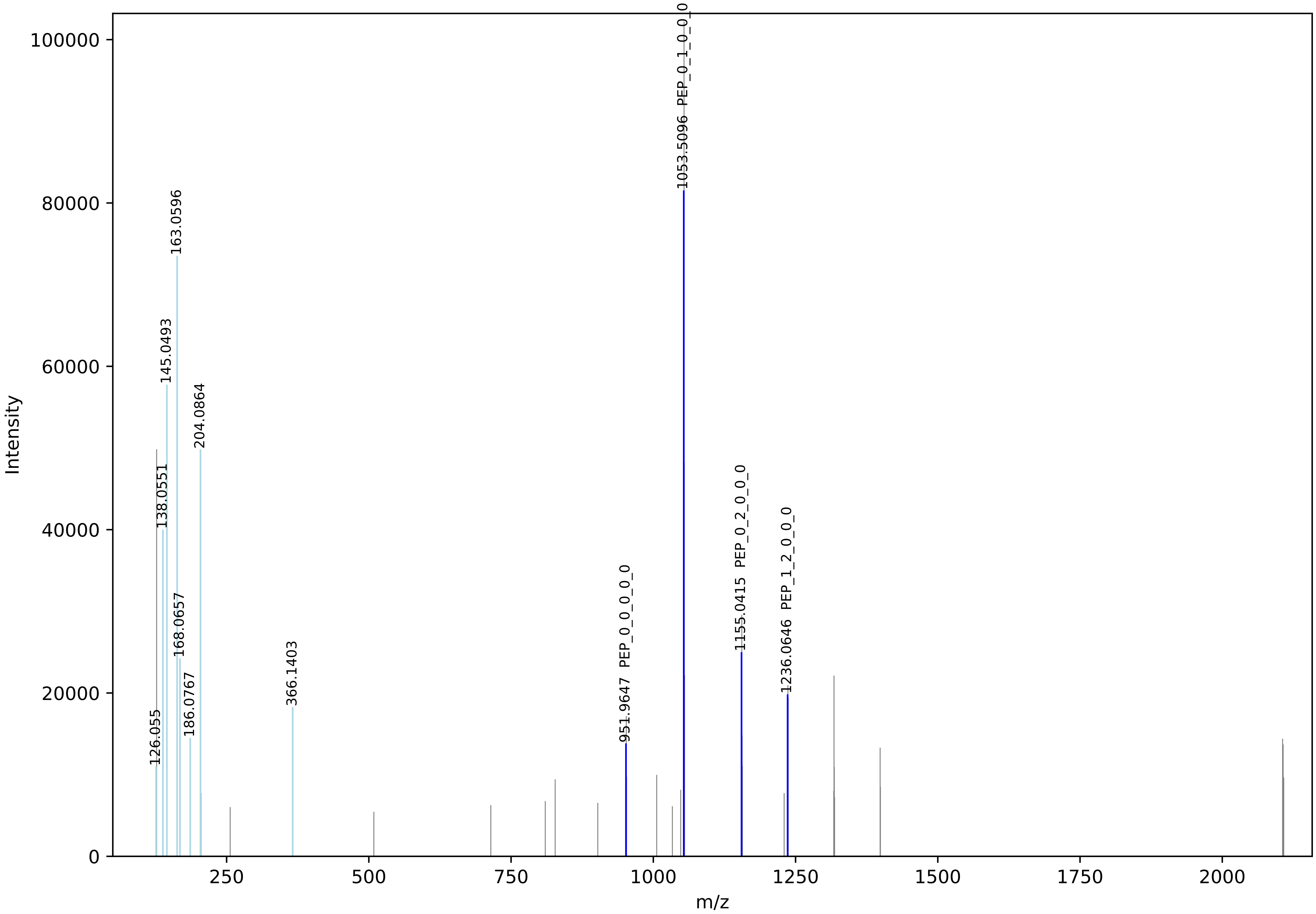

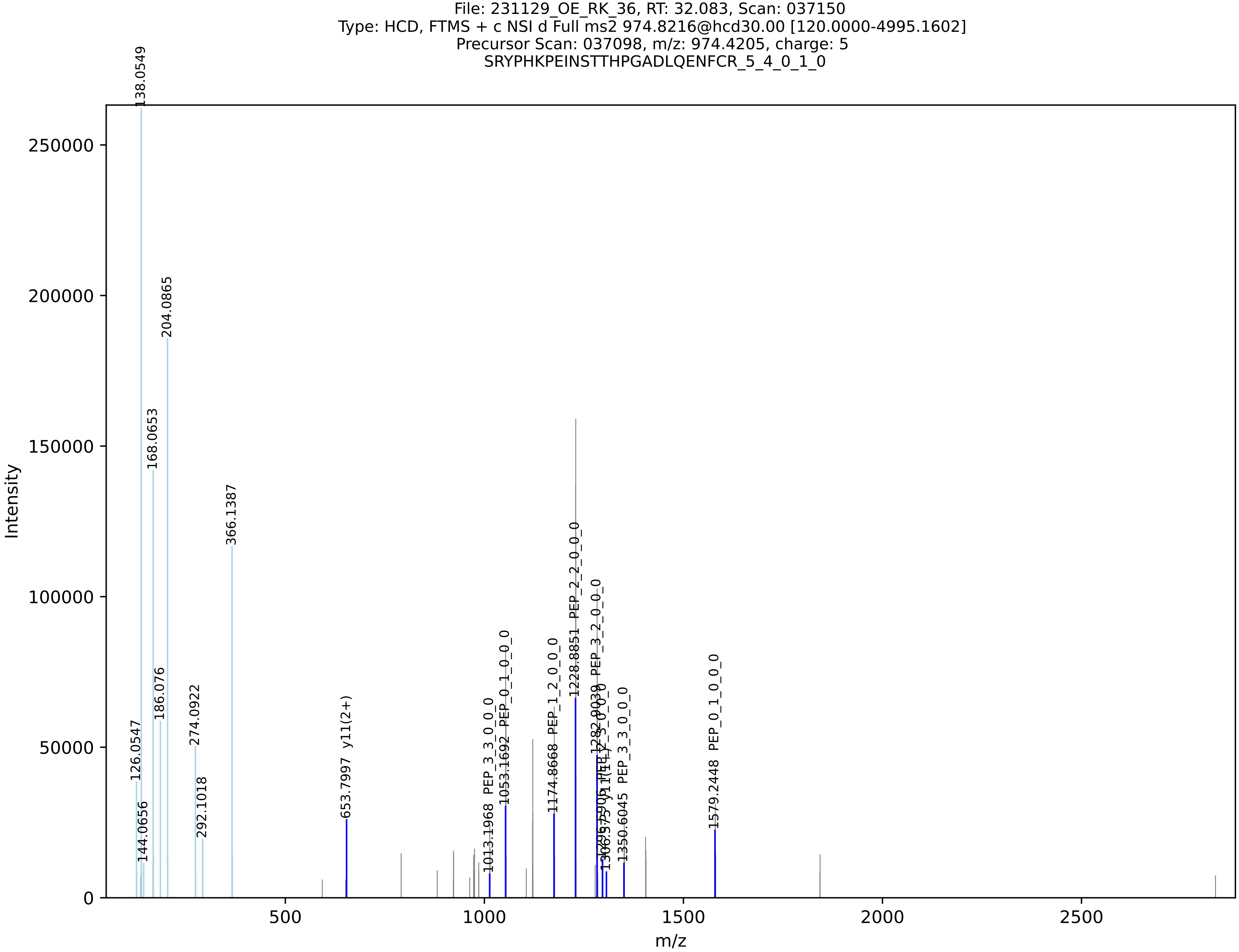
File: 231129_OE_RK_36, RT: 32.083, Scan: 037150
Type: HCD, FTMS + c NSI d Full ms2 974.8216@hcd30.00 [120.0000-4995.1602]
Precursor Scan: 037098, m/z: 974.4205, charge: 5
SRYPHKPEINSTTHPGADLQENFCR_5_4_0_1_0
Intensity
m/z
138.0549
204.0865
168.0653
366.1387
186.076
274.0922
126.0547
292.1018
144.0656
653.7997 y11(2+)
1013.1968 PEP_3_3_0_0_0
1053.1692 PEP_0_1_0_0_0
1174.8668 PEP_1_2_0_0_0
1228.8851 PEP_2_2_0_0_0
1282.9039 PEP_3_2_0_0_0
1350.6045 PEP_3_3_0_0_0
1579.2448 PEP_0_1_0_0_0

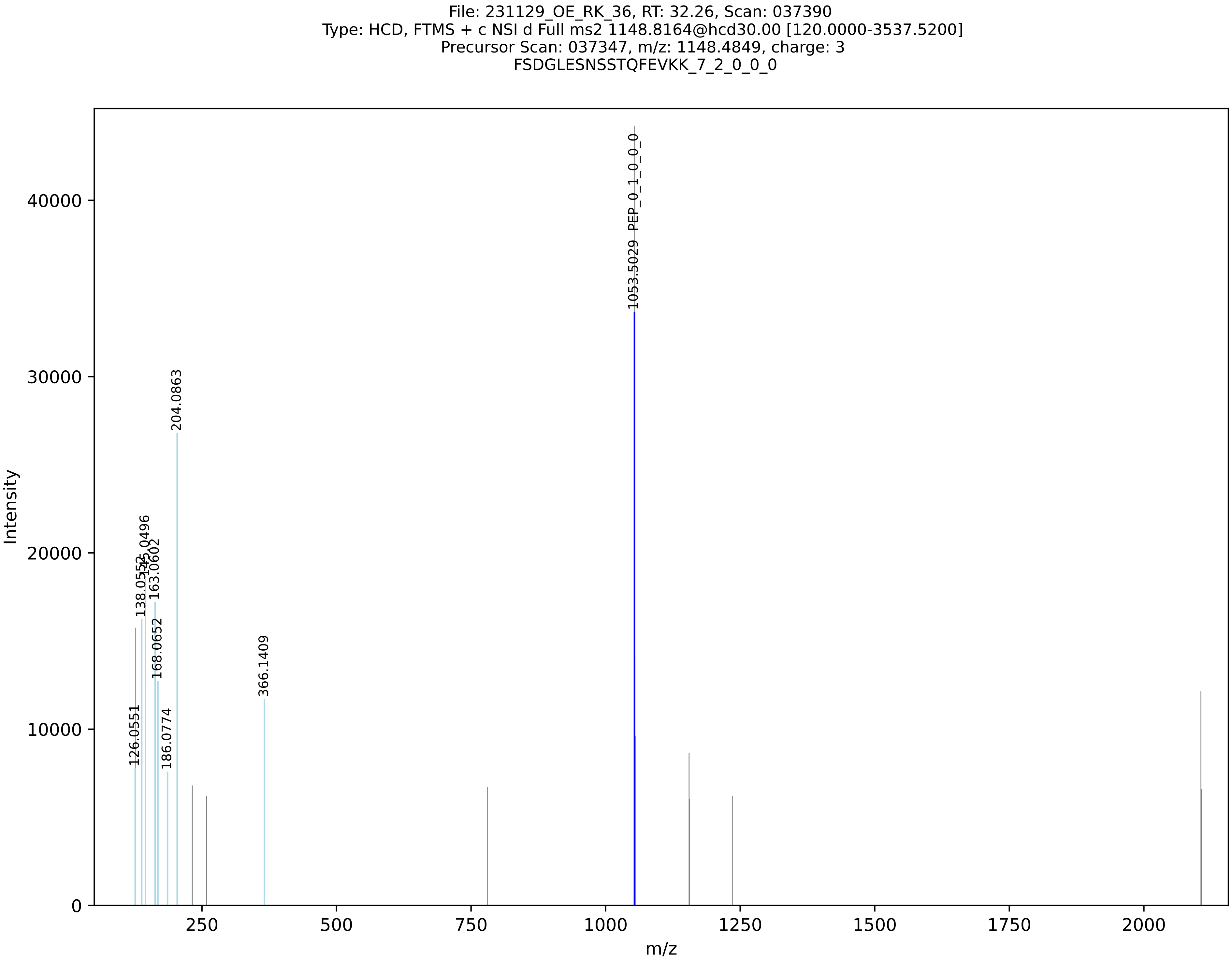
File: 231129_OE_RK_36, RT: 32.26, Scan: 037390
Type: HCD, FTMS + c NSI d Full ms2 1148.8164@hcd30.00 [120.0000-3537.5200]
Precursor Scan: 037347, m/z: 1148.4849, charge: 3
FSDGLESNSSTQFEVKK_7_2_0_0_0
Intensity
m/z
1053.5029_PEP_0_1_0_0_0
204.0863
138.0552
145.0496
163.0602
168.0652
366.1409
126.0551
186.0774

File: 231129_OE_RK_36, RT: 32.755, Scan: 038078
Type: HCD, FTMS + c NSI d Full ms2 1061.9222@hcd30.00 [120.0000-4355.4990]
Precursor Scan: 038045, m/z: 1061.6717, charge: 4
EHEGAIYPDNTTDFQR_5_4_1_2_0

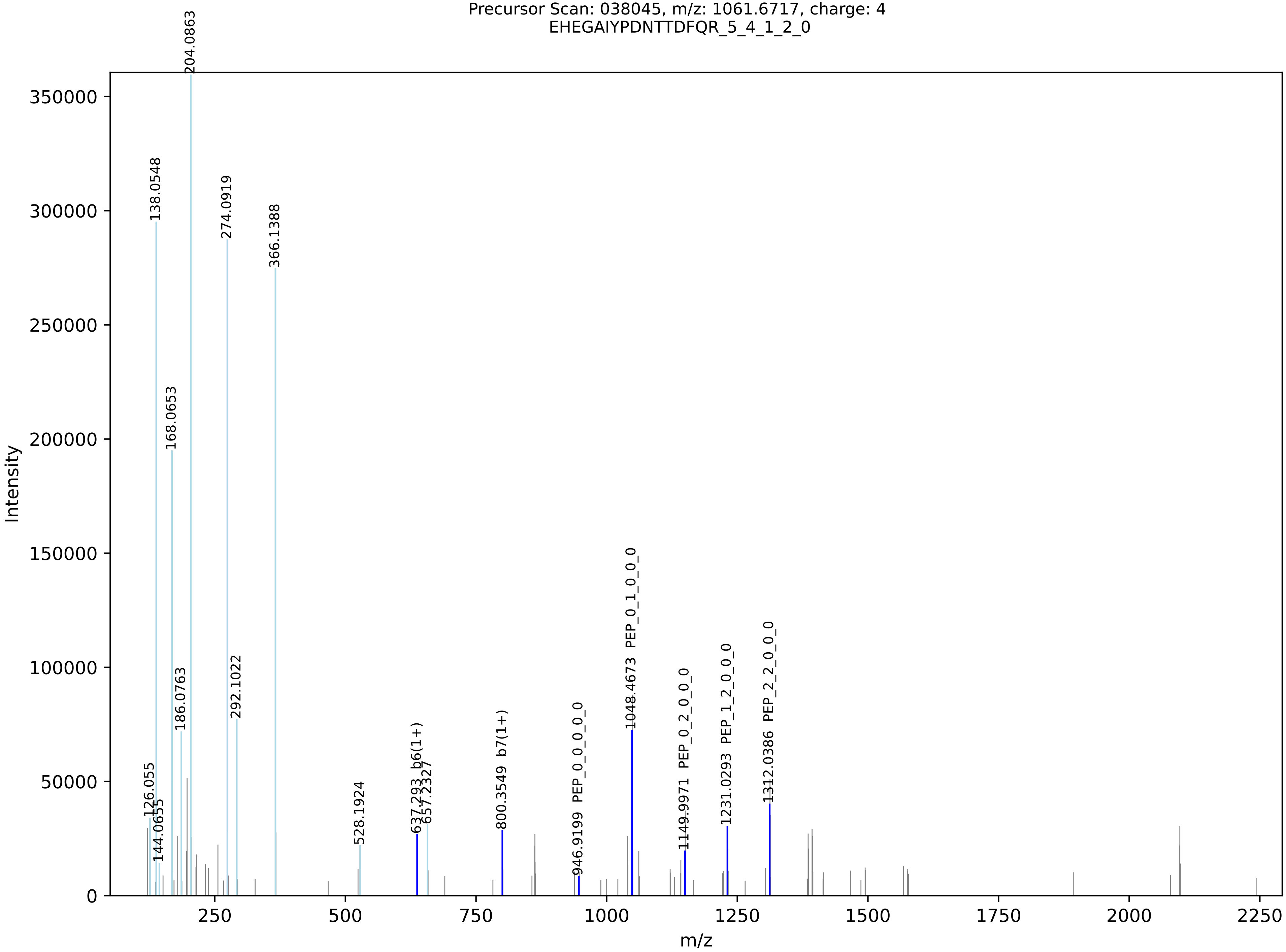

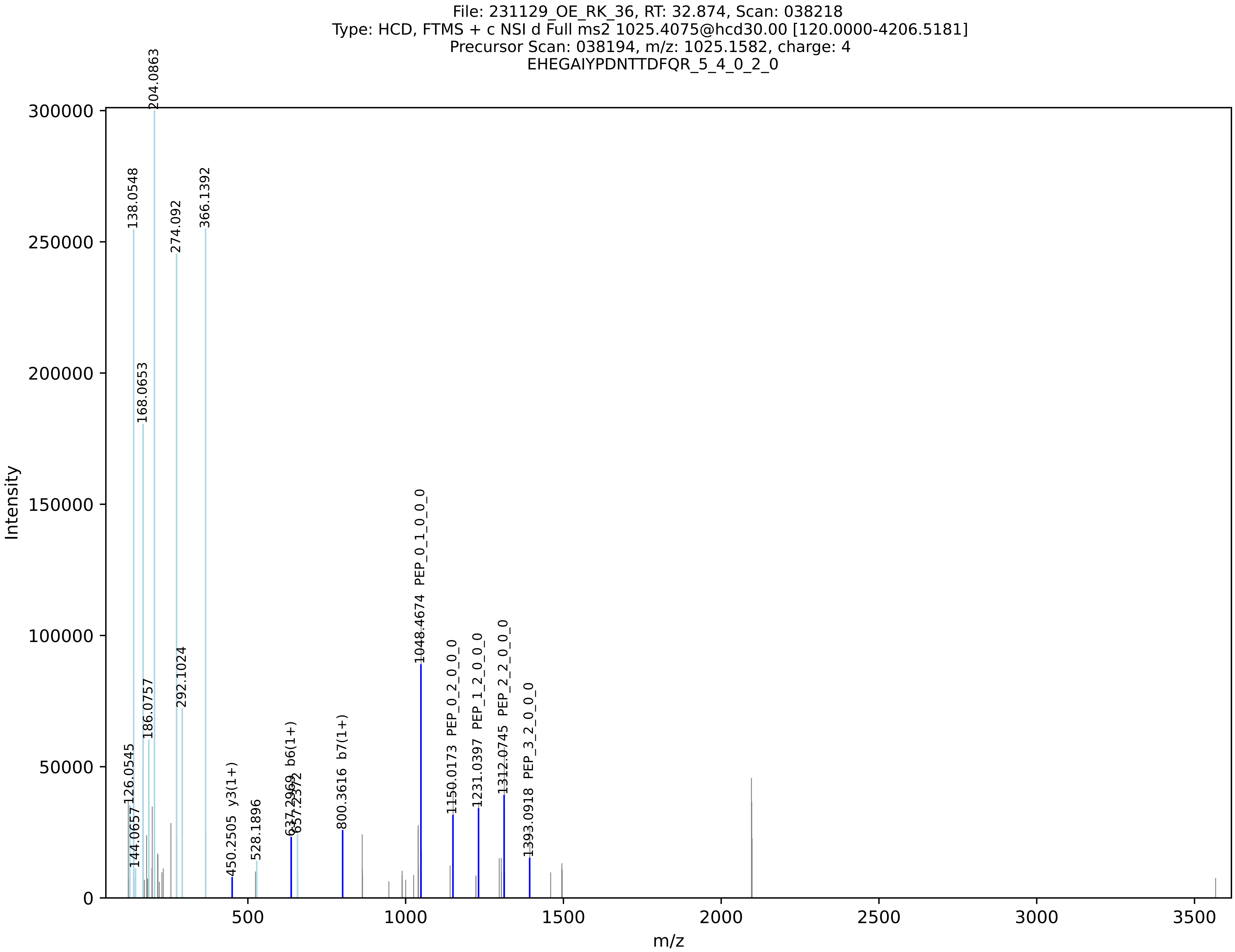

File: 231129_OE_RK_36, RT: 32.874, Scan: 038218
Type: HCD, FTMS + c NSI d Full ms2 1025.4075@hcd30.00 [120.0000-4206.5181]
Precursor Scan: 038194, m/z: 1025.1582, charge: 4
EHEGAIYPDNTTDFQR_5_4_0_2_0
Intensity
m/z
0
50000
100000
150000
200000
250000
300000
500
1000
1500
2000
2500
3000
3500
204.0863
138.0548
366.1392
274.092
168.0653
292.1024
186.0757
126.0545
144.0657
450.2505 y3(1+)
528.1896
637.2969 b6(1+)
657.2372
800.3616 b7(1+)
1048.4674 PEP_0_1_0_0_0
1150.0173 PEP_0_2_0_0_0
1231.0397 PEP_1_2_0_0_0
1312.0745 PEP_2_2_0_0_0
1393.0918 PEP_3_2_0_0_0

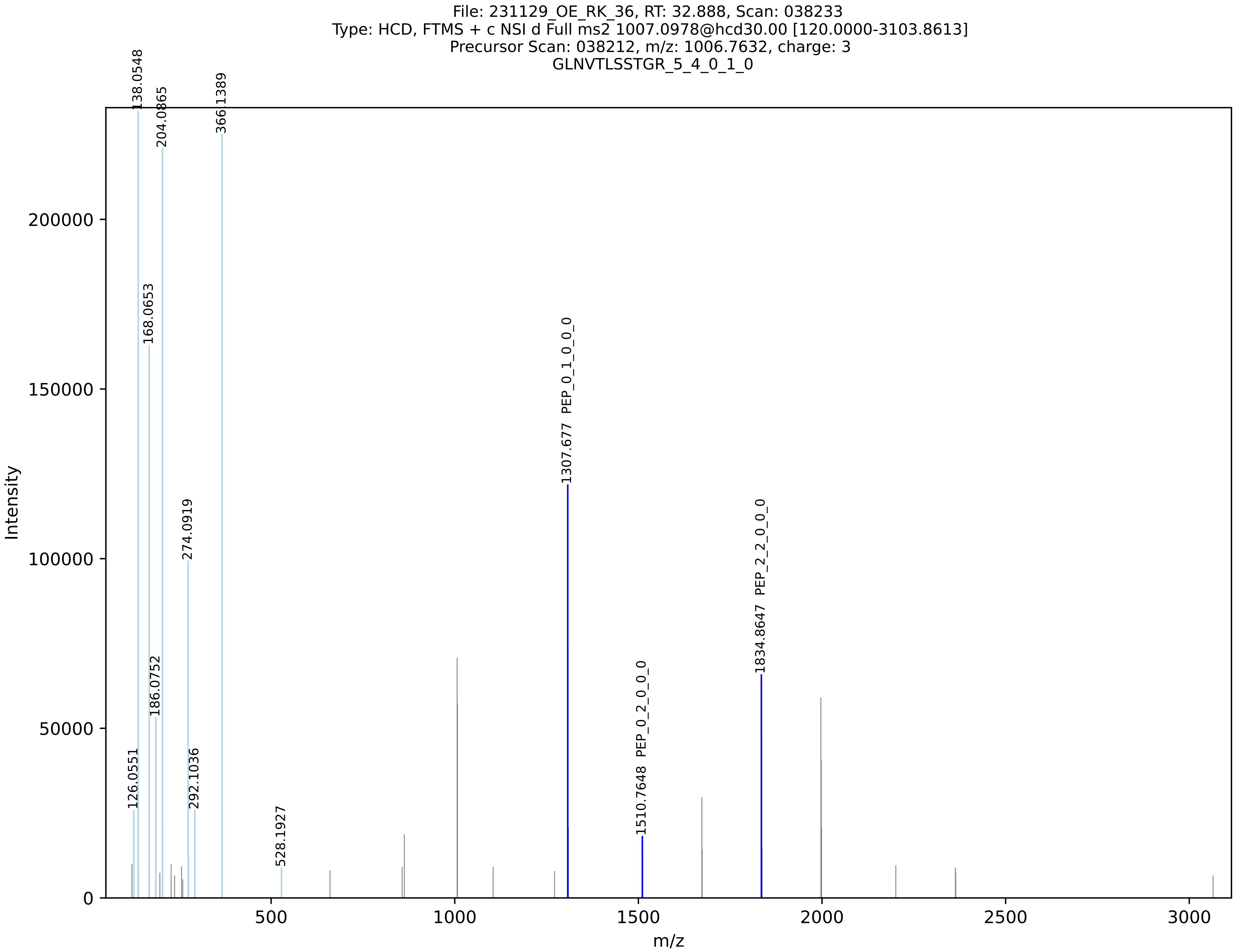
File: 231129_OE_RK_36, RT: 32.888, Scan: 038233
Type: HCD, FTMS + c NSI d Full ms2 1007.0978@hcd30.00 [120.0000-3103.8613]
Precursor Scan: 038212, m/z: 1006.7632, charge: 3
GLNVTLSSTGR_5_4_0_1_0
138.0548
204.0865
366.1389
168.0653
186.0752
274.0919
126.0551
292.1036
528.1927
1307.677 PEP_0_1_0_0_0
1510.7648 PEP_0_2_0_0_0
1834.8647 PEP_2_2_0_0_0
Intensity
m/z
0
50000
100000
150000
200000
500
1000
1500
2000
2500
3000

File: 231129_OE_RK_36, RT: 33.056, Scan: 038445
Type: HCD, FTMS + c NSI d Full ms2 1025.6588@hcd30.00 [120.0000-4207.5439]
Precursor Scan: 038404, m/z: 1025.1576, charge: 4
EHEGAIYPDNTTDFQR_5_4_0_2_0

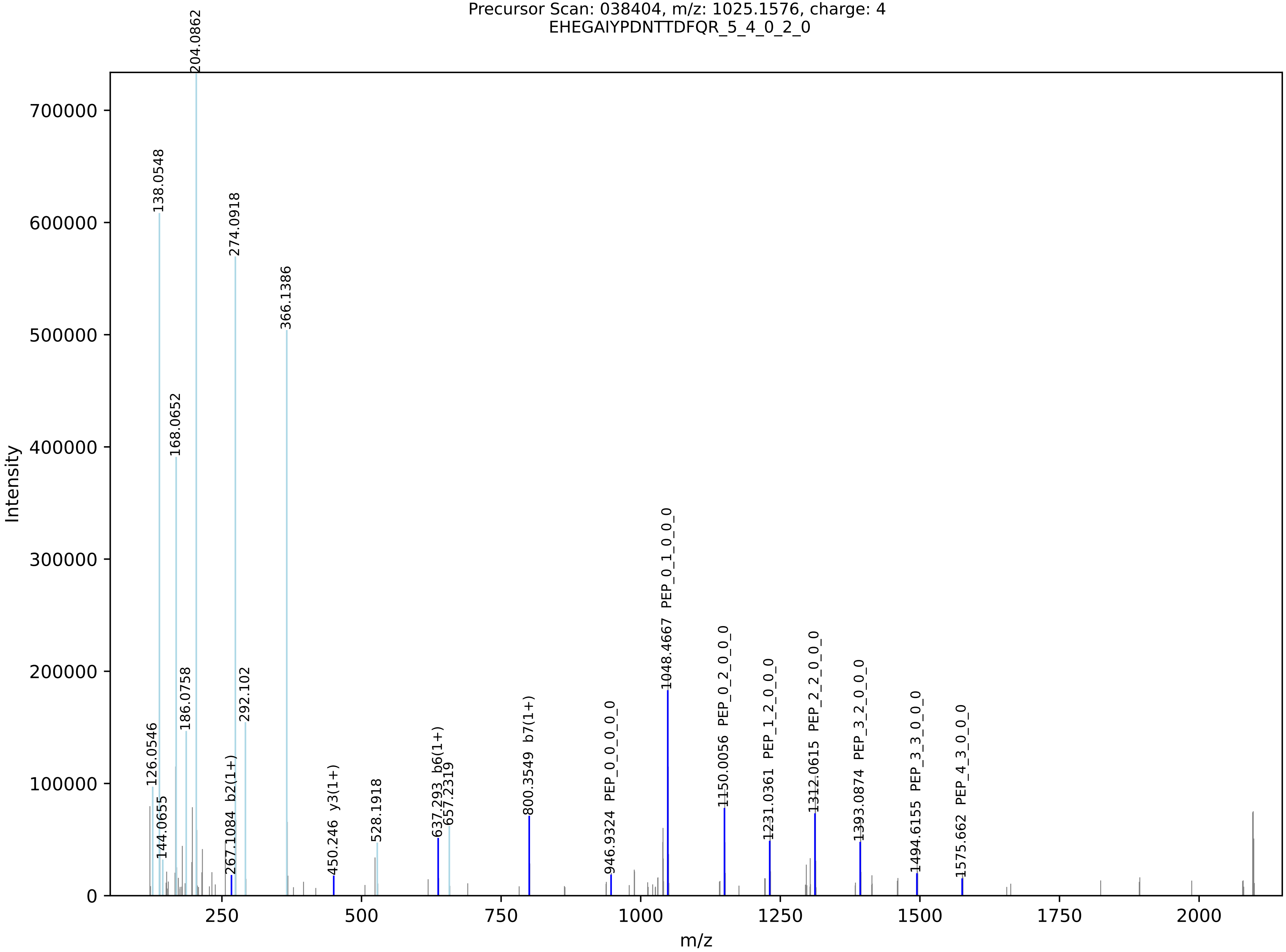

File: 231129_OE_RK_36, RT: 33.267, Scan: 038743
Type: HCD, FTMS + c NSI d Full ms2 1007.0977@hcd30.00 [120.0000-3103.8608]
Precursor Scan: 038676, m/z: 1006.7635, charge: 3
GLNVTLSSTGR_5_4_0_1_0

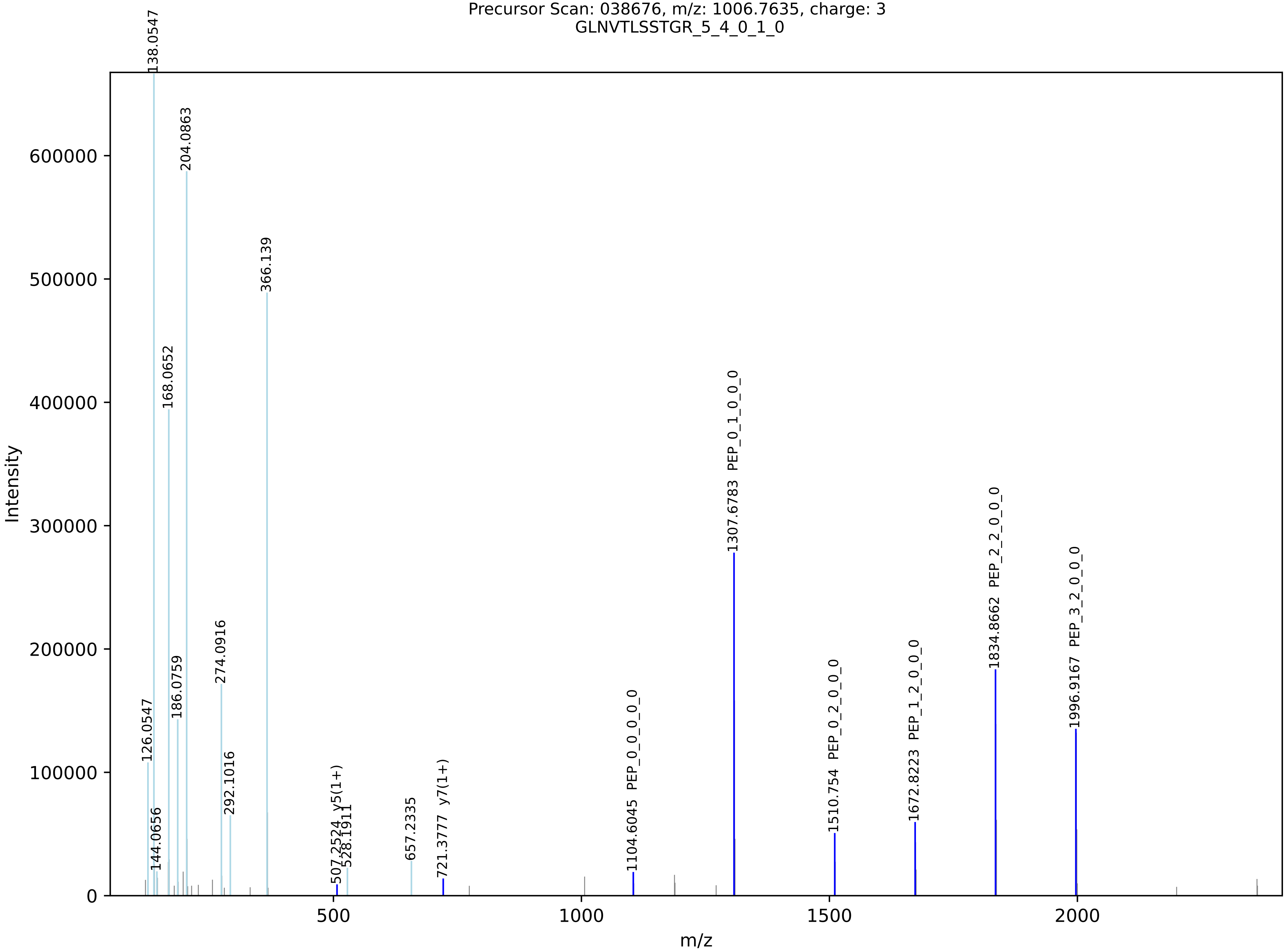

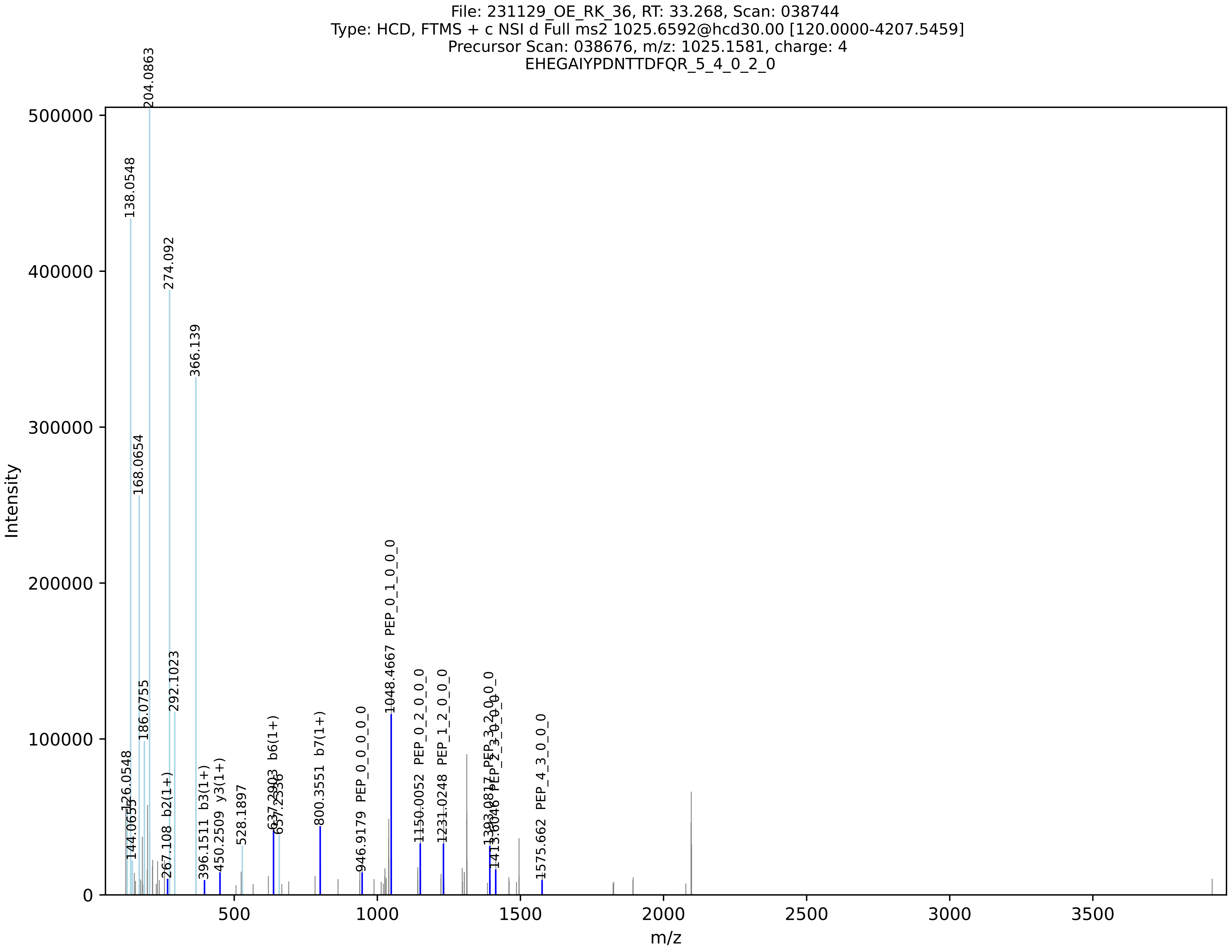

File: 231129_OE_RK_36, RT: 33.268, Scan: 038744
Type: HCD, FTMS + c NSI d Full ms2 1025.6592@hcd30.00 [120.0000-4207.5459]
Precursor Scan: 038676, m/z: 1025.1581, charge: 4
EHEGAIYPDNTTDFQR_5_4_0_2_0
Intensity
m/z
204.0863
138.0548
274.092
366.139
168.0654
292.1023
186.0755
126.0548
144.0655
267.108 b2(1+)
396.1511 b3(1+)
450.2509 y3(1+)
528.1897
637.2903 b6(1+)
657.2336
800.3551 b7(1+)
946.9179 PEP_0_0_0_0_0
1048.4667 PEP_0_1_0_0_0
1150.0052 PEP_0_2_0_0_0
1231.0248 PEP_1_2_0_0_0
1393.0817 PEP_3_2_0_0_0
1413.6046 PEP_2_3_0_0_0
1575.662 PEP_4_3_0_0_0

File: 231129_OE_RK_36, RT: 33.271, Scan: 038748
Type: HCD, FTMS + c NSI d Full ms2 1415.8954@hcd30.00 [120.0000-4354.7817]
Precursor Scan: 038676, m/z: 1415.2271, charge: 3
EHEGAIYPDNTTDFQR_5_4_1_2_0

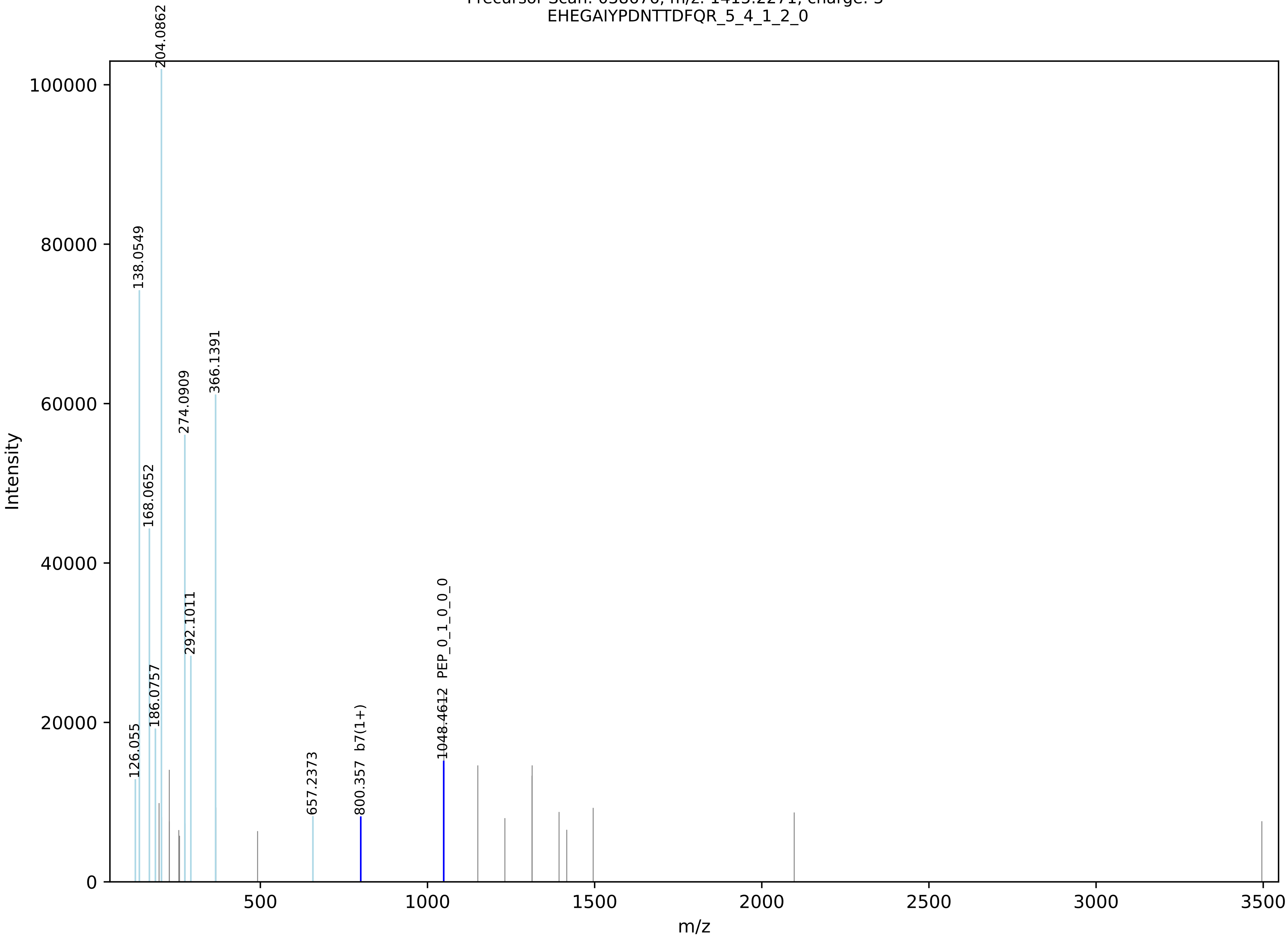

File: 231129_OE_RK_36, RT: 33.349, Scan: 038861
Type: HCD, FTMS + c NSI d Full ms2 1010.4206@hcd30.00 [120.0000-3114.0291]
Precursor Scan: 038803, m/z: 1009.7534, charge: 3
VLNFTTK_5_4_0_2_0

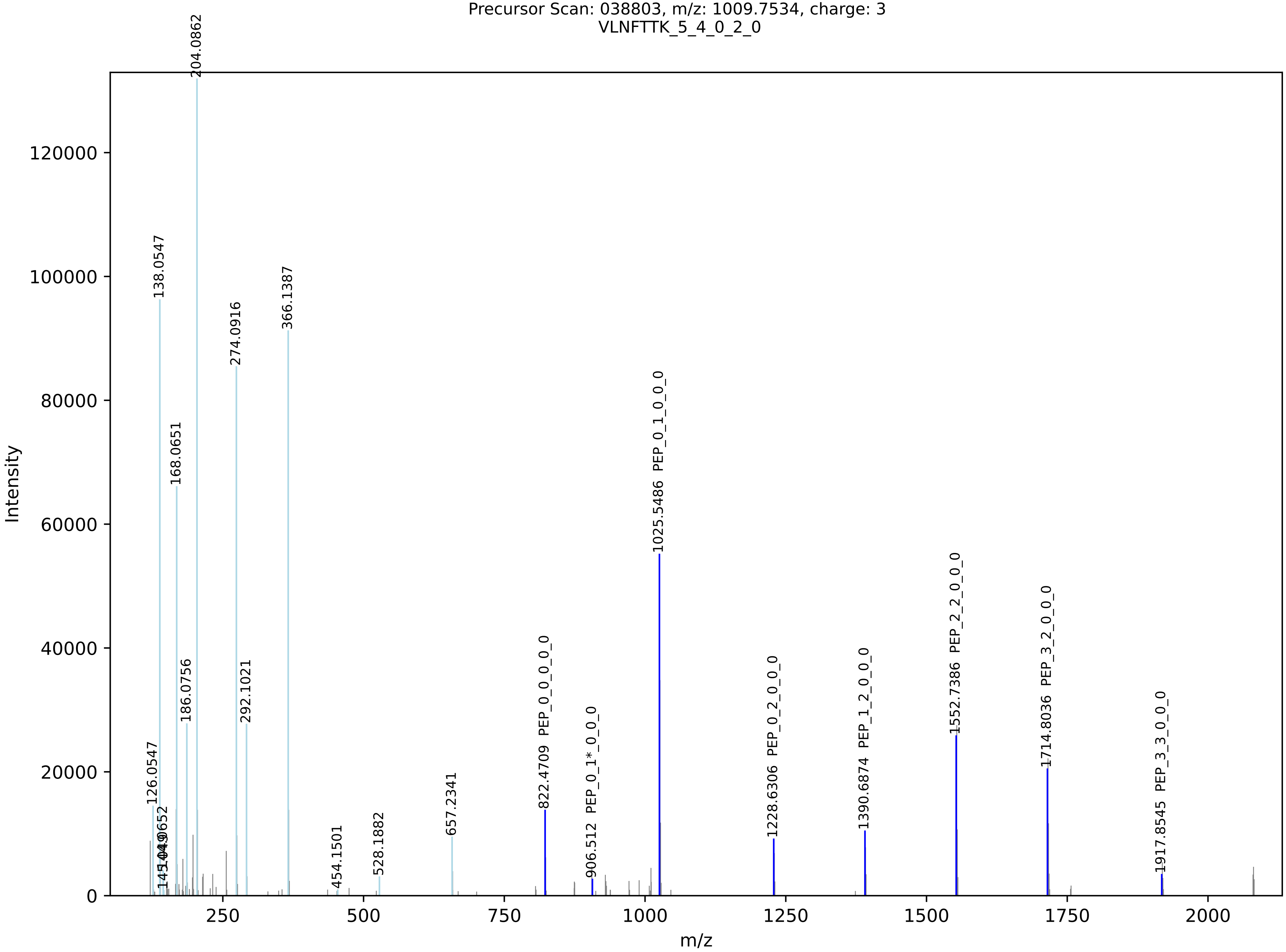

File: 231129_OE_RK_36, RT: 33.521, Scan: 039109
Type: HCD, FTMS + c NSI d Full ms2 1010.0870@hcd30.00 [120.0000-3113.0081]
Precursor Scan: 039039, m/z: 1009.7529, charge: 3
VLNFTTK_5_4_0_2_0

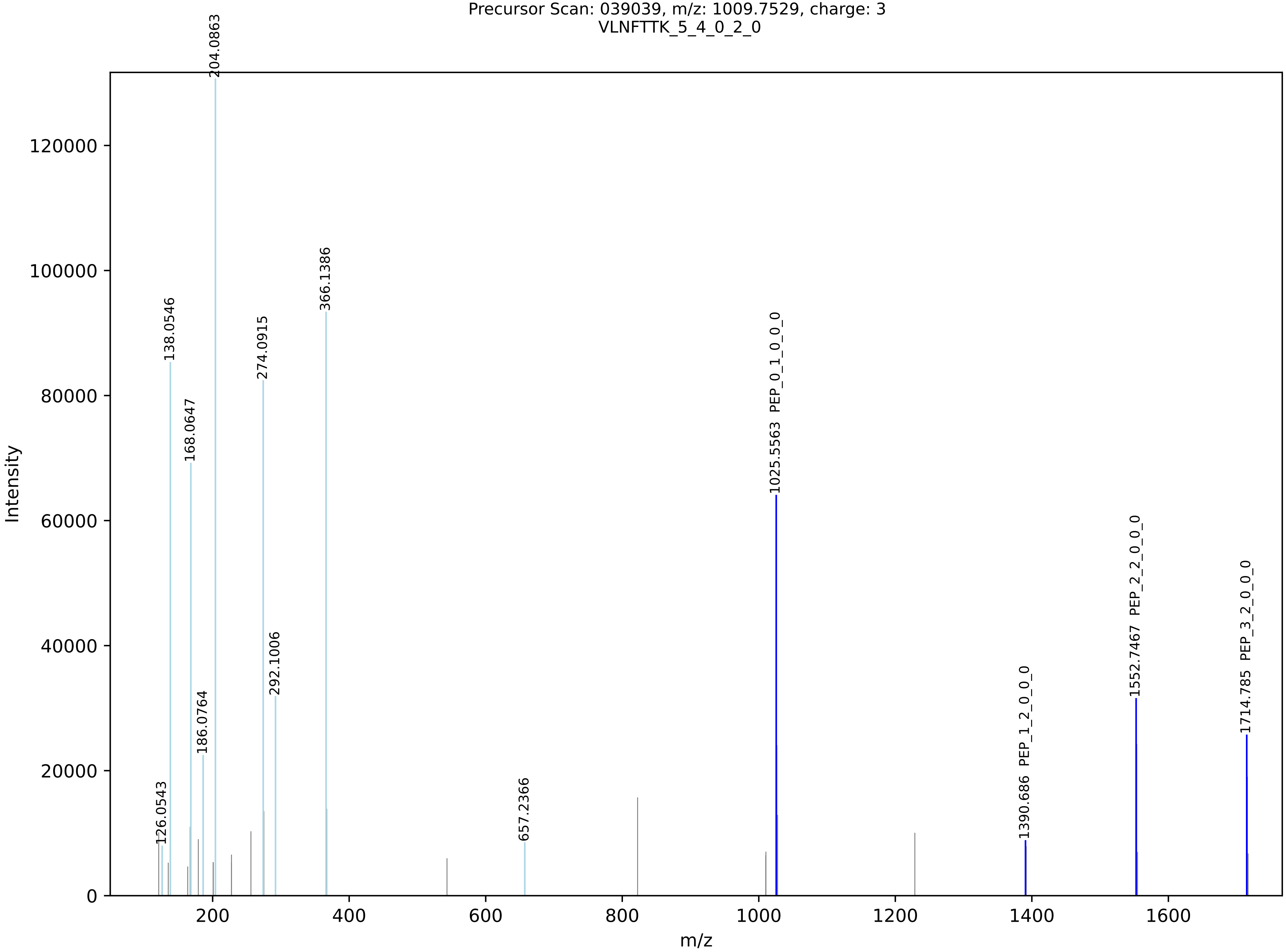

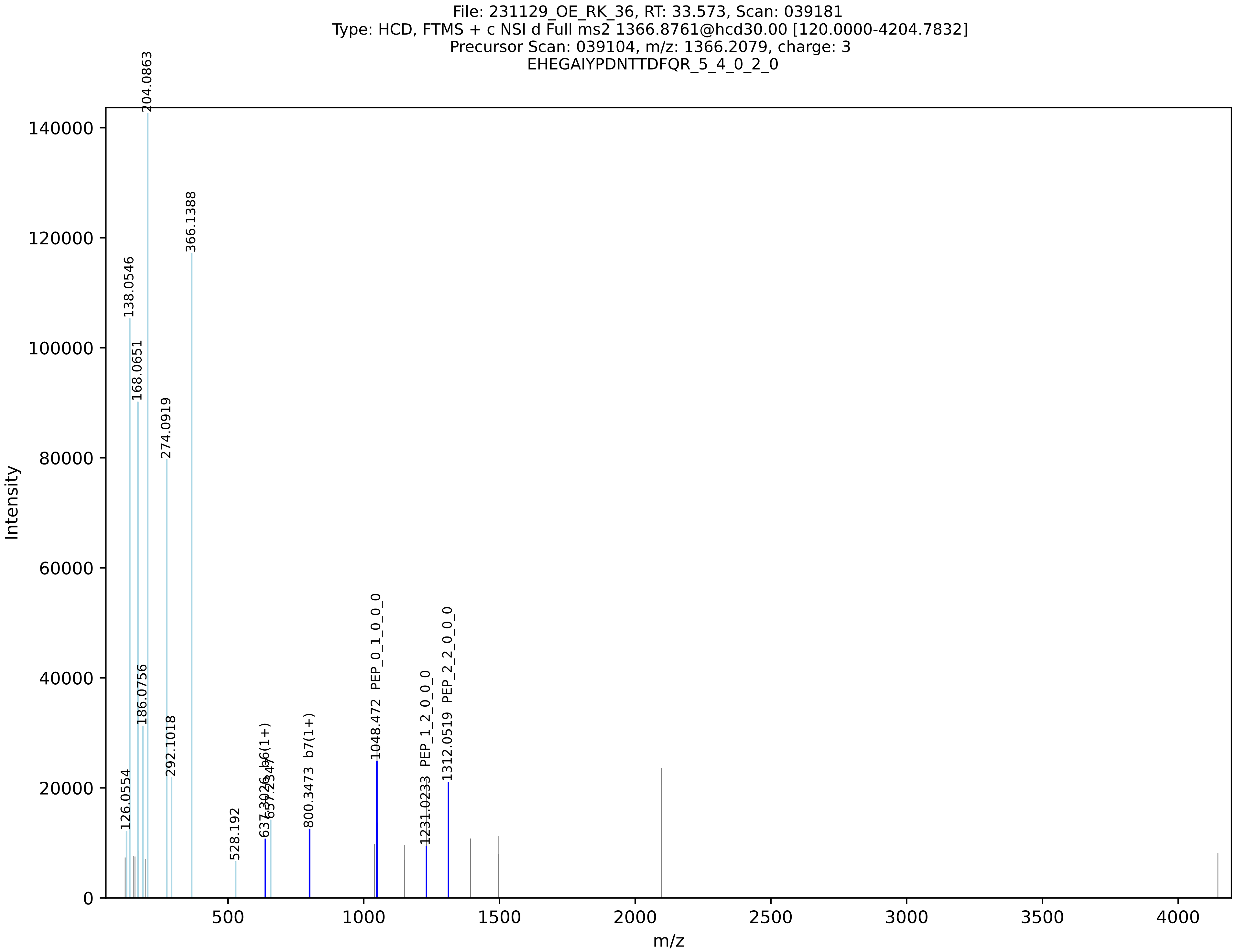
File: 231129_OE_RK_36, RT: 33.573, Scan: 039181
Type: HCD, FTMS + c NSI d Full ms2 1366.8761@hcd30.00 [120.0000-4204.7832]
Precursor Scan: 039104, m/z: 1366.2079, charge: 3
EHEGAIYPDNTTDFQR_5_4_0_2_0
Intensity
m/z
204.0863
366.1388
138.0546
168.0651
274.0919
186.0756
292.1018
126.0554
528.192
637.3026 b6(1+)
657.2347
800.3473 b7(1+)
1048.472 PEP_0_1_0_0_0
1231.0233 PEP_1_2_0_0_0
1312.0519 PEP_2_2_0_0_0

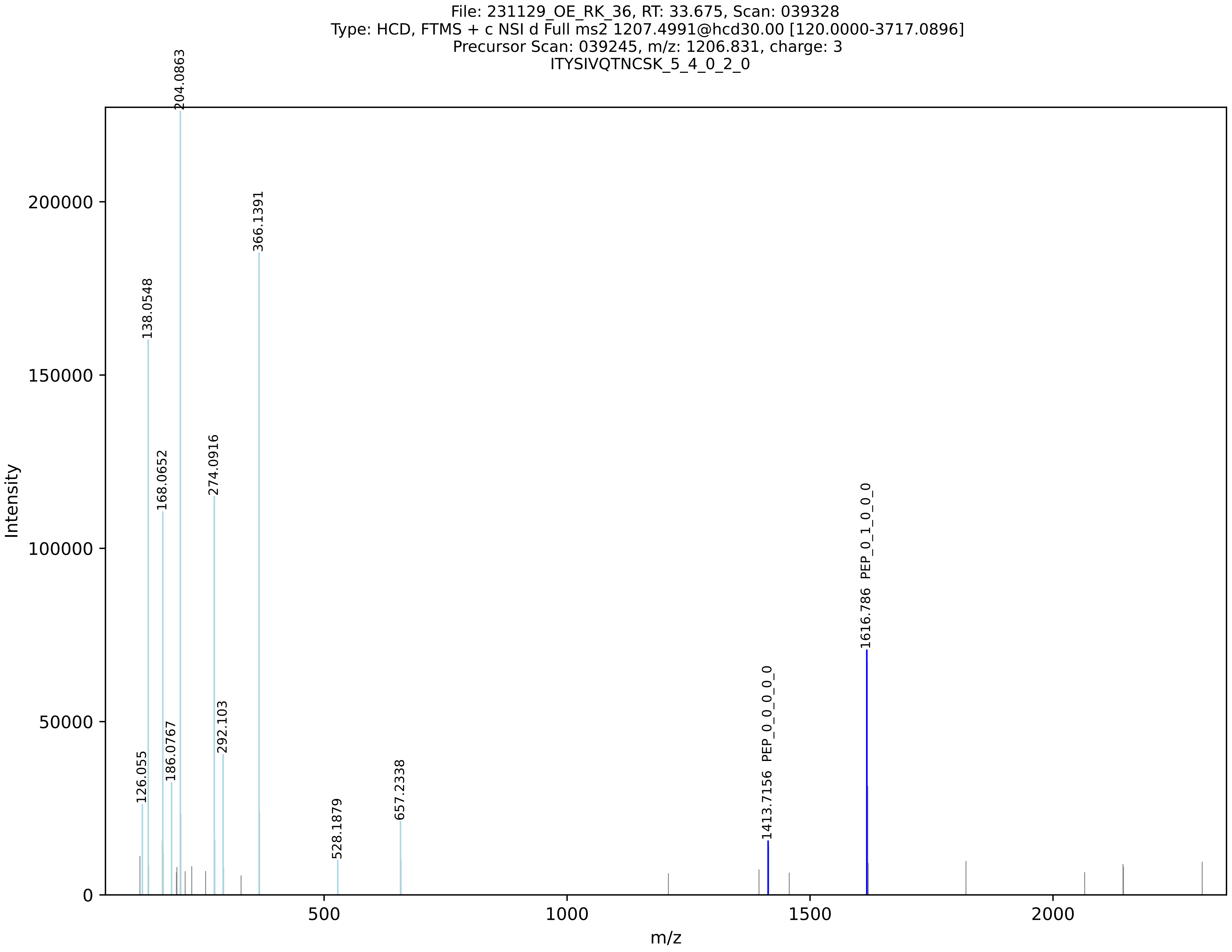

File: 231129_OE_RK_36, RT: 33.675, Scan: 039328
Type: HCD, FTMS + c NSI d Full ms2 1207.4991@hcd30.00 [120.0000-3717.0896]
Precursor Scan: 039245, m/z: 1206.831, charge: 3
ITYSIVQTNCSK_5_4_0_2_0
Intensity
m/z
0
50000
100000
150000
200000
500
1000
1500
2000
204.0863
366.1391
138.0548
168.0652
274.0916
292.103
186.0767
126.055
528.1879
657.2338
1413.7156 PEP_0_0_0_0_0
1616.786 PEP_0_1_0_0_0

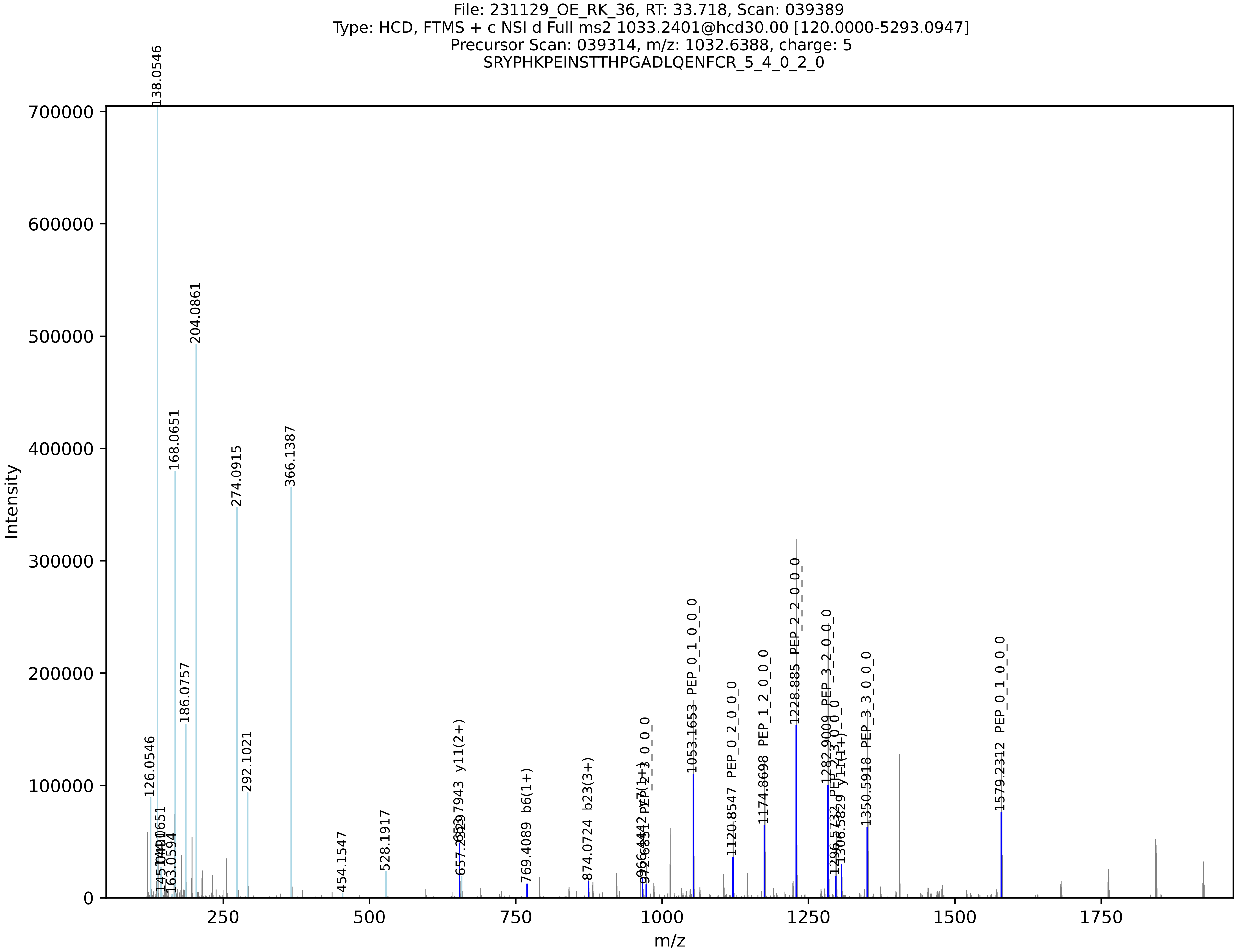
File: 231129_OE_RK_36, RT: 33.718, Scan: 039389
Type: HCD, FTMS + c NSI d Full ms2 1033.2401@hcd30.00 [120.0000-5293.0947]
Precursor Scan: 039314, m/z: 1032.6388, charge: 5
SRYPHKPEINSTTHPGADLQENFCR_5_4_0_2_0
Intensity
m/z
138.0546
204.0861
168.0651
274.0915
366.1387
186.0757
292.1021
126.0546
163.0594
454.1547
528.1917
657.7943 y11(2+)
769.4089 b6(1+)
874.0724 b23(3+)
1053.1653 PEP_0_1_0_0_0
1120.8547 PEP_0_2_0_0_0
1174.8698 PEP_1_2_0_0_0
1228.885 PEP_2_2_0_0_0
1282.9009 PEP_3_2_0_0_0
1350.5918 PEP_3_3_0_0_0
1579.2312 PEP_0_1_0_0_0
0
100000
200000
300000
400000
500000
600000
700000
250
500
750
1000
1250
1500
1750

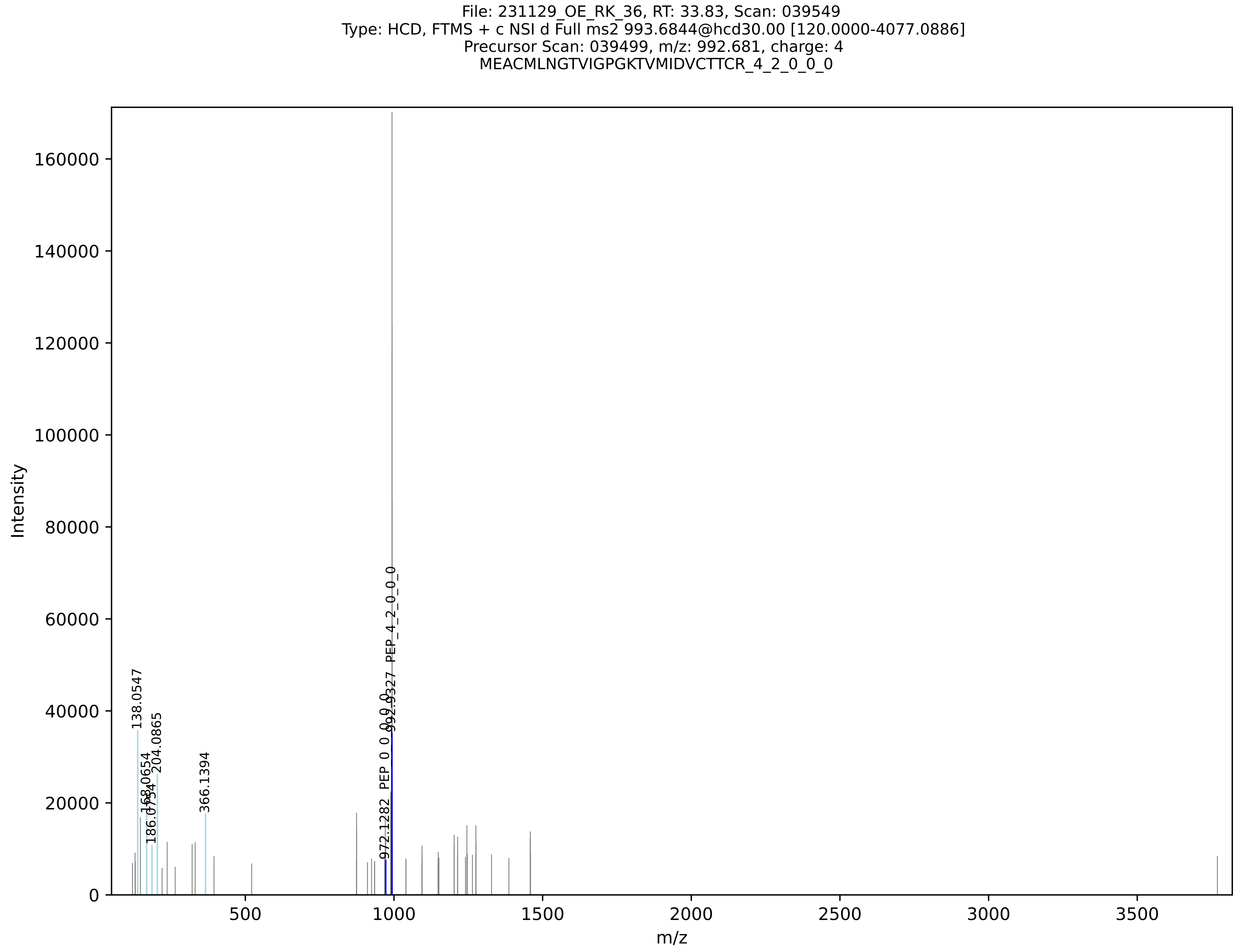

File: 231129_OE_RK_36, RT: 33.83, Scan: 039549
Type: HCD, FTMS + c NSI d Full ms2 993.6844@hcd30.00 [120.0000-4077.0886]
Precursor Scan: 039499, m/z: 992.681, charge: 4
MEACMLNGTVIGPGKTVMIDVCTTCR_4_2_0_0_0
Intensity
m/z
138.0547
168.0654
186.0754
204.0865
366.1394
972.1282 PEP_0_0_0_0_0
992.9327 PEP_4_2_0_0_0

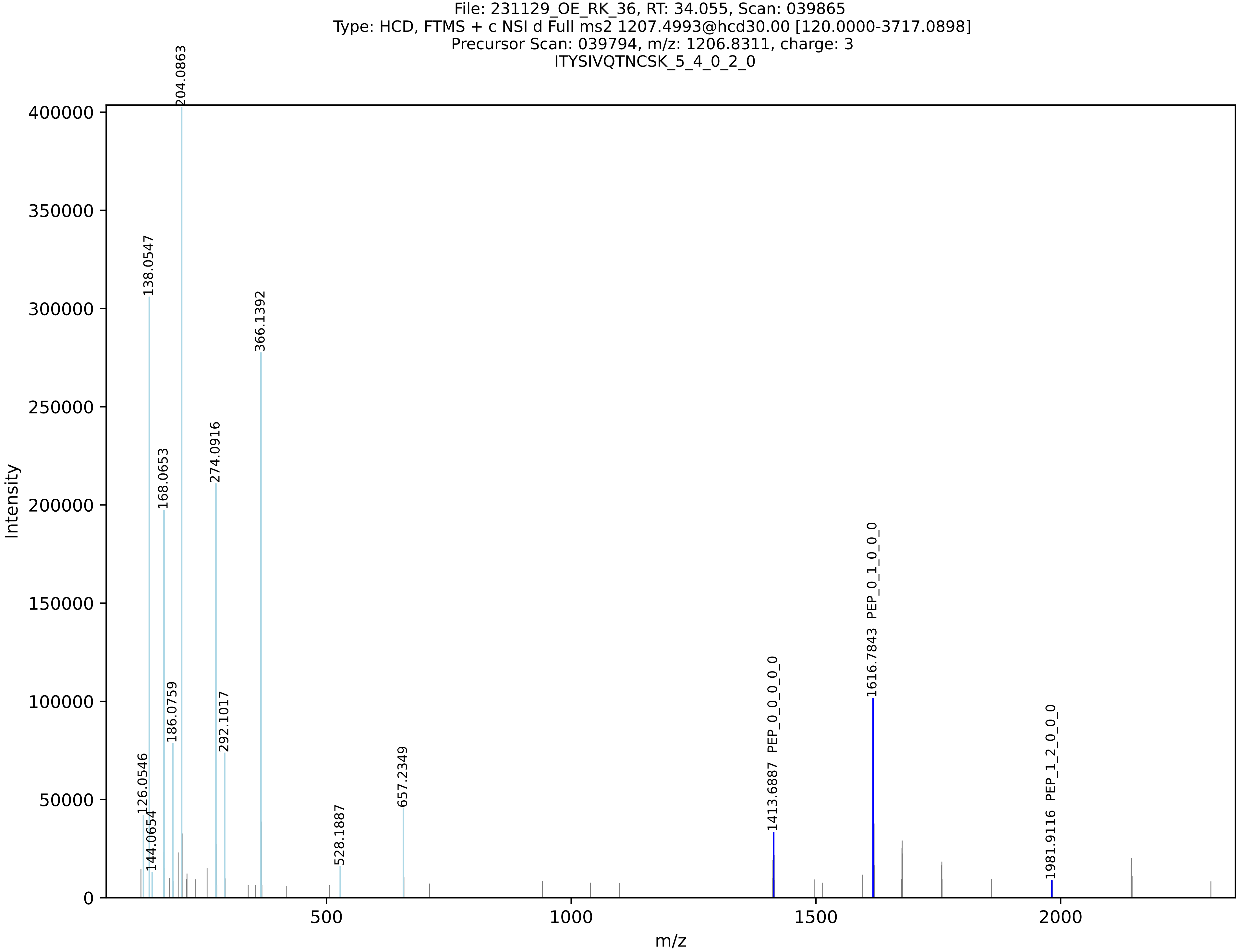

File: 231129_OE_RK_36, RT: 34.055, Scan: 039865
Type: HCD, FTMS + c NSI d Full ms2 1207.4993@hcd30.00 [120.0000-3717.0898]
Precursor Scan: 039794, m/z: 1206.8311, charge: 3
ITYSIVQTNCSK_5_4_0_2_0
Intensity
m/z
204.0863
138.0547
366.1392
274.0916
168.0653
186.0759
292.1017
126.0546
144.0654
528.1887
657.2349
1413.6887 PEP_0_0_0_0_0
1616.7843 PEP_0_1_0_0_0
1981.9116 PEP_1_2_0_0_0

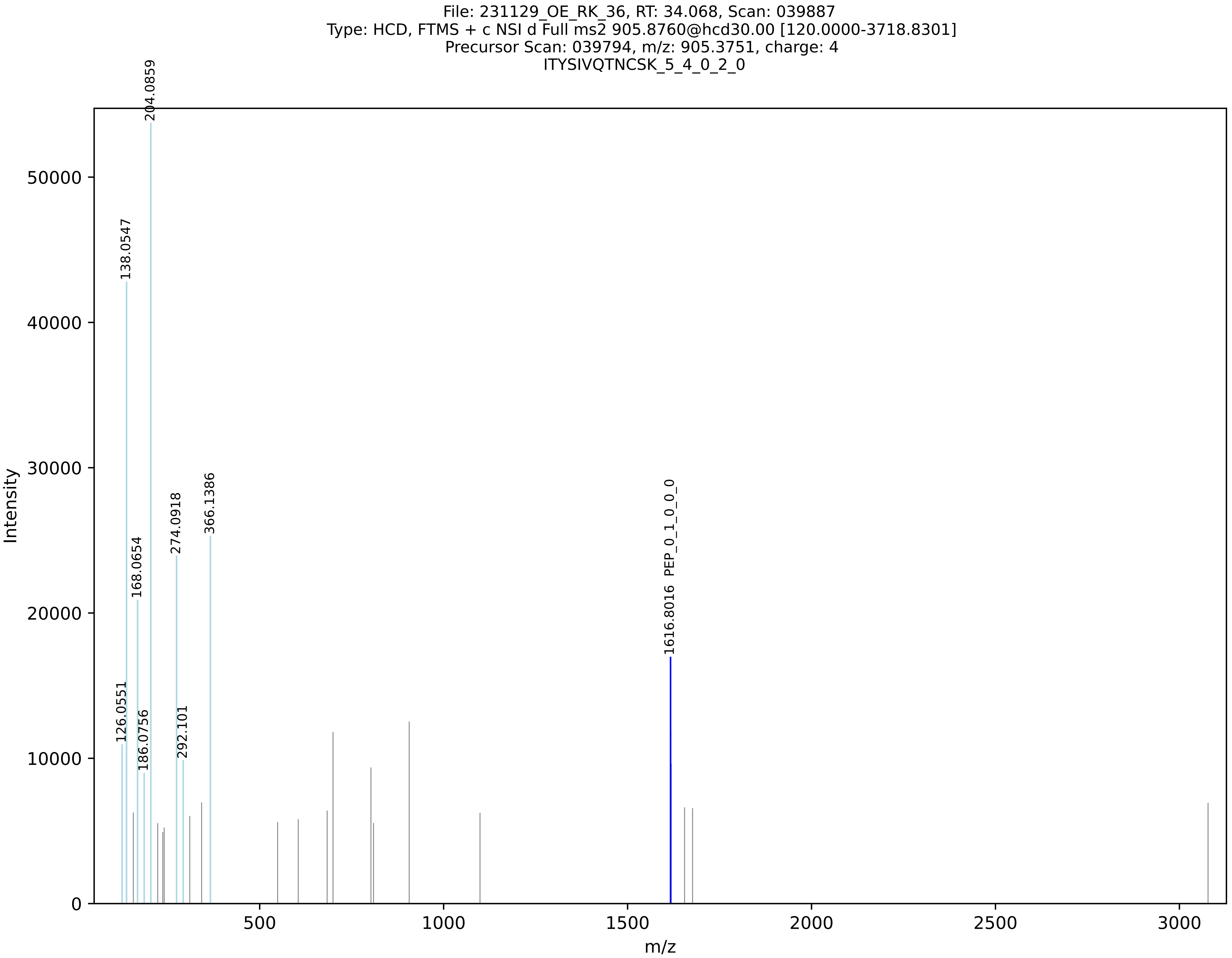
File: 231129_OE_RK_36, RT: 34.068, Scan: 039887
Type: HCD, FTMS + c NSI d Full ms2 905.8760@hcd30.00 [120.0000-3718.8301]
Precursor Scan: 039794, m/z: 905.3751, charge: 4
ITYSIVQTNCSK_5_4_0_2_0
204.0859
138.0547
168.0654
274.0918
366.1386
126.0551
186.0756
292.101
1616.8016 PEP_0_1_0_0_0
Intensity
m/z
0
10000
20000
30000
40000
50000
500
1000
1500
2000
2500
3000

File: 231129_OE_RK_36, RT: 34.138, Scan: 039986
Type: HCD, FTMS + c NSI d Full ms2 926.1943@hcd30.00 [120.0000-4747.1606]
Precursor Scan: 039922, m/z: 925.7933, charge: 5
YPHKPEINSTTHPGADLQENFCR_5_4_0_1_0

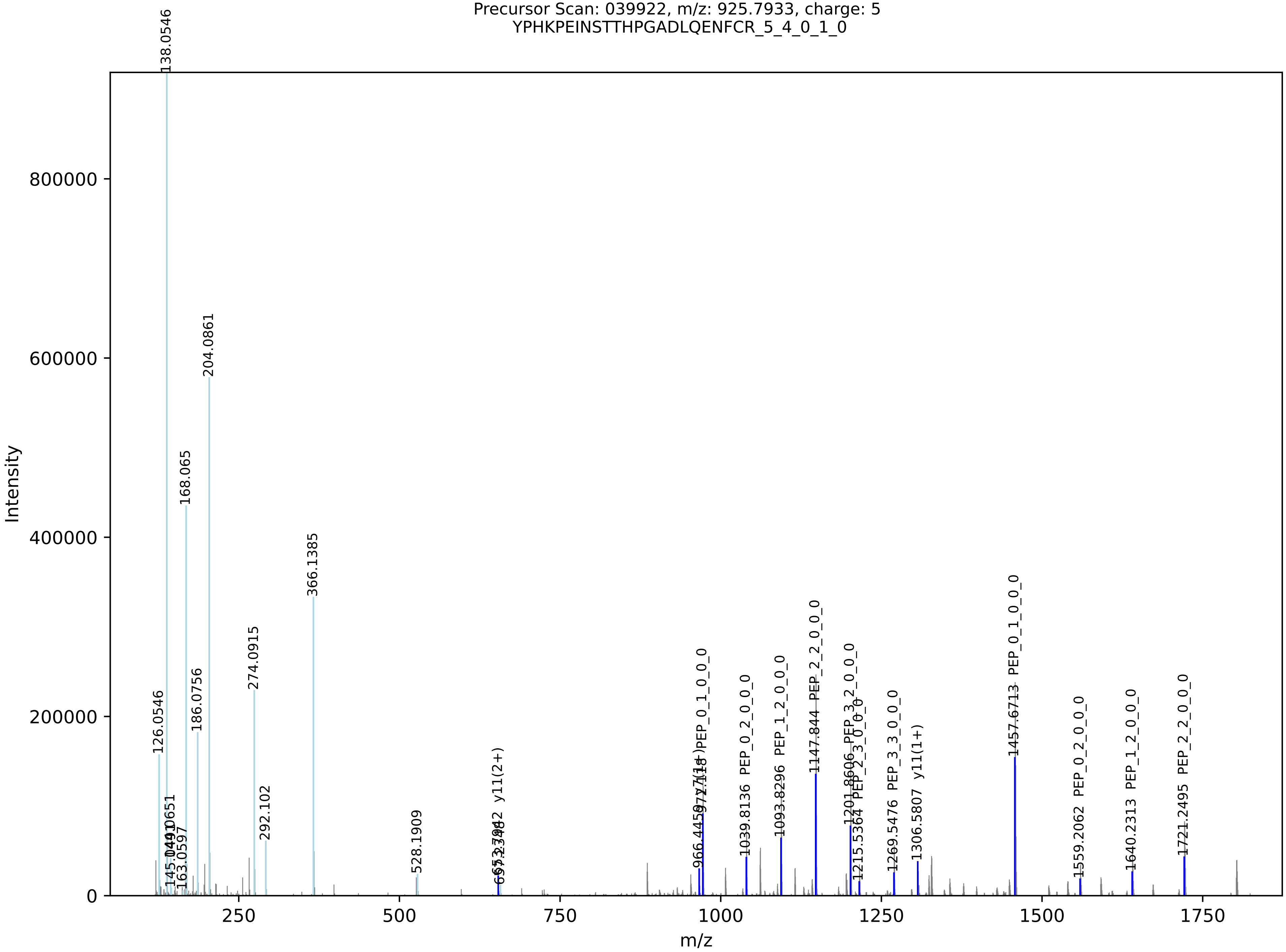

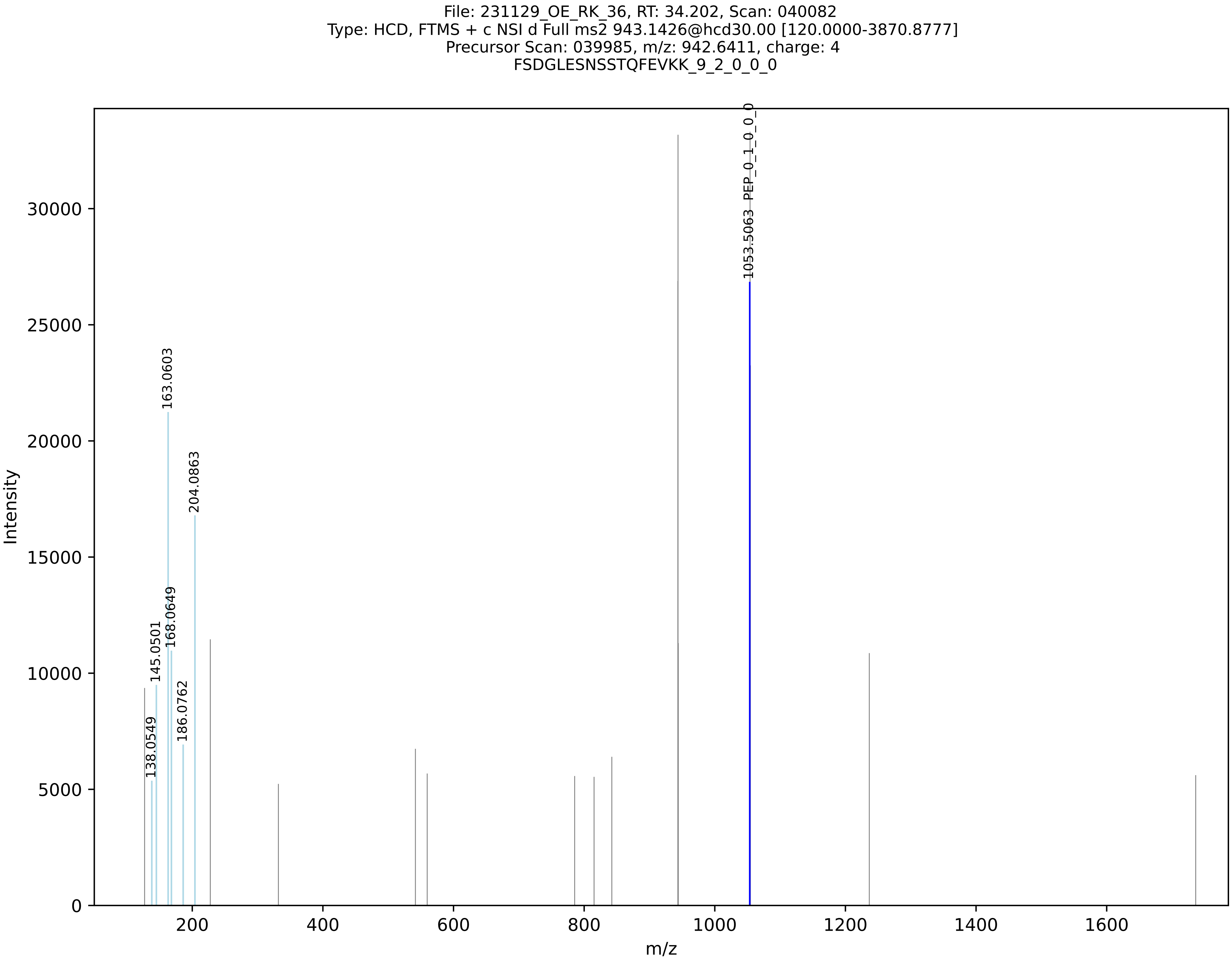
File: 231129_OE_RK_36, RT: 34.202, Scan: 040082
Type: HCD, FTMS + c NSI d Full ms2 943.1426@hcd30.00 [120.0000-3870.8777]
Precursor Scan: 039985, m/z: 942.6411, charge: 4
FSDGLESNSSTQFEVKK_9_2_0_0_0
1053.5063_PEP_0_1_0_0_0
163.0603
204.0863
168.0649
145.0501
186.0762
138.0549
Intensity
m/z
0
5000
10000
15000
20000
25000
30000
200
400
600
800
1000
1200
1400
1600

File: 231129_OE_RK_36, RT: 34.217, Scan: 040097
Type: HCD, FTMS + c NSI d Full ms2 1157.4913@hcd30.00 [120.0000-4745.4204]
Precursor Scan: 040039, m/z: 1156.9905, charge: 4
YPHKPEINSTTHPGADLQENFCR_5_4_0_1_0

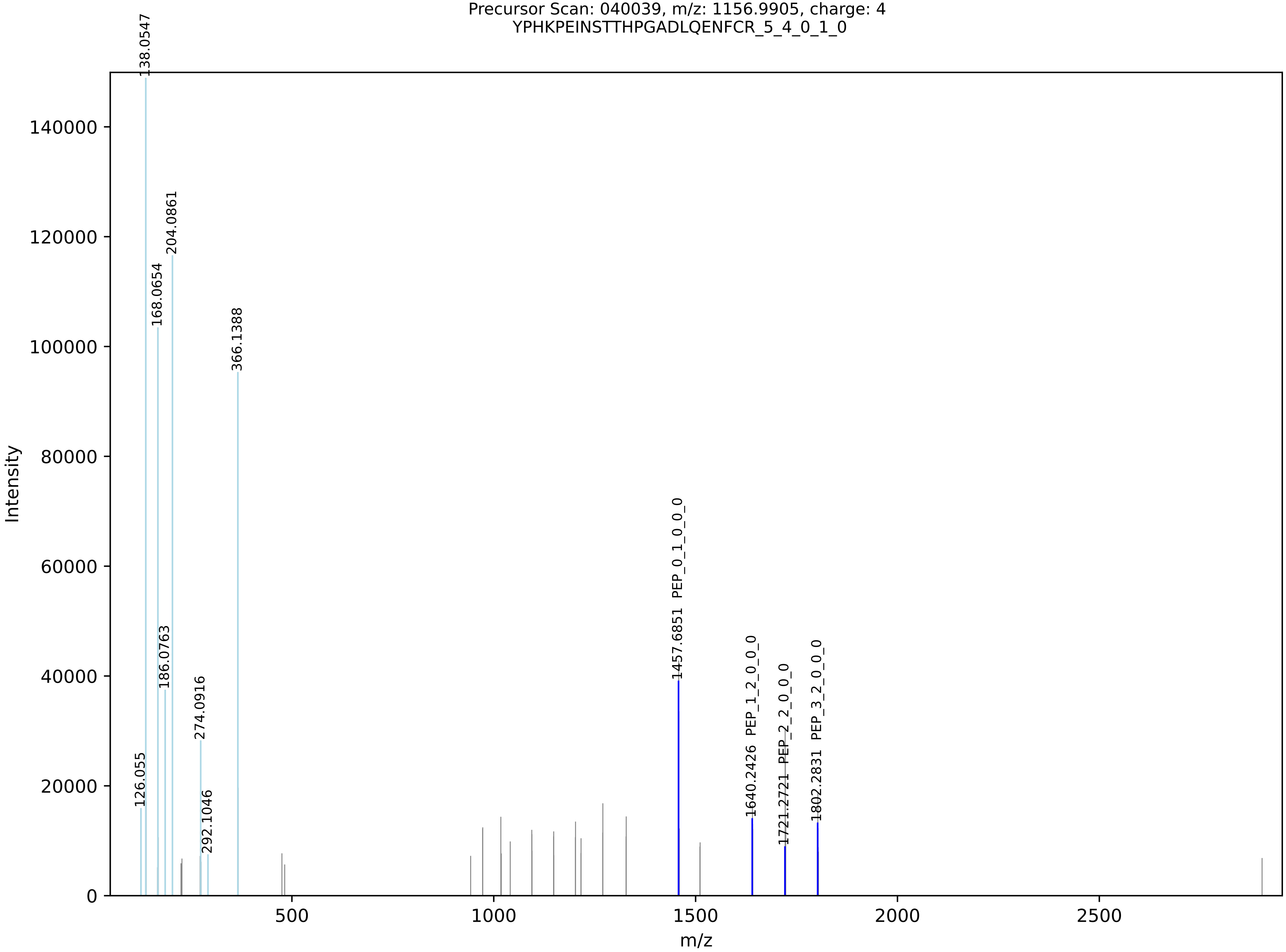

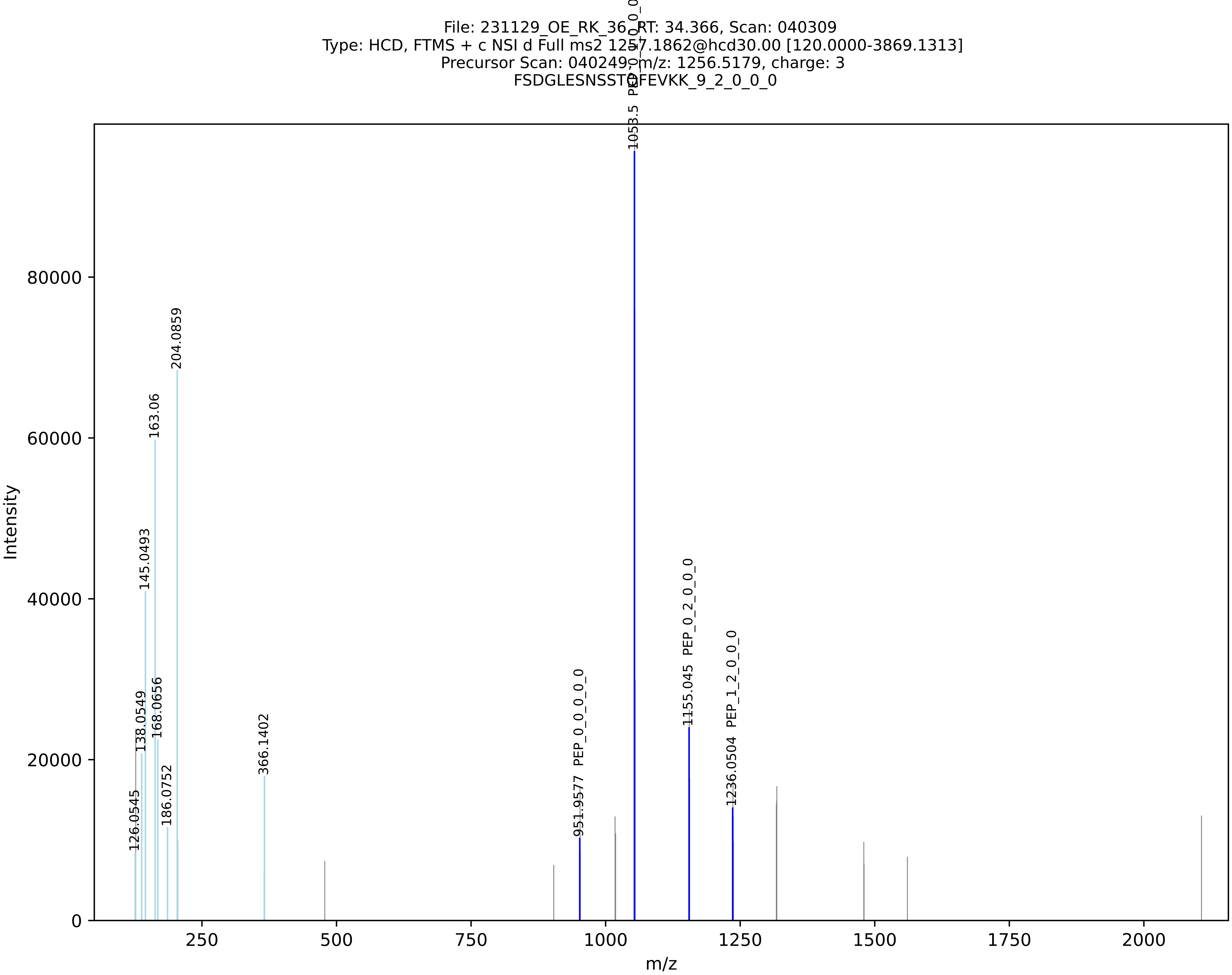
File: 231129_OE_RK_36, RT: 34.366, Scan: 040309
Type: HCD, FTMS + c NSI d Full ms2 1257.1862@hcd30.00 [120.0000-3869.1313]
Precursor Scan: 040249, m/z: 1256.5179, charge: 3
FSDGLESNSSTQFEVKK_9_2_0_0_0
1053.5
204.0859
163.06
145.0493
138.0549
168.0656
186.0752
126.0545
366.1402
951.9577 PEP_0_0_0_0_0
1155.045 PEP_0_2_0_0_0
1236.0504 PEP_1_2_0_0_0
Intensity
0
20000
40000
60000
80000
250
500
750
1000
1250
1500
1750
2000
m/z

File: 231129_OE_RK_36, RT: 34.628, Scan: 040685
Type: HCD, FTMS + c NSI d Full ms2 1074.9679@hcd30.00 [120.0000-4408.7251]
Precursor Scan: 040620, m/z: 1074.4671, charge: 4
NNATVHEQVGGPSLTSDLQAQSK_5_4_0_1_0

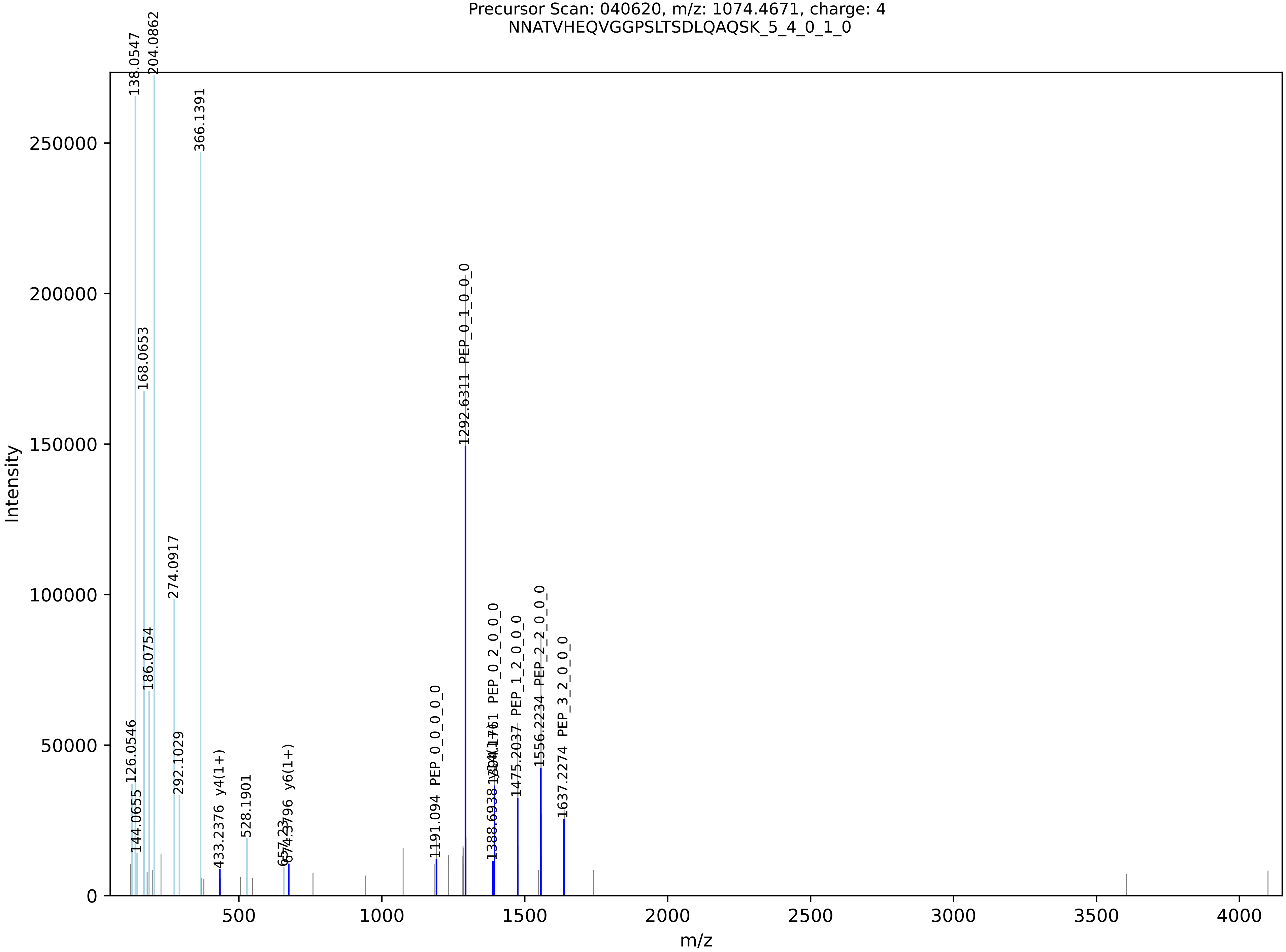

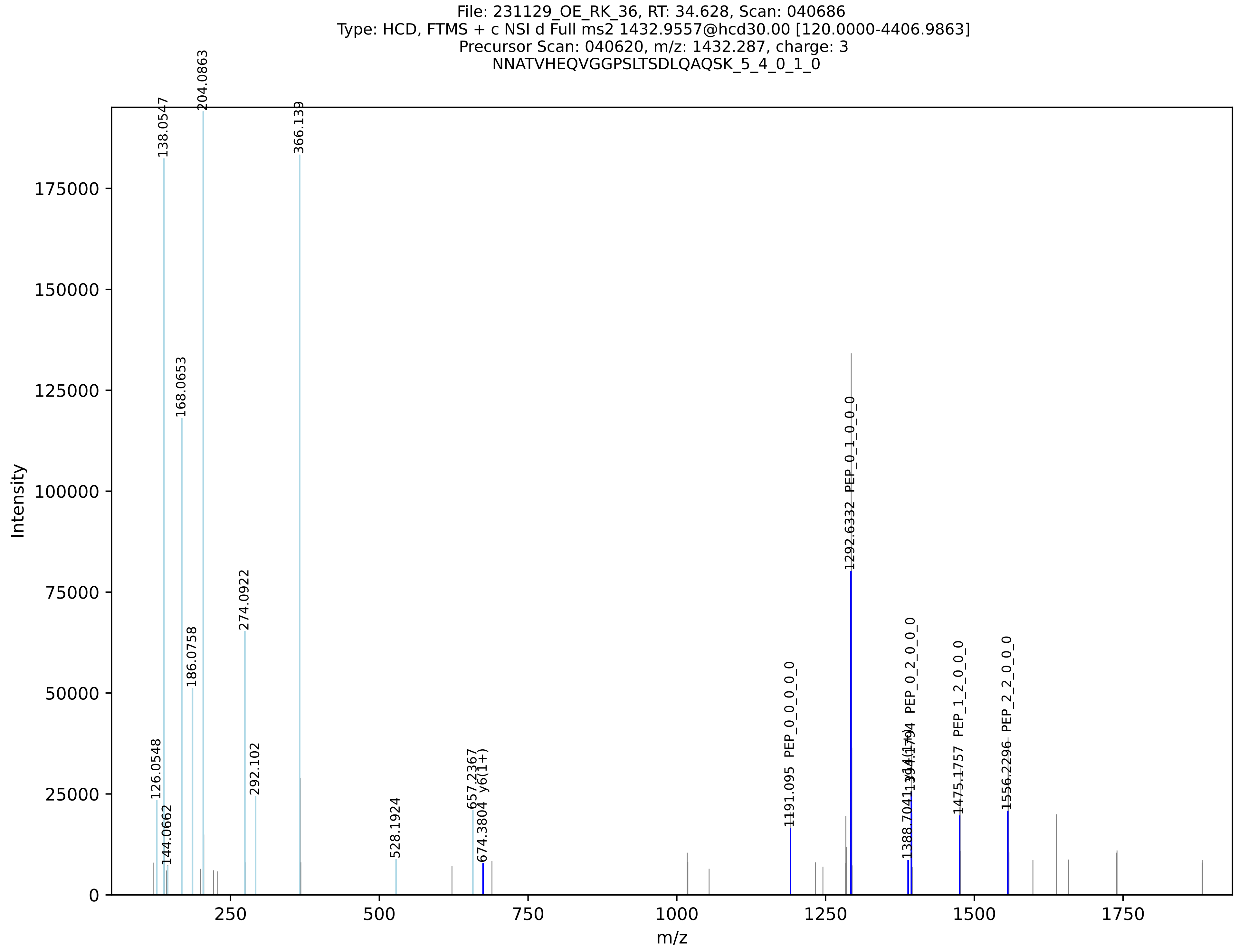

File: 231129_OE_RK_36, RT: 34.628, Scan: 040686
Type: HCD, FTMS + c NSI d Full ms2 1432.9557@hcd30.00 [120.0000-4406.9863]
Precursor Scan: 040620, m/z: 1432.287, charge: 3
NNATVHEQVGGPSLTSDLQAQSK_5_4_0_1_0
Intensity
m/z
0
25000
50000
75000
100000
125000
150000
175000
250
500
750
1000
1250
1500
1750
126.0548
138.0547
144.0662
168.0653
186.0758
204.0863
274.0922
292.102
366.139
528.1924
657.2367
674.3804 y6(1+)
1191.095 PEP_0_0_0_0_0
1292.6332 PEP_0_1_0_0_0
1388.7041
1394.1794 PEP_0_2_0_0_0
1475.1757 PEP_1_2_0_0_0
1556.2296 PEP_2_2_0_0_0

File: 231129_OE_RK_36, RT: 34.729, Scan: 040832
Type: HCD, FTMS + c NSI d Full ms2 1202.5056@hcd30.00 [120.0000-3701.8093]
Precursor Scan: 040744, m/z: 1201.8374, charge: 3
FLREGPNK_7_6_2_0_0

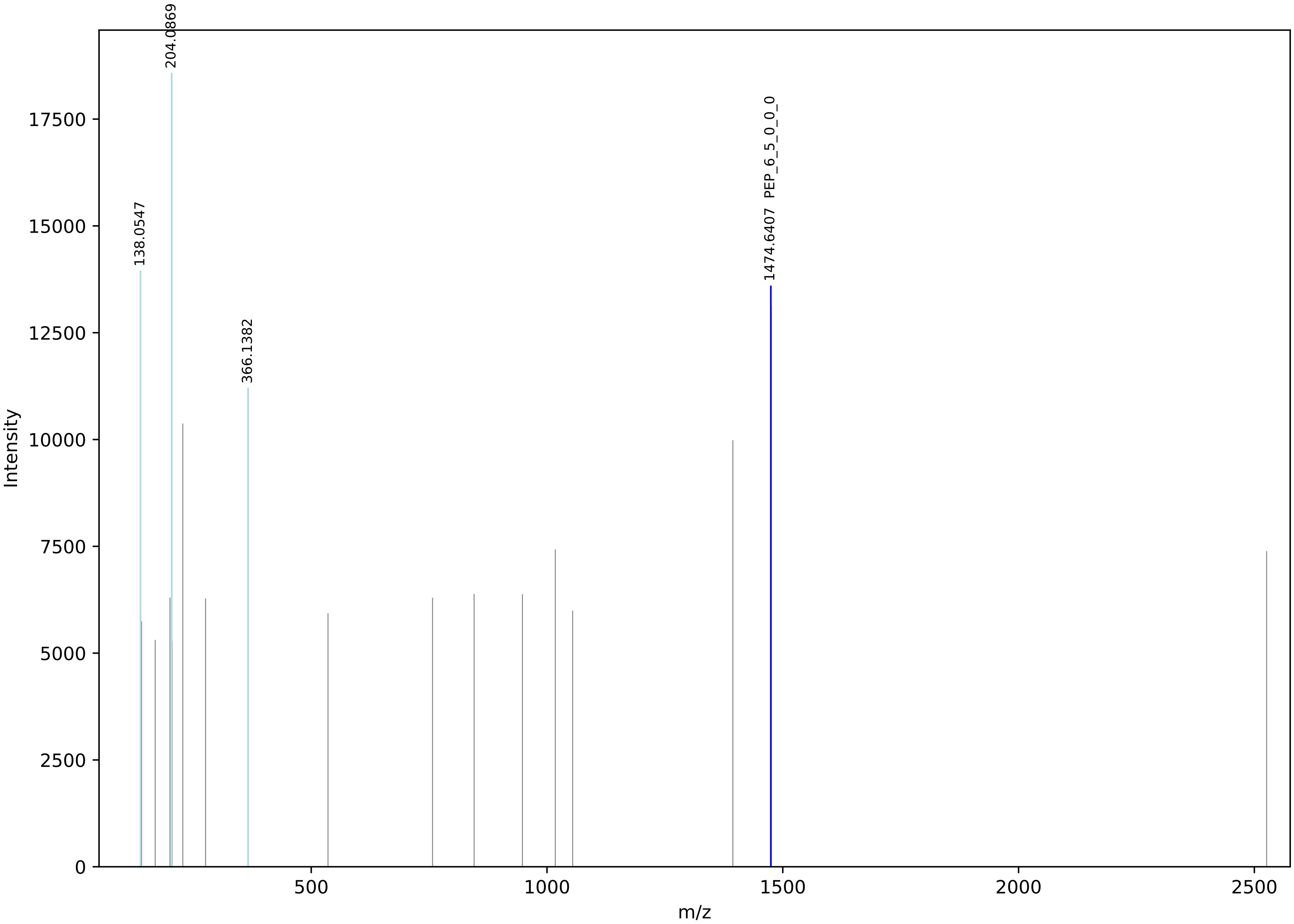

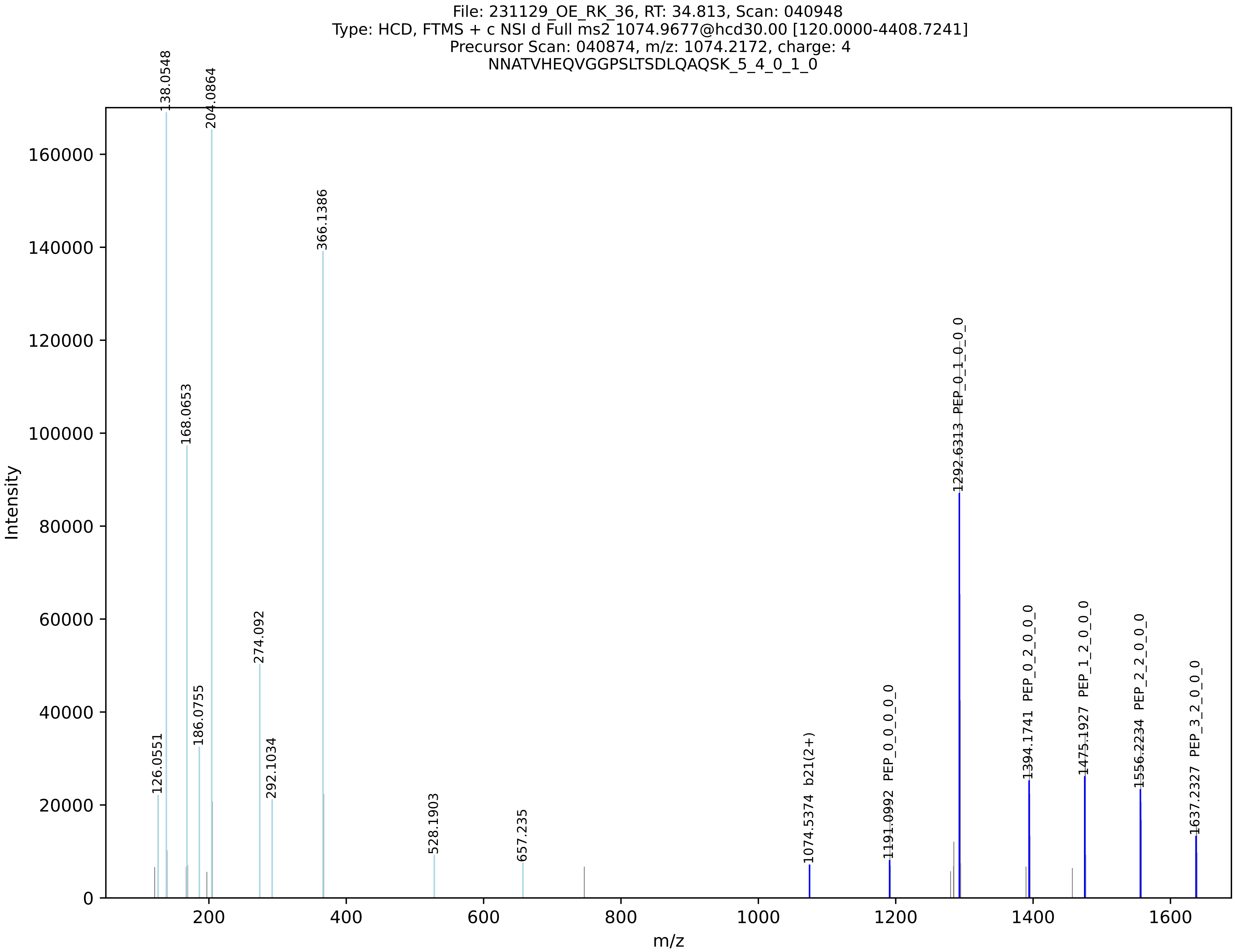
File: 231129_OE_RK_36, RT: 34.813, Scan: 040948
Type: HCD, FTMS + c NSI d Full ms2 1074.9677@hcd30.00 [120.0000-4408.7241]
Precursor Scan: 040874, m/z: 1074.2172, charge: 4
NNATVHEQVGGPSLTSDLQAQSK_5_4_0_1_0
Intensity
m/z
138.0548
204.0864
366.1386
168.0653
274.092
186.0755
126.0551
292.1034
528.1903
657.235
1074.5374 b21(2+)
1191.0992 PEP_0_0_0_0_0
1292.6313 PEP_0_1_0_0_0
1394.1741 PEP_0_2_0_0_0
1475.1927 PEP_1_2_0_0_0
1556.2234 PEP_2_2_0_0_0
1637.2327 PEP_3_2_0_0_0

File: 231129_OE_RK_36, RT: 34.907, Scan: 041086
Type: HCD, FTMS + c NSI d Full ms2 1104.1290@hcd30.00 [120.0000-3400.7769]
Precursor Scan: 041015, m/z: 1103.7949, charge: 3
GLNVTLSSTGR_5_4_0_2_0

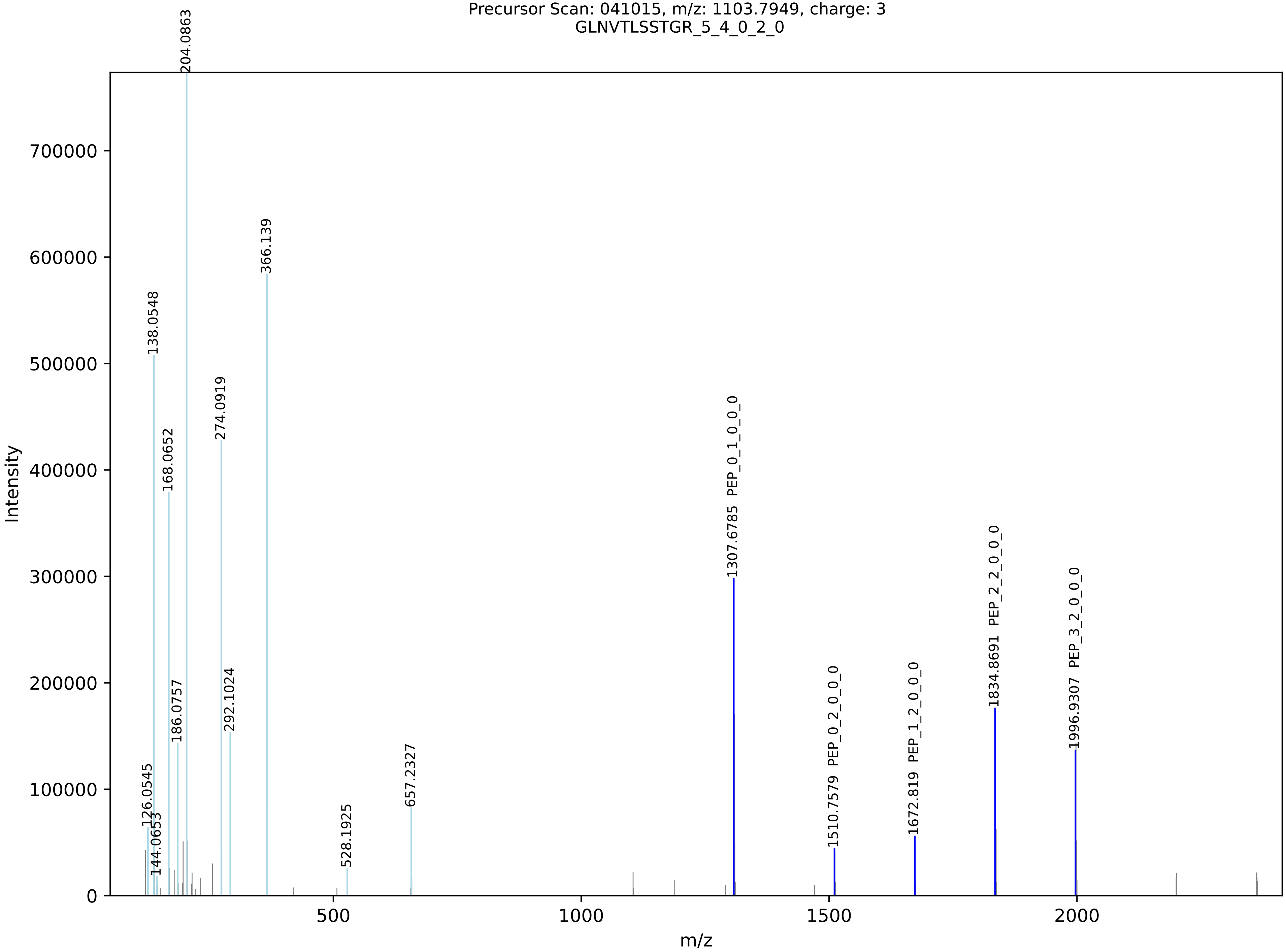

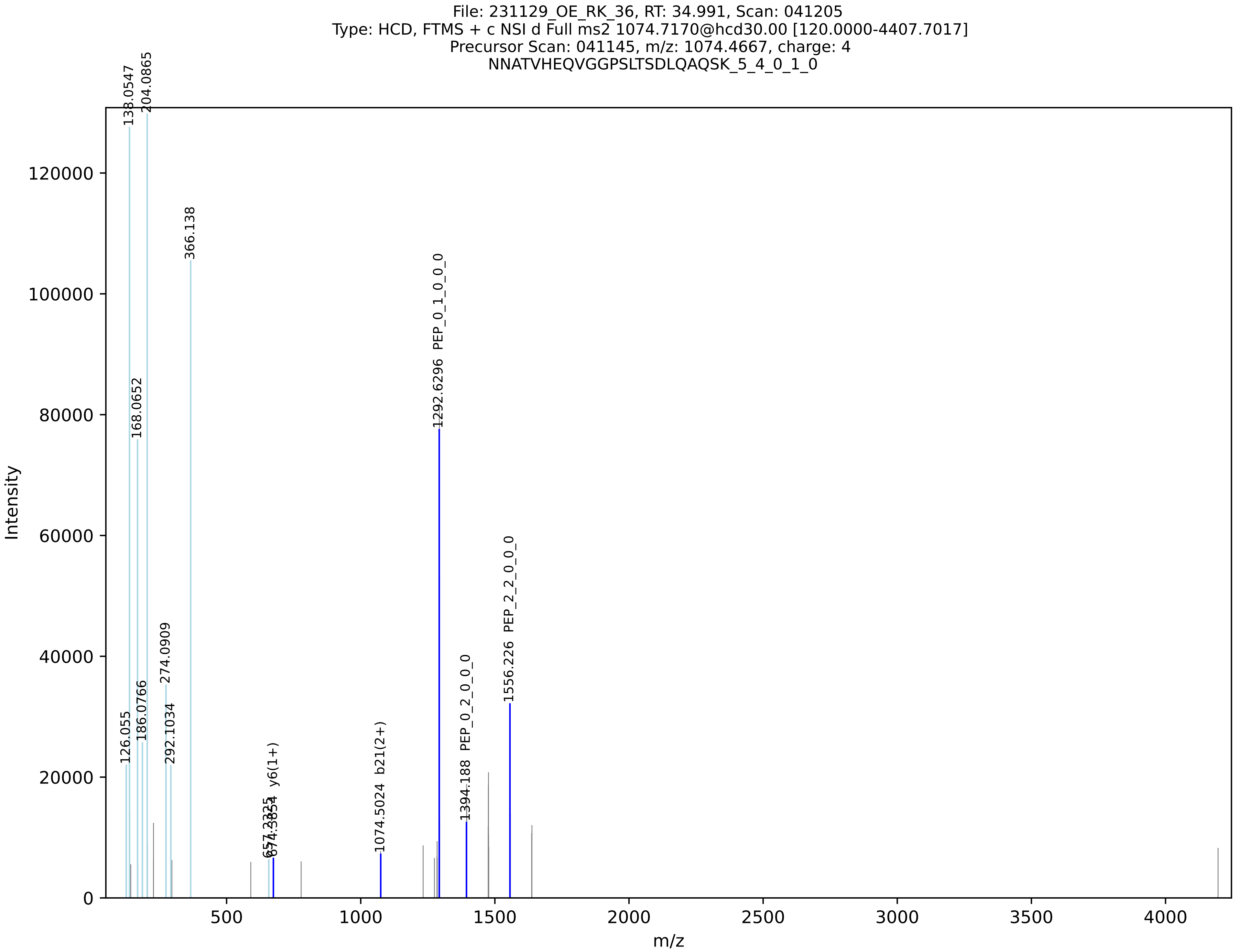
File: 231129_OE_RK_36, RT: 34.991, Scan: 041205
Type: HCD, FTMS + c NSI d Full ms2 1074.7170@hcd30.00 [120.0000-4407.7017]
Precursor Scan: 041145, m/z: 1074.4667, charge: 4
NNATVHEQVGGPSLTSDLQAQSK_5_4_0_1_0
Intensity
m/z
138.0547
204.0865
366.138
168.0652
274.0909
186.0766
126.055
292.1034
657.3325
674.3854 y6(1+)
1074.5024 b21(2+)
1292.6296 PEP_0_1_0_0_0
1394.188 PEP_0_2_0_0_0
1556.226 PEP_2_2_0_0_0
0
20000
40000
60000
80000
100000
120000
500
1000
1500
2000
2500
3000
3500
4000

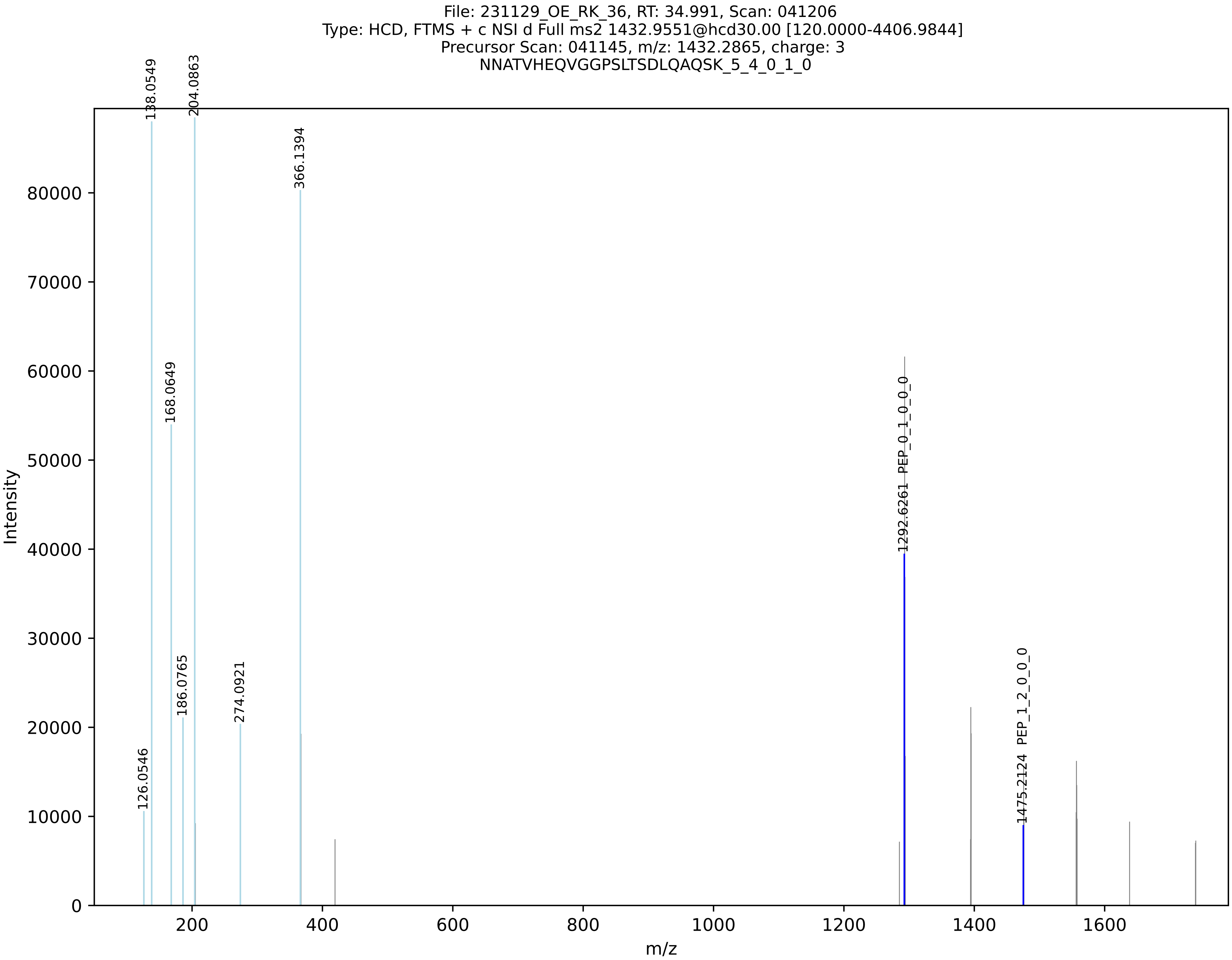
File: 231129_OE_RK_36, RT: 34.991, Scan: 041206
Type: HCD, FTMS + c NSI d Full ms2 1432.9551@hcd30.00 [120.0000-4406.9844]
Precursor Scan: 041145, m/z: 1432.2865, charge: 3
NNATVHEQVGGPSLTSDLQAQSK_5_4_0_1_0
Intensity
m/z
138.0549
204.0863
366.1394
168.0649
186.0765
274.0921
126.0546
1292.6261_PEP_0_1_0_0_0
1475.2124_PEP_1_2_0_0_0
0
10000
20000
30000
40000
50000
60000
70000
80000
200
400
600
800
1000
1200
1400
1600

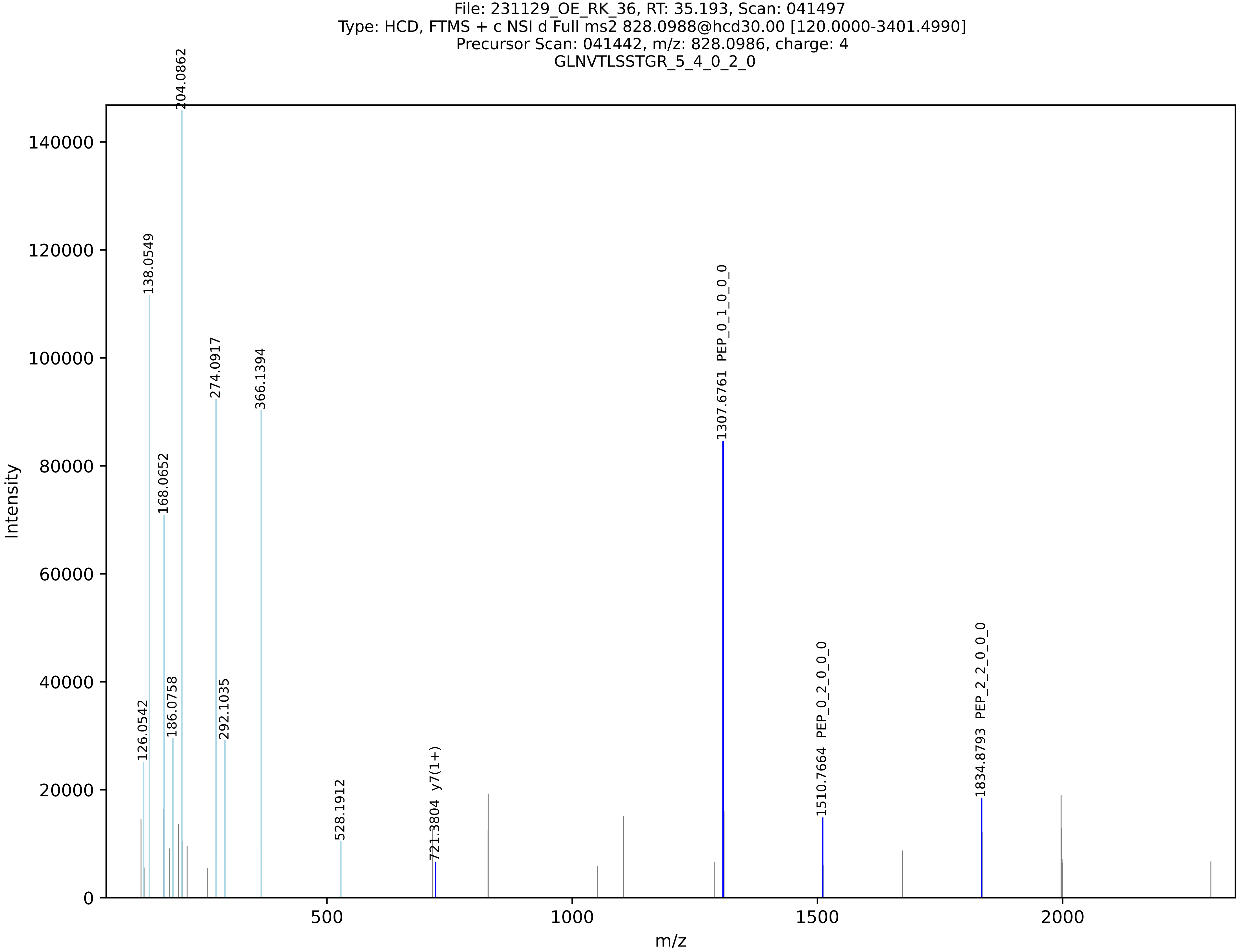

File: 231129_OE_RK_36, RT: 35.193, Scan: 041497
Type: HCD, FTMS + c NSI d Full ms2 828.0988@hcd30.00 [120.0000-3401.4990]
Precursor Scan: 041442, m/z: 828.0986, charge: 4
GLNVTLSSTGR_5_4_0_2_0
Intensity
m/z
204.0862
138.0549
168.0652
274.0917
366.1394
126.0542
186.0758
292.1035
528.1912
721.3804 y7(1+)
1307.6761 PEP_0_1_0_0_0
1510.7664 PEP_0_2_0_0_0
1834.8793 PEP_2_2_0_0_0
0
20000
40000
60000
80000
100000
120000
140000
500
1000
1500
2000

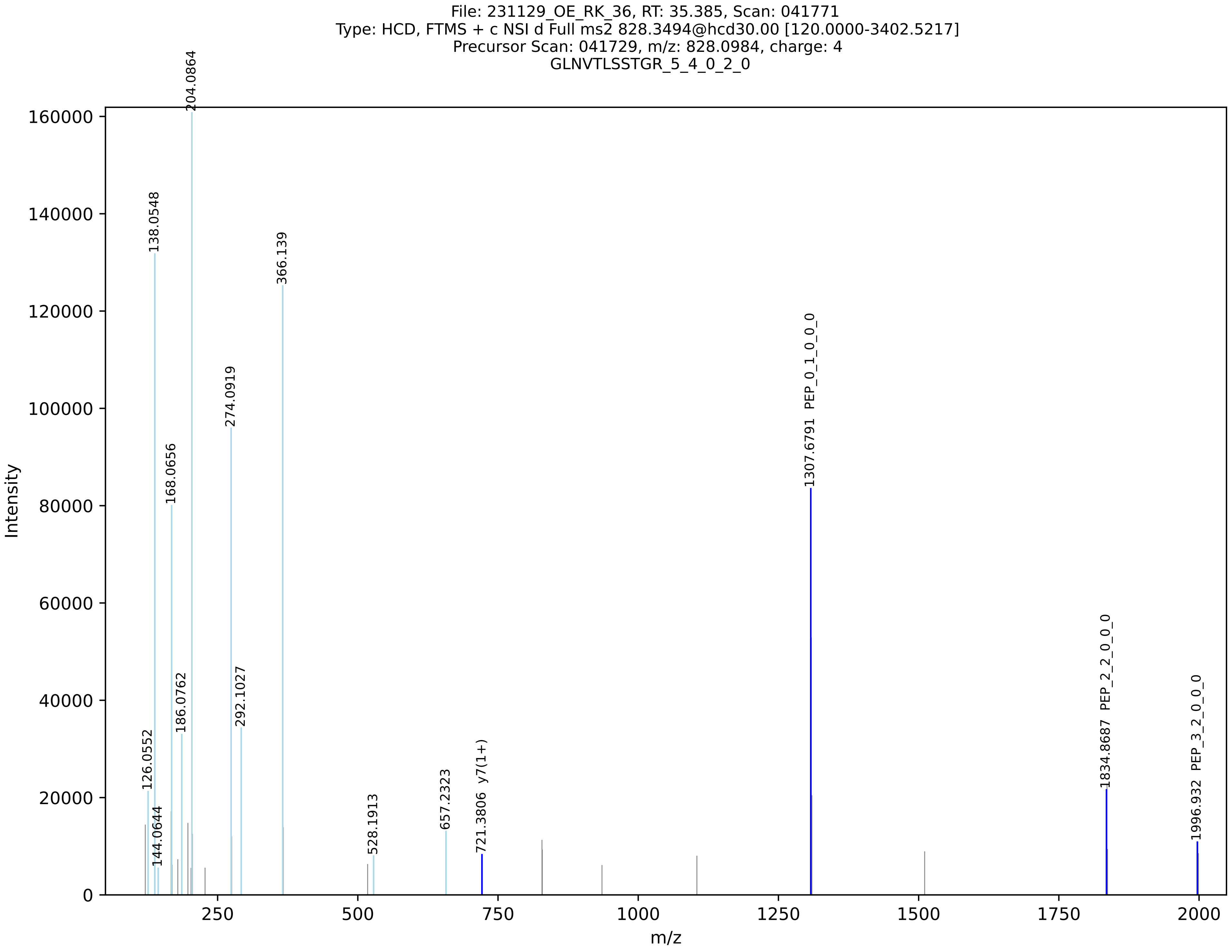

File: 231129_OE_RK_36, RT: 35.385, Scan: 041771
Type: HCD, FTMS + c NSI d Full ms2 828.3494@hcd30.00 [120.0000-3402.5217]
Precursor Scan: 041729, m/z: 828.0984, charge: 4
GLNVTLSSTGR_5_4_0_2_0
Intensity
m/z
204.0864
138.0548
366.139
274.0919
168.0656
186.0762
292.1027
126.0552
144.0644
528.1913
657.2323
721.3806 y7(1+)
1307.6791 PEP_0_1_0_0_0_
1834.8687 PEP_2_2_0_0_0_
1996.932 PEP_3_2_0_0_0_

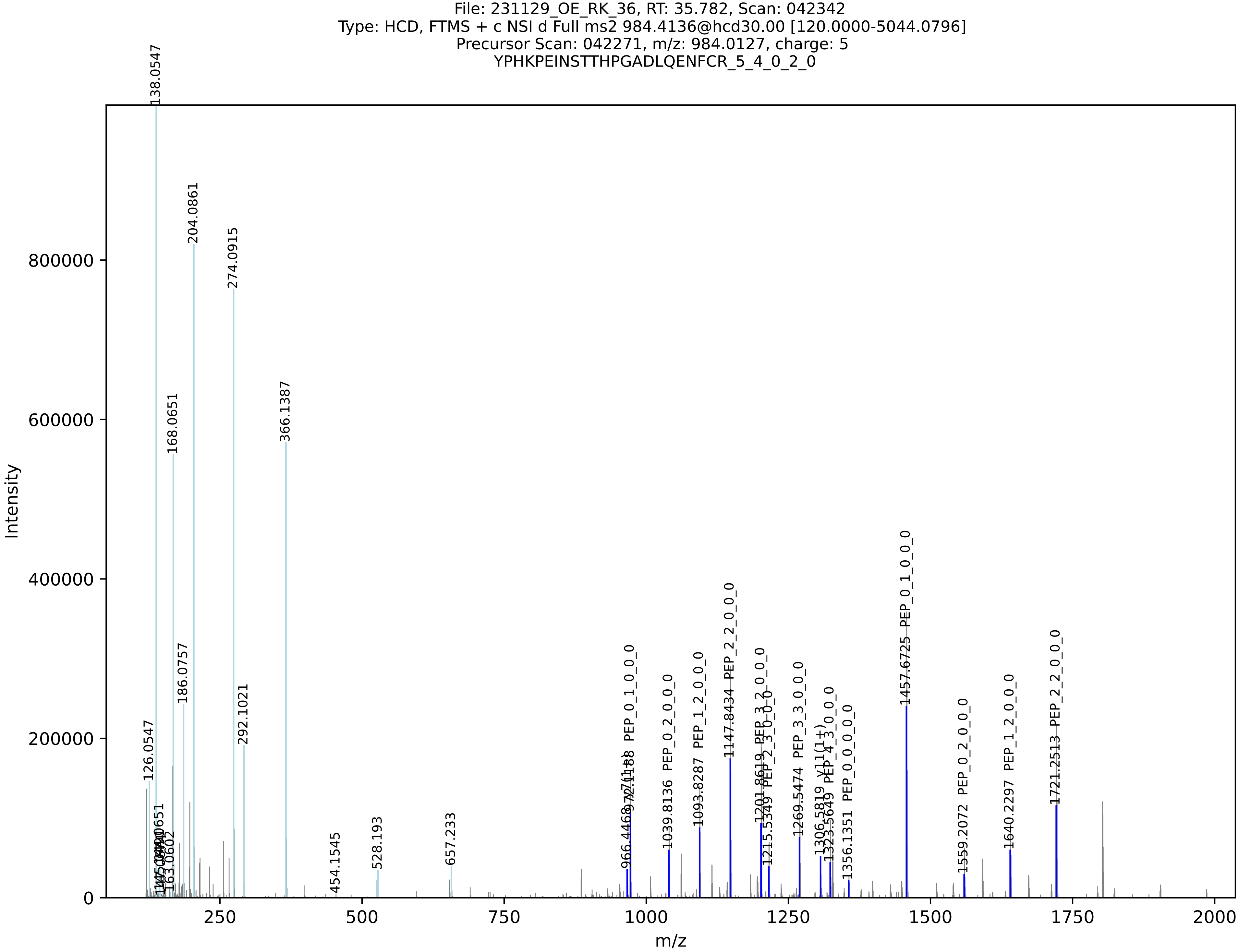
File: 231129_OE_RK_36, RT: 35.782, Scan: 042342
Type: HCD, FTMS + c NSI d Full ms2 984.4136@hcd30.00 [120.0000-5044.0796]
Precursor Scan: 042271, m/z: 984.0127, charge: 5
YPHKPEINSTTHPGADLQENFCR_5_4_0_2_0
Intensity
m/z
138.0547
204.0861
274.0915
366.1387
168.0651
186.0757
292.1021
126.0547
163.0602
454.1545
528.193
657.233
966.4468
PEP_0_1_0_0_0
1039.8136 PEP_0_2_0_0_0
1093.8287 PEP_1_2_0_0_0
1147.8434 PEP_2_2_0_0_0
1201.8619 PEP_3_2_0_0_0
1215.5349 PEP_2_3_0_0_0
1269.5474 PEP_3_3_0_0_0
1306.5819 y11(1+)
1323.5649 PEP_4_3_0_0_0
1356.1351 PEP_0_0_0_0_0
1457.6725 PEP_0_1_0_0_0
1559.2072 PEP_0_2_0_0_0
1640.2297 PEP_1_2_0_0_0
1721.2513 PEP_2_2_0_0_0

File: 231129_OE_RK_36, RT: 36.189, Scan: 042902
Type: HCD, FTMS + c NSI d Full ms2 985.1750@hcd30.00 [120.0000-4042.3701]
Precursor Scan: 042880, m/z: 984.6738, charge: 4
ELHHLQEQNVSNAFLDK_5_4_0_1_0

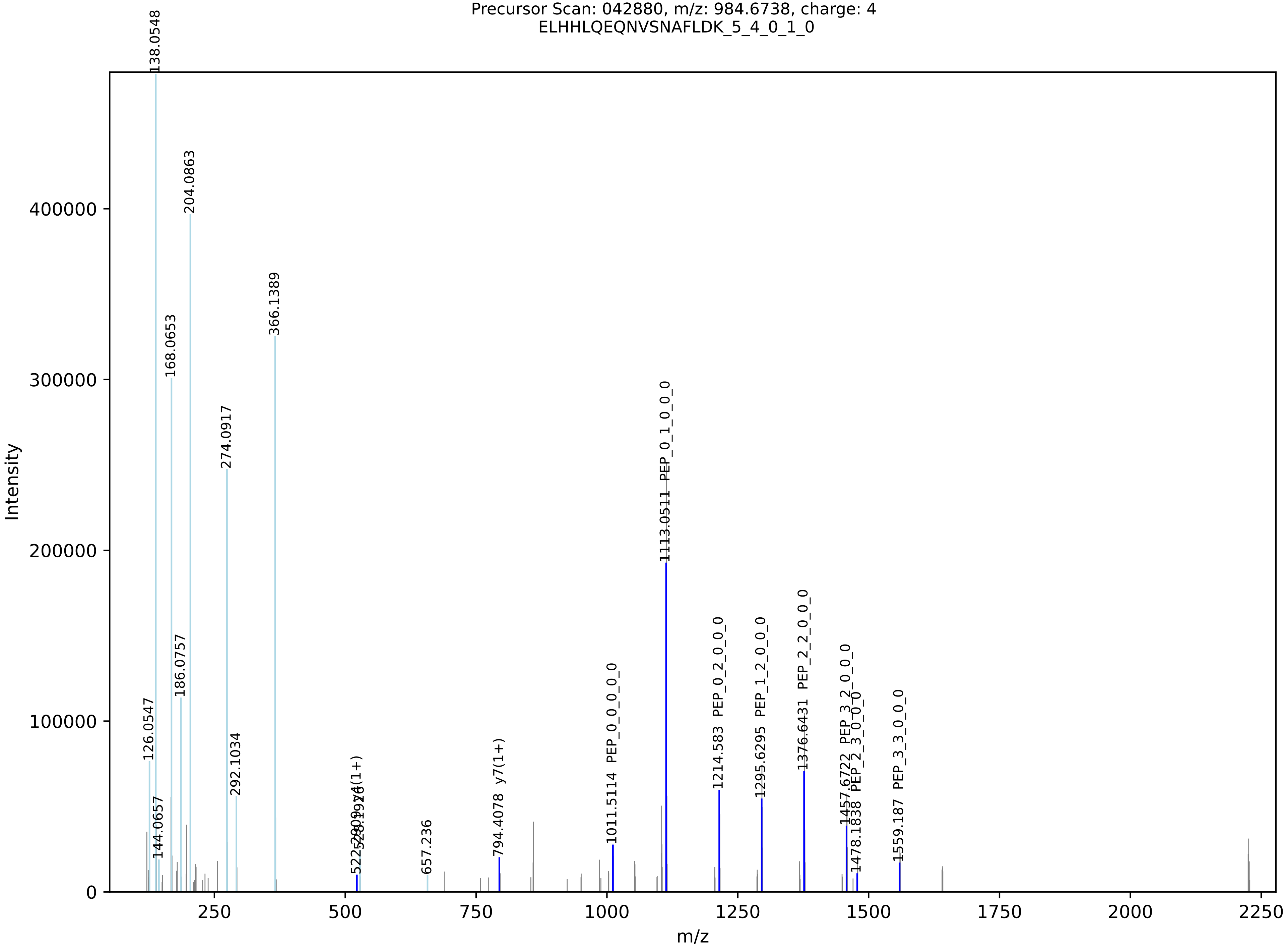

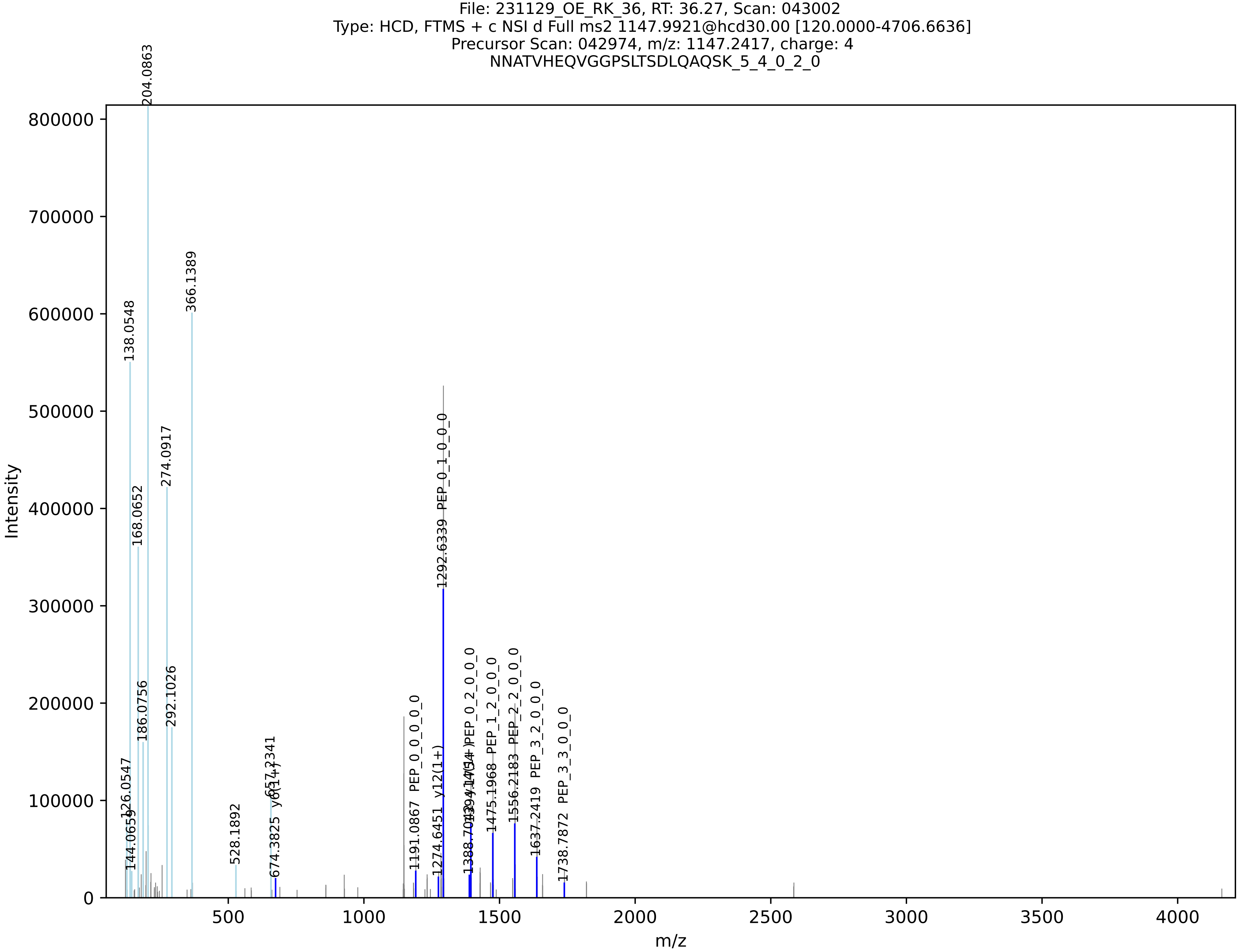

File: 231129_OE_RK_36, RT: 36.27, Scan: 043002
Type: HCD, FTMS + c NSI d Full ms2 1147.9921@hcd30.00 [120.0000-4706.6636]
Precursor Scan: 042974, m/z: 1147.2417, charge: 4
NNATVHEQVGGPSLTSDLQAQSK_5_4_0_2_0
Intensity
m/z
0
100000
200000
300000
400000
500000
600000
700000
800000
500
1000
1500
2000
2500
3000
3500
4000
204.0863
366.1389
138.0548
274.0917
168.0652
186.0756
292.1026
126.0547
144.0659
528.1892
657.2341
674.3825 y6(1+)
1191.0867 PEP_0_0_0_0_0
1274.6451 y12(1+)
1292.6339 PEP_0_1_0_0_0
PEP_0_2_0_0_0
1475.1968 PEP_1_2_0_0_0
1556.2183 PEP_2_2_0_0_0
1637.2419 PEP_3_2_0_0_0
1738.7872 PEP_3_3_0_0_0

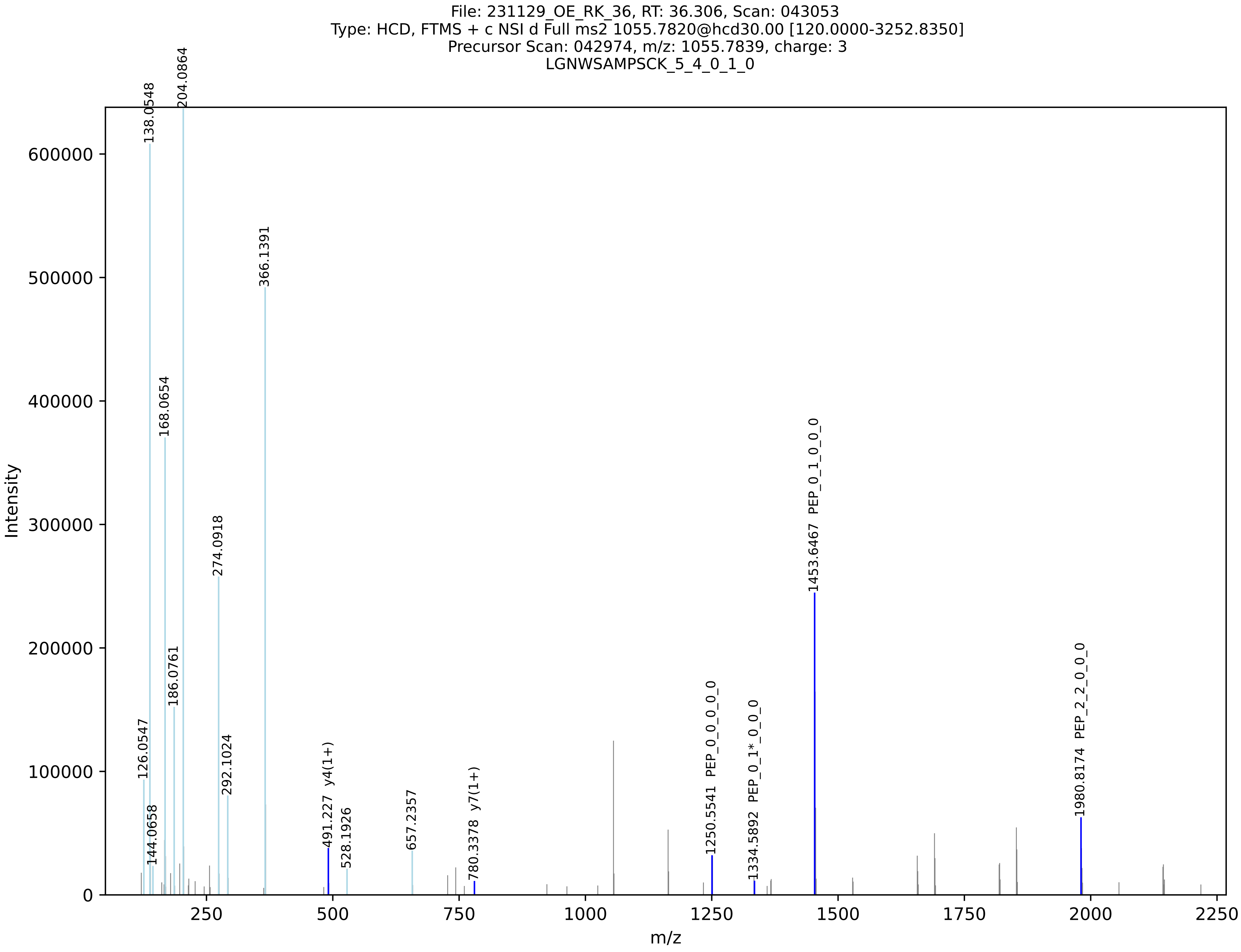
File: 231129_OE_RK_36, RT: 36.306, Scan: 043053
Type: HCD, FTMS + c NSI d Full ms2 1055.7820@hcd30.00 [120.0000-3252.8350]
Precursor Scan: 042974, m/z: 1055.7839, charge: 3
LGNWSAMPSCK_5_4_0_1_0
Intensity
m/z
138.0548
204.0864
366.1391
168.0654
274.0918
186.0761
126.0547
292.1024
144.0658
491.227 y4(1+)
528.1926
657.2357
780.3378 y7(1+)
1250.5541 PEP_0_0_0_0_0
1334.5892 PEP_0_1*_0_0_0
1453.6467 PEP_0_1_0_0_0
1980.8174 PEP_2_2_0_0_0

File: 231129_OE_RK_36, RT: 36.449, Scan: 043238
Type: HCD, FTMS + c NSI d Full ms2 1147.7404@hcd30.00 [120.0000-4705.6367]
Precursor Scan: 043204, m/z: 1147.2392, charge: 4
NNATVHEQVGGPSLTSDLQAQSK_5_4_0_2_0

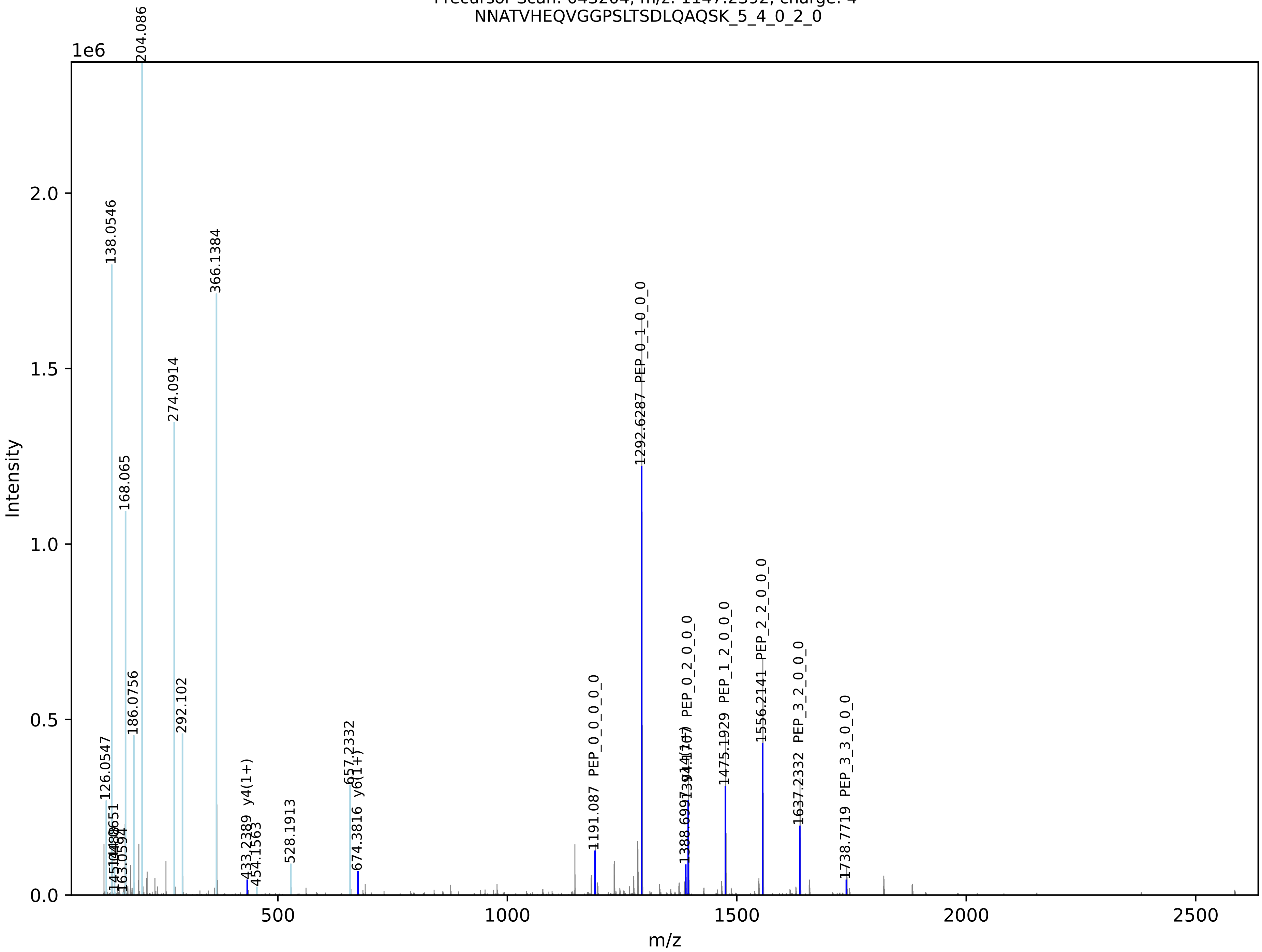

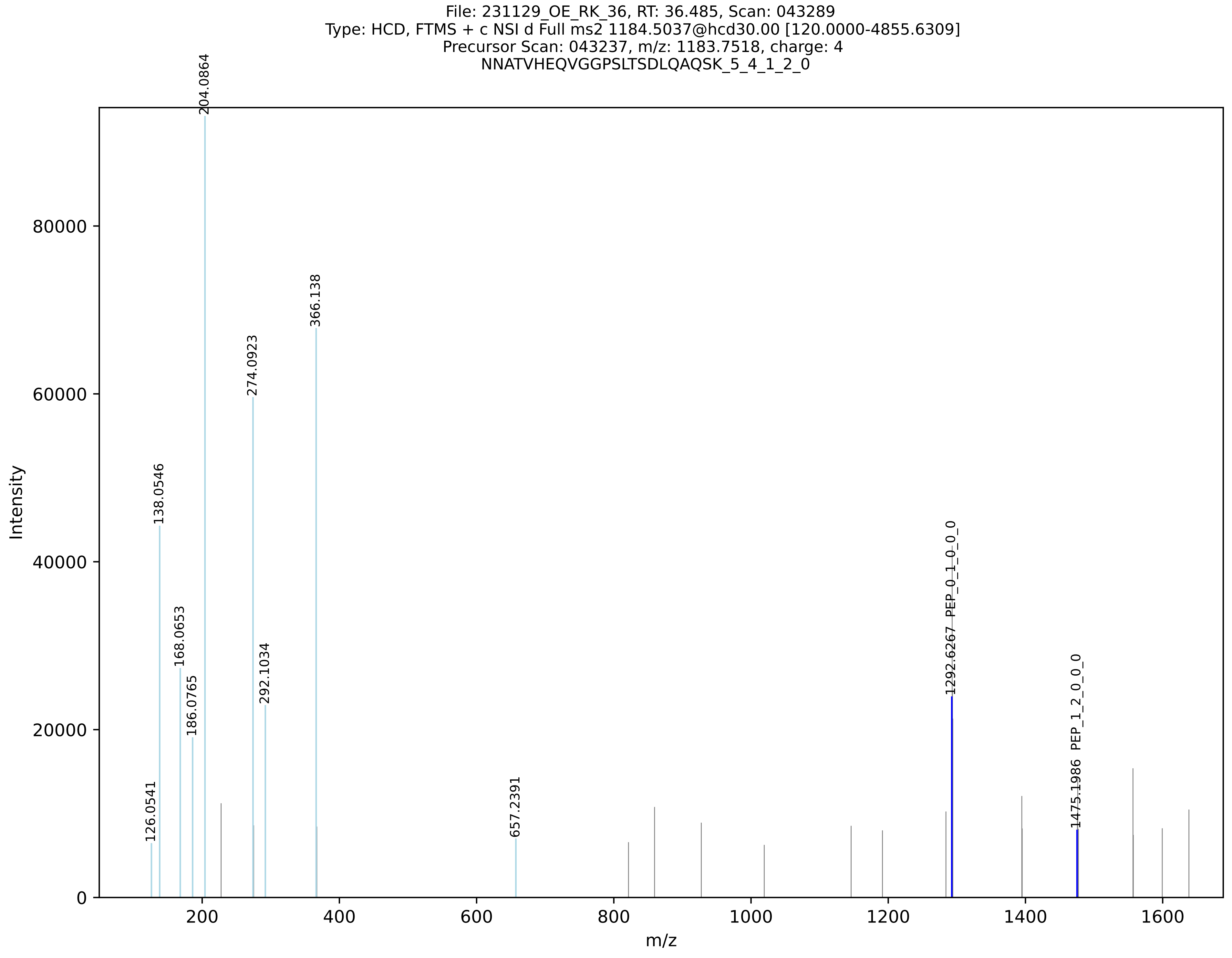
File: 231129_OE_RK_36, RT: 36.485, Scan: 043289
Type: HCD, FTMS + c NSI d Full ms2 1184.5037@hcd30.00 [120.0000-4855.6309]
Precursor Scan: 043237, m/z: 1183.7518, charge: 4
NNATVHEQVGGPSLTSDLQAQSK_5_4_1_2_0
204.0864
366.138
274.0923
138.0546
168.0653
186.0765
292.1034
126.0541
657.2391
1292.6267 PEP_0_1_0_0_0_
1475.1986 PEP_1_2_0_0_0_
Intensity
m/z
0
20000
40000
60000
80000
200
400
600
800
1000
1200
1400
1600

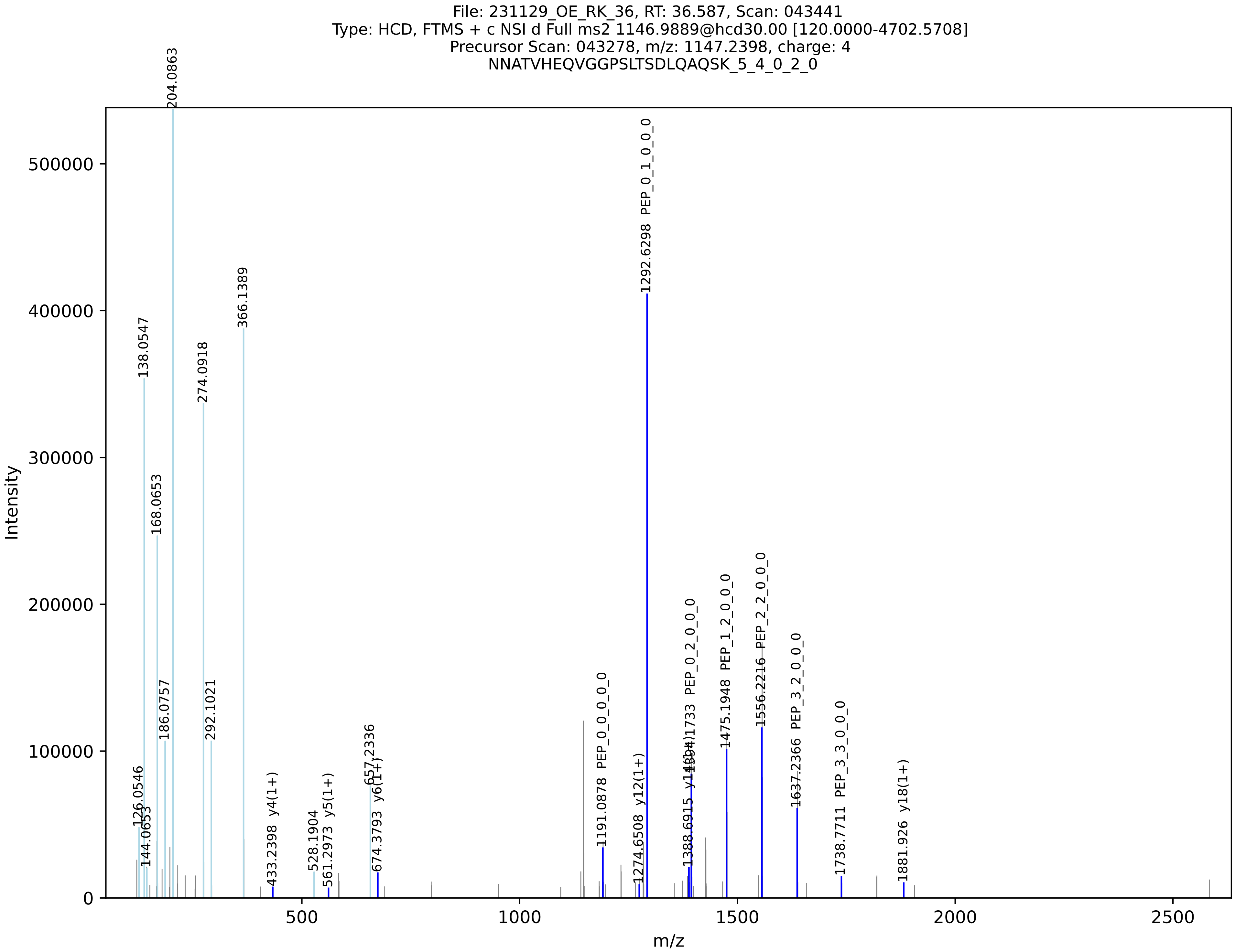
File: 231129_OE_RK_36, RT: 36.587, Scan: 043441
Type: HCD, FTMS + c NSI d Full ms2 1146.9889@hcd30.00 [120.0000-4702.5708]
Precursor Scan: 043278, m/z: 1147.2398, charge: 4
NNATVHEQVGGPSLTSDLQAQSK_5_4_0_2_0
Intensity
m/z
0
100000
200000
300000
400000
500000
500
1000
1500
2000
2500
204.0863
138.0547
274.0918
366.1389
168.0653
186.0757
292.1021
126.0546
144.0653
433.2398 y4(1+)
528.1904
561.2973 y5(1+)
657.2336
674.3793 y6(1+)
1191.0878 PEP_0_0_0_0_0
1274.6508 y12(1+)
1292.6298 PEP_0_1_0_0_0
1388.6915 y14(1+)
PEP_0_2_0_0_0
1475.1948 PEP_1_2_0_0_0
1556.2216 PEP_2_2_0_0_0
1637.2366 PEP_3_2_0_0_0
1738.7711 PEP_3_3_0_0_0
1881.926 y18(1+)

File: 231129_OE_RK_36, RT: 36.698, Scan: 043599
Type: HCD, FTMS + c NSI d Full ms2 1210.1526@hcd30.00 [120.0000-3725.2087]
Precursor Scan: 043520, m/z: 1209.1502, charge: 3
FTNITGEK_6_5_1_2_0

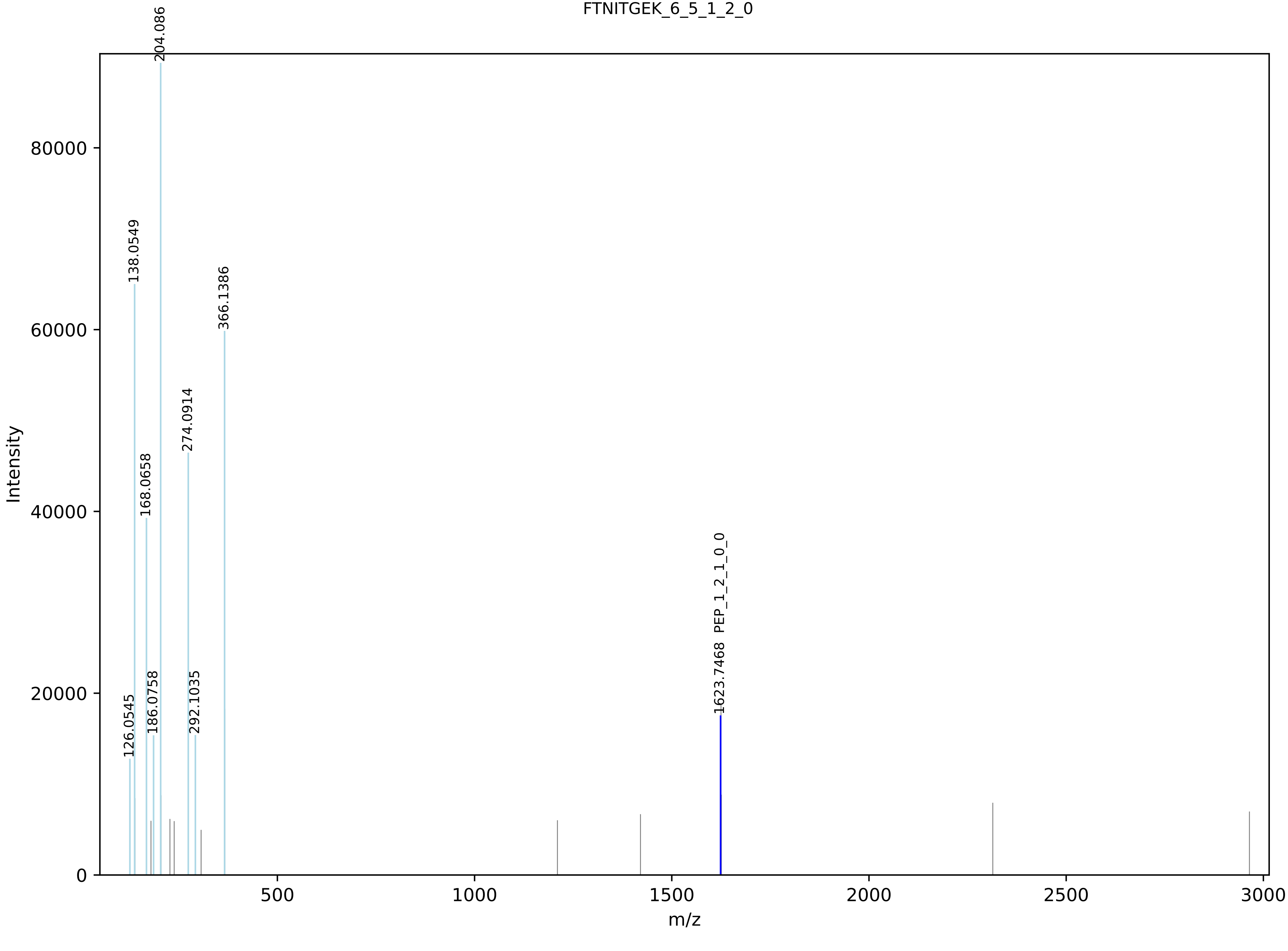

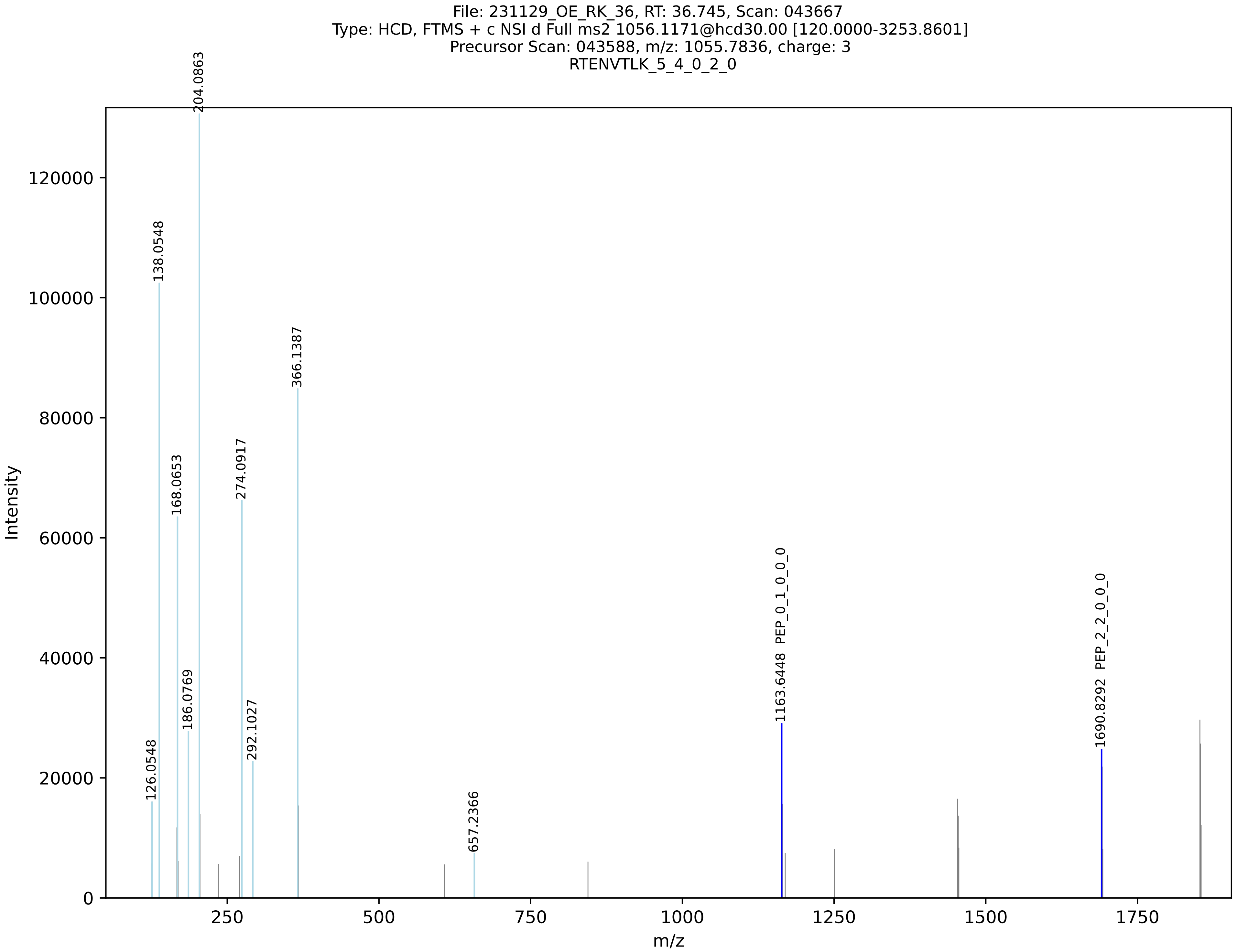

File: 231129_OE_RK_36, RT: 36.745, Scan: 043667
Type: HCD, FTMS + c NSI d Full ms2 1056.1171@hcd30.00 [120.0000-3253.8601]
Precursor Scan: 043588, m/z: 1055.7836, charge: 3
RTENVTLK_5_4_0_2_0
Intensity
m/z
204.0863
138.0548
366.1387
274.0917
168.0653
186.0769
292.1027
126.0548
657.2366
1163.6448 PEP_0_1_0_0_0
1690.8292 PEP_2_2_0_0_0

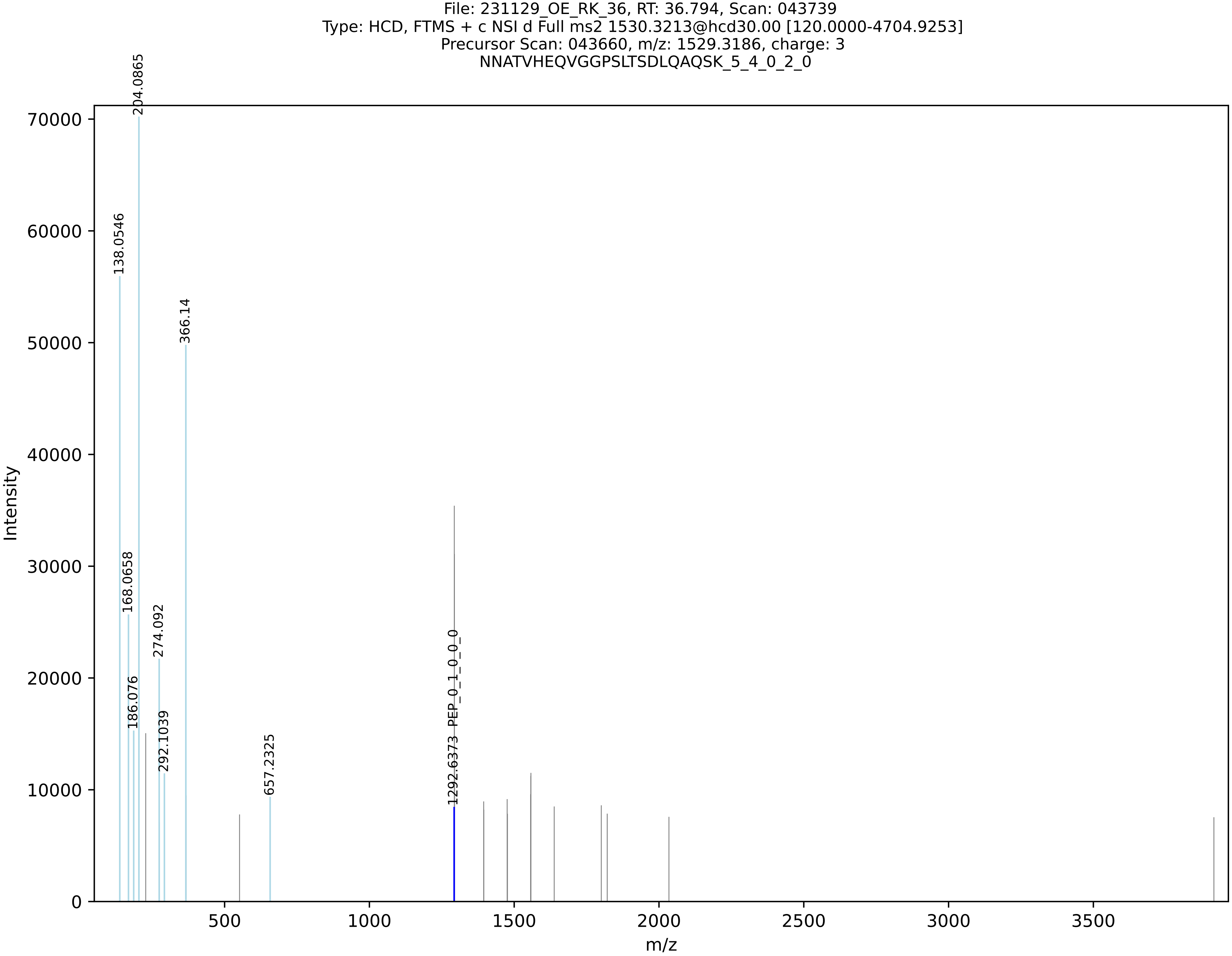
File: 231129_OE_RK_36, RT: 36.794, Scan: 043739
Type: HCD, FTMS + c NSI d Full ms2 1530.3213@hcd30.00 [120.0000-4704.9253]
Precursor Scan: 043660, m/z: 1529.3186, charge: 3
NNATVHEQVGGPSLTSDLQAQSK_5_4_0_2_0
Intensity
m/z
204.0865
138.0546
366.14
168.0658
274.092
186.076
292.1039
657.2325
1292.6373_PEP_0_1_0_0_0
0
10000
20000
30000
40000
50000
60000
70000
500
1000
1500
2000
2500
3000
3500

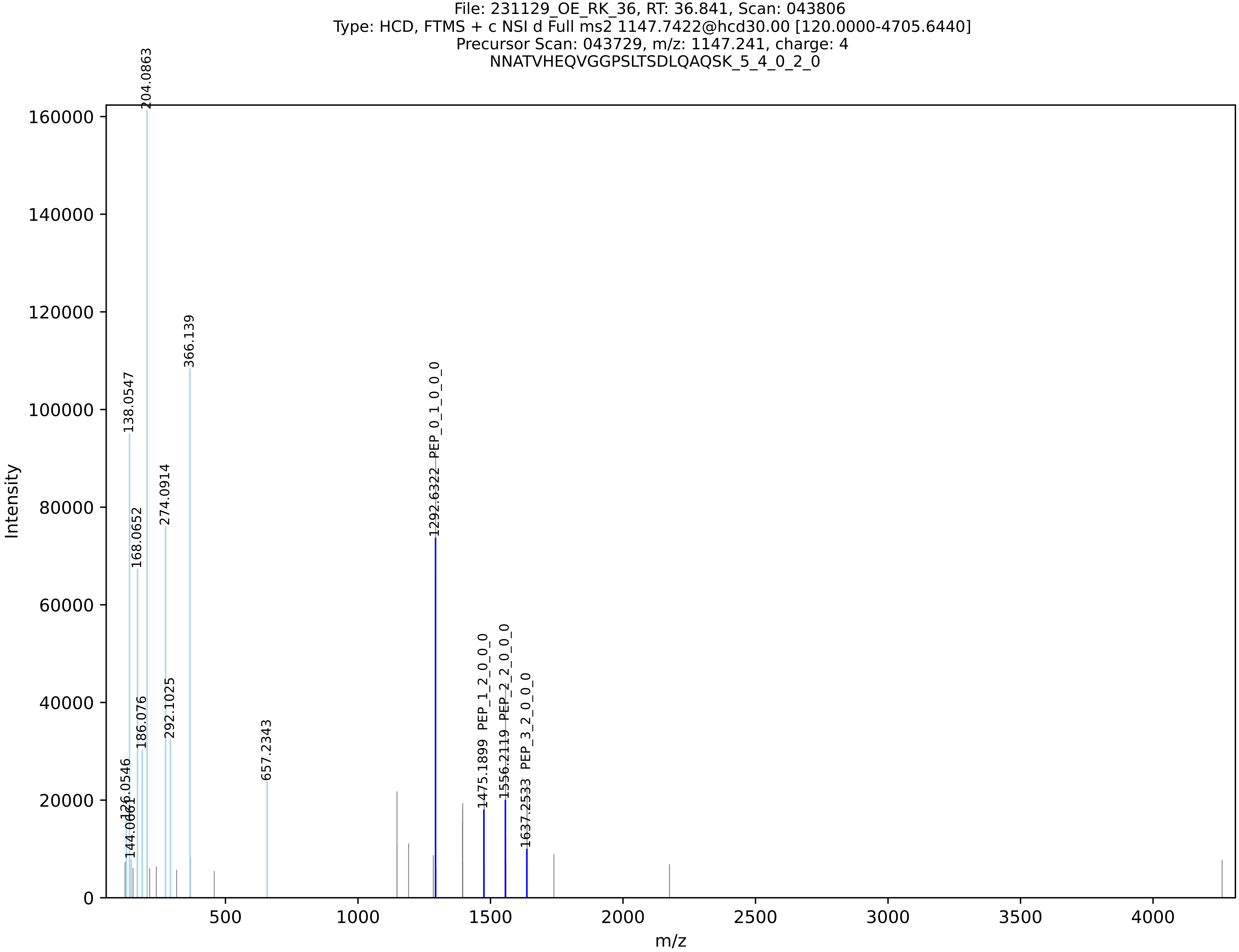
File: 231129_OE_RK_36, RT: 36.841, Scan: 043806
Type: HCD, FTMS + c NSI d Full ms2 1147.7422@hcd30.00 [120.0000-4705.6440]
Precursor Scan: 043729, m/z: 1147.241, charge: 4
NNATVHEQVGGPSLTSDLQAQSK_5_4_0_2_0
Intensity
m/z
204.0863
366.139
138.0547
274.0914
168.0652
292.1025
186.076
126.0546
144.0661
657.2343
1292.6322 PEP_0_1_0_0_0
1475.1899 PEP_1_2_0_0_0
1556.2119 PEP_2_2_0_0_0
1637.2533 PEP_3_2_0_0_0
0
20000
40000
60000
80000
100000
120000
140000
160000
500
1000
1500
2000
2500
3000
3500
4000

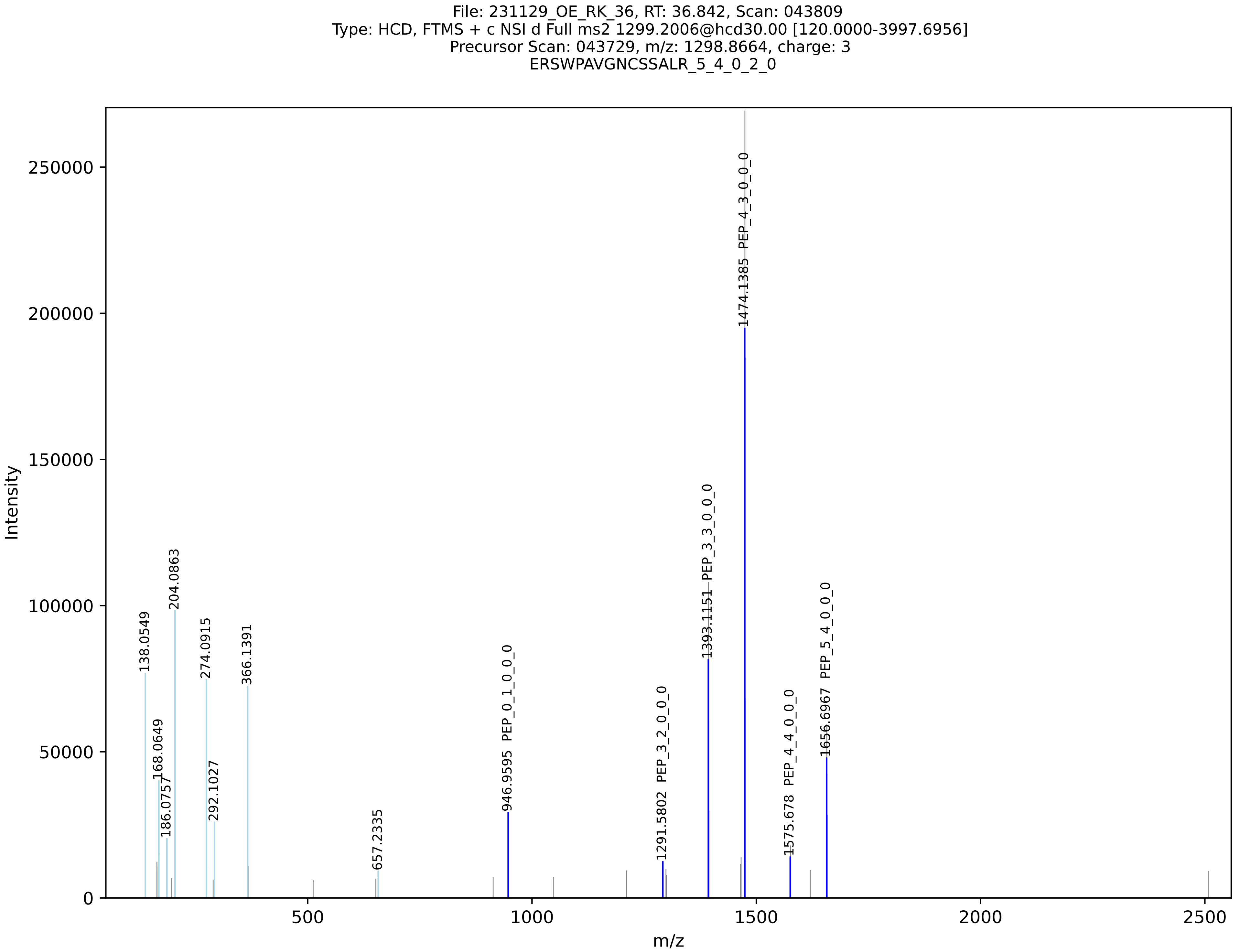
File: 231129_OE_RK_36, RT: 36.842, Scan: 043809
Type: HCD, FTMS + c NSI d Full ms2 1299.2006@hcd30.00 [120.0000-3997.6956]
Precursor Scan: 043729, m/z: 1298.8664, charge: 3
ERSWPAVGNCSSALR_5_4_0_2_0
Intensity
m/z
138.0549
168.0649
186.0757
204.0863
274.0915
292.1027
366.1391
657.2335
946.9595_PEP_0_1_0_0_0
1291.5802_PEP_3_2_0_0_0
1393.1151_PEP_3_3_0_0_0
1474.1385_PEP_4_3_0_0_0
1575.678_PEP_4_4_0_0_0
1656.6967_PEP_5_4_0_0_0

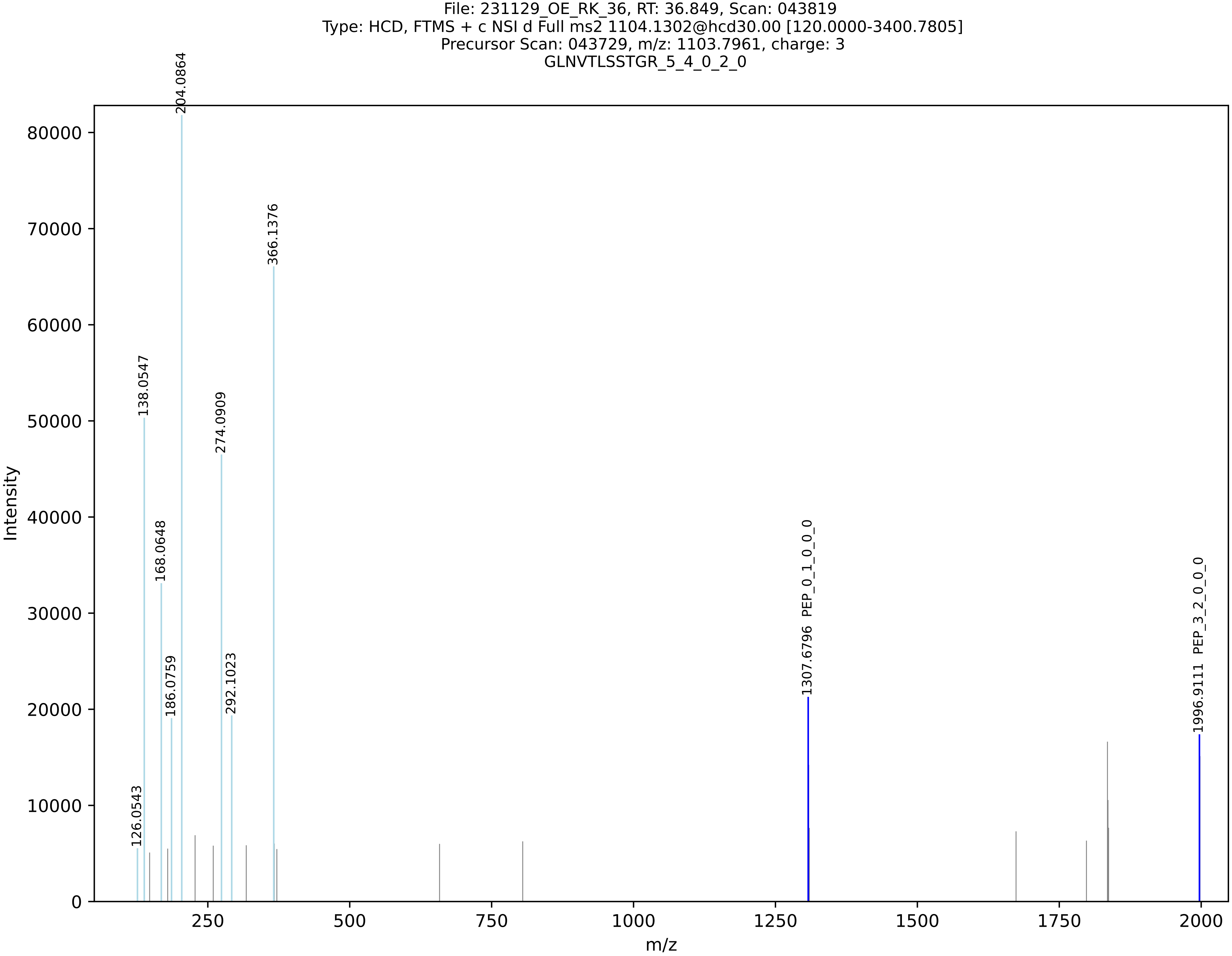
File: 231129_OE_RK_36, RT: 36.849, Scan: 043819
Type: HCD, FTMS + c NSI d Full ms2 1104.1302@hcd30.00 [120.0000-3400.7805]
Precursor Scan: 043729, m/z: 1103.7961, charge: 3
GLNVTLSSTGR_5_4_0_2_0
Intensity
m/z
204.0864
366.1376
138.0547
274.0909
168.0648
186.0759
292.1023
126.0543
1307.6796 PEP_0_1_0_0_0
1996.9111 PEP_3_2_0_0_0
0
10000
20000
30000
40000
50000
60000
70000
80000
250
500
750
1000
1250
1500
1750
2000

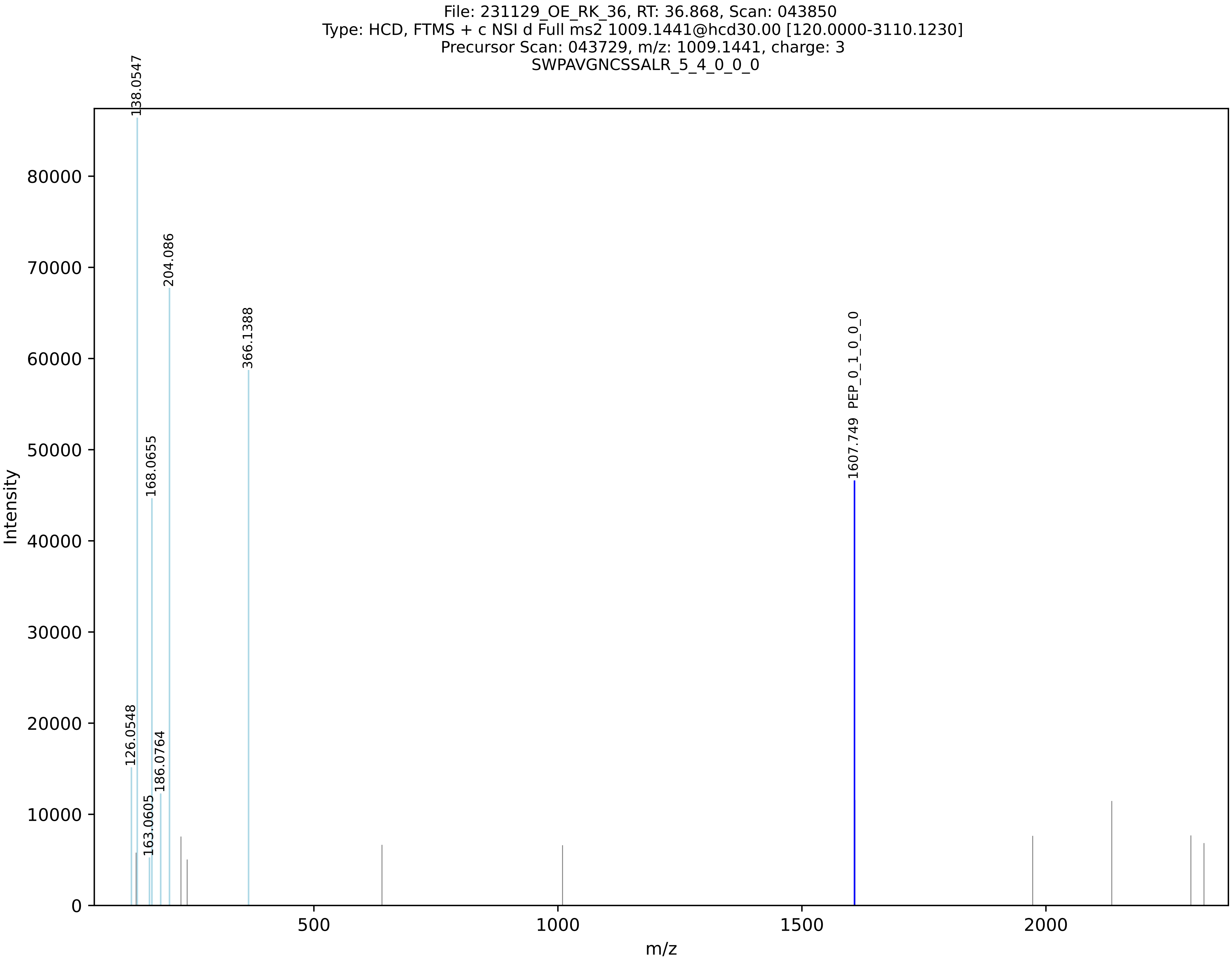
File: 231129_OE_RK_36, RT: 36.868, Scan: 043850
Type: HCD, FTMS + c NSI d Full ms2 1009.1441@hcd30.00 [120.0000-3110.1230]
Precursor Scan: 043729, m/z: 1009.1441, charge: 3
SWPAVGNCSSALR_5_4_0_0_0
138.0547
204.086
366.1388
168.0655
126.0548
186.0764
163.0605
1607.749 PEP_0_1_0_0_0
Intensity
m/z
0
10000
20000
30000
40000
50000
60000
70000
80000
500
1000
1500
2000

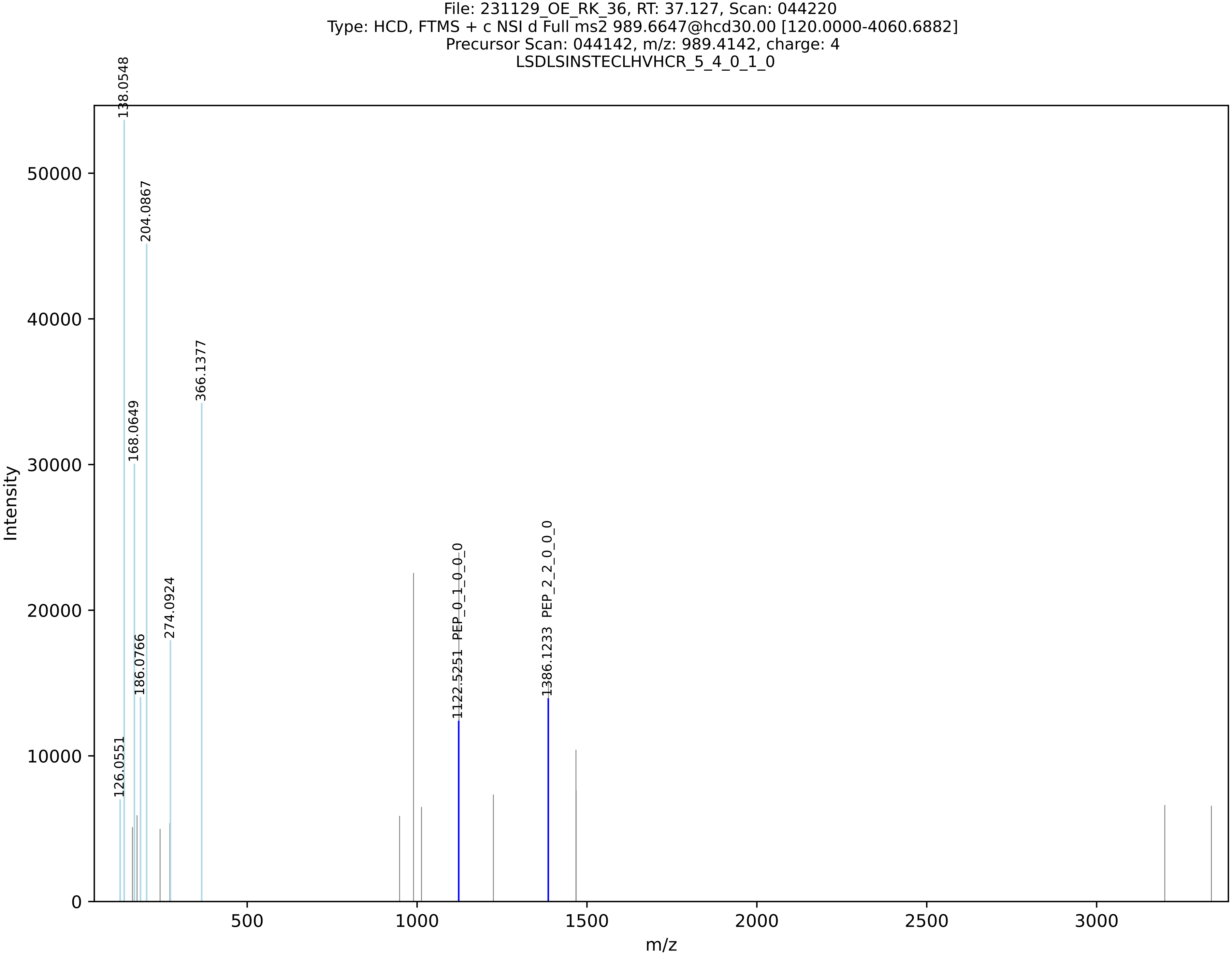
File: 231129_OE_RK_36, RT: 37.127, Scan: 044220
Type: HCD, FTMS + c NSI d Full ms2 989.6647@hcd30.00 [120.0000-4060.6882]
Precursor Scan: 044142, m/z: 989.4142, charge: 4
LSDLSINSTECLHVHCR_5_4_0_1_0
Intensity
m/z
138.0548
204.0867
366.1377
168.0649
274.0924
186.0766
126.0551
1122.5251_PEP_0_1_0_0_0
1386.1233_PEP_2_2_0_0_0
0
10000
20000
30000
40000
50000
500
1000
1500
2000
2500
3000

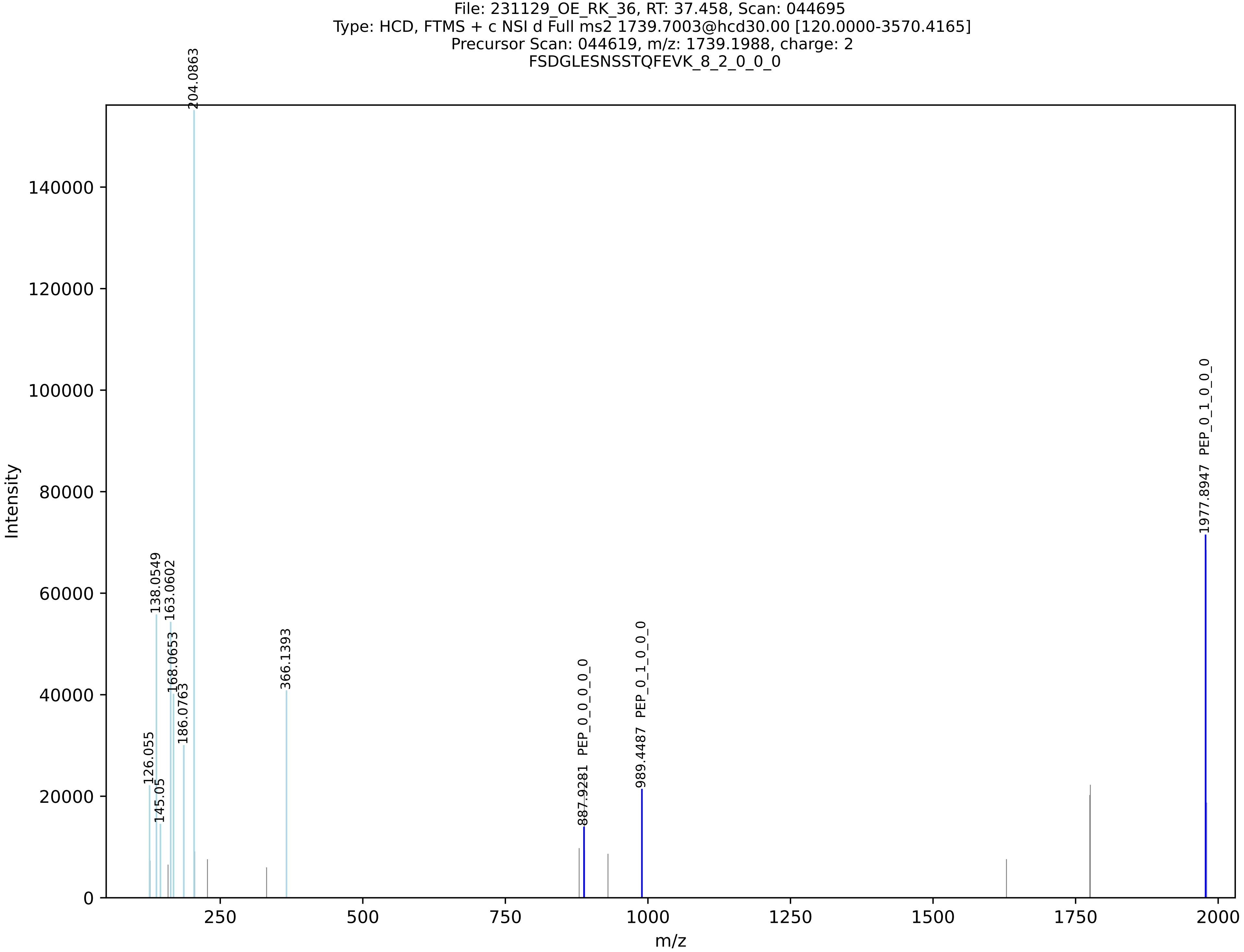
File: 231129_OE_RK_36, RT: 37.458, Scan: 044695
Type: HCD, FTMS + c NSI d Full ms2 1739.7003@hcd30.00 [120.0000-3570.4165]
Precursor Scan: 044619, m/z: 1739.1988, charge: 2
FSDGLESNSSTQFEVK_8_2_0_0_0
Intensity
m/z
204.0863
138.0549
163.0602
168.0653
186.0763
126.055
145.05
366.1393
887.9281 PEP_0_0_0_0_0
989.4487 PEP_0_1_0_0_0
1977.8947 PEP_0_1_0_0_
0
20000
40000
60000
80000
100000
120000
140000
250
500
750
1000
1250
1500
1750
2000

File: 231129_OE_RK_36, RT: 37.558, Scan: 044835
Type: HCD, FTMS + c NSI d Full ms2 1311.7963@hcd30.00 [120.0000-5374.9849]
Precursor Scan: 044775, m/z: 1311.2968, charge: 4
DVLWFRPTLLNDTGNYTCMLR_4_7_2_1_0

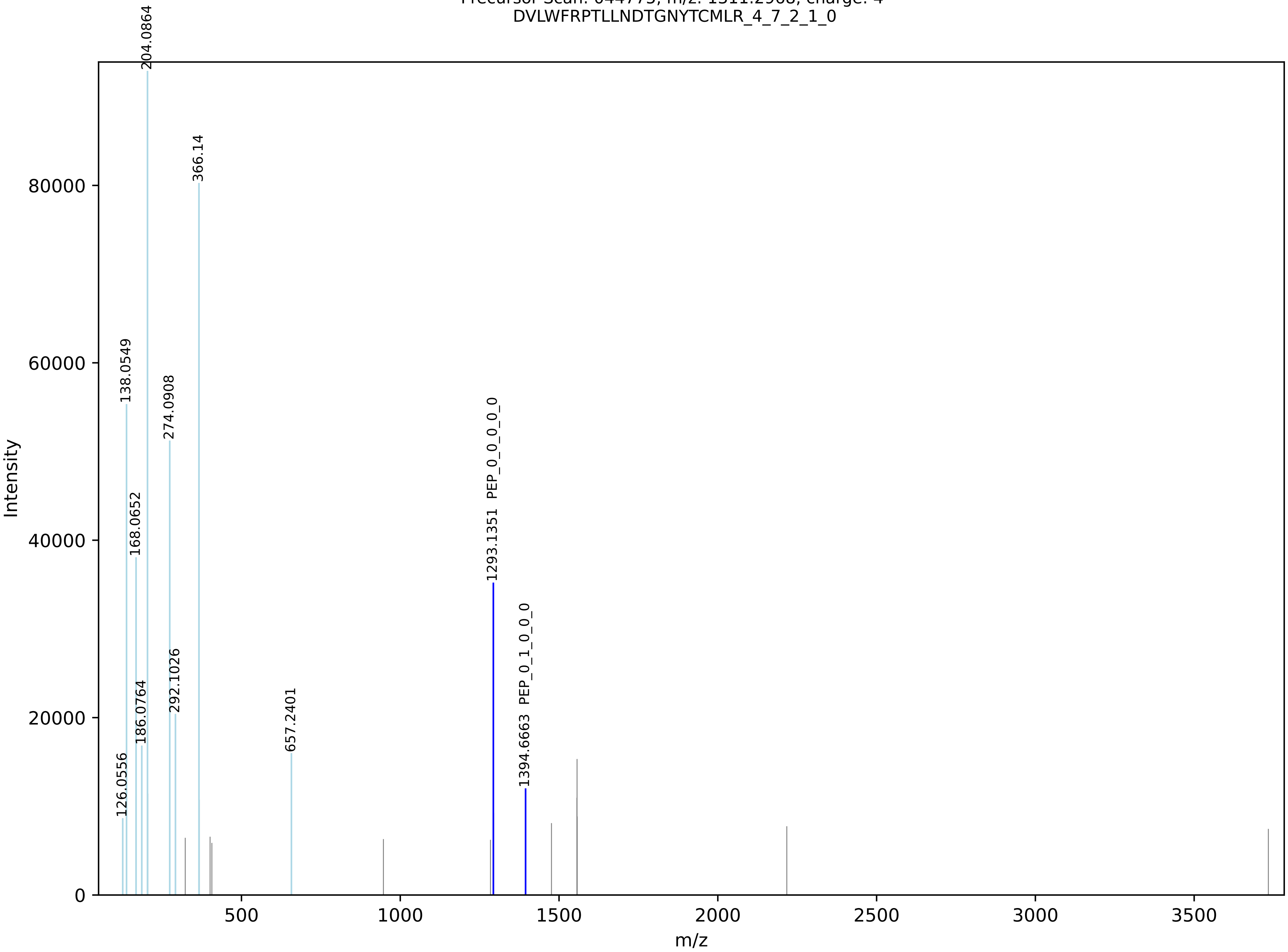

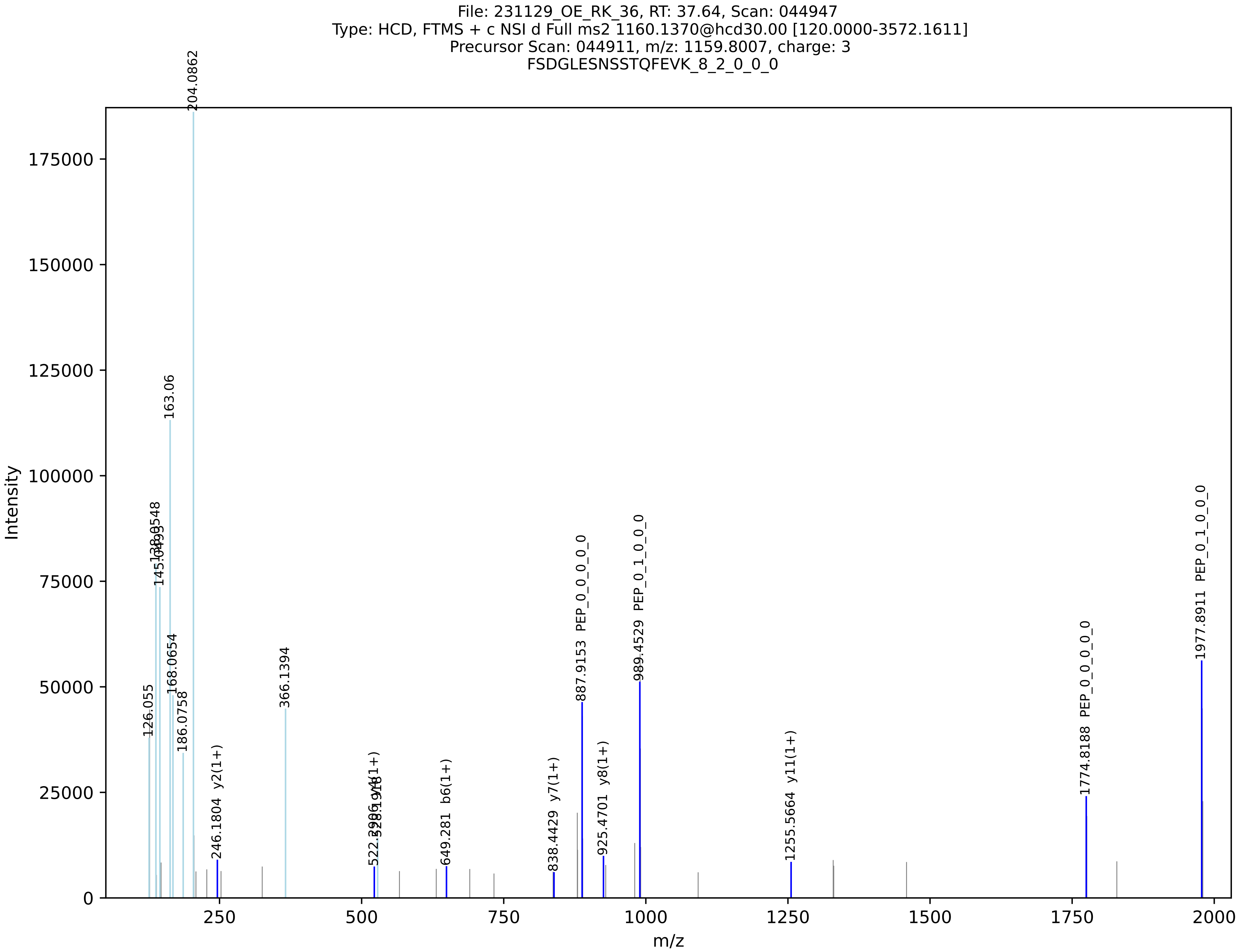
File: 231129_OE_RK_36, RT: 37.64, Scan: 044947
Type: HCD, FTMS + c NSI d Full ms2 1160.1370@hcd30.00 [120.0000-3572.1611]
Precursor Scan: 044911, m/z: 1159.8007, charge: 3
FSDGLESNSSTQFEVK_8_2_0_0_0
Intensity
m/z
204.0862
163.06
138.0548
145.0495
168.0654
126.055
186.0758
366.1394
246.1804 y2(1+)
522.2906 y4(1+)
528.1918
649.281 b6(1+)
838.4429 y7(1+)
887.9153 PEP_0_0_0_0_0
925.4701 y8(1+)
989.4529 PEP_0_1_0_0_0
1255.5664 y11(1+)
1774.8188 PEP_0_0_0_0_0
1977.8911 PEP_0_1_0_0_0
0
25000
50000
75000
100000
125000
150000
175000
250
500
750
1000
1250
1500
1750
2000

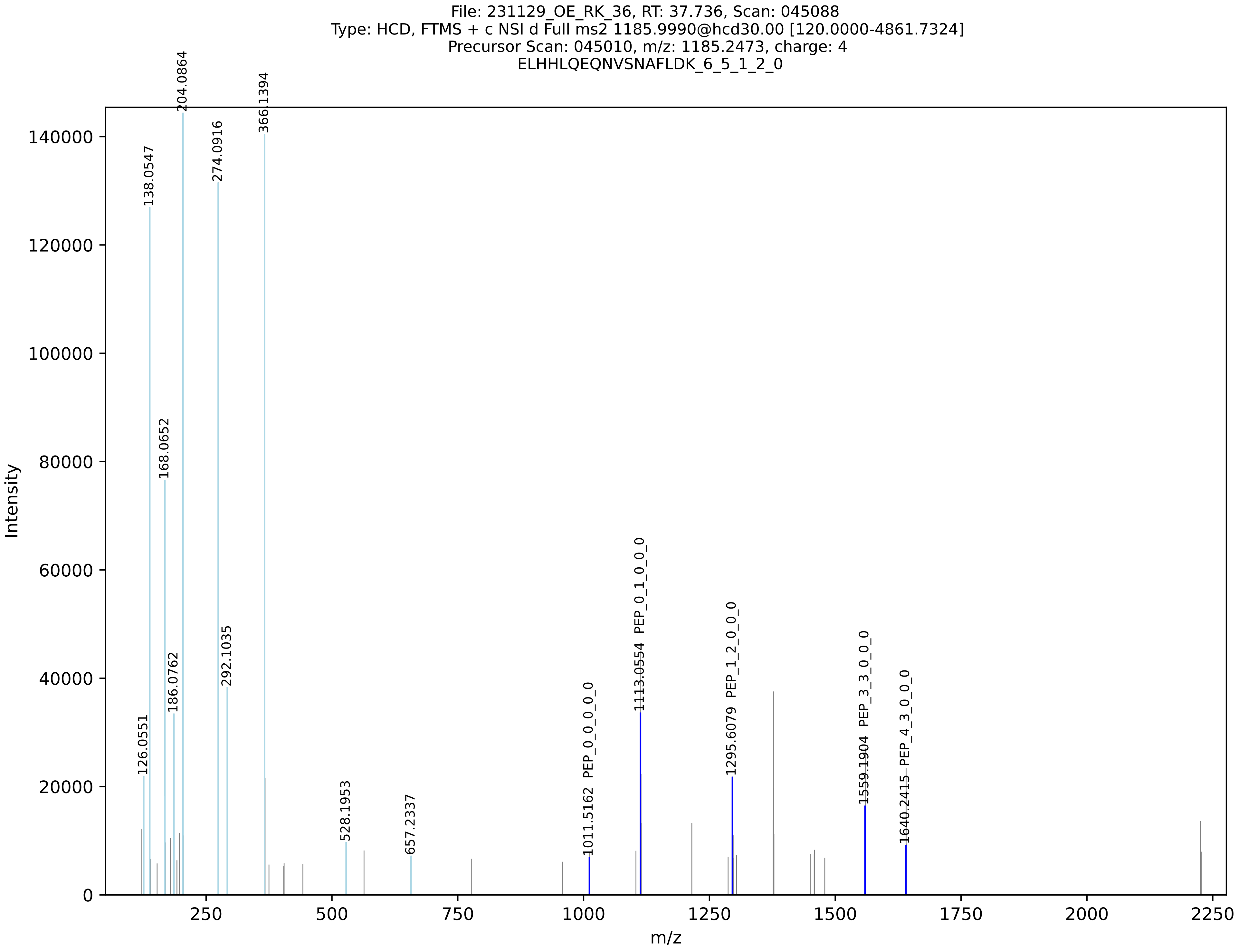

File: 231129_OE_RK_36, RT: 37.736, Scan: 045088
Type: HCD, FTMS + c NSI d Full ms2 1185.9990@hcd30.00 [120.0000-4861.7324]
Precursor Scan: 045010, m/z: 1185.2473, charge: 4
ELHHLQEQNVSNAFLDK_6_5_1_2_0
Intensity
m/z
204.0864
366.1394
274.0916
138.0547
168.0652
292.1035
186.0762
126.0551
528.1953
657.2337
1011.5162 PEP_0_0_0_0_0
1113.0554 PEP_0_1_0_0_0
1295.6079 PEP_1_2_0_0_0
1559.1904 PEP_3_3_0_0_0
1640.2415 PEP_4_3_0_0_0

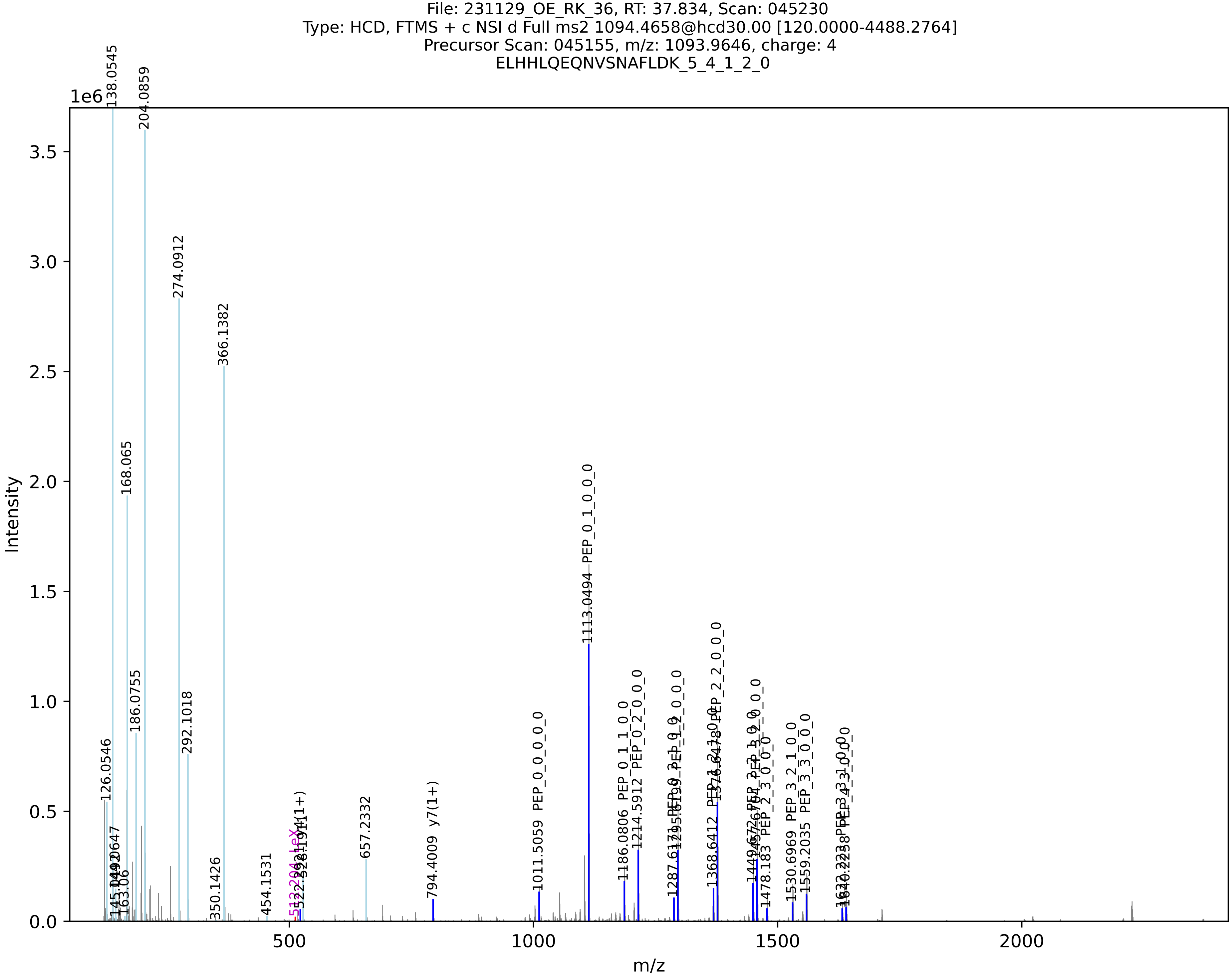

File: 231129_OE_RK_36, RT: 37.834, Scan: 045230
Type: HCD, FTMS + c NSI d Full ms2 1094.4658@hcd30.00 [120.0000-4488.2764]
Precursor Scan: 045155, m/z: 1093.9646, charge: 4
ELHHLQEQNVSNAFLDK_5_4_1_2_0
1e6
Intensity
m/z
138.0545
204.0859
274.0912
366.1382
168.065
186.0755
292.1018
126.0546
350.1426
454.1531
657.2332
794.4009 y7(1+)
1011.5059 PEP_0_0_0_0_0
1113.0494 PEP_0_1_0_0_0
1186.0806 PEP_0_1_1_0_0
1214.5912 PEP_0_2_0_0_0
1530.6969 PEP_3_2_1_0_0
1559.2035 PEP_3_3_0_0_0
1478.183 PEP_2_3_0_0_0

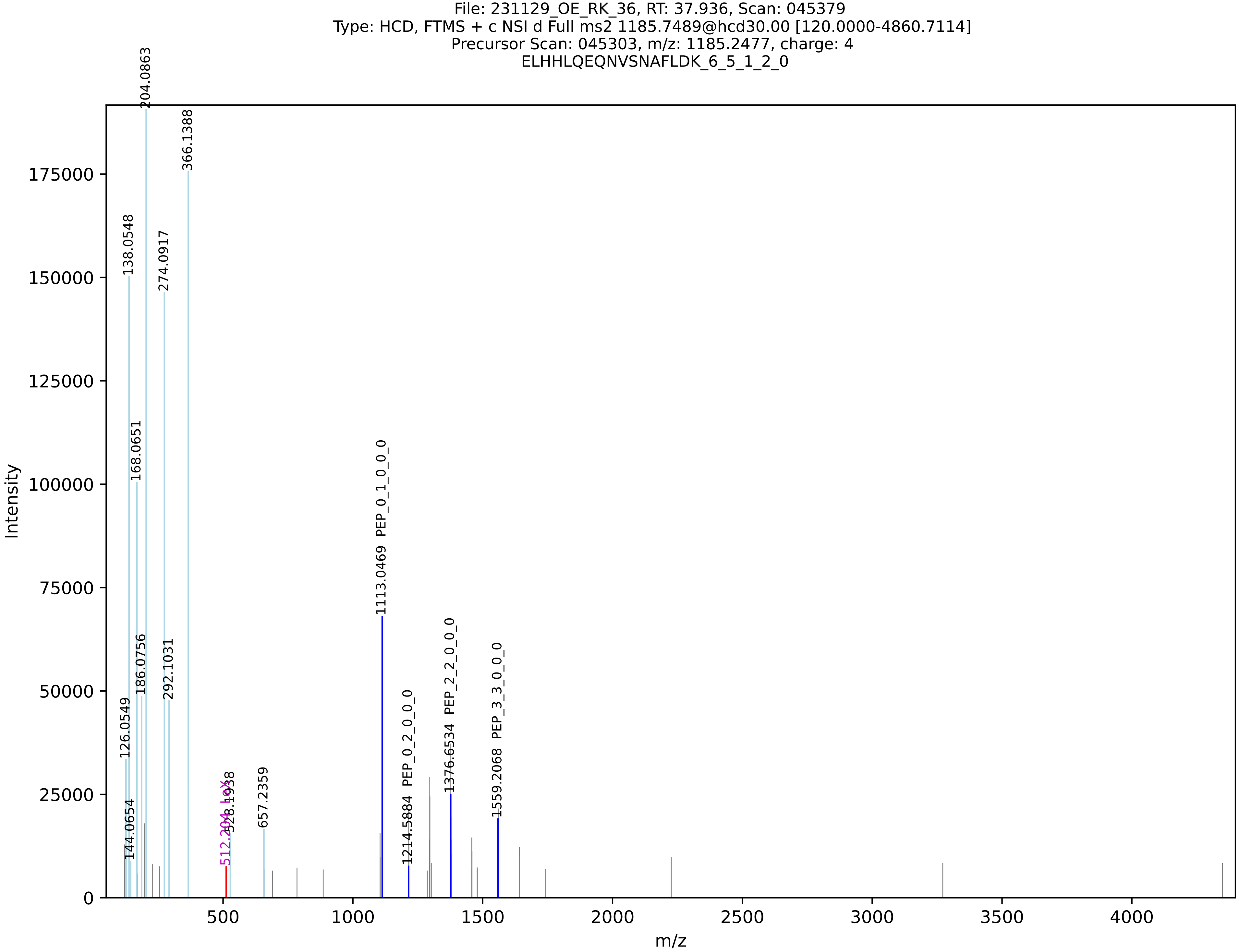

File: 231129_OE_RK_36, RT: 37.936, Scan: 045379
Type: HCD, FTMS + c NSI d Full ms2 1185.7489@hcd30.00 [120.0000-4860.7114]
Precursor Scan: 045303, m/z: 1185.2477, charge: 4
ELHHLQEQNVSNAFLDK_6_5_1_2_0
Intensity
m/z
204.0863
366.1388
138.0548
274.0917
168.0651
186.0756
292.1031
126.0549
144.0654
512.204 LeX
528.1938
657.2359
1113.0469 PEP_0_1_0_0_0
1214.5884 PEP_0_2_0_0_0
1376.6534 PEP_2_2_0_0_0
1559.2068 PEP_3_3_0_0_0

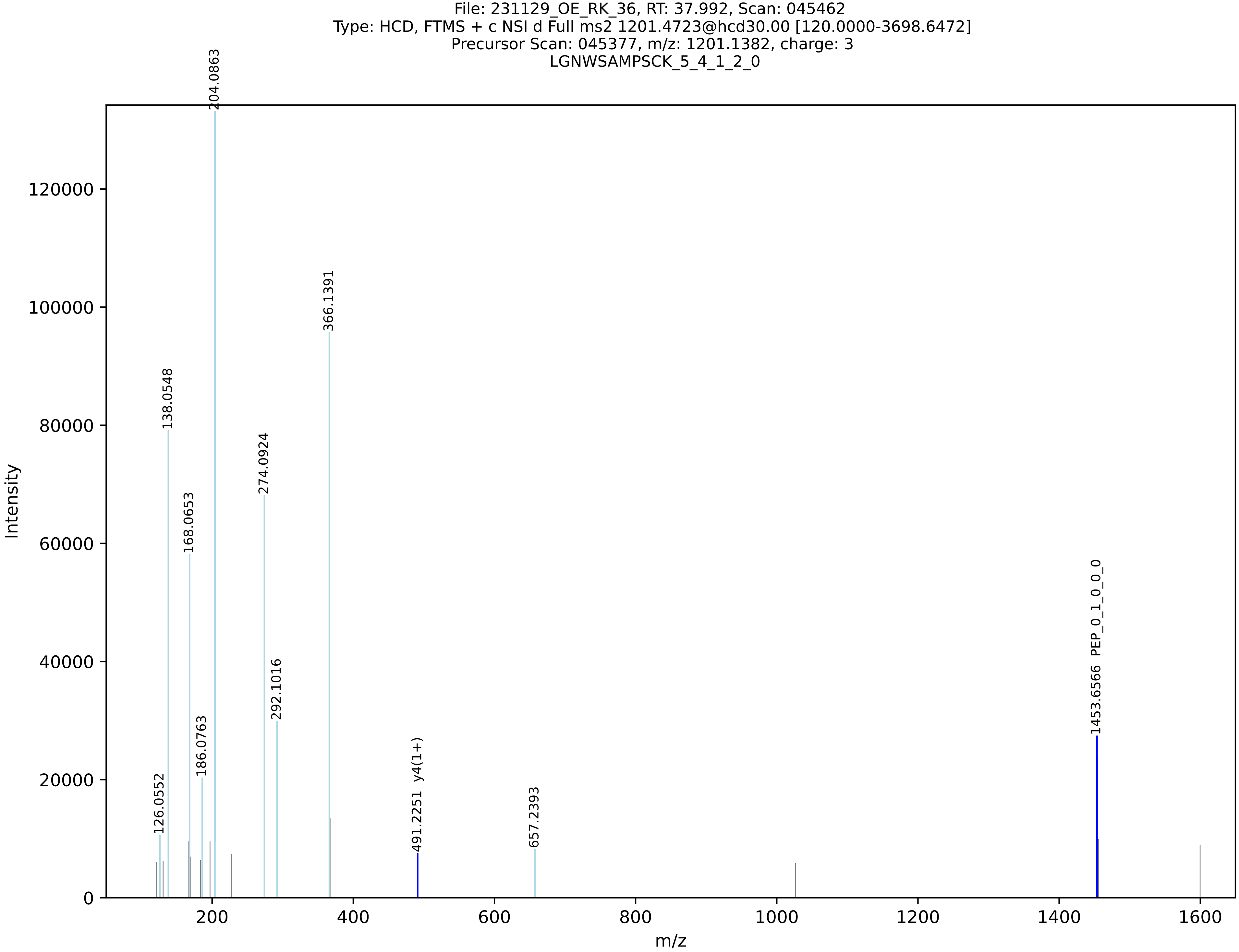
File: 231129_OE_RK_36, RT: 37.992, Scan: 045462
Type: HCD, FTMS + c NSI d Full ms2 1201.4723@hcd30.00 [120.0000-3698.6472]
Precursor Scan: 045377, m/z: 1201.1382, charge: 3
LGNWSAMPSCK_5_4_1_2_0
Intensity
m/z
204.0863
366.1391
138.0548
274.0924
168.0653
292.1016
186.0763
126.0552
491.2251 y4(1+)
657.2393
1453.6566 PEP_0_1_0_0_0
0
20000
40000
60000
80000
100000
120000
200
400
600
800
1000
1200
1400
1600

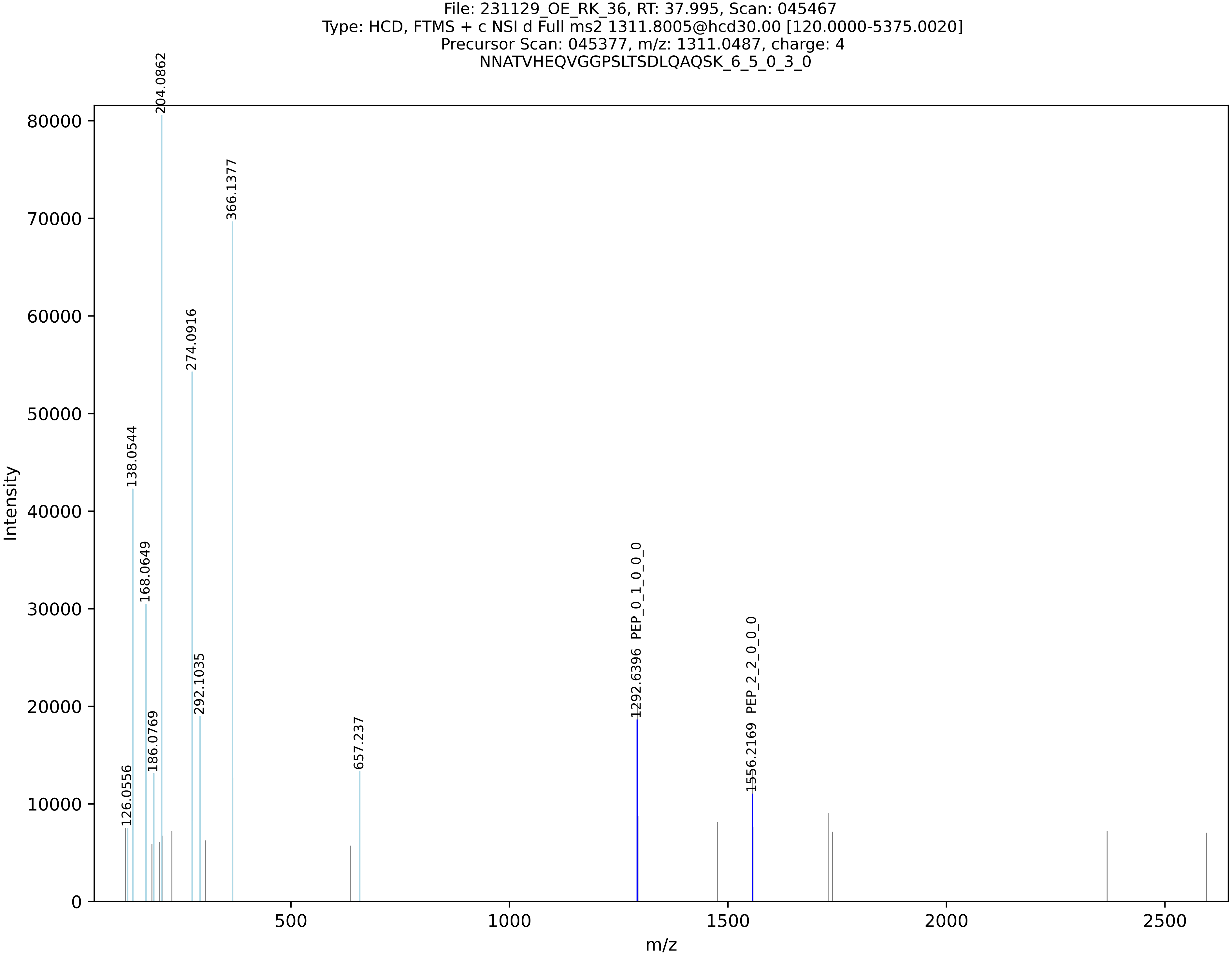
File: 231129_OE_RK_36, RT: 37.995, Scan: 045467
Type: HCD, FTMS + c NSI d Full ms2 1311.8005@hcd30.00 [120.0000-5375.0020]
Precursor Scan: 045377, m/z: 1311.0487, charge: 4
NNATVHEQVGGPSLTSDLQAQSK_6_5_0_3_0
Intensity
m/z
204.0862
366.1377
274.0916
138.0544
168.0649
292.1035
186.0769
126.0556
657.237
1292.6396 PEP_0_1_0_0_0_
1556.2169 PEP_2_2_0_0_0_
0
10000
20000
30000
40000
50000
60000
70000
80000
500
1000
1500
2000
2500

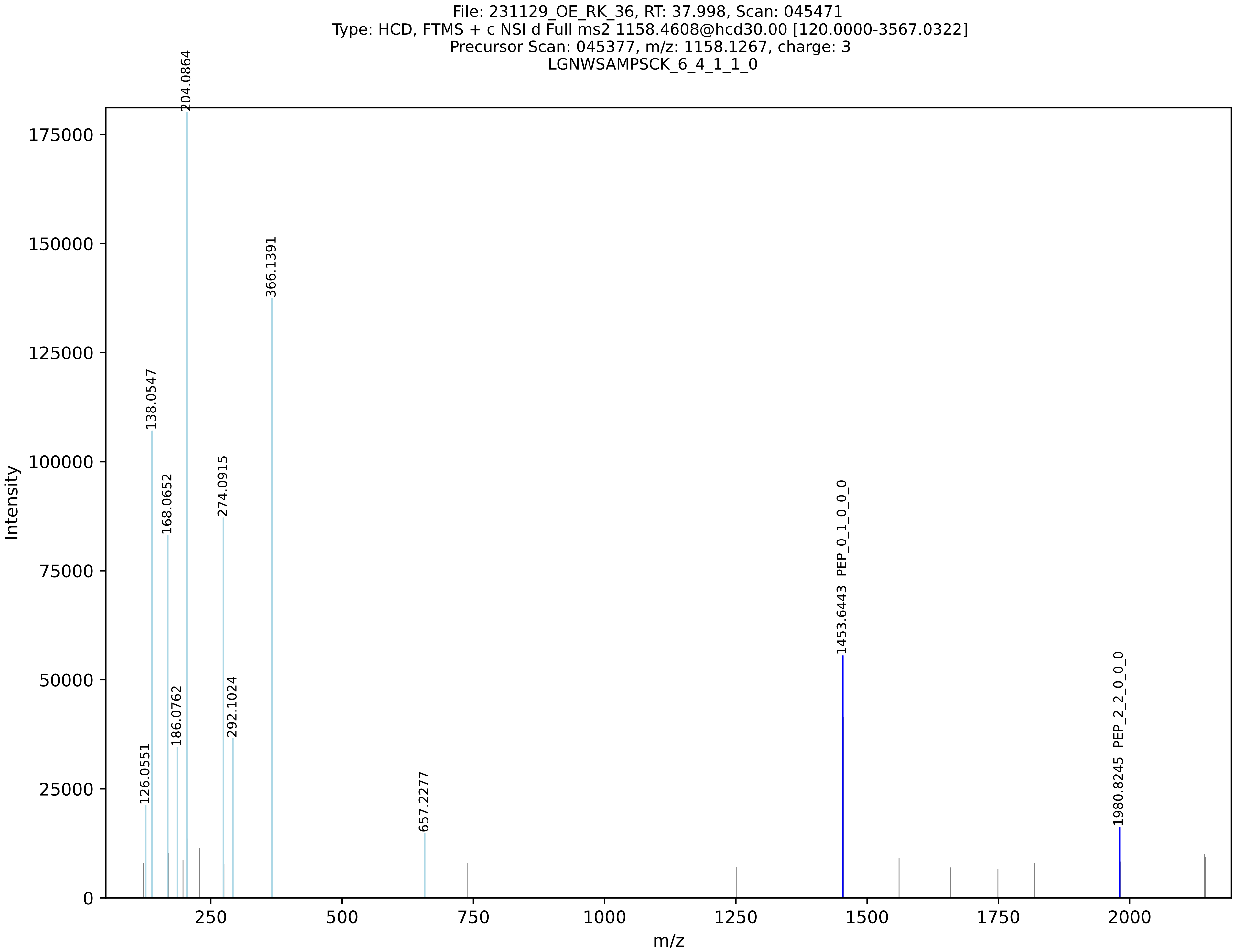
File: 231129_OE_RK_36, RT: 37.998, Scan: 045471
Type: HCD, FTMS + c NSI d Full ms2 1158.4608@hcd30.00 [120.0000-3567.0322]
Precursor Scan: 045377, m/z: 1158.1267, charge: 3
LGNWSAMPSCK_6_4_1_1_0
Intensity
m/z
204.0864
366.1391
138.0547
168.0652
274.0915
186.0762
292.1024
126.0551
657.2277
1453.6443 PEP_0_1_0_0_0
1980.8245 PEP_2_2_0_0_0
0
25000
50000
75000
100000
125000
150000
175000
250
500
750
1000
1250
1500
1750
2000

File: 231129_OE_RK_36, RT: 38.039, Scan: 045527
Type: HCD, FTMS + c NSI d Full ms2 1094.4657@hcd30.00 [120.0000-4488.2759]
Precursor Scan: 045451, m/z: 1093.9646, charge: 4
ELHHLQEQNVSNAFLDK_5_4_1_2_0

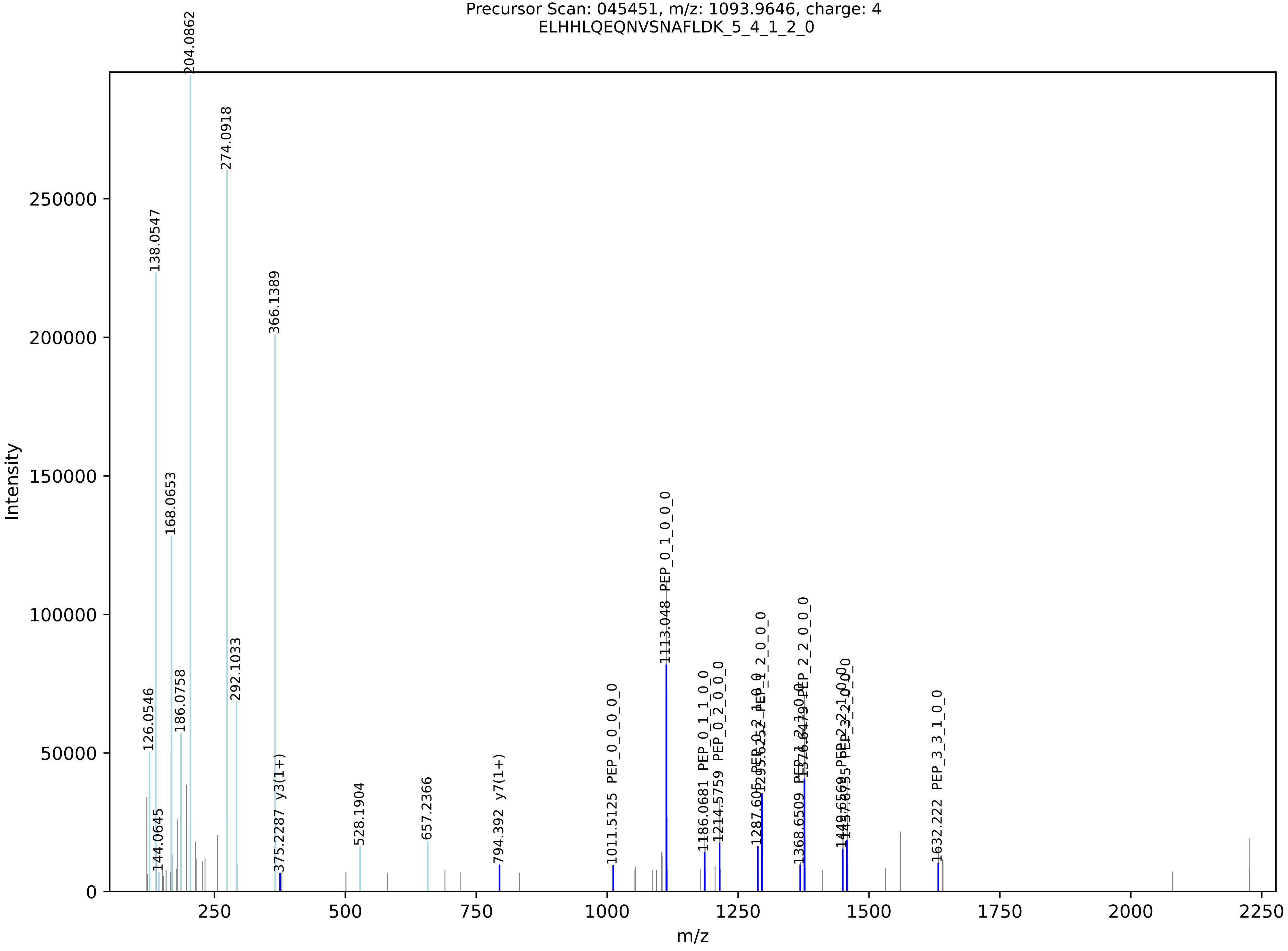

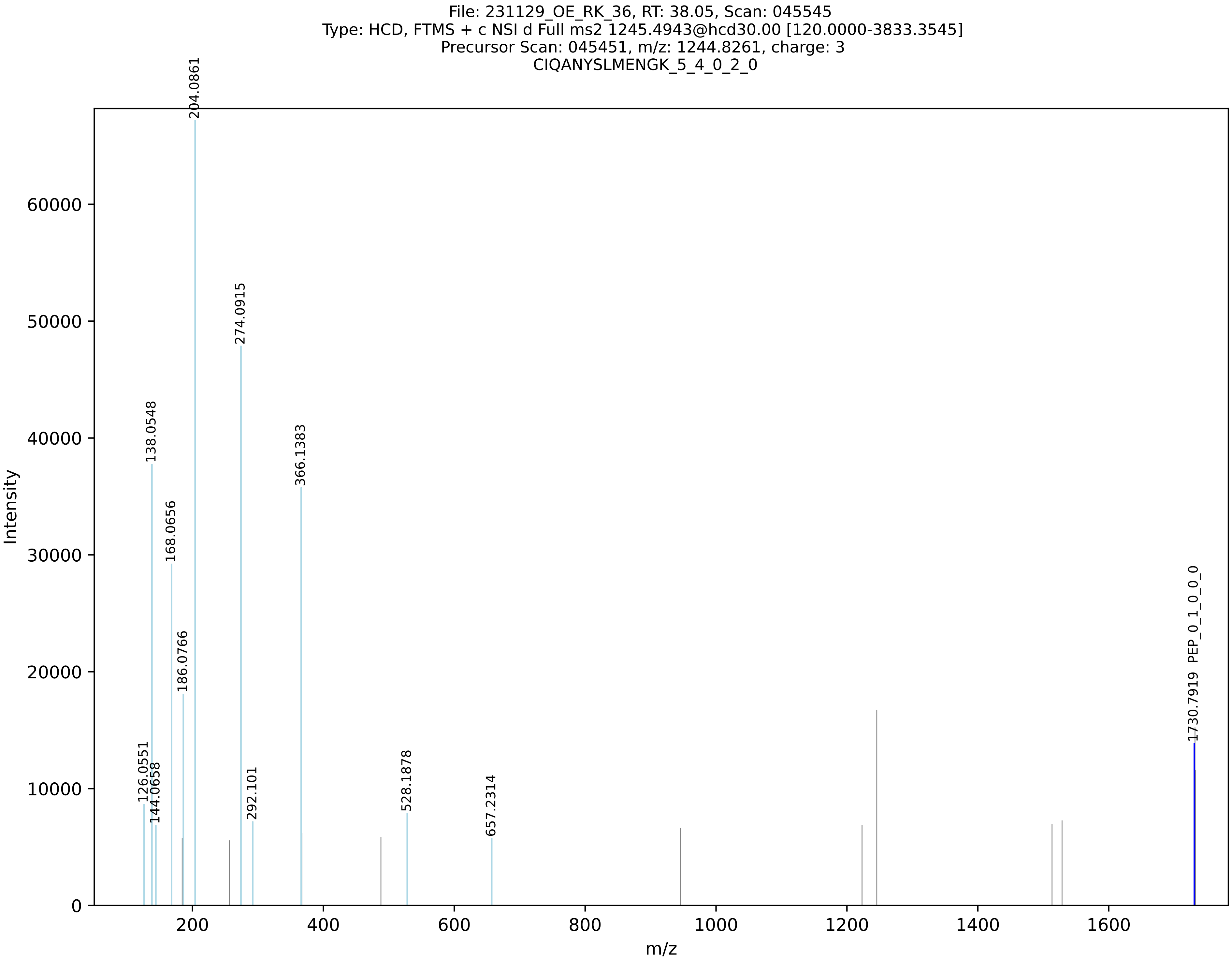

File: 231129_OE_RK_36, RT: 38.05, Scan: 045545
Type: HCD, FTMS + c NSI d Full ms2 1245.4943@hcd30.00 [120.0000-3833.3545]
Precursor Scan: 045451, m/z: 1244.8261, charge: 3
CIQANYSLMENGK_5_4_0_2_0
Intensity
m/z
204.0861
274.0915
138.0548
366.1383
168.0656
186.0766
126.0551
144.0658
292.101
528.1878
657.2314
1730.7919 PEP_0_1_0_0_0

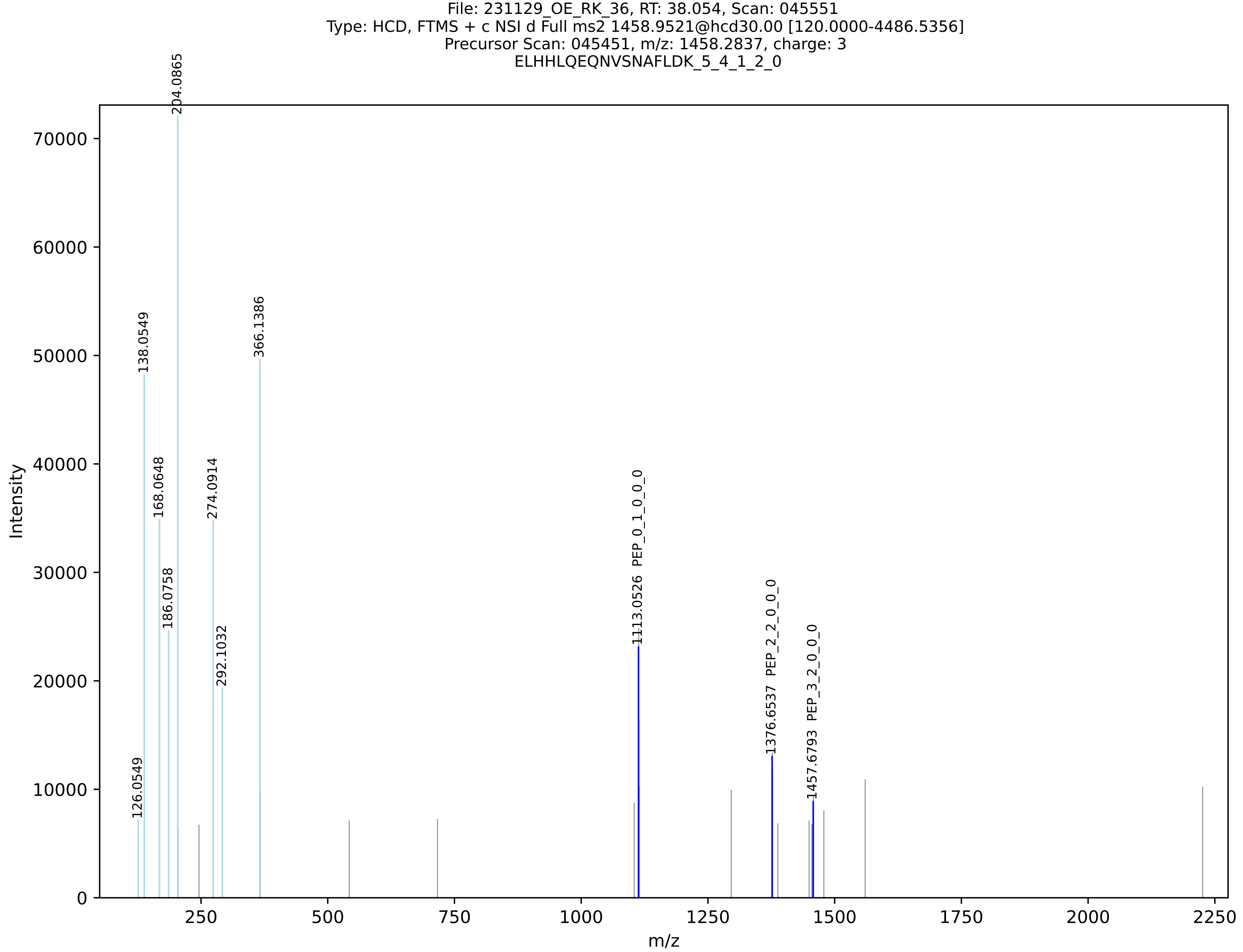

File: 231129_OE_RK_36, RT: 38.054, Scan: 045551
Type: HCD, FTMS + c NSI d Full ms2 1458.9521@hcd30.00 [120.0000-4486.5356]
Precursor Scan: 045451, m/z: 1458.2837, charge: 3
ELHHLQEQNVSNAFLDK_5_4_1_2_0
204.0865
138.0549
366.1386
168.0648
274.0914
186.0758
292.1032
126.0549
1113.0526 PEP_0_1_0_0_0
1376.6537 PEP_2_2_0_0_0
1457.6793 PEP_3_2_0_0_0
Intensity
m/z
0
10000
20000
30000
40000
50000
60000
70000
250
500
750
1000
1250
1500
1750
2000
2250

File: 231129_OE_RK_36, RT: 38.138, Scan: 045668
Type: HCD, FTMS + c NSI d Full ms2 1153.1189@hcd30.00 [120.0000-3550.6858]
Precursor Scan: 045596, m/z: 1152.4506, charge: 3
LGNWSAMPSCK_5_4_0_2_0

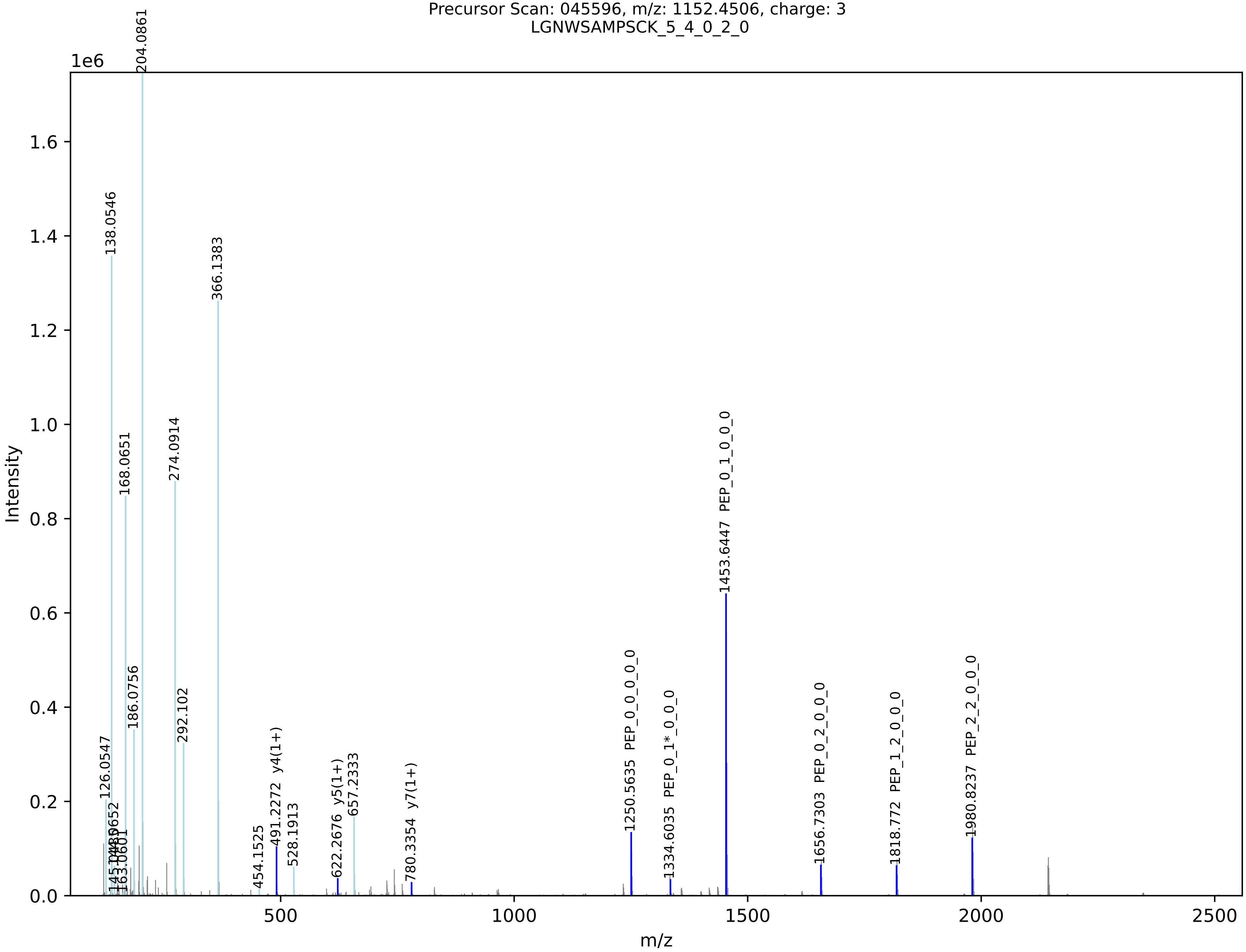

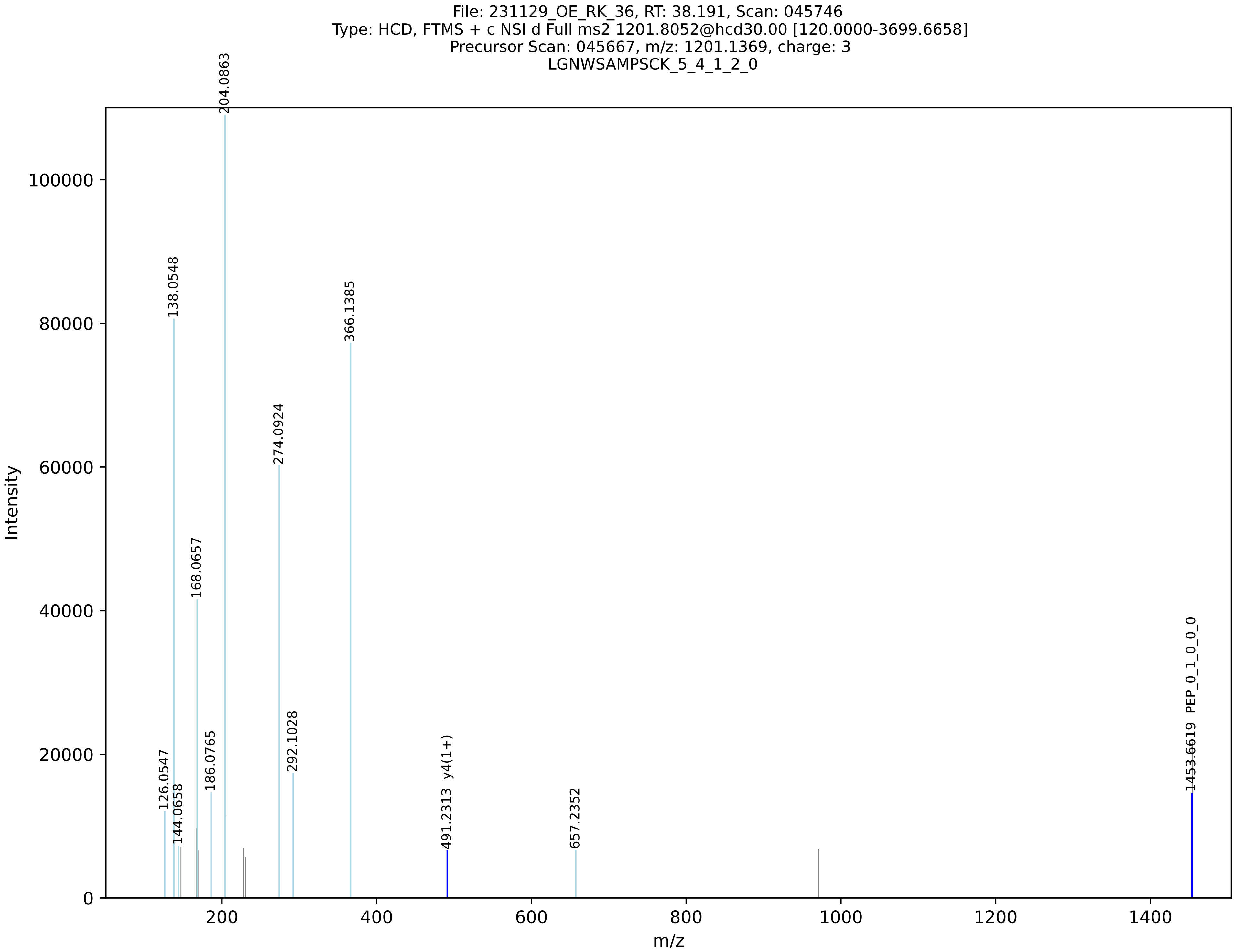
File: 231129_OE_RK_36, RT: 38.191, Scan: 045746
Type: HCD, FTMS + c NSI d Full ms2 1201.8052@hcd30.00 [120.0000-3699.6658]
Precursor Scan: 045667, m/z: 1201.1369, charge: 3
LGNWSAMPSCK_5_4_1_2_0
Intensity
m/z
204.0863
138.0548
366.1385
274.0924
168.0657
292.1028
186.0765
126.0547
144.0658
491.2313 y4(1+)
657.2352
1453.6619 PEP_0_1_0_0_0
0
20000
40000
60000
80000
100000
200
400
600
800
1000
1200
1400

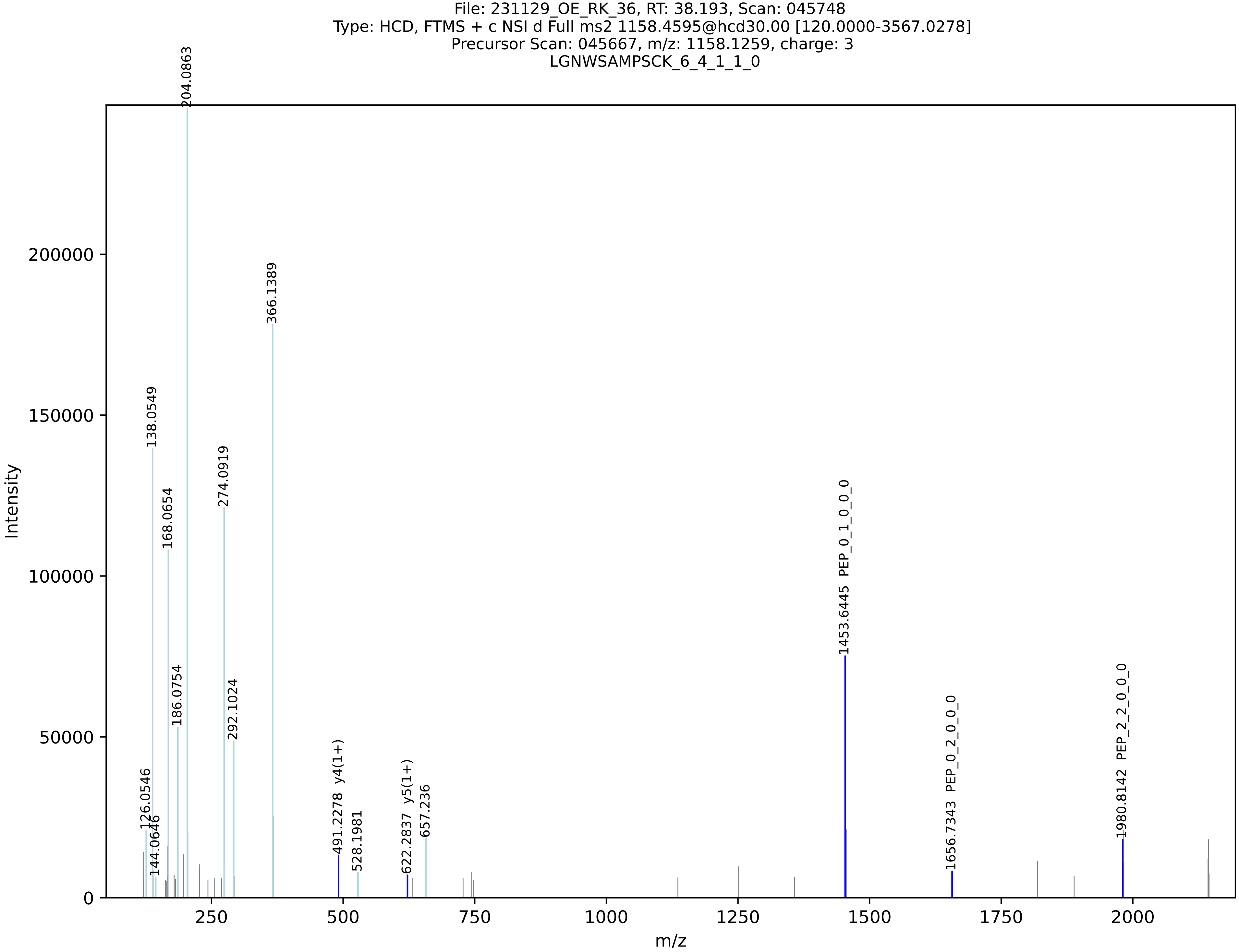
File: 231129_OE_RK_36, RT: 38.193, Scan: 045748
Type: HCD, FTMS + c NSI d Full ms2 1158.4595@hcd30.00 [120.0000-3567.0278]
Precursor Scan: 045667, m/z: 1158.1259, charge: 3
LGNWSAMPSCK_6_4_1_1_0
Intensity
m/z
204.0863
366.1389
138.0549
274.0919
168.0654
186.0754
292.1024
126.0546
144.0646
491.2278 y4(1+)
528.1981
622.2837 y5(1+)
657.236
1453.6445 PEP_0_1_0_0_0
1656.7343 PEP_0_2_0_0_0
1980.8142 PEP_2_2_0_0_0
0
50000
100000
150000
200000
250
500
750
1000
1250
1500
1750
2000

File: 231129_OE_RK_36, RT: 38.335, Scan: 045954
Type: HCD, FTMS + c NSI d Full ms2 1152.7847@hcd30.00 [120.0000-3549.6631]
Precursor Scan: 045879, m/z: 1152.4506, charge: 3
LGNWSAMPSCK_5_4_0_2_0

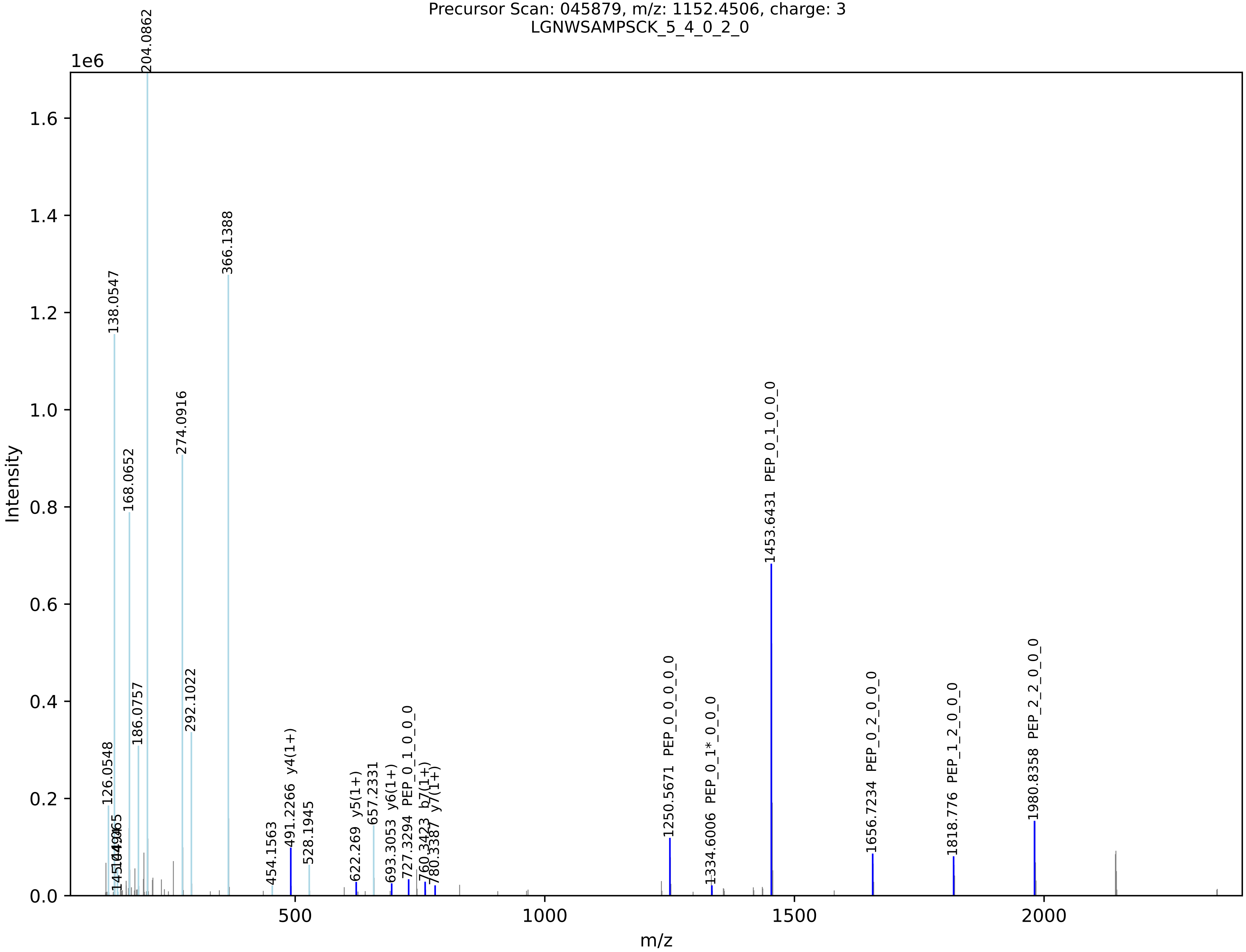

File: 231129_OE_RK_36, RT: 38.387, Scan: 046027
Type: HCD, FTMS + c NSI d Full ms2 1410.2650@hcd30.00 [120.0000-4337.5527]
Precursor Scan: 045951, m/z: 1409.5968, charge: 3
ELHHLQEQNVSNAFLDK_5_4_0_2_0

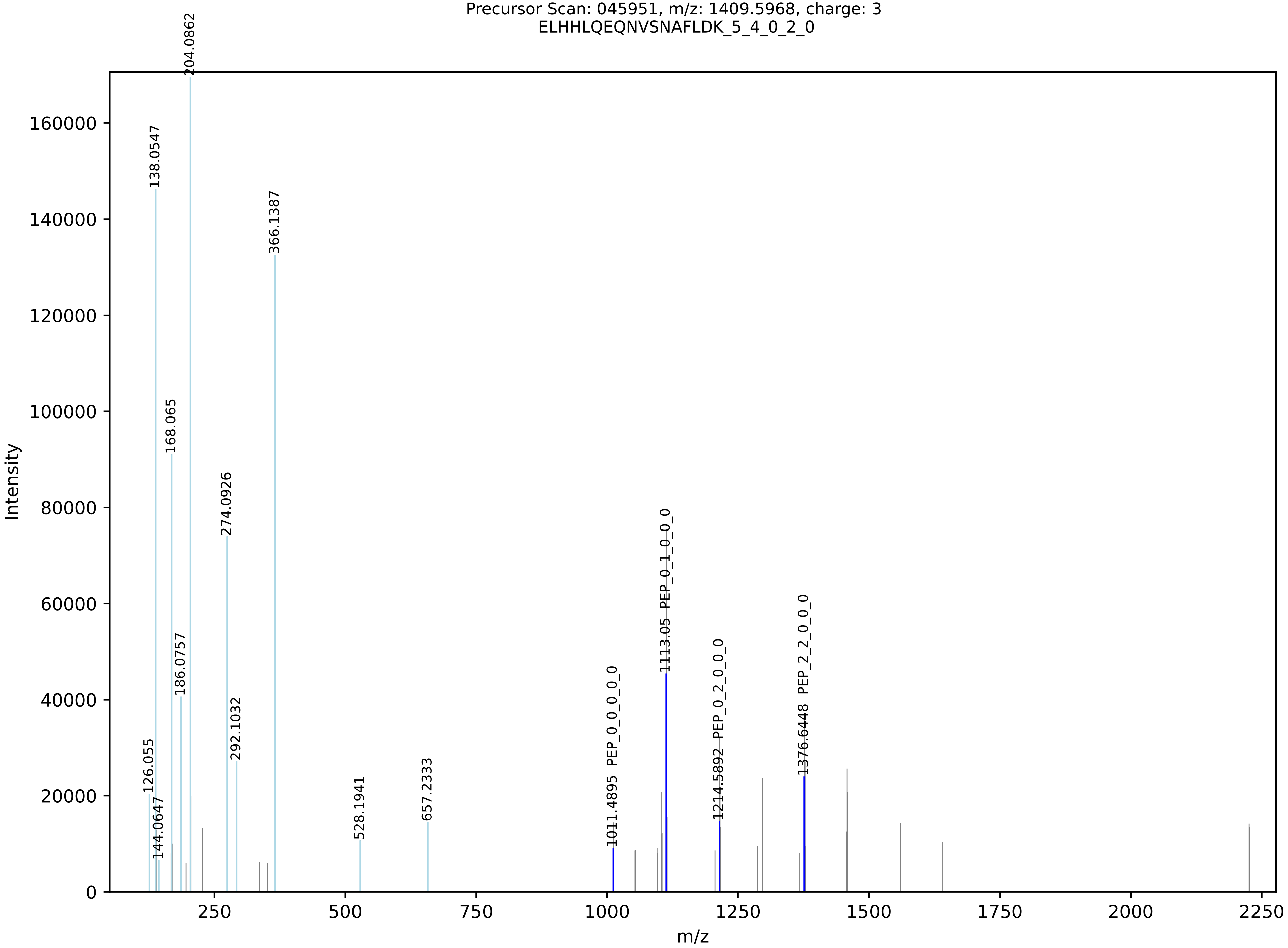

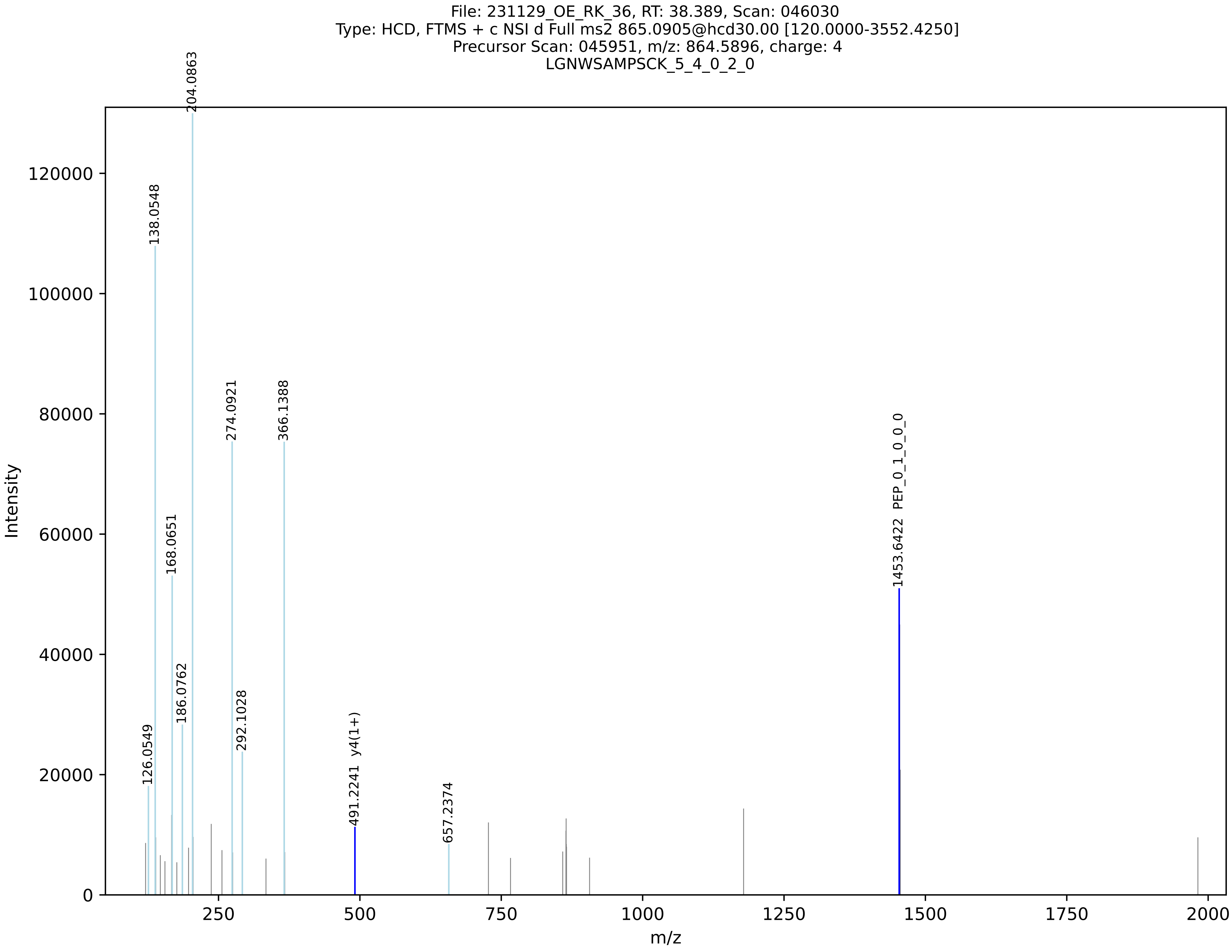

File: 231129_OE_RK_36, RT: 38.389, Scan: 046030
Type: HCD, FTMS + c NSI d Full ms2 865.0905@hcd30.00 [120.0000-3552.4250]
Precursor Scan: 045951, m/z: 864.5896, charge: 4
LGNWSAMPSCK_5_4_0_2_0
204.0863
138.0548
168.0651
186.0762
126.0549
274.0921
292.1028
366.1388
491.2241 y4(1+)
657.2374
1453.6422 PEP_0_1_0_0_0
Intensity
m/z

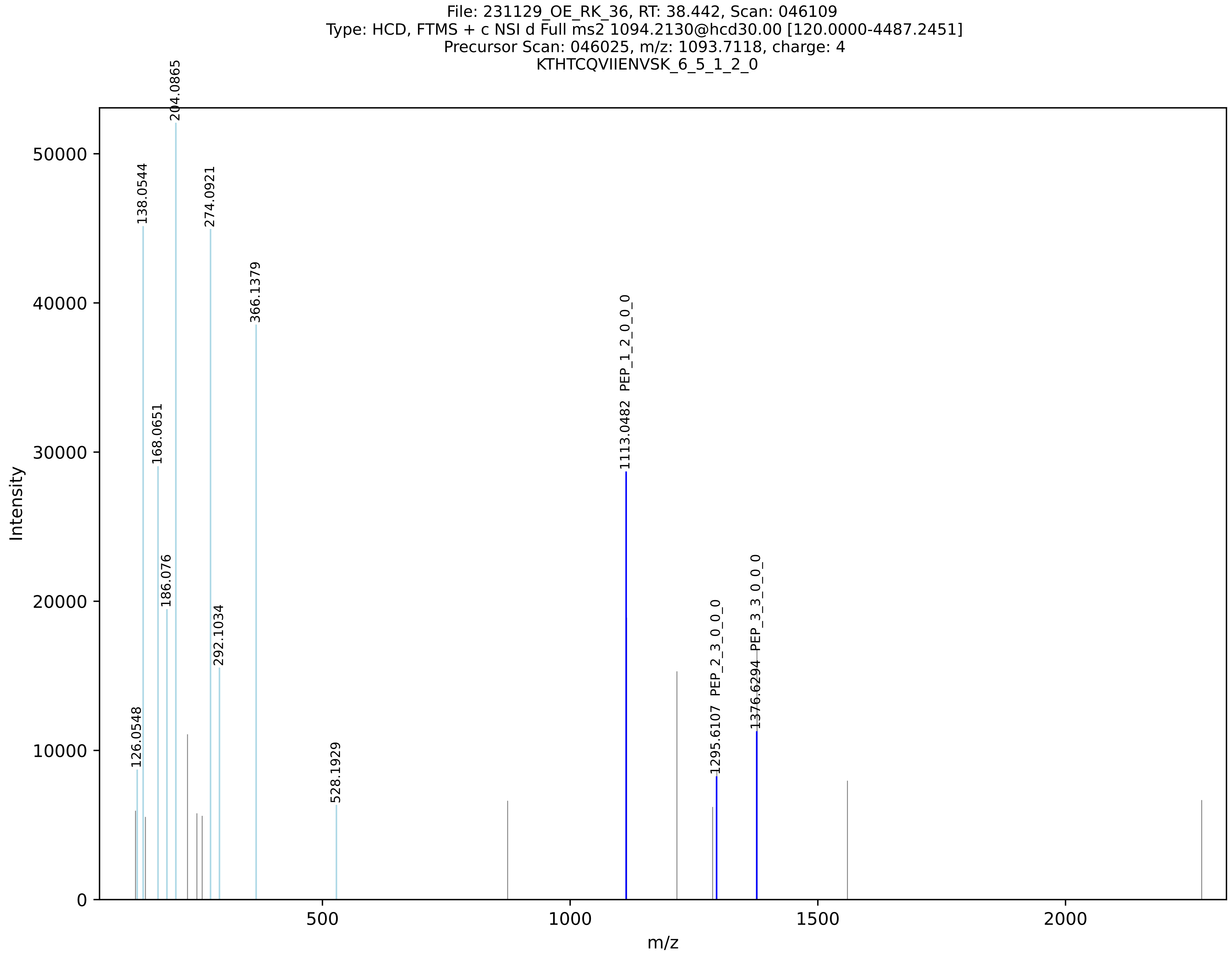
File: 231129_OE_RK_36, RT: 38.442, Scan: 046109
Type: HCD, FTMS + c NSI d Full ms2 1094.2130@hcd30.00 [120.0000-4487.2451]
Precursor Scan: 046025, m/z: 1093.7118, charge: 4
KTHTCQVIIENVSK_6_5_1_2_0
Intensity
m/z
0
10000
20000
30000
40000
50000
500
1000
1500
2000
204.0865
138.0544
274.0921
366.1379
168.0651
186.076
292.1034
126.0548
528.1929
1113.0482 PEP_1_2_0_0_0
1295.6107 PEP_2_3_0_0_0
1376.6294 PEP_3_3_0_0_0

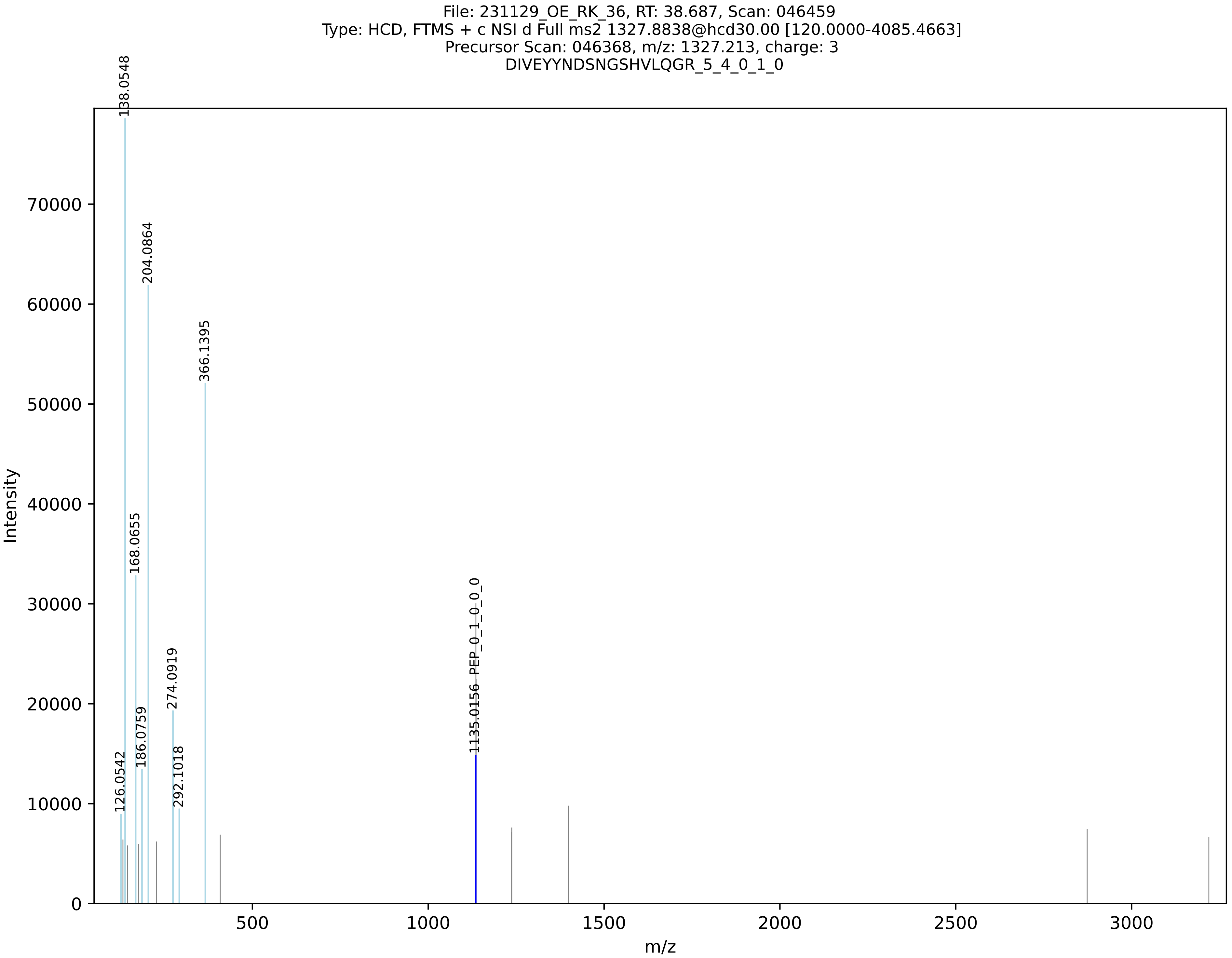
File: 231129_OE_RK_36, RT: 38.687, Scan: 046459
Type: HCD, FTMS + c NSI d Full ms2 1327.8838@hcd30.00 [120.0000-4085.4663]
Precursor Scan: 046368, m/z: 1327.213, charge: 3
DIVEYYNDSNGSHVLQGR_5_4_0_1_0
138.0548
204.0864
366.1395
168.0655
274.0919
186.0759
126.0542
292.1018
1135.0156_PEP_0_1_0_0_0_
Intensity
m/z
0
10000
20000
30000
40000
50000
60000
70000
500
1000
1500
2000
2500
3000

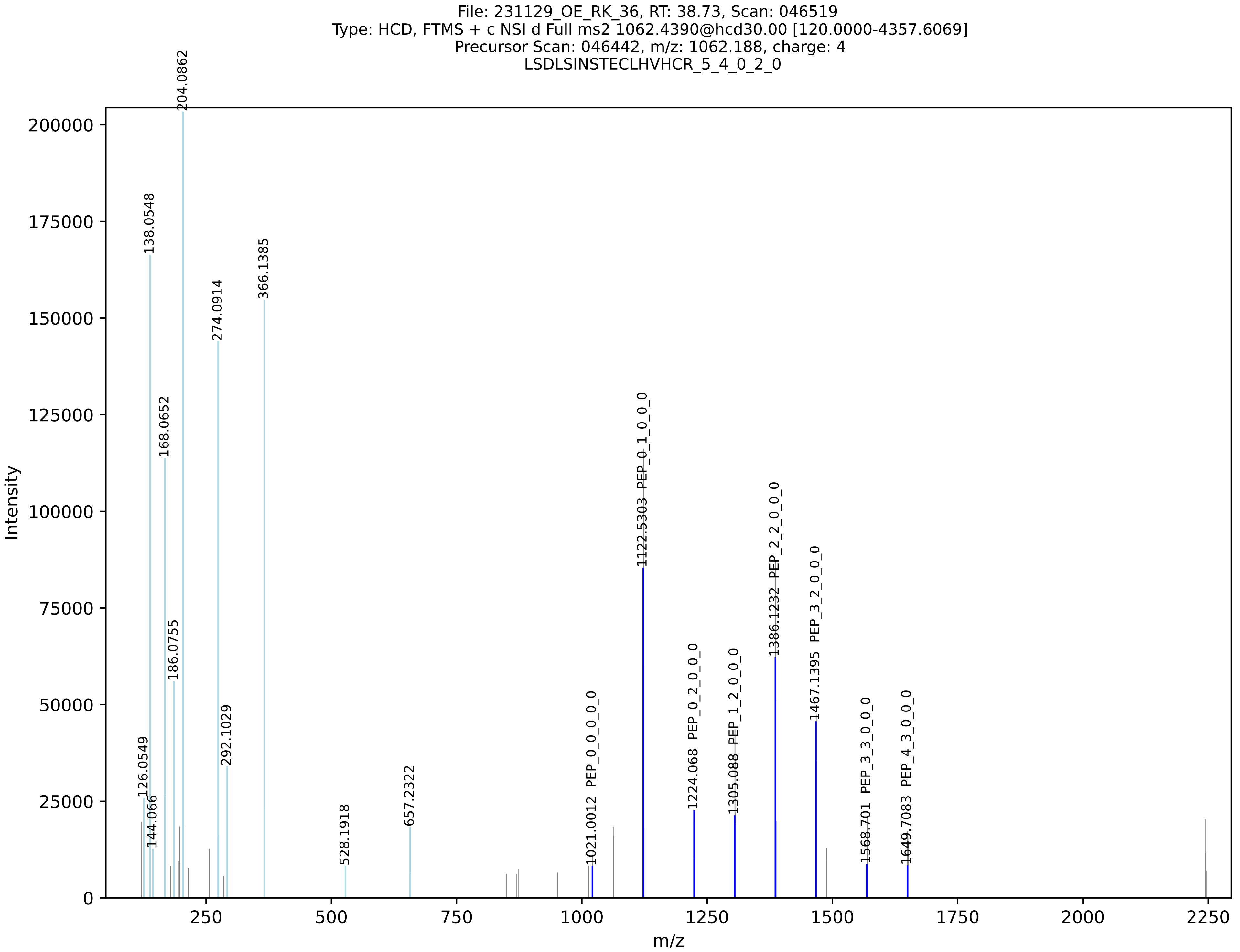
File: 231129_OE_RK_36, RT: 38.73, Scan: 046519
Type: HCD, FTMS + c NSI d Full ms2 1062.4390@hcd30.00 [120.0000-4357.6069]
Precursor Scan: 046442, m/z: 1062.188, charge: 4
LSDLSINSTECLHVHCR_5_4_0_2_0
Intensity
m/z
204.0862
138.0548
366.1385
274.0914
168.0652
186.0755
292.1029
126.0549
144.066
528.1918
657.2322
1021.0012 PEP_0_0_0_0_0_
1122.5303 PEP_0_1_0_0_0_
1224.068 PEP_0_2_0_0_0_
1305.088 PEP_1_2_0_0_0_
1386.1232 PEP_2_2_0_0_0_
1467.1395 PEP_3_2_0_0_0_
1568.701 PEP_3_3_0_0_0_
1649.7083 PEP_4_3_0_0_0_

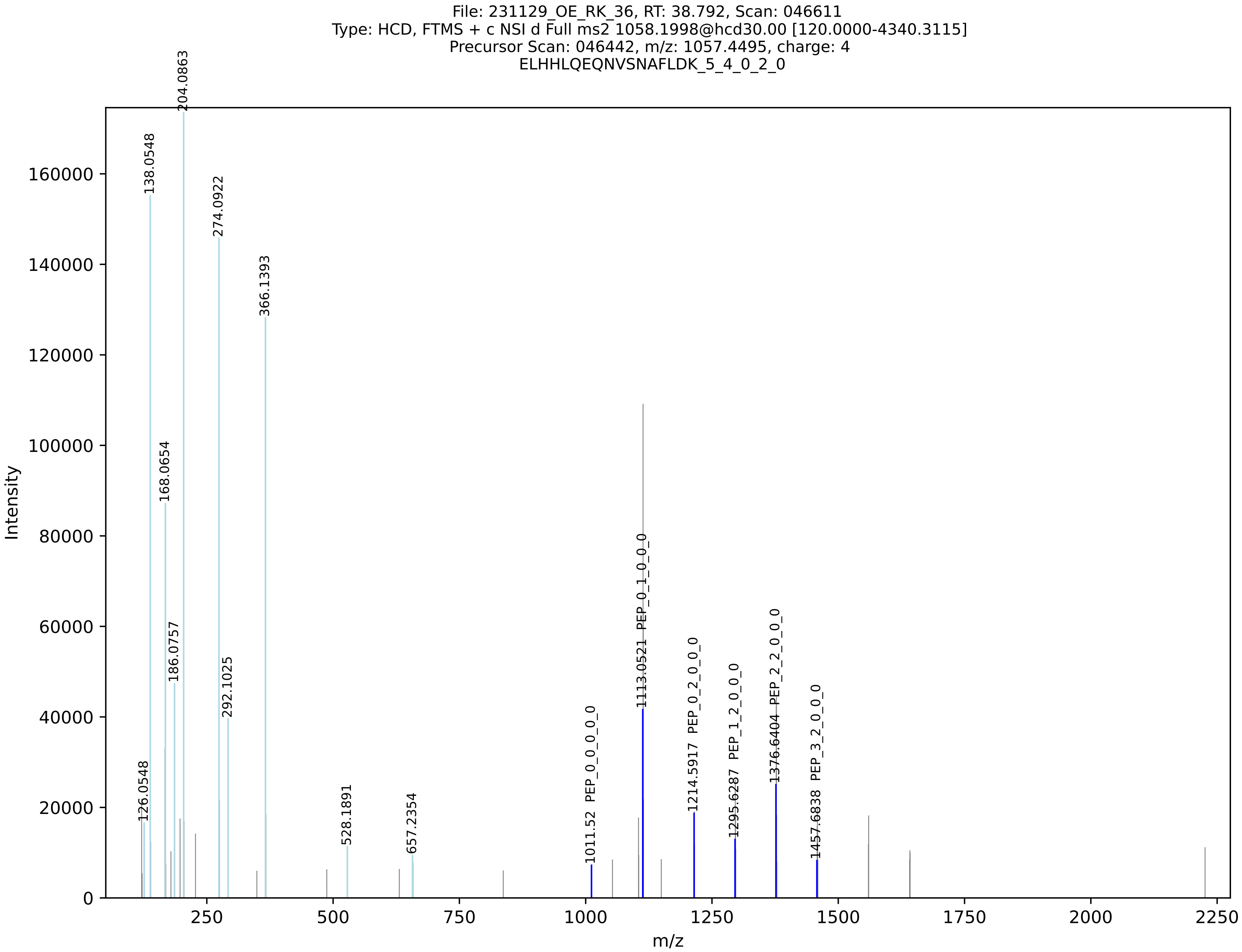

File: 231129_OE_RK_36, RT: 38.792, Scan: 046611
Type: HCD, FTMS + c NSI d Full ms2 1058.1998@hcd30.00 [120.0000-4340.3115]
Precursor Scan: 046442, m/z: 1057.4495, charge: 4
ELHHLQEQNVSNAFLDK_5_4_0_2_0
204.0863
138.0548
274.0922
366.1393
168.0654
186.0757
292.1025
126.0548
528.1891
657.2354
1011.52 PEP_0_0_0_0_0
1113.0521 PEP_0_1_0_0_0
1214.5917 PEP_0_2_0_0_0
1295.6287 PEP_1_2_0_0_0
1376.6404 PEP_2_2_0_0_0
1457.6838 PEP_3_2_0_0_0
Intensity
m/z

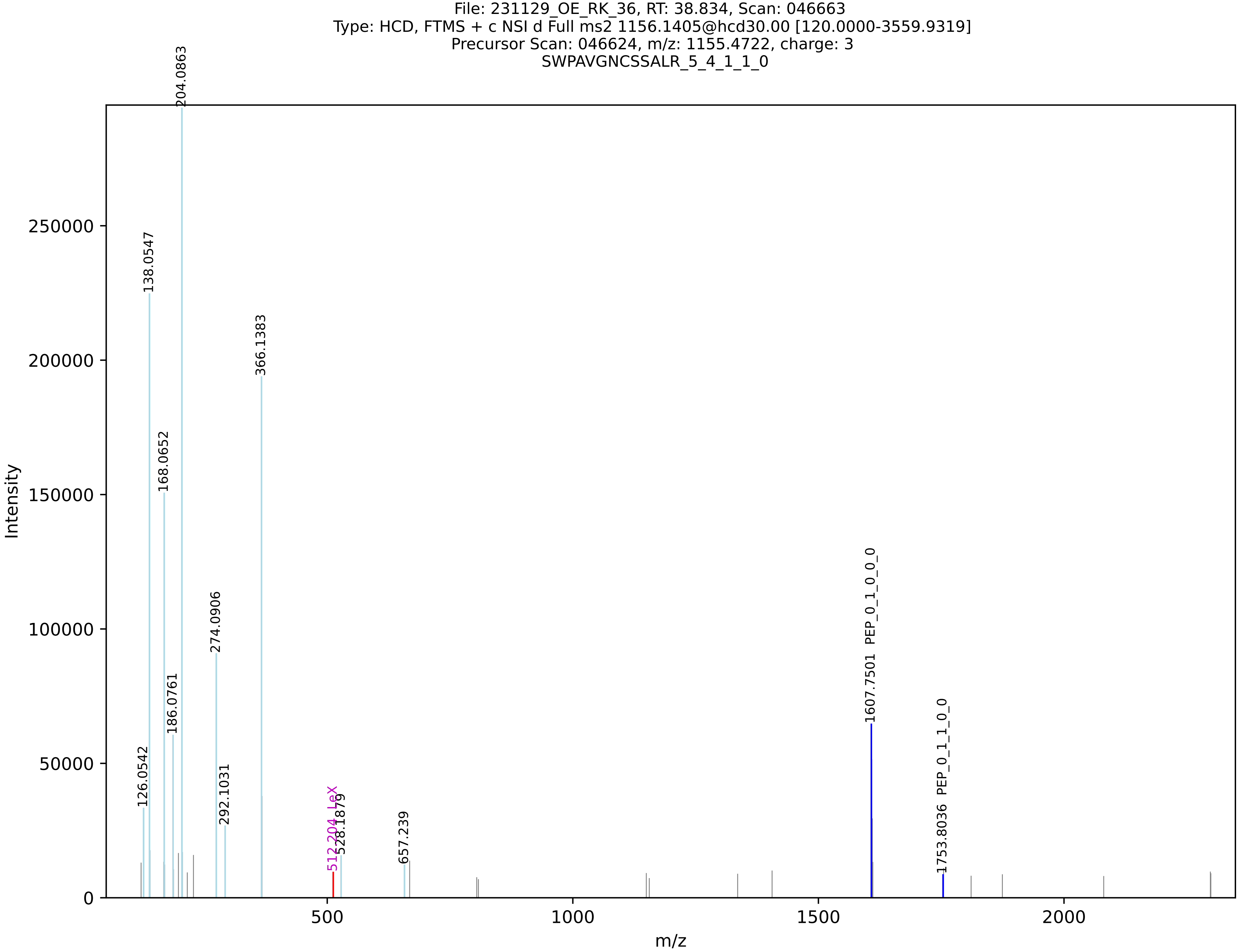

File: 231129_OE_RK_36, RT: 38.834, Scan: 046663
Type: HCD, FTMS + c NSI d Full ms2 1156.1405@hcd30.00 [120.0000-3559.9319]
Precursor Scan: 046624, m/z: 1155.4722, charge: 3
SWPAVGNCSSALR_5_4_1_1_0
Intensity
m/z
204.0863
138.0547
366.1383
168.0652
274.0906
186.0761
126.0542
292.1031
512.204_LeX
528.1879
657.239
1607.7501 PEP_0_1_0_0_0
1753.8036 PEP_0_1_1_0_0

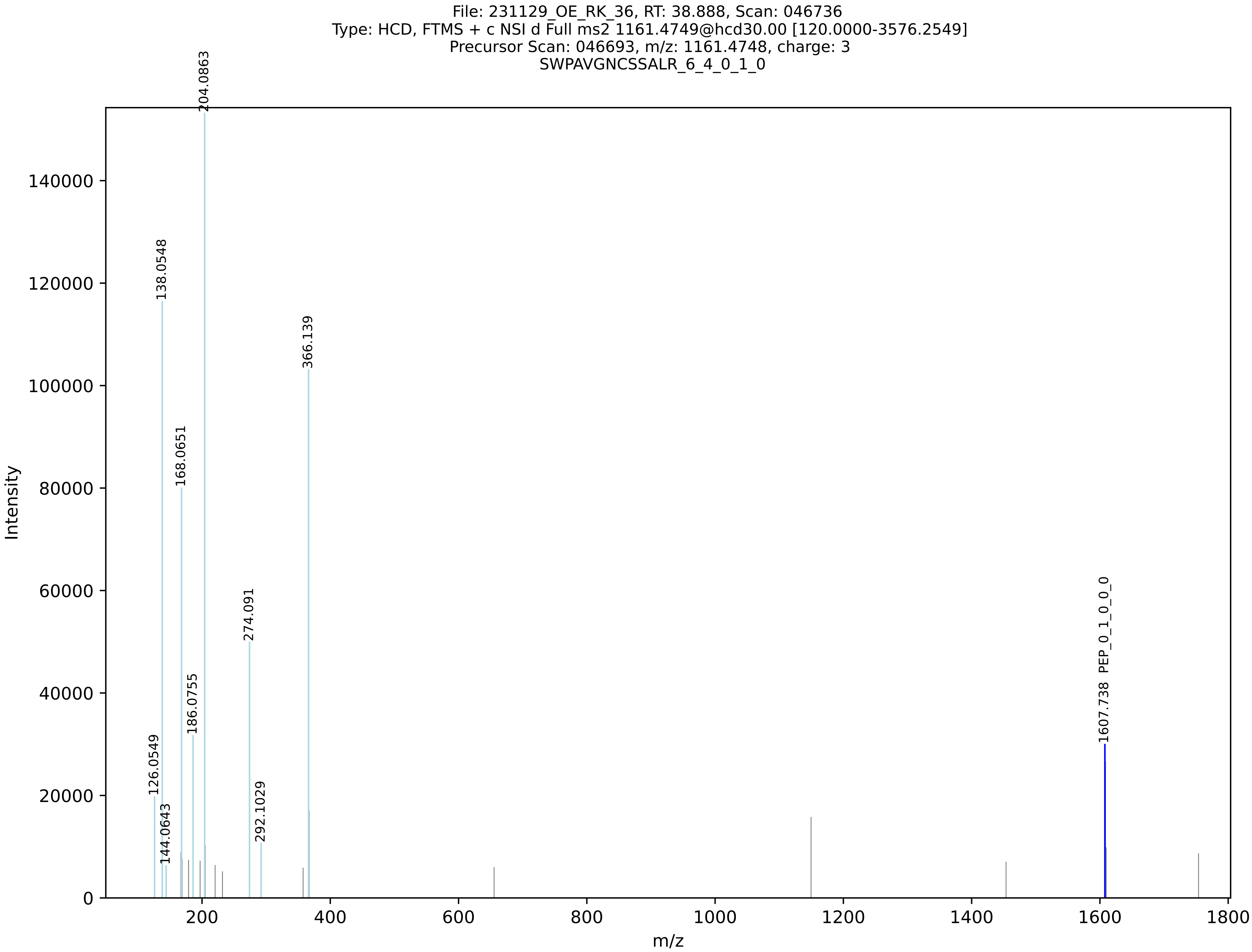
File: 231129_OE_RK_36, RT: 38.888, Scan: 046736
Type: HCD, FTMS + c NSI d Full ms2 1161.4749@hcd30.00 [120.0000-3576.2549]
Precursor Scan: 046693, m/z: 1161.4748, charge: 3
SWPAVGNCSSALR_6_4_0_1_0
204.0863
138.0548
366.139
168.0651
274.091
186.0755
126.0549
144.0643
292.1029
1607.738 PEP_0_1_0_0_0
Intensity
m/z
0
20000
40000
60000
80000
100000
120000
140000
200
400
600
800
1000
1200
1400
1600
1800

File: 231129_OE_RK_36, RT: 38.934, Scan: 046795
Type: HCD, FTMS + c NSI d Full ms2 1062.6882@hcd30.00 [120.0000-4358.6240]
Precursor Scan: 046751, m/z: 1062.1871, charge: 4
LSDLSINSTECLHVHCR_5_4_0_2_0

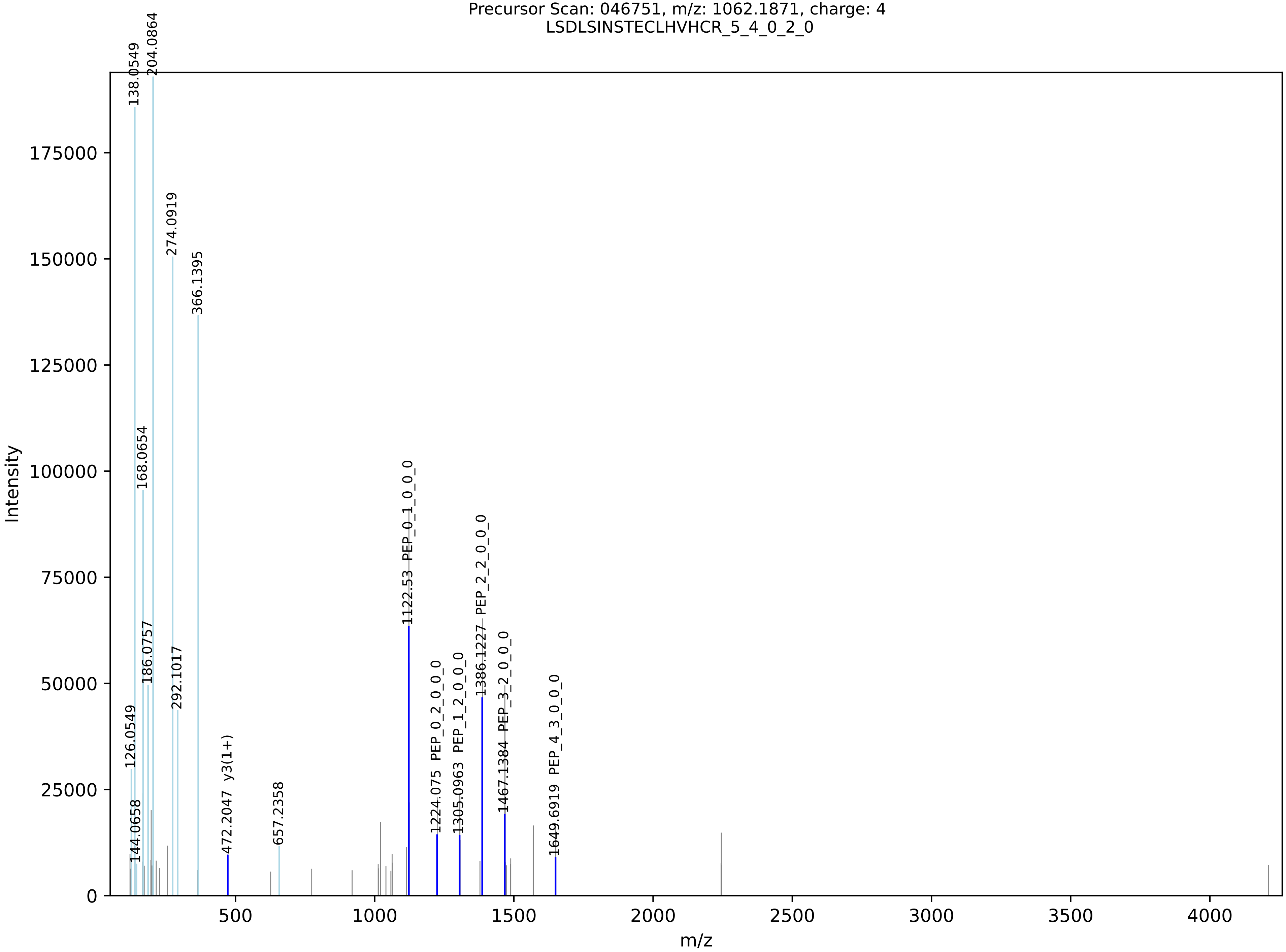

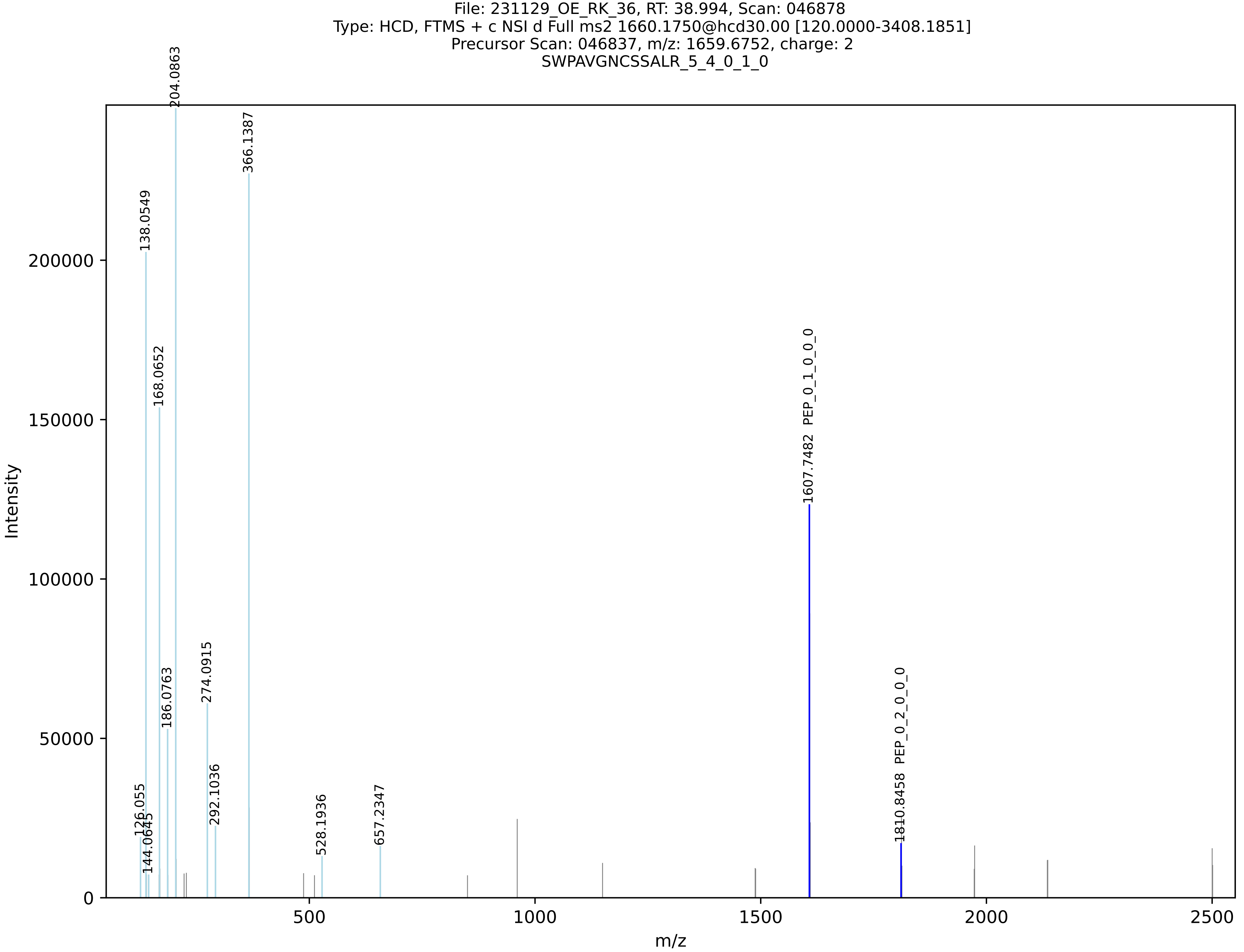
File: 231129_OE_RK_36, RT: 38.994, Scan: 046878
Type: HCD, FTMS + c NSI d Full ms2 1660.1750@hcd30.00 [120.0000-3408.1851]
Precursor Scan: 046837, m/z: 1659.6752, charge: 2
SWPAVGNCSSALR_5_4_0_1_0
Intensity
m/z
0
50000
100000
150000
200000
500
1000
1500
2000
2500
204.0863
366.1387
138.0549
168.0652
186.0763
274.0915
292.1036
126.055
144.0645
528.1936
657.2347
1607.7482 PEP_0_1_0_0_0
1810.8458 PEP_0_2_0_0_0

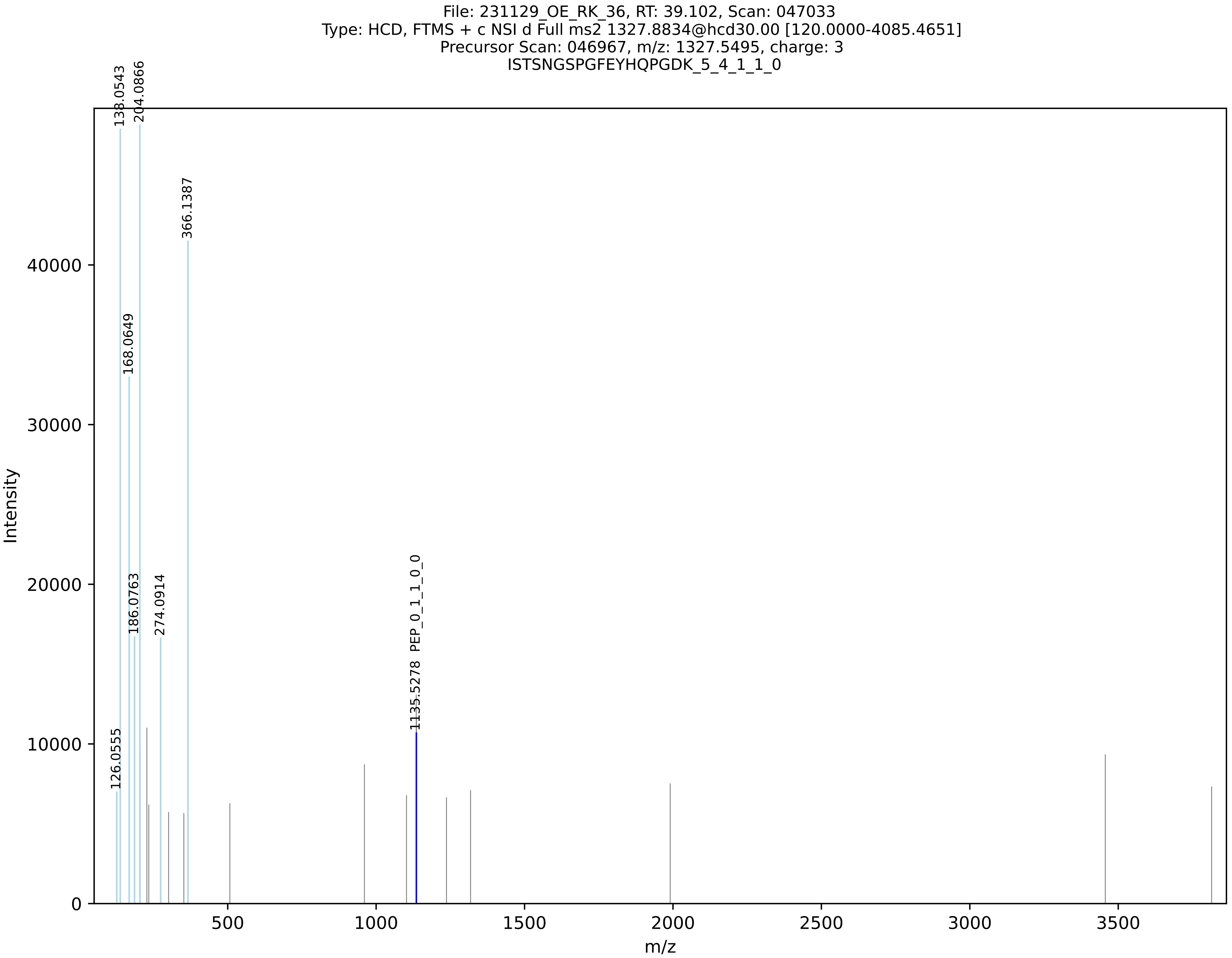

File: 231129_OE_RK_36, RT: 39.102, Scan: 047033
Type: HCD, FTMS + c NSI d Full ms2 1327.8834@hcd30.00 [120.0000-4085.4651]
Precursor Scan: 046967, m/z: 1327.5495, charge: 3
ISTSNGSPGFEYHQPGDK_5_4_1_1_0
138.0543
204.0866
366.1387
168.0649
186.0763
274.0914
126.0555
1135.5278 PEP_0_1_1_0_0
Intensity
m/z
0
10000
20000
30000
40000
500
1000
1500
2000
2500
3000
3500

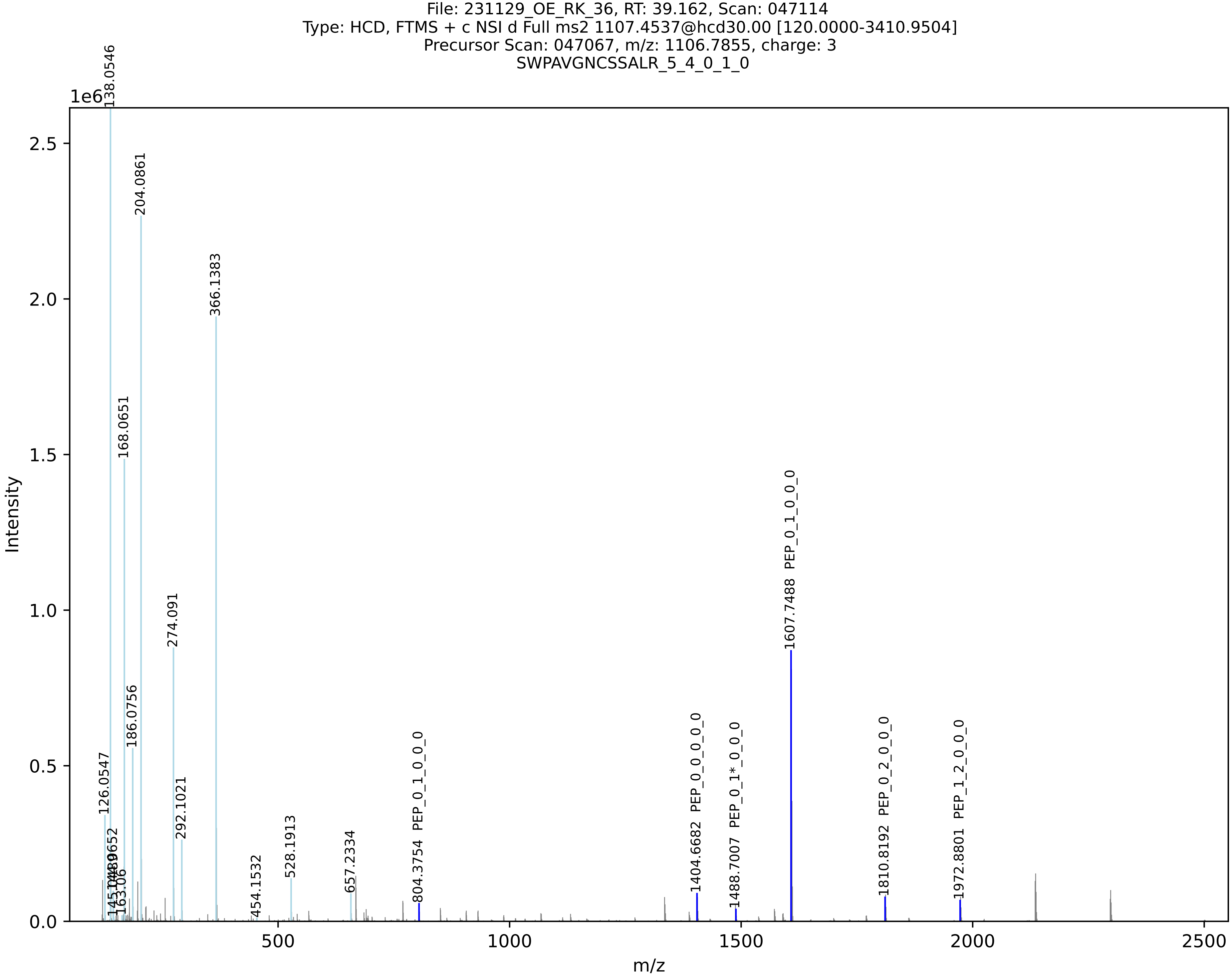
File: 231129_OE_RK_36, RT: 39.162, Scan: 047114
Type: HCD, FTMS + c NSI d Full ms2 1107.4537@hcd30.00 [120.0000-3410.9504]
Precursor Scan: 047067, m/z: 1106.7855, charge: 3
SWPAVGNCSSALR_5_4_0_1_0
1e6
Intensity
m/z
138.0546
204.0861
366.1383
168.0651
274.091
186.0756
126.0547
292.1021
163.06
454.1532
528.1913
657.2334
804.3754 PEP_0_1_0_0_0
1404.6682 PEP_0_0_0_0_0
1488.7007 PEP_0_1*_0_0_0
1607.7488 PEP_0_1_0_0_0
1810.8192 PEP_0_2_0_0_0
1972.8801 PEP_1_2_0_0_0

File: 231129_OE_RK_36, RT: 39.165, Scan: 047119
Type: HCD, FTMS + c NSI d Full ms2 1067.7150@hcd30.00 [120.0000-5468.9165]
Precursor Scan: 047067, m/z: 1067.5155, charge: 5
VSEGNHDIALIKLQAPLNYTEFQKPICLPSK_4_5_1_0_0

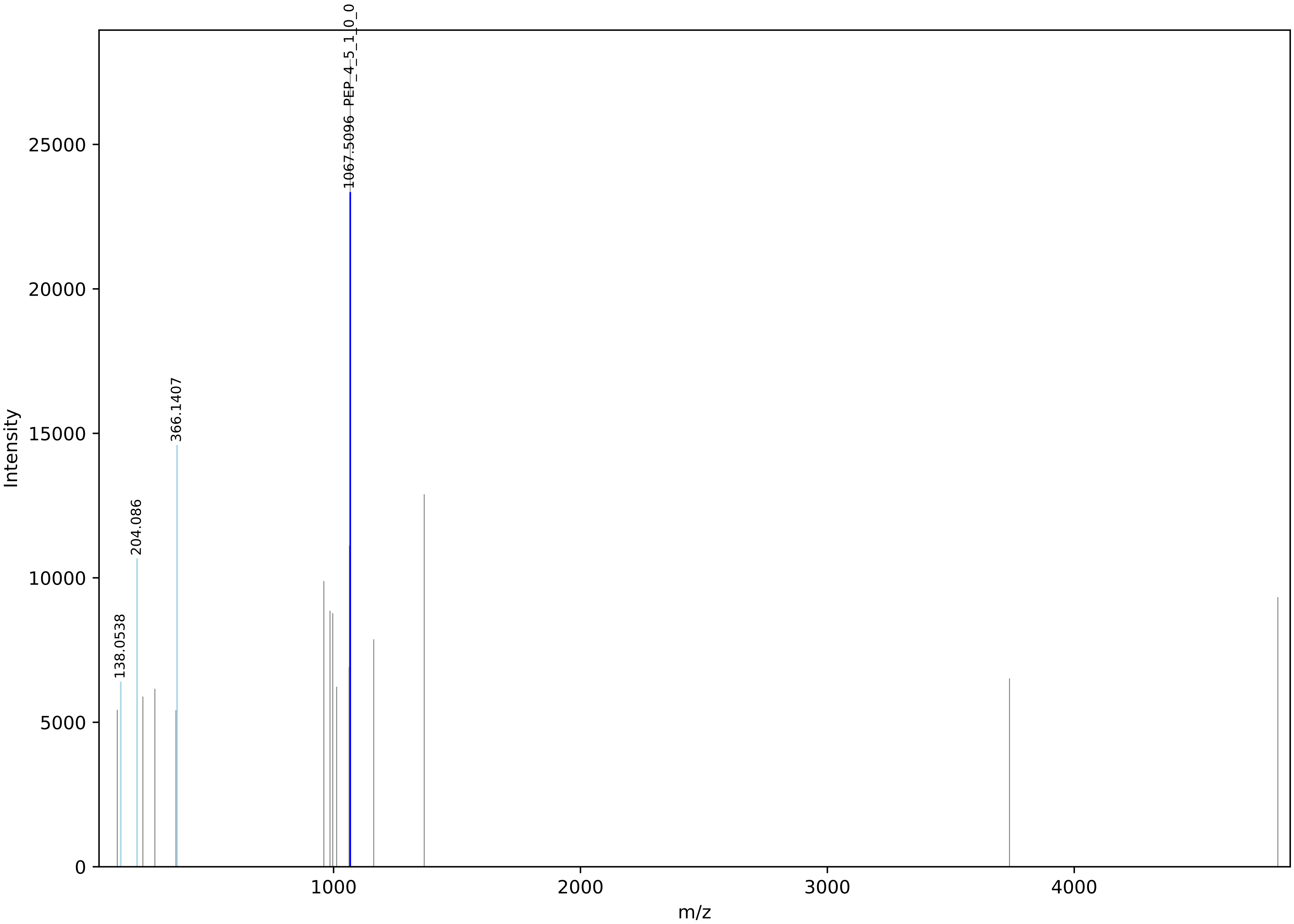

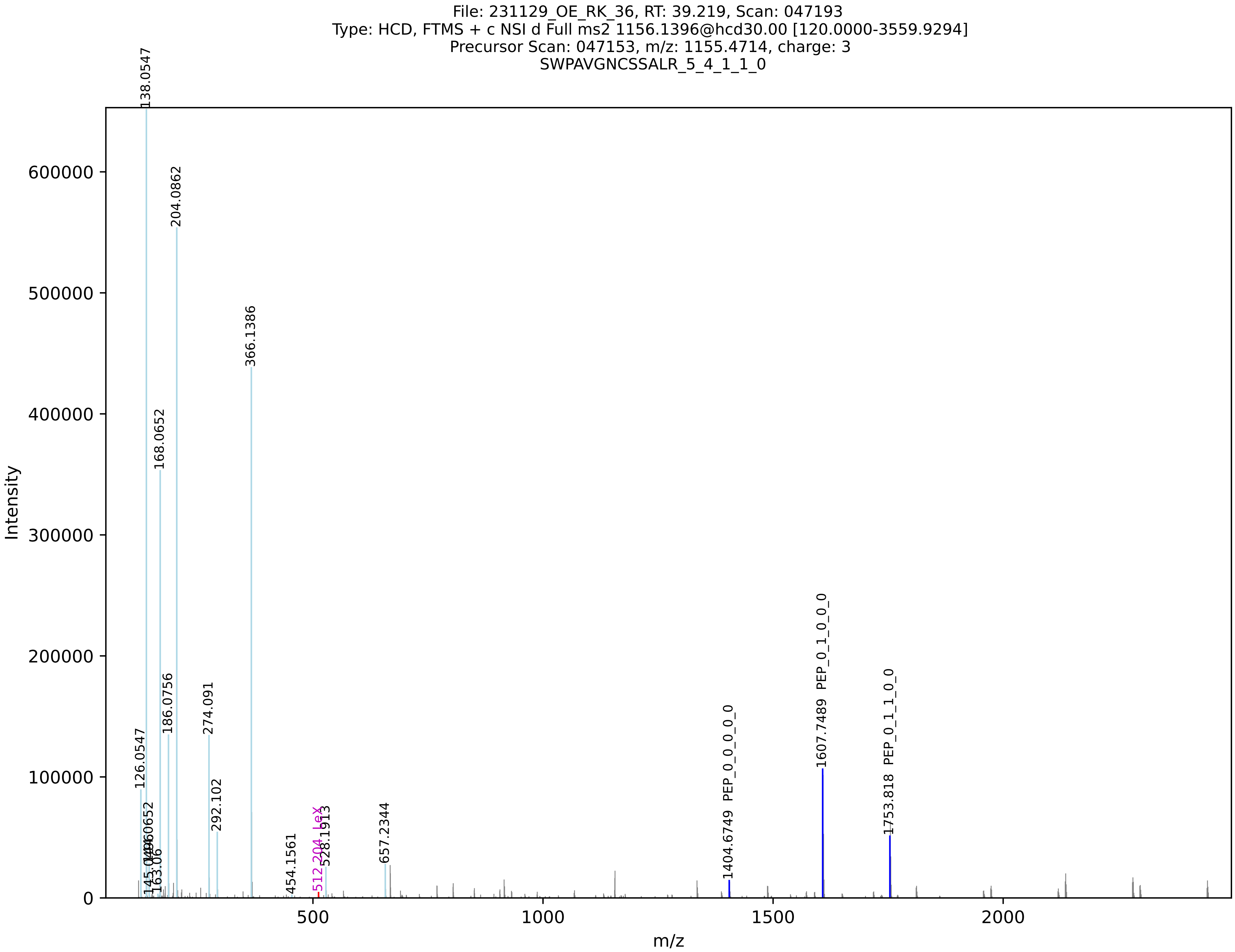
File: 231129_OE_RK_36, RT: 39.219, Scan: 047193
Type: HCD, FTMS + c NSI d Full ms2 1156.1396@hcd30.00 [120.0000-3559.9294]
Precursor Scan: 047153, m/z: 1155.4714, charge: 3
SWPAVGNCSSALR_5_4_1_1_0
138.0547
204.0862
366.1386
168.0652
186.0756
274.091
126.0547
292.102
145.0496
163.06
454.1561
512.204_LeX
528.1913
657.2344
1404.6749 PEP_0_0_0_0_0
1607.7489 PEP_0_1_0_0_0
1753.818 PEP_0_1_1_0_0
Intensity
m/z
0
100000
200000
300000
400000
500000
600000
500
1000
1500
2000

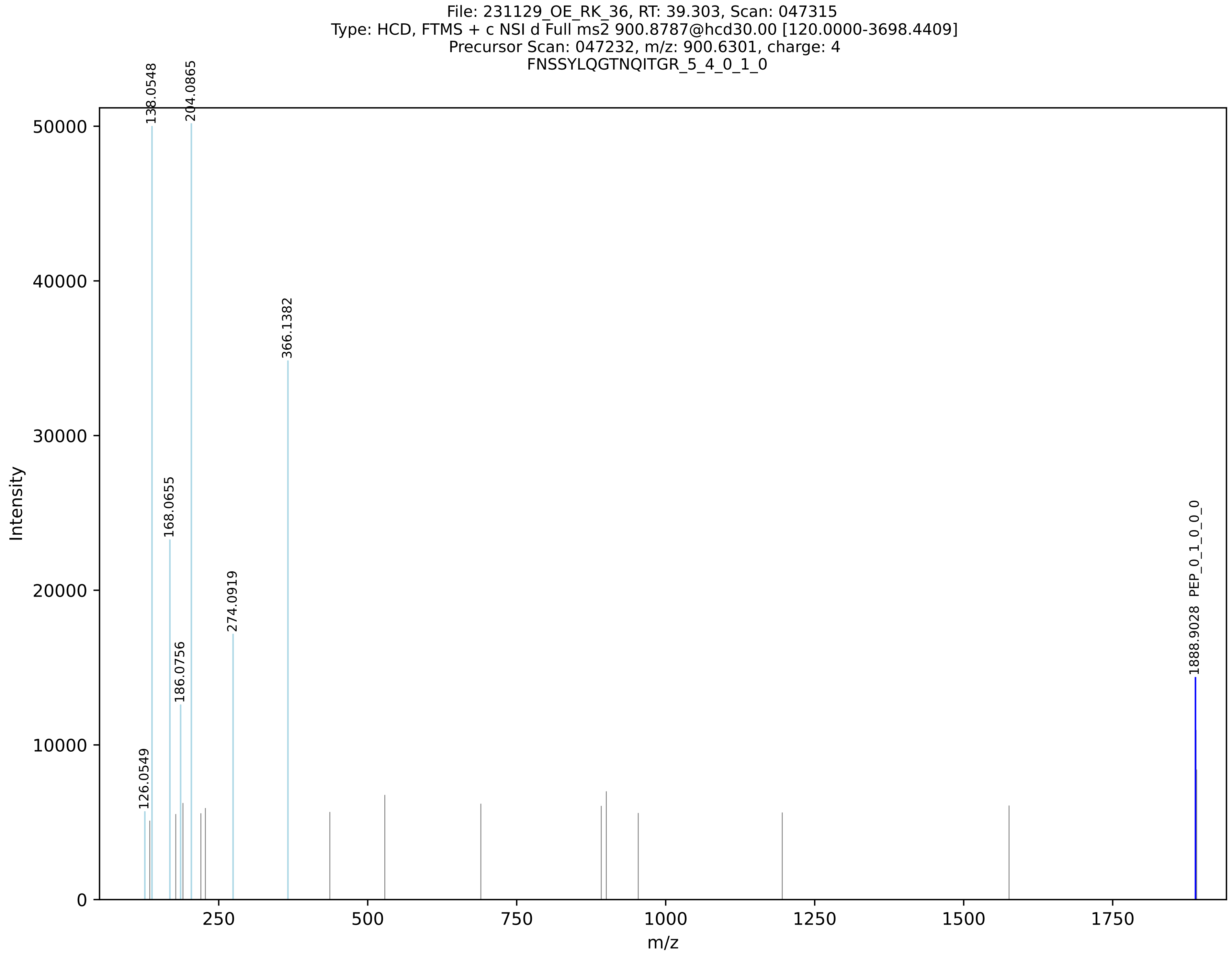

File: 231129_OE_RK_36, RT: 39.303, Scan: 047315
Type: HCD, FTMS + c NSI d Full ms2 900.8787@hcd30.00 [120.0000-3698.4409]
Precursor Scan: 047232, m/z: 900.6301, charge: 4
FNSSYLQGTNQITGR_5_4_0_1_0
138.0548
204.0865
366.1382
168.0655
274.0919
186.0756
126.0549
1888.9028 PEP_0_1_0_0_0
Intensity
m/z
0
10000
20000
30000
40000
50000
250
500
750
1000
1250
1500
1750

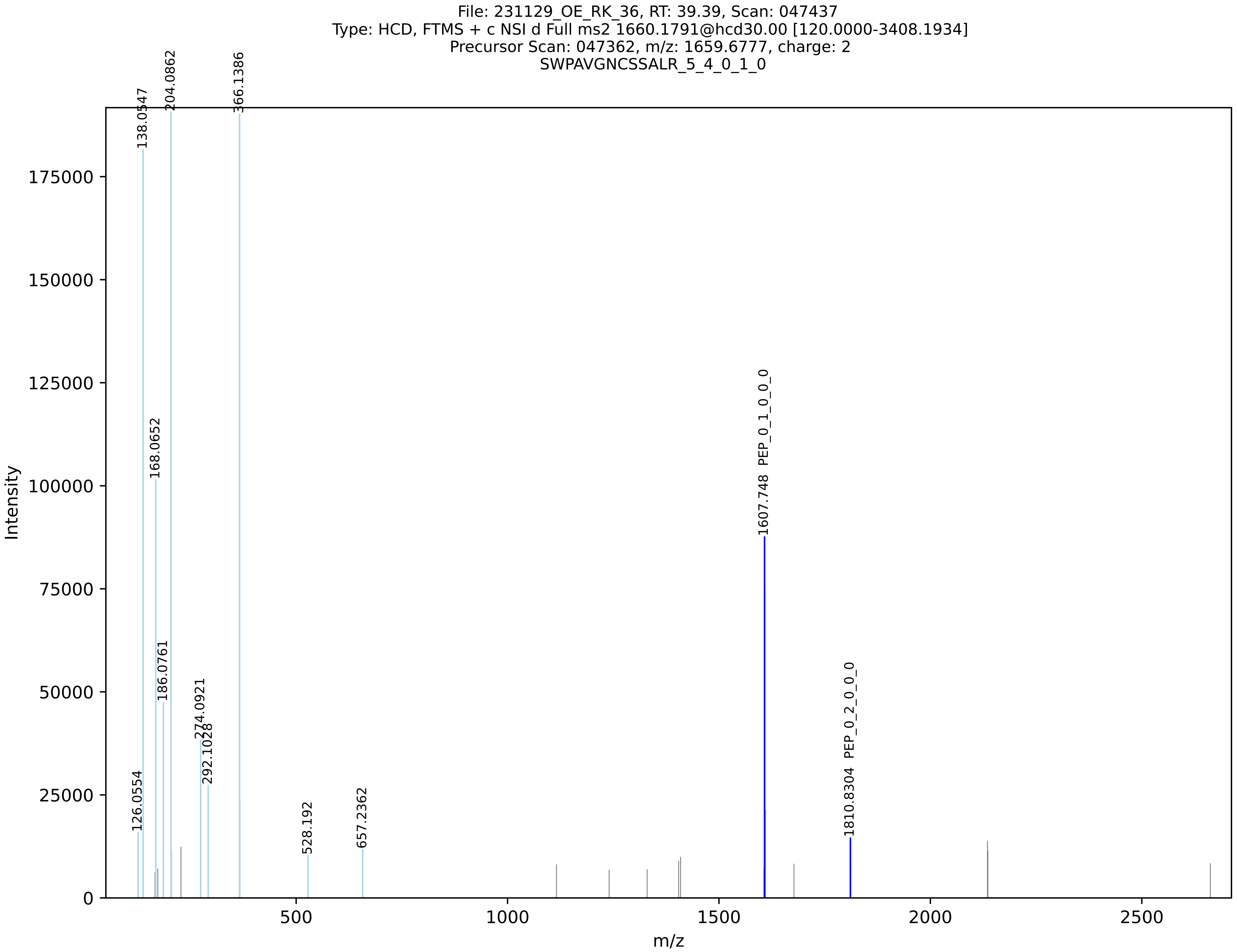
File: 231129_OE_RK_36, RT: 39.39, Scan: 047437
Type: HCD, FTMS + c NSI d Full ms2 1660.1791@hcd30.00 [120.0000-3408.1934]
Precursor Scan: 047362, m/z: 1659.6777, charge: 2
SWPAVGNCSSALR_5_4_0_1_0
Intensity
m/z
138.0547
204.0862
366.1386
168.0652
186.0761
274.0921
292.1028
126.0554
528.192
657.2362
1607.748 PEP_0_1_0_0_0
1810.8304 PEP_0_2_0_0_0
0
25000
50000
75000
100000
125000
150000
175000
500
1000
1500
2000
2500

File: 231129_OE_RK_36, RT: 39.454, Scan: 047532
Type: HCD, FTMS + c NSI d Full ms2 993.7667@hcd30.00 [120.0000-3063.0681]
Precursor Scan: 047435, m/z: 993.4321, charge: 3
VKSLMASLTNMSR_7_2_0_0_0

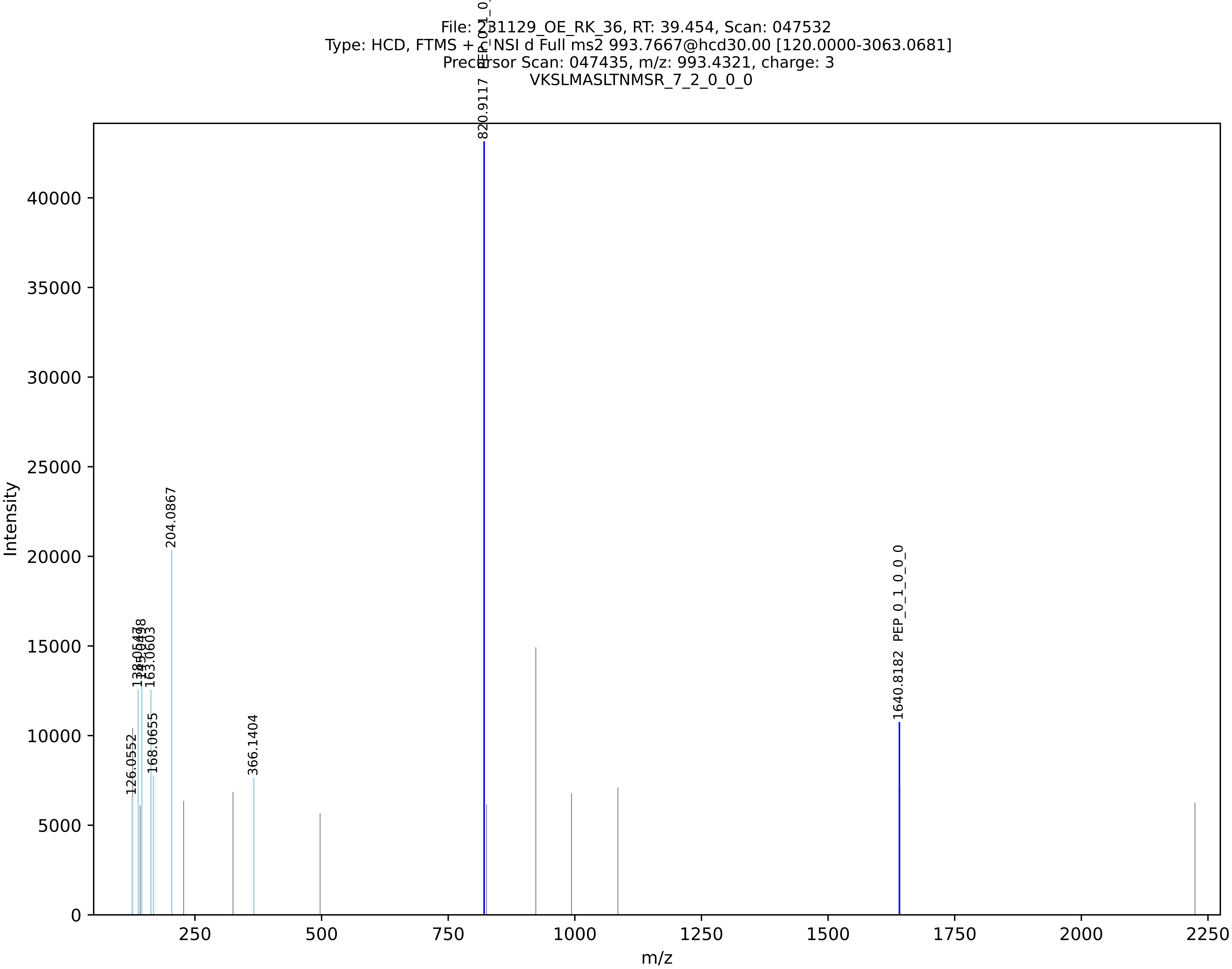

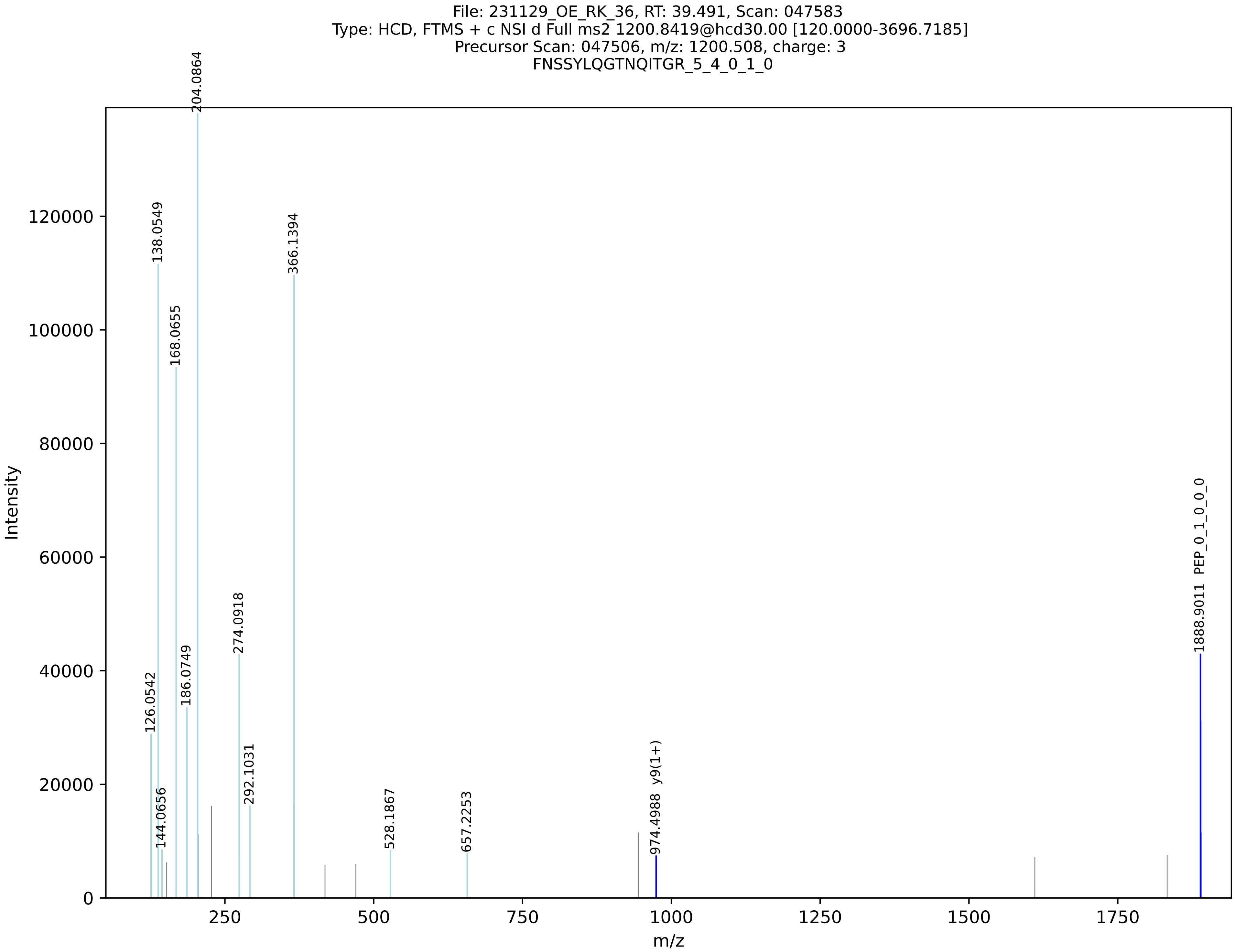

File: 231129_OE_RK_36, RT: 39.491, Scan: 047583
Type: HCD, FTMS + c NSI d Full ms2 1200.8419@hcd30.00 [120.0000-3696.7185]
Precursor Scan: 047506, m/z: 1200.508, charge: 3
FNSSYLQGTNQITGR_5_4_0_1_0
204.0864
138.0549
366.1394
168.0655
274.0918
186.0749
126.0542
292.1031
144.0656
528.1867
657.2253
974.4988 y9(1+)
1888.9011 PEP_0_1_0_0_0
Intensity
m/z
0
20000
40000
60000
80000
100000
120000
250
500
750
1000
1250
1500
1750

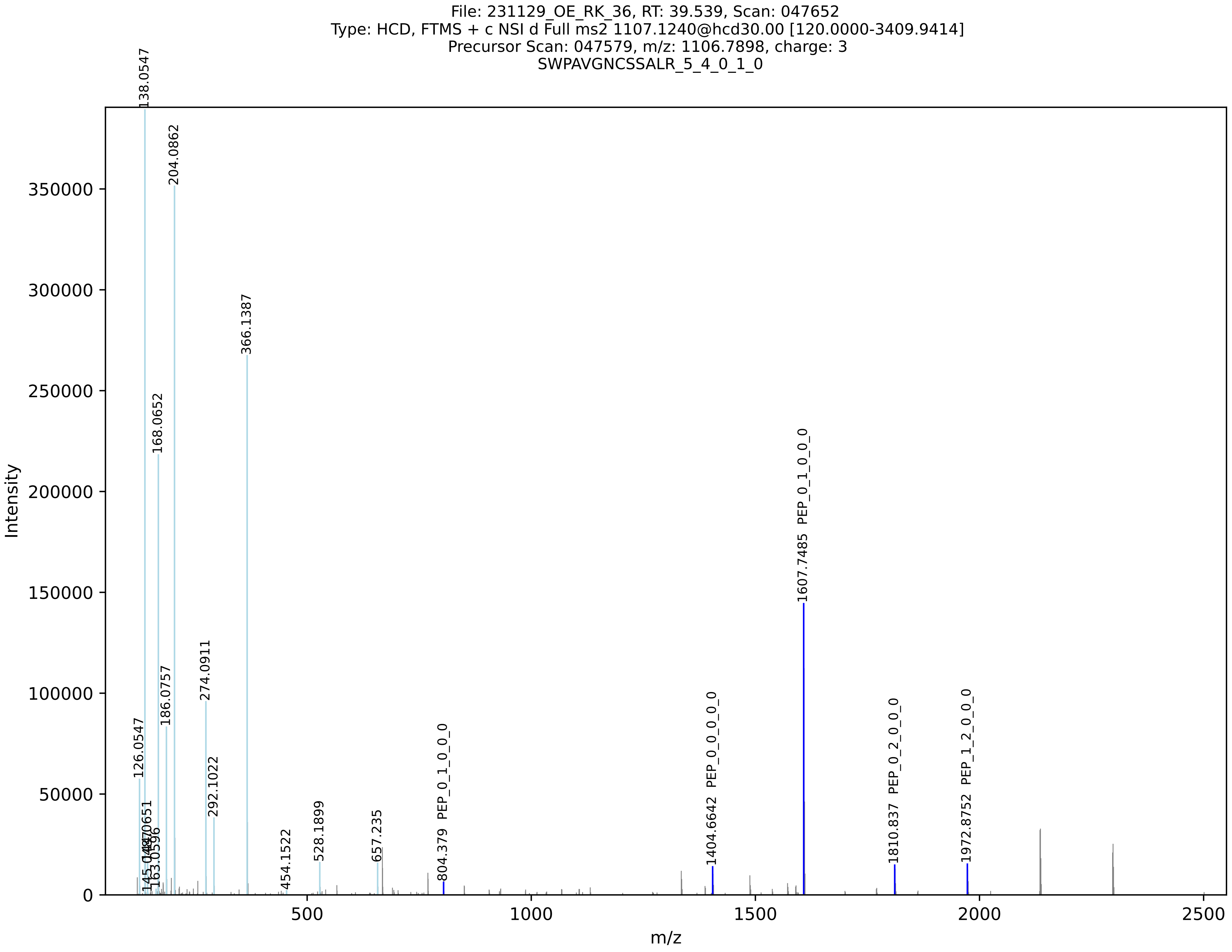
File: 231129_OE_RK_36, RT: 39.539, Scan: 047652
Type: HCD, FTMS + c NSI d Full ms2 1107.1240@hcd30.00 [120.0000-3409.9414]
Precursor Scan: 047579, m/z: 1106.7898, charge: 3
SWPAVGNCSSALR_5_4_0_1_0
138.0547
204.0862
366.1387
168.0652
274.0911
186.0757
126.0547
292.1022
163.0596
454.1522
528.1899
657.235
804.379 PEP_0_1_0_0_0_
1404.6642 PEP_0_0_0_0_0_
1607.7485 PEP_0_1_0_0_0_
1810.837 PEP_0_2_0_0_0_
1972.8752 PEP_1_2_0_0_0_
Intensity
m/z
0
50000
100000
150000
200000
250000
300000
350000
500
1000
1500
2000
2500

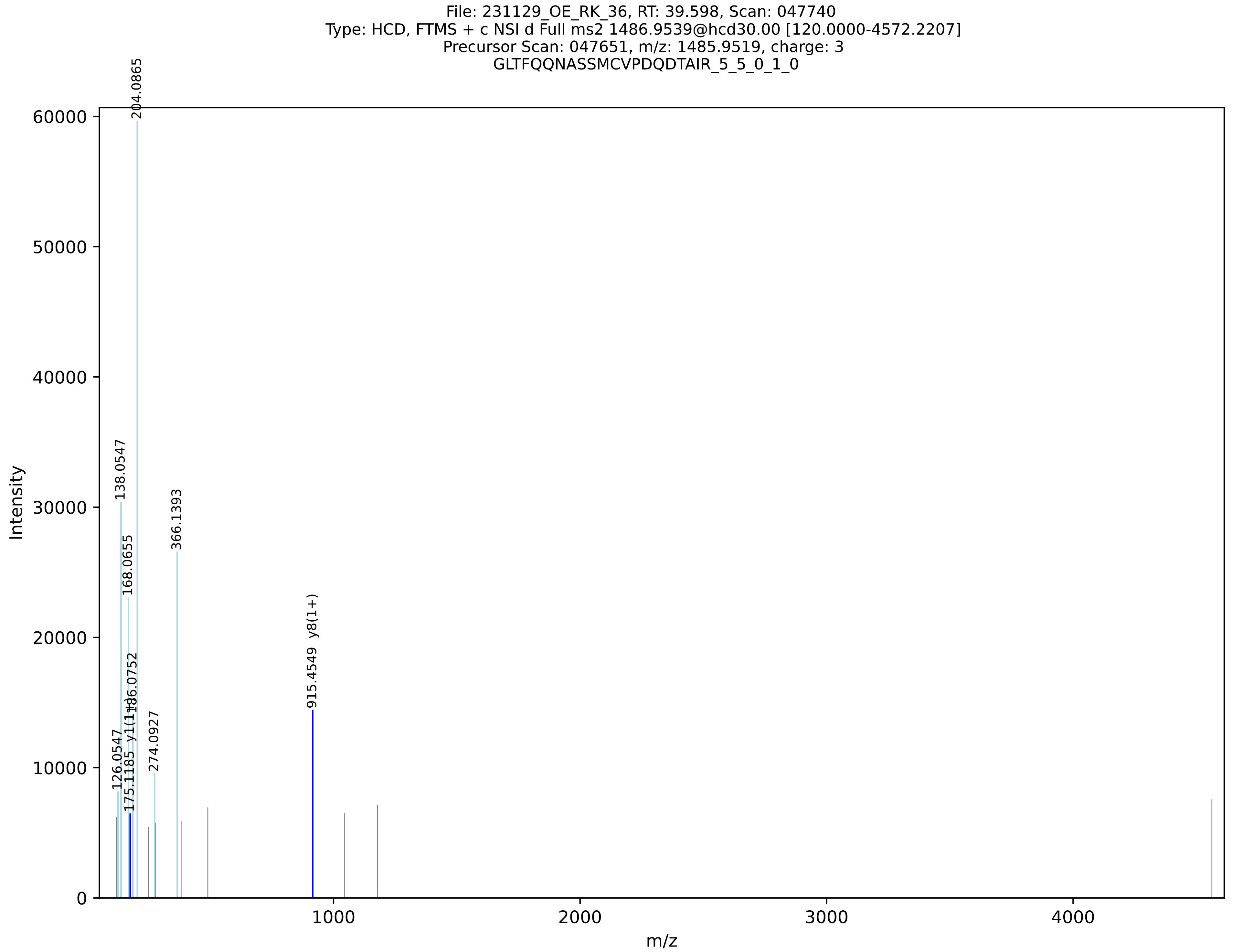
File: 231129_OE_RK_36, RT: 39.598, Scan: 047740
Type: HCD, FTMS + c NSI d Full ms2 1486.9539@hcd30.00 [120.0000-4572.2207]
Precursor Scan: 047651, m/z: 1485.9519, charge: 3
GLTFQQNASSMCVPDQDTAIR_5_5_0_1_0
Intensity
m/z
204.0865
138.0547
168.0655
366.1393
915.4549 y8(1+)
186.0752
175.1185 y1(1+)
126.0547
274.0927
0
10000
20000
30000
40000
50000
60000
1000
2000
3000
4000

File: 231129_OE_RK_36, RT: 39.641, Scan: 047798
Type: HCD, FTMS + c NSI d Full ms2 1258.5236@hcd30.00 [120.0000-5157.6323]
Precursor Scan: 047724, m/z: 1258.0225, charge: 4
ELHHLQEQNVSNAFLDK_6_5_1_3_0

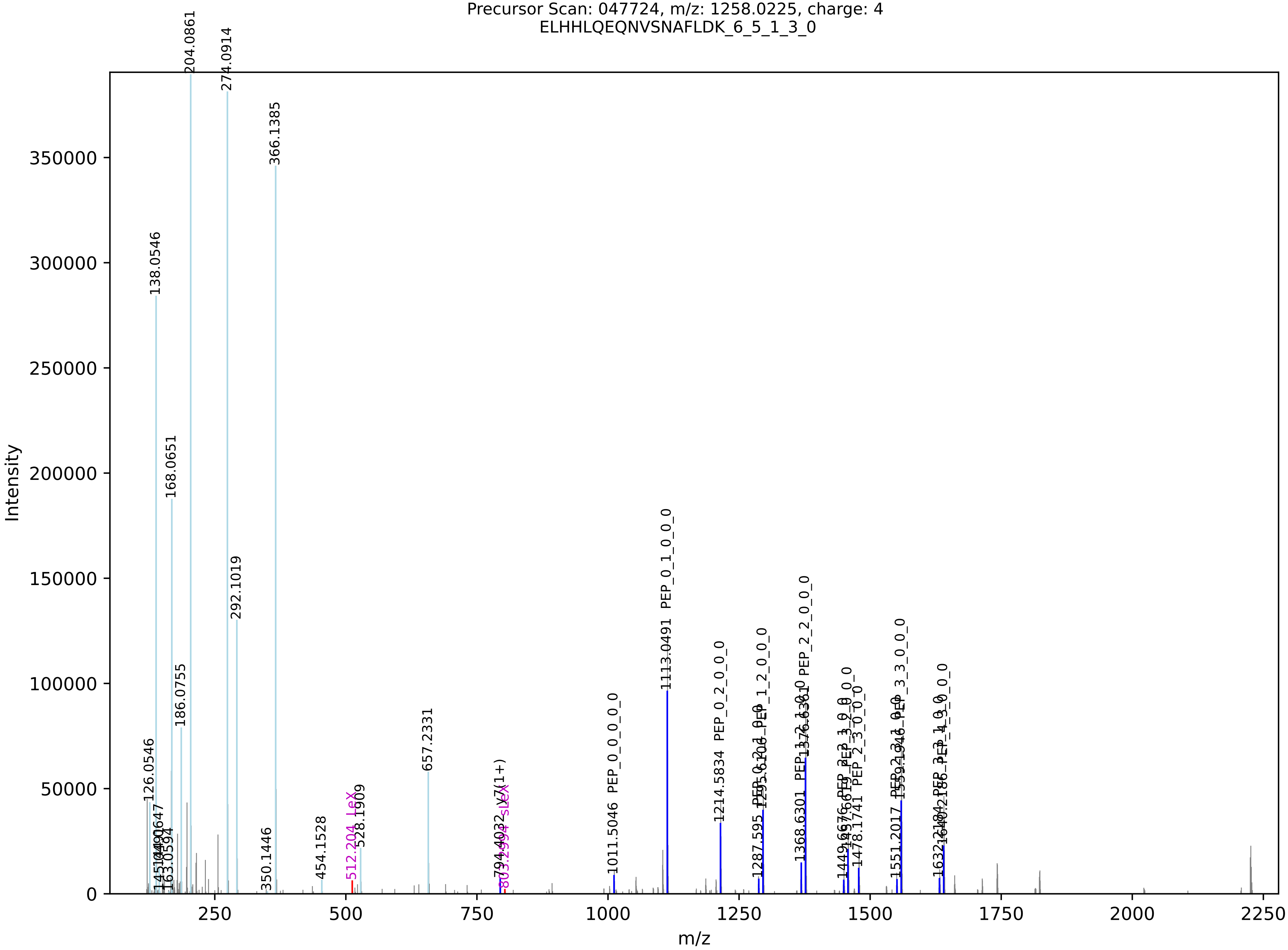

File: 231129_OE_RK_36, RT: 39.654, Scan: 047819
Type: HCD, FTMS + c NSI d Full ms2 985.7459@hcd30.00 [120.0000-3038.5244]
Precursor Scan: 047724, m/z: 985.0777, charge: 3
SWPAVGNCSSALR_4_3_0_1_0

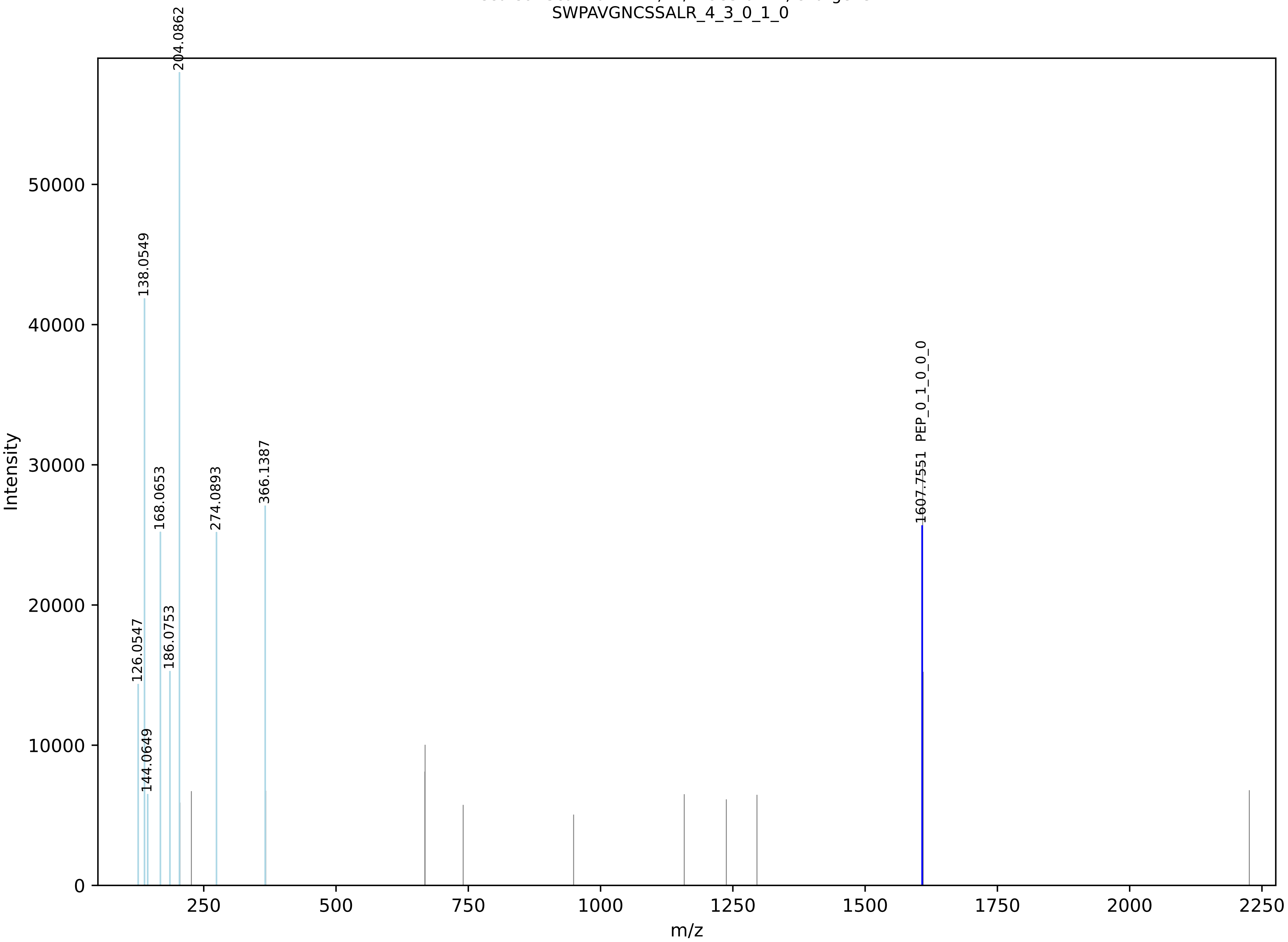

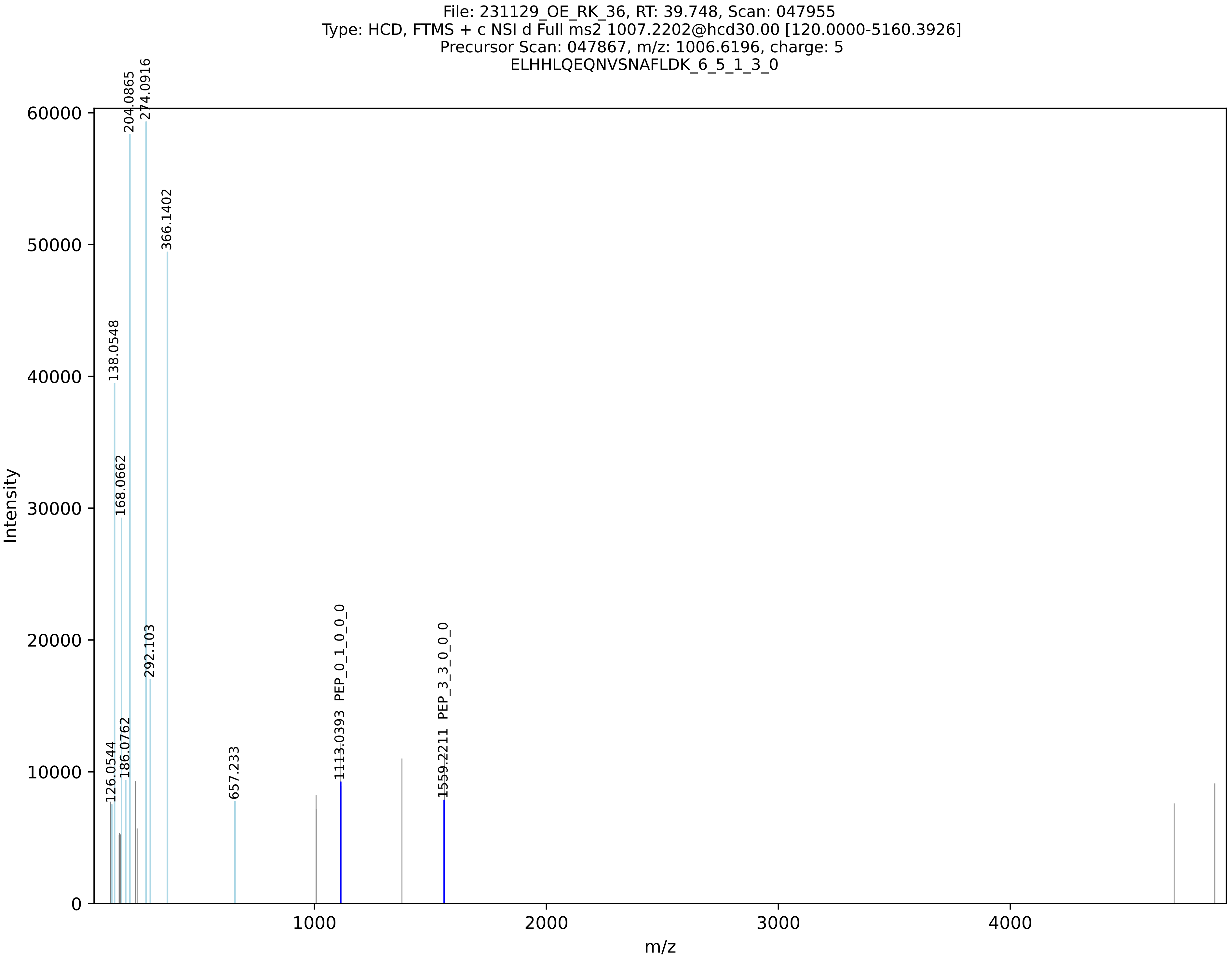

File: 231129_OE_RK_36, RT: 39.748, Scan: 047955
Type: HCD, FTMS + c NSI d Full ms2 1007.2202@hcd30.00 [120.0000-5160.3926]
Precursor Scan: 047867, m/z: 1006.6196, charge: 5
ELHHLQEQNVSNAFLDK_6_5_1_3_0
Intensity
m/z
204.0865
274.0916
366.1402
138.0548
168.0662
292.103
126.0544
186.0762
657.233
1113.0393 PEP_0_1_0_0_0
1559.2211 PEP_3_3_0_0_0

File: 231129_OE_RK_36, RT: 39.828, Scan: 048075
Type: HCD, FTMS + c NSI d Full ms2 1078.7975@hcd30.00 [120.0000-3323.2625]
Precursor Scan: 047940, m/z: 1078.7975, charge: 3
FNSSYLQGTNQITGR_4_3_0_1_0

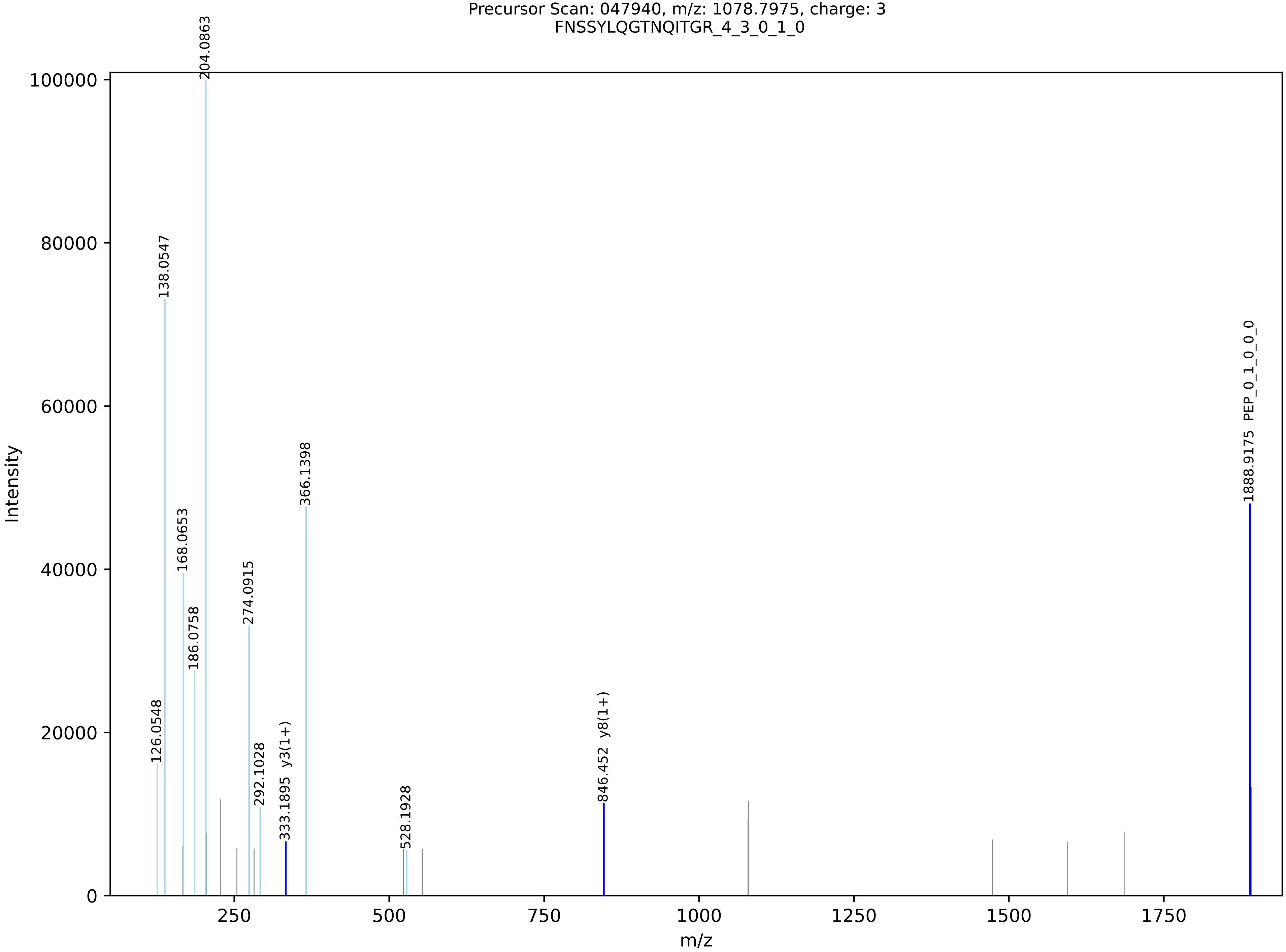

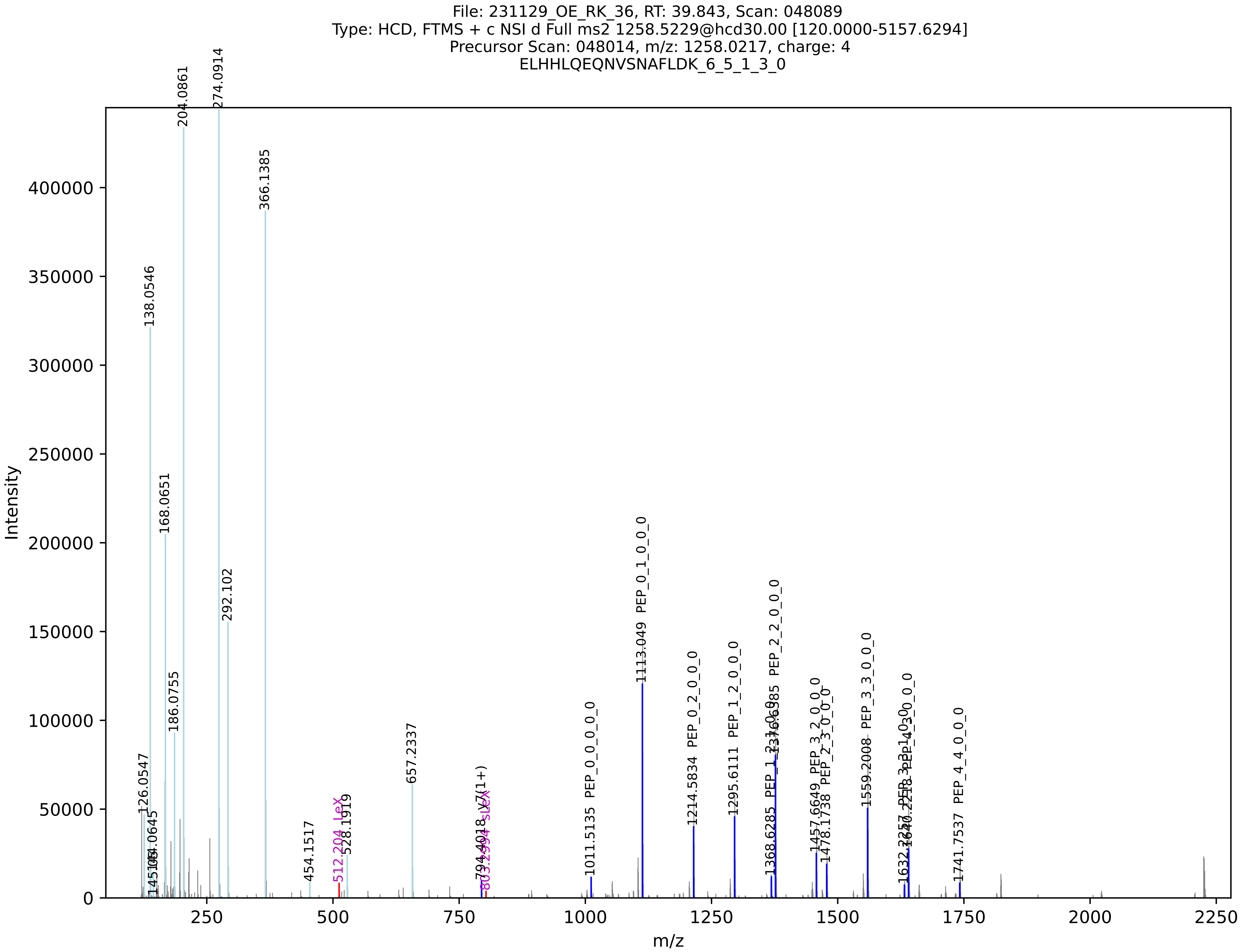
File: 231129_OE_RK_36, RT: 39.843, Scan: 048089
Type: HCD, FTMS + c NSI d Full ms2 1258.5229@hcd30.00 [120.0000-5157.6294]
Precursor Scan: 048014, m/z: 1258.0217, charge: 4
ELHHLQEQNVSNAFLDK_6_5_1_3_0
Intensity
m/z
274.0914
204.0861
366.1385
138.0546
168.0651
292.102
186.0755
126.0547
657.2337
454.1517
512.204_LeX
528.1919
794.4018_y7(1+)
803.2994_sLeX
1011.5135_PEP_0_0_0_0_0
1113.049_PEP_0_1_0_0_0
1214.5834_PEP_0_2_0_0_0
1295.6111_PEP_1_2_0_0_0
1368.6285_PEP_1_2_1_0_0
1376.6385_PEP_2_2_0_0_0
1457.6649_PEP_3_2_0_0_0
1478.1738_PEP_2_3_0_0_0
1559.2008_PEP_3_3_0_0_0
1741.7537_PEP_4_4_0_0_0

File: 231129_OE_RK_36, RT: 39.858, Scan: 048114
Type: HCD, FTMS + c NSI d Full ms2 1032.4492@hcd30.00 [120.0000-3181.4365]
Precursor Scan: 048014, m/z: 1032.115, charge: 3
HANWTLTPLK_5_4_0_1_0

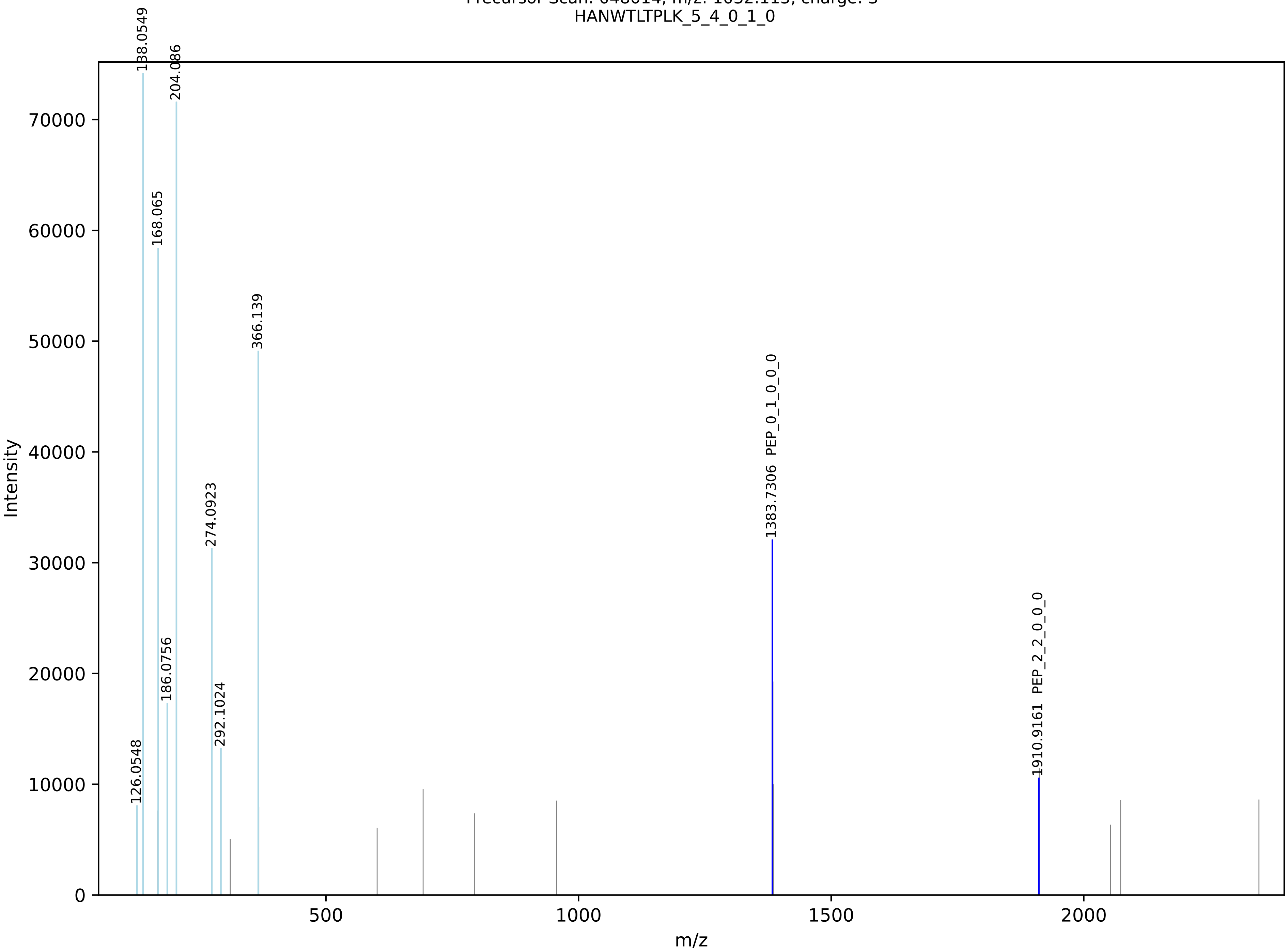

File: 231129_OE_RK_36, RT: 40.008, Scan: 048332
Type: HCD, FTMS + c NSI d Full ms2 1153.4575@hcd30.00 [120.0000-3551.7219]
Precursor Scan: 048233, m/z: 1152.7893, charge: 3
HNFSHCCSKVDAQR_5_3_0_1_0

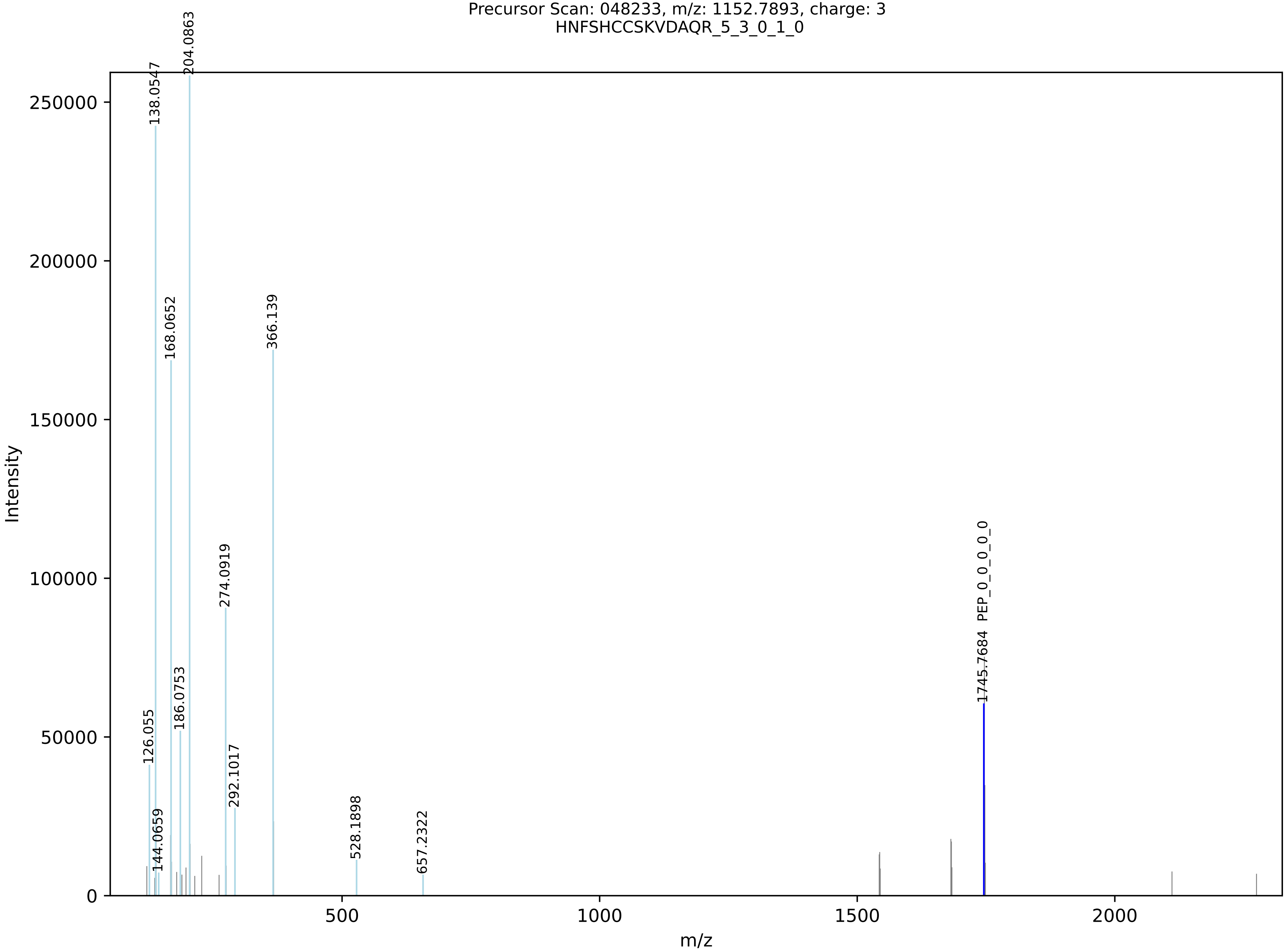

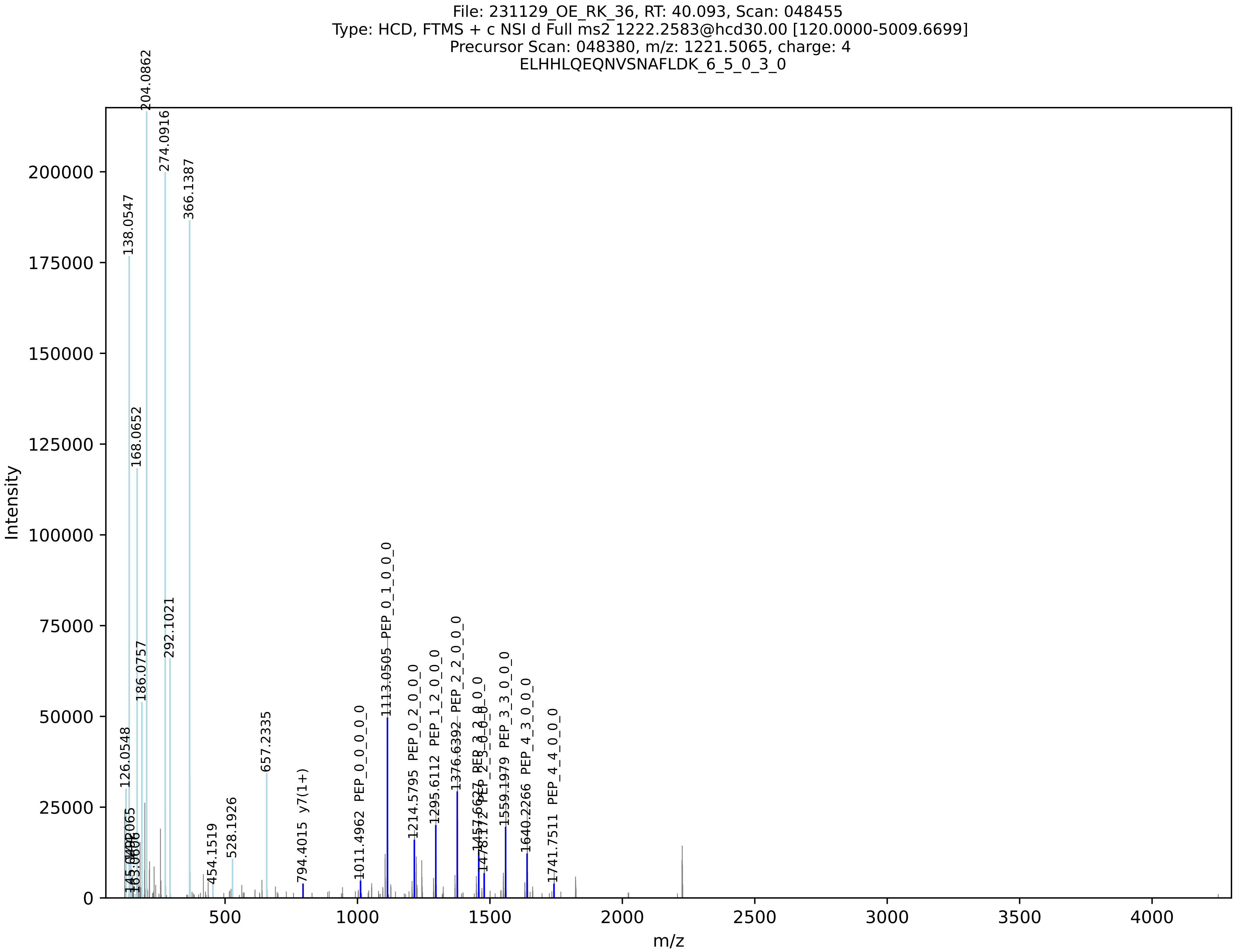
File: 231129_OE_RK_36, RT: 40.093, Scan: 048455
Type: HCD, FTMS + c NSI d Full ms2 1222.2583@hcd30.00 [120.0000-5009.6699]
Precursor Scan: 048380, m/z: 1221.5065, charge: 4
ELHHLQEQNVSNAFLDK_6_5_0_3_0
Intensity
m/z
204.0862
274.0916
366.1387
138.0547
168.0652
186.0757
292.1021
126.0548
657.2335
528.1926
454.1519
794.4015 y7(1+)
1011.4962 PEP_0_0_0_0_0
1113.0505 PEP_0_1_0_0_0
1214.5795 PEP_0_2_0_0_0
1295.6112 PEP_1_2_0_0_0
1376.6392 PEP_2_2_0_0_0
1559.1979 PEP_3_3_0_0_0
1640.2266 PEP_4_3_0_0_0
1741.7511 PEP_4_4_0_0_0

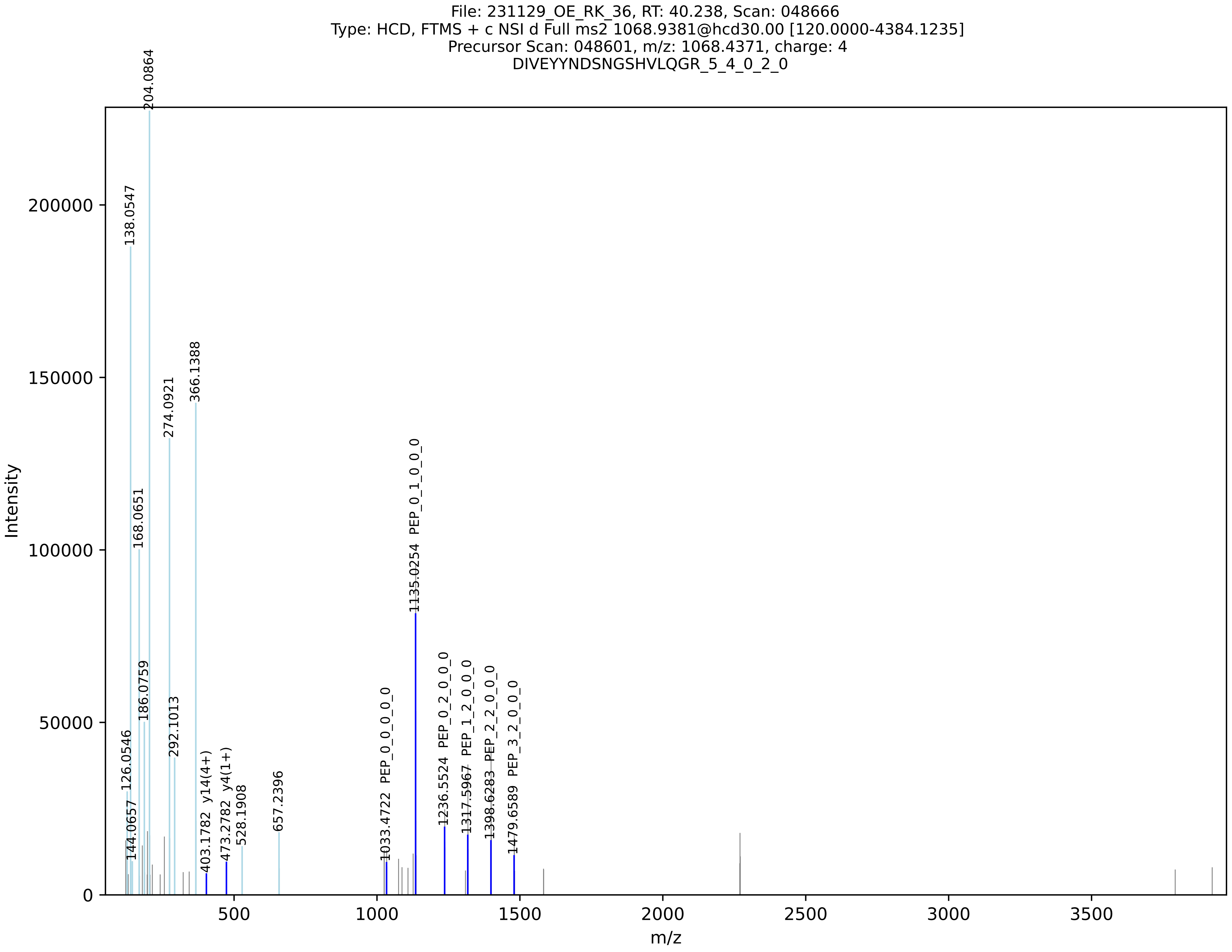
File: 231129_OE_RK_36, RT: 40.238, Scan: 048666
Type: HCD, FTMS + c NSI d Full ms2 1068.9381@hcd30.00 [120.0000-4384.1235]
Precursor Scan: 048601, m/z: 1068.4371, charge: 4
DIVEYYNDSNGSHVLQGR_5_4_0_2_0
Intensity
m/z
204.0864
138.0547
366.1388
274.0921
168.0651
186.0759
292.1013
126.0546
144.0657
403.1782 y14(4+)
473.2782 y4(1+)
528.1908
657.2396
1033.4722 PEP_0_0_0_0_0
1135.0254 PEP_0_1_0_0_0
1236.5524 PEP_0_2_0_0_0
1317.5967 PEP_1_2_0_0_0
1398.6283 PEP_2_2_0_0_0
1479.6589 PEP_3_2_0_0_0
0
50000
100000
150000
200000
500
1000
1500
2000
2500
3000
3500

File: 231129_OE_RK_36, RT: 40.287, Scan: 048737
Type: HCD, FTMS + c NSI d Full ms2 1105.2024@hcd30.00 [120.0000-4532.0815]
Precursor Scan: 048658, m/z: 1104.9528, charge: 4
DIVEYYNDSNGSHVLQGR_5_4_1_2_0

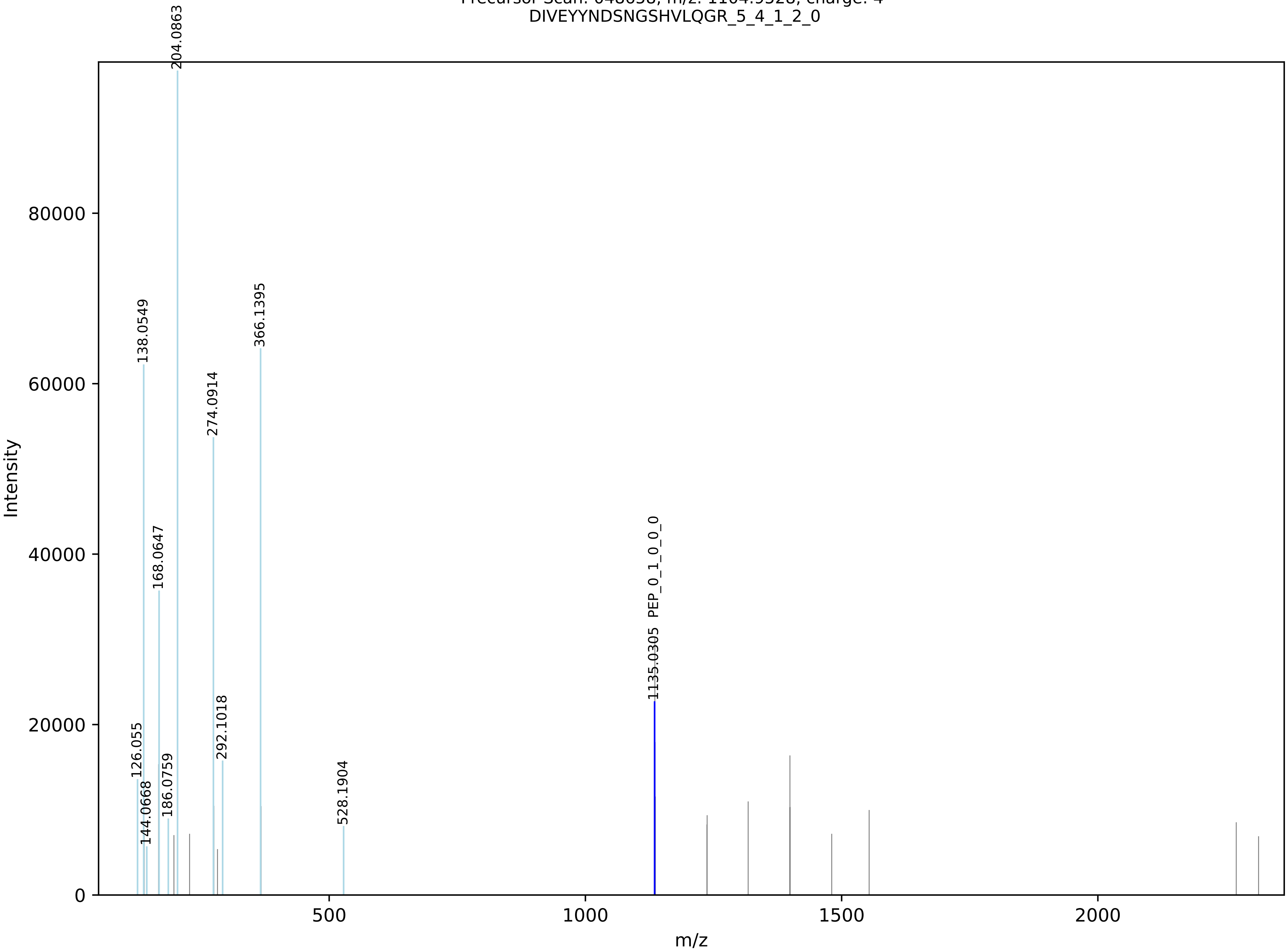

File: 231129_OE_RK_36, RT: 40.342, Scan: 048820
Type: HCD, FTMS + c NSI d Full ms2 964.4203@hcd30.00 [120.0000-2973.2681]
Precursor Scan: 048732, m/z: 964.4203, charge: 3
HANWTLTPLK_5_3_0_1_0

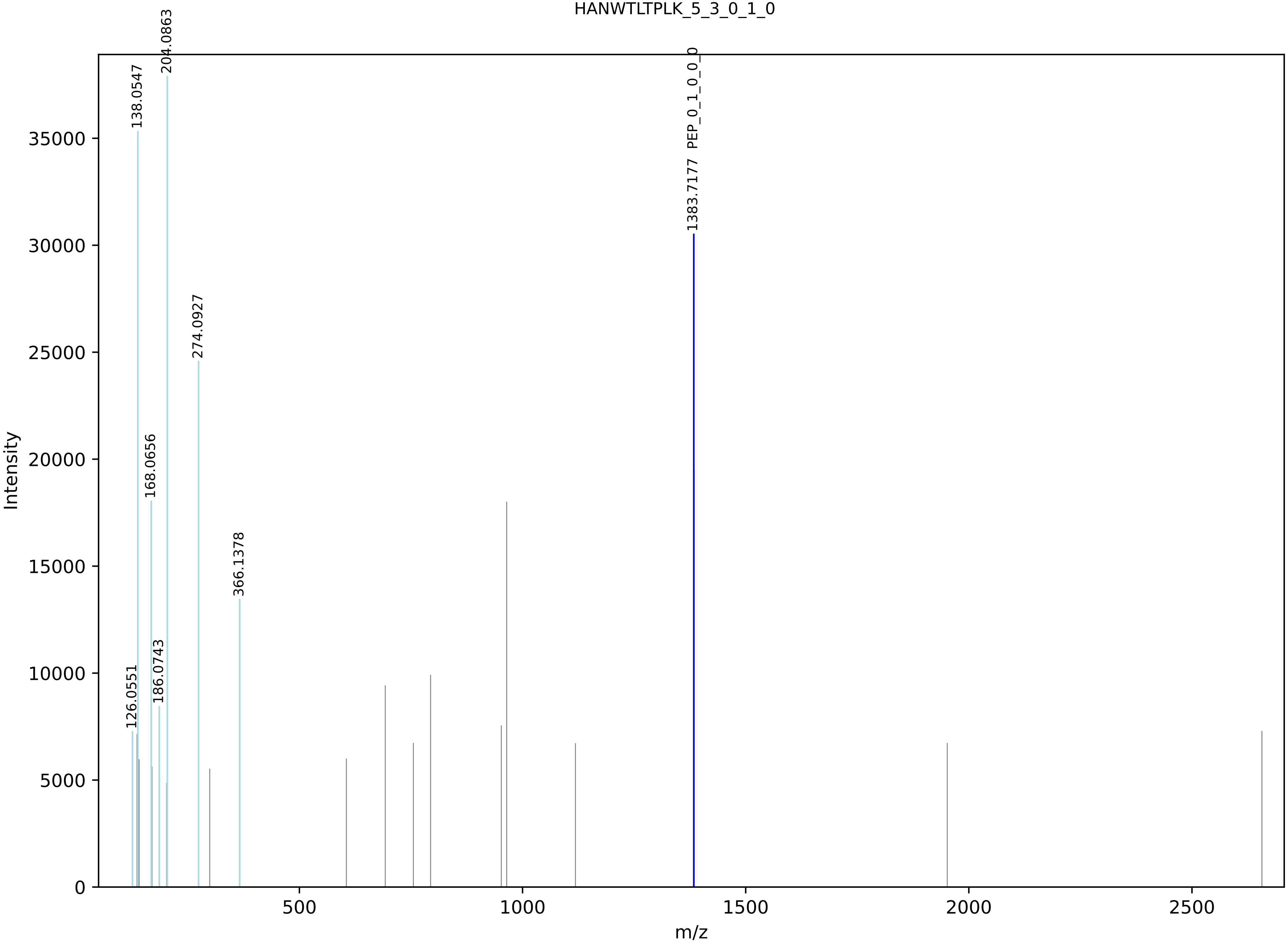

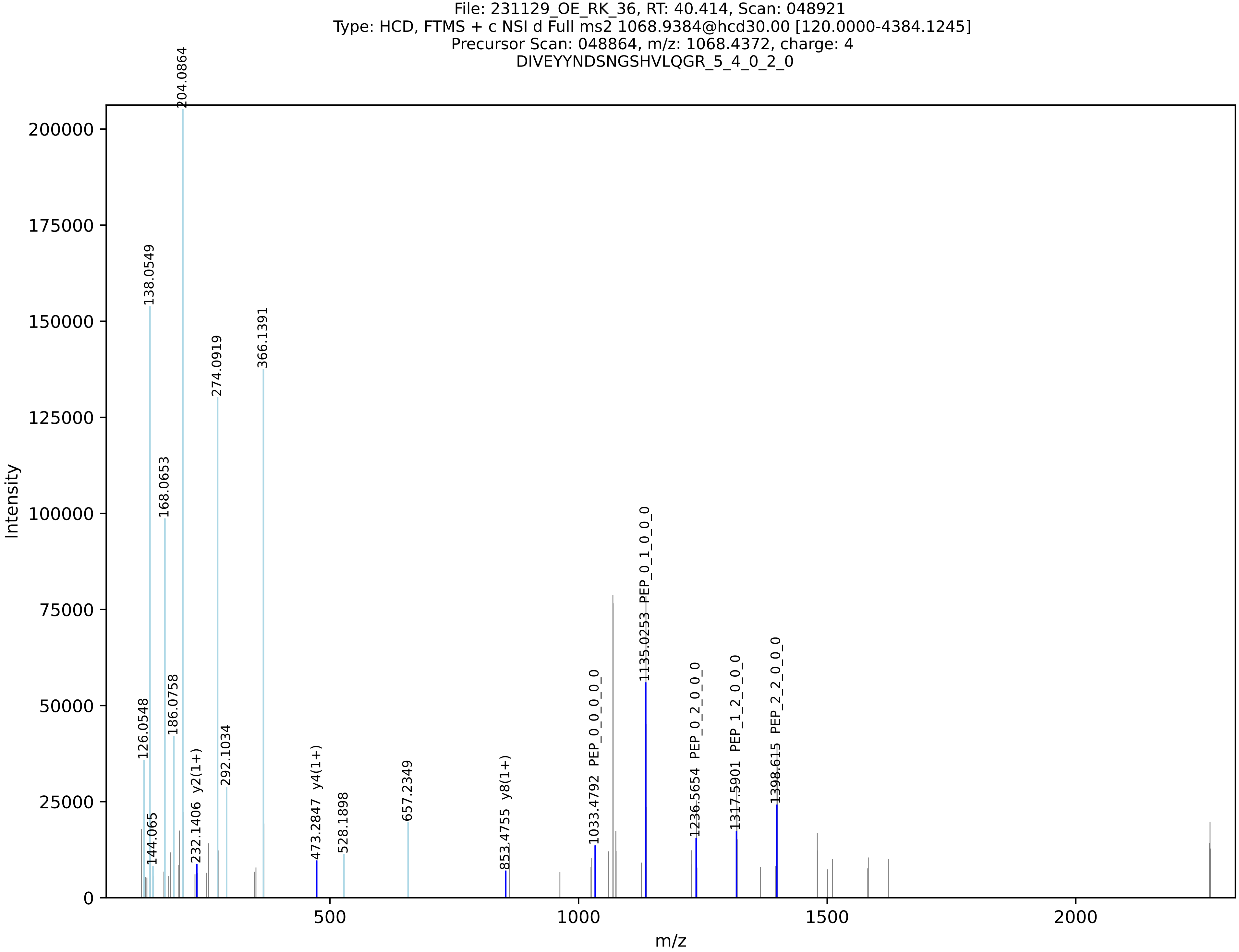

File: 231129_OE_RK_36, RT: 40.414, Scan: 048921
Type: HCD, FTMS + c NSI d Full ms2 1068.9384@hcd30.00 [120.0000-4384.1245]
Precursor Scan: 048864, m/z: 1068.4372, charge: 4
DIVEYYNDSNGSHVLQGR_5_4_0_2_0
Intensity
m/z
204.0864
138.0549
366.1391
274.0919
168.0653
126.0548
186.0758
292.1034
144.065
232.1406 y2(1+)
473.2847 y4(1+)
528.1898
657.2349
853.4755 y8(1+)
1033.4792 PEP_0_0_0_0_0_
1135.0253 PEP_0_1_0_0_0_
1236.5654 PEP_0_2_0_0_0_
1317.5901 PEP_1_2_0_0_0_
1398.615 PEP_2_2_0_0_0_

File: 231129_OE_RK_36, RT: 40.552, Scan: 049121
Type: HCD, FTMS + c NSI d Full ms2 1424.9156@hcd30.00 [120.0000-4382.3838]
Precursor Scan: 049057, m/z: 1424.2475, charge: 3
DIVEYYNDSNGSHVLQGR_5_4_0_2_0

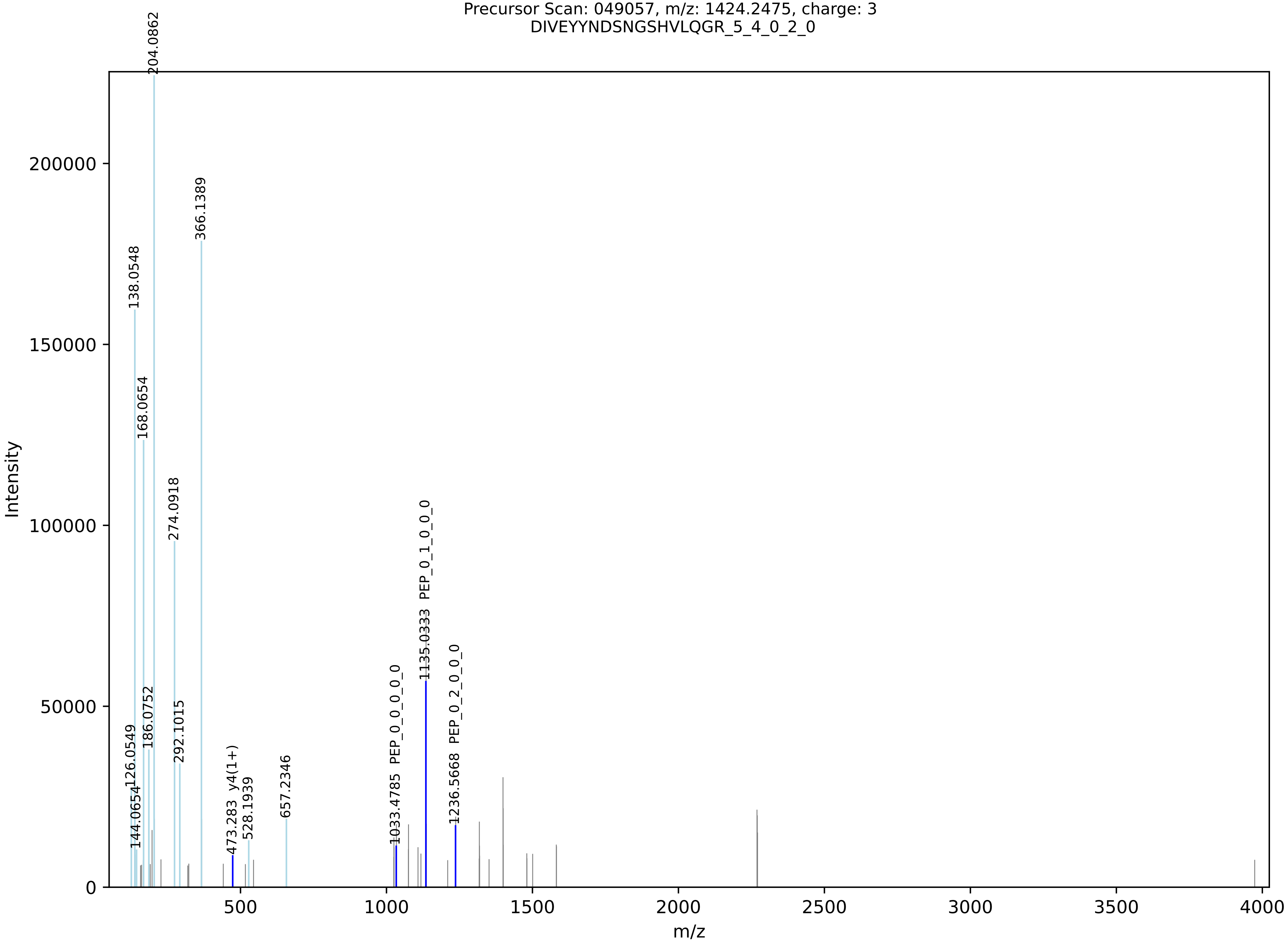

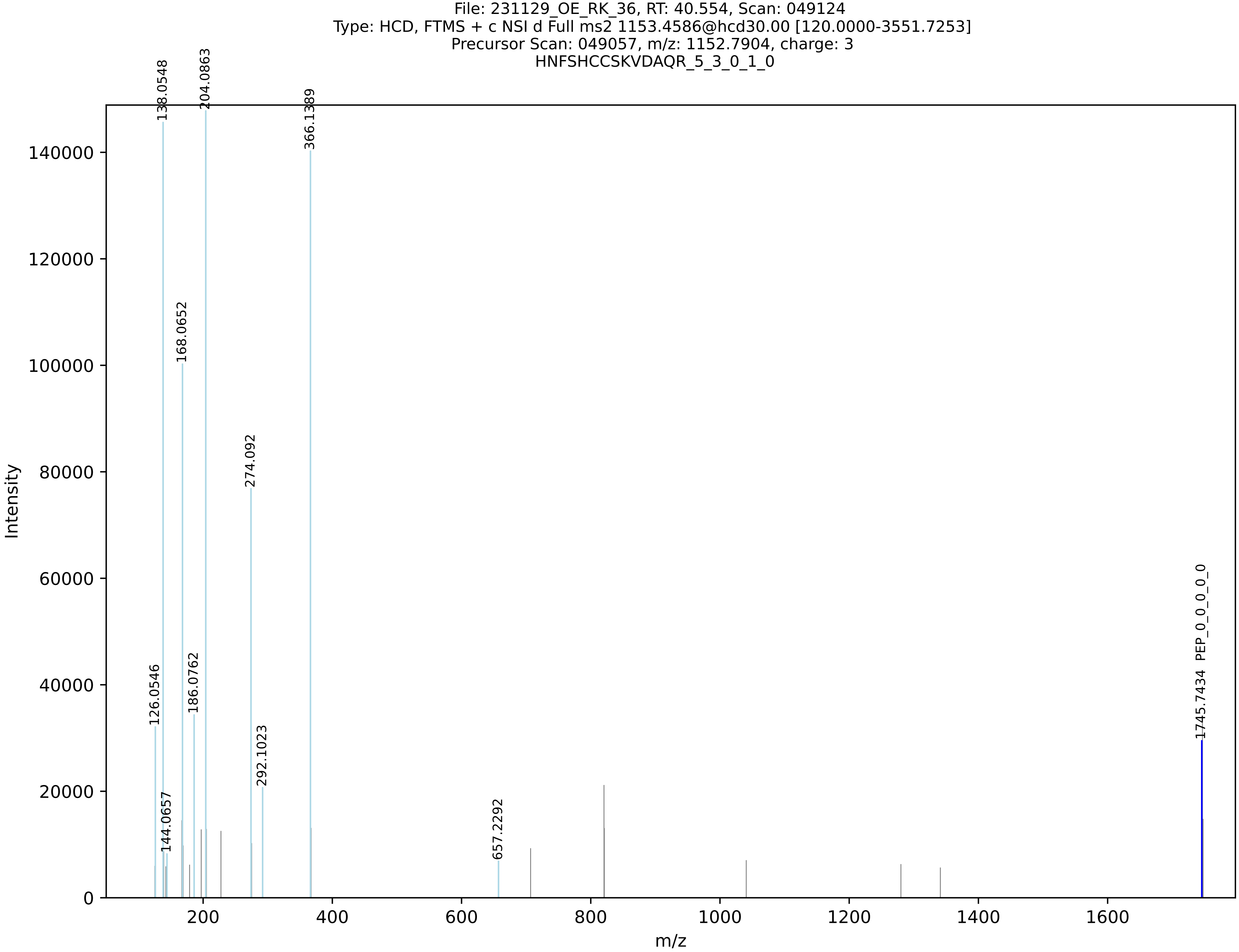
File: 231129_OE_RK_36, RT: 40.554, Scan: 049124
Type: HCD, FTMS + c NSI d Full ms2 1153.4586@hcd30.00 [120.0000-3551.7253]
Precursor Scan: 049057, m/z: 1152.7904, charge: 3
HNFSHCCSKVDAQR_5_3_0_1_0
Intensity
m/z
138.0548
204.0863
366.1389
168.0652
274.092
126.0546
186.0762
292.1023
144.0657
657.2292
1745.7434 PEP_0_0_0_0_0

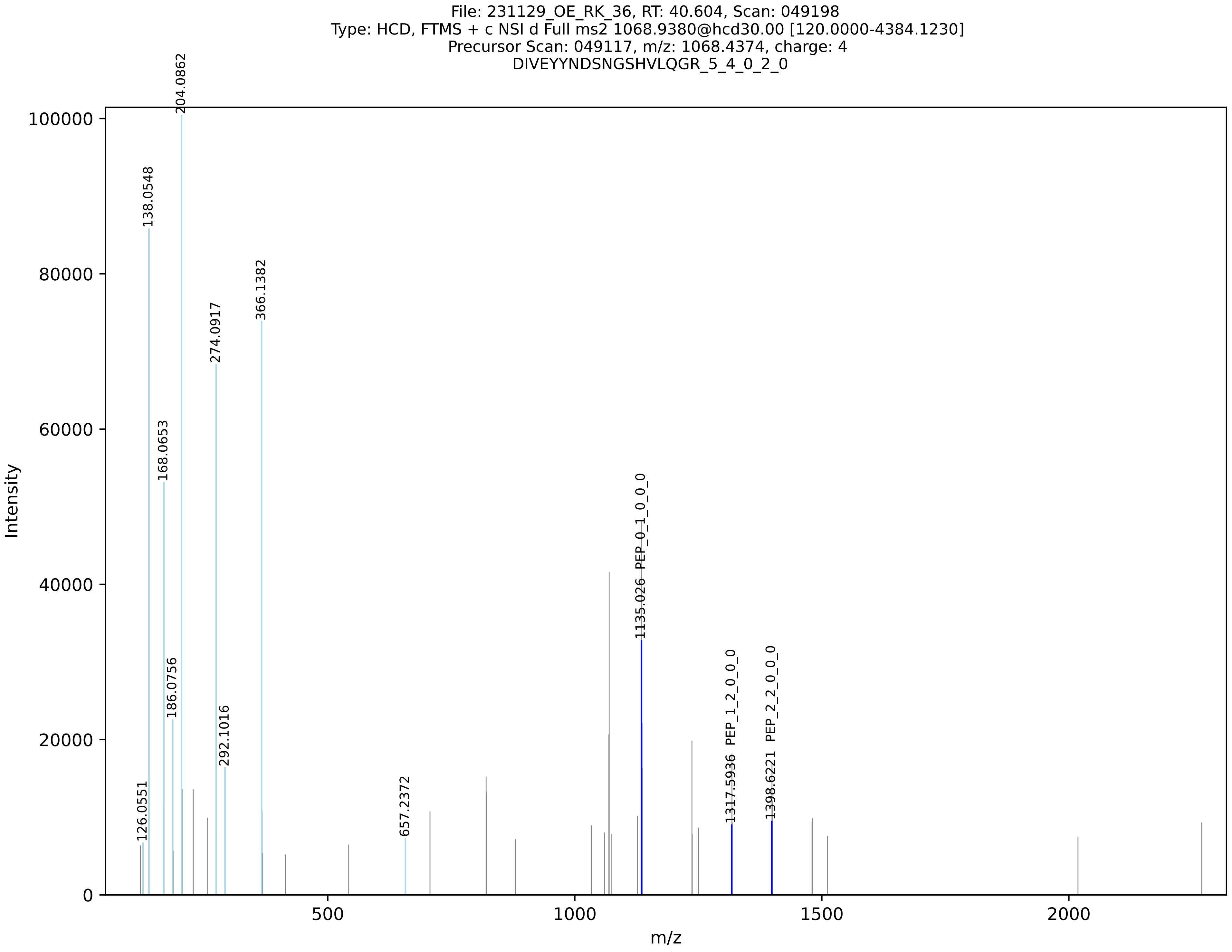
File: 231129_OE_RK_36, RT: 40.604, Scan: 049198
Type: HCD, FTMS + c NSI d Full ms2 1068.9380@hcd30.00 [120.0000-4384.1230]
Precursor Scan: 049117, m/z: 1068.4374, charge: 4
DIVEYYNDSNGSHVLQGR_5_4_0_2_0
Intensity
m/z
0
20000
40000
60000
80000
100000
500
1000
1500
2000
204.0862
138.0548
168.0653
186.0756
274.0917
292.1016
366.1382
126.0551
657.2372
1135.026 PEP_0_1_0_0_0
1317.5936 PEP_1_2_0_0_0
1398.6221 PEP_2_2_0_0_0

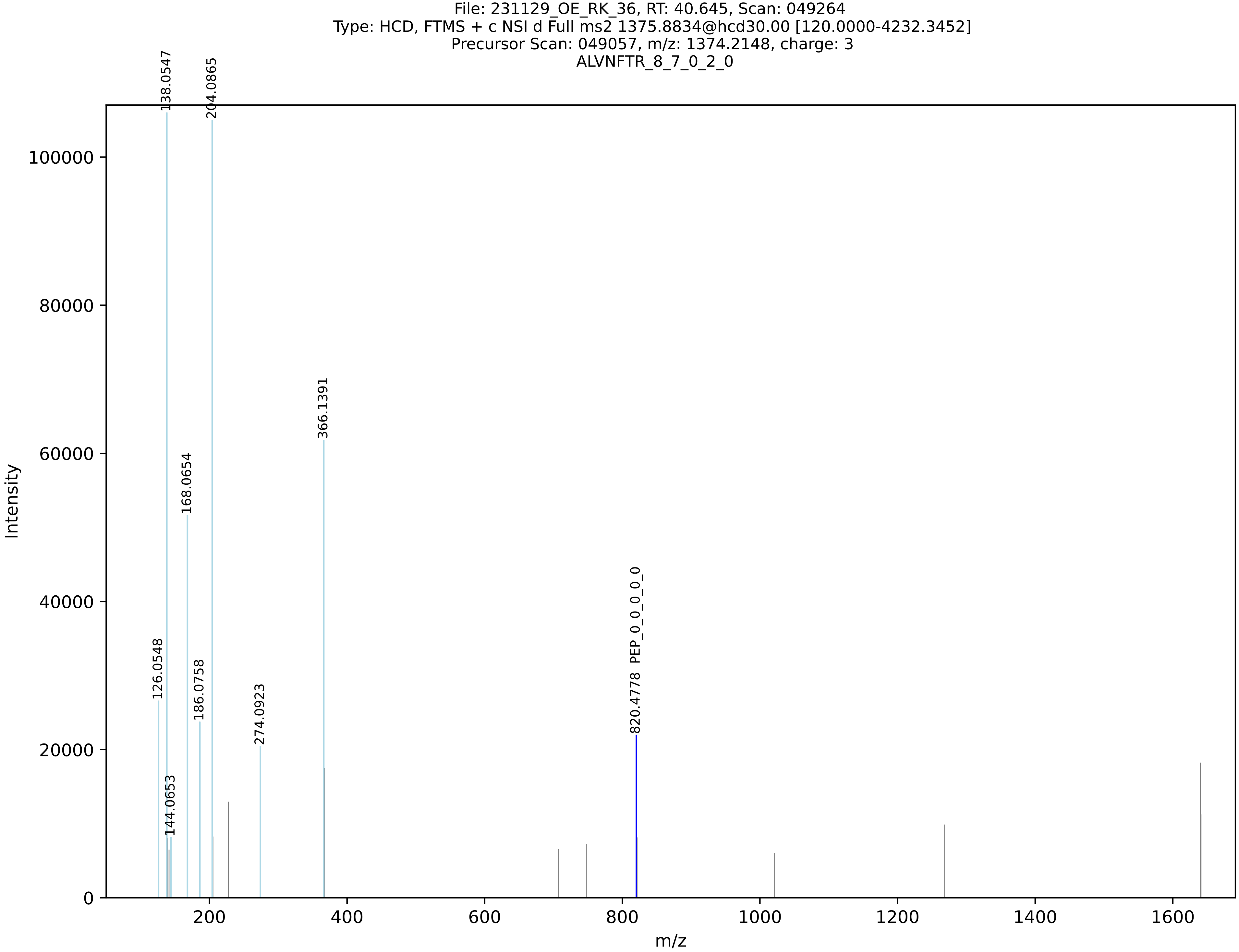
File: 231129_OE_RK_36, RT: 40.645, Scan: 049264
Type: HCD, FTMS + c NSI d Full ms2 1375.8834@hcd30.00 [120.0000-4232.3452]
Precursor Scan: 049057, m/z: 1374.2148, charge: 3
ALVNFTR_8_7_0_2_0
138.0547
204.0865
366.1391
168.0654
126.0548
186.0758
274.0923
144.0653
820.4778 PEP_0_0_0_0_0
Intensity
m/z
0
20000
40000
60000
80000
100000
200
400
600
800
1000
1200
1400
1600

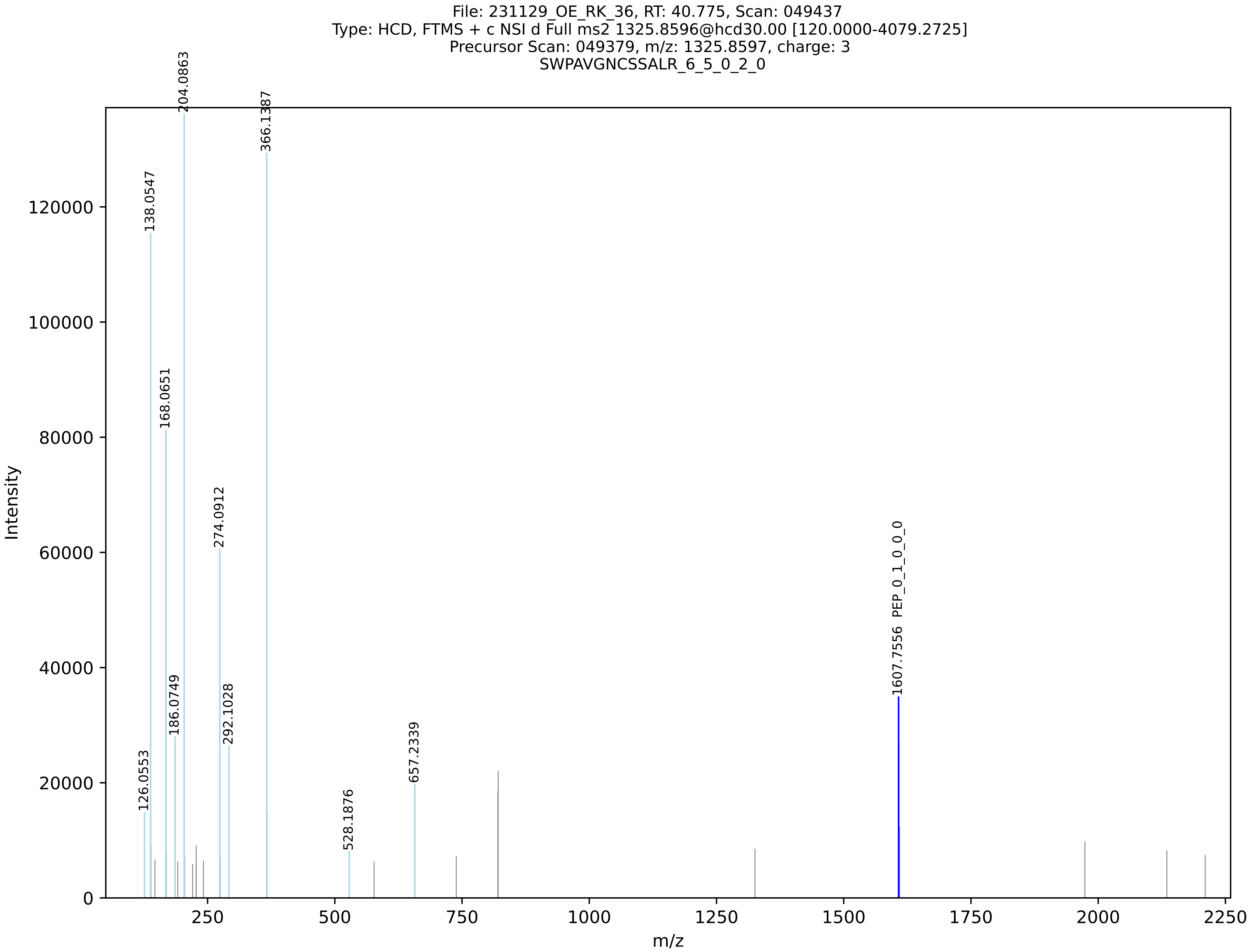
File: 231129_OE_RK_36, RT: 40.775, Scan: 049437
Type: HCD, FTMS + c NSI d Full ms2 1325.8596@hcd30.00 [120.0000-4079.2725]
Precursor Scan: 049379, m/z: 1325.8597, charge: 3
SWPAVGNCSSALR_6_5_0_2_0
204.0863
366.1387
138.0547
168.0651
274.0912
186.0749
292.1028
126.0553
528.1876
657.2339
1607.7556 PEP_0_1_0_0_0
Intensity
m/z
0
20000
40000
60000
80000
100000
120000
250
500
750
1000
1250
1500
1750
2000
2250

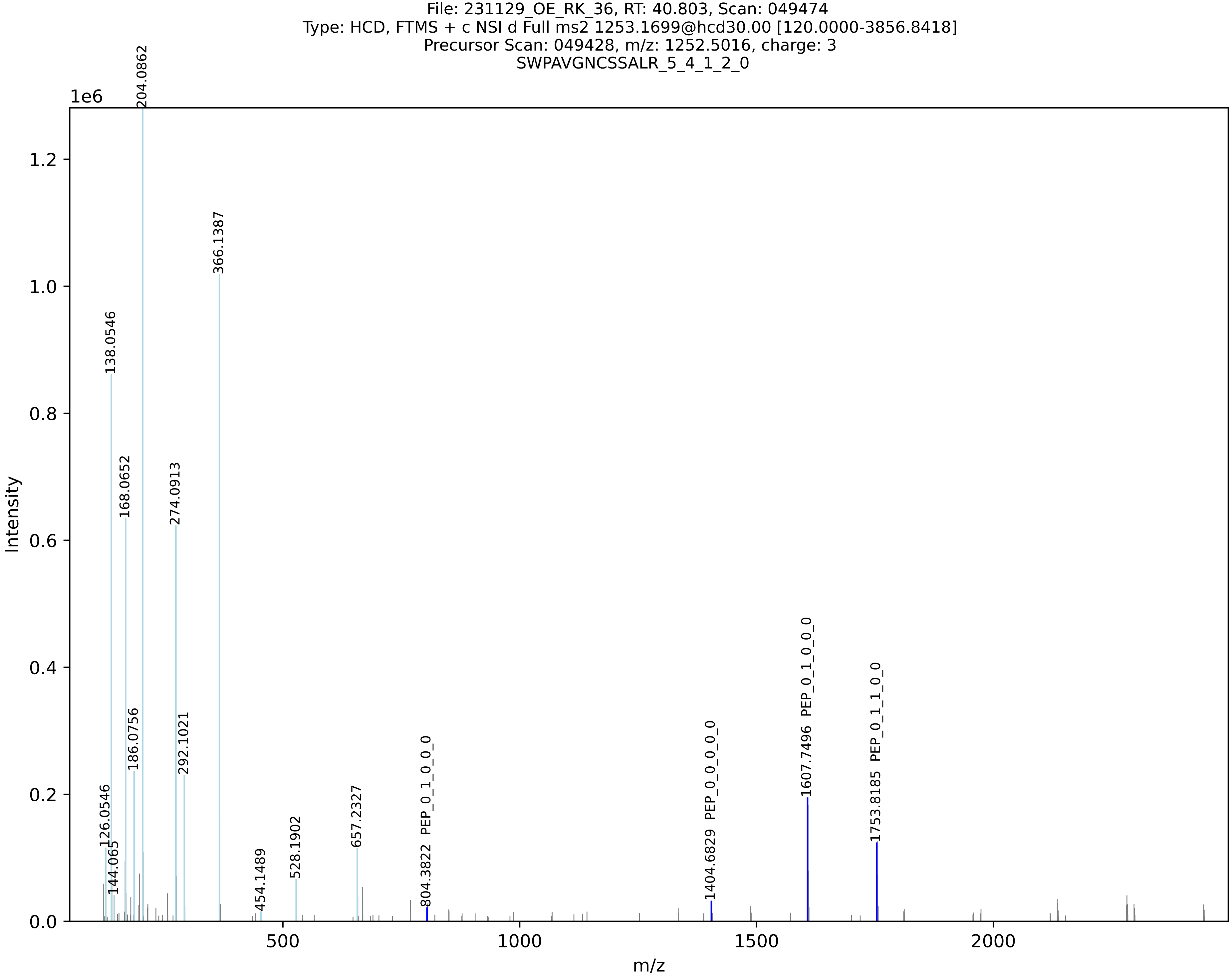
File: 231129_OE_RK_36, RT: 40.803, Scan: 049474
Type: HCD, FTMS + c NSI d Full ms2 1253.1699@hcd30.00 [120.0000-3856.8418]
Precursor Scan: 049428, m/z: 1252.5016, charge: 3
SWPAVGNCSSALR_5_4_1_2_0
1e6
Intensity
m/z
204.0862
366.1387
138.0546
168.0652
274.0913
186.0756
292.1021
126.0546
144.065
454.1489
528.1902
657.2327
804.3822 PEP_0_1_0_0_0_
1404.6829 PEP_0_0_0_0_0_
1607.7496 PEP_0_1_0_0_0_
1753.8185 PEP_0_1_1_0_0_

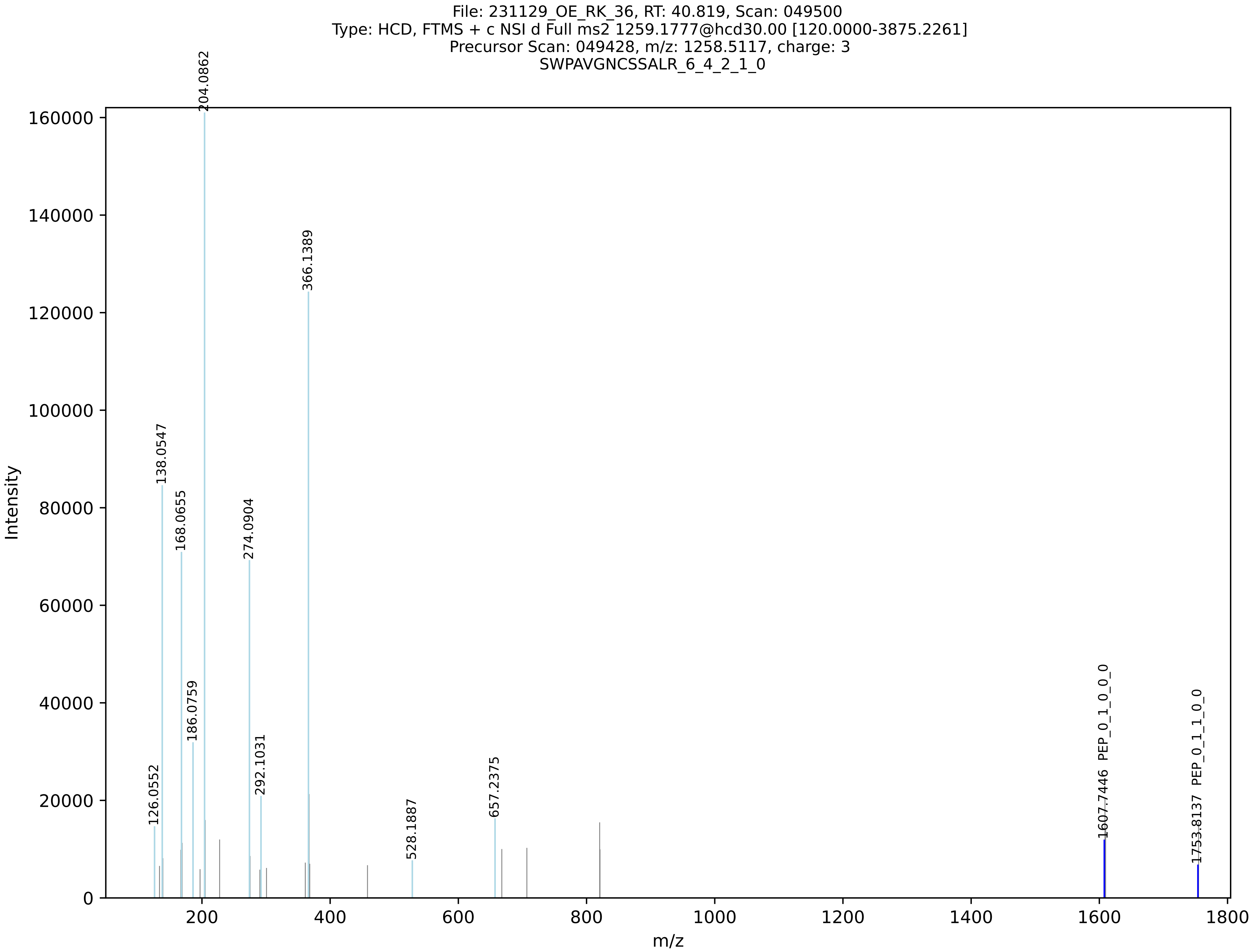
File: 231129_OE_RK_36, RT: 40.819, Scan: 049500
Type: HCD, FTMS + c NSI d Full ms2 1259.1777@hcd30.00 [120.0000-3875.2261]
Precursor Scan: 049428, m/z: 1258.5117, charge: 3
SWPAVGNCSSALR_6_4_2_1_0
Intensity
m/z
204.0862
366.1389
138.0547
168.0655
274.0904
186.0759
292.1031
126.0552
528.1887
657.2375
1607.7446 PEP_0_1_0_0_0
1753.8137 PEP_0_1_1_0_0
0
20000
40000
60000
80000
100000
120000
140000
160000
200
400
600
800
1000
1200
1400
1600
1800

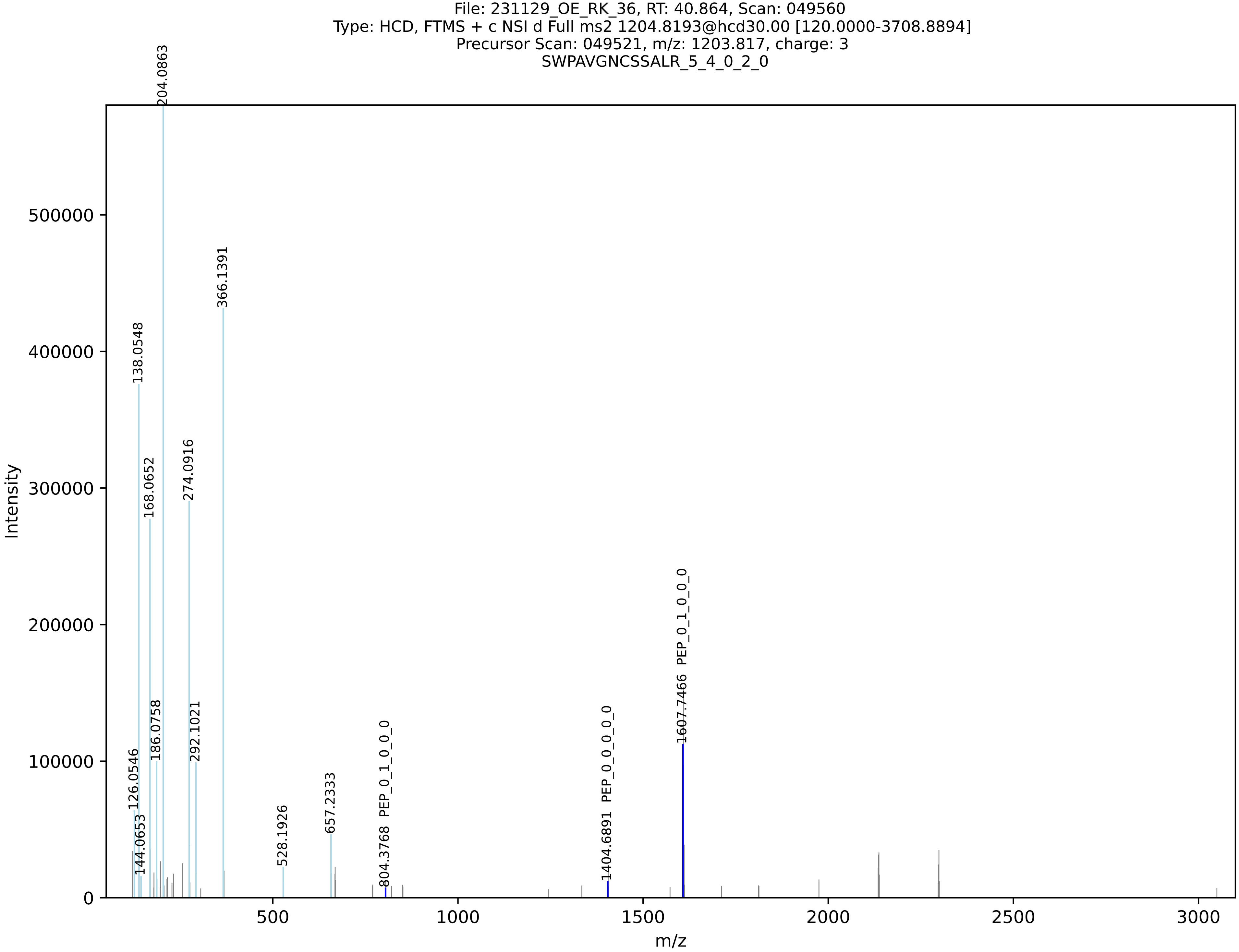

File: 231129_OE_RK_36, RT: 40.864, Scan: 049560
Type: HCD, FTMS + c NSI d Full ms2 1204.8193@hcd30.00 [120.0000-3708.8894]
Precursor Scan: 049521, m/z: 1203.817, charge: 3
SWPAVGNCSSALR_5_4_0_2_0
204.0863
366.1391
138.0548
274.0916
168.0652
186.0758
292.1021
126.0546
144.0653
528.1926
657.2333
804.3768 PEP_0_1_0_0_0
1404.6891 PEP_0_0_0_0_0
1607.7466 PEP_0_1_0_0_0
Intensity
m/z
0
100000
200000
300000
400000
500000
500
1000
1500
2000
2500
3000

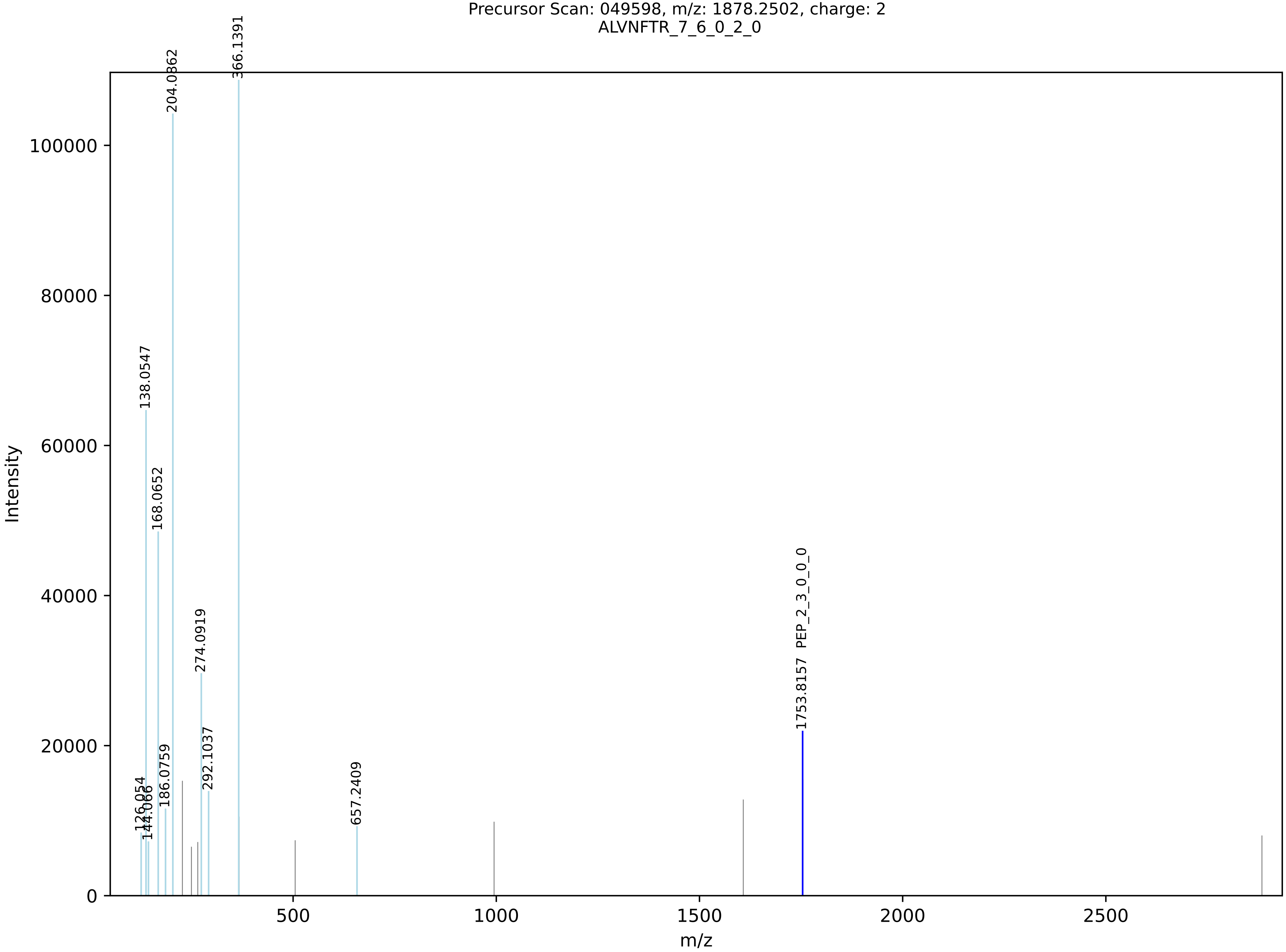
File: 231129_OE_RK_36, RT: 40.925, Scan: 049645
Type: HCD, FTMS + c NSI d Full ms2 1878.7532@hcd30.00 [120.0000-3854.0845]
Precursor Scan: 049598, m/z: 1878.2502, charge: 2
ALVNFTR_7_6_0_2_0
Intensity
m/z
366.1391
204.0362
138.0547
168.0652
274.0919
292.1037
186.0759
126.054
144.066
657.2409
1753.8157 PEP_2_3_0_0_0

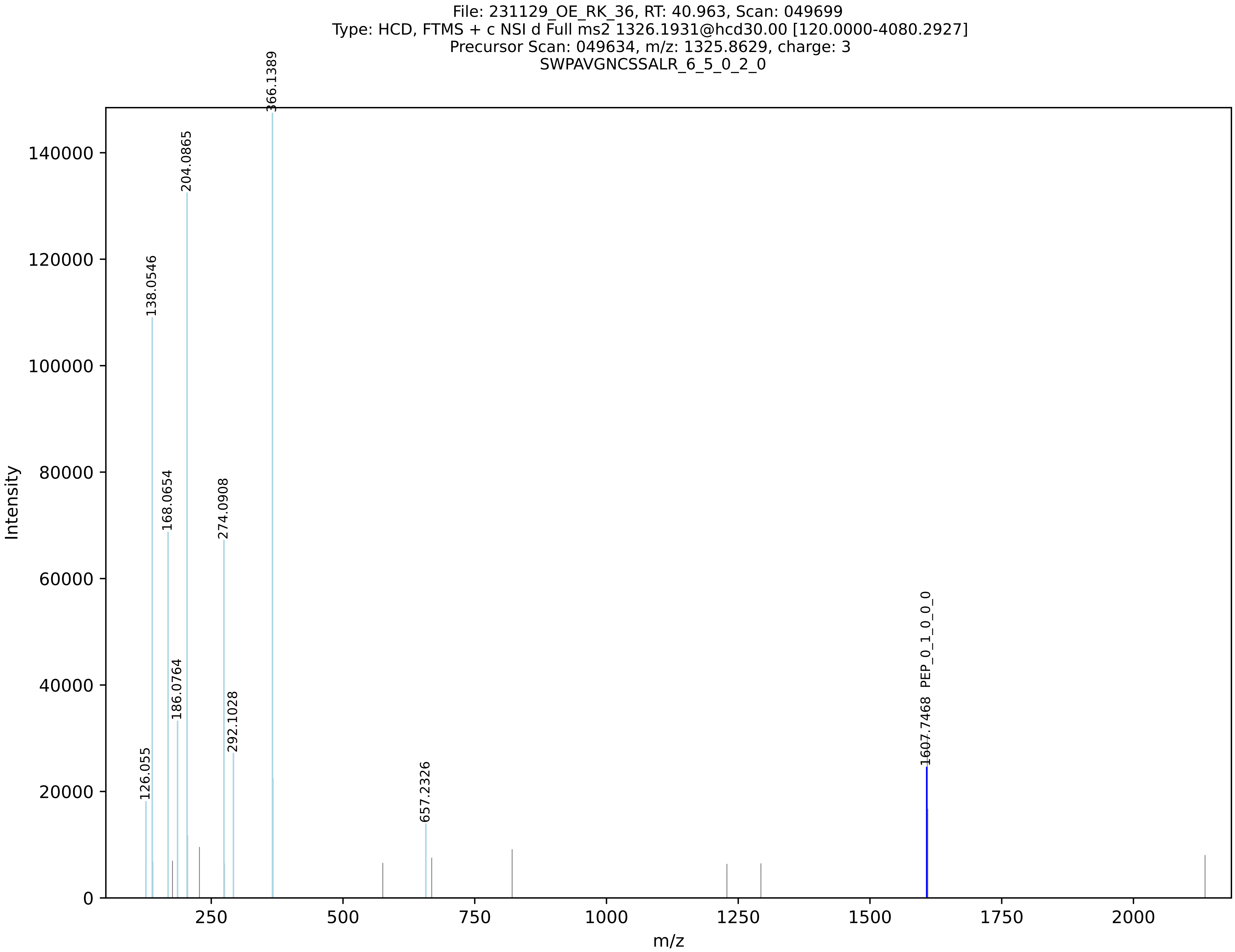
File: 231129_OE_RK_36, RT: 40.963, Scan: 049699
Type: HCD, FTMS + c NSI d Full ms2 1326.1931@hcd30.00 [120.0000-4080.2927]
Precursor Scan: 049634, m/z: 1325.8629, charge: 3
SWPAVGNCSSALR_6_5_0_2_0
366.1389
204.0865
138.0546
168.0654
274.0908
186.0764
292.1028
126.055
657.2326
1607.7468 PEP_0_1_0_0_0
Intensity
m/z
0
20000
40000
60000
80000
100000
120000
140000
250
500
750
1000
1250
1500
1750
2000

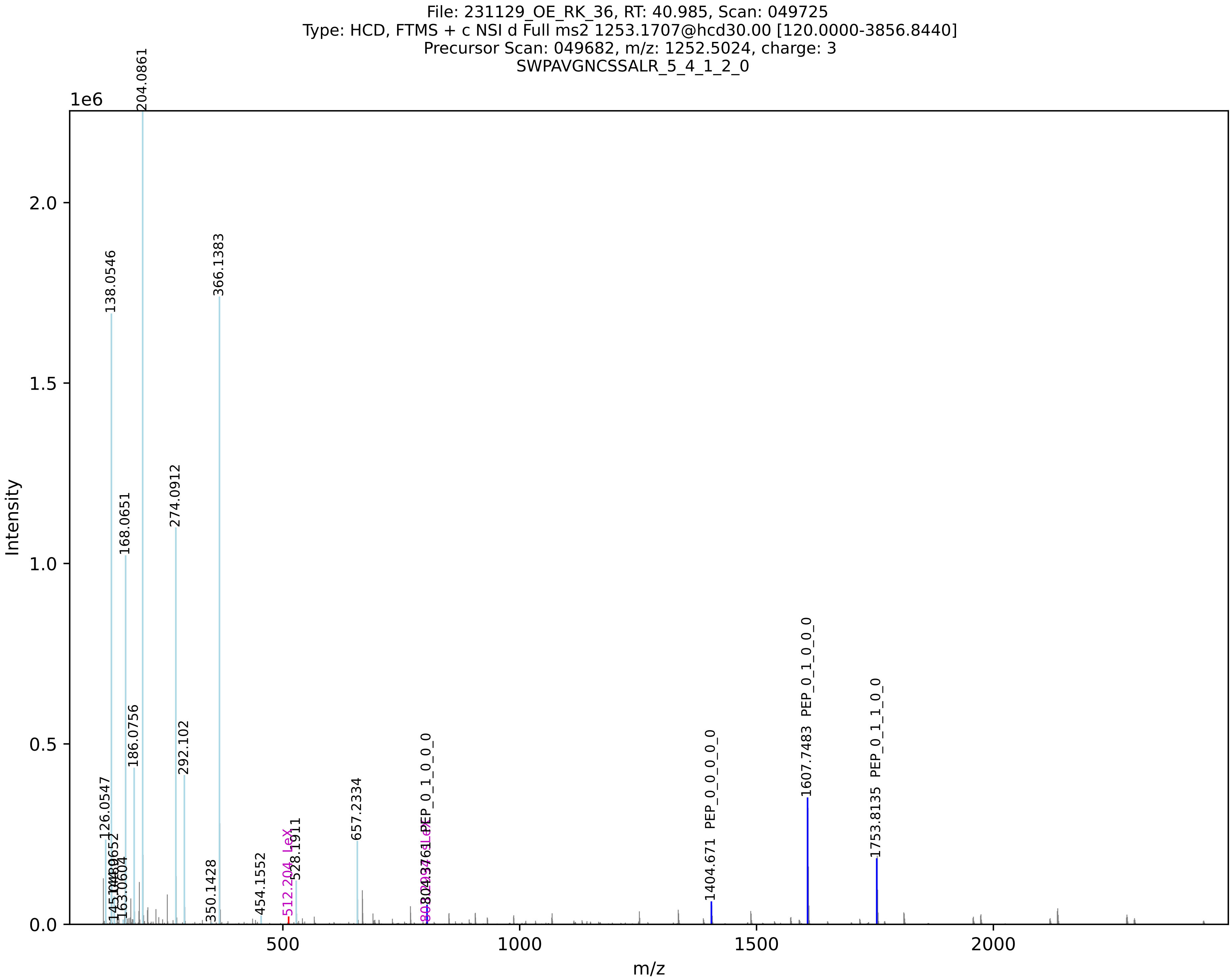
File: 231129_OE_RK_36, RT: 40.985, Scan: 049725
Type: HCD, FTMS + c NSI d Full ms2 1253.1707@hcd30.00 [120.0000-3856.8440]
Precursor Scan: 049682, m/z: 1252.5024, charge: 3
SWPAVGNCSSALR_5_4_1_2_0
1e6
Intensity
m/z
204.0861
138.0546
366.1383
274.0912
168.0651
186.0756
292.102
126.0547
163.0604
350.1428
454.1552
512.204_LeX
528.1911
657.2334
PEP_0_1_0_0_0
1404.671 PEP_0_0_0_0_0
1607.7483 PEP_0_1_0_0_0
1753.8135 PEP_0_1_1_0_0

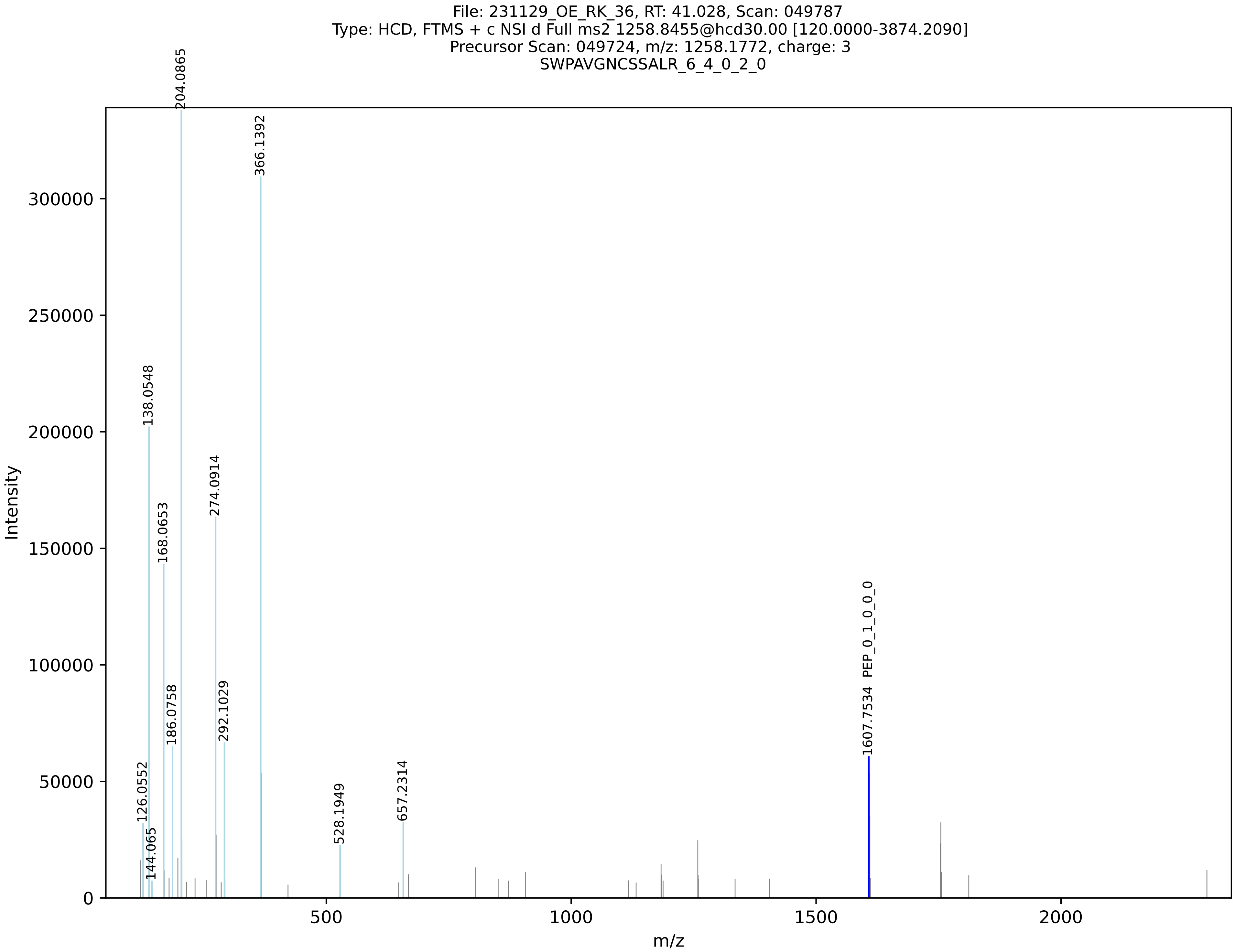
File: 231129_OE_RK_36, RT: 41.028, Scan: 049787
Type: HCD, FTMS + c NSI d Full ms2 1258.8455@hcd30.00 [120.0000-3874.2090]
Precursor Scan: 049724, m/z: 1258.1772, charge: 3
SWPAVGNCSSALR_6_4_0_2_0
Intensity
m/z
204.0865
366.1392
138.0548
274.0914
168.0653
186.0758
292.1029
126.0552
144.065
528.1949
657.2314
1607.7534 PEP_0_1_0_0_0
0
50000
100000
150000
200000
250000
300000
500
1000
1500
2000

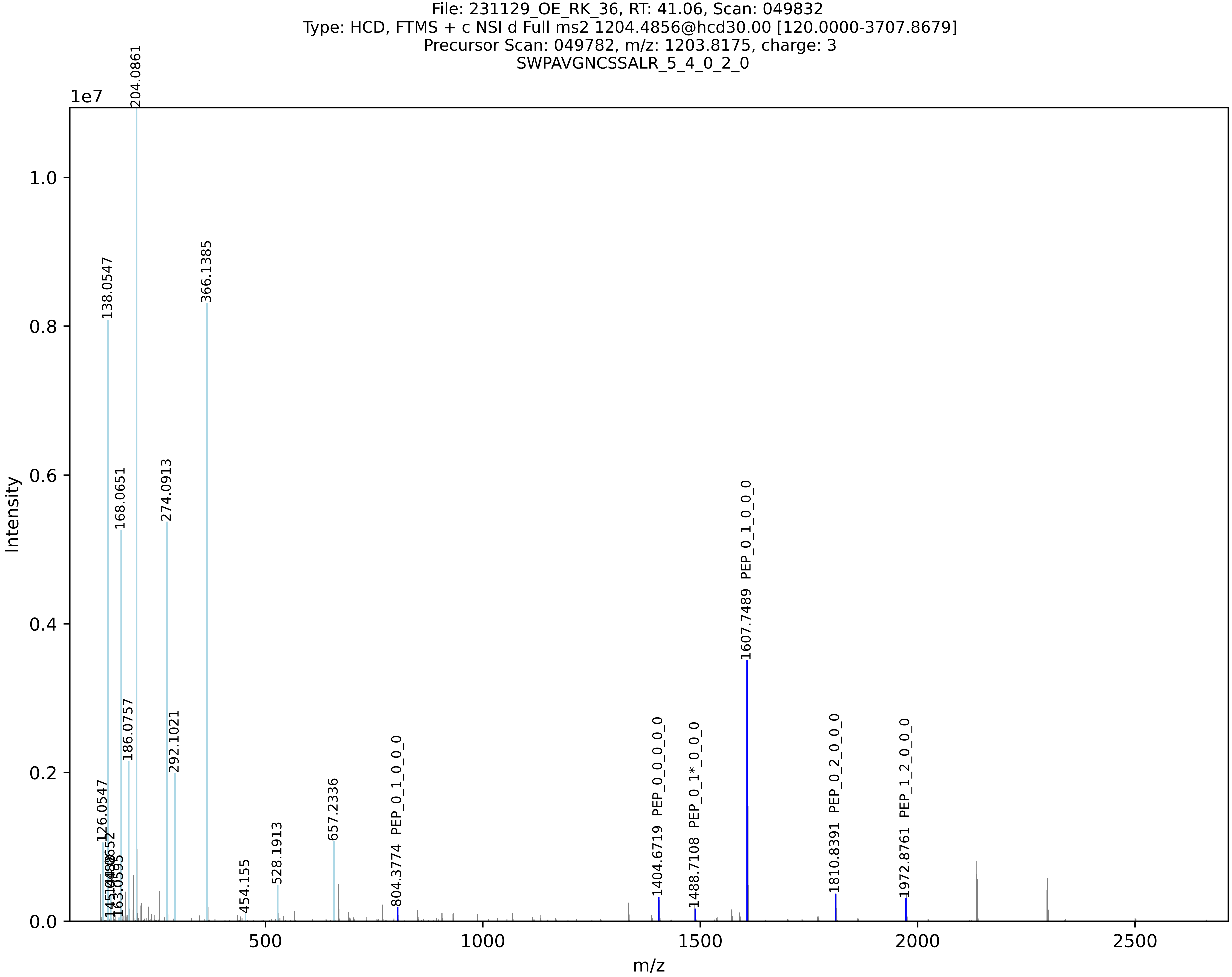
File: 231129_OE_RK_36, RT: 41.06, Scan: 049832
Type: HCD, FTMS + c NSI d Full ms2 1204.4856@hcd30.00 [120.0000-3707.8679]
Precursor Scan: 049782, m/z: 1203.8175, charge: 3
SWPAVGNCSSALR_5_4_0_2_0
1e7
Intensity
m/z
0.0
0.2
0.4
0.6
0.8
1.0
500
1000
1500
2000
2500
204.0861
138.0547
366.1385
168.0651
274.0913
186.0757
292.1021
126.0547
163.0595
454.155
528.1913
657.2336
804.3774 PEP_0_1_0_0_0
1404.6719 PEP_0_0_0_0_0
1488.7108 PEP_0_1*_0_0_0
1607.7489 PEP_0_1_0_0_0
1810.8391 PEP_0_2_0_0_0
1972.8761 PEP_1_2_0_0_0

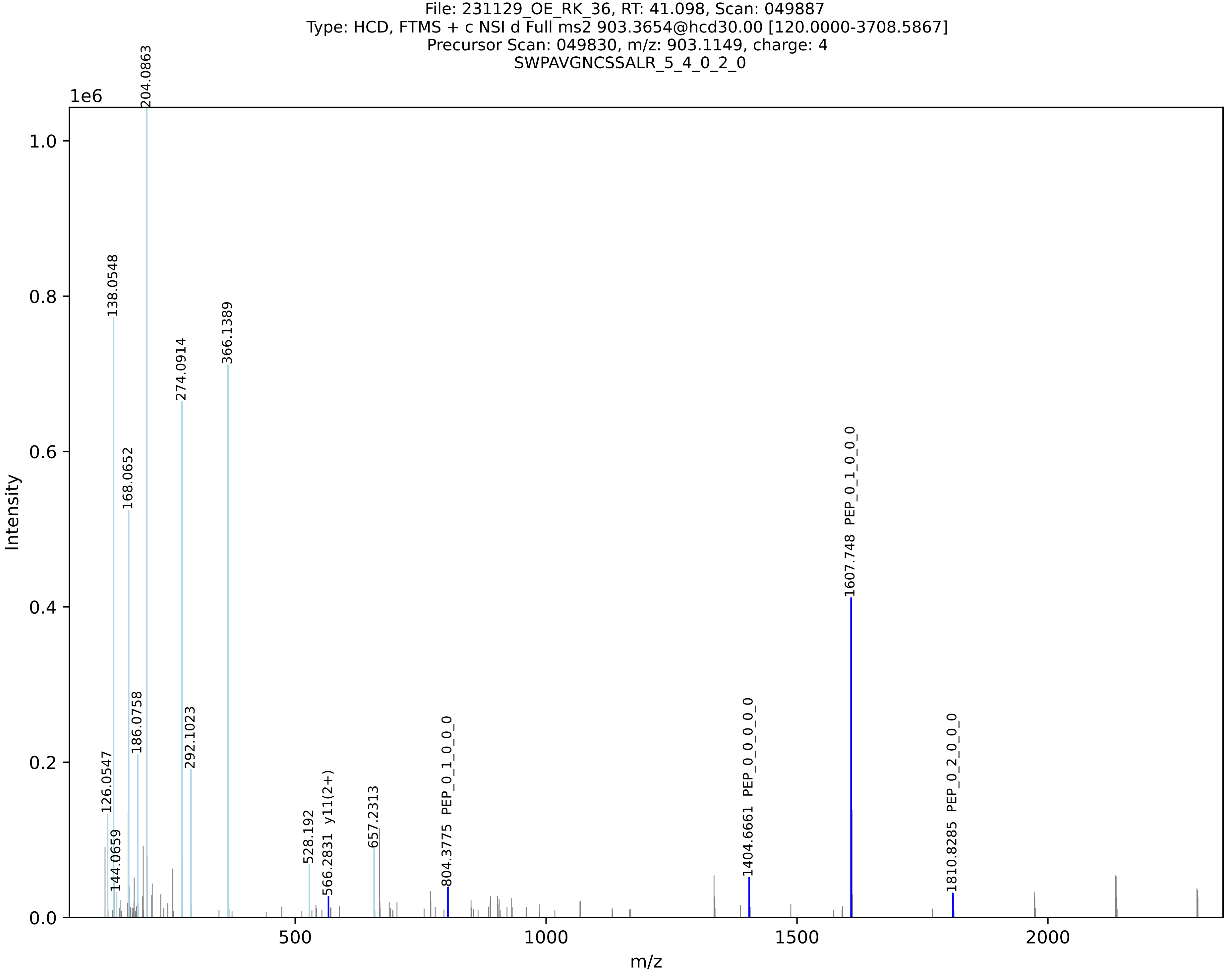
File: 231129_OE_RK_36, RT: 41.098, Scan: 049887
Type: HCD, FTMS + c NSI d Full ms2 903.3654@hcd30.00 [120.0000-3708.5867]
Precursor Scan: 049830, m/z: 903.1149, charge: 4
SWPAVGNCSSALR_5_4_0_2_0
1e6
Intensity
m/z
204.0863
138.0548
366.1389
274.0914
168.0652
186.0758
292.1023
126.0547
144.0659
528.192
566.2831 y11(2+)
657.2313
804.3775 PEP_0_1_0_0_0
1404.6661 PEP_0_0_0_0_0
1607.748 PEP_0_1_0_0_0
1810.8285 PEP_0_2_0_0_0

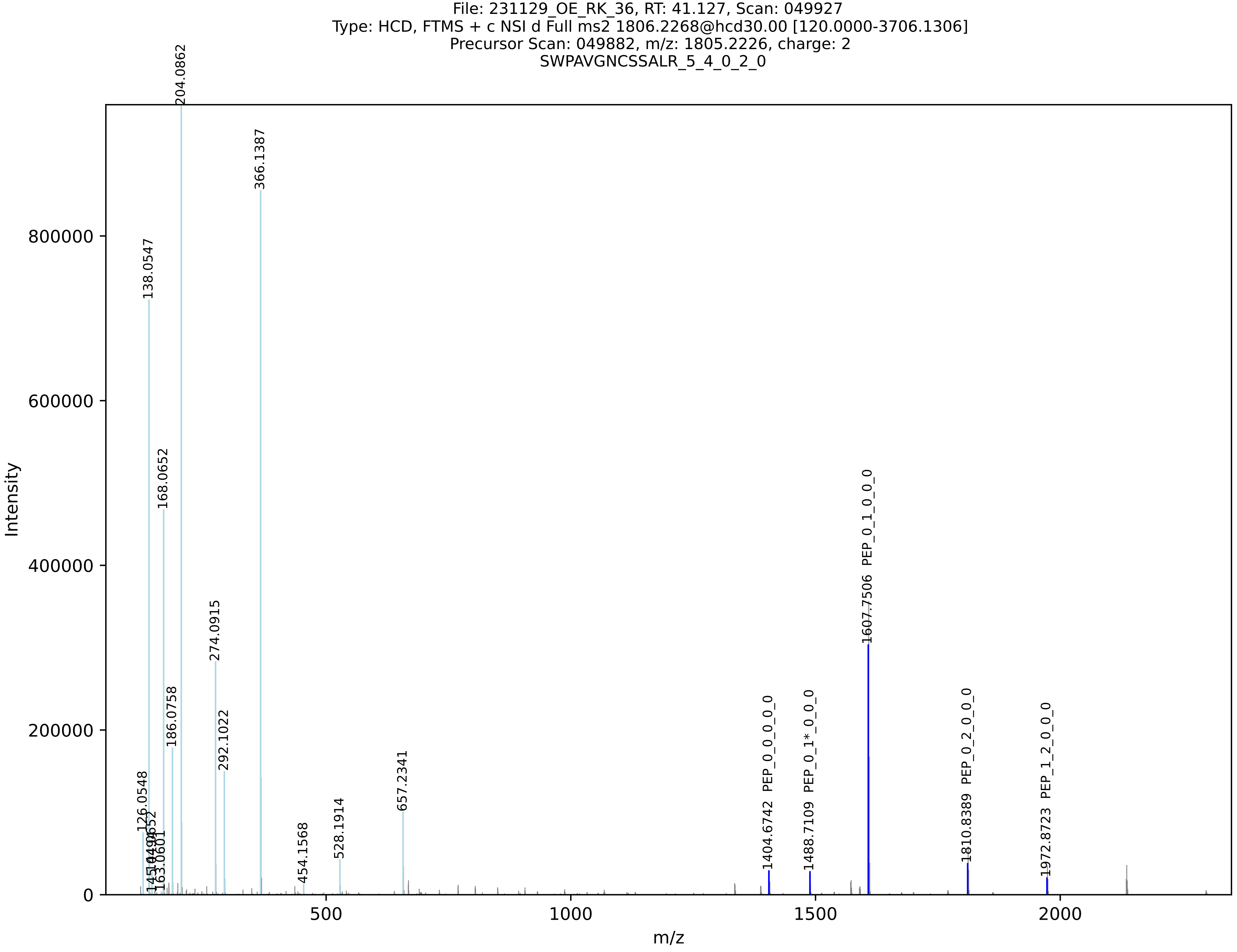
File: 231129_OE_RK_36, RT: 41.127, Scan: 049927
Type: HCD, FTMS + c NSI d Full ms2 1806.2268@hcd30.00 [120.0000-3706.1306]
Precursor Scan: 049882, m/z: 1805.2226, charge: 2
SWPAVGNCSSALR_5_4_0_2_0
Intensity
m/z
0
200000
400000
600000
800000
500
1000
1500
2000
204.0862
366.1387
138.0547
168.0652
274.0915
186.0758
292.1022
126.0548
163.0601
454.1568
528.1914
657.2341
1404.6742 PEP_0_0_0_0_0
1488.7109 PEP_0_1*_0_0_0
1607.7506 PEP_0_1_0_0_0
1810.8389 PEP_0_2_0_0_0
1972.8723 PEP_1_2_0_0_0

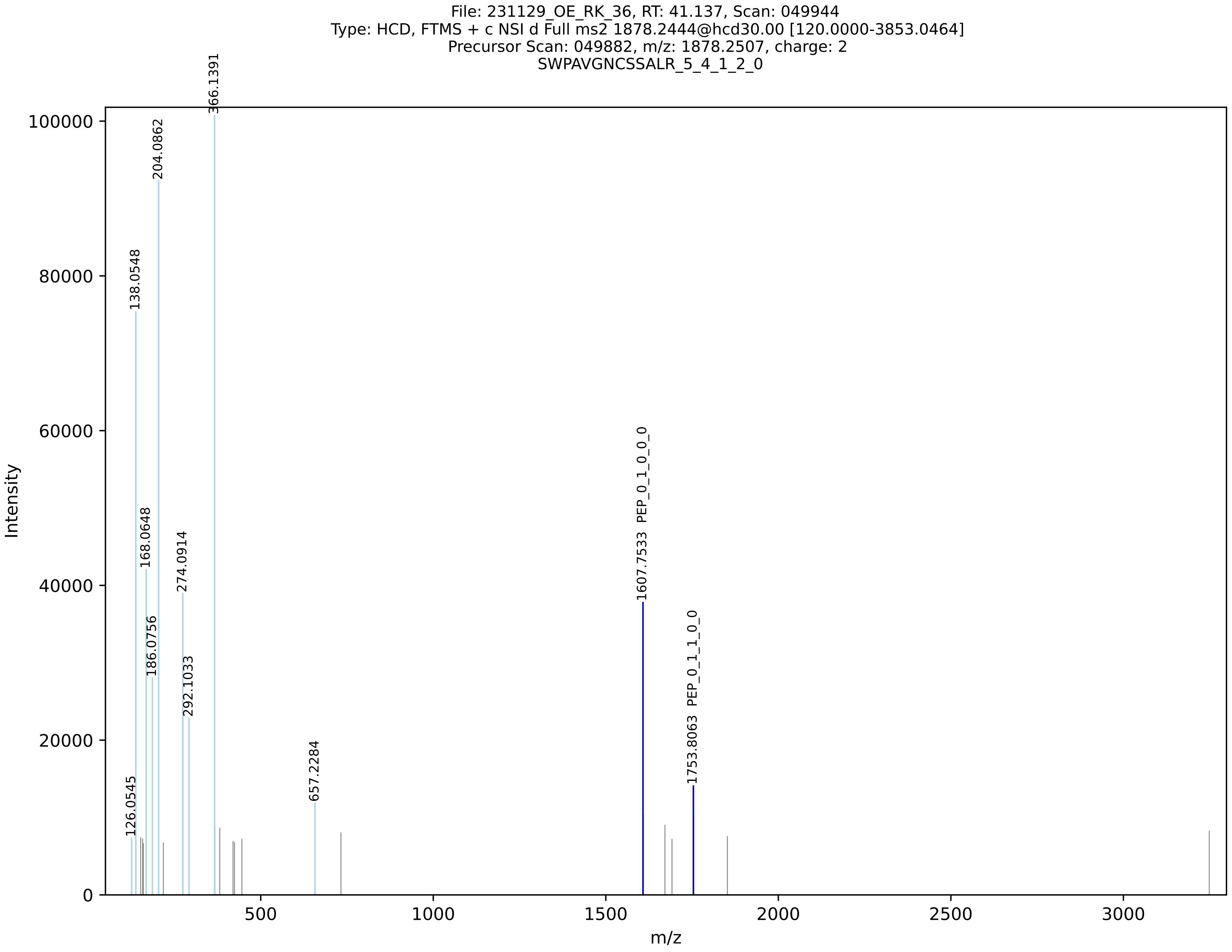

File: 231129_OE_RK_36, RT: 41.137, Scan: 049944
Type: HCD, FTMS + c NSI d Full ms2 1878.2444@hcd30.00 [120.0000-3853.0464]
Precursor Scan: 049882, m/z: 1878.2507, charge: 2
SWPAVGNCSSALR_5_4_1_2_0
366.1391
204.0862
138.0548
168.0648
274.0914
186.0756
292.1033
126.0545
657.2284
1607.7533 PEP_0_1_0_0_0
1753.8063 PEP_0_1_1_0_0
Intensity
m/z
0
20000
40000
60000
80000
100000
500
1000
1500
2000
2500
3000

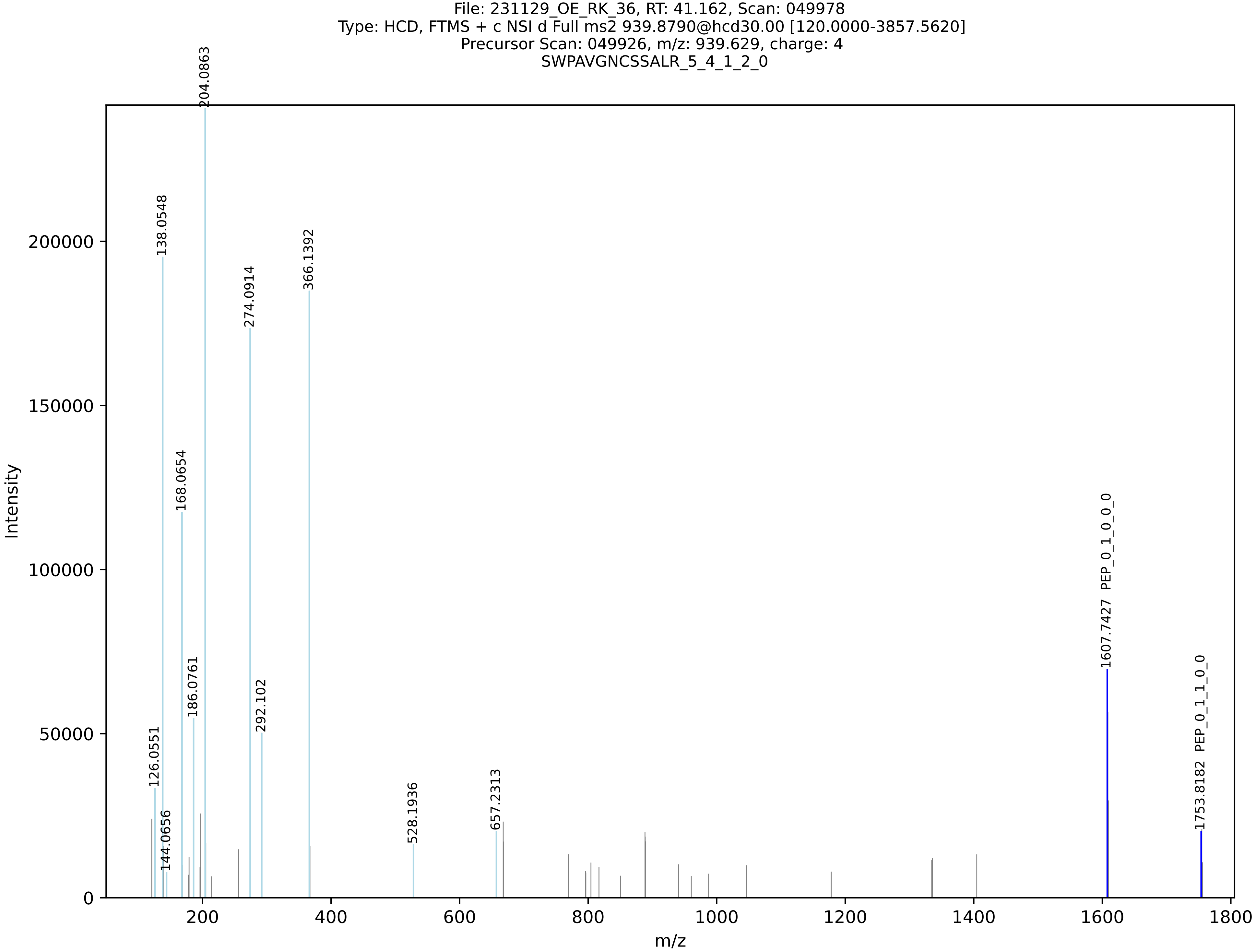
File: 231129_OE_RK_36, RT: 41.162, Scan: 049978
Type: HCD, FTMS + c NSI d Full ms2 939.8790@hcd30.00 [120.0000-3857.5620]
Precursor Scan: 049926, m/z: 939.629, charge: 4
SWPAVGNCSSALR_5_4_1_2_0
Intensity
m/z
204.0863
138.0548
366.1392
274.0914
168.0654
186.0761
292.102
126.0551
144.0656
528.1936
657.2313
1607.7427 PEP_0_1_0_0_0
1753.8182 PEP_0_1_1_0_0
0
50000
100000
150000
200000
200
400
600
800
1000
1200
1400
1600
1800

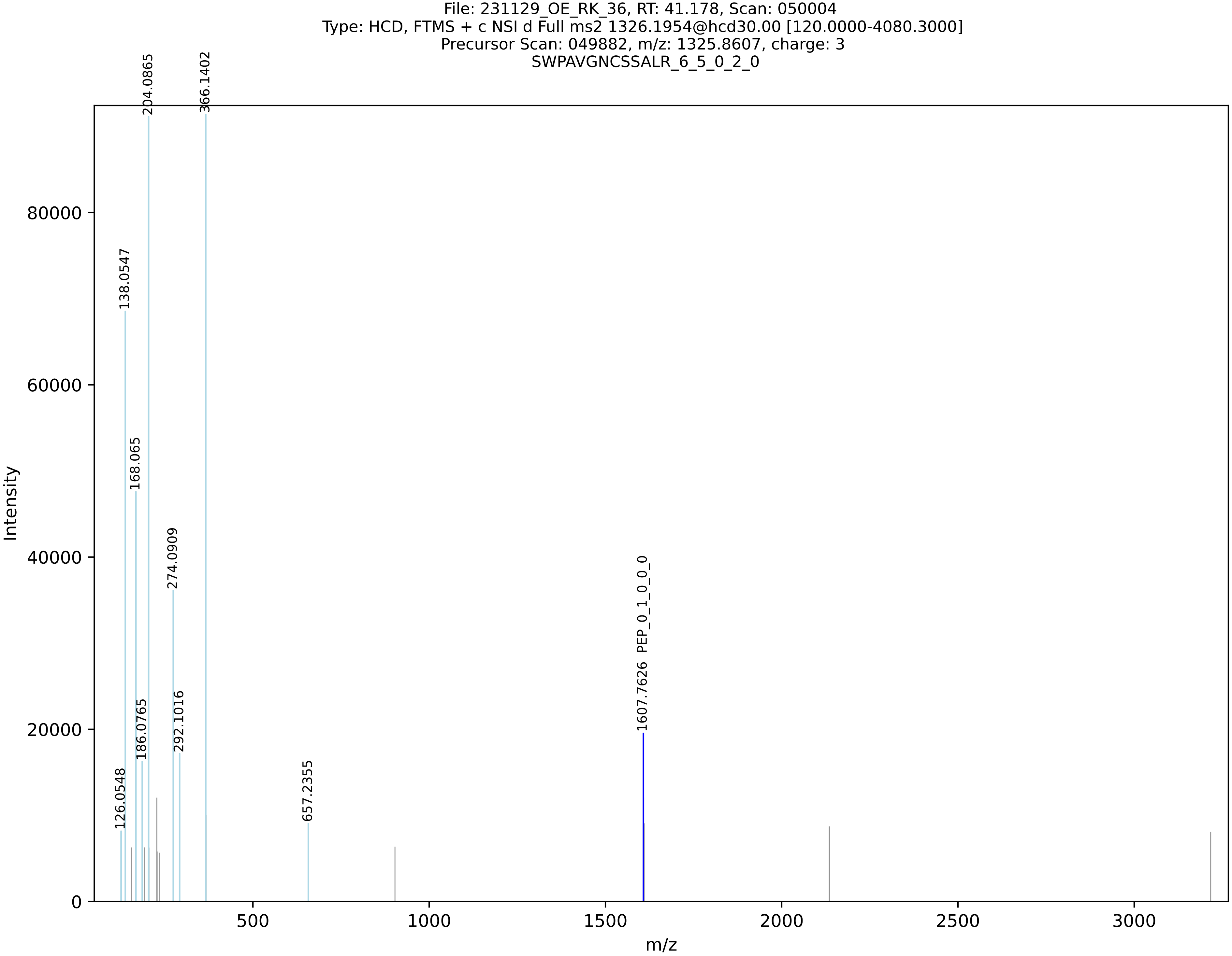
File: 231129_OE_RK_36, RT: 41.178, Scan: 050004
Type: HCD, FTMS + c NSI d Full ms2 1326.1954@hcd30.00 [120.0000-4080.3000]
Precursor Scan: 049882, m/z: 1325.8607, charge: 3
SWPAVGNCSSALR_6_5_0_2_0
Intensity
m/z
0
20000
40000
60000
80000
500
1000
1500
2000
2500
3000
126.0548
138.0547
168.065
186.0765
204.0865
274.0909
292.1016
366.1402
657.2355
1607.7626 PEP_0_1_0_0_0

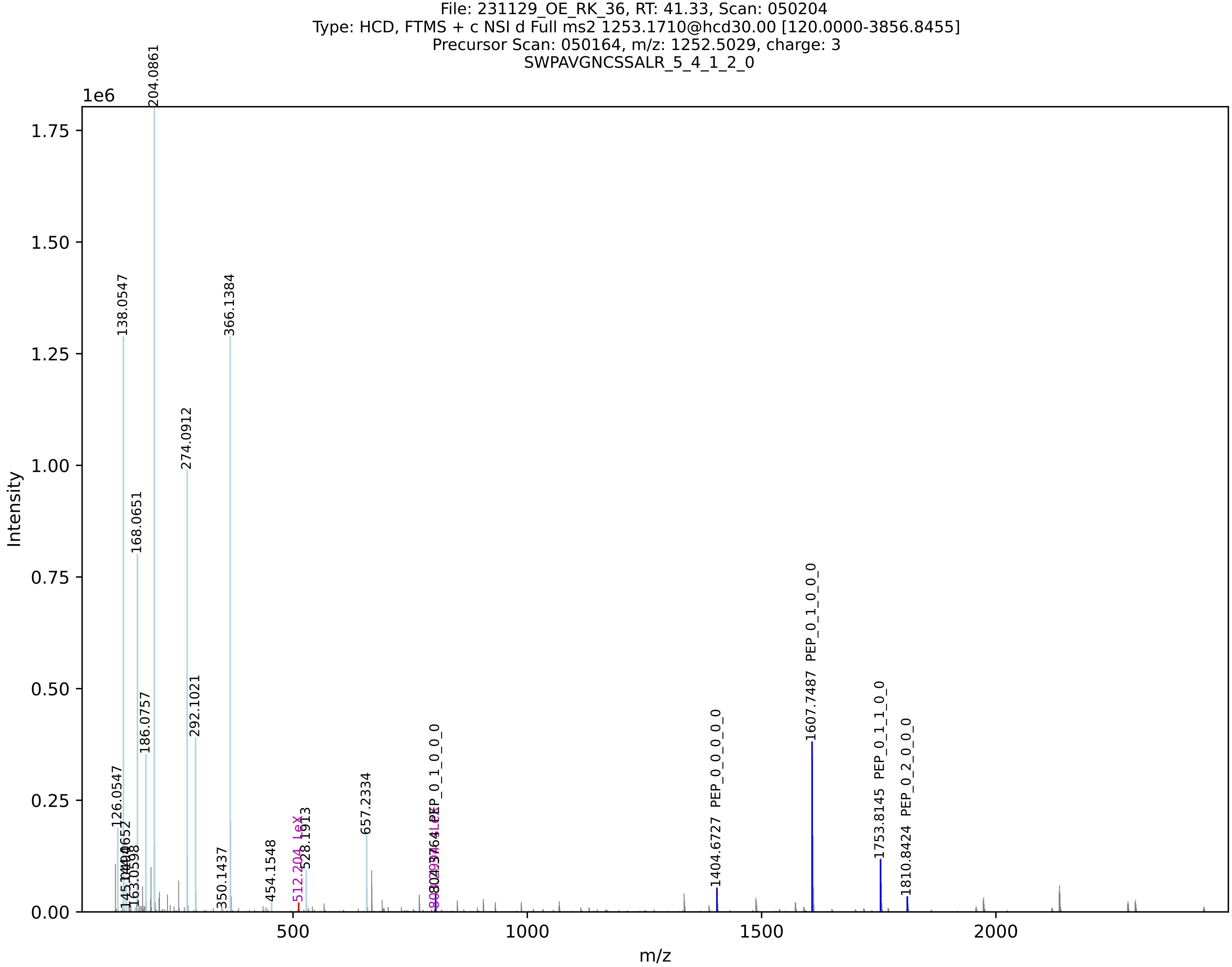

File: 231129_OE_RK_36, RT: 41.33, Scan: 050204
Type: HCD, FTMS + c NSI d Full ms2 1253.1710@hcd30.00 [120.0000-3856.8455]
Precursor Scan: 050164, m/z: 1252.5029, charge: 3
SWPAVGNCSSALR_5_4_1_2_0
1e6
Intensity
m/z
0.00
0.25
0.50
0.75
1.00
1.25
1.50
1.75
500
1000
1500
2000
204.0861
138.0547
366.1384
274.0912
168.0651
292.1021
186.0757
126.0547
163.0598
350.1437
454.1548
512.204_LeX
528.1913
657.2334
PEP_0_1_0_0_0
1404.6727 PEP_0_0_0_0_0
1607.7487 PEP_0_1_0_0_0
1753.8145 PEP_0_1_1_0_0
1810.8424 PEP_0_2_0_0_0

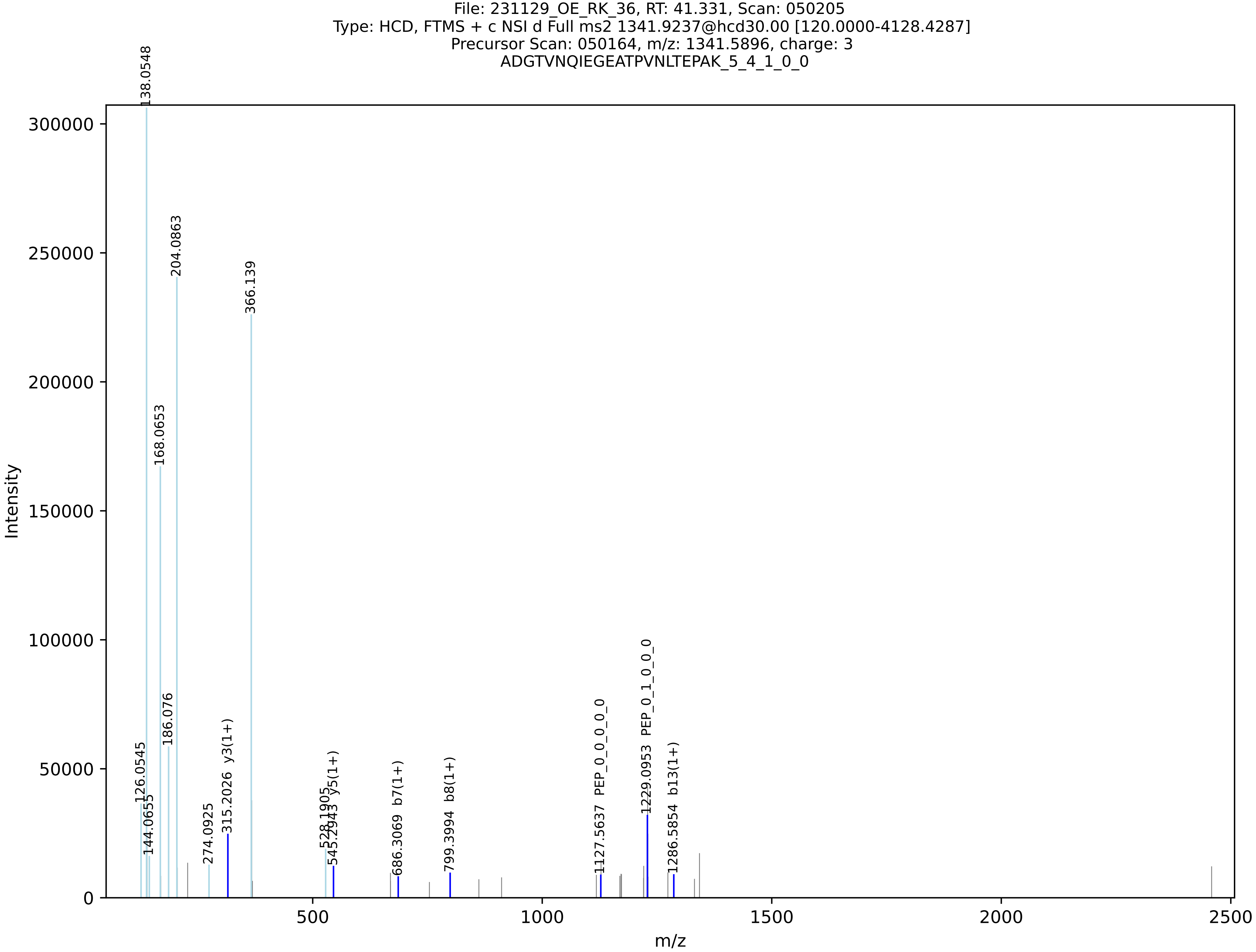
File: 231129_OE_RK_36, RT: 41.331, Scan: 050205
Type: HCD, FTMS + c NSI d Full ms2 1341.9237@hcd30.00 [120.0000-4128.4287]
Precursor Scan: 050164, m/z: 1341.5896, charge: 3
ADGTVNQIEGEATPVNLTEPAK_5_4_1_0_0
Intensity
m/z
0
50000
100000
150000
200000
250000
300000
500
1000
1500
2000
2500
138.0548
204.0863
366.139
168.0653
186.076
126.0545
144.0655
274.0925
315.2026 y3(1+)
528.1905
545.2943 y5(1+)
686.3069 b7(1+)
799.3994 b8(1+)
1127.5637 PEP_0_0_0_0_0
1229.0953 PEP_0_1_0_0_0
1286.5854 b13(1+)

File: 231129_OE_RK_36, RT: 41.484, Scan: 050424
Type: HCD, FTMS + c NSI d Full ms2 903.6168@hcd30.00 [120.0000-3709.6123]
Precursor Scan: 050357, m/z: 903.1157, charge: 4
SWPAVGNCSSALR_5_4_0_2_0

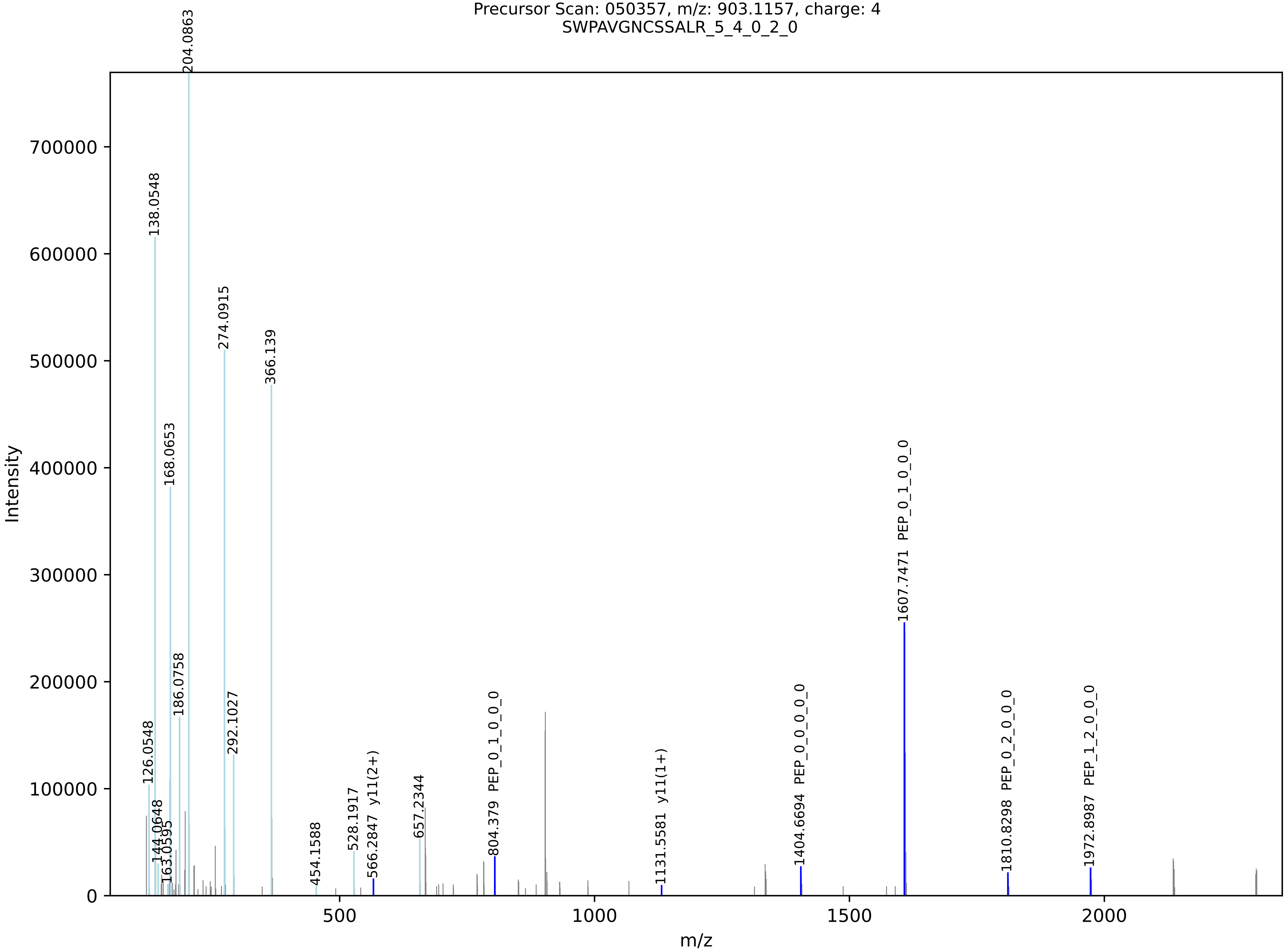

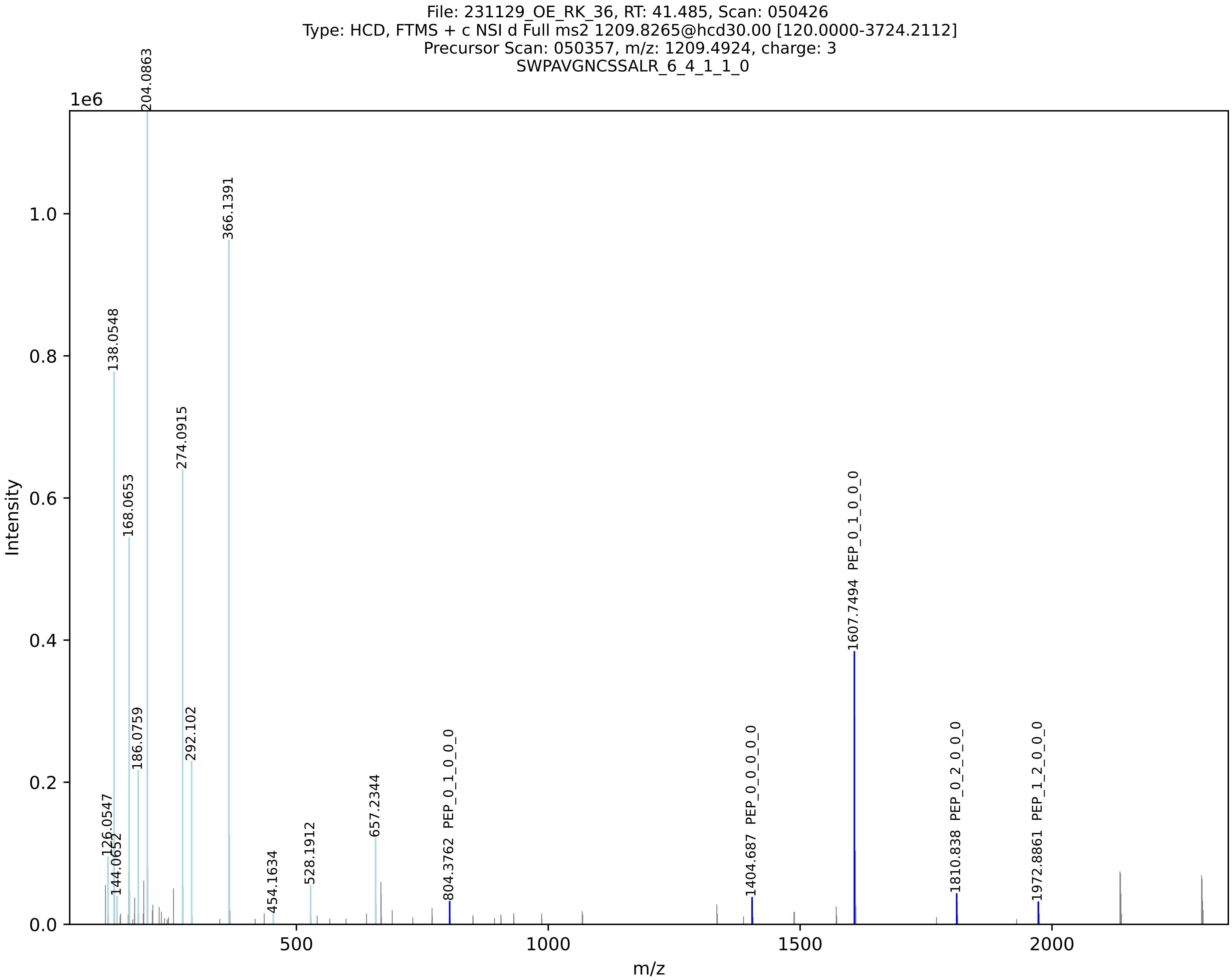

File: 231129_OE_RK_36, RT: 41.485, Scan: 050426
Type: HCD, FTMS + c NSI d Full ms2 1209.8265@hcd30.00 [120.0000-3724.2112]
Precursor Scan: 050357, m/z: 1209.4924, charge: 3
SWPAVGNCSSALR_6_4_1_1_0
1e6
Intensity
m/z
204.0863
366.1391
138.0548
274.0915
168.0653
186.0759
292.102
126.0547
144.0652
454.1634
528.1912
657.2344
804.3762 PEP_0_1_0_0_0
1404.687 PEP_0_0_0_0_0
1607.7494 PEP_0_1_0_0_0
1810.838 PEP_0_2_0_0_0
1972.8861 PEP_1_2_0_0_0

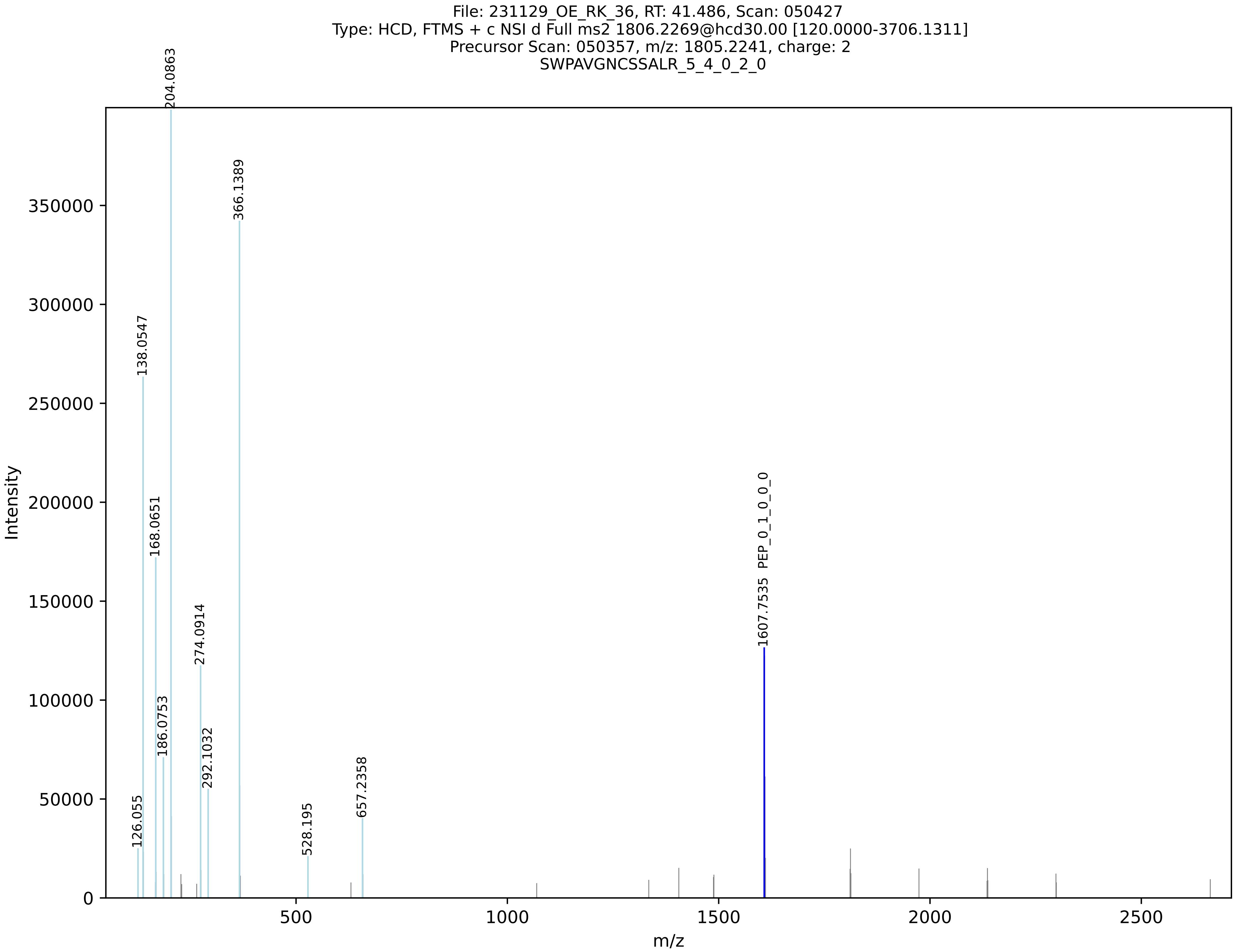
File: 231129_OE_RK_36, RT: 41.486, Scan: 050427
Type: HCD, FTMS + c NSI d Full ms2 1806.2269@hcd30.00 [120.0000-3706.1311]
Precursor Scan: 050357, m/z: 1805.2241, charge: 2
SWPAVGNCSSALR_5_4_0_2_0
204.0863
366.1389
138.0547
168.0651
274.0914
186.0753
292.1032
126.055
528.195
657.2358
1607.7535 PEP_0_1_0_0_0
Intensity
m/z
0
50000
100000
150000
200000
250000
300000
350000
500
1000
1500
2000
2500

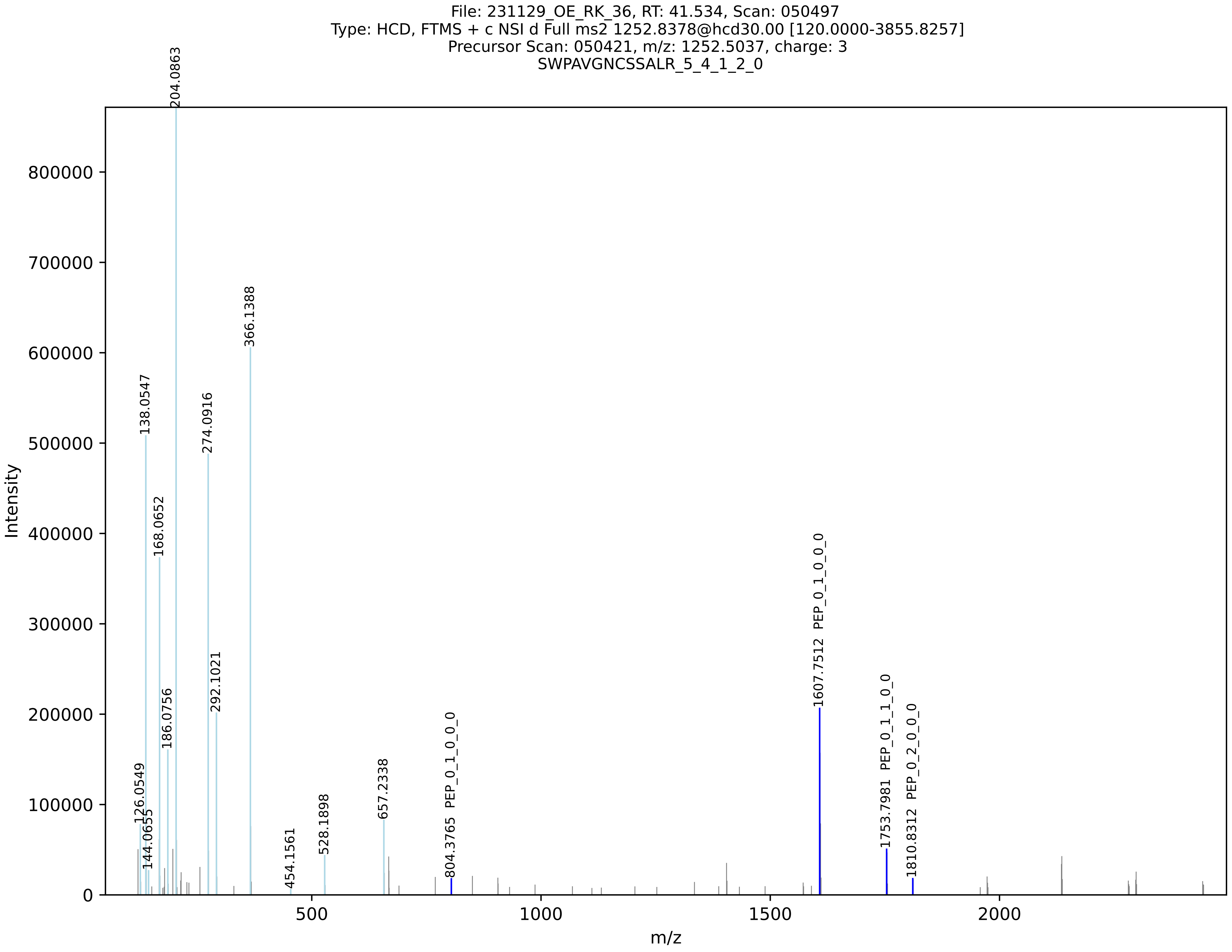
File: 231129_OE_RK_36, RT: 41.534, Scan: 050497
Type: HCD, FTMS + c NSI d Full ms2 1252.8378@hcd30.00 [120.0000-3855.8257]
Precursor Scan: 050421, m/z: 1252.5037, charge: 3
SWPAVGNCSSALR_5_4_1_2_0
Intensity
m/z
204.0863
366.1388
138.0547
274.0916
168.0652
292.1021
186.0756
126.0549
144.0655
454.1561
528.1898
657.2338
804.3765 PEP_0_1_0_0_0
1607.7512 PEP_0_1_0_0_0
1753.7981 PEP_0_1_1_0_0
1810.8312 PEP_0_2_0_0_0
0
100000
200000
300000
400000
500000
600000
700000
800000
500
1000
1500
2000

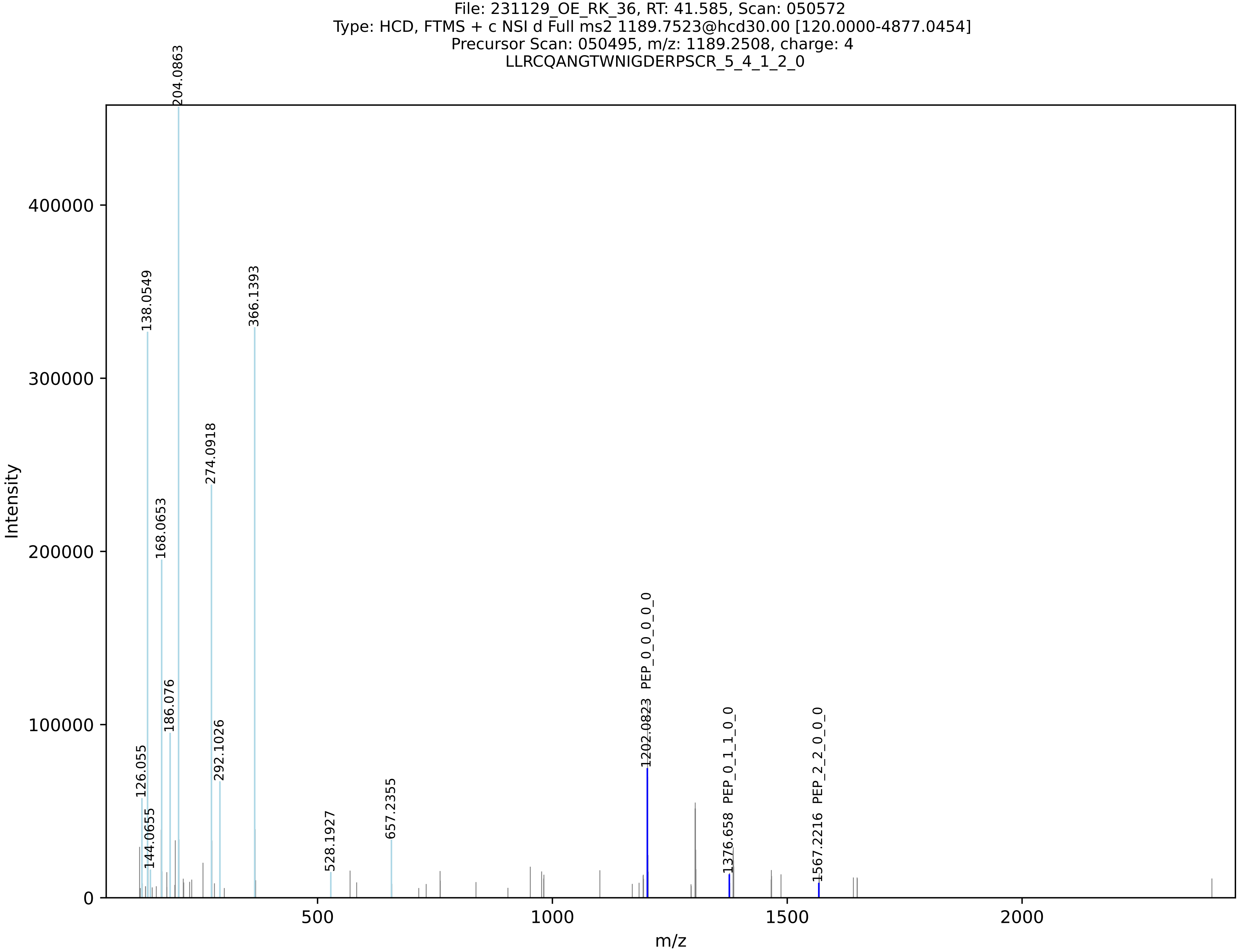

File: 231129_OE_RK_36, RT: 41.585, Scan: 050572
Type: HCD, FTMS + c NSI d Full ms2 1189.7523@hcd30.00 [120.0000-4877.0454]
Precursor Scan: 050495, m/z: 1189.2508, charge: 4
LLRCQANGTWNIGDERPSCR_5_4_1_2_0
Intensity
m/z
204.0863
138.0549
366.1393
274.0918
168.0653
186.076
126.055
292.1026
144.0655
528.1927
657.2355
1202.0823 PEP_0_0_0_0_0
1376.658 PEP_0_1_1_0_0
1567.2216 PEP_2_2_0_0_0

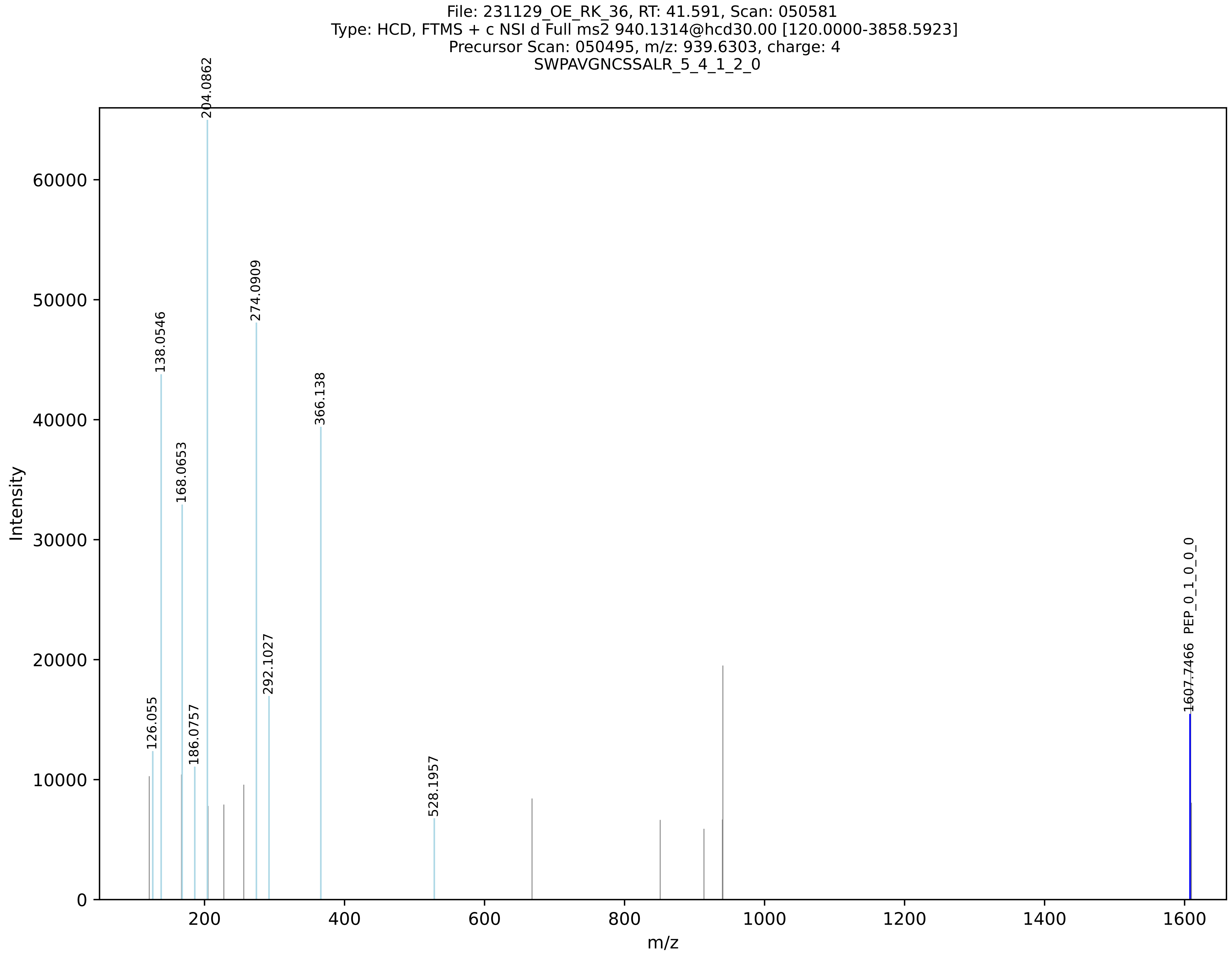
File: 231129_OE_RK_36, RT: 41.591, Scan: 050581
Type: HCD, FTMS + c NSI d Full ms2 940.1314@hcd30.00 [120.0000-3858.5923]
Precursor Scan: 050495, m/z: 939.6303, charge: 4
SWPAVGNCSSALR_5_4_1_2_0
Intensity
m/z
204.0862
274.0909
138.0546
366.138
168.0653
292.1027
126.055
186.0757
528.1957
1607.7466 PEP_0_1_0_0_
0
10000
20000
30000
40000
50000
60000
200
400
600
800
1000
1200
1400
1600

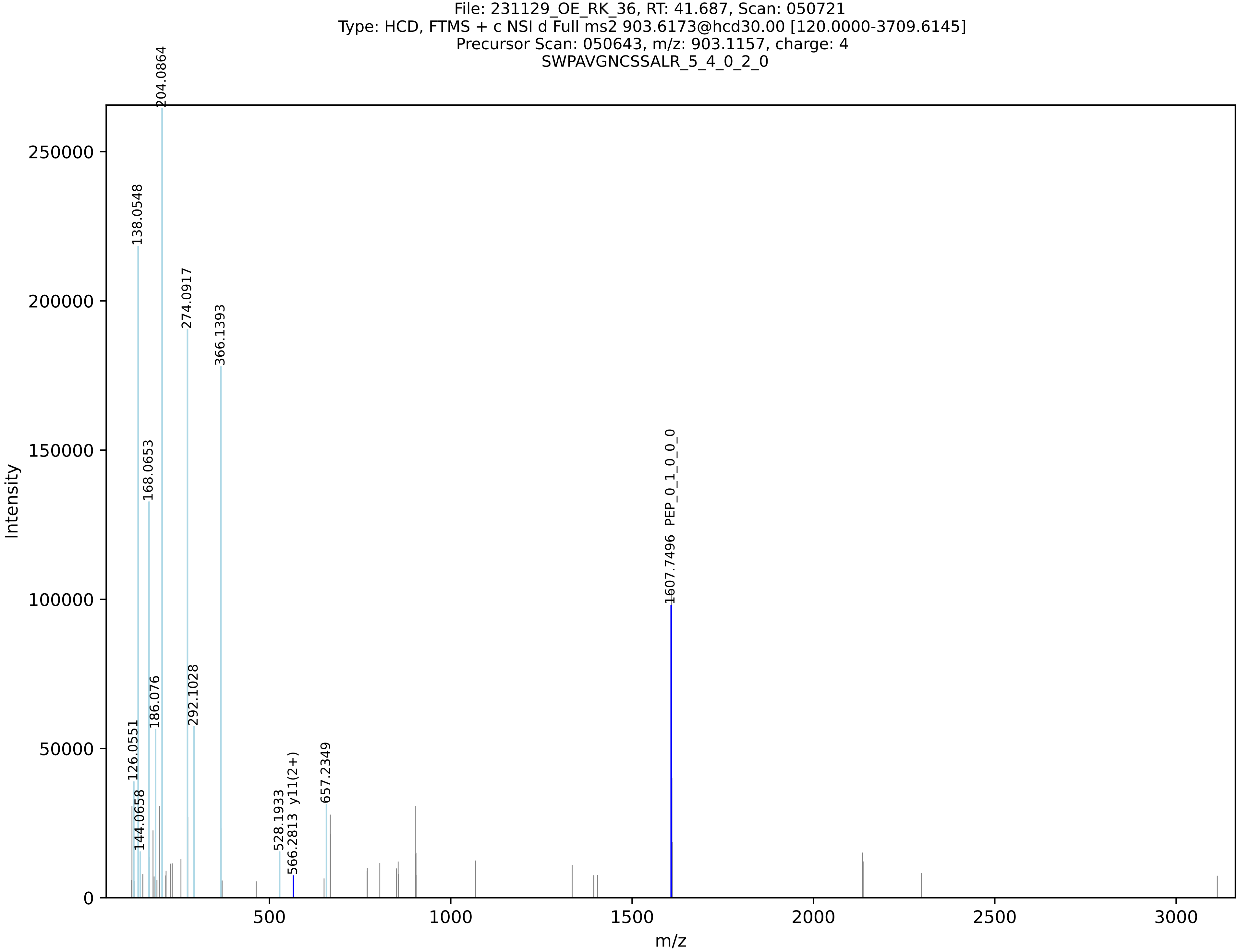

File: 231129_OE_RK_36, RT: 41.687, Scan: 050721
Type: HCD, FTMS + c NSI d Full ms2 903.6173@hcd30.00 [120.0000-3709.6145]
Precursor Scan: 050643, m/z: 903.1157, charge: 4
SWPAVGNCSSALR_5_4_0_2_0
Intensity
m/z
204.0864
138.0548
274.0917
366.1393
168.0653
186.076
292.1028
126.0551
144.0658
528.1933
566.2813 y11(2+)
657.2349
1607.7496 PEP_0_1_0_0_0
0
50000
100000
150000
200000
250000
500
1000
1500
2000
2500
3000

File: 231129_OE_RK_36, RT: 41.735, Scan: 050790
Type: HCD, FTMS + c NSI d Full ms2 1253.1722@hcd30.00 [120.0000-3856.8491]
Precursor Scan: 050714, m/z: 1252.504, charge: 3
SWPAVGNCSSALR_5_4_1_2_0

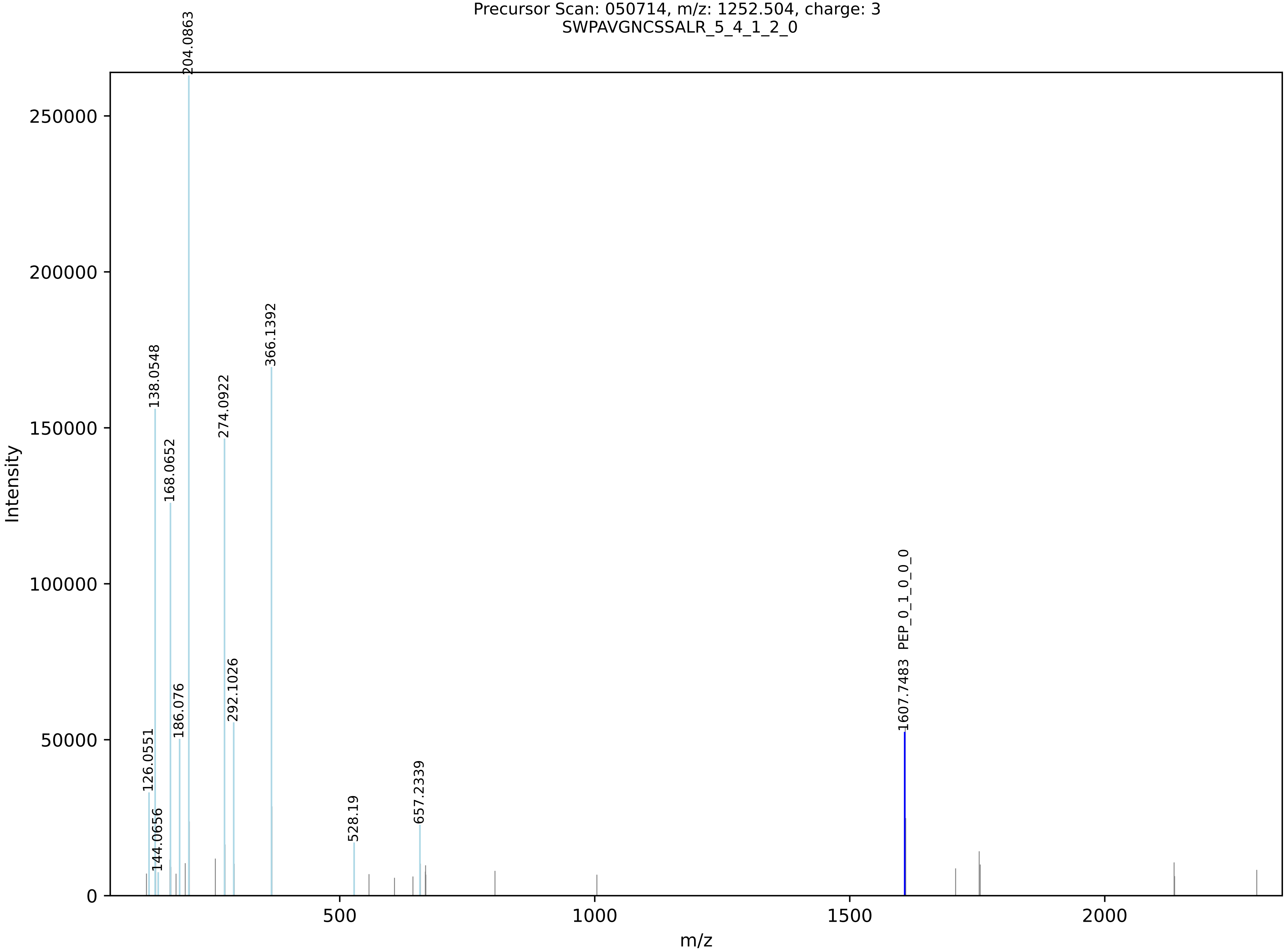

File: 231129_OE_RK_36, RT: 41.769, Scan: 050844
Type: HCD, FTMS + c NSI d Full ms2 1299.5813@hcd30.00 [120.0000-3998.8608]
Precursor Scan: 050643, m/z: 1298.5842, charge: 3
EVNETYKLLFNFVEQNMK_3_5_1_0_0

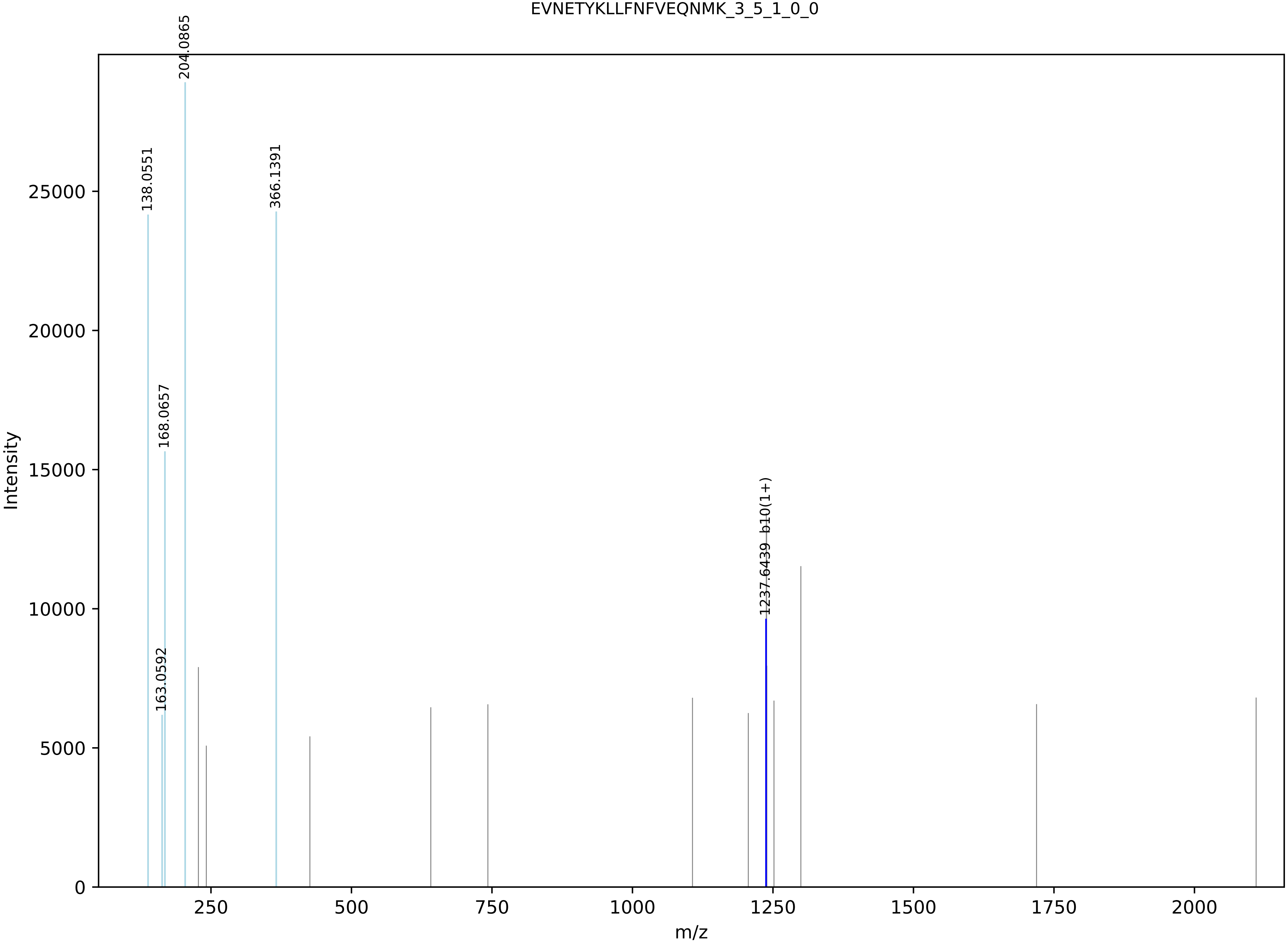

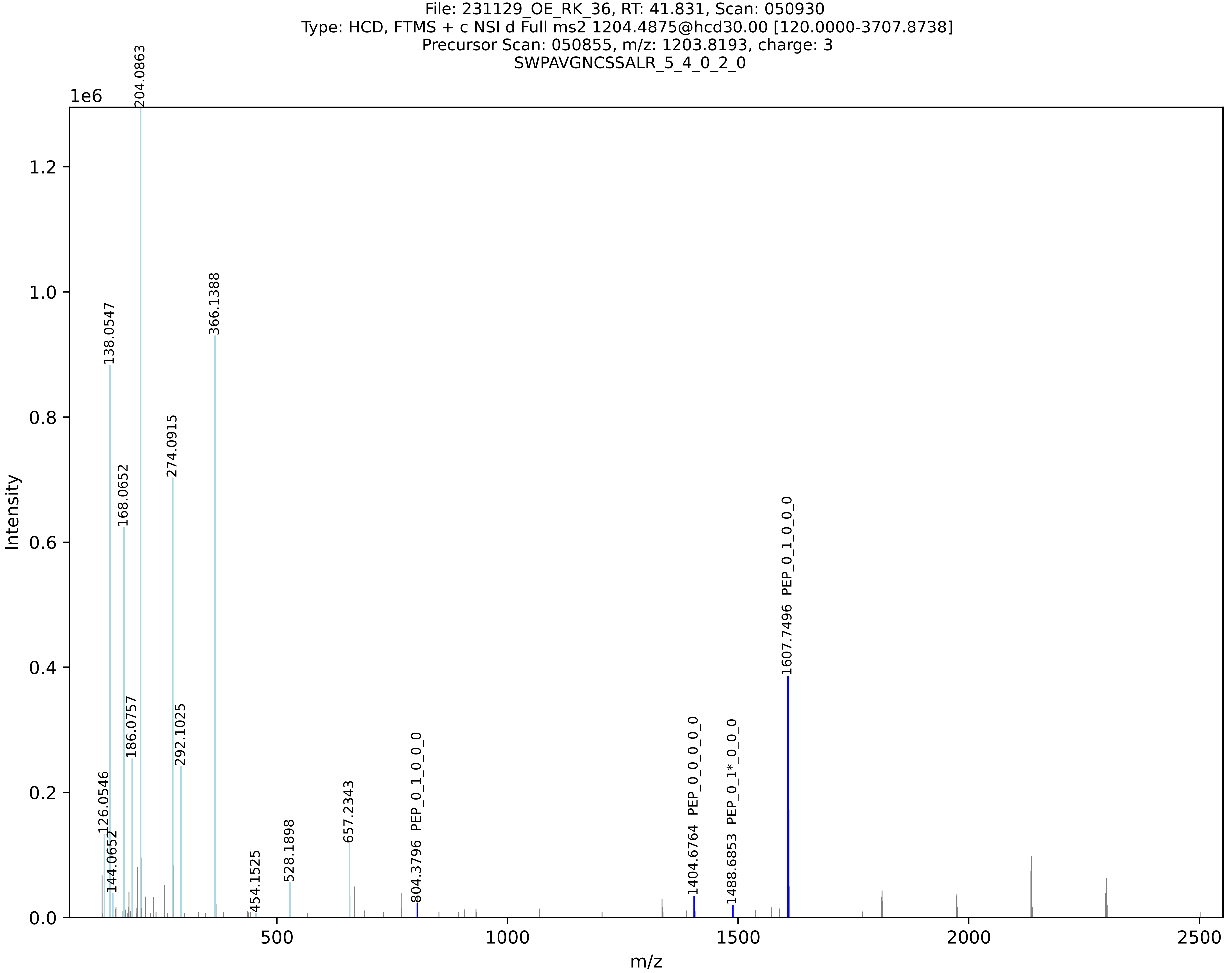

File: 231129_OE_RK_36, RT: 41.831, Scan: 050930
Type: HCD, FTMS + c NSI d Full ms2 1204.4875@hcd30.00 [120.0000-3707.8738]
Precursor Scan: 050855, m/z: 1203.8193, charge: 3
SWPAVGNCSSALR_5_4_0_2_0
1e6
Intensity
m/z
204.0863
138.0547
366.1388
274.0915
168.0652
186.0757
292.1025
126.0546
144.0652
454.1525
528.1898
657.2343
804.3796 PEP_0_1_0_0_0
1404.6764 PEP_0_0_0_0_0
1488.6853 PEP_0_1*_0_0_0
1607.7496 PEP_0_1_0_0_0

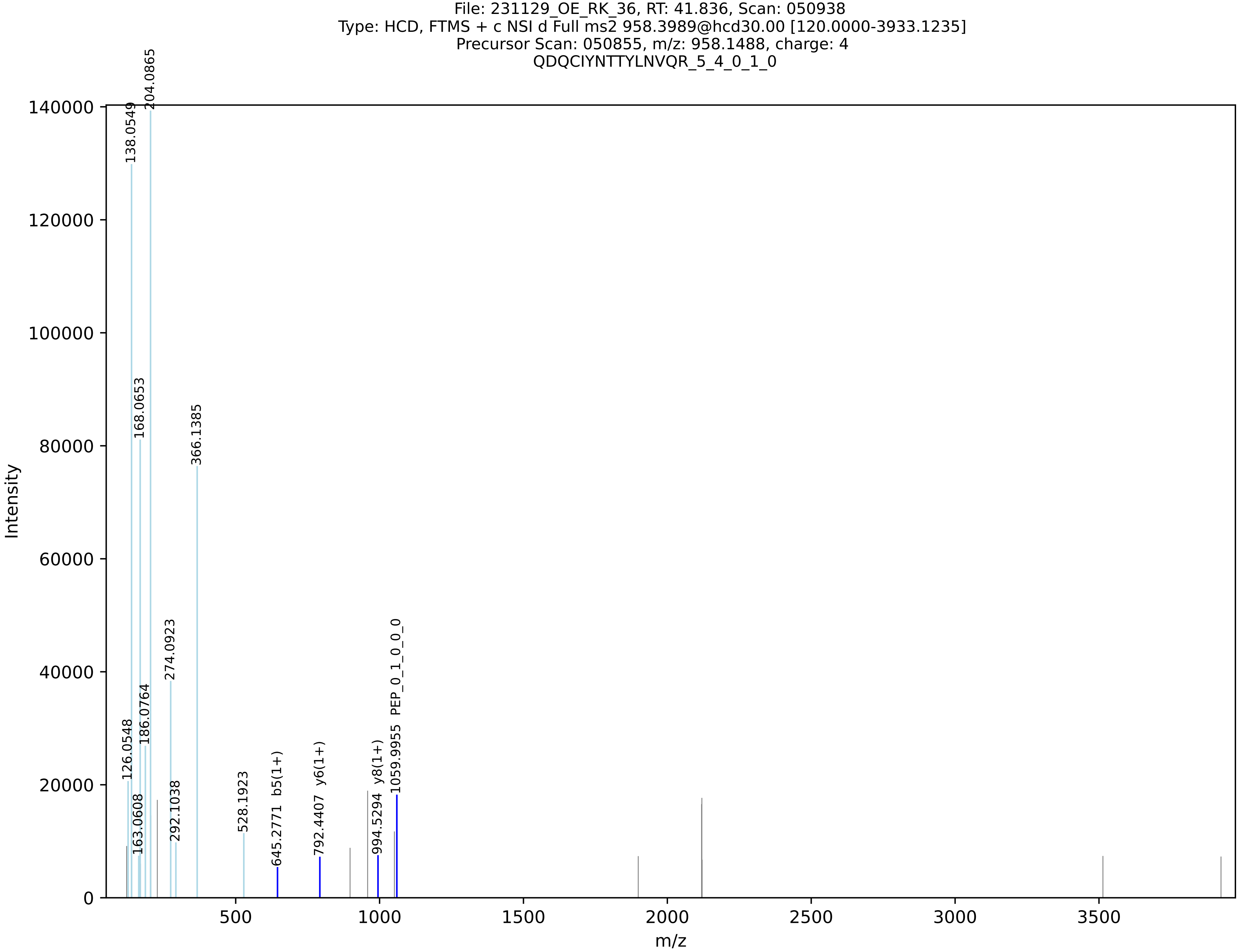

File: 231129_OE_RK_36, RT: 41.836, Scan: 050938
Type: HCD, FTMS + c NSI d Full ms2 958.3989@hcd30.00 [120.0000-3933.1235]
Precursor Scan: 050855, m/z: 958.1488, charge: 4
QDQCIYNTTYLNVQR_5_4_0_1_0
Intensity
m/z
204.0865
138.0549
168.0653
366.1385
274.0923
186.0764
126.0548
163.0608
292.1038
528.1923
645.2771 b5(1+)
792.4407 y6(1+)
994.5294 y8(1+)
1059.9955 PEP_0_1_0_0_0

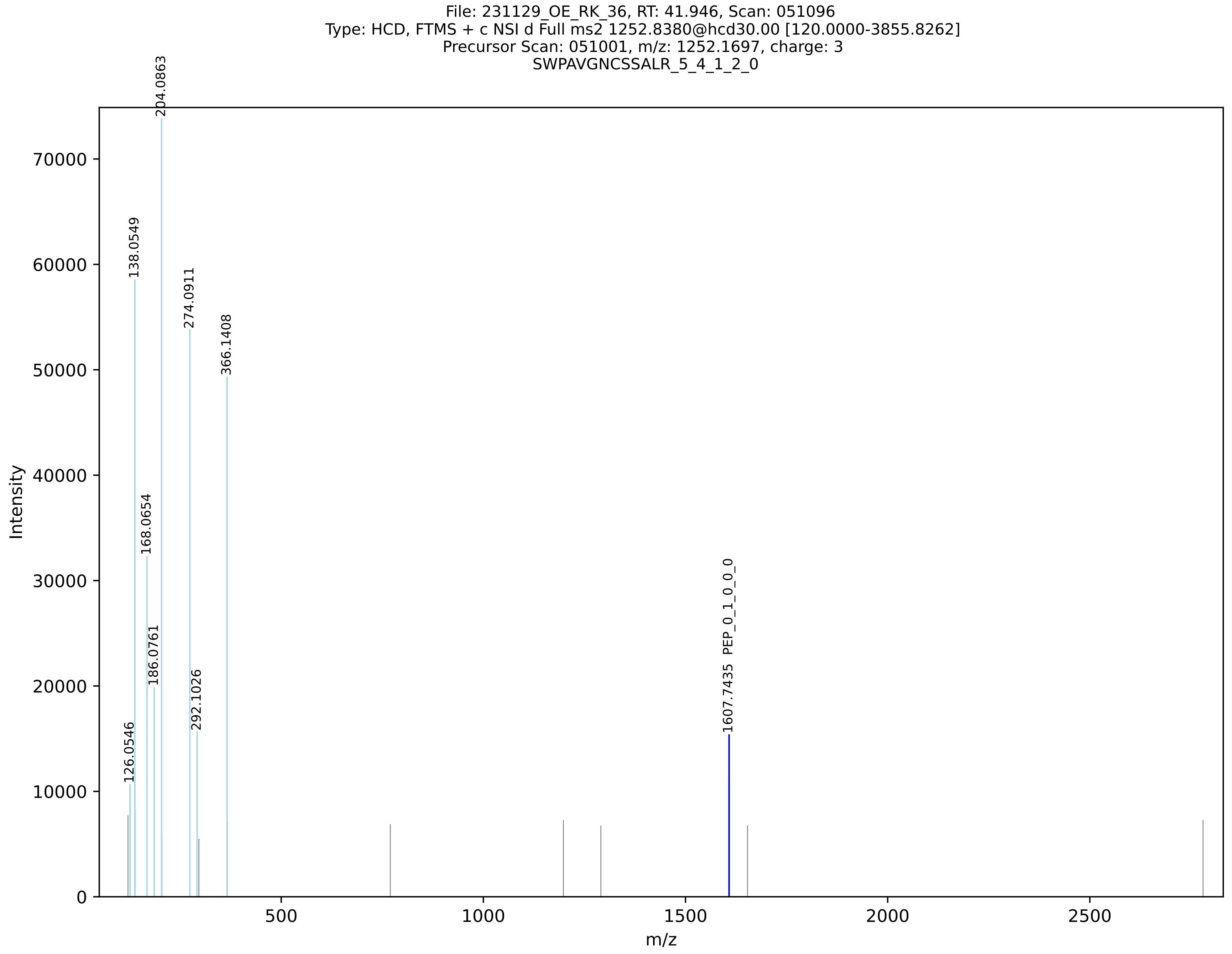
File: 231129_OE_RK_36, RT: 41.946, Scan: 051096
Type: HCD, FTMS + c NSI d Full ms2 1252.8380@hcd30.00 [120.0000-3855.8262]
Precursor Scan: 051001, m/z: 1252.1697, charge: 3
SWPAVGNCSSALR_5_4_1_2_0
Intensity
m/z
204.0863
138.0549
274.0911
366.1408
168.0654
186.0761
292.1026
126.0546
1607.7435 PEP_0_1_0_0_0
0
10000
20000
30000
40000
50000
60000
70000
500
1000
1500
2000
2500

File: 231129_OE_RK_36, RT: 42.007, Scan: 051186
Type: HCD, FTMS + c NSI d Full ms2 1104.1903@hcd30.00 [120.0000-4527.9526]
Precursor Scan: 051075, m/z: 1103.6879, charge: 4
SSPSELSLNDSLR_6_5_1_3_0

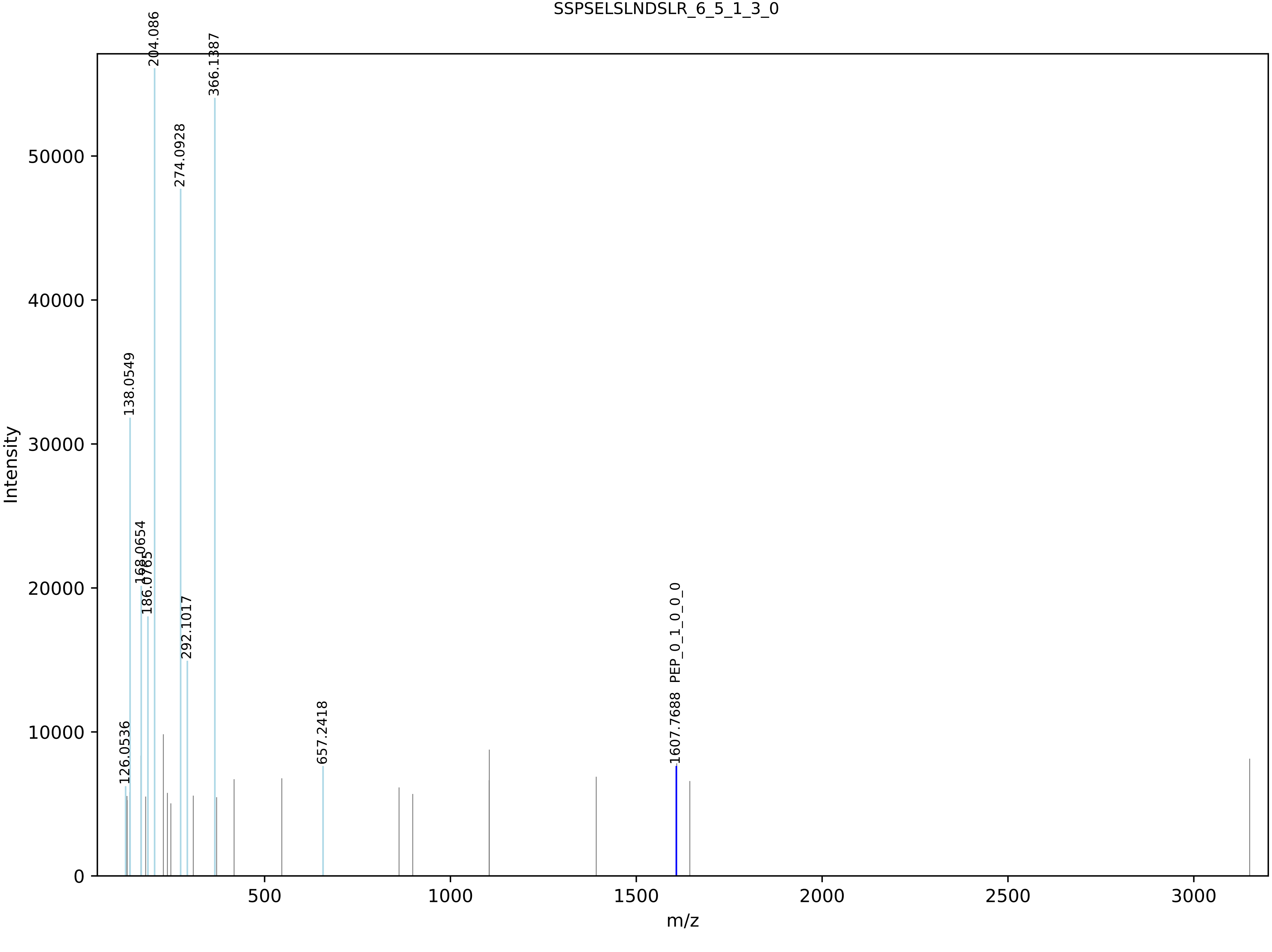

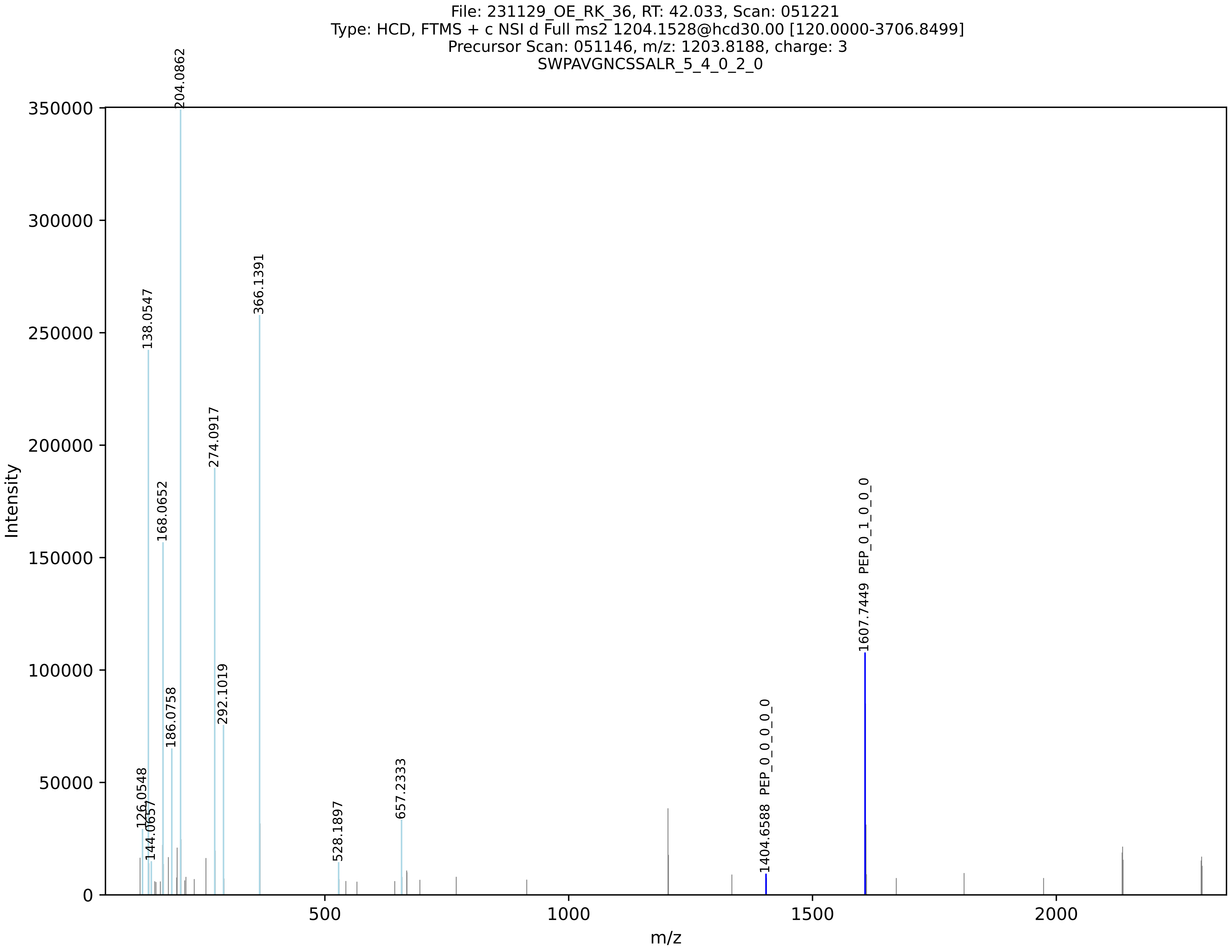
File: 231129_OE_RK_36, RT: 42.033, Scan: 051221
Type: HCD, FTMS + c NSI d Full ms2 1204.1528@hcd30.00 [120.0000-3706.8499]
Precursor Scan: 051146, m/z: 1203.8188, charge: 3
SWPAVGNCSSALR_5_4_0_2_0
Intensity
m/z
204.0862
138.0547
366.1391
274.0917
168.0652
186.0758
292.1019
126.0548
144.0657
528.1897
657.2333
1404.6588 PEP_0_0_0_0_0
1607.7449 PEP_0_1_0_0_0
350000
300000
250000
200000
150000
100000
50000
0
500
1000
1500
2000

File: 231129_OE_RK_36, RT: 42.293, Scan: 051599
Type: HCD, FTMS + c NSI d Full ms2 1423.2290@hcd30.00 [120.0000-4377.2227]
Precursor Scan: 051514, m/z: 1422.5607, charge: 3
SWPAVGNCSSALR_6_5_0_3_0

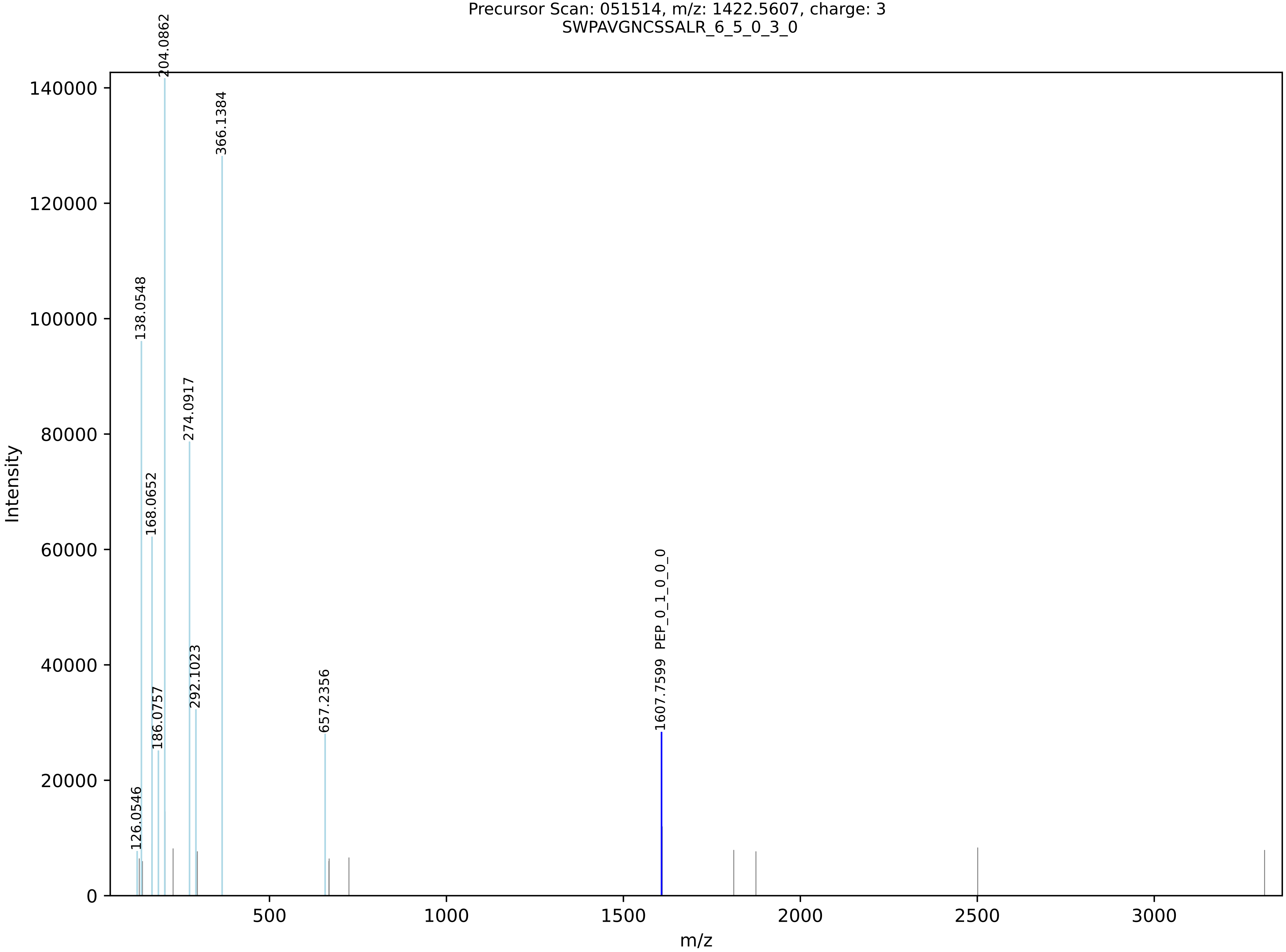

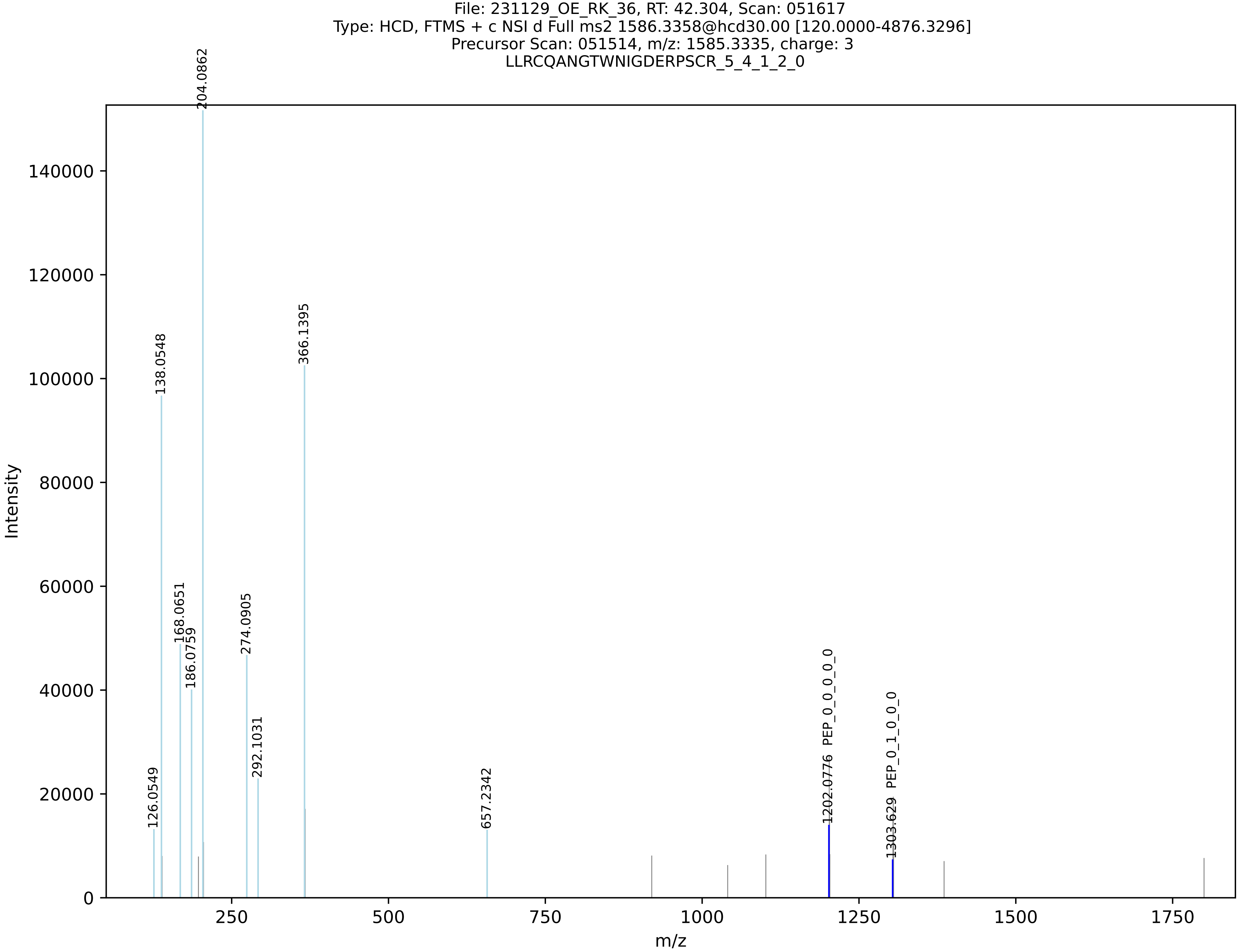
File: 231129_OE_RK_36, RT: 42.304, Scan: 051617
Type: HCD, FTMS + c NSI d Full ms2 1586.3358@hcd30.00 [120.0000-4876.3296]
Precursor Scan: 051514, m/z: 1585.3335, charge: 3
LLRCQANGTWNIGDERPSCR_5_4_1_2_0
Intensity
m/z
0
20000
40000
60000
80000
100000
120000
140000
250
500
750
1000
1250
1500
1750
126.0549
138.0548
168.0651
186.0759
204.0862
274.0905
292.1031
366.1395
657.2342
1202.0776 PEP_0_0_0_0_0_
1303.629 PEP_0_1_0_0_0_

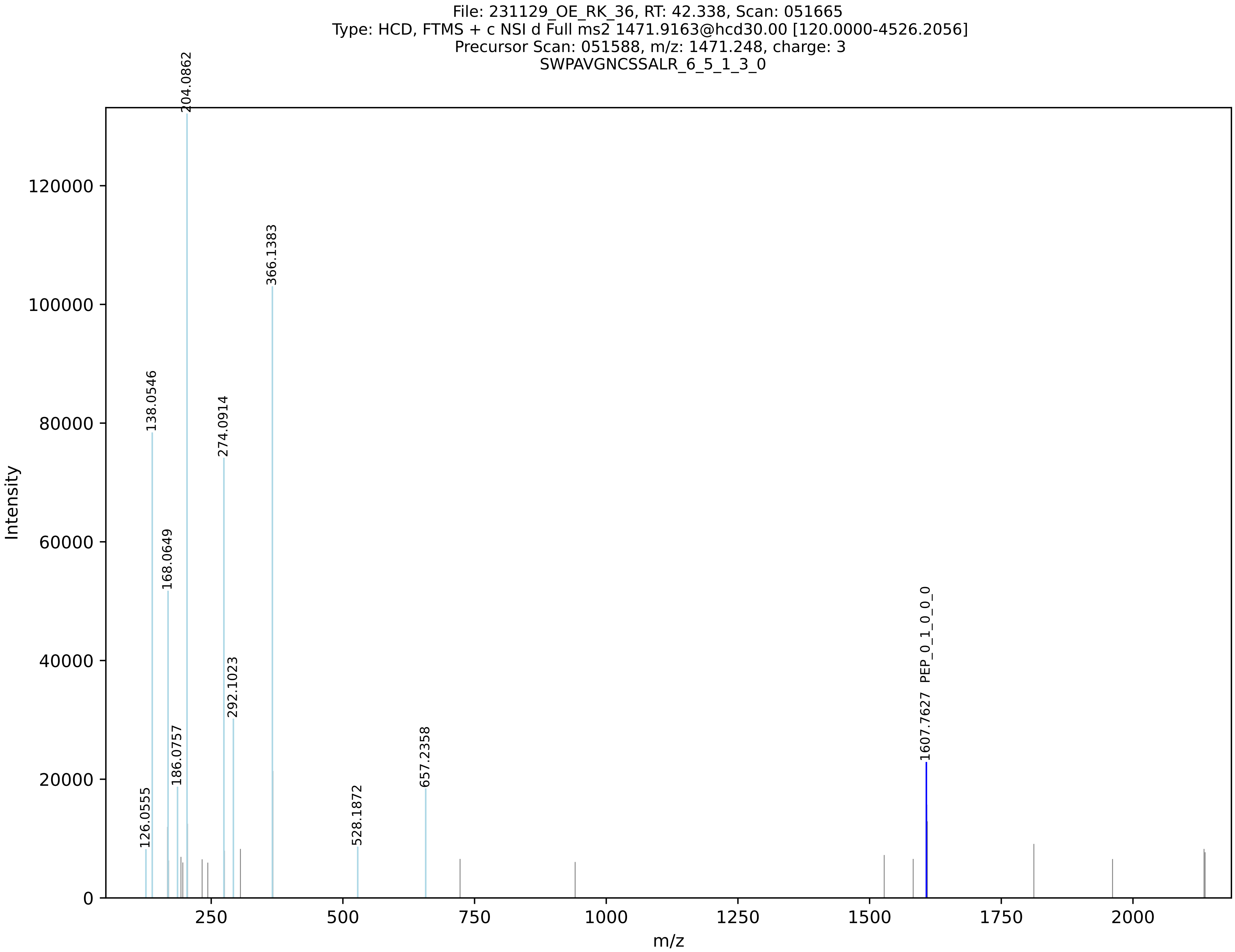
File: 231129_OE_RK_36, RT: 42.338, Scan: 051665
Type: HCD, FTMS + c NSI d Full ms2 1471.9163@hcd30.00 [120.0000-4526.2056]
Precursor Scan: 051588, m/z: 1471.248, charge: 3
SWPAVGNCSSALR_6_5_1_3_0
Intensity
m/z
204.0862
366.1383
138.0546
274.0914
168.0649
292.1023
186.0757
126.0555
528.1872
657.2358
1607.7627 PEP_0_1_0_0_0
0
20000
40000
60000
80000
100000
120000
250
500
750
1000
1250
1500
1750
2000

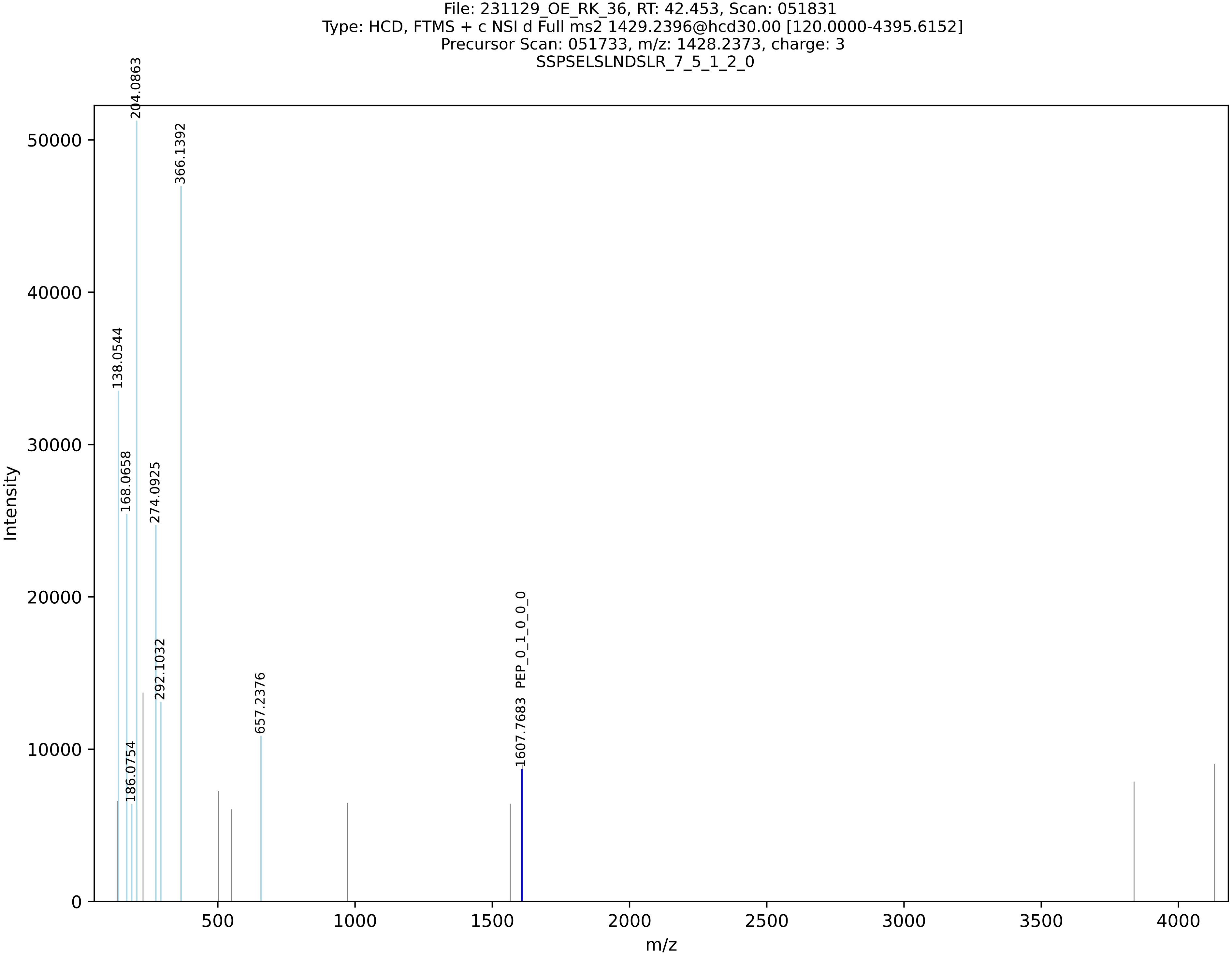

File: 231129_OE_RK_36, RT: 42.453, Scan: 051831
Type: HCD, FTMS + c NSI d Full ms2 1429.2396@hcd30.00 [120.0000-4395.6152]
Precursor Scan: 051733, m/z: 1428.2373, charge: 3
SSPSELSLNDSLR_7_5_1_2_0
Intensity
m/z
204.0863
366.1392
138.0544
168.0658
274.0925
292.1032
186.0754
657.2376
1607.7683 PEP_0_1_0_0_

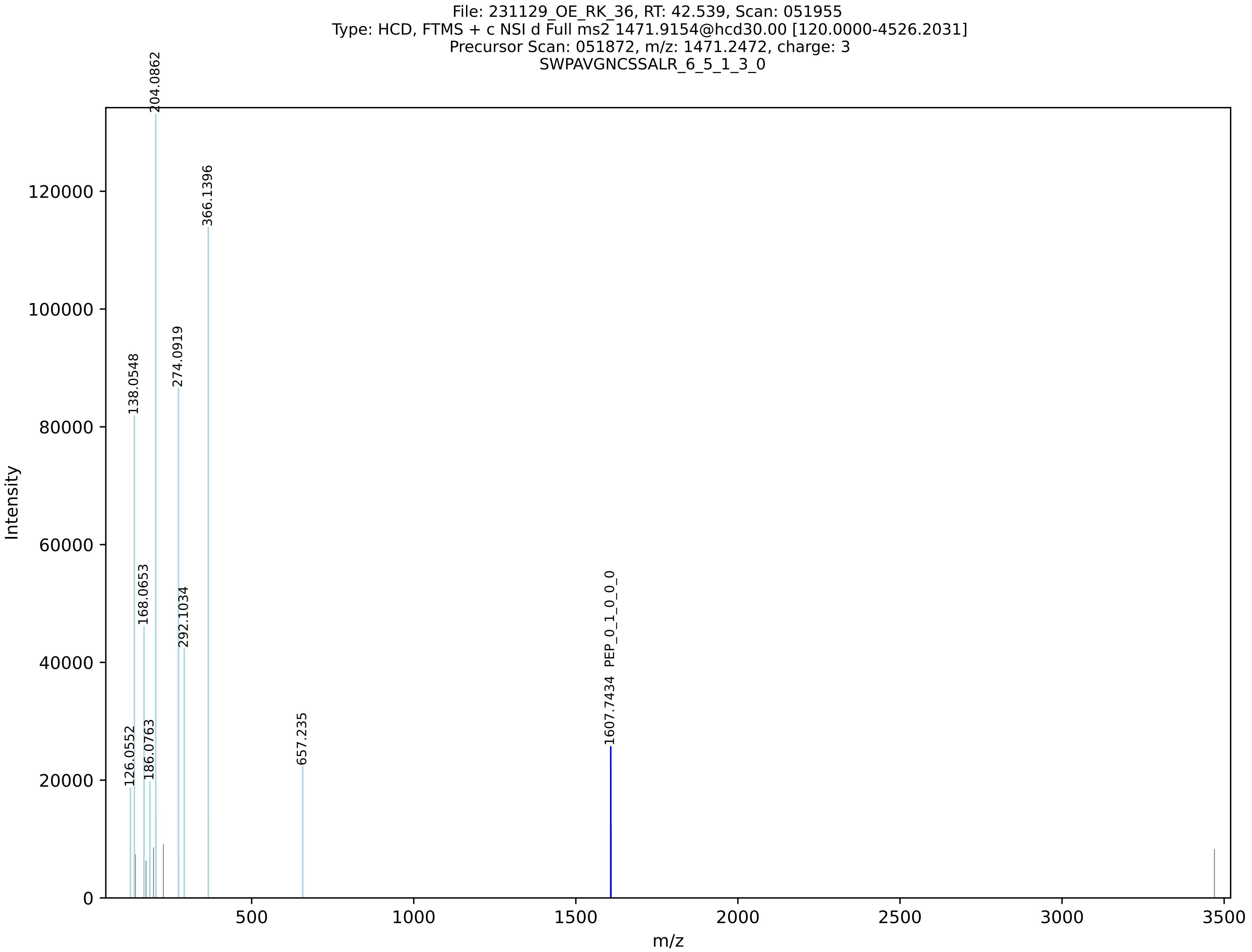
File: 231129_OE_RK_36, RT: 42.539, Scan: 051955
Type: HCD, FTMS + c NSI d Full ms2 1471.9154@hcd30.00 [120.0000-4526.2031]
Precursor Scan: 051872, m/z: 1471.2472, charge: 3
SWPAVGNCSSALR_6_5_1_3_0
Intensity
m/z
204.0862
366.1396
274.0919
138.0548
168.0653
292.1034
PEP_0_1_0_0_0
1607.7434
657.235
126.0552
186.0763
0
20000
40000
60000
80000
100000
120000
500
1000
1500
2000
2500
3000
3500

File: 231129_OE_RK_36, RT: 42.547, Scan: 051968
Type: HCD, FTMS + c NSI d Full ms2 1103.2820@hcd30.00 [120.0000-4524.2466]
Precursor Scan: 051872, m/z: 1102.7808, charge: 4
IRPTAVTLEHVPKALSPNSTISSAPK_4_3_1_1_0

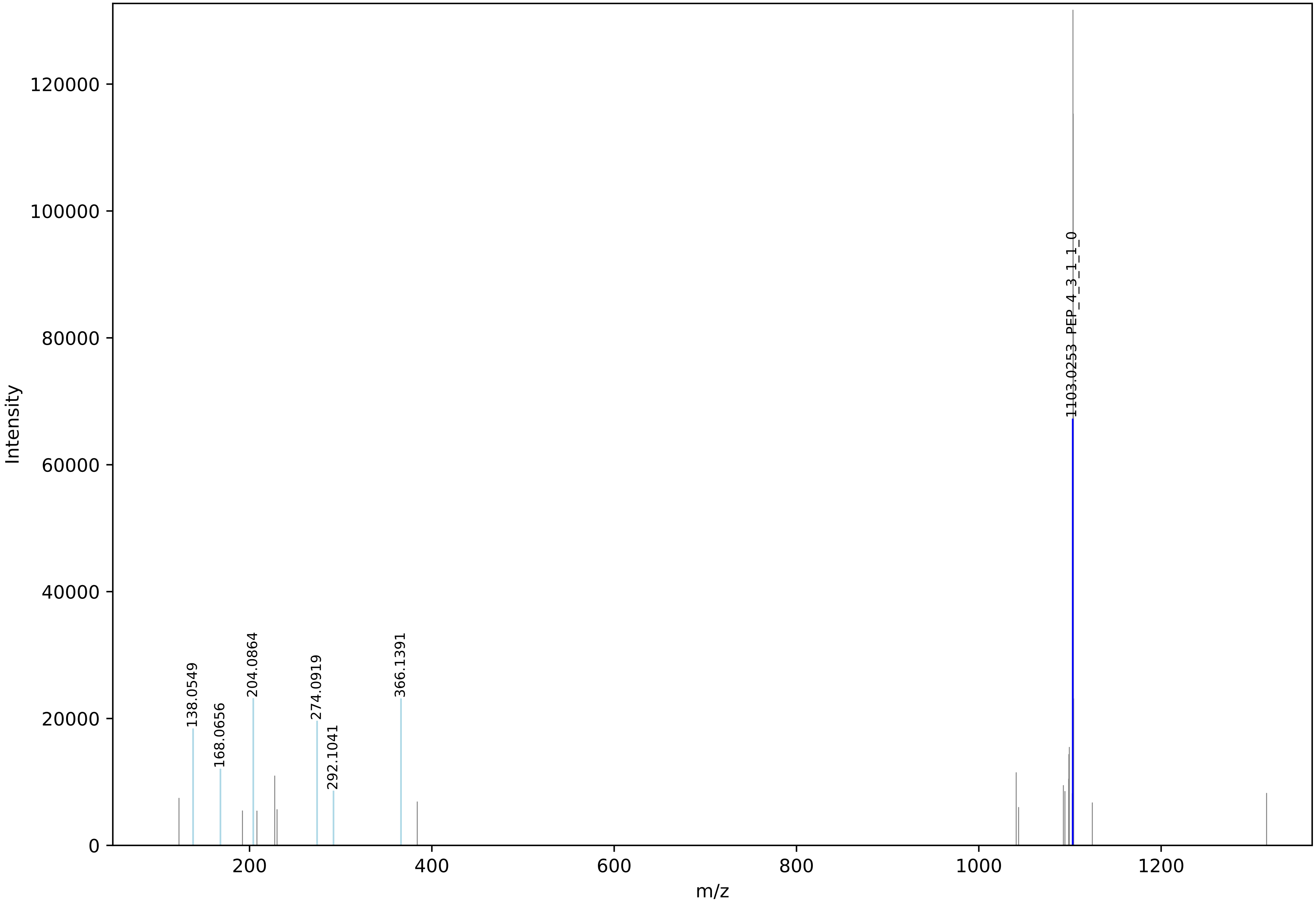

File: 231129_OE_RK_36, RT: 42.732, Scan: 052234
Type: HCD, FTMS + c NSI d Full ms2 1213.9899@hcd30.00 [120.0000-4975.9346]
Precursor Scan: 052156, m/z: 1213.4887, charge: 4
QDQCIYNTTYLNVQR_7_6_0_2_0

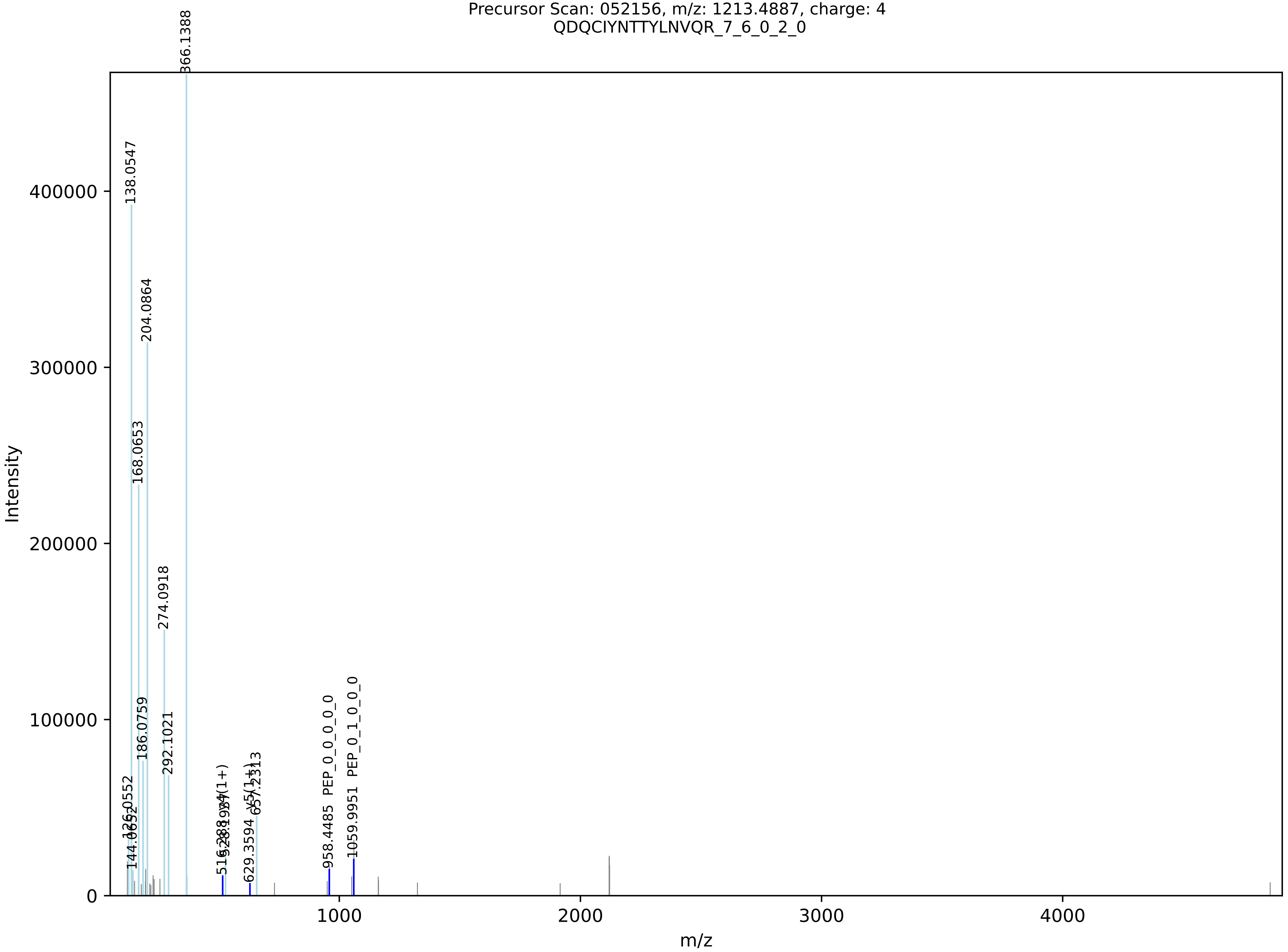

File: 231129_OE_RK_36, RT: 42.833, Scan: 052382
Type: HCD, FTMS + c NSI d Full ms2 1200.1752@hcd30.00 [120.0000-3694.6780]
Precursor Scan: 052301, m/z: 1199.8418, charge: 3
LANLTQGEDQYYLR_5_4_0_1_0

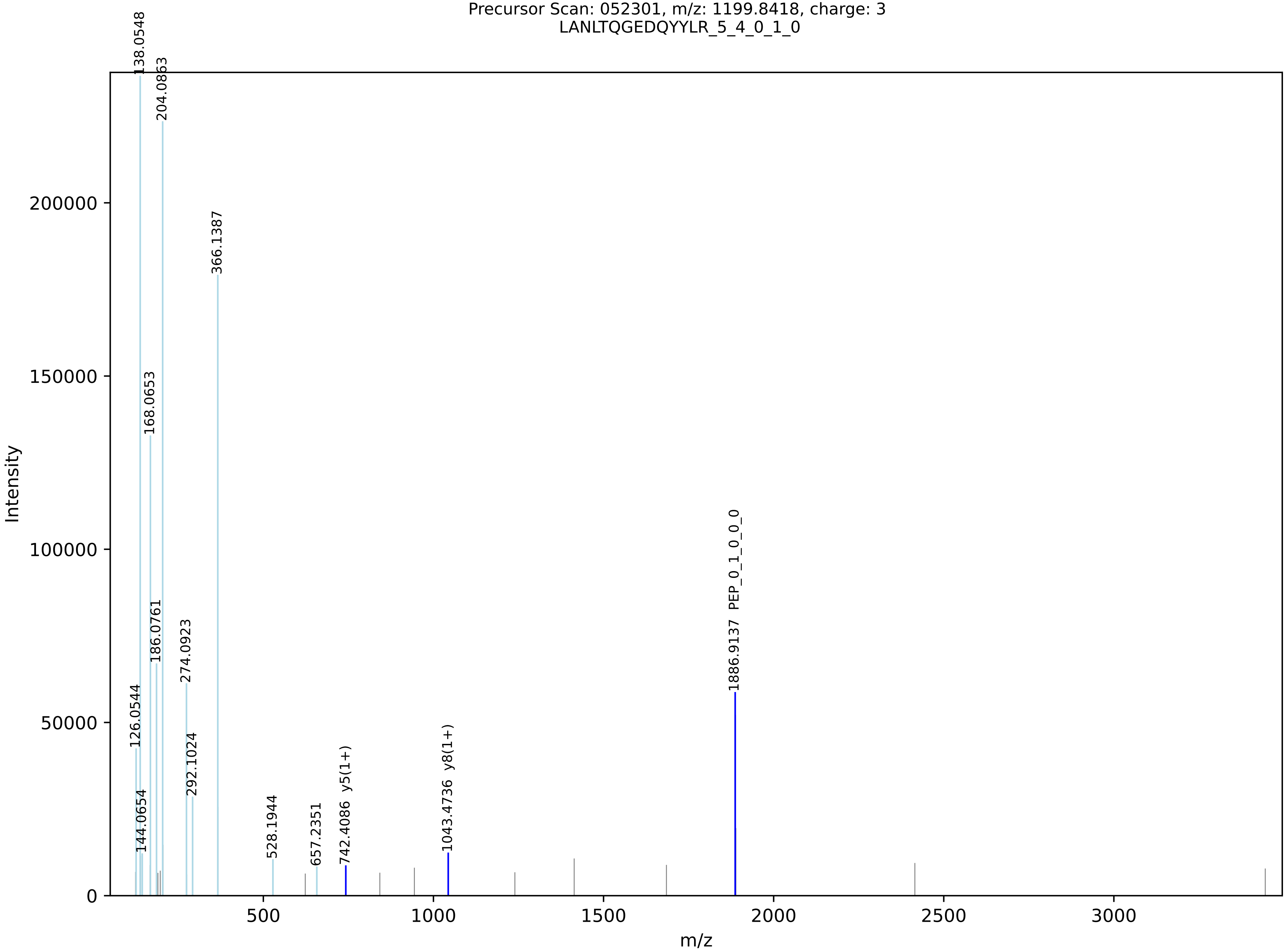

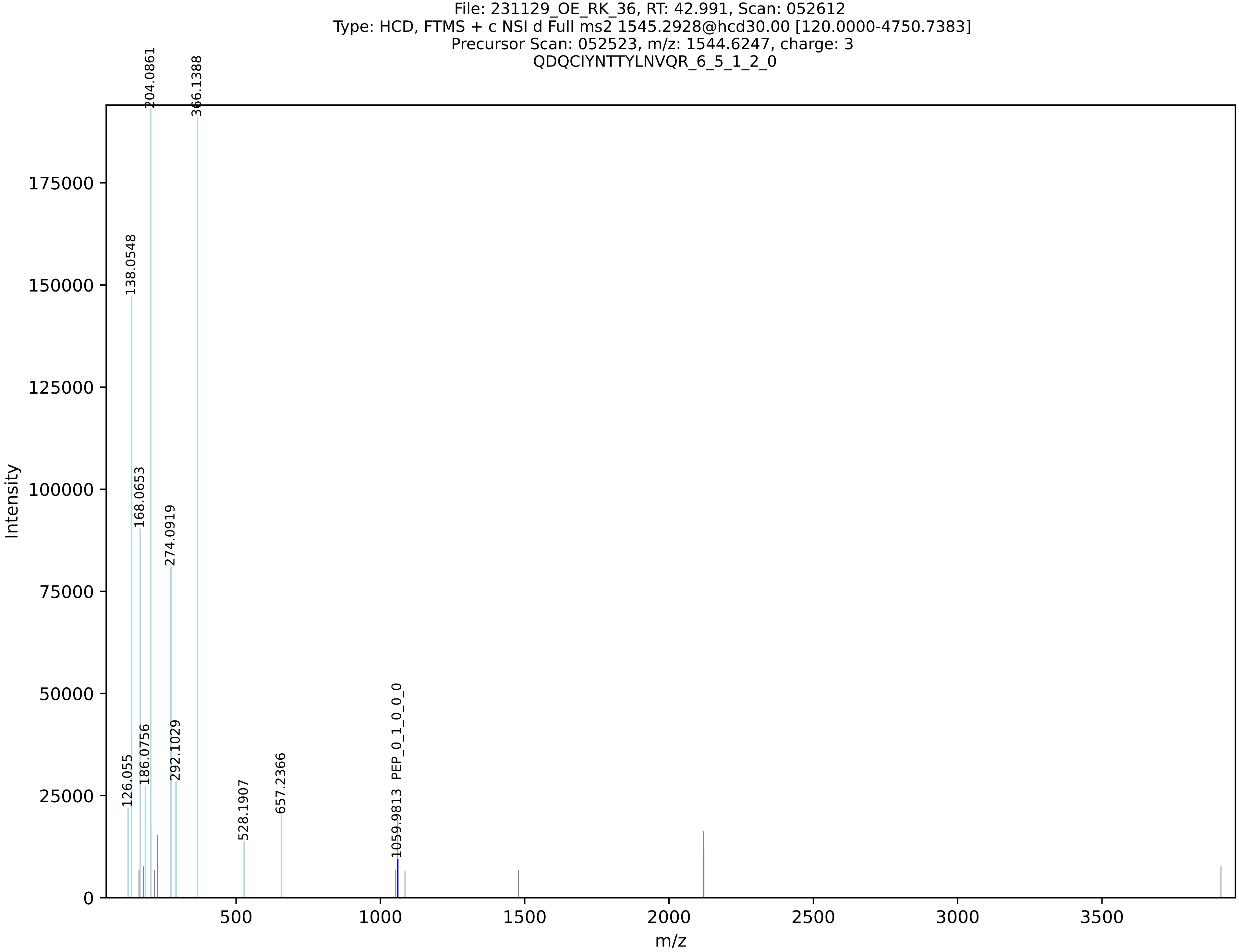
File: 231129_OE_RK_36, RT: 42.991, Scan: 052612
Type: HCD, FTMS + c NSI d Full ms2 1545.2928@hcd30.00 [120.0000-4750.7383]
Precursor Scan: 052523, m/z: 1544.6247, charge: 3
QDQCIYNTTYLNVQR_6_5_1_2_0
204.0861
366.1388
138.0548
168.0653
274.0919
126.055
186.0756
292.1029
528.1907
657.2366
1059.9813
PEP_0_1_0_0_0_
Intensity
m/z
0
25000
50000
75000
100000
125000
150000
175000
500
1000
1500
2000
2500
3000
3500

File: 231129_OE_RK_36, RT: 42.994, Scan: 052616
Type: HCD, FTMS + c NSI d Full ms2 1471.9169@hcd30.00 [120.0000-4526.2075]
Precursor Scan: 052523, m/z: 1471.2478, charge: 3
SWPAVGNCSSALR_6_5_1_3_0

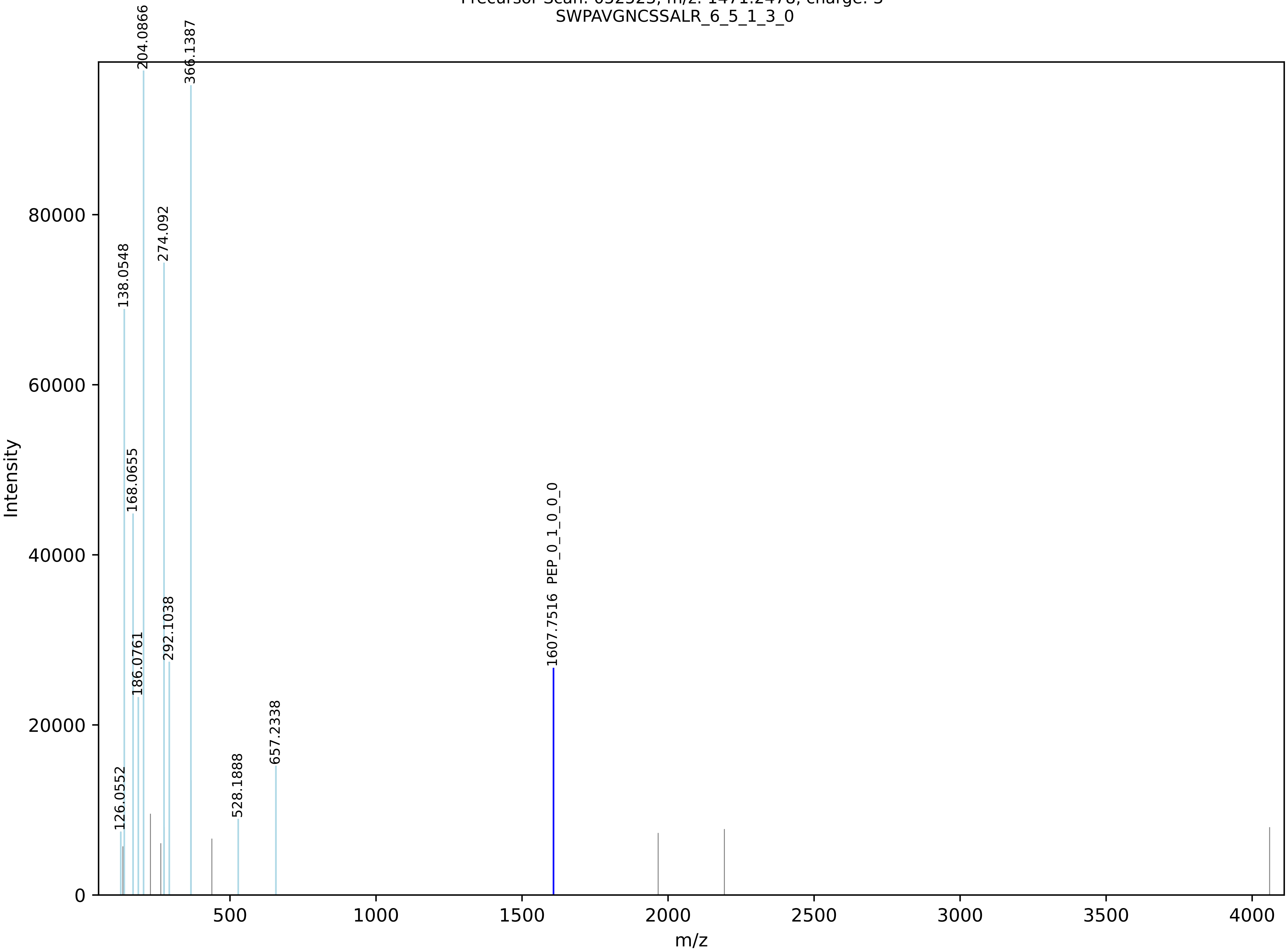

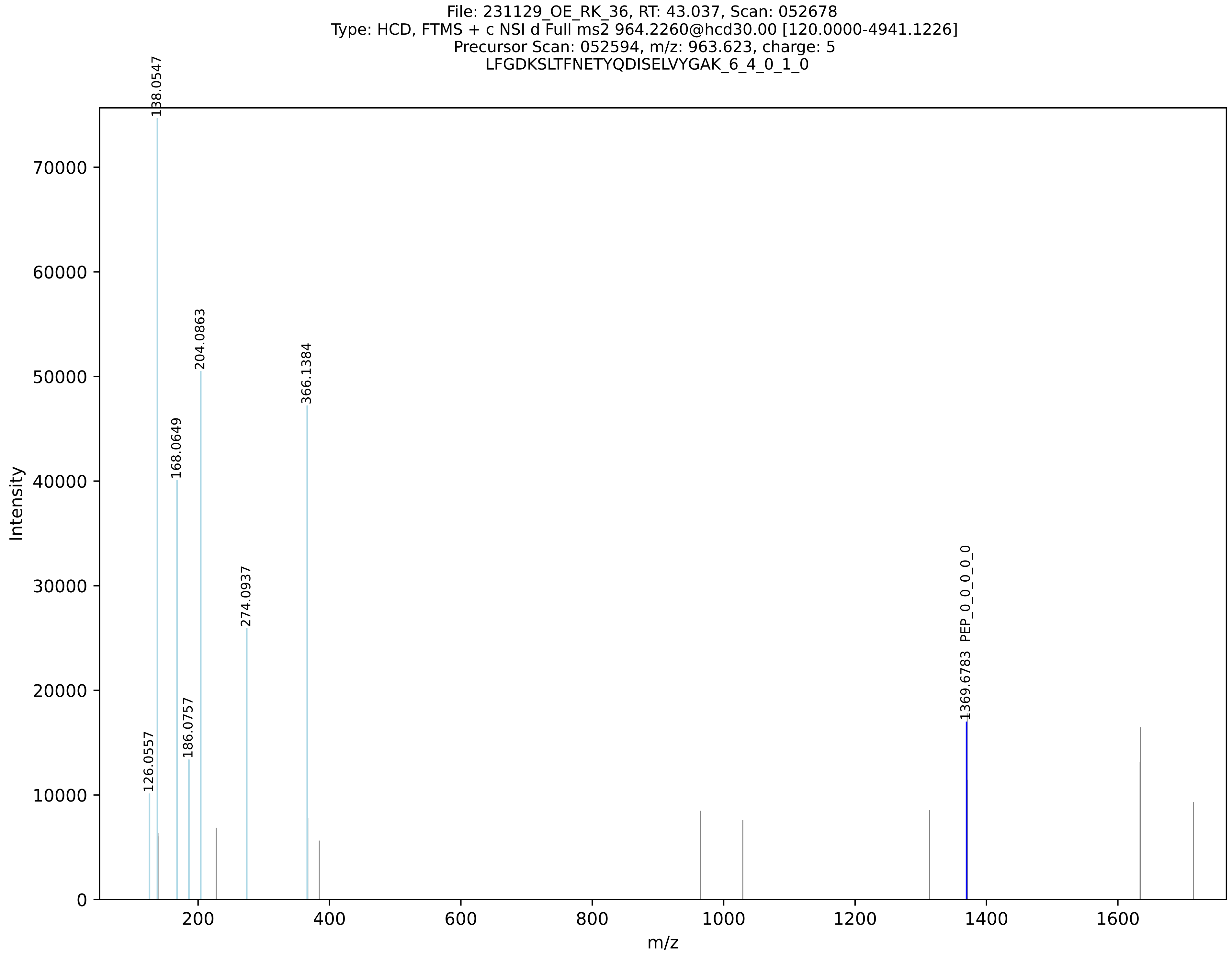
File: 231129_OE_RK_36, RT: 43.037, Scan: 052678
Type: HCD, FTMS + c NSI d Full ms2 964.2260@hcd30.00 [120.0000-4941.1226]
Precursor Scan: 052594, m/z: 963.623, charge: 5
LFGDKSLTFNETYQDISELVYGAK_6_4_0_1_0
138.0547
204.0863
366.1384
168.0649
274.0937
186.0757
126.0557
1369.6783 PEP_0_0_0_0_0_
Intensity
m/z
0
10000
20000
30000
40000
50000
60000
70000
200
400
600
800
1000
1200
1400
1600

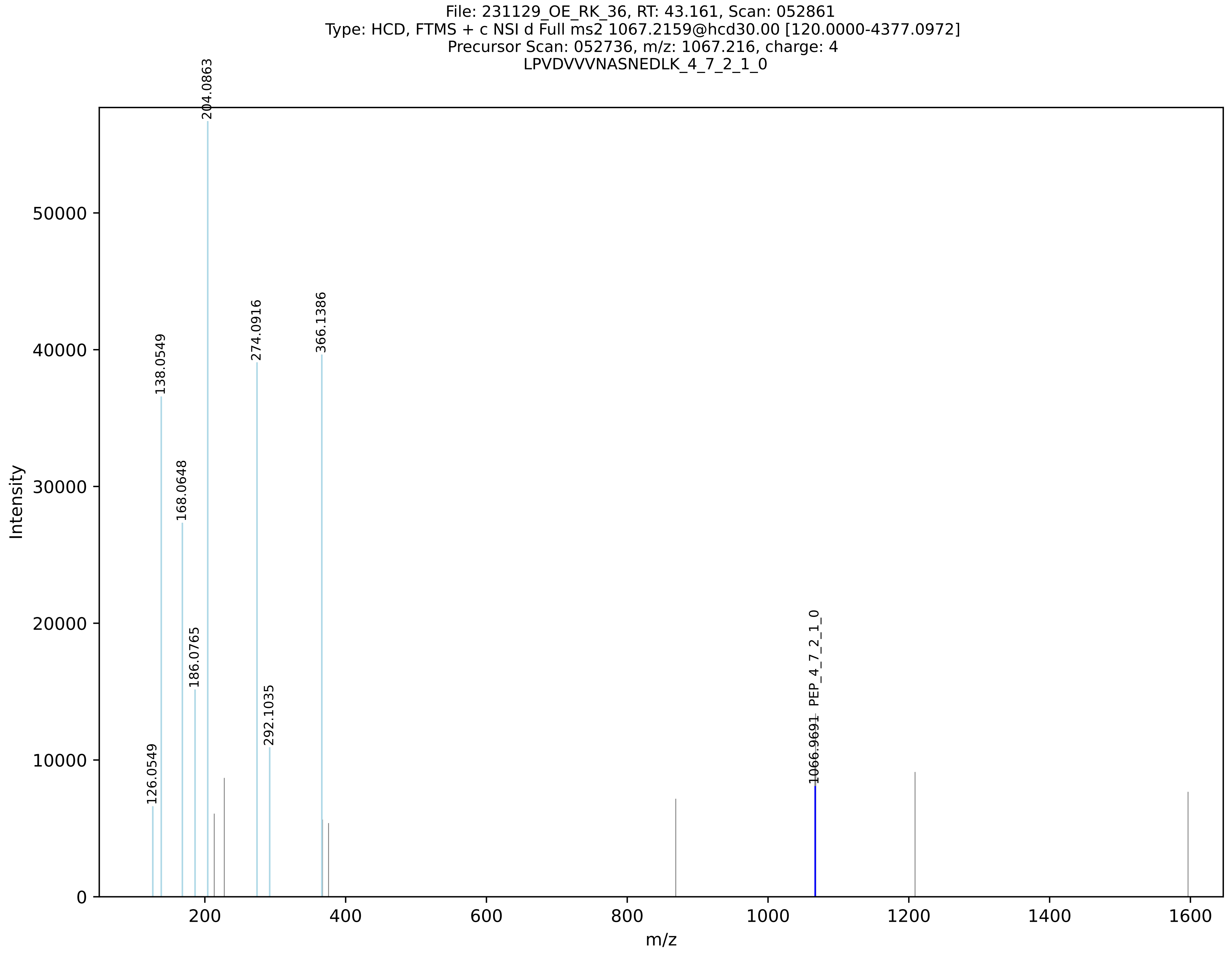

File: 231129_OE_RK_36, RT: 43.161, Scan: 052861
Type: HCD, FTMS + c NSI d Full ms2 1067.2159@hcd30.00 [120.0000-4377.0972]
Precursor Scan: 052736, m/z: 1067.216, charge: 4
LPVDVVVNASNEDLK_4_7_2_1_0
Intensity
m/z
204.0863
138.0549
168.0648
186.0765
126.0549
274.0916
292.1035
366.1386
1066.9691_PEP_4_7_2_1_0
0
10000
20000
30000
40000
50000
200
400
600
800
1000
1200
1400
1600

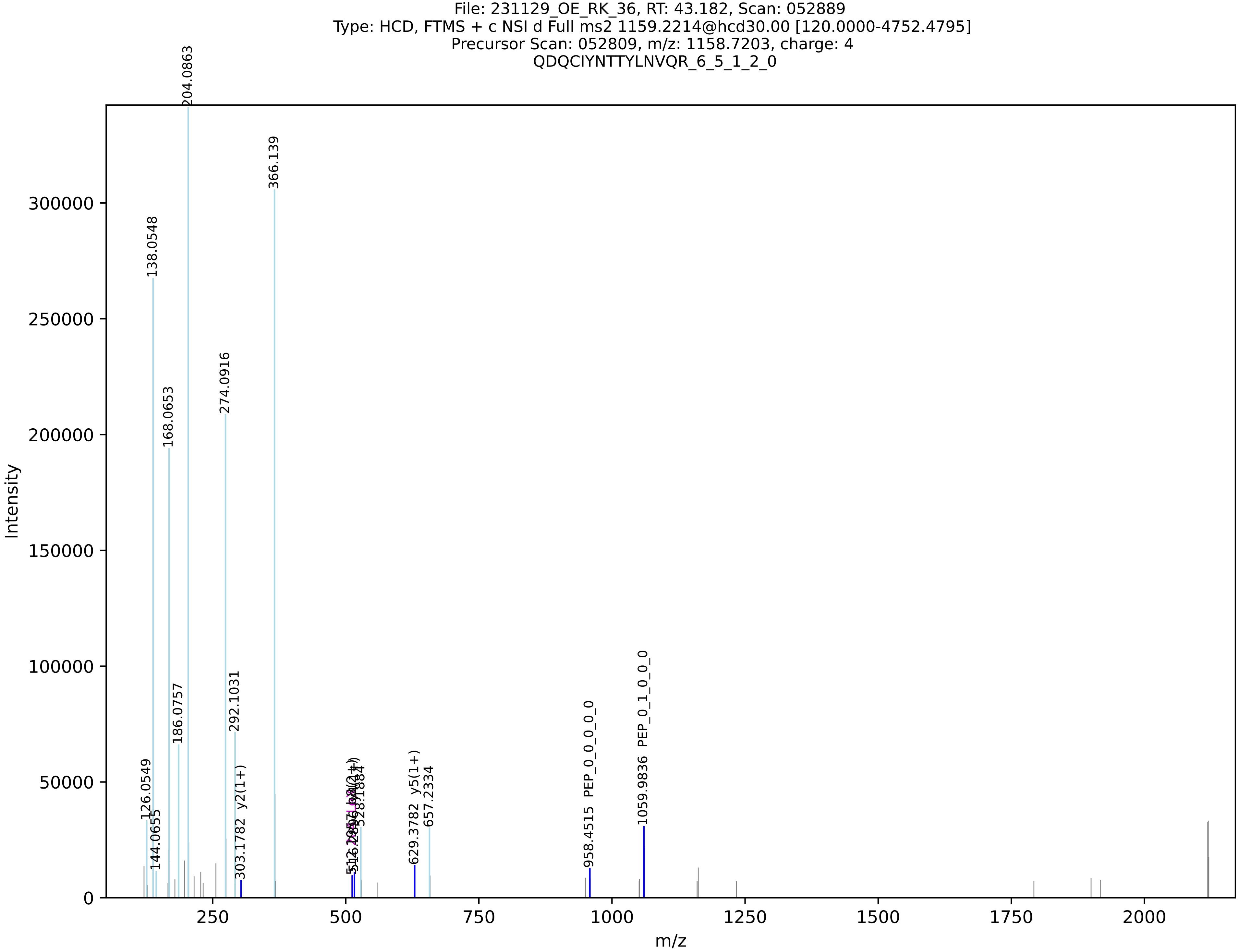
File: 231129_OE_RK_36, RT: 43.182, Scan: 052889
Type: HCD, FTMS + c NSI d Full ms2 1159.2214@hcd30.00 [120.0000-4752.4795]
Precursor Scan: 052809, m/z: 1158.7203, charge: 4
QDQCIYNTTYLNVQR_6_5_1_2_0
Intensity
m/z
204.0863
366.139
138.0548
274.0916
168.0653
186.0757
292.1031
126.0549
144.0655
303.1782 y2(1+)
528.1884
629.3782 y5(1+)
657.2334
958.4515 PEP_0_0_0_0_0_
1059.9836 PEP_0_1_0_0_0_

File: 231129_OE_RK_36, RT: 43.187, Scan: 052897
Type: HCD, FTMS + c NSI d Full ms2 1439.2928@hcd30.00 [120.0000-4426.3784]
Precursor Scan: 052809, m/z: 1438.6248, charge: 3
ADGTVNQIEGEATPVNLTEPAK_5_4_1_1_0

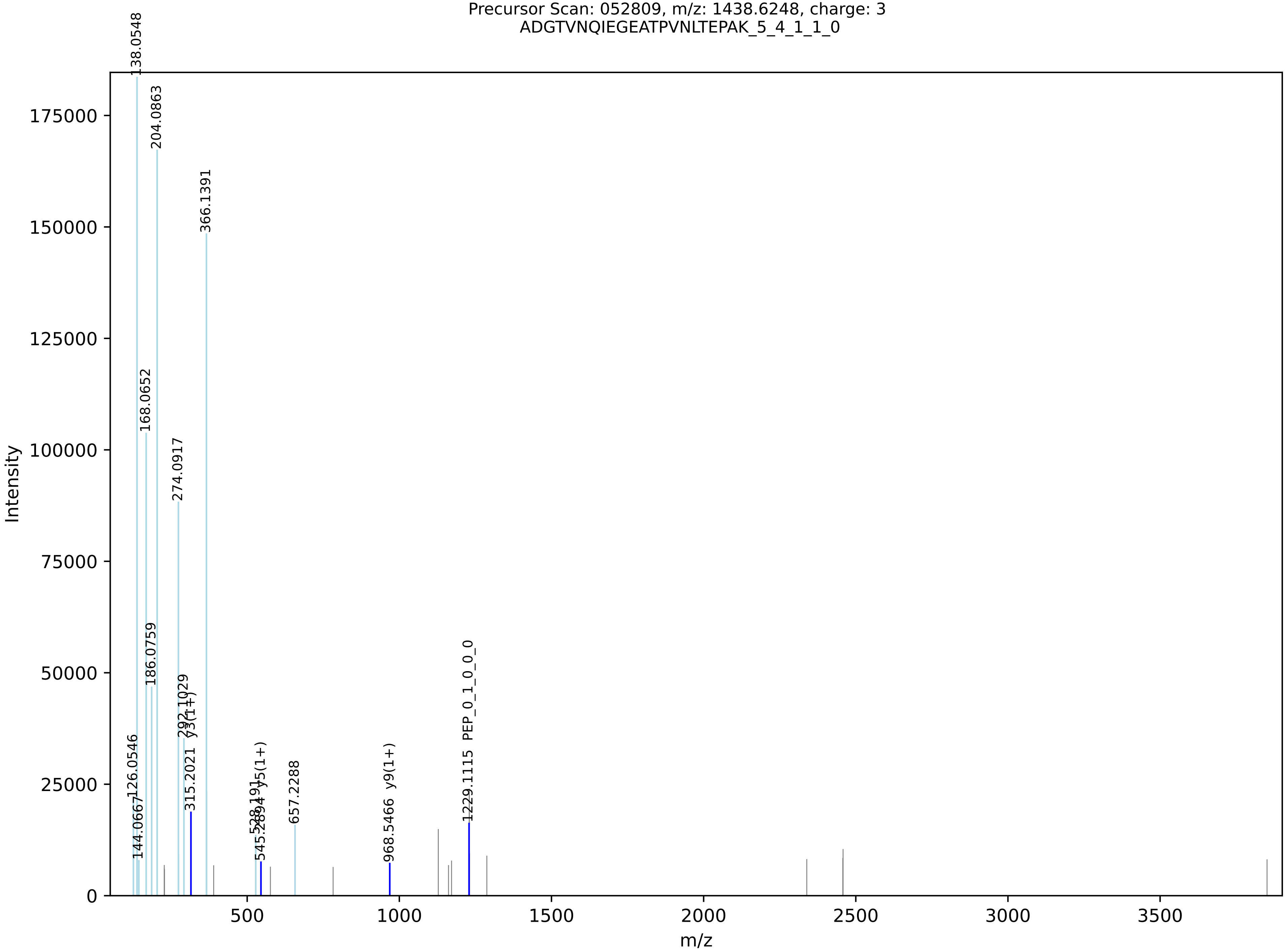

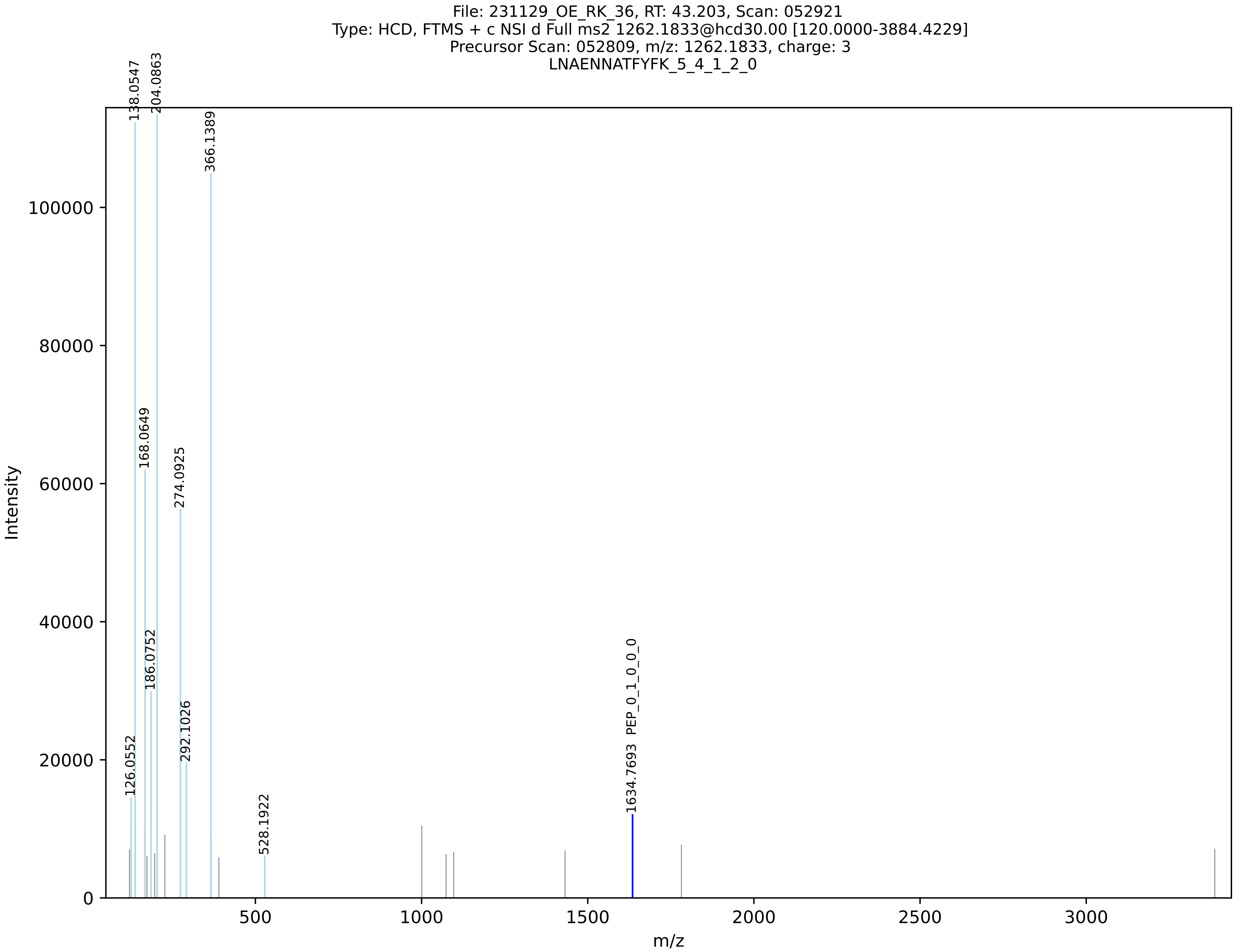

File: 231129_OE_RK_36, RT: 43.203, Scan: 052921
Type: HCD, FTMS + c NSI d Full ms2 1262.1833@hcd30.00 [120.0000-3884.4229]
Precursor Scan: 052809, m/z: 1262.1833, charge: 3
LNAENNATFYFK_5_4_1_2_0
138.0547
204.0863
366.1389
168.0649
274.0925
186.0752
292.1026
126.0552
528.1922
1634.7693 PEP_0_1_0_0_0
Intensity
m/z
0
20000
40000
60000
80000
100000
500
1000
1500
2000
2500
3000

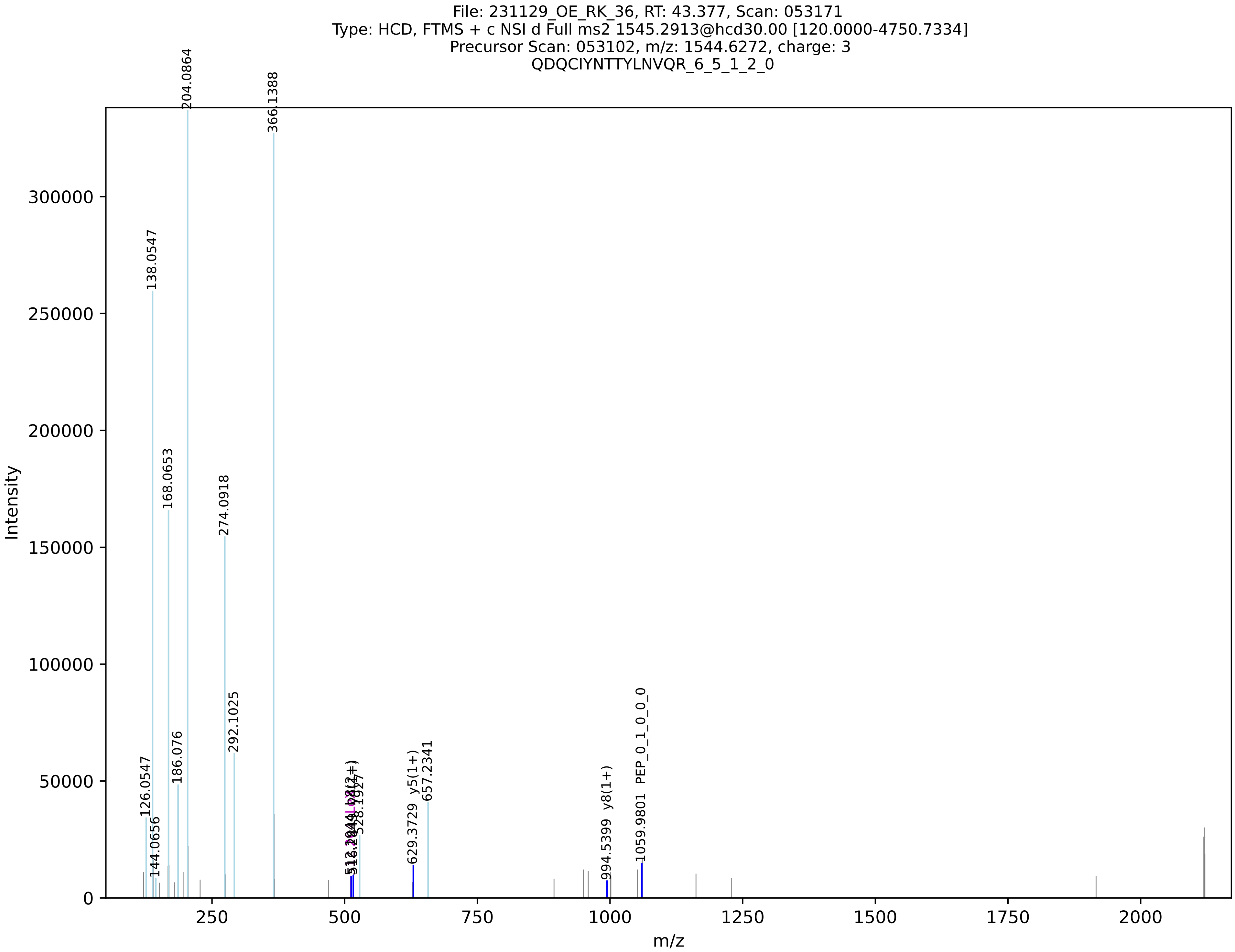
File: 231129_OE_RK_36, RT: 43.377, Scan: 053171
Type: HCD, FTMS + c NSI d Full ms2 1545.2913@hcd30.00 [120.0000-4750.7334]
Precursor Scan: 053102, m/z: 1544.6272, charge: 3
QDQCIYNTTYLNVQR_6_5_1_2_0
Intensity
m/z
204.0864
366.1388
138.0547
168.0653
274.0918
292.1025
186.076
126.0547
144.0656
528.1927
629.3729 y5(1+)
657.2341
994.5399 y8(1+)
1059.9801 PEP_0_1_0_0_0

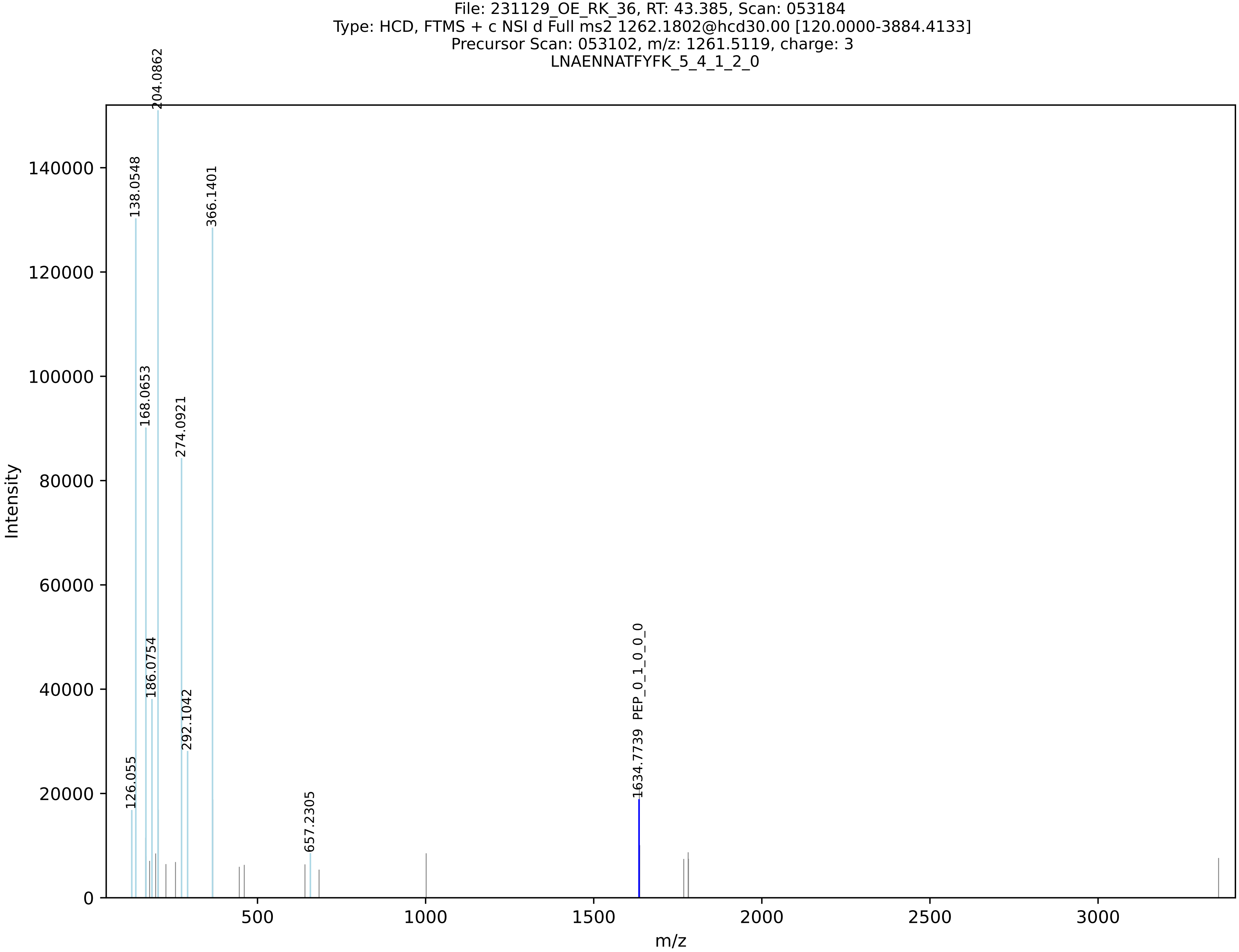

File: 231129_OE_RK_36, RT: 43.385, Scan: 053184
Type: HCD, FTMS + c NSI d Full ms2 1262.1802@hcd30.00 [120.0000-3884.4133]
Precursor Scan: 053102, m/z: 1261.5119, charge: 3
LNAENNATFYFK_5_4_1_2_0
Intensity
m/z
204.0862
138.0548
366.1401
168.0653
274.0921
186.0754
292.1042
126.055
657.2305
1634.7739 PEP_0_1_0_0_0

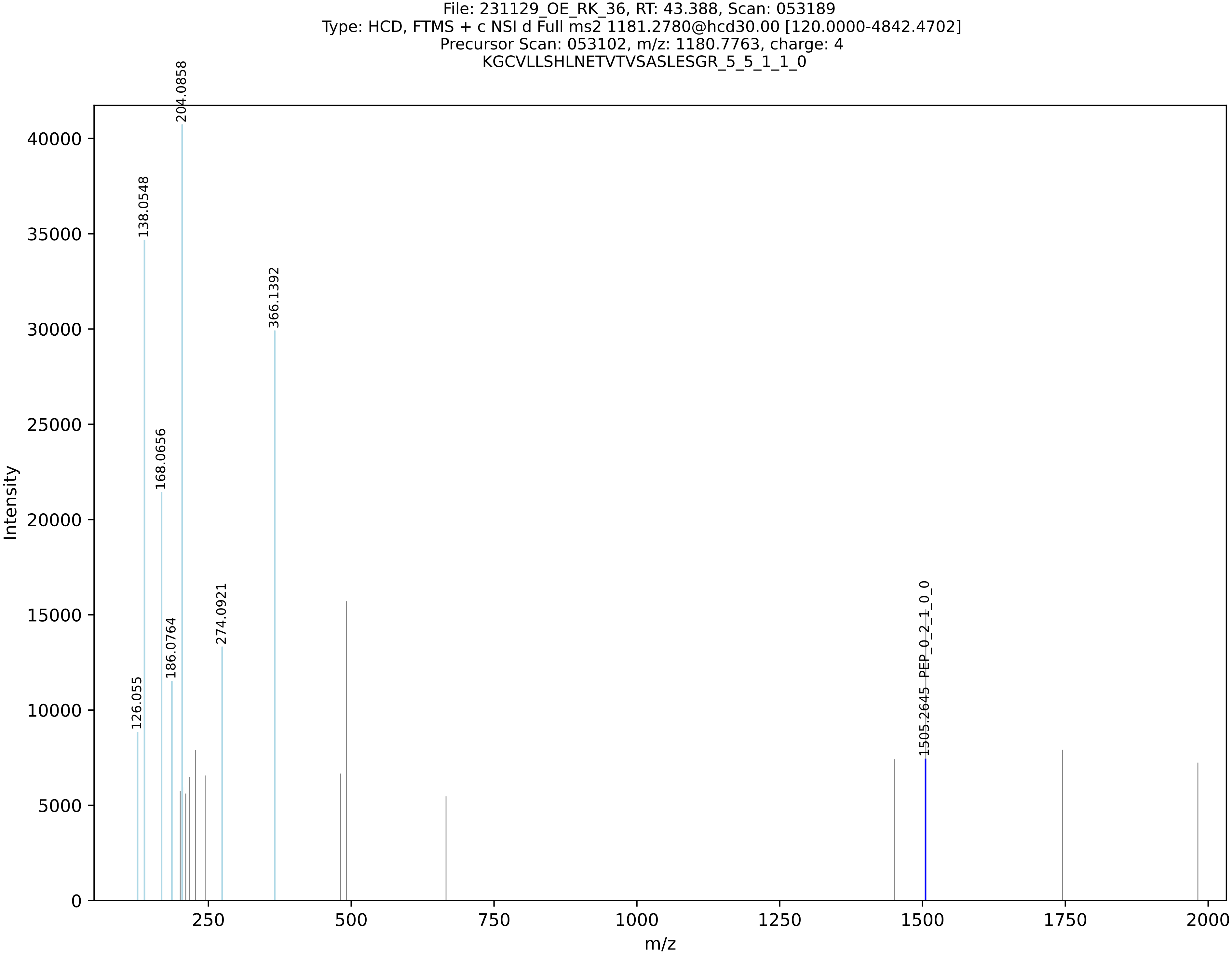

File: 231129_OE_RK_36, RT: 43.388, Scan: 053189
Type: HCD, FTMS + c NSI d Full ms2 1181.2780@hcd30.00 [120.0000-4842.4702]
Precursor Scan: 053102, m/z: 1180.7763, charge: 4
KGCVLLSHLNETVTVSASLESGR_5_5_1_1_0
204.0858
138.0548
366.1392
168.0656
186.0764
274.0921
126.055
1505.2645_PEP_0_2_1_0_0
Intensity
m/z
0
5000
10000
15000
20000
25000
30000
35000
40000
250
500
750
1000
1250
1500
1750
2000

File: 231129_OE_RK_36, RT: 43.465, Scan: 053300
Type: HCD, FTMS + c NSI d Full ms2 1496.6067@hcd30.00 [120.0000-4601.7588]
Precursor Scan: 053241, m/z: 1495.9385, charge: 3
QDQCIYNTTYLNVQR_6_5_0_2_0

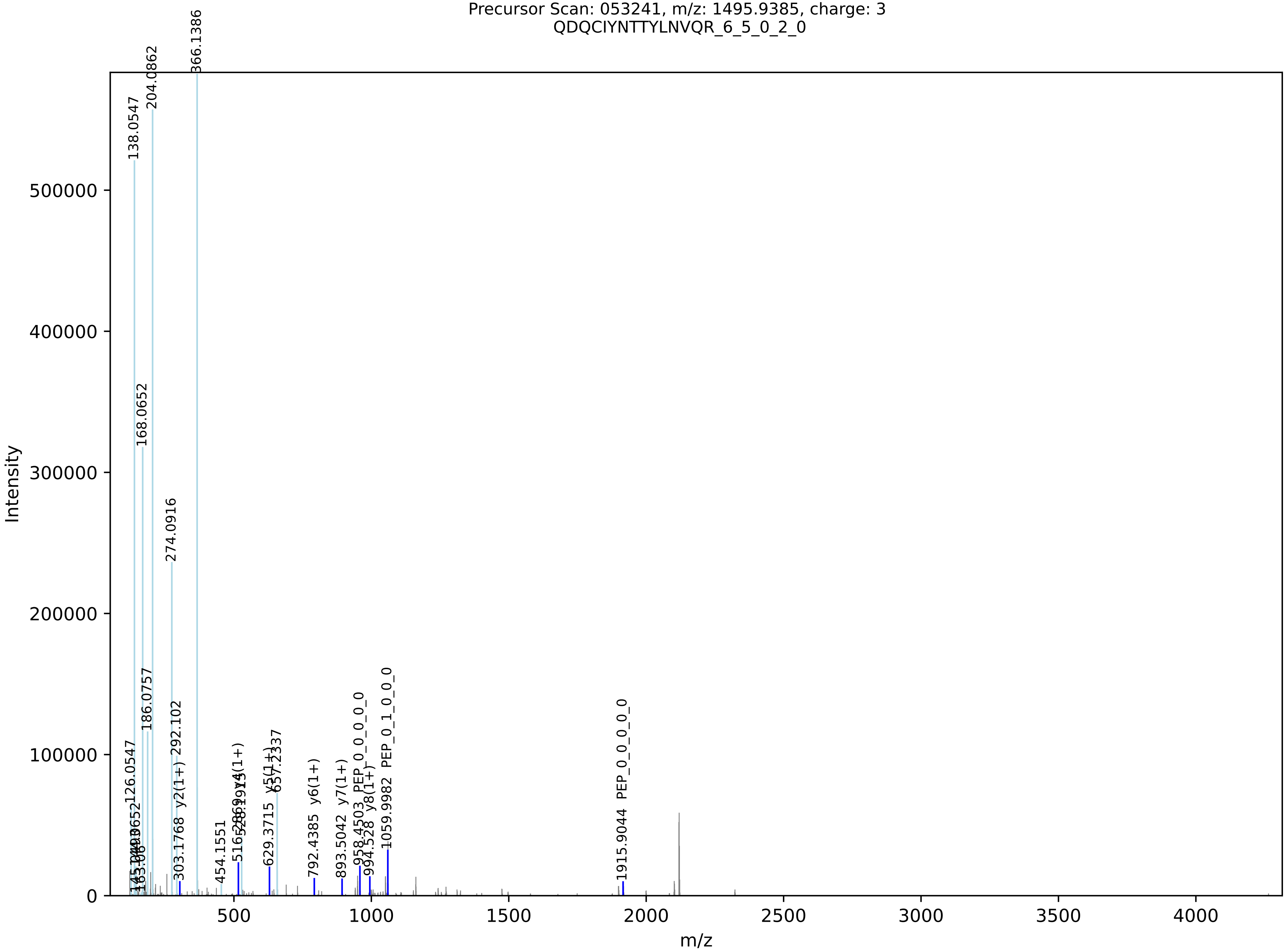

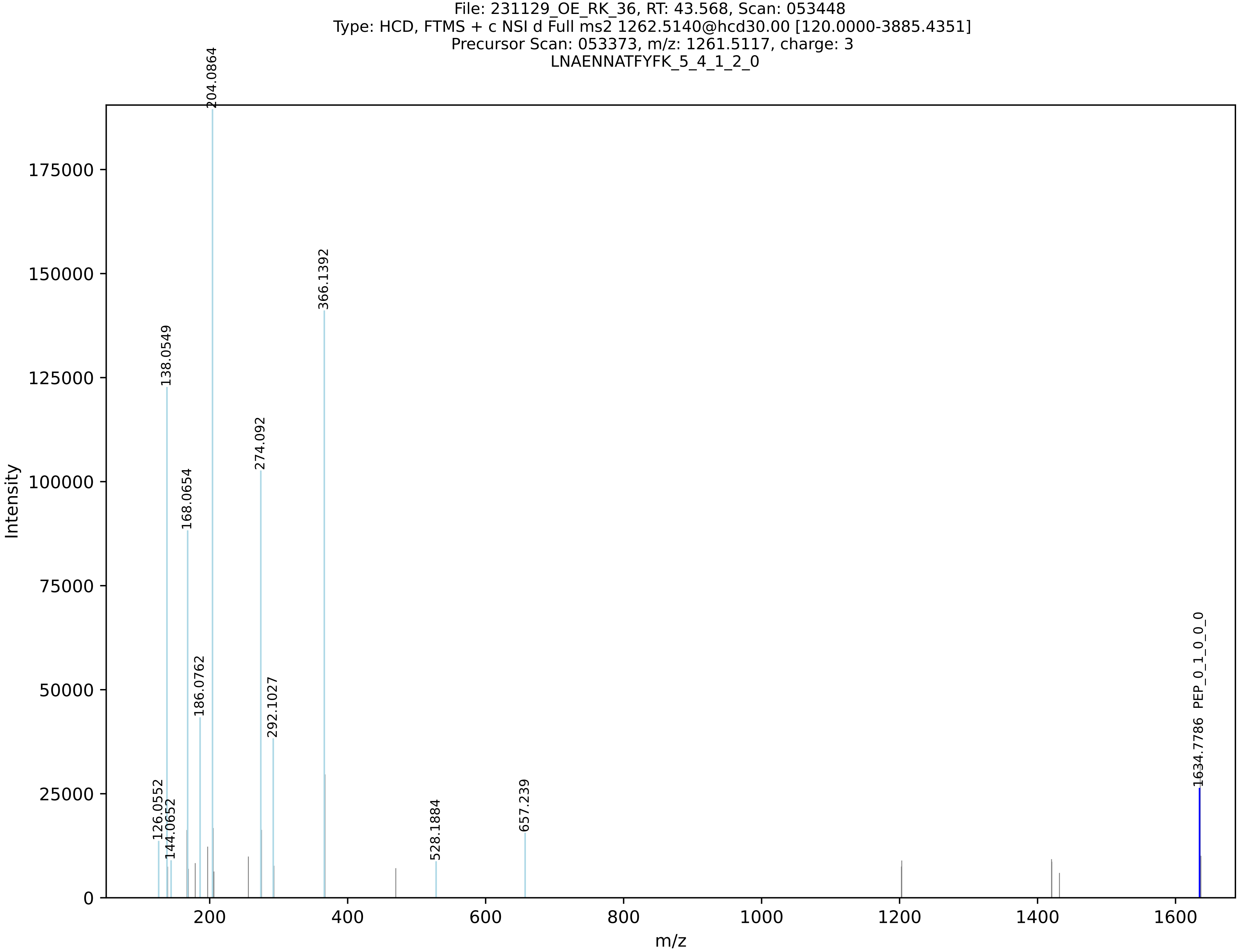

File: 231129_OE_RK_36, RT: 43.568, Scan: 053448
Type: HCD, FTMS + c NSI d Full ms2 1262.5140@hcd30.00 [120.0000-3885.4351]
Precursor Scan: 053373, m/z: 1261.5117, charge: 3
LNAENNATFYFK_5_4_1_2_0
Intensity
m/z
204.0864
366.1392
138.0549
274.092
168.0654
186.0762
292.1027
126.0552
144.0652
528.1884
657.239
1634.7786 PEP_0_1_0_0_0

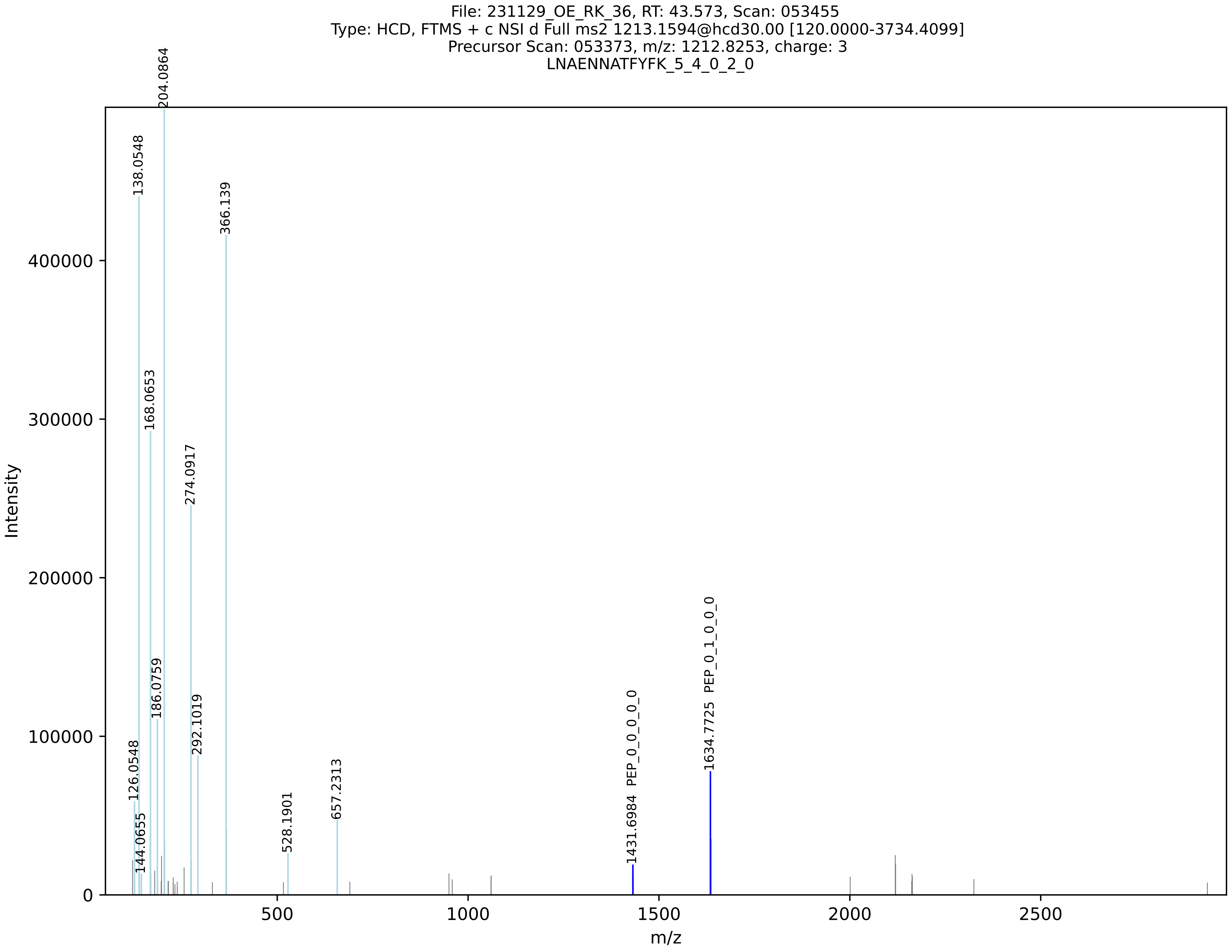

File: 231129_OE_RK_36, RT: 43.573, Scan: 053455
Type: HCD, FTMS + c NSI d Full ms2 1213.1594@hcd30.00 [120.0000-3734.4099]
Precursor Scan: 053373, m/z: 1212.8253, charge: 3
LNAENNATFYFK_5_4_0_2_0
Intensity
m/z
204.0864
138.0548
366.139
168.0653
274.0917
186.0759
292.1019
126.0548
144.0655
528.1901
657.2313
1431.6984 PEP_0_0_0_0_0
1634.7725 PEP_0_1_0_0_0
0
100000
200000
300000
400000
500
1000
1500
2000
2500

File: 231129_OE_RK_36, RT: 43.617, Scan: 053518
Type: HCD, FTMS + c NSI d Full ms2 1159.2216@hcd30.00 [120.0000-4752.4800]
Precursor Scan: 053438, m/z: 1158.7199, charge: 4
QDQCIYNTTYLNVQR_6_5_1_2_0

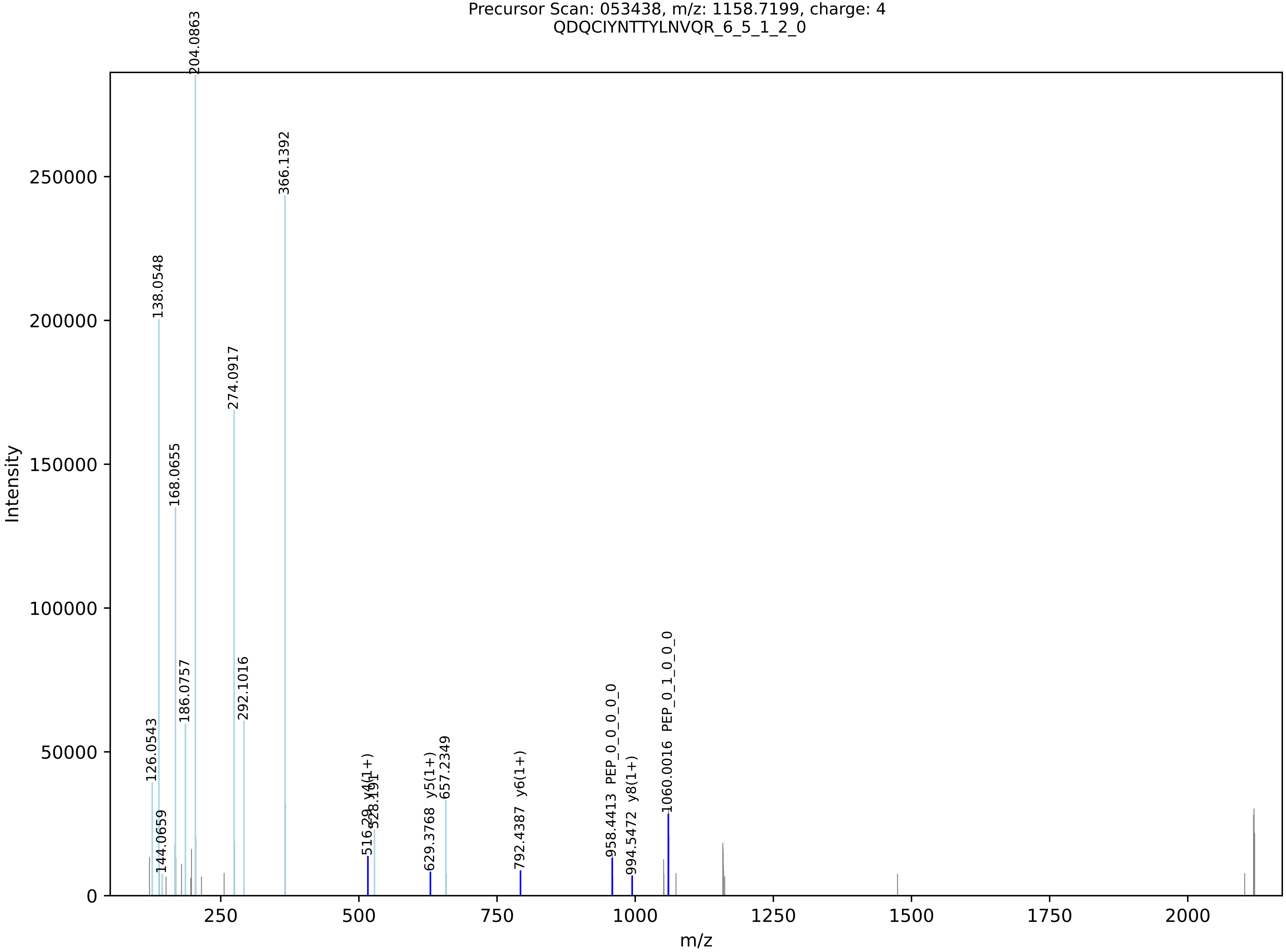

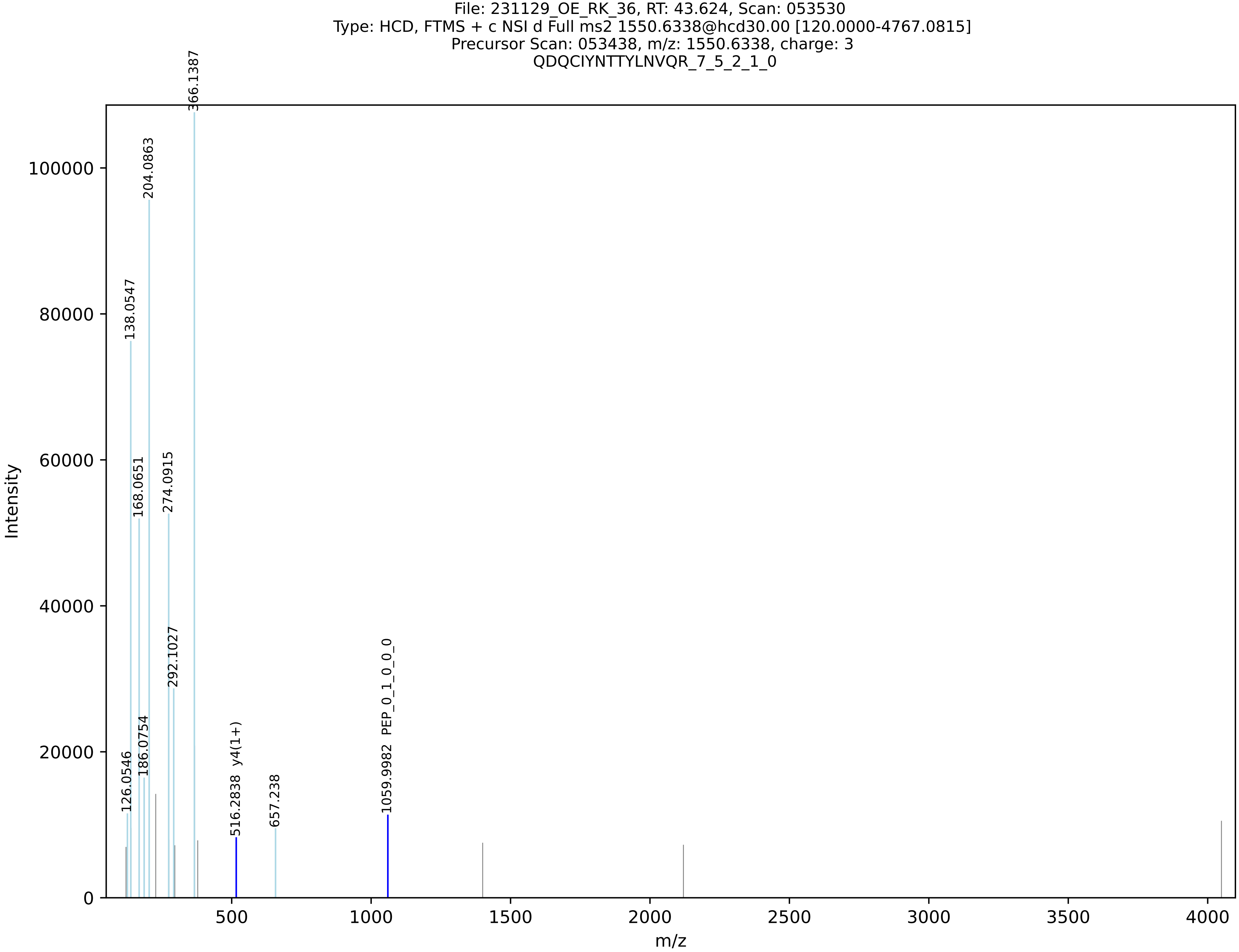
File: 231129_OE_RK_36, RT: 43.624, Scan: 053530
Type: HCD, FTMS + c NSI d Full ms2 1550.6338@hcd30.00 [120.0000-4767.0815]
Precursor Scan: 053438, m/z: 1550.6338, charge: 3
QDQCIYNTTYLNVQR_7_5_2_1_0
Intensity
m/z
366.1387
204.0863
138.0547
168.0651
274.0915
292.1027
186.0754
126.0546
516.2838 y4(1+)
657.238
1059.9982 PEP_0_1_0_0_0

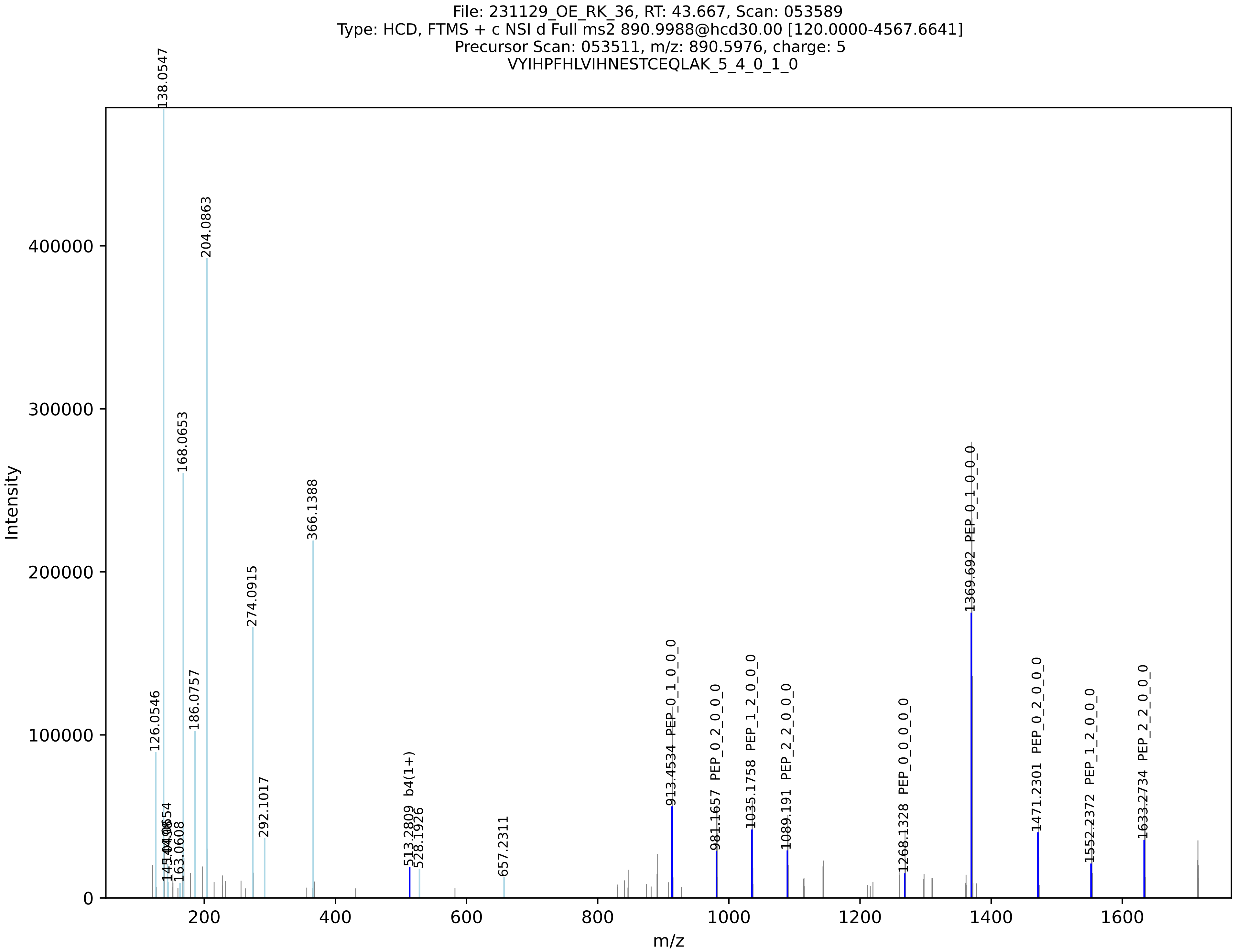
File: 231129_OE_RK_36, RT: 43.667, Scan: 053589
Type: HCD, FTMS + c NSI d Full ms2 890.9988@hcd30.00 [120.0000-4567.6641]
Precursor Scan: 053511, m/z: 890.5976, charge: 5
VYIHPFHLVIHNESTCEQLAK_5_4_0_1_0
Intensity
m/z
138.0547
204.0863
168.0653
366.1388
274.0915
186.0757
126.0546
292.1017
163.0608
513.2809 b4(1+)
528.1926
657.2311
913.4534 PEP_0_1_0_0_0
981.1657 PEP_0_2_0_0_0
1035.1758 PEP_1_2_0_0_0
1089.191 PEP_2_2_0_0_0
1268.1328 PEP_0_0_0_0_0
1369.692 PEP_0_1_0_0_
1471.2301 PEP_0_2_0_0_0
1552.2372 PEP_1_2_0_0_0
1633.2734 PEP_2_2_0_0_0

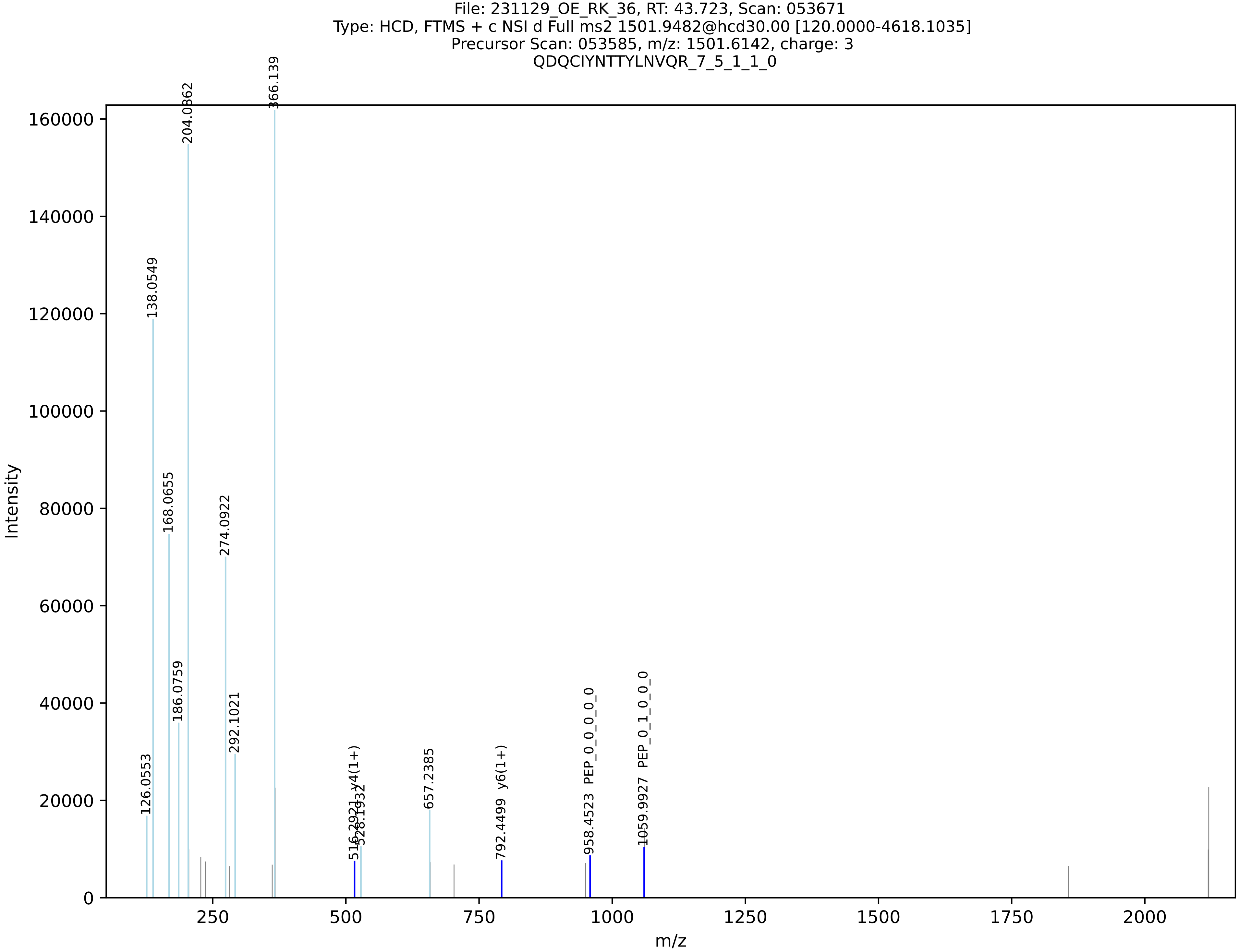
File: 231129_OE_RK_36, RT: 43.723, Scan: 053671
Type: HCD, FTMS + c NSI d Full ms2 1501.9482@hcd30.00 [120.0000-4618.1035]
Precursor Scan: 053585, m/z: 1501.6142, charge: 3
QDQCIYNTTYLNVQR_7_5_1_1_0
Intensity
m/z
366.139
204.0862
138.0549
168.0655
274.0922
186.0759
292.1021
126.0553
516.2921 y4(1+)
528.1932
657.2385
792.4499 y6(1+)
958.4523 PEP_0_0_0_0_0
1059.9927 PEP_0_1_0_0_0
0
20000
40000
60000
80000
100000
120000
140000
160000
250
500
750
1000
1250
1500
1750
2000

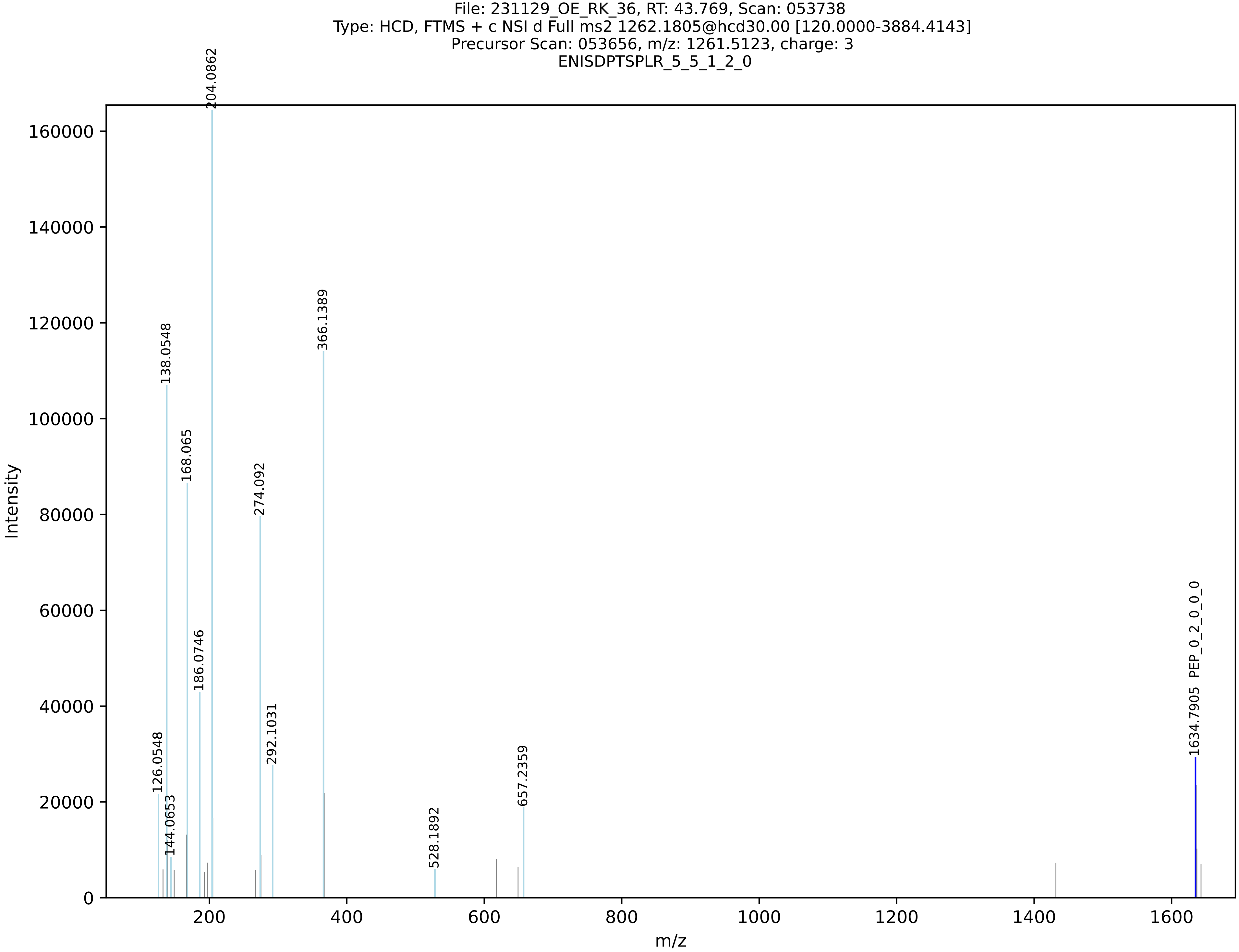
File: 231129_OE_RK_36, RT: 43.769, Scan: 053738
Type: HCD, FTMS + c NSI d Full ms2 1262.1805@hcd30.00 [120.0000-3884.4143]
Precursor Scan: 053656, m/z: 1261.5123, charge: 3
ENISDPTSPLR_5_5_1_2_0
Intensity
m/z
204.0862
138.0548
168.065
186.0746
126.0548
144.0653
274.092
292.1031
366.1389
528.1892
657.2359
1634.7905 PEP_0_2_0_0_0
0
20000
40000
60000
80000
100000
120000
140000
160000
200
400
600
800
1000
1200
1400
1600

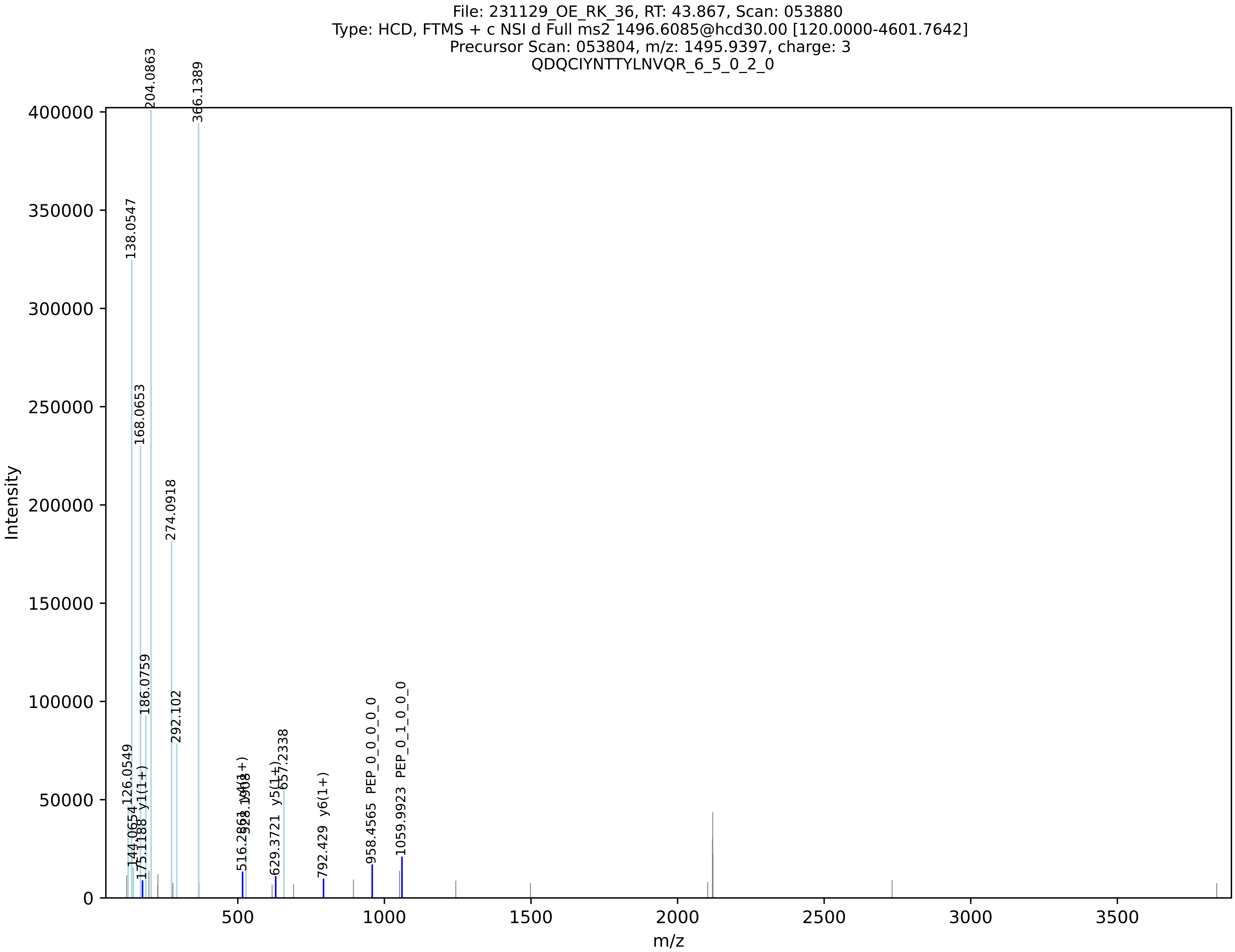
File: 231129_OE_RK_36, RT: 43.867, Scan: 053880
Type: HCD, FTMS + c NSI d Full ms2 1496.6085@hcd30.00 [120.0000-4601.7642]
Precursor Scan: 053804, m/z: 1495.9397, charge: 3
QDQCIYNTTYLNVQR_6_5_0_2_0
204.0863
366.1389
138.0547
168.0653
274.0918
186.0759
292.102
126.0549
144.0654
175.1188 y1(1+)
516.2861 y4(1+)
528.1908
629.3721 y5(1+)
657.2338
792.429 y6(1+)
958.4565 PEP_0_0_0_0_0_
1059.9923 PEP_0_1_0_0_0_
Intensity
m/z
0
50000
100000
150000
200000
250000
300000
350000
400000
500
1000
1500
2000
2500
3000
3500

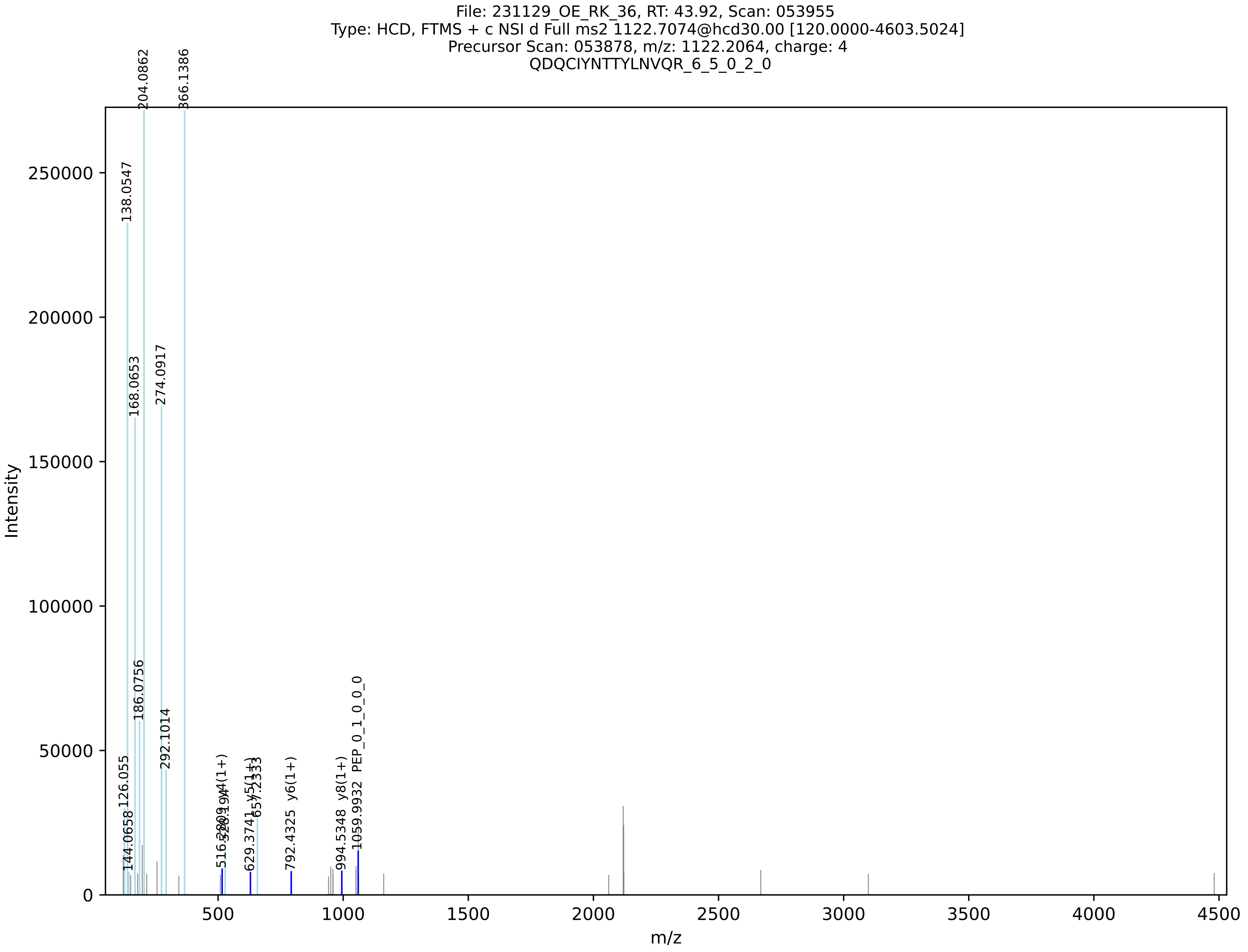

File: 231129_OE_RK_36, RT: 43.92, Scan: 053955
Type: HCD, FTMS + c NSI d Full ms2 1122.7074@hcd30.00 [120.0000-4603.5024]
Precursor Scan: 053878, m/z: 1122.2064, charge: 4
QDQCIYNTTYLNVQR_6_5_0_2_0
Intensity
m/z
204.0862
366.1386
138.0547
168.0653
274.0917
186.0756
292.1014
126.055
144.0658
y4(1+)
y5(1+)
657.2333
792.4325 y6(1+)
994.5348 y8(1+)
1059.9932 PEP_0_1_0_0_0
629.3741

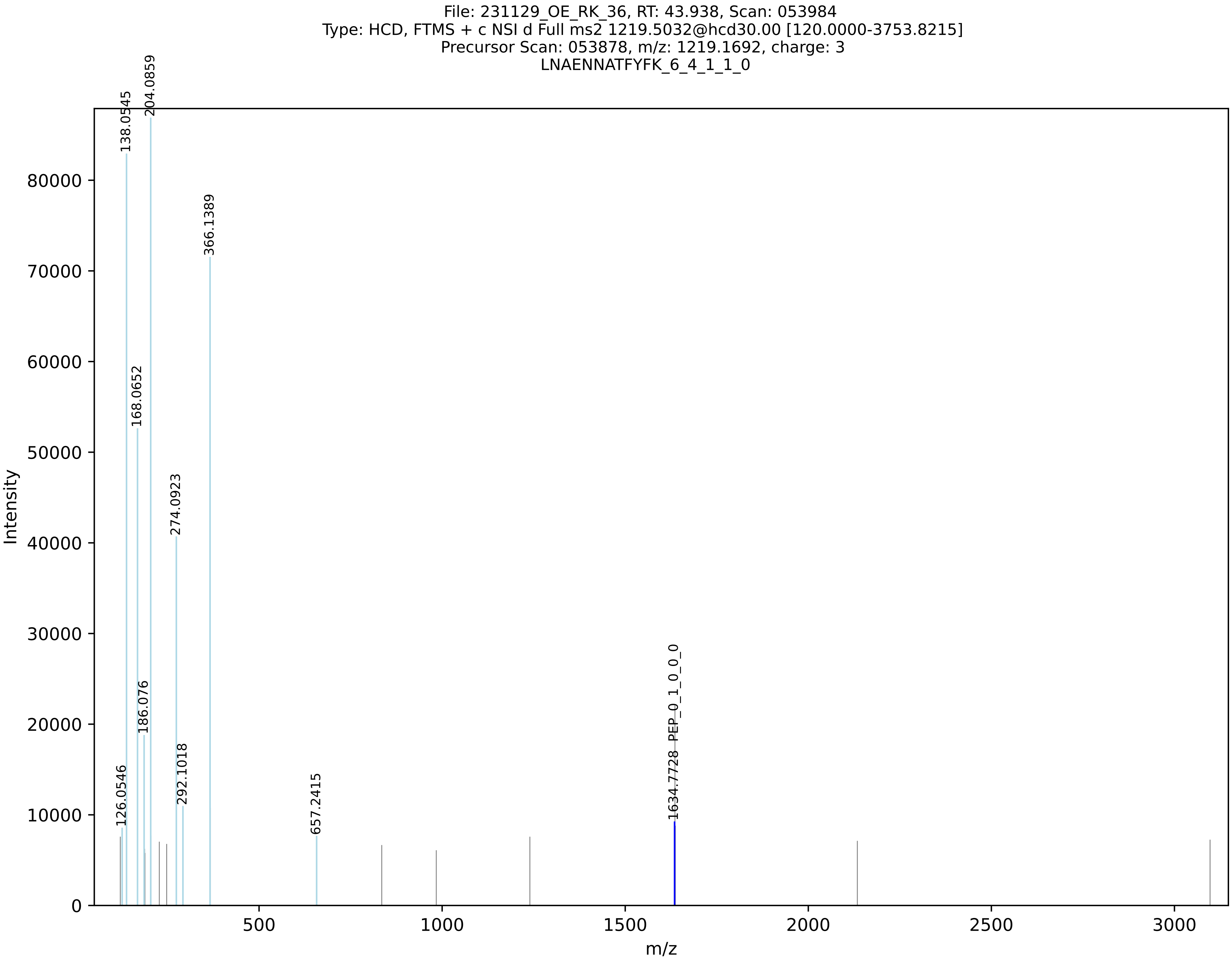
File: 231129_OE_RK_36, RT: 43.938, Scan: 053984
Type: HCD, FTMS + c NSI d Full ms2 1219.5032@hcd30.00 [120.0000-3753.8215]
Precursor Scan: 053878, m/z: 1219.1692, charge: 3
LNAENNATFYFK_6_4_1_1_0
Intensity
m/z
138.0545
204.0859
366.1389
168.0652
274.0923
186.076
126.0546
292.1018
657.2415
1634.7728_PEP_0_1_0_0_0
0
10000
20000
30000
40000
50000
60000
70000
80000
500
1000
1500
2000
2500
3000

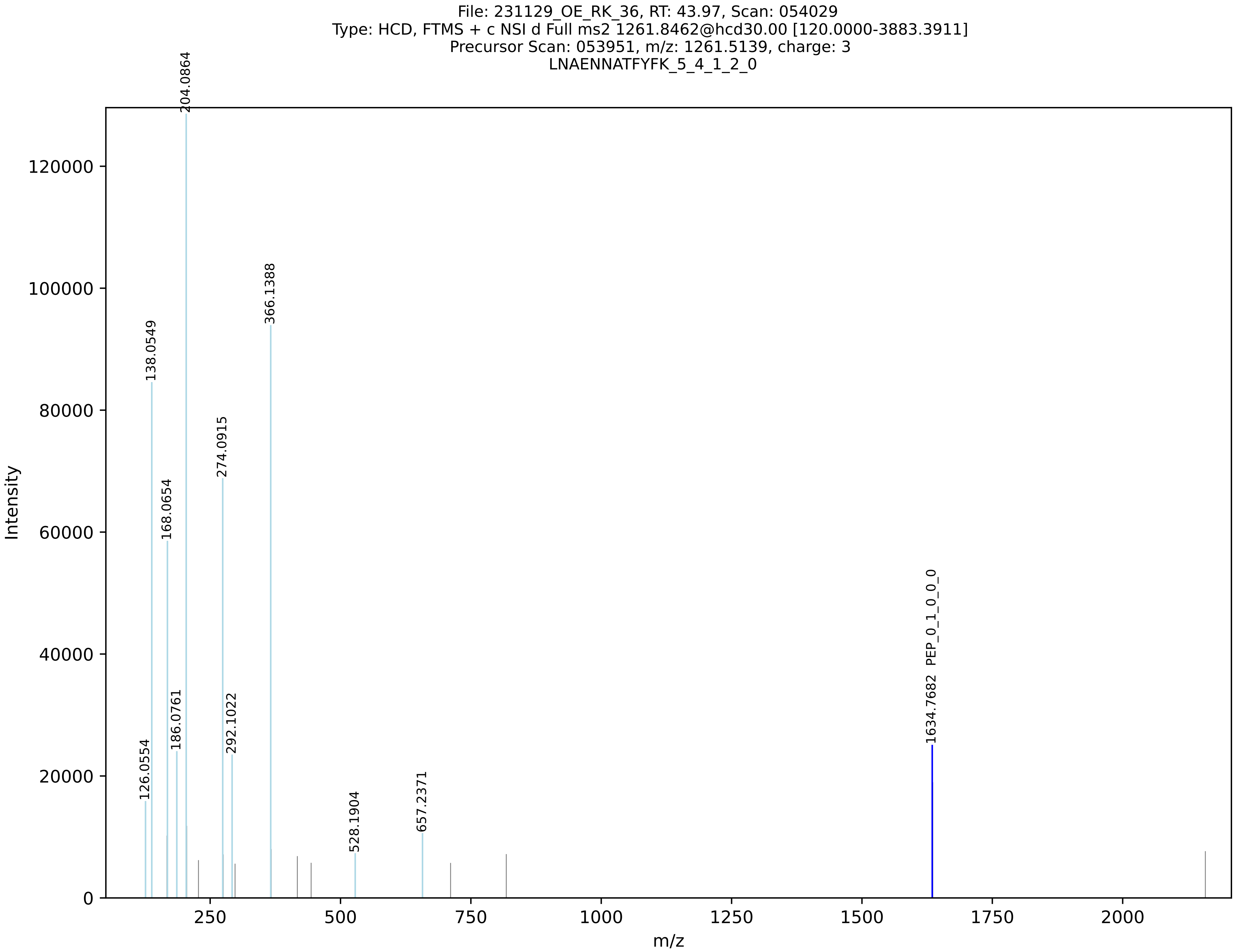
File: 231129_OE_RK_36, RT: 43.97, Scan: 054029
Type: HCD, FTMS + c NSI d Full ms2 1261.8462@hcd30.00 [120.0000-3883.3911]
Precursor Scan: 053951, m/z: 1261.5139, charge: 3
LNAENNATFYFK_5_4_1_2_0
Intensity
m/z
204.0864
366.1388
138.0549
274.0915
168.0654
186.0761
292.1022
126.0554
528.1904
657.2371
1634.7682 PEP_0_1_0_0_0_
0
20000
40000
60000
80000
100000
120000
250
500
750
1000
1250
1500
1750
2000

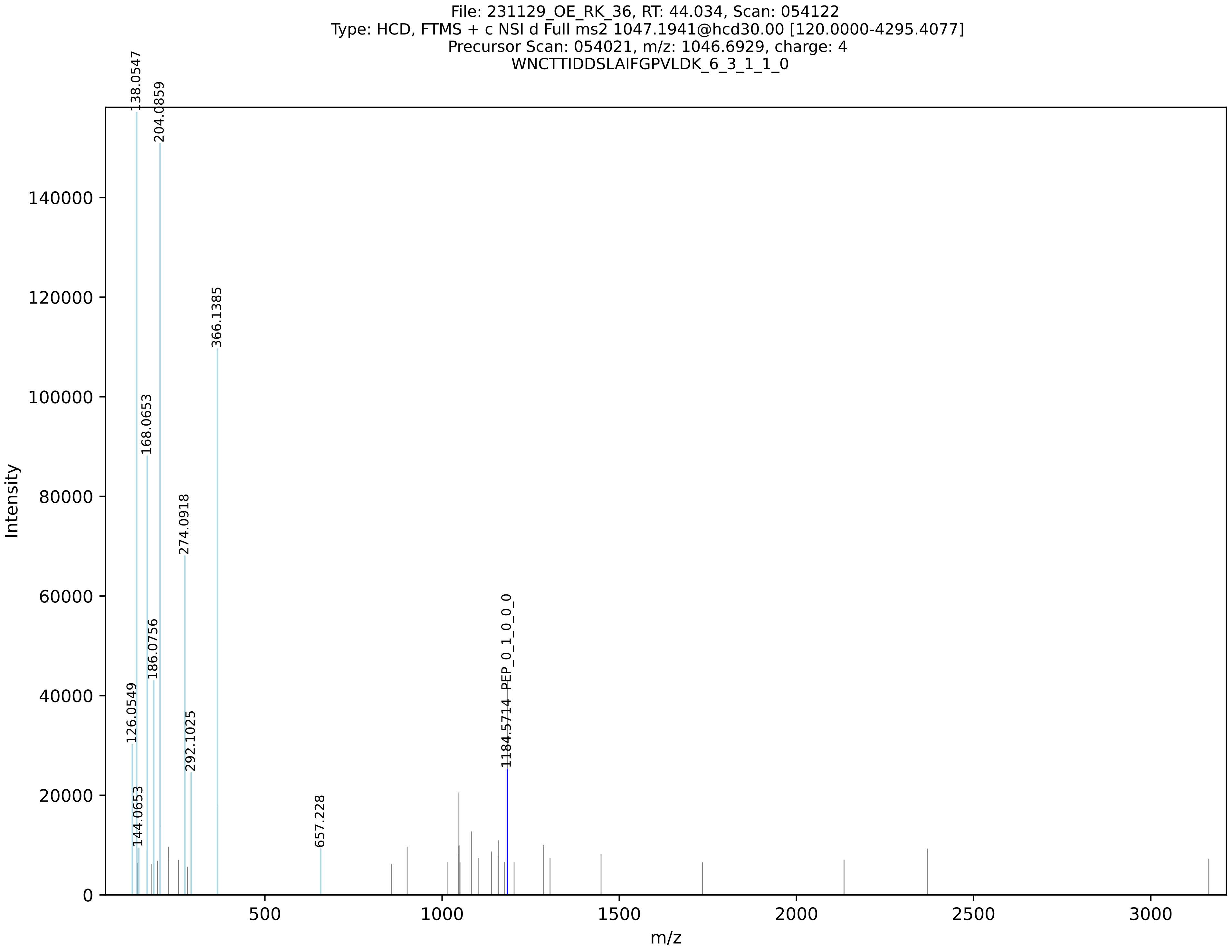

File: 231129_OE_RK_36, RT: 44.034, Scan: 054122
Type: HCD, FTMS + c NSI d Full ms2 1047.1941@hcd30.00 [120.0000-4295.4077]
Precursor Scan: 054021, m/z: 1046.6929, charge: 4
WNCTTIDDSLAIFGPVLDK_6_3_1_1_0
138.0547
204.0859
366.1385
168.0653
274.0918
186.0756
126.0549
292.1025
144.0653
657.228
1184.5714_PEP_0_1_0_0_0
Intensity
m/z
0
20000
40000
60000
80000
100000
120000
140000
500
1000
1500
2000
2500
3000

File: 231129_OE_RK_36, RT: 44.066, Scan: 054166
Type: HCD, FTMS + c NSI d Full ms2 1031.4242@hcd30.00 [120.0000-4231.0669]
Precursor Scan: 054095, m/z: 1030.9227, charge: 4
QDQCIYNTTYLNVQR_5_4_0_2_0

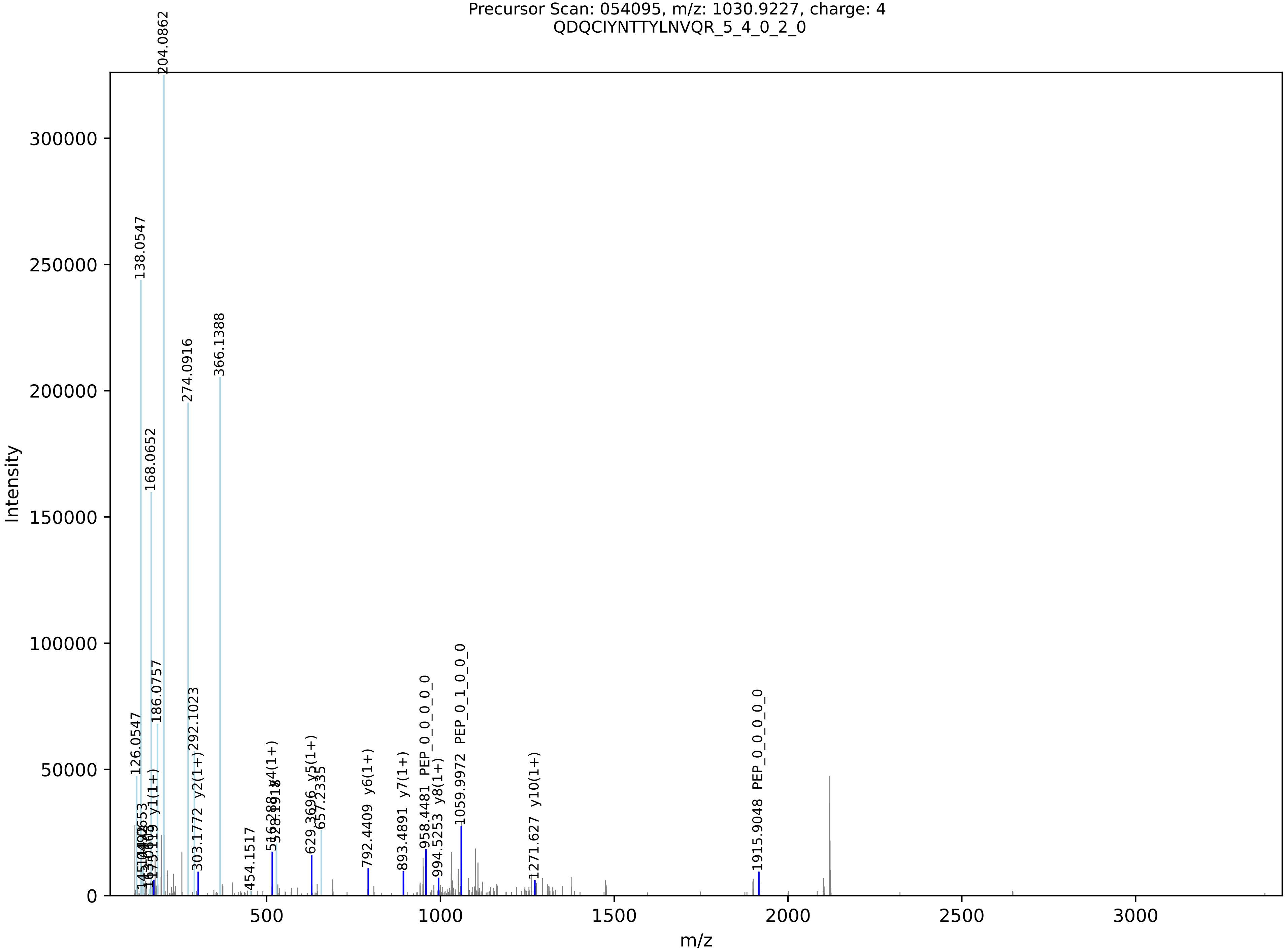

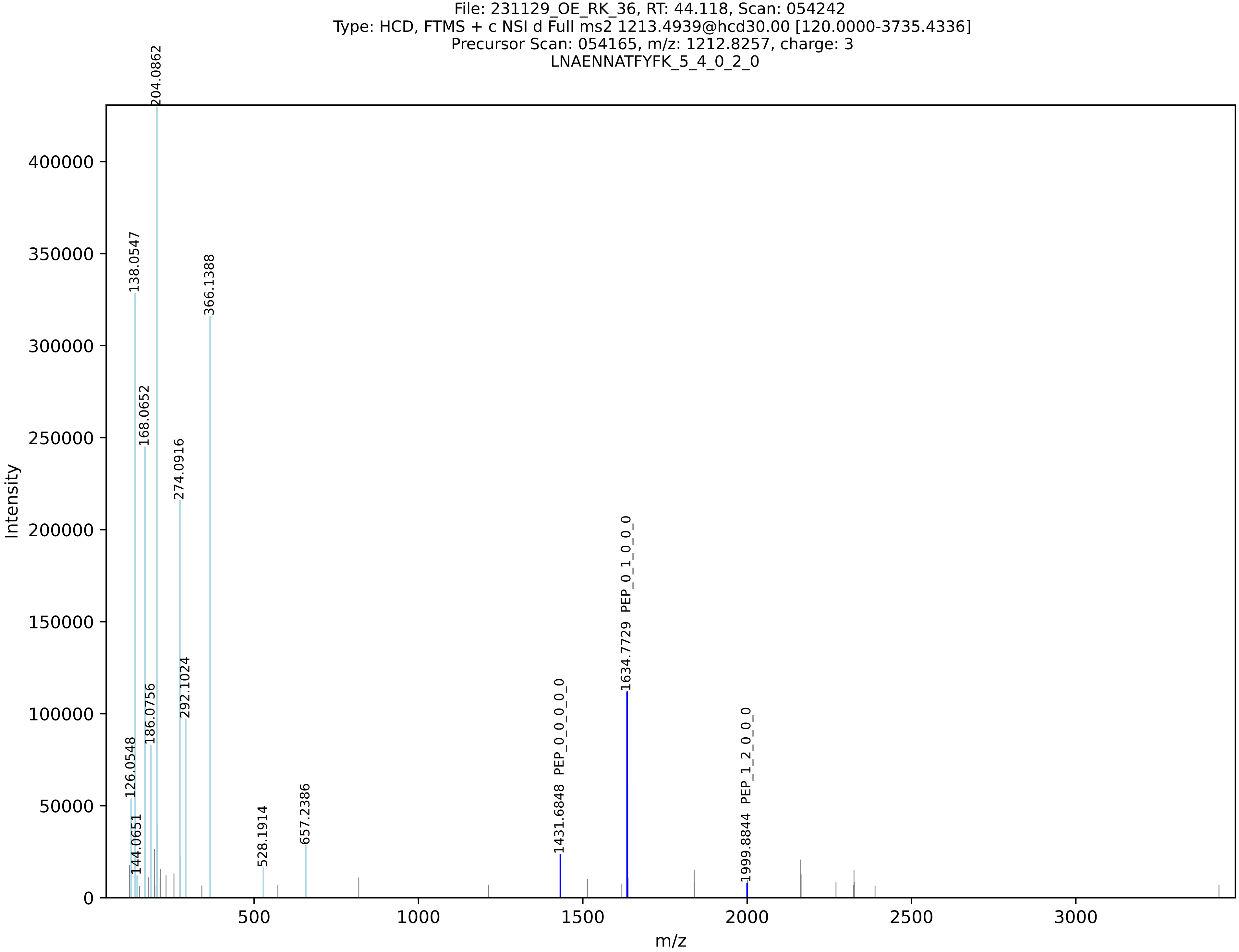
File: 231129_OE_RK_36, RT: 44.118, Scan: 054242
Type: HCD, FTMS + c NSI d Full ms2 1213.4939@hcd30.00 [120.0000-3735.4336]
Precursor Scan: 054165, m/z: 1212.8257, charge: 3
LNAENNATFYFK_5_4_0_2_0
Intensity
m/z
204.0862
138.0547
366.1388
168.0652
274.0916
292.1024
186.0756
126.0548
144.0651
528.1914
657.2386
1431.6848 PEP_0_0_0_0_0
1634.7729 PEP_0_1_0_0_0
1999.8844 PEP_1_2_0_0_0

File: 231129_OE_RK_36, RT: 44.121, Scan: 054246
Type: HCD, FTMS + c NSI d Full ms2 1122.9574@hcd30.00 [120.0000-4604.5220]
Precursor Scan: 054165, m/z: 1122.2056, charge: 4
QDQCIYNTTYLNVQR_6_5_0_2_0

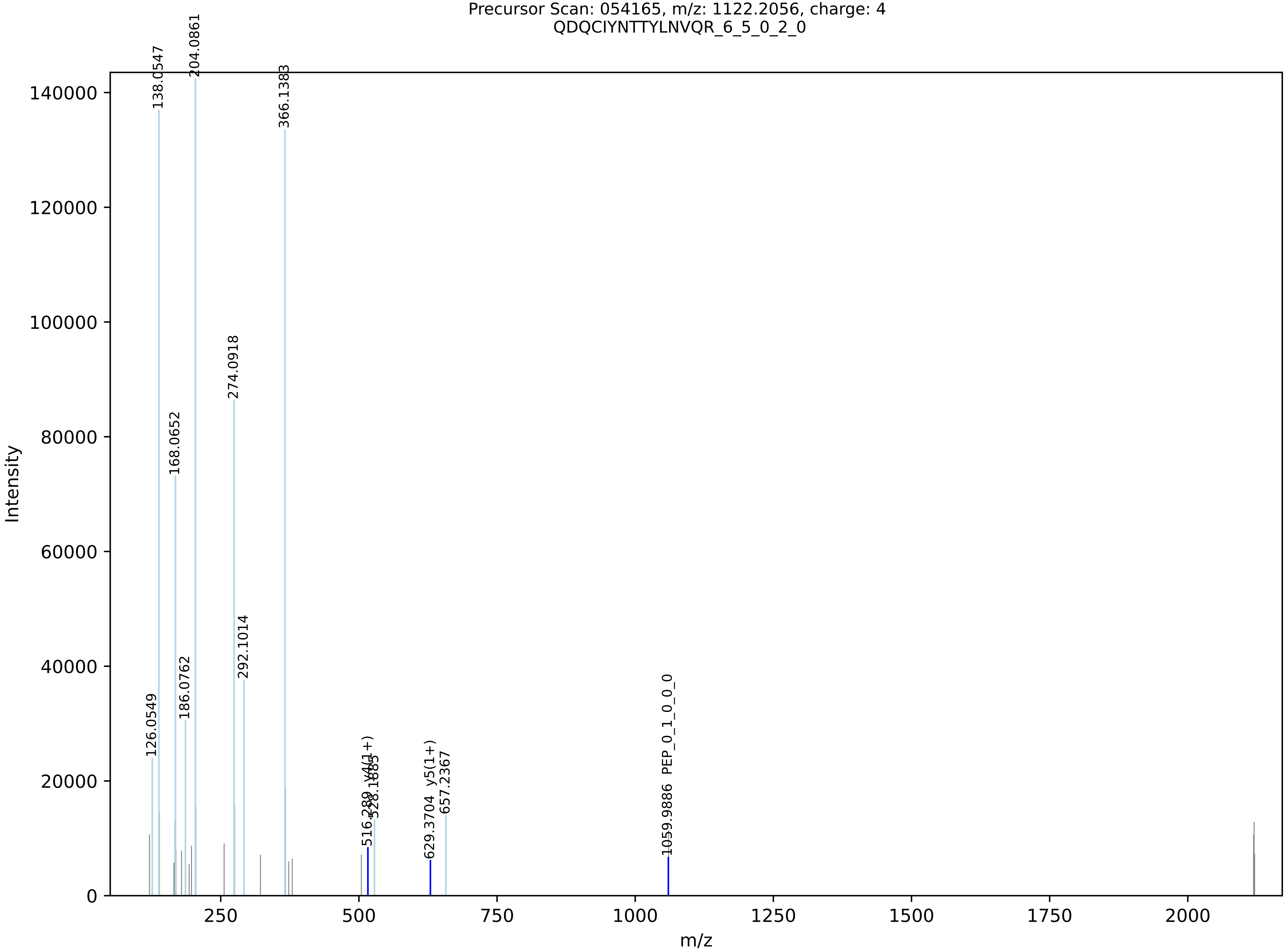

File: 231129_OE_RK_36, RT: 44.321, Scan: 054535
Type: HCD, FTMS + c NSI d Full ms2 1213.4938@hcd30.00 [120.0000-3735.4331]
Precursor Scan: 054460, m/z: 1212.8256, charge: 3
LNAENNATFYFK_5_4_0_2_0

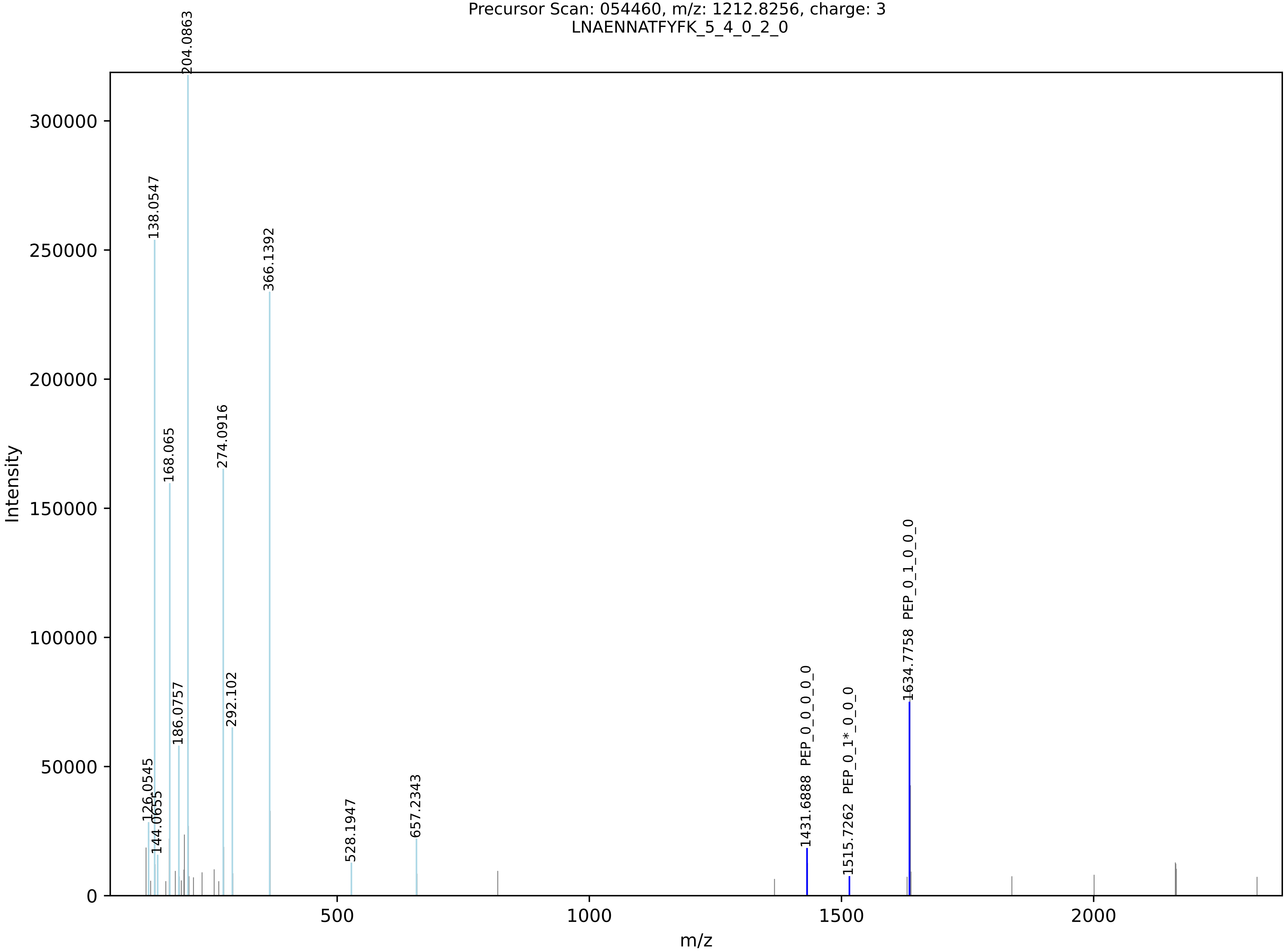

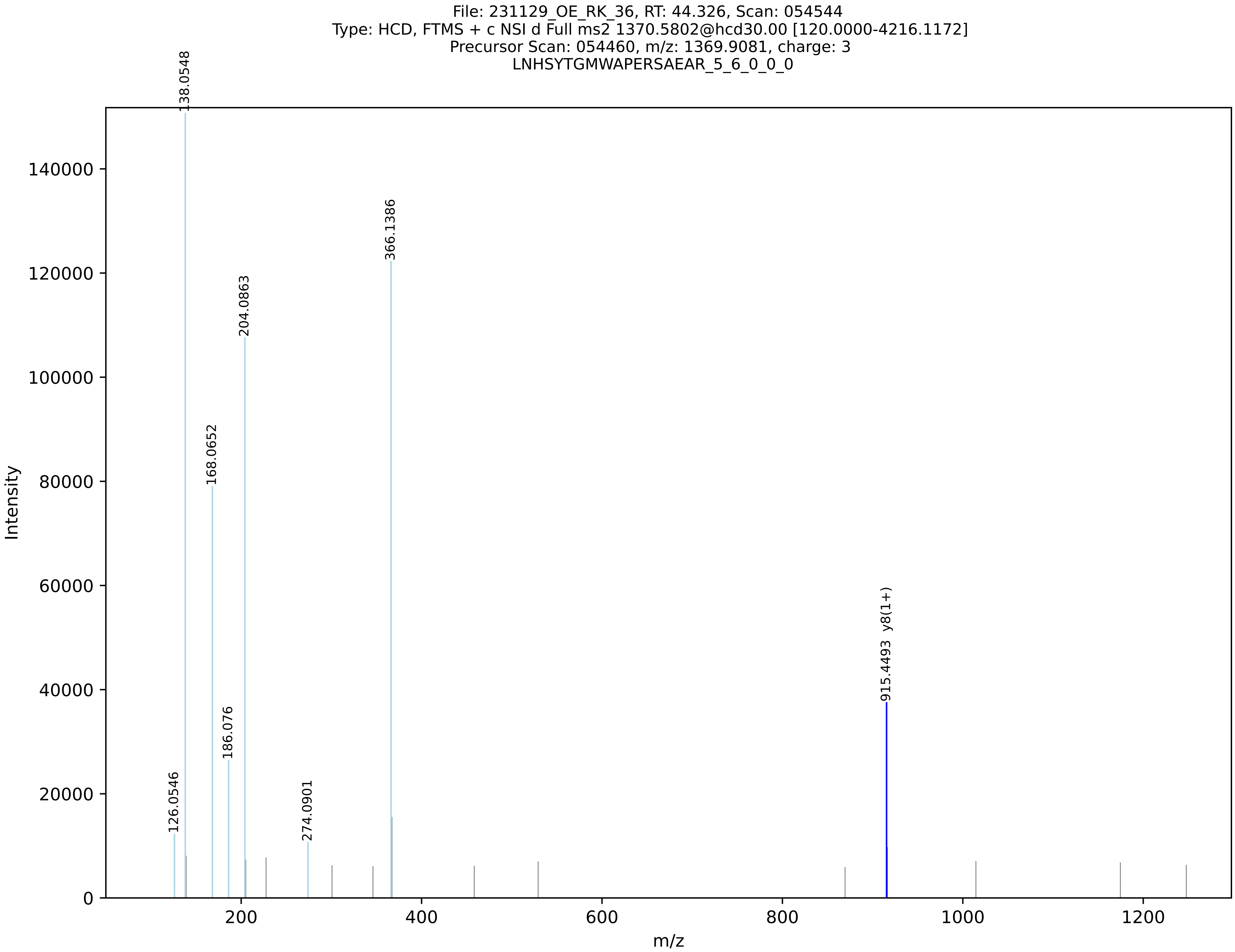
File: 231129_OE_RK_36, RT: 44.326, Scan: 054544
Type: HCD, FTMS + c NSI d Full ms2 1370.5802@hcd30.00 [120.0000-4216.1172]
Precursor Scan: 054460, m/z: 1369.9081, charge: 3
LNHSYTGMWAPERSAEAR_5_6_0_0_0
Intensity
m/z
138.0548
366.1386
204.0863
168.0652
915.4493 y8(1+)
186.076
126.0546
274.0901
0
20000
40000
60000
80000
100000
120000
140000
200
400
600
800
1000
1200

File: 231129_OE_RK_36, RT: 44.387, Scan: 054634
Type: HCD, FTMS + c NSI d Full ms2 1297.2092@hcd30.00 [120.0000-3991.6023]
Precursor Scan: 054533, m/z: 1296.8789, charge: 3
LANLTQGEDQYYLR_5_4_0_2_0

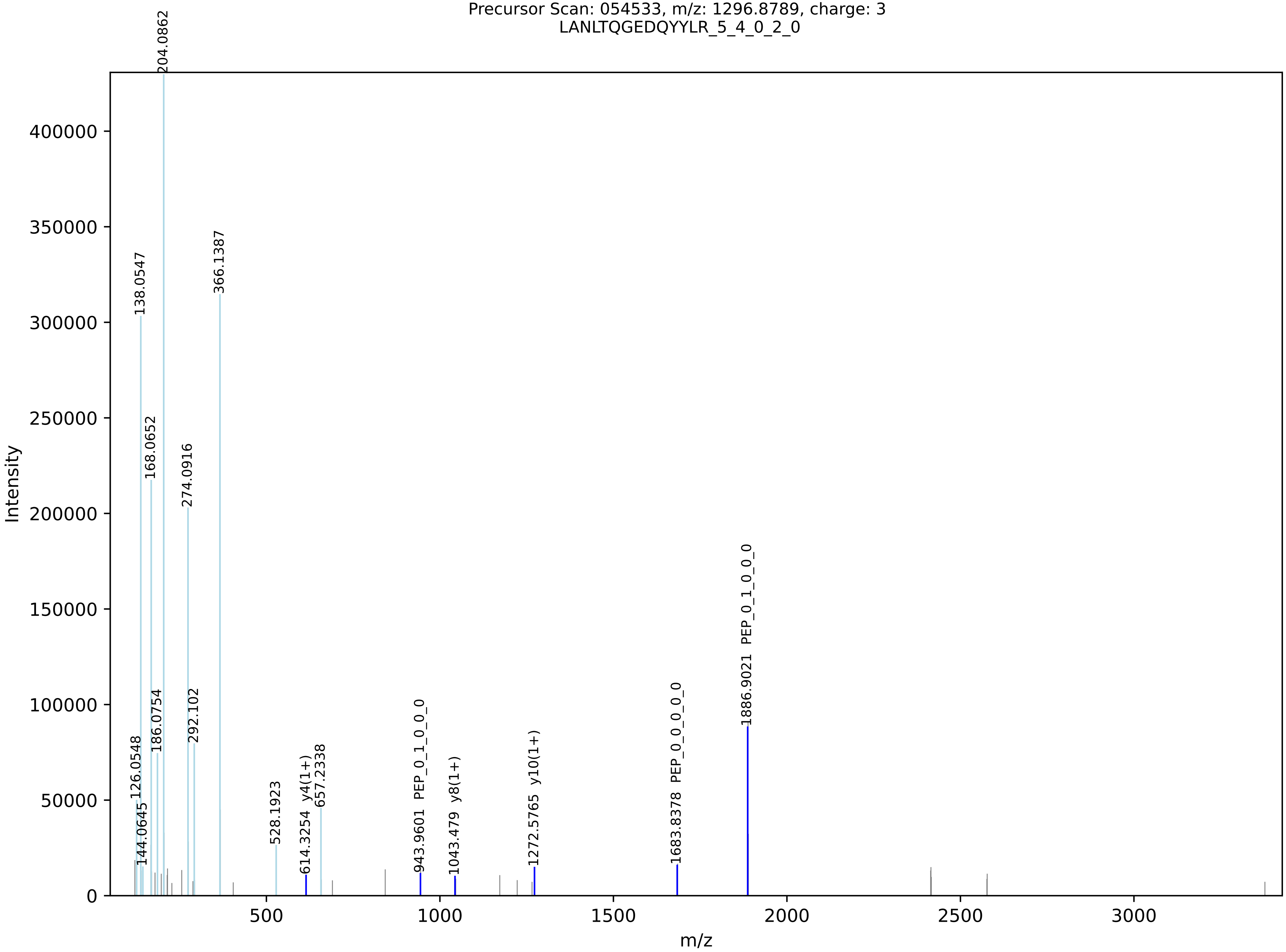

File: 231129_OE_RK_36, RT: 44.472, Scan: 054757
Type: HCD, FTMS + c NSI d Full ms2 1360.0443@hcd30.00 [120.0000-5571.8369]
Precursor Scan: 054678, m/z: 1359.2926, charge: 4
QDQCIYNTTYLNVQR_7_6_2_3_0

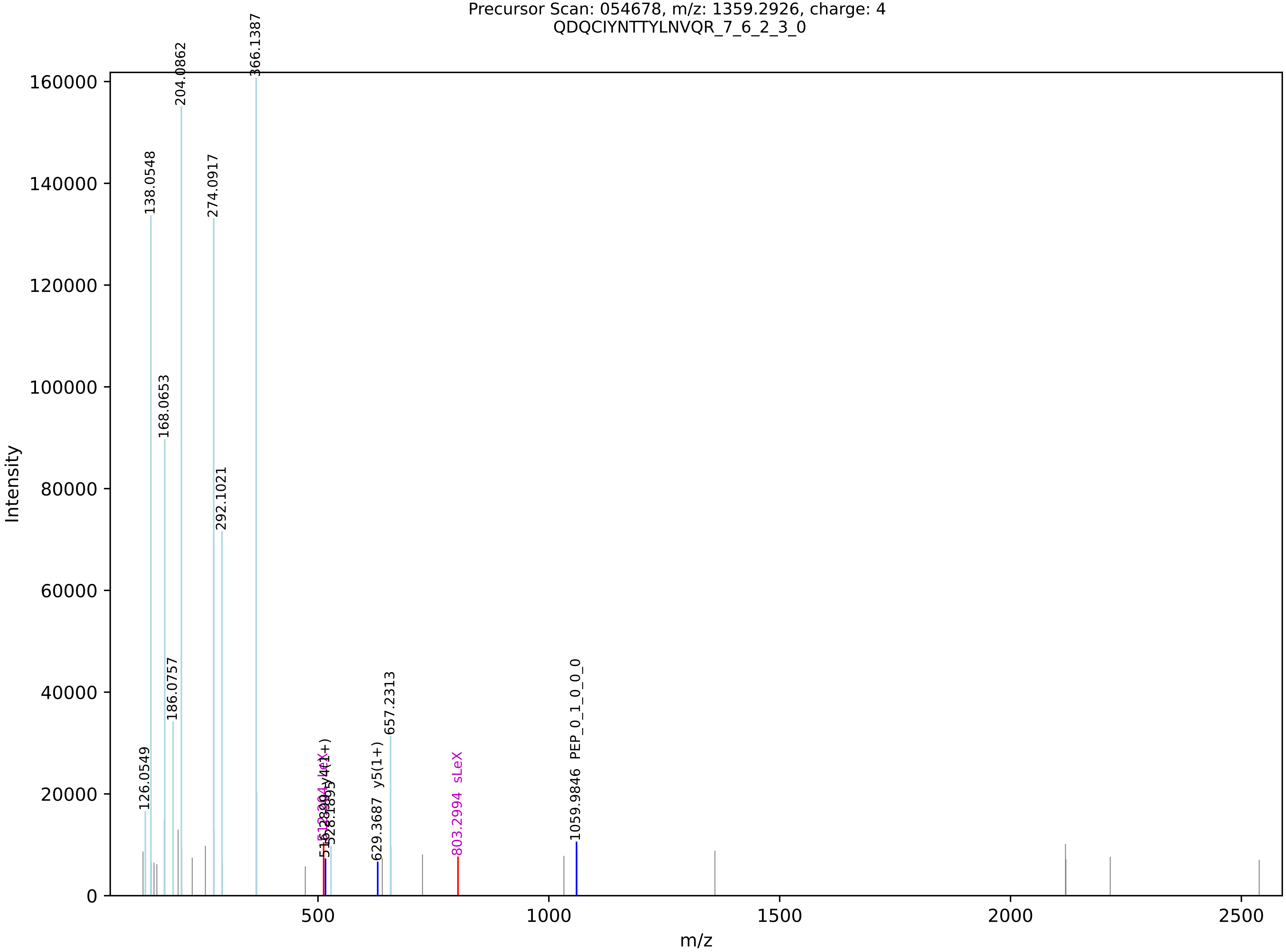

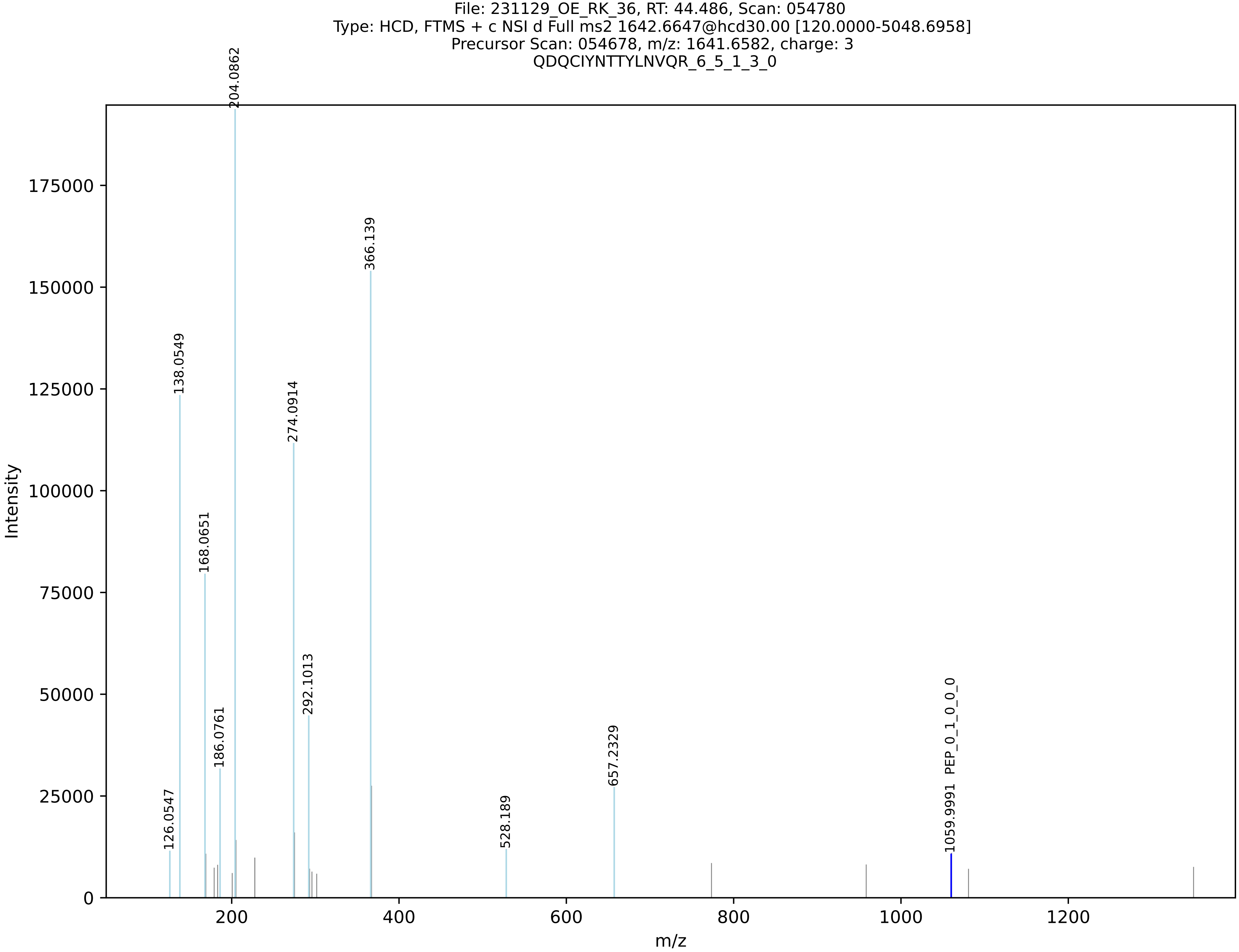
File: 231129_OE_RK_36, RT: 44.486, Scan: 054780
Type: HCD, FTMS + c NSI d Full ms2 1642.6647@hcd30.00 [120.0000-5048.6958]
Precursor Scan: 054678, m/z: 1641.6582, charge: 3
QDQCIYNTTYLNVQR_6_5_1_3_0
Intensity
m/z
204.0862
366.139
138.0549
274.0914
168.0651
292.1013
186.0761
126.0547
528.189
657.2329
1059.9991 PEP_0_1_0_0_0
0
25000
50000
75000
100000
125000
150000
175000
200
400
600
800
1000
1200

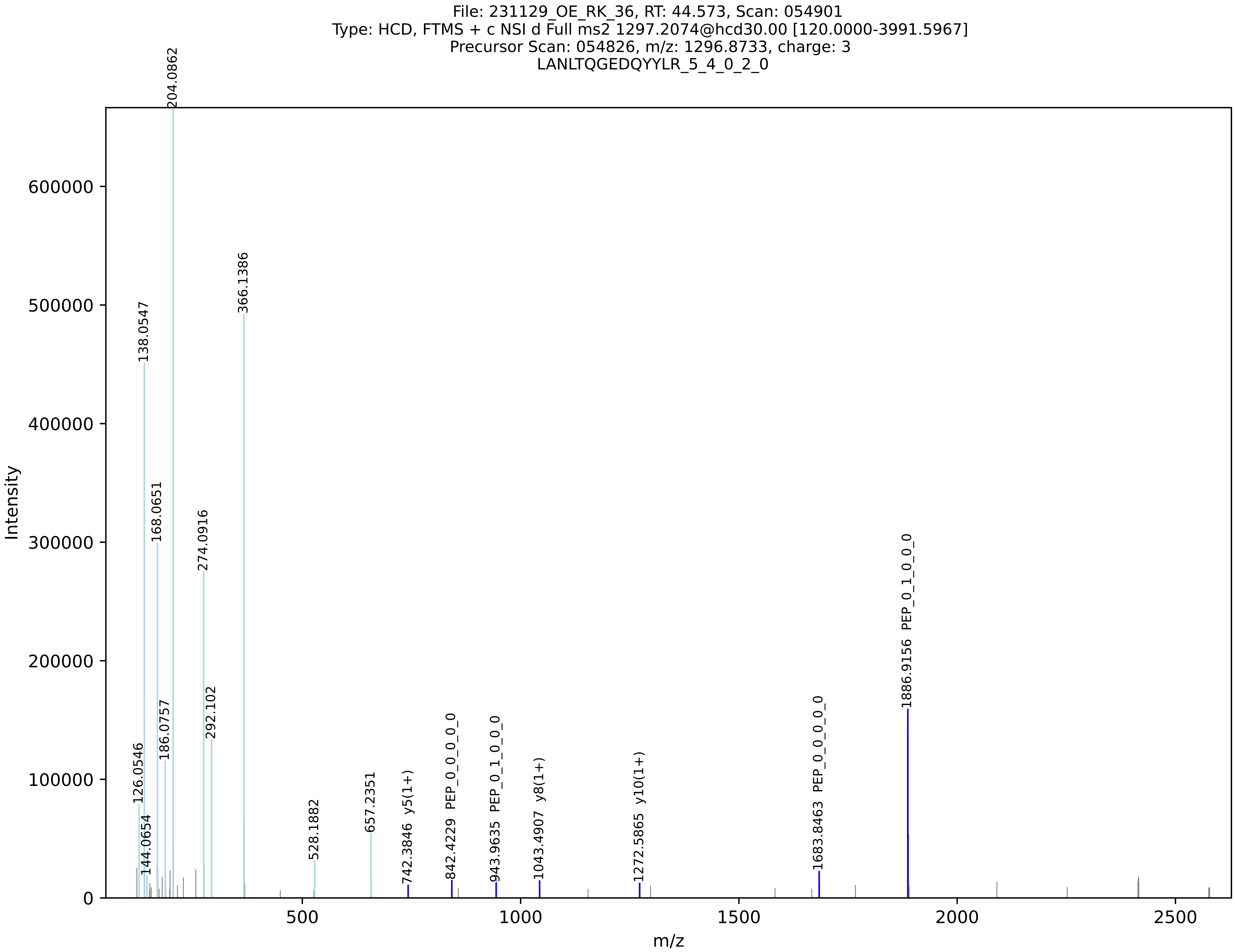
File: 231129_OE_RK_36, RT: 44.573, Scan: 054901
Type: HCD, FTMS + c NSI d Full ms2 1297.2074@hcd30.00 [120.0000-3991.5967]
Precursor Scan: 054826, m/z: 1296.8733, charge: 3
LANLTQGEDQYYLR_5_4_0_2_0
Intensity
m/z
0
100000
200000
300000
400000
500000
600000
500
1000
1500
2000
2500
204.0862
366.1386
138.0547
168.0651
274.0916
292.102
186.0757
126.0546
144.0654
528.1882
657.2351
742.3846 y5(1+)
842.4229 PEP_0_0_0_0_0
943.9635 PEP_0_1_0_0_0
1043.4907 y8(1+)
1272.5865 y10(1+)
1683.8463 PEP_0_0_0_0_0
1886.9156 PEP_0_1_0_0_0

File: 231129_OE_RK_36, RT: 44.623, Scan: 054975
Type: HCD, FTMS + c NSI d Full ms2 995.9674@hcd30.00 [120.0000-4086.4028]
Precursor Scan: 054897, m/z: 995.4662, charge: 4
VVTVAALGTNISIHKDEIGK_5_4_0_1_0

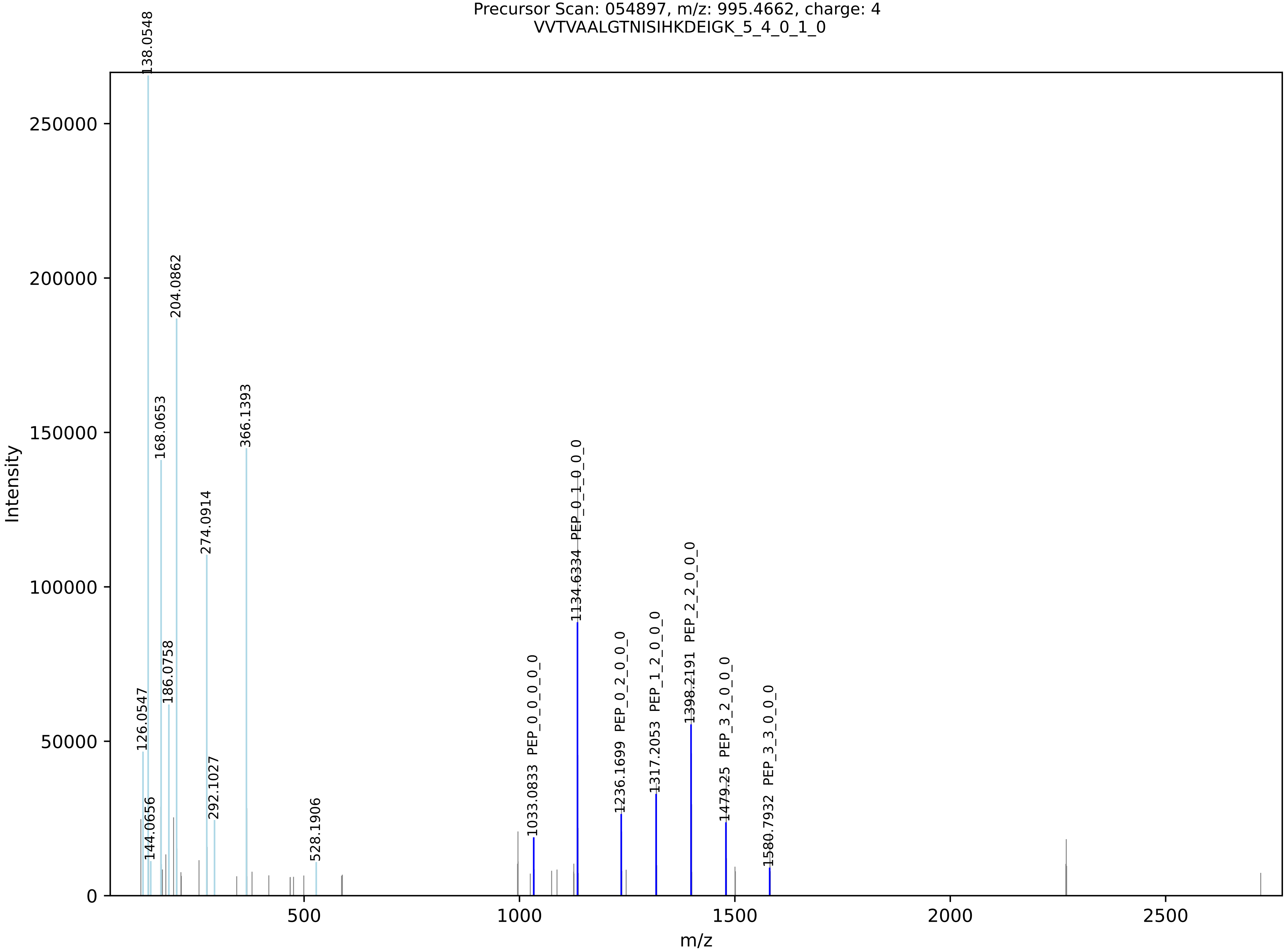

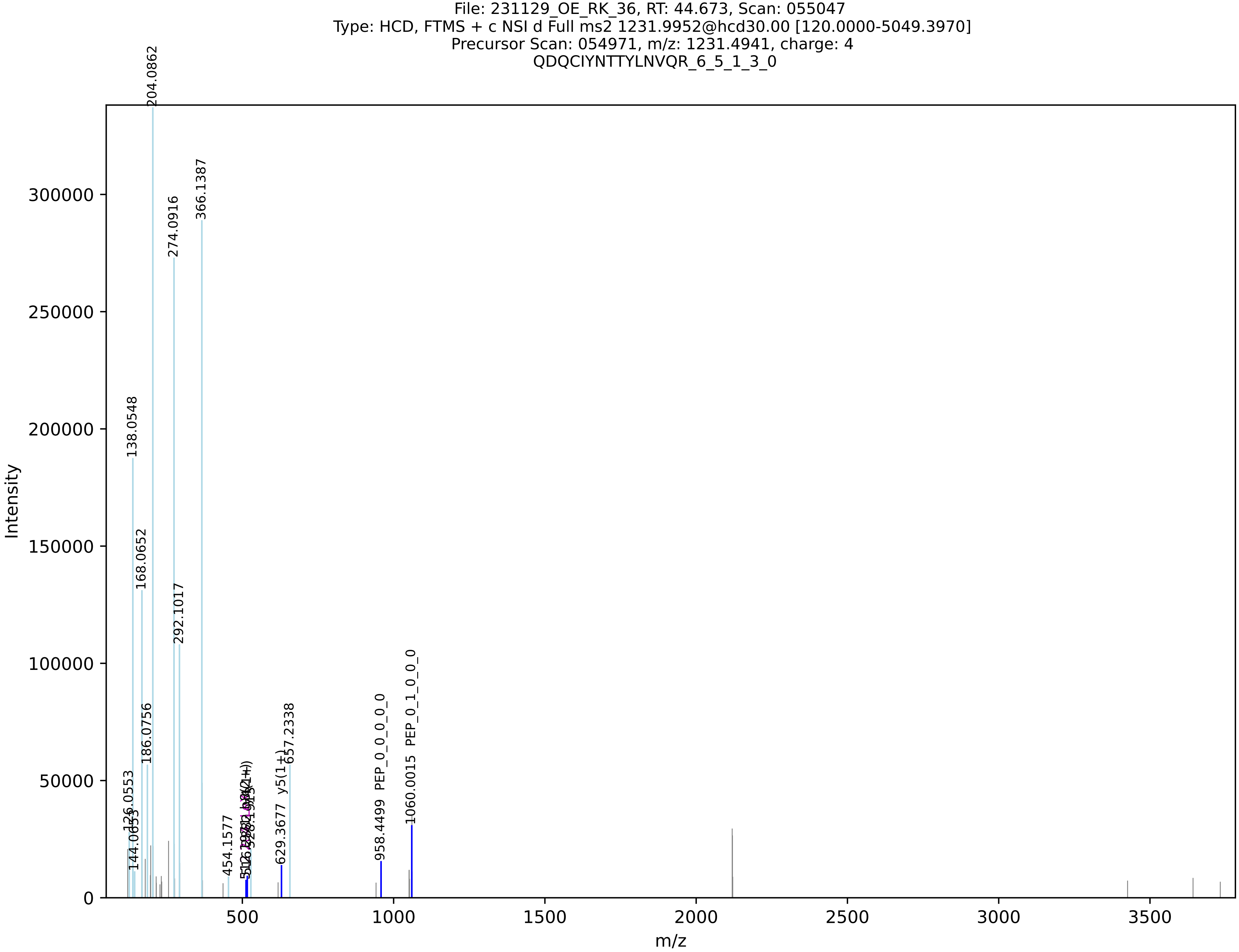
File: 231129_OE_RK_36, RT: 44.673, Scan: 055047
Type: HCD, FTMS + c NSI d Full ms2 1231.9952@hcd30.00 [120.0000-5049.3970]
Precursor Scan: 054971, m/z: 1231.4941, charge: 4
QDQCIYNTTYLNVQR_6_5_1_3_0
204.0862
366.1387
274.0916
138.0548
168.0652
292.1017
186.0756
126.0553
144.0653
454.1577
629.3677
y5(1+)
657.2338
958.4499
PEP_0_0_0_0_0
1060.0015
PEP_0_1_0_0_0
Intensity
m/z

File: 231129_OE_RK_36, RT: 44.69, Scan: 055076
Type: HCD, FTMS + c NSI d Full ms2 1363.7971@hcd30.00 [120.0000-5587.1484]
Precursor Scan: 054971, m/z: 1363.296, charge: 4
ALGFENATQALGR_9_8_1_3_0

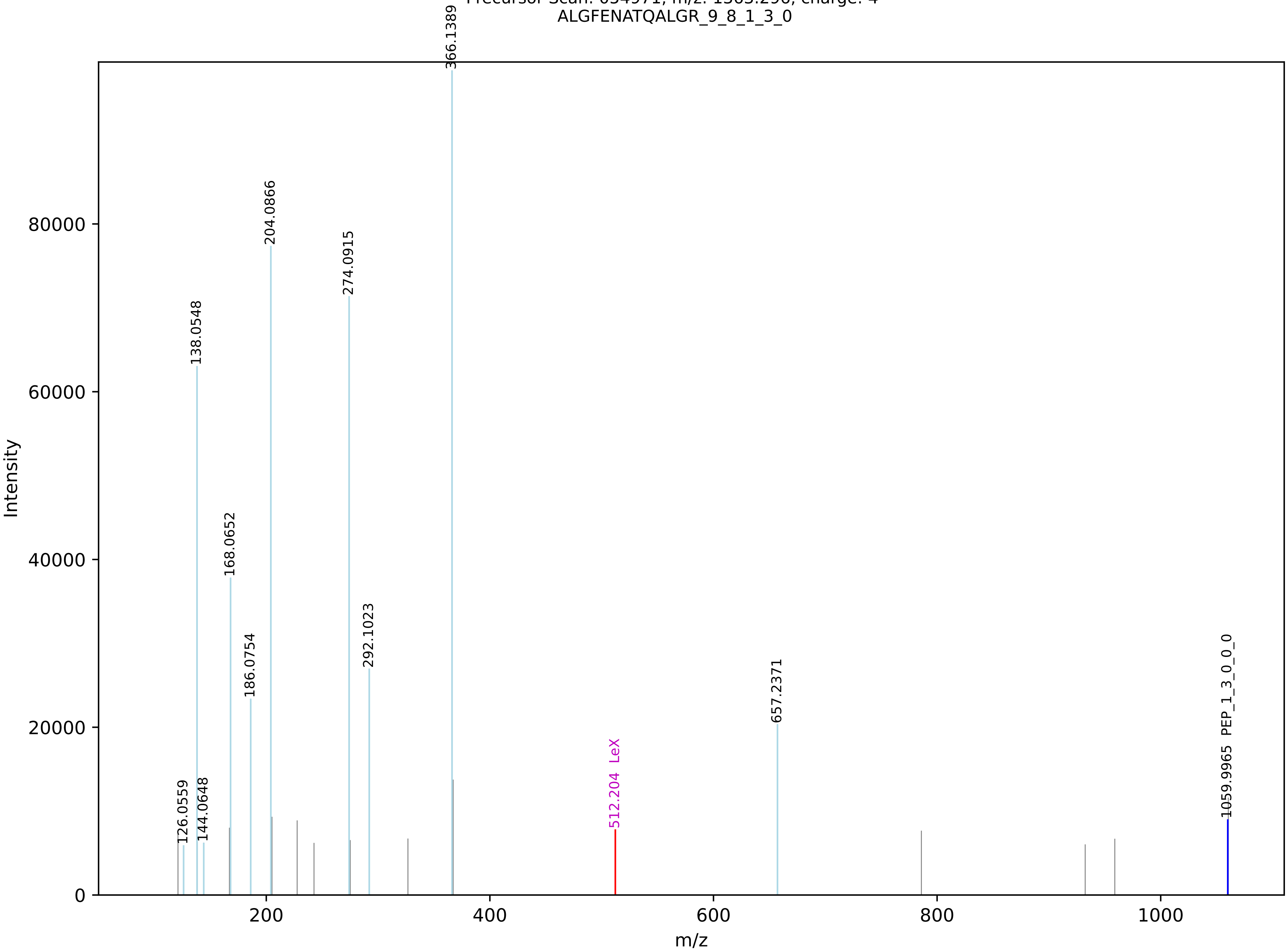

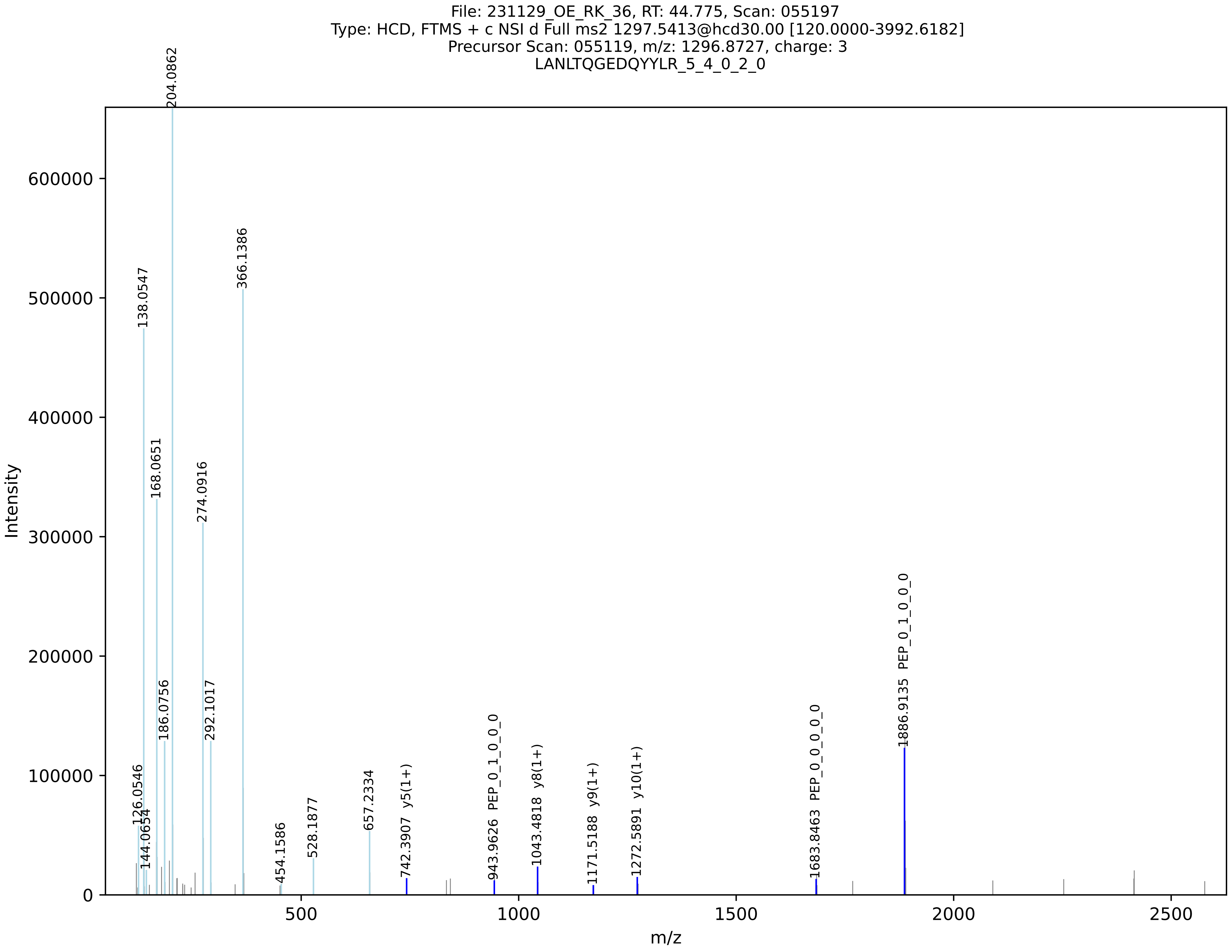
File: 231129_OE_RK_36, RT: 44.775, Scan: 055197
Type: HCD, FTMS + c NSI d Full ms2 1297.5413@hcd30.00 [120.0000-3992.6182]
Precursor Scan: 055119, m/z: 1296.8727, charge: 3
LANLTQGEDQYYLR_5_4_0_2_0
Intensity
m/z
204.0862
138.0547
366.1386
168.0651
274.0916
186.0756
292.1017
126.0546
144.0654
454.1586
528.1877
657.2334
742.3907 y5(1+)
943.9626 PEP_0_1_0_0_0
1043.4818 y8(1+)
1171.5188 y9(1+)
1272.5891 y10(1+)
1683.8463 PEP_0_0_0_0_0
1886.9135 PEP_0_1_0_0_0
0
100000
200000
300000
400000
500000
600000
500
1000
1500
2000
2500

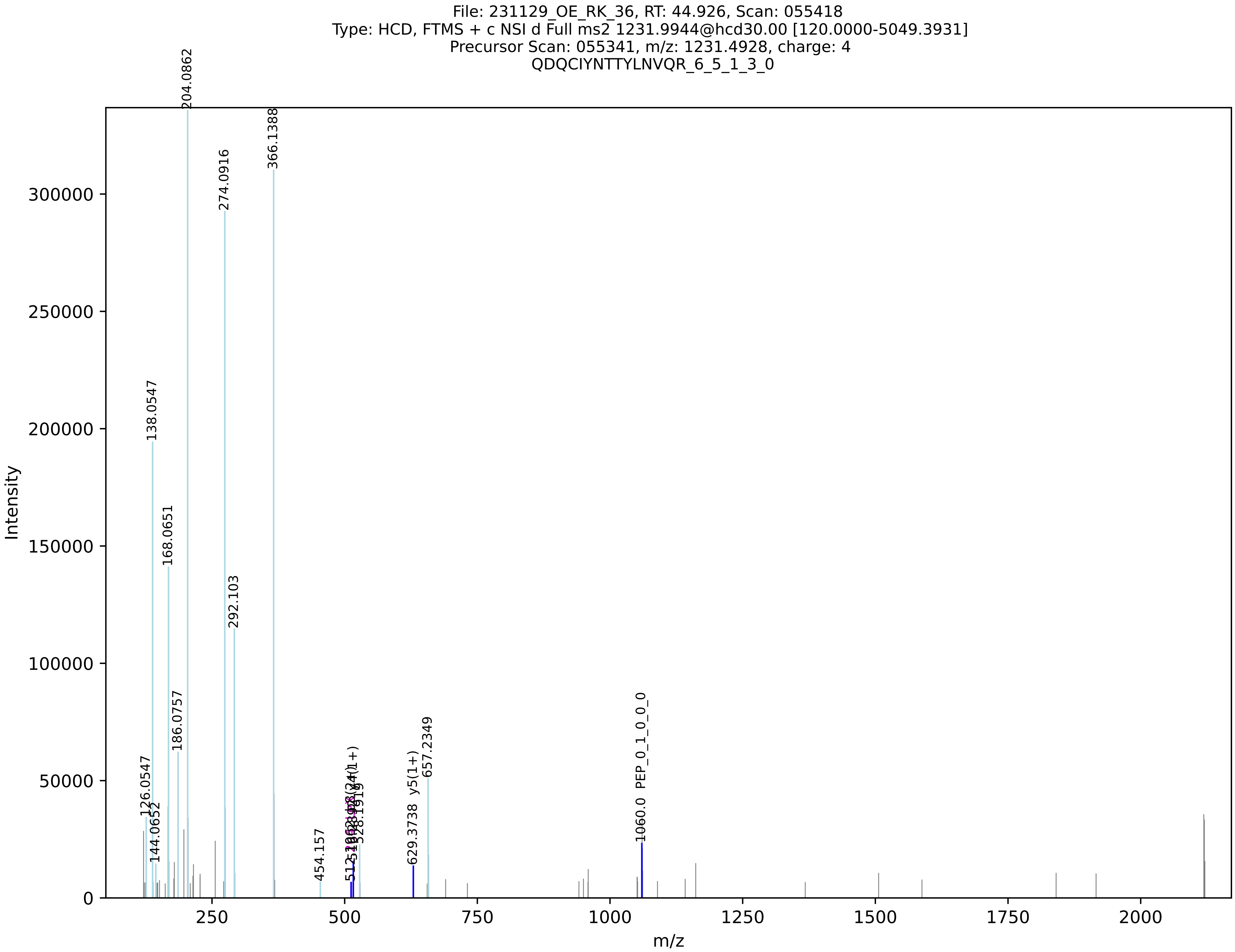
File: 231129_OE_RK_36, RT: 44.926, Scan: 055418
Type: HCD, FTMS + c NSI d Full ms2 1231.9944@hcd30.00 [120.0000-5049.3931]
Precursor Scan: 055341, m/z: 1231.4928, charge: 4
QDQCIYNTTYLNVQR_6_5_1_3_0
Intensity
m/z
204.0862
366.1388
274.0916
138.0547
168.0651
292.103
186.0757
126.0547
144.0652
454.157
629.3738
y5(1+)
657.2349
1060.0
PEP_0_1_0_0_0_

File: 231129_OE_RK_36, RT: 44.976, Scan: 055491
Type: HCD, FTMS + c NSI d Full ms2 1287.0127@hcd30.00 [120.0000-5273.8677]
Precursor Scan: 055415, m/z: 1286.2613, charge: 4
QDQCIYNTTYLNVQR_7_6_0_3_0

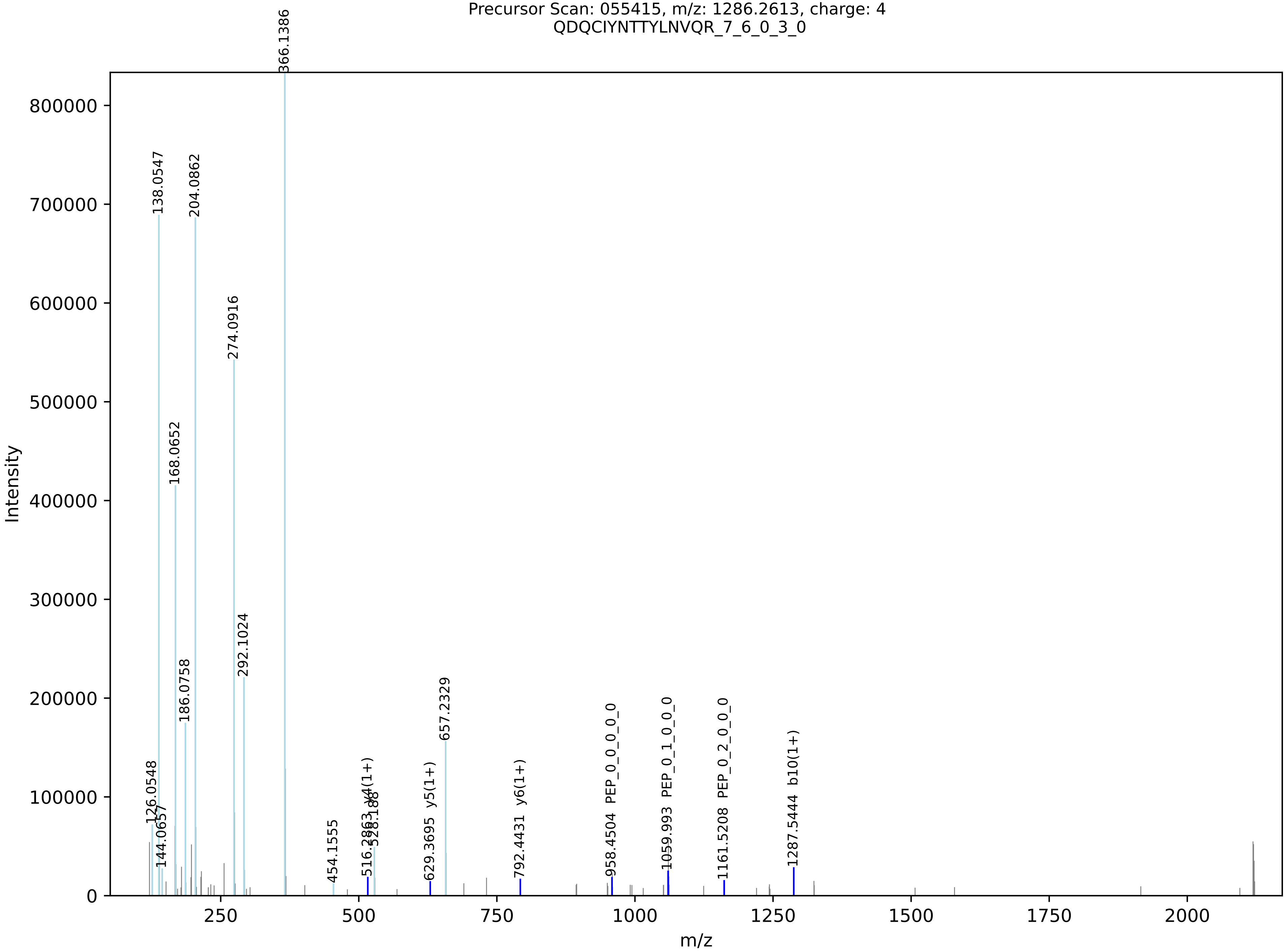

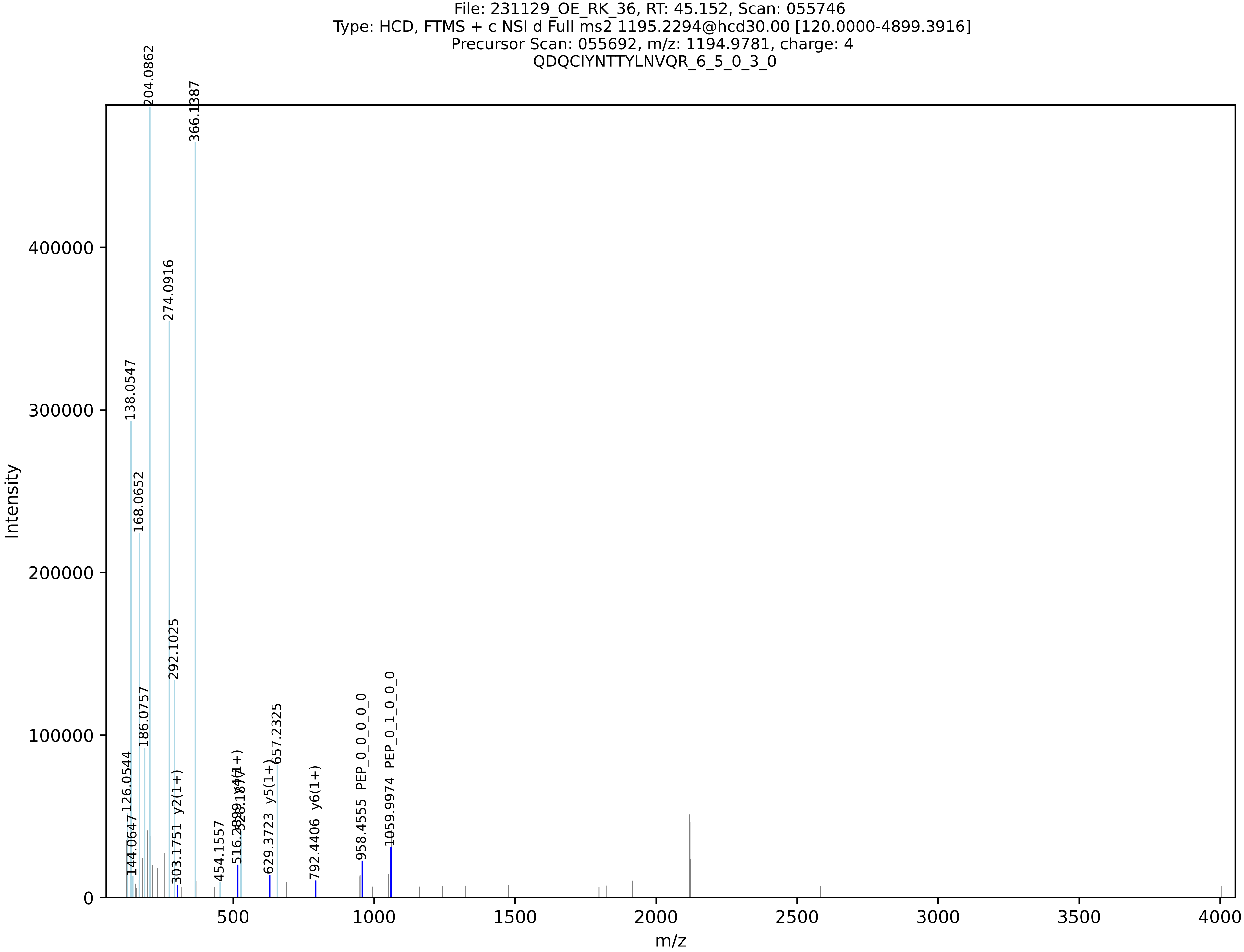
File: 231129_OE_RK_36, RT: 45.152, Scan: 055746
Type: HCD, FTMS + c NSI d Full ms2 1195.2294@hcd30.00 [120.0000-4899.3916]
Precursor Scan: 055692, m/z: 1194.9781, charge: 4
QDQCIYNTTYLNVQR_6_5_0_3_0
204.0862
366.1387
274.0916
138.0547
168.0652
292.1025
186.0757
126.0544
144.0647
303.1751 y2(1+)
454.1557
516.2890 y4(1+)
528.1870
629.3723 y5(1+)
657.2325
792.4406 y6(1+)
958.4555 PEP_0_0_0_0_0
1059.9974 PEP_0_1_0_0_0
Intensity
m/z
0
100000
200000
300000
400000
500
1000
1500
2000
2500
3000
3500
4000

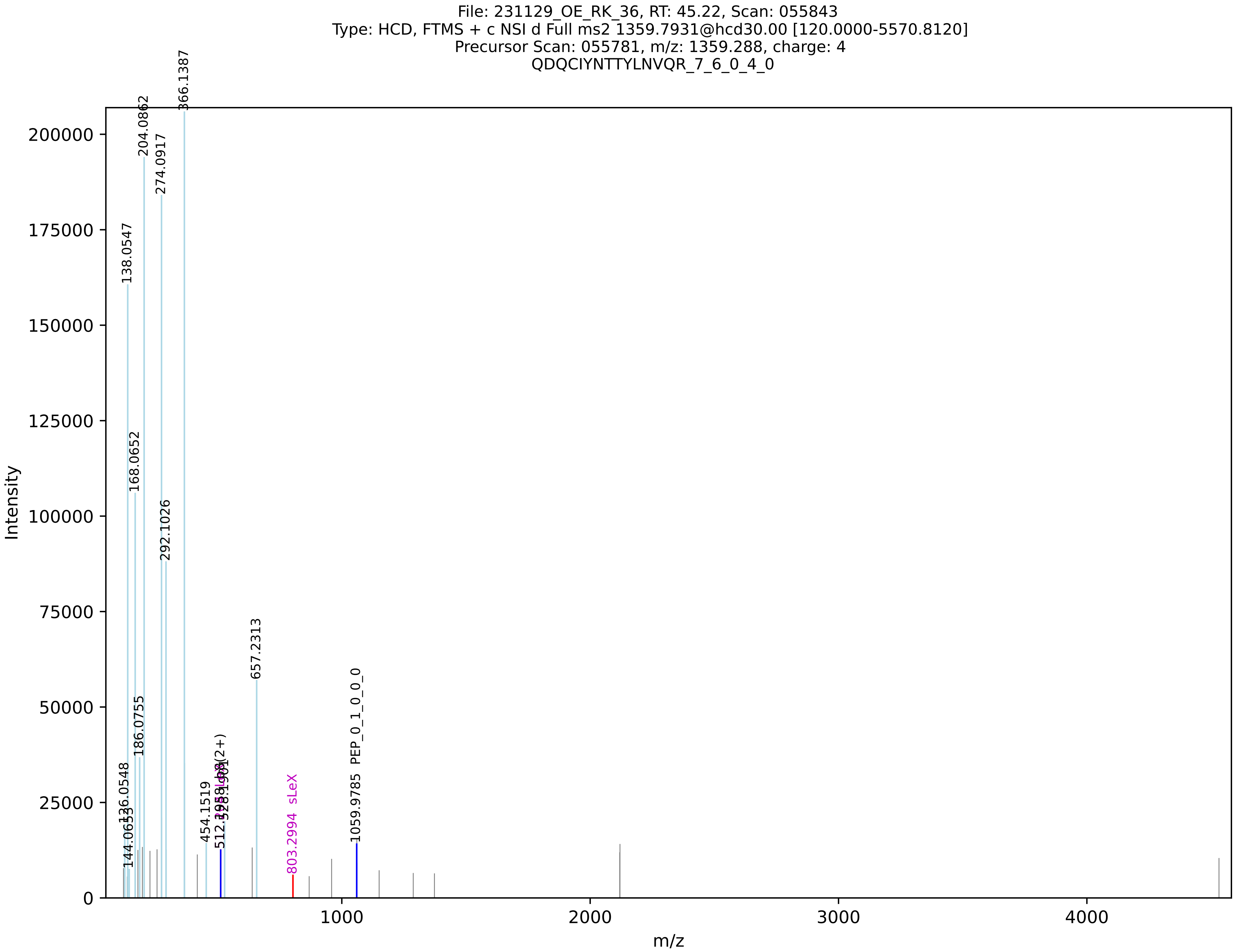
File: 231129_OE_RK_36, RT: 45.22, Scan: 055843
Type: HCD, FTMS + c NSI d Full ms2 1359.7931@hcd30.00 [120.0000-5570.8120]
Precursor Scan: 055781, m/z: 1359.288, charge: 4
QDQCIYNTTYLNVQR_7_6_0_4_0
Intensity
m/z
0
25000
50000
75000
100000
125000
150000
175000
200000
1000
2000
3000
4000
366.1387
204.0862
274.0917
138.0547
168.0652
292.1026
186.0755
126.0548
144.0653
454.1519
528.1901
657.2313
803.2994 sLeX
1059.9785 PEP_0_1_0_0_0_

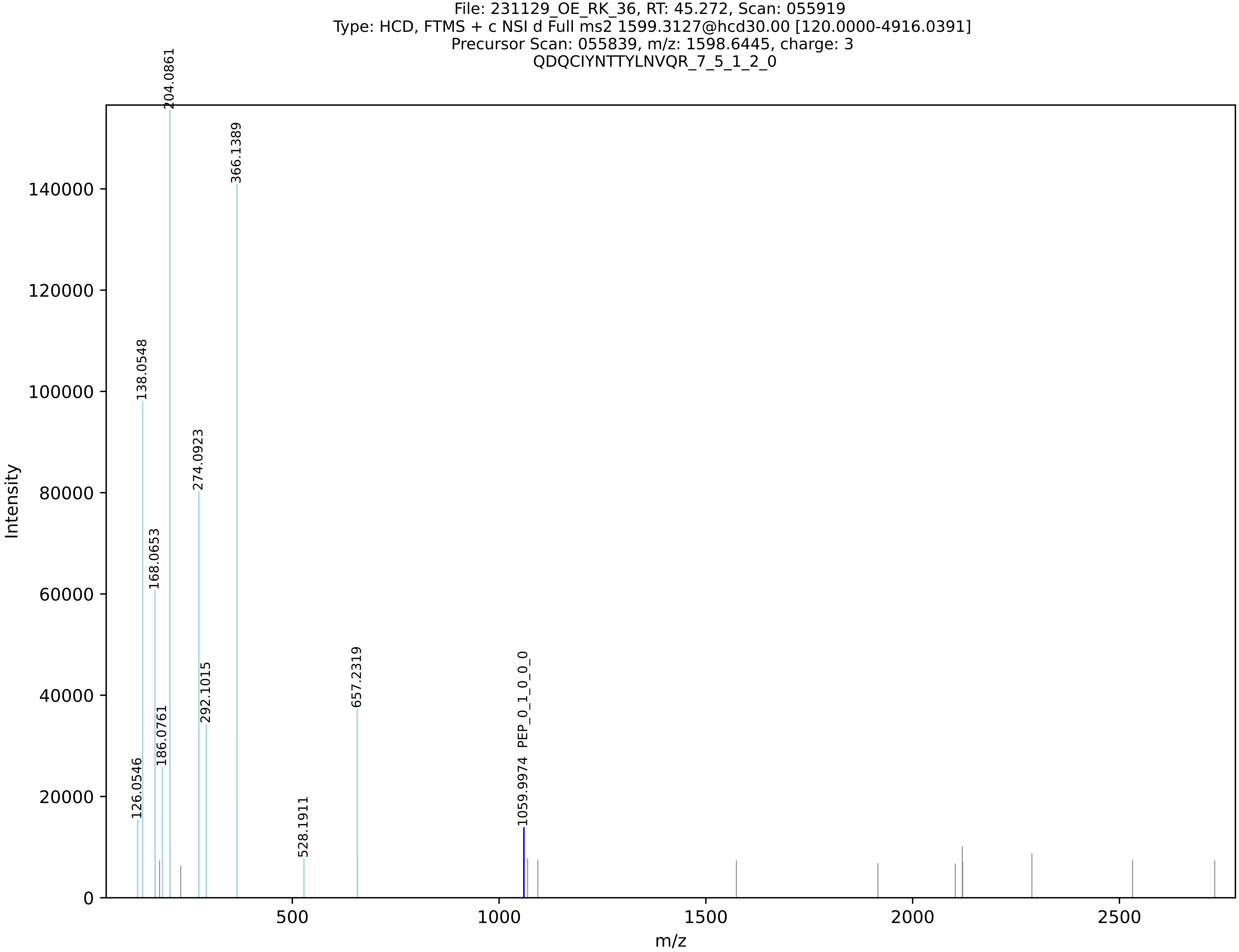
File: 231129_OE_RK_36, RT: 45.272, Scan: 055919
Type: HCD, FTMS + c NSI d Full ms2 1599.3127@hcd30.00 [120.0000-4916.0391]
Precursor Scan: 055839, m/z: 1598.6445, charge: 3
QDQCIYNTTYLNVQR_7_5_1_2_0
Intensity
m/z
204.0861
366.1389
138.0548
274.0923
168.0653
292.1015
186.0761
126.0546
657.2319
528.1911
1059.9974 PEP_0_1_0_0_0
0
20000
40000
60000
80000
100000
120000
140000
500
1000
1500
2000
2500

File: 231129_OE_RK_36, RT: 45.323, Scan: 055990
Type: HCD, FTMS + c NSI d Full ms2 1287.0135@hcd30.00 [120.0000-5273.8711]
Precursor Scan: 055913, m/z: 1286.2619, charge: 4
QDQCIYNTTYLNVQR_7_6_0_3_0

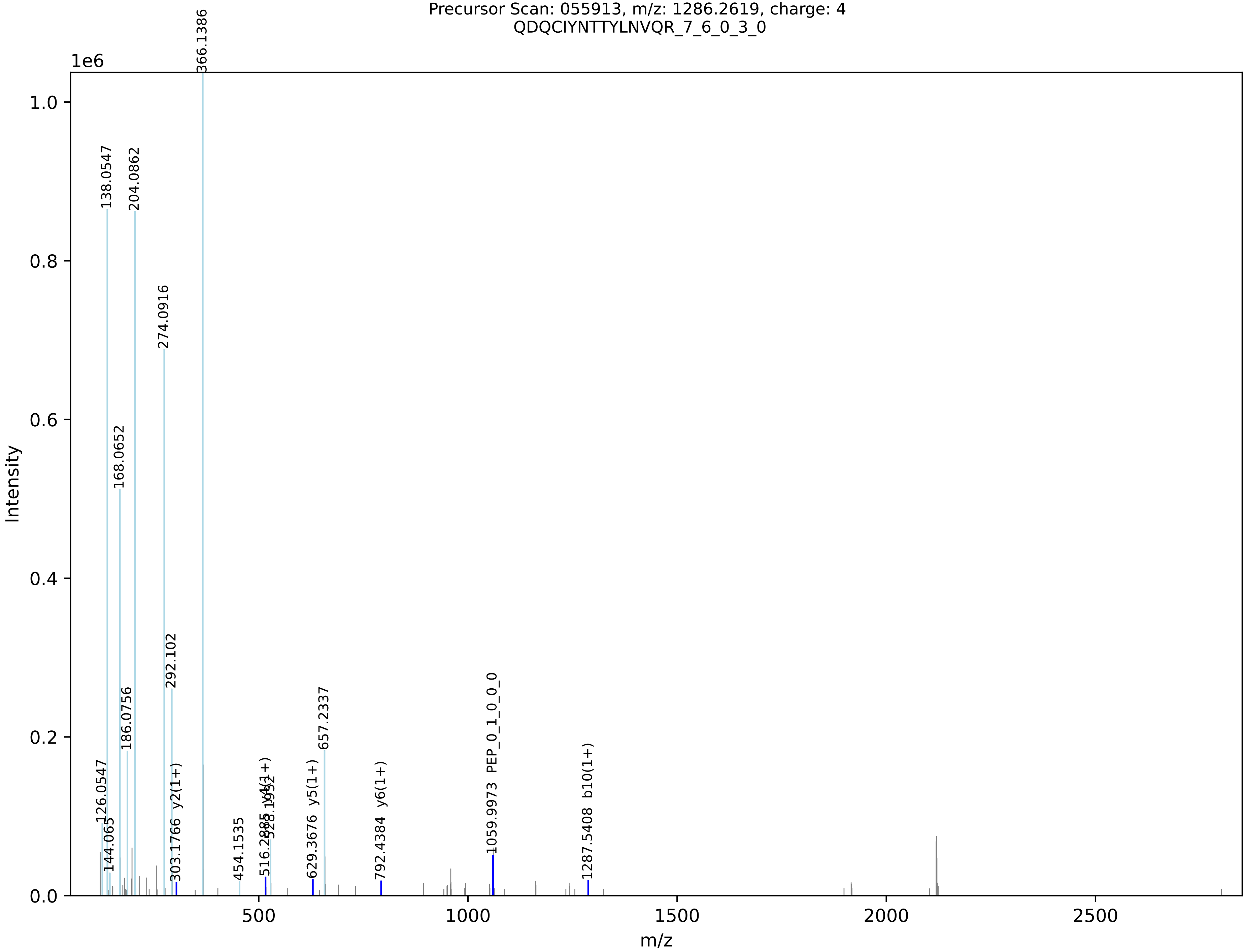

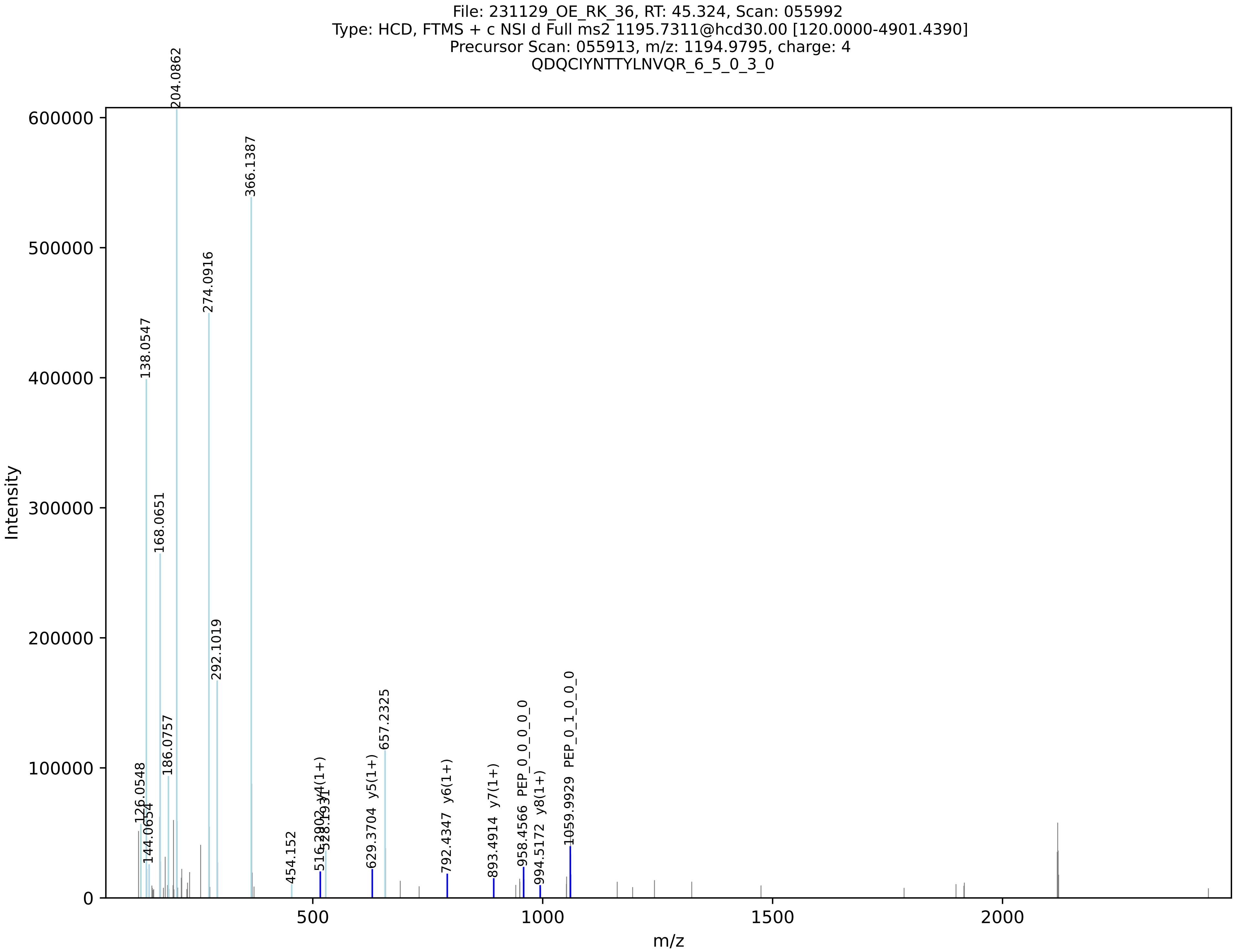

File: 231129_OE_RK_36, RT: 45.324, Scan: 055992
Type: HCD, FTMS + c NSI d Full ms2 1195.7311@hcd30.00 [120.0000-4901.4390]
Precursor Scan: 055913, m/z: 1194.9795, charge: 4
QDQCIYNTTYLNVQR_6_5_0_3_0
Intensity
m/z
204.0862
366.1387
274.0916
138.0547
168.0651
292.1019
186.0757
126.0548
144.0654
454.152
516.2902 y4(1+)
528.1931
629.3704 y5(1+)
657.2325
792.4347 y6(1+)
893.4914 y7(1+)
958.4566 PEP_0_0_0_0_0
994.5172 y8(1+)
1059.9929 PEP_0_1_0_0_0

File: 231129_OE_RK_36, RT: 45.328, Scan: 055998
Type: HCD, FTMS + c NSI d Full ms2 1641.9904@hcd30.00 [120.0000-5046.6323]
Precursor Scan: 055913, m/z: 1641.656, charge: 3
QDQCIYNTTYLNVQR_6_5_1_3_0

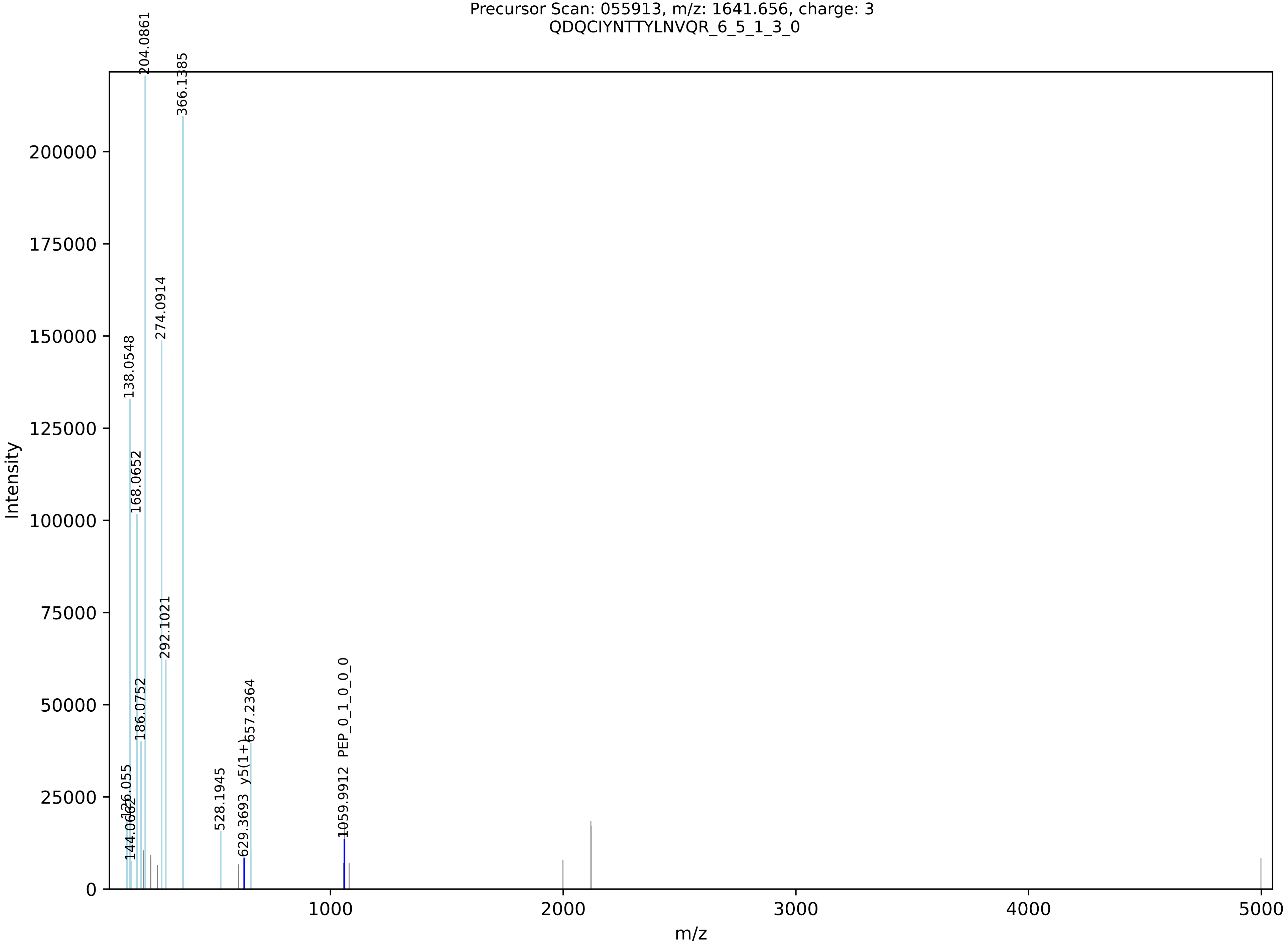

File: 231129_OE_RK_36, RT: 45.441, Scan: 056165
Type: HCD, FTMS + c NSI d Full ms2 1413.5807@hcd30.00 [120.0000-2905.1326]
Precursor Scan: 056058, m/z: 1413.0782, charge: 2
NGSLFAFR_5_4_0_1_0

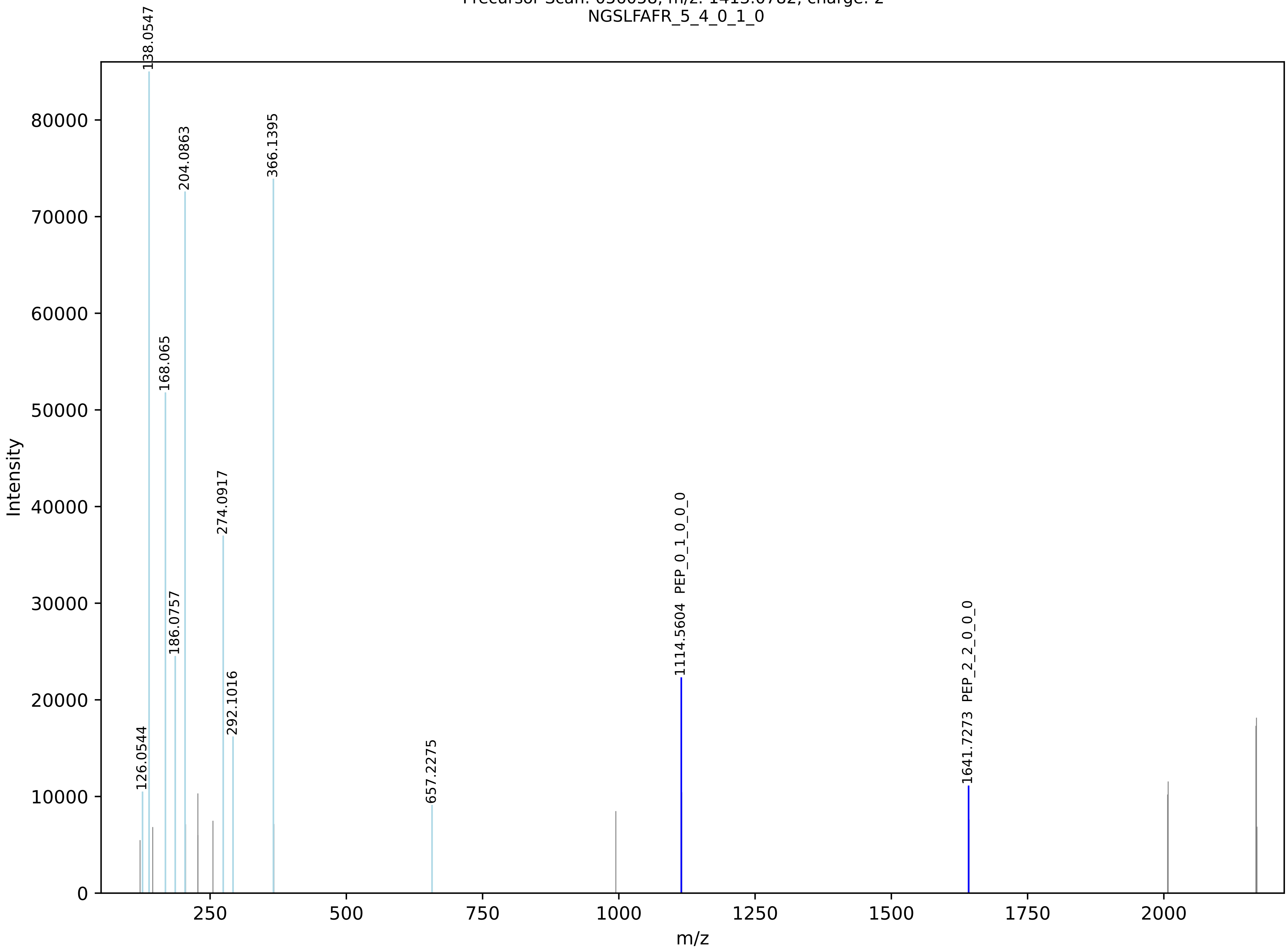

File: 231129_OE_RK_36, RT: 45.441, Scan: 056166
Type: HCD, FTMS + c NSI d Full ms2 1034.4493@hcd30.00 [120.0000-3187.5569]
Precursor Scan: 056058, m/z: 1033.7798, charge: 3
CGLVPVLAENYNK_5_4_0_0_0

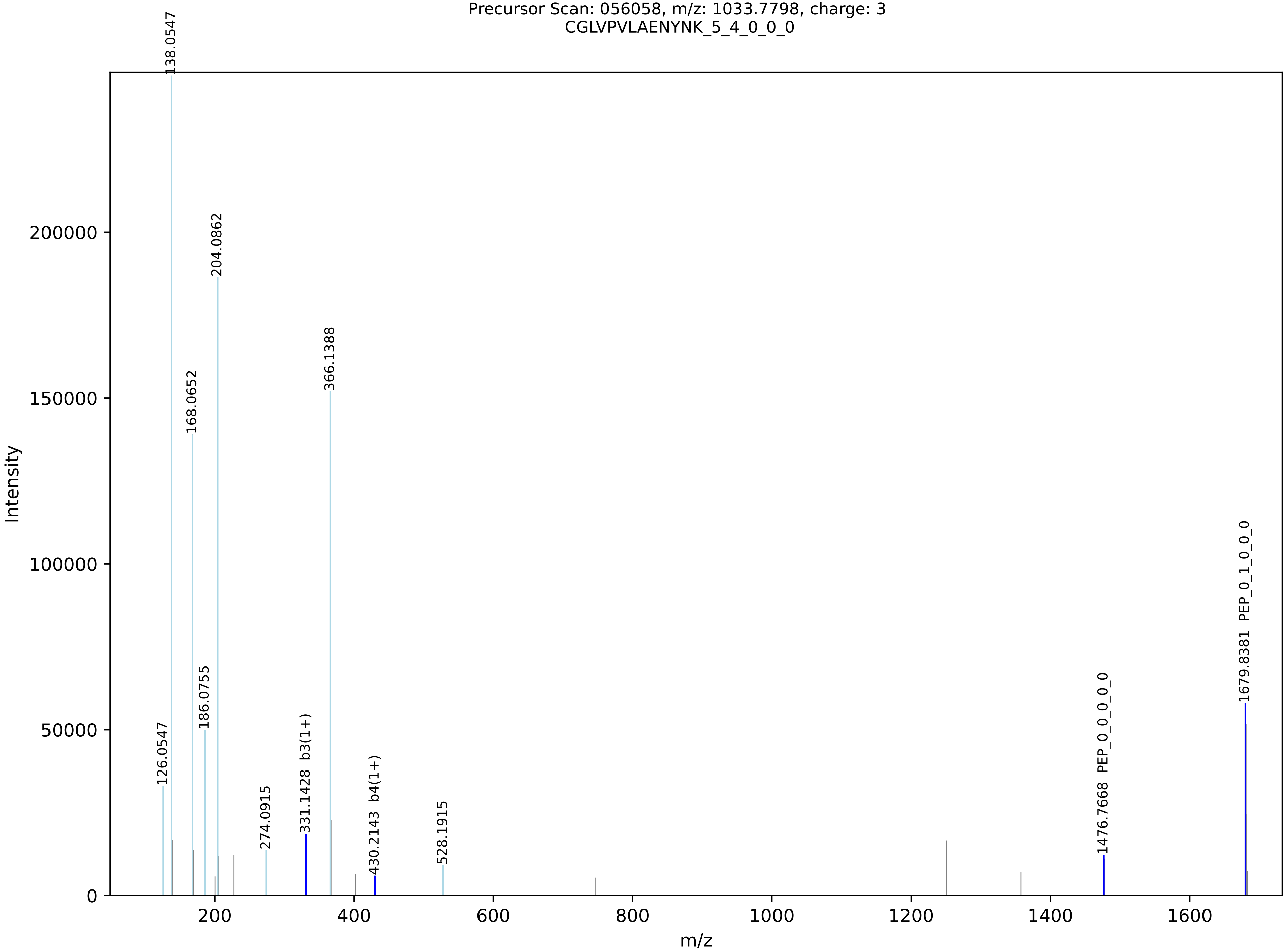

File: 231129_OE_RK_36, RT: 45.523, Scan: 056282
Type: HCD, FTMS + c NSI d Full ms2 1195.4801@hcd30.00 [120.0000-4900.4150]
Precursor Scan: 056205, m/z: 1194.979, charge: 4
QDQCIYNTTYLNVQR_6_5_0_3_0

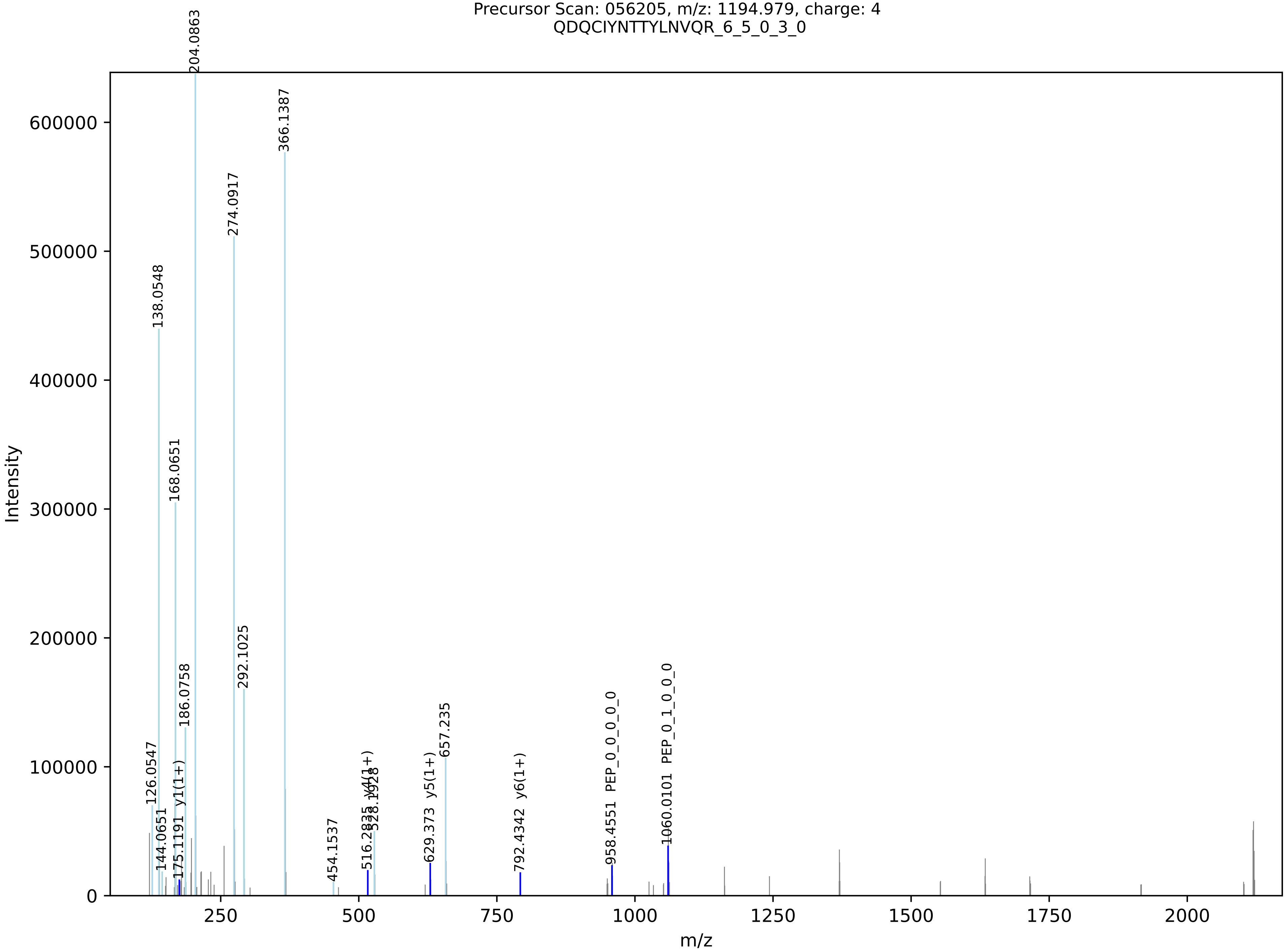

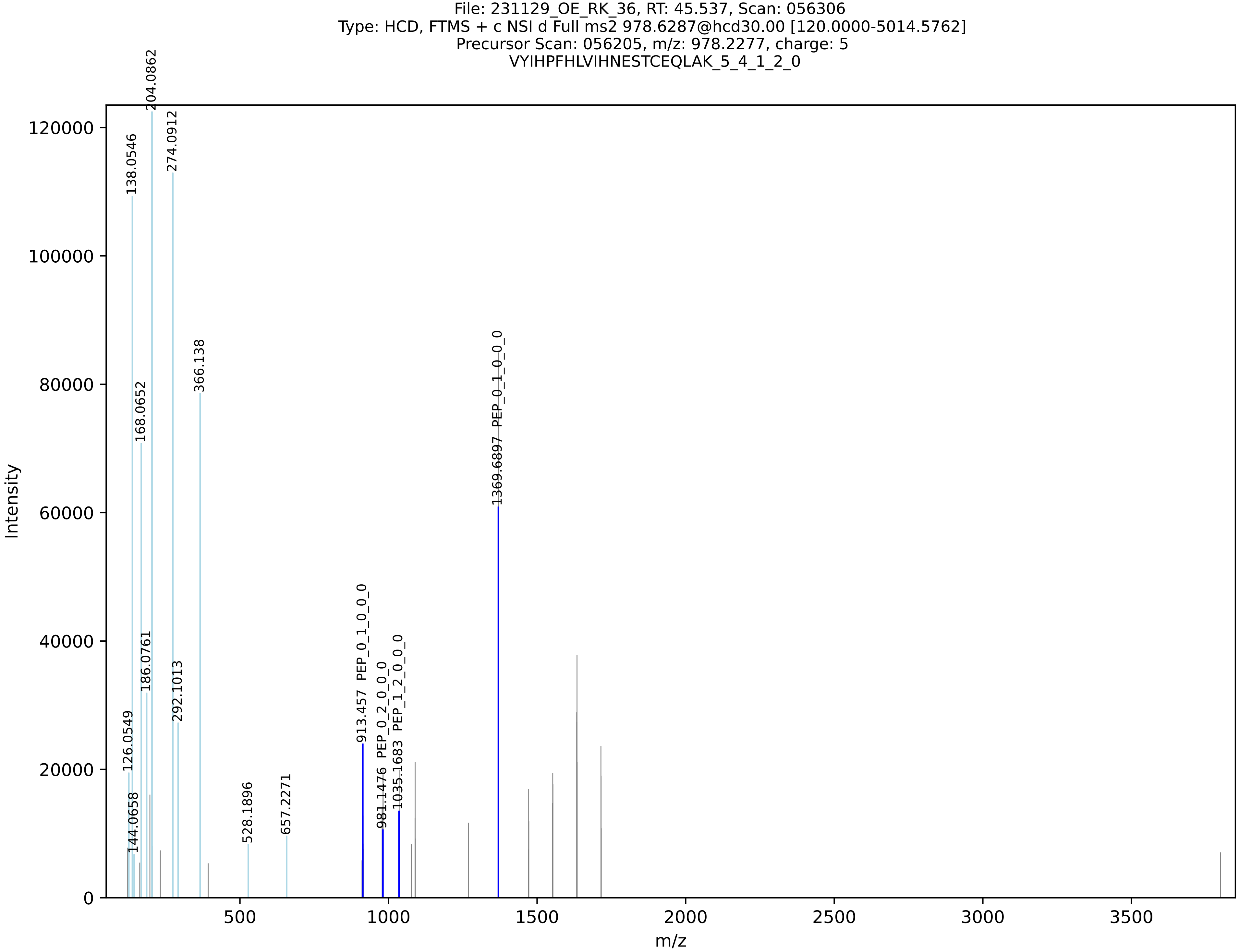

File: 231129_OE_RK_36, RT: 45.537, Scan: 056306
Type: HCD, FTMS + c NSI d Full ms2 978.6287@hcd30.00 [120.0000-5014.5762]
Precursor Scan: 056205, m/z: 978.2277, charge: 5
VYIHPFHLVIHNESTCEQLAK_5_4_1_2_0
Intensity
m/z
204.0862
274.0912
138.0546
366.138
168.0652
1369.6897 PEP_0_1_0_0_
913.457 PEP_0_1_0_0_0
981.1476 PEP_0_2_0_0_0
1035.1683 PEP_1_2_0_0_0
186.0761
292.1013
126.0549
144.0658
528.1896
657.2271
0
20000
40000
60000
80000
100000
120000
500
1000
1500
2000
2500
3000
3500

File: 231129_OE_RK_36, RT: 45.581, Scan: 056369
Type: HCD, FTMS + c NSI d Full ms2 1072.9739@hcd30.00 [120.0000-4400.5894]
Precursor Scan: 056279, m/z: 1072.2221, charge: 4
INVTDSLDLTTLSIK_4_7_2_1_0

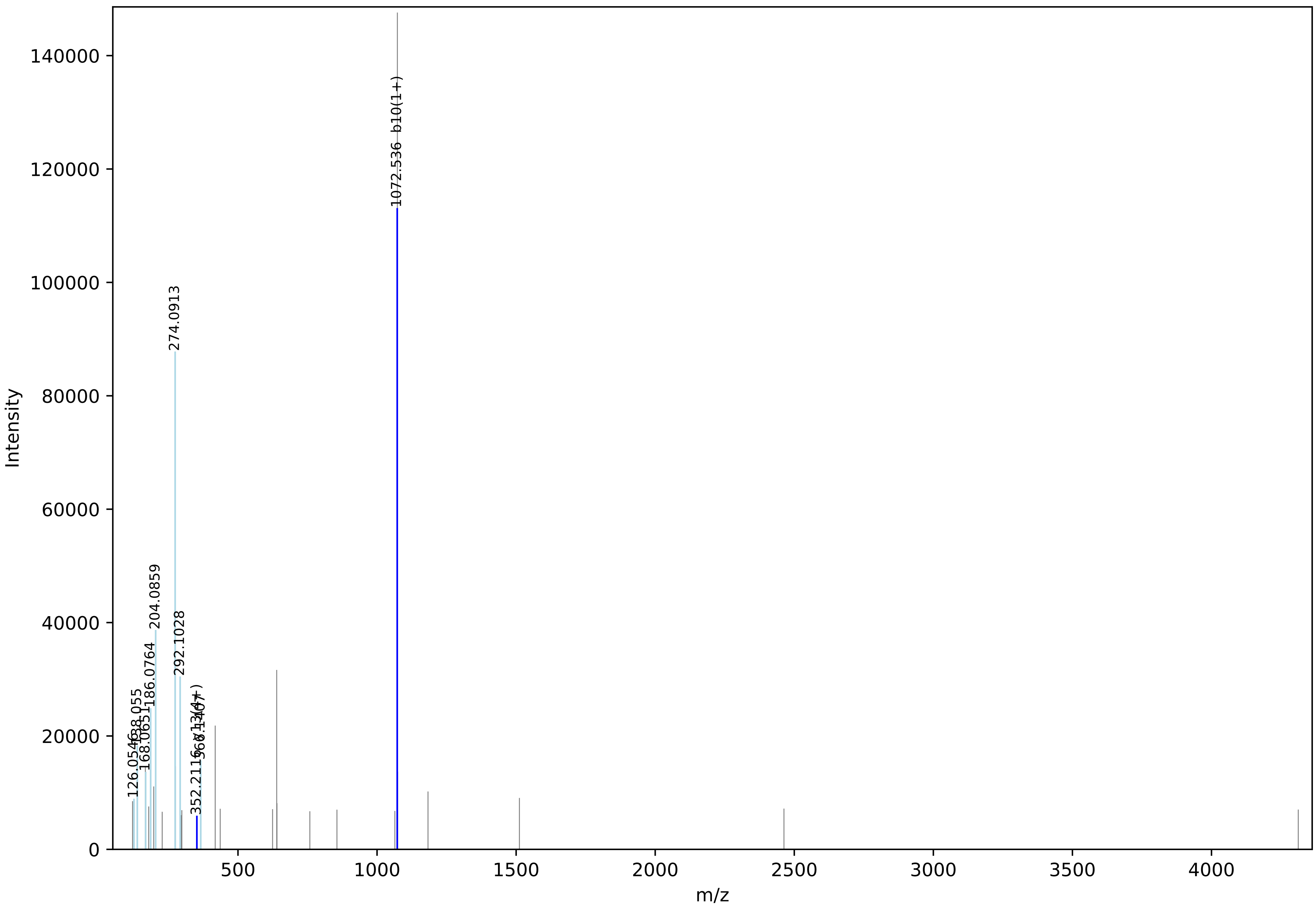

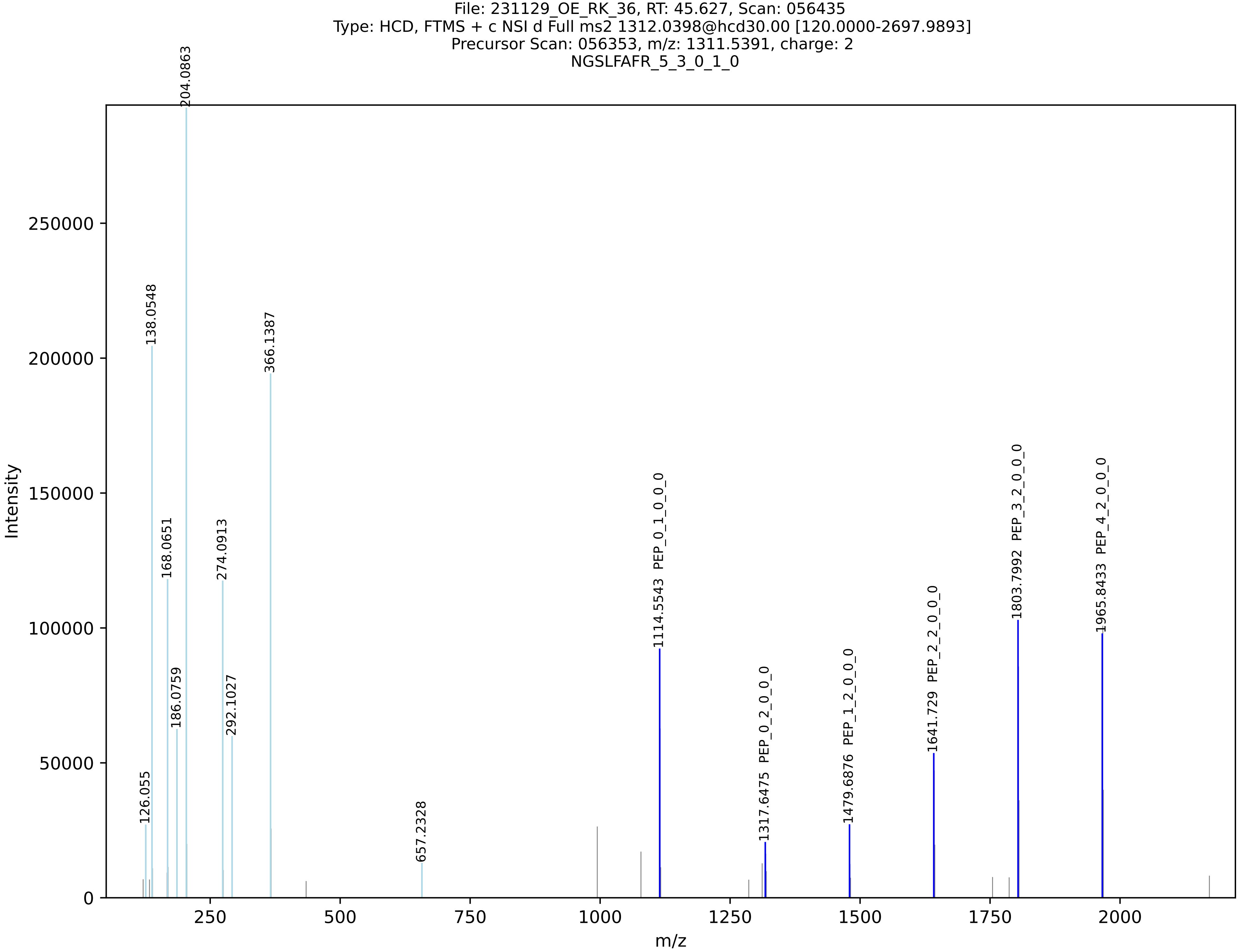

File: 231129_OE_RK_36, RT: 45.627, Scan: 056435
Type: HCD, FTMS + c NSI d Full ms2 1312.0398@hcd30.00 [120.0000-2697.9893]
Precursor Scan: 056353, m/z: 1311.5391, charge: 2
NGSLFAFR_5_3_0_1_0
Intensity
m/z
204.0863
138.0548
366.1387
168.0651
274.0913
186.0759
292.1027
126.055
657.2328
1114.5543 PEP_0_1_0_0_
1317.6475 PEP_0_2_0_0_
1479.6876 PEP_1_2_0_0_
1641.729 PEP_2_2_0_0_
1803.7992 PEP_3_2_0_0_
1965.8433 PEP_4_2_0_0_

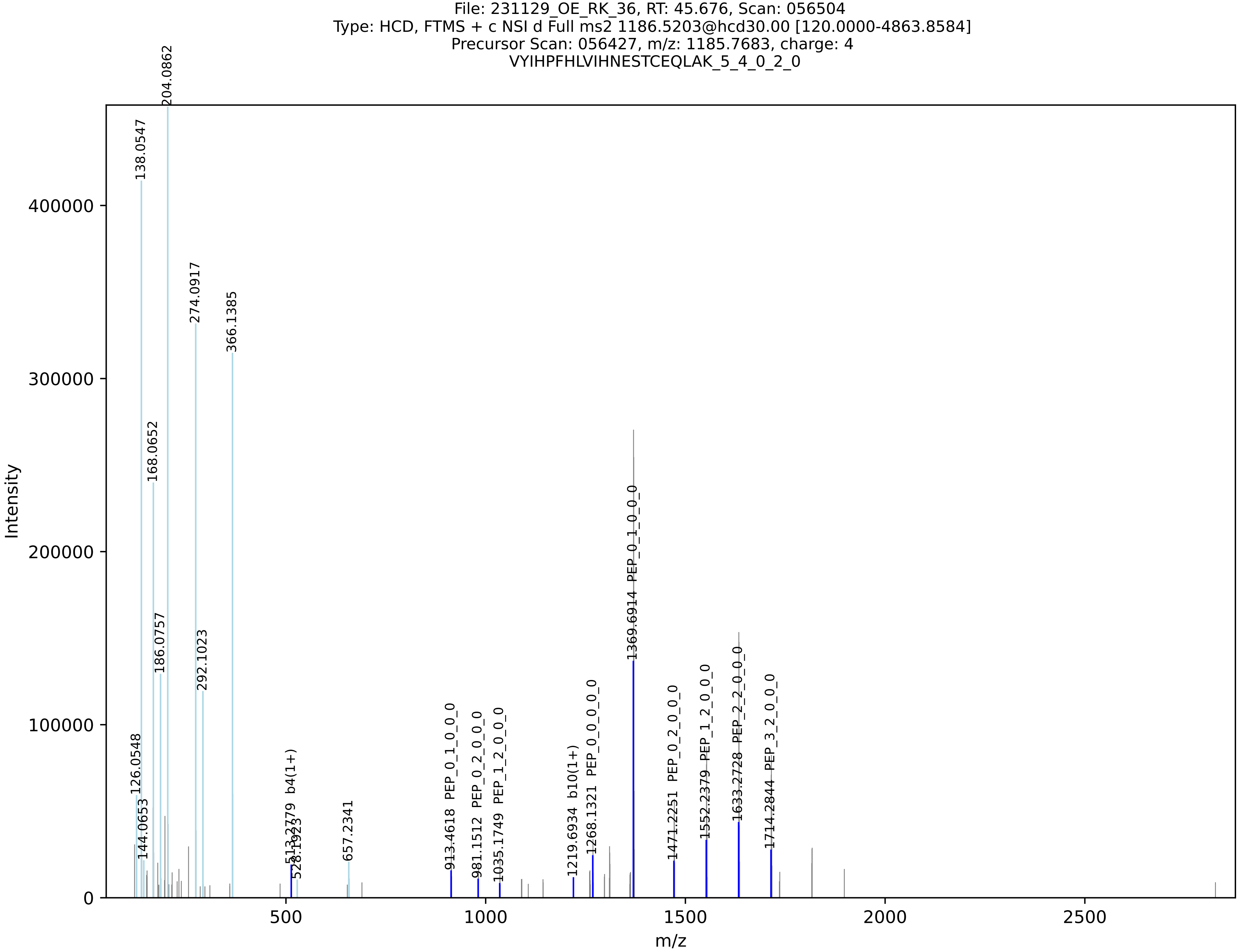
File: 231129_OE_RK_36, RT: 45.676, Scan: 056504
Type: HCD, FTMS + c NSI d Full ms2 1186.5203@hcd30.00 [120.0000-4863.8584]
Precursor Scan: 056427, m/z: 1185.7683, charge: 4
VYIHPFHLVIHNESTCEQLAK_5_4_0_2_0
Intensity
m/z
204.0862
138.0547
274.0917
366.1385
168.0652
186.0757
292.1023
126.0548
144.0653
513.2279 b4(1+)
528.1923
657.2341
913.4618 PEP_0_1_0_0_0
981.1512 PEP_0_2_0_0_0
1035.1749 PEP_1_2_0_0_0
1219.6934 b10(1+)
1268.1321 PEP_0_0_0_0_0
1369.6914 PEP_0_1_0_0_0
1471.2251 PEP_0_2_0_0_0
1552.2379 PEP_1_2_0_0_0
1633.2728 PEP_2_2_0_0_0
1714.2844 PEP_3_2_0_0_0

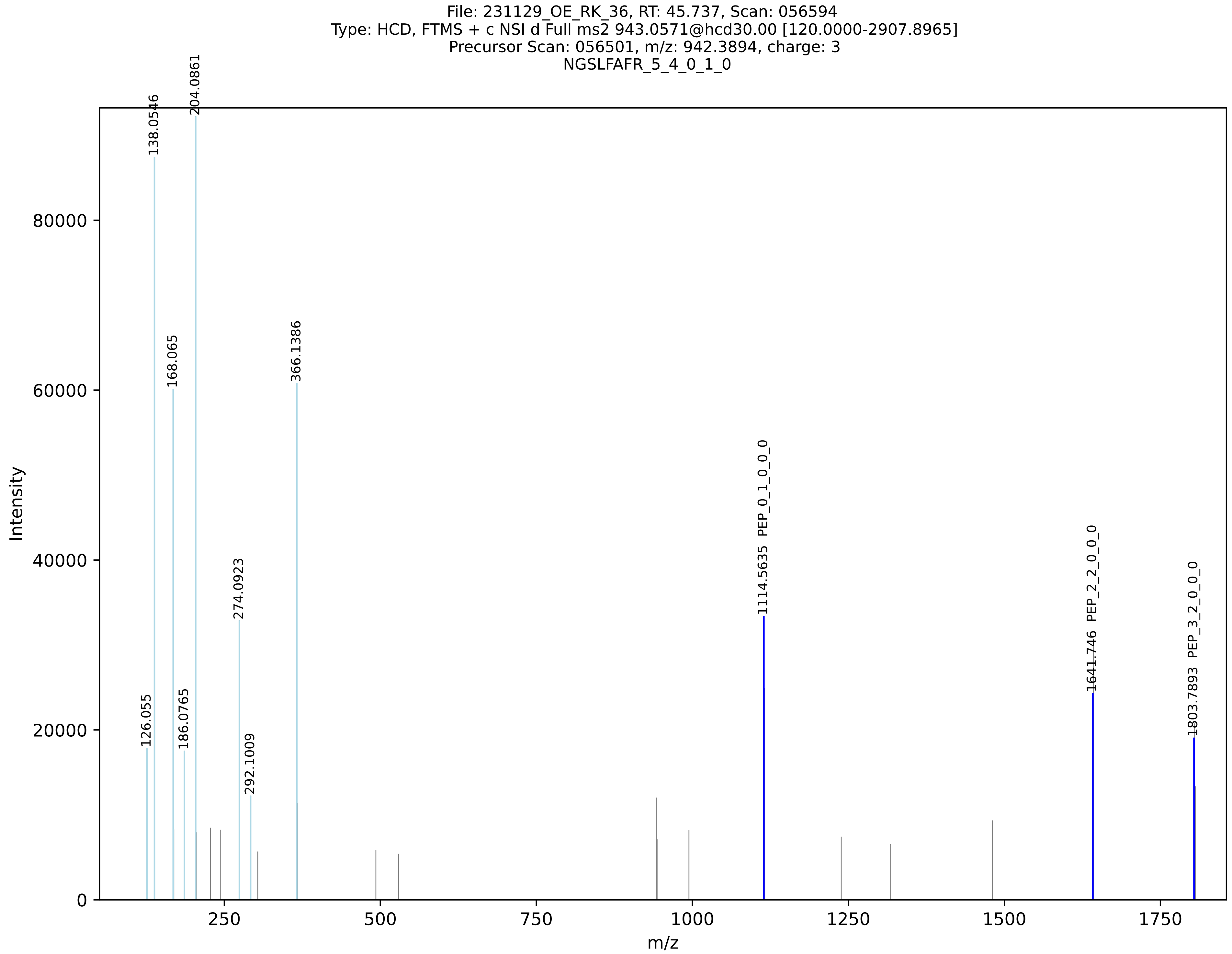
File: 231129_OE_RK_36, RT: 45.737, Scan: 056594
Type: HCD, FTMS + c NSI d Full ms2 943.0571@hcd30.00 [120.0000-2907.8965]
Precursor Scan: 056501, m/z: 942.3894, charge: 3
NGSLFAFR_5_4_0_1_0
Intensity
m/z
126.055
138.0546
168.065
186.0765
204.0861
274.0923
292.1009
366.1386
1114.5635 PEP_0_1_0_0_0
1641.746 PEP_2_2_0_0_0
1803.7893 PEP_3_2_0_0_0

File: 231129_OE_RK_36, RT: 45.741, Scan: 056600
Type: HCD, FTMS + c NSI d Full ms2 1509.9786@hcd30.00 [120.0000-4642.6768]
Precursor Scan: 056501, m/z: 1508.9777, charge: 3
ERTLLIEAHNETFANR_5_6_0_2_0

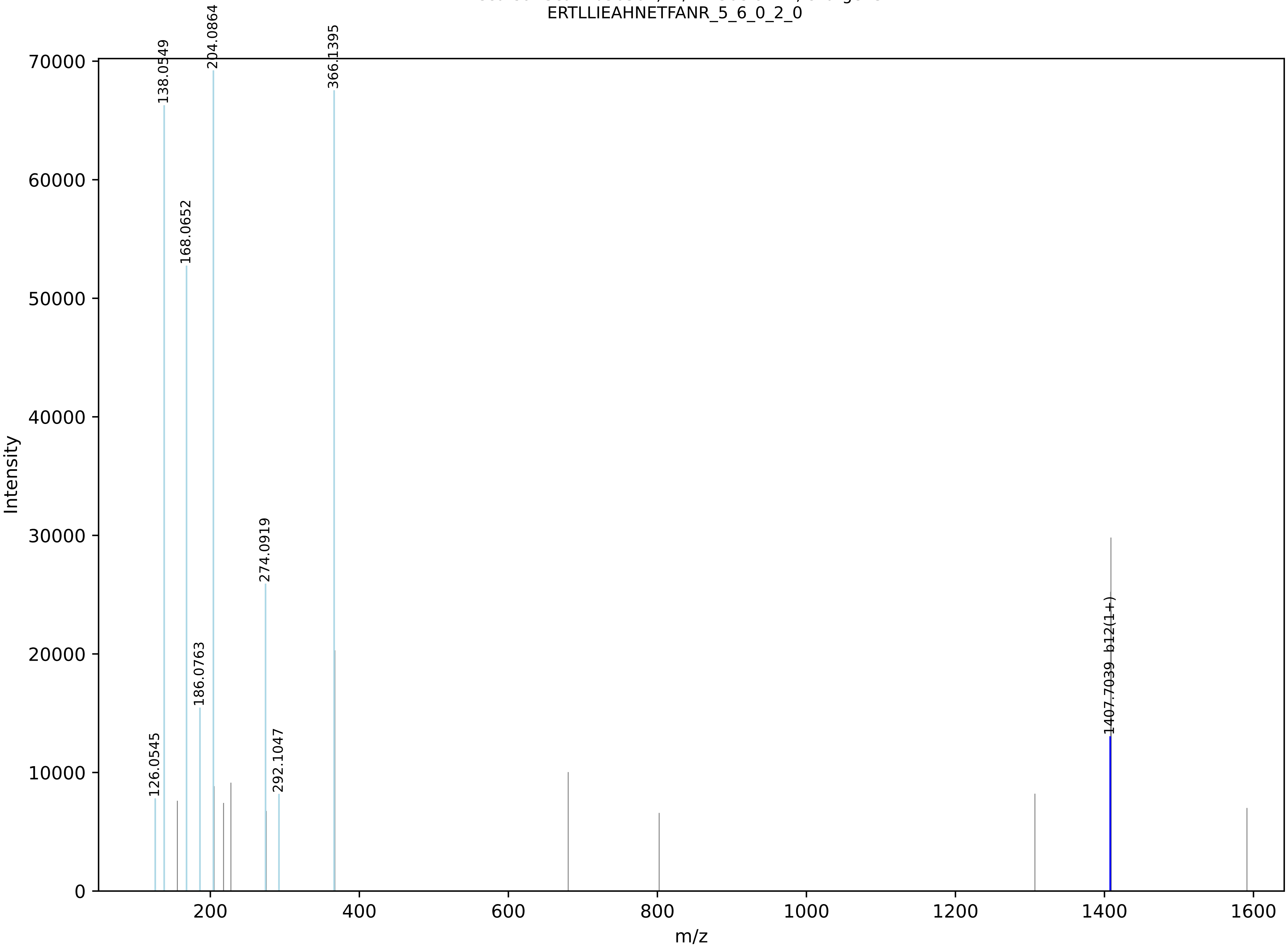

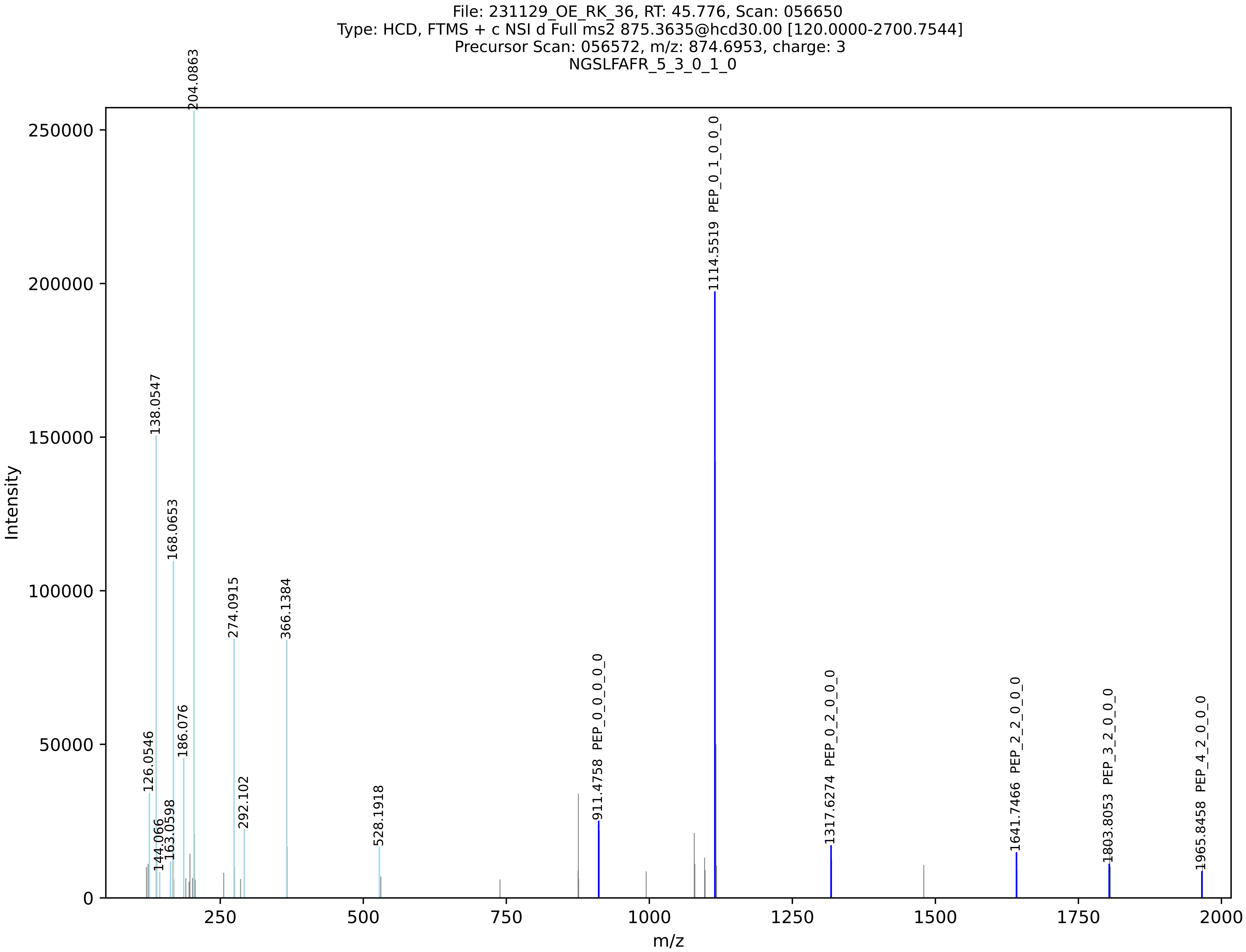

File: 231129_OE_RK_36, RT: 45.776, Scan: 056650
Type: HCD, FTMS + c NSI d Full ms2 875.3635@hcd30.00 [120.0000-2700.7544]
Precursor Scan: 056572, m/z: 874.6953, charge: 3
NGSLFAFR_5_3_0_1_0
Intensity
m/z
204.0863
138.0547
168.0653
274.0915
366.1384
186.076
126.0546
144.066
163.0598
292.102
528.1918
911.4758 PEP_0_0_0_0_0
1114.5519 PEP_0_1_0_0_0
1317.6274 PEP_0_2_0_0_0
1641.7466 PEP_2_2_0_0_0
1803.8053 PEP_3_2_0_0_0
1965.8458 PEP_4_2_0_0_0

File: 231129_OE_RK_36, RT: 45.831, Scan: 056727
Type: HCD, FTMS + c NSI d Full ms2 1312.0402@hcd30.00 [120.0000-2697.9900]
Precursor Scan: 056645, m/z: 1311.5394, charge: 2
NGSLFAFR_5_3_0_1_0

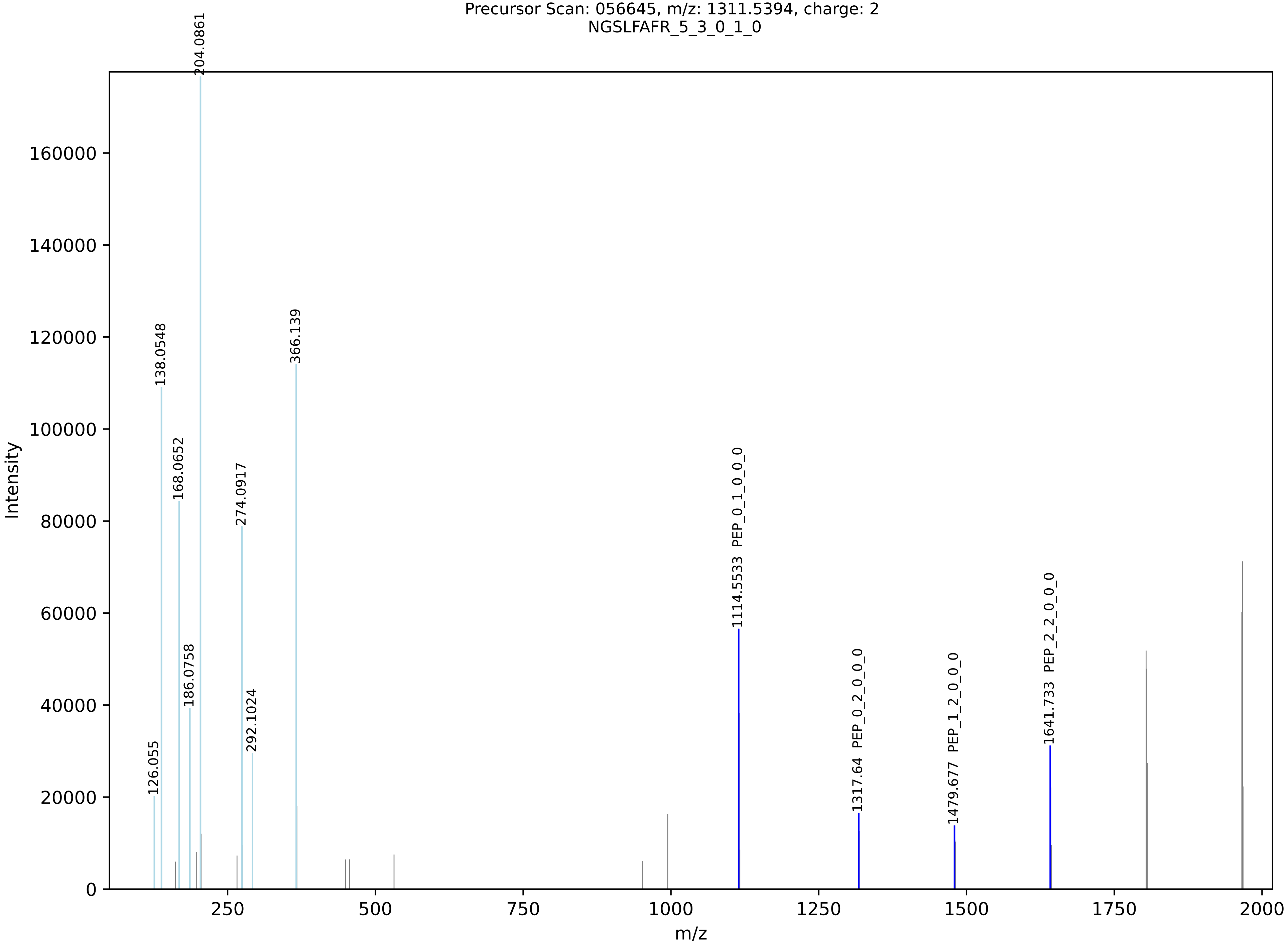

File: 231129_OE_RK_36, RT: 45.882, Scan: 056803
Type: HCD, FTMS + c NSI d Full ms2 1535.6356@hcd30.00 [120.0000-4721.1870]
Precursor Scan: 056719, m/z: 1534.6348, charge: 3
GLTFQQNASSMCVPDQDTAIR_5_5_1_1_0

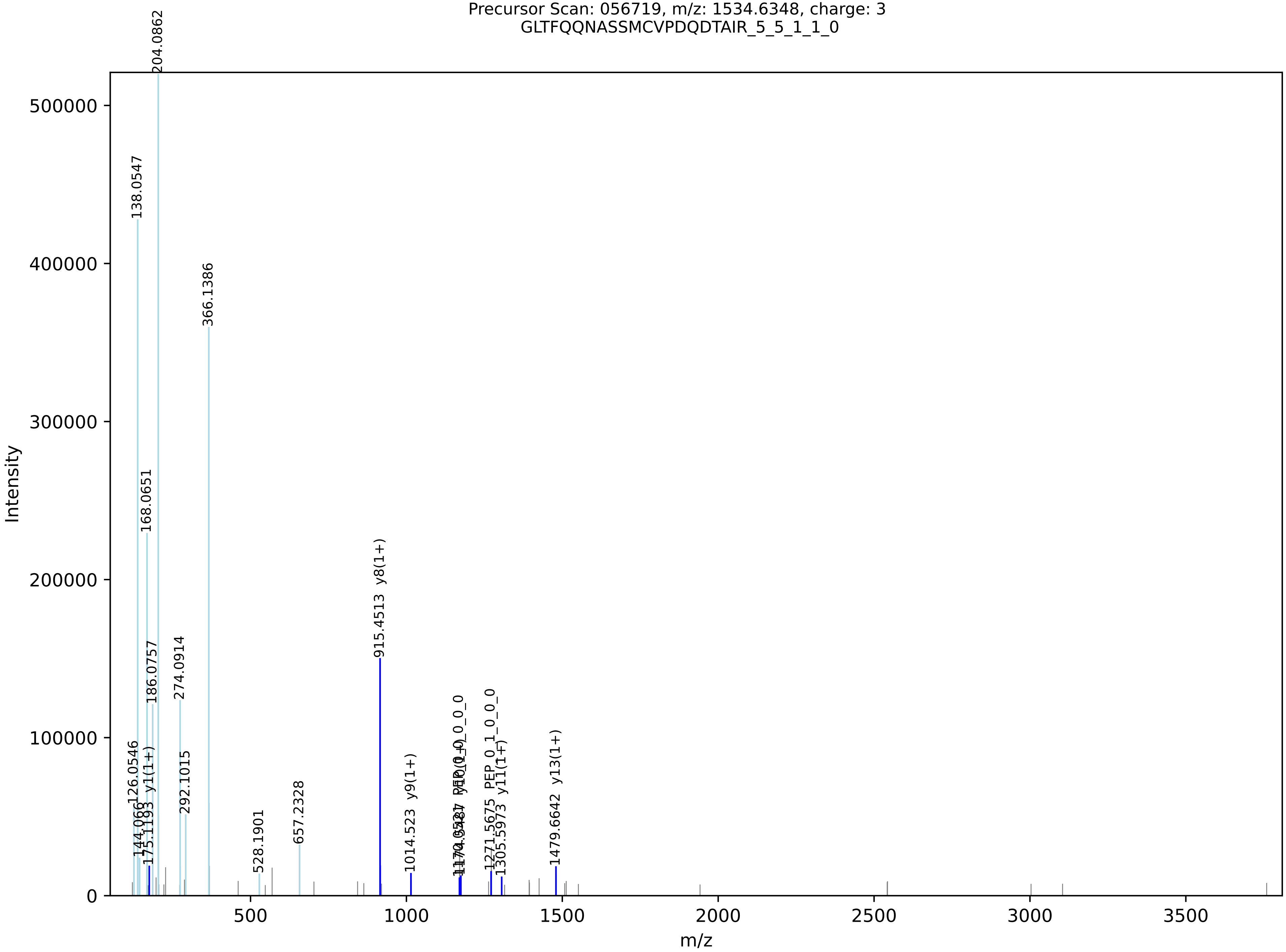

File: 231129_OE_RK_36, RT: 45.949, Scan: 056902
Type: HCD, FTMS + c NSI d Full ms2 954.8160@hcd30.00 [120.0000-4893.1313]
Precursor Scan: 056790, m/z: 954.415, charge: 5
ELCTISHIFIPAMGNITYDFSFK_5_4_1_1_0

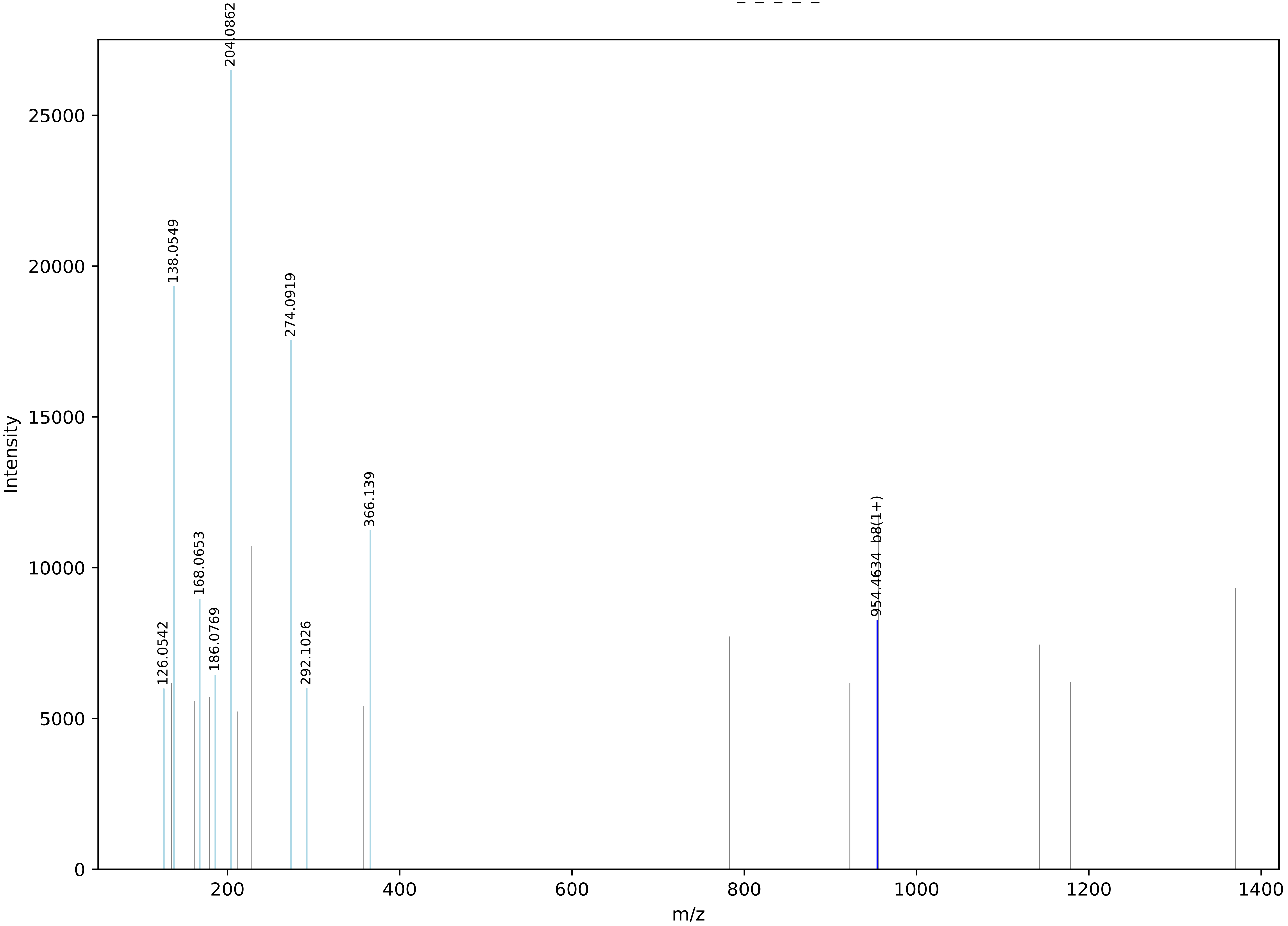

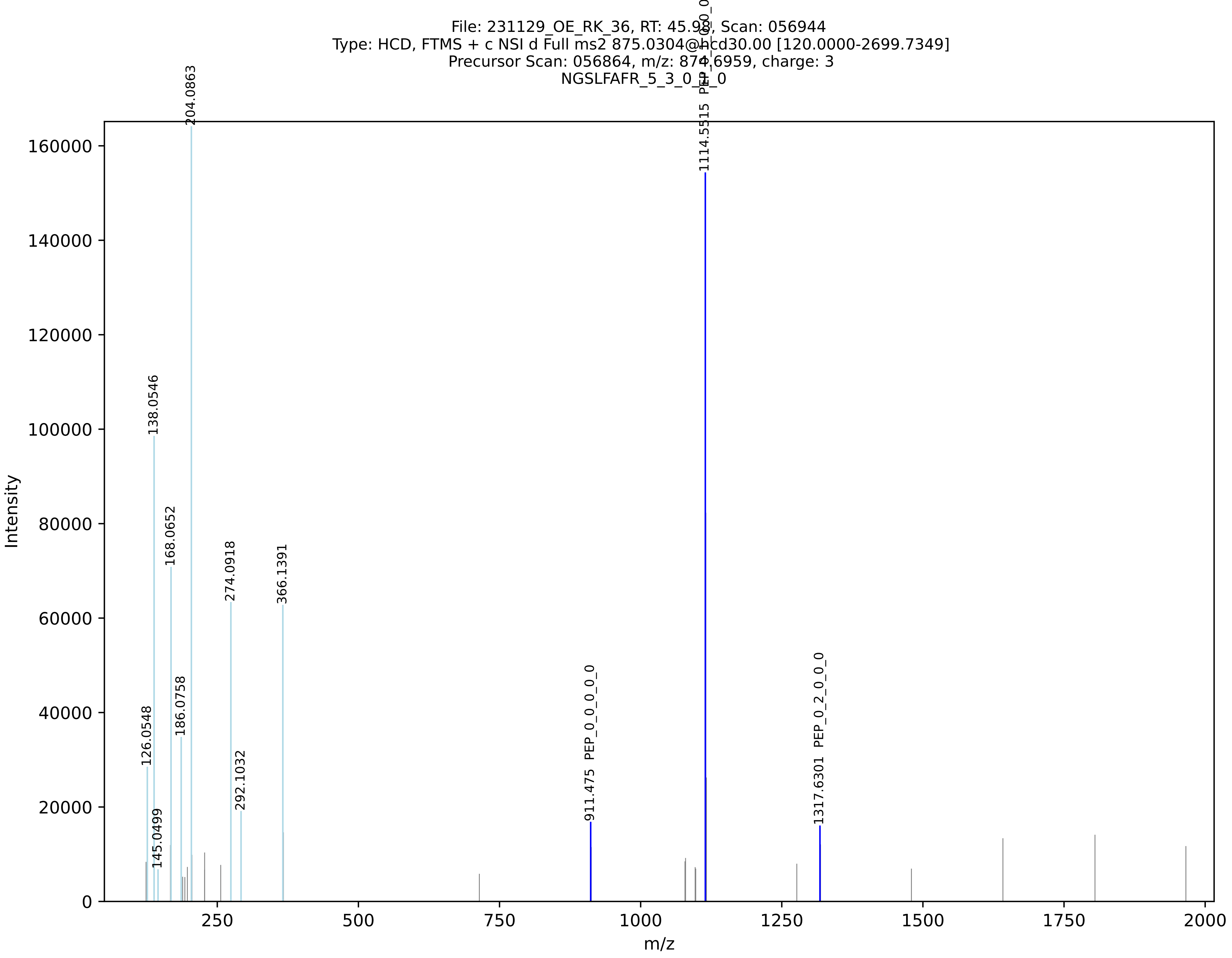
File: 231129_OE_RK_36, RT: 45.9, Scan: 056944
Type: HCD, FTMS + c NSI d Full ms2 875.0304@hcd30.00 [120.0000-2699.7349]
Precursor Scan: 056864, m/z: 874.6959, charge: 3
NGSLFAFR_5_3_0_0
Intensity
m/z
204.0863
138.0546
168.0652
274.0918
366.1391
186.0758
126.0548
292.1032
145.0499
911.475 PEP_0_0_0_0_0
1114.5515
1317.6301 PEP_0_2_0_0_0
160000
140000
120000
100000
80000
60000
40000
20000
0
250
500
750
1000
1250
1500
1750
2000

File: 231129_OE_RK_36, RT: 46.029, Scan: 057014
Type: HCD, FTMS + c NSI d Full ms2 1360.0431@hcd30.00 [120.0000-5571.8315]
Precursor Scan: 056938, m/z: 1359.2864, charge: 4
QDQCIYNTTYLNVQR_7_6_0_4_0

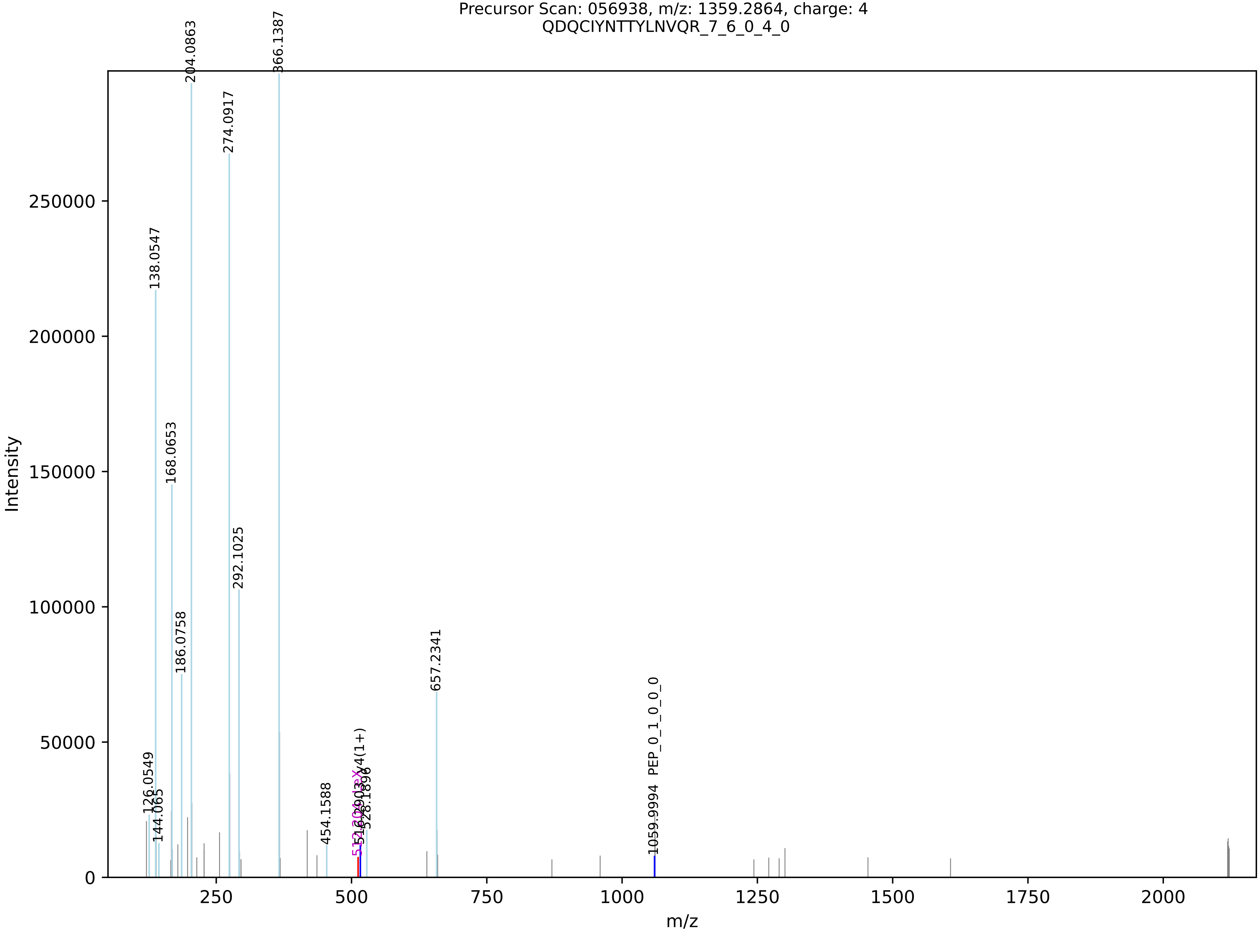

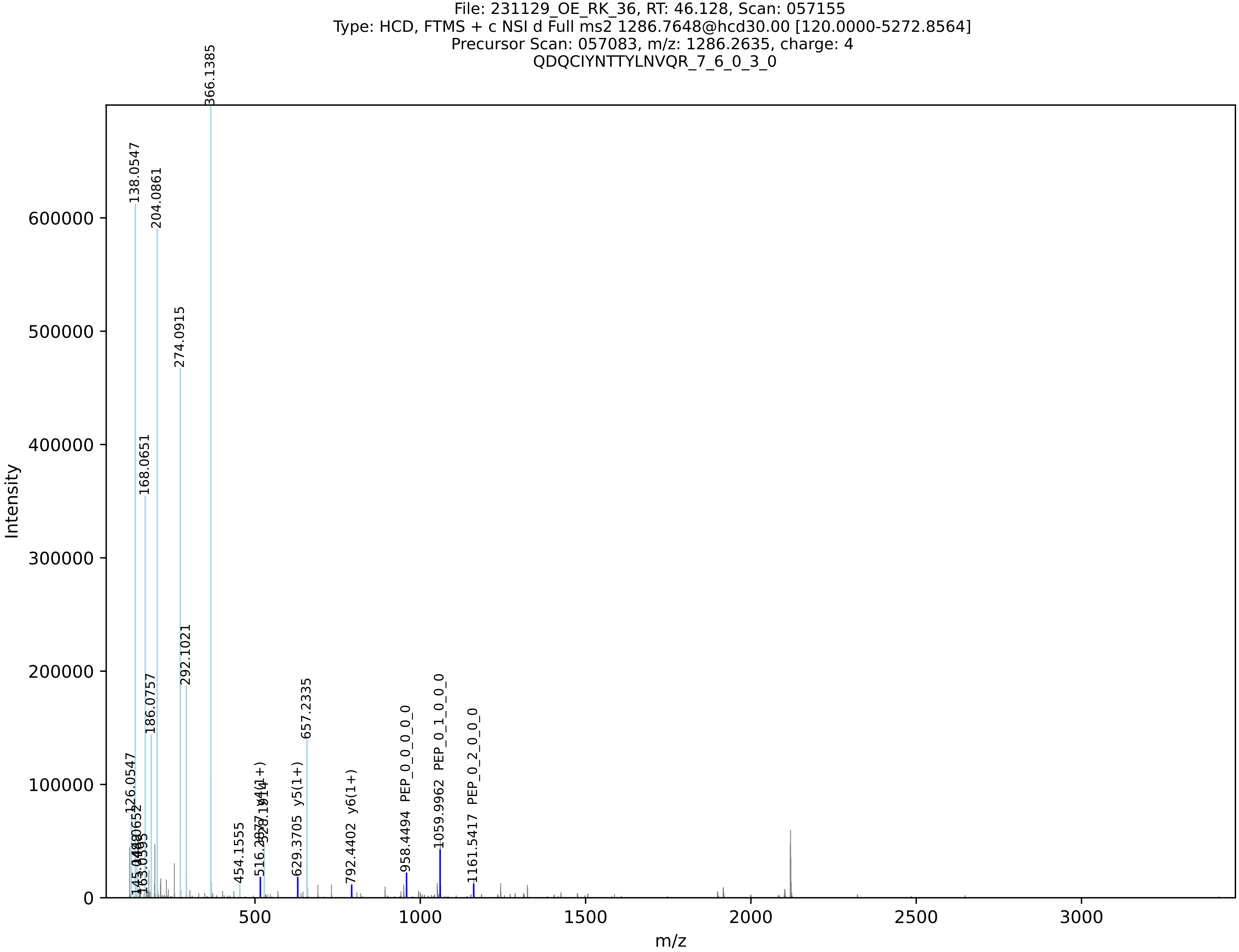
File: 231129_OE_RK_36, RT: 46.128, Scan: 057155
Type: HCD, FTMS + c NSI d Full ms2 1286.7648@hcd30.00 [120.0000-5272.8564]
Precursor Scan: 057083, m/z: 1286.2635, charge: 4
QDQCIYNTTYLNVQR_7_6_0_3_0
366.1385
138.0547
204.0861
274.0915
168.0651
292.1021
186.0757
126.0547
657.2335
454.1555
629.3705 y5(1+)
792.4402 y6(1+)
958.4494 PEP_0_0_0_0_0
1059.9962 PEP_0_1_0_0_0
1161.5417 PEP_0_2_0_0_0
Intensity
m/z
0
100000
200000
300000
400000
500000
600000
500
1000
1500
2000
2500
3000

File: 231129_OE_RK_36, RT: 46.132, Scan: 057161
Type: HCD, FTMS + c NSI d Full ms2 1396.3033@hcd30.00 [120.0000-5719.7734]
Precursor Scan: 057083, m/z: 1395.5516, charge: 4
QDQCIYNTTYLNVQR_7_6_1_4_0

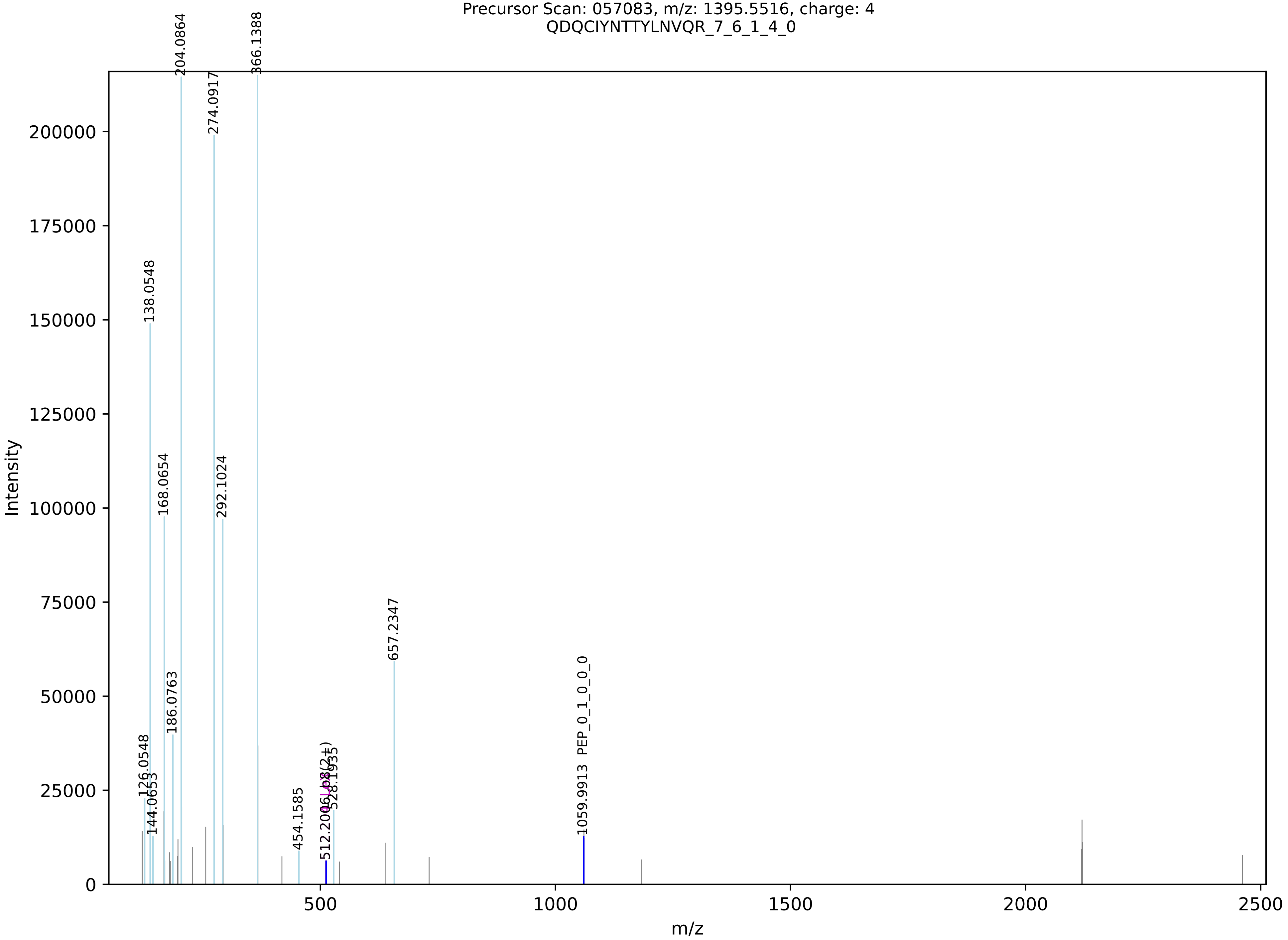

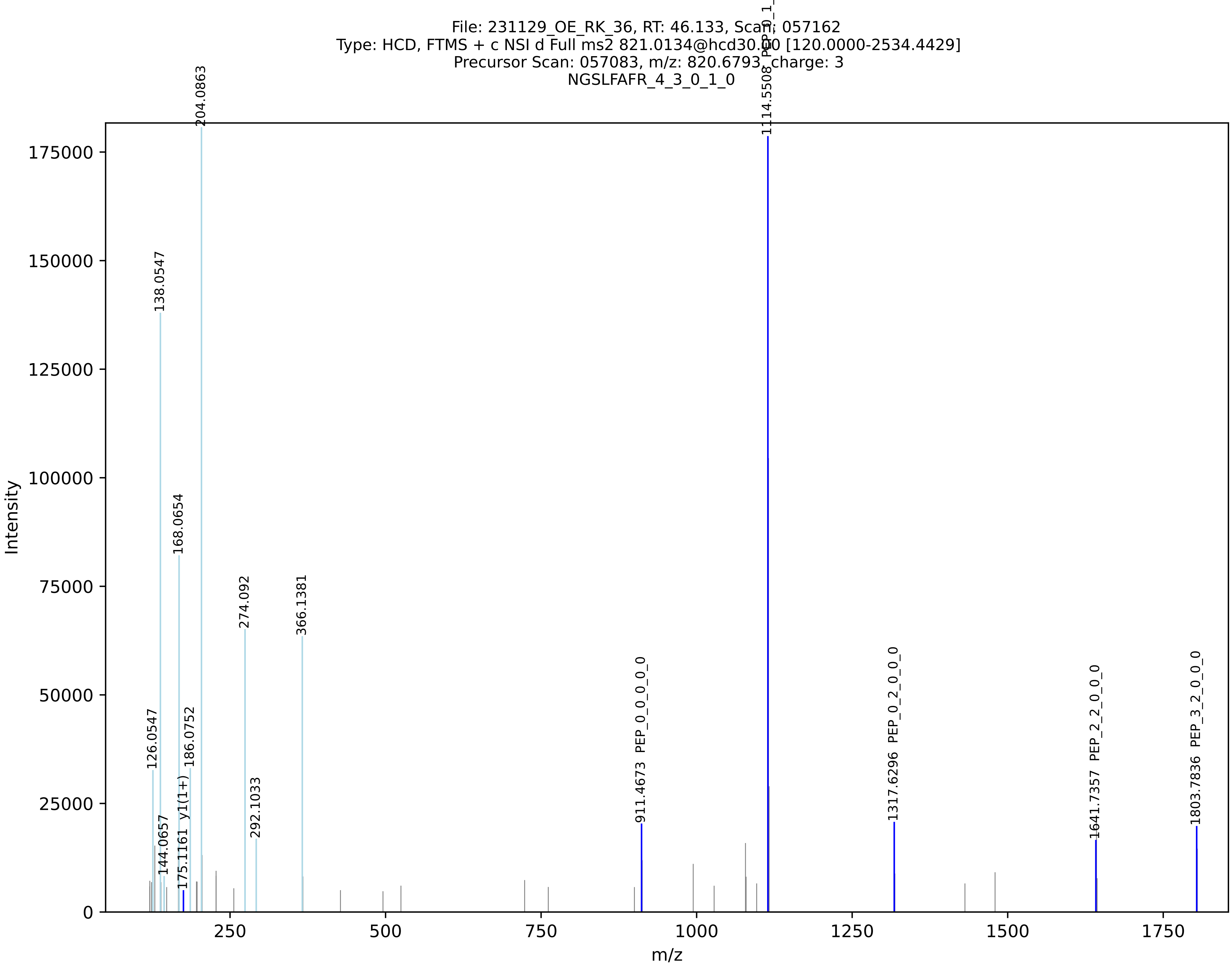
File: 231129_OE_RK_36, RT: 46.133, Scan: 057162
Type: HCD, FTMS + c NSI d Full ms2 821.0134@hcd30.00 [120.0000-2534.4429]
Precursor Scan: 057083, m/z: 820.6793, charge: 3
NGSLFAFR_4_3_0_1_0
Intensity
m/z
204.0863
1114.5508 PEP_0_1_
138.0547
168.0654
274.092
366.1381
126.0547
186.0752
144.0657
175.1161 y1(1+)
292.1033
911.4673 PEP_0_0_0_0_0
1317.6296 PEP_0_2_0_0_0
1641.7357 PEP_2_2_0_0_0
1803.7836 PEP_3_2_0_0_0
0
25000
50000
75000
100000
125000
150000
175000
250
500
750
1000
1250
1500
1750

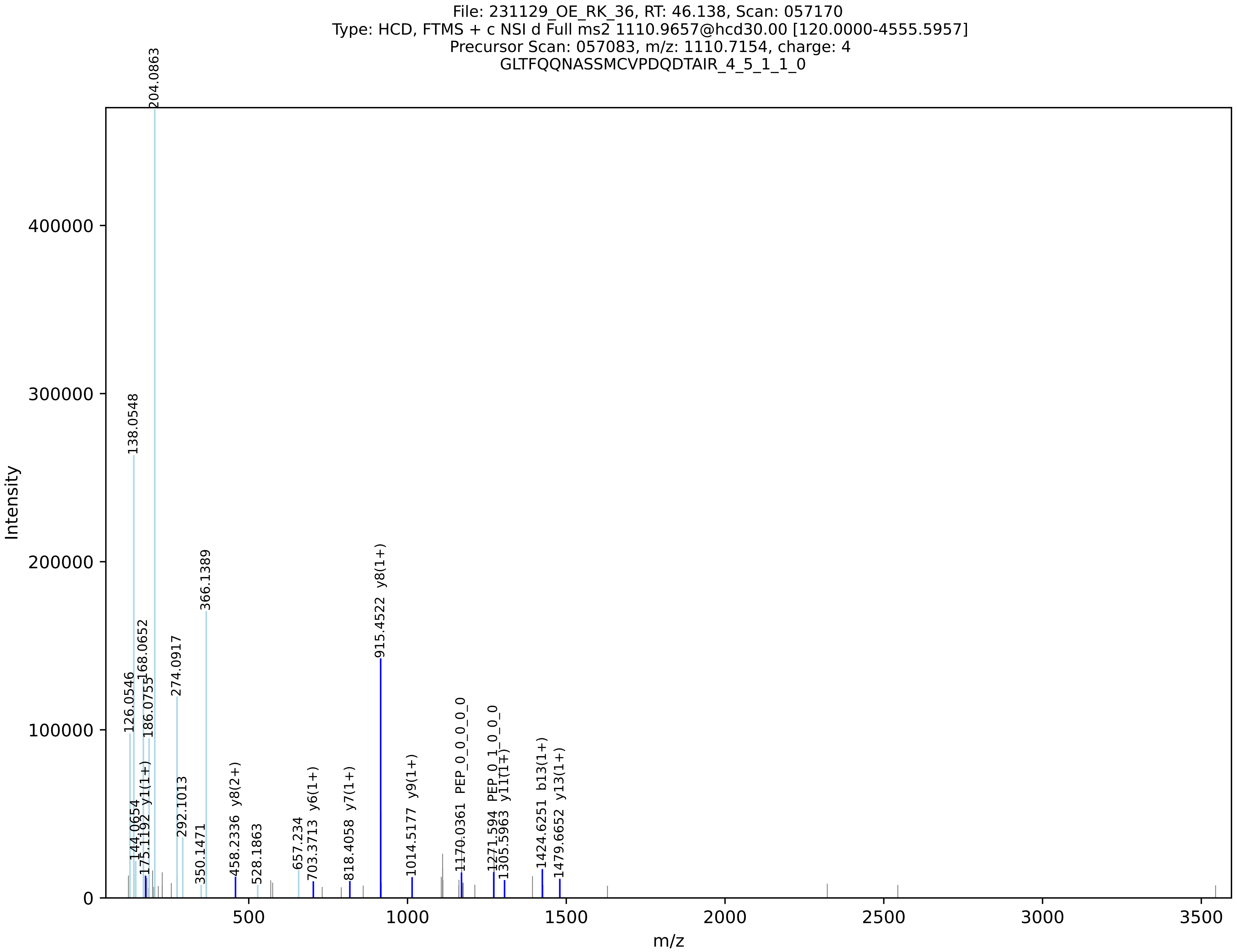

File: 231129_OE_RK_36, RT: 46.138, Scan: 057170
Type: HCD, FTMS + c NSI d Full ms2 1110.9657@hcd30.00 [120.0000-4555.5957]
Precursor Scan: 057083, m/z: 1110.7154, charge: 4
GLTFQQNASSMCVPDQDTAIR_4_5_1_1_0
Intensity
m/z
204.0863
138.0548
366.1389
168.0652
126.0546
186.0755
274.0917
915.4522 y8(1+)
144.0654
175.1192 y1(1+)
292.1013
350.1471
458.2336 y8(2+)
528.1863
657.234
703.3713 y6(1+)
818.4058 y7(1+)
1014.5177 y9(1+)
1170.0361 PEP_0_0_0_0_0
1271.594 PEP_0_1_0_0_0
1305.5963 y11(1+)
1424.6251 b13(1+)
1479.6652 y13(1+)

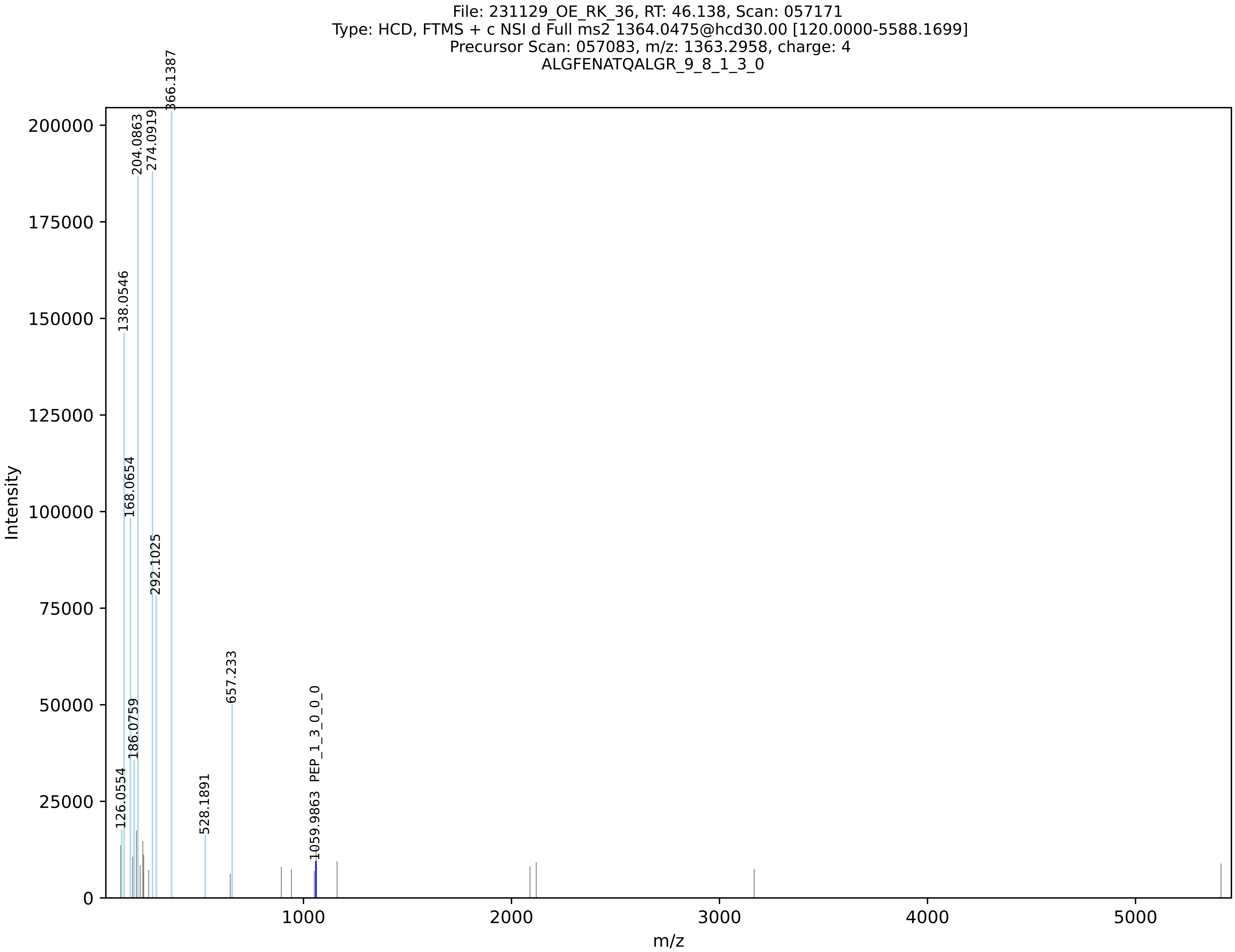
File: 231129_OE_RK_36, RT: 46.138, Scan: 057171
Type: HCD, FTMS + c NSI d Full ms2 1364.0475@hcd30.00 [120.0000-5588.1699]
Precursor Scan: 057083, m/z: 1363.2958, charge: 4
ALGFENATQALGR_9_8_1_3_0
Intensity
m/z
366.1387
204.0863
274.0919
138.0546
168.0654
292.1025
657.233
186.0759
126.0554
528.1891
1059.9863 PEP_1_3_0_0_0
200000
175000
150000
125000
100000
75000
50000
25000
0
1000
2000
3000
4000
5000

File: 231129_OE_RK_36, RT: 46.141, Scan: 057175
Type: HCD, FTMS + c NSI d Full ms2 1481.2871@hcd30.00 [120.0000-4554.8804]
Precursor Scan: 057083, m/z: 1480.6181, charge: 3
GLTFQQNASSMCVPDQDTAIR_4_5_1_1_0

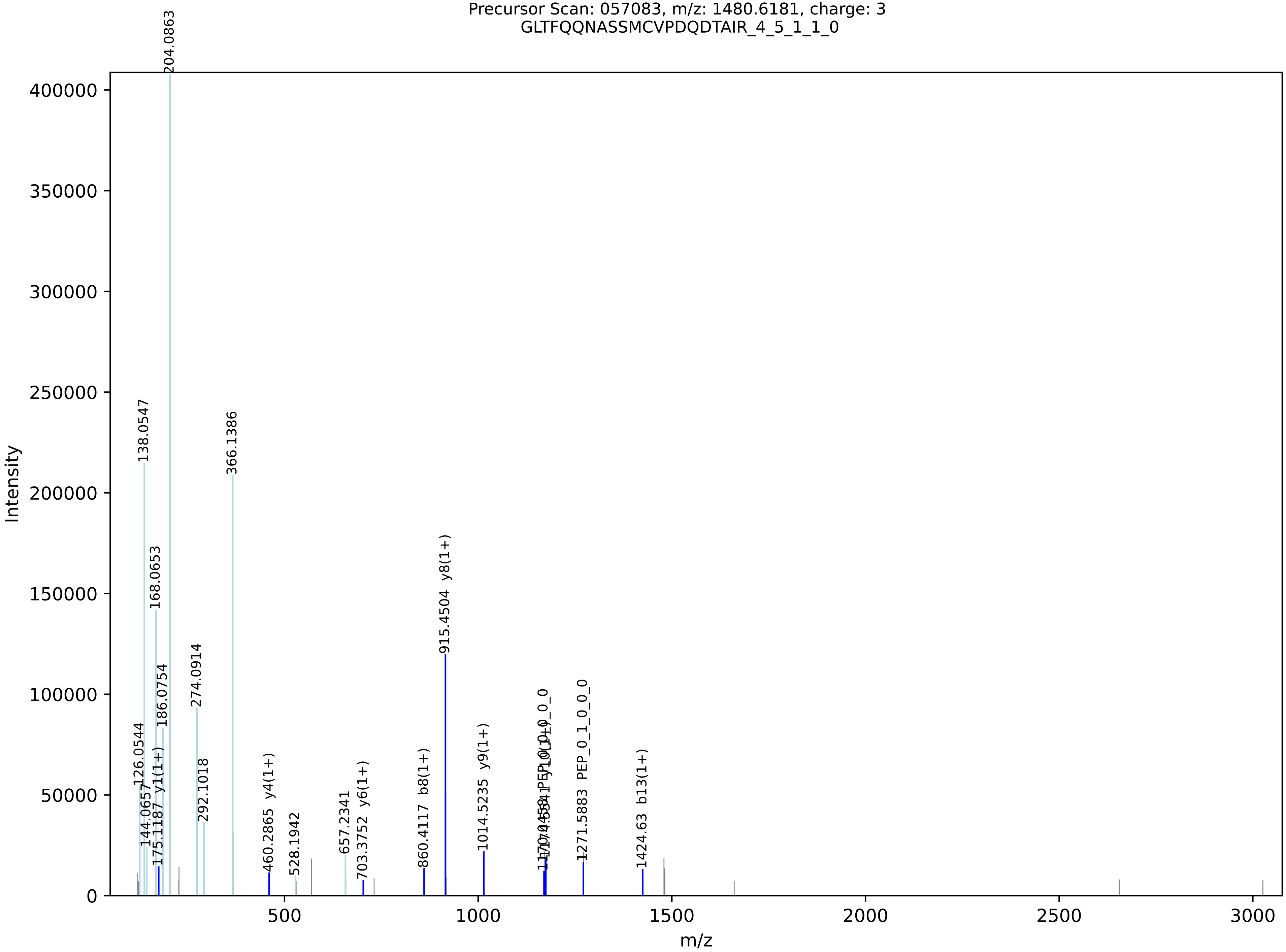

File: 231129_OE_RK_36, RT: 46.198, Scan: 057256
Type: HCD, FTMS + c NSI d Full ms2 1156.4468@hcd30.00 [120.0000-3560.8691]
Precursor Scan: 057154, m/z: 1155.7814, charge: 3
NQSVR_6_5_2_2_0

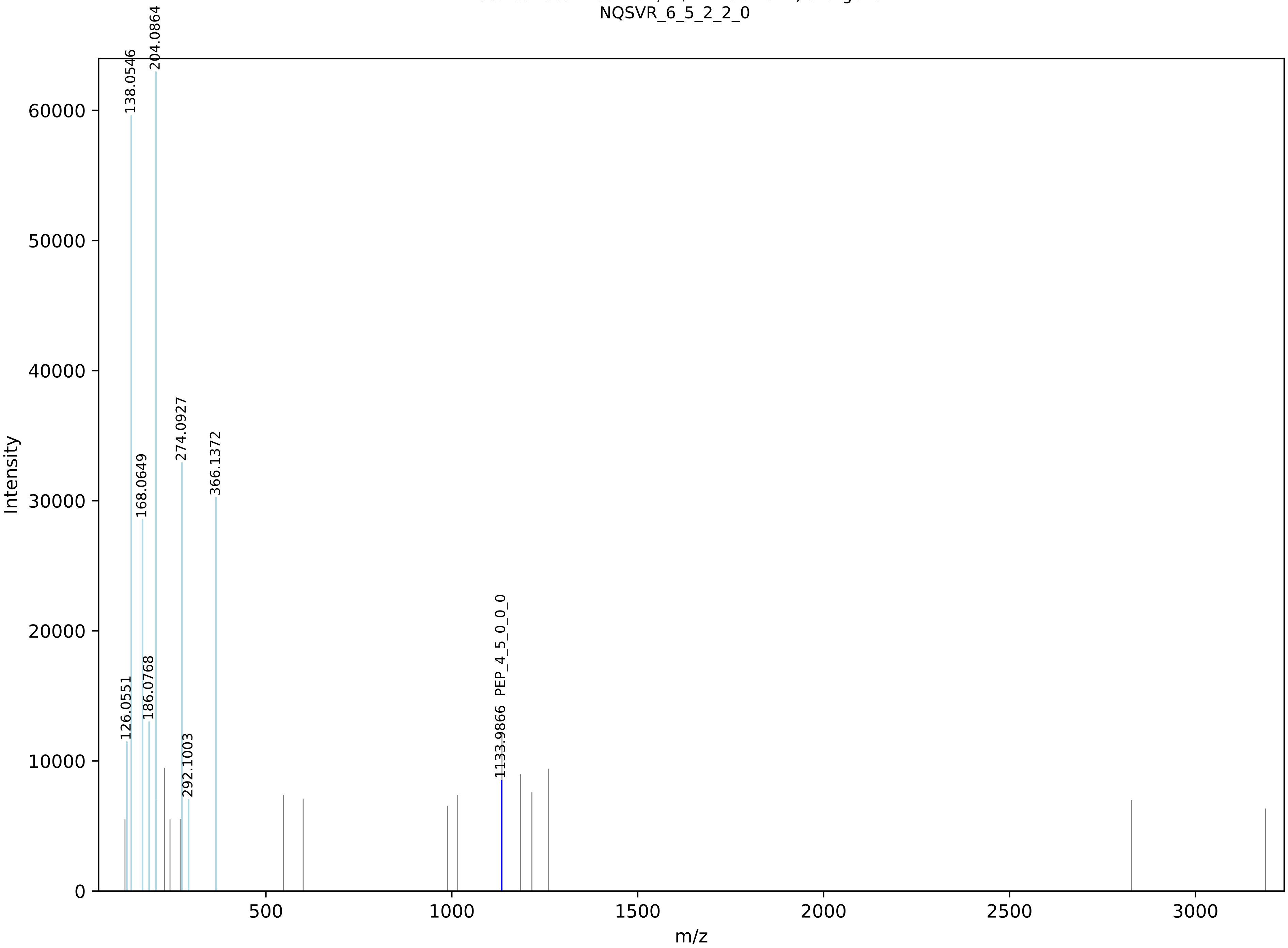

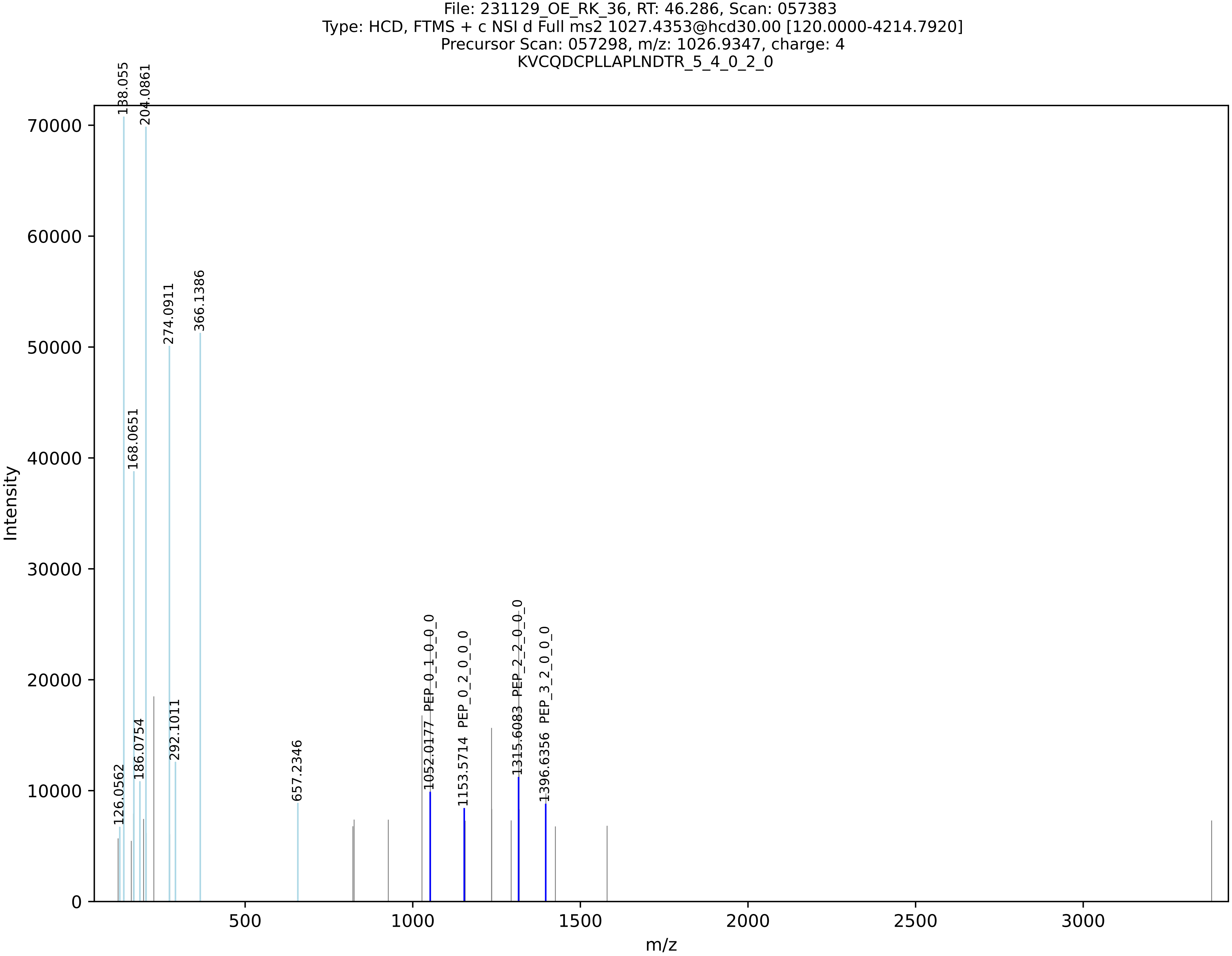
File: 231129_OE_RK_36, RT: 46.286, Scan: 057383
Type: HCD, FTMS + c NSI d Full ms2 1027.4353@hcd30.00 [120.0000-4214.7920]
Precursor Scan: 057298, m/z: 1026.9347, charge: 4
KVCQDCPLLAPLNDTR_5_4_0_2_0
138.055
204.0861
274.0911
366.1386
168.0651
186.0754
292.1011
126.0562
657.2346
1052.0177 PEP_0_1_0_0_0
1153.5714 PEP_0_2_0_0_0
1315.6083 PEP_2_2_0_0_0
1396.6356 PEP_3_2_0_0_0
Intensity
m/z
0
10000
20000
30000
40000
50000
60000
70000
500
1000
1500
2000
2500
3000

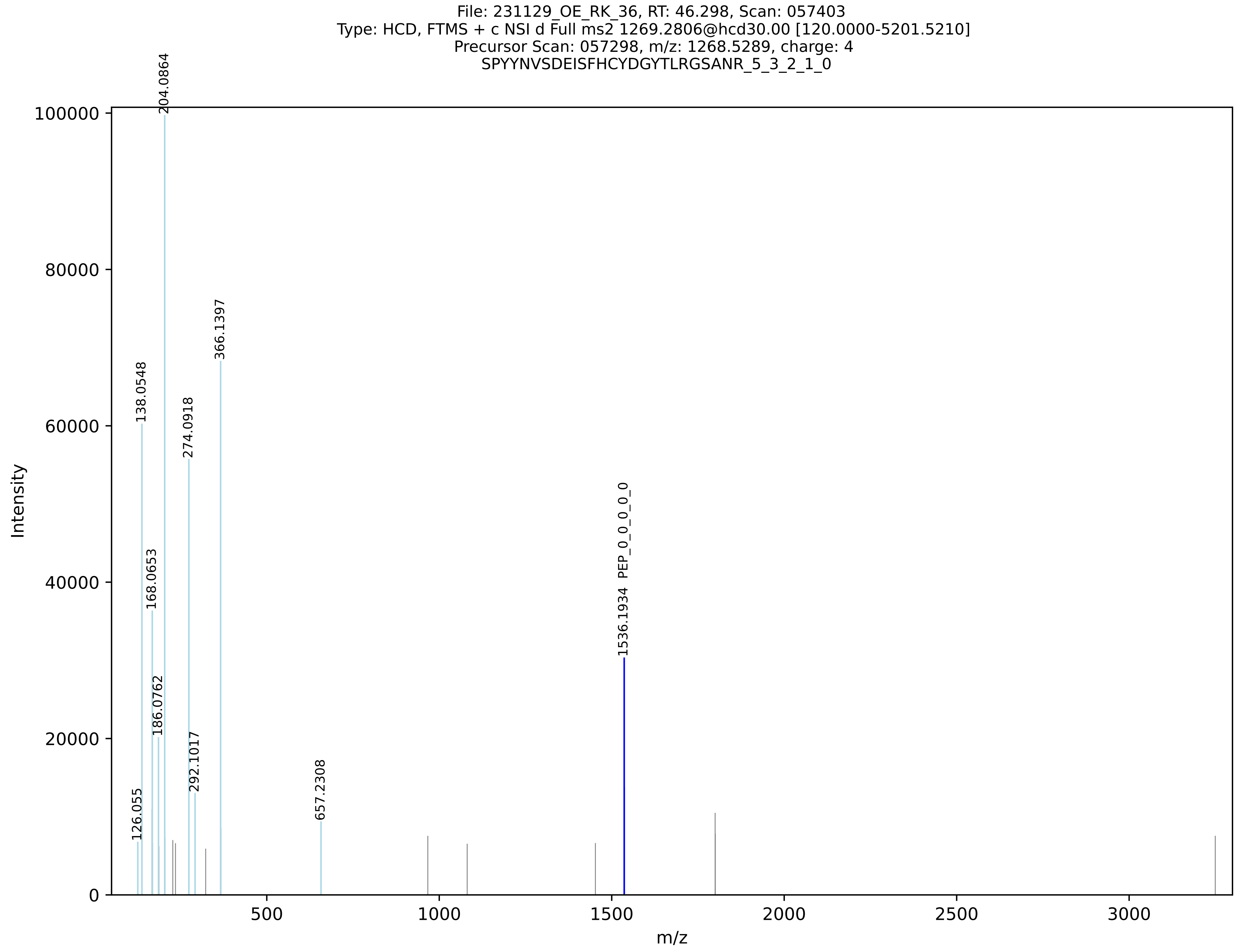

File: 231129_OE_RK_36, RT: 46.298, Scan: 057403
Type: HCD, FTMS + c NSI d Full ms2 1269.2806@hcd30.00 [120.0000-5201.5210]
Precursor Scan: 057298, m/z: 1268.5289, charge: 4
SPYYNVSDEISFHCYDGYTLRGSANR_5_3_2_1_0
Intensity
m/z
204.0864
366.1397
138.0548
274.0918
168.0653
186.0762
292.1017
126.055
657.2308
1536.1934 PEP_0_0_0_0_
0
20000
40000
60000
80000
100000
500
1000
1500
2000
2500
3000

File: 231129_OE_RK_36, RT: 46.333, Scan: 057448
Type: HCD, FTMS + c NSI d Full ms2 1593.6394@hcd30.00 [120.0000-4898.6787]
Precursor Scan: 057372, m/z: 1592.9723, charge: 3
QDQCIYNTTYLNVQR_6_5_0_3_0

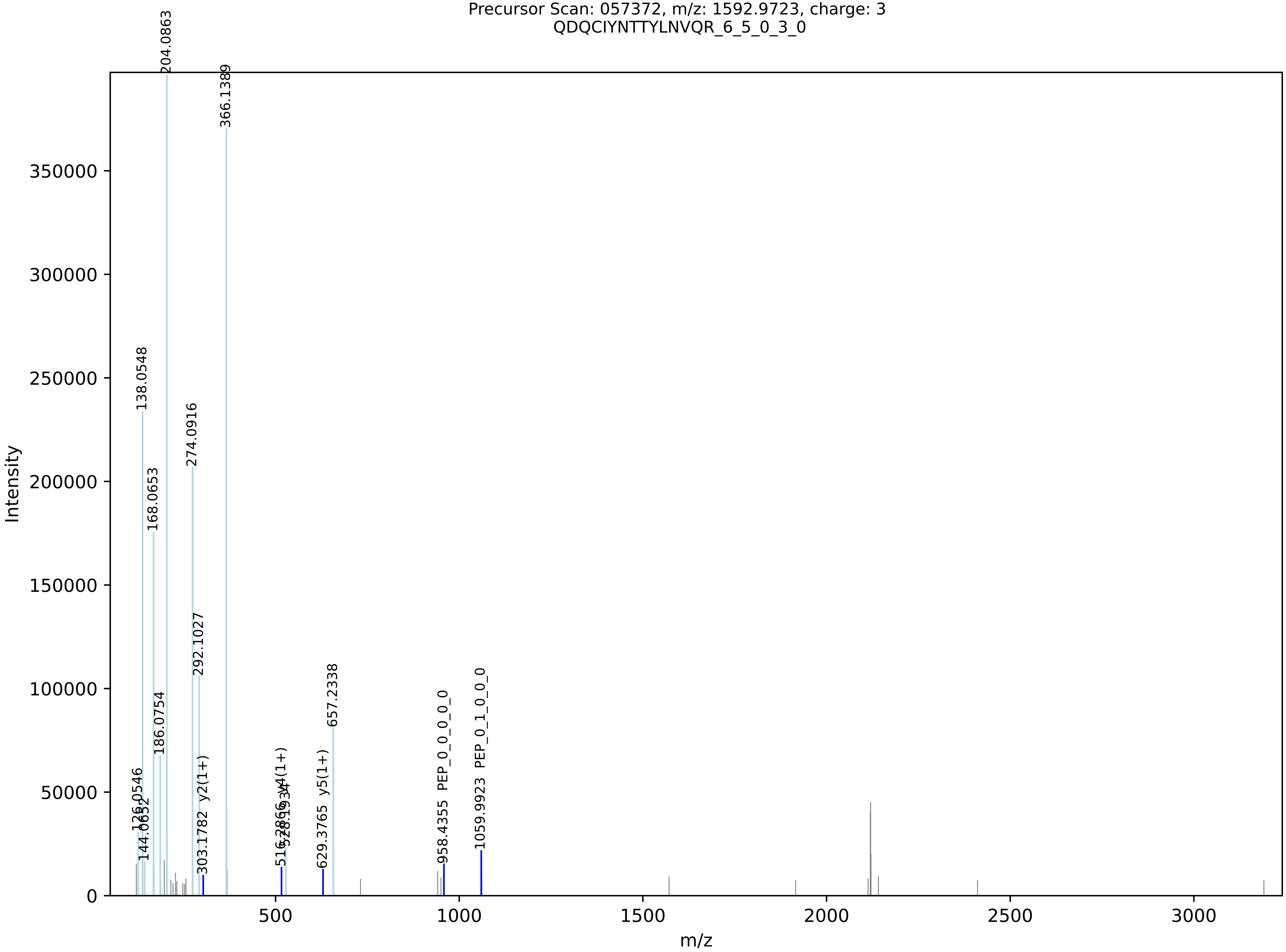

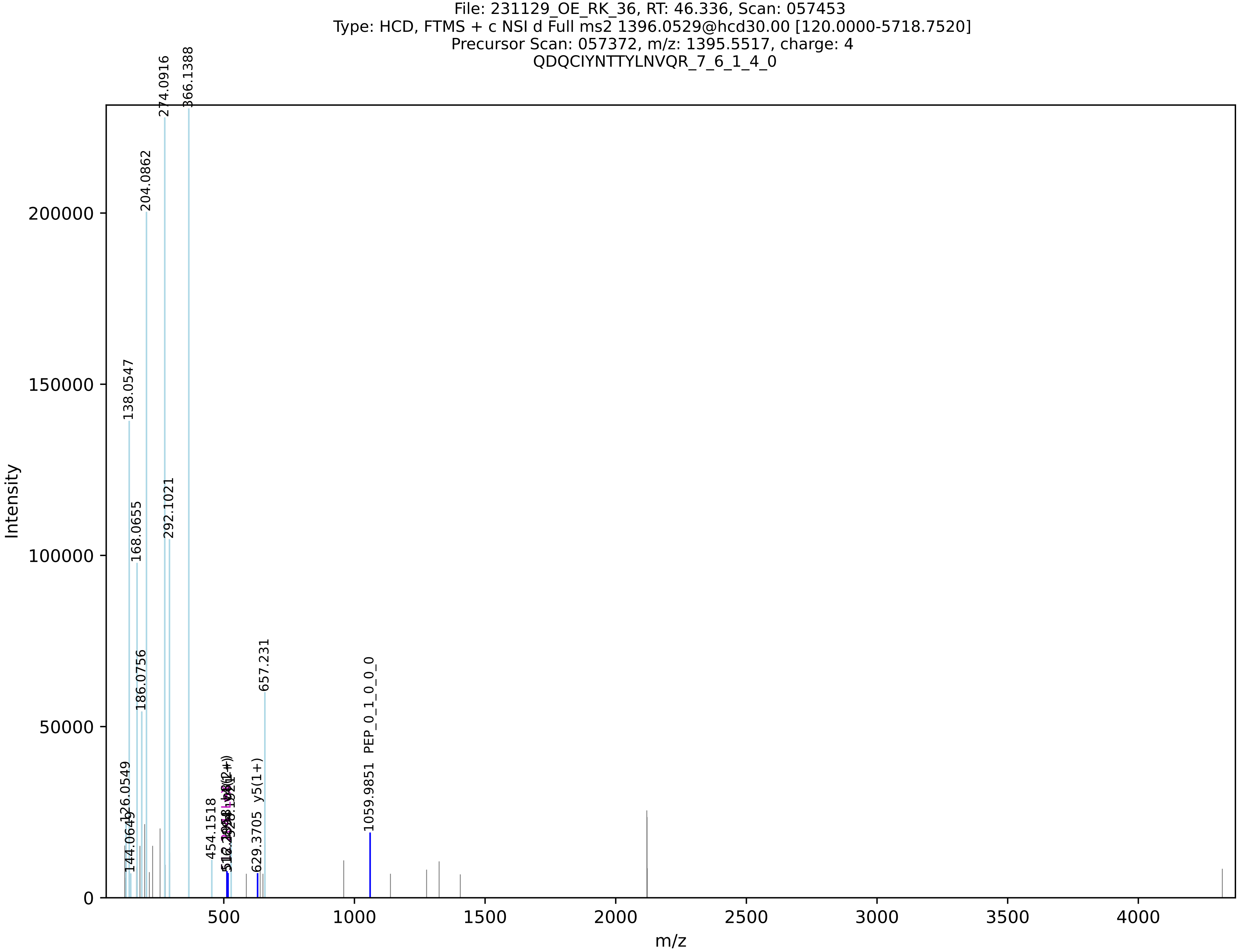
File: 231129_OE_RK_36, RT: 46.336, Scan: 057453
Type: HCD, FTMS + c NSI d Full ms2 1396.0529@hcd30.00 [120.0000-5718.7520]
Precursor Scan: 057372, m/z: 1395.5517, charge: 4
QDQCIYNTTYLNVQR_7_6_1_4_0
274.0916
366.1388
204.0862
138.0547
292.1021
168.0655
186.0756
657.231
PEP_0_1_0_0_0_
1059.9851
y5(1+)
629.3705
454.1518
126.0549
144.0649
Intensity
m/z
0
50000
100000
150000
200000
500
1000
1500
2000
2500
3000
3500
4000

File: 231129_OE_RK_36, RT: 46.393, Scan: 057538
Type: HCD, FTMS + c NSI d Full ms2 1199.7377@hcd30.00 [120.0000-4917.7856]
Precursor Scan: 057446, m/z: 1198.9859, charge: 4
QDQCIYNTTYLNVQR_7_5_1_2_0

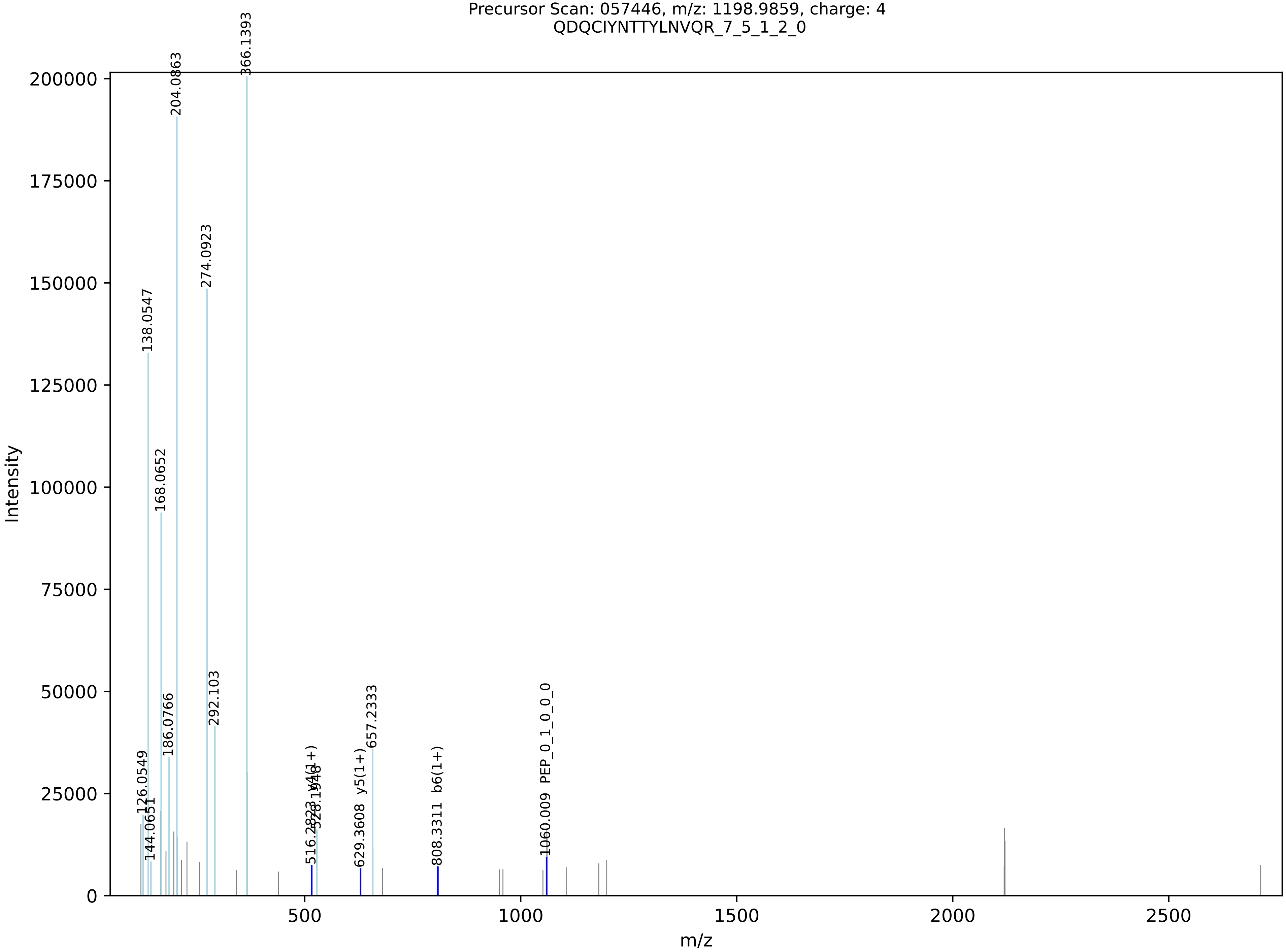

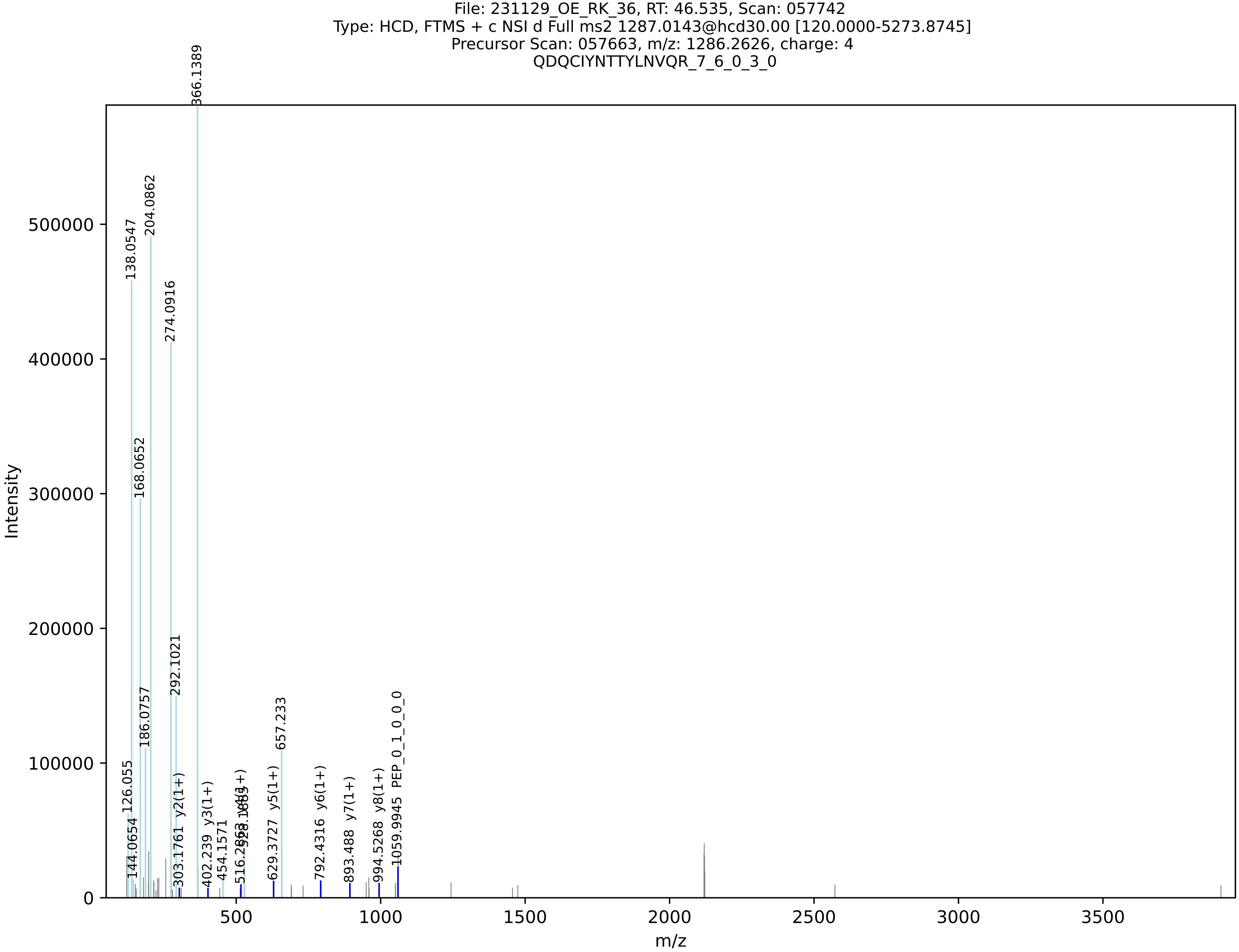
File: 231129_OE_RK_36, RT: 46.535, Scan: 057742
Type: HCD, FTMS + c NSI d Full ms2 1287.0143@hcd30.00 [120.0000-5273.8745]
Precursor Scan: 057663, m/z: 1286.2626, charge: 4
QDQCIYNTTYLNVQR_7_6_0_3_0
Intensity
m/z
366.1389
204.0862
138.0547
274.0916
168.0652
292.1021
186.0757
657.233
126.055
144.0654
303.1761 y2(1+)
402.239 y3(1+)
454.1571
516.2863 y4(1+)
528.1885
629.3727 y5(1+)
792.4316 y6(1+)
893.488 y7(1+)
994.5268 y8(1+)
1059.9945 PEP_0_1_0_0_0
0
100000
200000
300000
400000
500000
500
1000
1500
2000
2500
3000
3500

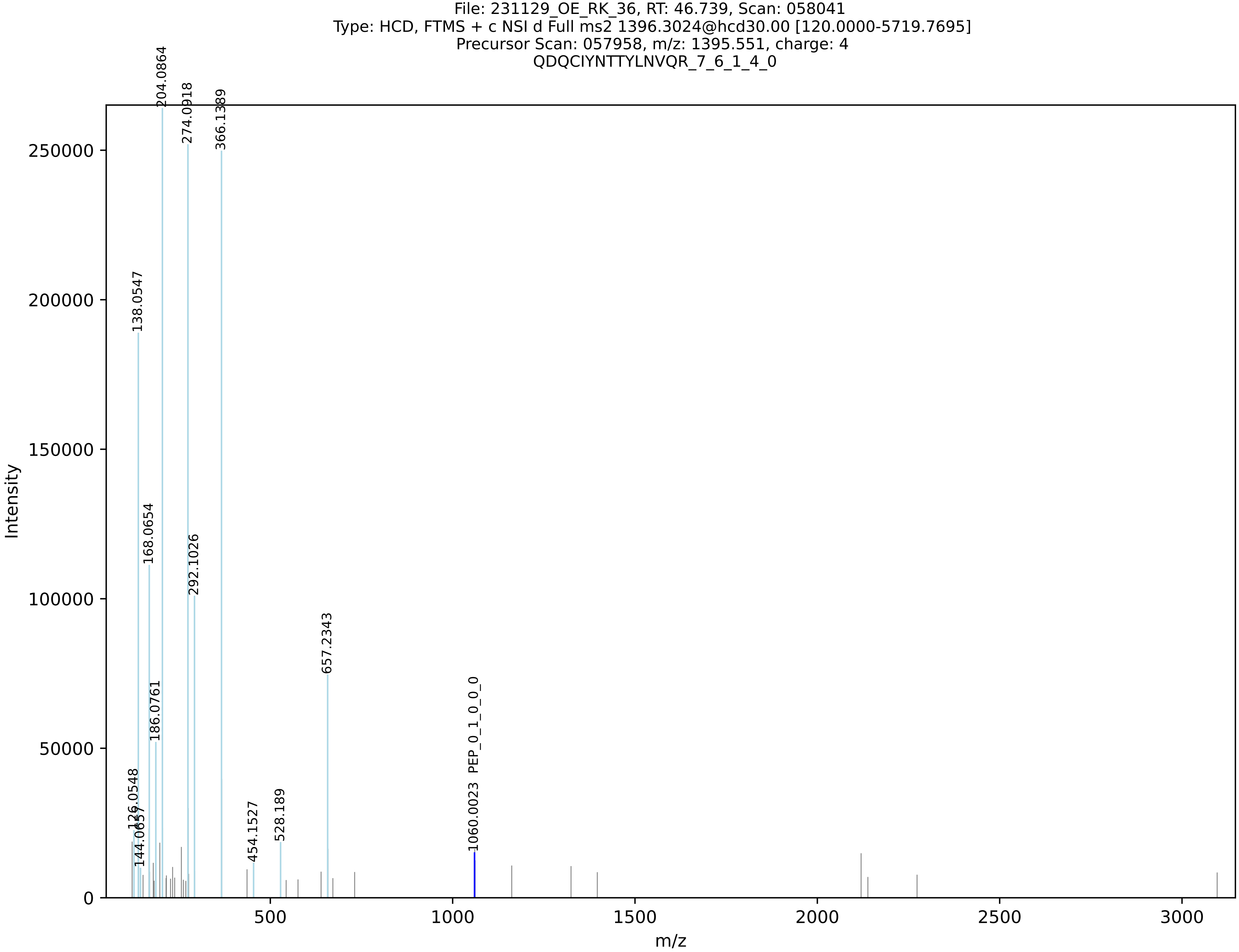
File: 231129_OE_RK_36, RT: 46.739, Scan: 058041
Type: HCD, FTMS + c NSI d Full ms2 1396.3024@hcd30.00 [120.0000-5719.7695]
Precursor Scan: 057958, m/z: 1395.551, charge: 4
QDQCIYNTTYLNVQR_7_6_1_4_0
Intensity
m/z
0
50000
100000
150000
200000
250000
500
1000
1500
2000
2500
3000
204.0864
274.0918
366.1389
138.0547
168.0654
292.1026
657.2343
186.0761
126.0548
144.0657
454.1527
528.189
1060.0023 PEP_0_1_0_0_0

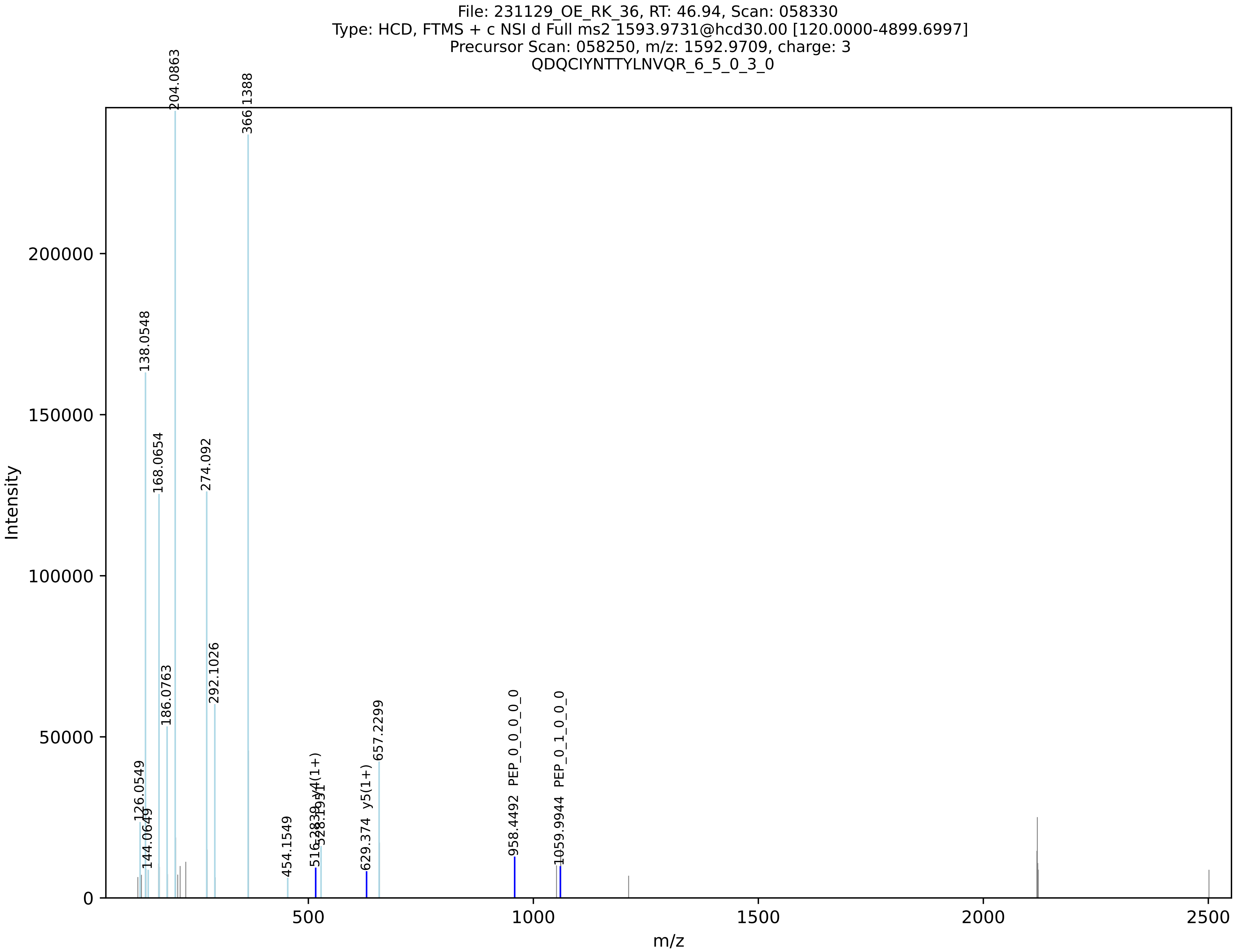
File: 231129_OE_RK_36, RT: 46.94, Scan: 058330
Type: HCD, FTMS + c NSI d Full ms2 1593.9731@hcd30.00 [120.0000-4899.6997]
Precursor Scan: 058250, m/z: 1592.9709, charge: 3
QDQCIYNTTYLNVQR_6_5_0_3_0
Intensity
m/z
204.0863
366.1388
138.0548
168.0654
274.092
186.0763
292.1026
126.0549
144.0649
454.1549
516.2839 y4(1+)
528.1951
629.374 y5(1+)
657.2299
958.4492 PEP_0_0_0_0_0
1059.9944 PEP_0_1_0_0_0
0
50000
100000
150000
200000
500
1000
1500
2000
2500

File: 231129_OE_RK_36, RT: 46.941, Scan: 058331
Type: HCD, FTMS + c NSI d Full ms2 1396.3048@hcd30.00 [120.0000-5719.7798]
Precursor Scan: 058250, m/z: 1395.5517, charge: 4
QDQCIYNTTYLNVQR_7_6_1_4_0

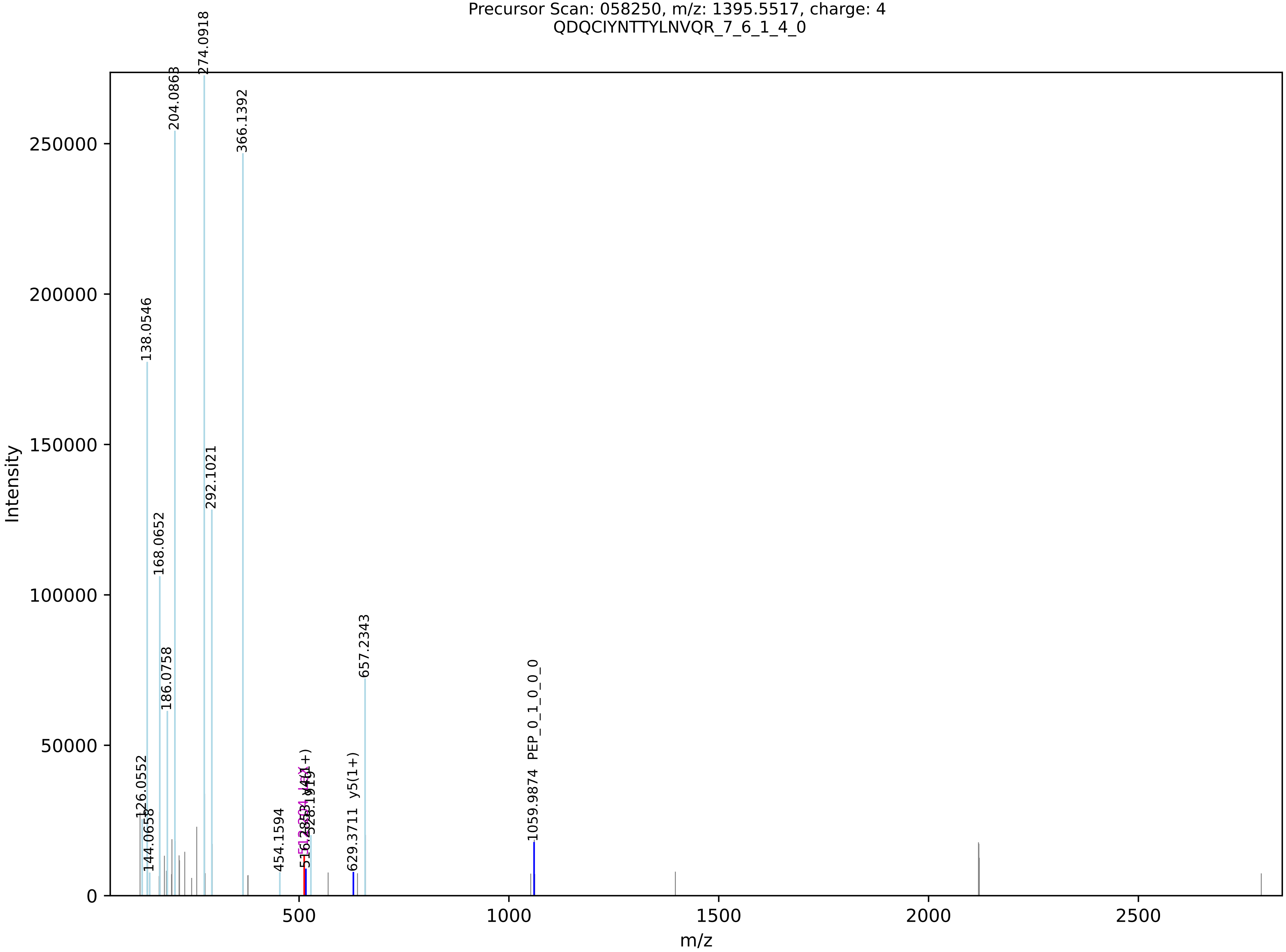

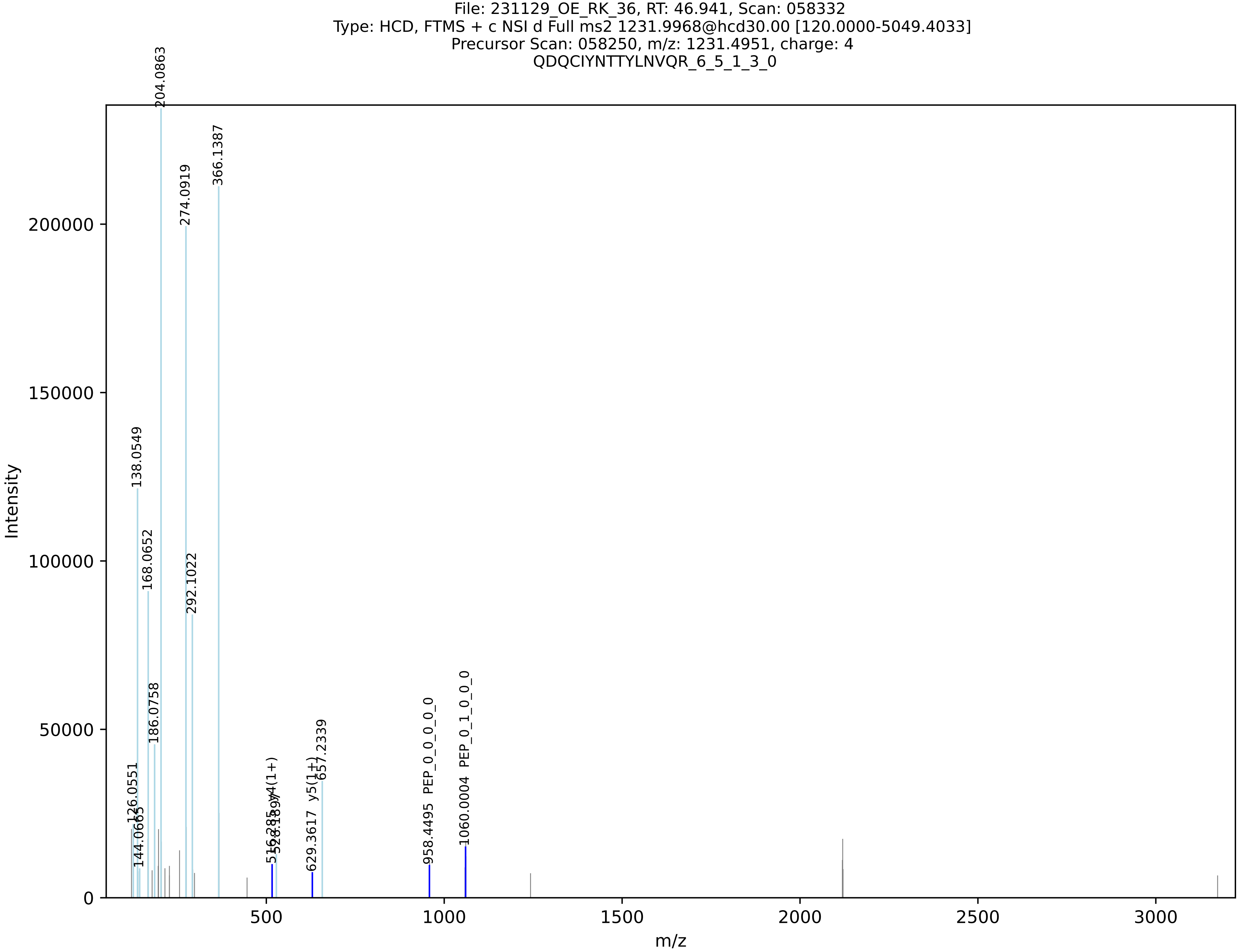
File: 231129_OE_RK_36, RT: 46.941, Scan: 058332
Type: HCD, FTMS + c NSI d Full ms2 1231.9968@hcd30.00 [120.0000-5049.4033]
Precursor Scan: 058250, m/z: 1231.4951, charge: 4
QDQCIYNTTYLNVQR_6_5_1_3_0
Intensity
m/z
204.0863
366.1387
274.0919
138.0549
168.0652
292.1022
186.0758
126.0551
144.0665
516.285 y4(1+)
528.1897
629.3617 y5(1+)
657.2339
958.4495 PEP_0_0_0_0_0
1060.0004 PEP_0_1_0_0_0

File: 231129_OE_RK_36, RT: 46.943, Scan: 058335
Type: HCD, FTMS + c NSI d Full ms2 1027.6863@hcd30.00 [120.0000-4215.8159]
Precursor Scan: 058250, m/z: 1026.9355, charge: 4
KVCQDCPLLAPLNDTR_5_4_0_2_0

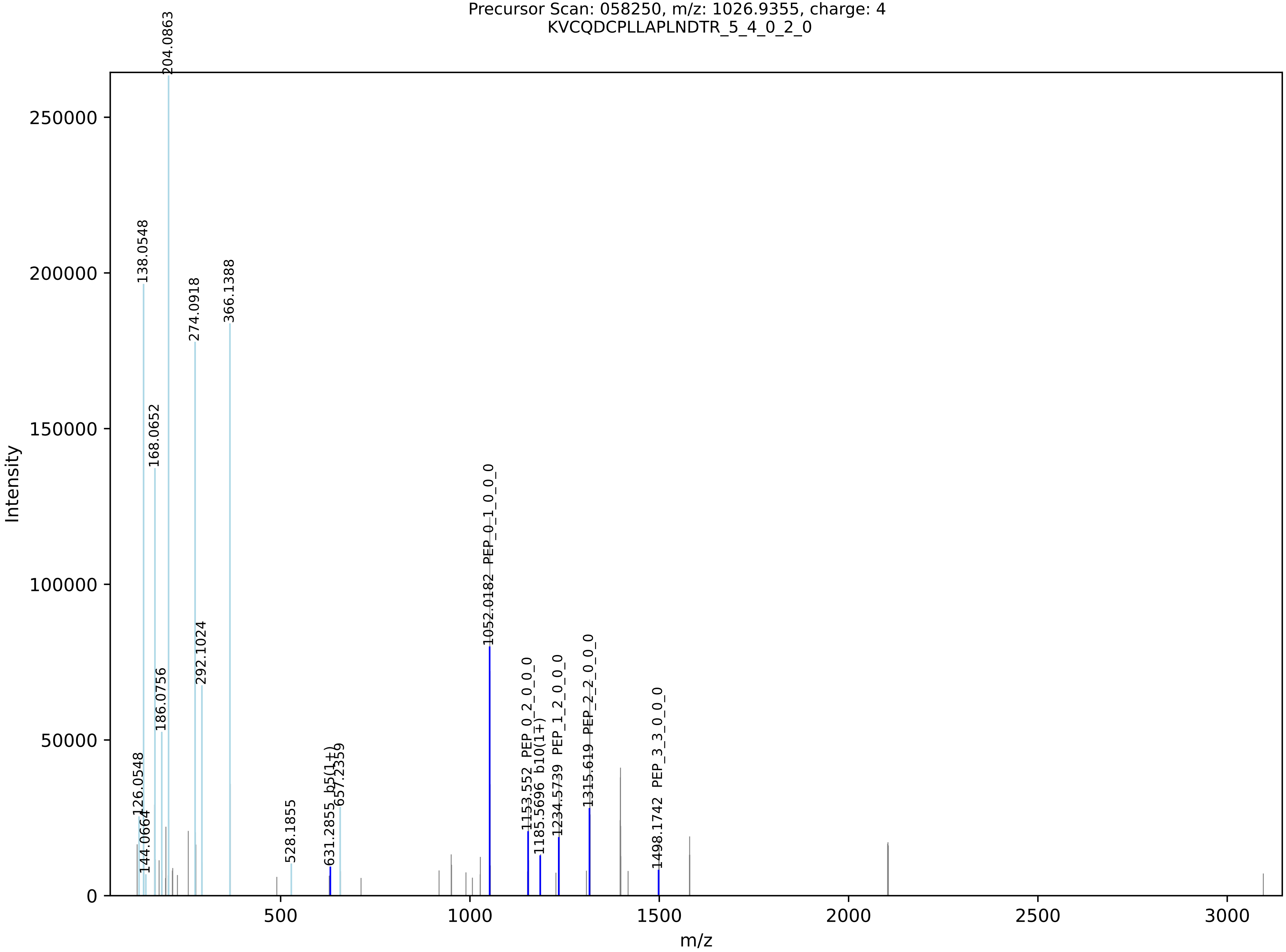

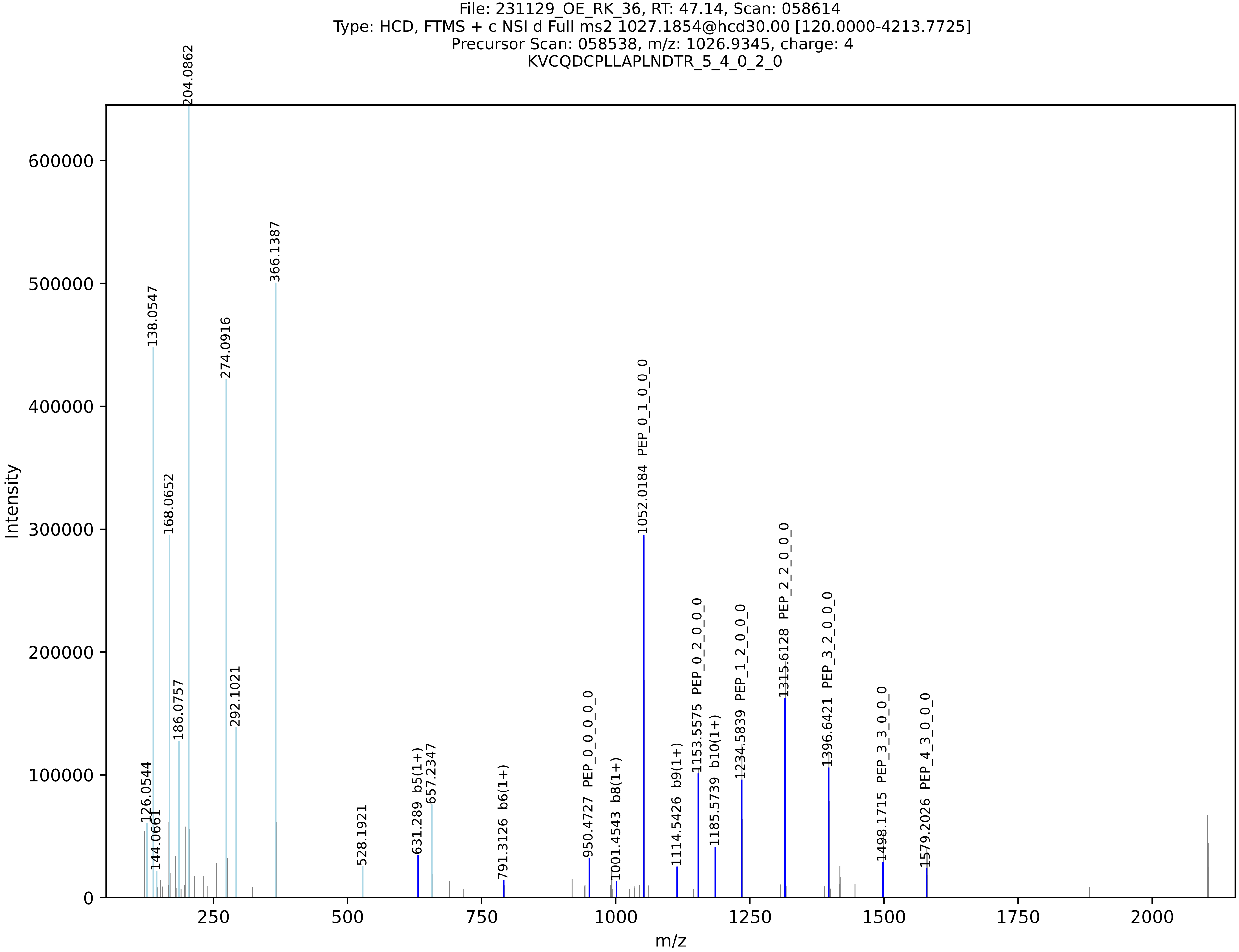

File: 231129_OE_RK_36, RT: 47.14, Scan: 058614
Type: HCD, FTMS + c NSI d Full ms2 1027.1854@hcd30.00 [120.0000-4213.7725]
Precursor Scan: 058538, m/z: 1026.9345, charge: 4
KVCQDCPLLAPLNDTR_5_4_0_2_0
Intensity
m/z
204.0862
366.1387
138.0547
274.0916
168.0652
186.0757
292.1021
126.0544
144.0661
528.1921
631.289 b5(1+)
657.2347
791.3126 b6(1+)
950.4727 PEP_0_0_0_0_0
1001.4543 b8(1+)
1052.0184 PEP_0_1_0_0_0
1114.5426 b9(1+)
1153.5575 PEP_0_2_0_0_0
1185.5739 b10(1+)
1234.5839 PEP_1_2_0_0_0
1315.6128 PEP_2_2_0_0_0
1396.6421 PEP_3_2_0_0_0
1498.1715 PEP_3_3_0_0_0
1579.2026 PEP_4_3_0_0_0

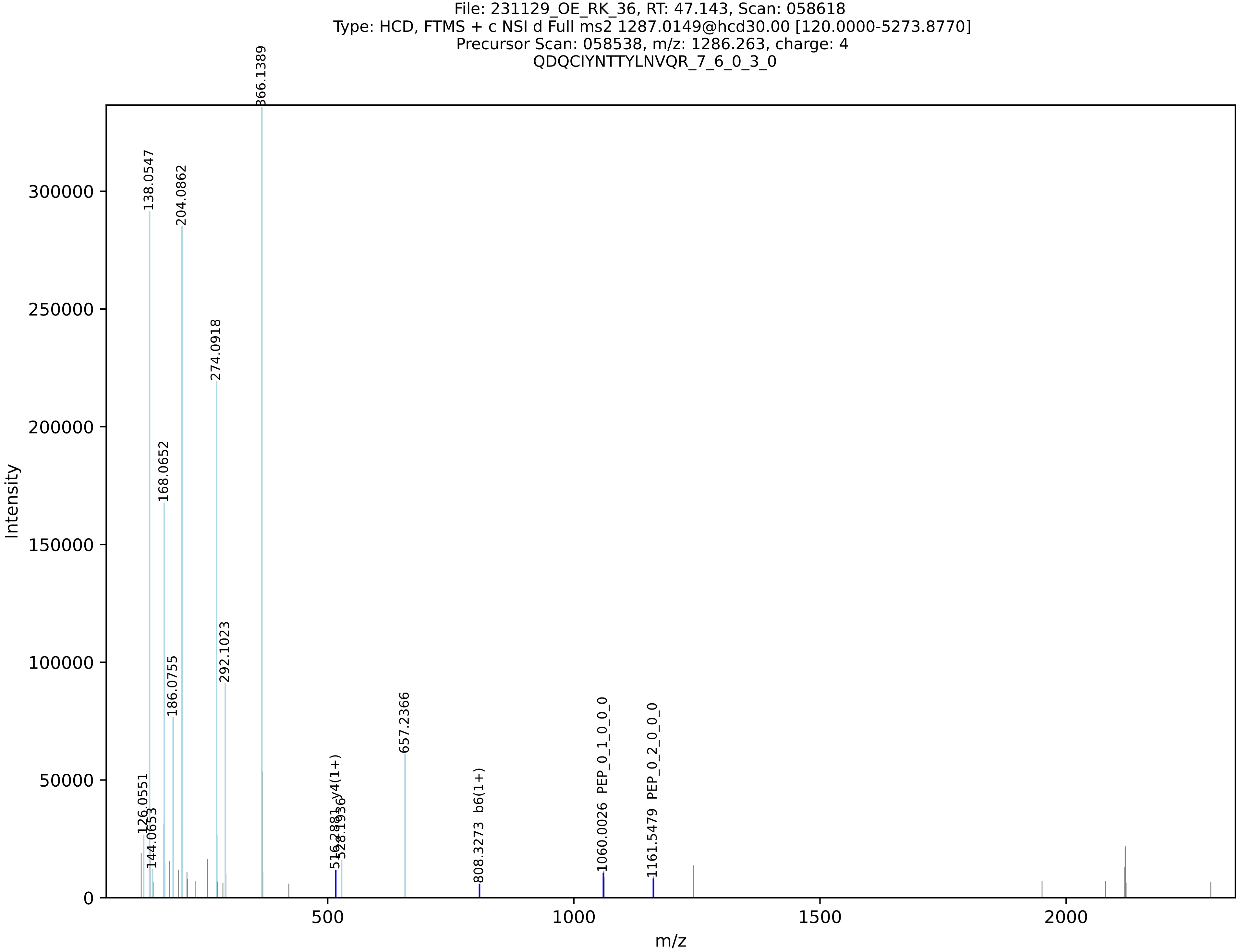
File: 231129_OE_RK_36, RT: 47.143, Scan: 058618
Type: HCD, FTMS + c NSI d Full ms2 1287.0149@hcd30.00 [120.0000-5273.8770]
Precursor Scan: 058538, m/z: 1286.263, charge: 4
QDQCIYNTTYLNVQR_7_6_0_3_0
Intensity
m/z
0
50000
100000
150000
200000
250000
300000
500
1000
1500
2000
366.1389
138.0547
204.0862
274.0918
168.0652
292.1023
186.0755
126.0551
144.0653
516.2881 y4(1+)
528.1936
657.2366
808.3273 b6(1+)
1060.0026 PEP_0_1_0_0_0_
1161.5479 PEP_0_2_0_0_0_

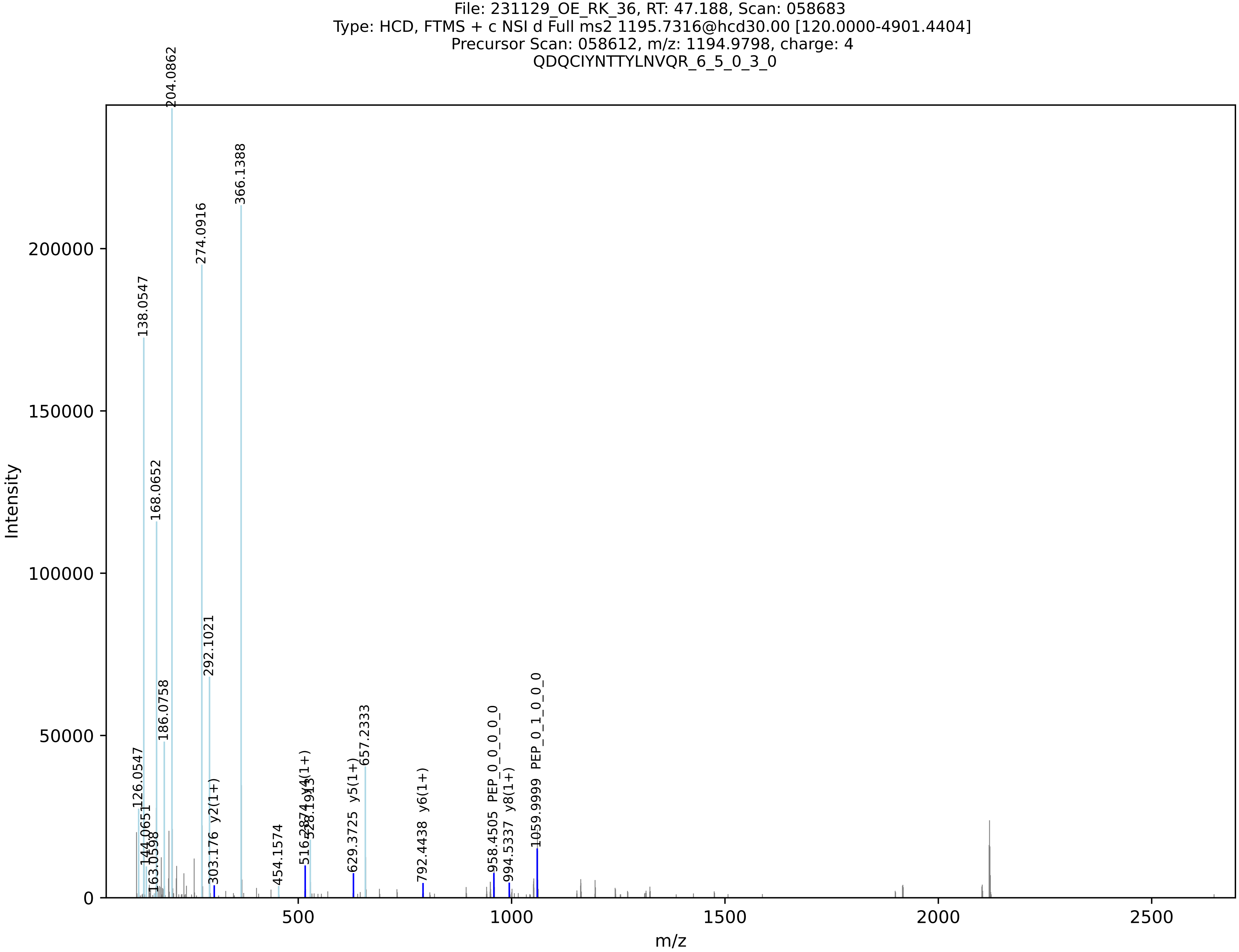

File: 231129_OE_RK_36, RT: 47.188, Scan: 058683
Type: HCD, FTMS + c NSI d Full ms2 1195.7316@hcd30.00 [120.0000-4901.4404]
Precursor Scan: 058612, m/z: 1194.9798, charge: 4
QDQCIYNTTYLNVQR_6_5_0_3_0
Intensity
m/z
204.0862
366.1388
274.0916
138.0547
168.0652
292.1021
186.0758
126.0547
144.0651
163.0598
303.176 y2(1+)
454.1574
516.2874 y4(1+)
528.1913
629.3725 y5(1+)
657.2333
792.4438 y6(1+)
958.4505 PEP_0_0_0_0_0
994.5337 y8(1+)
1059.9999 PEP_0_1_0_0_0

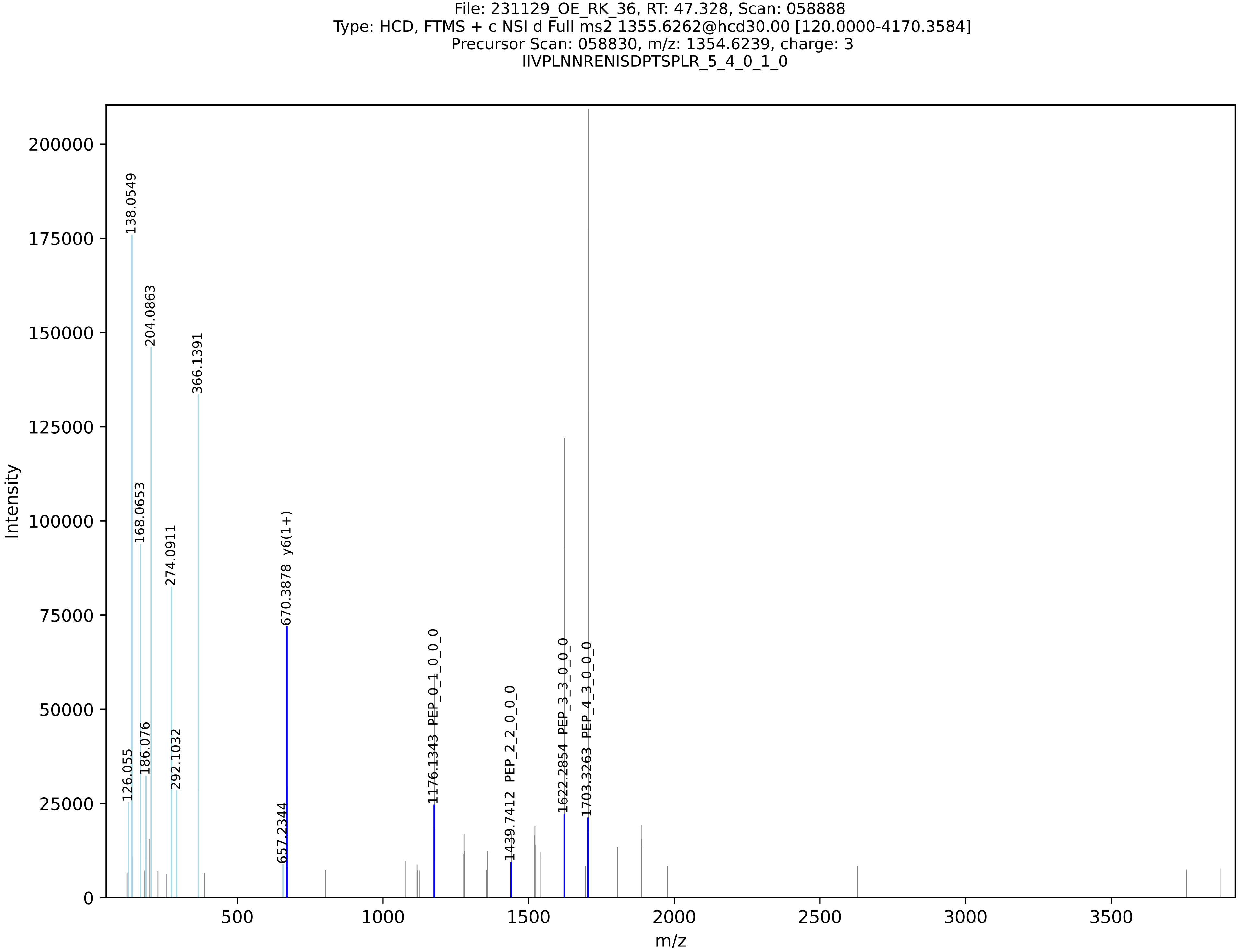
File: 231129_OE_RK_36, RT: 47.328, Scan: 058888
Type: HCD, FTMS + c NSI d Full ms2 1355.6262@hcd30.00 [120.0000-4170.3584]
Precursor Scan: 058830, m/z: 1354.6239, charge: 3
IIVPLNNRENISDPTSPLR_5_4_0_1_0
Intensity
m/z
138.0549
204.0863
366.1391
168.0653
274.0911
670.3878 y6(1+)
186.076
126.055
292.1032
657.2344
1176.1343 PEP_0_1_0_0_0
1439.7412 PEP_2_2_0_0_0
1622.2854 PEP_3_3_0_0_0
1703.3263 PEP_4_3_0_0_0

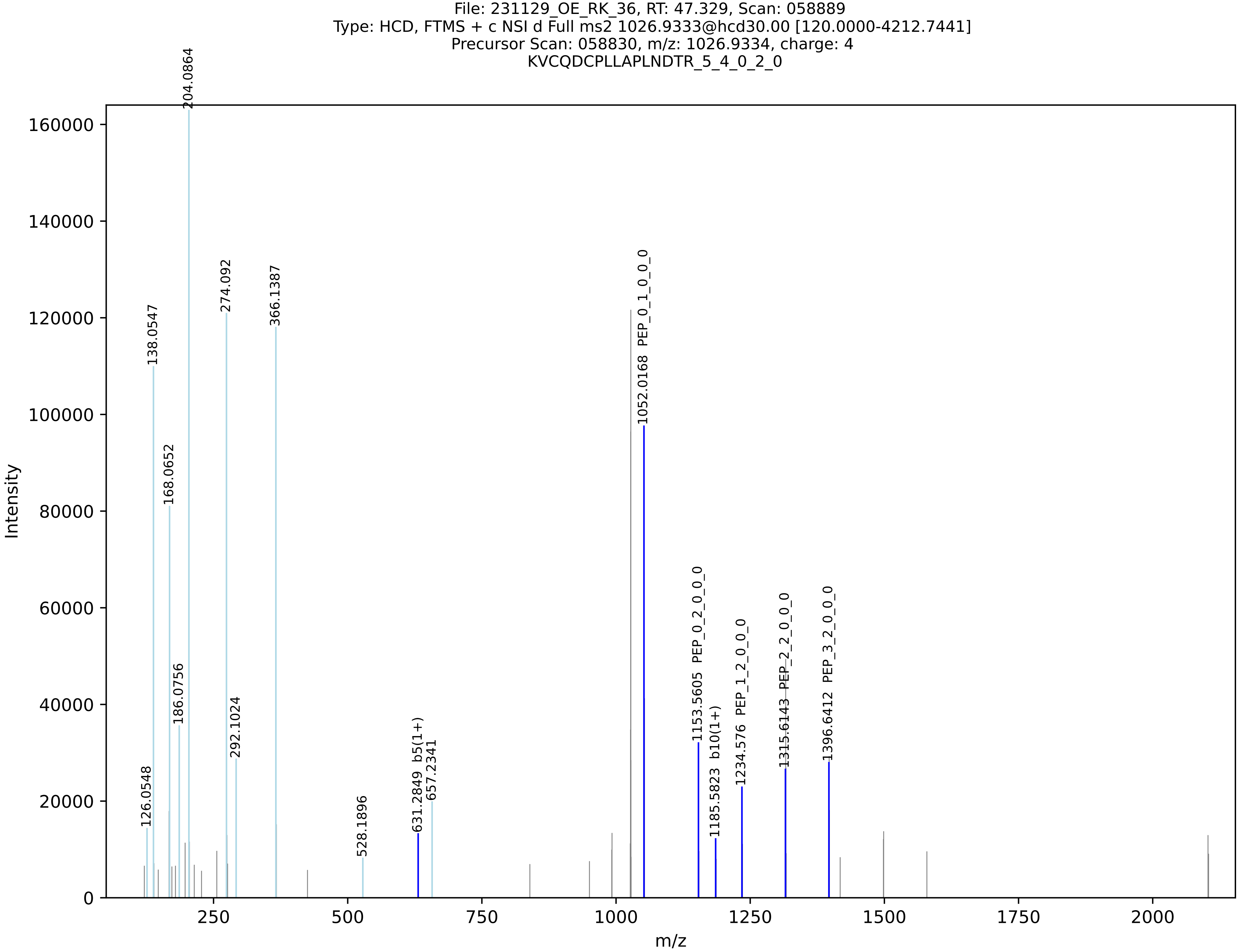
File: 231129_OE_RK_36, RT: 47.329, Scan: 058889
Type: HCD, FTMS + c NSI d Full ms2 1026.9333@hcd30.00 [120.0000-4212.7441]
Precursor Scan: 058830, m/z: 1026.9334, charge: 4
KVCQDCPLLAPLNDTR_5_4_0_2_0
Intensity
m/z
126.0548
138.0547
168.0652
186.0756
204.0864
274.092
292.1024
366.1387
528.1896
631.2849 b5(1+)
657.2341
1052.0168 PEP_0_1_0_0_0
1153.5605 PEP_0_2_0_0_0
1185.5823 b10(1+)
1234.576 PEP_1_2_0_0_0
1315.6143 PEP_2_2_0_0_0
1396.6412 PEP_3_2_0_0_0

File: 231129_OE_RK_36, RT: 47.626, Scan: 059317
Type: HCD, FTMS + c NSI d Full ms2 1131.1482@hcd30.00 [120.0000-3483.4553]
Precursor Scan: 059227, m/z: 1130.814, charge: 3
CGLVPVLAENYNK_5_4_0_1_0

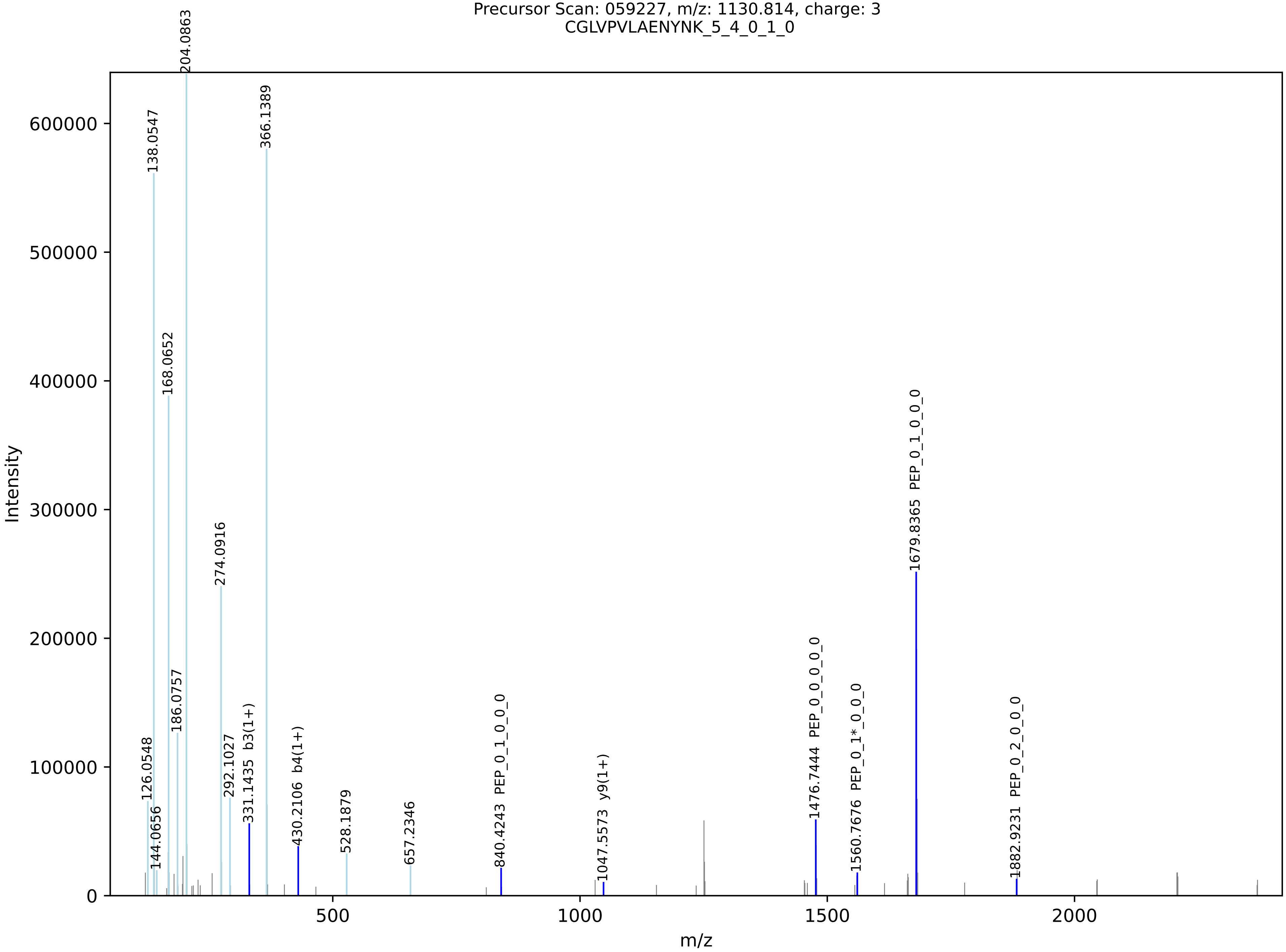

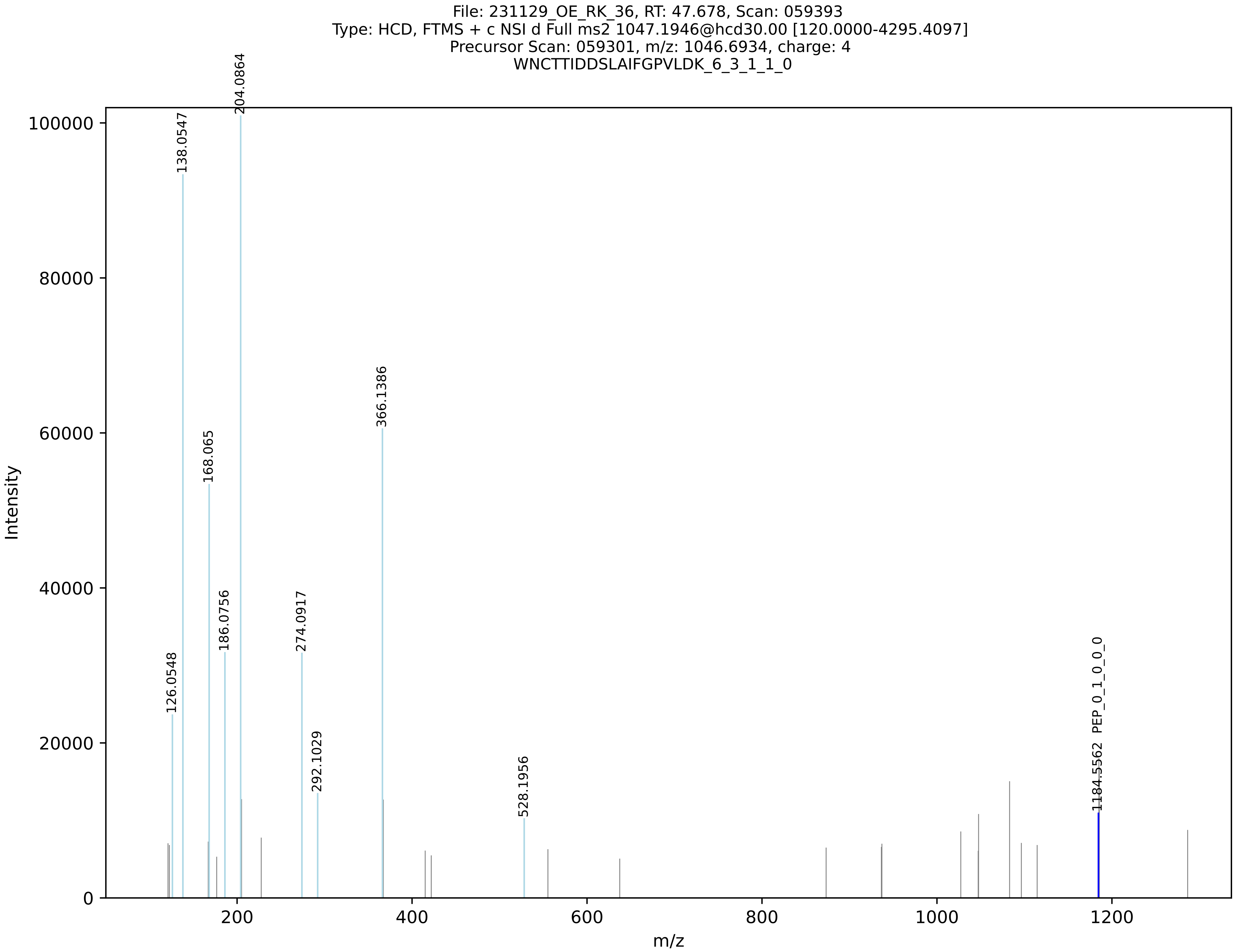
File: 231129_OE_RK_36, RT: 47.678, Scan: 059393
Type: HCD, FTMS + c NSI d Full ms2 1047.1946@hcd30.00 [120.0000-4295.4097]
Precursor Scan: 059301, m/z: 1046.6934, charge: 4
WNCTTIDDSLAIFGPVLDK_6_3_1_1_0
204.0864
138.0547
168.065
366.1386
186.0756
274.0917
126.0548
292.1029
528.1956
1184.5562 PEP_0_1_0_0_0
Intensity
m/z
0
20000
40000
60000
80000
100000
200
400
600
800
1000
1200

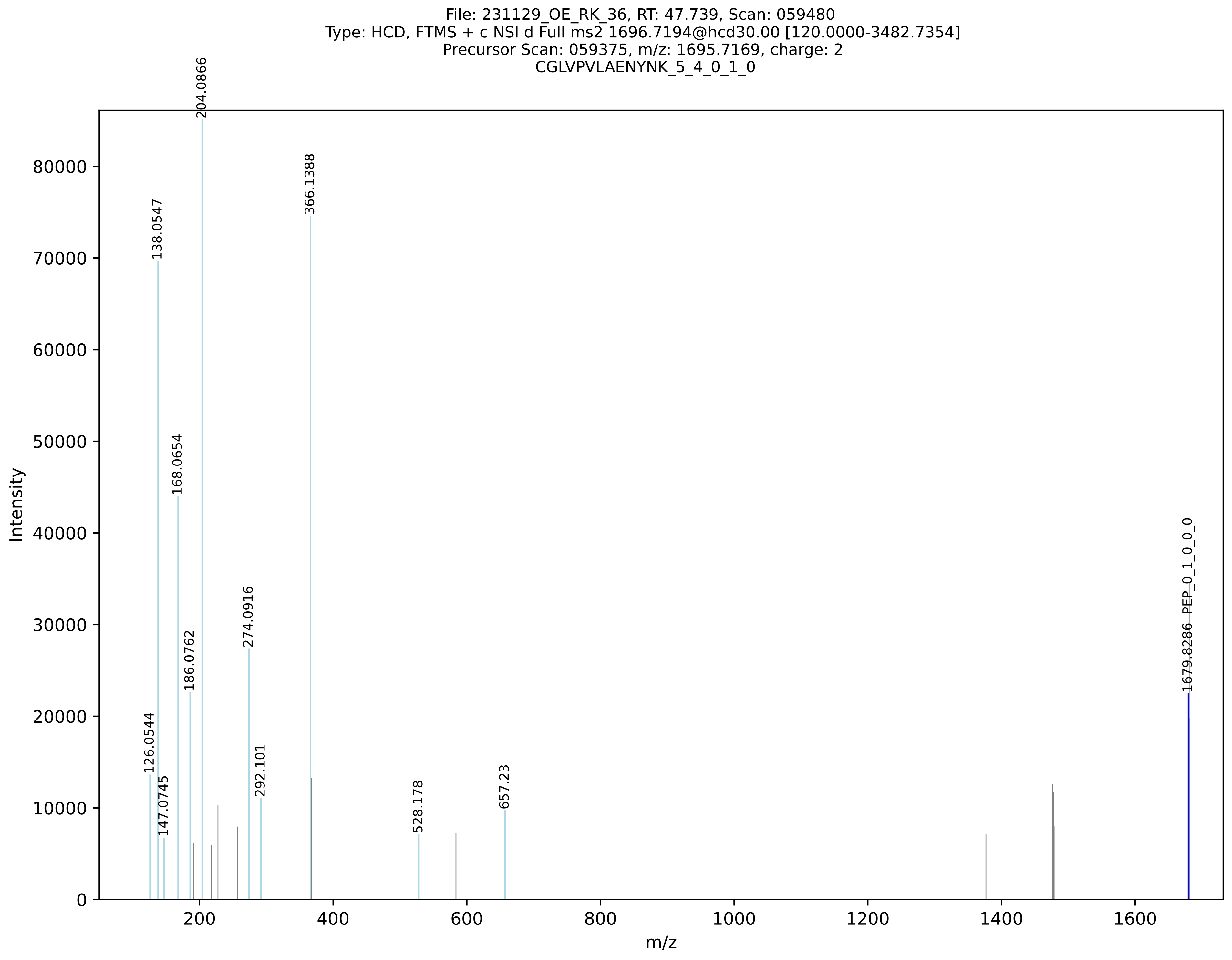
File: 231129_OE_RK_36, RT: 47.739, Scan: 059480
Type: HCD, FTMS + c NSI d Full ms2 1696.7194@hcd30.00 [120.0000-3482.7354]
Precursor Scan: 059375, m/z: 1695.7169, charge: 2
CGLVPVLAENYNK_5_4_0_1_0
Intensity
m/z
204.0866
366.1388
138.0547
168.0654
186.0762
274.0916
126.0544
147.0745
292.101
528.178
657.23
1679.8286_PEP_0_1_0_0_0
0
10000
20000
30000
40000
50000
60000
70000
80000
200
400
600
800
1000
1200
1400
1600

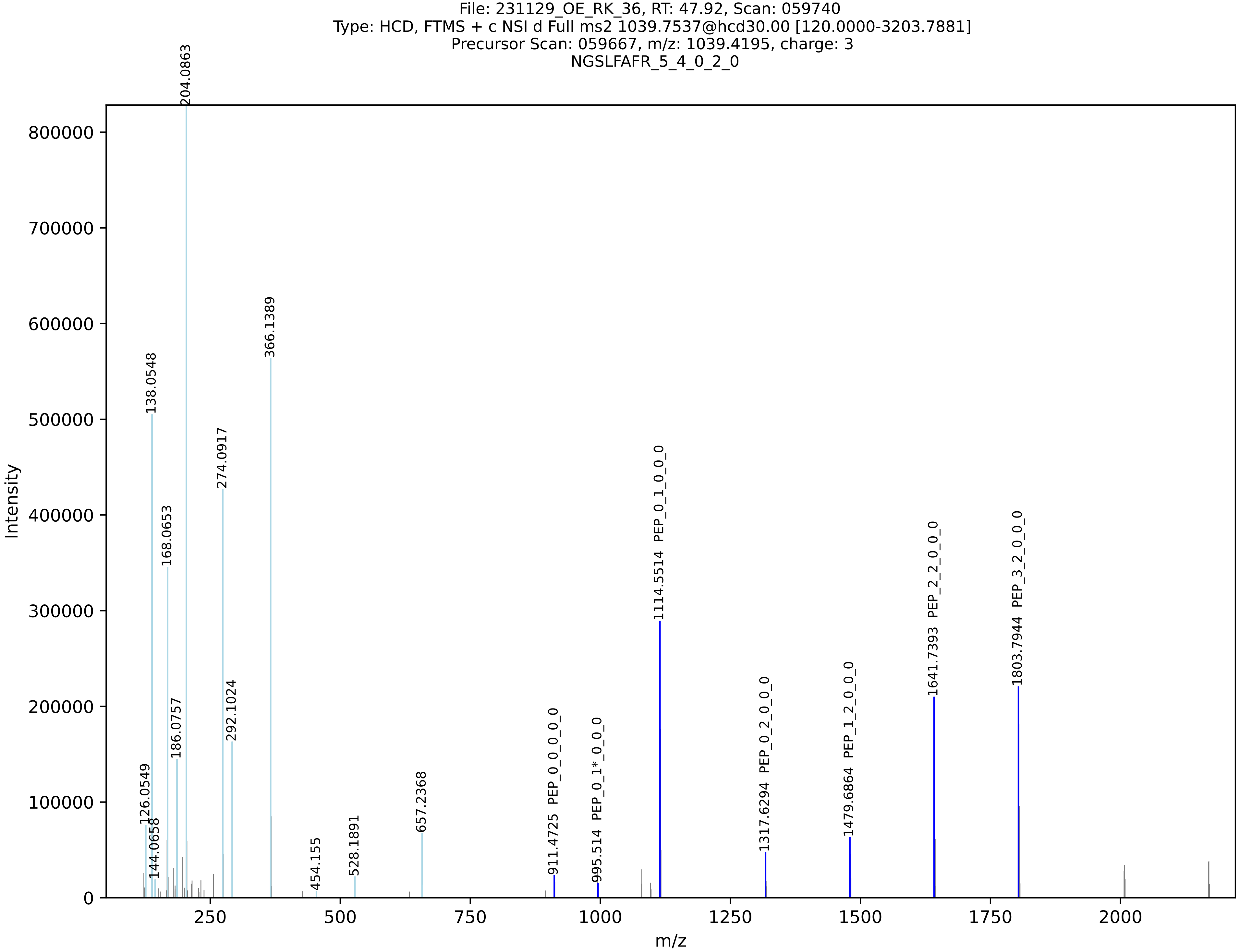

File: 231129_OE_RK_36, RT: 47.92, Scan: 059740
Type: HCD, FTMS + c NSI d Full ms2 1039.7537@hcd30.00 [120.0000-3203.7881]
Precursor Scan: 059667, m/z: 1039.4195, charge: 3
NGSLFAFR_5_4_0_2_0
Intensity
m/z
204.0863
366.1389
138.0548
274.0917
168.0653
292.1024
186.0757
126.0549
144.0658
454.155
528.1891
657.2368
911.4725 PEP_0_0_0_0_0
995.514 PEP_0_1*_0_0_0
1114.5514 PEP_0_1_0_0_0
1317.6294 PEP_0_2_0_0_0
1479.6864 PEP_1_2_0_0_0
1641.7393 PEP_2_2_0_0_0
1803.7944 PEP_3_2_0_0_0

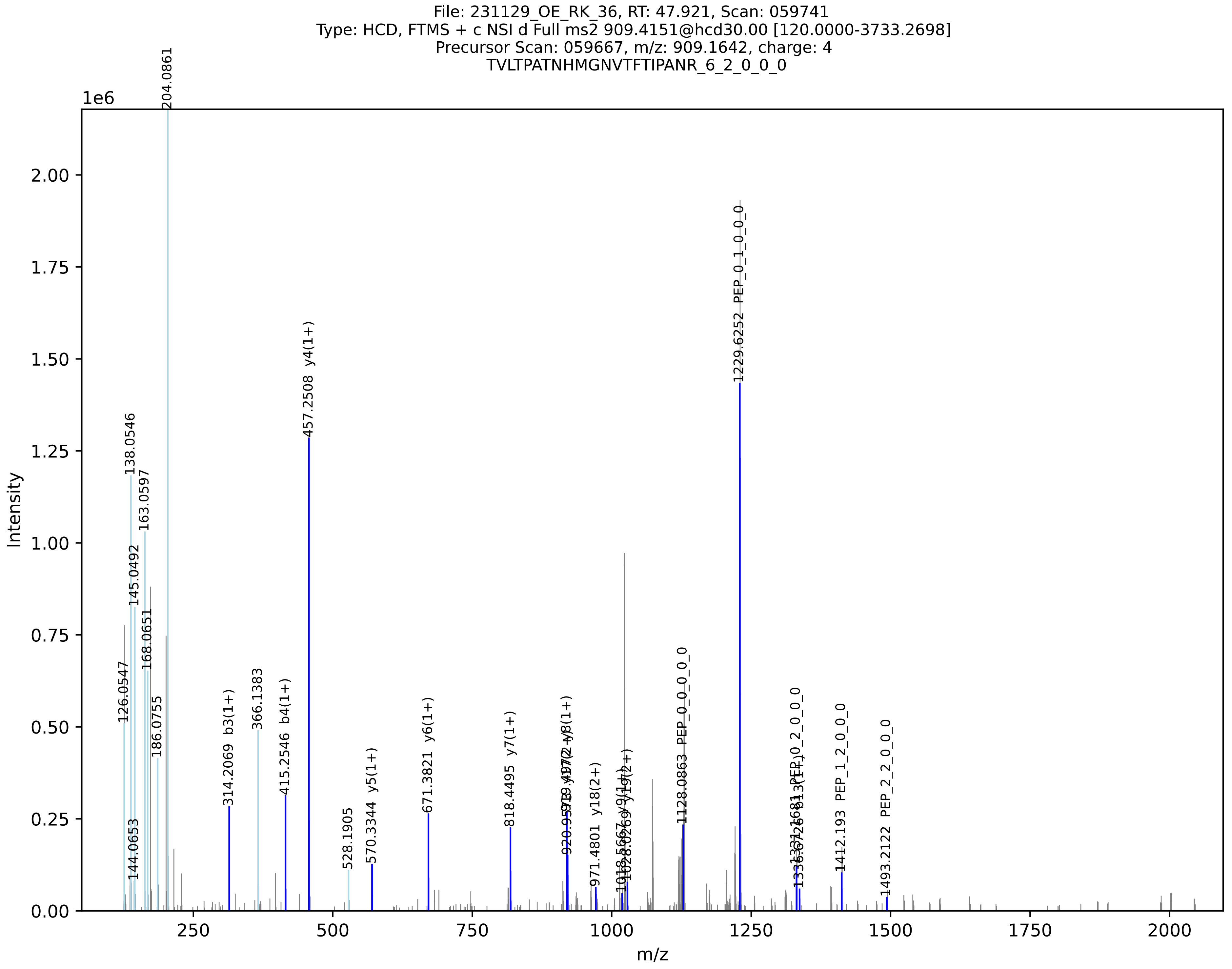

File: 231129_OE_RK_36, RT: 47.921, Scan: 059741
Type: HCD, FTMS + c NSI d Full ms2 909.4151@hcd30.00 [120.0000-3733.2698]
Precursor Scan: 059667, m/z: 909.1642, charge: 4
TVLTPATNHMGNVTFTIPANR_6_2_0_0_0
1e6
Intensity
m/z
204.0861
138.0546
163.0597
145.0492
168.0651
126.0547
186.0755
144.0653
314.2069 b3(1+)
366.1383
415.2546 b4(1+)
457.2508 y4(1+)
528.1905
570.3344 y5(1+)
671.3821 y6(1+)
818.4495 y7(1+)
971.4801 y18(2+)
1128.0863 PEP_0_0_0_0_0
1229.6252 PEP_0_1_0_0_0
1412.193 PEP_1_2_0_0_0
1493.2122 PEP_2_2_0_0_0

File: 231129_OE_RK_36, RT: 47.926, Scan: 059749
Type: HCD, FTMS + c NSI d Full ms2 1275.5673@hcd30.00 [120.0000-3925.3779]
Precursor Scan: 059667, m/z: 1275.2332, charge: 3
QDNTTQLVQDDQVKGPLR_5_4_1_0_0

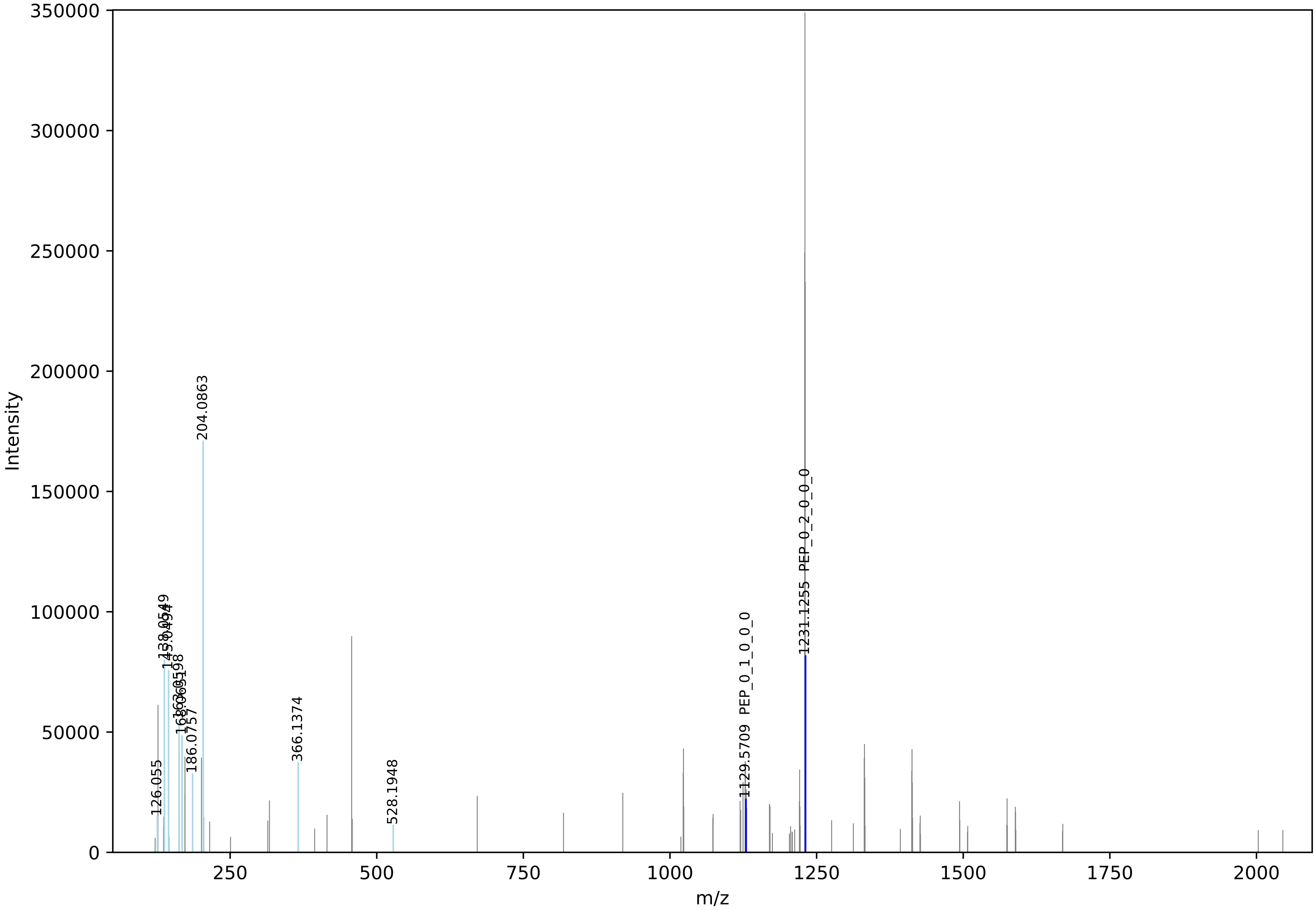

File: 231129_OE_RK_36, RT: 47.958, Scan: 059801
Type: HCD, FTMS + c NSI d Full ms2 1252.5538@hcd30.00 [120.0000-3854.9565]
Precursor Scan: 059667, m/z: 1252.5538, charge: 3
KEGVILTNESAASTGQPDNDVTEGQR_3_2_1_0_0

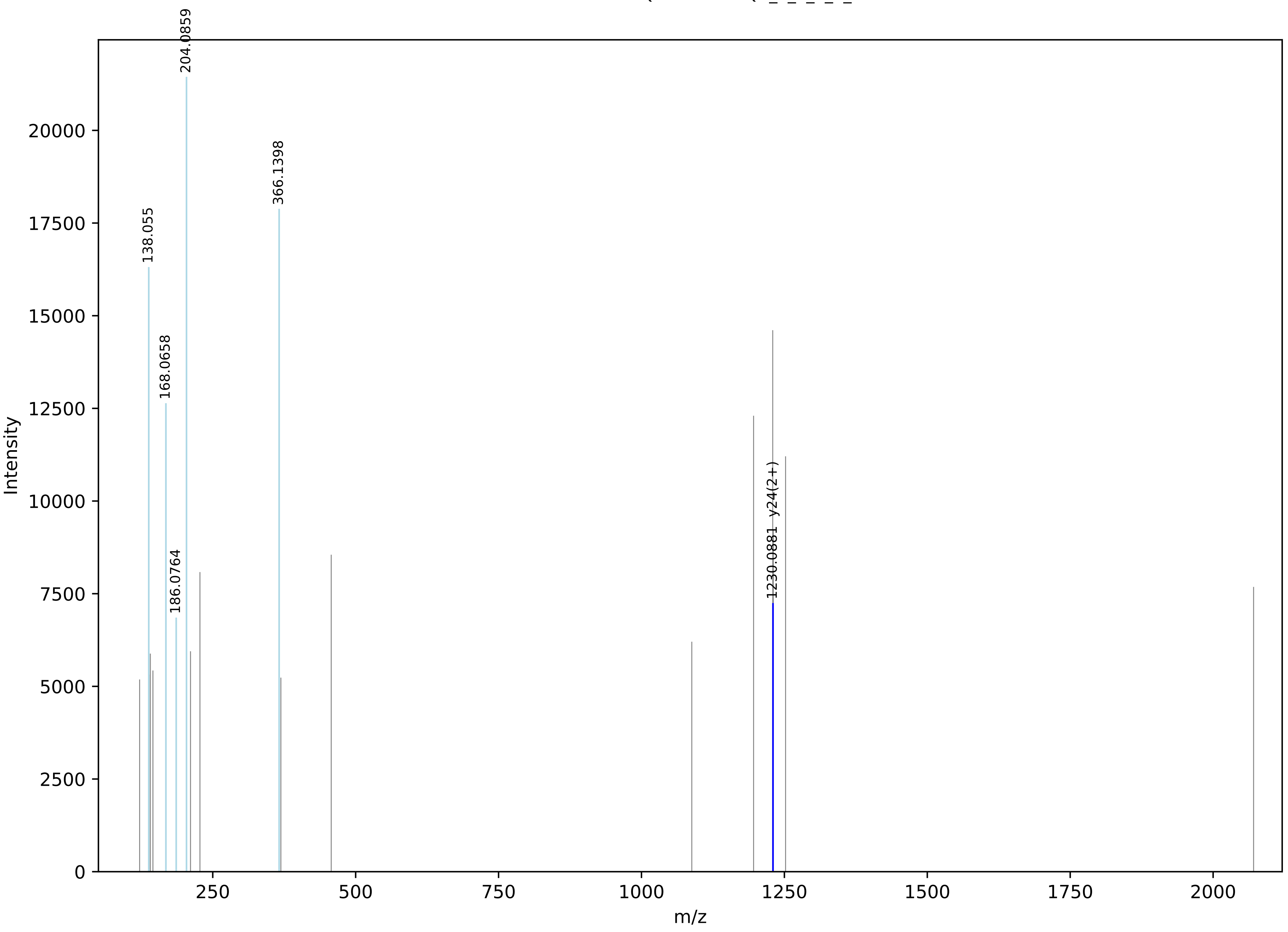

File: 231129_OE_RK_36, RT: 47.972, Scan: 059813
Type: HCD, FTMS + c NSI d Full ms2 1266.5675@hcd30.00 [120.0000-3897.8386]
Precursor Scan: 059738, m/z: 1265.8994, charge: 3
TVLTPATNHMGNVTFTIPANR_7_2_0_0_0

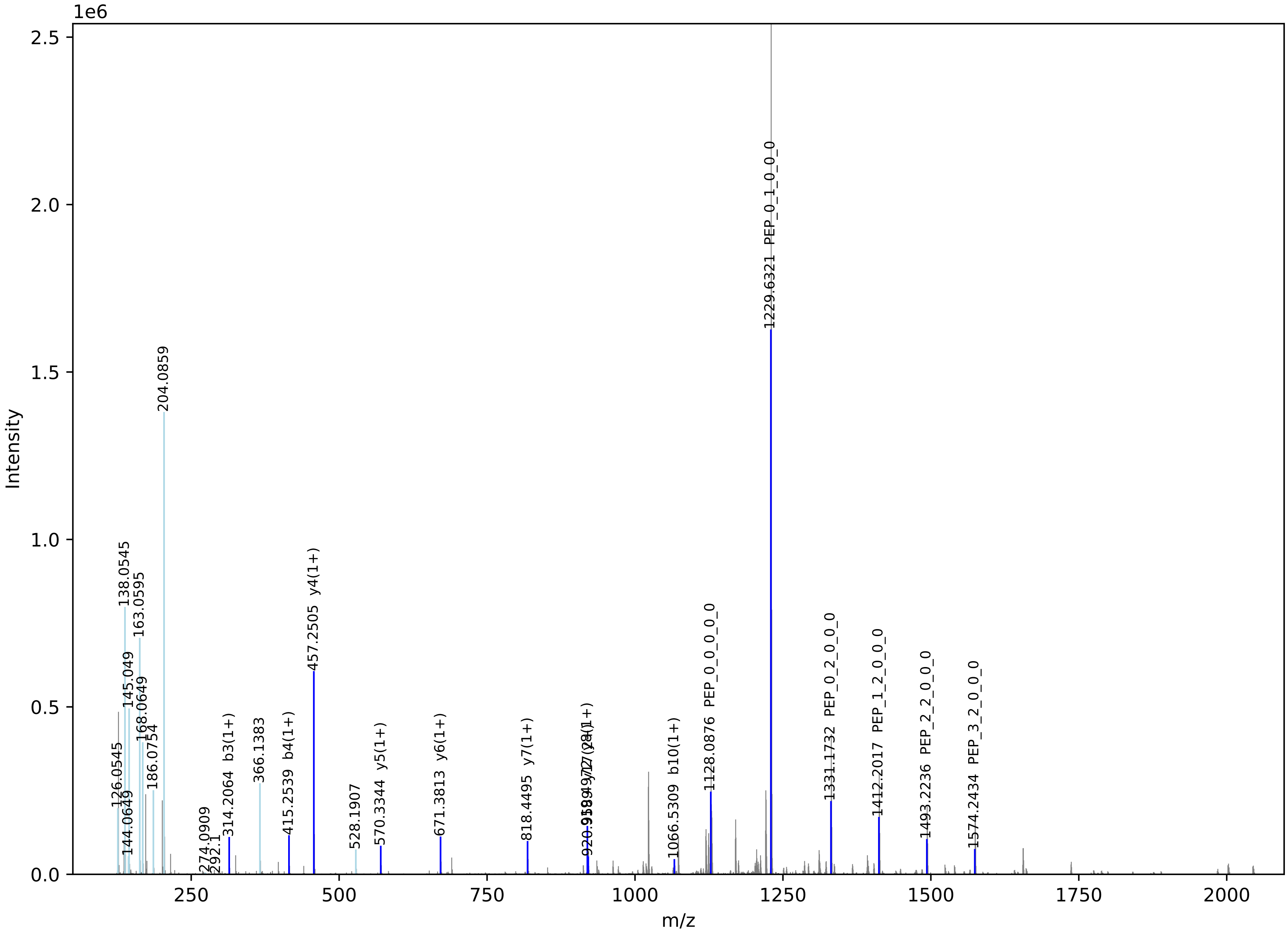

File: 231129_OE_RK_36, RT: 47.983, Scan: 059831
Type: HCD, FTMS + c NSI d Full ms2 1013.4739@hcd30.00 [120.0000-3123.3721]
Precursor Scan: 059738, m/z: 1012.8067, charge: 3
VFHIHNESWVLLTPK_5_2_0_0_0

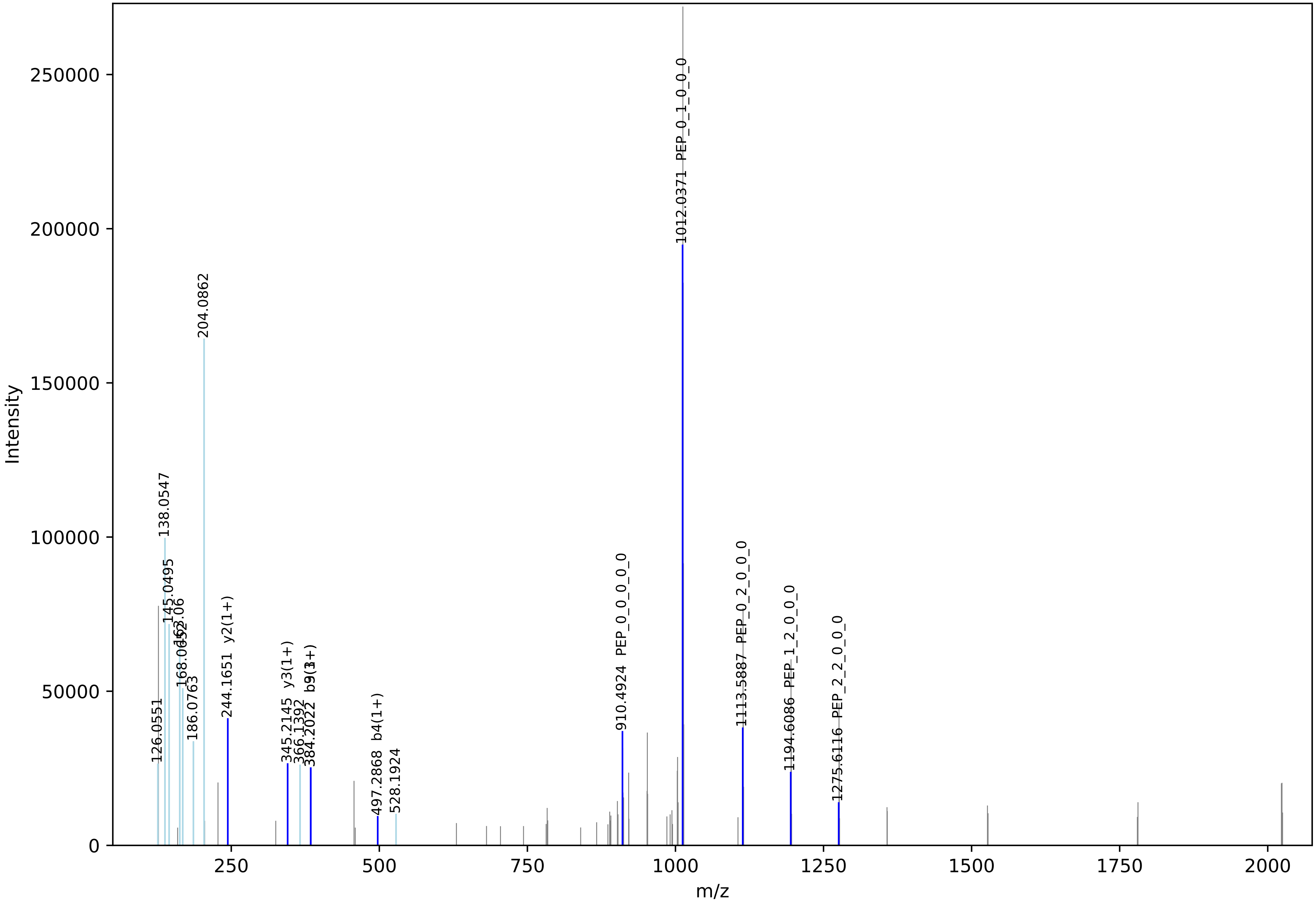

File: 231129_OE_RK_36, RT: 47.993, Scan: 059848
Type: HCD, FTMS + c NSI d Full ms2 1265.5626@hcd30.00 [120.0000-3894.7637]
Precursor Scan: 059738, m/z: 1265.5626, charge: 3
TVLTPATNHMGNVTFTIPANR_7_2_0_0_0

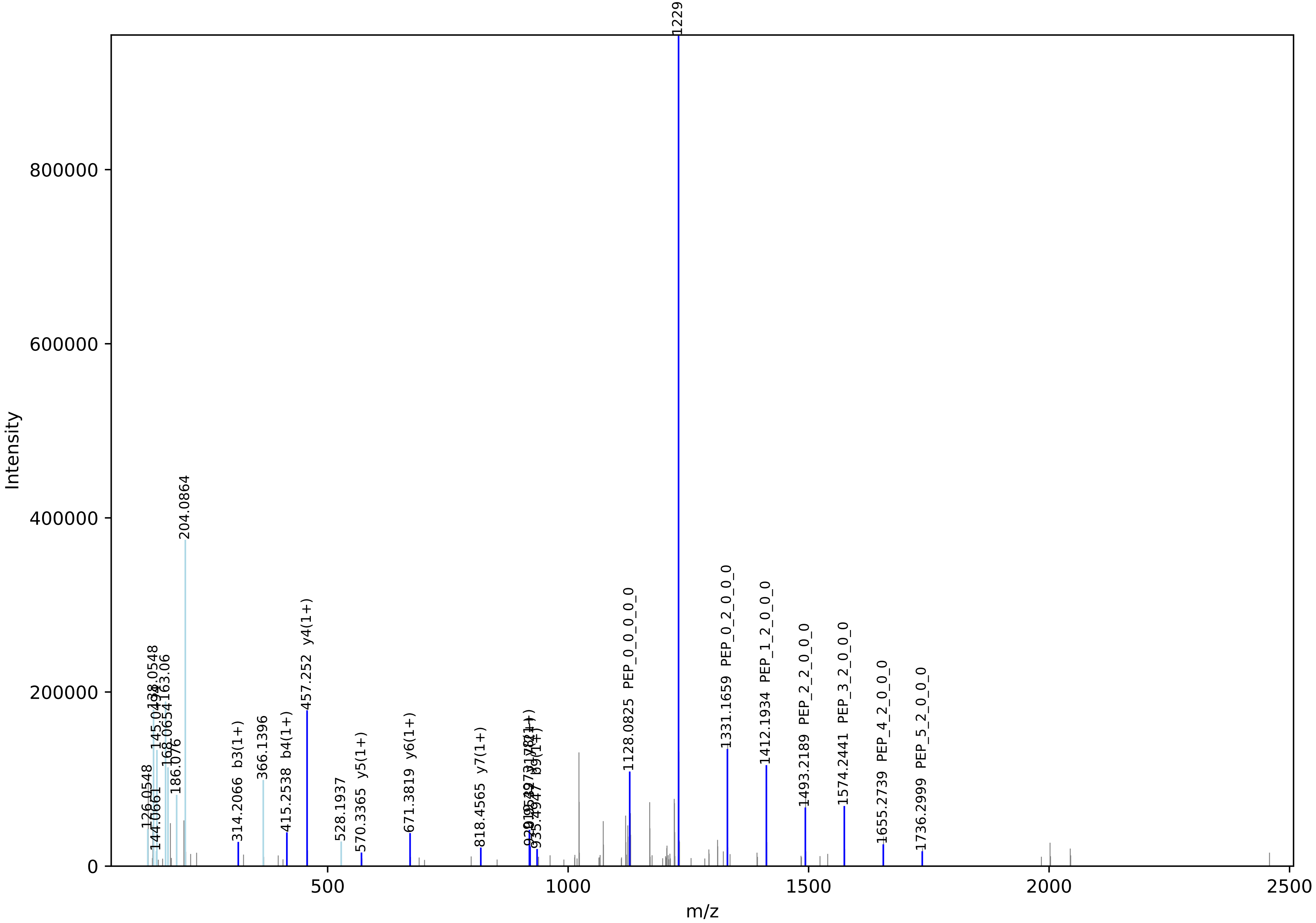

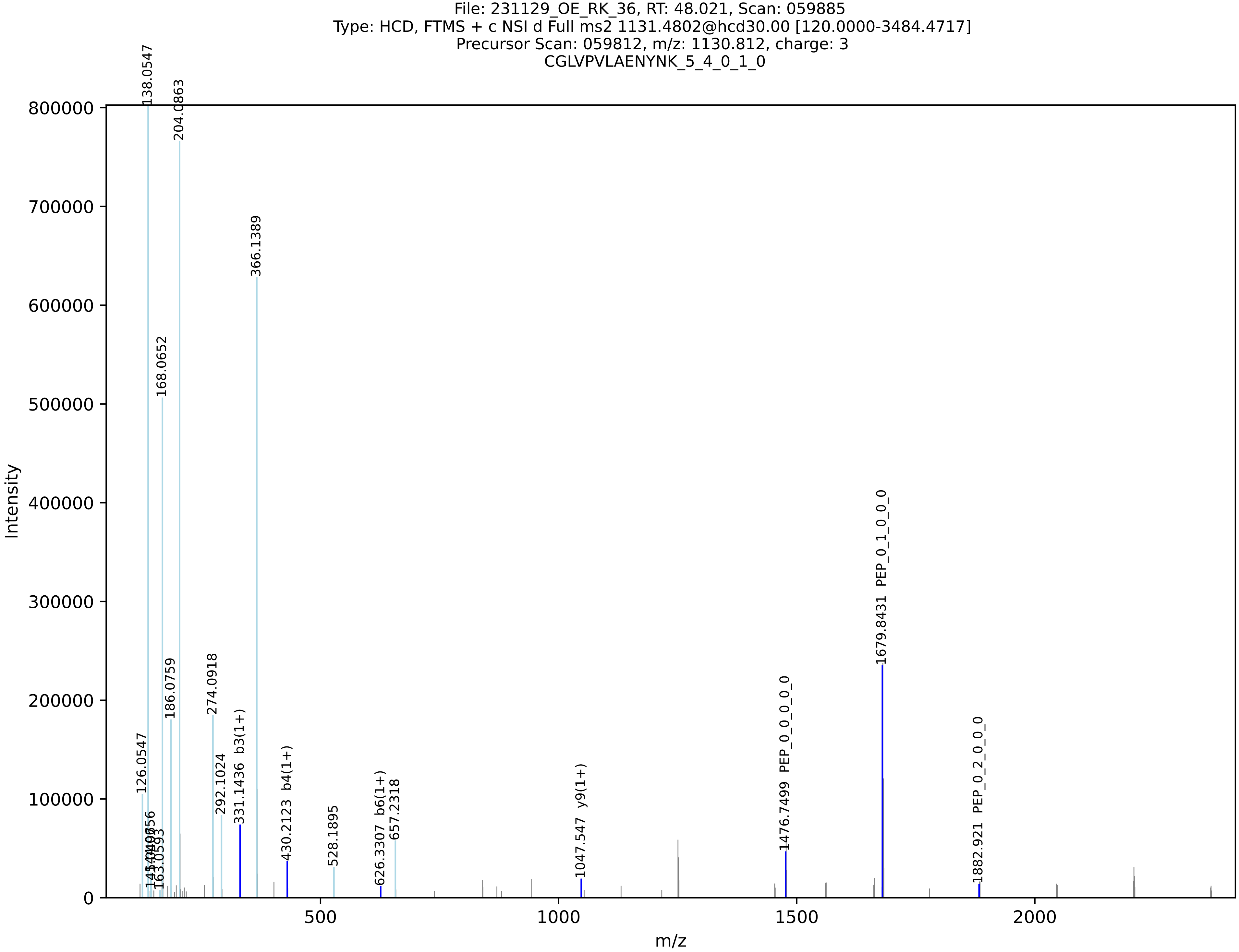

File: 231129_OE_RK_36, RT: 48.021, Scan: 059885
Type: HCD, FTMS + c NSI d Full ms2 1131.4802@hcd30.00 [120.0000-3484.4717]
Precursor Scan: 059812, m/z: 1130.812, charge: 3
CGLVPVLAENYNK_5_4_0_1_0
Intensity
m/z
138.0547
204.0863
366.1389
168.0652
186.0759
274.0918
126.0547
292.1024
331.1436 b3(1+)
430.2123 b4(1+)
528.1895
626.3307 b6(1+)
657.2318
163.0593
1047.547 y9(1+)
1476.7499 PEP_0_0_0_0_0
1679.8431 PEP_0_1_0_0_0
1882.921 PEP_0_2_0_0_0

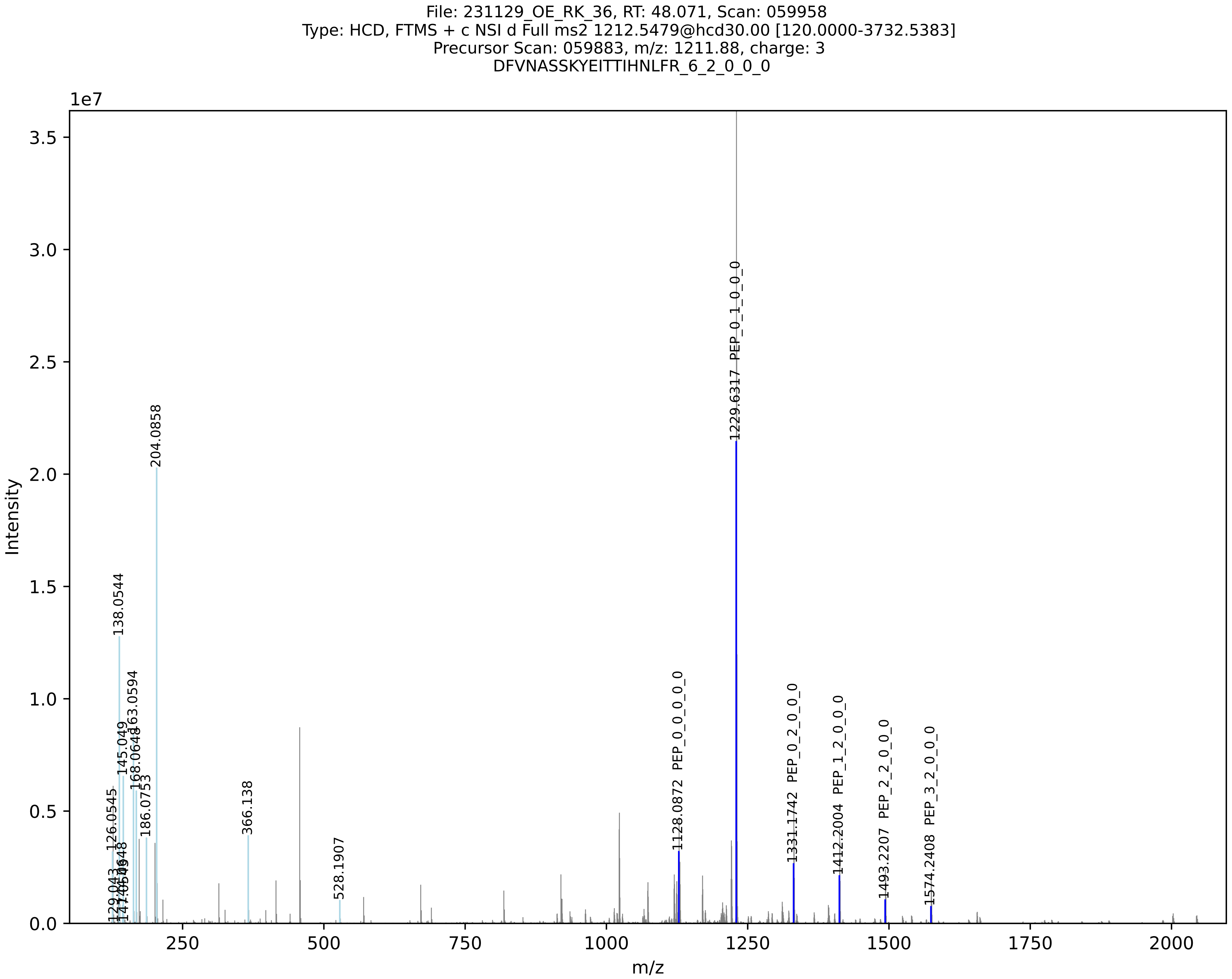

File: 231129_OE_RK_36, RT: 48.071, Scan: 059958
Type: HCD, FTMS + c NSI d Full ms2 1212.5479@hcd30.00 [120.0000-3732.5383]
Precursor Scan: 059883, m/z: 1211.88, charge: 3
DFVNASSKYEITTIHNLFR_6_2_0_0_0
1e7
Intensity
m/z
1229.6317 PEP_0_1_0_0_0
204.0858
138.0544
163.0594
145.049
168.0648
186.0753
126.0545
366.138
528.1907
1128.0872 PEP_0_0_0_0_0
1331.1742 PEP_0_2_0_0_0
1412.2004 PEP_1_2_0_0_0
1493.2207 PEP_2_2_0_0_0
1574.2408 PEP_3_2_0_0_0

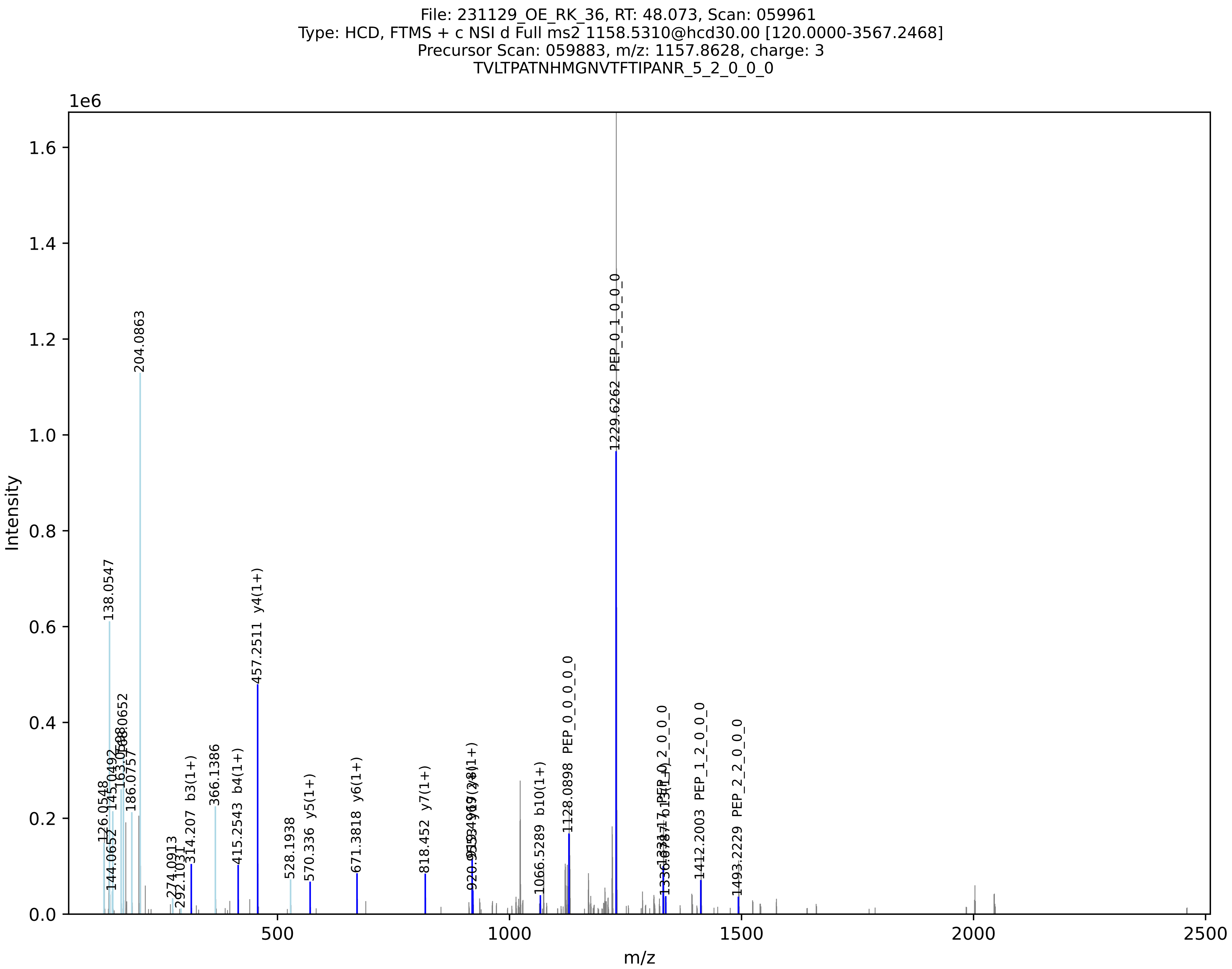
File: 231129_OE_RK_36, RT: 48.073, Scan: 059961
Type: HCD, FTMS + c NSI d Full ms2 1158.5310@hcd30.00 [120.0000-3567.2468]
Precursor Scan: 059883, m/z: 1157.8628, charge: 3
TVLTPATNHMGNVTFTIPANR_5_2_0_0_0
1e6
Intensity
m/z
204.0863
138.0547
457.2511 y4(1+)
1229.6262 PEP_0_1_0_0_0
1128.0898 PEP_0_0_0_0_0
366.1386
415.2543 b4(1+)
314.207 b3(1+)
186.0757
528.1938
570.336 y5(1+)
671.3818 y6(1+)
818.452 y7(1+)
1066.5289 b10(1+)
1412.2003 PEP_1_2_0_0_0
1493.2229 PEP_2_2_0_0_0
274.0913
292.1031
126.0548
144.0652

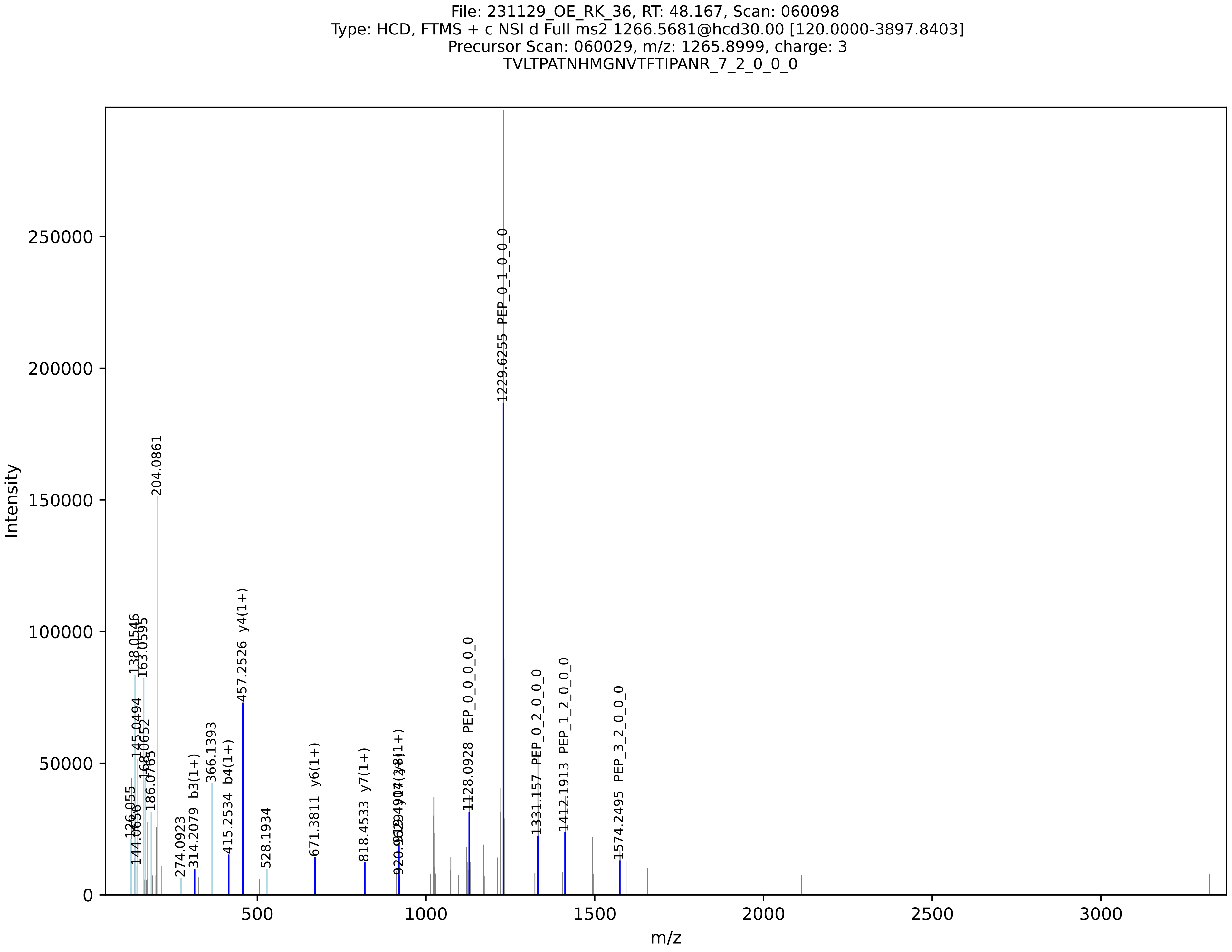
File: 231129_OE_RK_36, RT: 48.167, Scan: 060098
Type: HCD, FTMS + c NSI d Full ms2 1266.5681@hcd30.00 [120.0000-3897.8403]
Precursor Scan: 060029, m/z: 1265.8999, charge: 3
TVLTPATNHMGNVTFTIPANR_7_2_0_0_0
Intensity
m/z
1229.6255 PEP_0_1_0_0_0
204.0861
138.0546
163.0595
145.0494
168.0652
186.0765
126.055
144.0656
274.0923
314.2079 b3(1+)
366.1393
415.2534 b4(1+)
457.2526 y4(1+)
528.1934
671.3811 y6(1+)
818.4533 y7(1+)
1128.0928 PEP_0_0_0_0_0
1331.157 PEP_0_2_0_0_0
1412.1913 PEP_1_2_0_0_0
1574.2495 PEP_3_2_0_0_0
0
50000
100000
150000
200000
250000
500
1000
1500
2000
2500
3000

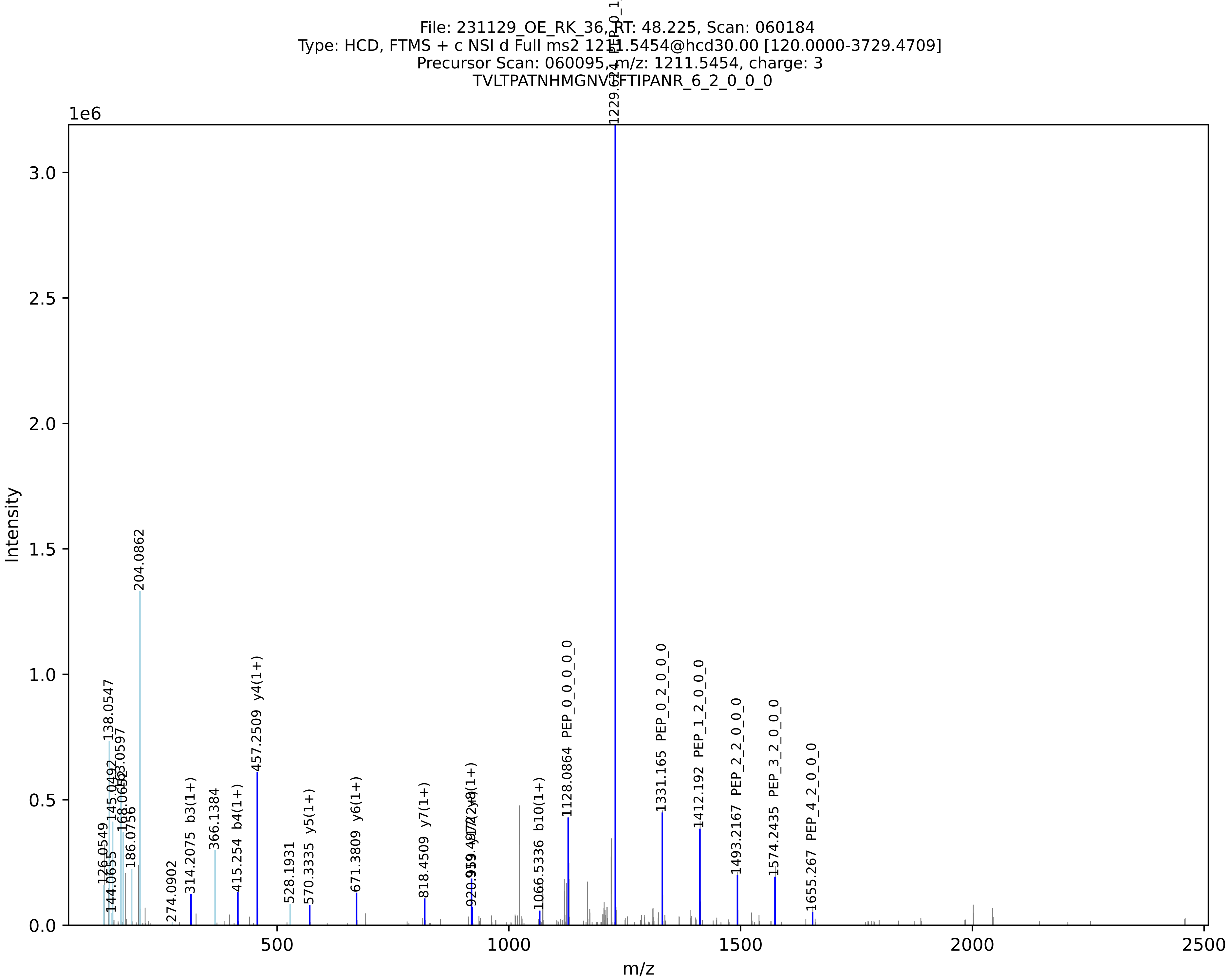
File: 231129_OE_RK_36, RT: 48.225, Scan: 060184
Type: HCD, FTMS + c NSI d Full ms2 1211.5454@hcd30.00 [120.0000-3729.4709]
Precursor Scan: 060095, m/z: 1211.5454, charge: 3
TVLTPATNHMGNVTFTIPANR_6_2_0_0_0
1e6
Intensity
m/z
204.0862
138.0547
145.0492
126.0549
144.0655
186.0756
274.0902
314.2075 b3(1+)
366.1384
415.254 b4(1+)
457.2509 y4(1+)
528.1931
570.3335 y5(1+)
671.3809 y6(1+)
818.4509 y7(1+)
1066.5336 b10(1+)
1128.0864 PEP_0_0_0_0_0
1331.165 PEP_0_2_0_0_0
1412.192 PEP_1_2_0_0_0
1493.2167 PEP_2_2_0_0_0
1574.2435 PEP_3_2_0_0_0
1655.267 PEP_4_2_0_0_0

File: 231129_OE_RK_36, RT: 48.267, Scan: 060245
Type: HCD, FTMS + c NSI d Full ms2 1212.2164@hcd30.00 [120.0000-3731.5242]
Precursor Scan: 060169, m/z: 1211.8823, charge: 3
EIRPGGNYTITCVGNTPHLR_6_2_0_0_0

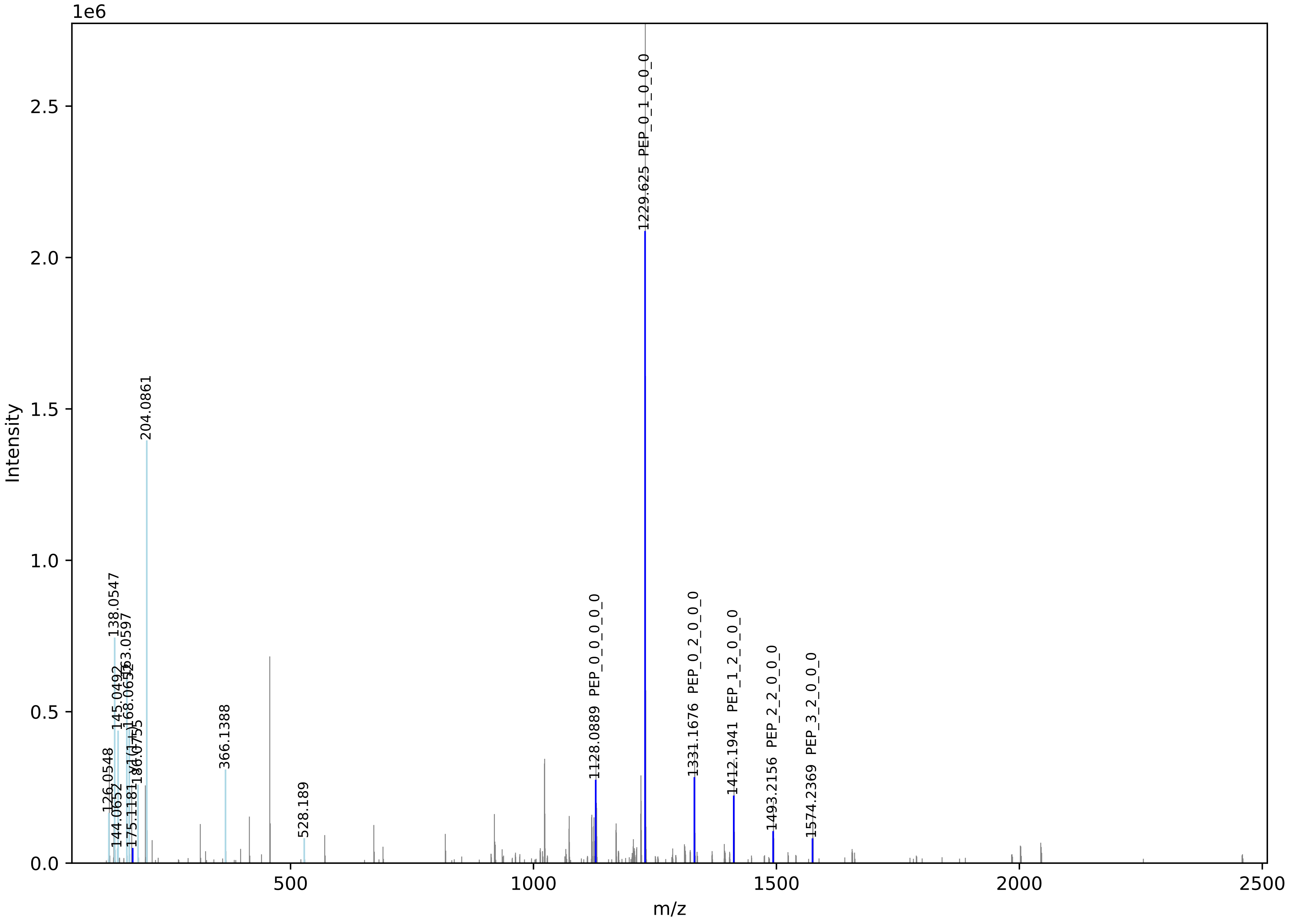

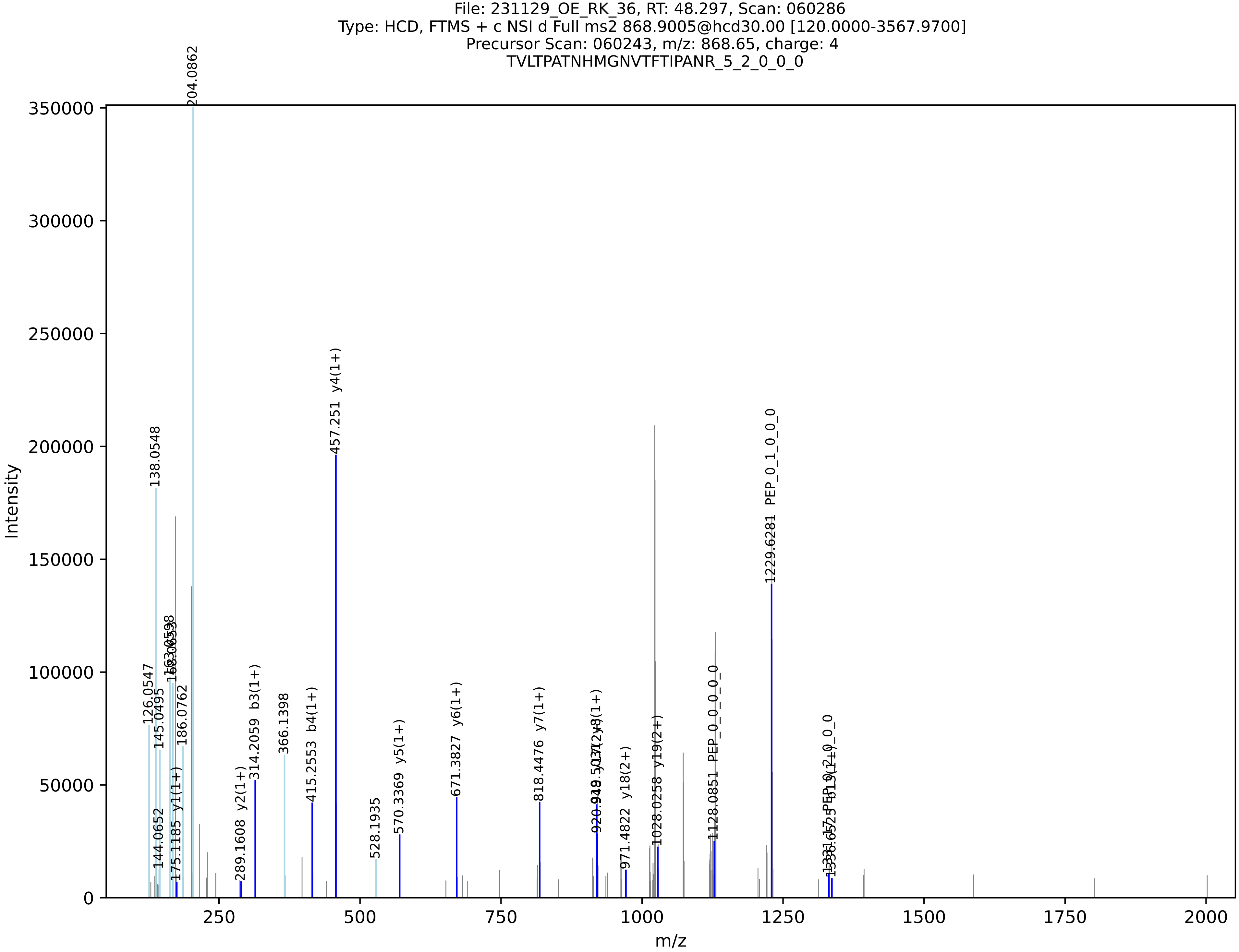

File: 231129_OE_RK_36, RT: 48.297, Scan: 060286
Type: HCD, FTMS + c NSI d Full ms2 868.9005@hcd30.00 [120.0000-3567.9700]
Precursor Scan: 060243, m/z: 868.65, charge: 4
TVLTPATNHMGNVTFTIPANR_5_2_0_0_0
Intensity
m/z
204.0862
138.0548
126.0547
145.0495
144.0652
186.0762
175.1185 y1(1+)
289.1608 y2(1+)
314.2059 b3(1+)
366.1398
415.2553 b4(1+)
457.251 y4(1+)
528.1935
570.3369 y5(1+)
671.3827 y6(1+)
818.4476 y7(1+)
971.4822 y18(2+)
1028.0258 y19(2+)
1128.0851 PEP_0_0_0_0_0
1229.6281 PEP_0_1_0_0_0

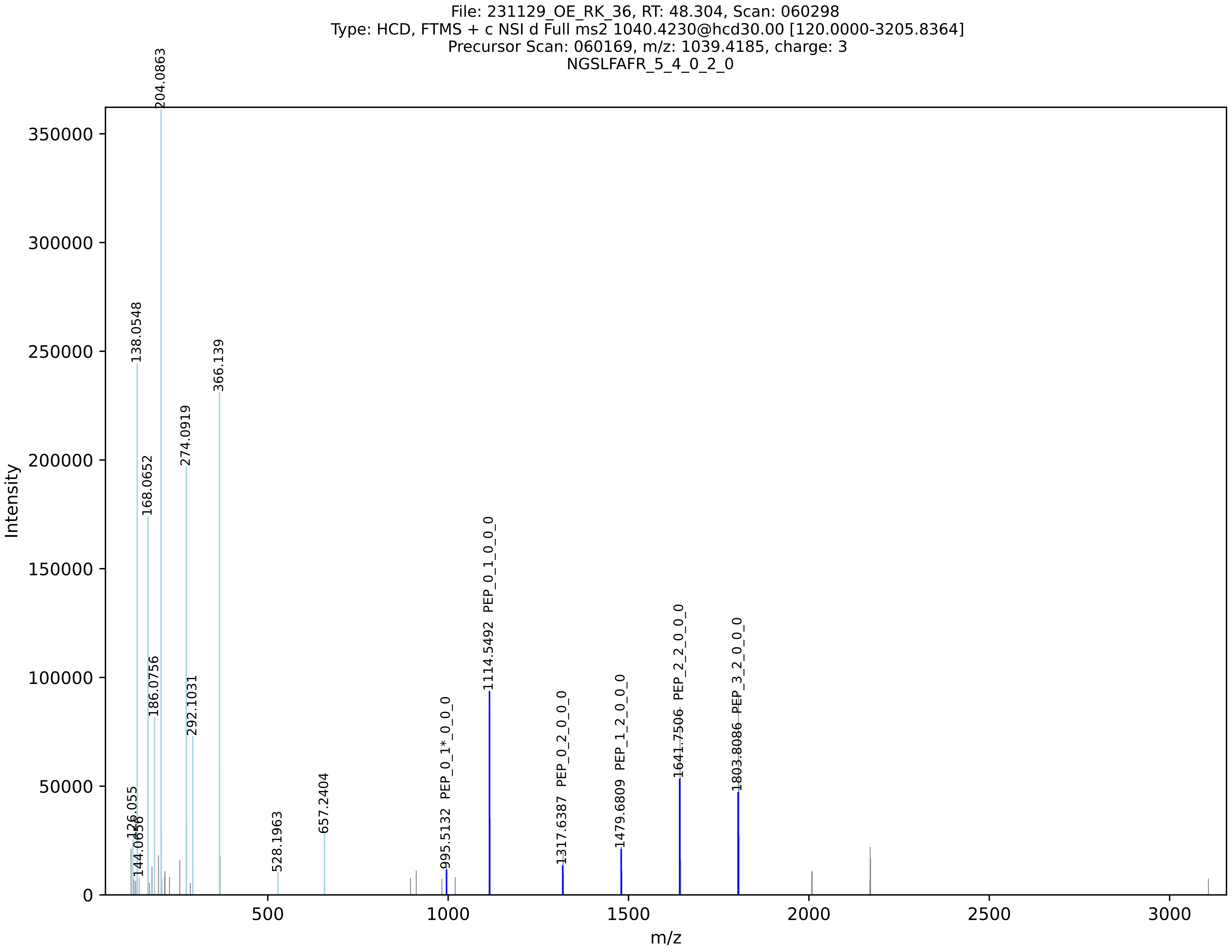

File: 231129_OE_RK_36, RT: 48.304, Scan: 060298
Type: HCD, FTMS + c NSI d Full ms2 1040.4230@hcd30.00 [120.0000-3205.8364]
Precursor Scan: 060169, m/z: 1039.4185, charge: 3
NGSLFAFR_5_4_0_2_0
Intensity
m/z
204.0863
138.0548
366.139
274.0919
168.0652
186.0756
292.1031
126.055
144.0656
528.1963
657.2404
995.5132 PEP_0_1*_0_0_0
1114.5492 PEP_0_1_0_0_0
1317.6387 PEP_0_2_0_0_0
1479.6809 PEP_1_2_0_0_0
1641.7506 PEP_2_2_0_0_0
1803.8086 PEP_3_2_0_0_0

File: 231129_OE_RK_36, RT: 48.398, Scan: 060403
Type: HCD, FTMS + c NSI d Full ms2 1131.1466@hcd30.00 [120.0000-3483.4507]
Precursor Scan: 060370, m/z: 1130.8125, charge: 3
CGLVPVLAENYNK_5_4_0_1_0

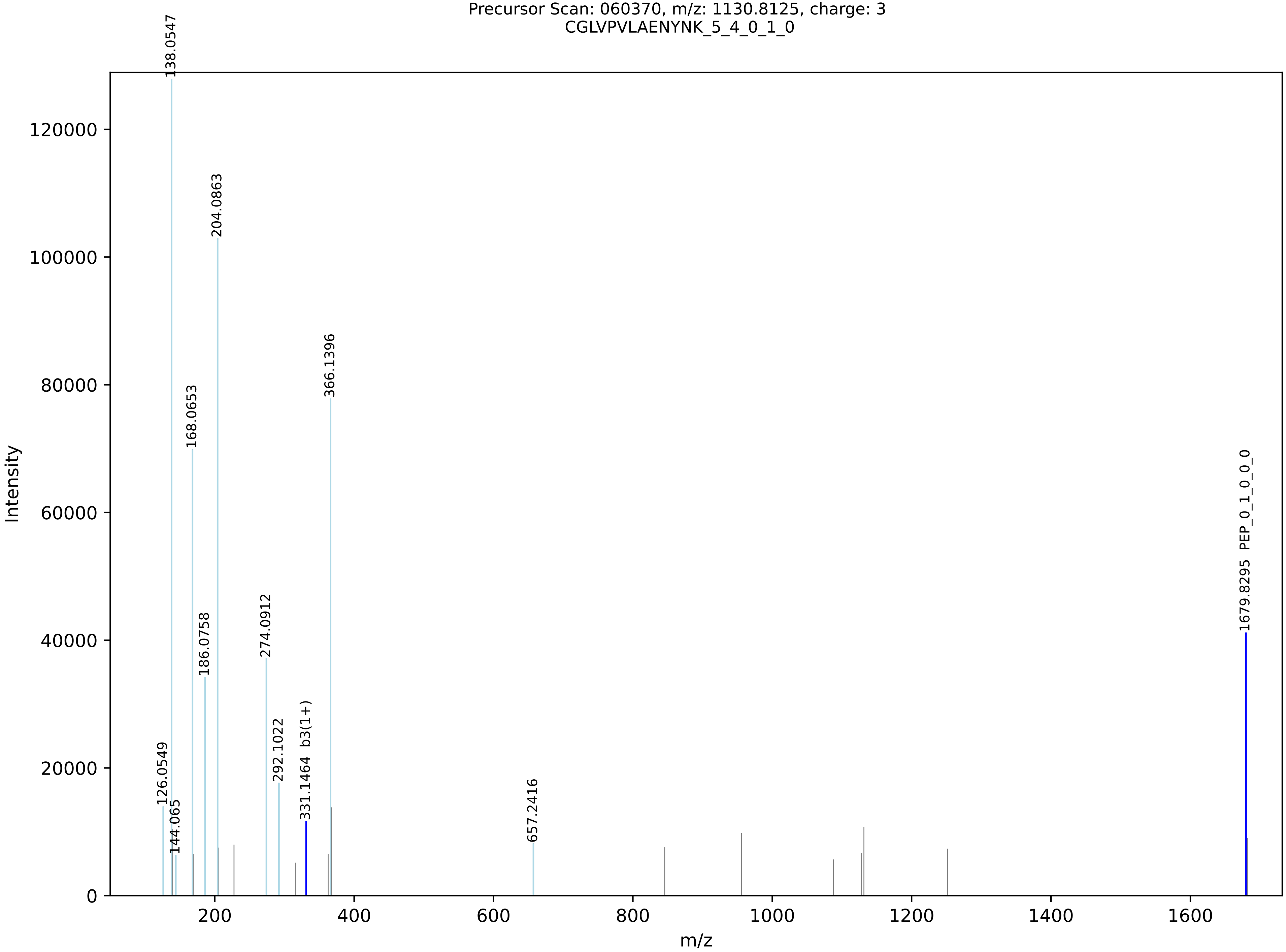

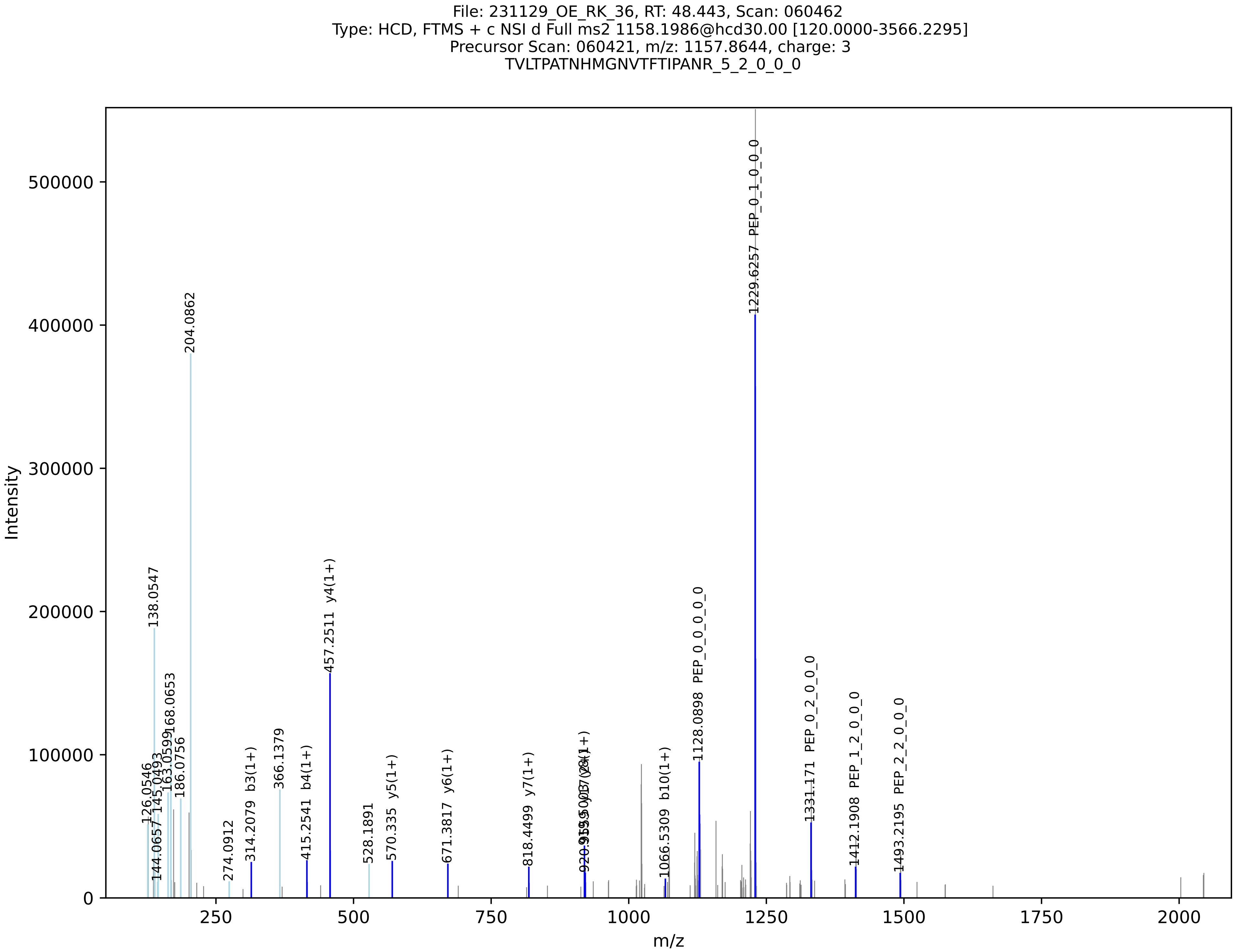
File: 231129_OE_RK_36, RT: 48.443, Scan: 060462
Type: HCD, FTMS + c NSI d Full ms2 1158.1986@hcd30.00 [120.0000-3566.2295]
Precursor Scan: 060421, m/z: 1157.8644, charge: 3
TVLTPATNHMGNVTFTIPANR_5_2_0_0_0
Intensity
m/z
0
100000
200000
300000
400000
500000
250
500
750
1000
1250
1500
1750
2000
126.0546
144.0657
145.0493
138.0547
163.0599
168.0653
186.0756
204.0862
274.0912
314.2079 b3(1+)
366.1379
415.2541 b4(1+)
457.2511 y4(1+)
528.1891
570.335 y5(1+)
671.3817 y6(1+)
818.4499 y7(1+)
1066.5309 b10(1+)
1128.0898 PEP_0_0_0_0_0
1229.6257 PEP_0_1_0_0_0
1331.171 PEP_0_2_0_0_0
1412.1908 PEP_1_2_0_0_0
1493.2195 PEP_2_2_0_0_0

File: 231129_OE_RK_36, RT: 48.444, Scan: 060463
Type: HCD, FTMS + c NSI d Full ms2 1212.5500@hcd30.00 [120.0000-3732.5449]
Precursor Scan: 060421, m/z: 1211.8818, charge: 3
EIRPGGNYTITCVGNTPHLR_6_2_0_0_0

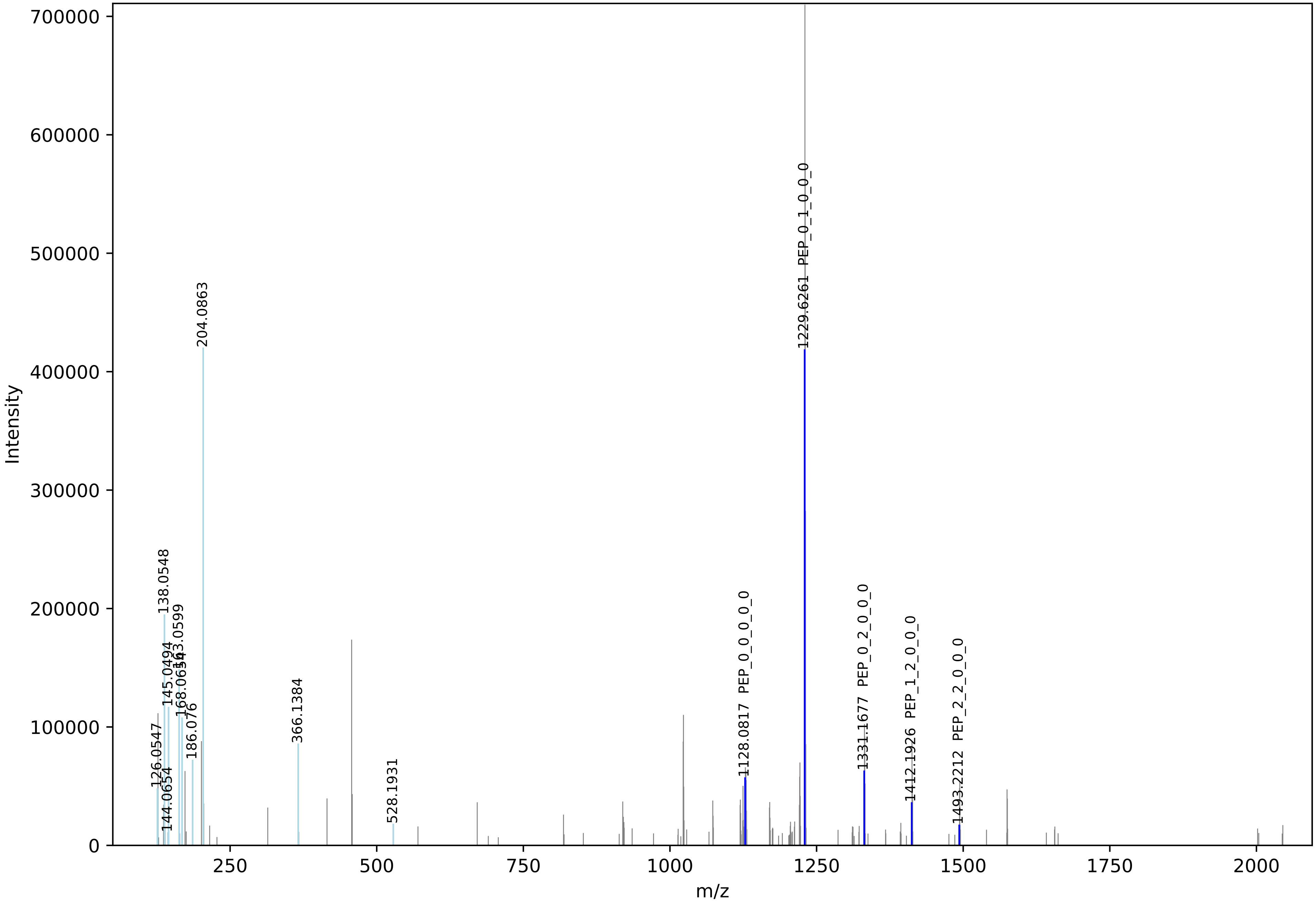

File: 231129_OE_RK_36, RT: 48.472, Scan: 060502
Type: HCD, FTMS + c NSI d Full ms2 1359.5367@hcd30.00 [120.0000-5569.7656]
Precursor Scan: 060460, m/z: 1358.7849, charge: 4
QDQCIYNTTYLNVQR_7_6_2_3_0

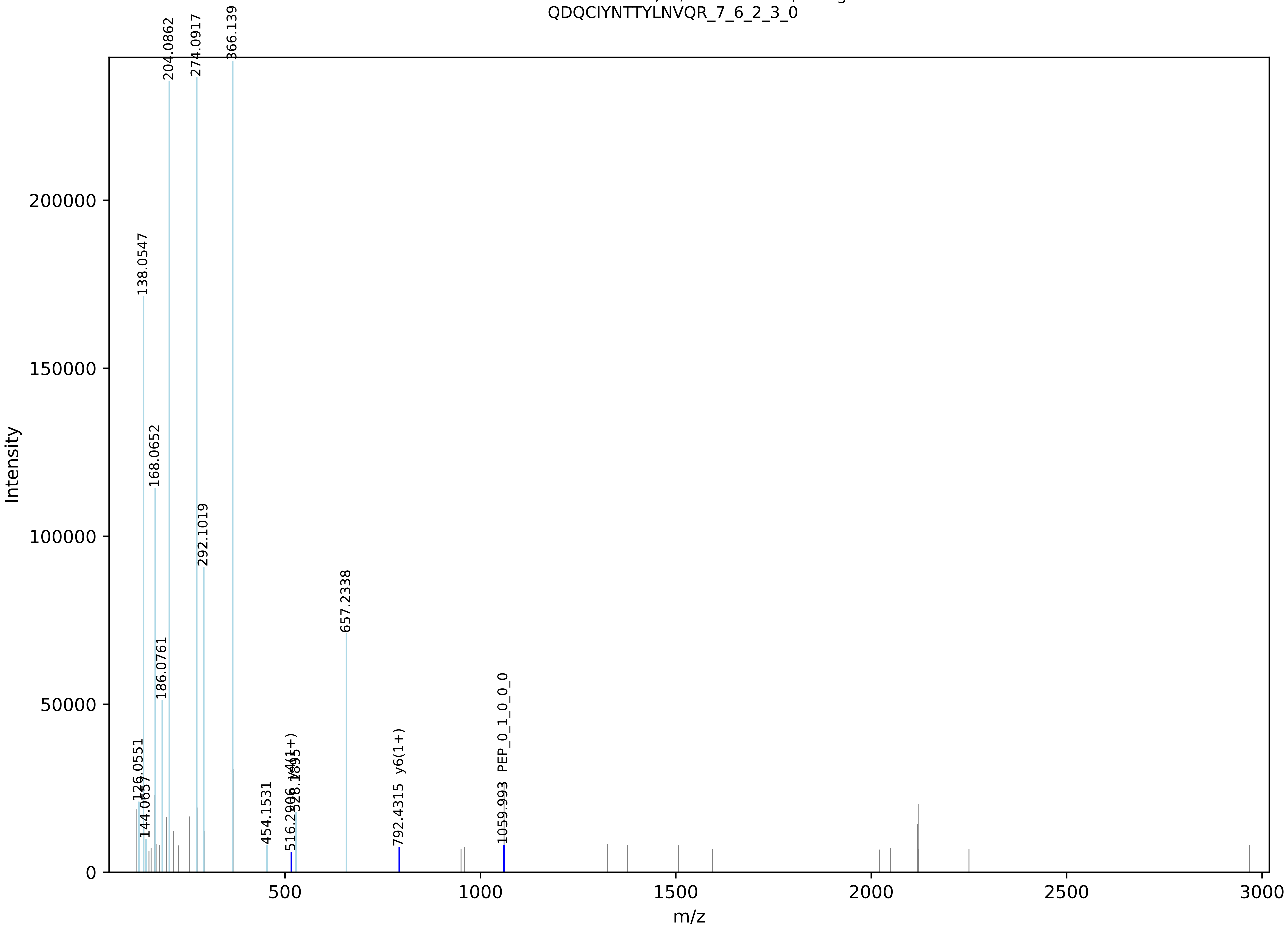

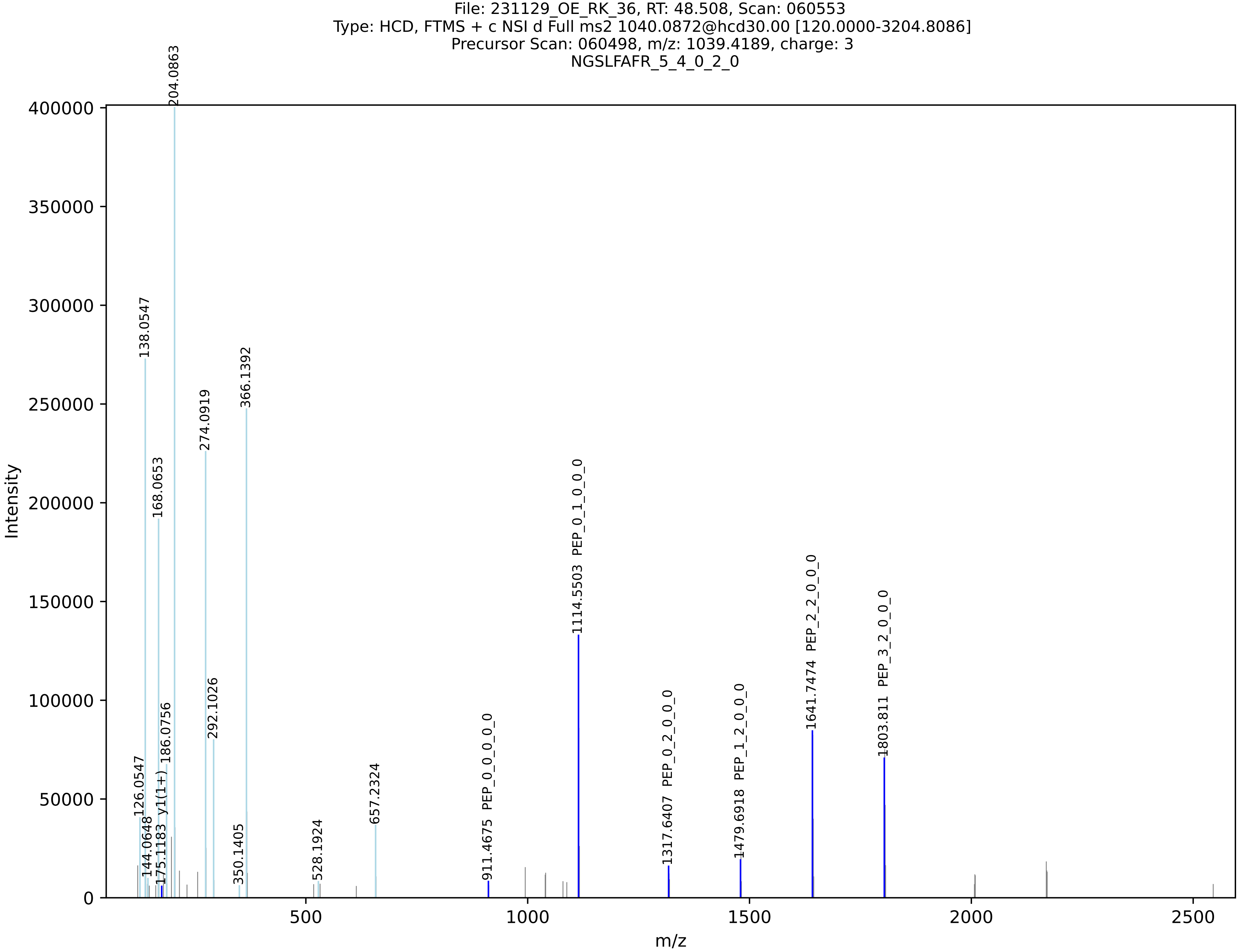
File: 231129_OE_RK_36, RT: 48.508, Scan: 060553
Type: HCD, FTMS + c NSI d Full ms2 1040.0872@hcd30.00 [120.0000-3204.8086]
Precursor Scan: 060498, m/z: 1039.4189, charge: 3
NGSLFAFR_5_4_0_2_0
Intensity
m/z
204.0863
138.0547
168.0653
274.0919
366.1392
292.1026
186.0756
126.0547
y1(1+)
144.0648
175.1183
350.1405
528.1924
657.2324
911.4675 PEP_0_0_0_0_0
1114.5503 PEP_0_1_0_0_0
1317.6407 PEP_0_2_0_0_0
1479.6918 PEP_1_2_0_0_0
1641.7474 PEP_2_2_0_0_0
1803.811 PEP_3_2_0_0_0

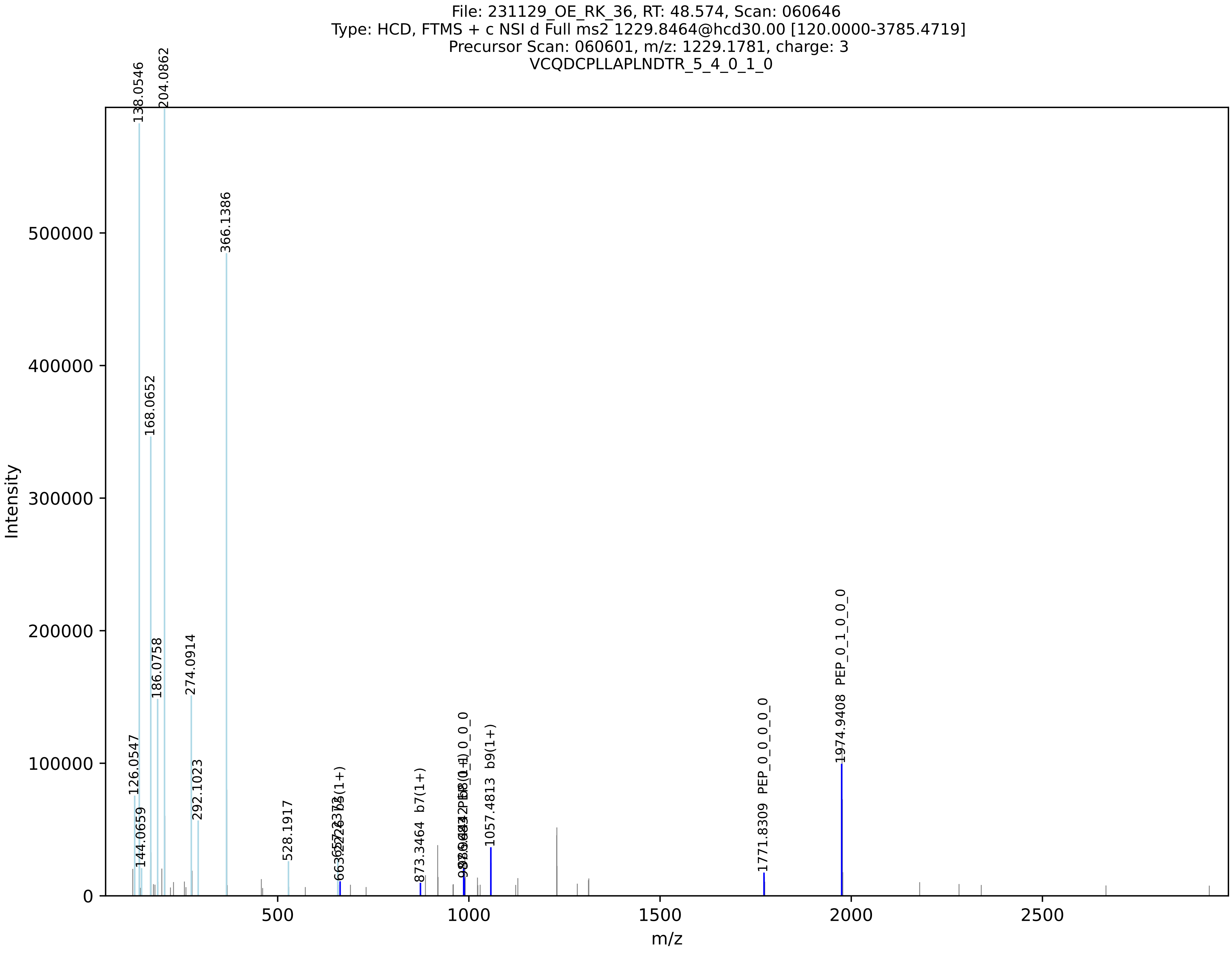

File: 231129_OE_RK_36, RT: 48.574, Scan: 060646
Type: HCD, FTMS + c NSI d Full ms2 1229.8464@hcd30.00 [120.0000-3785.4719]
Precursor Scan: 060601, m/z: 1229.1781, charge: 3
VCQDCPLLAPLNDTR_5_4_0_1_0
138.0546
204.0862
366.1386
168.0652
186.0758
274.0914
126.0547
292.1023
144.0659
528.1917
873.3464 b7(1+)
1057.4813 b9(1+)
1771.8309 PEP_0_0_0_0_0
1974.9408 PEP_0_1_0_0_0
Intensity
m/z
0
100000
200000
300000
400000
500000
500
1000
1500
2000
2500

File: 231129_OE_RK_36, RT: 48.719, Scan: 060858
Type: HCD, FTMS + c NSI d Full ms2 1266.2345@hcd30.00 [120.0000-3896.8196]
Precursor Scan: 060767, m/z: 1265.9004, charge: 3
TVLTPATNHMGNVTFTIPANR_7_2_0_0_0

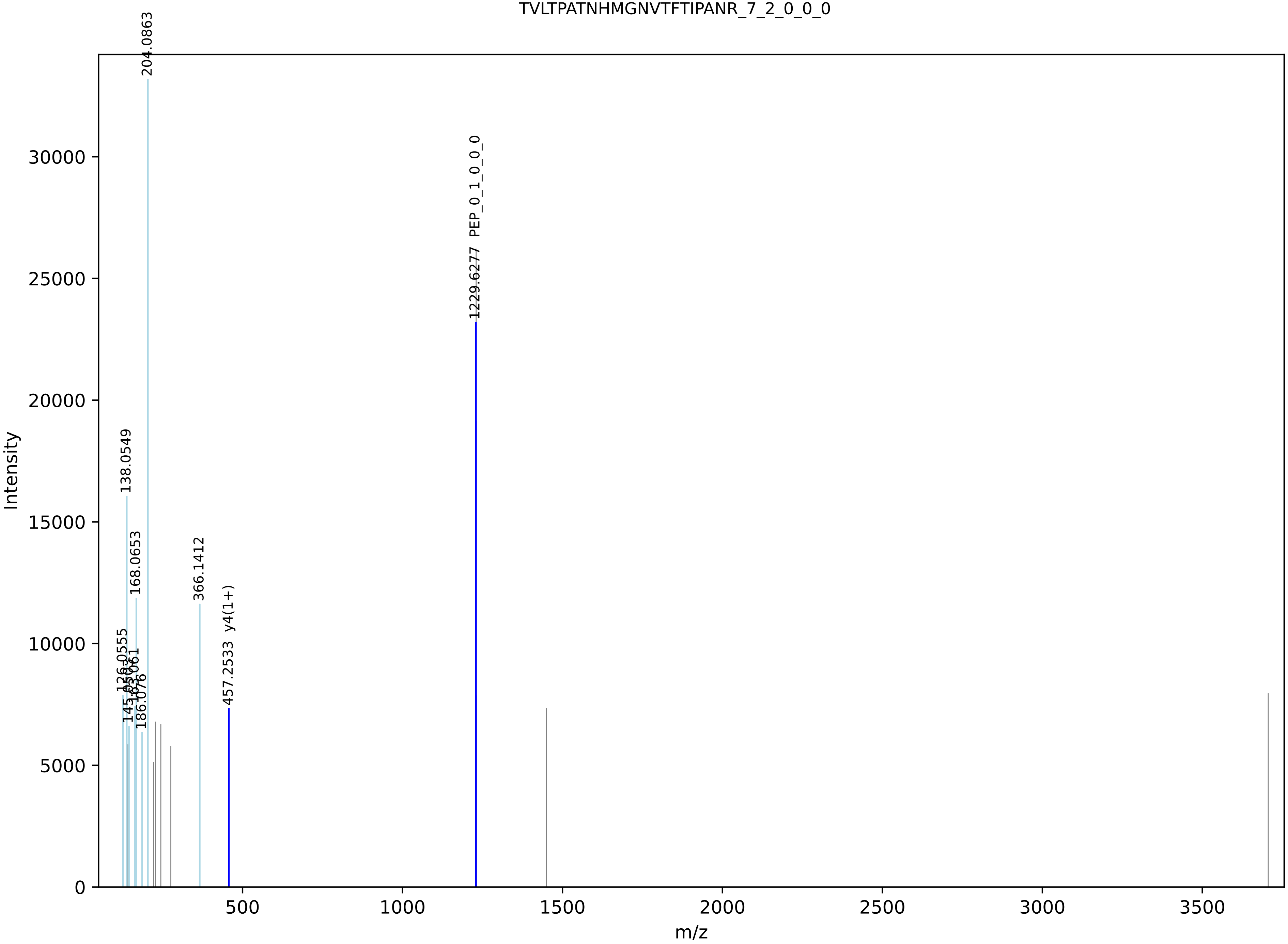

File: 231129_OE_RK_36, RT: 48.76, Scan: 060916
Type: HCD, FTMS + c NSI d Full ms2 1396.0519@hcd30.00 [120.0000-5718.7476]
Precursor Scan: 060835, m/z: 1395.5509, charge: 4
QDQCIYNTTYLNVQR_7_6_1_4_0

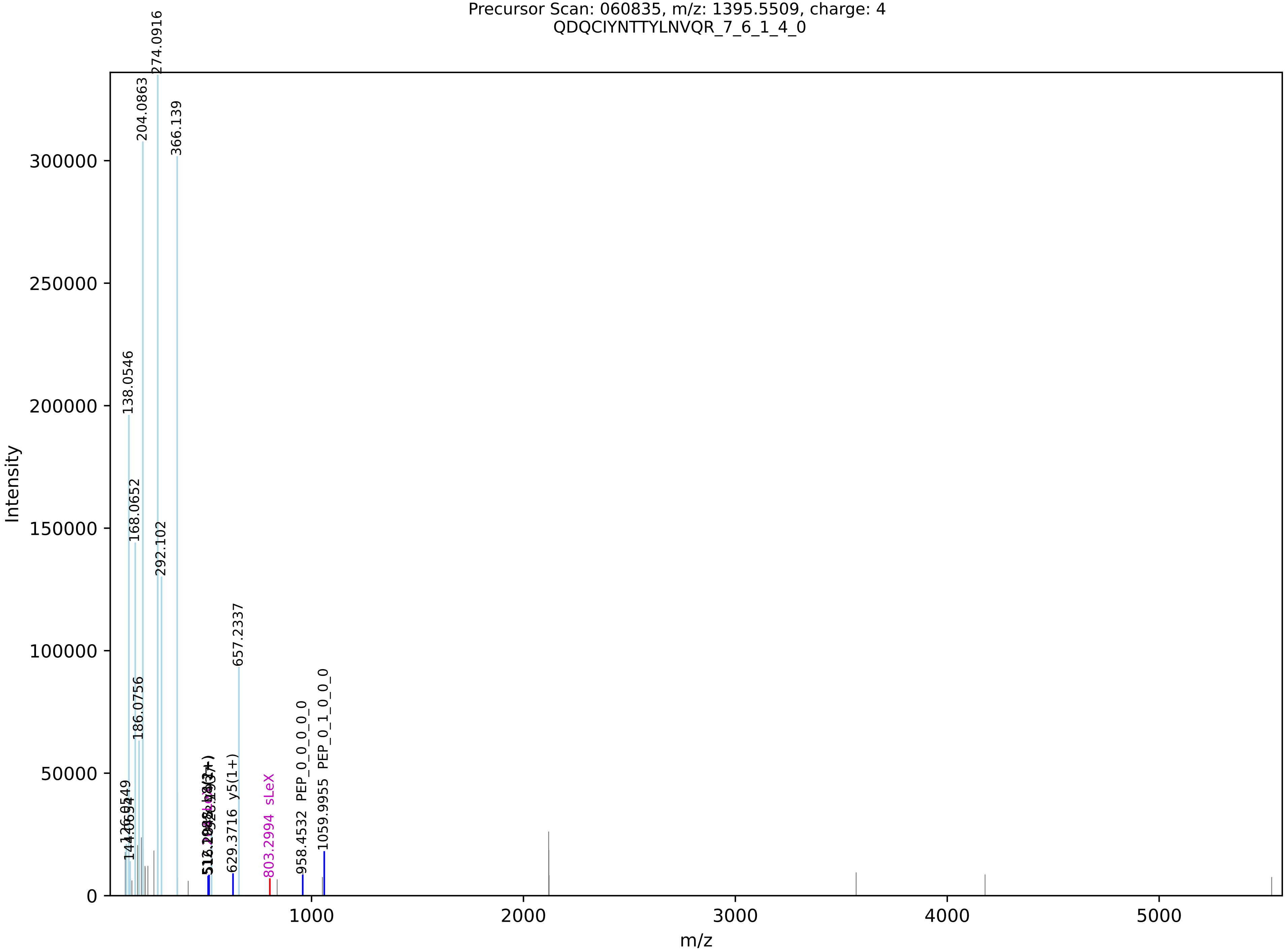

File: 231129_OE_RK_36, RT: 48.858, Scan: 061059
Type: HCD, FTMS + c NSI d Full ms2 1359.7877@hcd30.00 [120.0000-5570.7896]
Precursor Scan: 060982, m/z: 1359.0359, charge: 4
QDQCIYNTTYLNVQR_7_6_0_4_0

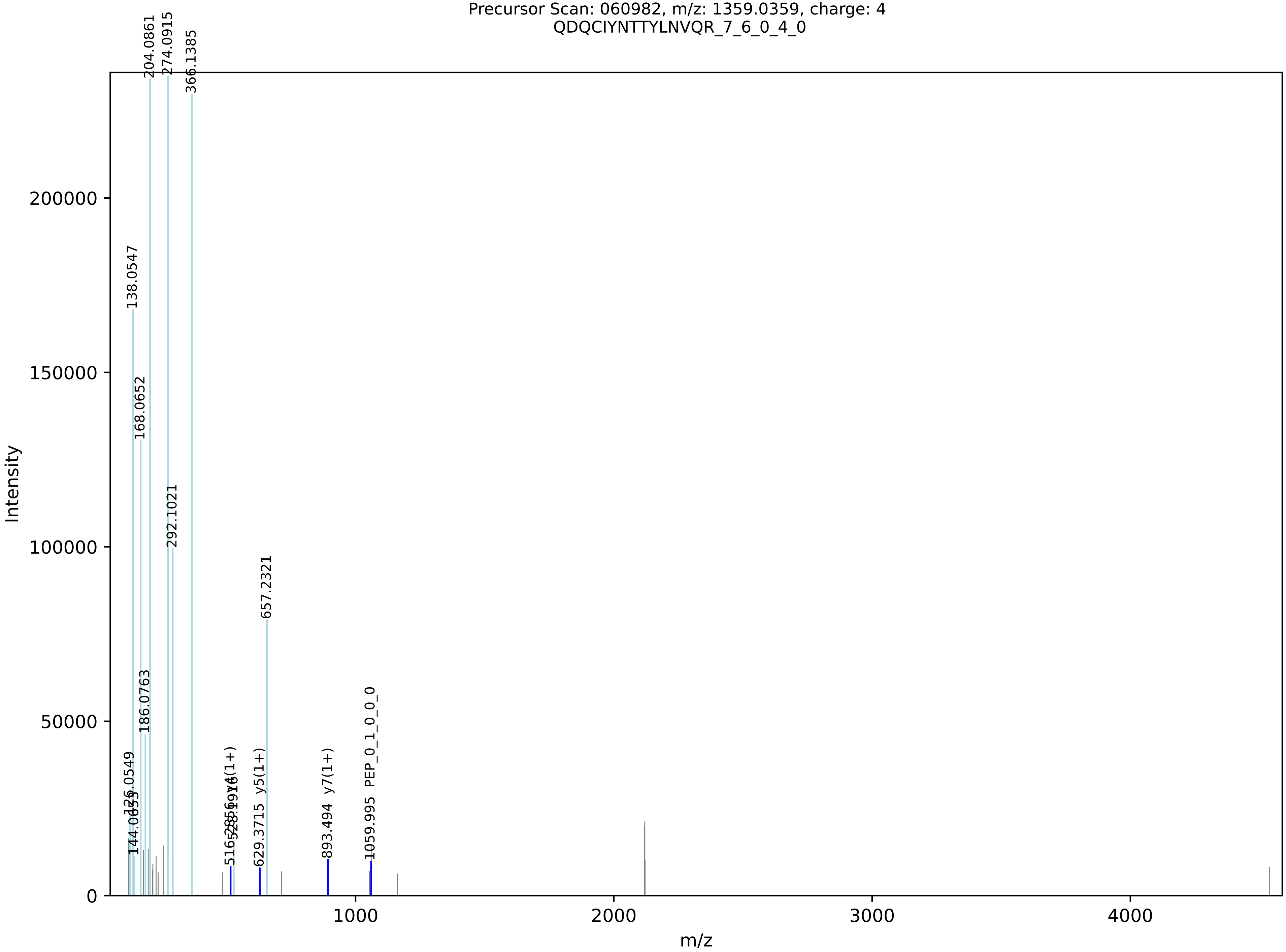

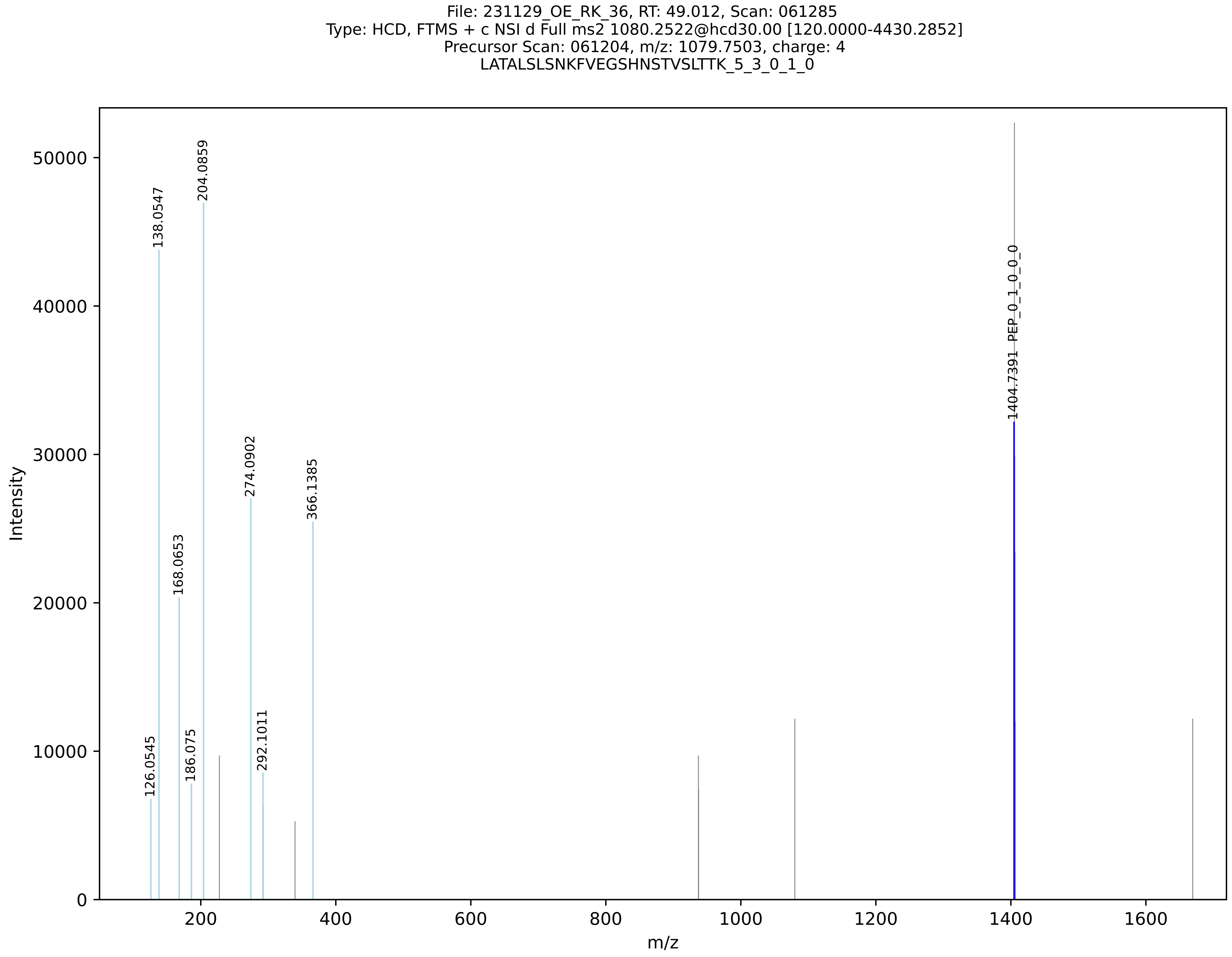
File: 231129_OE_RK_36, RT: 49.012, Scan: 061285
Type: HCD, FTMS + c NSI d Full ms2 1080.2522@hcd30.00 [120.0000-4430.2852]
Precursor Scan: 061204, m/z: 1079.7503, charge: 4
LATALSLSNKFVEGSHNSTVSLTTK_5_3_0_1_0
Intensity
m/z
126.0545
138.0547
168.0653
186.075
204.0859
274.0902
292.1011
366.1385
1404.7391_PEP_0_1_0_0_0
0
10000
20000
30000
40000
50000
200
400
600
800
1000
1200
1400
1600

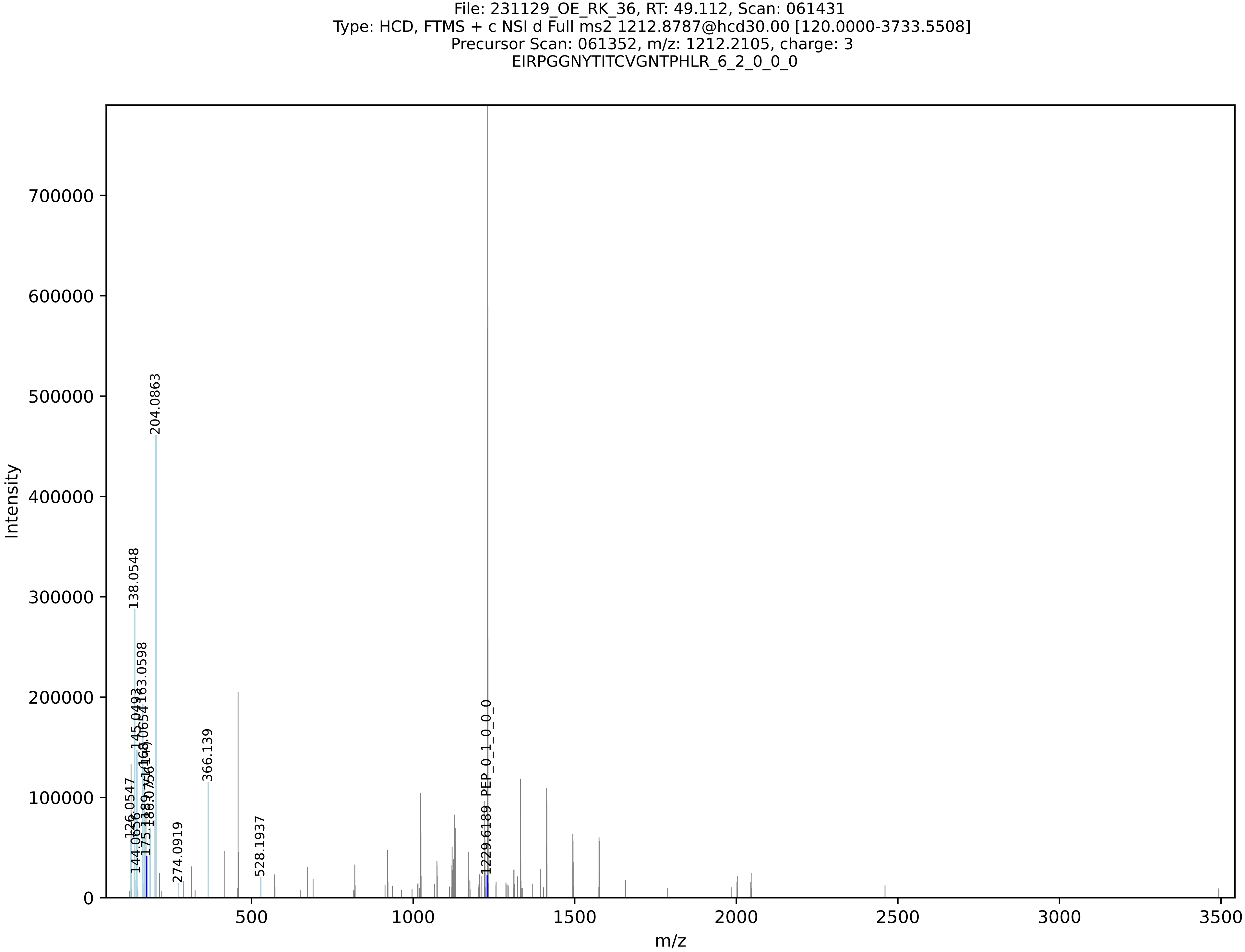
File: 231129_OE_RK_36, RT: 49.112, Scan: 061431
Type: HCD, FTMS + c NSI d Full ms2 1212.8787@hcd30.00 [120.0000-3733.5508]
Precursor Scan: 061352, m/z: 1212.2105, charge: 3
EIRPGGNYTITCVGNTPHLR_6_2_0_0_0
Intensity
m/z
204.0863
138.0548
366.139
274.0919
528.1937
1229.6189_PEP_0_1_0_0_0
126.0547
144.0656
145.0493
175.1189
0
100000
200000
300000
400000
500000
600000
700000
500
1000
1500
2000
2500
3000
3500

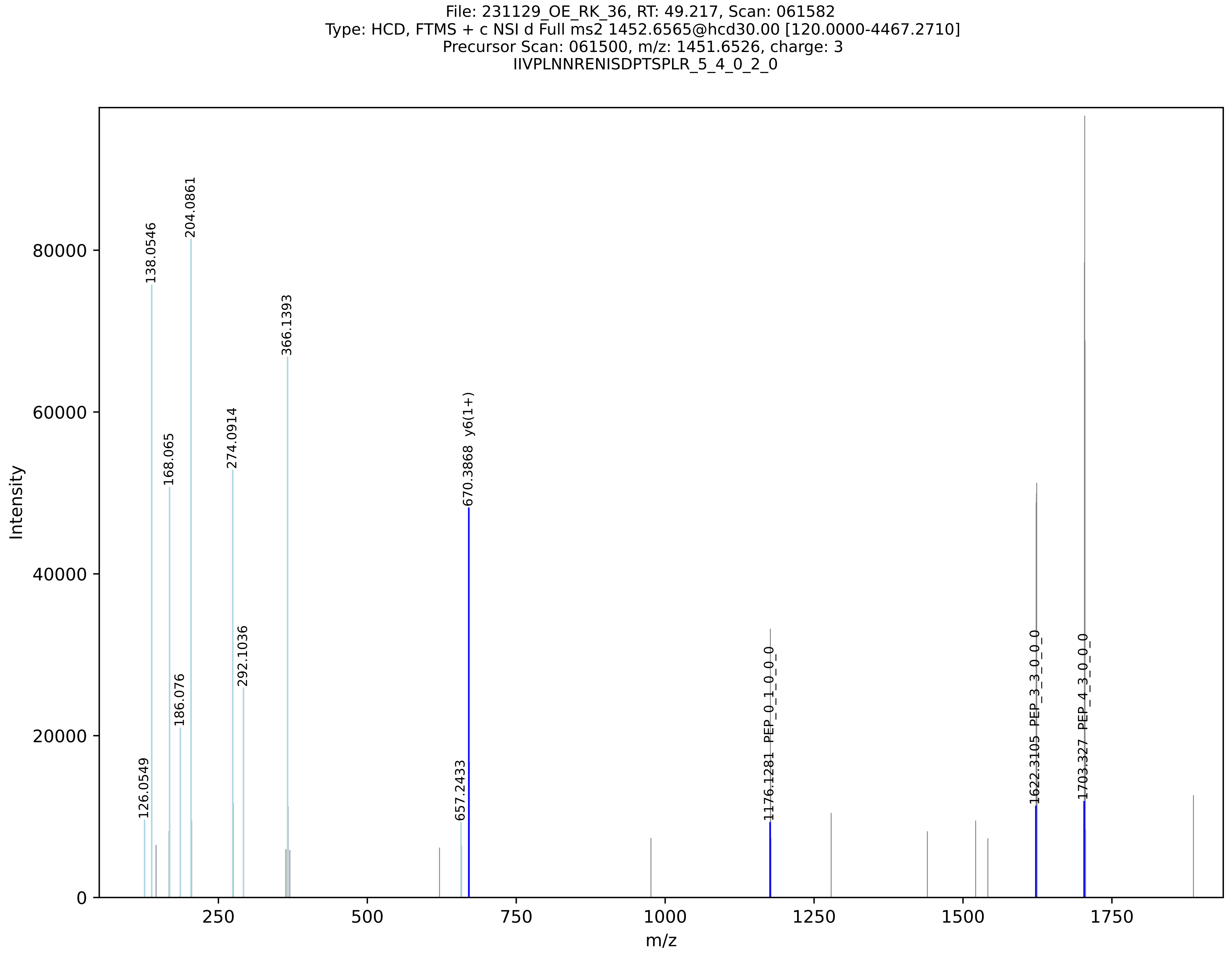
File: 231129_OE_RK_36, RT: 49.217, Scan: 061582
Type: HCD, FTMS + c NSI d Full ms2 1452.6565@hcd30.00 [120.0000-4467.2710]
Precursor Scan: 061500, m/z: 1451.6526, charge: 3
IIVPLNNRENISDPTSPLR_5_4_0_2_0
Intensity
m/z
126.0549
138.0546
168.065
186.076
204.0861
274.0914
292.1036
366.1393
657.2433
670.3868 y6(1+)
1176.1281 PEP_0_1_0_0_0
1622.3105 PEP_3_3_0_0_0
1703.327 PEP_4_3_0_0_0

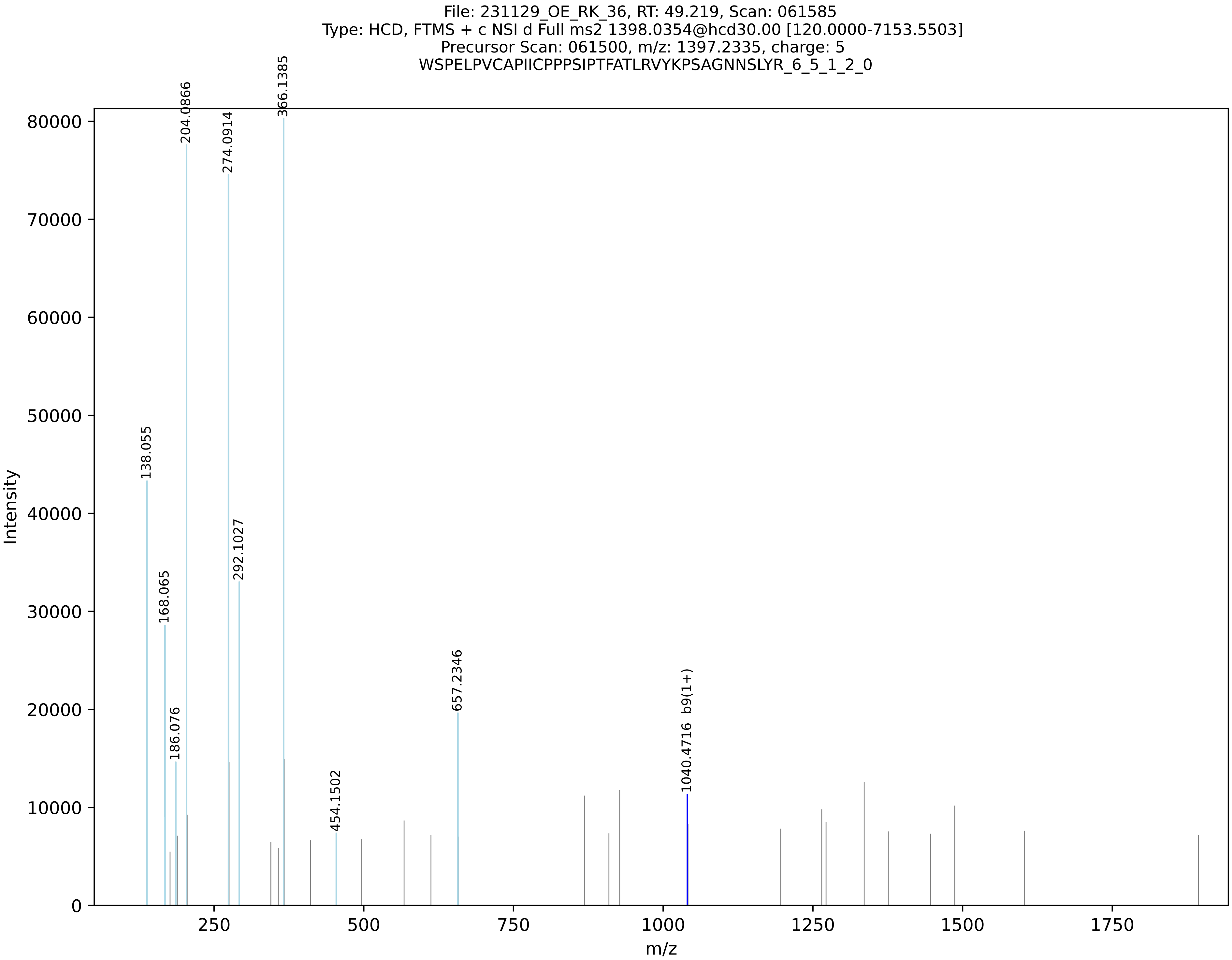
File: 231129_OE_RK_36, RT: 49.219, Scan: 061585
Type: HCD, FTMS + c NSI d Full ms2 1398.0354@hcd30.00 [120.0000-7153.5503]
Precursor Scan: 061500, m/z: 1397.2335, charge: 5
WSPELPVCAPIICPPPSIPTFATLRVYKPSAGNNSLYR_6_5_1_2_0
Intensity
m/z
138.055
168.065
186.076
204.0866
274.0914
292.1027
366.1385
454.1502
657.2346
1040.4716 b9(1+)

File: 231129_OE_RK_36, RT: 49.273, Scan: 061667
Type: HCD, FTMS + c NSI d Full ms2 1108.4788@hcd30.00 [120.0000-4545.4492]
Precursor Scan: 061571, m/z: 1107.9775, charge: 4
HCNTTRHSKPTDKPTGNSK_5_5_1_1_0

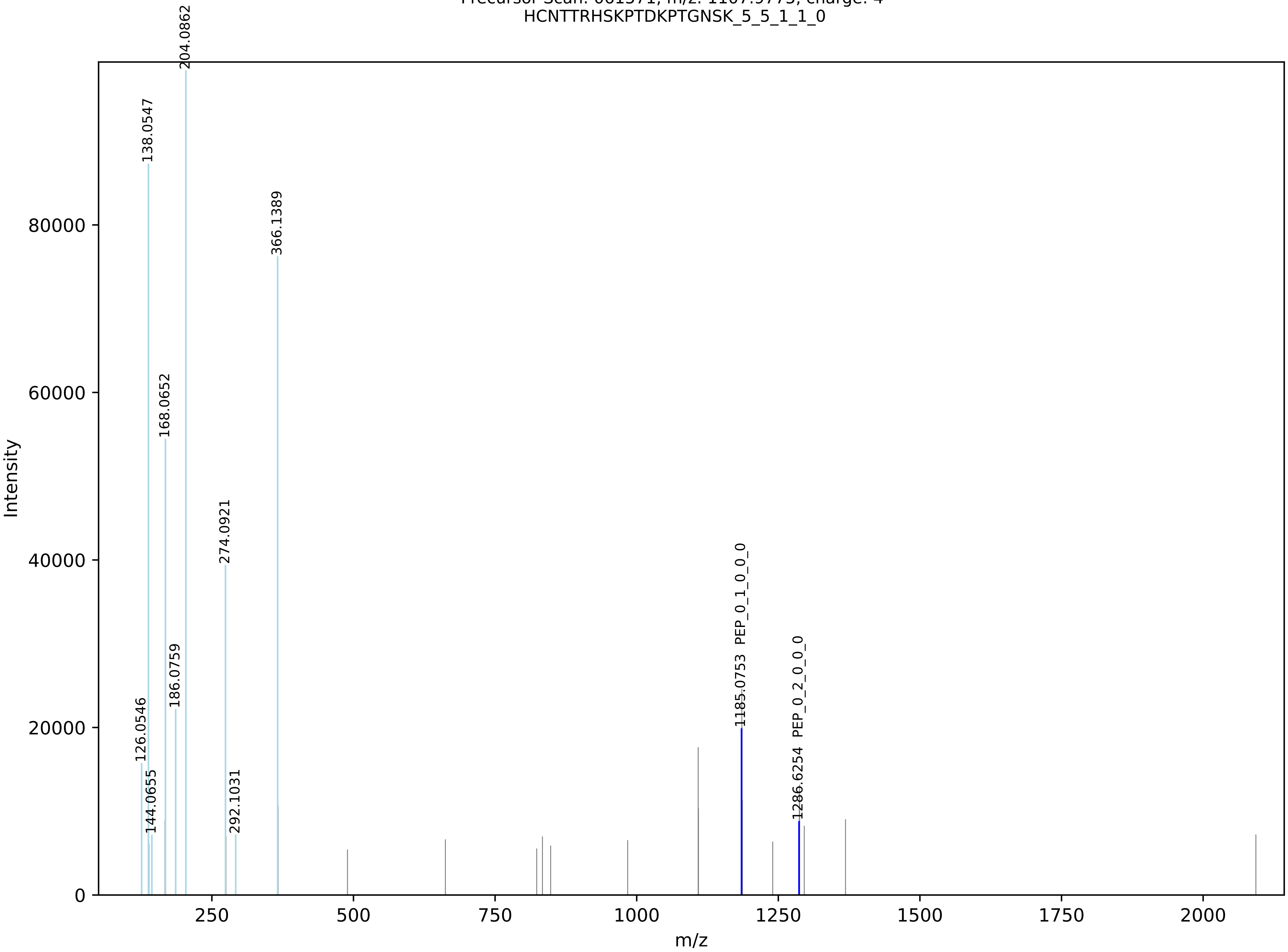

File: 231129_OE_RK_36, RT: 49.437, Scan: 061903
Type: HCD, FTMS + c NSI d Full ms2 1051.2289@hcd30.00 [120.0000-4311.8701]
Precursor Scan: 061849, m/z: 1050.7277, charge: 4
TVLTPATNHMGNVTFTIPANREFK_7_2_0_0_0

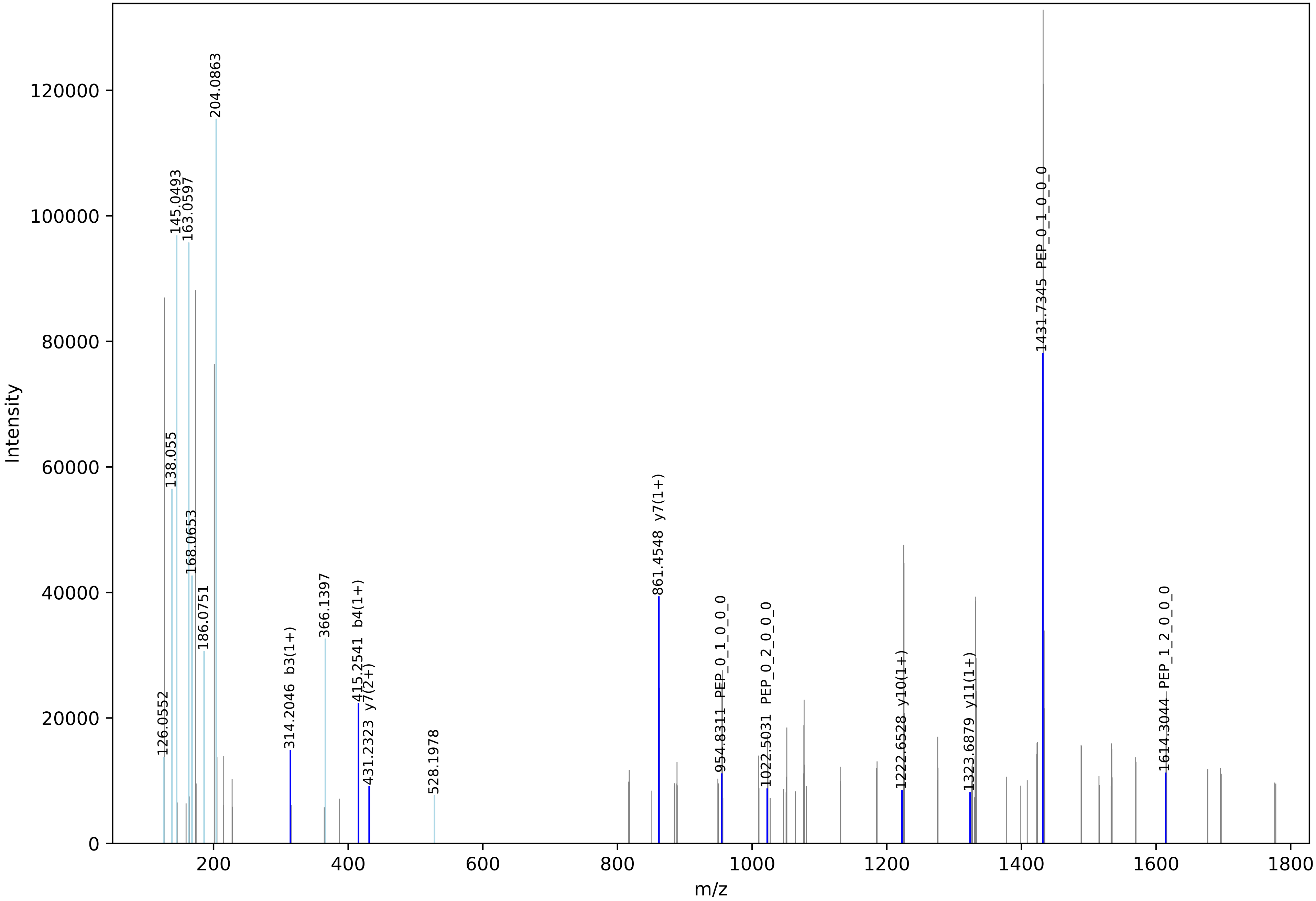

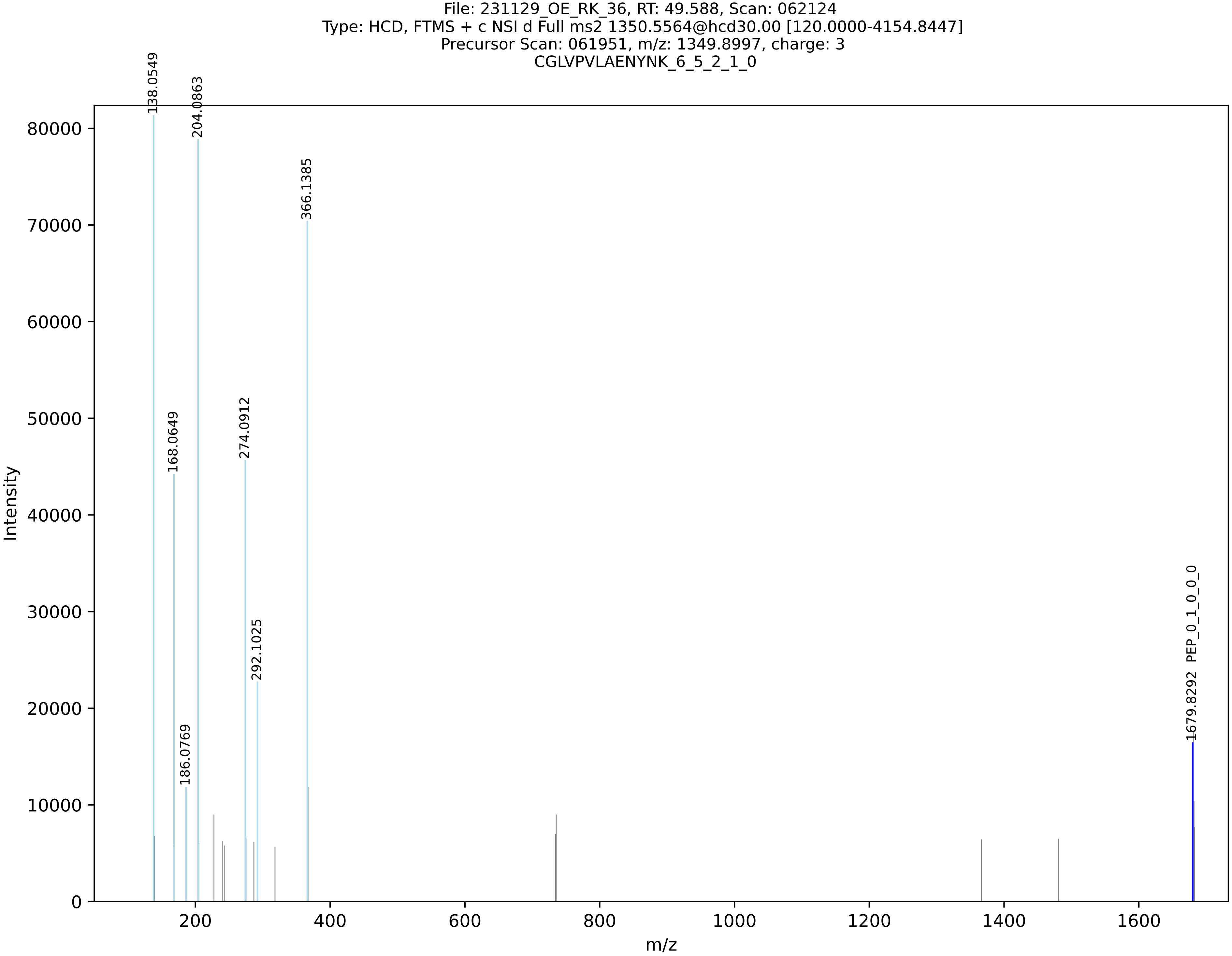
File: 231129_OE_RK_36, RT: 49.588, Scan: 062124
Type: HCD, FTMS + c NSI d Full ms2 1350.5564@hcd30.00 [120.0000-4154.8447]
Precursor Scan: 061951, m/z: 1349.8997, charge: 3
CGLVPVLAENYNK_6_5_2_1_0
138.0549
204.0863
366.1385
168.0649
274.0912
292.1025
186.0769
1679.8292 PEP_0_1_0_0_0
Intensity
m/z
0
10000
20000
30000
40000
50000
60000
70000
80000
200
400
600
800
1000
1200
1400
1600

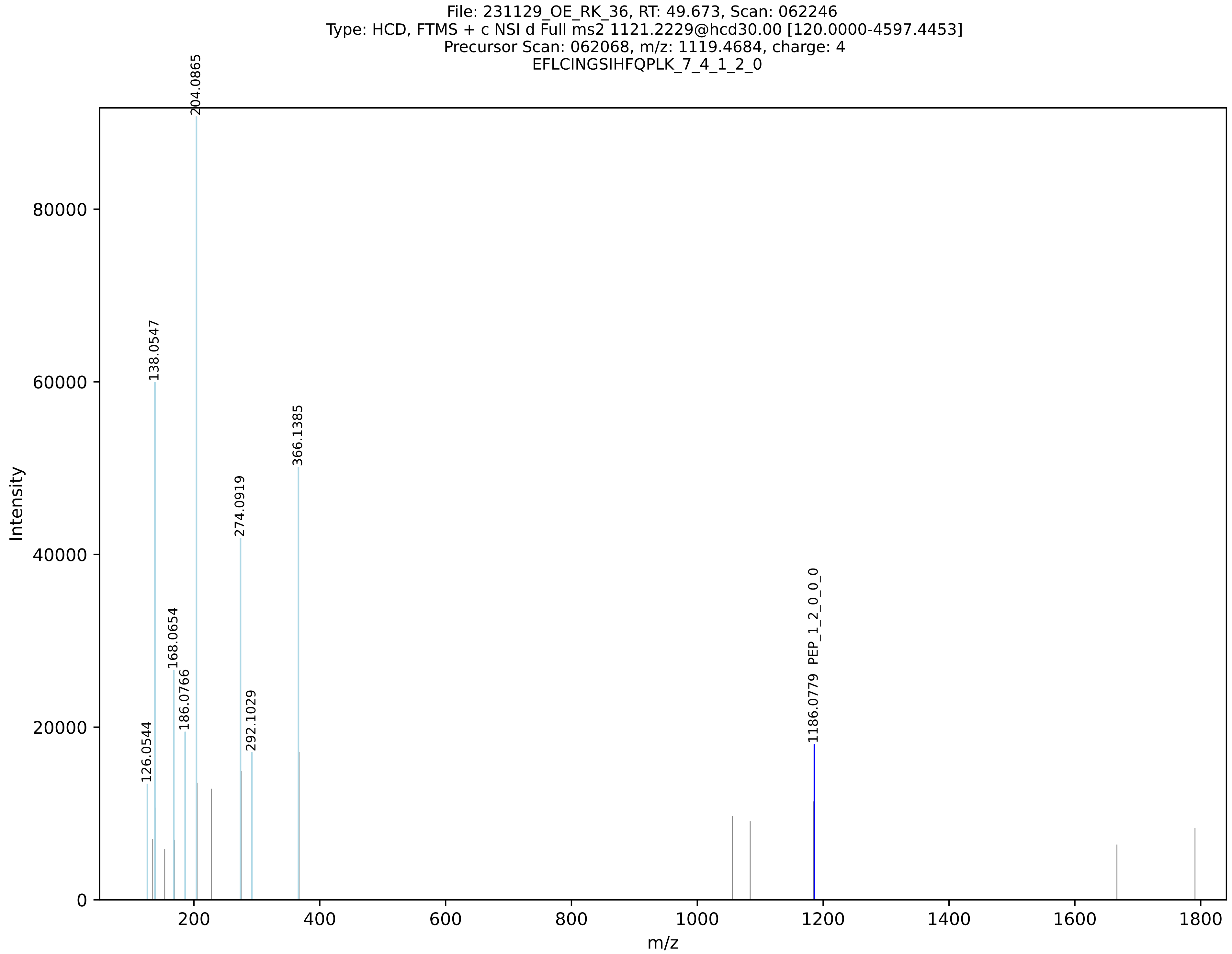
File: 231129_OE_RK_36, RT: 49.673, Scan: 062246
Type: HCD, FTMS + c NSI d Full ms2 1121.2229@hcd30.00 [120.0000-4597.4453]
Precursor Scan: 062068, m/z: 1119.4684, charge: 4
EFLCINGSIHFQPLK_7_4_1_2_0
204.0865
138.0547
366.1385
274.0919
168.0654
186.0766
126.0544
292.1029
1186.0779 PEP_1_2_0_0_0
Intensity
m/z
0
20000
40000
60000
80000
200
400
600
800
1000
1200
1400
1600
1800

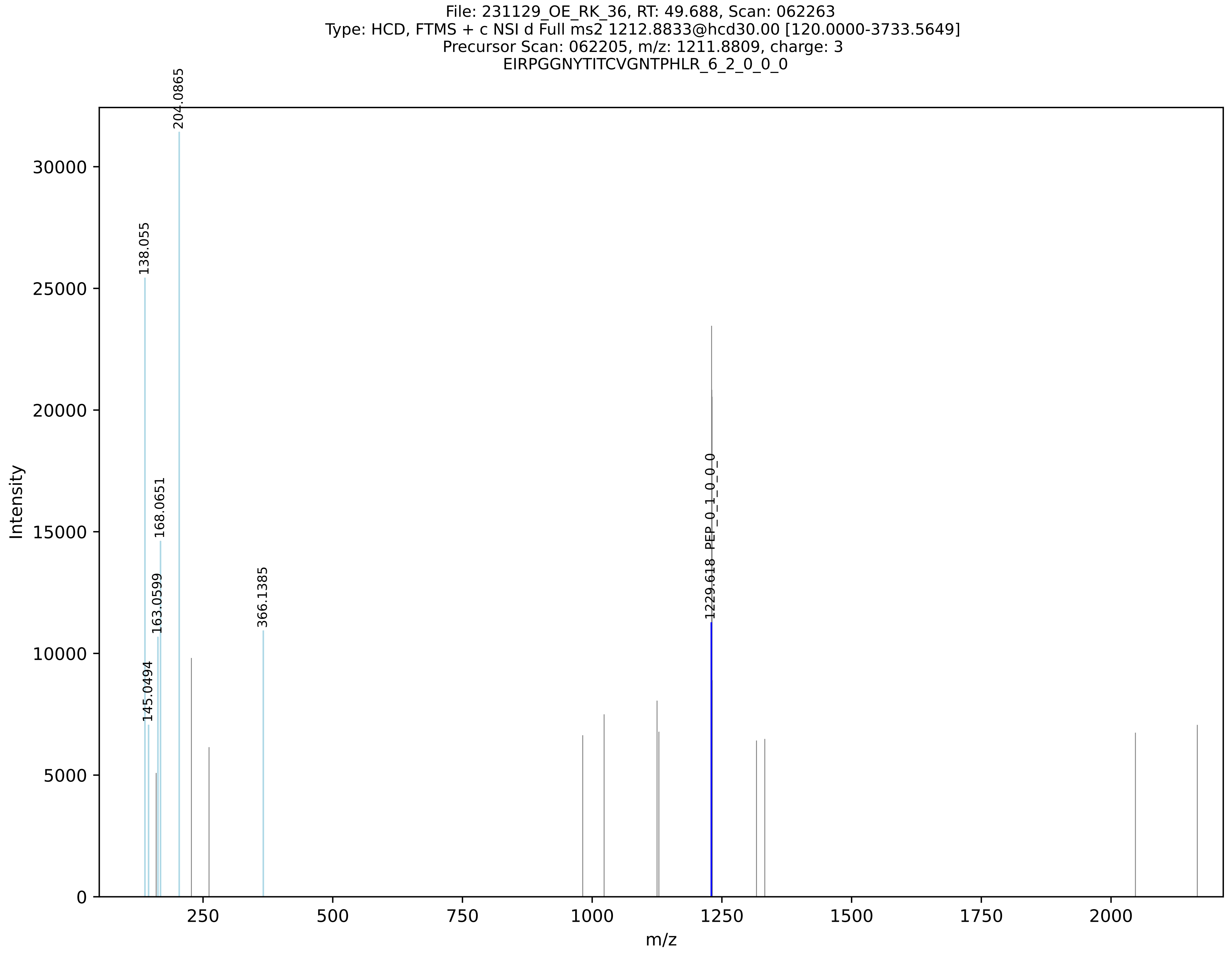
File: 231129_OE_RK_36, RT: 49.688, Scan: 062263
Type: HCD, FTMS + c NSI d Full ms2 1212.8833@hcd30.00 [120.0000-3733.5649]
Precursor Scan: 062205, m/z: 1211.8809, charge: 3
EIRPGGNYTITCVGNTPHLR_6_2_0_0_0
204.0865
138.055
168.0651
163.0599
145.0494
366.1385
1229.618_PEP_0_1_0_0_0
Intensity
m/z
0
5000
10000
15000
20000
25000
30000
250
500
750
1000
1250
1500
1750
2000

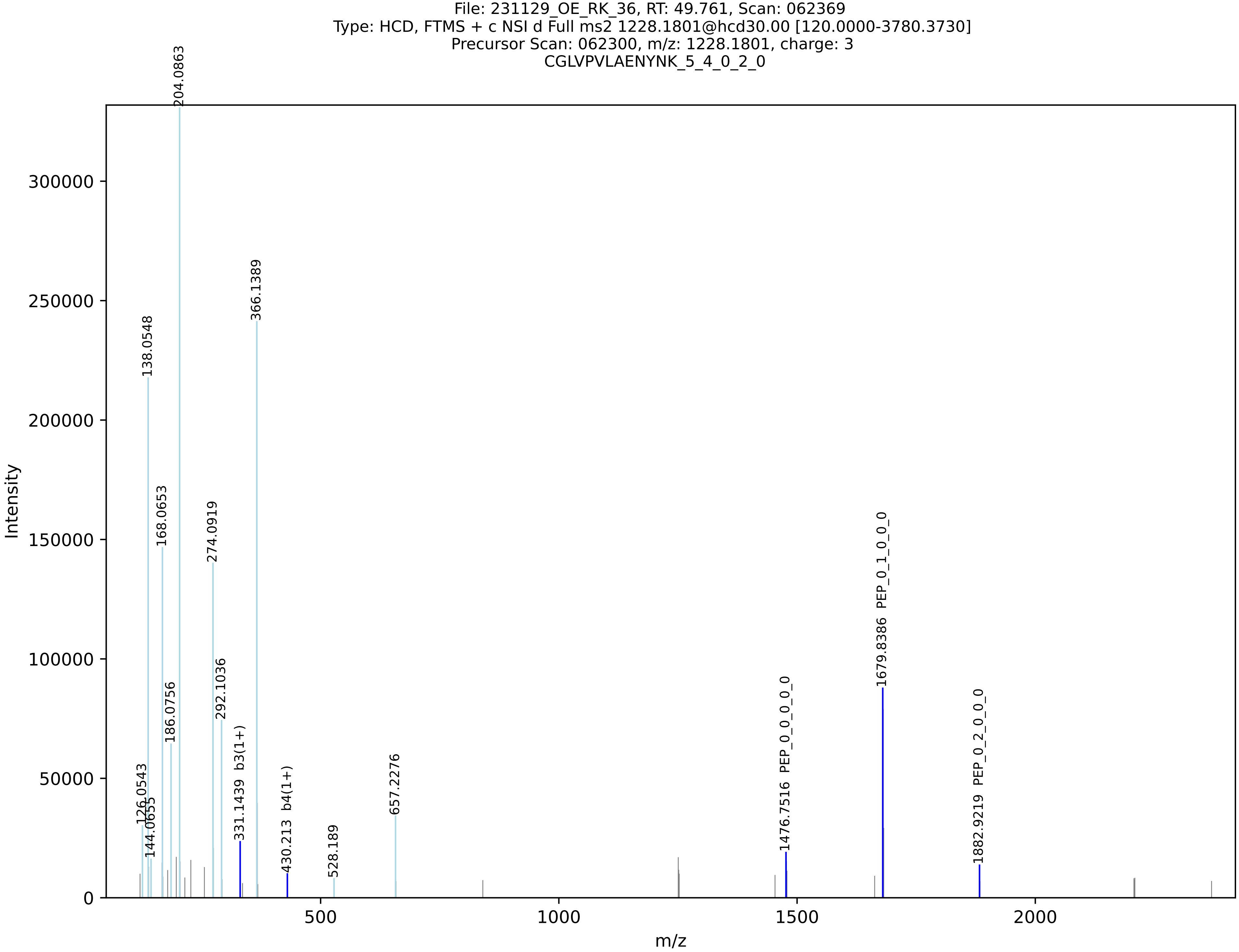

File: 231129_OE_RK_36, RT: 49.761, Scan: 062369
Type: HCD, FTMS + c NSI d Full ms2 1228.1801@hcd30.00 [120.0000-3780.3730]
Precursor Scan: 062300, m/z: 1228.1801, charge: 3
CGLVPVLAENYNK_5_4_0_2_0
Intensity
m/z
204.0863
366.1389
138.0548
168.0653
274.0919
292.1036
186.0756
126.0543
144.0655
331.1439 b3(1+)
430.213 b4(1+)
528.189
657.2276
1476.7516 PEP_0_0_0_0_0_
1679.8386 PEP_0_1_0_0_0_
1882.9219 PEP_0_2_0_0_0_

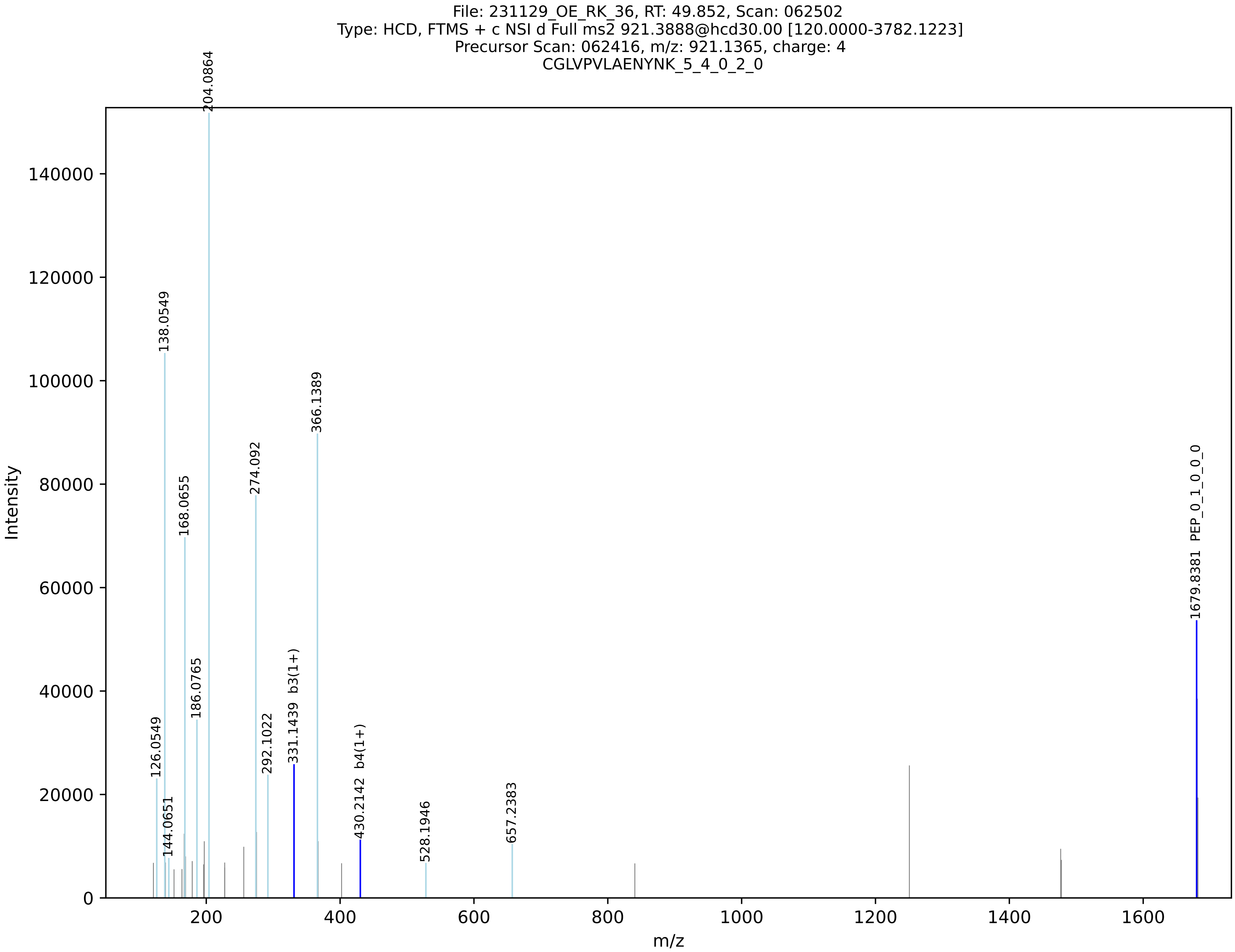

File: 231129_OE_RK_36, RT: 49.852, Scan: 062502
Type: HCD, FTMS + c NSI d Full ms2 921.3888@hcd30.00 [120.0000-3782.1223]
Precursor Scan: 062416, m/z: 921.1365, charge: 4
CGLVPVLAENYNK_5_4_0_2_0
Intensity
m/z
204.0864
138.0549
366.1389
274.092
168.0655
186.0765
126.0549
144.0651
292.1022
331.1439 b3(1+)
430.2142 b4(1+)
528.1946
657.2383
1679.8381 PEP_0_1_0_0_0

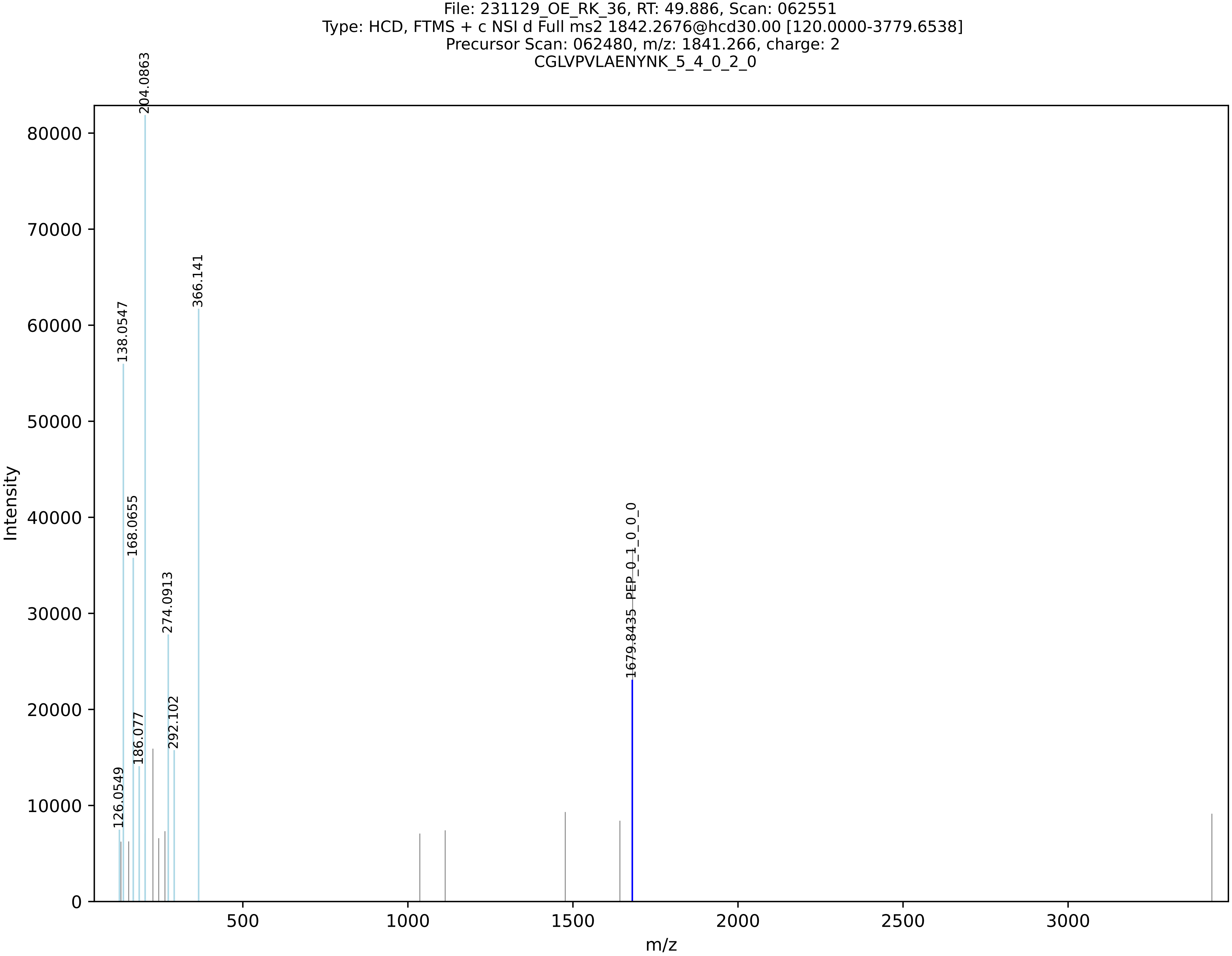
File: 231129_OE_RK_36, RT: 49.886, Scan: 062551
Type: HCD, FTMS + c NSI d Full ms2 1842.2676@hcd30.00 [120.0000-3779.6538]
Precursor Scan: 062480, m/z: 1841.266, charge: 2
CGLVPVLAENYNK_5_4_0_2_0
Intensity
m/z
204.0863
366.141
138.0547
168.0655
274.0913
292.102
186.077
126.0549
1679.8435_PEP_0_1_0_0_0
0
10000
20000
30000
40000
50000
60000
70000
80000
500
1000
1500
2000
2500
3000

File: 231129_OE_RK_36, RT: 50.138, Scan: 062911
Type: HCD, FTMS + c NSI d Full ms2 924.4183@hcd30.00 [120.0000-3794.4827]
Precursor Scan: 062831, m/z: 923.9171, charge: 4
VFHIHNESWVLLTPK_6_3_0_1_0

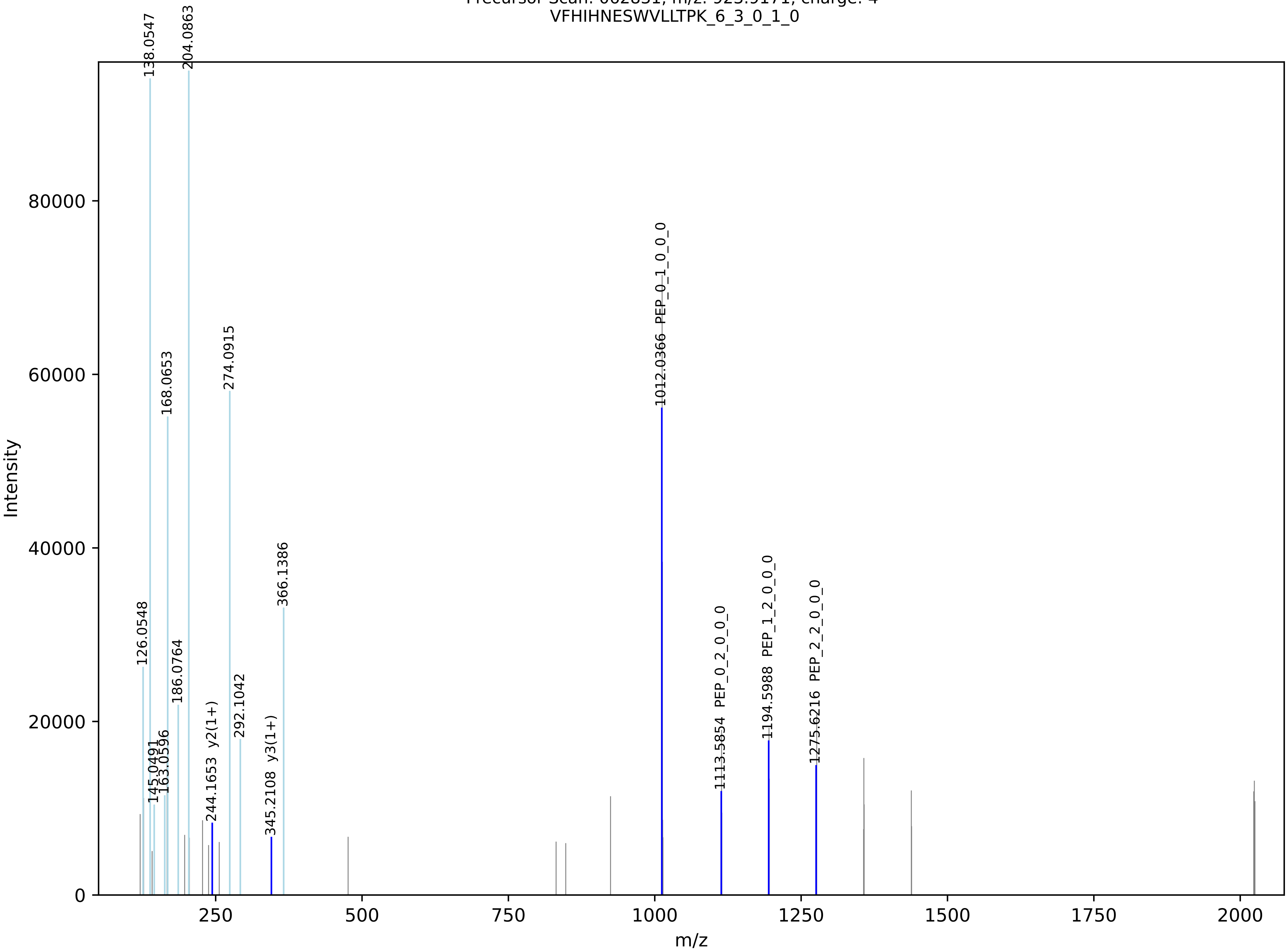

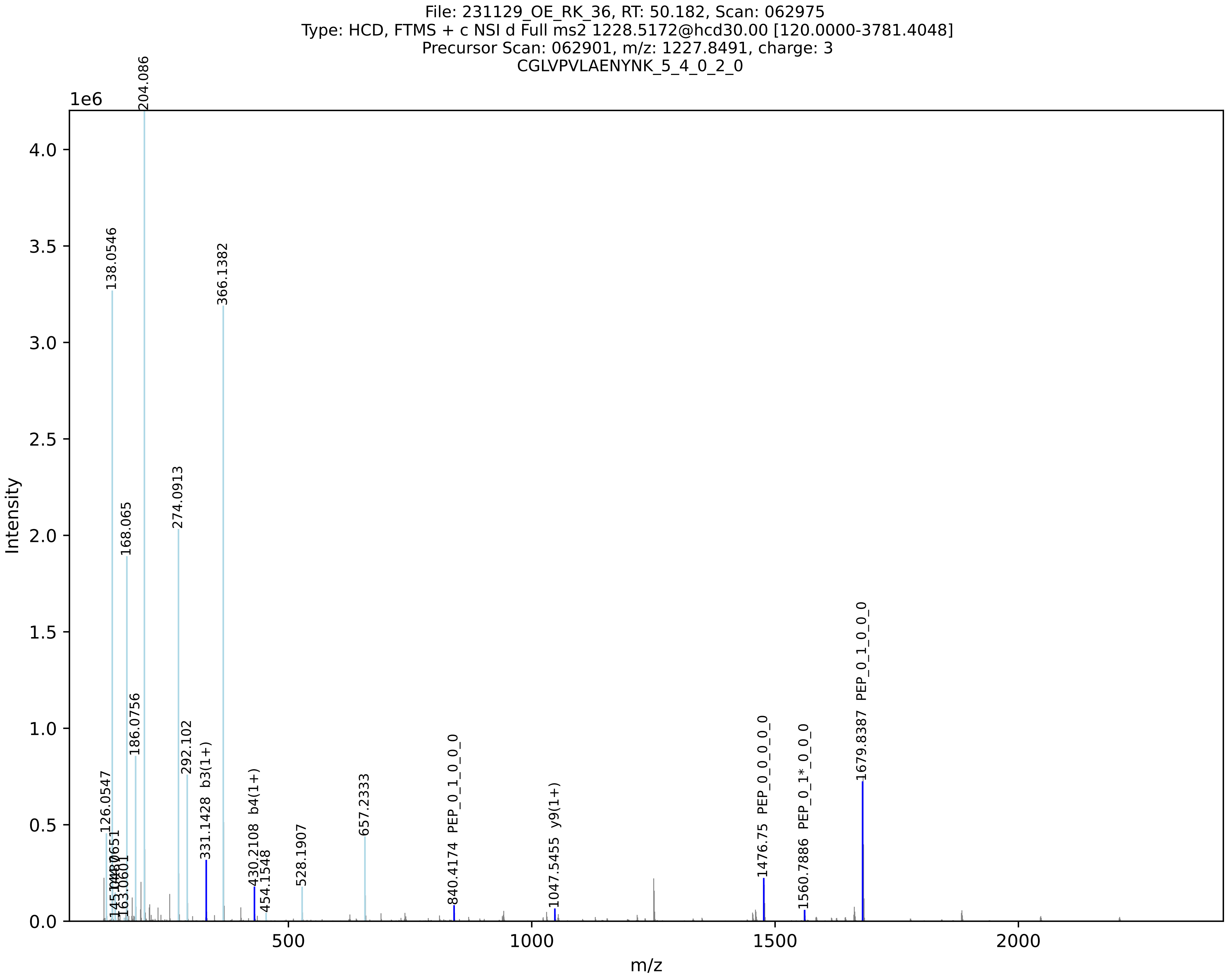
File: 231129_OE_RK_36, RT: 50.182, Scan: 062975
Type: HCD, FTMS + c NSI d Full ms2 1228.5172@hcd30.00 [120.0000-3781.4048]
Precursor Scan: 062901, m/z: 1227.8491, charge: 3
CGLVPVLAENYNK_5_4_0_2_0
1e6
Intensity
m/z
204.086
138.0546
366.1382
274.0913
168.065
186.0756
292.102
126.0547
163.0601
331.1428 b3(1+)
430.2108 b4(1+)
454.1548
528.1907
657.2333
840.4174 PEP_0_1_0_0_0
1047.5455 y9(1+)
1476.75 PEP_0_0_0_0_0
1560.7886 PEP_0_1*_0_0_0
1679.8387 PEP_0_1_0_0_0

File: 231129_OE_RK_36, RT: 50.286, Scan: 063127
Type: HCD, FTMS + c NSI d Full ms2 1234.1917@hcd30.00 [120.0000-3798.7686]
Precursor Scan: 063048, m/z: 1233.5235, charge: 3
CGLVPVLAENYNK_6_4_1_1_0

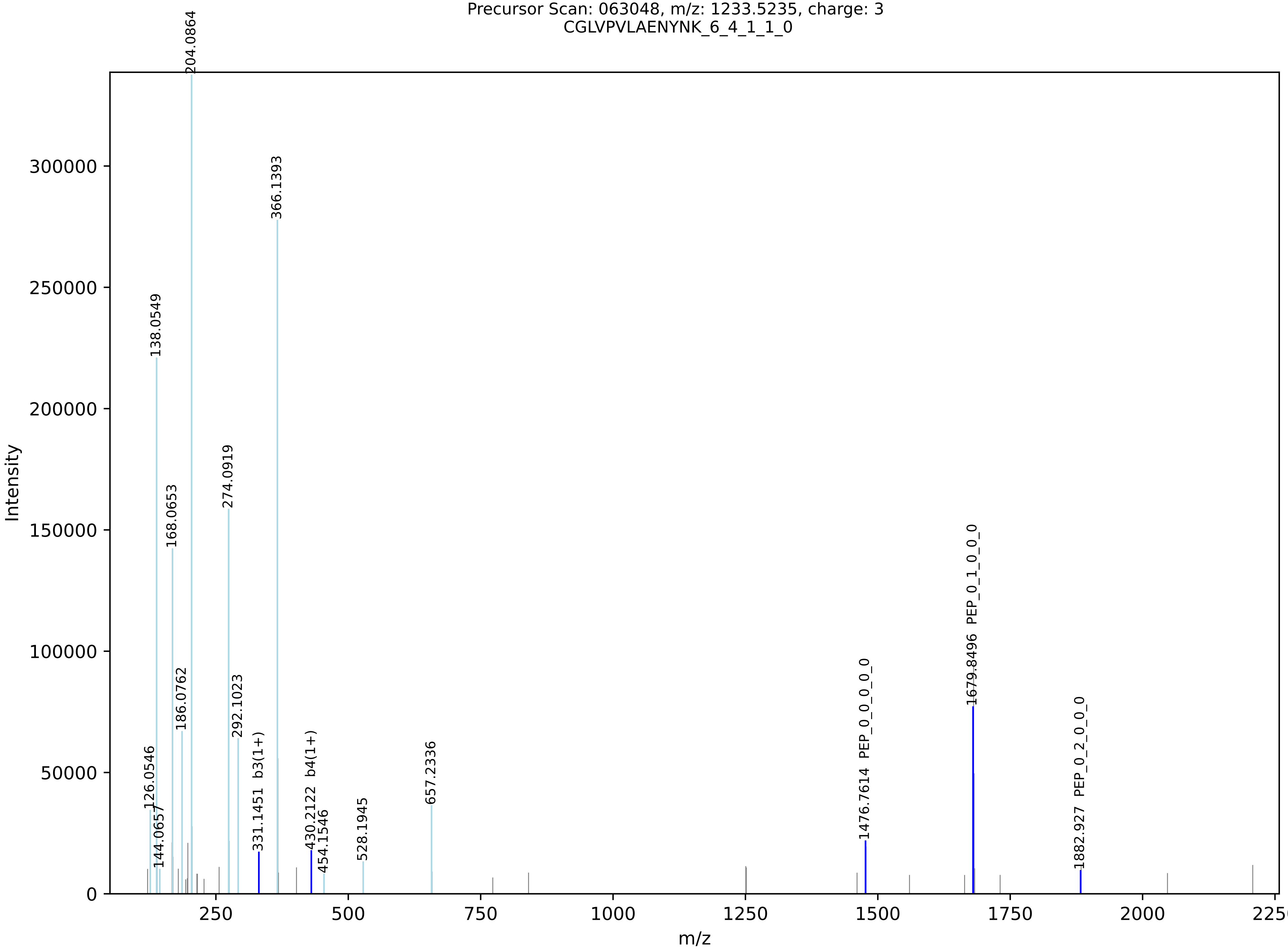

File: 231129_OE_RK_36, RT: 50.288, Scan: 063131
Type: HCD, FTMS + c NSI d Full ms2 1033.4622@hcd30.00 [120.0000-4239.3813]
Precursor Scan: 063048, m/z: 1032.7103, charge: 4
TVLTPATNHMGNVTFTIPANR_6_3_0_1_0

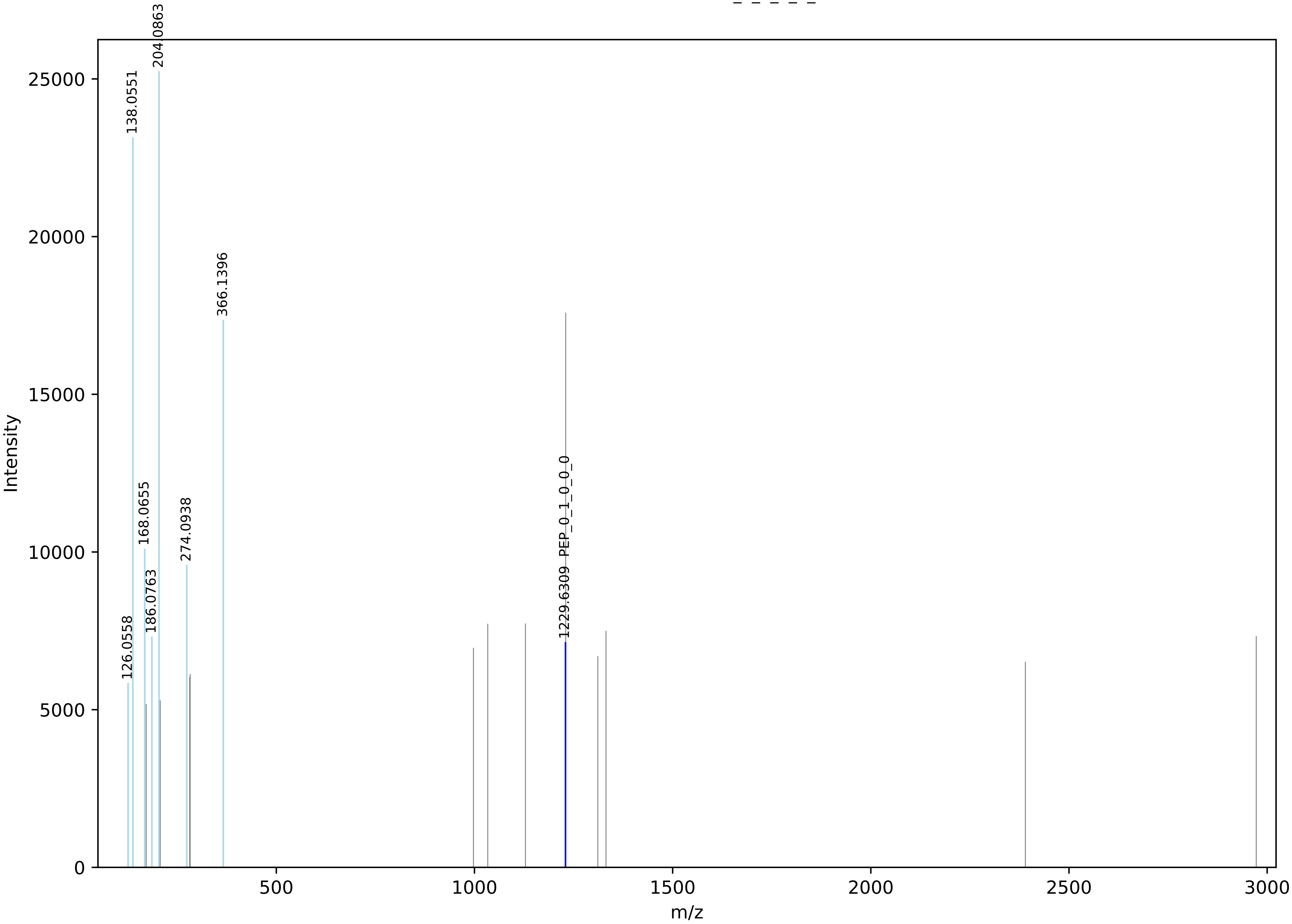

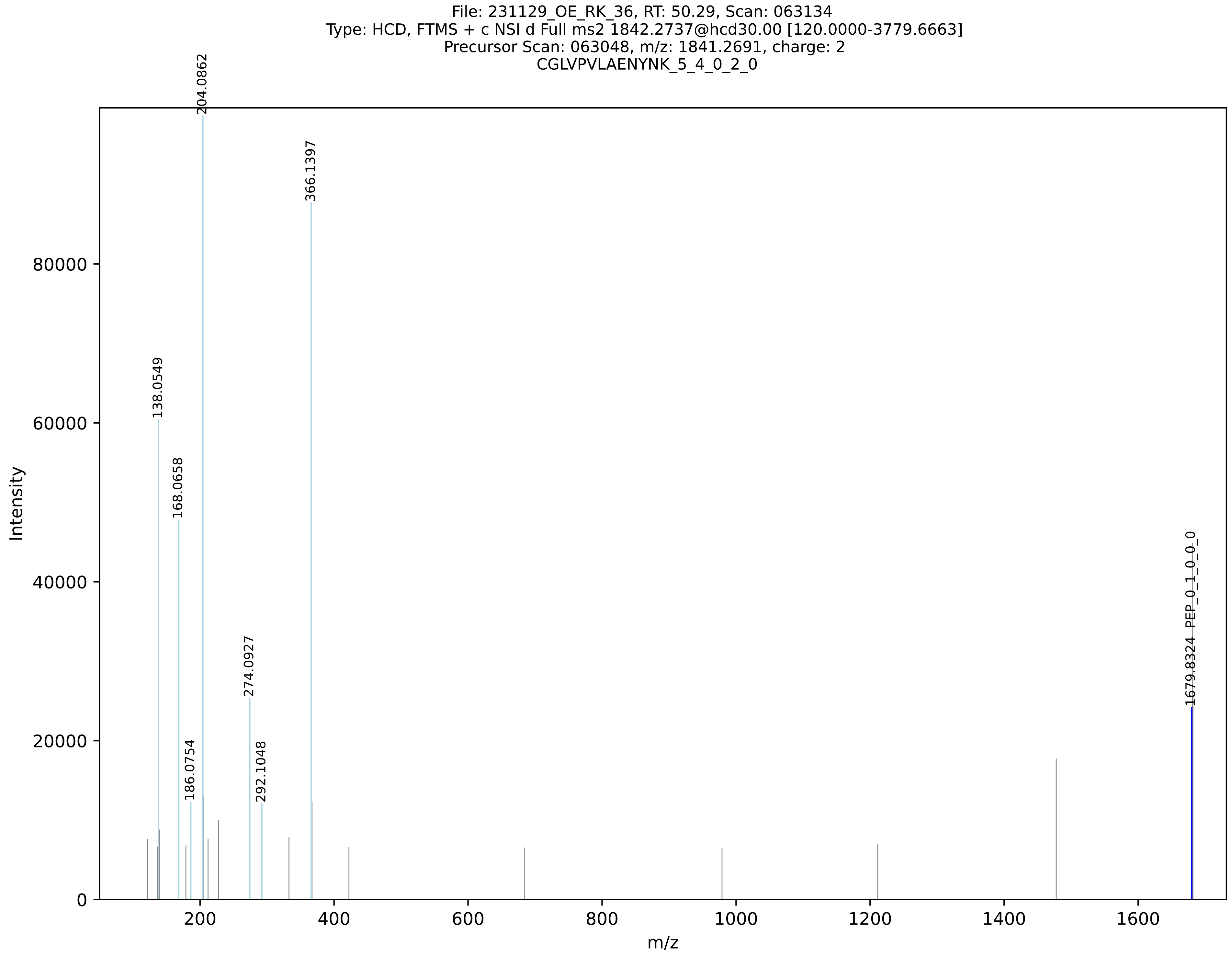
File: 231129_OE_RK_36, RT: 50.29, Scan: 063134
Type: HCD, FTMS + c NSI d Full ms2 1842.2737@hcd30.00 [120.0000-3779.6663]
Precursor Scan: 063048, m/z: 1841.2691, charge: 2
CGLVPVLAENYNK_5_4_0_2_0
204.0862
366.1397
138.0549
168.0658
274.0927
186.0754
292.1048
1679.8324_PEP_0_1_0_0_0
Intensity
m/z
0
20000
40000
60000
80000
200
400
600
800
1000
1200
1400
1600

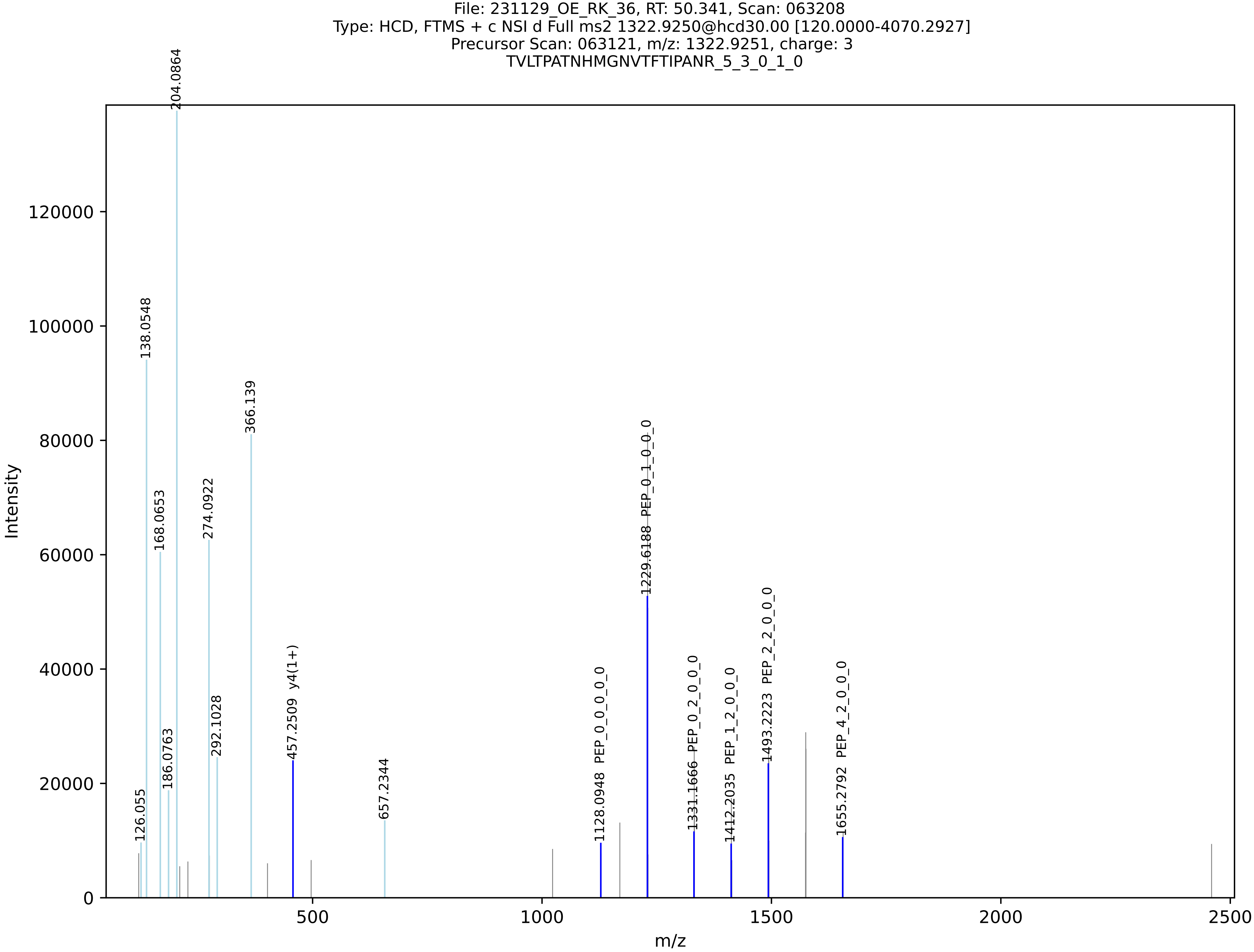
File: 231129_OE_RK_36, RT: 50.341, Scan: 063208
Type: HCD, FTMS + c NSI d Full ms2 1322.9250@hcd30.00 [120.0000-4070.2927]
Precursor Scan: 063121, m/z: 1322.9251, charge: 3
TVLTPATNHMGNVTFTIPANR_5_3_0_1_0
Intensity
m/z
204.0864
138.0548
168.0653
186.0763
126.055
274.0922
292.1028
366.139
457.2509 y4(1+)
657.2344
1128.0948 PEP_0_0_0_0_0
1229.6188 PEP_0_1_0_0_0
1331.1666 PEP_0_2_0_0_0
1412.2035 PEP_1_2_0_0_0
1493.2223 PEP_2_2_0_0_0
1655.2792 PEP_4_2_0_0_0

File: 231129_OE_RK_36, RT: 50.383, Scan: 063269
Type: HCD, FTMS + c NSI d Full ms2 1228.5138@hcd30.00 [120.0000-3781.3943]
Precursor Scan: 063194, m/z: 1227.8456, charge: 3
CGLVPVLAENYNK_5_4_0_2_0

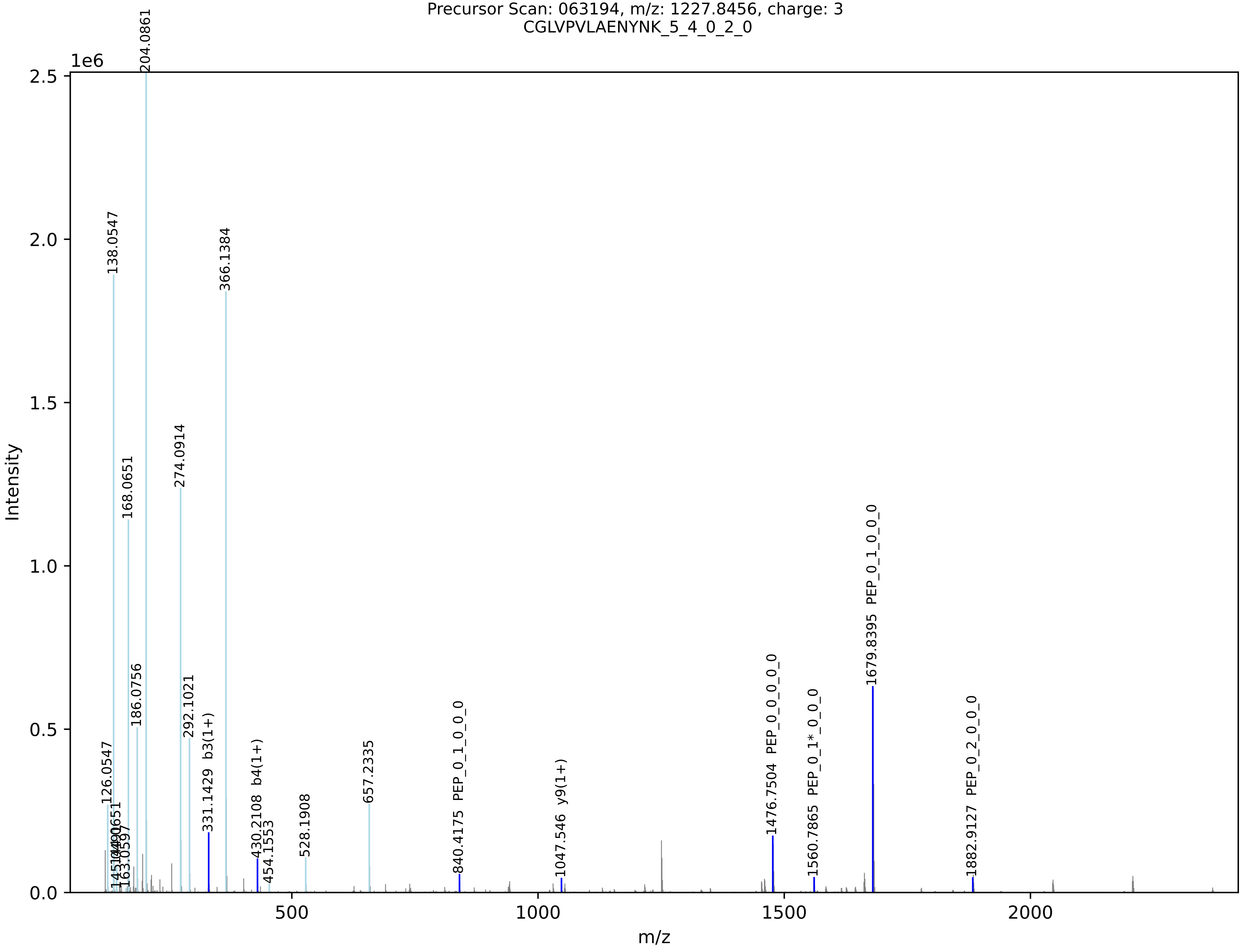

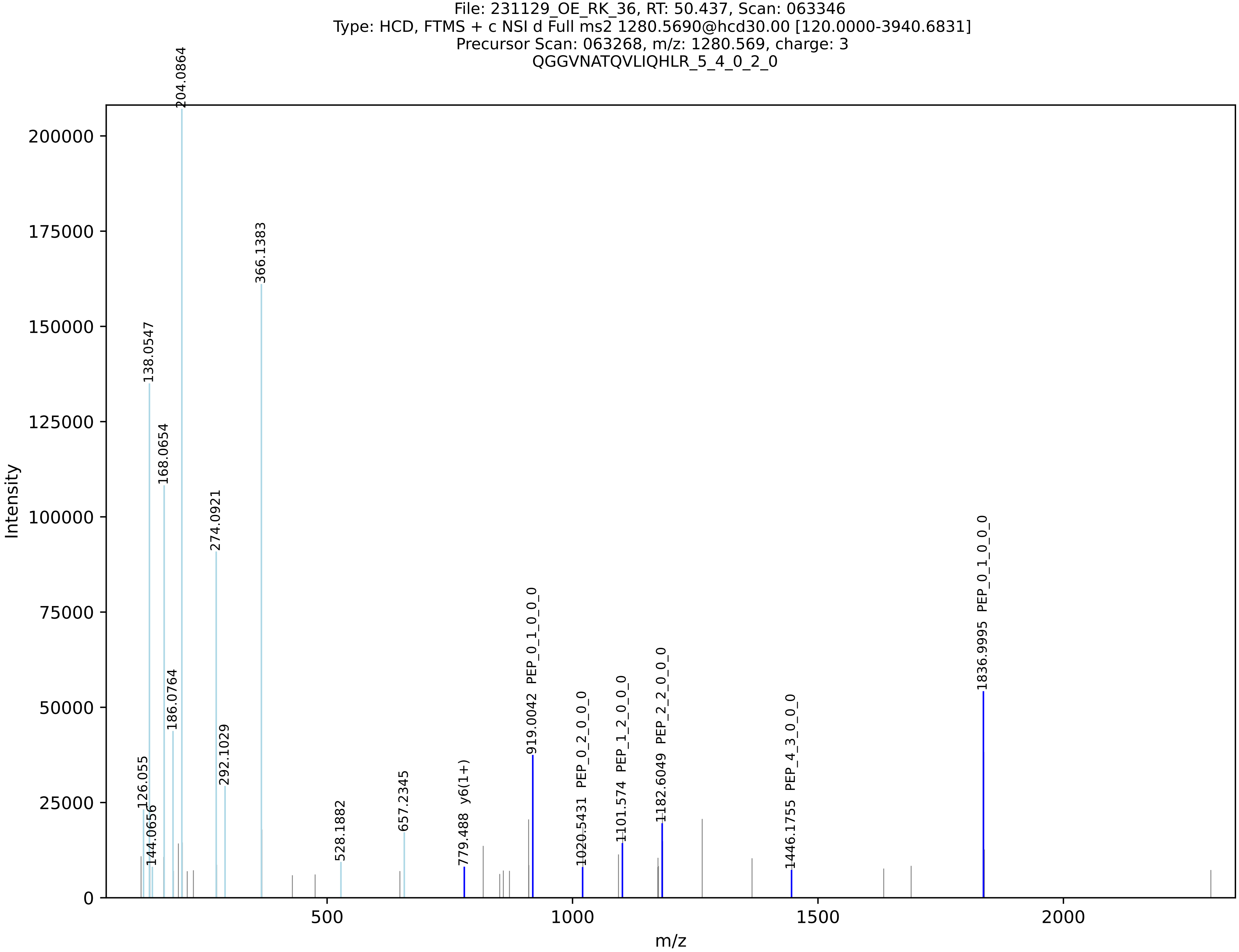

File: 231129_OE_RK_36, RT: 50.437, Scan: 063346
Type: HCD, FTMS + c NSI d Full ms2 1280.5690@hcd30.00 [120.0000-3940.6831]
Precursor Scan: 063268, m/z: 1280.569, charge: 3
QGGVNATQVLIQHLR_5_4_0_2_0
Intensity
m/z
204.0864
366.1383
138.0547
168.0654
274.0921
186.0764
292.1029
126.055
144.0656
528.1882
657.2345
779.488 y6(1+)
919.0042 PEP_0_1_0_0_0
1020.5431 PEP_0_2_0_0_0
1101.574 PEP_1_2_0_0_0
1182.6049 PEP_2_2_0_0_0
1446.1755 PEP_4_3_0_0_0
1836.9995 PEP_0_1_0_0_0

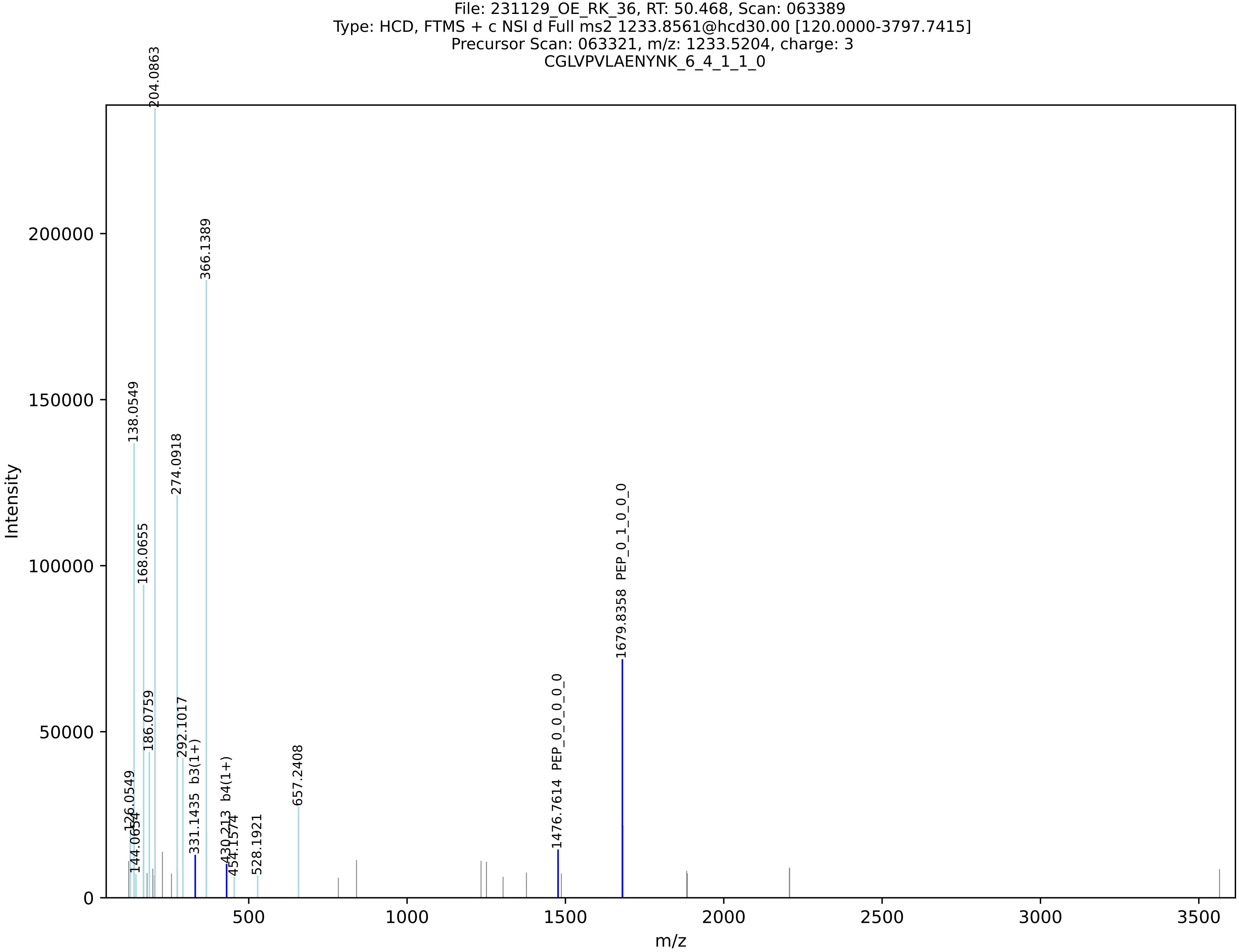

File: 231129_OE_RK_36, RT: 50.468, Scan: 063389
Type: HCD, FTMS + c NSI d Full ms2 1233.8561@hcd30.00 [120.0000-3797.7415]
Precursor Scan: 063321, m/z: 1233.5204, charge: 3
CGLVPVLAENYNK_6_4_1_1_0
Intensity
m/z
204.0863
366.1389
138.0549
274.0918
168.0655
186.0759
292.1017
331.1435 b3(1+)
430.213 b4(1+)
454.1574
528.1921
657.2408
126.0549
144.0654
1476.7614 PEP_0_0_0_0_0
1679.8358 PEP_0_1_0_0_0

File: 231129_OE_RK_36, RT: 50.628, Scan: 063619
Type: HCD, FTMS + c NSI d Full ms2 1326.8822@hcd30.00 [120.0000-4082.4016]
Precursor Scan: 063522, m/z: 1326.214, charge: 3
VCQDCPLLAPLNDTR_5_4_0_2_0

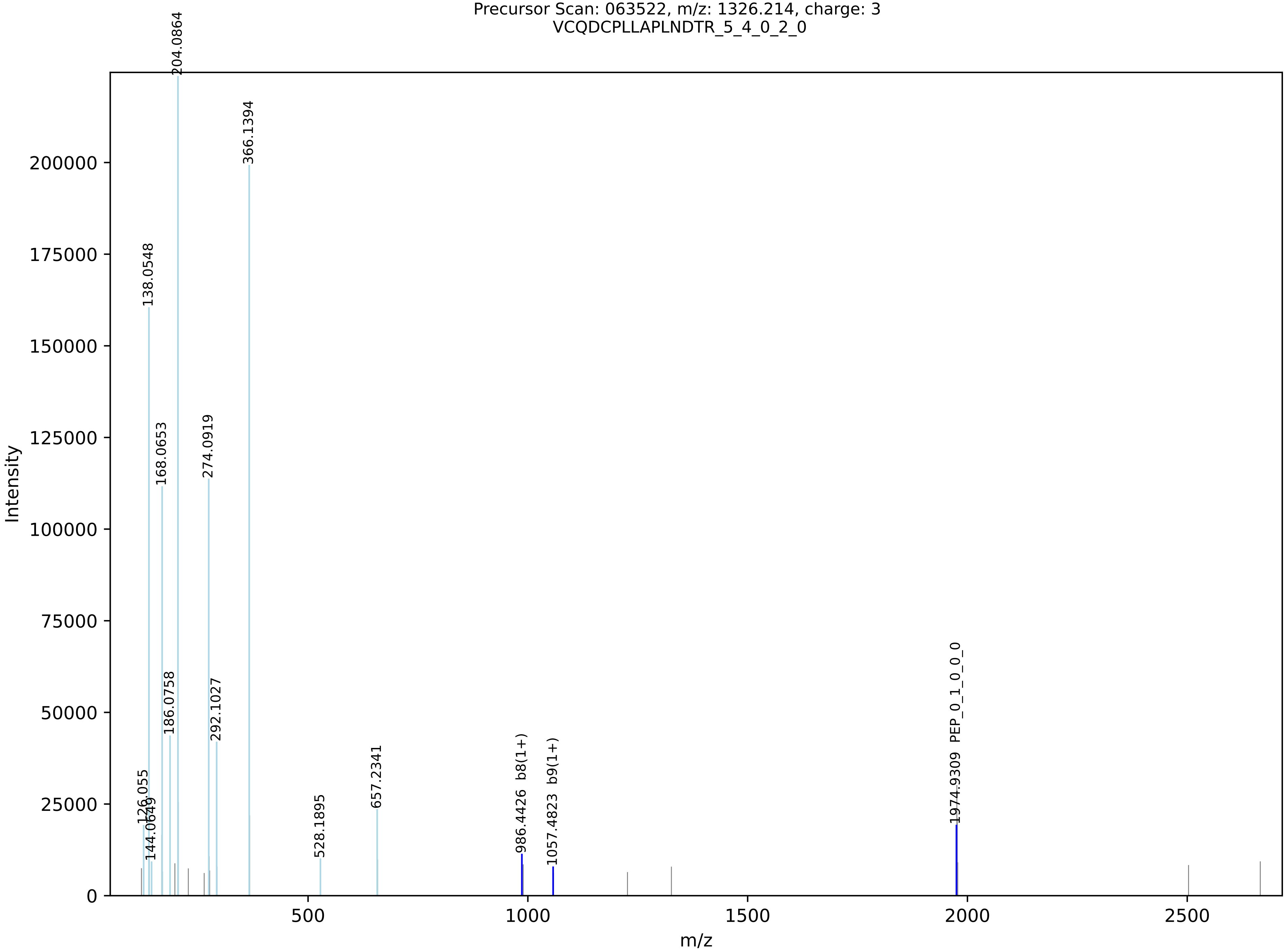

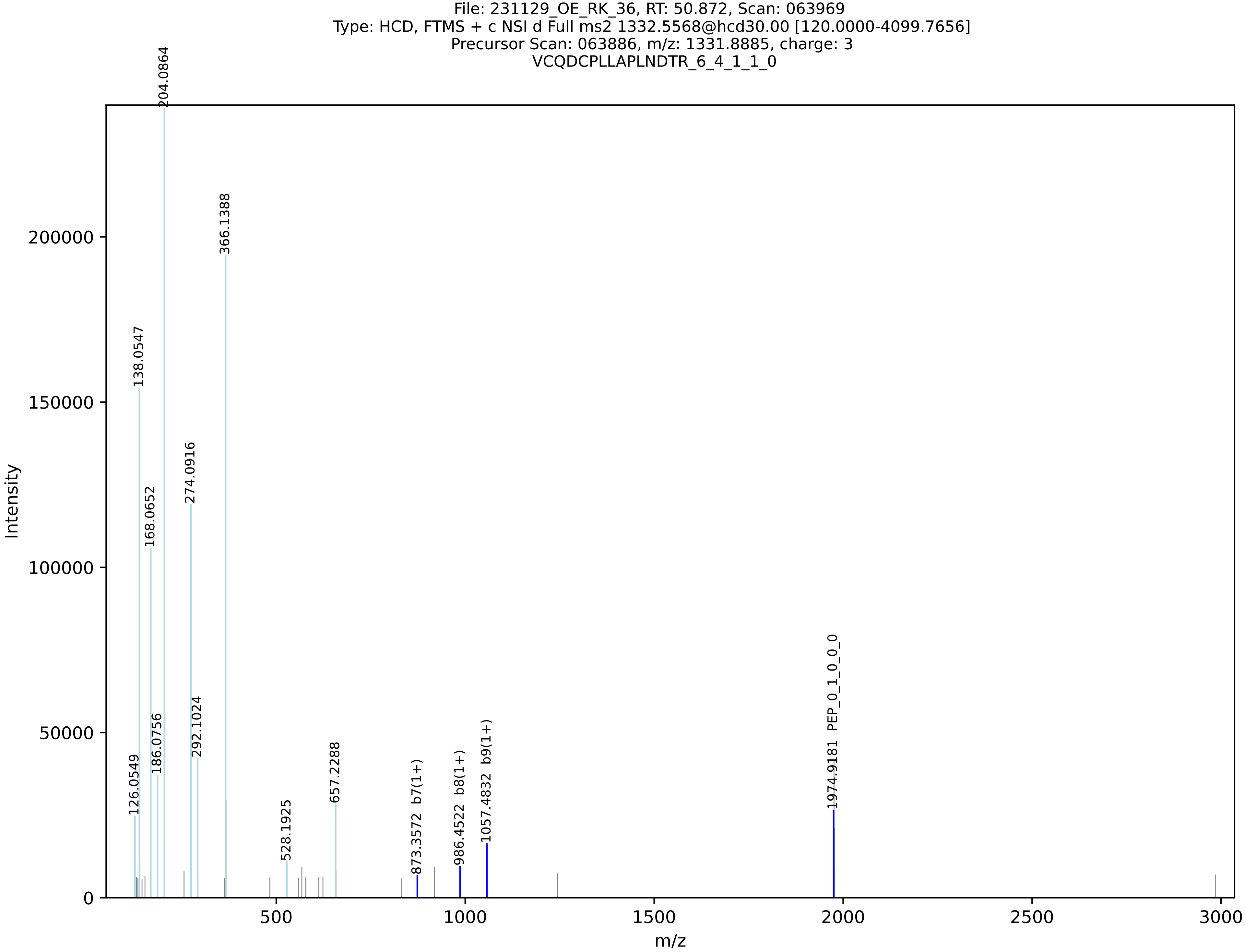

File: 231129_OE_RK_36, RT: 50.872, Scan: 063969
Type: HCD, FTMS + c NSI d Full ms2 1332.5568@hcd30.00 [120.0000-4099.7656]
Precursor Scan: 063886, m/z: 1331.8885, charge: 3
VCQDCPLLAPLNDTR_6_4_1_1_0
Intensity
m/z
204.0864
366.1388
138.0547
274.0916
168.0652
292.1024
186.0756
126.0549
657.2288
528.1925
873.3572 b7(1+)
986.4522 b8(1+)
1057.4832 b9(1+)
1974.9181 PEP_0_1_0_0_0

File: 231129_OE_RK_36, RT: 50.917, Scan: 064034
Type: HCD, FTMS + c NSI d Full ms2 1120.4946@hcd30.00 [120.0000-3450.8557]
Precursor Scan: 063960, m/z: 1120.1605, charge: 3
ALPQPQNVTSLLGCTH_5_4_0_0_0

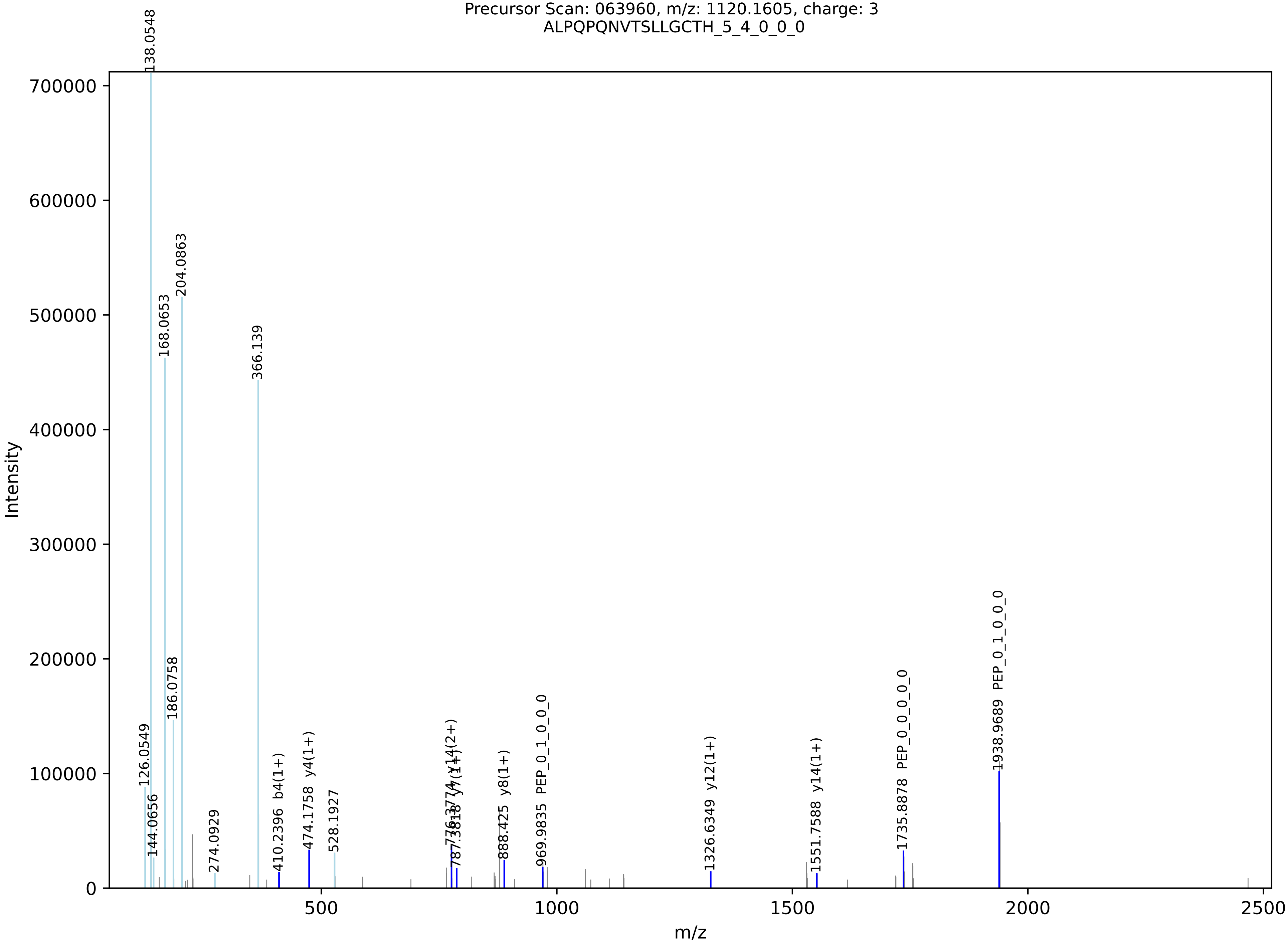

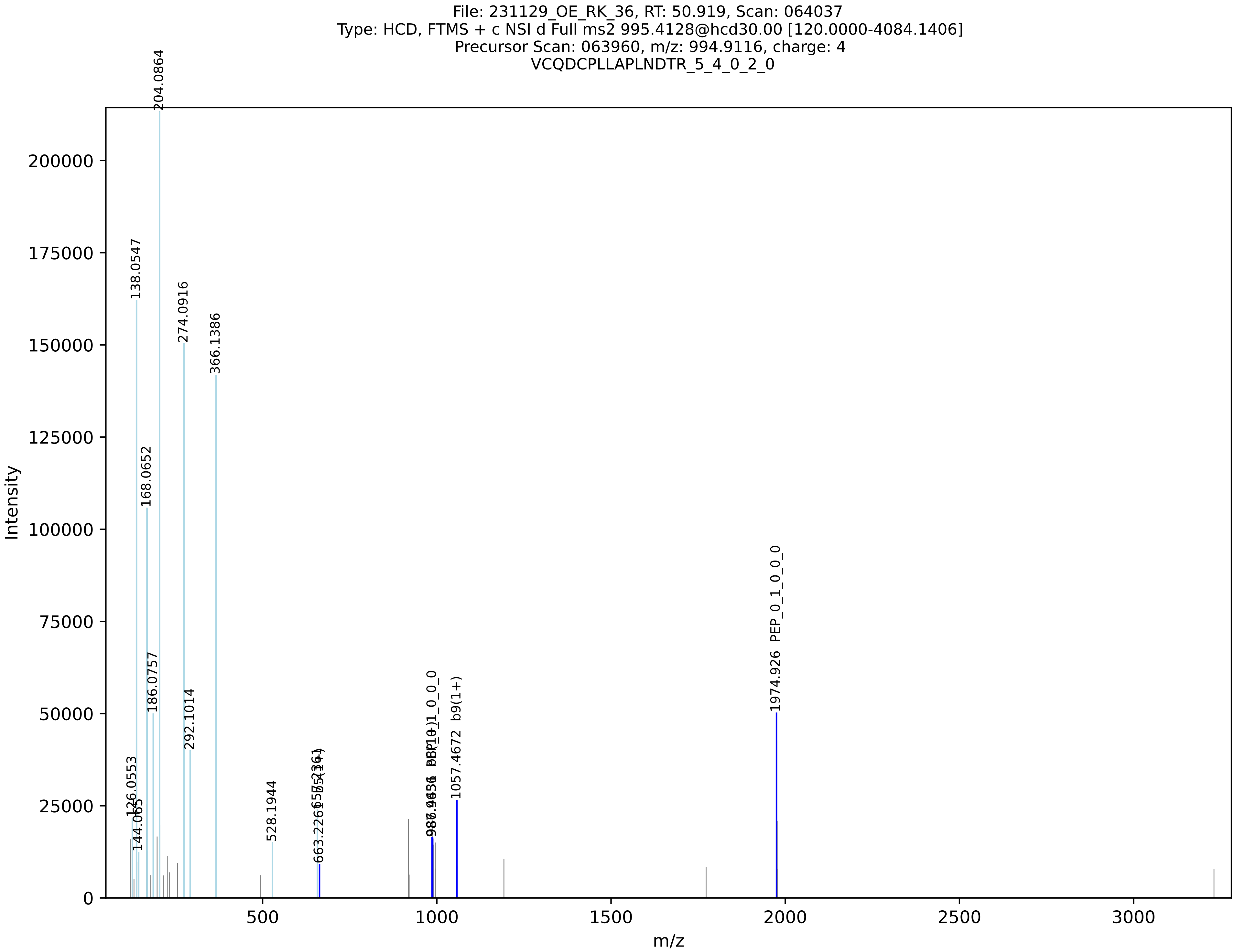

File: 231129_OE_RK_36, RT: 50.919, Scan: 064037
Type: HCD, FTMS + c NSI d Full ms2 995.4128@hcd30.00 [120.0000-4084.1406]
Precursor Scan: 063960, m/z: 994.9116, charge: 4
VCQDCPLLAPLNDTR_5_4_0_2_0
Intensity
m/z
204.0864
138.0547
274.0916
366.1386
168.0652
186.0757
292.1014
126.0553
144.065
528.1944
663.2261
1057.4672 b9(1+)
1974.926 PEP_0_1_0_0_0

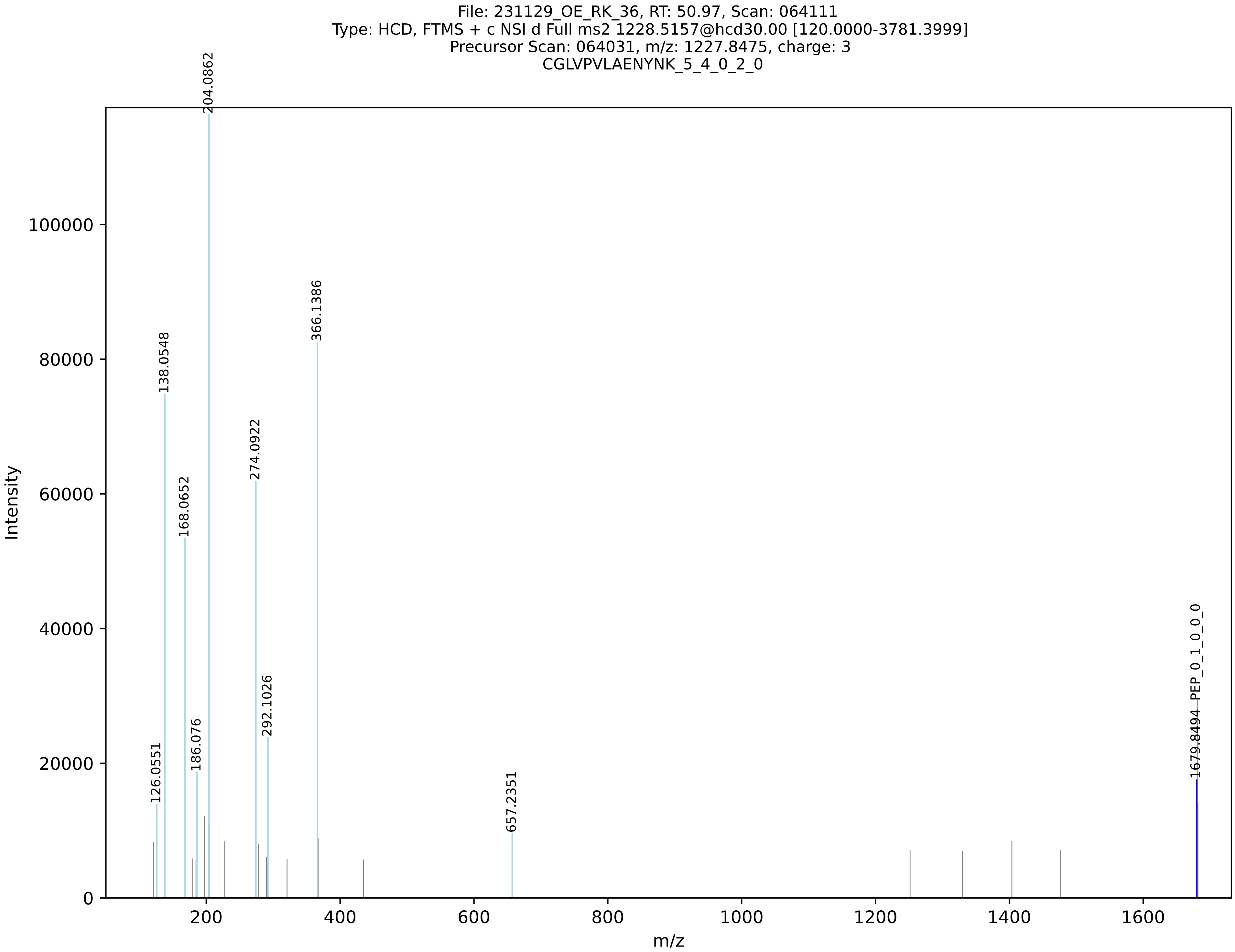
File: 231129_OE_RK_36, RT: 50.97, Scan: 064111
Type: HCD, FTMS + c NSI d Full ms2 1228.5157@hcd30.00 [120.0000-3781.3999]
Precursor Scan: 064031, m/z: 1227.8475, charge: 3
CGLVPVLAENYNK_5_4_0_2_0
204.0862
366.1386
138.0548
274.0922
168.0652
292.1026
186.076
126.0551
657.2351
1679.8494_PEP_0_1_0_0_0
Intensity
m/z
0
20000
40000
60000
80000
100000
200
400
600
800
1000
1200
1400
1600

File: 231129_OE_RK_36, RT: 51.017, Scan: 064179
Type: HCD, FTMS + c NSI d Full ms2 1326.8813@hcd30.00 [120.0000-4082.3989]
Precursor Scan: 064104, m/z: 1326.2131, charge: 3
VCQDCPLLAPLNDTR_5_4_0_2_0

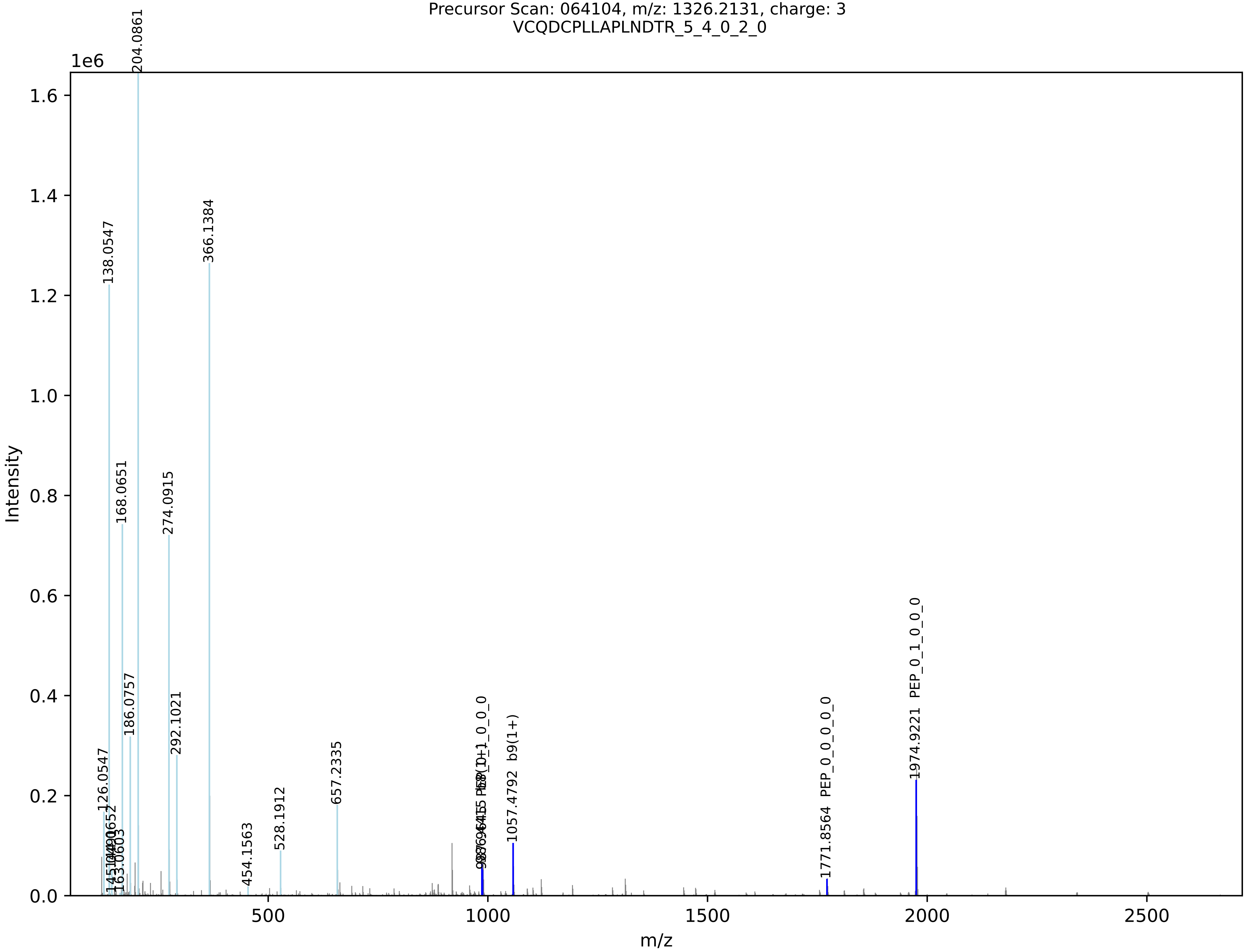

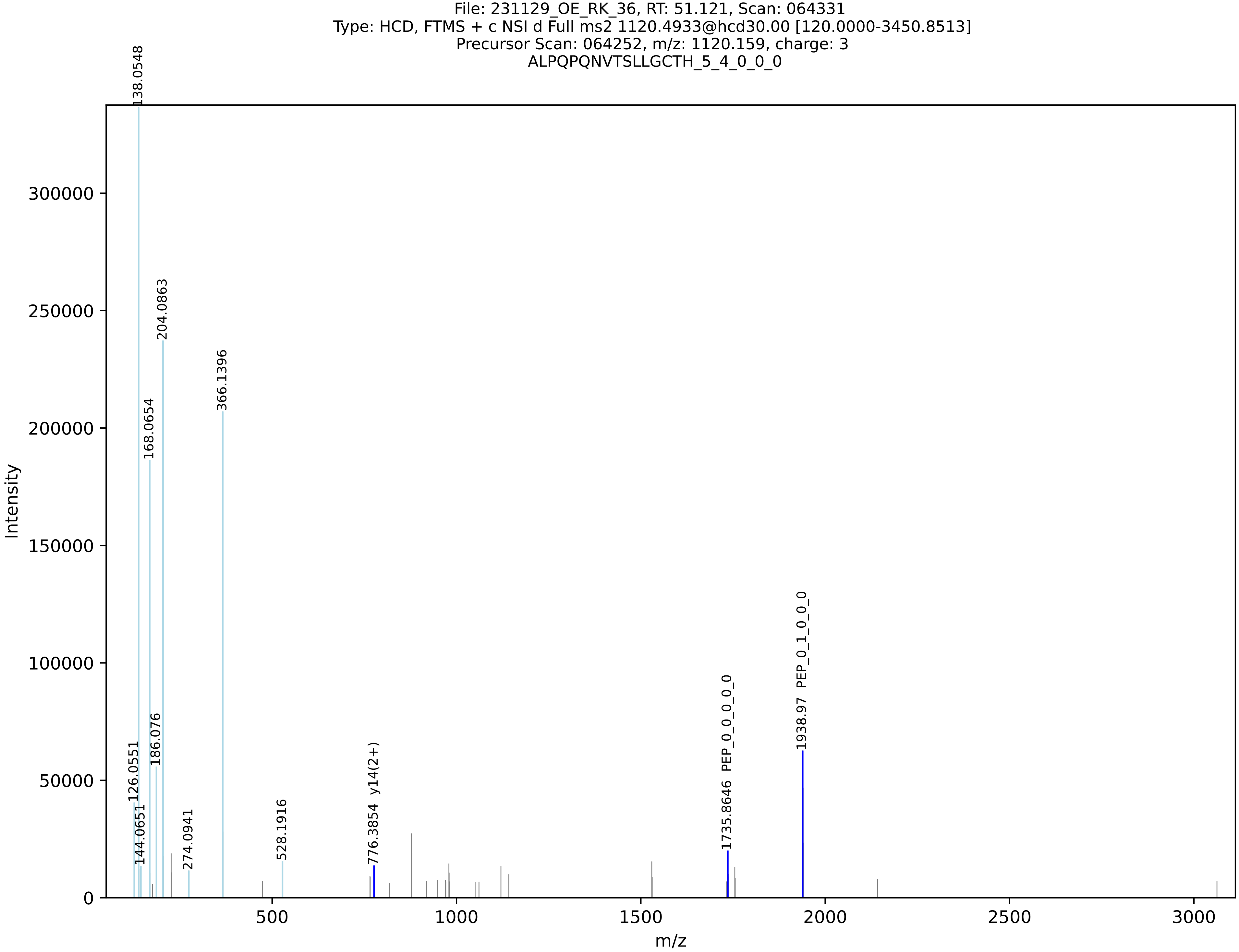

File: 231129_OE_RK_36, RT: 51.121, Scan: 064331
Type: HCD, FTMS + c NSI d Full ms2 1120.4933@hcd30.00 [120.0000-3450.8513]
Precursor Scan: 064252, m/z: 1120.159, charge: 3
ALPQPQNVTSLLGCTH_5_4_0_0_0
138.0548
204.0863
366.1396
168.0654
186.076
126.0551
144.0651
274.0941
528.1916
776.3854 y14(2+)
1735.8646 PEP_0_0_0_0_0
1938.97 PEP_0_1_0_0_0
Intensity
m/z

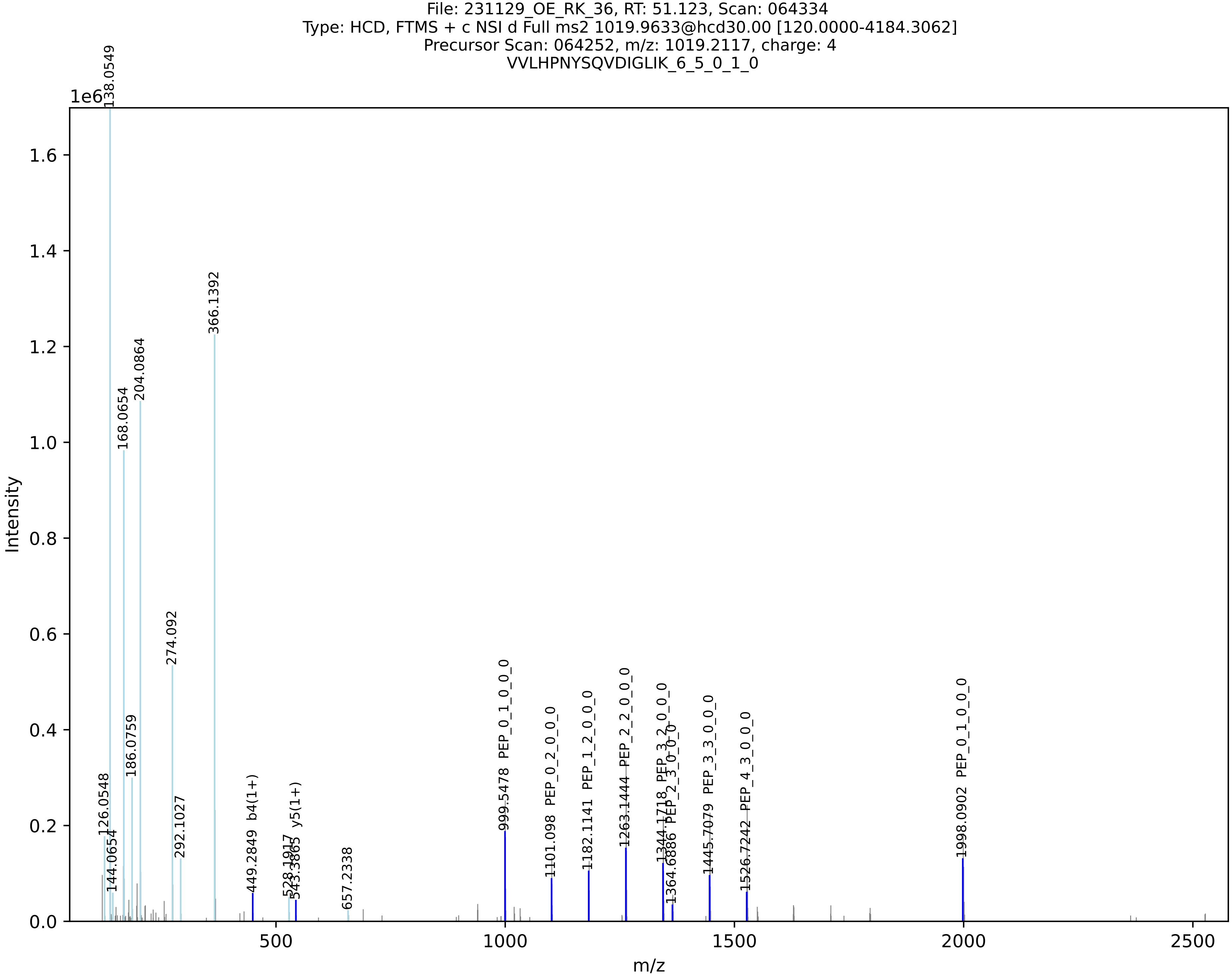
File: 231129_OE_RK_36, RT: 51.123, Scan: 064334
Type: HCD, FTMS + c NSI d Full ms2 1019.9633@hcd30.00 [120.0000-4184.3062]
Precursor Scan: 064252, m/z: 1019.2117, charge: 4
VVLHPNYSQVDIGLIK_6_5_0_1_0
1e6
Intensity
m/z
138.0549
366.1392
204.0864
168.0654
274.092
186.0759
126.0548
144.0654
292.1027
449.2849 b4(1+)
528.1917
543.3865 y5(1+)
657.2338
999.5478 PEP_0_1_0_0_
1101.098 PEP_0_2_0_0_
1182.1141 PEP_1_2_0_0_
1263.1444 PEP_2_2_0_0_
1344.1718 PEP_3_2_0_0_
1364.6886 PEP_2_3_0_0_
1445.7079 PEP_3_3_0_0_
1526.7242 PEP_4_3_0_0_
1998.0902 PEP_0_1_0_0_

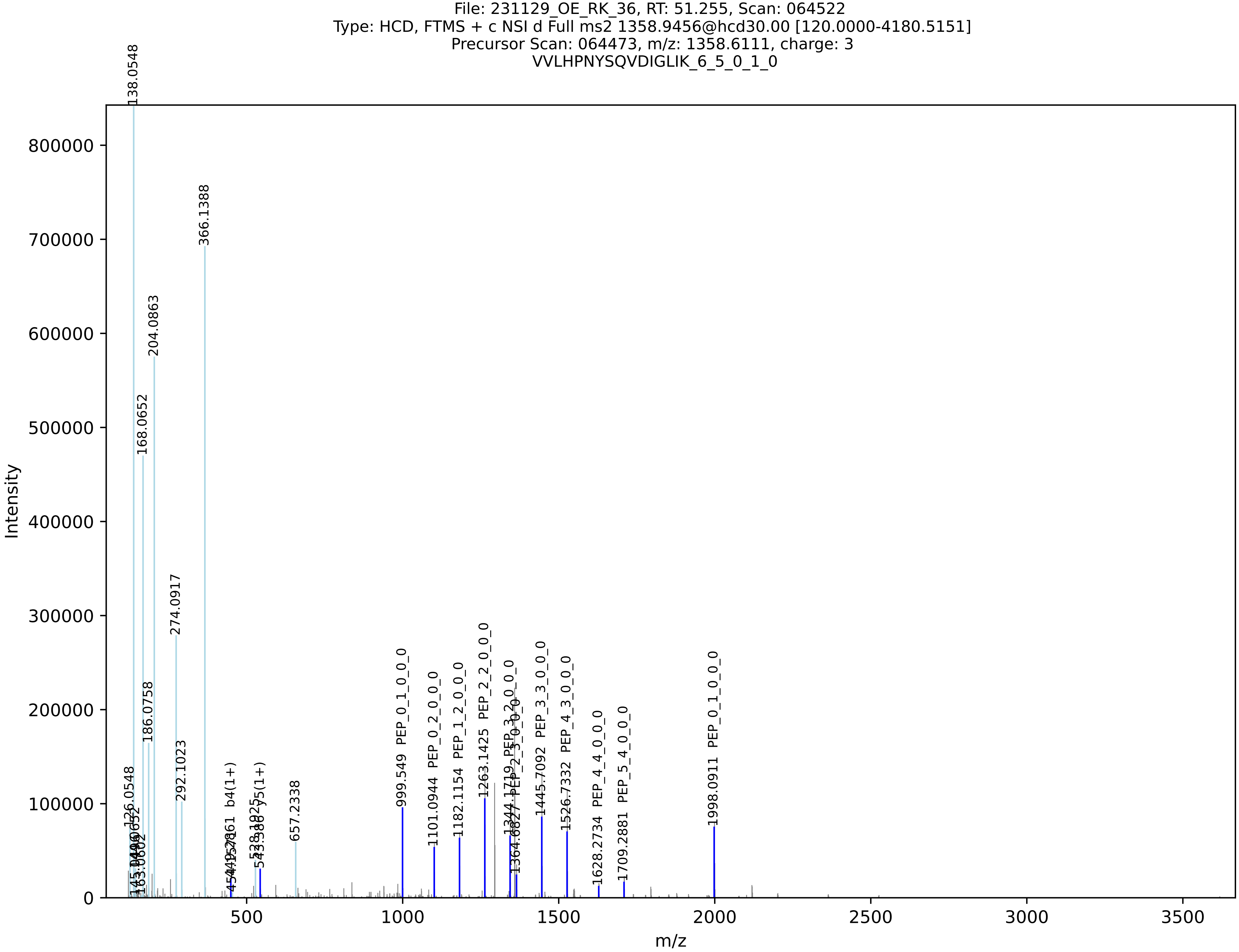
File: 231129_OE_RK_36, RT: 51.255, Scan: 064522
Type: HCD, FTMS + c NSI d Full ms2 1358.9456@hcd30.00 [120.0000-4180.5151]
Precursor Scan: 064473, m/z: 1358.6111, charge: 3
VVLHPNYSQVDIGLIK_6_5_0_1_0
Intensity
m/z
138.0548
366.1388
204.0863
168.0652
274.0917
186.0758
292.1023
126.0548
657.2338
b4(1+)
y5(1+)
999.549 PEP_0_1_0_0_0
1101.0944 PEP_0_2_0_0_0
1182.1154 PEP_1_2_0_0_0
1263.1425 PEP_2_2_0_0_0
1344.1719 PEP_3_2_0_0_0
1445.7092 PEP_3_3_0_0_0
1526.7332 PEP_4_3_0_0_0
1628.2734 PEP_4_4_0_0_0
1709.2881 PEP_5_4_0_0_0
1998.0911 PEP_0_1_0_0_0

File: 231129_OE_RK_36, RT: 51.305, Scan: 064596
Type: HCD, FTMS + c NSI d Full ms2 1019.4603@hcd30.00 [120.0000-4182.2539]
Precursor Scan: 064521, m/z: 1019.211, charge: 4
VVLHPNYSQVDIGLIK_6_5_0_1_0

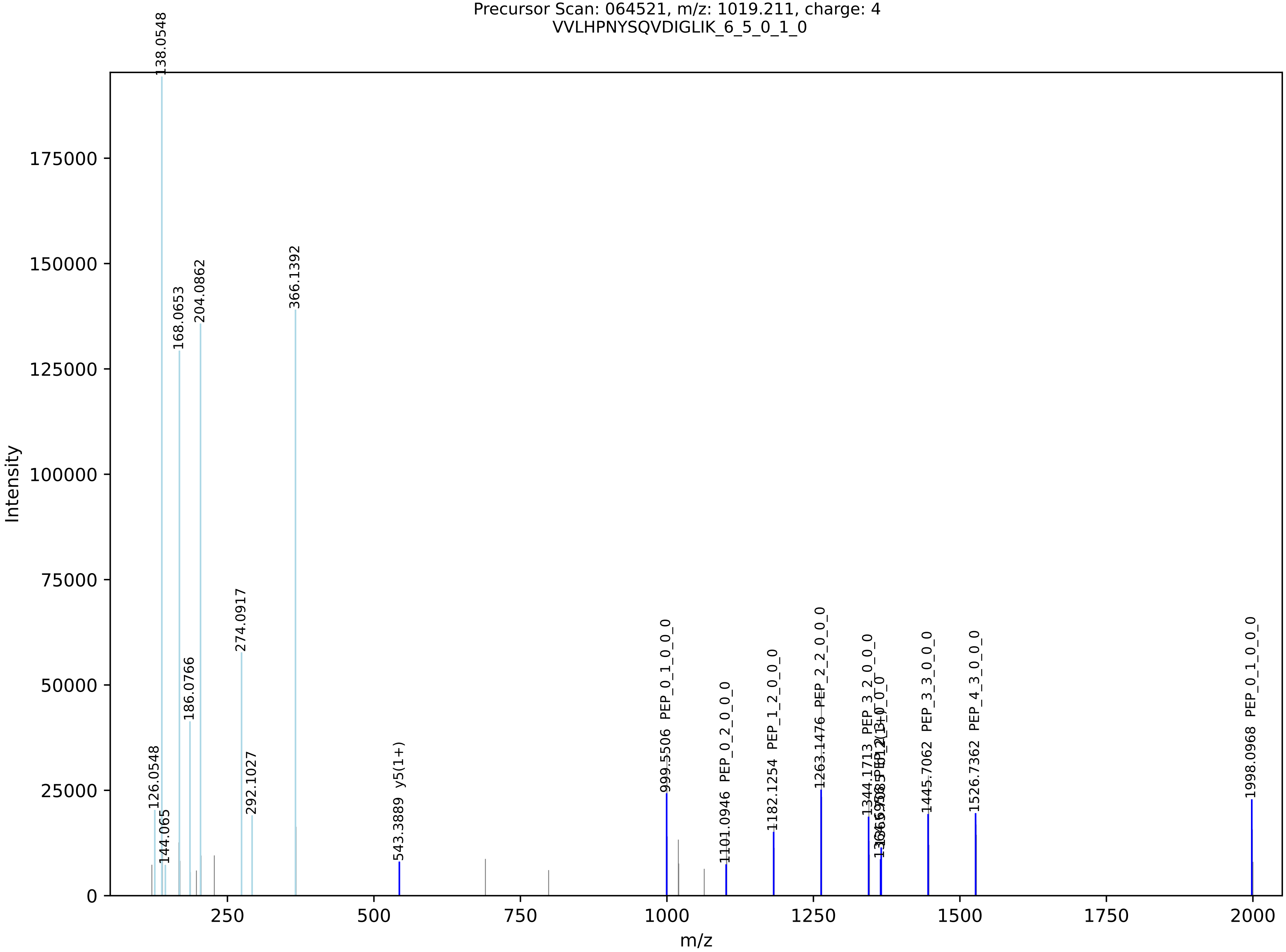

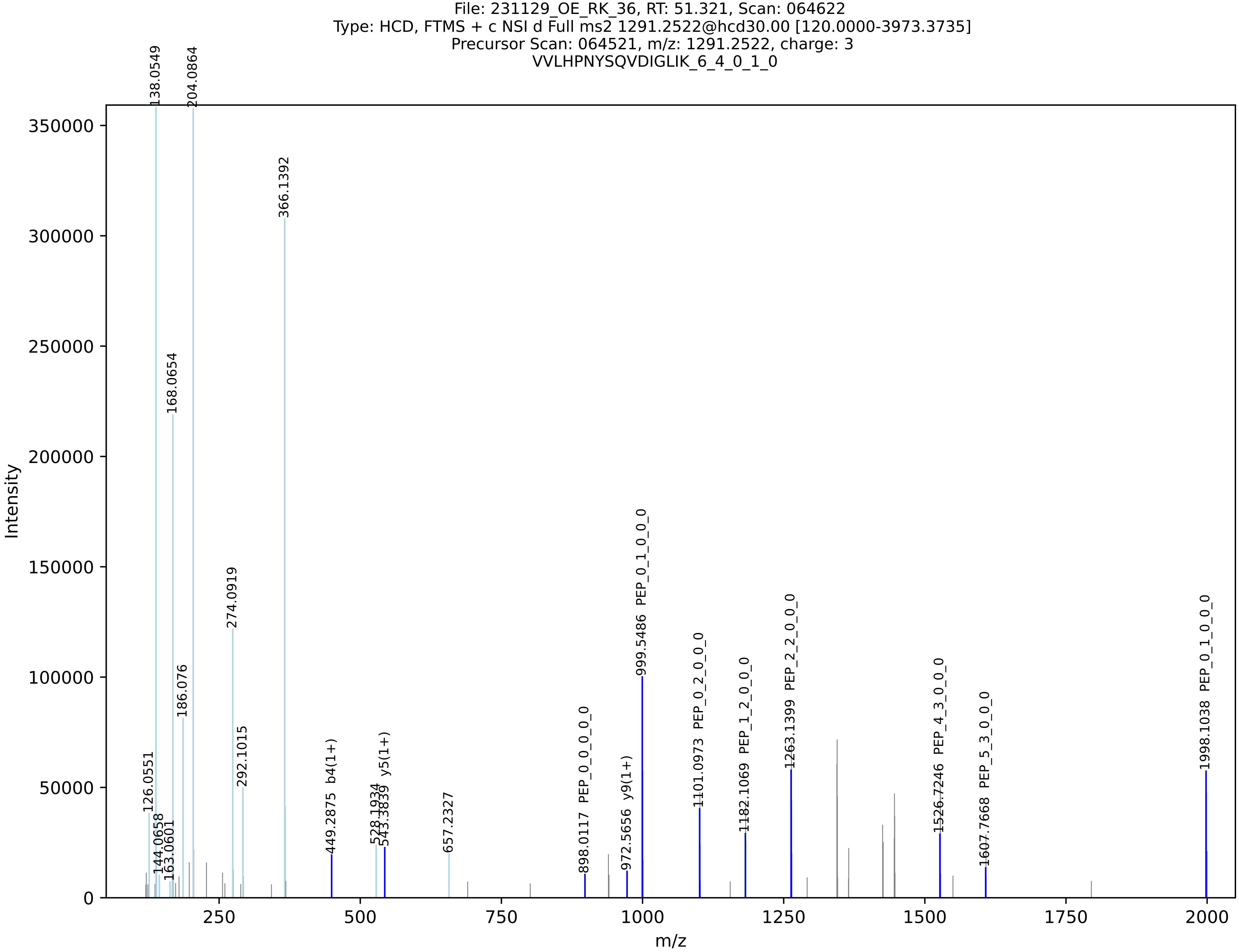

File: 231129_OE_RK_36, RT: 51.321, Scan: 064622
Type: HCD, FTMS + c NSI d Full ms2 1291.2522@hcd30.00 [120.0000-3973.3735]
Precursor Scan: 064521, m/z: 1291.2522, charge: 3
VVLHPNYSQVDIGLIK_6_4_0_1_0
Intensity
m/z
138.0549
204.0864
366.1392
168.0654
274.0919
186.076
292.1015
126.0551
144.0658
163.0601
449.2875 b4(1+)
528.1934
543.3839 y5(1+)
657.2327
898.0117 PEP_0_0_0_0_0
972.5656 y9(1+)
999.5486 PEP_0_1_0_0_0
1101.0973 PEP_0_2_0_0_0
1182.1069 PEP_1_2_0_0_0
1263.1399 PEP_2_2_0_0_0
1526.7246 PEP_4_3_0_0_0
1607.7668 PEP_5_3_0_0_0
1998.1038 PEP_0_1_0_0_0

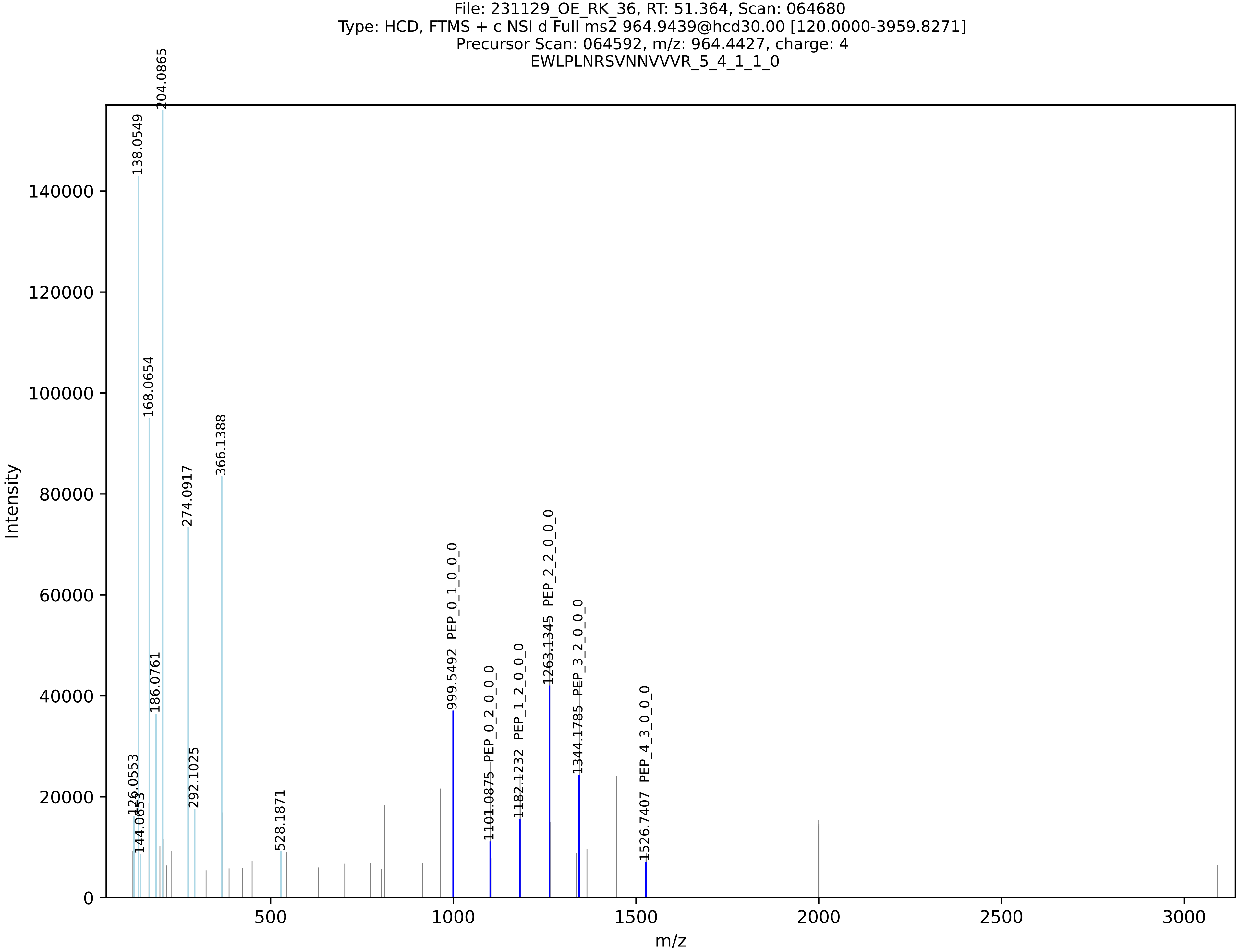

File: 231129_OE_RK_36, RT: 51.364, Scan: 064680
Type: HCD, FTMS + c NSI d Full ms2 964.9439@hcd30.00 [120.0000-3959.8271]
Precursor Scan: 064592, m/z: 964.4427, charge: 4
EWLPLNRSVNNVVVR_5_4_1_1_0
Intensity
m/z
204.0865
138.0549
168.0654
366.1388
274.0917
186.0761
292.1025
126.0553
144.0653
528.1871
999.5492 PEP_0_1_0_0_0
1101.0875 PEP_0_2_0_0_0
1182.1232 PEP_1_2_0_0_0
1263.1345 PEP_2_2_0_0_0
1344.1785 PEP_3_2_0_0_0
1526.7407 PEP_4_3_0_0_0

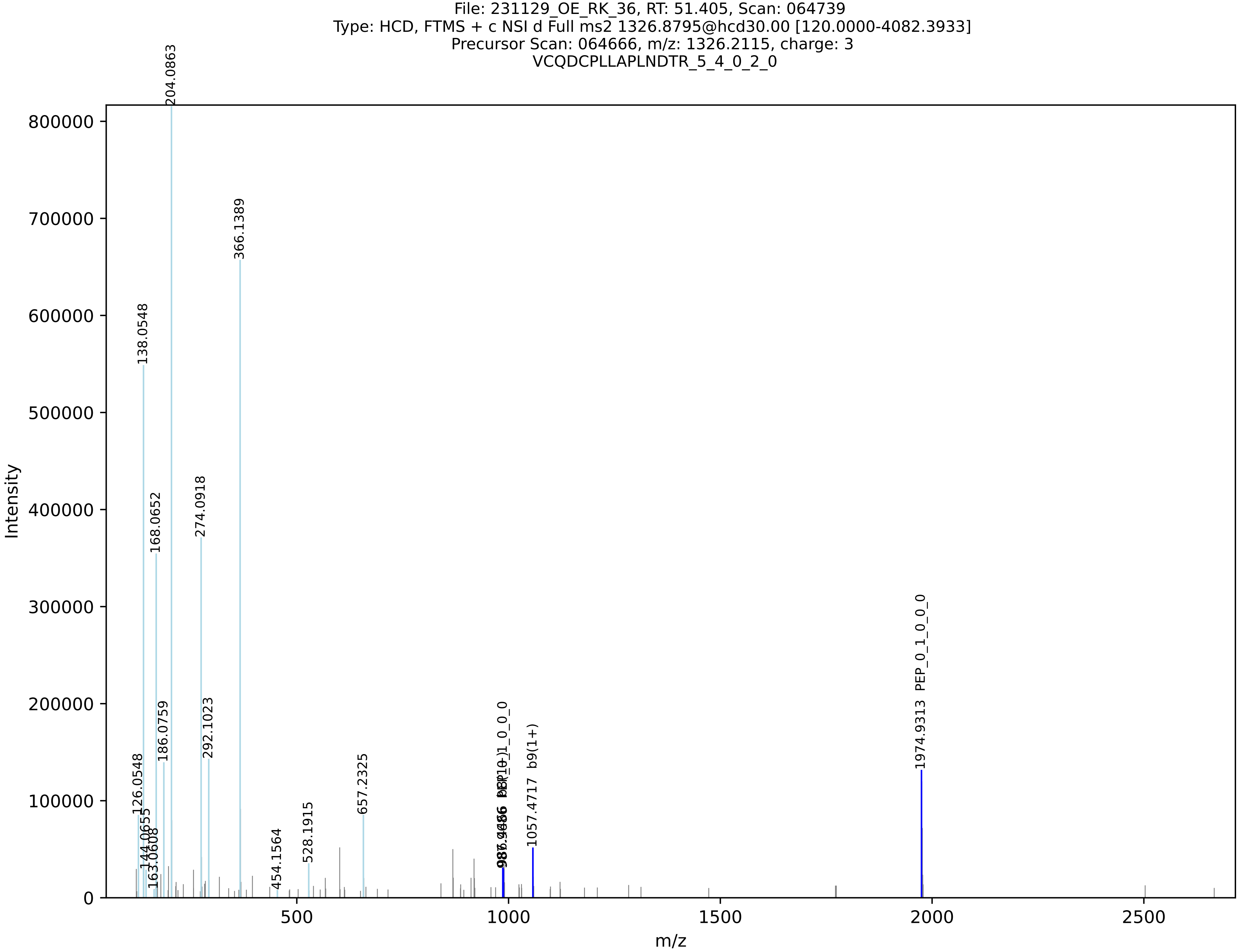
File: 231129_OE_RK_36, RT: 51.405, Scan: 064739
Type: HCD, FTMS + c NSI d Full ms2 1326.8795@hcd30.00 [120.0000-4082.3933]
Precursor Scan: 064666, m/z: 1326.2115, charge: 3
VCQDCPLLAPLNDTR_5_4_0_2_0
Intensity
m/z
204.0863
366.1389
138.0548
168.0652
274.0918
186.0759
292.1023
126.0548
144.0655
163.0608
454.1564
528.1915
657.2325
1057.4717 b9(1+)
1974.9313 PEP_0_1_0_0_0

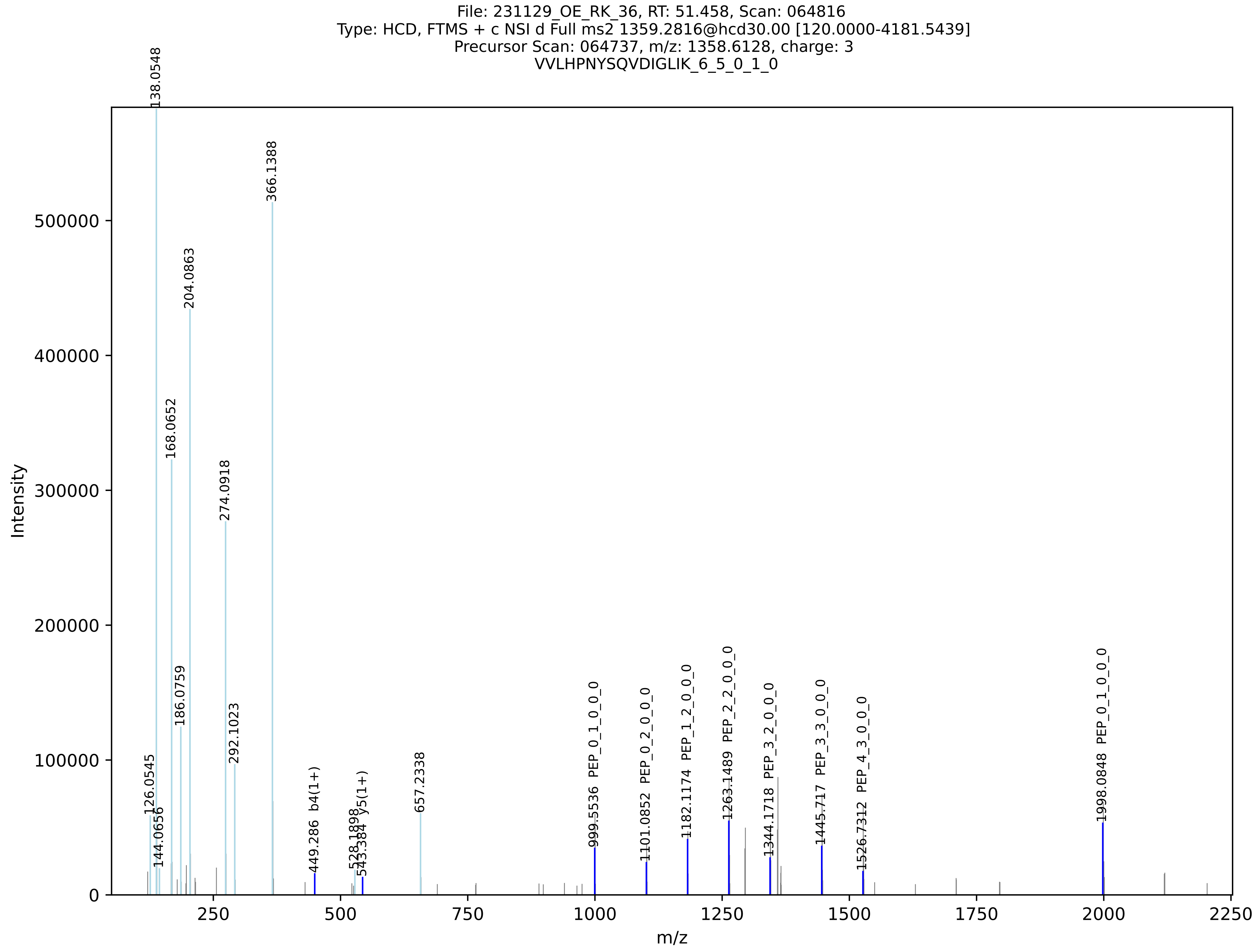
File: 231129_OE_RK_36, RT: 51.458, Scan: 064816
Type: HCD, FTMS + c NSI d Full ms2 1359.2816@hcd30.00 [120.0000-4181.5439]
Precursor Scan: 064737, m/z: 1358.6128, charge: 3
VVLHPNYSQVDIGLIK_6_5_0_1_0
138.0548
366.1388
204.0863
168.0652
274.0918
186.0759
292.1023
126.0545
144.0656
449.286 b4(1+)
528.1898
543.384 y5(1+)
657.2338
999.5536 PEP_0_1_0_0_0
1101.0852 PEP_0_2_0_0_0
1182.1174 PEP_1_2_0_0_0
1263.1489 PEP_2_2_0_0_0
1344.1718 PEP_3_2_0_0_0
1445.717 PEP_3_3_0_0_0
1526.7312 PEP_4_3_0_0_0
1998.0848 PEP_0_1_0_0_0
Intensity
m/z
0
100000
200000
300000
400000
500000
250
500
750
1000
1250
1500
1750
2000
2250

File: 231129_OE_RK_36, RT: 51.507, Scan: 064886
Type: HCD, FTMS + c NSI d Full ms2 1237.5685@hcd30.00 [120.0000-3809.1016]
Precursor Scan: 064811, m/z: 1236.9003, charge: 3
VVLHPNYSQVDIGLIK_5_4_0_1_0

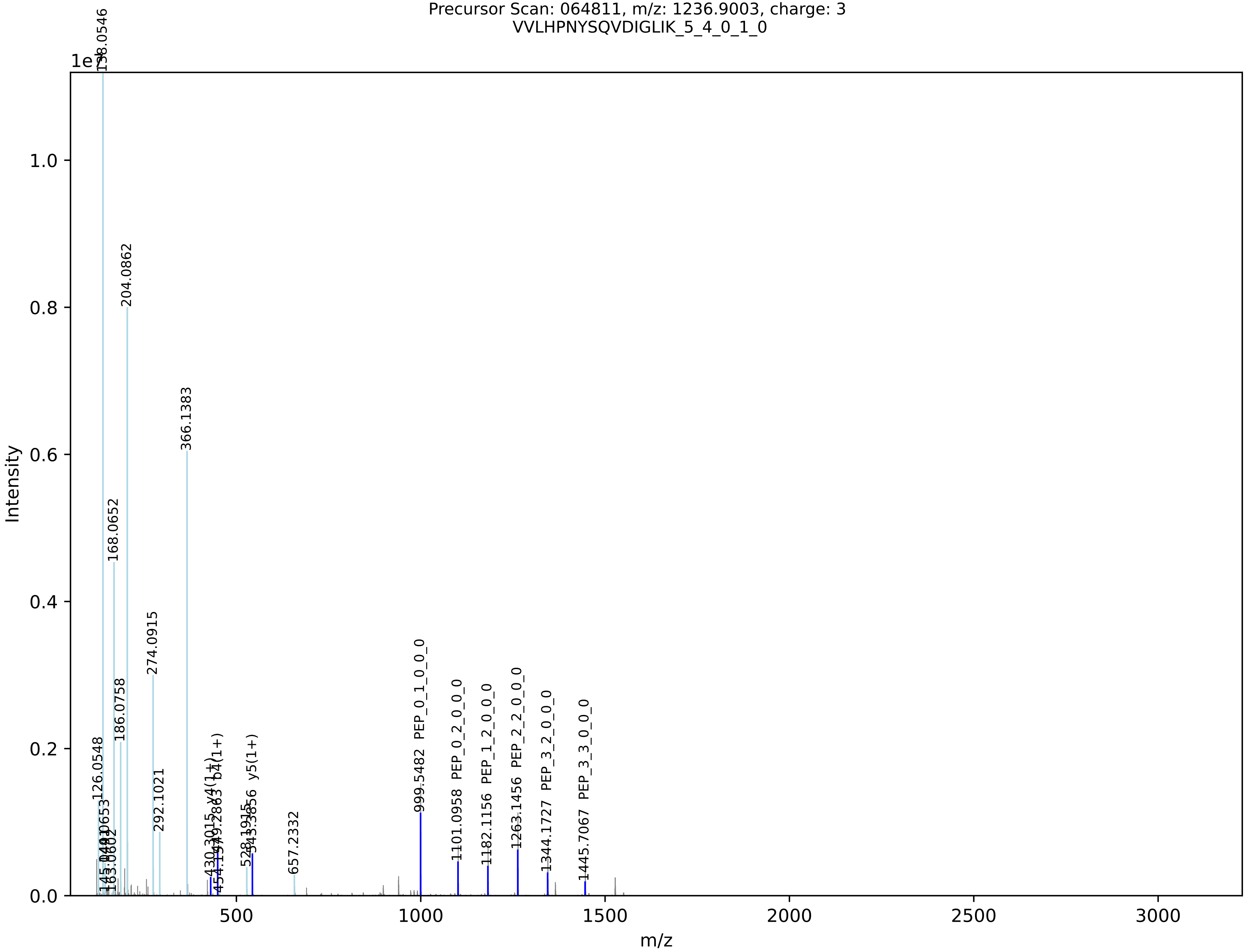

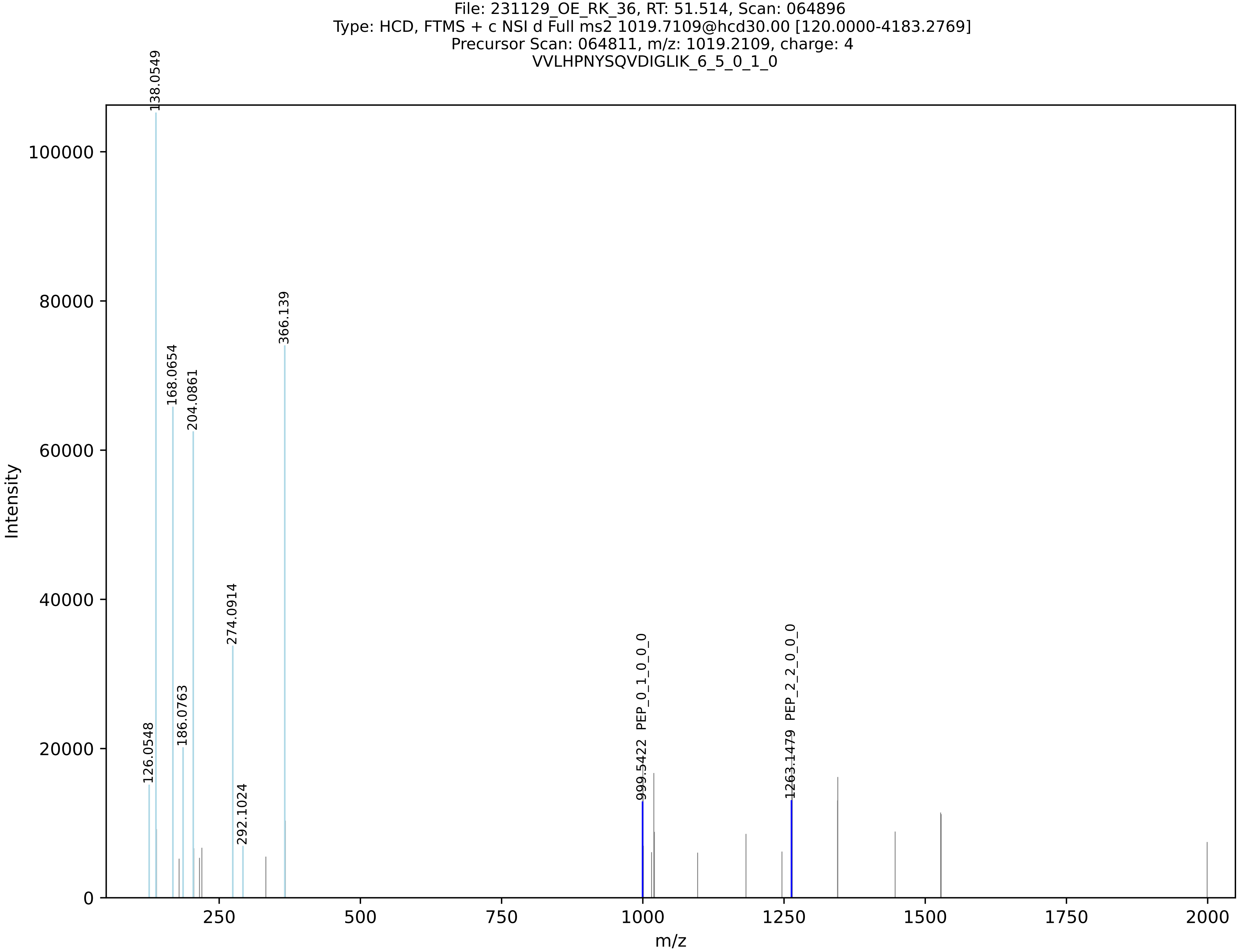
File: 231129_OE_RK_36, RT: 51.514, Scan: 064896
Type: HCD, FTMS + c NSI d Full ms2 1019.7109@hcd30.00 [120.0000-4183.2769]
Precursor Scan: 064811, m/z: 1019.2109, charge: 4
VVLHPNYSQVDIGLIK_6_5_0_1_0
138.0549
366.139
168.0654
204.0861
274.0914
186.0763
126.0548
292.1024
999.5422 PEP_0_1_0_0_0
1263.1479 PEP_2_2_0_0_0
Intensity
m/z
0
20000
40000
60000
80000
100000
250
500
750
1000
1250
1500
1750
2000

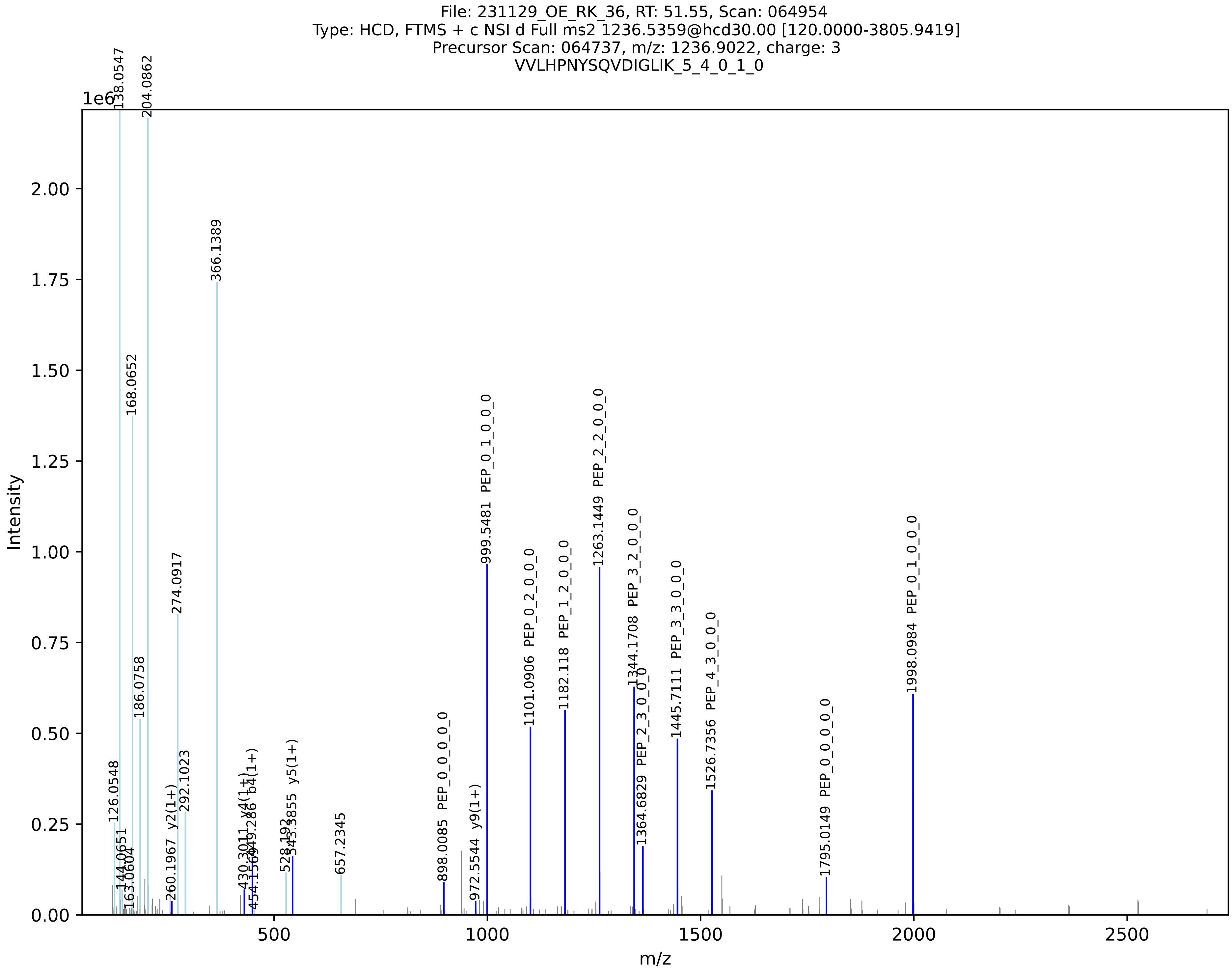

File: 231129_OE_RK_36, RT: 51.55, Scan: 064954
Type: HCD, FTMS + c NSI d Full ms2 1236.5359@hcd30.00 [120.0000-3805.9419]
Precursor Scan: 064737, m/z: 1236.9022, charge: 3
VVLHPNYSQVDIGLIK_5_4_0_1_0
1e6
Intensity
m/z
138.0547
204.0862
366.1389
168.0652
186.0758
274.0917
292.1023
126.0548
144.0651
163.0604
260.1967 y2(1+)
430.3011 y4(1+)
449.286 b4(1+)
454.1569
528.192
543.3855 y5(1+)
657.2345
898.0085 PEP_0_0_0_0_0
972.5544 y9(1+)
999.5481 PEP_0_1_0_0_0
1101.0906 PEP_0_2_0_0_0
1182.118 PEP_1_2_0_0_0
1263.1449 PEP_2_2_0_0_0
1344.1708 PEP_3_2_0_0_0
1364.6829 PEP_2_3_0_0_0
1445.7111 PEP_3_3_0_0_0
1526.7356 PEP_4_3_0_0_0
1795.0149 PEP_0_0_0_0_0
1998.0984 PEP_0_1_0_0_0

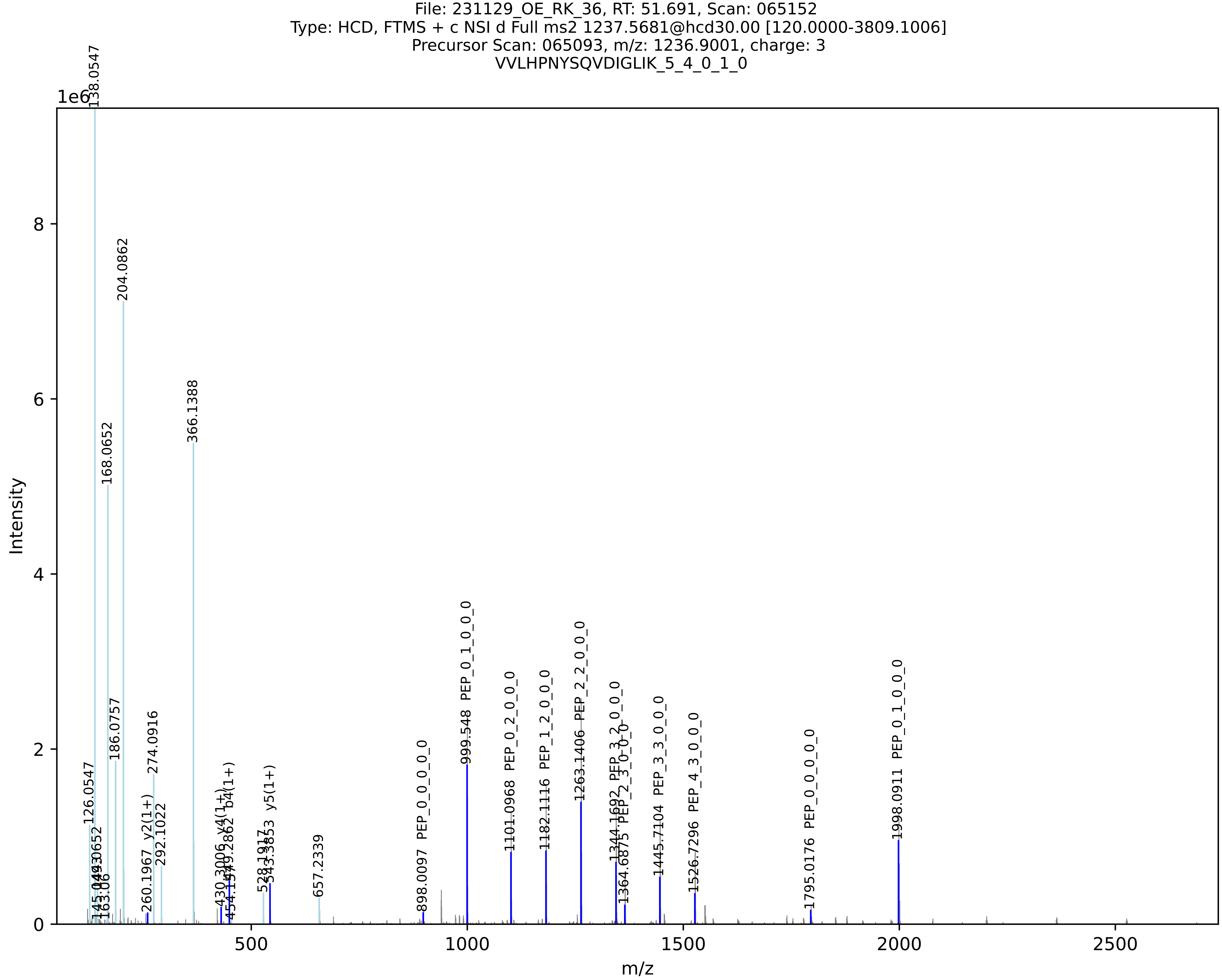
File: 231129_OE_RK_36, RT: 51.691, Scan: 065152
Type: HCD, FTMS + c NSI d Full ms2 1237.5681@hcd30.00 [120.0000-3809.1006]
Precursor Scan: 065093, m/z: 1236.9001, charge: 3
VVLHPNYSQVDIGLIK_5_4_0_1_0
1e6
Intensity
m/z
138.0547
204.0862
366.1388
168.0652
186.0757
274.0916
126.0547
292.1022
260.1967 y2(1+)
163.06
543.3853 y5(1+)
528.1917
657.2339
898.0097 PEP_0_0_0_0_0
999.548 PEP_0_1_0_0_0
1101.0968 PEP_0_2_0_0_0
1182.1116 PEP_1_2_0_0_0
1263.1406 PEP_2_2_0_0_0
1344.1692 PEP_3_2_0_0_0
1364.6875 PEP_2_3_0_0_0
1445.7104 PEP_3_3_0_0_0
1526.7296 PEP_4_3_0_0_0
1795.0176 PEP_0_0_0_0_0
1998.0911 PEP_0_1_0_0_0

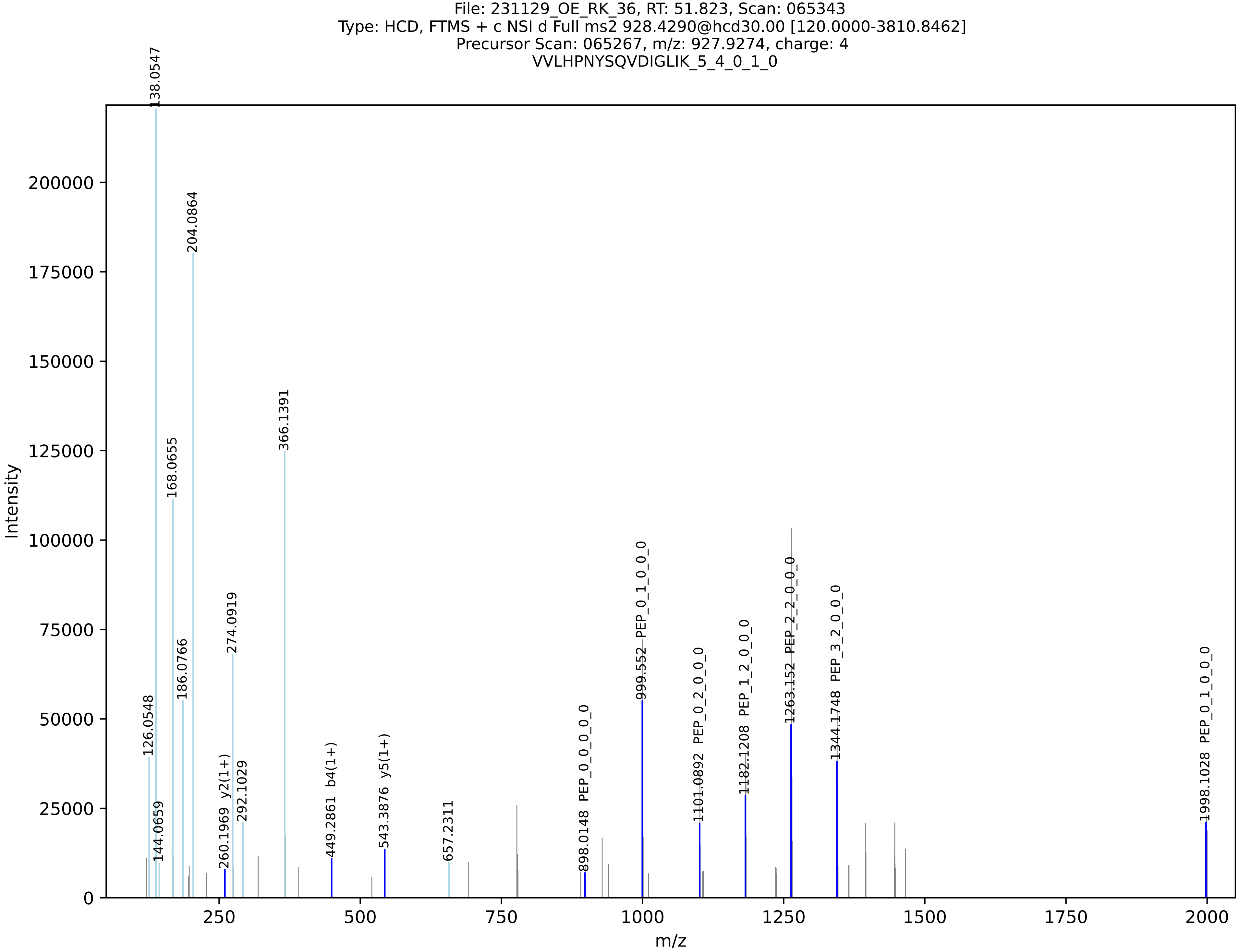

File: 231129_OE_RK_36, RT: 51.823, Scan: 065343
Type: HCD, FTMS + c NSI d Full ms2 928.4290@hcd30.00 [120.0000-3810.8462]
Precursor Scan: 065267, m/z: 927.9274, charge: 4
VVLHPNYSQVDIGLIK_5_4_0_1_0
Intensity
m/z
138.0547
204.0864
366.1391
168.0655
274.0919
186.0766
126.0548
144.0659
260.1969 y2(1+)
292.1029
449.2861 b4(1+)
543.3876 y5(1+)
657.2311
898.0148 PEP_0_0_0_0_0
999.552 PEP_0_1_0_0_0
1101.0892 PEP_0_2_0_0_0
1182.1208 PEP_1_2_0_0_0
1263.152 PEP_2_2_0_0_0
1344.1748 PEP_3_2_0_0_0
1998.1028 PEP_0_1_0_0_0

File: 231129_OE_RK_36, RT: 52.275, Scan: 065998
Type: HCD, FTMS + c NSI d Full ms2 1098.7744@hcd30.00 [120.0000-4505.8560]
Precursor Scan: 065917, m/z: 1098.2733, charge: 4
LVLSSEKTVLTPATNHMGNVTFTIPANR_6_2_0_0_0

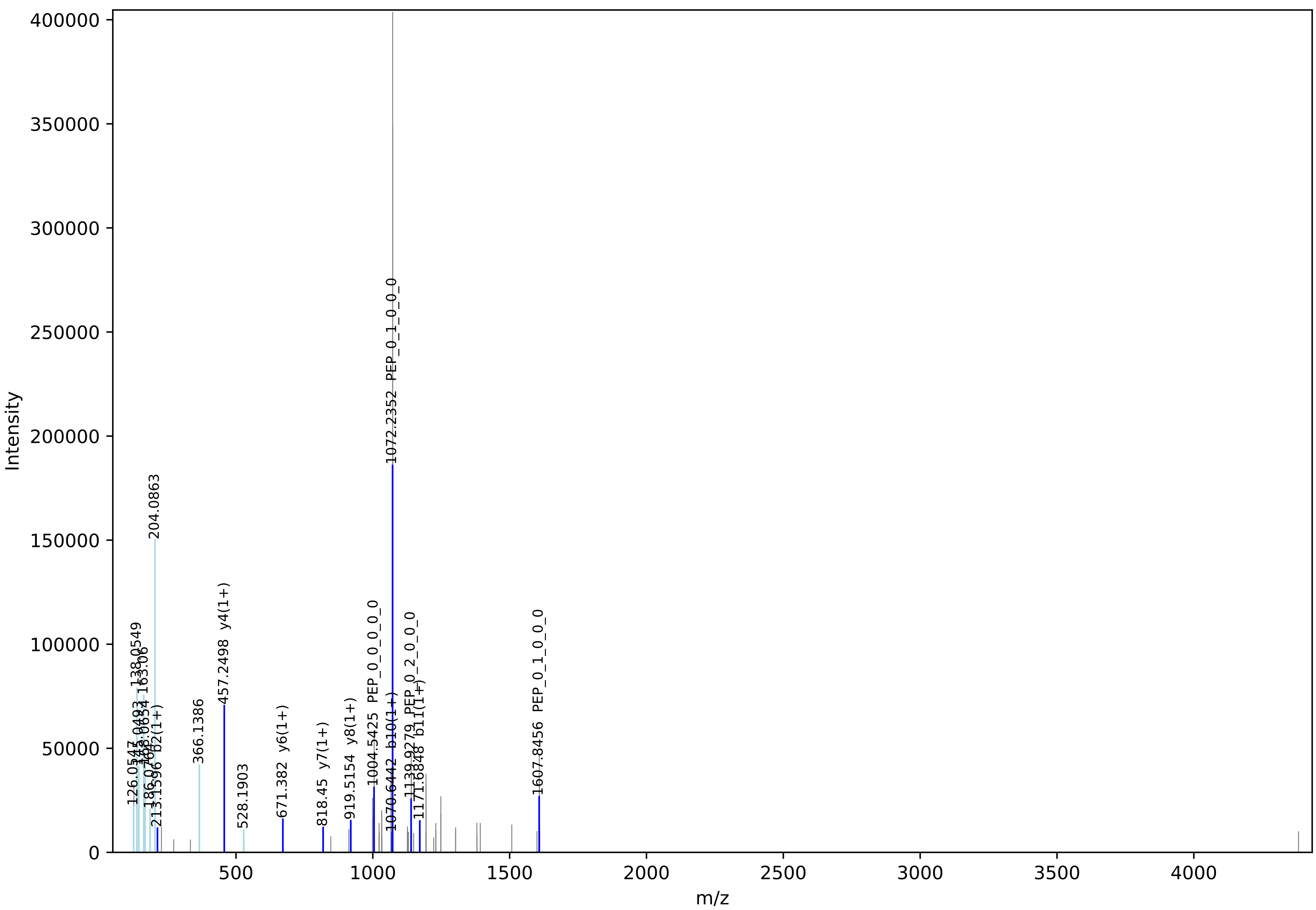

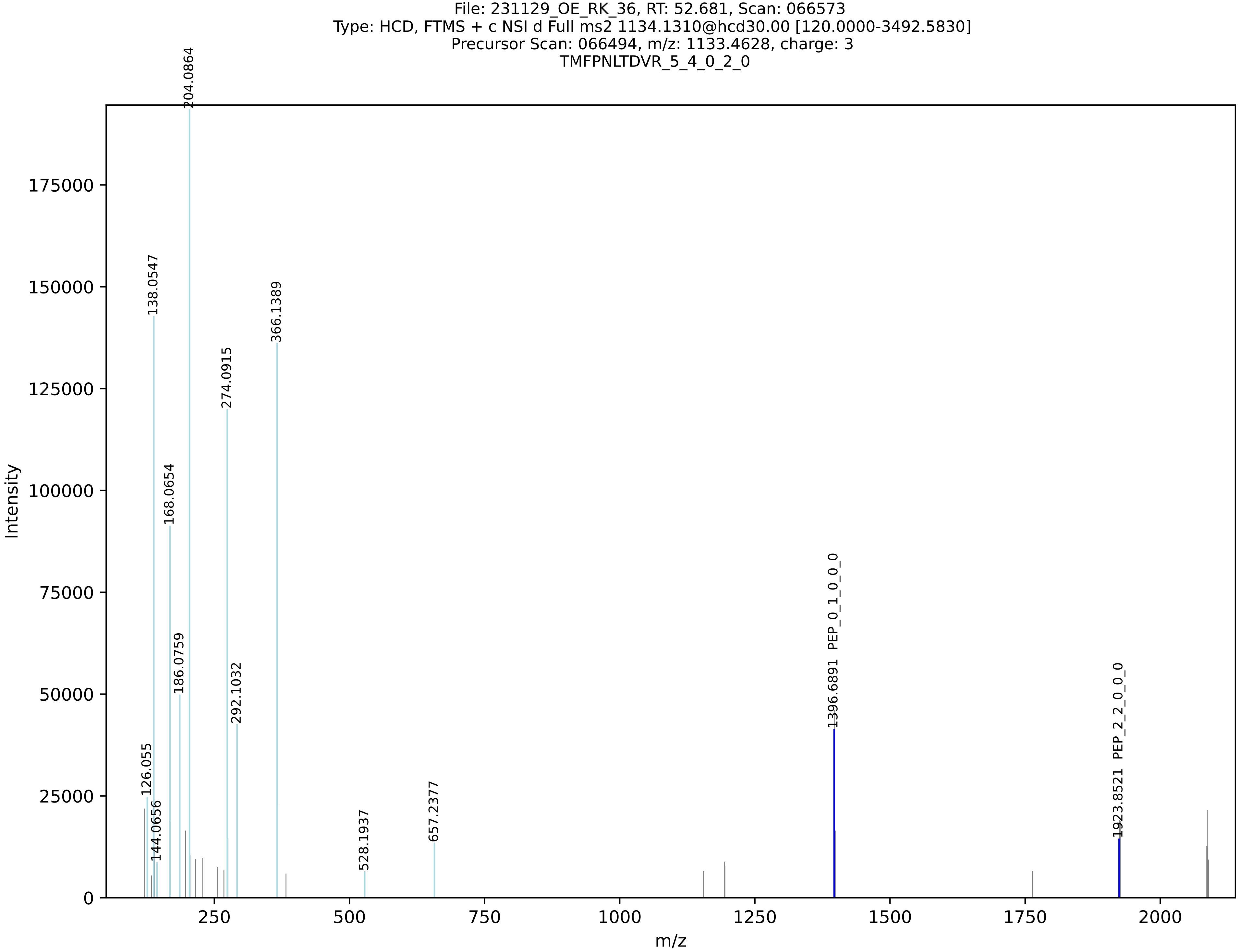

File: 231129_OE_RK_36, RT: 52.681, Scan: 066573
Type: HCD, FTMS + c NSI d Full ms2 1134.1310@hcd30.00 [120.0000-3492.5830]
Precursor Scan: 066494, m/z: 1133.4628, charge: 3
TMFPNLTDVR_5_4_0_2_0
Intensity
m/z
204.0864
138.0547
366.1389
274.0915
168.0654
186.0759
292.1032
126.055
144.0656
528.1937
657.2377
1396.6891 PEP_0_1_0_0_
1923.8521 PEP_2_2_0_0_0
0
25000
50000
75000
100000
125000
150000
175000
250
500
750
1000
1250
1500
1750
2000

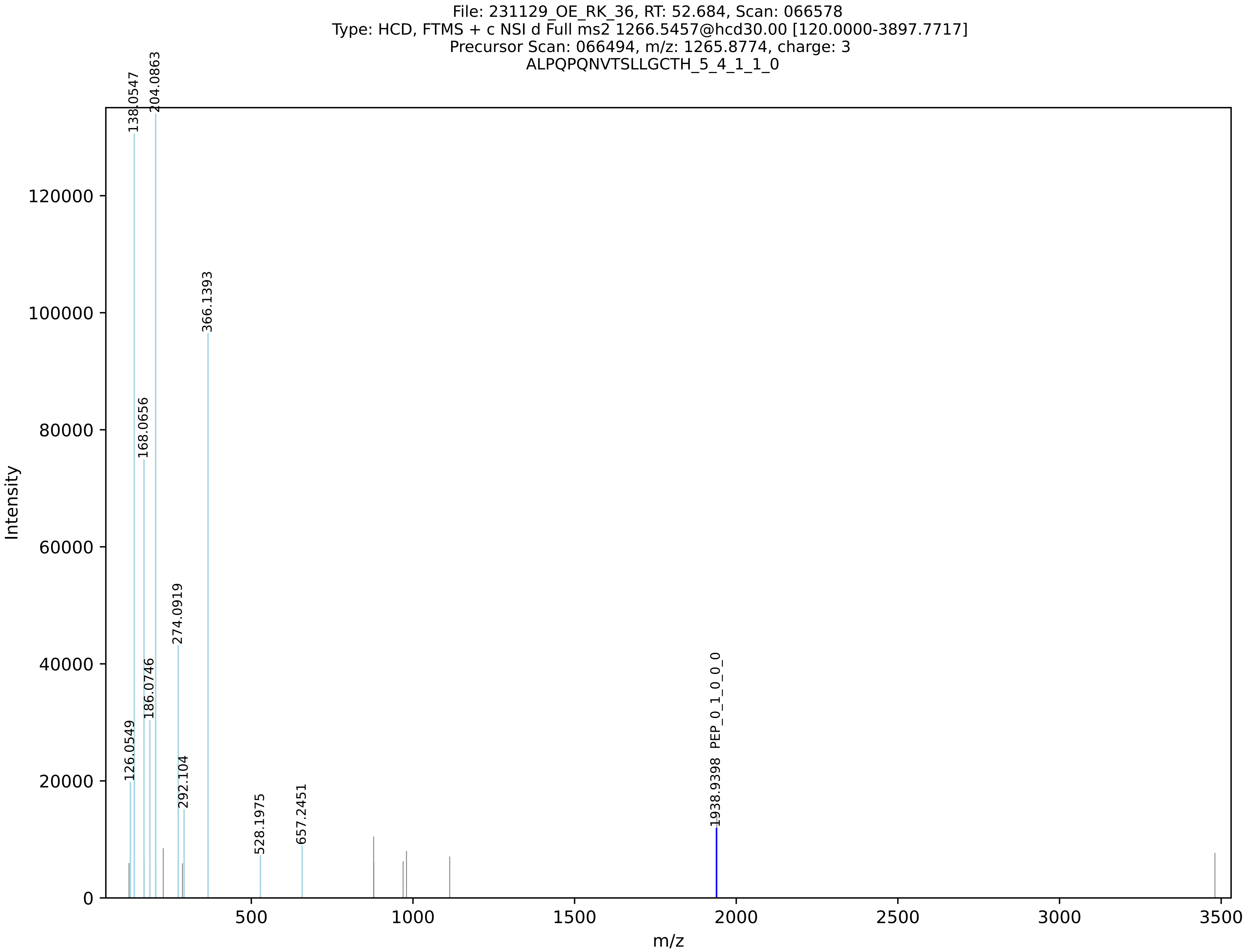
File: 231129_OE_RK_36, RT: 52.684, Scan: 066578
Type: HCD, FTMS + c NSI d Full ms2 1266.5457@hcd30.00 [120.0000-3897.7717]
Precursor Scan: 066494, m/z: 1265.8774, charge: 3
ALPQPQNVTSLLGCTH_5_4_1_1_0
138.0547
204.0863
366.1393
168.0656
274.0919
186.0746
126.0549
292.104
528.1975
657.2451
1938.9398 PEP_0_1_0_0_0
Intensity
m/z
0
20000
40000
60000
80000
100000
120000
500
1000
1500
2000
2500
3000
3500

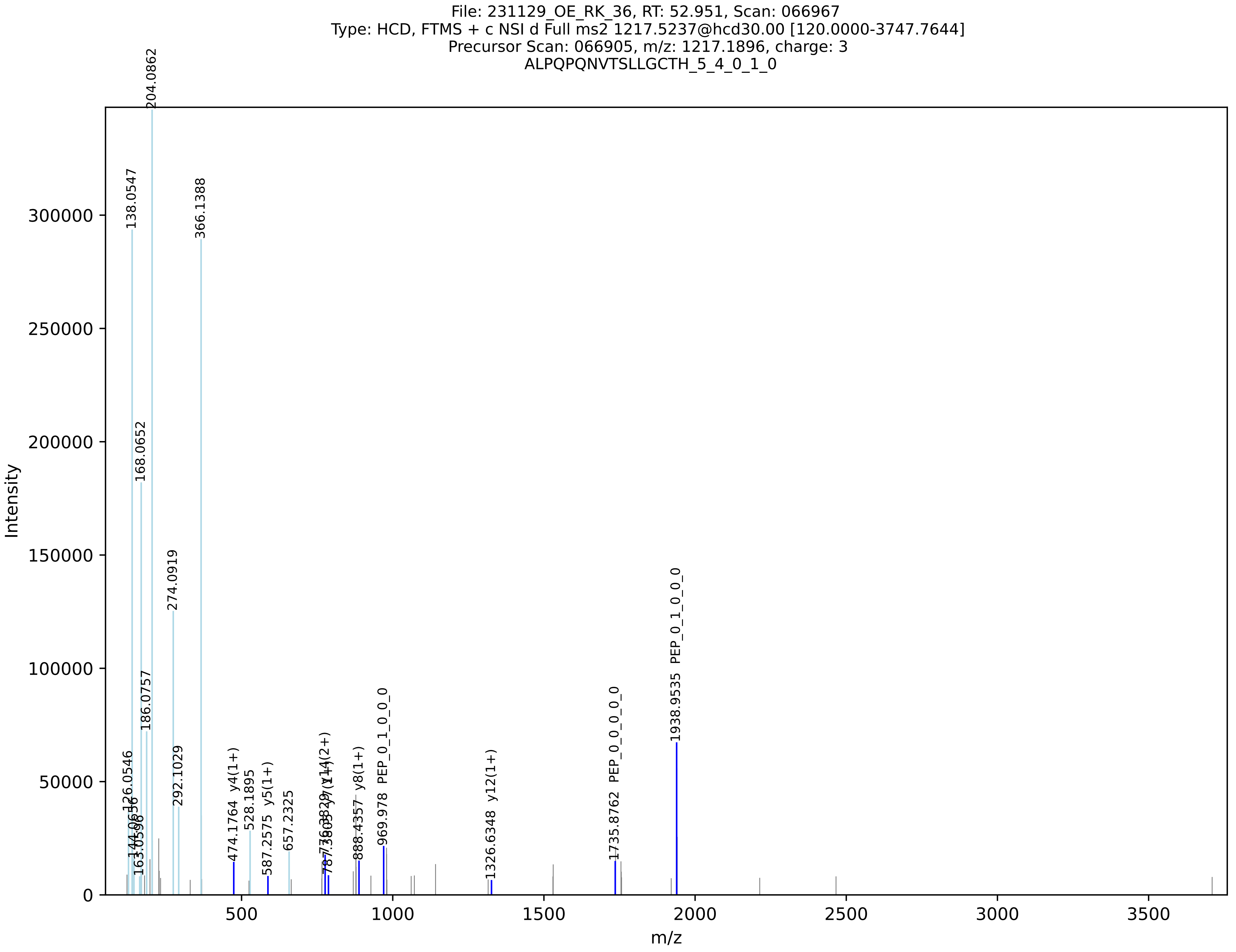

File: 231129_OE_RK_36, RT: 52.951, Scan: 066967
Type: HCD, FTMS + c NSI d Full ms2 1217.5237@hcd30.00 [120.0000-3747.7644]
Precursor Scan: 066905, m/z: 1217.1896, charge: 3
ALPQPQNVTSLLGCTH_5_4_0_1_0
Intensity
m/z
204.0862
138.0547
366.1388
168.0652
274.0919
186.0757
126.0546
144.0656
163.0598
292.1029
474.1764 y4(1+)
528.1895
587.2575 y5(1+)
657.2325
776.3829 y14(2+)
787.3803 y7(1+)
888.4357 y8(1+)
969.978 PEP_0_1_0_0_0_
1326.6348 y12(1+)
1735.8762 PEP_0_0_0_0_0_
1938.9535 PEP_0_1_0_0_0_

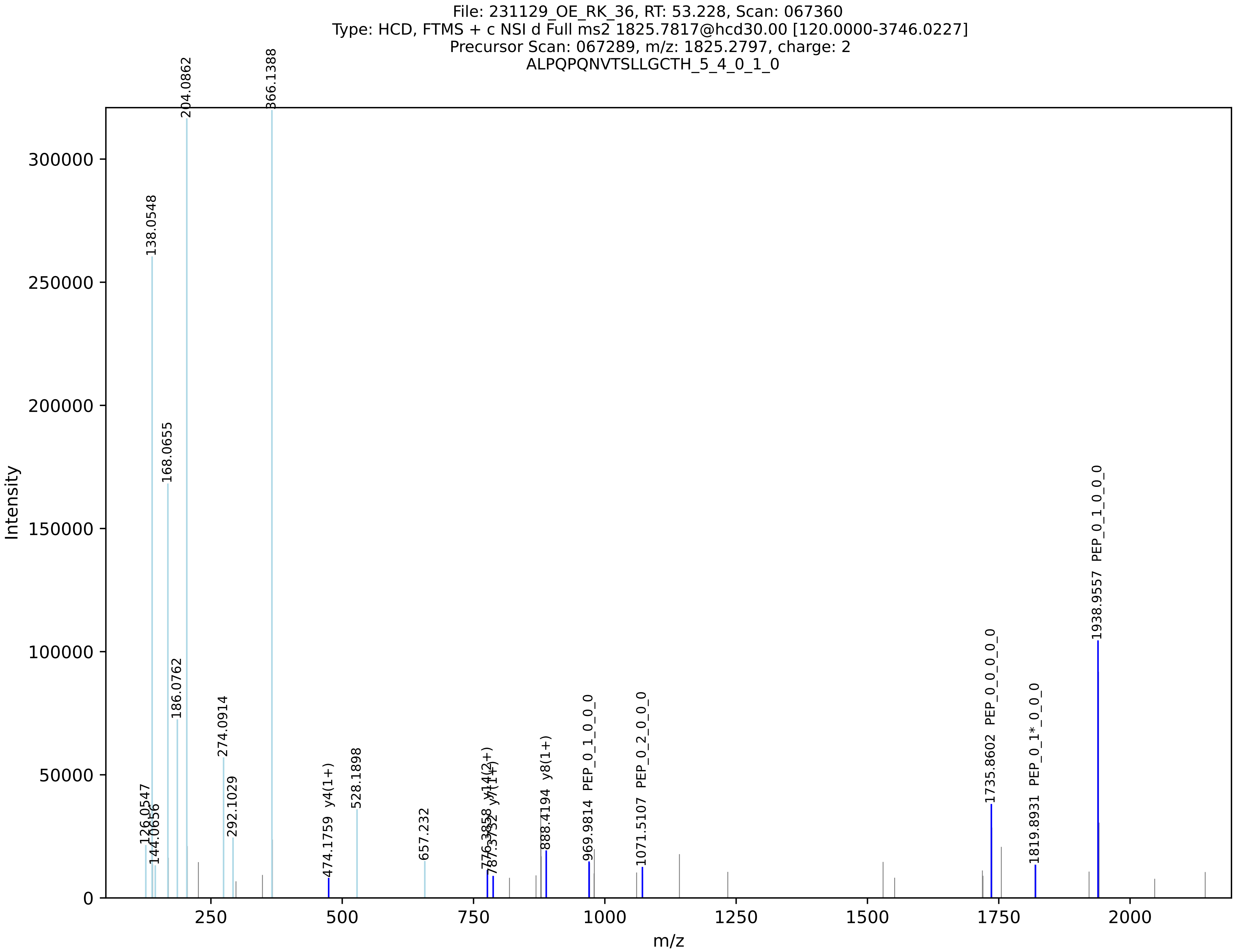
File: 231129_OE_RK_36, RT: 53.228, Scan: 067360
Type: HCD, FTMS + c NSI d Full ms2 1825.7817@hcd30.00 [120.0000-3746.0227]
Precursor Scan: 067289, m/z: 1825.2797, charge: 2
ALPQPQNVTSLLGCTH_5_4_0_1_0
Intensity
m/z
204.0862
366.1388
138.0548
168.0655
186.0762
274.0914
292.1029
126.0547
144.0656
474.1759 y4(1+)
528.1898
657.232
776.3858 y14(2+)
787.3752 y7(1+)
888.4194 y8(1+)
969.9814 PEP_0_1_0_0_0
1071.5107 PEP_0_2_0_0_0
1735.8602 PEP_0_0_0_0_0
1819.8931 PEP_0_1*_0_0_0
1938.9557 PEP_0_1_0_0_0

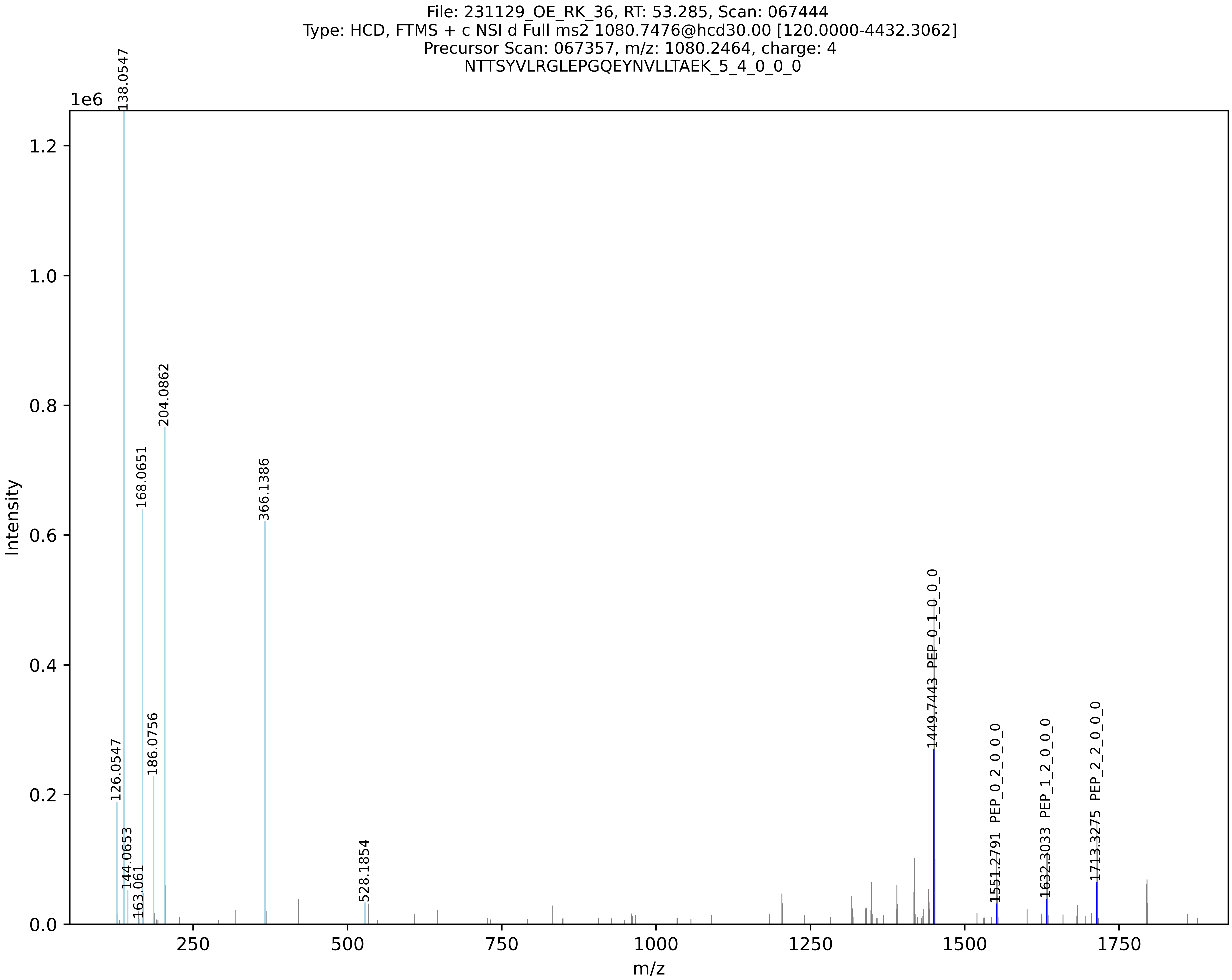
File: 231129_OE_RK_36, RT: 53.285, Scan: 067444
Type: HCD, FTMS + c NSI d Full ms2 1080.7476@hcd30.00 [120.0000-4432.3062]
Precursor Scan: 067357, m/z: 1080.2464, charge: 4
NTTSYVLRGLEPGQEYNVLLTAEK_5_4_0_0_0
1e6
Intensity
m/z
138.0547
204.0862
168.0651
366.1386
126.0547
186.0756
144.0653
163.061
528.1854
1449.7443 PEP_0_1_0_0_
1551.2791 PEP_0_2_0_0_0
1632.3033 PEP_1_2_0_0_0
1713.3275 PEP_2_2_0_0_0

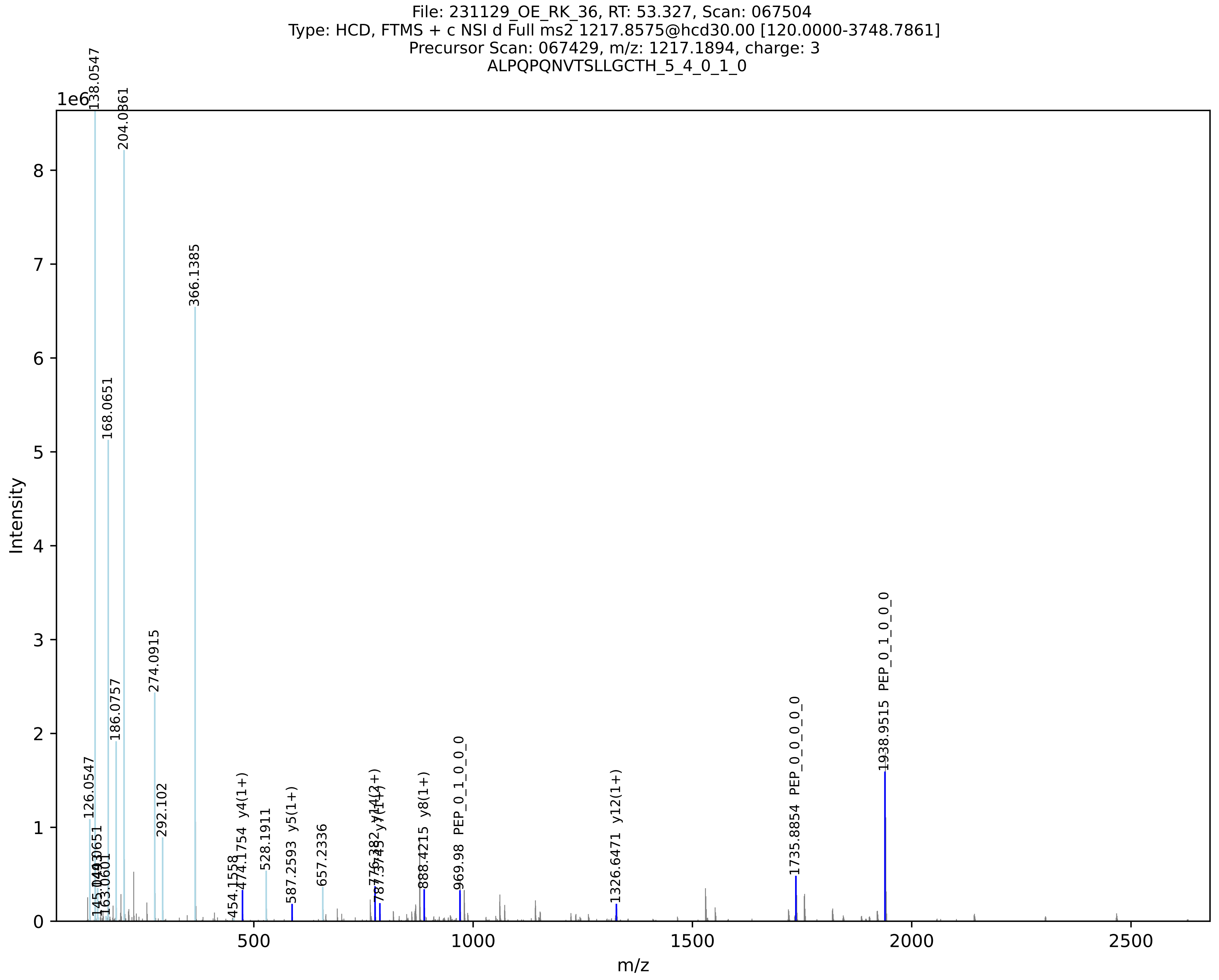
File: 231129_OE_RK_36, RT: 53.327, Scan: 067504
Type: HCD, FTMS + c NSI d Full ms2 1217.8575@hcd30.00 [120.0000-3748.7861]
Precursor Scan: 067429, m/z: 1217.1894, charge: 3
ALPQPQNVTSLLGCTH_5_4_0_1_0
1e6
Intensity
m/z
138.0547
204.0861
366.1385
168.0651
274.0915
186.0757
126.0547
292.102
163.0601
454.1558
474.1754 y4(1+)
528.1911
587.2593 y5(1+)
657.2336
888.4215 y8(1+)
969.98 PEP_0_1_0_0_0
1326.6471 y12(1+)
1735.8854 PEP_0_0_0_0_0
1938.9515 PEP_0_1_0_0_0
0
1
2
3
4
5
6
7
8
500
1000
1500
2000
2500

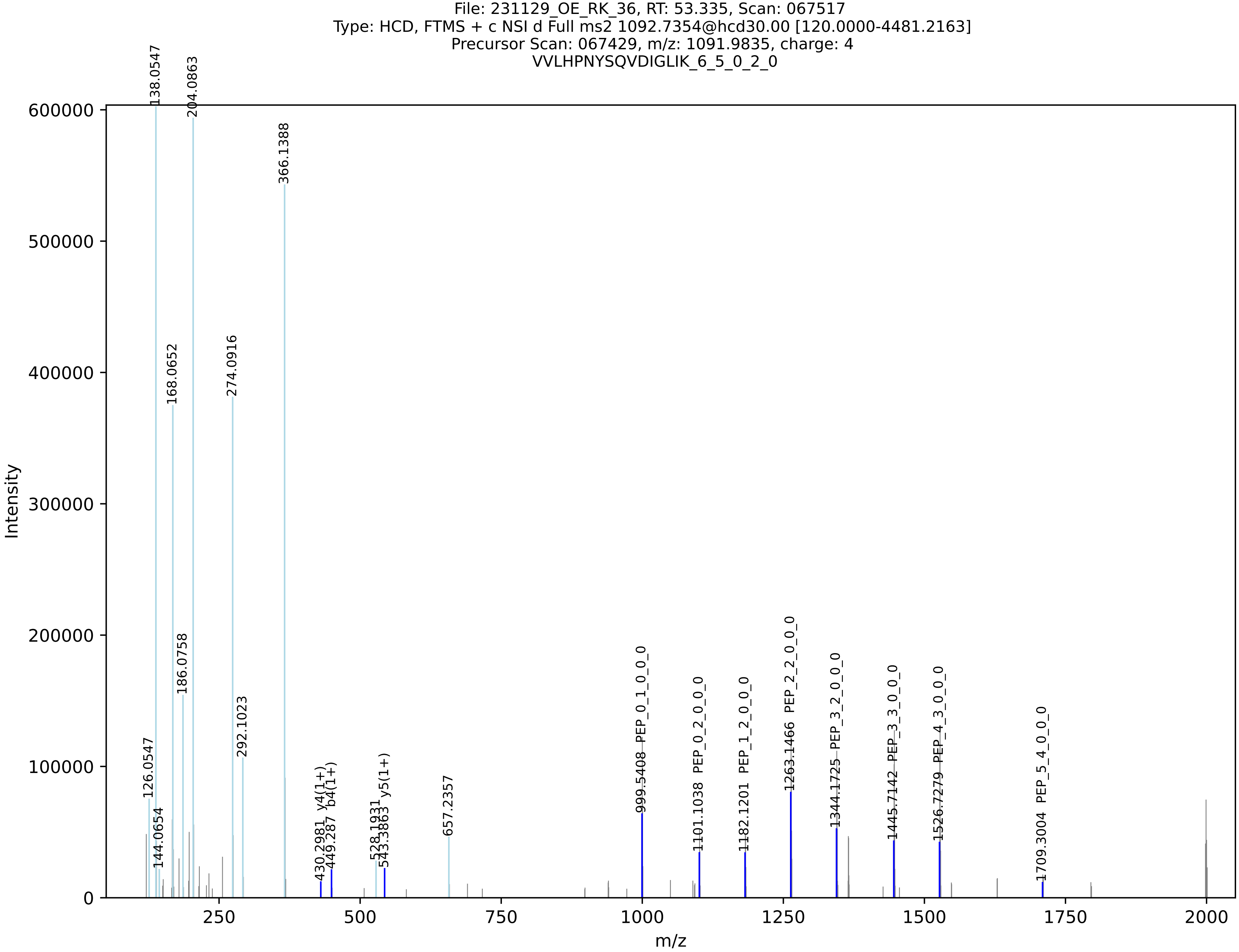

File: 231129_OE_RK_36, RT: 53.335, Scan: 067517
Type: HCD, FTMS + c NSI d Full ms2 1092.7354@hcd30.00 [120.0000-4481.2163]
Precursor Scan: 067429, m/z: 1091.9835, charge: 4
VVLHPNYSQVDIGLIK_6_5_0_2_0
Intensity
m/z
138.0547
204.0863
366.1388
168.0652
274.0916
186.0758
292.1023
126.0547
144.0654
430.2981 y4(1+)
449.287 b4(1+)
528.1931
543.3863 y5(1+)
657.2357
999.5408 PEP_0_1_0_0_0
1101.1038 PEP_0_2_0_0_0
1182.1201 PEP_1_2_0_0_0
1263.1466 PEP_2_2_0_0_0
1344.1725 PEP_3_2_0_0_0
1445.7142 PEP_3_3_0_0_0
1526.7279 PEP_4_3_0_0_0
1709.3004 PEP_5_4_0_0_0

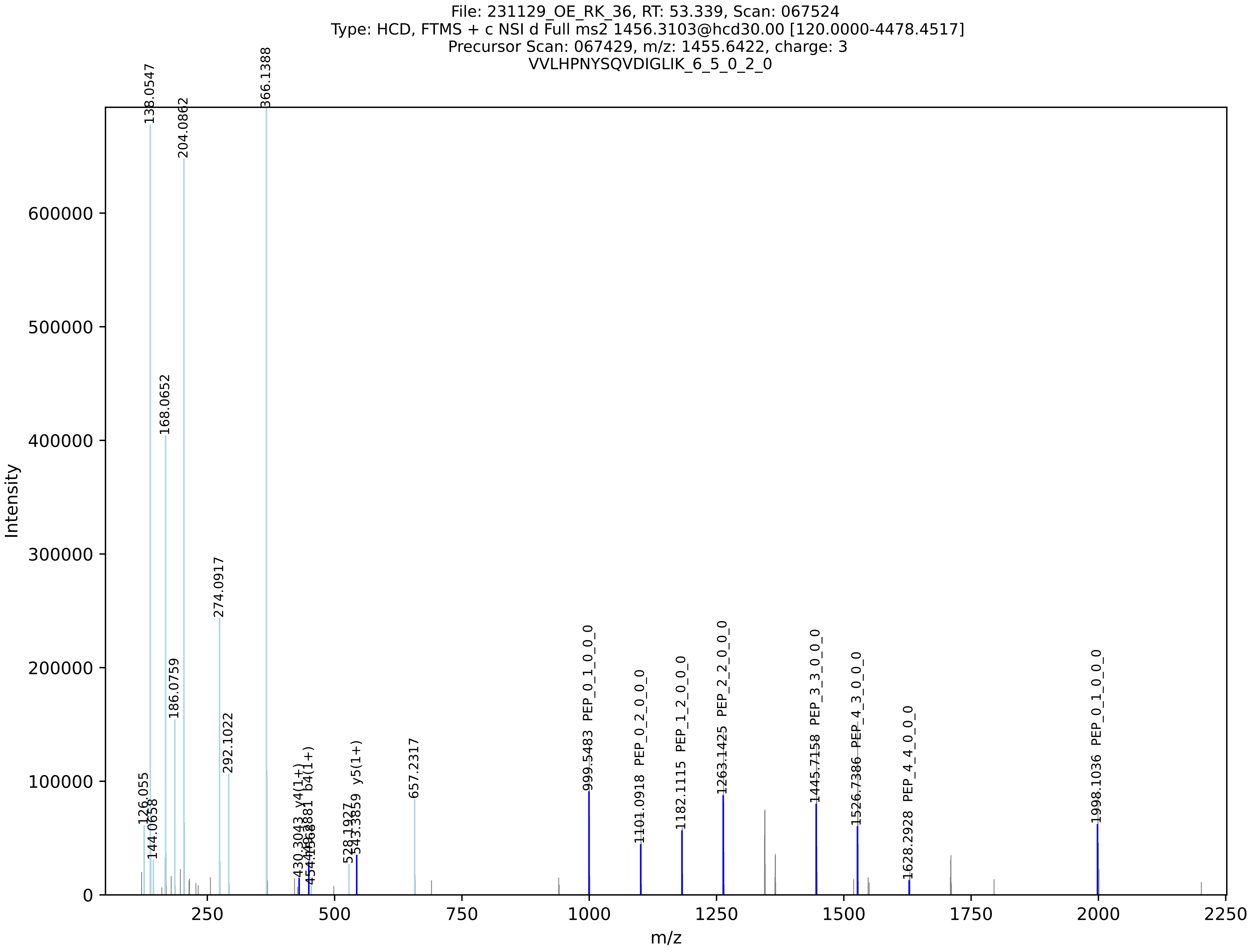
File: 231129_OE_RK_36, RT: 53.339, Scan: 067524
Type: HCD, FTMS + c NSI d Full ms2 1456.3103@hcd30.00 [120.0000-4478.4517]
Precursor Scan: 067429, m/z: 1455.6422, charge: 3
VVLHPNYSQVDIGLIK_6_5_0_2_0
138.0547
366.1388
204.0862
168.0652
274.0917
186.0759
292.1022
126.055
144.0658
430.3043 y4(1+)
b4(1+)
528.1927
543.3859 y5(1+)
657.2317
999.5483 PEP_0_1_0_0_
1101.0918 PEP_0_2_0_0_
1182.1115 PEP_1_2_0_0_
1263.1425 PEP_2_2_0_0_
1445.7158 PEP_3_3_0_0_
1526.7386 PEP_4_3_0_0_
1628.2928 PEP_4_4_0_0_
1998.1036 PEP_0_1_0_0_
Intensity
m/z
0
100000
200000
300000
400000
500000
600000
250
500
750
1000
1250
1500
1750
2000
2250

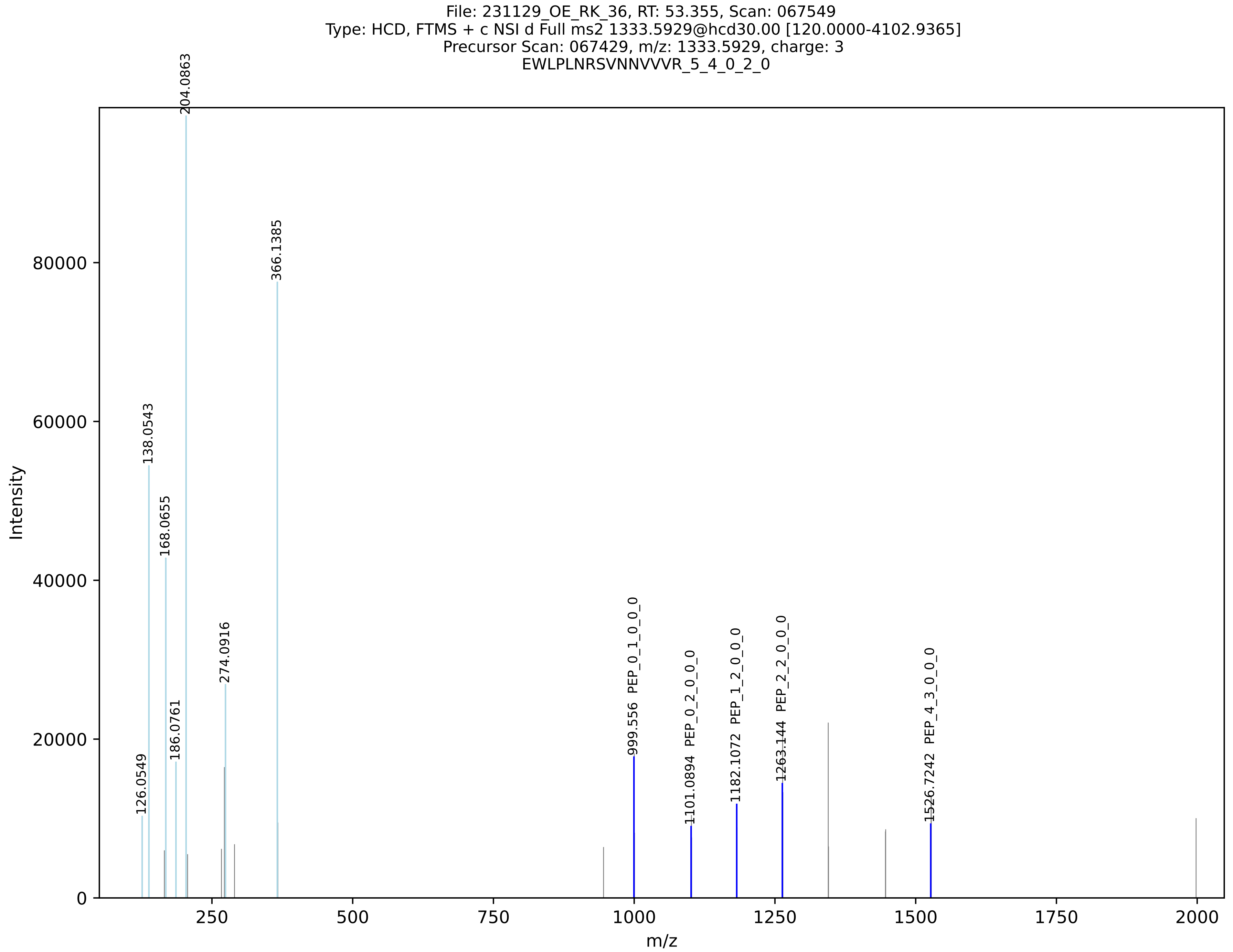

File: 231129_OE_RK_36, RT: 53.355, Scan: 067549
Type: HCD, FTMS + c NSI d Full ms2 1333.5929@hcd30.00 [120.0000-4102.9365]
Precursor Scan: 067429, m/z: 1333.5929, charge: 3
EWLPLNRSVNNVVVR_5_4_0_2_0
Intensity
m/z
204.0863
366.1385
138.0543
168.0655
274.0916
186.0761
126.0549
999.556 PEP_0_1_0_0_0
1101.0894 PEP_0_2_0_0_0
1182.1072 PEP_1_2_0_0_0
1263.144 PEP_2_2_0_0_0
1526.7242 PEP_4_3_0_0_0

File: 231129_OE_RK_36, RT: 53.43, Scan: 067655
Type: HCD, FTMS + c NSI d Full ms2 1185.8287@hcd30.00 [120.0000-3650.7781]
Precursor Scan: 067576, m/z: 1185.1605, charge: 3
AGLQAFFQVQECNK_5_4_0_1_0

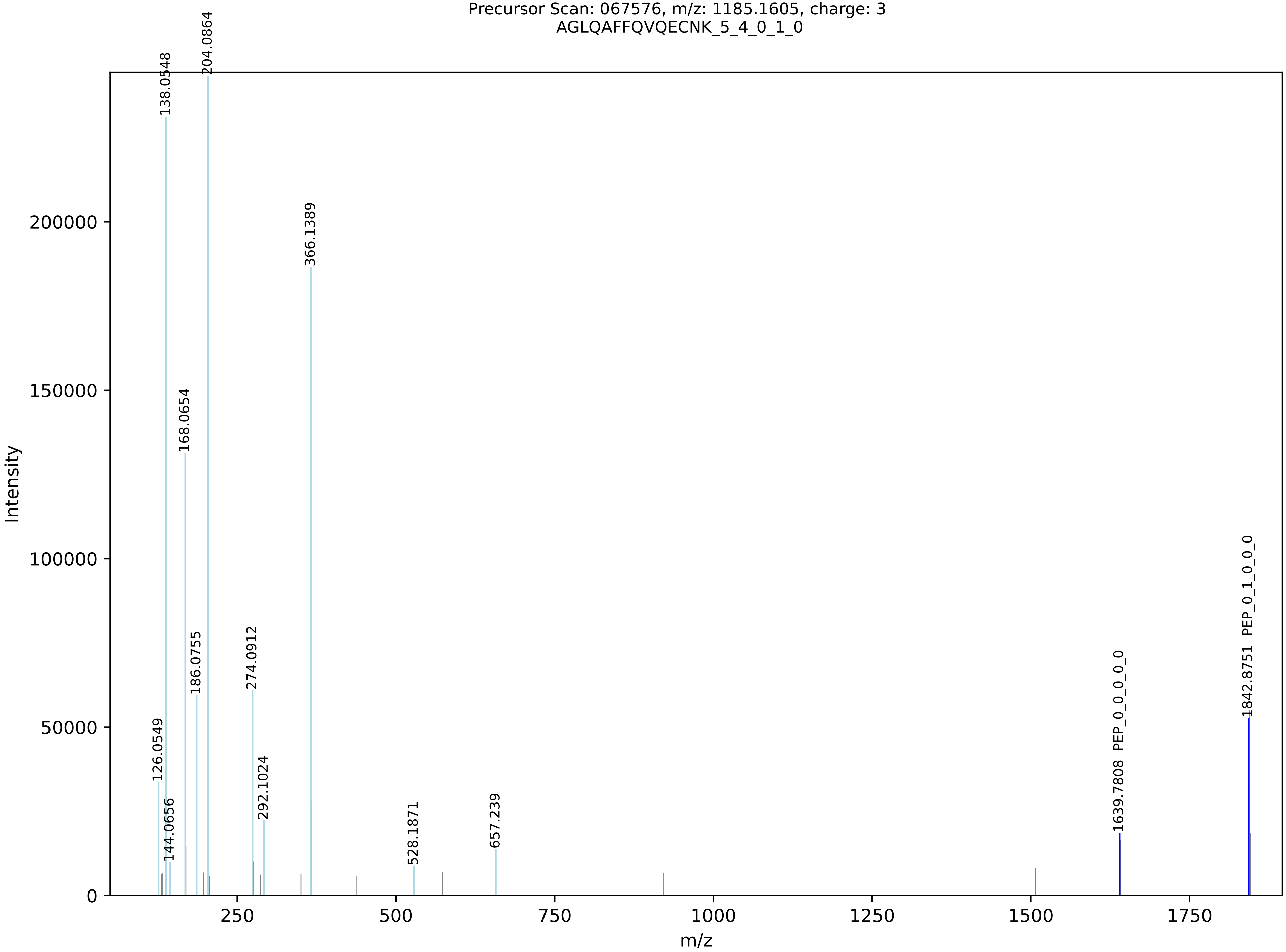

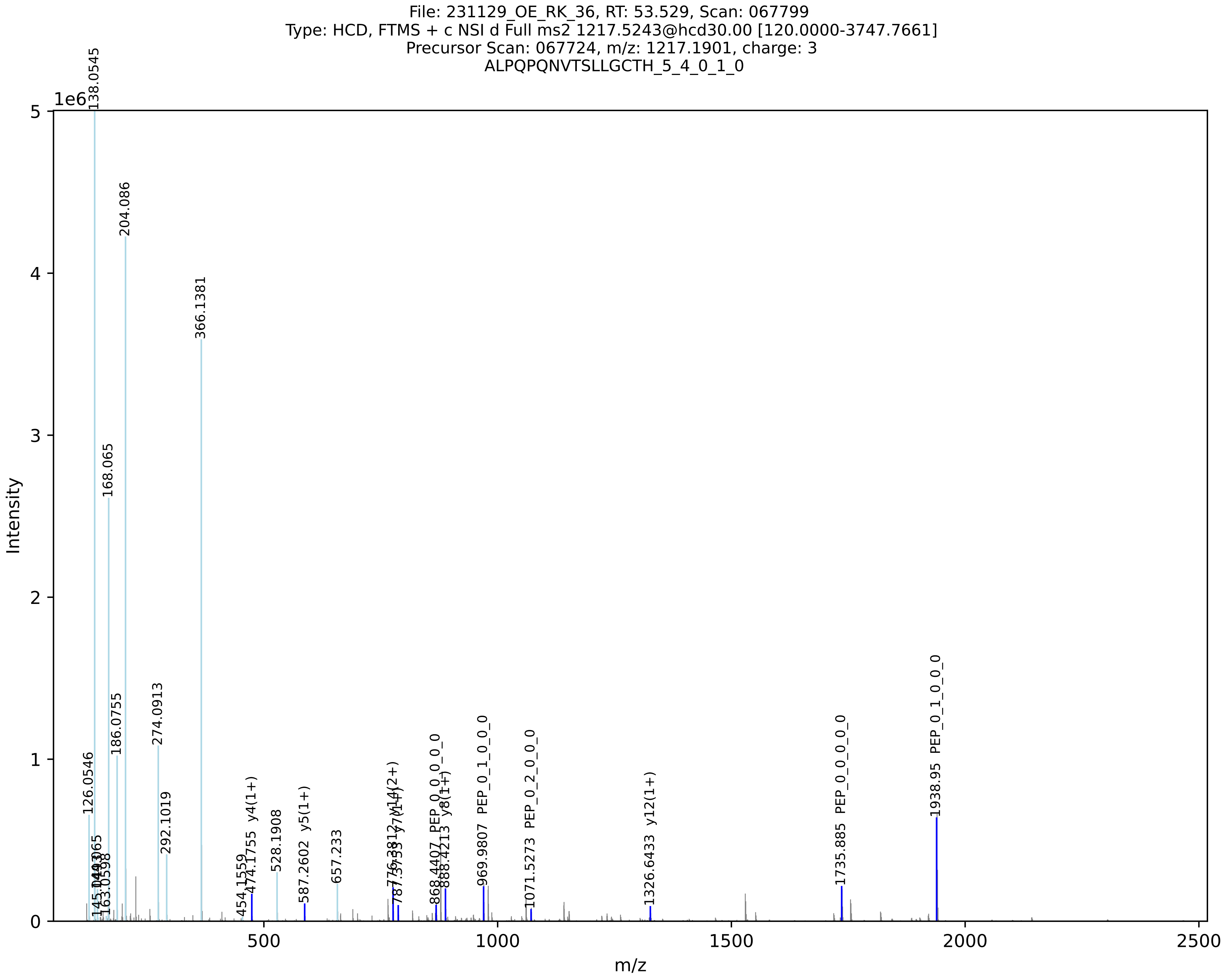
File: 231129_OE_RK_36, RT: 53.529, Scan: 067799
Type: HCD, FTMS + c NSI d Full ms2 1217.5243@hcd30.00 [120.0000-3747.7661]
Precursor Scan: 067724, m/z: 1217.1901, charge: 3
ALPQPQNVTSLLGCTH_5_4_0_1_0
1e6
Intensity
m/z
138.0545
204.086
366.1381
168.065
186.0755
274.0913
126.0546
292.1019
163.0598
454.1559
474.1755 y4(1+)
528.1908
587.2602 y5(1+)
657.233
787.3753 y7(1+)
868.4407 PEP_0_0_0_0_0
888.4213 y8(1+)
969.9807 PEP_0_1_0_0_0
1071.5273 PEP_0_2_0_0_0
1326.6433 y12(1+)
1735.885 PEP_0_0_0_0_0
1938.95 PEP_0_1_0_0_0

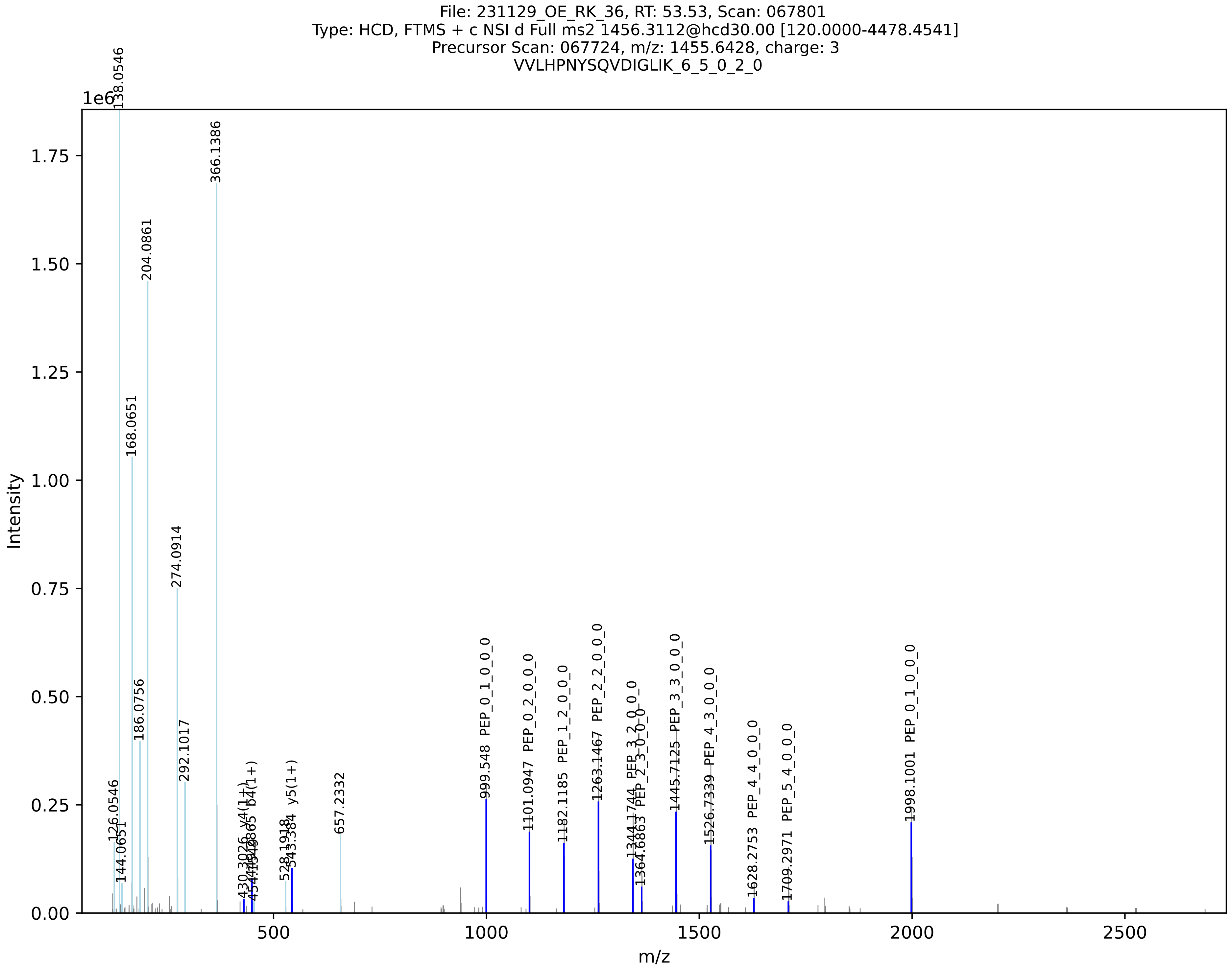
File: 231129_OE_RK_36, RT: 53.53, Scan: 067801
Type: HCD, FTMS + c NSI d Full ms2 1456.3112@hcd30.00 [120.0000-4478.4541]
Precursor Scan: 067724, m/z: 1455.6428, charge: 3
VVLHPNYSQVDIGLIK_6_5_0_2_0
1e6
Intensity
m/z
138.0546
366.1386
204.0861
168.0651
274.0914
186.0756
292.1017
126.0546
144.0651
y5(1+)
528.1918
543.384
657.2332
999.548 PEP_0_1_0_0_0
1101.0947 PEP_0_2_0_0_0
1182.1185 PEP_1_2_0_0_0
1263.1467 PEP_2_2_0_0_0
1344.1744 PEP_3_2_0_0_0
1364.6863 PEP_2_3_0_0_0
1445.7125 PEP_3_3_0_0_0
1526.7339 PEP_4_3_0_0_0
1628.2753 PEP_4_4_0_0_0
1709.2971 PEP_5_4_0_0_0
1998.1001 PEP_0_1_0_0_0

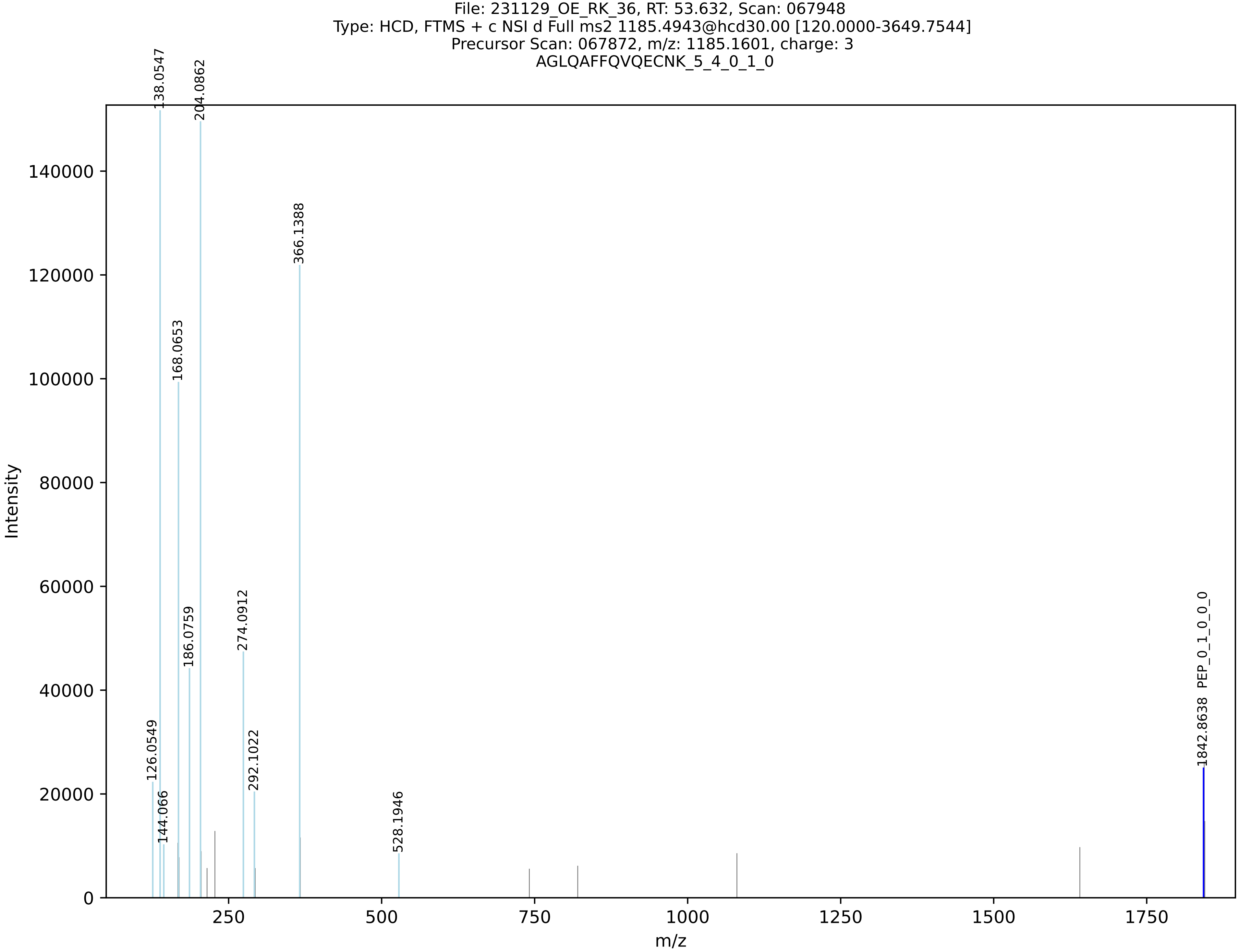
File: 231129_OE_RK_36, RT: 53.632, Scan: 067948
Type: HCD, FTMS + c NSI d Full ms2 1185.4943@hcd30.00 [120.0000-3649.7544]
Precursor Scan: 067872, m/z: 1185.1601, charge: 3
AGLQAFFQVQECNK_5_4_0_1_0
Intensity
m/z
138.0547
204.0862
366.1388
168.0653
186.0759
274.0912
126.0549
292.1022
144.066
528.1946
1842.8638 PEP_0_1_0_0_0
0
20000
40000
60000
80000
100000
120000
140000
250
500
750
1000
1250
1500
1750

File: 231129_OE_RK_36, RT: 53.68, Scan: 068017
Type: HCD, FTMS + c NSI d Full ms2 1000.9518@hcd30.00 [120.0000-4106.7393]
Precursor Scan: 067946, m/z: 1000.7011, charge: 4
VVLHPNYSQVDIGLIK_5_4_0_2_0

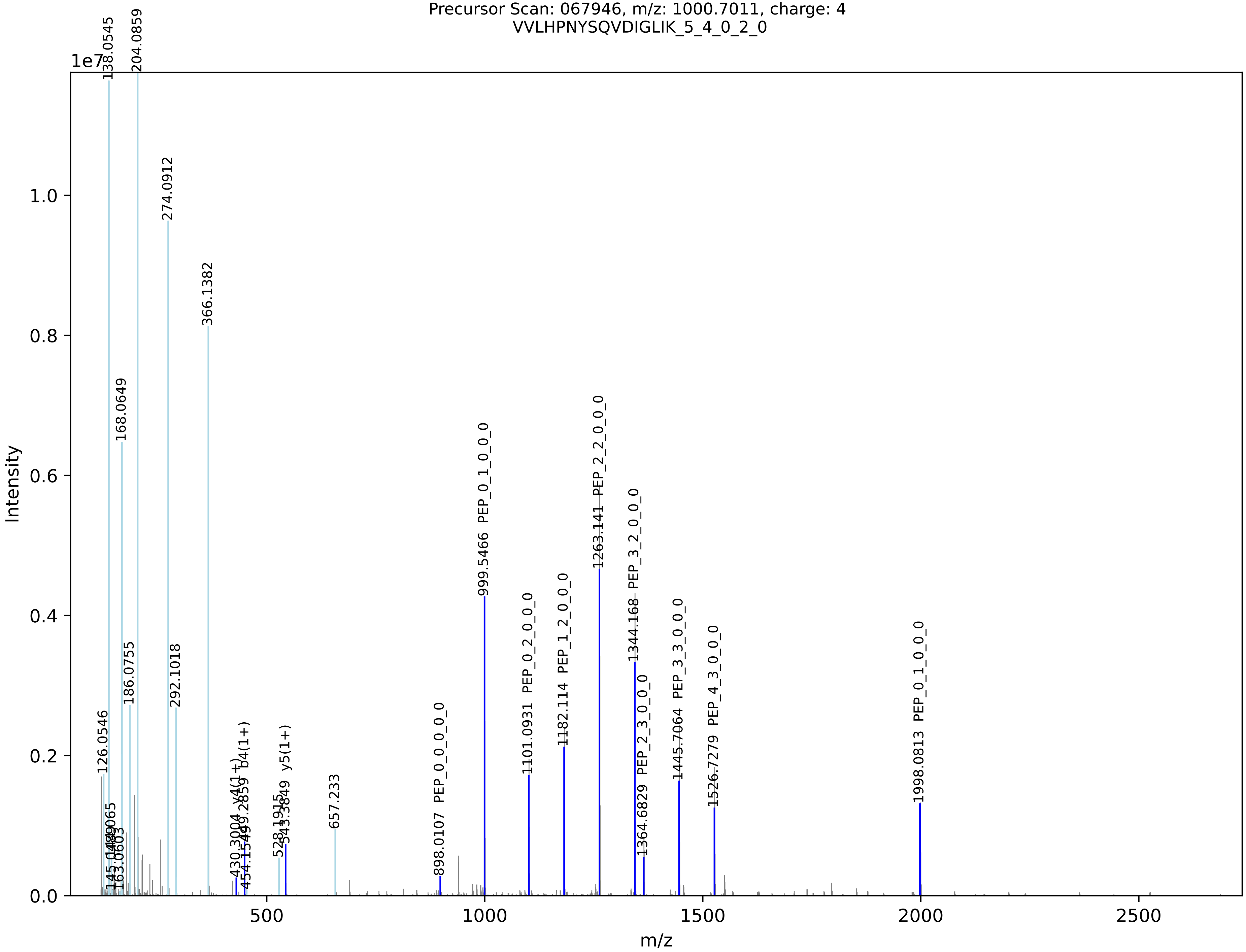

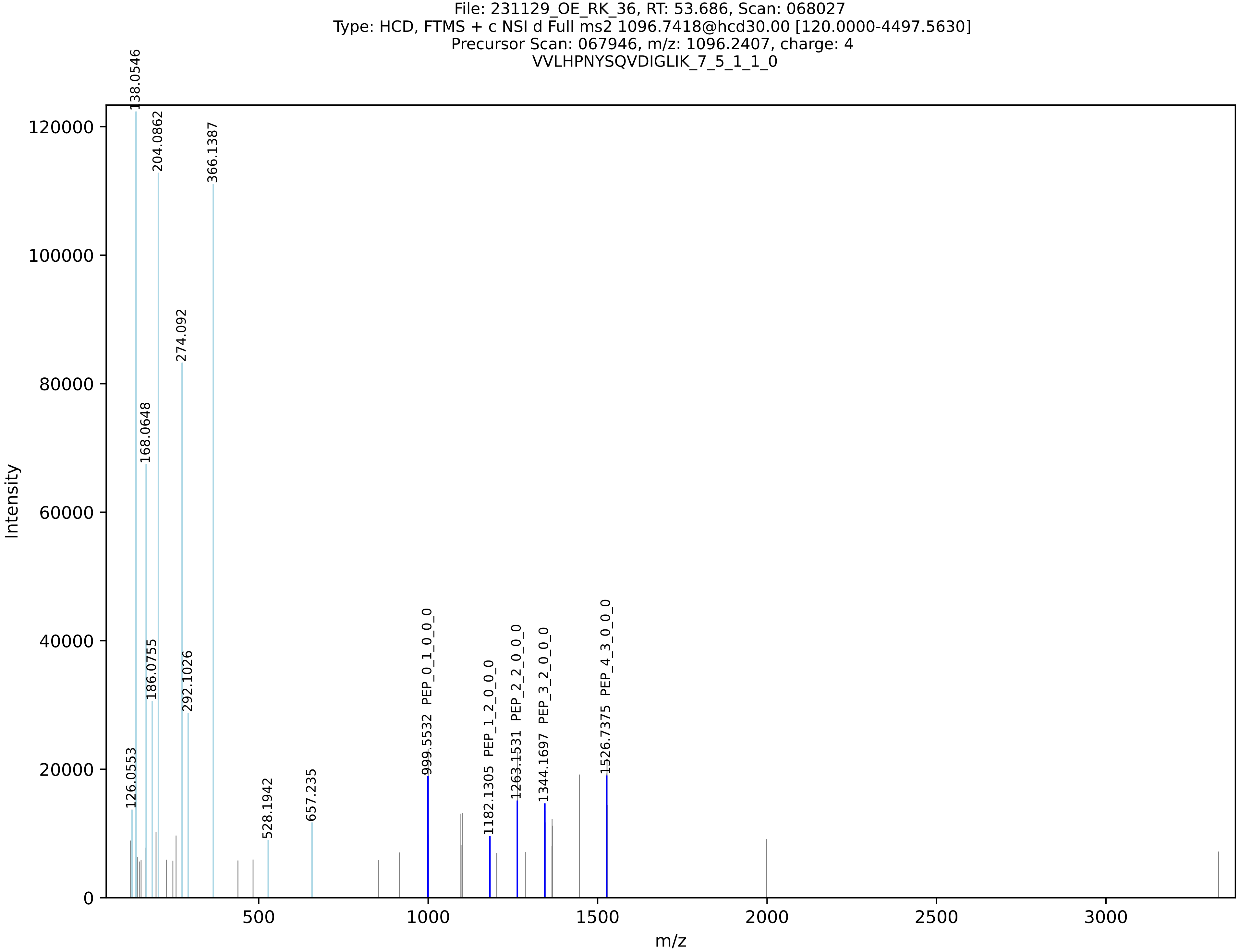

File: 231129_OE_RK_36, RT: 53.686, Scan: 068027
Type: HCD, FTMS + c NSI d Full ms2 1096.7418@hcd30.00 [120.0000-4497.5630]
Precursor Scan: 067946, m/z: 1096.2407, charge: 4
VVLHPNYSQVDIGLIK_7_5_1_1_0
138.0546
204.0862
366.1387
274.092
168.0648
186.0755
292.1026
126.0553
528.1942
657.235
999.5532 PEP_0_1_0_0_0
1182.1305 PEP_1_2_0_0_0
1263.1531 PEP_2_2_0_0_0
1344.1697 PEP_3_2_0_0_0
1526.7375 PEP_4_3_0_0_0
Intensity
m/z

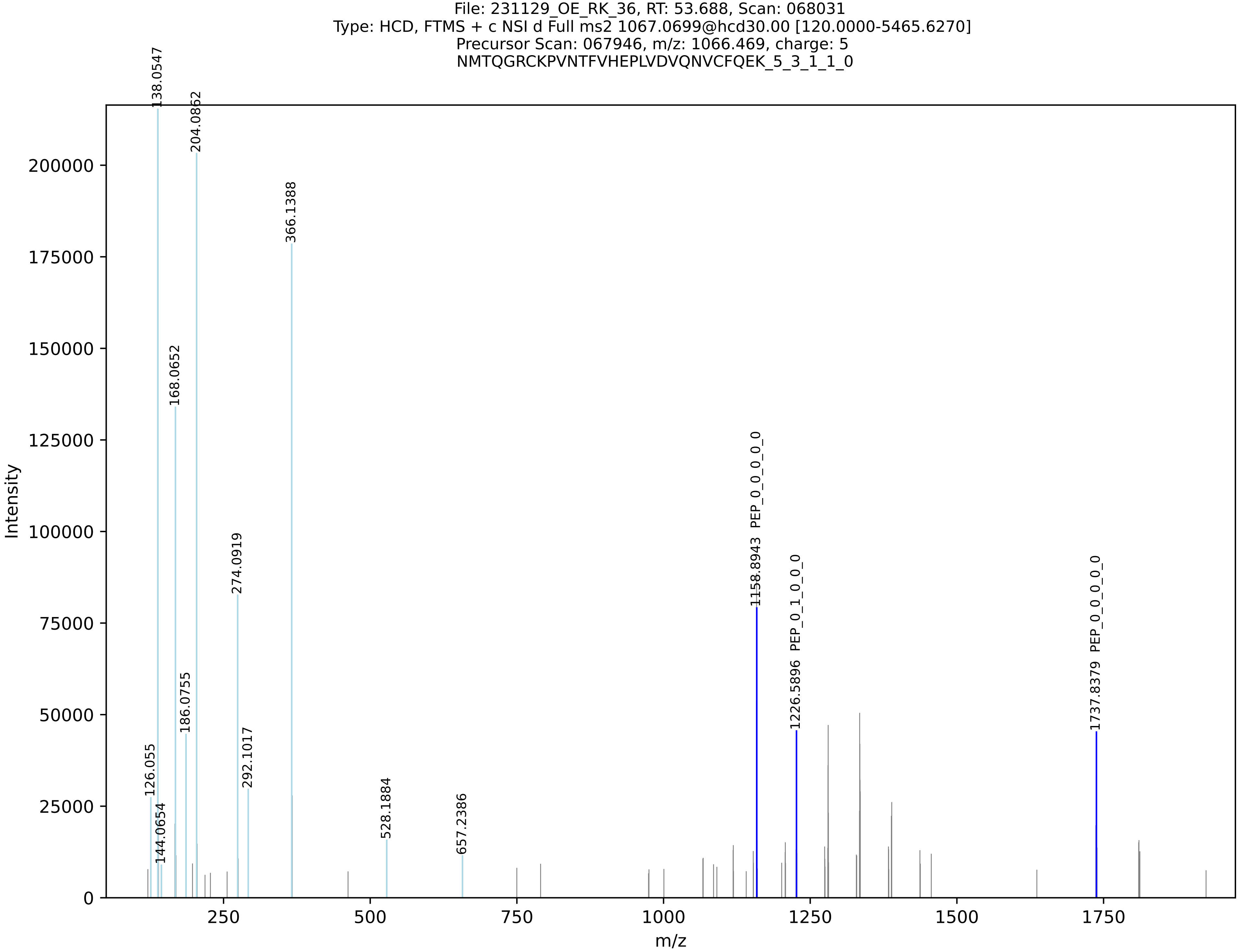

File: 231129_OE_RK_36, RT: 53.688, Scan: 068031
Type: HCD, FTMS + c NSI d Full ms2 1067.0699@hcd30.00 [120.0000-5465.6270]
Precursor Scan: 067946, m/z: 1066.469, charge: 5
NMTQGRCKPVNTFVHEPLVDVQNVCFQEK_5_3_1_1_0
Intensity
m/z
138.0547
204.0862
366.1388
168.0652
274.0919
186.0755
126.055
292.1017
144.0654
528.1884
657.2386
1158.8943 PEP_0_0_0_0_0
1226.5896 PEP_0_1_0_0_0
1737.8379 PEP_0_0_0_0_0

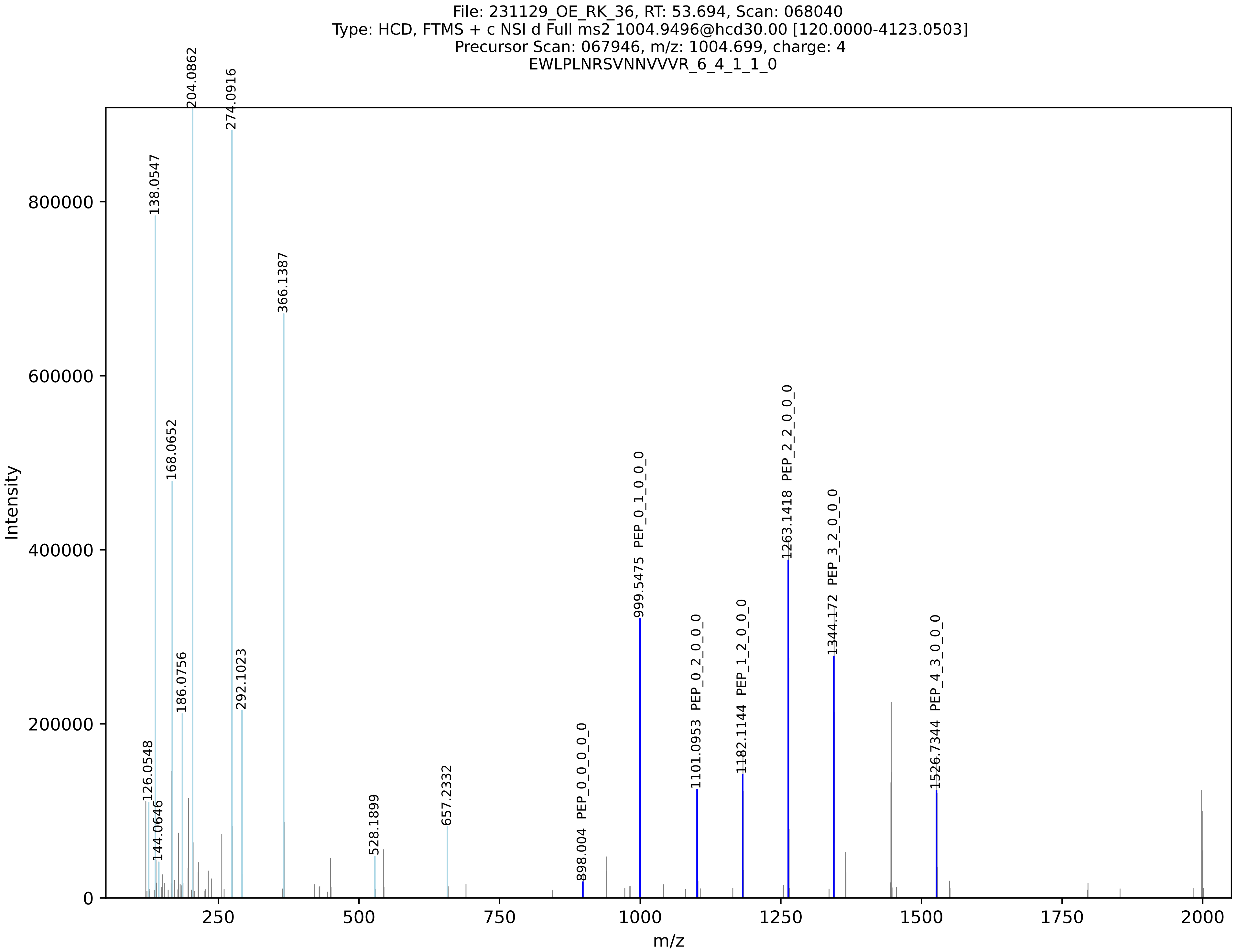

File: 231129_OE_RK_36, RT: 53.694, Scan: 068040
Type: HCD, FTMS + c NSI d Full ms2 1004.9496@hcd30.00 [120.0000-4123.0503]
Precursor Scan: 067946, m/z: 1004.699, charge: 4
EWLPLNRSVNNVVVR_6_4_1_1_0
Intensity
m/z
0
200000
400000
600000
800000
250
500
750
1000
1250
1500
1750
2000
204.0862
274.0916
138.0547
366.1387
168.0652
186.0756
292.1023
126.0548
144.0646
528.1899
657.2332
898.004 PEP_0_0_0_0_
999.5475 PEP_0_1_0_0_0
1101.0953 PEP_0_2_0_0_0
1182.1144 PEP_1_2_0_0_0
1263.1418 PEP_2_2_0_0_0
1344.172 PEP_3_2_0_0_0
1526.7344 PEP_4_3_0_0_0

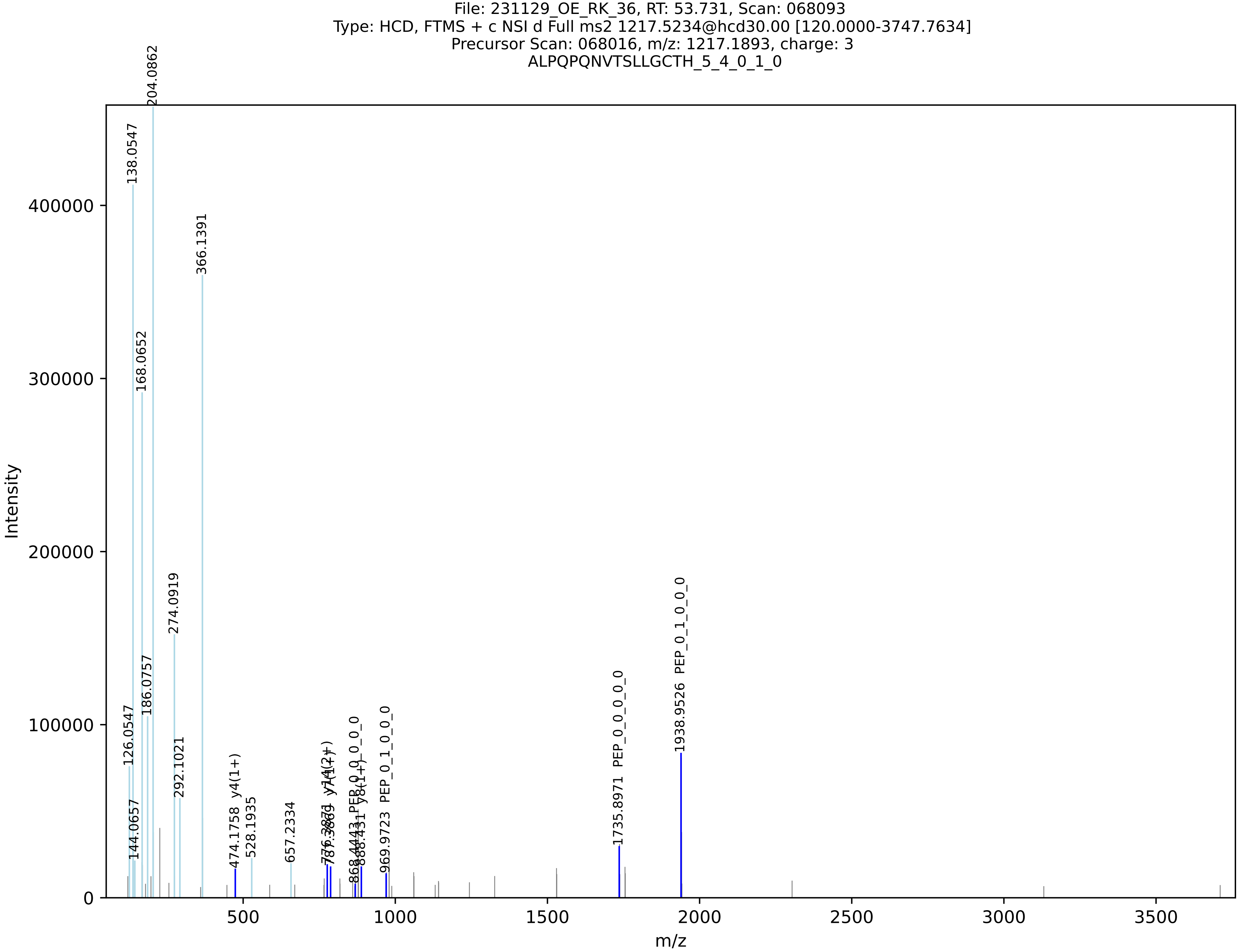
File: 231129_OE_RK_36, RT: 53.731, Scan: 068093
Type: HCD, FTMS + c NSI d Full ms2 1217.5234@hcd30.00 [120.0000-3747.7634]
Precursor Scan: 068016, m/z: 1217.1893, charge: 3
ALPQPQNVTSLLGCTH_5_4_0_1_0
Intensity
m/z
204.0862
138.0547
366.1391
168.0652
274.0919
186.0757
126.0547
292.1021
144.0657
474.1758 y4(1+)
528.1935
657.2334
969.9723 PEP_0_1_0_0_0
1735.8971 PEP_0_0_0_0_0
1938.9526 PEP_0_1_0_0_0

File: 231129_OE_RK_36, RT: 53.734, Scan: 068097
Type: HCD, FTMS + c NSI d Full ms2 1092.4844@hcd30.00 [120.0000-4480.1924]
Precursor Scan: 068016, m/z: 1091.9832, charge: 4
VVLHPNYSQVDIGLIK_6_5_0_2_0

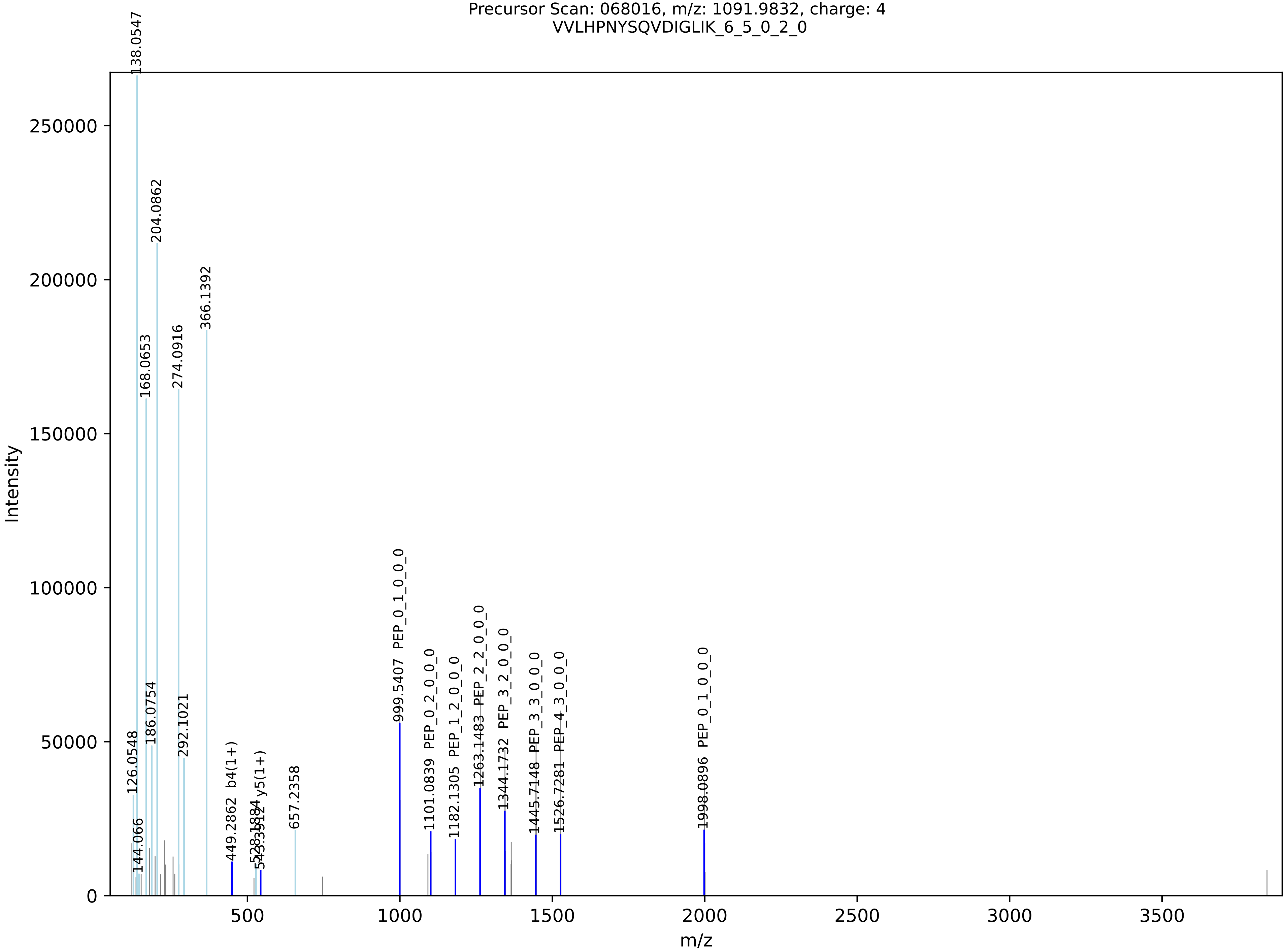

File: 231129_OE_RK_36, RT: 53.735, Scan: 068098
Type: HCD, FTMS + c NSI d Full ms2 1456.3108@hcd30.00 [120.0000-4478.4531]
Precursor Scan: 068016, m/z: 1455.6418, charge: 3
VVLHPNYSQVDIGLIK_6_5_0_2_0

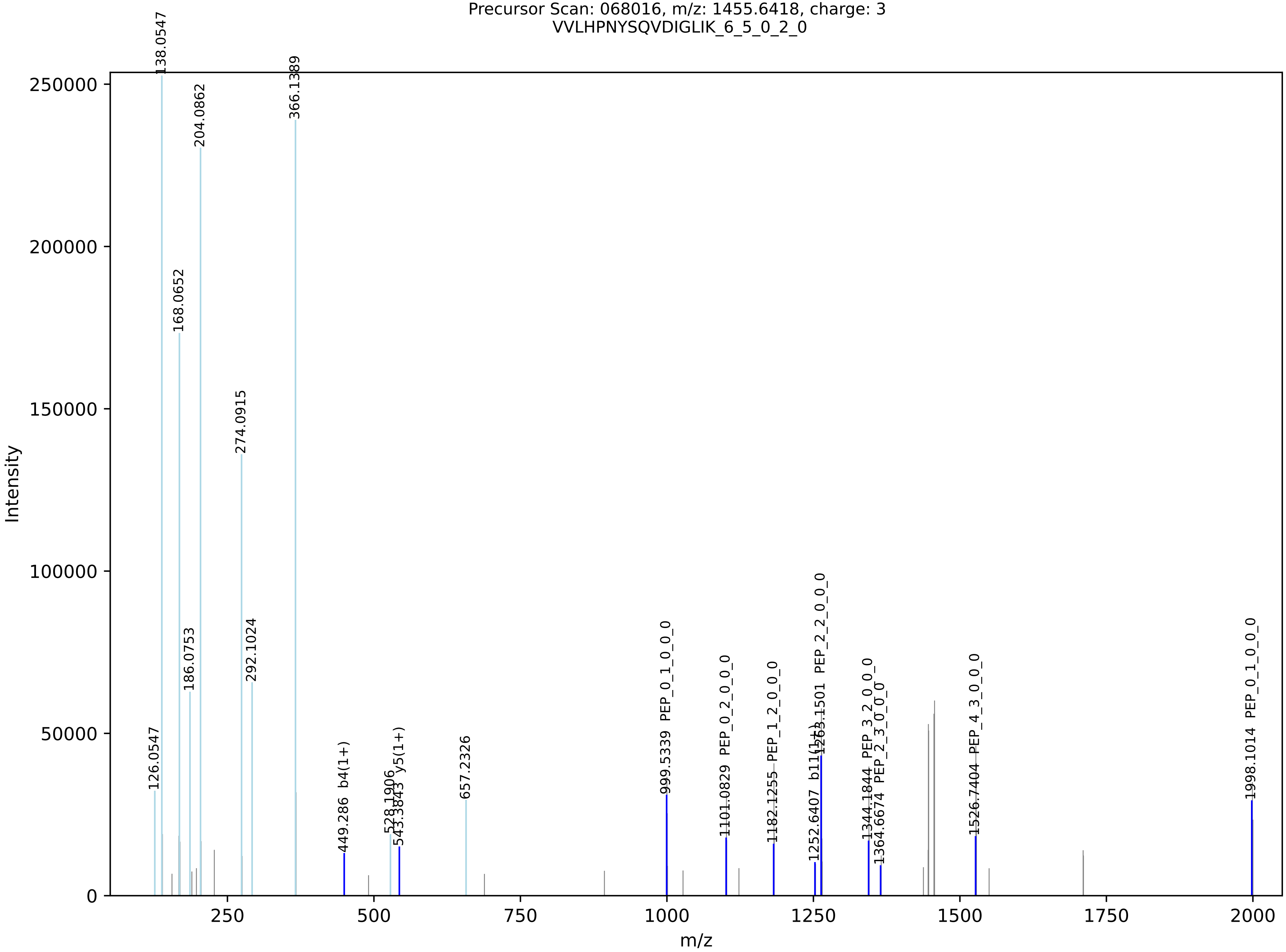

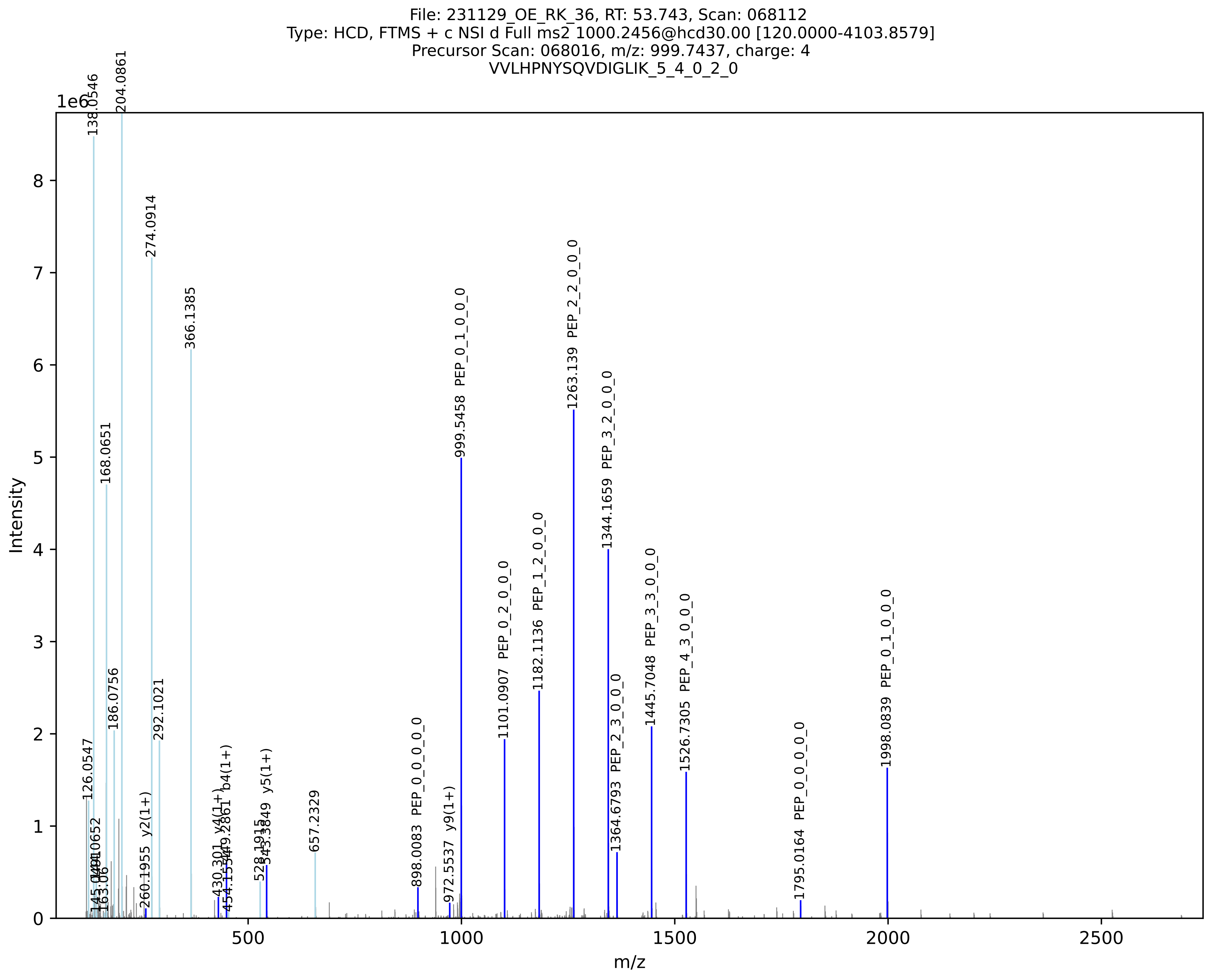

File: 231129_OE_RK_36, RT: 53.743, Scan: 068112
Type: HCD, FTMS + c NSI d Full ms2 1000.2456@hcd30.00 [120.0000-4103.8579]
Precursor Scan: 068016, m/z: 999.7437, charge: 4
VVLHPNYSQVDIGLIK_5_4_0_2_0
1e6
Intensity
m/z
0
1
2
3
4
5
6
7
8
500
1000
1500
2000
2500
204.0861
274.0914
366.1385
168.0651
186.0756
292.1021
126.0547
260.1955 y2(1+)
449.2861 b4(1+)
528.1915
543.3849 y5(1+)
657.2329
898.0083 PEP_0_0_0_0_0
972.5537 y9(1+)
999.5458 PEP_0_1_0_0_0
1101.0907 PEP_0_2_0_0_0
1182.1136 PEP_1_2_0_0_0
1263.139 PEP_2_2_0_0_0
1344.1659 PEP_3_2_0_0_0
1364.6793 PEP_2_3_0_0_0
1445.7048 PEP_3_3_0_0_0
1526.7305 PEP_4_3_0_0_0
1795.0164 PEP_0_0_0_0_0
1998.0839 PEP_0_1_0_0_0

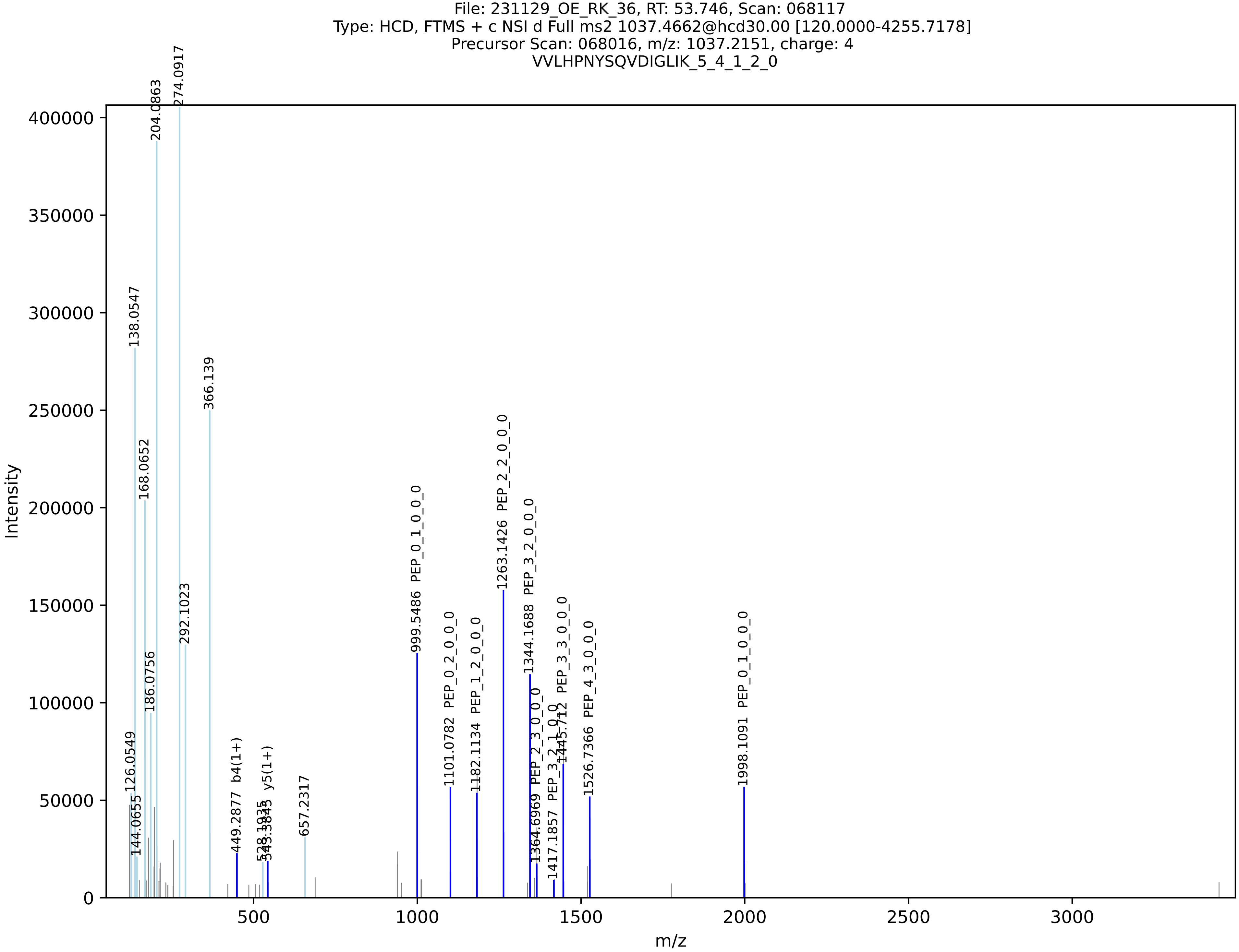
File: 231129_OE_RK_36, RT: 53.746, Scan: 068117
Type: HCD, FTMS + c NSI d Full ms2 1037.4662@hcd30.00 [120.0000-4255.7178]
Precursor Scan: 068016, m/z: 1037.2151, charge: 4
VVLHPNYSQVDIGLIK_5_4_1_2_0
Intensity
m/z
274.0917
204.0863
138.0547
366.139
168.0652
292.1023
186.0756
126.0549
144.0655
449.2877 b4(1+)
528.1935
543.3843 y5(1+)
657.2317
999.5486 PEP_0_1_0_0_0
1101.0782 PEP_0_2_0_0_0
1182.1134 PEP_1_2_0_0_0
1263.1426 PEP_2_2_0_0_0
1344.1688 PEP_3_2_0_0_0
1364.6969 PEP_2_3_0_0_0
1417.1857 PEP_3_2_1_0_0
1445.712 PEP_3_3_0_0_0
1526.7366 PEP_4_3_0_0_0
1998.1091 PEP_0_1_0_0_0

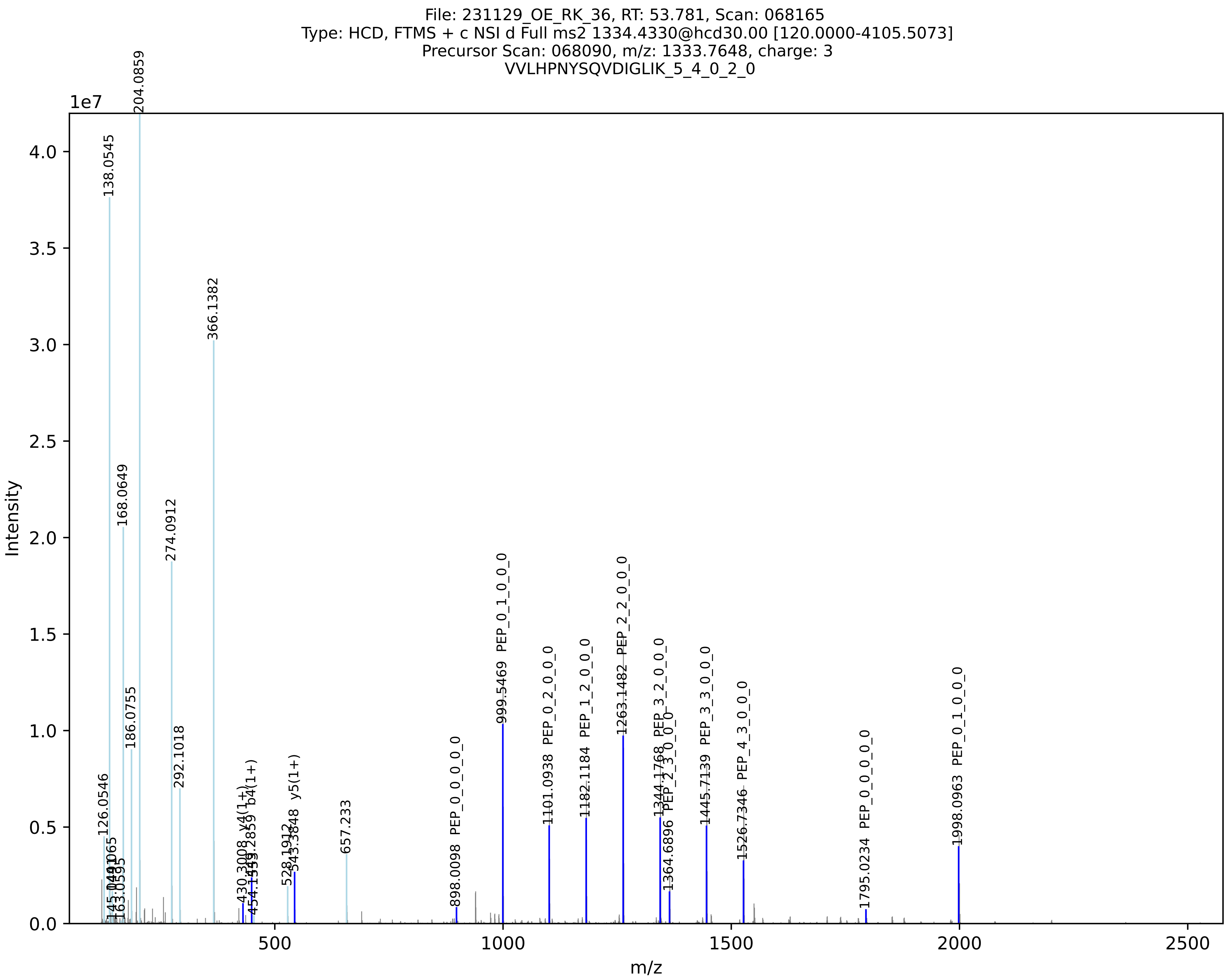
File: 231129_OE_RK_36, RT: 53.781, Scan: 068165
Type: HCD, FTMS + c NSI d Full ms2 1334.4330@hcd30.00 [120.0000-4105.5073]
Precursor Scan: 068090, m/z: 1333.7648, charge: 3
VVLHPNYSQVDIGLIK_5_4_0_2_0
1e7
Intensity
m/z
204.0859
138.0545
366.1382
168.0649
274.0912
186.0755
292.1018
126.0546
657.233
543.3848 y5(1+)
430.3008 y4(1+)
454.1559
528.1912
898.0098 PEP_0_0_0_0_0
999.5469 PEP_0_1_0_0_0
1101.0938 PEP_0_2_0_0_0
1182.1184 PEP_1_2_0_0_0
1263.1482 PEP_2_2_0_0_0
1344.1768 PEP_3_2_0_0_0
1364.6896 PEP_2_3_0_0_0
1445.7139 PEP_3_3_0_0_0
1526.7346 PEP_4_3_0_0_0
1795.0234 PEP_0_0_0_0_0
1998.0963 PEP_0_1_0_0_0

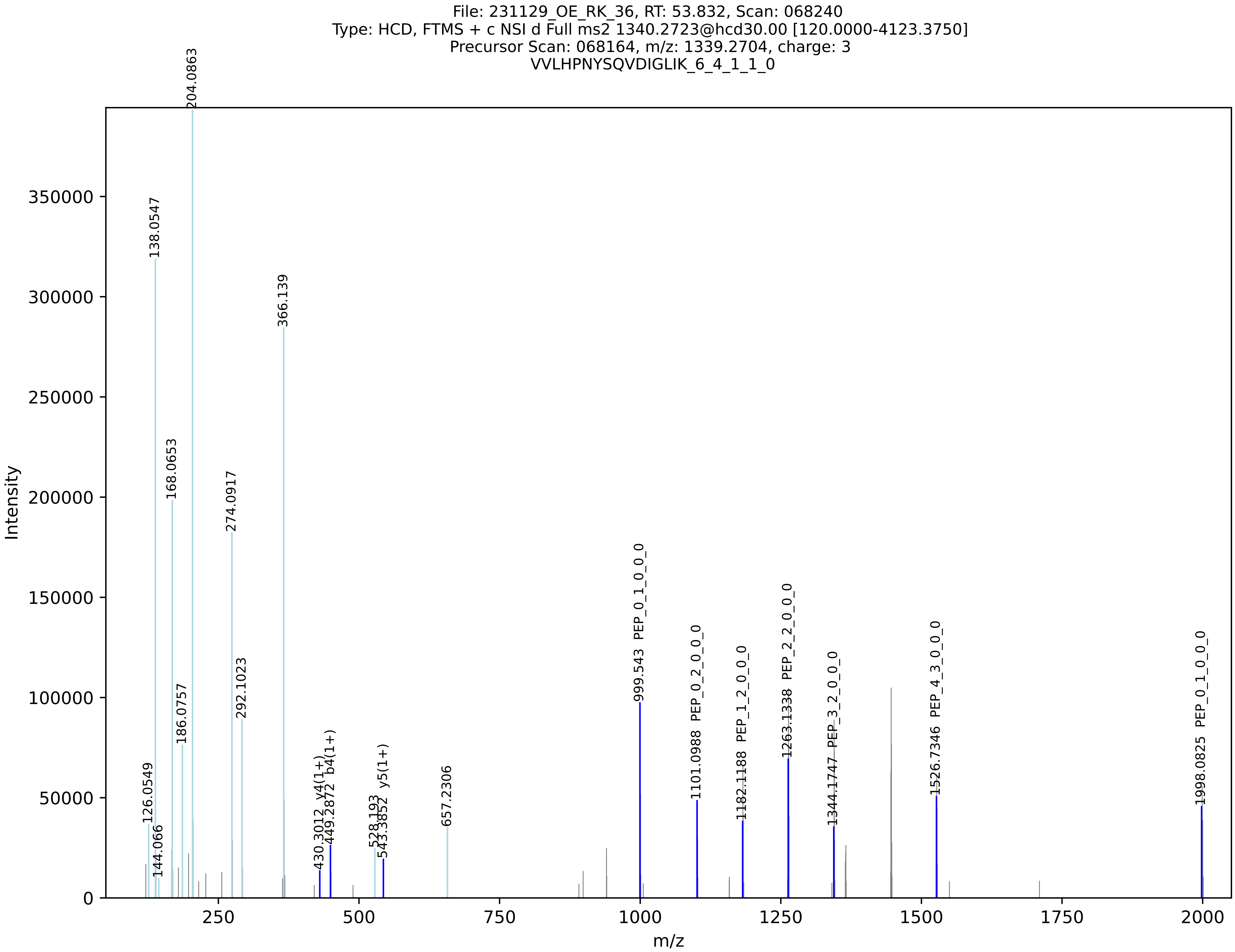

File: 231129_OE_RK_36, RT: 53.832, Scan: 068240
Type: HCD, FTMS + c NSI d Full ms2 1340.2723@hcd30.00 [120.0000-4123.3750]
Precursor Scan: 068164, m/z: 1339.2704, charge: 3
VVLHPNYSQVDIGLIK_6_4_1_1_0
Intensity
m/z
204.0863
138.0547
366.139
168.0653
274.0917
292.1023
186.0757
126.0549
144.066
430.3012 y4(1+)
449.2872 b4(1+)
528.193
543.3852 y5(1+)
657.2306
999.543 PEP_0_1_0_0_0
1101.0988 PEP_0_2_0_0_0
1182.1188 PEP_1_2_0_0_0
1263.1338 PEP_2_2_0_0_0
1344.1747 PEP_3_2_0_0_0
1526.7346 PEP_4_3_0_0_0
1998.0825 PEP_0_1_0_0_0

File: 231129_OE_RK_36, RT: 53.866, Scan: 068294
Type: HCD, FTMS + c NSI d Full ms2 1338.9229@hcd30.00 [120.0000-4119.2456]
Precursor Scan: 068090, m/z: 1339.2654, charge: 3
EWLPLNRSVNNVVVR_6_4_1_1_0

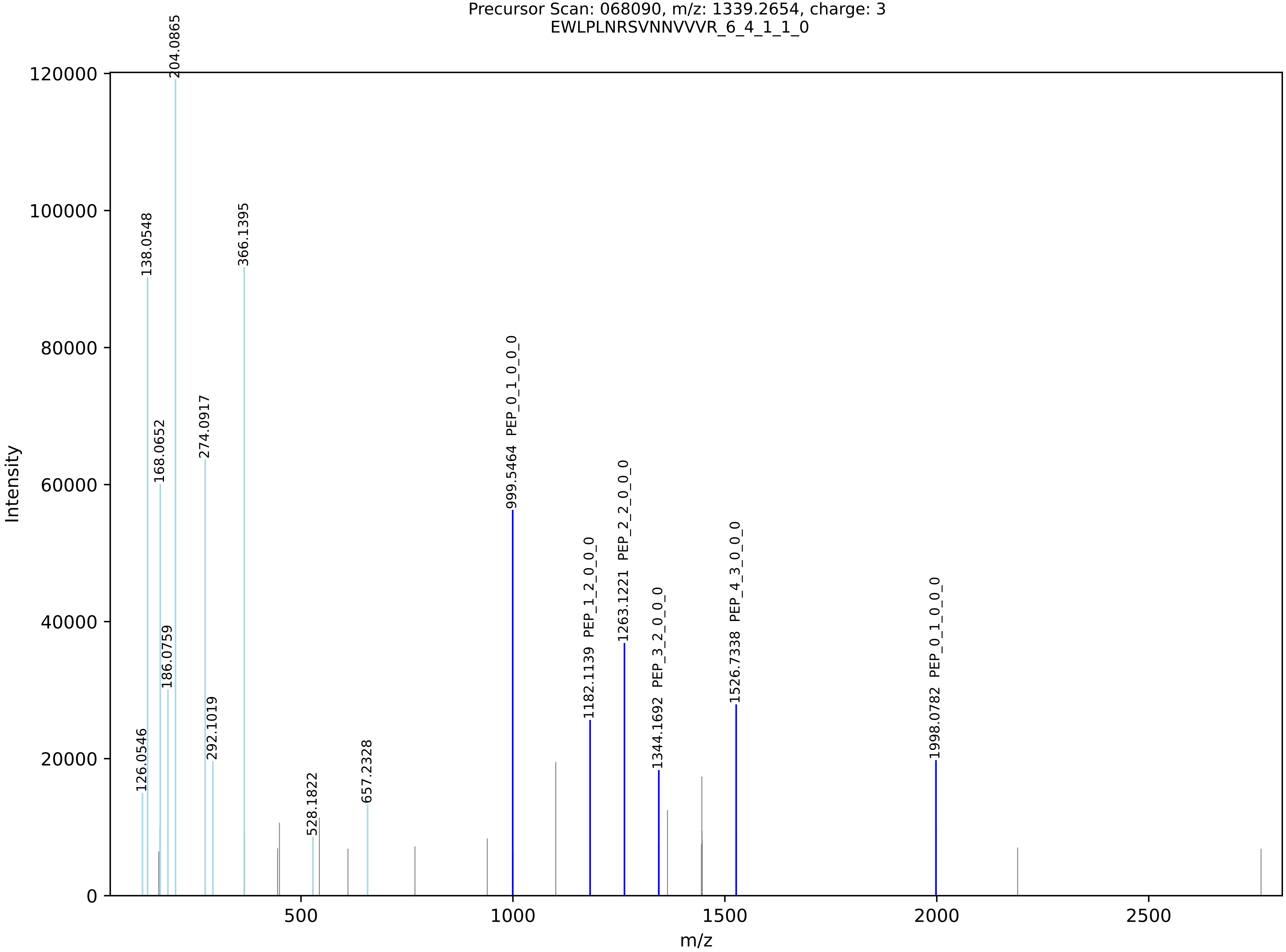

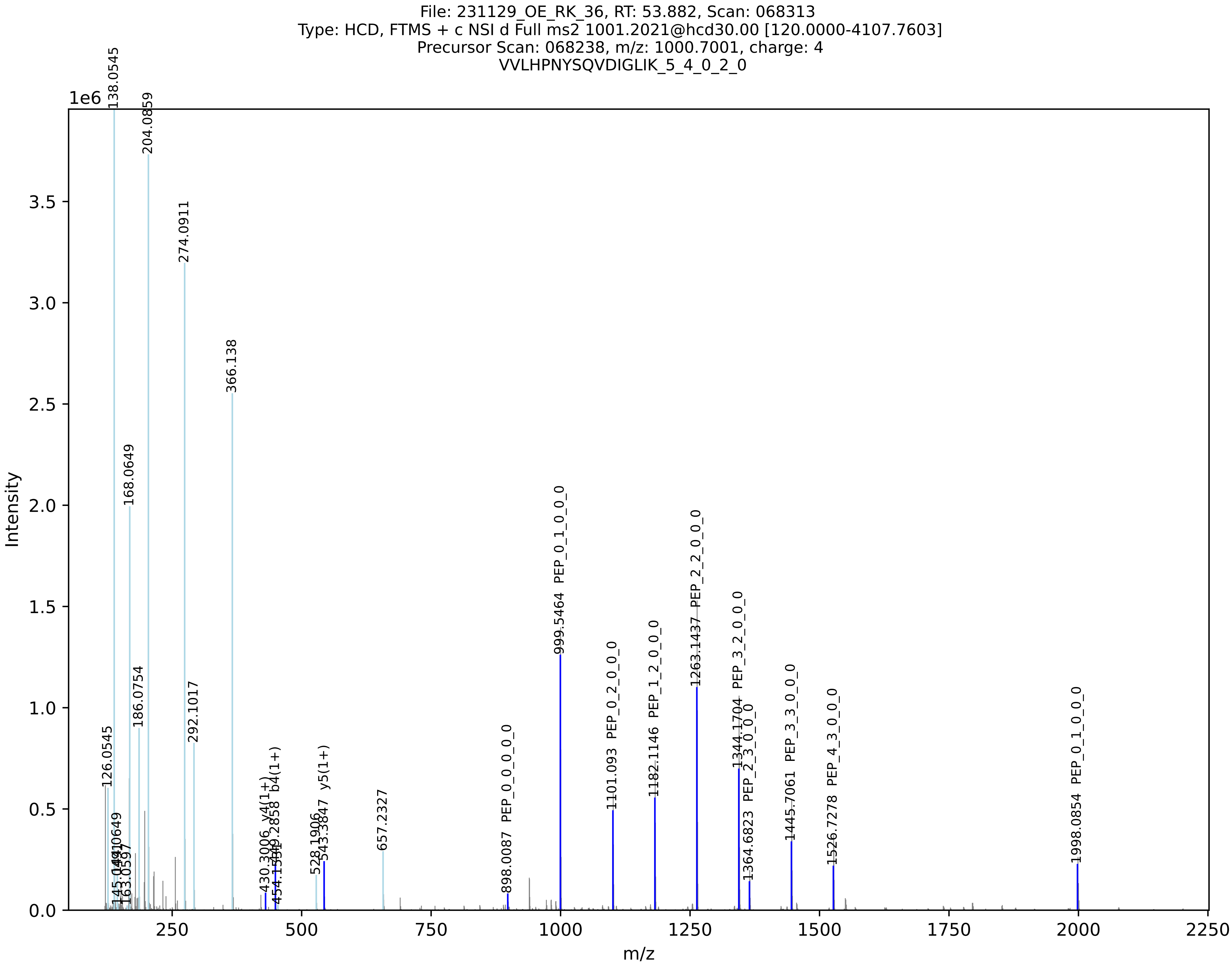
File: 231129_OE_RK_36, RT: 53.882, Scan: 068313
Type: HCD, FTMS + c NSI d Full ms2 1001.2021@hcd30.00 [120.0000-4107.7603]
Precursor Scan: 068238, m/z: 1000.7001, charge: 4
VVLHPNYSQVDIGLIK_5_4_0_2_0
1e6
Intensity
m/z
138.0545
204.0859
274.0911
366.138
168.0649
186.0754
292.1017
126.0545
y4(1+)
b4(1+)
528.1906
543.3847 y5(1+)
657.2327
898.0087 PEP_0_0_0_0_0
999.5464 PEP_0_1_0_0_0
1101.093 PEP_0_2_0_0_0
1182.1146 PEP_1_2_0_0_0
1263.1437 PEP_2_2_0_0_0
1344.1704 PEP_3_2_0_0_0
1364.6823 PEP_2_3_0_0_0
1445.7061 PEP_3_3_0_0_0
1526.7278 PEP_4_3_0_0_0
1998.0854 PEP_0_1_0_0_0

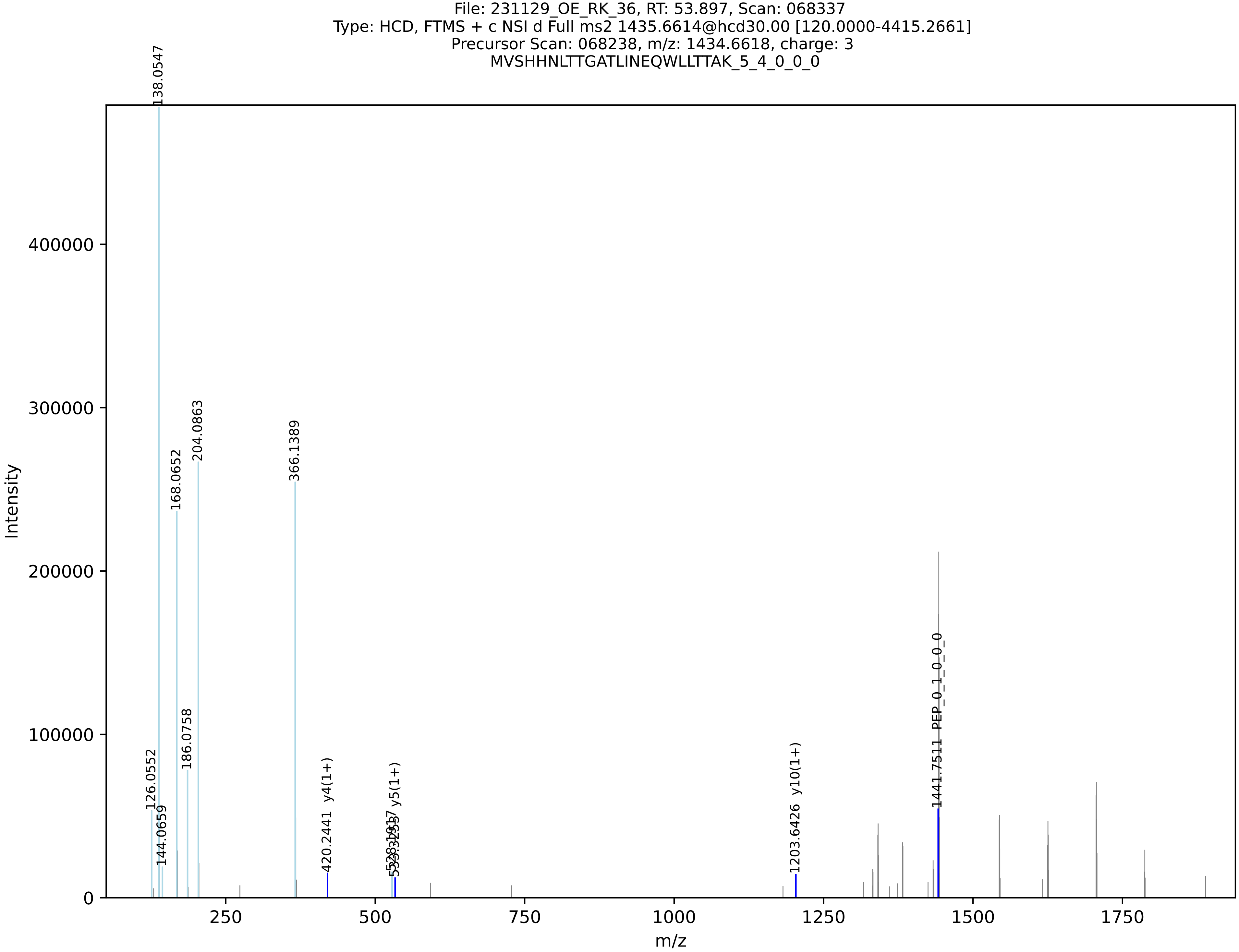
File: 231129_OE_RK_36, RT: 53.897, Scan: 068337
Type: HCD, FTMS + c NSI d Full ms2 1435.6614@hcd30.00 [120.0000-4415.2661]
Precursor Scan: 068238, m/z: 1434.6618, charge: 3
MVSHHNLTTGATLINEQWLLTTAK_5_4_0_0_0
138.0547
204.0863
168.0652
366.1389
186.0758
126.0552
144.0659
420.2441 y4(1+)
y5(1+)
1203.6426 y10(1+)
1441.7511 PEP_0_1_0_0_0
Intensity
m/z
0
100000
200000
300000
400000
250
500
750
1000
1250
1500
1750

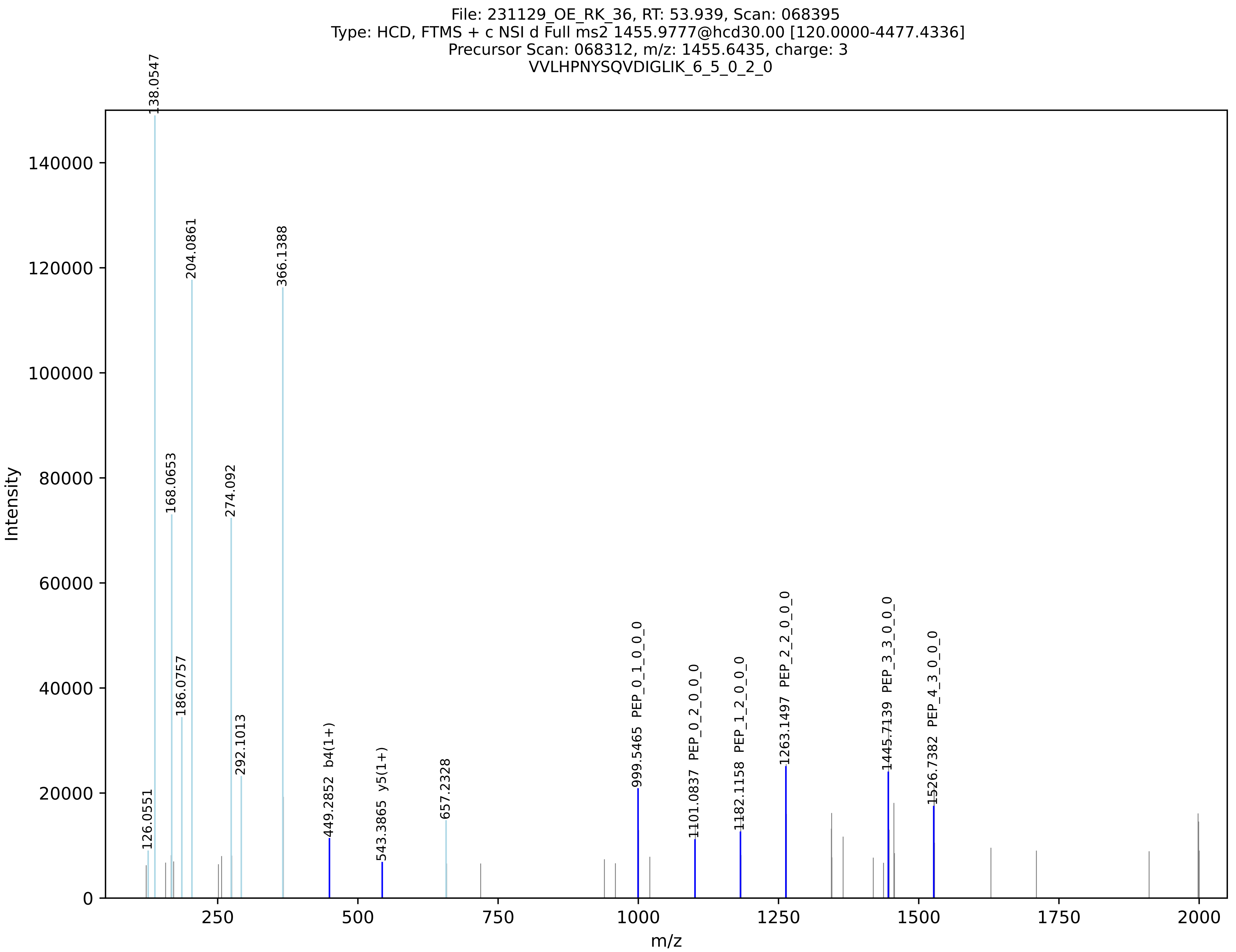

File: 231129_OE_RK_36, RT: 53.939, Scan: 068395
Type: HCD, FTMS + c NSI d Full ms2 1455.9777@hcd30.00 [120.0000-4477.4336]
Precursor Scan: 068312, m/z: 1455.6435, charge: 3
VVLHPNYSQVDIGLIK_6_5_0_2_0
Intensity
m/z
138.0547
204.0861
366.1388
168.0653
274.092
186.0757
292.1013
126.0551
449.2852 b4(1+)
543.3865 y5(1+)
657.2328
999.5465 PEP_0_1_0_0_0
1101.0837 PEP_0_2_0_0_0
1182.1158 PEP_1_2_0_0_0
1263.1497 PEP_2_2_0_0_0
1445.7139 PEP_3_3_0_0_0
1526.7382 PEP_4_3_0_0_0

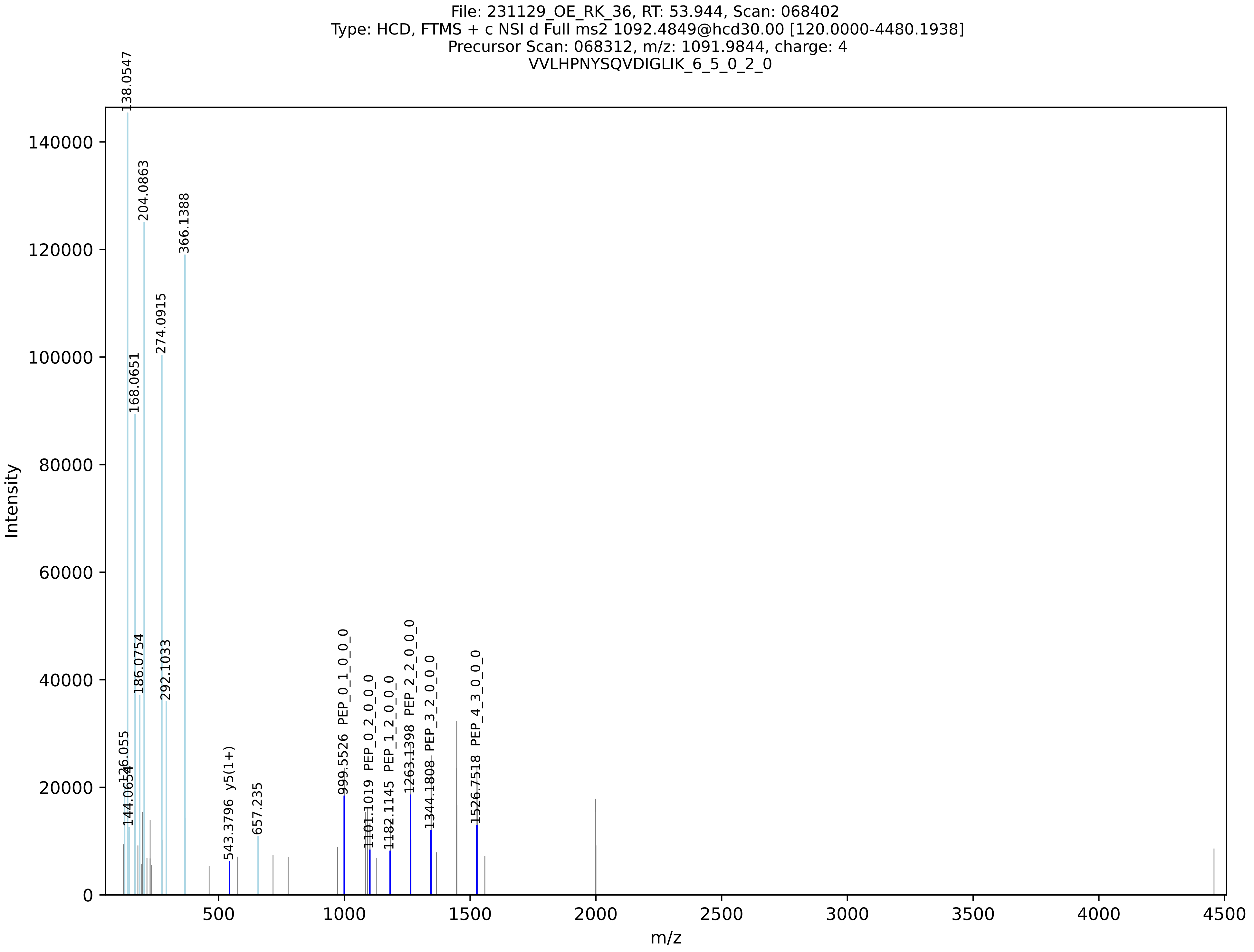
File: 231129_OE_RK_36, RT: 53.944, Scan: 068402
Type: HCD, FTMS + c NSI d Full ms2 1092.4849@hcd30.00 [120.0000-4480.1938]
Precursor Scan: 068312, m/z: 1091.9844, charge: 4
VVLHPNYSQVDIGLIK_6_5_0_2_0
Intensity
m/z
138.0547
204.0863
366.1388
274.0915
168.0651
186.0754
292.1033
126.055
144.0654
543.3796 y5(1+)
657.235
999.5526 PEP_0_1_0_0_0
1101.1019 PEP_0_2_0_0_0
1182.1145 PEP_1_2_0_0_0
1263.1398 PEP_2_2_0_0_0
1344.1808 PEP_3_2_0_0_0
1526.7518 PEP_4_3_0_0_0

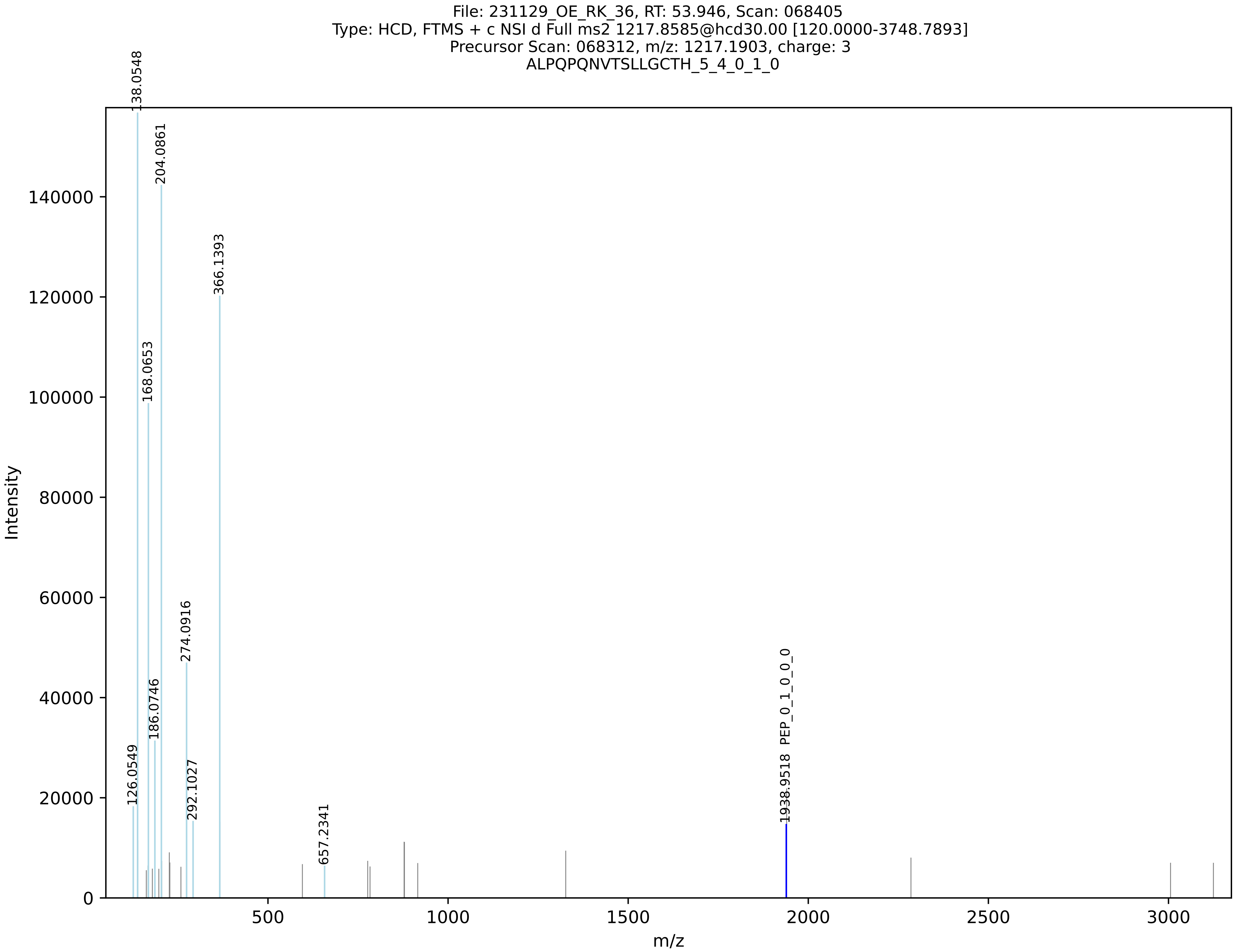

File: 231129_OE_RK_36, RT: 53.946, Scan: 068405
Type: HCD, FTMS + c NSI d Full ms2 1217.8585@hcd30.00 [120.0000-3748.7893]
Precursor Scan: 068312, m/z: 1217.1903, charge: 3
ALPQPQNVTSLLGCTH_5_4_0_1_0
Intensity
m/z
138.0548
204.0861
366.1393
168.0653
274.0916
186.0746
126.0549
292.1027
657.2341
1938.9518 PEP_0_1_0_0_0

File: 231129_OE_RK_36, RT: 53.989, Scan: 068467
Type: HCD, FTMS + c NSI d Full ms2 1383.6198@hcd30.00 [120.0000-4256.0186]
Precursor Scan: 068386, m/z: 1382.6175, charge: 3
VVLHPNYSQVDIGLIK_5_4_1_2_0

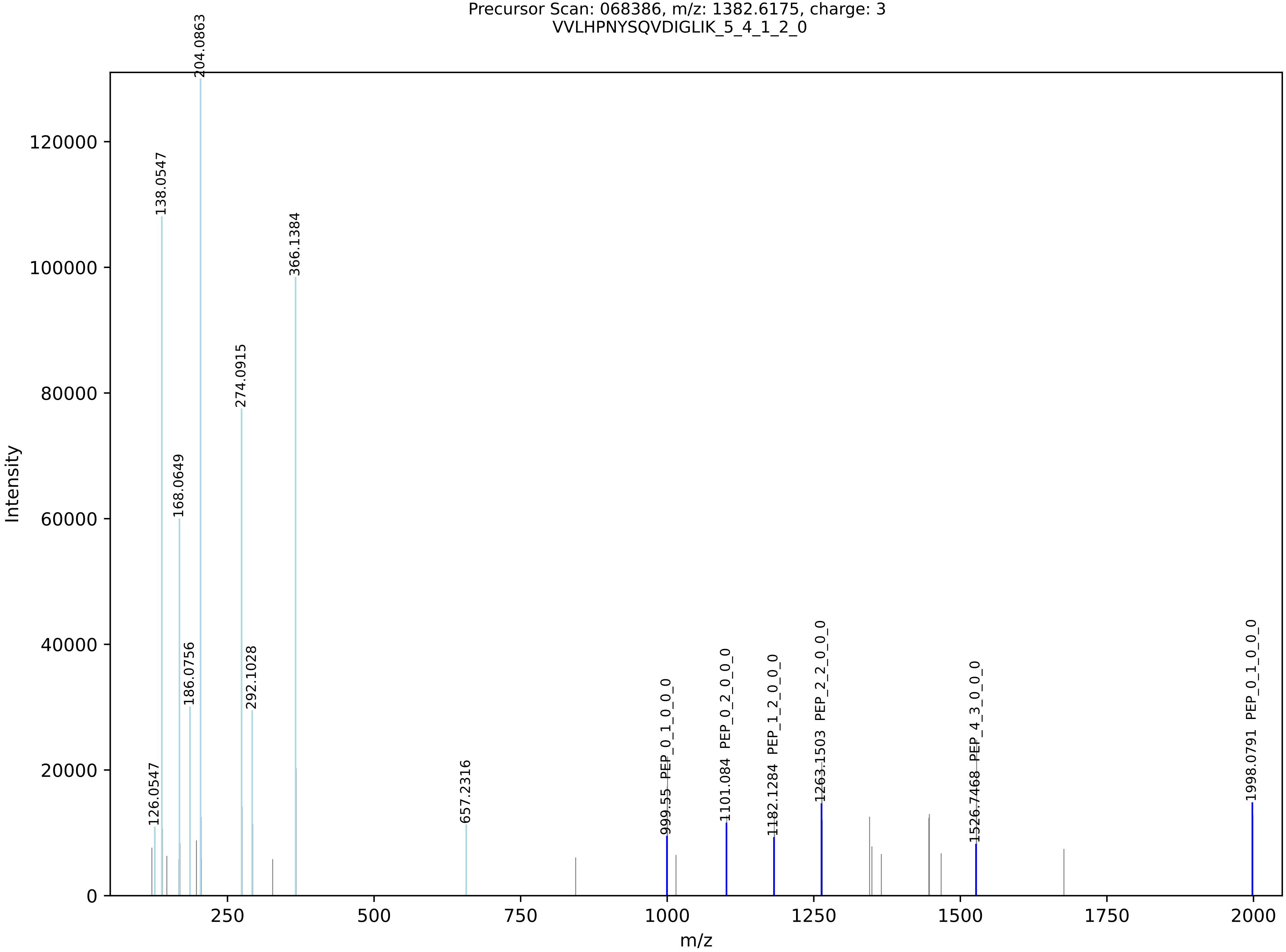

File: 231129_OE_RK_36, RT: 54.037, Scan: 068537
Type: HCD, FTMS + c NSI d Full ms2 1339.6045@hcd30.00 [120.0000-4121.3320]
Precursor Scan: 068457, m/z: 1339.2704, charge: 3
EWLPLNRSVNNVVVR_6_4_1_1_0

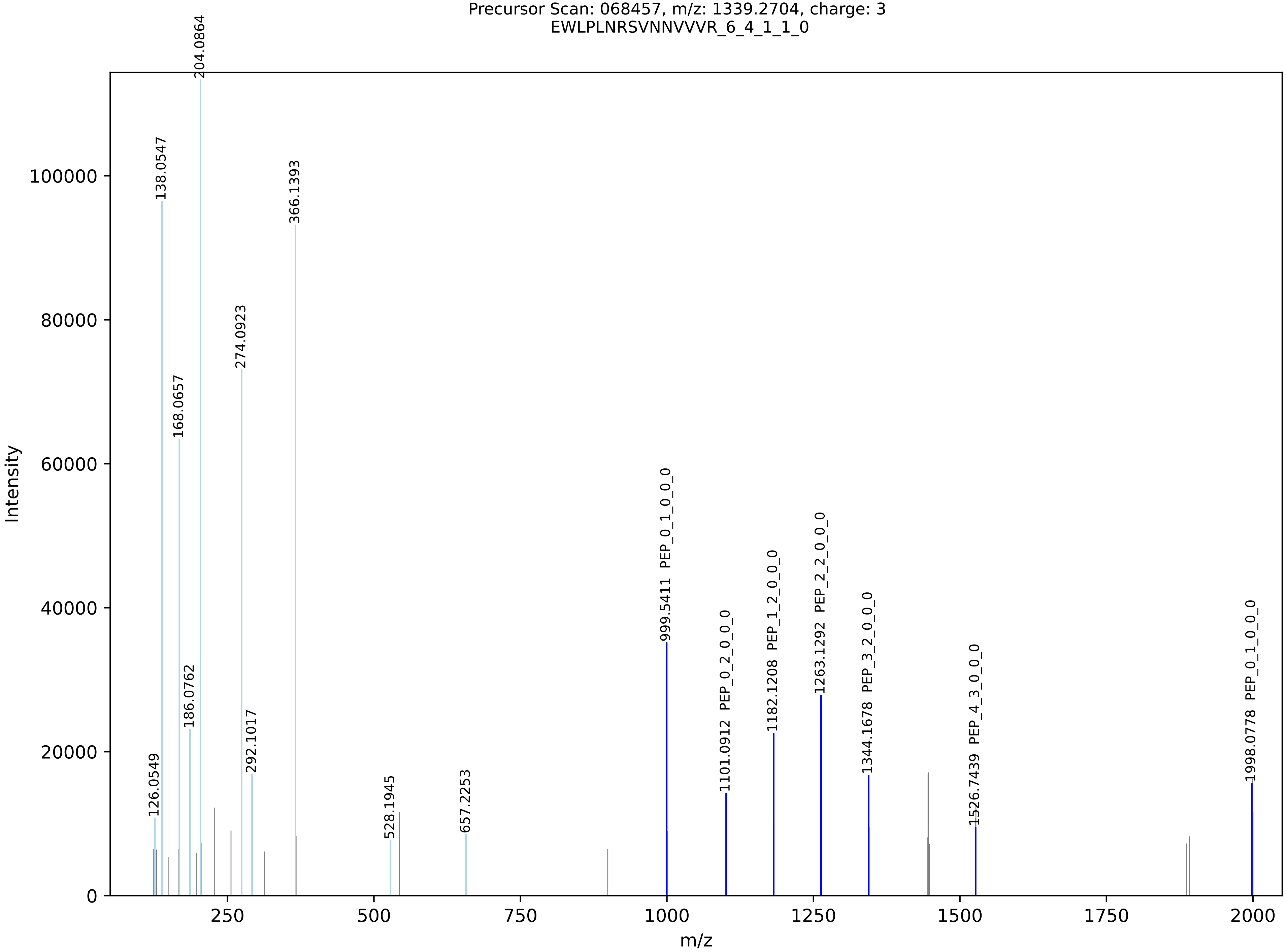

File: 231129_OE_RK_36, RT: 54.086, Scan: 068605
Type: HCD, FTMS + c NSI d Full ms2 1001.2029@hcd30.00 [120.0000-4107.7637]
Precursor Scan: 068530, m/z: 1000.7015, charge: 4
VVLHPNYSQVDIGLIK_5_4_0_2_0

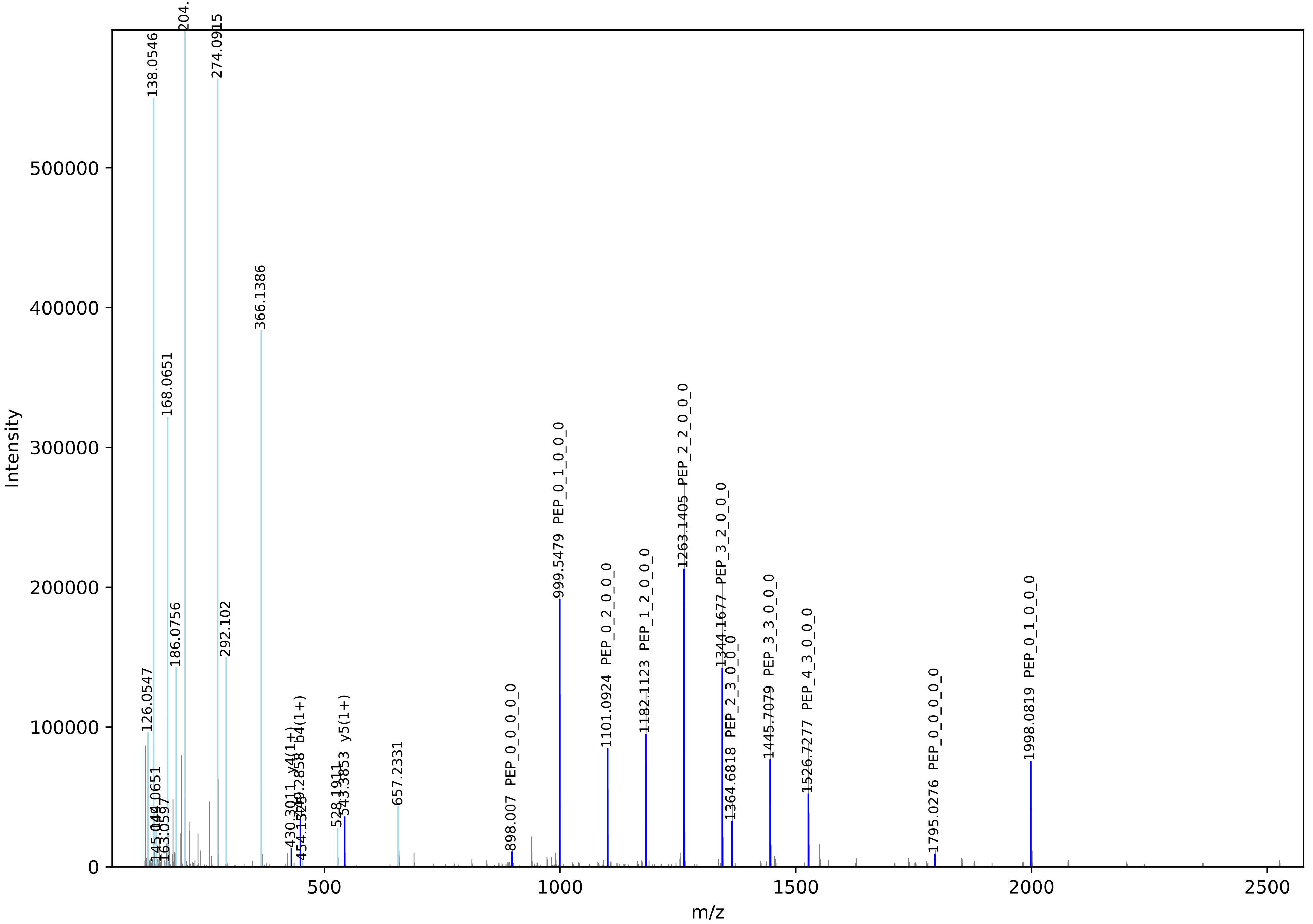

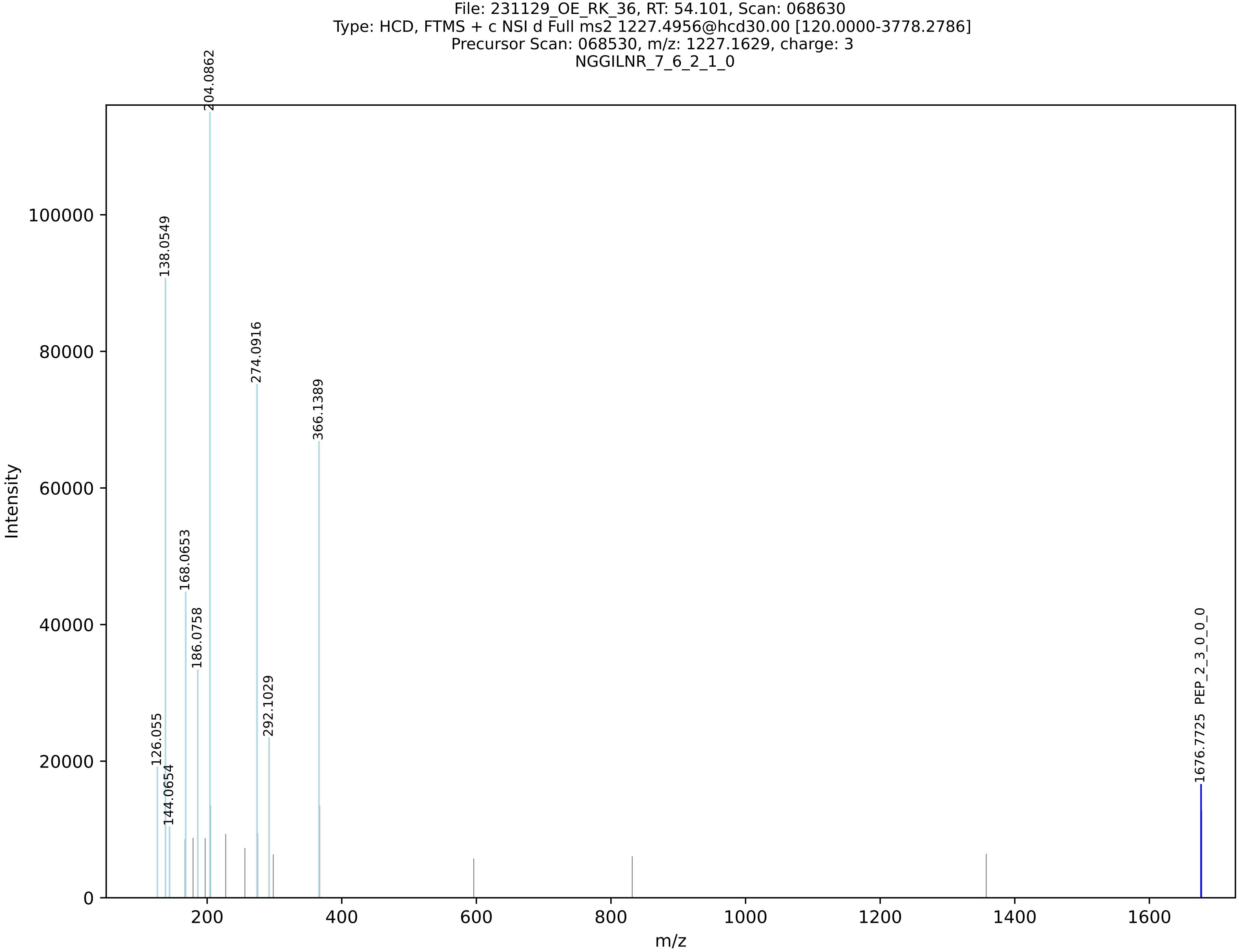
File: 231129_OE_RK_36, RT: 54.101, Scan: 068630
Type: HCD, FTMS + c NSI d Full ms2 1227.4956@hcd30.00 [120.0000-3778.2786]
Precursor Scan: 068530, m/z: 1227.1629, charge: 3
NGGILNR_7_6_2_1_0
Intensity
m/z
204.0862
138.0549
274.0916
366.1389
168.0653
186.0758
292.1029
126.055
144.0654
1676.7725 PEP_2_3_0_0_0
0
20000
40000
60000
80000
100000
200
400
600
800
1000
1200
1400
1600

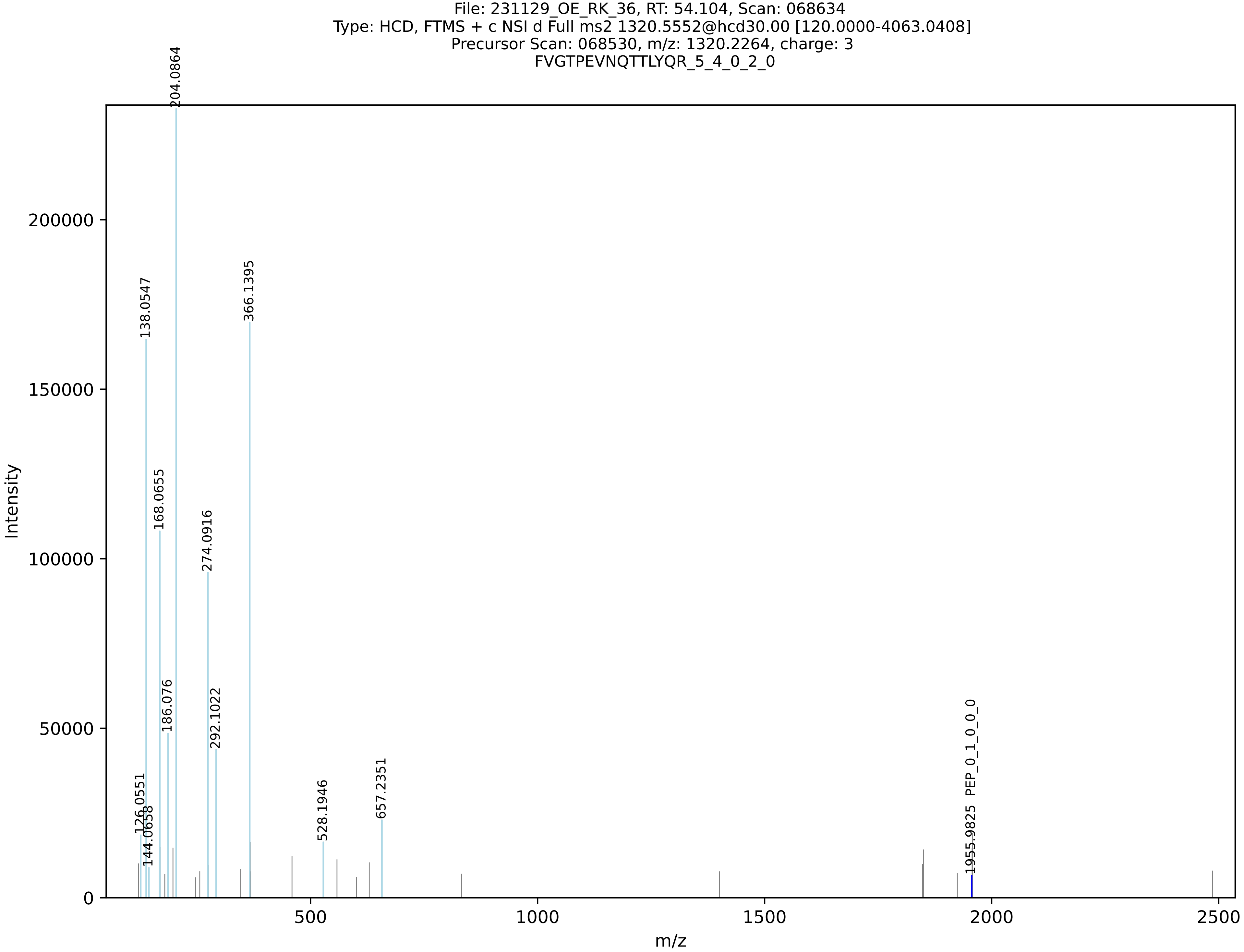

File: 231129_OE_RK_36, RT: 54.104, Scan: 068634
Type: HCD, FTMS + c NSI d Full ms2 1320.5552@hcd30.00 [120.0000-4063.0408]
Precursor Scan: 068530, m/z: 1320.2264, charge: 3
FVGTPEVNQTTLYQR_5_4_0_2_0
204.0864
138.0547
366.1395
168.0655
274.0916
186.076
292.1022
126.0551
144.0658
528.1946
657.2351
1955.9825 PEP_0_1_0_0_0_
Intensity
m/z
0
50000
100000
150000
200000
500
1000
1500
2000
2500

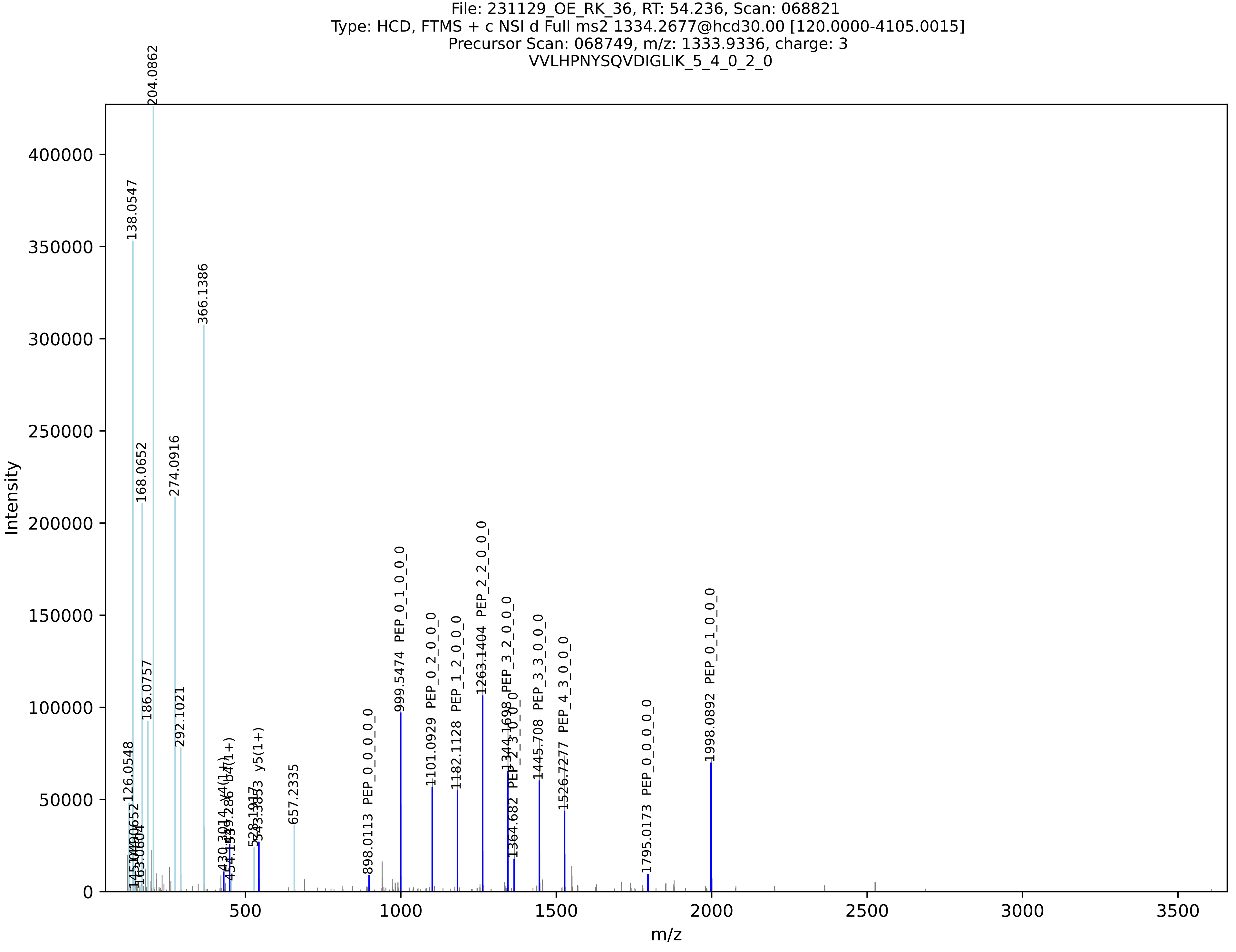
File: 231129_OE_RK_36, RT: 54.236, Scan: 068821
Type: HCD, FTMS + c NSI d Full ms2 1334.2677@hcd30.00 [120.0000-4105.0015]
Precursor Scan: 068749, m/z: 1333.9336, charge: 3
VVLHPNYSQVDIGLIK_5_4_0_2_0
Intensity
m/z
204.0862
138.0547
366.1386
168.0652
274.0916
186.0757
292.1021
126.0548
657.2335
y5(1+)
898.0113 PEP_0_0_0_0_0
999.5474 PEP_0_1_0_0_0
1101.0929 PEP_0_2_0_0_0
1182.1128 PEP_1_2_0_0_0
1263.1404 PEP_2_2_0_0_0
1344.1698 PEP_3_2_0_0_0
1364.682 PEP_2_3_0_0
1445.708 PEP_3_3_0_0_0
1526.7277 PEP_4_3_0_0_0
1795.0173 PEP_0_0_0_0_0
1998.0892 PEP_0_1_0_0_0

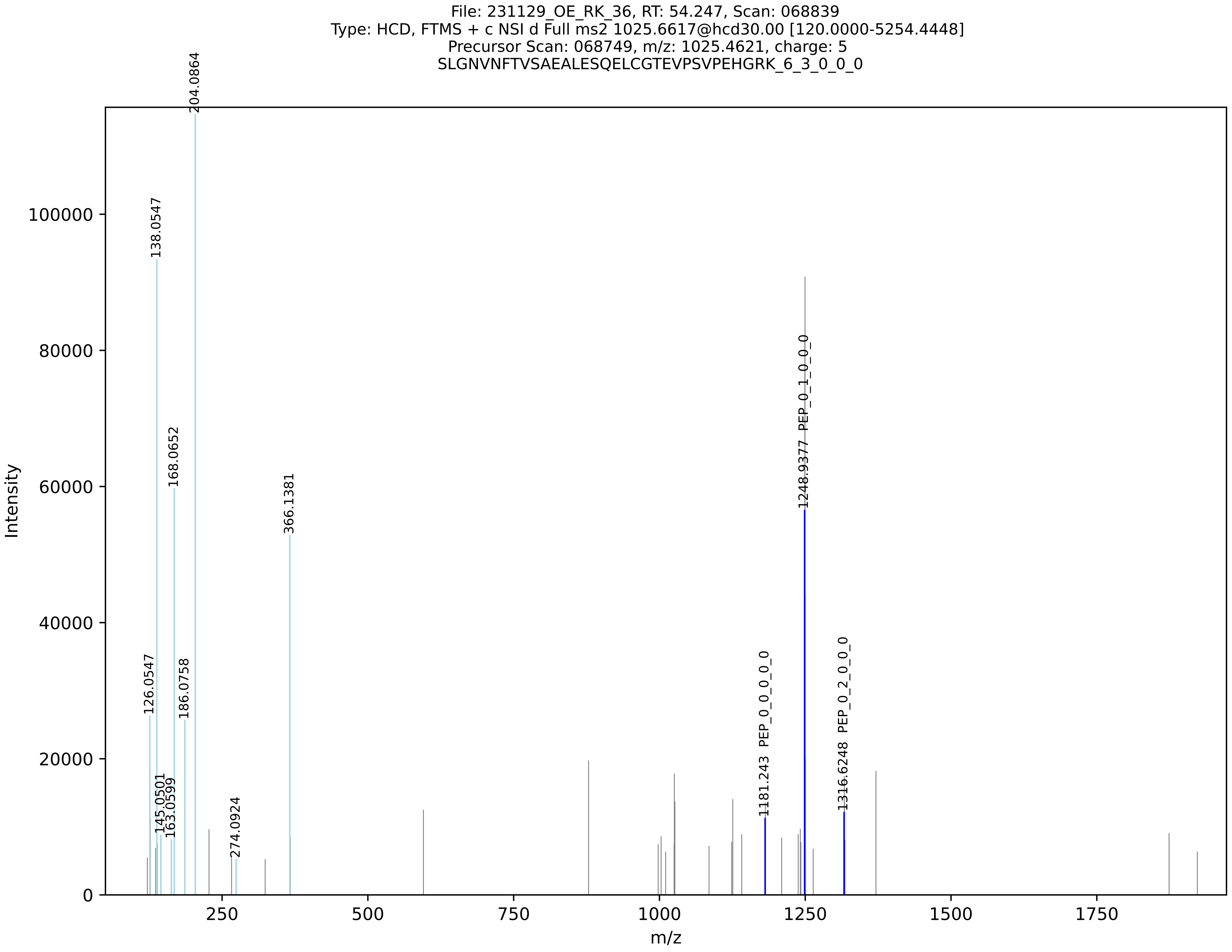
File: 231129_OE_RK_36, RT: 54.247, Scan: 068839
Type: HCD, FTMS + c NSI d Full ms2 1025.6617@hcd30.00 [120.0000-5254.4448]
Precursor Scan: 068749, m/z: 1025.4621, charge: 5
SLGNVNFTVSAEALESQELCGTEVPSVPEHGRK_6_3_0_0_0
204.0864
138.0547
168.0652
366.1381
126.0547
186.0758
145.0501
163.0599
274.0924
1248.9377 PEP_0_1_0_0_0
1181.243 PEP_0_0_0_0_0
1316.6248 PEP_0_2_0_0_0
Intensity
m/z
0
20000
40000
60000
80000
100000
250
500
750
1000
1250
1500
1750

File: 231129_OE_RK_36, RT: 54.348, Scan: 068988
Type: HCD, FTMS + c NSI d Full ms2 1223.8618@hcd30.00 [120.0000-3767.1592]
Precursor Scan: 068894, m/z: 1223.1941, charge: 3
YTGNASALFILPDQDK_5_4_0_1_0

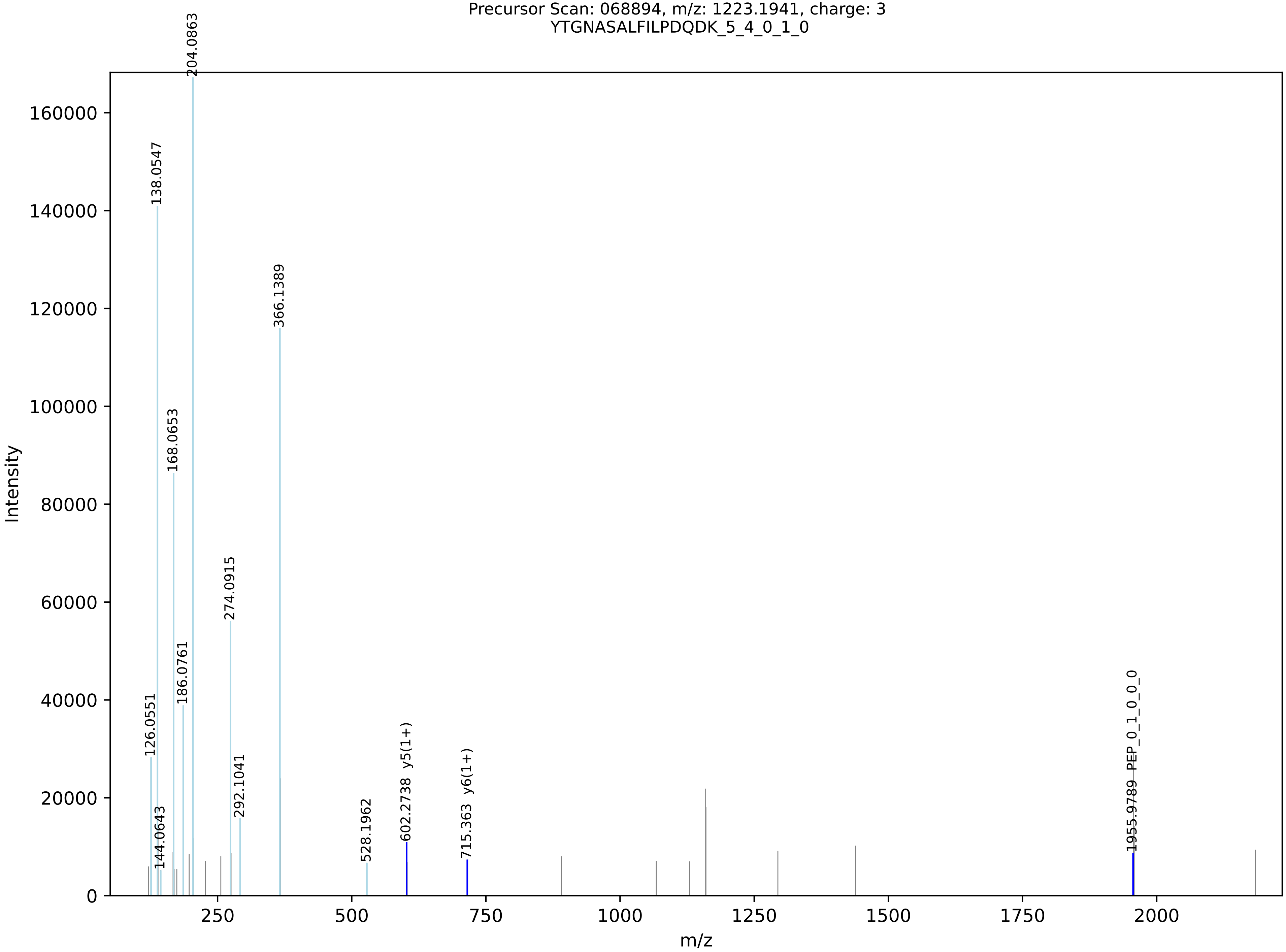

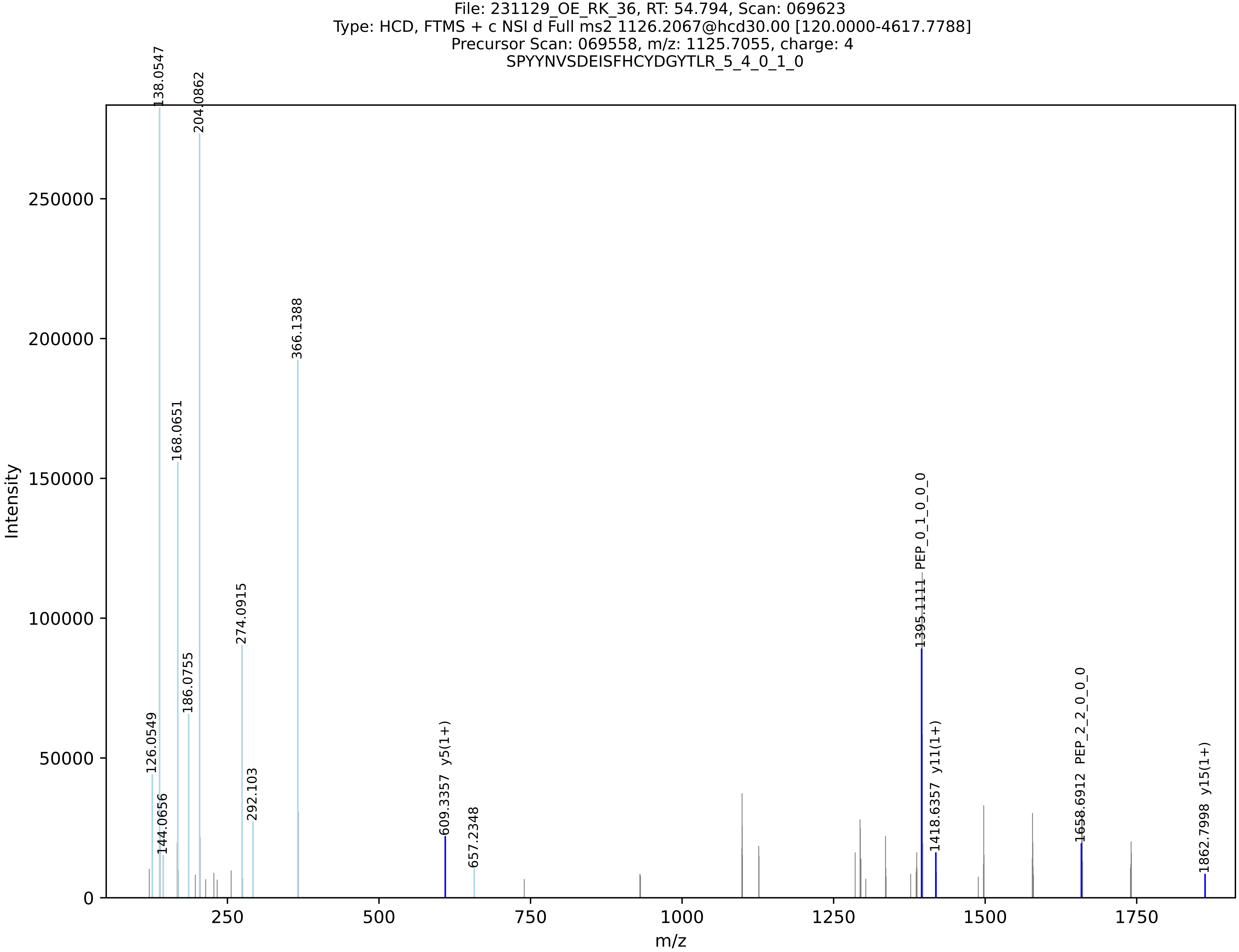

File: 231129_OE_RK_36, RT: 54.794, Scan: 069623
Type: HCD, FTMS + c NSI d Full ms2 1126.2067@hcd30.00 [120.0000-4617.7788]
Precursor Scan: 069558, m/z: 1125.7055, charge: 4
SPYYNVSDEISFHCYDGYTLR_5_4_0_1_0
Intensity
m/z
138.0547
204.0862
366.1388
168.0651
274.0915
186.0755
126.0549
144.0656
292.103
609.3357 y5(1+)
657.2348
1395.1111 PEP_0_1_0_0_0_
1418.6357 y11(1+)
1658.6912 PEP_2_2_0_0_0_
1862.7998 y15(1+)

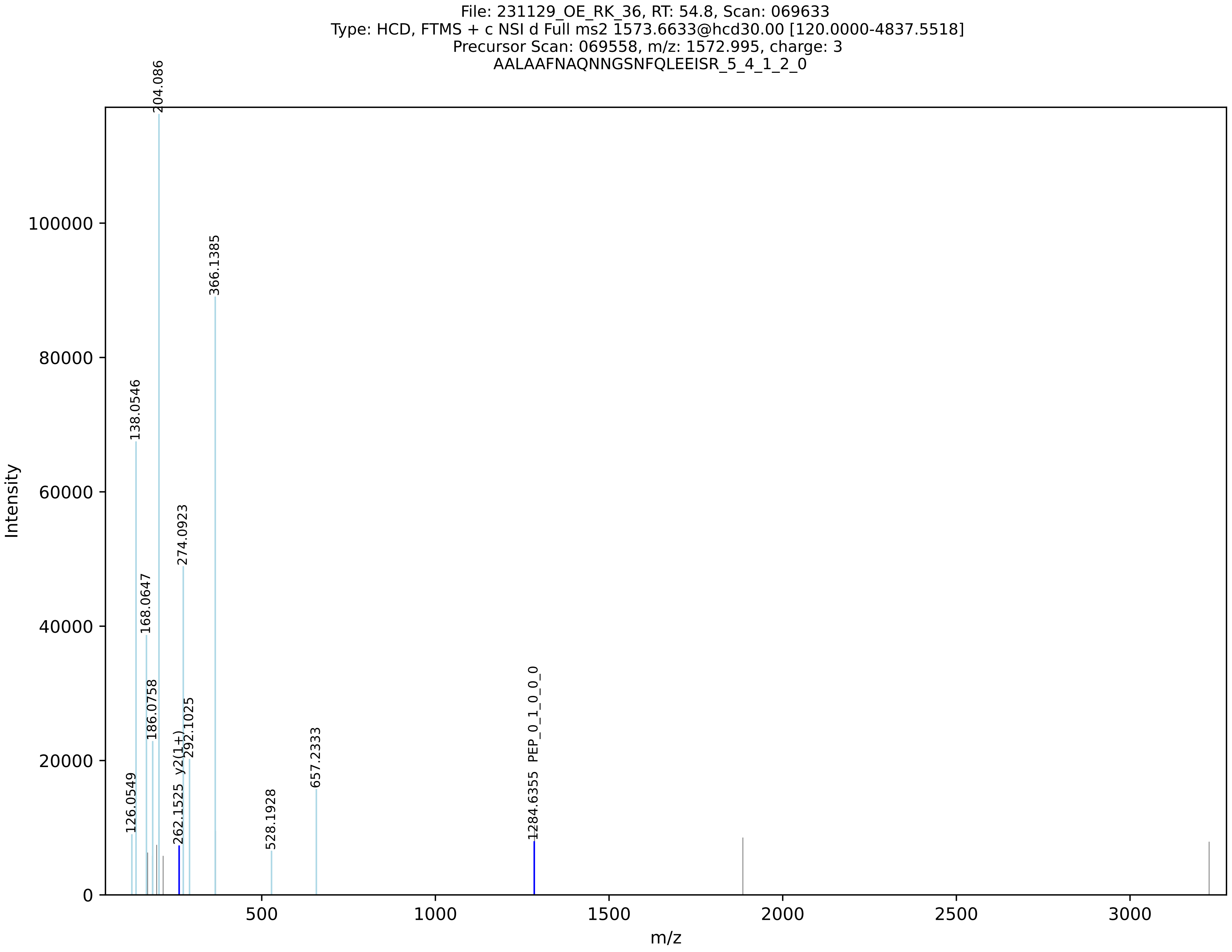

File: 231129_OE_RK_36, RT: 54.8, Scan: 069633
Type: HCD, FTMS + c NSI d Full ms2 1573.6633@hcd30.00 [120.0000-4837.5518]
Precursor Scan: 069558, m/z: 1572.995, charge: 3
AALAAFNAQNNGSNFQLEEISR_5_4_1_2_0
Intensity
m/z
0
20000
40000
60000
80000
100000
500
1000
1500
2000
2500
3000
204.086
366.1385
138.0546
274.0923
168.0647
186.0758
292.1025
y2(1+)
262.1525
126.0549
528.1928
657.2333
PEP_0_1_0_0_0
1284.6355

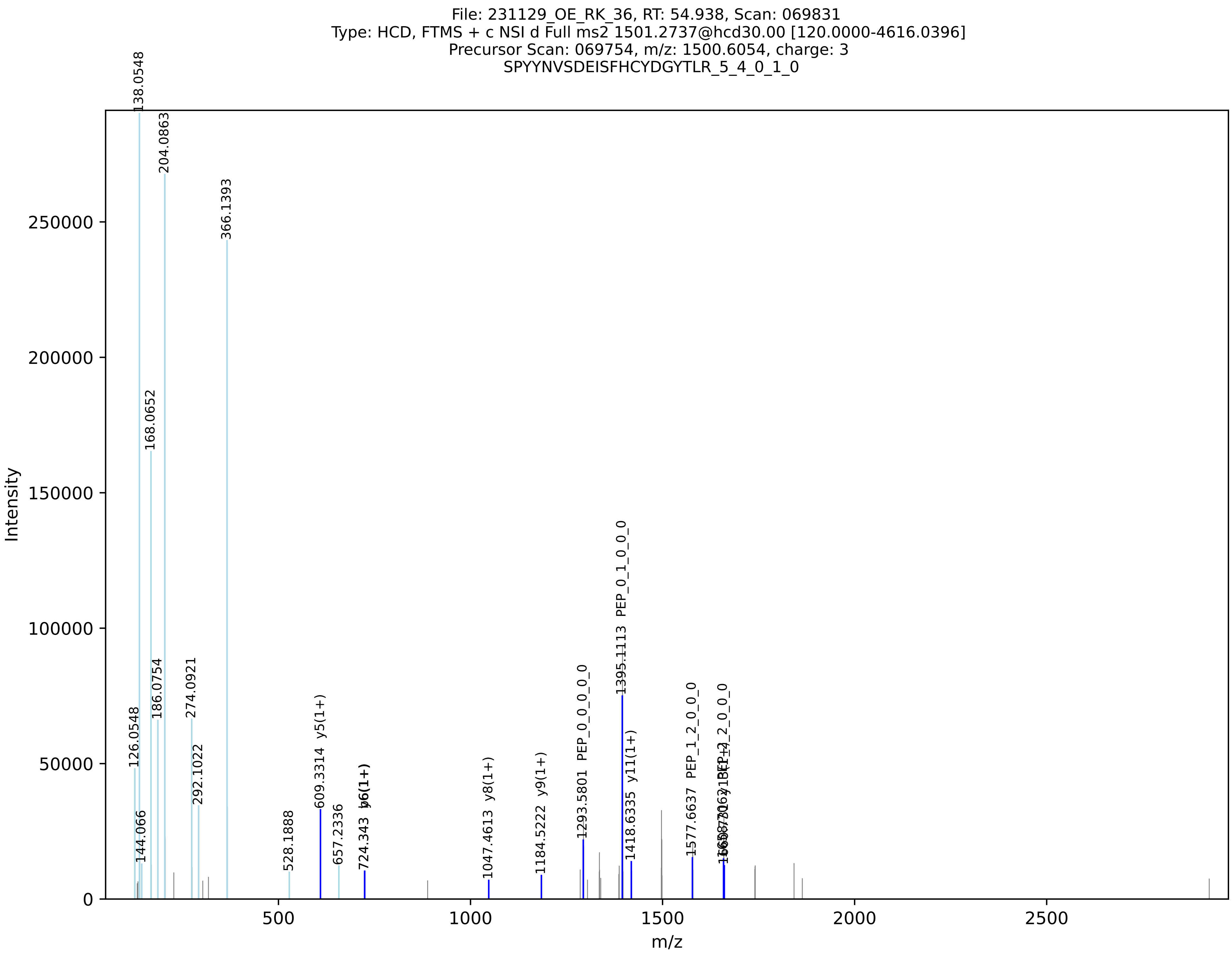
File: 231129_OE_RK_36, RT: 54.938, Scan: 069831
Type: HCD, FTMS + c NSI d Full ms2 1501.2737@hcd30.00 [120.0000-4616.0396]
Precursor Scan: 069754, m/z: 1500.6054, charge: 3
SPYYNVSDEISFHCYDGYTLR_5_4_0_1_0
Intensity
m/z
138.0548
204.0863
366.1393
168.0652
186.0754
274.0921
126.0548
292.1022
144.066
528.1888
609.3314 y5(1+)
657.2336
724.343 y6(1+)
1047.4613 y8(1+)
1184.5222 y9(1+)
1293.5801 PEP_0_0_0_0_0
1395.1113 PEP_0_1_0_0_0
1418.6335 y11(1+)
1577.6637 PEP_1_2_0_0_0
PEP_2_2_0_0_0
y13(1+)

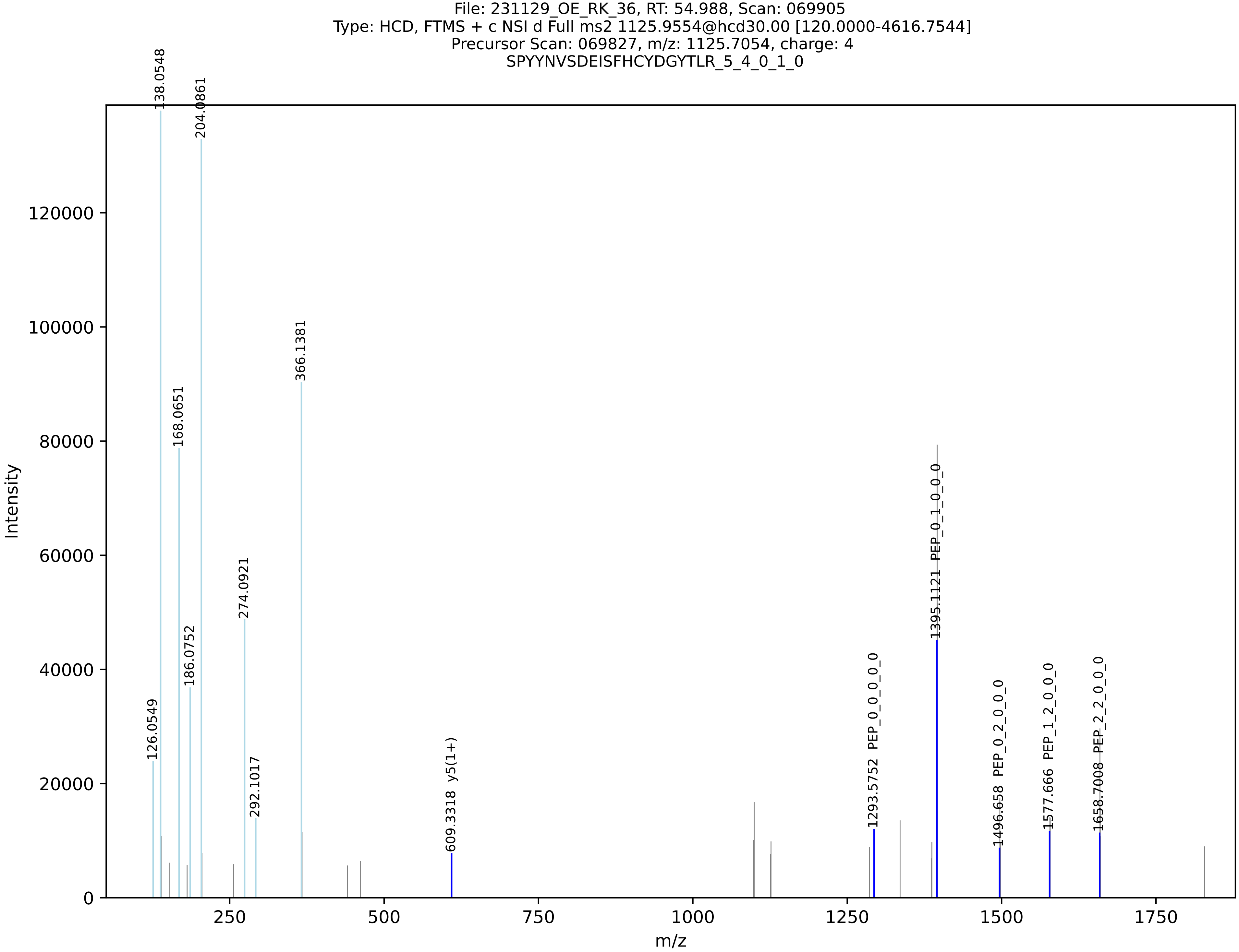

File: 231129_OE_RK_36, RT: 54.988, Scan: 069905
Type: HCD, FTMS + c NSI d Full ms2 1125.9554@hcd30.00 [120.0000-4616.7544]
Precursor Scan: 069827, m/z: 1125.7054, charge: 4
SPYYNVSDEISFHCYDGYTLR_5_4_0_1_0
Intensity
m/z
138.0548
204.0861
168.0651
186.0752
126.0549
274.0921
292.1017
366.1381
609.3318 y5(1+)
1293.5752 PEP_0_0_0_0_0
1395.1121 PEP_0_1_0_0_0
1496.658 PEP_0_2_0_0_0
1577.666 PEP_1_2_0_0_0
1658.7008 PEP_2_2_0_0_0

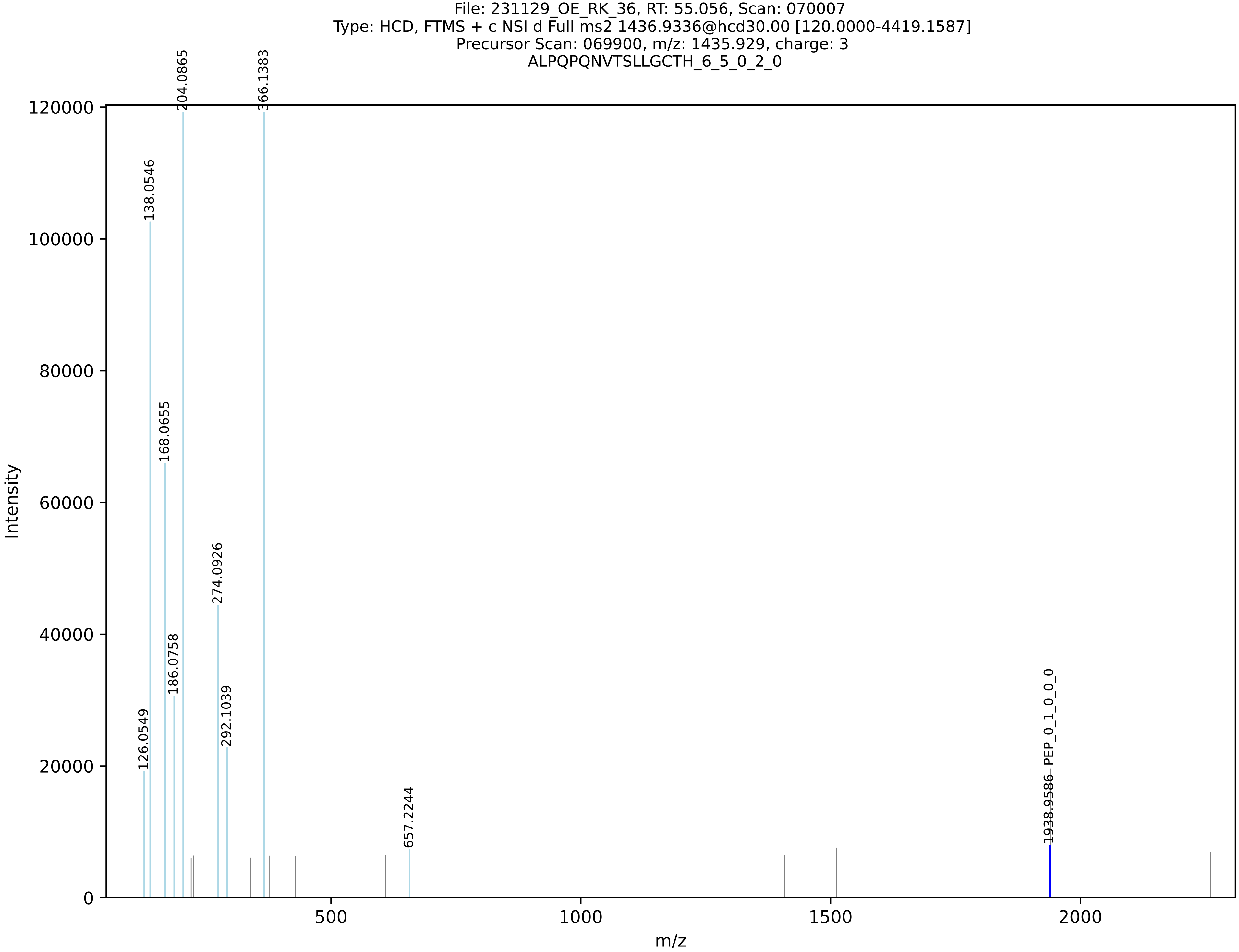
File: 231129_OE_RK_36, RT: 55.056, Scan: 070007
Type: HCD, FTMS + c NSI d Full ms2 1436.9336@hcd30.00 [120.0000-4419.1587]
Precursor Scan: 069900, m/z: 1435.929, charge: 3
ALPQPQNVTSLLGCTH_6_5_0_2_0
Intensity
m/z
204.0865
366.1383
138.0546
168.0655
274.0926
186.0758
292.1039
126.0549
657.2244
1938.9586 PEP_0_1_0_0_0
0
20000
40000
60000
80000
100000
120000
500
1000
1500
2000

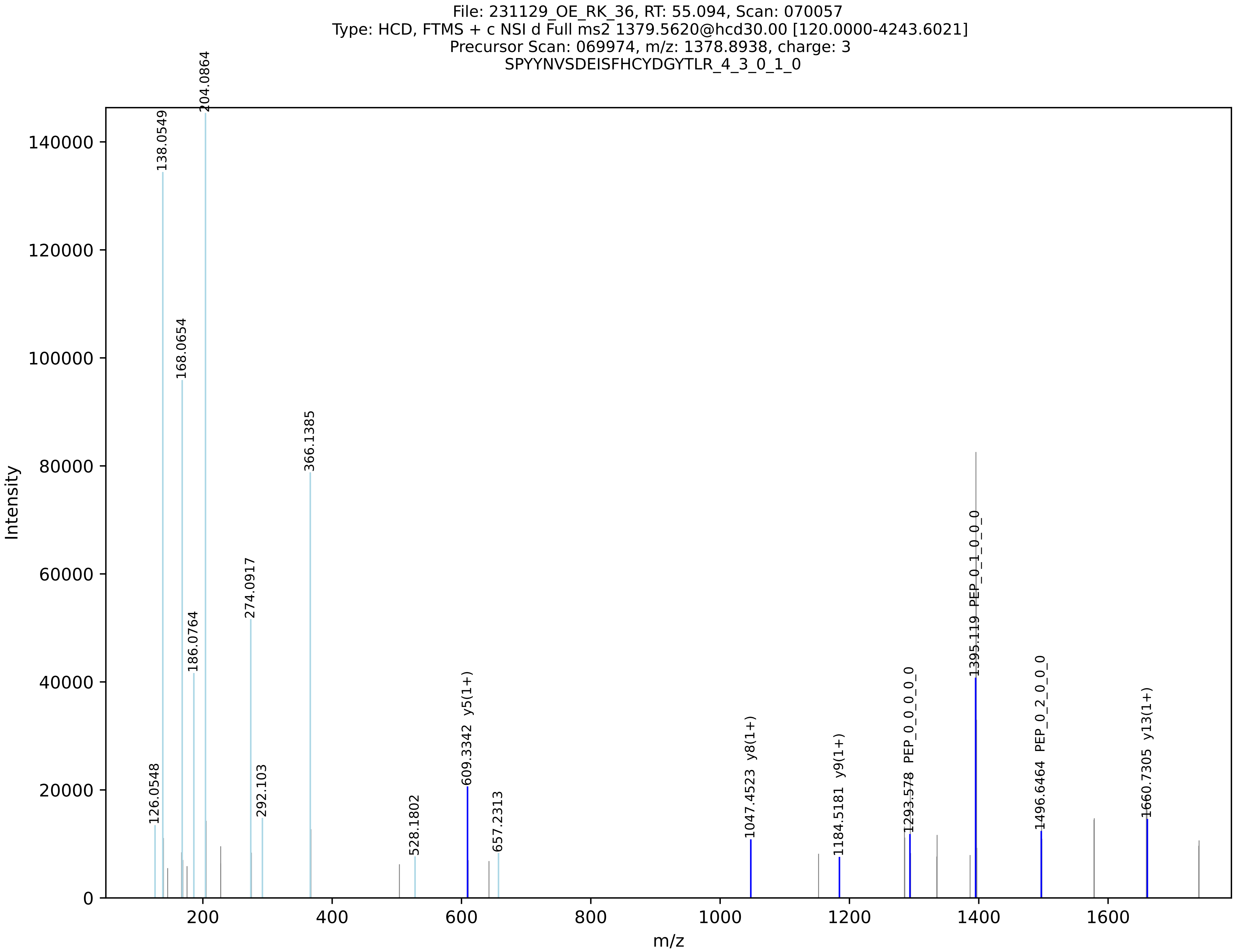
File: 231129_OE_RK_36, RT: 55.094, Scan: 070057
Type: HCD, FTMS + c NSI d Full ms2 1379.5620@hcd30.00 [120.0000-4243.6021]
Precursor Scan: 069974, m/z: 1378.8938, charge: 3
SPYYNVSDEISFHCYDGYTLR_4_3_0_1_0
Intensity
m/z
204.0864
138.0549
168.0654
366.1385
274.0917
186.0764
126.0548
292.103
528.1802
609.3342 y5(1+)
657.2313
1047.4523 y8(1+)
1184.5181 y9(1+)
1293.578 PEP_0_0_0_0_0
1395.119 PEP_0_1_0_0_0
1496.6464 PEP_0_2_0_0_0
1660.7305 y13(1+)
0
20000
40000
60000
80000
100000
120000
140000
200
400
600
800
1000
1200
1400
1600

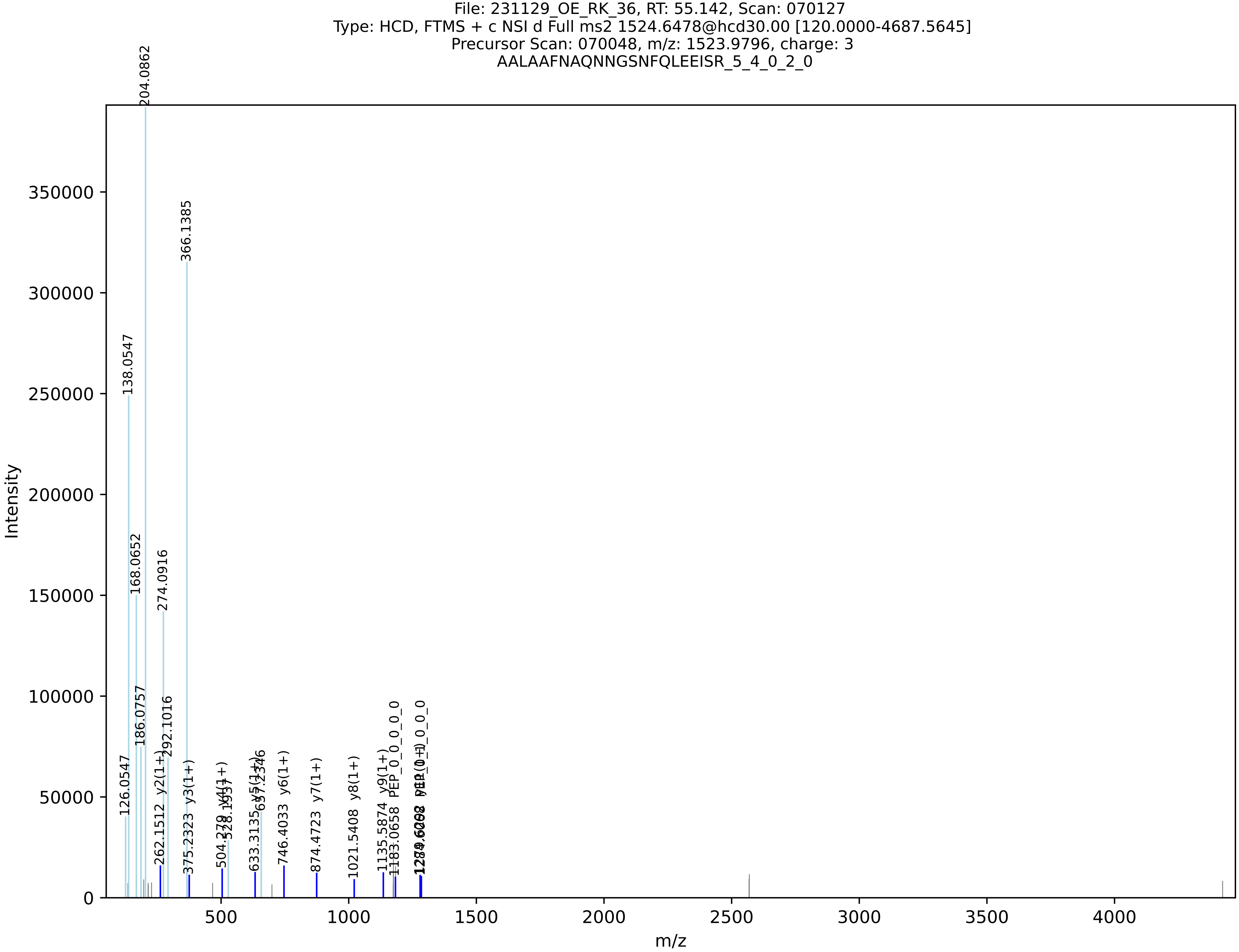

File: 231129_OE_RK_36, RT: 55.142, Scan: 070127
Type: HCD, FTMS + c NSI d Full ms2 1524.6478@hcd30.00 [120.0000-4687.5645]
Precursor Scan: 070048, m/z: 1523.9796, charge: 3
AALAAFNAQNNGSNFQLEEISR_5_4_0_2_0
Intensity
m/z
204.0862
366.1385
138.0547
168.0652
274.0916
186.0757
292.1016
126.0547
262.1512 y2(1+)
375.2323 y3(1+)
504.279 y4(1+)
528.1937
633.3135 y5(1+)
657.2346
746.4033 y6(1+)
874.4723 y7(1+)
1021.5408 y8(1+)
1135.5874 y9(1+)
1183.0658 PEP_0_0_0_0_0

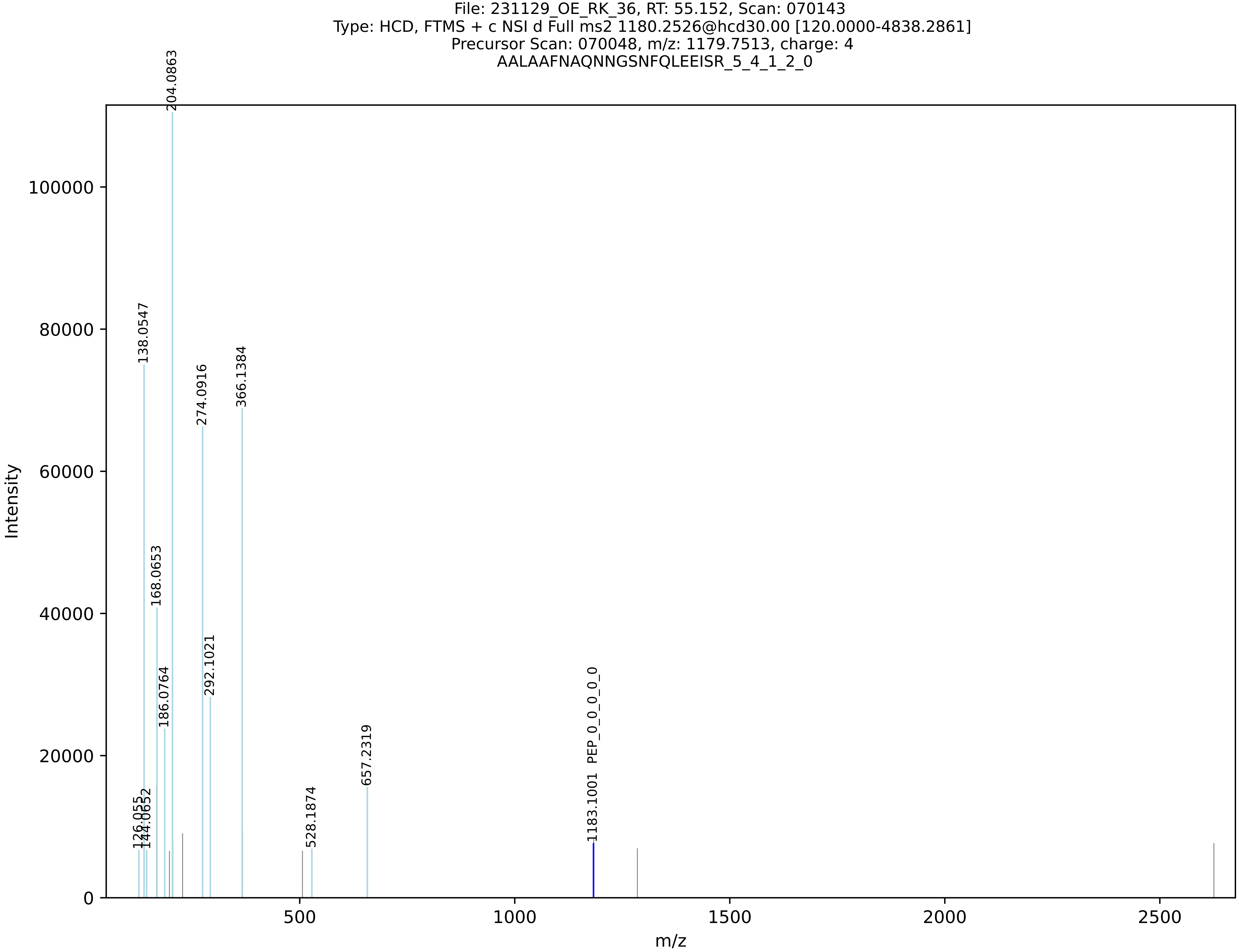

File: 231129_OE_RK_36, RT: 55.152, Scan: 070143
Type: HCD, FTMS + c NSI d Full ms2 1180.2526@hcd30.00 [120.0000-4838.2861]
Precursor Scan: 070048, m/z: 1179.7513, charge: 4
AALAAFNAQNNGSNFQLEEISR_5_4_1_2_0
Intensity
m/z
204.0863
138.0547
274.0916
366.1384
168.0653
292.1021
186.0764
126.055
144.0652
528.1874
657.2319
1183.1001 PEP_0_0_0_0_0
0
20000
40000
60000
80000
100000
500
1000
1500
2000
2500

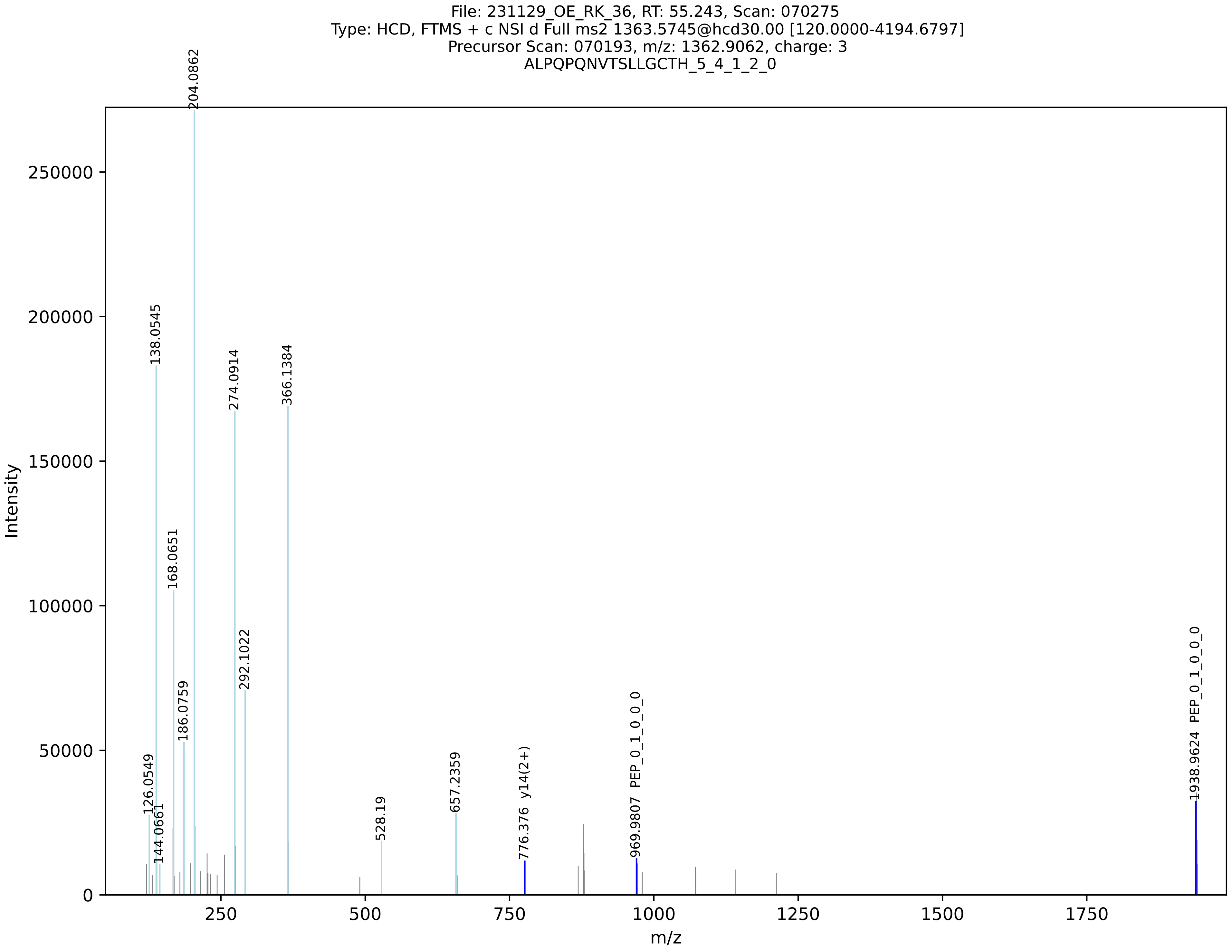

File: 231129_OE_RK_36, RT: 55.243, Scan: 070275
Type: HCD, FTMS + c NSI d Full ms2 1363.5745@hcd30.00 [120.0000-4194.6797]
Precursor Scan: 070193, m/z: 1362.9062, charge: 3
ALPQPQNVTSLLGCTH_5_4_1_2_0
Intensity
m/z
204.0862
138.0545
274.0914
366.1384
168.0651
292.1022
186.0759
126.0549
144.0661
528.19
657.2359
776.376 y14(2+)
969.9807 PEP_0_1_0_0_0
1938.9624 PEP_0_1_0_0_0

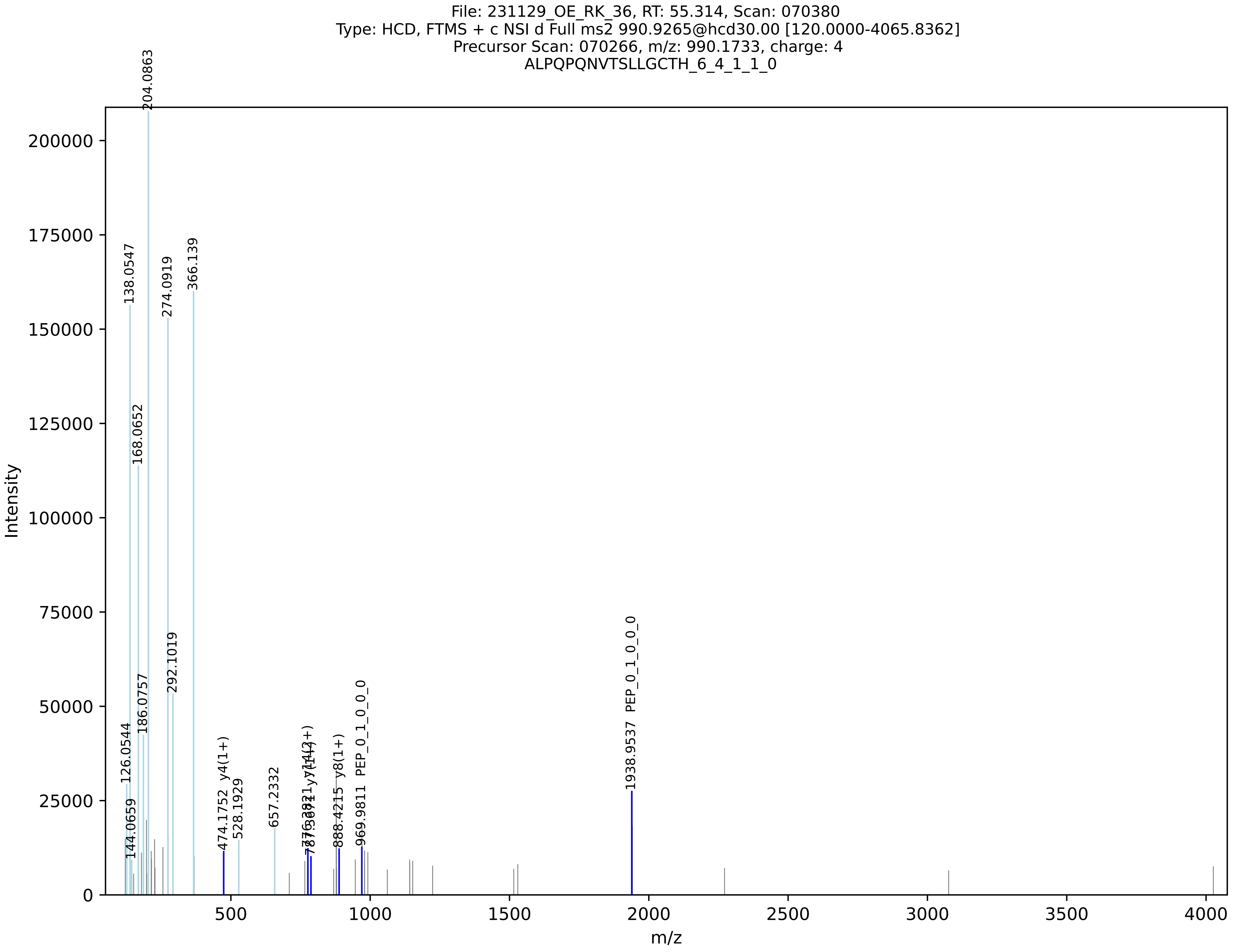
File: 231129_OE_RK_36, RT: 55.314, Scan: 070380
Type: HCD, FTMS + c NSI d Full ms2 990.9265@hcd30.00 [120.0000-4065.8362]
Precursor Scan: 070266, m/z: 990.1733, charge: 4
ALPQPQNVTSLLGCTH_6_4_1_1_0
204.0863
138.0547
274.0919
366.139
168.0652
292.1019
186.0757
126.0544
144.0659
474.1752 y4(1+)
528.1929
657.2332
888.4215 y8(1+)
969.9811 PEP_0_1_0_0_0
1938.9537 PEP_0_1_0_0_0
Intensity
m/z
0
25000
50000
75000
100000
125000
150000
175000
200000
500
1000
1500
2000
2500
3000
3500
4000

File: 231129_OE_RK_36, RT: 55.361, Scan: 070447
Type: HCD, FTMS + c NSI d Full ms2 1313.8867@hcd30.00 [120.0000-4042.6353]
Precursor Scan: 070337, m/z: 1313.5501, charge: 3
ALPQPQNVTSLLGCTH_5_4_0_2_0

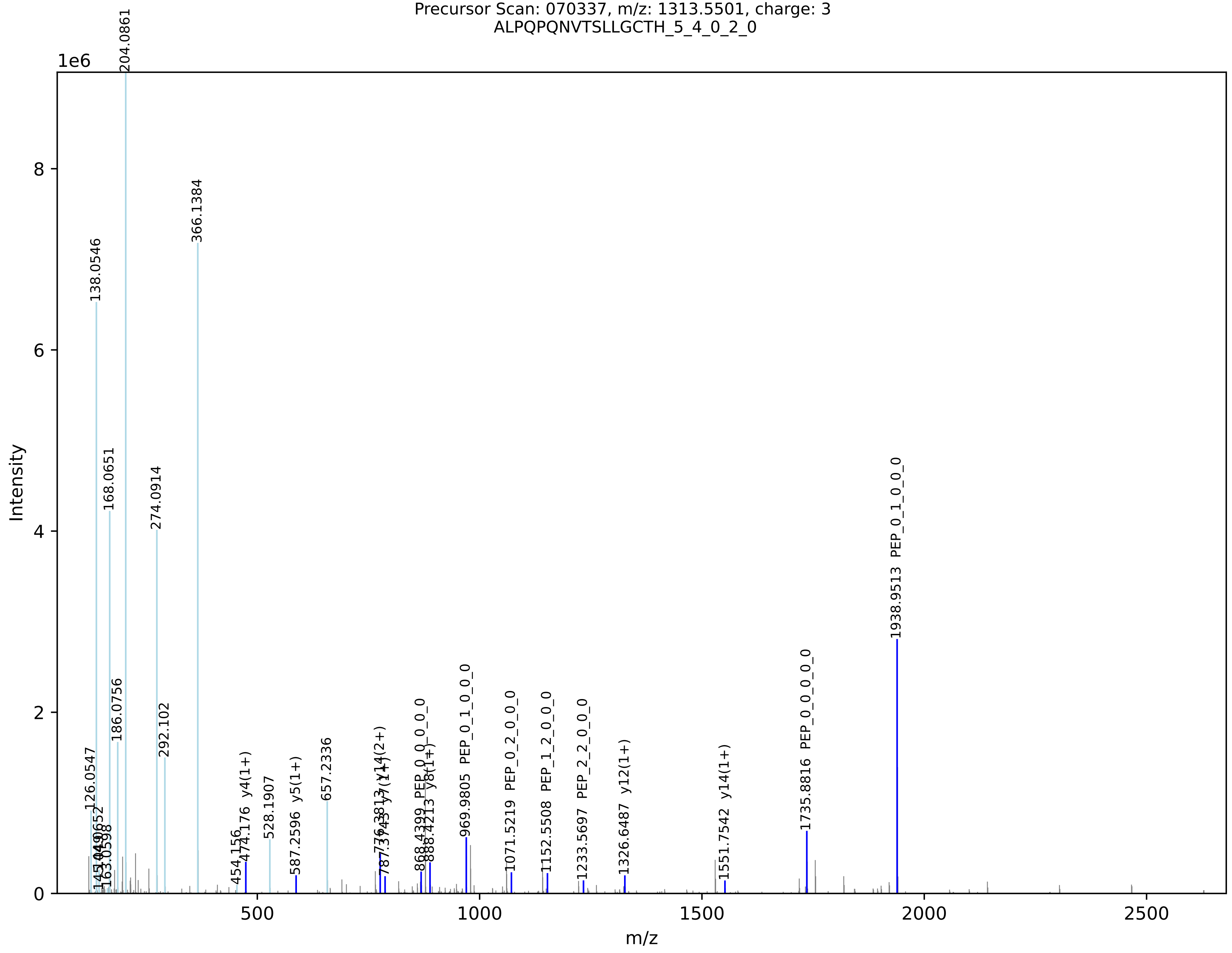

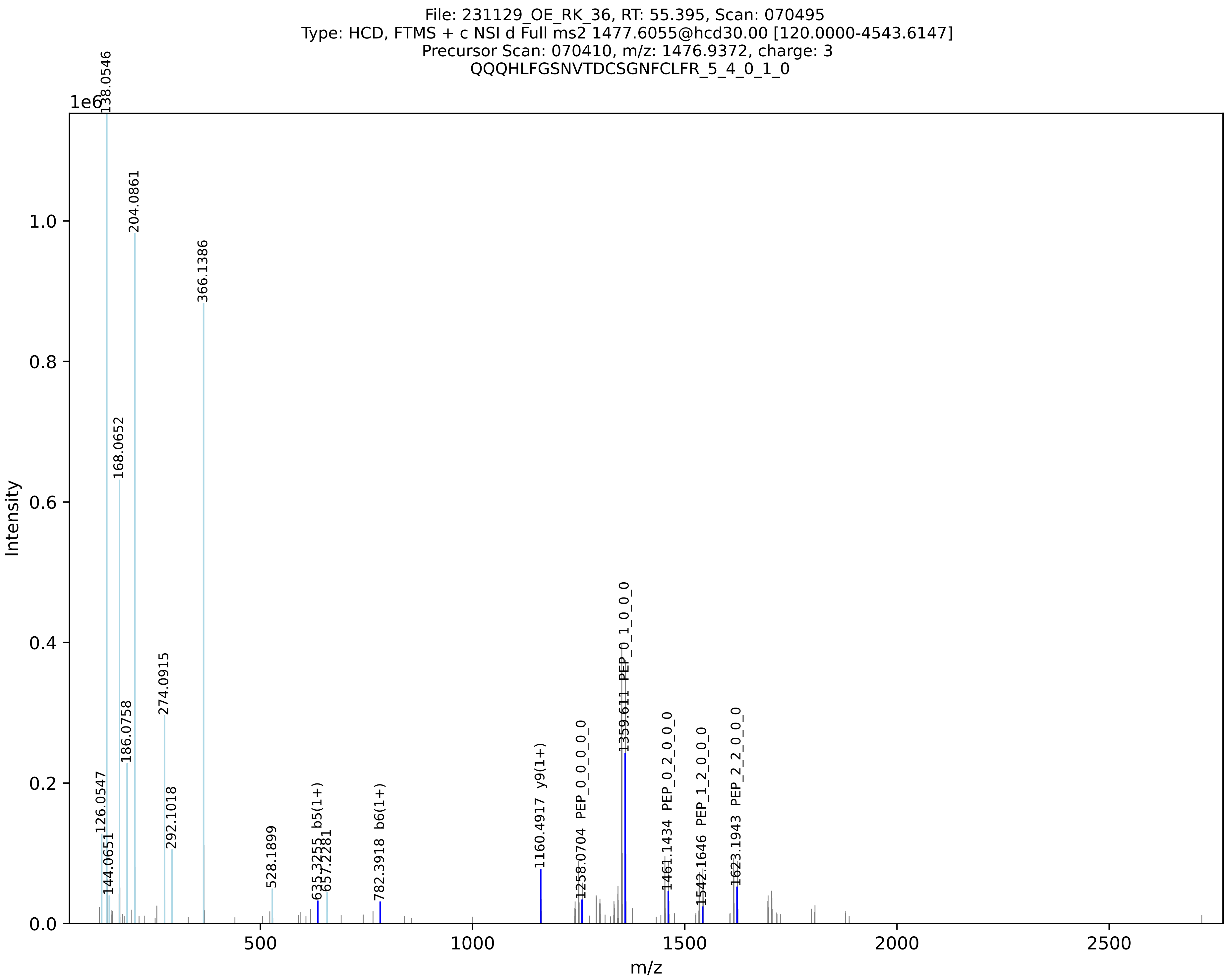
File: 231129_OE_RK_36, RT: 55.395, Scan: 070495
Type: HCD, FTMS + c NSI d Full ms2 1477.6055@hcd30.00 [120.0000-4543.6147]
Precursor Scan: 070410, m/z: 1476.9372, charge: 3
QQQHLFGSNVTDCSGNFCLFR_5_4_0_1_0
1e6
Intensity
m/z
138.0546
204.0861
366.1386
168.0652
274.0915
186.0758
292.1018
126.0547
144.0651
528.1899
635.3255 b5(1+)
657.2281
782.3918 b6(1+)
1160.4917 y9(1+)
1258.0704 PEP_0_0_0_0_0
1359.611 PEP_0_1_0_0_0
1461.1434 PEP_0_2_0_0_0
1542.1646 PEP_1_2_0_0_0
1623.1943 PEP_2_2_0_0_0

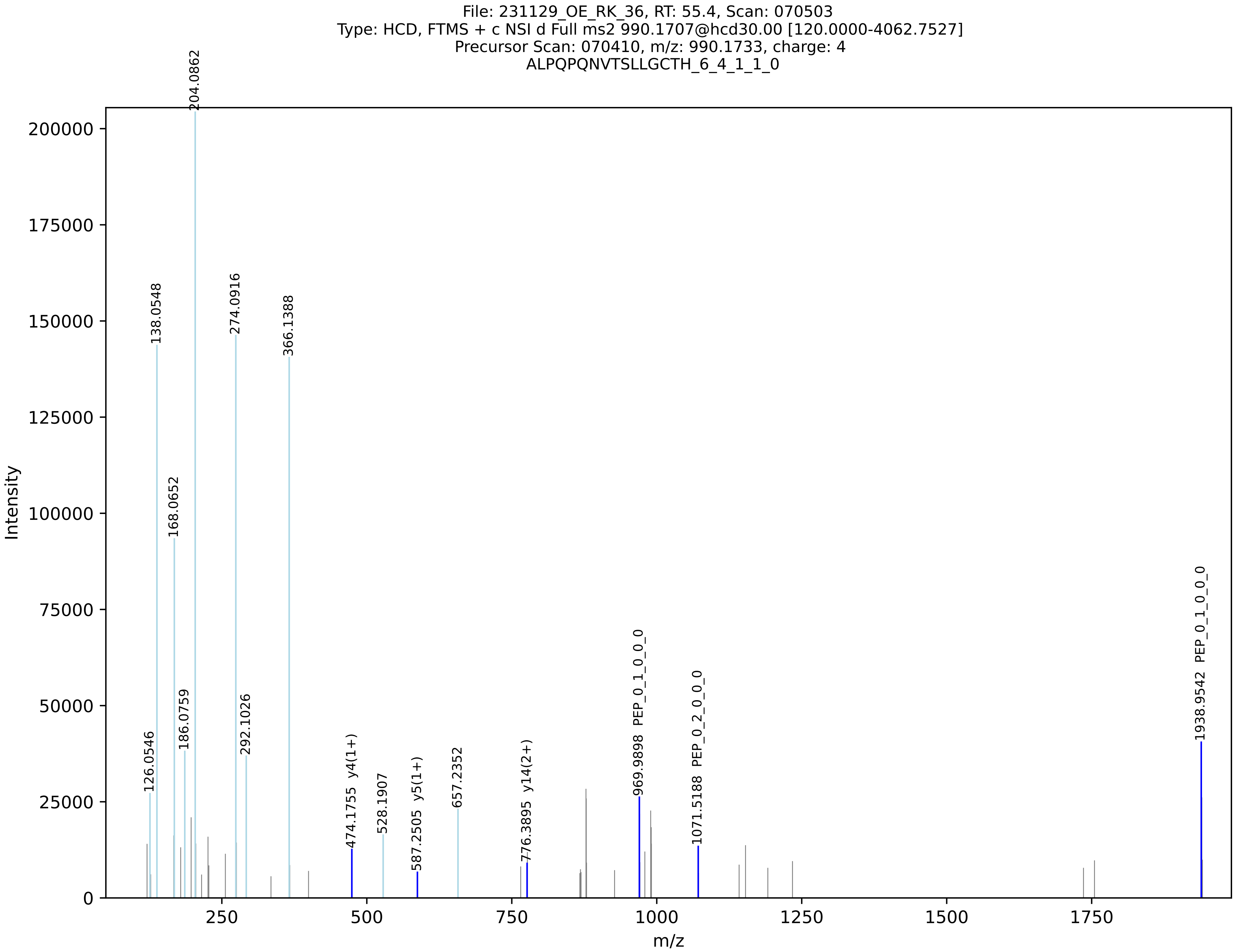
File: 231129_OE_RK_36, RT: 55.4, Scan: 070503
Type: HCD, FTMS + c NSI d Full ms2 990.1707@hcd30.00 [120.0000-4062.7527]
Precursor Scan: 070410, m/z: 990.1733, charge: 4
ALPQPQNVTSLLGCTH_6_4_1_1_0
Intensity
m/z
204.0862
138.0548
274.0916
366.1388
168.0652
186.0759
292.1026
126.0546
474.1755 y4(1+)
528.1907
587.2505 y5(1+)
657.2352
776.3895 y14(2+)
969.9898 PEP_0_1_0_0_0
1071.5188 PEP_0_2_0_0_0
1938.9542 PEP_0_1_0_0_0

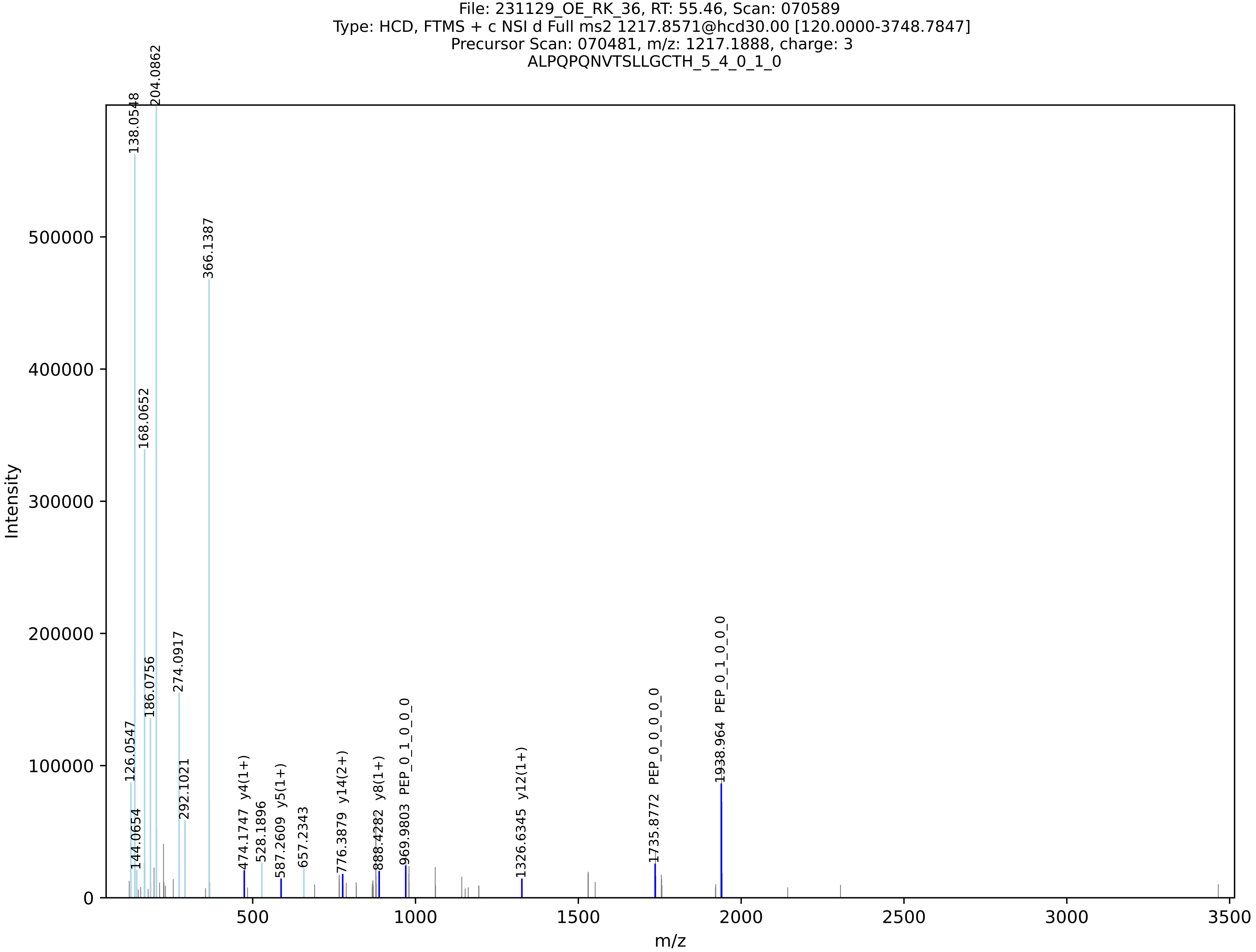
File: 231129_OE_RK_36, RT: 55.46, Scan: 070589
Type: HCD, FTMS + c NSI d Full ms2 1217.8571@hcd30.00 [120.0000-3748.7847]
Precursor Scan: 070481, m/z: 1217.1888, charge: 3
ALPQPQNVTSLLGCTH_5_4_0_1_0
Intensity
m/z
0
100000
200000
300000
400000
500000
500
1000
1500
2000
2500
3000
3500
204.0862
138.0548
366.1387
168.0652
274.0917
186.0756
126.0547
292.1021
144.0654
474.1747 y4(1+)
528.1896
587.2609 y5(1+)
657.2343
776.3879 y14(2+)
888.4282 y8(1+)
969.9803 PEP_0_1_0_0_0
1326.6345 y12(1+)
1735.8772 PEP_0_0_0_0_0
1938.964 PEP_0_1_0_0_0

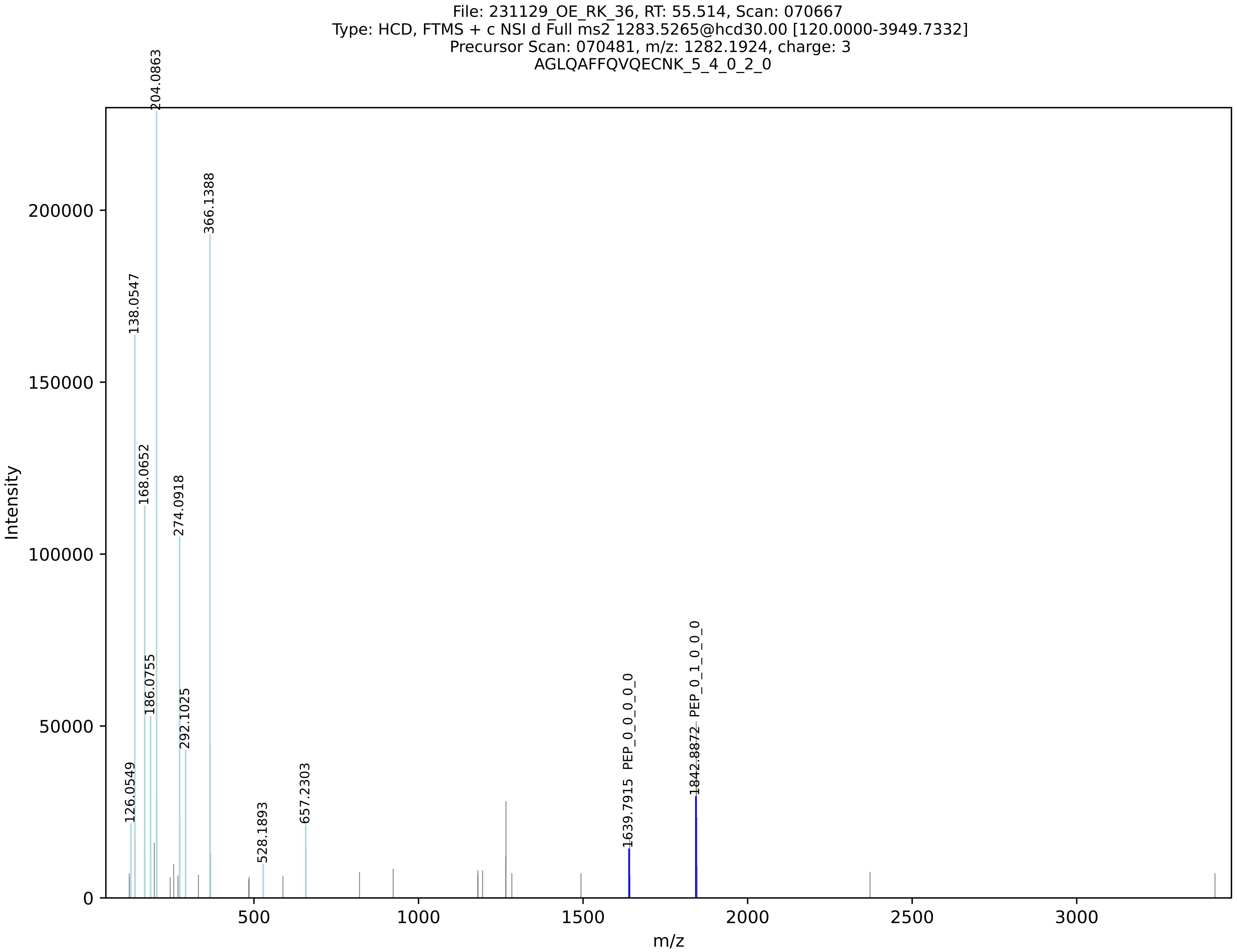

File: 231129_OE_RK_36, RT: 55.514, Scan: 070667
Type: HCD, FTMS + c NSI d Full ms2 1283.5265@hcd30.00 [120.0000-3949.7332]
Precursor Scan: 070481, m/z: 1282.1924, charge: 3
AGLQAFFQVQECNK_5_4_0_2_0
Intensity
m/z
204.0863
366.1388
138.0547
168.0652
274.0918
186.0755
292.1025
126.0549
528.1893
657.2303
1639.7915 PEP_0_0_0_0_0
1842.8872 PEP_0_1_0_0_0
0
50000
100000
150000
200000
500
1000
1500
2000
2500
3000

File: 231129_OE_RK_36, RT: 55.602, Scan: 070793
Type: HCD, FTMS + c NSI d Full ms2 1149.8445@hcd30.00 [120.0000-3540.6660]
Precursor Scan: 070700, m/z: 1149.5096, charge: 3
QVLFLDTVYGNCSTHFTVK_5_2_0_0_0

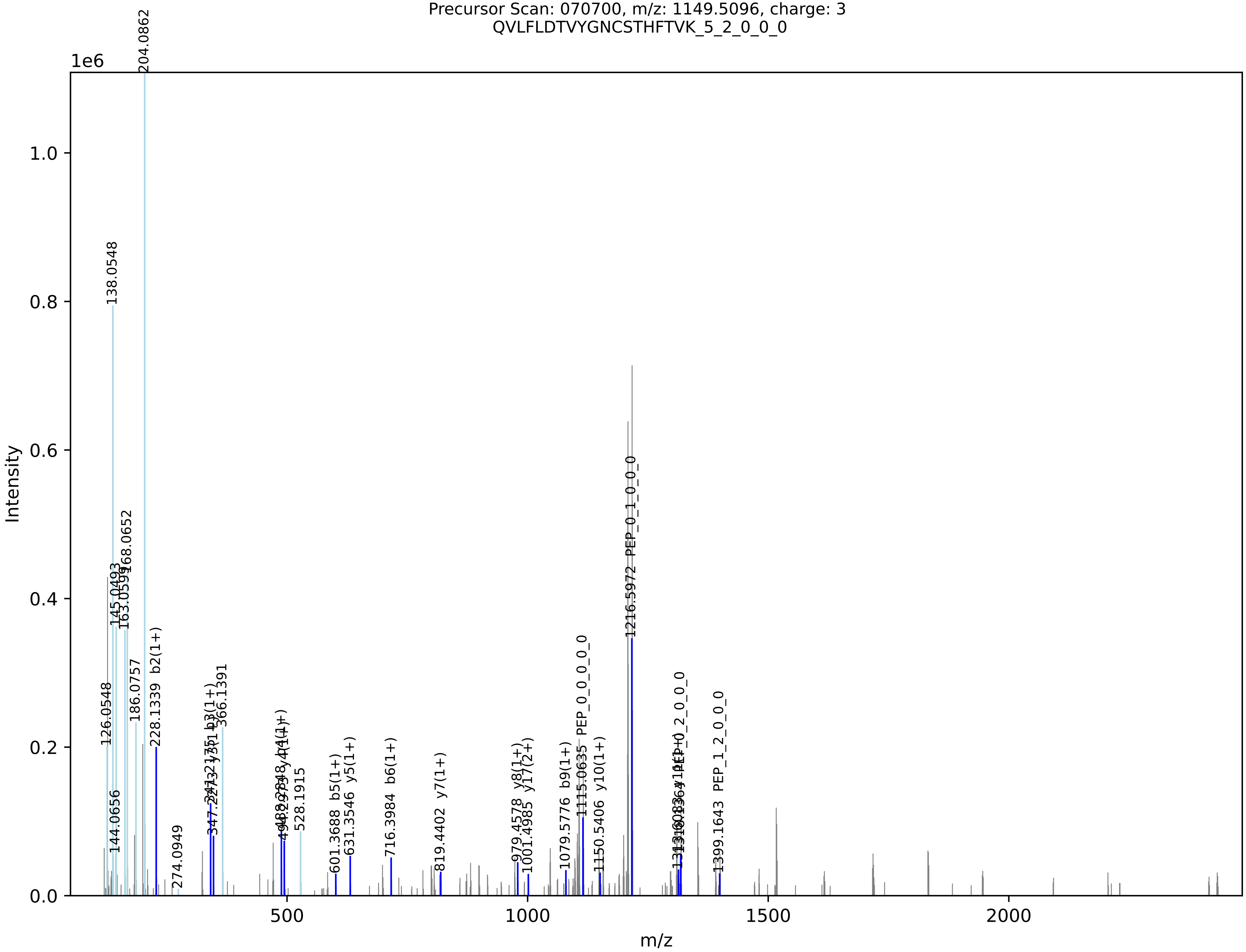

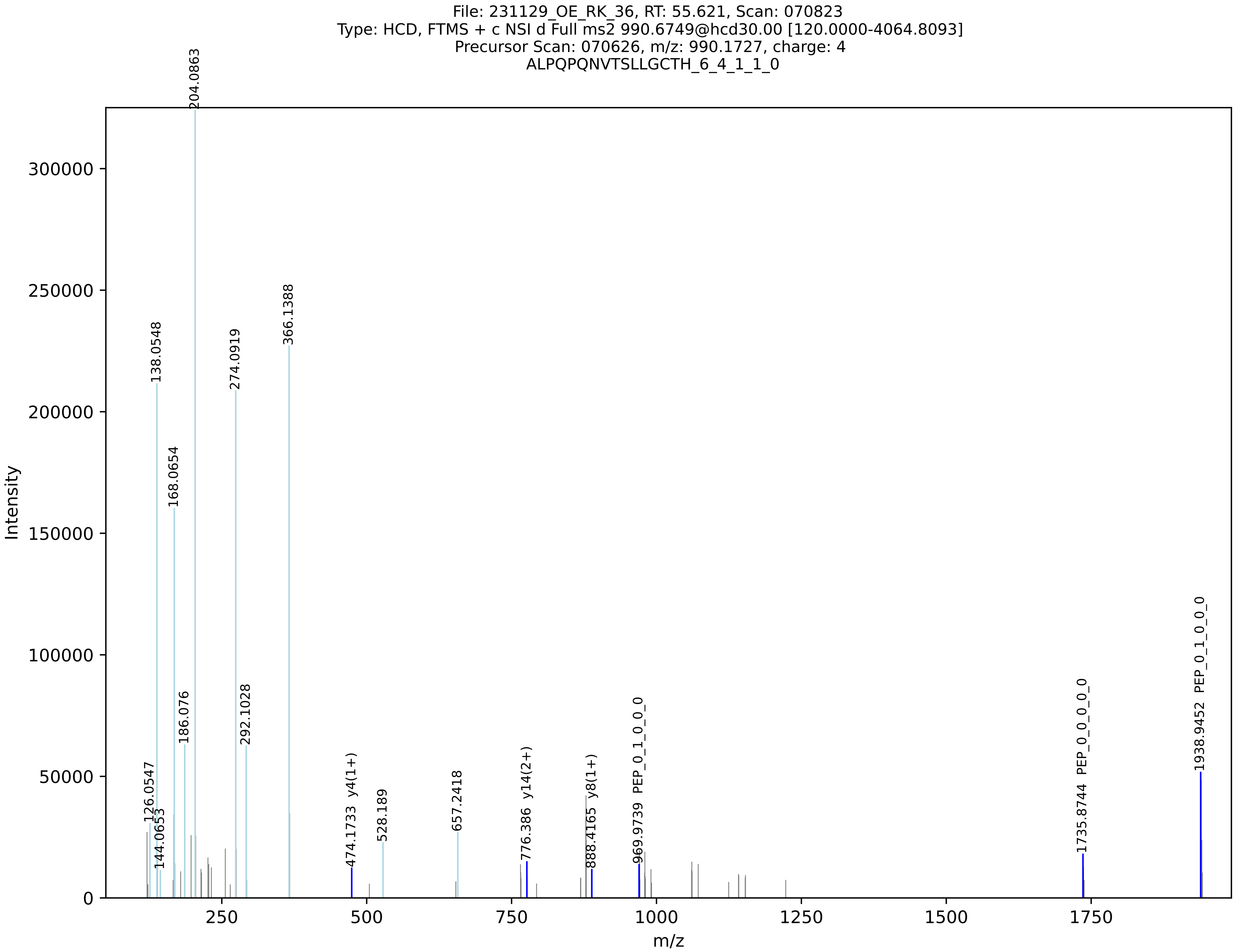
File: 231129_OE_RK_36, RT: 55.621, Scan: 070823
Type: HCD, FTMS + c NSI d Full ms2 990.6749@hcd30.00 [120.0000-4064.8093]
Precursor Scan: 070626, m/z: 990.1727, charge: 4
ALPQPQNVTSLLGCTH_6_4_1_1_0
Intensity
m/z
204.0863
138.0548
274.0919
366.1388
168.0654
186.076
292.1028
126.0547
144.0653
474.1733 y4(1+)
528.189
657.2418
776.386 y14(2+)
888.4165 y8(1+)
969.9739 PEP_0_1_0_0_0
1735.8744 PEP_0_0_0_0_0
1938.9452 PEP_0_1_0_0_0
0
50000
100000
150000
200000
250000
300000
250
500
750
1000
1250
1500
1750

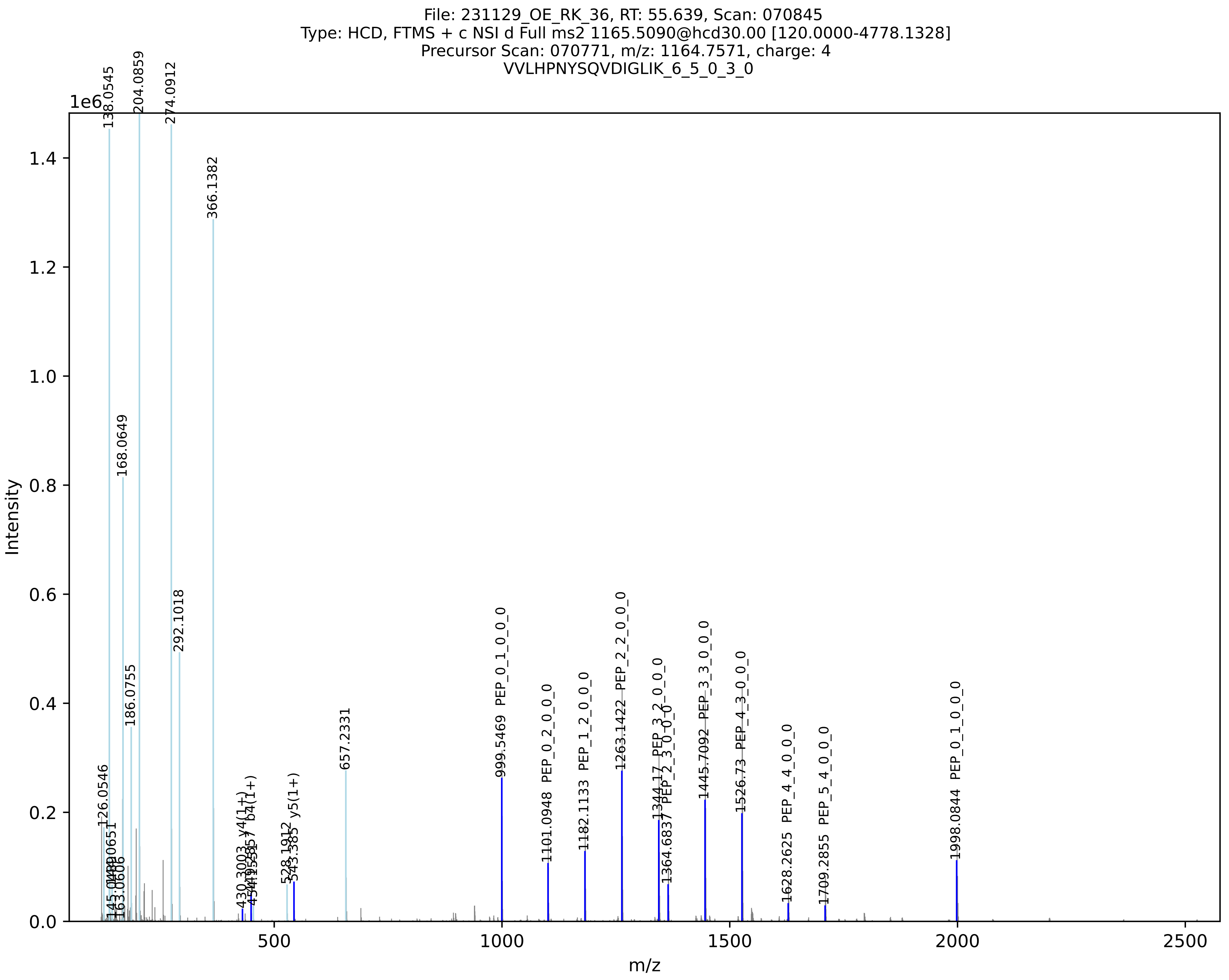

File: 231129_OE_RK_36, RT: 55.639, Scan: 070845
Type: HCD, FTMS + c NSI d Full ms2 1165.5090@hcd30.00 [120.0000-4778.1328]
Precursor Scan: 070771, m/z: 1164.7571, charge: 4
VVLHPNYSQVDIGLIK_6_5_0_3_0
1e6
Intensity
m/z
138.0545
204.0859
274.0912
366.1382
168.0649
292.1018
186.0755
126.0546
657.2331
430.3003 y4(1+)
b4(1+)
528.1912
543.385 y5(1+)
999.5469 PEP_0_1_0_0_0
1101.0948 PEP_0_2_0_0_0
1182.1133 PEP_1_2_0_0_0
1263.1422 PEP_2_2_0_0_0
1344.17 PEP_3_2_0_0_0
1364.6837 PEP_2_3_0_0_0
1445.7092 PEP_3_3_0_0_0
1526.73 PEP_4_3_0_0_0
1628.2625 PEP_4_4_0_0_0
1709.2855 PEP_5_4_0_0_0
1998.0844 PEP_0_1_0_0_0

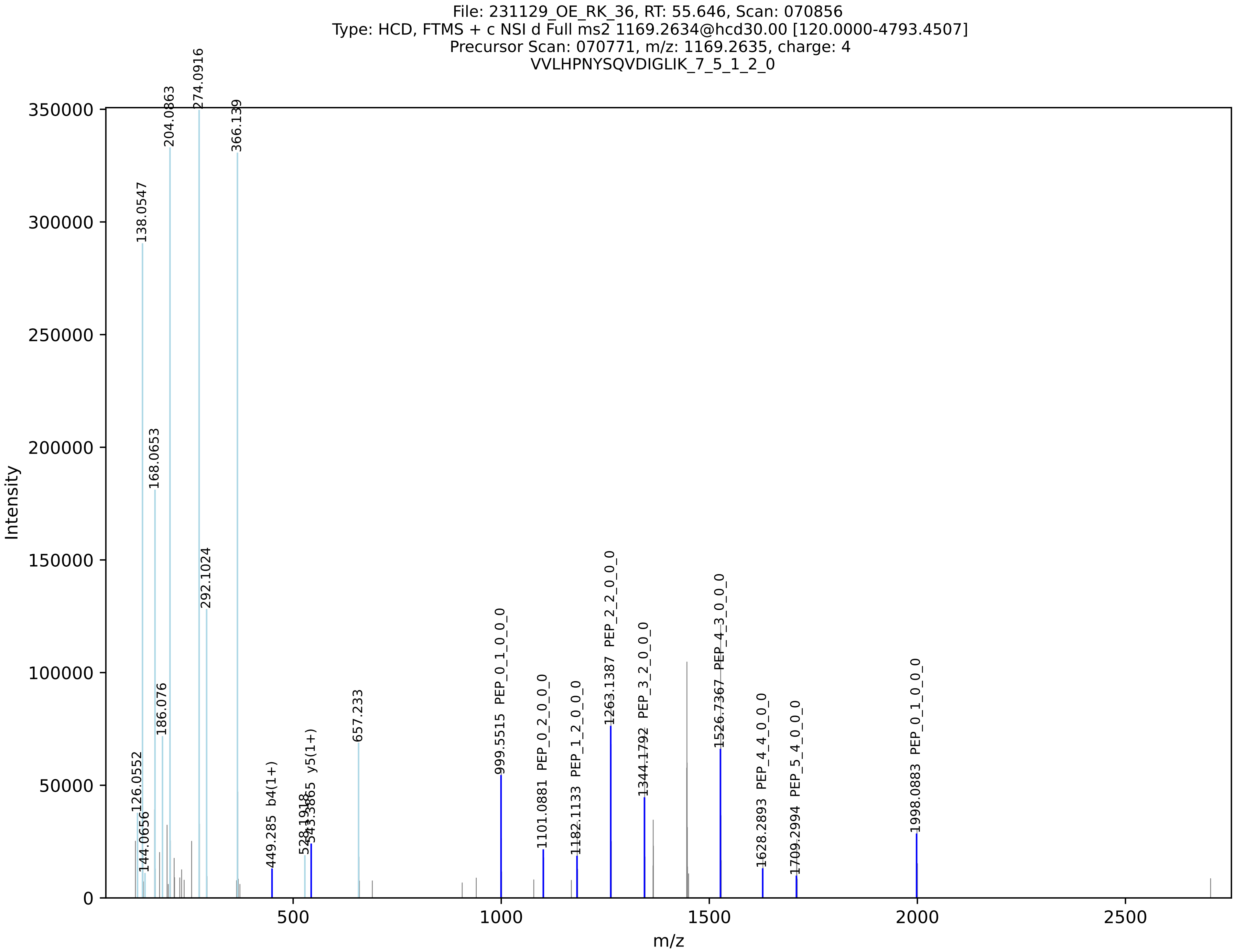

File: 231129_OE_RK_36, RT: 55.646, Scan: 070856
Type: HCD, FTMS + c NSI d Full ms2 1169.2634@hcd30.00 [120.0000-4793.4507]
Precursor Scan: 070771, m/z: 1169.2635, charge: 4
VVLHPNYSQVDIGLIK_7_5_1_2_0
Intensity
m/z
138.0547
168.0653
186.076
126.0552
144.0656
204.0863
274.0916
292.1024
366.139
449.285 b4(1+)
528.1918
543.3865 y5(1+)
657.233
999.5515 PEP_0_1_0_0_0
1101.0881 PEP_0_2_0_0_0
1182.1133 PEP_1_2_0_0_0
1263.1387 PEP_2_2_0_0_0
1344.1792 PEP_3_2_0_0_0
1526.7367 PEP_4_3_0_0_0
1628.2893 PEP_4_4_0_0_0
1709.2994 PEP_5_4_0_0_0
1998.0883 PEP_0_1_0_0_0

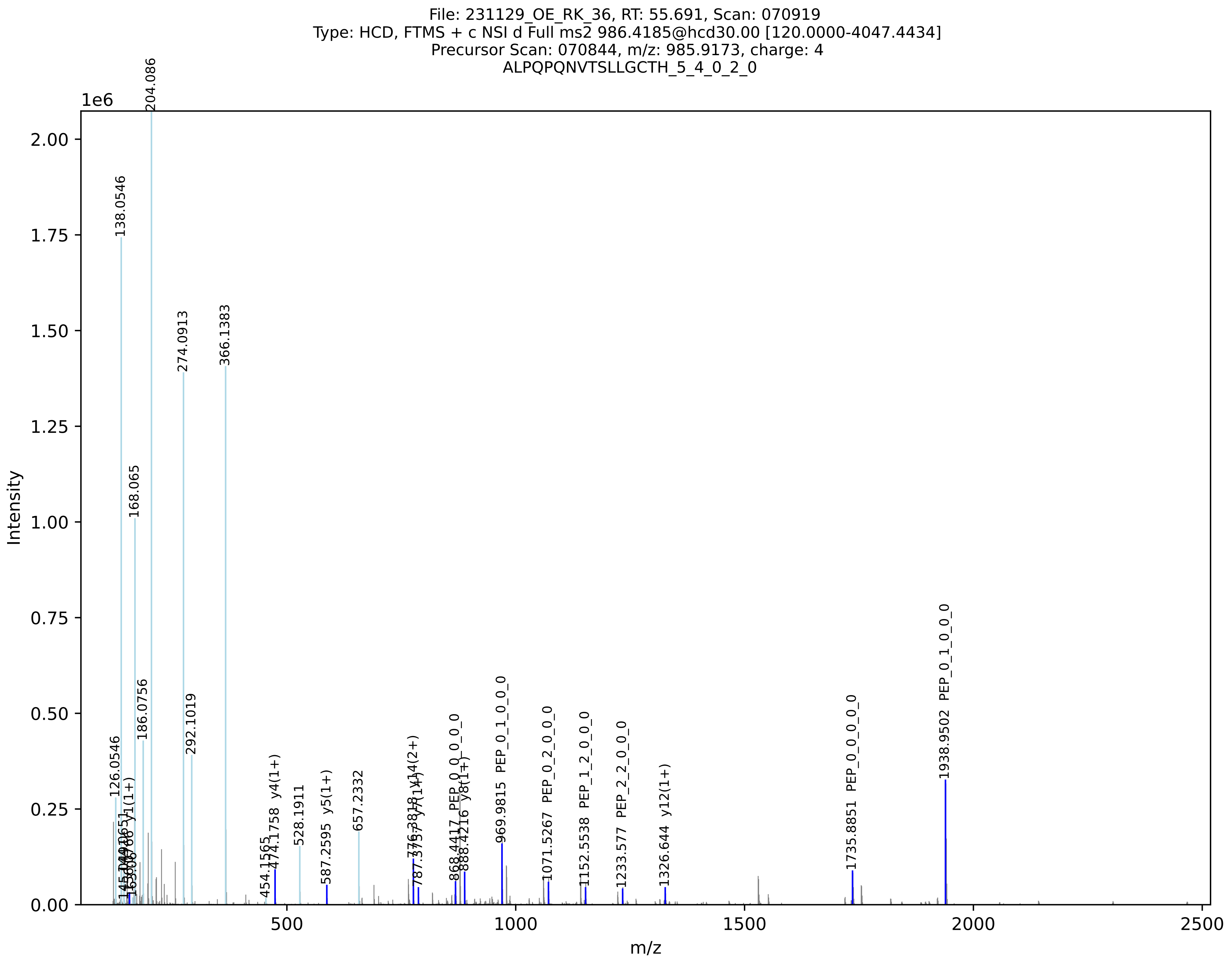

File: 231129_OE_RK_36, RT: 55.691, Scan: 070919
Type: HCD, FTMS + c NSI d Full ms2 986.4185@hcd30.00 [120.0000-4047.4434]
Precursor Scan: 070844, m/z: 985.9173, charge: 4
ALPQPQNVTSLLGCTH_5_4_0_2_0
1e6
Intensity
m/z
204.086
138.0546
274.0913
366.1383
168.065
186.0756
292.1019
126.0546
454.1565
474.1758 y4(1+)
528.1911
587.2595 y5(1+)
657.2332
868.4417 PEP_0_0_0_0_0
888.4216 y8(1+)
969.9815 PEP_0_1_0_0_0
1071.5267 PEP_0_2_0_0_0
1152.5538 PEP_1_2_0_0_0
1233.577 PEP_2_2_0_0_0
1326.644 y12(1+)
1735.8851 PEP_0_0_0_0_0
1938.9502 PEP_0_1_0_0_0

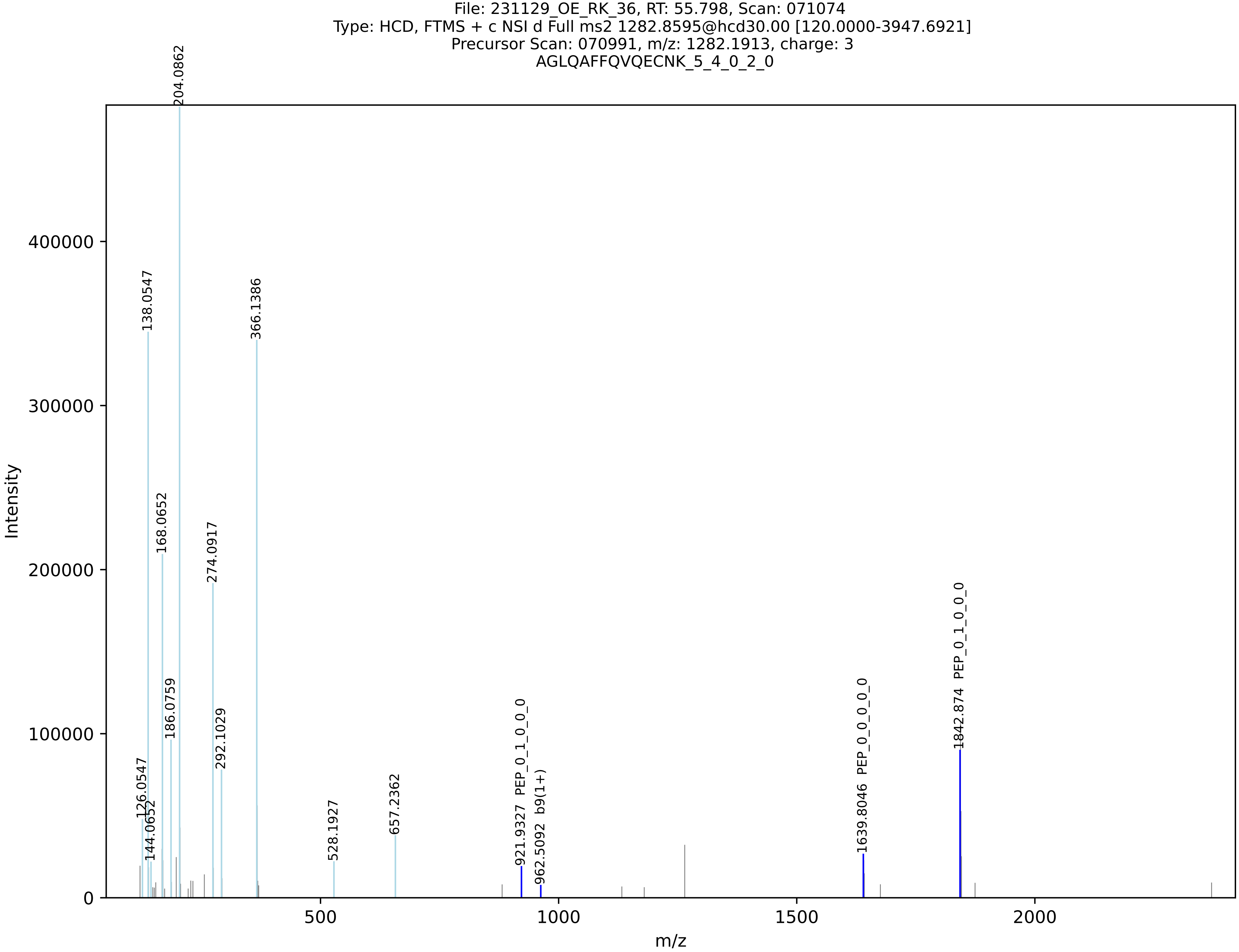

File: 231129_OE_RK_36, RT: 55.798, Scan: 071074
Type: HCD, FTMS + c NSI d Full ms2 1282.8595@hcd30.00 [120.0000-3947.6921]
Precursor Scan: 070991, m/z: 1282.1913, charge: 3
AGLQAFFQVQECNK_5_4_0_2_0
Intensity
m/z
204.0862
138.0547
366.1386
168.0652
274.0917
186.0759
292.1029
126.0547
144.0652
528.1927
657.2362
921.9327 PEP_0_1_0_0_0
962.5092 b9(1+)
1639.8046 PEP_0_0_0_0_0
1842.874 PEP_0_1_0_0_0

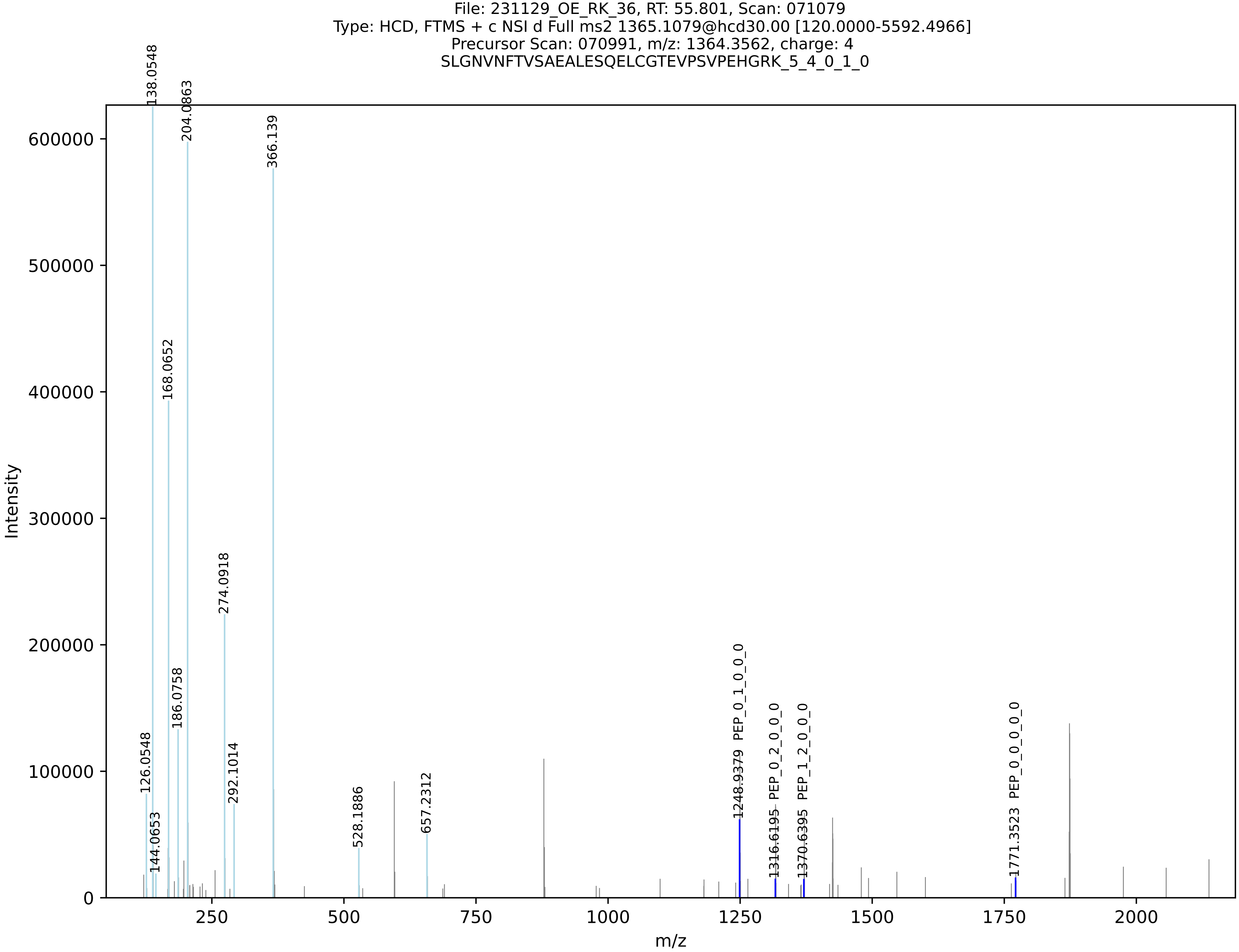
File: 231129_OE_RK_36, RT: 55.801, Scan: 071079
Type: HCD, FTMS + c NSI d Full ms2 1365.1079@hcd30.00 [120.0000-5592.4966]
Precursor Scan: 070991, m/z: 1364.3562, charge: 4
SLGNVNFTVSAEALESQELCGTEVPSVPEHGRK_5_4_0_1_0
Intensity
m/z
138.0548
204.0863
366.139
168.0652
274.0918
186.0758
126.0548
292.1014
144.0653
528.1886
657.2312
1248.9379 PEP_0_1_0_0_0
1316.6195 PEP_0_2_0_0_0
1370.6395 PEP_1_2_0_0_0
1771.3523 PEP_0_0_0_0_0

File: 231129_OE_RK_36, RT: 55.844, Scan: 071140
Type: HCD, FTMS + c NSI d Full ms2 1169.5143@hcd30.00 [120.0000-4794.4741]
Precursor Scan: 071065, m/z: 1169.0131, charge: 4
VVLHPNYSQVDIGLIK_7_5_1_2_0

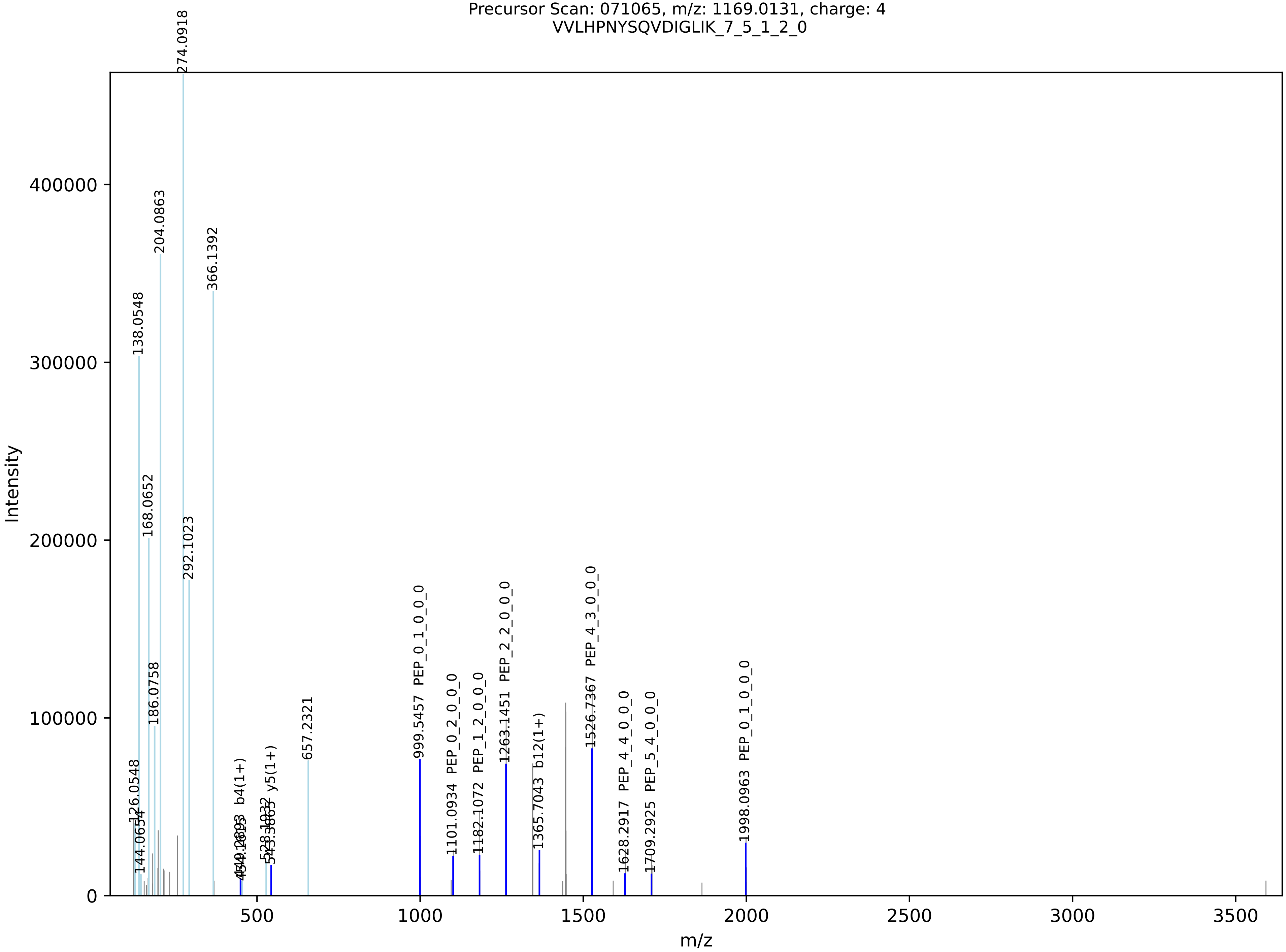

File: 231129_OE_RK_36, RT: 55.871, Scan: 071183
Type: HCD, FTMS + c NSI d Full ms2 1170.2664@hcd30.00 [120.0000-4797.5425]
Precursor Scan: 070991, m/z: 1169.0135, charge: 4
VVLHPNYSQVDIGLIK_7_5_1_2_0

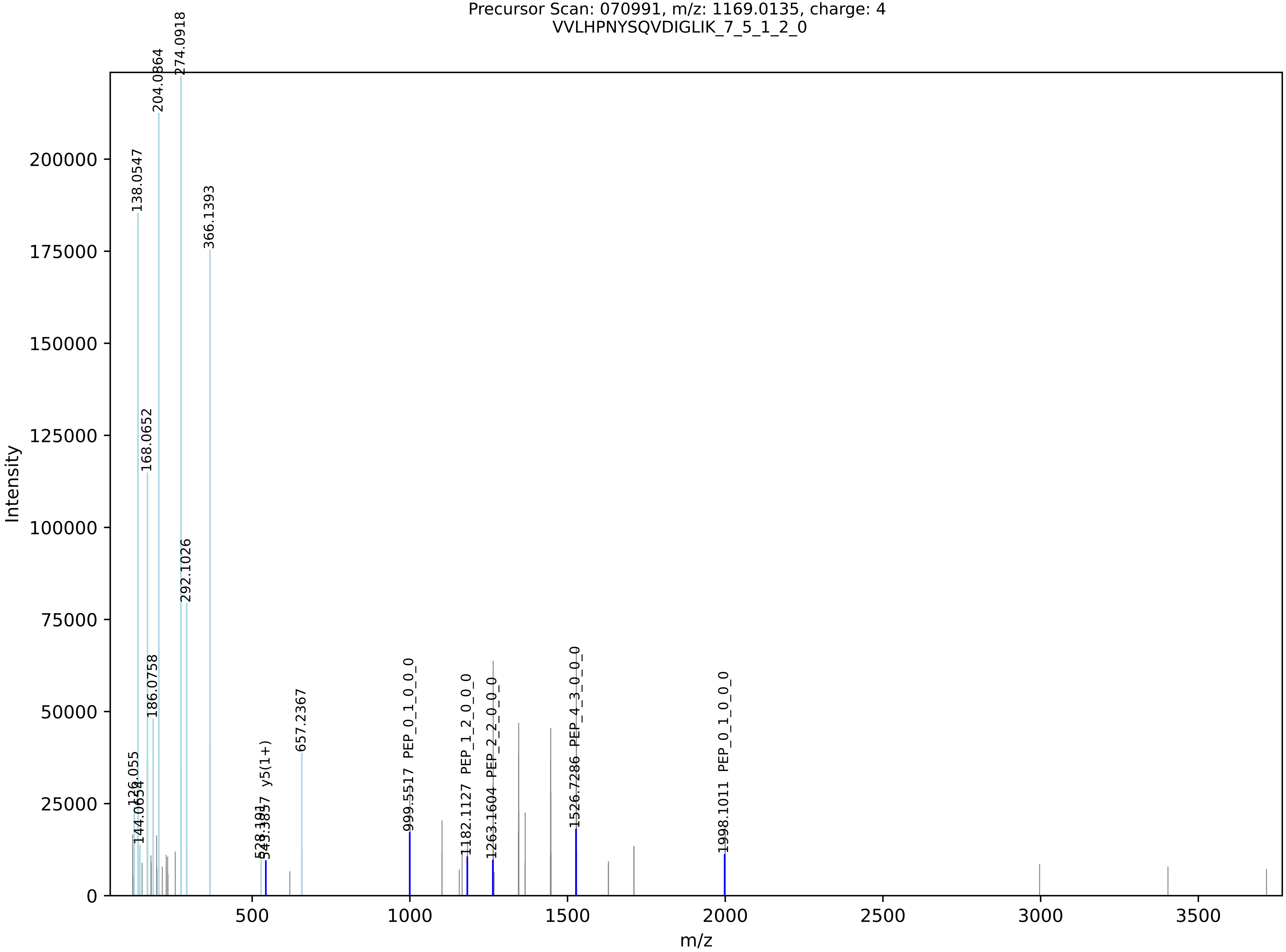

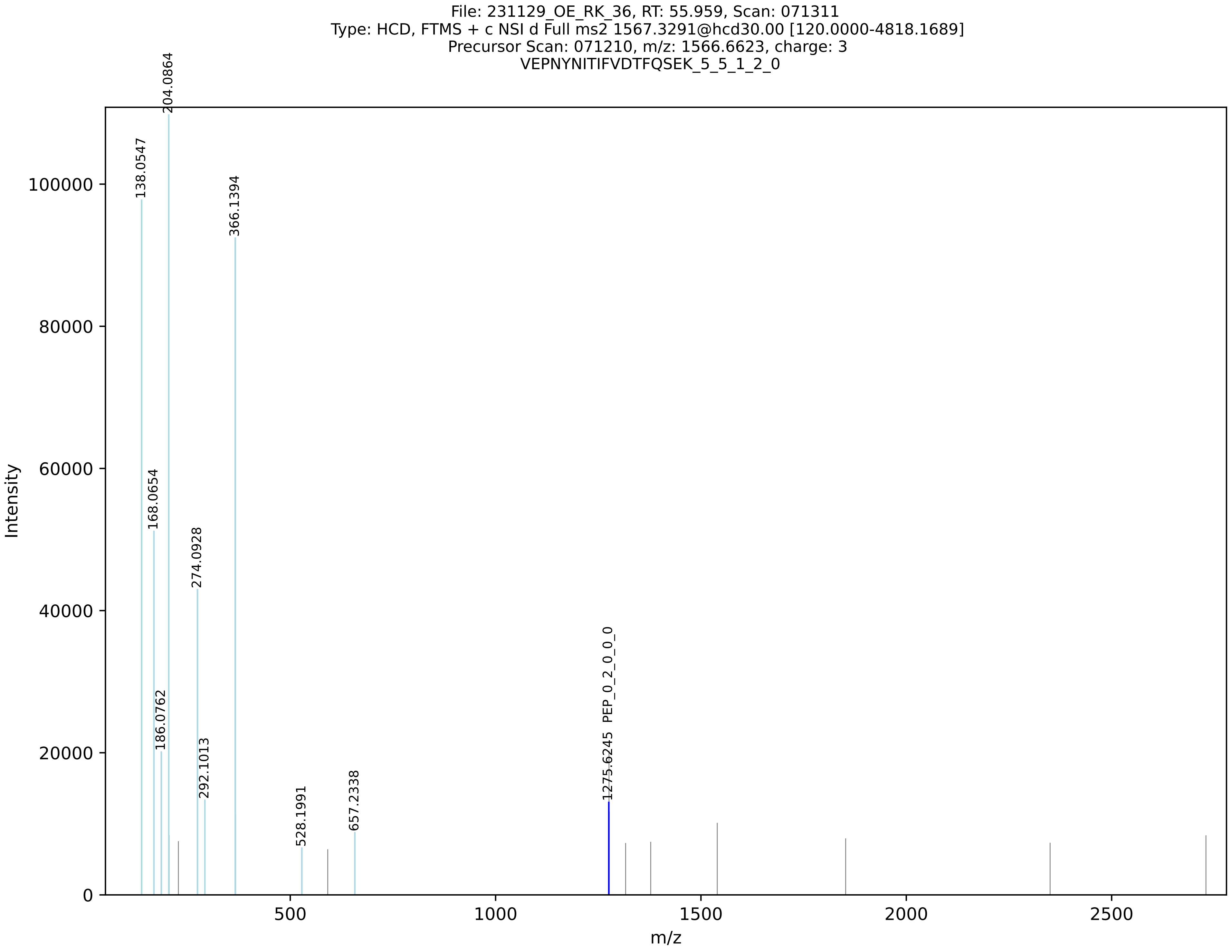
File: 231129_OE_RK_36, RT: 55.959, Scan: 071311
Type: HCD, FTMS + c NSI d Full ms2 1567.3291@hcd30.00 [120.0000-4818.1689]
Precursor Scan: 071210, m/z: 1566.6623, charge: 3
VEPNYNITIFVDTFQSEK_5_5_1_2_0
Intensity
m/z
204.0864
138.0547
366.1394
168.0654
274.0928
186.0762
292.1013
528.1991
657.2338
1275.6245 PEP_0_2_0_0_0
0
20000
40000
60000
80000
100000
500
1000
1500
2000
2500

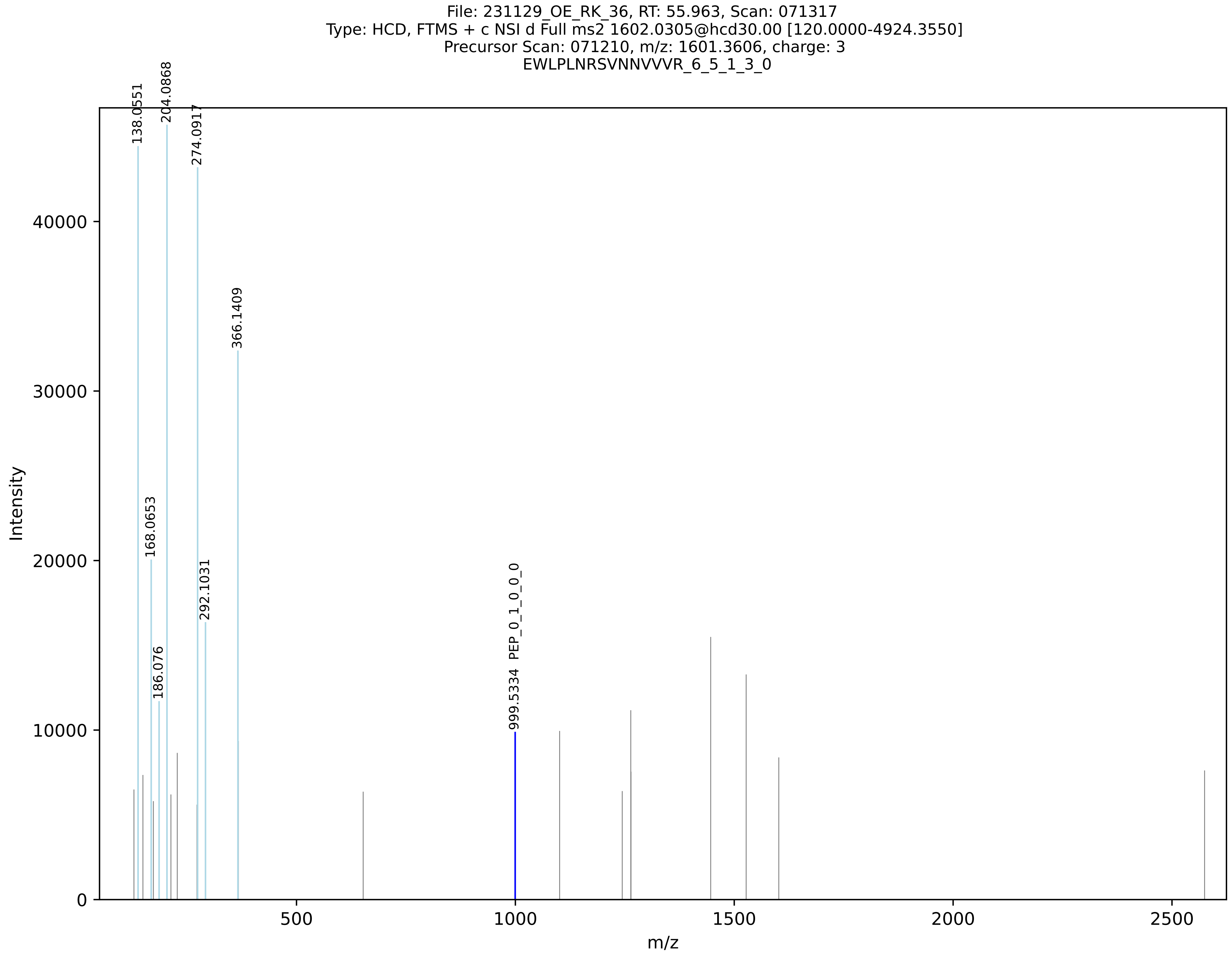

File: 231129_OE_RK_36, RT: 55.963, Scan: 071317
Type: HCD, FTMS + c NSI d Full ms2 1602.0305@hcd30.00 [120.0000-4924.3550]
Precursor Scan: 071210, m/z: 1601.3606, charge: 3
EWLPLNRSVNNVVVR_6_5_1_3_0
138.0551
204.0868
274.0917
366.1409
168.0653
292.1031
186.076
999.5334 PEP_0_1_0_0_0_
Intensity
40000
30000
20000
10000
0
500
1000
1500
2000
2500
m/z

File: 231129_OE_RK_36, RT: 55.976, Scan: 071338
Type: HCD, FTMS + c NSI d Full ms2 1331.8754@hcd30.00 [120.0000-4097.6807]
Precursor Scan: 071210, m/z: 1331.543, charge: 3
SYPPDLRCNYSIR_5_4_1_2_0

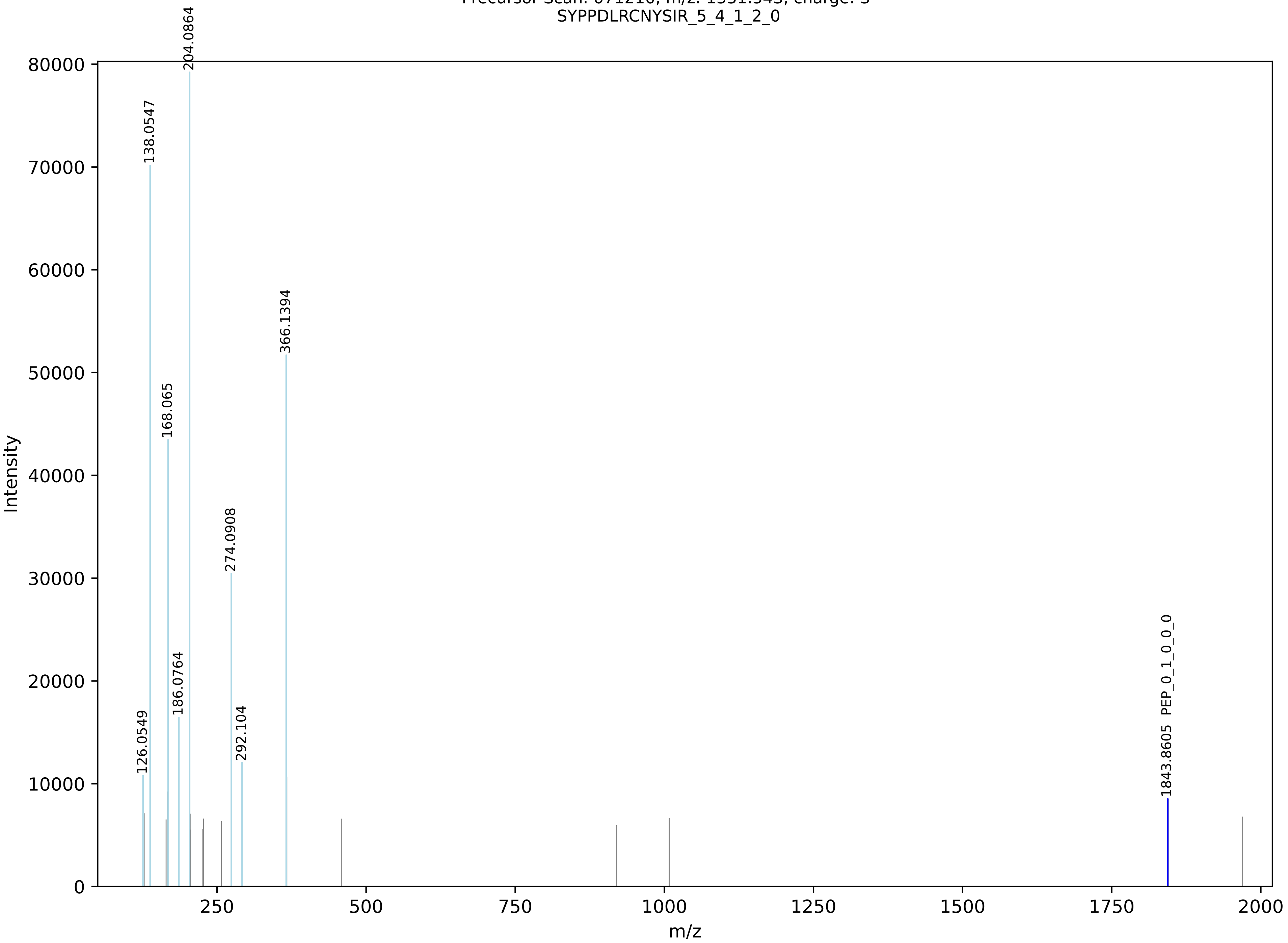

File: 231129_OE_RK_36, RT: 56.045, Scan: 071433
Type: HCD, FTMS + c NSI d Full ms2 1165.2593@hcd30.00 [120.0000-4777.1138]
Precursor Scan: 071357, m/z: 1164.7584, charge: 4
EWLPLNRSVNNVVVR_6_5_0_3_0

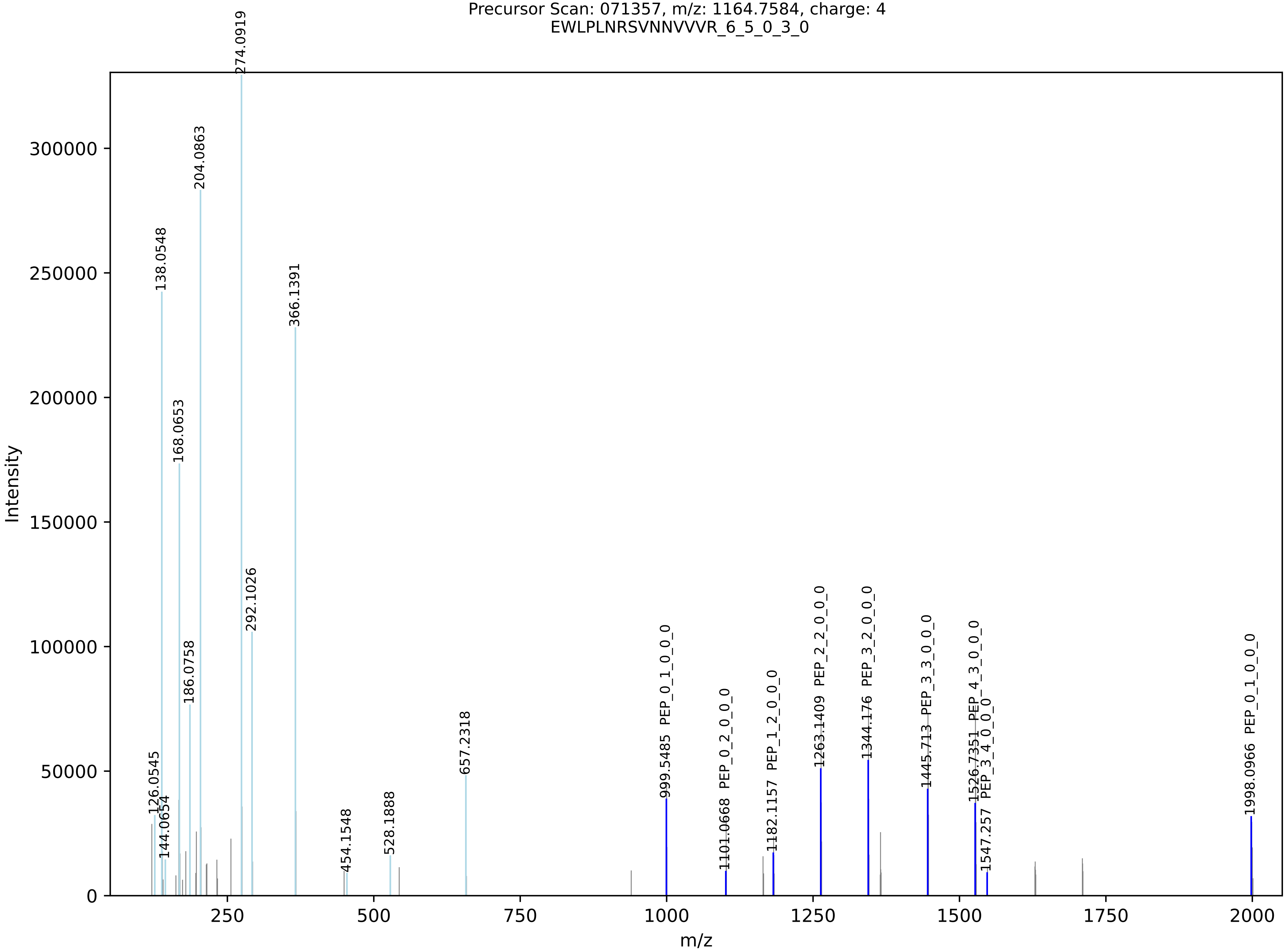

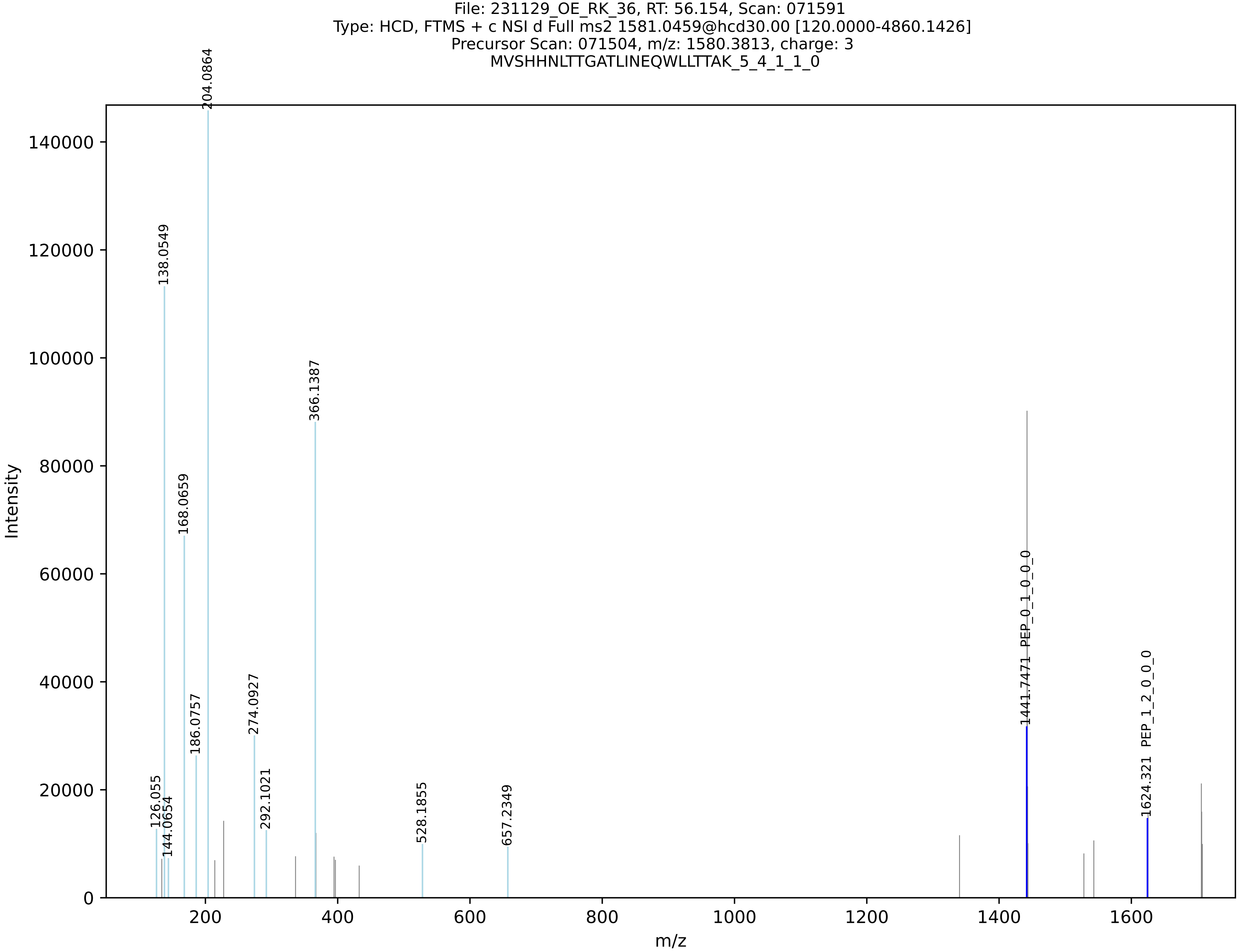
File: 231129_OE_RK_36, RT: 56.154, Scan: 071591
Type: HCD, FTMS + c NSI d Full ms2 1581.0459@hcd30.00 [120.0000-4860.1426]
Precursor Scan: 071504, m/z: 1580.3813, charge: 3
MVSHHNLTTGATLINEQWLLTTAK_5_4_1_1_0
Intensity
m/z
204.0864
138.0549
366.1387
168.0659
186.0757
274.0927
292.1021
126.055
144.0654
528.1855
657.2349
1441.7471 PEP_0_1_0_0_0_
1624.321 PEP_1_2_0_0_0
0
20000
40000
60000
80000
100000
120000
140000
200
400
600
800
1000
1200
1400
1600

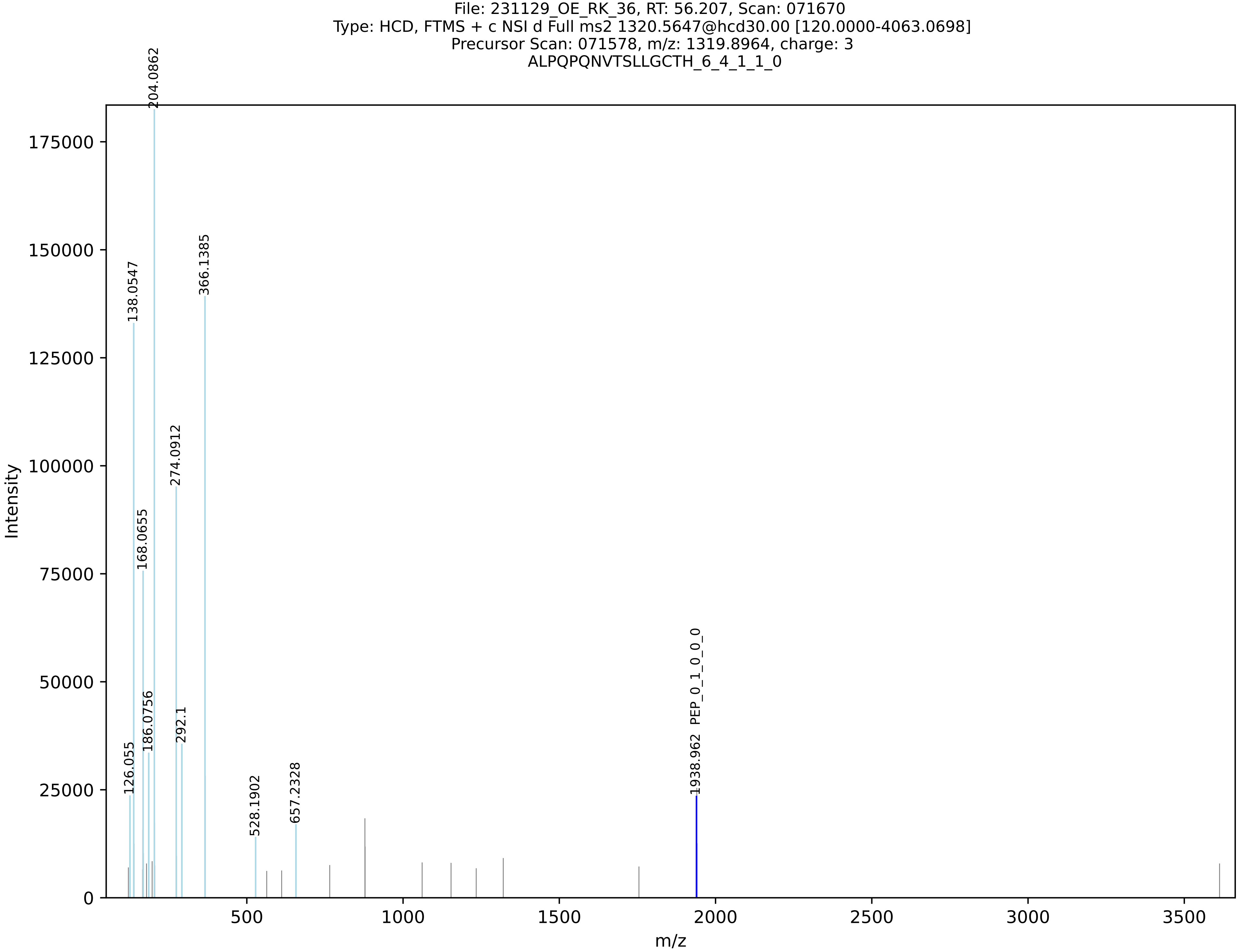
File: 231129_OE_RK_36, RT: 56.207, Scan: 071670
Type: HCD, FTMS + c NSI d Full ms2 1320.5647@hcd30.00 [120.0000-4063.0698]
Precursor Scan: 071578, m/z: 1319.8964, charge: 3
ALPQPQNVTSLLGCTH_6_4_1_1_0
Intensity
m/z
204.0862
138.0547
366.1385
274.0912
168.0655
186.0756
292.1
126.055
528.1902
657.2328
1938.962 PEP_0_1_0_0_0
175000
150000
125000
100000
75000
50000
25000
0
500
1000
1500
2000
2500
3000
3500

File: 231129_OE_RK_36, RT: 56.249, Scan: 071727
Type: HCD, FTMS + c NSI d Full ms2 1201.0497@hcd30.00 [120.0000-4923.1387]
Precursor Scan: 071649, m/z: 1200.5455, charge: 4
SLGNVNFTVSAEALESQELCGTEVPSVPEHGRK_4_3_0_0_0

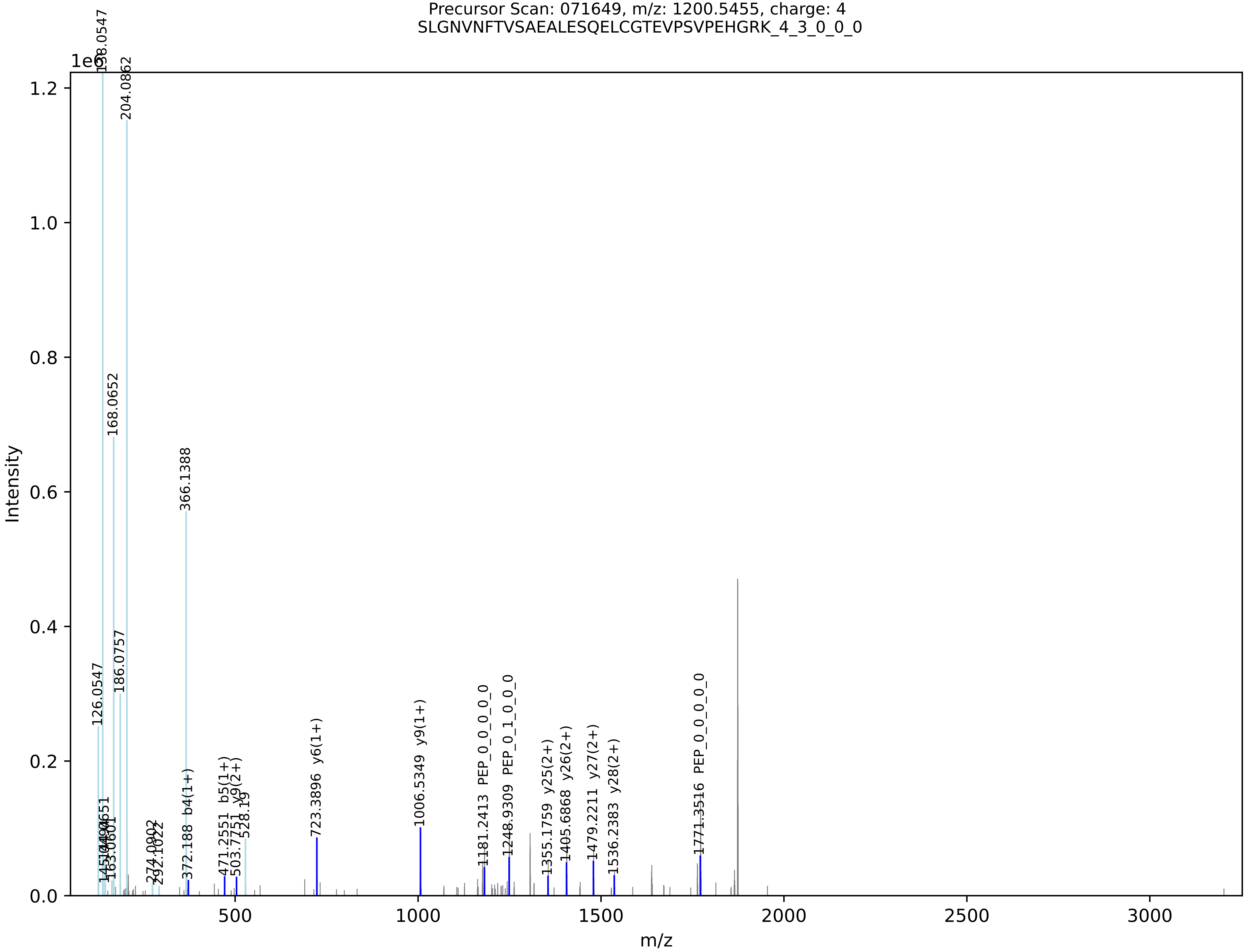

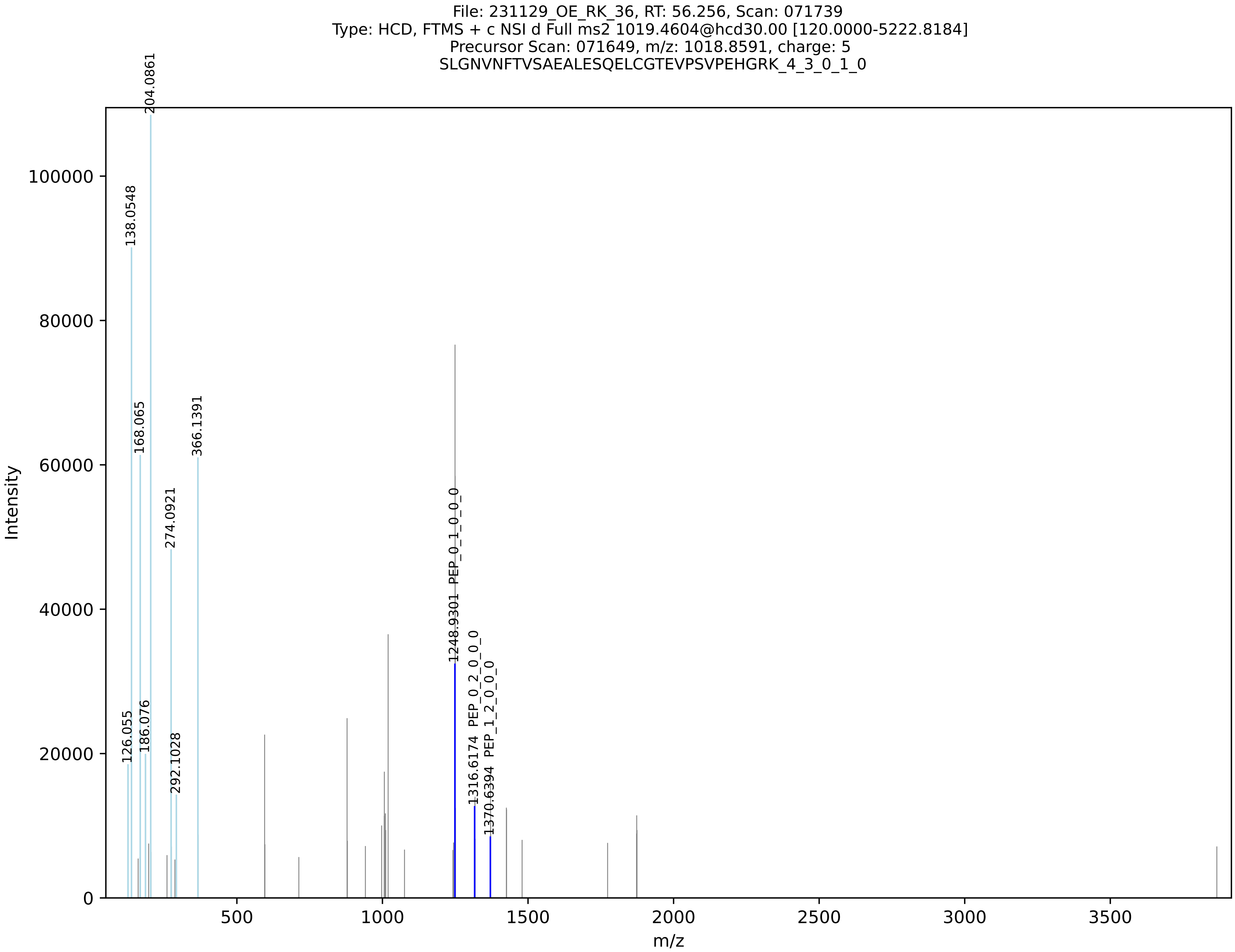

File: 231129_OE_RK_36, RT: 56.256, Scan: 071739
Type: HCD, FTMS + c NSI d Full ms2 1019.4604@hcd30.00 [120.0000-5222.8184]
Precursor Scan: 071649, m/z: 1018.8591, charge: 5
SLGNVNFTVSAEALESQELCGTEVPSVPEHGRK_4_3_0_1_0
Intensity
m/z
0
20000
40000
60000
80000
100000
500
1000
1500
2000
2500
3000
3500
204.0861
138.0548
168.065
366.1391
274.0921
126.055
186.076
292.1028
1248.9301 PEP_0_1_0_0_0
1316.6174 PEP_0_2_0_0_0
1370.6394 PEP_1_2_0_0_0

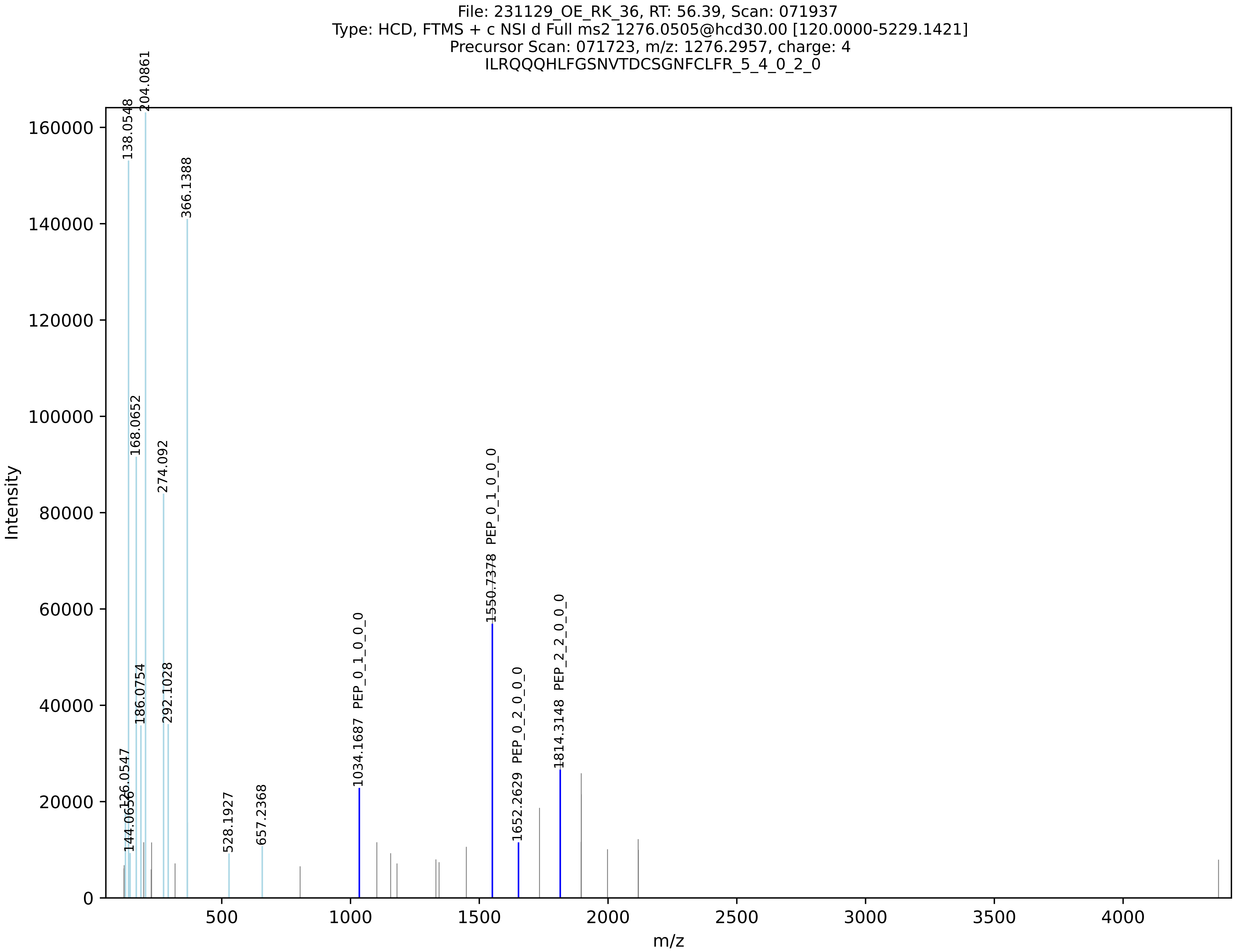

File: 231129_OE_RK_36, RT: 56.39, Scan: 071937
Type: HCD, FTMS + c NSI d Full ms2 1276.0505@hcd30.00 [120.0000-5229.1421]
Precursor Scan: 071723, m/z: 1276.2957, charge: 4
ILRQQQHLFGSNVTDCSGNFCLFR_5_4_0_2_0
Intensity
m/z
204.0861
138.0548
366.1388
168.0652
274.092
186.0754
292.1028
126.0547
144.0656
528.1927
657.2368
1034.1687 PEP_0_1_0_0_0
1550.7378 PEP_0_1_0_0_0
1652.2629 PEP_0_2_0_0_0
1814.3148 PEP_2_2_0_0_0

File: 231129_OE_RK_36, RT: 56.472, Scan: 072053
Type: HCD, FTMS + c NSI d Full ms2 1320.5590@hcd30.00 [120.0000-4063.0525]
Precursor Scan: 071940, m/z: 1320.559, charge: 3
YTGNASALFILPDQDK_5_4_0_2_0

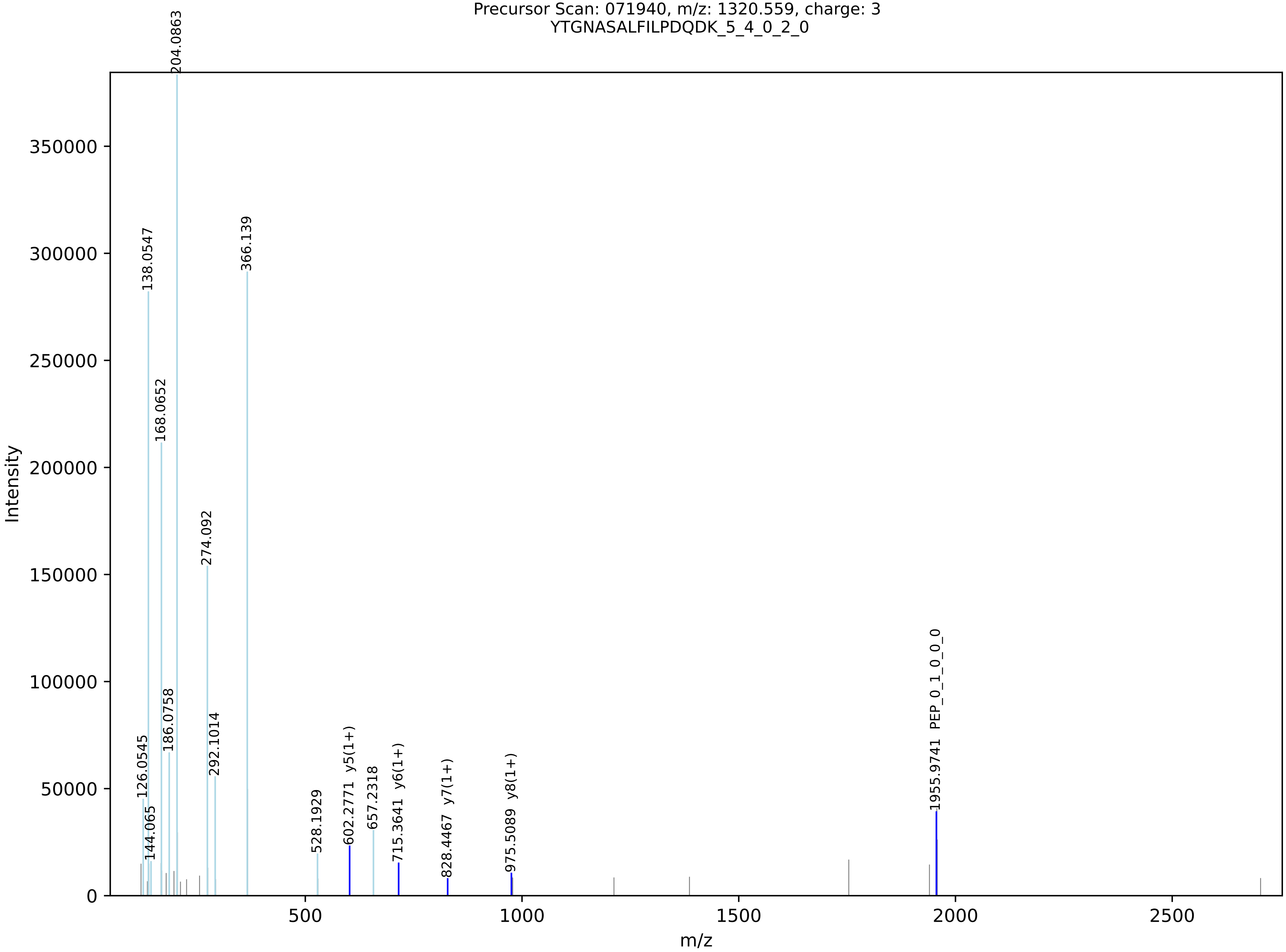

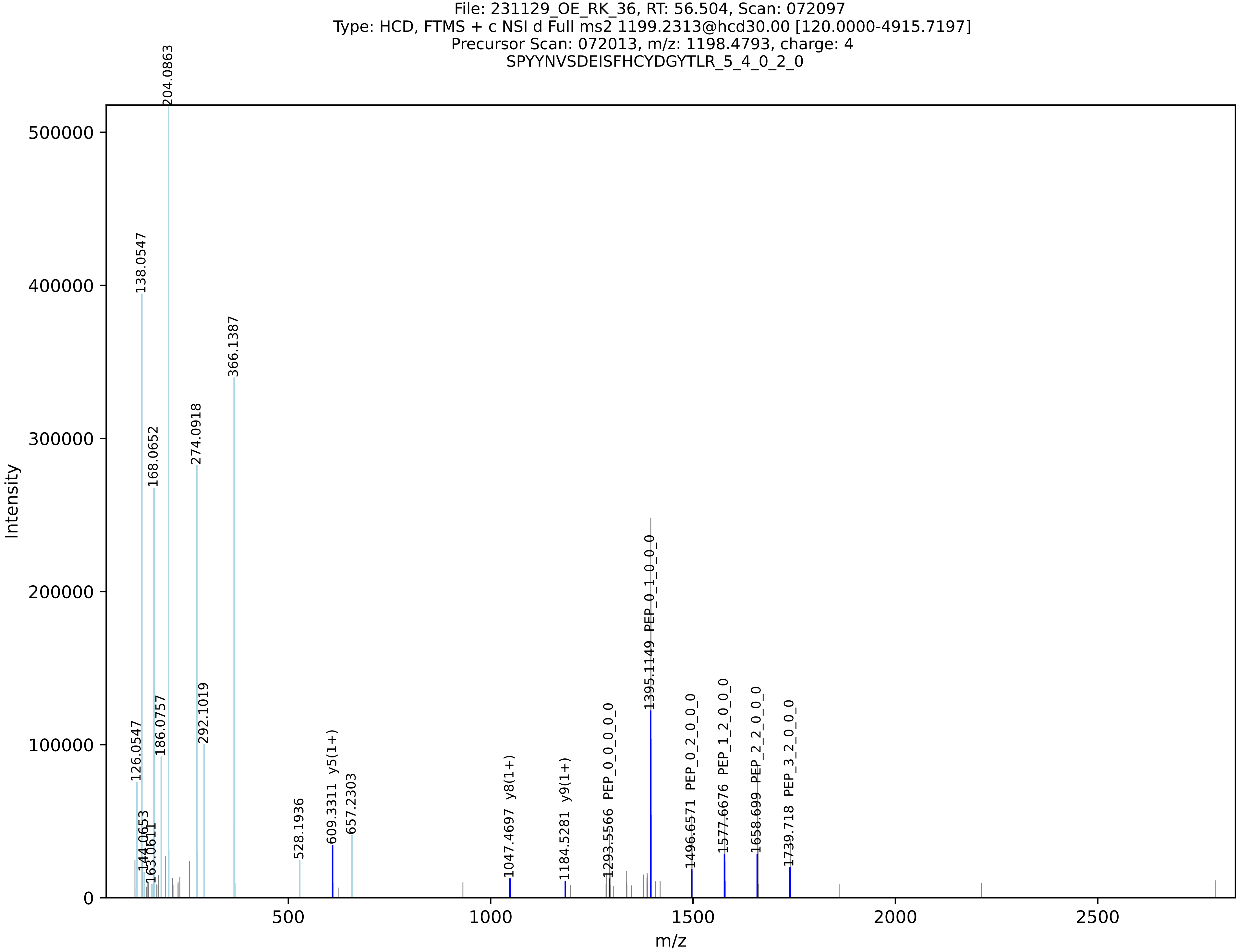
File: 231129_OE_RK_36, RT: 56.504, Scan: 072097
Type: HCD, FTMS + c NSI d Full ms2 1199.2313@hcd30.00 [120.0000-4915.7197]
Precursor Scan: 072013, m/z: 1198.4793, charge: 4
SPYYNVSDEISFHCYDGYTLR_5_4_0_2_0
Intensity
m/z
204.0863
138.0547
366.1387
274.0918
168.0652
292.1019
186.0757
126.0547
144.0653
163.0611
528.1936
609.3311 y5(1+)
657.2303
1047.4697 y8(1+)
1184.5281 y9(1+)
1293.5566 PEP_0_0_0_0_0
1395.1149 PEP_0_1_0_0_0
1496.6571 PEP_0_2_0_0_0
1577.6676 PEP_1_2_0_0_0
1658.699 PEP_2_2_0_0_0
1739.718 PEP_3_2_0_0_0

File: 231129_OE_RK_36, RT: 56.511, Scan: 072108
Type: HCD, FTMS + c NSI d Full ms2 1598.3044@hcd30.00 [120.0000-4912.9536]
Precursor Scan: 072013, m/z: 1597.6366, charge: 3
SPYYNVSDEISFHCYDGYTLR_5_4_0_2_0

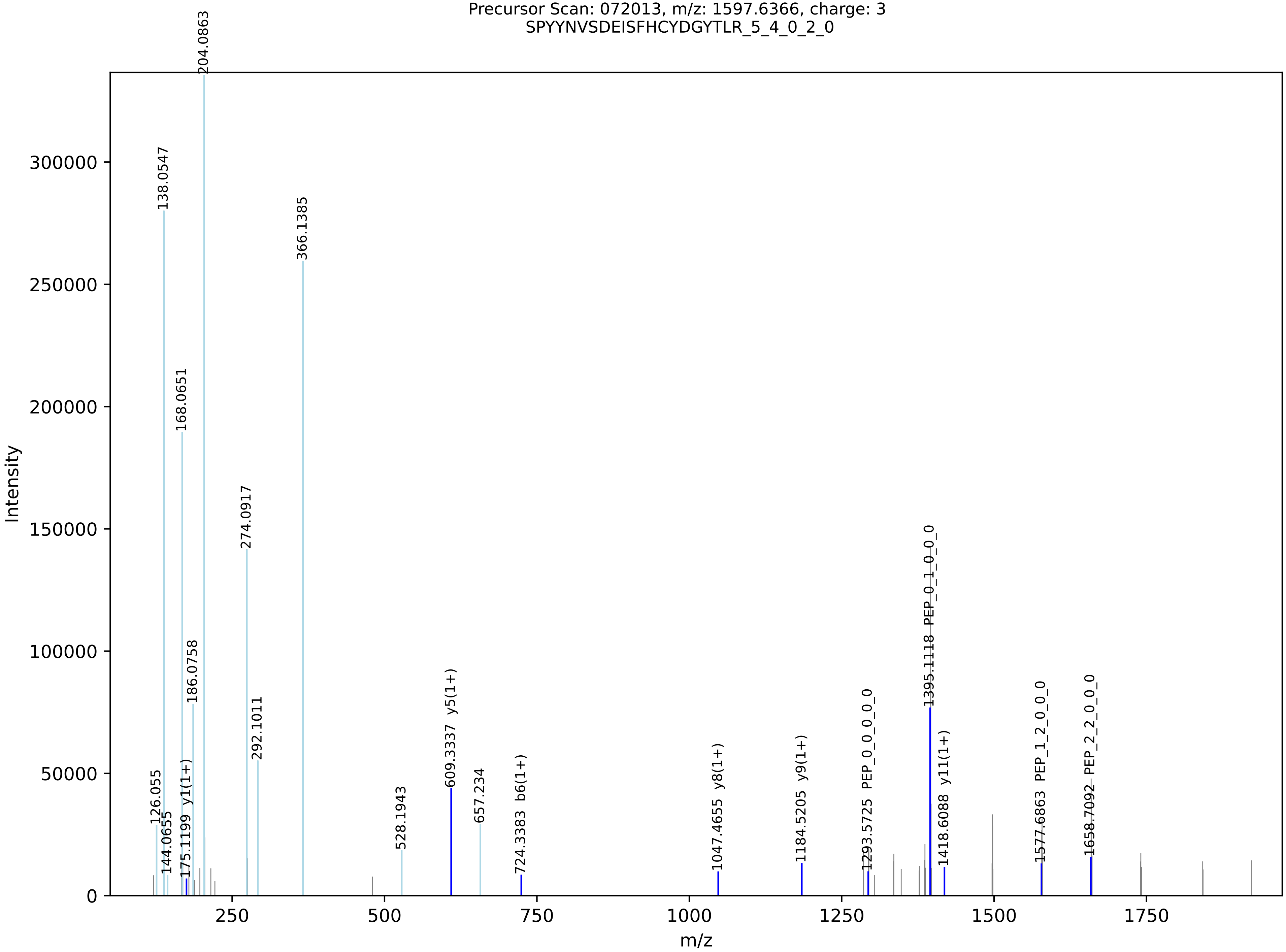

File: 231129_OE_RK_36, RT: 56.653, Scan: 072310
Type: HCD, FTMS + c NSI d Full ms2 1532.6962@hcd30.00 [120.0000-4712.1924]
Precursor Scan: 072231, m/z: 1531.6927, charge: 3
MVSHHNLTTGATLINEQWLLTTAK_5_4_0_1_0

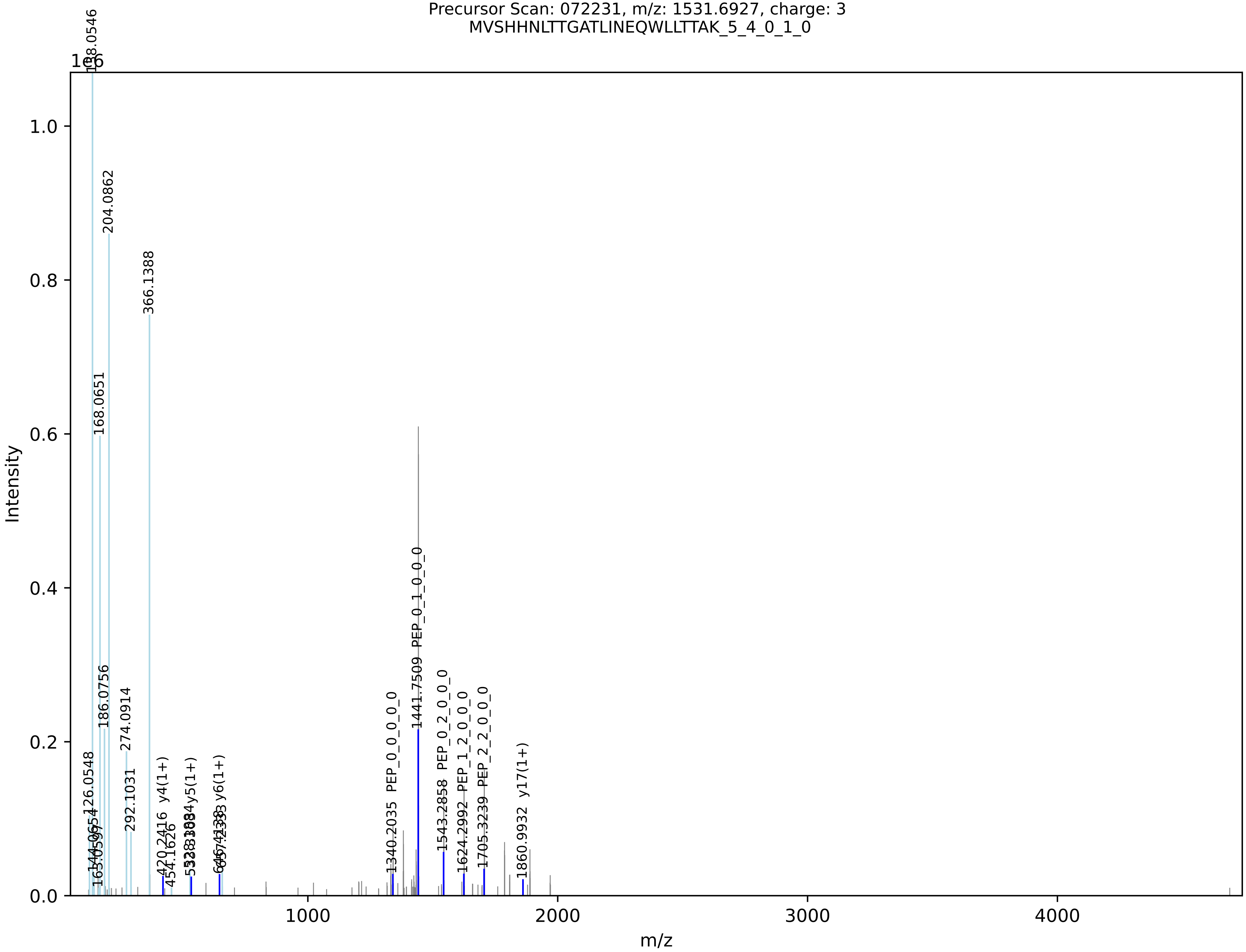

File: 231129_OE_RK_36, RT: 56.654, Scan: 072312
Type: HCD, FTMS + c NSI d Full ms2 1320.5568@hcd30.00 [120.0000-4063.0457]
Precursor Scan: 072231, m/z: 1319.8885, charge: 3
YTGNASALFILPDQDK_5_4_0_2_0

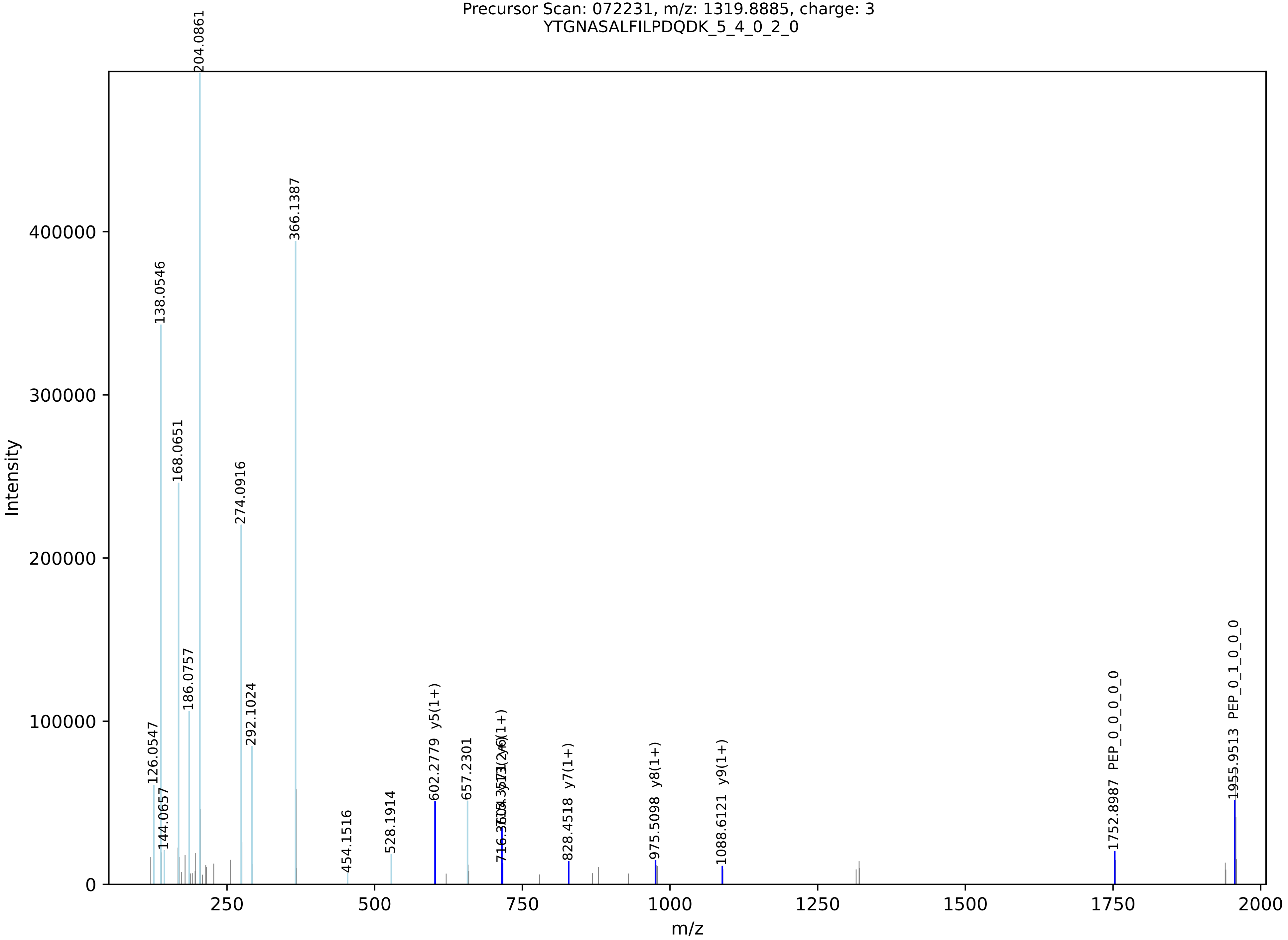

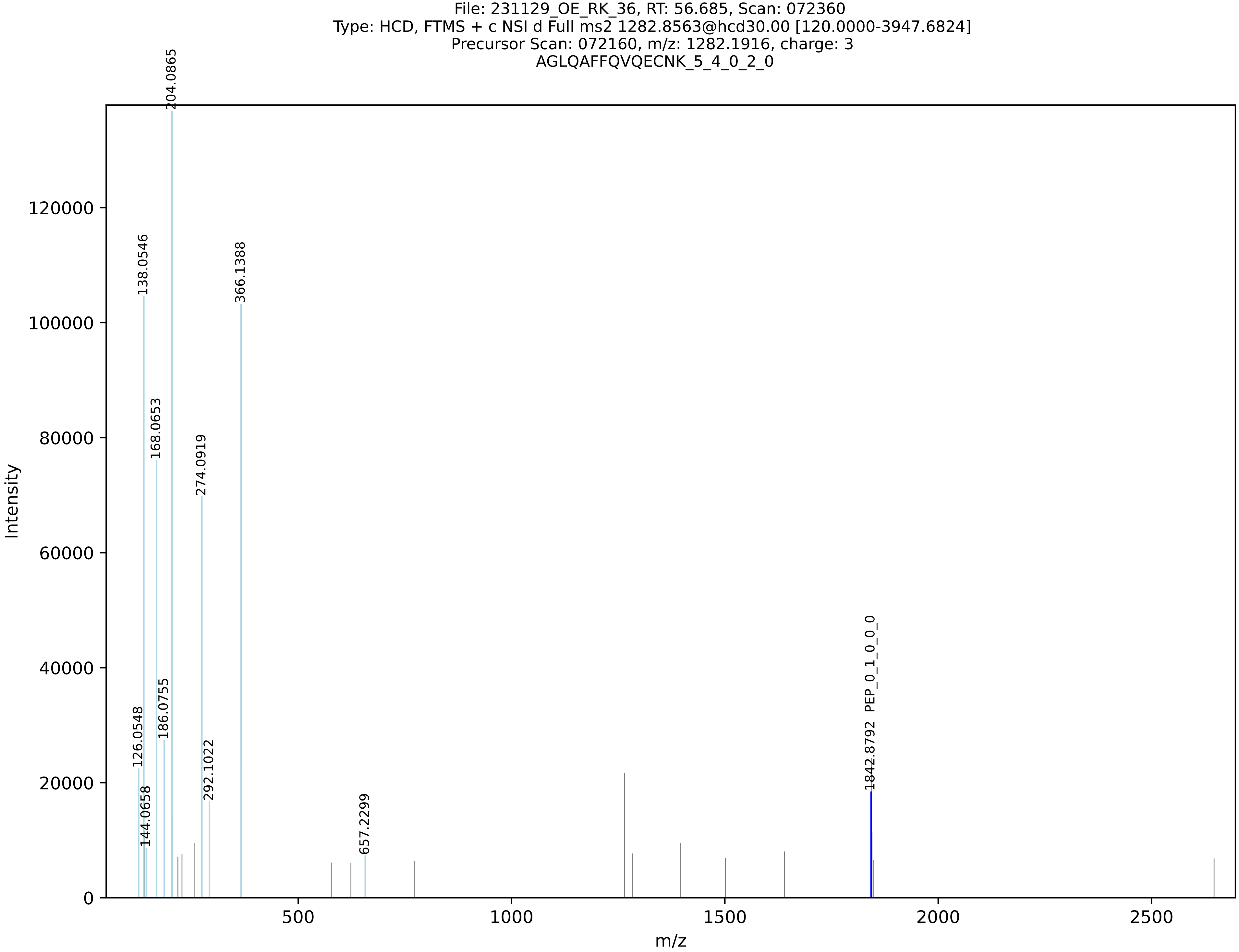
File: 231129_OE_RK_36, RT: 56.685, Scan: 072360
Type: HCD, FTMS + c NSI d Full ms2 1282.8563@hcd30.00 [120.0000-3947.6824]
Precursor Scan: 072160, m/z: 1282.1916, charge: 3
AGLQAFFQVQECNK_5_4_0_2_0
Intensity
m/z
204.0865
138.0546
366.1388
168.0653
274.0919
186.0755
126.0548
292.1022
144.0658
657.2299
1842.8792 PEP_0_1_0_0_0
0
20000
40000
60000
80000
100000
120000
500
1000
1500
2000
2500

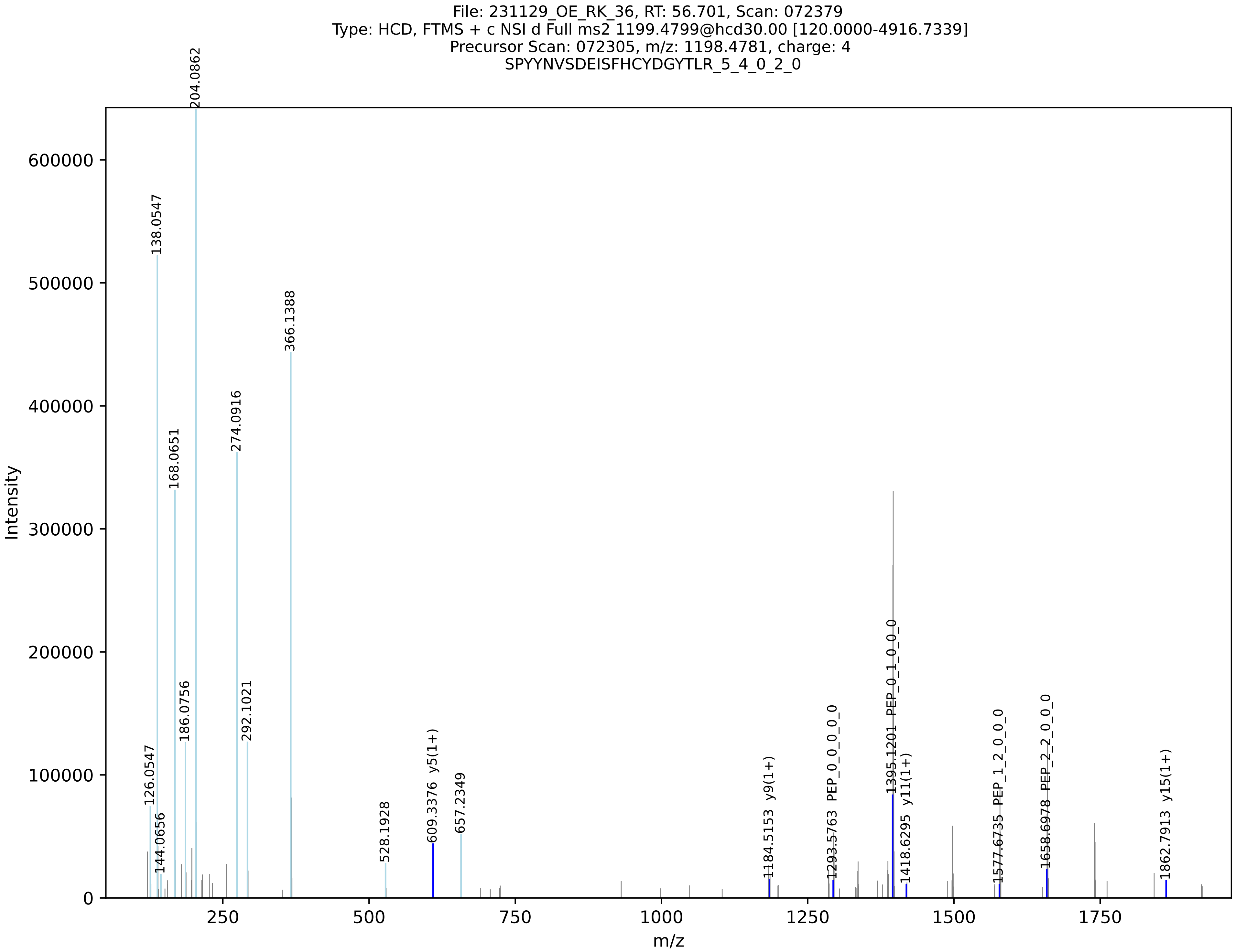
File: 231129_OE_RK_36, RT: 56.701, Scan: 072379
Type: HCD, FTMS + c NSI d Full ms2 1199.4799@hcd30.00 [120.0000-4916.7339]
Precursor Scan: 072305, m/z: 1198.4781, charge: 4
SPYYNVSDEISFHCYDGYTLR_5_4_0_2_0
Intensity
m/z
204.0862
138.0547
366.1388
274.0916
168.0651
186.0756
292.1021
126.0547
144.0656
528.1928
609.3376 y5(1+)
657.2349
1184.5153 y9(1+)
1293.5763 PEP_0_0_0_0_0
1395.1201 PEP_0_1_0_0_0
1418.6295 y11(1+)
1577.6735 PEP_1_2_0_0_0
1658.6978 PEP_2_2_0_0_0
1862.7913 y15(1+)

File: 231129_OE_RK_36, RT: 56.99, Scan: 072798
Type: HCD, FTMS + c NSI d Full ms2 1149.2727@hcd30.00 [120.0000-4711.8887]
Precursor Scan: 072723, m/z: 1148.7715, charge: 4
MVSHHNLTTGATLINEQWLLTTAK_5_4_0_1_0

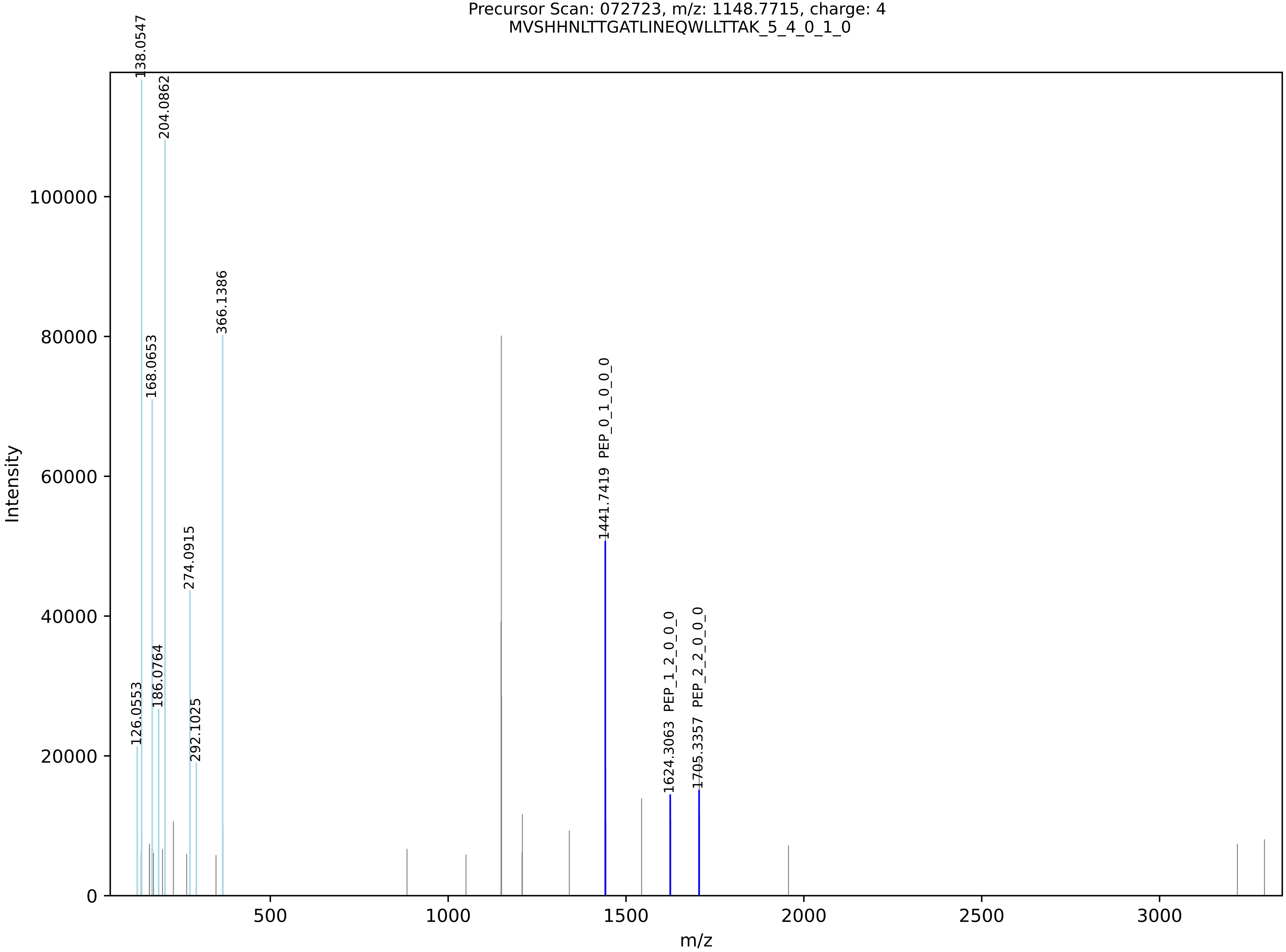

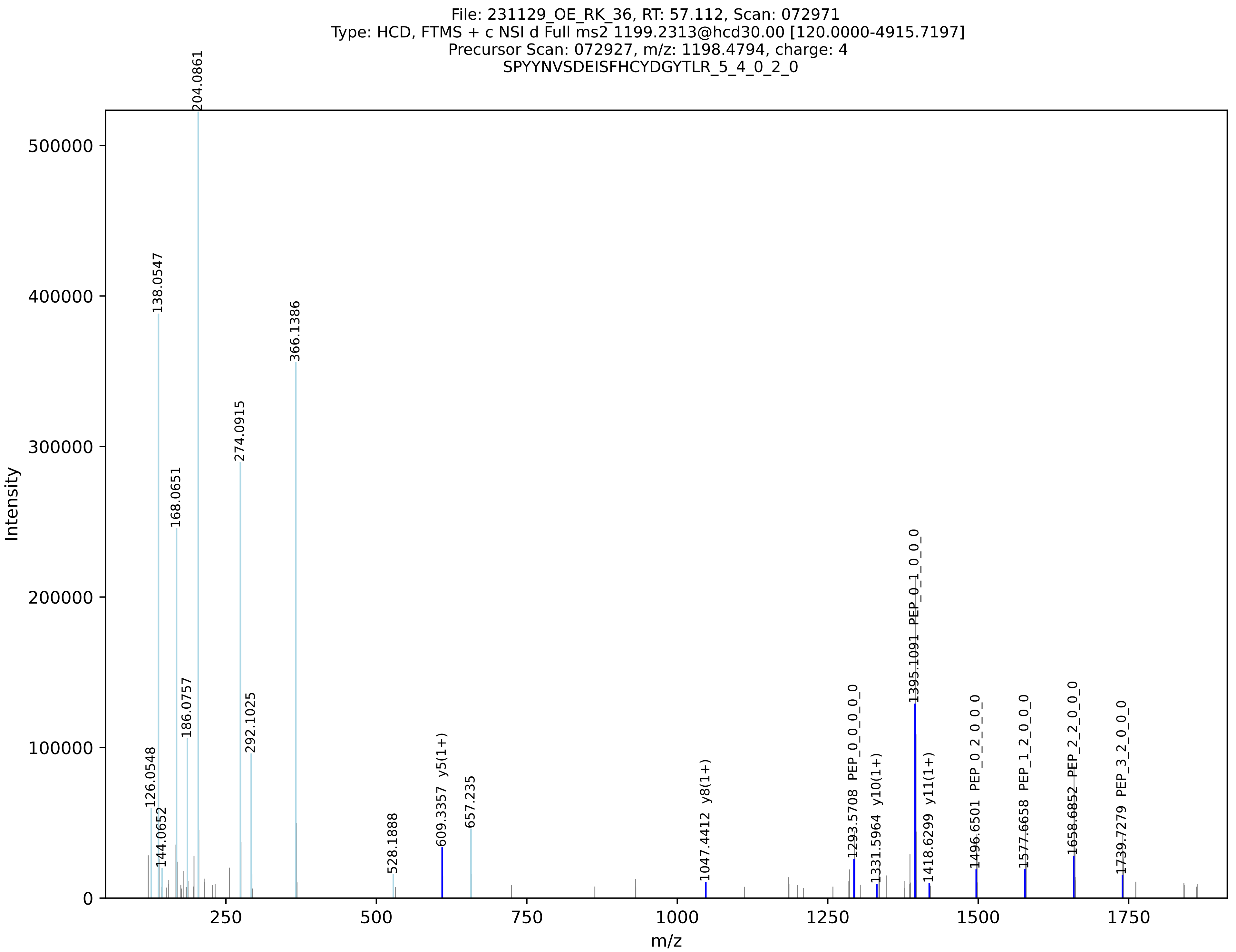
File: 231129_OE_RK_36, RT: 57.112, Scan: 072971
Type: HCD, FTMS + c NSI d Full ms2 1199.2313@hcd30.00 [120.0000-4915.7197]
Precursor Scan: 072927, m/z: 1198.4794, charge: 4
SPYYNVSDEISFHCYDGYTLR_5_4_0_2_0
Intensity
m/z
204.0861
138.0547
366.1386
274.0915
168.0651
186.0757
292.1025
126.0548
144.0652
528.1888
609.3357 y5(1+)
657.235
1047.4412 y8(1+)
1293.5708 PEP_0_0_0_0_0_
1331.5964 y10(1+)
1395.1091 PEP_0_1_0_0_0_
1418.6299 y11(1+)
1496.6501 PEP_0_2_0_0_0_
1577.6658 PEP_1_2_0_0_0_
1658.6852 PEP_2_2_0_0_0_
1739.7279 PEP_3_2_0_0_0_

File: 231129_OE_RK_36, RT: 57.199, Scan: 073096
Type: HCD, FTMS + c NSI d Full ms2 977.2986@hcd30.00 [120.0000-6005.3521]
Precursor Scan: 073022, m/z: 977.2987, charge: 6
LTAQVASLTSELTTLNATIQQQDQELAGLKQQAK_4_7_1_0_0

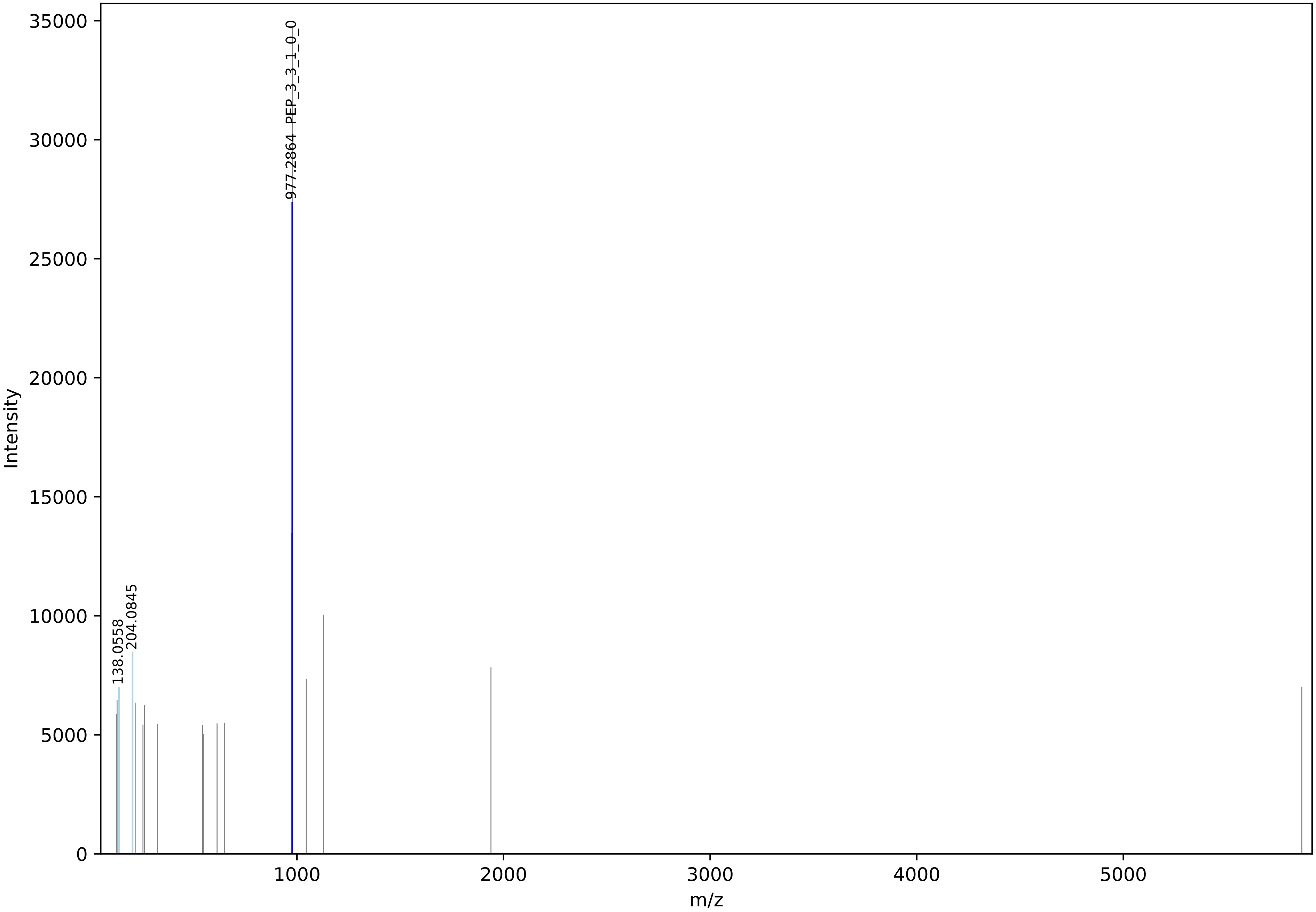

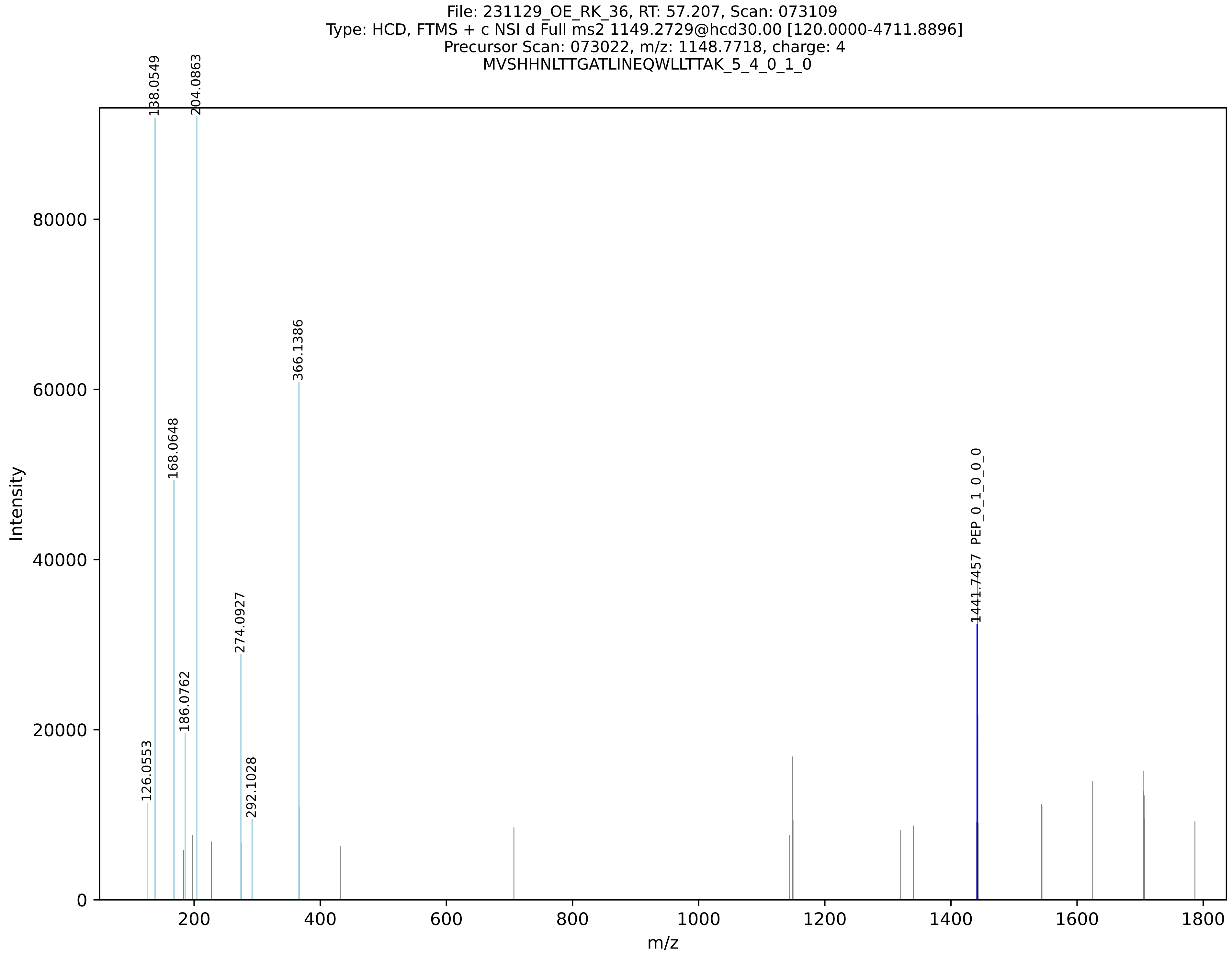
File: 231129_OE_RK_36, RT: 57.207, Scan: 073109
Type: HCD, FTMS + c NSI d Full ms2 1149.2729@hcd30.00 [120.0000-4711.8896]
Precursor Scan: 073022, m/z: 1148.7718, charge: 4
MVSHHNLTTGATLINEQWLLTTAK_5_4_0_1_0
138.0549
204.0863
168.0648
366.1386
186.0762
274.0927
126.0553
292.1028
1441.7457 PEP_0_1_0_0_0
Intensity
m/z
0
20000
40000
60000
80000
200
400
600
800
1000
1200
1400
1600
1800

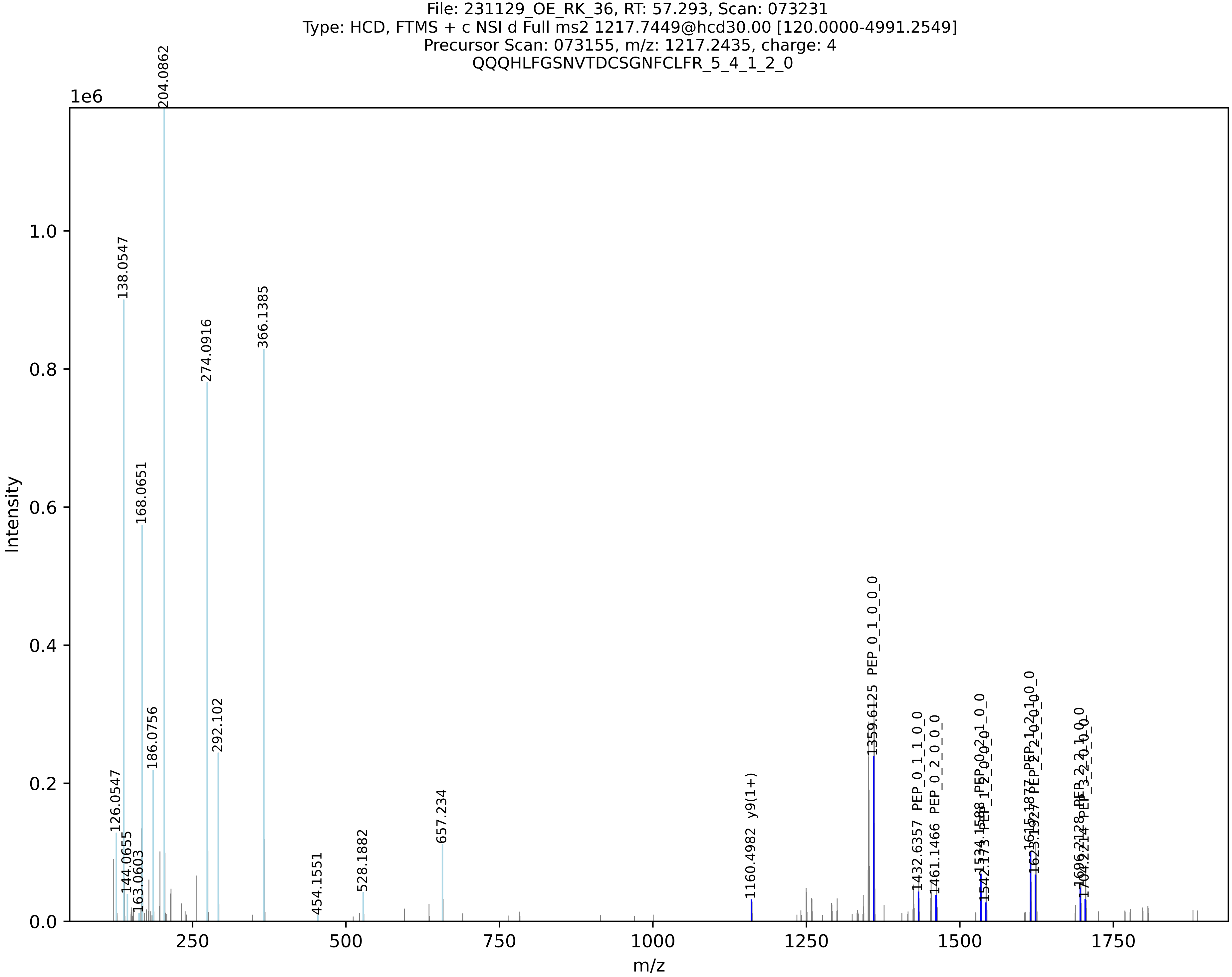
File: 231129_OE_RK_36, RT: 57.293, Scan: 073231
Type: HCD, FTMS + c NSI d Full ms2 1217.7449@hcd30.00 [120.0000-4991.2549]
Precursor Scan: 073155, m/z: 1217.2435, charge: 4
QQQHLFGSNVTDCSGNFCLFR_5_4_1_2_0
1e6
Intensity
m/z
204.0862
138.0547
366.1385
274.0916
168.0651
186.0756
292.102
126.0547
144.0655
163.0603
454.1551
528.1882
657.234
1160.4982 y9(1+)
1359.6125 PEP_0_1_0_0_0
1432.6357 PEP_0_1_1_0_0
1461.1466 PEP_0_2_0_0_0
1534.1588
1542.173
1696.2128
1704.2214

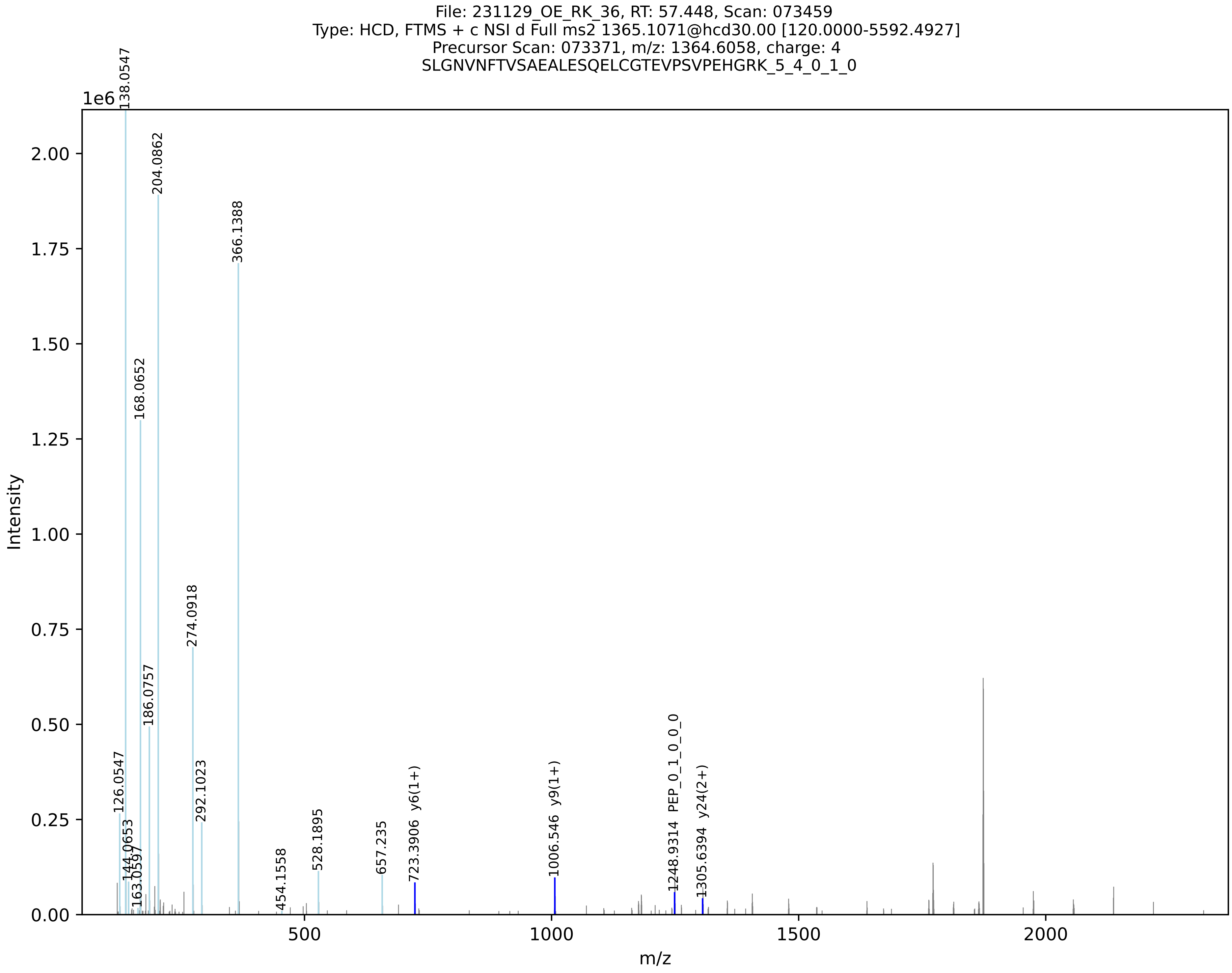
File: 231129_OE_RK_36, RT: 57.448, Scan: 073459
Type: HCD, FTMS + c NSI d Full ms2 1365.1071@hcd30.00 [120.0000-5592.4927]
Precursor Scan: 073371, m/z: 1364.6058, charge: 4
SLGNVNFTVSAEALESQELCGTEVPSVPEHGRK_5_4_0_1_0
1e6
Intensity
m/z
138.0547
204.0862
366.1388
168.0652
274.0918
186.0757
126.0547
292.1023
144.0653
163.0597
454.1558
528.1895
657.235
723.3906 y6(1+)
1006.546 y9(1+)
1248.9314 PEP_0_1_0_0_0
1305.6394 y24(2+)

File: 231129_OE_RK_36, RT: 57.449, Scan: 073461
Type: HCD, FTMS + c NSI d Full ms2 1485.3239@hcd30.00 [120.0000-4567.2329]
Precursor Scan: 073371, m/z: 1484.6556, charge: 3
LQAPLNYTEFQKPICLPSK_5_4_0_2_0

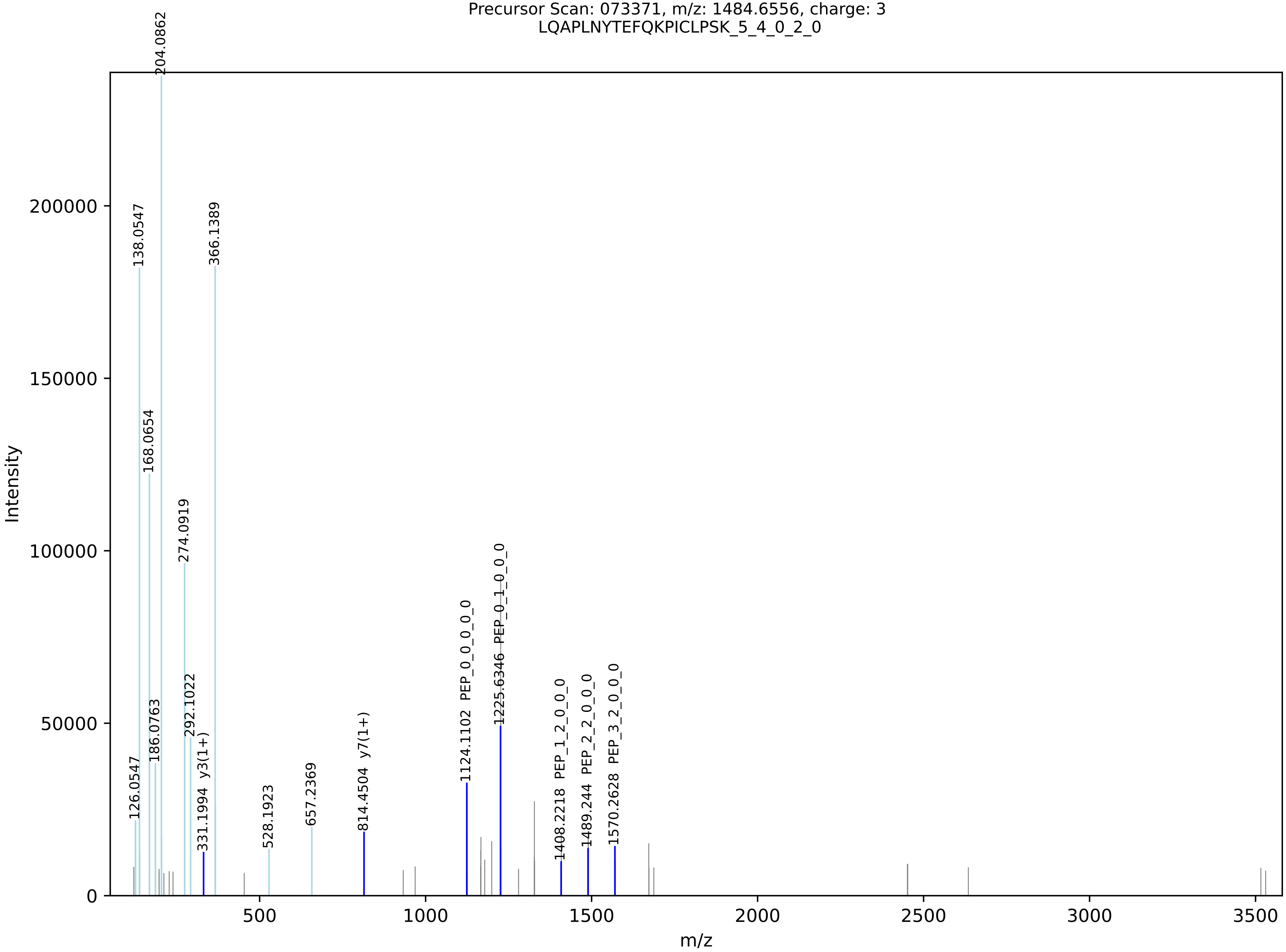

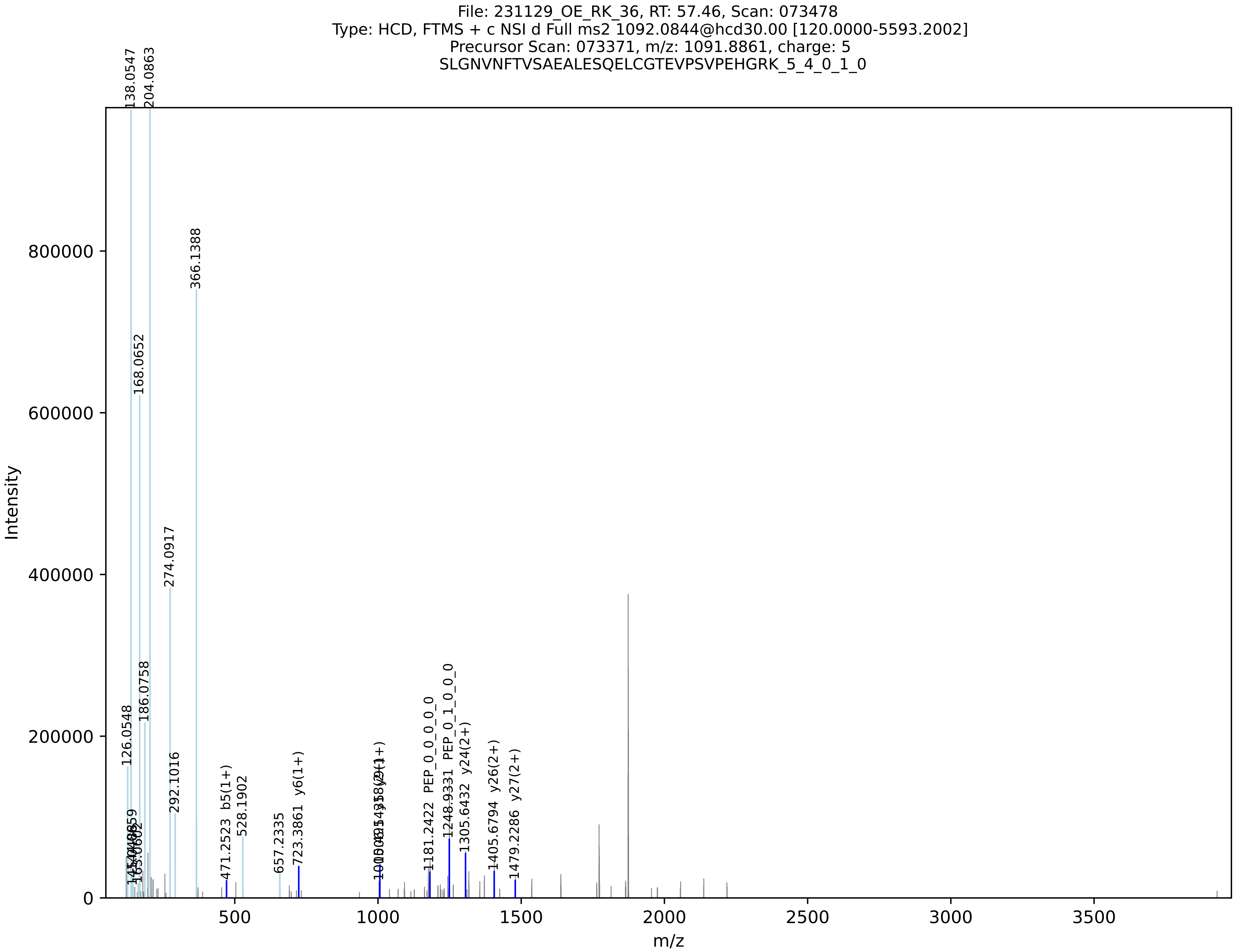

File: 231129_OE_RK_36, RT: 57.46, Scan: 073478
Type: HCD, FTMS + c NSI d Full ms2 1092.0844@hcd30.00 [120.0000-5593.2002]
Precursor Scan: 073371, m/z: 1091.8861, charge: 5
SLGNVNFTVSAEALESQELCGTEVPSVPEHGRK_5_4_0_1_0
138.0547
204.0863
366.1388
168.0652
274.0917
186.0758
126.0548
292.1016
471.2523 b5(1+)
528.1902
657.2335
723.3861 y6(1+)
1181.2422 PEP_0_0_0_0_0
1248.9331 PEP_0_1_0_0_0
1305.6432 y24(2+)
1405.6794 y26(2+)
1479.2286 y27(2+)
Intensity
m/z
800000
600000
400000
200000
0
500
1000
1500
2000
2500
3000
3500

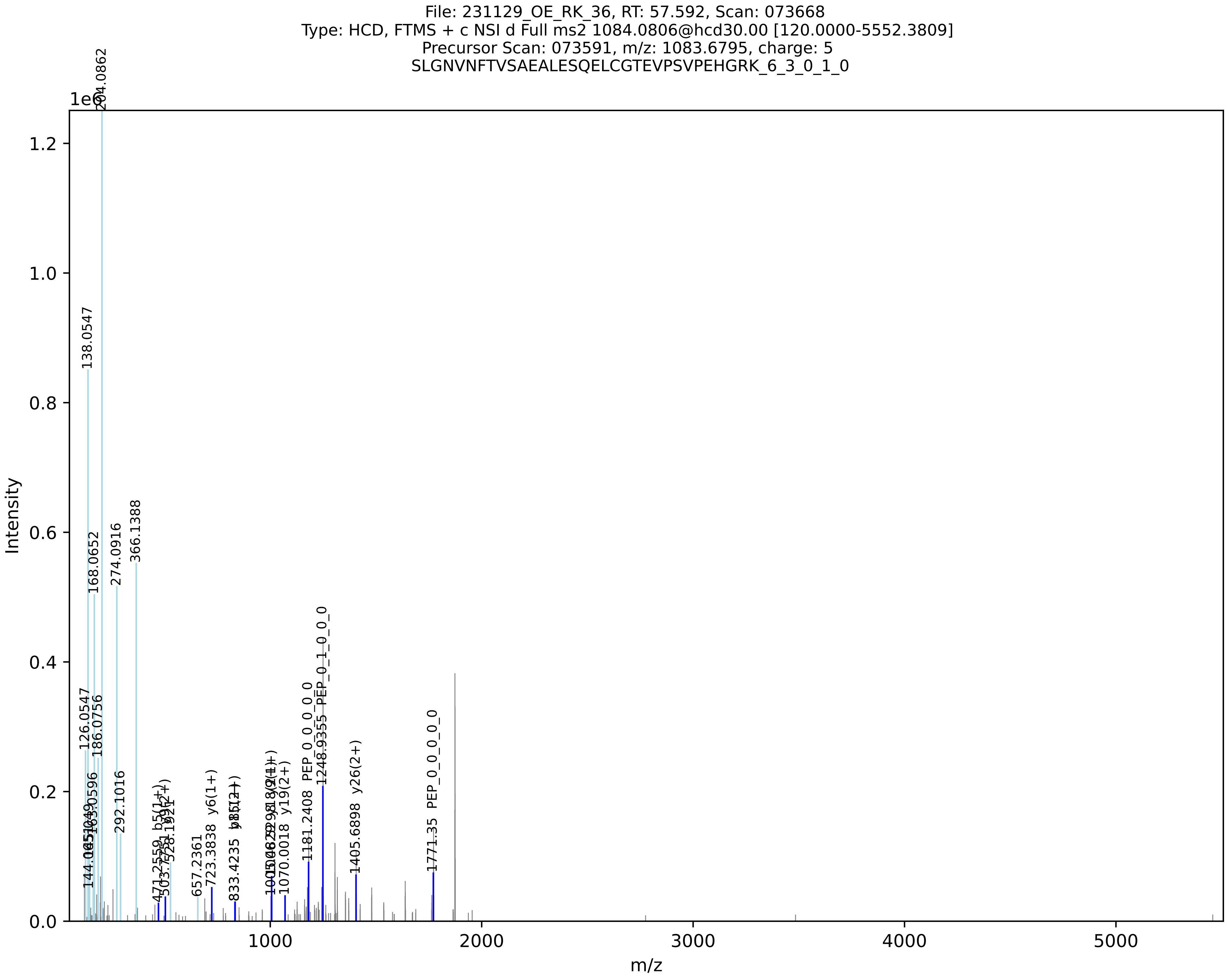
File: 231129_OE_RK_36, RT: 57.592, Scan: 073668
Type: HCD, FTMS + c NSI d Full ms2 1084.0806@hcd30.00 [120.0000-5552.3809]
Precursor Scan: 073591, m/z: 1083.6795, charge: 5
SLGNVNFTVSAEALESQELCGTEVPSVPEHGRK_6_3_0_1_0
1e6
Intensity
m/z
138.0547
168.0652
274.0916
366.1388
126.0547
186.0756
292.1016
657.2361
723.3838 y6(1+)
833.4235
1070.0018 y19(2+)
1181.2408 PEP_0_0_0_0_0
1248.9355 PEP_0_1_0_0_0
1405.6898 y26(2+)
1771.35 PEP_0_0_0_0_0
0.0
0.2
0.4
0.6
0.8
1.0
1.2
1000
2000
3000
4000
5000

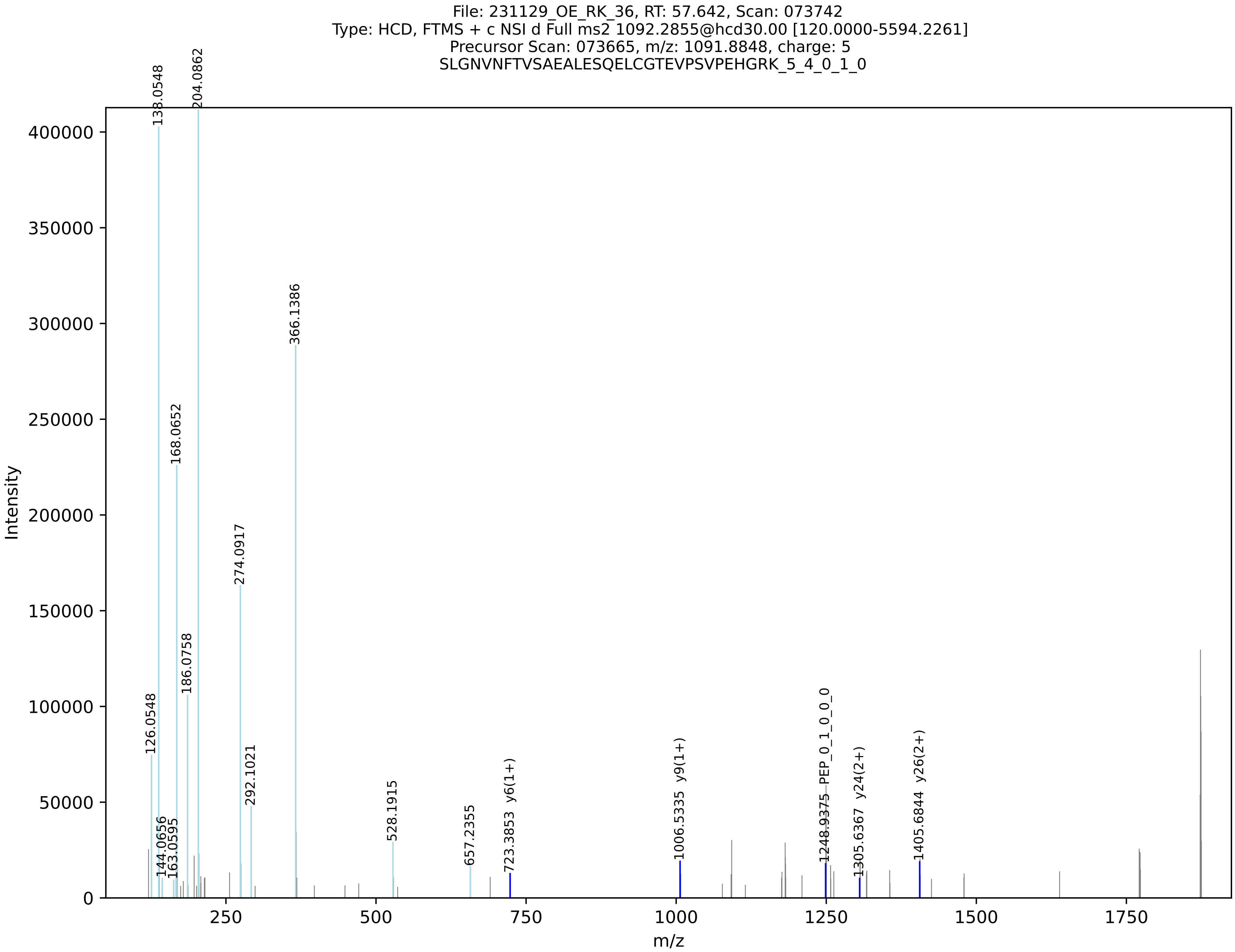

File: 231129_OE_RK_36, RT: 57.642, Scan: 073742
Type: HCD, FTMS + c NSI d Full ms2 1092.2855@hcd30.00 [120.0000-5594.2261]
Precursor Scan: 073665, m/z: 1091.8848, charge: 5
SLGNVNFTVSAEALESQELCGTEVPSVPEHGRK_5_4_0_1_0
Intensity
m/z
138.0548
204.0862
366.1386
168.0652
274.0917
186.0758
126.0548
292.1021
144.0656
163.0595
528.1915
657.2355
723.3853 y6(1+)
1006.5335 y9(1+)
1248.9375 PEP_0_1_0_0_0
1305.6367 y24(2+)
1405.6844 y26(2+)

File: 231129_OE_RK_36, RT: 57.693, Scan: 073816
Type: HCD, FTMS + c NSI d Full ms2 1198.9790@hcd30.00 [120.0000-4914.6904]
Precursor Scan: 073739, m/z: 1198.4778, charge: 4
SPYYNVSDEISFHCYDGYTLR_5_4_0_2_0

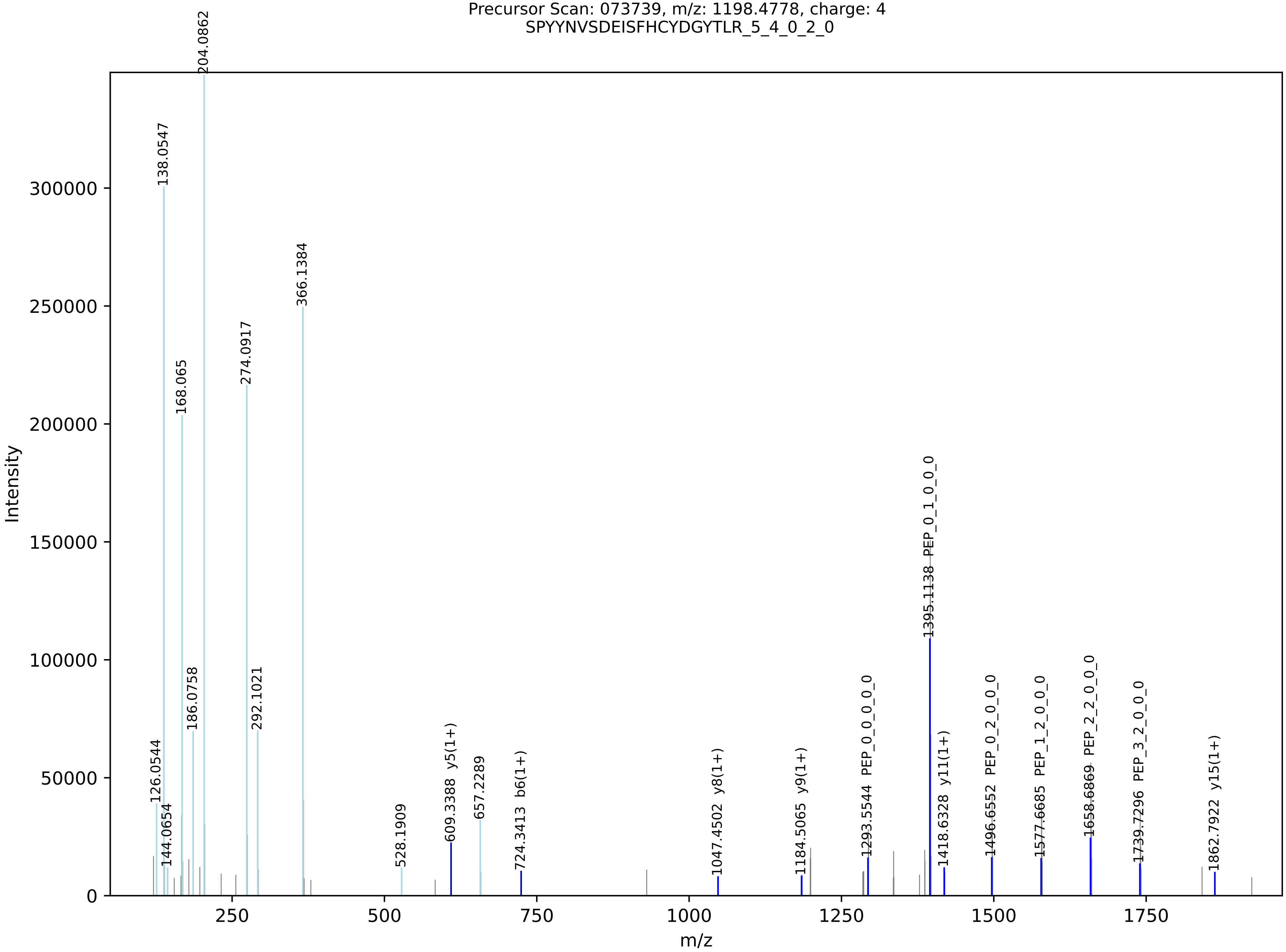

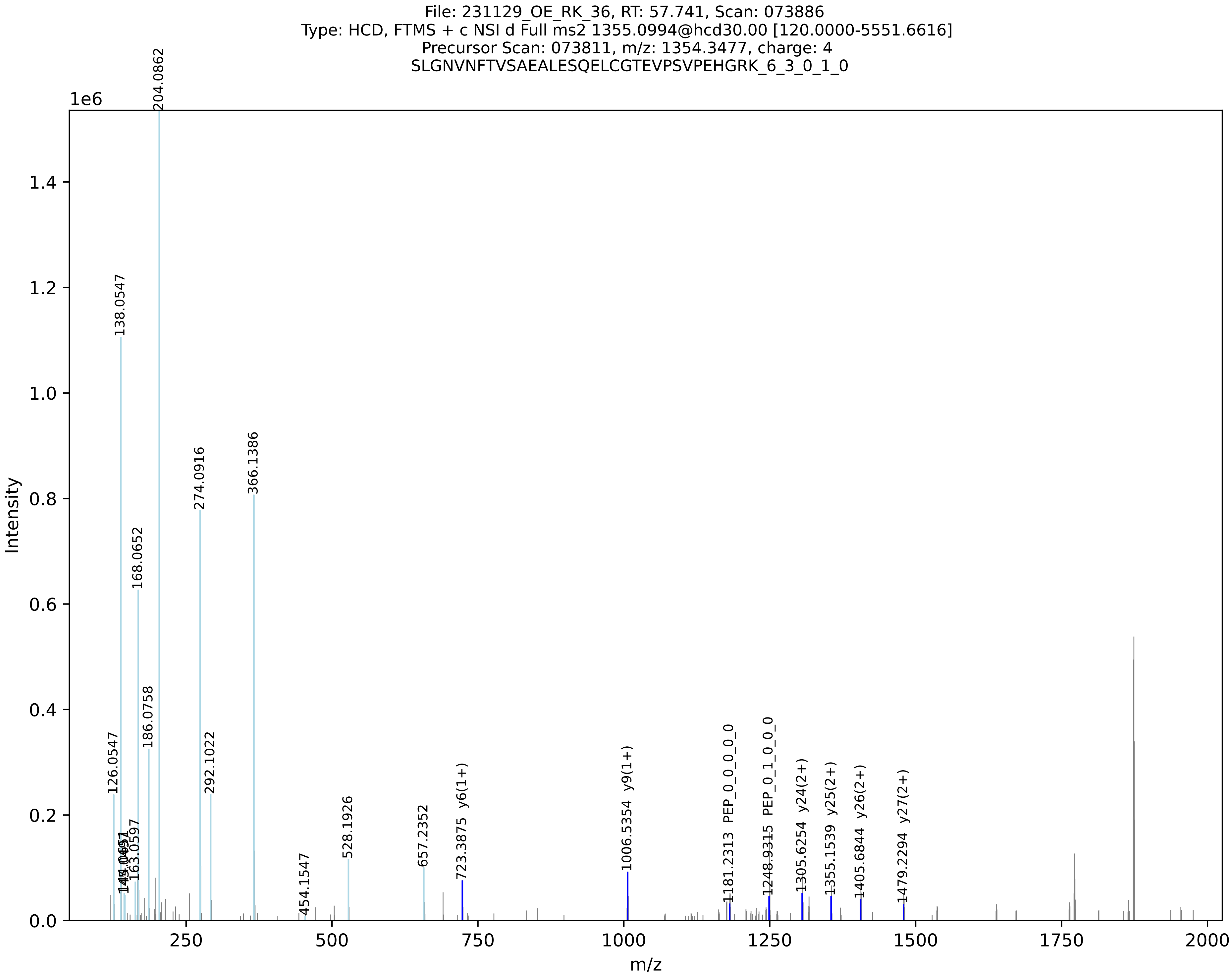
File: 231129_OE_RK_36, RT: 57.741, Scan: 073886
Type: HCD, FTMS + c NSI d Full ms2 1355.0994@hcd30.00 [120.0000-5551.6616]
Precursor Scan: 073811, m/z: 1354.3477, charge: 4
SLGNVNFTVSAEALESQELCGTEVPSVPEHGRK_6_3_0_1_0
1e6
Intensity
m/z
204.0862
138.0547
366.1386
274.0916
168.0652
186.0758
126.0547
292.1022
163.0597
528.1926
454.1547
657.2352
723.3875 y6(1+)
1006.5354 y9(1+)
1181.2313 PEP_0_0_0_0_0
1248.9315 PEP_0_1_0_0_0
1305.6254 y24(2+)
1355.1539 y25(2+)
1405.6844 y26(2+)
1479.2294 y27(2+)

File: 231129_OE_RK_36, RT: 57.742, Scan: 073887
Type: HCD, FTMS + c NSI d Full ms2 1181.2300@hcd30.00 [120.0000-4842.2744]
Precursor Scan: 073811, m/z: 1180.7288, charge: 4
QQQHLFGSNVTDCSGNFCLFR_5_4_0_2_0

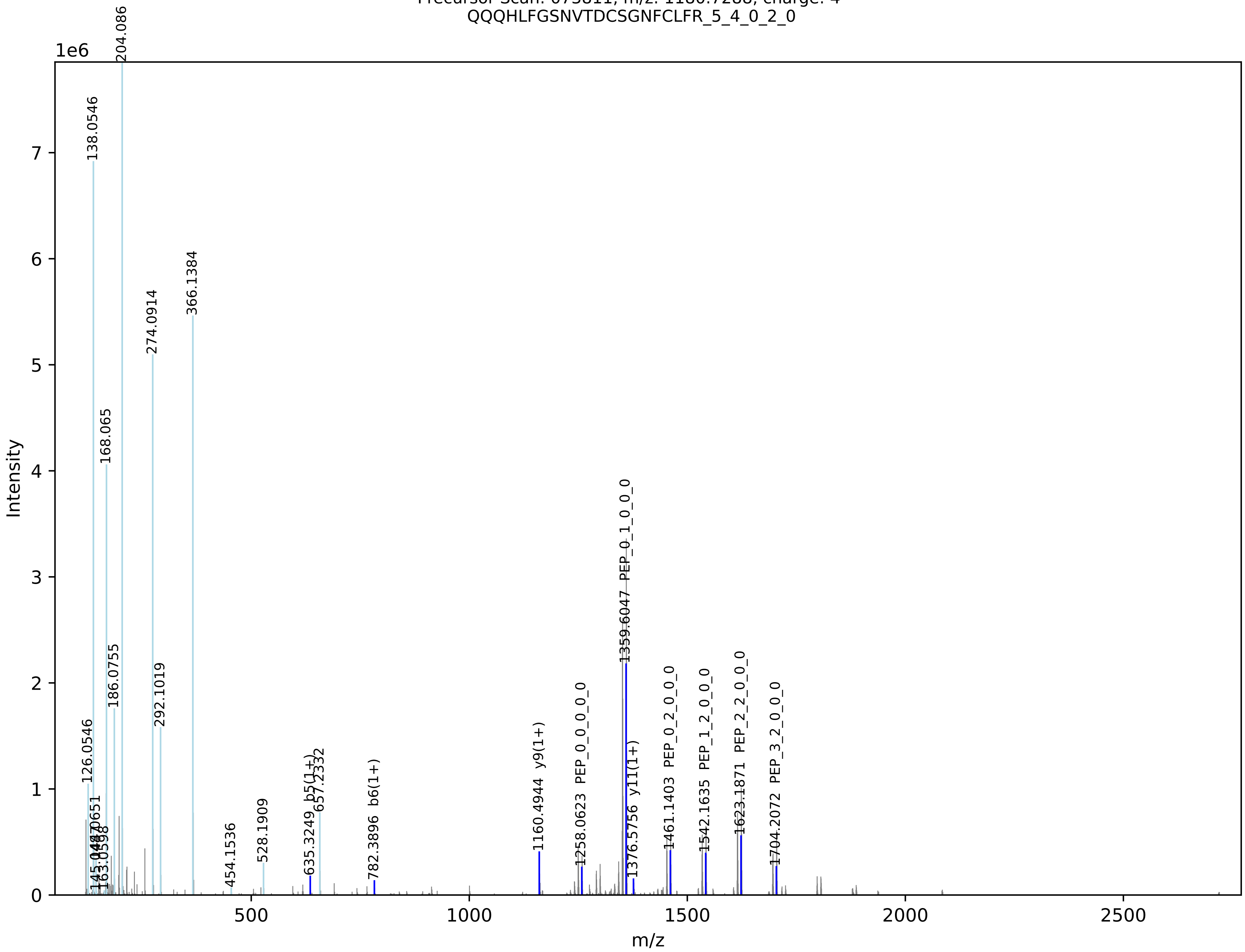

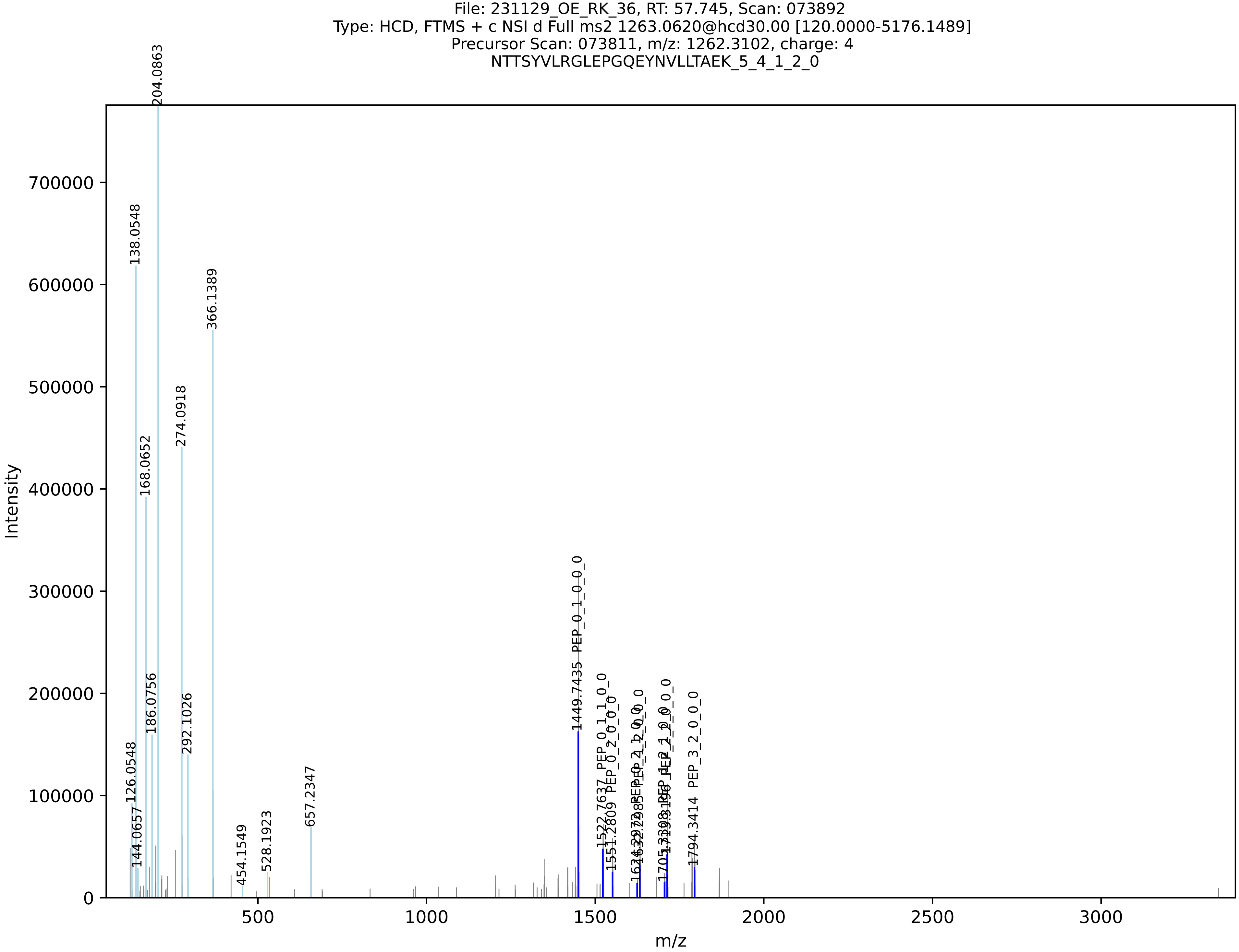

File: 231129_OE_RK_36, RT: 57.745, Scan: 073892
Type: HCD, FTMS + c NSI d Full ms2 1263.0620@hcd30.00 [120.0000-5176.1489]
Precursor Scan: 073811, m/z: 1262.3102, charge: 4
NTTSYVLRGLEPGQEYNVLLTAEK_5_4_1_2_0
Intensity
m/z
204.0863
138.0548
366.1389
274.0918
168.0652
186.0756
292.1026
126.0548
144.0657
454.1549
528.1923
657.2347
1449.7435_PEP_0_1_0_0_0
1522.7637_PEP_0_1_1_0_0
1551.2809_PEP_0_2_0_0_0
1794.3414_PEP_3_2_0_0_0

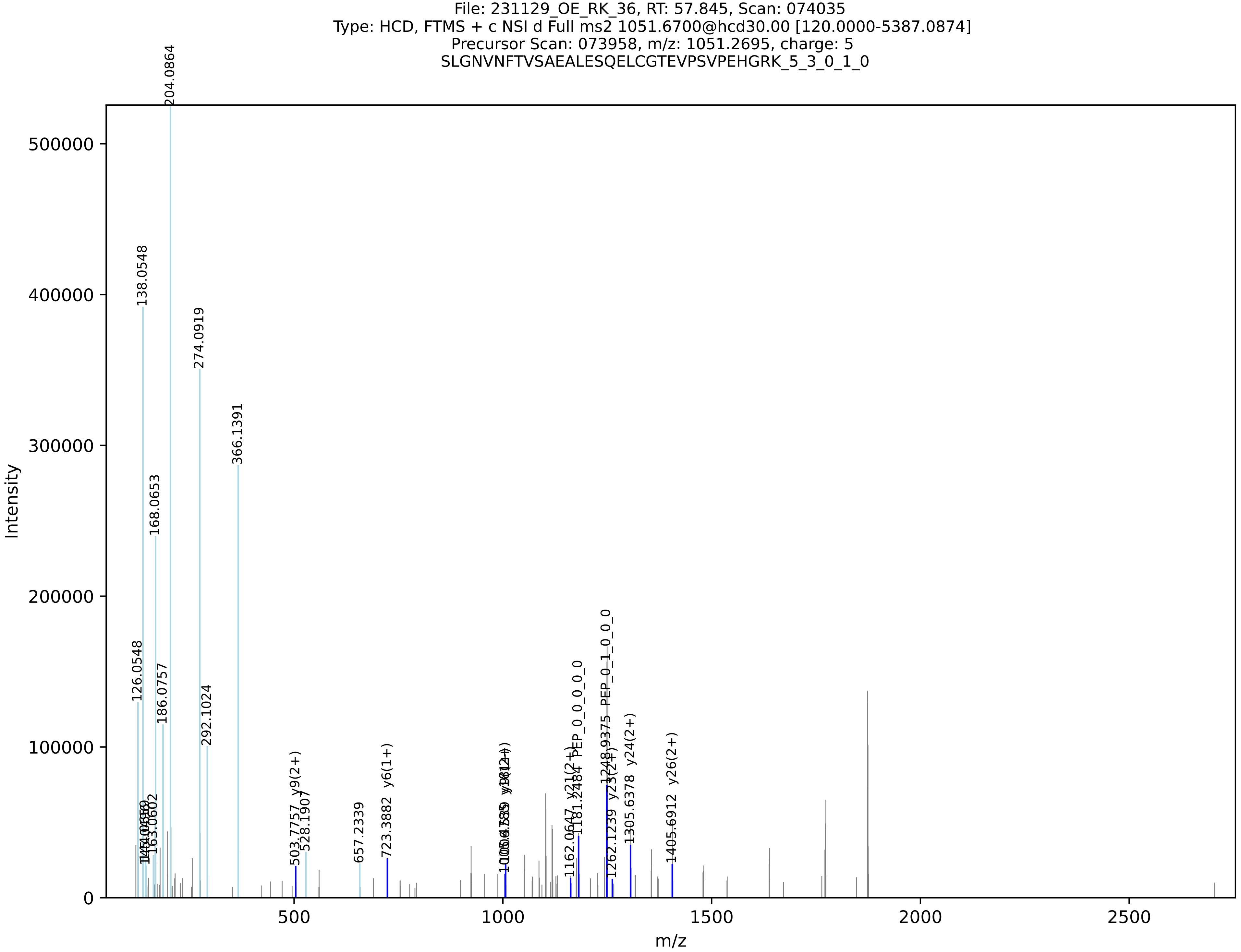
File: 231129_OE_RK_36, RT: 57.845, Scan: 074035
Type: HCD, FTMS + c NSI d Full ms2 1051.6700@hcd30.00 [120.0000-5387.0874]
Precursor Scan: 073958, m/z: 1051.2695, charge: 5
SLGNVNFTVSAEALESQELCGTEVPSVPEHGRK_5_3_0_1_0
204.0864
138.0548
274.0919
366.1391
168.0653
126.0548
186.0757
292.1024
163.0602
503.7757 y9(2+)
528.1907
657.2339
723.3882 y6(1+)
1162.0647 y21(2+)
1181.2484 PEP_0_0_0_0_0
1248.9375 PEP_0_1_0_0_0
1262.1239 y23(2+)
1305.6378 y24(2+)
1405.6912 y26(2+)
Intensity
m/z
0
100000
200000
300000
400000
500000
500
1000
1500
2000
2500

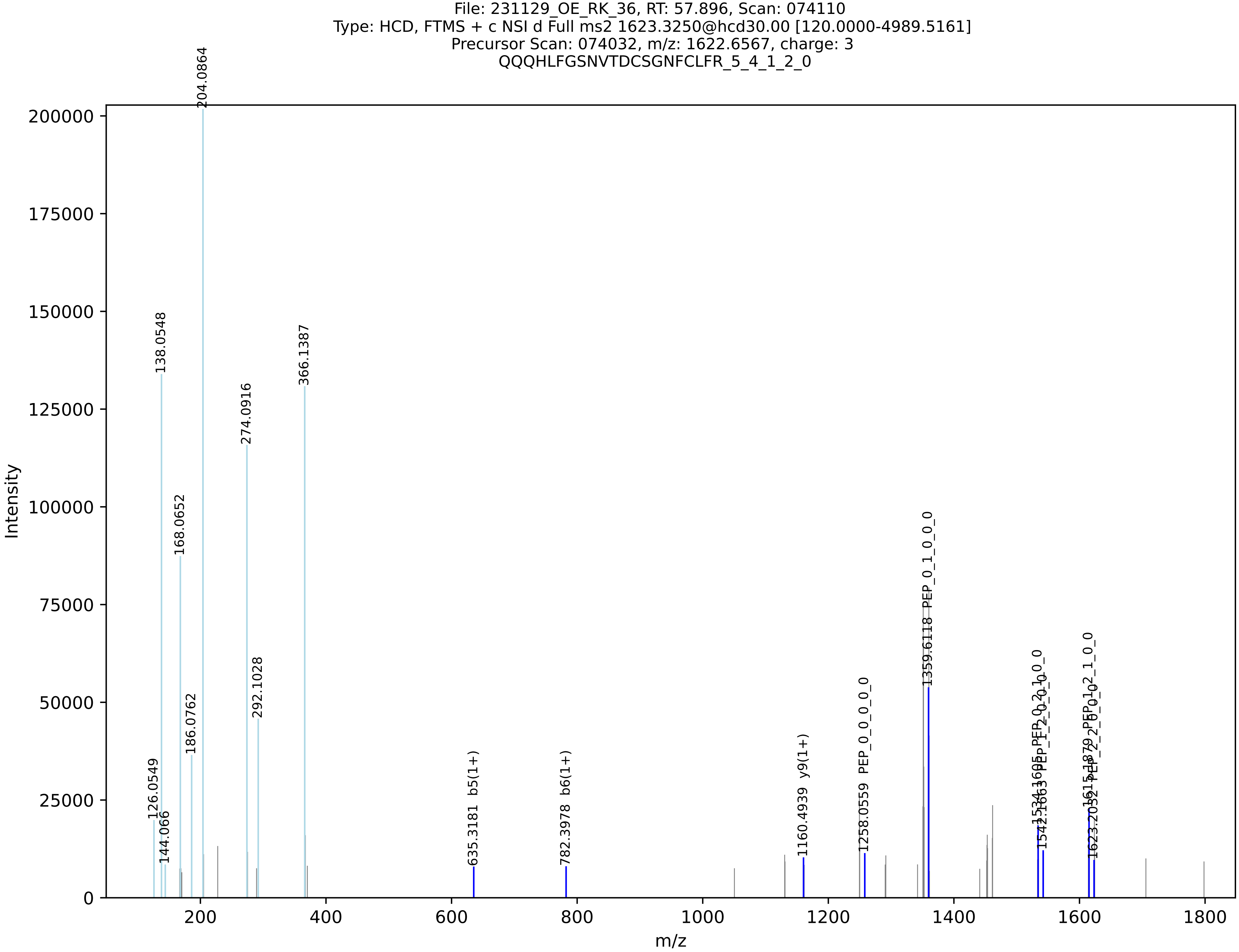

File: 231129_OE_RK_36, RT: 57.896, Scan: 074110
Type: HCD, FTMS + c NSI d Full ms2 1623.3250@hcd30.00 [120.0000-4989.5161]
Precursor Scan: 074032, m/z: 1622.6567, charge: 3
QQQHLFGSNVTDCSGNFCLFR_5_4_1_2_0
Intensity
m/z
204.0864
138.0548
366.1387
274.0916
168.0652
292.1028
186.0762
126.0549
144.066
635.3181 b5(1+)
782.3978 b6(1+)
1160.4939 y9(1+)
1258.0559 PEP_0_0_0_0_0
1359.6118 PEP_0_1_0_0_0

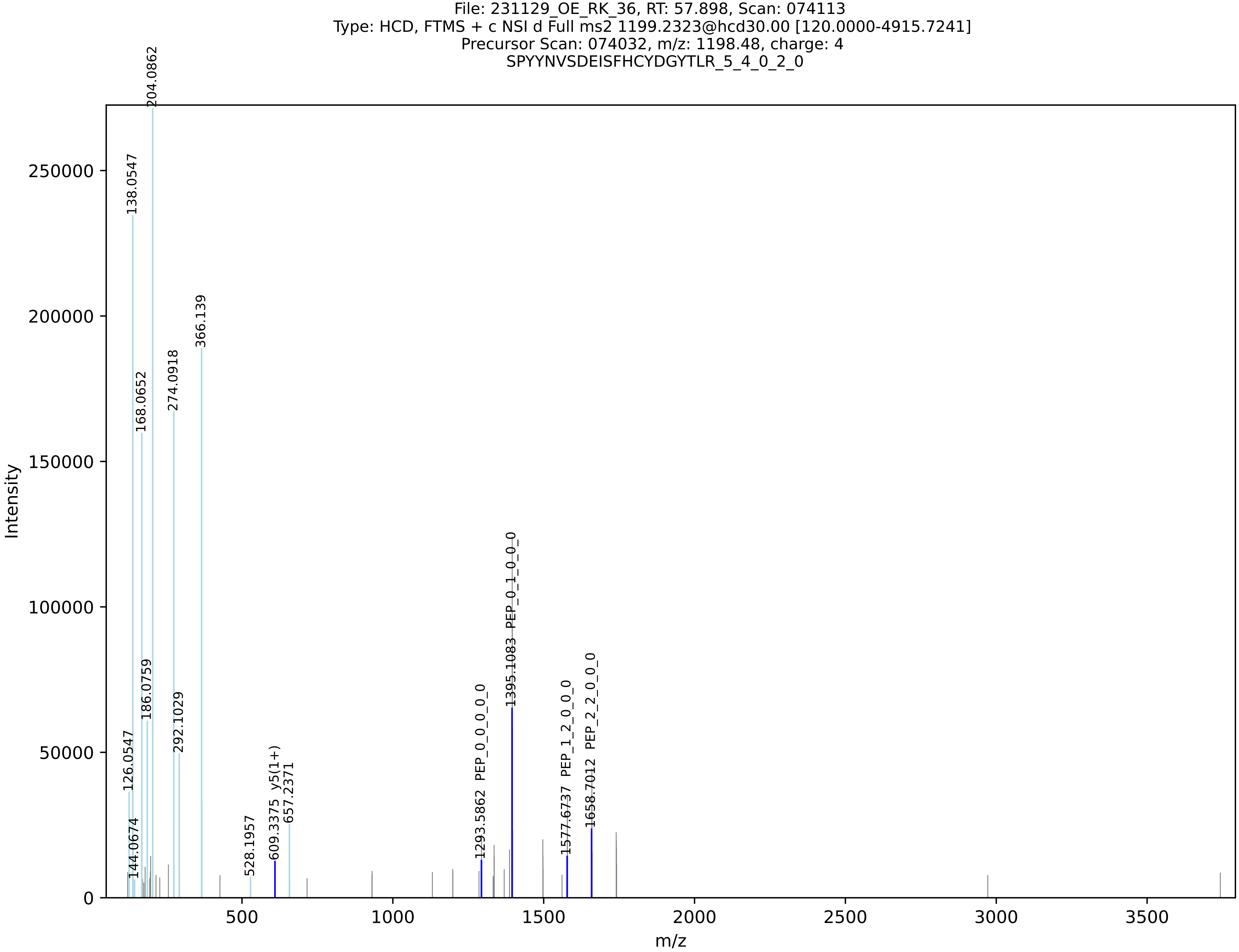

File: 231129_OE_RK_36, RT: 57.898, Scan: 074113
Type: HCD, FTMS + c NSI d Full ms2 1199.2323@hcd30.00 [120.0000-4915.7241]
Precursor Scan: 074032, m/z: 1198.48, charge: 4
SPYYNVSDEISFHCYDGYTLR_5_4_0_2_0
Intensity
m/z
204.0862
138.0547
366.139
274.0918
168.0652
186.0759
292.1029
126.0547
144.0674
528.1957
609.3375 y5(1+)
657.2371
1293.5862 PEP_0_0_0_0_0_
1395.1083 PEP_0_1_0_0_0_
1577.6737 PEP_1_2_0_0_0_
1658.7012 PEP_2_2_0_0_0_

File: 231129_OE_RK_36, RT: 57.944, Scan: 074180
Type: HCD, FTMS + c NSI d Full ms2 1226.2955@hcd30.00 [120.0000-5026.1416]
Precursor Scan: 074103, m/z: 1225.7934, charge: 4
QSGVNATLPEENQPVVFNHVYNIK_5_4_2_1_0

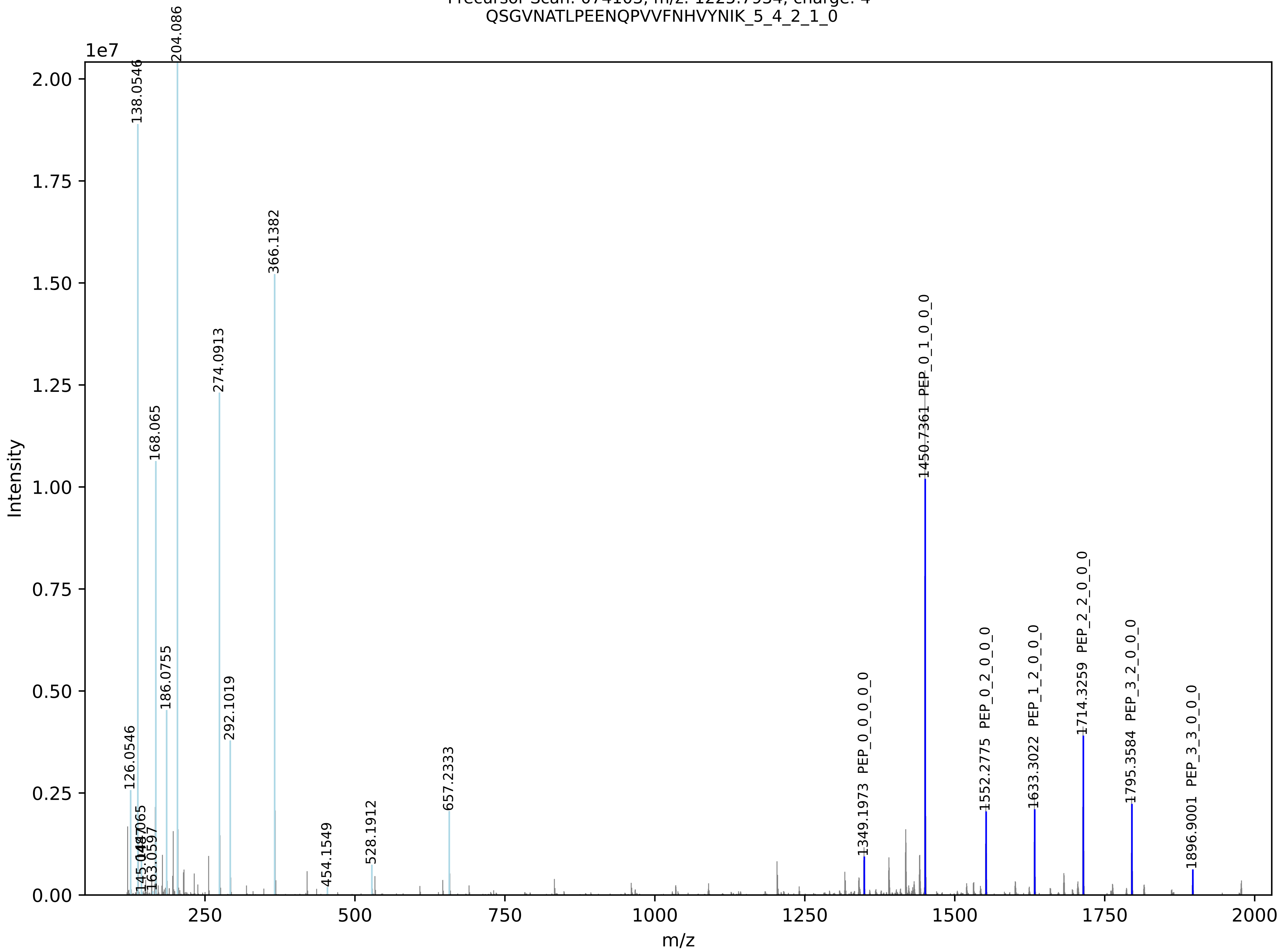

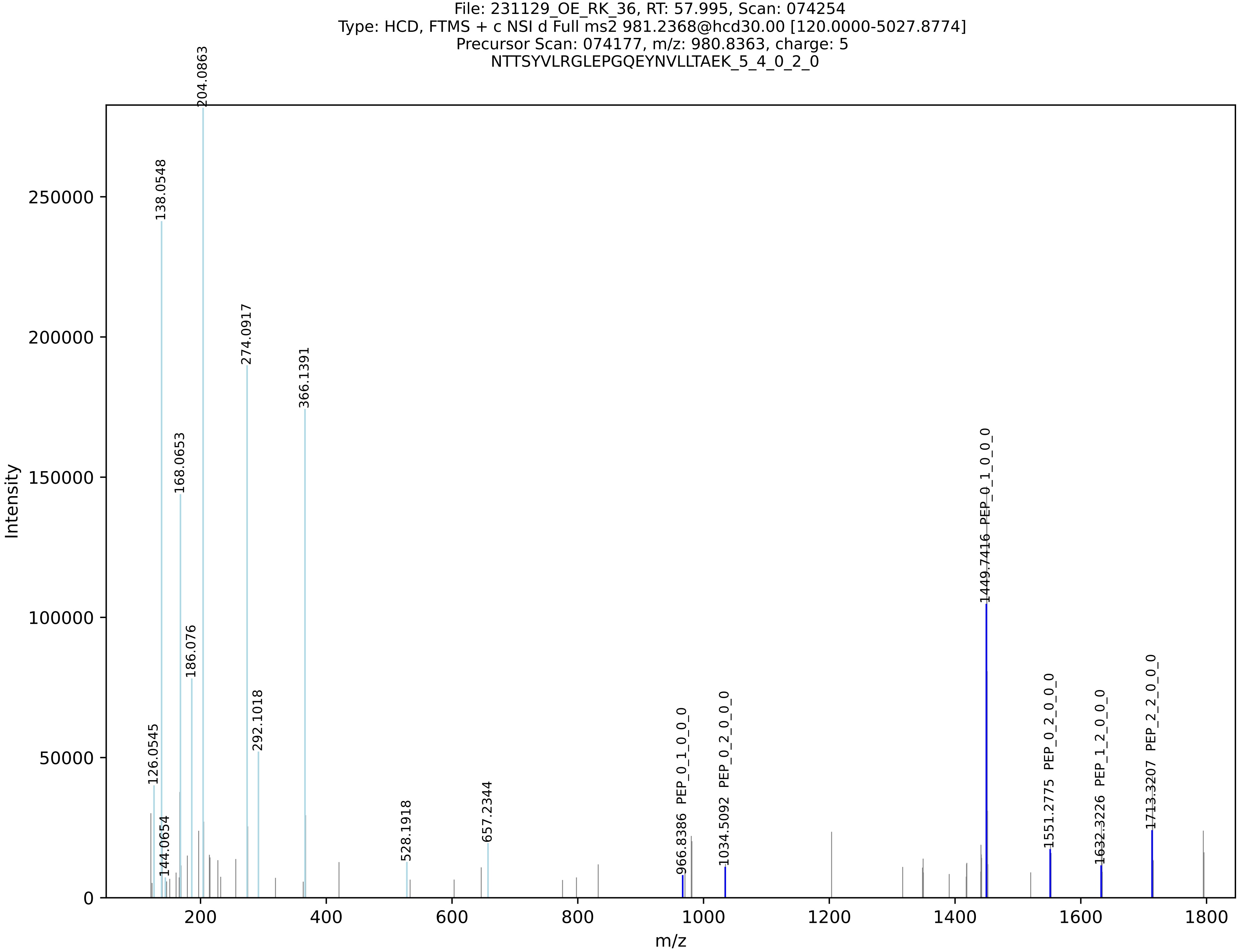
File: 231129_OE_RK_36, RT: 57.995, Scan: 074254
Type: HCD, FTMS + c NSI d Full ms2 981.2368@hcd30.00 [120.0000-5027.8774]
Precursor Scan: 074177, m/z: 980.8363, charge: 5
NTTSYVLRGLEPGQEYNVLLTAEK_5_4_0_2_0
Intensity
m/z
204.0863
138.0548
274.0917
366.1391
168.0653
186.076
292.1018
126.0545
144.0654
528.1918
657.2344
966.8386 PEP_0_1_0_0_0
1034.5092 PEP_0_2_0_0_0
1449.7416 PEP_0_1_0_0_0
1551.2775 PEP_0_2_0_0_0
1632.3226 PEP_1_2_0_0_0
1713.3207 PEP_2_2_0_0_0

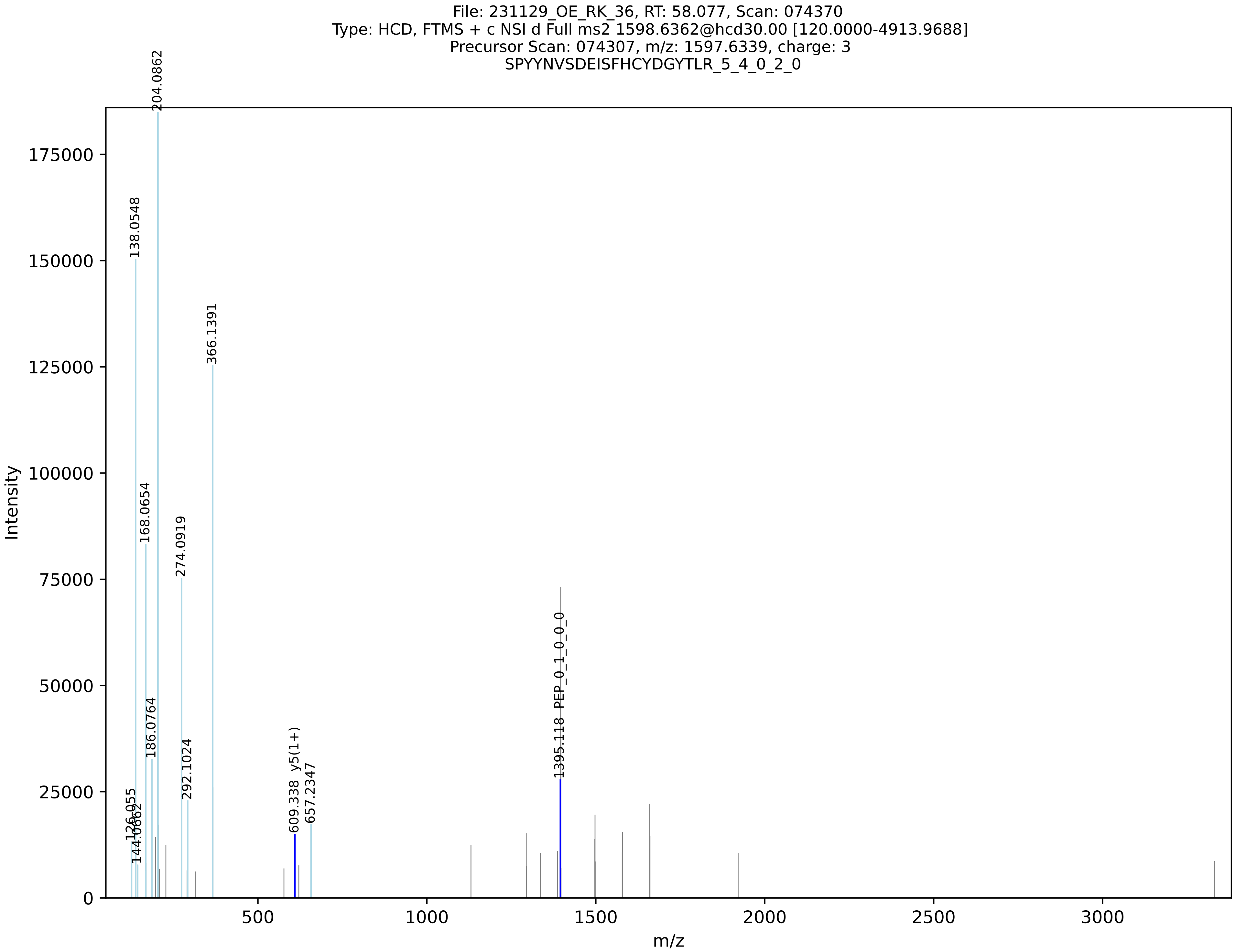

File: 231129_OE_RK_36, RT: 58.077, Scan: 074370
Type: HCD, FTMS + c NSI d Full ms2 1598.6362@hcd30.00 [120.0000-4913.9688]
Precursor Scan: 074307, m/z: 1597.6339, charge: 3
SPYYNVSDEISFHCYDGYTLR_5_4_0_2_0
204.0862
138.0548
366.1391
168.0654
274.0919
186.0764
292.1024
126.055
144.0662
609.338 y5(1+)
657.2347
1395.118 PEP_0_1_0_0_0_
Intensity
m/z
0
25000
50000
75000
100000
125000
150000
175000
500
1000
1500
2000
2500
3000

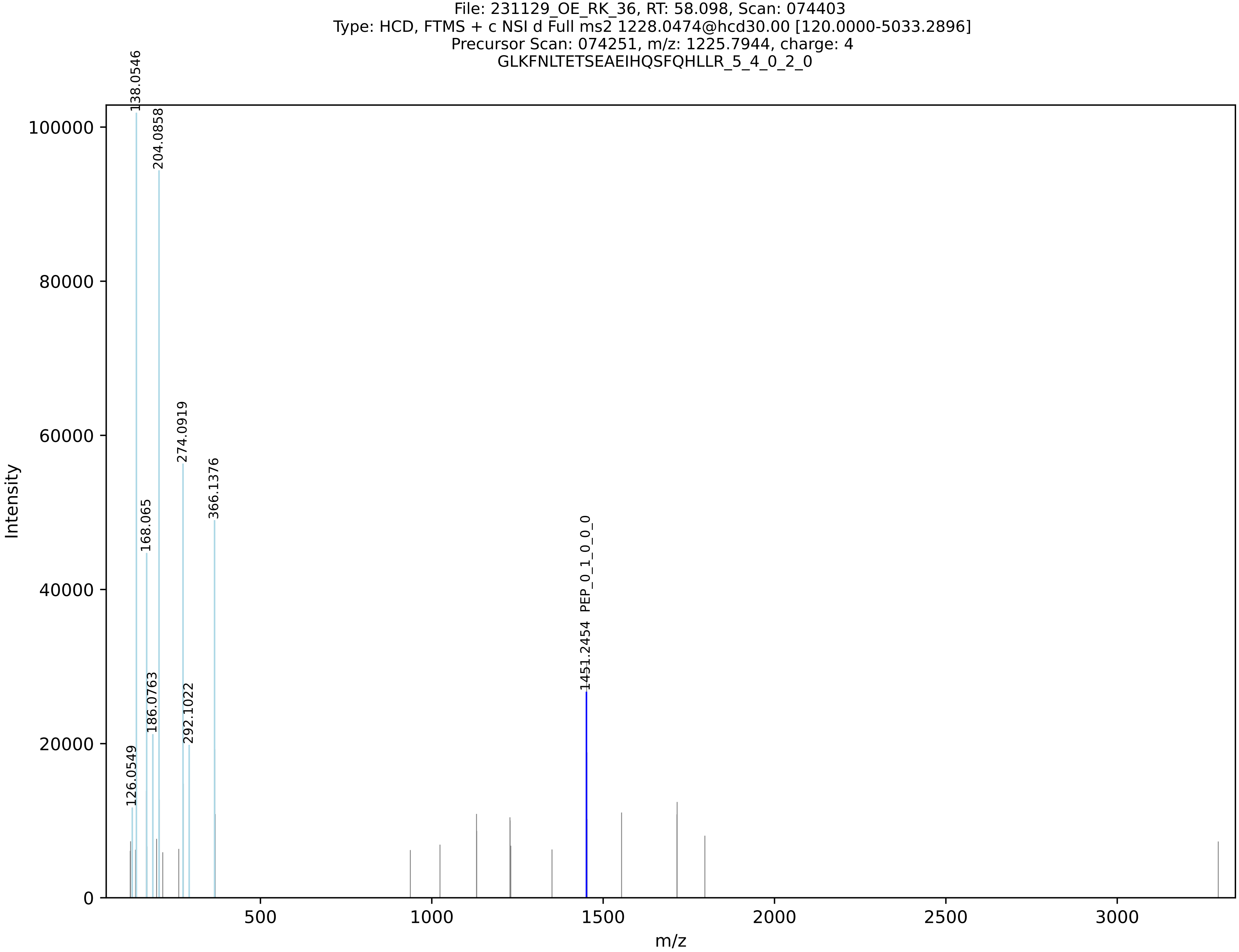

File: 231129_OE_RK_36, RT: 58.098, Scan: 074403
Type: HCD, FTMS + c NSI d Full ms2 1228.0474@hcd30.00 [120.0000-5033.2896]
Precursor Scan: 074251, m/z: 1225.7944, charge: 4
GLKFNLTETSEAEIHQSFQHLLR_5_4_0_2_0
Intensity
m/z
138.0546
204.0858
274.0919
366.1376
168.065
186.0763
292.1022
126.0549
1451.2454 PEP_0_1_0_0_0

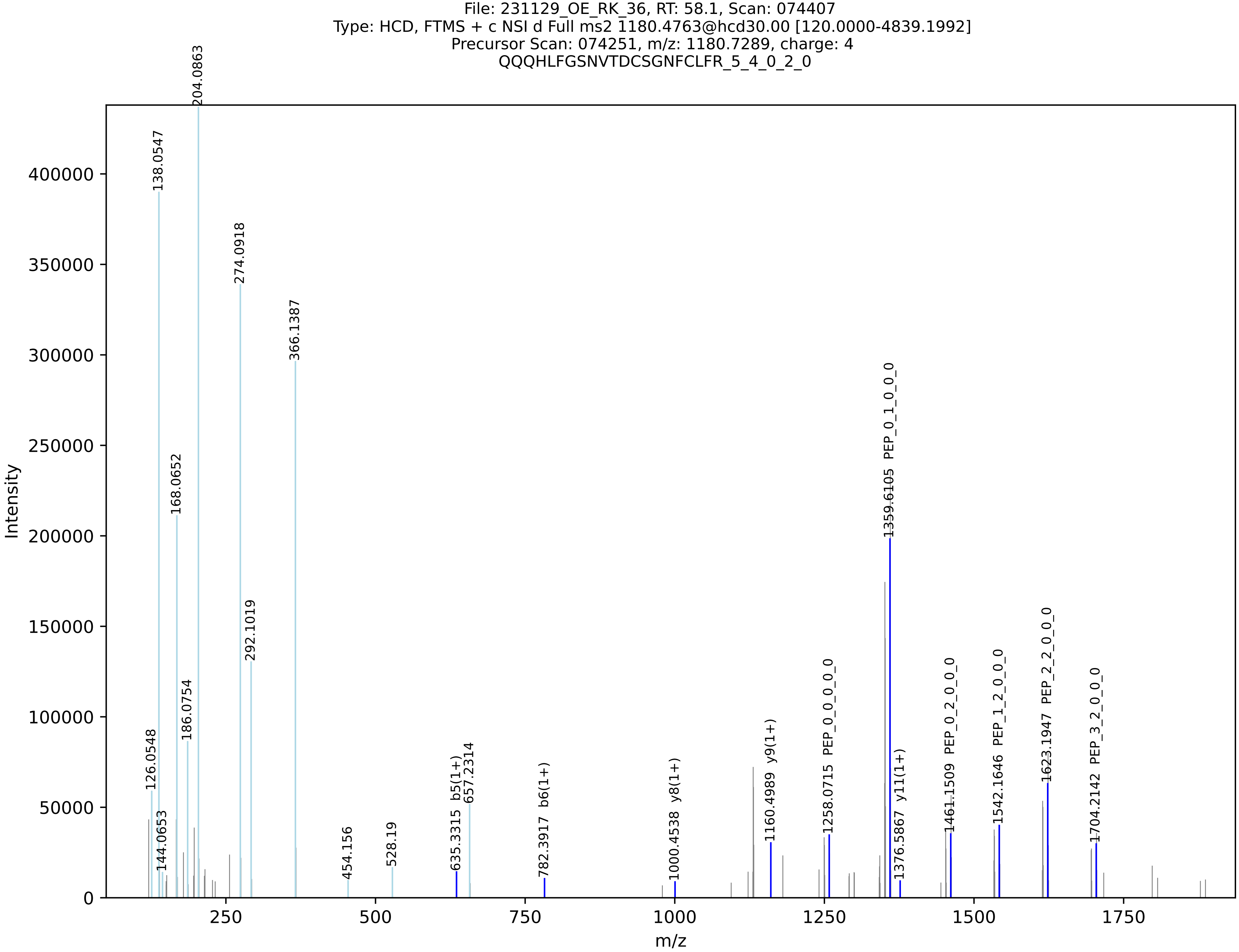
File: 231129_OE_RK_36, RT: 58.1, Scan: 074407
Type: HCD, FTMS + c NSI d Full ms2 1180.4763@hcd30.00 [120.0000-4839.1992]
Precursor Scan: 074251, m/z: 1180.7289, charge: 4
QQQHLFGSNVTDCSGNFCLFR_5_4_0_2_0
Intensity
m/z
204.0863
138.0547
274.0918
366.1387
168.0652
292.1019
186.0754
126.0548
144.0653
454.156
528.19
635.3315 b5(1+)
657.2314
782.3917 b6(1+)
1000.4538 y8(1+)
1160.4989 y9(1+)
1258.0715 PEP_0_0_0_0_0
1359.6105 PEP_0_1_0_0_0
1376.5867 y11(1+)
1461.1509 PEP_0_2_0_0_0
1542.1646 PEP_1_2_0_0_0
1623.1947 PEP_2_2_0_0_0
1704.2142 PEP_3_2_0_0_0

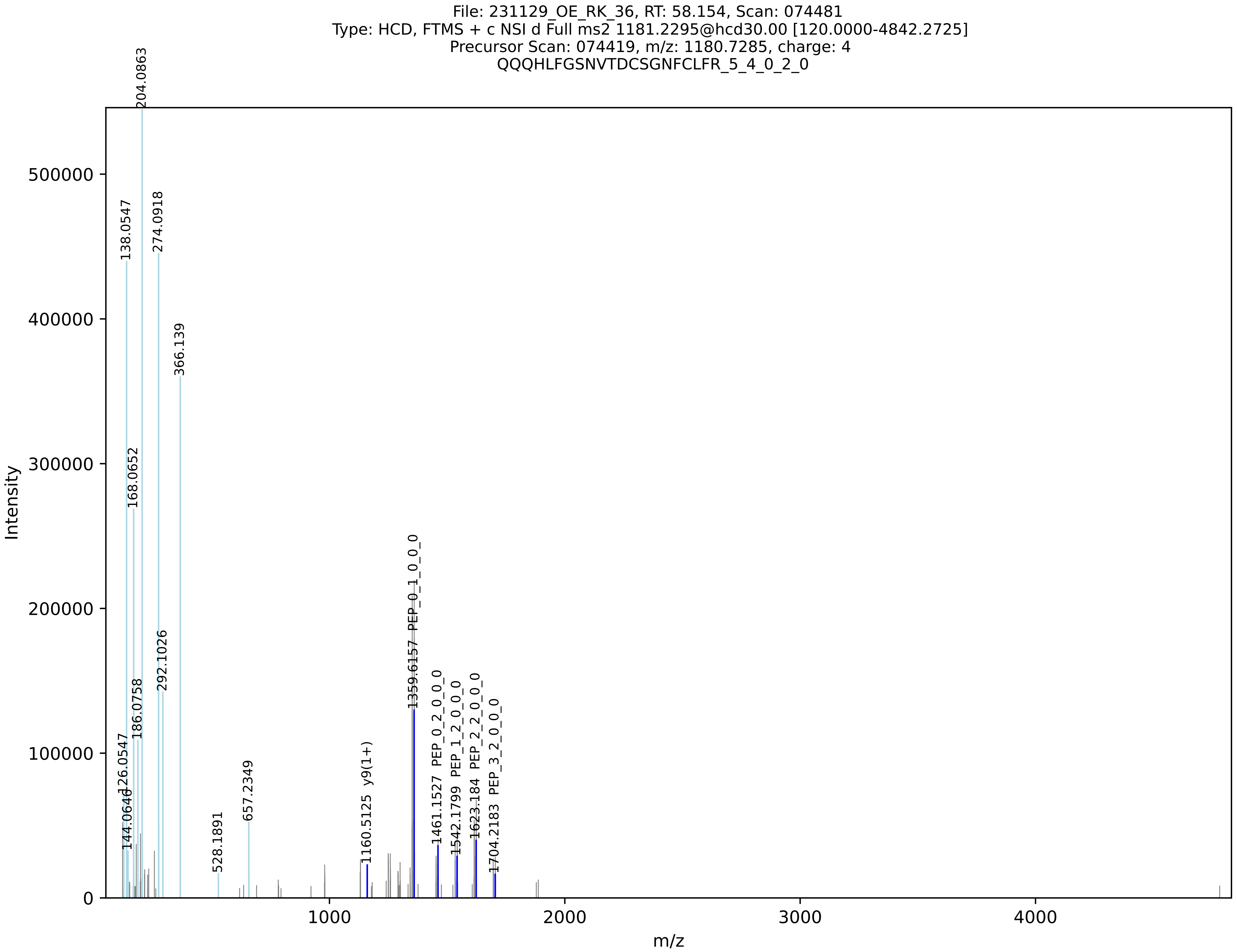
File: 231129_OE_RK_36, RT: 58.154, Scan: 074481
Type: HCD, FTMS + c NSI d Full ms2 1181.2295@hcd30.00 [120.0000-4842.2725]
Precursor Scan: 074419, m/z: 1180.7285, charge: 4
QQQHLFGSNVTDCSGNFCLFR_5_4_0_2_0
Intensity
m/z
204.0863
138.0547
274.0918
366.139
168.0652
292.1026
186.0758
126.0547
144.0646
528.1891
657.2349
1160.5125 y9(1+)
1359.6157 PEP_0_1_0_0_0
1461.1527 PEP_0_2_0_0_0
1542.1799 PEP_1_2_0_0_0
1623.184 PEP_2_2_0_0_0
1704.2183 PEP_3_2_0_0_0
0
100000
200000
300000
400000
500000
1000
2000
3000
4000

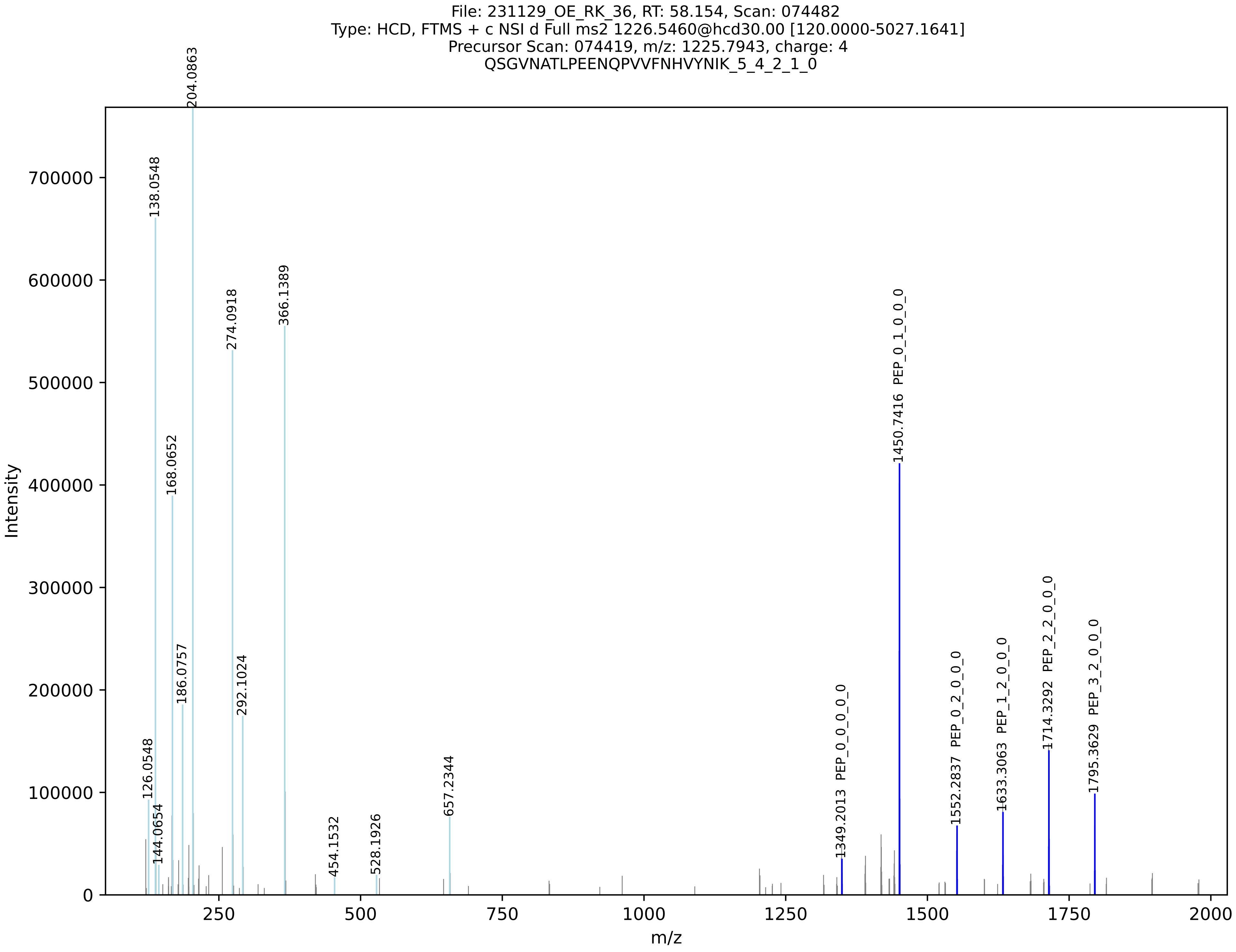
File: 231129_OE_RK_36, RT: 58.154, Scan: 074482
Type: HCD, FTMS + c NSI d Full ms2 1226.5460@hcd30.00 [120.0000-5027.1641]
Precursor Scan: 074419, m/z: 1225.7943, charge: 4
QSGVNATLPEENQPVVFNHVYNIK_5_4_2_1_0
Intensity
m/z
204.0863
138.0548
168.0652
186.0757
126.0548
144.0654
274.0918
292.1024
366.1389
454.1532
528.1926
657.2344
1349.2013 PEP_0_0_0_0_0
1450.7416 PEP_0_1_0_0_0
1552.2837 PEP_0_2_0_0_0
1633.3063 PEP_1_2_0_0_0
1714.3292 PEP_2_2_0_0_0
1795.3629 PEP_3_2_0_0_0

File: 231129_OE_RK_36, RT: 58.226, Scan: 074582
Type: HCD, FTMS + c NSI d Full ms2 1377.2493@hcd30.00 [120.0000-4236.5244]
Precursor Scan: 074537, m/z: 1376.2469, charge: 3
TLNQSSDELQLSMGNAMFVK_5_4_0_1_0

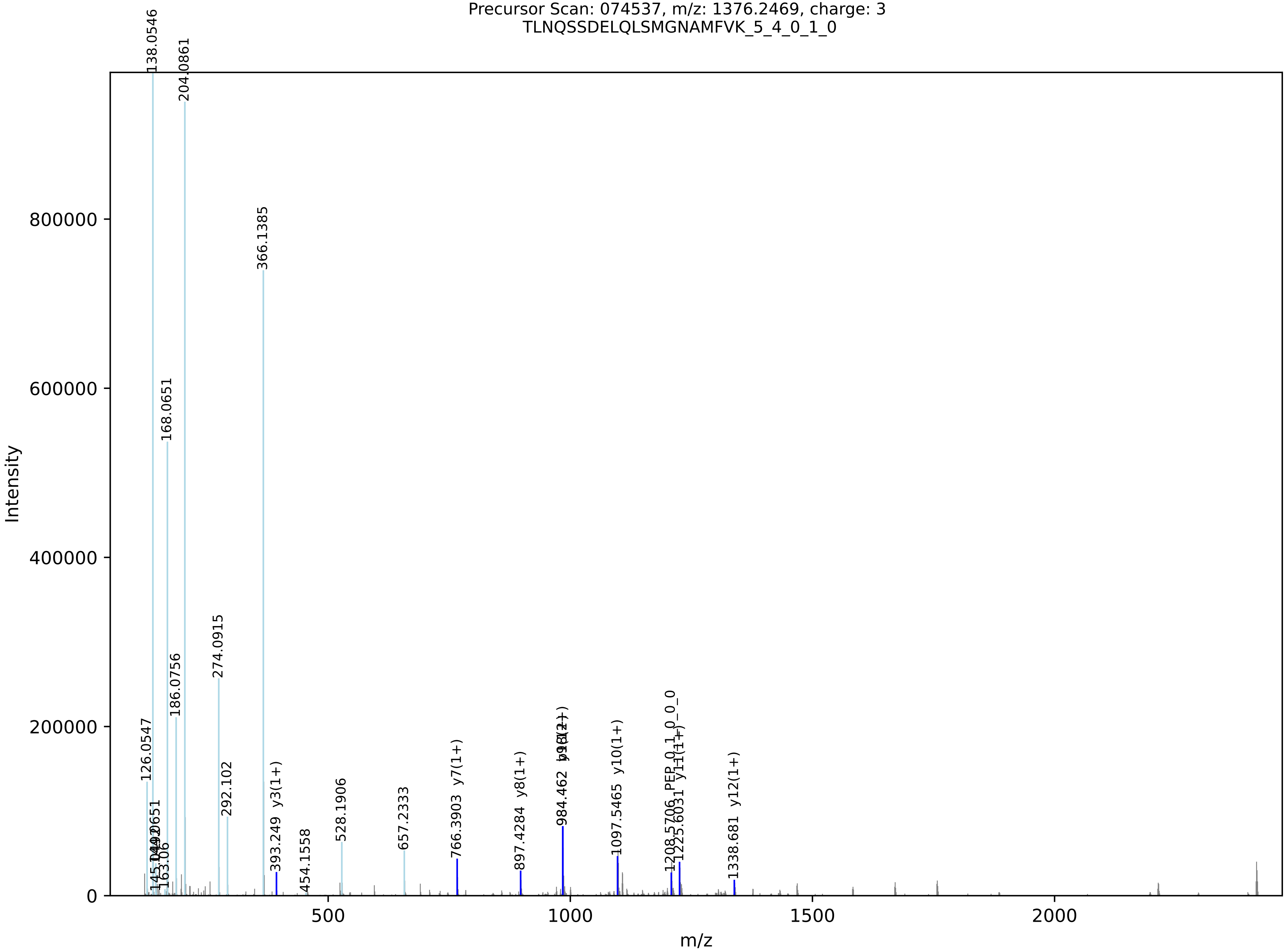

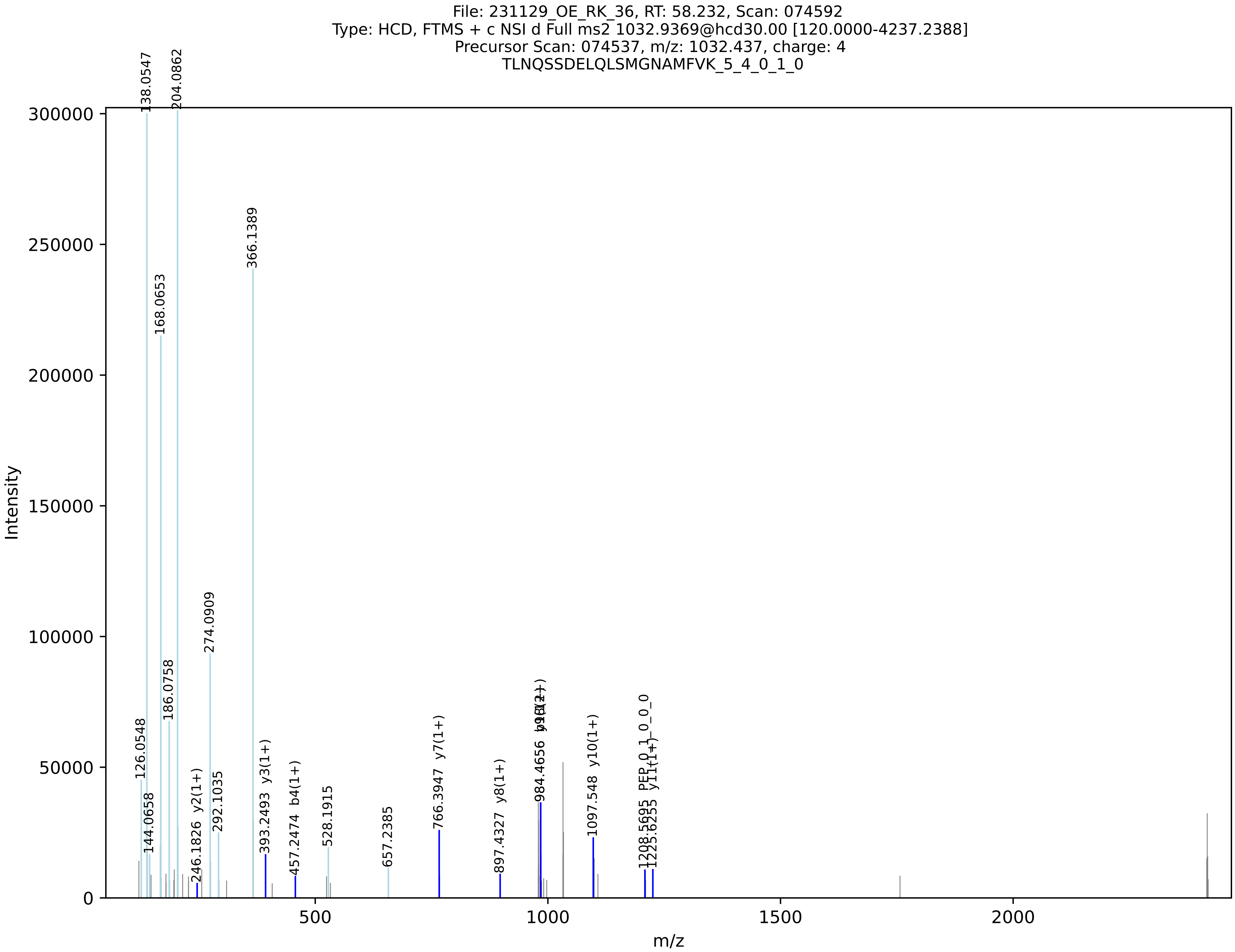
File: 231129_OE_RK_36, RT: 58.232, Scan: 074592
Type: HCD, FTMS + c NSI d Full ms2 1032.9369@hcd30.00 [120.0000-4237.2388]
Precursor Scan: 074537, m/z: 1032.437, charge: 4
TLNQSSDELQLSMGNAMFVK_5_4_0_1_0
Intensity
m/z
138.0547
204.0862
366.1389
168.0653
274.0909
186.0758
126.0548
144.0658
246.1826 y2(1+)
292.1035
393.2493 y3(1+)
457.2474 b4(1+)
528.1915
657.2385
766.3947 y7(1+)
897.4327 y8(1+)
984.4656 y9(1+)
1097.548 y10(1+)
1208.5695 PEP_0_1_0_0_0
1225.6255 y11(1+)

File: 231129_OE_RK_36, RT: 58.428, Scan: 074854
Type: HCD, FTMS + c NSI d Full ms2 1377.2515@hcd30.00 [120.0000-4236.5313]
Precursor Scan: 074807, m/z: 1376.2491, charge: 3
TLNQSSDELQLSMGNAMFVK_5_4_0_1_0

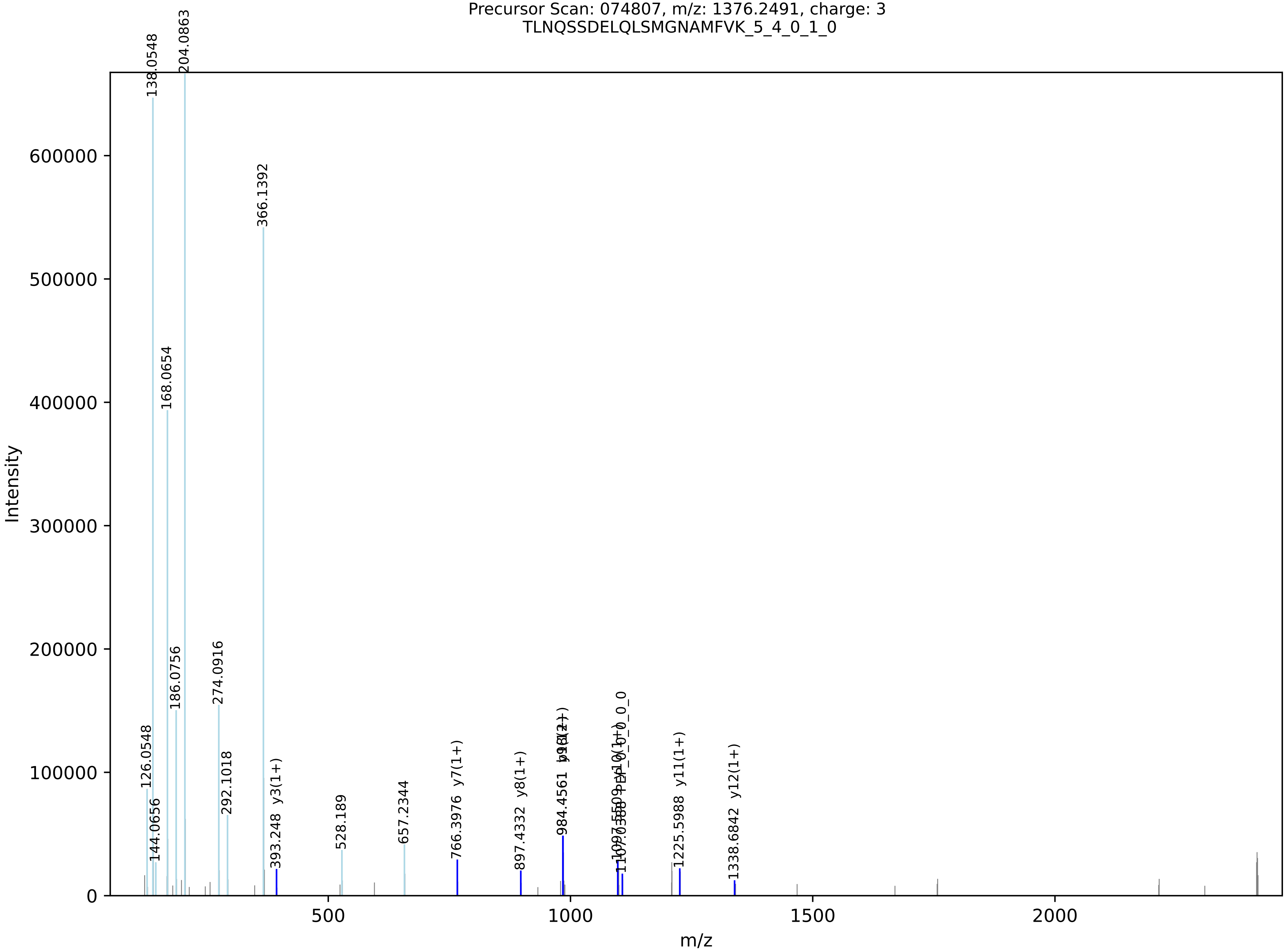

File: 231129_OE_RK_36, RT: 58.529, Scan: 074997
Type: HCD, FTMS + c NSI d Full ms2 1222.0454@hcd30.00 [120.0000-5008.8013]
Precursor Scan: 074947, m/z: 1221.5442, charge: 4
MVSHHNLTTGATLINEQWLLTTAK_5_4_0_2_0

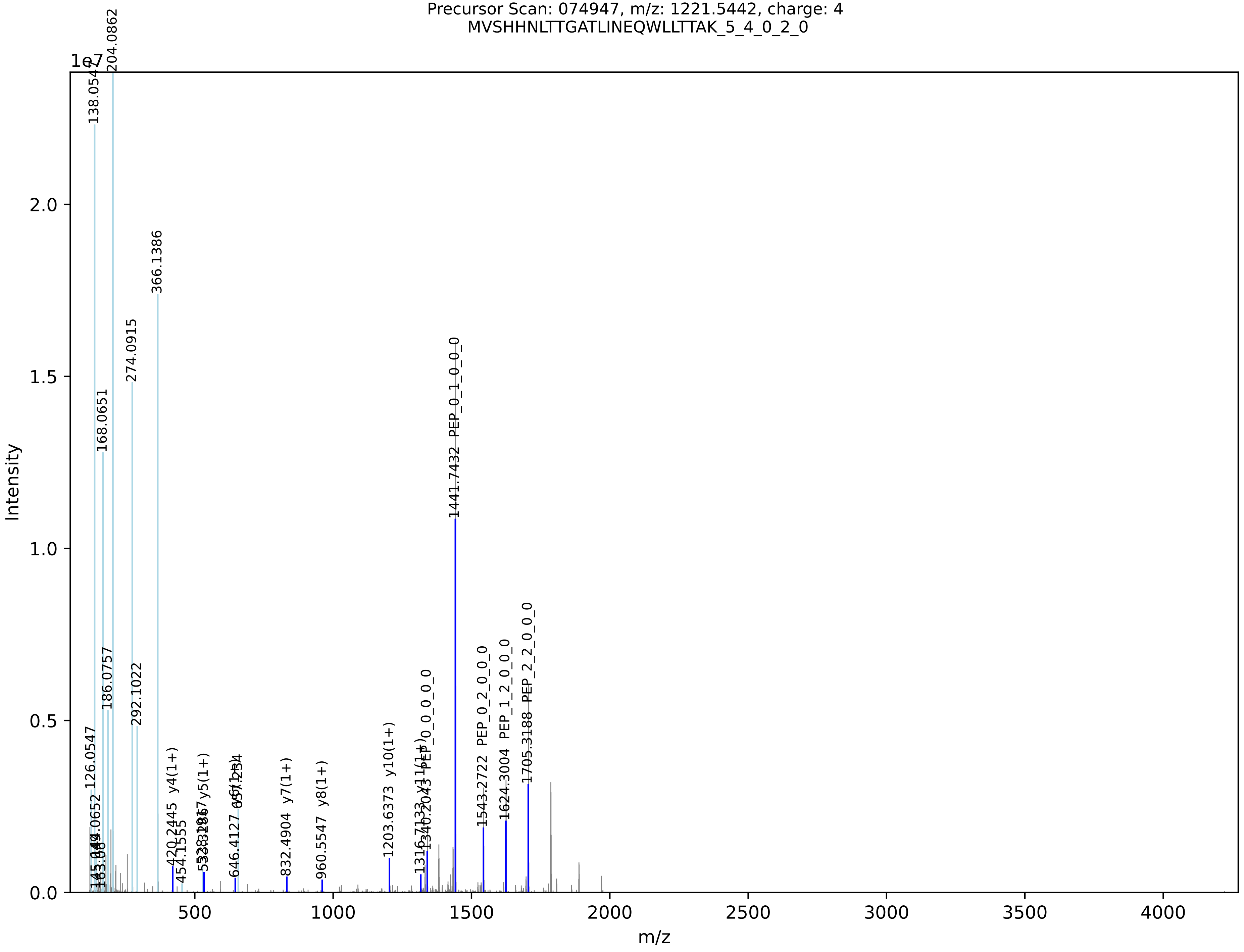

File: 231129_OE_RK_36, RT: 58.622, Scan: 075134
Type: HCD, FTMS + c NSI d Full ms2 1376.5796@hcd30.00 [120.0000-4234.4756]
Precursor Scan: 075050, m/z: 1376.2473, charge: 3
TLNQSSDELQLSMGNAMFVK_5_4_0_1_0

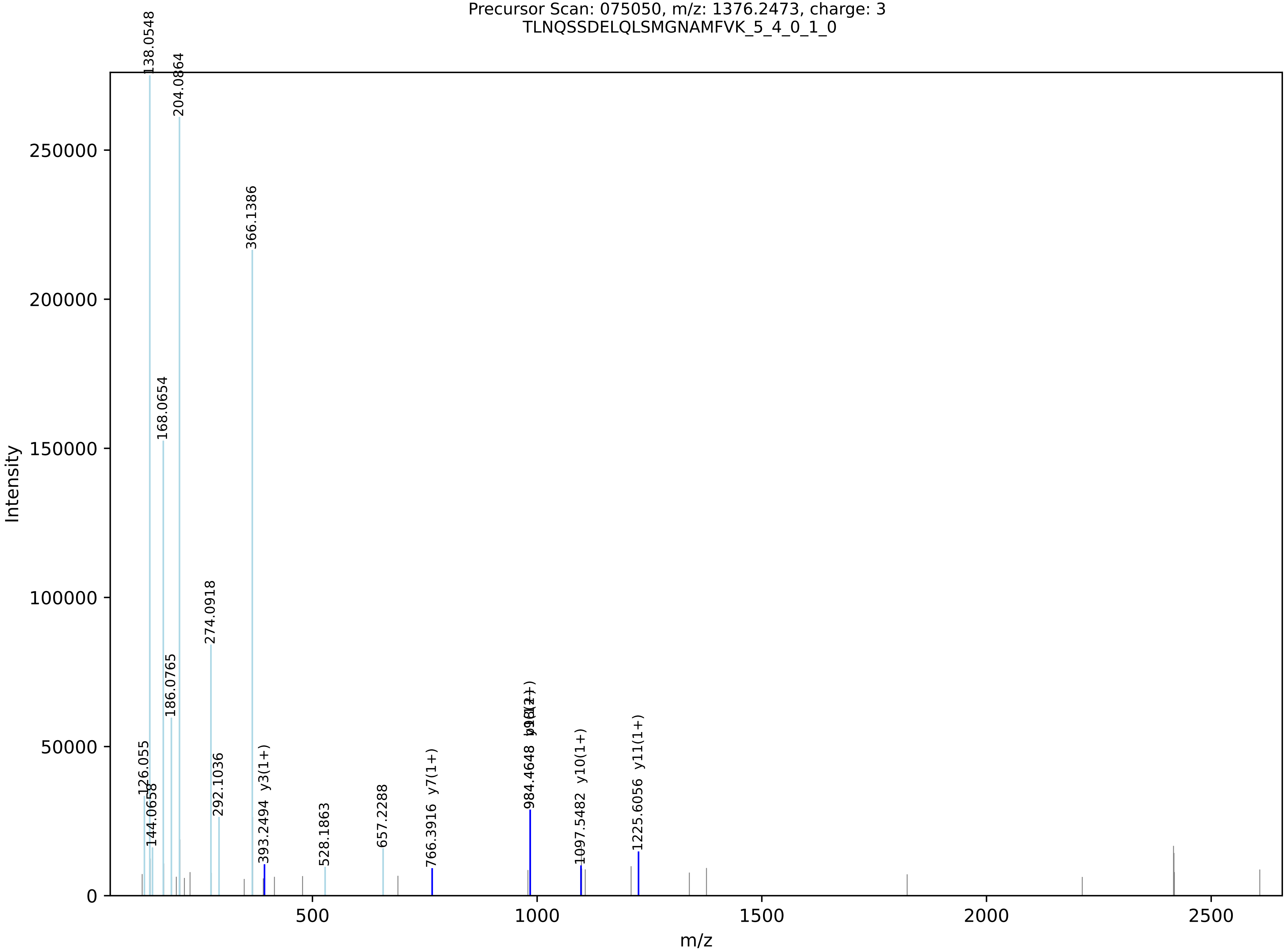

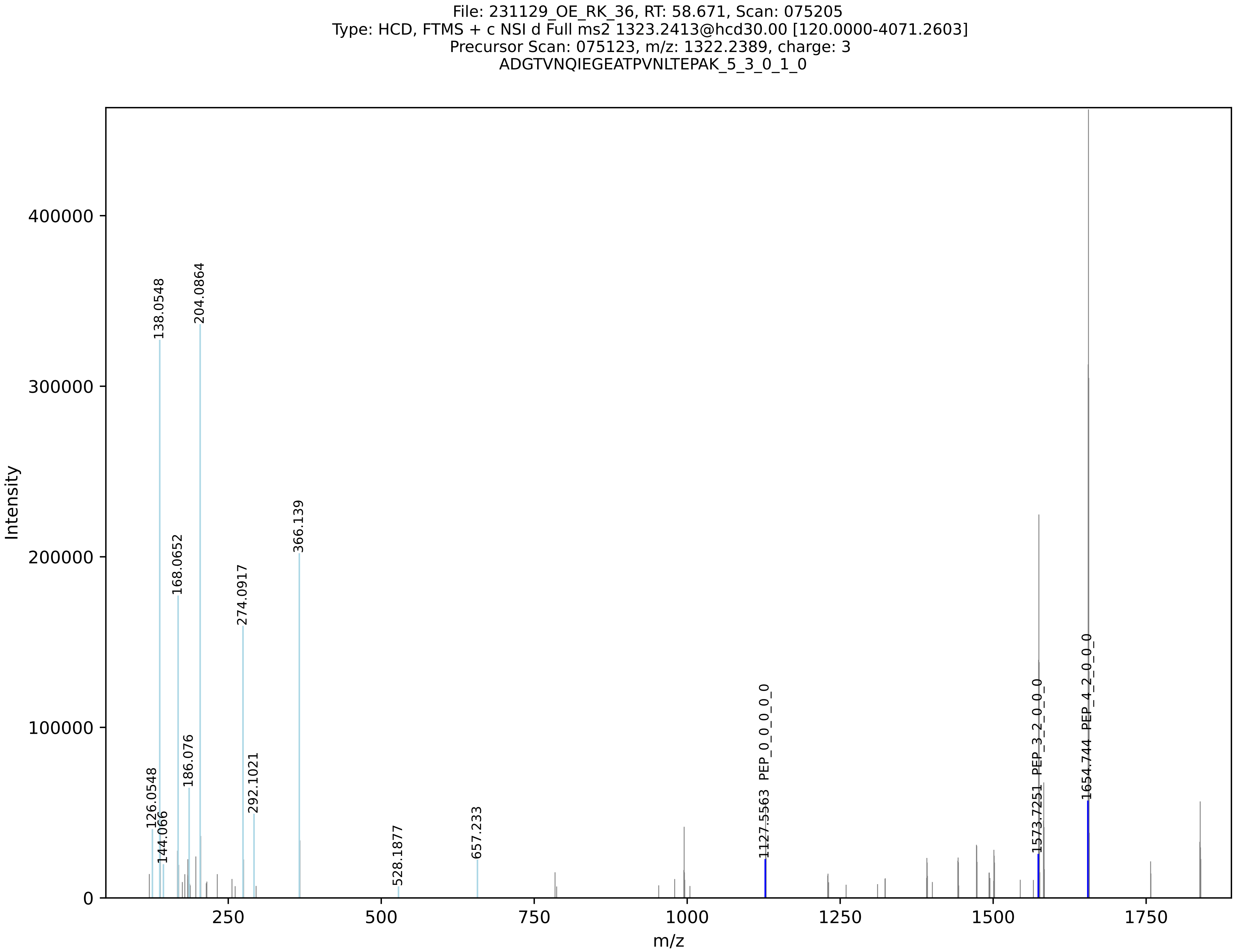
File: 231129_OE_RK_36, RT: 58.671, Scan: 075205
Type: HCD, FTMS + c NSI d Full ms2 1323.2413@hcd30.00 [120.0000-4071.2603]
Precursor Scan: 075123, m/z: 1322.2389, charge: 3
ADGTVNQIEGEATPVNLTEPAK_5_3_0_1_0
Intensity
m/z
138.0548
204.0864
168.0652
186.076
126.0548
144.066
274.0917
292.1021
366.139
528.1877
657.233
1127.5563 PEP_0_0_0_0_0_
1573.7251 PEP_3_2_0_0_0_
1654.744 PEP_4_2_0_0_0_
0
100000
200000
300000
400000
250
500
750
1000
1250
1500
1750

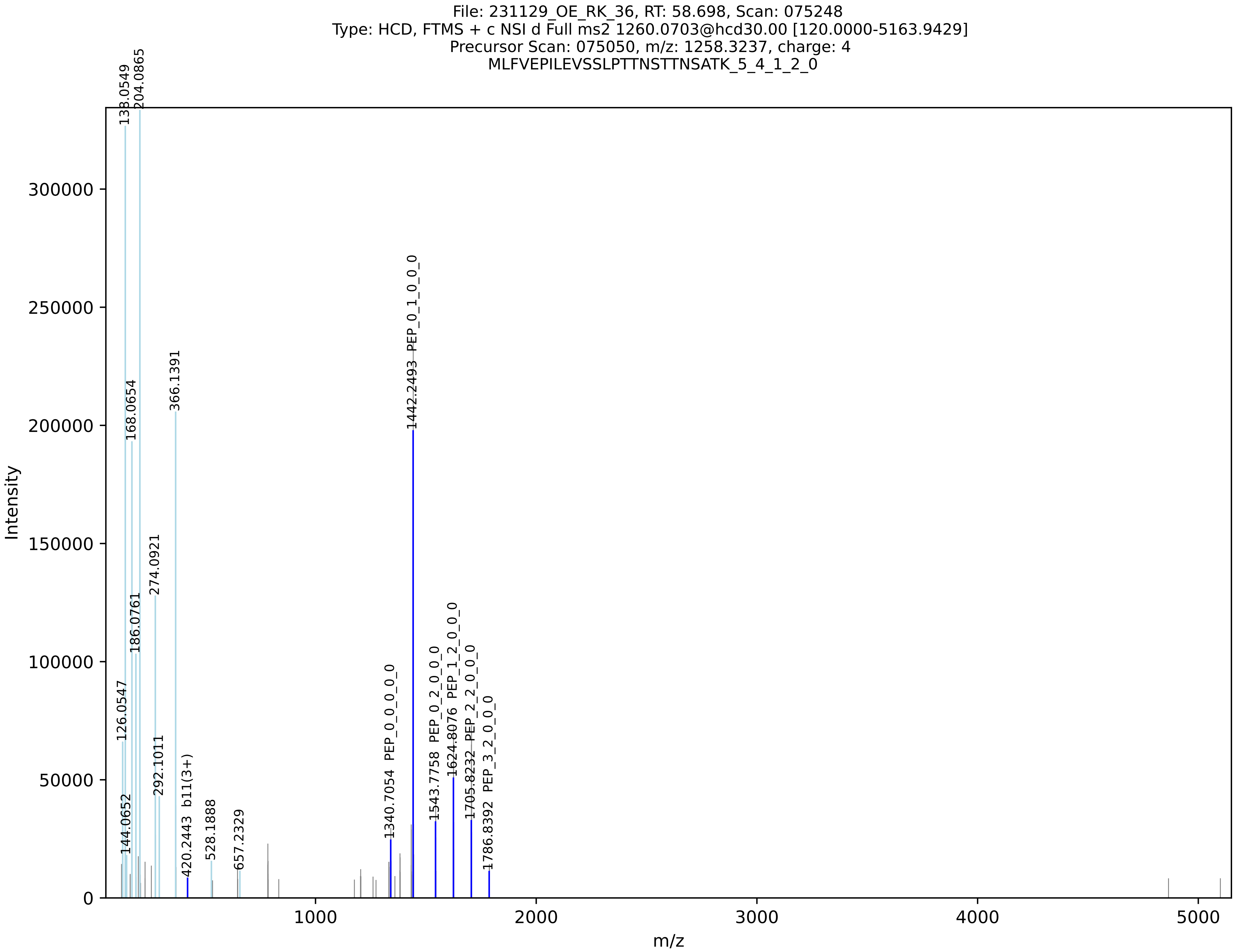

File: 231129_OE_RK_36, RT: 58.698, Scan: 075248
Type: HCD, FTMS + c NSI d Full ms2 1260.0703@hcd30.00 [120.0000-5163.9429]
Precursor Scan: 075050, m/z: 1258.3237, charge: 4
MLFVEPILEVSSLPTTNSTTNSATK_5_4_1_2_0
Intensity
m/z
138.0549
204.0865
168.0654
366.1391
274.0921
186.0761
126.0547
292.1011
144.0652
420.2443 b11(3+)
528.1888
657.2329
1442.2493 PEP_0_1_0_0_0
1340.7054 PEP_0_0_0_0_0
1543.7758 PEP_0_2_0_0_0
1624.8076 PEP_1_2_0_0_0
1705.8232 PEP_2_2_0_0_0
1786.8392 PEP_3_2_0_0_0

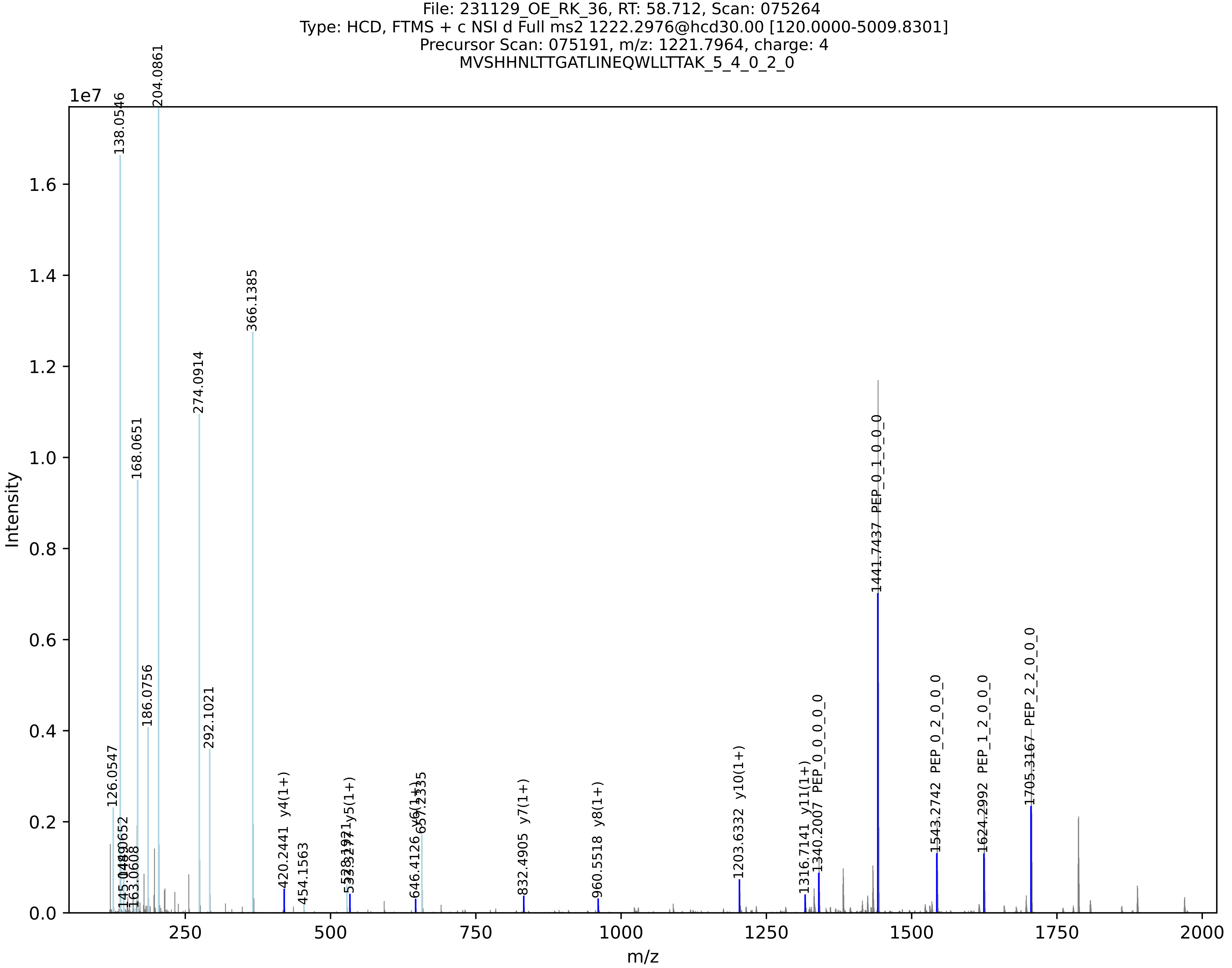
File: 231129_OE_RK_36, RT: 58.712, Scan: 075264
Type: HCD, FTMS + c NSI d Full ms2 1222.2976@hcd30.00 [120.0000-5009.8301]
Precursor Scan: 075191, m/z: 1221.7964, charge: 4
MVSHHNLTTGATLINEQWLLTTAK_5_4_0_2_0
1e7
Intensity
m/z
204.0861
138.0546
168.0651
186.0756
126.0547
163.0608
274.0914
292.1021
366.1385
420.2441 y4(1+)
454.1563
533.3177 y5(1+)
646.4126 y6(1+)
657.2335
832.4905 y7(1+)
960.5518 y8(1+)
1203.6332 y10(1+)
1316.7141 y11(1+)
1340.2007 PEP_0_0_0_0_0
1441.7437 PEP_0_1_0_0_0
1543.2742 PEP_0_2_0_0_0
1624.2992 PEP_1_2_0_0_0
1705.3167 PEP_2_2_0_0_0

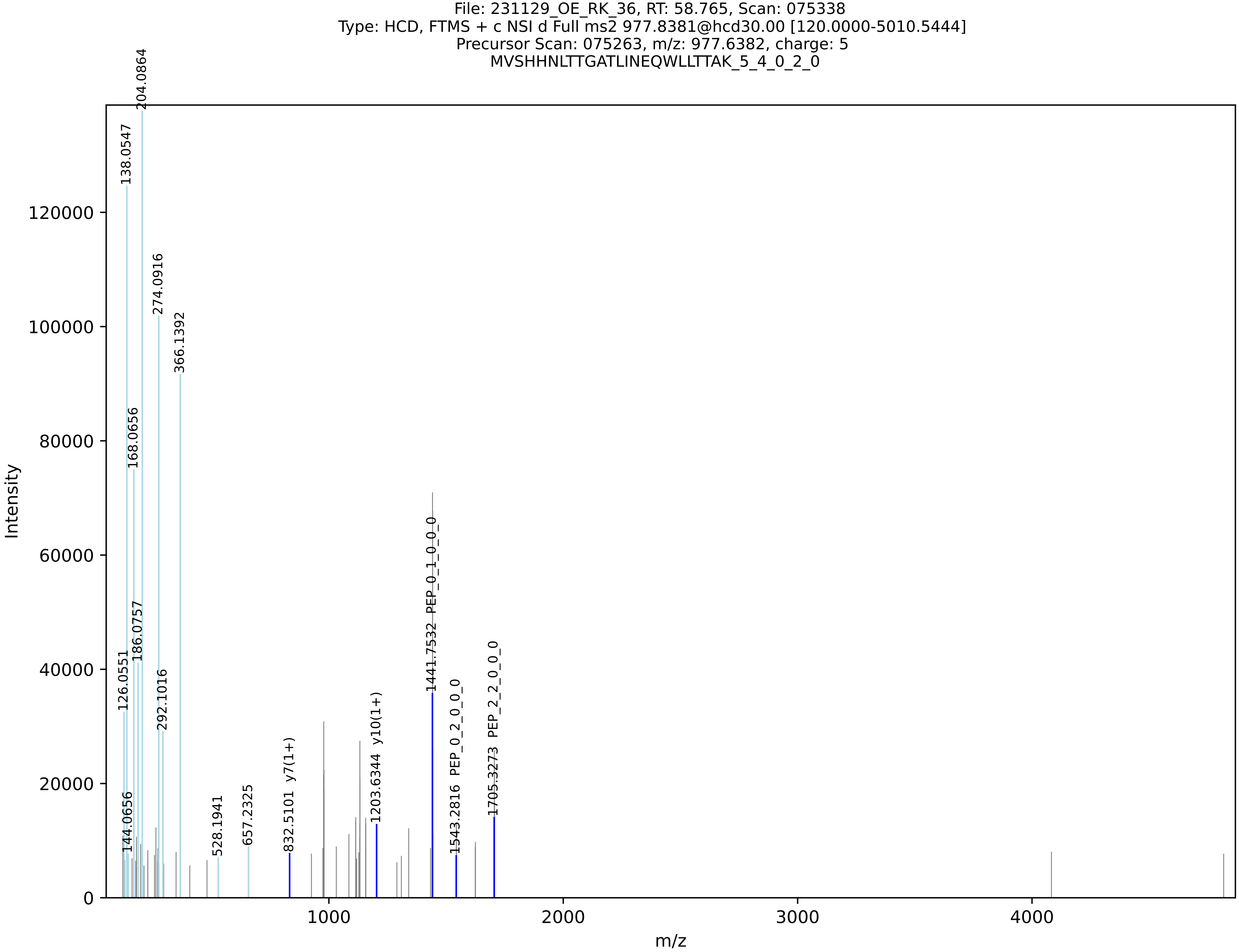

File: 231129_OE_RK_36, RT: 58.765, Scan: 075338
Type: HCD, FTMS + c NSI d Full ms2 977.8381@hcd30.00 [120.0000-5010.5444]
Precursor Scan: 075263, m/z: 977.6382, charge: 5
MVSHHNLTTGATLINEQWLLTTAK_5_4_0_2_0
Intensity
m/z
204.0864
138.0547
274.0916
366.1392
168.0656
186.0757
126.0551
292.1016
144.0656
528.1941
657.2325
832.5101 y7(1+)
1203.6344 y10(1+)
1441.7532 PEP_0_1_0_0_0
1543.2816 PEP_0_2_0_0_0
1705.3273 PEP_2_2_0_0_0

File: 231129_OE_RK_36, RT: 58.848, Scan: 075459
Type: HCD, FTMS + c NSI d Full ms2 1322.9089@hcd30.00 [120.0000-4070.2434]
Precursor Scan: 075400, m/z: 1322.2407, charge: 3
ADGTVNQIEGEATPVNLTEPAK_5_3_0_1_0

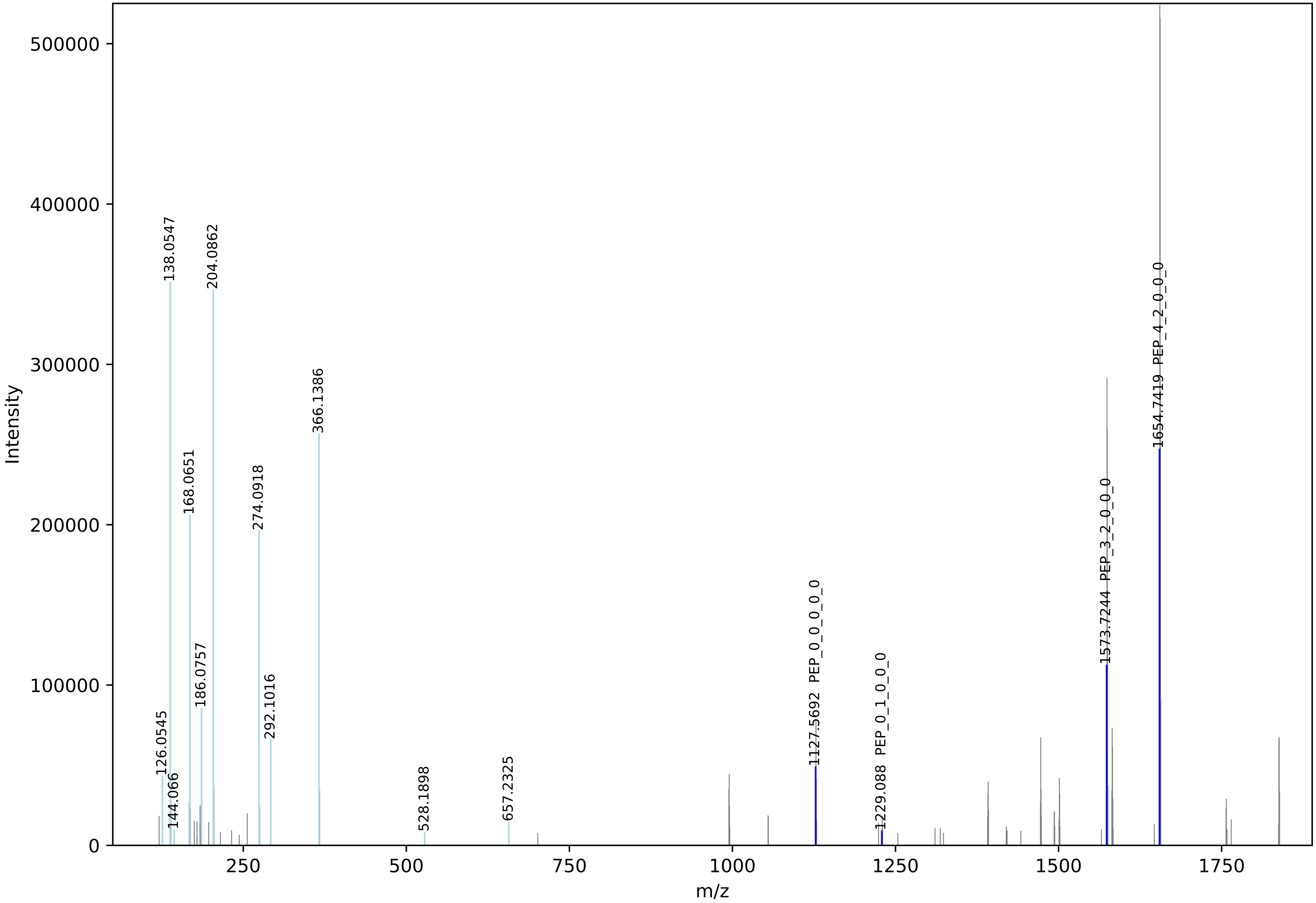

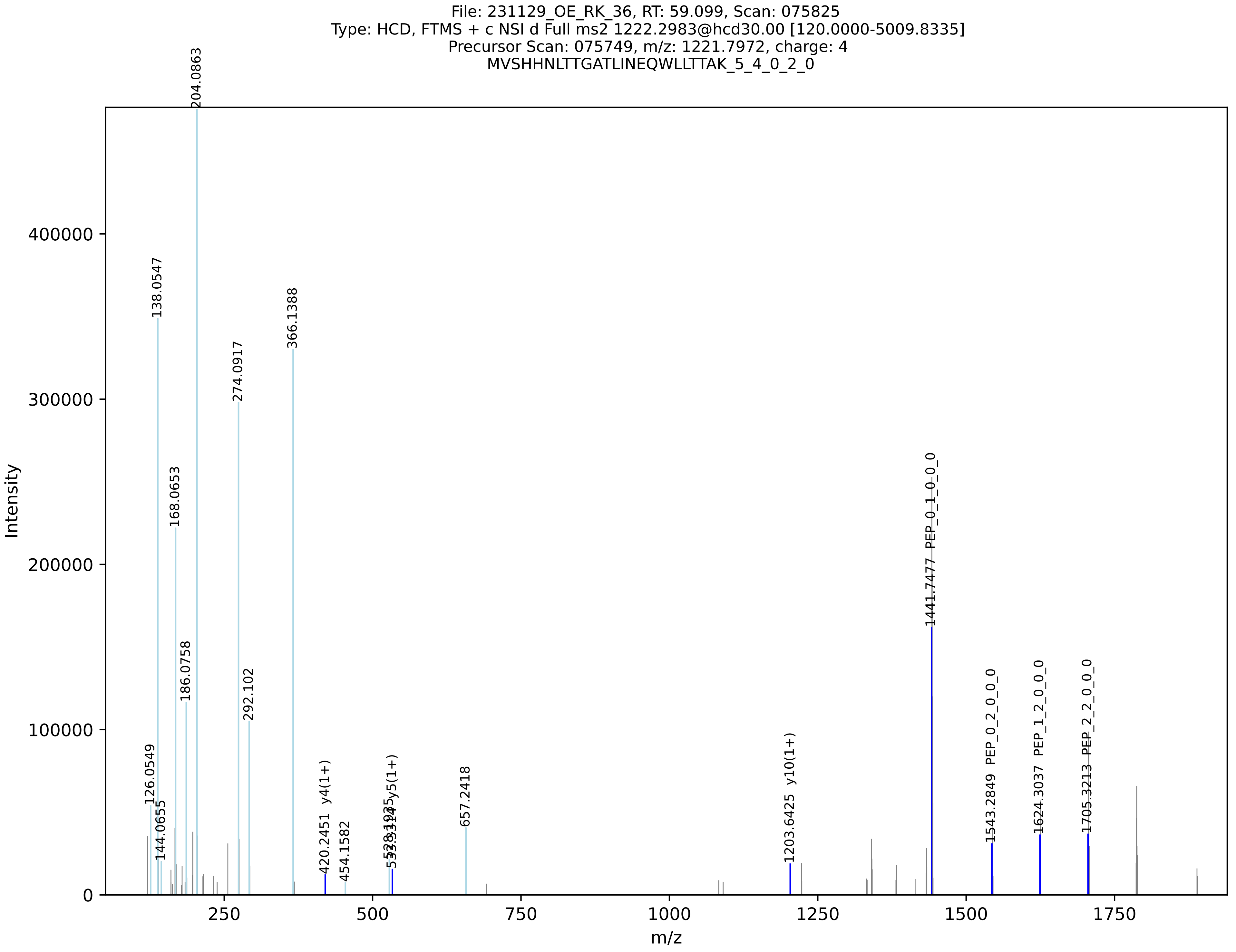
File: 231129_OE_RK_36, RT: 59.099, Scan: 075825
Type: HCD, FTMS + c NSI d Full ms2 1222.2983@hcd30.00 [120.0000-5009.8335]
Precursor Scan: 075749, m/z: 1221.7972, charge: 4
MVSHHNLTTGATLINEQWLLTTAK_5_4_0_2_0
Intensity
m/z
204.0863
138.0547
366.1388
274.0917
168.0653
186.0758
292.102
126.0549
144.0655
420.2451 y4(1+)
454.1582
528.1925
533.3114 y5(1+)
657.2418
1203.6425 y10(1+)
1441.7477 PEP_0_1_0_0_0
1543.2849 PEP_0_2_0_0_0
1624.3037 PEP_1_2_0_0_0
1705.3213 PEP_2_2_0_0_0

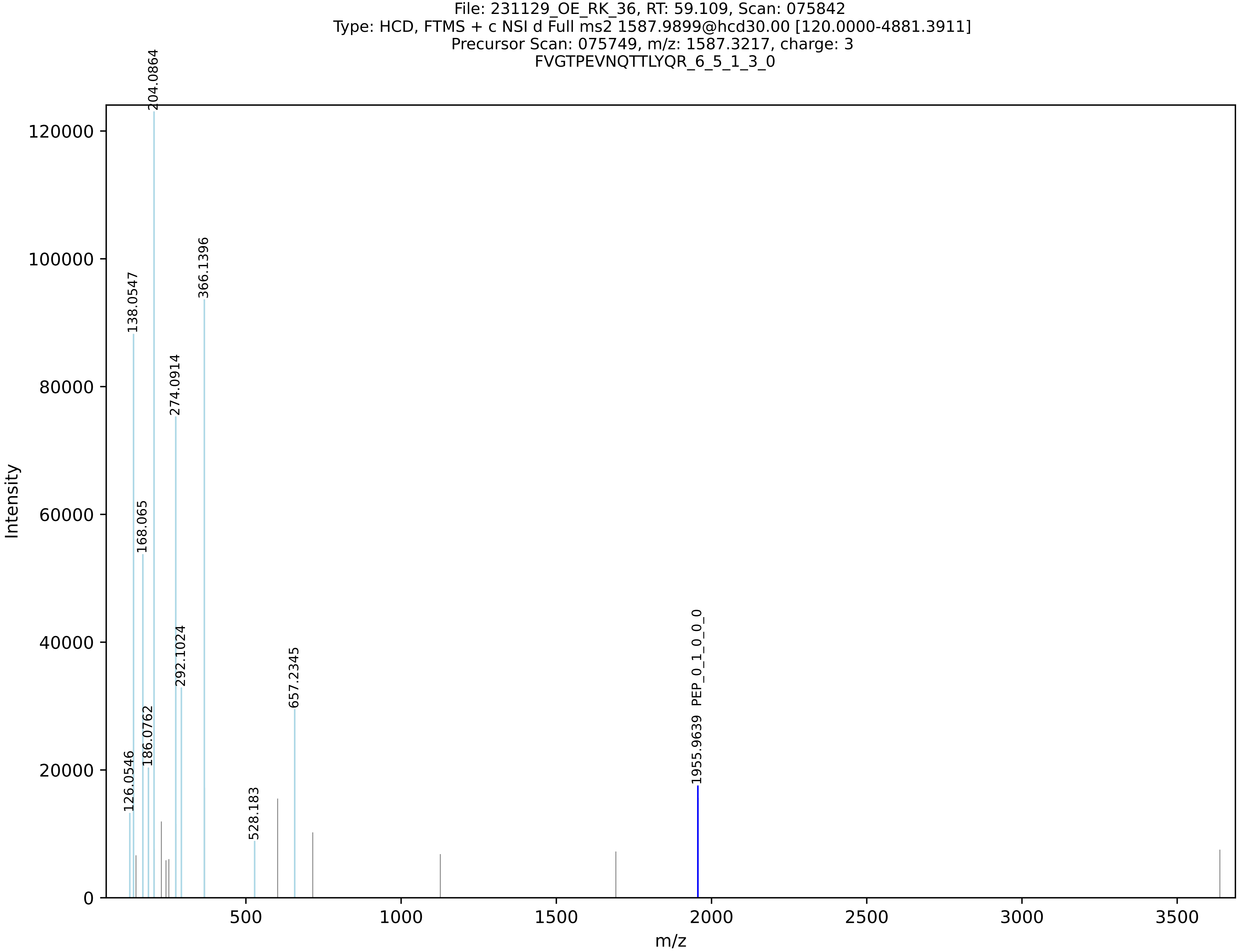
File: 231129_OE_RK_36, RT: 59.109, Scan: 075842
Type: HCD, FTMS + c NSI d Full ms2 1587.9899@hcd30.00 [120.0000-4881.3911]
Precursor Scan: 075749, m/z: 1587.3217, charge: 3
FVGTPEVNQTTLYQR_6_5_1_3_0
Intensity
m/z
204.0864
138.0547
366.1396
274.0914
168.065
292.1024
657.2345
186.0762
126.0546
528.183
1955.9639 PEP_0_1_0_0_0
0
20000
40000
60000
80000
100000
120000
500
1000
1500
2000
2500
3000
3500

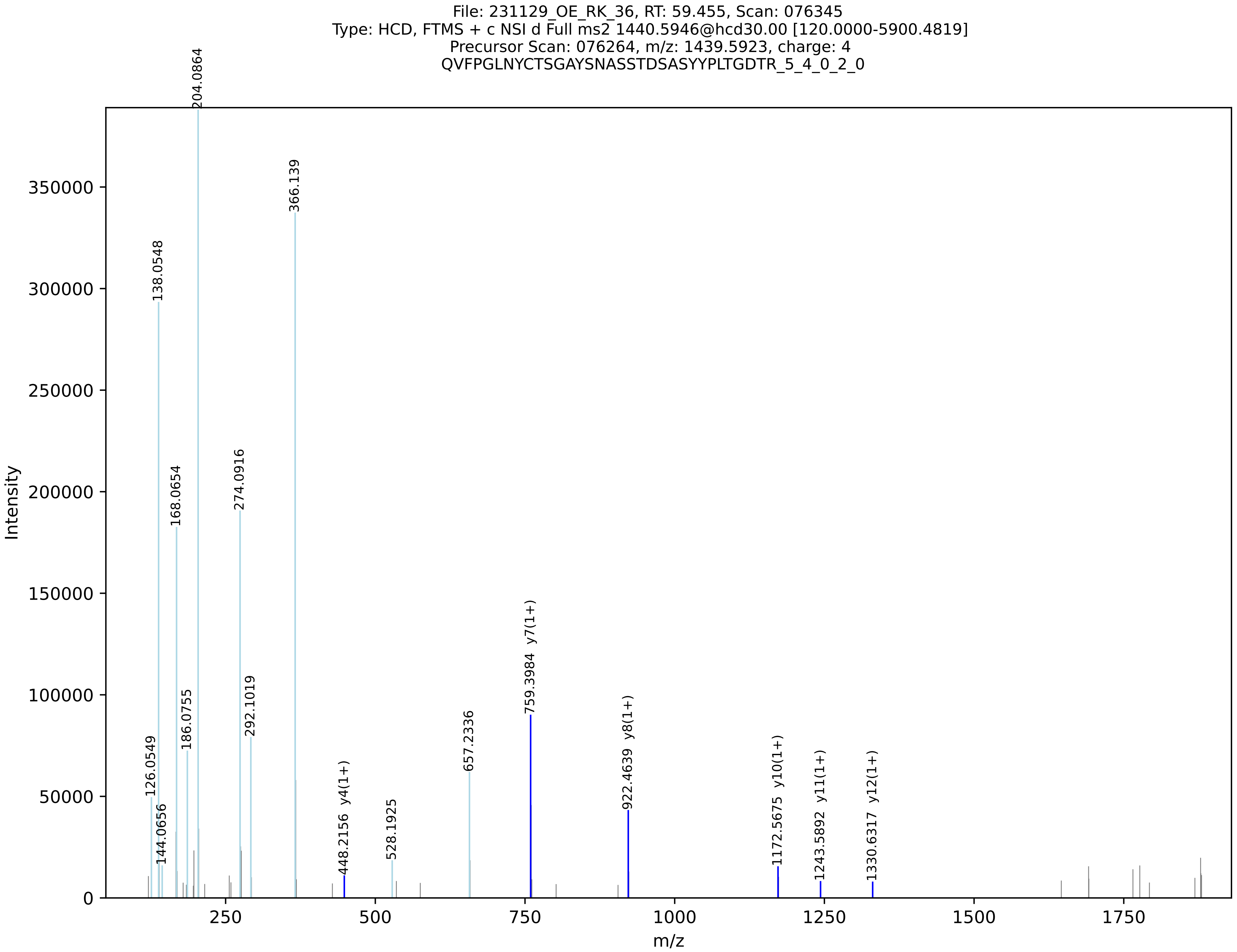

File: 231129_OE_RK_36, RT: 59.455, Scan: 076345
Type: HCD, FTMS + c NSI d Full ms2 1440.5946@hcd30.00 [120.0000-5900.4819]
Precursor Scan: 076264, m/z: 1439.5923, charge: 4
QVFPGLNYCTSGAYSNASSTDSASYYPLTGDTR_5_4_0_2_0
Intensity
m/z
204.0864
366.139
138.0548
168.0654
274.0916
186.0755
292.1019
126.0549
144.0656
448.2156 y4(1+)
528.1925
657.2336
759.3984 y7(1+)
922.4639 y8(1+)
1172.5675 y10(1+)
1243.5892 y11(1+)
1330.6317 y12(1+)

File: 231129_OE_RK_36, RT: 59.51, Scan: 076426
Type: HCD, FTMS + c NSI d Full ms2 1222.5470@hcd30.00 [120.0000-5010.8481]
Precursor Scan: 076338, m/z: 1221.7978, charge: 4
MVSHHNLTTGATLINEQWLLTTAK_5_4_0_2_0

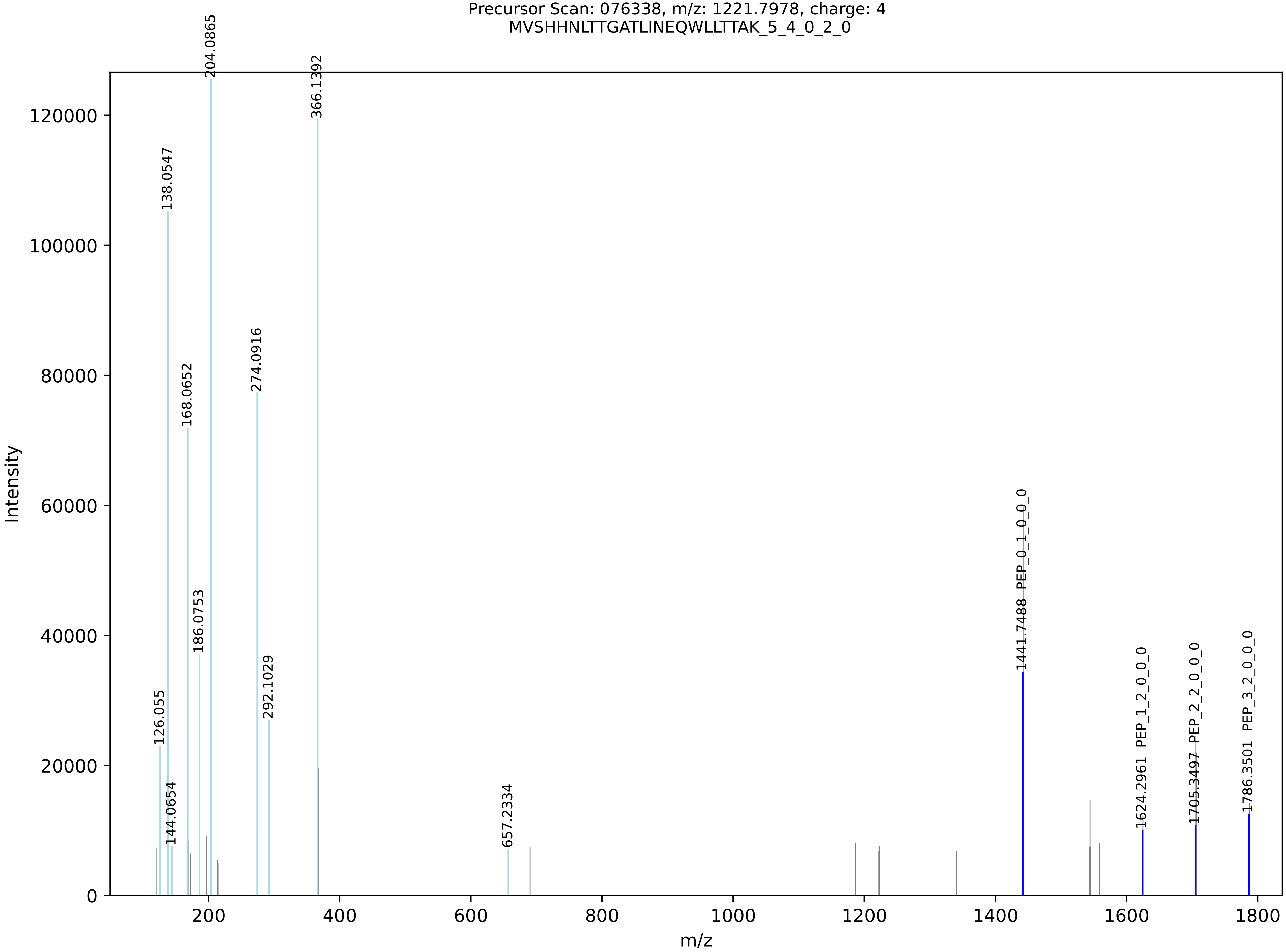

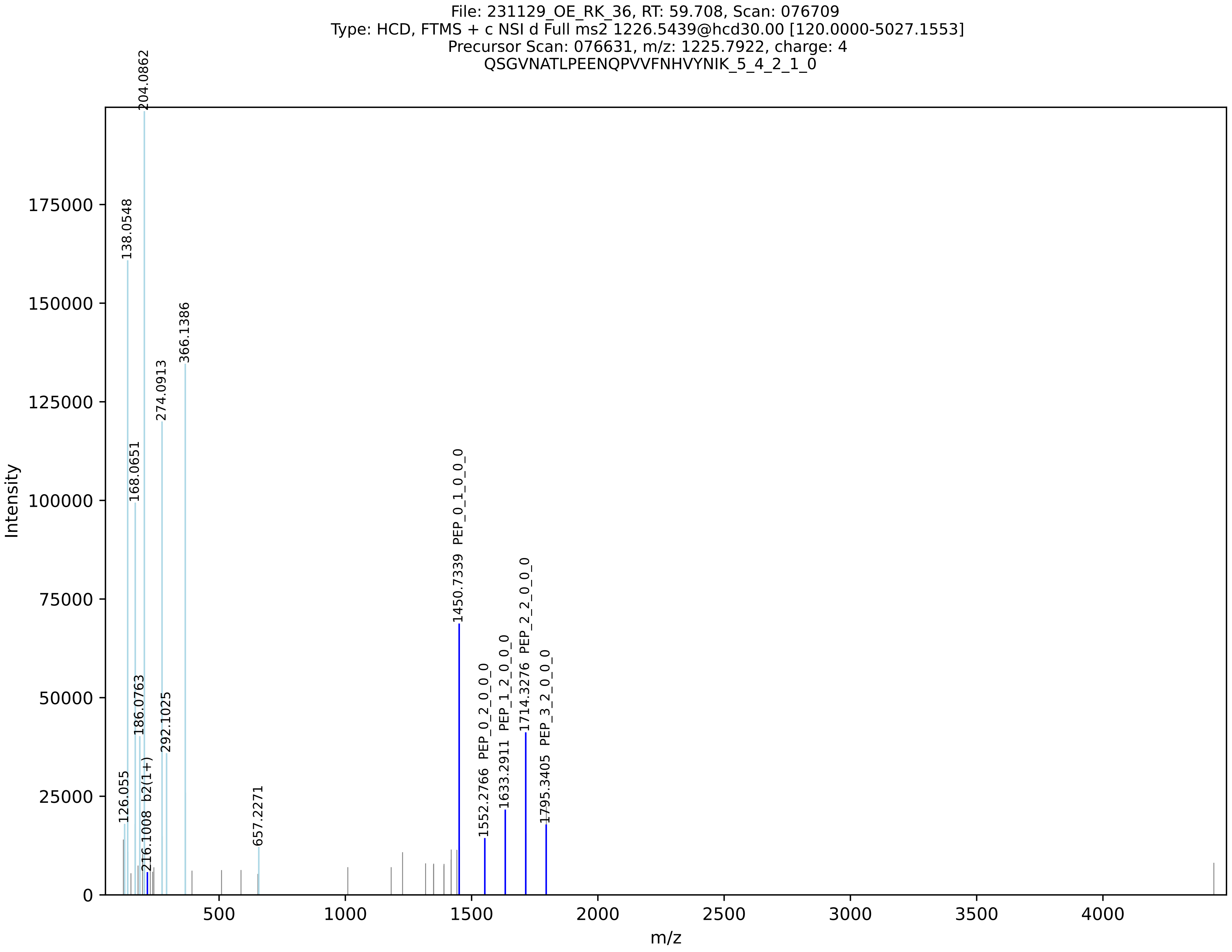

File: 231129_OE_RK_36, RT: 59.708, Scan: 076709
Type: HCD, FTMS + c NSI d Full ms2 1226.5439@hcd30.00 [120.0000-5027.1553]
Precursor Scan: 076631, m/z: 1225.7922, charge: 4
QSGVNATLPEENQPVVFNHVYNIK_5_4_2_1_0
204.0862
138.0548
366.1386
274.0913
168.0651
186.0763
292.1025
126.055
216.1008 b2(1+)
657.2271
1450.7339 PEP_0_1_0_0_0
1552.2766 PEP_0_2_0_0_0
1633.2911 PEP_1_2_0_0_0
1714.3276 PEP_2_2_0_0_0
1795.3405 PEP_3_2_0_0_0
Intensity
m/z
0
25000
50000
75000
100000
125000
150000
175000
500
1000
1500
2000
2500
3000
3500
4000

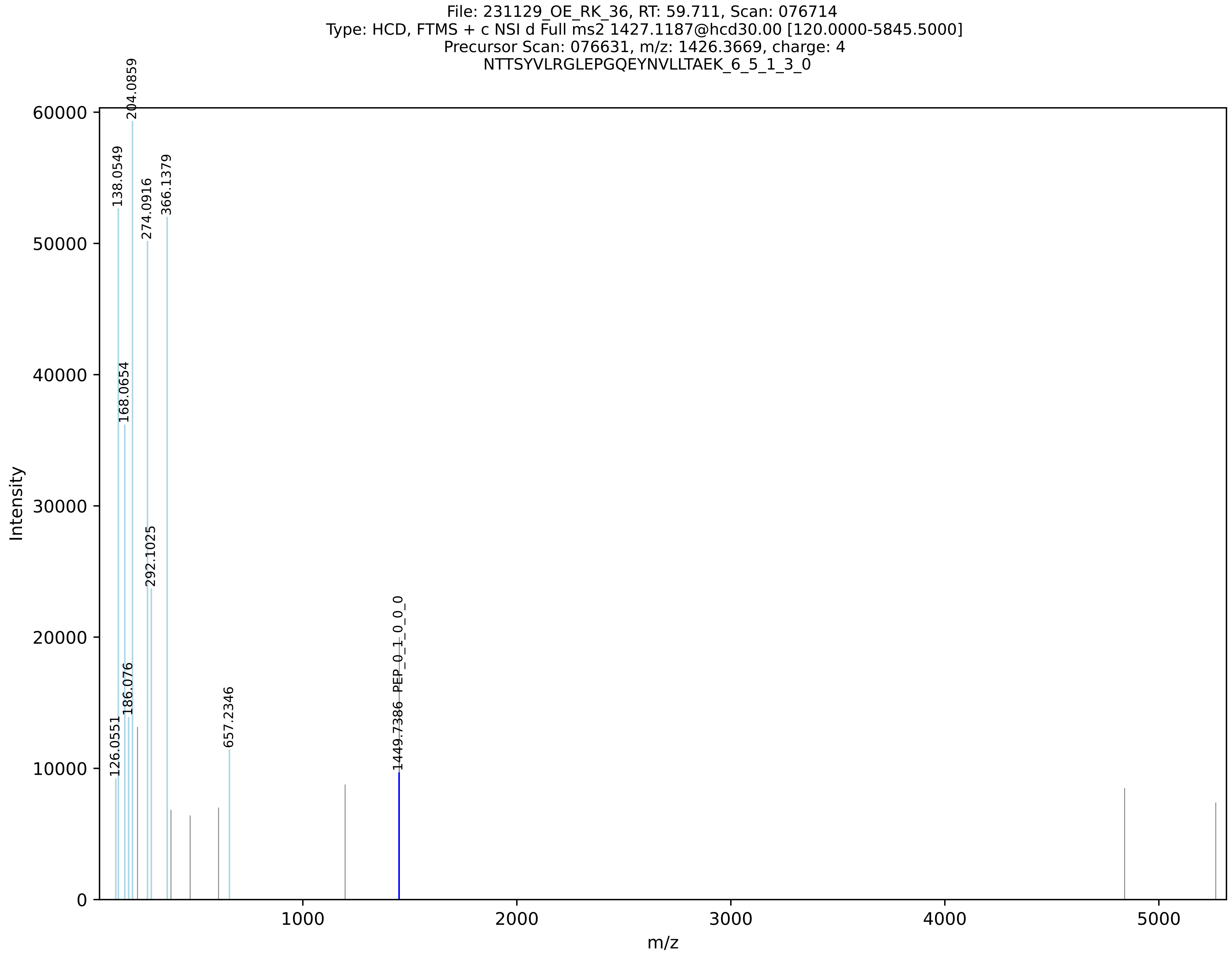
File: 231129_OE_RK_36, RT: 59.711, Scan: 076714
Type: HCD, FTMS + c NSI d Full ms2 1427.1187@hcd30.00 [120.0000-5845.5000]
Precursor Scan: 076631, m/z: 1426.3669, charge: 4
NTTSYVLRGLEPGQEYNVLLTAEK_6_5_1_3_0
204.0859
138.0549
366.1379
274.0916
168.0654
292.1025
186.076
126.0551
657.2346
1449.7386_PEP_0_1_0_0_0
Intensity
m/z
0
10000
20000
30000
40000
50000
60000
1000
2000
3000
4000
5000

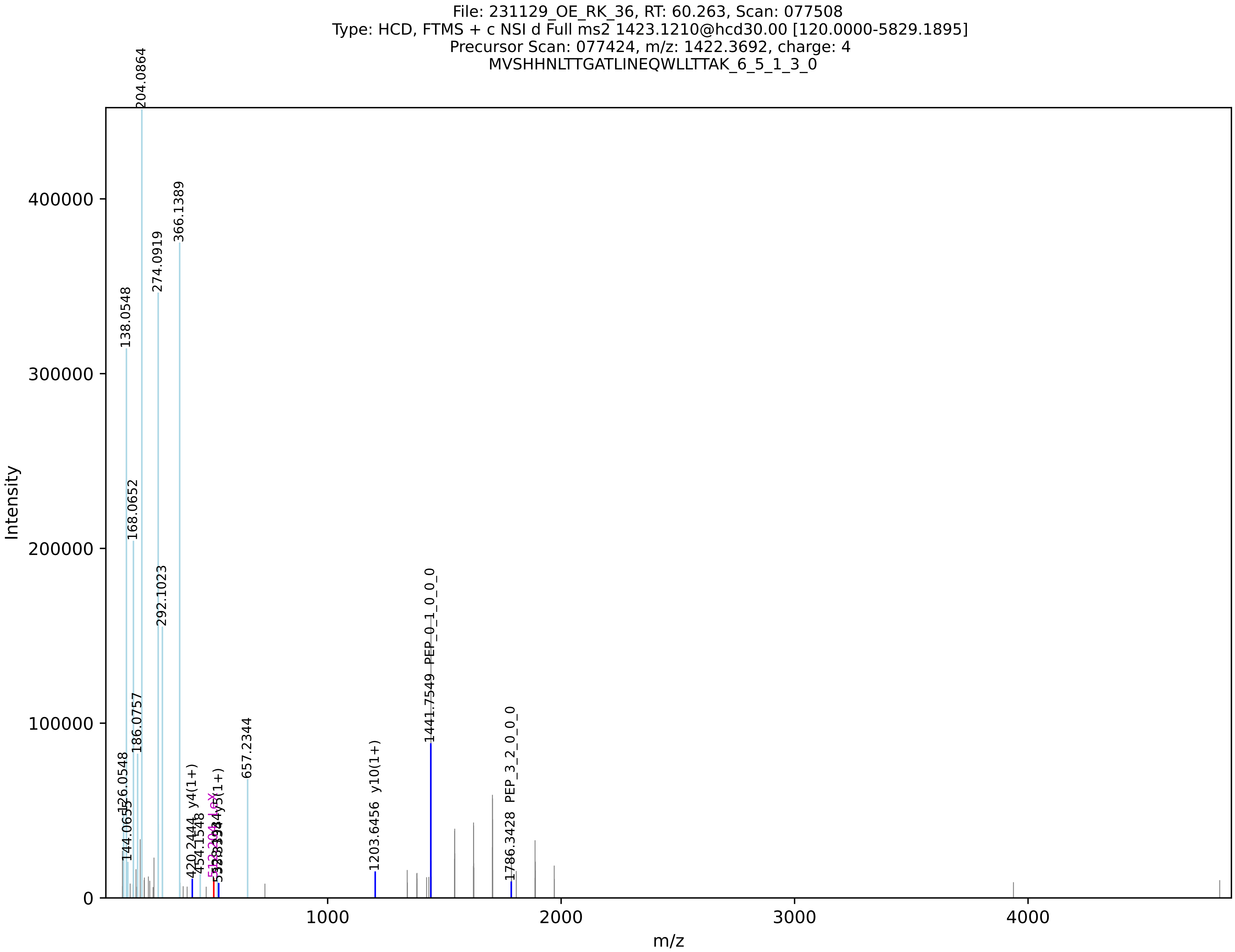
File: 231129_OE_RK_36, RT: 60.263, Scan: 077508
Type: HCD, FTMS + c NSI d Full ms2 1423.1210@hcd30.00 [120.0000-5829.1895]
Precursor Scan: 077424, m/z: 1422.3692, charge: 4
MVSHHNLTTGATLINEQWLLTTAK_6_5_1_3_0
Intensity
m/z
204.0864
366.1389
274.0919
138.0548
168.0652
292.1023
186.0757
126.0548
144.0655
420.2444 y4(1+)
454.1548
657.2344
1203.6456 y10(1+)
1441.7549 PEP_0_1_0_0_0
1786.3428 PEP_3_2_0_0_0

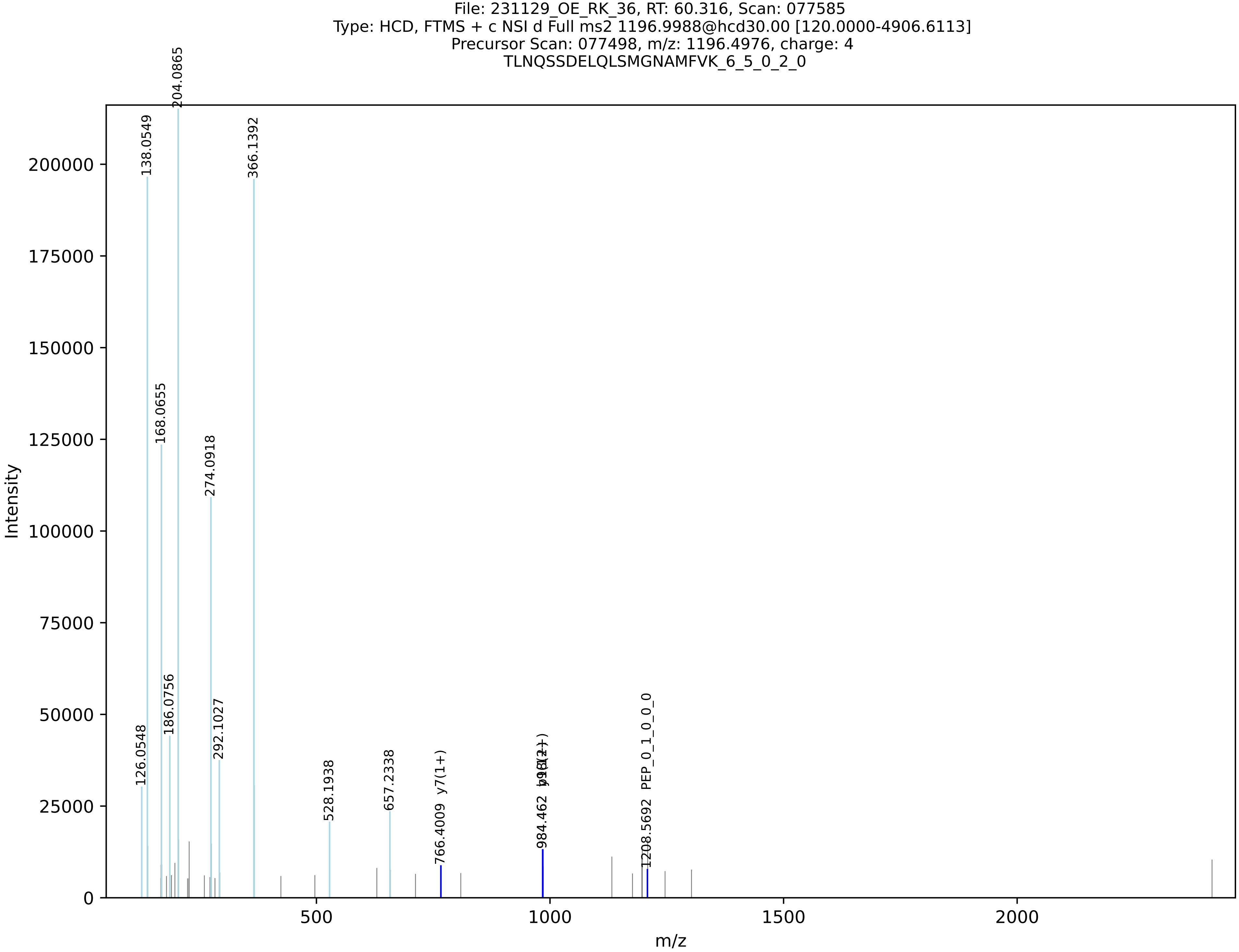
File: 231129_OE_RK_36, RT: 60.316, Scan: 077585
Type: HCD, FTMS + c NSI d Full ms2 1196.9988@hcd30.00 [120.0000-4906.6113]
Precursor Scan: 077498, m/z: 1196.4976, charge: 4
TLNQSSDELQLSMGNAMFVK_6_5_0_2_0
Intensity
m/z
204.0865
138.0549
366.1392
168.0655
274.0918
186.0756
292.1027
126.0548
528.1938
657.2338
766.4009 y7(1+)
984.462
1208.5692 PEP_0_1_0_0_0
0
25000
50000
75000
100000
125000
150000
175000
200000
500
1000
1500
2000

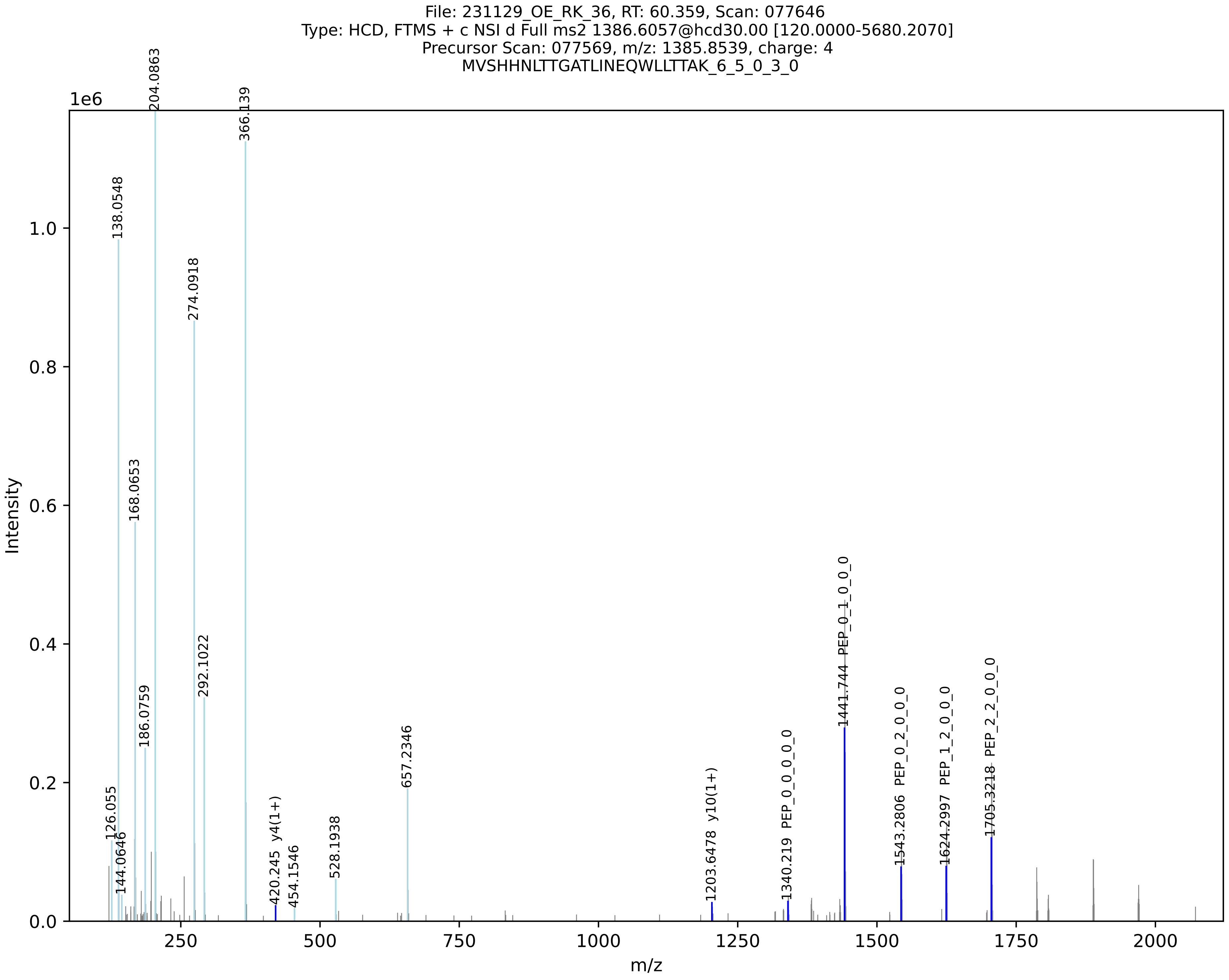

File: 231129_OE_RK_36, RT: 60.359, Scan: 077646
Type: HCD, FTMS + c NSI d Full ms2 1386.6057@hcd30.00 [120.0000-5680.2070]
Precursor Scan: 077569, m/z: 1385.8539, charge: 4
MVSHHNLTTGATLINEQWLLTTAK_6_5_0_3_0
1e6
Intensity
m/z
204.0863
366.139
138.0548
274.0918
168.0653
292.1022
186.0759
126.055
144.0646
420.245 y4(1+)
454.1546
528.1938
657.2346
1203.6478 y10(1+)
1340.219 PEP_0_0_0_0_0
1441.744 PEP_0_1_0_0_0
1543.2806 PEP_0_2_0_0_0
1624.2997 PEP_1_2_0_0_0
1705.3218 PEP_2_2_0_0_0

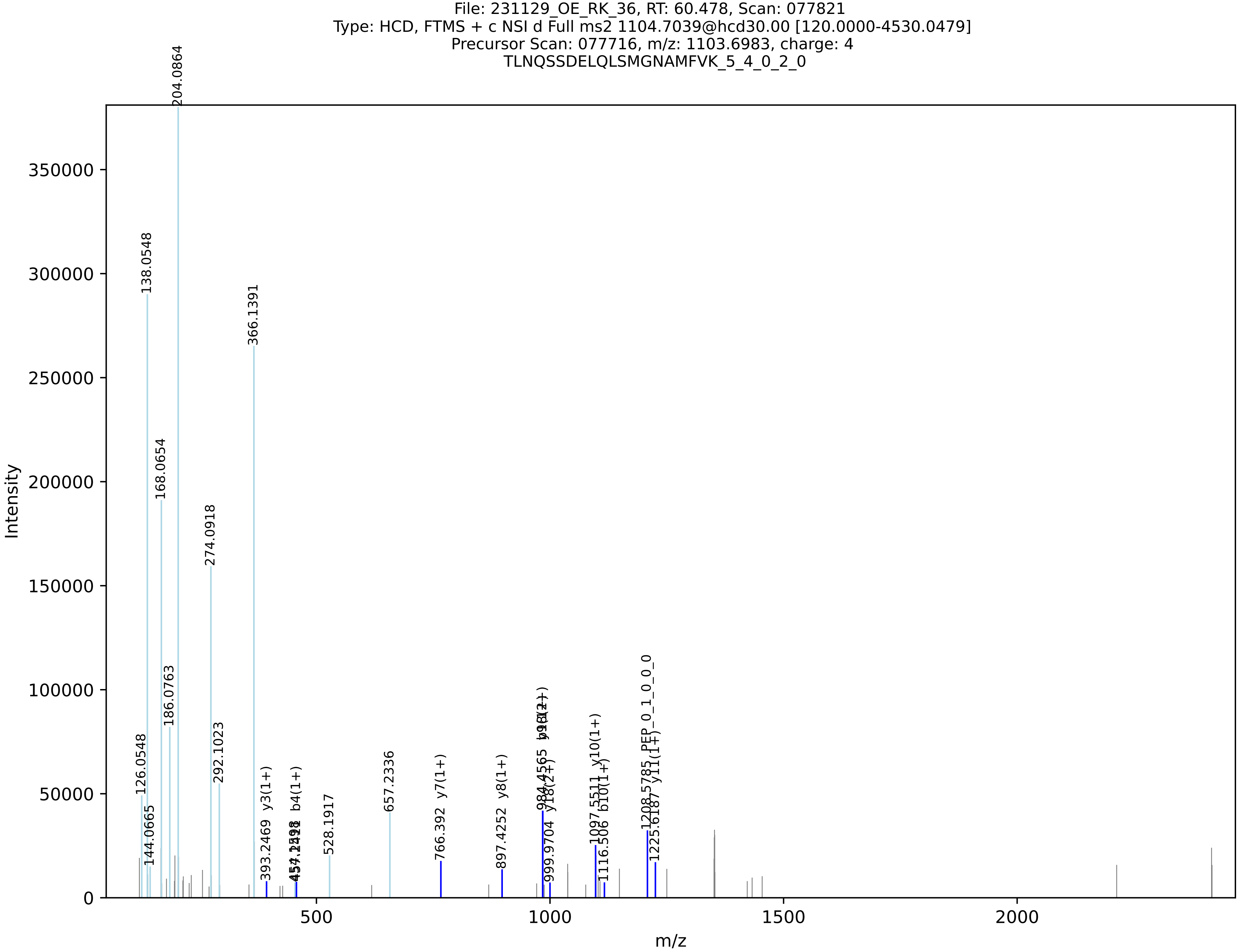
File: 231129_OE_RK_36, RT: 60.478, Scan: 077821
Type: HCD, FTMS + c NSI d Full ms2 1104.7039@hcd30.00 [120.0000-4530.0479]
Precursor Scan: 077716, m/z: 1103.6983, charge: 4
TLNQSSDELQLSMGNAMFVK_5_4_0_2_0
Intensity
m/z
204.0864
138.0548
168.0654
274.0918
366.1391
186.0763
292.1023
126.0548
144.0665
393.2469 y3(1+)
b4(1+)
528.1917
657.2336
766.392 y7(1+)
897.4252 y8(1+)
984.4565
y18(2+)
999.9704
1097.5511 y10(1+)
1116.506 b10(1+)
1208.5785 PEP_0_1_0_0_0
1225.6187 y11(1+)
0
50000
100000
150000
200000
250000
300000
350000
500
1000
1500
2000

File: 231129_OE_RK_36, RT: 60.978, Scan: 078512
Type: HCD, FTMS + c NSI d Full ms2 1474.2844@hcd30.00 [120.0000-4533.4526]
Precursor Scan: 078463, m/z: 1473.2821, charge: 3
TLNQSSDELQLSMGNAMFVK_5_4_0_2_0

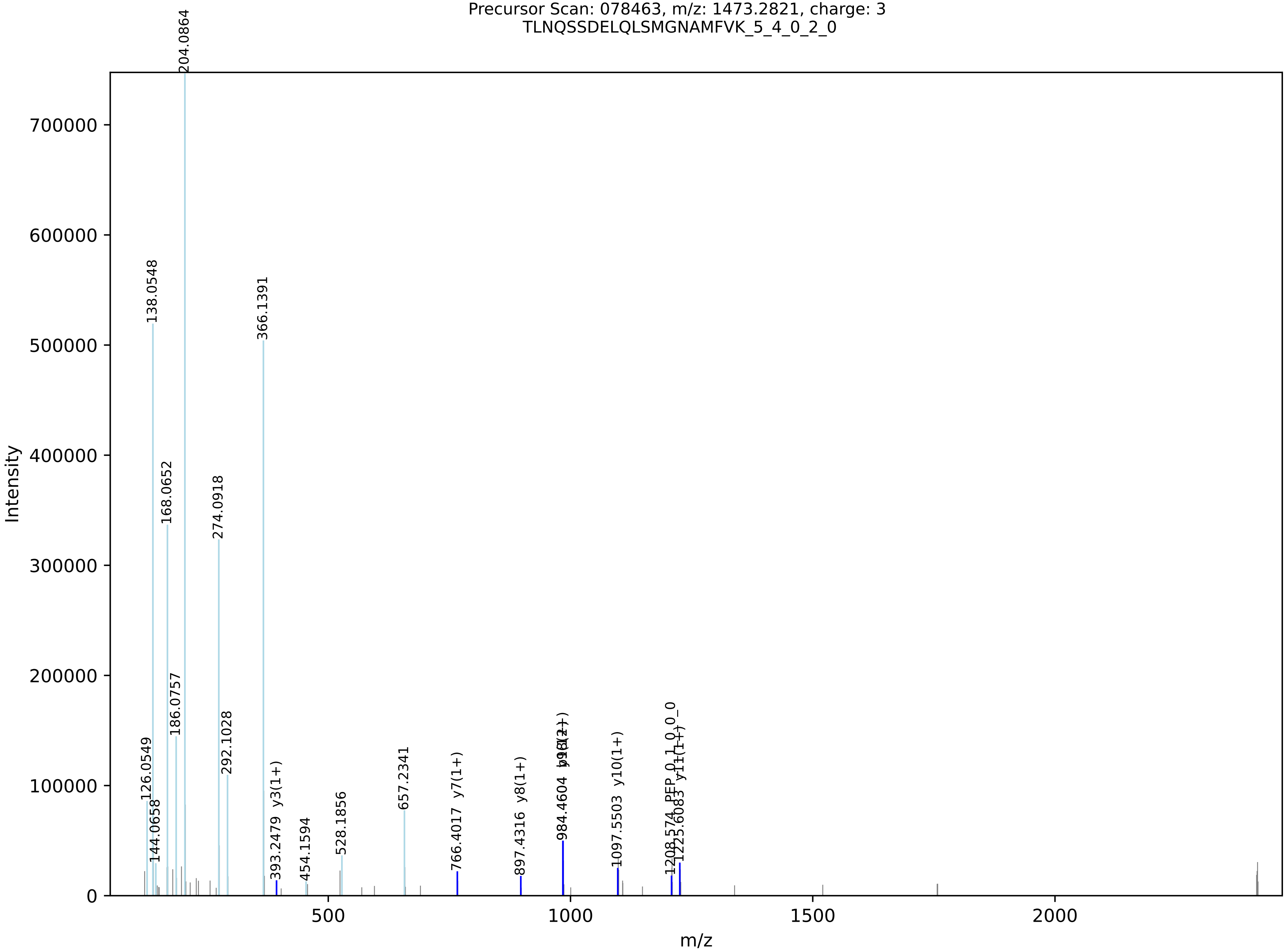

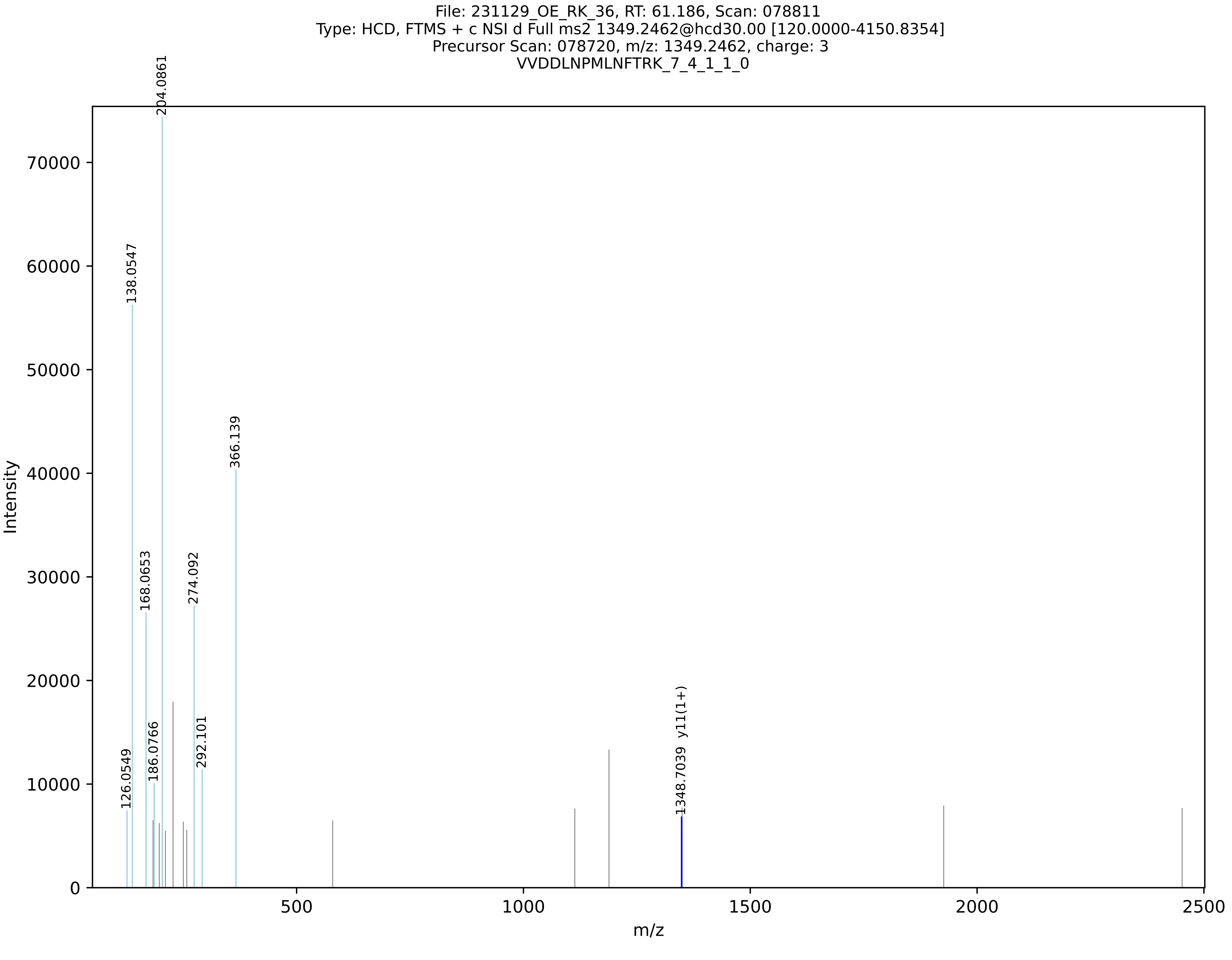
File: 231129_OE_RK_36, RT: 61.186, Scan: 078811
Type: HCD, FTMS + c NSI d Full ms2 1349.2462@hcd30.00 [120.0000-4150.8354]
Precursor Scan: 078720, m/z: 1349.2462, charge: 3
VVDDLNPMLNFTRK_7_4_1_1_0
204.0861
138.0547
366.139
168.0653
274.092
292.101
186.0766
126.0549
1348.7039 y11(1+)
Intensity
m/z
0
10000
20000
30000
40000
50000
60000
70000
500
1000
1500
2000
2500

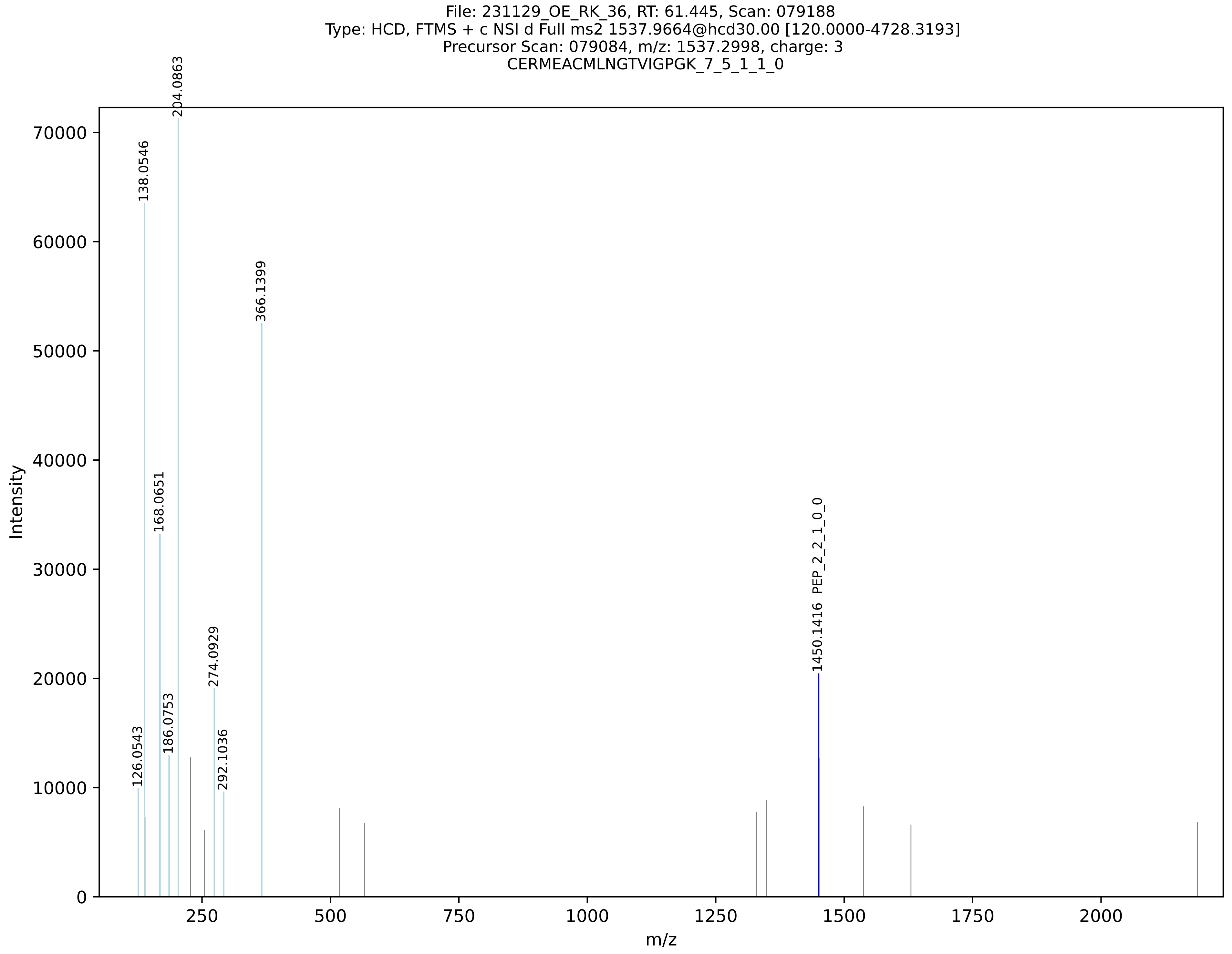
File: 231129_OE_RK_36, RT: 61.445, Scan: 079188
Type: HCD, FTMS + c NSI d Full ms2 1537.9664@hcd30.00 [120.0000-4728.3193]
Precursor Scan: 079084, m/z: 1537.2998, charge: 3
CERMEACMLNGTVIGPGK_7_5_1_1_0
Intensity
m/z
204.0863
138.0546
366.1399
168.0651
1450.1416 PEP_2_2_1_0_0
274.0929
186.0753
126.0543
292.1036

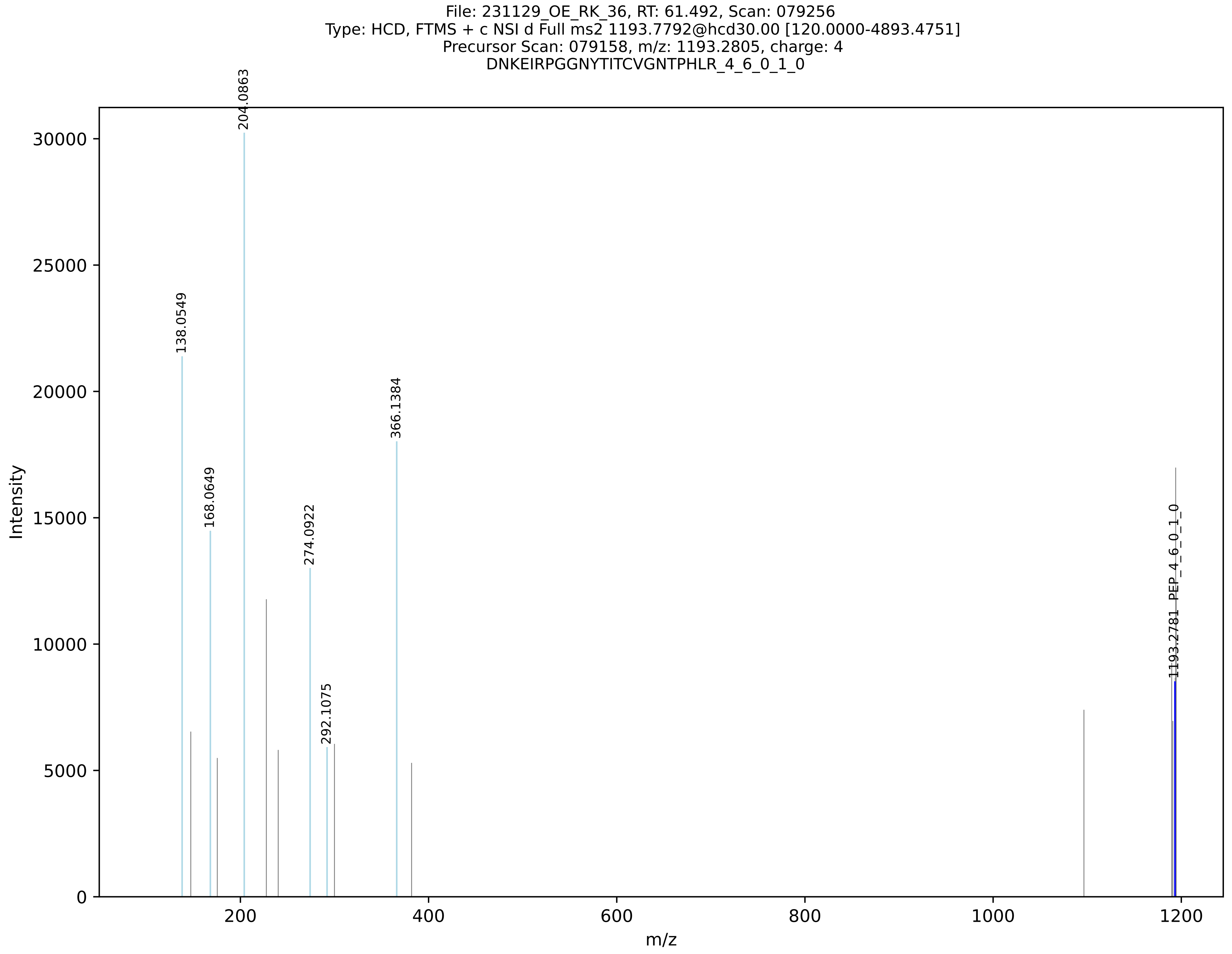
File: 231129_OE_RK_36, RT: 61.492, Scan: 079256
Type: HCD, FTMS + c NSI d Full ms2 1193.7792@hcd30.00 [120.0000-4893.4751]
Precursor Scan: 079158, m/z: 1193.2805, charge: 4
DNKEIRPGGNYTITCVGNTPHLR_4_6_0_1_0
204.0863
138.0549
168.0649
274.0922
292.1075
366.1384
1193.2781_PEP_4_6_0_1_0
Intensity
m/z

File: 231129_OE_RK_36, RT: 62.825, Scan: 081171
Type: HCD, FTMS + c NSI d Full ms2 1436.6294@hcd30.00 [120.0000-4418.2280]
Precursor Scan: 081090, m/z: 1435.9612, charge: 3
VLSNNSDANLELINTWVAK_5_4_0_2_0

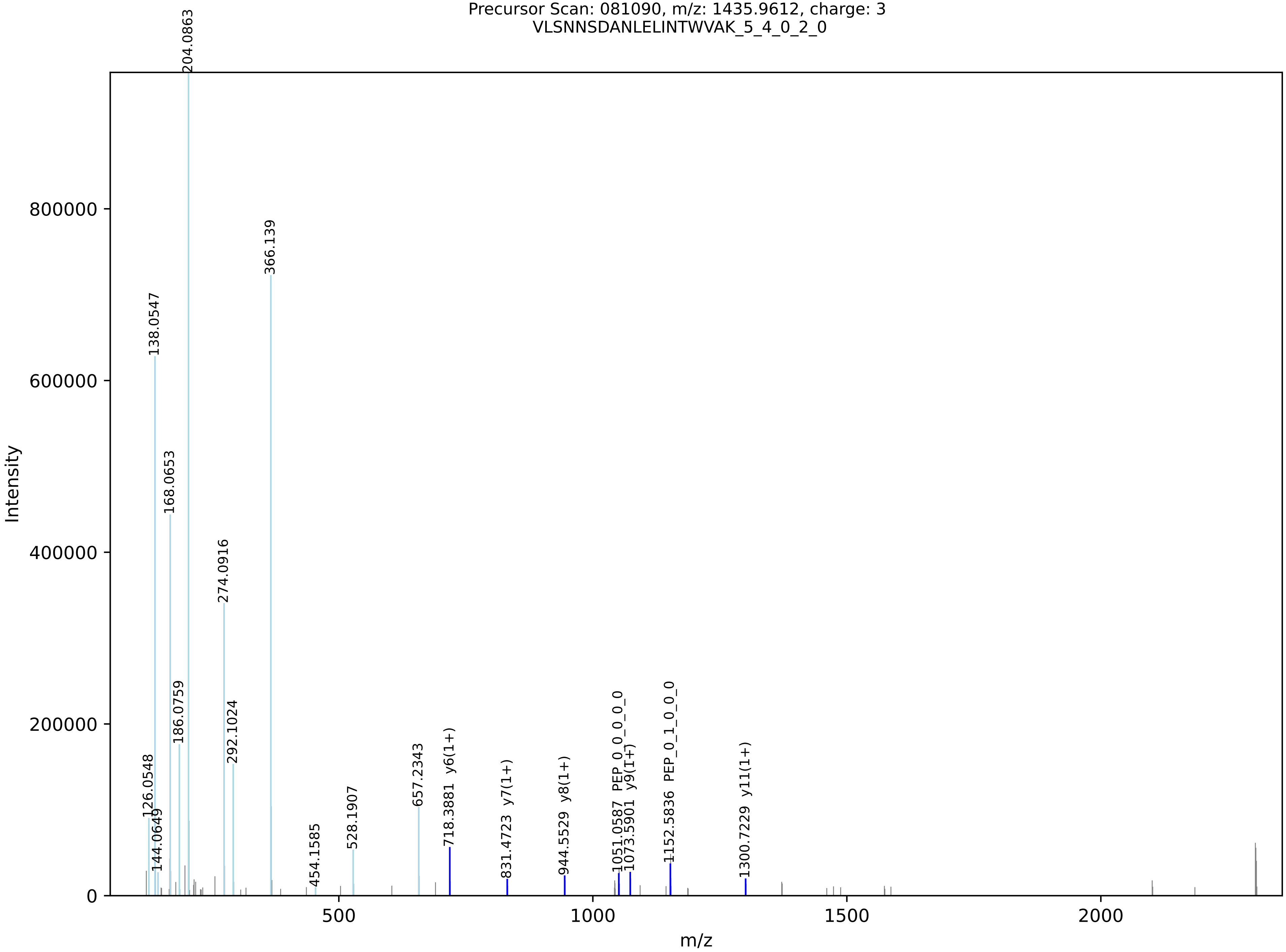

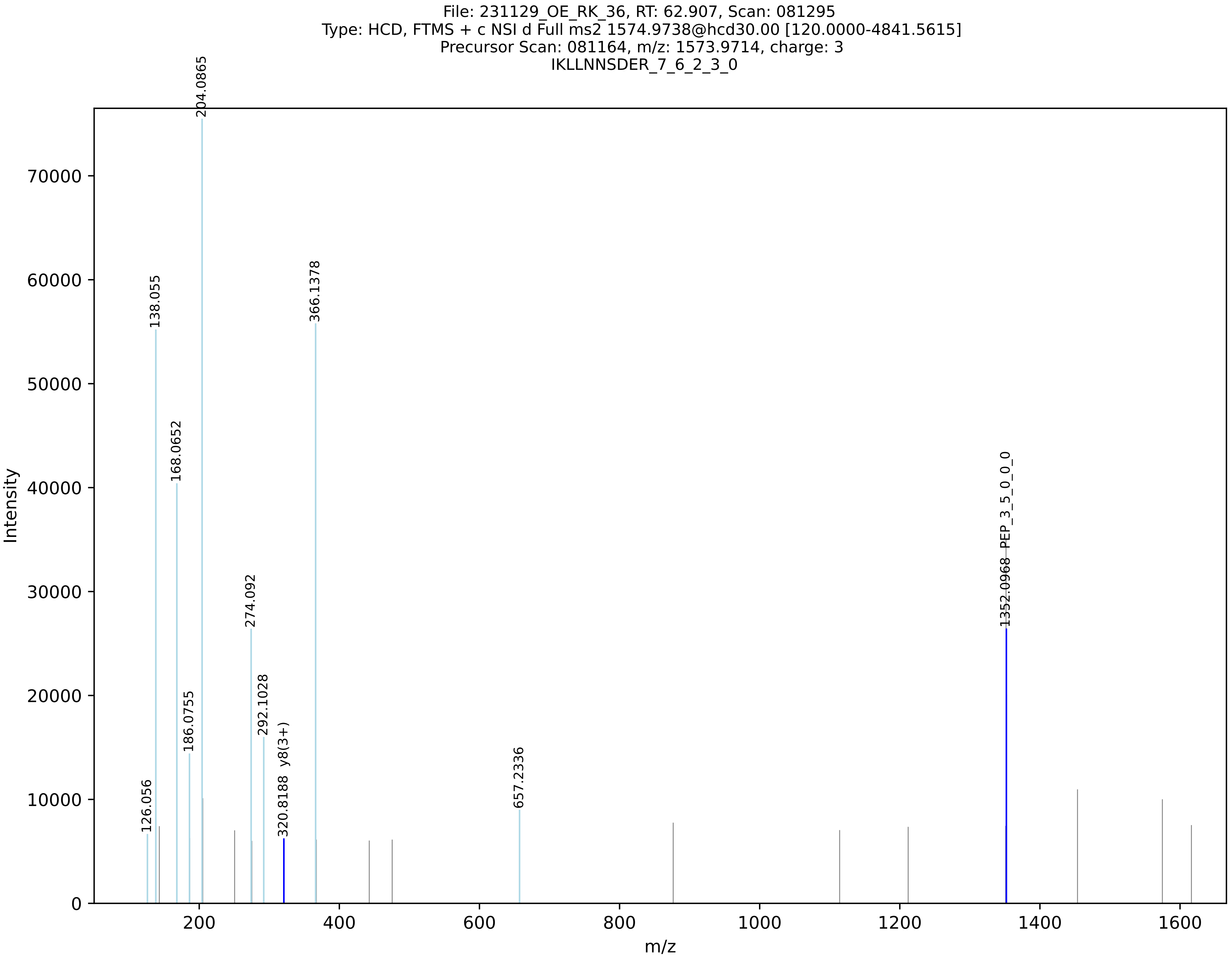
File: 231129_OE_RK_36, RT: 62.907, Scan: 081295
Type: HCD, FTMS + c NSI d Full ms2 1574.9738@hcd30.00 [120.0000-4841.5615]
Precursor Scan: 081164, m/z: 1573.9714, charge: 3
IKLLNNSDER_7_6_2_3_0
Intensity
m/z
204.0865
138.055
366.1378
168.0652
274.092
292.1028
186.0755
y8(3+)
320.8188
126.056
657.2336
1352.0968_PEP_3_5_0_0_0
0
10000
20000
30000
40000
50000
60000
70000
200
400
600
800
1000
1200
1400
1600

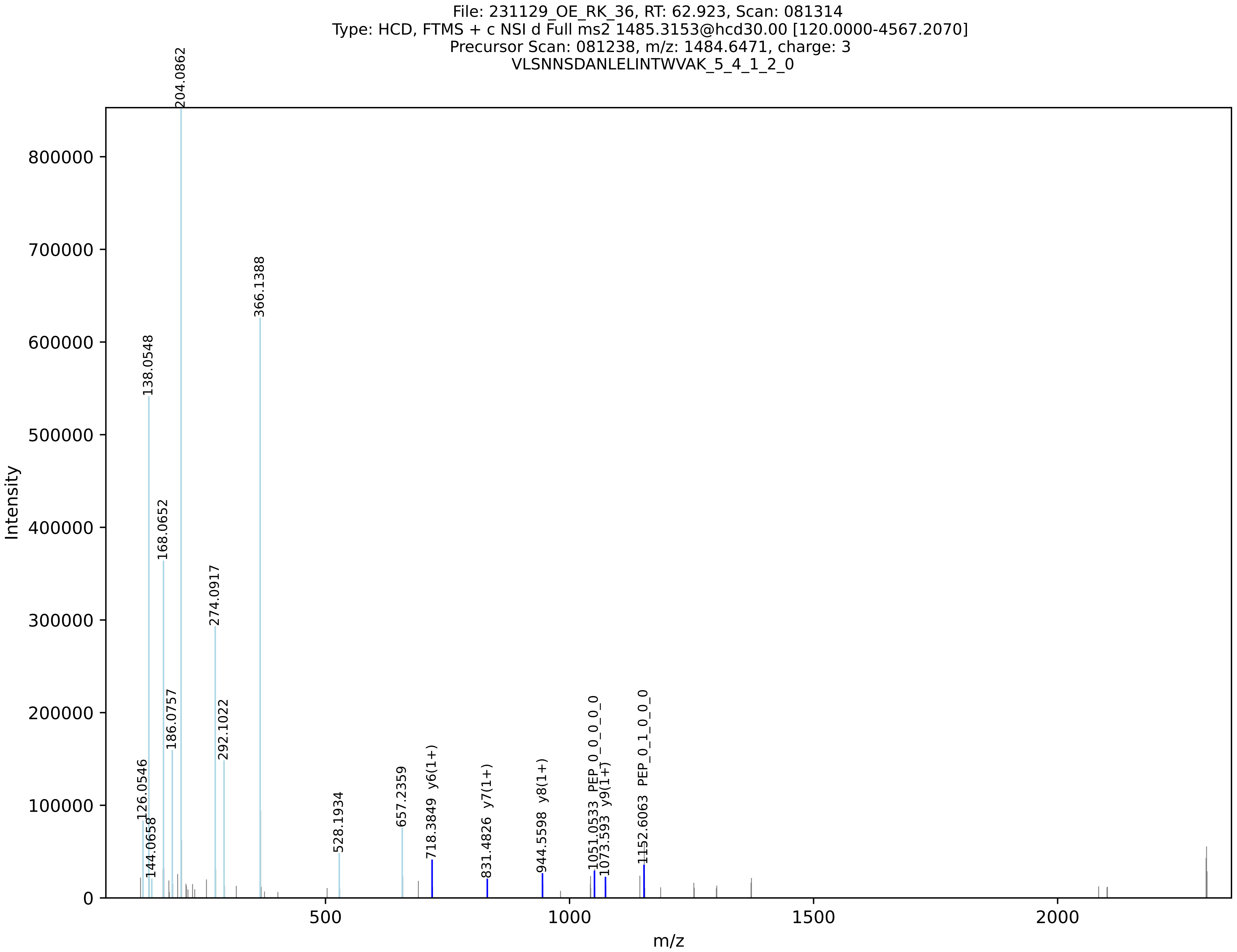
File: 231129_OE_RK_36, RT: 62.923, Scan: 081314
Type: HCD, FTMS + c NSI d Full ms2 1485.3153@hcd30.00 [120.0000-4567.2070]
Precursor Scan: 081238, m/z: 1484.6471, charge: 3
VLSNNSDANLELINTWVAK_5_4_1_2_0
Intensity
m/z
204.0862
366.1388
138.0548
168.0652
274.0917
186.0757
292.1022
126.0546
144.0658
528.1934
657.2359
718.3849 y6(1+)
831.4826 y7(1+)
944.5598 y8(1+)
1051.0533 PEP_0_0_0_0_0
1073.593 y9(1+)
1152.6063 PEP_0_1_0_0_0

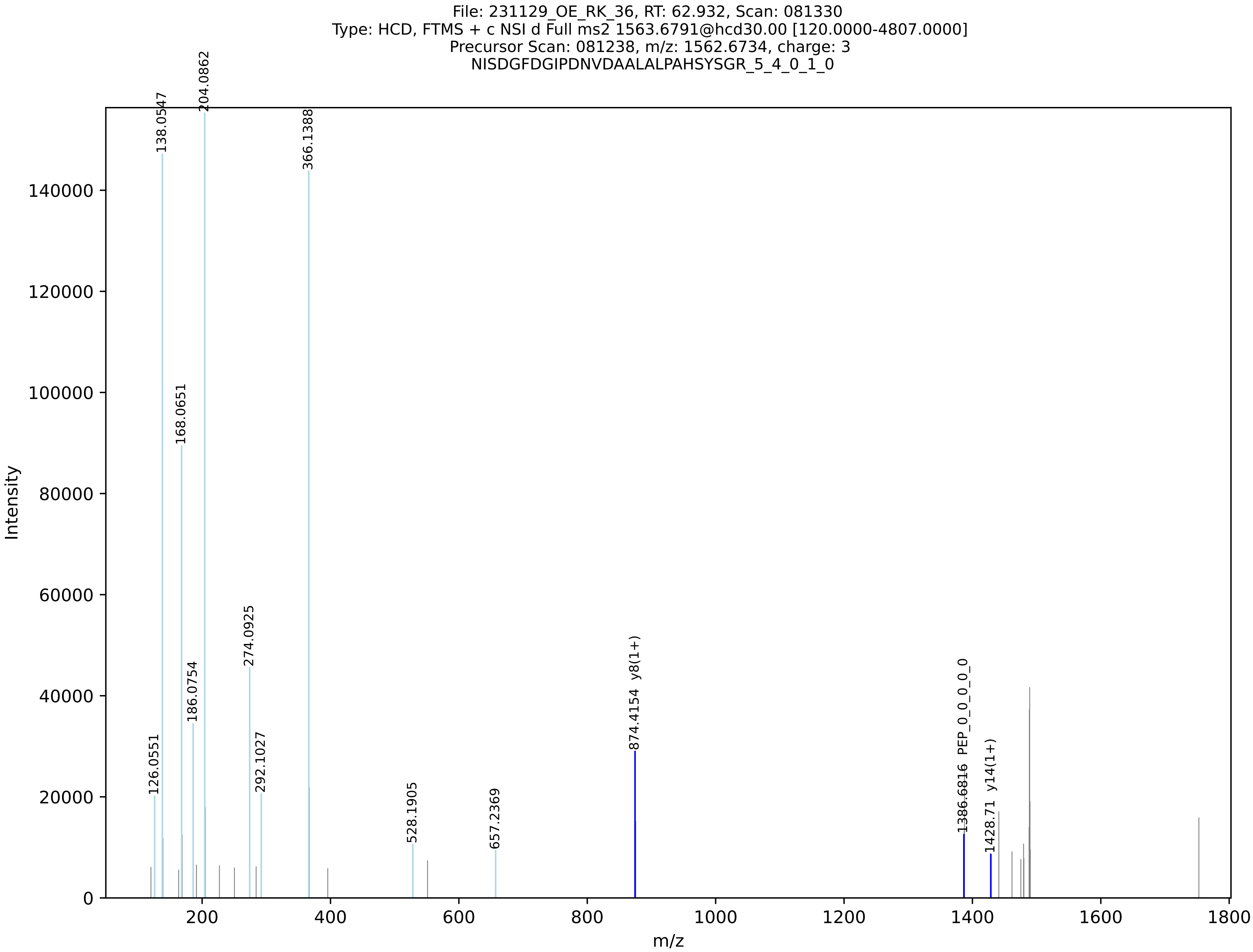

File: 231129_OE_RK_36, RT: 62.932, Scan: 081330
Type: HCD, FTMS + c NSI d Full ms2 1563.6791@hcd30.00 [120.0000-4807.0000]
Precursor Scan: 081238, m/z: 1562.6734, charge: 3
NISDGFDGIPDNVDAALALPAHSYSGR_5_4_0_1_0
Intensity
m/z
138.0547
204.0862
366.1388
168.0651
186.0754
126.0551
274.0925
292.1027
528.1905
657.2369
874.4154 y8(1+)
1386.6816 PEP_0_0_0_0_0
1428.71 y14(1+)

File: 231129_OE_RK_36, RT: 62.975, Scan: 081391
Type: HCD, FTMS + c NSI d Full ms2 1114.2371@hcd30.00 [120.0000-4568.9434]
Precursor Scan: 081312, m/z: 1113.7367, charge: 4
VLSNNSDANLELINTWVAK_5_4_1_2_0

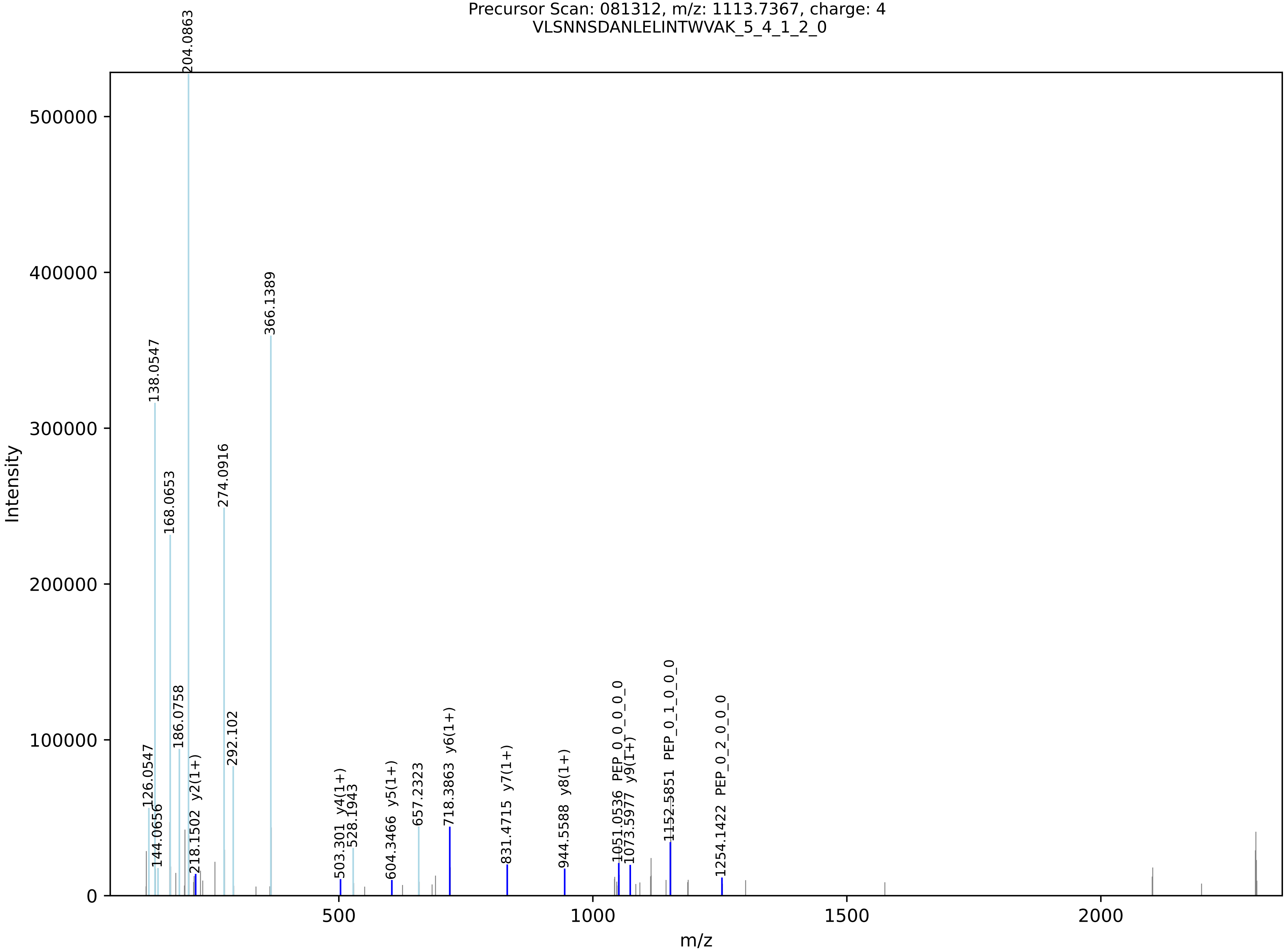

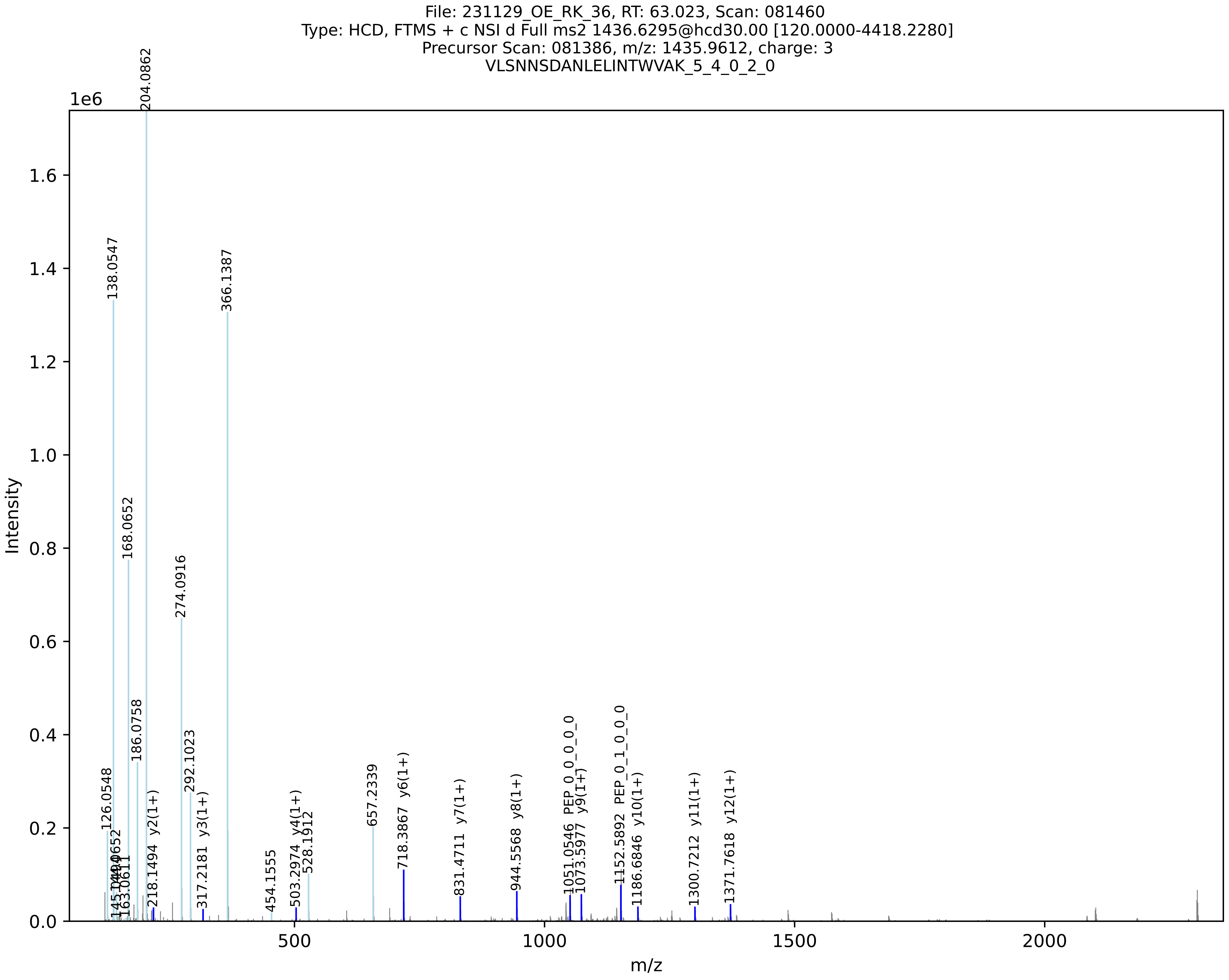

File: 231129_OE_RK_36, RT: 63.023, Scan: 081460
Type: HCD, FTMS + c NSI d Full ms2 1436.6295@hcd30.00 [120.0000-4418.2280]
Precursor Scan: 081386, m/z: 1435.9612, charge: 3
VLSNNSDANLELINTWVAK_5_4_0_2_0
1e6
Intensity
m/z
204.0862
138.0547
366.1387
168.0652
274.0916
186.0758
292.1023
126.0548
218.1494 y2(1+)
317.2181 y3(1+)
454.1555
503.2974 y4(1+)
528.1912
657.2339
718.3867 y6(1+)
831.4711 y7(1+)
944.5568 y8(1+)
1051.0546 PEP_0_0_0_0_0
1073.5977 y9(1+)
1152.5892 PEP_0_1_0_0_0
1186.6846 y10(1+)
1300.7212 y11(1+)
1371.7618 y12(1+)
163.0611

File: 231129_OE_RK_36, RT: 63.024, Scan: 081461
Type: HCD, FTMS + c NSI d Full ms2 1077.4731@hcd30.00 [120.0000-4418.9463]
Precursor Scan: 081386, m/z: 1077.2227, charge: 4
VLSNNSDANLELINTWVAK_5_4_0_2_0

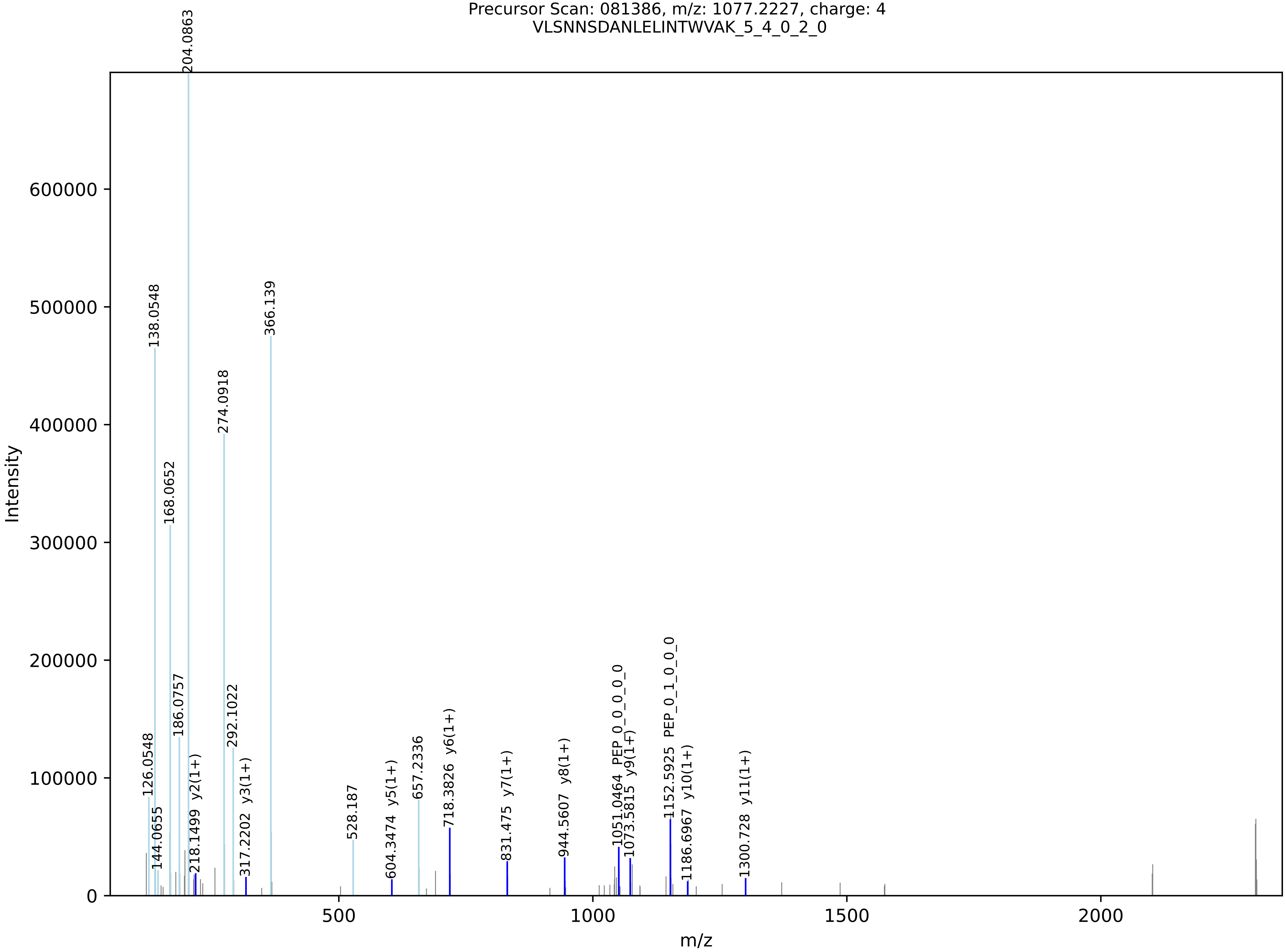

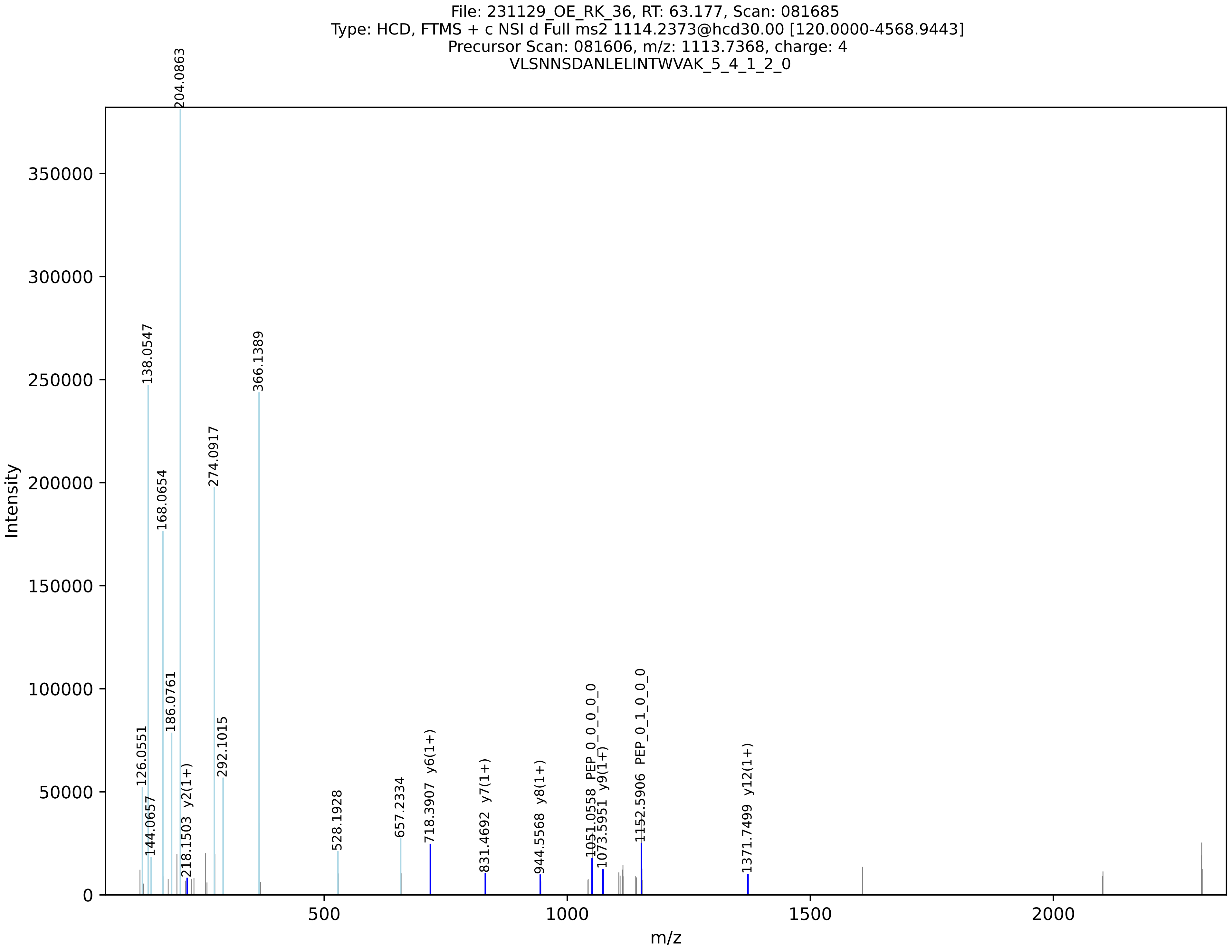
File: 231129_OE_RK_36, RT: 63.177, Scan: 081685
Type: HCD, FTMS + c NSI d Full ms2 1114.2373@hcd30.00 [120.0000-4568.9443]
Precursor Scan: 081606, m/z: 1113.7368, charge: 4
VLSNNSDANLELINTWVAK_5_4_1_2_0
Intensity
m/z
204.0863
138.0547
366.1389
274.0917
168.0654
186.0761
126.0551
292.1015
144.0657
218.1503 y2(1+)
528.1928
657.2334
718.3907 y6(1+)
831.4692 y7(1+)
944.5568 y8(1+)
1051.0558 PEP_0_0_0_0_0_
1073.5951 y9(1+)
1152.5906 PEP_0_1_0_0_0_
1371.7499 y12(1+)

File: 231129_OE_RK_36, RT: 63.226, Scan: 081756
Type: HCD, FTMS + c NSI d Full ms2 1436.6292@hcd30.00 [120.0000-4418.2271]
Precursor Scan: 081680, m/z: 1435.9601, charge: 3
VLSNNSDANLELINTWVAK_5_4_0_2_0

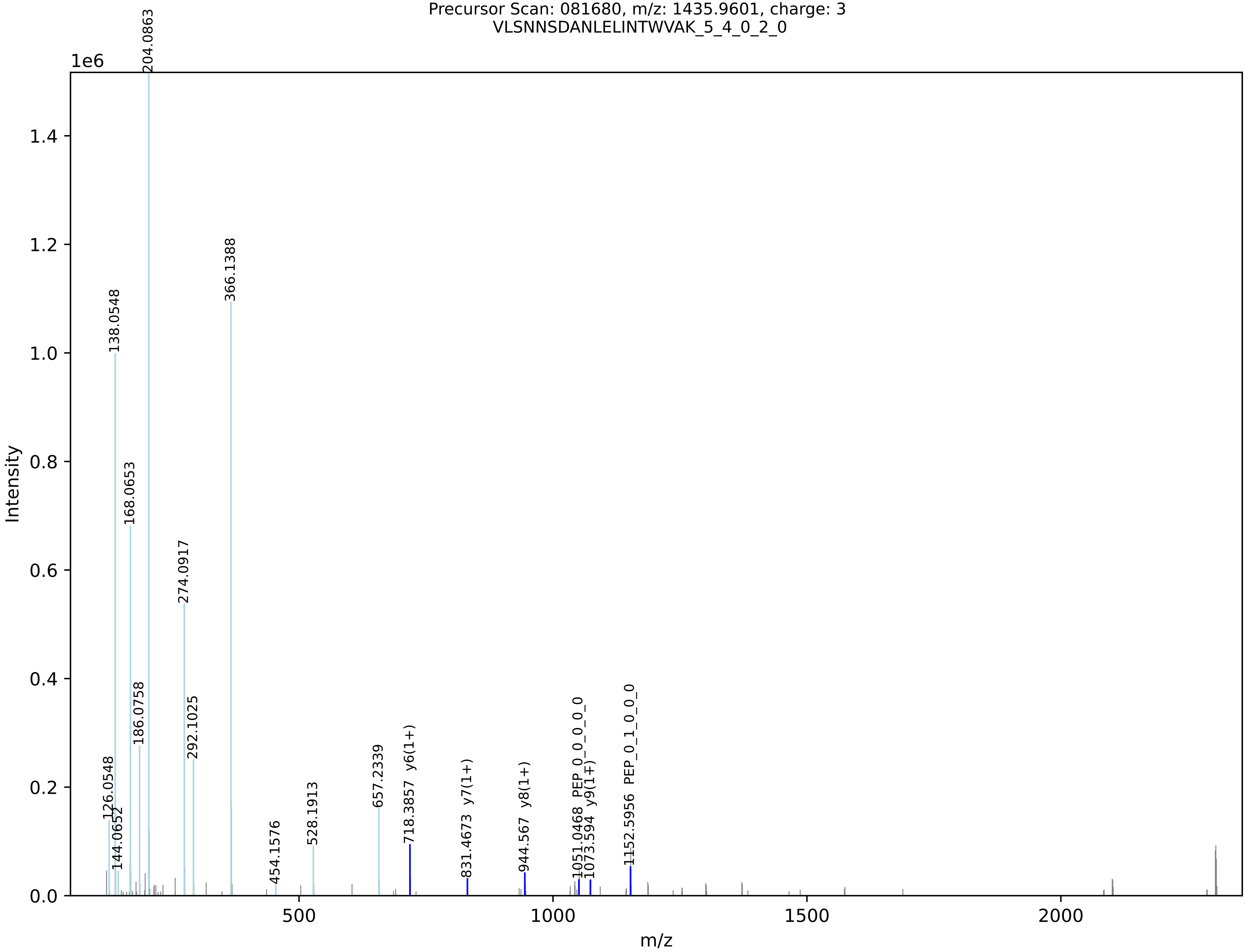

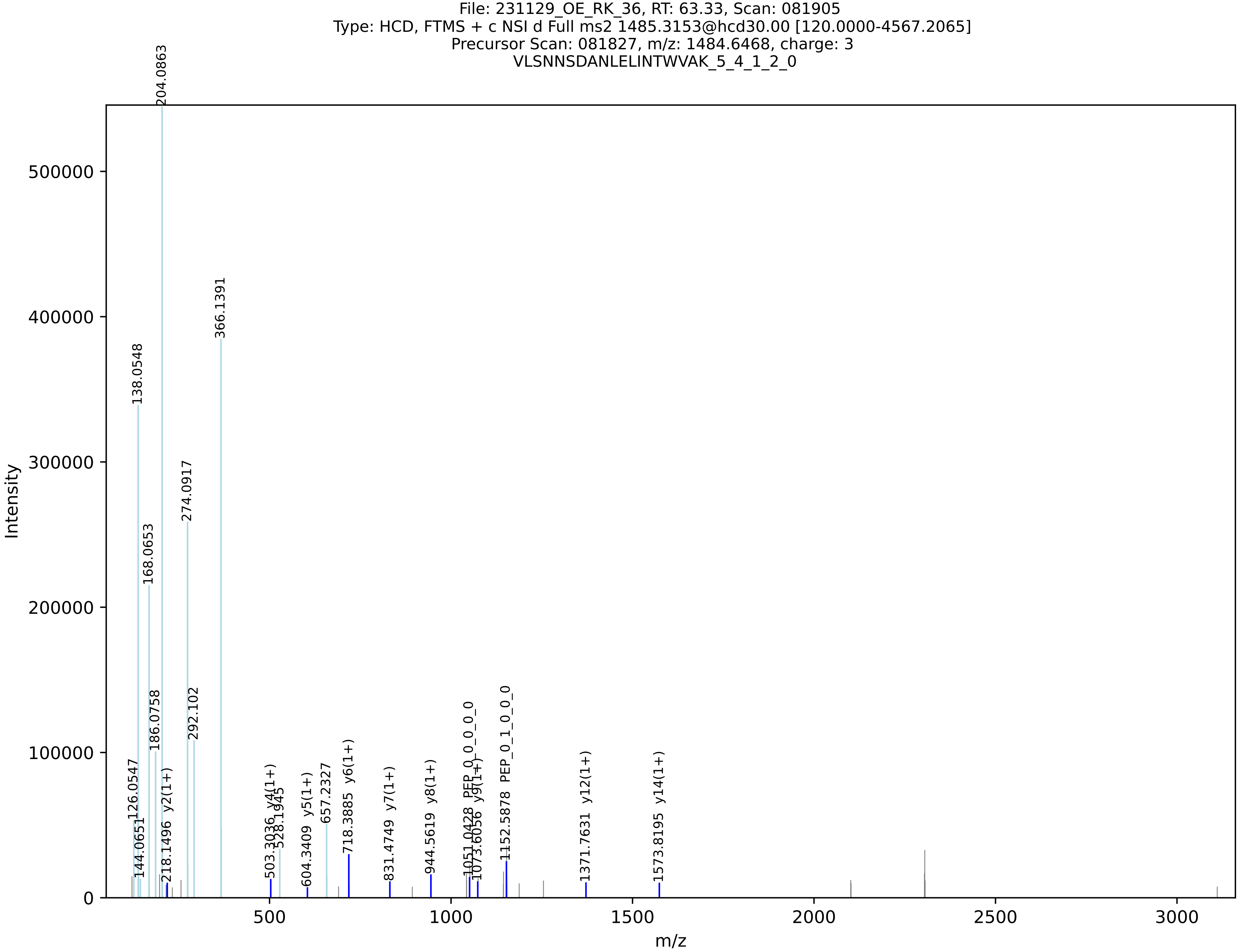
File: 231129_OE_RK_36, RT: 63.33, Scan: 081905
Type: HCD, FTMS + c NSI d Full ms2 1485.3153@hcd30.00 [120.0000-4567.2065]
Precursor Scan: 081827, m/z: 1484.6468, charge: 3
VLSNNSDANLELINTWVAK_5_4_1_2_0
Intensity
m/z
204.0863
366.1391
138.0548
274.0917
168.0653
186.0758
292.102
126.0547
144.0651
218.1496 y2(1+)
503.3036 y4(1+)
528.1945
604.3409 y5(1+)
657.2327
718.3885 y6(1+)
831.4749 y7(1+)
944.5619 y8(1+)
1051.0428 PEP_0_0_0_0_0
1073.6056 y9(1+)
1152.5878 PEP_0_1_0_0_0
1371.7631 y12(1+)
1573.8195 y14(1+)

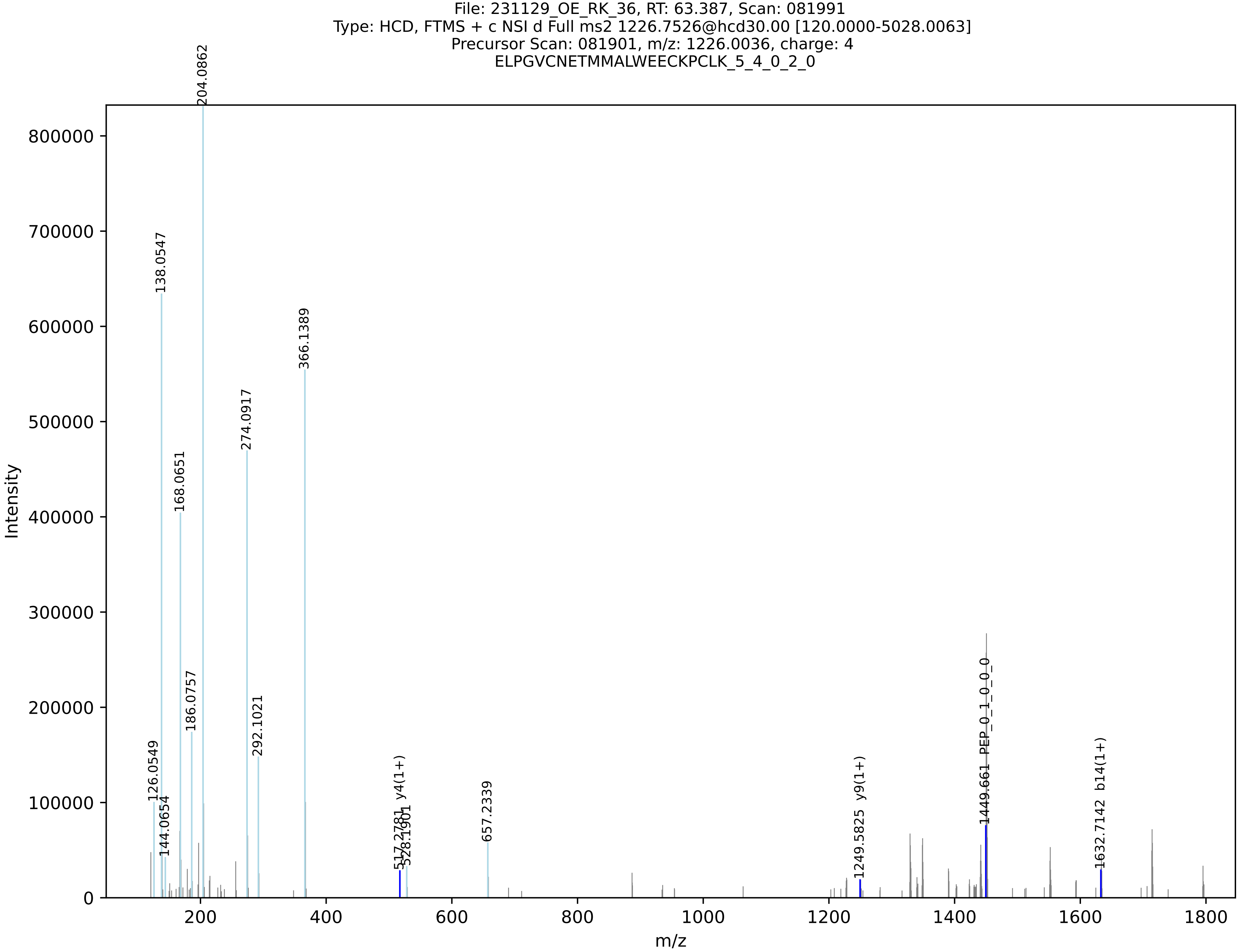

File: 231129_OE_RK_36, RT: 63.387, Scan: 081991
Type: HCD, FTMS + c NSI d Full ms2 1226.7526@hcd30.00 [120.0000-5028.0063]
Precursor Scan: 081901, m/z: 1226.0036, charge: 4
ELPGVCNETMMALWEECKPCLK_5_4_0_2_0
Intensity
m/z
204.0862
138.0547
366.1389
274.0917
168.0651
186.0757
292.1021
126.0549
144.0654
517.2781 y4(1+)
528.1901
657.2339
1249.5825 y9(1+)
1449.661 PEP_0_1_0_0_0_
1632.7142 b14(1+)

File: 231129_OE_RK_36, RT: 63.58, Scan: 082270
Type: HCD, FTMS + c NSI d Full ms2 1226.5018@hcd30.00 [120.0000-5026.9834]
Precursor Scan: 082194, m/z: 1225.7501, charge: 4
ELPGVCNETMMALWEECKPCLK_5_4_0_2_0

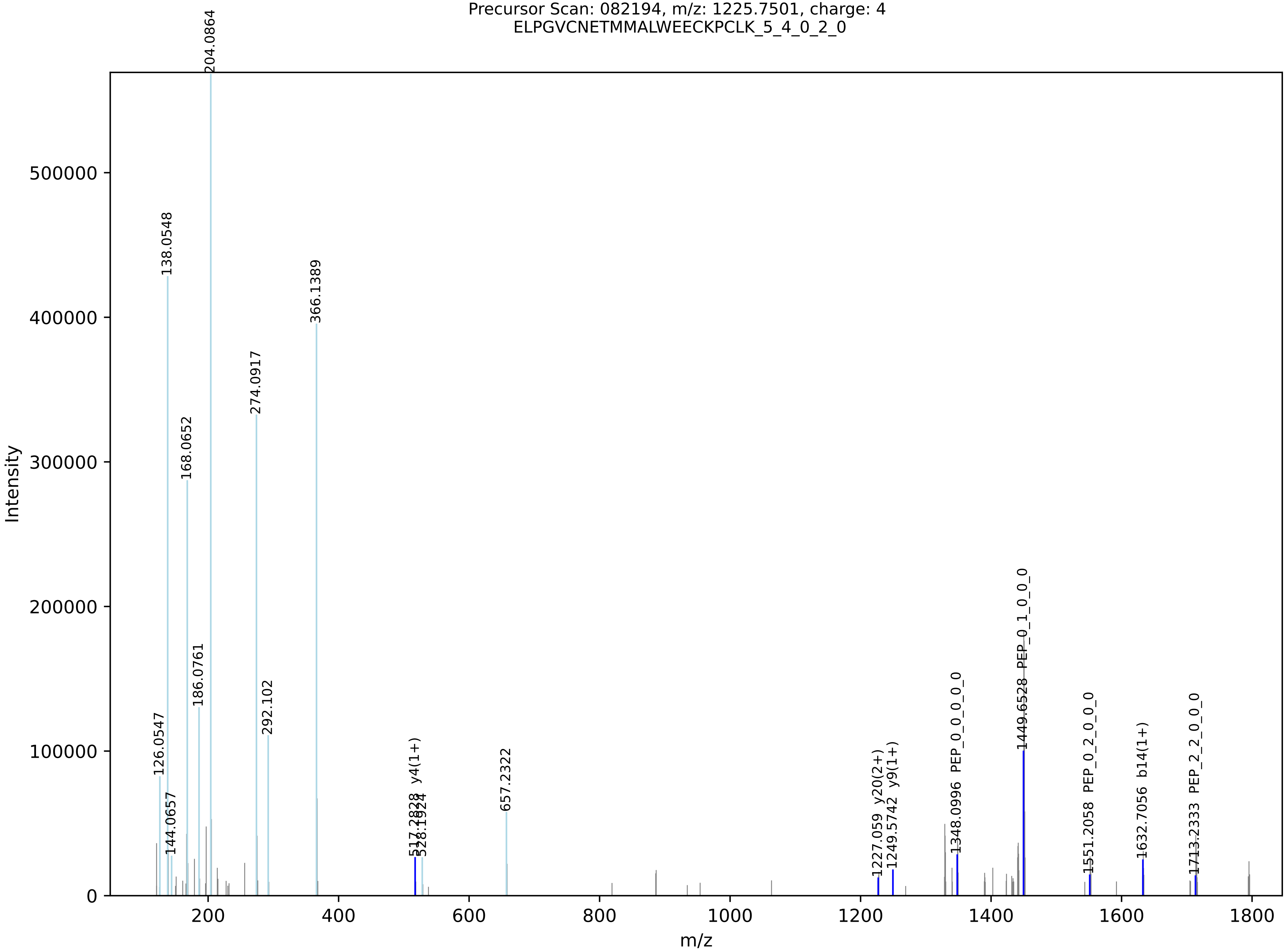

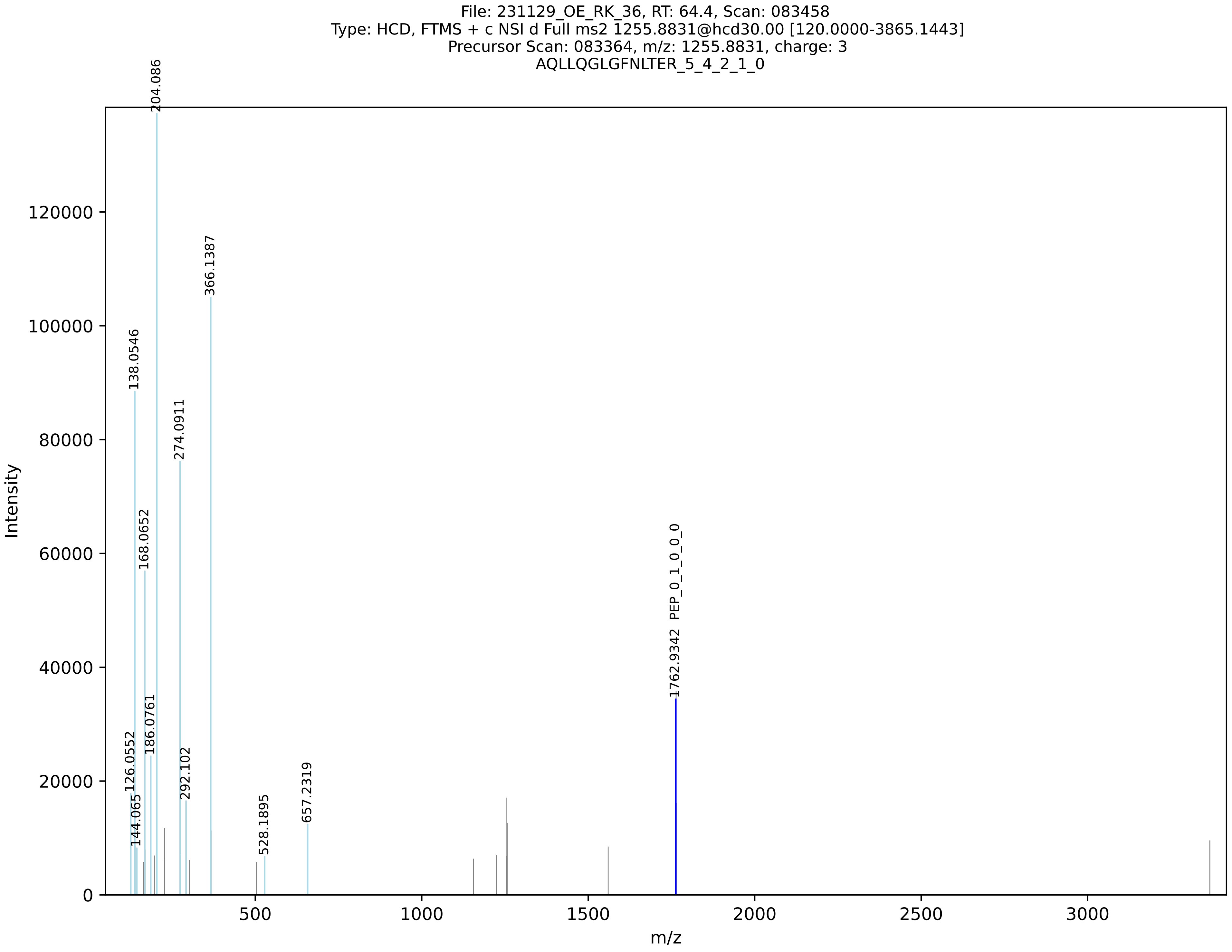

File: 231129_OE_RK_36, RT: 64.4, Scan: 083458
Type: HCD, FTMS + c NSI d Full ms2 1255.8831@hcd30.00 [120.0000-3865.1443]
Precursor Scan: 083364, m/z: 1255.8831, charge: 3
AQLLQGLGFNLTER_5_4_2_1_0
Intensity
m/z
204.086
366.1387
138.0546
274.0911
168.0652
186.0761
126.0552
144.065
292.102
528.1895
657.2319
1762.9342 PEP_0_1_0_0_0_
0
20000
40000
60000
80000
100000
120000
500
1000
1500
2000
2500
3000

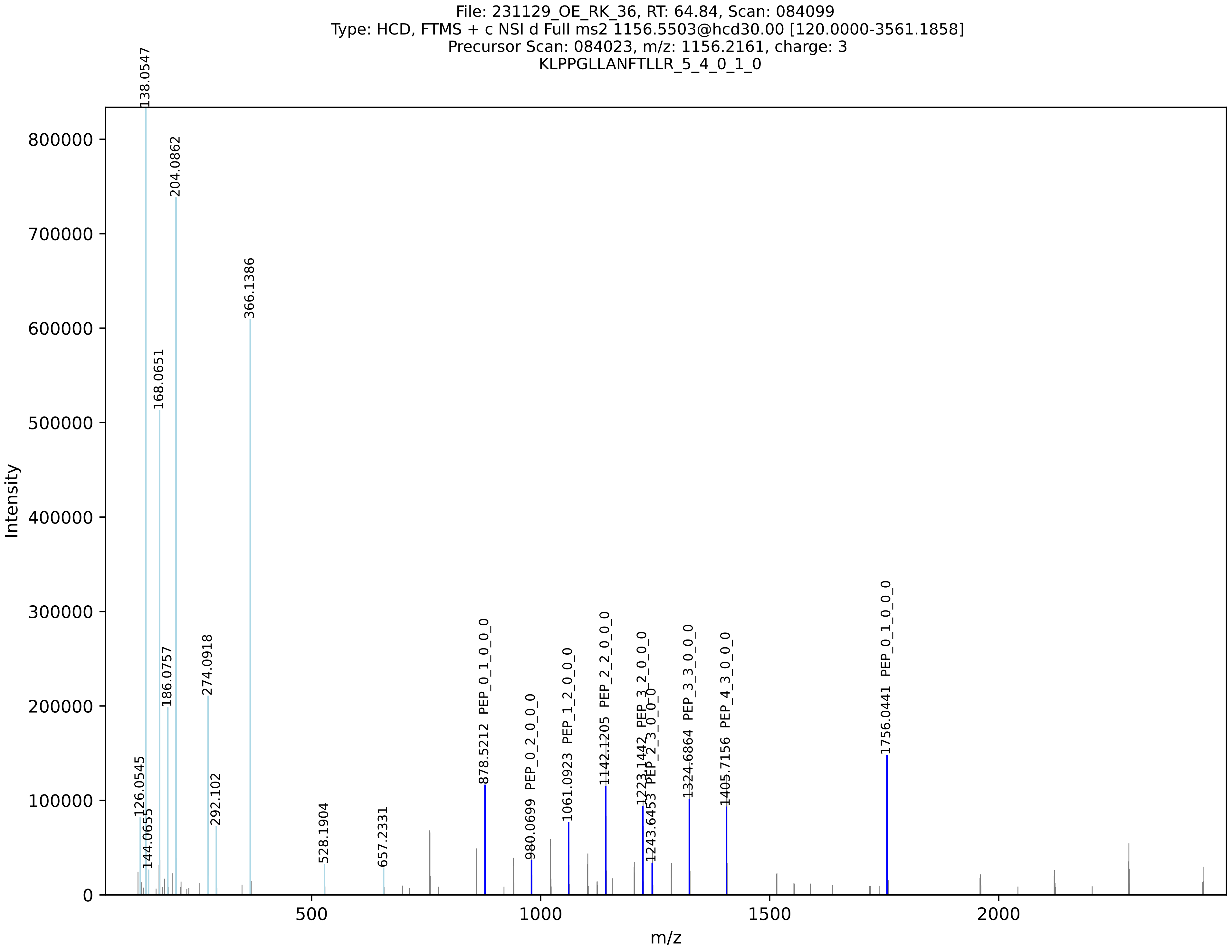
File: 231129_OE_RK_36, RT: 64.84, Scan: 084099
Type: HCD, FTMS + c NSI d Full ms2 1156.5503@hcd30.00 [120.0000-3561.1858]
Precursor Scan: 084023, m/z: 1156.2161, charge: 3
KLPPGLLANFTLLR_5_4_0_1_0
Intensity
m/z
138.0547
204.0862
366.1386
168.0651
186.0757
274.0918
126.0545
144.0655
292.102
528.1904
657.2331
878.5212 PEP_0_1_0_0_0
980.0699 PEP_0_2_0_0_0
1061.0923 PEP_1_2_0_0_0
1142.1205 PEP_2_2_0_0_0
1223.1442 PEP_3_2_0_0_0
1243.6453 PEP_2_3_0_0_0
1324.6864 PEP_3_3_0_0_0
1405.7156 PEP_4_3_0_0_0
1756.0441 PEP_0_1_0_0_0

File: 231129_OE_RK_36, RT: 64.901, Scan: 084189
Type: HCD, FTMS + c NSI d Full ms2 1250.0371@hcd30.00 [120.0000-5123.0073]
Precursor Scan: 084097, m/z: 1249.2853, charge: 4
NISDGFDGIPDNVDAALALPAHSYSGR_6_4_1_1_0

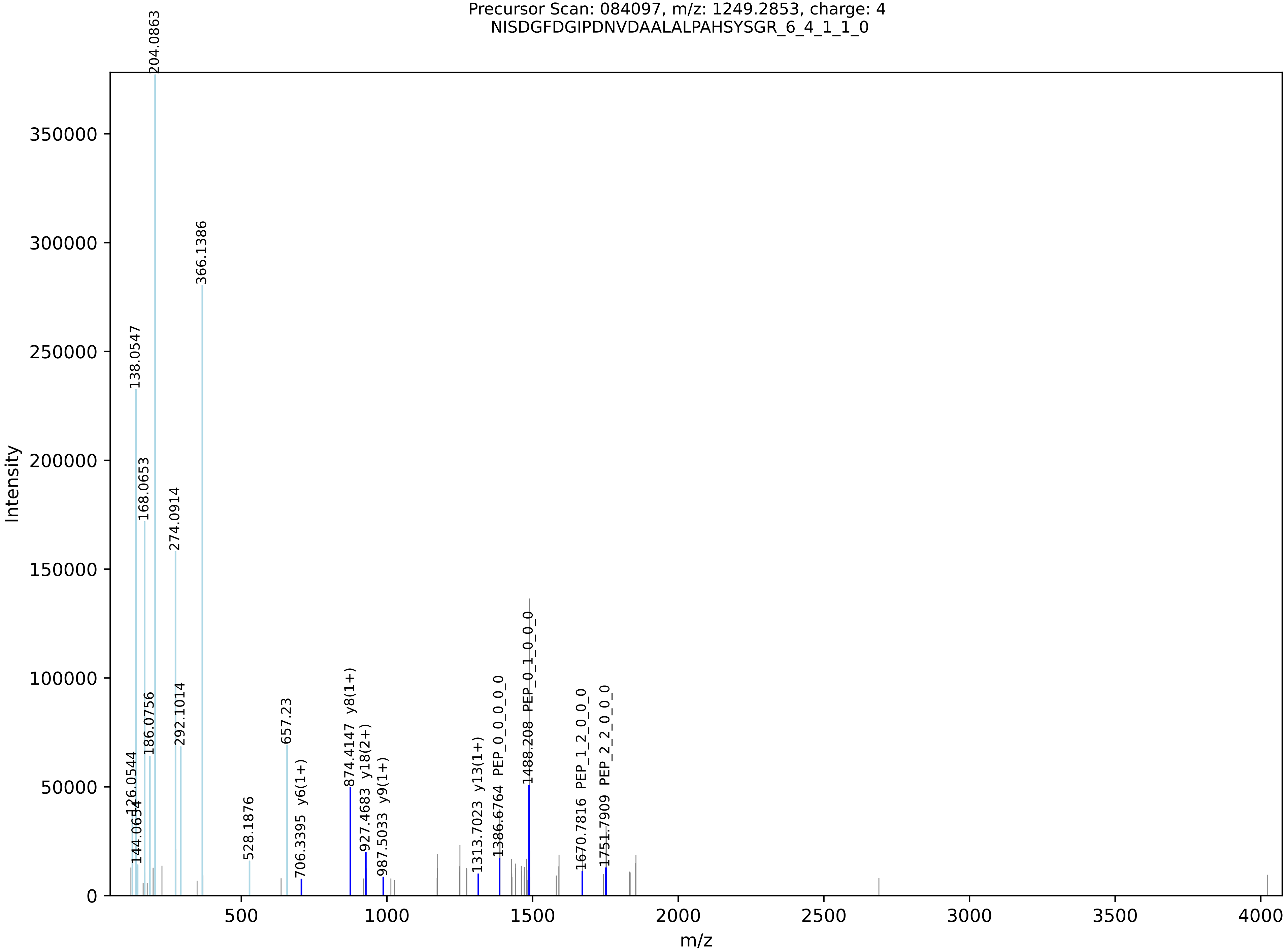

File: 231129_OE_RK_36, RT: 65.045, Scan: 084398
Type: HCD, FTMS + c NSI d Full ms2 1272.8810@hcd30.00 [120.0000-3917.1580]
Precursor Scan: 084319, m/z: 1272.5469, charge: 3
YLGNATAIFFLPDEGK_5_4_1_1_0

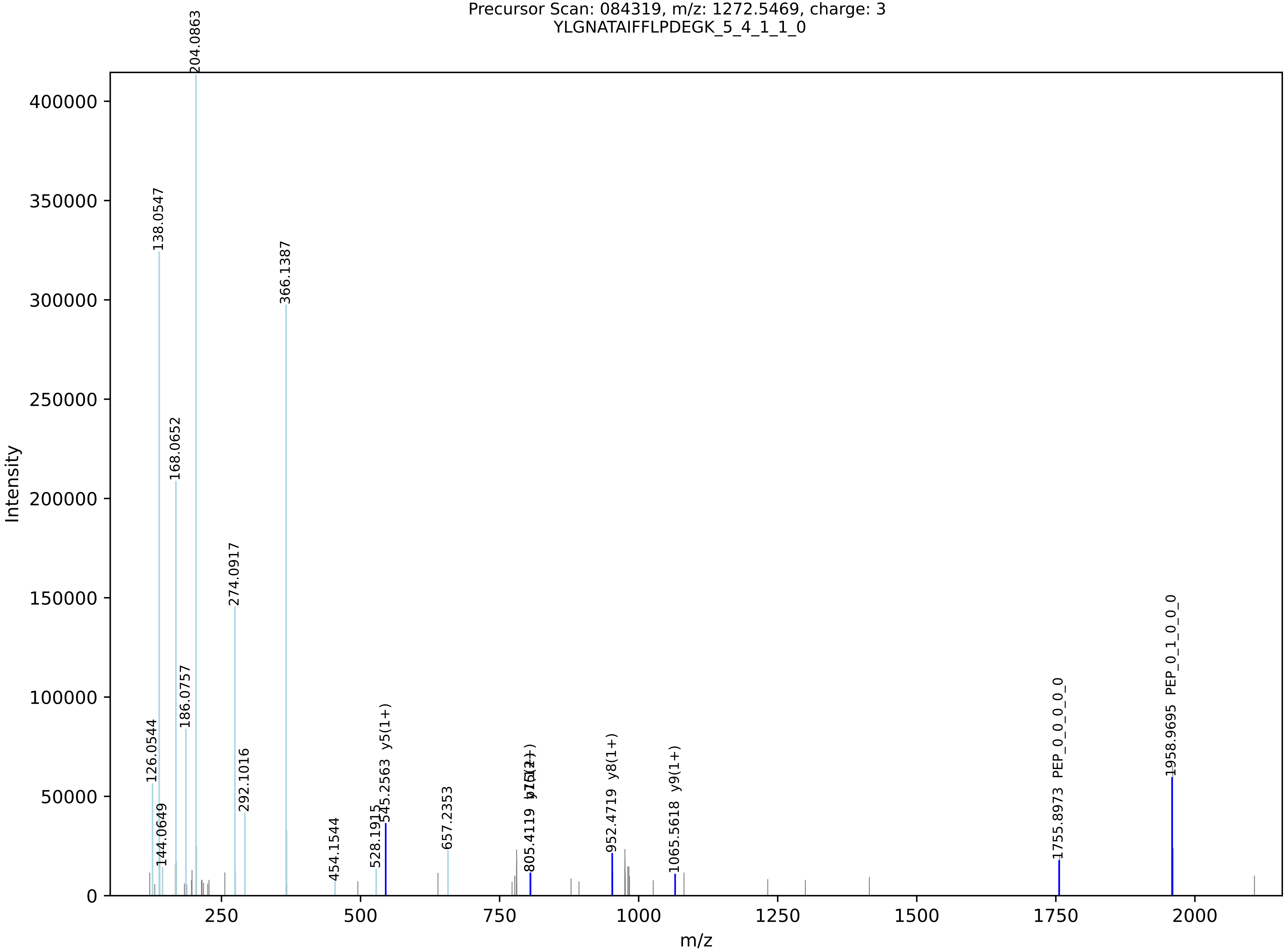

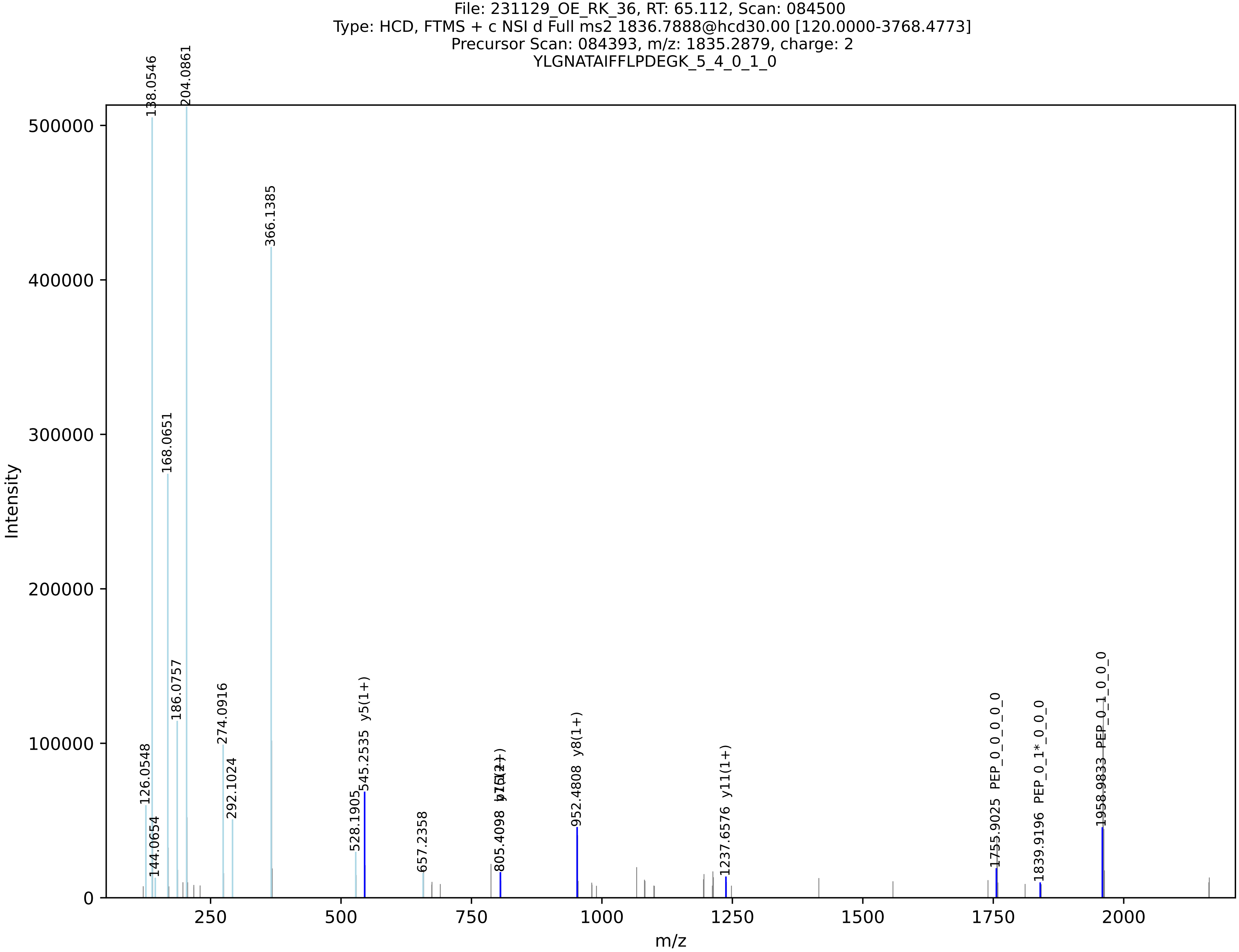
File: 231129_OE_RK_36, RT: 65.112, Scan: 084500
Type: HCD, FTMS + c NSI d Full ms2 1836.7888@hcd30.00 [120.0000-3768.4773]
Precursor Scan: 084393, m/z: 1835.2879, charge: 2
YLGNATAIFFLPDEGK_5_4_0_1_0
Intensity
m/z
138.0546
204.0861
366.1385
168.0651
186.0757
274.0916
292.1024
126.0548
144.0654
545.2535 y5(1+)
528.1905
657.2358
805.4098
952.4808 y8(1+)
1237.6576 y11(1+)
1755.9025 PEP_0_0_0_0_0
1839.9196 PEP_0_1*_0_0_0
1958.9833 PEP_0_1_0_0_0

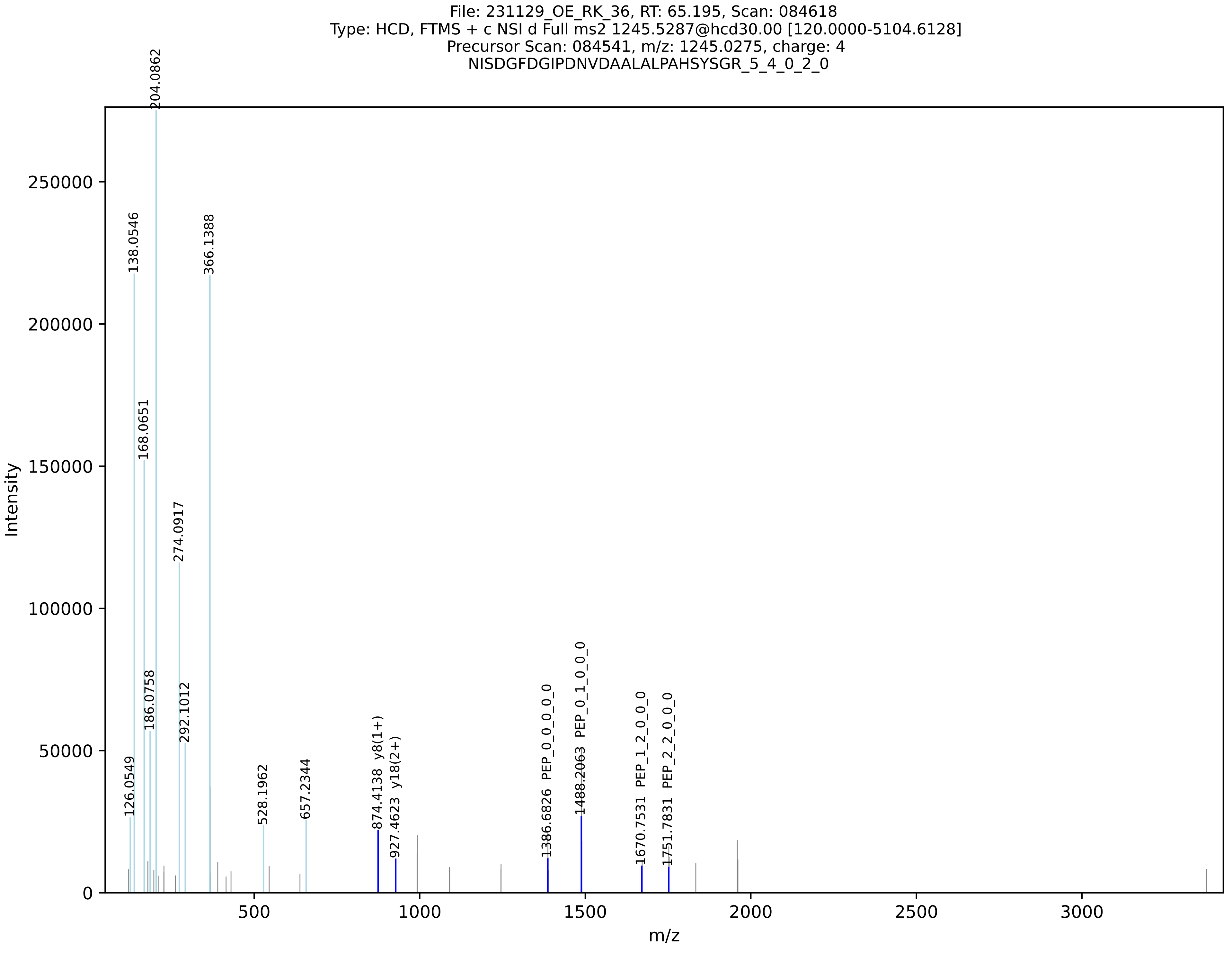
File: 231129_OE_RK_36, RT: 65.195, Scan: 084618
Type: HCD, FTMS + c NSI d Full ms2 1245.5287@hcd30.00 [120.0000-5104.6128]
Precursor Scan: 084541, m/z: 1245.0275, charge: 4
NISDGFDGIPDNVDAALALPAHSYSGR_5_4_0_2_0
Intensity
m/z
204.0862
138.0546
366.1388
168.0651
274.0917
186.0758
292.1012
126.0549
528.1962
657.2344
874.4138 y8(1+)
927.4623 y18(2+)
1386.6826 PEP_0_0_0_0_0
1488.2063 PEP_0_1_0_0_0
1670.7531 PEP_1_2_0_0_0
1751.7831 PEP_2_2_0_0_0
0
50000
100000
150000
200000
250000
500
1000
1500
2000
2500
3000

File: 231129_OE_RK_36, RT: 65.288, Scan: 084755
Type: HCD, FTMS + c NSI d Full ms2 1252.5754@hcd30.00 [120.0000-5133.3638]
Precursor Scan: 084676, m/z: 1252.3282, charge: 4
EVREFLGENISNFLSLAGNTYQLTR_6_5_1_0_0

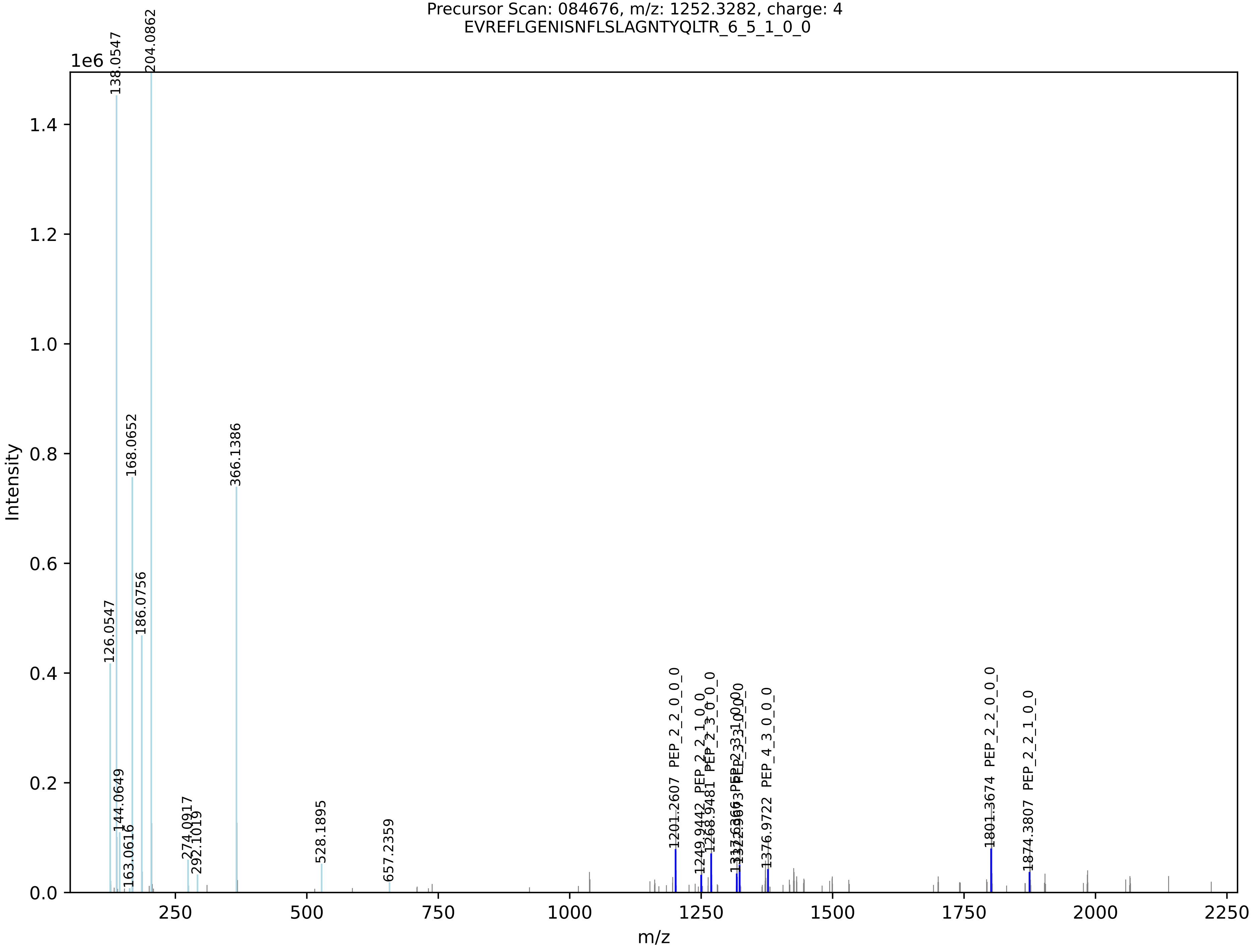

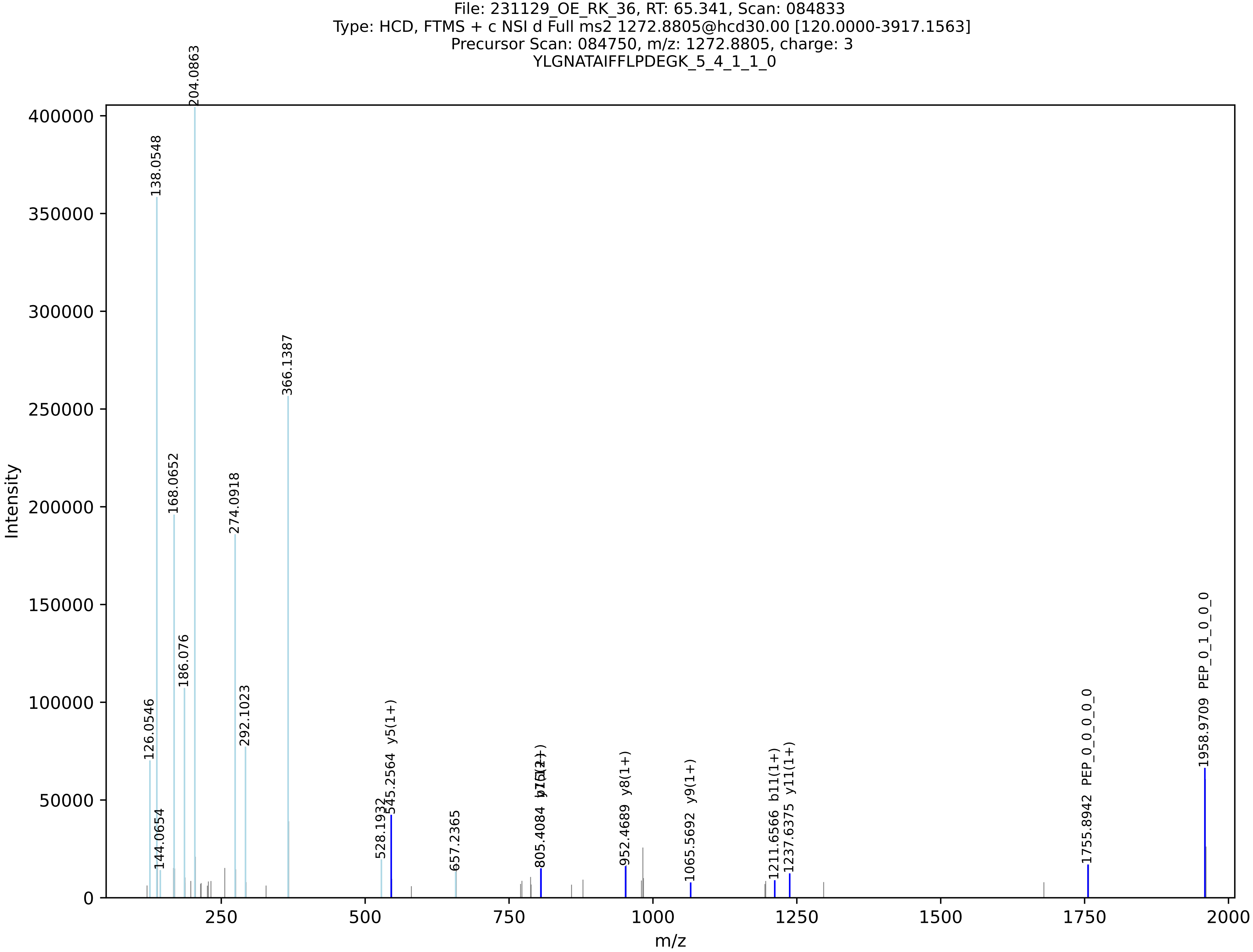

File: 231129_OE_RK_36, RT: 65.341, Scan: 084833
Type: HCD, FTMS + c NSI d Full ms2 1272.8805@hcd30.00 [120.0000-3917.1563]
Precursor Scan: 084750, m/z: 1272.8805, charge: 3
YLGNATAIFFLPDEGK_5_4_1_1_0
Intensity
m/z
0
50000
100000
150000
200000
250000
300000
350000
400000
250
500
750
1000
1250
1500
1750
2000
204.0863
138.0548
366.1387
168.0652
274.0918
186.076
292.1023
126.0546
144.0654
528.1932
545.2564 y5(1+)
657.2365
805.4084
952.4689 y8(1+)
1065.5692 y9(1+)
1211.6566 b11(1+)
1237.6375 y11(1+)
1755.8942 PEP_0_0_0_0_0
1958.9709 PEP_0_1_0_0_0

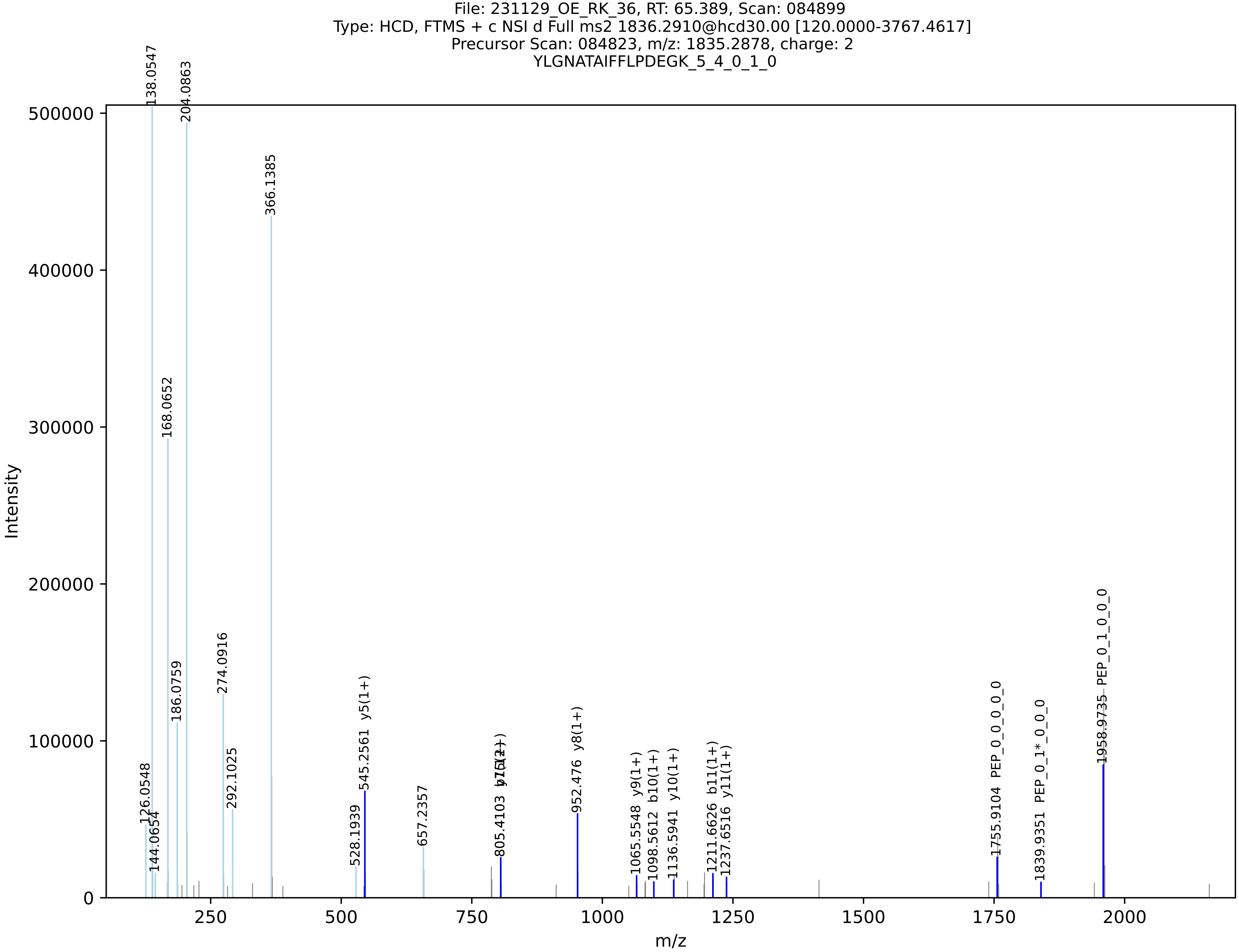

File: 231129_OE_RK_36, RT: 65.389, Scan: 084899
Type: HCD, FTMS + c NSI d Full ms2 1836.2910@hcd30.00 [120.0000-3767.4617]
Precursor Scan: 084823, m/z: 1835.2878, charge: 2
YLGNATAIFFLPDEGK_5_4_0_1_0
Intensity
m/z
138.0547
204.0863
366.1385
168.0652
186.0759
274.0916
292.1025
126.0548
144.0654
528.1939
545.2561 y5(1+)
657.2357
805.4103
952.476 y8(1+)
1065.5548 y9(1+)
1098.5612 b10(1+)
1136.5941 y10(1+)
1211.6626 b11(1+)
1237.6516 y11(1+)
1755.9104 PEP_0_0_0_0_0
1839.9351 PEP_0_1*_0_0_0
1958.9735 PEP_0_1_0_0_0

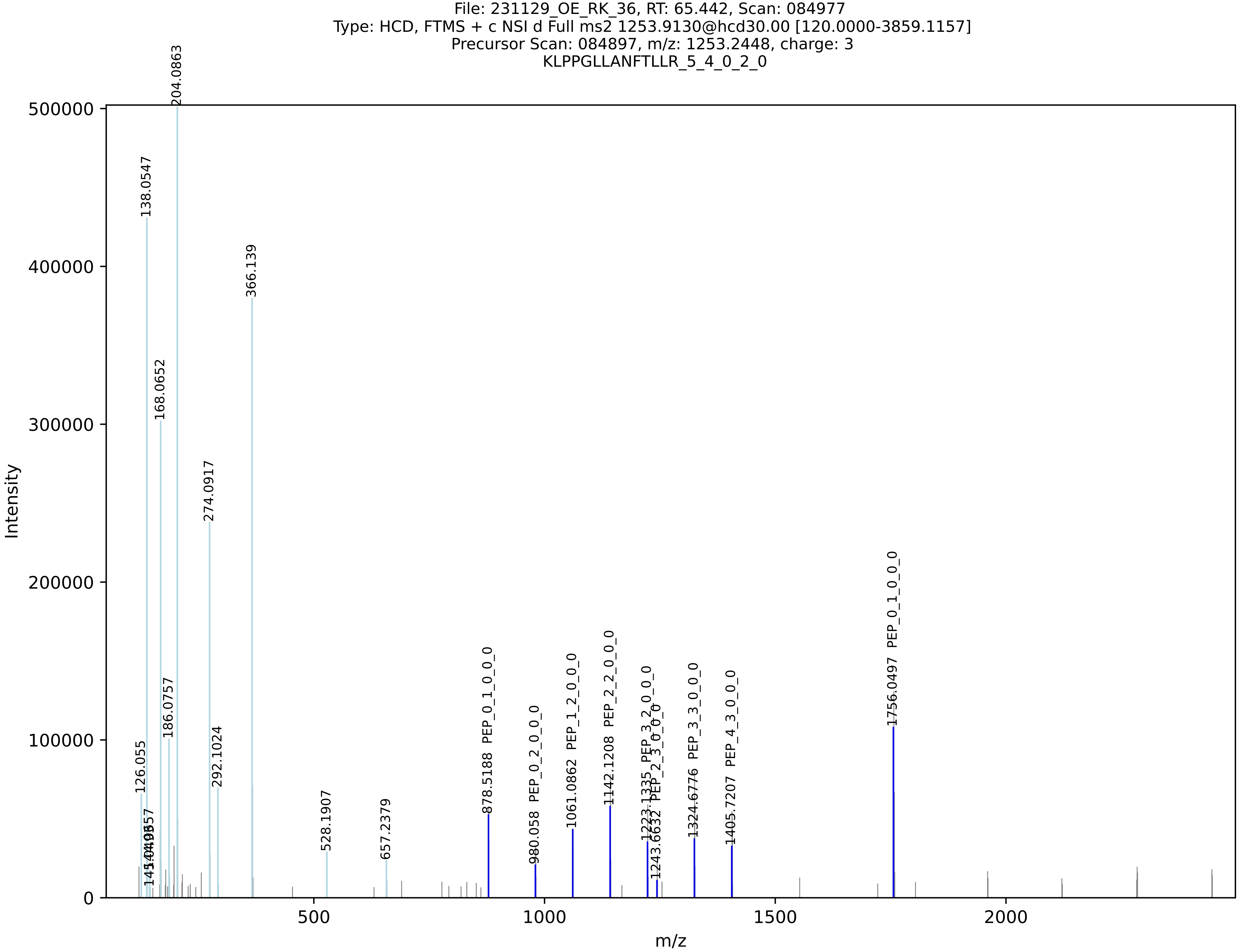
File: 231129_OE_RK_36, RT: 65.442, Scan: 084977
Type: HCD, FTMS + c NSI d Full ms2 1253.9130@hcd30.00 [120.0000-3859.1157]
Precursor Scan: 084897, m/z: 1253.2448, charge: 3
KLPPGLLANFTLLR_5_4_0_2_0
204.0863
138.0547
366.139
168.0652
274.0917
186.0757
126.055
292.1024
528.1907
657.2379
878.5188 PEP_0_1_0_0_0_
980.058 PEP_0_2_0_0_0_
1061.0862 PEP_1_2_0_0_0_
1142.1208 PEP_2_2_0_0_0_
1223.1335 PEP_3_2_0_0_0_
1243.6632 PEP_2_3_0_0_0_
1324.6776 PEP_3_3_0_0_0_
1405.7207 PEP_4_3_0_0_0_
1756.0497 PEP_0_1_0_0_0_
Intensity
m/z
0
100000
200000
300000
400000
500000
500
1000
1500
2000

File: 231129_OE_RK_36, RT: 65.524, Scan: 085096
Type: HCD, FTMS + c NSI d Full ms2 1028.2440@hcd30.00 [120.0000-5267.6147]
Precursor Scan: 085031, m/z: 1027.6433, charge: 5
YNFSFRYIYPLDSLTWIEYWPR_5_4_0_2_0

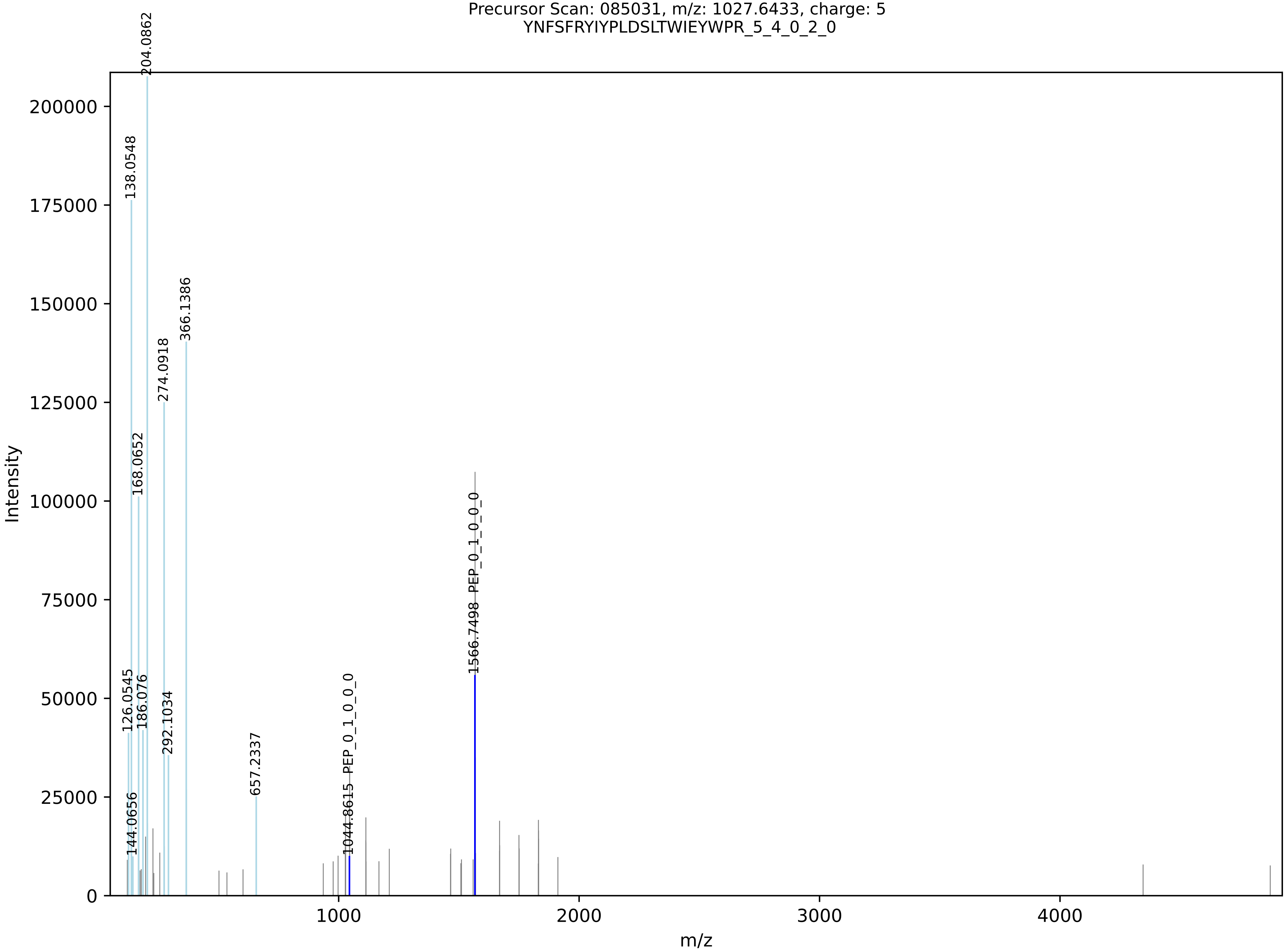

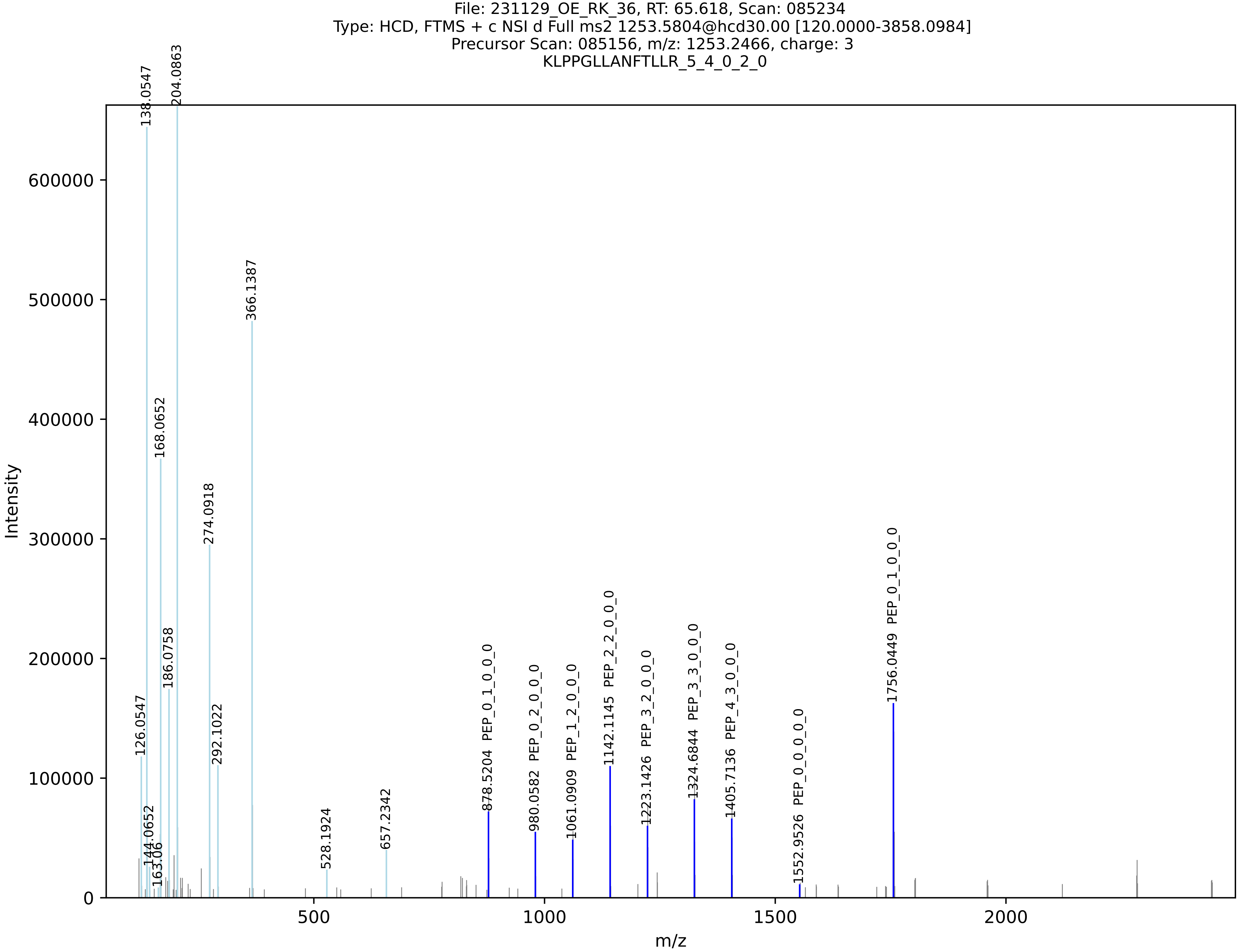
File: 231129_OE_RK_36, RT: 65.618, Scan: 085234
Type: HCD, FTMS + c NSI d Full ms2 1253.5804@hcd30.00 [120.0000-3858.0984]
Precursor Scan: 085156, m/z: 1253.2466, charge: 3
KLPPGLLANFTLLR_5_4_0_2_0
Intensity
m/z
138.0547
204.0863
366.1387
168.0652
274.0918
186.0758
126.0547
292.1022
144.0652
163.06
528.1924
657.2342
878.5204 PEP_0_1_0_0_0
980.0582 PEP_0_2_0_0_0
1061.0909 PEP_1_2_0_0_0
1142.1145 PEP_2_2_0_0_0
1223.1426 PEP_3_2_0_0_0
1324.6844 PEP_3_3_0_0_0
1405.7136 PEP_4_3_0_0_0
1552.9526 PEP_0_0_0_0_0
1756.0449 PEP_0_1_0_0_

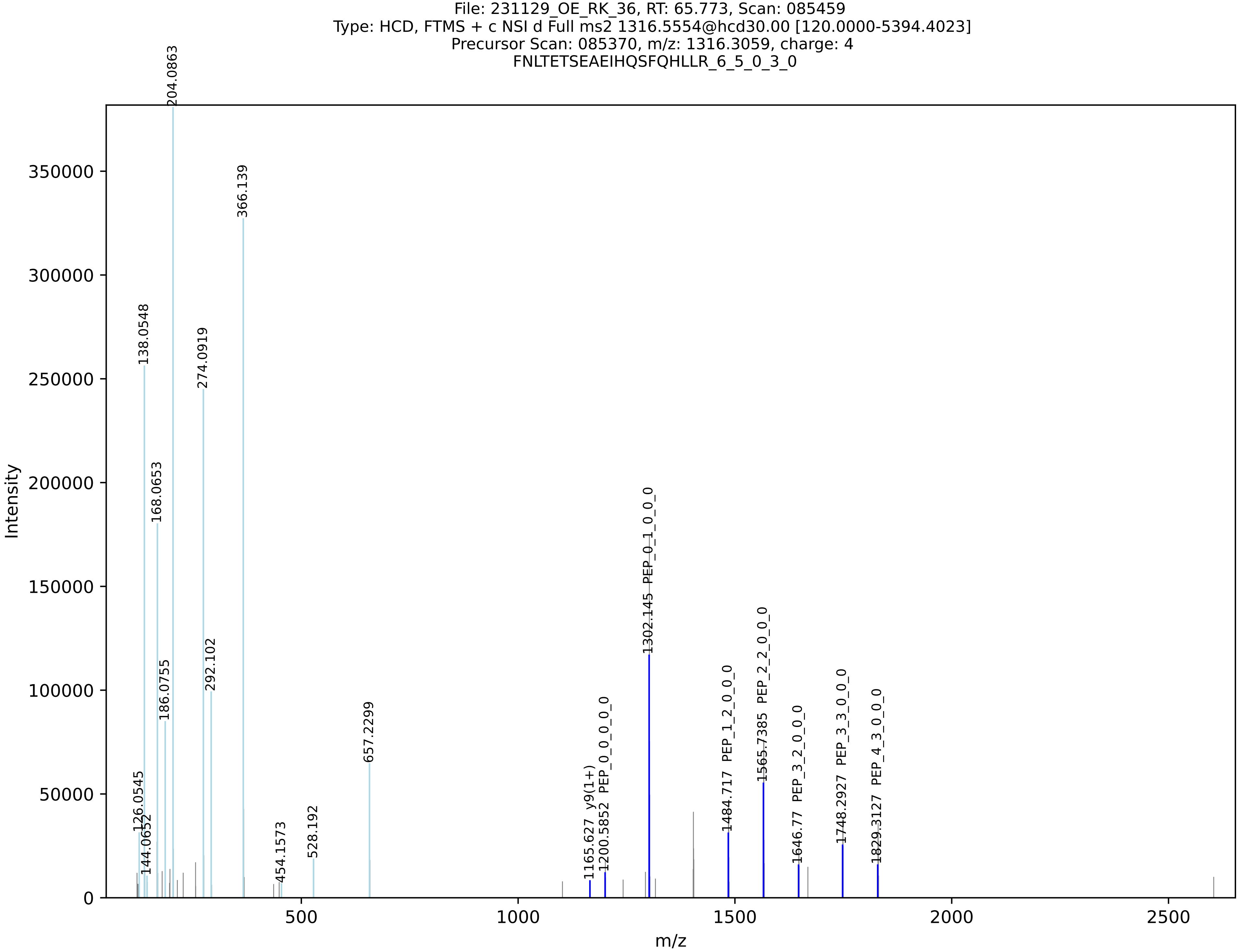
File: 231129_OE_RK_36, RT: 65.773, Scan: 085459
Type: HCD, FTMS + c NSI d Full ms2 1316.5554@hcd30.00 [120.0000-5394.4023]
Precursor Scan: 085370, m/z: 1316.3059, charge: 4
FNLTETSEAEIHQSFQHLLR_6_5_0_3_0
Intensity
m/z
204.0863
366.139
138.0548
274.0919
168.0653
186.0755
292.102
126.0545
144.0652
454.1573
528.192
657.2299
1165.627 y9(1+)
1200.5852 PEP_0_0_0_0_0
1302.145 PEP_0_1_0_0_0
1484.717 PEP_1_2_0_0_0
1565.7385 PEP_2_2_0_0_0
1646.77 PEP_3_2_0_0_0
1748.2927 PEP_3_3_0_0_0
1829.3127 PEP_4_3_0_0_0

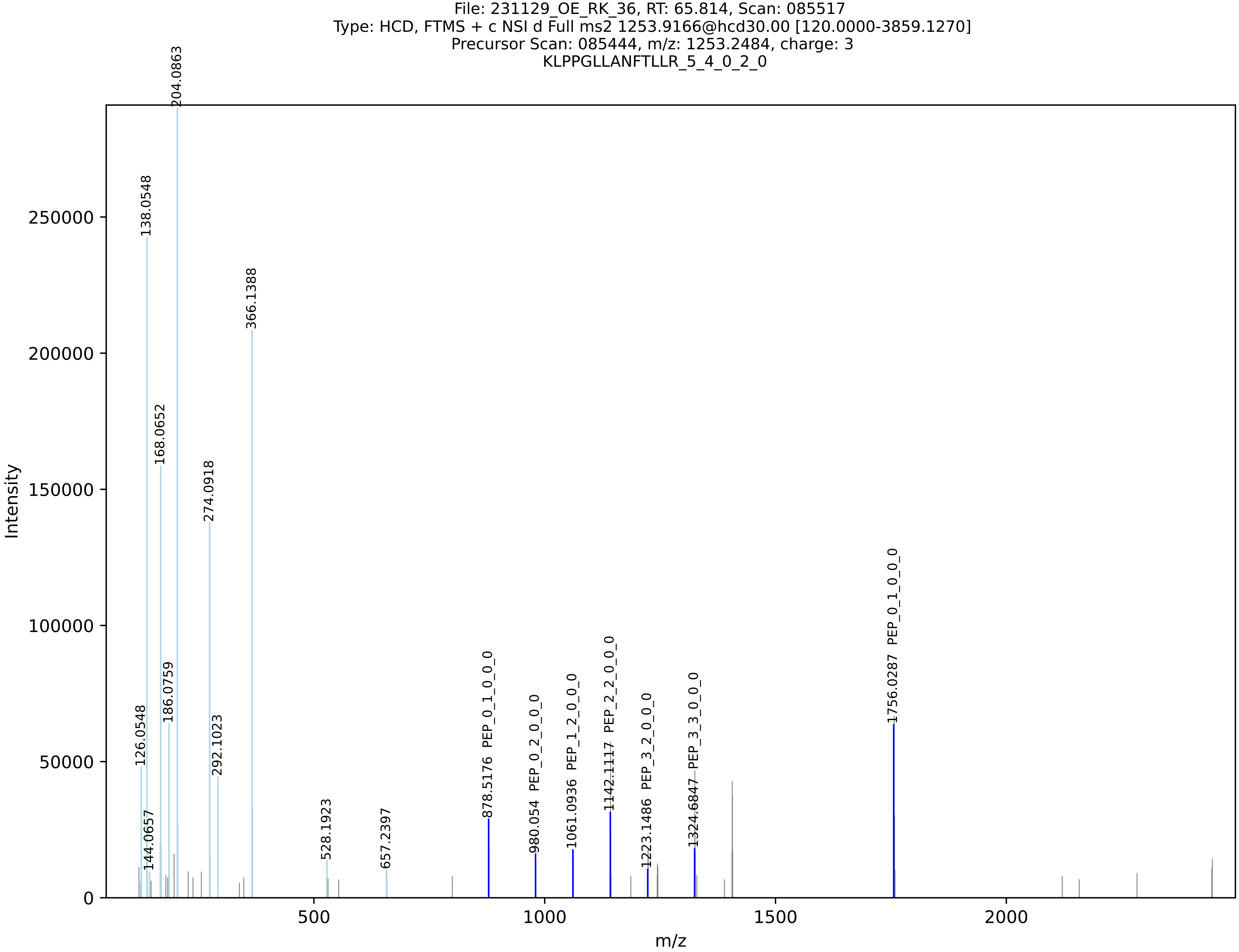

File: 231129_OE_RK_36, RT: 65.814, Scan: 085517
Type: HCD, FTMS + c NSI d Full ms2 1253.9166@hcd30.00 [120.0000-3859.1270]
Precursor Scan: 085444, m/z: 1253.2484, charge: 3
KLPPGLLANFTLLR_5_4_0_2_0
Intensity
m/z
0
50000
100000
150000
200000
250000
500
1000
1500
2000
204.0863
138.0548
366.1388
168.0652
274.0918
186.0759
126.0548
292.1023
144.0657
528.1923
657.2397
878.5176 PEP_0_1_0_0_0
980.054 PEP_0_2_0_0_0
1061.0936 PEP_1_2_0_0_0
1142.1117 PEP_2_2_0_0_0
1223.1486 PEP_3_2_0_0_0
1324.6847 PEP_3_3_0_0_0
1756.0287 PEP_0_1_0_0_0

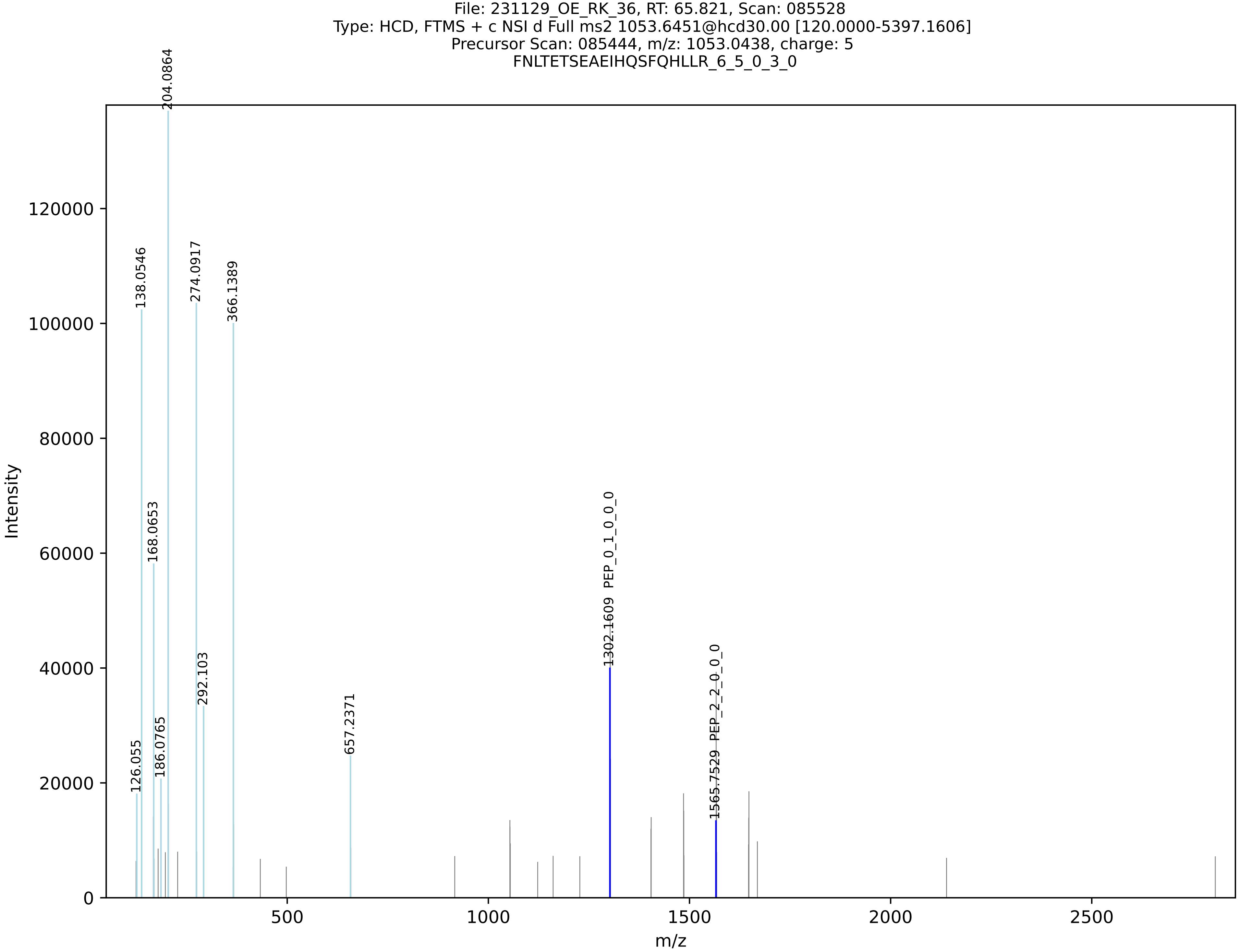
File: 231129_OE_RK_36, RT: 65.821, Scan: 085528
Type: HCD, FTMS + c NSI d Full ms2 1053.6451@hcd30.00 [120.0000-5397.1606]
Precursor Scan: 085444, m/z: 1053.0438, charge: 5
FNLTETSEAEIHQSFQHLLR_6_5_0_3_0
204.0864
138.0546
274.0917
366.1389
168.0653
292.103
186.0765
126.055
657.2371
1302.1609 PEP_0_1_0_0_0
1565.7529 PEP_2_2_0_0_0
Intensity
m/z
0
20000
40000
60000
80000
100000
120000
500
1000
1500
2000
2500

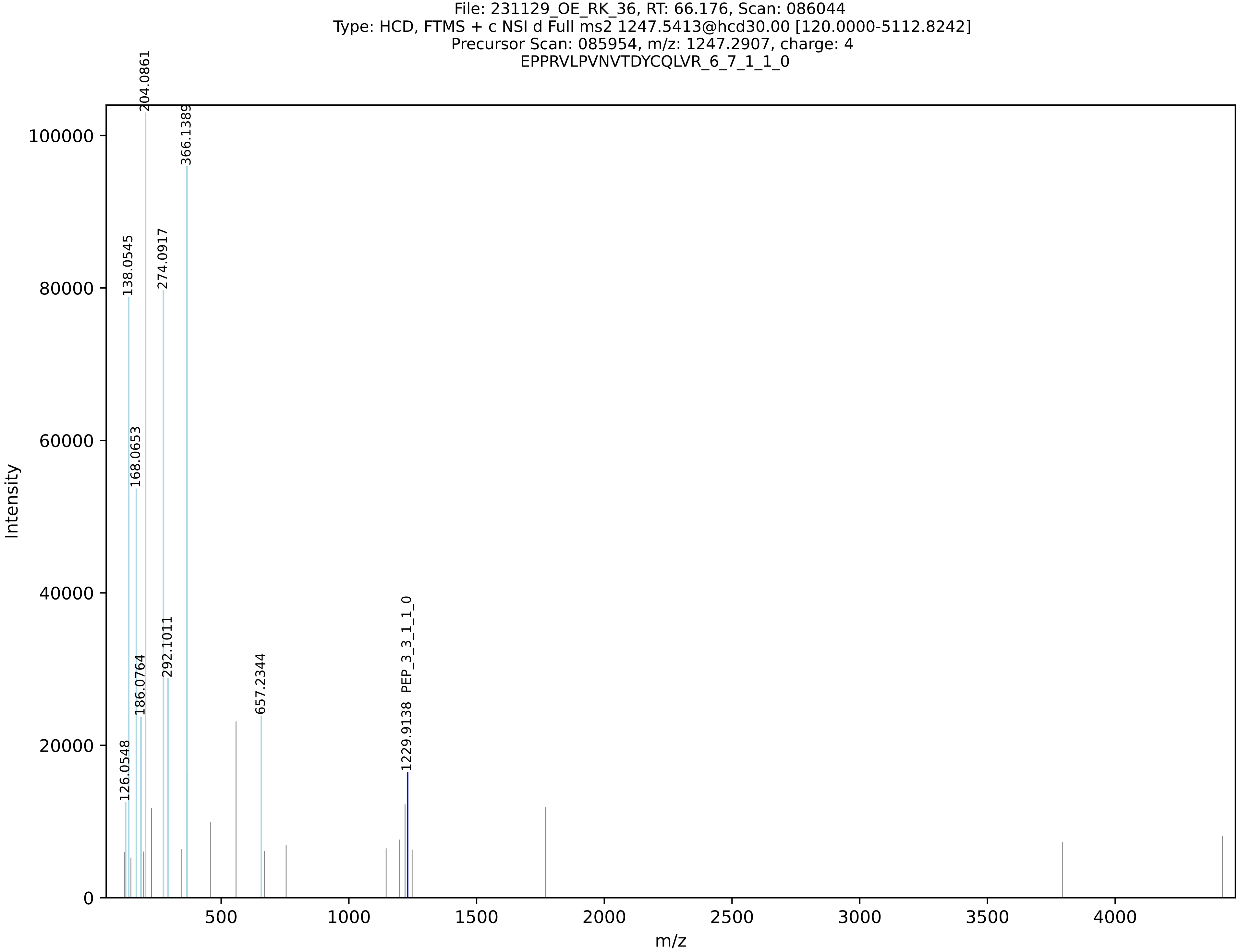
File: 231129_OE_RK_36, RT: 66.176, Scan: 086044
Type: HCD, FTMS + c NSI d Full ms2 1247.5413@hcd30.00 [120.0000-5112.8242]
Precursor Scan: 085954, m/z: 1247.2907, charge: 4
EPPRVLPVNVTDYCQLVR_6_7_1_1_0
204.0861
366.1389
138.0545
274.0917
168.0653
292.1011
186.0764
657.2344
PEP_3_3_1_1_0
1229.9138
126.0548
Intensity
m/z
0
20000
40000
60000
80000
100000
500
1000
1500
2000
2500
3000
3500
4000

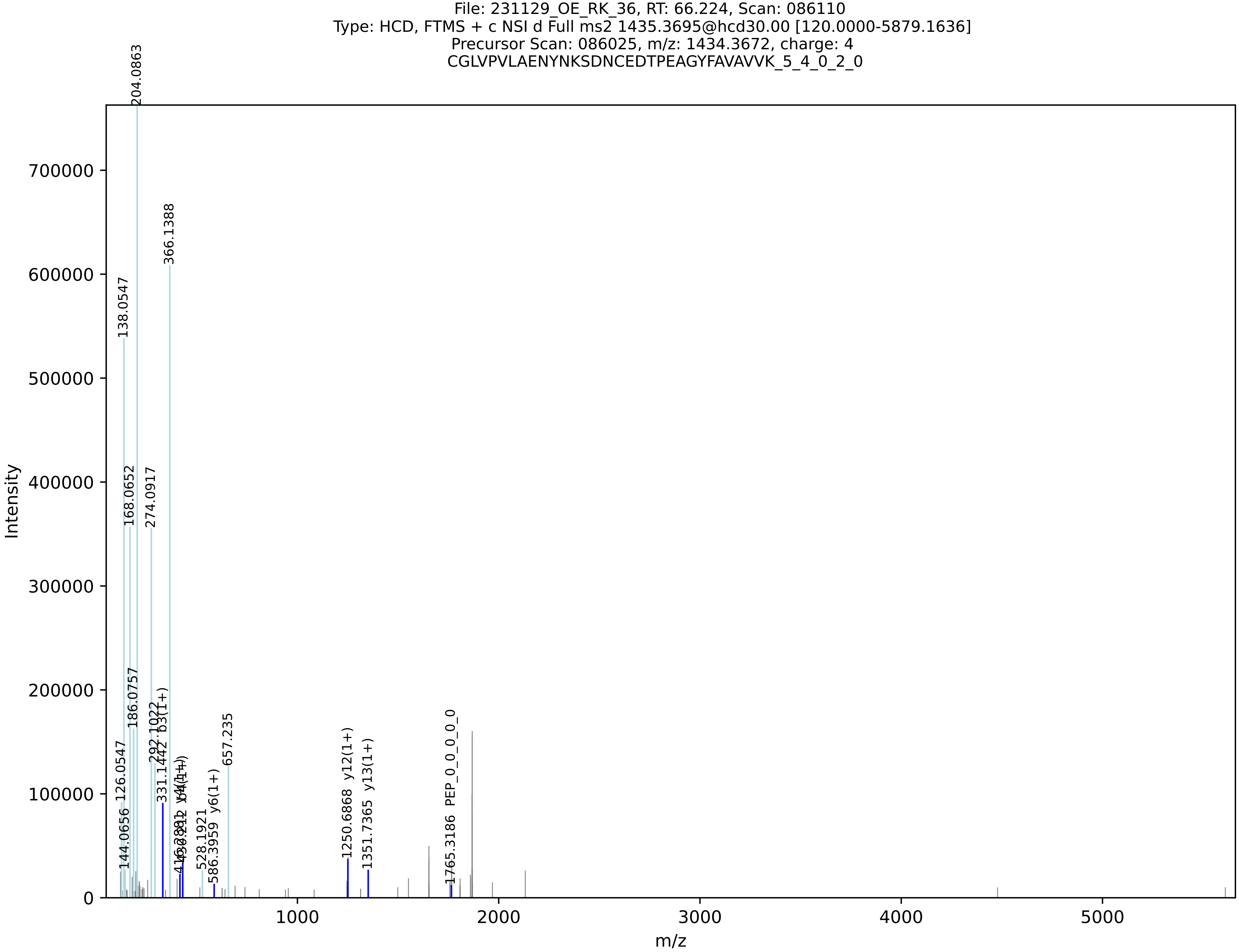
File: 231129_OE_RK_36, RT: 66.224, Scan: 086110
Type: HCD, FTMS + c NSI d Full ms2 1435.3695@hcd30.00 [120.0000-5879.1636]
Precursor Scan: 086025, m/z: 1434.3672, charge: 4
CGLVPVLAENYNKSDNCEDTPEAGYFAVAVVK_5_4_0_2_0
204.0863
366.1388
138.0547
168.0652
274.0917
186.0757
292.1022
331.1442 b3(1+)
126.0547
144.0656
657.235
528.1921
586.3959 y6(1+)
1250.6868 y12(1+)
1351.7365 y13(1+)
1765.3186 PEP_0_0_0_0_0
Intensity
m/z

File: 231129_OE_RK_36, RT: 66.33, Scan: 086262
Type: HCD, FTMS + c NSI d Full ms2 1345.8351@hcd30.00 [120.0000-5513.8628]
Precursor Scan: 086170, m/z: 1345.0833, charge: 4
DKICDLLVANNHFAHFFAPQNLTNMNK_5_4_0_2_0

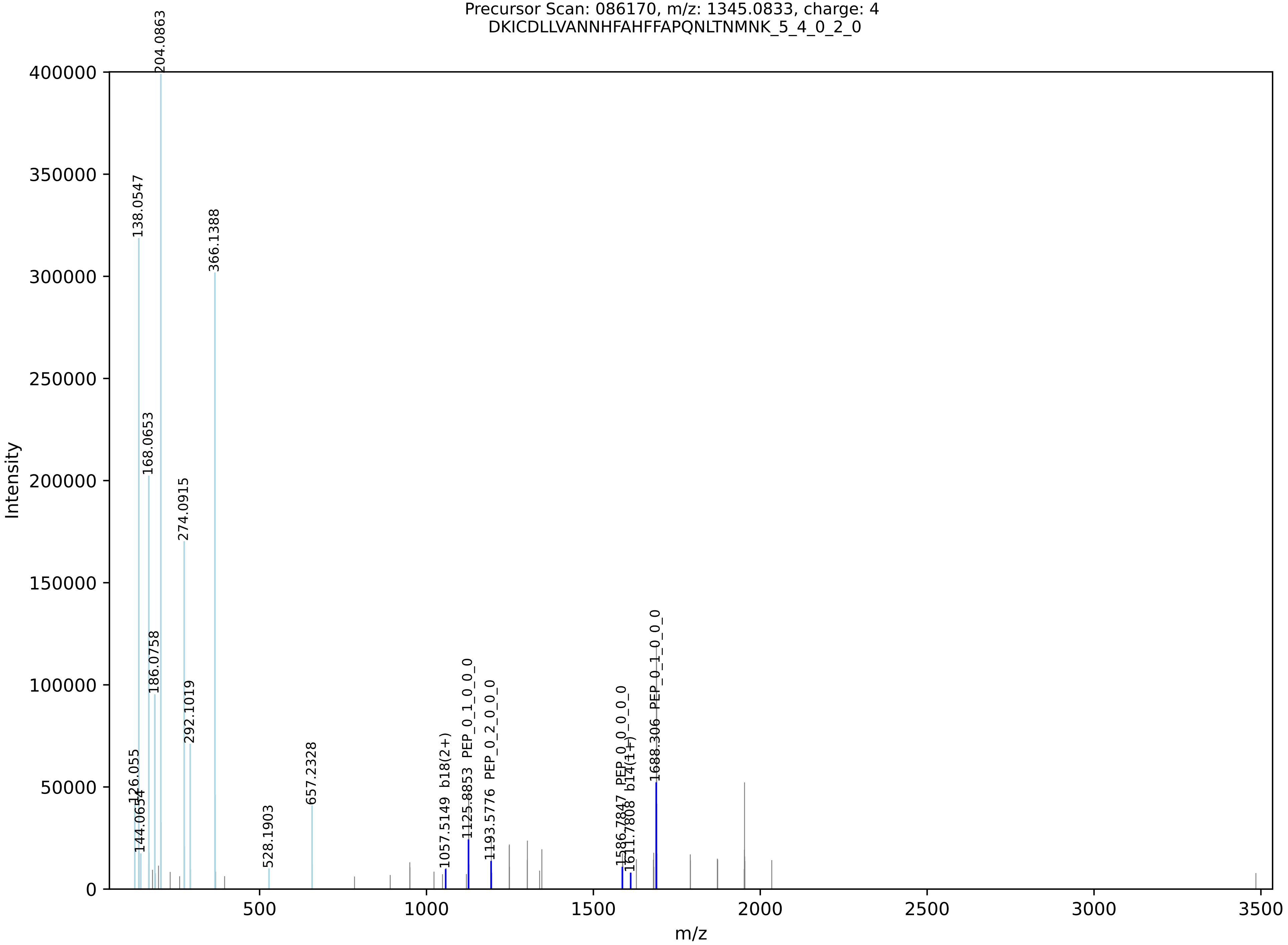

File: 231129_OE_RK_36, RT: 67.042, Scan: 087276
Type: HCD, FTMS + c NSI d Full ms2 1459.0419@hcd30.00 [120.0000-4486.8101]
Precursor Scan: 087184, m/z: 1458.3721, charge: 3
VGQLQLSHNLSLVILVPQNLK_5_4_1_1_0

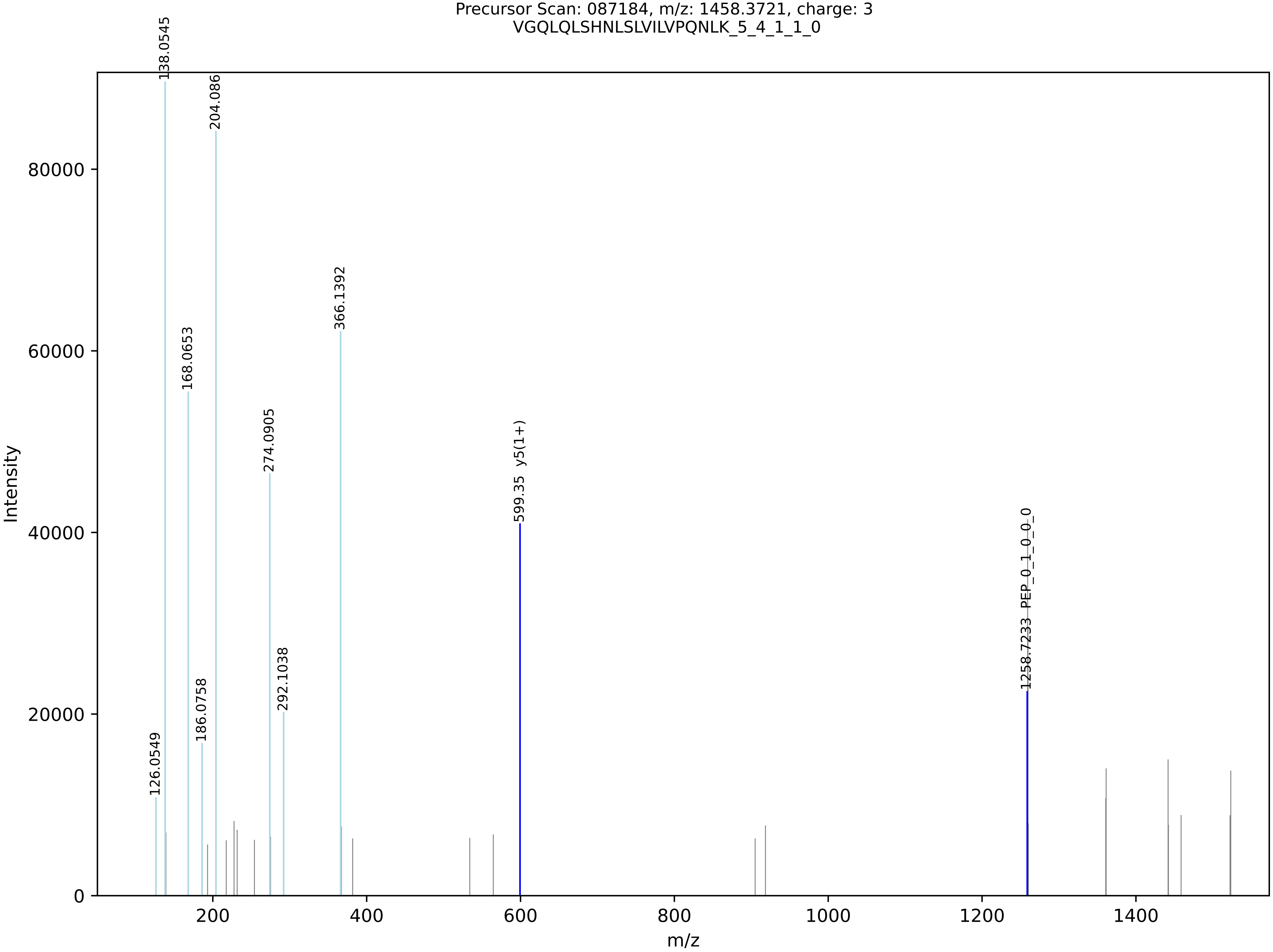

File: 231129_OE_RK_36, RT: 72.222, Scan: 094780
Type: HCD, FTMS + c NSI d Full ms2 1386.6080@hcd30.00 [120.0000-5680.2168]
Precursor Scan: 094701, m/z: 1385.6056, charge: 4
SVQEIQATFFYFTPNKTEDTIFLR_7_6_0_1_0

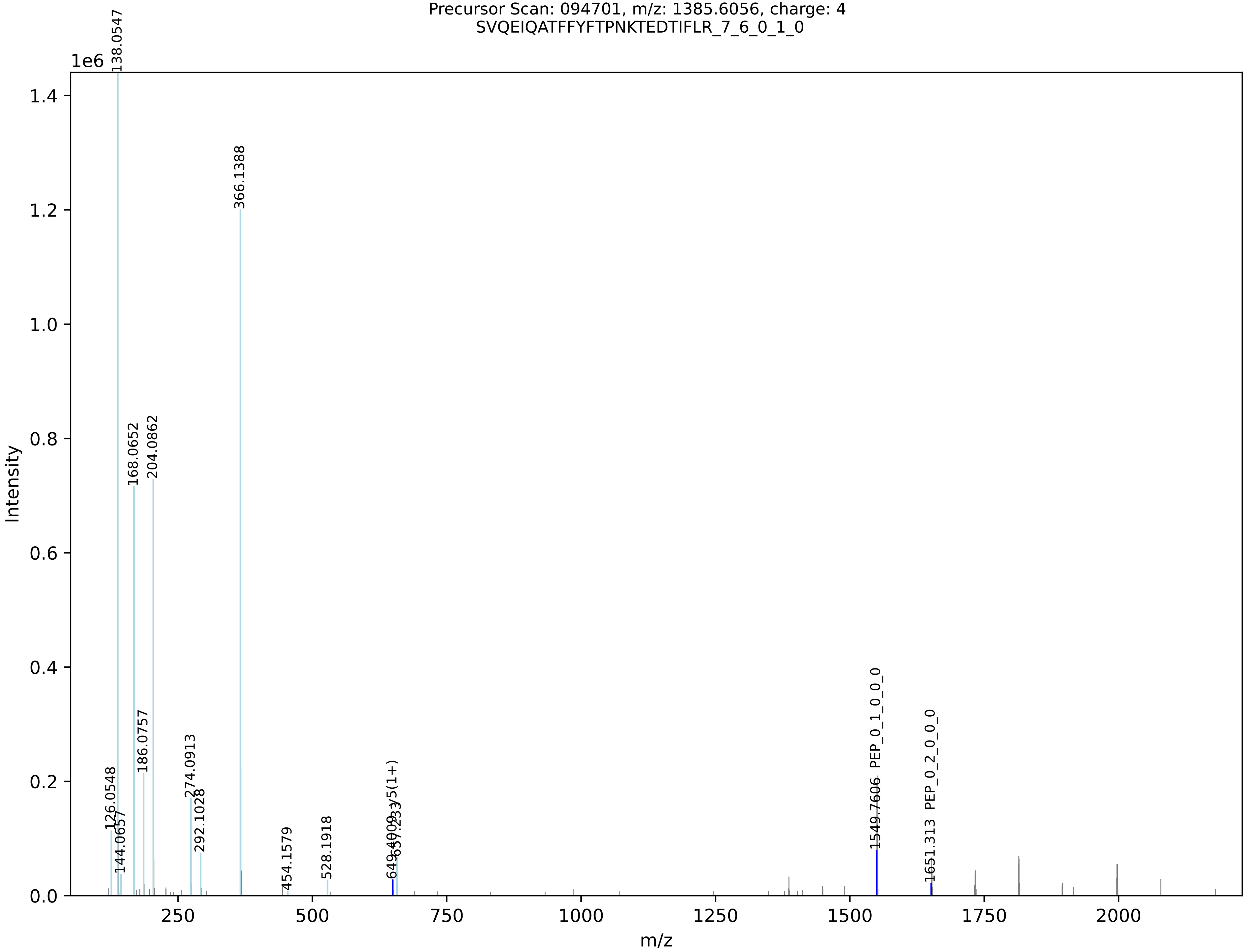

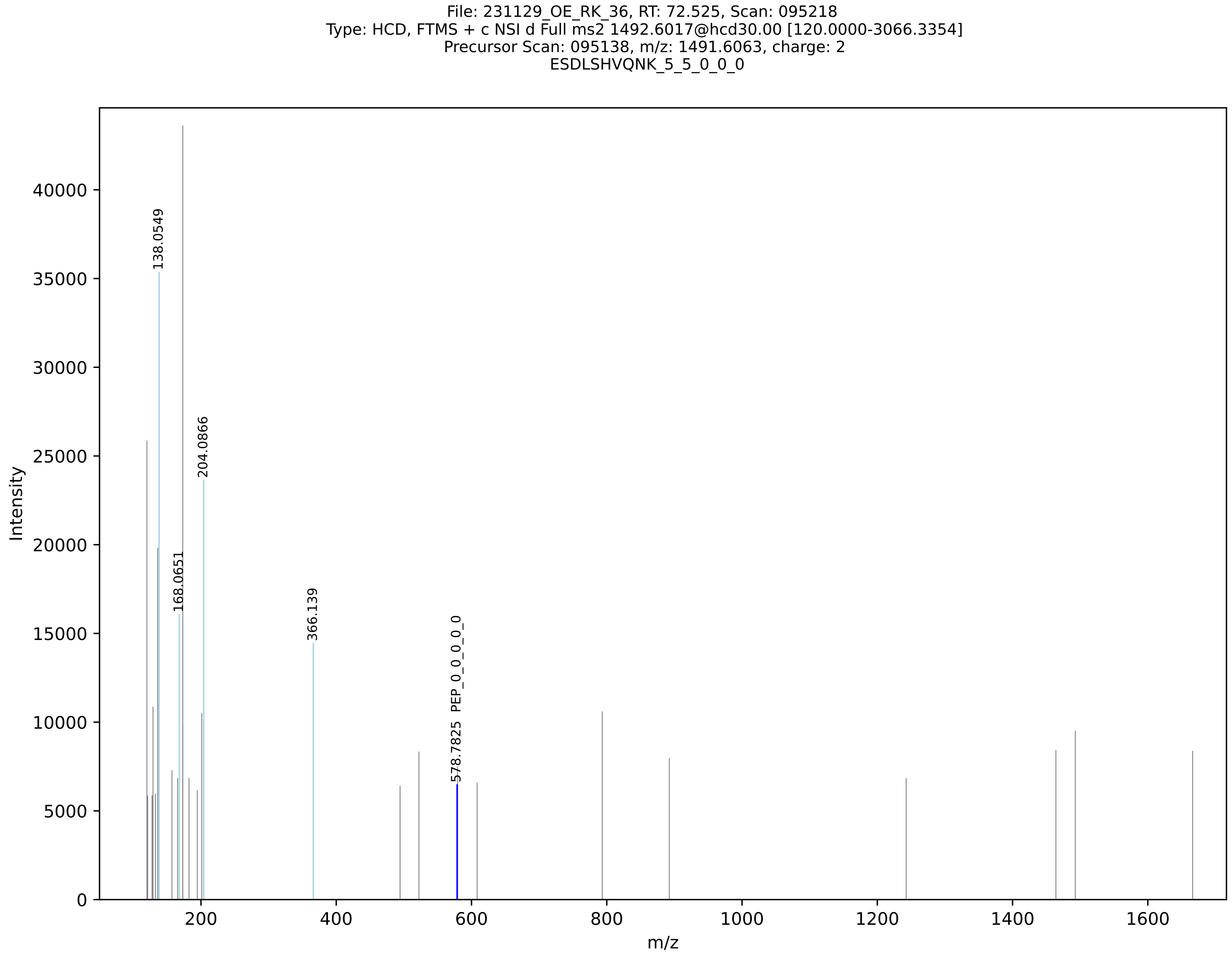
File: 231129_OE_RK_36, RT: 72.525, Scan: 095218
Type: HCD, FTMS + c NSI d Full ms2 1492.6017@hcd30.00 [120.0000-3066.3354]
Precursor Scan: 095138, m/z: 1491.6063, charge: 2
ESDLSHVQNK_5_5_0_0_0
Intensity
m/z
138.0549
204.0866
168.0651
366.139
578.7825 PEP_0_0_0_0_0
0
5000
10000
15000
20000
25000
30000
35000
40000
200
400
600
800
1000
1200
1400
1600

File: 231129_OE_RK_36, RT: 72.574, Scan: 095290
Type: HCD, FTMS + c NSI d Full ms2 1295.0724@hcd30.00 [120.0000-5306.7515]
Precursor Scan: 095212, m/z: 1294.3206, charge: 4
SVQEIQATFFYFTPNKTEDTIFLR_6_5_0_1_0

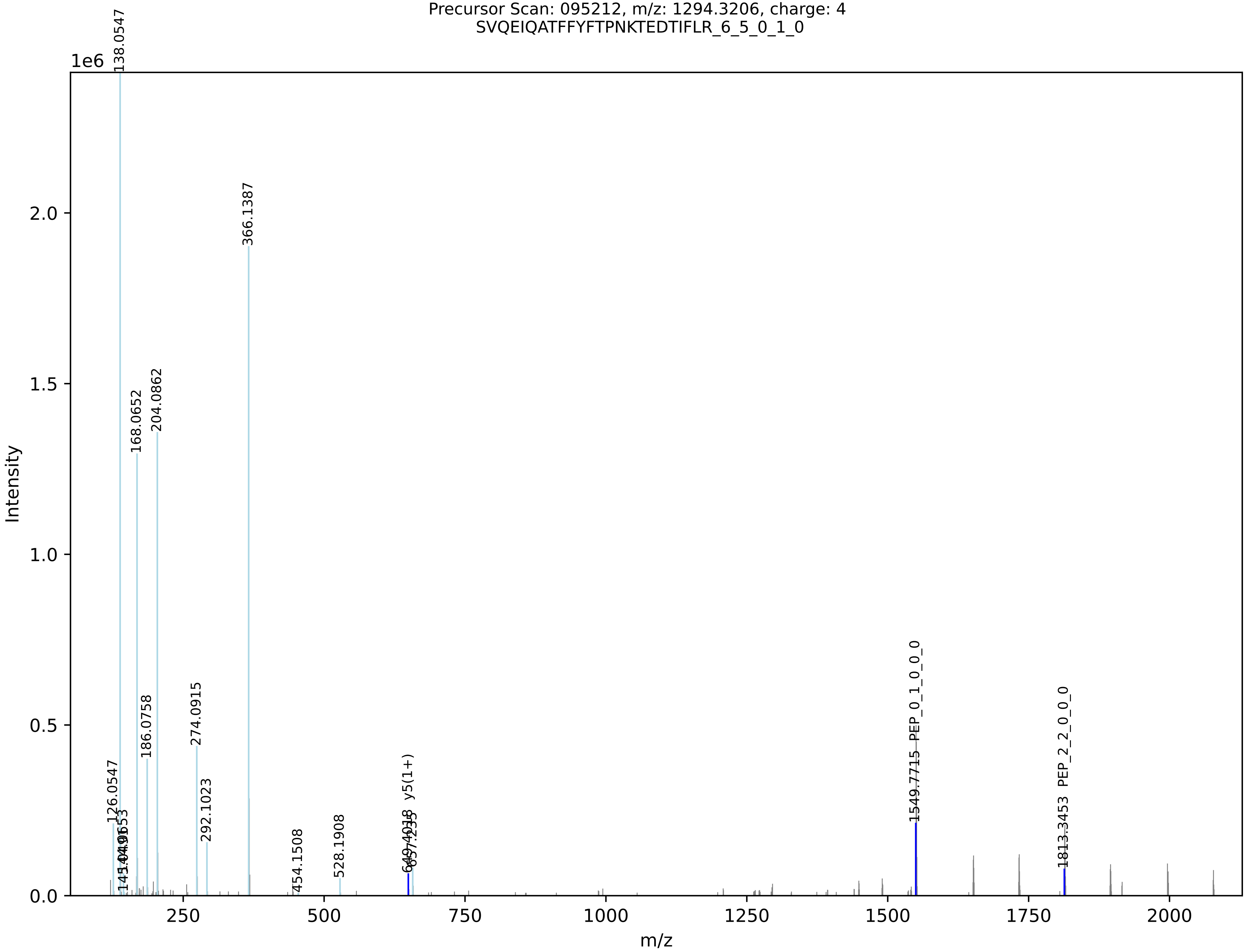

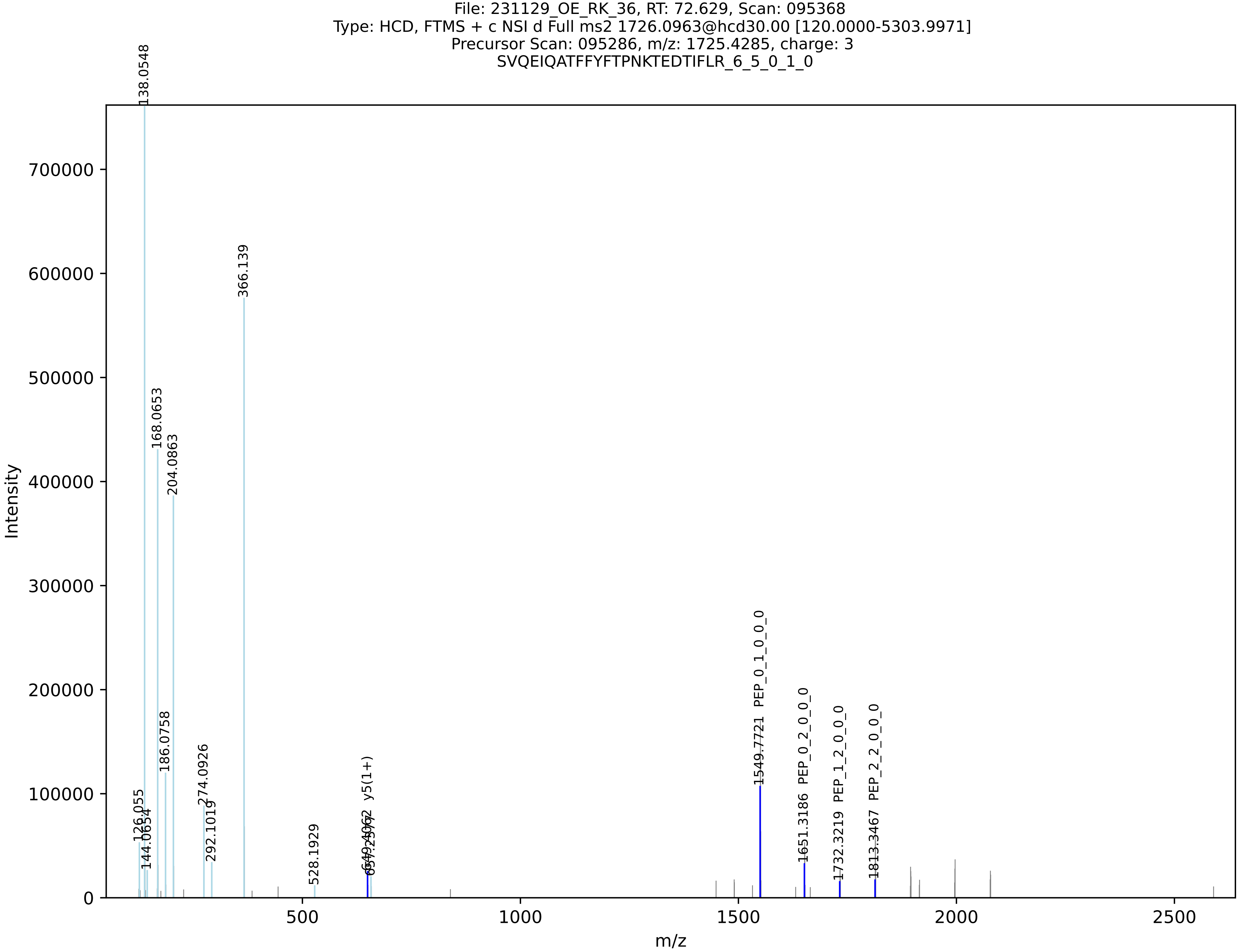
File: 231129_OE_RK_36, RT: 72.629, Scan: 095368
Type: HCD, FTMS + c NSI d Full ms2 1726.0963@hcd30.00 [120.0000-5303.9971]
Precursor Scan: 095286, m/z: 1725.4285, charge: 3
SVQEIQATFFYFTPNKTEDTIFLR_6_5_0_1_0
138.0548
366.139
168.0653
204.0863
186.0758
274.0926
126.055
144.0654
292.1019
528.1929
y5(1+)
1549.7721 PEP_0_1_0_0_0
1651.3186 PEP_0_2_0_0_0
1732.3219 PEP_1_2_0_0_0
1813.3467 PEP_2_2_0_0_0
Intensity
m/z
0
100000
200000
300000
400000
500000
600000
700000
500
1000
1500
2000
2500

File: 231129_OE_RK_36, RT: 72.83, Scan: 095658
Type: HCD, FTMS + c NSI d Full ms2 1543.7422@hcd30.00 [120.0000-4745.9932]
Precursor Scan: 095578, m/z: 1543.0745, charge: 3
LSLHRPALEDLLLGSEANLTCTLTGLR_4_5_0_0_0

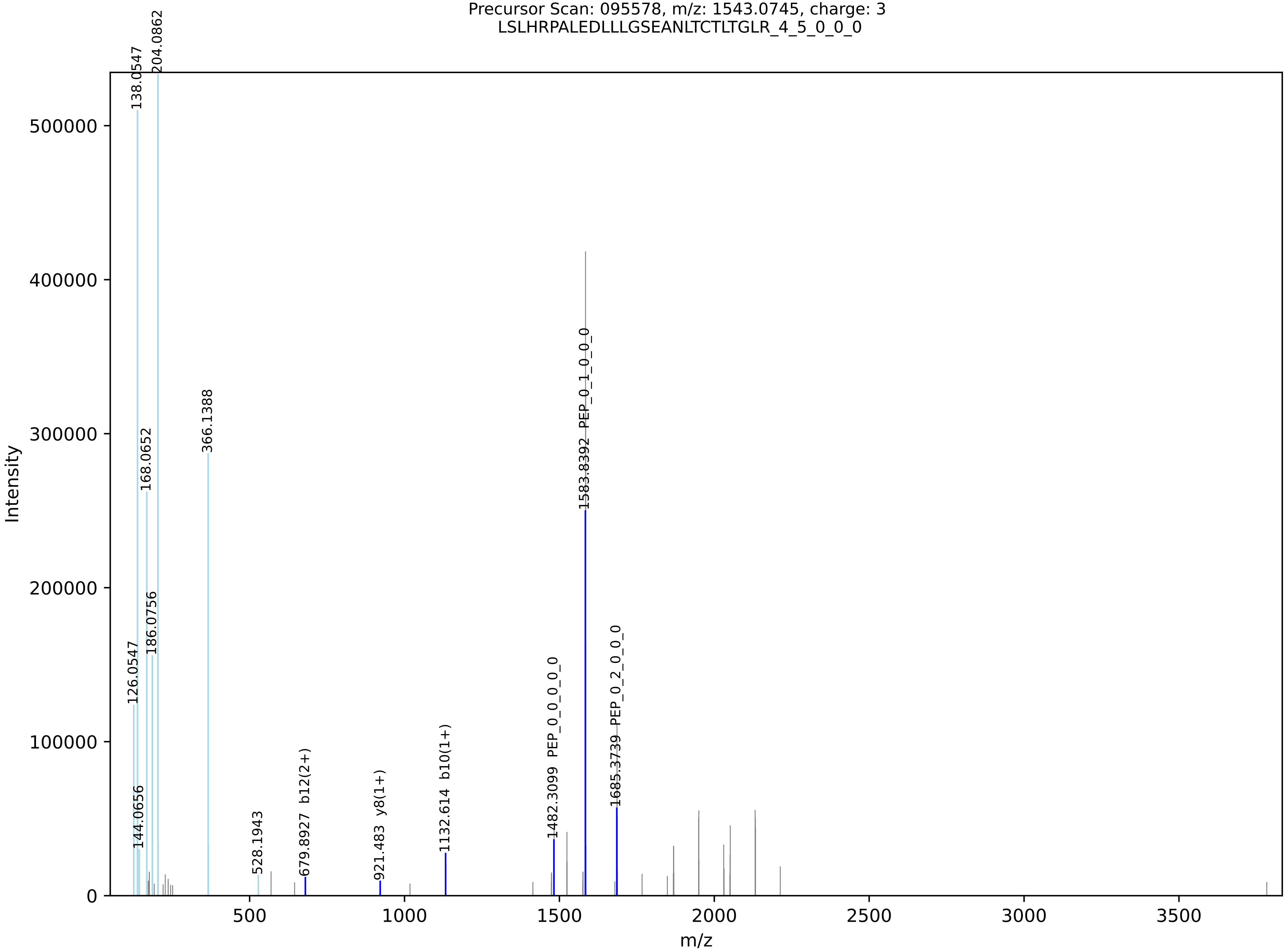

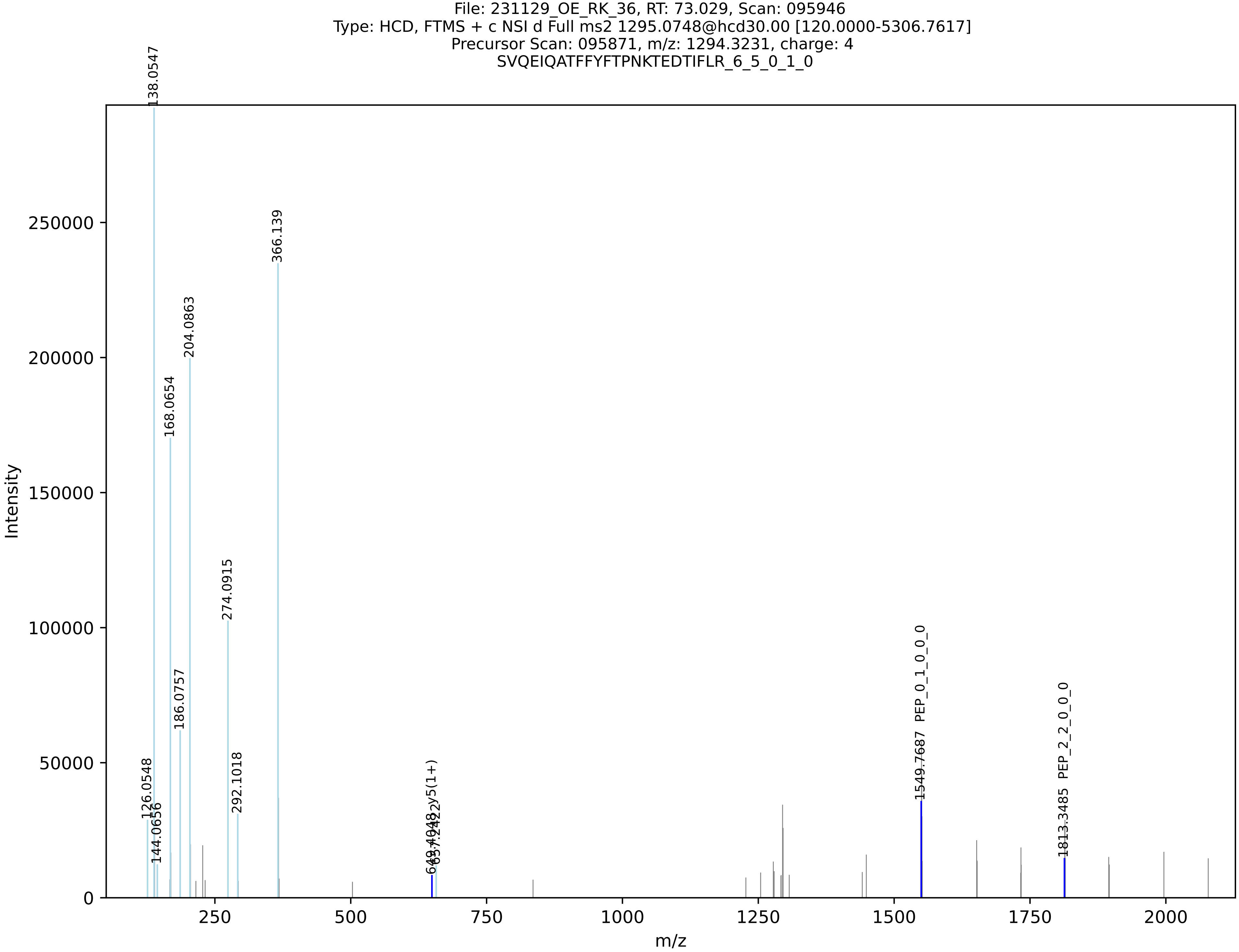
File: 231129_OE_RK_36, RT: 73.029, Scan: 095946
Type: HCD, FTMS + c NSI d Full ms2 1295.0748@hcd30.00 [120.0000-5306.7617]
Precursor Scan: 095871, m/z: 1294.3231, charge: 4
SVQEIQATFFYFTPNKTEDTIFLR_6_5_0_1_0
Intensity
m/z
0
50000
100000
150000
200000
250000
250
500
750
1000
1250
1500
1750
2000
138.0547
366.139
204.0863
168.0654
274.0915
186.0757
126.0548
144.0656
292.1018
649.4048 y5(1+)
657.2422
1549.7687 PEP_0_1_0_0_0
1813.3485 PEP_2_2_0_0_0

File: 231129_OE_RK_36, RT: 73.039, Scan: 095963
Type: HCD, FTMS + c NSI d Full ms2 1543.7391@hcd30.00 [120.0000-4745.9834]
Precursor Scan: 095871, m/z: 1543.074, charge: 3
NLTVTETQDAVFTVELTHPNVKGVQWIK_4_4_0_0_0

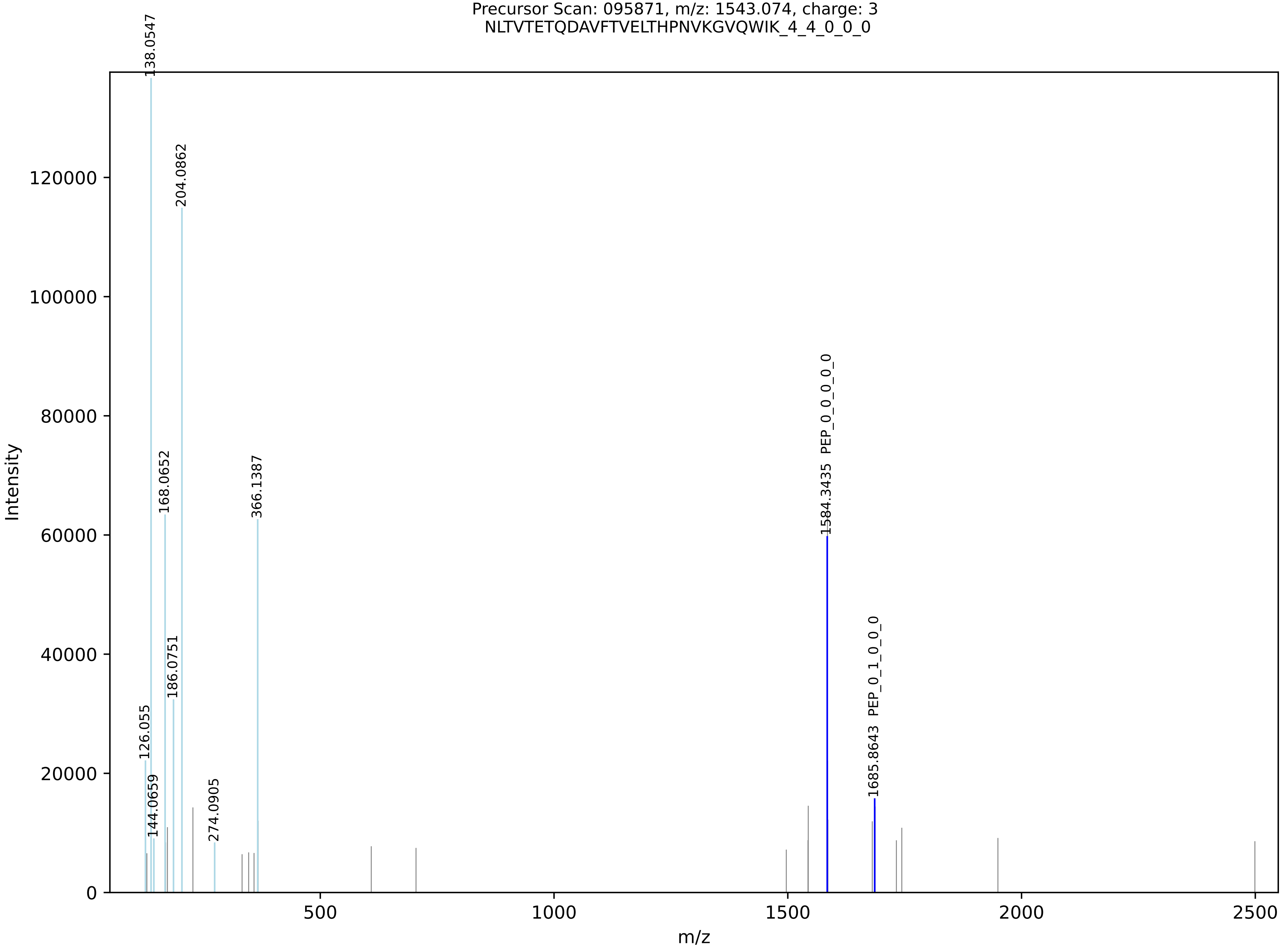

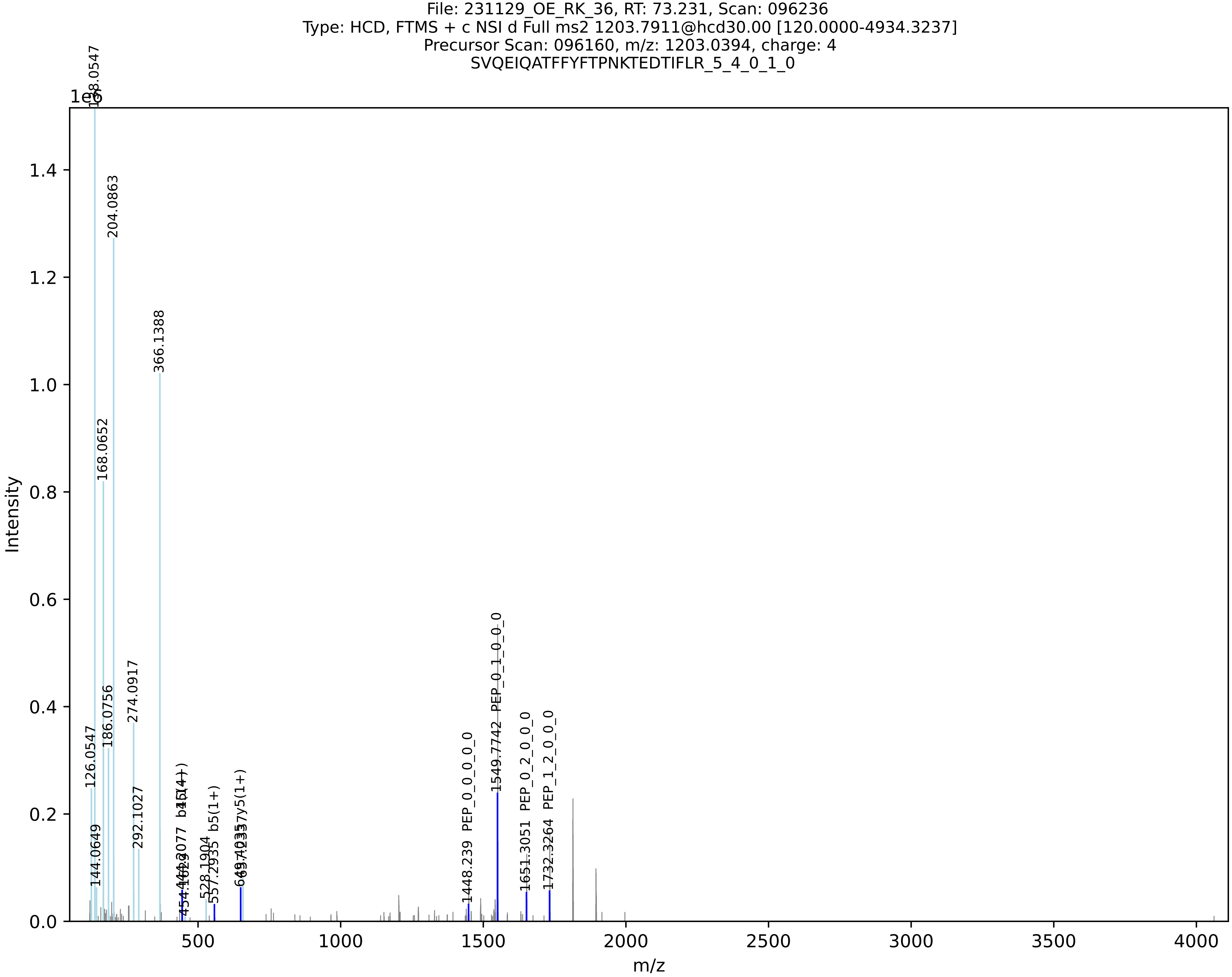

File: 231129_OE_RK_36, RT: 73.231, Scan: 096236
Type: HCD, FTMS + c NSI d Full ms2 1203.7911@hcd30.00 [120.0000-4934.3237]
Precursor Scan: 096160, m/z: 1203.0394, charge: 4
SVQEIQATFFYFTPNKTEDTIFLR_5_4_0_1_0
1e6
Intensity
m/z
204.0863
366.1388
168.0652
274.0917
186.0756
126.0547
292.1027
144.0649
b5(1+)
557.2935
528.1904
PEP_0_0_0_0_0_
1448.239
PEP_0_1_0_0_0_
1549.7742
PEP_0_2_0_0_0_
1651.3051
PEP_1_2_0_0_0_
1732.3264

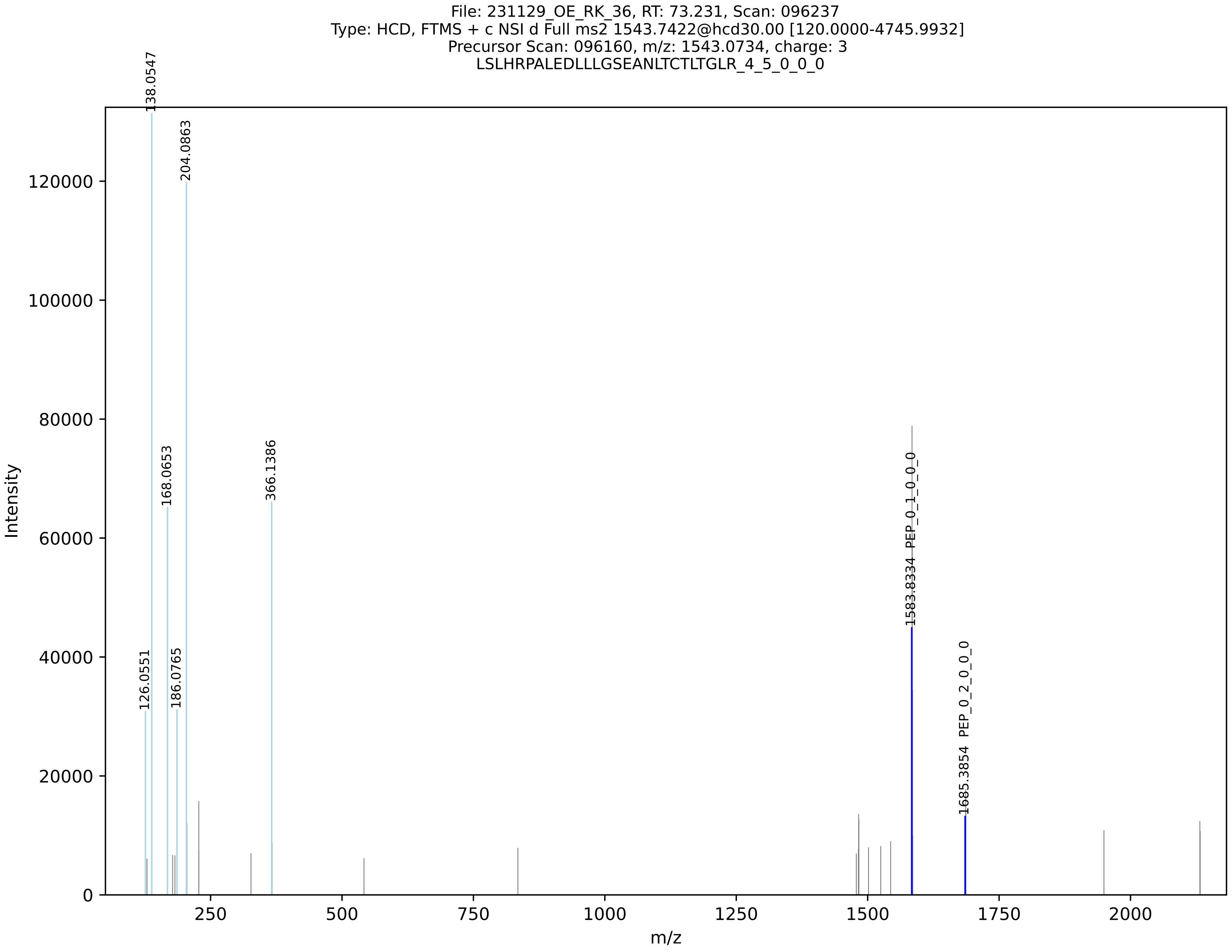
File: 231129_OE_RK_36, RT: 73.231, Scan: 096237
Type: HCD, FTMS + c NSI d Full ms2 1543.7422@hcd30.00 [120.0000-4745.9932]
Precursor Scan: 096160, m/z: 1543.0734, charge: 3
LSLHRPALEDLLLGSEANLTCTLTGLR_4_5_0_0_0
138.0547
204.0863
168.0653
366.1386
126.0551
186.0765
1583.8334 PEP_0_1_0_0_0
1685.3854 PEP_0_2_0_0_0
Intensity
m/z
0
20000
40000
60000
80000
100000
120000
250
500
750
1000
1250
1500
1750
2000

File: 231129_OE_RK_36, RT: 73.236, Scan: 096244
Type: HCD, FTMS + c NSI d Full ms2 1394.6813@hcd30.00 [120.0000-4289.8667]
Precursor Scan: 096160, m/z: 1394.0119, charge: 3
LSLHRPALEDLLLGSEANLTCTLTGLR_5_2_0_0_0

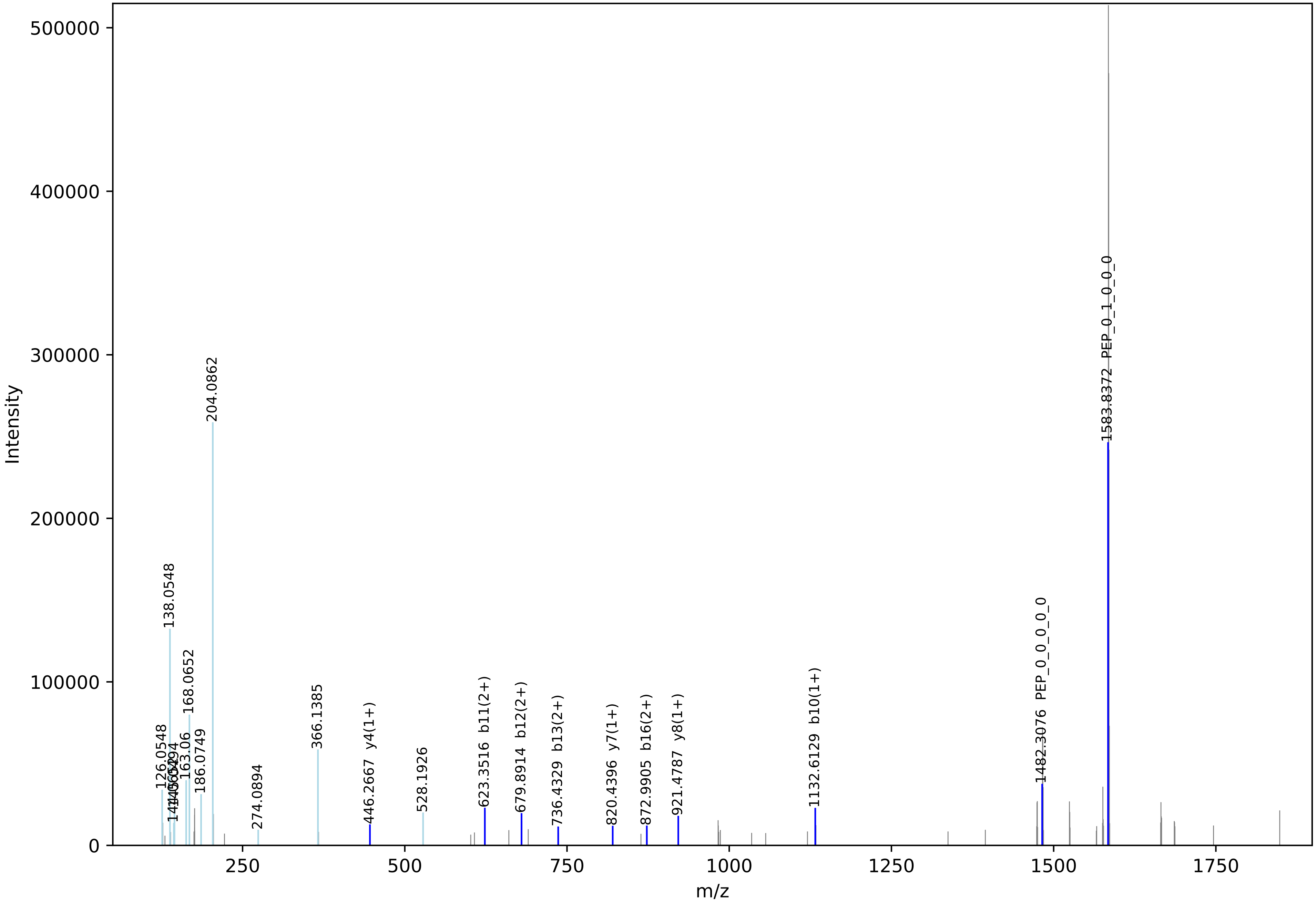

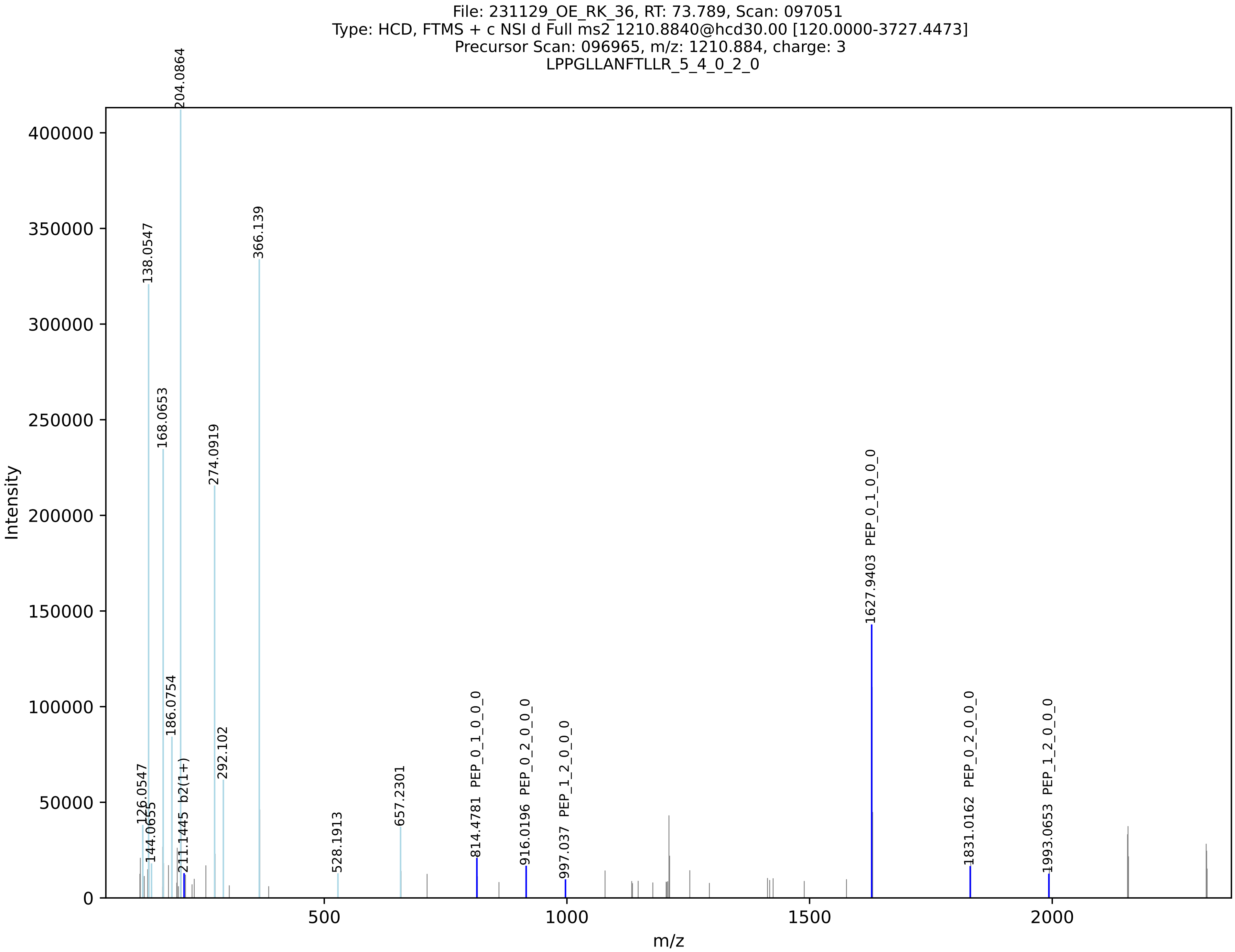
File: 231129_OE_RK_36, RT: 73.789, Scan: 097051
Type: HCD, FTMS + c NSI d Full ms2 1210.8840@hcd30.00 [120.0000-3727.4473]
Precursor Scan: 096965, m/z: 1210.884, charge: 3
LPPGLLANFTLLR_5_4_0_2_0
Intensity
m/z
204.0864
138.0547
366.139
168.0653
274.0919
186.0754
292.102
126.0547
144.0655
211.1445 b2(1+)
528.1913
657.2301
814.4781 PEP_0_1_0_0_0
916.0196 PEP_0_2_0_0_0
997.037 PEP_1_2_0_0_0
1627.9403 PEP_0_1_0_0_0
1831.0162 PEP_0_2_0_0_0
1993.0653 PEP_1_2_0_0_0

File: 231129_OE_RK_36, RT: 74.038, Scan: 097412
Type: HCD, FTMS + c NSI d Full ms2 1345.6018@hcd30.00 [120.0000-5512.9111]
Precursor Scan: 097331, m/z: 1344.85, charge: 4
WVLTAAHCLLYPPWDKNFTENDLLVR_5_4_0_2_0

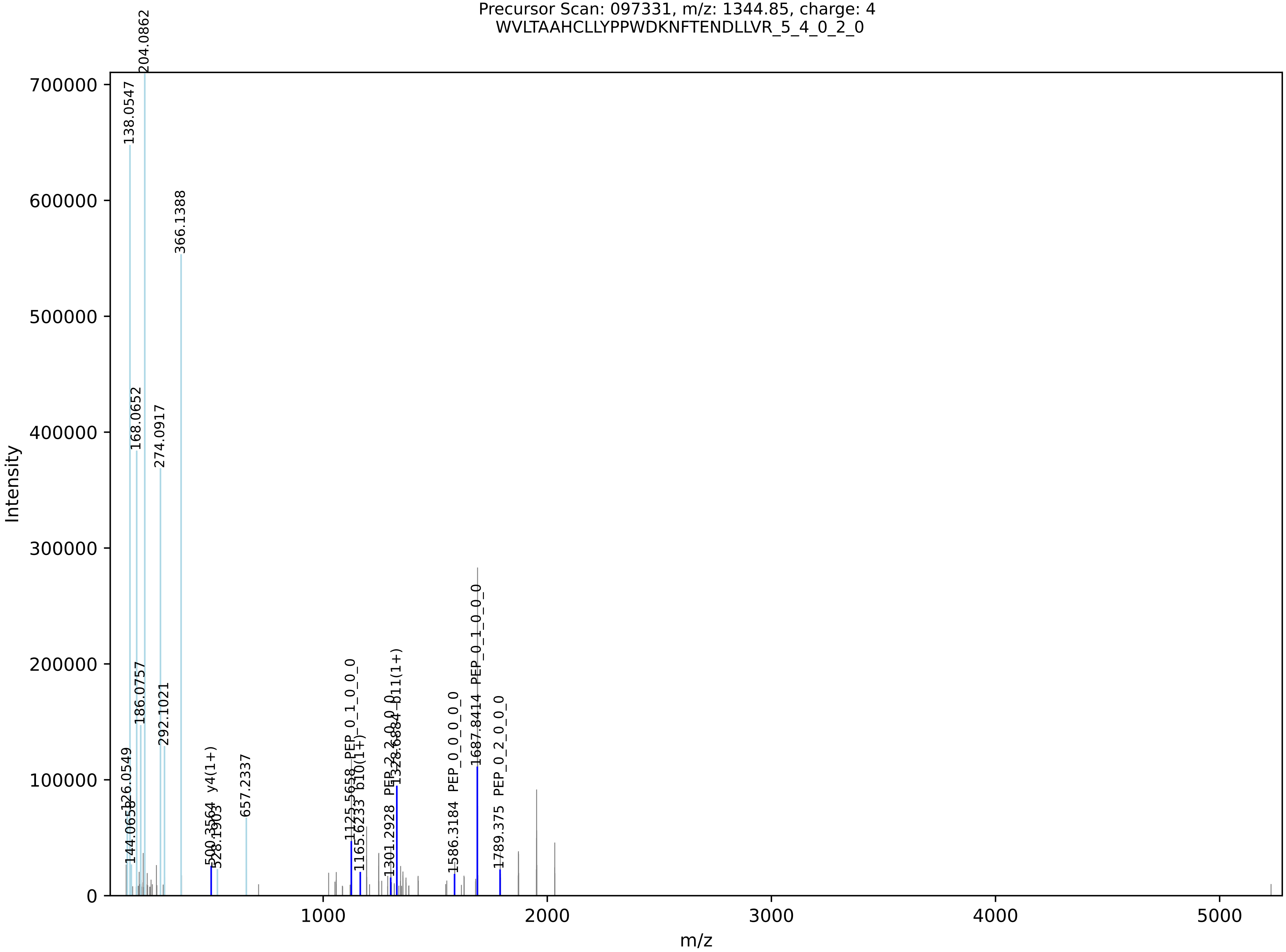

File: 231129_OE_RK_36, RT: 74.237, Scan: 097701
Type: HCD, FTMS + c NSI d Full ms2 1345.3527@hcd30.00 [120.0000-5511.8950]
Precursor Scan: 097625, m/z: 1344.8519, charge: 4
WVLTAAHCLLYPPWDKNFTENDLLVR_5_4_0_2_0

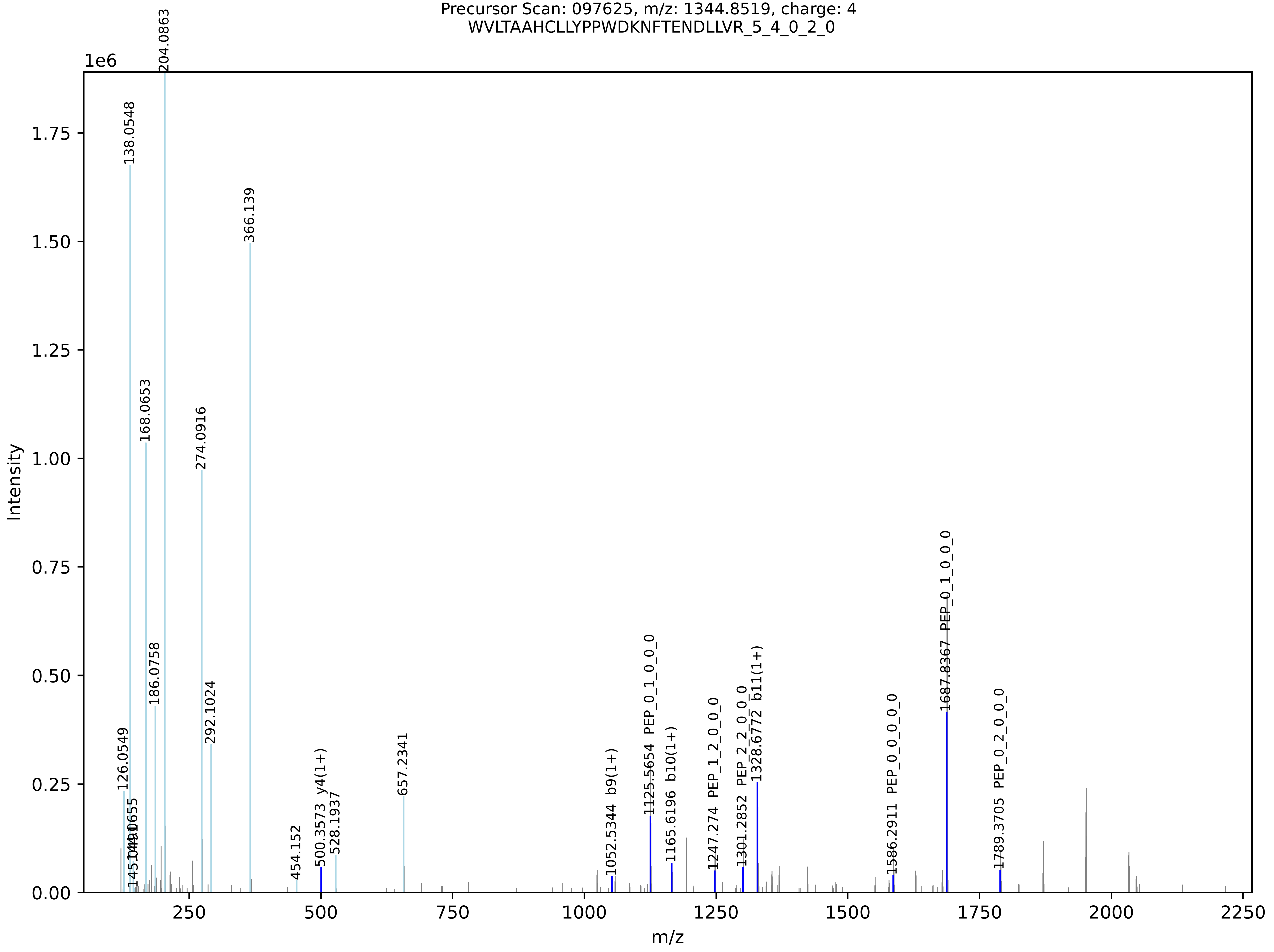

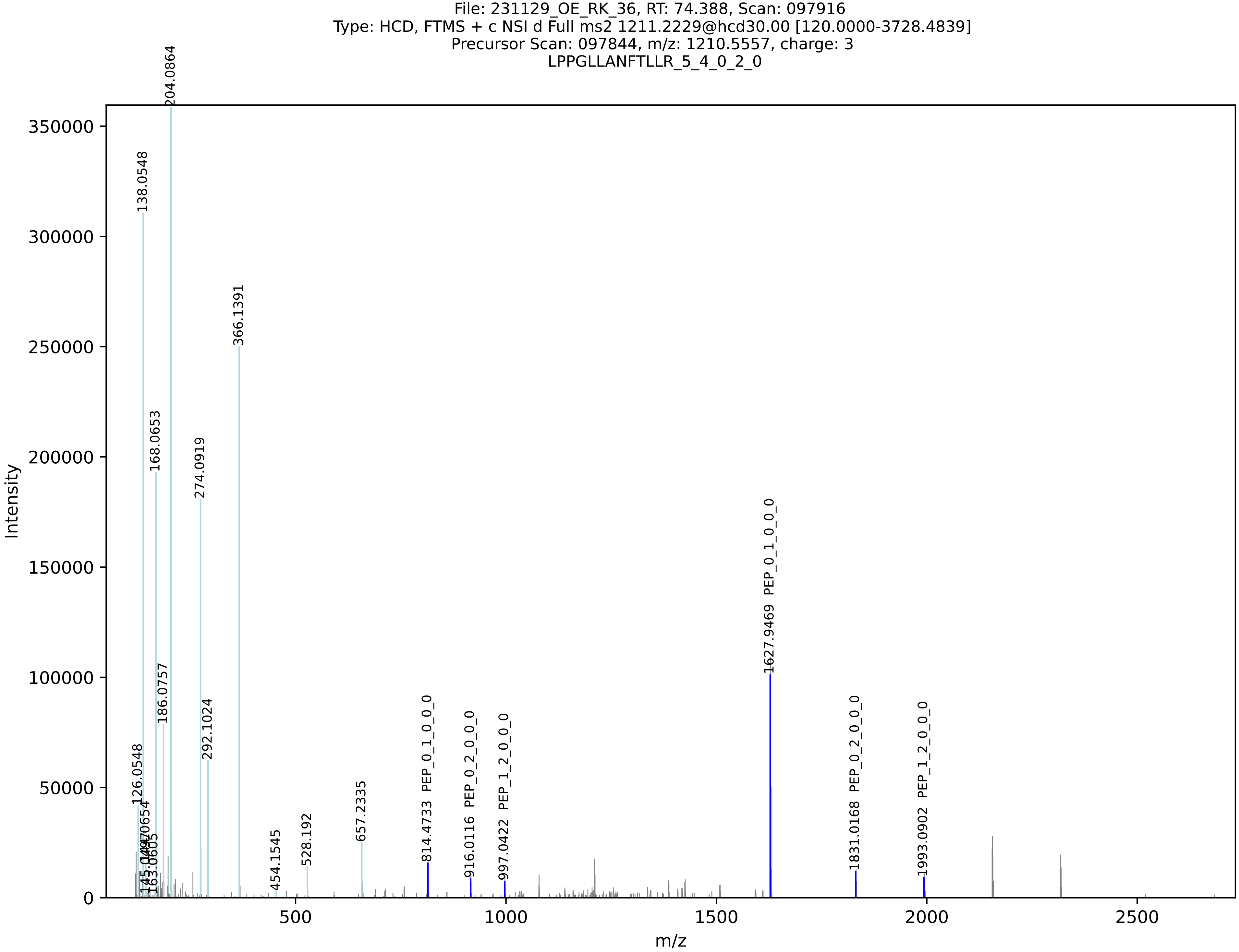
File: 231129_OE_RK_36, RT: 74.388, Scan: 097916
Type: HCD, FTMS + c NSI d Full ms2 1211.2229@hcd30.00 [120.0000-3728.4839]
Precursor Scan: 097844, m/z: 1210.5557, charge: 3
LPPGLLANFTLLR_5_4_0_2_0
Intensity
m/z
204.0864
138.0548
366.1391
168.0653
274.0919
186.0757
292.1024
126.0548
163.0605
454.1545
528.192
657.2335
814.4733 PEP_0_1_0_0_0
916.0116 PEP_0_2_0_0_0
997.0422 PEP_1_2_0_0_0
1627.9469 PEP_0_1_0_0_0
1831.0168 PEP_0_2_0_0_0
1993.0902 PEP_1_2_0_0_0

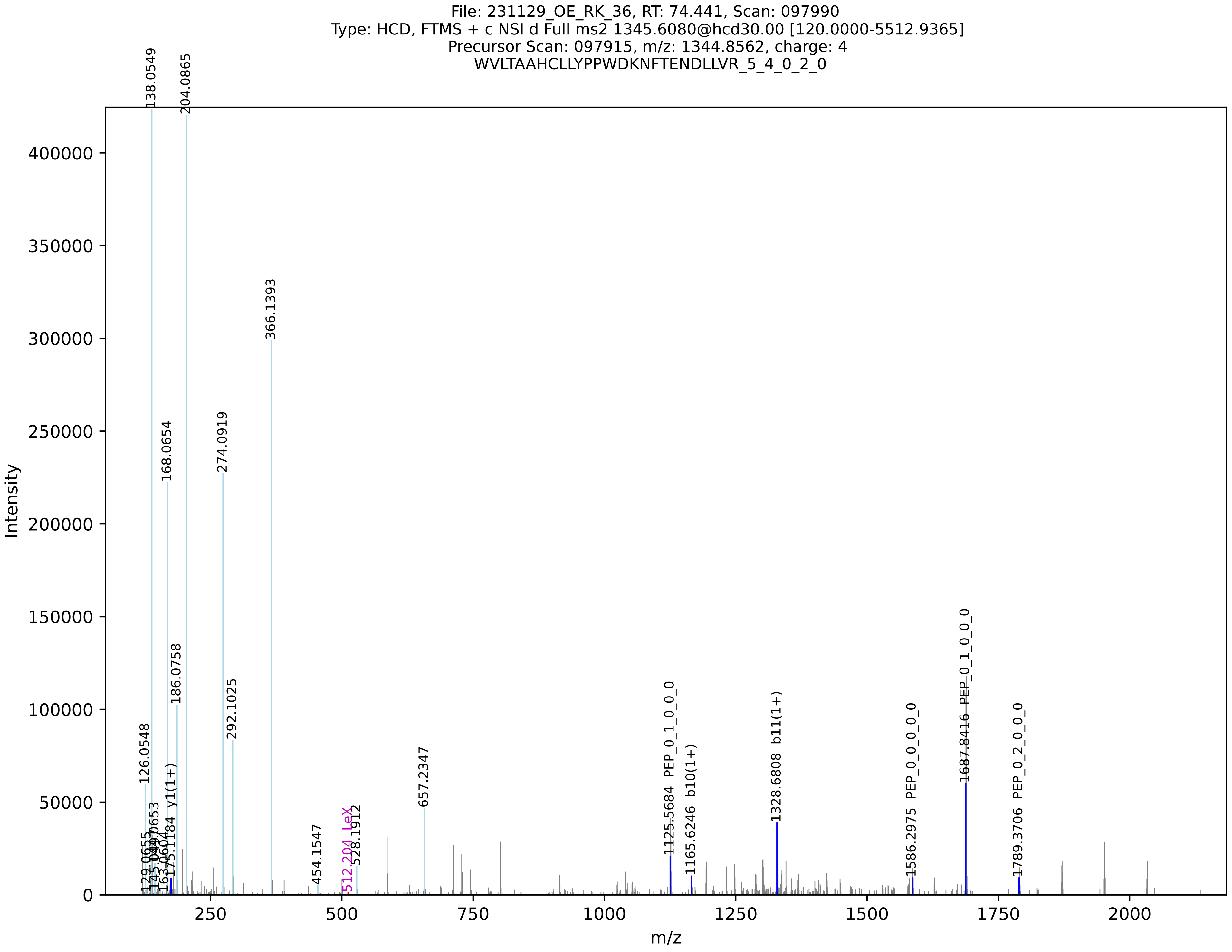
File: 231129_OE_RK_36, RT: 74.441, Scan: 097990
Type: HCD, FTMS + c NSI d Full ms2 1345.6080@hcd30.00 [120.0000-5512.9365]
Precursor Scan: 097915, m/z: 1344.8562, charge: 4
WVLTAAHCLLYPPWDKNFTENDLLVR_5_4_0_2_0
Intensity
m/z
138.0549
204.0865
366.1393
168.0654
274.0919
186.0758
292.1025
126.0548
175.1184 y1(1+)
454.1547
512.204_LeX
528.1912
657.2347
1125.5684 PEP_0_1_0_0_0
1165.6246 b10(1+)
1328.6808 b11(1+)
1586.2975 PEP_0_0_0_0_0
1687.8416 PEP_0_1_0_0_0
1789.3706 PEP_0_2_0_0_0

File: 231129_OE_RK_36, RT: 74.602, Scan: 098228
Type: HCD, FTMS + c NSI d Full ms2 1531.9094@hcd30.00 [120.0000-6273.0464]
Precursor Scan: 098133, m/z: 1531.1577, charge: 4
SVQEIQATFFYFTPNKTEDTIFLR_7_6_0_3_0

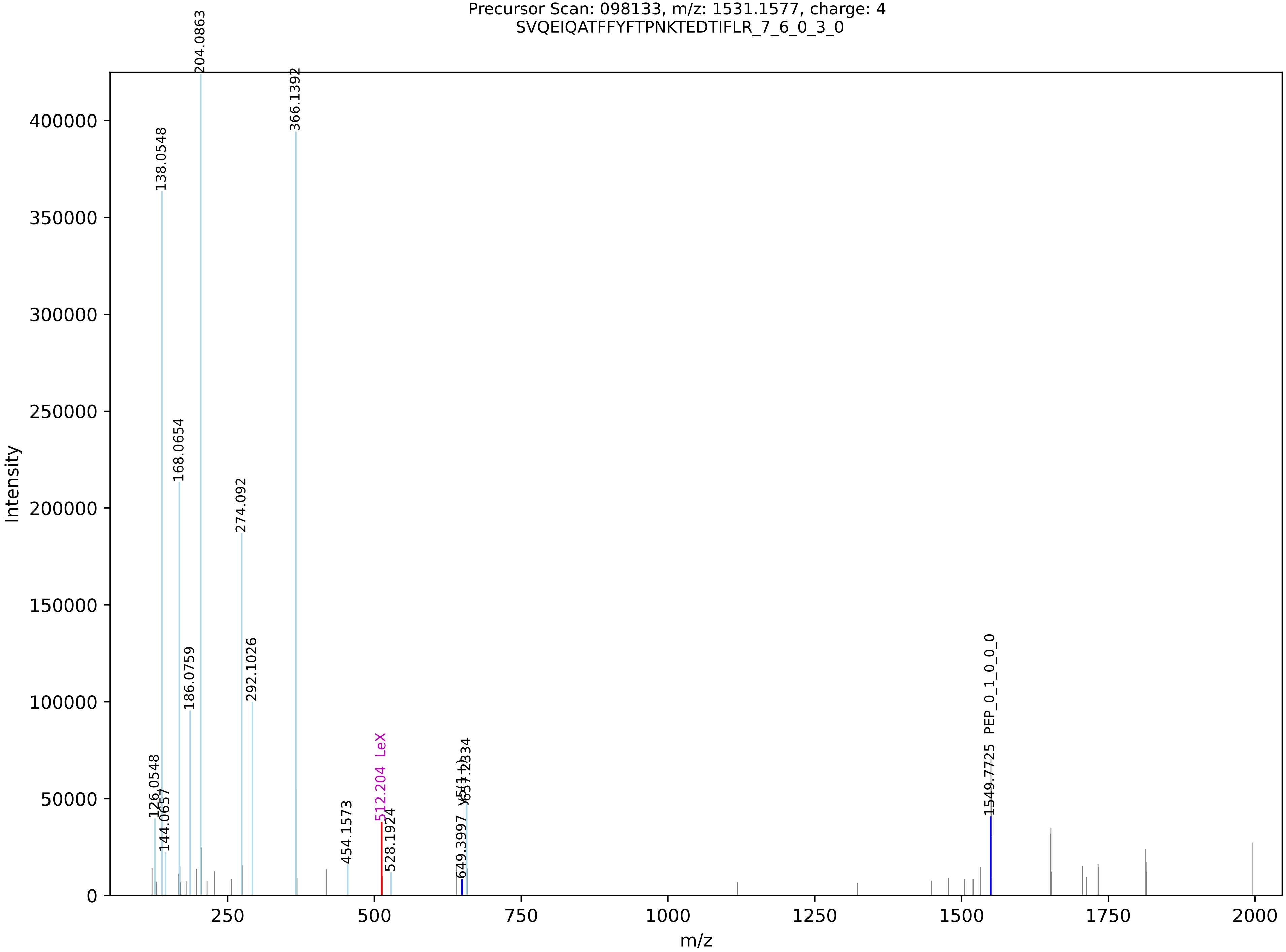

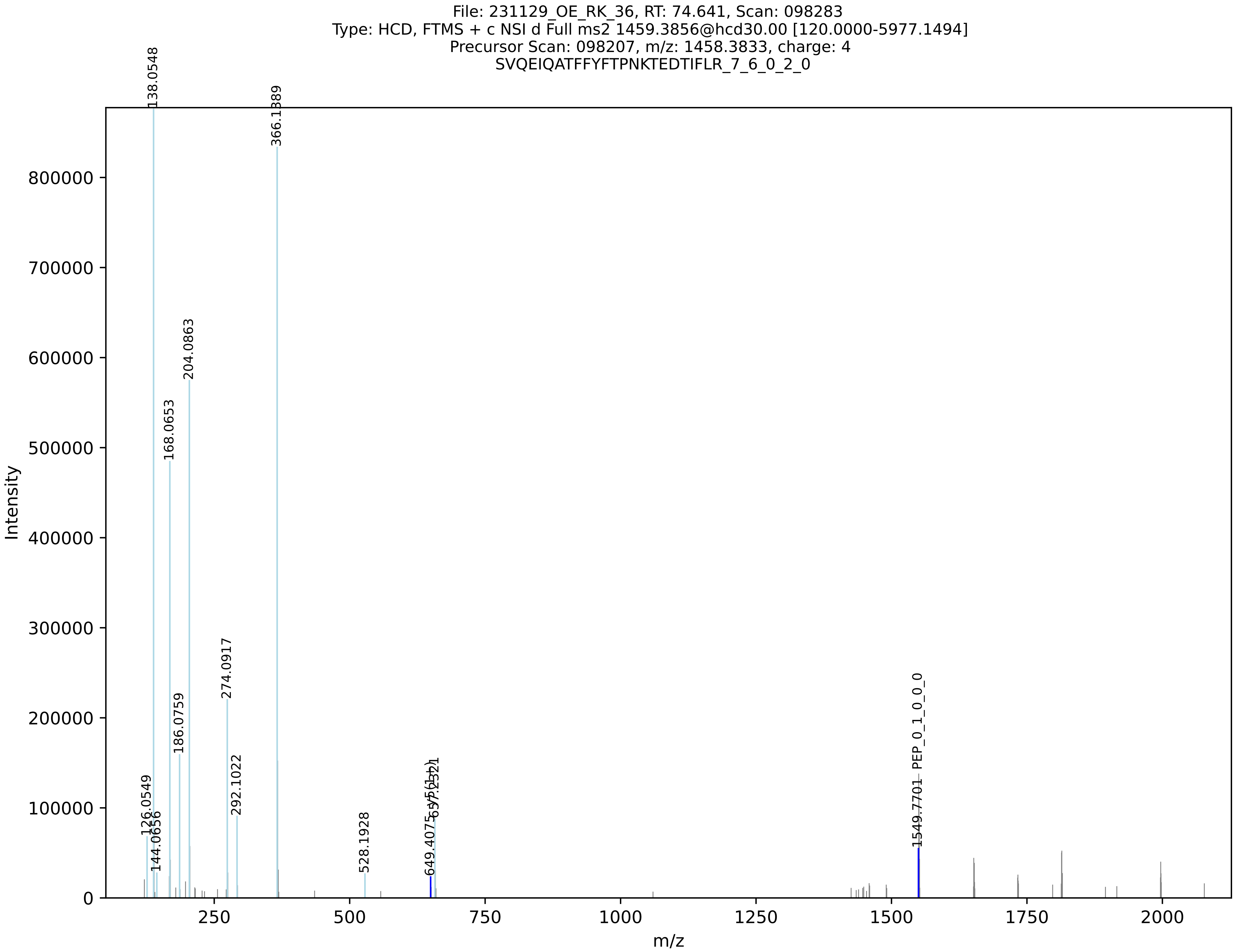

File: 231129_OE_RK_36, RT: 74.641, Scan: 098283
Type: HCD, FTMS + c NSI d Full ms2 1459.3856@hcd30.00 [120.0000-5977.1494]
Precursor Scan: 098207, m/z: 1458.3833, charge: 4
SVQEIQATFFYFTPNKTEDTIFLR_7_6_0_2_0
138.0548
366.1389
204.0863
168.0653
274.0917
186.0759
292.1022
126.0549
144.0656
528.1928
649.4075
657.2321
1549.7701 PEP_0_1_0_0_0
Intensity
m/z
0
100000
200000
300000
400000
500000
600000
700000
800000
250
500
750
1000
1250
1500
1750
2000

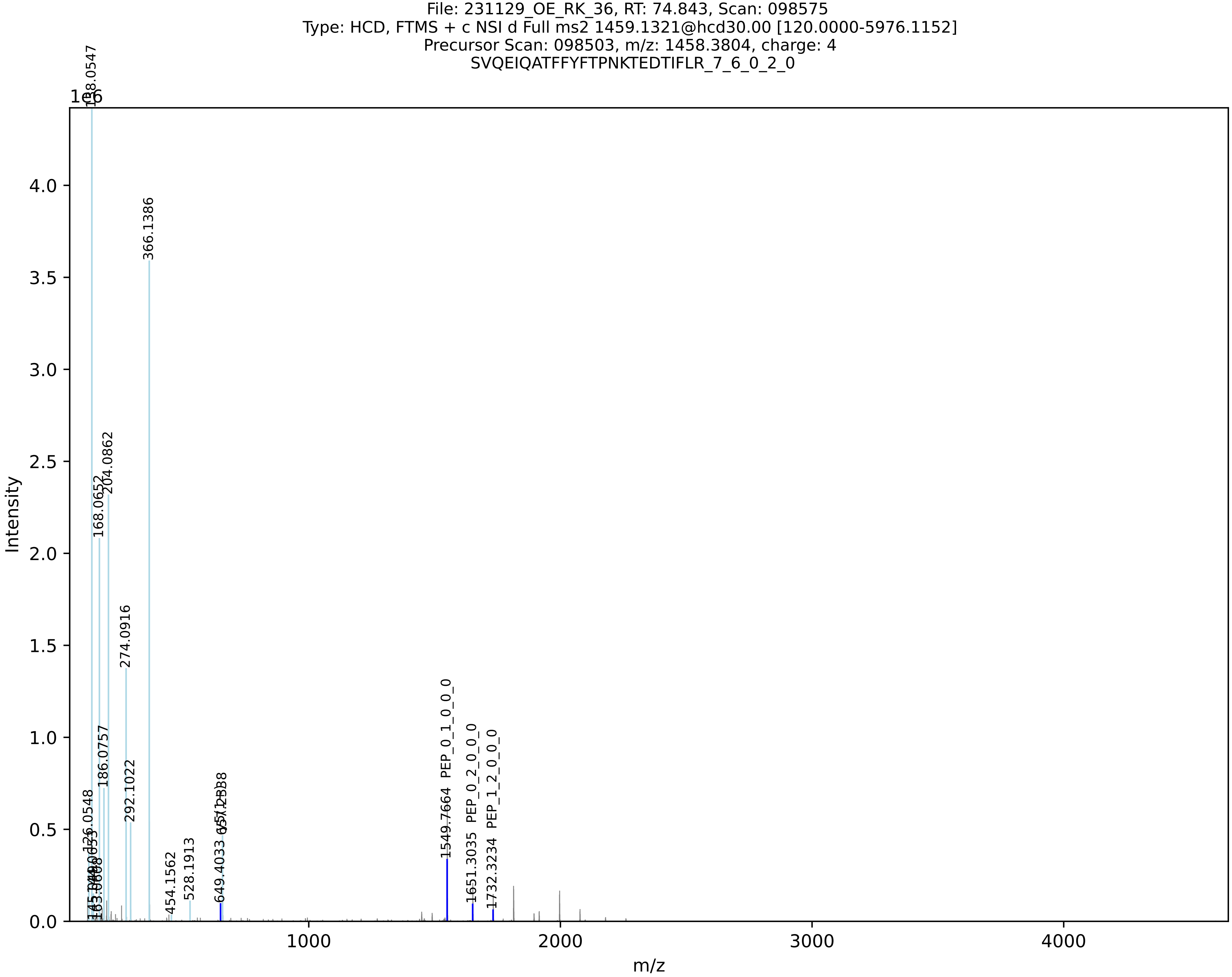

File: 231129_OE_RK_36, RT: 74.843, Scan: 098575
Type: HCD, FTMS + c NSI d Full ms2 1459.1321@hcd30.00 [120.0000-5976.1152]
Precursor Scan: 098503, m/z: 1458.3804, charge: 4
SVQEIQATFFYFTPNKTEDTIFLR_7_6_0_2_0
1e6
Intensity
m/z
138.0547
366.1386
204.0862
168.0652
274.0916
186.0757
292.1022
126.0548
454.1562
528.1913
649.4033
1549.7664 PEP_0_1_0_0_0
1651.3035 PEP_0_2_0_0_0
1732.3234 PEP_1_2_0_0_0

File: 231129_OE_RK_36, RT: 75.043, Scan: 098866
Type: HCD, FTMS + c NSI d Full ms2 1459.1321@hcd30.00 [120.0000-5976.1152]
Precursor Scan: 098794, m/z: 1458.3804, charge: 4
SVQEIQATFFYFTPNKTEDTIFLR_7_6_0_2_0

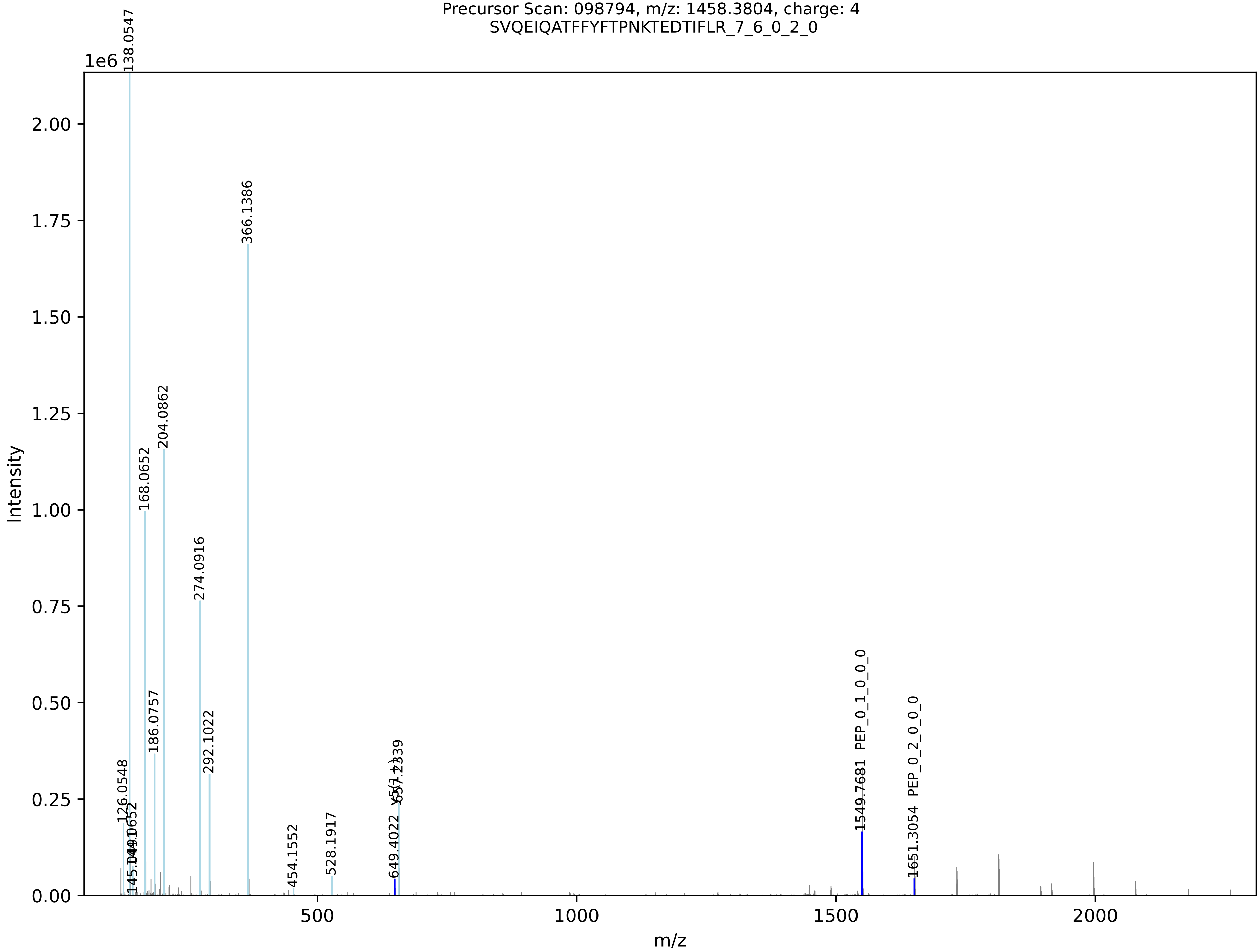

File: 231129_OE_RK_36, RT: 75.153, Scan: 099028
Type: HCD, FTMS + c NSI d Full ms2 1367.8470@hcd30.00 [120.0000-5603.6719]
Precursor Scan: 098939, m/z: 1367.0953, charge: 4
SVQEIQATFFYFTPNKTEDTIFLR_6_5_0_2_0

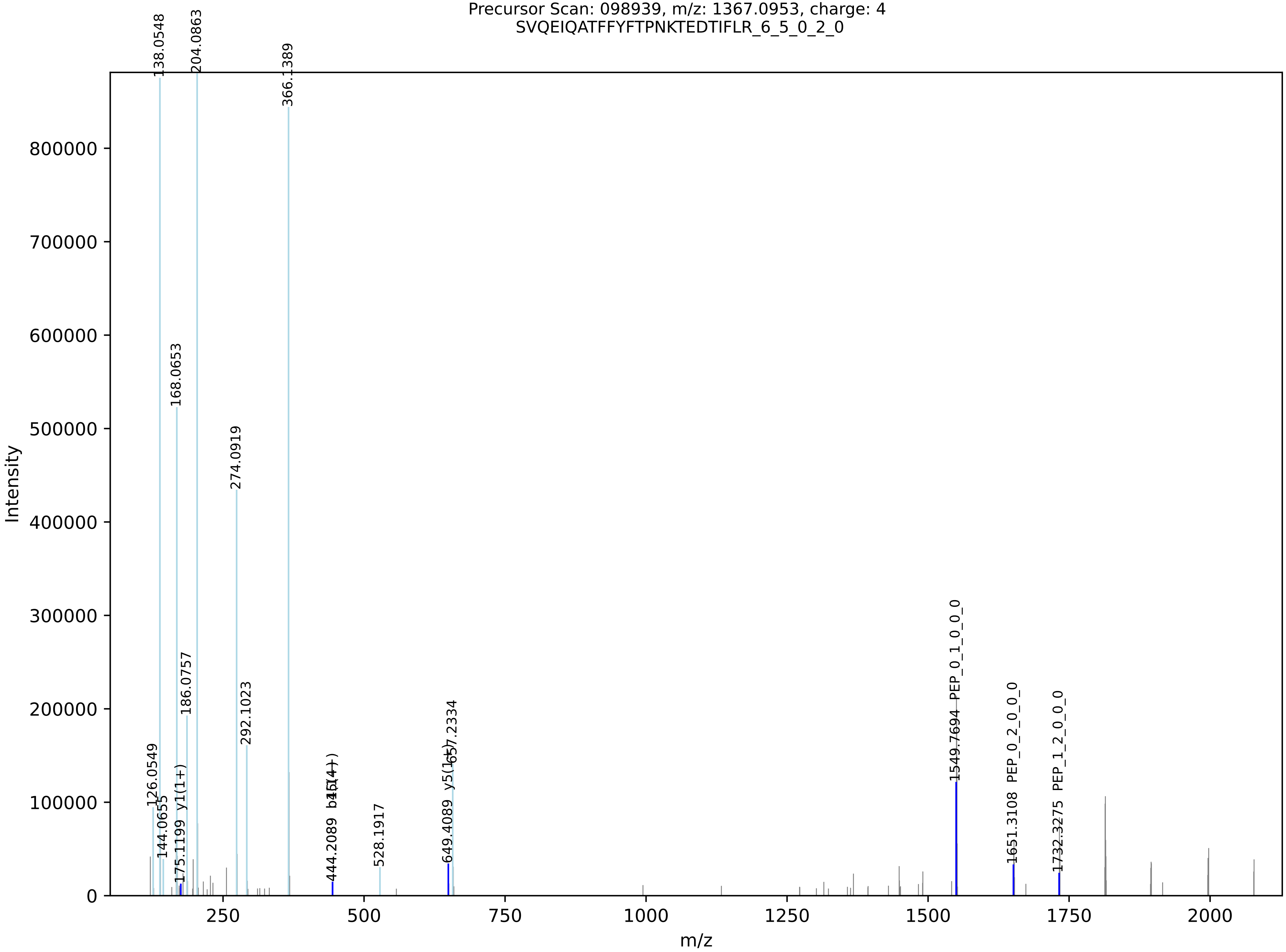

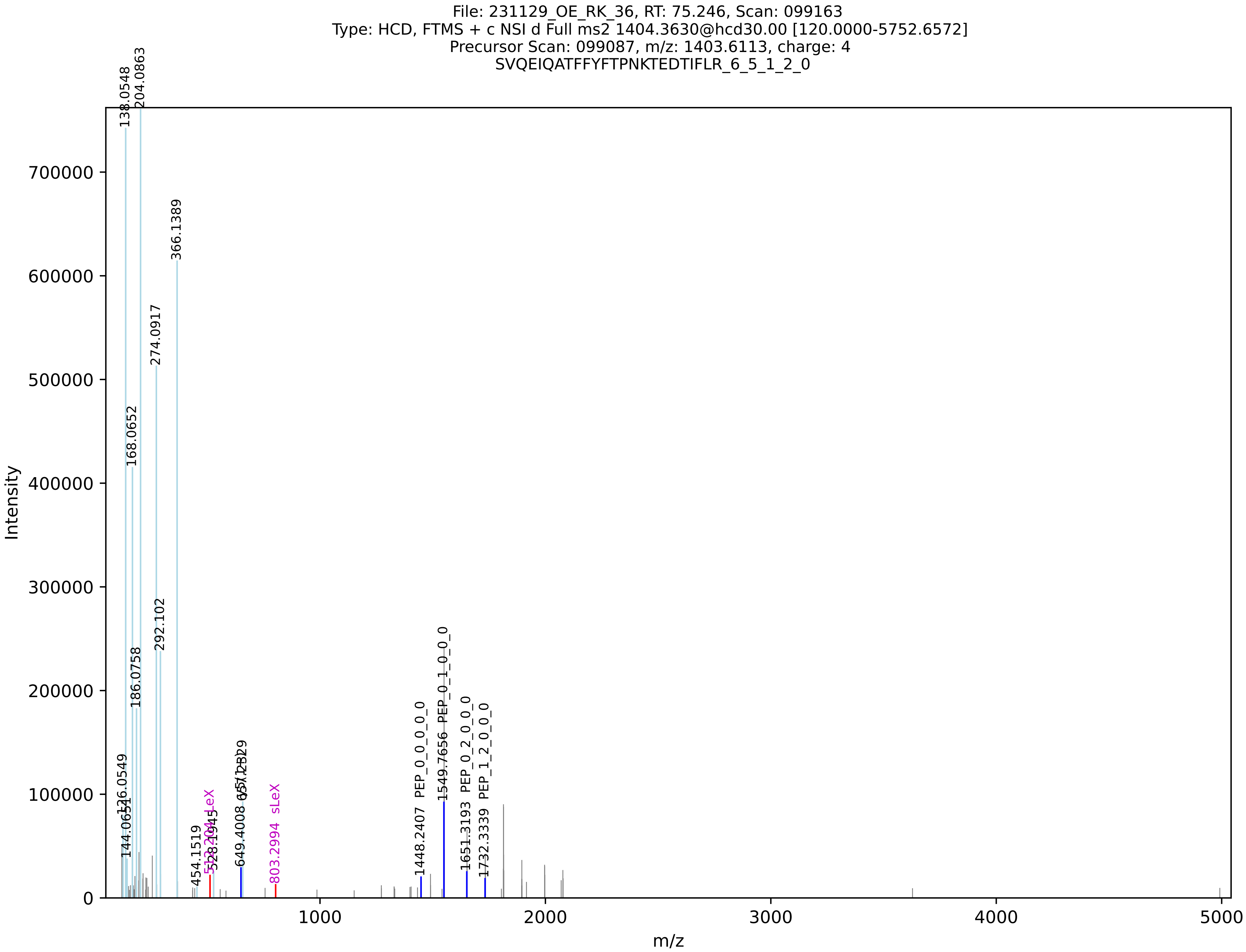

File: 231129_OE_RK_36, RT: 75.246, Scan: 099163
Type: HCD, FTMS + c NSI d Full ms2 1404.3630@hcd30.00 [120.0000-5752.6572]
Precursor Scan: 099087, m/z: 1403.6113, charge: 4
SVQEIQATFFYFTPNKTEDTIFLR_6_5_1_2_0
138.0548
204.0863
366.1389
274.0917
168.0652
292.102
186.0758
126.0549
144.0651
454.1519
528.1945
649.4008
803.2994 sLeX
1448.2407 PEP_0_0_0_0_0
1549.7656 PEP_0_1_0_0_0
1651.3193 PEP_0_2_0_0_0
1732.3339 PEP_1_2_0_0_0
Intensity
m/z

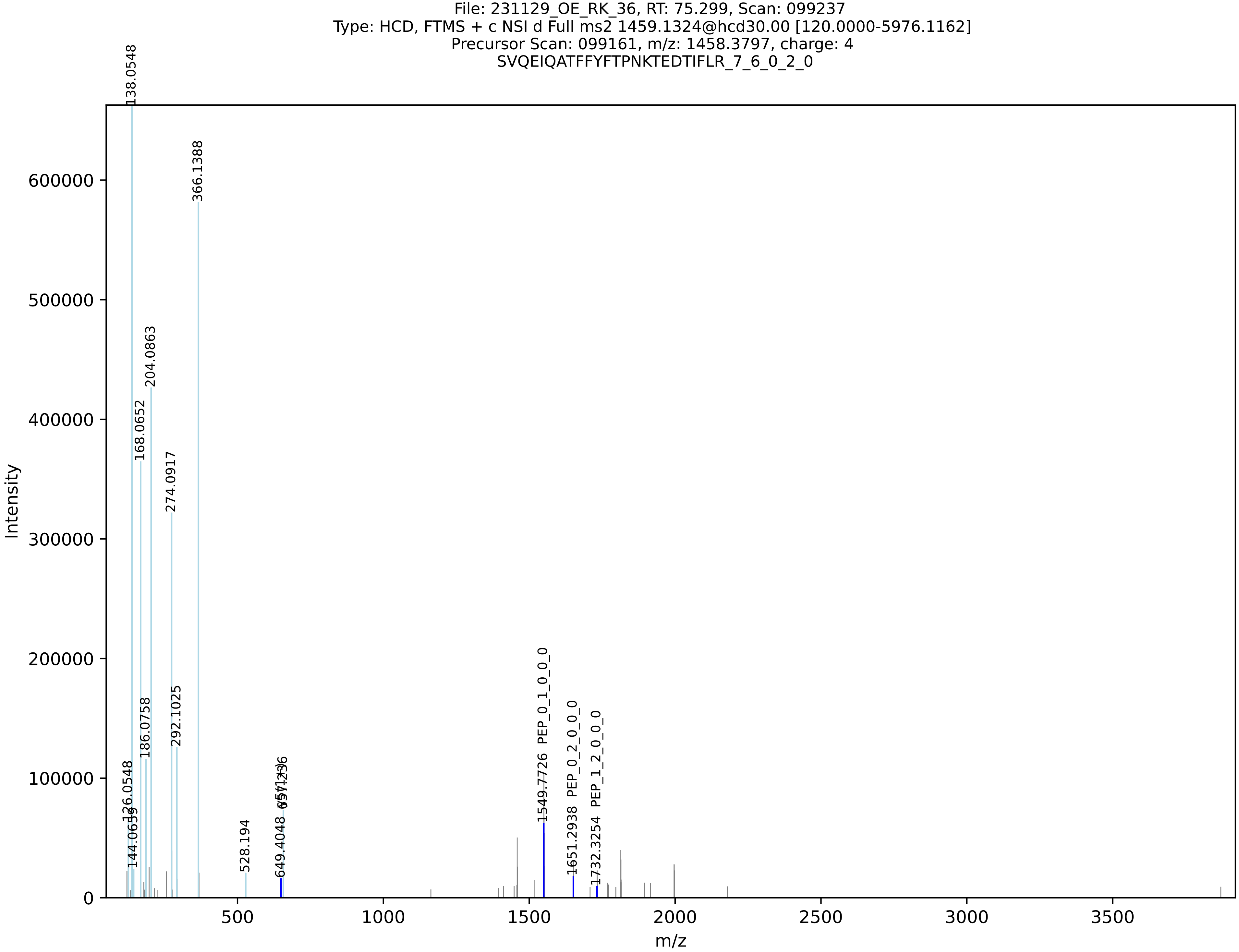

File: 231129_OE_RK_36, RT: 75.299, Scan: 099237
Type: HCD, FTMS + c NSI d Full ms2 1459.1324@hcd30.00 [120.0000-5976.1162]
Precursor Scan: 099161, m/z: 1458.3797, charge: 4
SVQEIQATFFYFTPNKTEDTIFLR_7_6_0_2_0
Intensity
m/z
0
100000
200000
300000
400000
500000
600000
500
1000
1500
2000
2500
3000
3500
138.0548
366.1388
204.0863
168.0652
274.0917
292.1025
186.0758
126.0548
144.0659
528.194
649.4048
657.1236
1549.7726 PEP_0_1_0_0_0
1651.2938 PEP_0_2_0_0_0
1732.3254 PEP_1_2_0_0_0

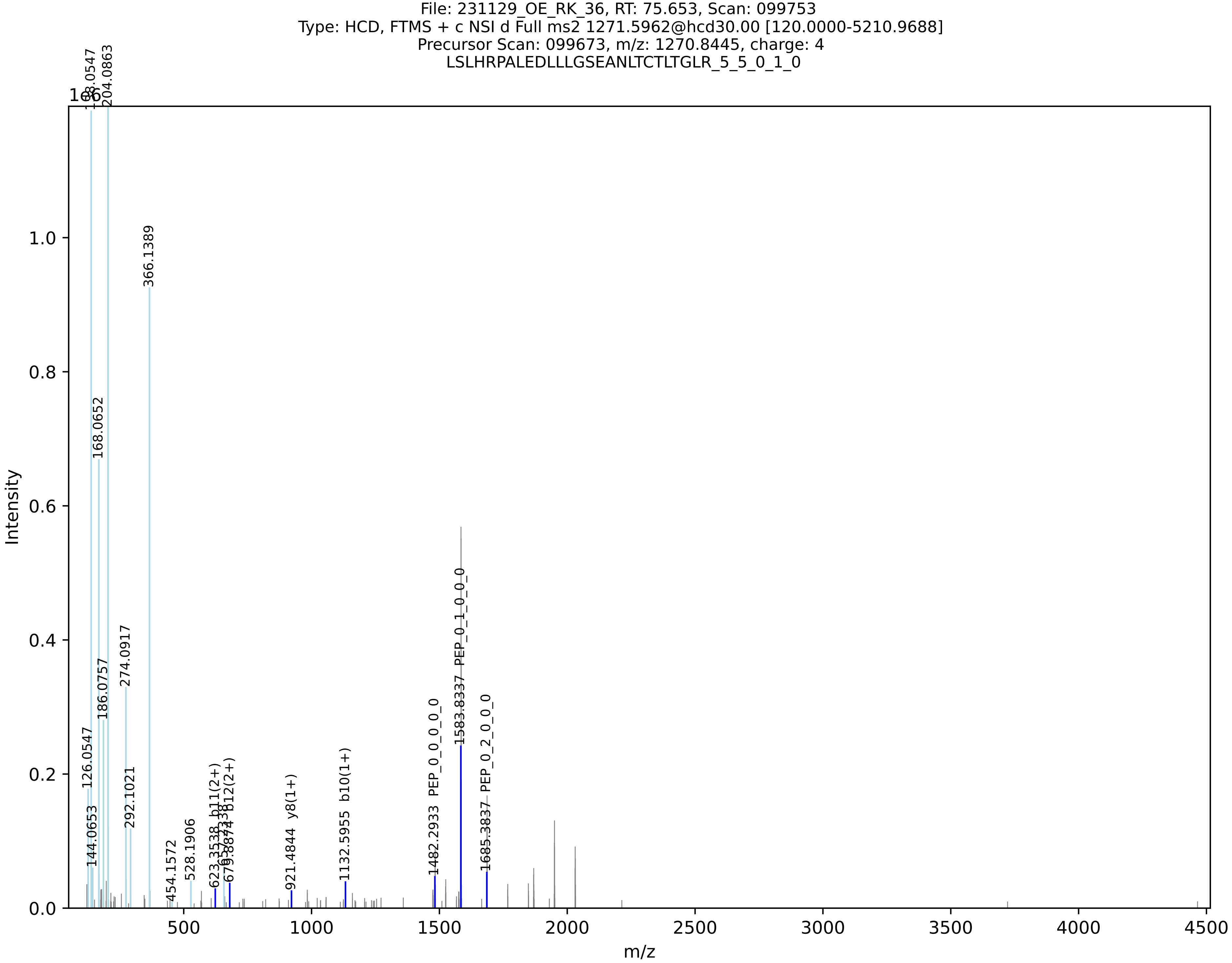

File: 231129_OE_RK_36, RT: 75.653, Scan: 099753
Type: HCD, FTMS + c NSI d Full ms2 1271.5962@hcd30.00 [120.0000-5210.9688]
Precursor Scan: 099673, m/z: 1270.8445, charge: 4
LSLHRPALEDLLLGSEANLTCTLTGLR_5_5_0_1_0
204.0863
366.1389
168.0652
274.0917
186.0757
126.0547
292.1021
144.0653
454.1572
528.1906
623.3538 b11(2+)
679.8874 b12(2+)
921.4844 y8(1+)
1132.5955 b10(1+)
1482.2933 PEP_0_0_0_0_0
1583.8337 PEP_0_1_0_0_0
1685.3837 PEP_0_2_0_0_0
Intensity
m/z
0.0
0.2
0.4
0.6
0.8
1.0
500
1000
1500
2000
2500
3000
3500
4000
4500

File: 231129_OE_RK_36, RT: 75.708, Scan: 099834
Type: HCD, FTMS + c NSI d Full ms2 1017.6795@hcd30.00 [120.0000-5213.7354]
Precursor Scan: 099747, m/z: 1016.8777, charge: 5
LSLHRPALEDLLLGSEANLTCTLTGLR_5_5_0_1_0

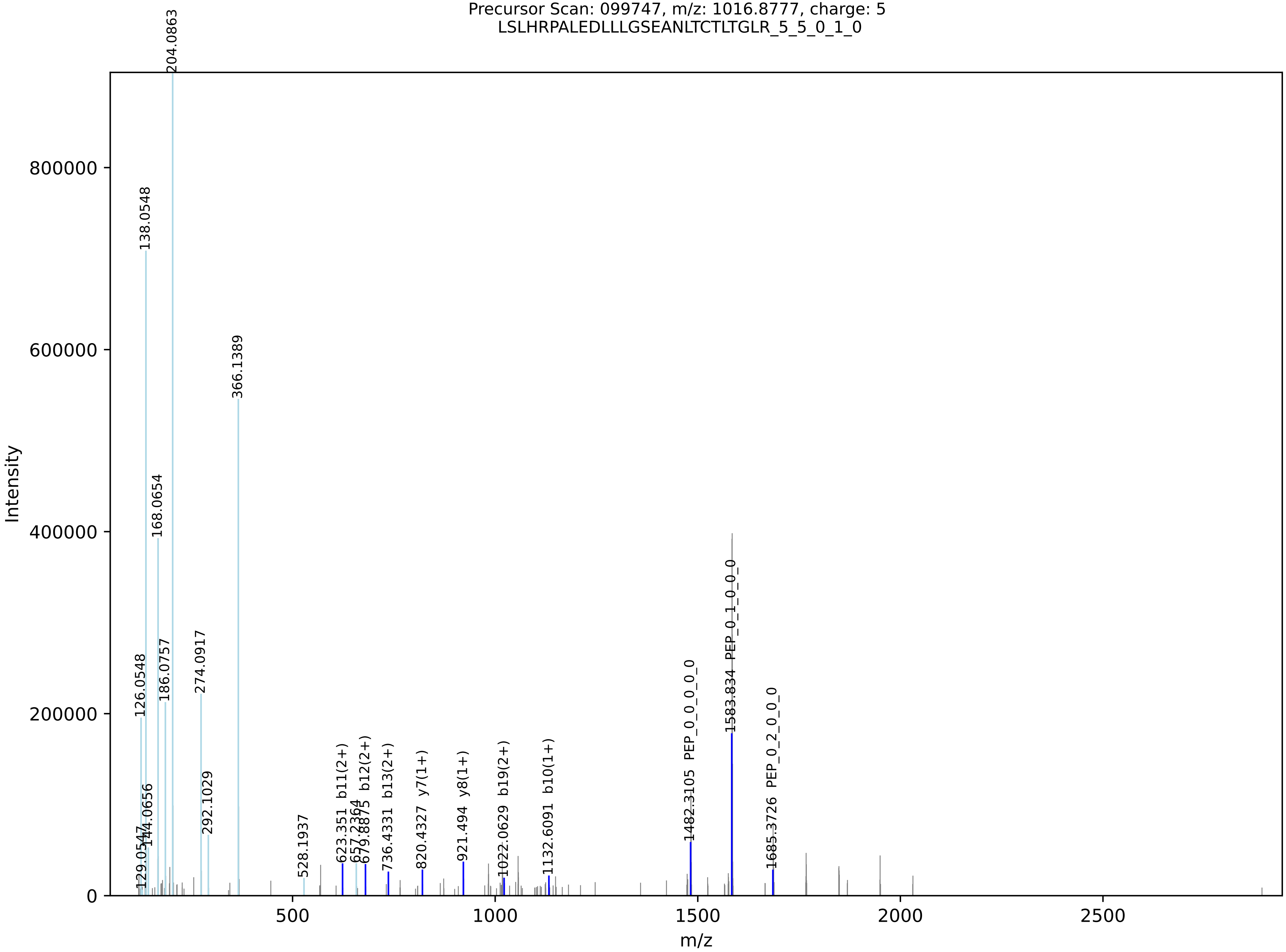

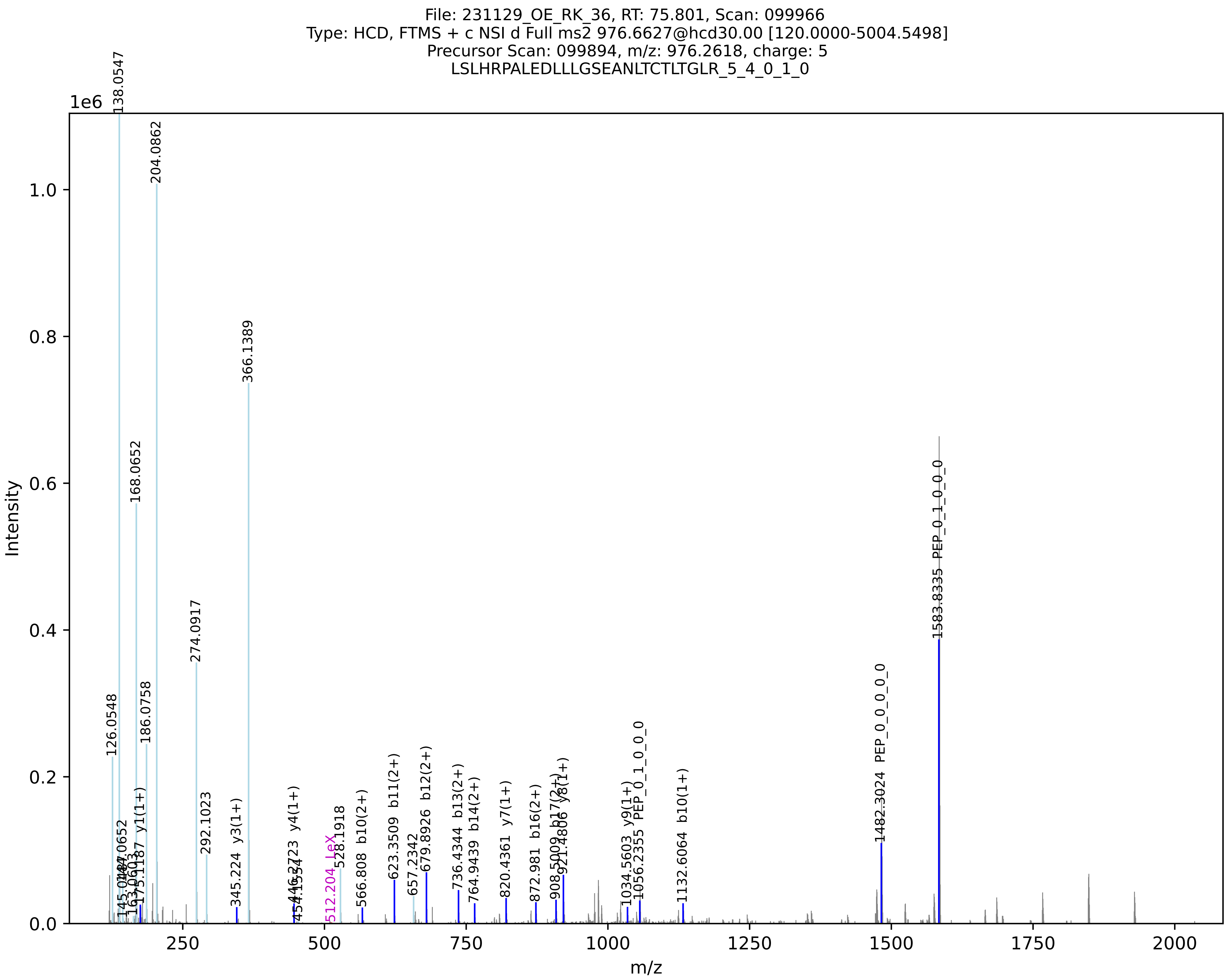

File: 231129_OE_RK_36, RT: 75.801, Scan: 099966
Type: HCD, FTMS + c NSI d Full ms2 976.6627@hcd30.00 [120.0000-5004.5498]
Precursor Scan: 099894, m/z: 976.2618, charge: 5
LSLHRPALEDLLLGSEANLTCTLTGLR_5_4_0_1_0
1e6
Intensity
m/z
138.0547
204.0862
366.1389
168.0652
274.0917
126.0548
186.0758
292.1023
175.1187 y1(1+)
345.224 y3(1+)
446.2723 y4(1+)
512.204_LeX
528.1918
566.808 b10(2+)
623.3509 b11(2+)
657.2342
679.8926 b12(2+)
736.4344 b13(2+)
764.9439 b14(2+)
820.4361 y7(1+)
872.981 b16(2+)
908.5009 b17(2+)
1034.5603 y9(1+)
1056.2355 PEP_0_1_0_0_0
1132.6064 b10(1+)
1482.3024 PEP_0_0_0_0_0
1583.8335 PEP_0_1_0_0_0

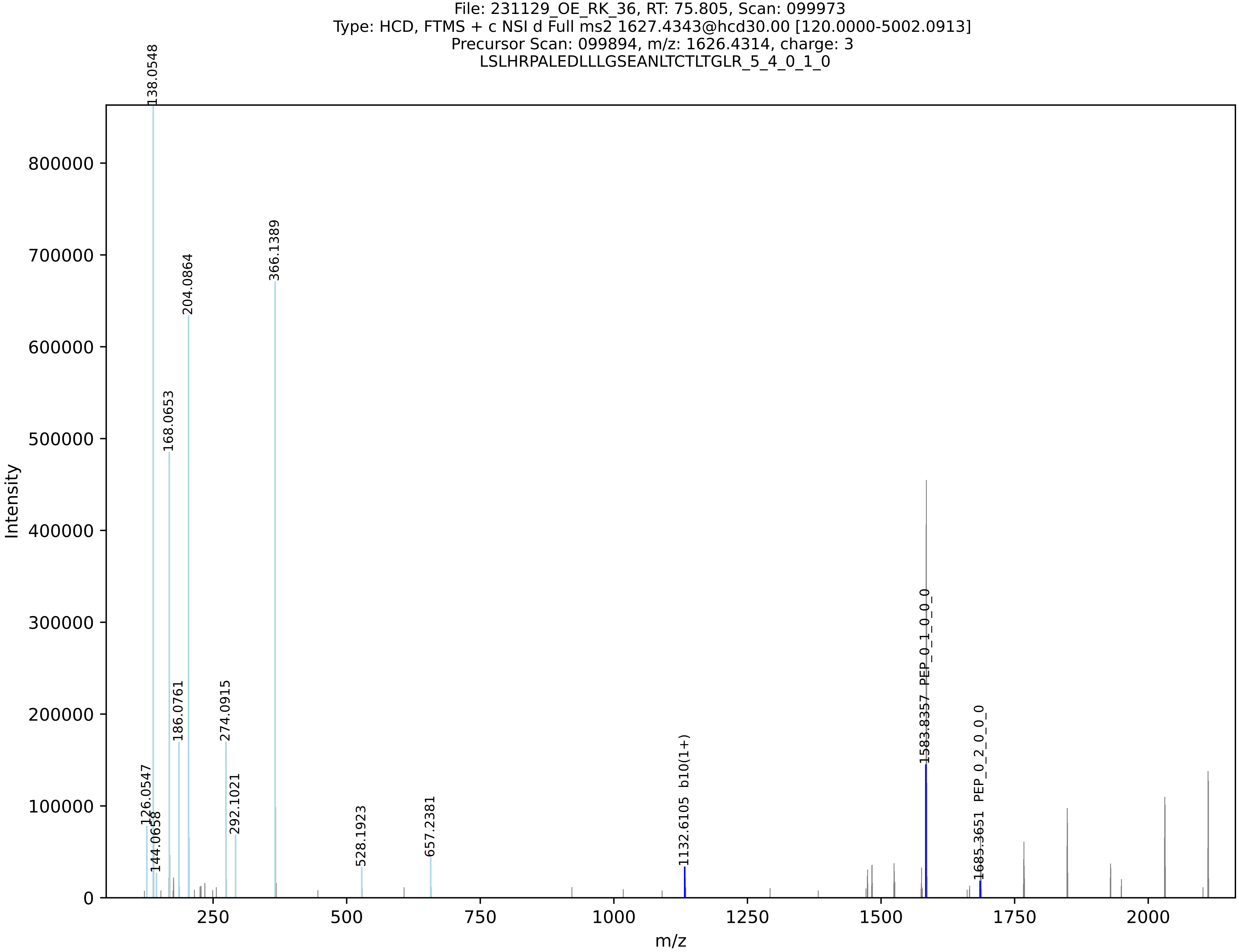
File: 231129_OE_RK_36, RT: 75.805, Scan: 099973
Type: HCD, FTMS + c NSI d Full ms2 1627.4343@hcd30.00 [120.0000-5002.0913]
Precursor Scan: 099894, m/z: 1626.4314, charge: 3
LSLHRPALEDLLLGSEANLTCTLTGLR_5_4_0_1_0
138.0548
366.1389
204.0864
168.0653
186.0761
274.0915
126.0547
144.0658
292.1021
528.1923
657.2381
1132.6105 b10(1+)
1583.8357 PEP_0_1_0_0_0
1685.3651 PEP_0_2_0_0_0
Intensity
m/z
0
100000
200000
300000
400000
500000
600000
700000
800000
250
500
750
1000
1250
1500
1750
2000

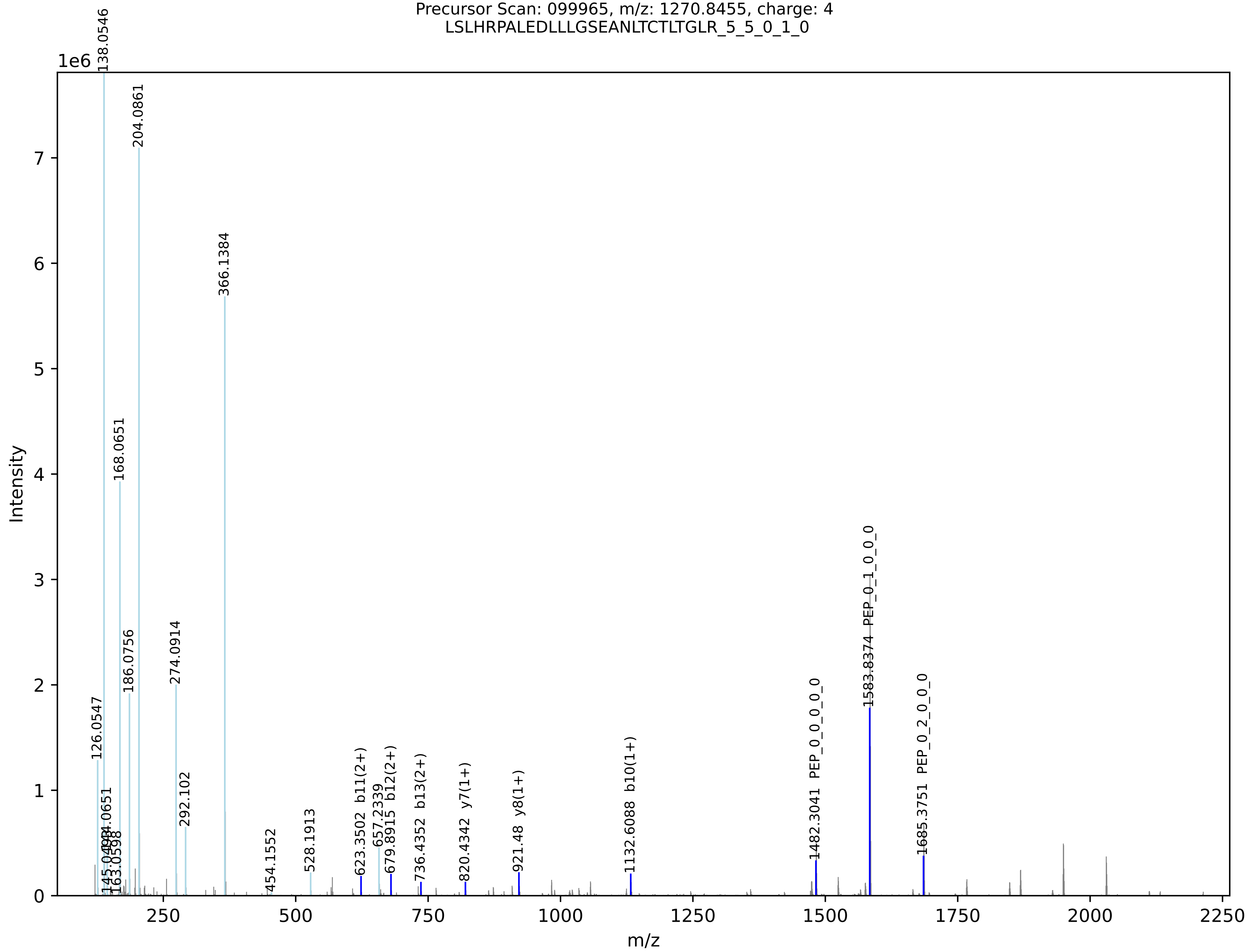
File: 231129_OE_RK_36, RT: 75.851, Scan: 100040
Type: HCD, FTMS + c NSI d Full ms2 1271.3467@hcd30.00 [120.0000-5209.9502]
Precursor Scan: 099965, m/z: 1270.8455, charge: 4
LSLHRPALEDLLLGSEANLTCTLTGLR_5_5_0_1_0
1e6
Intensity
m/z
138.0546
204.0861
366.1384
168.0651
186.0756
274.0914
126.0547
292.102
454.1552
528.1913
623.3502 b11(2+)
657.2339
679.8915 b12(2+)
736.4352 b13(2+)
820.4342 y7(1+)
921.48 y8(1+)
1132.6088 b10(1+)
1482.3041 PEP_0_0_0_0_0
1583.8374 PEP_0_1_0_0_0
1685.3751 PEP_0_2_0_0_0

File: 231129_OE_RK_36, RT: 75.903, Scan: 100113
Type: HCD, FTMS + c NSI d Full ms2 1017.2777@hcd30.00 [120.0000-5211.6865]
Precursor Scan: 100039, m/z: 1016.8769, charge: 5
LSLHRPALEDLLLGSEANLTCTLTGLR_5_5_0_1_0

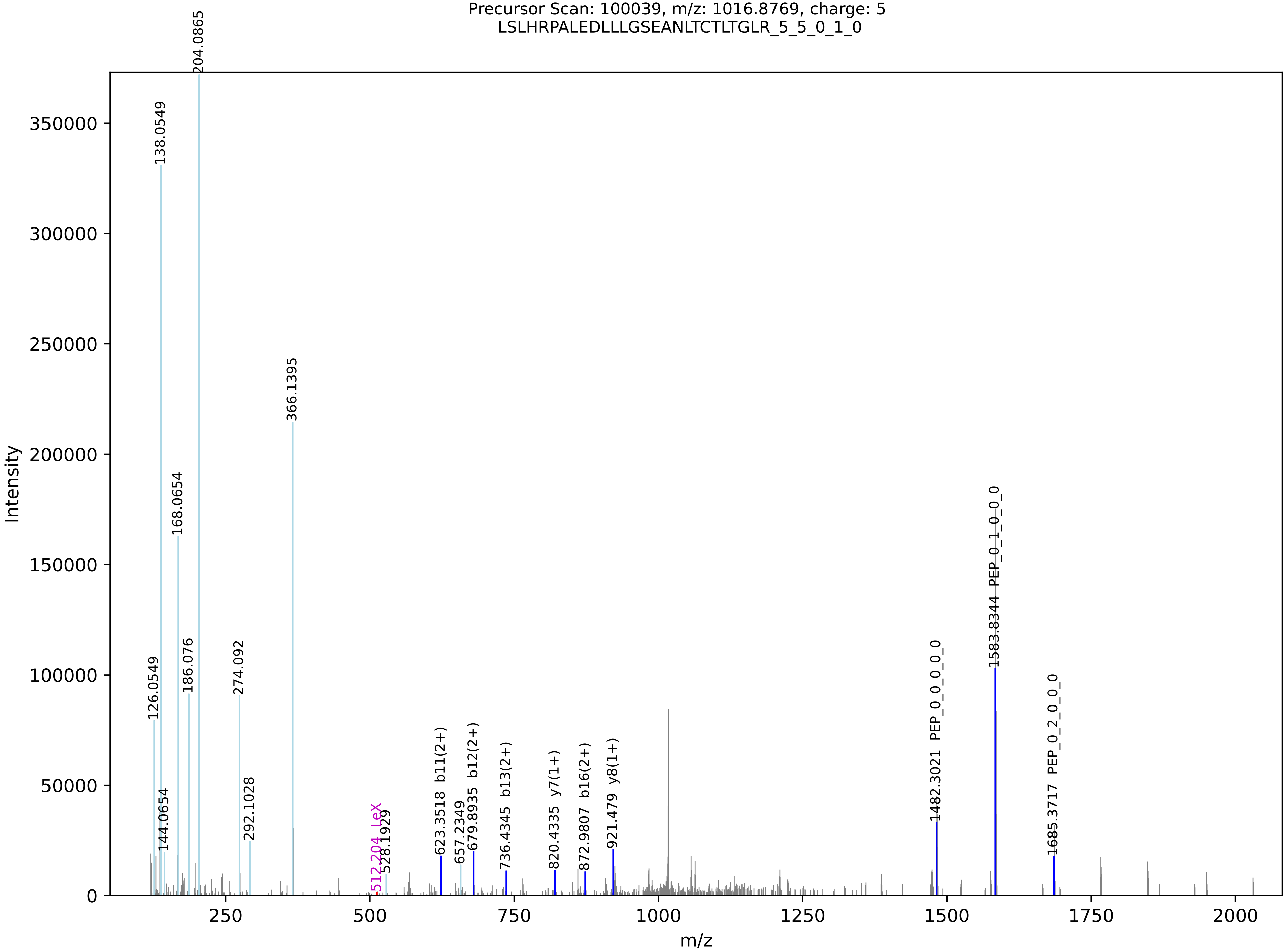

File: 231129_OE_RK_36, RT: 75.904, Scan: 100114
Type: HCD, FTMS + c NSI d Full ms2 1280.8209@hcd30.00 [120.0000-5248.6055]
Precursor Scan: 100039, m/z: 1280.0692, charge: 4
SVQEIQATFFYFTPNKTEDTIFLR_6_4_1_1_0

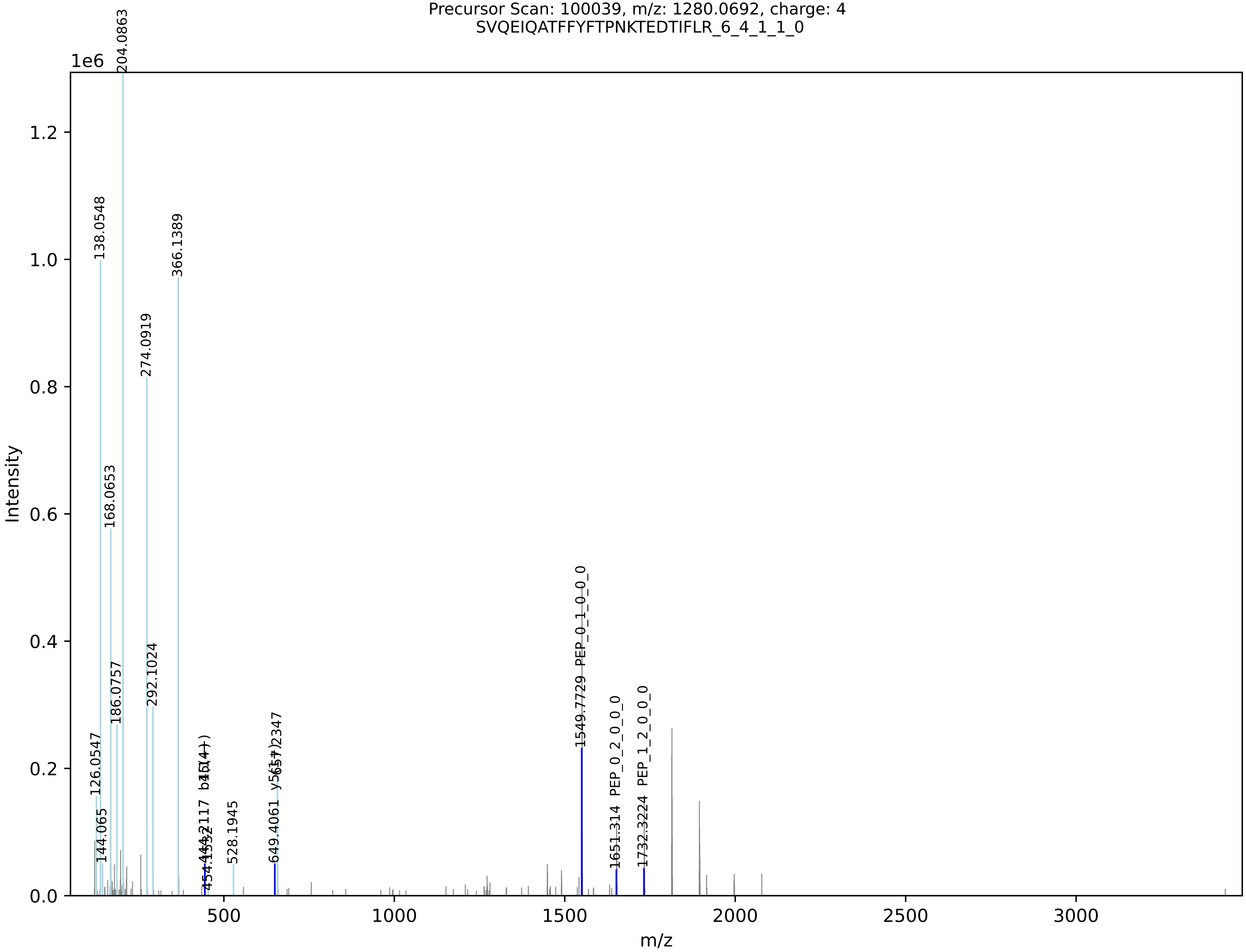

File: 231129_OE_RK_36, RT: 75.952, Scan: 100184
Type: HCD, FTMS + c NSI d Full ms2 1220.5765@hcd30.00 [120.0000-5002.8081]
Precursor Scan: 100112, m/z: 1220.0753, charge: 4
LSLHRPALEDLLLGSEANLTCTLTGLR_5_4_0_1_0

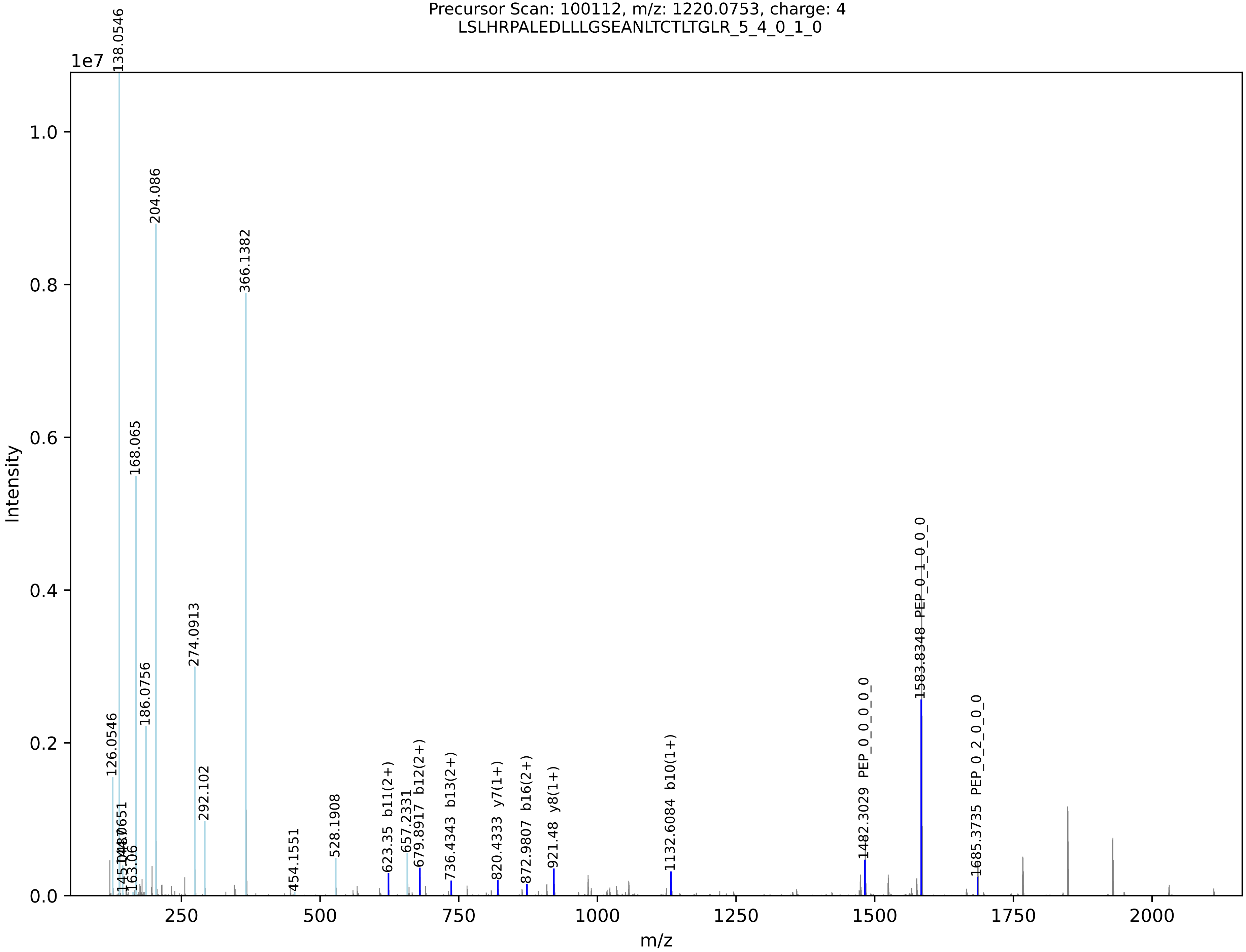

File: 231129_OE_RK_36, RT: 76.002, Scan: 100258
Type: HCD, FTMS + c NSI d Full ms2 976.8639@hcd30.00 [120.0000-5005.5757]
Precursor Scan: 100183, m/z: 976.2622, charge: 5
LSLHRPALEDLLLGSEANLTCTLTGLR_5_4_0_1_0

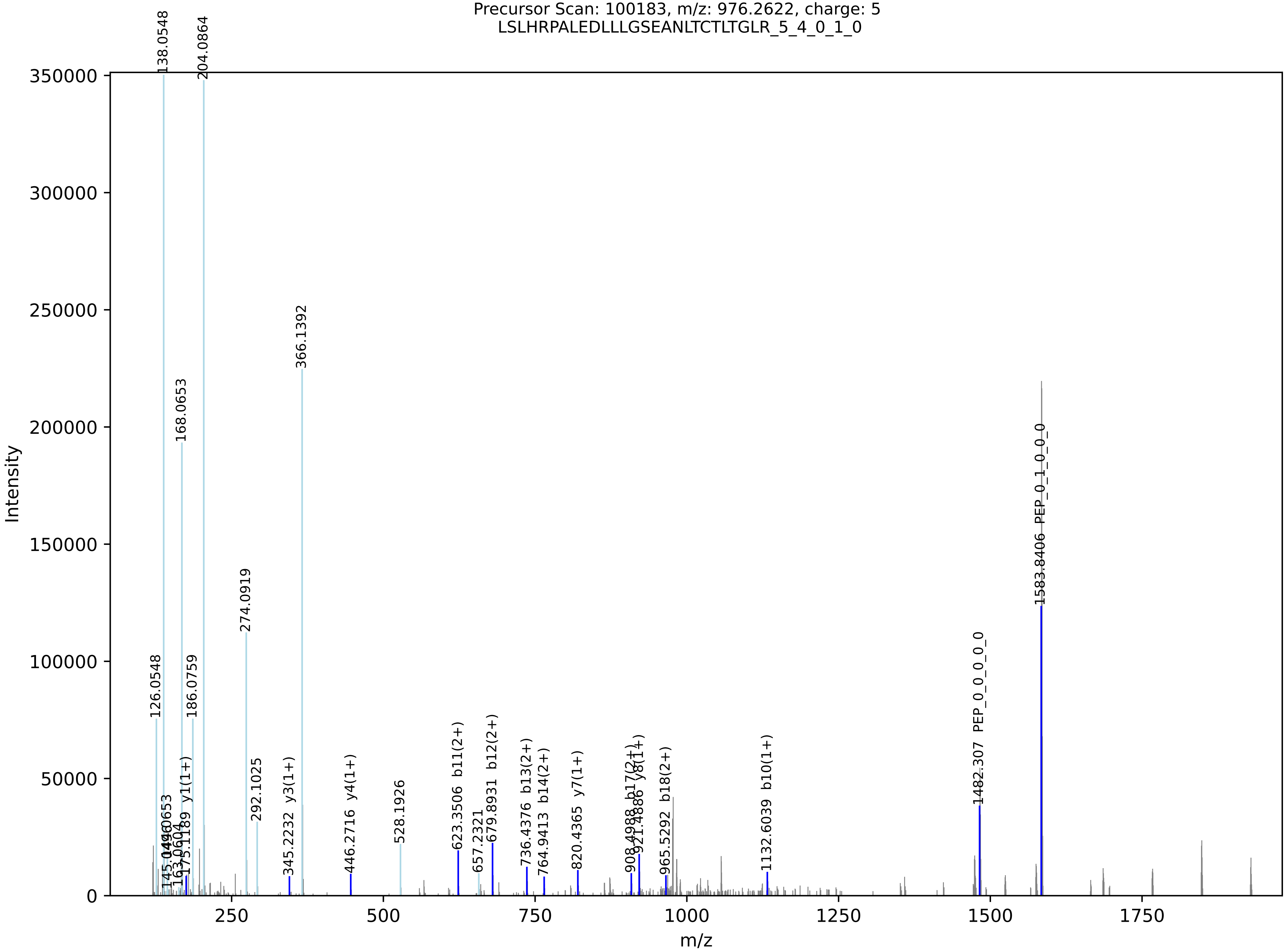

File: 231129_OE_RK_36, RT: 76.108, Scan: 100410
Type: HCD, FTMS + c NSI d Full ms2 1280.5704@hcd30.00 [120.0000-5247.5835]
Precursor Scan: 100331, m/z: 1280.0661, charge: 4
SVQEIQATFFYFTPNKTEDTIFLR_6_4_1_1_0

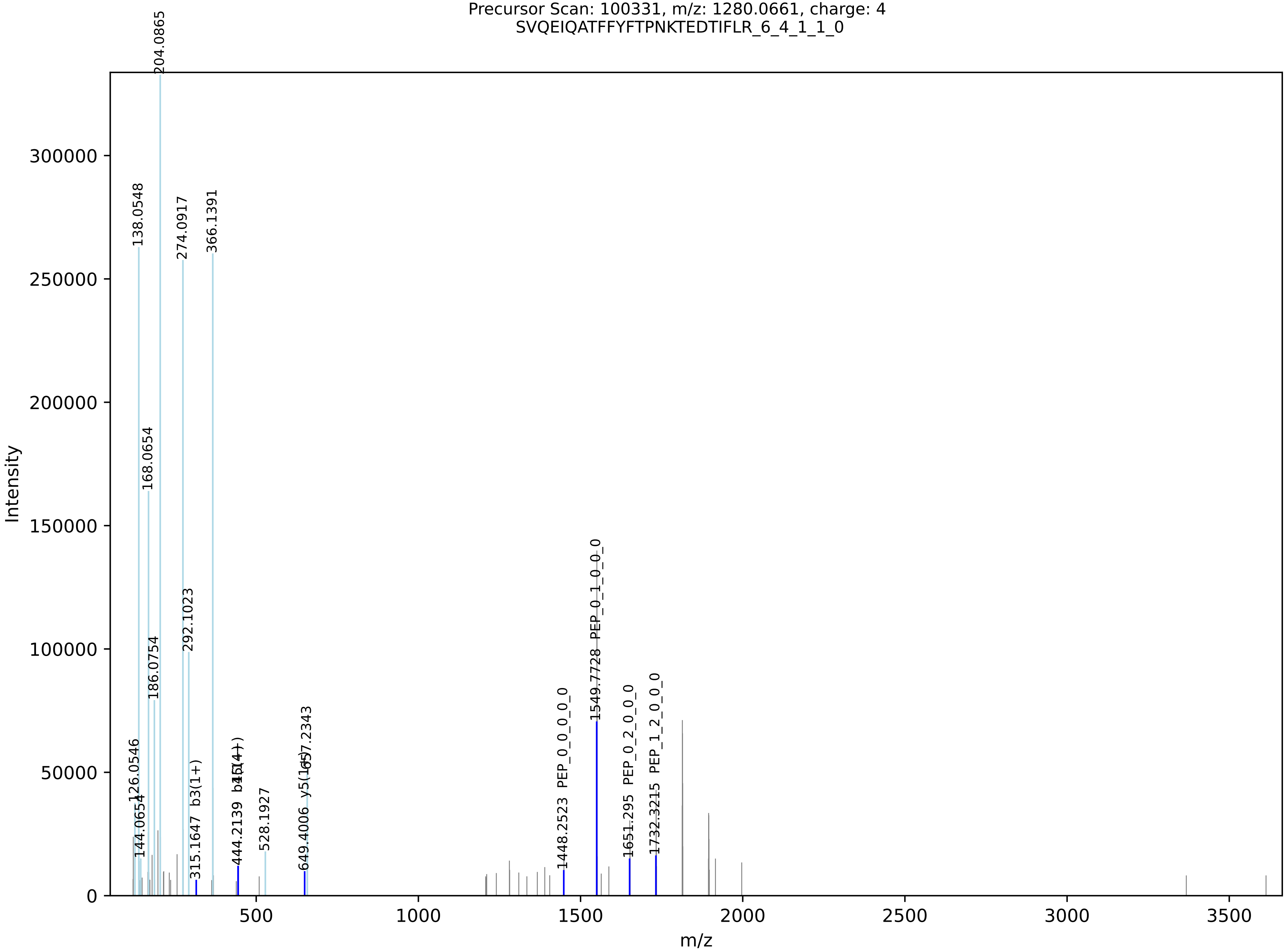

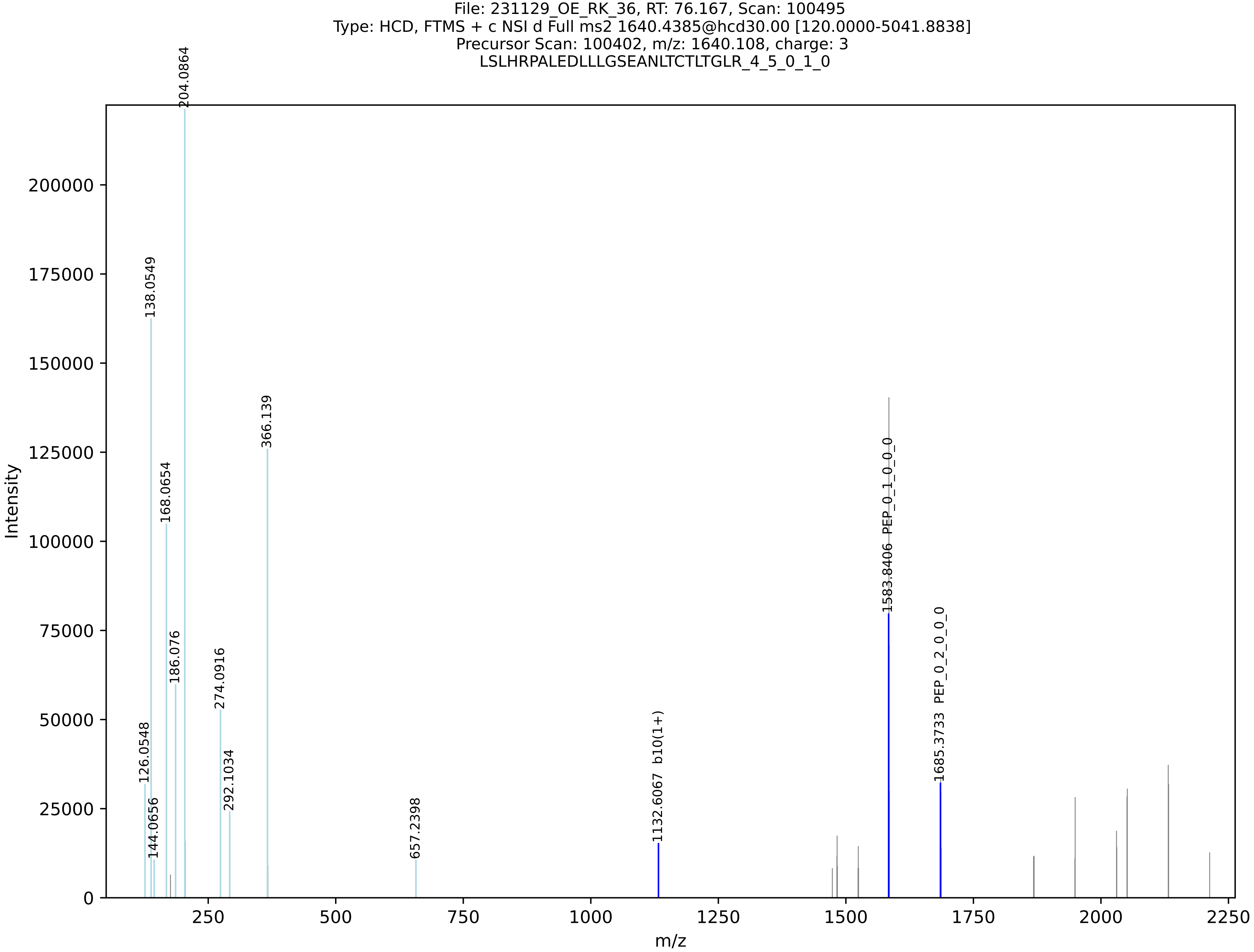

File: 231129_OE_RK_36, RT: 76.167, Scan: 100495
Type: HCD, FTMS + c NSI d Full ms2 1640.4385@hcd30.00 [120.0000-5041.8838]
Precursor Scan: 100402, m/z: 1640.108, charge: 3
LSLHRPALEDLLLGSEANLTCTLTGLR_4_5_0_1_0
Intensity
m/z
204.0864
138.0549
366.139
168.0654
186.076
274.0916
126.0548
292.1034
144.0656
657.2398
1132.6067 b10(1+)
1583.8406 PEP_0_1_0_0_0
1685.3733 PEP_0_2_0_0_0
0
25000
50000
75000
100000
125000
150000
175000
200000
250
500
750
1000
1250
1500
1750
2000
2250

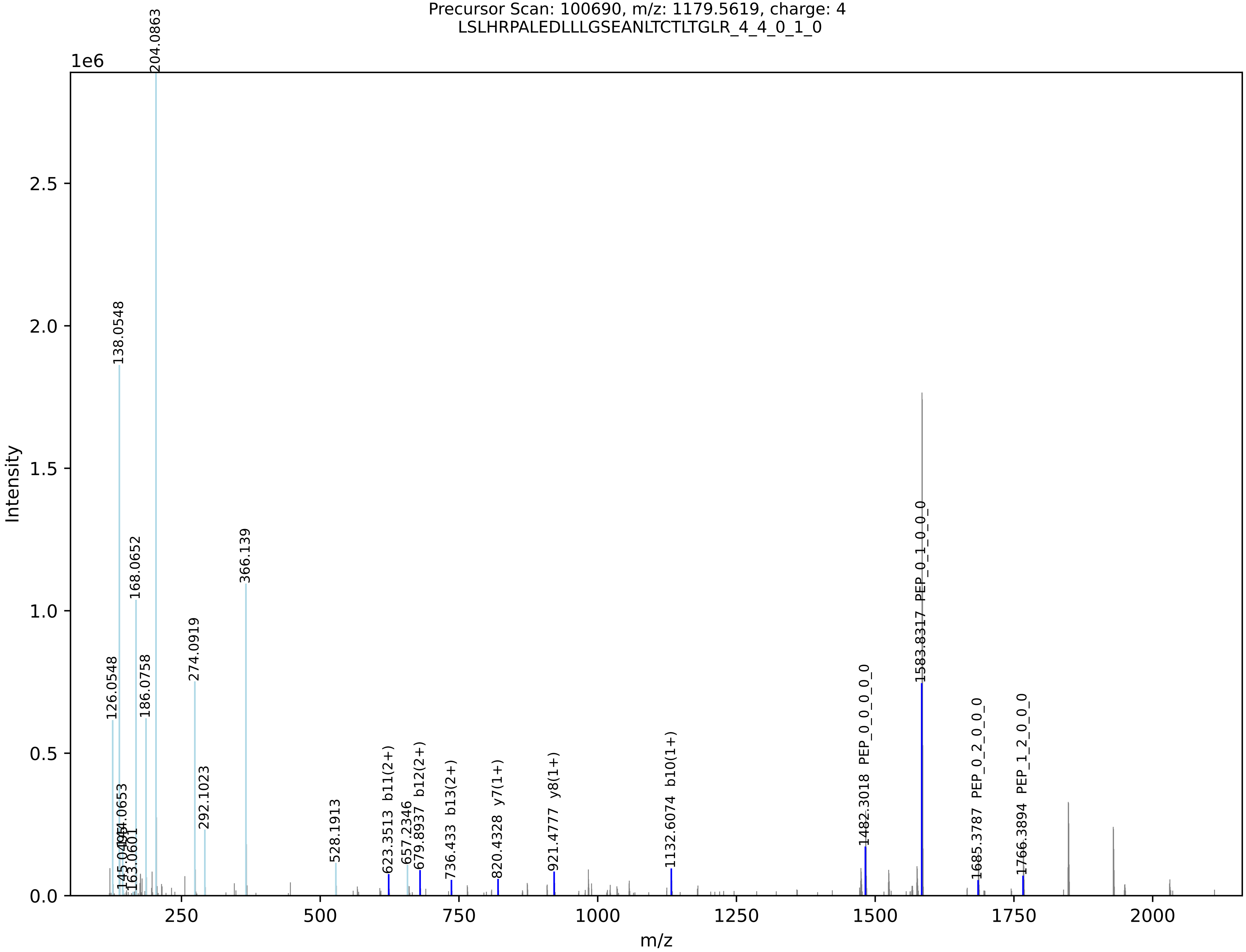

File: 231129_OE_RK_36, RT: 76.356, Scan: 100766
Type: HCD, FTMS + c NSI d Full ms2 1180.3136@hcd30.00 [120.0000-4838.5352]
Precursor Scan: 100690, m/z: 1179.5619, charge: 4
LSLHRPALEDLLLGSEANLTCTLTGLR_4_4_0_1_0
1e6
Intensity
m/z
204.0863
138.0548
168.0652
366.139
274.0919
126.0548
186.0758
292.1023
528.1913
623.3513 b11(2+)
657.2346
679.8937 b12(2+)
736.433 b13(2+)
820.4328 y7(1+)
921.4777 y8(1+)
1132.6074 b10(1+)
1482.3018 PEP_0_0_0_0_0
1583.8317 PEP_0_1_0_0_0
1685.3787 PEP_0_2_0_0_0
1766.3894 PEP_1_2_0_0_0
163.0601

File: 231129_OE_RK_36, RT: 76.61, Scan: 101132
Type: HCD, FTMS + c NSI d Full ms2 1129.5435@hcd30.00 [120.0000-4631.3931]
Precursor Scan: 101056, m/z: 1128.7917, charge: 4
LSLHRPALEDLLLGSEANLTCTLTGLR_4_3_0_1_0

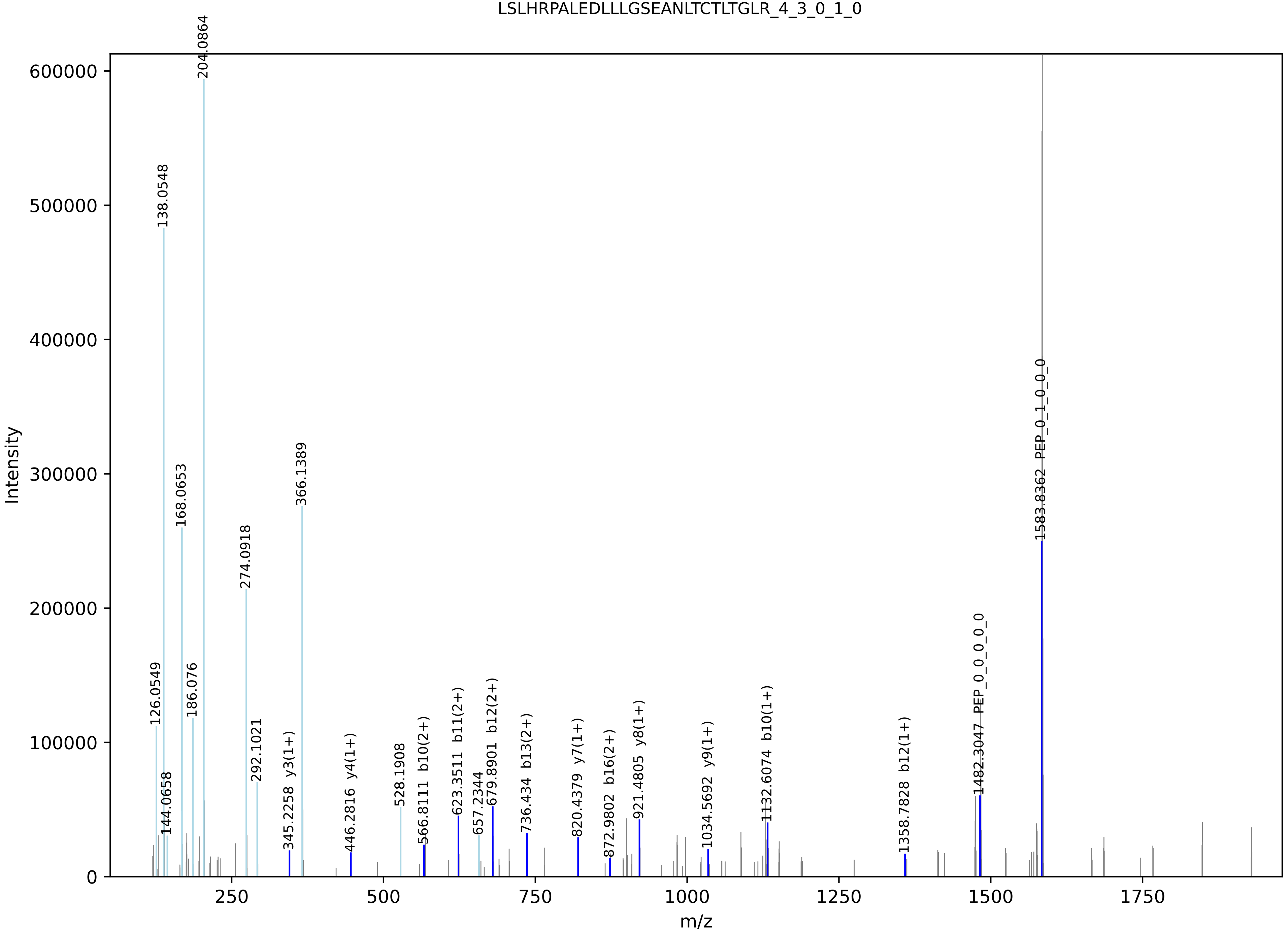

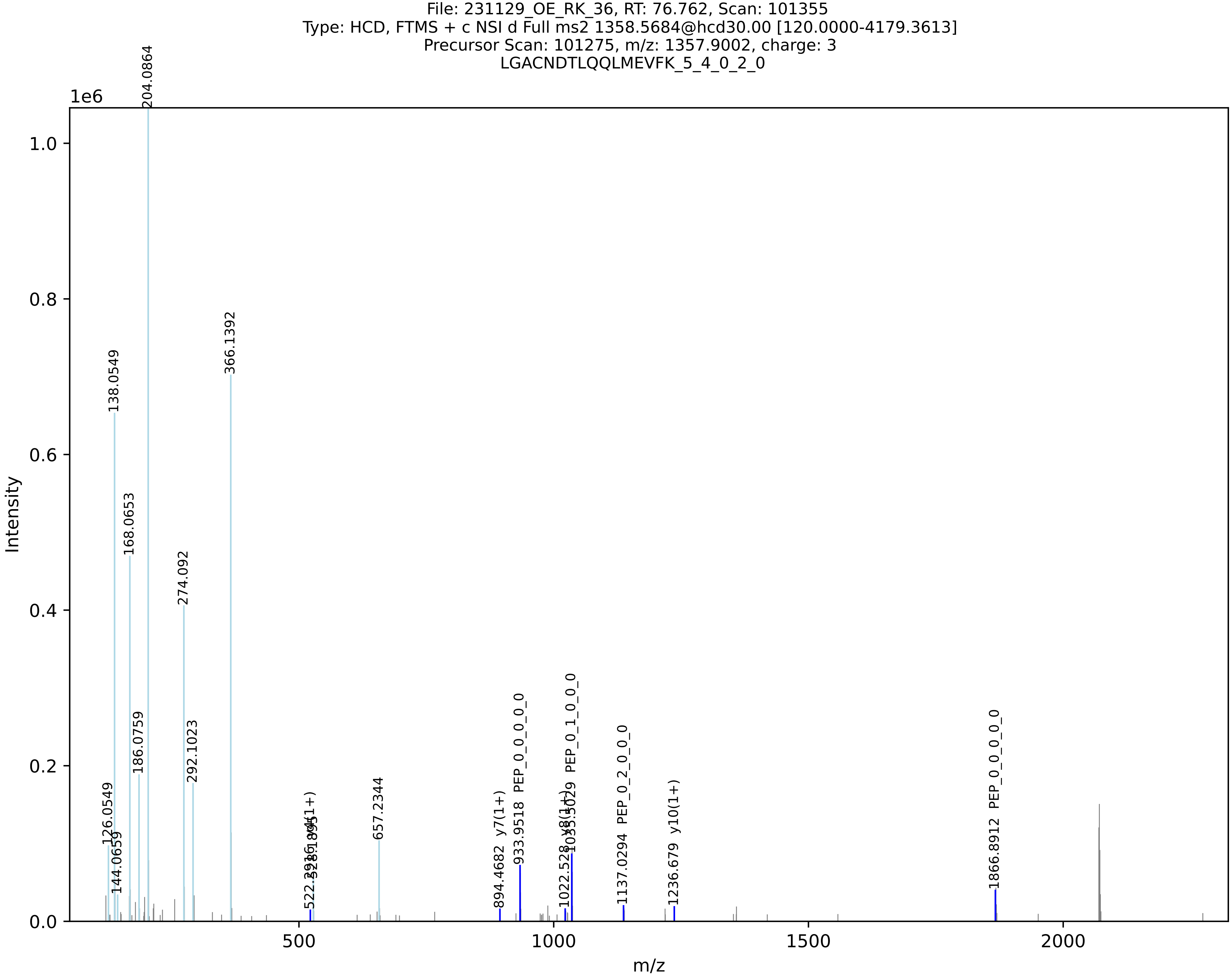

File: 231129_OE_RK_36, RT: 76.762, Scan: 101355
Type: HCD, FTMS + c NSI d Full ms2 1358.5684@hcd30.00 [120.0000-4179.3613]
Precursor Scan: 101275, m/z: 1357.9002, charge: 3
LGACNDTLQQLMEVFK_5_4_0_2_0
1e6
Intensity
m/z
204.0864
138.0549
366.1392
168.0653
274.092
292.1023
186.0759
126.0549
144.0659
657.2344
894.4682 y7(1+)
933.9518 PEP_0_0_0_0_0
PEP_0_1_0_0_0
1137.0294 PEP_0_2_0_0_0
1236.679 y10(1+)
1866.8912 PEP_0_0_0_0_0

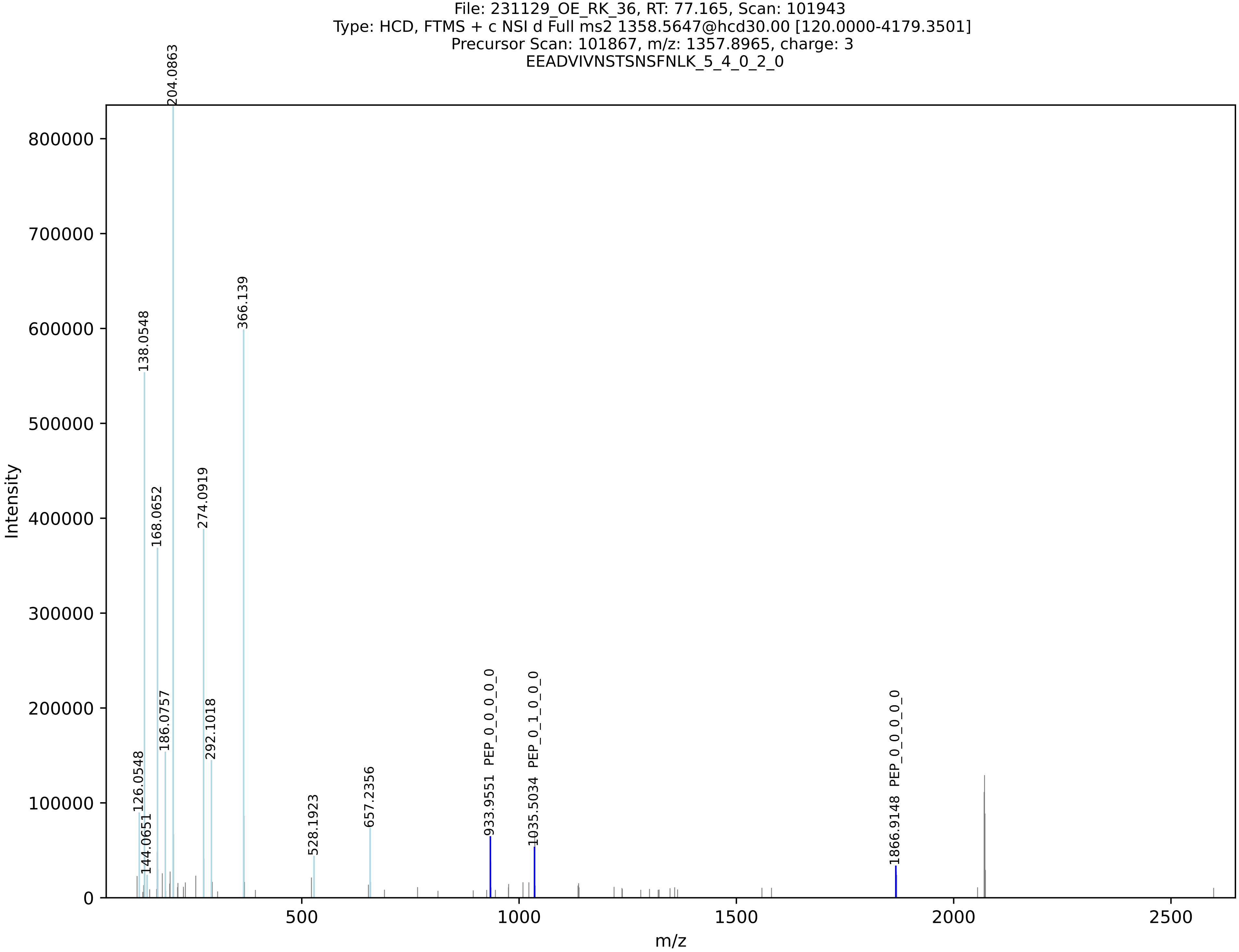

File: 231129_OE_RK_36, RT: 77.165, Scan: 101943
Type: HCD, FTMS + c NSI d Full ms2 1358.5647@hcd30.00 [120.0000-4179.3501]
Precursor Scan: 101867, m/z: 1357.8965, charge: 3
EEADVIVNSTSNSFNLK_5_4_0_2_0
Intensity
m/z
204.0863
366.139
138.0548
274.0919
168.0652
186.0757
292.1018
126.0548
144.0651
528.1923
657.2356
933.9551 PEP_0_0_0_0_0
1035.5034 PEP_0_1_0_0_0
1866.9148 PEP_0_0_0_0_0

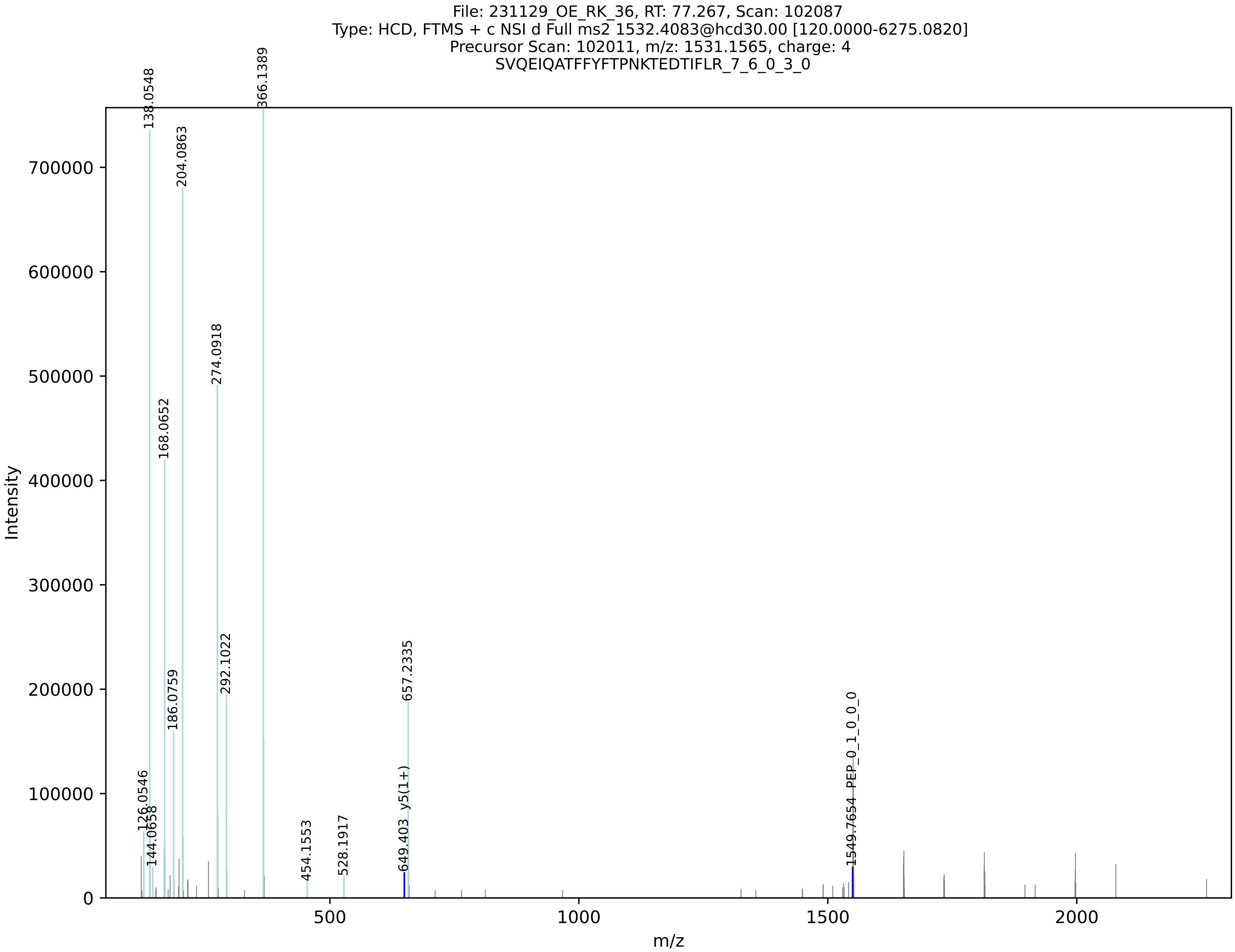
File: 231129_OE_RK_36, RT: 77.267, Scan: 102087
Type: HCD, FTMS + c NSI d Full ms2 1532.4083@hcd30.00 [120.0000-6275.0820]
Precursor Scan: 102011, m/z: 1531.1565, charge: 4
SVQEIQATFFYFTPNKTEDTIFLR_7_6_0_3_0
Intensity
m/z
138.0548
366.1389
204.0863
274.0918
168.0652
292.1022
186.0759
657.2335
126.0546
144.0658
454.1553
528.1917
649.403 y5(1+)
1549.7654 PEP_0_1_0_0_0
0
100000
200000
300000
400000
500000
600000
700000
500
1000
1500
2000

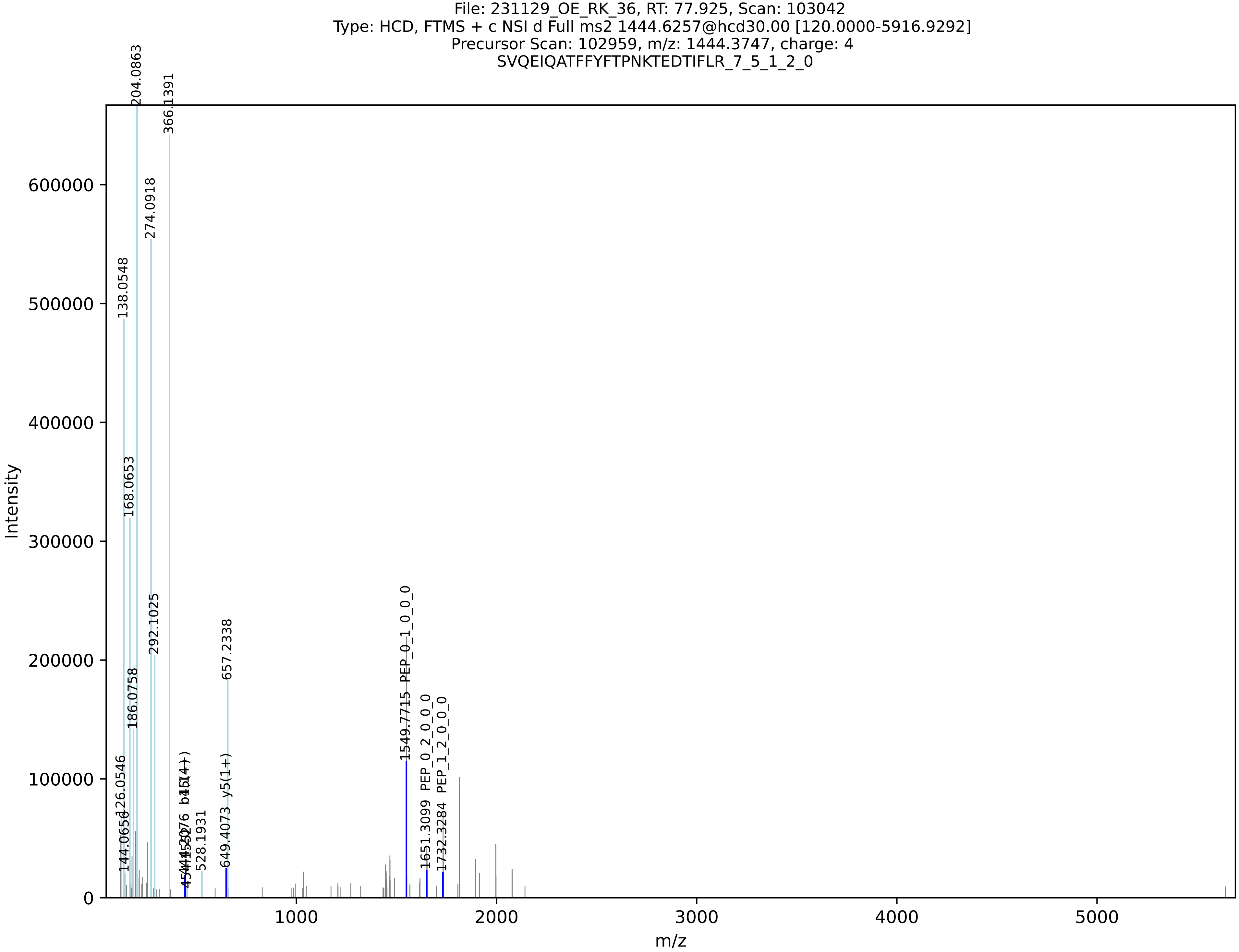
File: 231129_OE_RK_36, RT: 77.925, Scan: 103042
Type: HCD, FTMS + c NSI d Full ms2 1444.6257@hcd30.00 [120.0000-5916.9292]
Precursor Scan: 102959, m/z: 1444.3747, charge: 4
SVQEIQATFFYFTPNKTEDTIFLR_7_5_1_2_0
Intensity
m/z
204.0863
366.1391
274.0918
138.0548
168.0653
292.1025
657.2338
186.0758
126.0546
144.0650
528.1931
649.4073 y5(1+)
1549.7715 PEP_0_1_0_0_0
1651.3099 PEP_0_2_0_0_0
1732.3284 PEP_1_2_0_0_0
0
100000
200000
300000
400000
500000
600000
1000
2000
3000
4000
5000

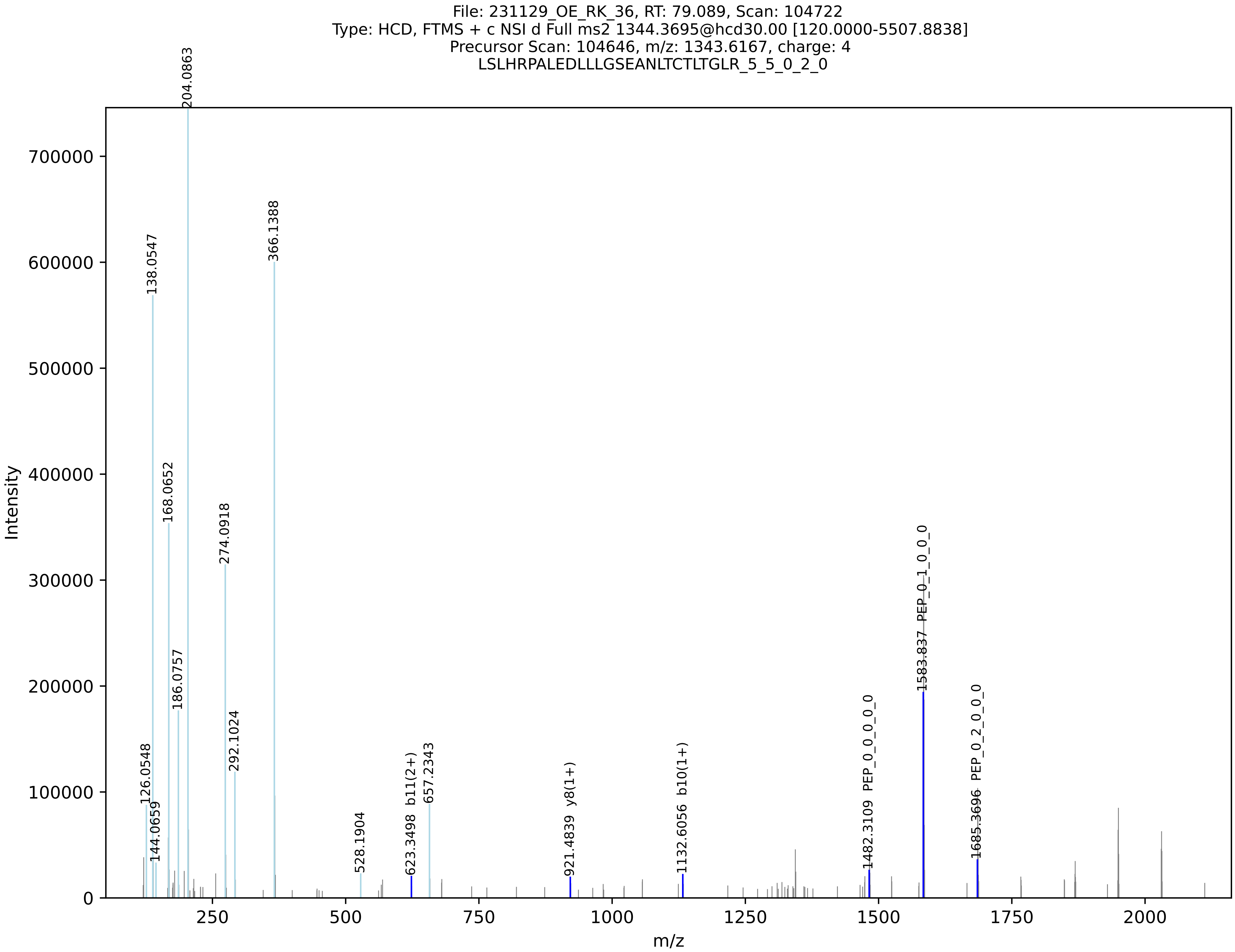
File: 231129_OE_RK_36, RT: 79.089, Scan: 104722
Type: HCD, FTMS + c NSI d Full ms2 1344.3695@hcd30.00 [120.0000-5507.8838]
Precursor Scan: 104646, m/z: 1343.6167, charge: 4
LSLHRPALEDLLLGSEANLTCTLTGLR_5_5_0_2_0
Intensity
m/z
204.0863
138.0547
366.1388
168.0652
274.0918
186.0757
292.1024
126.0548
144.0659
528.1904
623.3498 b11(2+)
657.2343
921.4839 y8(1+)
1132.6056 b10(1+)
1482.3109 PEP_0_0_0_0_0
1583.837 PEP_0_1_0_0_0
1685.3696 PEP_0_2_0_0_0

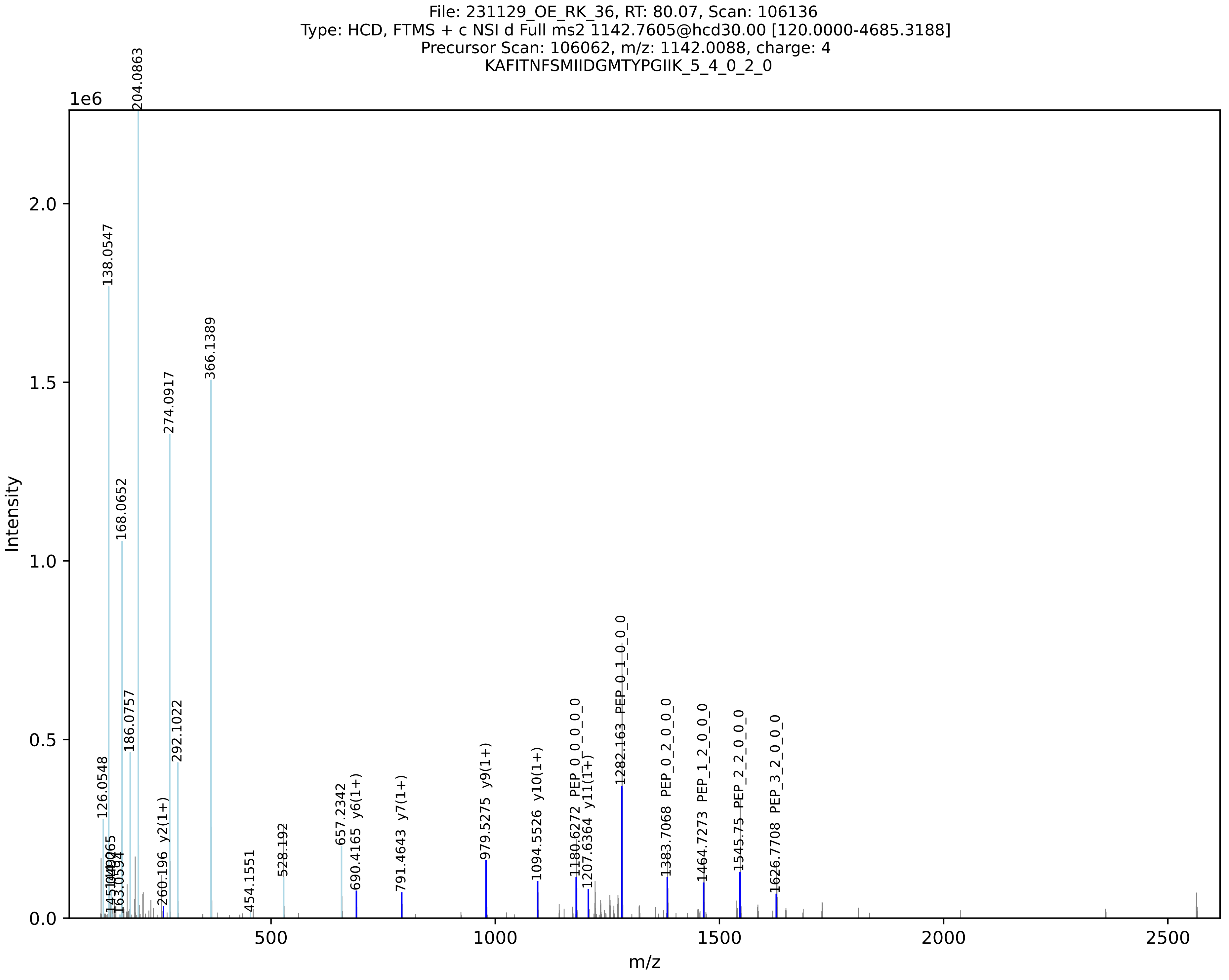

File: 231129_OE_RK_36, RT: 80.07, Scan: 106136
Type: HCD, FTMS + c NSI d Full ms2 1142.7605@hcd30.00 [120.0000-4685.3188]
Precursor Scan: 106062, m/z: 1142.0088, charge: 4
KAFITNFSMIIDGMTYPGIIK_5_4_0_2_0
1e6
Intensity
m/z
204.0863
138.0547
366.1389
274.0917
168.0652
186.0757
292.1022
126.0548
163.0594
260.196 y2(1+)
454.1551
528.192
657.2342
690.4165 y6(1+)
791.4643 y7(1+)
979.5275 y9(1+)
1094.5526 y10(1+)
1180.6272 PEP_0_0_0_0_0
1207.6364 y11(1+)
1282.163 PEP_0_1_0_0_0
1383.7068 PEP_0_2_0_0_0
1464.7273 PEP_1_2_0_0_0
1545.75 PEP_2_2_0_0_0
1626.7708 PEP_3_2_0_0_0

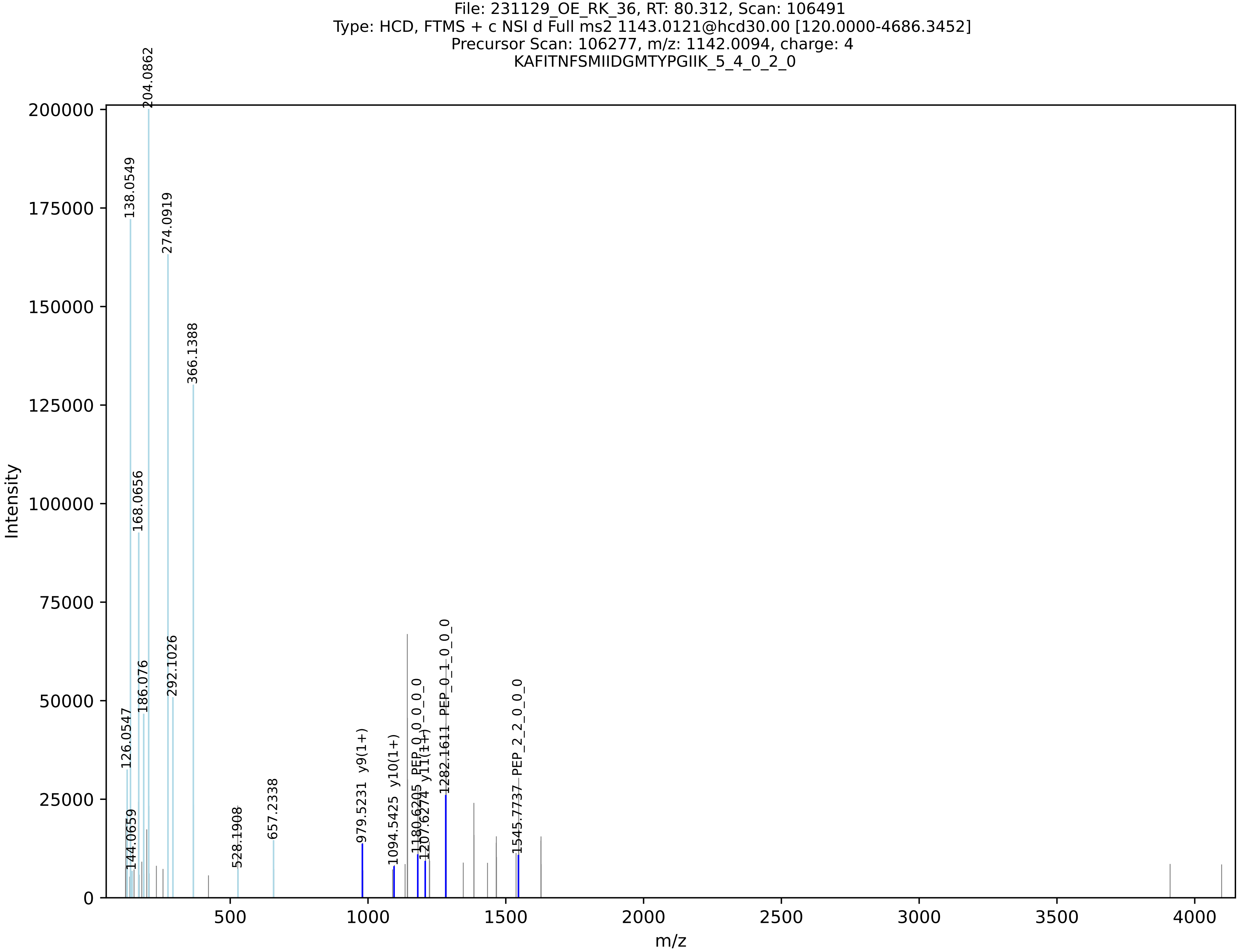

File: 231129_OE_RK_36, RT: 80.312, Scan: 106491
Type: HCD, FTMS + c NSI d Full ms2 1143.0121@hcd30.00 [120.0000-4686.3452]
Precursor Scan: 106277, m/z: 1142.0094, charge: 4
KAFITNFSMIIDGMTYPGIIK_5_4_0_2_0
Intensity
m/z
204.0862
138.0549
274.0919
366.1388
168.0656
186.076
292.1026
126.0547
144.0659
528.1908
657.2338
979.5231 y9(1+)
1094.5425 y10(1+)
1180.6205 PEP_0_0_0_0_0
1207.6274 y11(1+)
1282.1611 PEP_0_1_0_0_0
1545.7737 PEP_2_2_0_0_0

File: 231129_OE_RK_36, RT: 80.322, Scan: 106496
Type: HCD, FTMS + c NSI d Full ms2 1092.1117@hcd30.00 [120.0000-5593.3398]
Precursor Scan: 106422, m/z: 1091.7105, charge: 5
YLGNATAIFFLPDEGKLQHLENELTHDIITK_5_4_0_1_0

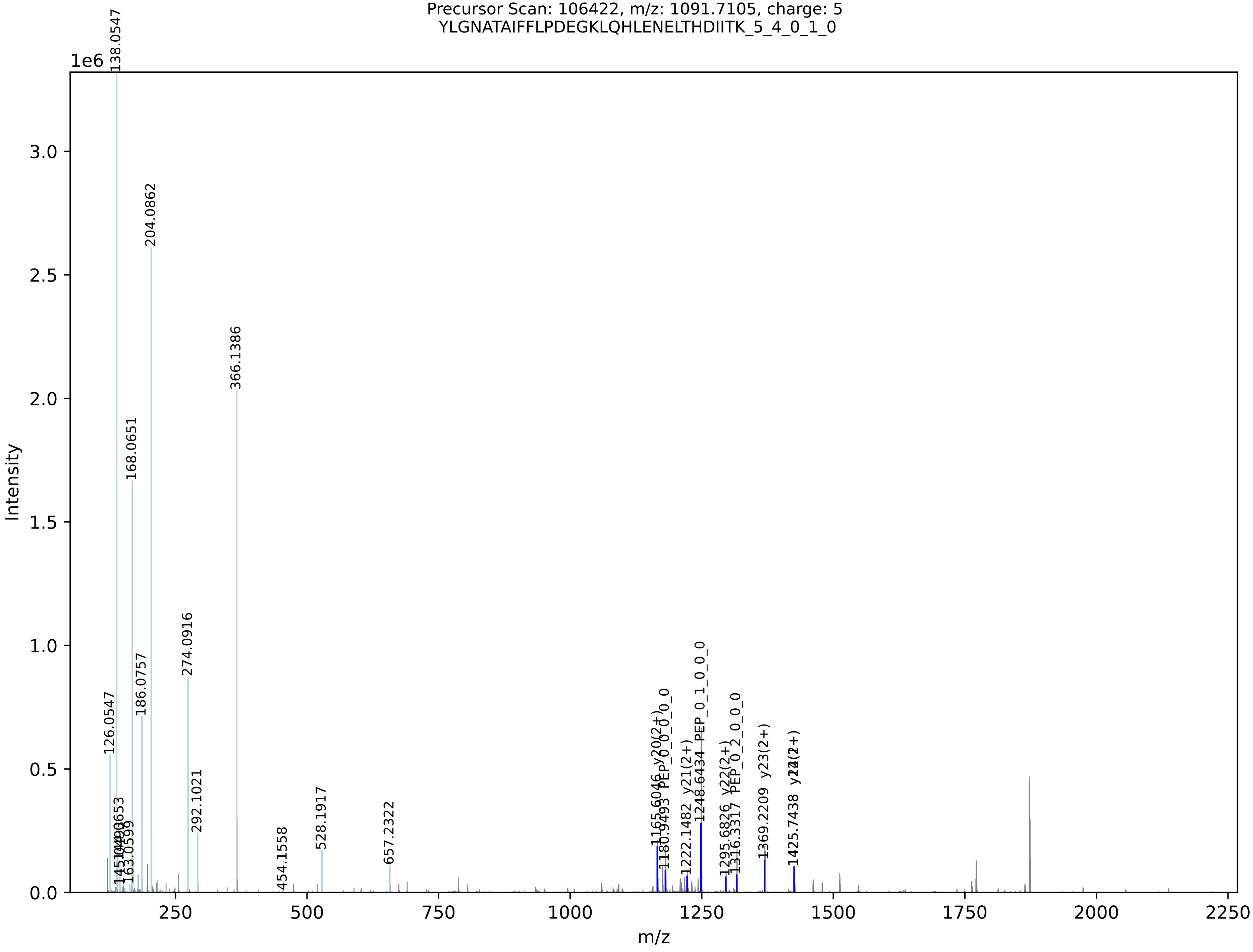

File: 231129_OE_RK_36, RT: 80.371, Scan: 106569
Type: HCD, FTMS + c NSI d Full ms2 1142.2616@hcd30.00 [120.0000-4683.2832]
Precursor Scan: 106495, m/z: 1142.011, charge: 4
KAFITNFSMIIDGMTYPGIIK_5_4_0_2_0

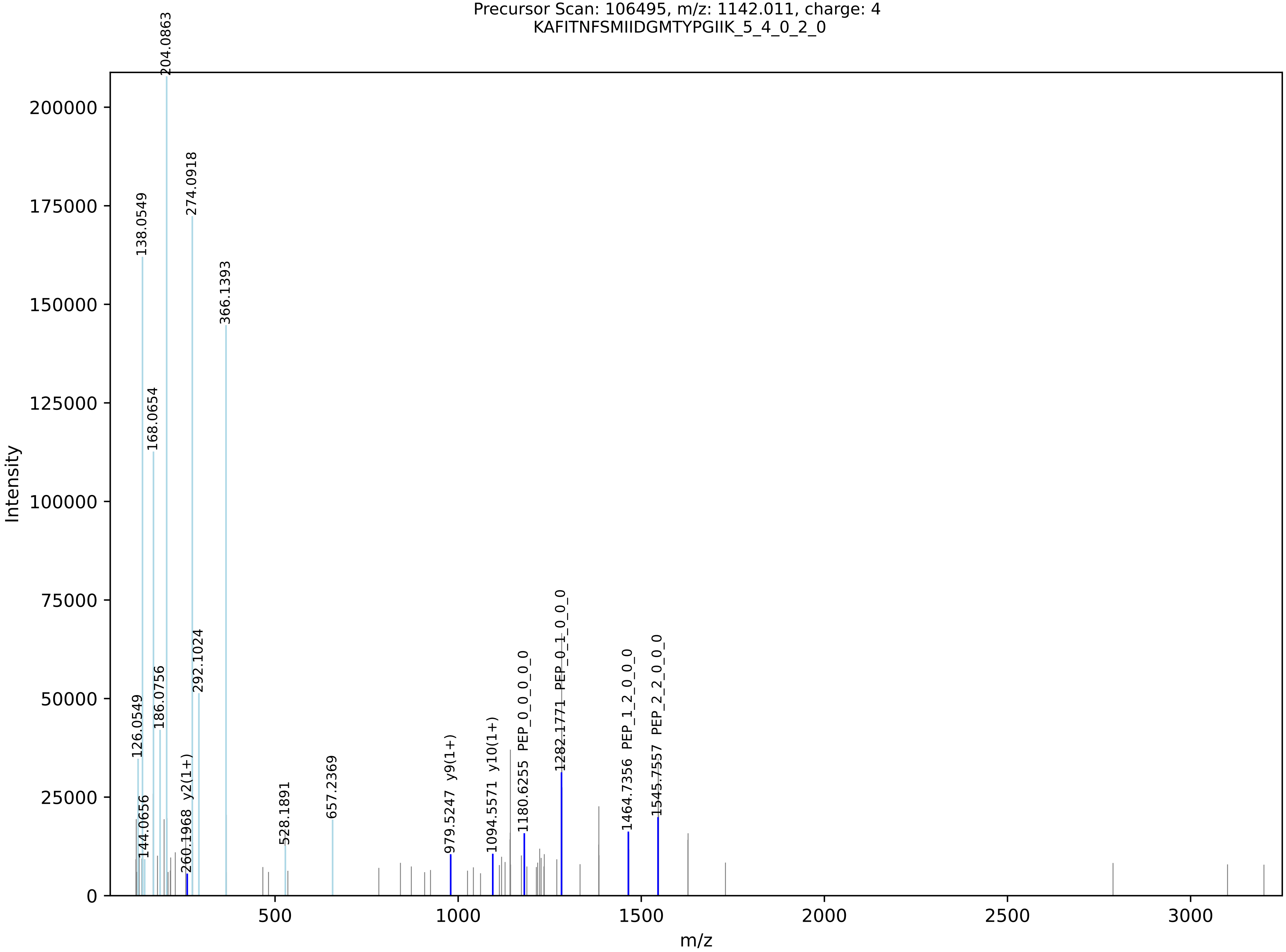

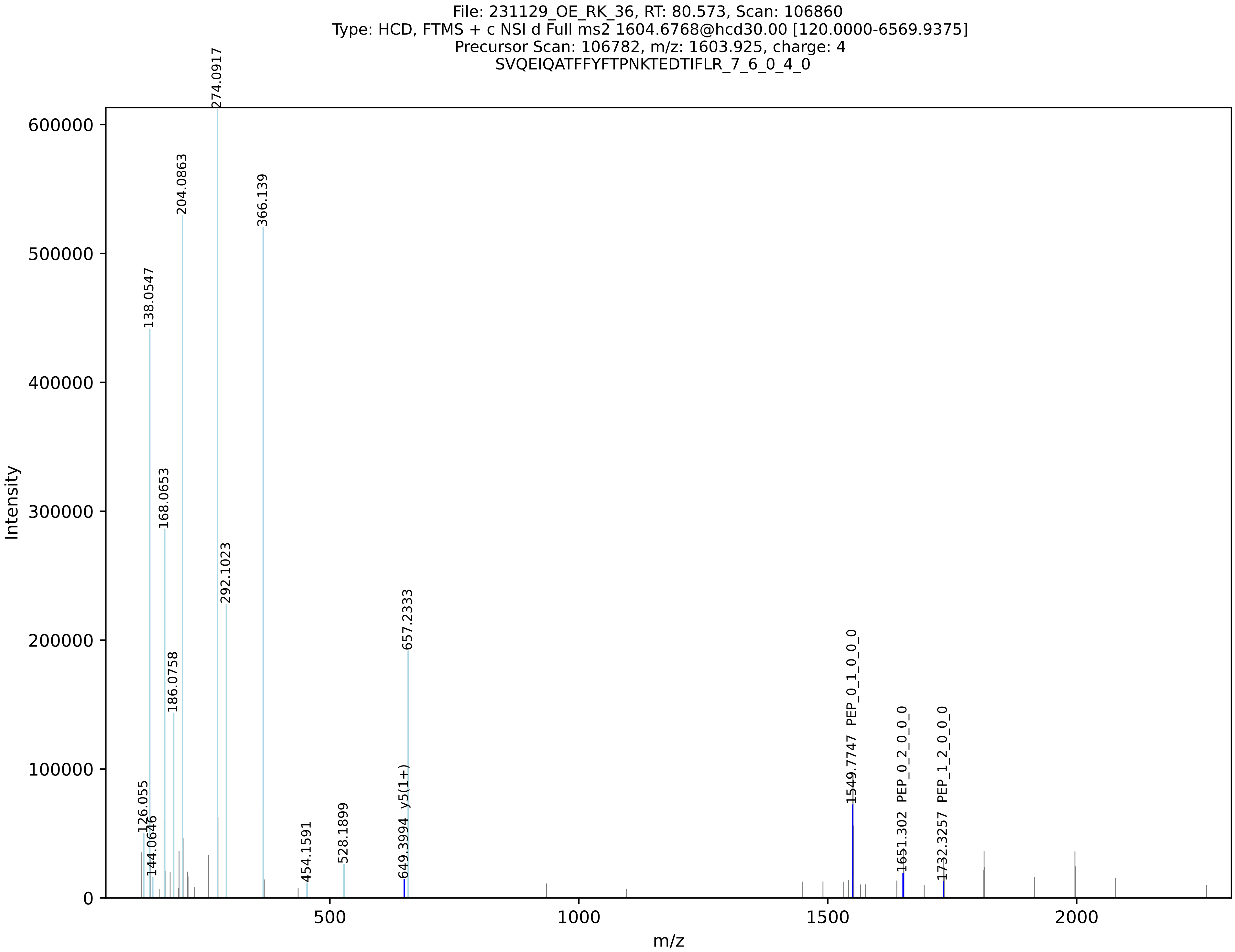
File: 231129_OE_RK_36, RT: 80.573, Scan: 106860
Type: HCD, FTMS + c NSI d Full ms2 1604.6768@hcd30.00 [120.0000-6569.9375]
Precursor Scan: 106782, m/z: 1603.925, charge: 4
SVQEIQATFFYFTPNKTEDTIFLR_7_6_0_4_0
Intensity
m/z
274.0917
204.0863
366.139
138.0547
168.0653
292.1023
186.0758
657.2333
126.055
144.0646
454.1591
528.1899
649.3994 y5(1+)
1549.7747 PEP_0_1_0_0_0
1651.302 PEP_0_2_0_0_0
1732.3257 PEP_1_2_0_0_0
600000
500000
400000
300000
200000
100000
0
500
1000
1500
2000

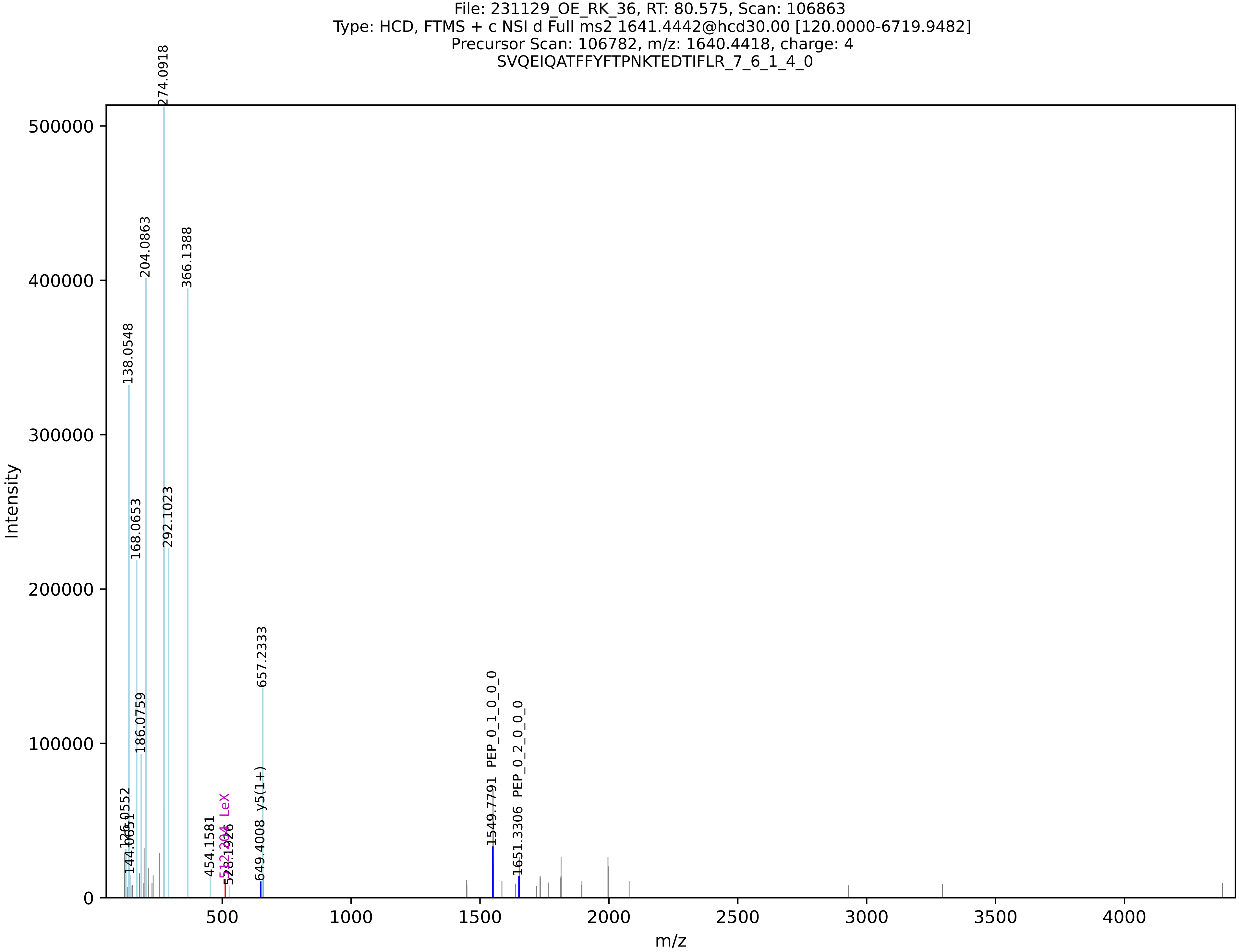

File: 231129_OE_RK_36, RT: 80.575, Scan: 106863
Type: HCD, FTMS + c NSI d Full ms2 1641.4442@hcd30.00 [120.0000-6719.9482]
Precursor Scan: 106782, m/z: 1640.4418, charge: 4
SVQEIQATFFYFTPNKTEDTIFLR_7_6_1_4_0
274.0918
204.0863
366.1388
138.0548
168.0653
292.1023
657.2333
186.0759
126.0552
144.0651
454.1581
512.204 LeX
528.1926
649.4008 y5(1+)
1549.7791 PEP_0_1_0_0_0_
1651.3306 PEP_0_2_0_0_0_
Intensity
m/z
500000
400000
300000
200000
100000
0
500
1000
1500
2000
2500
3000
3500
4000

File: 231129_OE_RK_36, RT: 81.582, Scan: 108321
Type: HCD, FTMS + c NSI d Full ms2 1142.7620@hcd30.00 [120.0000-4685.3247]
Precursor Scan: 108245, m/z: 1142.0097, charge: 4
KAFITNFSMIIDGMTYPGIIK_5_4_0_2_0

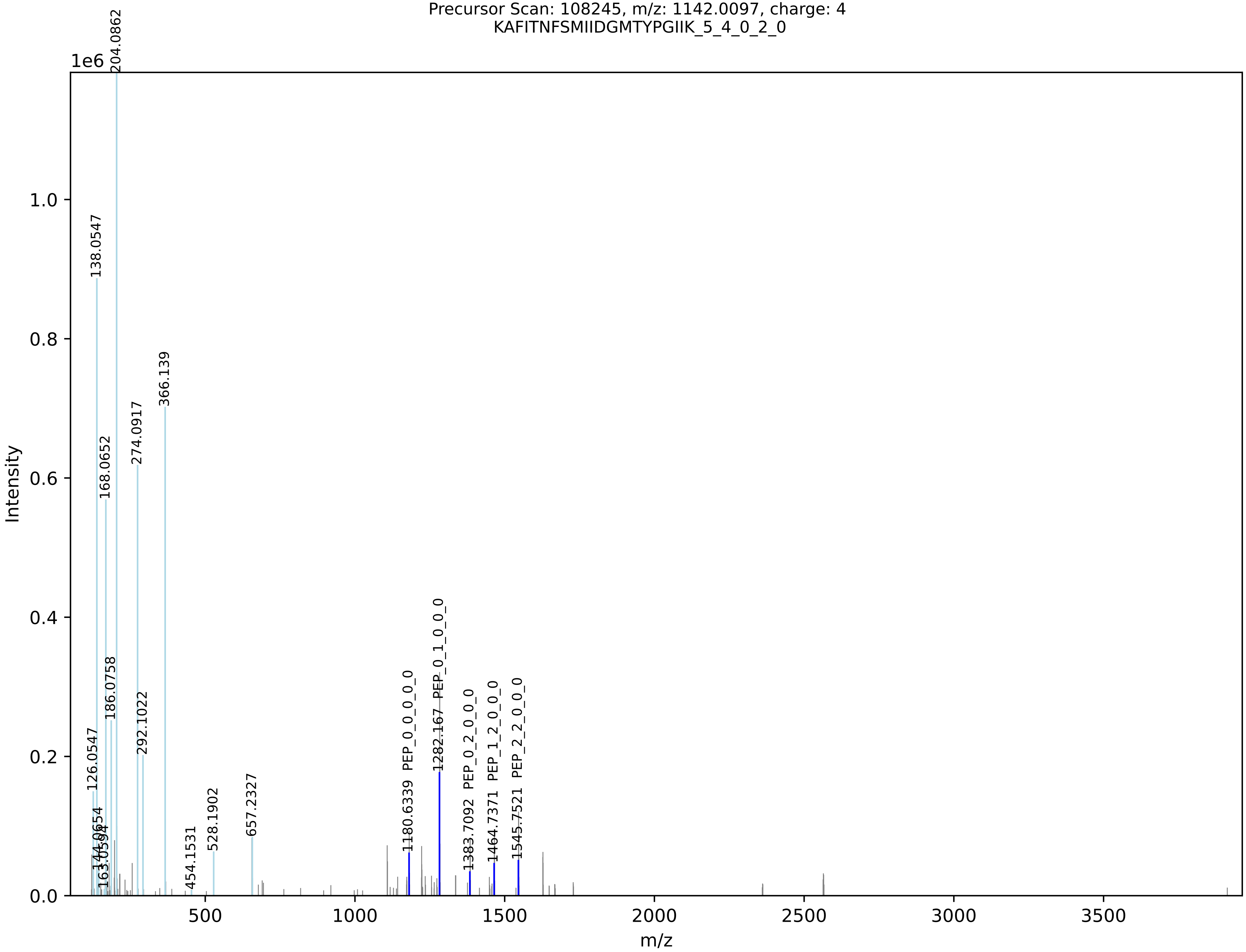

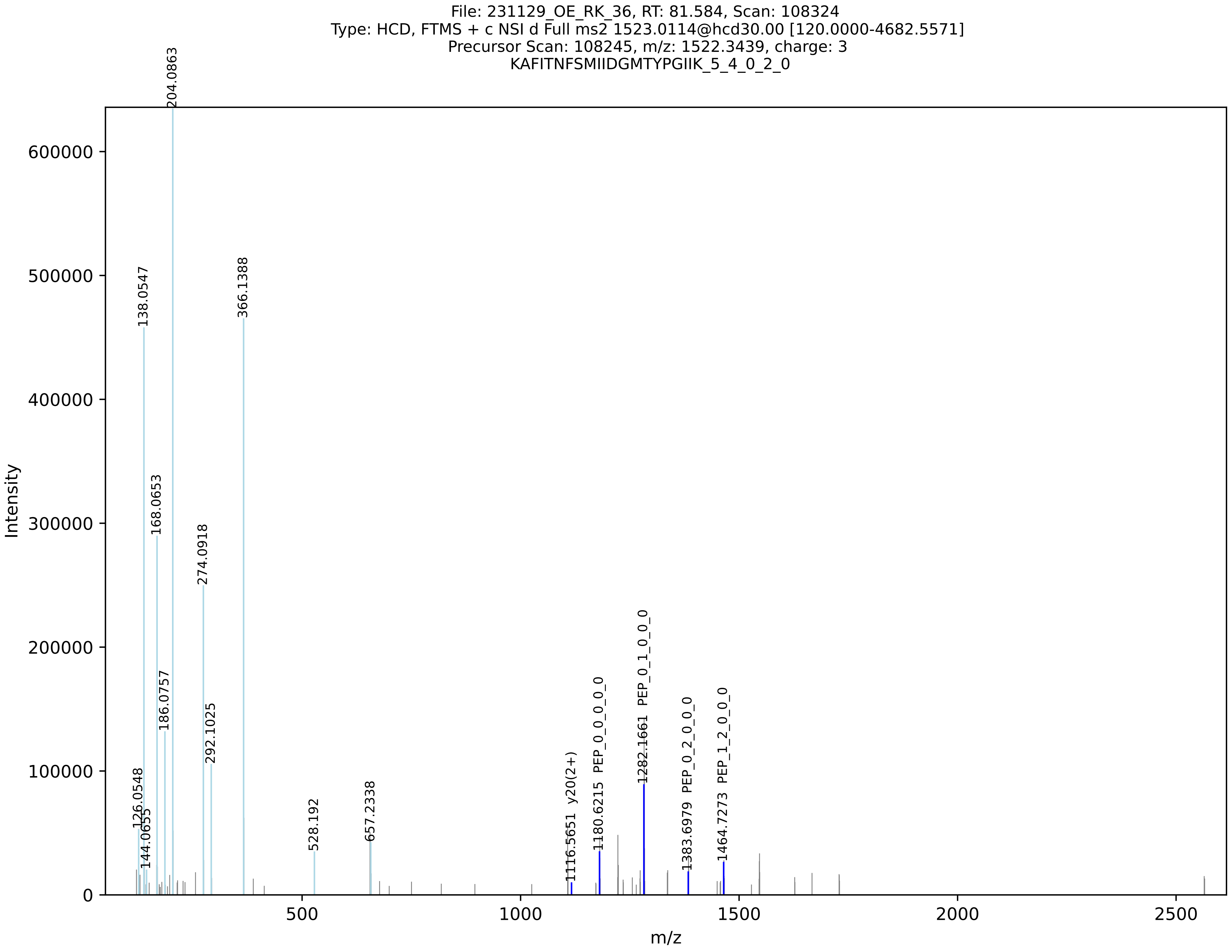

File: 231129_OE_RK_36, RT: 81.584, Scan: 108324
Type: HCD, FTMS + c NSI d Full ms2 1523.0114@hcd30.00 [120.0000-4682.5571]
Precursor Scan: 108245, m/z: 1522.3439, charge: 3
KAFITNFSMIIDGMTYPGIIK_5_4_0_2_0
Intensity
m/z
204.0863
138.0547
366.1388
168.0653
274.0918
186.0757
292.1025
126.0548
144.0655
528.192
657.2338
1116.5651 y20(2+)
1180.6215 PEP_0_0_0_0_0
1282.1661 PEP_0_1_0_0_0
1383.6979 PEP_0_2_0_0_0
1464.7273 PEP_1_2_0_0_0

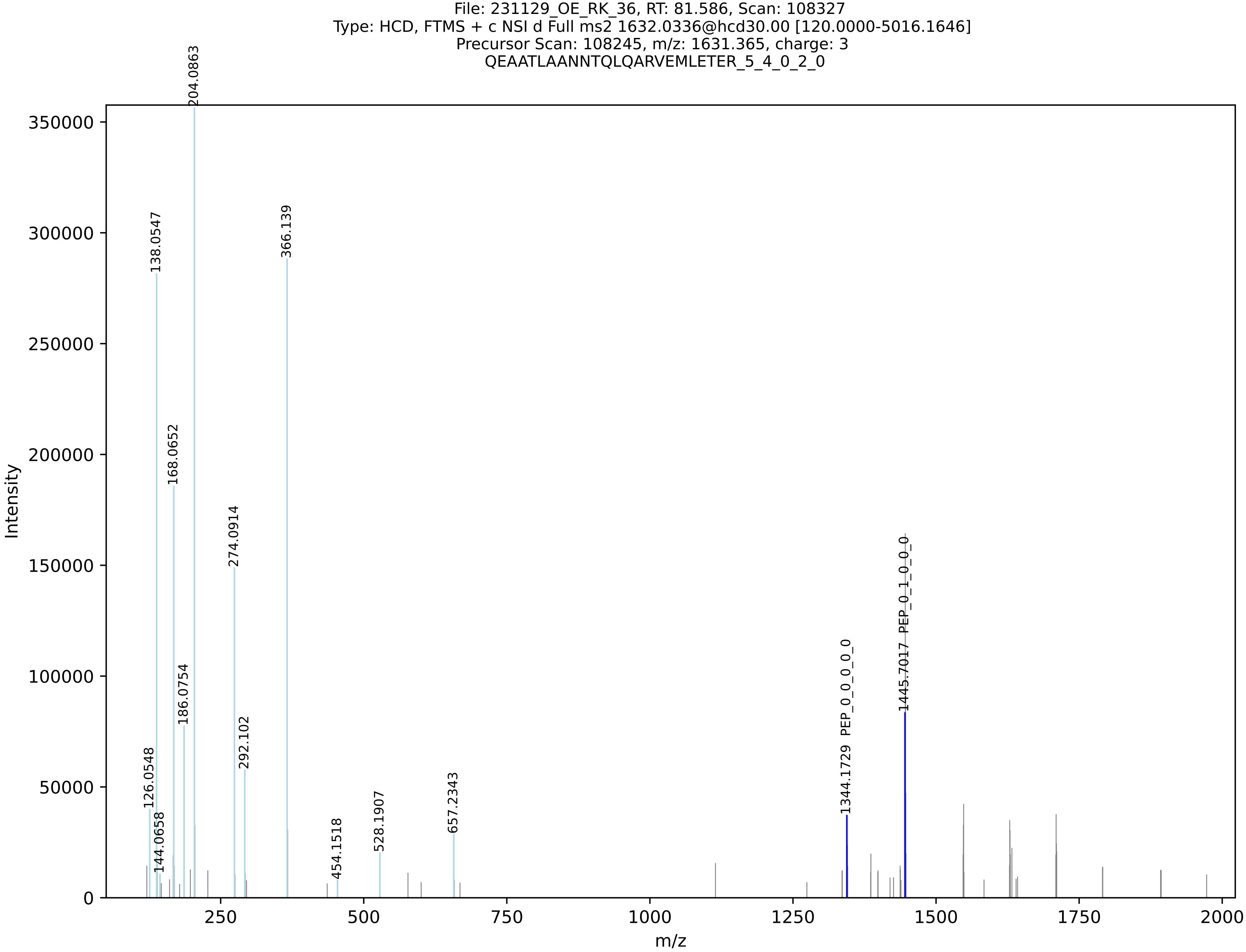

File: 231129_OE_RK_36, RT: 81.586, Scan: 108327
Type: HCD, FTMS + c NSI d Full ms2 1632.0336@hcd30.00 [120.0000-5016.1646]
Precursor Scan: 108245, m/z: 1631.365, charge: 3
QEAATLAANNTQLQARVEMLETER_5_4_0_2_0
Intensity
m/z
204.0863
138.0547
366.139
168.0652
274.0914
186.0754
292.102
126.0548
144.0658
454.1518
528.1907
657.2343
1344.1729 PEP_0_0_0_0_0
1445.7017 PEP_0_1_0_0_0

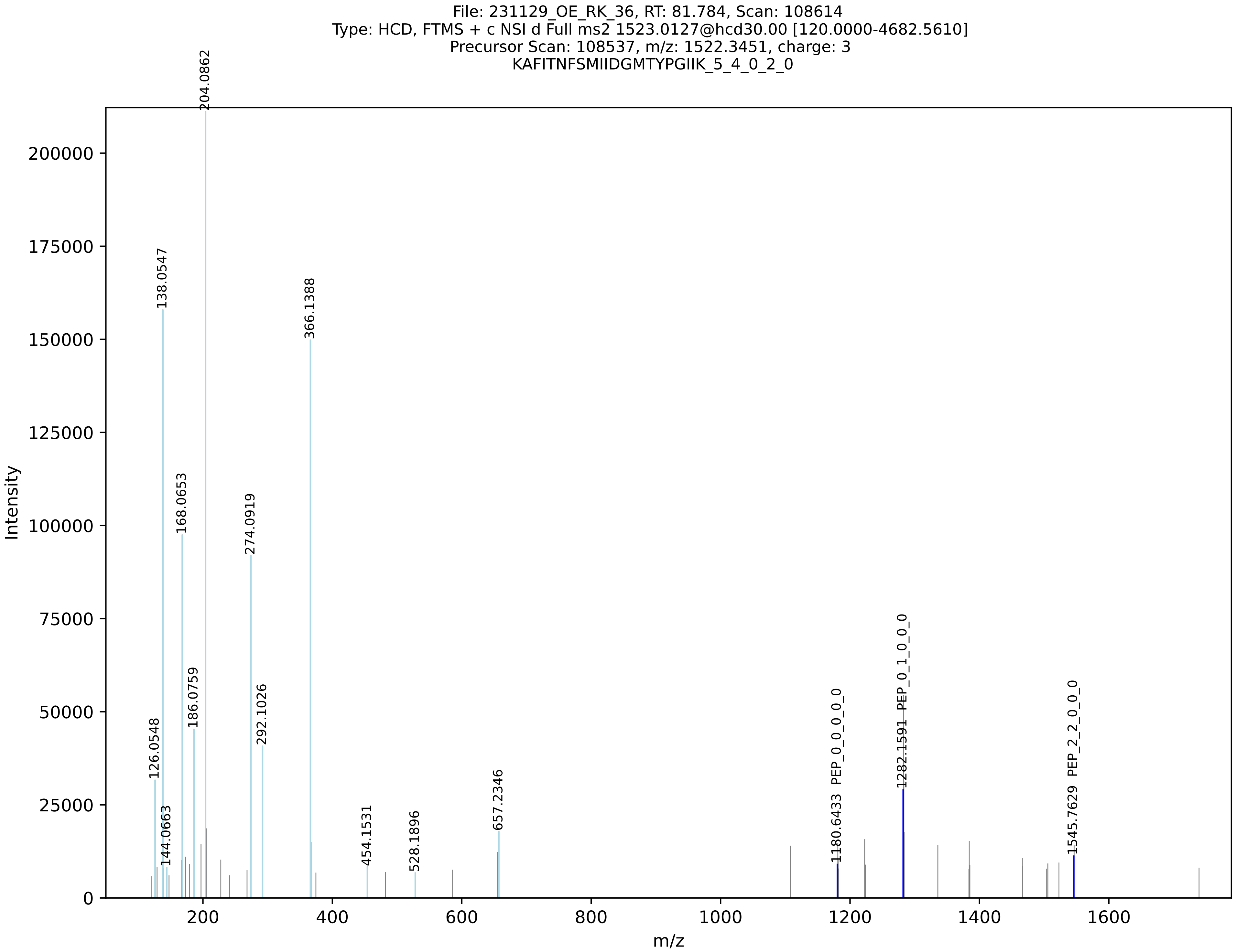
File: 231129_OE_RK_36, RT: 81.784, Scan: 108614
Type: HCD, FTMS + c NSI d Full ms2 1523.0127@hcd30.00 [120.0000-4682.5610]
Precursor Scan: 108537, m/z: 1522.3451, charge: 3
KAFITNFSMIIDGMTYPGIIK_5_4_0_2_0
Intensity
m/z
204.0862
138.0547
366.1388
168.0653
274.0919
186.0759
292.1026
126.0548
144.0663
454.1531
528.1896
657.2346
1180.6433 PEP_0_0_0_0_0
1282.1591 PEP_0_1_0_0_0
1545.7629 PEP_2_2_0_0_0

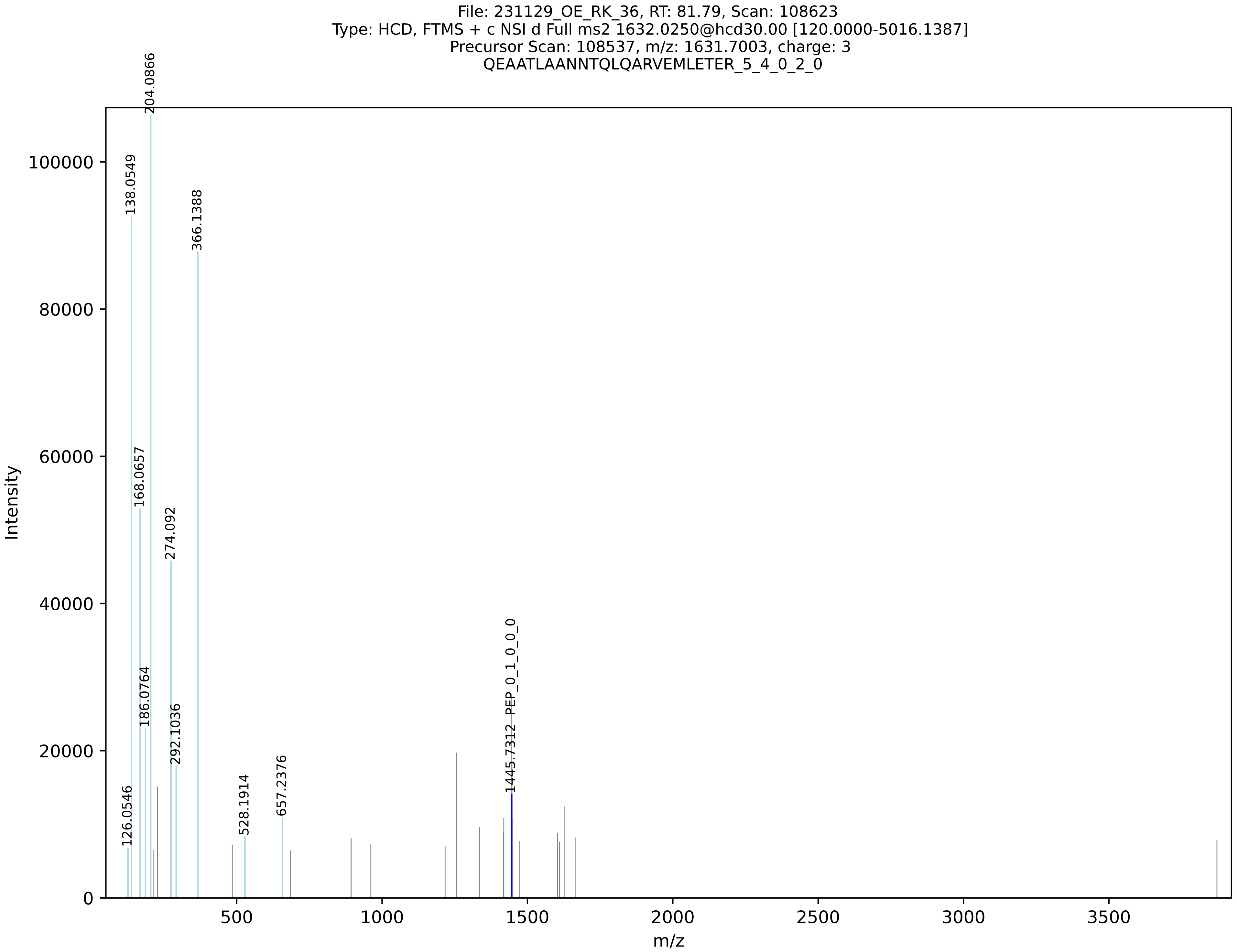

File: 231129_OE_RK_36, RT: 81.79, Scan: 108623
Type: HCD, FTMS + c NSI d Full ms2 1632.0250@hcd30.00 [120.0000-5016.1387]
Precursor Scan: 108537, m/z: 1631.7003, charge: 3
QEAATLAANNTQLQARVEMLETER_5_4_0_2_0
204.0866
138.0549
366.1388
168.0657
274.092
186.0764
292.1036
126.0546
528.1914
657.2376
1445.7312_PEP_0_1_0_0_0
Intensity
m/z
0
20000
40000
60000
80000
100000
500
1000
1500
2000
2500
3000
3500

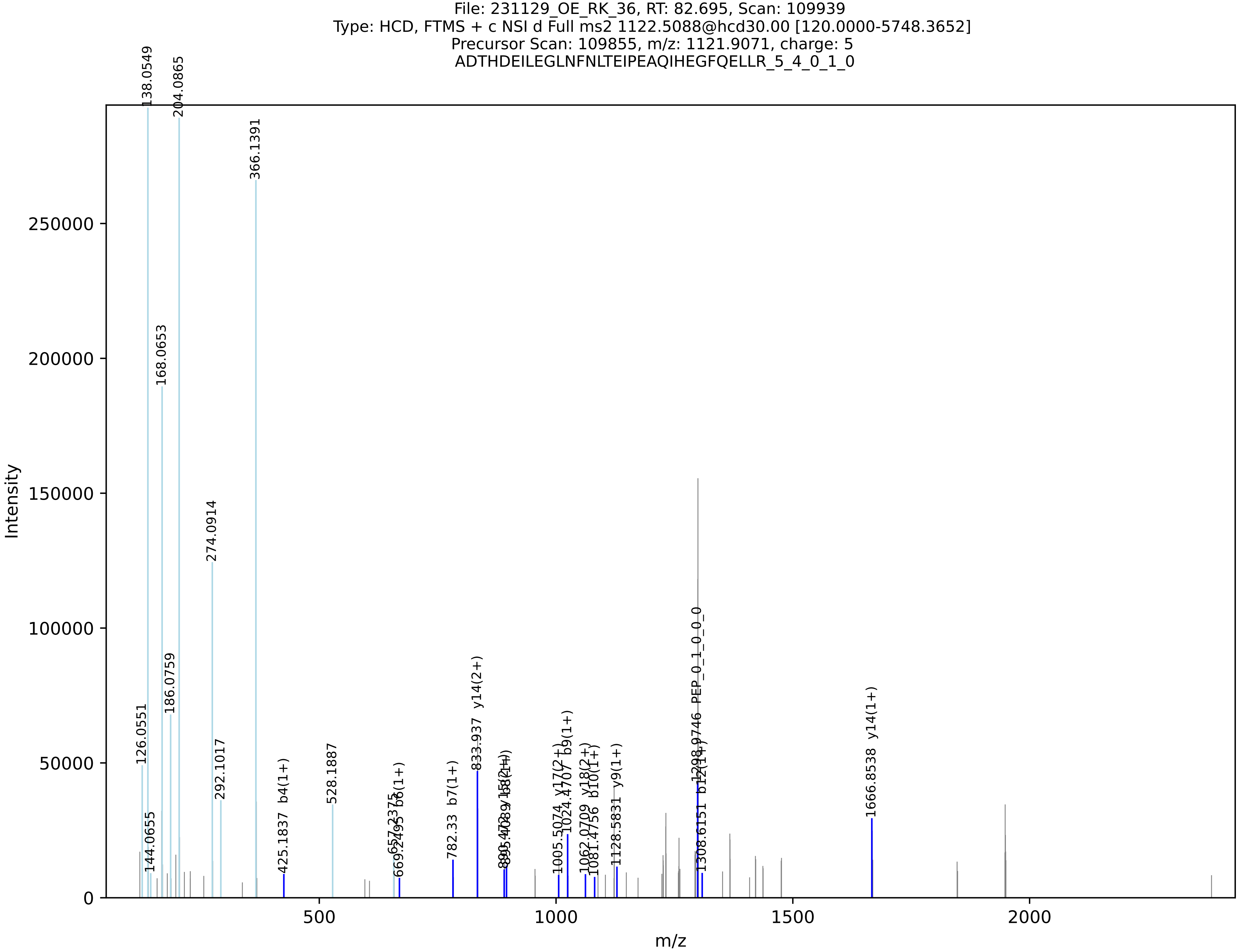

File: 231129_OE_RK_36, RT: 82.695, Scan: 109939
Type: HCD, FTMS + c NSI d Full ms2 1122.5088@hcd30.00 [120.0000-5748.3652]
Precursor Scan: 109855, m/z: 1121.9071, charge: 5
ADTHDEILEGLNFNLTEIPEAQIHEGFQELLR_5_4_0_1_0
Intensity
m/z
0
50000
100000
150000
200000
250000
500
1000
1500
2000
138.0549
204.0865
366.1391
168.0653
274.0914
186.0759
126.0551
292.1017
144.0655
425.1837 b4(1+)
528.1887
669.2495 b6(1+)
782.33 b7(1+)
833.937 y14(2+)
1005.5074 y17(2+)
1024.4707 b9(1+)
1062.0709 y18(2+)
1081.4756 b10(1+)
1128.5831 y9(1+)
1298.9746 PEP_0_1_0_0_0
1308.6151 b12(1+)
1666.8538 y14(1+)

File: 231129_OE_RK_36, RT: 83.043, Scan: 110448
Type: HCD, FTMS + c NSI d Full ms2 1402.8813@hcd30.00 [120.0000-5746.6118]
Precursor Scan: 110372, m/z: 1402.1296, charge: 4
ADTHDEILEGLNFNLTEIPEAQIHEGFQELLR_5_4_0_1_0

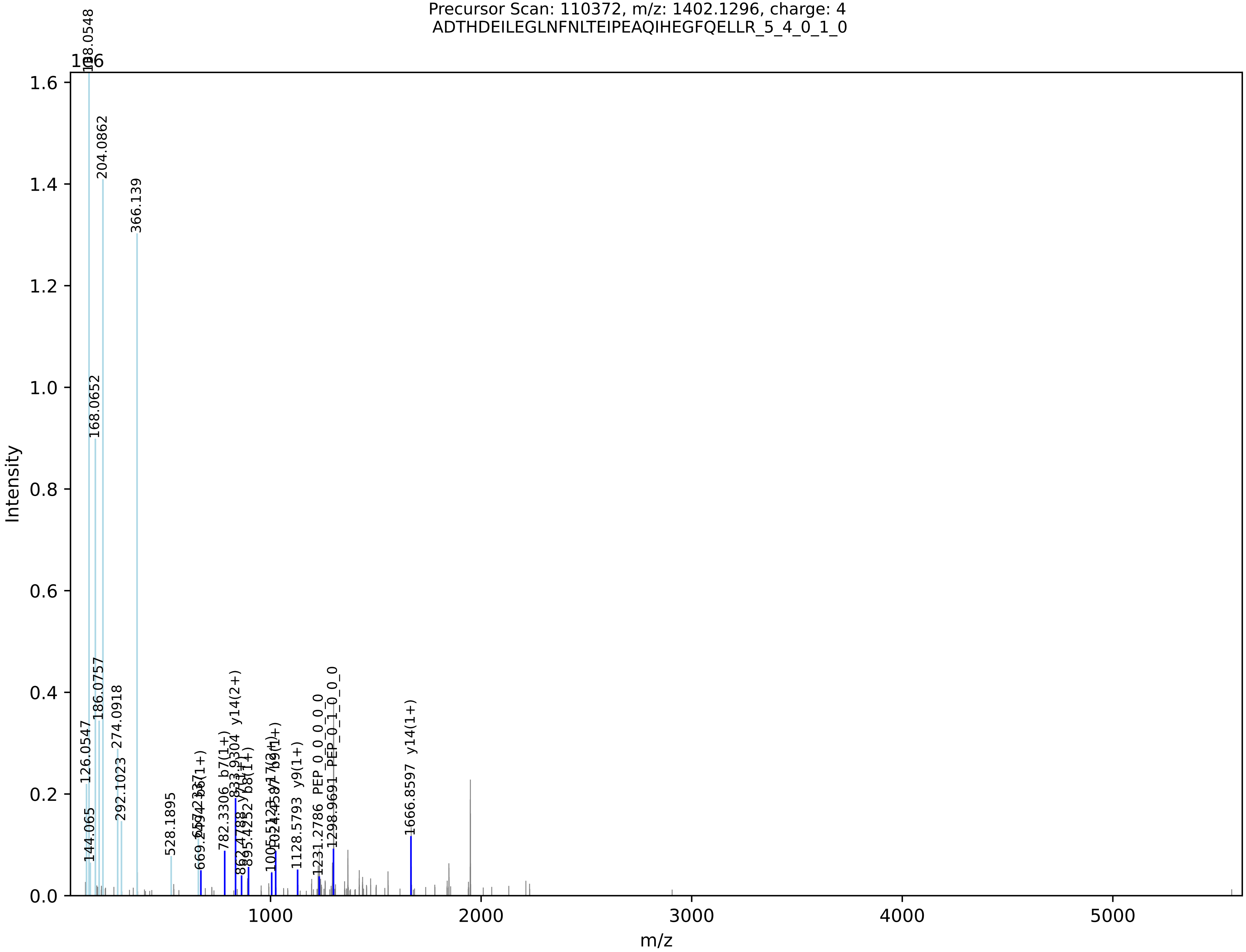

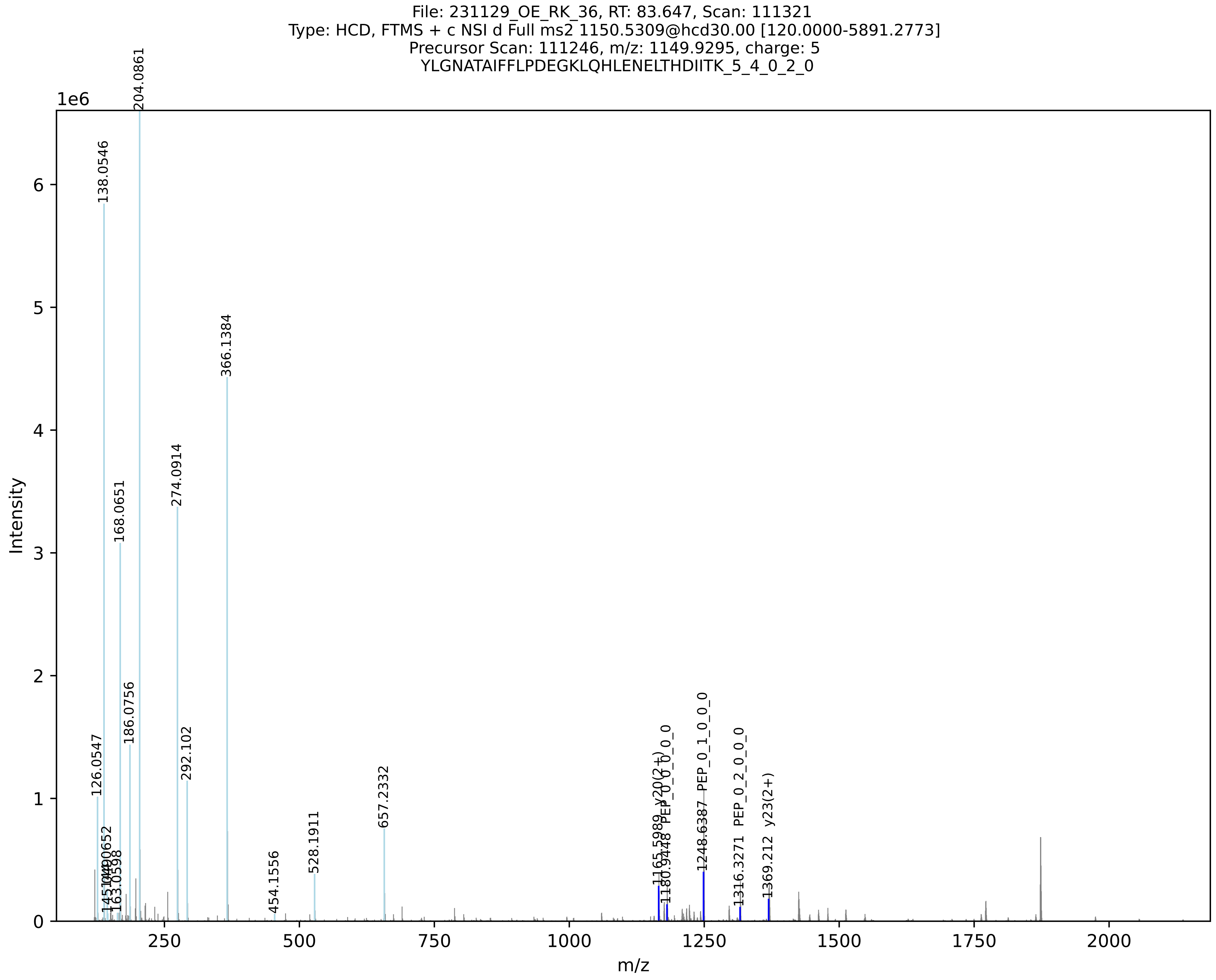
File: 231129_OE_RK_36, RT: 83.647, Scan: 111321
Type: HCD, FTMS + c NSI d Full ms2 1150.5309@hcd30.00 [120.0000-5891.2773]
Precursor Scan: 111246, m/z: 1149.9295, charge: 5
YLGNATAIFFLPDEGKLQHLENELTHDIITK_5_4_0_2_0
1e6
Intensity
m/z
204.0861
138.0546
366.1384
274.0914
168.0651
186.0756
126.0547
292.102
657.2332
528.1911
454.1556
163.0598
1165.5989 y20(2+)
1180.9448 PEP_0_0_0_0_0
1248.6387 PEP_0_1_0_0_0
1316.3271 PEP_0_2_0_0_0
1369.212 y23(2+)
0
1
2
3
4
5
6
250
500
750
1000
1250
1500
1750
2000

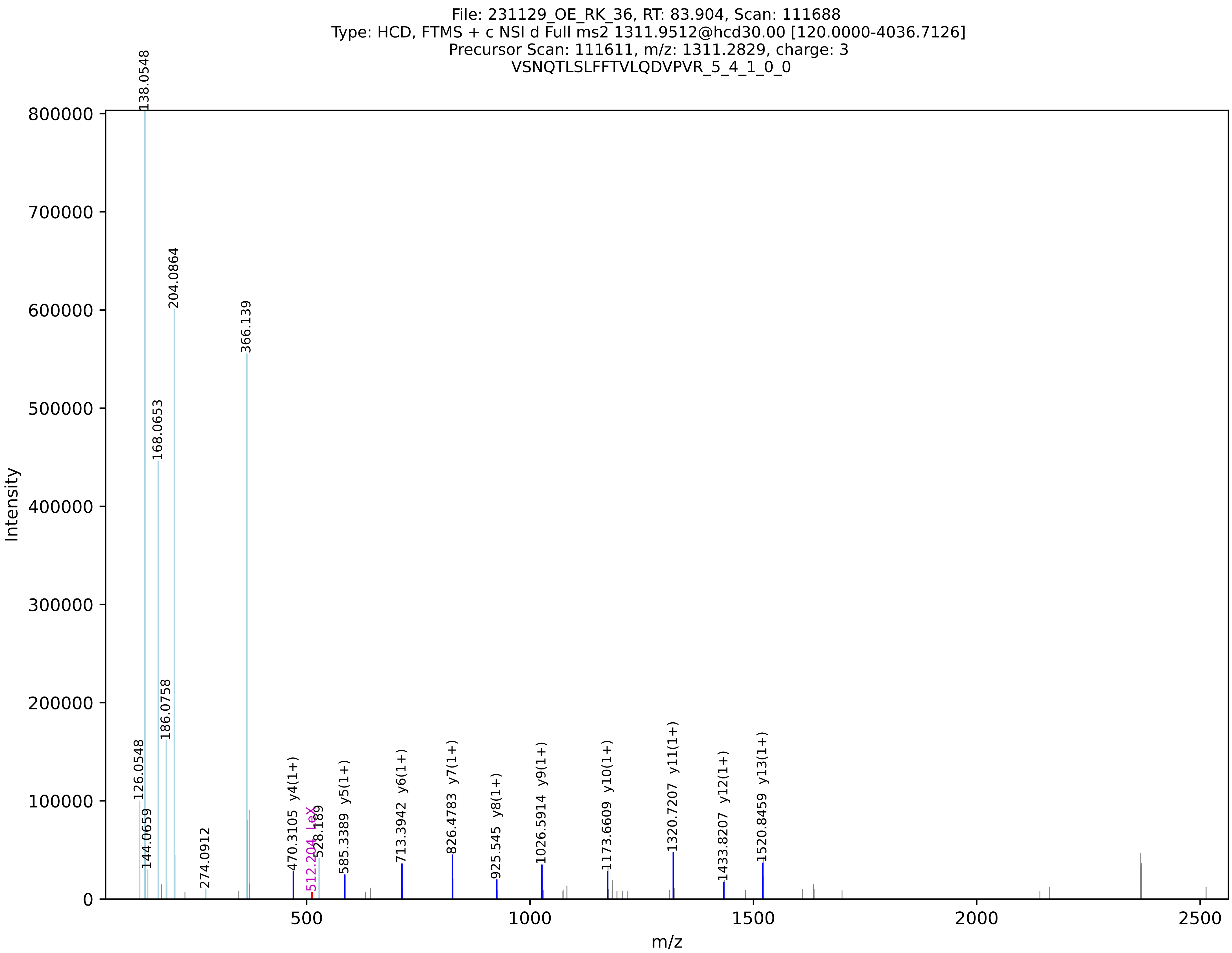
File: 231129_OE_RK_36, RT: 83.904, Scan: 111688
Type: HCD, FTMS + c NSI d Full ms2 1311.9512@hcd30.00 [120.0000-4036.7126]
Precursor Scan: 111611, m/z: 1311.2829, charge: 3
VSNQTLSLFFTVLQDVPVR_5_4_1_0_0
Intensity
m/z
138.0548
204.0864
366.139
168.0653
186.0758
126.0548
144.0659
274.0912
470.3105 y4(1+)
512.204 LeX
528.189
585.3389 y5(1+)
713.3942 y6(1+)
826.4783 y7(1+)
925.545 y8(1+)
1026.5914 y9(1+)
1173.6609 y10(1+)
1320.7207 y11(1+)
1433.8207 y12(1+)
1520.8459 y13(1+)
800000
700000
600000
500000
400000
300000
200000
100000
0
500
1000
1500
2000
2500

File: 231129_OE_RK_36, RT: 85.56, Scan: 114084
Type: HCD, FTMS + c NSI d Full ms2 1180.7299@hcd30.00 [120.0000-6045.2925]
Precursor Scan: 114007, m/z: 1180.3286, charge: 5
ADTHDEILEGLNFNLTEIPEAQIHEGFQELLR_5_4_0_2_0

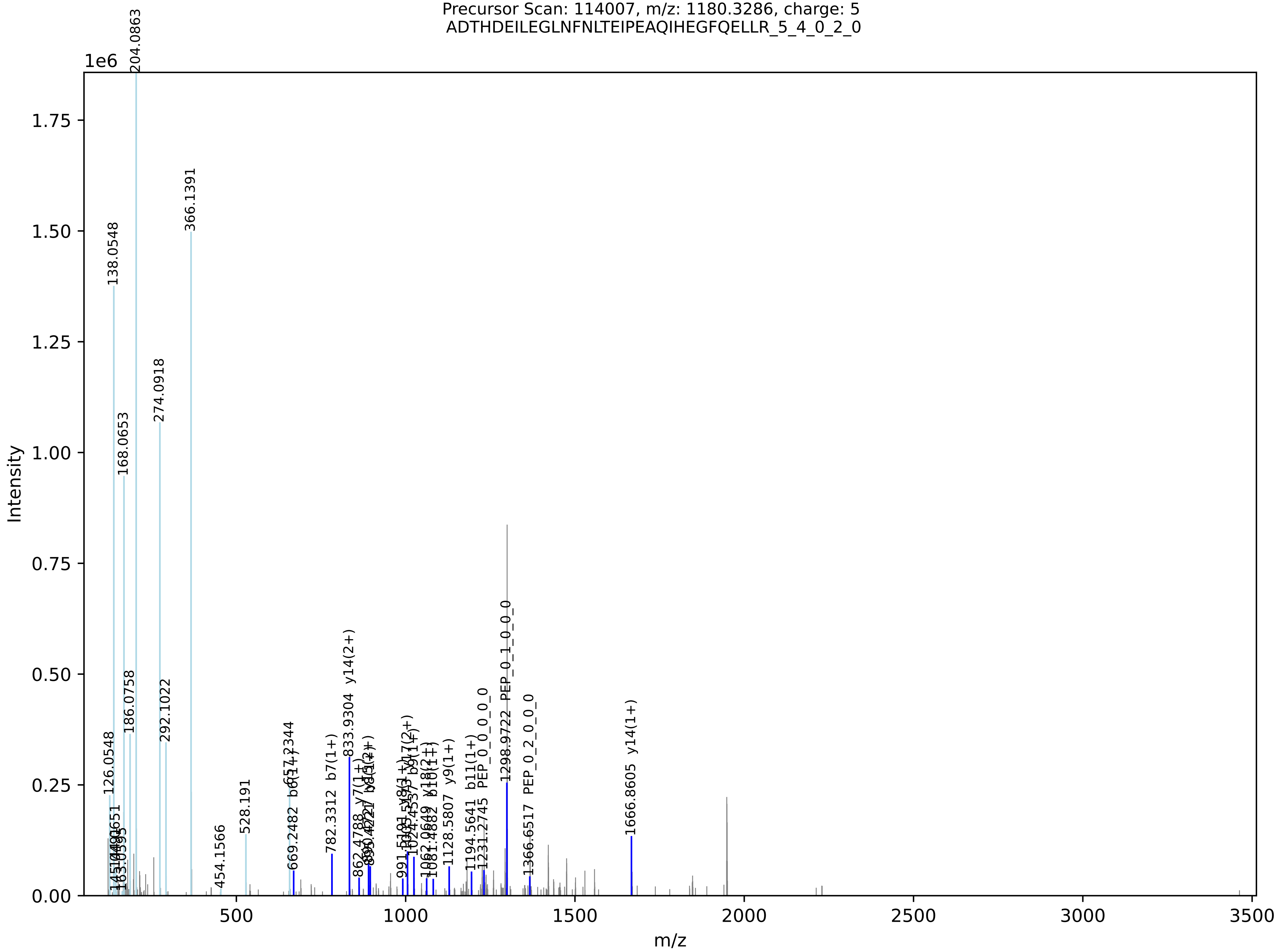

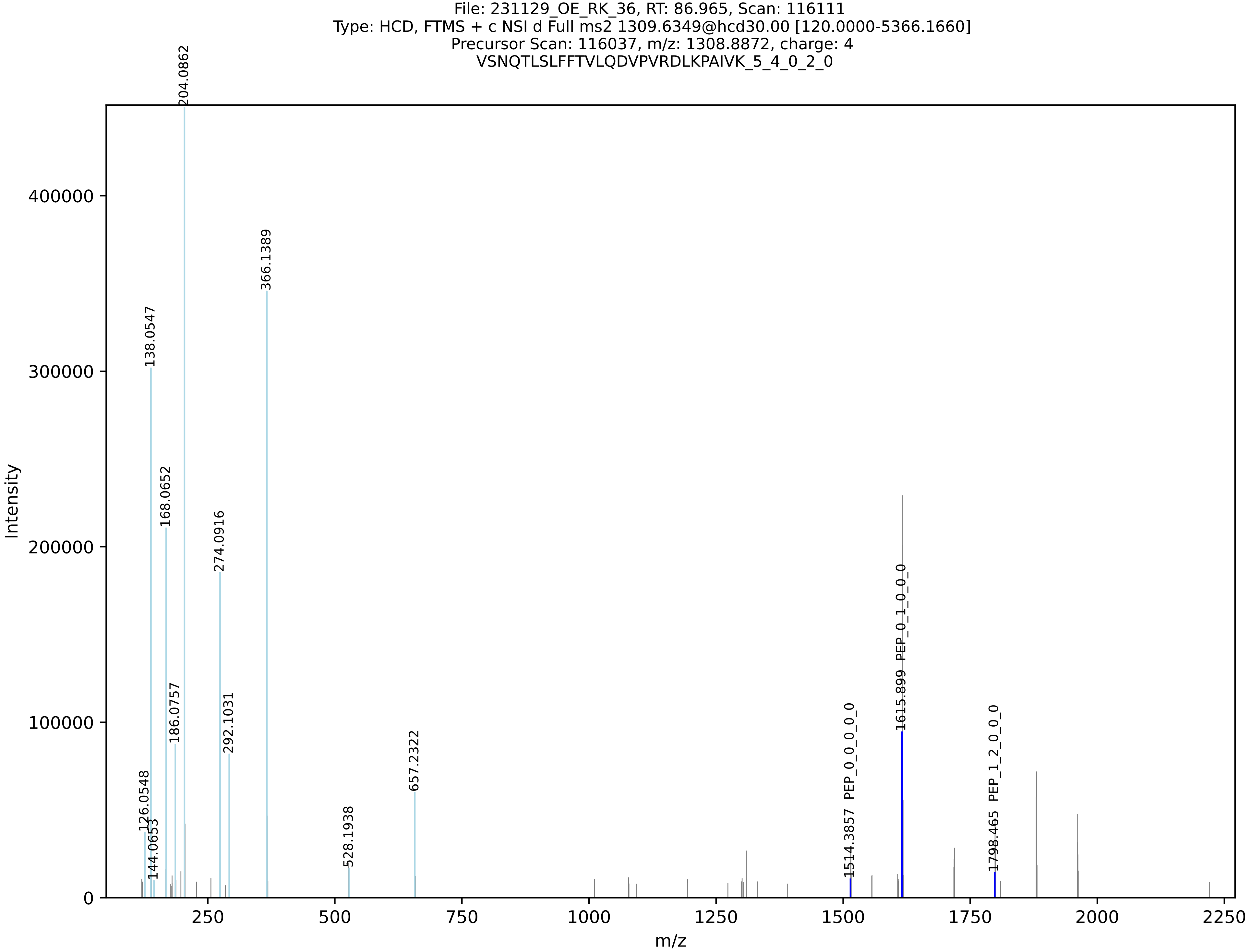
File: 231129_OE_RK_36, RT: 86.965, Scan: 116111
Type: HCD, FTMS + c NSI d Full ms2 1309.6349@hcd30.00 [120.0000-5366.1660]
Precursor Scan: 116037, m/z: 1308.8872, charge: 4
VSNQTLSLFFTVLQDVPVRDLKPAIVK_5_4_0_2_0
Intensity
m/z
0
100000
200000
300000
400000
250
500
750
1000
1250
1500
1750
2000
2250
204.0862
366.1389
138.0547
168.0652
274.0916
186.0757
292.1031
126.0548
144.0653
528.1938
657.2322
1514.3857 PEP_0_0_0_0_0_
1615.899 PEP_0_1_0_0_0_
1798.465 PEP_1_2_0_0_0_

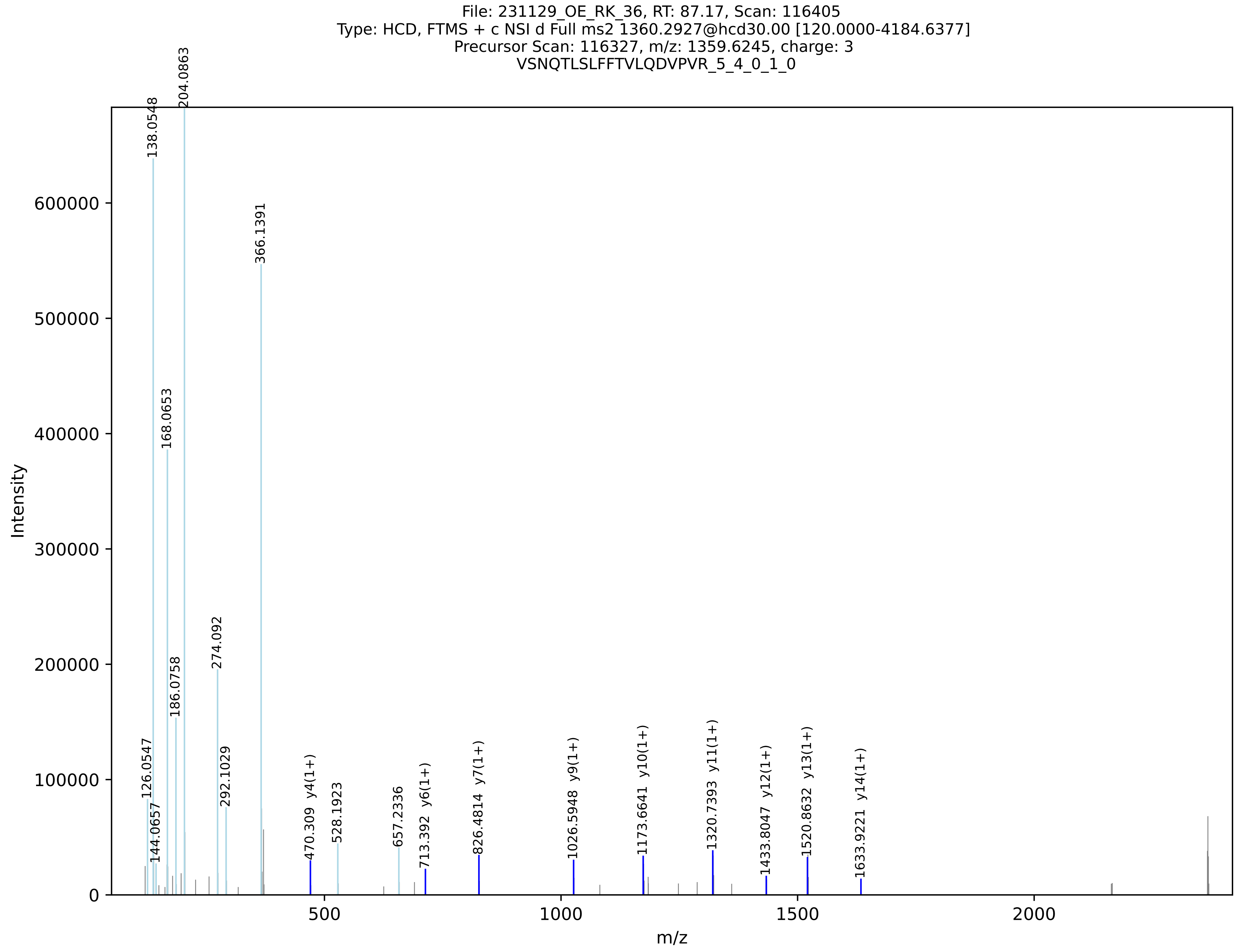
File: 231129_OE_RK_36, RT: 87.17, Scan: 116405
Type: HCD, FTMS + c NSI d Full ms2 1360.2927@hcd30.00 [120.0000-4184.6377]
Precursor Scan: 116327, m/z: 1359.6245, charge: 3
VSNQTLSLFFTVLQDVPVR_5_4_0_1_0
Intensity
m/z
204.0863
138.0548
366.1391
168.0653
274.092
186.0758
126.0547
292.1029
144.0657
470.309 y4(1+)
528.1923
657.2336
713.392 y6(1+)
826.4814 y7(1+)
1026.5948 y9(1+)
1173.6641 y10(1+)
1320.7393 y11(1+)
1433.8047 y12(1+)
1520.8632 y13(1+)
1633.9221 y14(1+)
0
100000
200000
300000
400000
500000
600000
500
1000
1500
2000

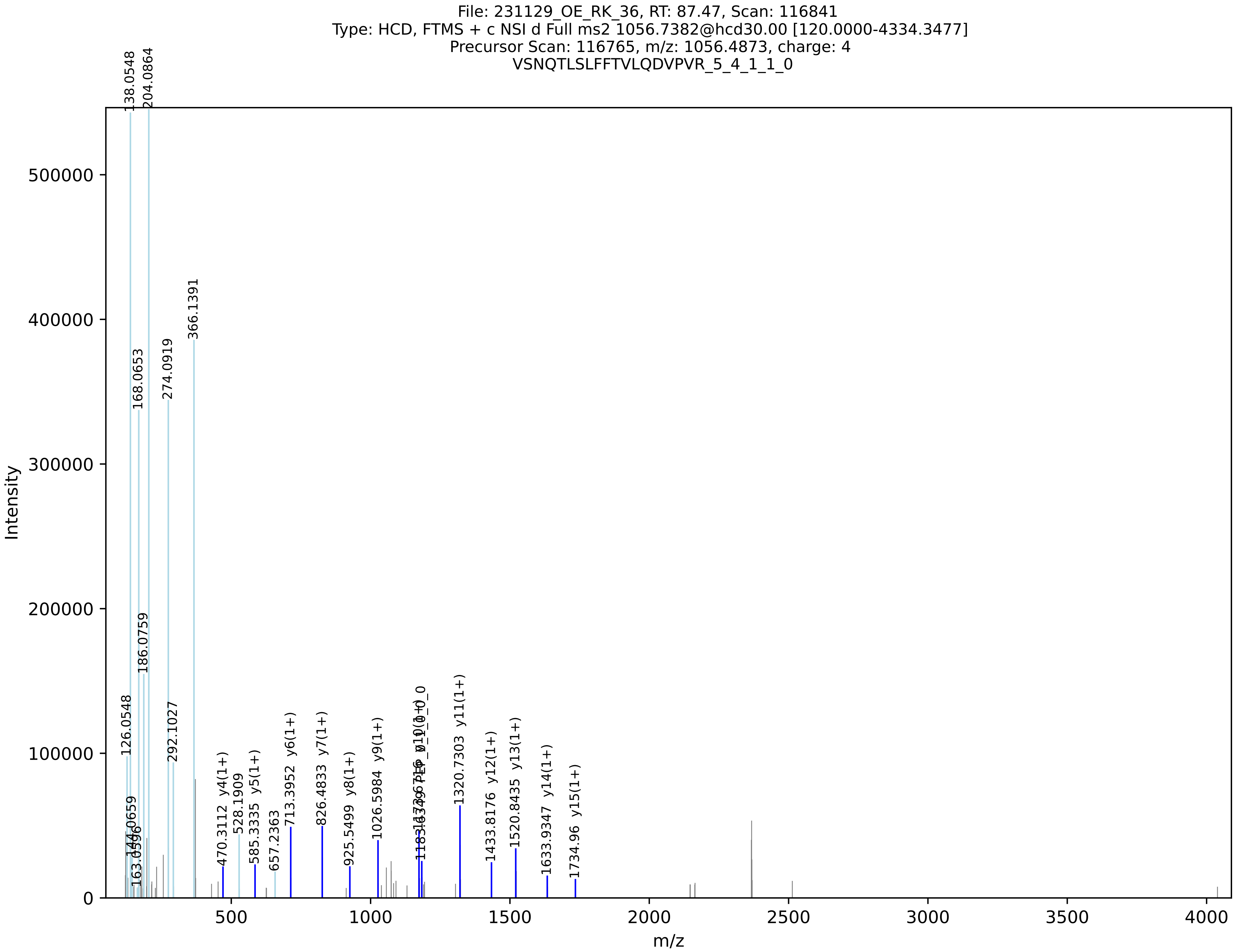

File: 231129_OE_RK_36, RT: 87.47, Scan: 116841
Type: HCD, FTMS + c NSI d Full ms2 1056.7382@hcd30.00 [120.0000-4334.3477]
Precursor Scan: 116765, m/z: 1056.4873, charge: 4
VSNQTLSLFFTVLQDVPVR_5_4_1_1_0
138.0548
204.0864
366.1391
168.0653
274.0919
186.0759
126.0548
292.1027
470.3112 y4(1+)
528.1909
585.3335 y5(1+)
657.2363
713.3952 y6(1+)
826.4833 y7(1+)
925.5499 y8(1+)
1026.5984 y9(1+)
1320.7303 y11(1+)
1433.8176 y12(1+)
1520.8435 y13(1+)
1633.9347 y14(1+)
1734.96 y15(1+)
Intensity
m/z
0
100000
200000
300000
400000
500000
500
1000
1500
2000
2500
3000
3500
4000

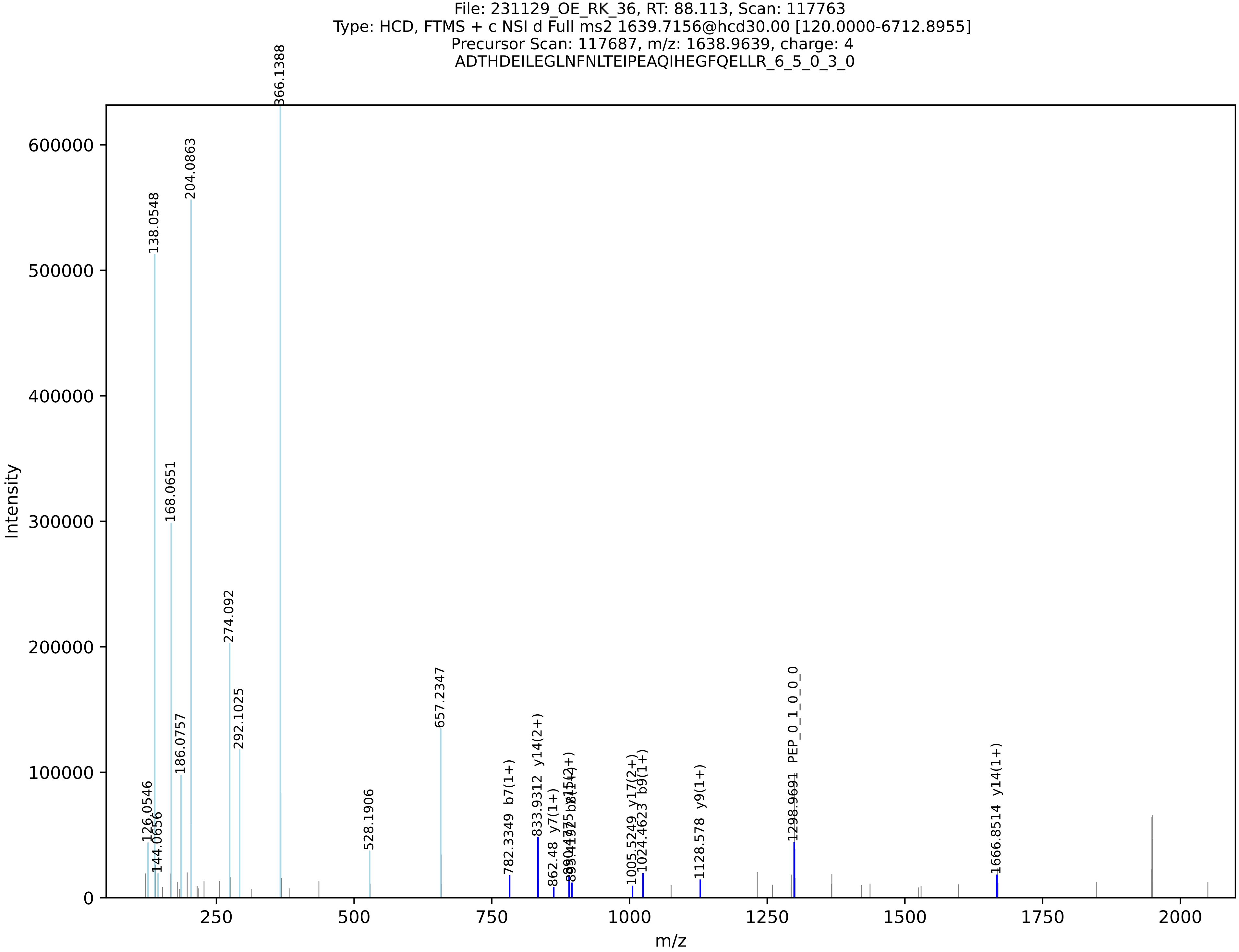
File: 231129_OE_RK_36, RT: 88.113, Scan: 117763
Type: HCD, FTMS + c NSI d Full ms2 1639.7156@hcd30.00 [120.0000-6712.8955]
Precursor Scan: 117687, m/z: 1638.9639, charge: 4
ADTHDEILEGLNFNLTEIPEAQIHEGFQELLR_6_5_0_3_0
Intensity
m/z
366.1388
204.0863
138.0548
168.0651
274.092
292.1025
186.0757
126.0546
144.0656
528.1906
657.2347
782.3349 b7(1+)
833.9312 y14(2+)
862.48 y7(1+)
1005.5249 y17(2+)
1024.4623 b9(1+)
1128.578 y9(1+)
1298.9691 PEP_0_1_0_0_0
1666.8514 y14(1+)

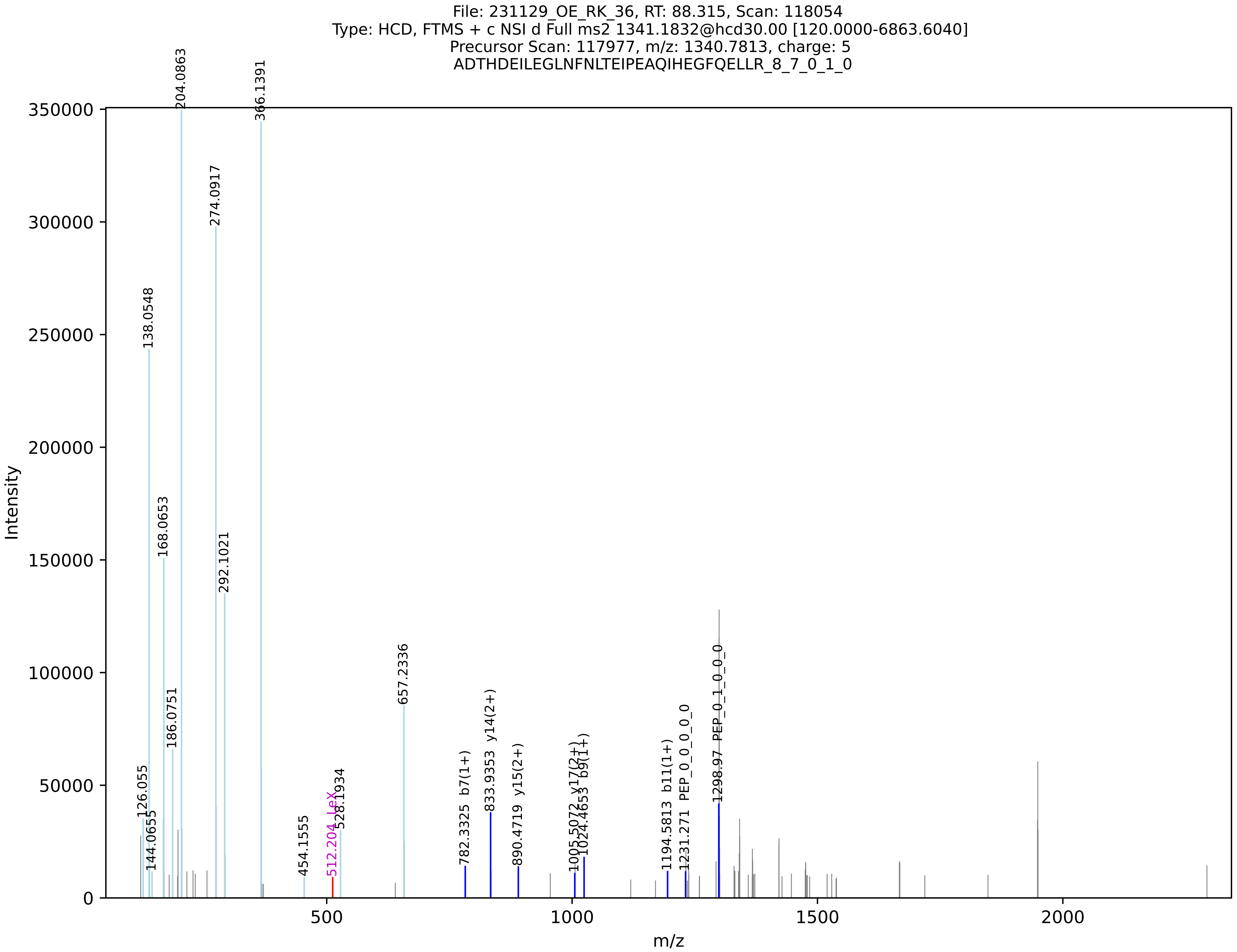

File: 231129_OE_RK_36, RT: 88.315, Scan: 118054
Type: HCD, FTMS + c NSI d Full ms2 1341.1832@hcd30.00 [120.0000-6863.6040]
Precursor Scan: 117977, m/z: 1340.7813, charge: 5
ADTHDEILEGLNFNLTEIPEAQIHEGFQELLR_8_7_0_1_0
Intensity
m/z
204.0863
366.1391
274.0917
138.0548
168.0653
292.1021
186.0751
126.055
144.0655
454.1555
512.204_LeX
528.1934
657.2336
782.3325 b7(1+)
833.9353 y14(2+)
890.4719 y15(2+)
1005.5072 y17(2+)
1024.4653 b9(1+)
1194.5813 b11(1+)
1231.271 PEP_0_0_0_0_0
1298.97 PEP_0_1_0_0_0

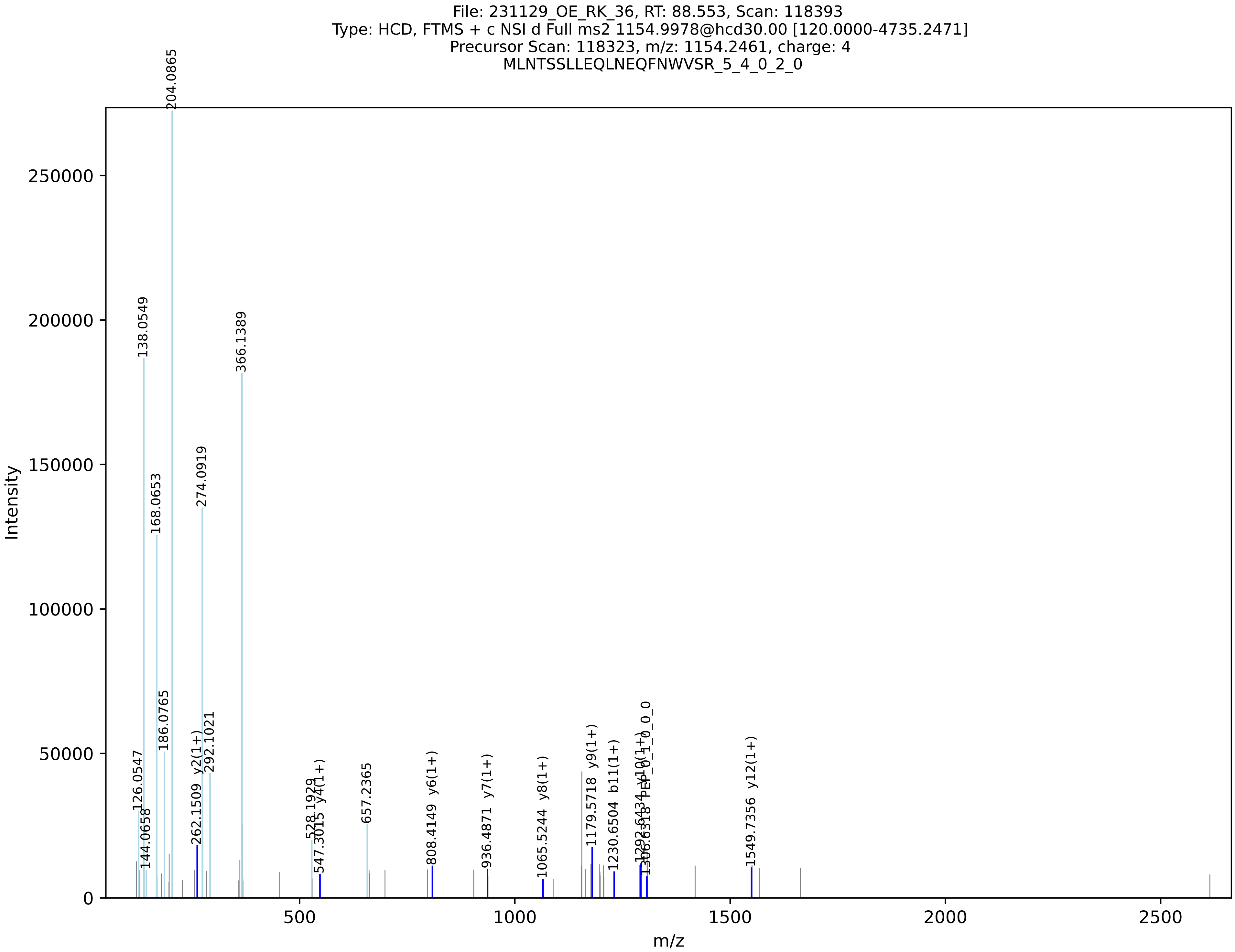

File: 231129_OE_RK_36, RT: 88.553, Scan: 118393
Type: HCD, FTMS + c NSI d Full ms2 1154.9978@hcd30.00 [120.0000-4735.2471]
Precursor Scan: 118323, m/z: 1154.2461, charge: 4
MLNTSSLLEQLNEQFNWVSR_5_4_0_2_0
Intensity
m/z
204.0865
138.0549
366.1389
274.0919
168.0653
186.0765
292.1021
126.0547
144.0658
262.1509 y2(1+)
528.1929
547.3015 y4(1+)
657.2365
808.4149 y6(1+)
936.4871 y7(1+)
1065.5244 y8(1+)
1179.5718 y9(1+)
1230.6504 b11(1+)
y10(1+)
1549.7356 y12(1+)

File: 231129_OE_RK_36, RT: 94.567, Scan: 126884
Type: HCD, FTMS + c NSI d Full ms2 1643.5748@hcd30.00 [120.0000-5051.4810]
Precursor Scan: 126831, m/z: 1643.5748, charge: 3
SGTRHTQNSSSGQAASSHEQAR_7_6_2_0_0

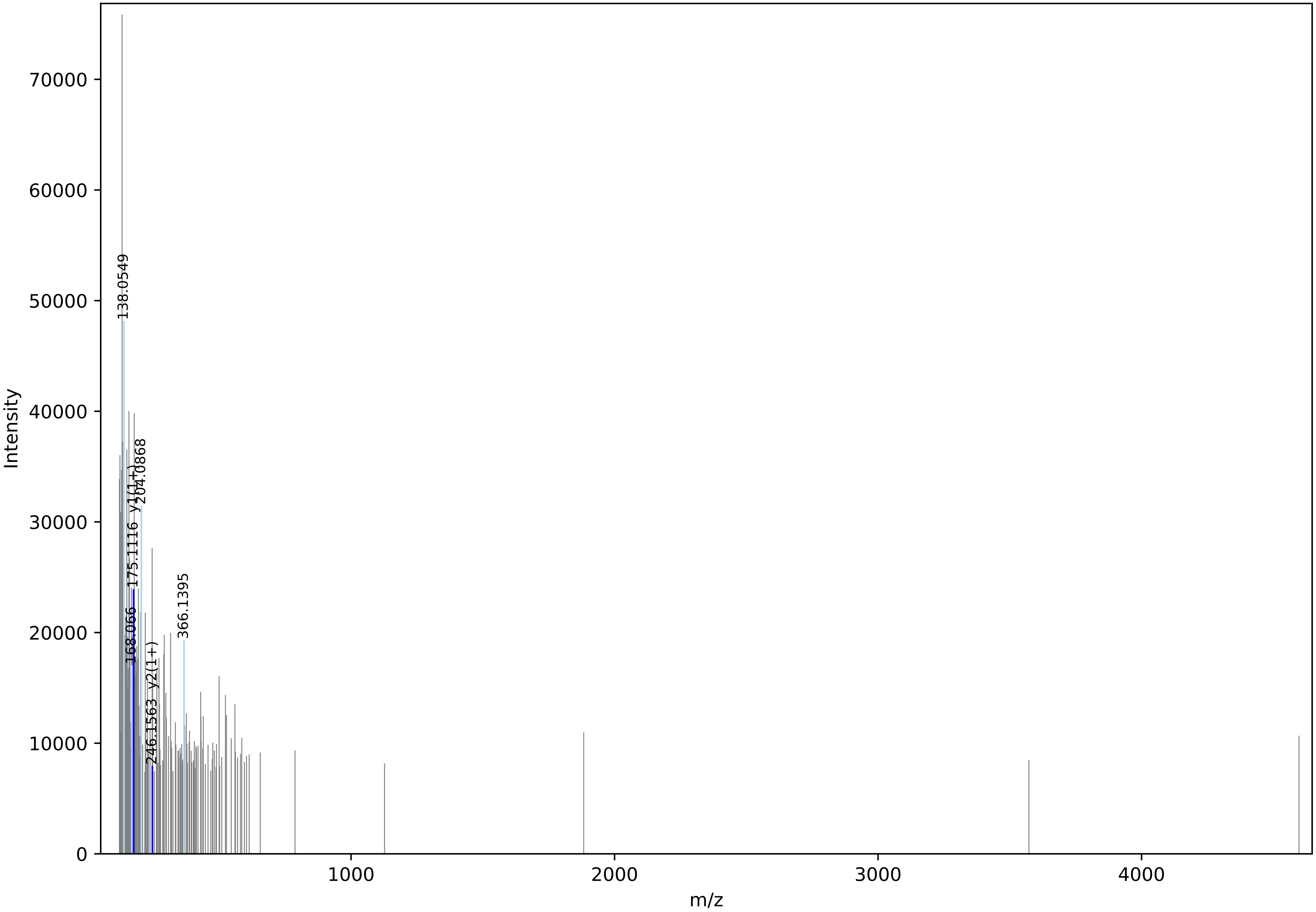

File: 231129_OE_RK_36, RT: 95.136, Scan: 127715
Type: HCD, FTMS + c NSI d Full ms2 1454.5278@hcd30.00 [120.0000-4472.9971]
Precursor Scan: 127613, m/z: 1454.5278, charge: 3
VSNQTLSLFFTVLQDVPVR_5_4_0_2_0

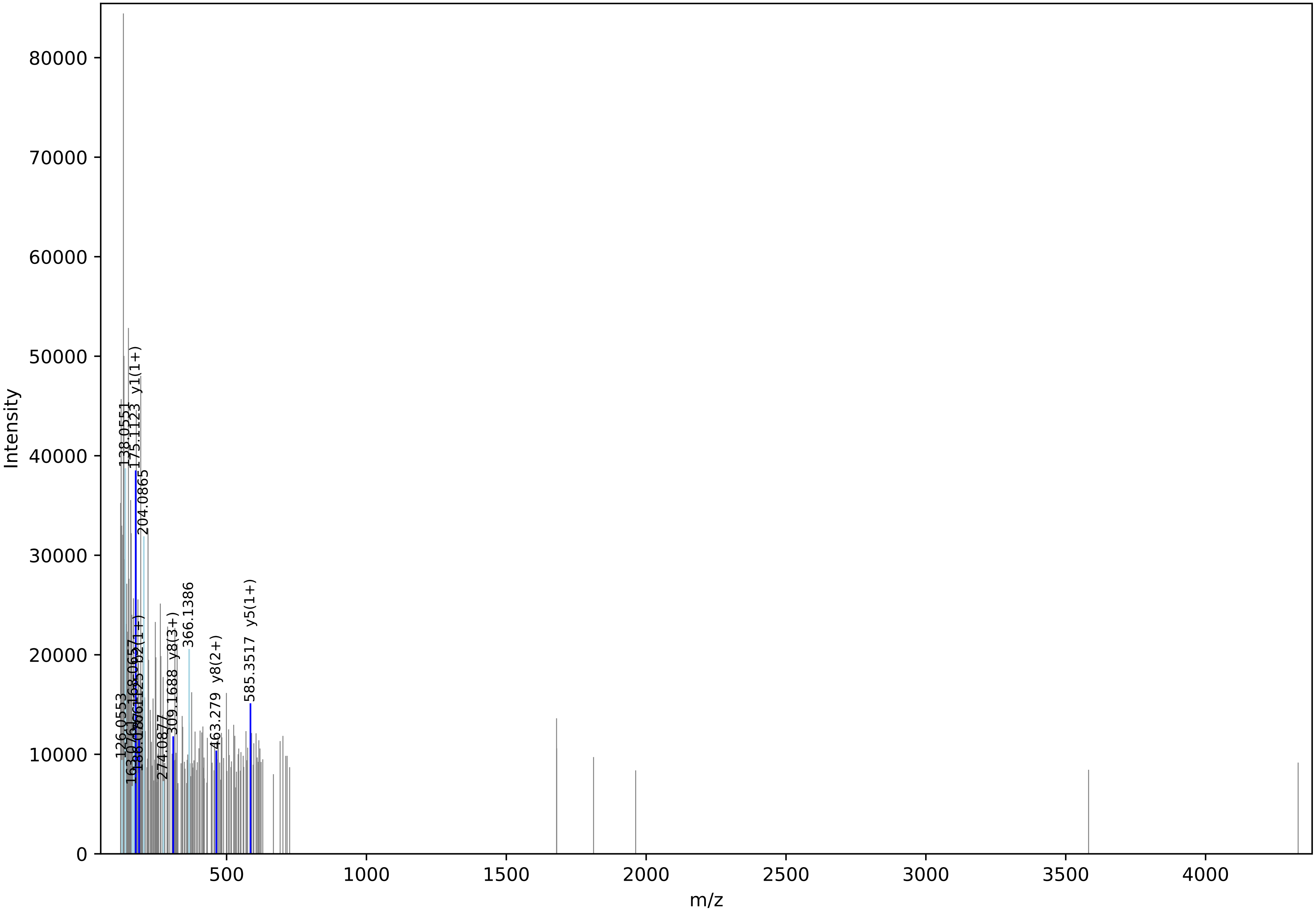

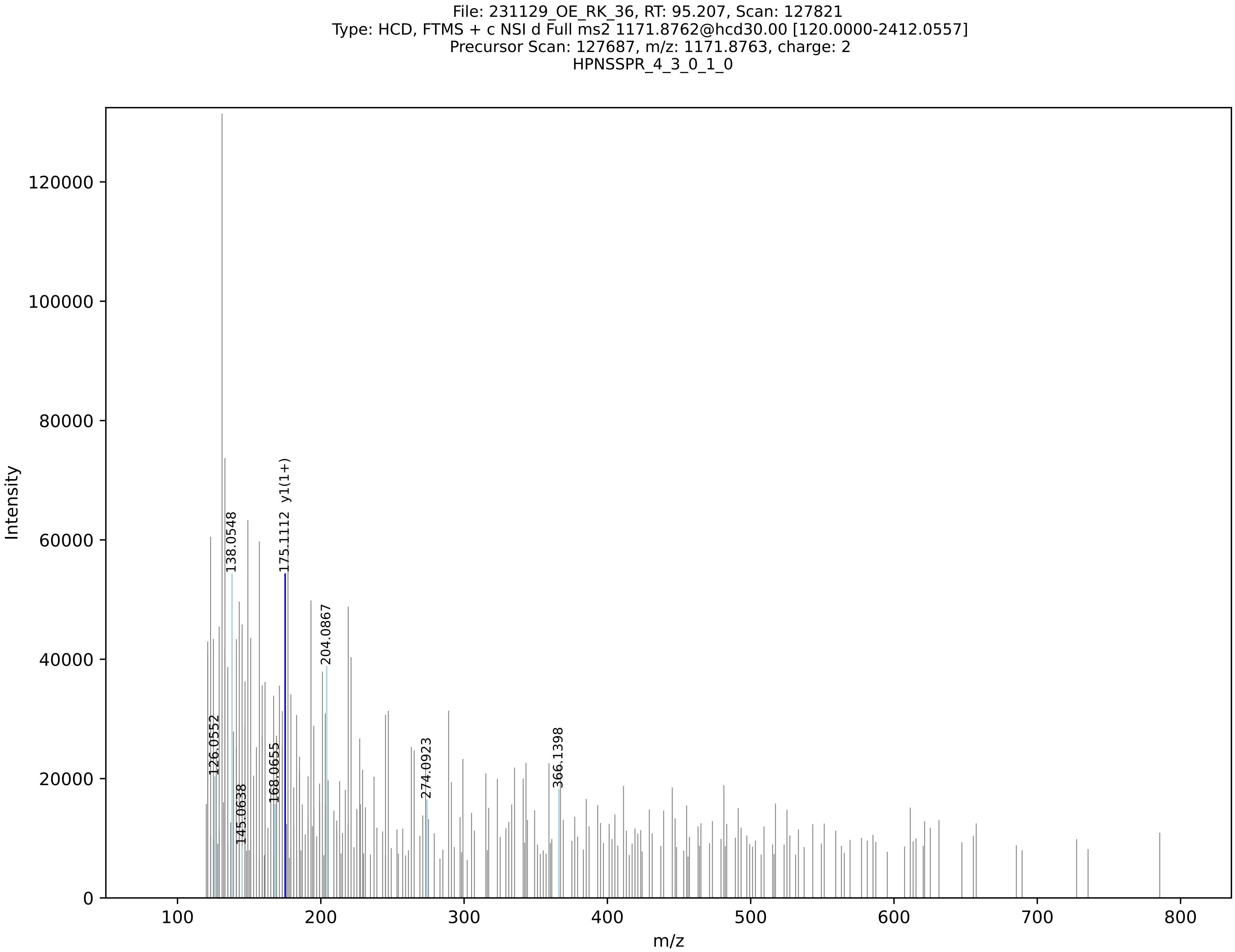
File: 231129_OE_RK_36, RT: 95.207, Scan: 127821
Type: HCD, FTMS + c NSI d Full ms2 1171.8762@hcd30.00 [120.0000-2412.0557]
Precursor Scan: 127687, m/z: 1171.8763, charge: 2
HPNSSPR_4_3_0_1_0
Intensity
m/z
126.0552
138.0548
145.0638
168.0655
175.1112 y1(1+)
204.0867
274.0923
366.1398
0
20000
40000
60000
80000
100000
120000
100
200
300
400
500
600
700
800

File: 231129_OE_RK_36, RT: 95.388, Scan: 128082
Type: HCD, FTMS + c NSI d Full ms2 1411.3632@hcd30.00 [120.0000-4340.9131]
Precursor Scan: 127981, m/z: 1411.3631, charge: 3
ANHTPESCPETKYK_6_5_0_2_0

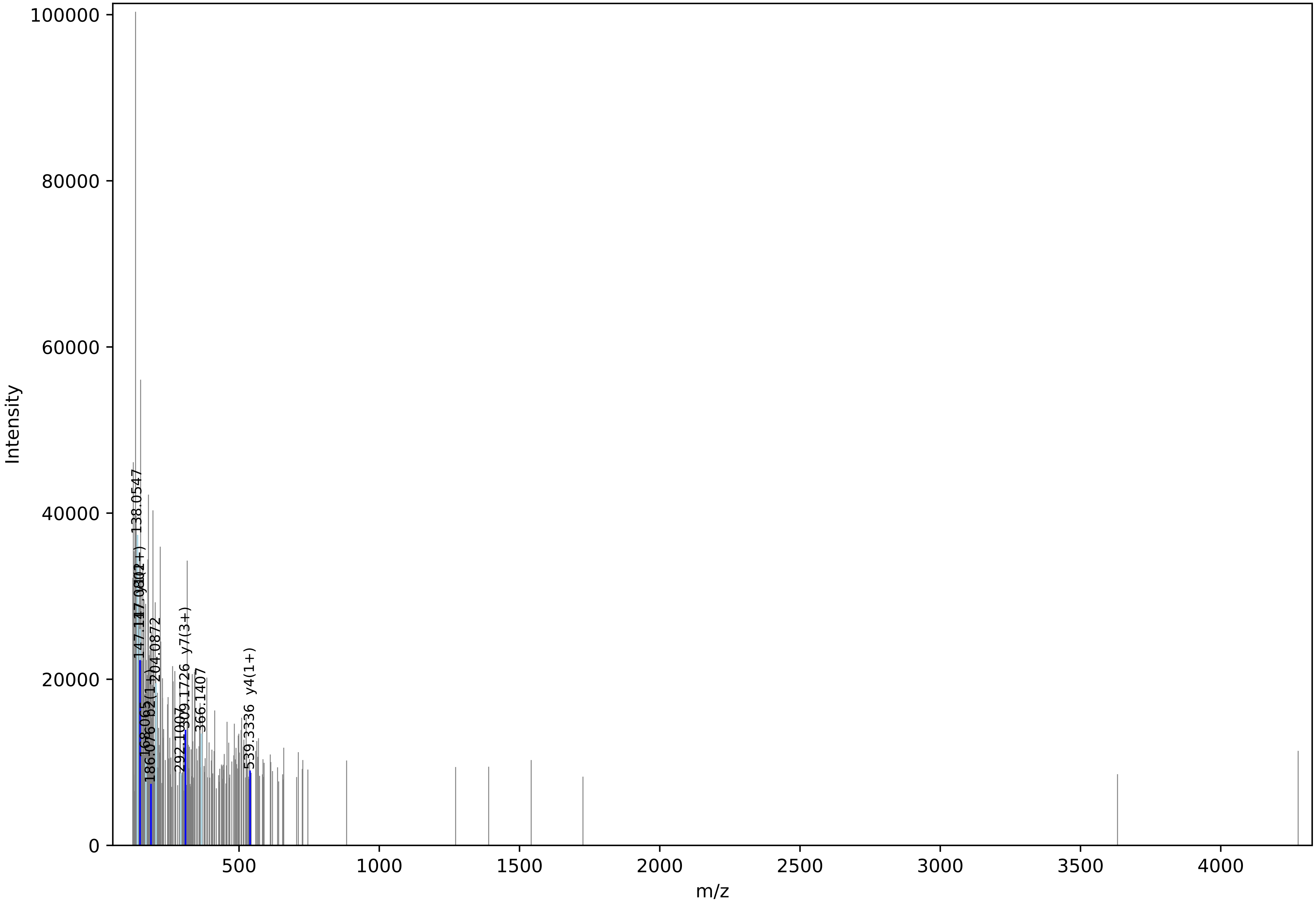

File: 231129_OE_RK_36, RT: 95.758, Scan: 128627
Type: HCD, FTMS + c NSI d Full ms2 1374.6027@hcd30.00 [120.0000-2825.6174]
Precursor Scan: 128498, m/z: 1374.6026, charge: 2
FNSTEYQVVTR_4_3_1_0_0

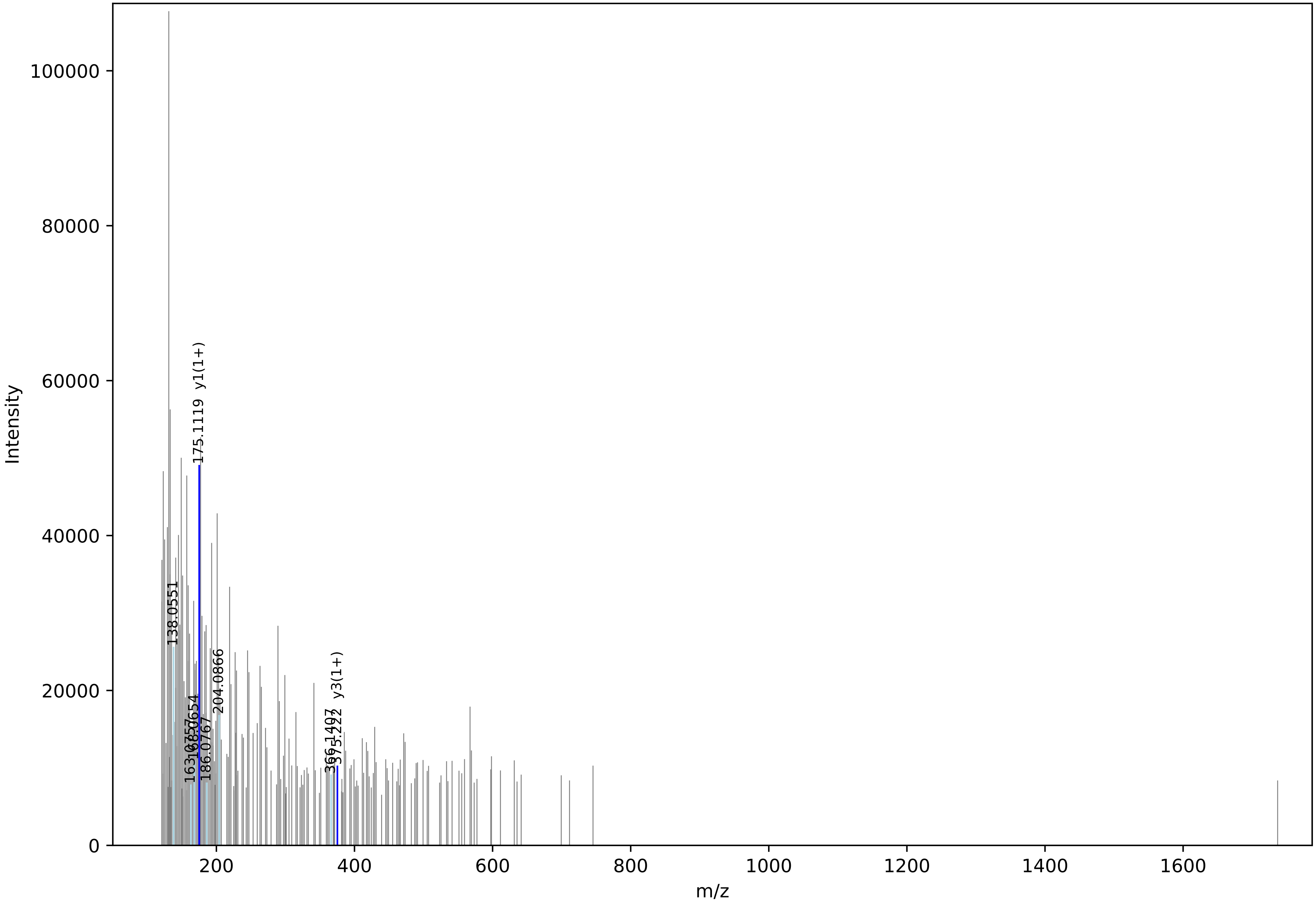

File: 231129_OE_RK_36, RT: 95.804, Scan: 128694
Type: HCD, FTMS + c NSI d Full ms2 1389.4684@hcd30.00 [120.0000-4273.9150]
Precursor Scan: 128572, m/z: 1389.4683, charge: 3
EEQYNSTFR_7_7_1_1_0

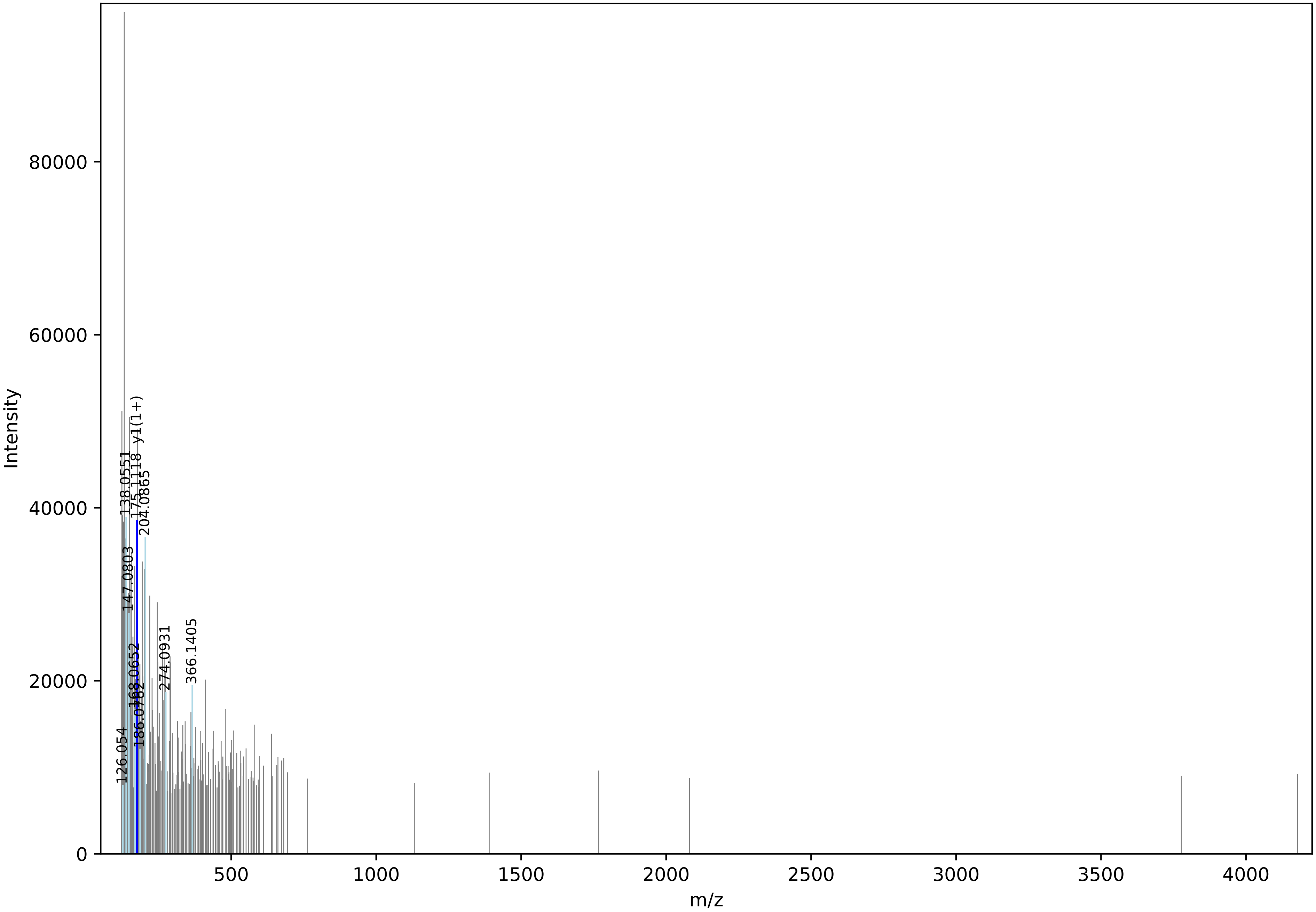

File: 231129_OE_RK_36, RT: 95.878, Scan: 128799
Type: HCD, FTMS + c NSI d Full ms2 1604.2588@hcd30.00 [120.0000-3294.1160]
Precursor Scan: 128720, m/z: 1603.2564, charge: 2
RTENVTLK_4_5_2_1_0

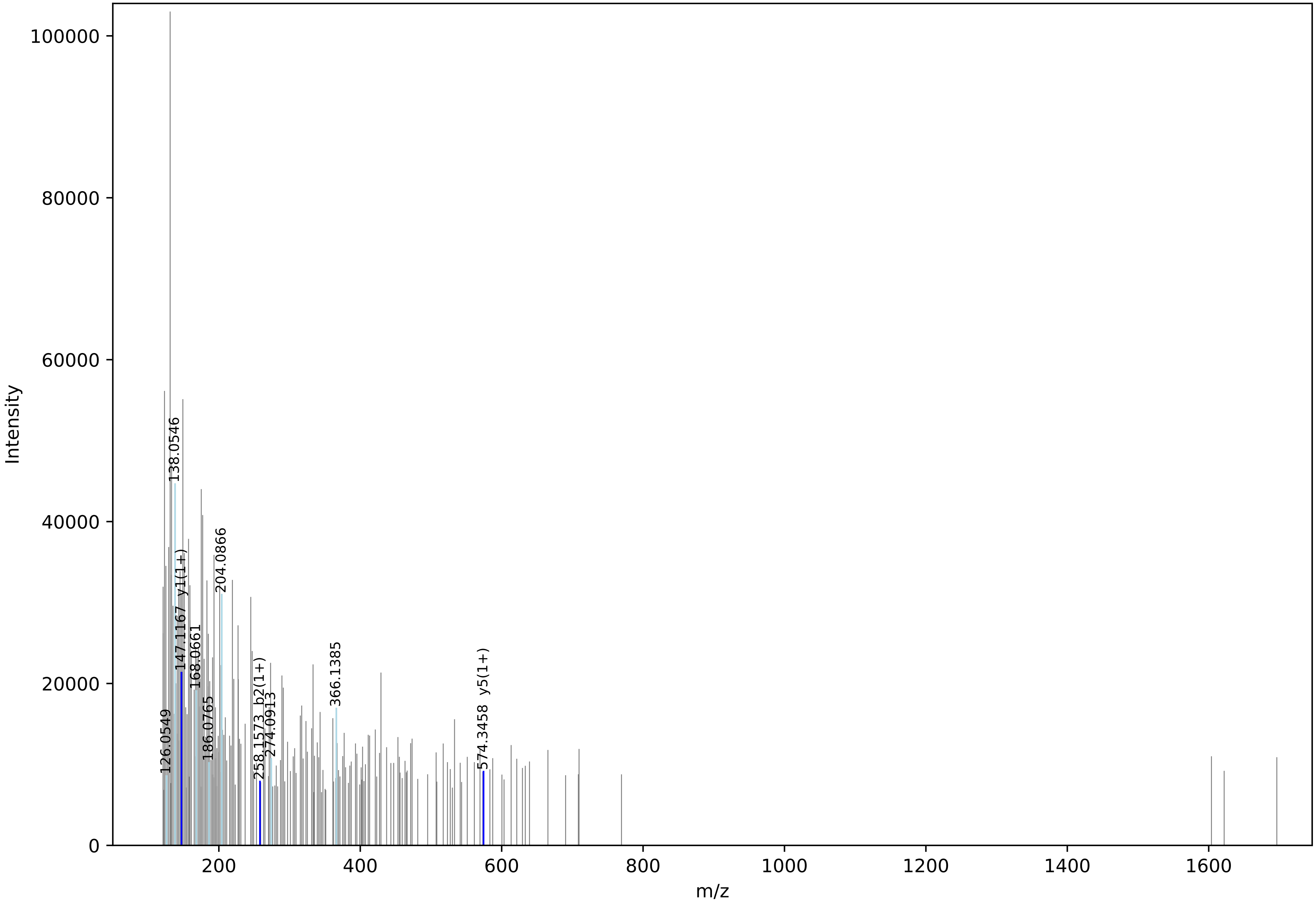

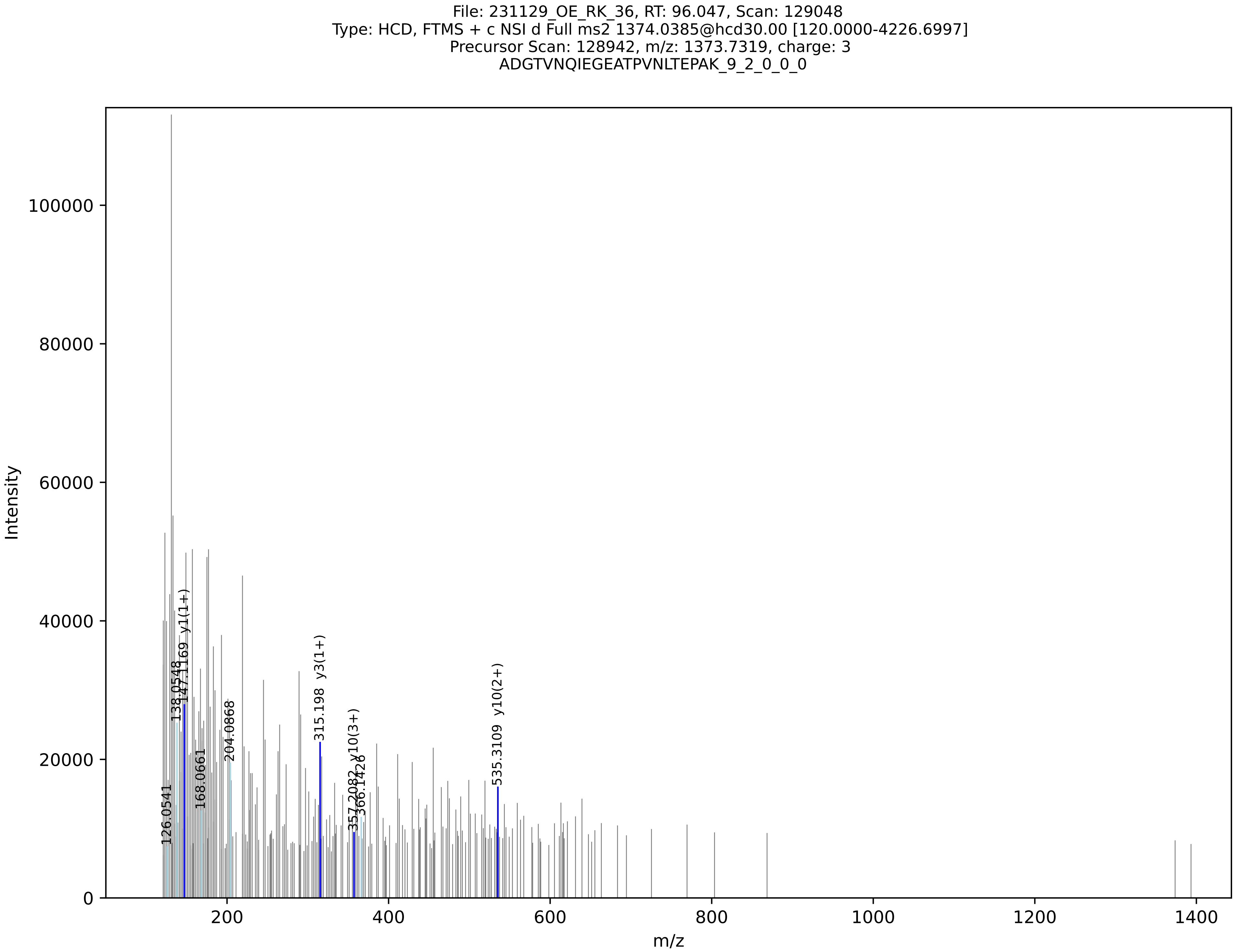

File: 231129_OE_RK_36, RT: 96.047, Scan: 129048
Type: HCD, FTMS + c NSI d Full ms2 1374.0385@hcd30.00 [120.0000-4226.6997]
Precursor Scan: 128942, m/z: 1373.7319, charge: 3
ADGTVNQIEGEATPVNLTEPAK_9_2_0_0_0
Intensity
m/z
126.0541
138.0548
147.1169 y1(1+)
168.0661
204.0868
315.198 y3(1+)
357.2082 y10(3+)
366.1426
535.3109 y10(2+)

File: 231129_OE_RK_36, RT: 96.099, Scan: 129124
Type: HCD, FTMS + c NSI d Full ms2 1599.5469@hcd30.00 [120.0000-4916.7554]
Precursor Scan: 129016, m/z: 1599.1814, charge: 3
MVGHSNATCR_9_8_0_2_0

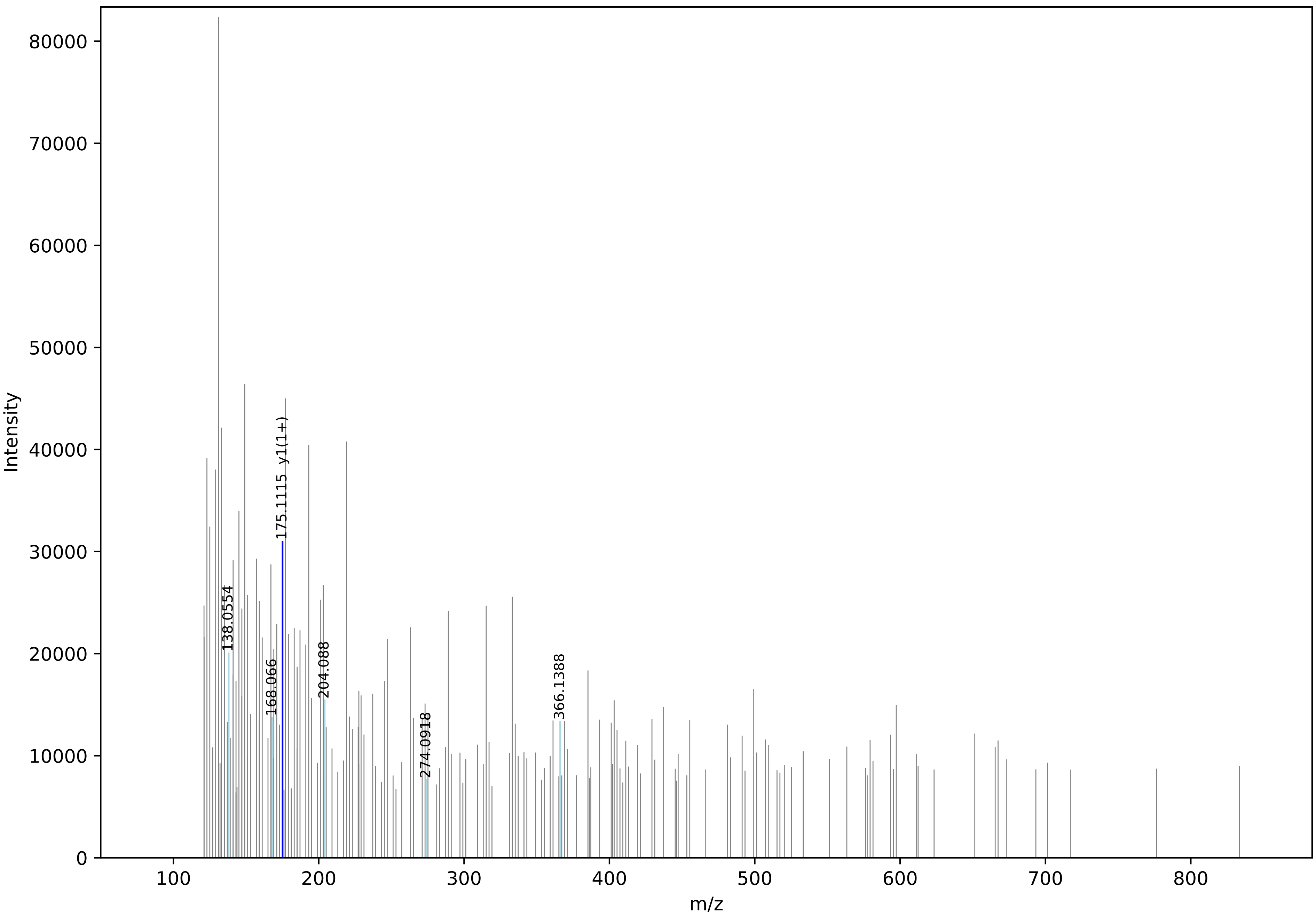

File: 231129_OE_RK_36, RT: 96.32, Scan: 129443
Type: HCD, FTMS + c NSI d Full ms2 1355.9103@hcd30.00 [120.0000-4171.2275]
Precursor Scan: 129365, m/z: 1355.5494, charge: 3
VKSLMASLTNMSR_6_6_1_1_0

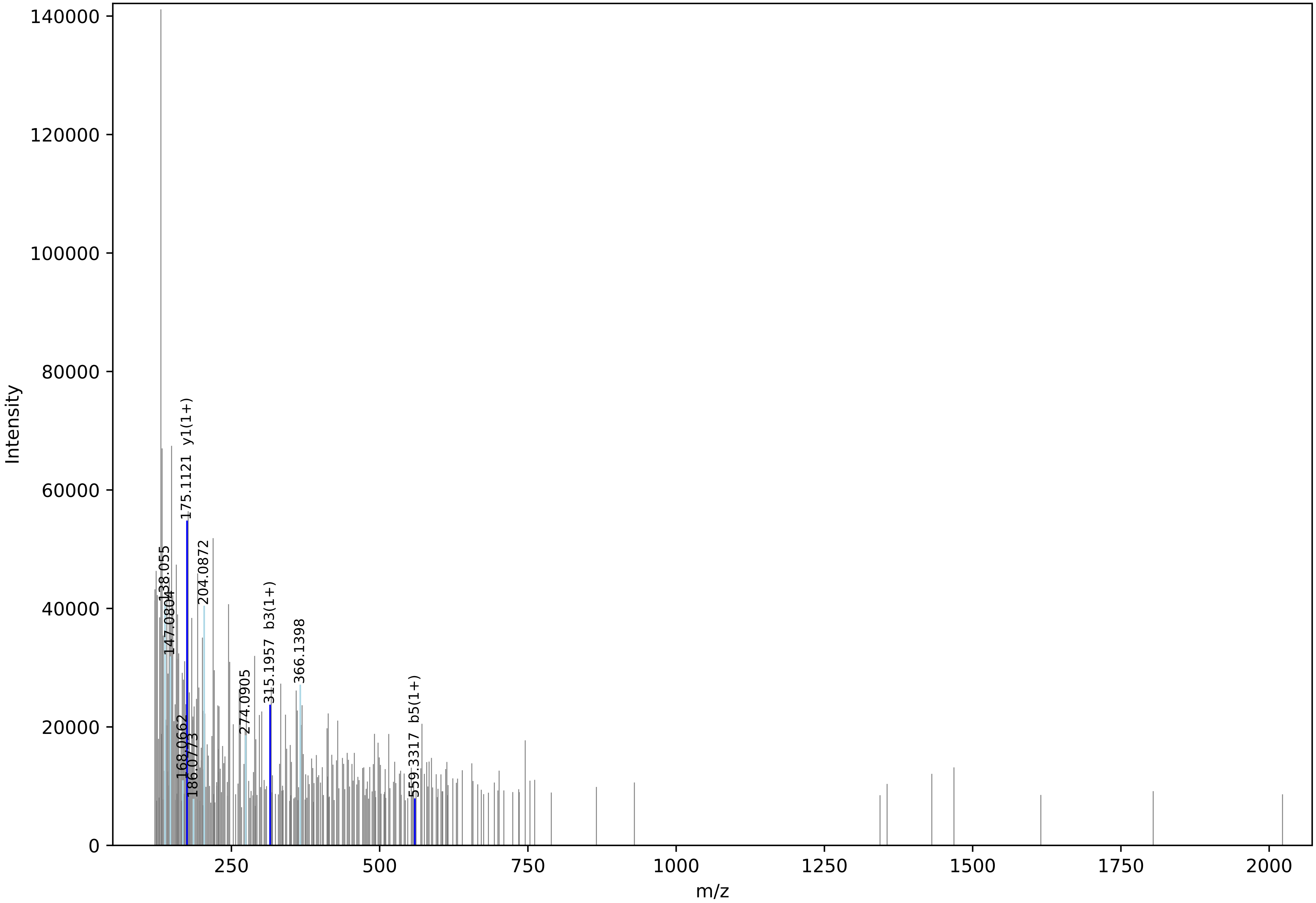

File: 231129_OE_RK_36, RT: 96.398, Scan: 129559
Type: HCD, FTMS + c NSI d Full ms2 1356.3416@hcd30.00 [120.0000-4172.5469]
Precursor Scan: 129365, m/z: 1356.0116, charge: 3
NISSEEKASWTRPEK_4_6_1_1_0

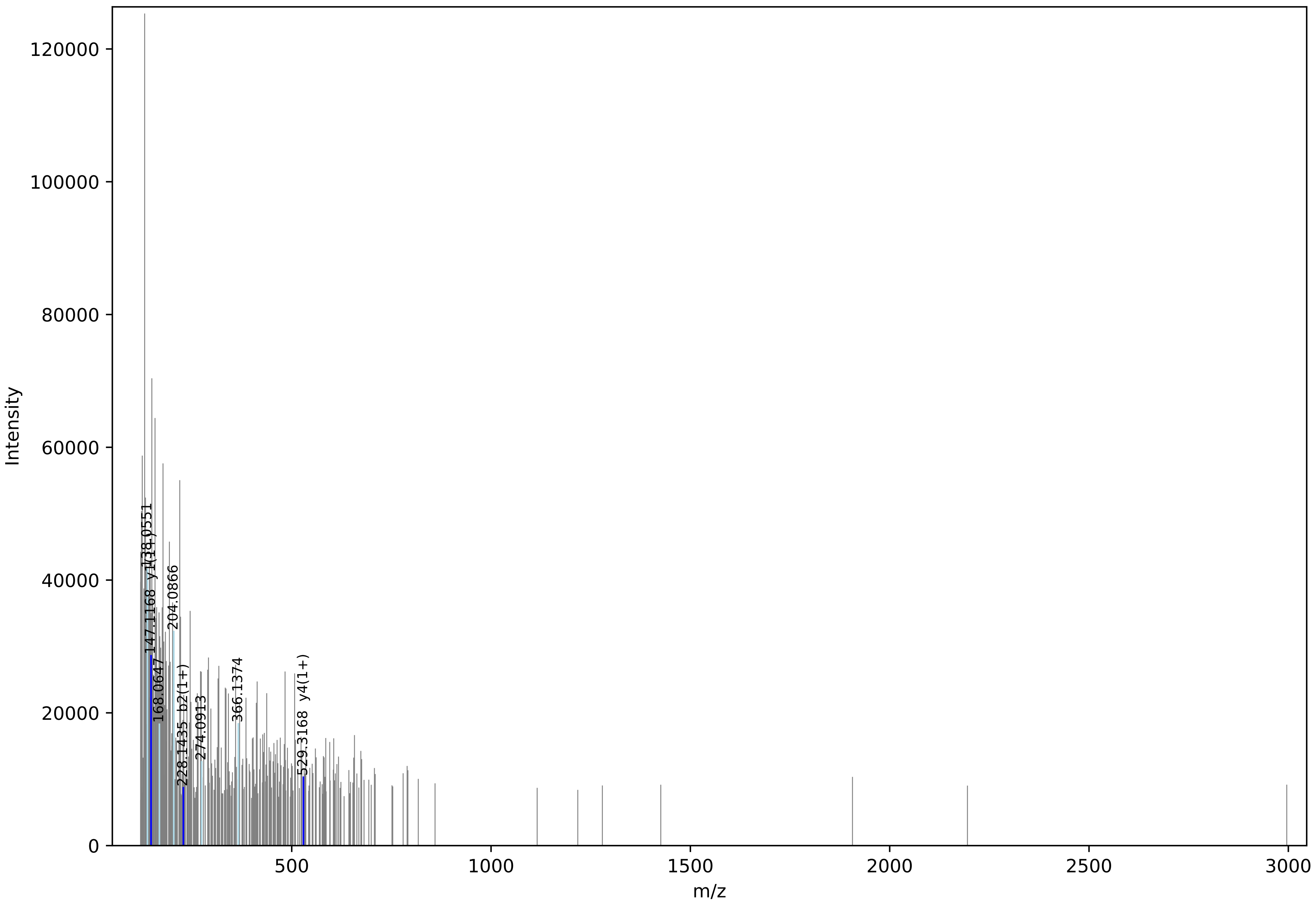

File: 231129_OE_RK_36, RT: 96.651, Scan: 129922
Type: HCD, FTMS + c NSI d Full ms2 1325.6942@hcd30.00 [120.0000-4078.7661]
Precursor Scan: 129860, m/z: 1325.6942, charge: 3
KYNSQNQSNNQFVLYR_5_5_1_0_0

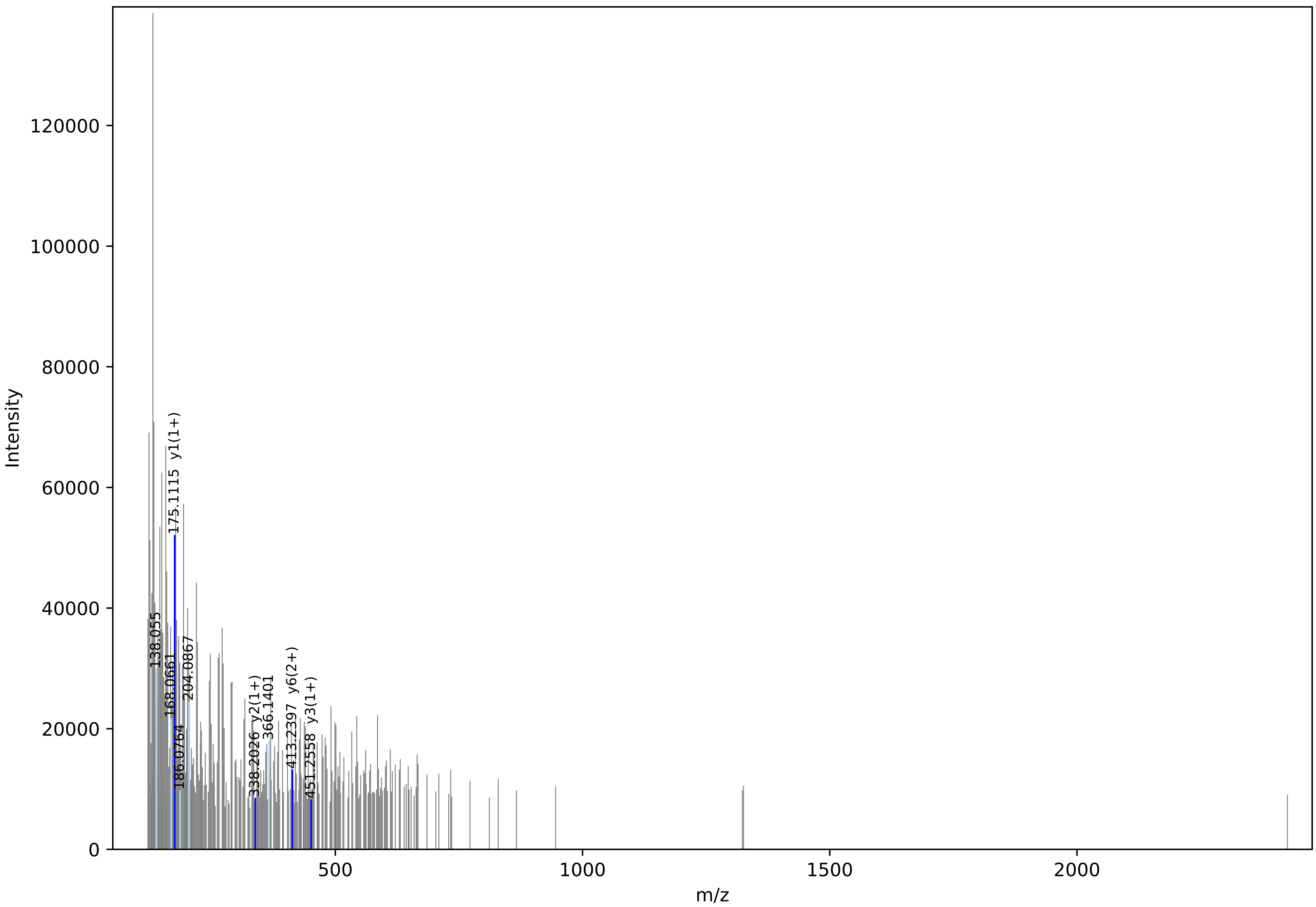

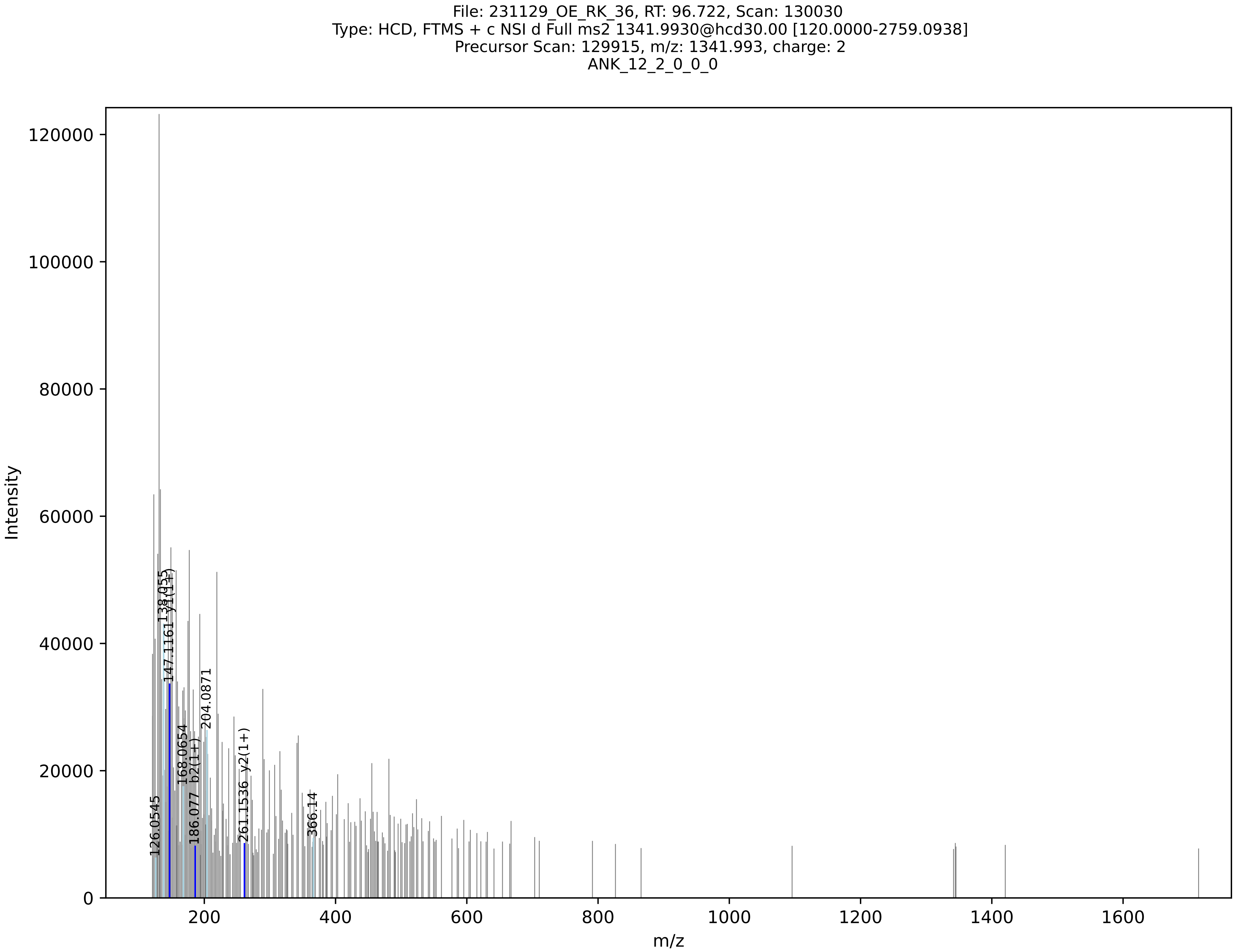

File: 231129_OE_RK_36, RT: 96.722, Scan: 130030
Type: HCD, FTMS + c NSI d Full ms2 1341.9930@hcd30.00 [120.0000-2759.0938]
Precursor Scan: 129915, m/z: 1341.993, charge: 2
ANK_12_2_0_0_0
Intensity
m/z
126.0545
138.055
147.1161_y1(1+)
168.0654
186.077_b2(1+)
204.0871
261.1536_y2(1+)
366.14

File: 231129_OE_RK_36, RT: 97.008, Scan: 130443
Type: HCD, FTMS + c NSI d Full ms2 1572.2069@hcd30.00 [120.0000-3228.7302]
Precursor Scan: 130356, m/z: 1572.207, charge: 2
NASVSDSGK_6_5_0_1_0

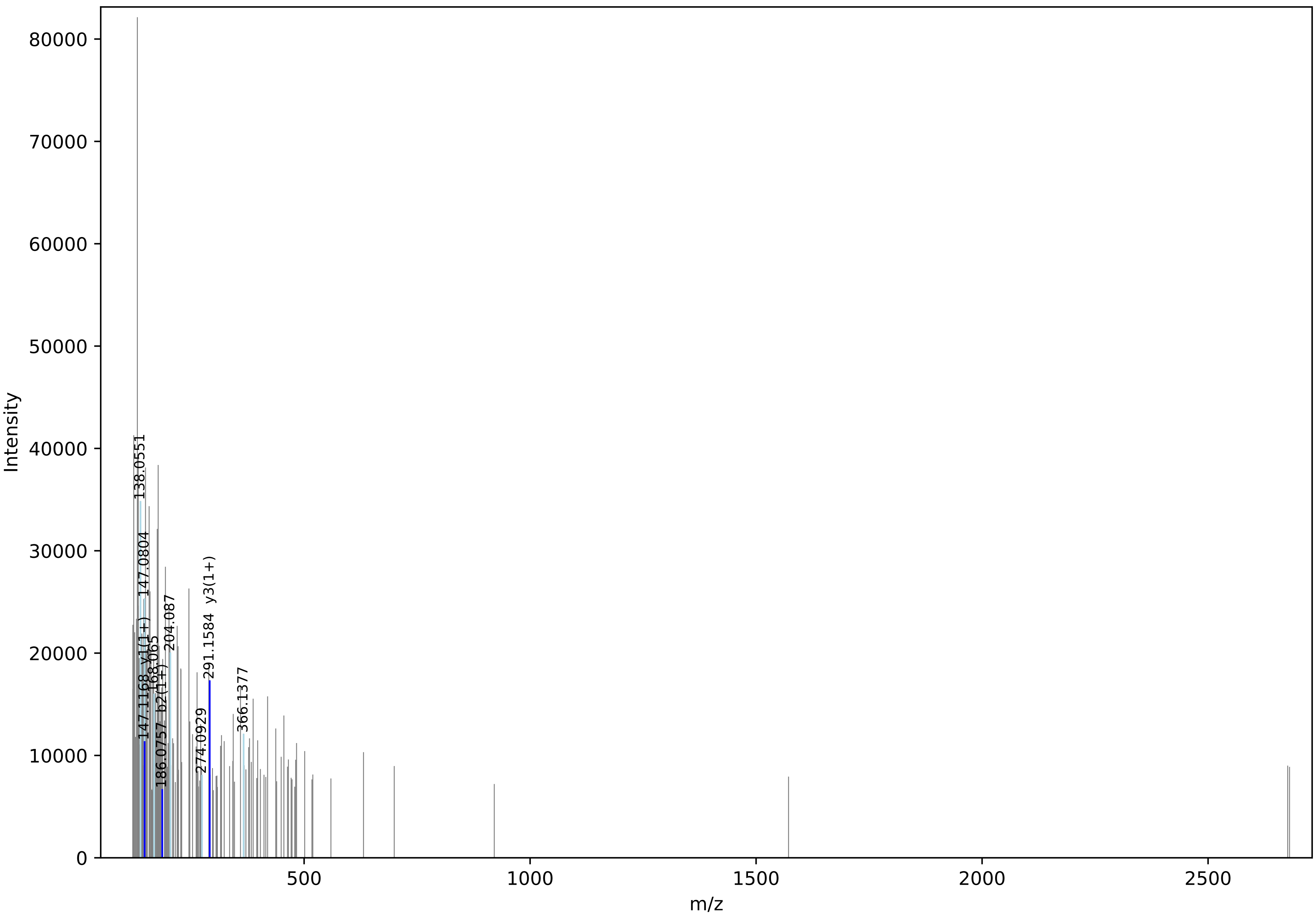

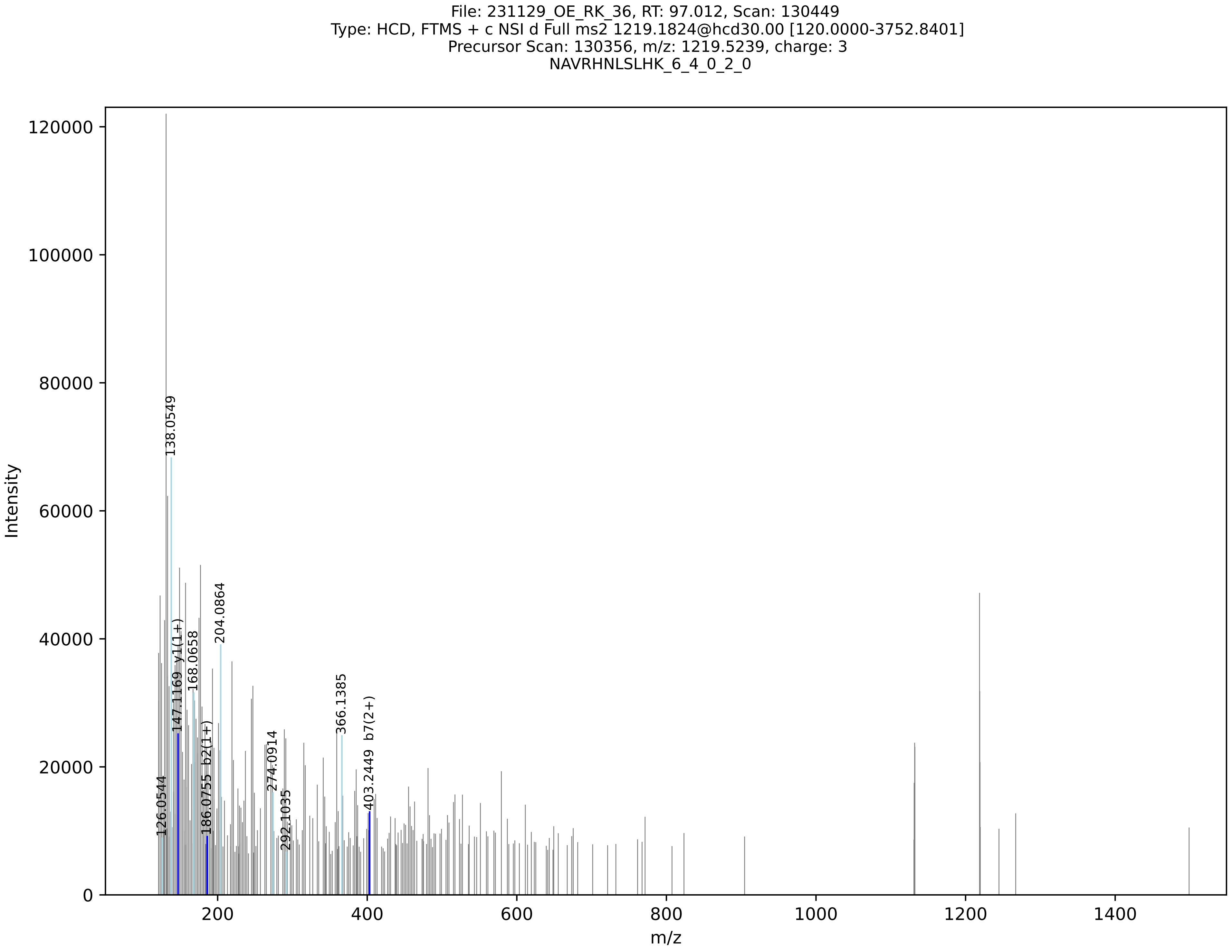
File: 231129_OE_RK_36, RT: 97.012, Scan: 130449
Type: HCD, FTMS + c NSI d Full ms2 1219.1824@hcd30.00 [120.0000-3752.8401]
Precursor Scan: 130356, m/z: 1219.5239, charge: 3
NAVRHNLSLHK_6_4_0_2_0
Intensity
m/z
138.0549
204.0864
147.1169 y1(1+)
168.0658
126.0544
186.0755 b2(1+)
274.0914
292.1035
366.1385
403.2449 b7(2+)

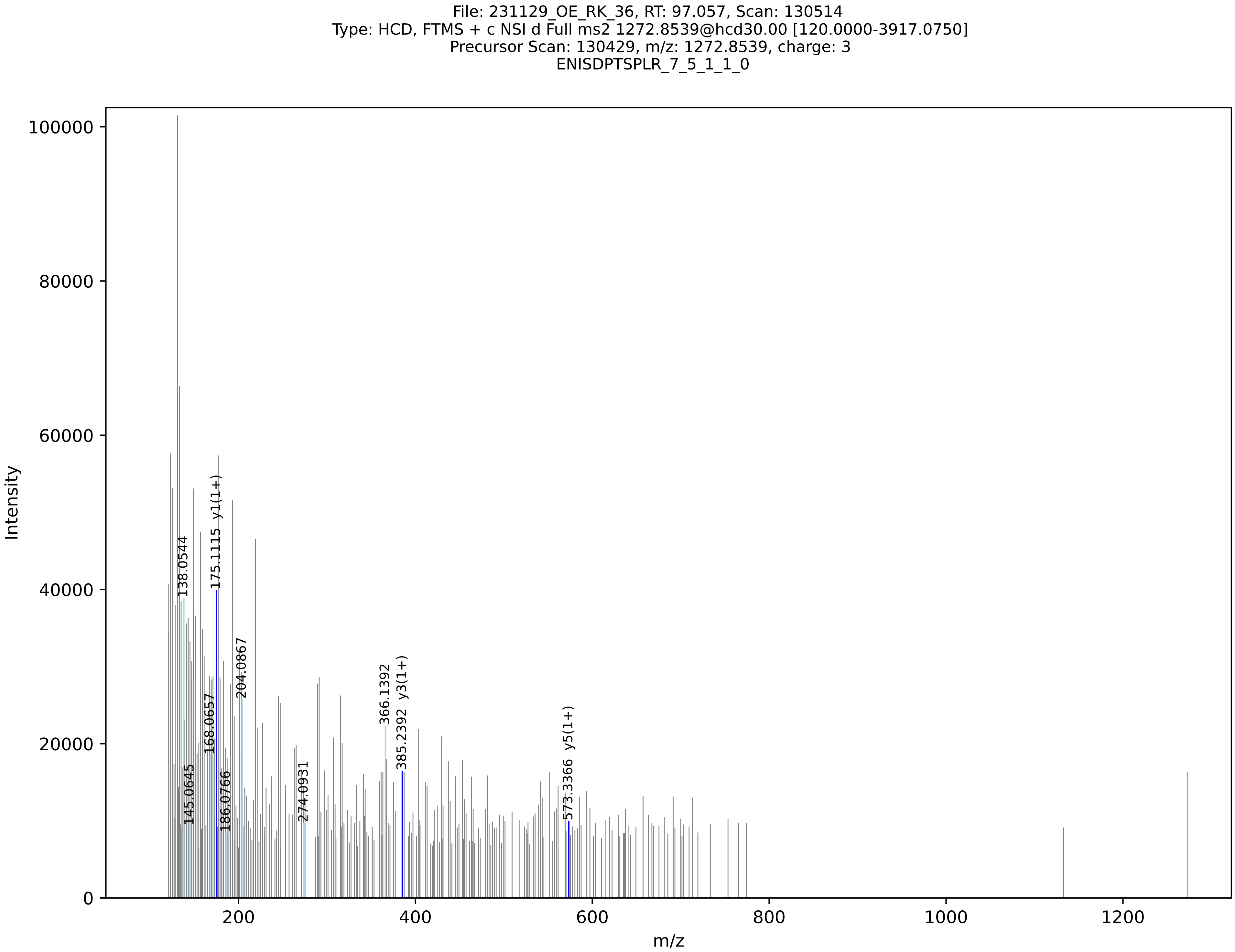
File: 231129_OE_RK_36, RT: 97.057, Scan: 130514
Type: HCD, FTMS + c NSI d Full ms2 1272.8539@hcd30.00 [120.0000-3917.0750]
Precursor Scan: 130429, m/z: 1272.8539, charge: 3
ENISDPTSPLR_7_5_1_1_0
Intensity
m/z
138.0544
145.0645
168.0657
175.1115 y1(1+)
186.0766
204.0867
274.0931
366.1392
385.2392 y3(1+)
573.3366 y5(1+)

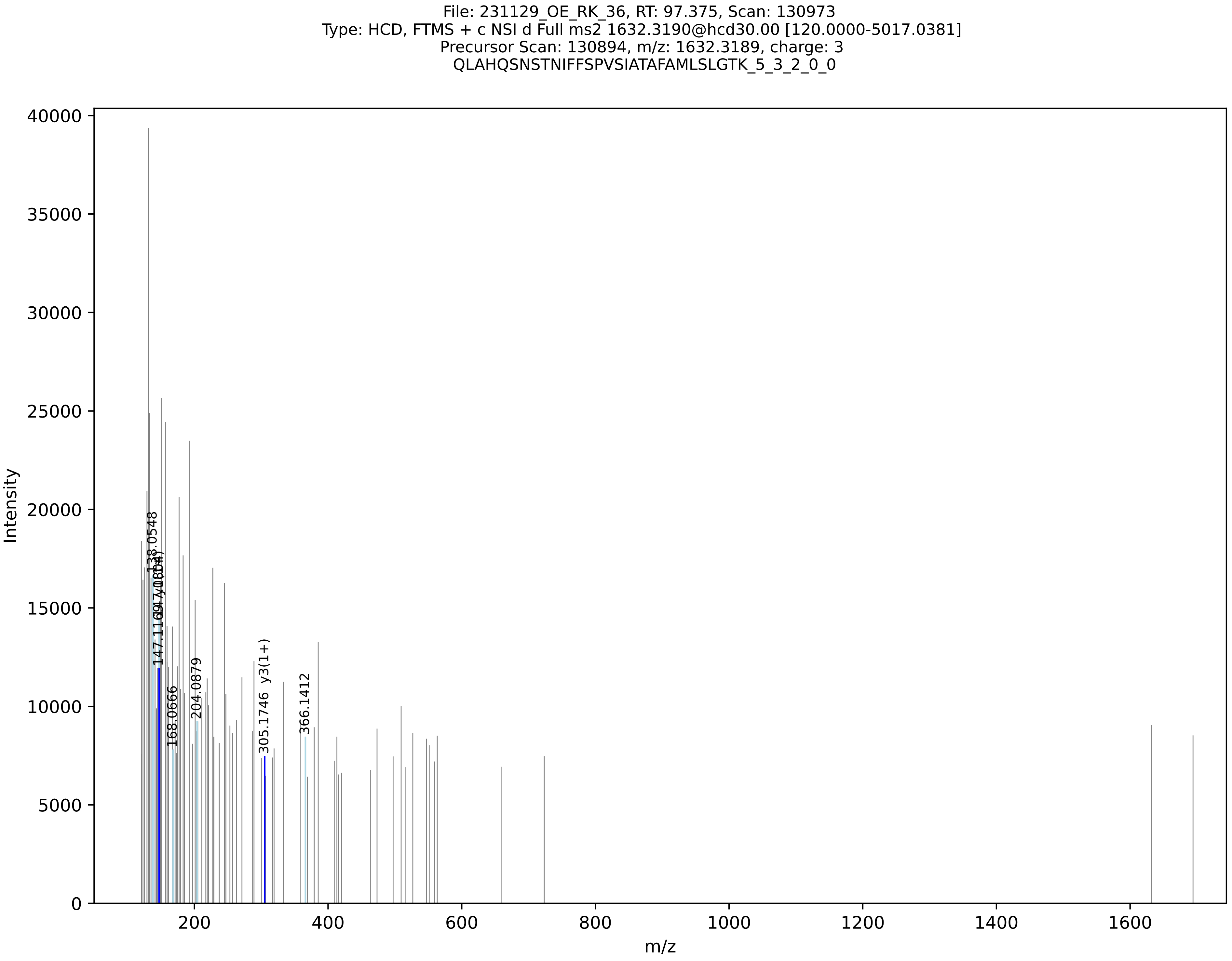
File: 231129_OE_RK_36, RT: 97.375, Scan: 130973
Type: HCD, FTMS + c NSI d Full ms2 1632.3190@hcd30.00 [120.0000-5017.0381]
Precursor Scan: 130894, m/z: 1632.3189, charge: 3
QLAHQSNSTNIFFSPVSIATAFAMLSLGTK_5_3_2_0_0
Intensity
m/z
0
5000
10000
15000
20000
25000
30000
35000
40000
200
400
600
800
1000
1200
1400
1600
138.0548
168.0666
204.0879
305.1746 y3(1+)
366.1412

File: 231129_OE_RK_36, RT: 97.478, Scan: 131123
Type: HCD, FTMS + c NSI d Full ms2 1593.6439@hcd30.00 [120.0000-3272.4617]
Precursor Scan: 131041, m/z: 1593.6439, charge: 2
ASPPSSSCNISSGEMQK_5_3_0_0_0

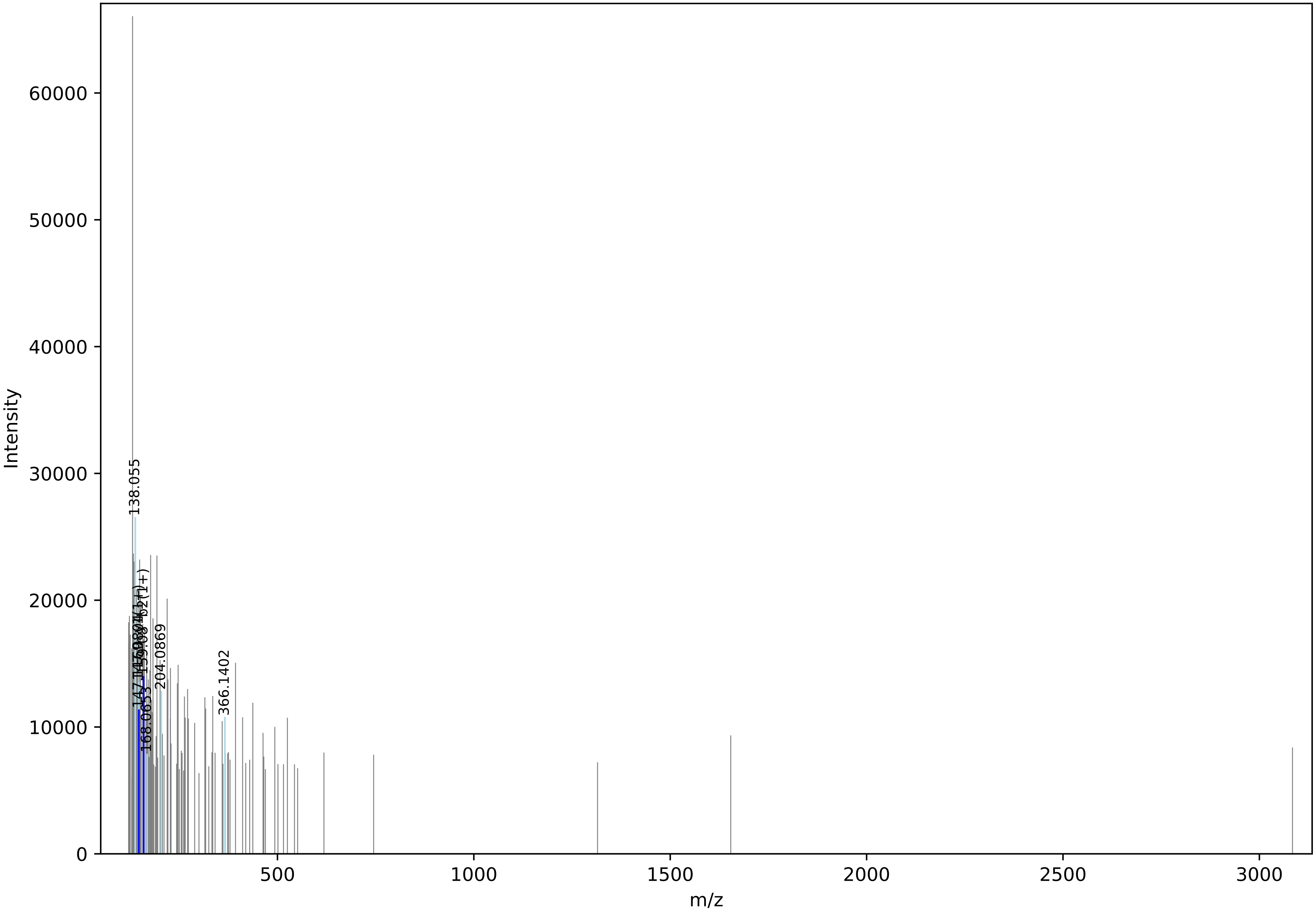

File: 231129_OE_RK_36, RT: 97.666, Scan: 131395
Type: HCD, FTMS + c NSI d Full ms2 1240.0898@hcd30.00 [120.0000-2551.2112]
Precursor Scan: 131334, m/z: 1240.0898, charge: 2
NVSWATGR_3_4_2_0_0

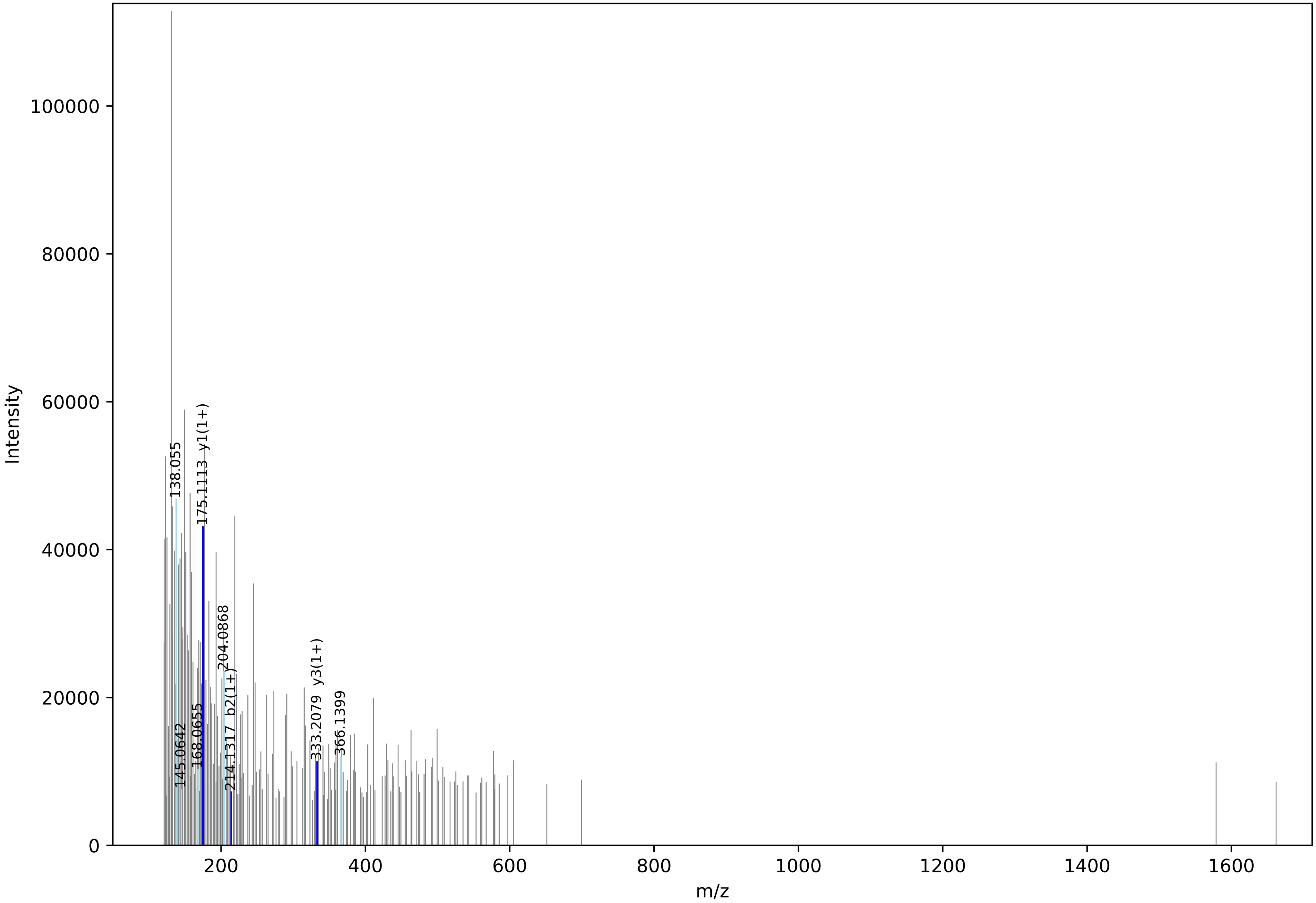

File: 231129_OE_RK_36, RT: 97.791, Scan: 131573
Type: HCD, FTMS + c NSI d Full ms2 1392.0800@hcd30.00 [120.0000-2861.2712]
Precursor Scan: 131504, m/z: 1392.08, charge: 2
NASLR_6_4_1_1_0

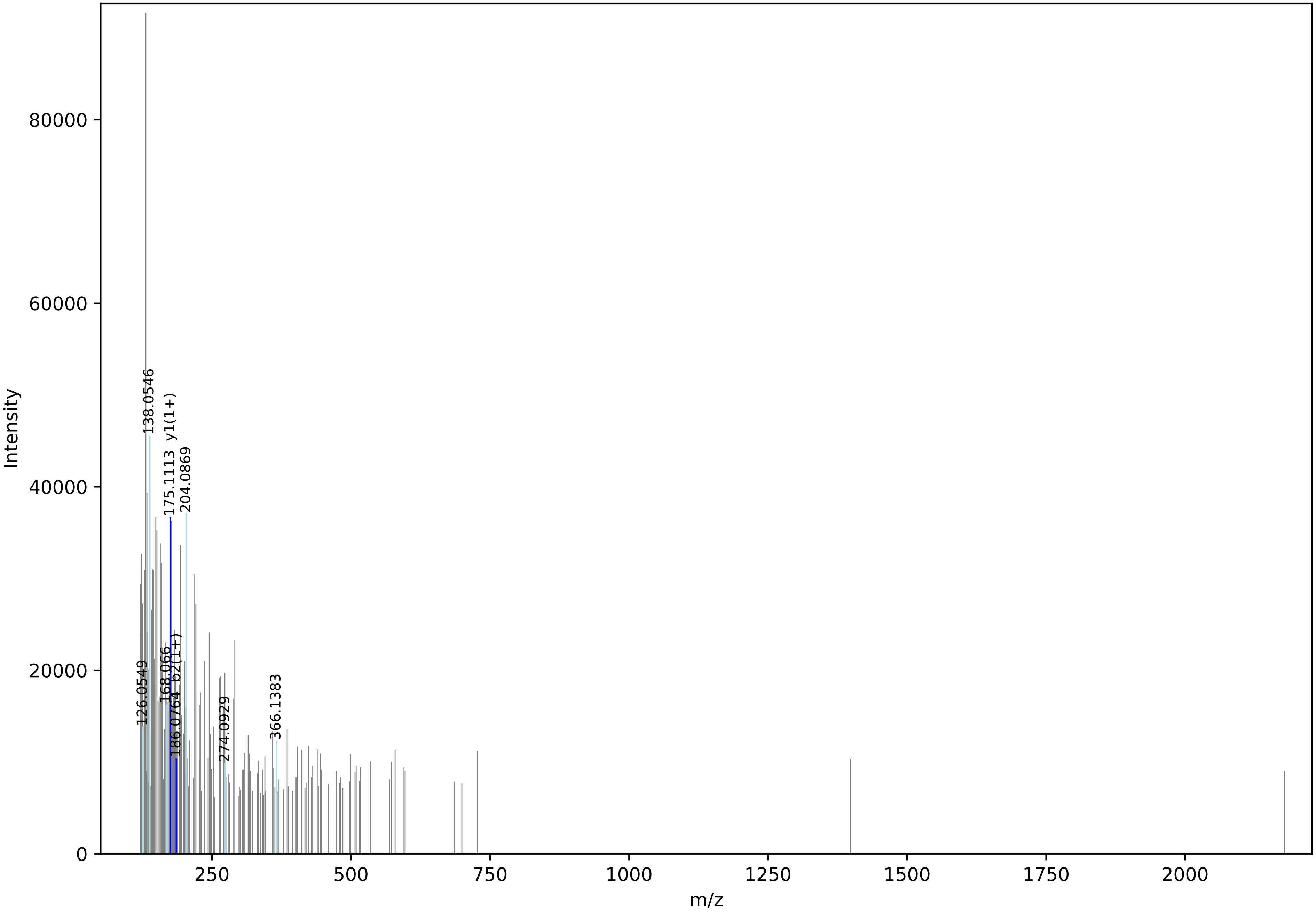

File: 231129_OE_RK_36, RT: 98.546, Scan: 132657
Type: HCD, FTMS + c NSI d Full ms2 1366.6665@hcd30.00 [120.0000-2809.4277]
Precursor Scan: 132575, m/z: 1366.1964, charge: 2
RNQTAAR_5_4_0_1_0

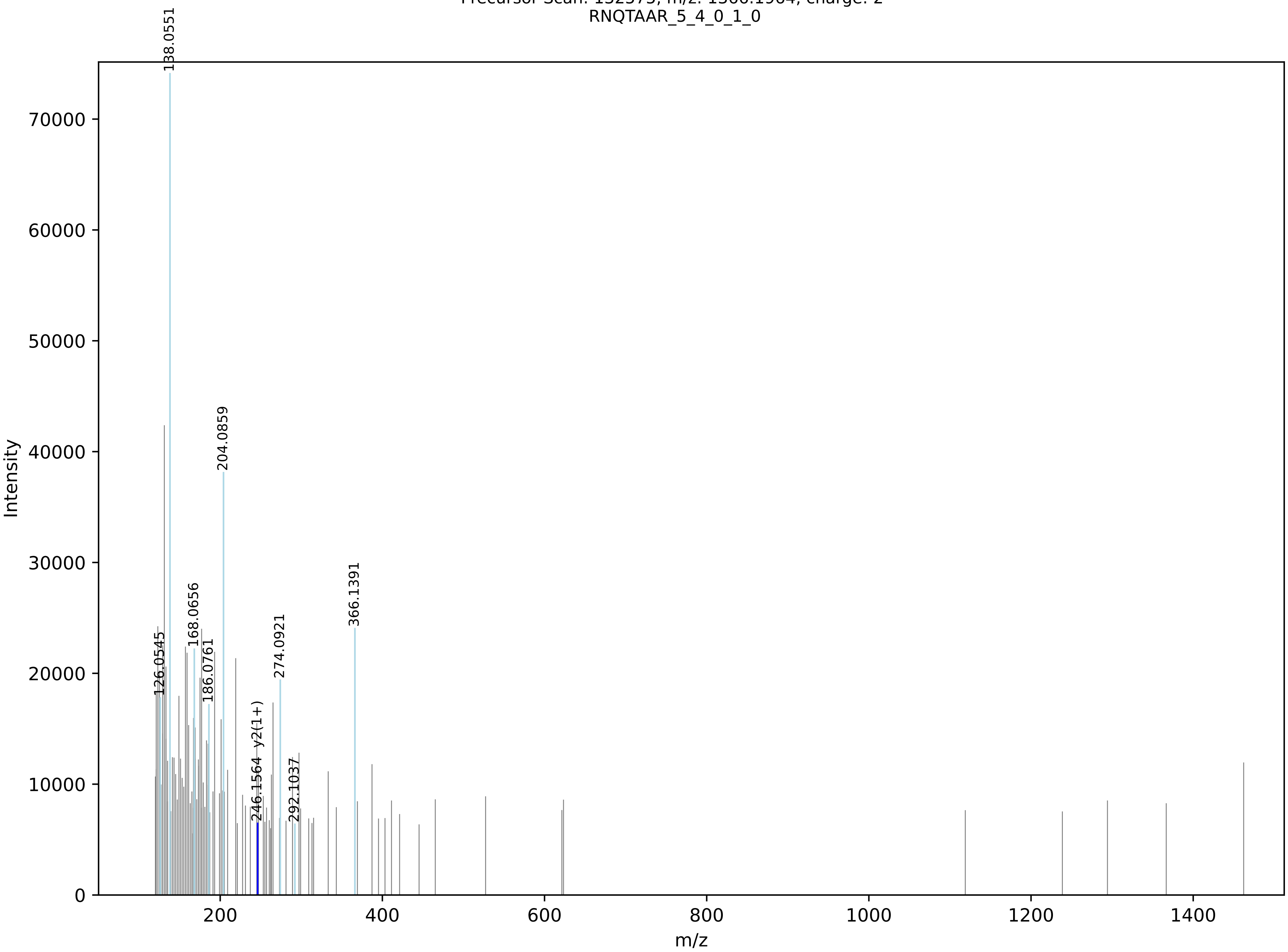

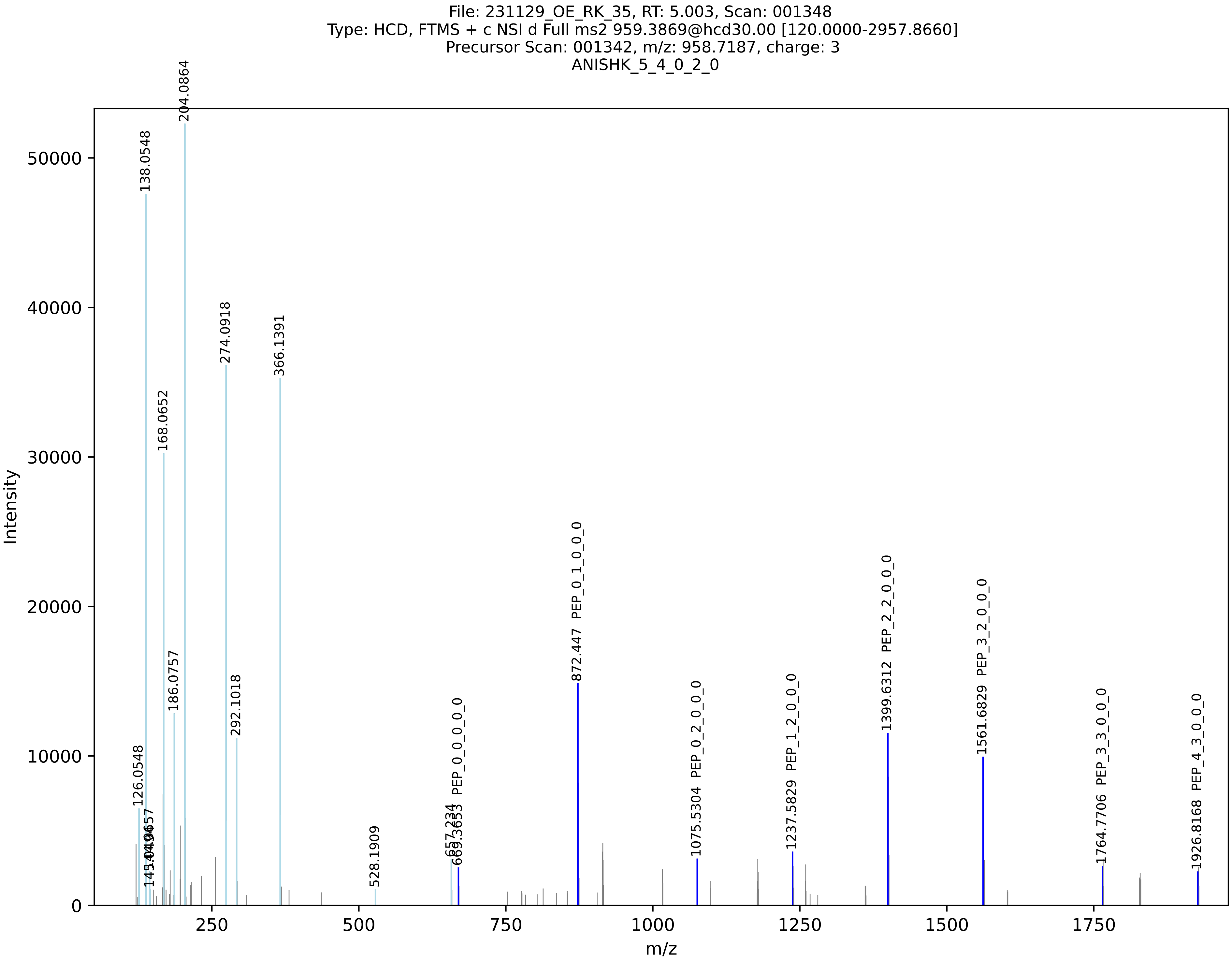

File: 231129_OE_RK_35, RT: 5.003, Scan: 001348
Type: HCD, FTMS + c NSI d Full ms2 959.3869@hcd30.00 [120.0000-2957.8660]
Precursor Scan: 001342, m/z: 958.7187, charge: 3
ANISHK_5_4_0_2_0
Intensity
m/z
204.0864
138.0548
168.0652
274.0918
366.1391
186.0757
292.1018
126.0548
528.1909
657.234
669.3653 PEP_0_0_0_0_0_
872.447 PEP_0_1_0_0_0_
1075.5304 PEP_0_2_0_0_0_
1237.5829 PEP_1_2_0_0_0_
1399.6312 PEP_2_2_0_0_0_
1561.6829 PEP_3_2_0_0_0_
1764.7706 PEP_3_3_0_0_0_
1926.8168 PEP_4_3_0_0_0_

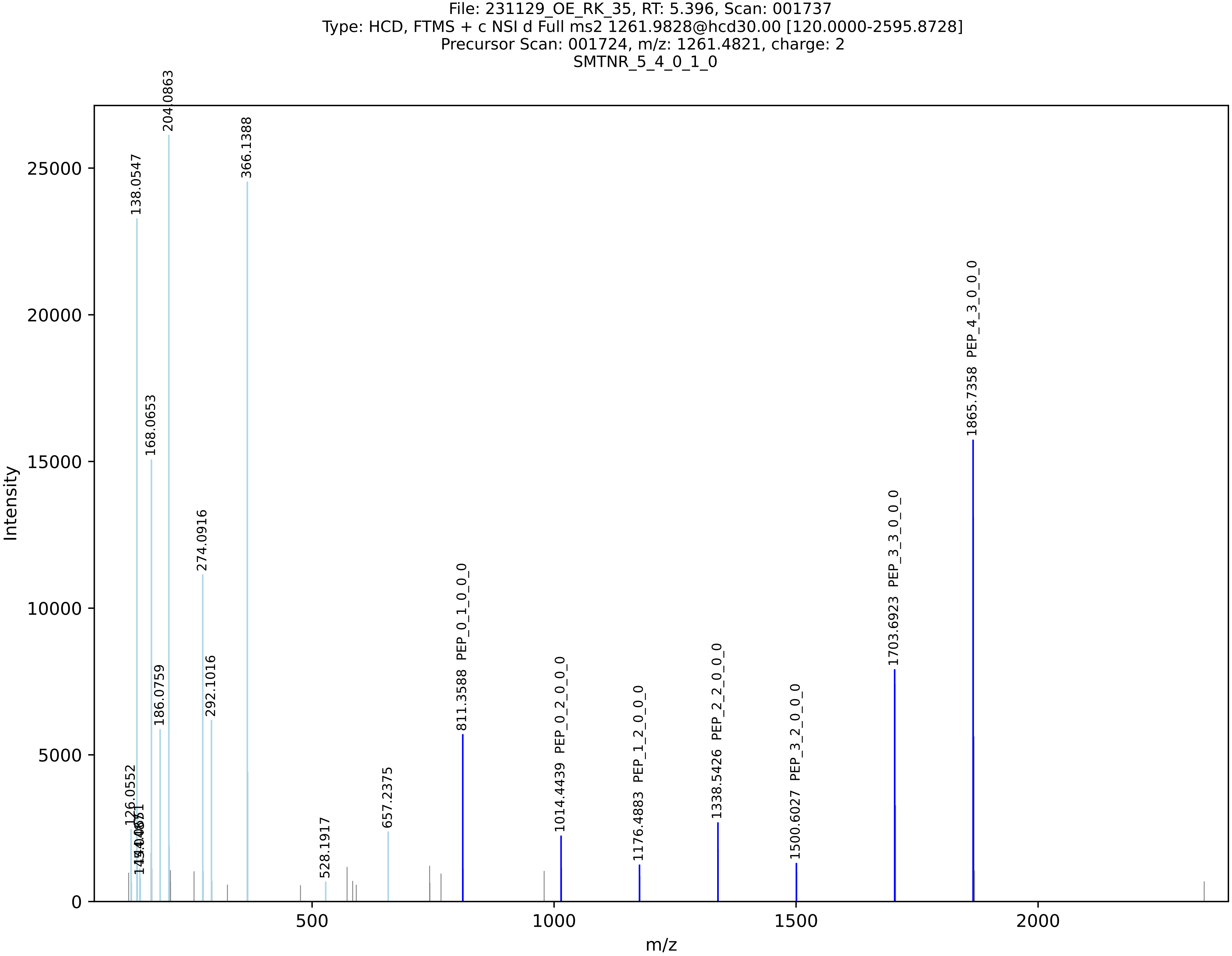
File: 231129_OE_RK_35, RT: 5.396, Scan: 001737
Type: HCD, FTMS + c NSI d Full ms2 1261.9828@hcd30.00 [120.0000-2595.8728]
Precursor Scan: 001724, m/z: 1261.4821, charge: 2
SMTNR_5_4_0_1_0
Intensity
m/z
0
5000
10000
15000
20000
25000
500
1000
1500
2000
204.0863
138.0547
366.1388
168.0653
274.0916
292.1016
186.0759
126.0552
528.1917
657.2375
811.3588 PEP_0_1_0_0_0
1014.4439 PEP_0_2_0_0_0
1176.4883 PEP_1_2_0_0_0
1338.5426 PEP_2_2_0_0_0
1500.6027 PEP_3_2_0_0_0
1703.6923 PEP_3_3_0_0_0
1865.7358 PEP_4_3_0_0_0

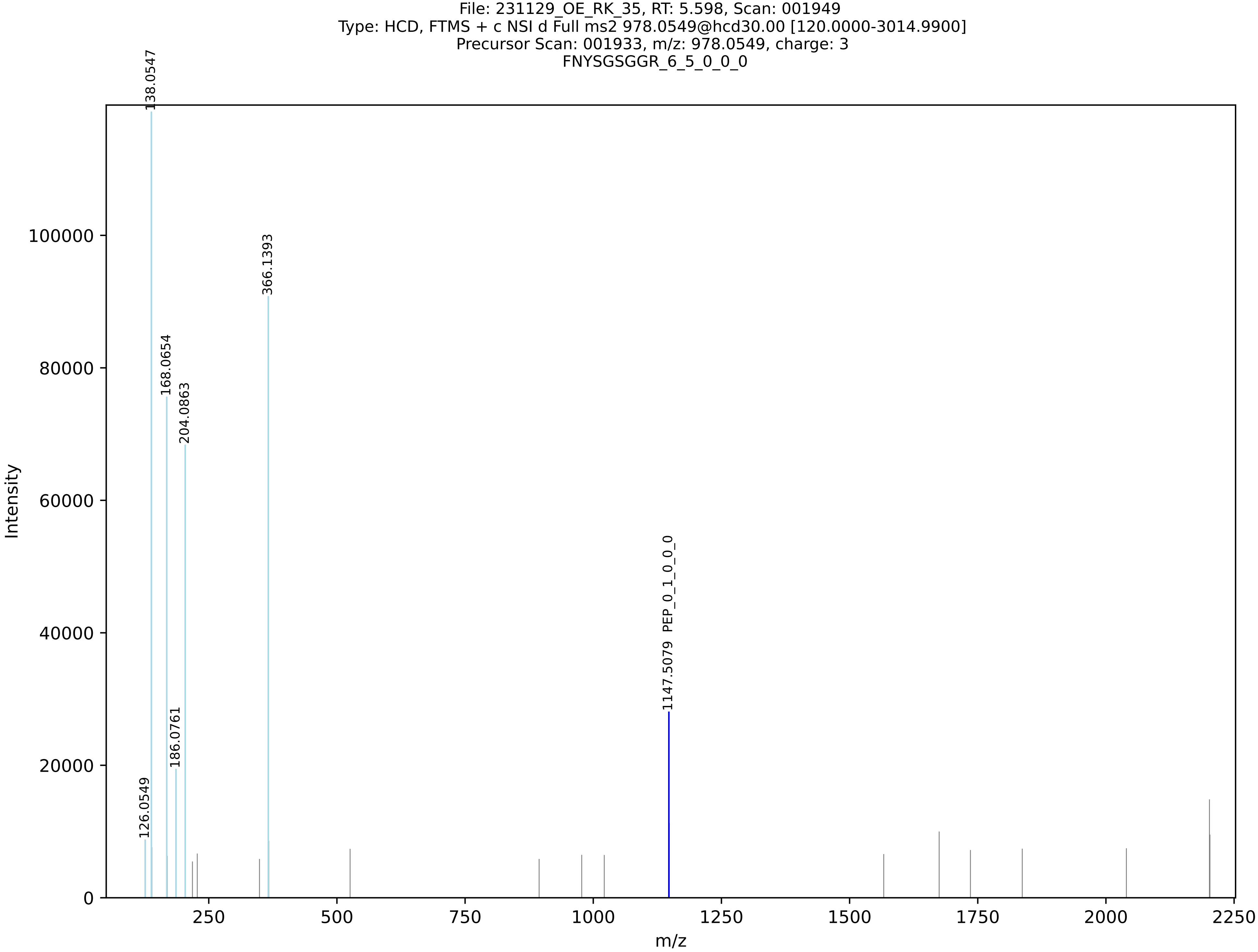
File: 231129_OE_RK_35, RT: 5.598, Scan: 001949
Type: HCD, FTMS + c NSI d Full ms2 978.0549@hcd30.00 [120.0000-3014.9900]
Precursor Scan: 001933, m/z: 978.0549, charge: 3
FNYSGSGGR_6_5_0_0_0
138.0547
366.1393
168.0654
204.0863
1147.5079 PEP_0_1_0_0_0
186.0761
126.0549
Intensity
m/z
0
20000
40000
60000
80000
100000
250
500
750
1000
1250
1500
1750
2000
2250

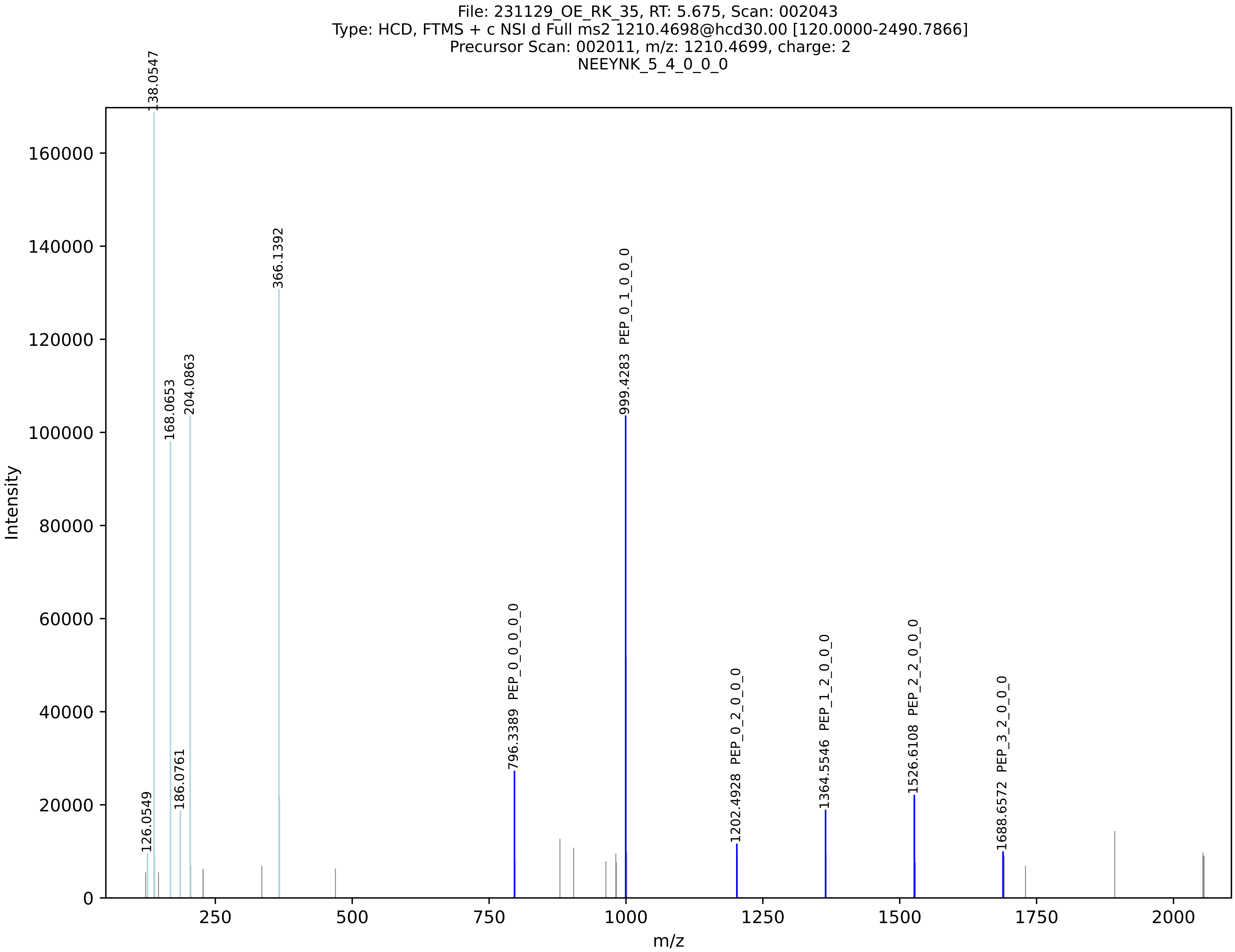
File: 231129_OE_RK_35, RT: 5.675, Scan: 002043
Type: HCD, FTMS + c NSI d Full ms2 1210.4698@hcd30.00 [120.0000-2490.7866]
Precursor Scan: 002011, m/z: 1210.4699, charge: 2
NEEYNK_5_4_0_0_0
Intensity
m/z
138.0547
366.1392
168.0653
204.0863
186.0761
126.0549
999.4283 PEP_0_1_0_0_0
796.3389 PEP_0_0_0_0_0
1202.4928 PEP_0_2_0_0_0
1364.5546 PEP_1_2_0_0_0
1526.6108 PEP_2_2_0_0_0
1688.6572 PEP_3_2_0_0_0

File: 231129_OE_RK_35, RT: 6.015, Scan: 002472
Type: HCD, FTMS + c NSI d Full ms2 1124.4392@hcd30.00 [120.0000-3462.9260]
Precursor Scan: 002465, m/z: 1123.771, charge: 3
HNSTGCLR_6_5_1_1_0

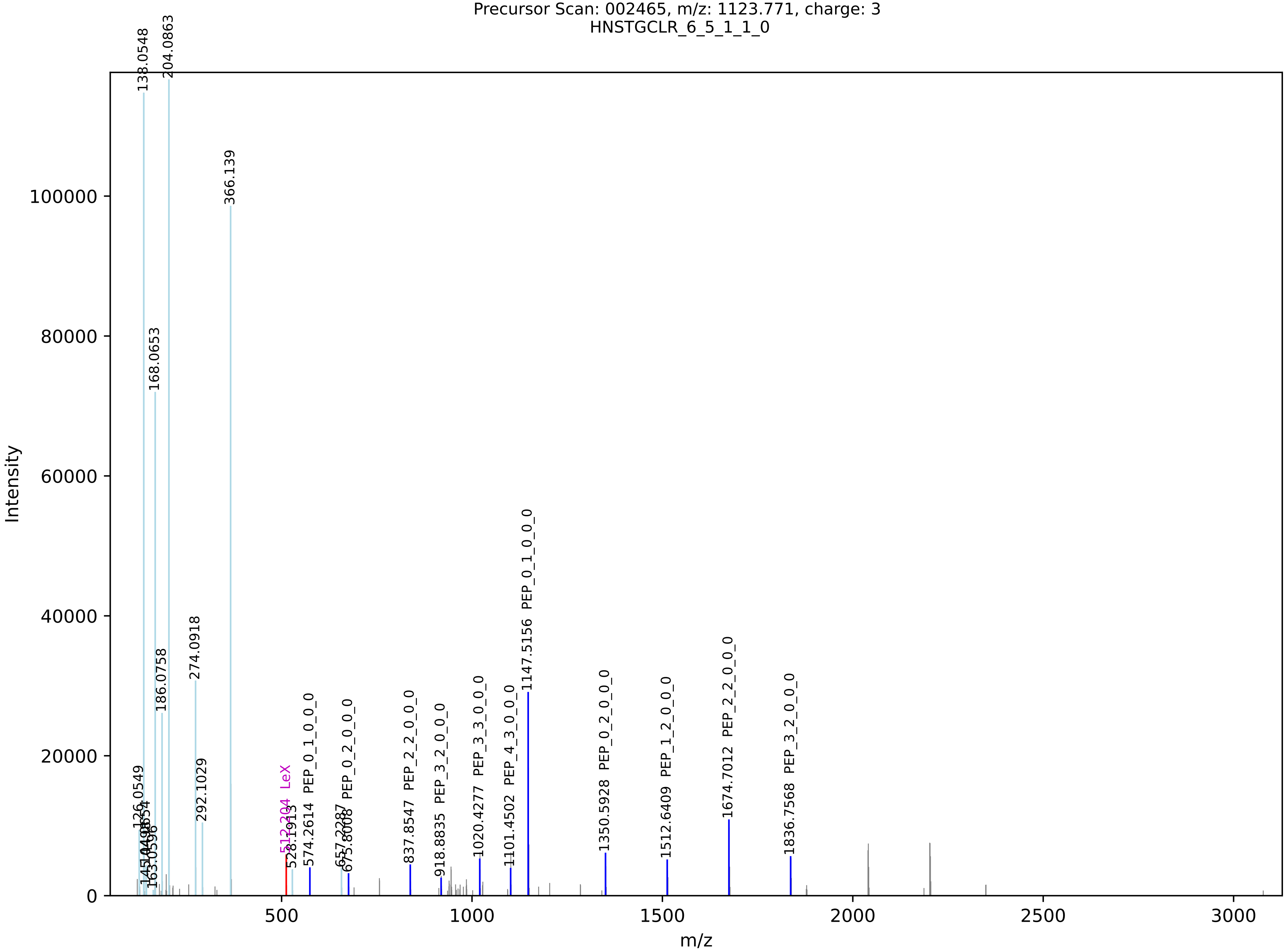

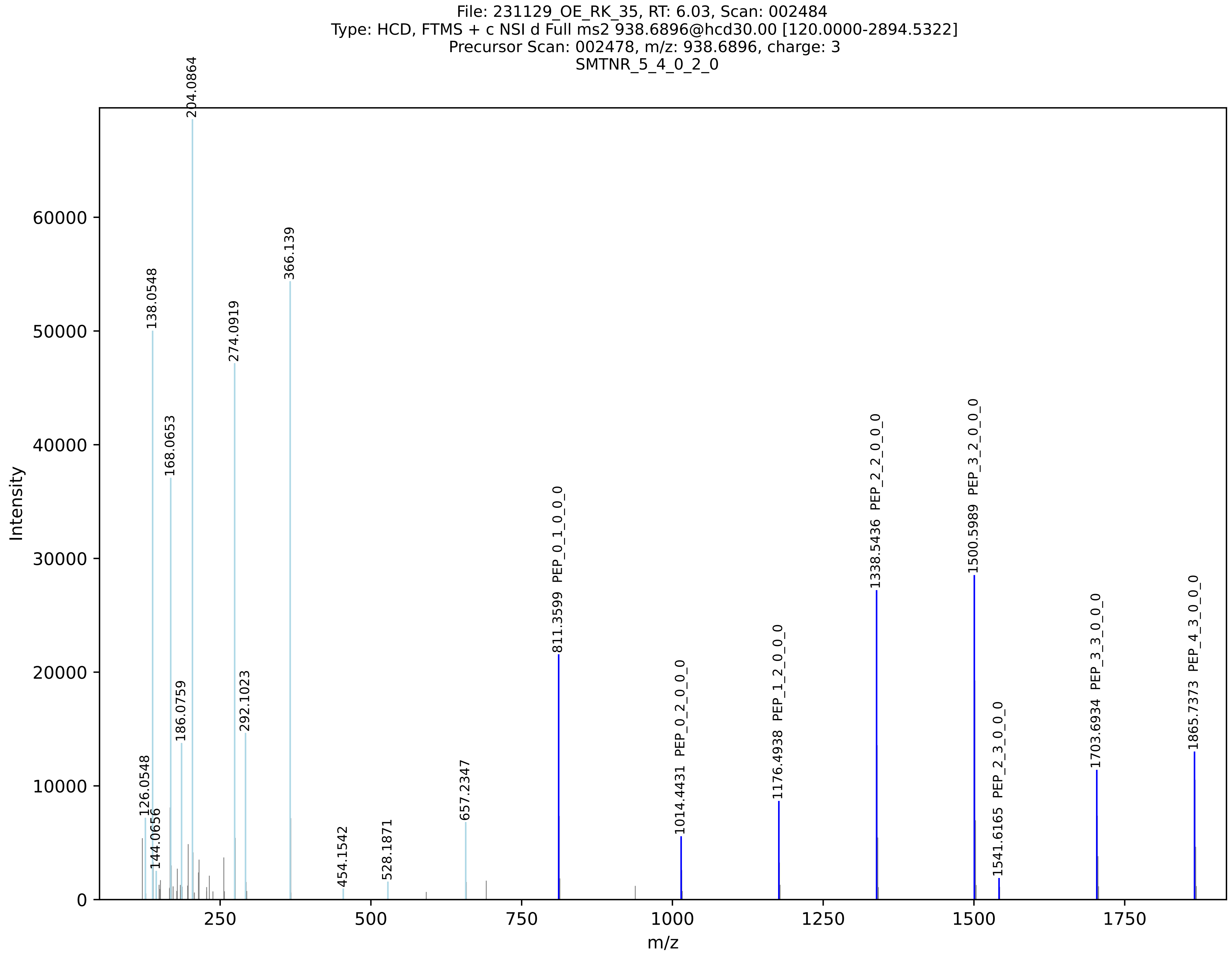
File: 231129_OE_RK_35, RT: 6.03, Scan: 002484
Type: HCD, FTMS + c NSI d Full ms2 938.6896@hcd30.00 [120.0000-2894.5322]
Precursor Scan: 002478, m/z: 938.6896, charge: 3
SMTNR_5_4_0_2_0
Intensity
m/z
204.0864
366.139
138.0548
274.0919
168.0653
186.0759
292.1023
126.0548
144.0656
454.1542
528.1871
657.2347
811.3599 PEP_0_1_0_0_0
1014.4431 PEP_0_2_0_0_0
1176.4938 PEP_1_2_0_0_0
1338.5436 PEP_2_2_0_0_0
1500.5989 PEP_3_2_0_0_0
1541.6165 PEP_2_3_0_0_0
1703.6934 PEP_3_3_0_0_0
1865.7373 PEP_4_3_0_0_0

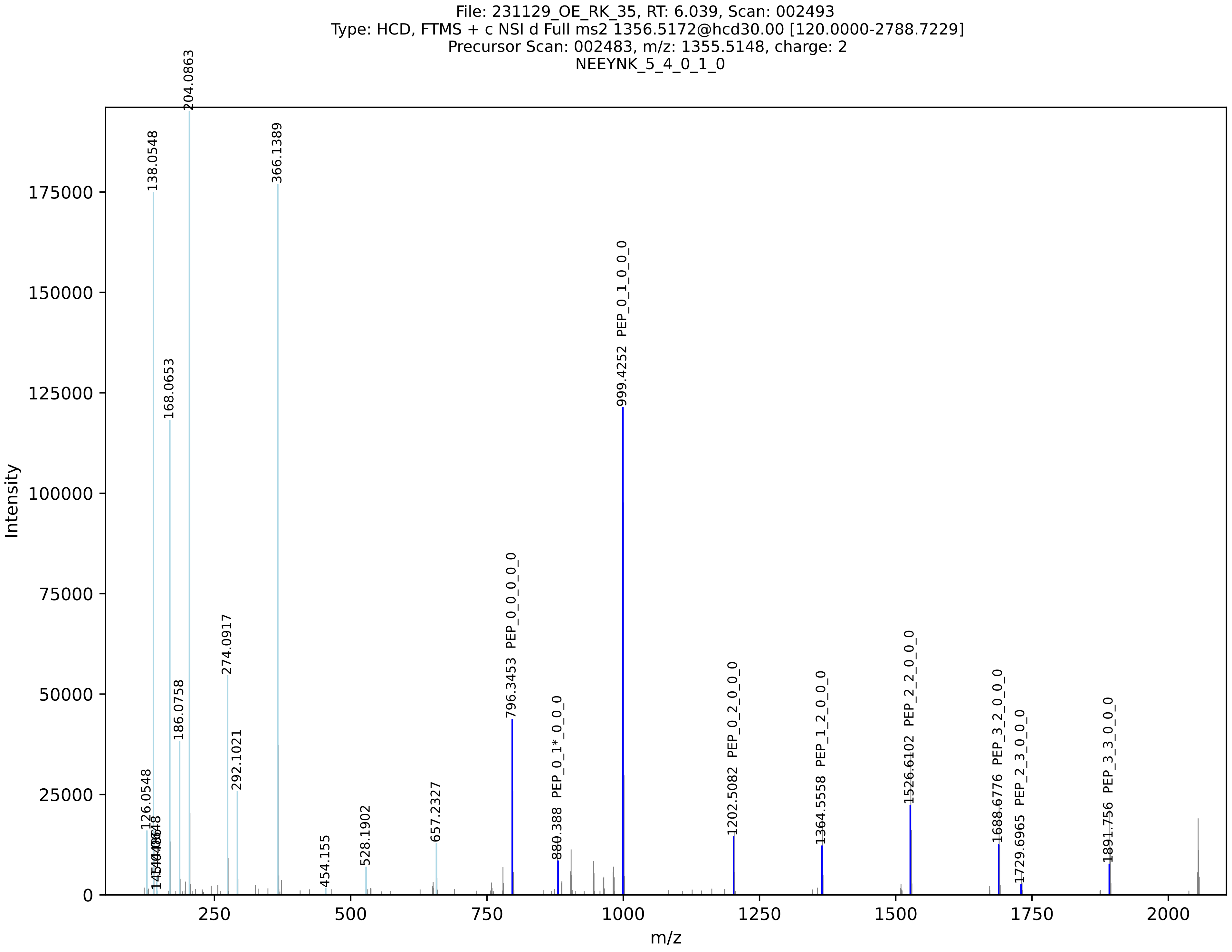
File: 231129_OE_RK_35, RT: 6.039, Scan: 002493
Type: HCD, FTMS + c NSI d Full ms2 1356.5172@hcd30.00 [120.0000-2788.7229]
Precursor Scan: 002483, m/z: 1355.5148, charge: 2
NEEYNK_5_4_0_1_0
Intensity
m/z
204.0863
138.0548
366.1389
168.0653
186.0758
274.0917
292.1021
126.0548
454.155
528.1902
657.2327
796.3453 PEP_0_0_0_0_0
880.388 PEP_0_1*_0_0_0
999.4252 PEP_0_1_0_0_0
1202.5082 PEP_0_2_0_0_0
1364.5558 PEP_1_2_0_0_0
1526.6102 PEP_2_2_0_0_0
1688.6776 PEP_3_2_0_0_0
1729.6965 PEP_2_3_0_0_0
1891.756 PEP_3_3_0_0_0

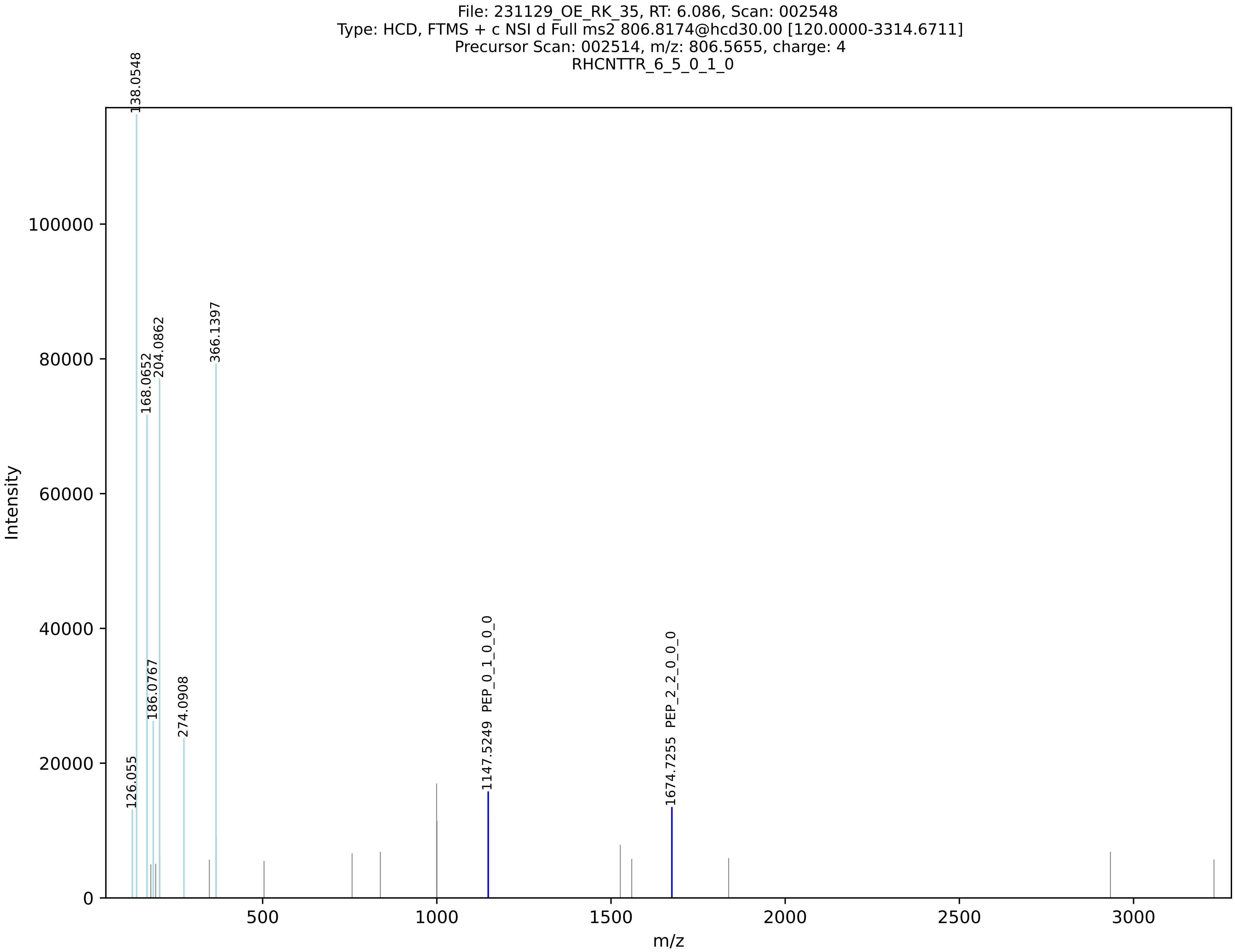
File: 231129_OE_RK_35, RT: 6.086, Scan: 002548
Type: HCD, FTMS + c NSI d Full ms2 806.8174@hcd30.00 [120.0000-3314.6711]
Precursor Scan: 002514, m/z: 806.5655, charge: 4
RHCNTTR_6_5_0_1_0
138.0548
168.0652
204.0862
366.1397
186.0767
274.0908
126.055
1147.5249 PEP_0_1_0_0_0
1674.7255 PEP_2_2_0_0_0
Intensity
0
20000
40000
60000
80000
100000
500
1000
1500
2000
2500
3000
m/z

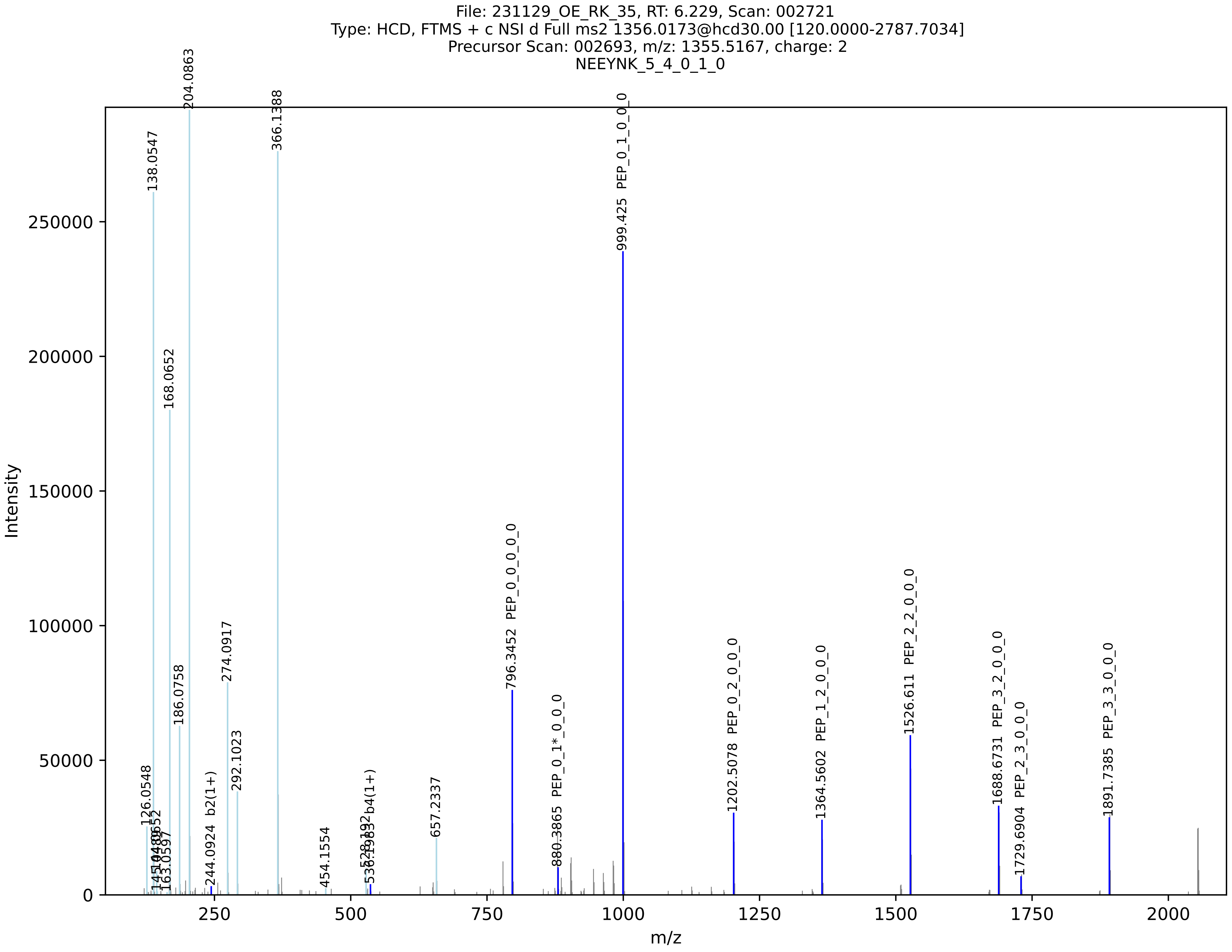
File: 231129_OE_RK_35, RT: 6.229, Scan: 002721
Type: HCD, FTMS + c NSI d Full ms2 1356.0173@hcd30.00 [120.0000-2787.7034]
Precursor Scan: 002693, m/z: 1355.5167, charge: 2
NEEYNK_5_4_0_1_0
Intensity
m/z
204.0863
366.1388
138.0547
168.0652
186.0758
126.0548
163.0597
244.0924 b2(1+)
274.0917
292.1023
454.1554
b4(1+)
657.2337
796.3452 PEP_0_0_0_0_0
880.3865 PEP_0_1*_0_0_0
999.425 PEP_0_1_0_0_0
1202.5078 PEP_0_2_0_0_0
1364.5602 PEP_1_2_0_0_0
1526.611 PEP_2_2_0_0_0
1688.6731 PEP_3_2_0_0_0
1729.6904 PEP_2_3_0_0_0
1891.7385 PEP_3_3_0_0_0

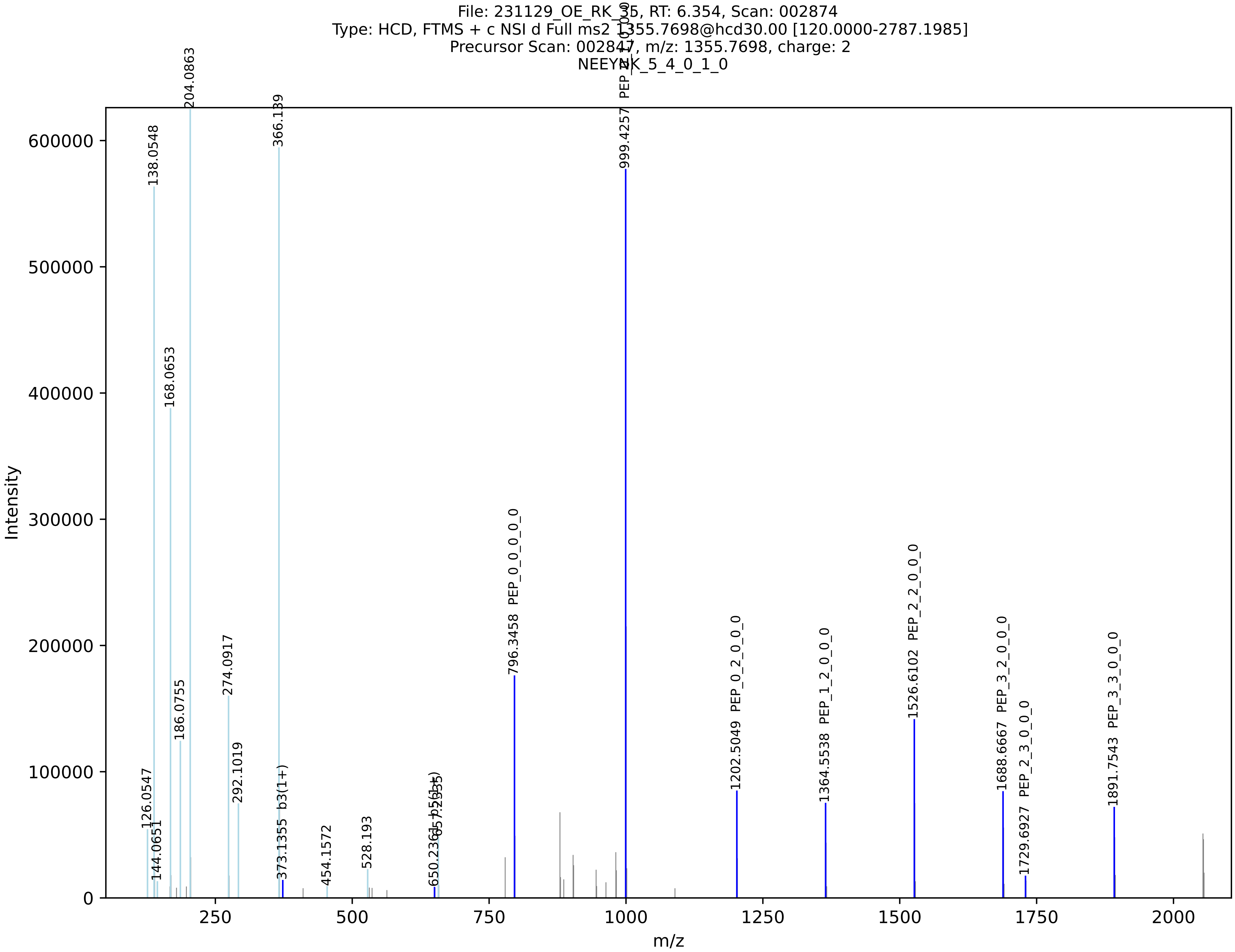
File: 231129_OE_RK_35, RT: 6.354, Scan: 002874
Type: HCD, FTMS + c NSI d Full ms2 1355.7698@hcd30.00 [120.0000-2787.1985]
Precursor Scan: 002847, m/z: 1355.7698, charge: 2
NEEYNK_5_4_0_1_0
Intensity
m/z
0
100000
200000
300000
400000
500000
600000
250
500
750
1000
1250
1500
1750
2000
126.0547
138.0548
144.0651
168.0653
186.0755
204.0863
274.0917
292.1019
366.139
373.1355 b3(1+)
454.1572
528.193
650.2361
657.2535
796.3458 PEP_0_0_0_0_0
999.4257
1202.5049 PEP_0_2_0_0_0
1364.5538 PEP_1_2_0_0_0
1526.6102 PEP_2_2_0_0_0
1688.6667 PEP_3_2_0_0_0
1729.6927 PEP_2_3_0_0_0
1891.7543 PEP_3_3_0_0_0

File: 231129_OE_RK_35, RT: 6.39, Scan: 002917
Type: HCD, FTMS + c NSI d Full ms2 904.3471@hcd30.00 [120.0000-2789.4441]
Precursor Scan: 002892, m/z: 904.0129, charge: 3
NEEYNK_5_4_0_1_0

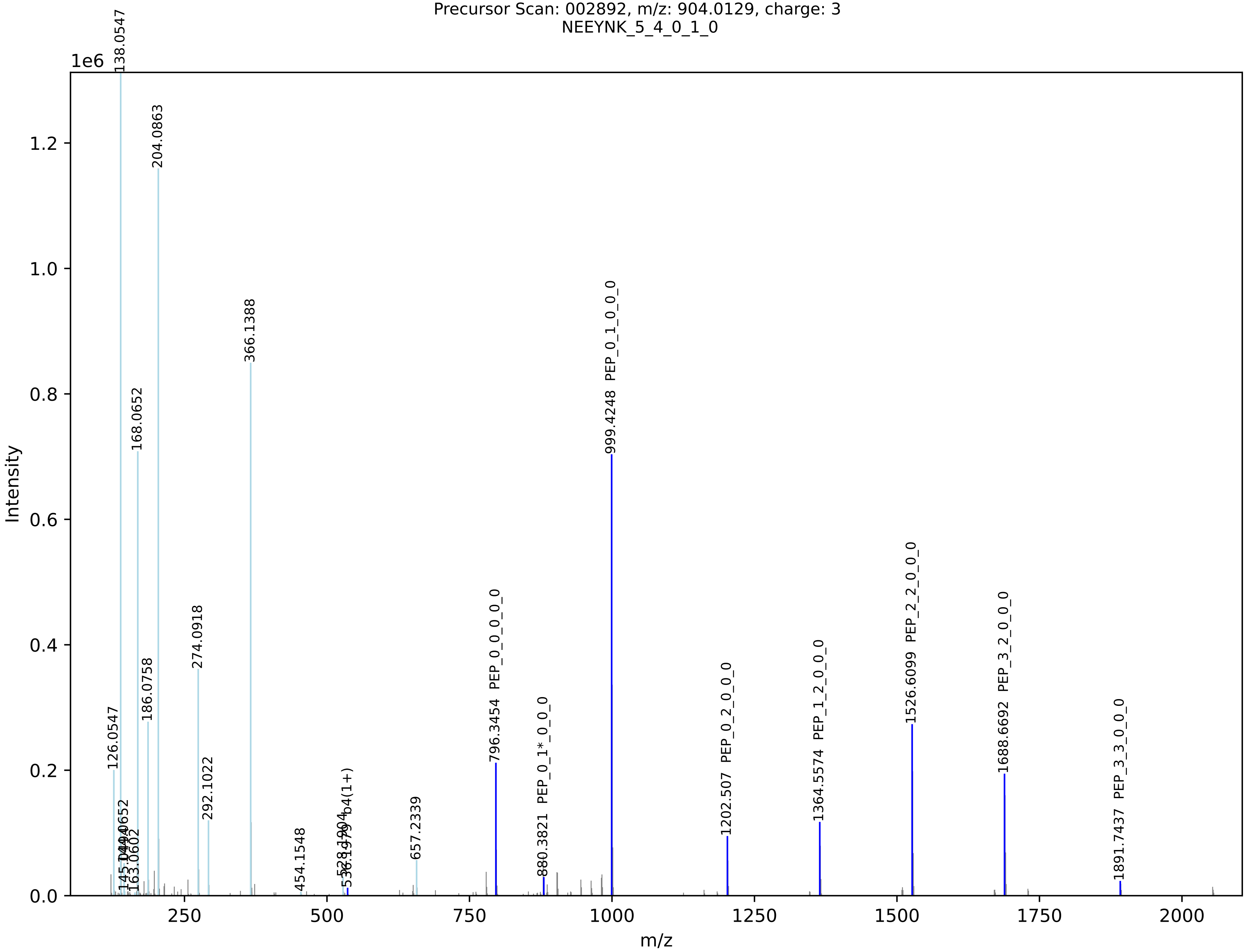

File: 231129_OE_RK_35, RT: 6.412, Scan: 002946
Type: HCD, FTMS + c NSI d Full ms2 1356.0164@hcd30.00 [120.0000-2787.7014]
Precursor Scan: 002916, m/z: 1355.5157, charge: 2
NEEYNK_5_4_0_1_0

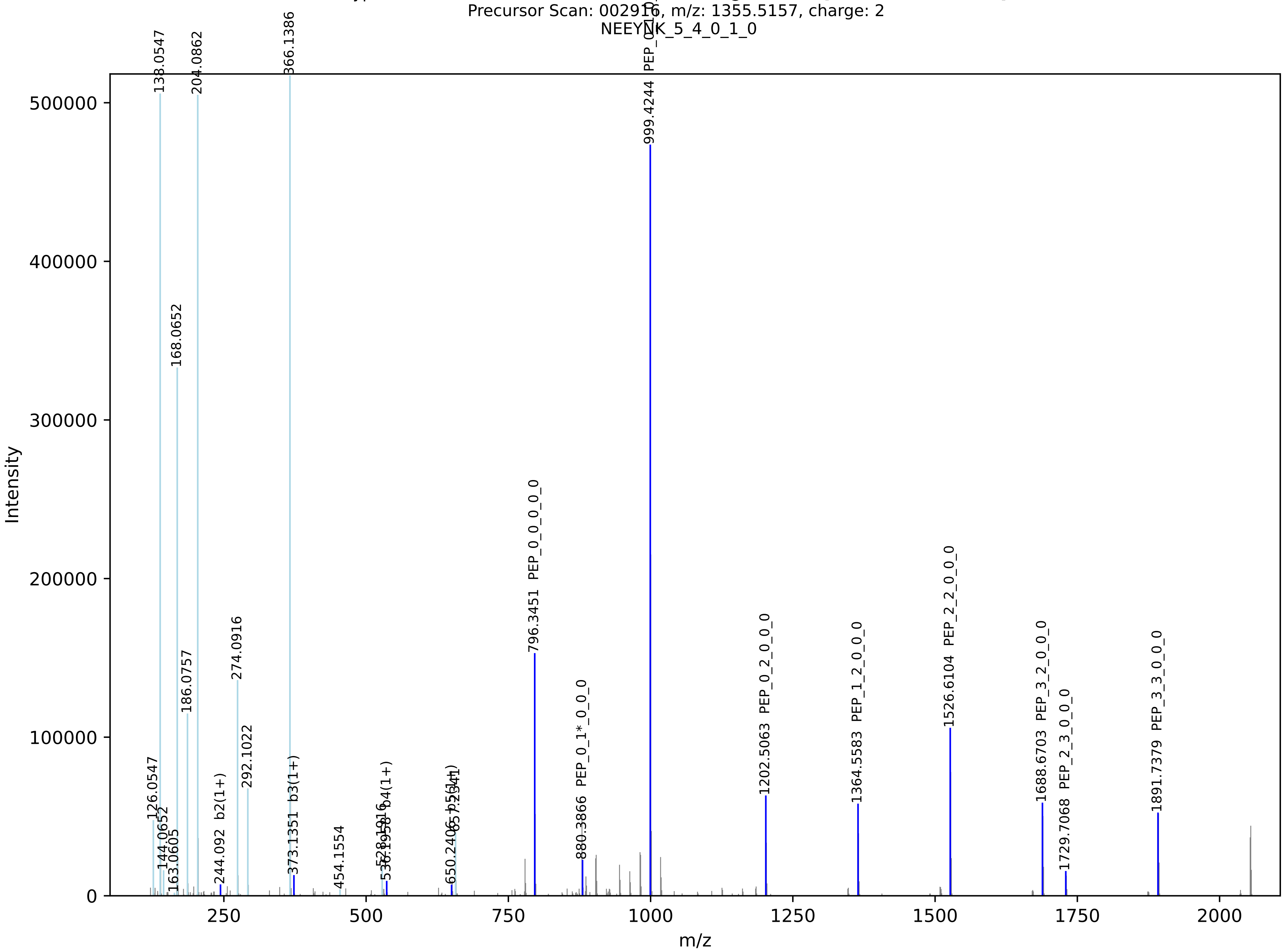

File: 231129_OE_RK_35, RT: 6.441, Scan: 002982
Type: HCD, FTMS + c NSI d Full ms2 846.3160@hcd30.00 [120.0000-3475.8254]
Precursor Scan: 002945, m/z: 846.0653, charge: 4
HNFSHCCSK_5_4_0_2_0

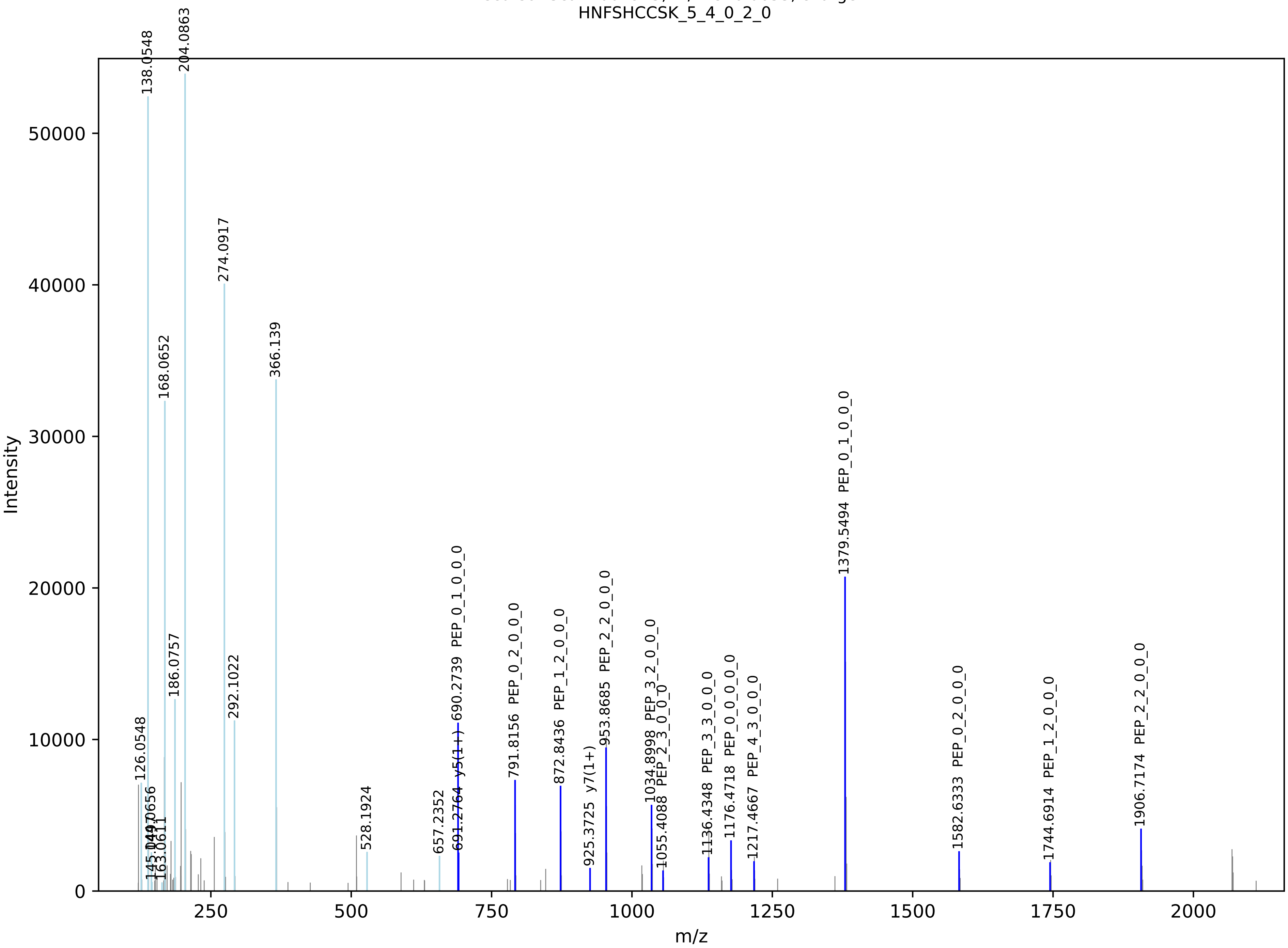

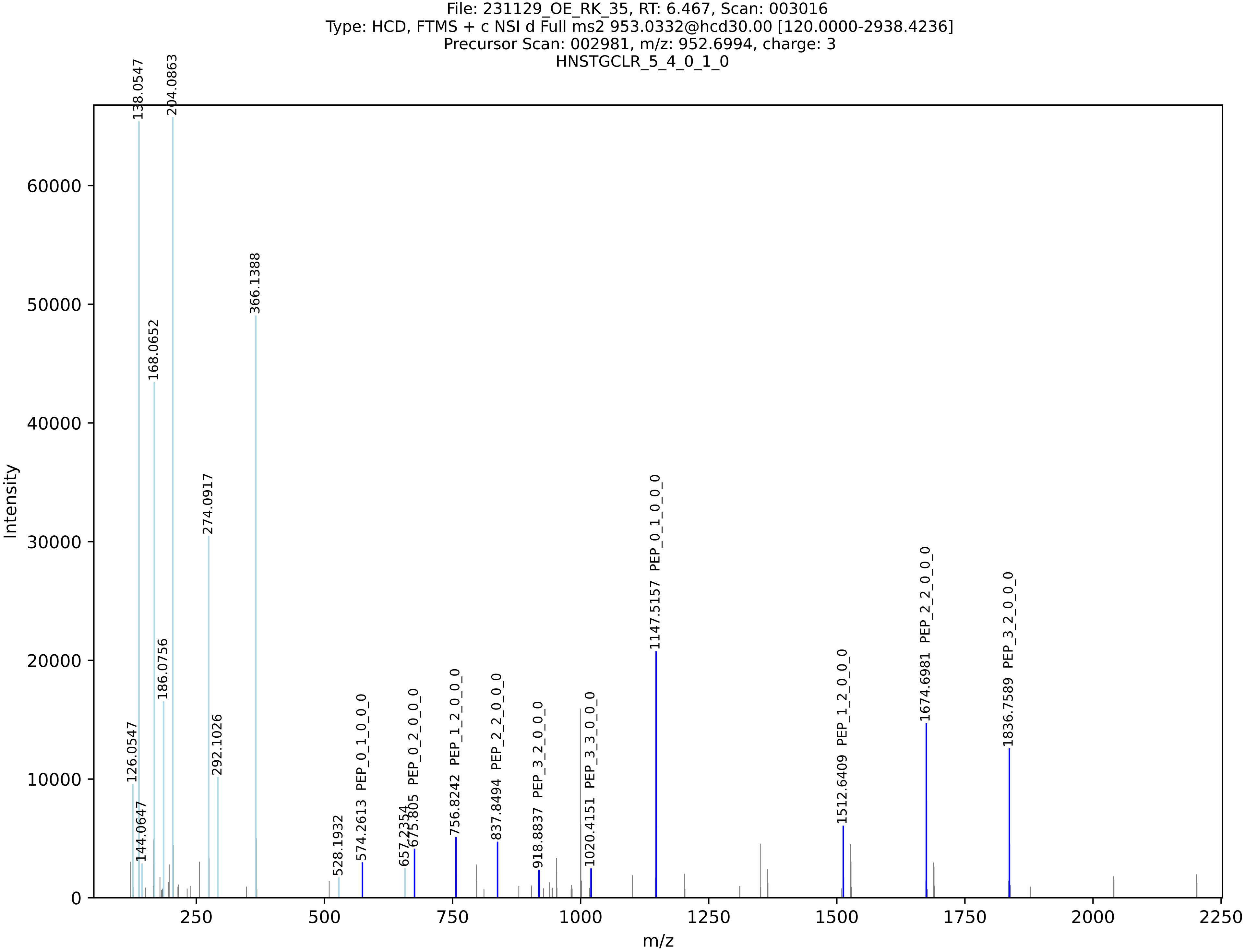
File: 231129_OE_RK_35, RT: 6.467, Scan: 003016
Type: HCD, FTMS + c NSI d Full ms2 953.0332@hcd30.00 [120.0000-2938.4236]
Precursor Scan: 002981, m/z: 952.6994, charge: 3
HNSTGCLR_5_4_0_1_0
Intensity
m/z
138.0547
204.0863
366.1388
168.0652
274.0917
186.0756
292.1026
126.0547
144.0647
528.1932
574.2613 PEP_0_1_0_0_0
657.2354
675.805 PEP_0_2_0_0_0
756.8242 PEP_1_2_0_0_0
837.8494 PEP_2_2_0_0_0
918.8837 PEP_3_2_0_0_0
1020.4151 PEP_3_3_0_0_0
1147.5157 PEP_0_1_0_0_0
1512.6409 PEP_1_2_0_0_0
1674.6981 PEP_2_2_0_0_0
1836.7589 PEP_3_2_0_0_0

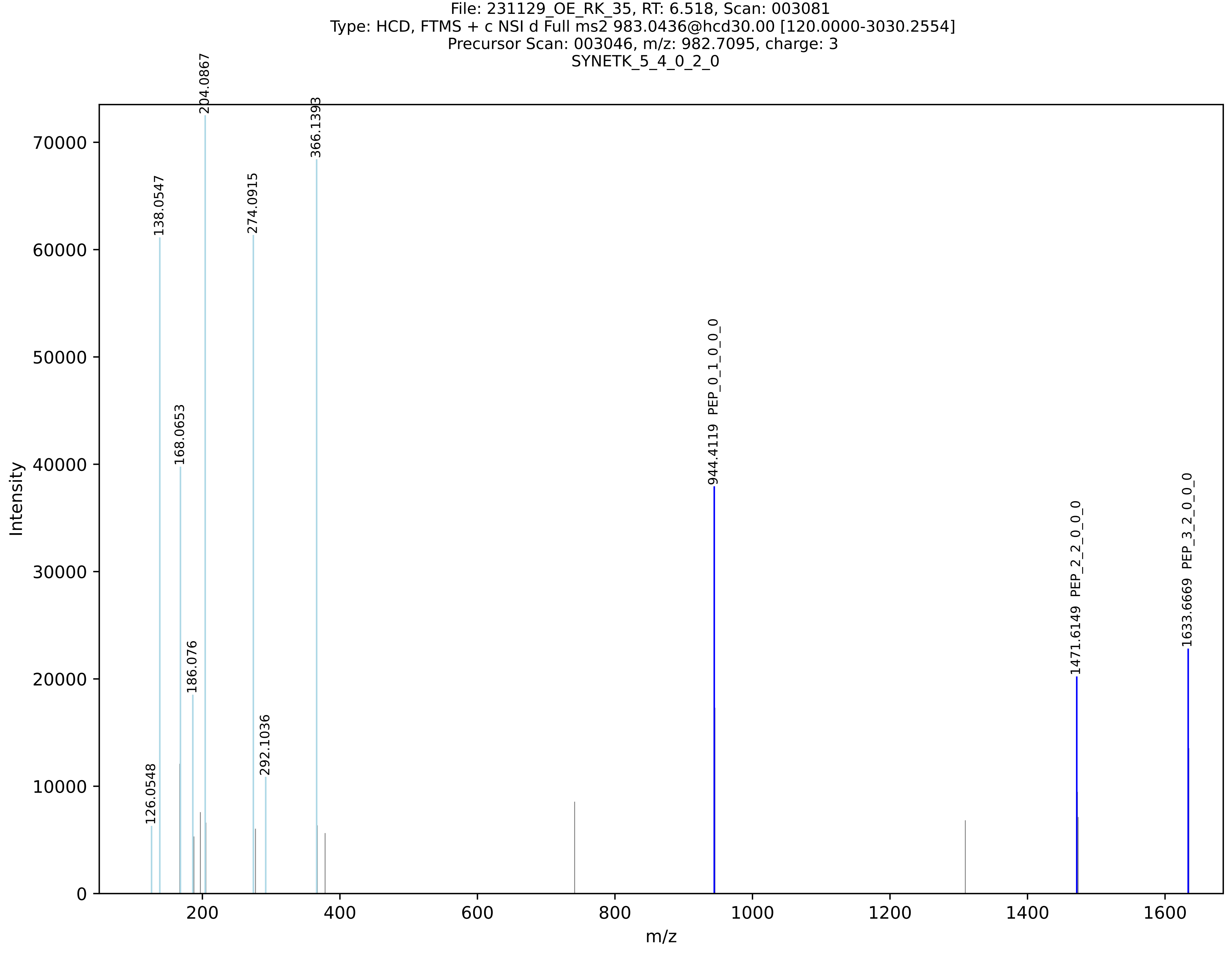
File: 231129_OE_RK_35, RT: 6.518, Scan: 003081
Type: HCD, FTMS + c NSI d Full ms2 983.0436@hcd30.00 [120.0000-3030.2554]
Precursor Scan: 003046, m/z: 982.7095, charge: 3
SYNETK_5_4_0_2_0
Intensity
m/z
126.0548
138.0547
168.0653
186.076
204.0867
274.0915
292.1036
366.1393
944.4119 PEP_0_1_0_0_0
1471.6149 PEP_2_2_0_0_0
1633.6669 PEP_3_2_0_0_0

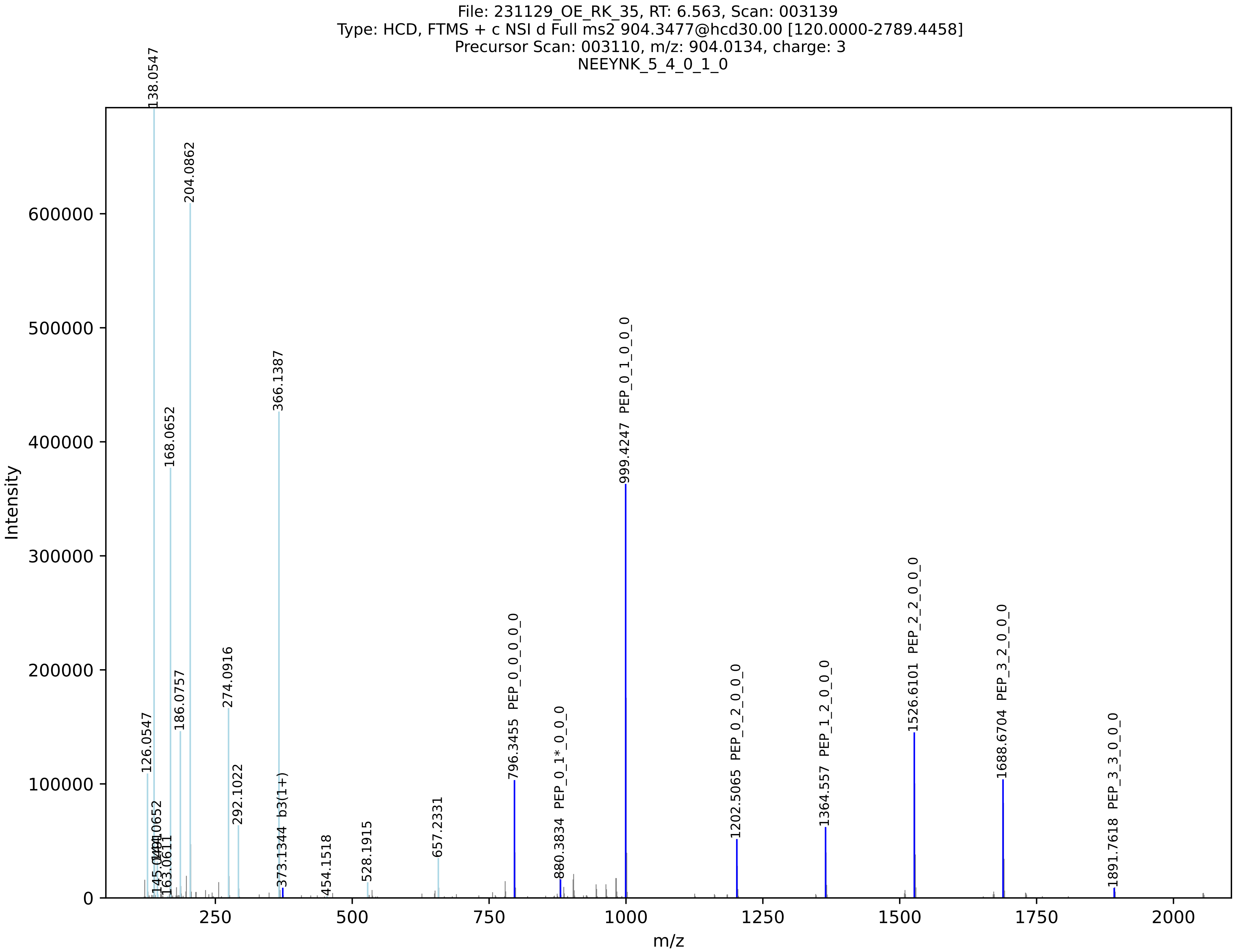
File: 231129_OE_RK_35, RT: 6.563, Scan: 003139
Type: HCD, FTMS + c NSI d Full ms2 904.3477@hcd30.00 [120.0000-2789.4458]
Precursor Scan: 003110, m/z: 904.0134, charge: 3
NEEYNK_5_4_0_1_0
Intensity
m/z
138.0547
204.0862
168.0652
366.1387
186.0757
274.0916
126.0547
292.1022
373.1344 b3(1+)
454.1518
528.1915
657.2331
796.3455 PEP_0_0_0_0_0
880.3834 PEP_0_1*_0_0_0
999.4247 PEP_0_1_0_0_0
1202.5065 PEP_0_2_0_0_0
1364.557 PEP_1_2_0_0_0
1526.6101 PEP_2_2_0_0_0
1688.6704 PEP_3_2_0_0_0
1891.7618 PEP_3_3_0_0_0
163.0611
145.0491

File: 231129_OE_RK_35, RT: 6.673, Scan: 003275
Type: HCD, FTMS + c NSI d Full ms2 1123.0909@hcd30.00 [120.0000-3458.8000]
Precursor Scan: 003251, m/z: 1122.7568, charge: 3
NEEYNK_6_5_0_2_0

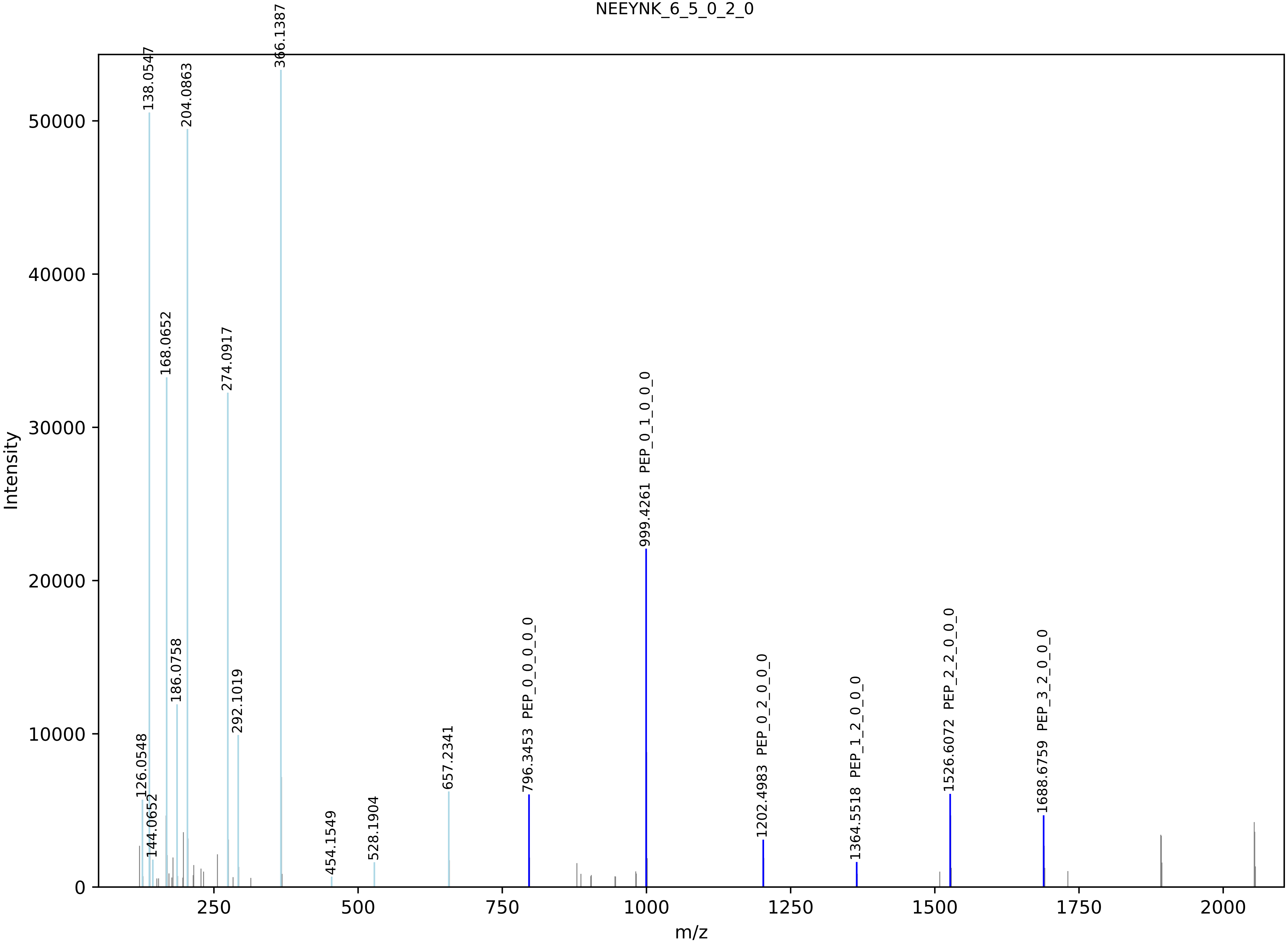

File: 231129_OE_RK_35, RT: 6.696, Scan: 003304
Type: HCD, FTMS + c NSI d Full ms2 948.7352@hcd30.00 [120.0000-2925.2717]
Precursor Scan: 003274, m/z: 948.0669, charge: 3
NGTAVCATNRR_5_4_0_0_0

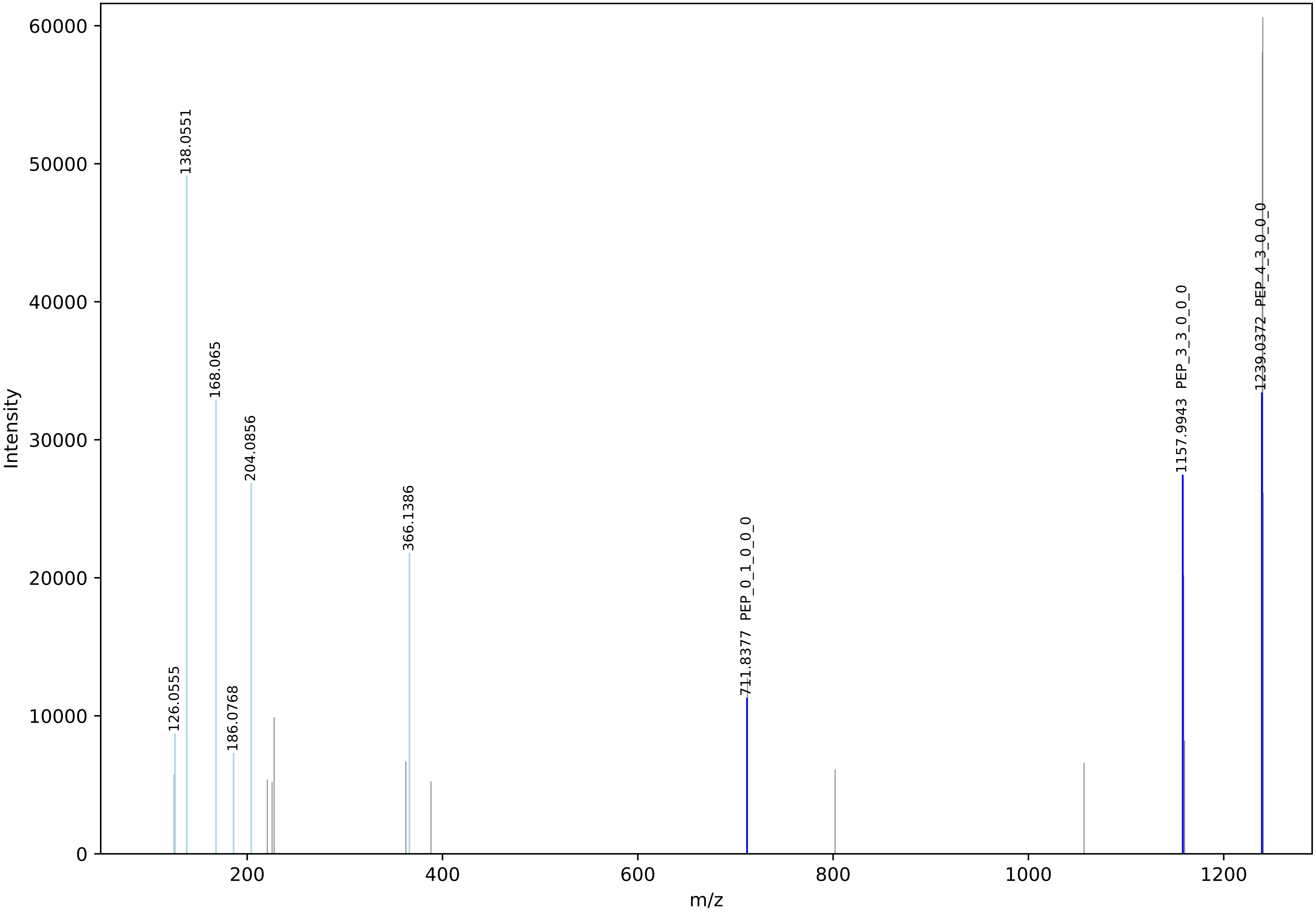

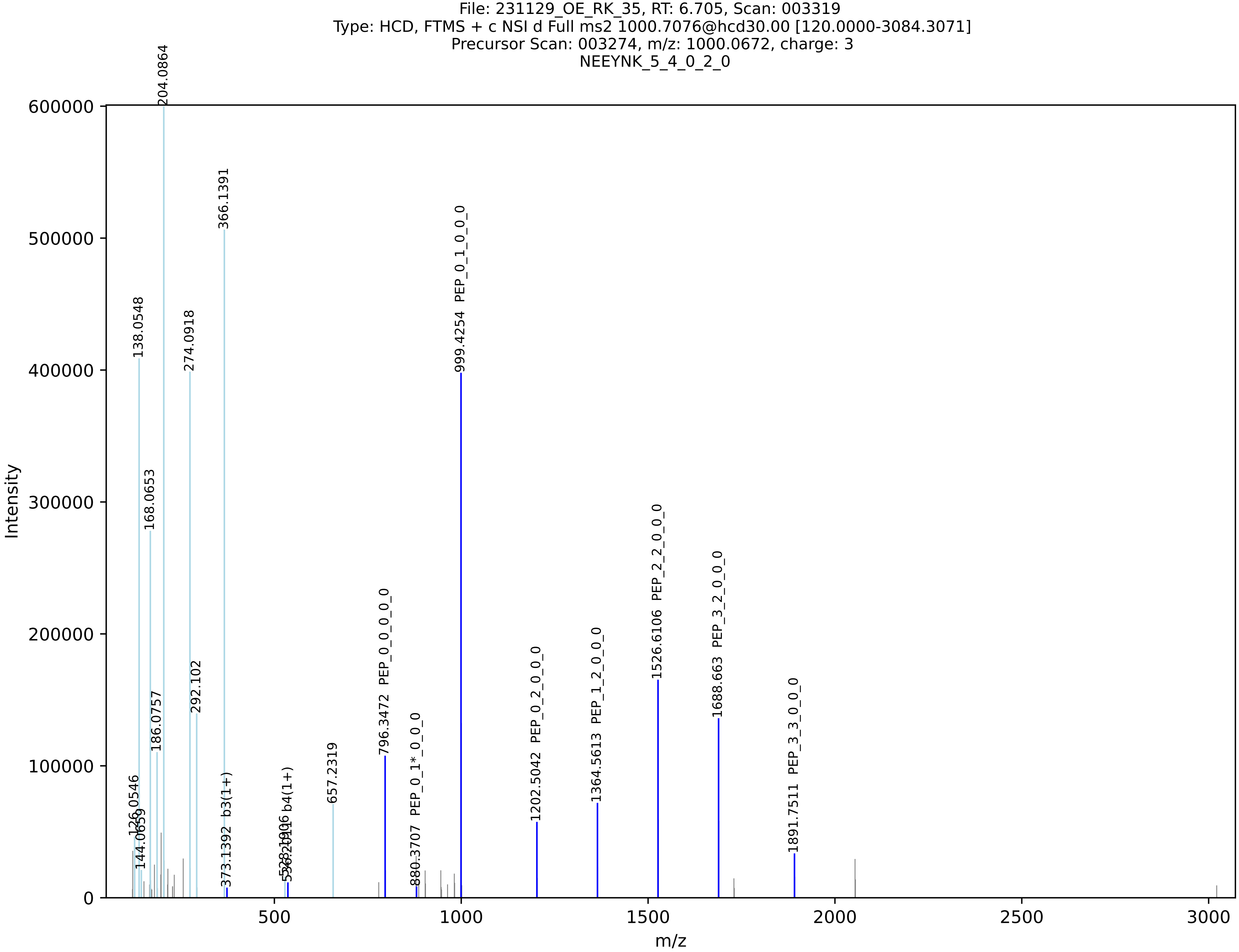
File: 231129_OE_RK_35, RT: 6.705, Scan: 003319
Type: HCD, FTMS + c NSI d Full ms2 1000.7076@hcd30.00 [120.0000-3084.3071]
Precursor Scan: 003274, m/z: 1000.0672, charge: 3
NEEYNK_5_4_0_2_0
Intensity
m/z
0
100000
200000
300000
400000
500000
600000
500
1000
1500
2000
2500
3000
204.0864
366.1391
138.0548
274.0918
168.0653
292.102
186.0757
126.0546
144.0659
373.1392 b3(1+)
b4(1+)
657.2319
796.3472 PEP_0_0_0_0_0
880.3707 PEP_0_1*_0_0_0
999.4254 PEP_0_1_0_0_0
1202.5042 PEP_0_2_0_0_0
1364.5613 PEP_1_2_0_0_0
1526.6106 PEP_2_2_0_0_0
1688.663 PEP_3_2_0_0_0
1891.7511 PEP_3_3_0_0_0

File: 231129_OE_RK_35, RT: 6.737, Scan: 003355
Type: HCD, FTMS + c NSI d Full ms2 904.3470@hcd30.00 [120.0000-2789.4438]
Precursor Scan: 003323, m/z: 904.0129, charge: 3
NEEYNK_5_4_0_1_0

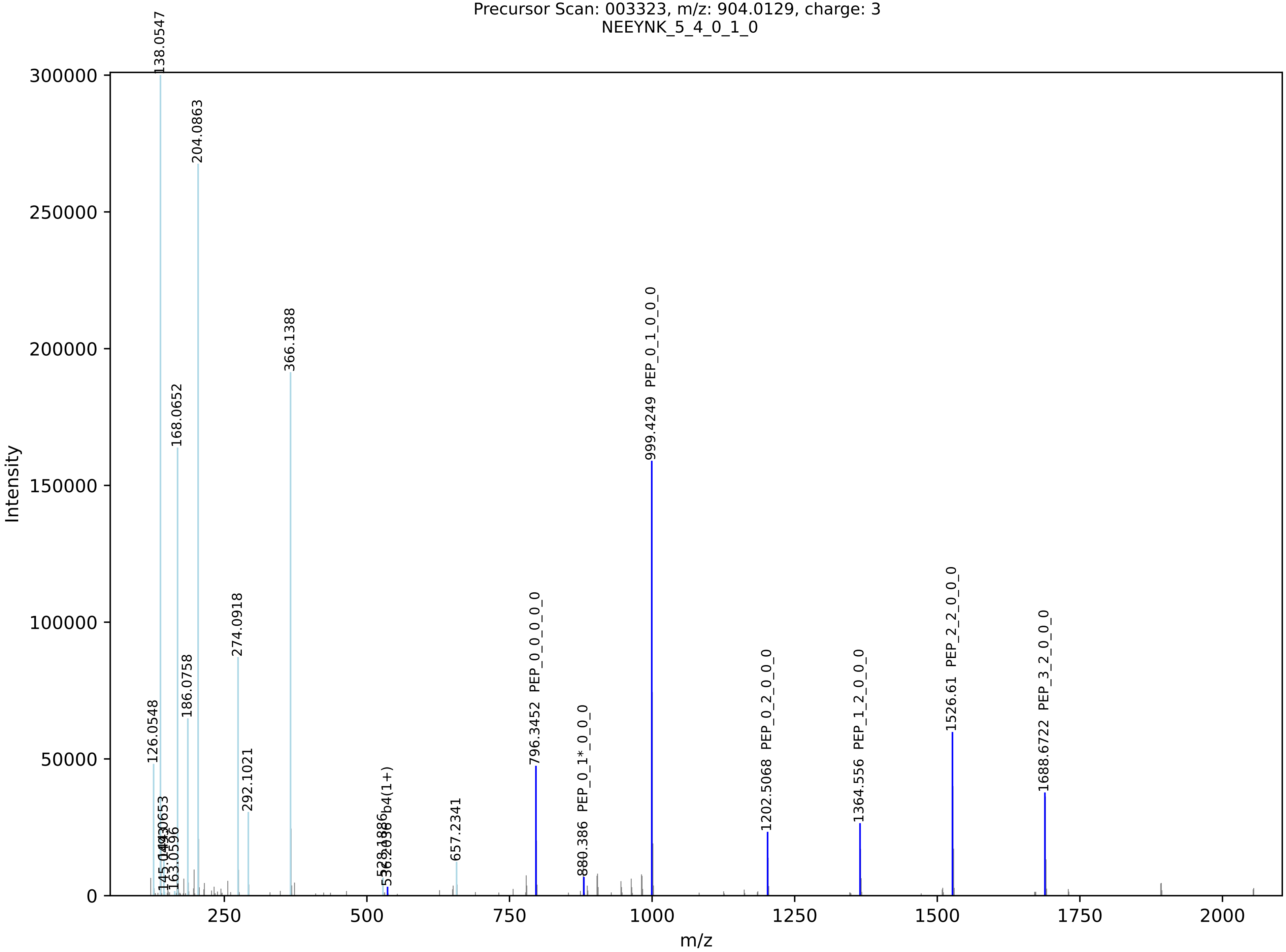

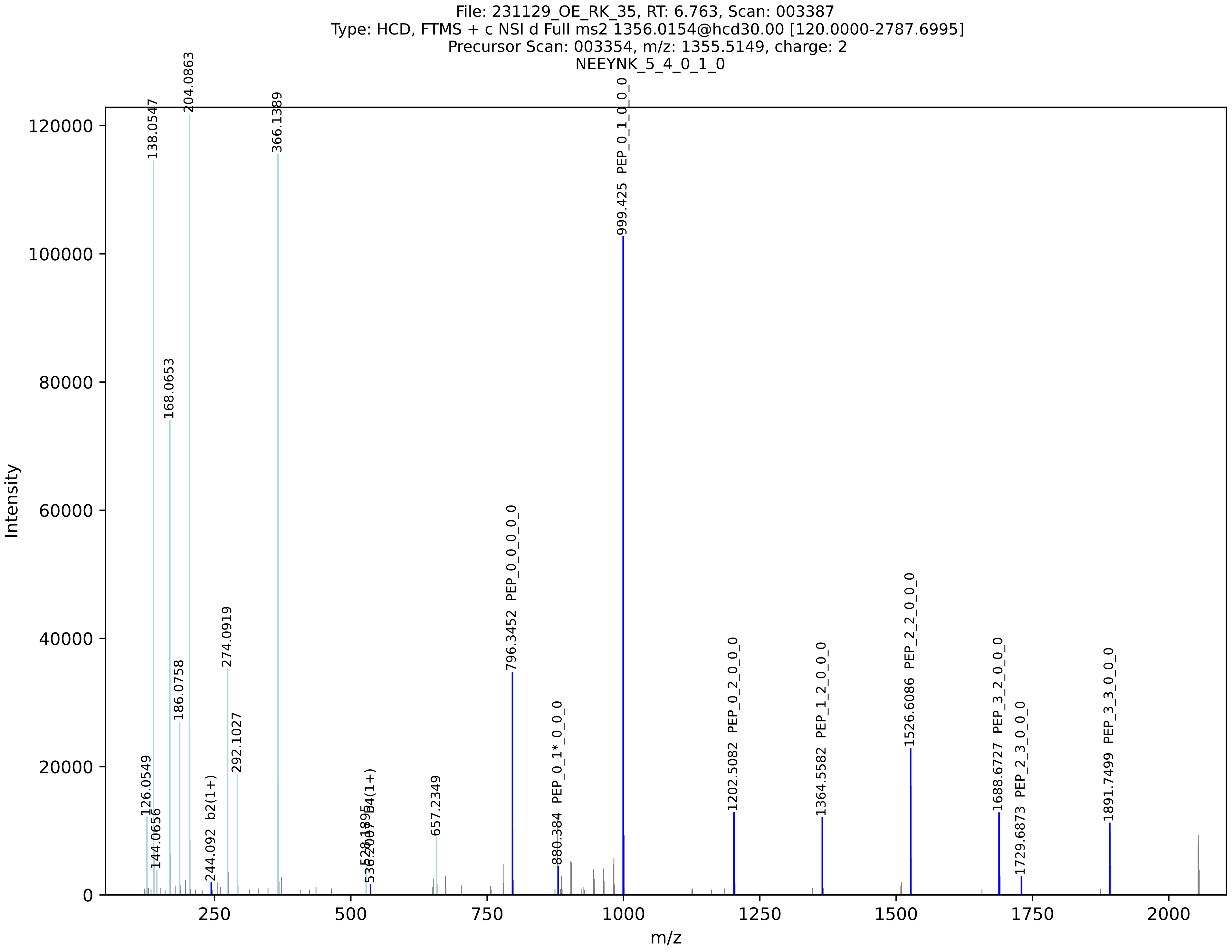

File: 231129_OE_RK_35, RT: 6.763, Scan: 003387
Type: HCD, FTMS + c NSI d Full ms2 1356.0154@hcd30.00 [120.0000-2787.6995]
Precursor Scan: 003354, m/z: 1355.5149, charge: 2
NEEYNK_5_4_0_1_0
Intensity
m/z
138.0547
204.0863
366.1389
168.0653
186.0758
274.0919
292.1027
126.0549
144.0656
244.092 b2(1+)
657.2349
796.3452 PEP_0_0_0_0_0
880.384 PEP_0_1*_0_0_0
999.425 PEP_0_1_0_0_0
1202.5082 PEP_0_2_0_0_0
1364.5582 PEP_1_2_0_0_0
1526.6086 PEP_2_2_0_0_0
1688.6727 PEP_3_2_0_0_0
1729.6873 PEP_2_3_0_0_0
1891.7499 PEP_3_3_0_0_0

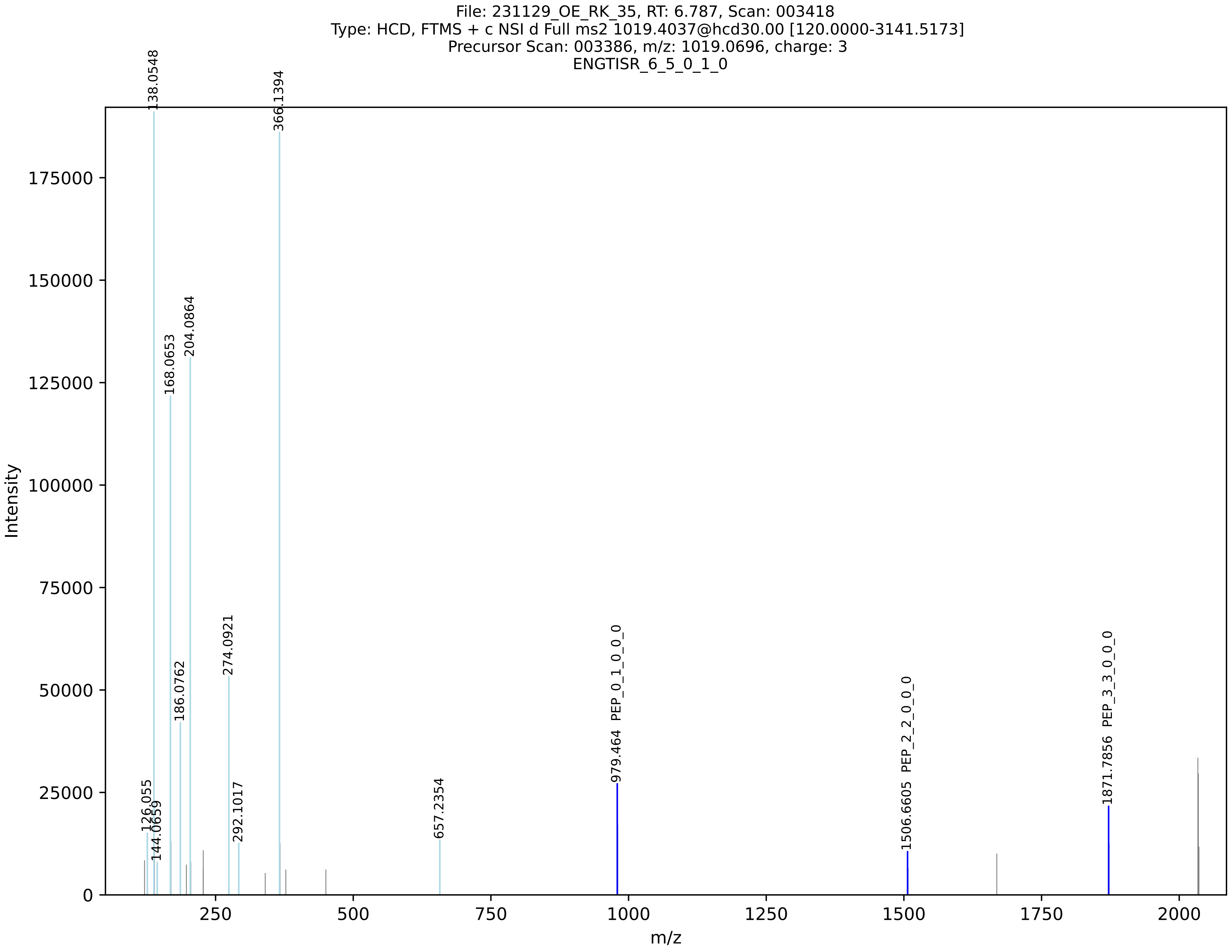
File: 231129_OE_RK_35, RT: 6.787, Scan: 003418
Type: HCD, FTMS + c NSI d Full ms2 1019.4037@hcd30.00 [120.0000-3141.5173]
Precursor Scan: 003386, m/z: 1019.0696, charge: 3
ENGTISR_6_5_0_1_0
Intensity
m/z
138.0548
366.1394
204.0864
168.0653
274.0921
186.0762
126.055
144.0659
292.1017
657.2354
979.464 PEP_0_1_0_0_0
1506.6605 PEP_2_2_0_0_0
1871.7856 PEP_3_3_0_0_0
0
25000
50000
75000
100000
125000
150000
175000
250
500
750
1000
1250
1500
1750
2000

File: 231129_OE_RK_35, RT: 6.88, Scan: 003536
Type: HCD, FTMS + c NSI d Full ms2 1501.5631@hcd30.00 [120.0000-3084.6167]
Precursor Scan: 003504, m/z: 1501.0622, charge: 2
NEEYNK_5_4_0_2_0

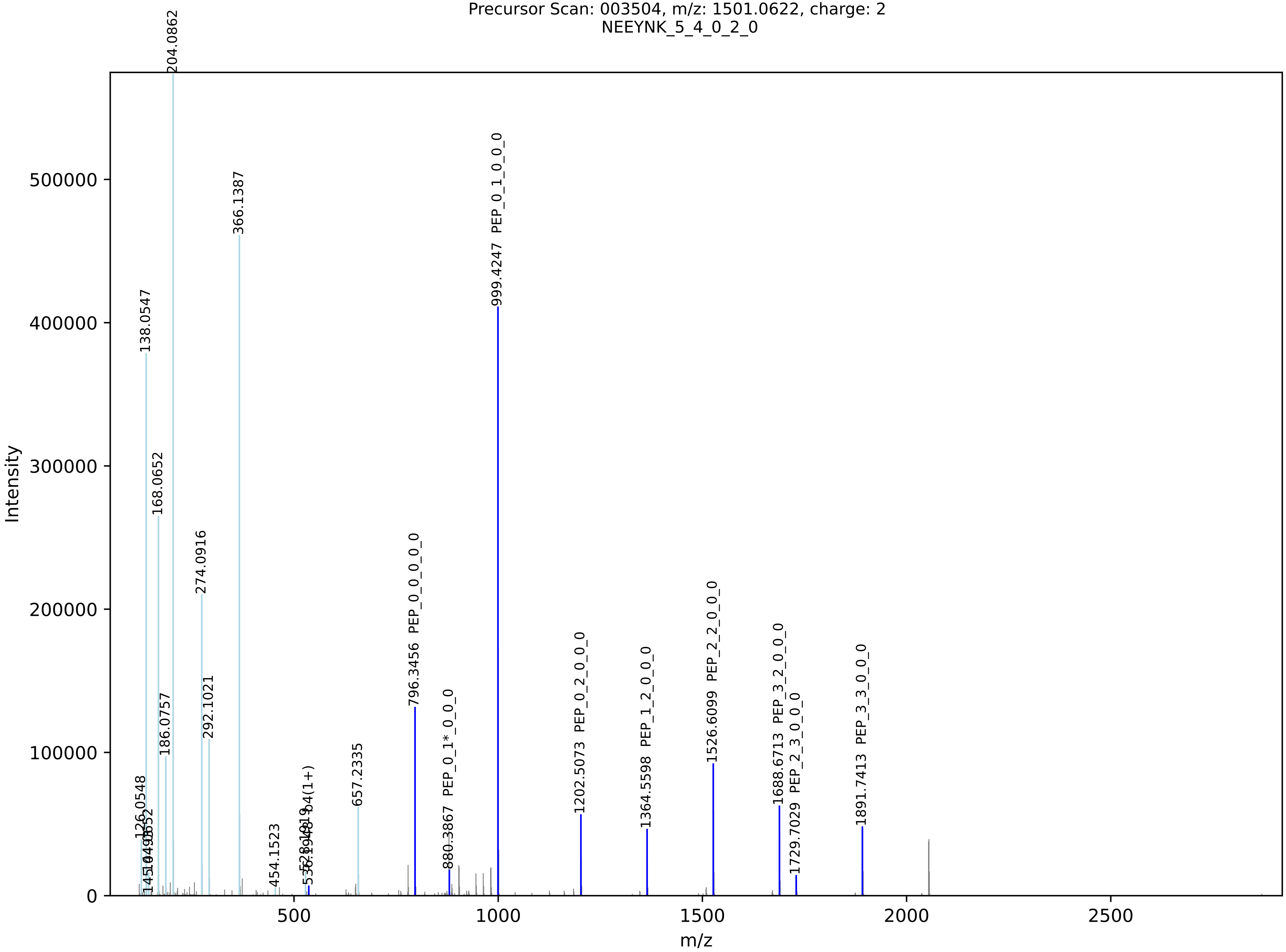

File: 231129_OE_RK_35, RT: 6.881, Scan: 003538
Type: HCD, FTMS + c NSI d Full ms2 983.0427@hcd30.00 [120.0000-3030.2527]
Precursor Scan: 003504, m/z: 982.7086, charge: 3
SYNETK_5_4_0_2_0

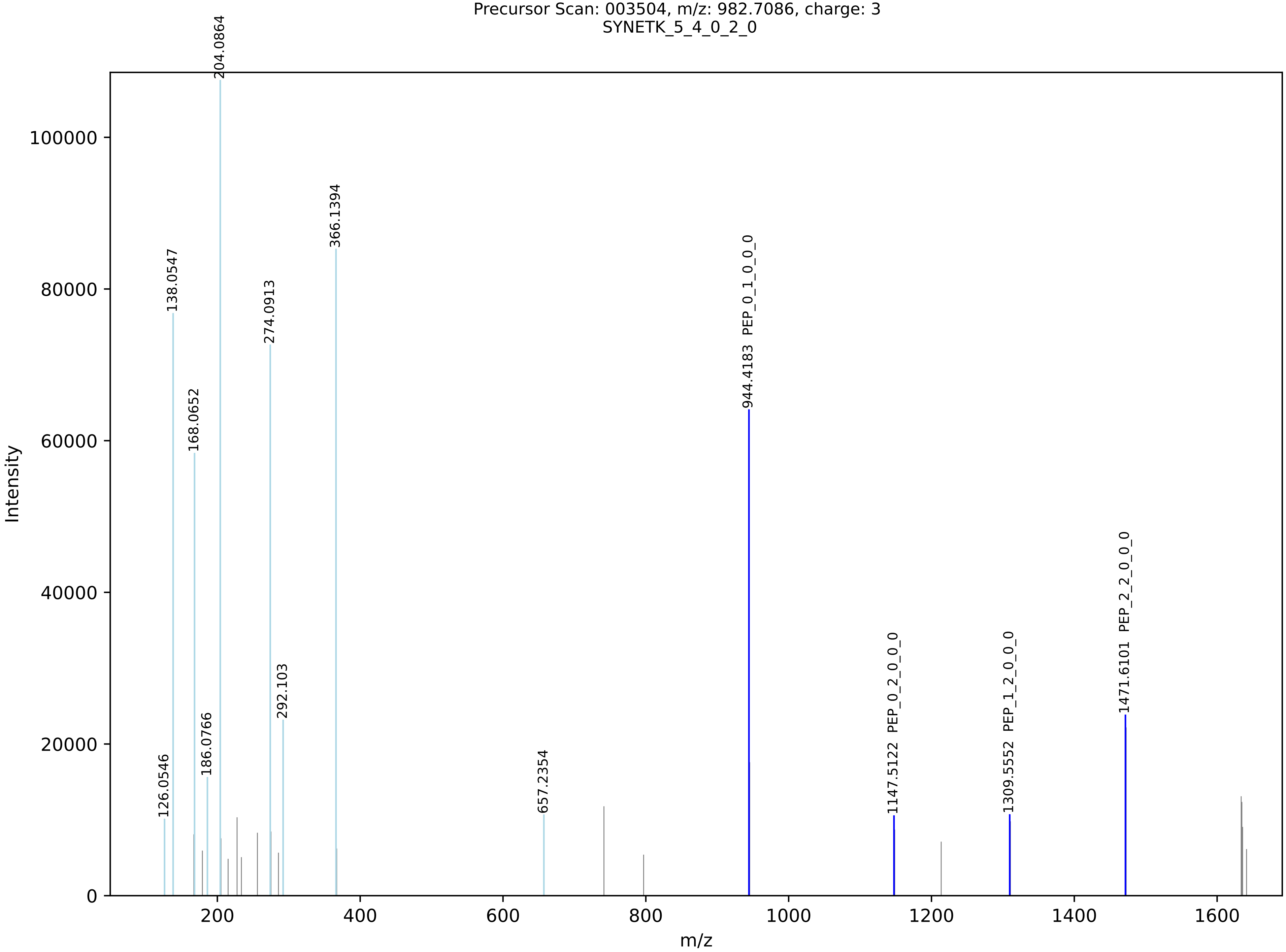

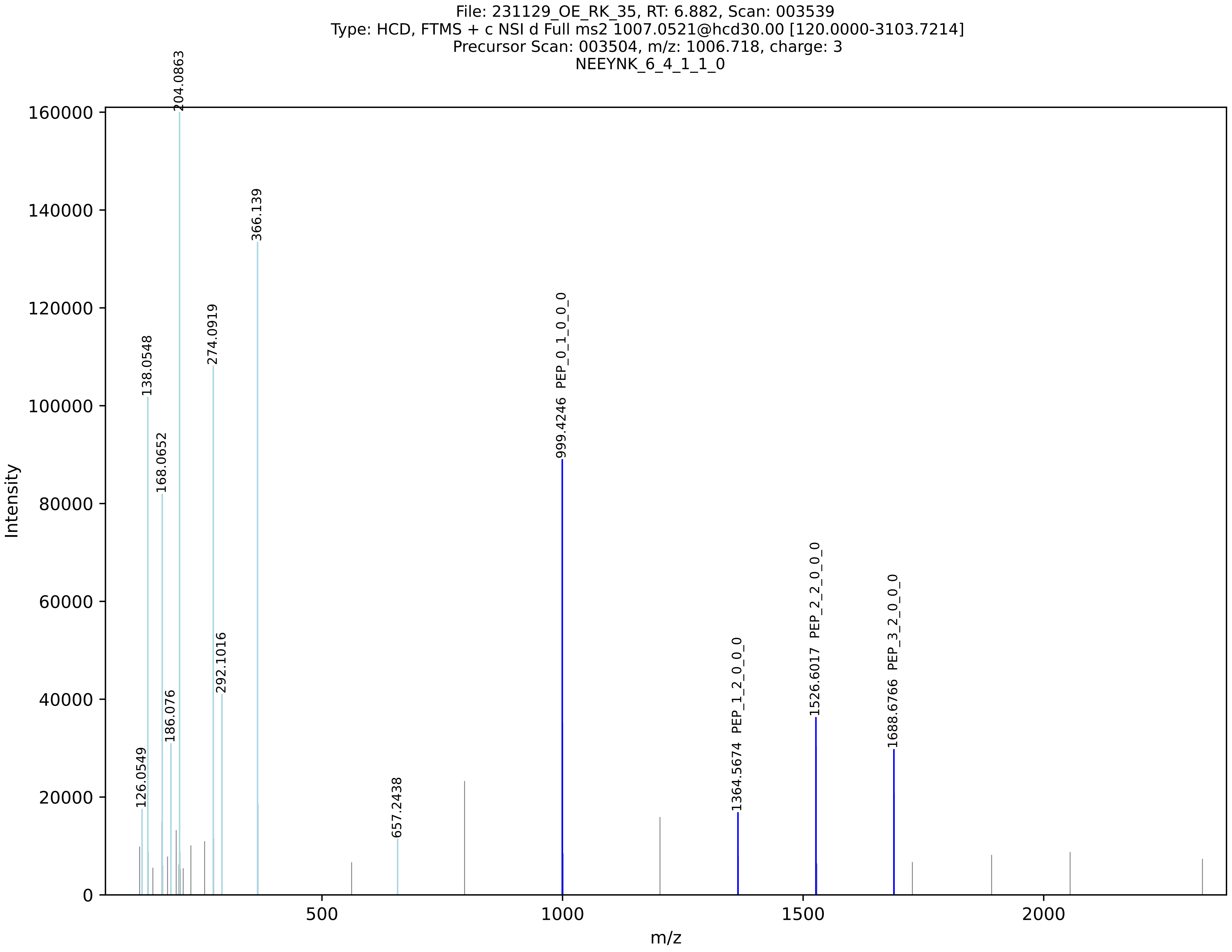
File: 231129_OE_RK_35, RT: 6.882, Scan: 003539
Type: HCD, FTMS + c NSI d Full ms2 1007.0521@hcd30.00 [120.0000-3103.7214]
Precursor Scan: 003504, m/z: 1006.718, charge: 3
NEEYNK_6_4_1_1_0
Intensity
m/z
204.0863
366.139
274.0919
138.0548
168.0652
292.1016
186.076
126.0549
657.2438
999.4246 PEP_0_1_0_0_0
1364.5674 PEP_1_2_0_0_0
1526.6017 PEP_2_2_0_0_0
1688.6766 PEP_3_2_0_0_0
0
20000
40000
60000
80000
100000
120000
140000
160000
500
1000
1500
2000

File: 231129_OE_RK_35, RT: 6.903, Scan: 003566
Type: HCD, FTMS + c NSI d Full ms2 1172.1099@hcd30.00 [120.0000-3608.7983]
Precursor Scan: 003535, m/z: 1172.1153, charge: 3
NEEYNK_6_5_1_2_0

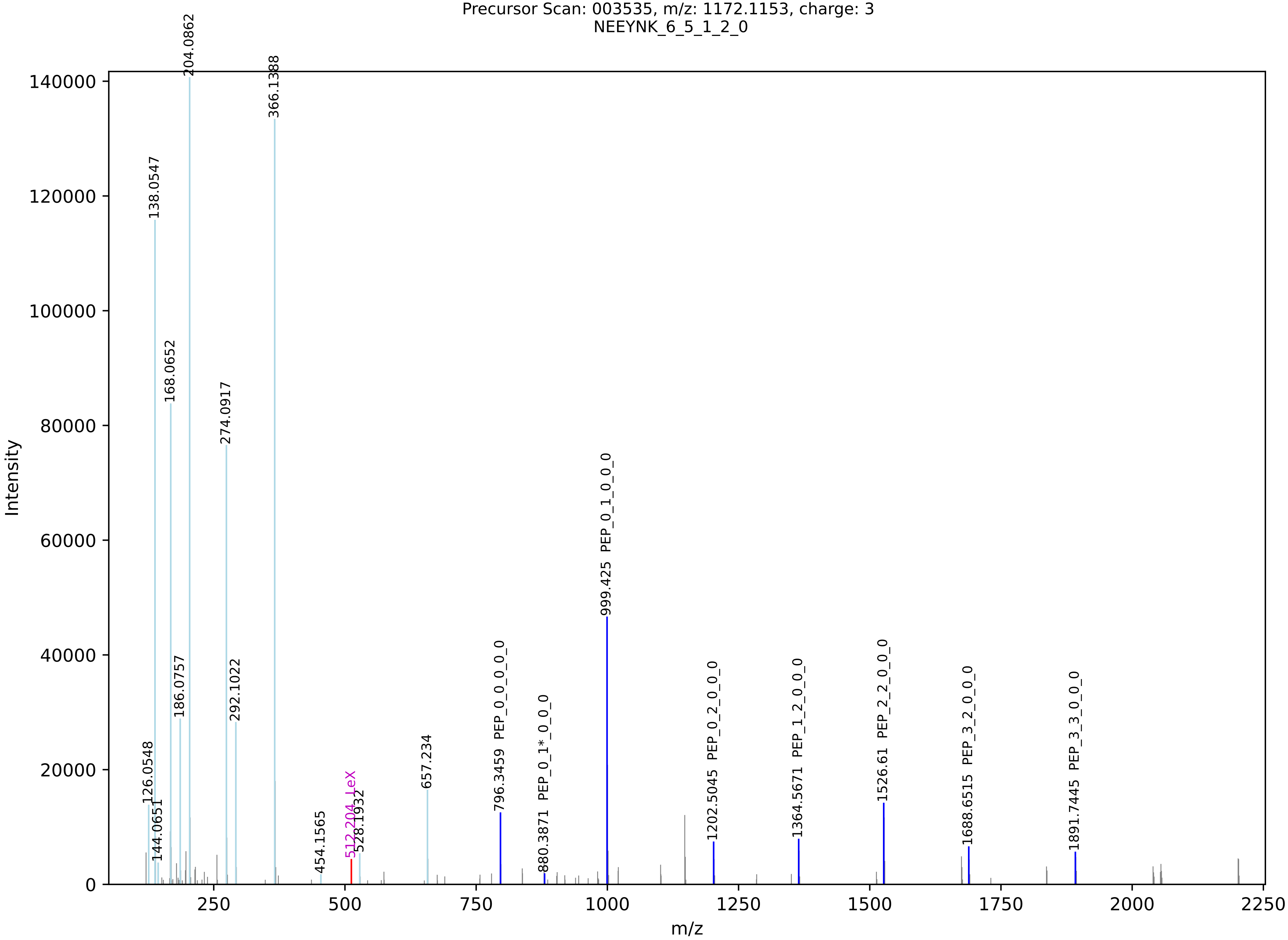

File: 231129_OE_RK_35, RT: 6.93, Scan: 003600
Type: HCD, FTMS + c NSI d Full ms2 904.3455@hcd30.00 [120.0000-2789.4392]
Precursor Scan: 003565, m/z: 904.0121, charge: 3
NEEYNK_5_4_0_1_0

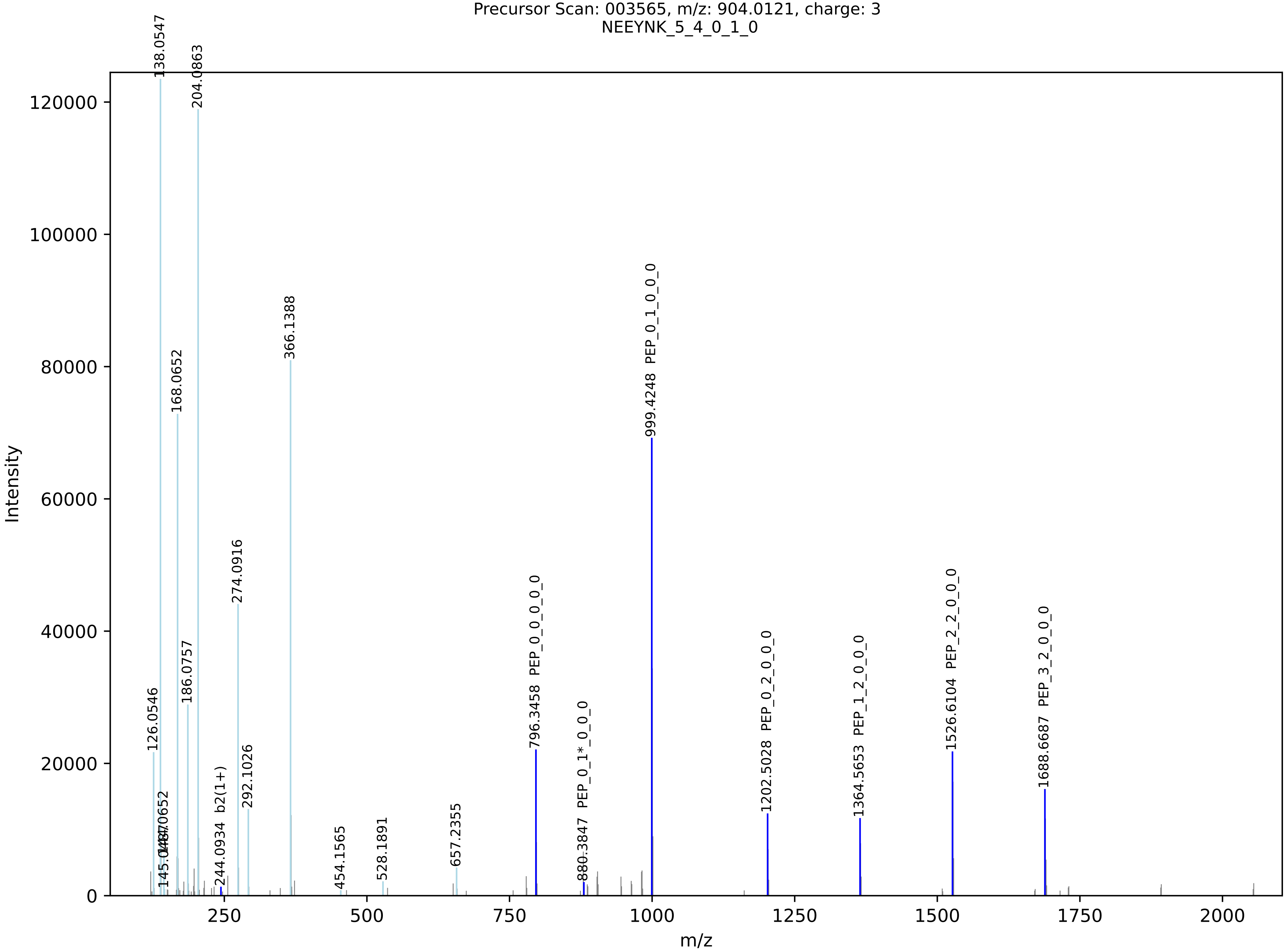

File: 231129_OE_RK_35, RT: 6.956, Scan: 003634
Type: HCD, FTMS + c NSI d Full ms2 1001.3782@hcd30.00 [120.0000-3086.3594]
Precursor Scan: 003599, m/z: 1001.0441, charge: 3
NEEYNK_5_4_0_2_0

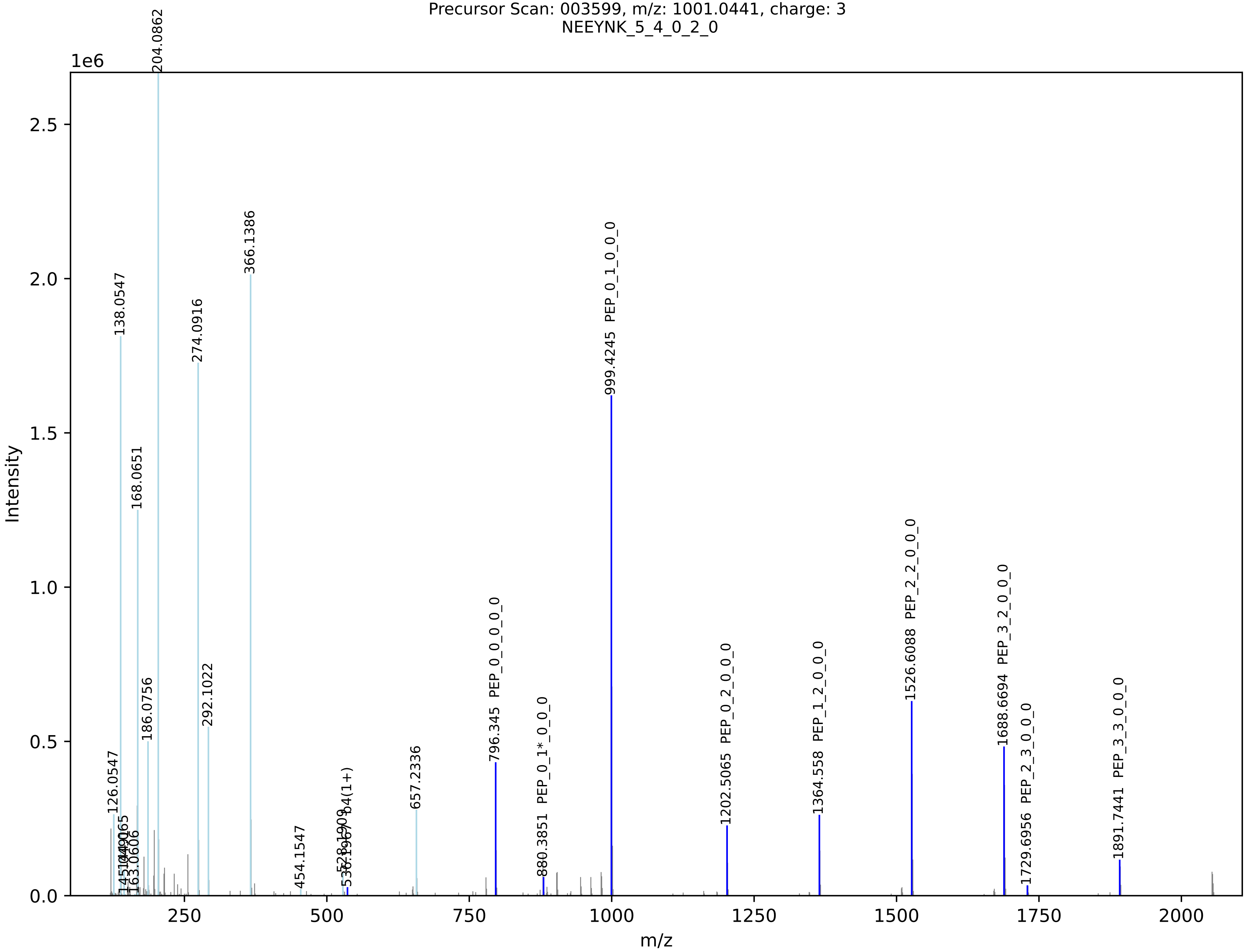

File: 231129_OE_RK_35, RT: 7.035, Scan: 003738
Type: HCD, FTMS + c NSI d Full ms2 1123.0884@hcd30.00 [120.0000-3458.7925]
Precursor Scan: 003711, m/z: 1122.7543, charge: 3
NEEYNK_6_5_0_2_0

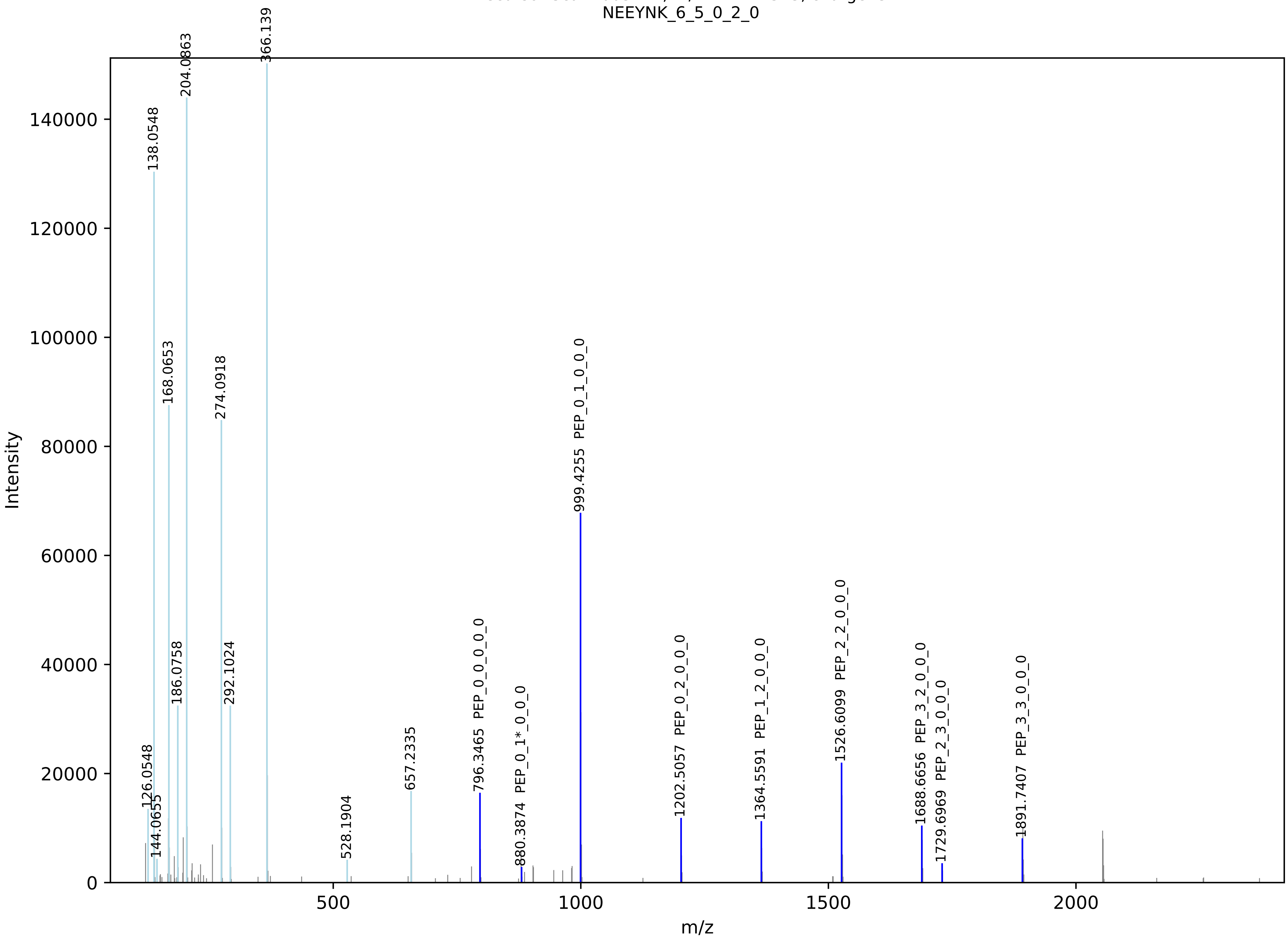

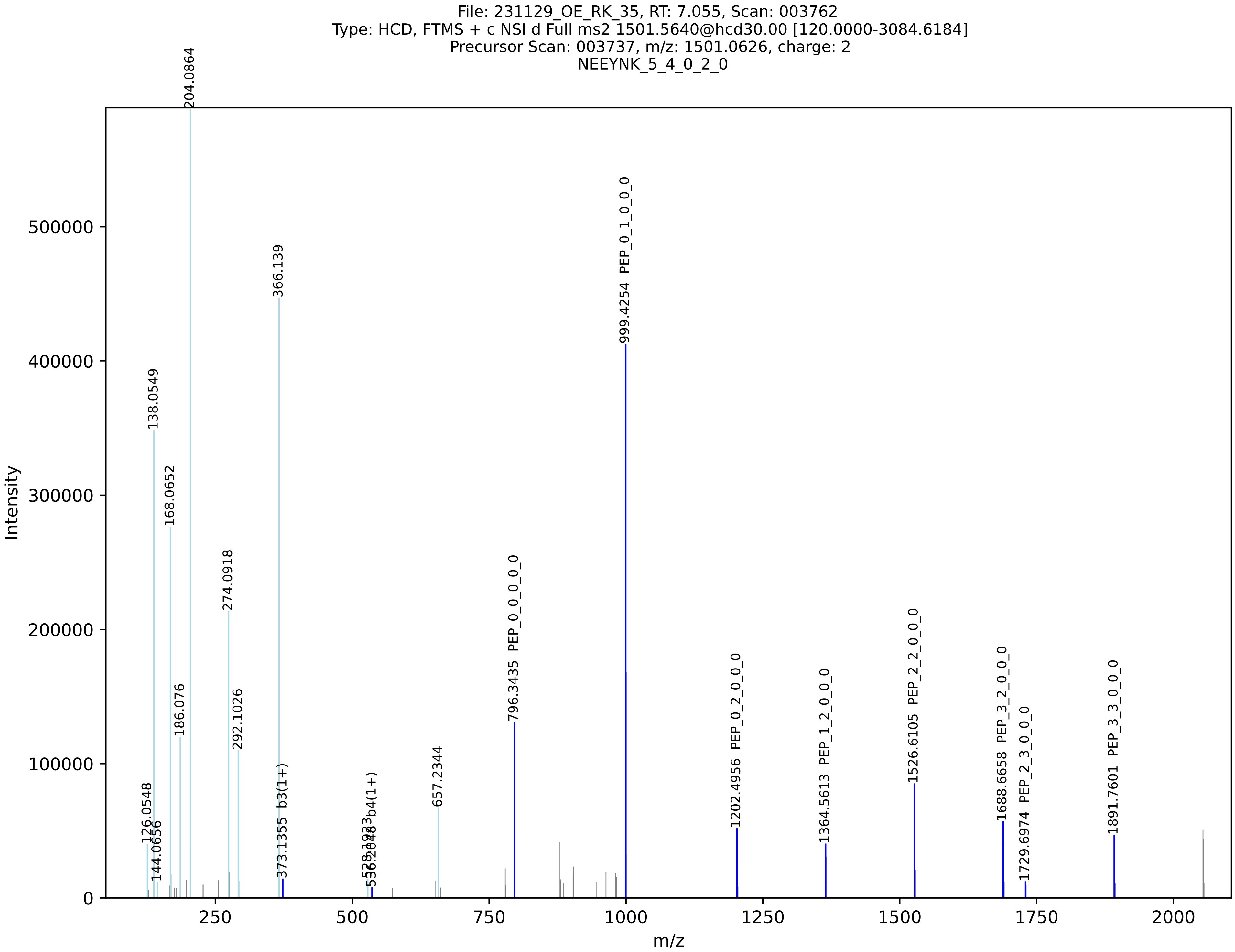
File: 231129_OE_RK_35, RT: 7.055, Scan: 003762
Type: HCD, FTMS + c NSI d Full ms2 1501.5640@hcd30.00 [120.0000-3084.6184]
Precursor Scan: 003737, m/z: 1501.0626, charge: 2
NEEYNK_5_4_0_2_0
Intensity
m/z
0
100000
200000
300000
400000
500000
250
500
750
1000
1250
1500
1750
2000
204.0864
138.0549
168.0652
186.076
126.0548
144.0656
274.0918
292.1026
366.139
373.1355 b3(1+)
528.1923
536.2048 b4(1+)
657.2344
796.3435 PEP_0_0_0_0_0
999.4254 PEP_0_1_0_0_0
1202.4956 PEP_0_2_0_0_0
1364.5613 PEP_1_2_0_0_0
1526.6105 PEP_2_2_0_0_0
1688.6658 PEP_3_2_0_0_0
1729.6974 PEP_2_3_0_0_0
1891.7601 PEP_3_3_0_0_0

File: 231129_OE_RK_35, RT: 7.057, Scan: 003765
Type: HCD, FTMS + c NSI d Full ms2 1007.0529@hcd30.00 [120.0000-3103.7236]
Precursor Scan: 003737, m/z: 1006.7187, charge: 3
NEEYNK_6_4_1_1_0

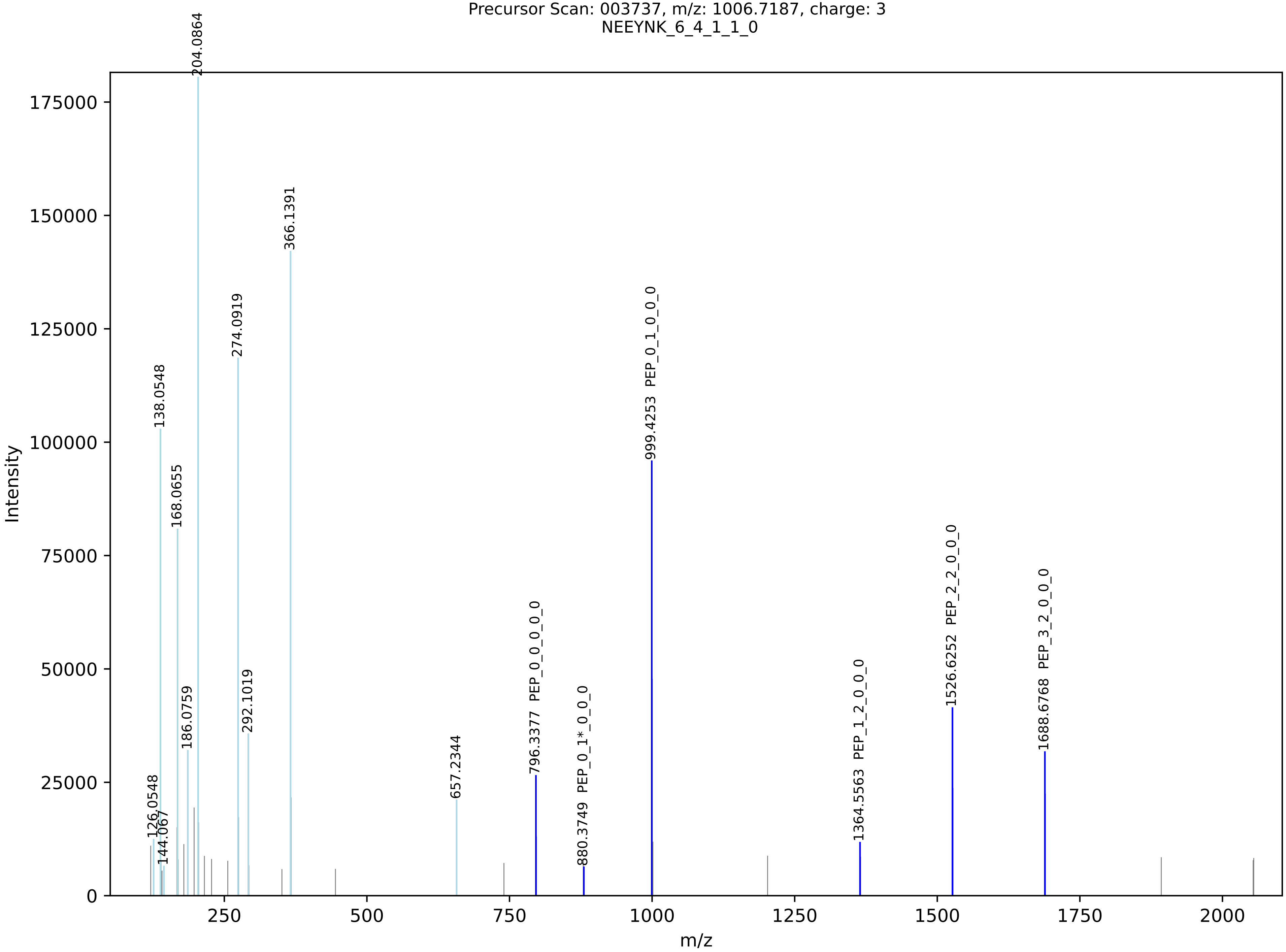

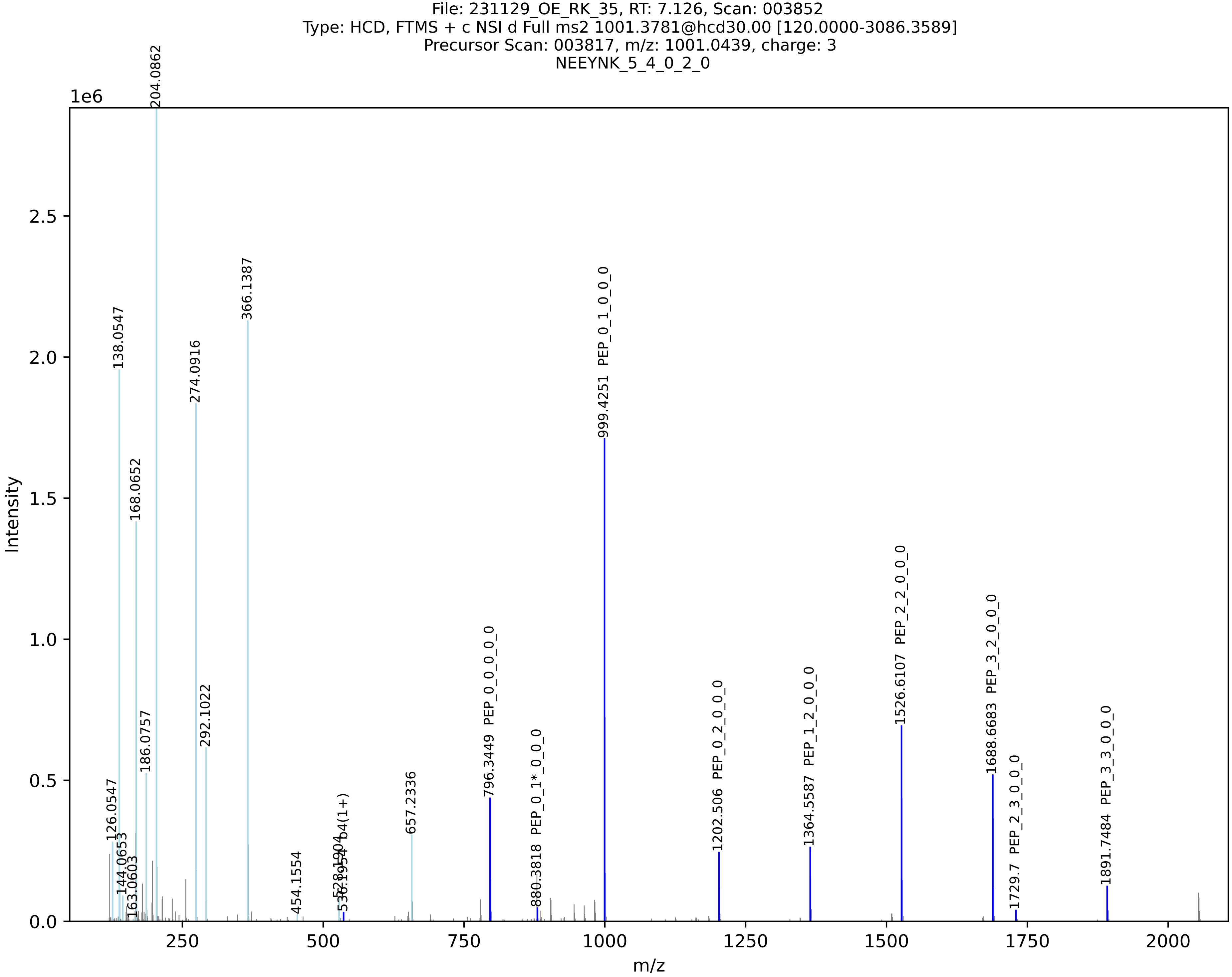

File: 231129_OE_RK_35, RT: 7.126, Scan: 003852
Type: HCD, FTMS + c NSI d Full ms2 1001.3781@hcd30.00 [120.0000-3086.3589]
Precursor Scan: 003817, m/z: 1001.0439, charge: 3
NEEYNK_5_4_0_2_0
1e6
Intensity
m/z
204.0862
138.0547
168.0652
186.0757
126.0547
144.0653
163.0603
274.0916
292.1022
366.1387
454.1554
528.1904
536.1954 b4(1+)
657.2336
796.3449 PEP_0_0_0_0_0
880.3818 PEP_0_1*_0_0_0
999.4251 PEP_0_1_0_0_0
1202.506 PEP_0_2_0_0_0
1364.5587 PEP_1_2_0_0_0
1526.6107 PEP_2_2_0_0_0
1688.6683 PEP_3_2_0_0_0
1729.7 PEP_2_3_0_0_0
1891.7484 PEP_3_3_0_0_0

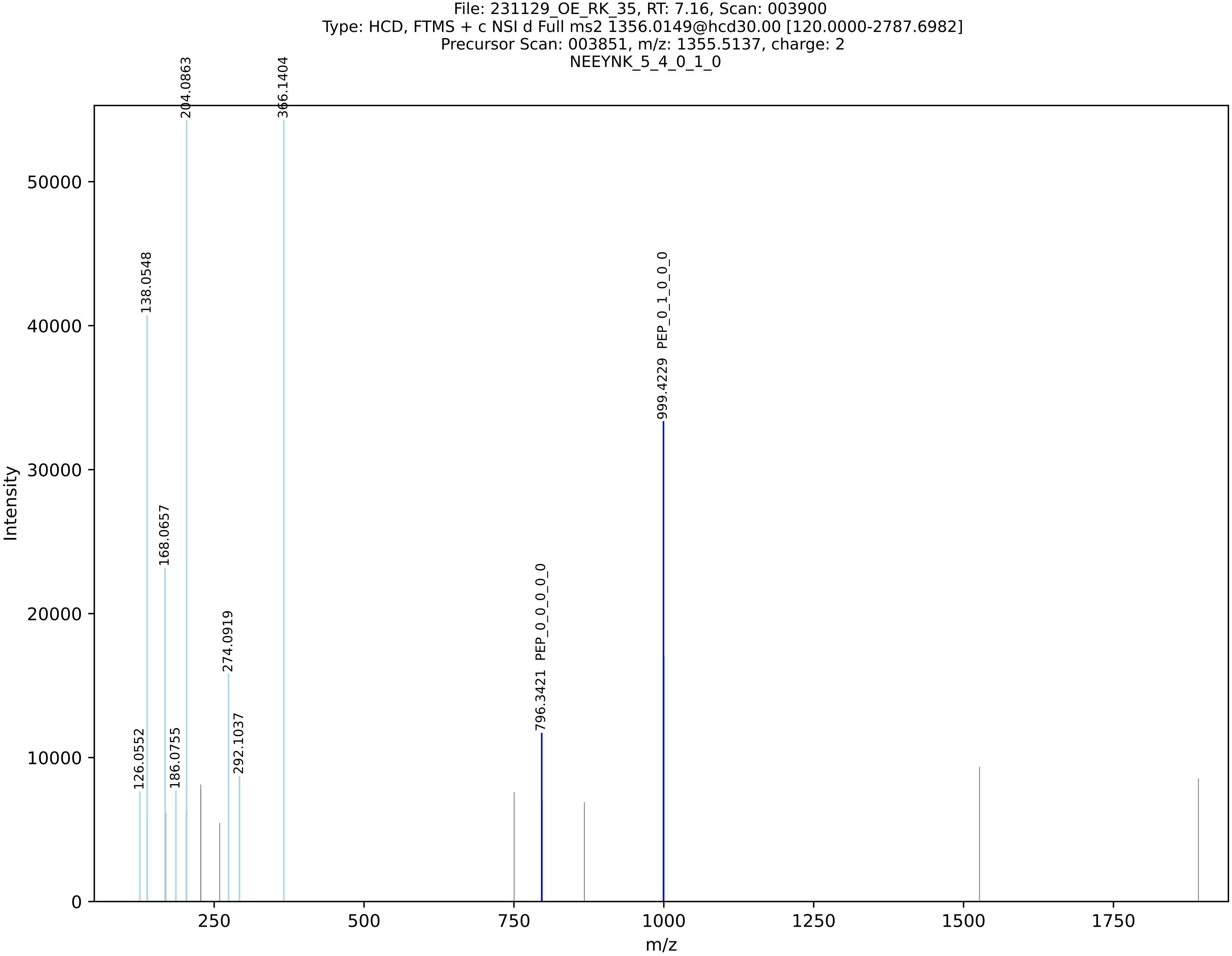

File: 231129_OE_RK_35, RT: 7.16, Scan: 003900
Type: HCD, FTMS + c NSI d Full ms2 1356.0149@hcd30.00 [120.0000-2787.6982]
Precursor Scan: 003851, m/z: 1355.5137, charge: 2
NEEYNK_5_4_0_1_0
204.0863
366.1404
138.0548
168.0657
274.0919
292.1037
126.0552
186.0755
796.3421 PEP_0_0_0_0_0_
999.4229 PEP_0_1_0_0_
Intensity
m/z
0
10000
20000
30000
40000
50000
250
500
750
1000
1250
1500
1750

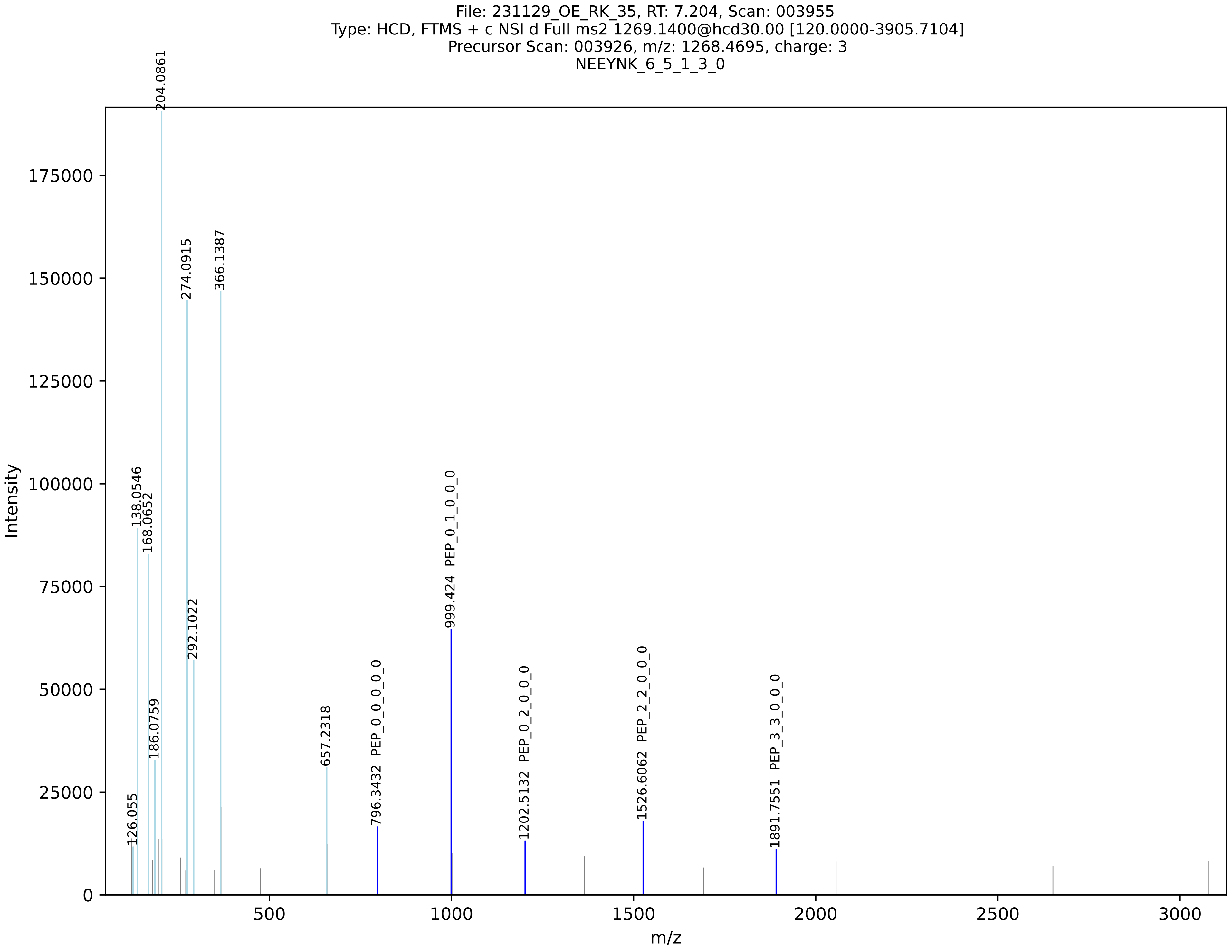
File: 231129_OE_RK_35, RT: 7.204, Scan: 003955
Type: HCD, FTMS + c NSI d Full ms2 1269.1400@hcd30.00 [120.0000-3905.7104]
Precursor Scan: 003926, m/z: 1268.4695, charge: 3
NEEYNK_6_5_1_3_0
Intensity
m/z
204.0861
274.0915
366.1387
138.0546
168.0652
292.1022
186.0759
126.055
657.2318
796.3432 PEP_0_0_0_0_0
999.424 PEP_0_1_0_0_0
1202.5132 PEP_0_2_0_0_0
1526.6062 PEP_2_2_0_0_0
1891.7551 PEP_3_3_0_0_0

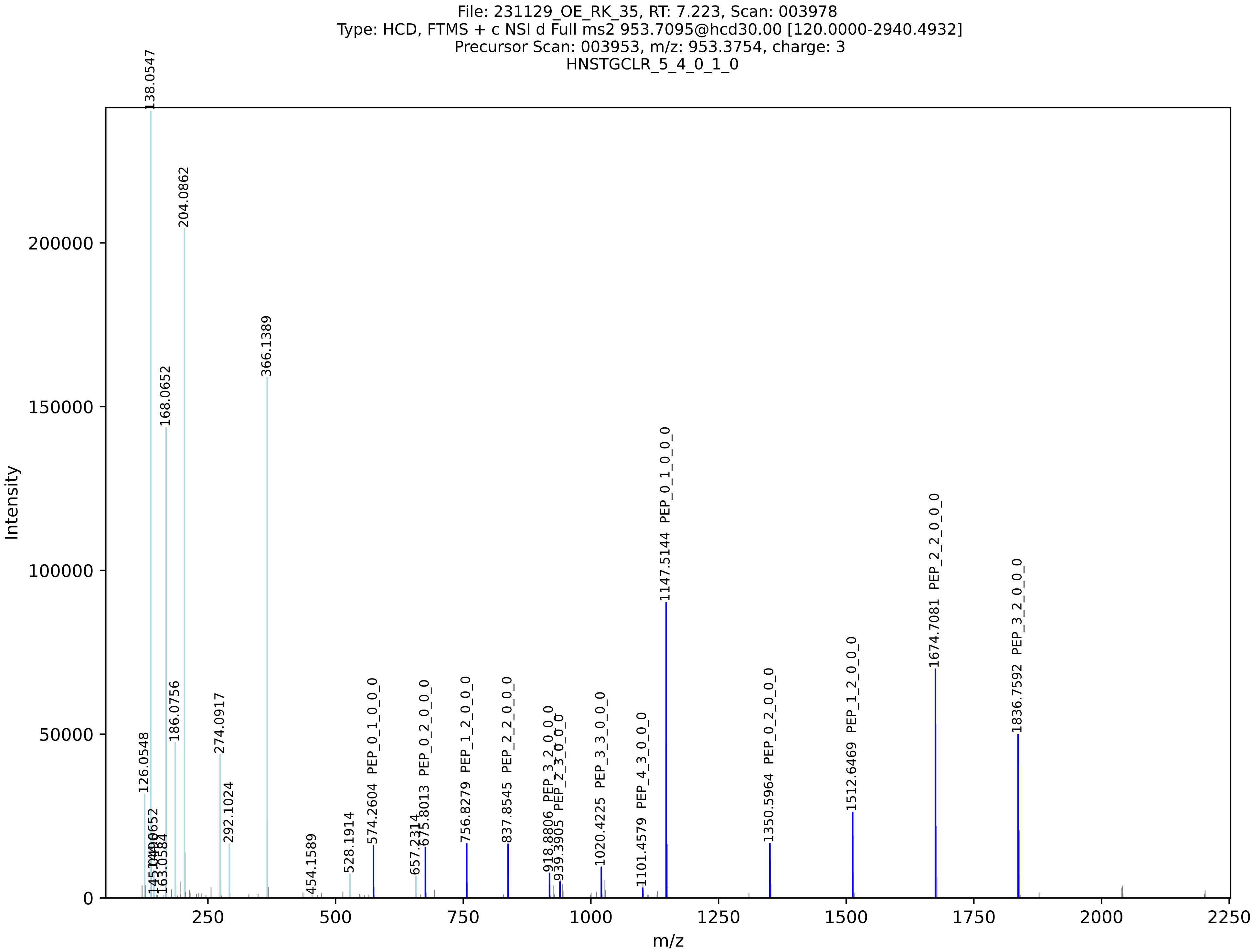
File: 231129_OE_RK_35, RT: 7.223, Scan: 003978
Type: HCD, FTMS + c NSI d Full ms2 953.7095@hcd30.00 [120.0000-2940.4932]
Precursor Scan: 003953, m/z: 953.3754, charge: 3
HNSTGCLR_5_4_0_1_0
Intensity
m/z
138.0547
204.0862
366.1389
168.0652
186.0756
274.0917
126.0548
292.1024
454.1589
528.1914
574.2604 PEP_0_1_0_0_
657.2314
675.8013 PEP_0_2_0_0_
756.8279 PEP_1_2_0_0_
837.8545 PEP_2_2_0_0_
918.8806 PEP_3_2_0_0_
939.3905 PEP_2_3_0_0_
1020.4225 PEP_3_3_0_0_
1101.4579 PEP_4_3_0_0_
1147.5144 PEP_0_1_0_0_
1350.5964 PEP_0_2_0_0_
1512.6469 PEP_1_2_0_0_
1674.7081 PEP_2_2_0_0_
1836.7592 PEP_3_2_0_0_

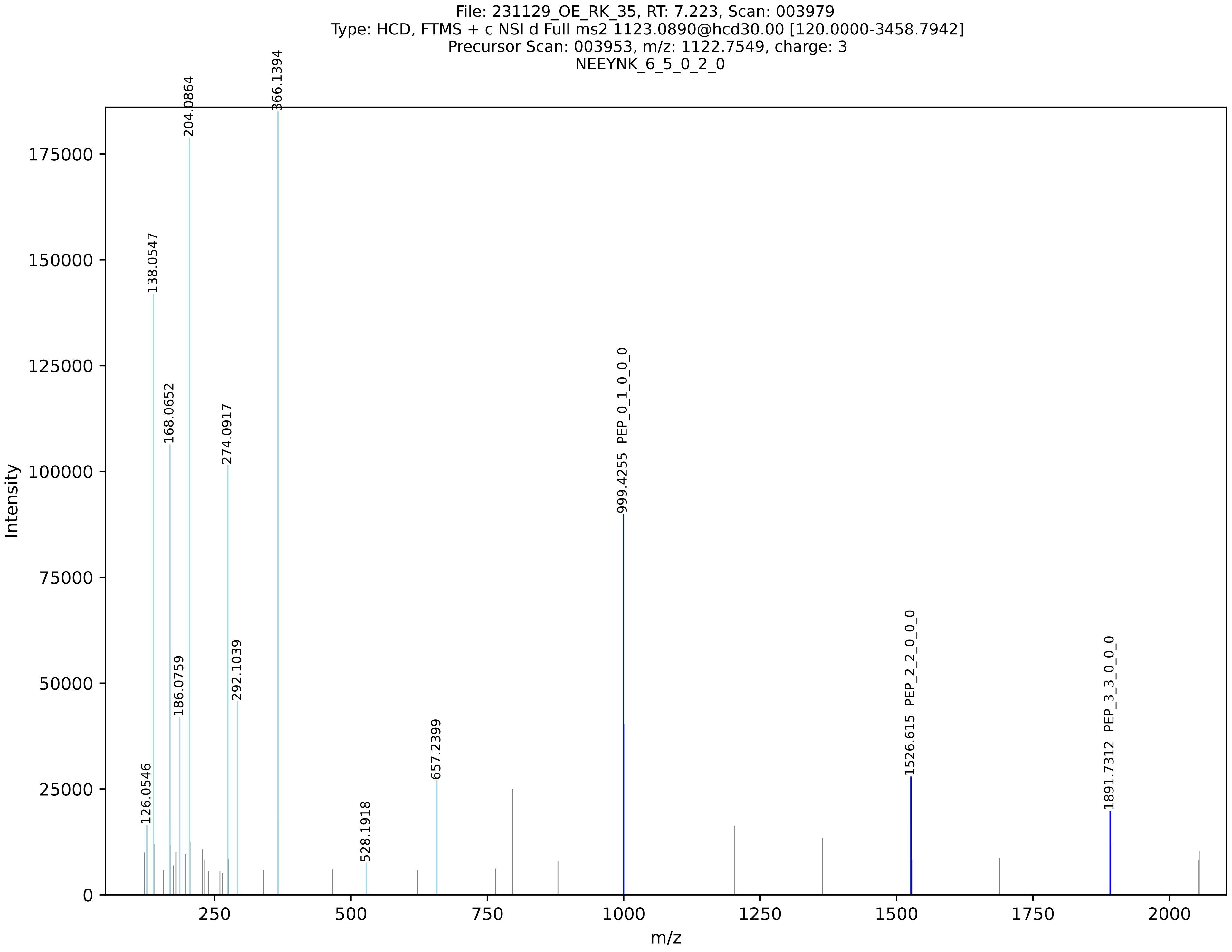
File: 231129_OE_RK_35, RT: 7.223, Scan: 003979
Type: HCD, FTMS + c NSI d Full ms2 1123.0890@hcd30.00 [120.0000-3458.7942]
Precursor Scan: 003953, m/z: 1122.7549, charge: 3
NEEYNK_6_5_0_2_0
366.1394
204.0864
138.0547
168.0652
274.0917
186.0759
292.1039
126.0546
528.1918
657.2399
999.4255 PEP_0_1_0_0_0
1526.615 PEP_2_2_0_0_0
1891.7312 PEP_3_3_0_0_0
Intensity
m/z
0
25000
50000
75000
100000
125000
150000
175000
250
500
750
1000
1250
1500
1750
2000

File: 231129_OE_RK_35, RT: 7.249, Scan: 004013
Type: HCD, FTMS + c NSI d Full ms2 1501.5636@hcd30.00 [120.0000-3084.6177]
Precursor Scan: 003977, m/z: 1501.0621, charge: 2
NEEYNK_5_4_0_2_0

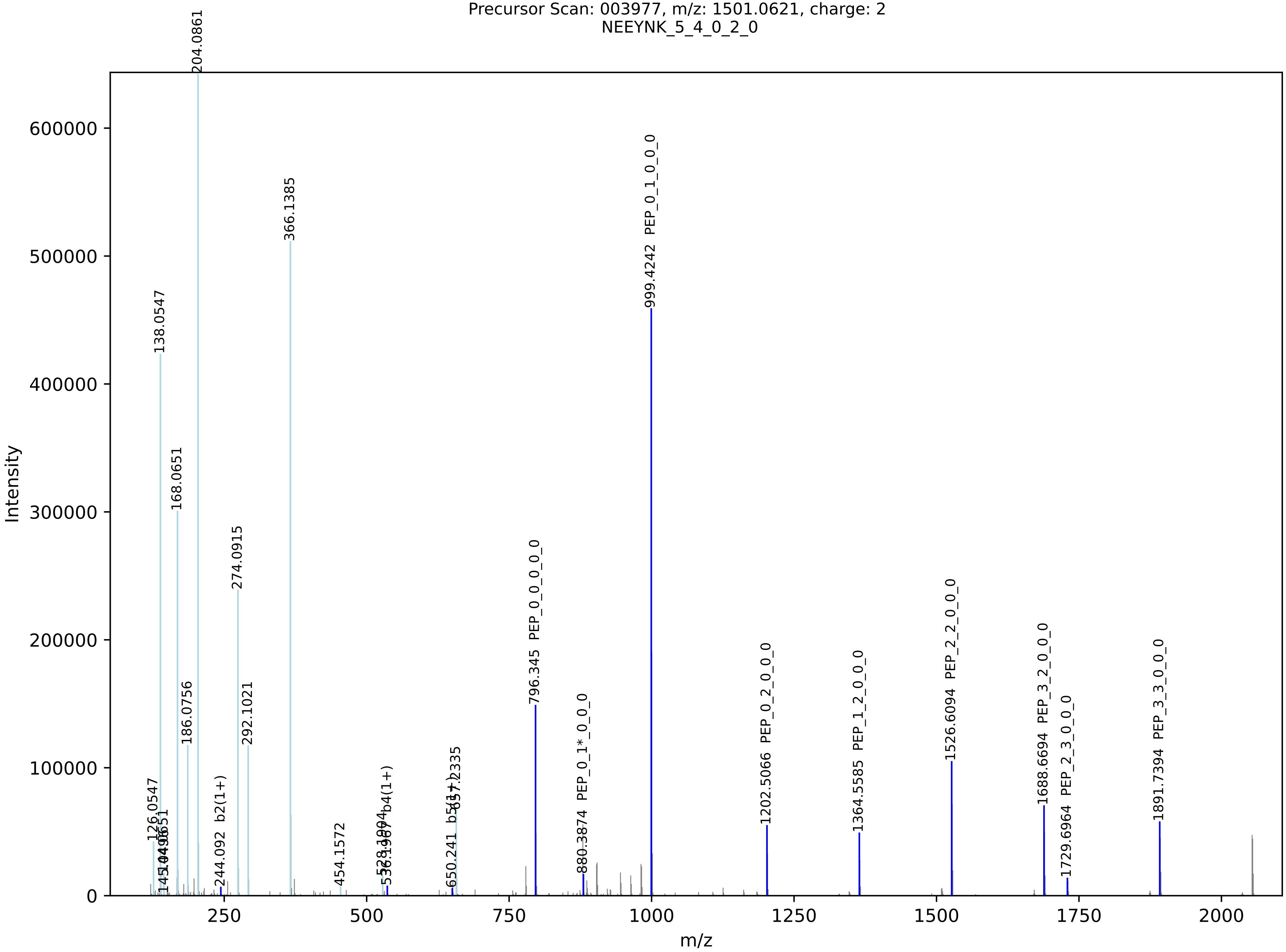

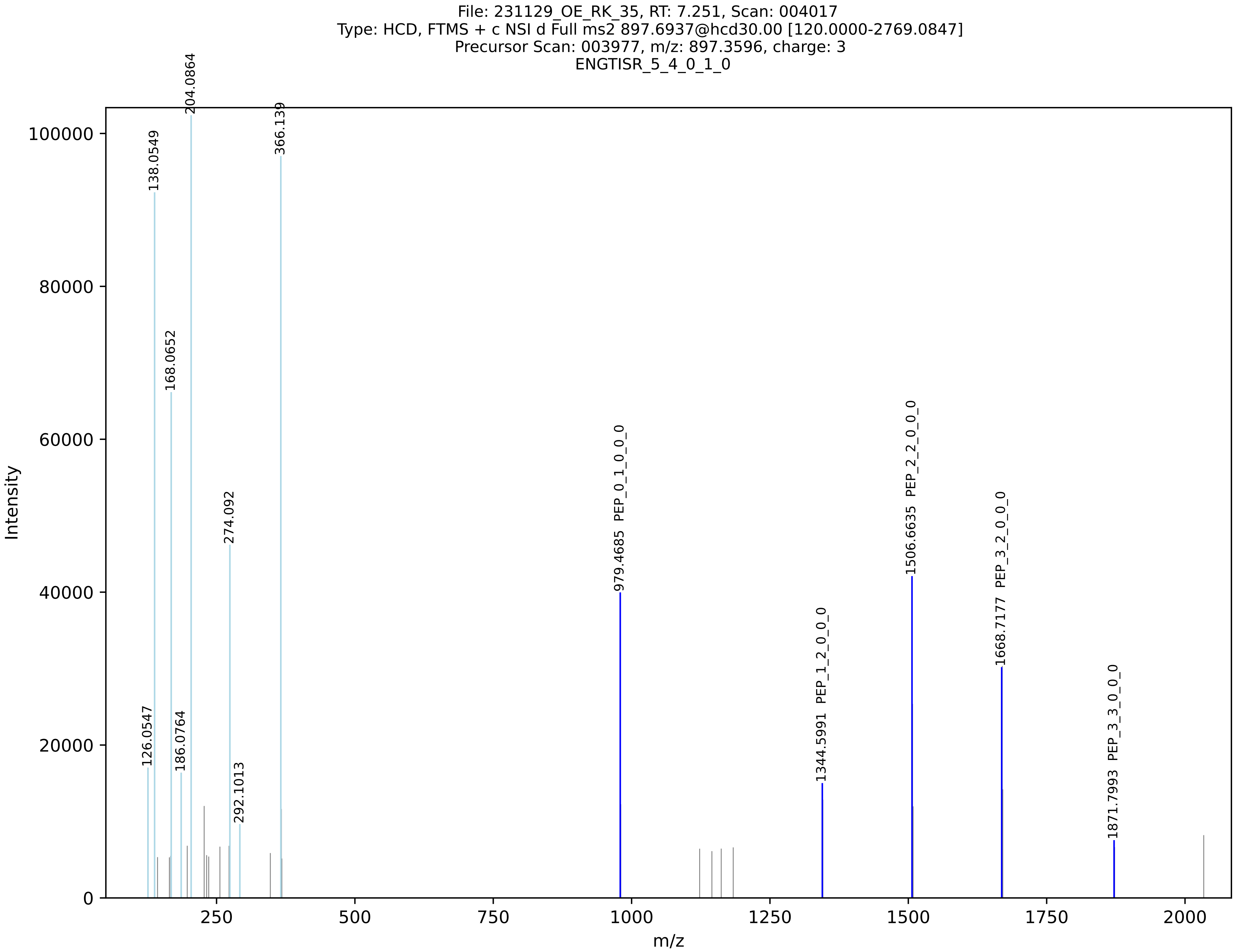
File: 231129_OE_RK_35, RT: 7.251, Scan: 004017
Type: HCD, FTMS + c NSI d Full ms2 897.6937@hcd30.00 [120.0000-2769.0847]
Precursor Scan: 003977, m/z: 897.3596, charge: 3
ENGTISR_5_4_0_1_0
Intensity
m/z
126.0547
138.0549
168.0652
186.0764
204.0864
274.092
292.1013
366.139
979.4685 PEP_0_1_0_0_0
1344.5991 PEP_1_2_0_0_0
1506.6635 PEP_2_2_0_0_0
1668.7177 PEP_3_2_0_0_0
1871.7993 PEP_3_3_0_0_0

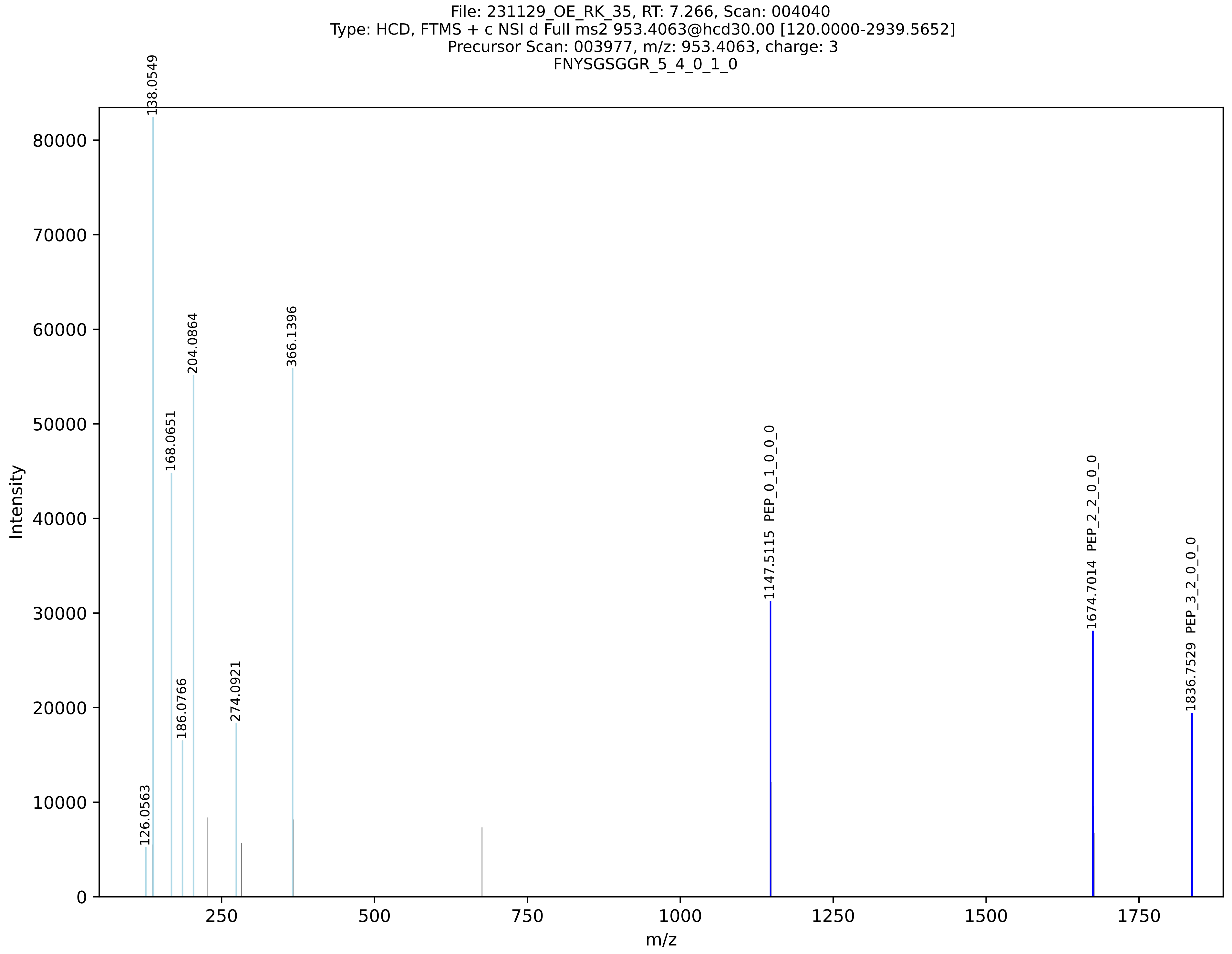
File: 231129_OE_RK_35, RT: 7.266, Scan: 004040
Type: HCD, FTMS + c NSI d Full ms2 953.4063@hcd30.00 [120.0000-2939.5652]
Precursor Scan: 003977, m/z: 953.4063, charge: 3
FNYSGSGGR_5_4_0_1_0
138.0549
204.0864
366.1396
168.0651
186.0766
274.0921
126.0563
1147.5115 PEP_0_1_0_0_0
1674.7014 PEP_2_2_0_0_0
1836.7529 PEP_3_2_0_0_0
Intensity
m/z
0
10000
20000
30000
40000
50000
60000
70000
80000
250
500
750
1000
1250
1500
1750

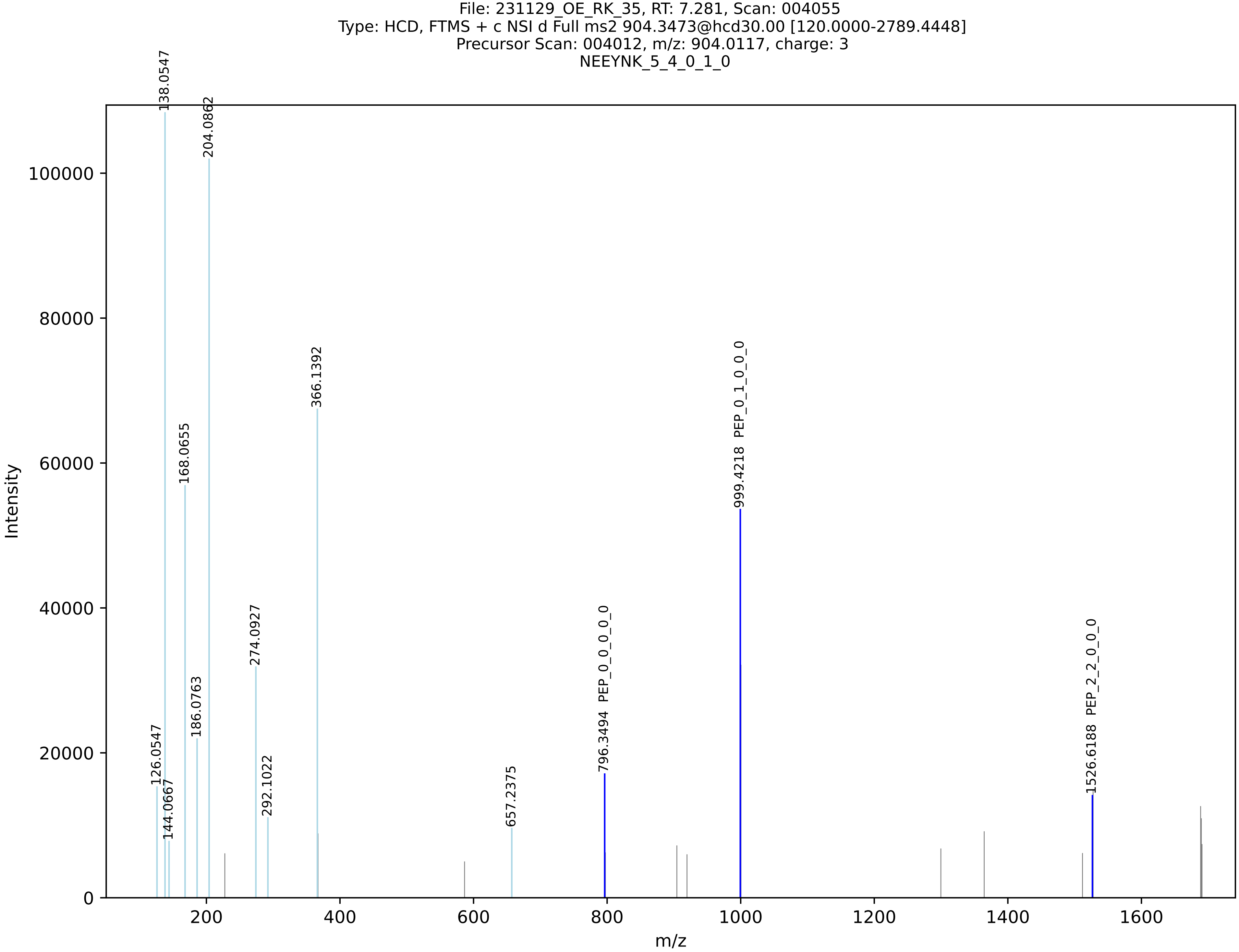
File: 231129_OE_RK_35, RT: 7.281, Scan: 004055
Type: HCD, FTMS + c NSI d Full ms2 904.3473@hcd30.00 [120.0000-2789.4448]
Precursor Scan: 004012, m/z: 904.0117, charge: 3
NEEYNK_5_4_0_1_0
Intensity
m/z
138.0547
204.0862
168.0655
366.1392
999.4218 PEP_0_1_0_0_0
274.0927
186.0763
126.0547
144.0667
292.1022
657.2375
796.3494 PEP_0_0_0_0_0
1526.6188 PEP_2_2_0_0_0

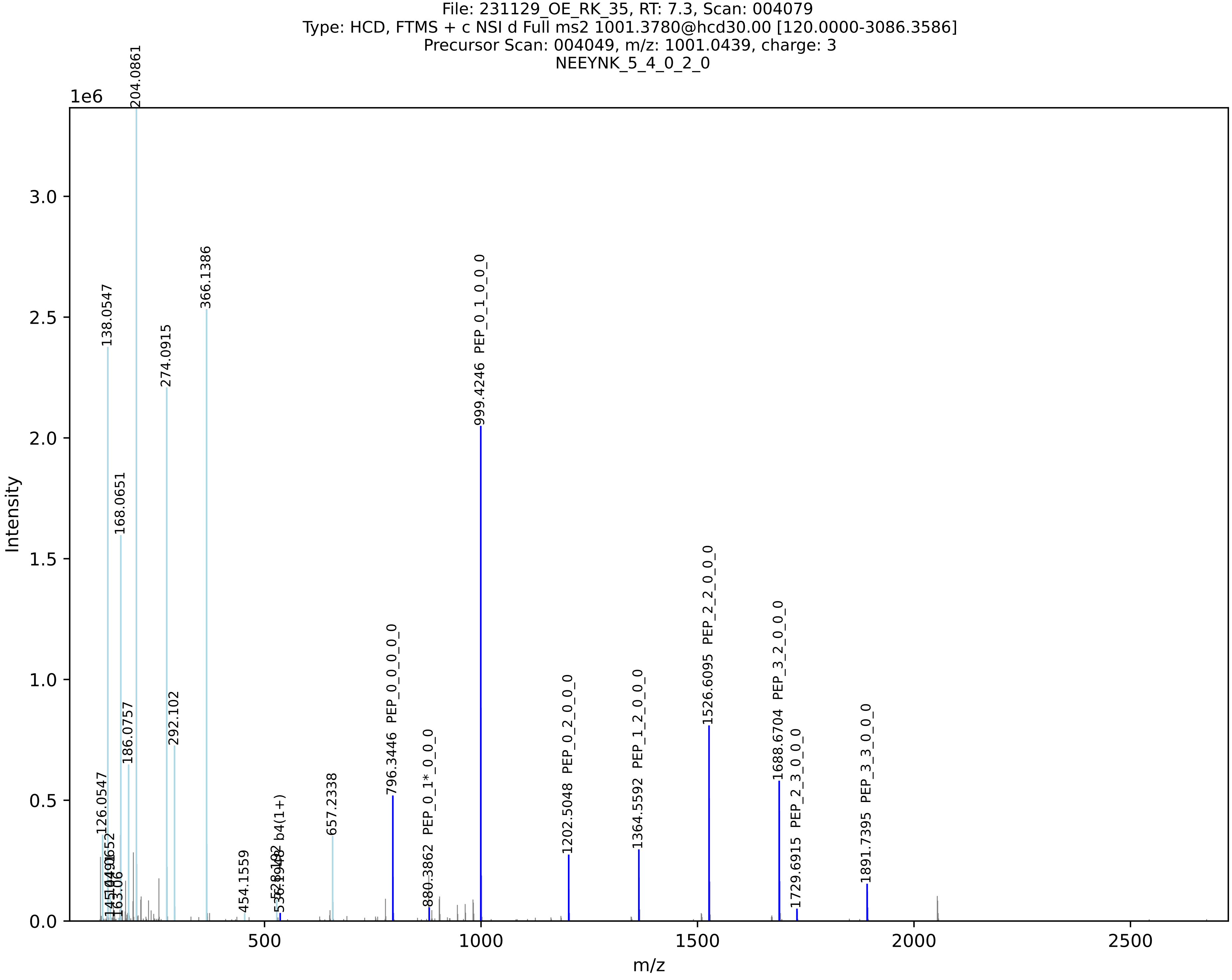

File: 231129_OE_RK_35, RT: 7.3, Scan: 004079
Type: HCD, FTMS + c NSI d Full ms2 1001.3780@hcd30.00 [120.0000-3086.3586]
Precursor Scan: 004049, m/z: 1001.0439, charge: 3
NEEYNK_5_4_0_2_0
1e6
Intensity
m/z
204.0861
138.0547
366.1386
274.0915
168.0651
186.0757
292.102
126.0547
454.1559
b4(1+)
657.2338
796.3446 PEP_0_0_0_0_0
880.3862 PEP_0_1*_0_0_0
999.4246 PEP_0_1_0_0_0
1202.5048 PEP_0_2_0_0_0
1364.5592 PEP_1_2_0_0_0
1526.6095 PEP_2_2_0_0_0
1688.6704 PEP_3_2_0_0_0
1729.6915 PEP_2_3_0_0_0
1891.7395 PEP_3_3_0_0_0

File: 231129_OE_RK_35, RT: 7.305, Scan: 004087
Type: HCD, FTMS + c NSI d Full ms2 1000.3749@hcd30.00 [120.0000-3083.2891]
Precursor Scan: 004049, m/z: 1000.3749, charge: 3
NEEYNK_5_4_0_2_0

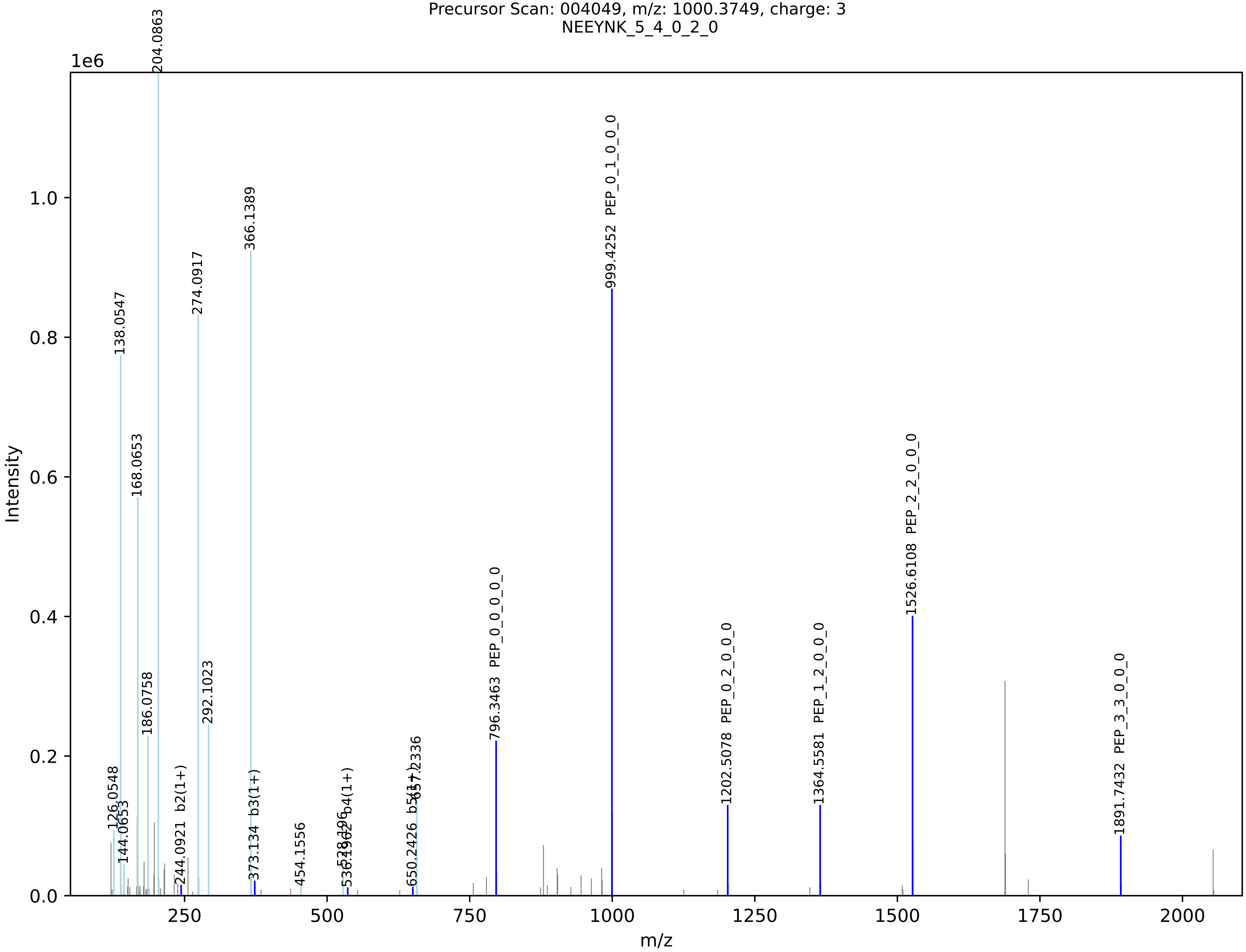

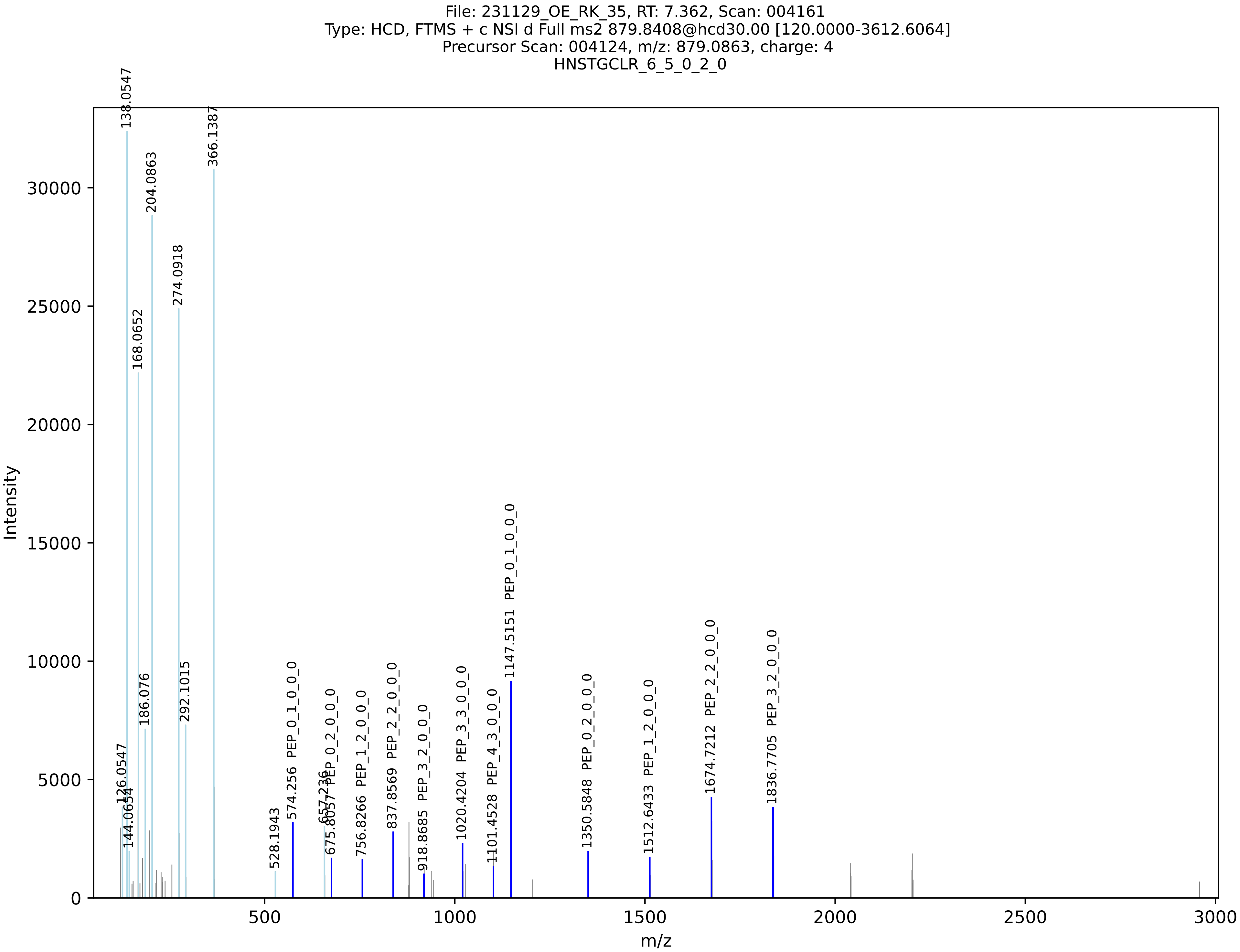
File: 231129_OE_RK_35, RT: 7.362, Scan: 004161
Type: HCD, FTMS + c NSI d Full ms2 879.8408@hcd30.00 [120.0000-3612.6064]
Precursor Scan: 004124, m/z: 879.0863, charge: 4
HNSTGCLR_6_5_0_2_0
Intensity
m/z
138.0547
366.1387
204.0863
274.0918
168.0652
186.076
292.1015
126.0547
144.0654
528.1943
574.256 PEP_0_1_0_0_0
657.236
675.8057 PEP_0_2_0_0_0
756.8266 PEP_1_2_0_0_0
837.8569 PEP_2_2_0_0_0
918.8685 PEP_3_2_0_0_0
1020.4204 PEP_3_3_0_0_0
1101.4528 PEP_4_3_0_0_0
1147.5151 PEP_0_1_0_0_0
1350.5848 PEP_0_2_0_0_0
1512.6433 PEP_1_2_0_0_0
1674.7212 PEP_2_2_0_0_0
1836.7705 PEP_3_2_0_0_0

File: 231129_OE_RK_35, RT: 7.385, Scan: 004191
Type: HCD, FTMS + c NSI d Full ms2 1269.1400@hcd30.00 [120.0000-3905.7104]
Precursor Scan: 004160, m/z: 1268.4726, charge: 3
NEEYNK_6_5_1_3_0

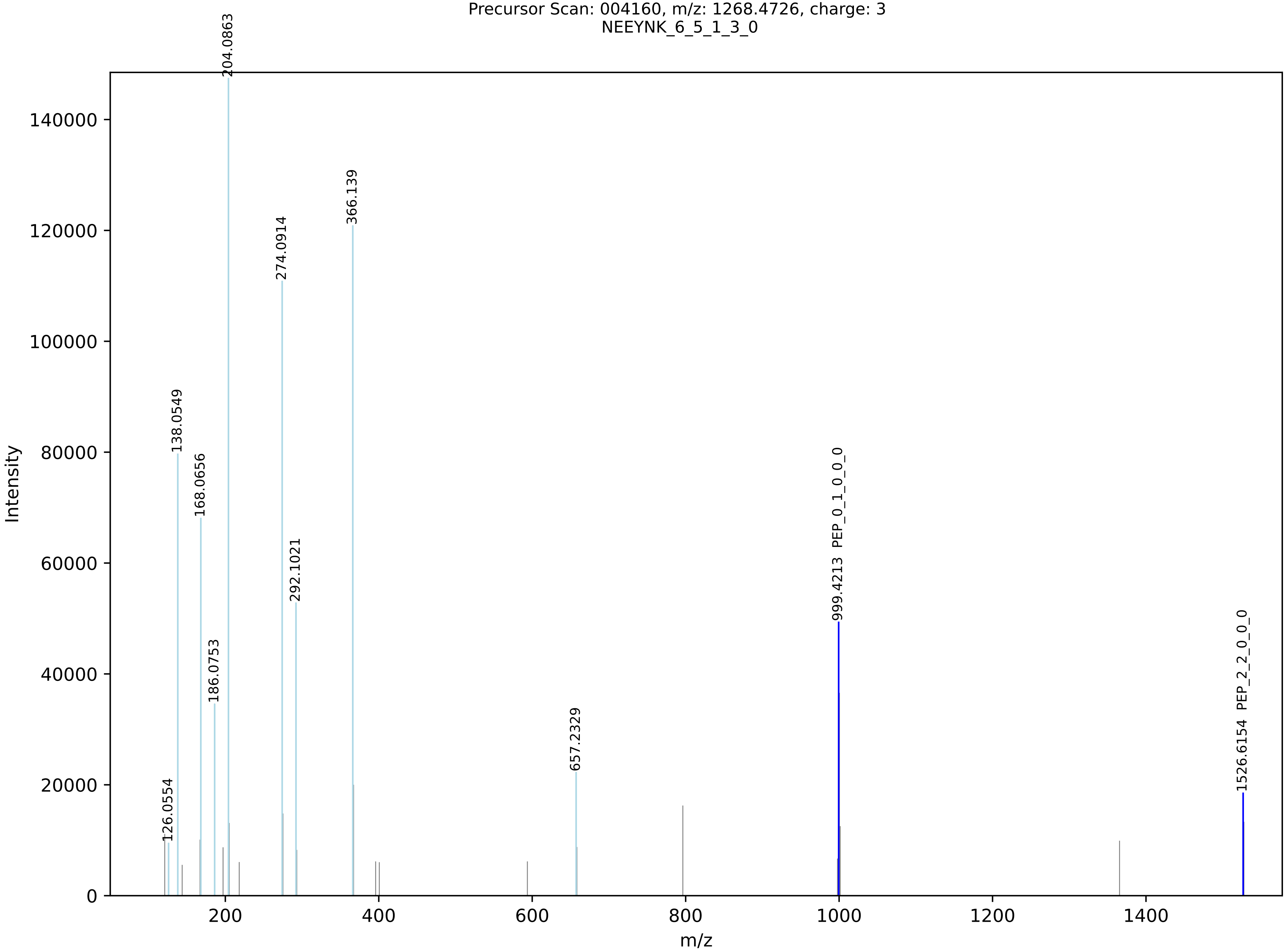

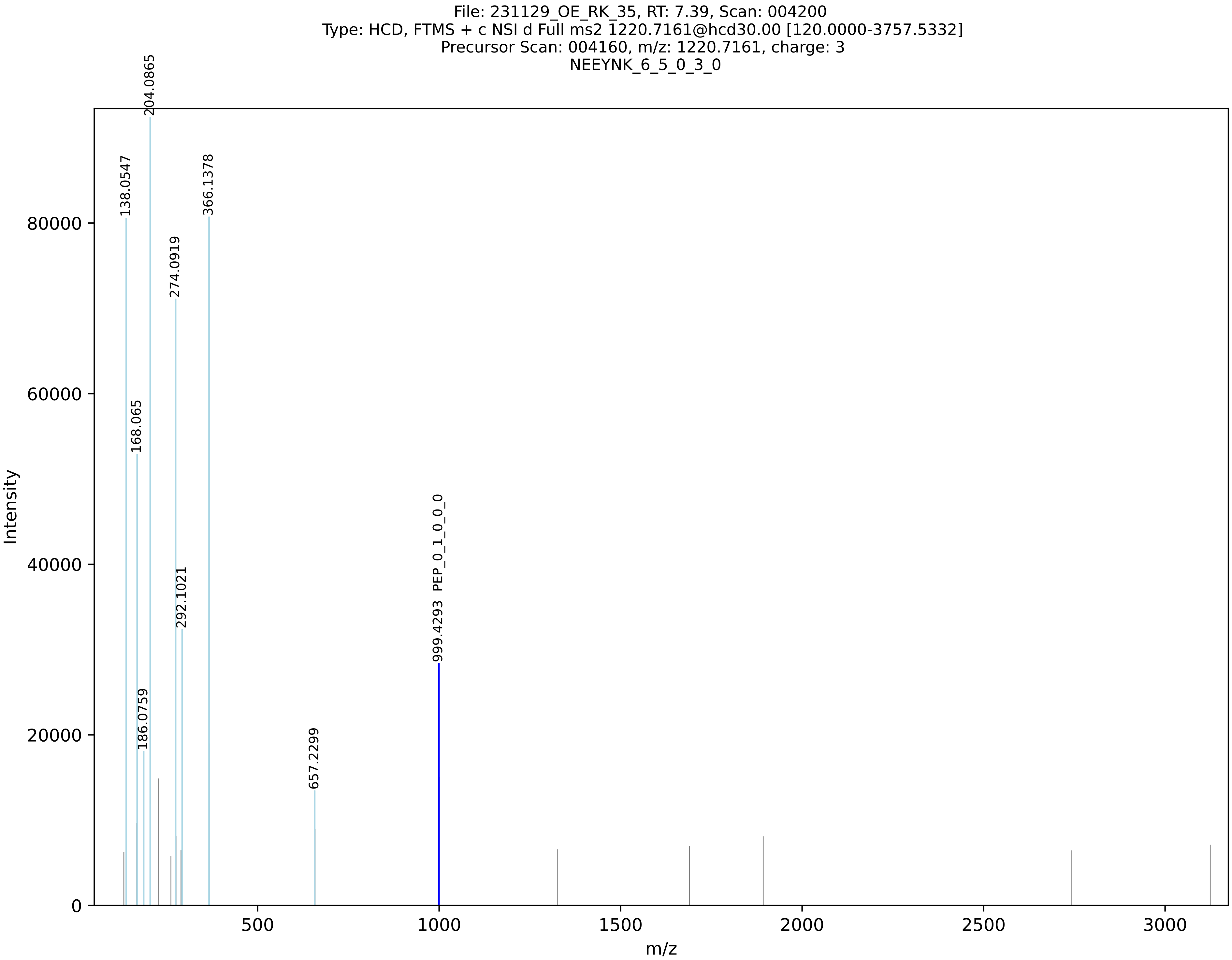
File: 231129_OE_RK_35, RT: 7.39, Scan: 004200
Type: HCD, FTMS + c NSI d Full ms2 1220.7161@hcd30.00 [120.0000-3757.5332]
Precursor Scan: 004160, m/z: 1220.7161, charge: 3
NEEYNK_6_5_0_3_0
204.0865
138.0547
366.1378
274.0919
168.065
292.1021
186.0759
657.2299
999.4293 PEP_0_1_0_0_0_
Intensity
m/z
0
20000
40000
60000
80000
500
1000
1500
2000
2500
3000

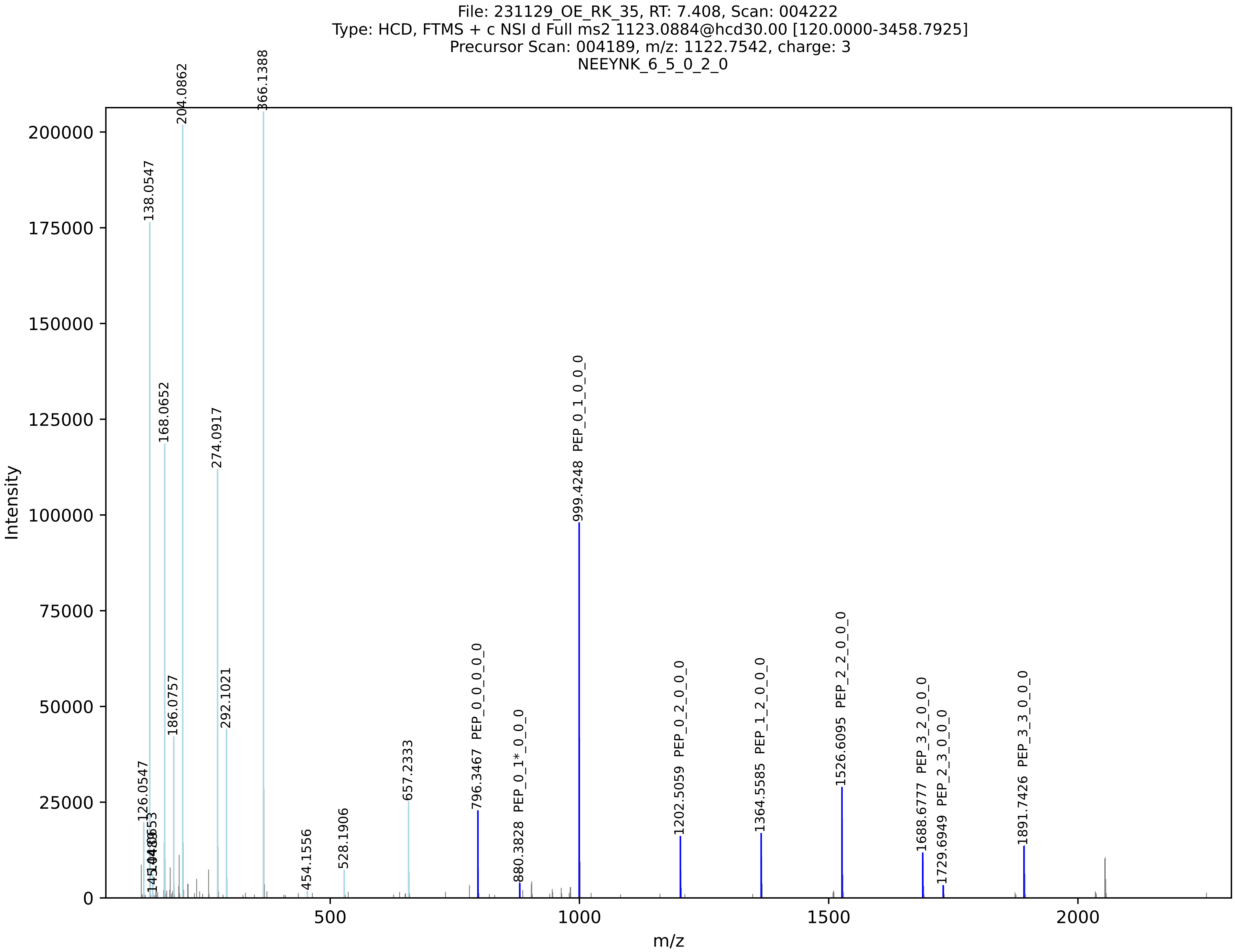

File: 231129_OE_RK_35, RT: 7.408, Scan: 004222
Type: HCD, FTMS + c NSI d Full ms2 1123.0884@hcd30.00 [120.0000-3458.7925]
Precursor Scan: 004189, m/z: 1122.7542, charge: 3
NEEYNK_6_5_0_2_0
Intensity
m/z
0
25000
50000
75000
100000
125000
150000
175000
200000
500
1000
1500
2000
126.0547
138.0547
168.0652
186.0757
204.0862
274.0917
292.1021
366.1388
454.1556
528.1906
657.2333
796.3467 PEP_0_0_0_0_0
880.3828 PEP_0_1*_0_0_0
999.4248 PEP_0_1_0_0_0
1202.5059 PEP_0_2_0_0_0
1364.5585 PEP_1_2_0_0_0
1526.6095 PEP_2_2_0_0_0
1688.6777 PEP_3_2_0_0_0
1729.6949 PEP_2_3_0_0_0
1891.7426 PEP_3_3_0_0_0

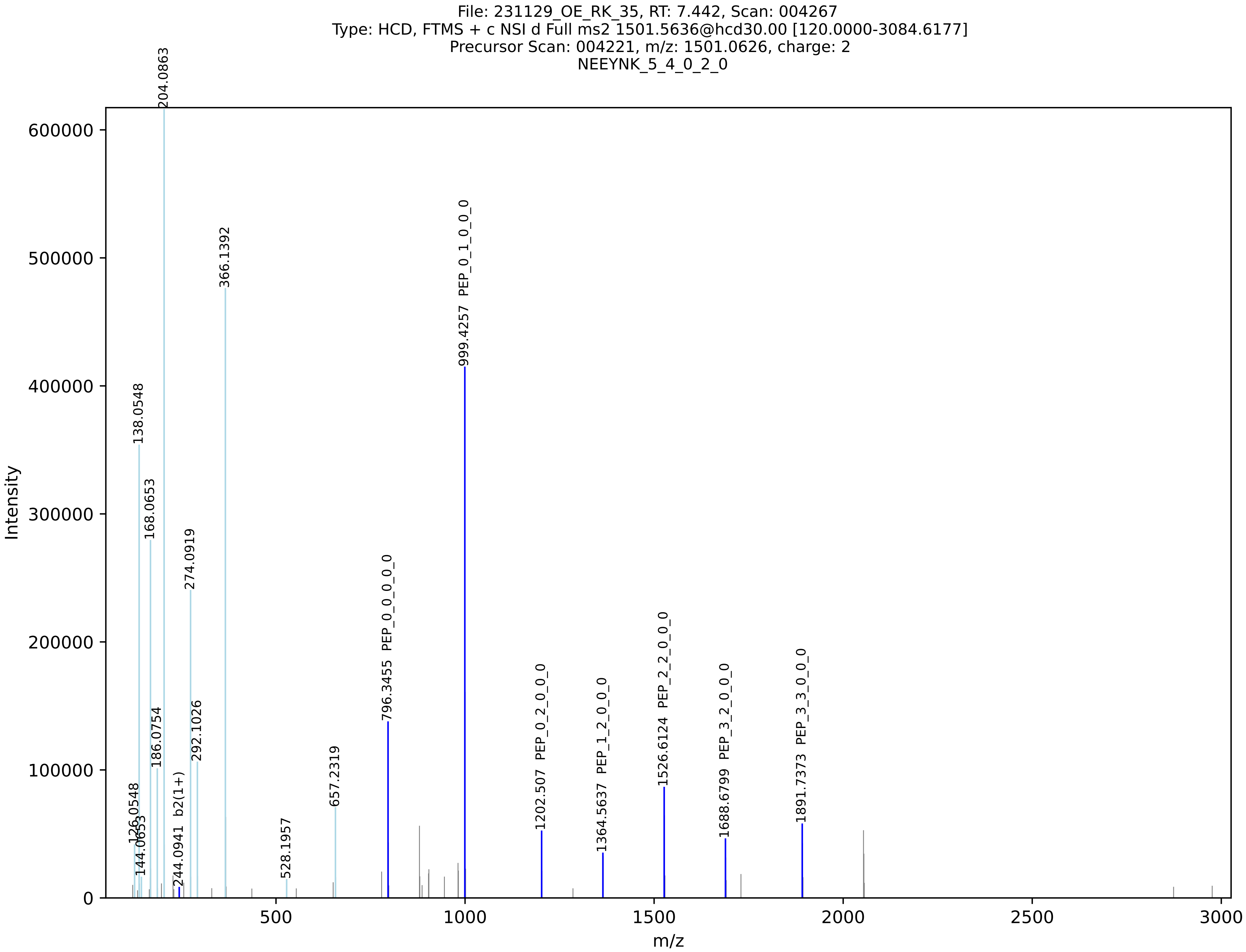
File: 231129_OE_RK_35, RT: 7.442, Scan: 004267
Type: HCD, FTMS + c NSI d Full ms2 1501.5636@hcd30.00 [120.0000-3084.6177]
Precursor Scan: 004221, m/z: 1501.0626, charge: 2
NEEYNK_5_4_0_2_0
Intensity
m/z
204.0863
366.1392
999.4257 PEP_0_1_0_0_0
138.0548
168.0653
274.0919
796.3455 PEP_0_0_0_0_0
186.0754
292.1026
1202.507 PEP_0_2_0_0_0
1364.5637 PEP_1_2_0_0_0
1526.6124 PEP_2_2_0_0_0
1688.6799 PEP_3_2_0_0_0
1891.7373 PEP_3_3_0_0_0
657.2319
126.0548
144.0653
244.0941 b2(1+)
528.1957

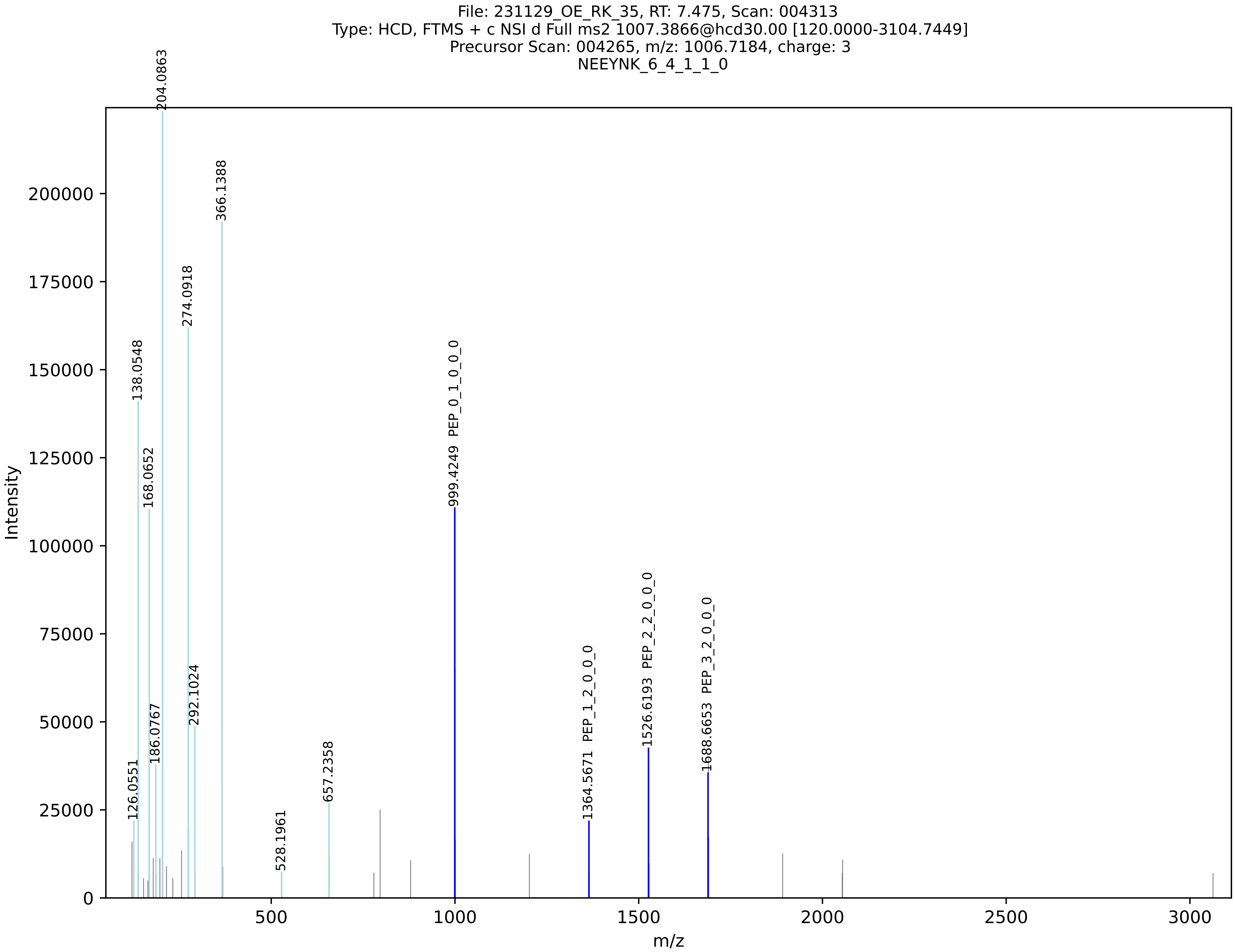

File: 231129_OE_RK_35, RT: 7.475, Scan: 004313
Type: HCD, FTMS + c NSI d Full ms2 1007.3866@hcd30.00 [120.0000-3104.7449]
Precursor Scan: 004265, m/z: 1006.7184, charge: 3
NEEYNK_6_4_1_1_0
204.0863
366.1388
274.0918
138.0548
168.0652
999.4249 PEP_0_1_0_0_0
292.1024
186.0767
126.0551
528.1961
657.2358
1364.5671 PEP_1_2_0_0_0
1526.6193 PEP_2_2_0_0_0
1688.6653 PEP_3_2_0_0_0
Intensity
m/z
0
25000
50000
75000
100000
125000
150000
175000
200000
500
1000
1500
2000
2500
3000

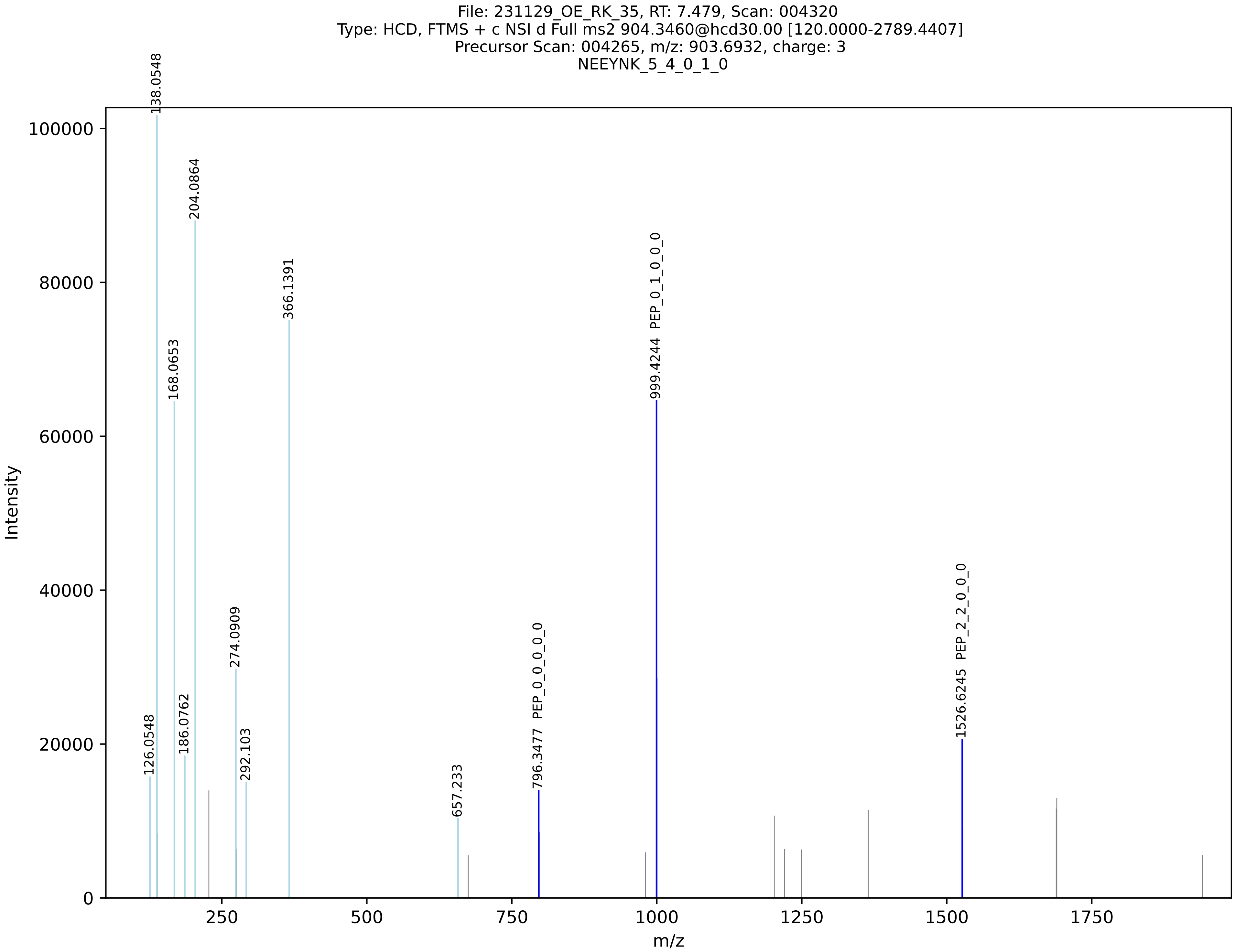
File: 231129_OE_RK_35, RT: 7.479, Scan: 004320
Type: HCD, FTMS + c NSI d Full ms2 904.3460@hcd30.00 [120.0000-2789.4407]
Precursor Scan: 004265, m/z: 903.6932, charge: 3
NEEYNK_5_4_0_1_0
Intensity
m/z
0
20000
40000
60000
80000
100000
250
500
750
1000
1250
1500
1750
138.0548
204.0864
366.1391
168.0653
999.4244 PEP_0_1_0_0_0
1526.6245 PEP_2_2_0_0_0
274.0909
796.3477 PEP_0_0_0_0_0
126.0548
186.0762
292.103
657.233

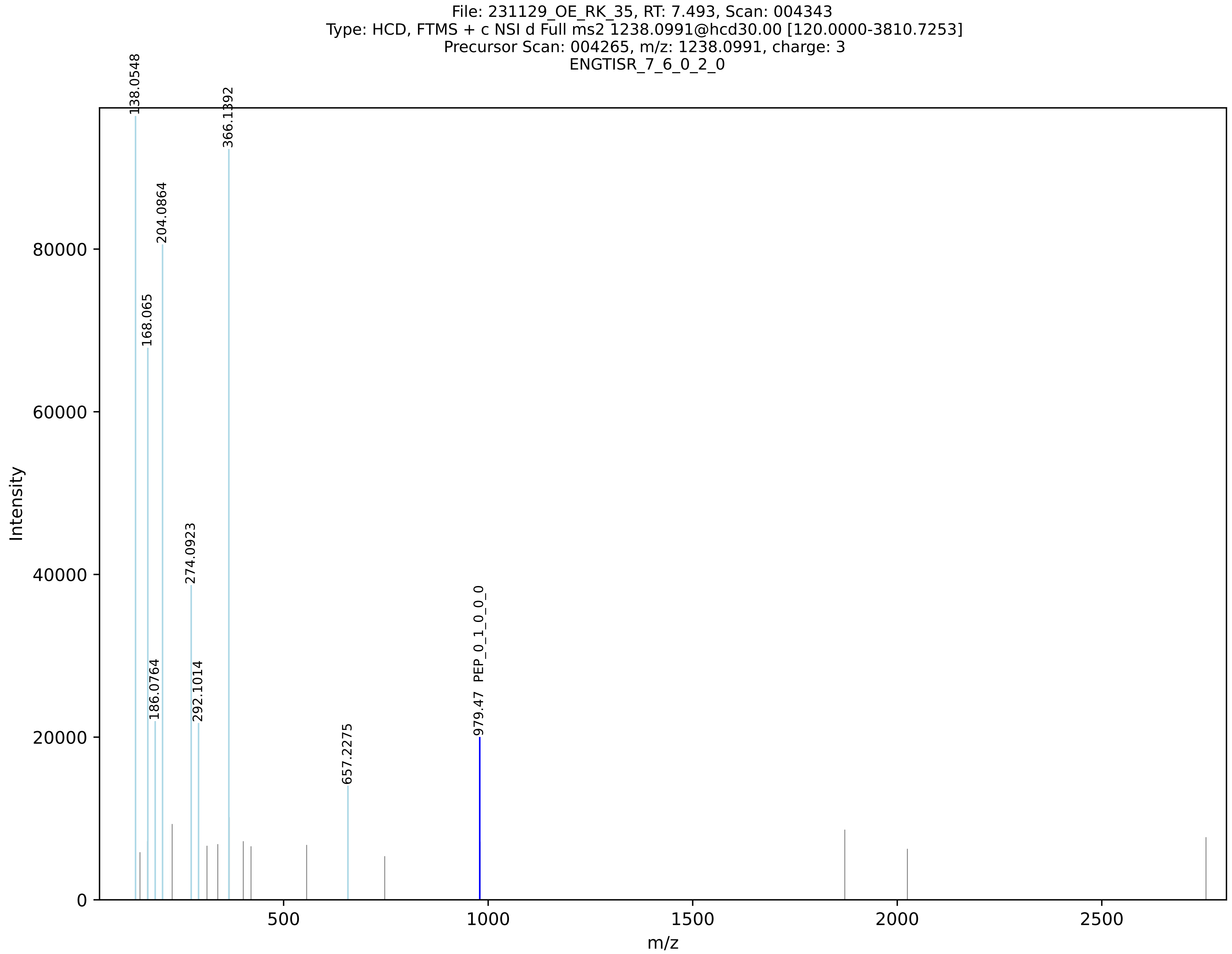
File: 231129_OE_RK_35, RT: 7.493, Scan: 004343
Type: HCD, FTMS + c NSI d Full ms2 1238.0991@hcd30.00 [120.0000-3810.7253]
Precursor Scan: 004265, m/z: 1238.0991, charge: 3
ENGTISR_7_6_0_2_0
138.0548
366.1392
204.0864
168.065
274.0923
186.0764
292.1014
657.2275
979.47 PEP_0_1_0_0_0
Intensity
m/z
0
20000
40000
60000
80000
500
1000
1500
2000
2500

File: 231129_OE_RK_35, RT: 7.544, Scan: 004409
Type: HCD, FTMS + c NSI d Full ms2 1238.4814@hcd30.00 [120.0000-3811.8953]
Precursor Scan: 004361, m/z: 1237.8133, charge: 3
ENGTISR_7_6_0_2_0

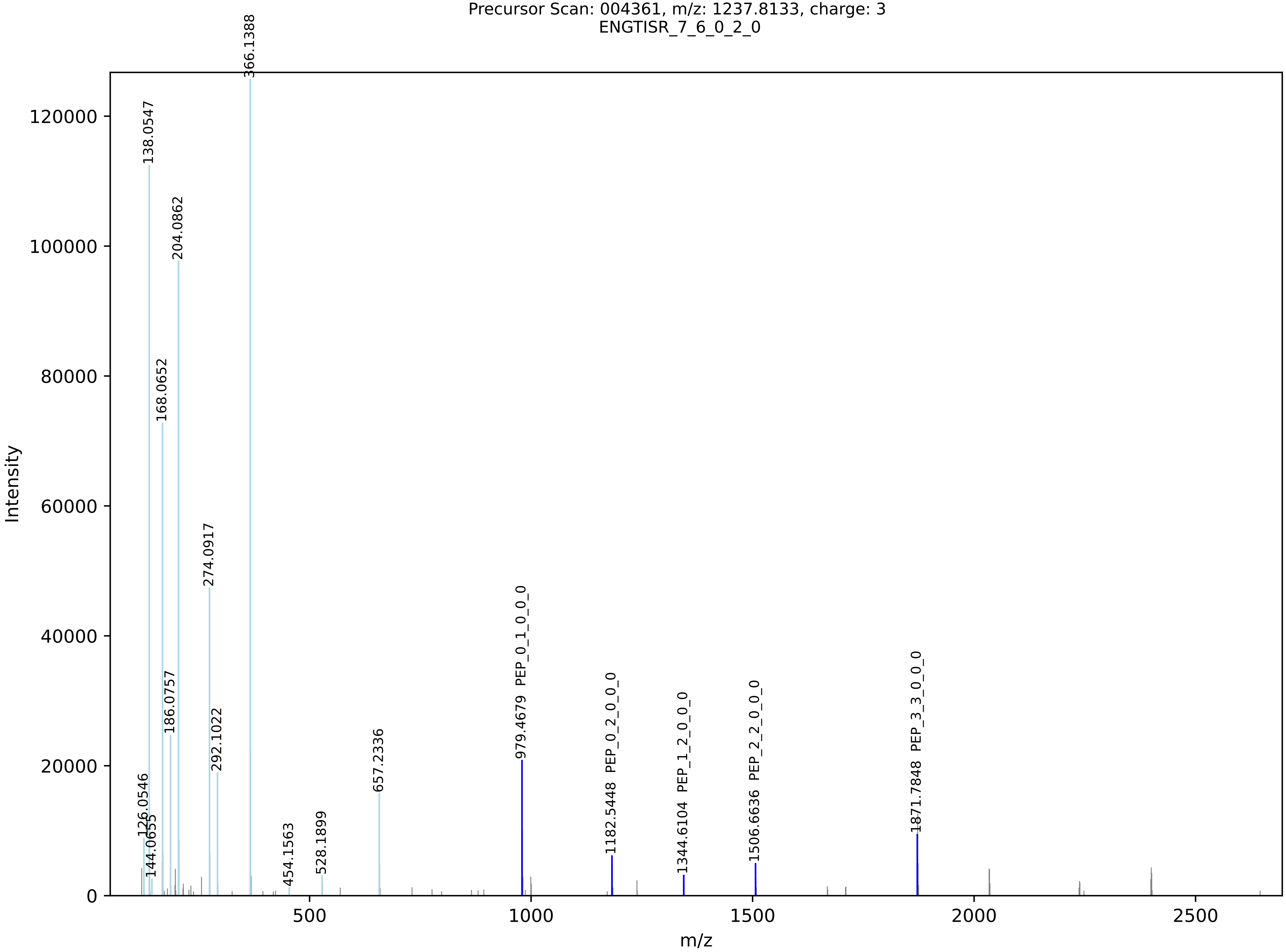

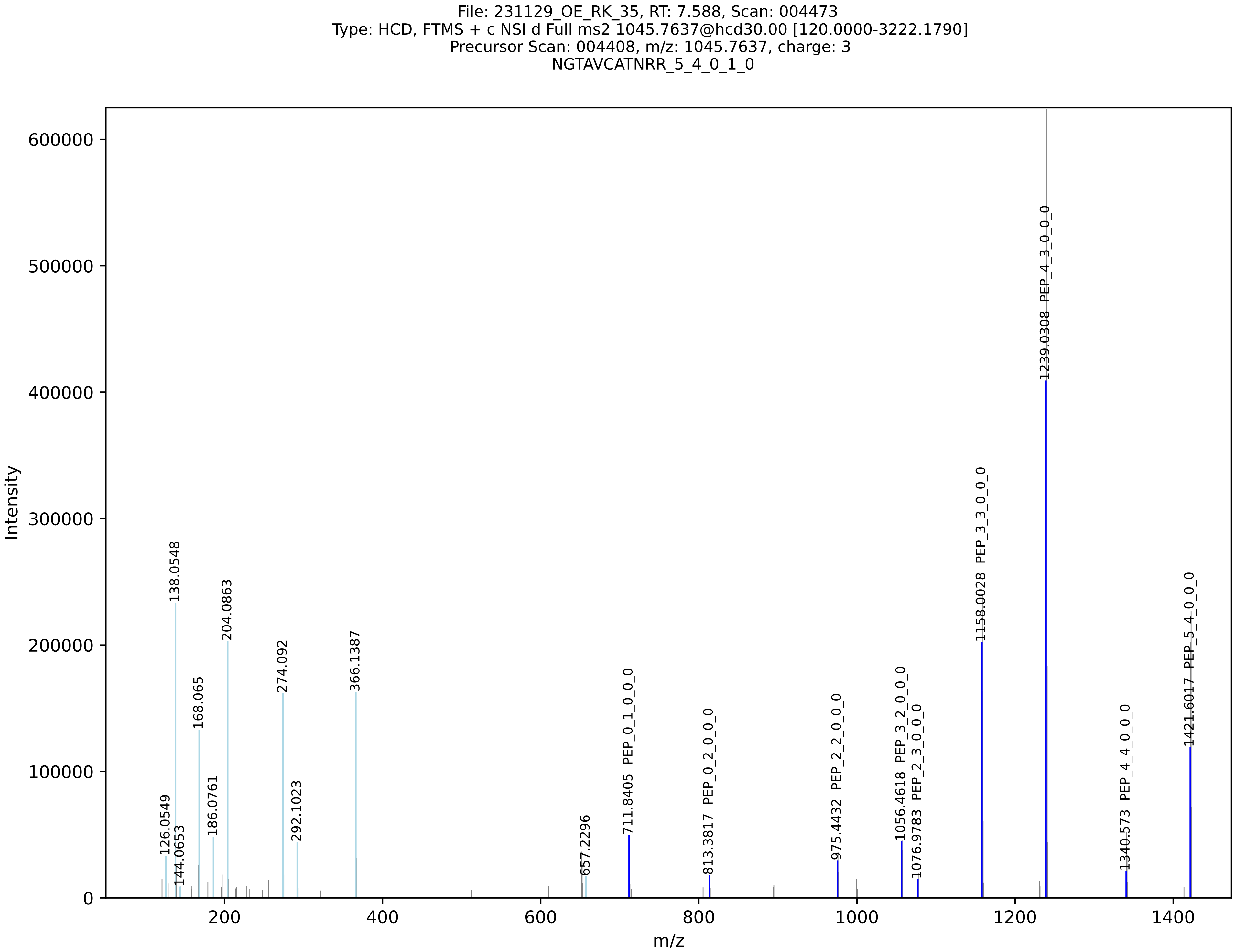
File: 231129_OE_RK_35, RT: 7.588, Scan: 004473
Type: HCD, FTMS + c NSI d Full ms2 1045.7637@hcd30.00 [120.0000-3222.1790]
Precursor Scan: 004408, m/z: 1045.7637, charge: 3
NGTAVCATNRR_5_4_0_1_0
Intensity
m/z
126.0549
138.0548
144.0653
168.065
186.0761
204.0863
274.092
292.1023
366.1387
657.2296
711.8405 PEP_0_1_0_0_0
813.3817 PEP_0_2_0_0_0
975.4432 PEP_2_2_0_0_0
1056.4618 PEP_3_2_0_0_0
1076.9783 PEP_2_3_0_0_0
1158.0028 PEP_3_3_0_0_0
1239.0308 PEP_4_3_0_0_0
1340.573 PEP_4_4_0_0_0
1421.6017 PEP_5_4_0_0_0

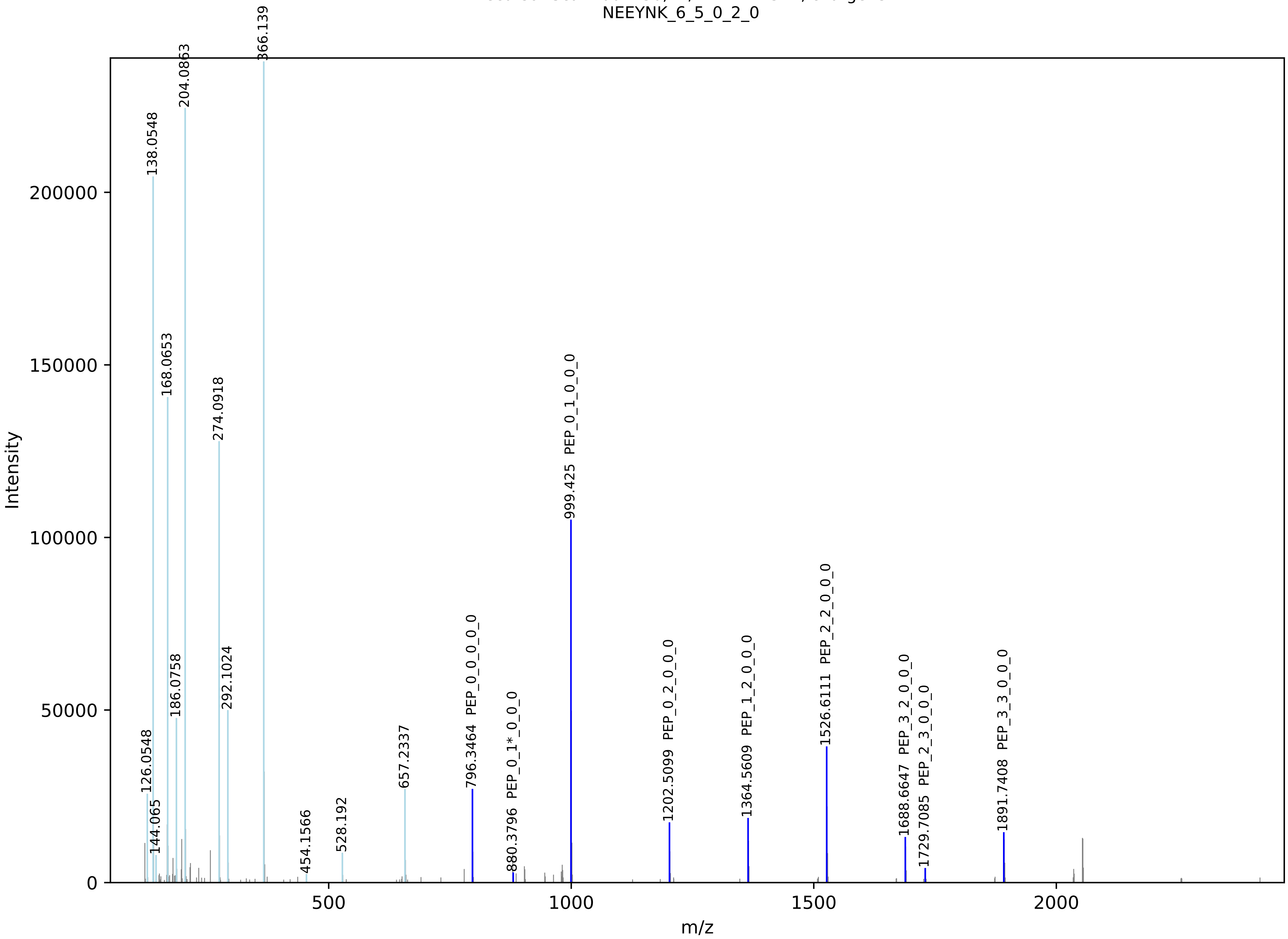

File: 231129_OE_RK_35, RT: 7.605, Scan: 004491
Type: HCD, FTMS + c NSI d Full ms2 1123.0883@hcd30.00 [120.0000-3458.7920]
Precursor Scan: 004450, m/z: 1122.7541, charge: 3
NEEYNK_6_5_0_2_0
Intensity
m/z
0
50000
100000
150000
200000
500
1000
1500
2000
366.139
204.0863
138.0548
168.0653
274.0918
292.1024
186.0758
126.0548
144.065
454.1566
528.192
657.2337
796.3464 PEP_0_0_0_0_0
880.3796 PEP_0_1*_0_0_0
999.425 PEP_0_1_0_0_0
1202.5099 PEP_0_2_0_0_0
1364.5609 PEP_1_2_0_0_0
1526.6111 PEP_2_2_0_0_0
1688.6647 PEP_3_2_0_0_0
1729.7085 PEP_2_3_0_0_0
1891.7408 PEP_3_3_0_0_0

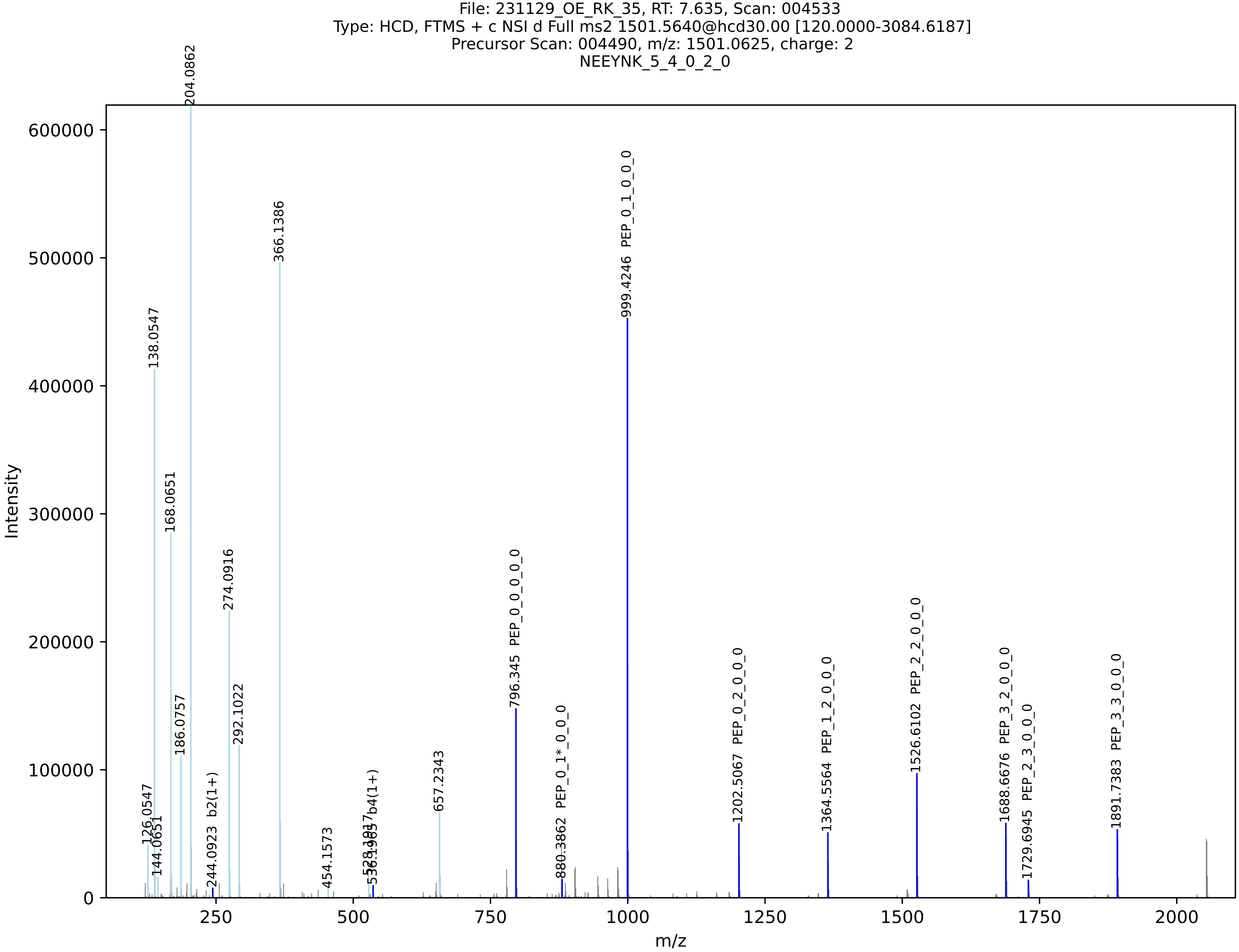

File: 231129_OE_RK_35, RT: 7.635, Scan: 004533
Type: HCD, FTMS + c NSI d Full ms2 1501.5640@hcd30.00 [120.0000-3084.6187]
Precursor Scan: 004490, m/z: 1501.0625, charge: 2
NEEYNK_5_4_0_2_0
Intensity
m/z
204.0862
366.1386
138.0547
168.0651
274.0916
292.1022
186.0757
126.0547
144.0651
244.0923 b2(1+)
454.1573
528.1917
536.1965 b4(1+)
657.2343
796.345 PEP_0_0_0_0_0
880.3862 PEP_0_1*_0_0_0
999.4246 PEP_0_1_0_0_0
1202.5067 PEP_0_2_0_0_0
1364.5564 PEP_1_2_0_0_0
1526.6102 PEP_2_2_0_0_0
1688.6676 PEP_3_2_0_0_0
1729.6945 PEP_2_3_0_0_0
1891.7383 PEP_3_3_0_0_0

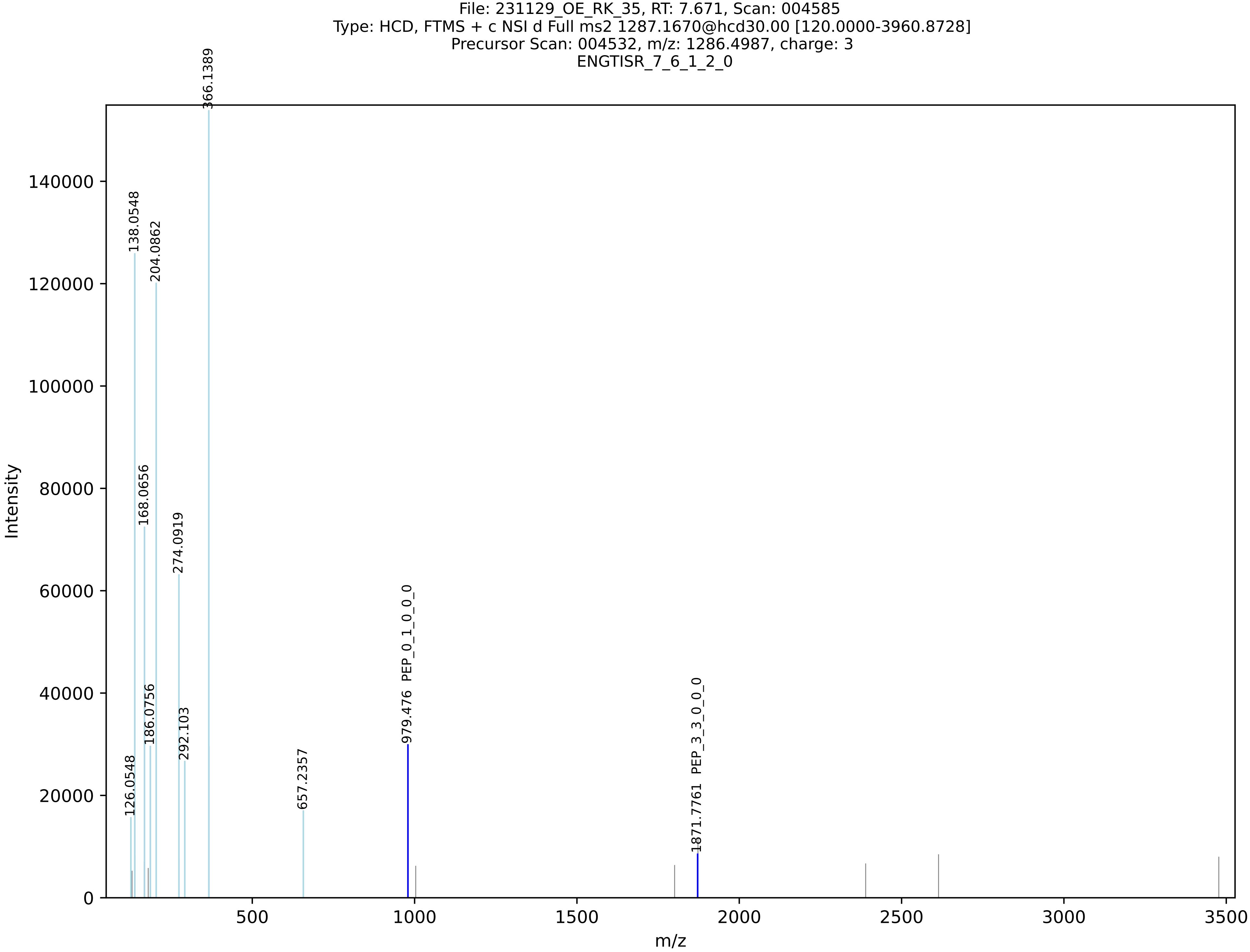
File: 231129_OE_RK_35, RT: 7.671, Scan: 004585
Type: HCD, FTMS + c NSI d Full ms2 1287.1670@hcd30.00 [120.0000-3960.8728]
Precursor Scan: 004532, m/z: 1286.4987, charge: 3
ENGTISR_7_6_1_2_0
Intensity
m/z
366.1389
138.0548
204.0862
168.0656
274.0919
979.476 PEP_0_1_0_0_0
186.0756
292.103
126.0548
657.2357
1871.7761 PEP_3_3_0_0_0
0
20000
40000
60000
80000
100000
120000
140000
500
1000
1500
2000
2500
3000
3500

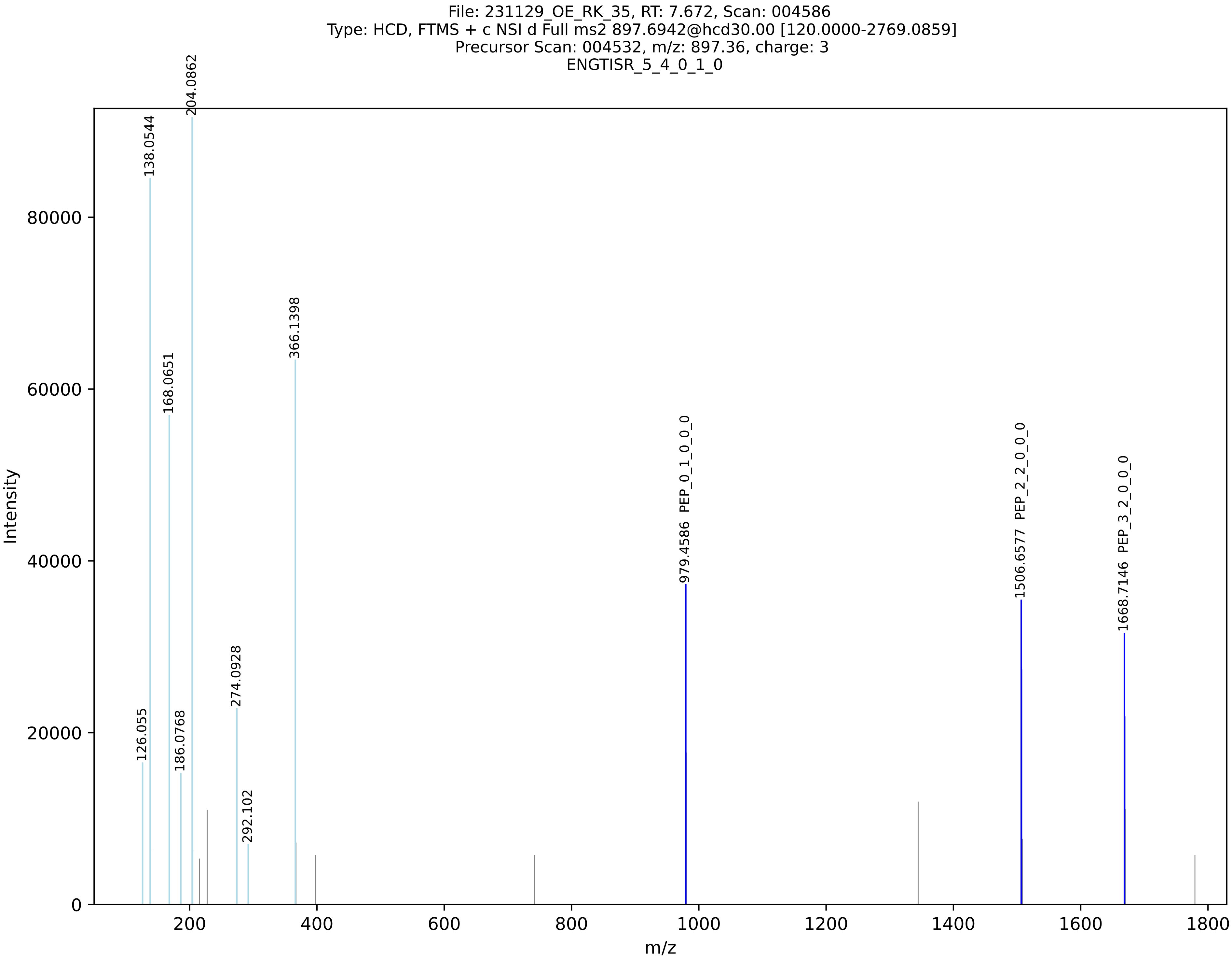

File: 231129_OE_RK_35, RT: 7.672, Scan: 004586
Type: HCD, FTMS + c NSI d Full ms2 897.6942@hcd30.00 [120.0000-2769.0859]
Precursor Scan: 004532, m/z: 897.36, charge: 3
ENGTISR_5_4_0_1_0
Intensity
m/z
126.055
138.0544
168.0651
186.0768
204.0862
274.0928
292.102
366.1398
979.4586 PEP_0_1_0_0_0
1506.6577 PEP_2_2_0_0_0
1668.7146 PEP_3_2_0_0_0
0
20000
40000
60000
80000
200
400
600
800
1000
1200
1400
1600
1800

File: 231129_OE_RK_35, RT: 7.725, Scan: 004663
Type: HCD, FTMS + c NSI d Full ms2 1050.0283@hcd30.00 [120.0000-3235.2285]
Precursor Scan: 004583, m/z: 1049.6955, charge: 3
HNSTGCLR_5_4_0_2_0

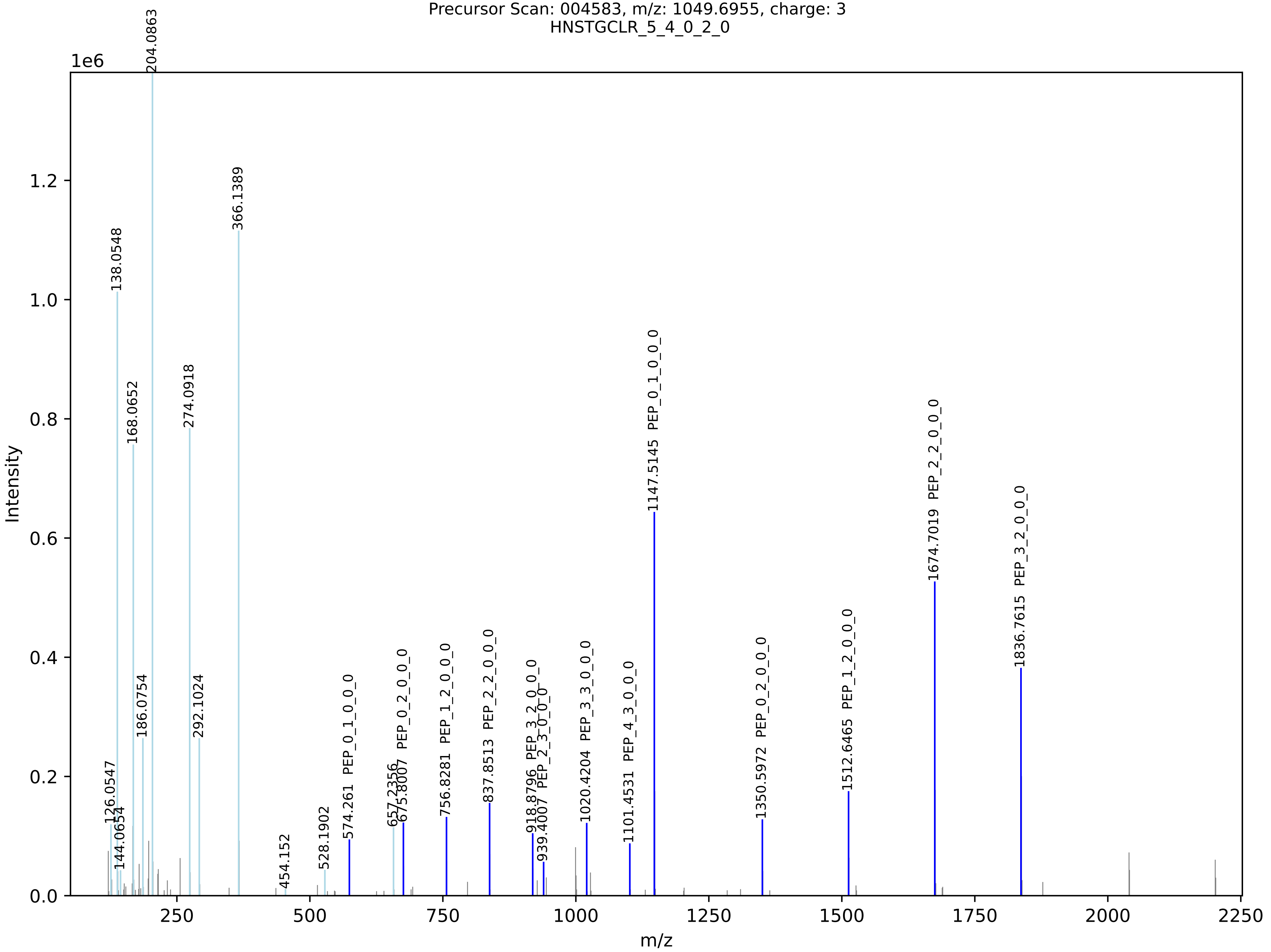

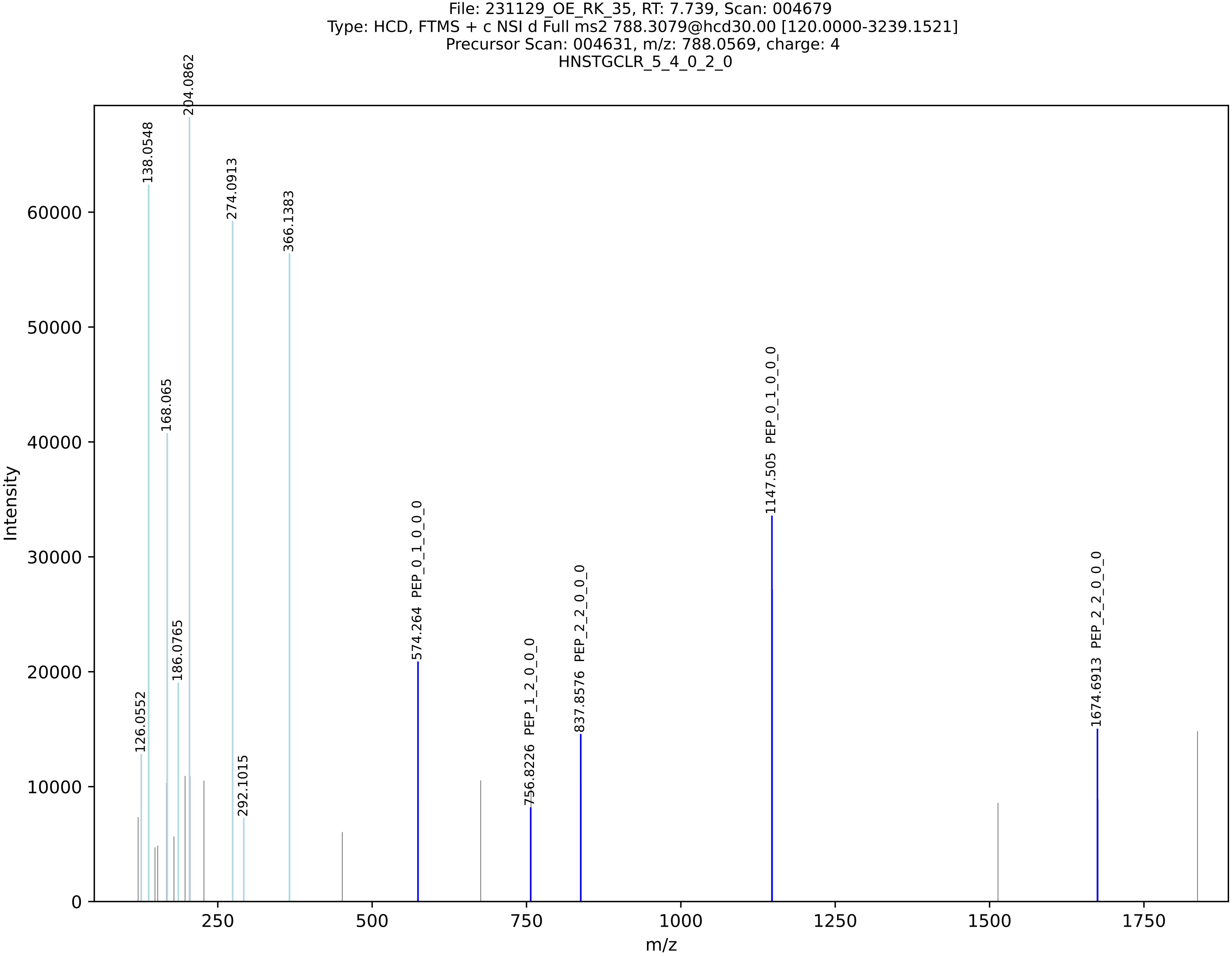
File: 231129_OE_RK_35, RT: 7.739, Scan: 004679
Type: HCD, FTMS + c NSI d Full ms2 788.3079@hcd30.00 [120.0000-3239.1521]
Precursor Scan: 004631, m/z: 788.0569, charge: 4
HNSTGCLR_5_4_0_2_0
Intensity
m/z
204.0862
138.0548
274.0913
366.1383
168.065
186.0765
126.0552
292.1015
574.264 PEP_0_1_0_0_0
756.8226 PEP_1_2_0_0_0
837.8576 PEP_2_2_0_0_0
1147.505 PEP_0_1_0_0_0
1674.6913 PEP_2_2_0_0_0

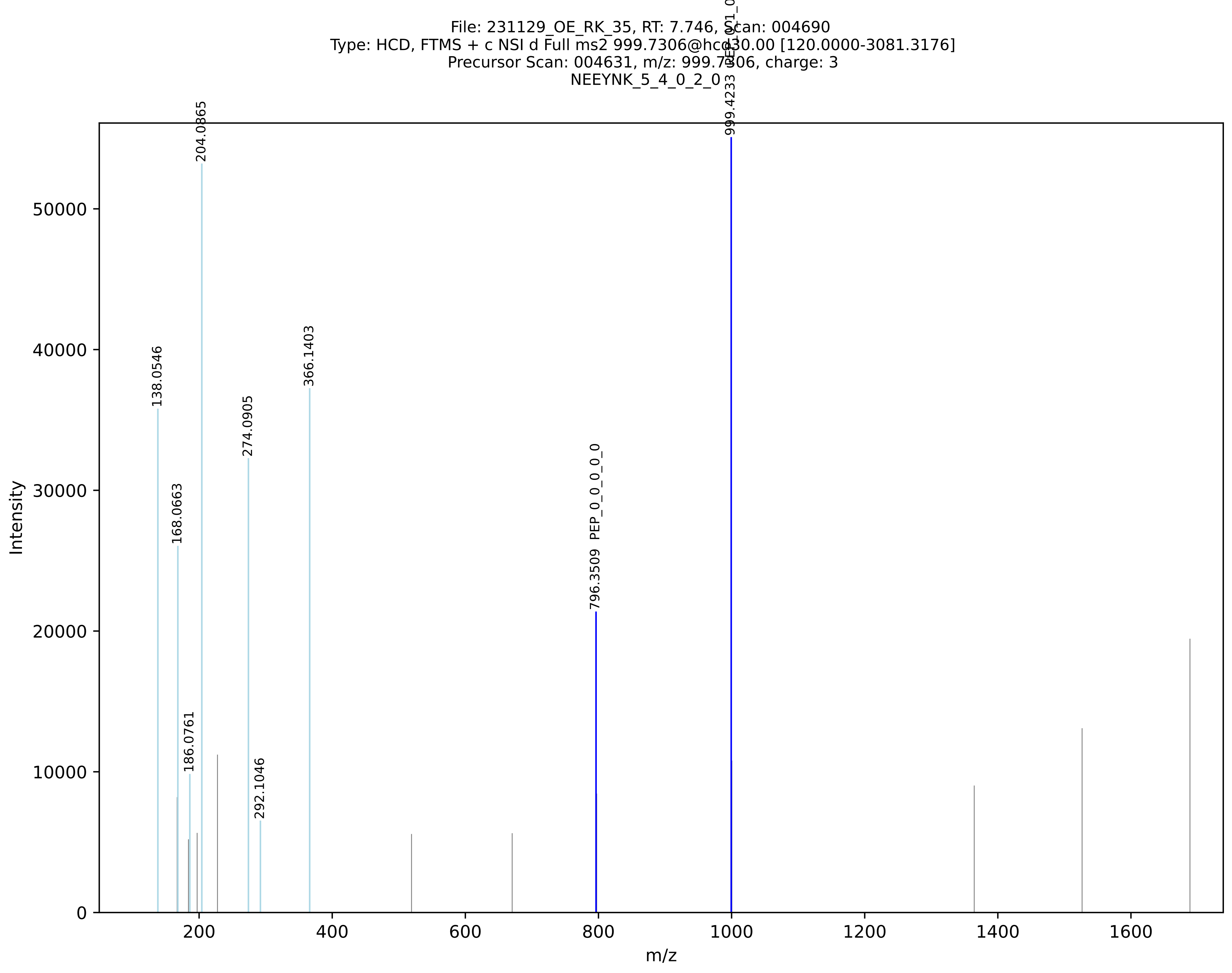
File: 231129_OE_RK_35, RT: 7.746, Scan: 004690
Type: HCD, FTMS + c NSI d Full ms2 999.7306@hcd30.00 [120.0000-3081.3176]
Precursor Scan: 004631, m/z: 999.7306, charge: 3
NEEYNK_5_4_0_2_0
Intensity
m/z
0
10000
20000
30000
40000
50000
200
400
600
800
1000
1200
1400
1600
138.0546
168.0663
186.0761
204.0865
274.0905
292.1046
366.1403
796.3509 PEP_0_0_0_0_0
999.4233

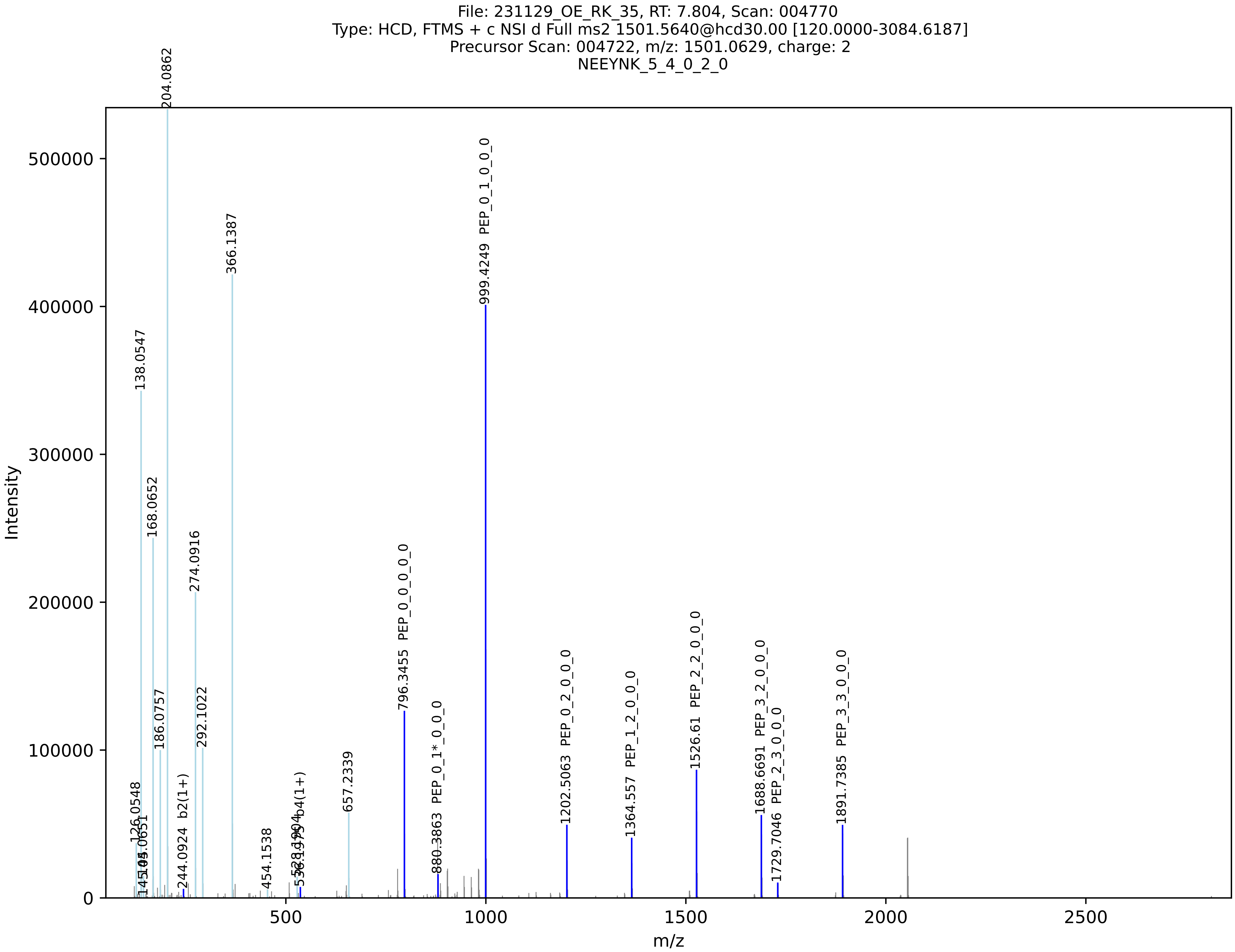
File: 231129_OE_RK_35, RT: 7.804, Scan: 004770
Type: HCD, FTMS + c NSI d Full ms2 1501.5640@hcd30.00 [120.0000-3084.6187]
Precursor Scan: 004722, m/z: 1501.0629, charge: 2
NEEYNK_5_4_0_2_0
Intensity
m/z
204.0862
366.1387
138.0547
168.0652
274.0916
186.0757
292.1022
126.0548
244.0924 b2(1+)
454.1538
536.1975 b4(1+)
657.2339
796.3455 PEP_0_0_0_0_0_
880.3863 PEP_0_1*_0_0_
999.4249 PEP_0_1_0_0_
1202.5063 PEP_0_2_0_0_
1364.557 PEP_1_2_0_0_
1526.61 PEP_2_2_0_0_
1688.6691 PEP_3_2_0_0_
1729.7046 PEP_2_3_0_0_
1891.7385 PEP_3_3_0_0_

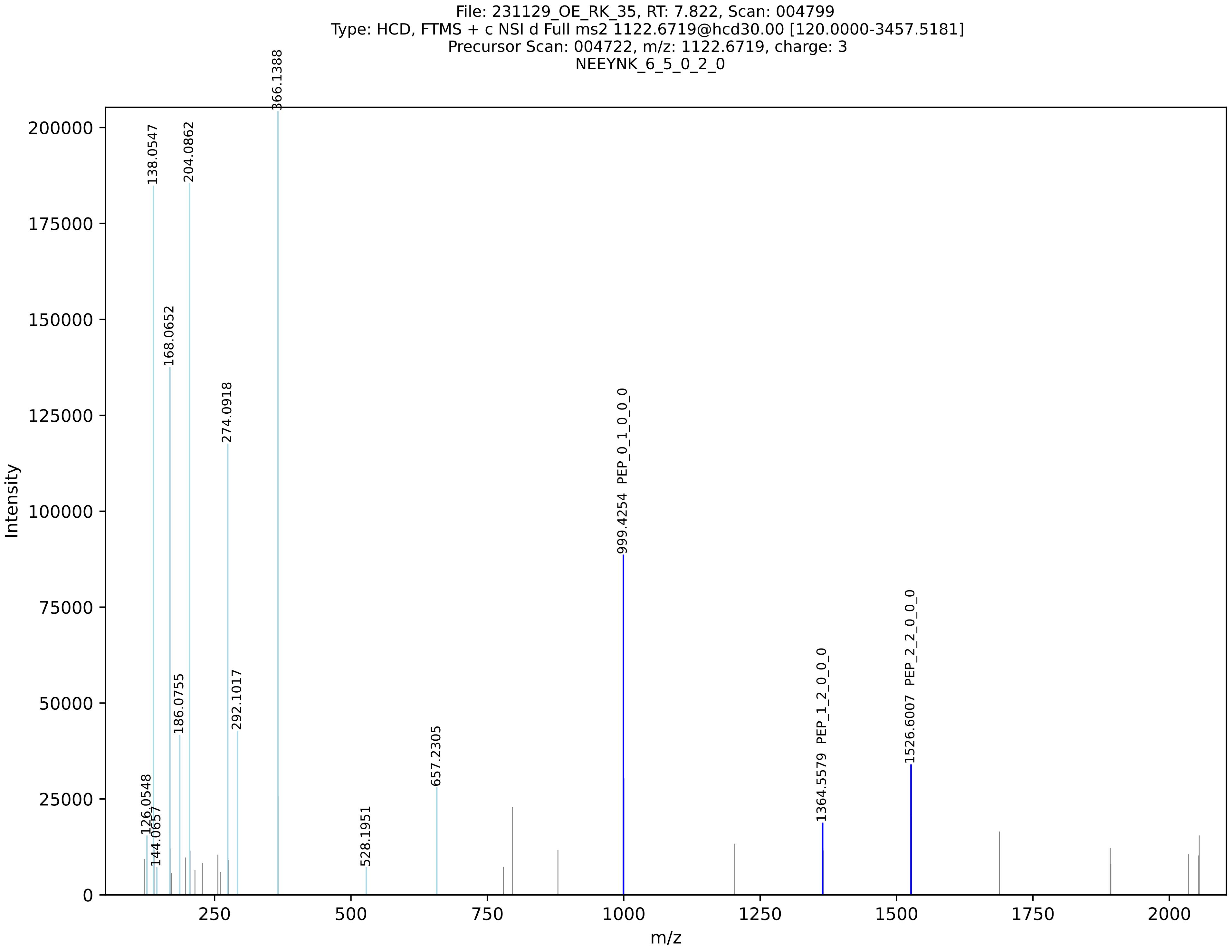

File: 231129_OE_RK_35, RT: 7.822, Scan: 004799
Type: HCD, FTMS + c NSI d Full ms2 1122.6719@hcd30.00 [120.0000-3457.5181]
Precursor Scan: 004722, m/z: 1122.6719, charge: 3
NEEYNK_6_5_0_2_0
Intensity
m/z
366.1388
138.0547
204.0862
168.0652
274.0918
186.0755
292.1017
126.0548
144.0657
528.1951
657.2305
999.4254 PEP_0_1_0_0_0
1364.5579 PEP_1_2_0_0_0
1526.6007 PEP_2_2_0_0_0

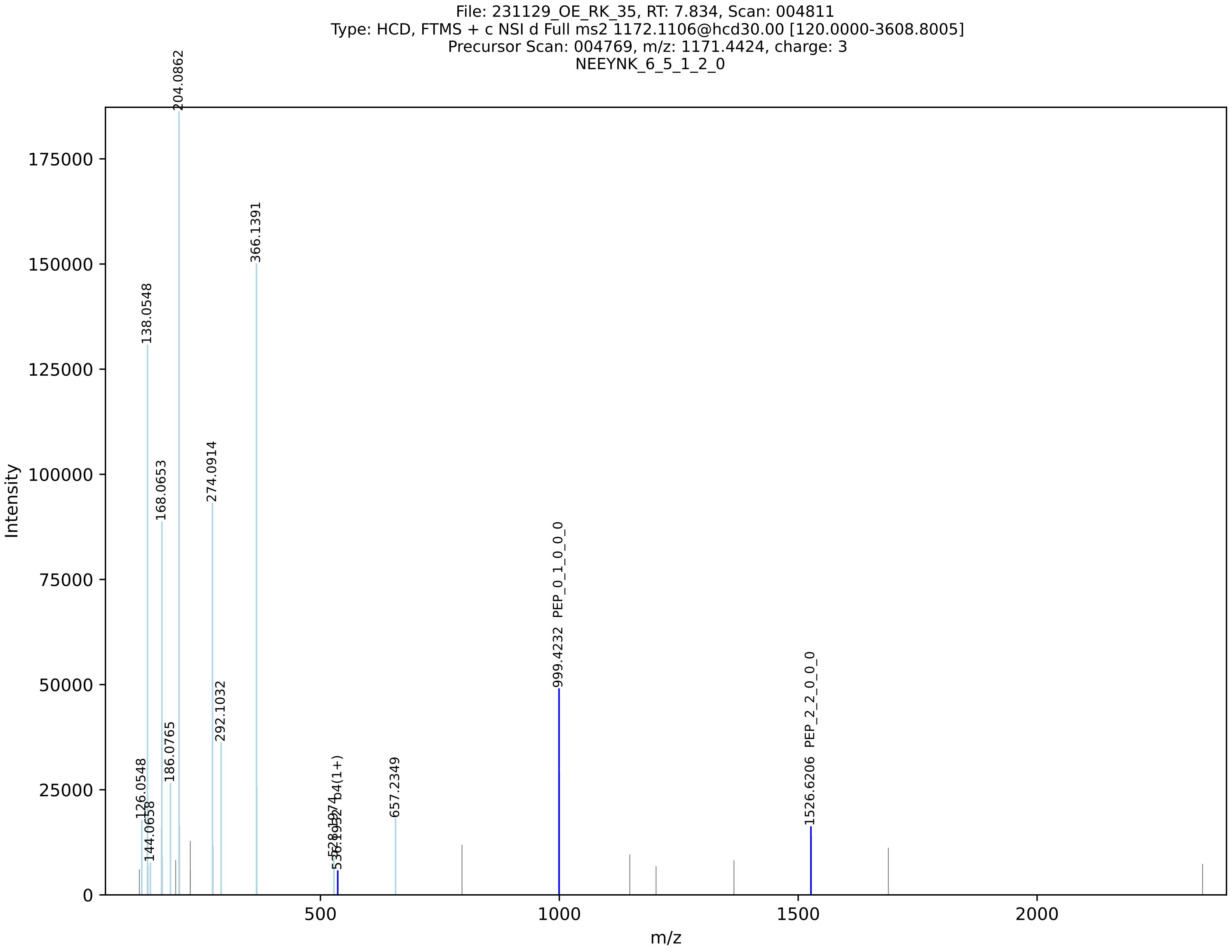
File: 231129_OE_RK_35, RT: 7.834, Scan: 004811
Type: HCD, FTMS + c NSI d Full ms2 1172.1106@hcd30.00 [120.0000-3608.8005]
Precursor Scan: 004769, m/z: 1171.4424, charge: 3
NEEYNK_6_5_1_2_0
Intensity
m/z
204.0862
366.1391
138.0548
274.0914
168.0653
292.1032
186.0765
126.0548
144.0658
528.1974
536.1932 b4(1+)
657.2349
999.4232 PEP_0_1_0_0_0_
1526.6206 PEP_2_2_0_0_0_
175000
150000
125000
100000
75000
50000
25000
0
500
1000
1500
2000

File: 231129_OE_RK_35, RT: 7.861, Scan: 004848
Type: HCD, FTMS + c NSI d Full ms2 1214.1421@hcd30.00 [120.0000-3737.4165]
Precursor Scan: 004807, m/z: 1213.4738, charge: 3
ENGTISR_6_5_0_3_0

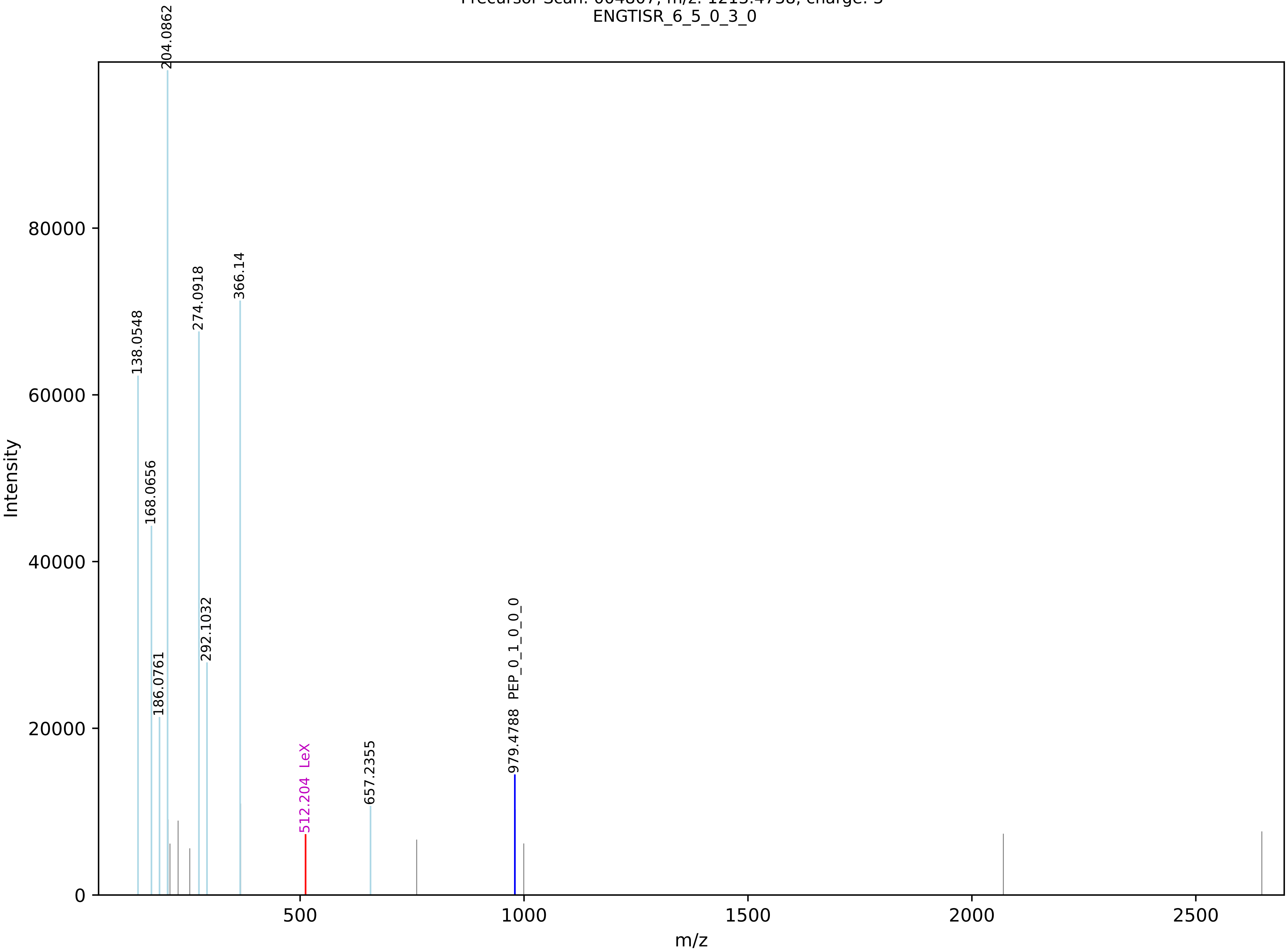

File: 231129_OE_RK_35, RT: 7.95, Scan: 004968
Type: HCD, FTMS + c NSI d Full ms2 1384.2010@hcd30.00 [120.0000-4257.7974]
Precursor Scan: 004919, m/z: 1383.5329, charge: 3
ENGTISR_7_6_1_3_0

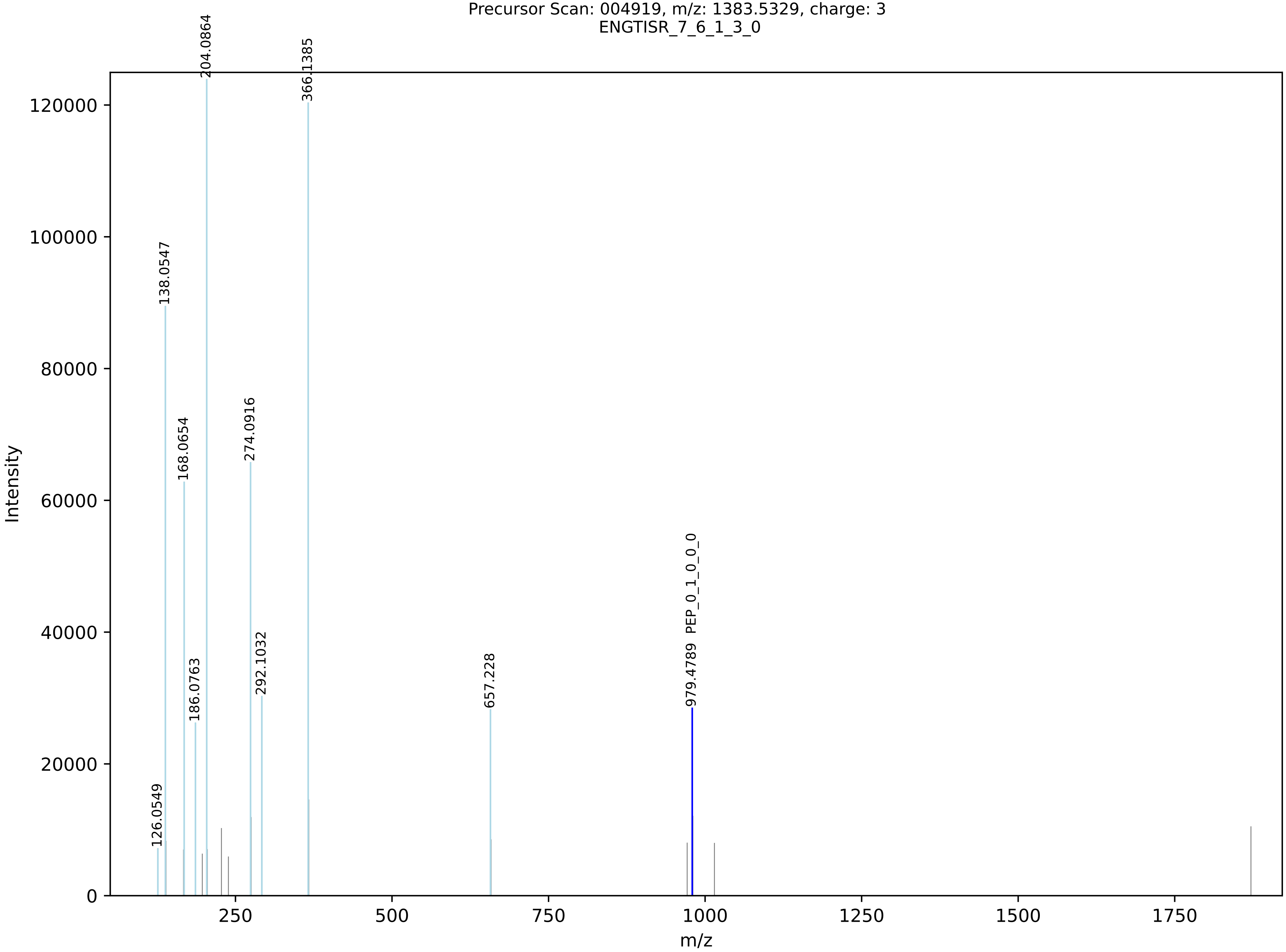

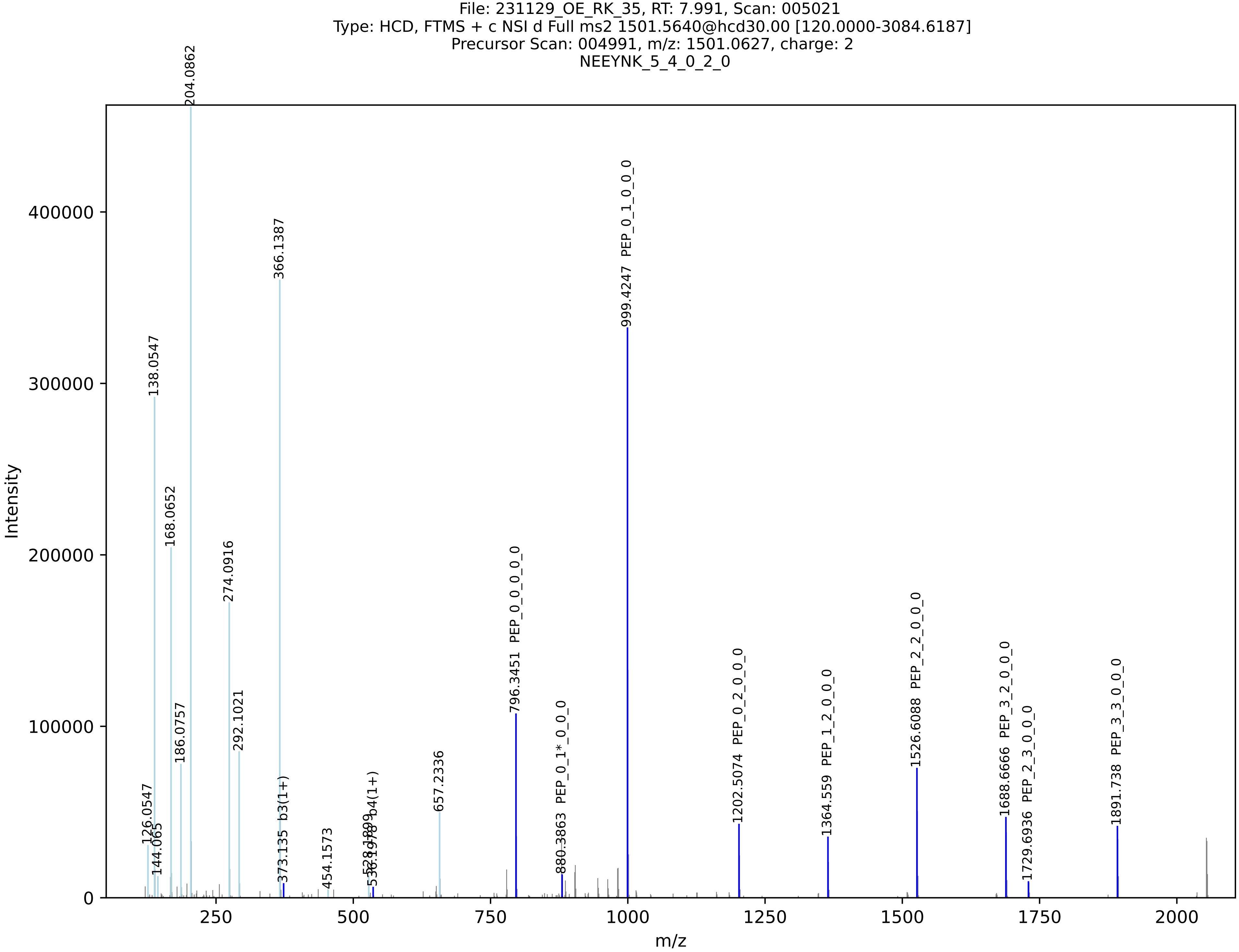
File: 231129_OE_RK_35, RT: 7.991, Scan: 005021
Type: HCD, FTMS + c NSI d Full ms2 1501.5640@hcd30.00 [120.0000-3084.6187]
Precursor Scan: 004991, m/z: 1501.0627, charge: 2
NEEYNK_5_4_0_2_0
Intensity
m/z
204.0862
366.1387
138.0547
168.0652
274.0916
292.1021
186.0757
126.0547
144.065
373.135 b3(1+)
454.1573
657.2336
796.3451 PEP_0_0_0_0_0
880.3863 PEP_0_1*_0_0_0
999.4247 PEP_0_1_0_0_0
1202.5074 PEP_0_2_0_0_0
1364.559 PEP_1_2_0_0_0
1526.6088 PEP_2_2_0_0_0
1688.6666 PEP_3_2_0_0_0
1729.6936 PEP_2_3_0_0_0
1891.738 PEP_3_3_0_0_0

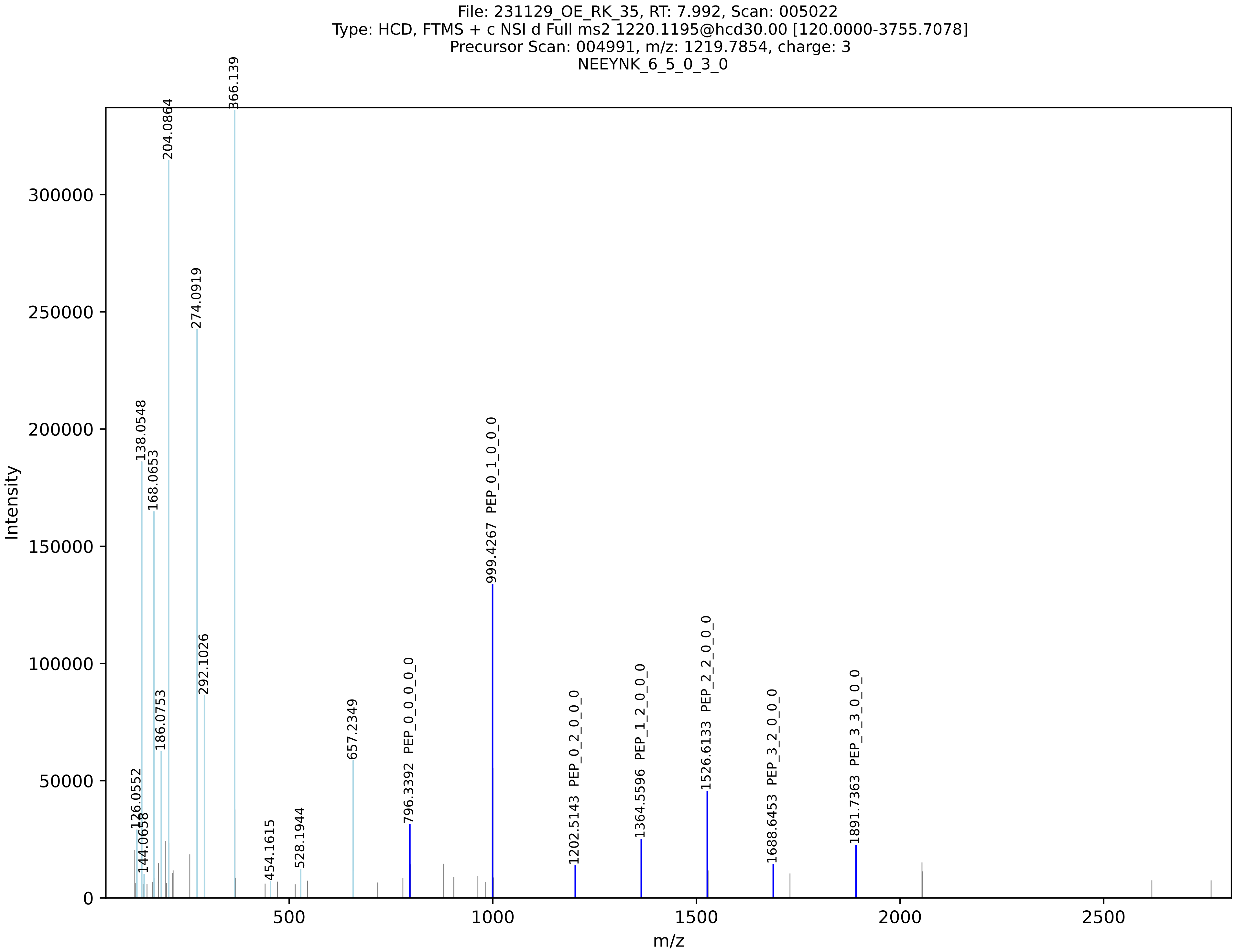
File: 231129_OE_RK_35, RT: 7.992, Scan: 005022
Type: HCD, FTMS + c NSI d Full ms2 1220.1195@hcd30.00 [120.0000-3755.7078]
Precursor Scan: 004991, m/z: 1219.7854, charge: 3
NEEYNK_6_5_0_3_0
Intensity
m/z
366.139
204.0864
274.0919
138.0548
168.0653
292.1026
186.0753
126.0552
144.0658
454.1615
528.1944
657.2349
796.3392 PEP_0_0_0_0_0
999.4267 PEP_0_1_0_0_0
1202.5143 PEP_0_2_0_0_0
1364.5596 PEP_1_2_0_0_0
1526.6133 PEP_2_2_0_0_0
1688.6453 PEP_3_2_0_0_0
1891.7363 PEP_3_3_0_0_0

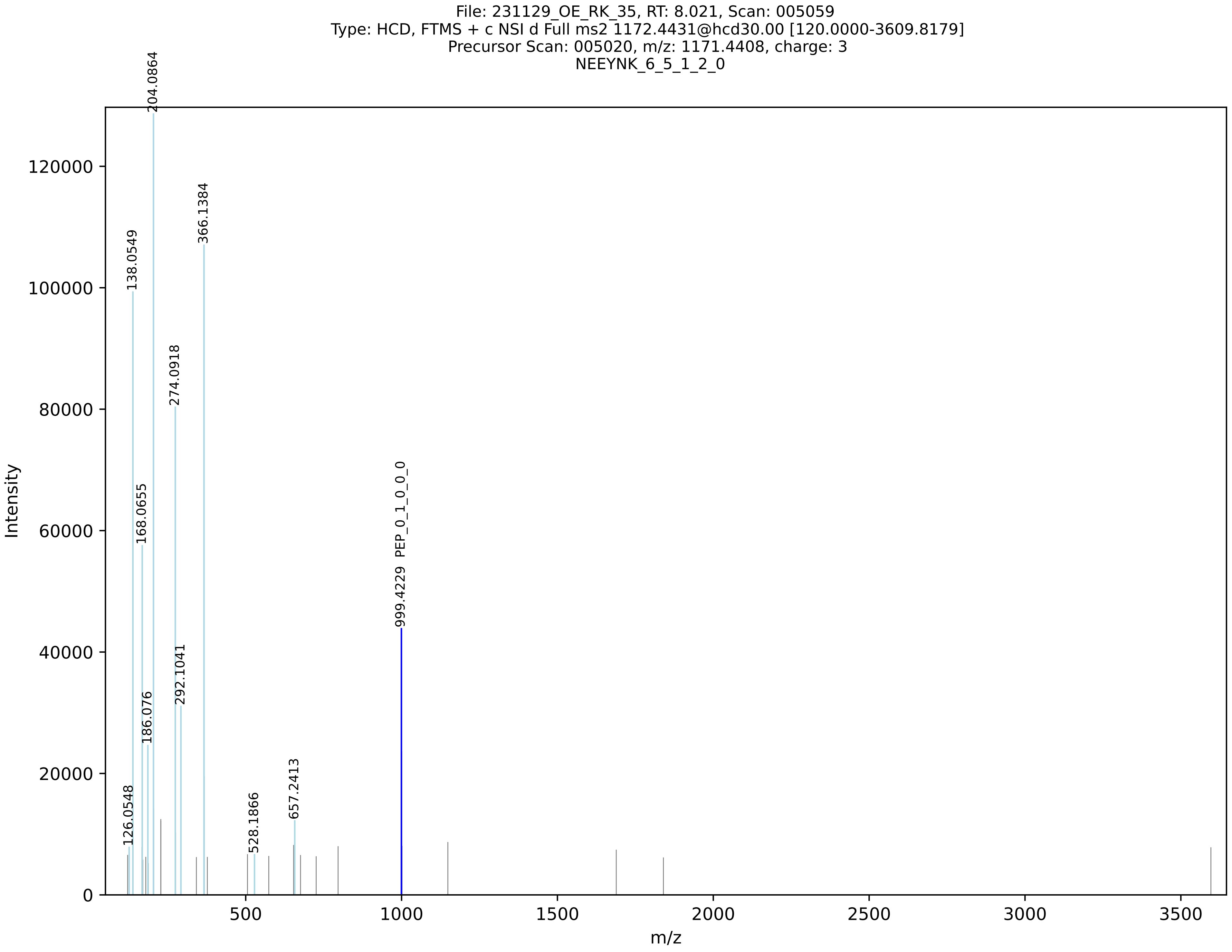
File: 231129_OE_RK_35, RT: 8.021, Scan: 005059
Type: HCD, FTMS + c NSI d Full ms2 1172.4431@hcd30.00 [120.0000-3609.8179]
Precursor Scan: 005020, m/z: 1171.4408, charge: 3
NEEYNK_6_5_1_2_0
204.0864
366.1384
138.0549
274.0918
168.0655
999.4229 PEP_0_1_0_0_0
292.1041
186.076
657.2413
528.1866
126.0548
Intensity
m/z
0
20000
40000
60000
80000
100000
120000
500
1000
1500
2000
2500
3000
3500

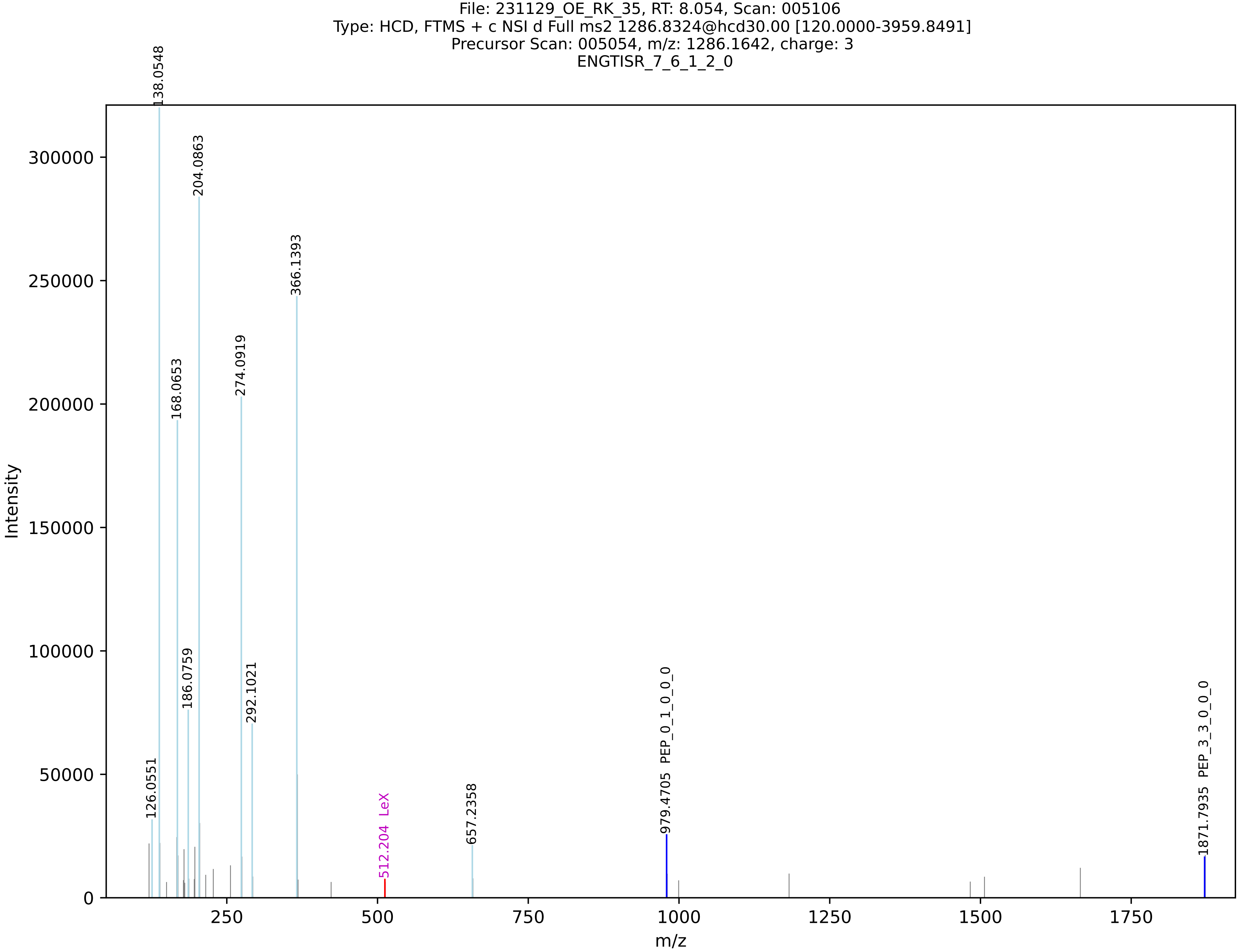

File: 231129_OE_RK_35, RT: 8.054, Scan: 005106
Type: HCD, FTMS + c NSI d Full ms2 1286.8324@hcd30.00 [120.0000-3959.8491]
Precursor Scan: 005054, m/z: 1286.1642, charge: 3
ENGTISR_7_6_1_2_0
138.0548
204.0863
366.1393
274.0919
168.0653
186.0759
292.1021
126.0551
512.204 LeX
657.2358
979.4705 PEP_0_1_0_0_0
1871.7935 PEP_3_3_0_0_0
Intensity
m/z
0
50000
100000
150000
200000
250000
300000
250
500
750
1000
1250
1500
1750

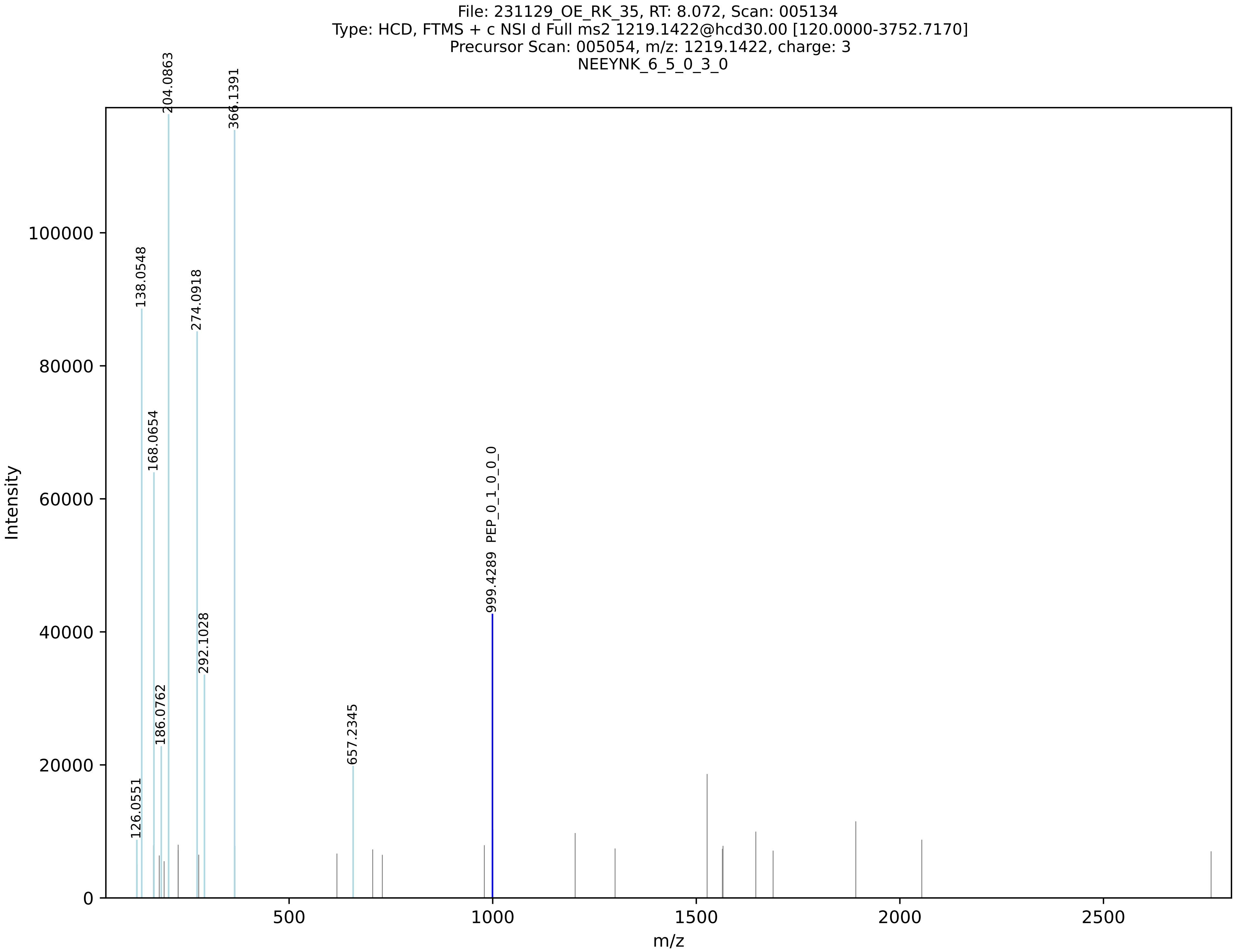
File: 231129_OE_RK_35, RT: 8.072, Scan: 005134
Type: HCD, FTMS + c NSI d Full ms2 1219.1422@hcd30.00 [120.0000-3752.7170]
Precursor Scan: 005054, m/z: 1219.1422, charge: 3
NEEYNK_6_5_0_3_0
204.0863
366.1391
138.0548
274.0918
168.0654
292.1028
186.0762
126.0551
657.2345
999.4289 PEP_0_1_0_0_0
Intensity
m/z
0
20000
40000
60000
80000
100000
500
1000
1500
2000
2500

File: 231129_OE_RK_35, RT: 8.088, Scan: 005153
Type: HCD, FTMS + c NSI d Full ms2 994.3922@hcd30.00 [120.0000-3064.9819]
Precursor Scan: 005102, m/z: 994.3921, charge: 3
ENGTISR_5_4_0_2_0

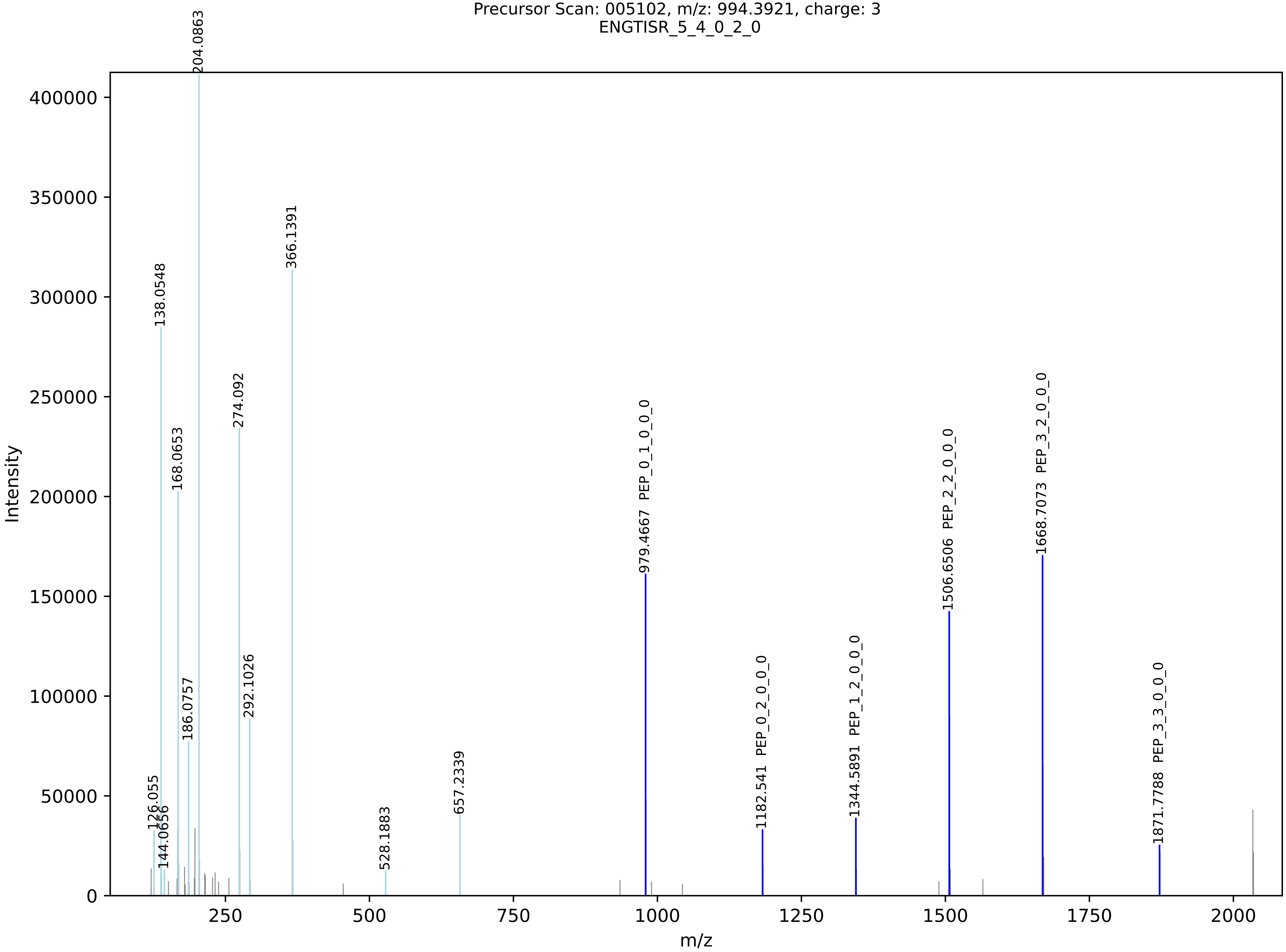

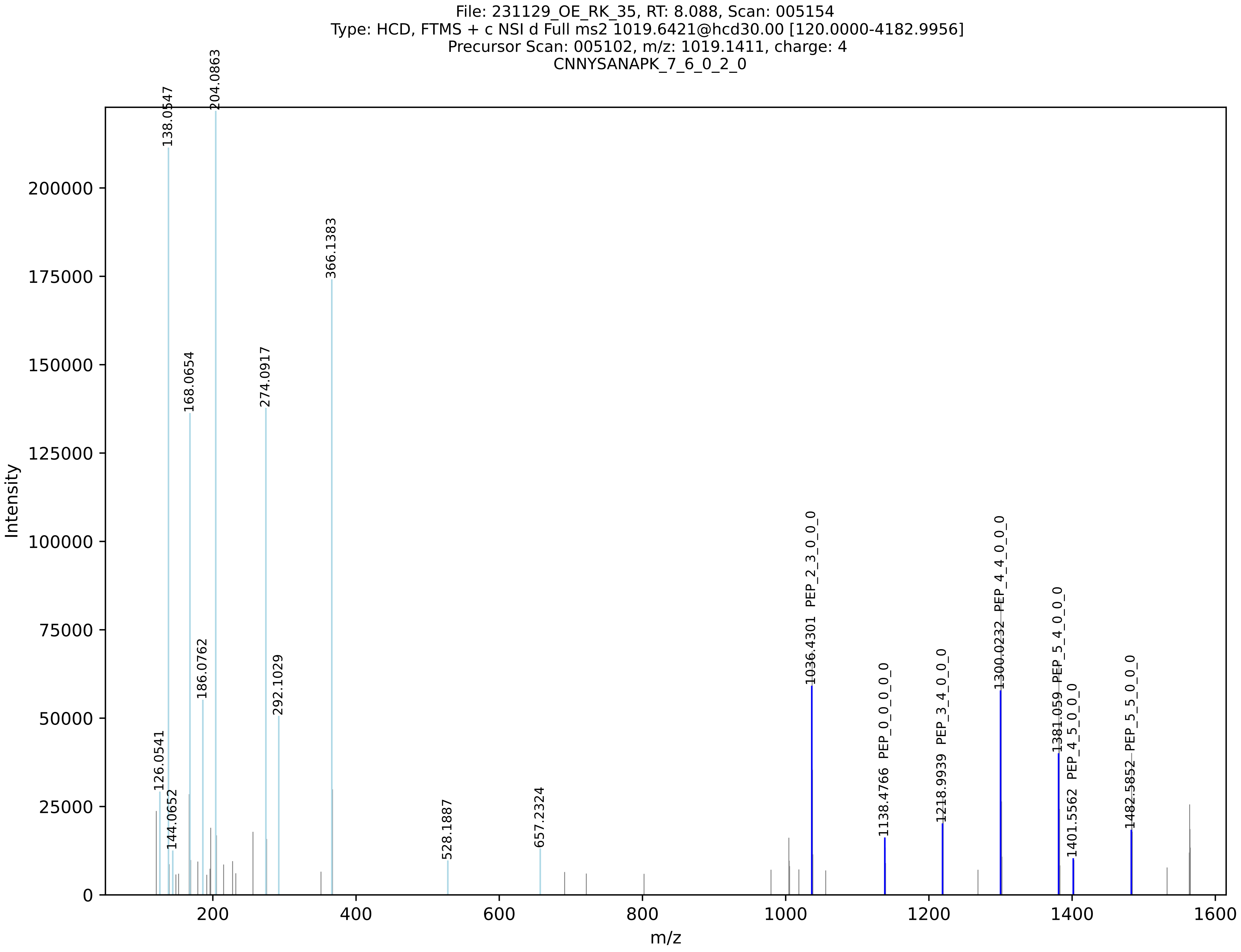
File: 231129_OE_RK_35, RT: 8.088, Scan: 005154
Type: HCD, FTMS + c NSI d Full ms2 1019.6421@hcd30.00 [120.0000-4182.9956]
Precursor Scan: 005102, m/z: 1019.1411, charge: 4
CNNYSANAPK_7_6_0_2_0
Intensity
m/z
138.0547
204.0863
168.0654
274.0917
366.1383
186.0762
292.1029
126.0541
144.0652
528.1887
657.2324
1036.4301 PEP_2_3_0_0_0
1138.4766 PEP_0_0_0_0_0
1218.9939 PEP_3_4_0_0_0
1300.0232 PEP_4_4_0_0_0
1381.059 PEP_5_4_0_0_0
1401.5562 PEP_4_5_0_0_0
1482.5852 PEP_5_5_0_0_0

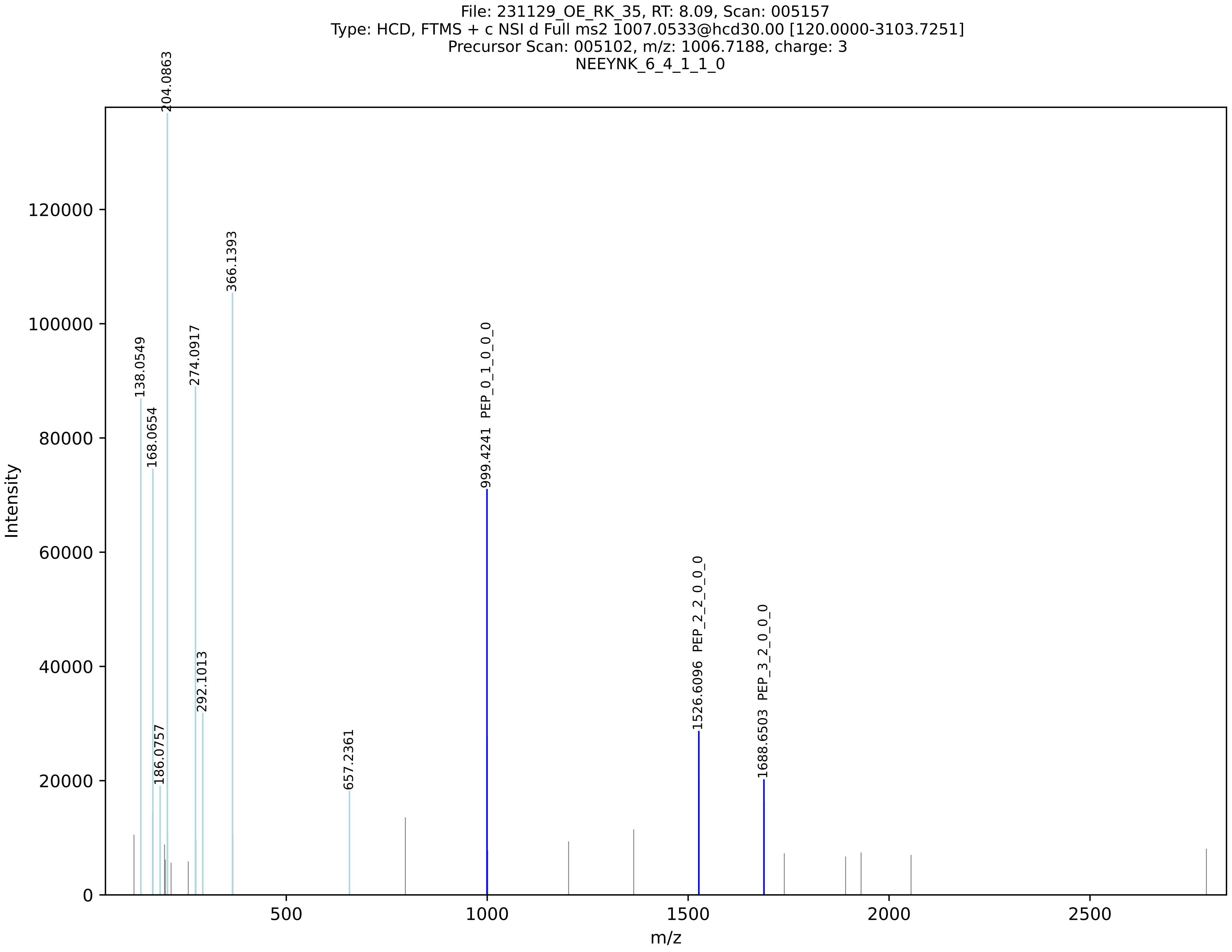
File: 231129_OE_RK_35, RT: 8.09, Scan: 005157
Type: HCD, FTMS + c NSI d Full ms2 1007.0533@hcd30.00 [120.0000-3103.7251]
Precursor Scan: 005102, m/z: 1006.7188, charge: 3
NEEYNK_6_4_1_1_0
204.0863
366.1393
138.0549
274.0917
168.0654
999.4241 PEP_0_1_0_0_0
1526.6096 PEP_2_2_0_0_0
1688.6503 PEP_3_2_0_0_0
292.1013
186.0757
657.2361
Intensity
m/z
0
20000
40000
60000
80000
100000
120000
500
1000
1500
2000
2500

File: 231129_OE_RK_35, RT: 8.12, Scan: 005196
Type: HCD, FTMS + c NSI d Full ms2 1116.4368@hcd30.00 [120.0000-3438.4385]
Precursor Scan: 005151, m/z: 1116.1026, charge: 3
ENGTISR_6_5_0_2_0

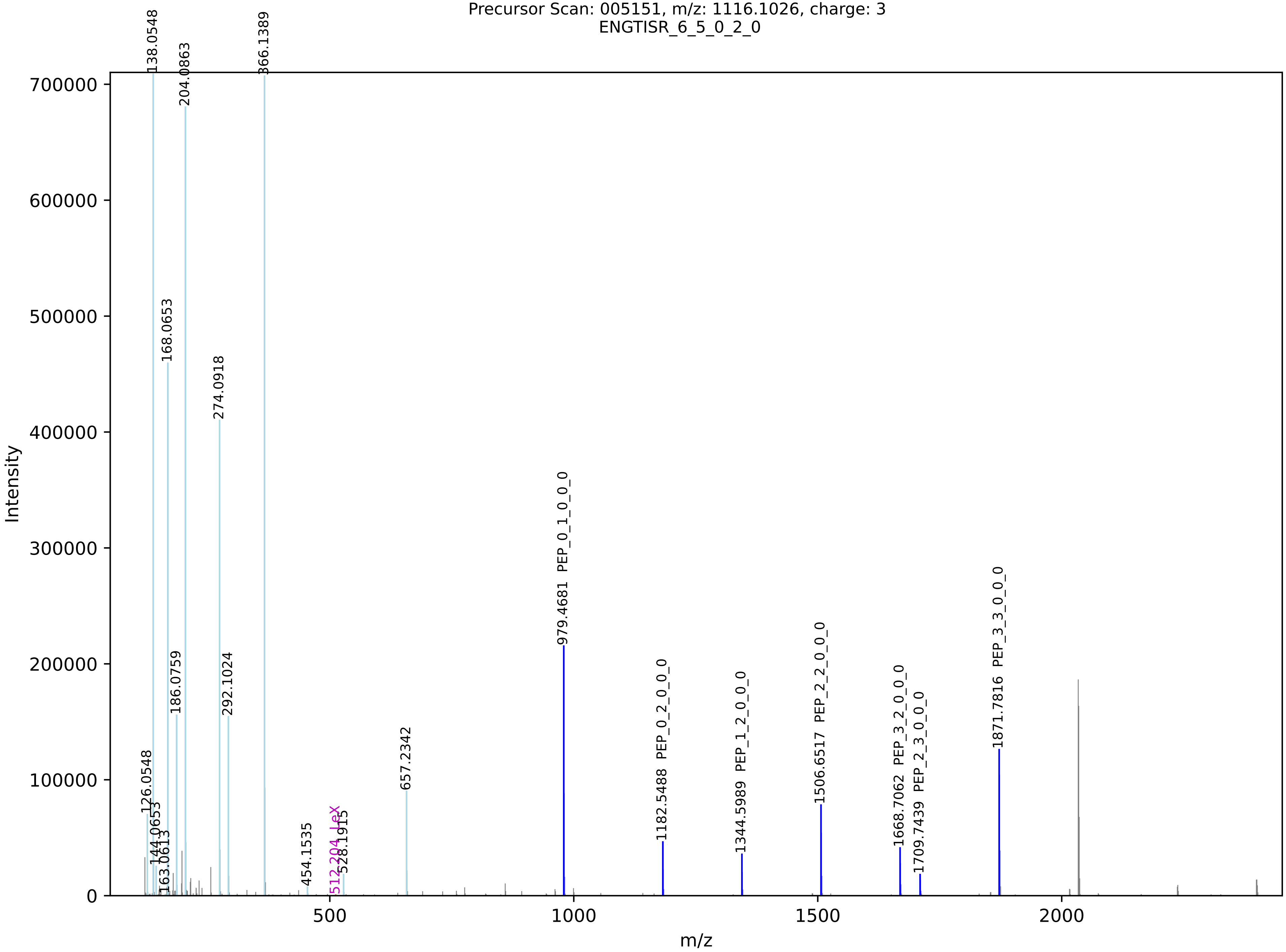

File: 231129_OE_RK_35, RT: 8.169, Scan: 005266
Type: HCD, FTMS + c NSI d Full ms2 1220.4547@hcd30.00 [120.0000-3756.7334]
Precursor Scan: 005195, m/z: 1219.7865, charge: 3
NEEYNK_6_5_0_3_0

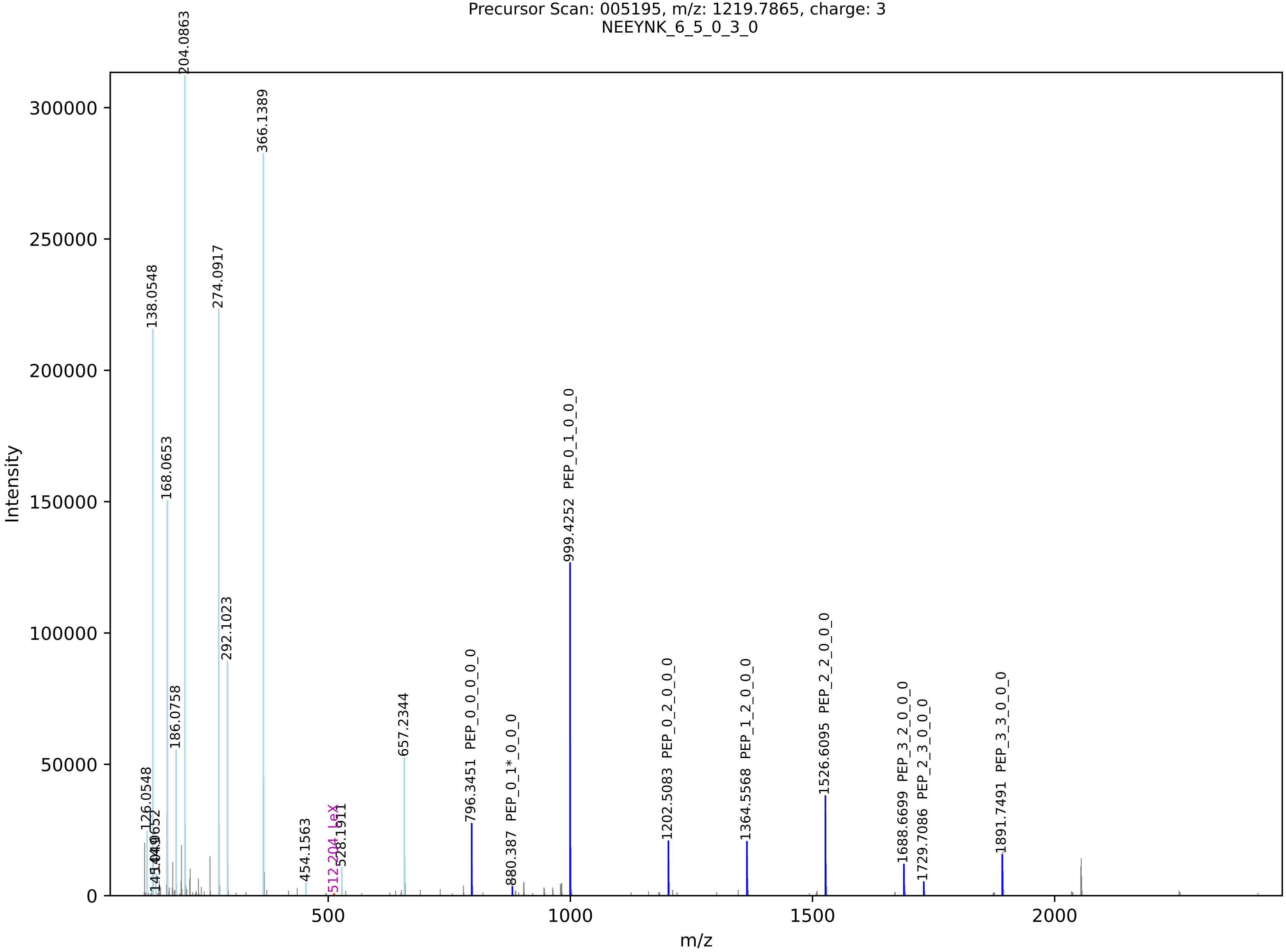

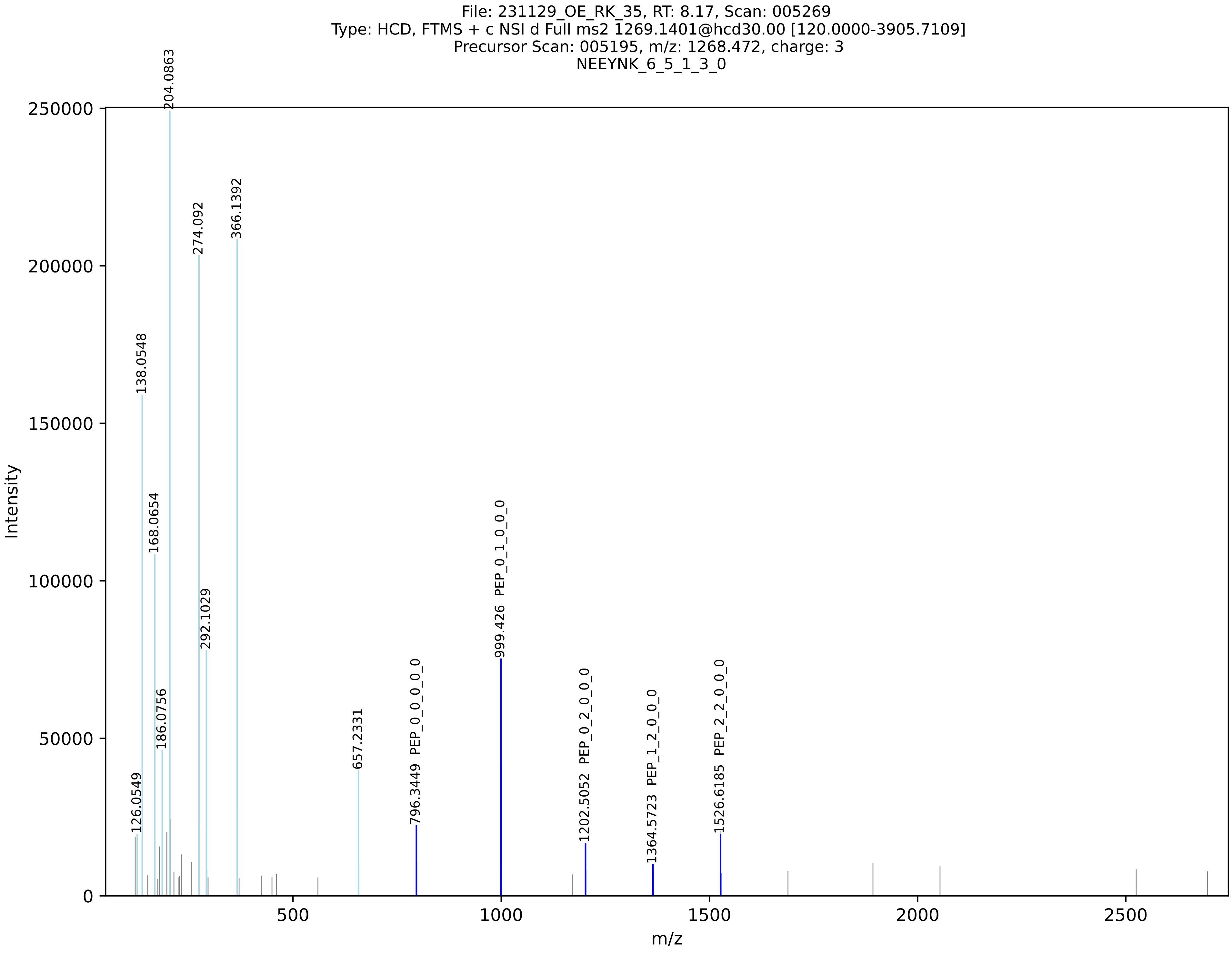
File: 231129_OE_RK_35, RT: 8.17, Scan: 005269
Type: HCD, FTMS + c NSI d Full ms2 1269.1401@hcd30.00 [120.0000-3905.7109]
Precursor Scan: 005195, m/z: 1268.472, charge: 3
NEEYNK_6_5_1_3_0
Intensity
m/z
0
50000
100000
150000
200000
250000
500
1000
1500
2000
2500
204.0863
366.1392
274.092
138.0548
168.0654
292.1029
186.0756
126.0549
657.2331
796.3449 PEP_0_0_0_0_0
999.426 PEP_0_1_0_0_0
1202.5052 PEP_0_2_0_0_0
1364.5723 PEP_1_2_0_0_0
1526.6185 PEP_2_2_0_0_0

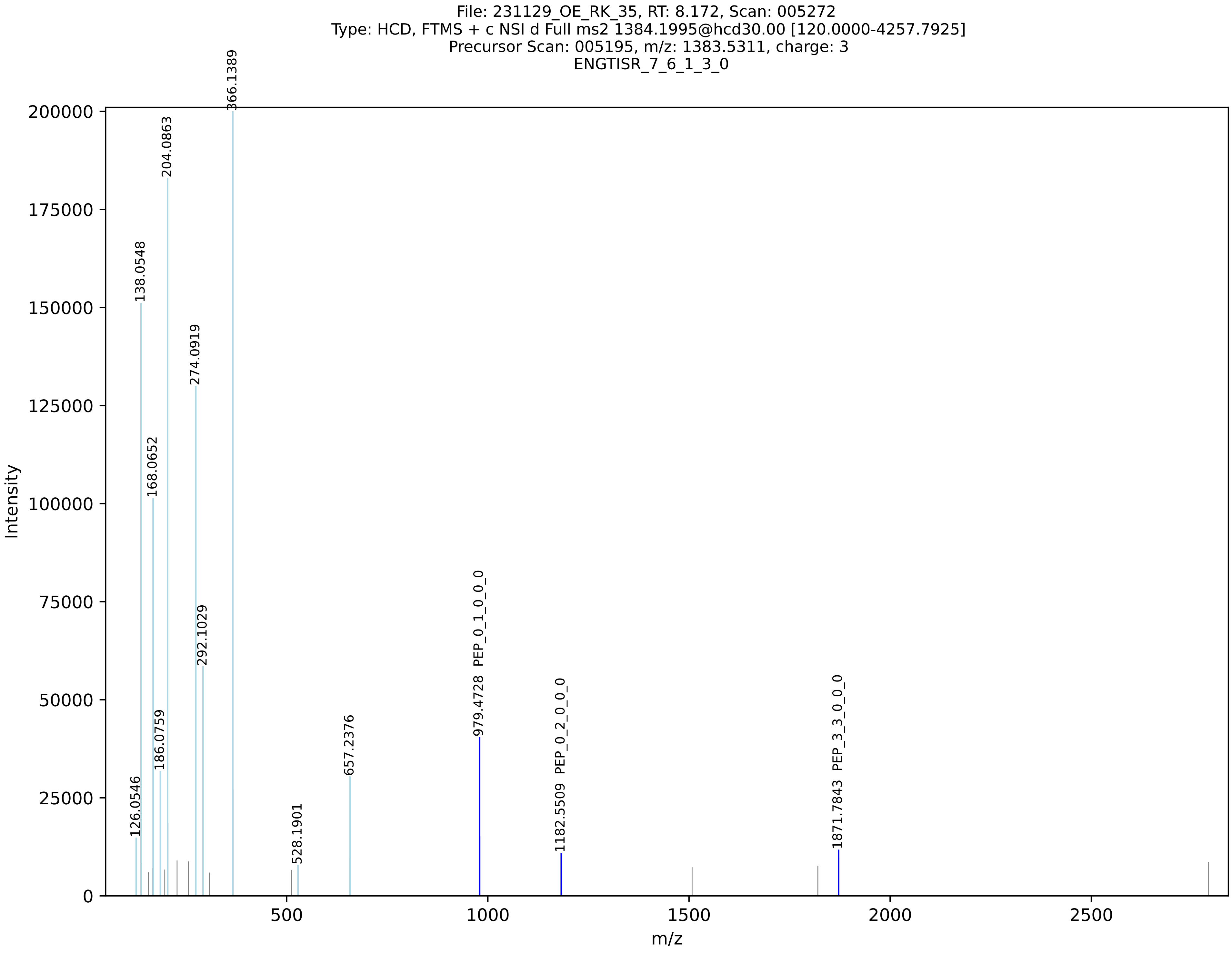
File: 231129_OE_RK_35, RT: 8.172, Scan: 005272
Type: HCD, FTMS + c NSI d Full ms2 1384.1995@hcd30.00 [120.0000-4257.7925]
Precursor Scan: 005195, m/z: 1383.5311, charge: 3
ENGTISR_7_6_1_3_0
Intensity
m/z
366.1389
204.0863
138.0548
274.0919
168.0652
292.1029
186.0759
126.0546
528.1901
657.2376
979.4728 PEP_0_1_0_0_0
1182.5509 PEP_0_2_0_0_0
1871.7843 PEP_3_3_0_0_0
0
25000
50000
75000
100000
125000
150000
175000
200000
500
1000
1500
2000
2500

File: 231129_OE_RK_35, RT: 8.173, Scan: 005273
Type: HCD, FTMS + c NSI d Full ms2 888.7036@hcd30.00 [120.0000-2741.5750]
Precursor Scan: 005195, m/z: 888.3694, charge: 3
YKNNSDISSTR_6_2_0_0_0

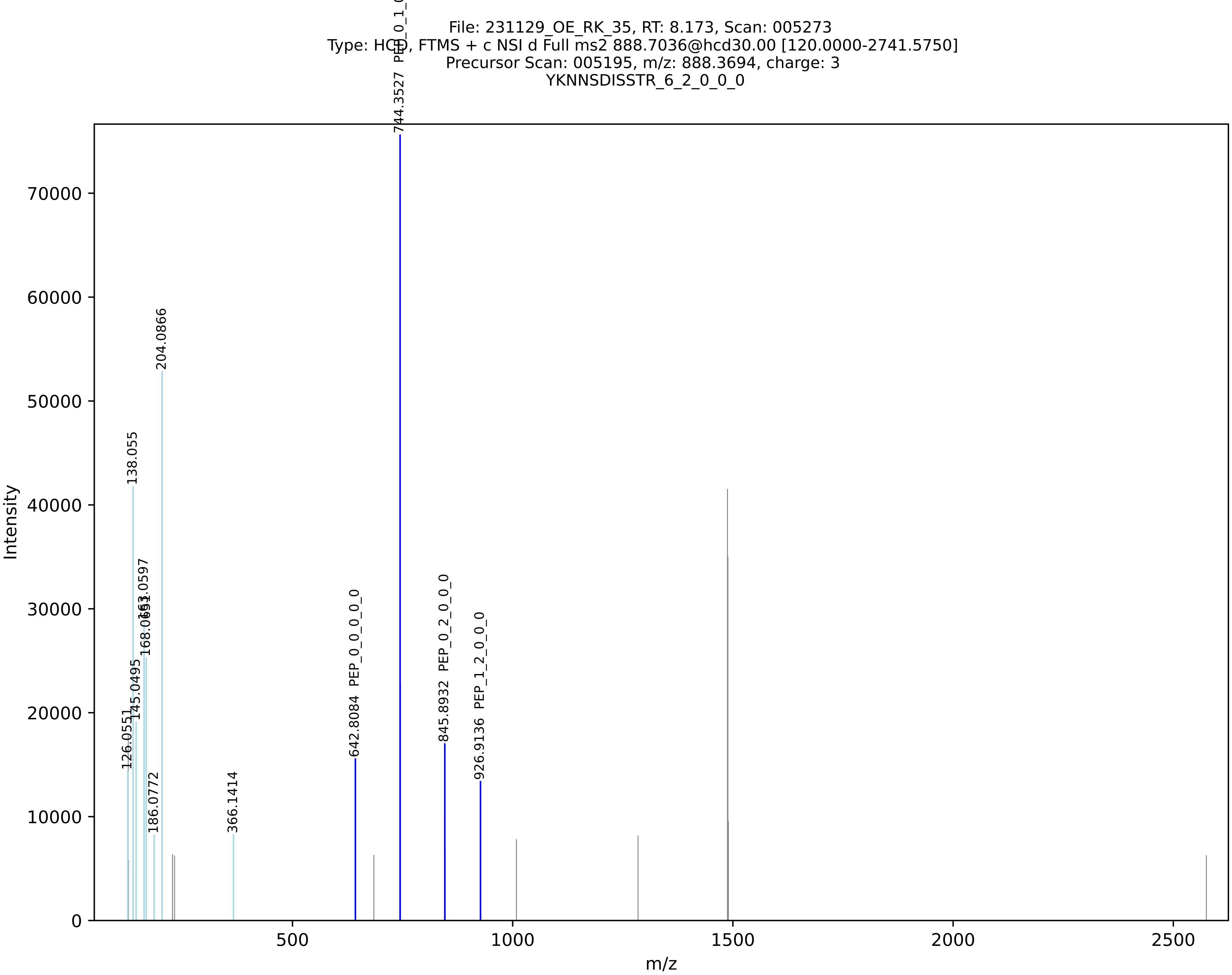

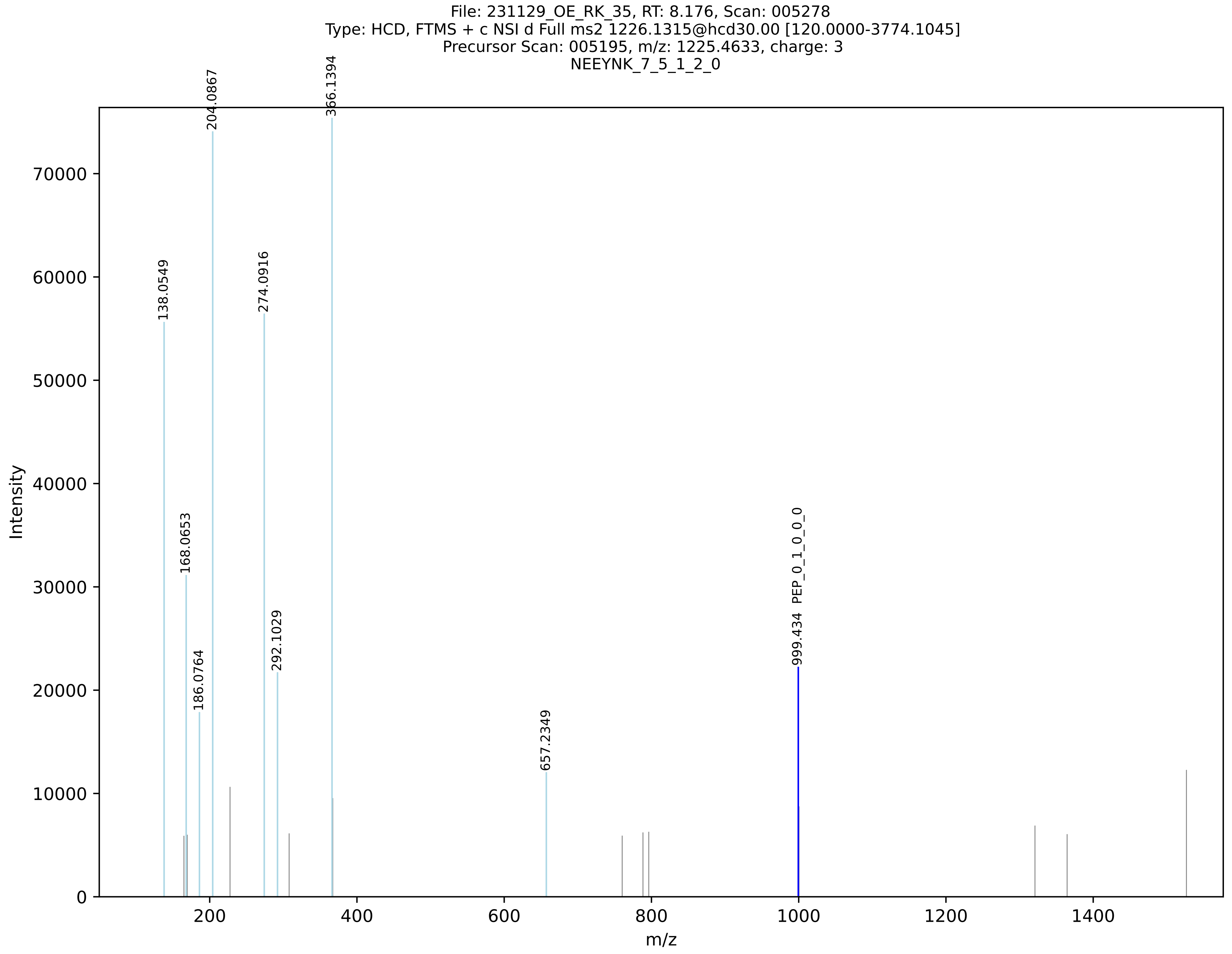

File: 231129_OE_RK_35, RT: 8.176, Scan: 005278
Type: HCD, FTMS + c NSI d Full ms2 1226.1315@hcd30.00 [120.0000-3774.1045]
Precursor Scan: 005195, m/z: 1225.4633, charge: 3
NEEYNK_7_5_1_2_0
Intensity
m/z
0
10000
20000
30000
40000
50000
60000
70000
200
400
600
800
1000
1200
1400
138.0549
168.0653
186.0764
204.0867
274.0916
292.1029
366.1394
657.2349
999.434 PEP_0_1_0_0_0

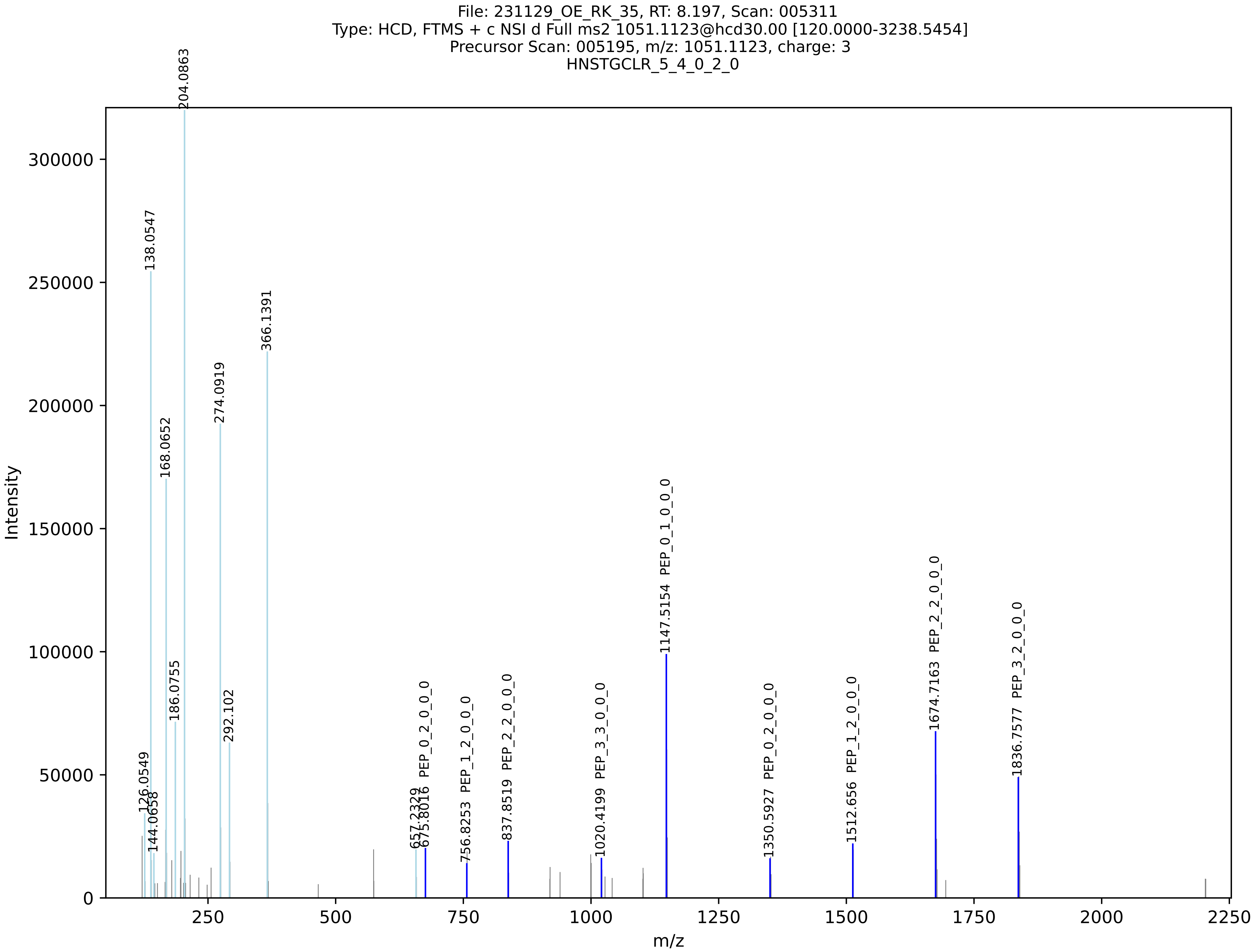
File: 231129_OE_RK_35, RT: 8.197, Scan: 005311
Type: HCD, FTMS + c NSI d Full ms2 1051.1123@hcd30.00 [120.0000-3238.5454]
Precursor Scan: 005195, m/z: 1051.1123, charge: 3
HNSTGCLR_5_4_0_2_0
Intensity
m/z
204.0863
138.0547
366.1391
274.0919
168.0652
186.0755
292.102
126.0549
144.0658
657.2329
675.8016 PEP_0_2_0_0_0
756.8253 PEP_1_2_0_0_0
837.8519 PEP_2_2_0_0_0
1020.4199 PEP_3_3_0_0_0
1147.5154 PEP_0_1_0_0_0
1350.5927 PEP_0_2_0_0_0
1512.656 PEP_1_2_0_0_0
1674.7163 PEP_2_2_0_0_0
1836.7577 PEP_3_2_0_0_0

File: 231129_OE_RK_35, RT: 8.268, Scan: 005407
Type: HCD, FTMS + c NSI d Full ms2 994.7267@hcd30.00 [120.0000-3066.0059]
Precursor Scan: 005334, m/z: 994.3926, charge: 3
ENGTISR_5_4_0_2_0

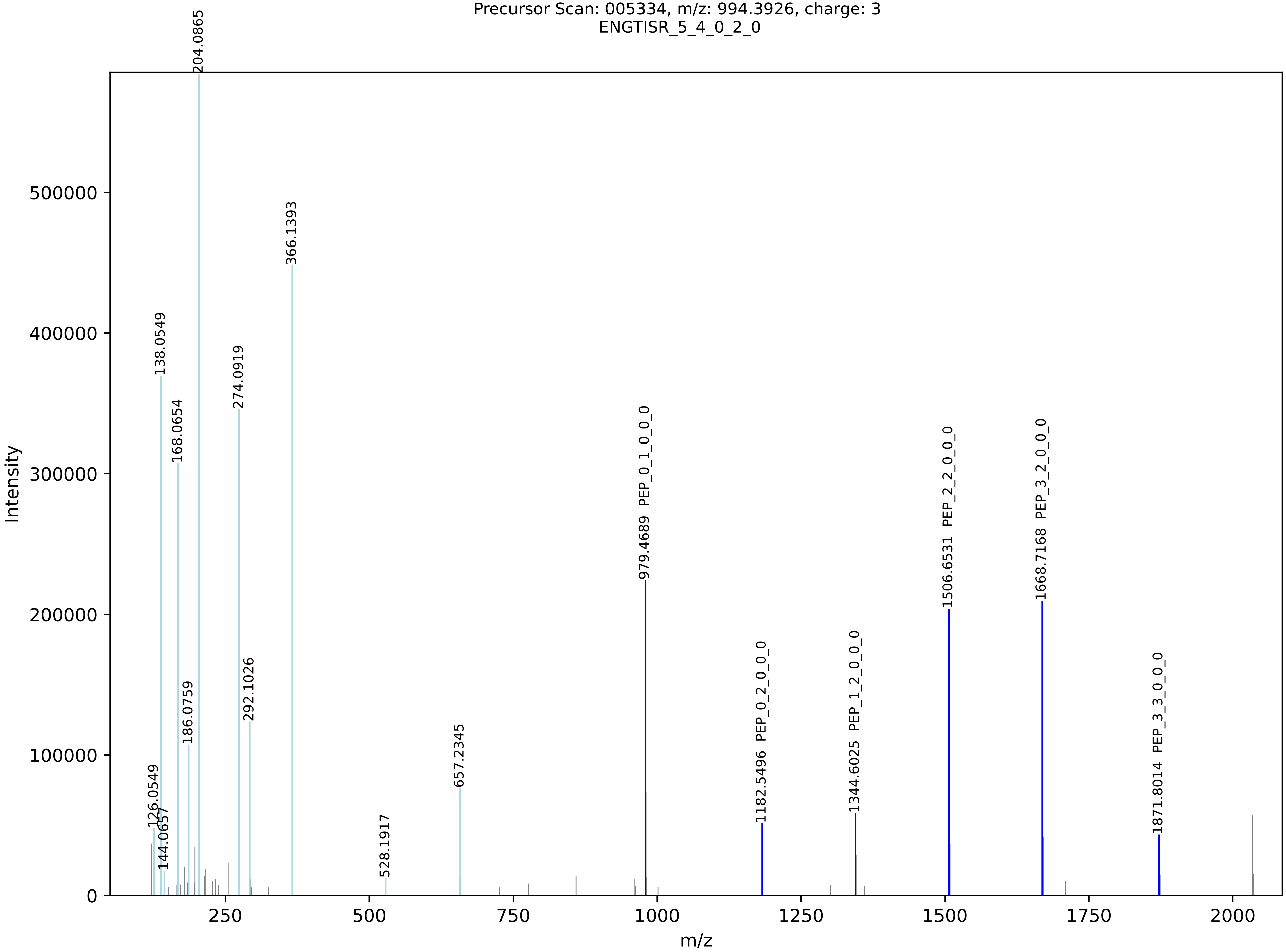

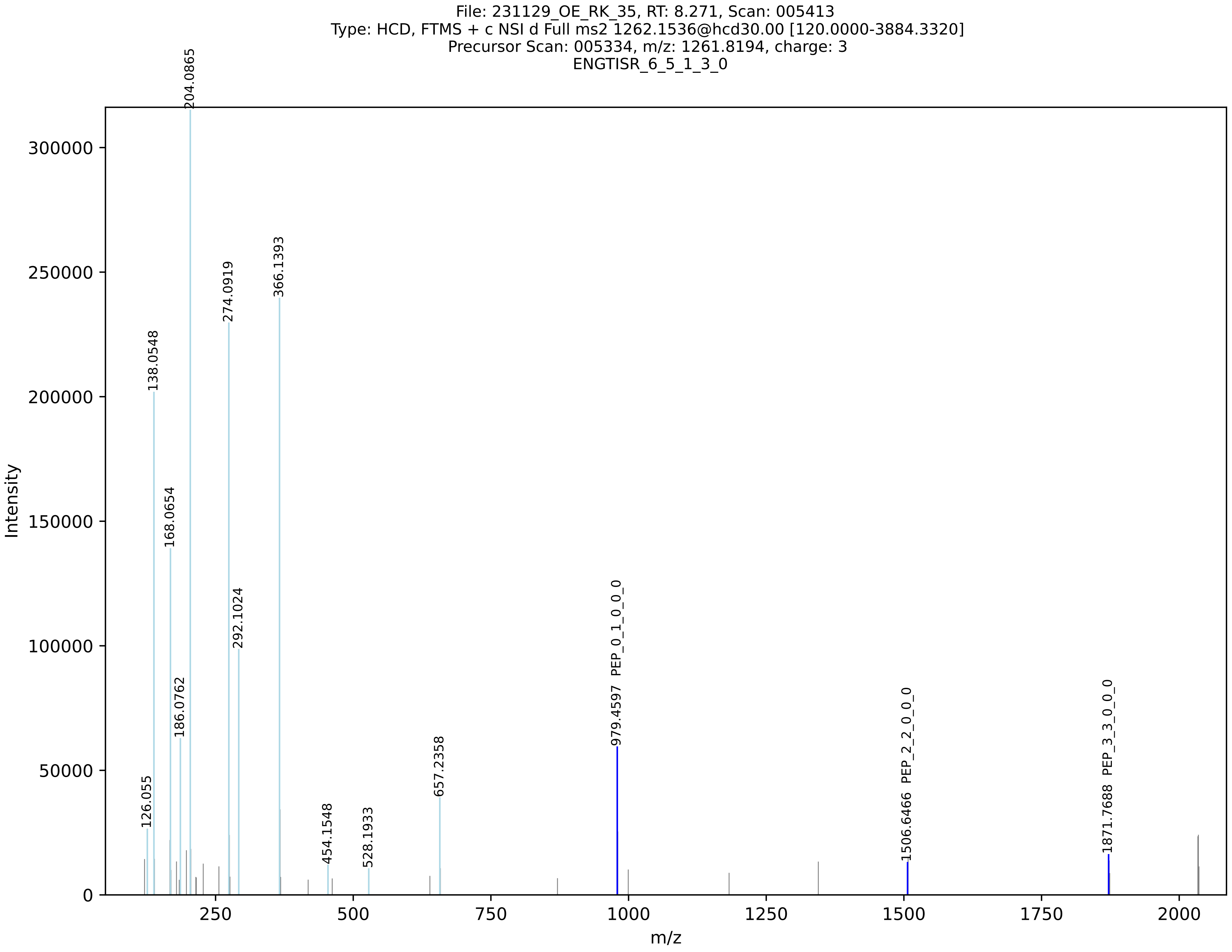
File: 231129_OE_RK_35, RT: 8.271, Scan: 005413
Type: HCD, FTMS + c NSI d Full ms2 1262.1536@hcd30.00 [120.0000-3884.3320]
Precursor Scan: 005334, m/z: 1261.8194, charge: 3
ENGTISR_6_5_1_3_0
204.0865
366.1393
274.0919
138.0548
168.0654
292.1024
186.0762
126.055
454.1548
528.1933
657.2358
979.4597 PEP_0_1_0_0_0_
1506.6466 PEP_2_2_0_0_0_
1871.7688 PEP_3_3_0_0_0_
Intensity
m/z
0
50000
100000
150000
200000
250000
300000
250
500
750
1000
1250
1500
1750
2000

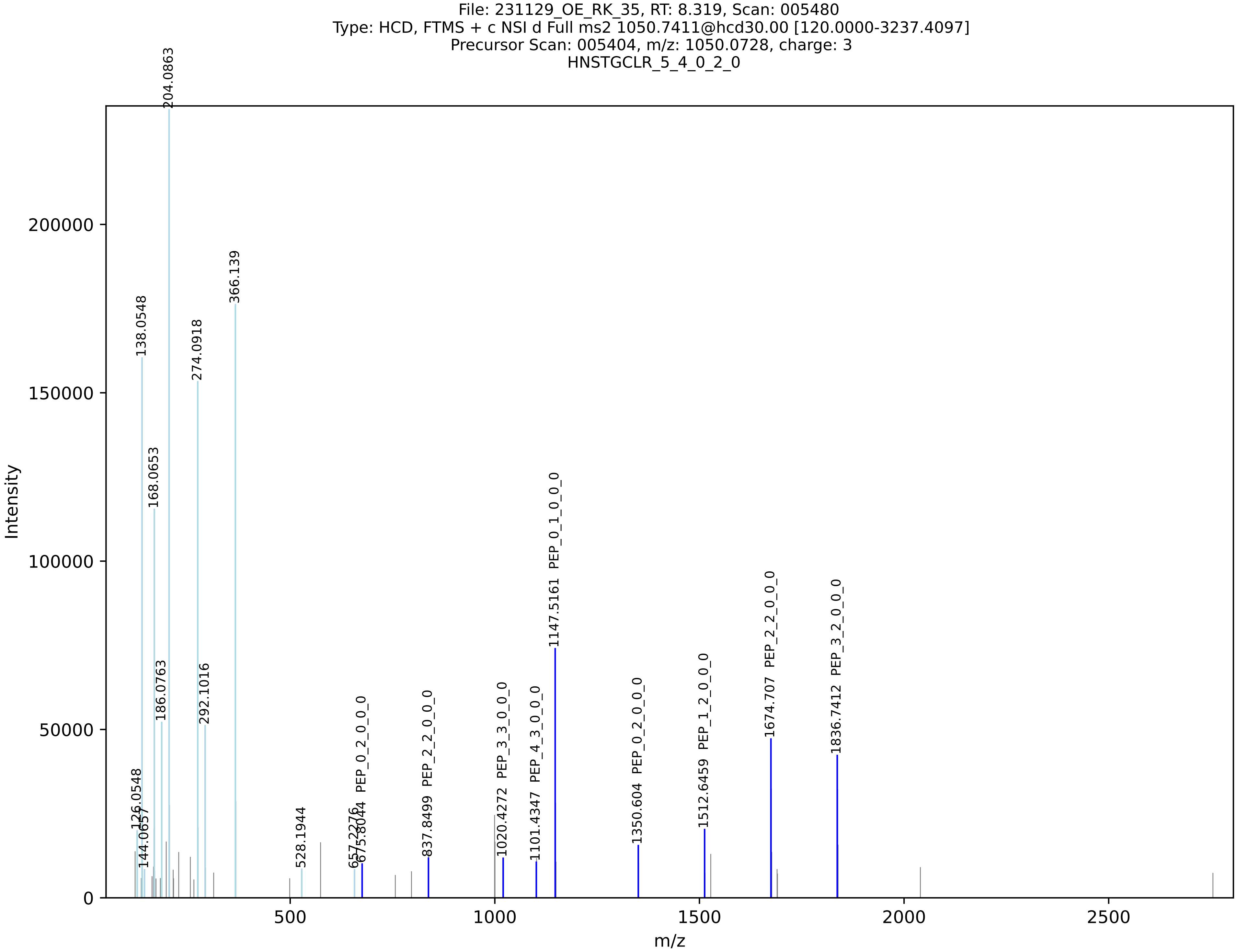
File: 231129_OE_RK_35, RT: 8.319, Scan: 005480
Type: HCD, FTMS + c NSI d Full ms2 1050.7411@hcd30.00 [120.0000-3237.4097]
Precursor Scan: 005404, m/z: 1050.0728, charge: 3
HNSTGCLR_5_4_0_2_0
Intensity
m/z
0
50000
100000
150000
200000
500
1000
1500
2000
2500
204.0863
366.139
138.0548
274.0918
168.0653
186.0763
292.1016
126.0548
144.0657
528.1944
657.2276
675.8044 PEP_0_2_0_0_
837.8499 PEP_2_2_0_0_
1020.4272 PEP_3_3_0_0_
1101.4347 PEP_4_3_0_0_
1147.5161 PEP_0_1_0_0_
1350.604 PEP_0_2_0_0_
1512.6459 PEP_1_2_0_0_
1674.707 PEP_2_2_0_0_
1836.7412 PEP_3_2_0_0_

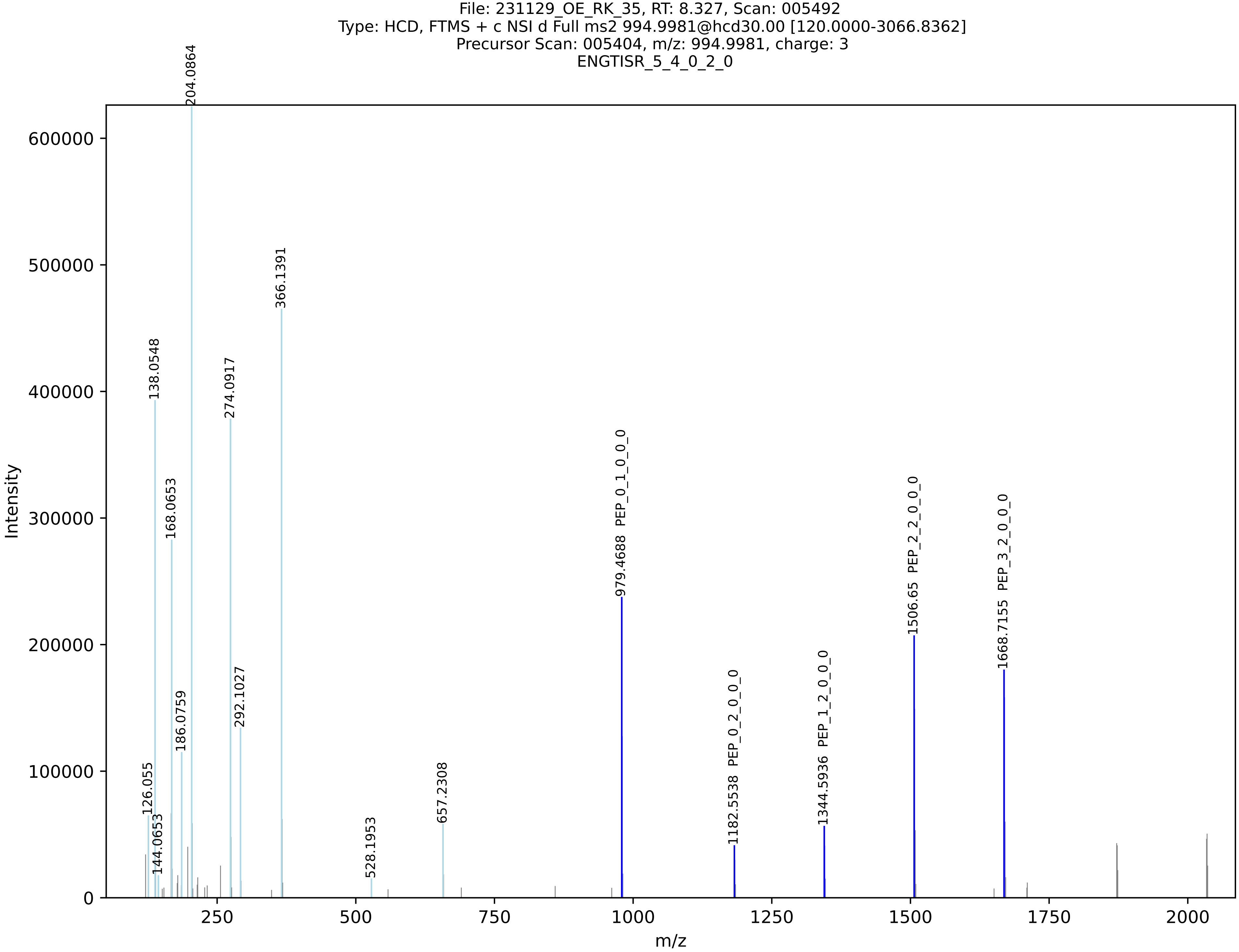
File: 231129_OE_RK_35, RT: 8.327, Scan: 005492
Type: HCD, FTMS + c NSI d Full ms2 994.9981@hcd30.00 [120.0000-3066.8362]
Precursor Scan: 005404, m/z: 994.9981, charge: 3
ENGTISR_5_4_0_2_0
Intensity
m/z
204.0864
366.1391
138.0548
274.0917
168.0653
186.0759
292.1027
126.055
144.0653
528.1953
657.2308
979.4688 PEP_0_1_0_0_
1182.5538 PEP_0_2_0_0_0
1344.5936 PEP_1_2_0_0_0
1506.65 PEP_2_2_0_0_0
1668.7155 PEP_3_2_0_0_0
0
100000
200000
300000
400000
500000
600000
250
500
750
1000
1250
1500
1750
2000

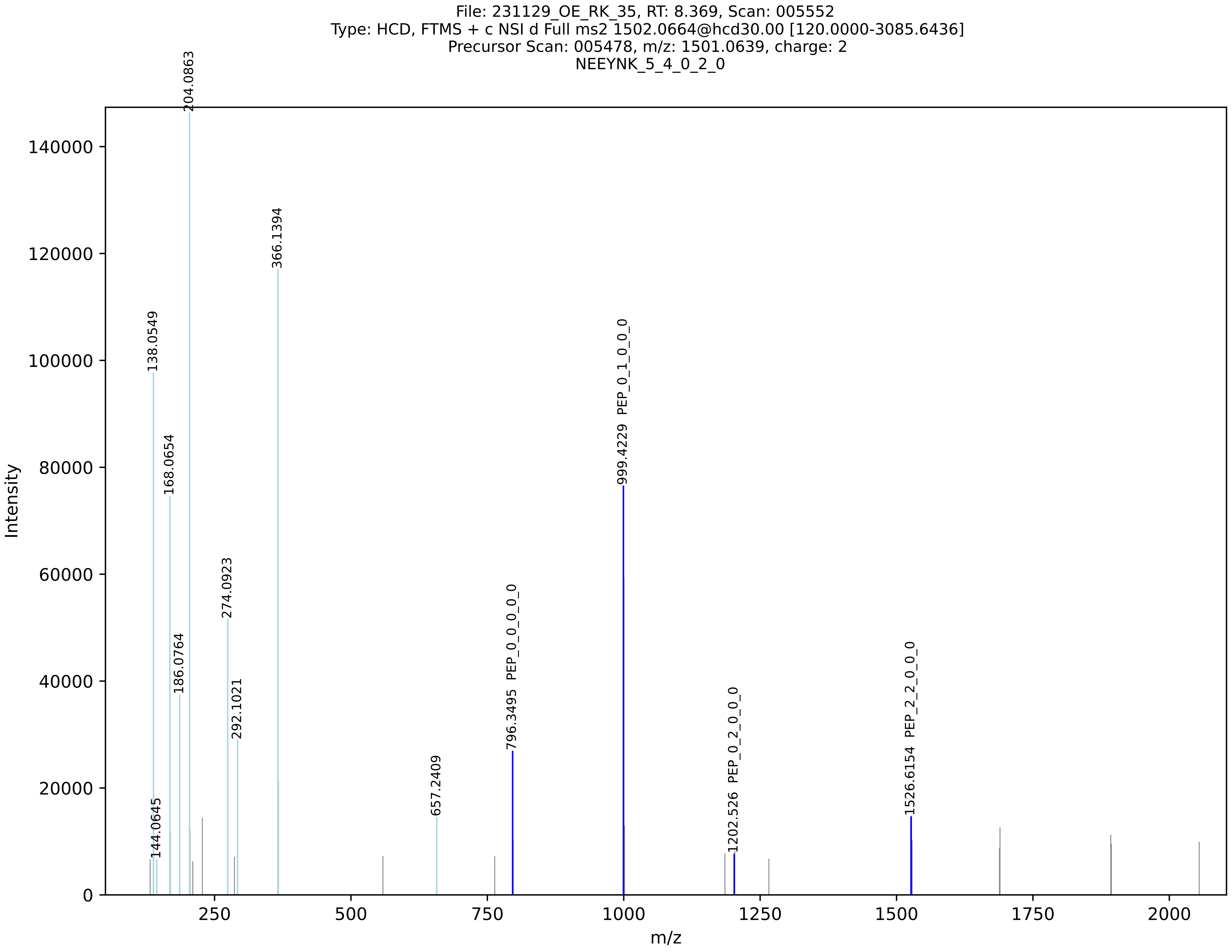
File: 231129_OE_RK_35, RT: 8.369, Scan: 005552
Type: HCD, FTMS + c NSI d Full ms2 1502.0664@hcd30.00 [120.0000-3085.6436]
Precursor Scan: 005478, m/z: 1501.0639, charge: 2
NEEYNK_5_4_0_2_0
Intensity
m/z
0
20000
40000
60000
80000
100000
120000
140000
250
500
750
1000
1250
1500
1750
2000
204.0863
366.1394
138.0549
168.0654
186.0764
144.0645
274.0923
292.1021
657.2409
796.3495 PEP_0_0_0_0_0
999.4229 PEP_0_1_0_0_0
1202.526 PEP_0_2_0_0_0
1526.6154 PEP_2_2_0_0_0

File: 231129_OE_RK_35, RT: 8.37, Scan: 005554
Type: HCD, FTMS + c NSI d Full ms2 1268.8057@hcd30.00 [120.0000-3904.6875]
Precursor Scan: 005478, m/z: 1268.8158, charge: 3
NEEYNK_6_5_1_3_0

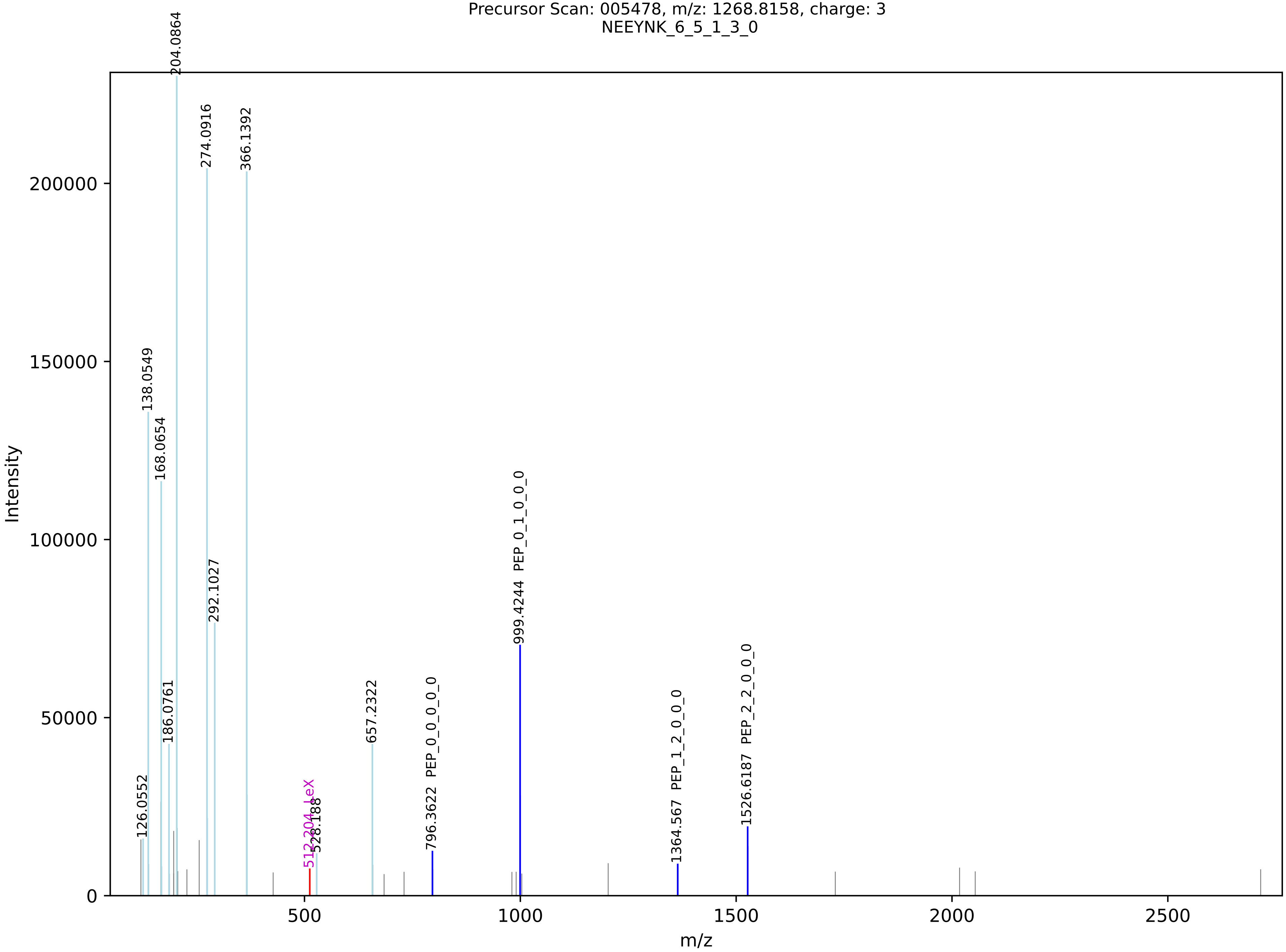

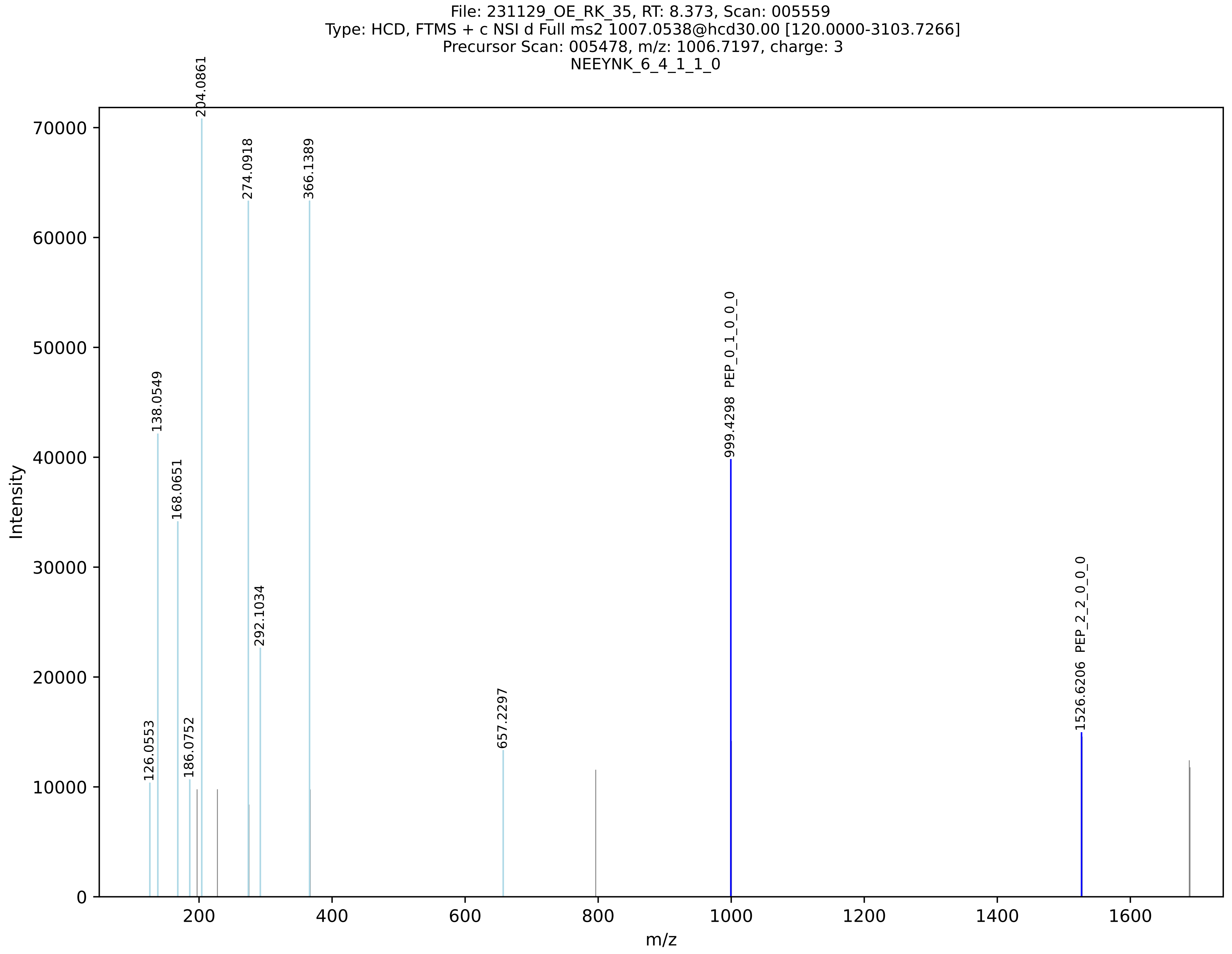
File: 231129_OE_RK_35, RT: 8.373, Scan: 005559
Type: HCD, FTMS + c NSI d Full ms2 1007.0538@hcd30.00 [120.0000-3103.7266]
Precursor Scan: 005478, m/z: 1006.7197, charge: 3
NEEYNK_6_4_1_1_0
Intensity
m/z
204.0861
274.0918
366.1389
138.0549
168.0651
292.1034
126.0553
186.0752
657.2297
999.4298 PEP_0_1_0_0_0
1526.6206 PEP_2_2_0_0_0
0
10000
20000
30000
40000
50000
60000
70000
200
400
600
800
1000
1200
1400
1600

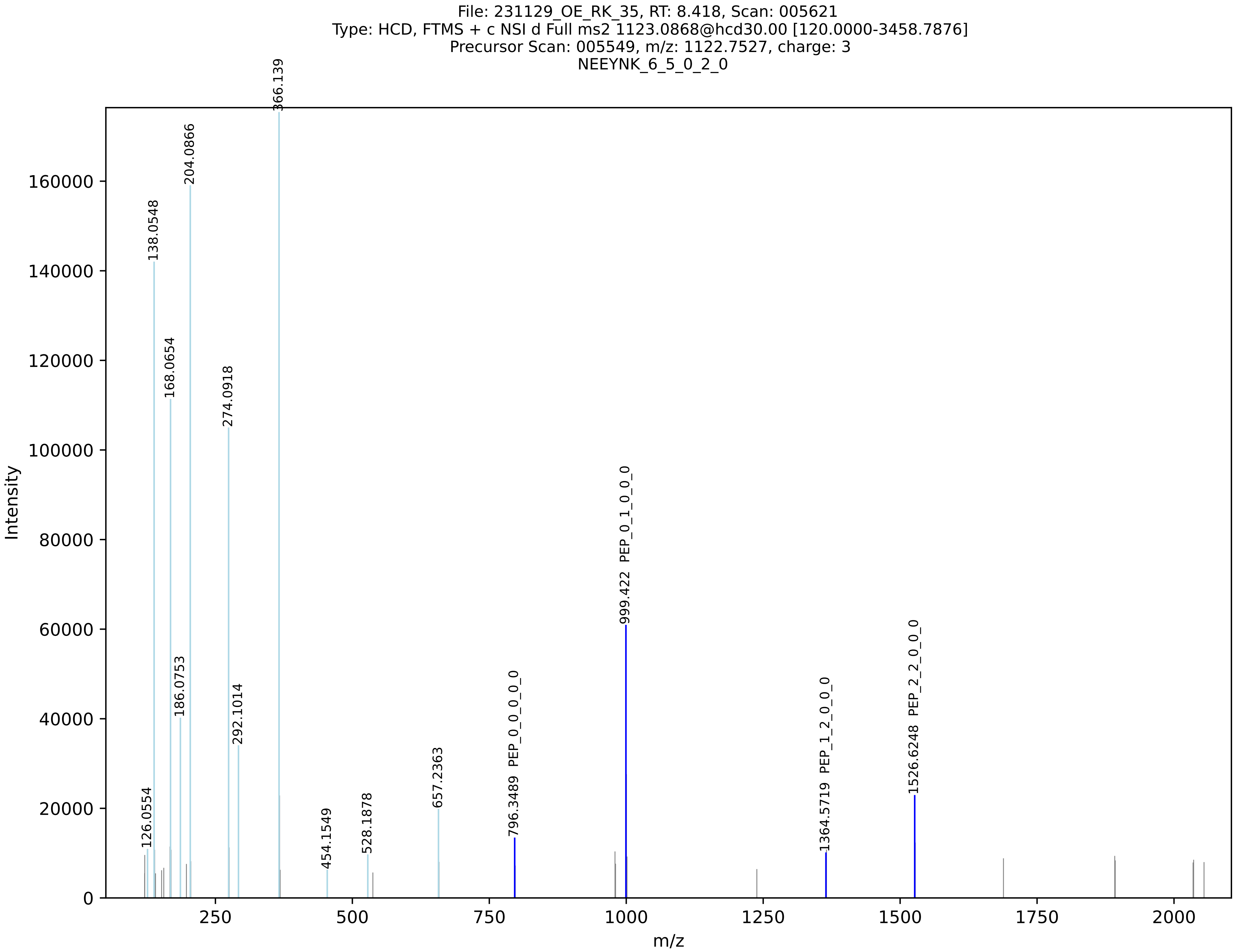

File: 231129_OE_RK_35, RT: 8.418, Scan: 005621
Type: HCD, FTMS + c NSI d Full ms2 1123.0868@hcd30.00 [120.0000-3458.7876]
Precursor Scan: 005549, m/z: 1122.7527, charge: 3
NEEYNK_6_5_0_2_0
Intensity
m/z
366.139
204.0866
138.0548
168.0654
274.0918
186.0753
292.1014
126.0554
454.1549
528.1878
657.2363
796.3489 PEP_0_0_0_0_0
999.422 PEP_0_1_0_0_0
1364.5719 PEP_1_2_0_0_0
1526.6248 PEP_2_2_0_0_0

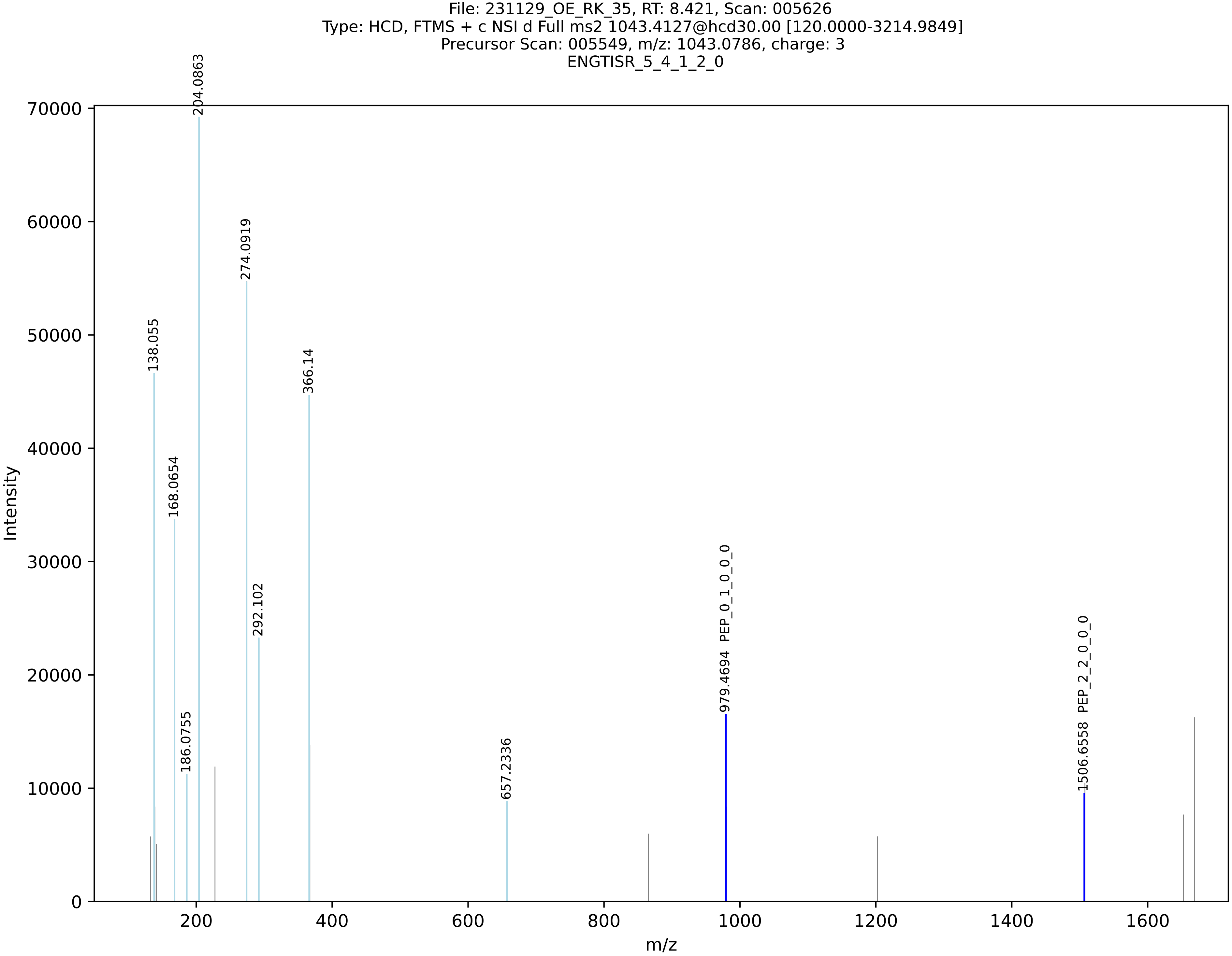

File: 231129_OE_RK_35, RT: 8.421, Scan: 005626
Type: HCD, FTMS + c NSI d Full ms2 1043.4127@hcd30.00 [120.0000-3214.9849]
Precursor Scan: 005549, m/z: 1043.0786, charge: 3
ENGTISR_5_4_1_2_0
204.0863
274.0919
138.055
366.14
168.0654
292.102
186.0755
657.2336
979.4694 PEP_0_1_0_0_0
1506.6558 PEP_2_2_0_0_0
Intensity
m/z
0
10000
20000
30000
40000
50000
60000
70000
200
400
600
800
1000
1200
1400
1600

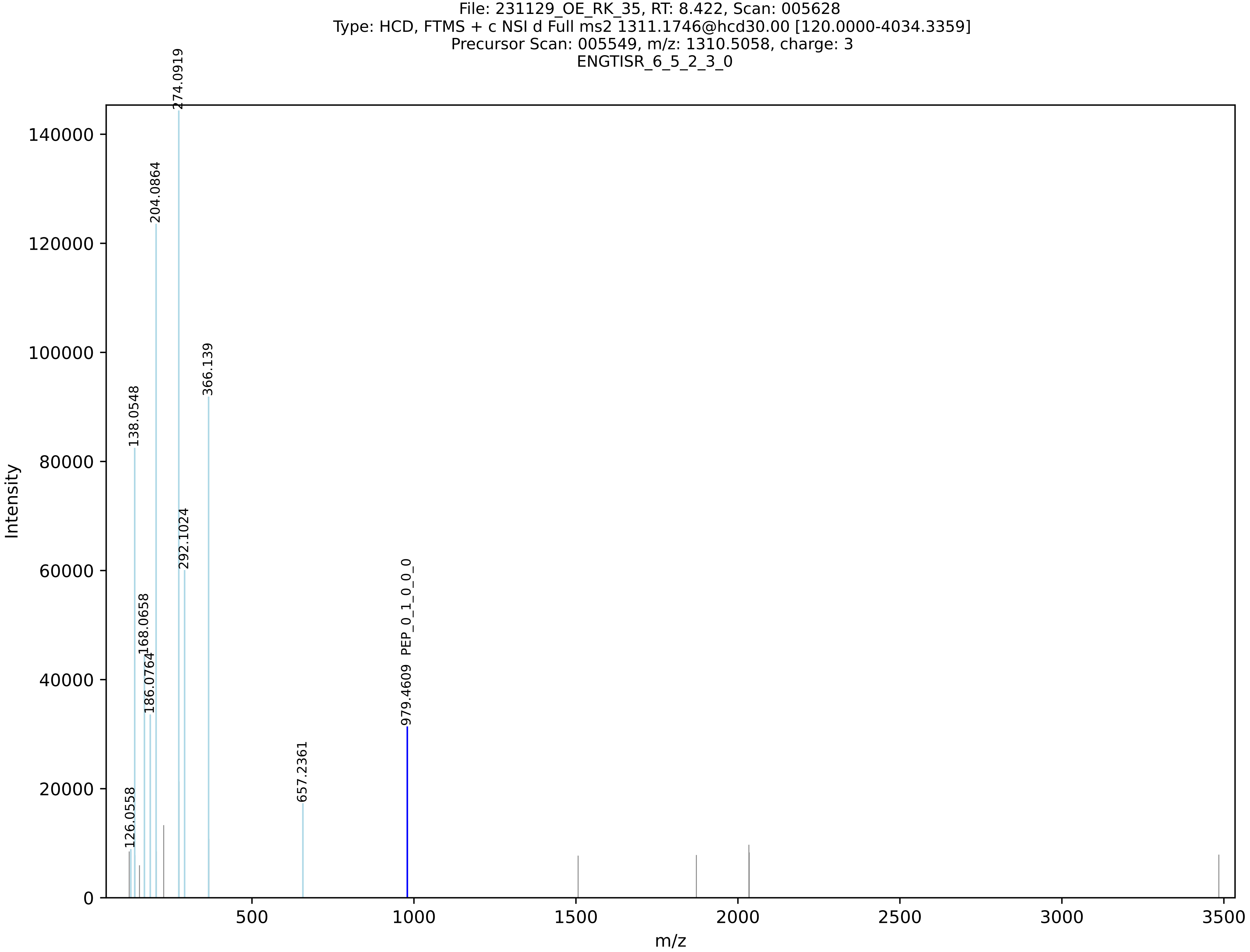
File: 231129_OE_RK_35, RT: 8.422, Scan: 005628
Type: HCD, FTMS + c NSI d Full ms2 1311.1746@hcd30.00 [120.0000-4034.3359]
Precursor Scan: 005549, m/z: 1310.5058, charge: 3
ENGTISR_6_5_2_3_0
Intensity
m/z
274.0919
204.0864
366.139
138.0548
292.1024
168.0658
186.0764
979.4609 PEP_0_1_0_0_0_
657.2361
126.0558
0
20000
40000
60000
80000
100000
120000
140000
500
1000
1500
2000
2500
3000
3500

File: 231129_OE_RK_35, RT: 8.464, Scan: 005688
Type: HCD, FTMS + c NSI d Full ms2 1001.3788@hcd30.00 [120.0000-3086.3611]
Precursor Scan: 005617, m/z: 1001.0447, charge: 3
NEEYNK_5_4_0_2_0

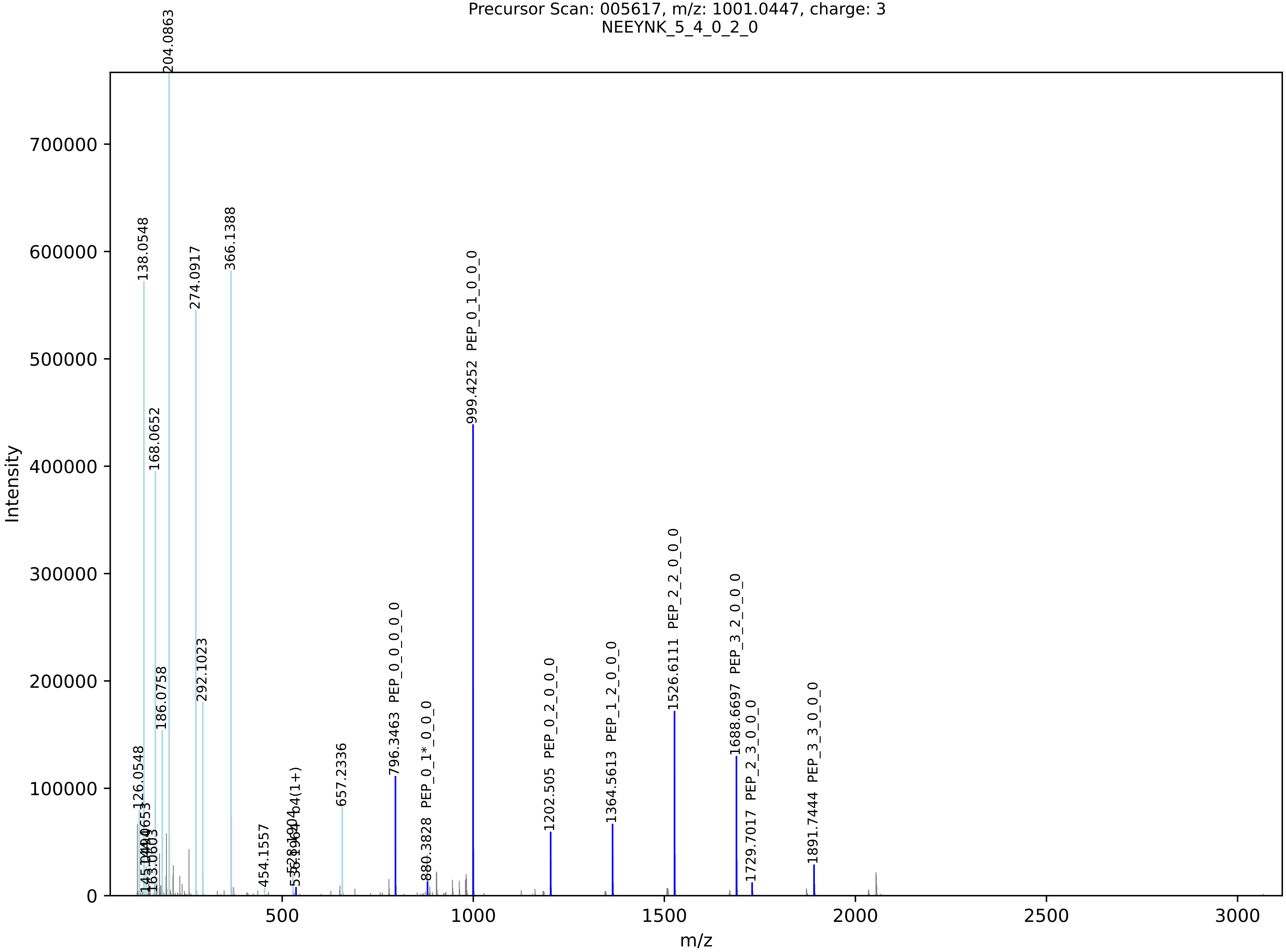

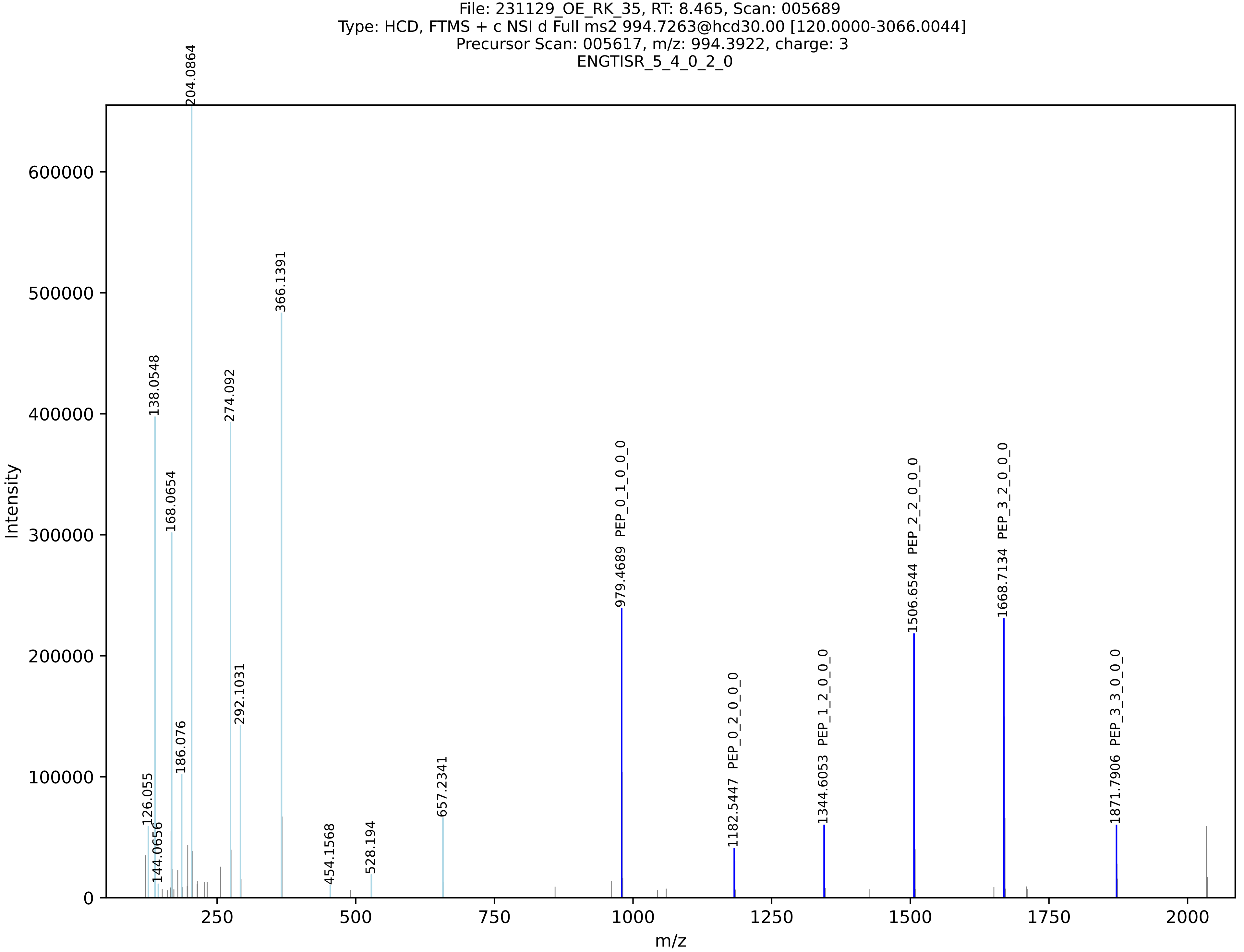

File: 231129_OE_RK_35, RT: 8.465, Scan: 005689
Type: HCD, FTMS + c NSI d Full ms2 994.7263@hcd30.00 [120.0000-3066.0044]
Precursor Scan: 005617, m/z: 994.3922, charge: 3
ENGTISR_5_4_0_2_0
Intensity
m/z
204.0864
366.1391
138.0548
274.092
168.0654
292.1031
186.076
126.055
144.0656
454.1568
528.194
657.2341
979.4689 PEP_0_1_0_0_0
1182.5447 PEP_0_2_0_0_0
1344.6053 PEP_1_2_0_0_0
1506.6544 PEP_2_2_0_0_0
1668.7134 PEP_3_2_0_0_0
1871.7906 PEP_3_3_0_0_0

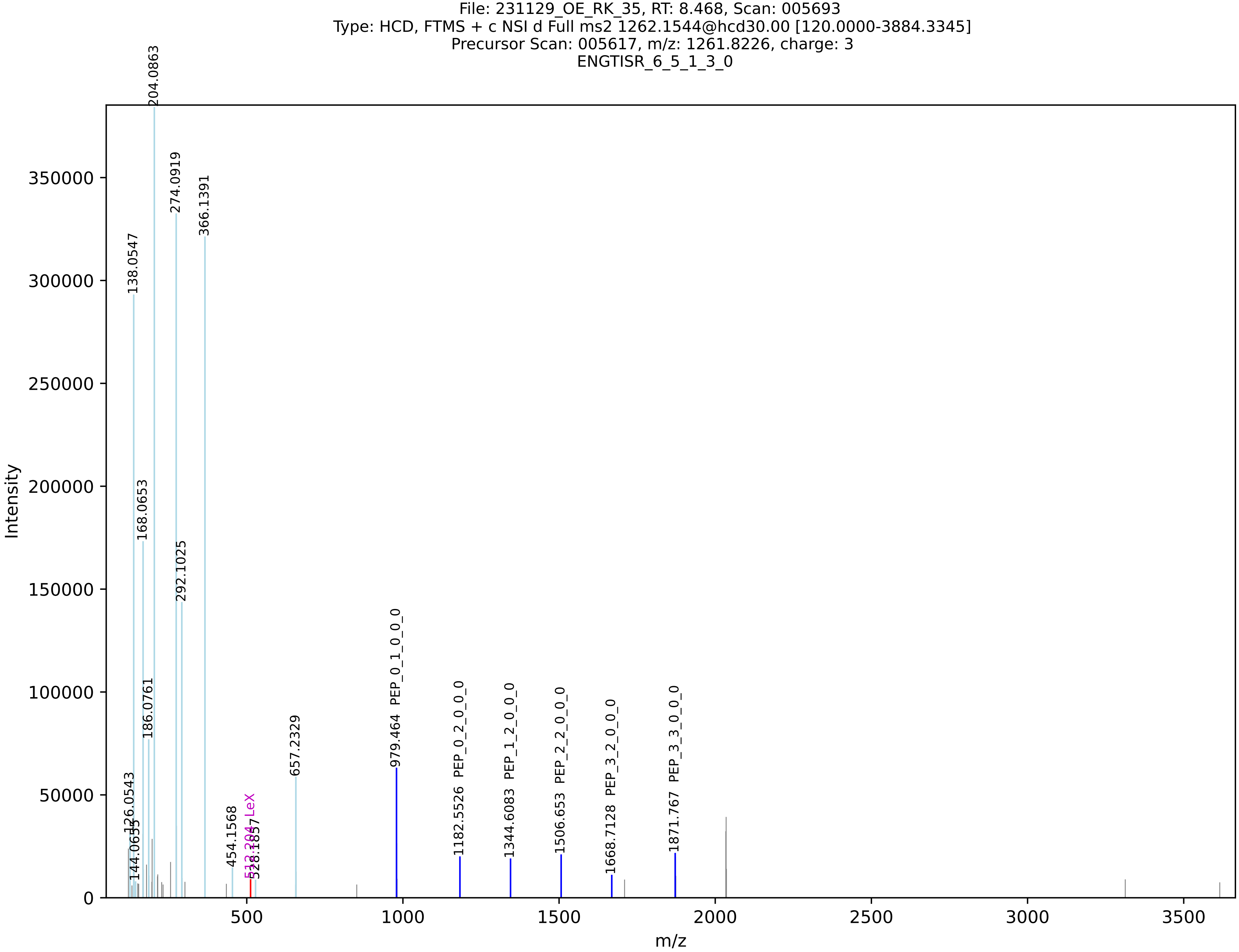
File: 231129_OE_RK_35, RT: 8.468, Scan: 005693
Type: HCD, FTMS + c NSI d Full ms2 1262.1544@hcd30.00 [120.0000-3884.3345]
Precursor Scan: 005617, m/z: 1261.8226, charge: 3
ENGTISR_6_5_1_3_0
204.0863
274.0919
366.1391
138.0547
168.0653
292.1025
186.0761
126.0543
144.0653
454.1568
512.204 LeX
528.1857
657.2329
979.464 PEP_0_1_0_0_0
1182.5526 PEP_0_2_0_0_0
1344.6083 PEP_1_2_0_0_0
1506.653 PEP_2_2_0_0_0
1668.7128 PEP_3_2_0_0_0
1871.767 PEP_3_3_0_0_0
Intensity
m/z

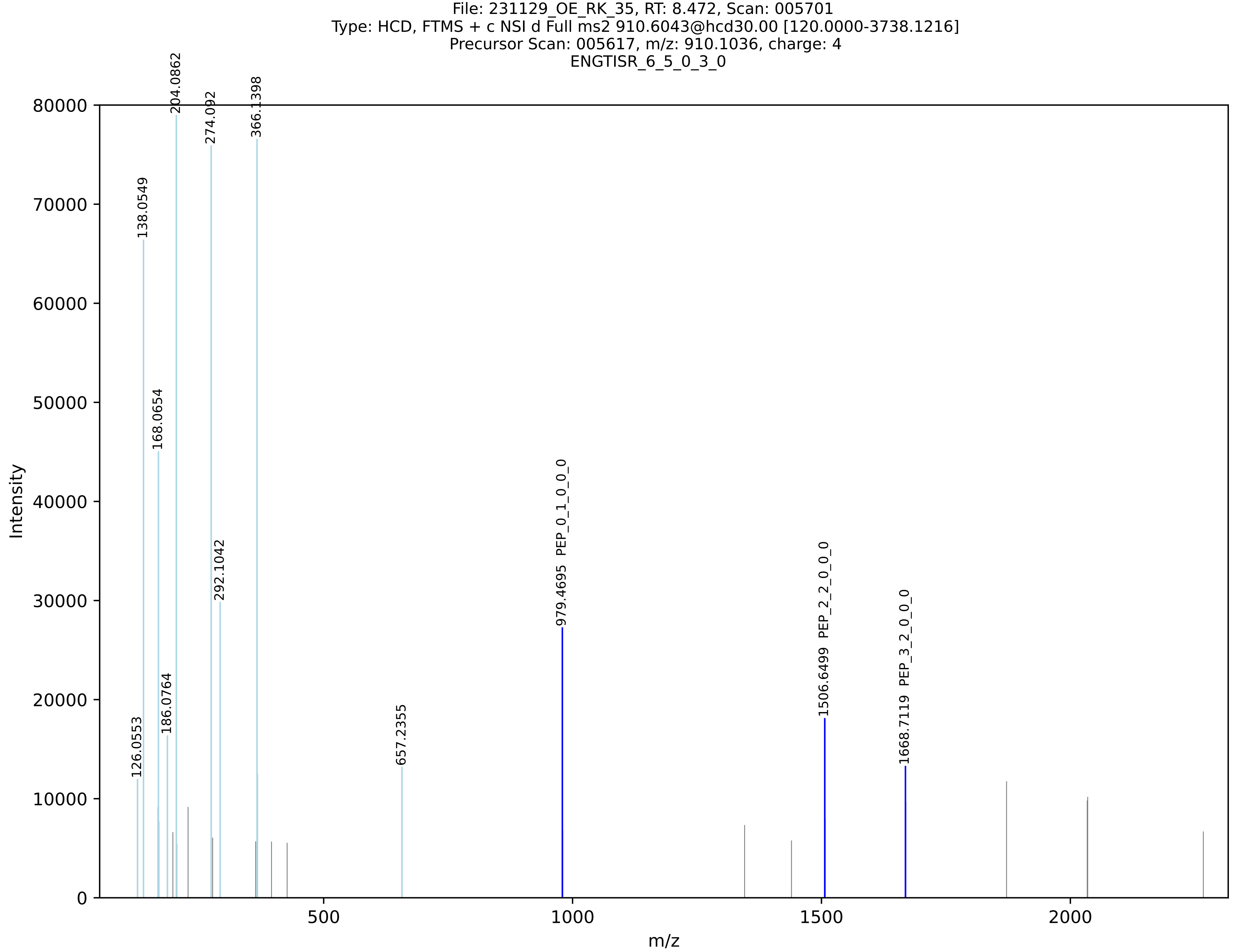
File: 231129_OE_RK_35, RT: 8.472, Scan: 005701
Type: HCD, FTMS + c NSI d Full ms2 910.6043@hcd30.00 [120.0000-3738.1216]
Precursor Scan: 005617, m/z: 910.1036, charge: 4
ENGTISR_6_5_0_3_0
Intensity
m/z
126.0553
138.0549
168.0654
186.0764
204.0862
274.092
292.1042
366.1398
657.2355
979.4695 PEP_0_1_0_0_0
1506.6499 PEP_2_2_0_0_0
1668.7119 PEP_3_2_0_0_0

File: 231129_OE_RK_35, RT: 8.475, Scan: 005705
Type: HCD, FTMS + c NSI d Full ms2 1074.9150@hcd30.00 [120.0000-4408.5093]
Precursor Scan: 005617, m/z: 1074.4142, charge: 4
ENGTISR_9_8_1_1_0

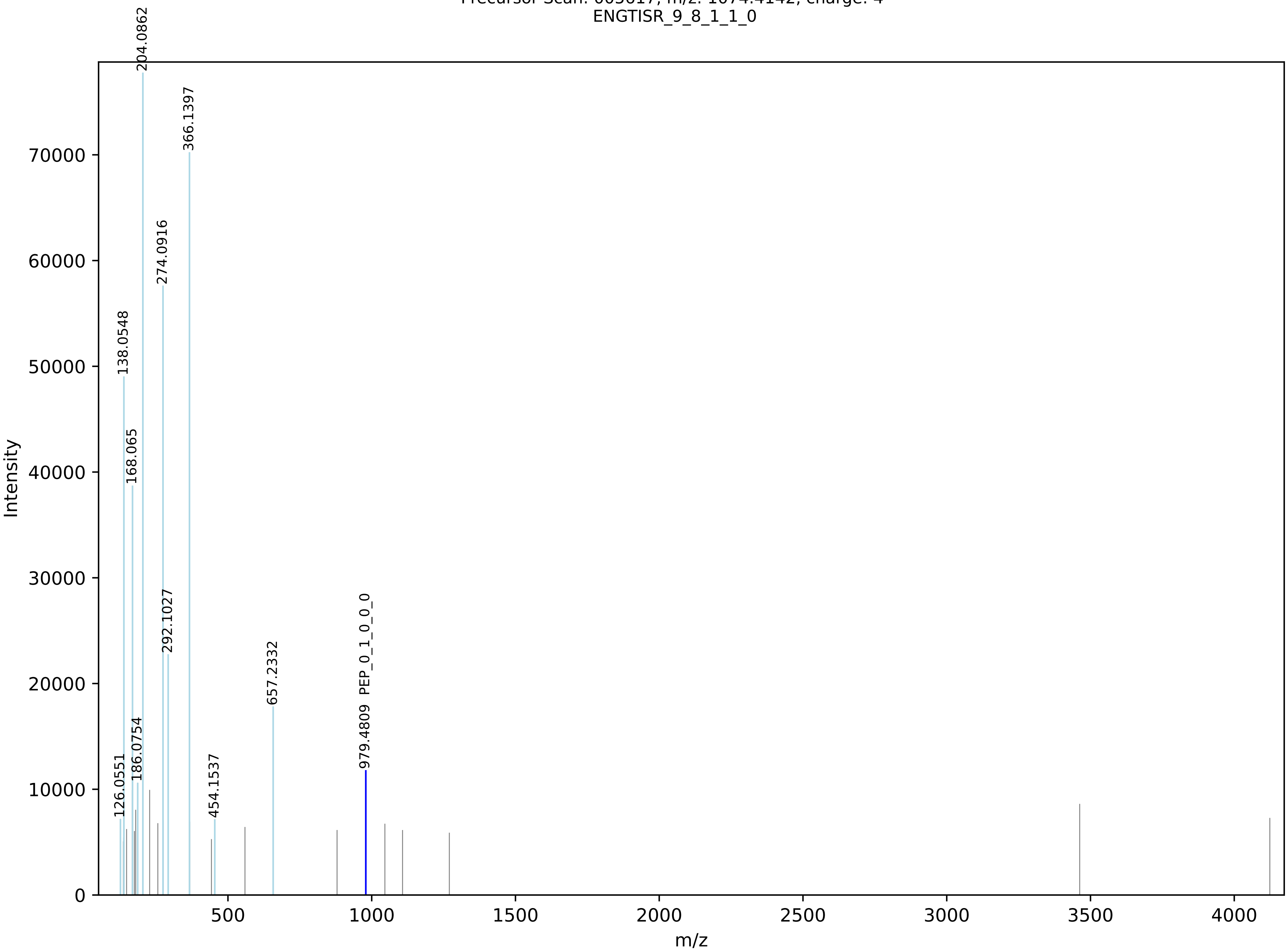

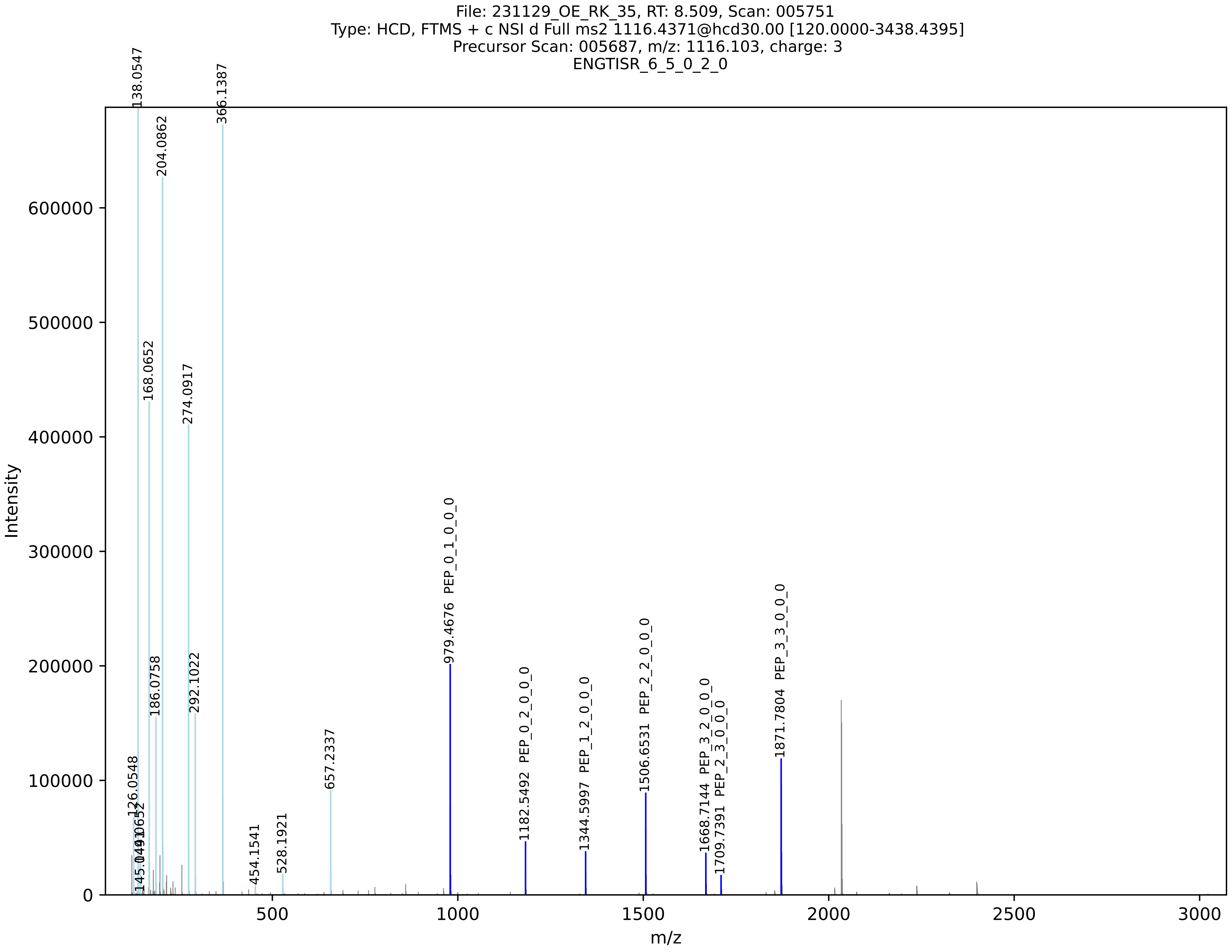

File: 231129_OE_RK_35, RT: 8.509, Scan: 005751
Type: HCD, FTMS + c NSI d Full ms2 1116.4371@hcd30.00 [120.0000-3438.4395]
Precursor Scan: 005687, m/z: 1116.103, charge: 3
ENGTISR_6_5_0_2_0
Intensity
m/z
138.0547
366.1387
204.0862
168.0652
274.0917
186.0758
292.1022
126.0548
145.0491
454.1541
528.1921
657.2337
979.4676 PEP_0_1_0_0_0
1182.5492 PEP_0_2_0_0_0
1344.5997 PEP_1_2_0_0_0
1506.6531 PEP_2_2_0_0_0
1668.7144 PEP_3_2_0_0_0
1709.7391 PEP_2_3_0_0_0
1871.7804 PEP_3_3_0_0_0

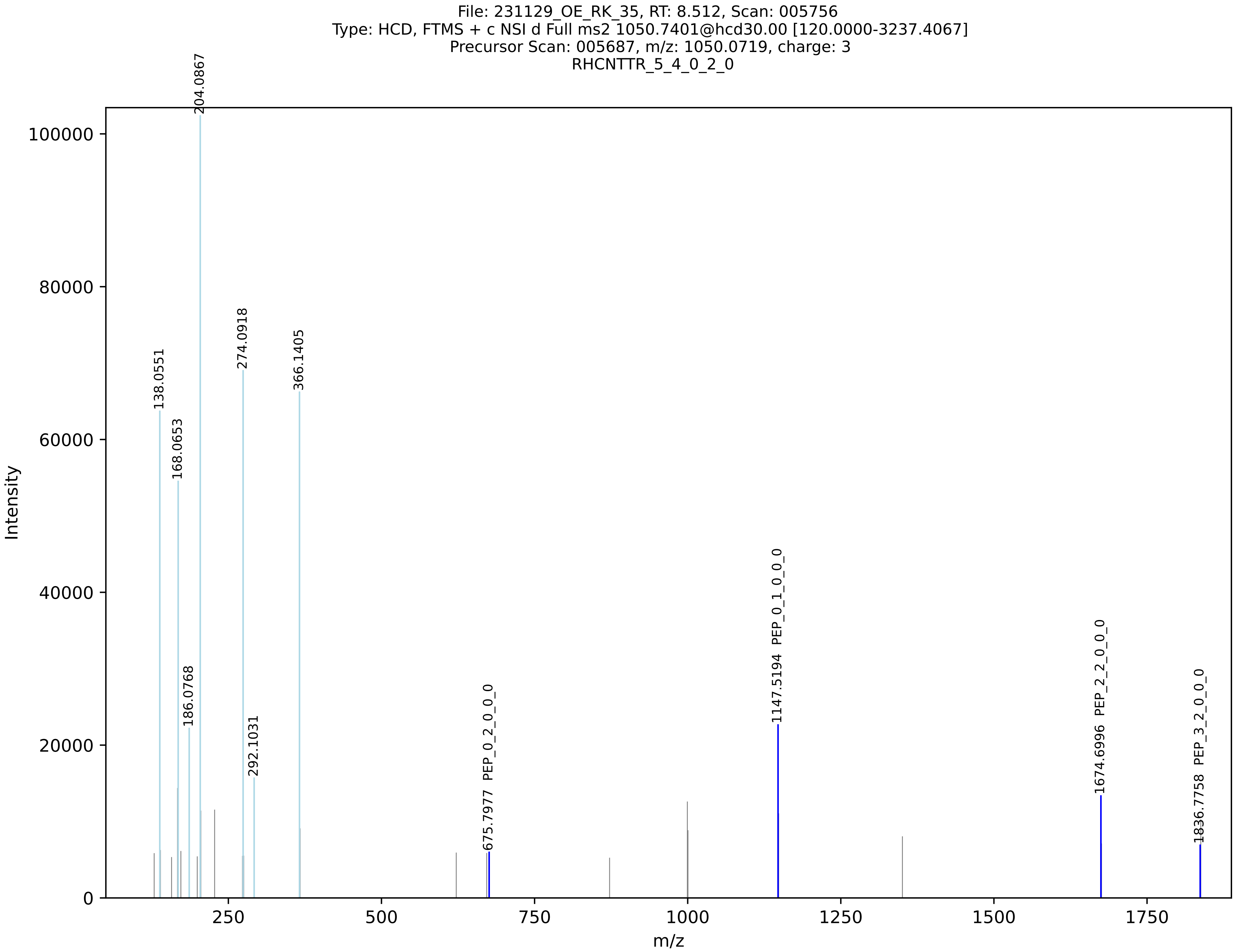
File: 231129_OE_RK_35, RT: 8.512, Scan: 005756
Type: HCD, FTMS + c NSI d Full ms2 1050.7401@hcd30.00 [120.0000-3237.4067]
Precursor Scan: 005687, m/z: 1050.0719, charge: 3
RHCNTTR_5_4_0_2_0
Intensity
m/z
204.0867
138.0551
168.0653
186.0768
274.0918
292.1031
366.1405
675.7977 PEP_0_2_0_0_0
1147.5194 PEP_0_1_0_0_0
1674.6996 PEP_2_2_0_0_0
1836.7758 PEP_3_2_0_0_0
0
20000
40000
60000
80000
100000
250
500
750
1000
1250
1500
1750

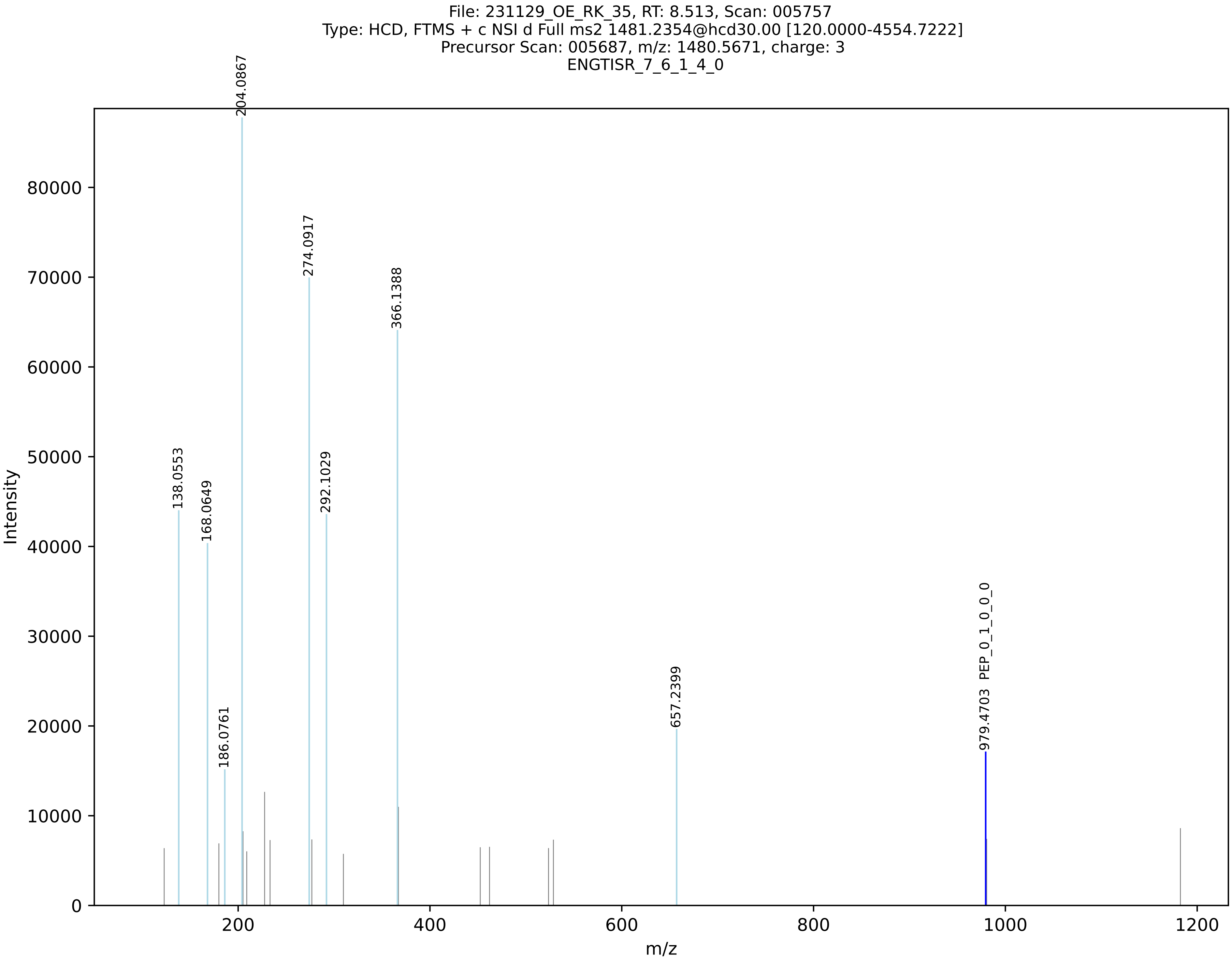

File: 231129_OE_RK_35, RT: 8.513, Scan: 005757
Type: HCD, FTMS + c NSI d Full ms2 1481.2354@hcd30.00 [120.0000-4554.7222]
Precursor Scan: 005687, m/z: 1480.5671, charge: 3
ENGTISR_7_6_1_4_0
Intensity
m/z
204.0867
274.0917
366.1388
138.0553
168.0649
292.1029
186.0761
657.2399
979.4703 PEP_0_1_0_0_0
0
10000
20000
30000
40000
50000
60000
70000
80000
200
400
600
800
1000
1200

File: 231129_OE_RK_35, RT: 8.53, Scan: 005785
Type: HCD, FTMS + c NSI d Full ms2 1117.4004@hcd30.00 [120.0000-3441.3872]
Precursor Scan: 005687, m/z: 1117.076, charge: 3
ENGTISR_6_5_0_2_0

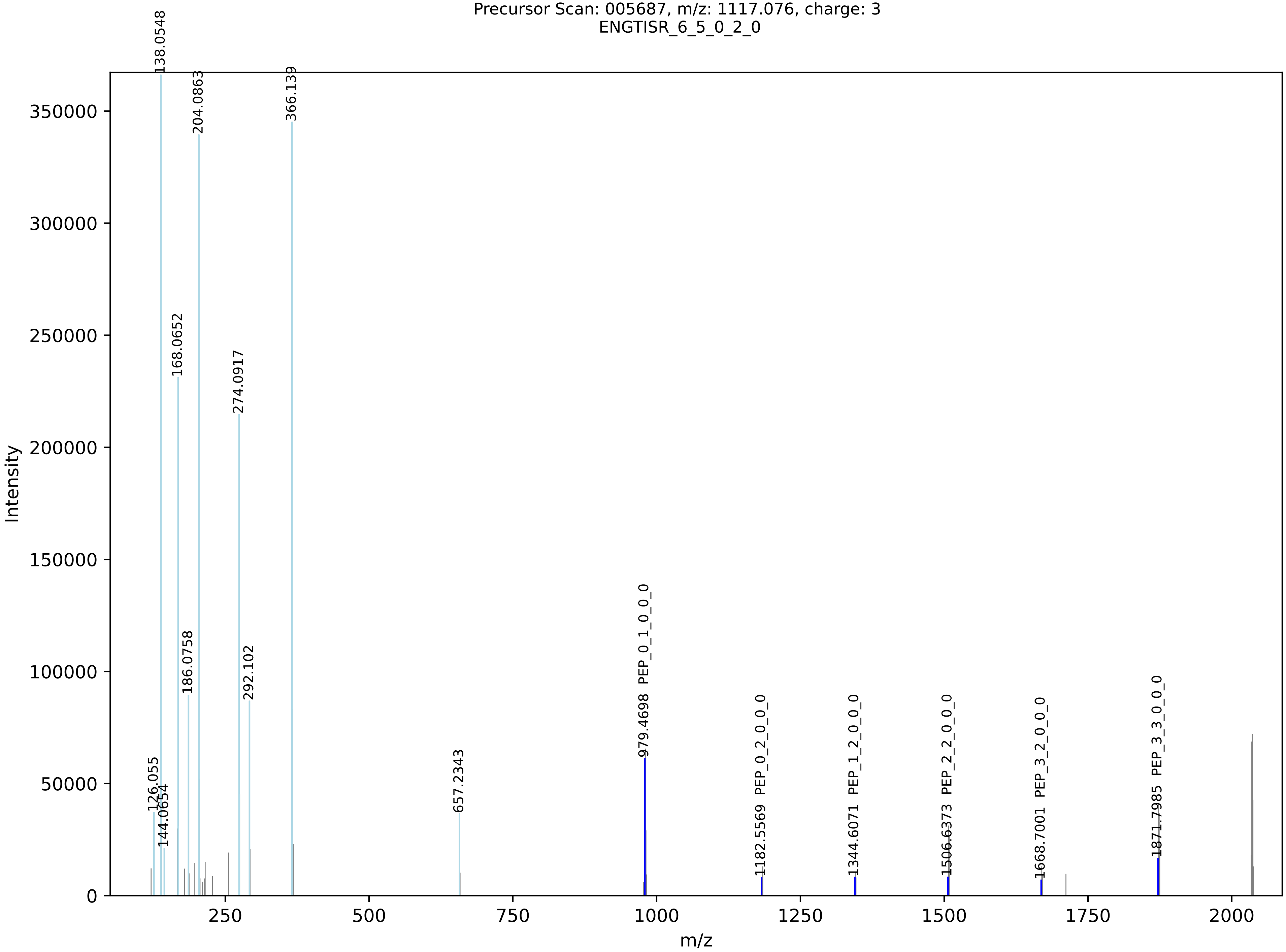

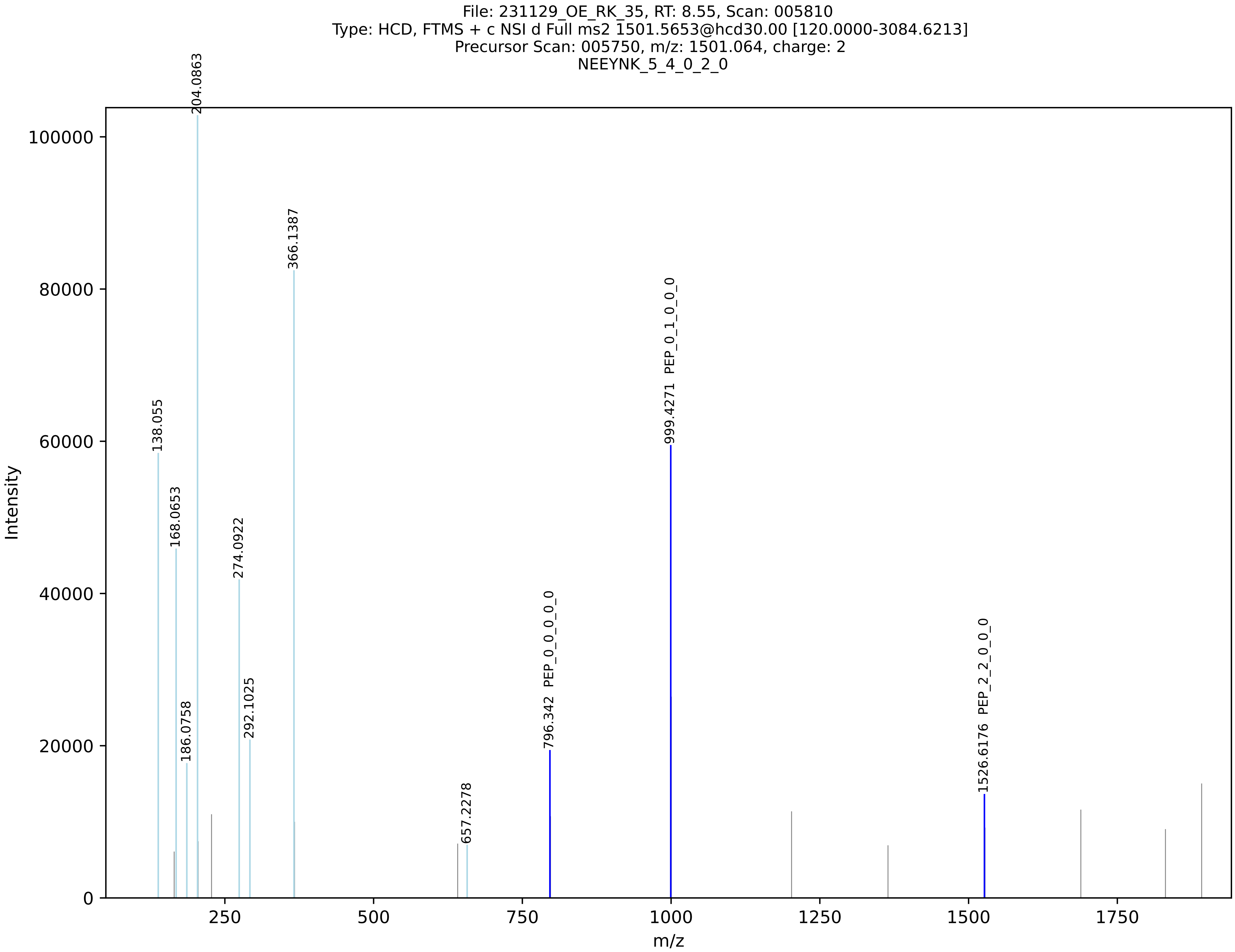
File: 231129_OE_RK_35, RT: 8.55, Scan: 005810
Type: HCD, FTMS + c NSI d Full ms2 1501.5653@hcd30.00 [120.0000-3084.6213]
Precursor Scan: 005750, m/z: 1501.064, charge: 2
NEEYNK_5_4_0_2_0
204.0863
366.1387
999.4271 PEP_0_1_0_0_0
138.055
168.0653
274.0922
292.1025
186.0758
796.342 PEP_0_0_0_0_0
1526.6176 PEP_2_2_0_0_0
657.2278
Intensity
m/z
0
20000
40000
60000
80000
100000
250
500
750
1000
1250
1500
1750

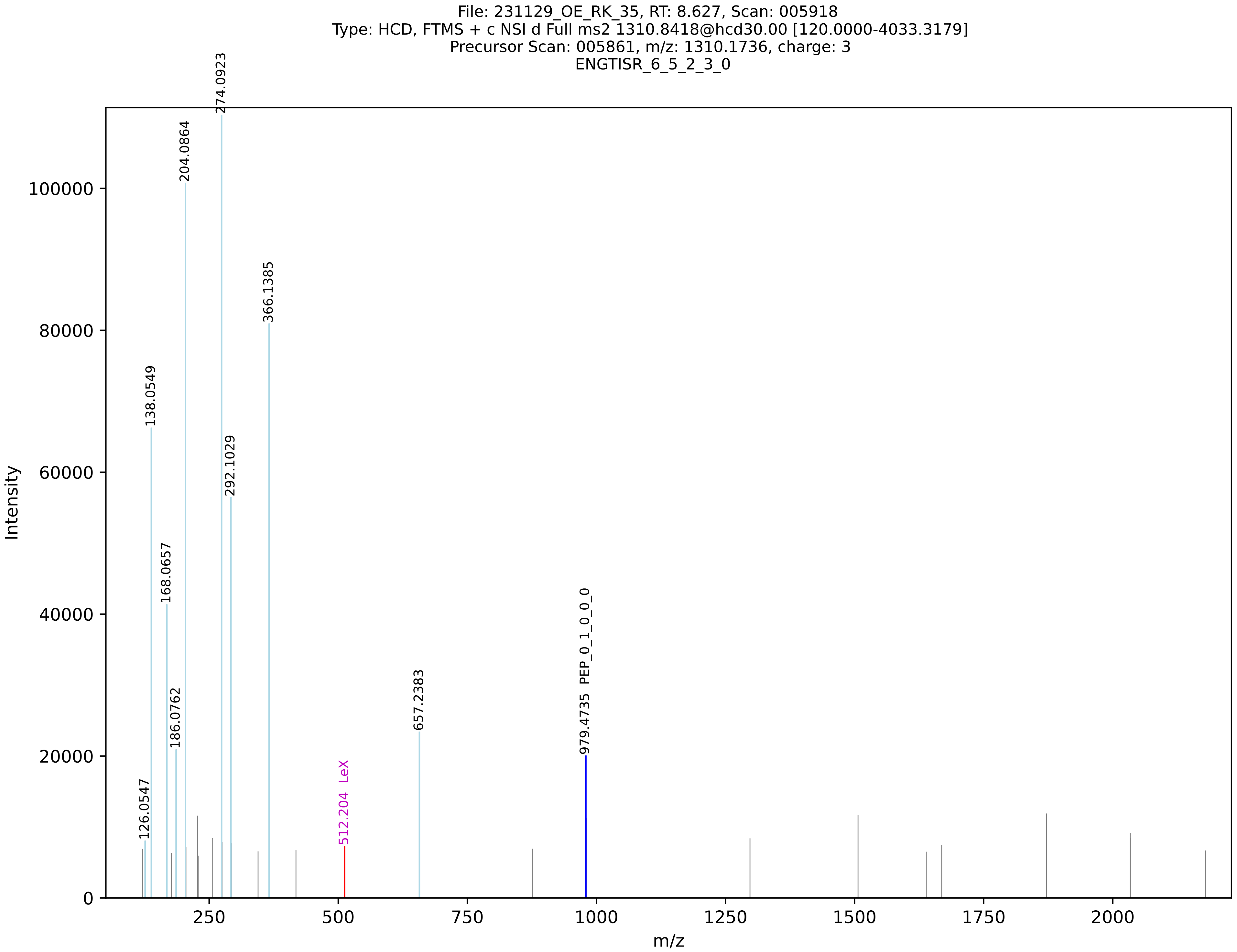
File: 231129_OE_RK_35, RT: 8.627, Scan: 005918
Type: HCD, FTMS + c NSI d Full ms2 1310.8418@hcd30.00 [120.0000-4033.3179]
Precursor Scan: 005861, m/z: 1310.1736, charge: 3
ENGTISR_6_5_2_3_0
274.0923
204.0864
366.1385
138.0549
292.1029
168.0657
186.0762
126.0547
512.204 LeX
657.2383
979.4735 PEP_0_1_0_0_0
Intensity
m/z
0
20000
40000
60000
80000
100000
250
500
750
1000
1250
1500
1750
2000

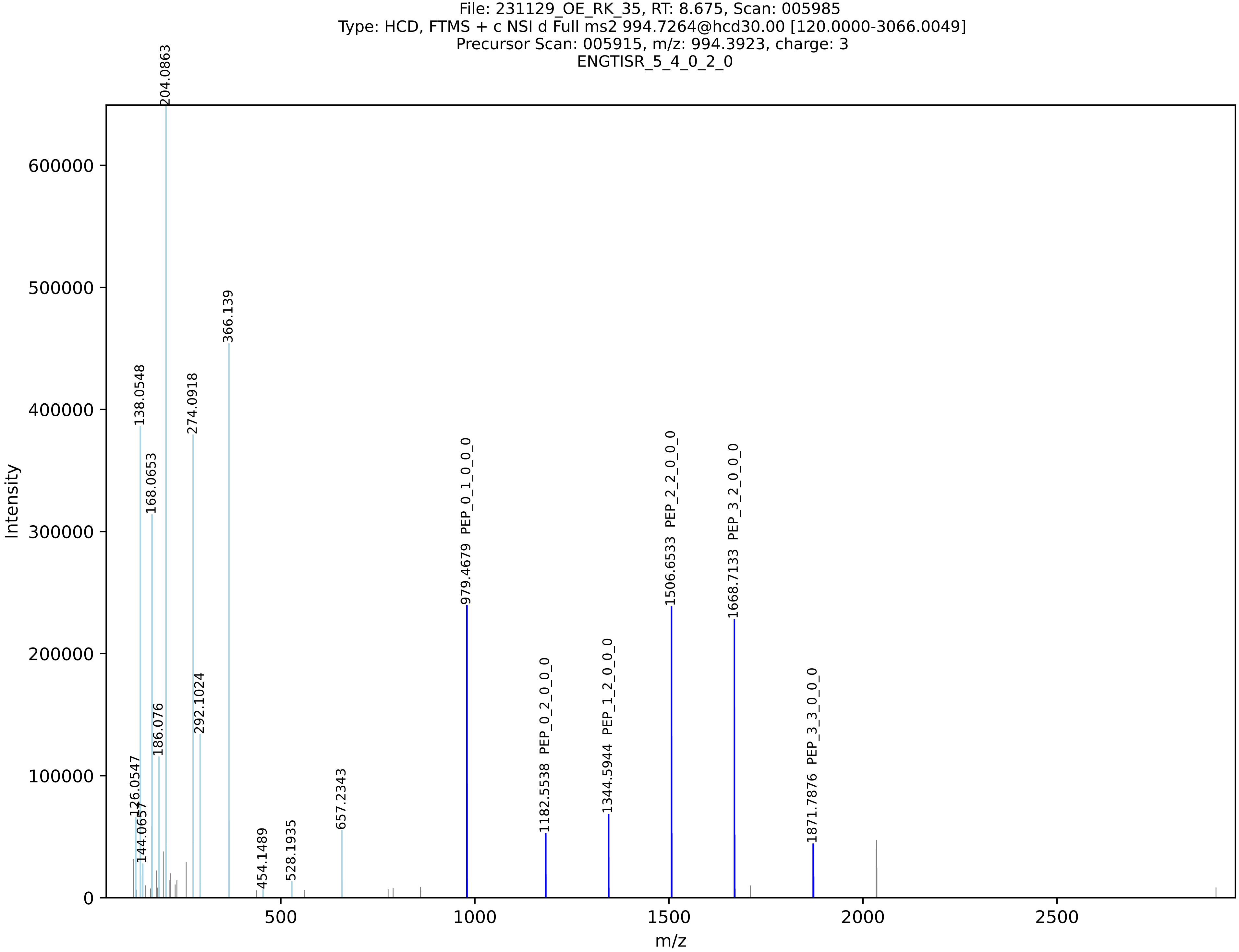

File: 231129_OE_RK_35, RT: 8.675, Scan: 005985
Type: HCD, FTMS + c NSI d Full ms2 994.7264@hcd30.00 [120.0000-3066.0049]
Precursor Scan: 005915, m/z: 994.3923, charge: 3
ENGTISR_5_4_0_2_0
Intensity
m/z
204.0863
366.139
138.0548
274.0918
168.0653
292.1024
186.076
126.0547
144.0657
454.1489
528.1935
657.2343
979.4679 PEP_0_1_0_0_0
1182.5538 PEP_0_2_0_0_0
1344.5944 PEP_1_2_0_0_0
1506.6533 PEP_2_2_0_0_0
1668.7133 PEP_3_2_0_0_0
1871.7876 PEP_3_3_0_0_0

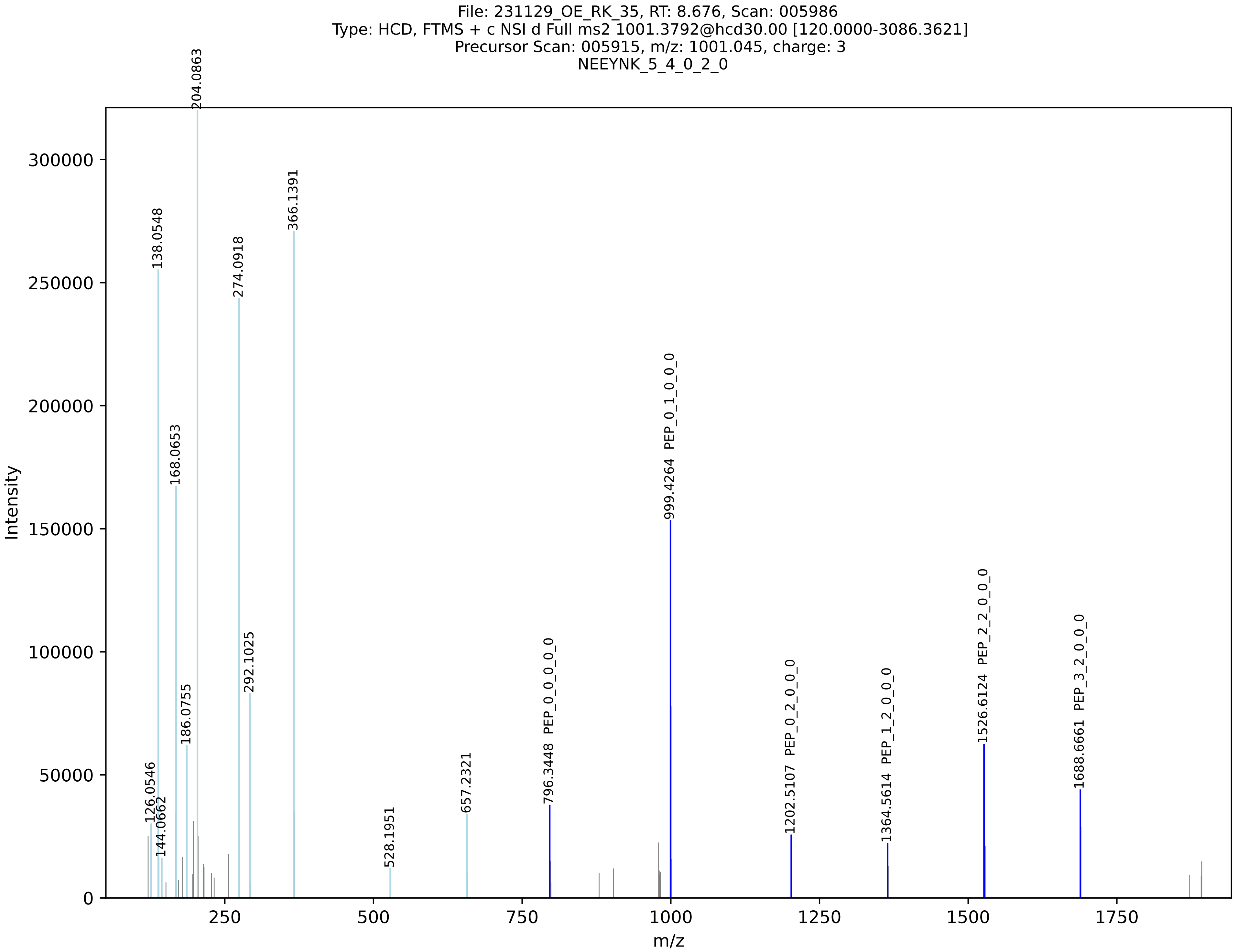

File: 231129_OE_RK_35, RT: 8.676, Scan: 005986
Type: HCD, FTMS + c NSI d Full ms2 1001.3792@hcd30.00 [120.0000-3086.3621]
Precursor Scan: 005915, m/z: 1001.045, charge: 3
NEEYNK_5_4_0_2_0
Intensity
m/z
204.0863
366.1391
138.0548
274.0918
168.0653
186.0755
292.1025
126.0546
144.0662
528.1951
657.2321
796.3448 PEP_0_0_0_0_0
999.4264 PEP_0_1_0_0_0
1202.5107 PEP_0_2_0_0_0
1364.5614 PEP_1_2_0_0_0
1526.6124 PEP_2_2_0_0_0
1688.6661 PEP_3_2_0_0_0
0
50000
100000
150000
200000
250000
300000
250
500
750
1000
1250
1500
1750

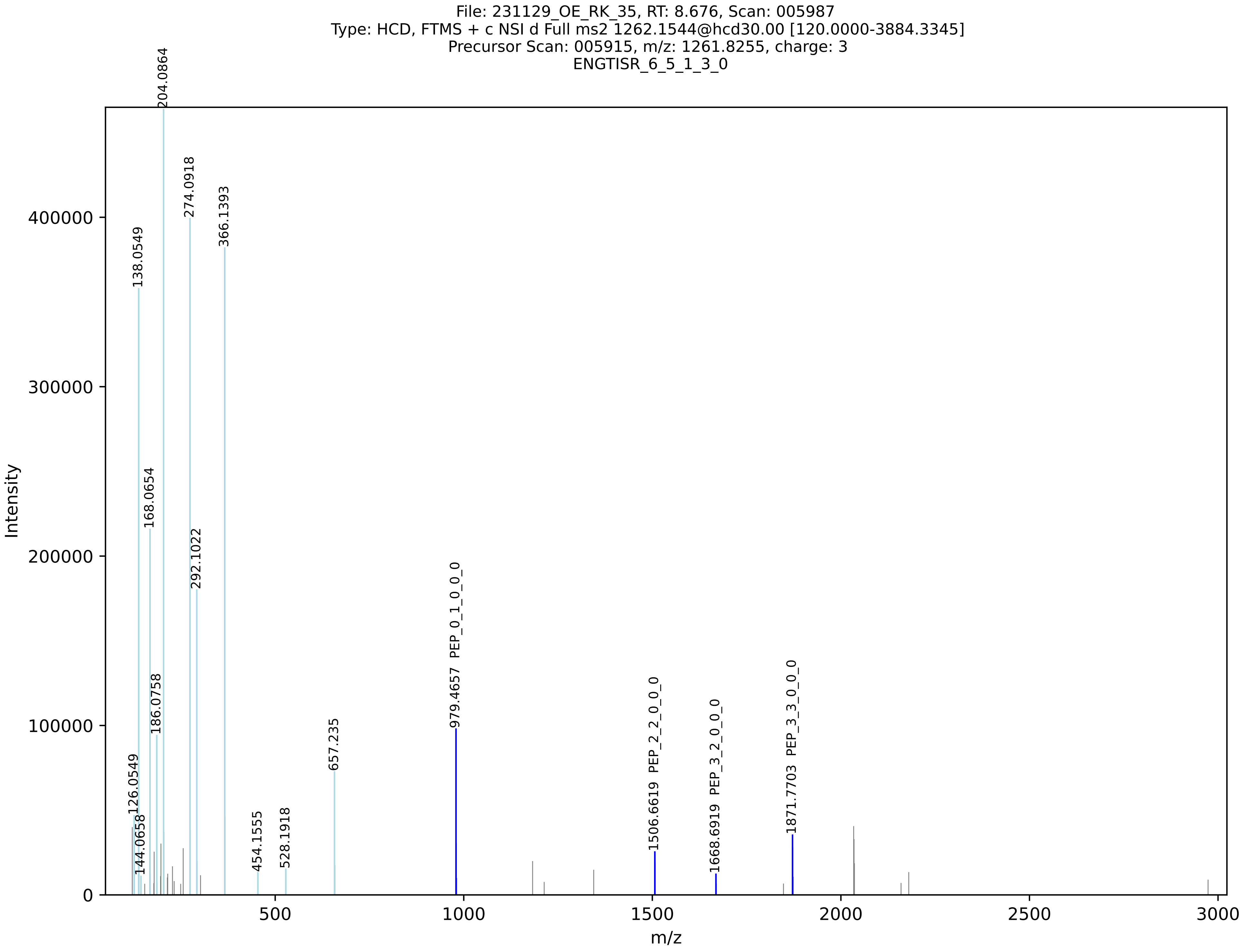
File: 231129_OE_RK_35, RT: 8.676, Scan: 005987
Type: HCD, FTMS + c NSI d Full ms2 1262.1544@hcd30.00 [120.0000-3884.3345]
Precursor Scan: 005915, m/z: 1261.8255, charge: 3
ENGTISR_6_5_1_3_0
204.0864
274.0918
366.1393
138.0549
168.0654
292.1022
186.0758
126.0549
144.0658
454.1555
528.1918
657.235
979.4657 PEP_0_1_0_0_0
1506.6619 PEP_2_2_0_0_0
1668.6919 PEP_3_2_0_0_0
1871.7703 PEP_3_3_0_0_0
Intensity
m/z
0
100000
200000
300000
400000
500
1000
1500
2000
2500
3000

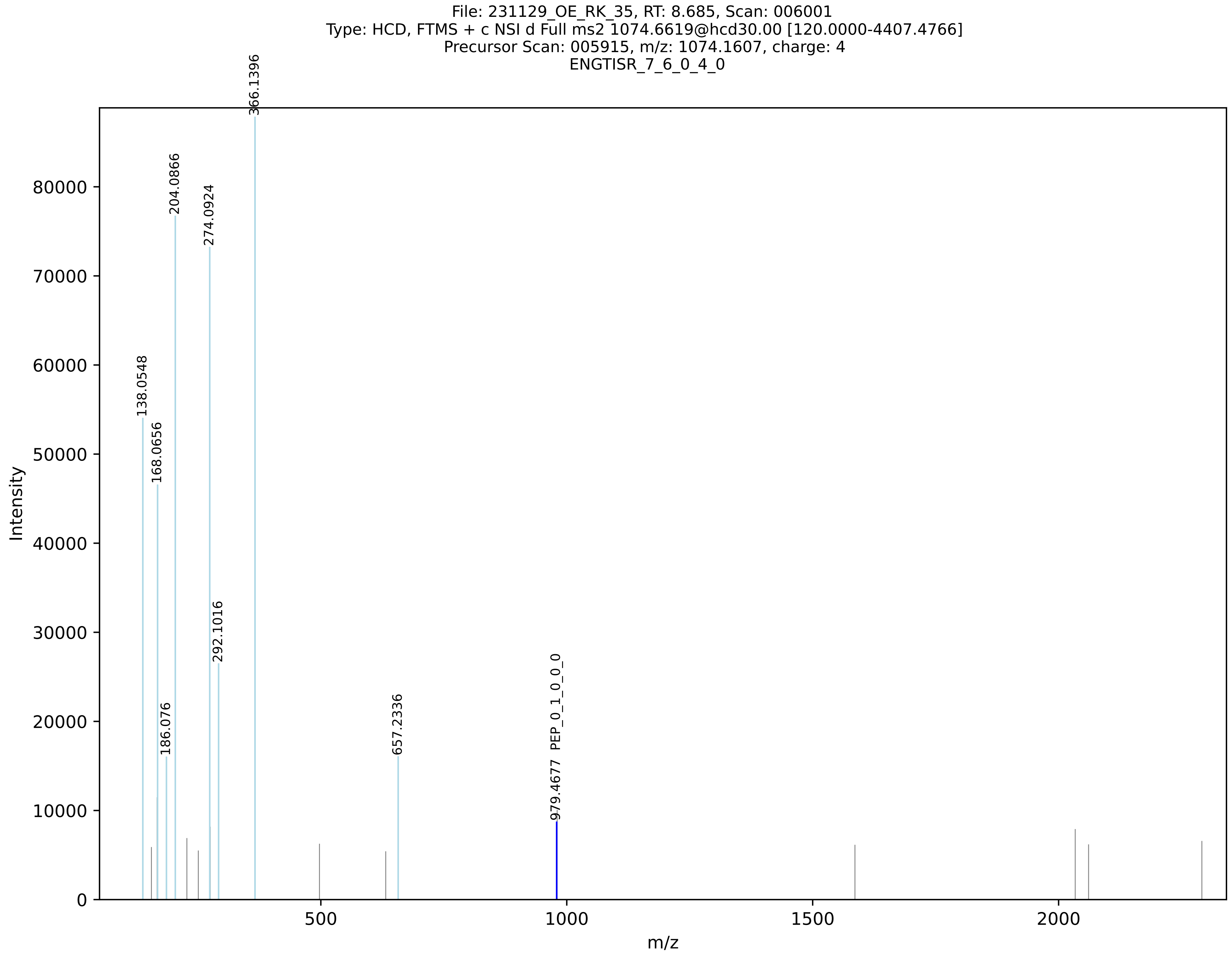
File: 231129_OE_RK_35, RT: 8.685, Scan: 006001
Type: HCD, FTMS + c NSI d Full ms2 1074.6619@hcd30.00 [120.0000-4407.4766]
Precursor Scan: 005915, m/z: 1074.1607, charge: 4
ENGTISR_7_6_0_4_0
Intensity
m/z
366.1396
204.0866
274.0924
138.0548
168.0656
292.1016
186.076
657.2336
979.4677 PEP_0_1_0_0_0
0
10000
20000
30000
40000
50000
60000
70000
80000
500
1000
1500
2000

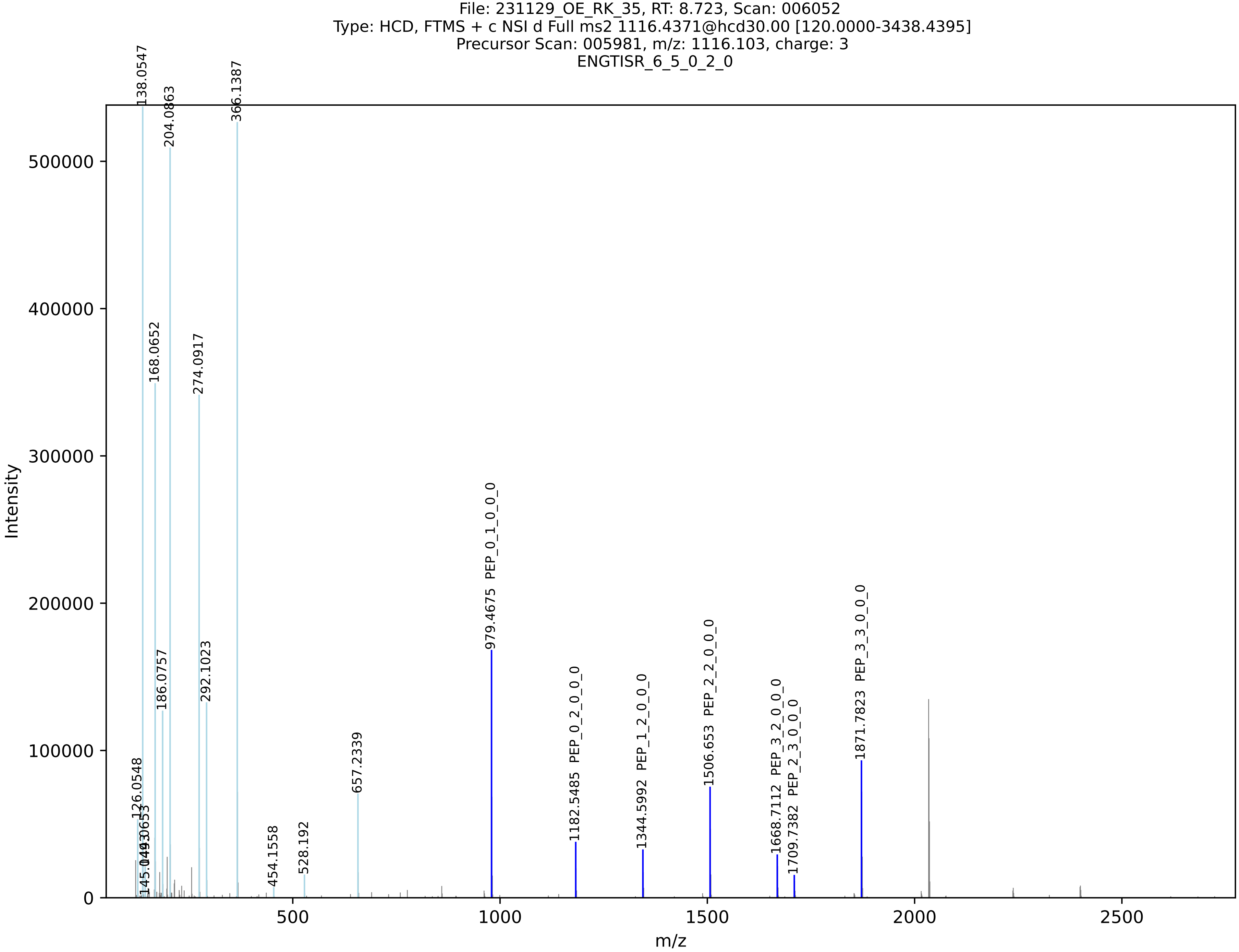

File: 231129_OE_RK_35, RT: 8.723, Scan: 006052
Type: HCD, FTMS + c NSI d Full ms2 1116.4371@hcd30.00 [120.0000-3438.4395]
Precursor Scan: 005981, m/z: 1116.103, charge: 3
ENGTISR_6_5_0_2_0
Intensity
m/z
138.0547
204.0863
366.1387
168.0652
274.0917
186.0757
292.1023
126.0548
454.1558
528.192
657.2339
979.4675 PEP_0_1_0_0_0
1182.5485 PEP_0_2_0_0_0
1344.5992 PEP_1_2_0_0_0
1506.653 PEP_2_2_0_0_0
1668.7112 PEP_3_2_0_0_0
1709.7382 PEP_2_3_0_0_0
1871.7823 PEP_3_3_0_0_0

File: 231129_OE_RK_35, RT: 8.73, Scan: 006063
Type: HCD, FTMS + c NSI d Full ms2 897.0479@hcd30.00 [120.0000-2767.1084]
Precursor Scan: 005981, m/z: 896.7137, charge: 3
YKNNSDISSTR_4_3_1_0_0

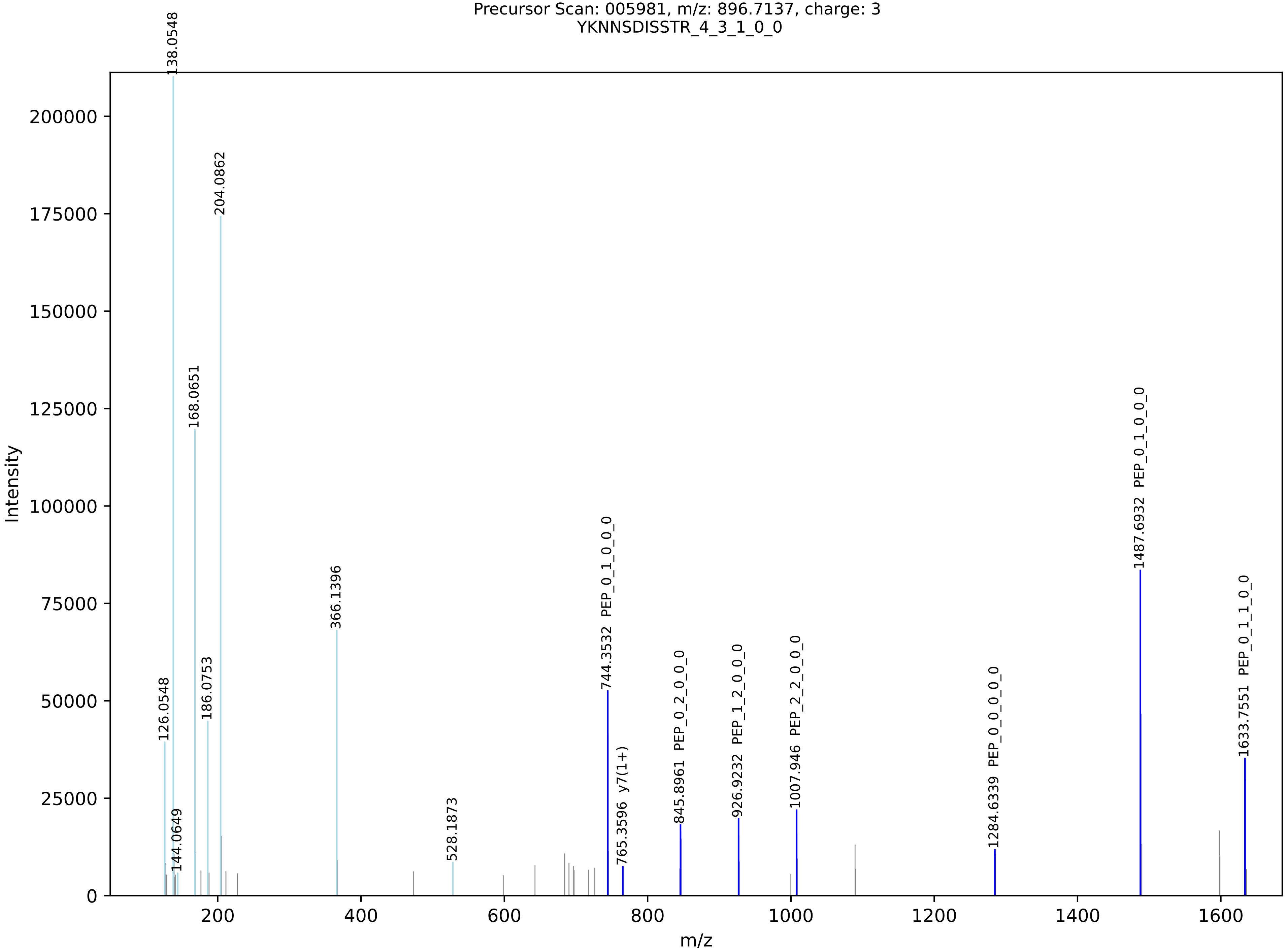

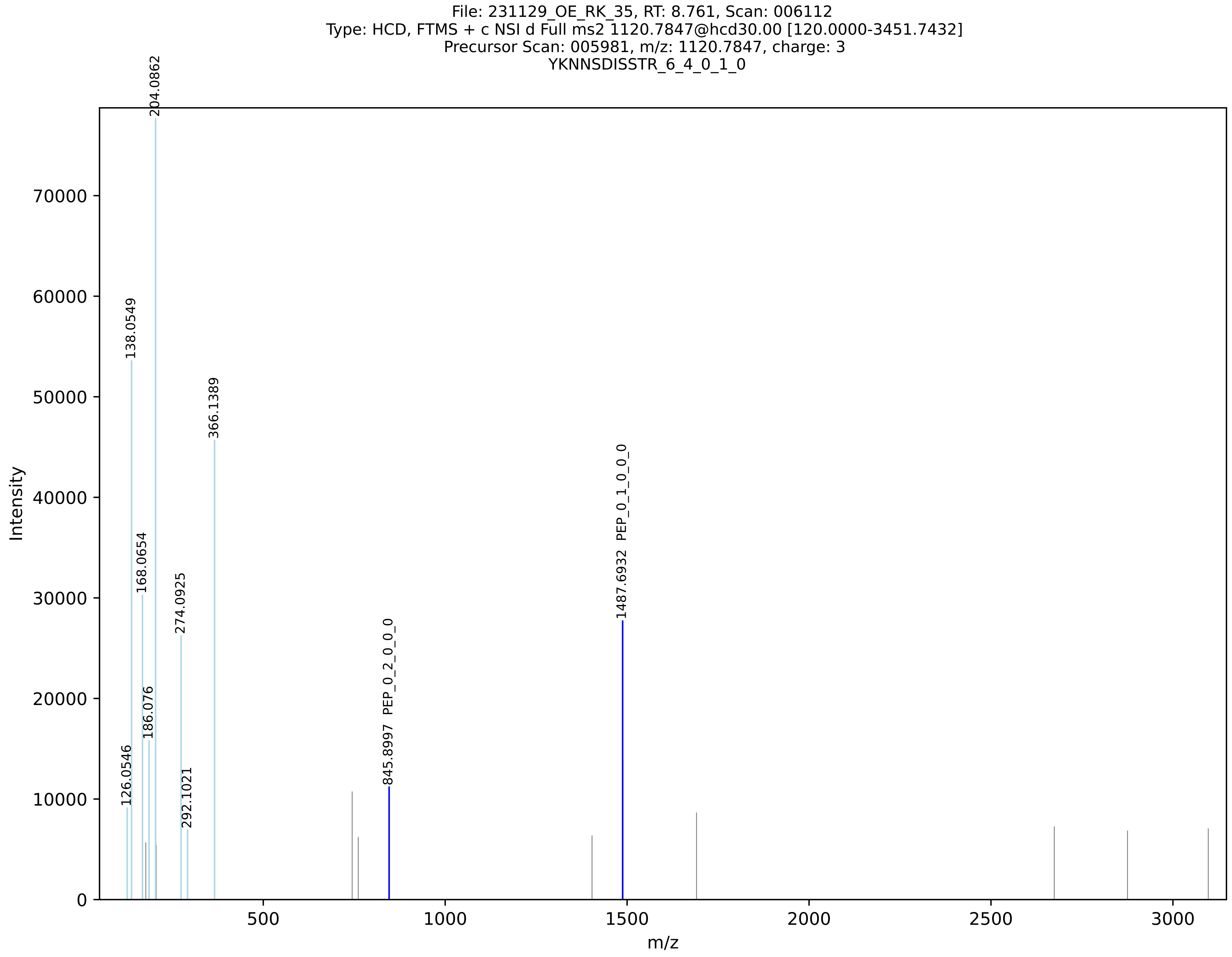
File: 231129_OE_RK_35, RT: 8.761, Scan: 006112
Type: HCD, FTMS + c NSI d Full ms2 1120.7847@hcd30.00 [120.0000-3451.7432]
Precursor Scan: 005981, m/z: 1120.7847, charge: 3
YKNNSDISSTR_6_4_0_1_0
204.0862
138.0549
366.1389
168.0654
274.0925
186.076
126.0546
292.1021
845.8997 PEP_0_2_0_0_0
1487.6932 PEP_0_1_0_0_0
Intensity
m/z
0
10000
20000
30000
40000
50000
60000
70000
500
1000
1500
2000
2500
3000

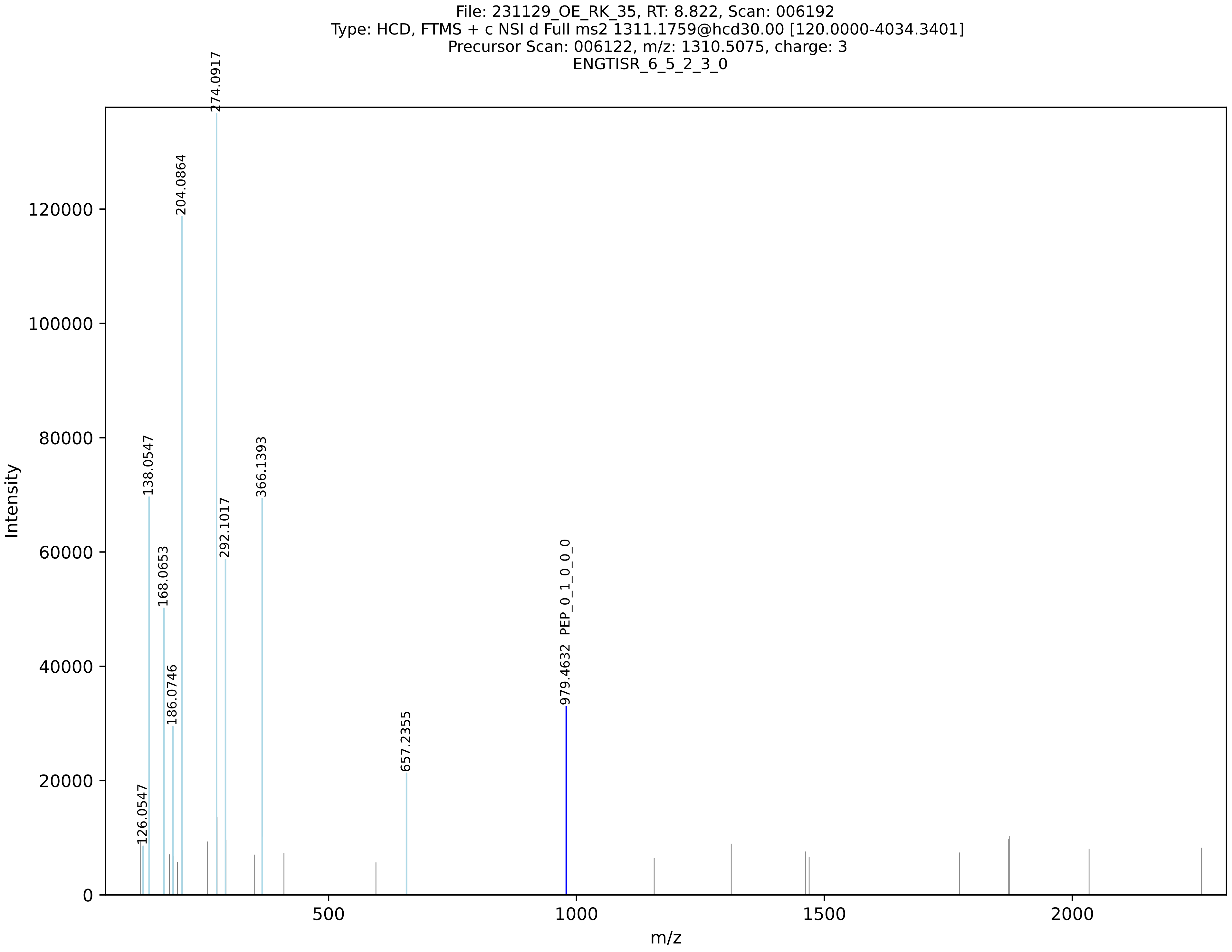
File: 231129_OE_RK_35, RT: 8.822, Scan: 006192
Type: HCD, FTMS + c NSI d Full ms2 1311.1759@hcd30.00 [120.0000-4034.3401]
Precursor Scan: 006122, m/z: 1310.5075, charge: 3
ENGTISR_6_5_2_3_0
274.0917
204.0864
138.0547
366.1393
292.1017
168.0653
186.0746
657.2355
979.4632 PEP_0_1_0_0_0
126.0547
Intensity
m/z
0
20000
40000
60000
80000
100000
120000
500
1000
1500
2000

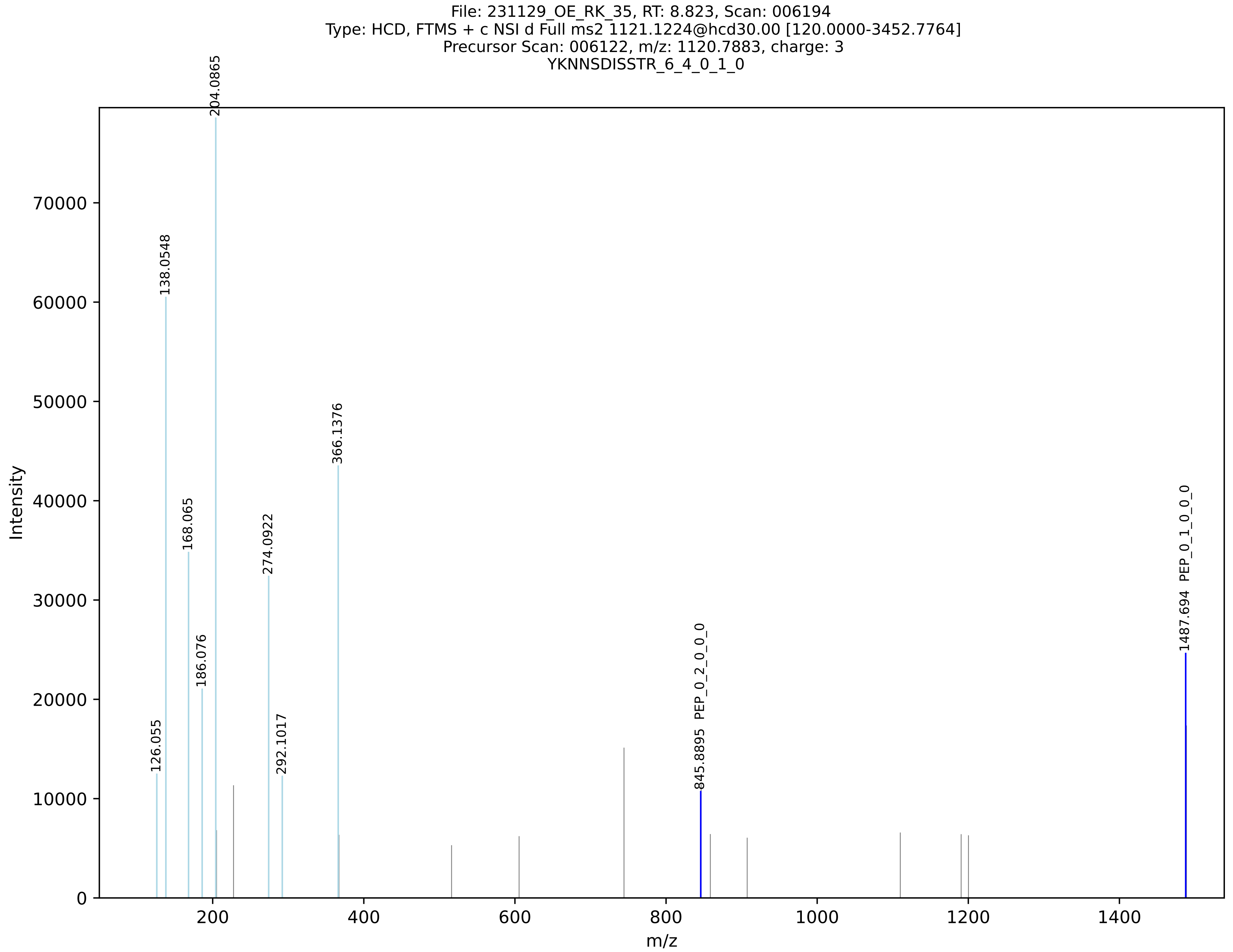
File: 231129_OE_RK_35, RT: 8.823, Scan: 006194
Type: HCD, FTMS + c NSI d Full ms2 1121.1224@hcd30.00 [120.0000-3452.7764]
Precursor Scan: 006122, m/z: 1120.7883, charge: 3
YKNNSDISSTR_6_4_0_1_0
Intensity
m/z
204.0865
138.0548
366.1376
168.065
274.0922
186.076
126.055
292.1017
845.8895 PEP_0_2_0_0_0
1487.694 PEP_0_1_0_0_0
0
10000
20000
30000
40000
50000
60000
70000
200
400
600
800
1000
1200
1400

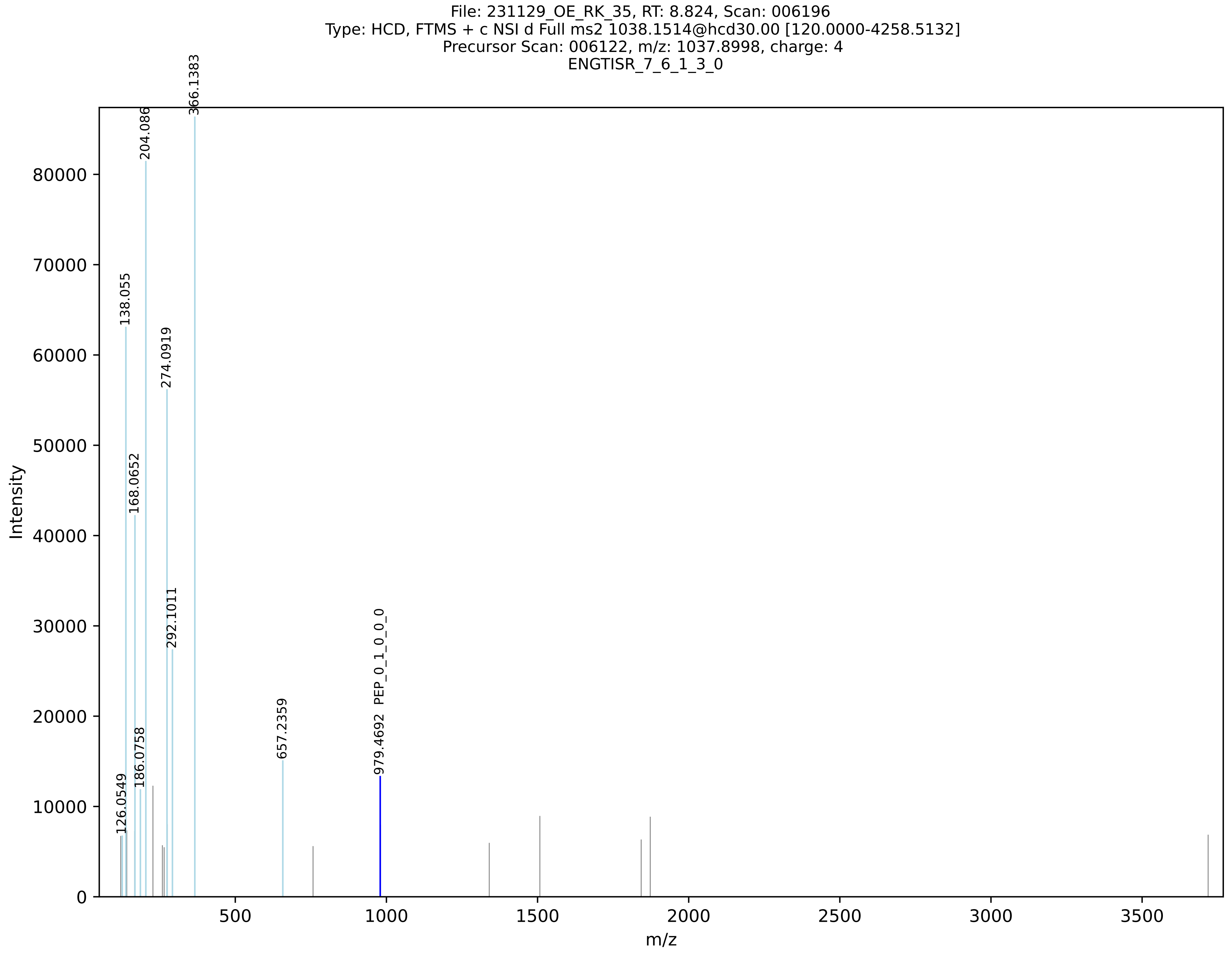
File: 231129_OE_RK_35, RT: 8.824, Scan: 006196
Type: HCD, FTMS + c NSI d Full ms2 1038.1514@hcd30.00 [120.0000-4258.5132]
Precursor Scan: 006122, m/z: 1037.8998, charge: 4
ENGTISR_7_6_1_3_0
Intensity
m/z
366.1383
204.086
138.055
274.0919
168.0652
292.1011
657.2359
979.4692 PEP_0_1_0_0_0
186.0758
126.0549

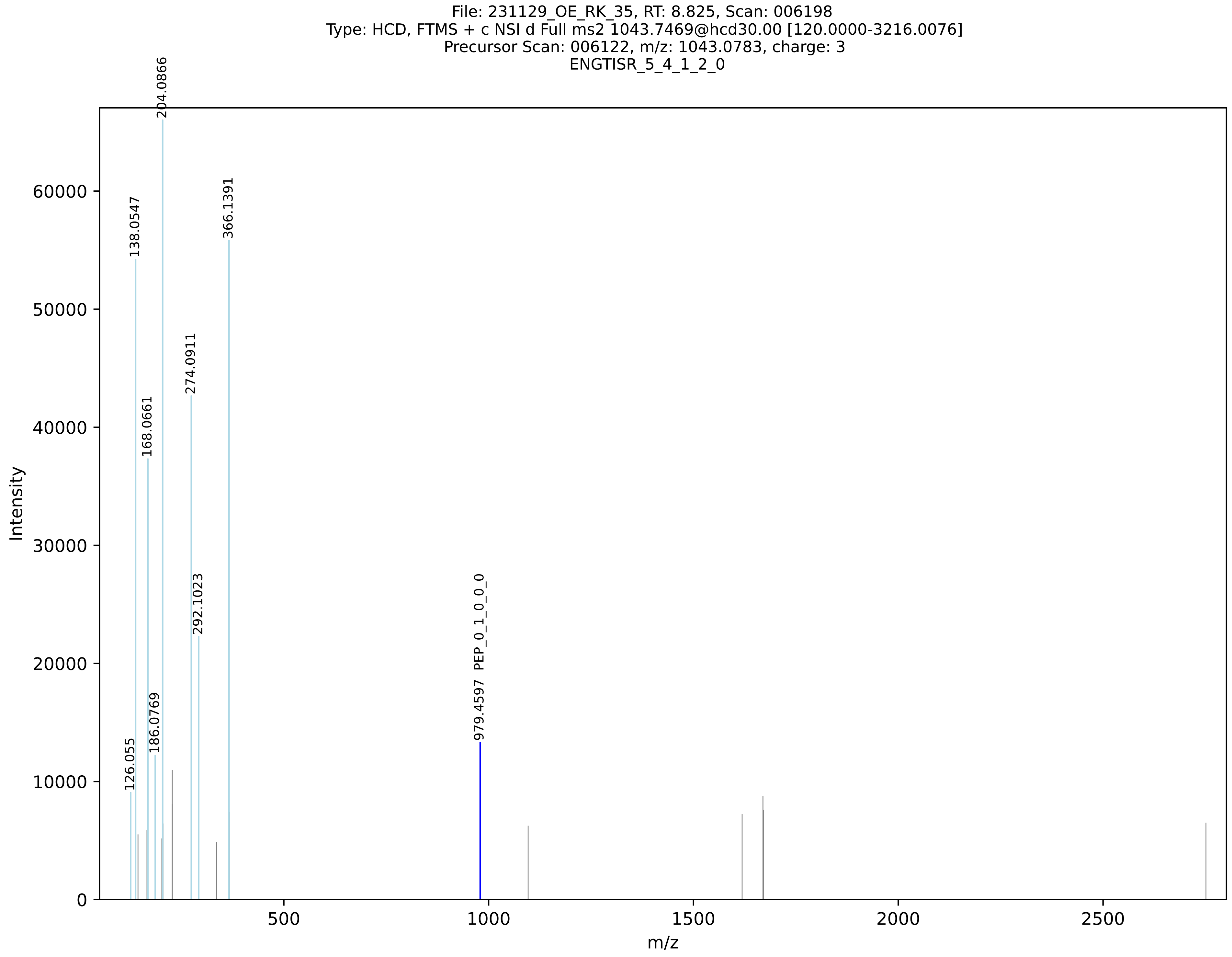
File: 231129_OE_RK_35, RT: 8.825, Scan: 006198
Type: HCD, FTMS + c NSI d Full ms2 1043.7469@hcd30.00 [120.0000-3216.0076]
Precursor Scan: 006122, m/z: 1043.0783, charge: 3
ENGTISR_5_4_1_2_0
Intensity
m/z
204.0866
366.1391
138.0547
274.0911
168.0661
292.1023
979.4597 PEP_0_1_0_0_0
186.0769
126.055
0
10000
20000
30000
40000
50000
60000
500
1000
1500
2000
2500

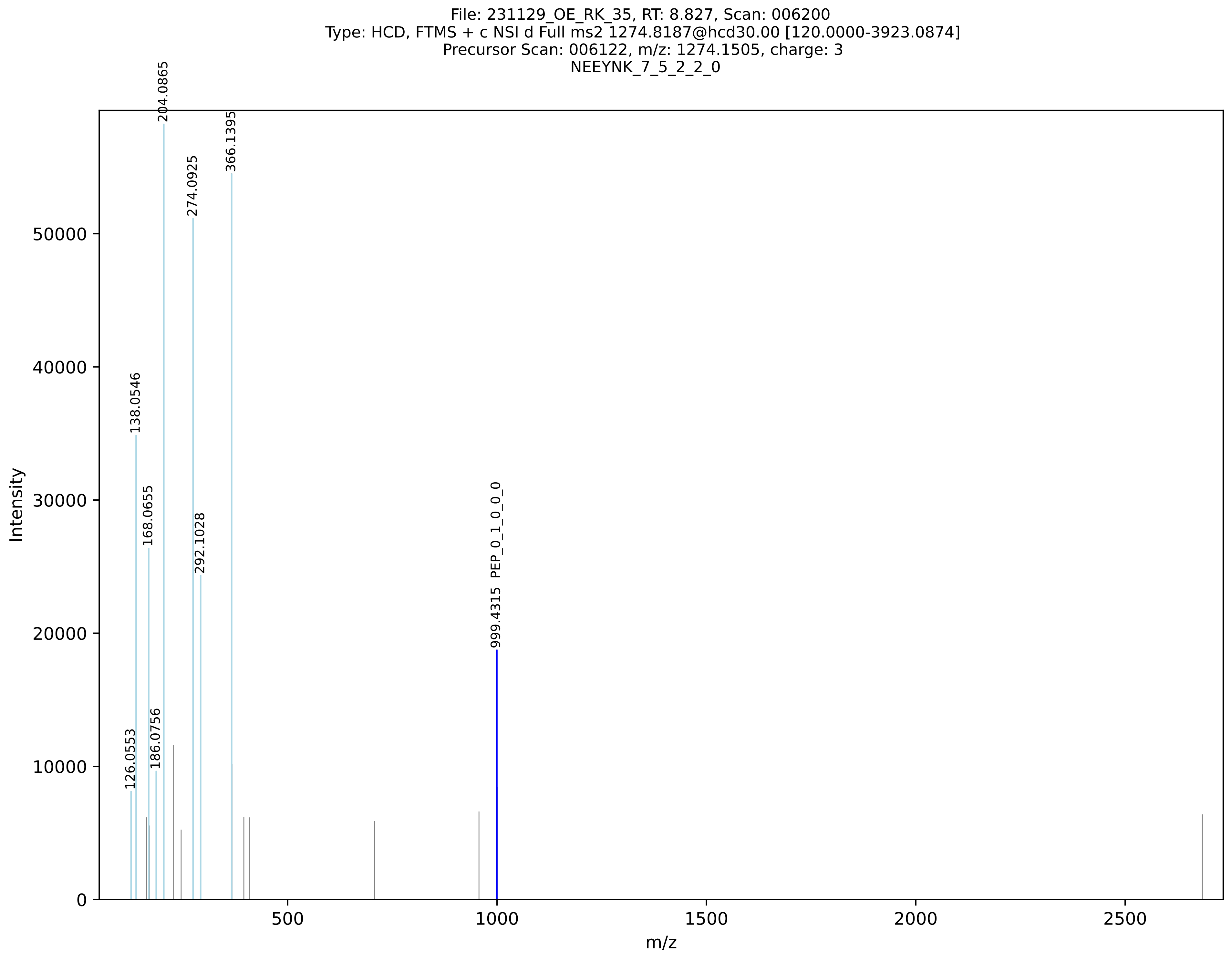
File: 231129_OE_RK_35, RT: 8.827, Scan: 006200
Type: HCD, FTMS + c NSI d Full ms2 1274.8187@hcd30.00 [120.0000-3923.0874]
Precursor Scan: 006122, m/z: 1274.1505, charge: 3
NEEYNK_7_5_2_2_0
204.0865
366.1395
274.0925
138.0546
168.0655
292.1028
999.4315 PEP_0_1_0_0_0
126.0553
186.0756
Intensity
m/z
0
10000
20000
30000
40000
50000
500
1000
1500
2000
2500

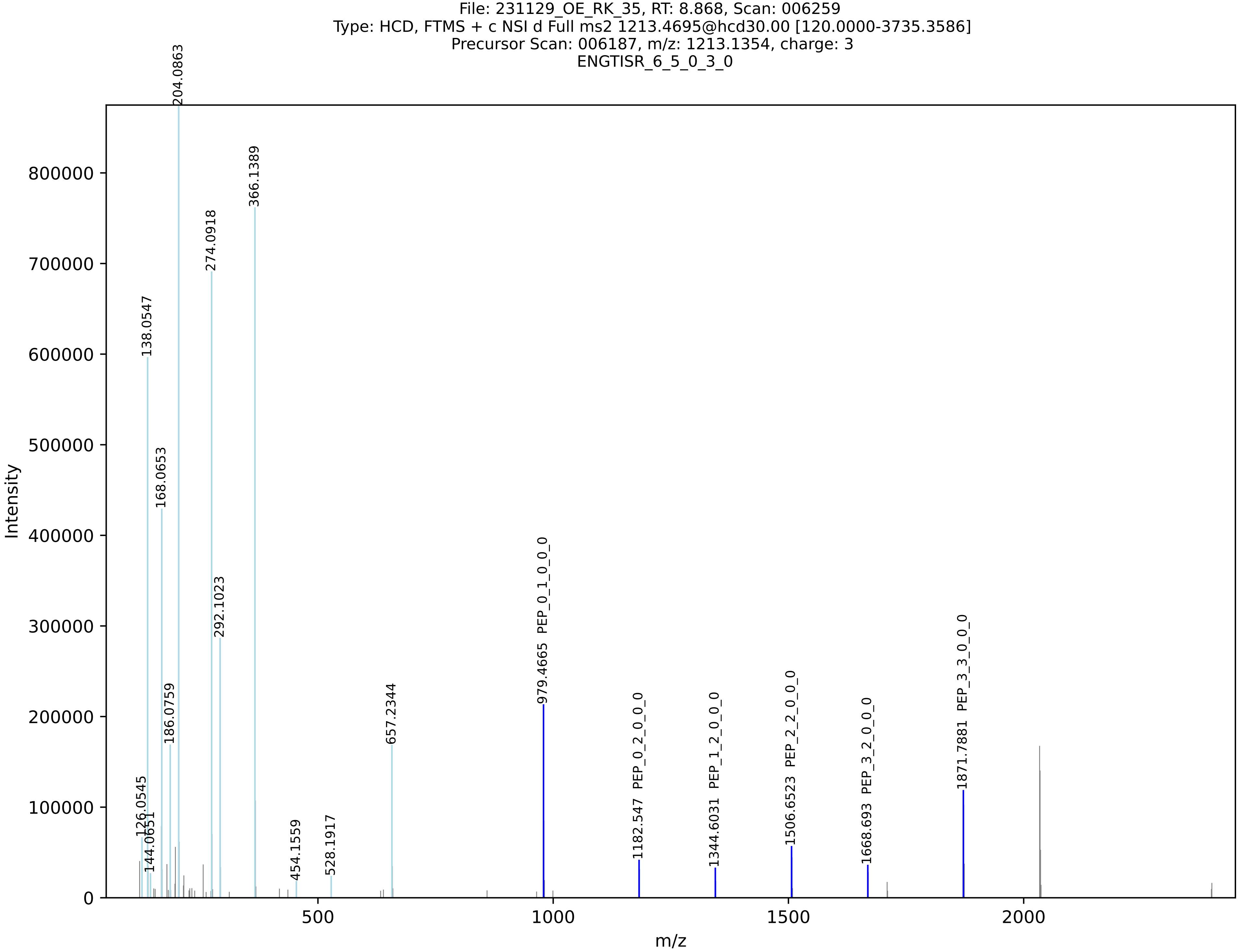
File: 231129_OE_RK_35, RT: 8.868, Scan: 006259
Type: HCD, FTMS + c NSI d Full ms2 1213.4695@hcd30.00 [120.0000-3735.3586]
Precursor Scan: 006187, m/z: 1213.1354, charge: 3
ENGTISR_6_5_0_3_0
Intensity
m/z
204.0863
366.1389
274.0918
138.0547
168.0653
292.1023
186.0759
126.0545
144.0651
454.1559
528.1917
657.2344
979.4665 PEP_0_1_0_0_
1182.547 PEP_0_2_0_0_
1344.6031 PEP_1_2_0_0_
1506.6523 PEP_2_2_0_0_
1668.693 PEP_3_2_0_0_
1871.7881 PEP_3_3_0_0_

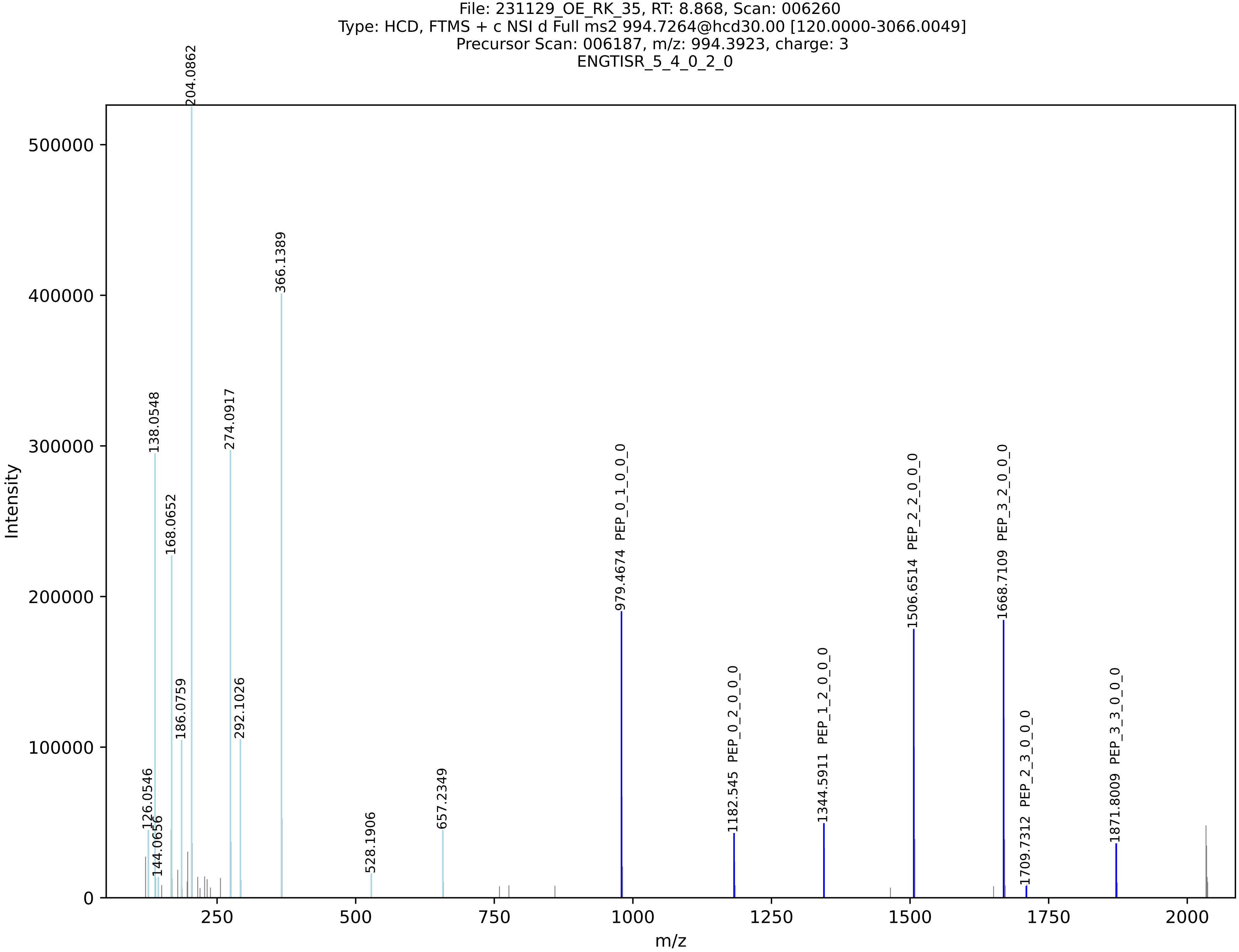
File: 231129_OE_RK_35, RT: 8.868, Scan: 006260
Type: HCD, FTMS + c NSI d Full ms2 994.7264@hcd30.00 [120.0000-3066.0049]
Precursor Scan: 006187, m/z: 994.3923, charge: 3
ENGTISR_5_4_0_2_0
Intensity
m/z
204.0862
366.1389
138.0548
274.0917
168.0652
186.0759
292.1026
126.0546
144.0656
528.1906
657.2349
979.4674 PEP_0_1_0_0_0
1182.545 PEP_0_2_0_0_0
1344.5911 PEP_1_2_0_0_0
1506.6514 PEP_2_2_0_0_0
1668.7109 PEP_3_2_0_0_0
1709.7312 PEP_2_3_0_0_0
1871.8009 PEP_3_3_0_0_0

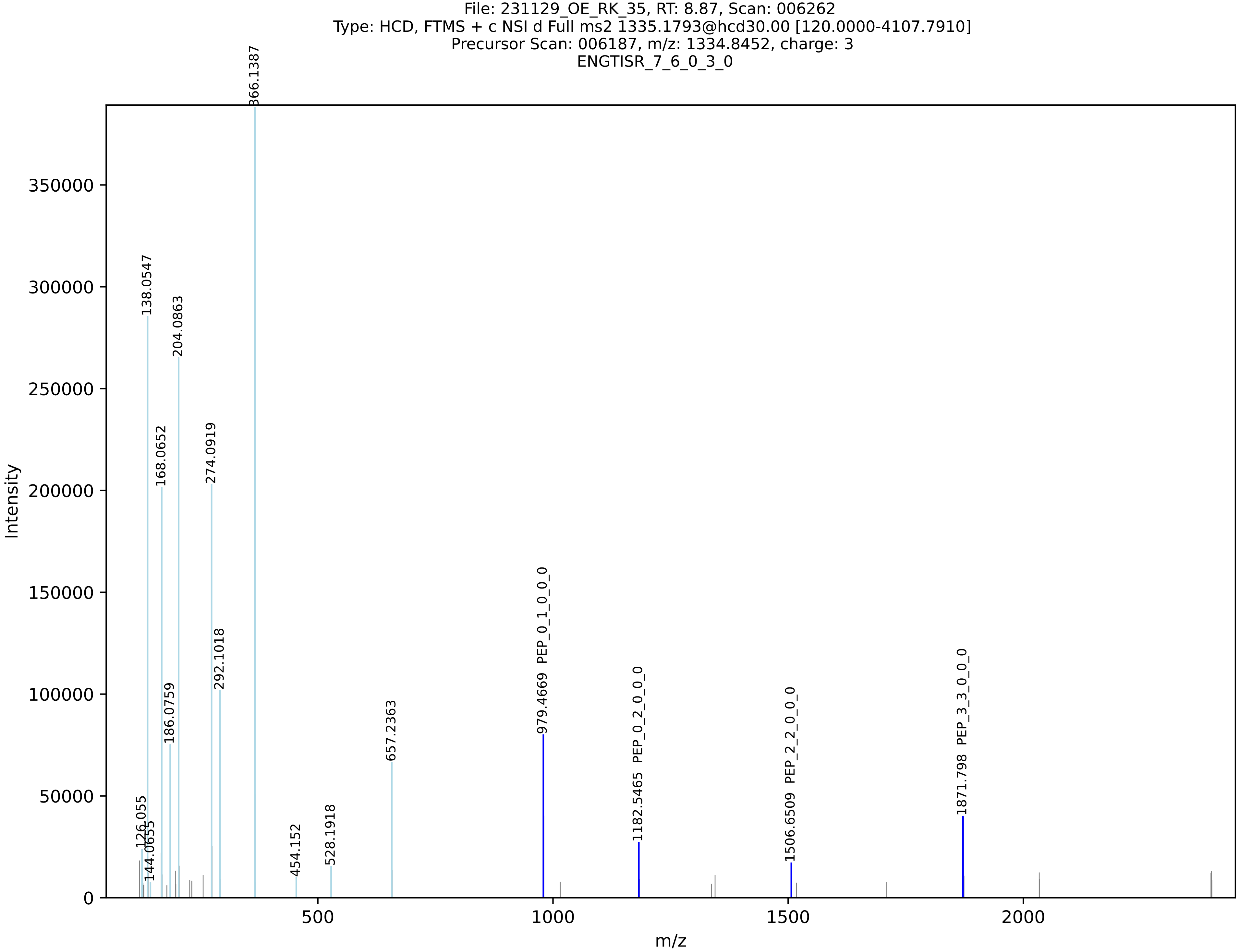

File: 231129_OE_RK_35, RT: 8.87, Scan: 006262
Type: HCD, FTMS + c NSI d Full ms2 1335.1793@hcd30.00 [120.0000-4107.7910]
Precursor Scan: 006187, m/z: 1334.8452, charge: 3
ENGTISR_7_6_0_3_0
Intensity
m/z
366.1387
138.0547
204.0863
168.0652
274.0919
292.1018
186.0759
126.055
144.0655
454.152
528.1918
657.2363
979.4669 PEP_0_1_0_0_0
1182.5465 PEP_0_2_0_0_0
1506.6509 PEP_2_2_0_0_0
1871.798 PEP_3_3_0_0_0

File: 231129_OE_RK_35, RT: 8.87, Scan: 006263
Type: HCD, FTMS + c NSI d Full ms2 1262.4896@hcd30.00 [120.0000-3885.3601]
Precursor Scan: 006187, m/z: 1261.8214, charge: 3
ENGTISR_6_5_1_3_0

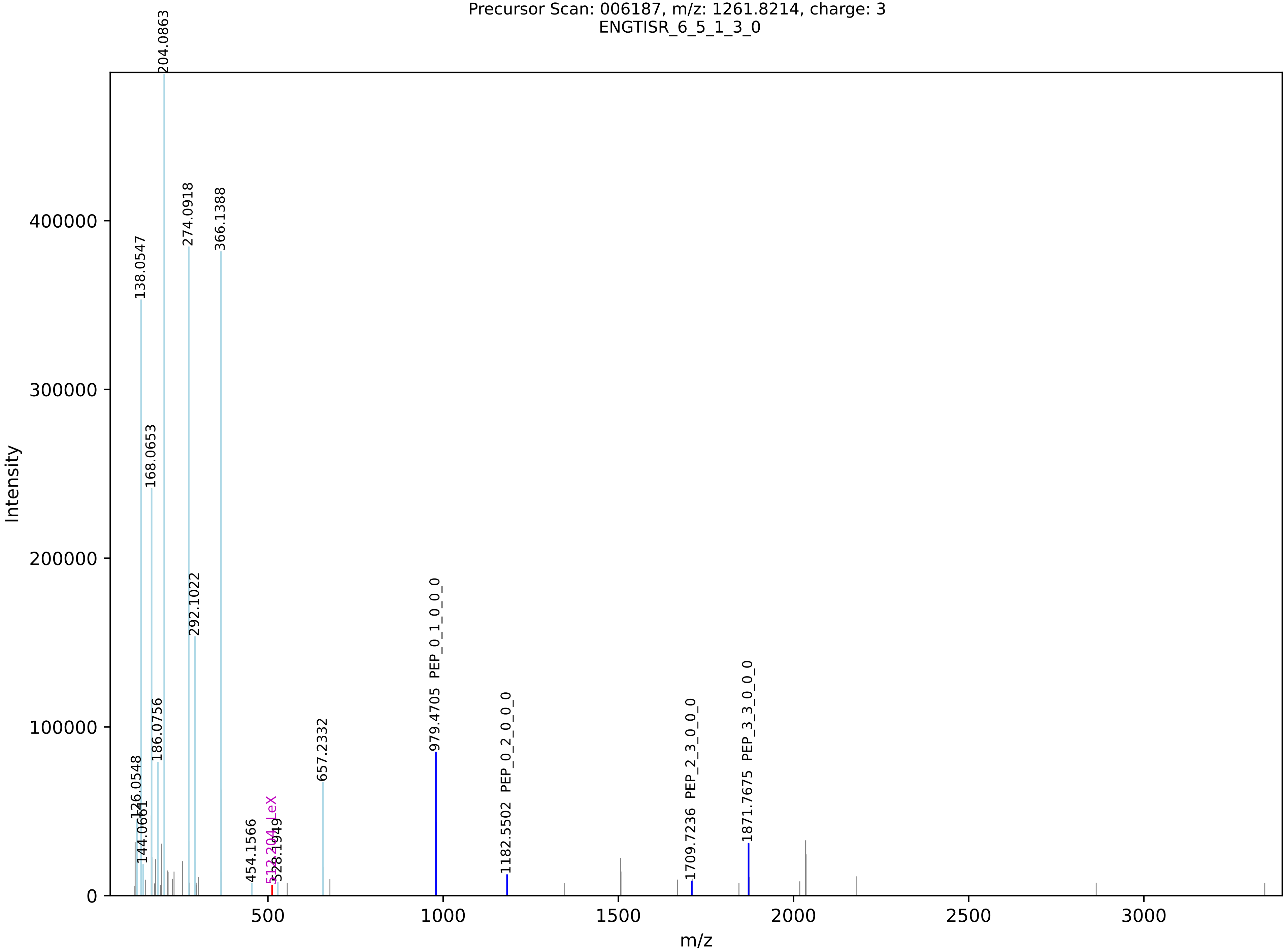

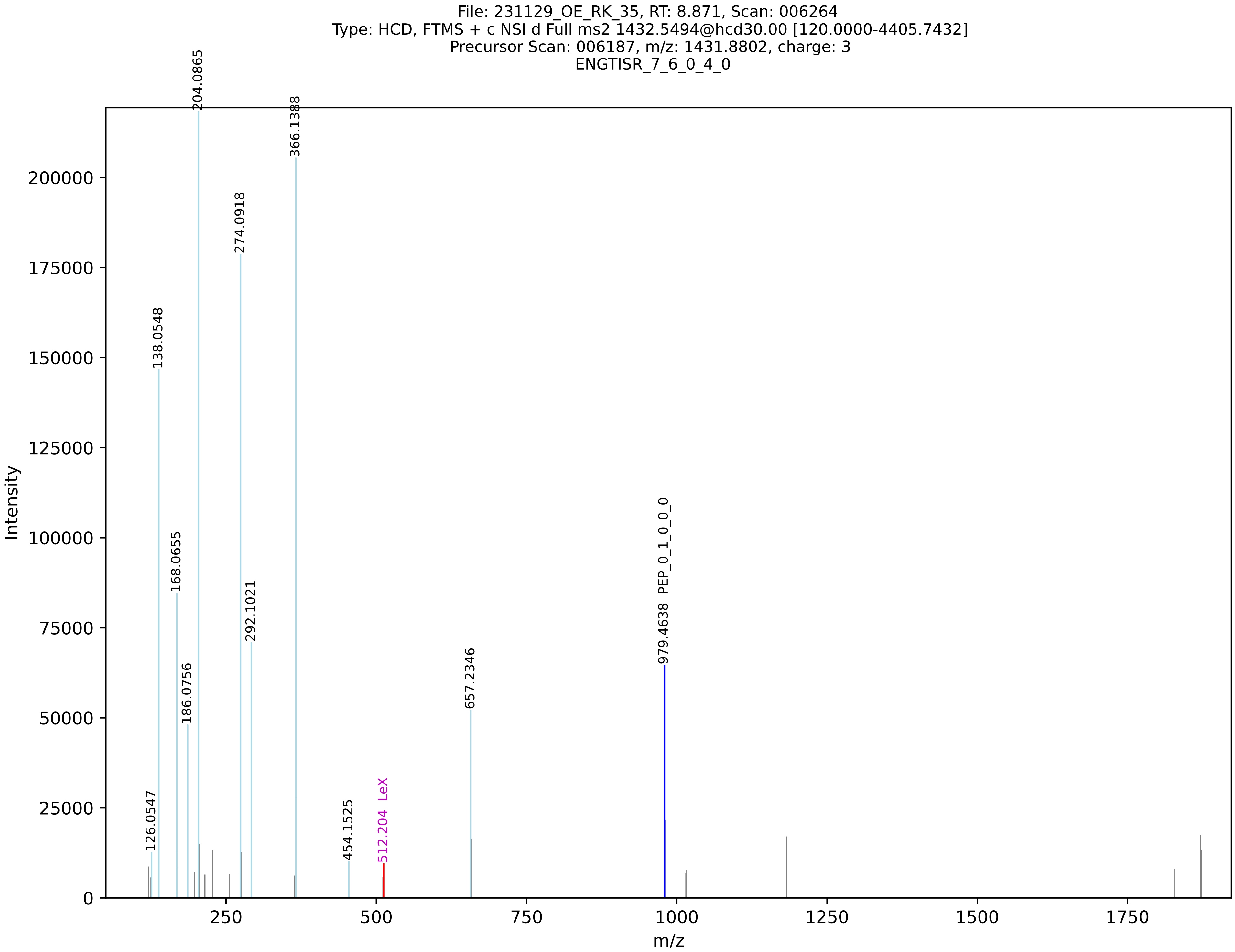
File: 231129_OE_RK_35, RT: 8.871, Scan: 006264
Type: HCD, FTMS + c NSI d Full ms2 1432.5494@hcd30.00 [120.0000-4405.7432]
Precursor Scan: 006187, m/z: 1431.8802, charge: 3
ENGTISR_7_6_0_4_0
Intensity
m/z
204.0865
366.1388
274.0918
138.0548
168.0655
292.1021
186.0756
126.0547
454.1525
512.204 LeX
657.2346
979.4638 PEP_0_1_0_0_0
0
25000
50000
75000
100000
125000
150000
175000
200000
250
500
750
1000
1250
1500
1750

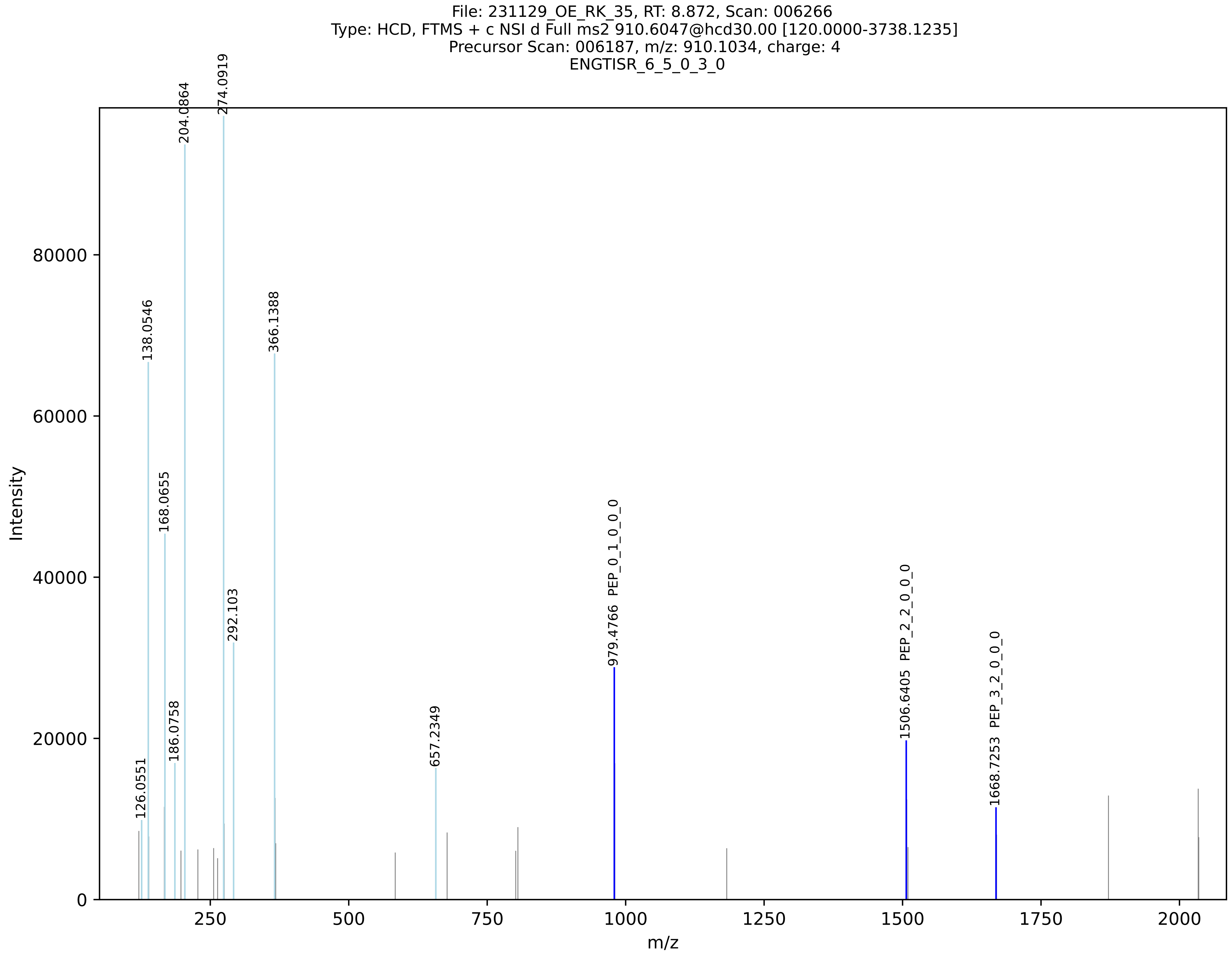
File: 231129_OE_RK_35, RT: 8.872, Scan: 006266
Type: HCD, FTMS + c NSI d Full ms2 910.6047@hcd30.00 [120.0000-3738.1235]
Precursor Scan: 006187, m/z: 910.1034, charge: 4
ENGTISR_6_5_0_3_0
274.0919
204.0864
138.0546
366.1388
168.0655
292.103
186.0758
126.0551
657.2349
979.4766 PEP_0_1_0_0_0
1506.6405 PEP_2_2_0_0_0
1668.7253 PEP_3_2_0_0_0
Intensity
m/z
0
20000
40000
60000
80000
250
500
750
1000
1250
1500
1750
2000

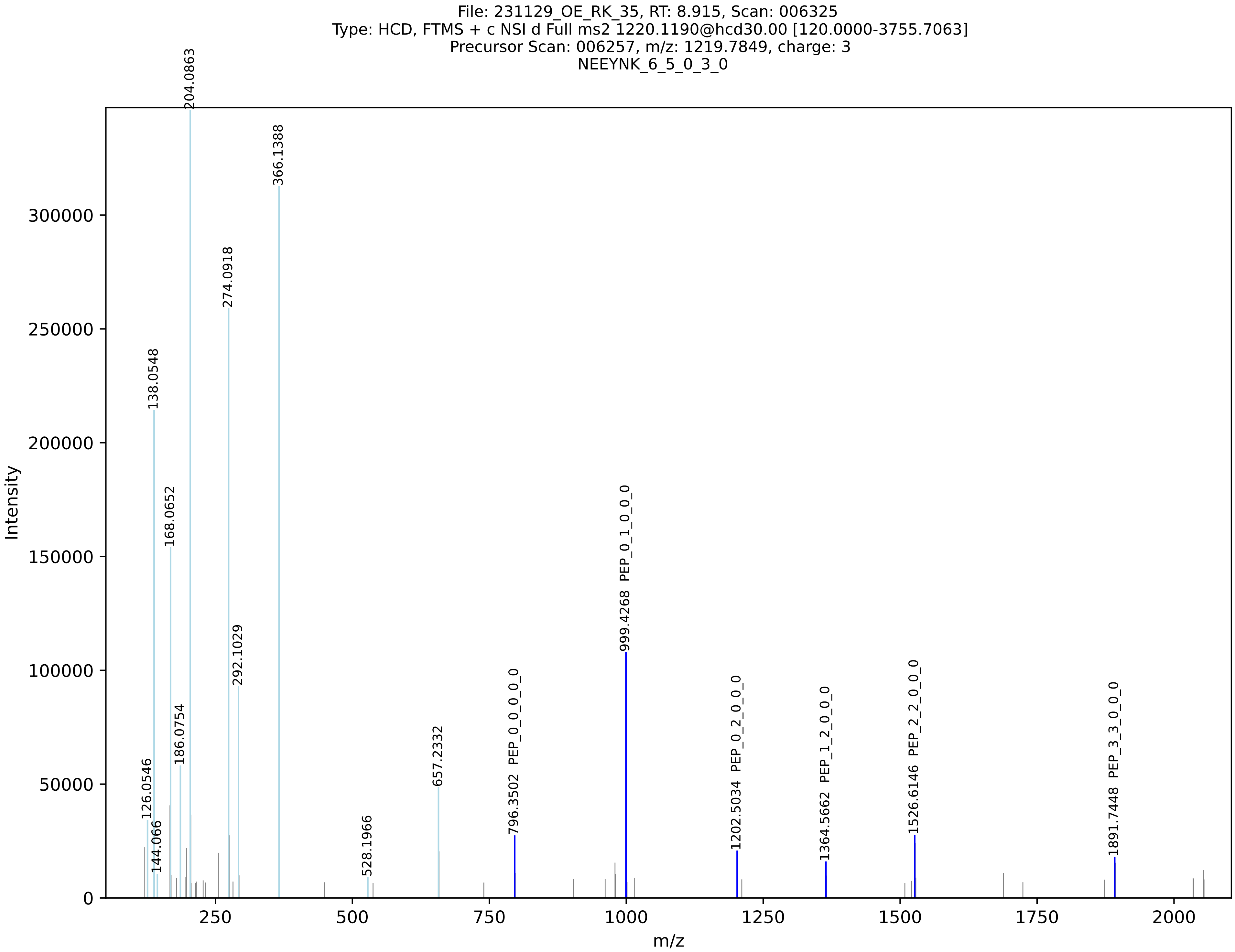
File: 231129_OE_RK_35, RT: 8.915, Scan: 006325
Type: HCD, FTMS + c NSI d Full ms2 1220.1190@hcd30.00 [120.0000-3755.7063]
Precursor Scan: 006257, m/z: 1219.7849, charge: 3
NEEYNK_6_5_0_3_0
Intensity
m/z
204.0863
366.1388
274.0918
138.0548
168.0652
292.1029
186.0754
126.0546
144.066
528.1966
657.2332
796.3502 PEP_0_0_0_0_0_
999.4268 PEP_0_1_0_0_0_
1202.5034 PEP_0_2_0_0_0_
1364.5662 PEP_1_2_0_0_0_
1526.6146 PEP_2_2_0_0_0_
1891.7448 PEP_3_3_0_0_0_

File: 231129_OE_RK_35, RT: 8.917, Scan: 006327
Type: HCD, FTMS + c NSI d Full ms2 1384.1995@hcd30.00 [120.0000-4257.7920]
Precursor Scan: 006257, m/z: 1383.5312, charge: 3
ENGTISR_7_6_1_3_0

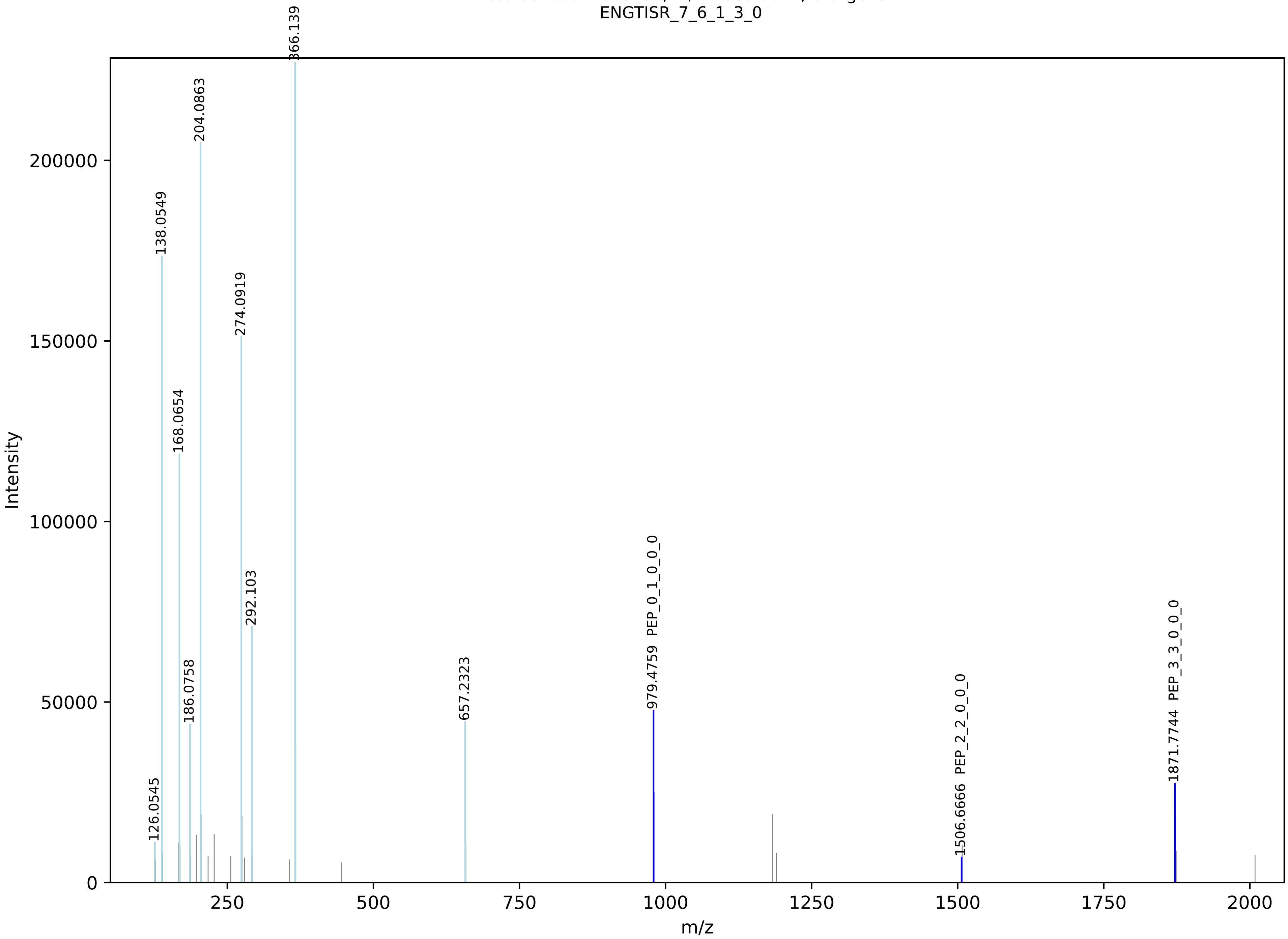

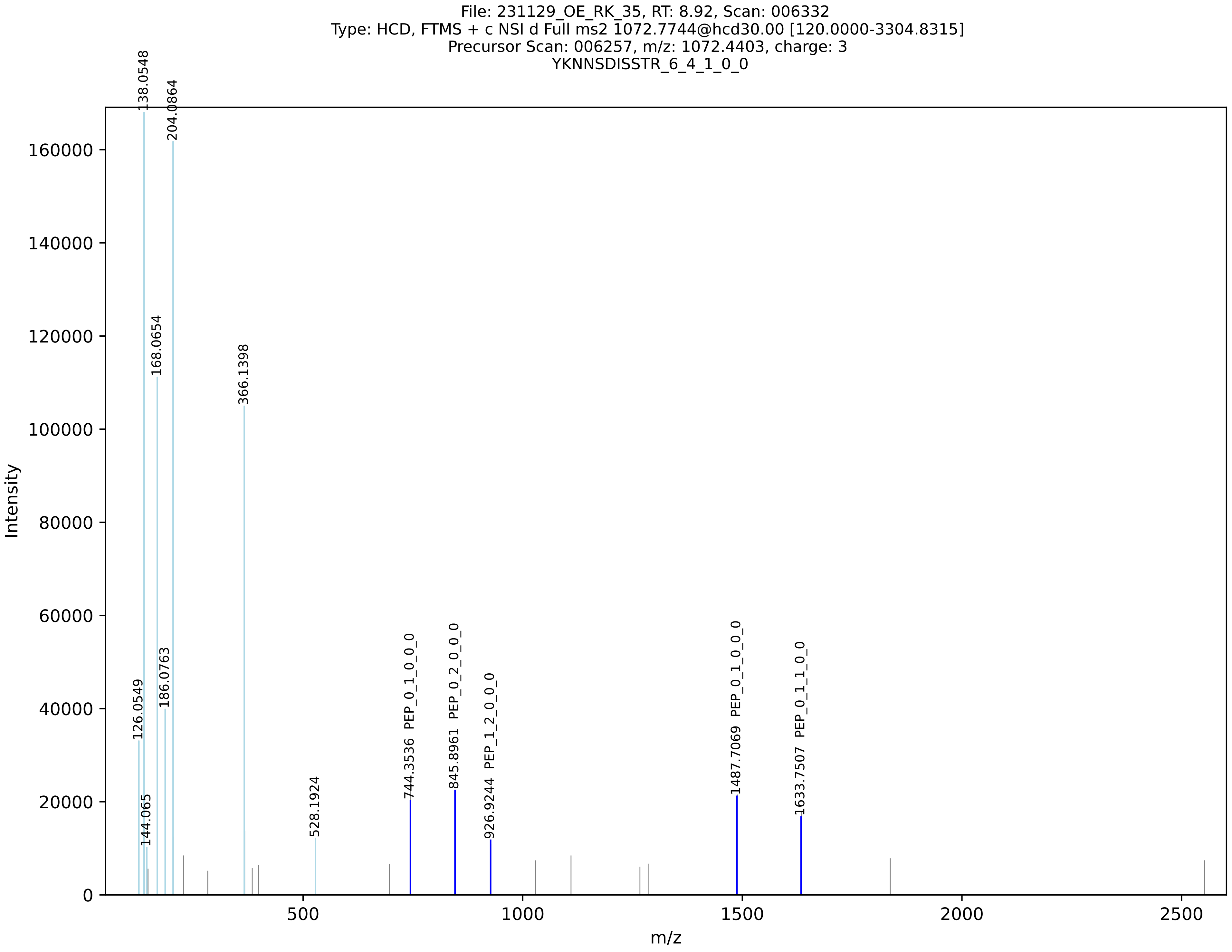
File: 231129_OE_RK_35, RT: 8.92, Scan: 006332
Type: HCD, FTMS + c NSI d Full ms2 1072.7744@hcd30.00 [120.0000-3304.8315]
Precursor Scan: 006257, m/z: 1072.4403, charge: 3
YKNNSDISSTR_6_4_1_0_0
Intensity
m/z
138.0548
204.0864
168.0654
366.1398
126.0549
186.0763
144.065
528.1924
744.3536 PEP_0_1_0_0_0
845.8961 PEP_0_2_0_0_0
926.9244 PEP_1_2_0_0_0
1487.7069 PEP_0_1_0_0_0
1633.7507 PEP_0_1_1_0_0
0
20000
40000
60000
80000
100000
120000
140000
160000
500
1000
1500
2000
2500

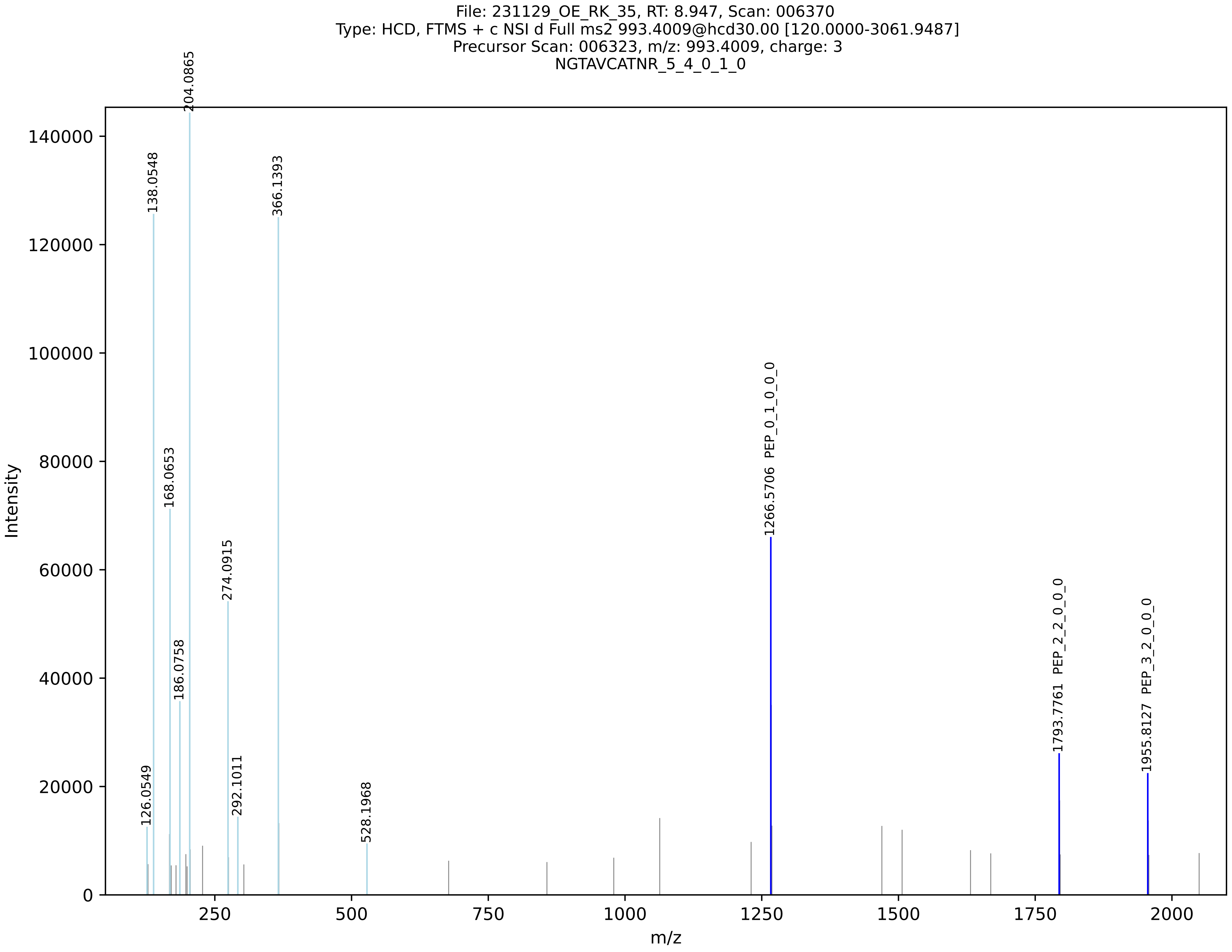
File: 231129_OE_RK_35, RT: 8.947, Scan: 006370
Type: HCD, FTMS + c NSI d Full ms2 993.4009@hcd30.00 [120.0000-3061.9487]
Precursor Scan: 006323, m/z: 993.4009, charge: 3
NGTAVCATNR_5_4_0_1_0
204.0865
138.0548
366.1393
168.0653
274.0915
186.0758
126.0549
292.1011
528.1968
1266.5706 PEP_0_1_0_0_0_
1793.7761 PEP_2_2_0_0_0_
1955.8127 PEP_3_2_0_0_0_
Intensity
m/z
0
20000
40000
60000
80000
100000
120000
140000
250
500
750
1000
1250
1500
1750
2000

File: 231129_OE_RK_35, RT: 9.013, Scan: 006455
Type: HCD, FTMS + c NSI d Full ms2 1311.5103@hcd30.00 [120.0000-4035.3633]
Precursor Scan: 006407, m/z: 1310.5063, charge: 3
ENGTISR_8_7_1_1_0

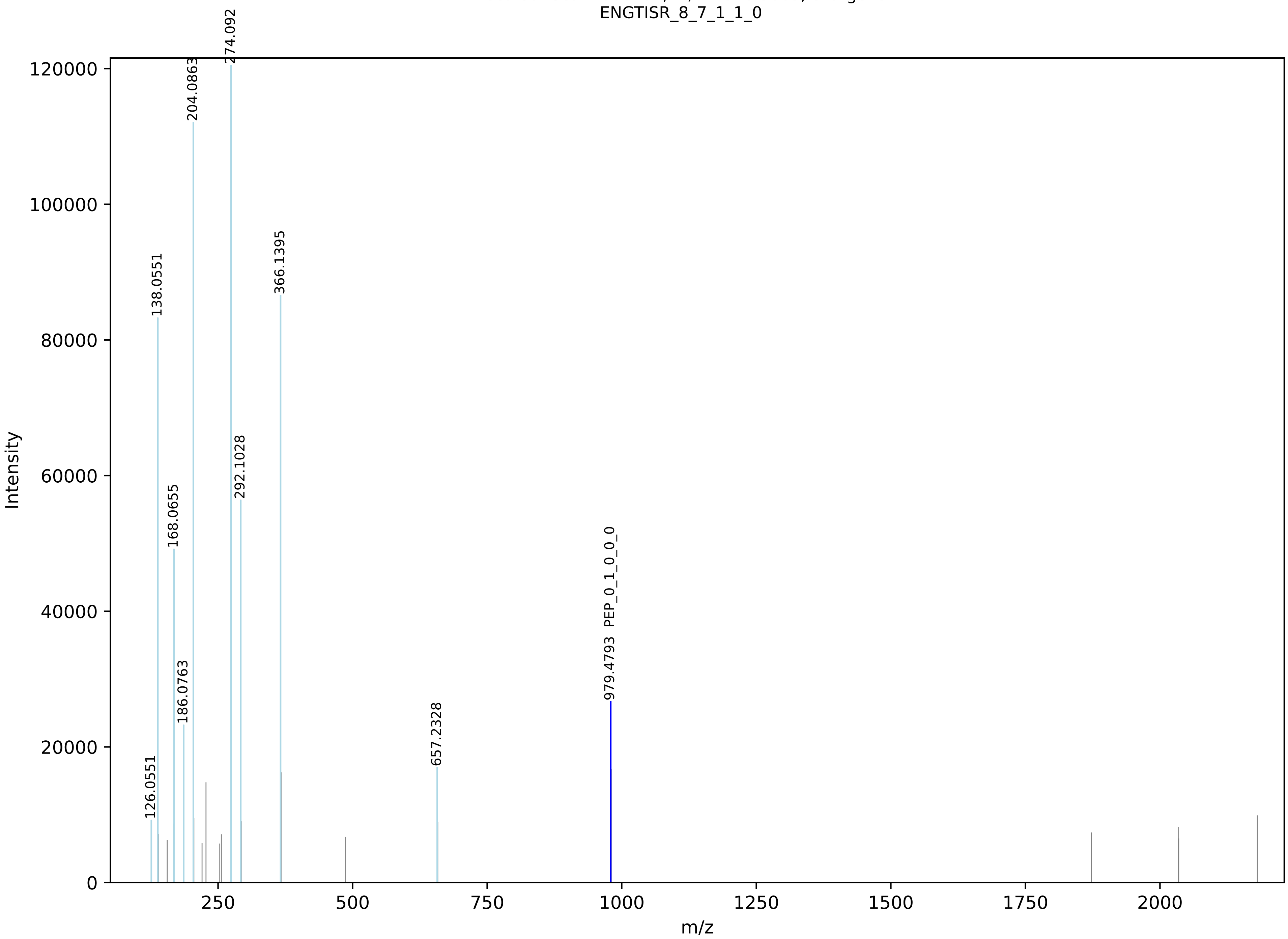

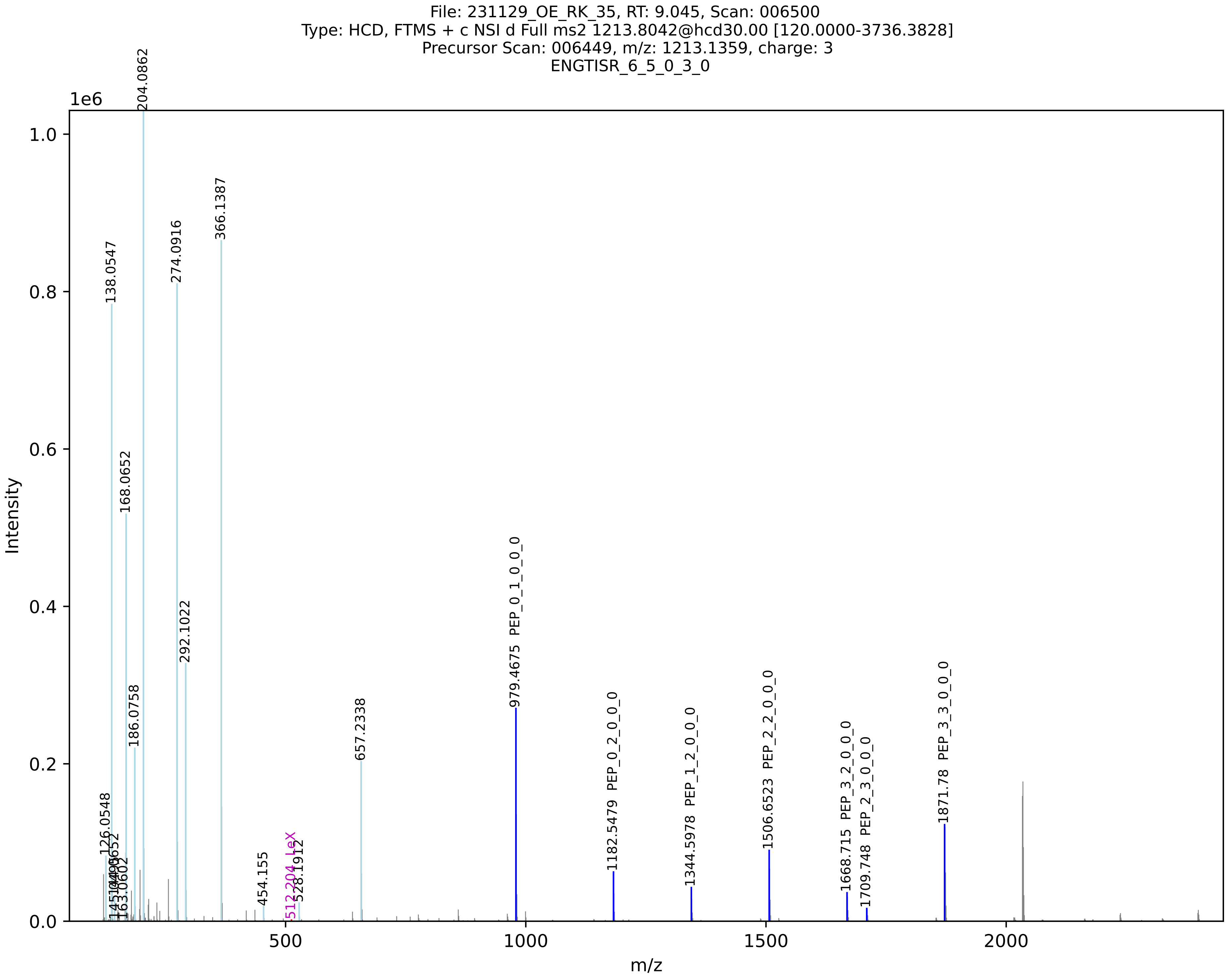
File: 231129_OE_RK_35, RT: 9.045, Scan: 006500
Type: HCD, FTMS + c NSI d Full ms2 1213.8042@hcd30.00 [120.0000-3736.3828]
Precursor Scan: 006449, m/z: 1213.1359, charge: 3
ENGTISR_6_5_0_3_0
1e6
Intensity
m/z
204.0862
366.1387
274.0916
138.0547
168.0652
292.1022
186.0758
126.0548
163.0602
657.2338
454.155
512.204_LeX
528.1912
979.4675 PEP_0_1_0_0_0
1182.5479 PEP_0_2_0_0_0
1344.5978 PEP_1_2_0_0_0
1506.6523 PEP_2_2_0_0_0
1668.715 PEP_3_2_0_0_0
1709.748 PEP_2_3_0_0_0
1871.78 PEP_3_3_0_0_0

File: 231129_OE_RK_35, RT: 9.046, Scan: 006501
Type: HCD, FTMS + c NSI d Full ms2 1262.4891@hcd30.00 [120.0000-3885.3589]
Precursor Scan: 006449, m/z: 1261.8209, charge: 3
ENGTISR_6_5_1_3_0

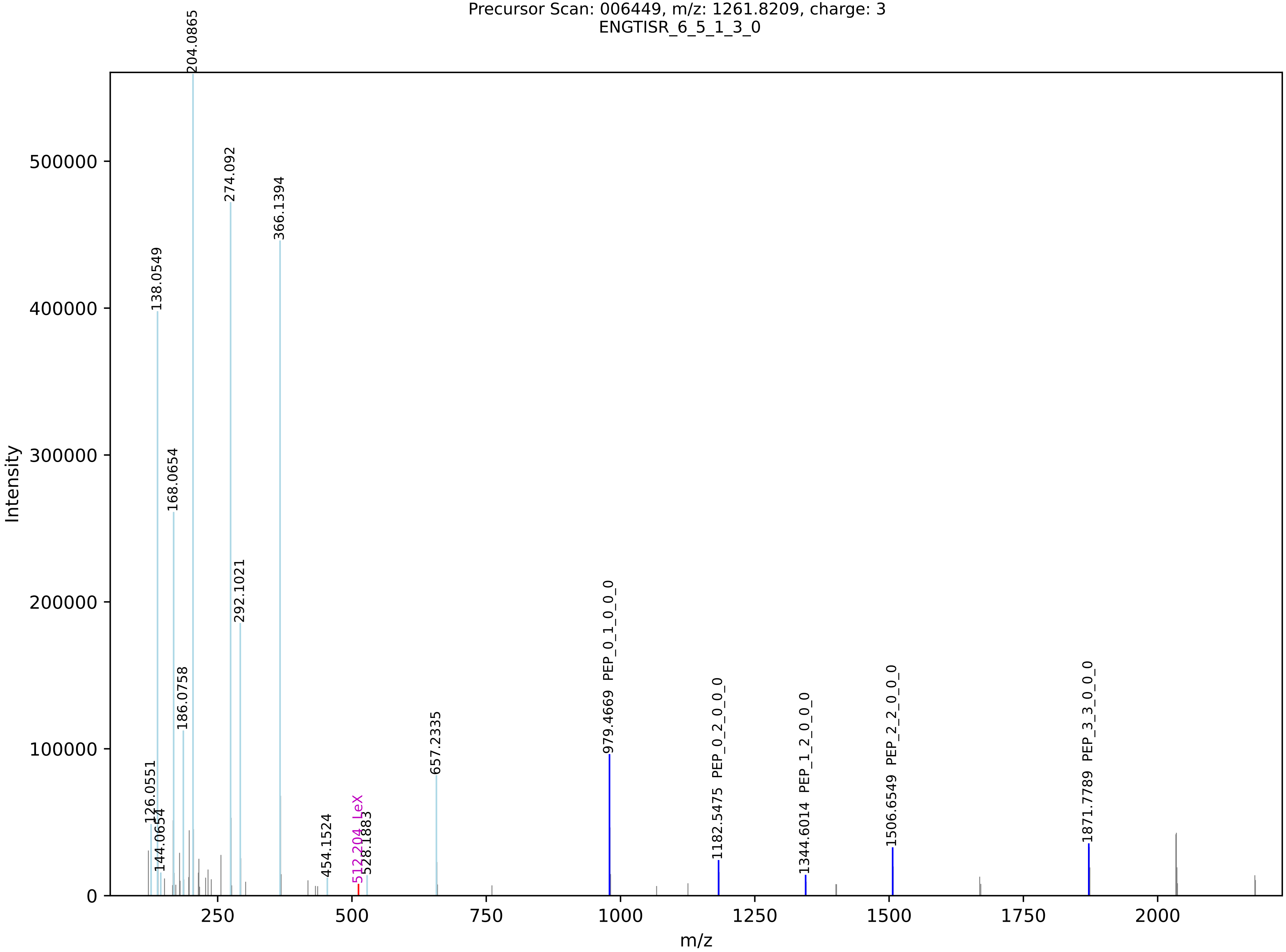

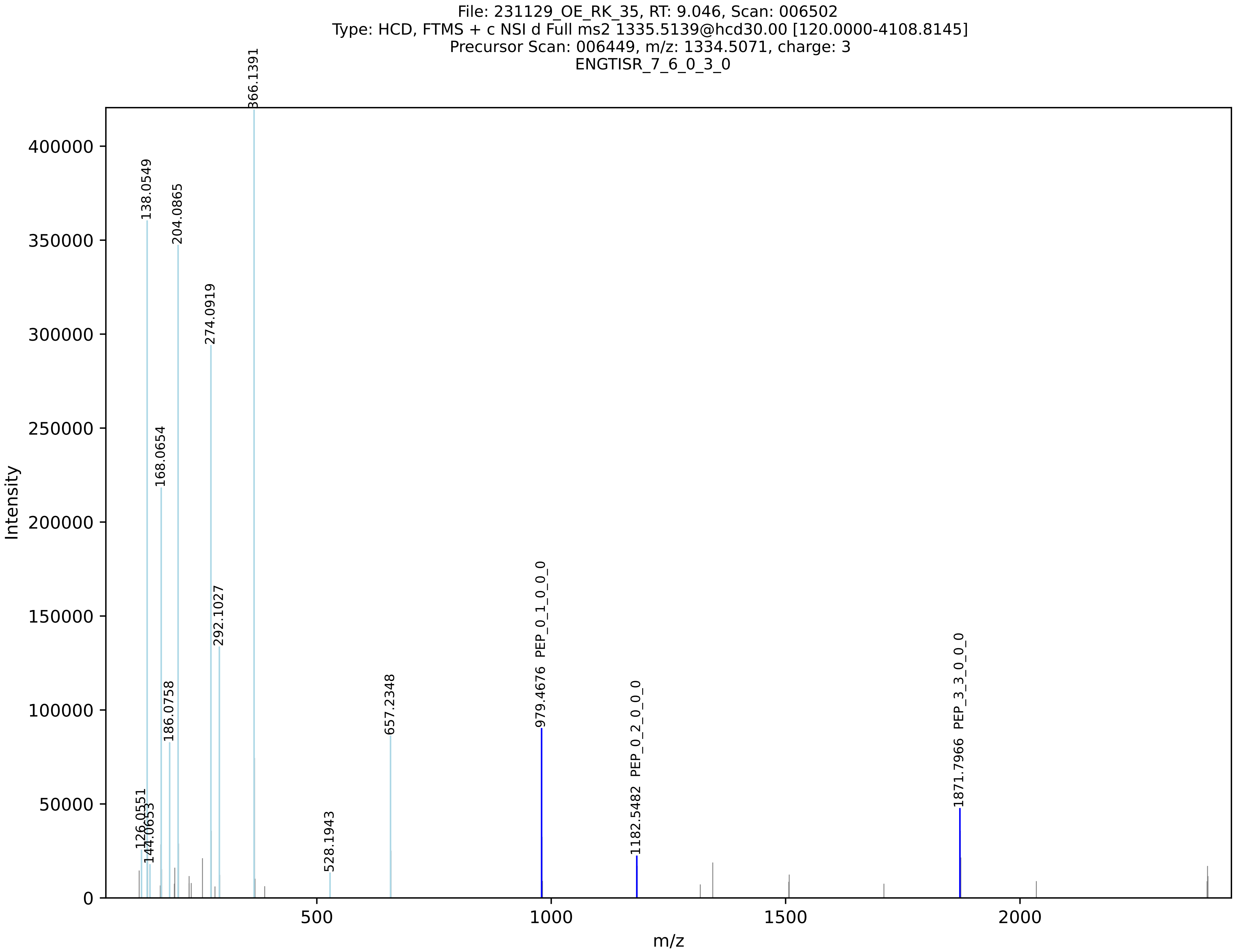

File: 231129_OE_RK_35, RT: 9.046, Scan: 006502
Type: HCD, FTMS + c NSI d Full ms2 1335.5139@hcd30.00 [120.0000-4108.8145]
Precursor Scan: 006449, m/z: 1334.5071, charge: 3
ENGTISR_7_6_0_3_0
Intensity
m/z
0
50000
100000
150000
200000
250000
300000
350000
400000
500
1000
1500
2000
366.1391
138.0549
204.0865
274.0919
168.0654
292.1027
186.0758
126.0551
144.0653
528.1943
657.2348
979.4676 PEP_0_1_0_0_0
1182.5482 PEP_0_2_0_0_0
1871.7966 PEP_3_3_0_0_0

File: 231129_OE_RK_35, RT: 9.048, Scan: 006505
Type: HCD, FTMS + c NSI d Full ms2 1432.5493@hcd30.00 [120.0000-4405.7427]
Precursor Scan: 006449, m/z: 1431.8794, charge: 3
ENGTISR_7_6_0_4_0

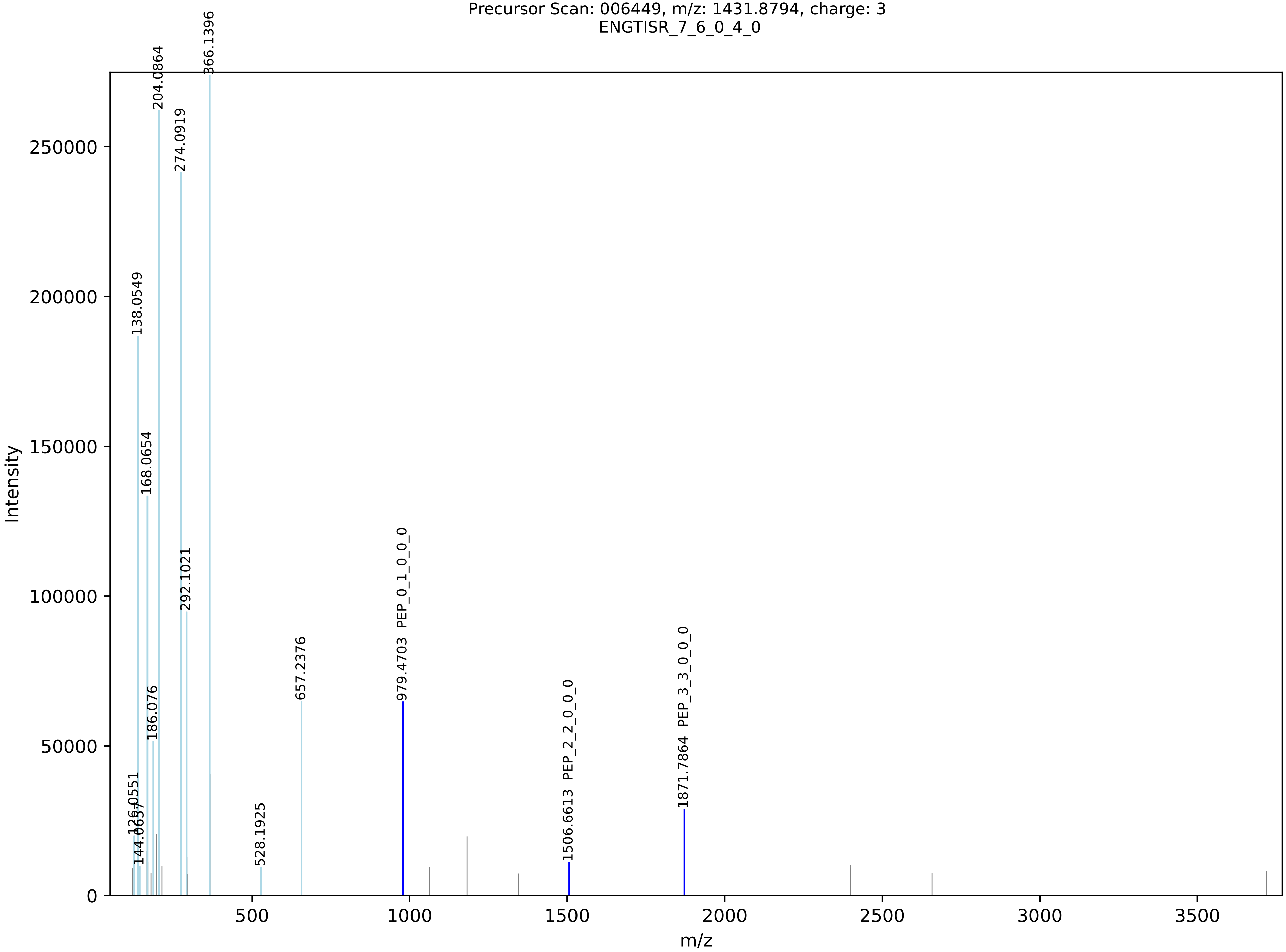

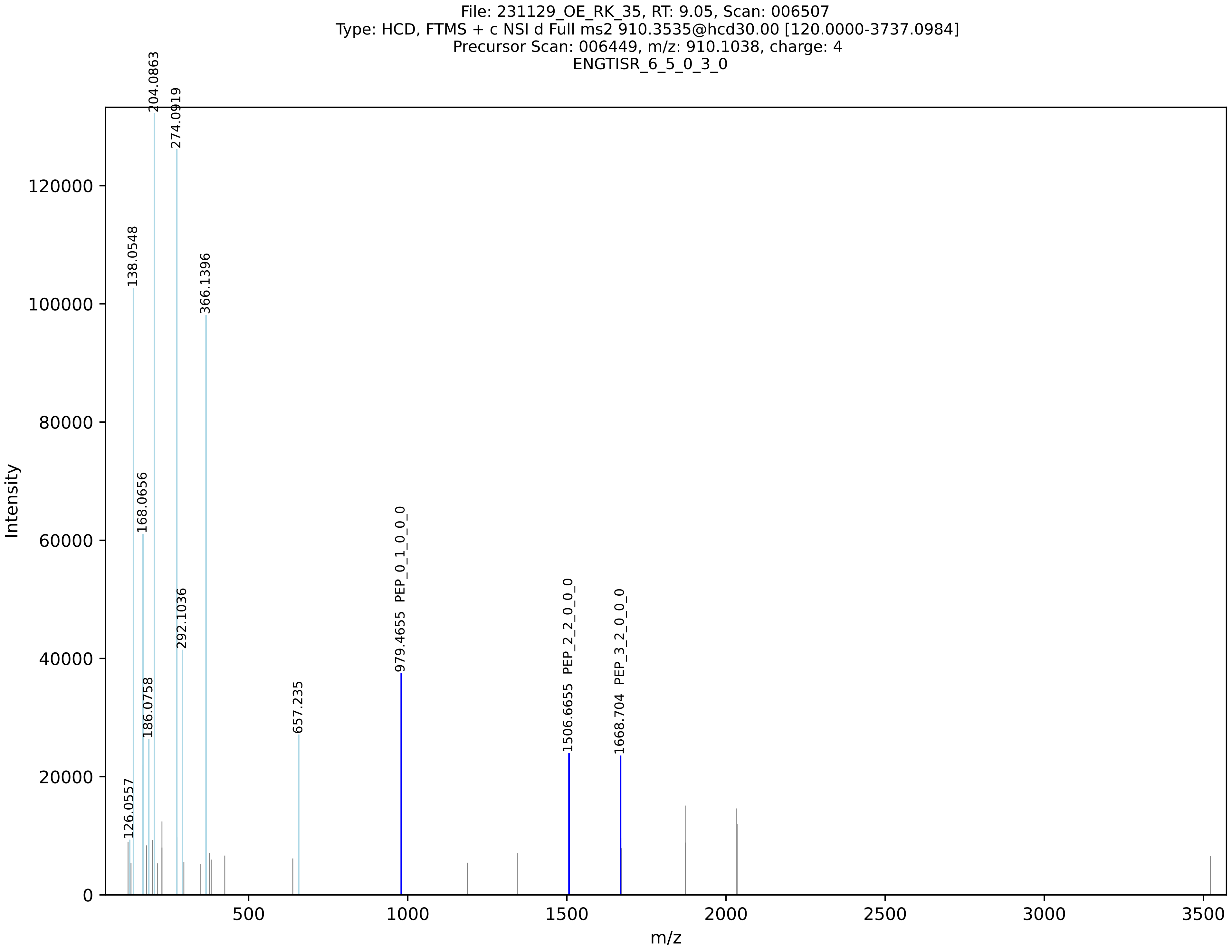
File: 231129_OE_RK_35, RT: 9.05, Scan: 006507
Type: HCD, FTMS + c NSI d Full ms2 910.3535@hcd30.00 [120.0000-3737.0984]
Precursor Scan: 006449, m/z: 910.1038, charge: 4
ENGTISR_6_5_0_3_0
Intensity
m/z
204.0863
274.0919
138.0548
366.1396
168.0656
292.1036
186.0758
126.0557
657.235
979.4655 PEP_0_1_0_0_0
1506.6655 PEP_2_2_0_0_0
1668.704 PEP_3_2_0_0_0
0
20000
40000
60000
80000
100000
120000
500
1000
1500
2000
2500
3000
3500

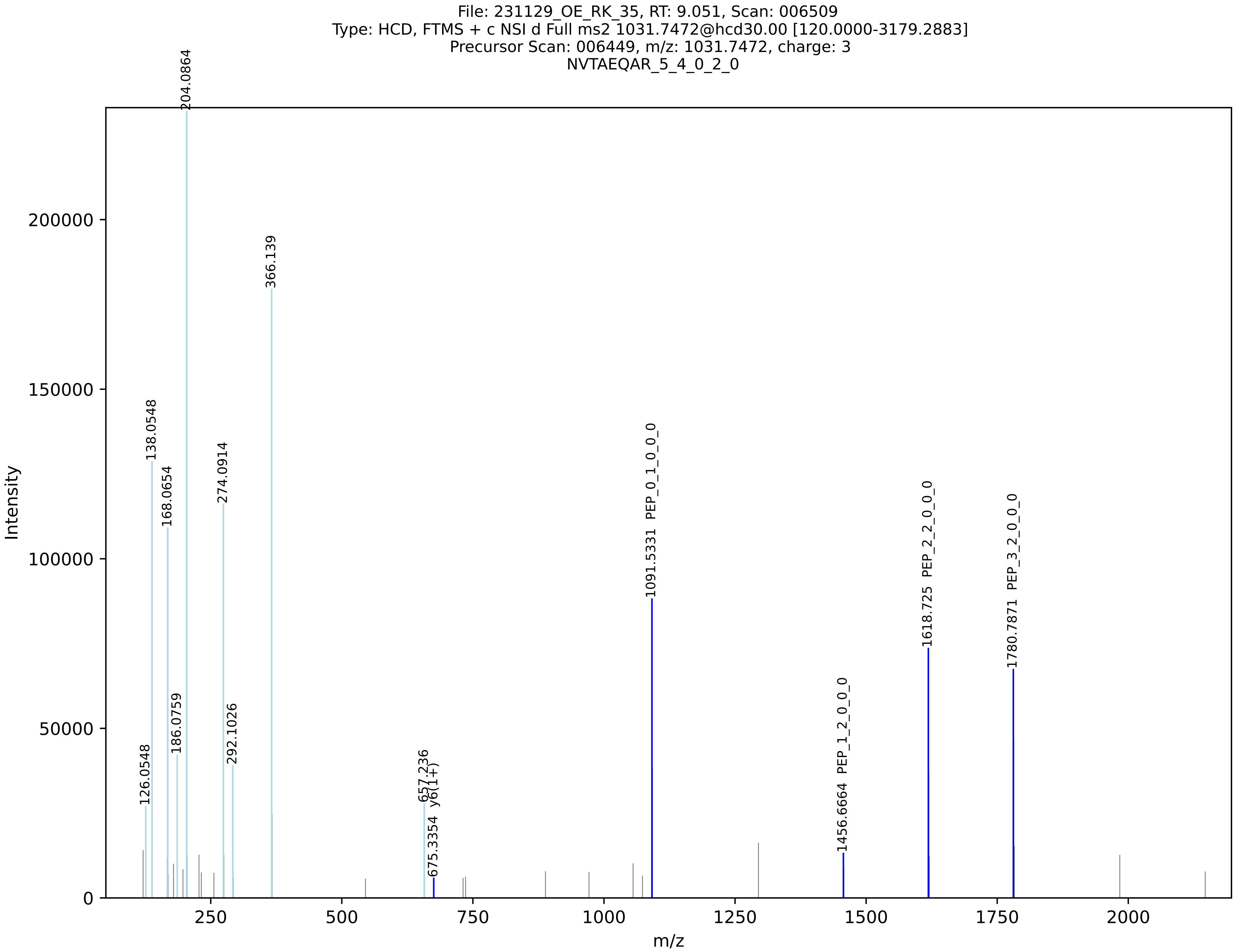
File: 231129_OE_RK_35, RT: 9.051, Scan: 006509
Type: HCD, FTMS + c NSI d Full ms2 1031.7472@hcd30.00 [120.0000-3179.2883]
Precursor Scan: 006449, m/z: 1031.7472, charge: 3
NVTAEQAR_5_4_0_2_0
Intensity
m/z
0
50000
100000
150000
200000
250
500
750
1000
1250
1500
1750
2000
204.0864
366.139
138.0548
168.0654
274.0914
186.0759
292.1026
126.0548
657.236
y6(1+)
675.3354
1091.5331 PEP_0_1_0_0_0
1456.6664 PEP_1_2_0_0_0
1618.725 PEP_2_2_0_0_0
1780.7871 PEP_3_2_0_0_0

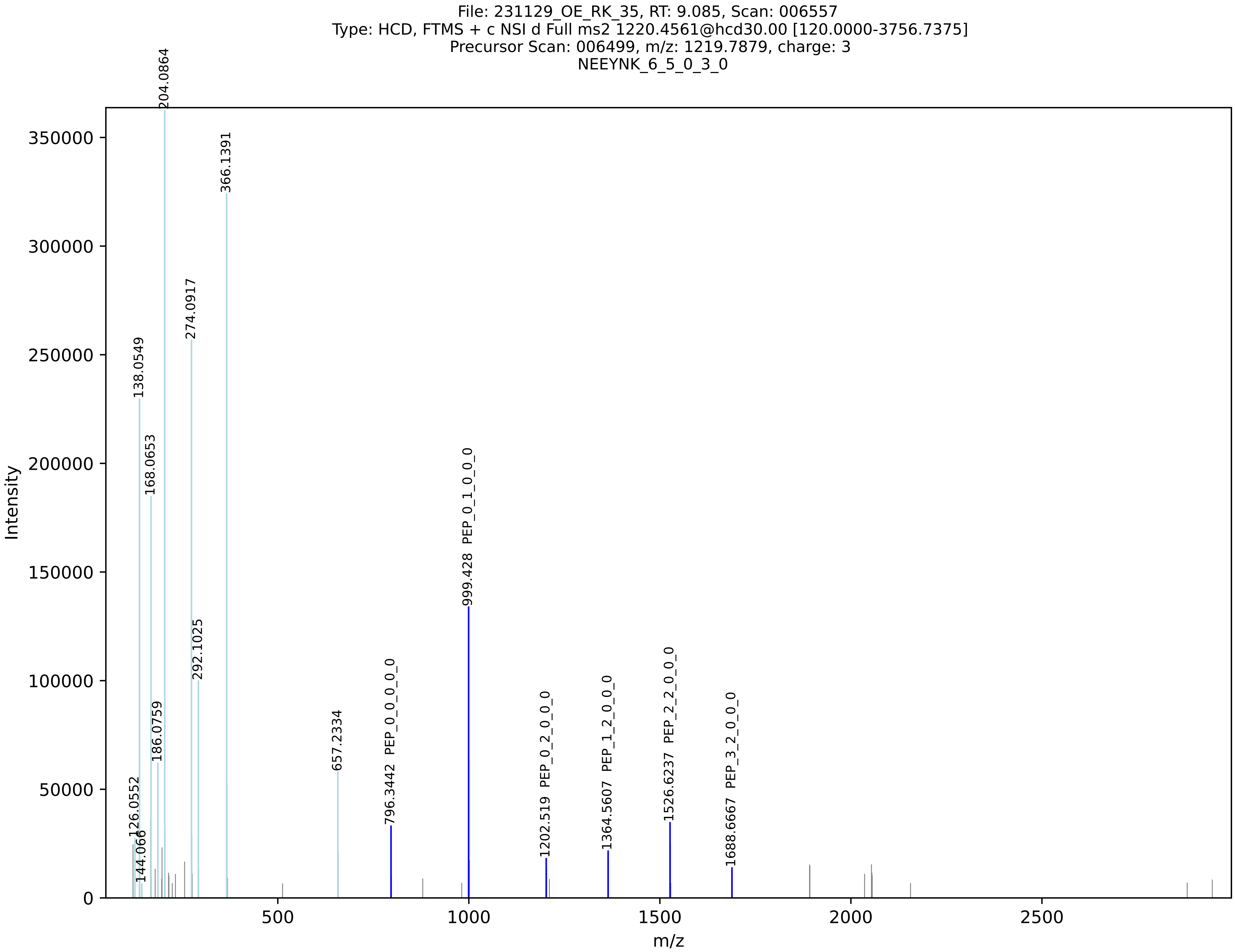
File: 231129_OE_RK_35, RT: 9.085, Scan: 006557
Type: HCD, FTMS + c NSI d Full ms2 1220.4561@hcd30.00 [120.0000-3756.7375]
Precursor Scan: 006499, m/z: 1219.7879, charge: 3
NEEYNK_6_5_0_3_0
Intensity
m/z
204.0864
366.1391
274.0917
138.0549
168.0653
292.1025
186.0759
126.0552
144.066
657.2334
796.3442 PEP_0_0_0_0_0
999.428 PEP_0_1_0_0_0
1202.519 PEP_0_2_0_0_0
1364.5607 PEP_1_2_0_0_0
1526.6237 PEP_2_2_0_0_0
1688.6667 PEP_3_2_0_0_0

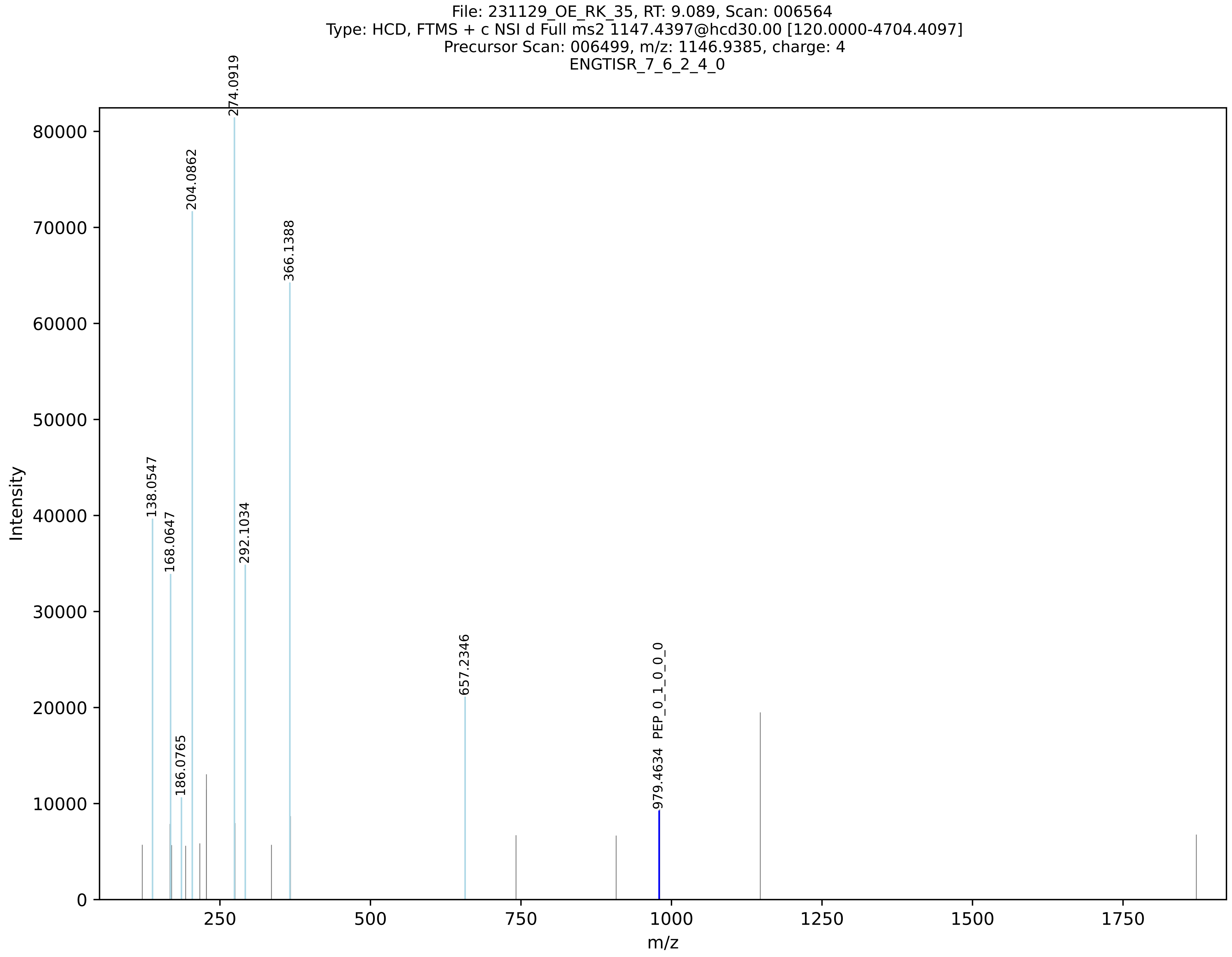

File: 231129_OE_RK_35, RT: 9.089, Scan: 006564
Type: HCD, FTMS + c NSI d Full ms2 1147.4397@hcd30.00 [120.0000-4704.4097]
Precursor Scan: 006499, m/z: 1146.9385, charge: 4
ENGTISR_7_6_2_4_0
Intensity
80000
70000
60000
50000
40000
30000
20000
10000
0
274.0919
204.0862
366.1388
138.0547
168.0647
292.1034
657.2346
PEP_0_1_0_0_0
186.0765
979.4634
250
500
750
1000
1250
1500
1750
m/z

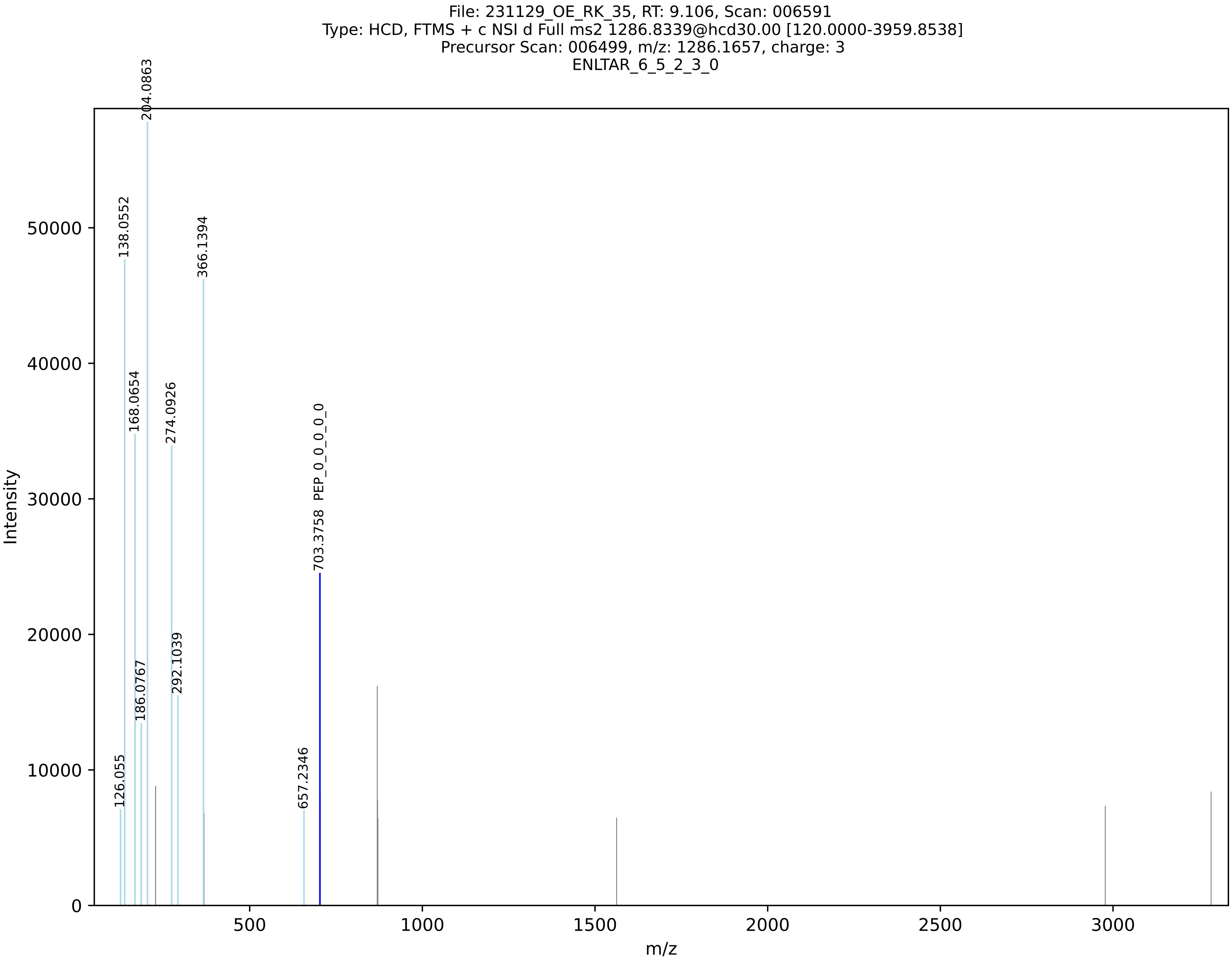

File: 231129_OE_RK_35, RT: 9.106, Scan: 006591
Type: HCD, FTMS + c NSI d Full ms2 1286.8339@hcd30.00 [120.0000-3959.8538]
Precursor Scan: 006499, m/z: 1286.1657, charge: 3
ENLTAR_6_5_2_3_0
204.0863
138.0552
366.1394
168.0654
274.0926
703.3758 PEP_0_0_0_0_0
292.1039
186.0767
126.055
657.2346
Intensity
m/z
0
10000
20000
30000
40000
50000
500
1000
1500
2000
2500
3000

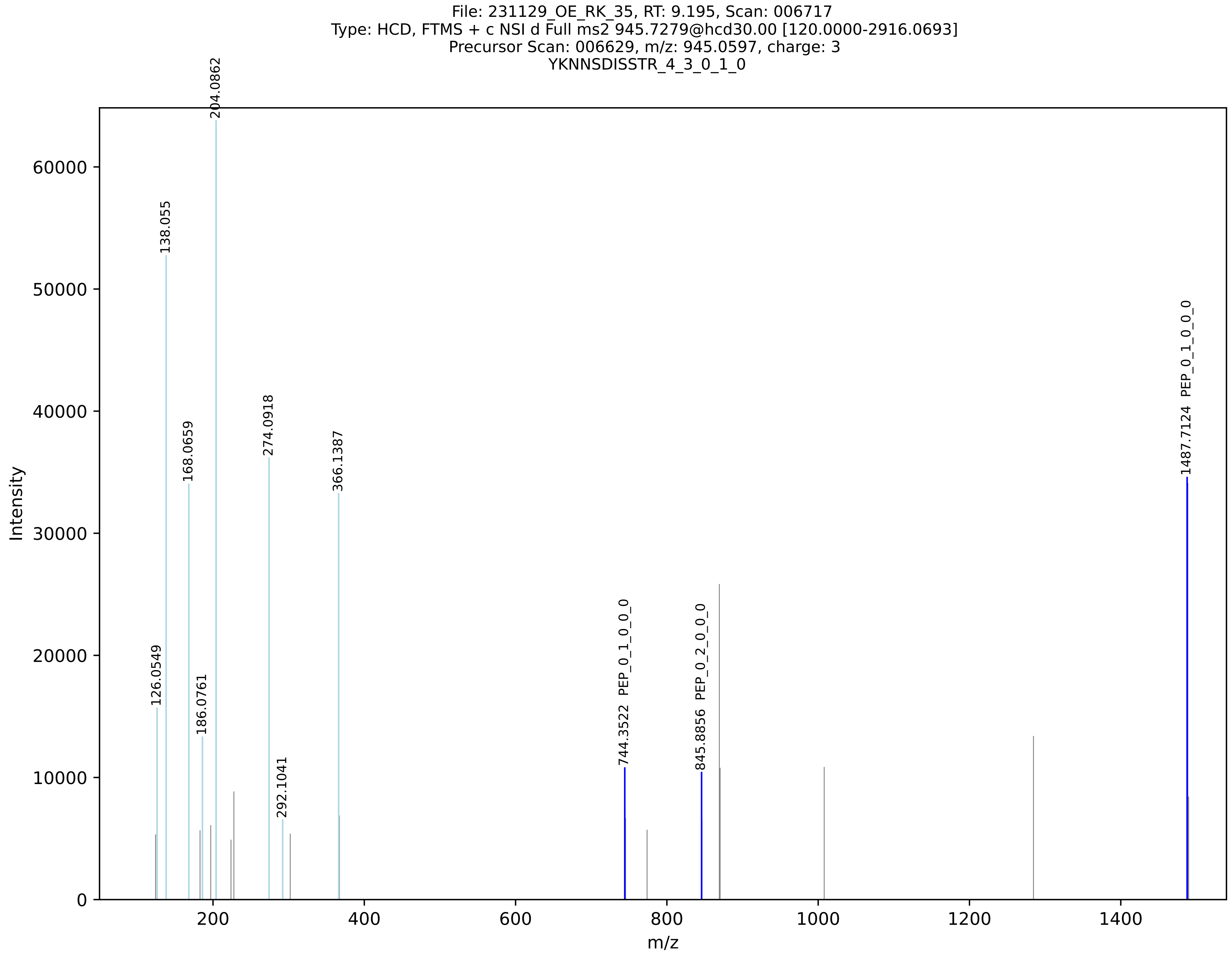
File: 231129_OE_RK_35, RT: 9.195, Scan: 006717
Type: HCD, FTMS + c NSI d Full ms2 945.7279@hcd30.00 [120.0000-2916.0693]
Precursor Scan: 006629, m/z: 945.0597, charge: 3
YKNNSDISSTR_4_3_0_1_0
Intensity
m/z
204.0862
138.055
168.0659
274.0918
366.1387
126.0549
186.0761
292.1041
744.3522 PEP_0_1_0_0_0
845.8856 PEP_0_2_0_0_0
1487.7124 PEP_0_1_0_0_0
0
10000
20000
30000
40000
50000
60000
200
400
600
800
1000
1200
1400

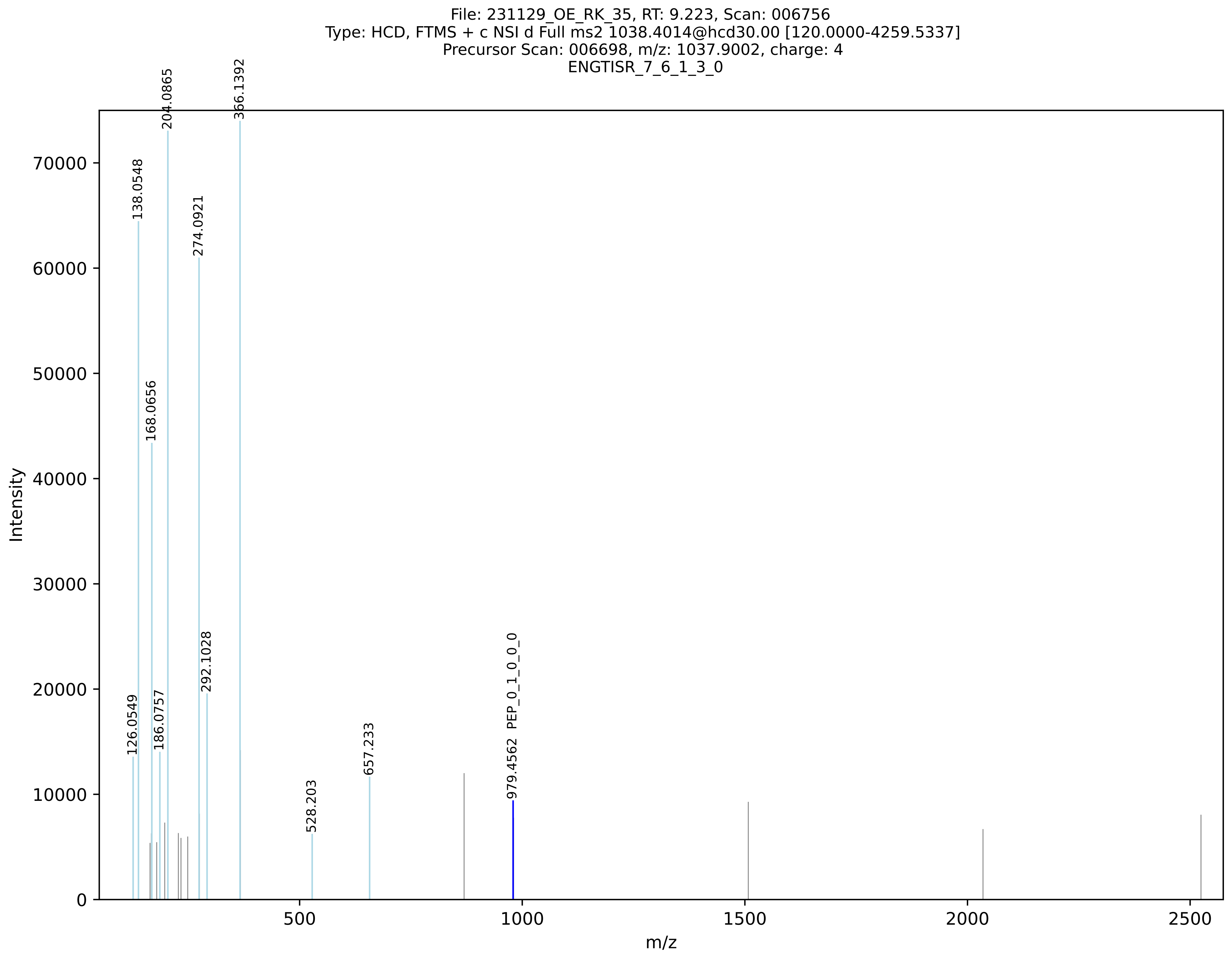
File: 231129_OE_RK_35, RT: 9.223, Scan: 006756
Type: HCD, FTMS + c NSI d Full ms2 1038.4014@hcd30.00 [120.0000-4259.5337]
Precursor Scan: 006698, m/z: 1037.9002, charge: 4
ENGTISR_7_6_1_3_0
366.1392
204.0865
138.0548
274.0921
168.0656
292.1028
126.0549
186.0757
528.203
657.233
979.4562 PEP_0_1_0_0_0_
Intensity
m/z
0
10000
20000
30000
40000
50000
60000
70000
500
1000
1500
2000
2500

File: 231129_OE_RK_35, RT: 9.254, Scan: 006799
Type: HCD, FTMS + c NSI d Full ms2 1432.5492@hcd30.00 [120.0000-4405.7427]
Precursor Scan: 006749, m/z: 1431.8799, charge: 3
ENGTISR_7_6_0_4_0

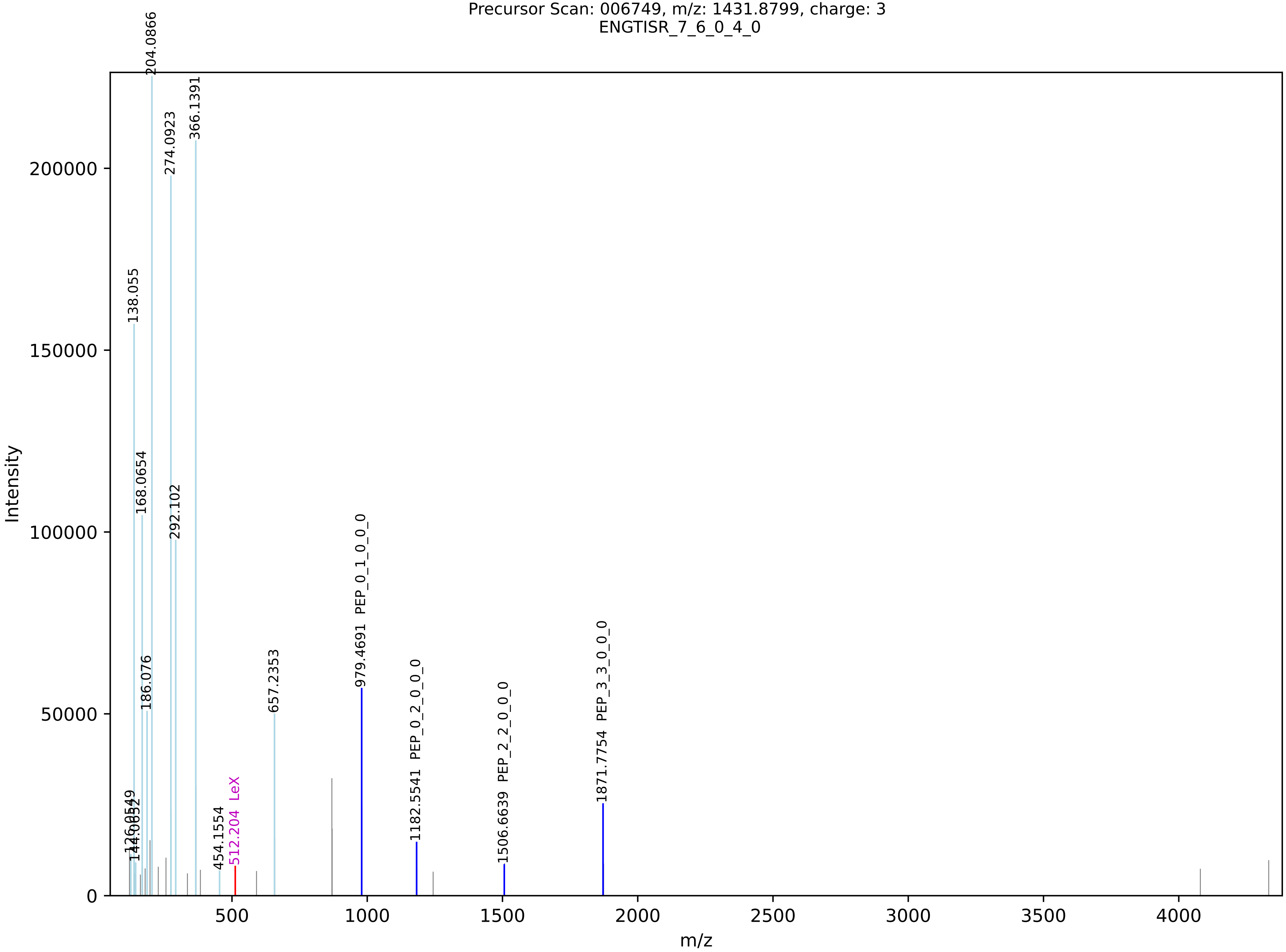

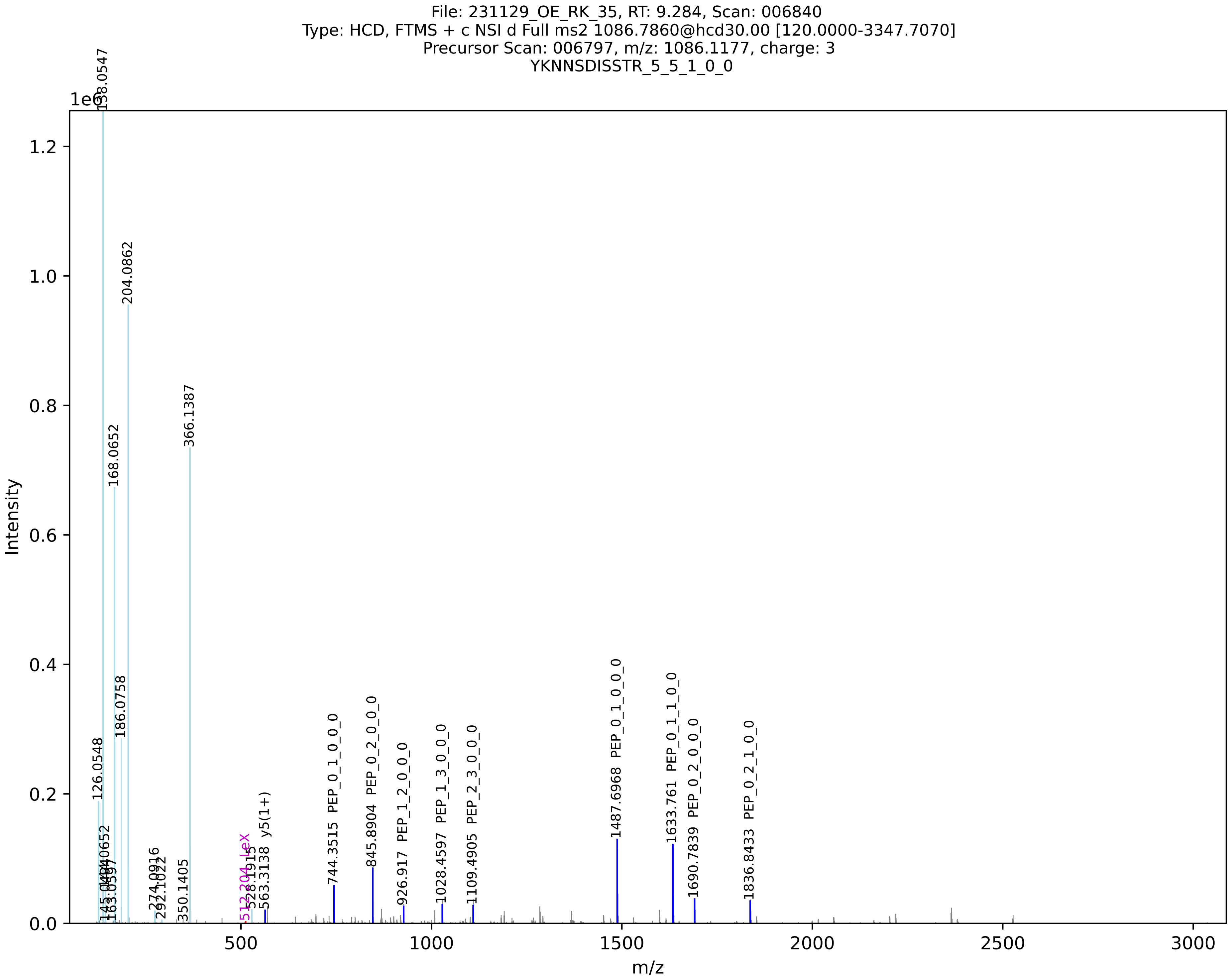

File: 231129_OE_RK_35, RT: 9.284, Scan: 006840
Type: HCD, FTMS + c NSI d Full ms2 1086.7860@hcd30.00 [120.0000-3347.7070]
Precursor Scan: 006797, m/z: 1086.1177, charge: 3
YKNNSDISSTR_5_5_1_0_0
1e6
Intensity
m/z
138.0547
204.0862
366.1387
168.0652
186.0758
126.0548
512.204 LeX
528.1915
563.3138 y5(1+)
744.3515 PEP_0_1_0_0_0
845.8904 PEP_0_2_0_0_0
926.917 PEP_1_2_0_0_0
1028.4597 PEP_1_3_0_0_0
1109.4905 PEP_2_3_0_0_0
1487.6968 PEP_0_1_0_0_0
1633.761 PEP_0_1_1_0_0
1690.7839 PEP_0_2_0_0_0
1836.8433 PEP_0_2_1_0_0
274.0916
292.1022
350.1405
163.0597

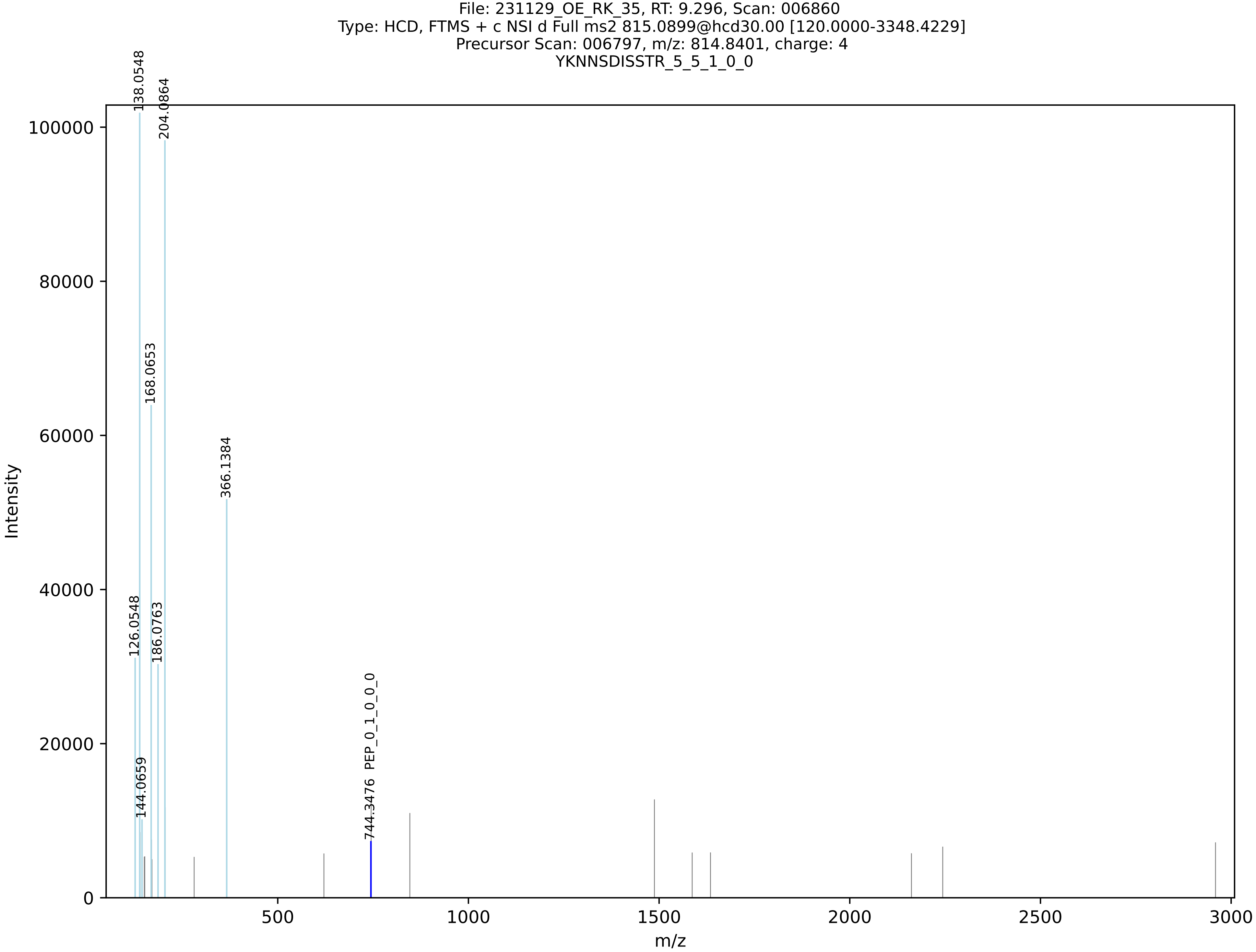
File: 231129_OE_RK_35, RT: 9.296, Scan: 006860
Type: HCD, FTMS + c NSI d Full ms2 815.0899@hcd30.00 [120.0000-3348.4229]
Precursor Scan: 006797, m/z: 814.8401, charge: 4
YKNNSDISSTR_5_5_1_0_0
138.0548
204.0864
168.0653
366.1384
126.0548
186.0763
144.0659
744.3476 PEP_0_1_0_0_0
Intensity
m/z
0
20000
40000
60000
80000
100000
500
1000
1500
2000
2500
3000

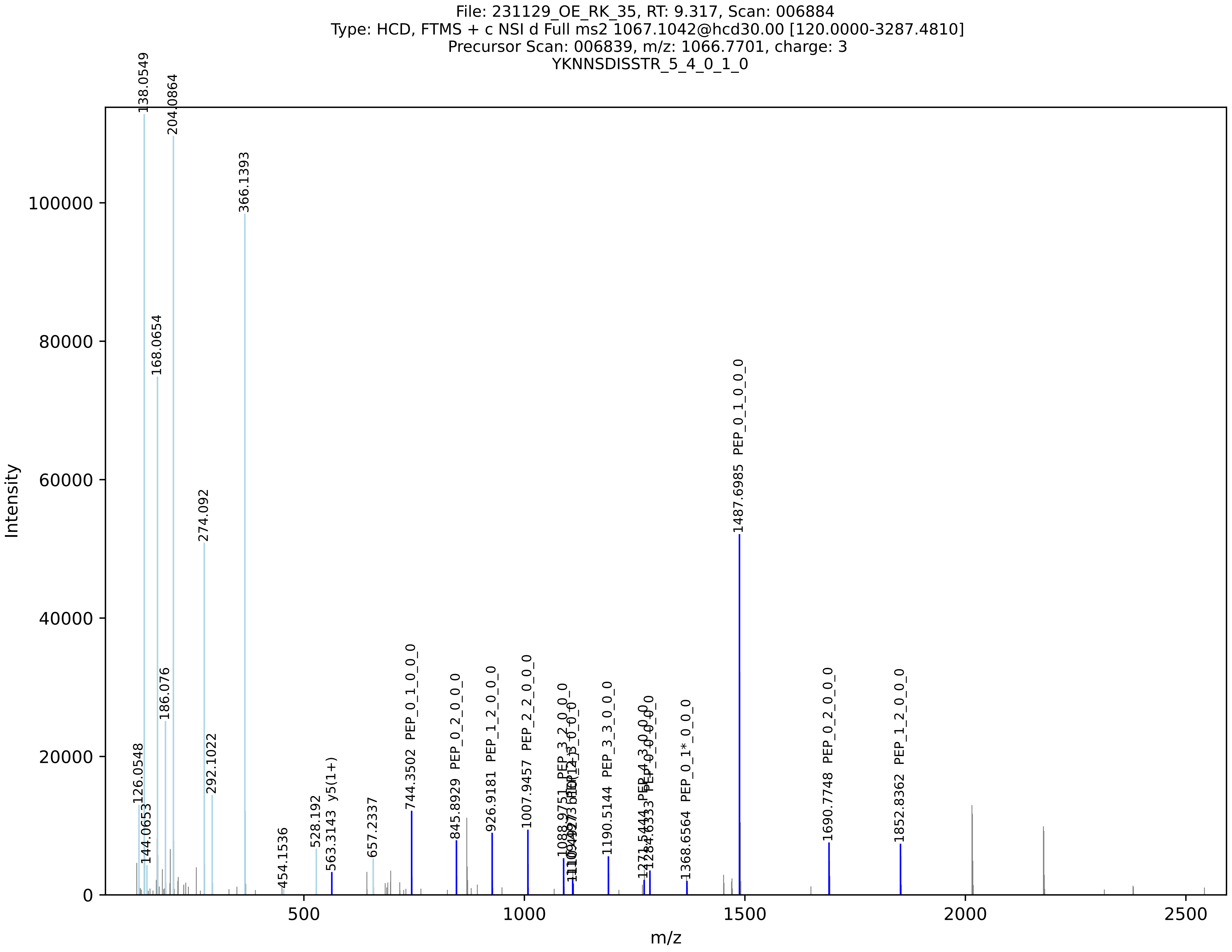
File: 231129_OE_RK_35, RT: 9.317, Scan: 006884
Type: HCD, FTMS + c NSI d Full ms2 1067.1042@hcd30.00 [120.0000-3287.4810]
Precursor Scan: 006839, m/z: 1066.7701, charge: 3
YKNNSDISSTR_5_4_0_1_0
Intensity
m/z
138.0549
204.0864
366.1393
168.0654
274.092
186.076
126.0548
144.0653
292.1022
454.1536
528.192
563.3143 y5(1+)
657.2337
744.3502 PEP_0_1_0_0_0
845.8929 PEP_0_2_0_0_0
926.9181 PEP_1_2_0_0_0
1007.9457 PEP_2_2_0_0_0
1190.5144 PEP_3_3_0_0_0
1368.6564 PEP_0_1*_0_0_0
1487.6985 PEP_0_1_0_0_0
1690.7748 PEP_0_2_0_0_0
1852.8362 PEP_1_2_0_0_0

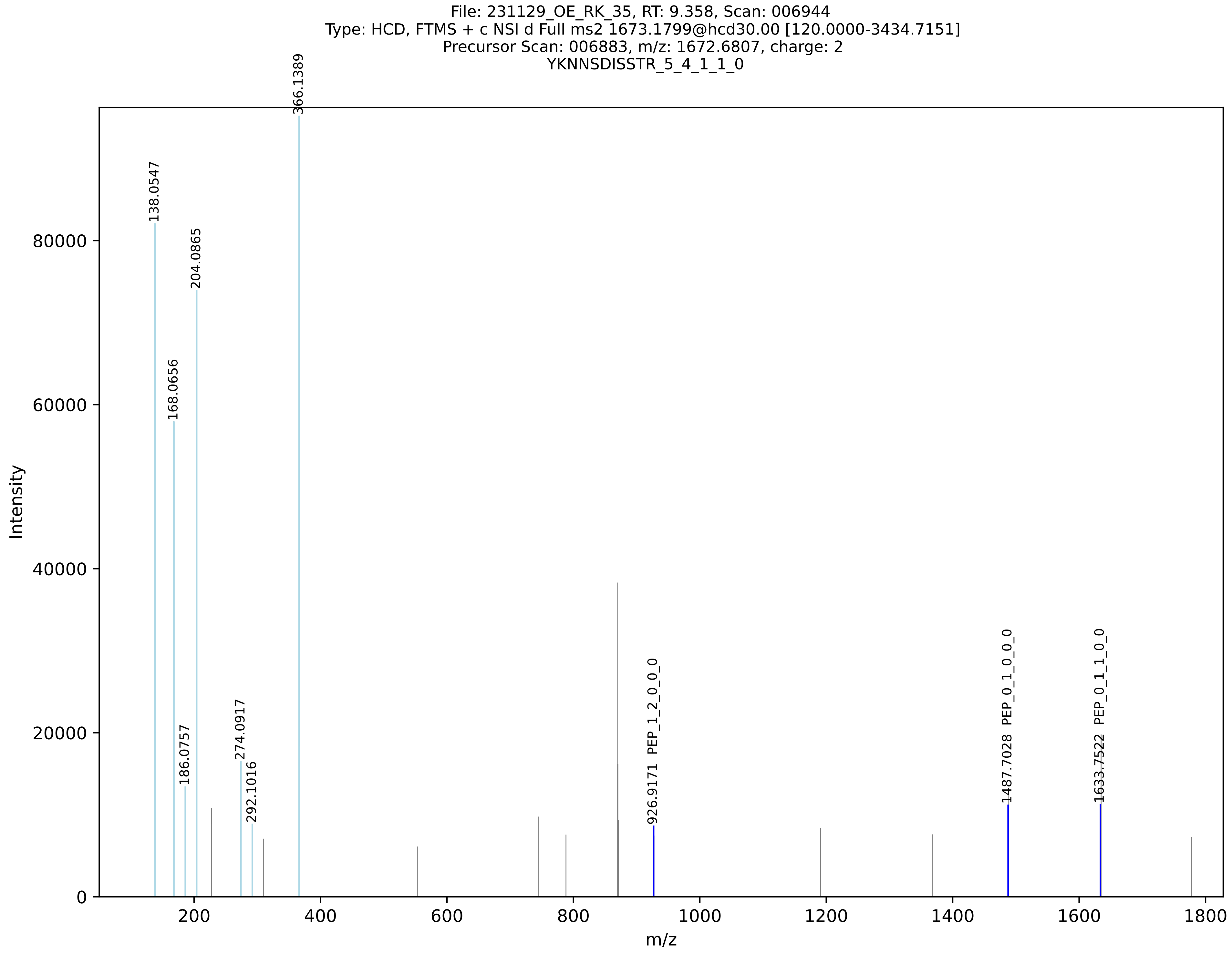

File: 231129_OE_RK_35, RT: 9.358, Scan: 006944
Type: HCD, FTMS + c NSI d Full ms2 1673.1799@hcd30.00 [120.0000-3434.7151]
Precursor Scan: 006883, m/z: 1672.6807, charge: 2
YKNNSDISSTR_5_4_1_1_0
366.1389
138.0547
204.0865
168.0656
186.0757
274.0917
292.1016
926.9171 PEP_1_2_0_0_0
1487.7028 PEP_0_1_0_0_0
1633.7522 PEP_0_1_1_0_0
Intensity
m/z
0
20000
40000
60000
80000
200
400
600
800
1000
1200
1400
1600
1800

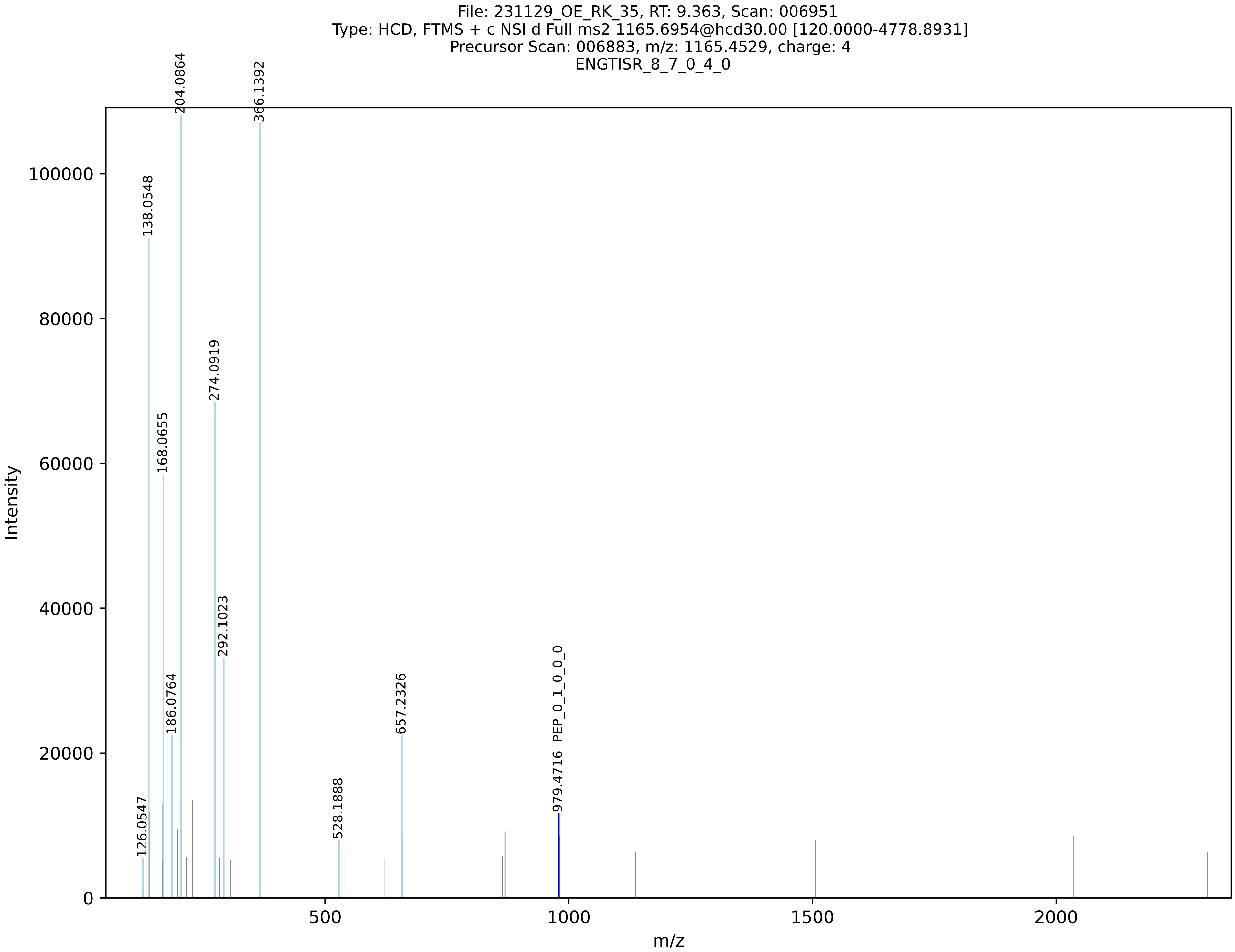

File: 231129_OE_RK_35, RT: 9.363, Scan: 006951
Type: HCD, FTMS + c NSI d Full ms2 1165.6954@hcd30.00 [120.0000-4778.8931]
Precursor Scan: 006883, m/z: 1165.4529, charge: 4
ENGTISR_8_7_0_4_0
Intensity
m/z
0
20000
40000
60000
80000
100000
500
1000
1500
2000
204.0864
366.1392
138.0548
274.0919
168.0655
292.1023
186.0764
657.2326
979.4716 PEP_0_1_0_0_0
528.1888
126.0547

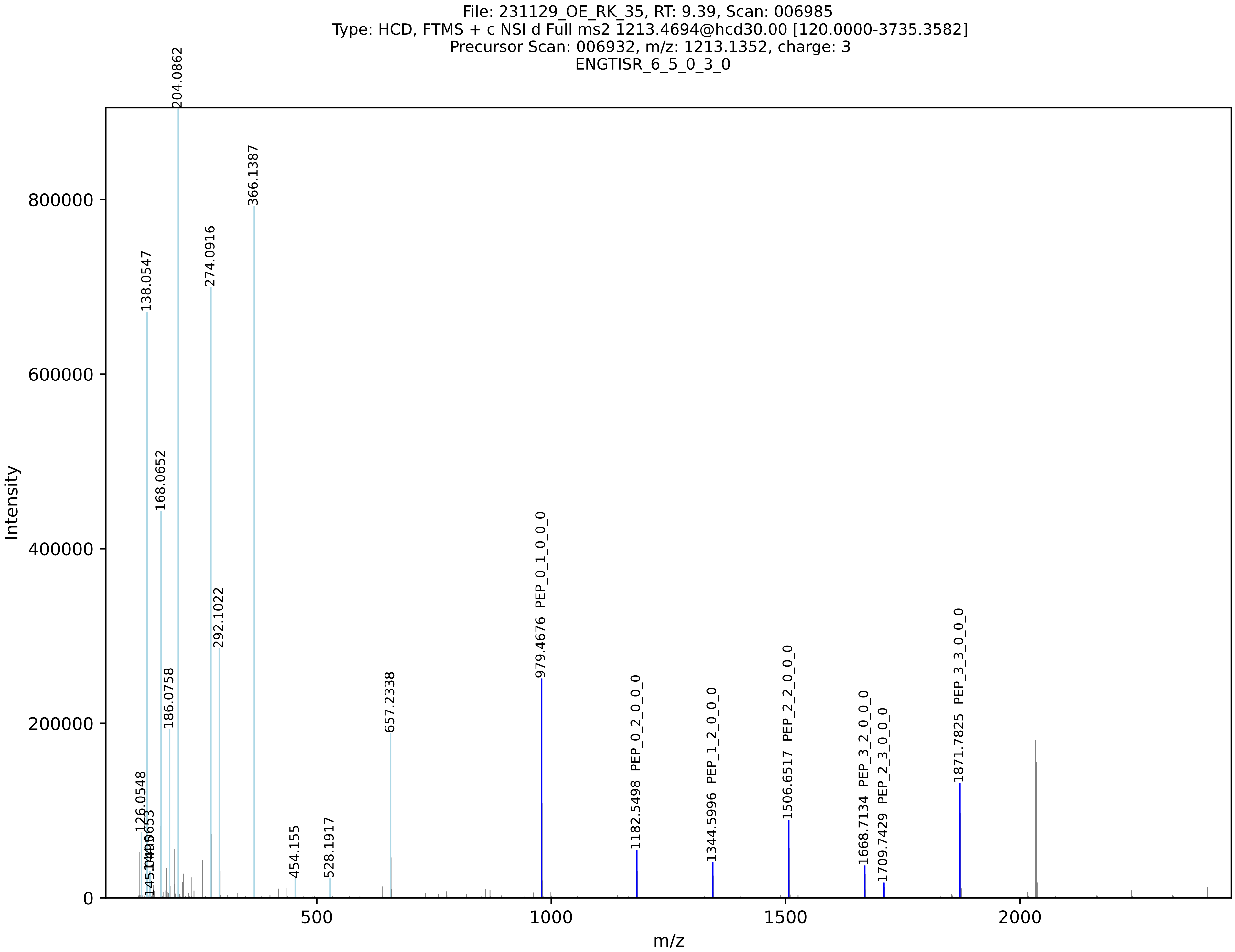
File: 231129_OE_RK_35, RT: 9.39, Scan: 006985
Type: HCD, FTMS + c NSI d Full ms2 1213.4694@hcd30.00 [120.0000-3735.3582]
Precursor Scan: 006932, m/z: 1213.1352, charge: 3
ENGTISR_6_5_0_3_0
Intensity
m/z
204.0862
366.1387
274.0916
138.0547
168.0652
292.1022
186.0758
126.0548
657.2338
454.155
528.1917
979.4676 PEP_0_1_0_0_0
1182.5498 PEP_0_2_0_0_0
1344.5996 PEP_1_2_0_0_0
1506.6517 PEP_2_2_0_0_0
1668.7134 PEP_3_2_0_0_0
1709.7429 PEP_2_3_0_0_0
1871.7825 PEP_3_3_0_0_0

File: 231129_OE_RK_35, RT: 9.39, Scan: 006986
Type: HCD, FTMS + c NSI d Full ms2 906.6258@hcd30.00 [120.0000-3721.8894]
Precursor Scan: 006932, m/z: 906.1246, charge: 4
EIRHNSTGCLR_6_5_0_1_0

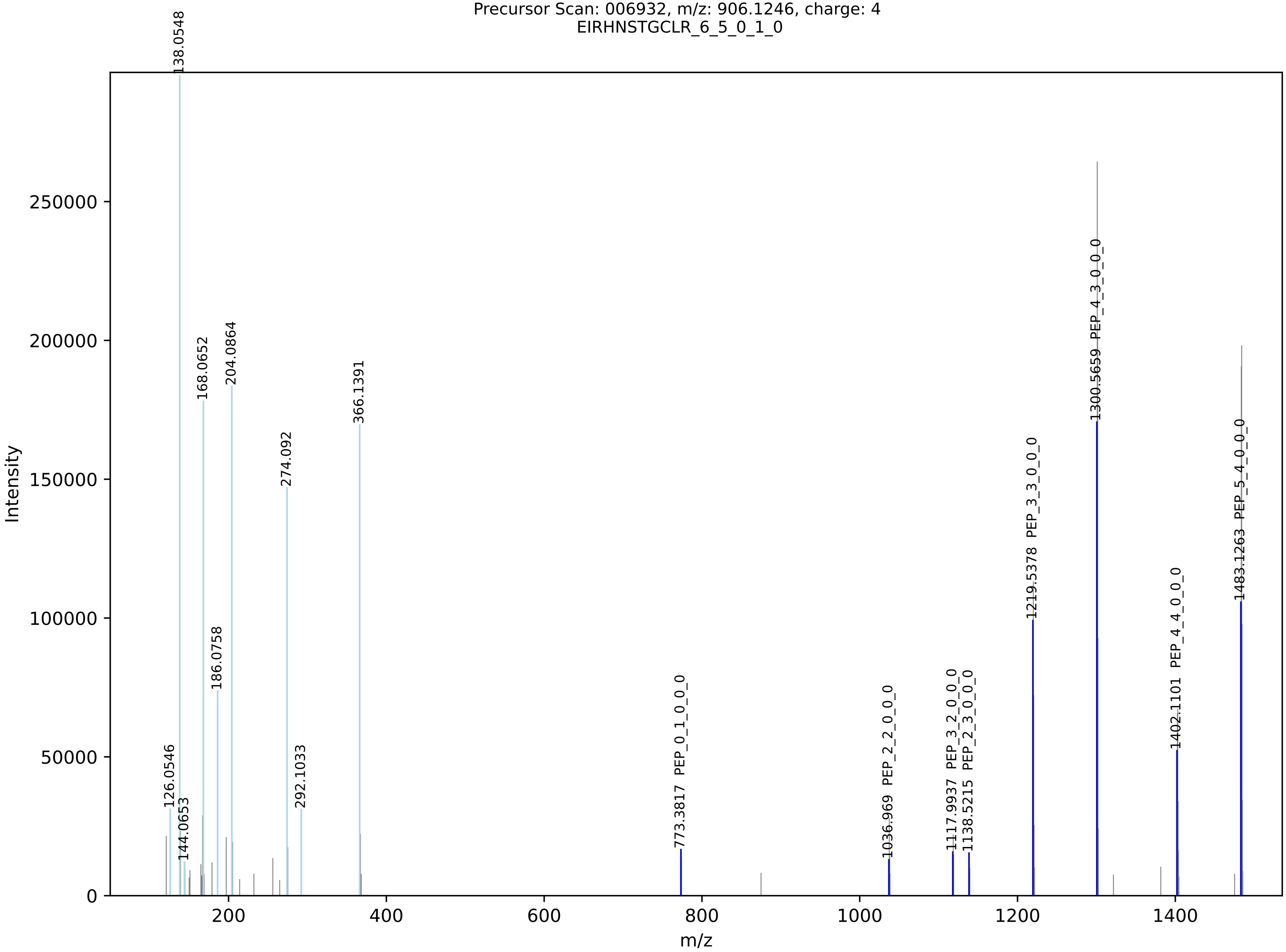

File: 231129_OE_RK_35, RT: 9.435, Scan: 007051
Type: HCD, FTMS + c NSI d Full ms2 850.0103@hcd30.00 [120.0000-2623.1736]
Precursor Scan: 006984, m/z: 849.6767, charge: 3
WVSNK_5_4_0_1_0

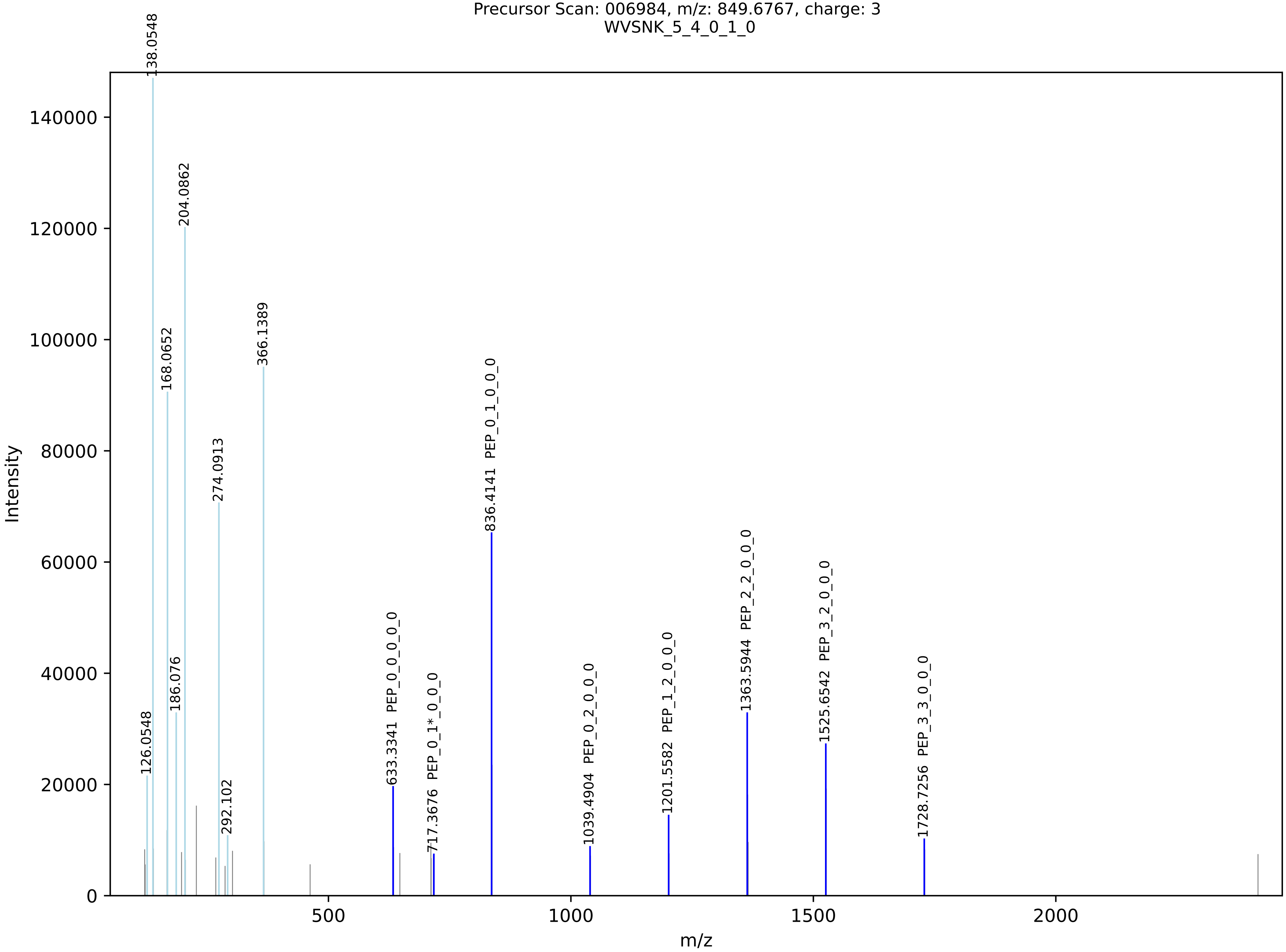

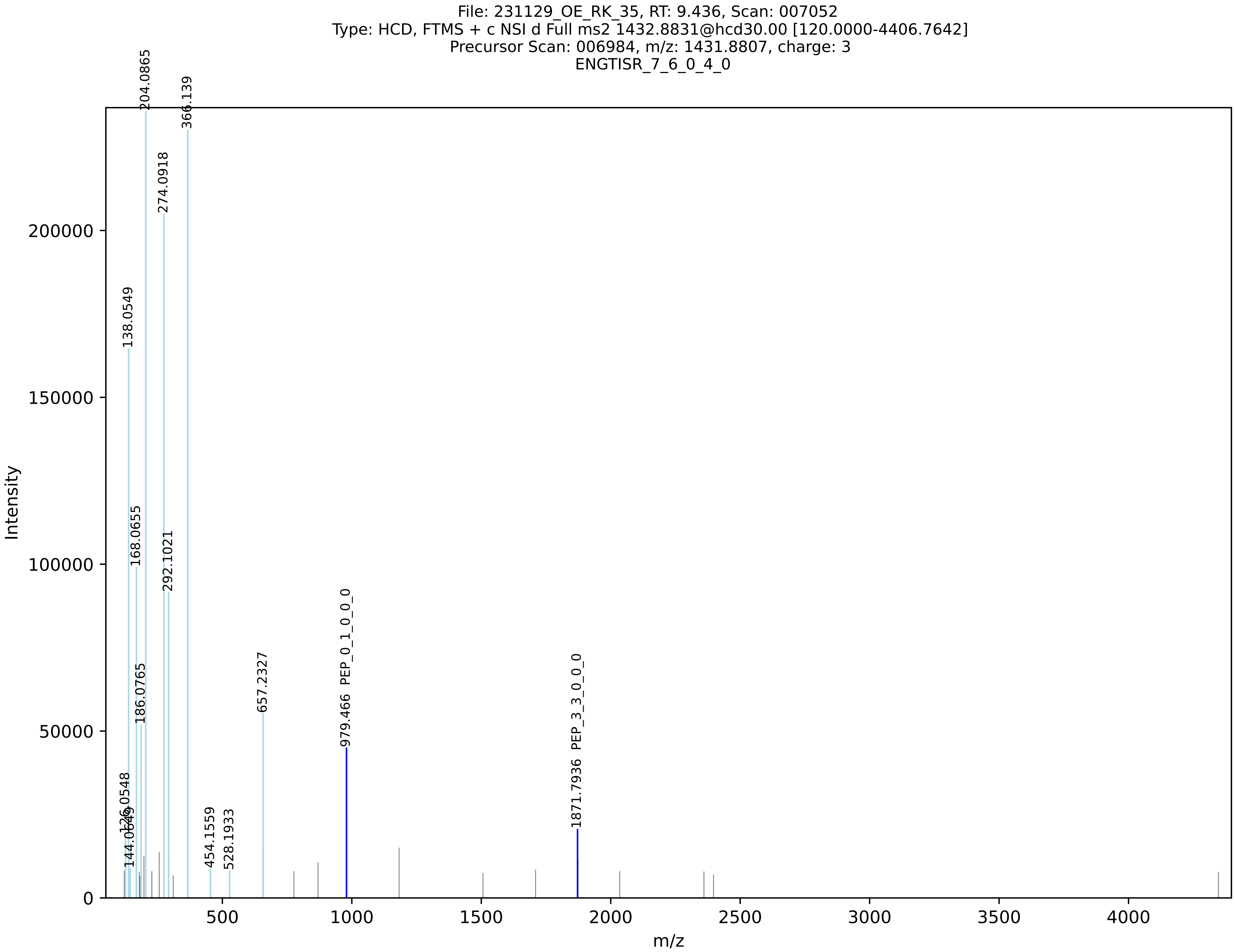

File: 231129_OE_RK_35, RT: 9.436, Scan: 007052
Type: HCD, FTMS + c NSI d Full ms2 1432.8831@hcd30.00 [120.0000-4406.7642]
Precursor Scan: 006984, m/z: 1431.8807, charge: 3
ENGTISR_7_6_0_4_0
204.0865
366.139
274.0918
138.0549
168.0655
292.1021
186.0765
657.2327
979.466 PEP_0_1_0_0_0
1871.7936 PEP_3_3_0_0_0
126.0548
144.0649
454.1559
528.1933
Intensity
m/z
0
50000
100000
150000
200000
500
1000
1500
2000
2500
3000
3500
4000

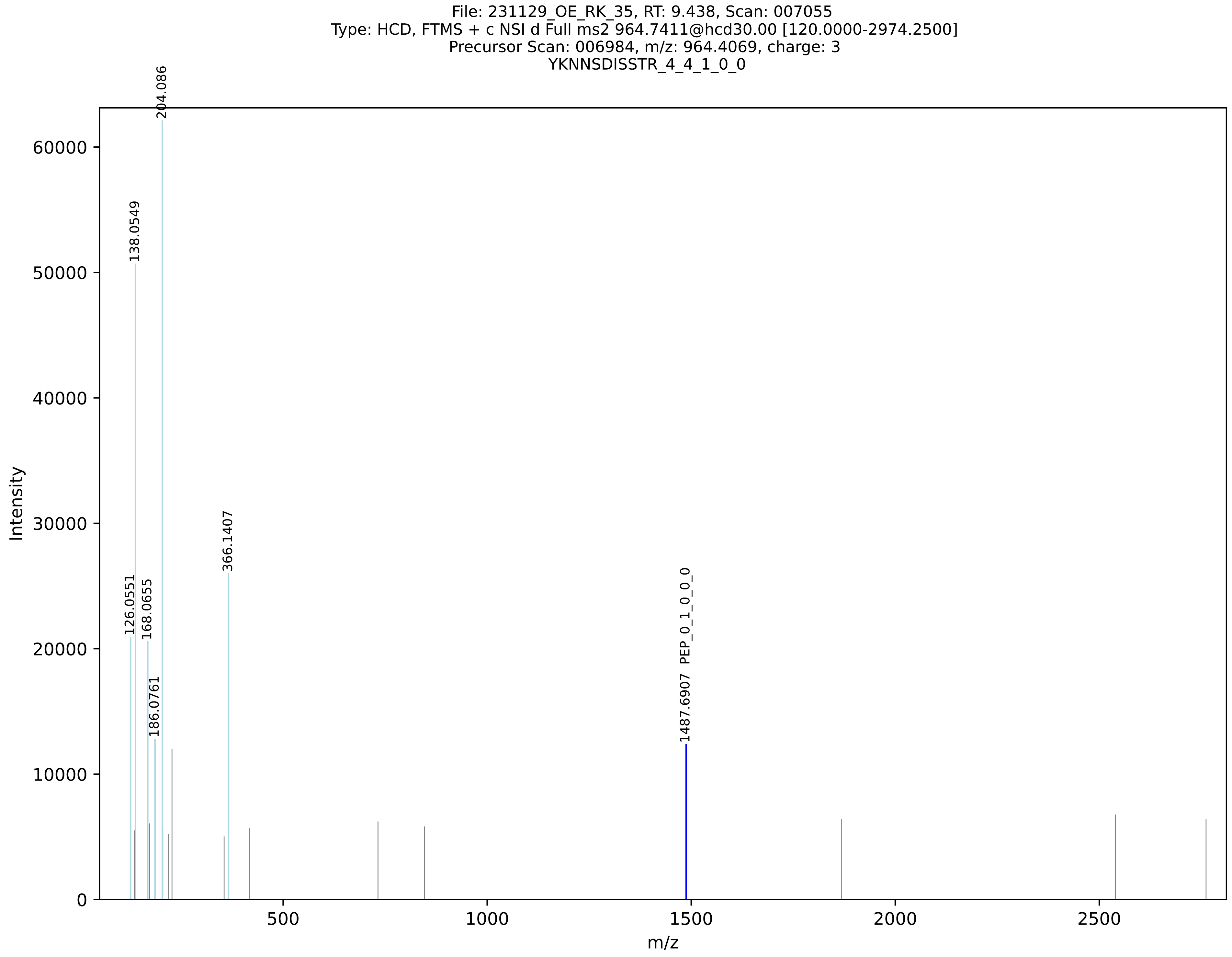
File: 231129_OE_RK_35, RT: 9.438, Scan: 007055
Type: HCD, FTMS + c NSI d Full ms2 964.7411@hcd30.00 [120.0000-2974.2500]
Precursor Scan: 006984, m/z: 964.4069, charge: 3
YKNNSDISSTR_4_4_1_0_0
204.086
138.0549
366.1407
126.0551
168.0655
186.0761
1487.6907 PEP_0_1_0_0_0
Intensity
m/z
0
10000
20000
30000
40000
50000
60000
500
1000
1500
2000
2500

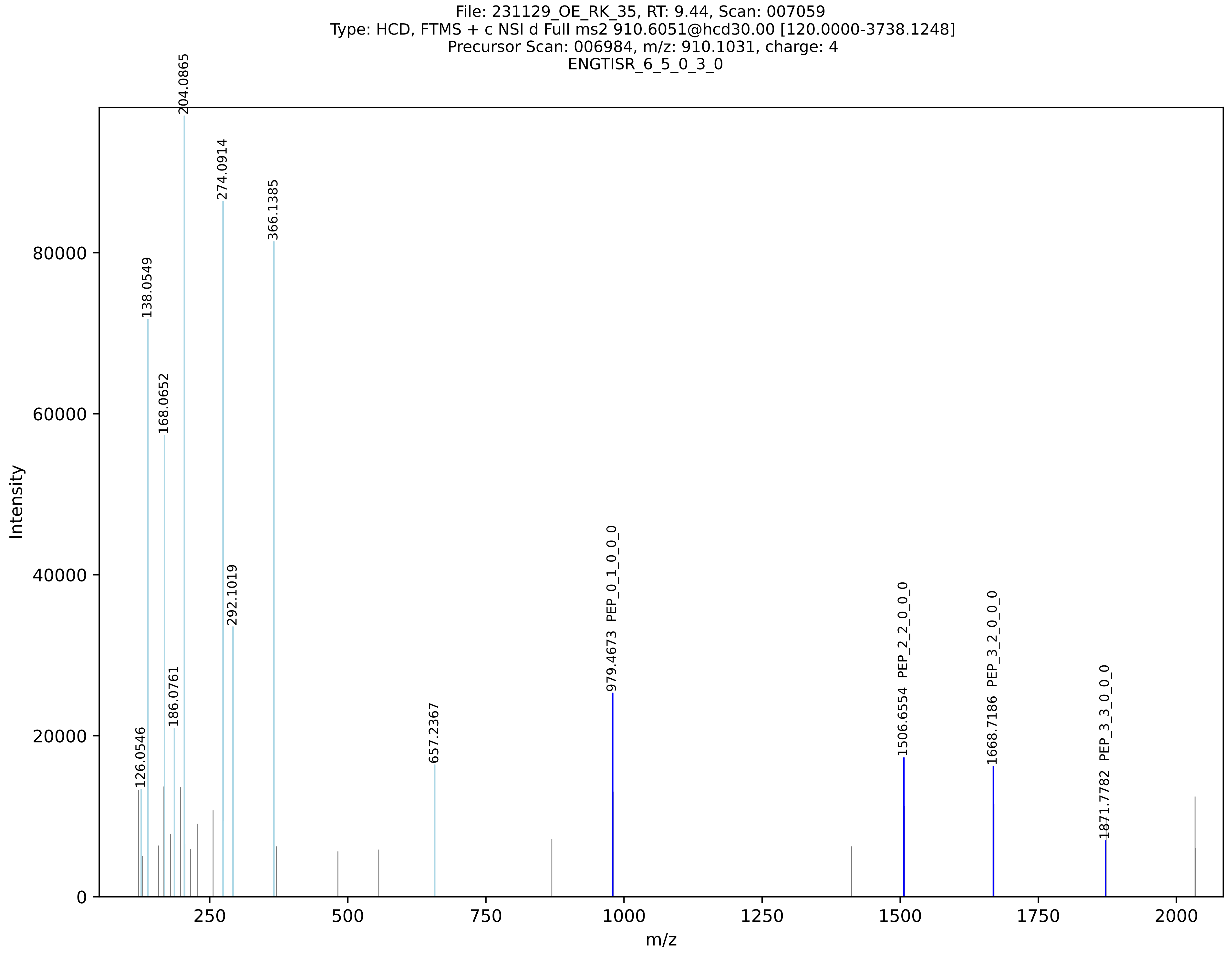
File: 231129_OE_RK_35, RT: 9.44, Scan: 007059
Type: HCD, FTMS + c NSI d Full ms2 910.6051@hcd30.00 [120.0000-3738.1248]
Precursor Scan: 006984, m/z: 910.1031, charge: 4
ENGTISR_6_5_0_3_0
204.0865
274.0914
366.1385
138.0549
168.0652
292.1019
186.0761
126.0546
657.2367
979.4673 PEP_0_1_0_0_0
1506.6554 PEP_2_2_0_0_0
1668.7186 PEP_3_2_0_0_0
1871.7782 PEP_3_3_0_0_0
Intensity
m/z
0
20000
40000
60000
80000
250
500
750
1000
1250
1500
1750
2000

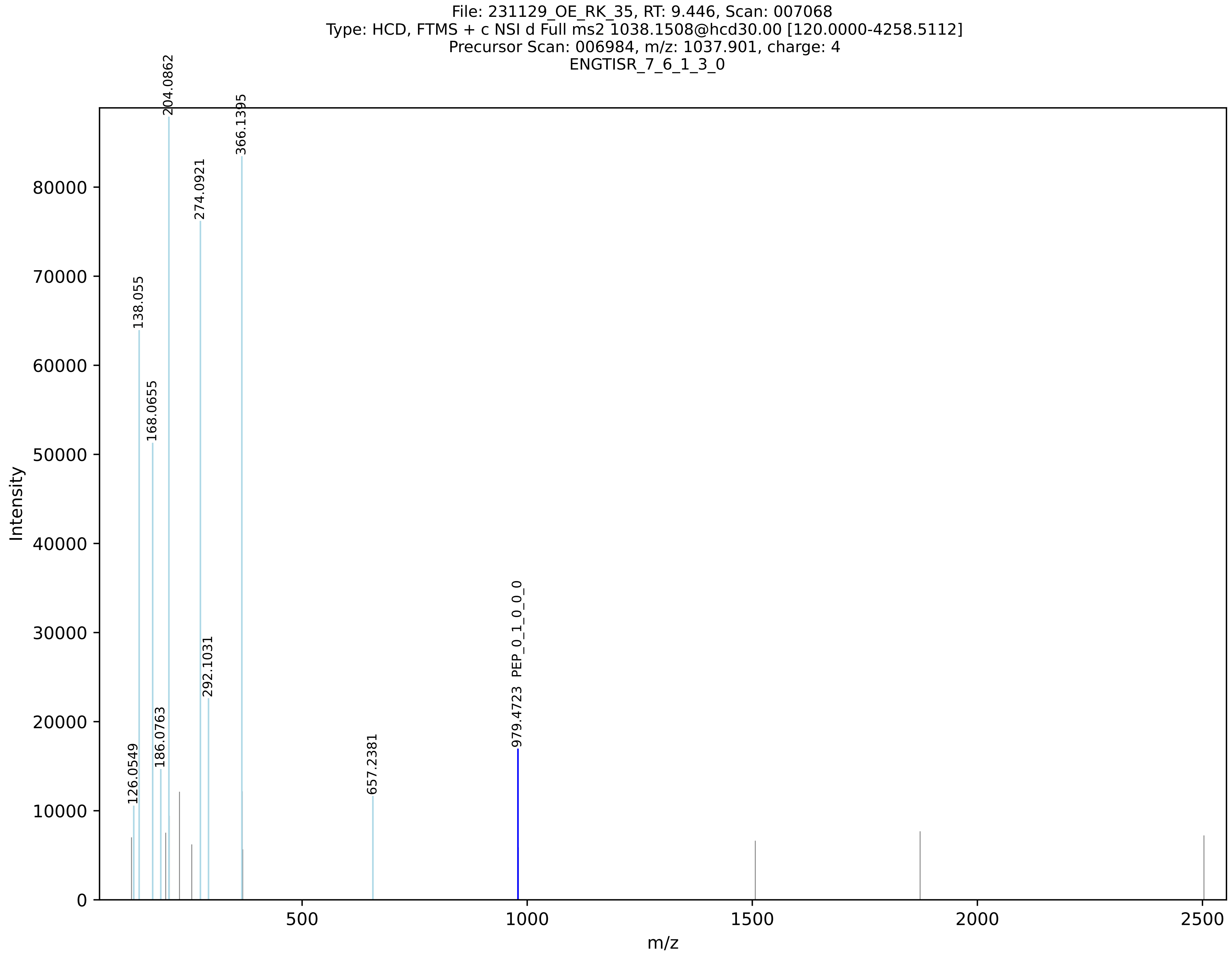

File: 231129_OE_RK_35, RT: 9.446, Scan: 007068
Type: HCD, FTMS + c NSI d Full ms2 1038.1508@hcd30.00 [120.0000-4258.5112]
Precursor Scan: 006984, m/z: 1037.901, charge: 4
ENGTISR_7_6_1_3_0
204.0862
366.1395
274.0921
138.055
168.0655
292.1031
186.0763
126.0549
657.2381
979.4723 PEP_0_1_0_0_0
Intensity
m/z
0
10000
20000
30000
40000
50000
60000
70000
80000
500
1000
1500
2000
2500

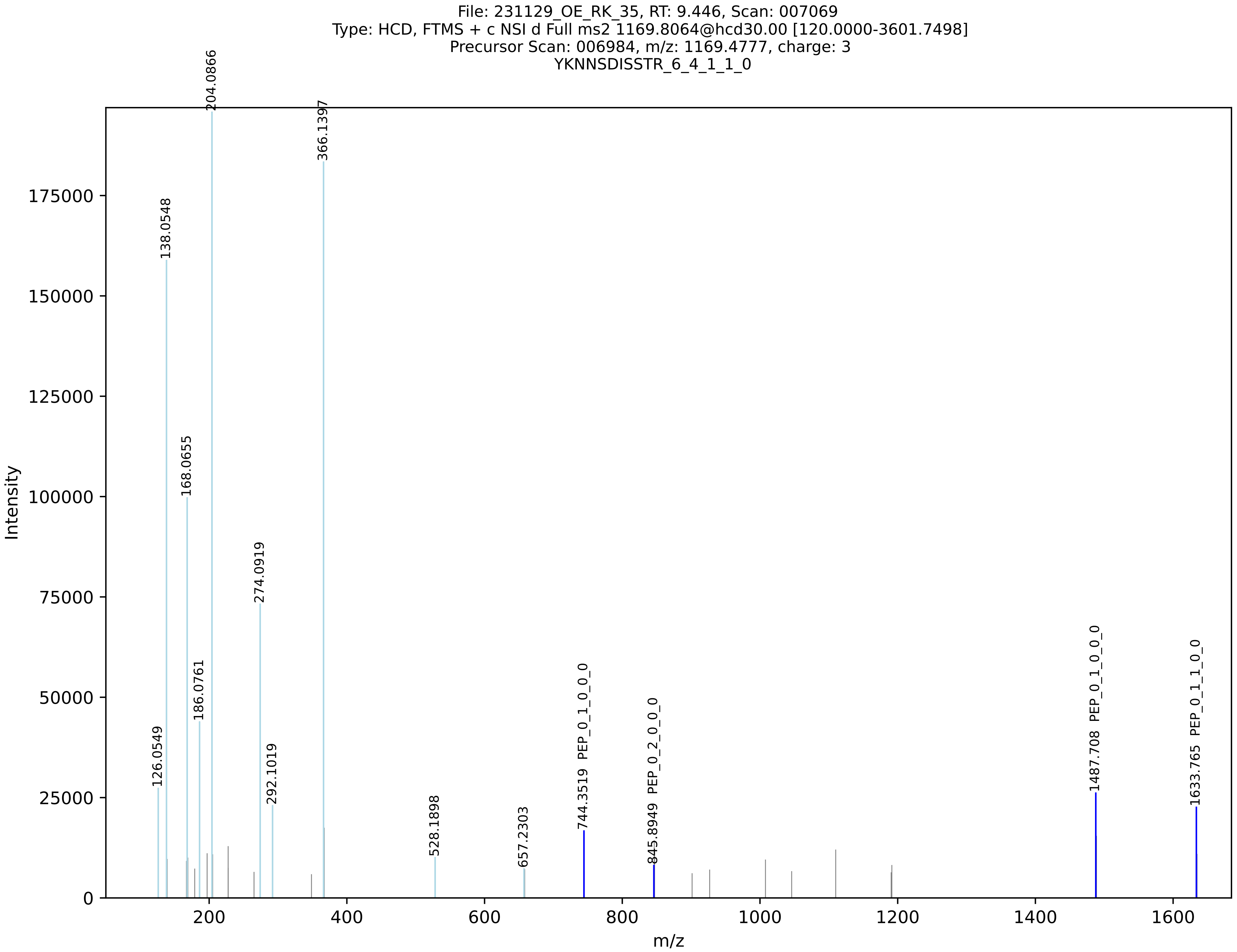
File: 231129_OE_RK_35, RT: 9.446, Scan: 007069
Type: HCD, FTMS + c NSI d Full ms2 1169.8064@hcd30.00 [120.0000-3601.7498]
Precursor Scan: 006984, m/z: 1169.4777, charge: 3
YKNNSDISSTR_6_4_1_1_0
Intensity
m/z
204.0866
366.1397
138.0548
168.0655
274.0919
186.0761
126.0549
292.1019
528.1898
657.2303
744.3519 PEP_0_1_0_0_0
845.8949 PEP_0_2_0_0_0
1487.708 PEP_0_1_0_0_
1633.765 PEP_0_1_1_0_0
0
25000
50000
75000
100000
125000
150000
175000
200
400
600
800
1000
1200
1400
1600

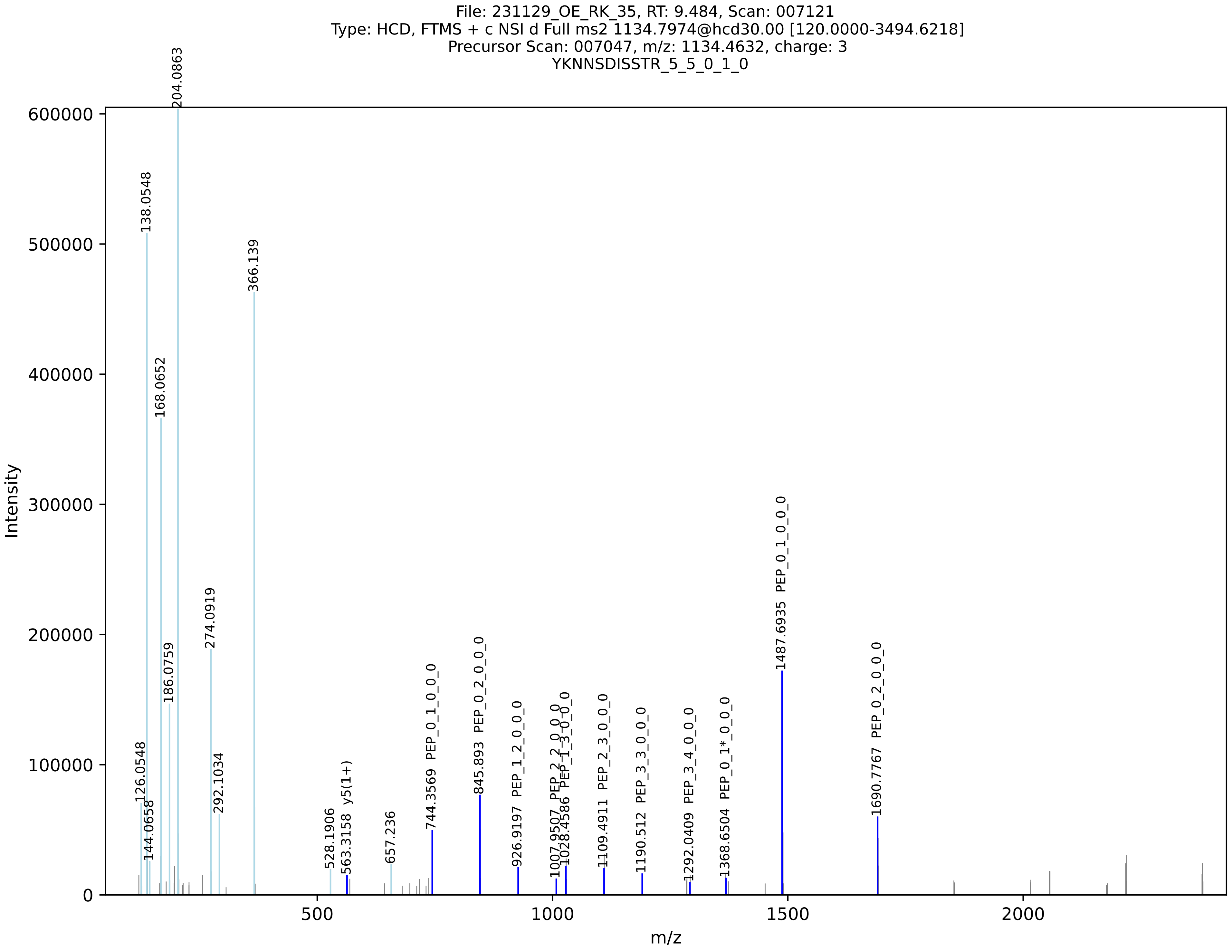

File: 231129_OE_RK_35, RT: 9.484, Scan: 007121
Type: HCD, FTMS + c NSI d Full ms2 1134.7974@hcd30.00 [120.0000-3494.6218]
Precursor Scan: 007047, m/z: 1134.4632, charge: 3
YKNNSDISSTR_5_5_0_1_0
Intensity
m/z
204.0863
138.0548
366.139
168.0652
274.0919
186.0759
126.0548
144.0658
292.1034
528.1906
563.3158 y5(1+)
657.236
744.3569 PEP_0_1_0_0_0
845.893 PEP_0_2_0_0_0
926.9197 PEP_1_2_0_0_0
1007.9507 PEP_2_2_0_0_0
1028.4586 PEP_1_3_0_0_0
1109.4911 PEP_2_3_0_0_0
1190.512 PEP_3_3_0_0_0
1292.0409 PEP_3_4_0_0_0
1368.6504 PEP_0_1*_0_0_0
1487.6935 PEP_0_1_0_0_0
1690.7767 PEP_0_2_0_0_0

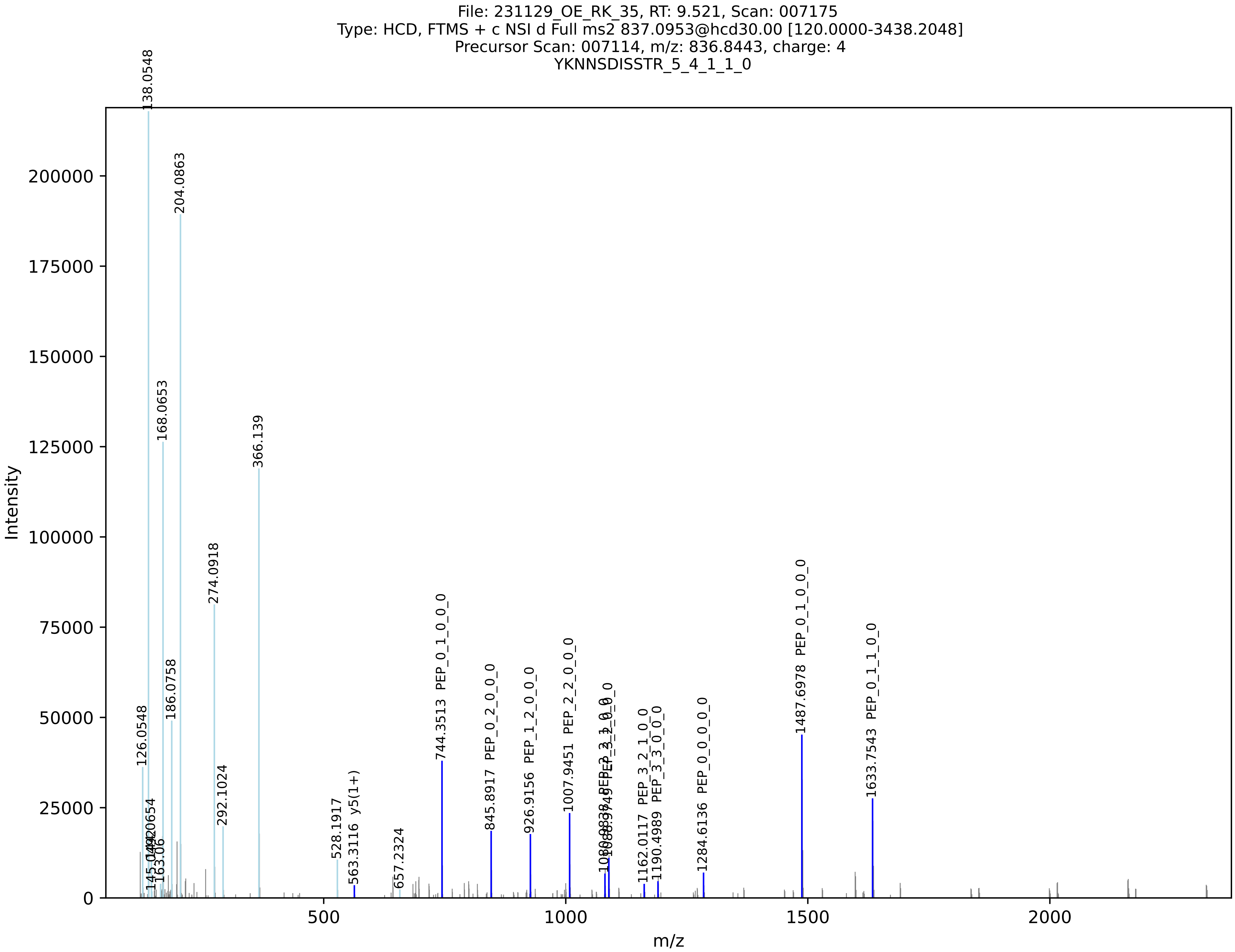
File: 231129_OE_RK_35, RT: 9.521, Scan: 007175
Type: HCD, FTMS + c NSI d Full ms2 837.0953@hcd30.00 [120.0000-3438.2048]
Precursor Scan: 007114, m/z: 836.8443, charge: 4
YKNNSDISSTR_5_4_1_1_0
Intensity
m/z
138.0548
204.0863
168.0653
366.139
274.0918
186.0758
126.0548
292.1024
163.06
528.1917
563.3116 y5(1+)
657.2324
744.3513 PEP_0_1_0_0_0
845.8917 PEP_0_2_0_0_0
926.9156 PEP_1_2_0_0_0
1007.9451 PEP_2_2_0_0_0
1162.0117 PEP_3_2_1_0_0
1190.4989 PEP_3_3_0_0_0
1284.6136 PEP_0_0_0_0_0
1487.6978 PEP_0_1_0_0_0
1633.7543 PEP_0_1_1_0_0
0
25000
50000
75000
100000
125000
150000
175000
200000
500
1000
1500
2000

File: 231129_OE_RK_35, RT: 9.522, Scan: 007177
Type: HCD, FTMS + c NSI d Full ms2 1383.8661@hcd30.00 [120.0000-4256.7720]
Precursor Scan: 007114, m/z: 1383.5337, charge: 3
ENGTISR_7_6_1_3_0

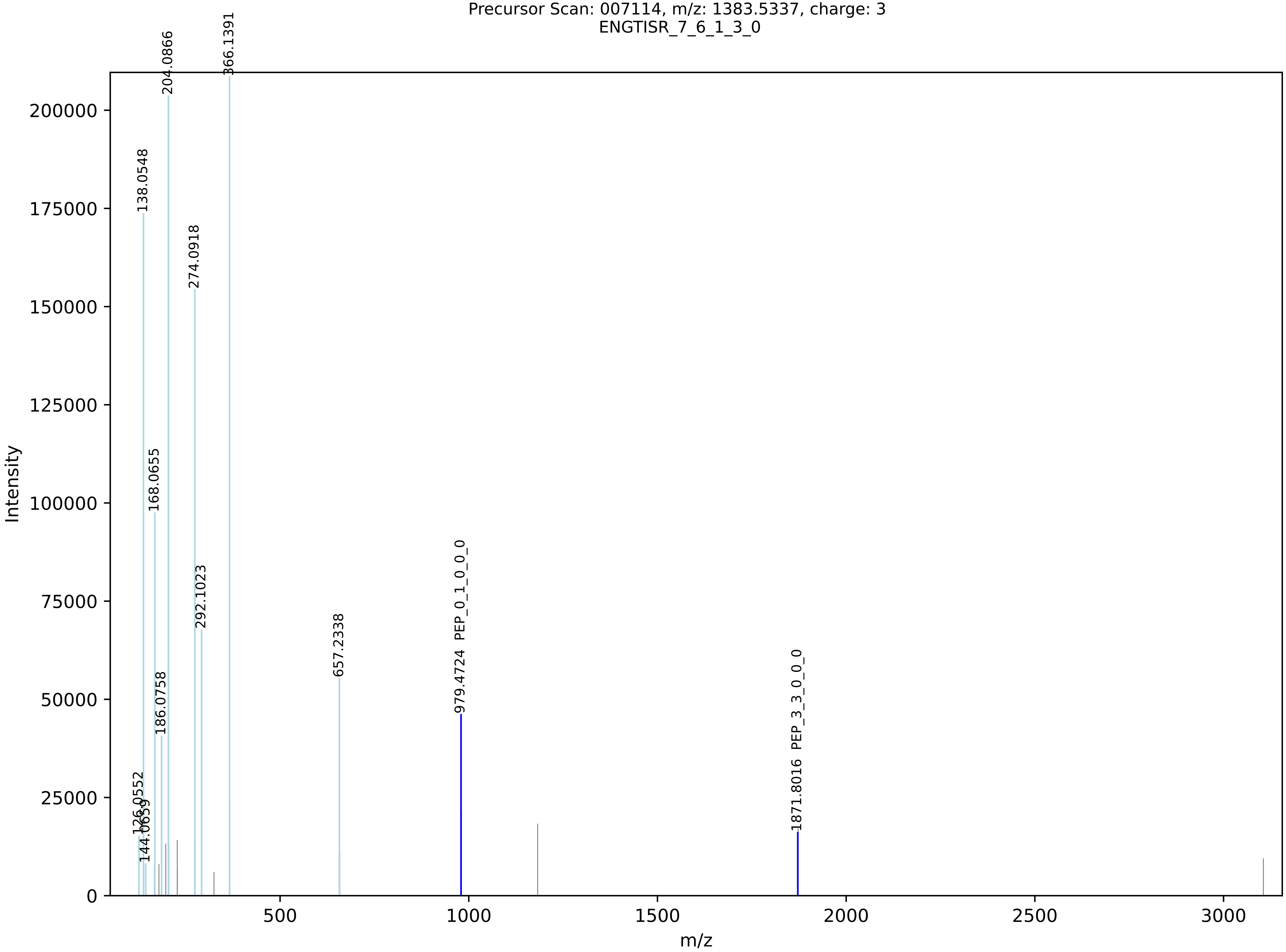

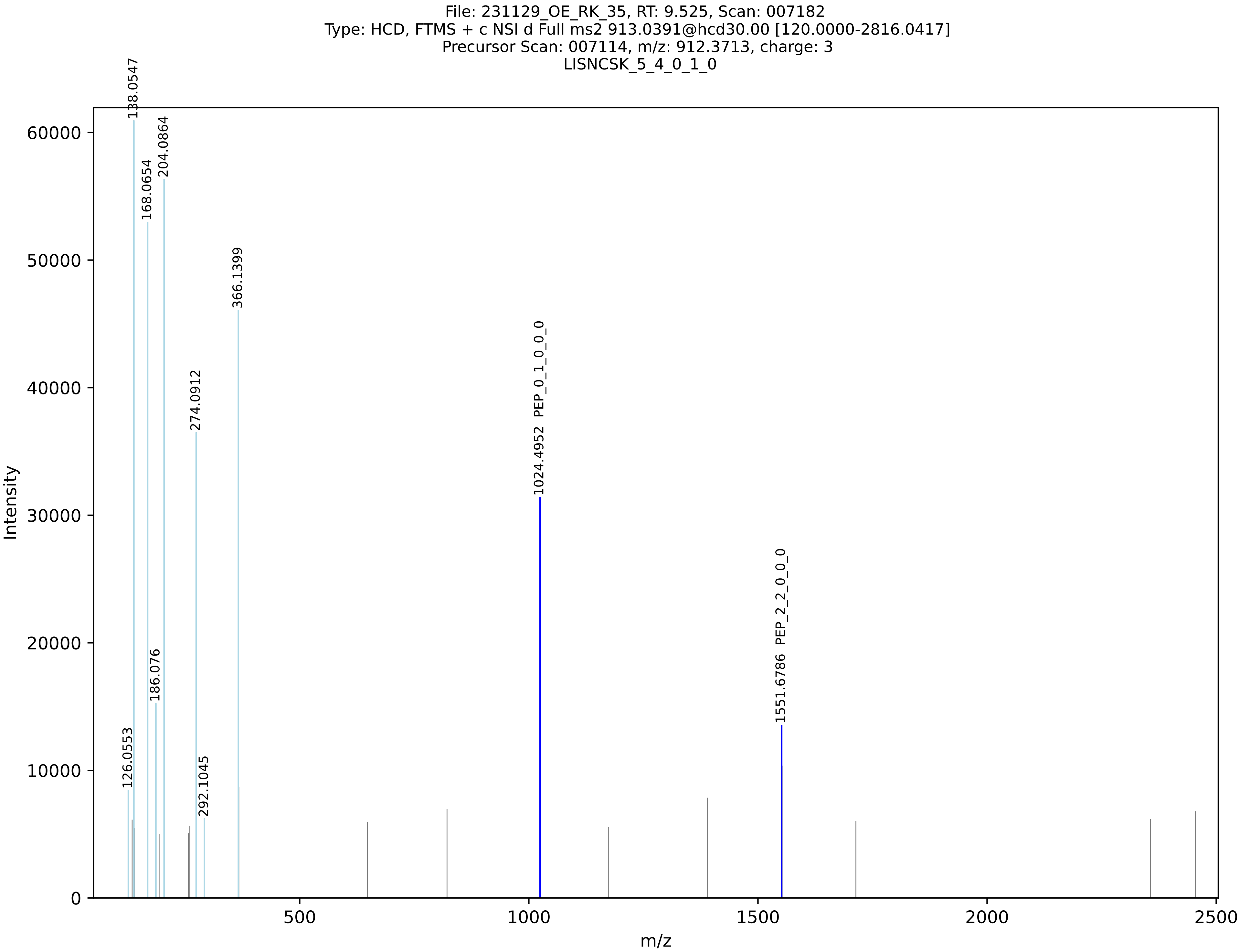
File: 231129_OE_RK_35, RT: 9.525, Scan: 007182
Type: HCD, FTMS + c NSI d Full ms2 913.0391@hcd30.00 [120.0000-2816.0417]
Precursor Scan: 007114, m/z: 912.3713, charge: 3
LISNCSK_5_4_0_1_0
138.0547
168.0654
204.0864
366.1399
274.0912
1024.4952 PEP_0_1_0_0_0
1551.6786 PEP_2_2_0_0_0
186.076
126.0553
292.1045
Intensity
m/z
0
10000
20000
30000
40000
50000
60000
500
1000
1500
2000
2500

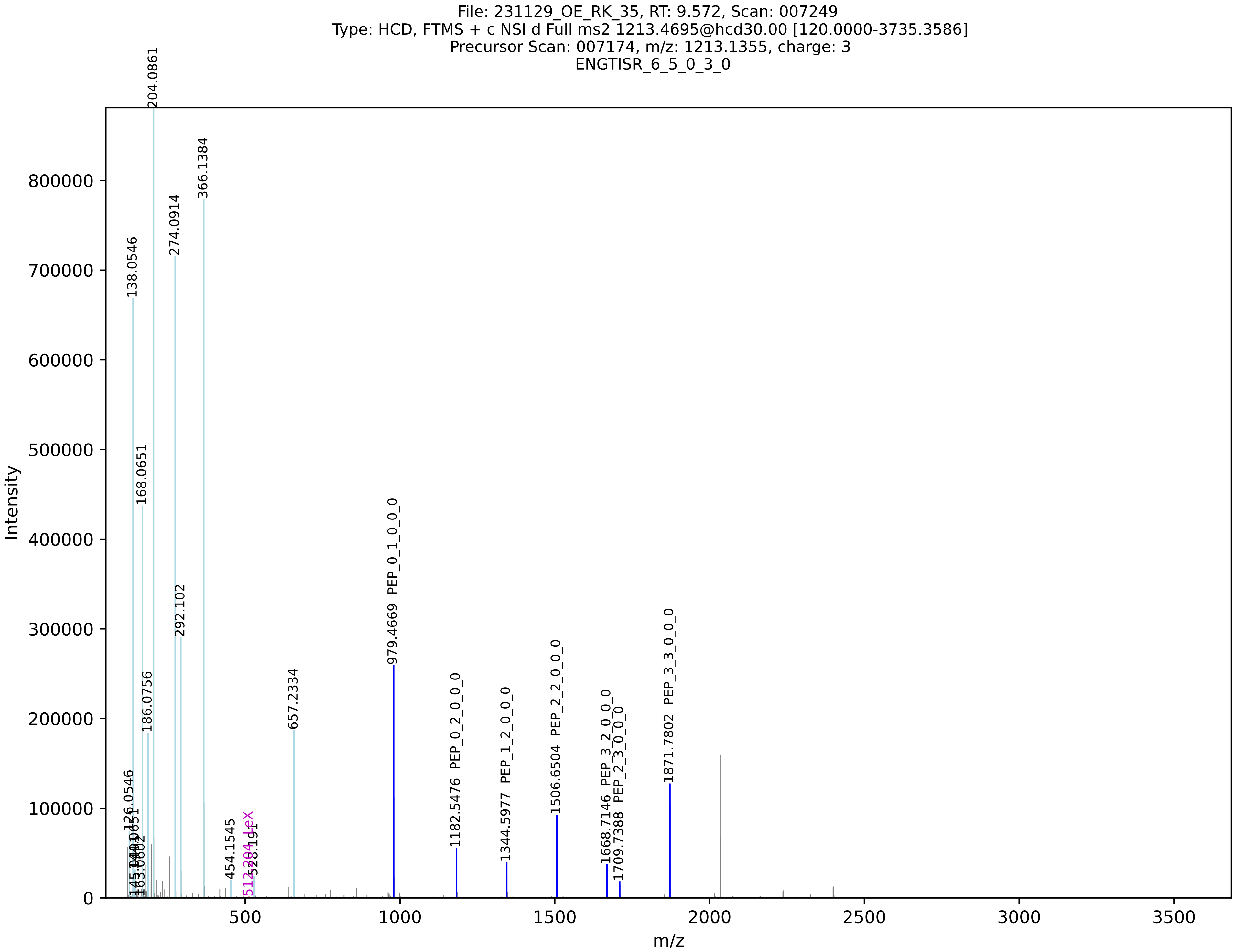
File: 231129_OE_RK_35, RT: 9.572, Scan: 007249
Type: HCD, FTMS + c NSI d Full ms2 1213.4695@hcd30.00 [120.0000-3735.3586]
Precursor Scan: 007174, m/z: 1213.1355, charge: 3
ENGTISR_6_5_0_3_0
204.0861
366.1384
274.0914
138.0546
168.0651
292.102
186.0756
657.2334
126.0546
454.1545
528.191
979.4669 PEP_0_1_0_0_0
1182.5476 PEP_0_2_0_0_0
1344.5977 PEP_1_2_0_0_0
1506.6504 PEP_2_2_0_0_0
1668.7146 PEP_3_2_0_0_0
1709.7388 PEP_2_3_0_0_0
1871.7802 PEP_3_3_0_0_0
Intensity
m/z
0
100000
200000
300000
400000
500000
600000
700000
800000
500
1000
1500
2000
2500
3000
3500

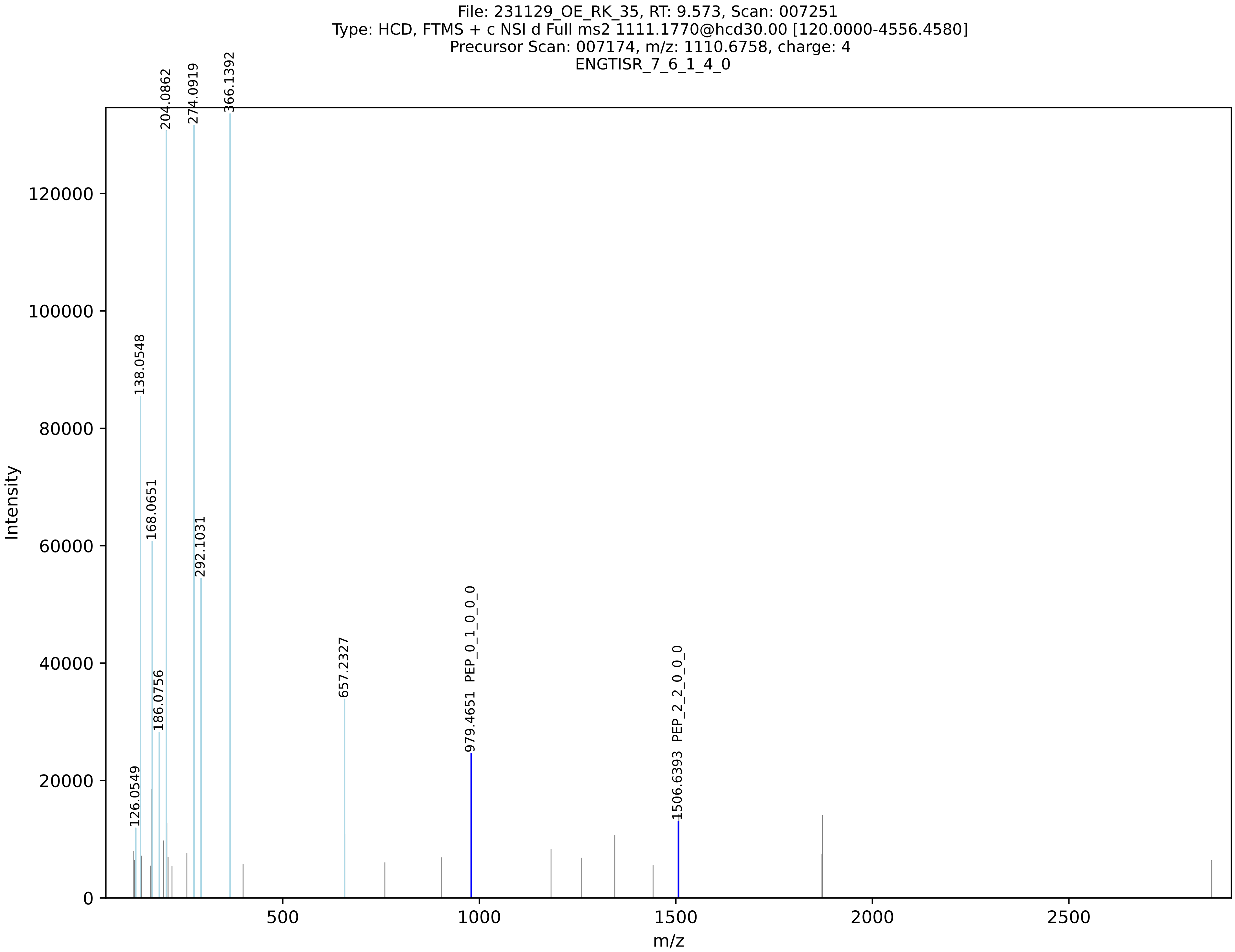
File: 231129_OE_RK_35, RT: 9.573, Scan: 007251
Type: HCD, FTMS + c NSI d Full ms2 1111.1770@hcd30.00 [120.0000-4556.4580]
Precursor Scan: 007174, m/z: 1110.6758, charge: 4
ENGTISR_7_6_1_4_0
Intensity
m/z
126.0549
138.0548
168.0651
186.0756
204.0862
274.0919
292.1031
366.1392
657.2327
979.4651 PEP_0_1_0_0_0
1506.6393 PEP_2_2_0_0_0
0
20000
40000
60000
80000
100000
120000
500
1000
1500
2000
2500

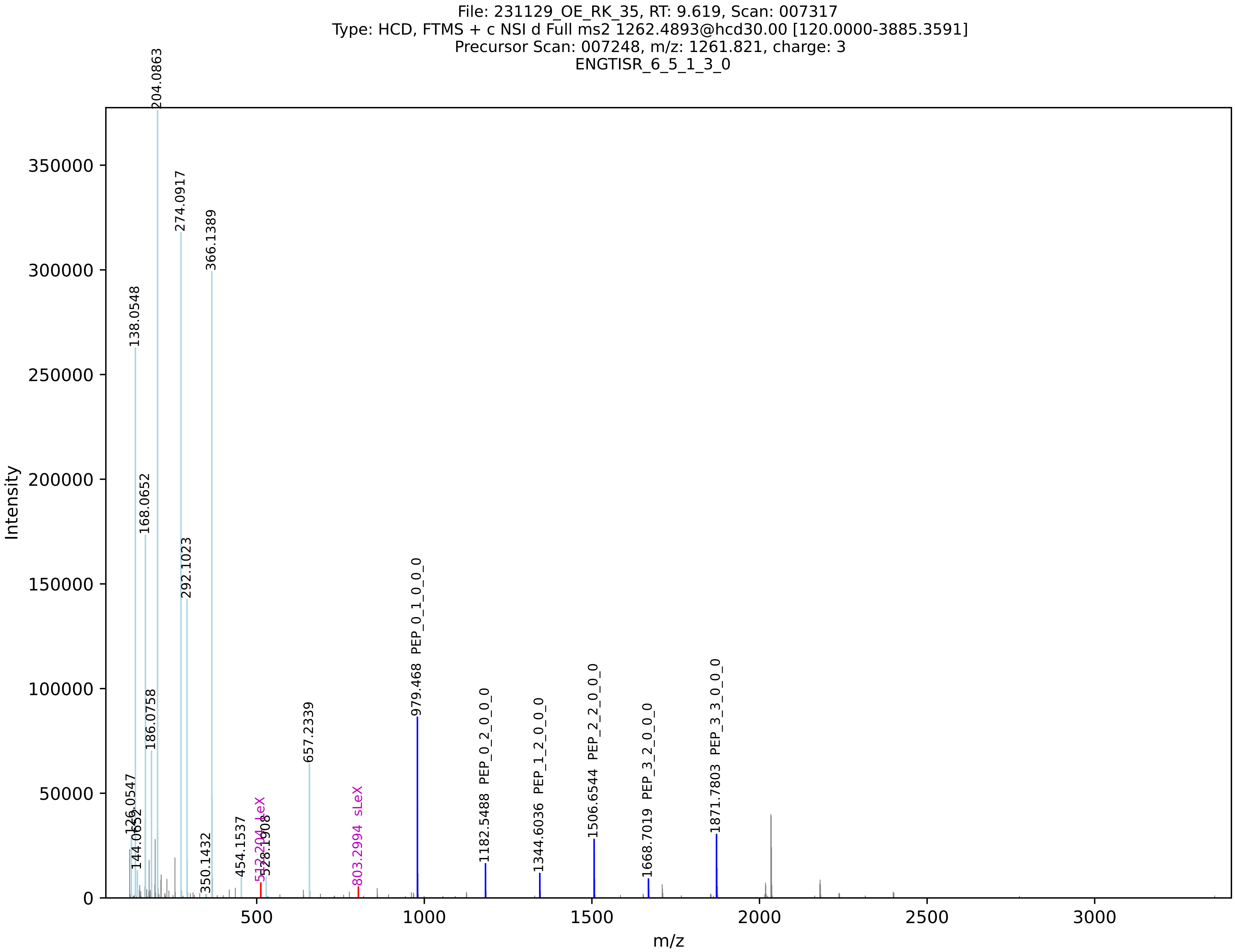
File: 231129_OE_RK_35, RT: 9.619, Scan: 007317
Type: HCD, FTMS + c NSI d Full ms2 1262.4893@hcd30.00 [120.0000-3885.3591]
Precursor Scan: 007248, m/z: 1261.821, charge: 3
ENGTISR_6_5_1_3_0
Intensity
m/z
204.0863
274.0917
366.1389
138.0548
168.0652
292.1023
186.0758
126.0547
144.0652
350.1432
454.1537
512.204 LeX
528.1908
657.2339
803.2994 sLeX
979.468 PEP_0_1_0_0_0
1182.5488 PEP_0_2_0_0_0
1344.6036 PEP_1_2_0_0_0
1506.6544 PEP_2_2_0_0_0
1668.7019 PEP_3_2_0_0_0
1871.7803 PEP_3_3_0_0_0

File: 231129_OE_RK_35, RT: 9.62, Scan: 007319
Type: HCD, FTMS + c NSI d Full ms2 1220.4557@hcd30.00 [120.0000-3756.7366]
Precursor Scan: 007248, m/z: 1219.7875, charge: 3
NEEYNK_6_5_0_3_0

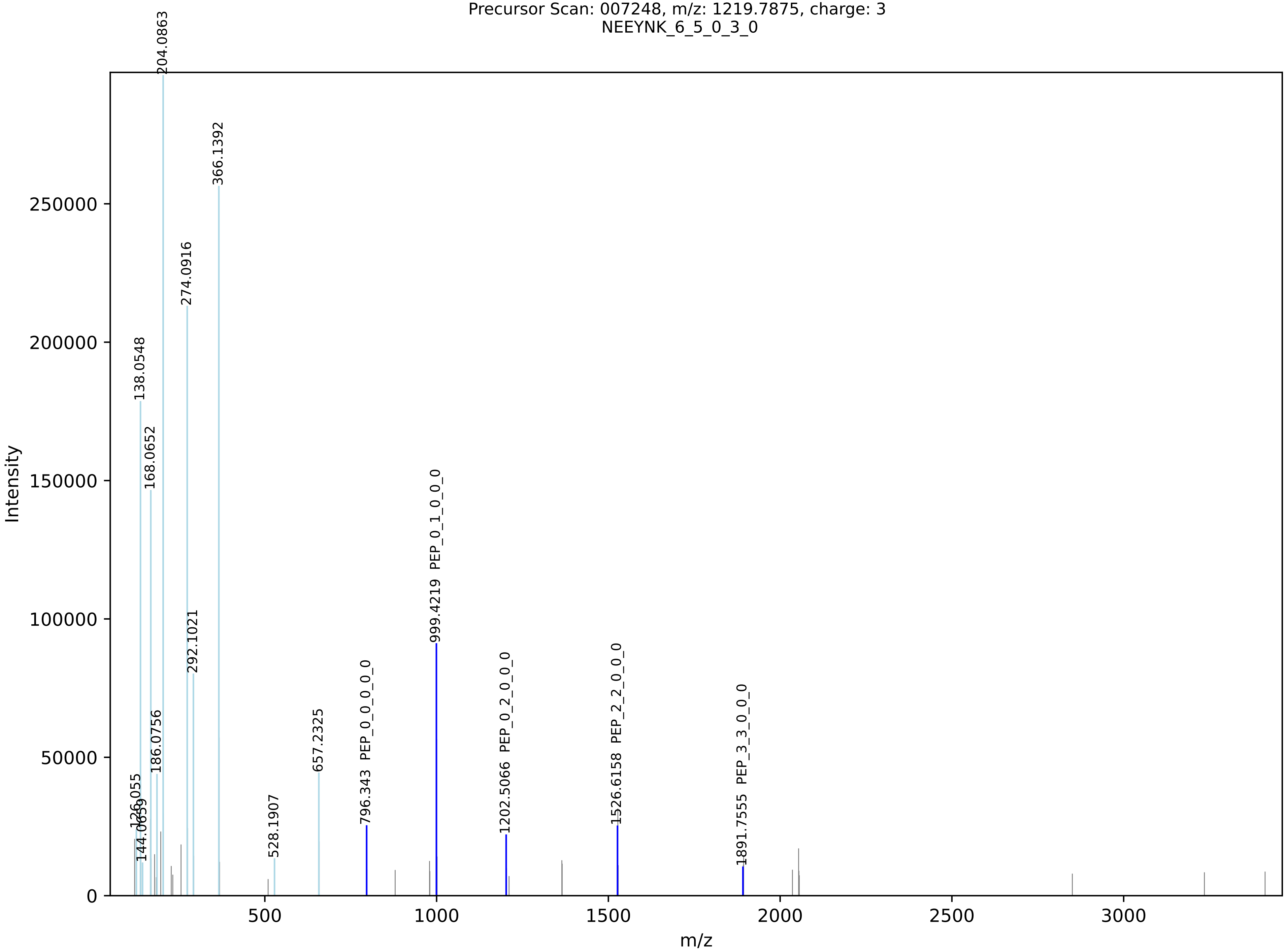

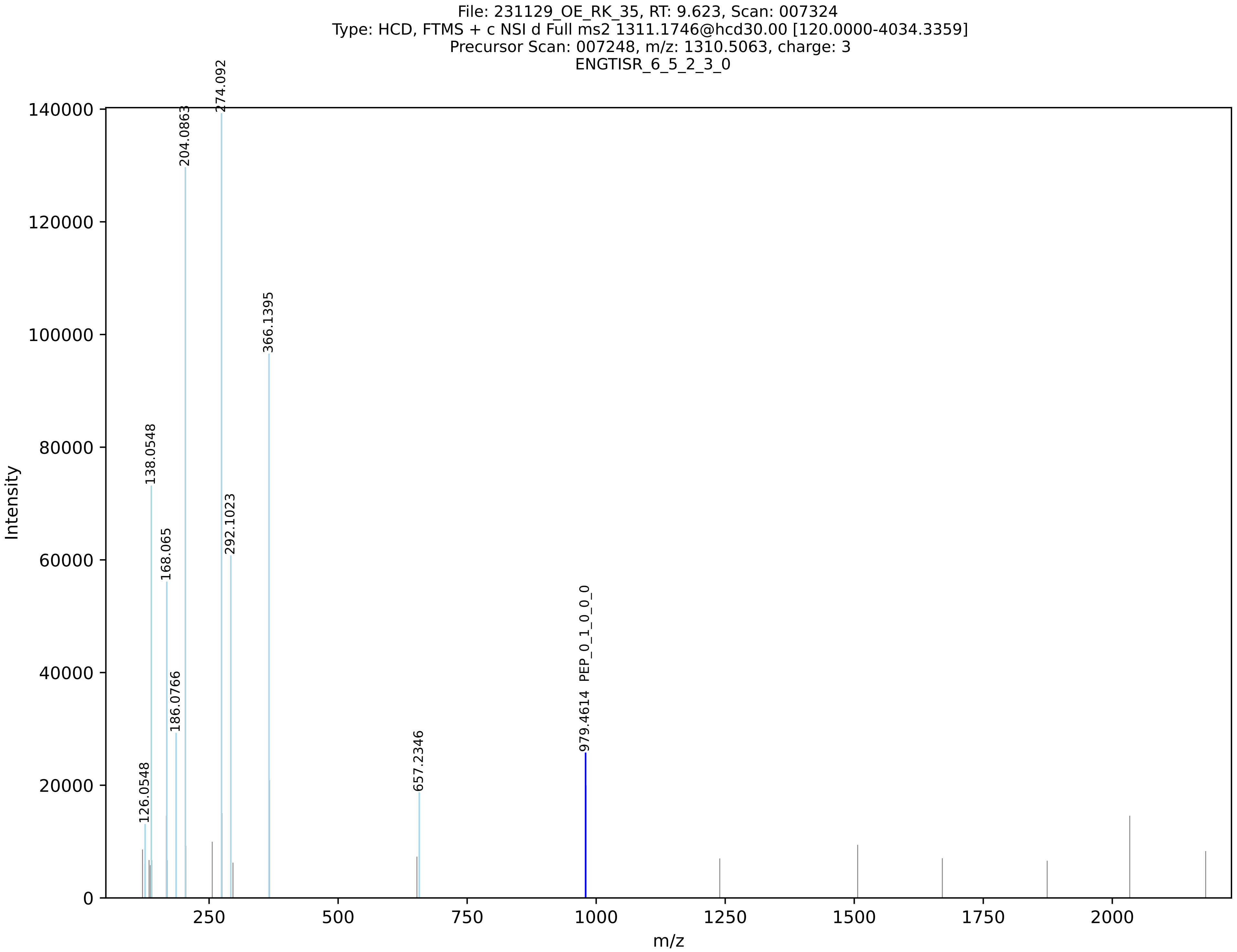
File: 231129_OE_RK_35, RT: 9.623, Scan: 007324
Type: HCD, FTMS + c NSI d Full ms2 1311.1746@hcd30.00 [120.0000-4034.3359]
Precursor Scan: 007248, m/z: 1310.5063, charge: 3
ENGTISR_6_5_2_3_0
274.092
204.0863
366.1395
138.0548
292.1023
168.065
186.0766
126.0548
657.2346
979.4614 PEP_0_1_0_0_0_
Intensity
m/z
0
20000
40000
60000
80000
100000
120000
140000
250
500
750
1000
1250
1500
1750
2000

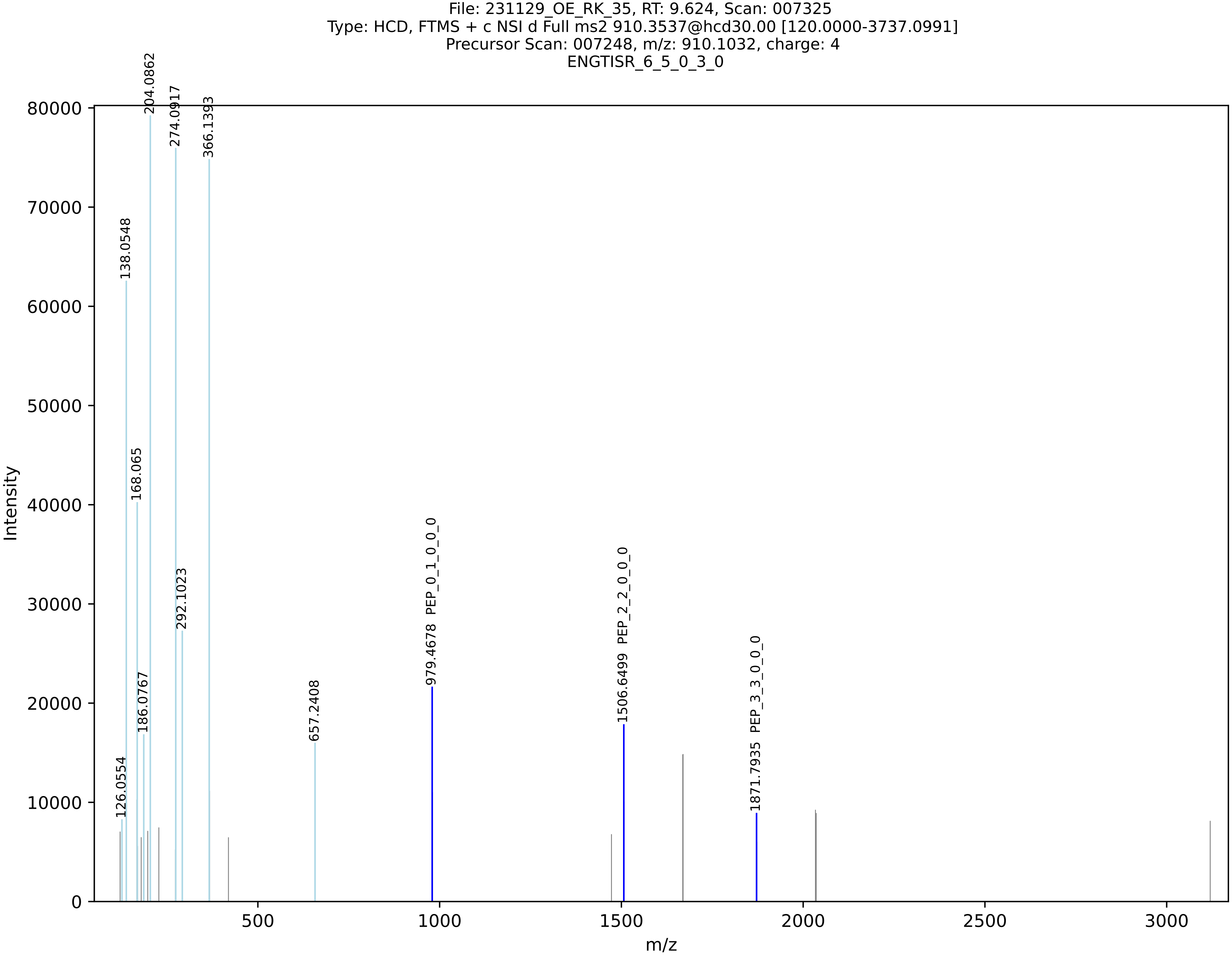

File: 231129_OE_RK_35, RT: 9.624, Scan: 007325
Type: HCD, FTMS + c NSI d Full ms2 910.3537@hcd30.00 [120.0000-3737.0991]
Precursor Scan: 007248, m/z: 910.1032, charge: 4
ENGTISR_6_5_0_3_0
Intensity
m/z
204.0862
274.0917
366.1393
138.0548
168.065
292.1023
186.0767
126.0554
657.2408
979.4678 PEP_0_1_0_0_0
1506.6499 PEP_2_2_0_0_0
1871.7935 PEP_3_3_0_0_0

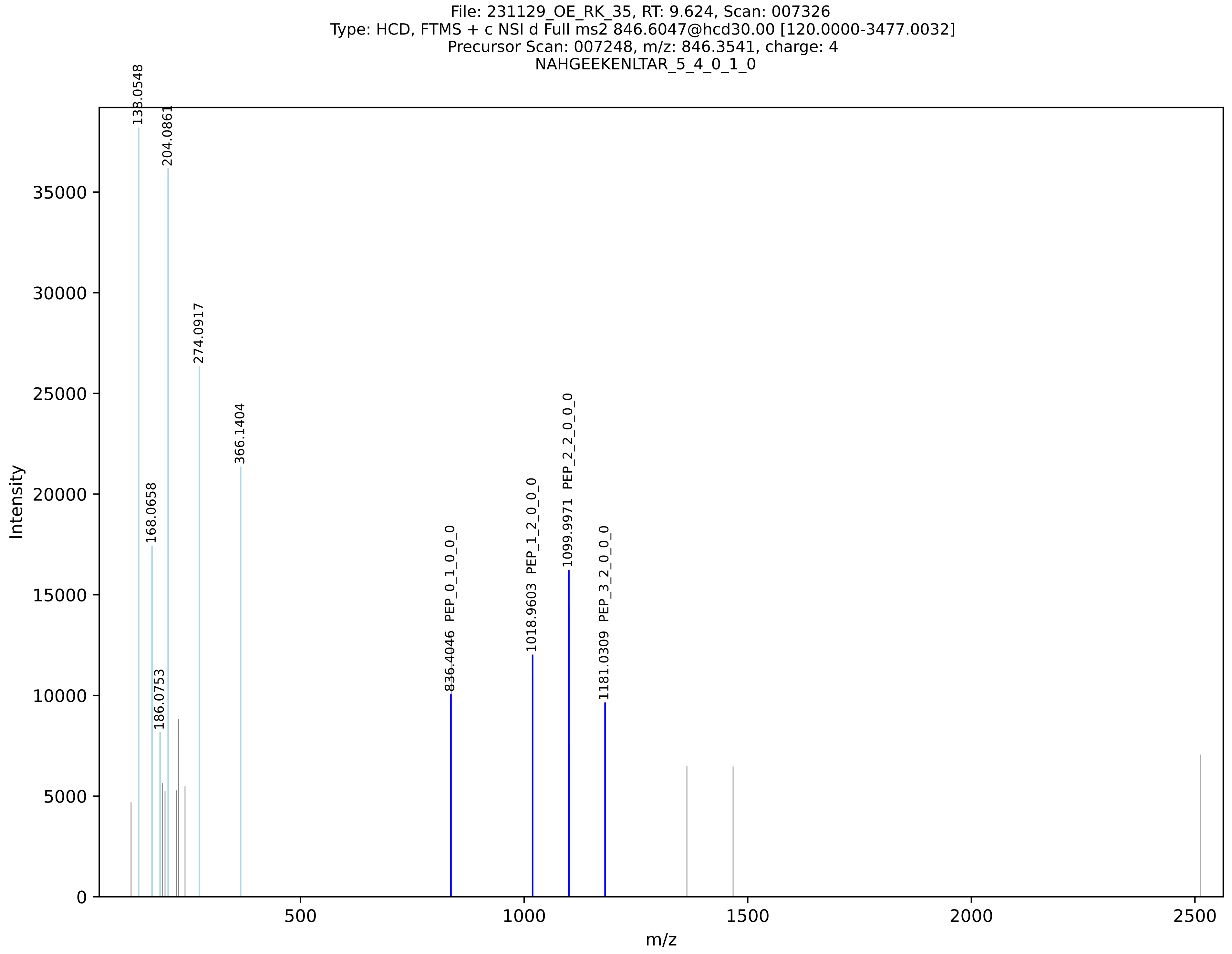

File: 231129_OE_RK_35, RT: 9.624, Scan: 007326
Type: HCD, FTMS + c NSI d Full ms2 846.6047@hcd30.00 [120.0000-3477.0032]
Precursor Scan: 007248, m/z: 846.3541, charge: 4
NAHGEEKENLTAR_5_4_0_1_0
138.0548
204.0861
274.0917
366.1404
168.0658
186.0753
836.4046 PEP_0_1_0_0_0
1018.9603 PEP_1_2_0_0_0
1099.9971 PEP_2_2_0_0_0
1181.0309 PEP_3_2_0_0_0
Intensity
m/z
0
5000
10000
15000
20000
25000
30000
35000
500
1000
1500
2000
2500

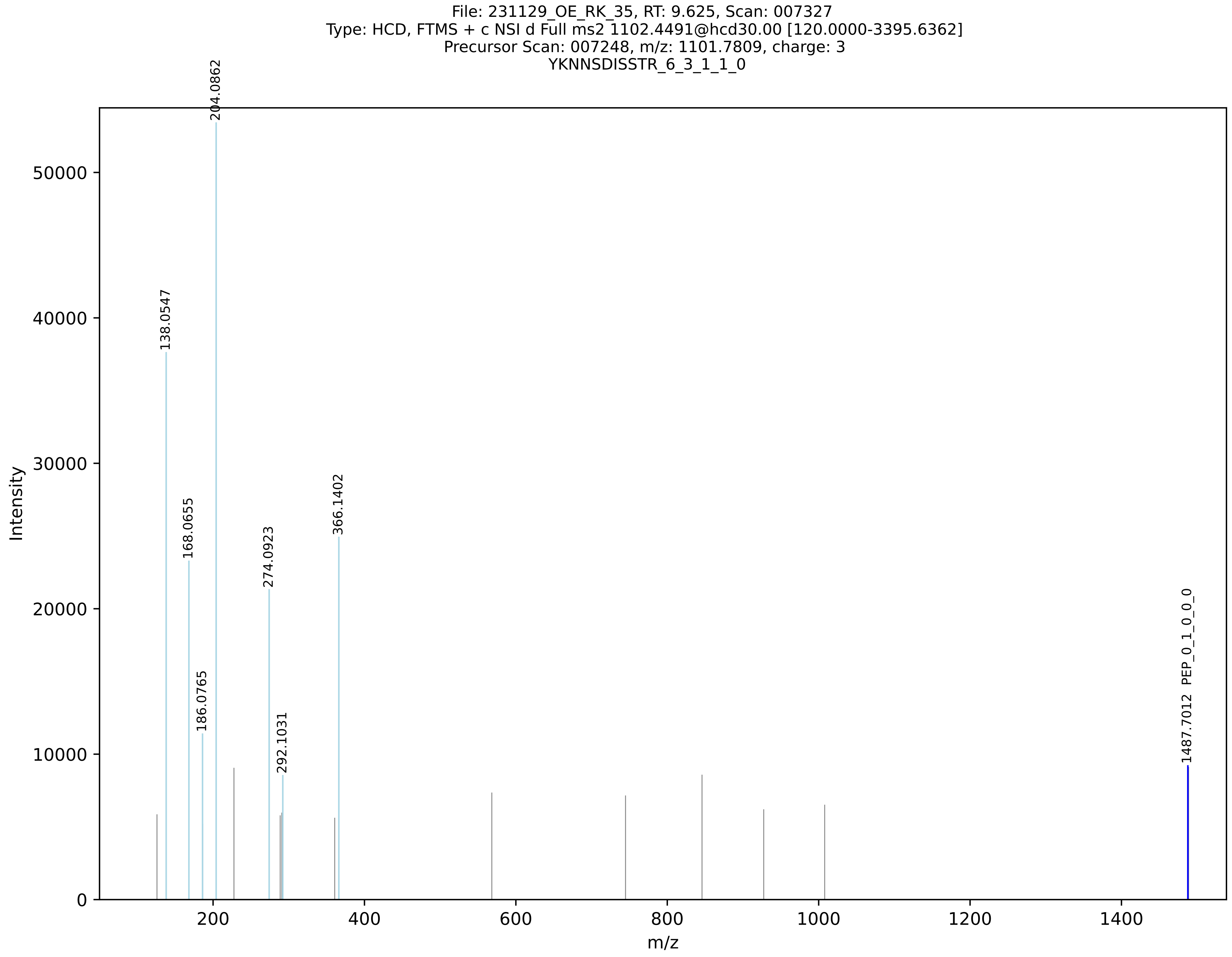
File: 231129_OE_RK_35, RT: 9.625, Scan: 007327
Type: HCD, FTMS + c NSI d Full ms2 1102.4491@hcd30.00 [120.0000-3395.6362]
Precursor Scan: 007248, m/z: 1101.7809, charge: 3
YKNNSDISSTR_6_3_1_1_0
204.0862
138.0547
168.0655
186.0765
274.0923
292.1031
366.1402
1487.7012 PEP_0_1_0_0_0
Intensity
m/z
0
10000
20000
30000
40000
50000
200
400
600
800
1000
1200
1400

File: 231129_OE_RK_35, RT: 9.666, Scan: 007385
Type: HCD, FTMS + c NSI d Full ms2 994.0797@hcd30.00 [120.0000-3064.0256]
Precursor Scan: 007316, m/z: 993.7455, charge: 3
YKNNSDISSTR_4_3_1_1_0

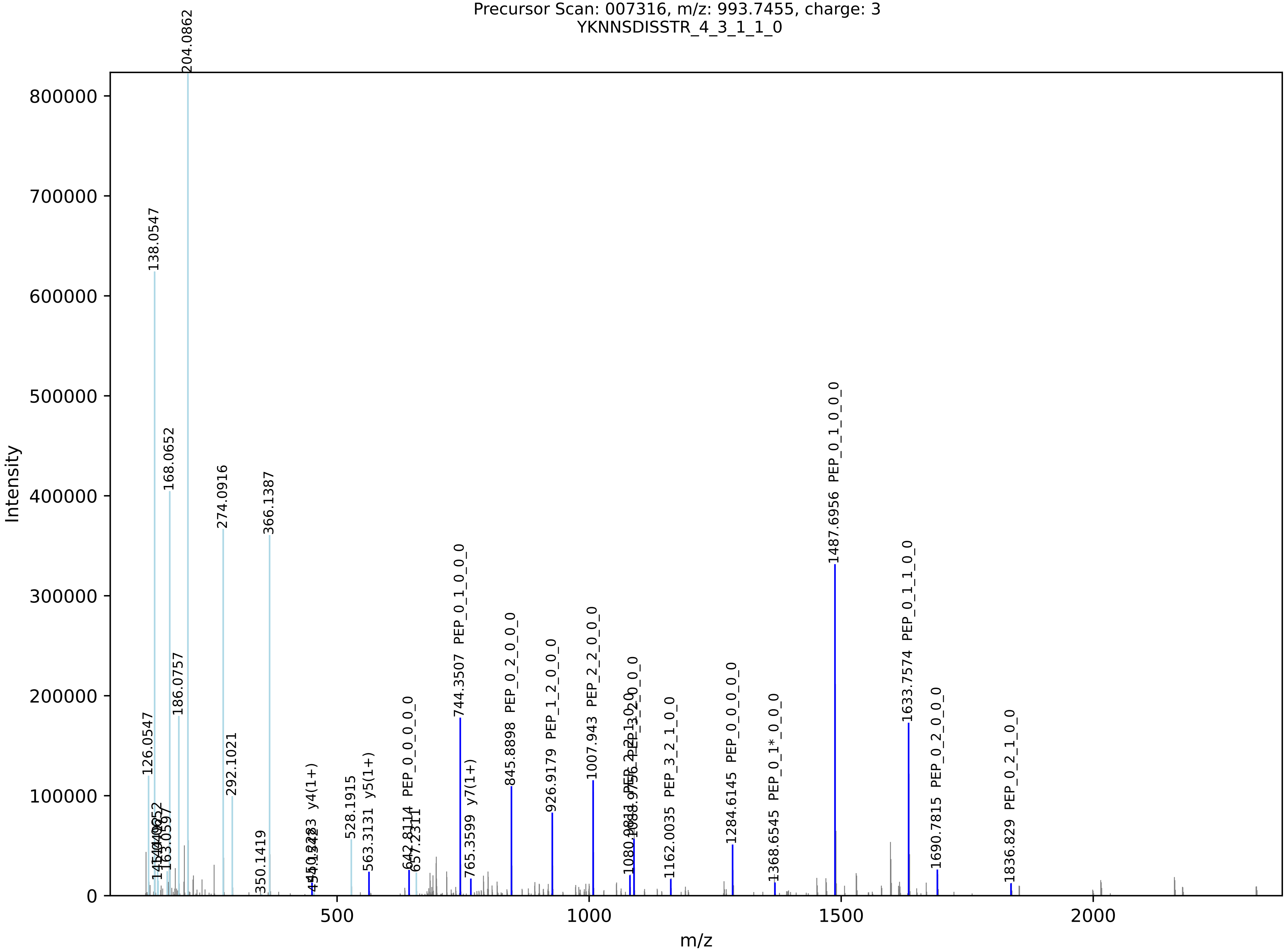

File: 231129_OE_RK_35, RT: 9.667, Scan: 007387
Type: HCD, FTMS + c NSI d Full ms2 1115.7904@hcd30.00 [120.0000-3436.4607]
Precursor Scan: 007316, m/z: 1115.4563, charge: 3
YKNNSDISSTR_5_4_1_1_0

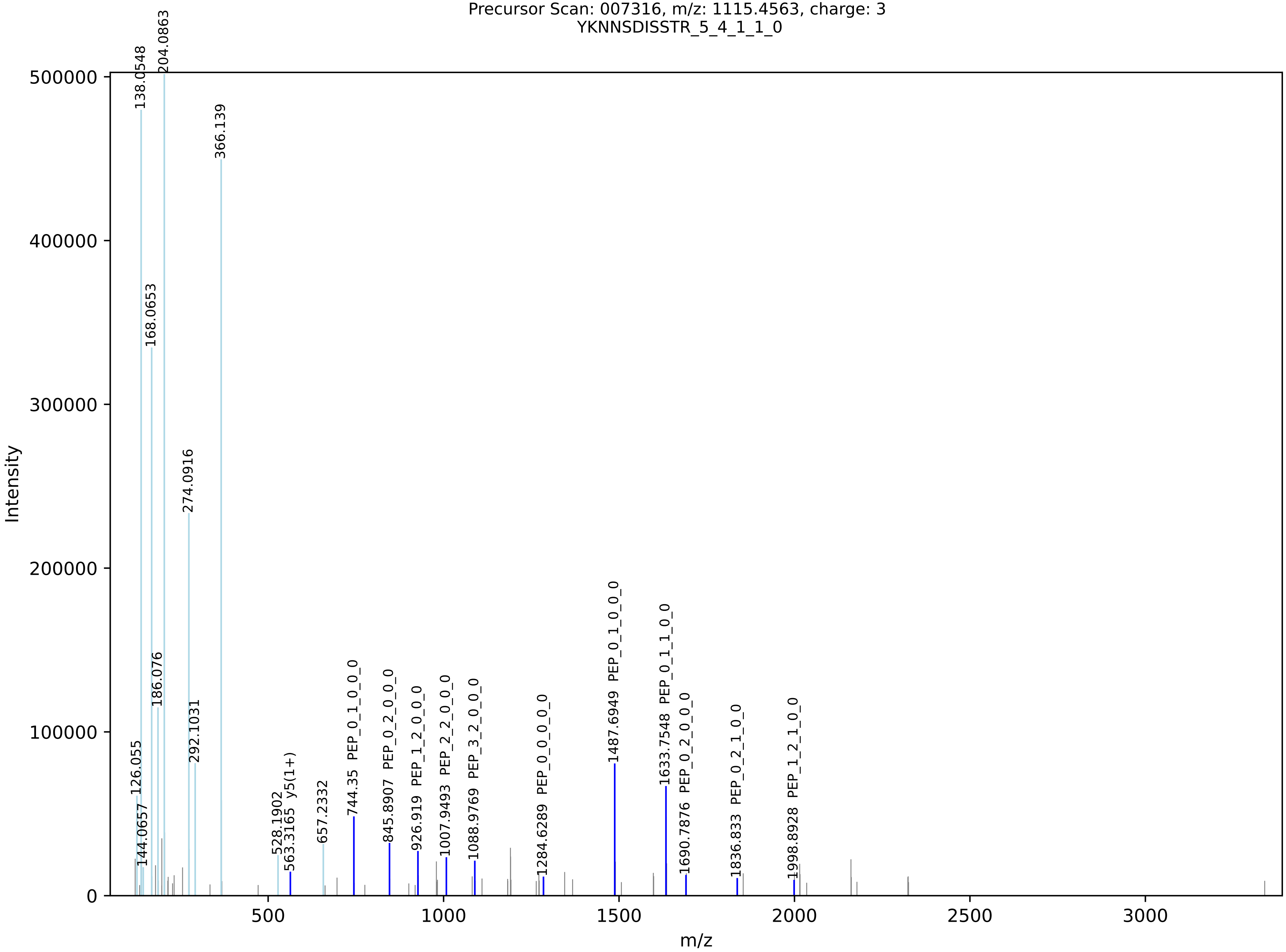

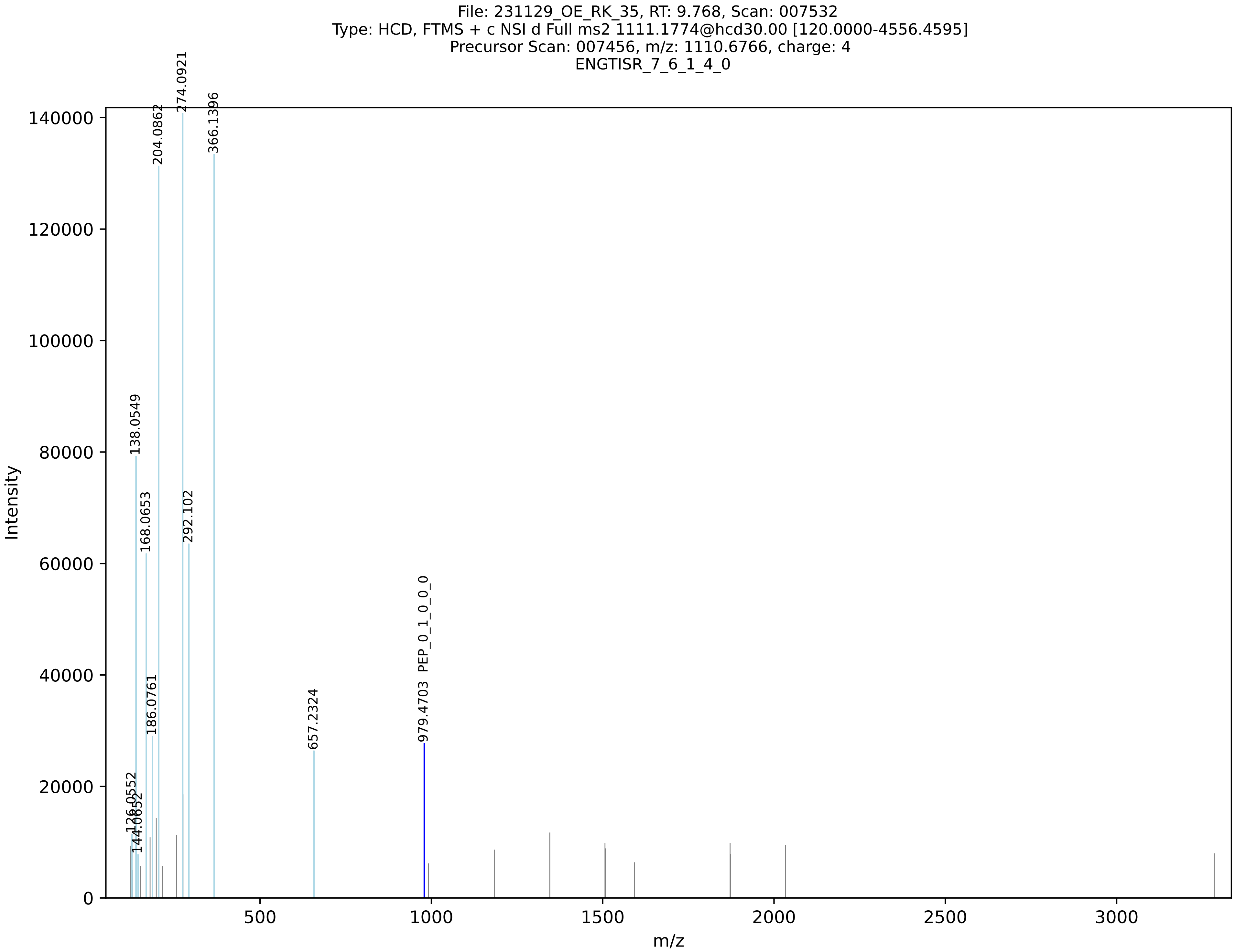
File: 231129_OE_RK_35, RT: 9.768, Scan: 007532
Type: HCD, FTMS + c NSI d Full ms2 1111.1774@hcd30.00 [120.0000-4556.4595]
Precursor Scan: 007456, m/z: 1110.6766, charge: 4
ENGTISR_7_6_1_4_0
Intensity
m/z
274.0921
366.1396
204.0862
138.0549
168.0653
292.102
186.0761
657.2324
979.4703 PEP_0_1_0_0_0
126.0552
144.0652

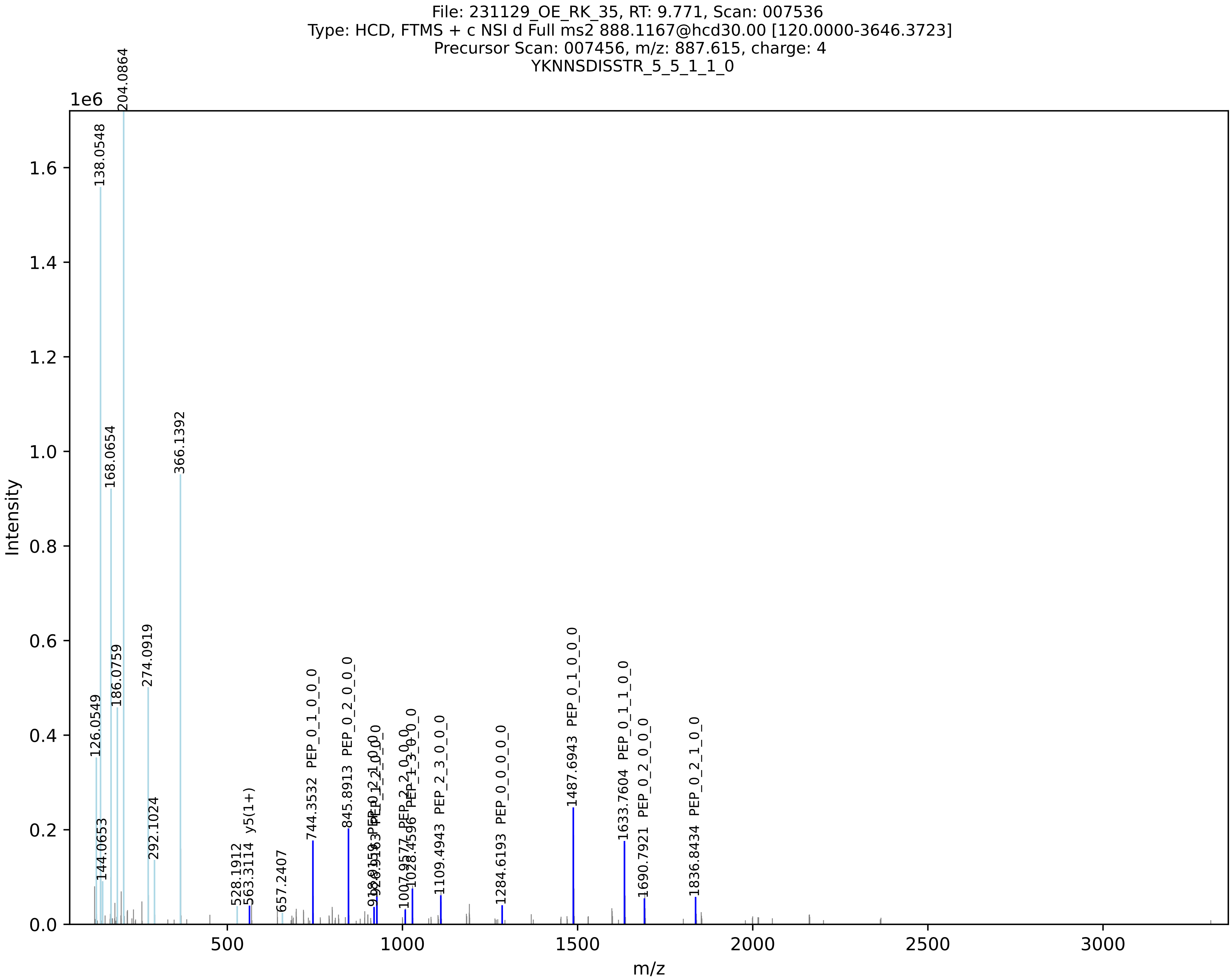

File: 231129_OE_RK_35, RT: 9.771, Scan: 007536
Type: HCD, FTMS + c NSI d Full ms2 888.1167@hcd30.00 [120.0000-3646.3723]
Precursor Scan: 007456, m/z: 887.615, charge: 4
YKNNSDISSTR_5_5_1_1_0
1e6
Intensity
m/z
204.0864
138.0548
168.0654
366.1392
186.0759
274.0919
126.0549
292.1024
144.0653
528.1912
563.3114 y5(1+)
657.2407
744.3532 PEP_0_1_0_0_0
845.8913 PEP_0_2_0_0_0
1007.9577 PEP_2_2_0_0_0
1028.4596 PEP_1_3_0_0_0
1109.4943 PEP_2_3_0_0_0
1284.6193 PEP_0_0_0_0_0
1487.6943 PEP_0_1_0_0_0
1633.7604 PEP_0_1_1_0_0
1690.7921 PEP_0_2_0_0_0
1836.8434 PEP_0_2_1_0_0

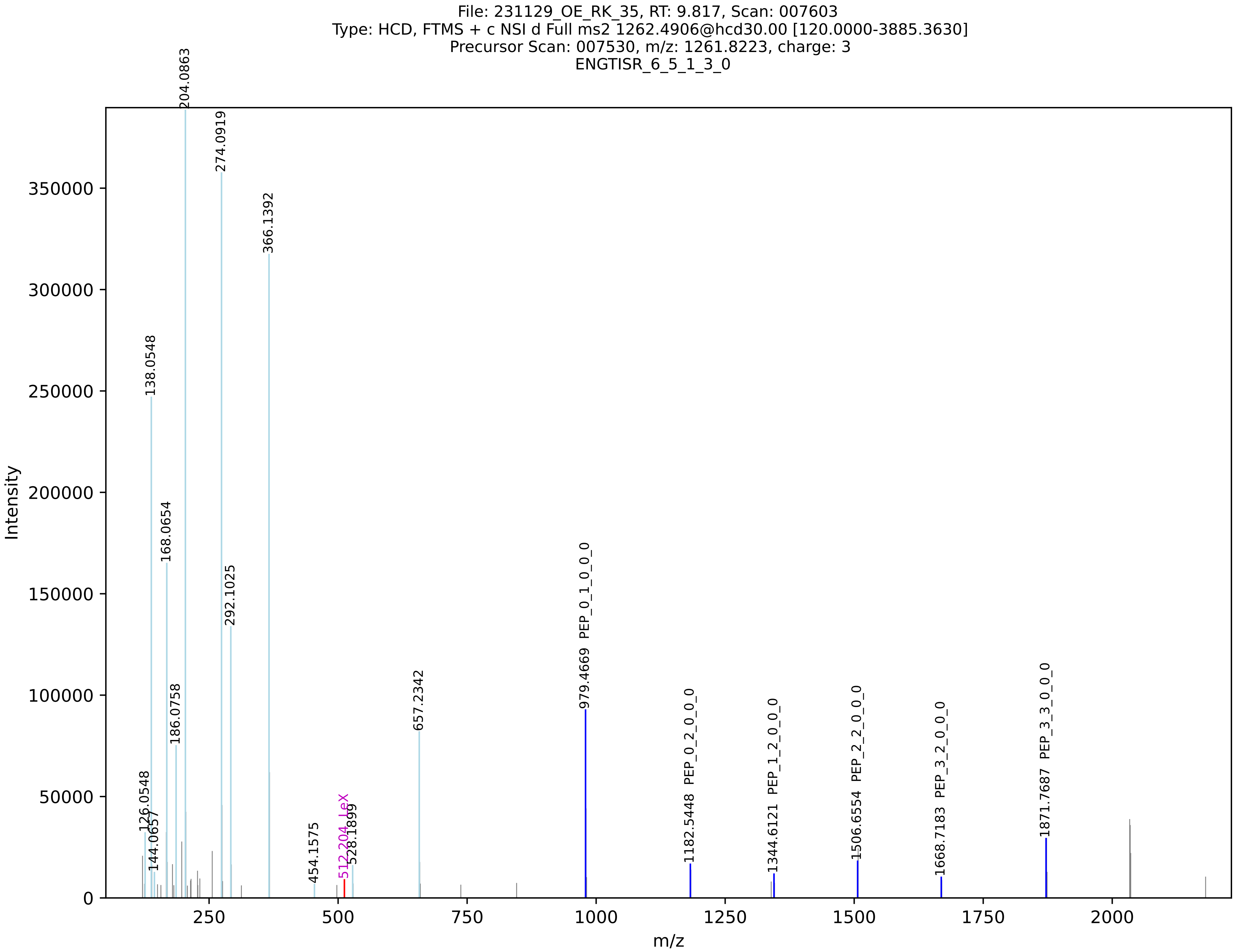
File: 231129_OE_RK_35, RT: 9.817, Scan: 007603
Type: HCD, FTMS + c NSI d Full ms2 1262.4906@hcd30.00 [120.0000-3885.3630]
Precursor Scan: 007530, m/z: 1261.8223, charge: 3
ENGTISR_6_5_1_3_0
Intensity
m/z
204.0863
274.0919
366.1392
138.0548
168.0654
292.1025
186.0758
126.0548
144.0657
454.1575
512.204_LeX
528.1899
657.2342
979.4669 PEP_0_1_0_0_0
1182.5448 PEP_0_2_0_0_0
1344.6121 PEP_1_2_0_0_0
1506.6554 PEP_2_2_0_0_0
1668.7183 PEP_3_2_0_0_0
1871.7687 PEP_3_3_0_0_0
0
50000
100000
150000
200000
250000
300000
350000
250
500
750
1000
1250
1500
1750
2000

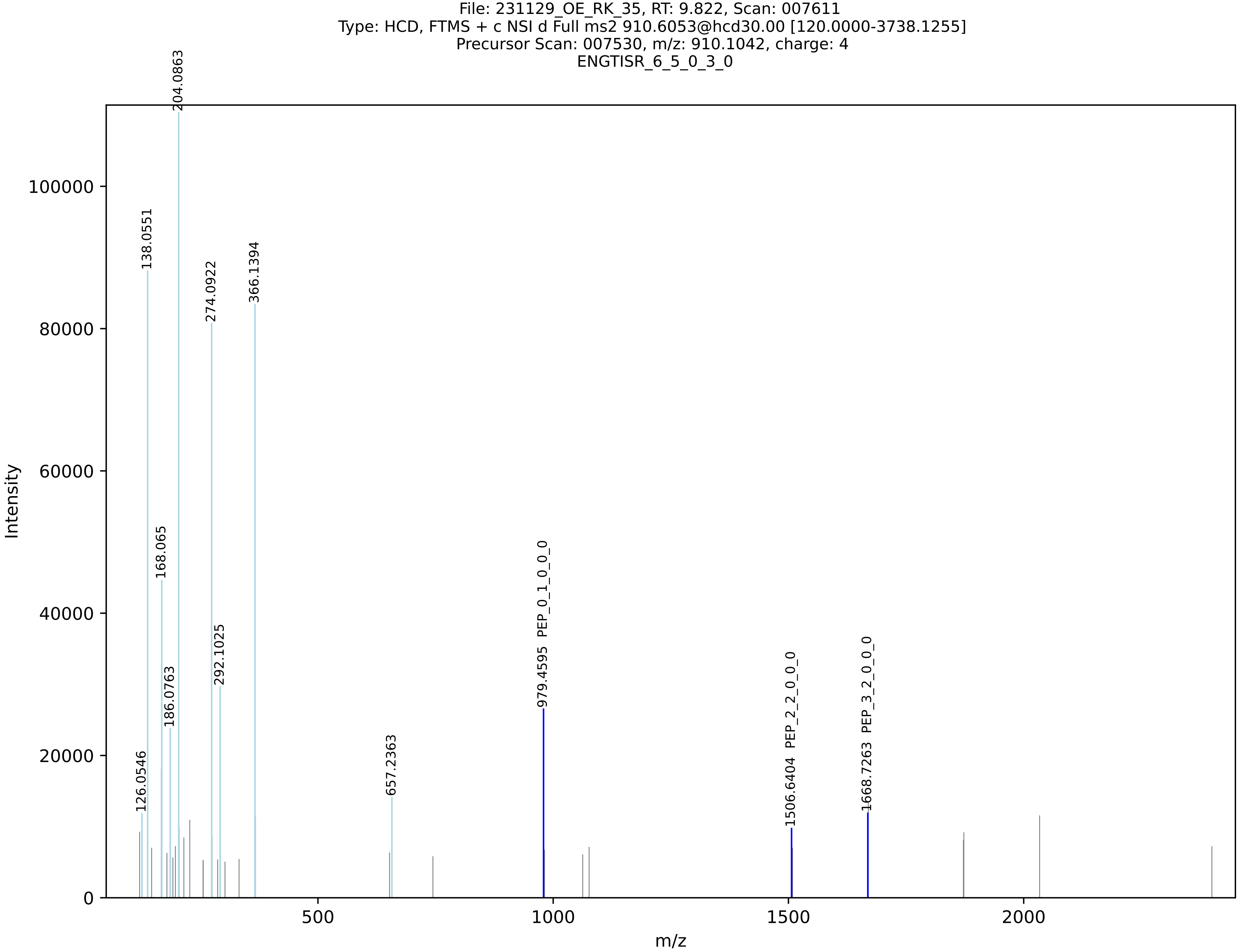

File: 231129_OE_RK_35, RT: 9.822, Scan: 007611
Type: HCD, FTMS + c NSI d Full ms2 910.6053@hcd30.00 [120.0000-3738.1255]
Precursor Scan: 007530, m/z: 910.1042, charge: 4
ENGTISR_6_5_0_3_0
204.0863
138.0551
274.0922
366.1394
168.065
292.1025
186.0763
126.0546
657.2363
979.4595 PEP_0_1_0_0_0
1506.6404 PEP_2_2_0_0_0
1668.7263 PEP_3_2_0_0_0
Intensity
m/z
0
20000
40000
60000
80000
100000
500
1000
1500
2000

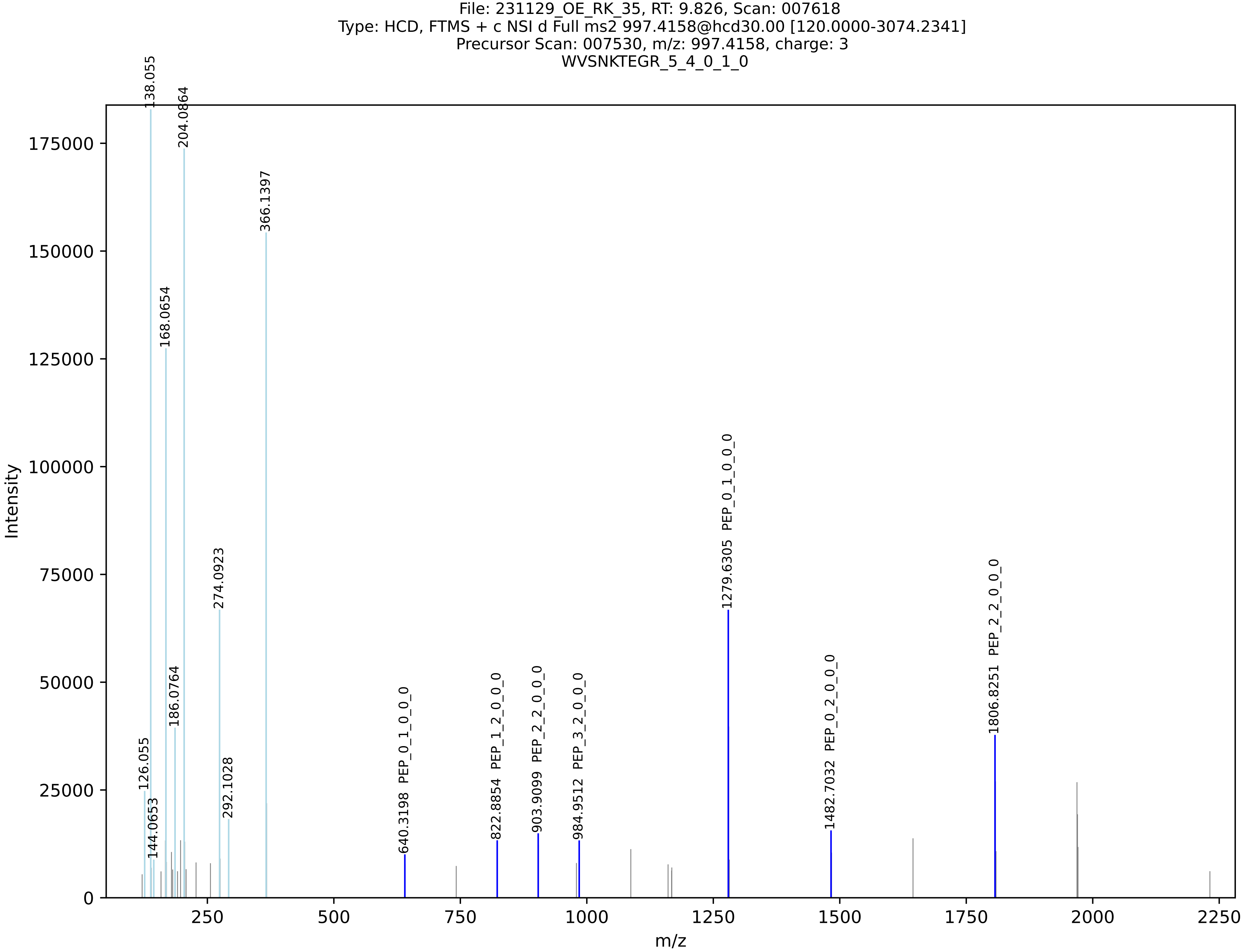

File: 231129_OE_RK_35, RT: 9.826, Scan: 007618
Type: HCD, FTMS + c NSI d Full ms2 997.4158@hcd30.00 [120.0000-3074.2341]
Precursor Scan: 007530, m/z: 997.4158, charge: 3
WVSNKTEGR_5_4_0_1_0
138.055
204.0864
366.1397
168.0654
274.0923
186.0764
126.055
144.0653
292.1028
640.3198 PEP_0_1_0_0_0
822.8854 PEP_1_2_0_0_0
903.9099 PEP_2_2_0_0_0
984.9512 PEP_3_2_0_0_0
1279.6305 PEP_0_1_0_0_0
1482.7032 PEP_0_2_0_0_0
1806.8251 PEP_2_2_0_0_0
Intensity
m/z
0
25000
50000
75000
100000
125000
150000
175000
250
500
750
1000
1250
1500
1750
2000
2250

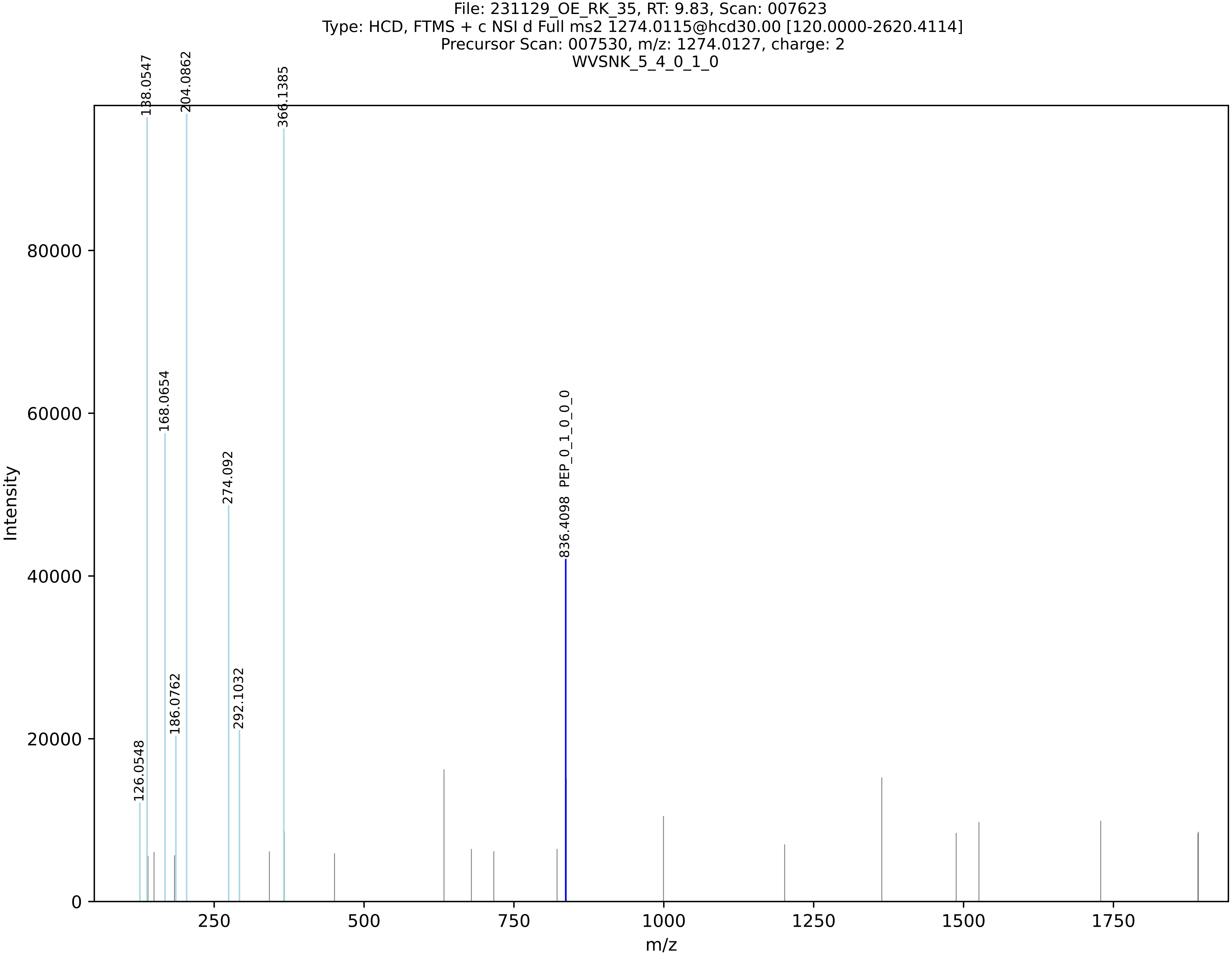
File: 231129_OE_RK_35, RT: 9.83, Scan: 007623
Type: HCD, FTMS + c NSI d Full ms2 1274.0115@hcd30.00 [120.0000-2620.4114]
Precursor Scan: 007530, m/z: 1274.0127, charge: 2
WVSNK_5_4_0_1_0
138.0547
204.0862
366.1385
168.0654
274.092
836.4098 PEP_0_1_0_0_0
186.0762
292.1032
126.0548
Intensity
m/z
0
20000
40000
60000
80000
250
500
750
1000
1250
1500
1750

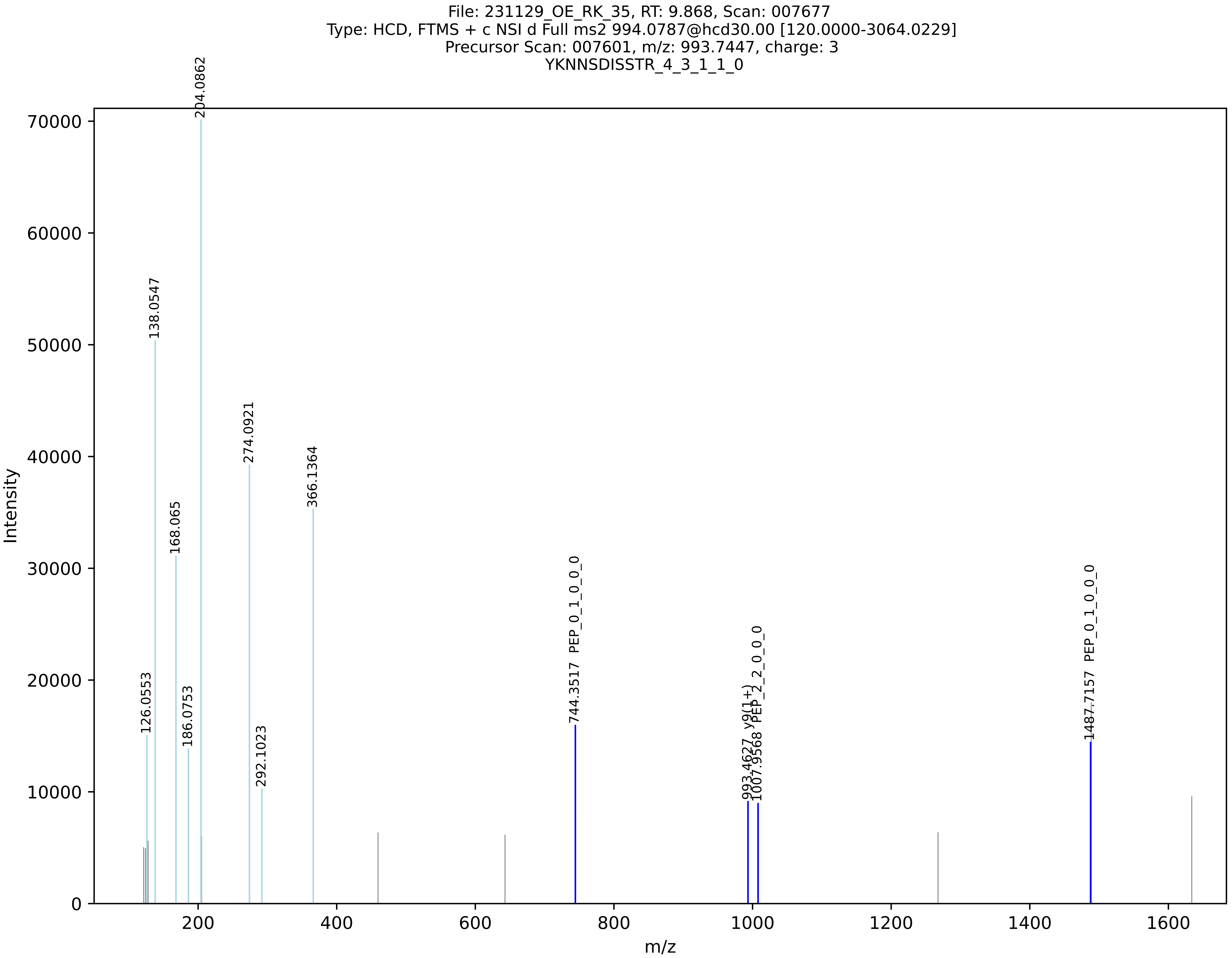

File: 231129_OE_RK_35, RT: 9.868, Scan: 007677
Type: HCD, FTMS + c NSI d Full ms2 994.0787@hcd30.00 [120.0000-3064.0229]
Precursor Scan: 007601, m/z: 993.7447, charge: 3
YKNNSDISSTR_4_3_1_1_0
Intensity
m/z
204.0862
138.0547
274.0921
366.1364
168.065
126.0553
186.0753
292.1023
744.3517 PEP_0_1_0_0_0
993.4627 y9(1+)
1007.9568 PEP_2_2_0_0_0
1487.7157 PEP_0_1_0_0_0

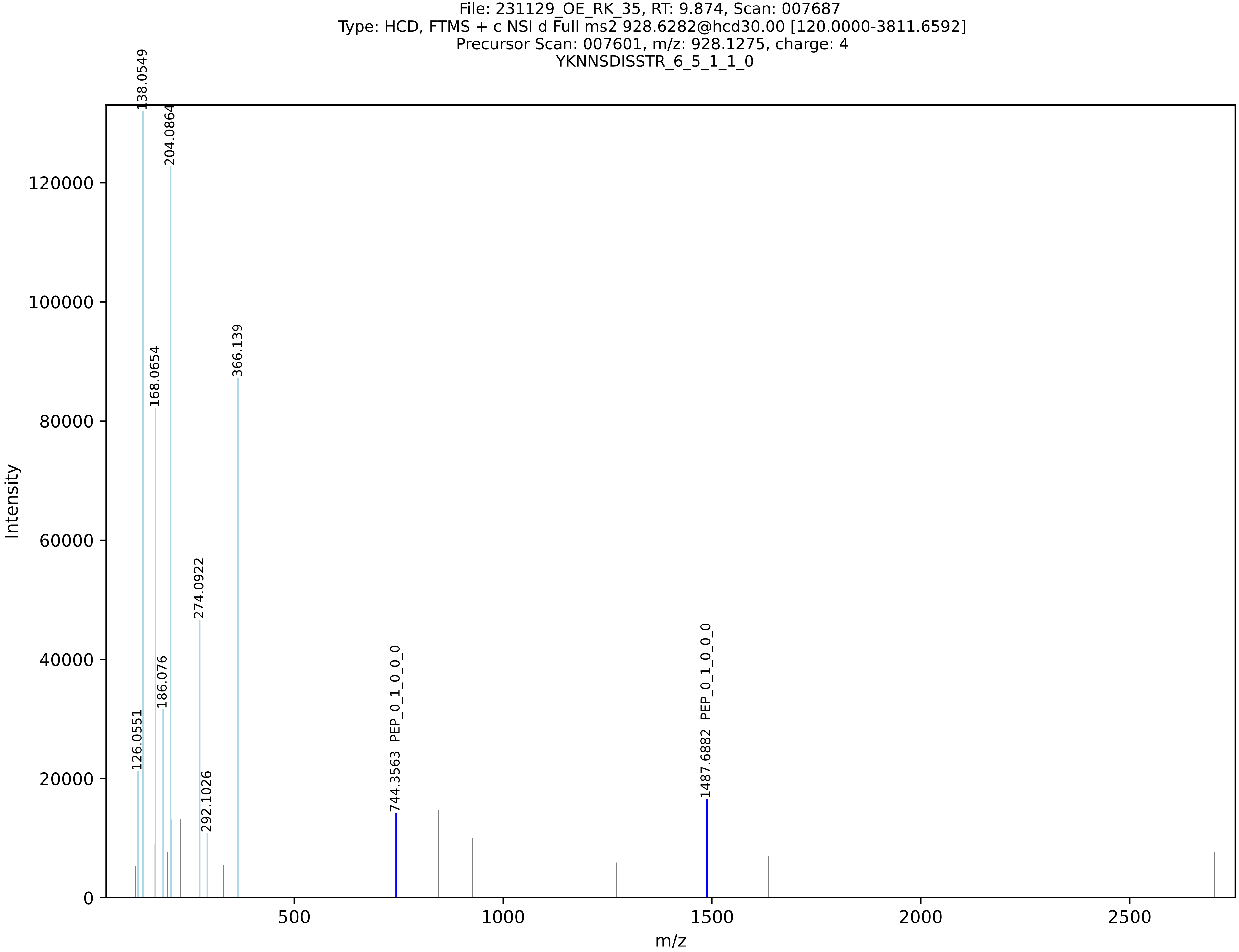
File: 231129_OE_RK_35, RT: 9.874, Scan: 007687
Type: HCD, FTMS + c NSI d Full ms2 928.6282@hcd30.00 [120.0000-3811.6592]
Precursor Scan: 007601, m/z: 928.1275, charge: 4
YKNNSDISSTR_6_5_1_1_0
Intensity
m/z
138.0549
204.0864
168.0654
366.139
274.0922
186.076
126.0551
292.1026
744.3563 PEP_0_1_0_0_0
1487.6882 PEP_0_1_0_0_0
0
20000
40000
60000
80000
100000
120000
500
1000
1500
2000
2500

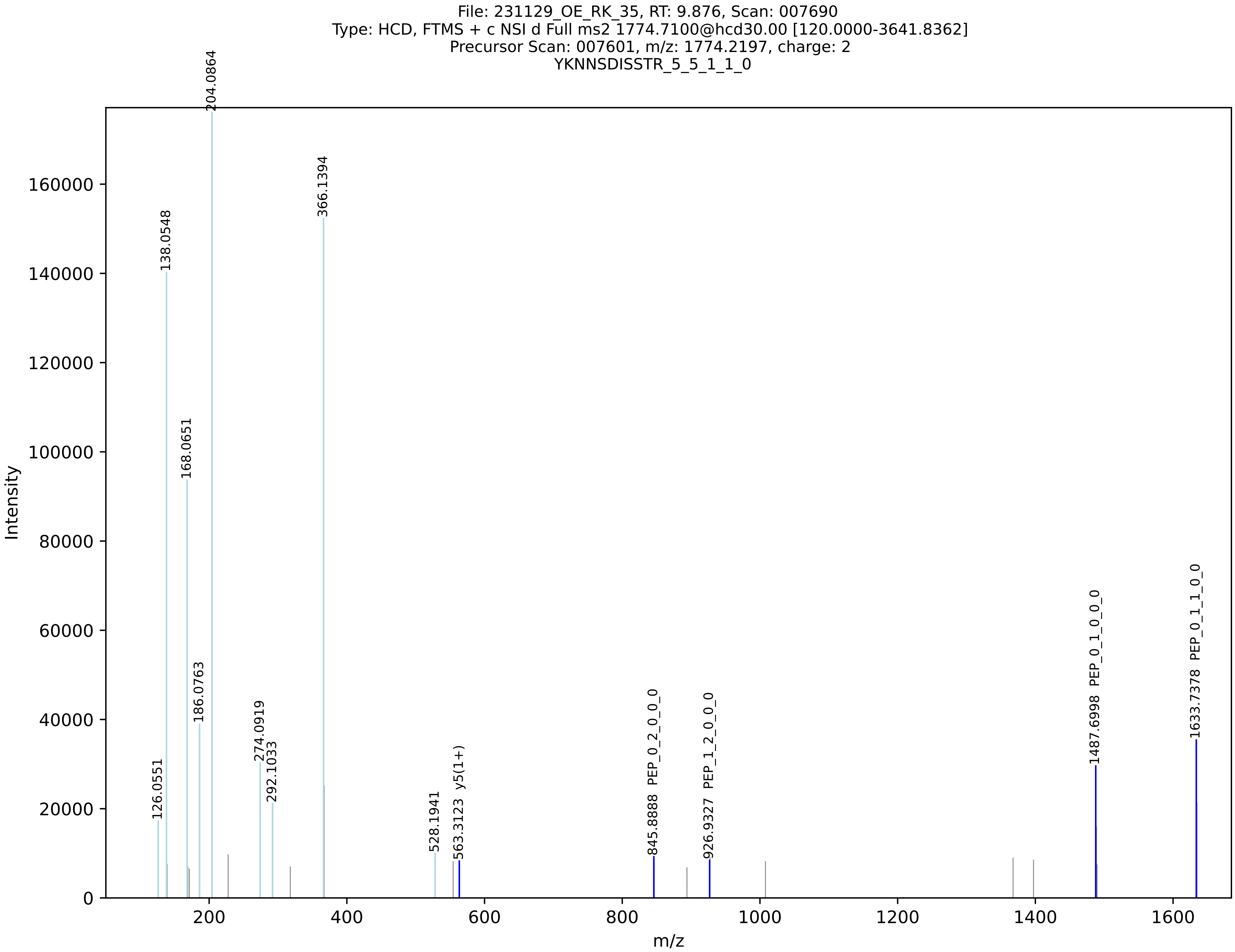
File: 231129_OE_RK_35, RT: 9.876, Scan: 007690
Type: HCD, FTMS + c NSI d Full ms2 1774.7100@hcd30.00 [120.0000-3641.8362]
Precursor Scan: 007601, m/z: 1774.2197, charge: 2
YKNNSDISSTR_5_5_1_1_0
Intensity
m/z
204.0864
366.1394
138.0548
168.0651
186.0763
274.0919
292.1033
126.0551
528.1941
563.3123 y5(1+)
845.8888 PEP_0_2_0_0_0
926.9327 PEP_1_2_0_0_0
1487.6998 PEP_0_1_0_0_0
1633.7378 PEP_0_1_1_0_0

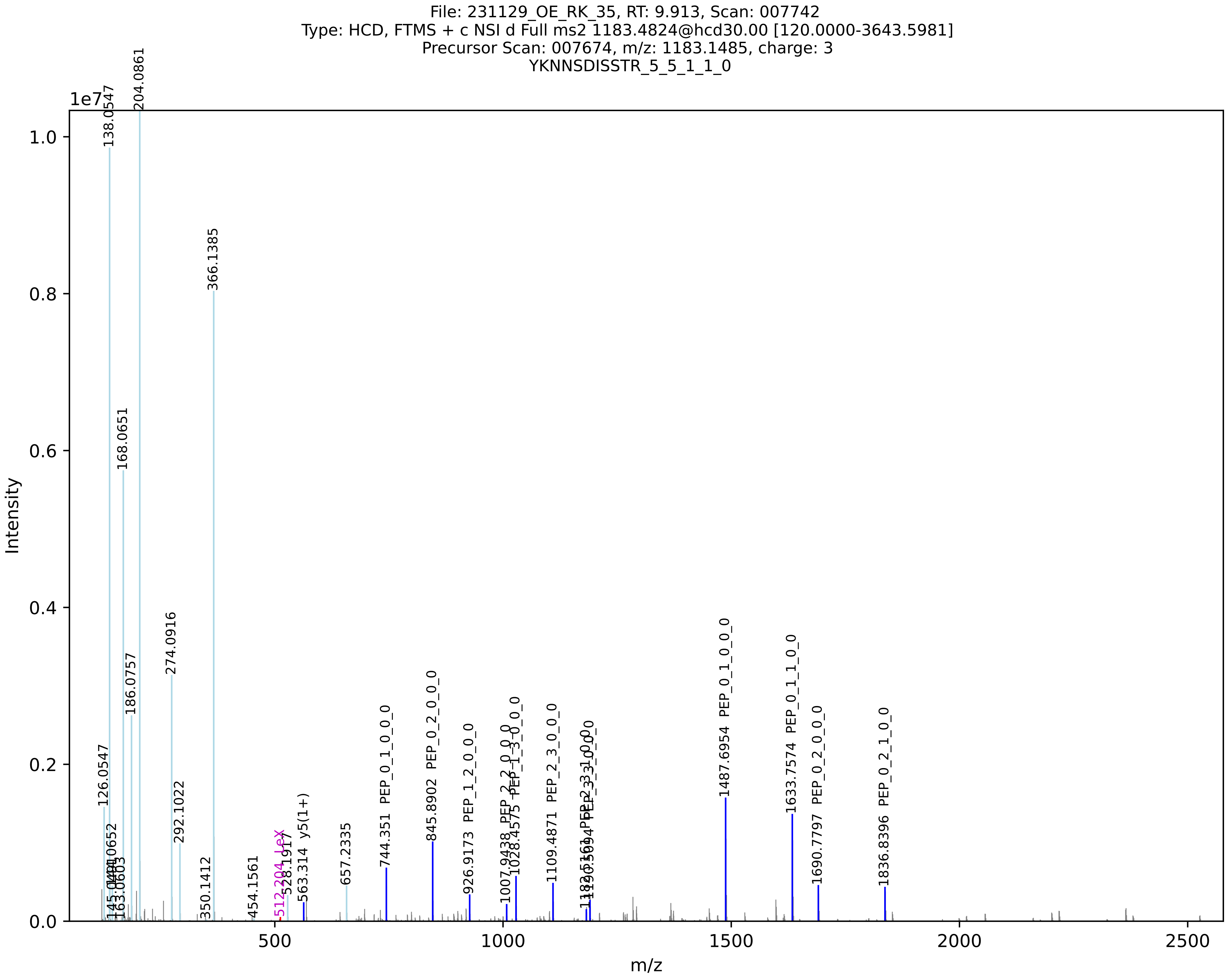
File: 231129_OE_RK_35, RT: 9.913, Scan: 007742
Type: HCD, FTMS + c NSI d Full ms2 1183.4824@hcd30.00 [120.0000-3643.5981]
Precursor Scan: 007674, m/z: 1183.1485, charge: 3
YKNNSDISSTR_5_5_1_1_0
1e7
Intensity
m/z
204.0861
138.0547
366.1385
168.0651
186.0757
274.0916
126.0547
292.1022
350.1412
454.1561
512.204_LeX
528.1917
563.314 y5(1+)
657.2335
744.351 PEP_0_1_0_0_0
845.8902 PEP_0_2_0_0_0
926.9173 PEP_1_2_0_0_0
1028.4575 PEP_1_3_0_0_0
1109.4871 PEP_2_3_0_0_0
1487.6954 PEP_0_1_0_0_0
1633.7574 PEP_0_1_1_0_0
1690.7797 PEP_0_2_0_0_0
1836.8396 PEP_0_2_1_0_0

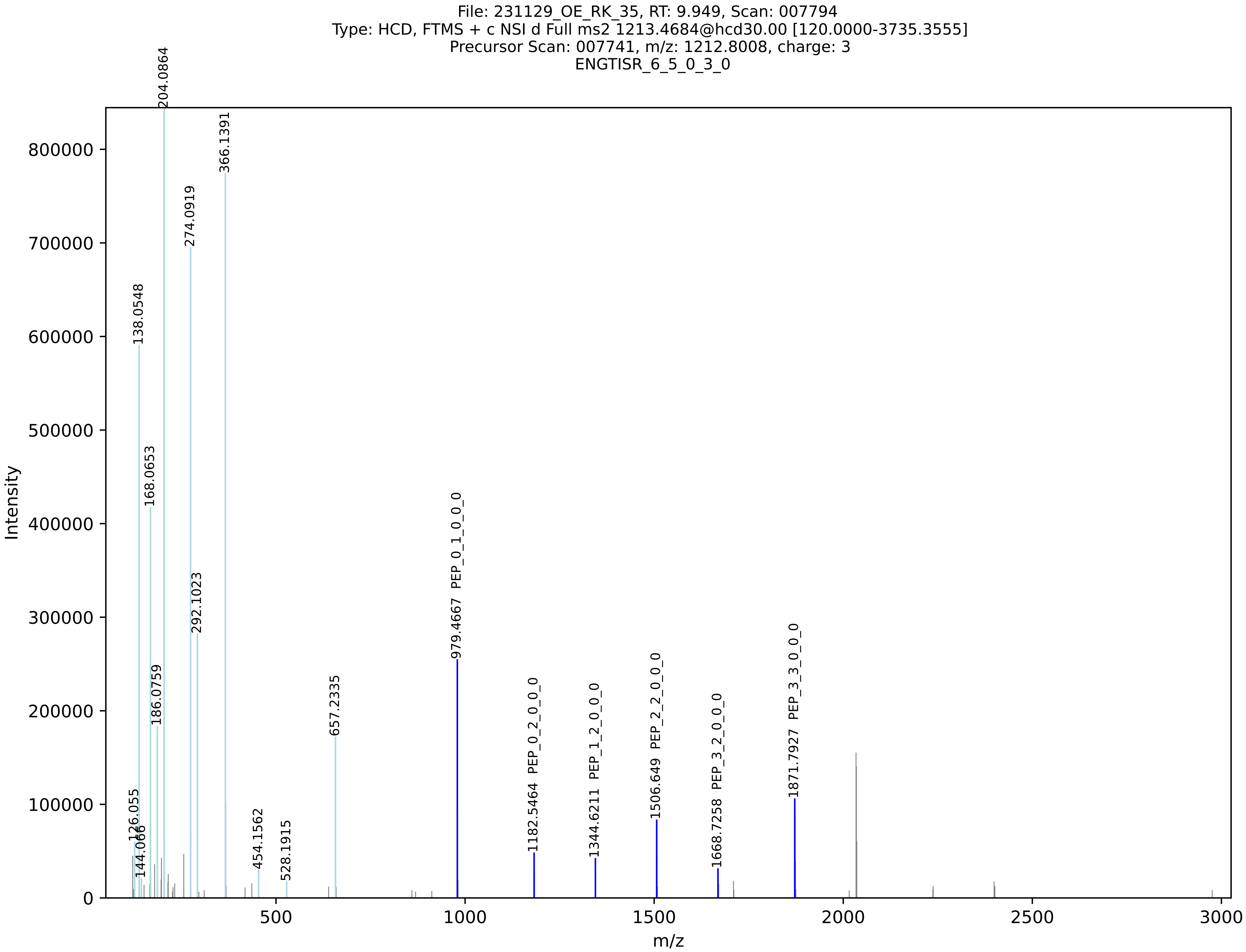

File: 231129_OE_RK_35, RT: 9.949, Scan: 007794
Type: HCD, FTMS + c NSI d Full ms2 1213.4684@hcd30.00 [120.0000-3735.3555]
Precursor Scan: 007741, m/z: 1212.8008, charge: 3
ENGTISR_6_5_0_3_0
204.0864
366.1391
274.0919
138.0548
168.0653
292.1023
186.0759
126.055
144.066
454.1562
528.1915
657.2335
979.4667 PEP_0_1_0_0_0_
1182.5464 PEP_0_2_0_0_0
1344.6211 PEP_1_2_0_0_0
1506.649 PEP_2_2_0_0_0
1668.7258 PEP_3_2_0_0_0
1871.7927 PEP_3_3_0_0_0
Intensity
m/z
0
100000
200000
300000
400000
500000
600000
700000
800000
500
1000
1500
2000
2500
3000

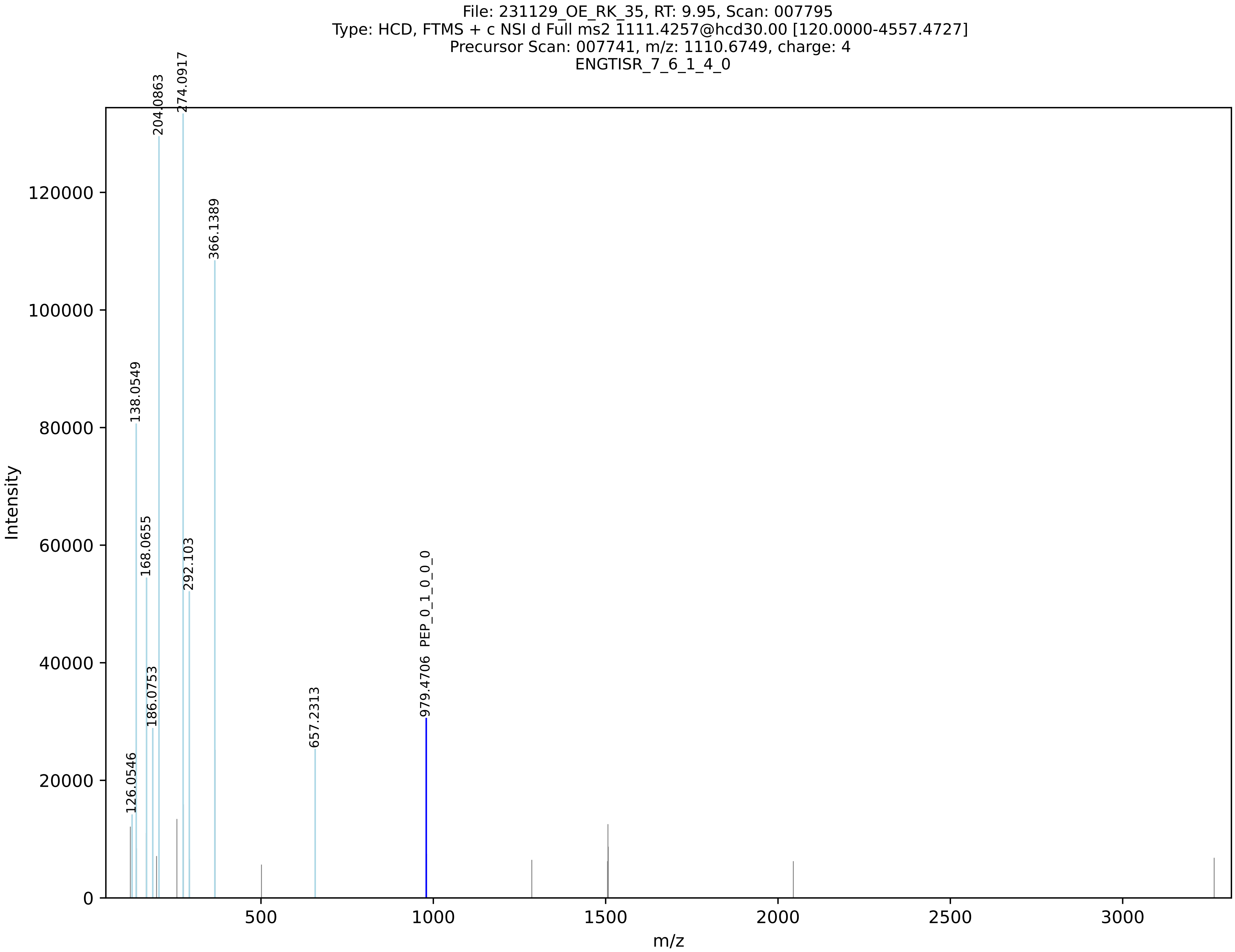

File: 231129_OE_RK_35, RT: 9.95, Scan: 007795
Type: HCD, FTMS + c NSI d Full ms2 1111.4257@hcd30.00 [120.0000-4557.4727]
Precursor Scan: 007741, m/z: 1110.6749, charge: 4
ENGTISR_7_6_1_4_0
Intensity
m/z
274.0917
204.0863
366.1389
138.0549
168.0655
292.103
186.0753
126.0546
657.2313
979.4706 PEP_0_1_0_0_0
0
20000
40000
60000
80000
100000
120000
500
1000
1500
2000
2500
3000

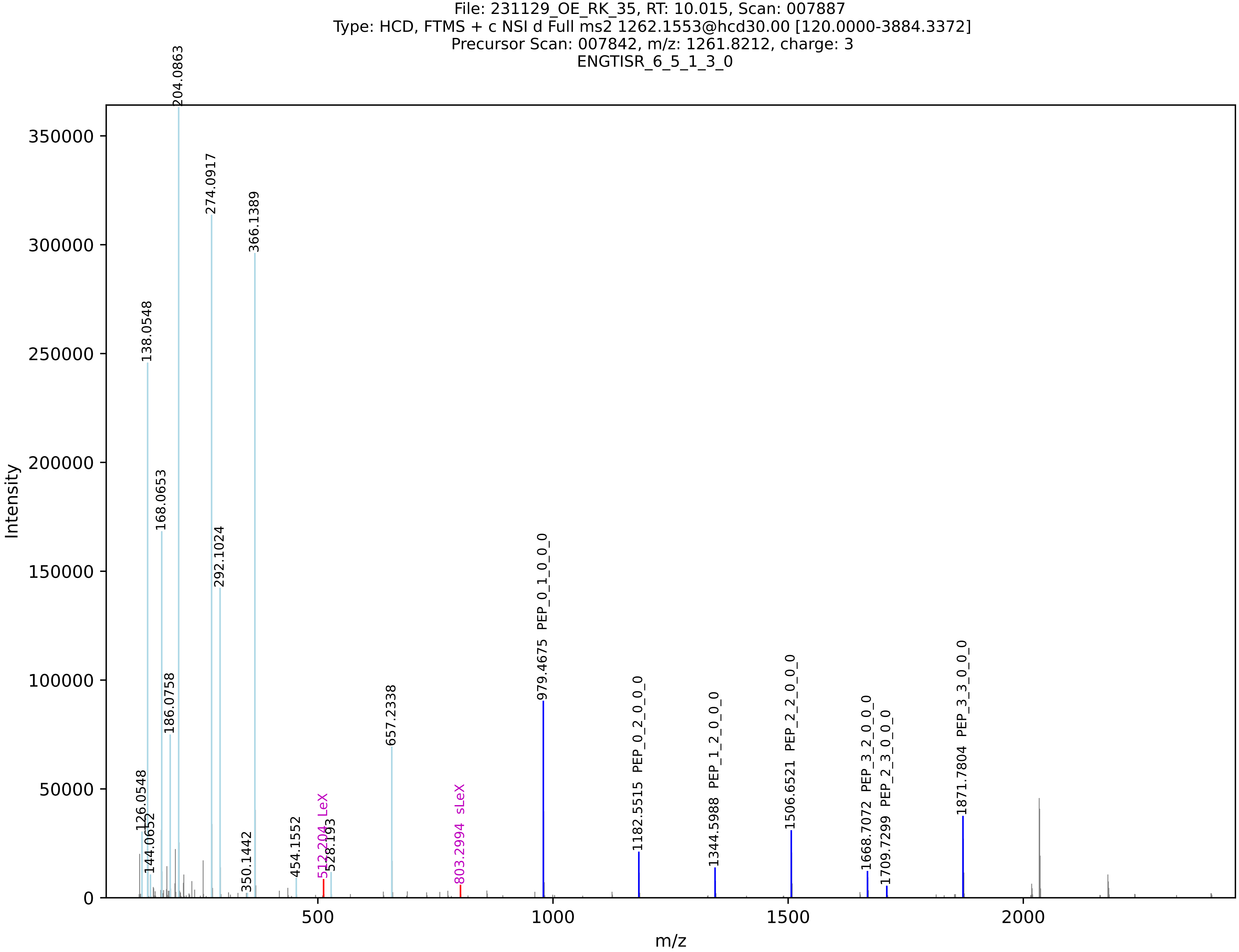

File: 231129_OE_RK_35, RT: 10.015, Scan: 007887
Type: HCD, FTMS + c NSI d Full ms2 1262.1553@hcd30.00 [120.0000-3884.3372]
Precursor Scan: 007842, m/z: 1261.8212, charge: 3
ENGTISR_6_5_1_3_0
Intensity
m/z
204.0863
274.0917
366.1389
138.0548
168.0653
292.1024
186.0758
126.0548
144.0652
350.1442
454.1552
512.204 LeX
528.193
657.2338
803.2994 sLeX
979.4675 PEP_0_1_0_0_0
1182.5515 PEP_0_2_0_0_0
1344.5988 PEP_1_2_0_0_0
1506.6521 PEP_2_2_0_0_0
1668.7072 PEP_3_2_0_0_0
1709.7299 PEP_2_3_0_0_0
1871.7804 PEP_3_3_0_0_0

File: 231129_OE_RK_35, RT: 10.02, Scan: 007894
Type: HCD, FTMS + c NSI d Full ms2 1311.1749@hcd30.00 [120.0000-4034.3374]
Precursor Scan: 007842, m/z: 1310.5067, charge: 3
ENGTISR_6_5_2_3_0

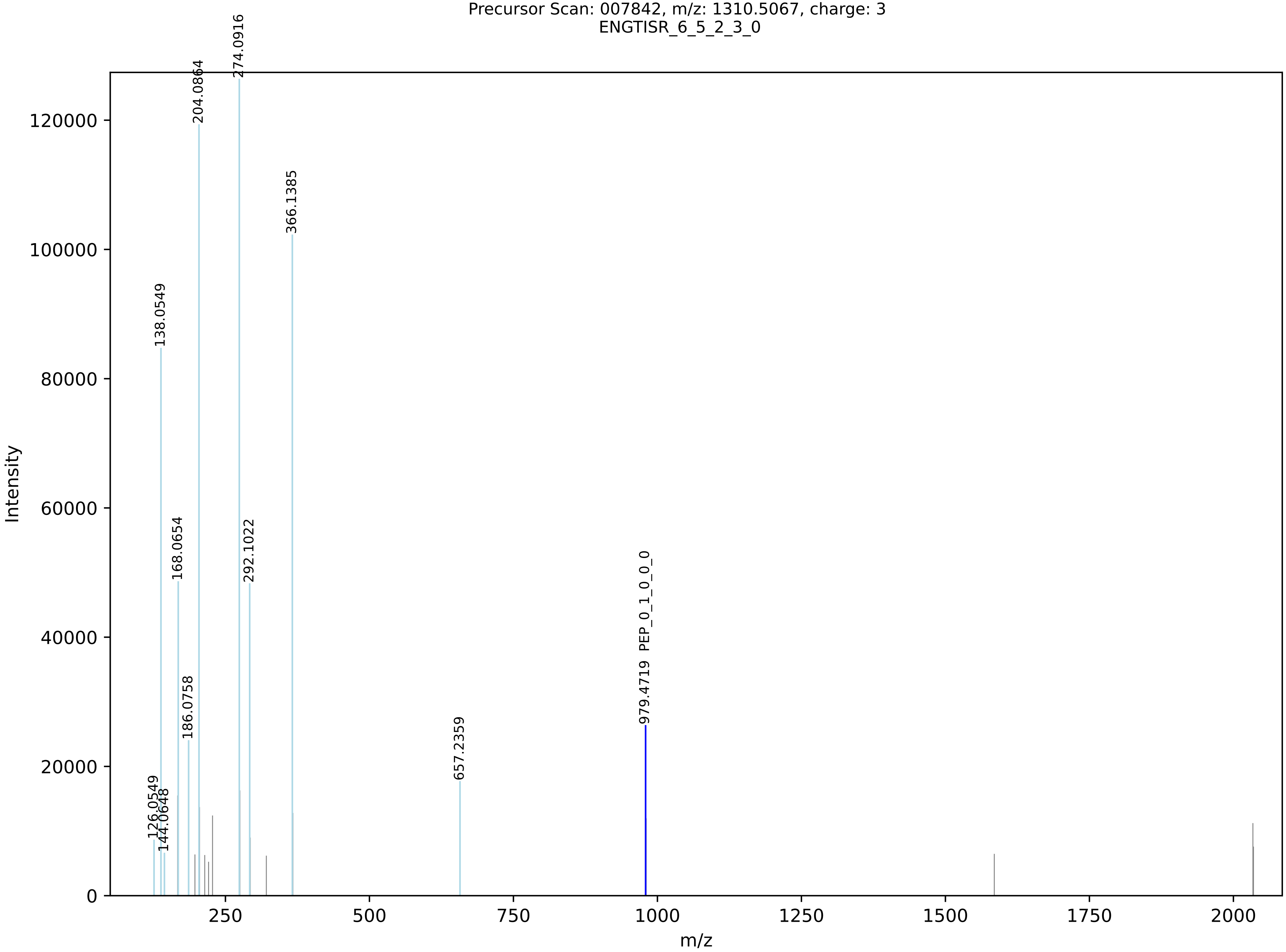

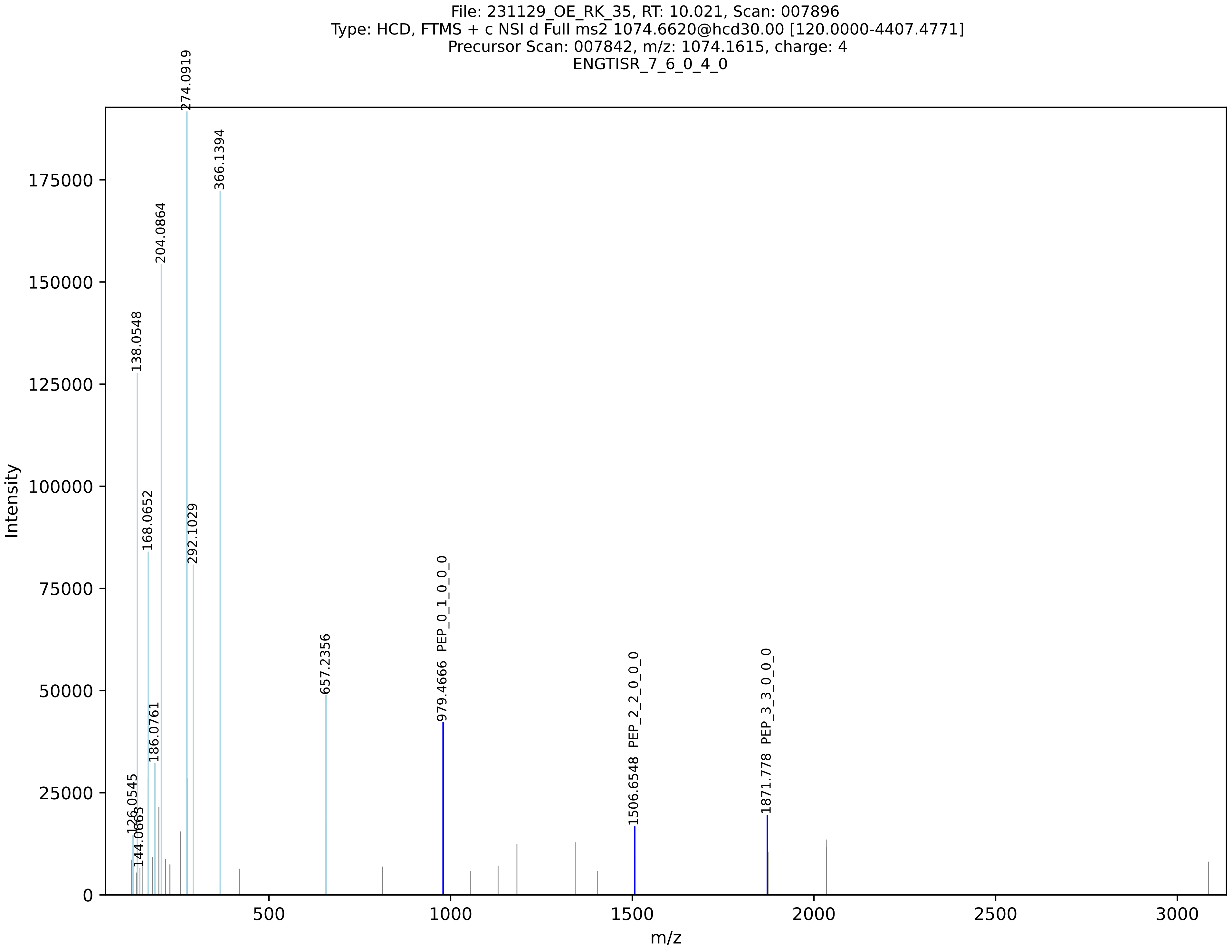
File: 231129_OE_RK_35, RT: 10.021, Scan: 007896
Type: HCD, FTMS + c NSI d Full ms2 1074.6620@hcd30.00 [120.0000-4407.4771]
Precursor Scan: 007842, m/z: 1074.1615, charge: 4
ENGTISR_7_6_0_4_0
Intensity
m/z
0
25000
50000
75000
100000
125000
150000
175000
500
1000
1500
2000
2500
3000
274.0919
366.1394
204.0864
138.0548
168.0652
292.1029
657.2356
979.4666 PEP_0_1_0_0_0
1506.6548 PEP_2_2_0_0_0
1871.778 PEP_3_3_0_0_0
186.0761
126.0545
144.0665

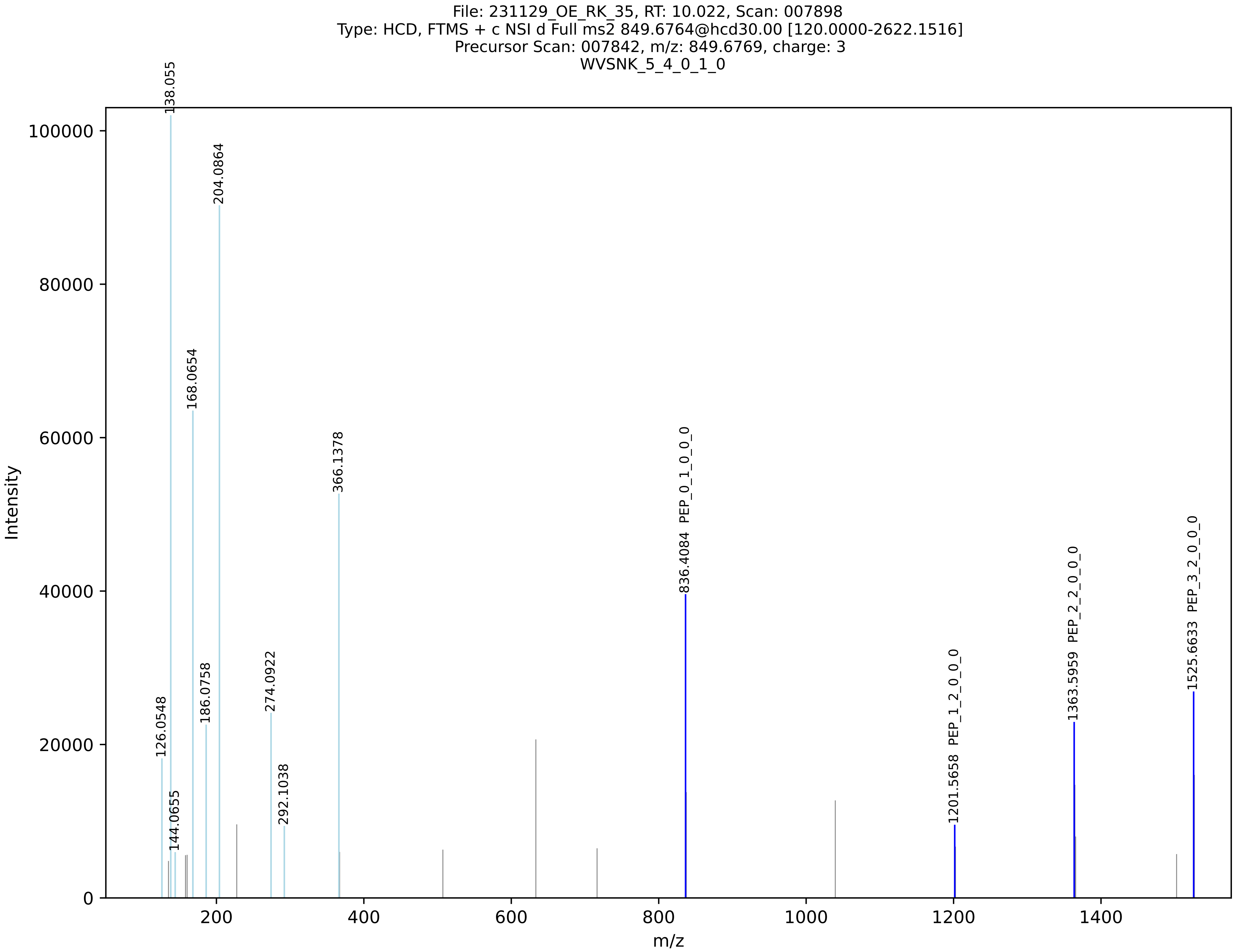
File: 231129_OE_RK_35, RT: 10.022, Scan: 007898
Type: HCD, FTMS + c NSI d Full ms2 849.6764@hcd30.00 [120.0000-2622.1516]
Precursor Scan: 007842, m/z: 849.6769, charge: 3
WVSNK_5_4_0_1_0
Intensity
m/z
138.055
204.0864
168.0654
366.1378
836.4084 PEP_0_1_0_0_0
1525.6633 PEP_3_2_0_0_0
1363.5959 PEP_2_2_0_0_0
1201.5658 PEP_1_2_0_0_0
274.0922
186.0758
126.0548
292.1038
144.0655

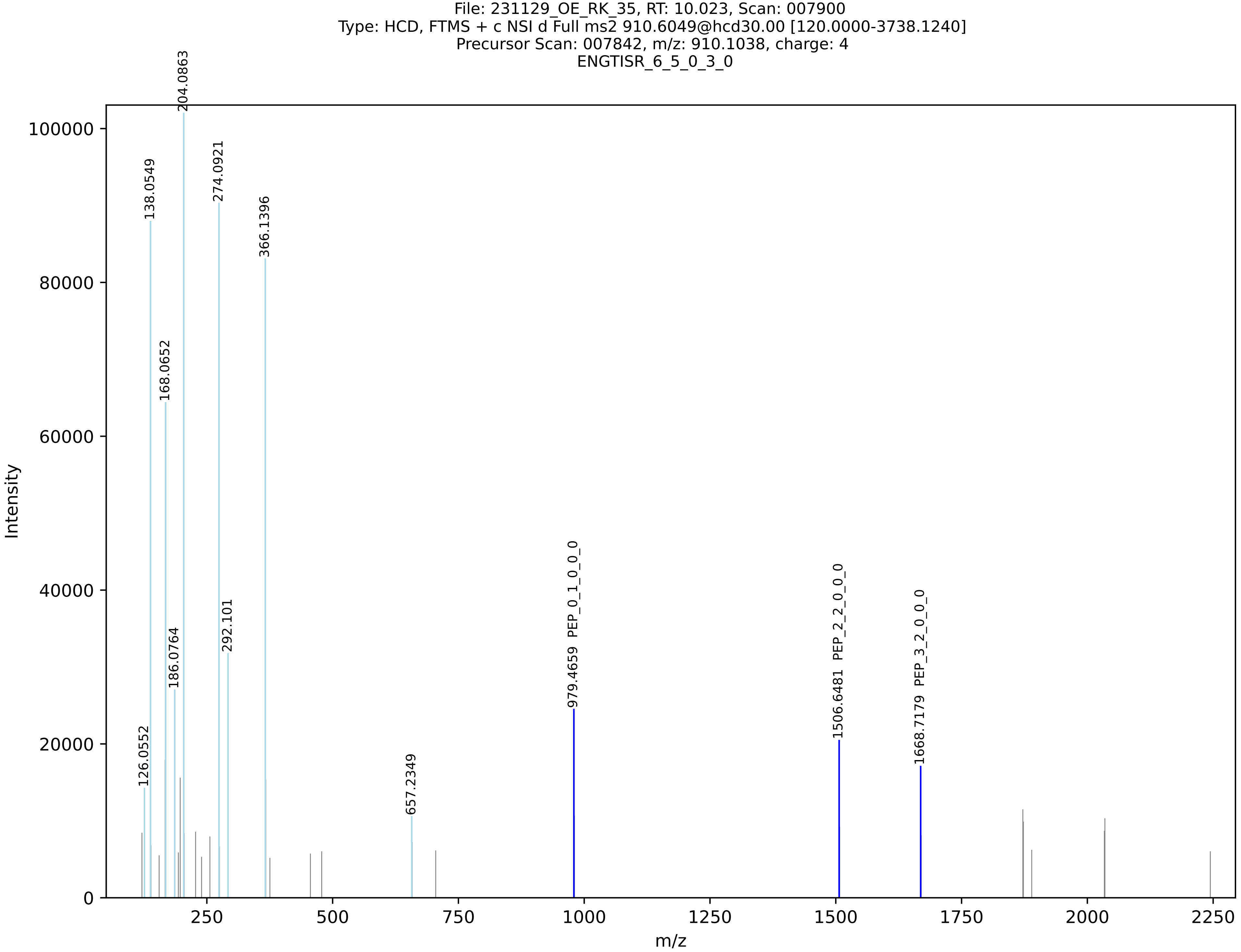

File: 231129_OE_RK_35, RT: 10.023, Scan: 007900
Type: HCD, FTMS + c NSI d Full ms2 910.6049@hcd30.00 [120.0000-3738.1240]
Precursor Scan: 007842, m/z: 910.1038, charge: 4
ENGTISR_6_5_0_3_0
Intensity
m/z
204.0863
138.0549
274.0921
366.1396
168.0652
292.101
186.0764
126.0552
657.2349
979.4659 PEP_0_1_0_0_0
1506.6481 PEP_2_2_0_0_0
1668.7179 PEP_3_2_0_0_0

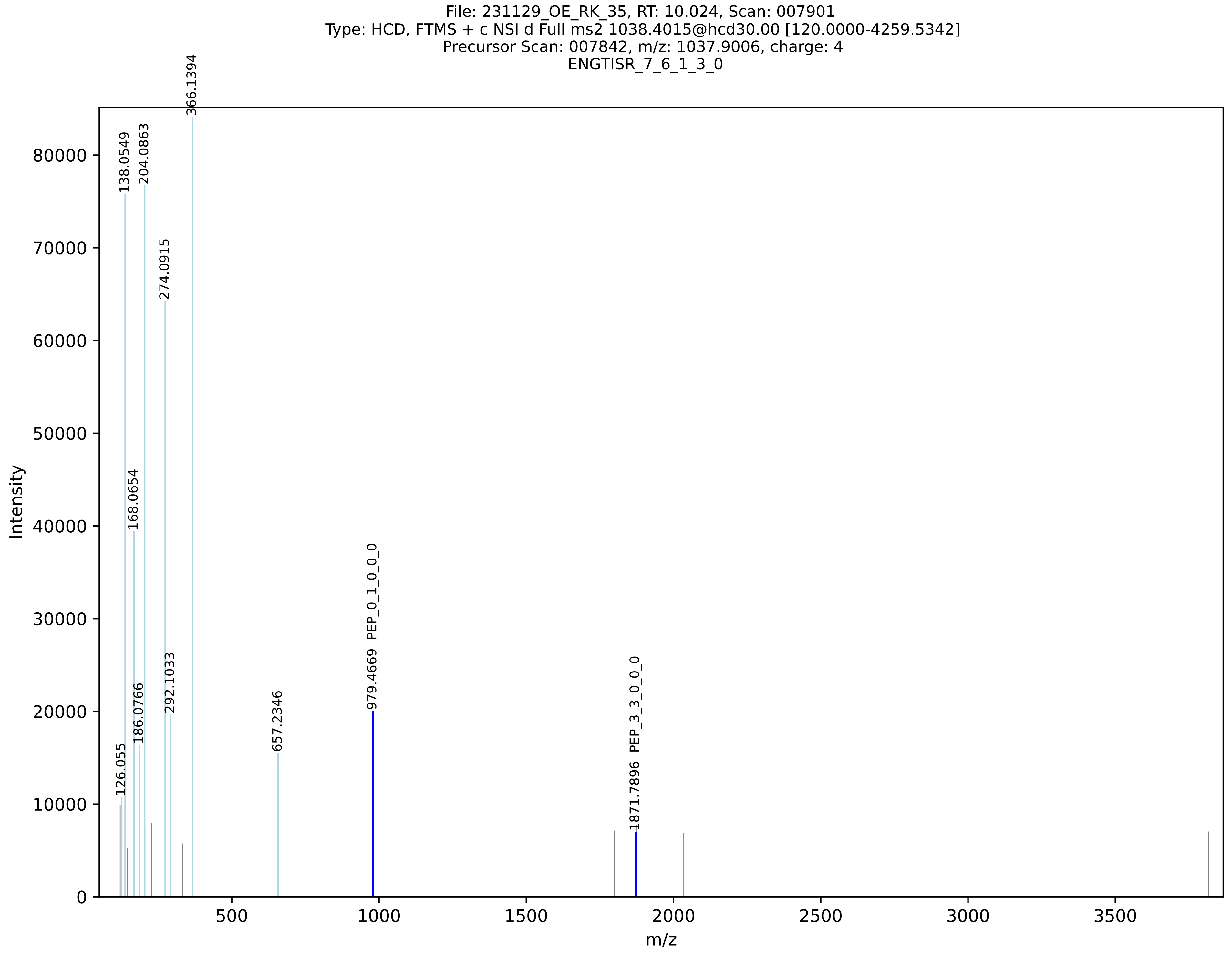
File: 231129_OE_RK_35, RT: 10.024, Scan: 007901
Type: HCD, FTMS + c NSI d Full ms2 1038.4015@hcd30.00 [120.0000-4259.5342]
Precursor Scan: 007842, m/z: 1037.9006, charge: 4
ENGTISR_7_6_1_3_0
366.1394
138.0549
204.0863
274.0915
168.0654
PEP_0_1_0_0_0
979.4669
PEP_3_3_0_0_0
1871.7896
292.1033
186.0766
657.2346
126.055
Intensity
m/z
0
10000
20000
30000
40000
50000
60000
70000
80000
500
1000
1500
2000
2500
3000
3500

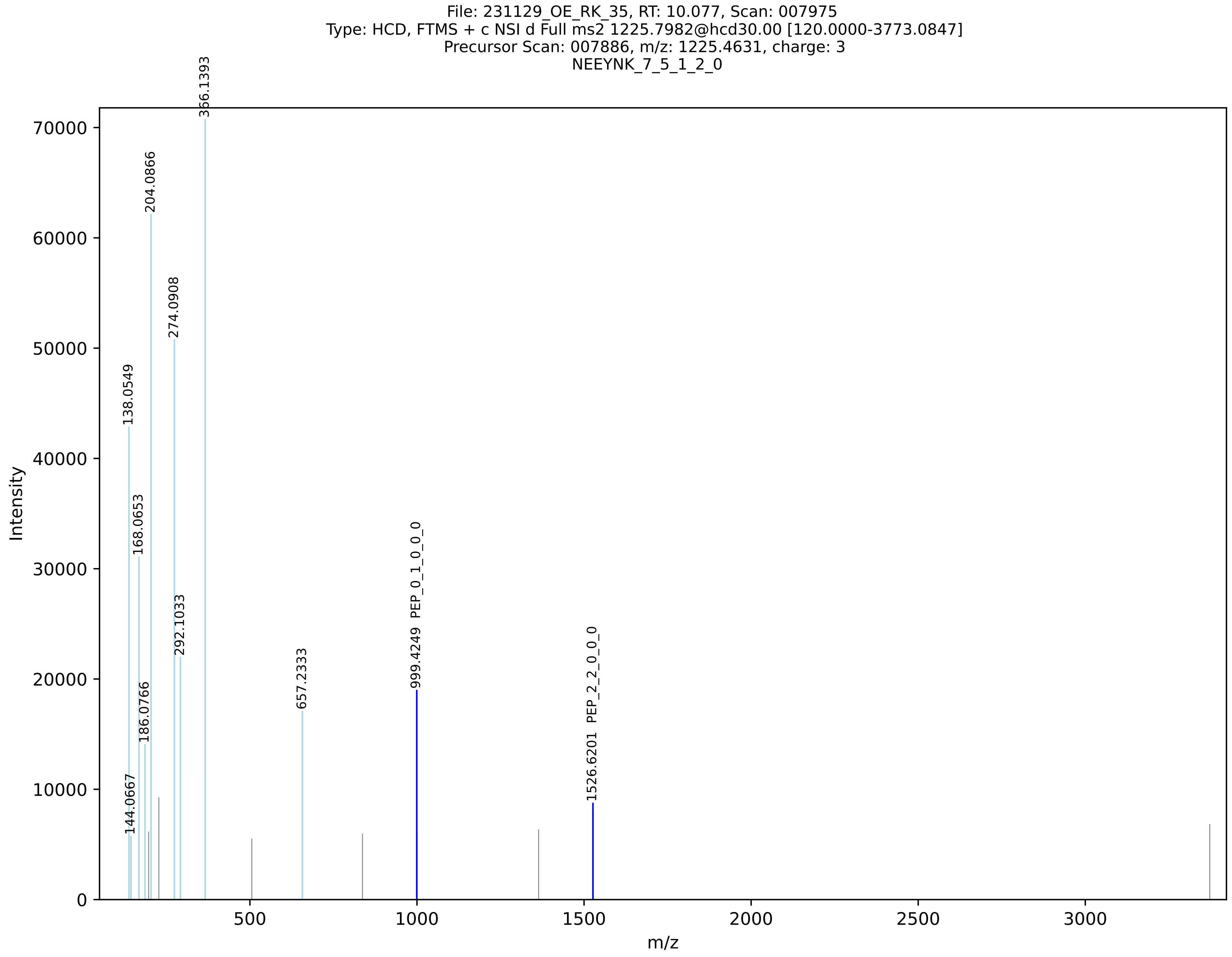
File: 231129_OE_RK_35, RT: 10.077, Scan: 007975
Type: HCD, FTMS + c NSI d Full ms2 1225.7982@hcd30.00 [120.0000-3773.0847]
Precursor Scan: 007886, m/z: 1225.4631, charge: 3
NEEYNK_7_5_1_2_0
Intensity
m/z
366.1393
204.0866
274.0908
138.0549
168.0653
292.1033
657.2333
999.4249 PEP_0_1_0_0_0
1526.6201 PEP_2_2_0_0_0
186.0766
144.0667
0
10000
20000
30000
40000
50000
60000
70000
500
1000
1500
2000
2500
3000

File: 231129_OE_RK_35, RT: 10.119, Scan: 008036
Type: HCD, FTMS + c NSI d Full ms2 1232.1722@hcd30.00 [120.0000-3792.5891]
Precursor Scan: 007958, m/z: 1231.8376, charge: 3
YKNNSDISSTR_5_5_0_2_0

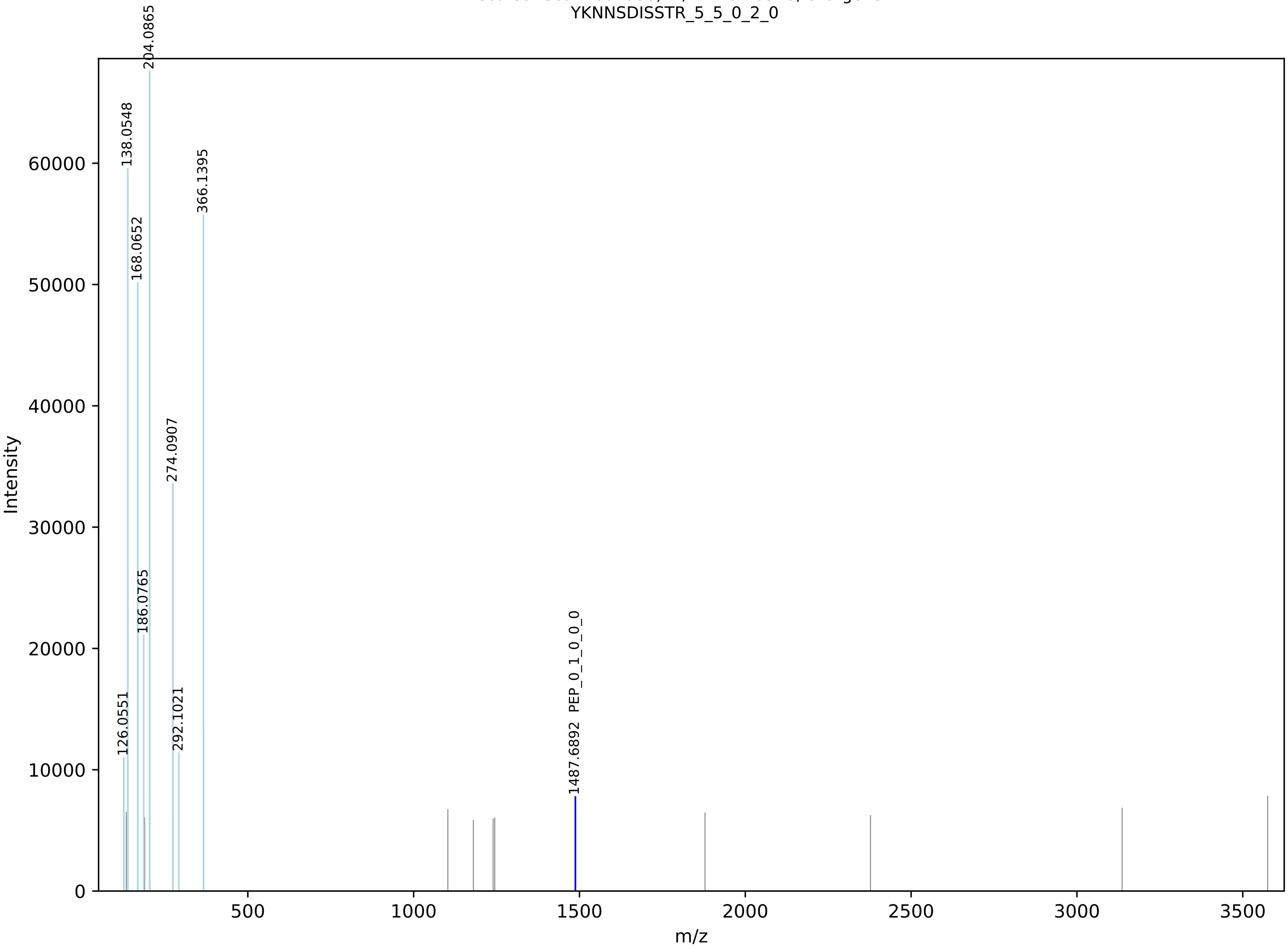

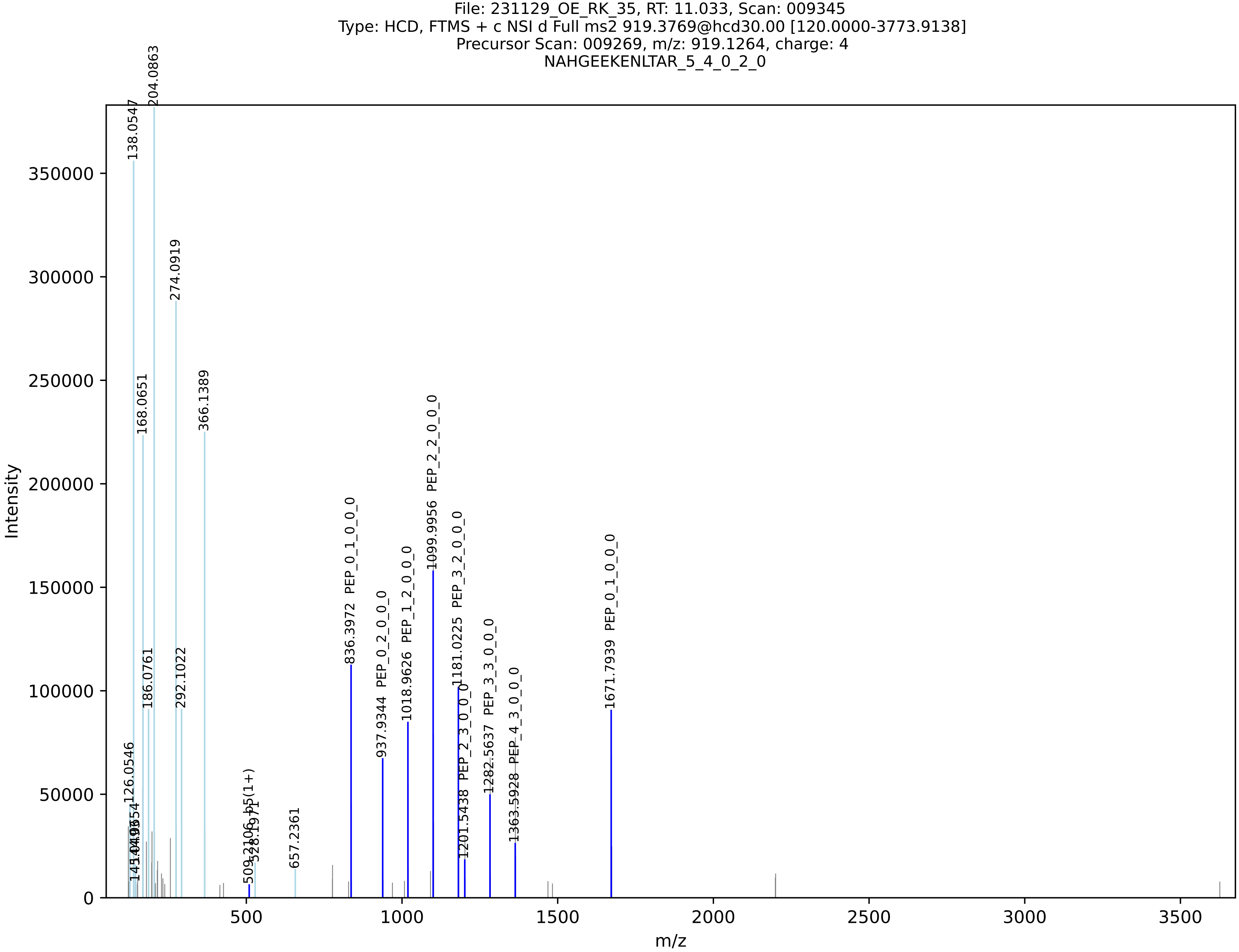

File: 231129_OE_RK_35, RT: 11.033, Scan: 009345
Type: HCD, FTMS + c NSI d Full ms2 919.3769@hcd30.00 [120.0000-3773.9138]
Precursor Scan: 009269, m/z: 919.1264, charge: 4
NAHGEEKENLTAR_5_4_0_2_0
Intensity
m/z
204.0863
138.0547
274.0919
168.0651
366.1389
186.0761
292.1022
126.0546
509.2106 b5(1+)
528.1971
657.2361
836.3972 PEP_0_1_0_0_0
937.9344 PEP_0_2_0_0_0
1018.9626 PEP_1_2_0_0_0
1099.9956 PEP_2_2_0_0_0
1181.0225 PEP_3_2_0_0_0
1201.5438 PEP_2_3_0_0_0
1282.5637 PEP_3_3_0_0_0
1363.5928 PEP_4_3_0_0_0
1671.7939 PEP_0_1_0_0_0

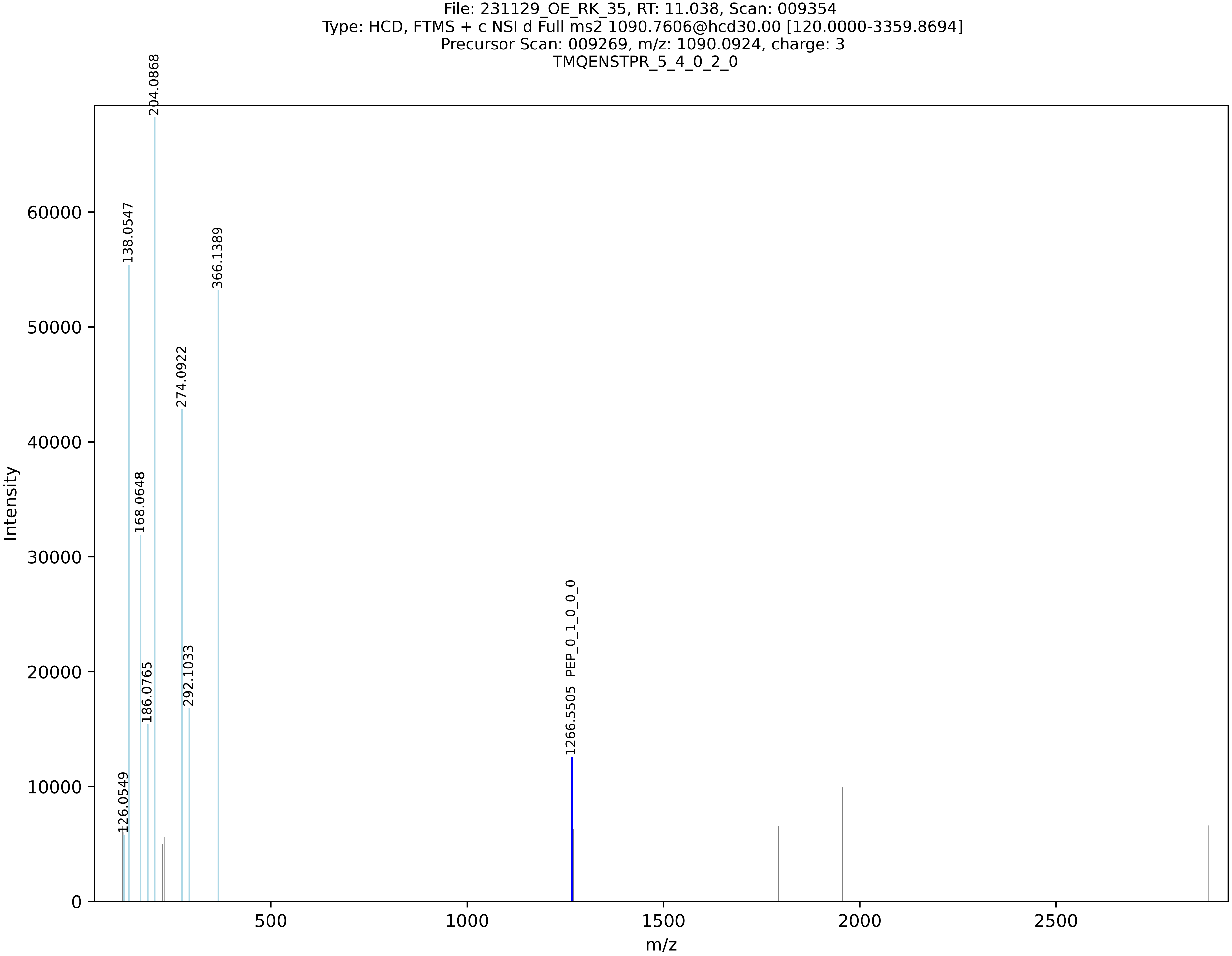
File: 231129_OE_RK_35, RT: 11.038, Scan: 009354
Type: HCD, FTMS + c NSI d Full ms2 1090.7606@hcd30.00 [120.0000-3359.8694]
Precursor Scan: 009269, m/z: 1090.0924, charge: 3
TMQENSTPR_5_4_0_2_0
Intensity
m/z
204.0868
138.0547
366.1389
274.0922
168.0648
186.0765
292.1033
126.0549
1266.5505 PEP_0_1_0_0_0_
0
10000
20000
30000
40000
50000
60000
500
1000
1500
2000
2500

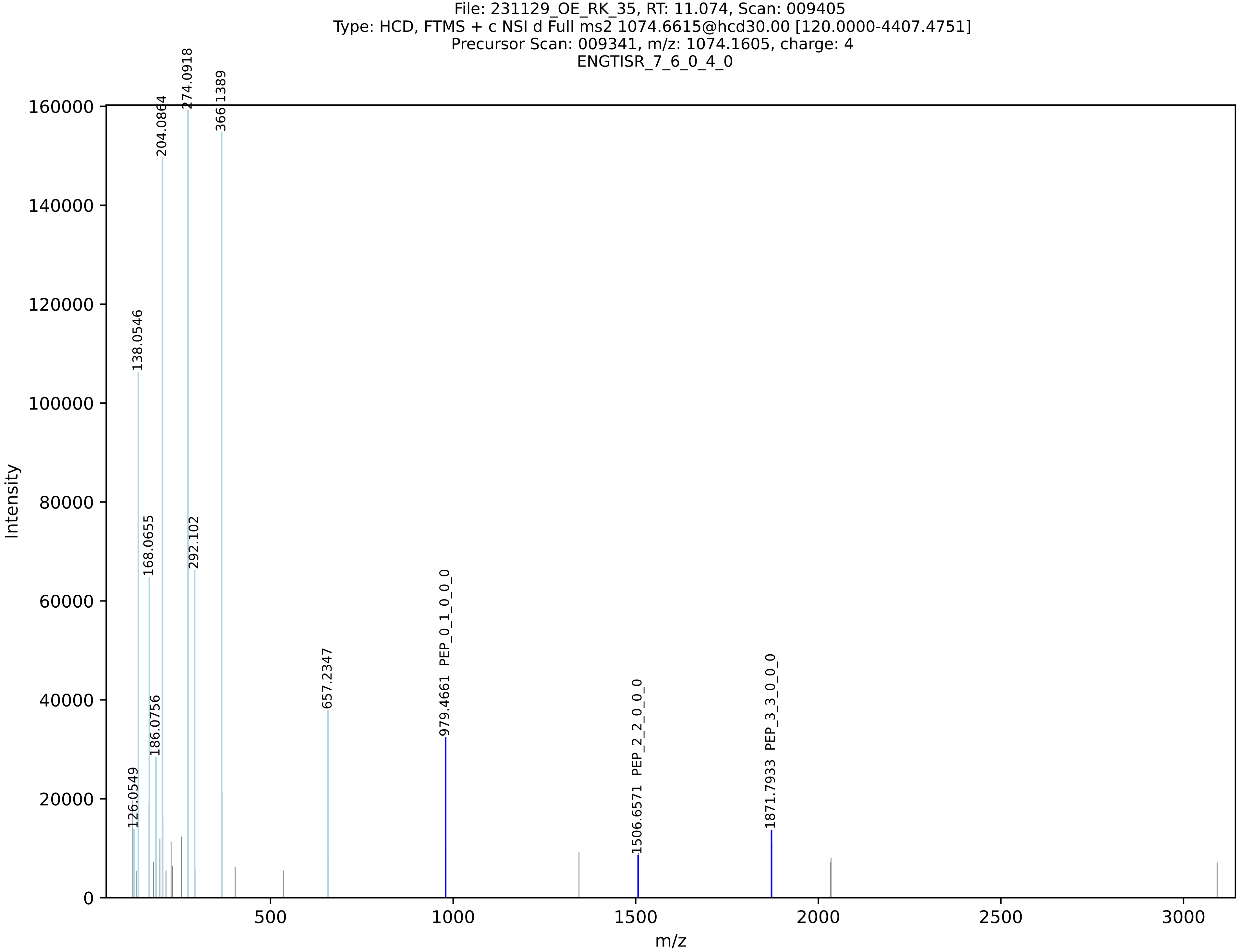
File: 231129_OE_RK_35, RT: 11.074, Scan: 009405
Type: HCD, FTMS + c NSI d Full ms2 1074.6615@hcd30.00 [120.0000-4407.4751]
Precursor Scan: 009341, m/z: 1074.1605, charge: 4
ENGTISR_7_6_0_4_0
Intensity
m/z
0
20000
40000
60000
80000
100000
120000
140000
160000
500
1000
1500
2000
2500
3000
126.0549
138.0546
168.0655
186.0756
204.0864
274.0918
292.102
366.1389
657.2347
979.4661 PEP_0_1_0_0_0
1506.6571 PEP_2_2_0_0_0
1871.7933 PEP_3_3_0_0_0

File: 231129_OE_RK_35, RT: 11.078, Scan: 009412
Type: HCD, FTMS + c NSI d Full ms2 910.3535@hcd30.00 [120.0000-3737.0984]
Precursor Scan: 009341, m/z: 910.1025, charge: 4
ENGTISR_6_5_0_3_0

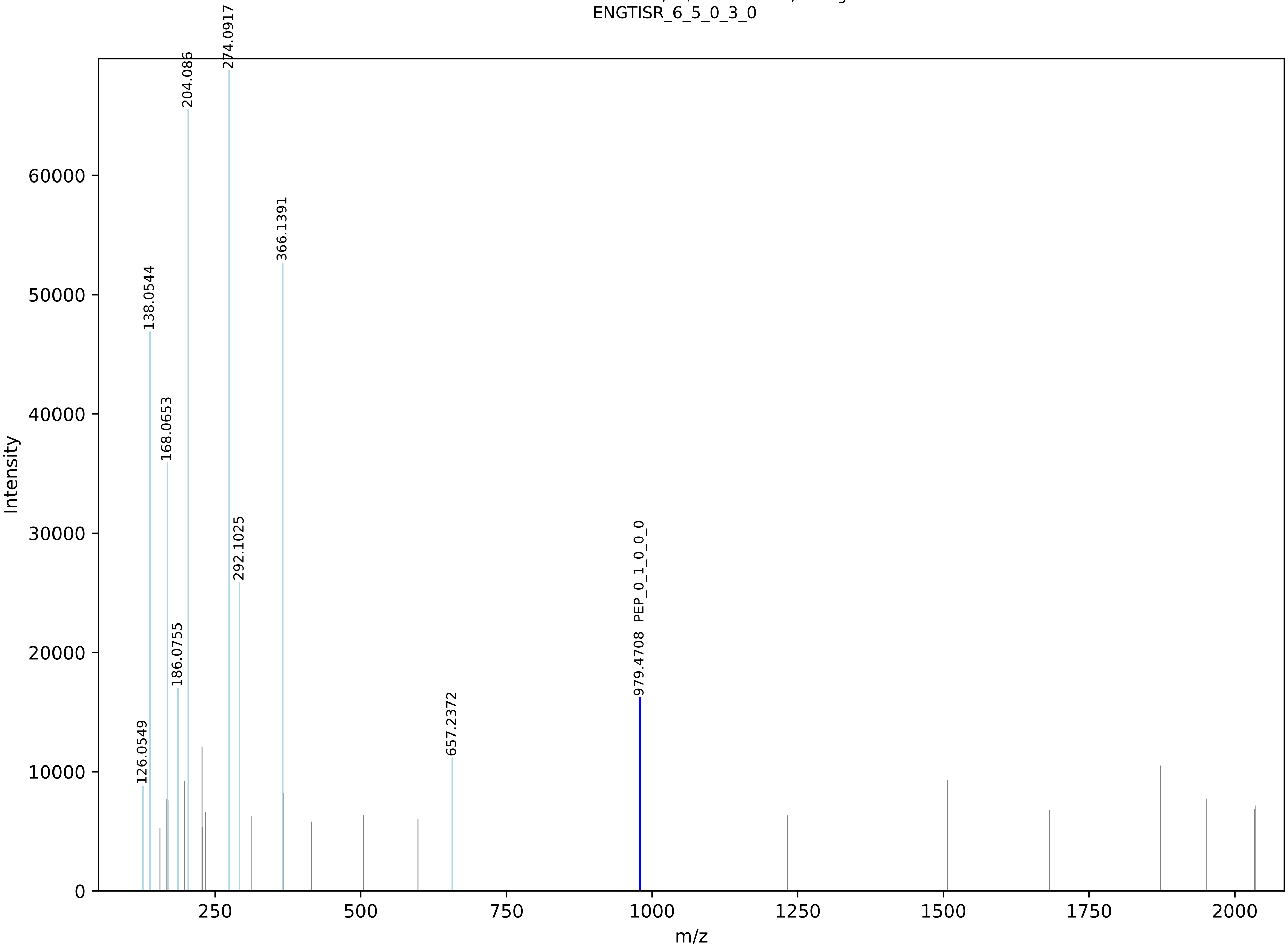

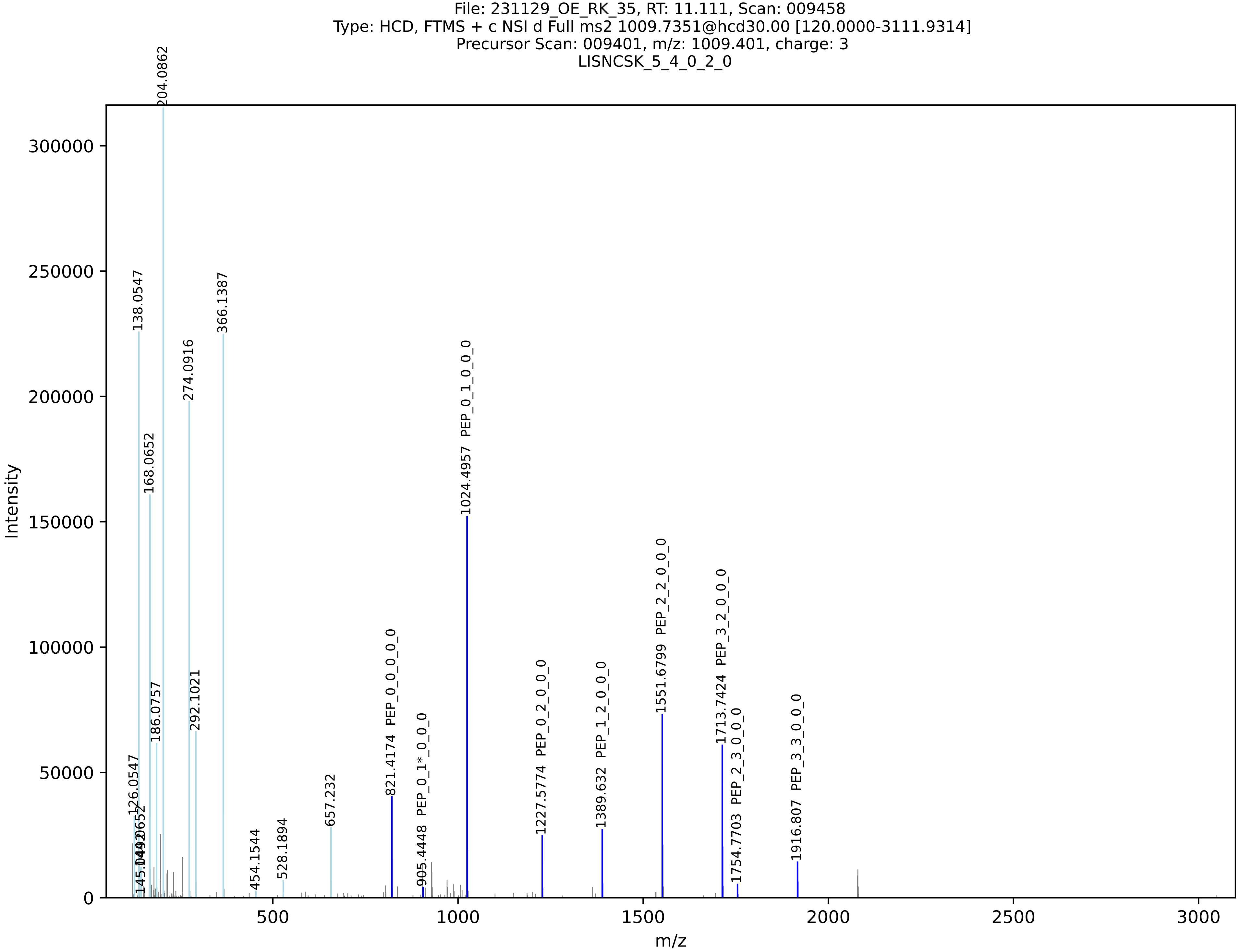

File: 231129_OE_RK_35, RT: 11.111, Scan: 009458
Type: HCD, FTMS + c NSI d Full ms2 1009.7351@hcd30.00 [120.0000-3111.9314]
Precursor Scan: 009401, m/z: 1009.401, charge: 3
LISNCSK_5_4_0_2_0
Intensity
m/z
204.0862
138.0547
366.1387
274.0916
168.0652
186.0757
292.1021
126.0547
454.1544
528.1894
657.232
821.4174 PEP_0_0_0_0_0
905.4448 PEP_0_1*_0_0_0
1024.4957 PEP_0_1_0_0_0
1227.5774 PEP_0_2_0_0_0
1389.632 PEP_1_2_0_0_0
1551.6799 PEP_2_2_0_0_0
1713.7424 PEP_3_2_0_0_0
1754.7703 PEP_2_3_0_0_0
1916.807 PEP_3_3_0_0_0

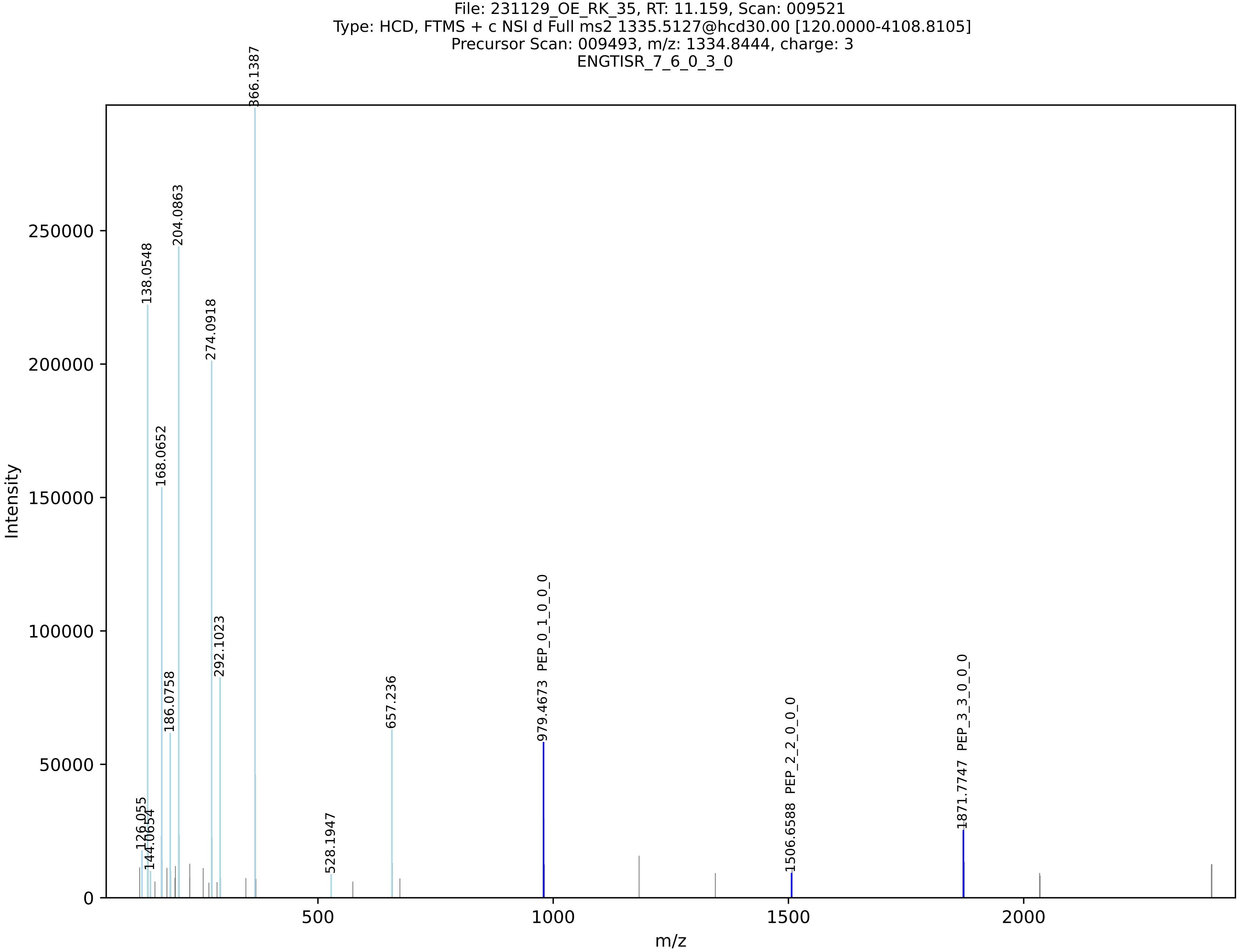
File: 231129_OE_RK_35, RT: 11.159, Scan: 009521
Type: HCD, FTMS + c NSI d Full ms2 1335.5127@hcd30.00 [120.0000-4108.8105]
Precursor Scan: 009493, m/z: 1334.8444, charge: 3
ENGTISR_7_6_0_3_0
366.1387
204.0863
138.0548
274.0918
168.0652
292.1023
186.0758
657.236
979.4673 PEP_0_1_0_0_0
1506.6588 PEP_2_2_0_0_0
1871.7747 PEP_3_3_0_0_0
126.055
144.0654
528.1947
Intensity
m/z
0
50000
100000
150000
200000
250000
500
1000
1500
2000

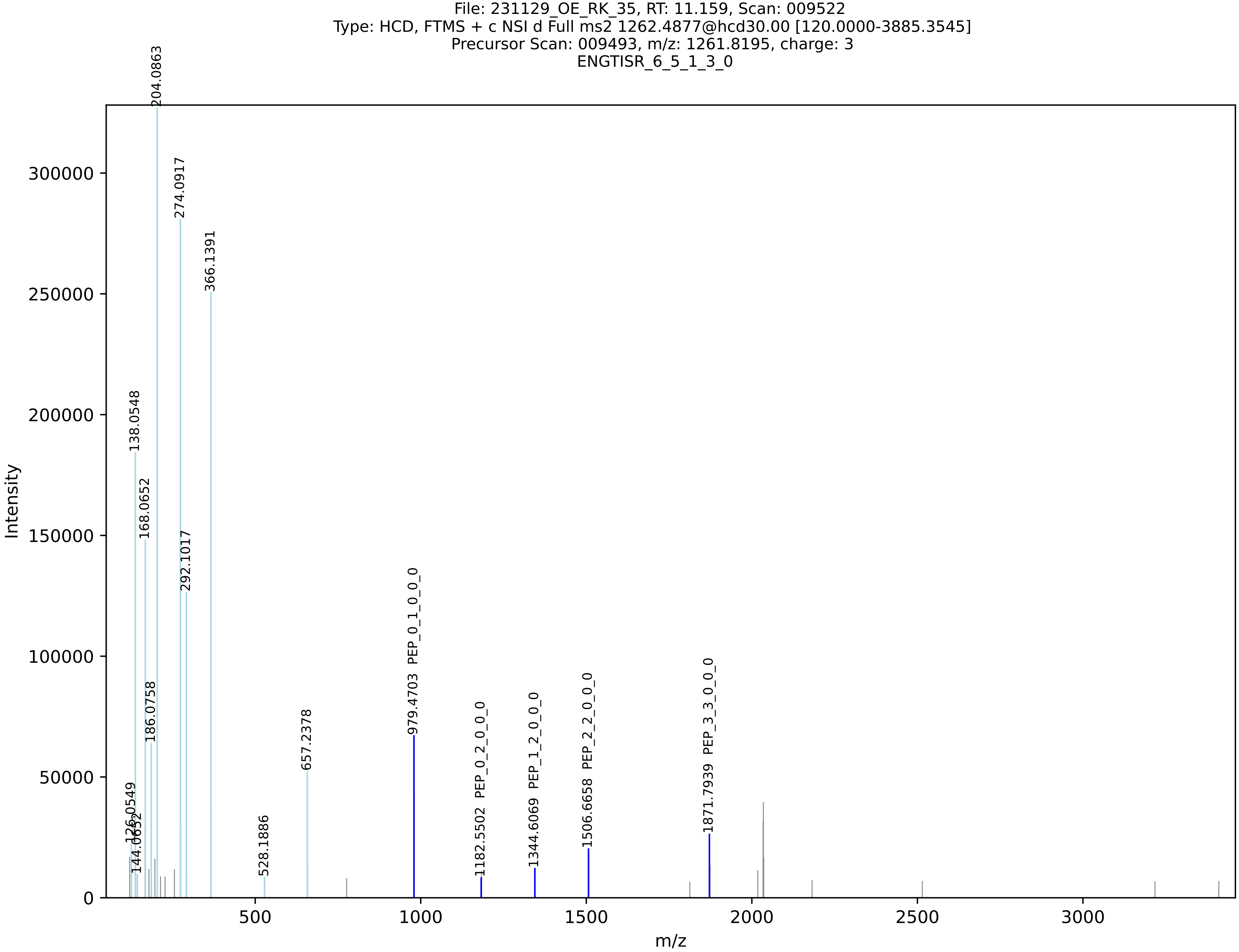

File: 231129_OE_RK_35, RT: 11.159, Scan: 009522
Type: HCD, FTMS + c NSI d Full ms2 1262.4877@hcd30.00 [120.0000-3885.3545]
Precursor Scan: 009493, m/z: 1261.8195, charge: 3
ENGTISR_6_5_1_3_0
204.0863
274.0917
366.1391
138.0548
168.0652
292.1017
186.0758
126.0549
144.0652
528.1886
657.2378
979.4703 PEP_0_1_0_0_0
1182.5502 PEP_0_2_0_0_0
1344.6069 PEP_1_2_0_0_0
1506.6658 PEP_2_2_0_0_0
1871.7939 PEP_3_3_0_0_0
Intensity
m/z

File: 231129_OE_RK_35, RT: 11.16, Scan: 009523
Type: HCD, FTMS + c NSI d Full ms2 1432.8815@hcd30.00 [120.0000-4406.7593]
Precursor Scan: 009493, m/z: 1431.8787, charge: 3
ENGTISR_7_6_0_4_0

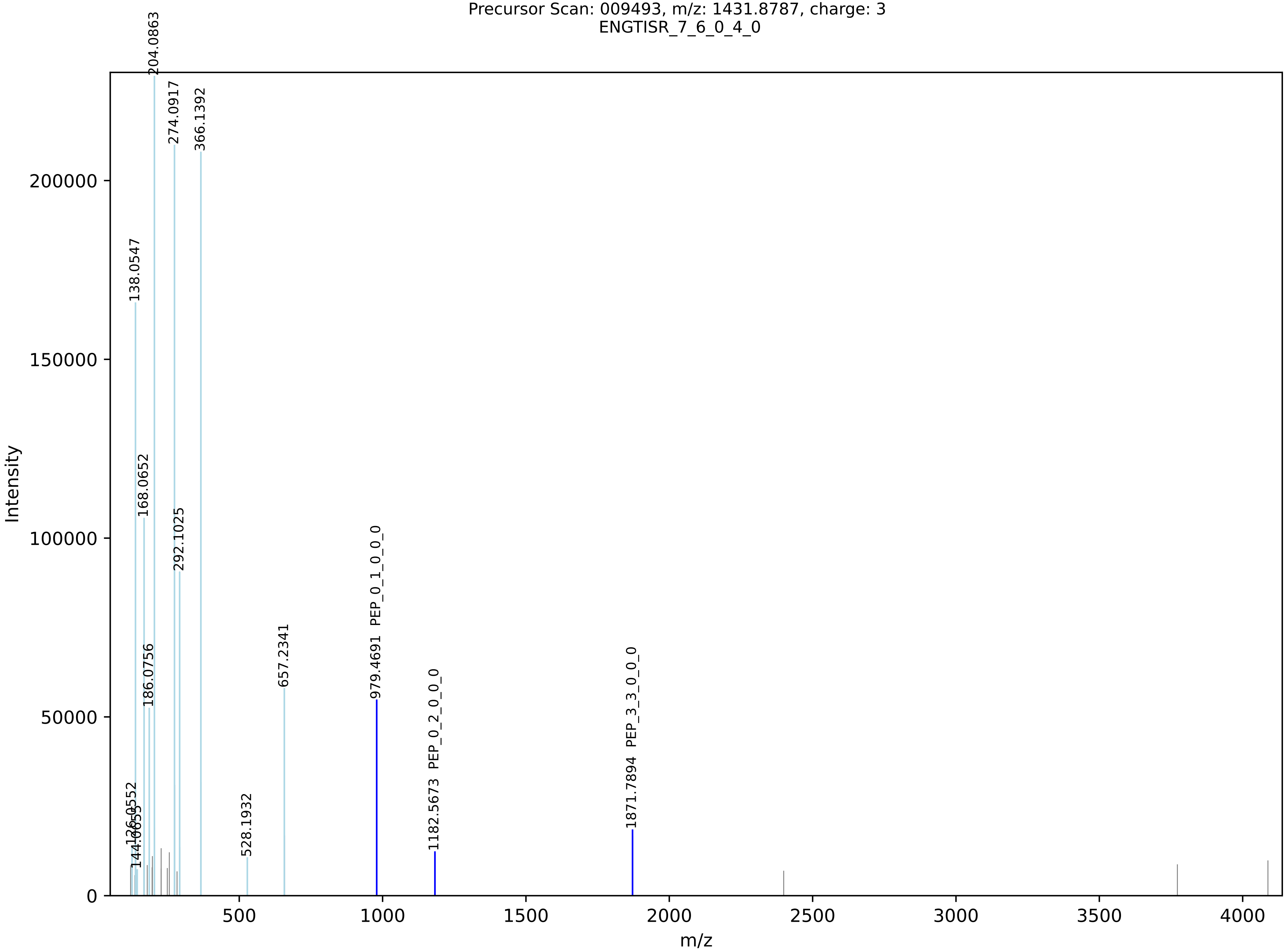

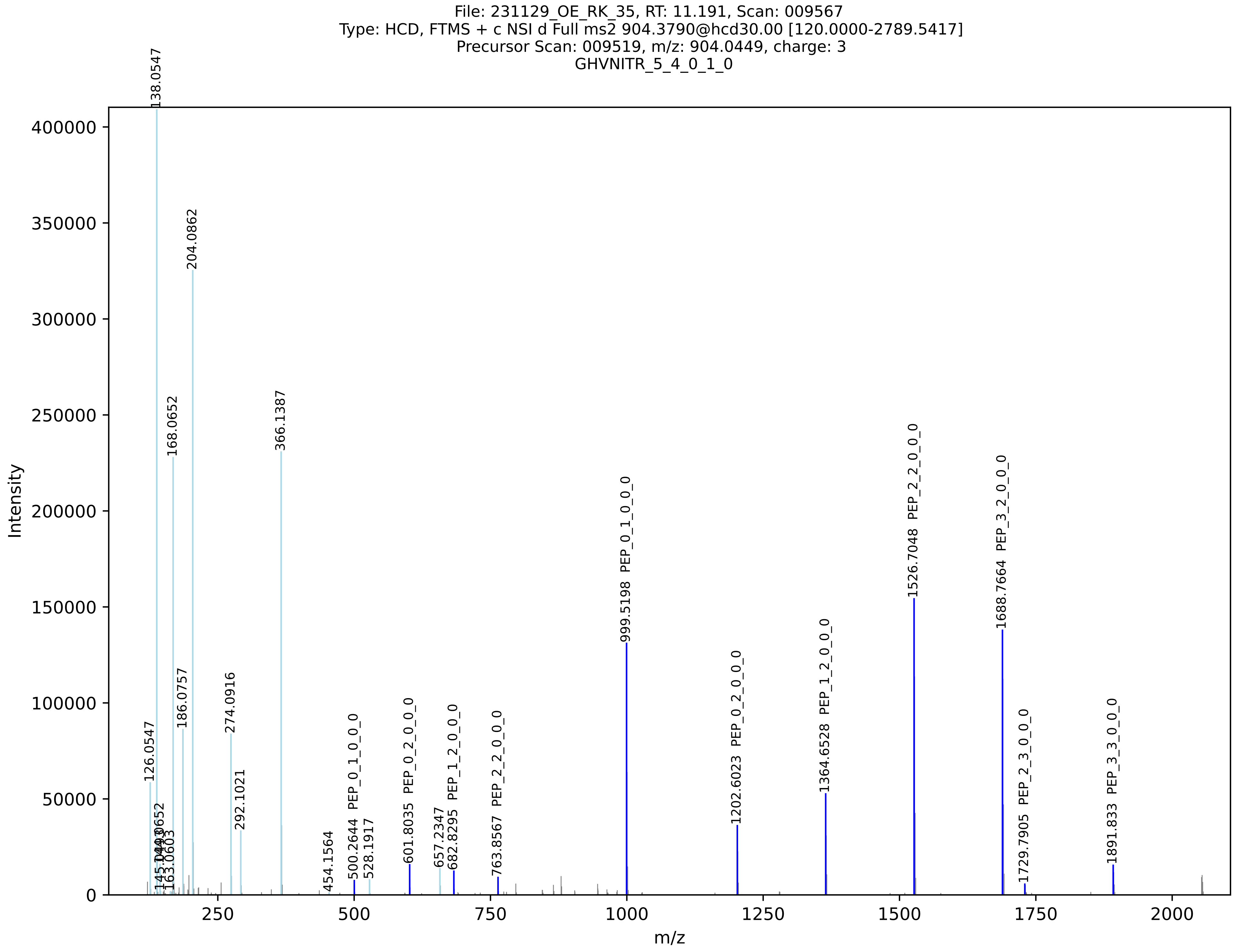

File: 231129_OE_RK_35, RT: 11.191, Scan: 009567
Type: HCD, FTMS + c NSI d Full ms2 904.3790@hcd30.00 [120.0000-2789.5417]
Precursor Scan: 009519, m/z: 904.0449, charge: 3
GHVNITR_5_4_0_1_0
Intensity
m/z
0
50000
100000
150000
200000
250000
300000
350000
400000
250
500
750
1000
1250
1500
1750
2000
138.0547
204.0862
168.0652
366.1387
186.0757
274.0916
126.0547
292.1021
163.0603
454.1564
500.2644 PEP_0_1_0_0_0
528.1917
601.8035 PEP_0_2_0_0_0
657.2347
682.8295 PEP_1_2_0_0_0
763.8567 PEP_2_2_0_0_0
999.5198 PEP_0_1_0_0_0
1202.6023 PEP_0_2_0_0_0
1364.6528 PEP_1_2_0_0_0
1526.7048 PEP_2_2_0_0_0
1688.7664 PEP_3_2_0_0_0
1729.7905 PEP_2_3_0_0_0
1891.833 PEP_3_3_0_0_0

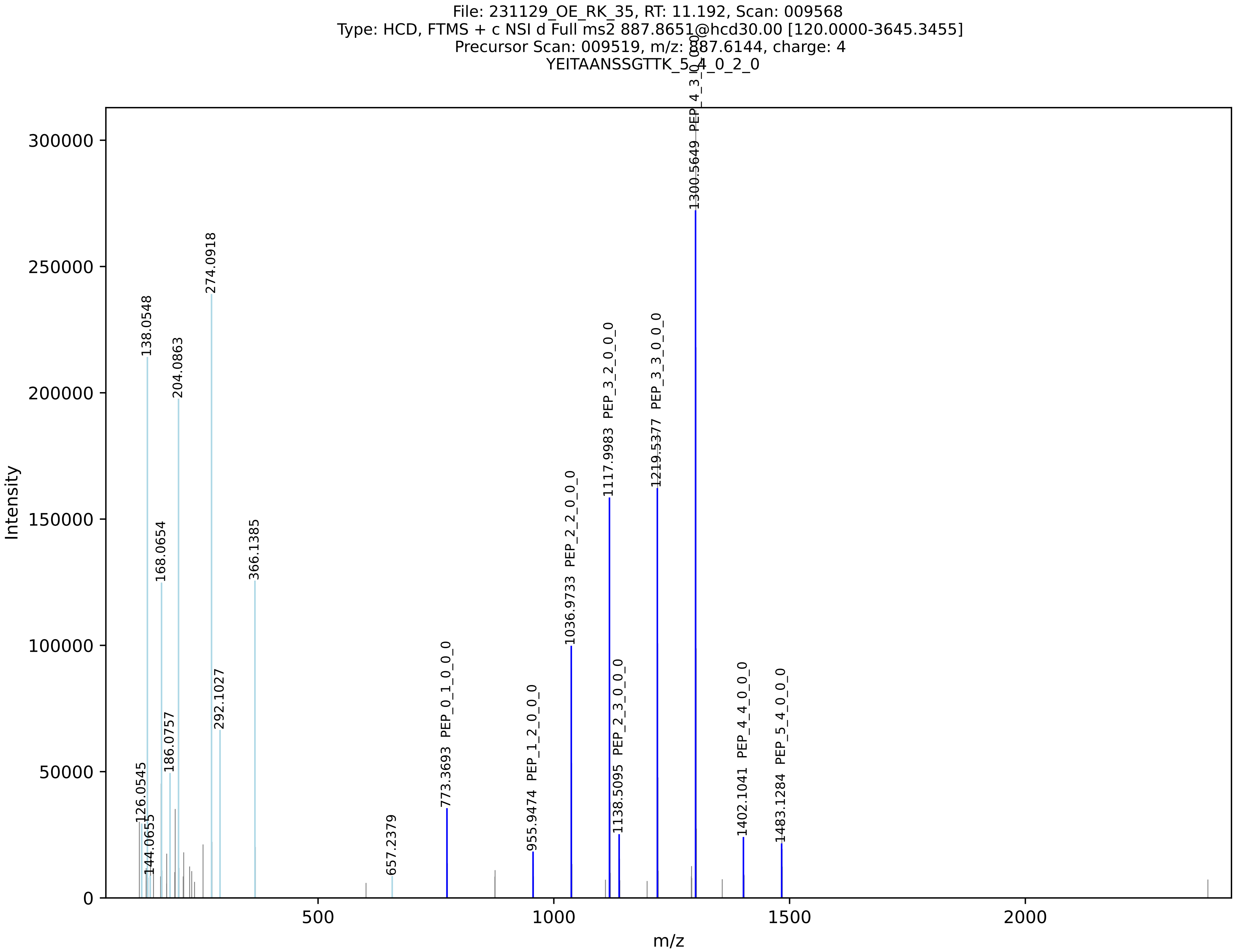
File: 231129_OE_RK_35, RT: 11.192, Scan: 009568
Type: HCD, FTMS + c NSI d Full ms2 887.8651@hcd30.00 [120.0000-3645.3455]
Precursor Scan: 009519, m/z: 887.6144, charge: 4
YEITAANSSGTTK_5_4_0_2_0
Intensity
m/z
0
50000
100000
150000
200000
250000
300000
500
1000
1500
2000
126.0545
144.0655
138.0548
168.0654
186.0757
204.0863
274.0918
292.1027
366.1385
657.2379
773.3693 PEP_0_1_0_0_0
955.9474 PEP_1_2_0_0_0
1036.9733 PEP_2_2_0_0_0
1117.9983 PEP_3_2_0_0_0
1138.5095 PEP_2_3_0_0_0
1219.5377 PEP_3_3_0_0_0
1300.5649 PEP_4_3_0_0_0
1402.1041 PEP_4_4_0_0_0
1483.1284 PEP_5_4_0_0_0

File: 231129_OE_RK_35, RT: 11.237, Scan: 009633
Type: HCD, FTMS + c NSI d Full ms2 1310.8402@hcd30.00 [120.0000-4033.3132]
Precursor Scan: 009566, m/z: 1310.5061, charge: 3
ENGTISR_6_5_2_3_0

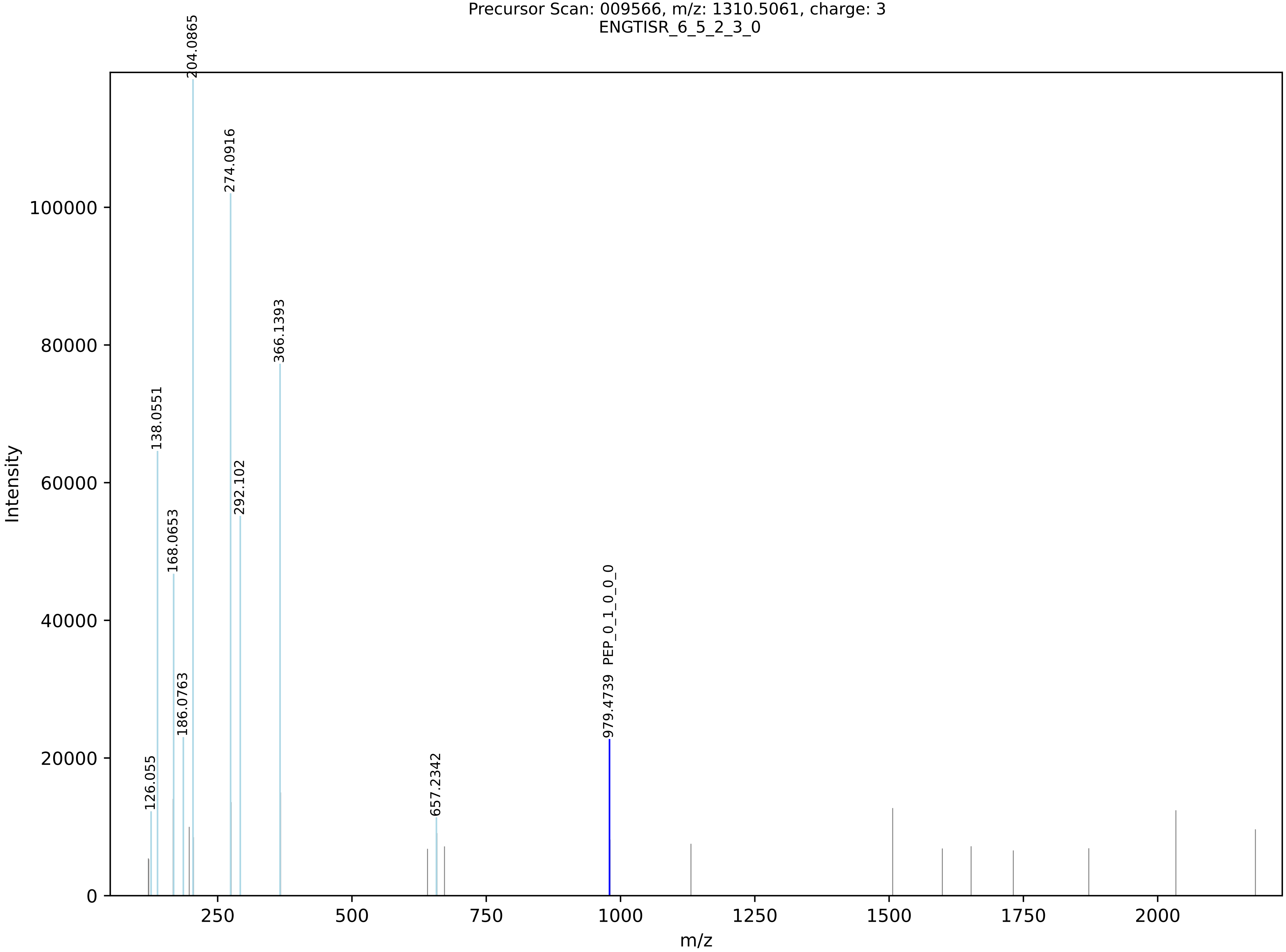

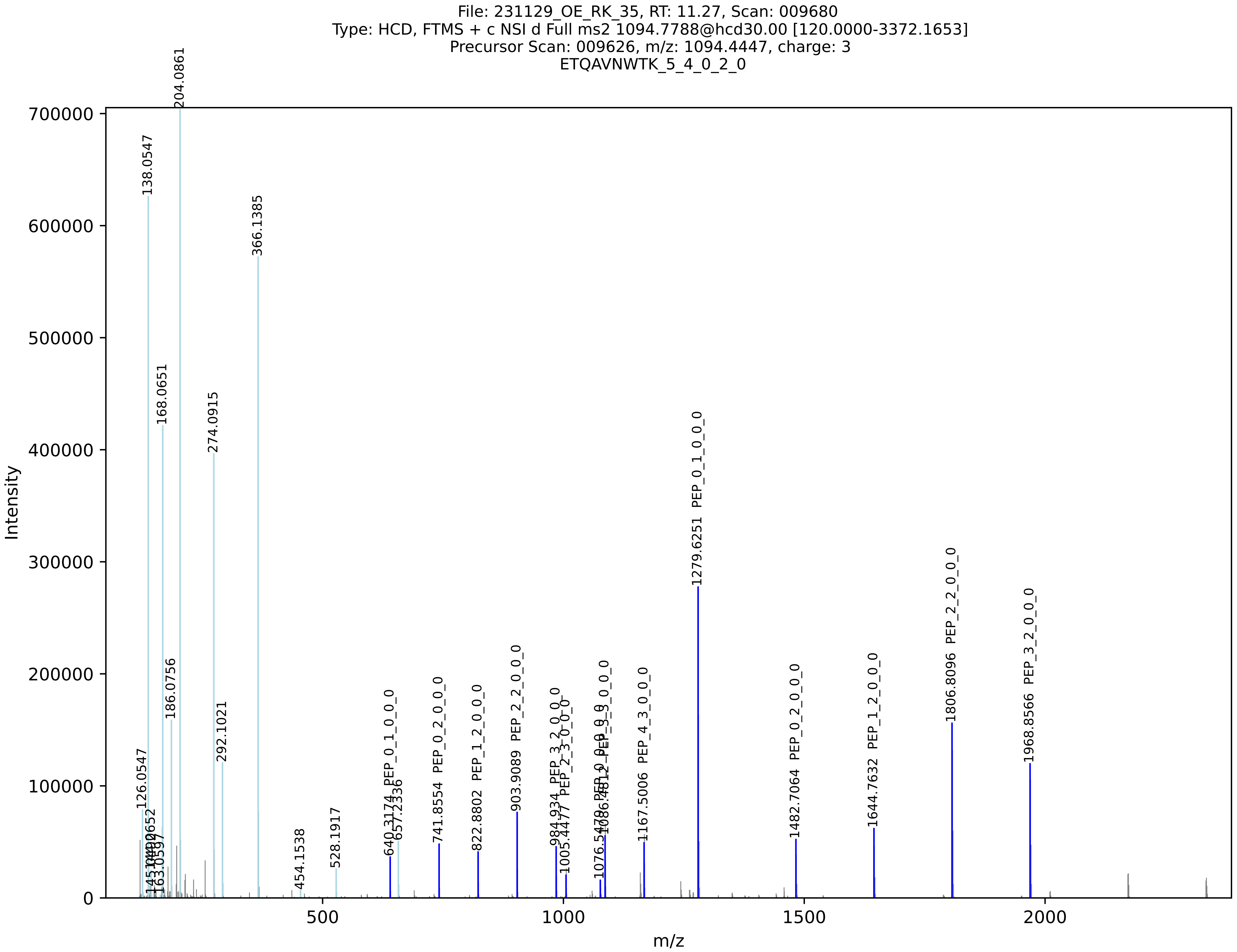
File: 231129_OE_RK_35, RT: 11.27, Scan: 009680
Type: HCD, FTMS + c NSI d Full ms2 1094.7788@hcd30.00 [120.0000-3372.1653]
Precursor Scan: 009626, m/z: 1094.4447, charge: 3
ETQAVNWTK_5_4_0_2_0
Intensity
m/z
204.0861
138.0547
366.1385
168.0651
274.0915
186.0756
292.1021
126.0547
454.1538
528.1917
640.3174 PEP_0_1_0_0_0
657.2336
741.8554 PEP_0_2_0_0_0
822.8802 PEP_1_2_0_0_0
903.9089 PEP_2_2_0_0_0
984.934 PEP_3_2_0_0_0
1005.4477 PEP_2_3_0_0_0
1167.5006 PEP_4_3_0_0_0
1279.6251 PEP_0_1_0_0_0
1482.7064 PEP_0_2_0_0_0
1644.7632 PEP_1_2_0_0_0
1806.8096 PEP_2_2_0_0_0
1968.8566 PEP_3_2_0_0_0

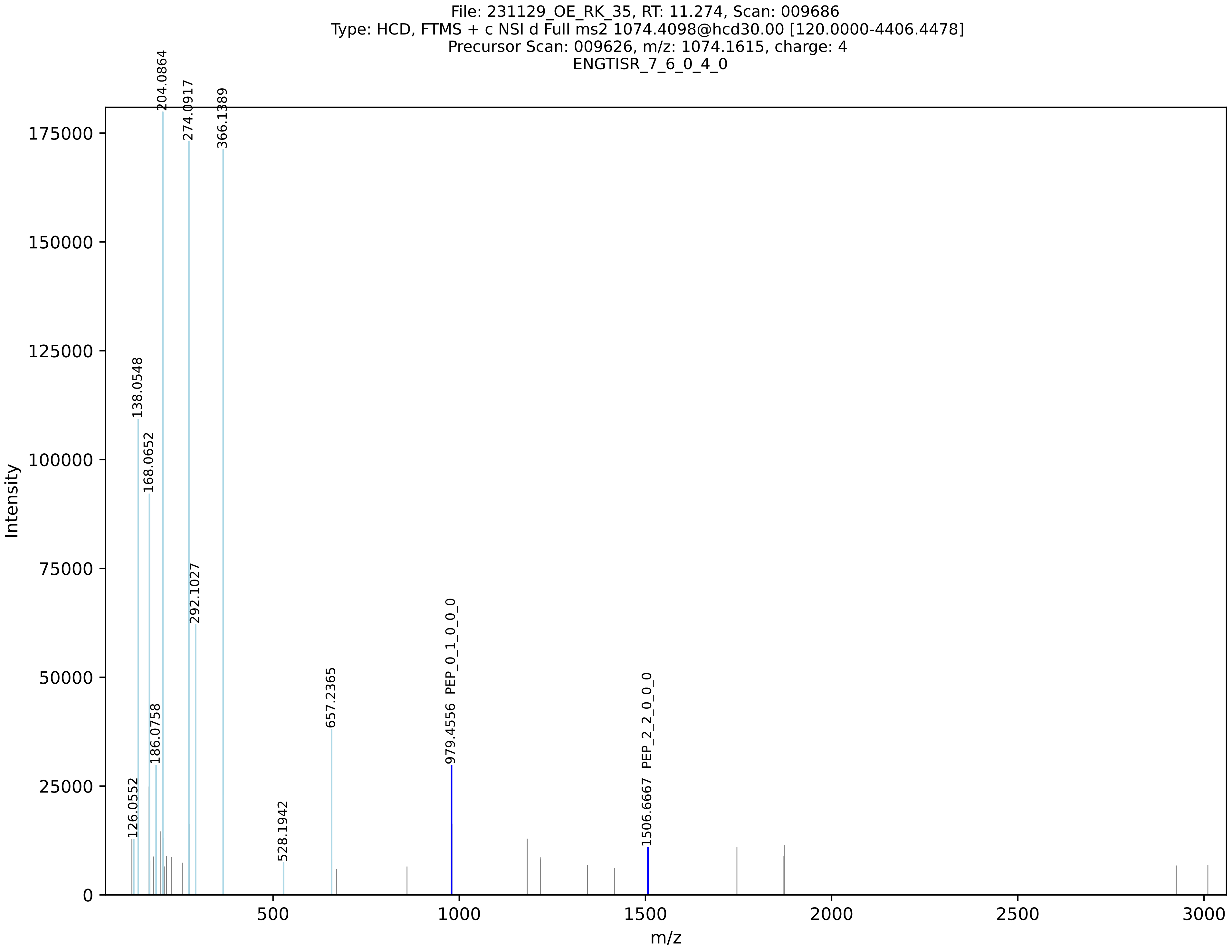
File: 231129_OE_RK_35, RT: 11.274, Scan: 009686
Type: HCD, FTMS + c NSI d Full ms2 1074.4098@hcd30.00 [120.0000-4406.4478]
Precursor Scan: 009626, m/z: 1074.1615, charge: 4
ENGTISR_7_6_0_4_0
Intensity
m/z
204.0864
274.0917
366.1389
138.0548
168.0652
292.1027
186.0758
126.0552
528.1942
657.2365
979.4556 PEP_0_1_0_0_0
1506.6667 PEP_2_2_0_0_0
0
25000
50000
75000
100000
125000
150000
175000
500
1000
1500
2000
2500
3000

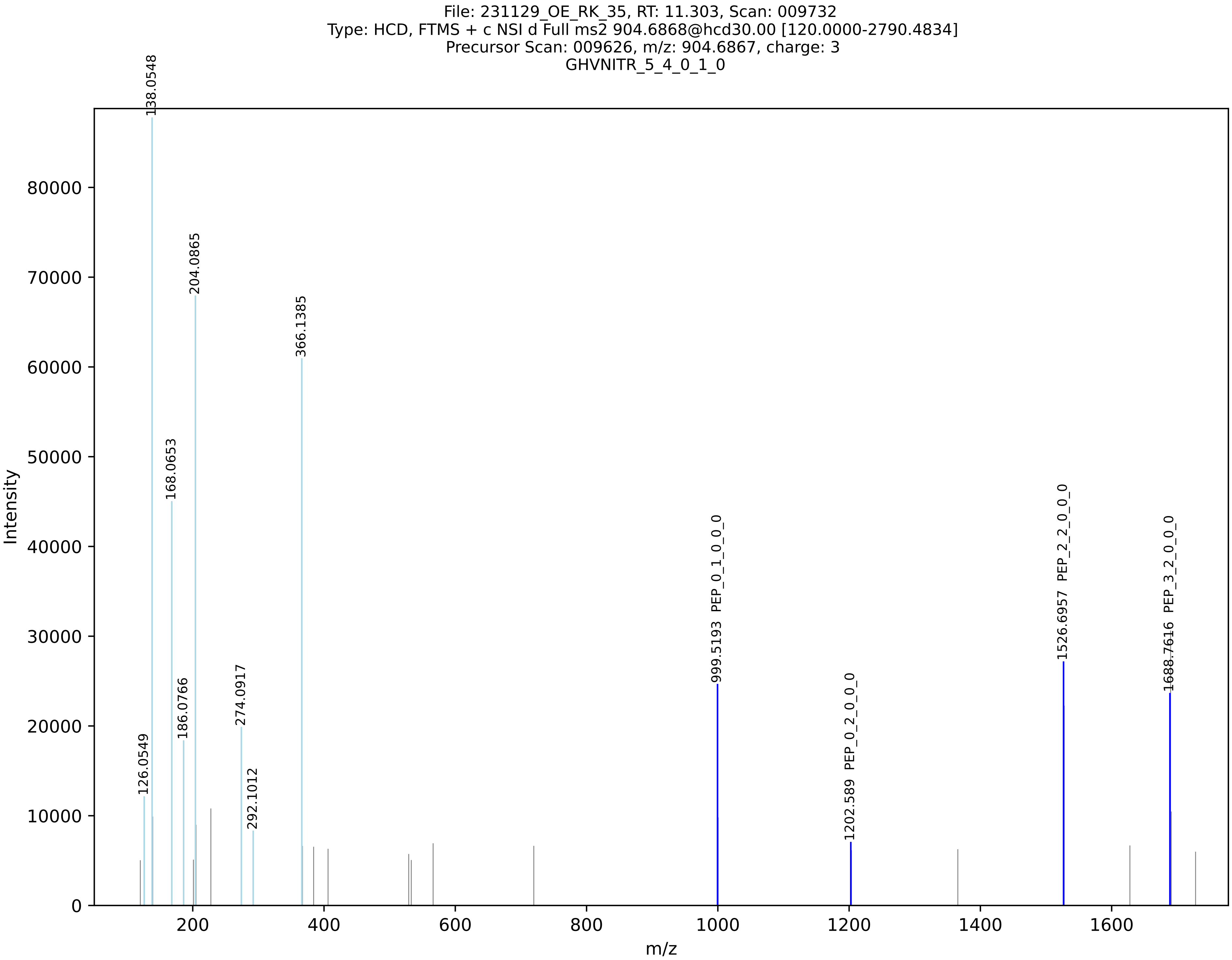
File: 231129_OE_RK_35, RT: 11.303, Scan: 009732
Type: HCD, FTMS + c NSI d Full ms2 904.6868@hcd30.00 [120.0000-2790.4834]
Precursor Scan: 009626, m/z: 904.6867, charge: 3
GHVNITR_5_4_0_1_0
Intensity
m/z
138.0548
204.0865
366.1385
168.0653
186.0766
274.0917
126.0549
292.1012
999.5193 PEP_0_1_0_0_
1202.589 PEP_0_2_0_0_
1526.6957 PEP_2_2_0_0_
1688.7616 PEP_3_2_0_0_

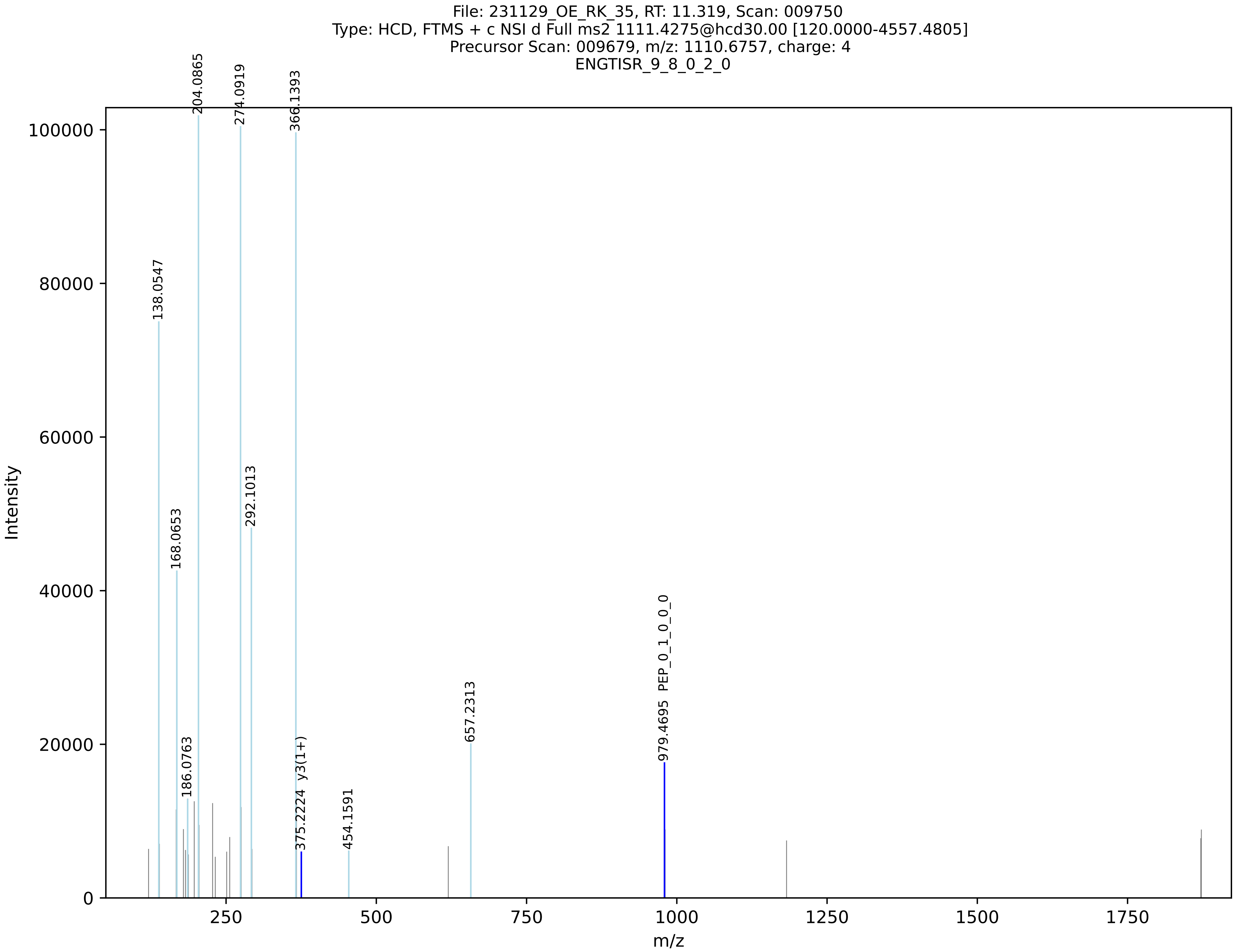
File: 231129_OE_RK_35, RT: 11.319, Scan: 009750
Type: HCD, FTMS + c NSI d Full ms2 1111.4275@hcd30.00 [120.0000-4557.4805]
Precursor Scan: 009679, m/z: 1110.6757, charge: 4
ENGTISR_9_8_0_2_0
Intensity
m/z
204.0865
274.0919
366.1393
138.0547
168.0653
292.1013
186.0763
375.2224 y3(1+)
454.1591
657.2313
979.4695 PEP_0_1_0_0_0
0
20000
40000
60000
80000
100000
250
500
750
1000
1250
1500
1750

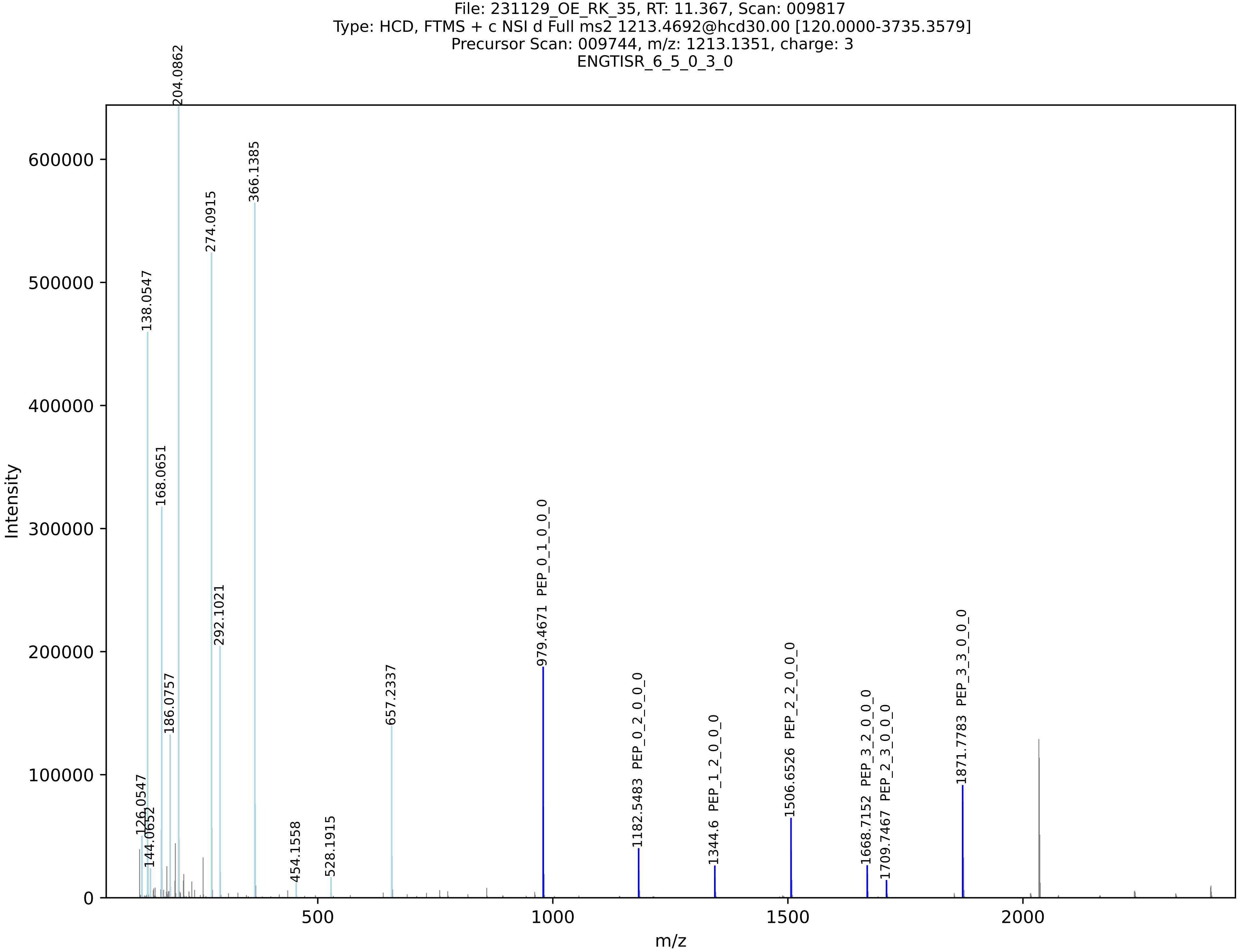

File: 231129_OE_RK_35, RT: 11.367, Scan: 009817
Type: HCD, FTMS + c NSI d Full ms2 1213.4692@hcd30.00 [120.0000-3735.3579]
Precursor Scan: 009744, m/z: 1213.1351, charge: 3
ENGTISR_6_5_0_3_0
Intensity
m/z
204.0862
366.1385
274.0915
138.0547
168.0651
292.1021
186.0757
126.0547
144.0652
454.1558
528.1915
657.2337
979.4671 PEP_0_1_0_0_0
1182.5483 PEP_0_2_0_0_0
1344.6 PEP_1_2_0_0_0
1506.6526 PEP_2_2_0_0_0
1668.7152 PEP_3_2_0_0_0
1709.7467 PEP_2_3_0_0_0
1871.7783 PEP_3_3_0_0_0

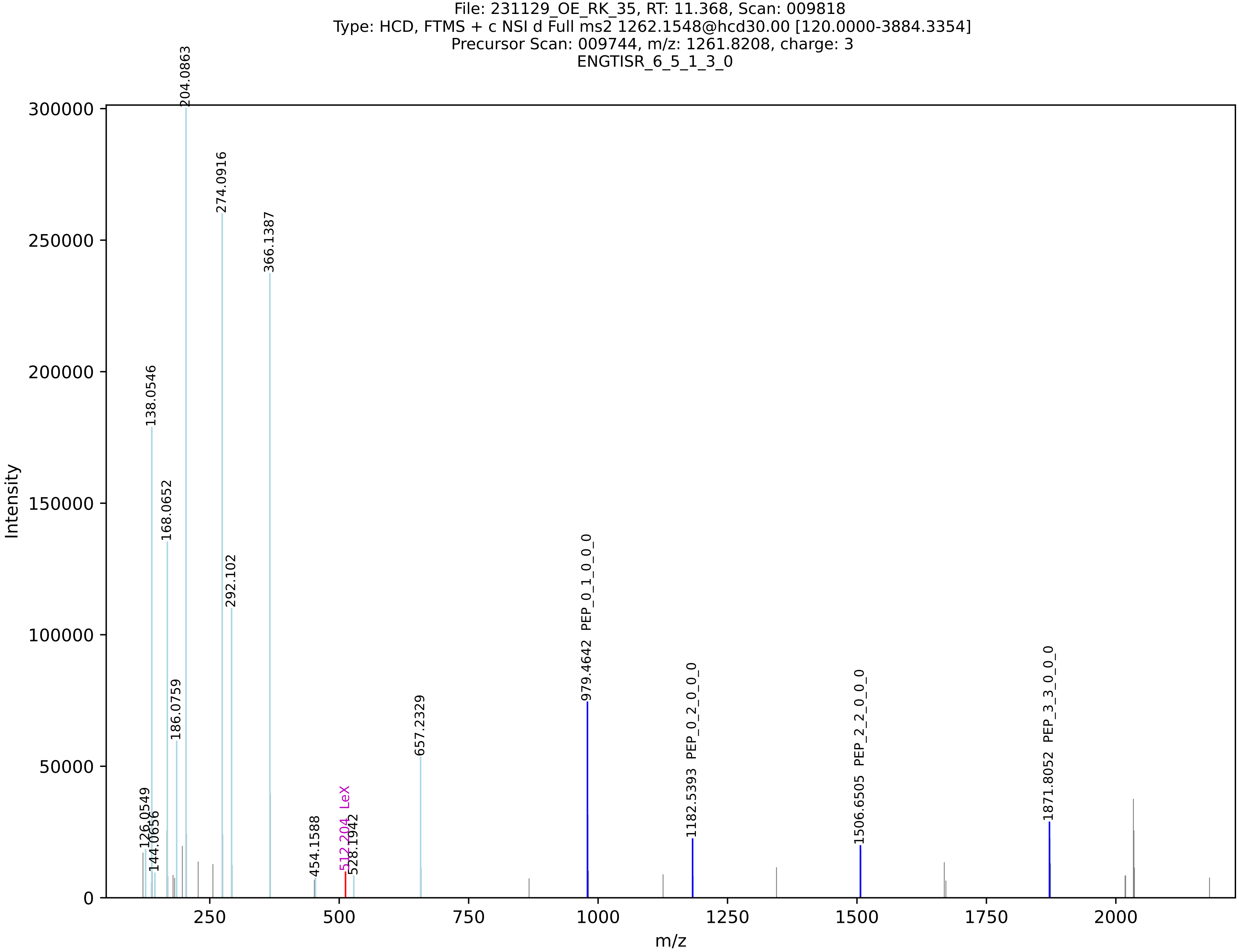

File: 231129_OE_RK_35, RT: 11.368, Scan: 009818
Type: HCD, FTMS + c NSI d Full ms2 1262.1548@hcd30.00 [120.0000-3884.3354]
Precursor Scan: 009744, m/z: 1261.8208, charge: 3
ENGTISR_6_5_1_3_0
Intensity
m/z
0
50000
100000
150000
200000
250000
300000
250
500
750
1000
1250
1500
1750
2000
204.0863
274.0916
366.1387
138.0546
168.0652
292.102
186.0759
126.0549
144.0656
454.1588
512.204 LeX
528.1942
657.2329
979.4642 PEP_0_1_0_0_0
1182.5393 PEP_0_2_0_0_0
1506.6505 PEP_2_2_0_0_0
1871.8052 PEP_3_3_0_0_0

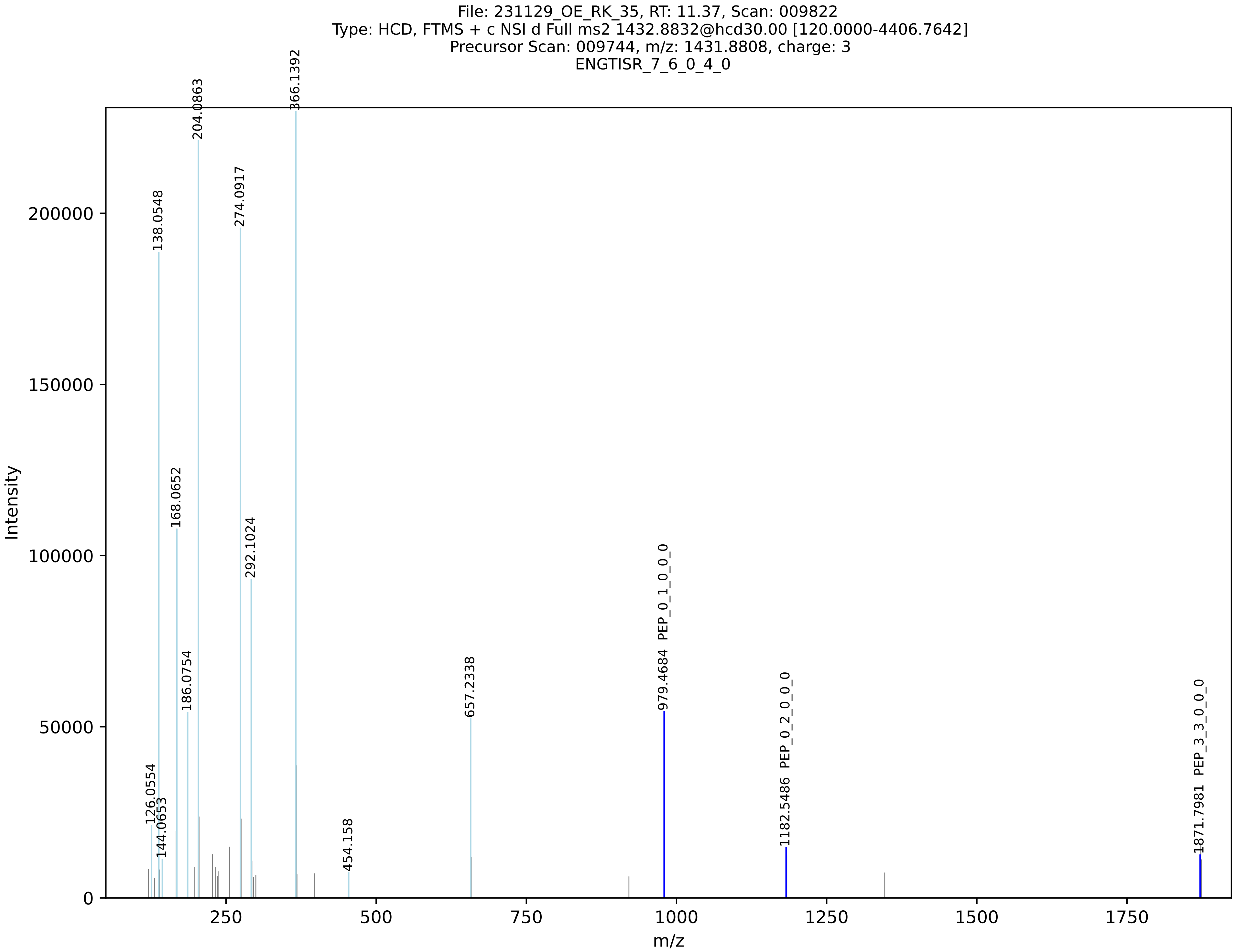
File: 231129_OE_RK_35, RT: 11.37, Scan: 009822
Type: HCD, FTMS + c NSI d Full ms2 1432.8832@hcd30.00 [120.0000-4406.7642]
Precursor Scan: 009744, m/z: 1431.8808, charge: 3
ENGTISR_7_6_0_4_0
Intensity
m/z
366.1392
204.0863
274.0917
138.0548
168.0652
292.1024
186.0754
126.0554
144.0653
454.158
657.2338
979.4684 PEP_0_1_0_0_0
1182.5486 PEP_0_2_0_0_0
1871.7981 PEP_3_3_0_0_0
0
50000
100000
150000
200000
250
500
750
1000
1250
1500
1750

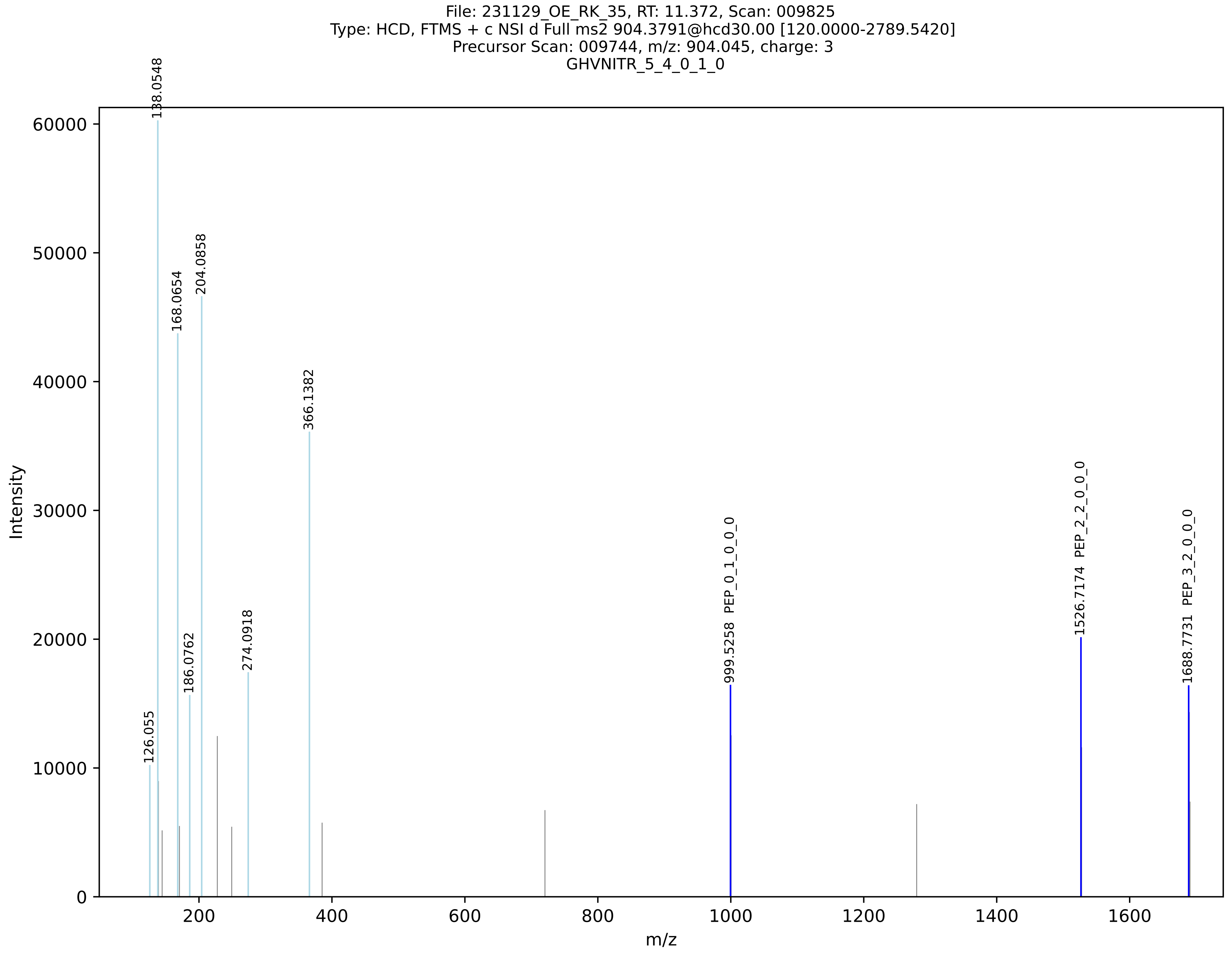
File: 231129_OE_RK_35, RT: 11.372, Scan: 009825
Type: HCD, FTMS + c NSI d Full ms2 904.3791@hcd30.00 [120.0000-2789.5420]
Precursor Scan: 009744, m/z: 904.045, charge: 3
GHVNITR_5_4_0_1_0
138.0548
204.0858
168.0654
366.1382
274.0918
186.0762
126.055
999.5258 PEP_0_1_0_0_0
1526.7174 PEP_2_2_0_0_0
1688.7731 PEP_3_2_0_0_0
Intensity
m/z
0
10000
20000
30000
40000
50000
60000
200
400
600
800
1000
1200
1400
1600

File: 231129_OE_RK_35, RT: 12.814, Scan: 011841
Type: HCD, FTMS + c NSI d Full ms2 904.0444@hcd30.00 [120.0000-2788.5176]
Precursor Scan: 011734, m/z: 904.0443, charge: 3
GHVNITR_5_4_0_1_0

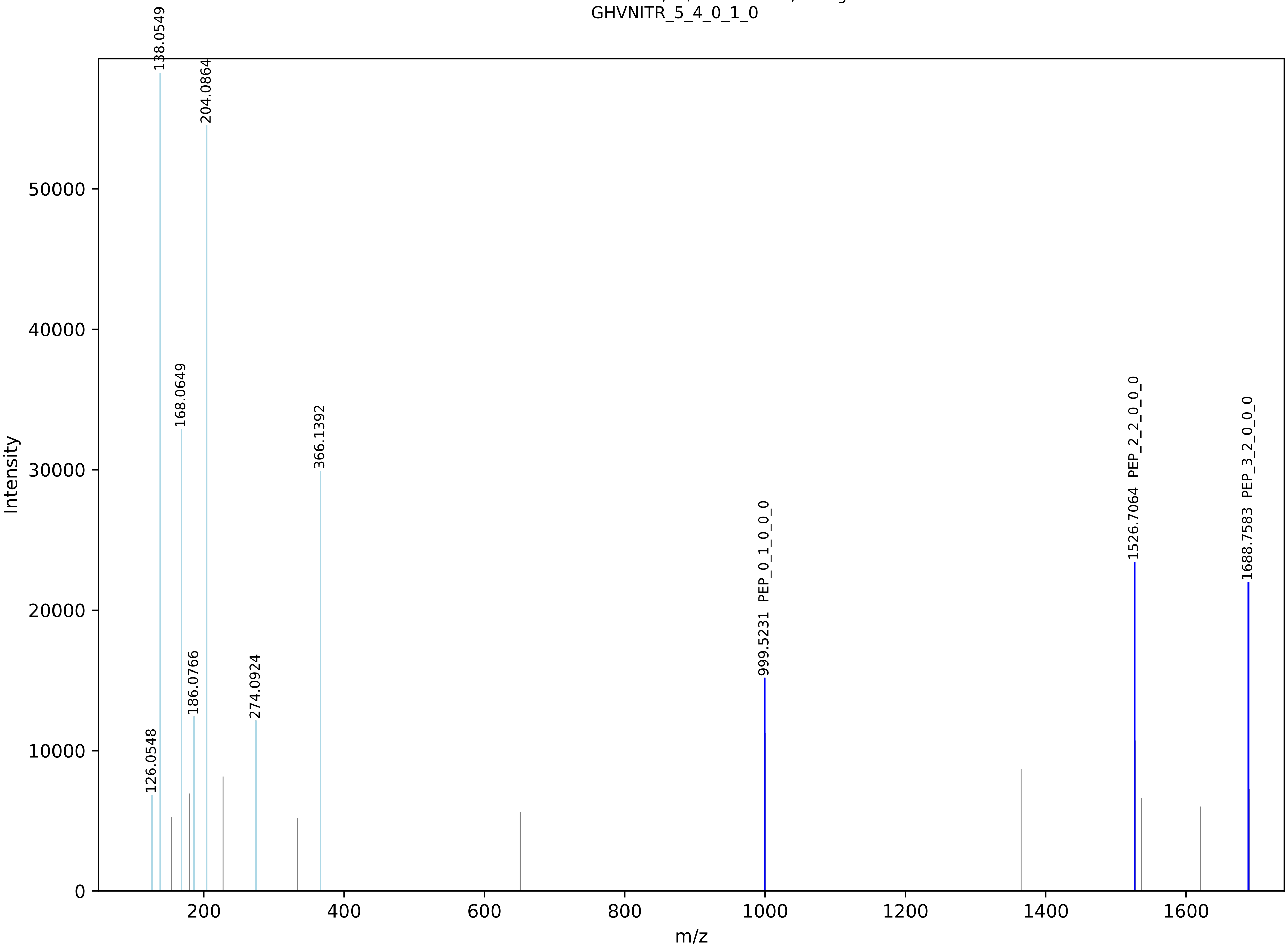

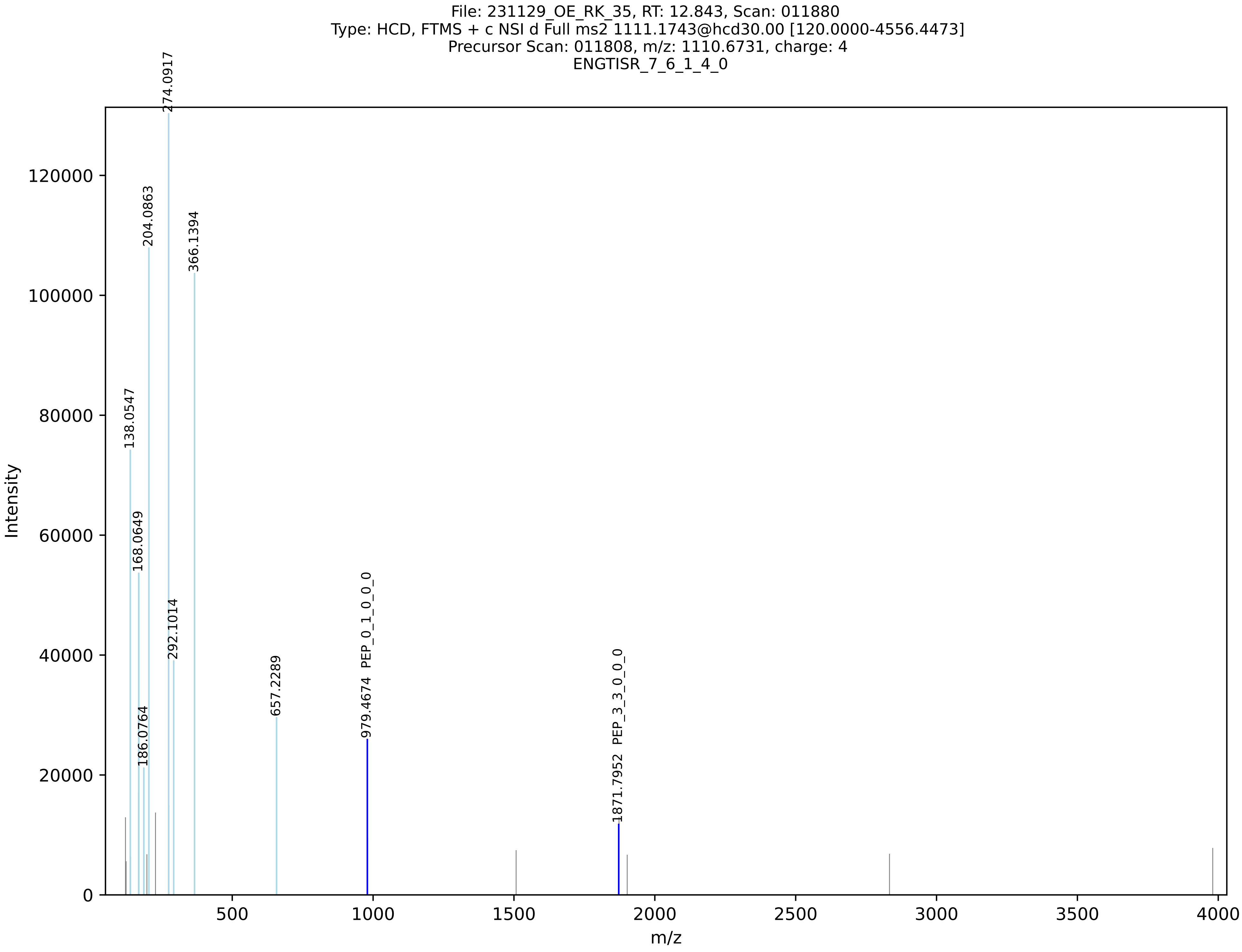
File: 231129_OE_RK_35, RT: 12.843, Scan: 011880
Type: HCD, FTMS + c NSI d Full ms2 1111.1743@hcd30.00 [120.0000-4556.4473]
Precursor Scan: 011808, m/z: 1110.6731, charge: 4
ENGTISR_7_6_1_4_0
274.0917
204.0863
366.1394
138.0547
168.0649
292.1014
657.2289
979.4674 PEP_0_1_0_0_0
1871.7952 PEP_3_3_0_0_0
186.0764
Intensity
m/z
0
20000
40000
60000
80000
100000
120000
500
1000
1500
2000
2500
3000
3500
4000

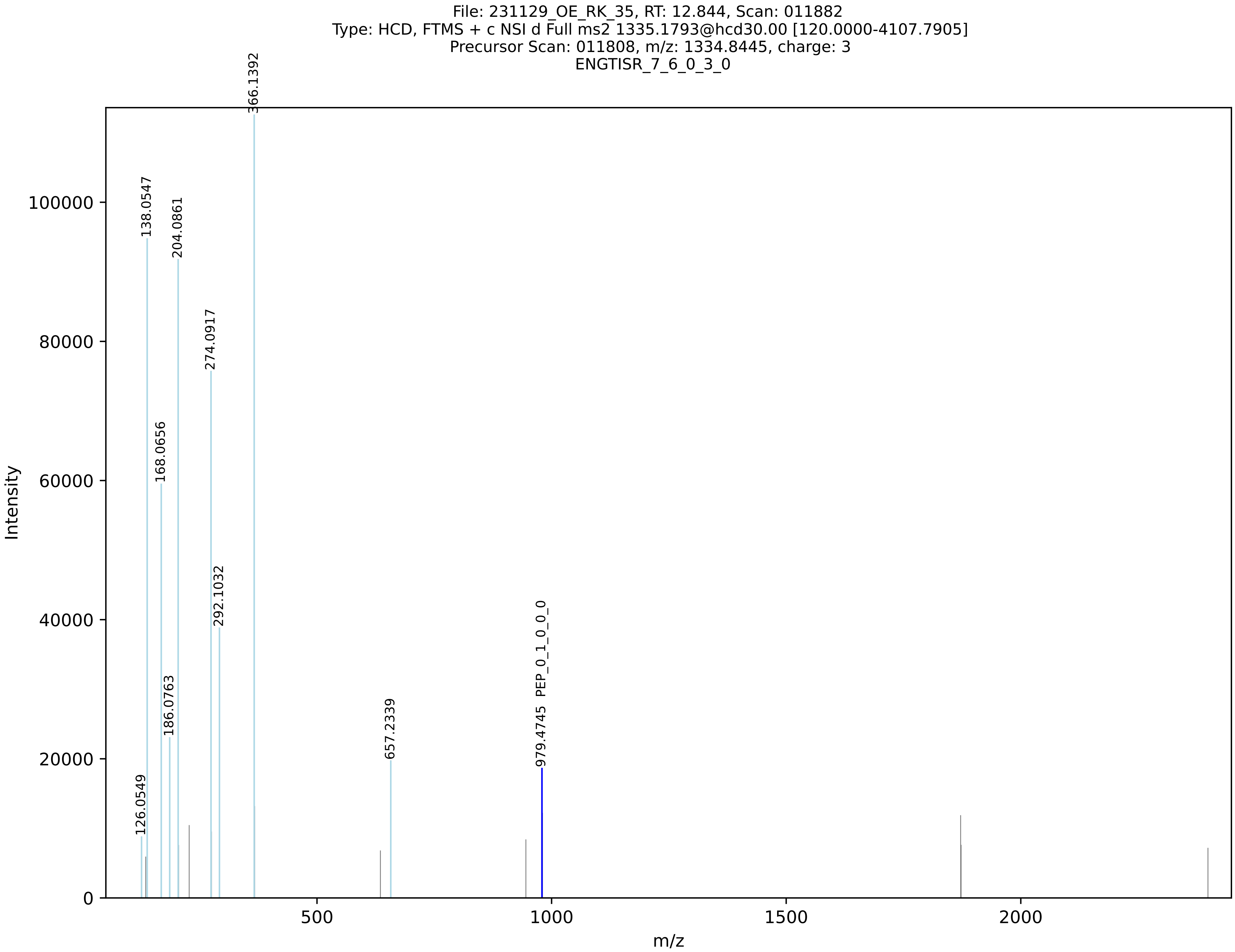

File: 231129_OE_RK_35, RT: 12.844, Scan: 011882
Type: HCD, FTMS + c NSI d Full ms2 1335.1793@hcd30.00 [120.0000-4107.7905]
Precursor Scan: 011808, m/z: 1334.8445, charge: 3
ENGTISR_7_6_0_3_0
366.1392
138.0547
204.0861
274.0917
168.0656
292.1032
186.0763
126.0549
657.2339
979.4745 PEP_0_1_0_0_0
Intensity
m/z
0
20000
40000
60000
80000
100000
500
1000
1500
2000

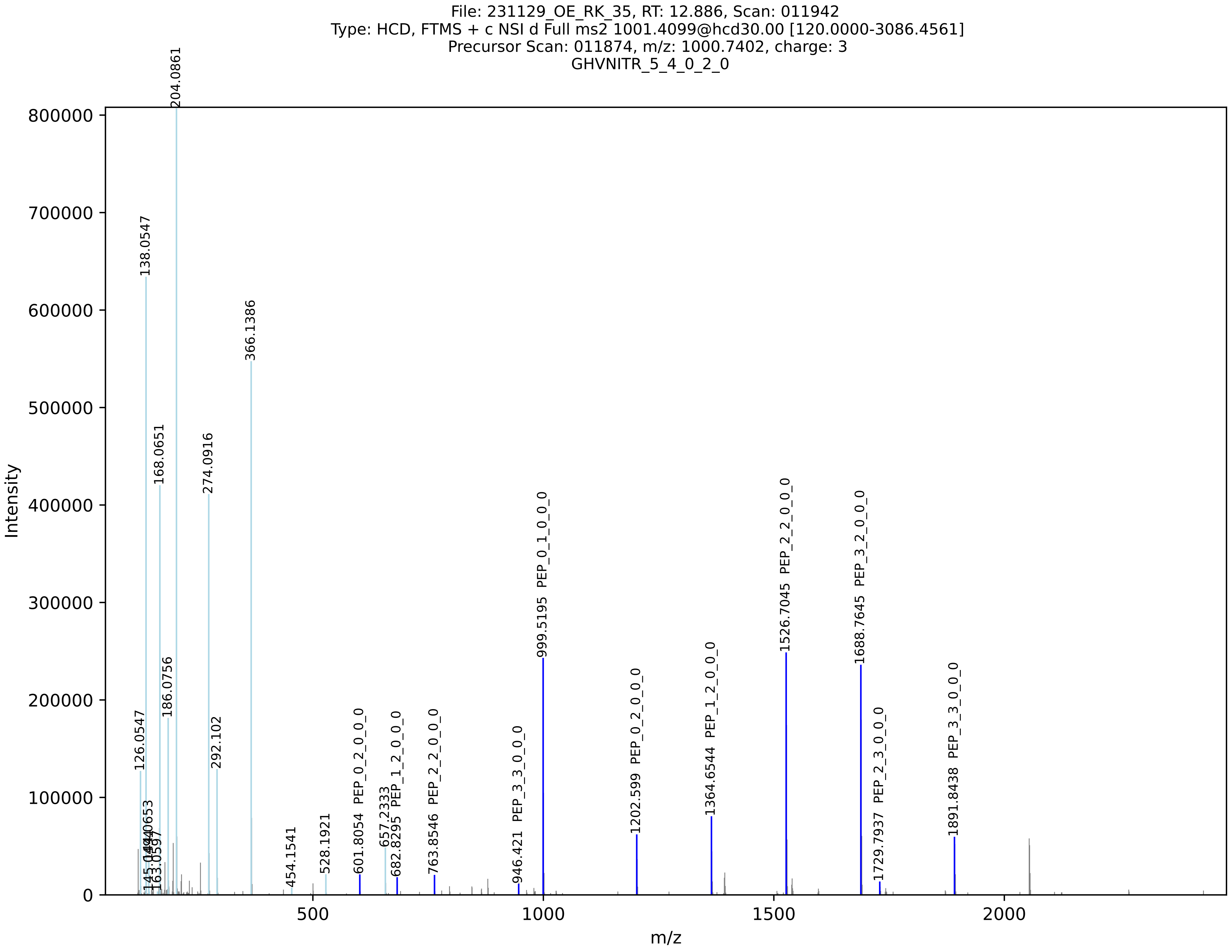

File: 231129_OE_RK_35, RT: 12.886, Scan: 011942
Type: HCD, FTMS + c NSI d Full ms2 1001.4099@hcd30.00 [120.0000-3086.4561]
Precursor Scan: 011874, m/z: 1000.7402, charge: 3
GHVNITR_5_4_0_2_0
Intensity
m/z
0
100000
200000
300000
400000
500000
600000
700000
800000
500
1000
1500
2000
204.0861
138.0547
366.1386
168.0651
274.0916
186.0756
126.0547
292.102
163.0597
454.1541
528.1921
601.8054 PEP_0_2_0_0_0
657.2333
682.8295 PEP_1_2_0_0_0
763.8546 PEP_2_2_0_0_0
946.421 PEP_3_3_0_0_0
999.5195 PEP_0_1_0_0_0
1202.599 PEP_0_2_0_0_0
1364.6544 PEP_1_2_0_0_0
1526.7045 PEP_2_2_0_0_0
1688.7645 PEP_3_2_0_0_0
1729.7937 PEP_2_3_0_0_0
1891.8438 PEP_3_3_0_0_0

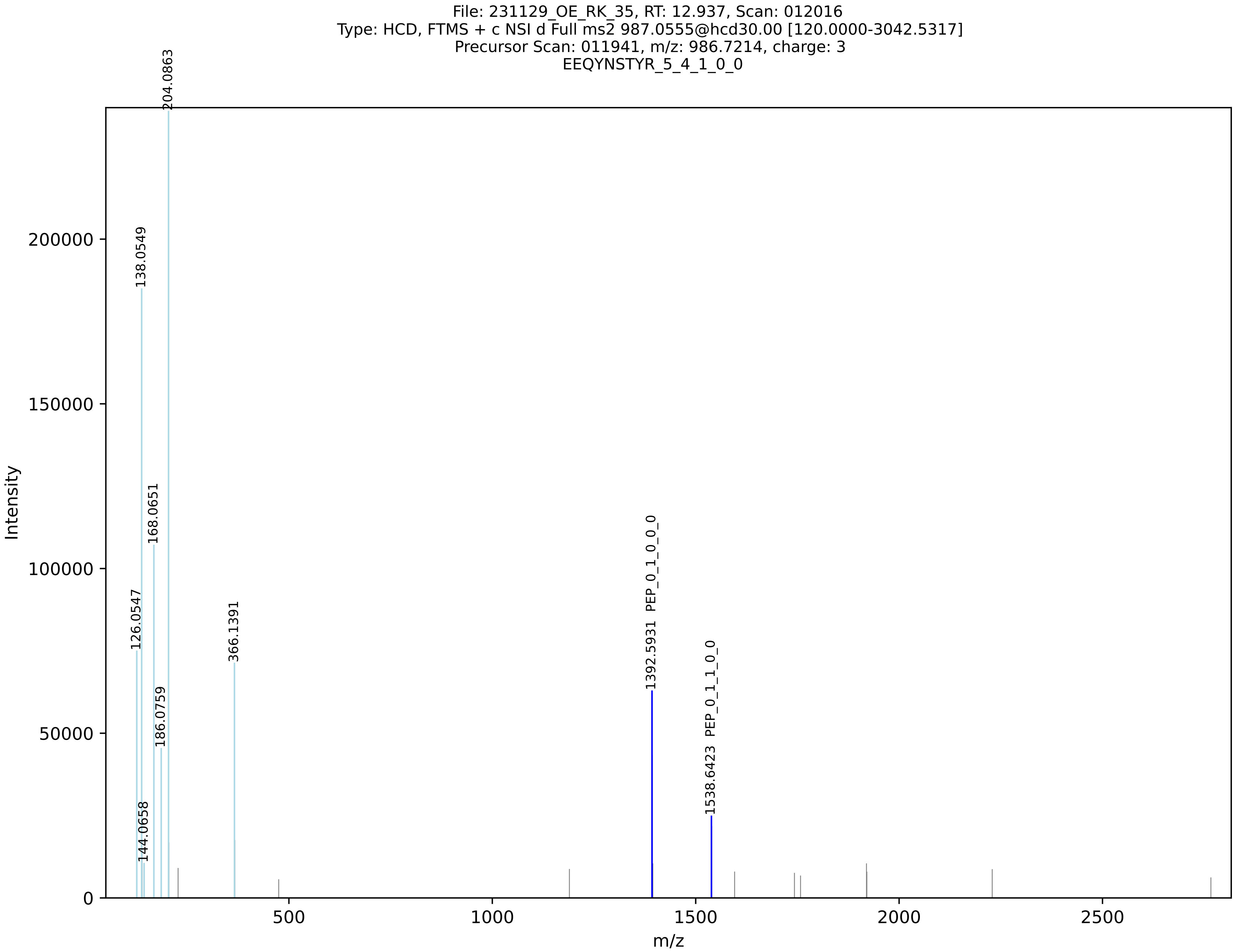
File: 231129_OE_RK_35, RT: 12.937, Scan: 012016
Type: HCD, FTMS + c NSI d Full ms2 987.0555@hcd30.00 [120.0000-3042.5317]
Precursor Scan: 011941, m/z: 986.7214, charge: 3
EEQYNSTYR_5_4_1_0_0
204.0863
138.0549
168.0651
126.0547
366.1391
186.0759
144.0658
1392.5931 PEP_0_1_0_0_0
1538.6423 PEP_0_1_1_0_0
Intensity
m/z
0
50000
100000
150000
200000
500
1000
1500
2000
2500

File: 231129_OE_RK_35, RT: 12.939, Scan: 012019
Type: HCD, FTMS + c NSI d Full ms2 1220.4552@hcd30.00 [120.0000-3756.7349]
Precursor Scan: 011941, m/z: 1218.8104, charge: 3
NEEYNK_6_5_2_2_0

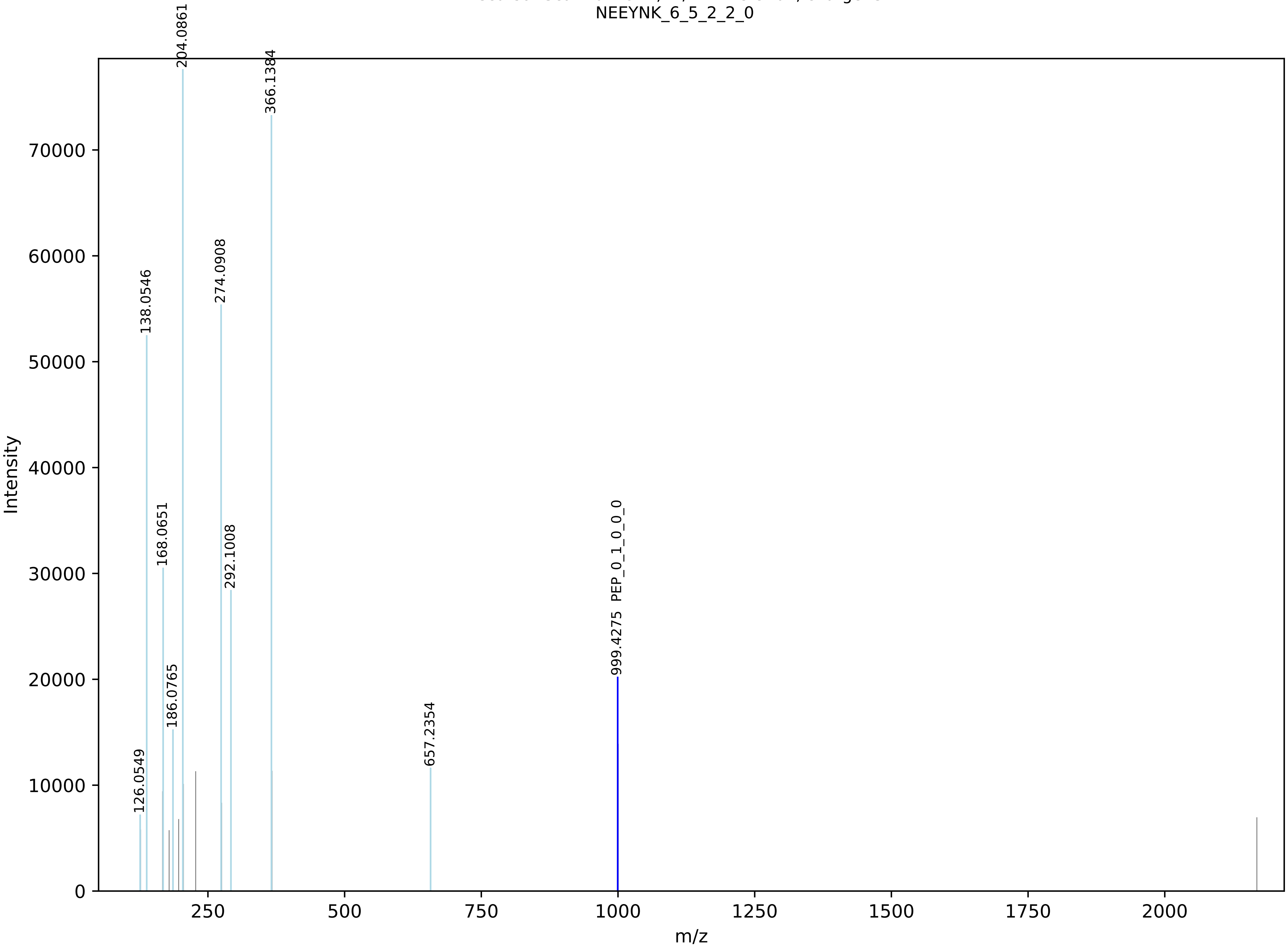

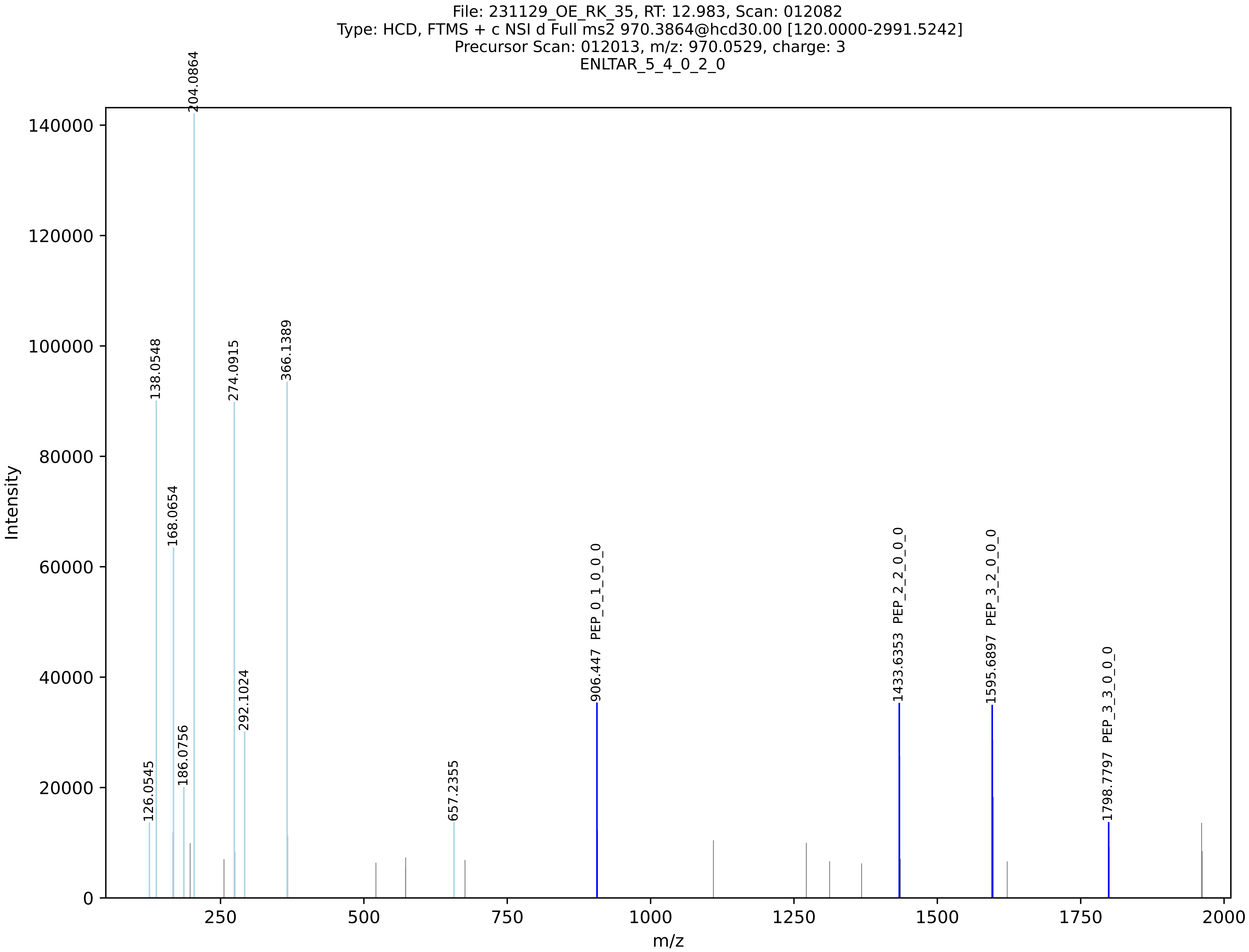

File: 231129_OE_RK_35, RT: 12.983, Scan: 012082
Type: HCD, FTMS + c NSI d Full ms2 970.3864@hcd30.00 [120.0000-2991.5242]
Precursor Scan: 012013, m/z: 970.0529, charge: 3
ENLTAR_5_4_0_2_0
Intensity
m/z
0
20000
40000
60000
80000
100000
120000
140000
250
500
750
1000
1250
1500
1750
2000
126.0545
138.0548
168.0654
186.0756
204.0864
274.0915
292.1024
366.1389
657.2355
906.447 PEP_0_1_0_0_0
1433.6353 PEP_2_2_0_0_0
1595.6897 PEP_3_2_0_0_0
1798.7797 PEP_3_3_0_0_0

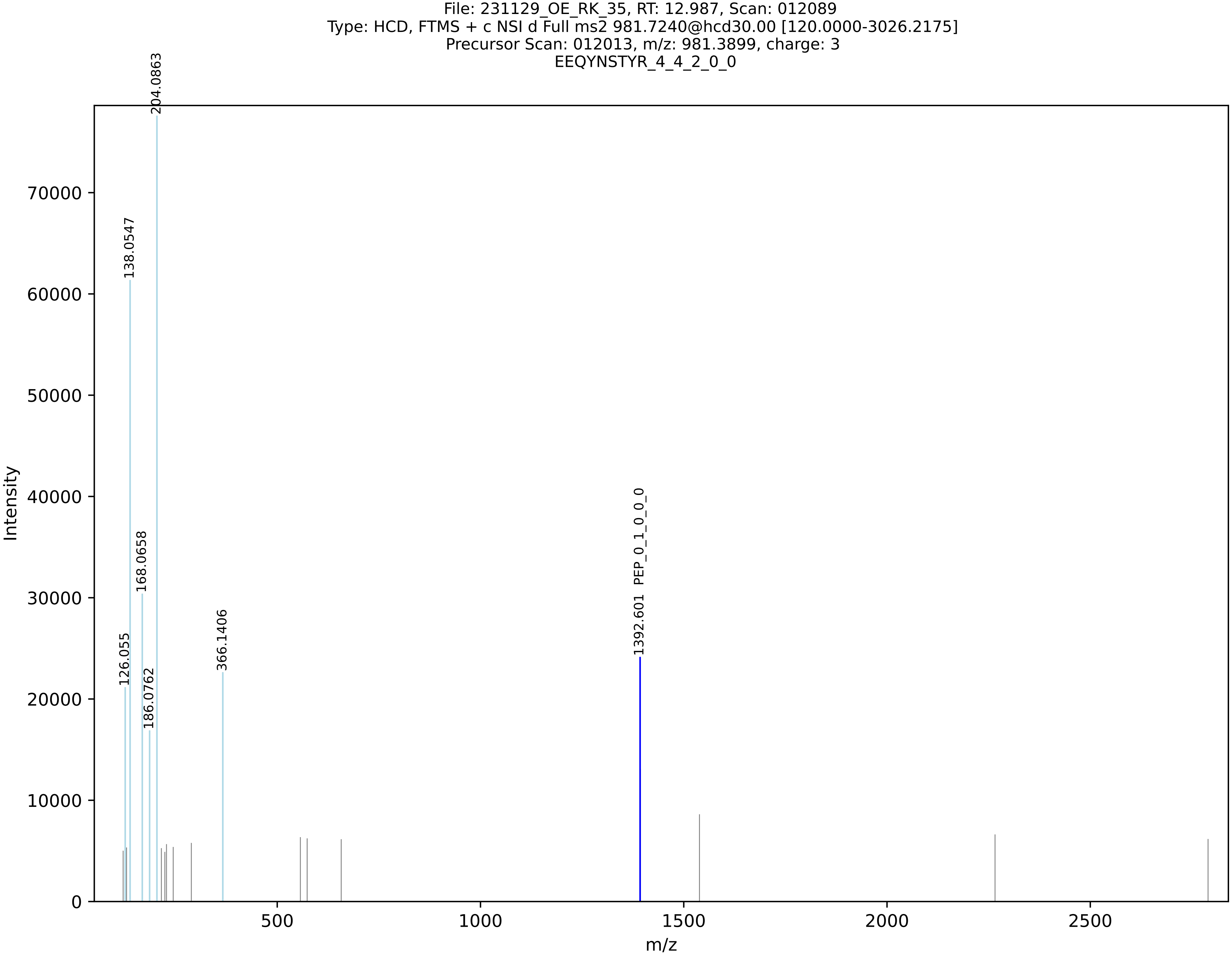

File: 231129_OE_RK_35, RT: 12.987, Scan: 012089
Type: HCD, FTMS + c NSI d Full ms2 981.7240@hcd30.00 [120.0000-3026.2175]
Precursor Scan: 012013, m/z: 981.3899, charge: 3
EEQYNSTYR_4_4_2_0_0
204.0863
138.0547
168.0658
126.055
186.0762
366.1406
1392.601 PEP_0_1_0_0_0
Intensity
m/z
0
10000
20000
30000
40000
50000
60000
70000
500
1000
1500
2000
2500

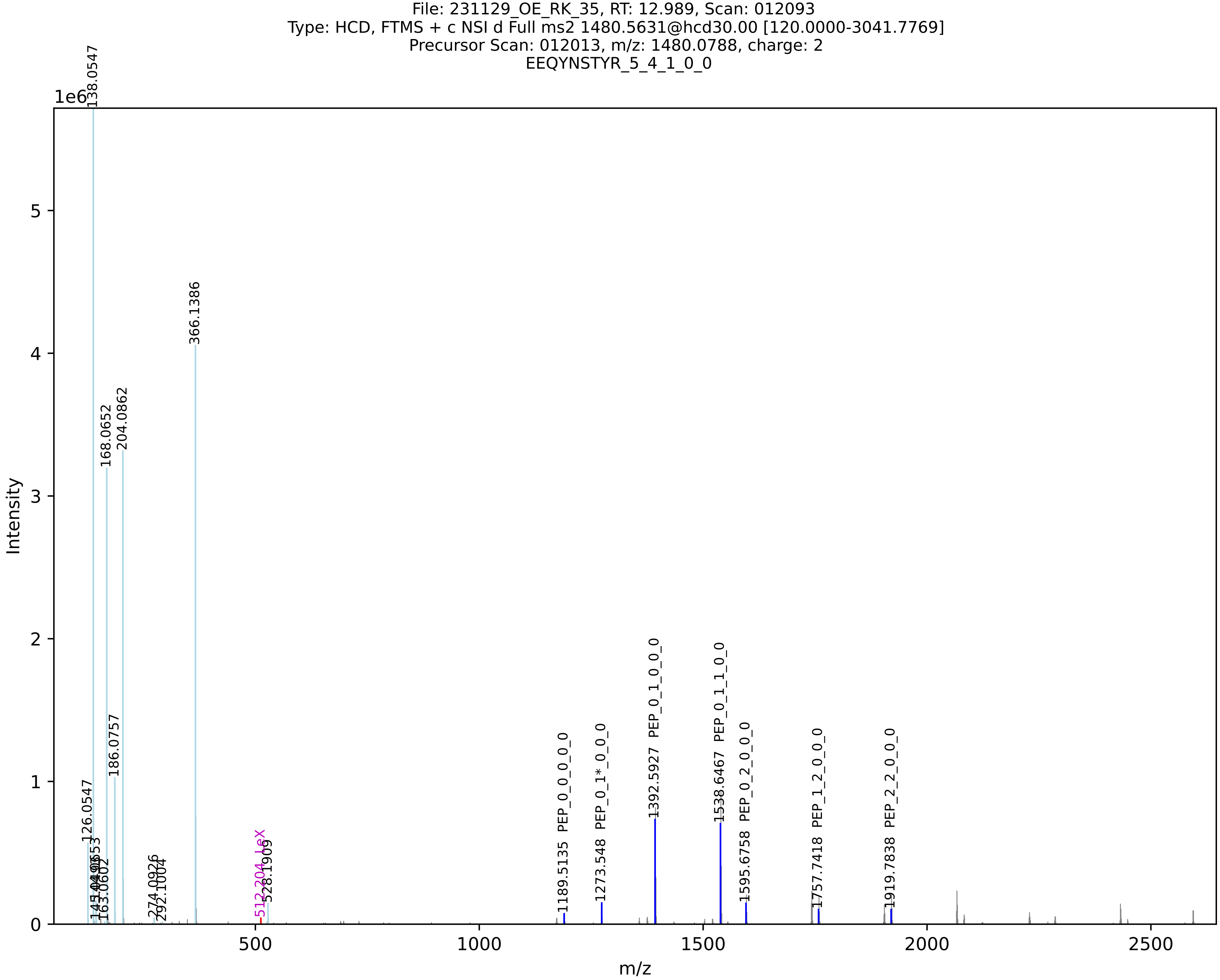

File: 231129_OE_RK_35, RT: 12.989, Scan: 012093
Type: HCD, FTMS + c NSI d Full ms2 1480.5631@hcd30.00 [120.0000-3041.7769]
Precursor Scan: 012013, m/z: 1480.0788, charge: 2
EEQYNSTYR_5_4_1_0_0
1e6
Intensity
m/z
138.0547
366.1386
168.0652
204.0862
186.0757
126.0547
163.0602
274.0926
292.1004
512.204_LeX
528.1909
1189.5135 PEP_0_0_0_0_0
1273.548 PEP_0_1*_0_0_0
1392.5927 PEP_0_1_0_0_0
1538.6467 PEP_0_1_1_0_0
1595.6758 PEP_0_2_0_0_0
1757.7418 PEP_1_2_0_0_0
1919.7838 PEP_2_2_0_0_0

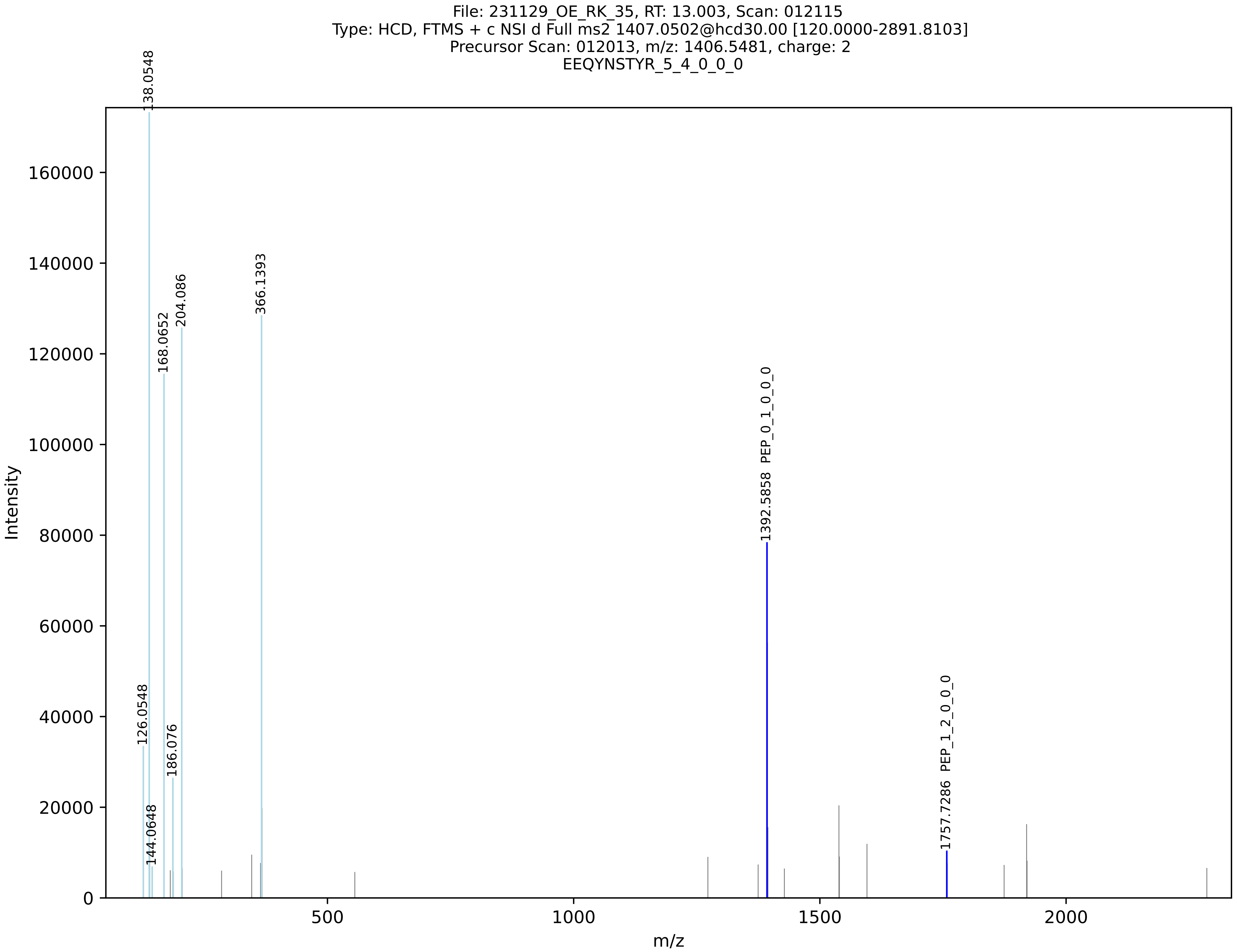
File: 231129_OE_RK_35, RT: 13.003, Scan: 012115
Type: HCD, FTMS + c NSI d Full ms2 1407.0502@hcd30.00 [120.0000-2891.8103]
Precursor Scan: 012013, m/z: 1406.5481, charge: 2
EEQYNSTYR_5_4_0_0_0
138.0548
366.1393
204.086
168.0652
1392.5858 PEP_0_1_0_0_
126.0548
186.076
144.0648
1757.7286 PEP_1_2_0_0_0
Intensity
m/z
0
20000
40000
60000
80000
100000
120000
140000
160000
500
1000
1500
2000

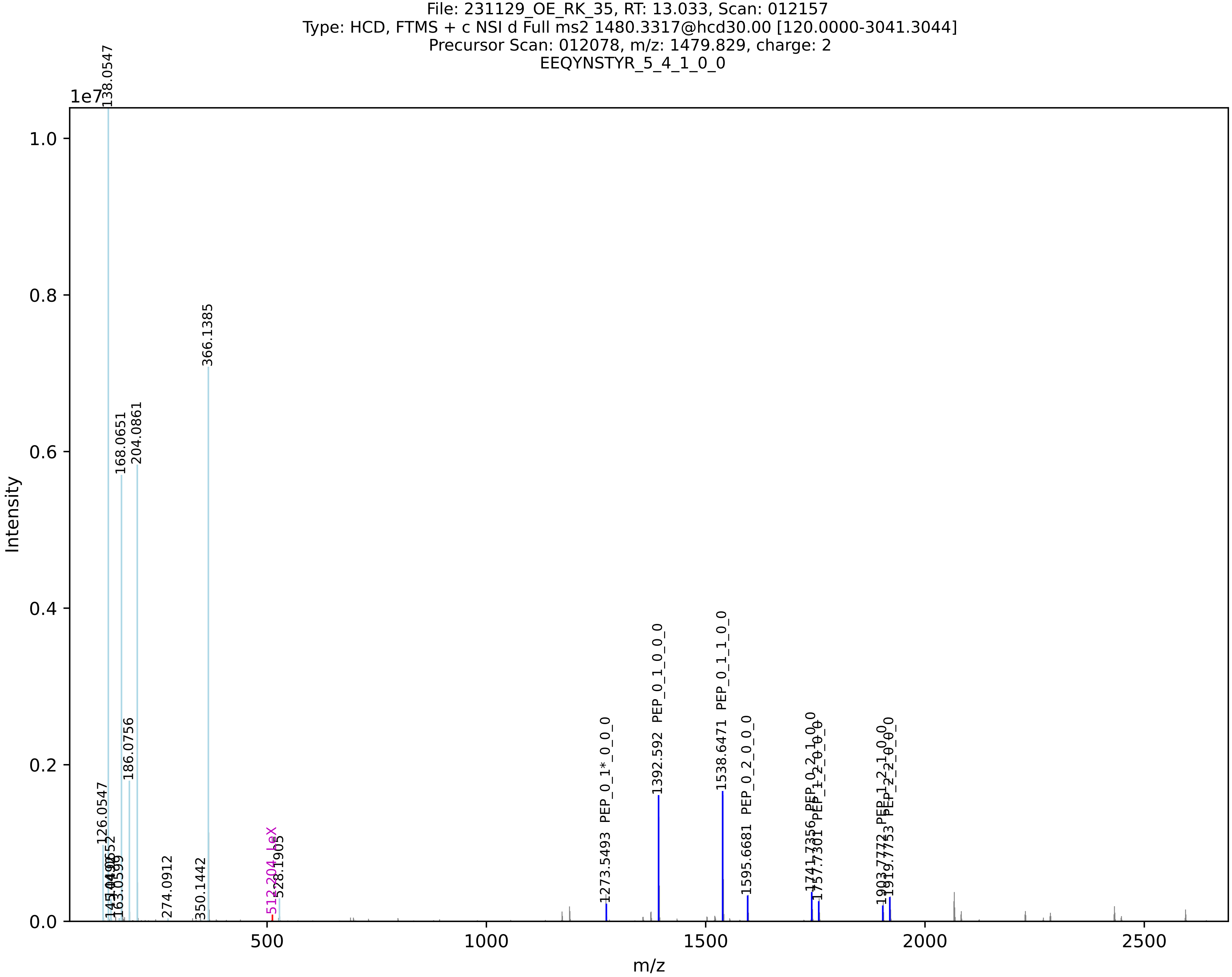

File: 231129_OE_RK_35, RT: 13.033, Scan: 012157
Type: HCD, FTMS + c NSI d Full ms2 1480.3317@hcd30.00 [120.0000-3041.3044]
Precursor Scan: 012078, m/z: 1479.829, charge: 2
EEQYNSTYR_5_4_1_0_0
1e7
Intensity
m/z
138.0547
366.1385
168.0651
204.0861
186.0756
126.0547
274.0912
350.1442
512.204 LeX
528.1905
1273.5493 PEP_0_1*_0_0_0
1392.592 PEP_0_1_0_0_0
1538.6471 PEP_0_1_1_0_0
1595.6681 PEP_0_2_0_0_0
1741.7356 PEP_0_2_1_0_0
1757.7301 PEP_1_2_0_0_0
1903.7772 PEP_1_2_1_0_0
1919.7753 PEP_2_2_0_0_0

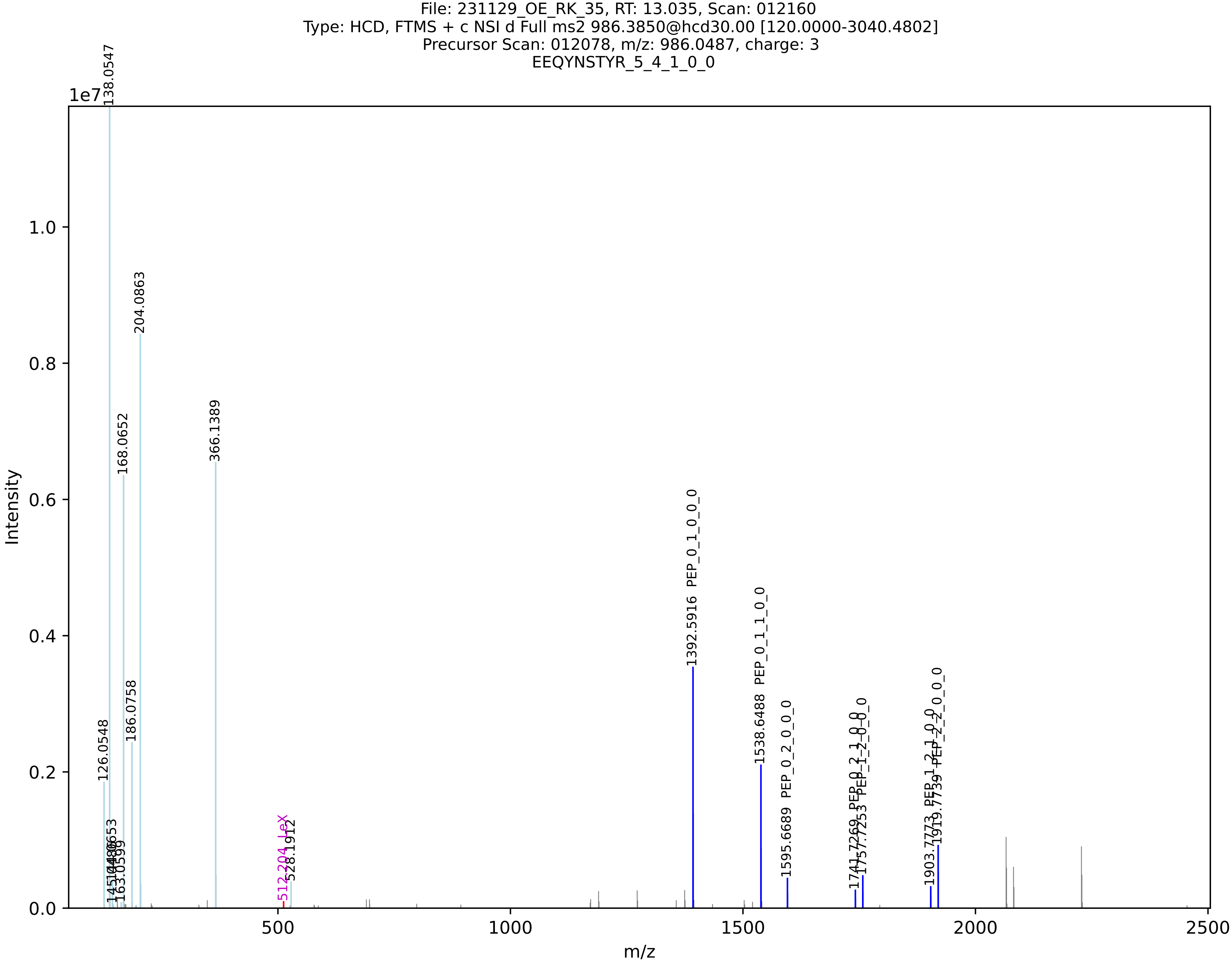

File: 231129_OE_RK_35, RT: 13.035, Scan: 012160
Type: HCD, FTMS + c NSI d Full ms2 986.3850@hcd30.00 [120.0000-3040.4802]
Precursor Scan: 012078, m/z: 986.0487, charge: 3
EEQYNSTYR_5_4_1_0_0
1e7
Intensity
m/z
138.0547
204.0863
168.0652
366.1389
186.0758
126.0548
163.0599
512.204 LeX
528.1912
1392.5916 PEP_0_1_0_0_0
1538.6488 PEP_0_1_1_0_0
1595.6689 PEP_0_2_0_0_0
1741.7269 PEP_0_2_1_0_0
1757.7253 PEP_1_2_0_0_0
1903.7773 PEP_1_2_1_0_0
1919.7739 PEP_2_2_0_0_0

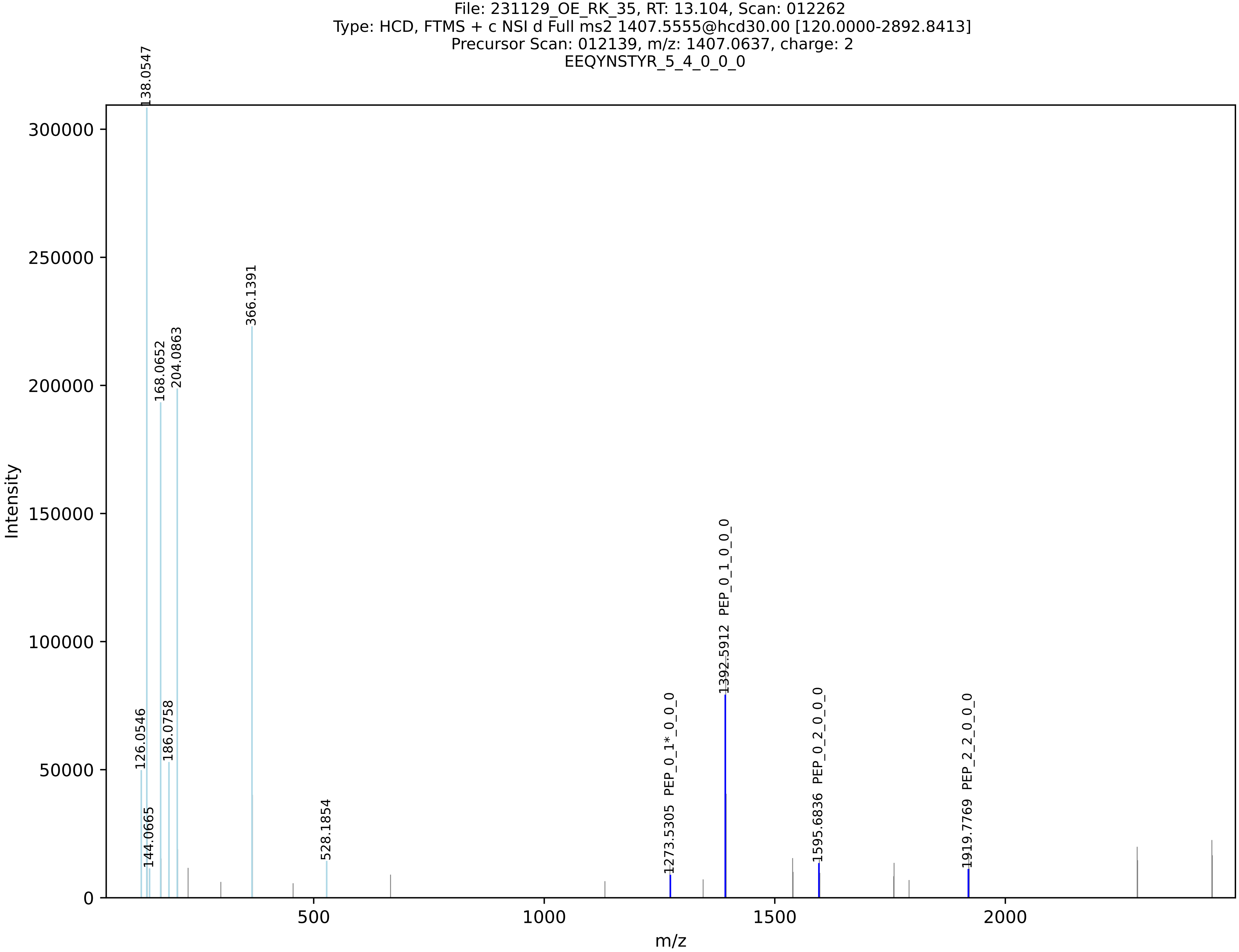
File: 231129_OE_RK_35, RT: 13.104, Scan: 012262
Type: HCD, FTMS + c NSI d Full ms2 1407.5555@hcd30.00 [120.0000-2892.8413]
Precursor Scan: 012139, m/z: 1407.0637, charge: 2
EEQYNSTYR_5_4_0_0_0
Intensity
m/z
138.0547
366.1391
168.0652
204.0863
126.0546
186.0758
144.0665
528.1854
1273.5305 PEP_0_1*_0_0_0
1392.5912 PEP_0_1_0_0_0
1595.6836 PEP_0_2_0_0_0
1919.7769 PEP_2_2_0_0_0
300000
250000
200000
150000
100000
50000
0
500
1000
1500
2000

File: 231129_OE_RK_35, RT: 13.121, Scan: 012282
Type: HCD, FTMS + c NSI d Full ms2 987.0549@hcd30.00 [120.0000-3042.5300]
Precursor Scan: 012210, m/z: 986.7208, charge: 3
EEQYNSTYR_5_4_1_0_0

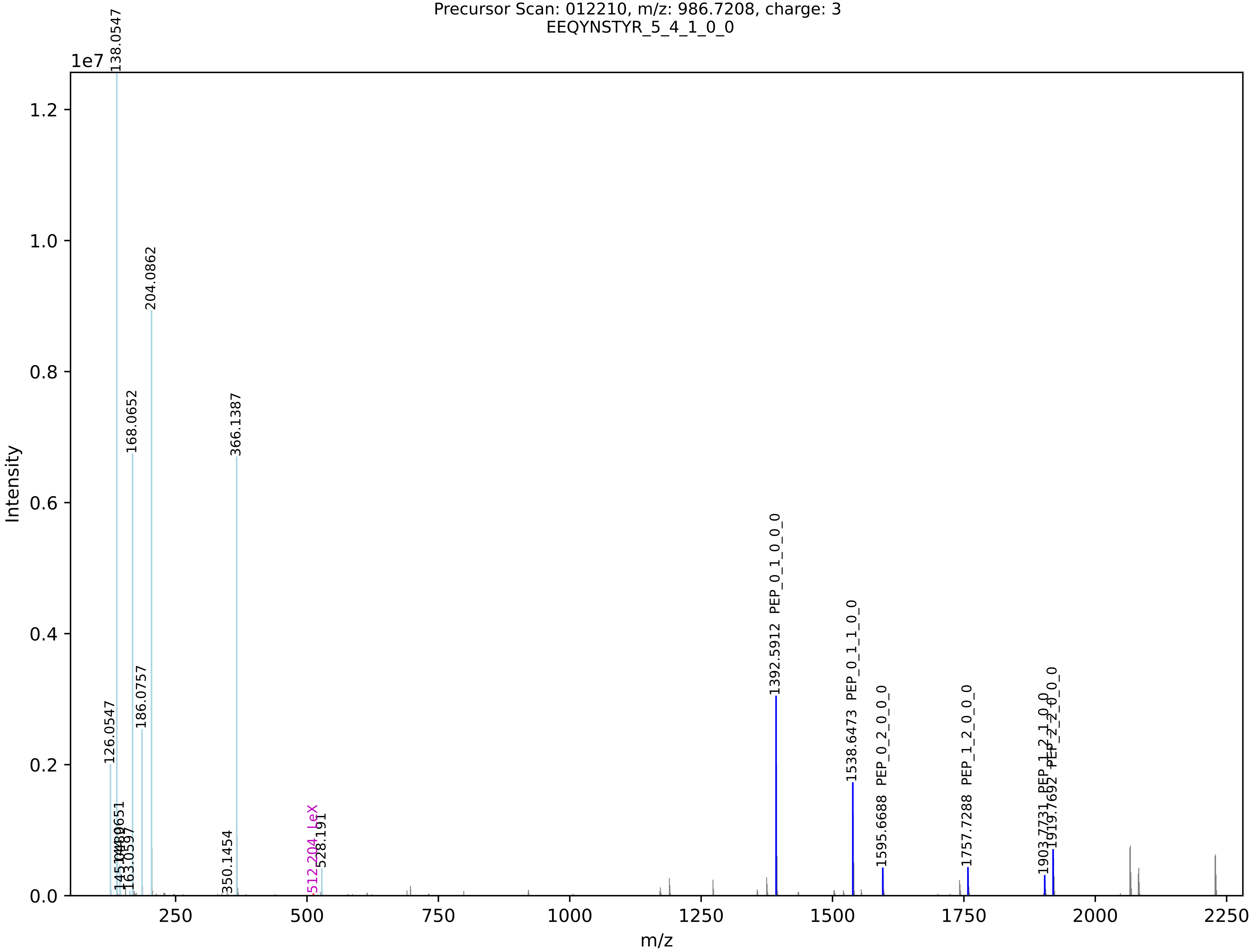

File: 231129_OE_RK_35, RT: 13.123, Scan: 012286
Type: HCD, FTMS + c NSI d Full ms2 1398.8838@hcd30.00 [120.0000-2875.1511]
Precursor Scan: 012210, m/z: 1398.8838, charge: 2
EEQYNSTYR_4_4_1_0_0

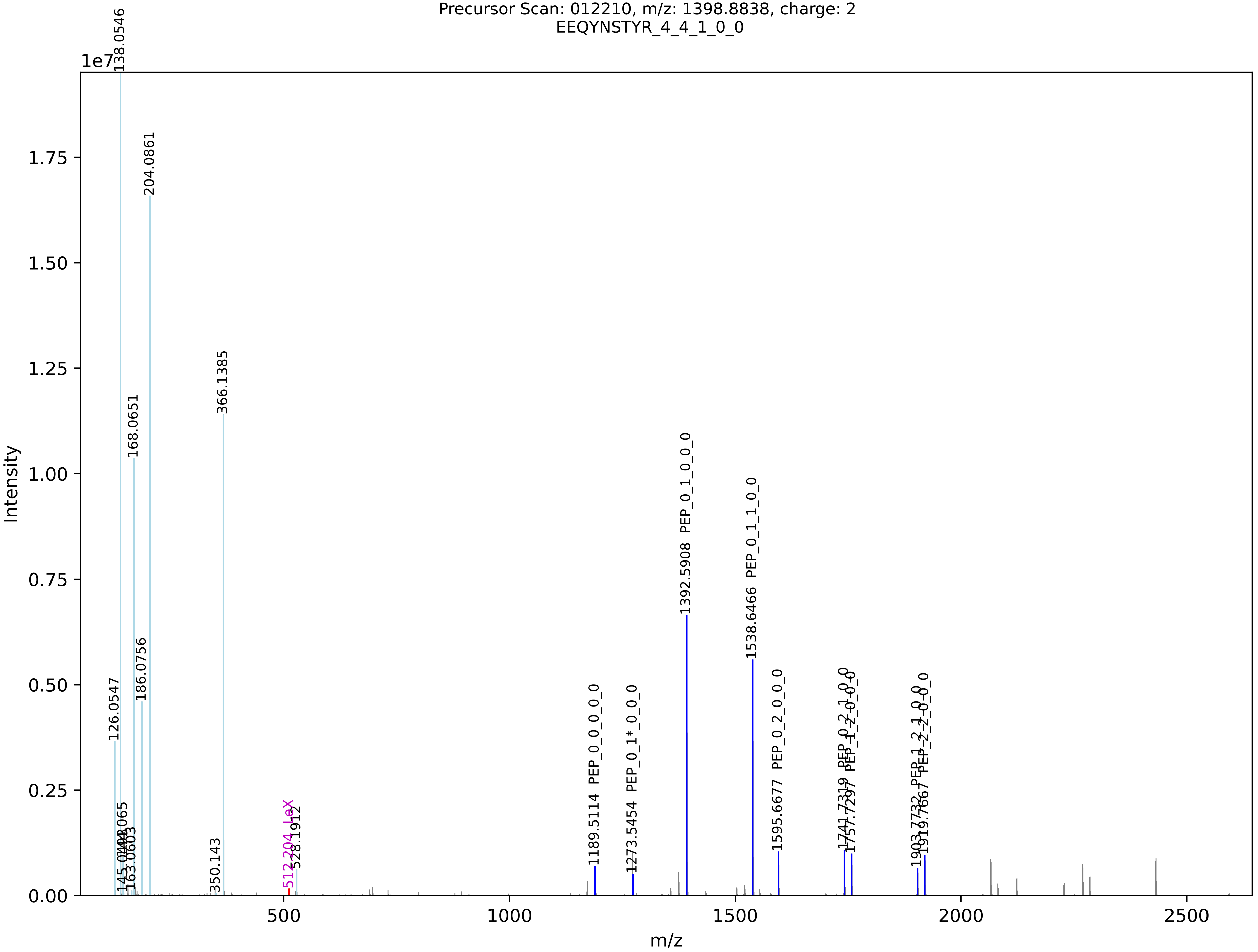

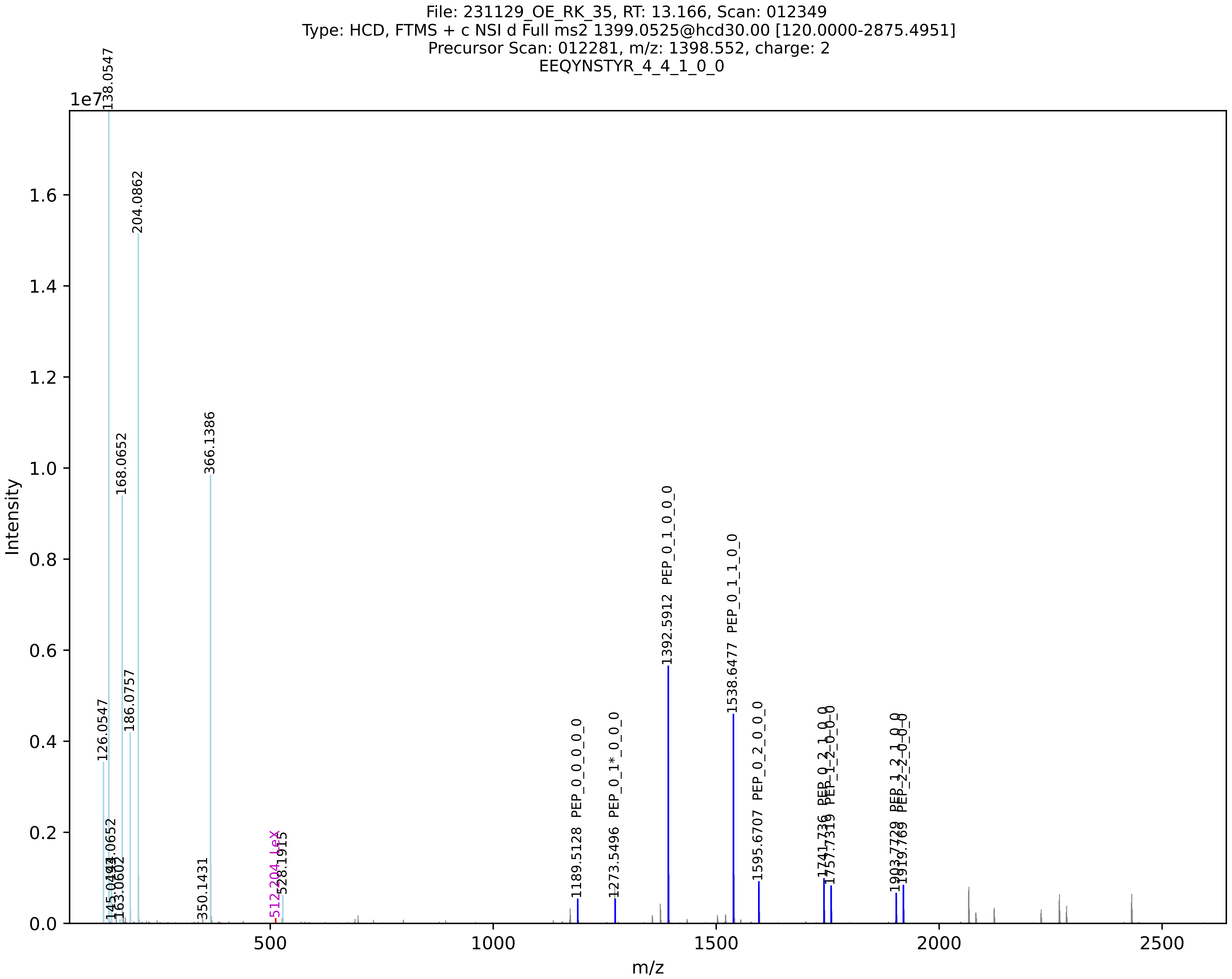
File: 231129_OE_RK_35, RT: 13.166, Scan: 012349
Type: HCD, FTMS + c NSI d Full ms2 1399.0525@hcd30.00 [120.0000-2875.4951]
Precursor Scan: 012281, m/z: 1398.552, charge: 2
EEQYNSTYR_4_4_1_0_0
1e7
Intensity
m/z
138.0547
204.0862
366.1386
168.0652
186.0757
126.0547
350.1431
512.204_LeX
528.1915
1189.5128 PEP_0_0_0_0_0
1273.5496 PEP_0_1*_0_0_0
1392.5912 PEP_0_1_0_0_0
1538.6477 PEP_0_1_1_0_0
1595.6707 PEP_0_2_0_0_0
1919.769 PEP_2_2_0_0_0

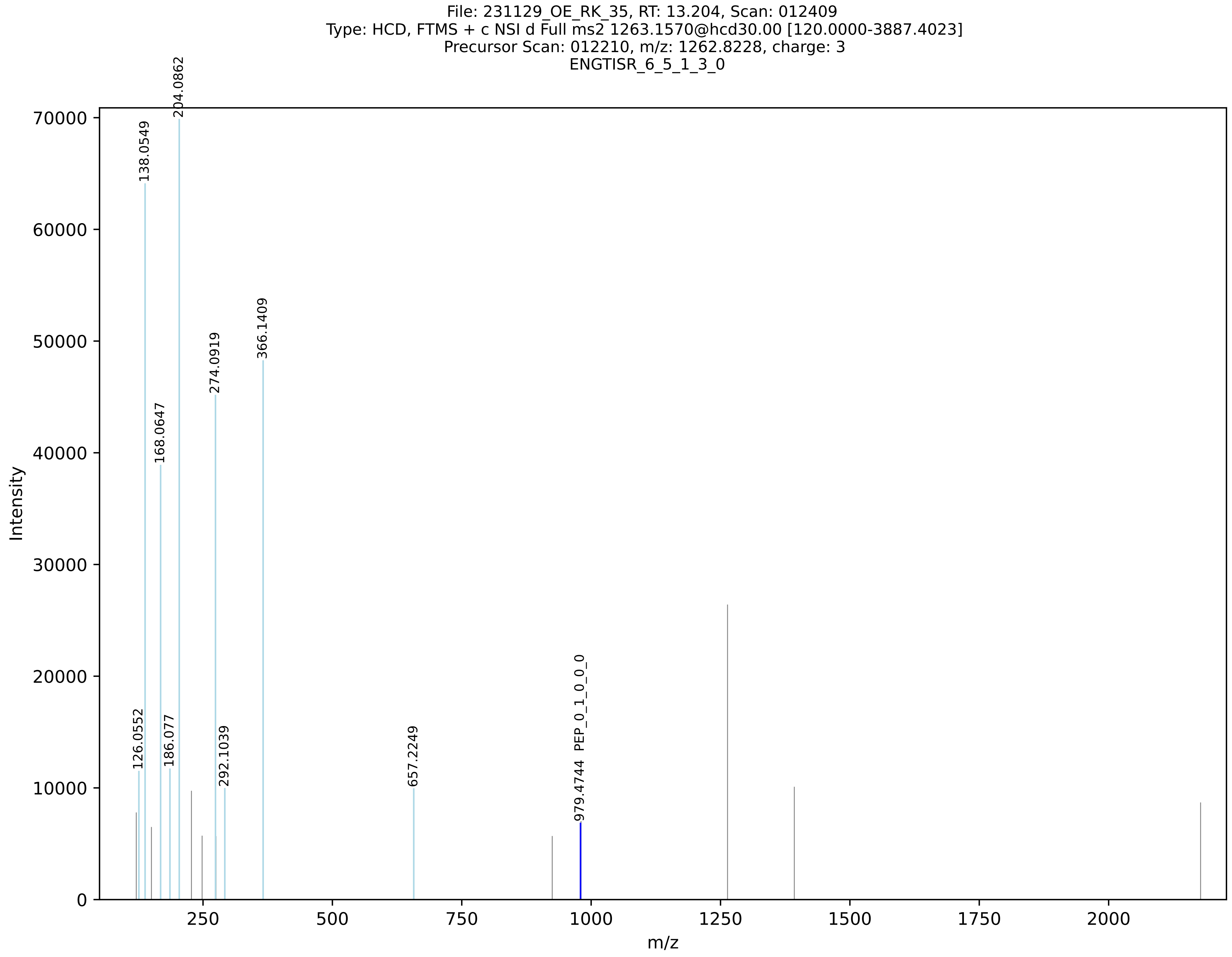

File: 231129_OE_RK_35, RT: 13.204, Scan: 012409
Type: HCD, FTMS + c NSI d Full ms2 1263.1570@hcd30.00 [120.0000-3887.4023]
Precursor Scan: 012210, m/z: 1262.8228, charge: 3
ENGTISR_6_5_1_3_0
Intensity
m/z
204.0862
138.0549
366.1409
274.0919
168.0647
126.0552
186.077
292.1039
657.2249
979.4744 PEP_0_1_0_0_0

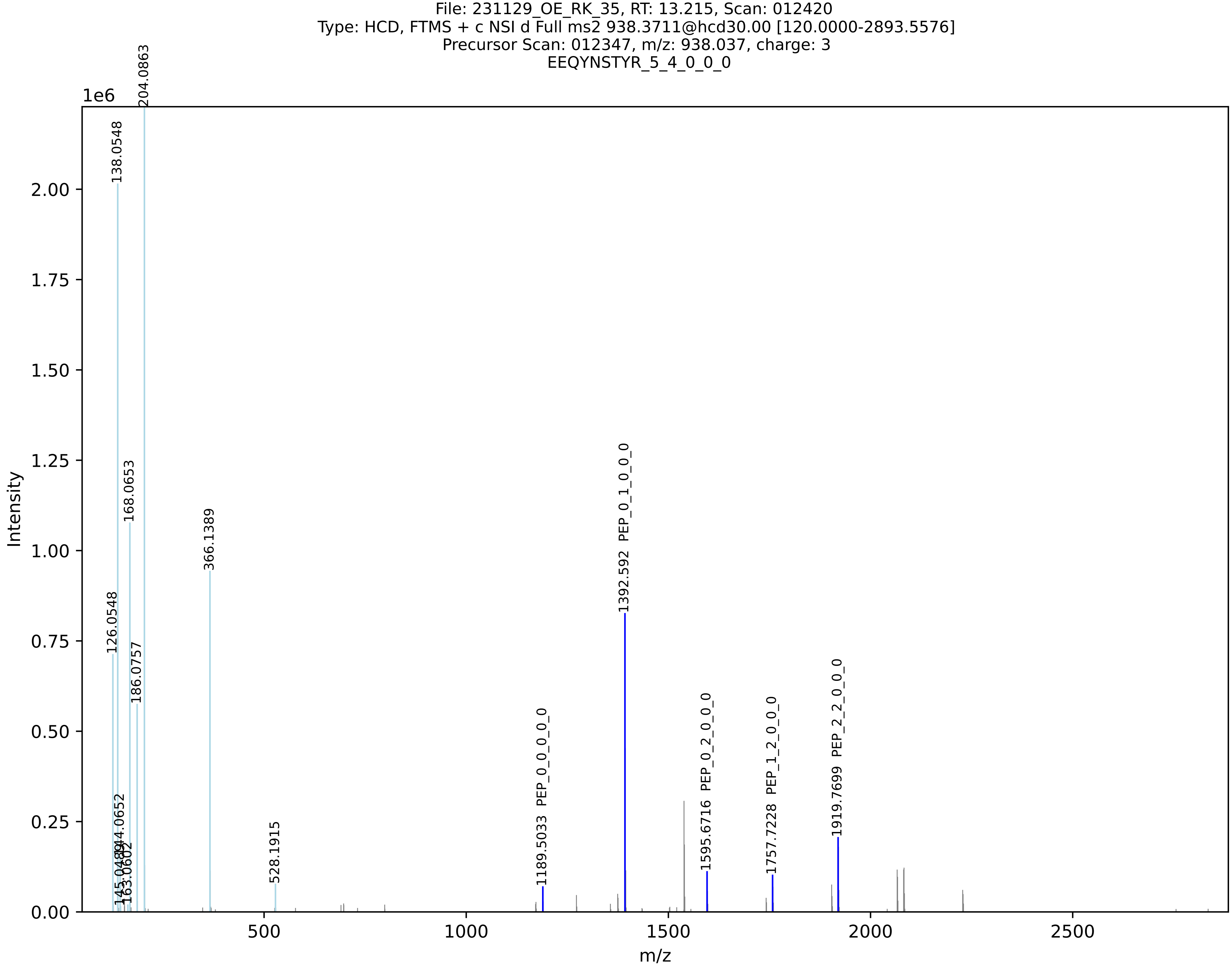

File: 231129_OE_RK_35, RT: 13.215, Scan: 012420
Type: HCD, FTMS + c NSI d Full ms2 938.3711@hcd30.00 [120.0000-2893.5576]
Precursor Scan: 012347, m/z: 938.037, charge: 3
EEQYNSTYR_5_4_0_0_0
1e6
Intensity
m/z
204.0863
138.0548
168.0653
366.1389
126.0548
186.0757
144.0652
145.0489
163.0602
528.1915
1189.5033 PEP_0_0_0_0_0
1392.592 PEP_0_1_0_0_0
1595.6716 PEP_0_2_0_0_0
1757.7228 PEP_1_2_0_0_0
1919.7699 PEP_2_2_0_0_0

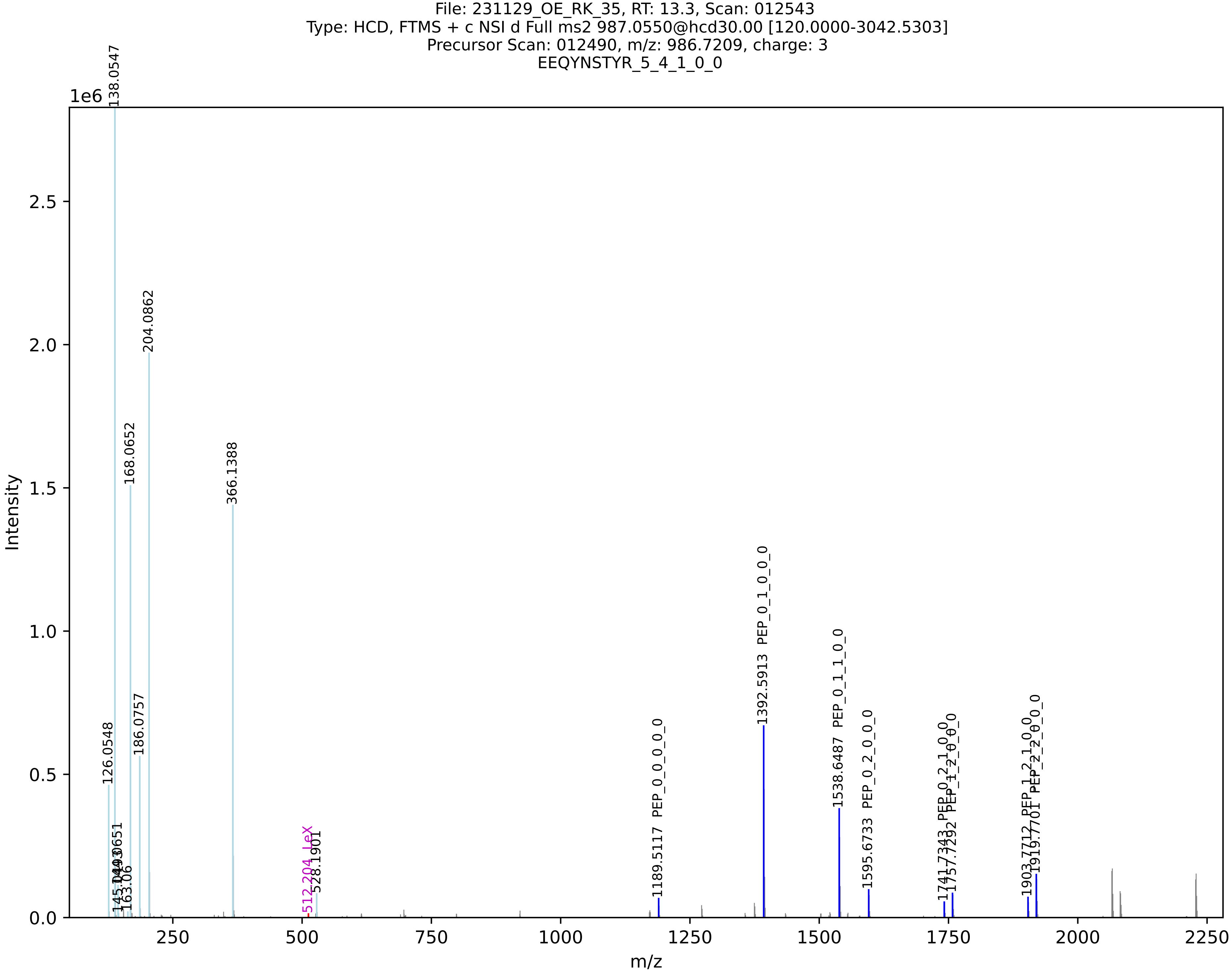
File: 231129_OE_RK_35, RT: 13.3, Scan: 012543
Type: HCD, FTMS + c NSI d Full ms2 987.0550@hcd30.00 [120.0000-3042.5303]
Precursor Scan: 012490, m/z: 986.7209, charge: 3
EEQYNSTYR_5_4_1_0_0
1e6
Intensity
m/z
0.0
0.5
1.0
1.5
2.0
2.5
250
500
750
1000
1250
1500
1750
2000
2250
138.0547
204.0862
168.0652
366.1388
186.0757
126.0548
163.06
512.204_LeX
528.1901
1189.5117 PEP_0_0_0_0_0_
1392.5913 PEP_0_1_0_0_0_
1538.6487 PEP_0_1_1_0_0_
1595.6733 PEP_0_2_0_0_0_
1741.7343 PEP_0_2_1_0_0_
1757.7292 PEP_1_2_0_0_0_
1903.7712 PEP_1_2_1_0_0_
1919.7701 PEP_2_2_0_0_0_

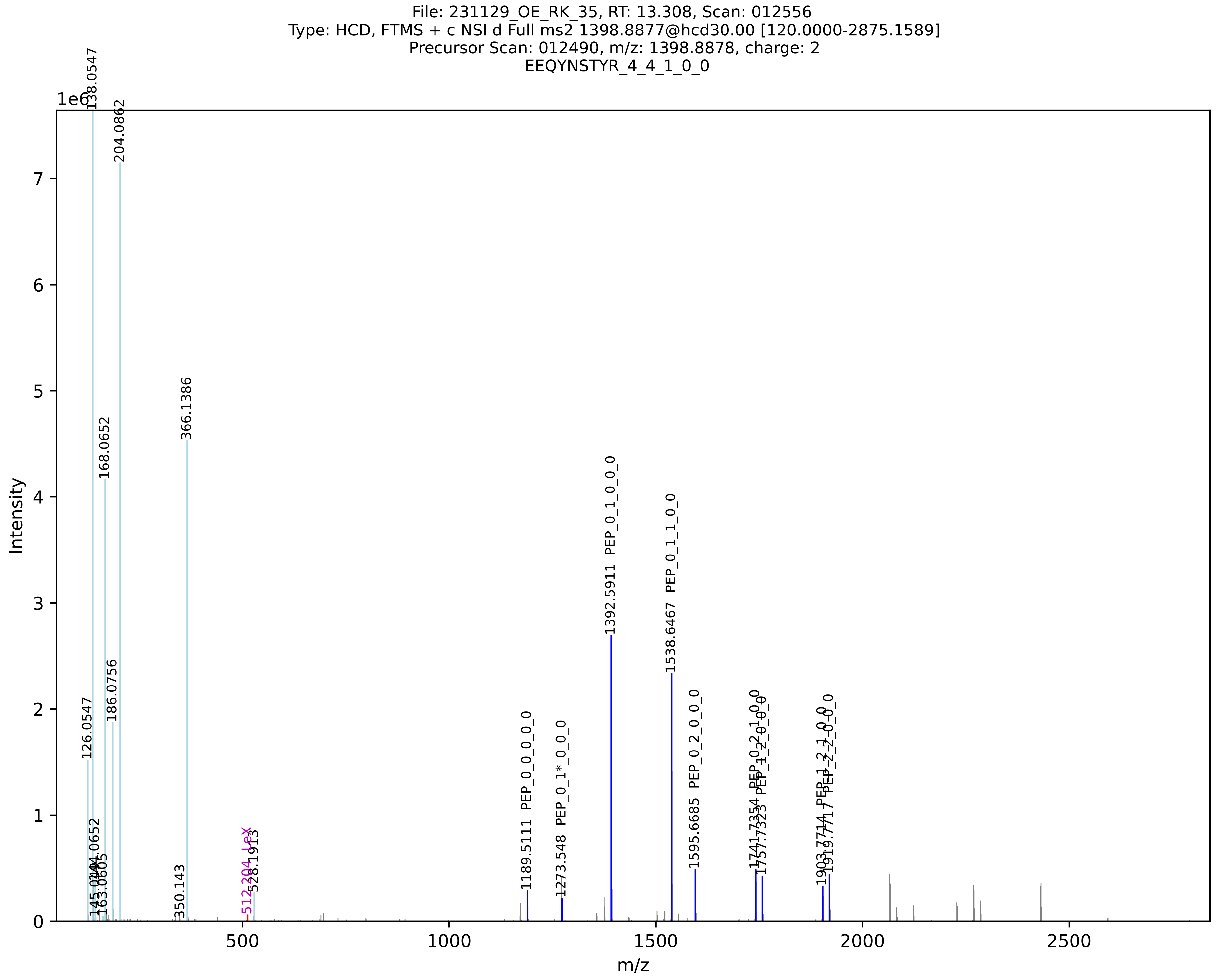
File: 231129_OE_RK_35, RT: 13.308, Scan: 012556
Type: HCD, FTMS + c NSI d Full ms2 1398.8877@hcd30.00 [120.0000-2875.1589]
Precursor Scan: 012490, m/z: 1398.8878, charge: 2
EEQYNSTYR_4_4_1_0_0
1e6
Intensity
m/z
138.0547
204.0862
366.1386
168.0652
186.0756
126.0547
350.143
528.1913
1189.5111 PEP_0_0_0_0_0
1273.548 PEP_0_1*_0_0_0
1392.5911 PEP_0_1_0_0_0
1538.6467 PEP_0_1_1_0_0
1595.6685 PEP_0_2_0_0_0
0
1
2
3
4
5
6
7
500
1000
1500
2000
2500

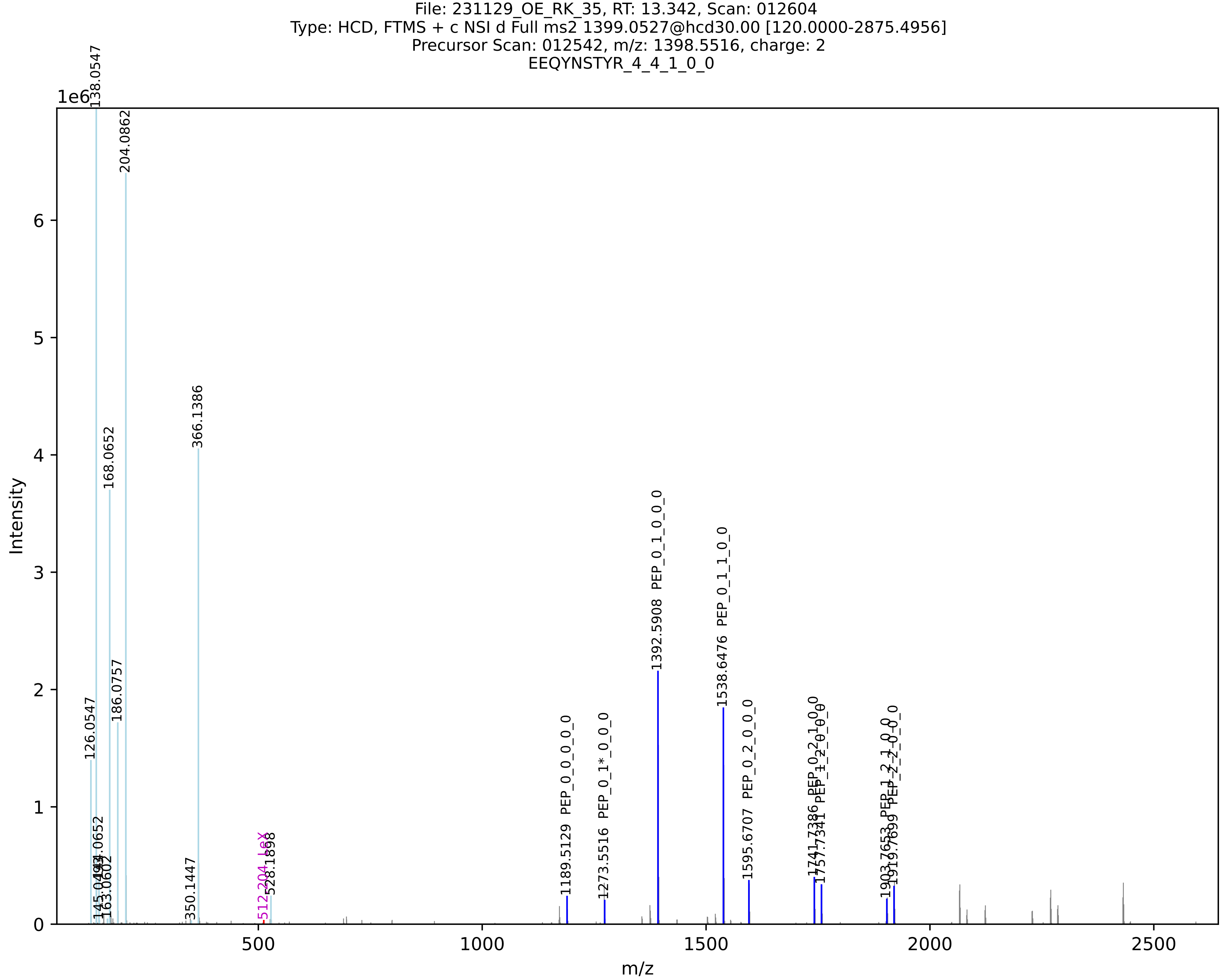
File: 231129_OE_RK_35, RT: 13.342, Scan: 012604
Type: HCD, FTMS + c NSI d Full ms2 1399.0527@hcd30.00 [120.0000-2875.4956]
Precursor Scan: 012542, m/z: 1398.5516, charge: 2
EEQYNSTYR_4_4_1_0_0
1e6
Intensity
m/z
138.0547
204.0862
366.1386
168.0652
186.0757
126.0547
350.1447
512.204_LeX
528.1898
1189.5129 PEP_0_0_0_0_0
1273.5516 PEP_0_1*_0_0_0
1392.5908 PEP_0_1_0_0_0
1538.6476 PEP_0_1_1_0_0
1595.6707 PEP_0_2_0_0_0
1903.7653 PEP_1_2_1_0_0
1919.7699 PEP_2_2_0_0_0

File: 231129_OE_RK_35, RT: 13.354, Scan: 012623
Type: HCD, FTMS + c NSI d Full ms2 1383.8696@hcd30.00 [120.0000-4256.7832]
Precursor Scan: 012542, m/z: 1383.8696, charge: 3
EEQYNSTYR_5_7_1_2_0

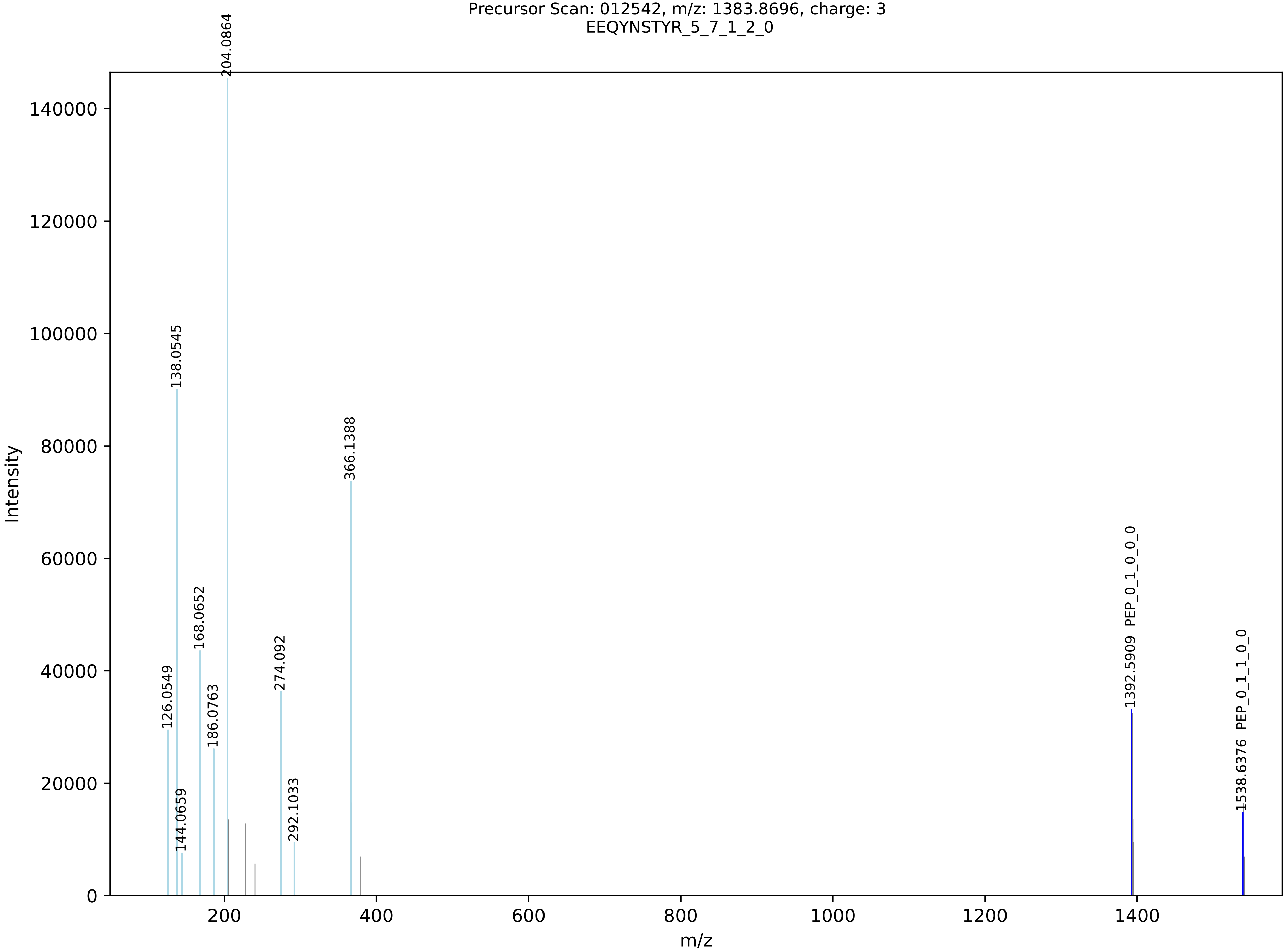

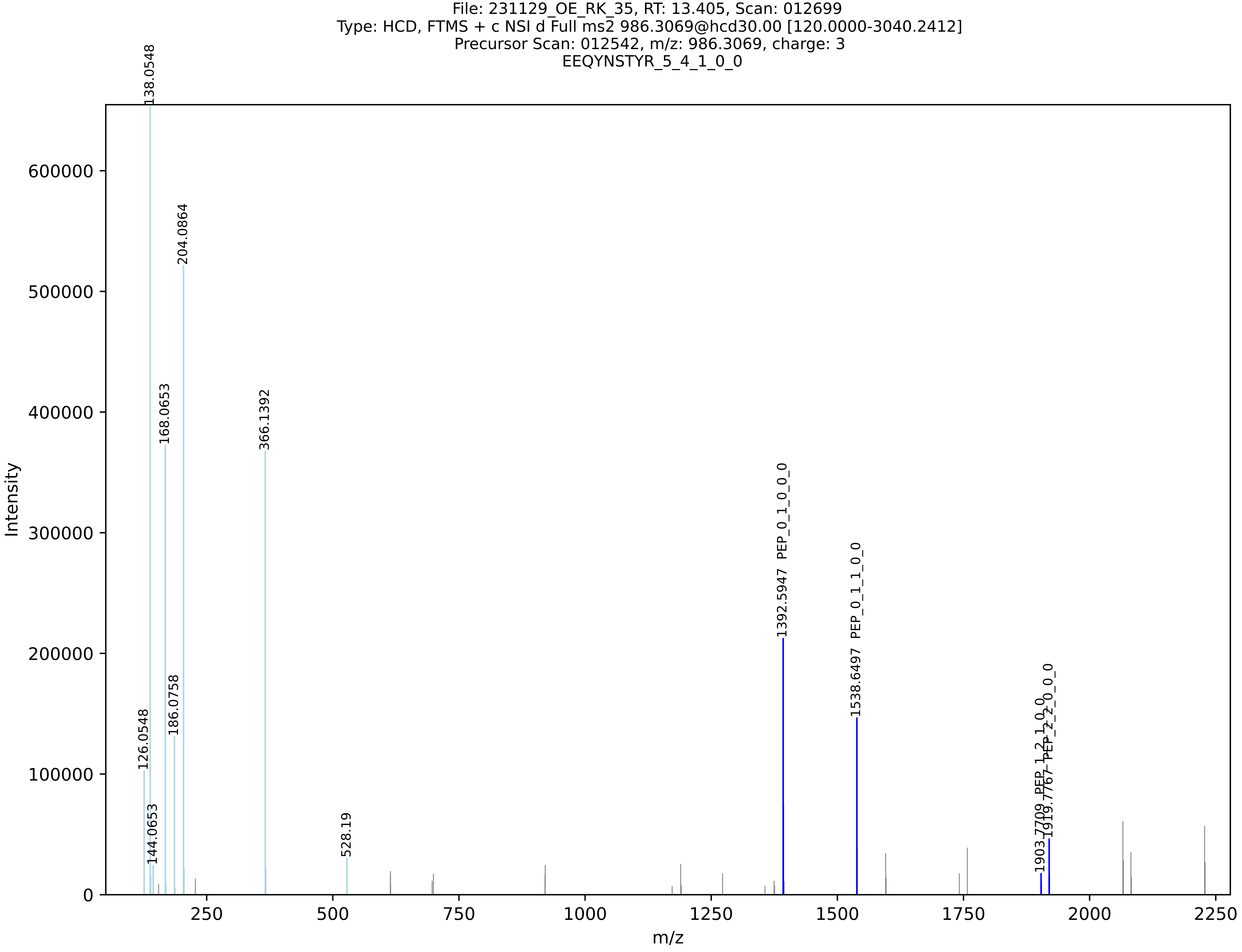

File: 231129_OE_RK_35, RT: 13.405, Scan: 012699
Type: HCD, FTMS + c NSI d Full ms2 986.3069@hcd30.00 [120.0000-3040.2412]
Precursor Scan: 012542, m/z: 986.3069, charge: 3
EEQYNSTYR_5_4_1_0_0
Intensity
m/z
138.0548
204.0864
168.0653
366.1392
186.0758
126.0548
144.0653
528.19
1392.5947 PEP_0_1_0_0_0
1538.6497 PEP_0_1_1_0_0
1903.7709 PEP_1_2_1_0_0
1919.7767 PEP_2_2_0_0_0

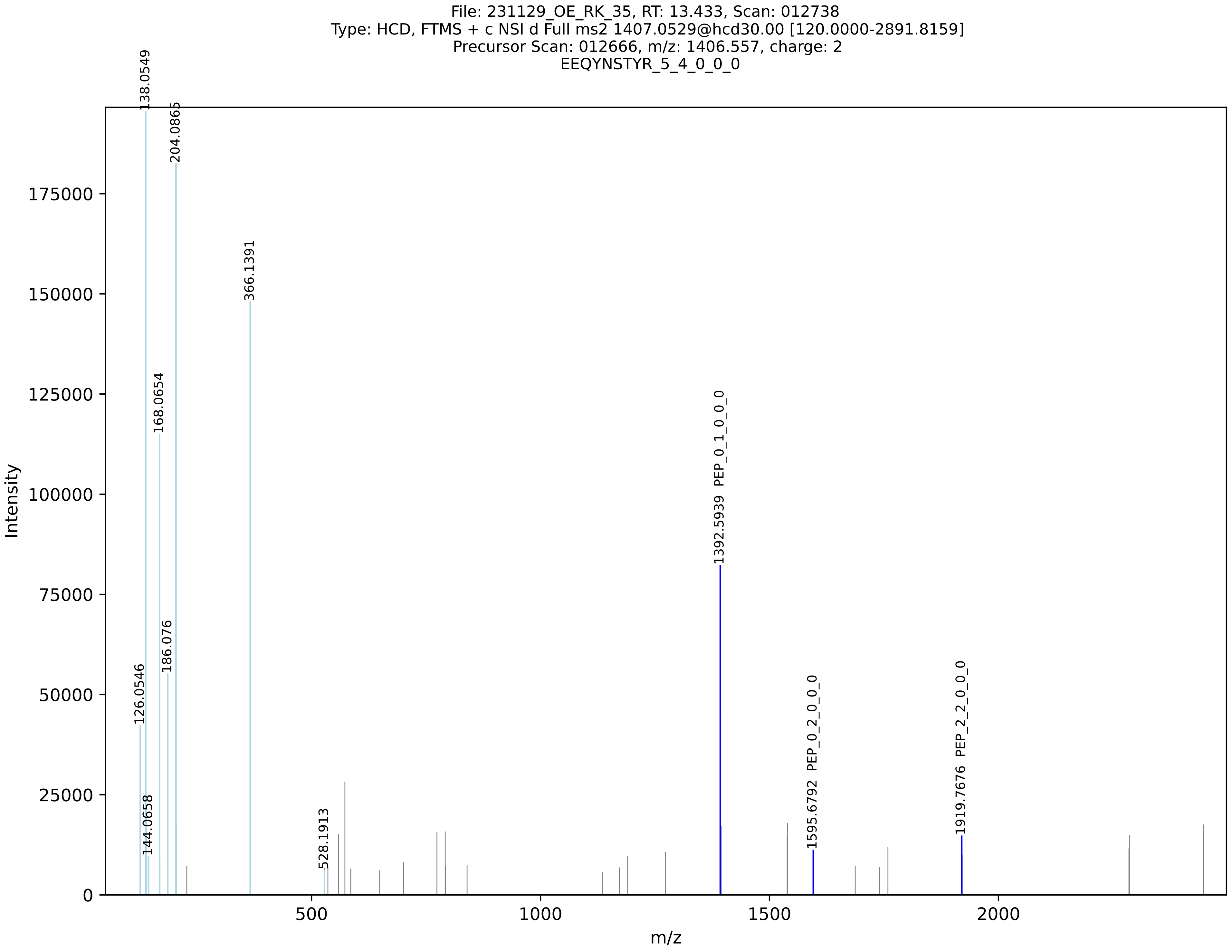
File: 231129_OE_RK_35, RT: 13.433, Scan: 012738
Type: HCD, FTMS + c NSI d Full ms2 1407.0529@hcd30.00 [120.0000-2891.8159]
Precursor Scan: 012666, m/z: 1406.557, charge: 2
EEQYNSTYR_5_4_0_0_0
Intensity
m/z
138.0549
204.0865
366.1391
168.0654
186.076
126.0546
144.0658
528.1913
1392.5939 PEP_0_1_0_0_0
1595.6792 PEP_0_2_0_0_0
1919.7676 PEP_2_2_0_0_0
175000
150000
125000
100000
75000
50000
25000
0
500
1000
1500
2000

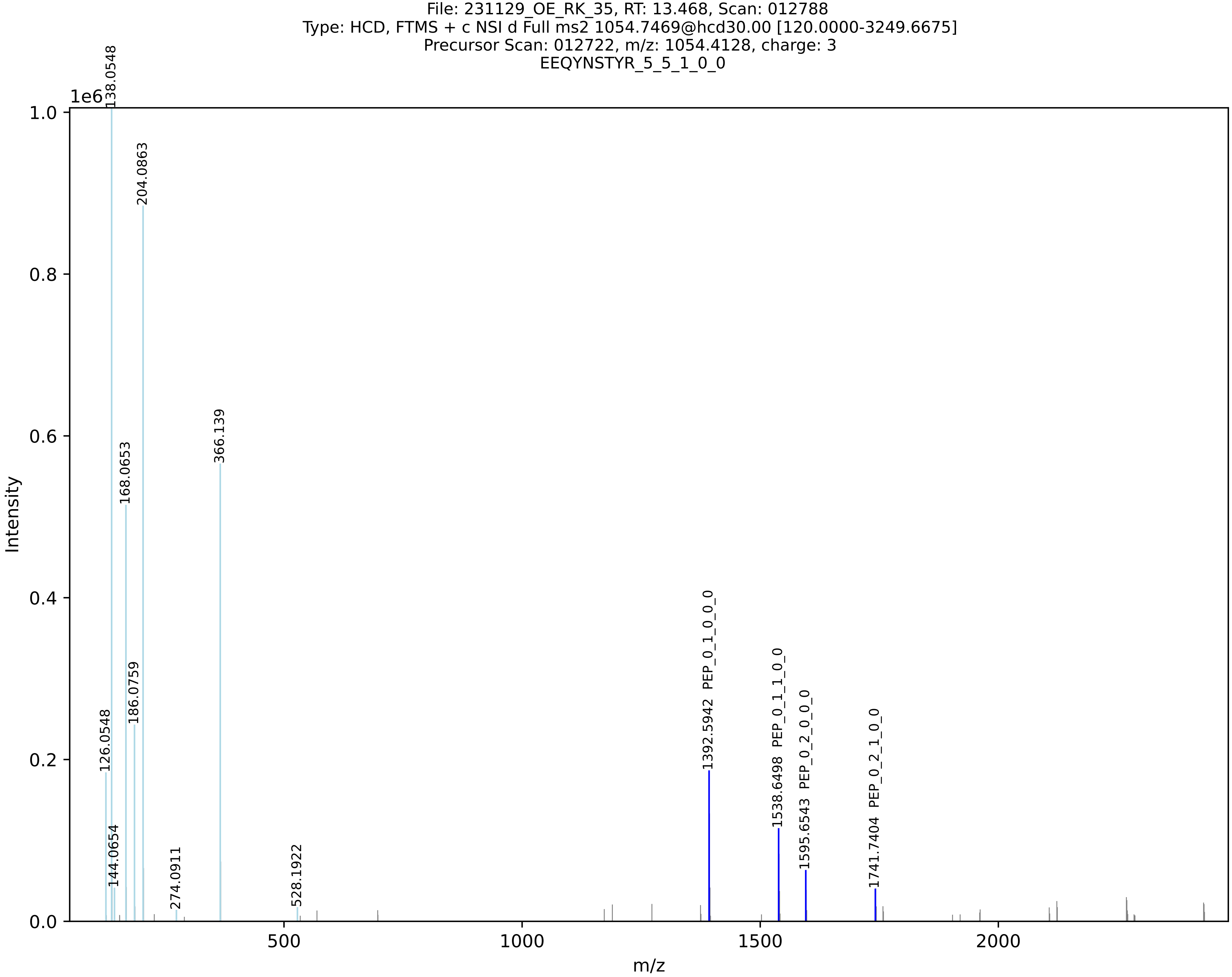

File: 231129_OE_RK_35, RT: 13.468, Scan: 012788
Type: HCD, FTMS + c NSI d Full ms2 1054.7469@hcd30.00 [120.0000-3249.6675]
Precursor Scan: 012722, m/z: 1054.4128, charge: 3
EEQYNSTYR_5_5_1_0_0
1e6
Intensity
m/z
138.0548
204.0863
366.139
168.0653
186.0759
126.0548
144.0654
274.0911
528.1922
1392.5942 PEP_0_1_0_0_0
1538.6498 PEP_0_1_1_0_0
1595.6543 PEP_0_2_0_0_0
1741.7404 PEP_0_2_1_0_0

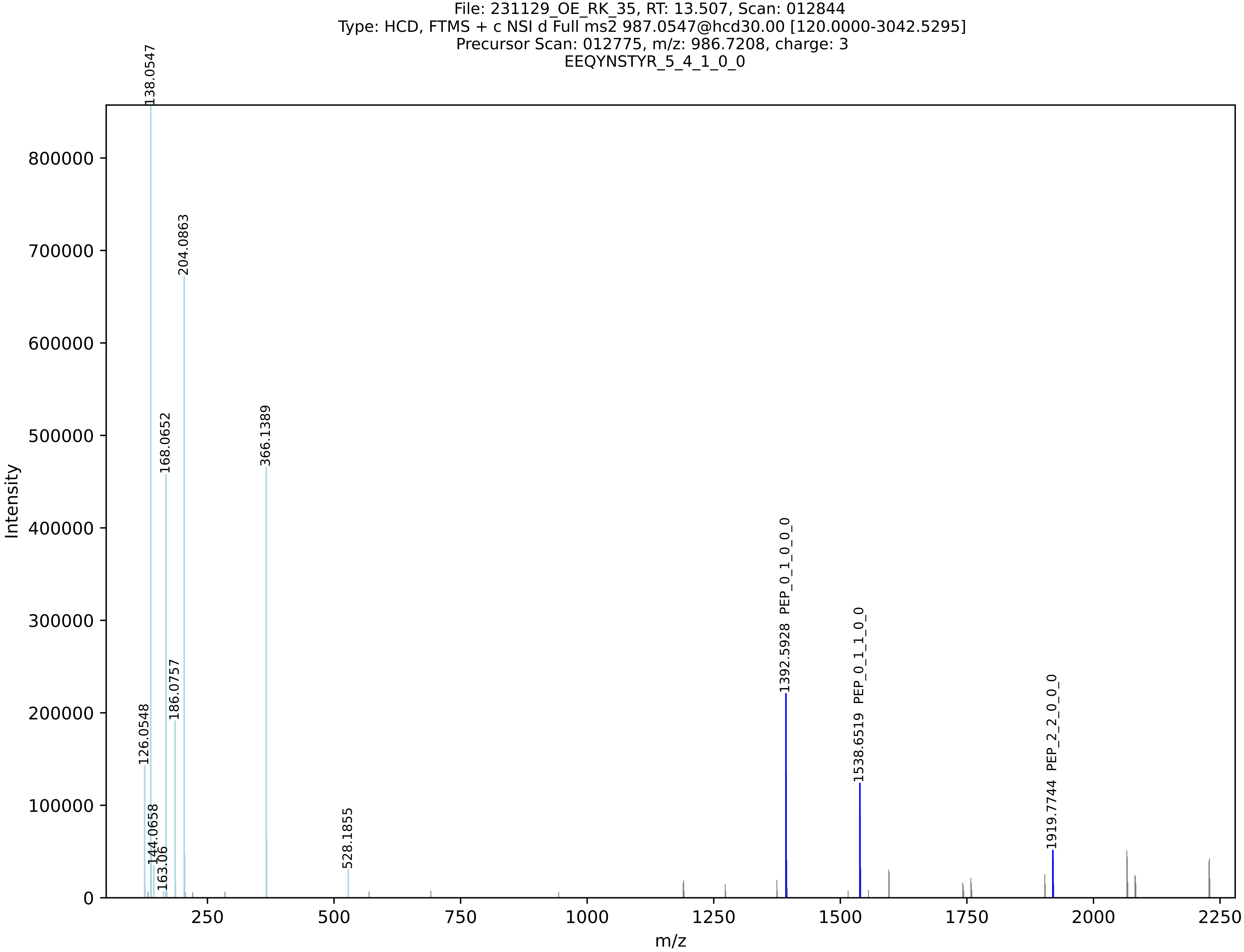

File: 231129_OE_RK_35, RT: 13.507, Scan: 012844
Type: HCD, FTMS + c NSI d Full ms2 987.0547@hcd30.00 [120.0000-3042.5295]
Precursor Scan: 012775, m/z: 986.7208, charge: 3
EEQYNSTYR_5_4_1_0_0
138.0547
204.0863
168.0652
366.1389
186.0757
126.0548
144.0658
163.06
528.1855
1392.5928 PEP_0_1_0_0_0
1538.6519 PEP_0_1_1_0_0
1919.7744 PEP_2_2_0_0_0
Intensity
m/z
0
100000
200000
300000
400000
500000
600000
700000
800000
250
500
750
1000
1250
1500
1750
2000
2250

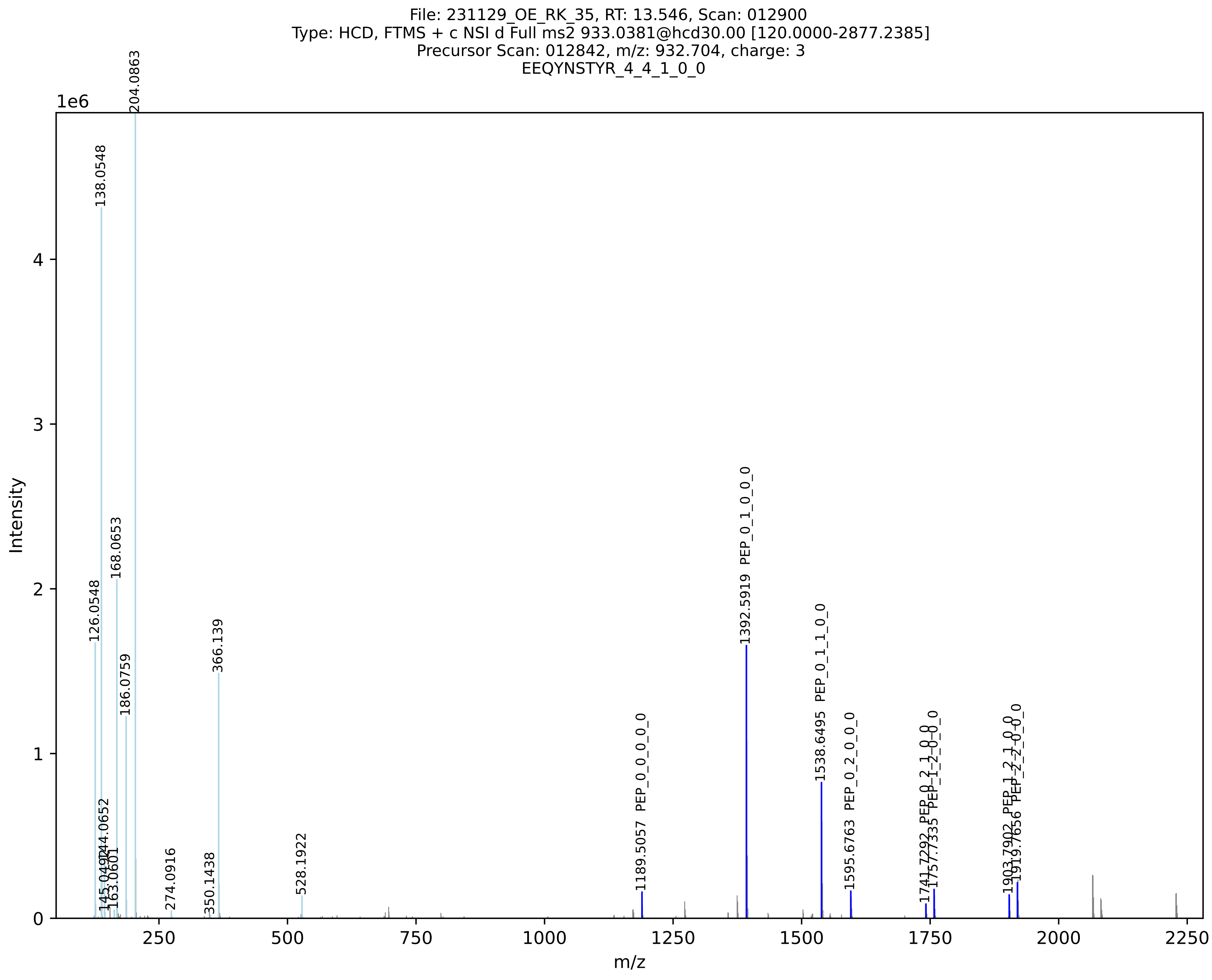

File: 231129_OE_RK_35, RT: 13.546, Scan: 012900
Type: HCD, FTMS + c NSI d Full ms2 933.0381@hcd30.00 [120.0000-2877.2385]
Precursor Scan: 012842, m/z: 932.704, charge: 3
EEQYNSTYR_4_4_1_0_0
1e6
Intensity
m/z
204.0863
138.0548
168.0653
126.0548
186.0759
366.139
144.0652
145.0491
163.0601
274.0916
350.1438
528.1922
1189.5057 PEP_0_0_0_0_0
1392.5919 PEP_0_1_0_0_0
1538.6495 PEP_0_1_1_0_0
1595.6763 PEP_0_2_0_0_0
1741.7292 PEP_0_2_1_0_0
1757.7335 PEP_1_2_0_0_0
1903.7902 PEP_1_2_1_0_0
1919.7656 PEP_2_2_0_0_0

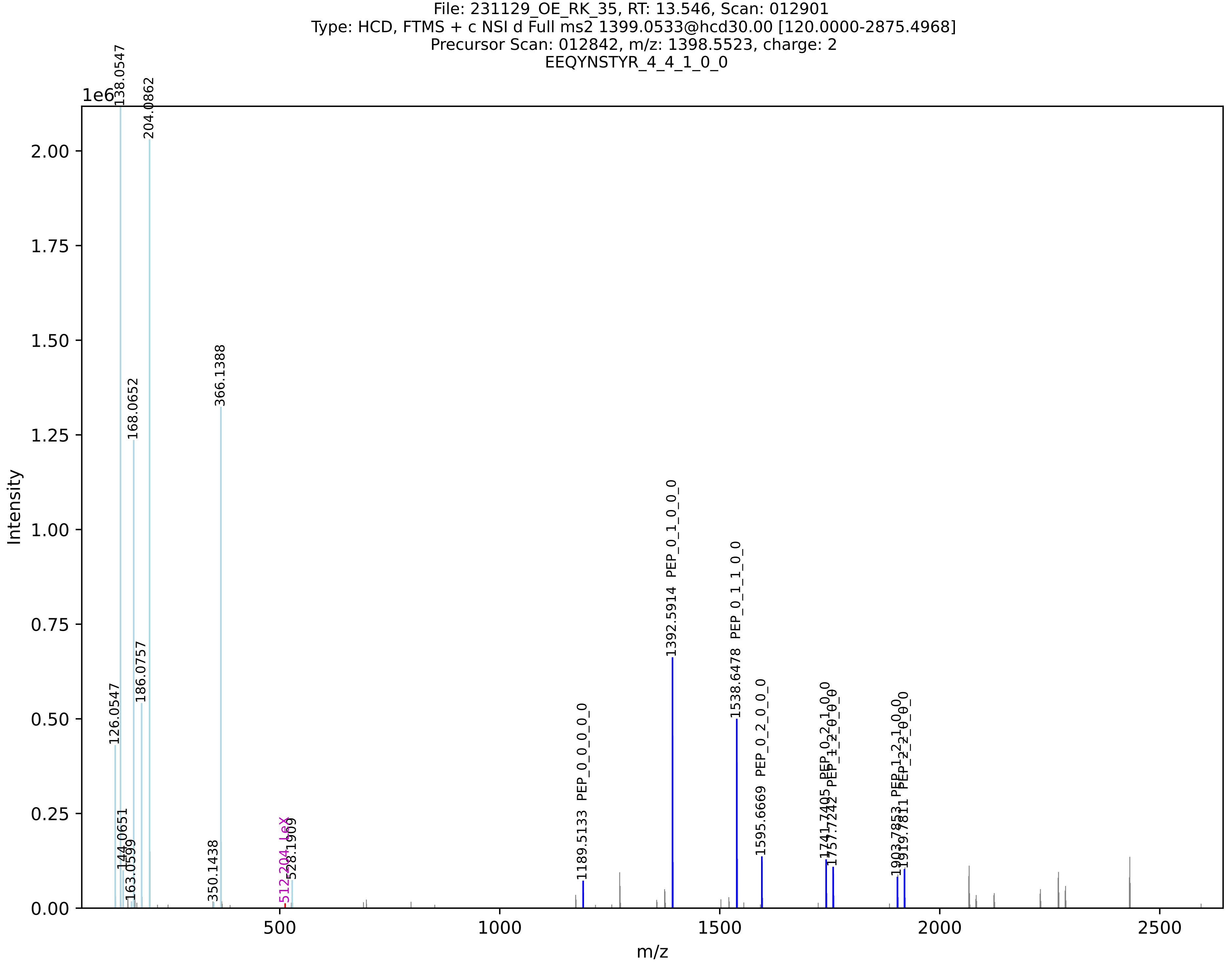

File: 231129_OE_RK_35, RT: 13.546, Scan: 012901
Type: HCD, FTMS + c NSI d Full ms2 1399.0533@hcd30.00 [120.0000-2875.4968]
Precursor Scan: 012842, m/z: 1398.5523, charge: 2
EEQYNSTYR_4_4_1_0_0
1e6
Intensity
m/z
138.0547
204.0862
366.1388
168.0652
186.0757
126.0547
144.0651
163.0599
350.1438
512.204_LeX
528.1909
1189.5133 PEP_0_0_0_0_0
1392.5914 PEP_0_1_0_0_0
1538.6478 PEP_0_1_1_0_0
1595.6669 PEP_0_2_0_0_0
1741.7405 PEP_0_2_1_0_0
1757.7242 PEP_1_2_0_0_0
1903.7853 PEP_1_2_1_0_0
1919.7811 PEP_2_2_0_0_0

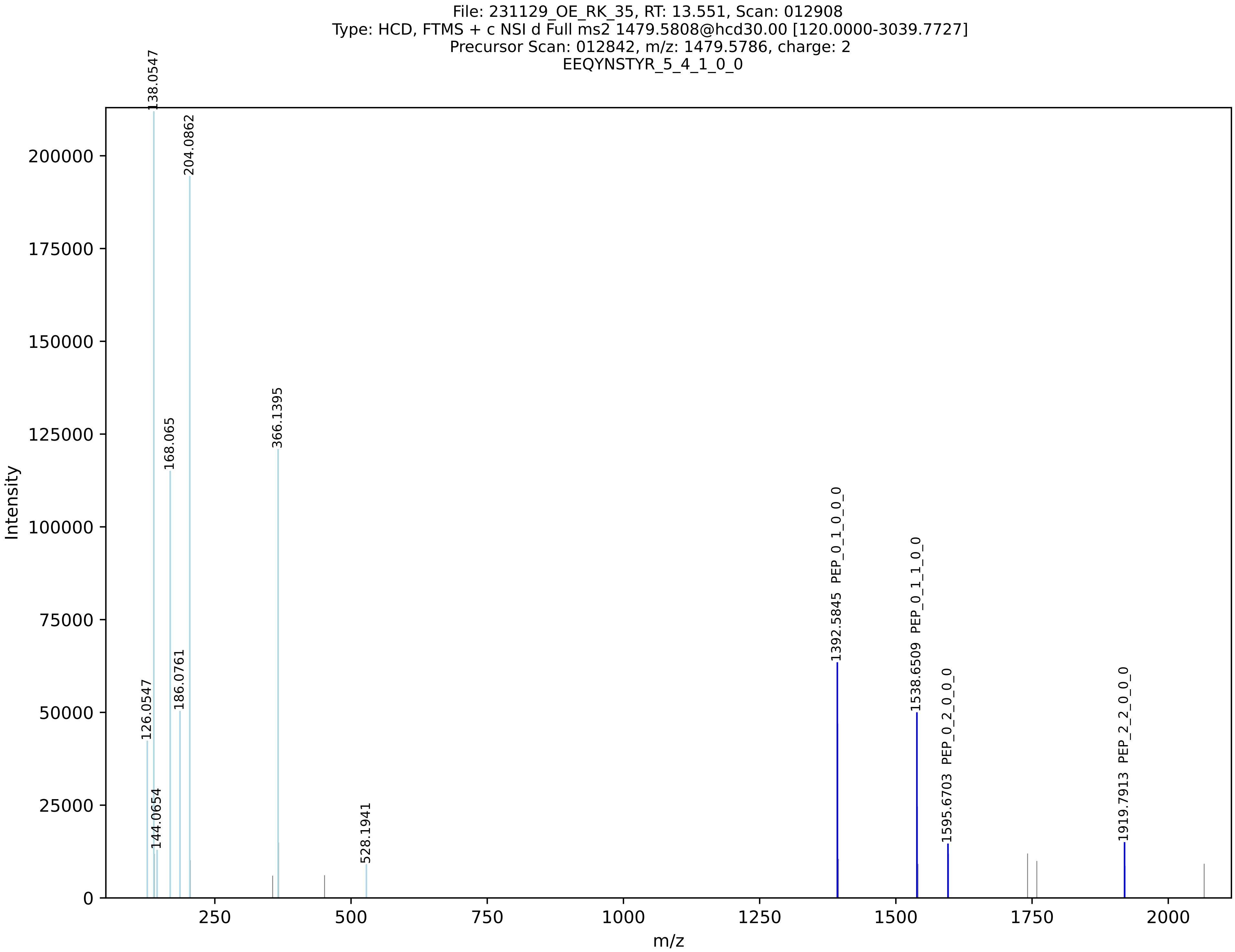
File: 231129_OE_RK_35, RT: 13.551, Scan: 012908
Type: HCD, FTMS + c NSI d Full ms2 1479.5808@hcd30.00 [120.0000-3039.7727]
Precursor Scan: 012842, m/z: 1479.5786, charge: 2
EEQYNSTYR_5_4_1_0_0
Intensity
m/z
138.0547
204.0862
168.065
366.1395
186.0761
126.0547
144.0654
528.1941
1392.5845 PEP_0_1_0_0_0
1538.6509 PEP_0_1_1_0_0
1595.6703 PEP_0_2_0_0_0
1919.7913 PEP_2_2_0_0_0
0
25000
50000
75000
100000
125000
150000
175000
200000
250
500
750
1000
1250
1500
1750
2000

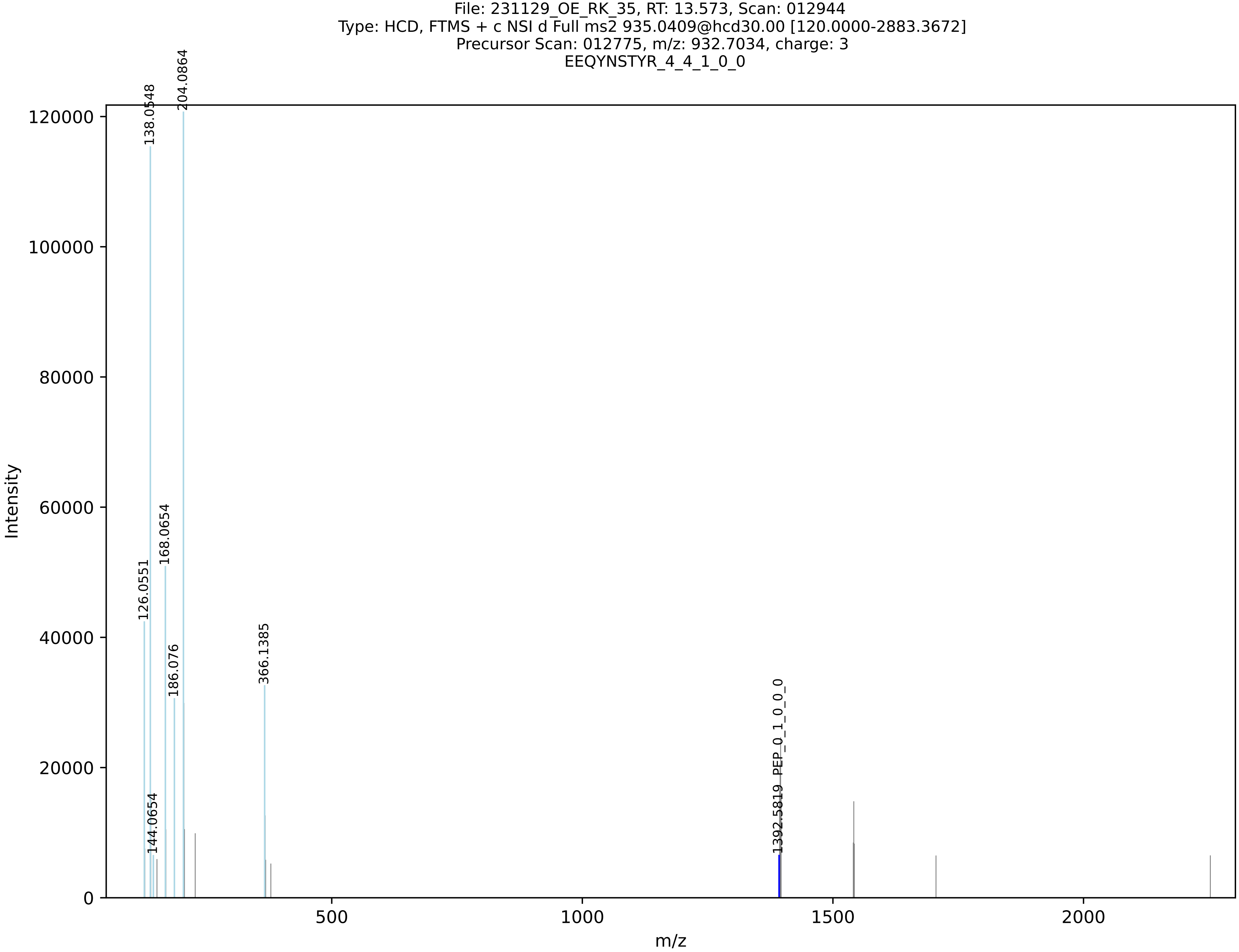
File: 231129_OE_RK_35, RT: 13.573, Scan: 012944
Type: HCD, FTMS + c NSI d Full ms2 935.0409@hcd30.00 [120.0000-2883.3672]
Precursor Scan: 012775, m/z: 932.7034, charge: 3
EEQYNSTYR_4_4_1_0_0
Intensity
m/z
138.0548
204.0864
126.0551
168.0654
186.076
144.0654
366.1385
1392.5819_PEP_0_1_0_0_0
0
20000
40000
60000
80000
100000
120000
500
1000
1500
2000

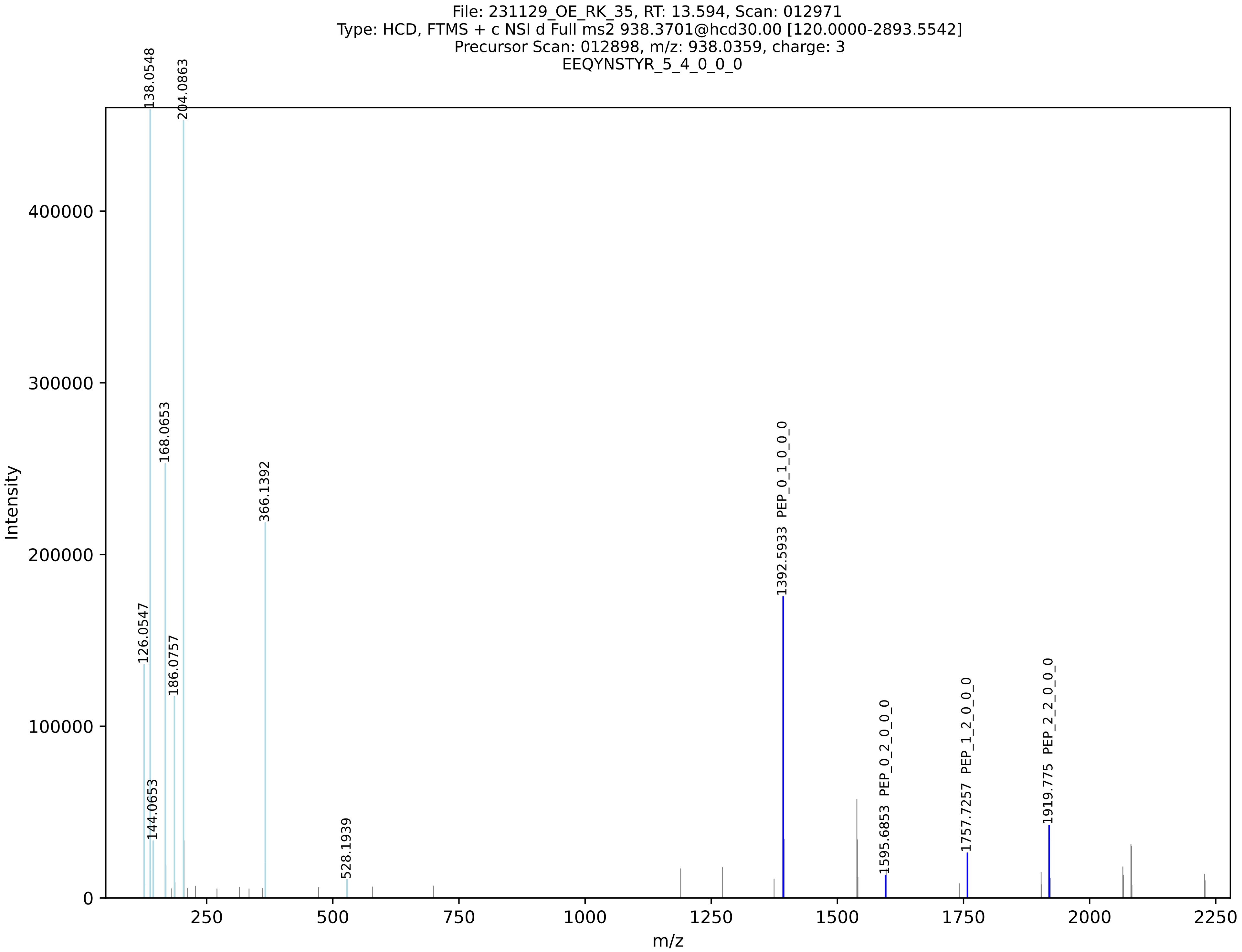

File: 231129_OE_RK_35, RT: 13.594, Scan: 012971
Type: HCD, FTMS + c NSI d Full ms2 938.3701@hcd30.00 [120.0000-2893.5542]
Precursor Scan: 012898, m/z: 938.0359, charge: 3
EEQYNSTYR_5_4_0_0_0
138.0548
204.0863
168.0653
366.1392
126.0547
186.0757
144.0653
528.1939
1392.5933 PEP_0_1_0_0_0
1595.6853 PEP_0_2_0_0_0
1757.7257 PEP_1_2_0_0_0
1919.775 PEP_2_2_0_0_0
Intensity
m/z

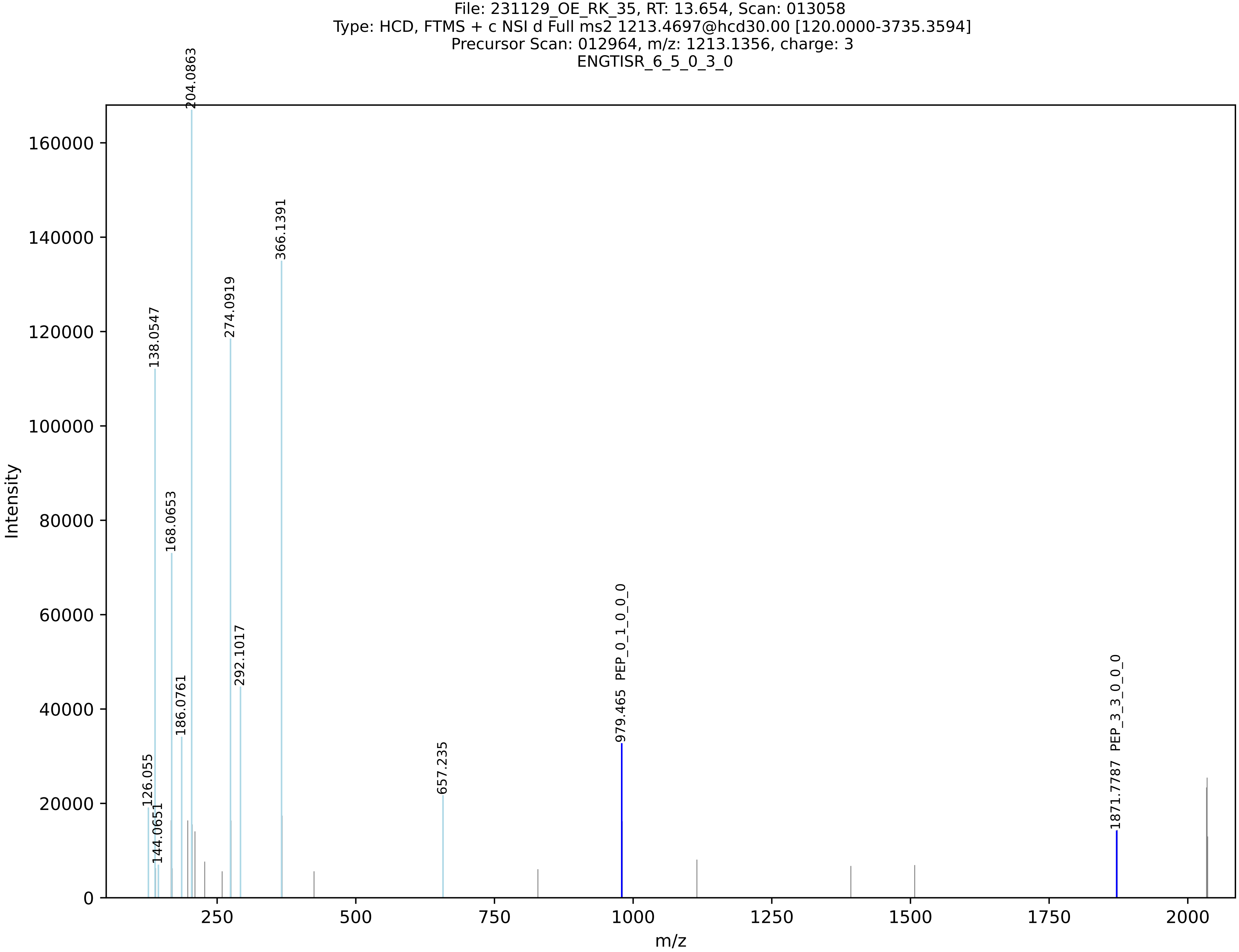

File: 231129_OE_RK_35, RT: 13.654, Scan: 013058
Type: HCD, FTMS + c NSI d Full ms2 1213.4697@hcd30.00 [120.0000-3735.3594]
Precursor Scan: 012964, m/z: 1213.1356, charge: 3
ENGTISR_6_5_0_3_0
Intensity
m/z
204.0863
366.1391
274.0919
138.0547
168.0653
292.1017
186.0761
126.055
144.0651
657.235
979.465 PEP_0_1_0_0_0
1871.7787 PEP_3_3_0_0_0

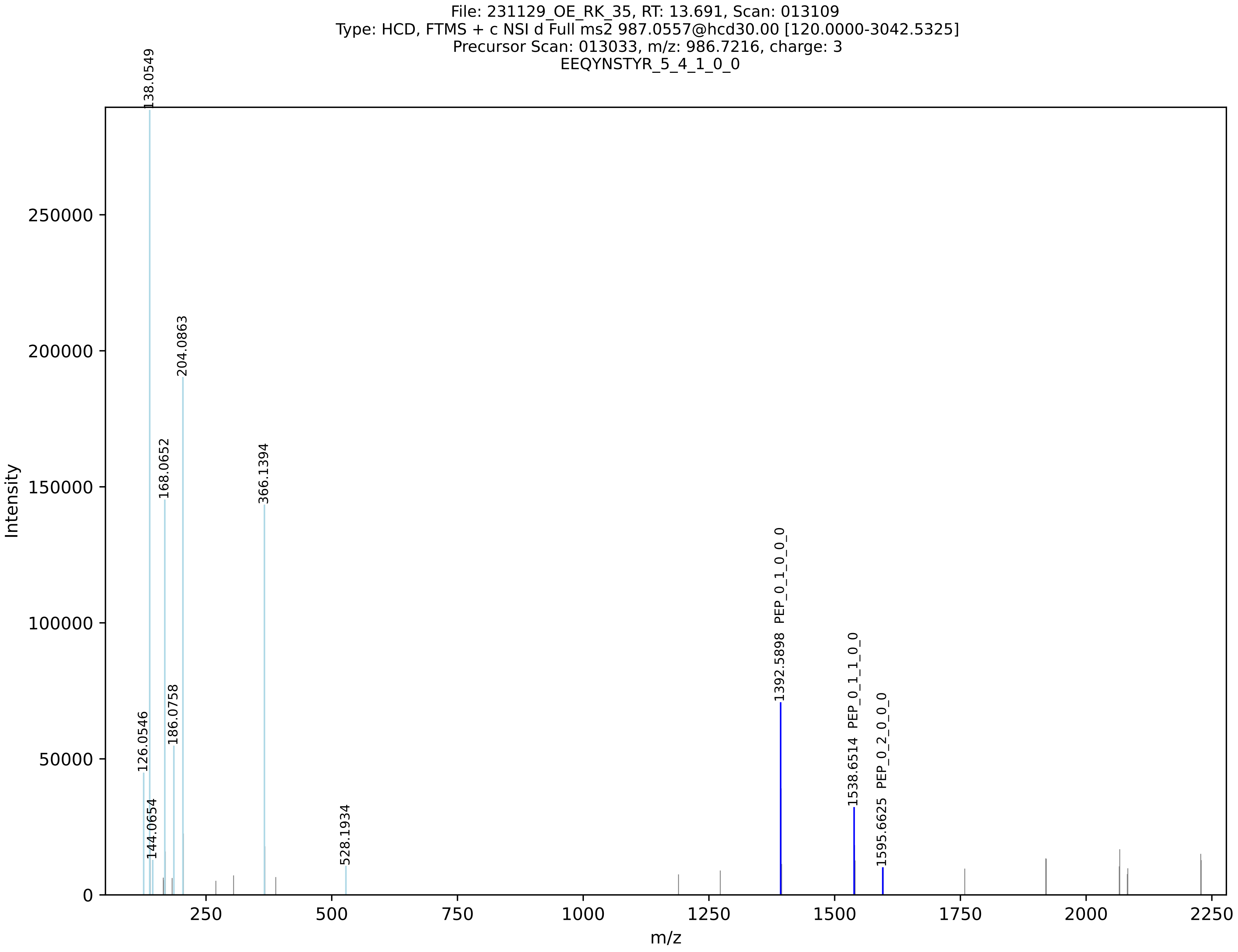

File: 231129_OE_RK_35, RT: 13.691, Scan: 013109
Type: HCD, FTMS + c NSI d Full ms2 987.0557@hcd30.00 [120.0000-3042.5325]
Precursor Scan: 013033, m/z: 986.7216, charge: 3
EEQYNSTYR_5_4_1_0_0
Intensity
m/z
138.0549
204.0863
168.0652
366.1394
126.0546
186.0758
144.0654
528.1934
1392.5898 PEP_0_1_0_0_0
1538.6514 PEP_0_1_1_0_0
1595.6625 PEP_0_2_0_0_0

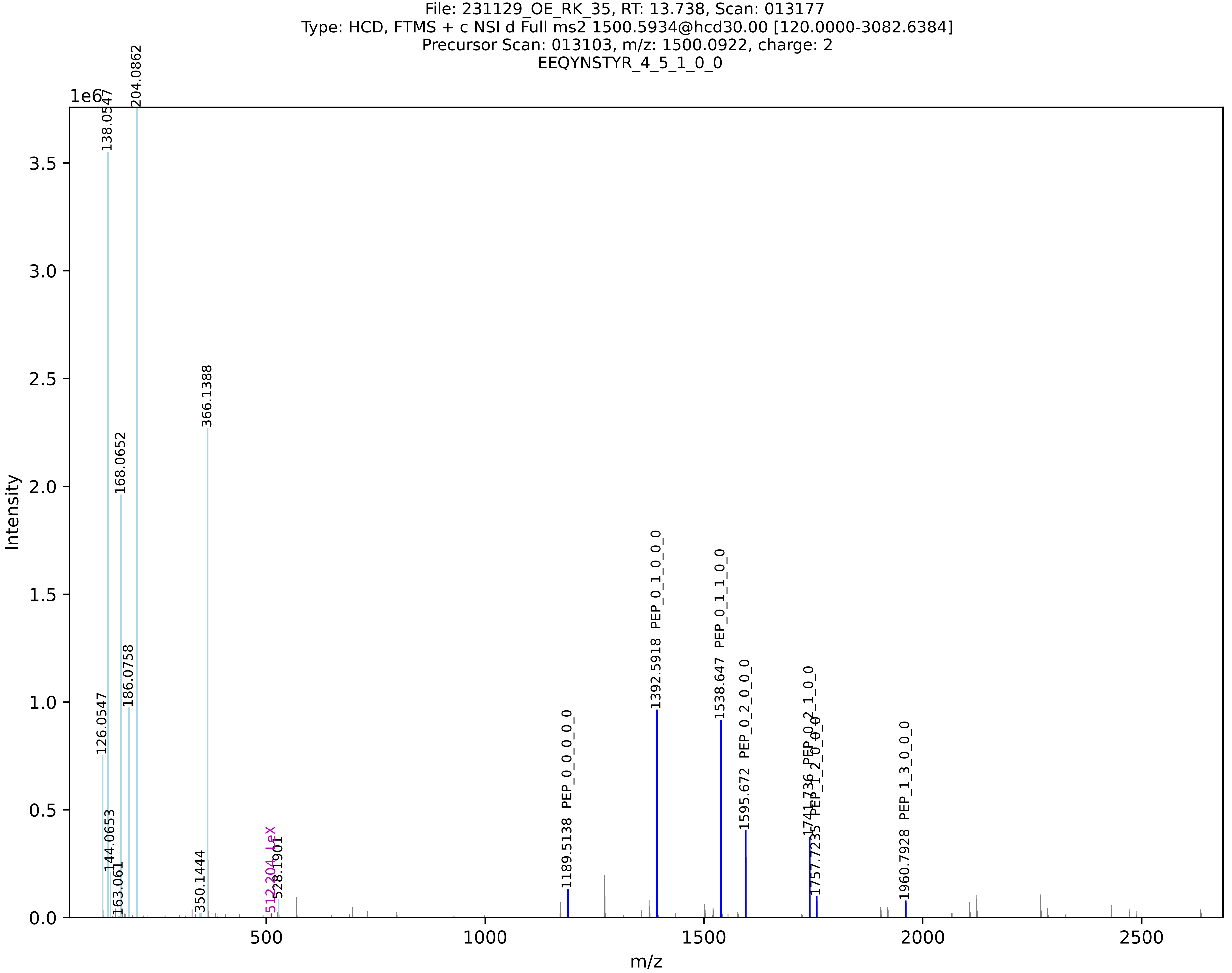
File: 231129_OE_RK_35, RT: 13.738, Scan: 013177
Type: HCD, FTMS + c NSI d Full ms2 1500.5934@hcd30.00 [120.0000-3082.6384]
Precursor Scan: 013103, m/z: 1500.0922, charge: 2
EEQYNSTYR_4_5_1_0_0
1e6
Intensity
m/z
204.0862
138.0547
168.0652
366.1388
186.0758
126.0547
144.0653
163.061
350.1444
512.204 LeX
528.1901
1189.5138 PEP_0_0_0_0_0
1392.5918 PEP_0_1_0_0_0
1538.647 PEP_0_1_1_0_0
1595.672 PEP_0_2_0_0_0
1741.736 PEP_0_2_1_0_0
1757.7235 PEP_1_2_0_0_0
1960.7928 PEP_1_3_0_0_0

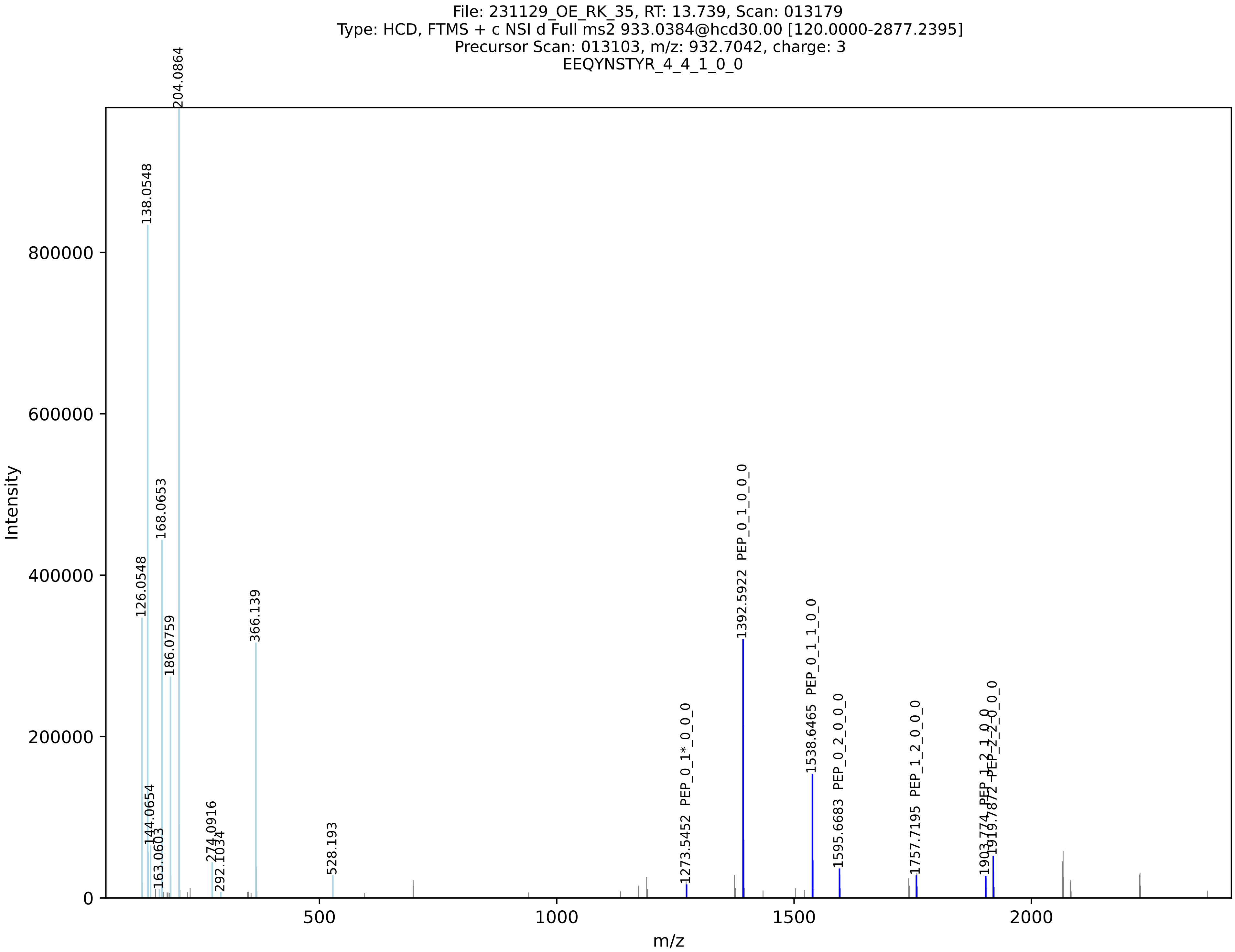
File: 231129_OE_RK_35, RT: 13.739, Scan: 013179
Type: HCD, FTMS + c NSI d Full ms2 933.0384@hcd30.00 [120.0000-2877.2395]
Precursor Scan: 013103, m/z: 932.7042, charge: 3
EEQYNSTYR_4_4_1_0_0
Intensity
m/z
204.0864
138.0548
168.0653
126.0548
186.0759
366.139
144.0654
163.0603
274.0916
292.1034
528.193
1273.5452 PEP_0_1*_0_0_0
1392.5922 PEP_0_1_0_0_0
1538.6465 PEP_0_1_1_0_0
1595.6683 PEP_0_2_0_0_0
1757.7195 PEP_1_2_0_0_0
1903.774 PEP_1_2_1_0_0
1919.7872 PEP_2_2_0_0_0

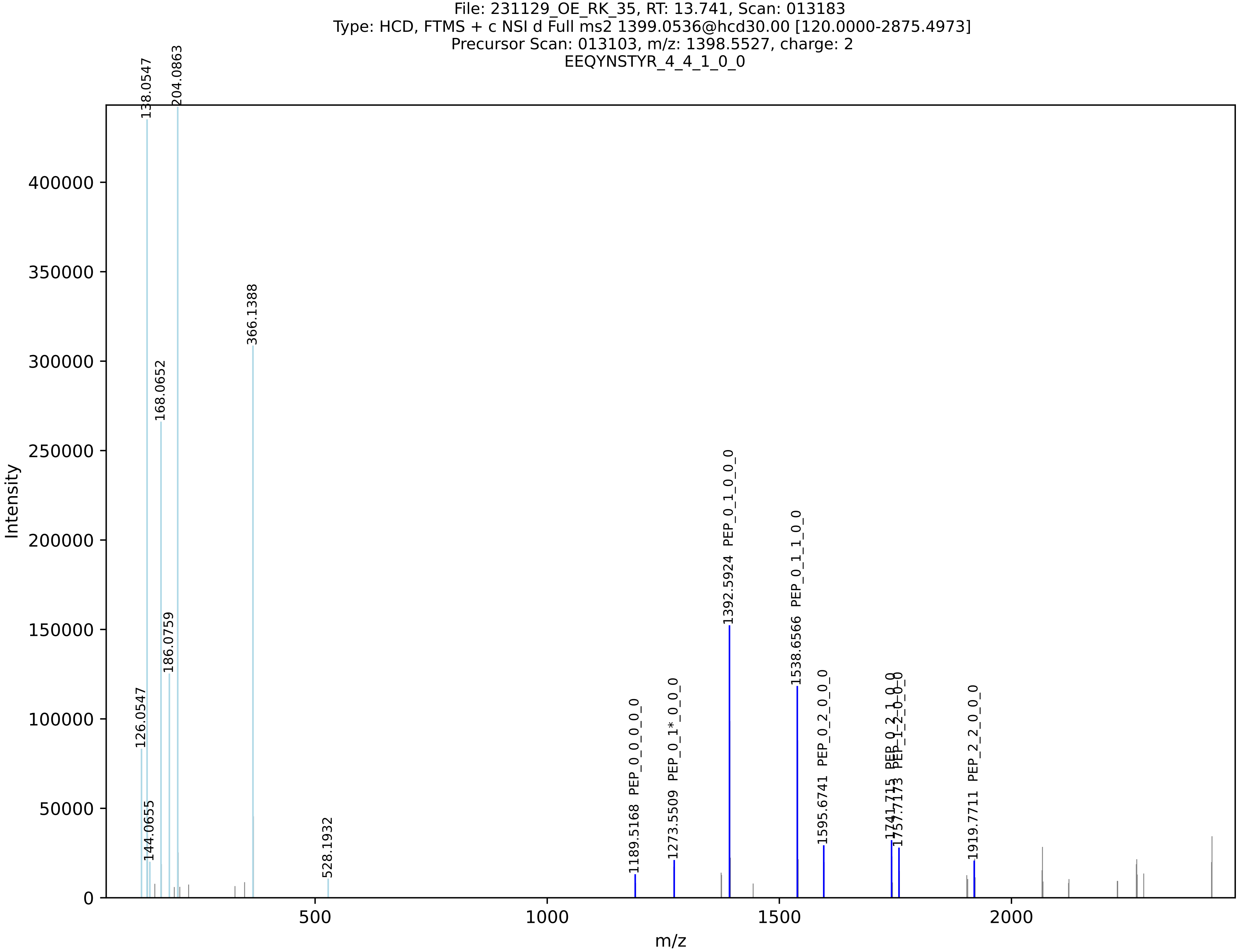

File: 231129_OE_RK_35, RT: 13.741, Scan: 013183
Type: HCD, FTMS + c NSI d Full ms2 1399.0536@hcd30.00 [120.0000-2875.4973]
Precursor Scan: 013103, m/z: 1398.5527, charge: 2
EEQYNSTYR_4_4_1_0_0
Intensity
m/z
138.0547
204.0863
366.1388
168.0652
186.0759
126.0547
144.0655
528.1932
1189.5168 PEP_0_0_0_0_0
1273.5509 PEP_0_1*_0_0_0
1392.5924 PEP_0_1_0_0_0
1538.6566 PEP_0_1_1_0_0
1595.6741 PEP_0_2_0_0_0
1741.715 PEP_0_2_1_0_0
1757.7173 PEP_1_2_0_0_0
1919.7711 PEP_2_2_0_0_0

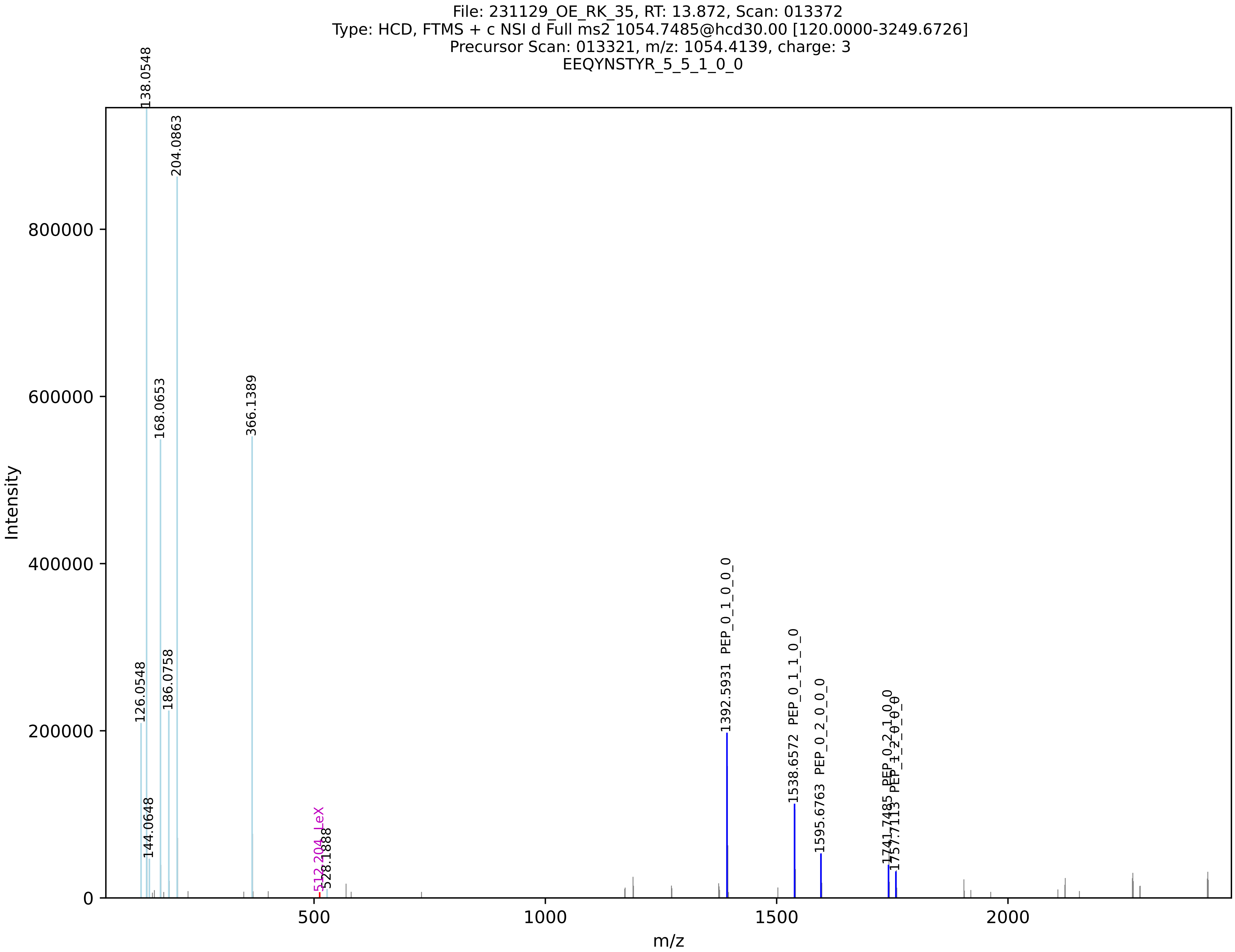

File: 231129_OE_RK_35, RT: 13.872, Scan: 013372
Type: HCD, FTMS + c NSI d Full ms2 1054.7485@hcd30.00 [120.0000-3249.6726]
Precursor Scan: 013321, m/z: 1054.4139, charge: 3
EEQYNSTYR_5_5_1_0_0
Intensity
m/z
138.0548
204.0863
168.0653
366.1389
126.0548
186.0758
144.0648
512.204_LeX
528.1888
1392.5931 PEP_0_1_0_0_0
1538.6572 PEP_0_1_1_0_0
1595.6763 PEP_0_2_0_0_0
1741.7485 PEP_0_2_1_0_0
1757.7113 PEP_1_2_0_0_0

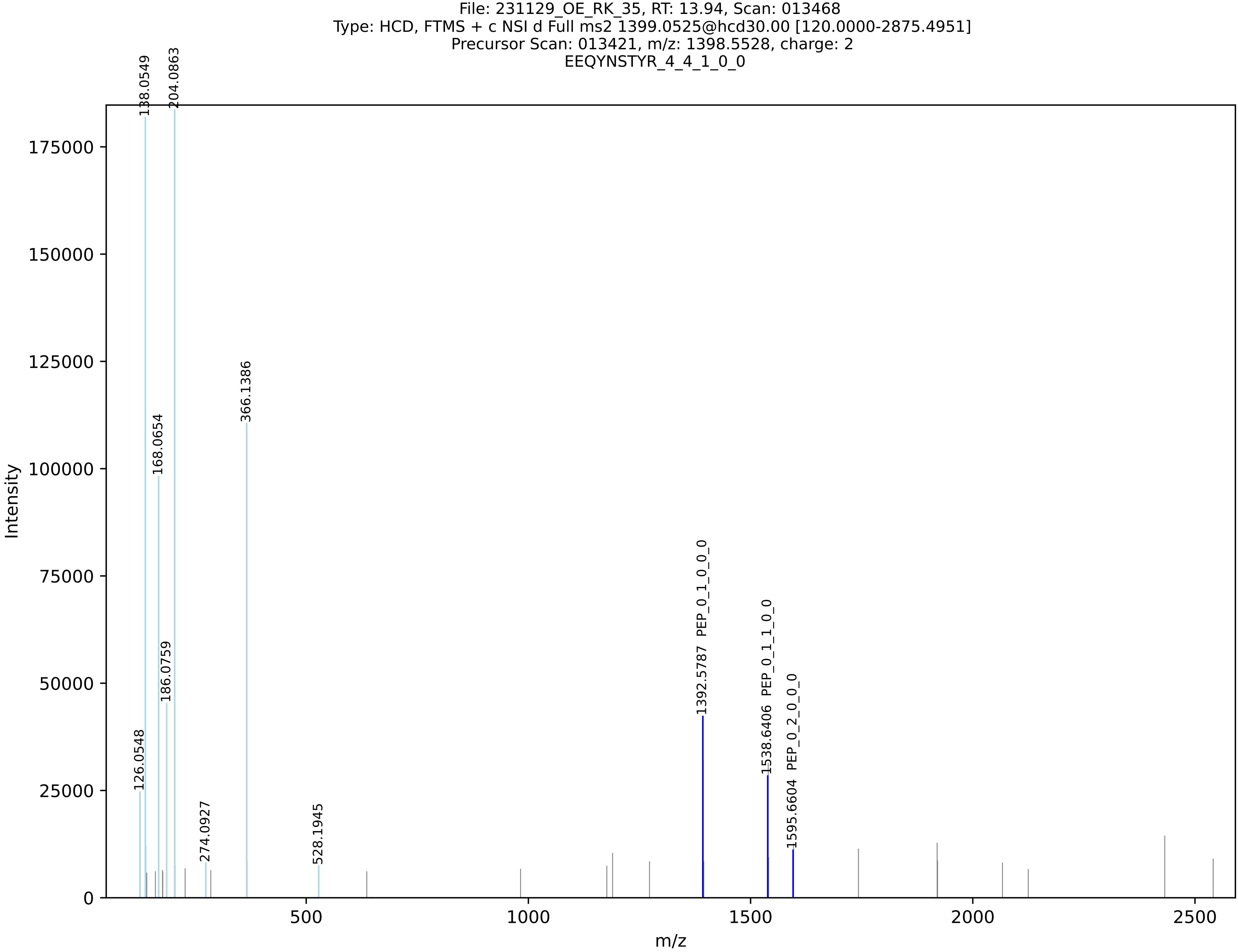

File: 231129_OE_RK_35, RT: 13.94, Scan: 013468
Type: HCD, FTMS + c NSI d Full ms2 1399.0525@hcd30.00 [120.0000-2875.4951]
Precursor Scan: 013421, m/z: 1398.5528, charge: 2
EEQYNSTYR_4_4_1_0_0
138.0549
204.0863
366.1386
168.0654
186.0759
126.0548
274.0927
528.1945
1392.5787 PEP_0_1_0_0_0
1538.6406 PEP_0_1_1_0_0
1595.6604 PEP_0_2_0_0_0
Intensity
m/z
175000
150000
125000
100000
75000
50000
25000
0
500
1000
1500
2000
2500

File: 231129_OE_RK_35, RT: 14.133, Scan: 013729
Type: HCD, FTMS + c NSI d Full ms2 933.0371@hcd30.00 [120.0000-2877.2356]
Precursor Scan: 013685, m/z: 932.703, charge: 3
EEQYNSTYR_4_4_1_0_0

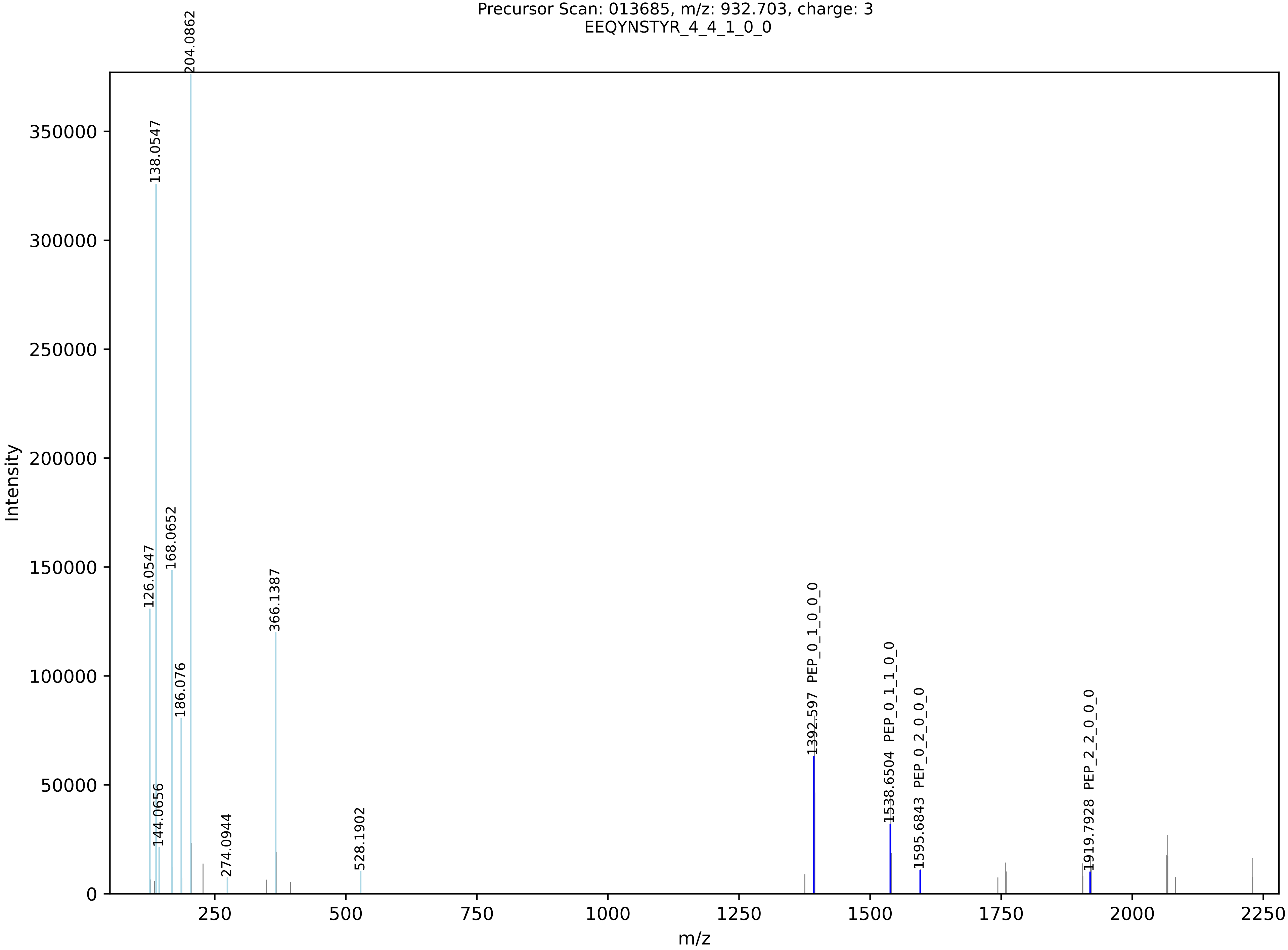

File: 231129_OE_RK_35, RT: 14.324, Scan: 013979
Type: HCD, FTMS + c NSI d Full ms2 1625.6243@hcd30.00 [120.0000-3337.7017]
Precursor Scan: 013930, m/z: 1625.1257, charge: 2
EEQYNSTYR_5_4_1_1_0

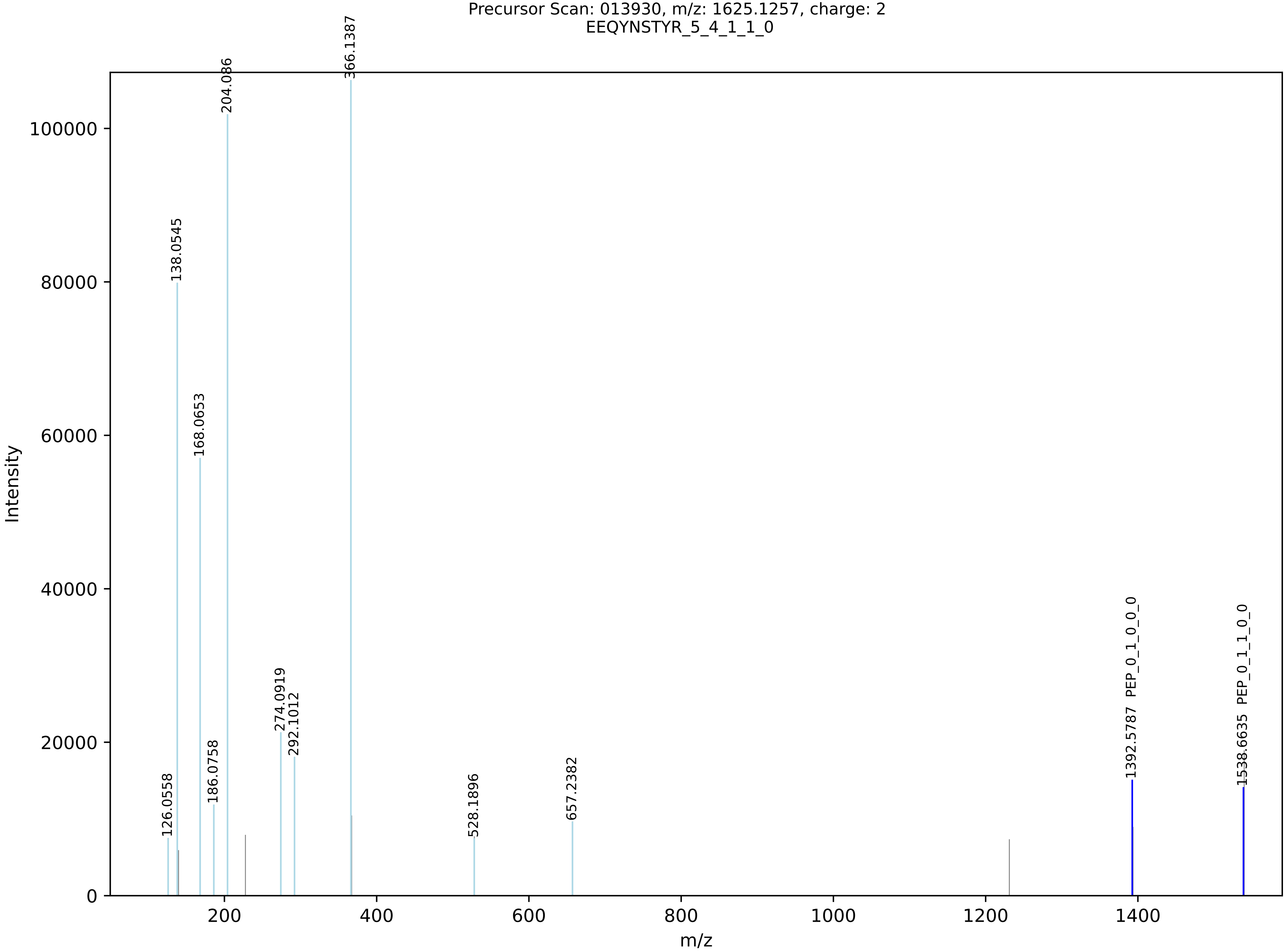

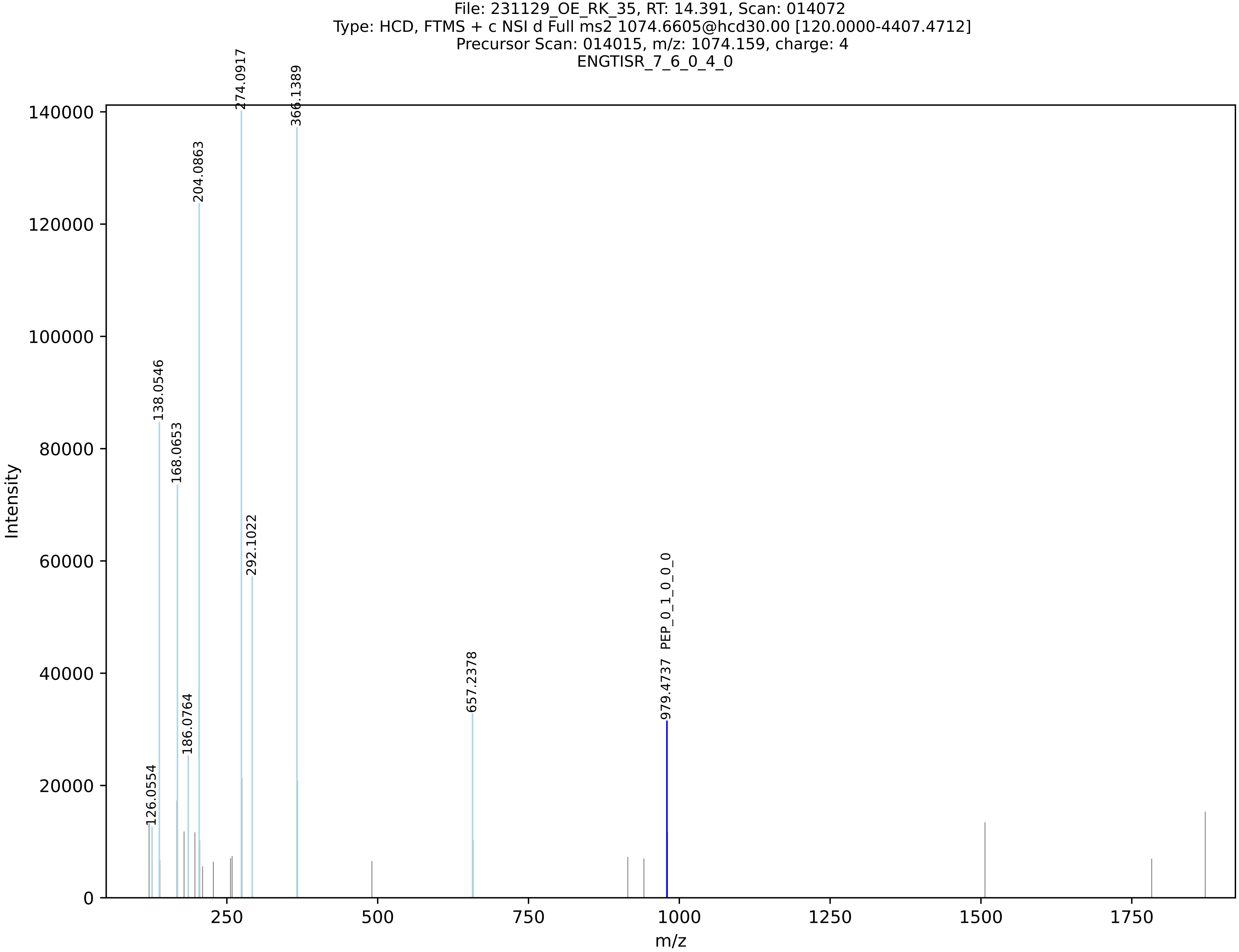
File: 231129_OE_RK_35, RT: 14.391, Scan: 014072
Type: HCD, FTMS + c NSI d Full ms2 1074.6605@hcd30.00 [120.0000-4407.4712]
Precursor Scan: 014015, m/z: 1074.159, charge: 4
ENGTISR_7_6_0_4_0
Intensity
m/z
0
20000
40000
60000
80000
100000
120000
140000
250
500
750
1000
1250
1500
1750
126.0554
138.0546
168.0653
186.0764
204.0863
274.0917
292.1022
366.1389
657.2378
979.4737 PEP_0_1_0_0_0

File: 231129_OE_RK_35, RT: 14.473, Scan: 014191
Type: HCD, FTMS + c NSI d Full ms2 1084.0878@hcd30.00 [120.0000-3339.4504]
Precursor Scan: 014129, m/z: 1083.7537, charge: 3
EEQYNSTYR_5_4_1_1_0

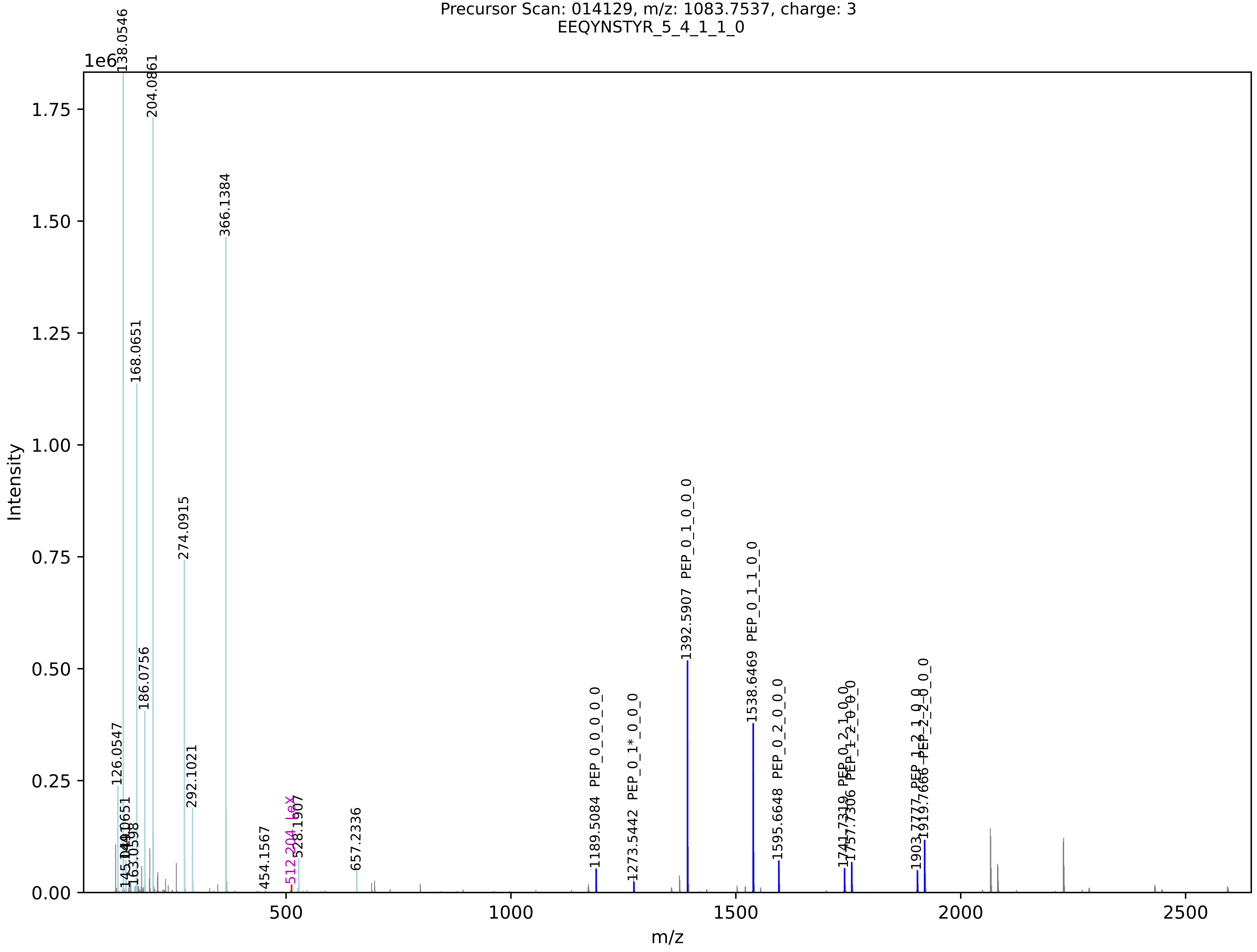

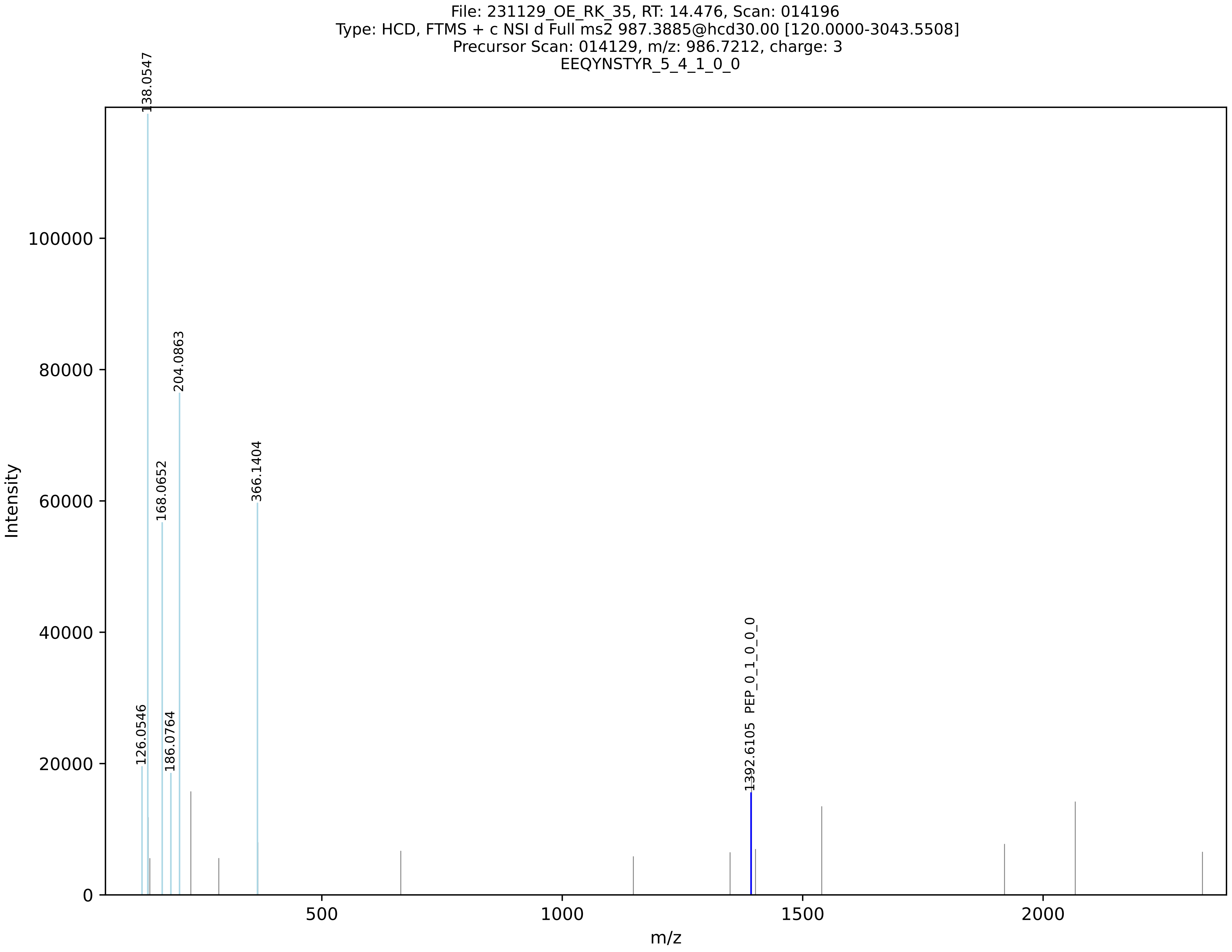
File: 231129_OE_RK_35, RT: 14.476, Scan: 014196
Type: HCD, FTMS + c NSI d Full ms2 987.3885@hcd30.00 [120.0000-3043.5508]
Precursor Scan: 014129, m/z: 986.7212, charge: 3
EEQYNSTYR_5_4_1_0_0
Intensity
m/z
138.0547
204.0863
366.1404
168.0652
126.0546
186.0764
1392.6105 PEP_0_1_0_0_0
0
20000
40000
60000
80000
100000
500
1000
1500
2000

File: 231129_OE_RK_35, RT: 14.518, Scan: 014257
Type: HCD, FTMS + c NSI d Full ms2 813.5686@hcd30.00 [120.0000-3342.2158]
Precursor Scan: 014190, m/z: 813.0672, charge: 4
EEQYNSTYR_5_4_1_1_0

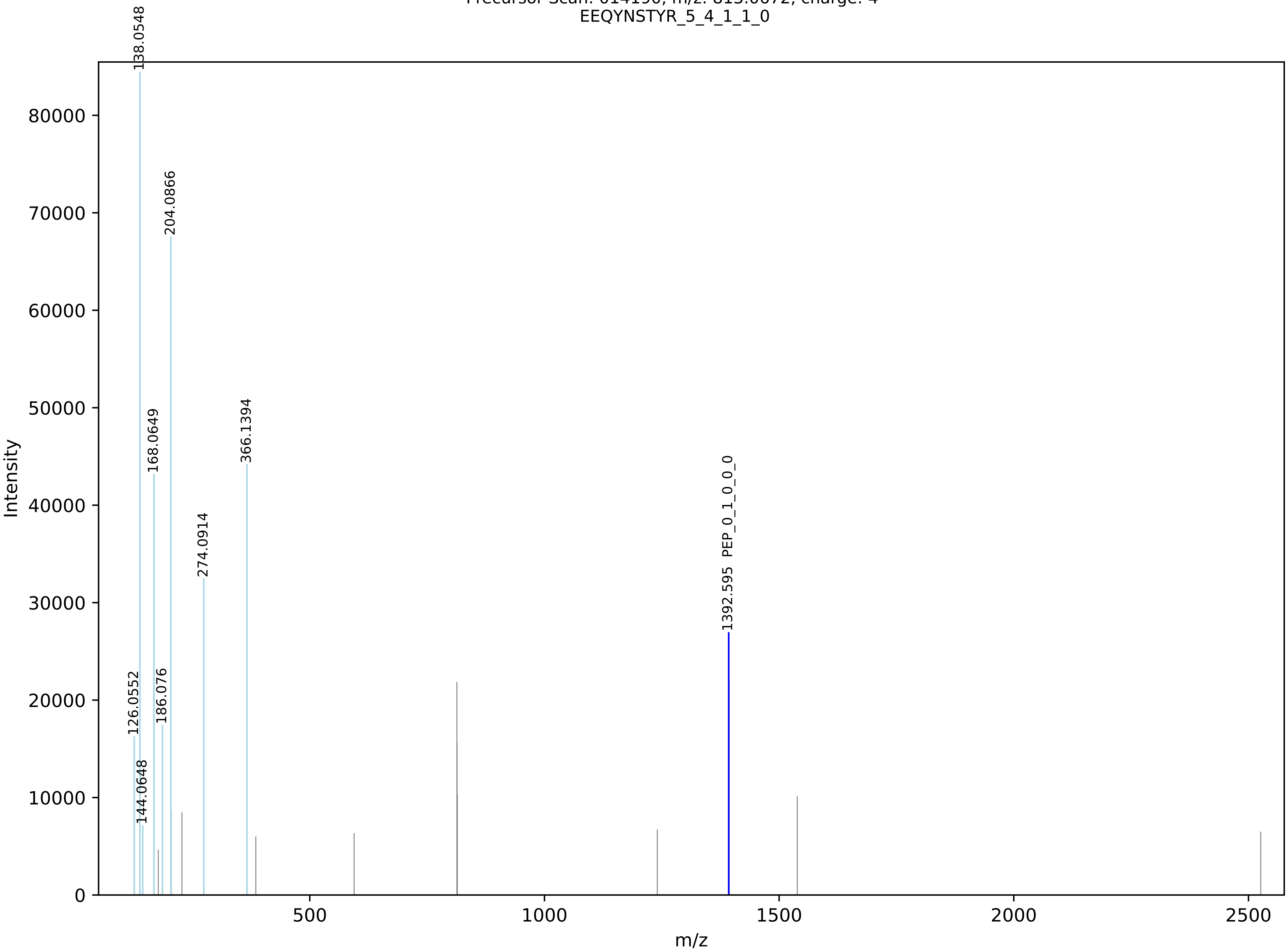

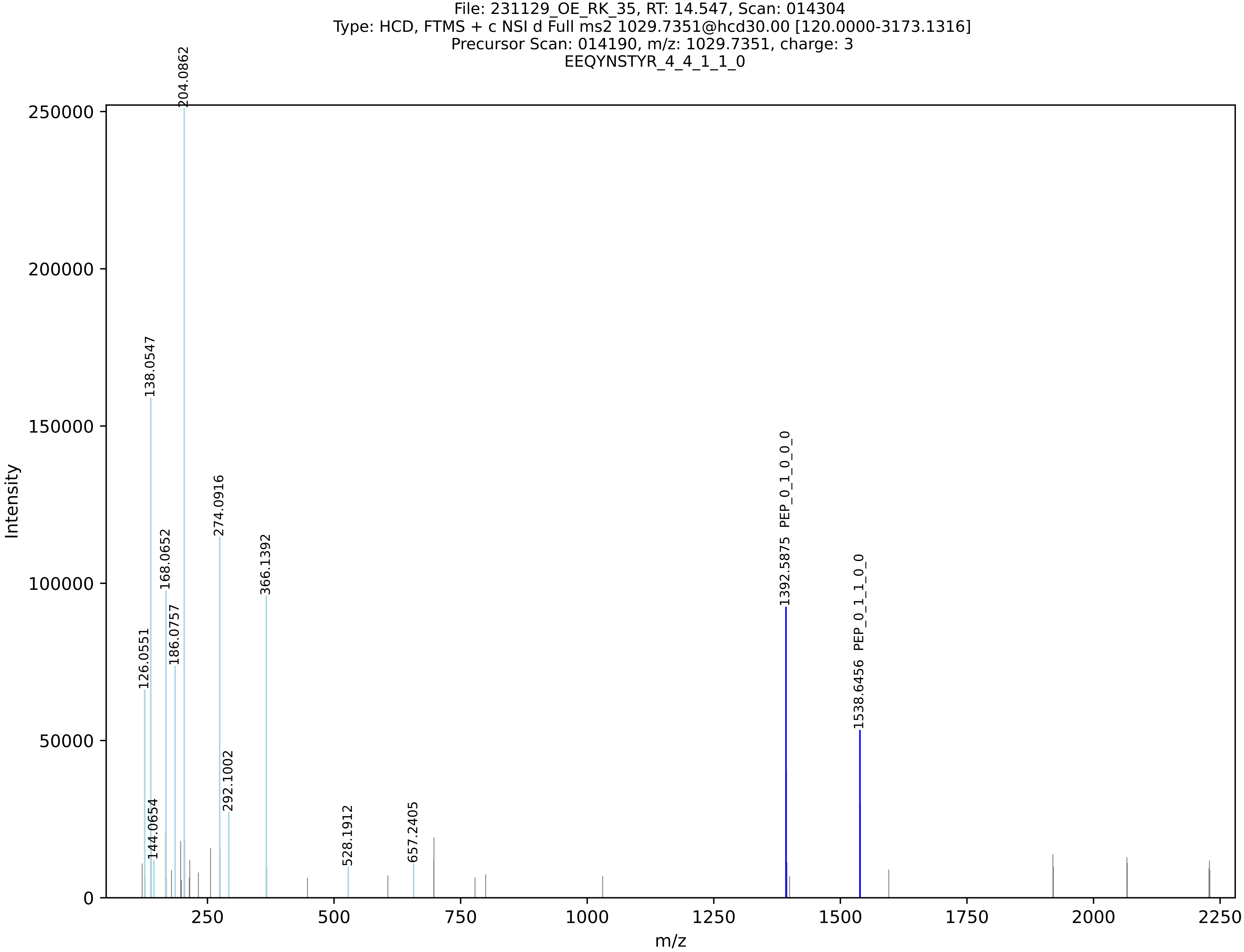

File: 231129_OE_RK_35, RT: 14.547, Scan: 014304
Type: HCD, FTMS + c NSI d Full ms2 1029.7351@hcd30.00 [120.0000-3173.1316]
Precursor Scan: 014190, m/z: 1029.7351, charge: 3
EEQYNSTYR_4_4_1_1_0
Intensity
m/z
204.0862
138.0547
274.0916
168.0652
366.1392
186.0757
126.0551
292.1002
144.0654
528.1912
657.2405
1392.5875 PEP_0_1_0_0_0
1538.6456 PEP_0_1_1_0_0

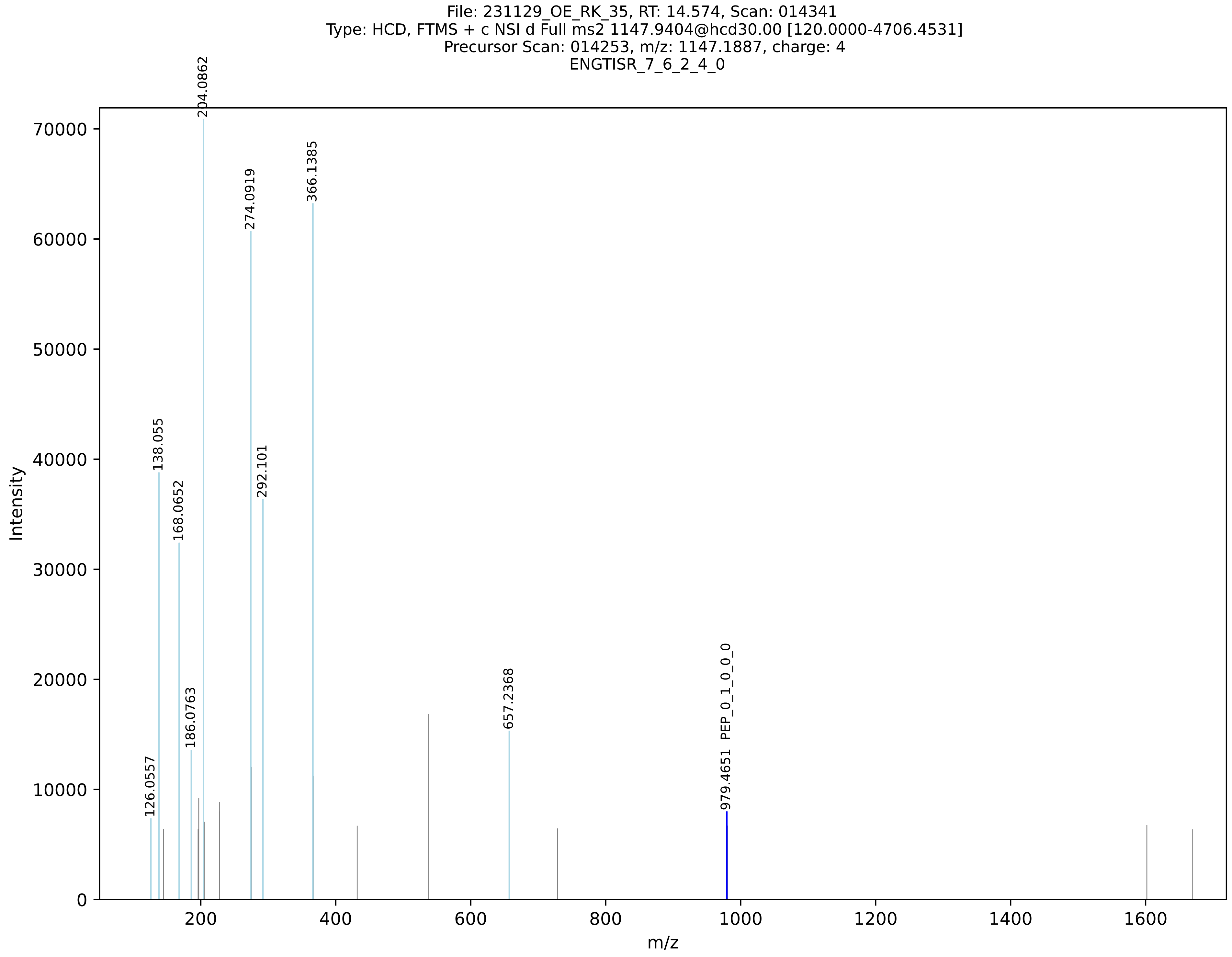
File: 231129_OE_RK_35, RT: 14.574, Scan: 014341
Type: HCD, FTMS + c NSI d Full ms2 1147.9404@hcd30.00 [120.0000-4706.4531]
Precursor Scan: 014253, m/z: 1147.1887, charge: 4
ENGTISR_7_6_2_4_0
Intensity
m/z
204.0862
366.1385
274.0919
138.055
292.101
168.0652
186.0763
126.0557
657.2368
979.4651 PEP_0_1_0_0_0
0
10000
20000
30000
40000
50000
60000
70000
200
400
600
800
1000
1200
1400
1600

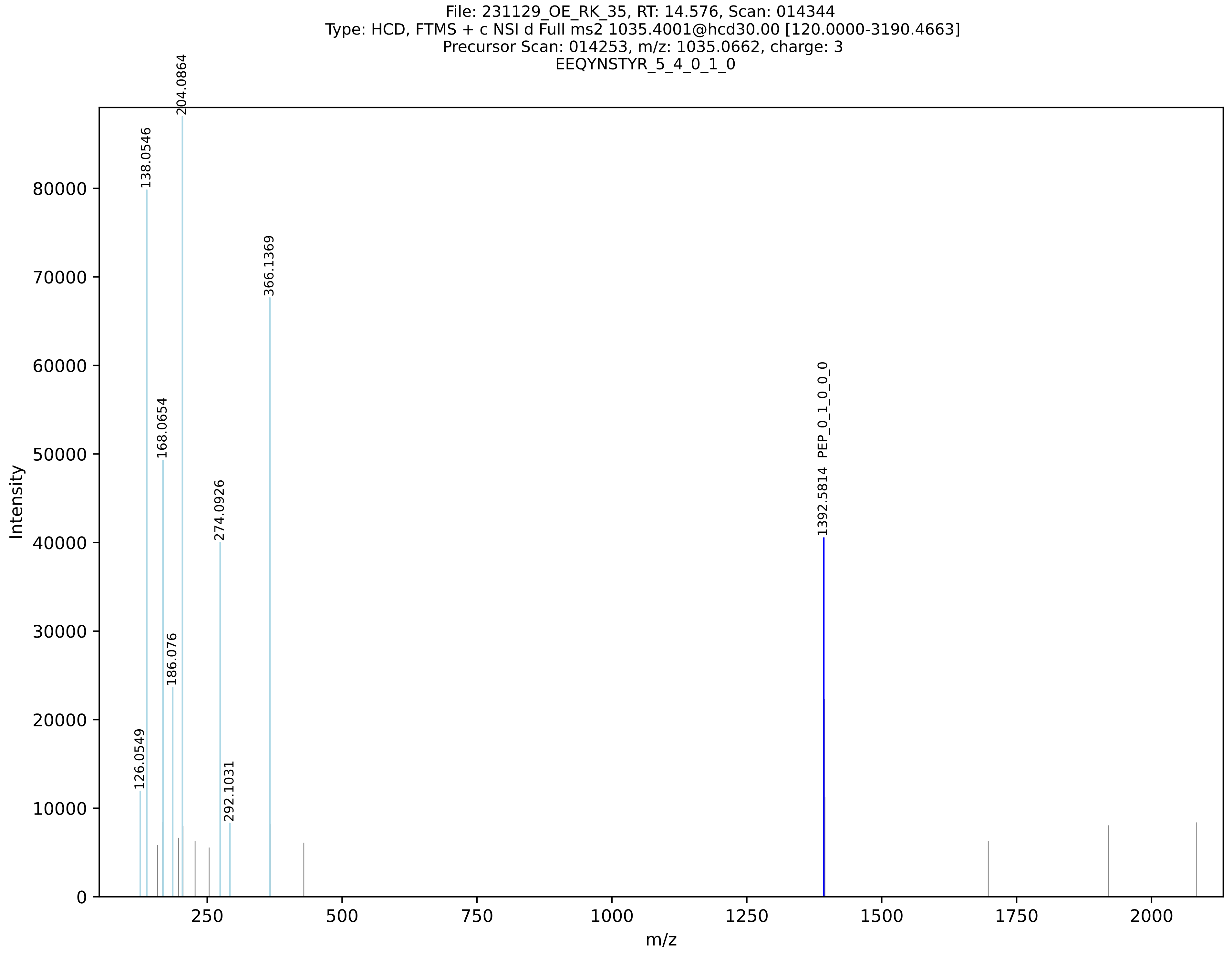

File: 231129_OE_RK_35, RT: 14.576, Scan: 014344
Type: HCD, FTMS + c NSI d Full ms2 1035.4001@hcd30.00 [120.0000-3190.4663]
Precursor Scan: 014253, m/z: 1035.0662, charge: 3
EEQYNSTYR_5_4_0_1_0
204.0864
138.0546
366.1369
PEP_0_1_0_0_0
168.0654
274.0926
1392.5814
186.076
126.0549
292.1031
Intensity
m/z

File: 231129_OE_RK_35, RT: 14.668, Scan: 014476
Type: HCD, FTMS + c NSI d Full ms2 1029.4493@hcd30.00 [120.0000-3172.2568]
Precursor Scan: 014394, m/z: 1028.7811, charge: 3
EEQYNSTYR_4_4_1_1_0

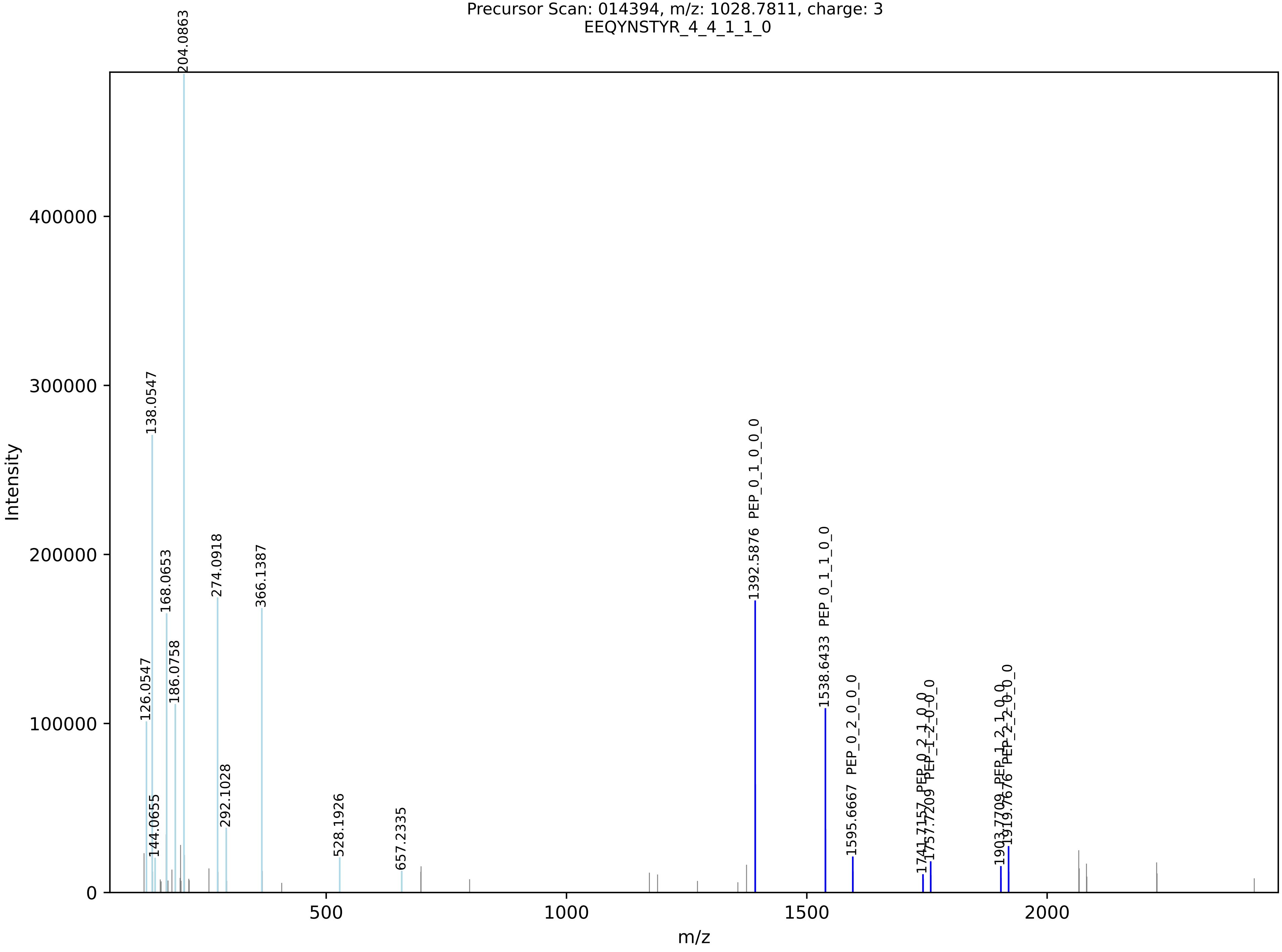

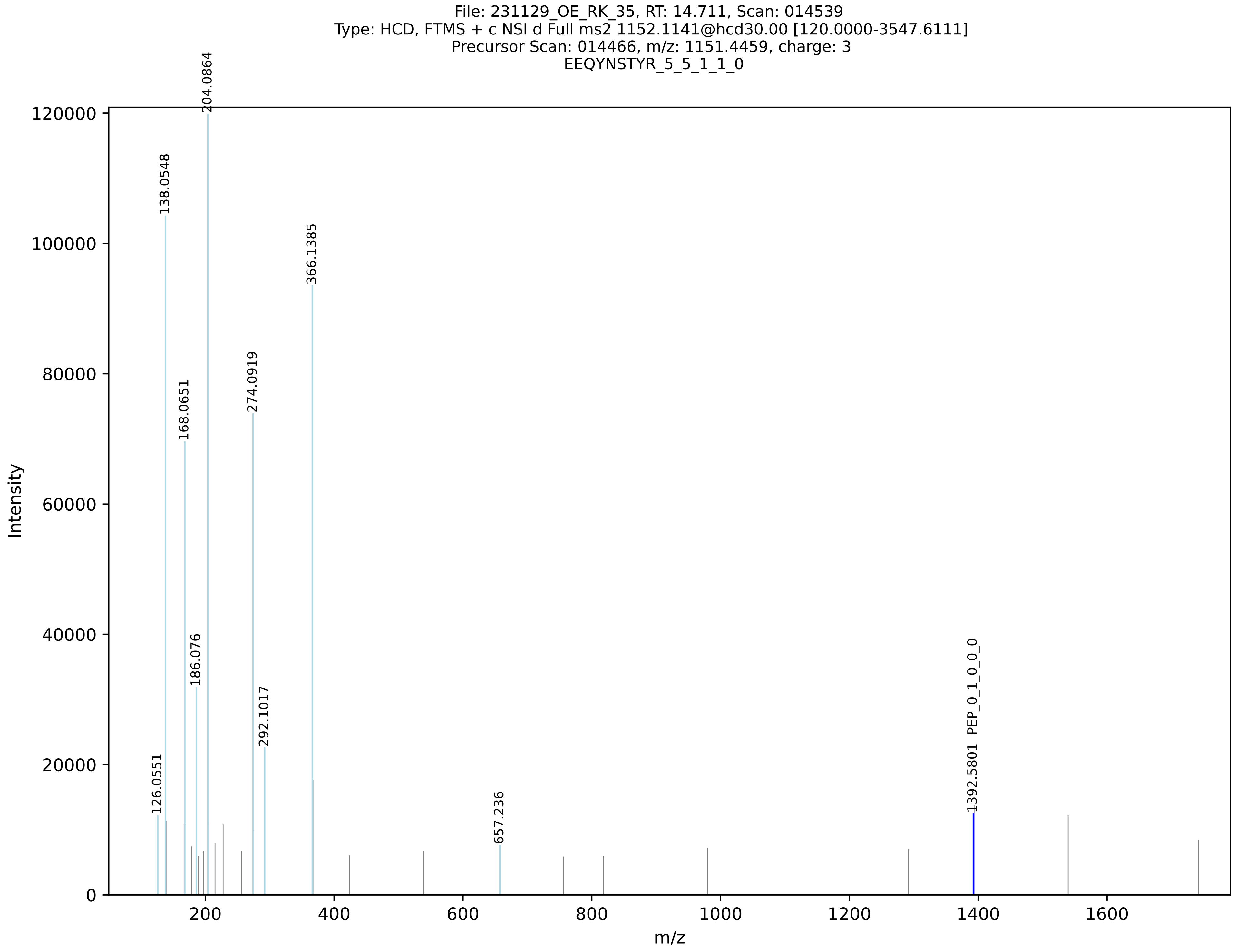

File: 231129_OE_RK_35, RT: 14.711, Scan: 014539
Type: HCD, FTMS + c NSI d Full ms2 1152.1141@hcd30.00 [120.0000-3547.6111]
Precursor Scan: 014466, m/z: 1151.4459, charge: 3
EEQYNSTYR_5_5_1_1_0
Intensity
m/z
204.0864
138.0548
366.1385
274.0919
168.0651
186.076
292.1017
126.0551
657.236
1392.5801 PEP_0_1_0_0_
0
20000
40000
60000
80000
100000
120000
200
400
600
800
1000
1200
1400
1600

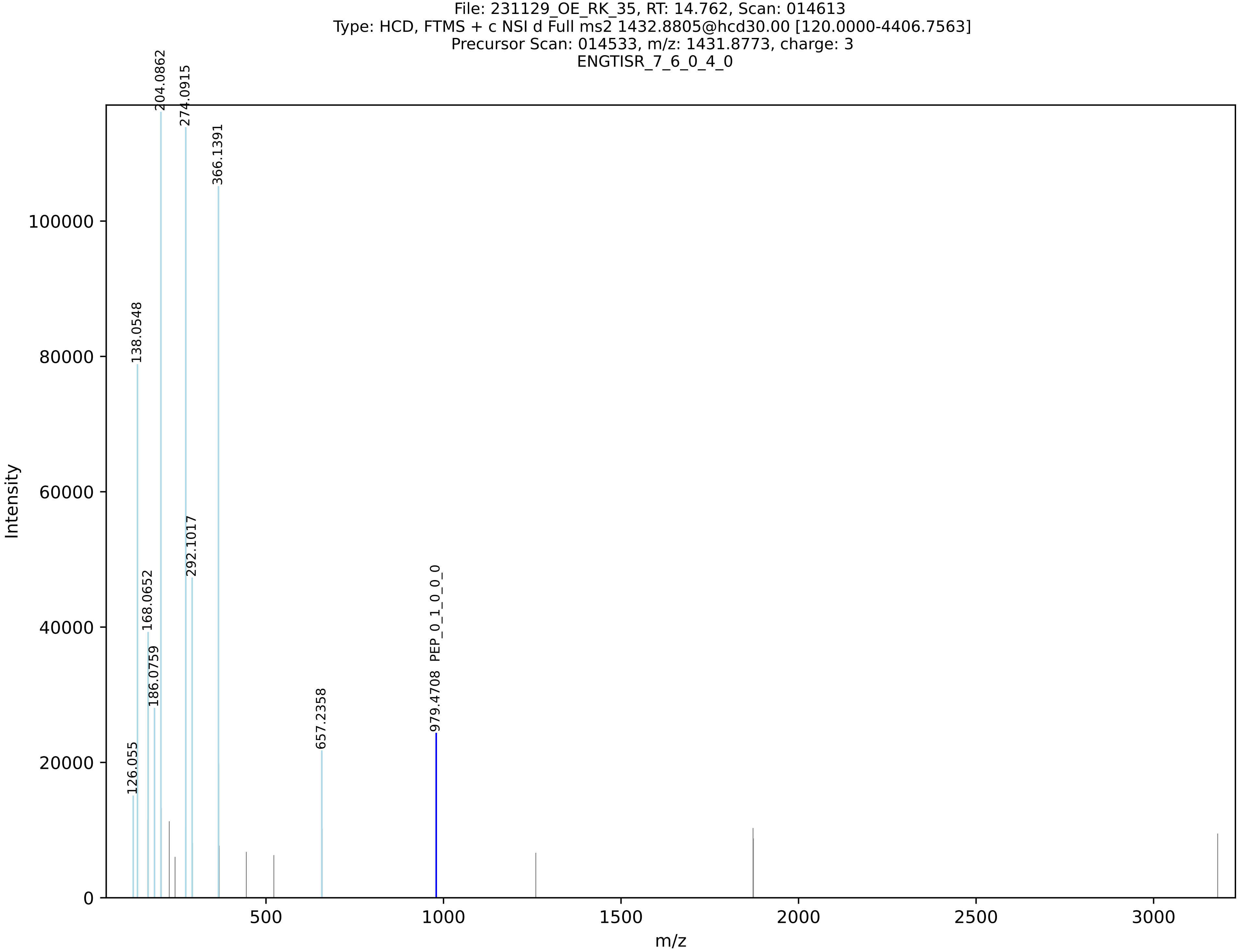
File: 231129_OE_RK_35, RT: 14.762, Scan: 014613
Type: HCD, FTMS + c NSI d Full ms2 1432.8805@hcd30.00 [120.0000-4406.7563]
Precursor Scan: 014533, m/z: 1431.8773, charge: 3
ENGTISR_7_6_0_4_0
204.0862
274.0915
366.1391
138.0548
292.1017
168.0652
186.0759
126.055
657.2358
979.4708 PEP_0_1_0_0_0
Intensity
m/z
0
20000
40000
60000
80000
100000
500
1000
1500
2000
2500
3000

File: 231129_OE_RK_35, RT: 14.766, Scan: 014619
Type: HCD, FTMS + c NSI d Full ms2 1072.0835@hcd30.00 [120.0000-3302.7175]
Precursor Scan: 014533, m/z: 1071.4153, charge: 3
GICNSSDVR_5_4_0_2_0

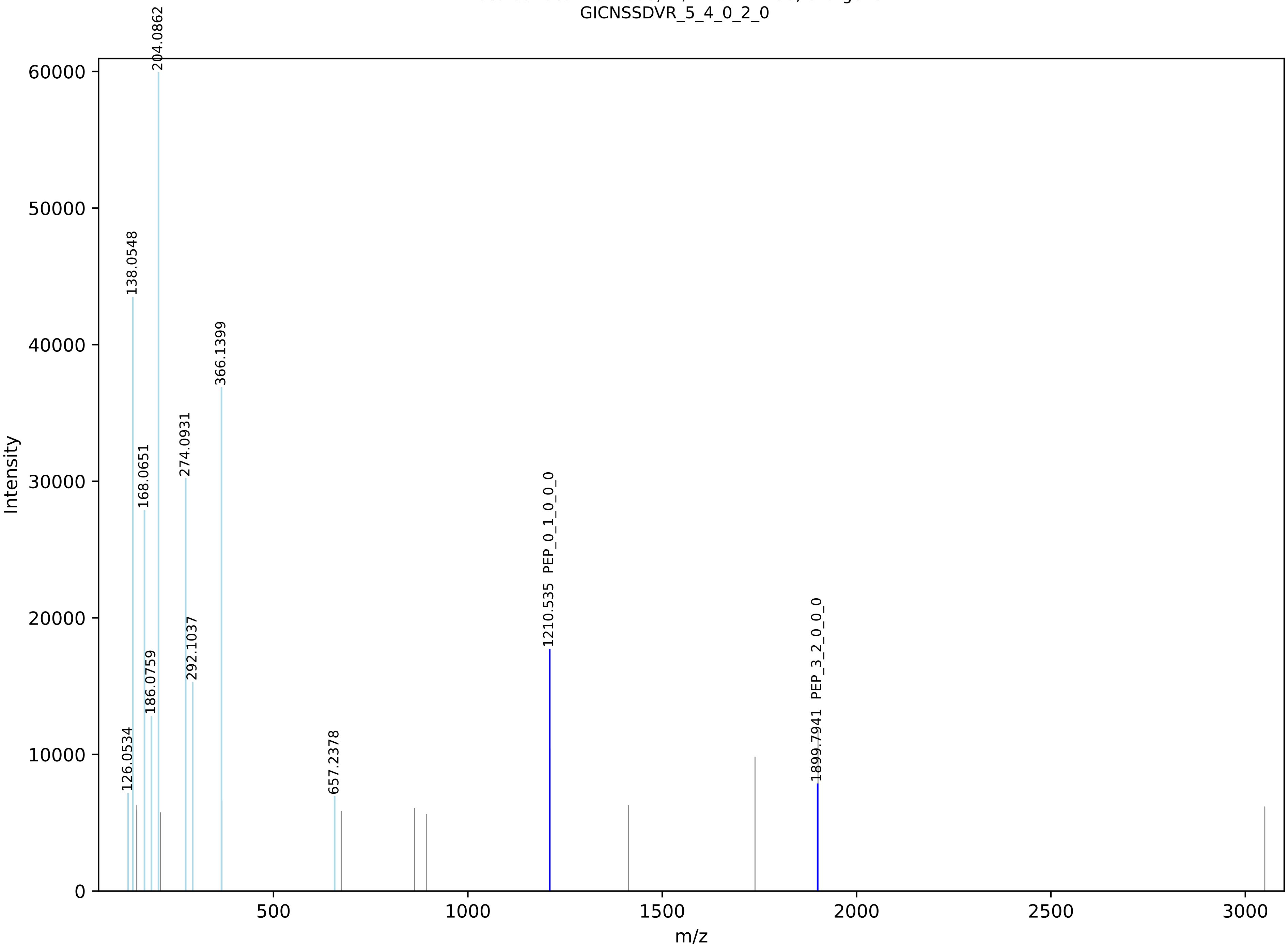

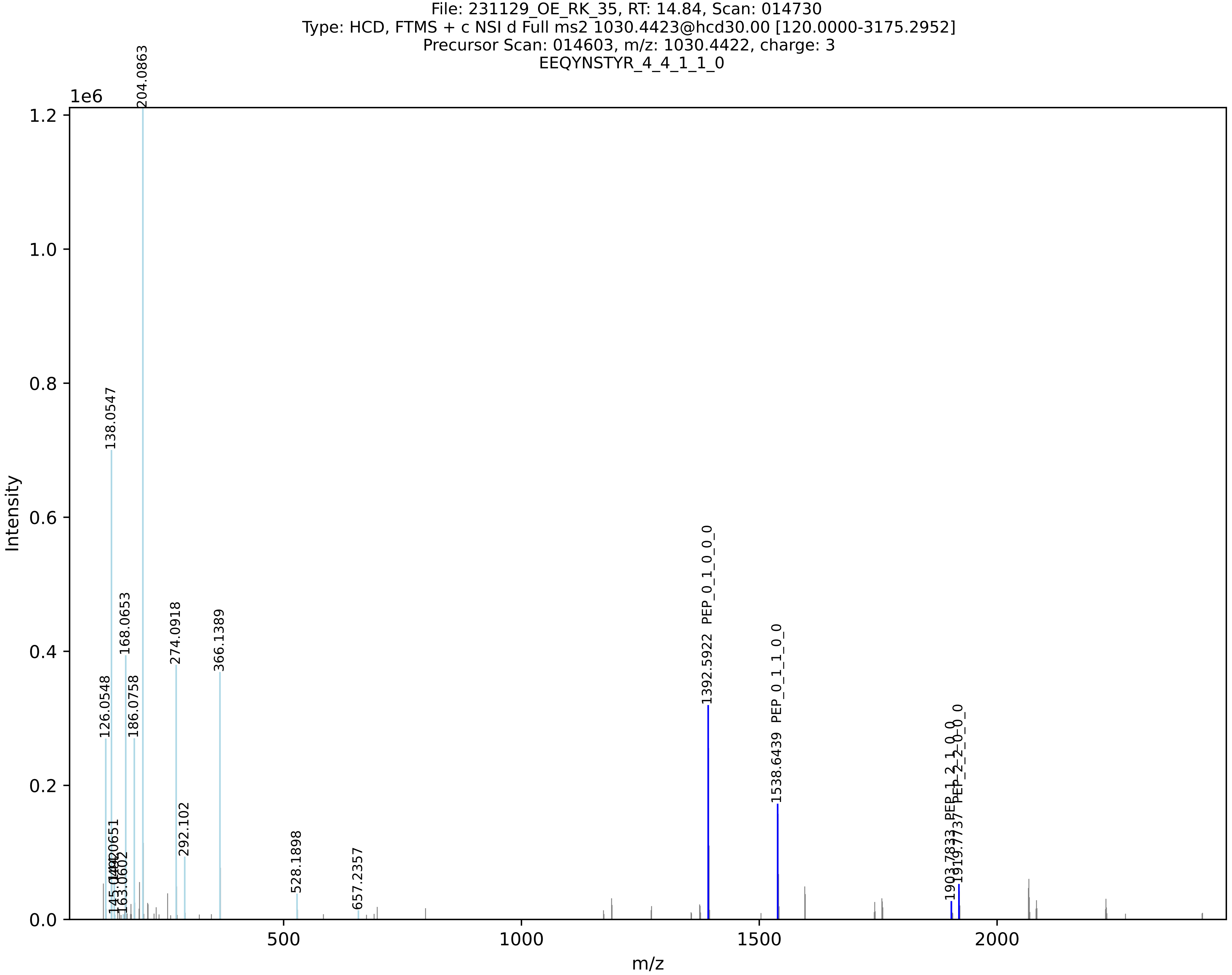

File: 231129_OE_RK_35, RT: 14.84, Scan: 014730
Type: HCD, FTMS + c NSI d Full ms2 1030.4423@hcd30.00 [120.0000-3175.2952]
Precursor Scan: 014603, m/z: 1030.4422, charge: 3
EEQYNSTYR_4_4_1_1_0
1e6
Intensity
m/z
0.0
0.2
0.4
0.6
0.8
1.0
1.2
500
1000
1500
2000
204.0863
138.0547
168.0653
126.0548
186.0758
274.0918
292.102
366.1389
145.0492
149.0651
163.0602
528.1898
657.2357
1392.5922 PEP_0_1_0_0_0
1538.6439 PEP_0_1_1_0_0
1903.7833 PEP_1_2_1_0_0
1919.7737 PEP_2_2_0_0_0

File: 231129_OE_RK_35, RT: 14.856, Scan: 014749
Type: HCD, FTMS + c NSI d Full ms2 1084.0879@hcd30.00 [120.0000-3339.4509]
Precursor Scan: 014675, m/z: 1083.7538, charge: 3
EEQYNSTYR_5_4_1_1_0

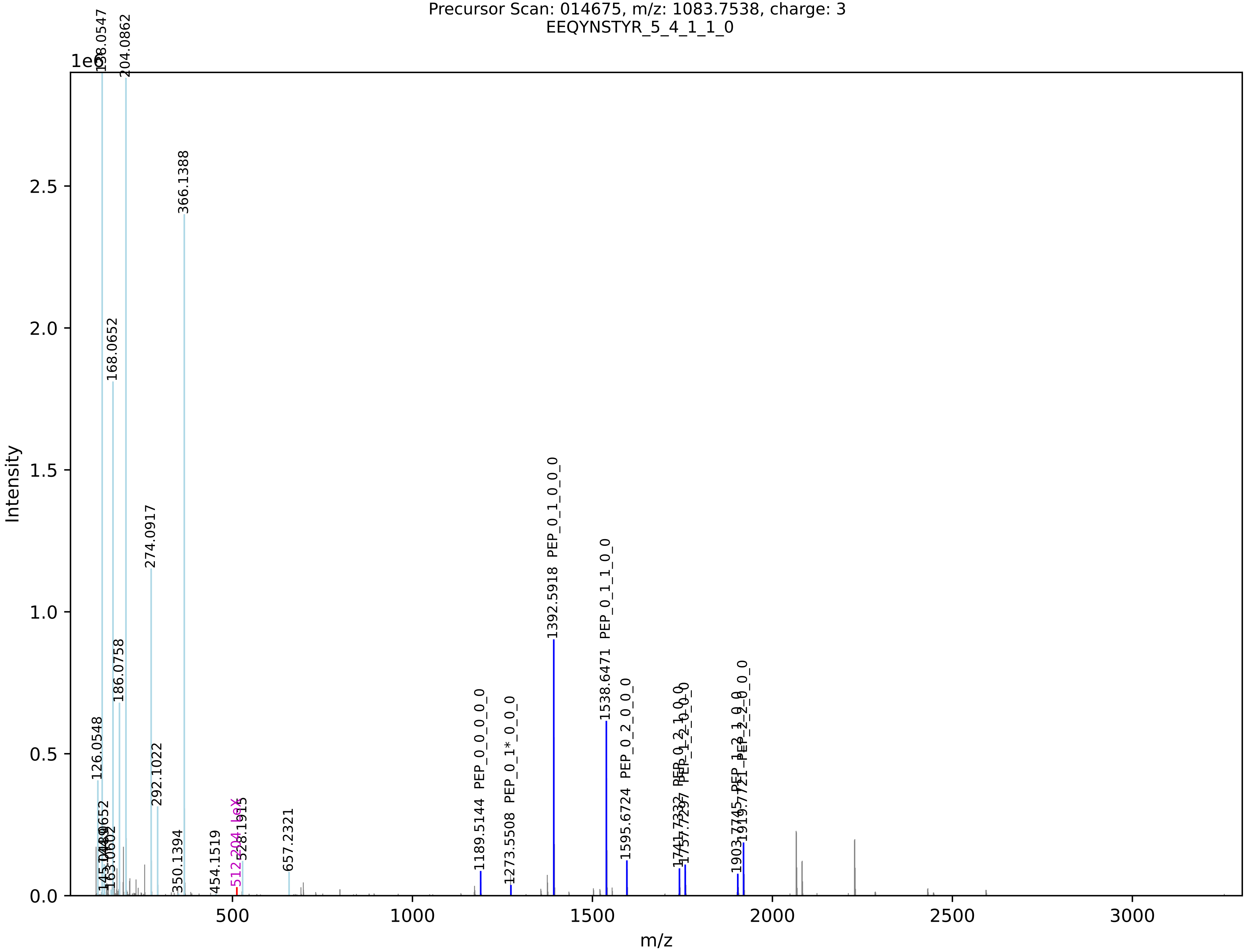

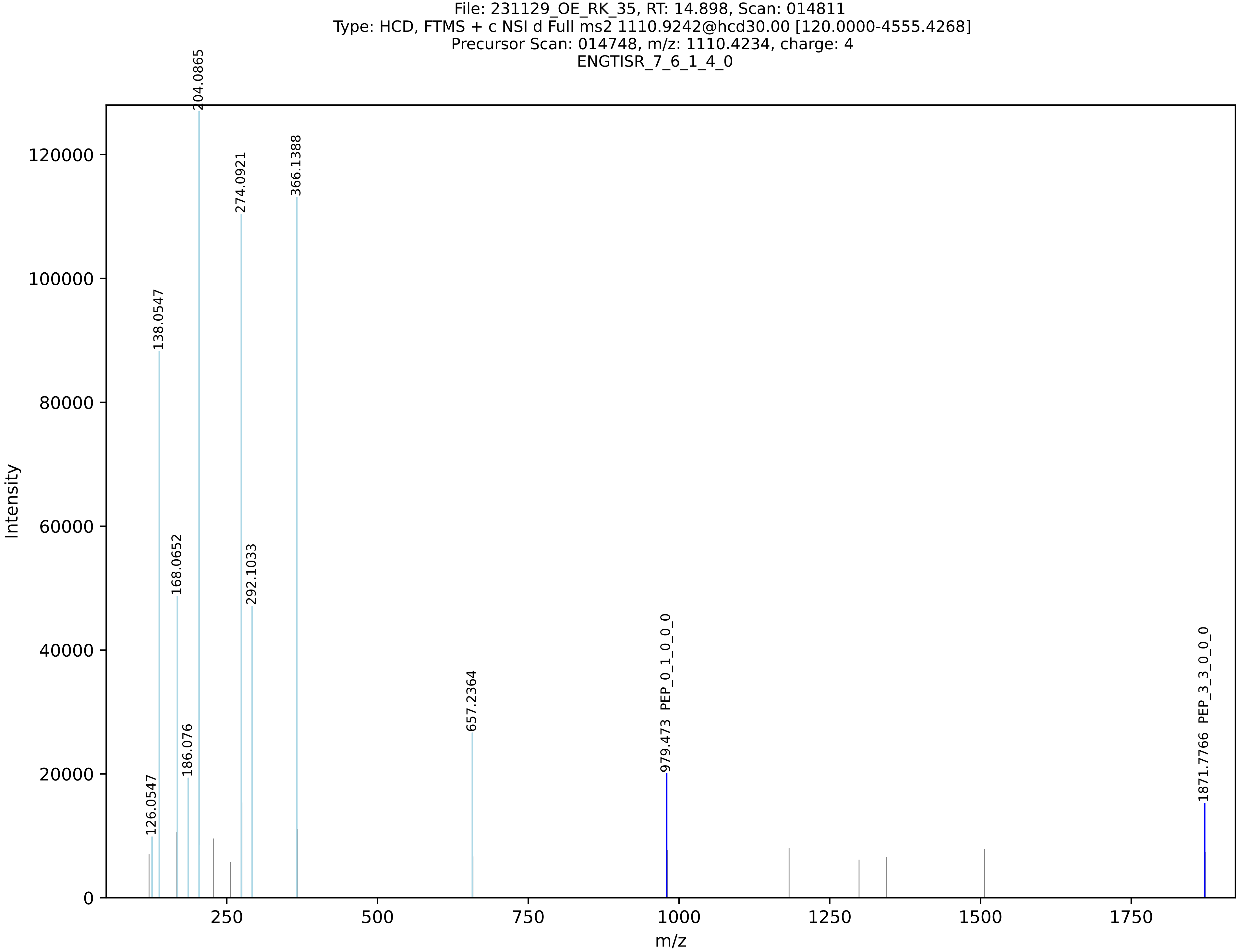
File: 231129_OE_RK_35, RT: 14.898, Scan: 014811
Type: HCD, FTMS + c NSI d Full ms2 1110.9242@hcd30.00 [120.0000-4555.4268]
Precursor Scan: 014748, m/z: 1110.4234, charge: 4
ENGTISR_7_6_1_4_0
Intensity
m/z
204.0865
366.1388
274.0921
138.0547
168.0652
292.1033
186.076
126.0547
657.2364
979.473 PEP_0_1_0_0_0
1871.7766 PEP_3_3_0_0_0
0
20000
40000
60000
80000
100000
120000
250
500
750
1000
1250
1500
1750

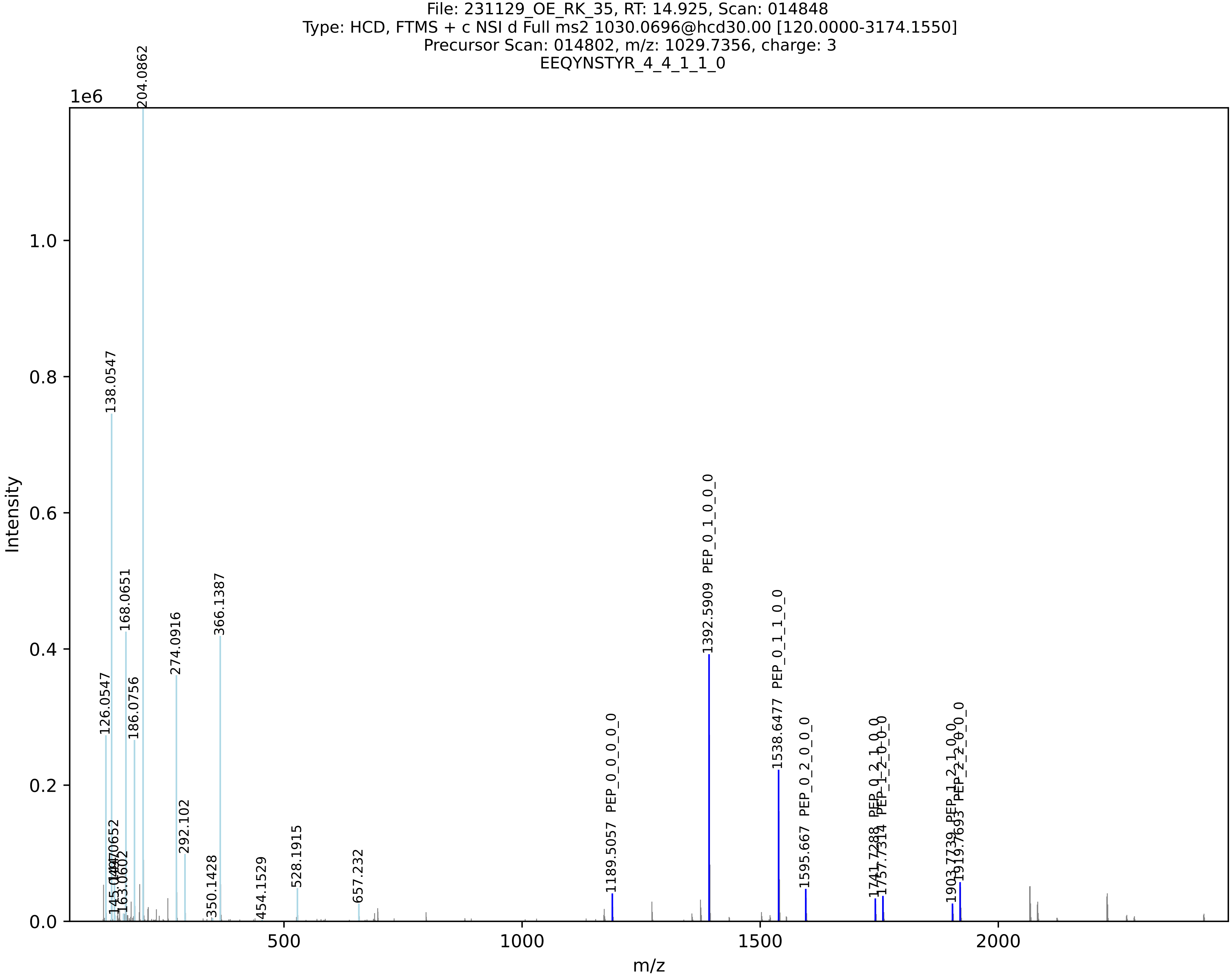
File: 231129_OE_RK_35, RT: 14.925, Scan: 014848
Type: HCD, FTMS + c NSI d Full ms2 1030.0696@hcd30.00 [120.0000-3174.1550]
Precursor Scan: 014802, m/z: 1029.7356, charge: 3
EEQYNSTYR_4_4_1_1_0
1e6
Intensity
m/z
0.0
0.2
0.4
0.6
0.8
1.0
500
1000
1500
2000
204.0862
138.0547
168.0651
126.0547
186.0756
274.0916
292.102
366.1387
350.1428
454.1529
528.1915
657.232
163.0602
1189.5057 PEP_0_0_0_0_0
1392.5909 PEP_0_1_0_0_0
1538.6477 PEP_0_1_1_0_0
1595.667 PEP_0_2_0_0_0
1741.7288 PEP_0_2_1_0_0
1757.7314 PEP_1_2_0_0_0

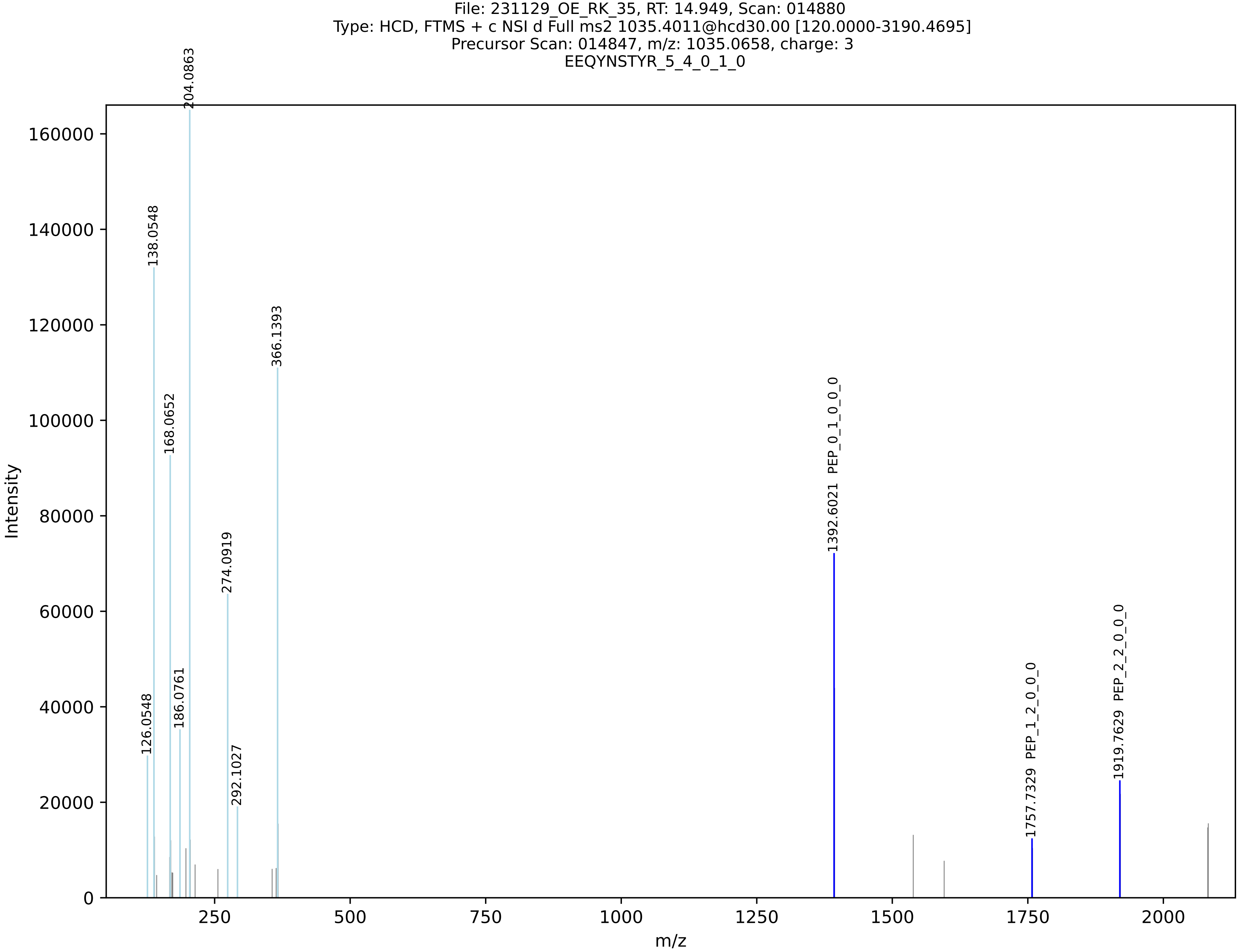

File: 231129_OE_RK_35, RT: 14.949, Scan: 014880
Type: HCD, FTMS + c NSI d Full ms2 1035.4011@hcd30.00 [120.0000-3190.4695]
Precursor Scan: 014847, m/z: 1035.0658, charge: 3
EEQYNSTYR_5_4_0_1_0
Intensity
m/z
204.0863
138.0548
366.1393
168.0652
274.0919
186.0761
126.0548
292.1027
1392.6021 PEP_0_1_0_0_
1757.7329 PEP_1_2_0_0_
1919.7629 PEP_2_2_0_0_

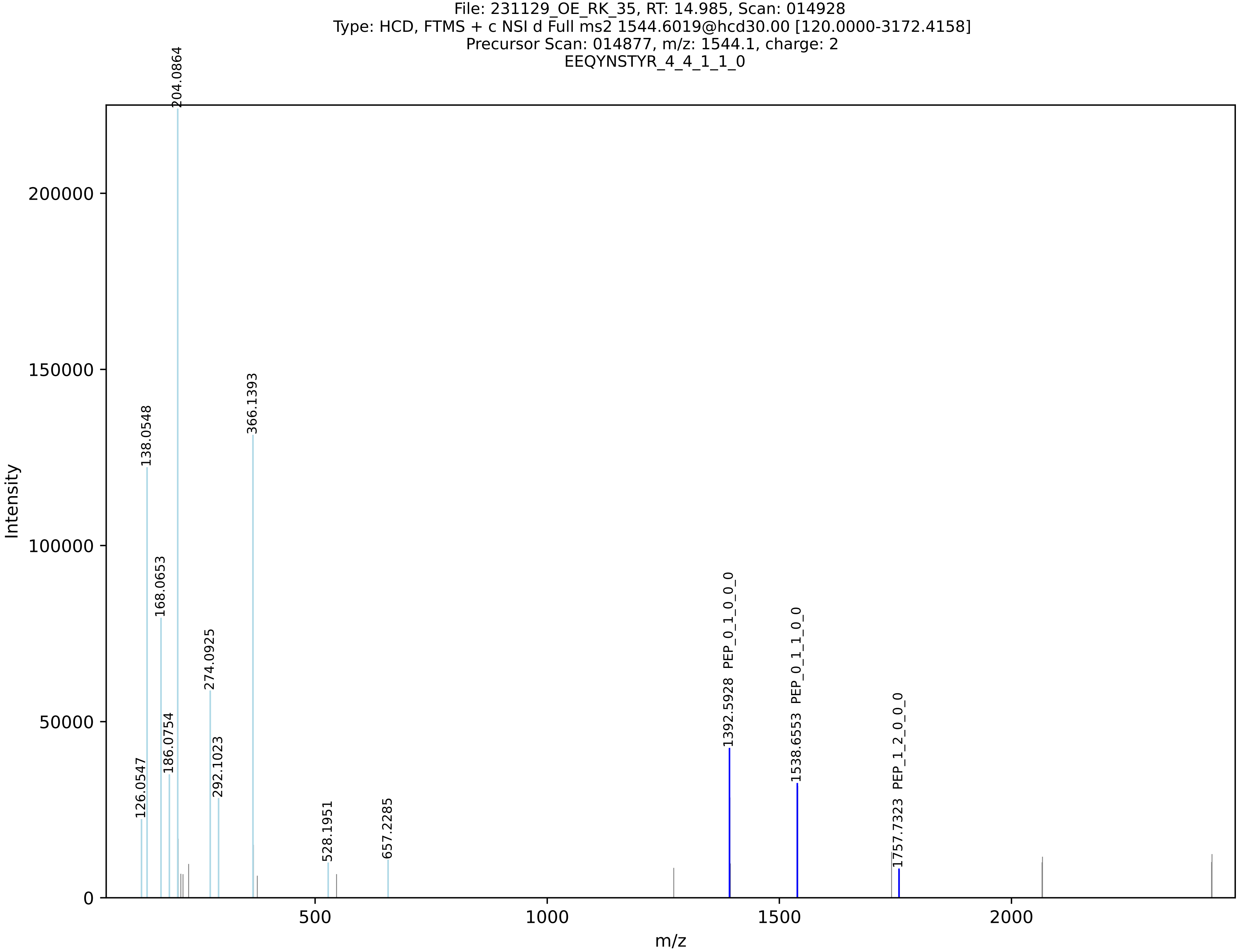

File: 231129_OE_RK_35, RT: 14.985, Scan: 014928
Type: HCD, FTMS + c NSI d Full ms2 1544.6019@hcd30.00 [120.0000-3172.4158]
Precursor Scan: 014877, m/z: 1544.1, charge: 2
EEQYNSTYR_4_4_1_1_0
204.0864
138.0548
366.1393
168.0653
274.0925
186.0754
292.1023
126.0547
528.1951
657.2285
1392.5928 PEP_0_1_0_0_
1538.6553 PEP_0_1_1_0_0
1757.7323 PEP_1_2_0_0_0
Intensity
m/z
0
50000
100000
150000
200000
500
1000
1500
2000

File: 231129_OE_RK_35, RT: 15.038, Scan: 014999
Type: HCD, FTMS + c NSI d Full ms2 1084.0875@hcd30.00 [120.0000-3339.4500]
Precursor Scan: 014963, m/z: 1083.7534, charge: 3
EEQYNSTYR_5_4_1_1_0

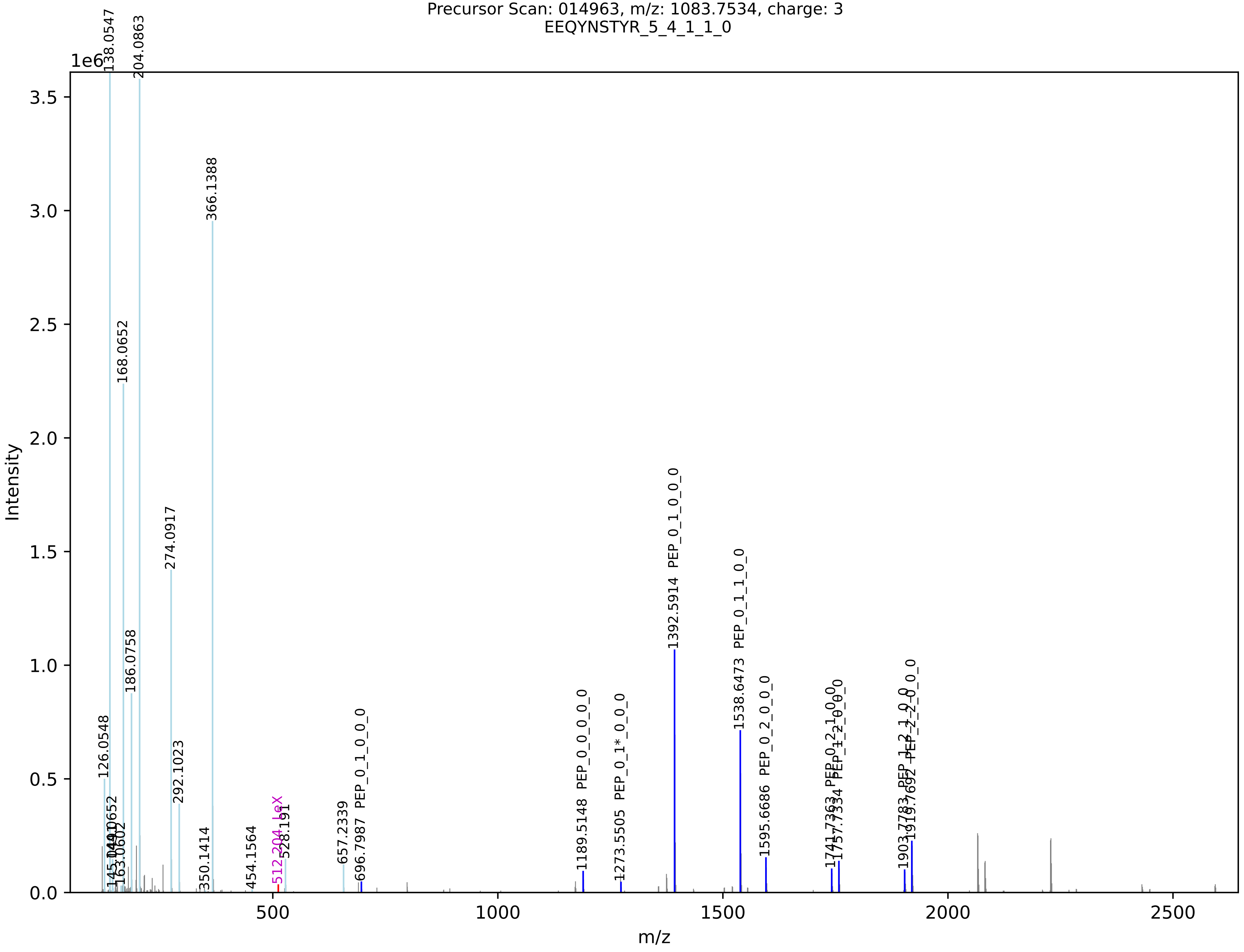

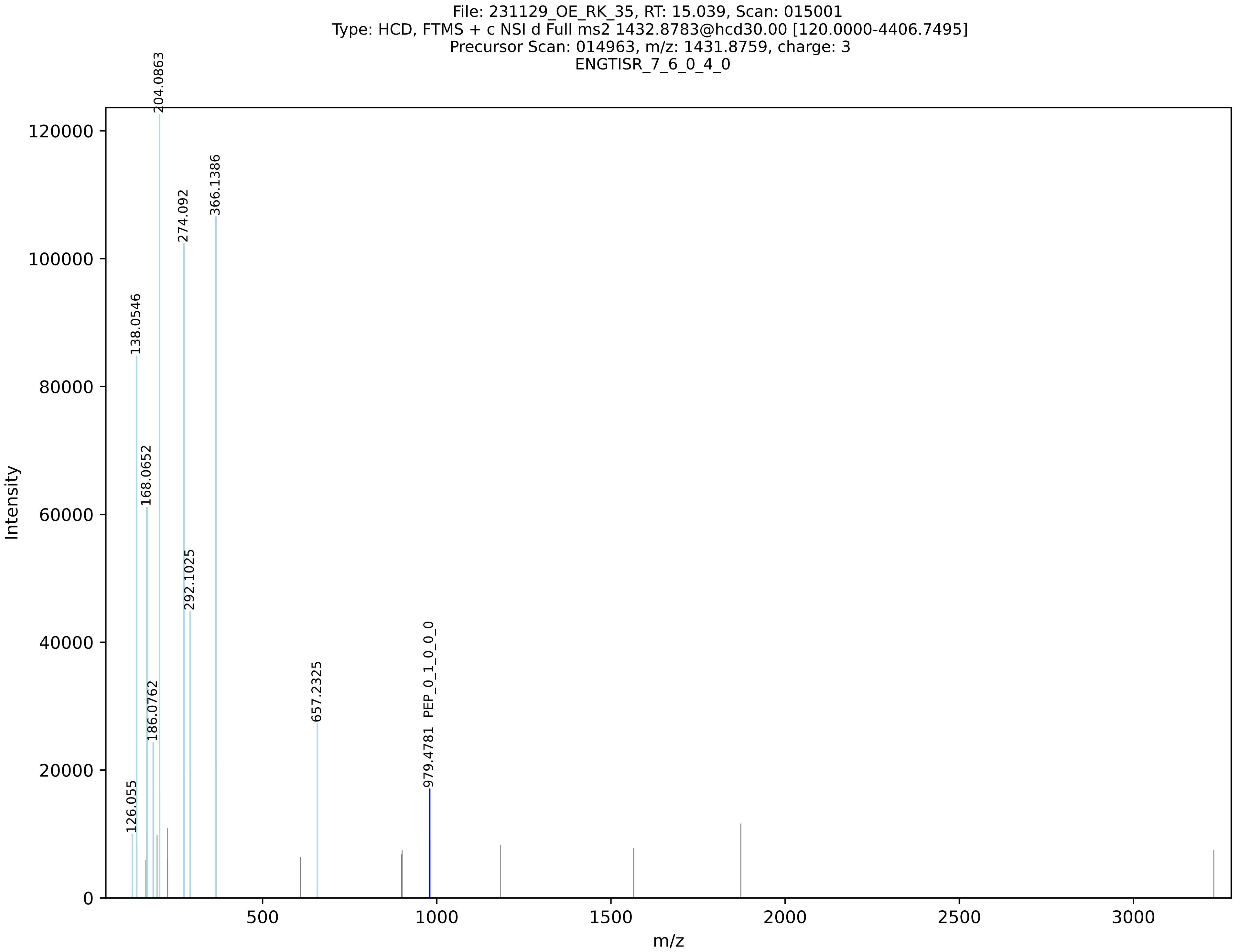
File: 231129_OE_RK_35, RT: 15.039, Scan: 015001
Type: HCD, FTMS + c NSI d Full ms2 1432.8783@hcd30.00 [120.0000-4406.7495]
Precursor Scan: 014963, m/z: 1431.8759, charge: 3
ENGTISR_7_6_0_4_0
Intensity
m/z
204.0863
366.1386
274.092
138.0546
168.0652
292.1025
186.0762
126.055
657.2325
979.4781 PEP_0_1_0_0_0
0
20000
40000
60000
80000
100000
120000
500
1000
1500
2000
2500
3000

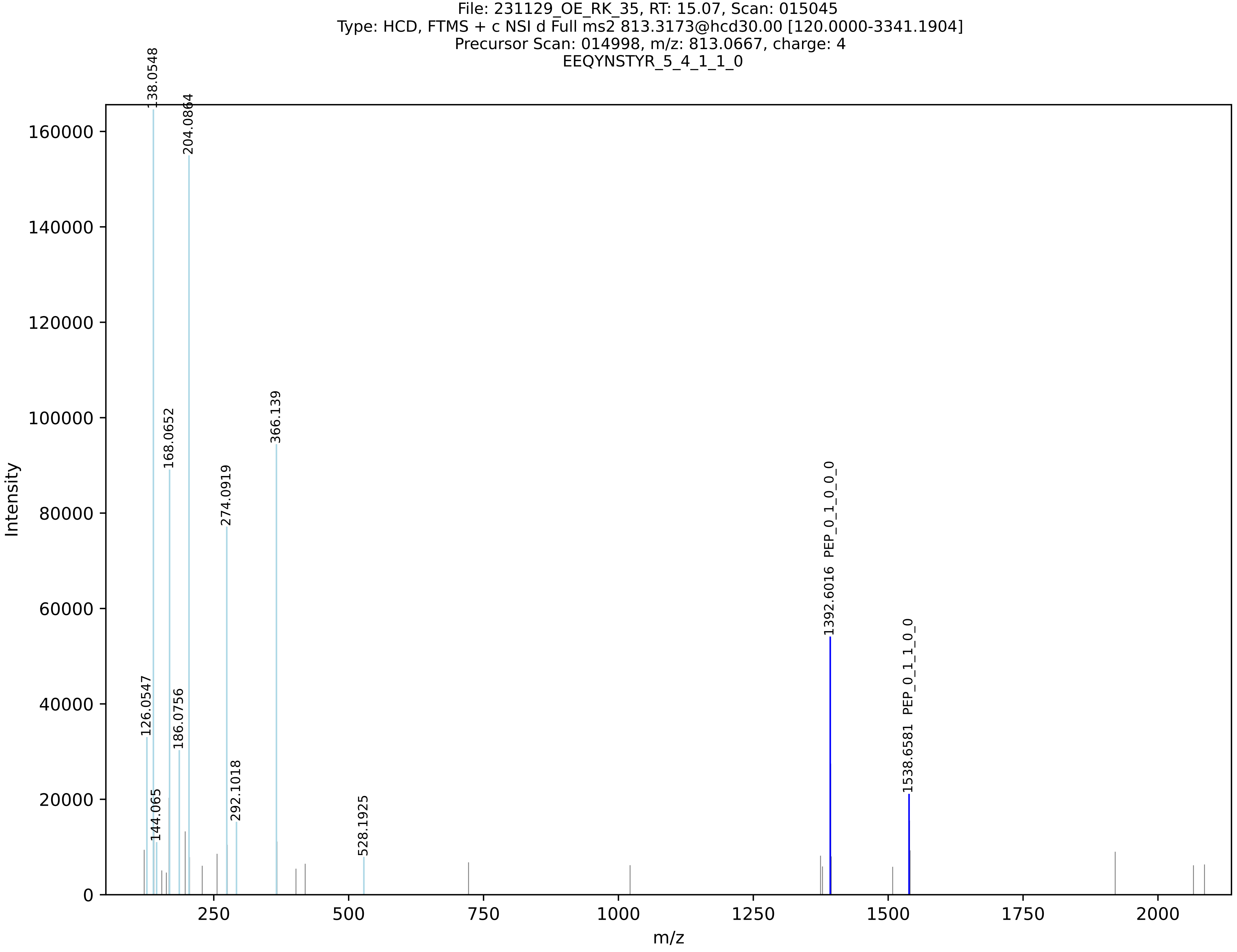
File: 231129_OE_RK_35, RT: 15.07, Scan: 015045
Type: HCD, FTMS + c NSI d Full ms2 813.3173@hcd30.00 [120.0000-3341.1904]
Precursor Scan: 014998, m/z: 813.0667, charge: 4
EEQYNSTYR_5_4_1_1_0
138.0548
204.0864
168.0652
366.139
274.0919
126.0547
186.0756
144.065
292.1018
528.1925
1392.6016 PEP_0_1_0_0_0
1538.6581 PEP_0_1_1_0_0
Intensity
m/z
0
20000
40000
60000
80000
100000
120000
140000
160000
250
500
750
1000
1250
1500
1750
2000

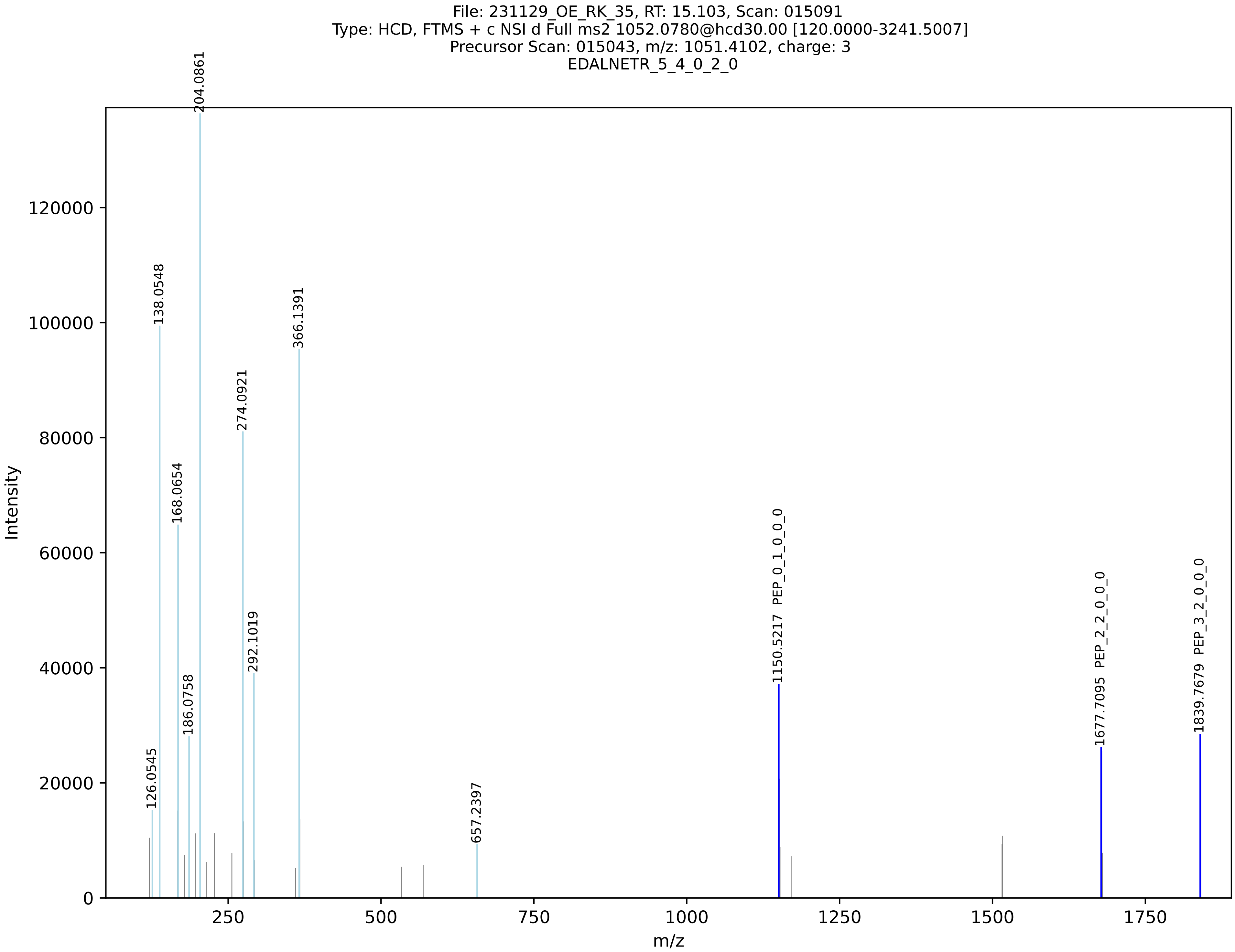
File: 231129_OE_RK_35, RT: 15.103, Scan: 015091
Type: HCD, FTMS + c NSI d Full ms2 1052.0780@hcd30.00 [120.0000-3241.5007]
Precursor Scan: 015043, m/z: 1051.4102, charge: 3
EDALNETR_5_4_0_2_0
204.0861
138.0548
366.1391
274.0921
168.0654
292.1019
186.0758
126.0545
657.2397
1150.5217 PEP_0_1_0_0_0
1677.7095 PEP_2_2_0_0_0
1839.7679 PEP_3_2_0_0_0
Intensity
m/z
0
20000
40000
60000
80000
100000
120000
250
500
750
1000
1250
1500
1750

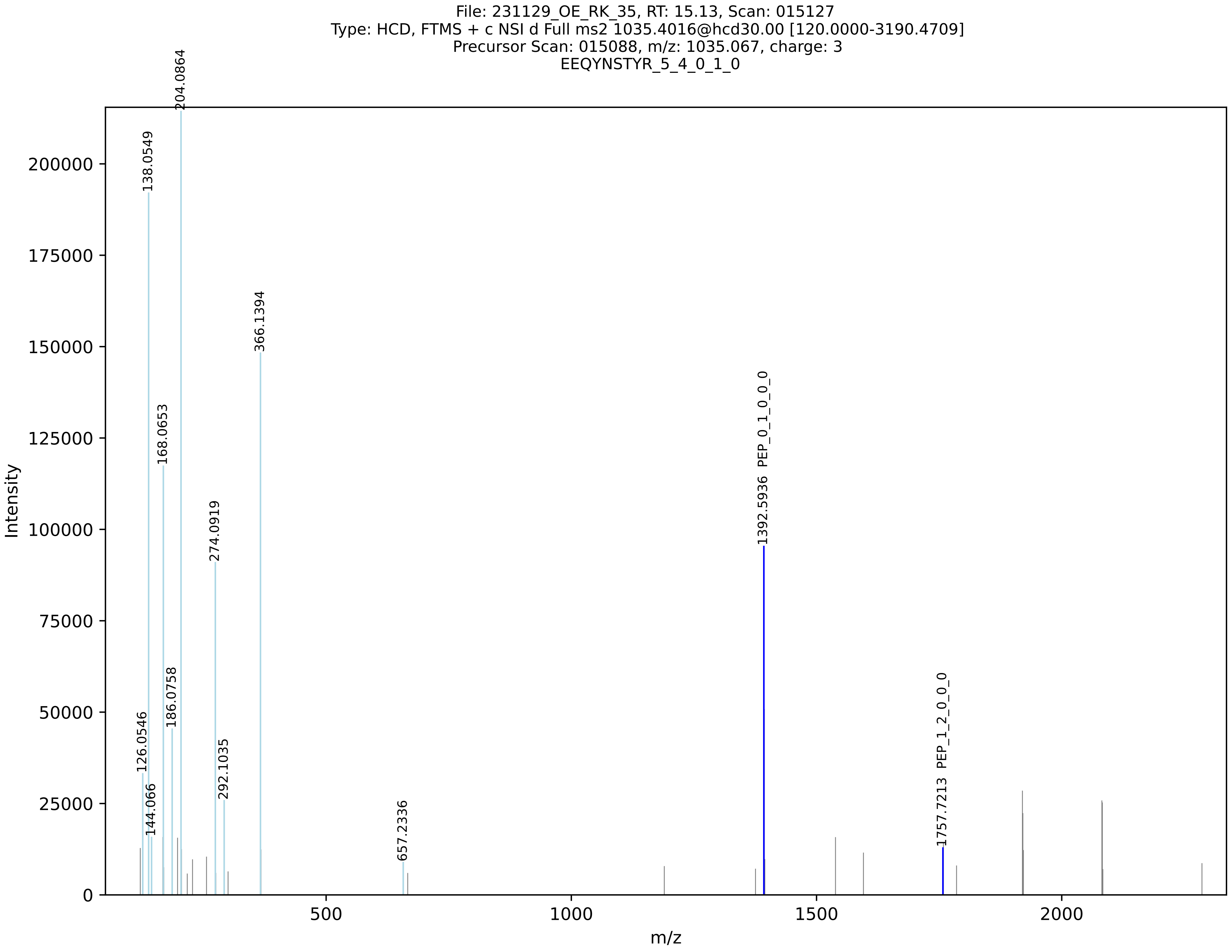

File: 231129_OE_RK_35, RT: 15.13, Scan: 015127
Type: HCD, FTMS + c NSI d Full ms2 1035.4016@hcd30.00 [120.0000-3190.4709]
Precursor Scan: 015088, m/z: 1035.067, charge: 3
EEQYNSTYR_5_4_0_1_0
Intensity
m/z
204.0864
138.0549
168.0653
366.1394
274.0919
186.0758
126.0546
144.066
292.1035
657.2336
1392.5936 PEP_0_1_0_0_0
1757.7213 PEP_1_2_0_0_0

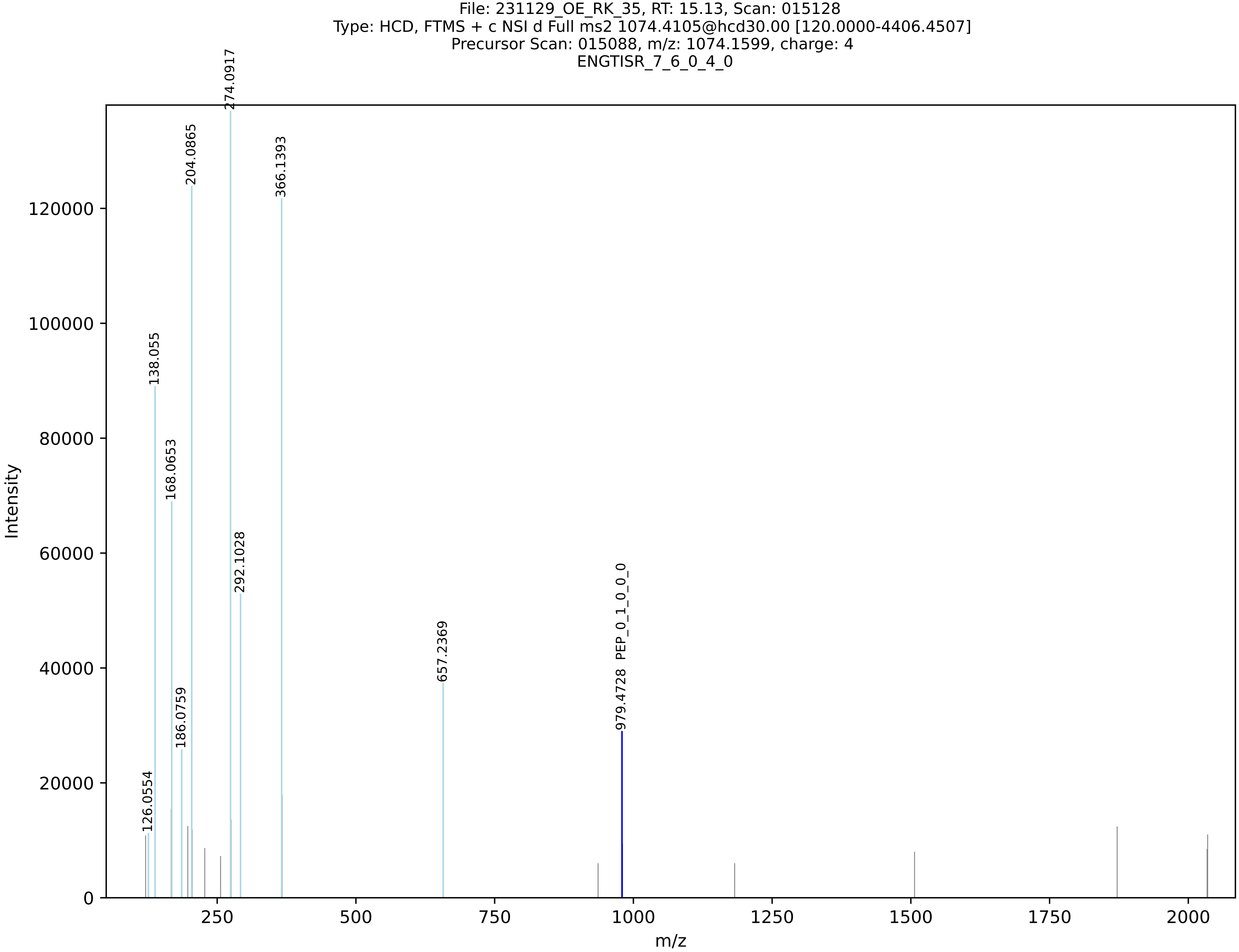
File: 231129_OE_RK_35, RT: 15.13, Scan: 015128
Type: HCD, FTMS + c NSI d Full ms2 1074.4105@hcd30.00 [120.0000-4406.4507]
Precursor Scan: 015088, m/z: 1074.1599, charge: 4
ENGTISR_7_6_0_4_0
274.0917
204.0865
366.1393
138.055
168.0653
292.1028
657.2369
979.4728 PEP_0_1_0_0_0_
186.0759
126.0554
Intensity
m/z
0
20000
40000
60000
80000
100000
120000
250
500
750
1000
1250
1500
1750
2000

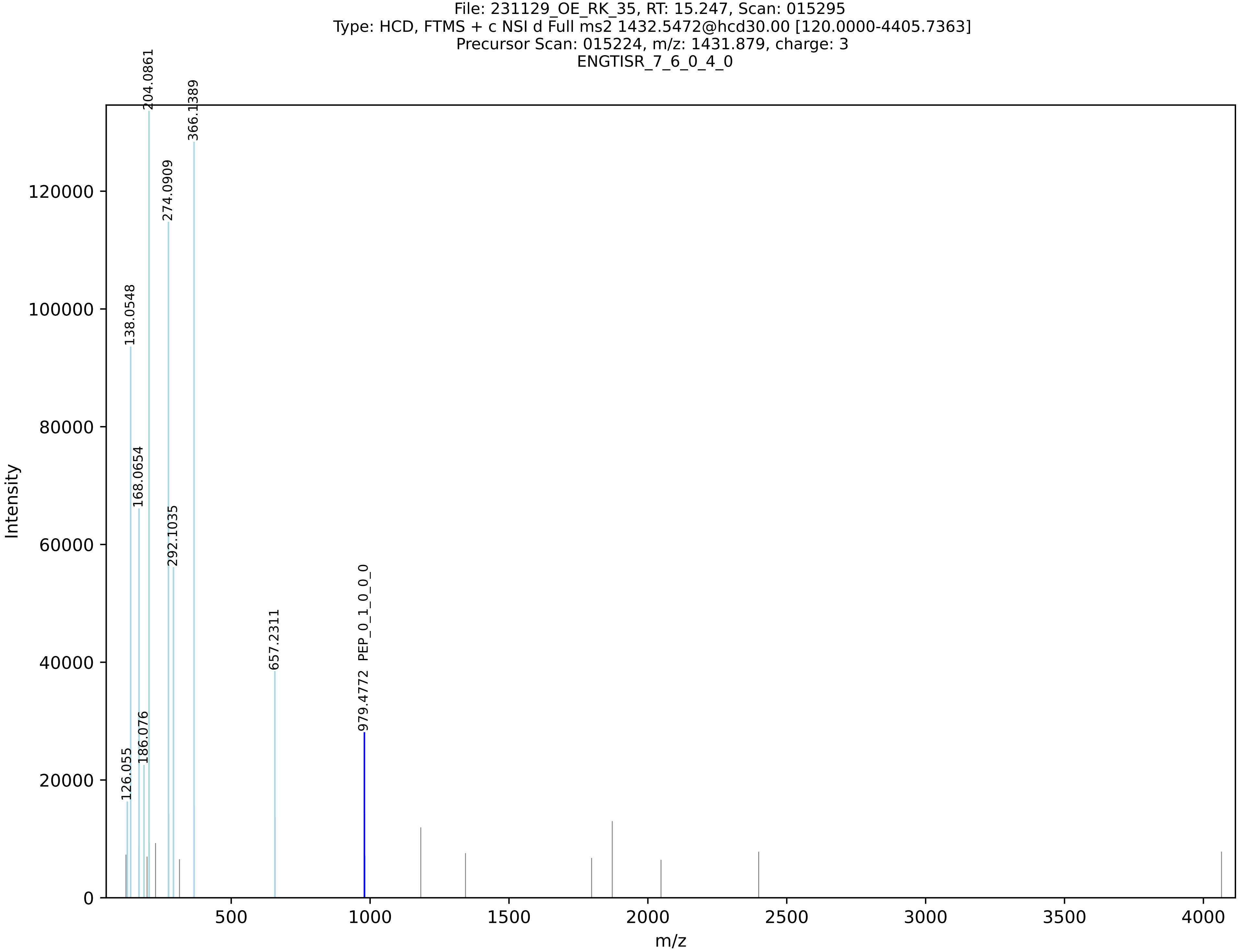
File: 231129_OE_RK_35, RT: 15.247, Scan: 015295
Type: HCD, FTMS + c NSI d Full ms2 1432.5472@hcd30.00 [120.0000-4405.7363]
Precursor Scan: 015224, m/z: 1431.879, charge: 3
ENGTISR_7_6_0_4_0
204.0861
366.1389
274.0909
138.0548
168.0654
292.1035
657.2311
979.4772 PEP_0_1_0_0_0
186.076
126.055
Intensity
m/z
0
20000
40000
60000
80000
100000
120000
500
1000
1500
2000
2500
3000
3500
4000

File: 231129_OE_RK_35, RT: 15.247, Scan: 015296
Type: HCD, FTMS + c NSI d Full ms2 813.3185@hcd30.00 [120.0000-3341.1958]
Precursor Scan: 015224, m/z: 813.0673, charge: 4
EEQYNSTYR_5_4_1_1_0

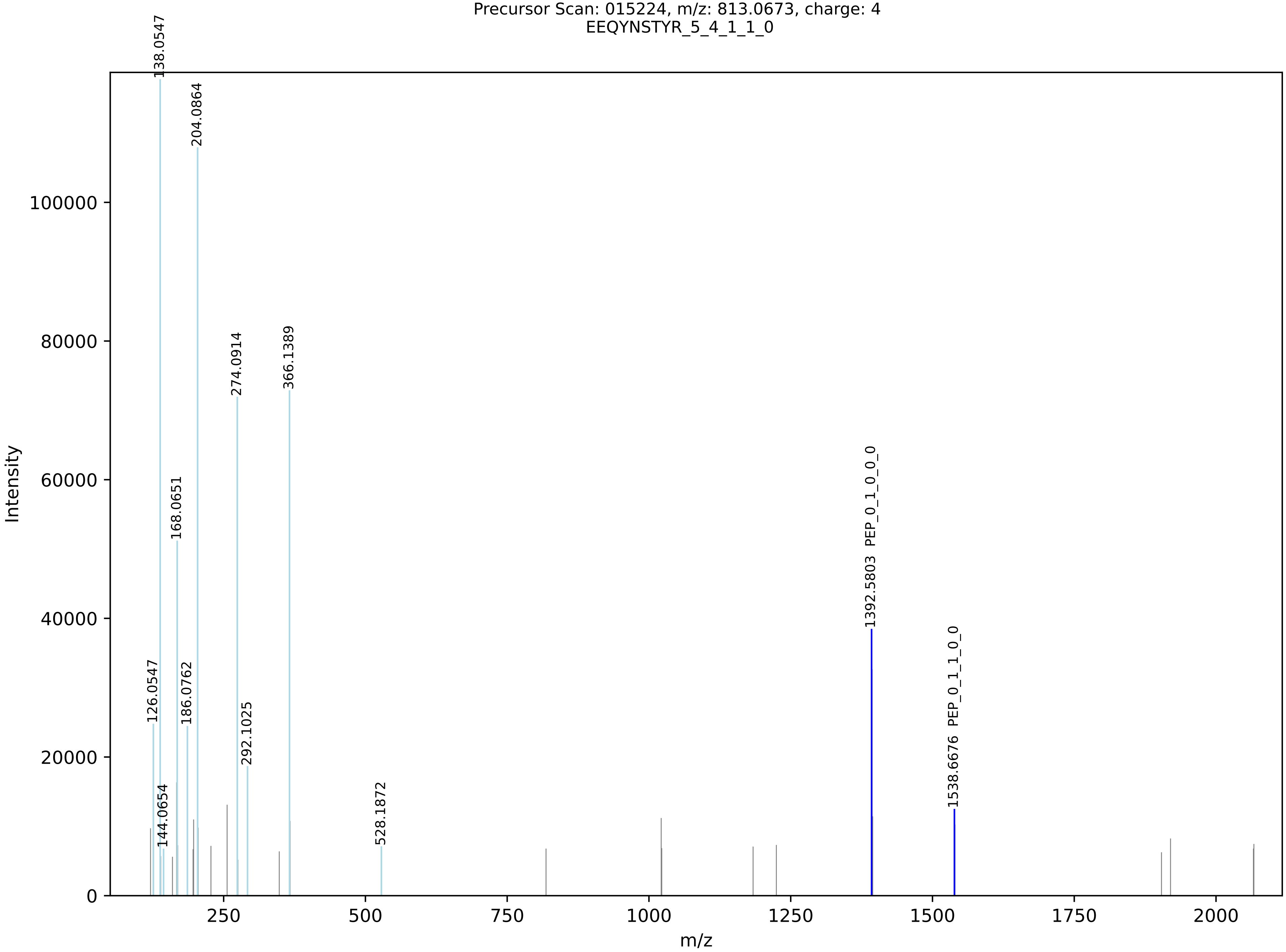

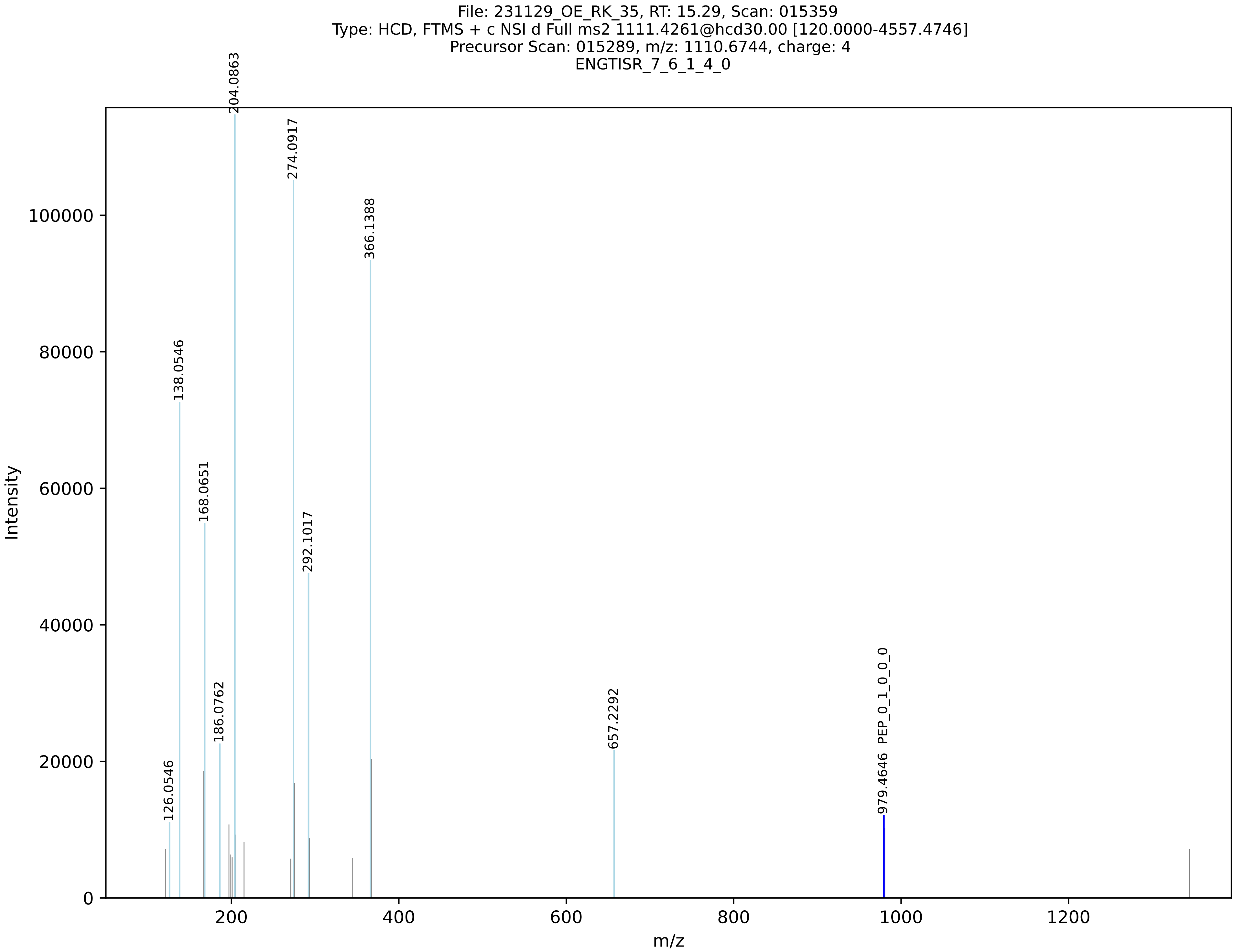
File: 231129_OE_RK_35, RT: 15.29, Scan: 015359
Type: HCD, FTMS + c NSI d Full ms2 1111.4261@hcd30.00 [120.0000-4557.4746]
Precursor Scan: 015289, m/z: 1110.6744, charge: 4
ENGTISR_7_6_1_4_0
204.0863
274.0917
366.1388
138.0546
168.0651
292.1017
186.0762
126.0546
657.2292
979.4646 PEP_0_1_0_0_0
Intensity
m/z
0
20000
40000
60000
80000
100000
200
400
600
800
1000
1200

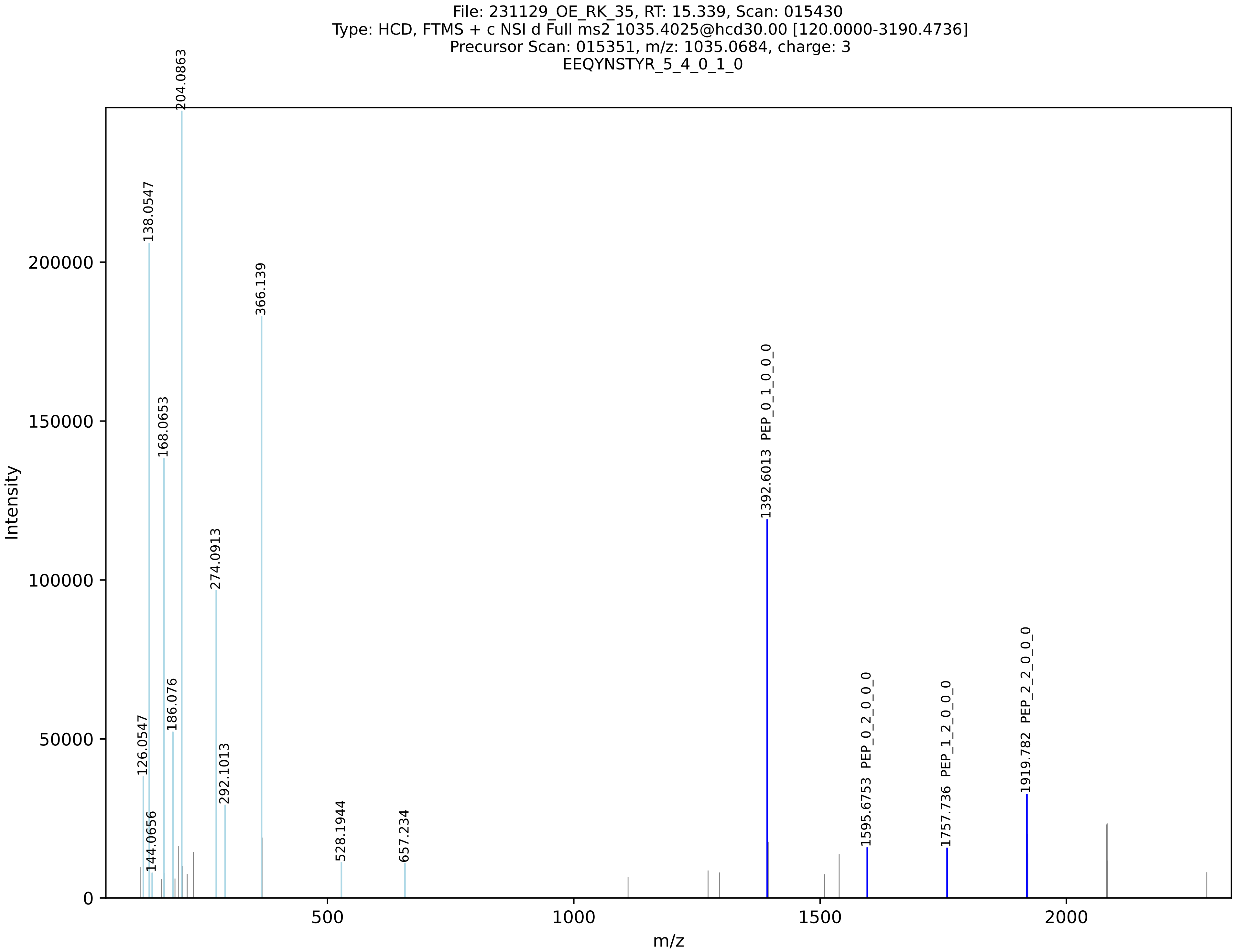
File: 231129_OE_RK_35, RT: 15.339, Scan: 015430
Type: HCD, FTMS + c NSI d Full ms2 1035.4025@hcd30.00 [120.0000-3190.4736]
Precursor Scan: 015351, m/z: 1035.0684, charge: 3
EEQYNSTYR_5_4_0_1_0
204.0863
138.0547
168.0653
366.139
274.0913
186.076
126.0547
292.1013
144.0656
528.1944
657.234
1392.6013 PEP_0_1_0_0_0
1595.6753 PEP_0_2_0_0_0
1757.736 PEP_1_2_0_0_0
1919.782 PEP_2_2_0_0_0
Intensity
m/z
0
50000
100000
150000
200000
500
1000
1500
2000

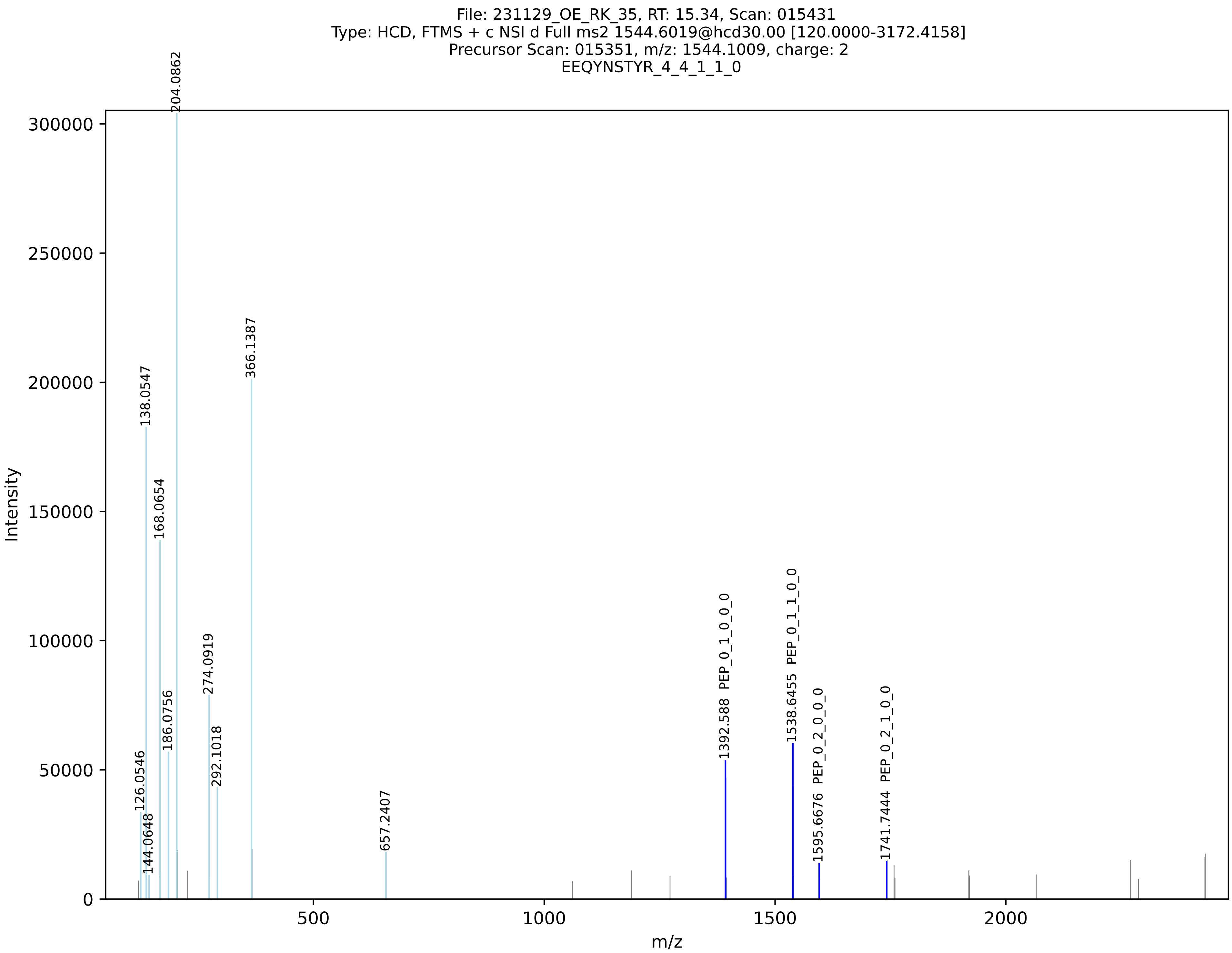
File: 231129_OE_RK_35, RT: 15.34, Scan: 015431
Type: HCD, FTMS + c NSI d Full ms2 1544.6019@hcd30.00 [120.0000-3172.4158]
Precursor Scan: 015351, m/z: 1544.1009, charge: 2
EEQYNSTYR_4_4_1_1_0
Intensity
m/z
204.0862
366.1387
138.0547
168.0654
274.0919
186.0756
292.1018
126.0546
144.0648
657.2407
1392.588 PEP_0_1_0_0_0
1538.6455 PEP_0_1_1_0_0
1595.6676 PEP_0_2_0_0_0
1741.7444 PEP_0_2_1_0_0
0
50000
100000
150000
200000
250000
300000
500
1000
1500
2000

File: 231129_OE_RK_35, RT: 15.372, Scan: 015482
Type: HCD, FTMS + c NSI d Full ms2 1052.0461@hcd30.00 [120.0000-3241.4031]
Precursor Scan: 015351, m/z: 1051.7088, charge: 3
EDALNETR_5_4_0_2_0

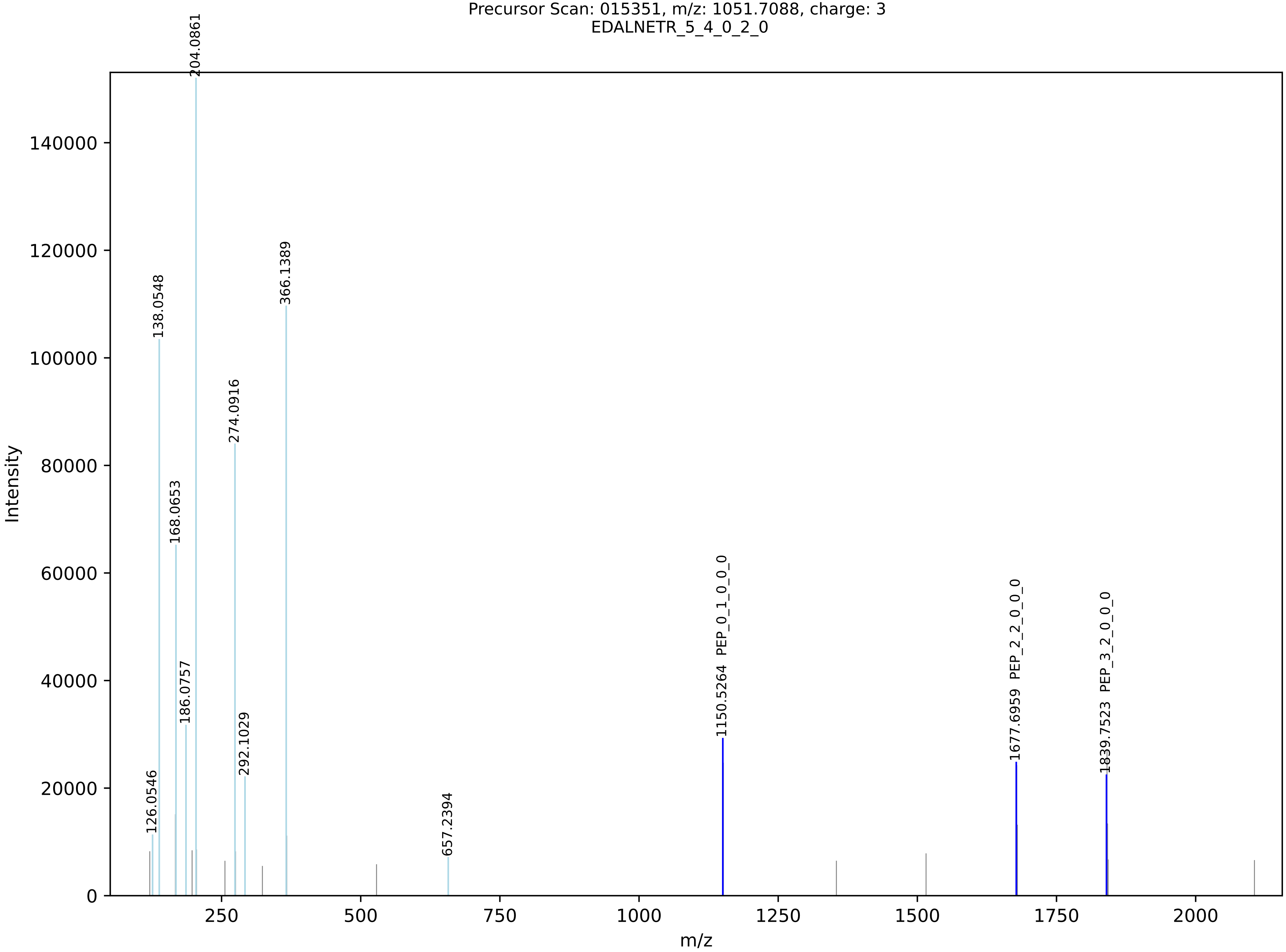

File: 231129_OE_RK_35, RT: 15.39, Scan: 015505
Type: HCD, FTMS + c NSI d Full ms2 1074.4109@hcd30.00 [120.0000-4406.4526]
Precursor Scan: 015425, m/z: 1074.1603, charge: 4
ENGTISR_7_6_0_4_0

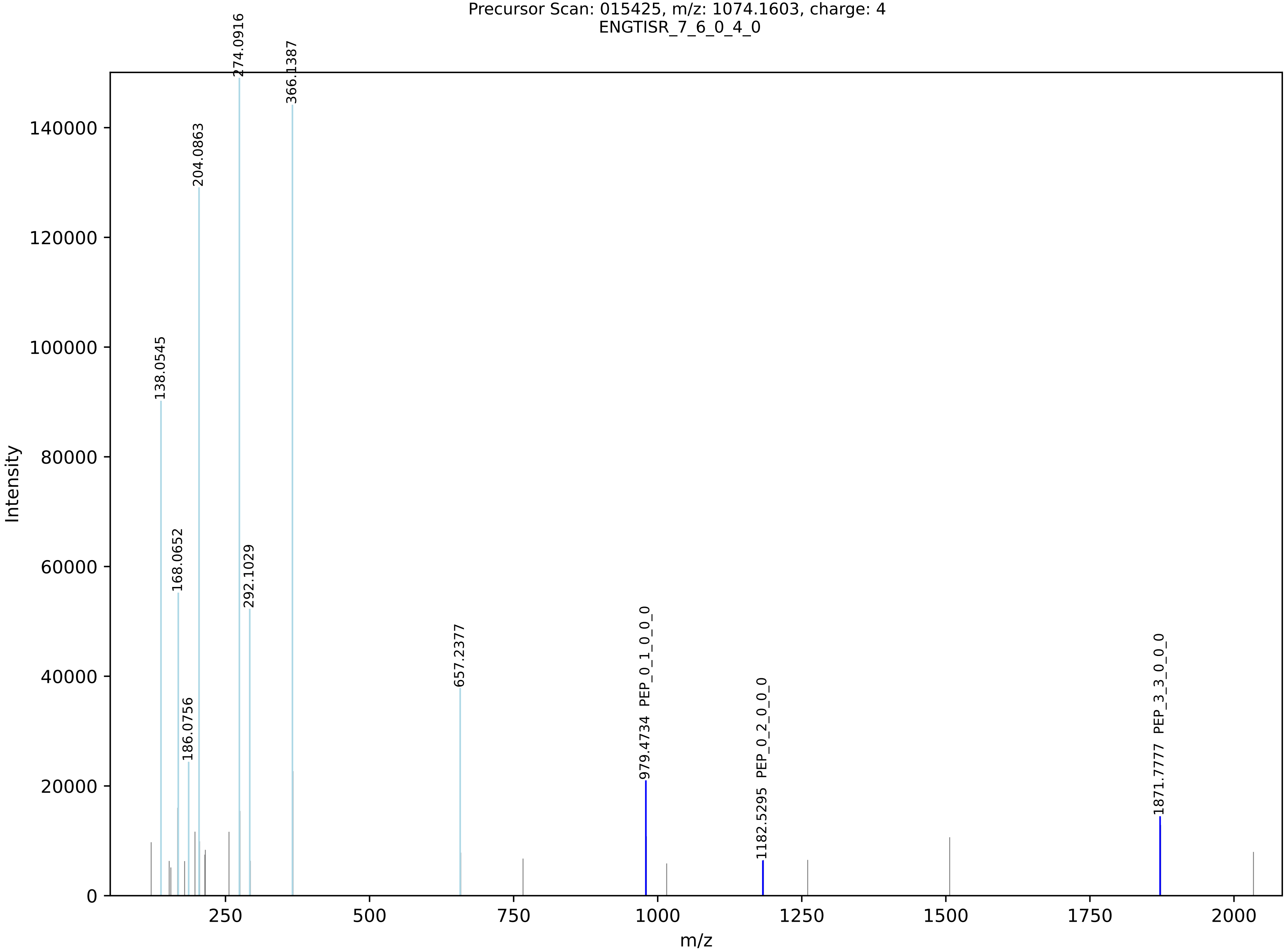

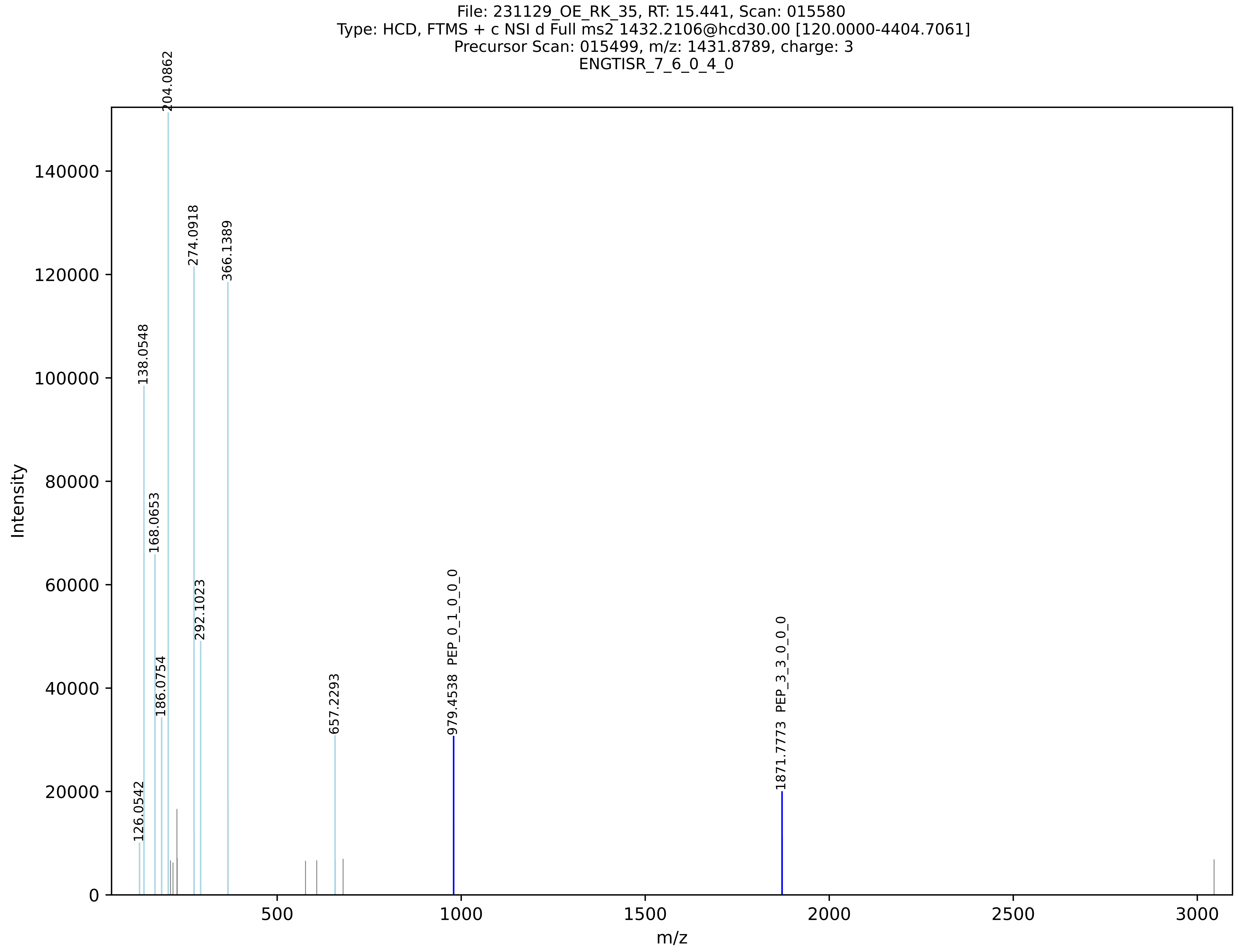

File: 231129_OE_RK_35, RT: 15.441, Scan: 015580
Type: HCD, FTMS + c NSI d Full ms2 1432.2106@hcd30.00 [120.0000-4404.7061]
Precursor Scan: 015499, m/z: 1431.8789, charge: 3
ENGTISR_7_6_0_4_0
Intensity
m/z
0
20000
40000
60000
80000
100000
120000
140000
500
1000
1500
2000
2500
3000
126.0542
138.0548
168.0653
186.0754
204.0862
274.0918
292.1023
366.1389
657.2293
979.4538 PEP_0_1_0_0_0
1871.7773 PEP_3_3_0_0_0

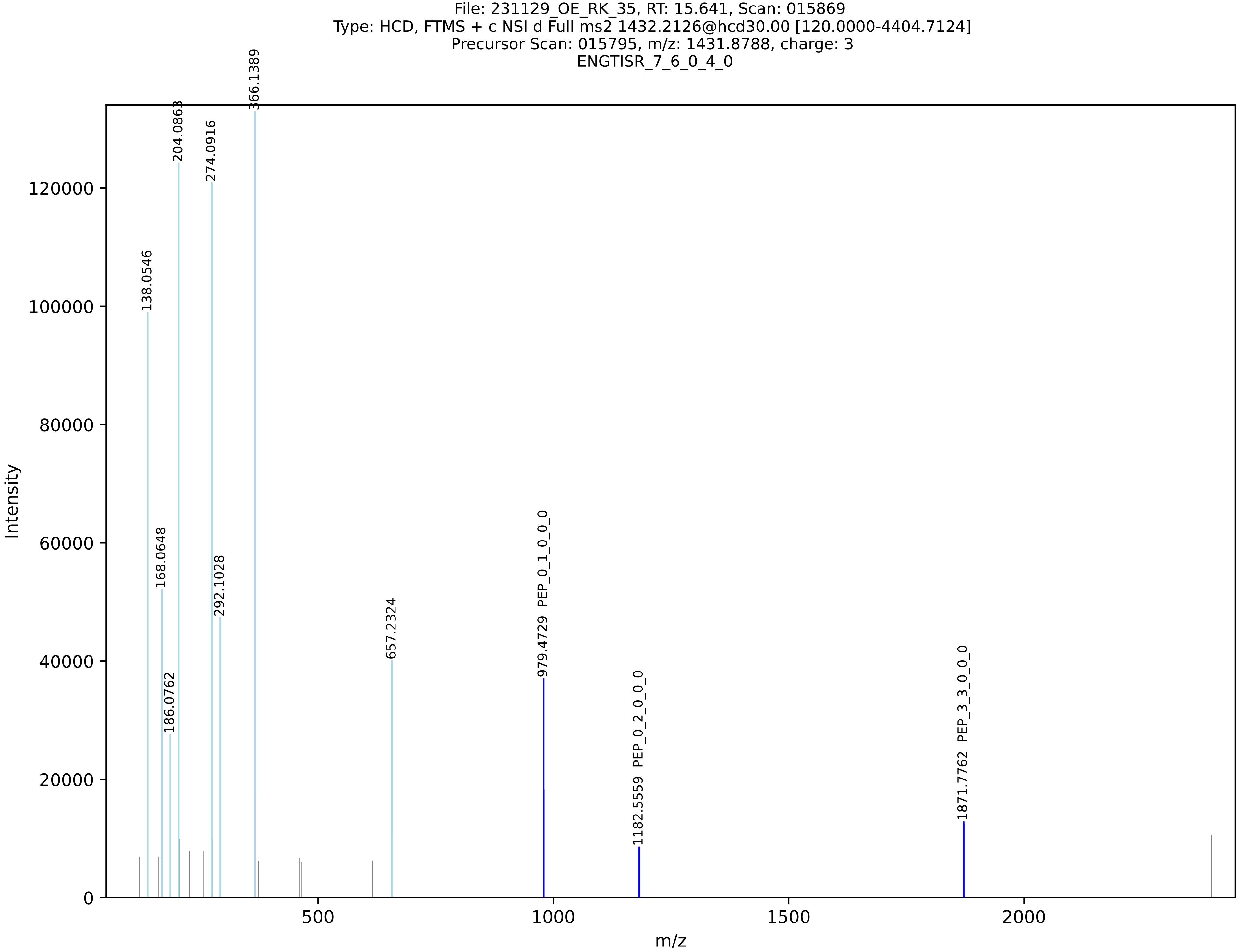
File: 231129_OE_RK_35, RT: 15.641, Scan: 015869
Type: HCD, FTMS + c NSI d Full ms2 1432.2126@hcd30.00 [120.0000-4404.7124]
Precursor Scan: 015795, m/z: 1431.8788, charge: 3
ENGTISR_7_6_0_4_0
Intensity
m/z
138.0546
168.0648
186.0762
204.0863
274.0916
292.1028
366.1389
657.2324
979.4729 PEP_0_1_0_0_0
1182.5559 PEP_0_2_0_0_0
1871.7762 PEP_3_3_0_0_0

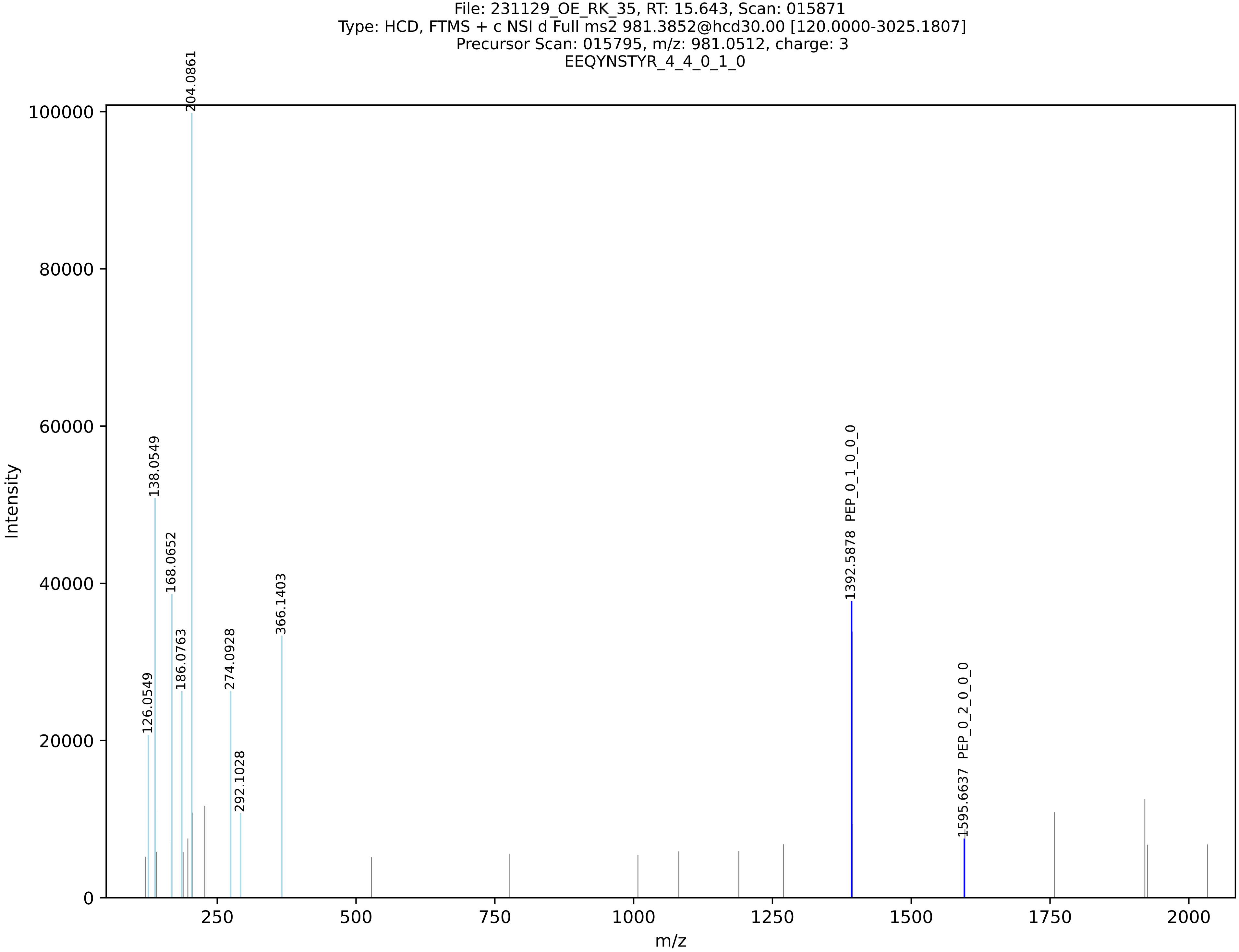
File: 231129_OE_RK_35, RT: 15.643, Scan: 015871
Type: HCD, FTMS + c NSI d Full ms2 981.3852@hcd30.00 [120.0000-3025.1807]
Precursor Scan: 015795, m/z: 981.0512, charge: 3
EEQYNSTYR_4_4_0_1_0
Intensity
m/z
0
20000
40000
60000
80000
100000
250
500
750
1000
1250
1500
1750
2000
204.0861
138.0549
168.0652
186.0763
126.0549
274.0928
292.1028
366.1403
1392.5878 PEP_0_1_0_0_0
1595.6637 PEP_0_2_0_0_0

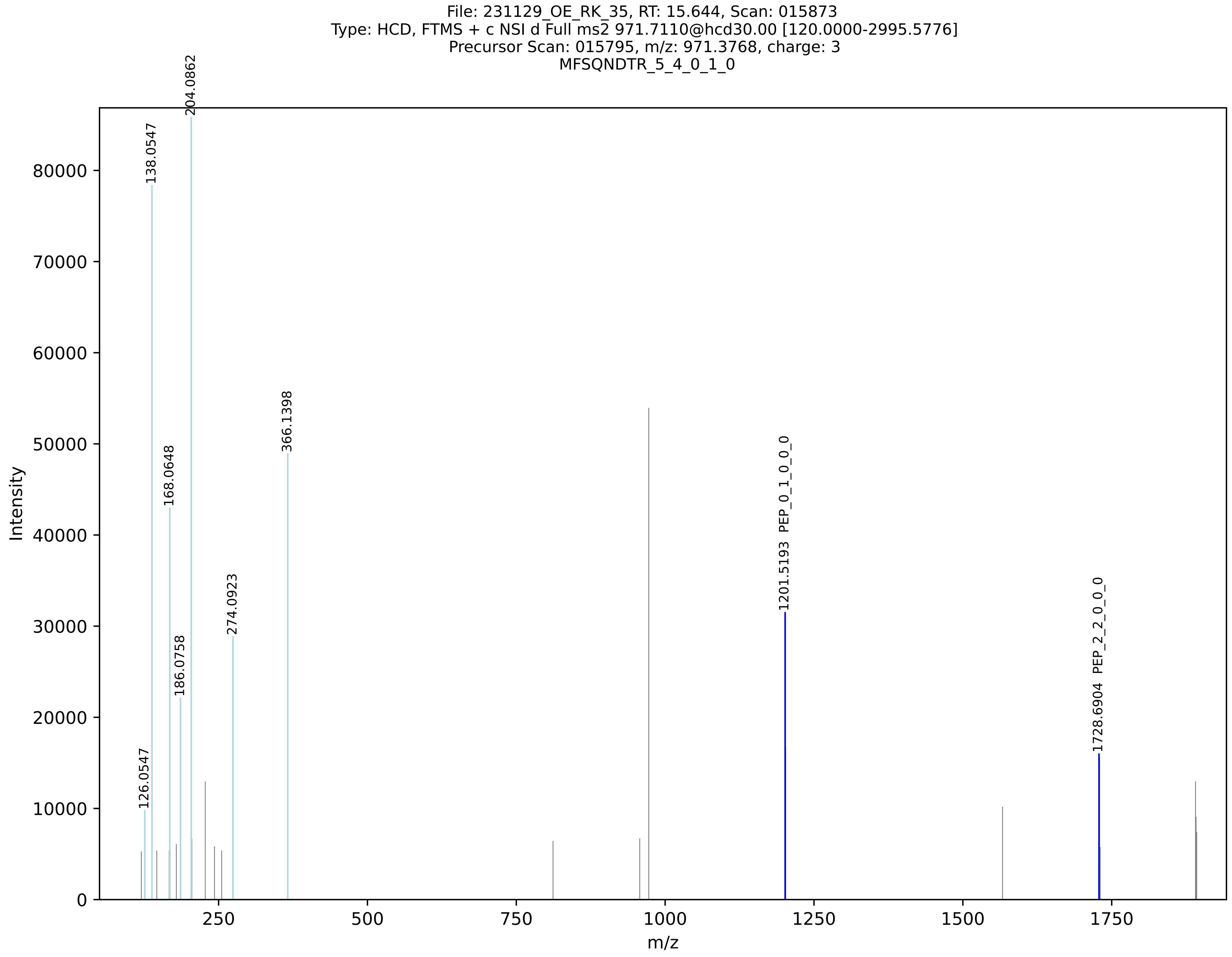
File: 231129_OE_RK_35, RT: 15.644, Scan: 015873
Type: HCD, FTMS + c NSI d Full ms2 971.7110@hcd30.00 [120.0000-2995.5776]
Precursor Scan: 015795, m/z: 971.3768, charge: 3
MFSQNDTR_5_4_0_1_0
Intensity
m/z
204.0862
138.0547
168.0648
186.0758
126.0547
274.0923
366.1398
1201.5193 PEP_0_1_0_0_0
1728.6904 PEP_2_2_0_0_0
0
10000
20000
30000
40000
50000
60000
70000
80000
250
500
750
1000
1250
1500
1750

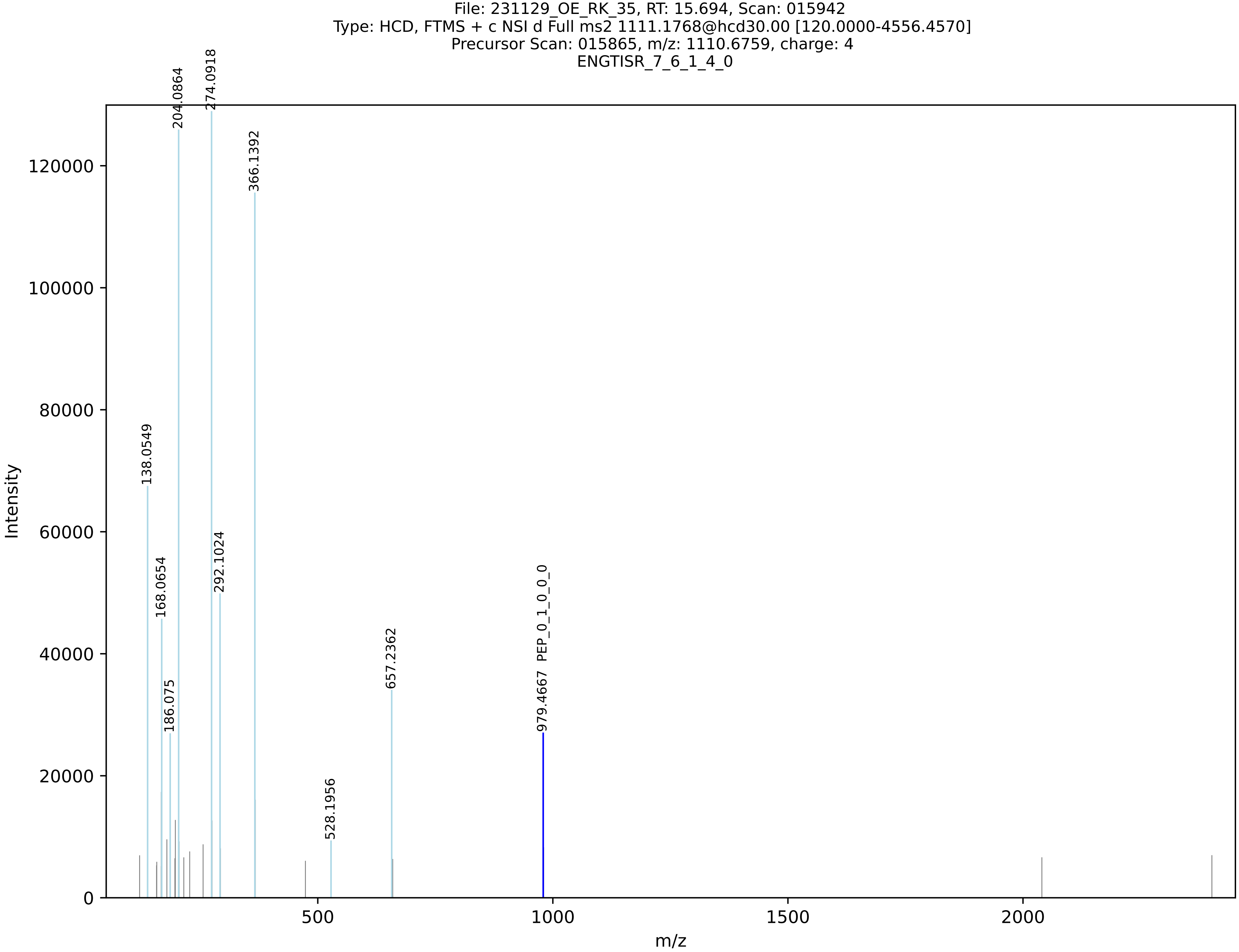

File: 231129_OE_RK_35, RT: 15.694, Scan: 015942
Type: HCD, FTMS + c NSI d Full ms2 1111.1768@hcd30.00 [120.0000-4556.4570]
Precursor Scan: 015865, m/z: 1110.6759, charge: 4
ENGTISR_7_6_1_4_0
204.0864
274.0918
366.1392
138.0549
168.0654
292.1024
186.075
657.2362
979.4667 PEP_0_1_0_0_0
528.1956
Intensity
m/z
0
20000
40000
60000
80000
100000
120000
500
1000
1500
2000

File: 231129_OE_RK_35, RT: 15.742, Scan: 016012
Type: HCD, FTMS + c NSI d Full ms2 1035.4032@hcd30.00 [120.0000-3190.4758]
Precursor Scan: 015939, m/z: 1035.0693, charge: 3
EEQYNSTYR_5_4_0_1_0

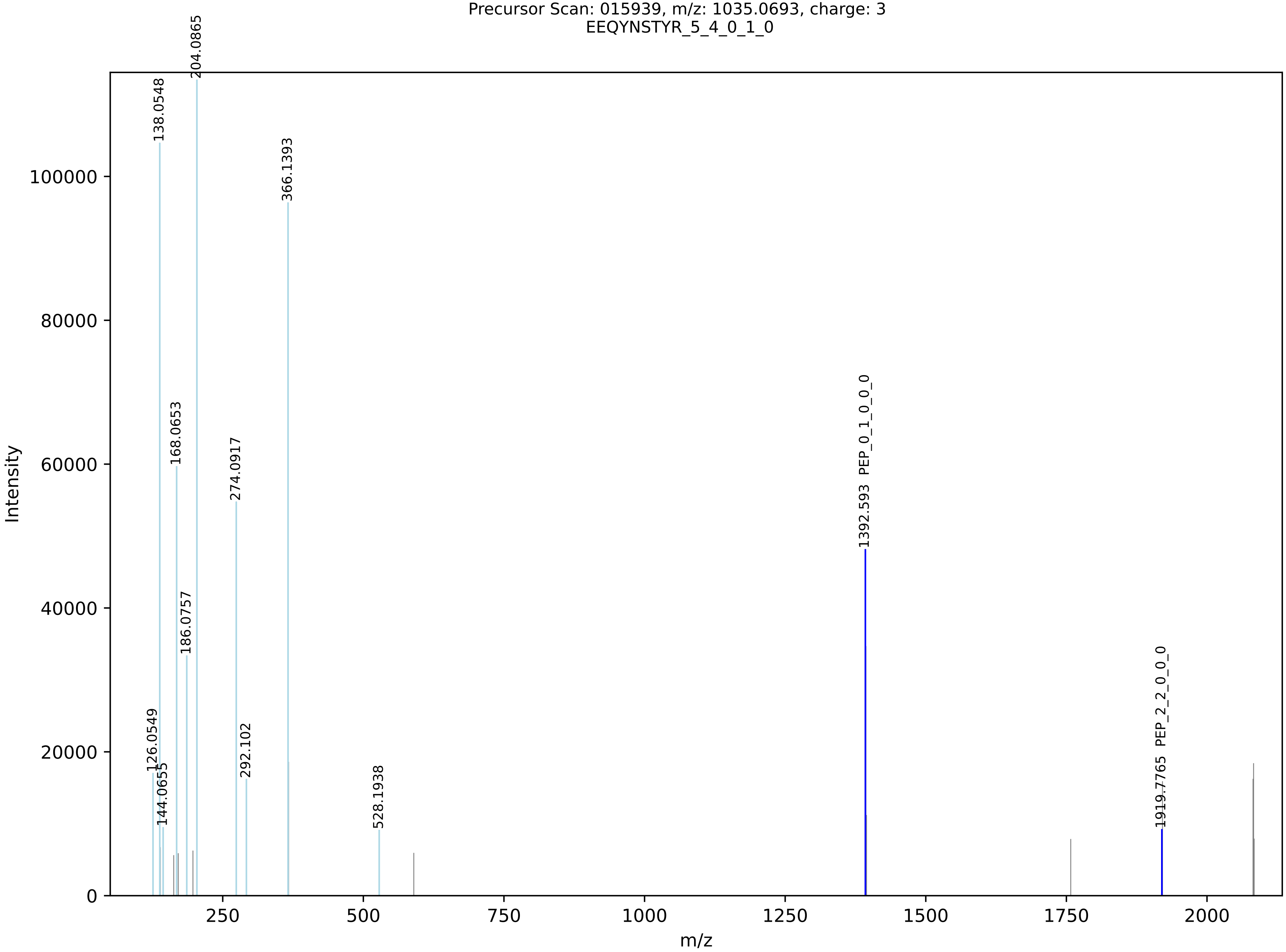

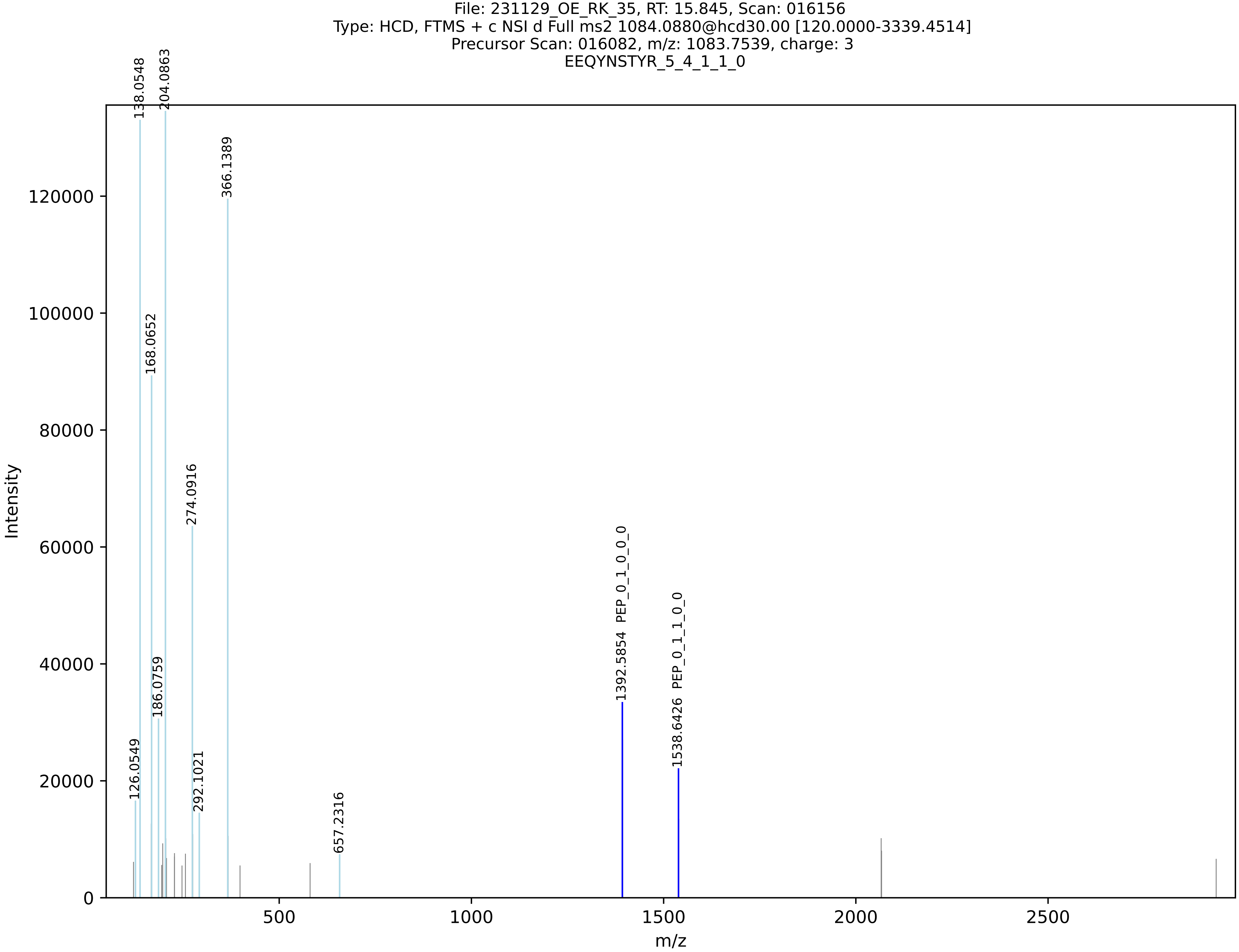

File: 231129_OE_RK_35, RT: 15.845, Scan: 016156
Type: HCD, FTMS + c NSI d Full ms2 1084.0880@hcd30.00 [120.0000-3339.4514]
Precursor Scan: 016082, m/z: 1083.7539, charge: 3
EEQYNSTYR_5_4_1_1_0
138.0548
204.0863
366.1389
168.0652
274.0916
186.0759
126.0549
292.1021
657.2316
1392.5854 PEP_0_1_0_0_0
1538.6426 PEP_0_1_1_0_0
Intensity
m/z
0
20000
40000
60000
80000
100000
120000
500
1000
1500
2000
2500

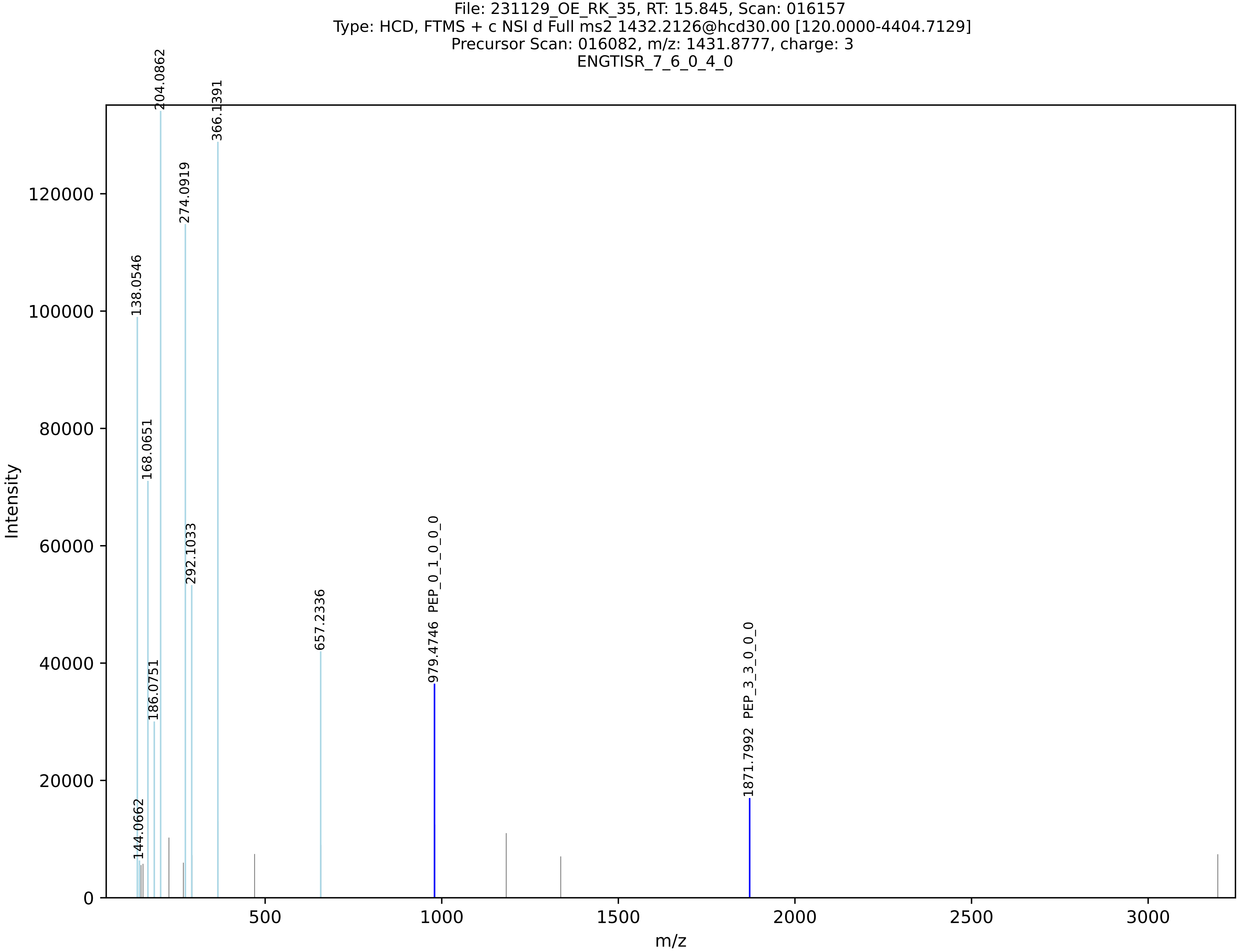
File: 231129_OE_RK_35, RT: 15.845, Scan: 016157
Type: HCD, FTMS + c NSI d Full ms2 1432.2126@hcd30.00 [120.0000-4404.7129]
Precursor Scan: 016082, m/z: 1431.8777, charge: 3
ENGTISR_7_6_0_4_0
204.0862
366.1391
274.0919
138.0546
168.0651
292.1033
657.2336
979.4746 PEP_0_1_0_0_0
186.0751
1871.7992 PEP_3_3_0_0_0
144.0662
Intensity
m/z
0
20000
40000
60000
80000
100000
120000
500
1000
1500
2000
2500
3000

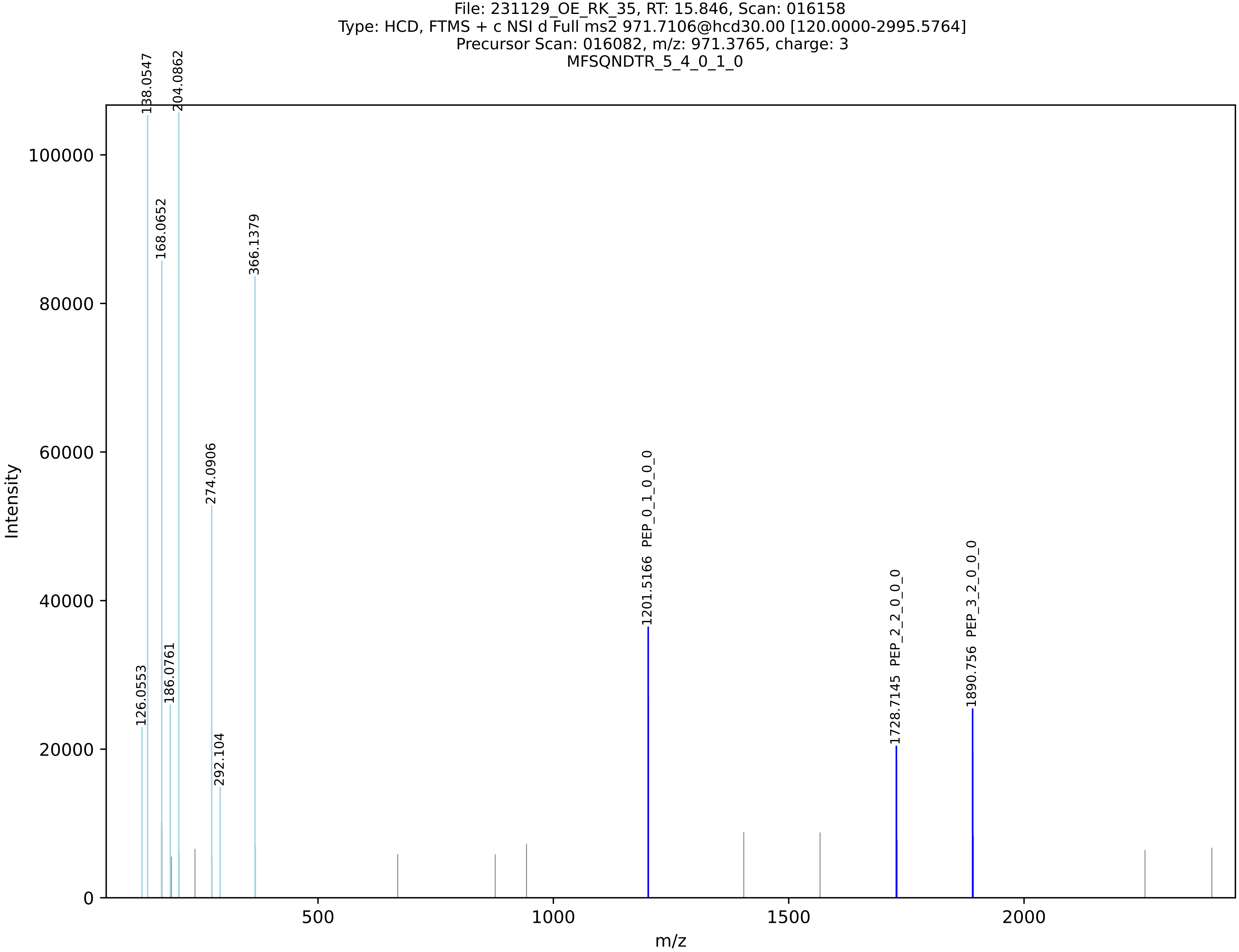
File: 231129_OE_RK_35, RT: 15.846, Scan: 016158
Type: HCD, FTMS + c NSI d Full ms2 971.7106@hcd30.00 [120.0000-2995.5764]
Precursor Scan: 016082, m/z: 971.3765, charge: 3
MFSQNDTR_5_4_0_1_0
Intensity
m/z
0
20000
40000
60000
80000
100000
500
1000
1500
2000
138.0547
204.0862
168.0652
366.1379
274.0906
126.0553
186.0761
292.104
1201.5166 PEP_0_1_0_0_0
1728.7145 PEP_2_2_0_0_0
1890.756 PEP_3_2_0_0_0

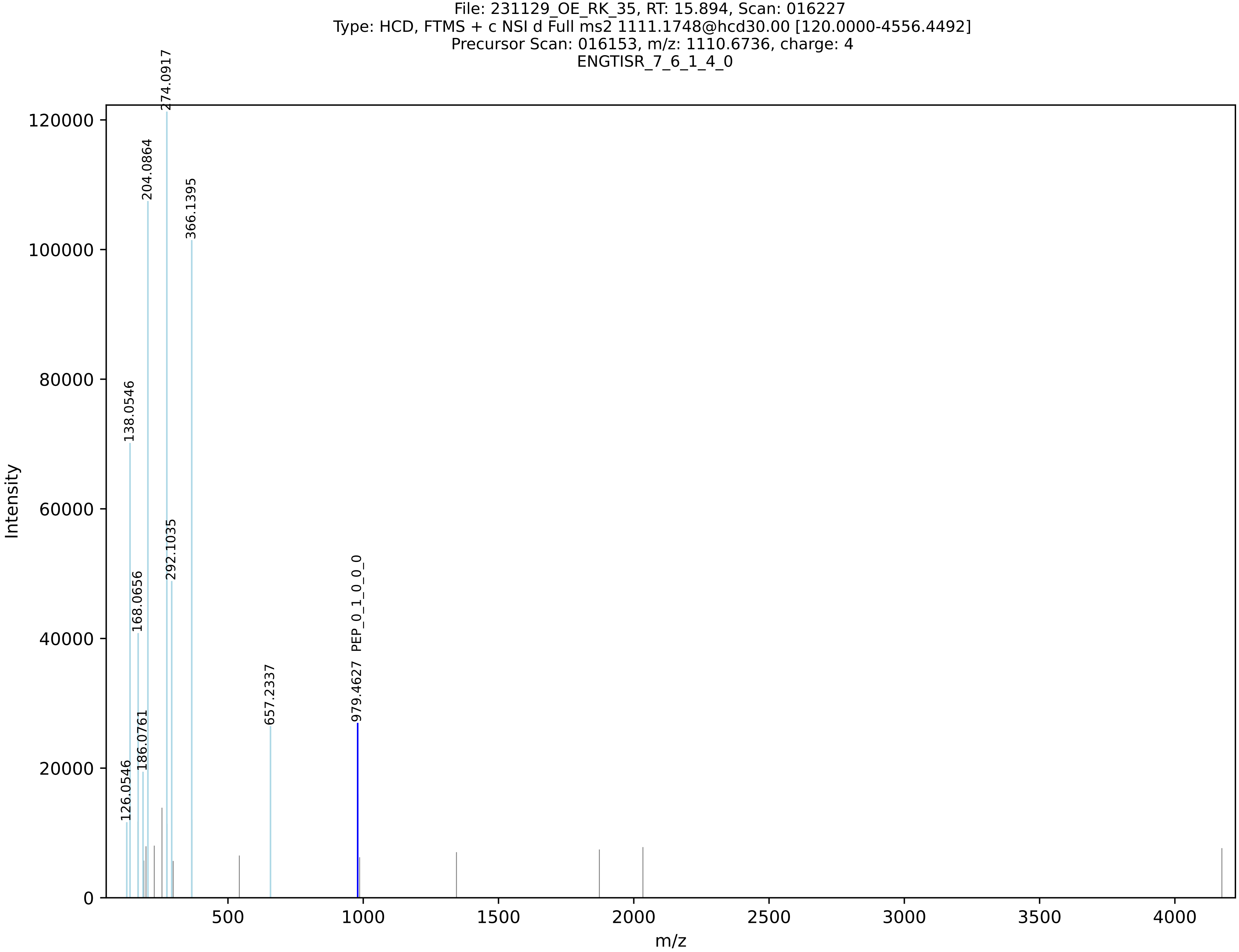
File: 231129_OE_RK_35, RT: 15.894, Scan: 016227
Type: HCD, FTMS + c NSI d Full ms2 1111.1748@hcd30.00 [120.0000-4556.4492]
Precursor Scan: 016153, m/z: 1110.6736, charge: 4
ENGTISR_7_6_1_4_0
Intensity
m/z
274.0917
204.0864
366.1395
138.0546
292.1035
168.0656
PEP_0_1_0_0_0
657.2337
979.4627
186.0761
126.0546
0
20000
40000
60000
80000
100000
120000
500
1000
1500
2000
2500
3000
3500
4000

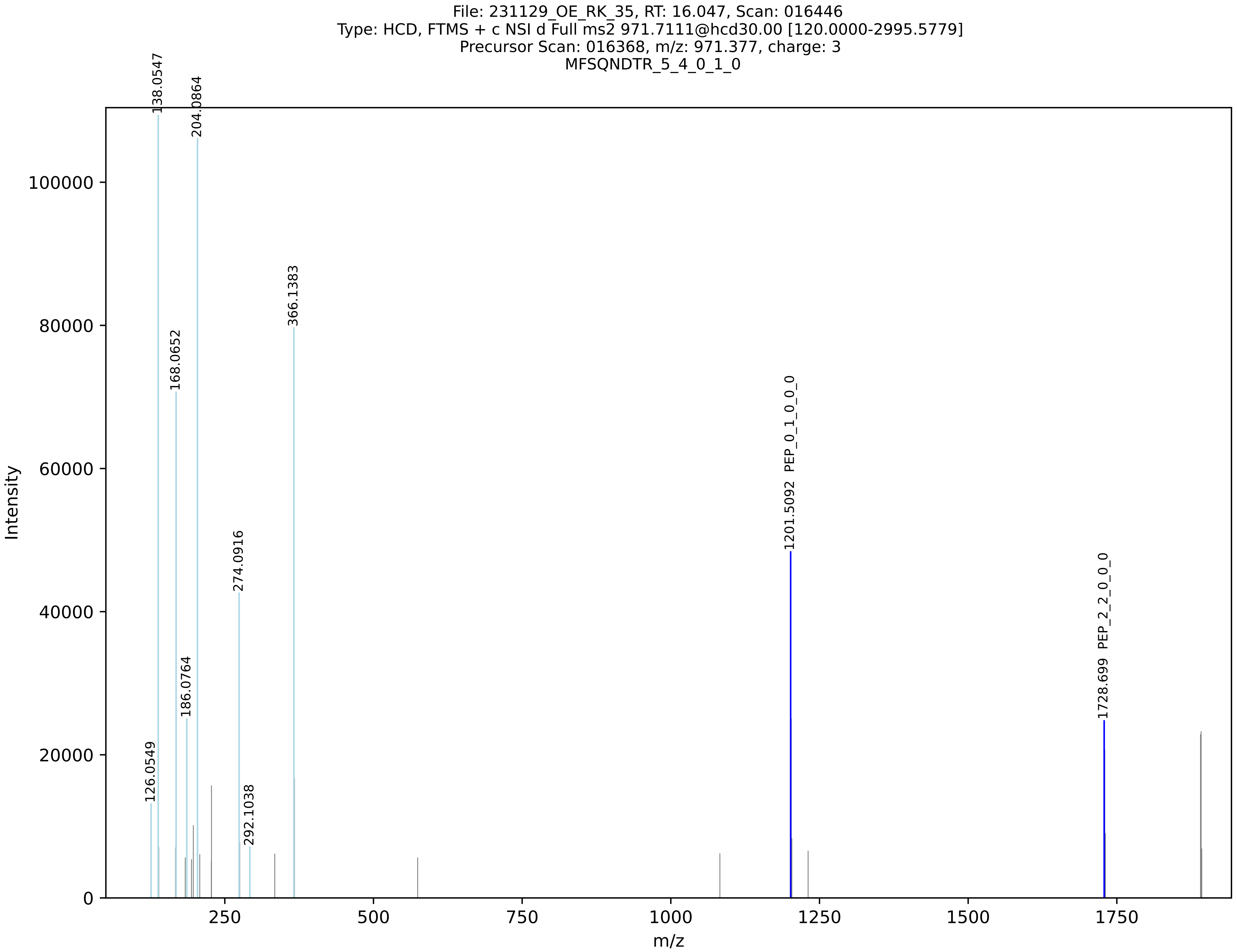
File: 231129_OE_RK_35, RT: 16.047, Scan: 016446
Type: HCD, FTMS + c NSI d Full ms2 971.7111@hcd30.00 [120.0000-2995.5779]
Precursor Scan: 016368, m/z: 971.377, charge: 3
MFSQNDTR_5_4_0_1_0
138.0547
204.0864
366.1383
168.0652
PEP_0_1_0_0_0_
1201.5092
274.0916
PEP_2_2_0_0_0_
1728.699
186.0764
126.0549
292.1038
Intensity
0
20000
40000
60000
80000
100000
250
500
750
1000
1250
1500
1750
m/z

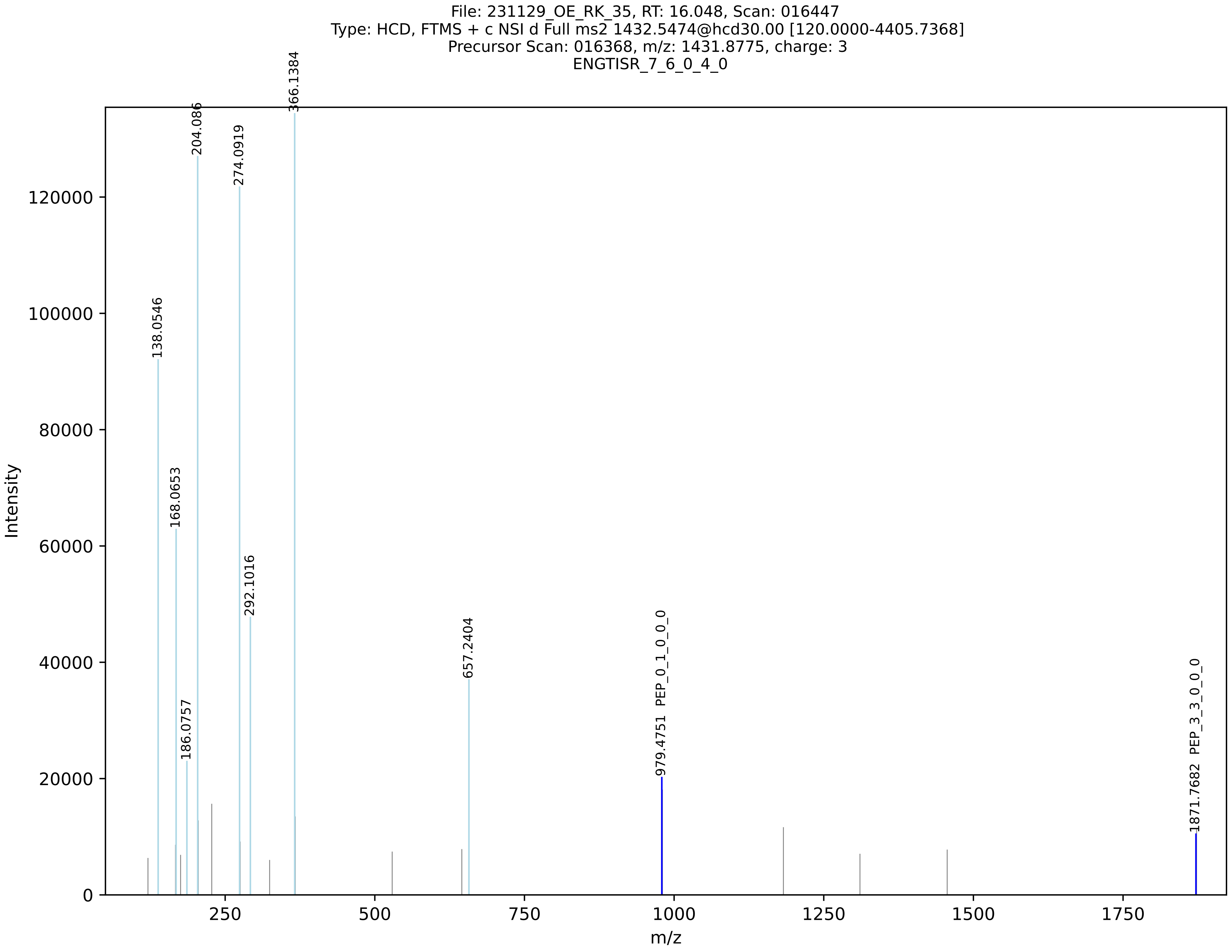
File: 231129_OE_RK_35, RT: 16.048, Scan: 016447
Type: HCD, FTMS + c NSI d Full ms2 1432.5474@hcd30.00 [120.0000-4405.7368]
Precursor Scan: 016368, m/z: 1431.8775, charge: 3
ENGTISR_7_6_0_4_0
366.1384
204.086
274.0919
138.0546
168.0653
292.1016
186.0757
657.2404
979.4751 PEP_0_1_0_0_0
1871.7682 PEP_3_3_0_0_0
Intensity
m/z
0
20000
40000
60000
80000
100000
120000
250
500
750
1000
1250
1500
1750

File: 231129_OE_RK_35, RT: 16.049, Scan: 016449
Type: HCD, FTMS + c NSI d Full ms2 1084.4207@hcd30.00 [120.0000-3340.4692]
Precursor Scan: 016368, m/z: 1083.7525, charge: 3
EEQYNSTYR_5_4_1_1_0

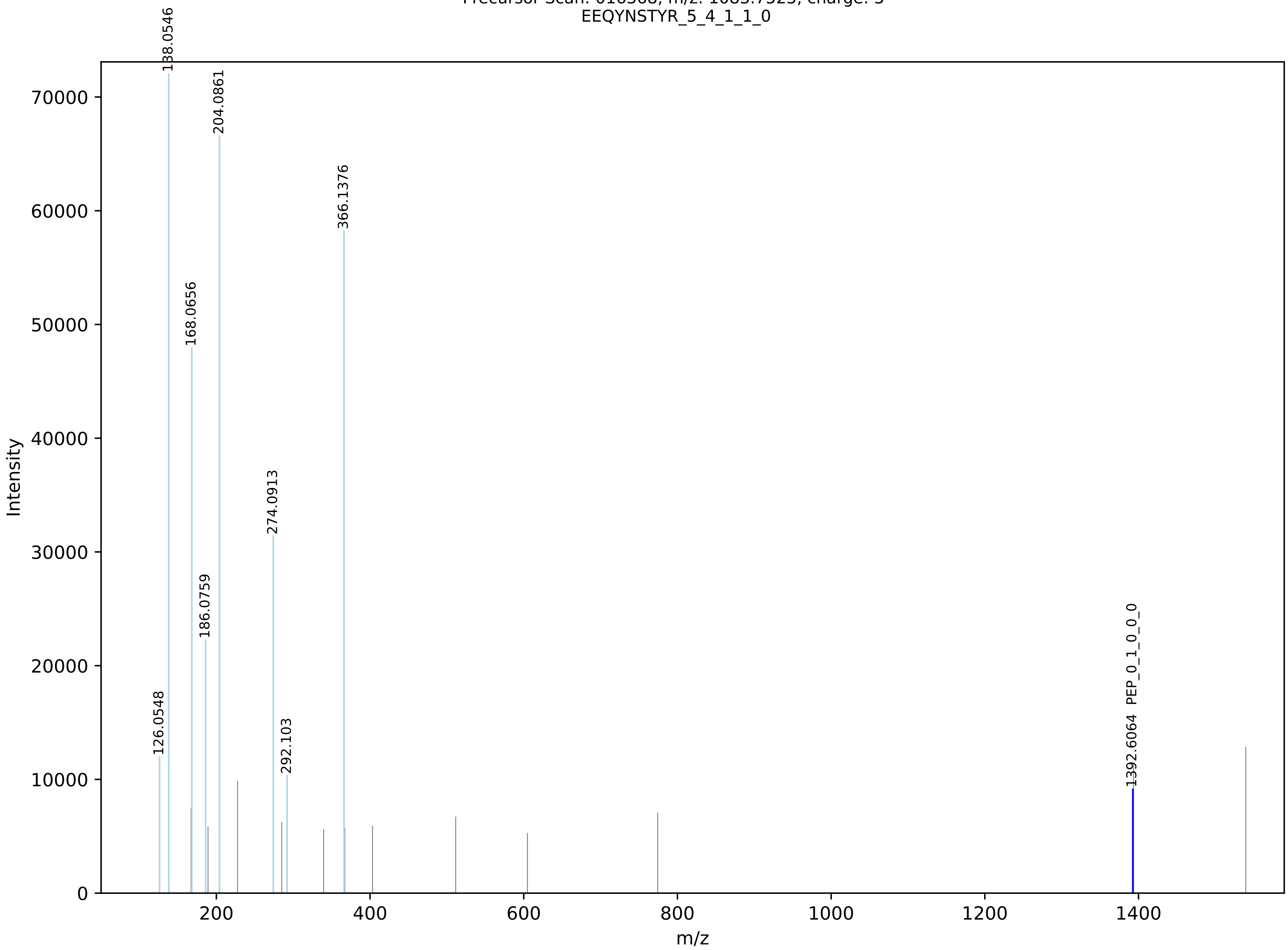

File: 231129_OE_RK_35, RT: 16.096, Scan: 016514
Type: HCD, FTMS + c NSI d Full ms2 1051.7452@hcd30.00 [120.0000-3240.4824]
Precursor Scan: 016442, m/z: 1051.4111, charge: 3
EDALNETR_5_4_0_2_0

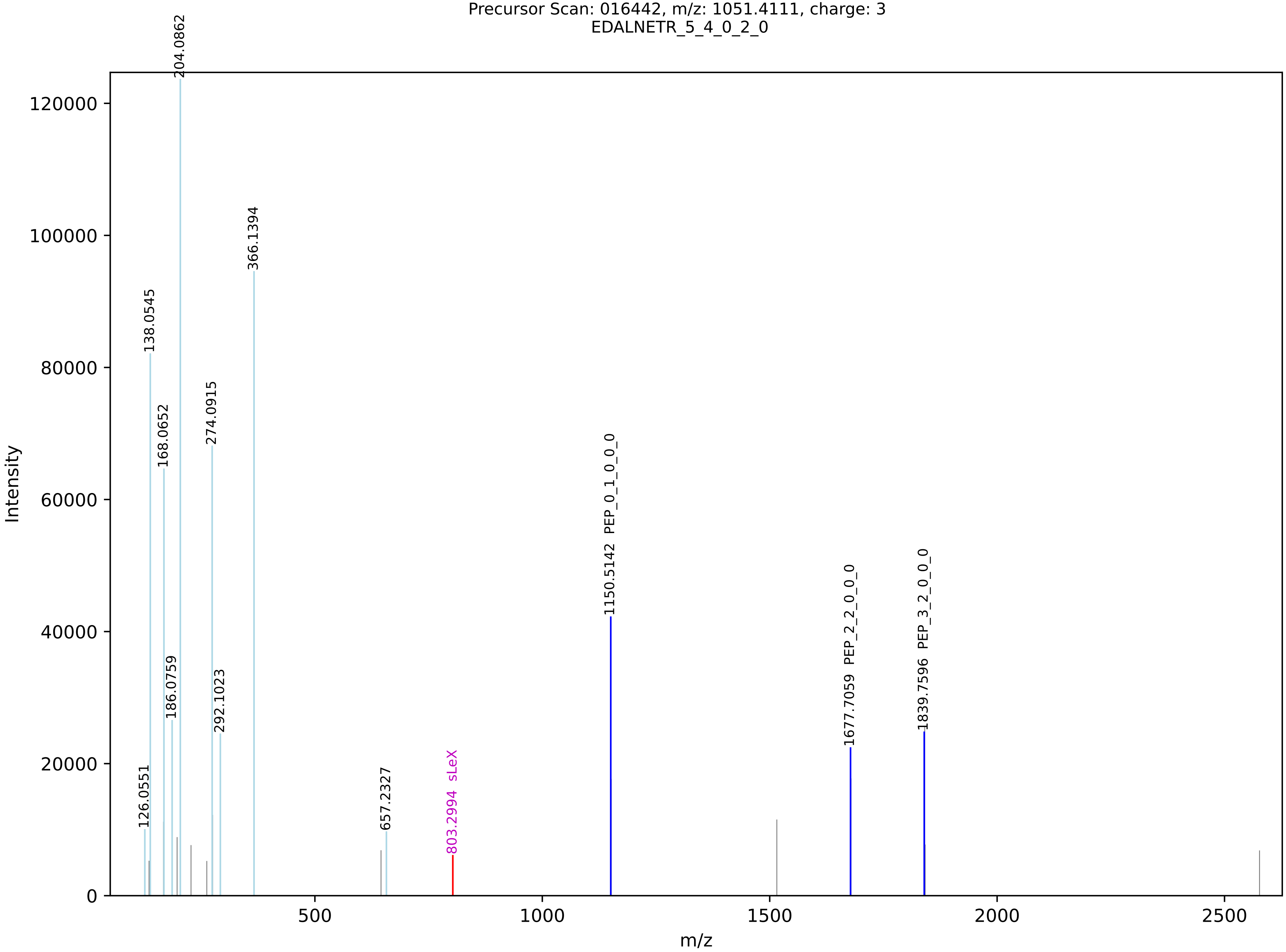

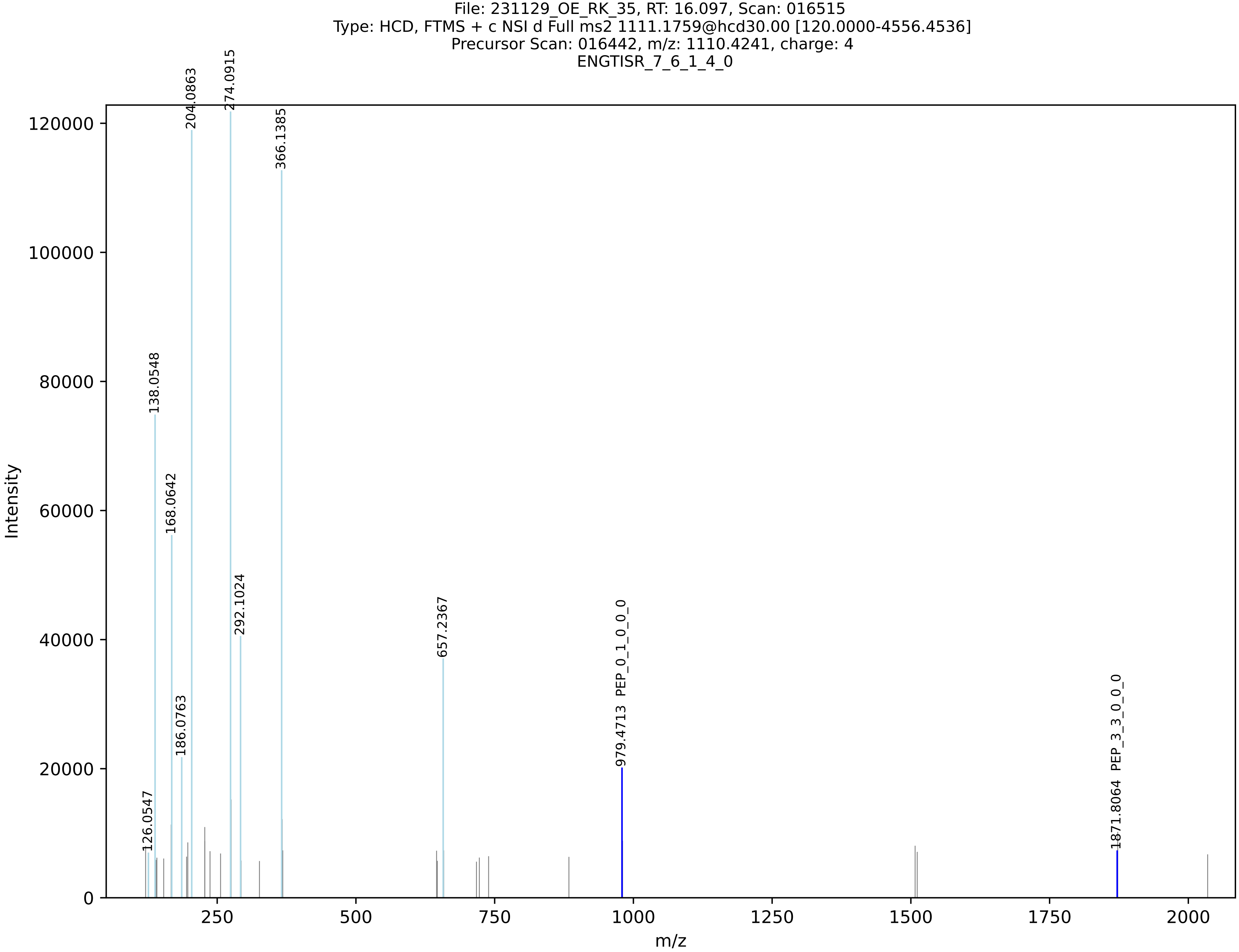

File: 231129_OE_RK_35, RT: 16.097, Scan: 016515
Type: HCD, FTMS + c NSI d Full ms2 1111.1759@hcd30.00 [120.0000-4556.4536]
Precursor Scan: 016442, m/z: 1110.4241, charge: 4
ENGTISR_7_6_1_4_0
Intensity
m/z
0
20000
40000
60000
80000
100000
120000
250
500
750
1000
1250
1500
1750
2000
126.0547
138.0548
168.0642
186.0763
204.0863
274.0915
292.1024
366.1385
657.2367
979.4713 PEP_0_1_0_0_0
1871.8064 PEP_3_3_0_0_0

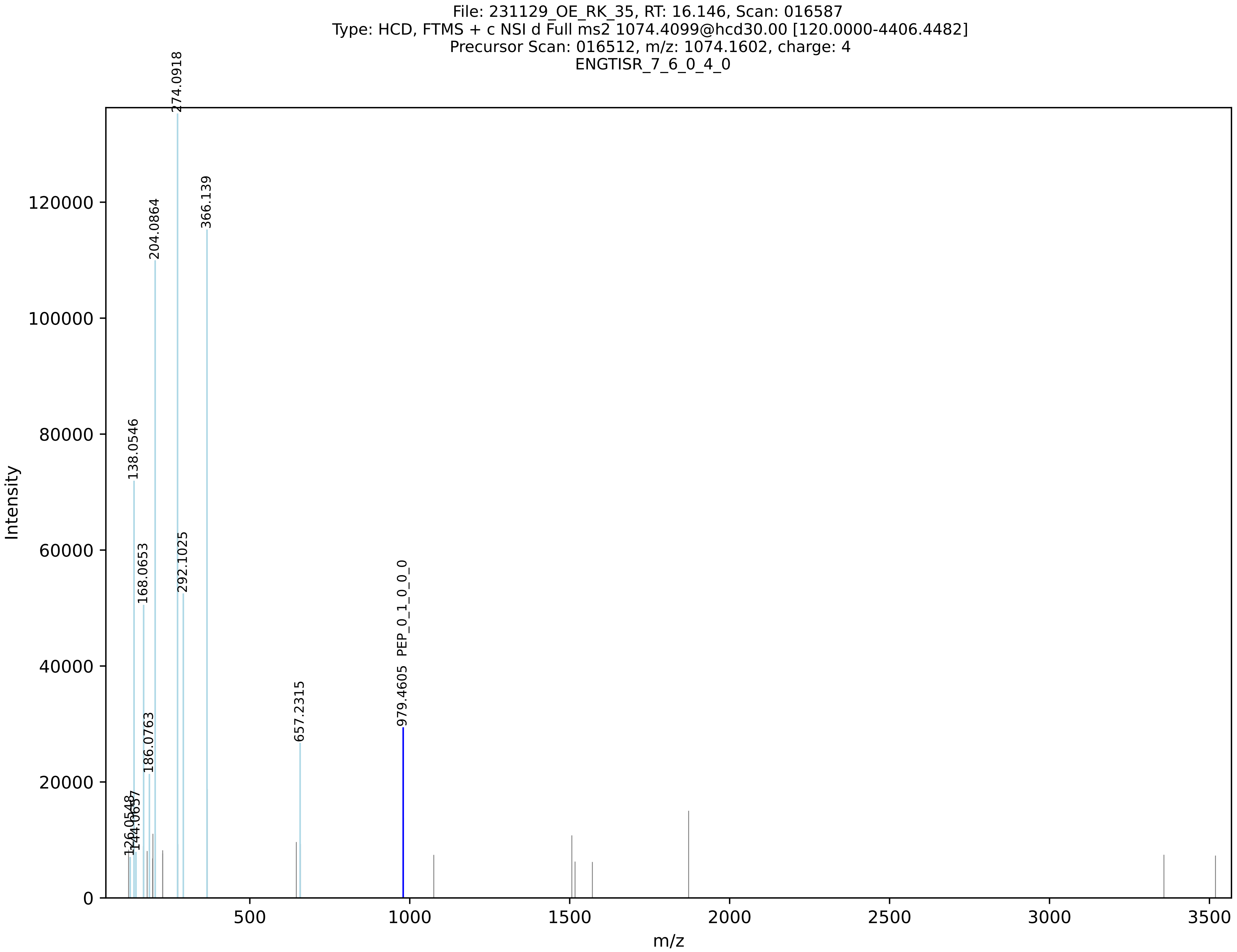
File: 231129_OE_RK_35, RT: 16.146, Scan: 016587
Type: HCD, FTMS + c NSI d Full ms2 1074.4099@hcd30.00 [120.0000-4406.4482]
Precursor Scan: 016512, m/z: 1074.1602, charge: 4
ENGTISR_7_6_0_4_0
Intensity
m/z
0
20000
40000
60000
80000
100000
120000
500
1000
1500
2000
2500
3000
3500
274.0918
366.139
204.0864
138.0546
168.0653
292.1025
186.0763
126.0548
144.0657
657.2315
979.4605 PEP_0_1_0_0_0_

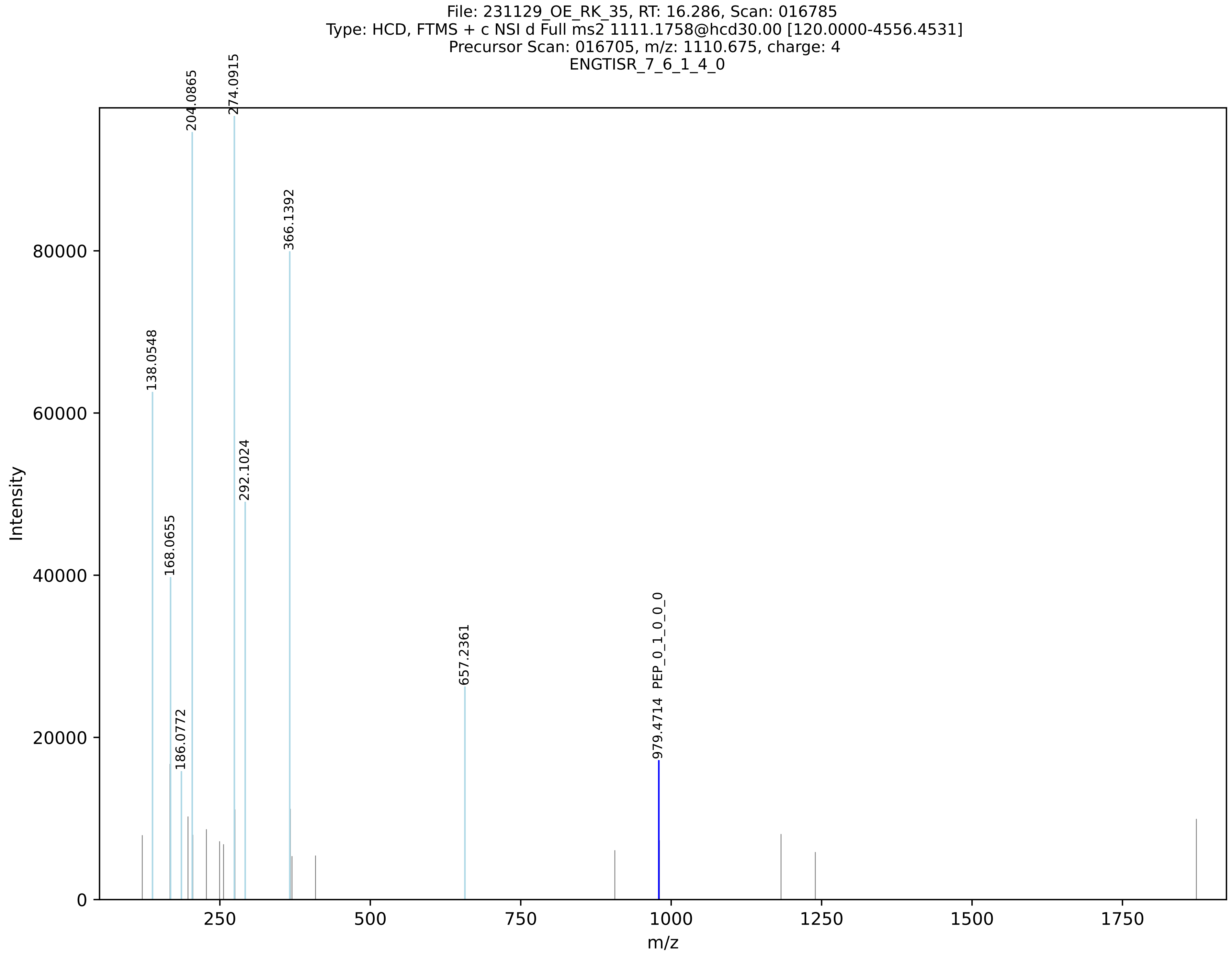
File: 231129_OE_RK_35, RT: 16.286, Scan: 016785
Type: HCD, FTMS + c NSI d Full ms2 1111.1758@hcd30.00 [120.0000-4556.4531]
Precursor Scan: 016705, m/z: 1110.675, charge: 4
ENGTISR_7_6_1_4_0
204.0865
274.0915
366.1392
138.0548
292.1024
168.0655
657.2361
979.4714 PEP_0_1_0_0_0
186.0772
Intensity
80000
60000
40000
20000
0
250
500
750
1000
1250
1500
1750
m/z

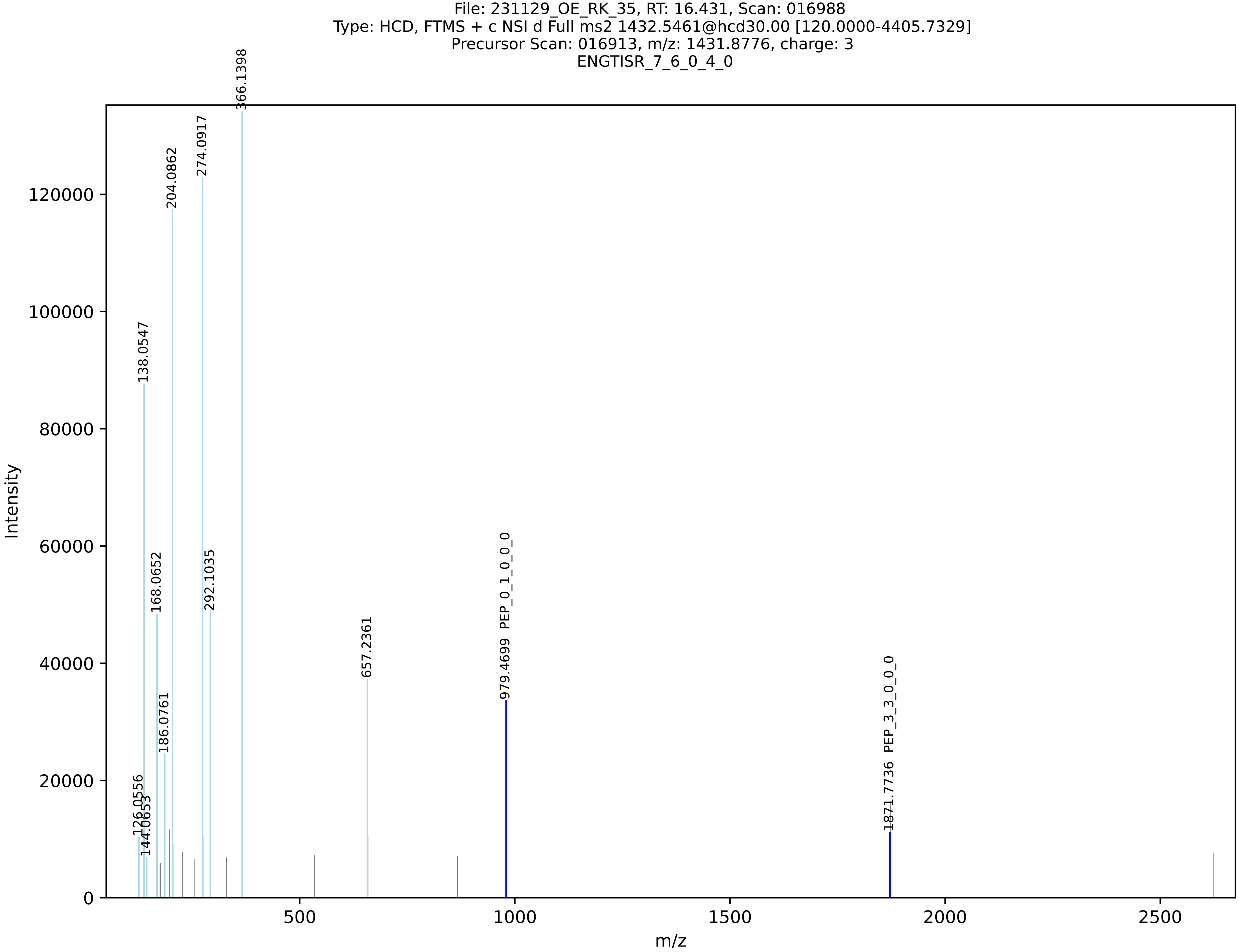
File: 231129_OE_RK_35, RT: 16.431, Scan: 016988
Type: HCD, FTMS + c NSI d Full ms2 1432.5461@hcd30.00 [120.0000-4405.7329]
Precursor Scan: 016913, m/z: 1431.8776, charge: 3
ENGTISR_7_6_0_4_0
366.1398
274.0917
204.0862
138.0547
168.0652
292.1035
186.0761
126.0556
144.0653
657.2361
979.4699 PEP_0_1_0_0_0
1871.7736 PEP_3_3_0_0_0
Intensity
m/z
0
20000
40000
60000
80000
100000
120000
500
1000
1500
2000
2500

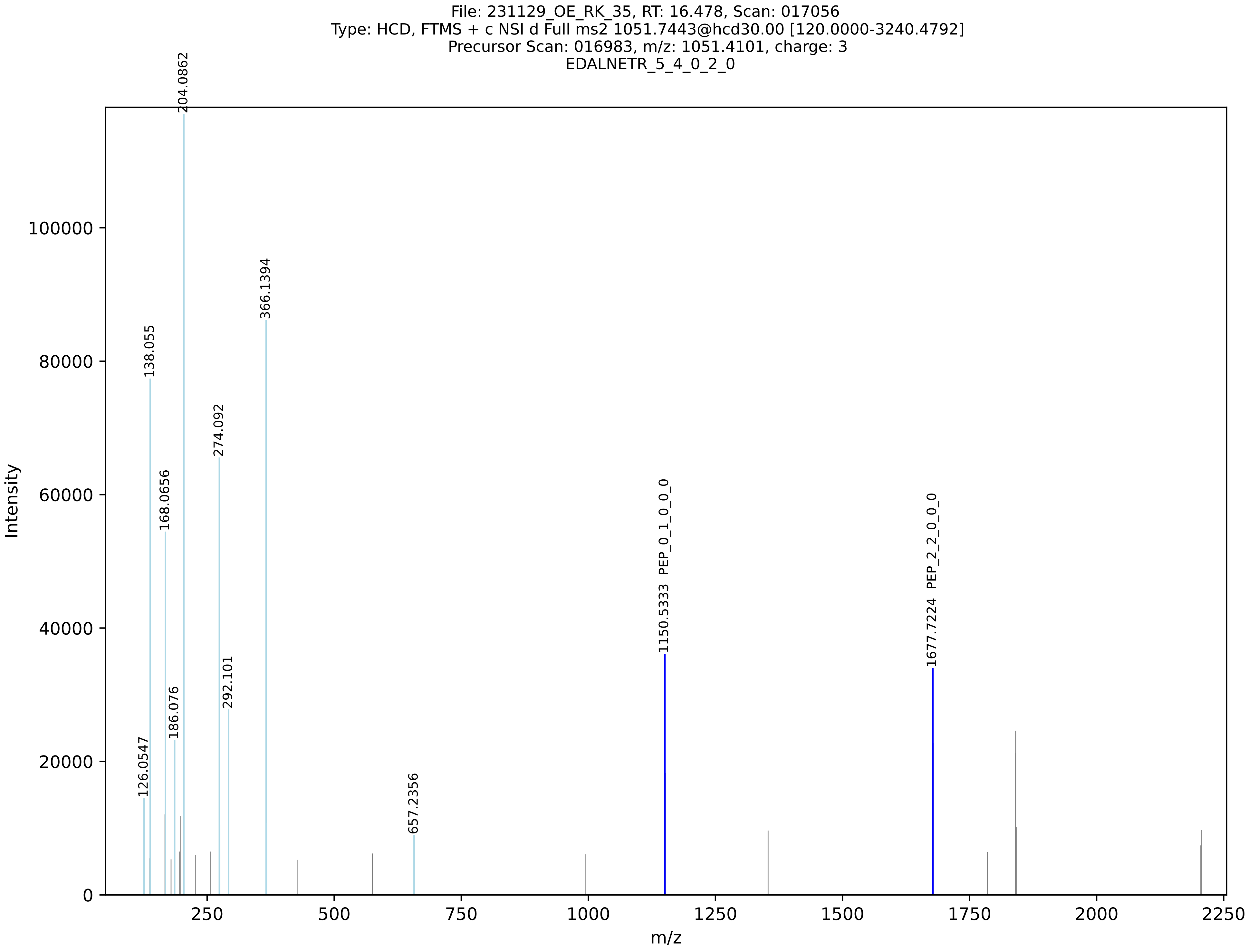
File: 231129_OE_RK_35, RT: 16.478, Scan: 017056
Type: HCD, FTMS + c NSI d Full ms2 1051.7443@hcd30.00 [120.0000-3240.4792]
Precursor Scan: 016983, m/z: 1051.4101, charge: 3
EDALNETR_5_4_0_2_0
204.0862
366.1394
138.055
274.092
168.0656
292.101
186.076
126.0547
657.2356
1150.5333 PEP_0_1_0_0_0
1677.7224 PEP_2_2_0_0_0
Intensity
m/z
0
20000
40000
60000
80000
100000
250
500
750
1000
1250
1500
1750
2000
2250

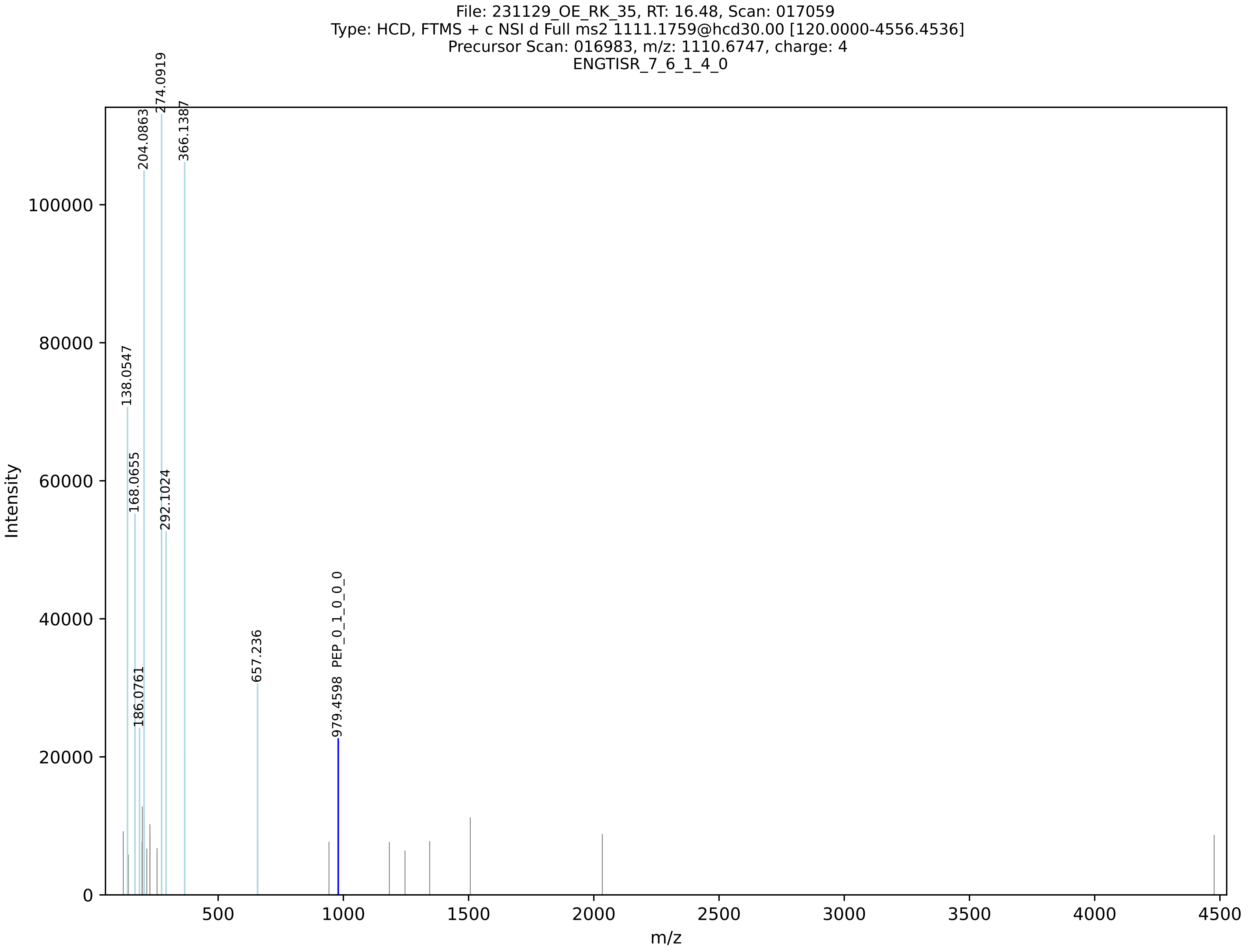
File: 231129_OE_RK_35, RT: 16.48, Scan: 017059
Type: HCD, FTMS + c NSI d Full ms2 1111.1759@hcd30.00 [120.0000-4556.4536]
Precursor Scan: 016983, m/z: 1110.6747, charge: 4
ENGTISR_7_6_1_4_0
274.0919
366.1387
204.0863
138.0547
168.0655
292.1024
186.0761
657.236
979.4598 PEP_0_1_0_0_0
Intensity
m/z
0
20000
40000
60000
80000
100000
500
1000
1500
2000
2500
3000
3500
4000
4500

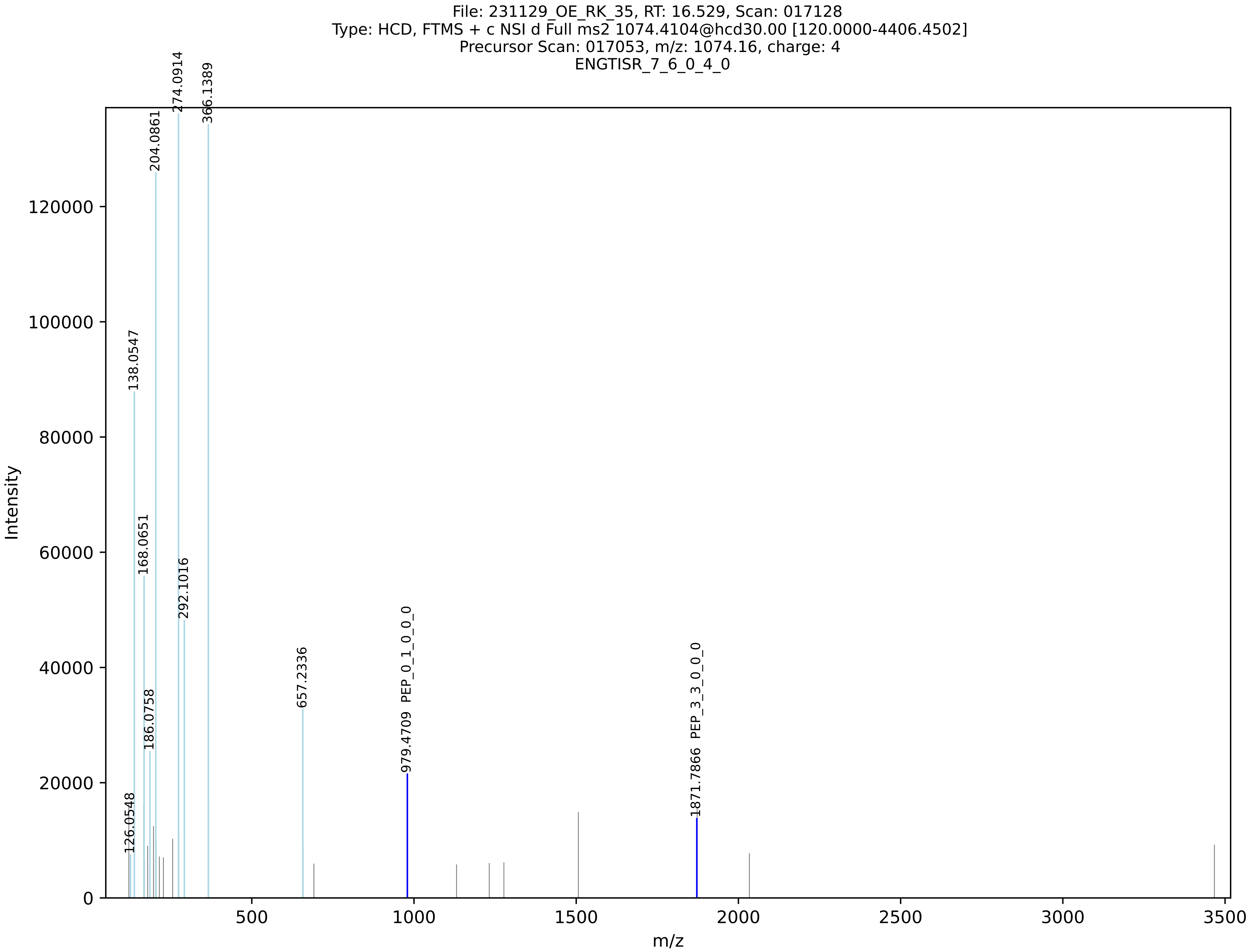
File: 231129_OE_RK_35, RT: 16.529, Scan: 017128
Type: HCD, FTMS + c NSI d Full ms2 1074.4104@hcd30.00 [120.0000-4406.4502]
Precursor Scan: 017053, m/z: 1074.16, charge: 4
ENGTISR_7_6_0_4_0
274.0914
366.1389
204.0861
138.0547
168.0651
292.1016
186.0758
126.0548
657.2336
979.4709 PEP_0_1_0_0_0
1871.7866 PEP_3_3_0_0_0
Intensity
0
20000
40000
60000
80000
100000
120000
500
1000
1500
2000
2500
3000
3500
m/z

File: 231129_OE_RK_35, RT: 16.632, Scan: 017274
Type: HCD, FTMS + c NSI d Full ms2 1432.5480@hcd30.00 [120.0000-4405.7388]
Precursor Scan: 017196, m/z: 1431.8789, charge: 3
ENGTISR_7_6_0_4_0

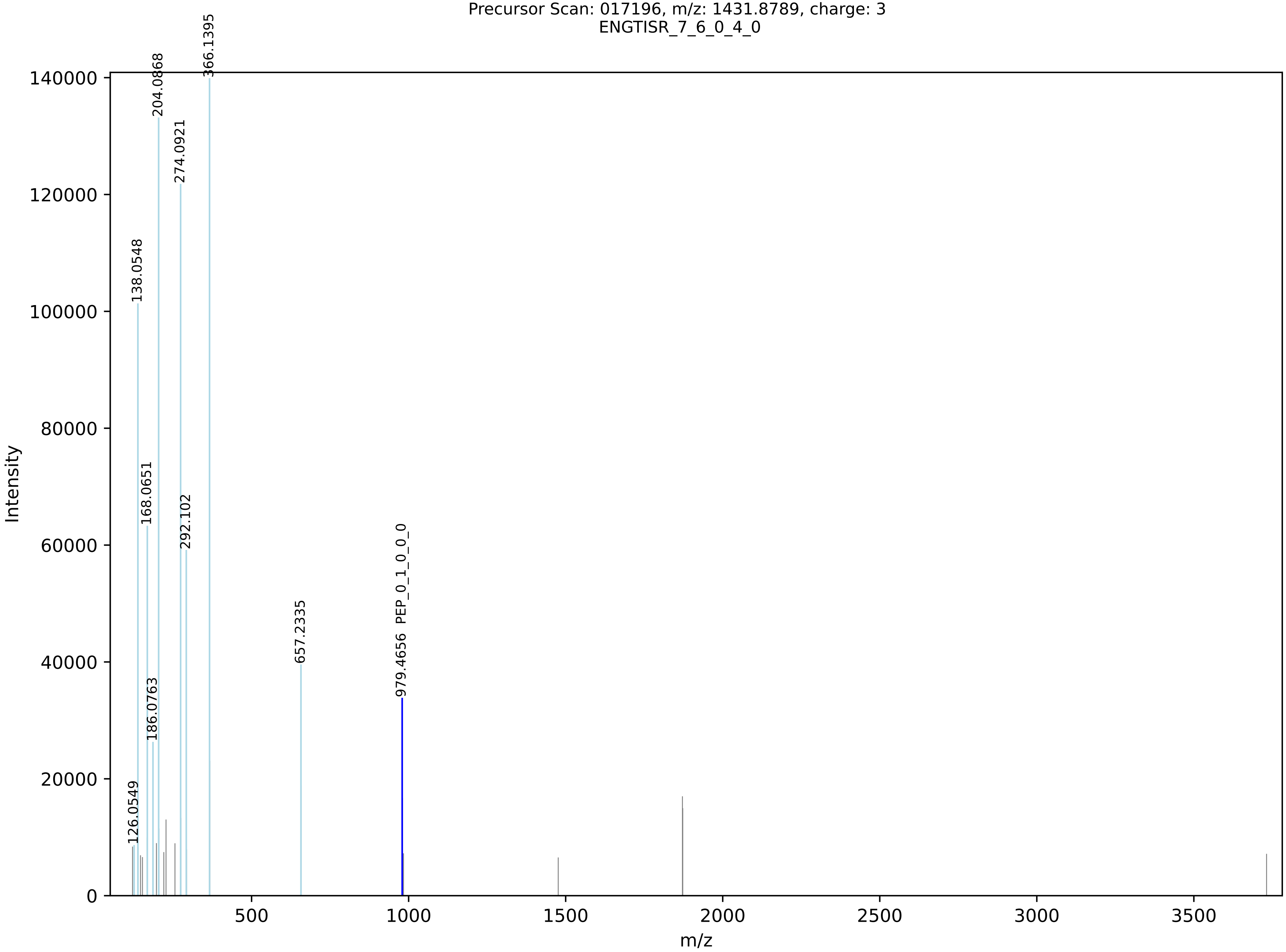

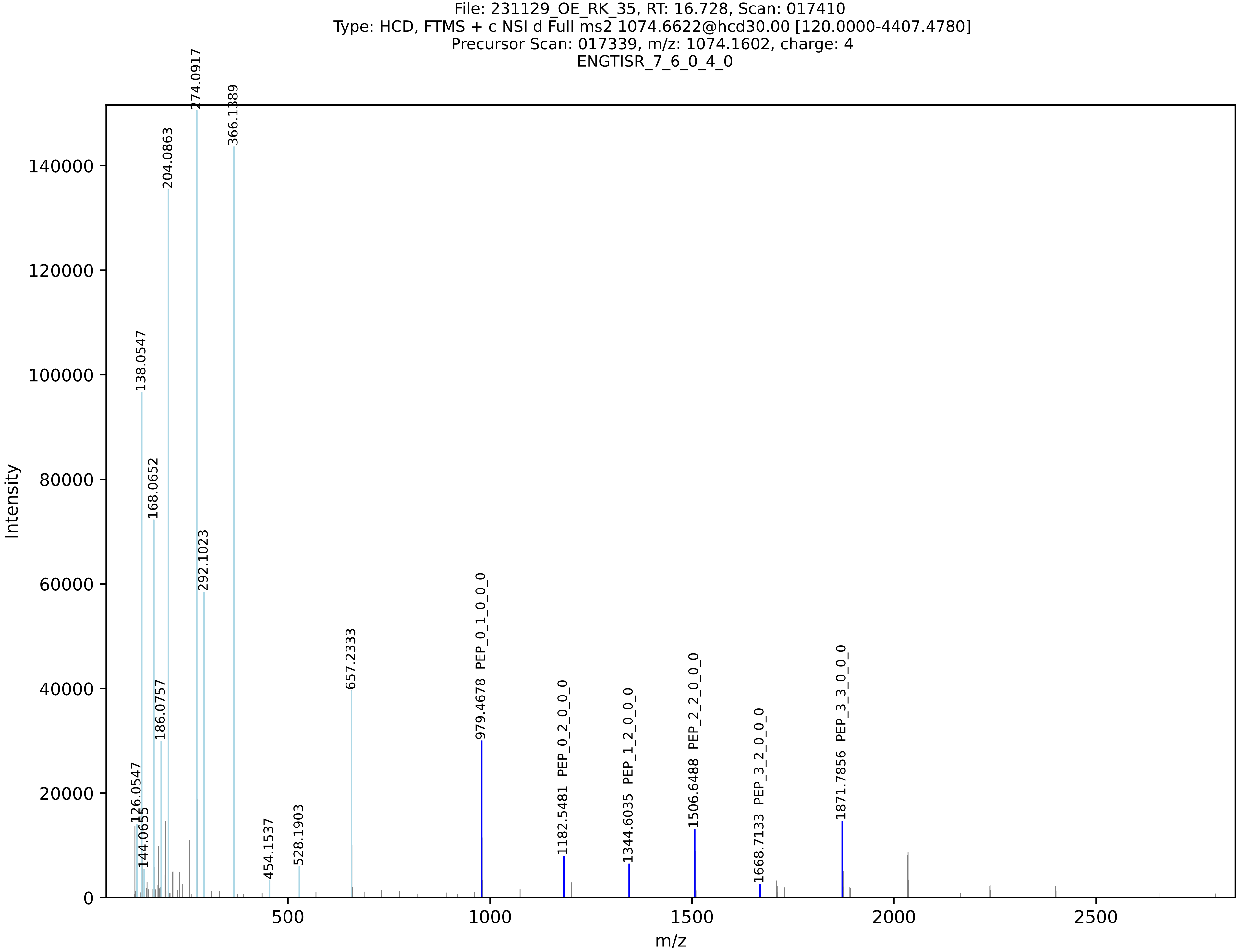
File: 231129_OE_RK_35, RT: 16.728, Scan: 017410
Type: HCD, FTMS + c NSI d Full ms2 1074.6622@hcd30.00 [120.0000-4407.4780]
Precursor Scan: 017339, m/z: 1074.1602, charge: 4
ENGTISR_7_6_0_4_0
Intensity
m/z
274.0917
366.1389
204.0863
138.0547
168.0652
292.1023
186.0757
126.0547
144.0655
454.1537
528.1903
657.2333
979.4678 PEP_0_1_0_0_0
1182.5481 PEP_0_2_0_0_0
1344.6035 PEP_1_2_0_0_0
1506.6488 PEP_2_2_0_0_0
1668.7133 PEP_3_2_0_0_0
1871.7856 PEP_3_3_0_0_0

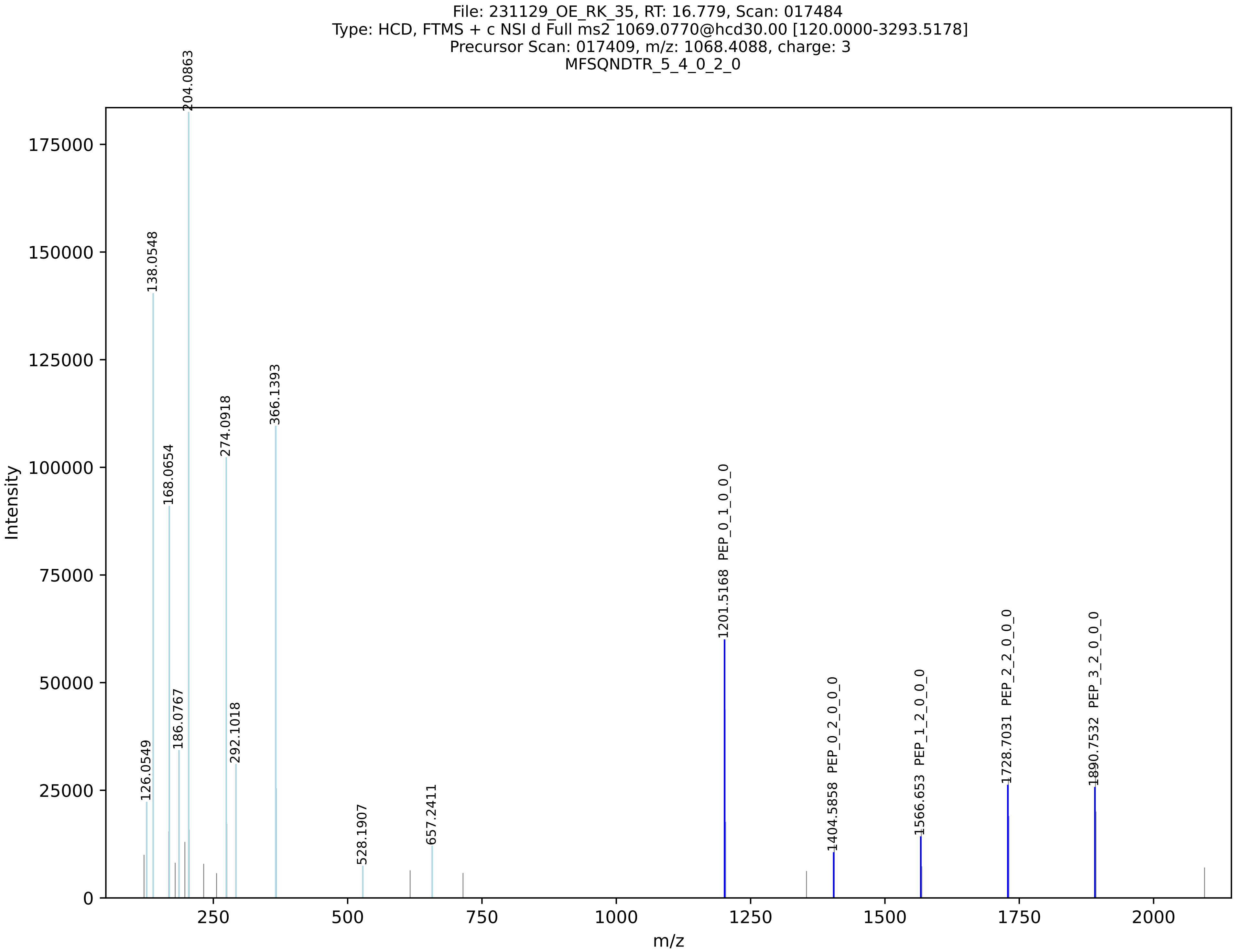
File: 231129_OE_RK_35, RT: 16.779, Scan: 017484
Type: HCD, FTMS + c NSI d Full ms2 1069.0770@hcd30.00 [120.0000-3293.5178]
Precursor Scan: 017409, m/z: 1068.4088, charge: 3
MFSQNDTR_5_4_0_2_0
Intensity
m/z
0
25000
50000
75000
100000
125000
150000
175000
250
500
750
1000
1250
1500
1750
2000
204.0863
138.0548
168.0654
186.0767
126.0549
274.0918
292.1018
366.1393
528.1907
657.2411
1201.5168 PEP_0_1_0_0_
1404.5858 PEP_0_2_0_0_0
1566.653 PEP_1_2_0_0_0
1728.7031 PEP_2_2_0_0_0
1890.7532 PEP_3_2_0_0_0

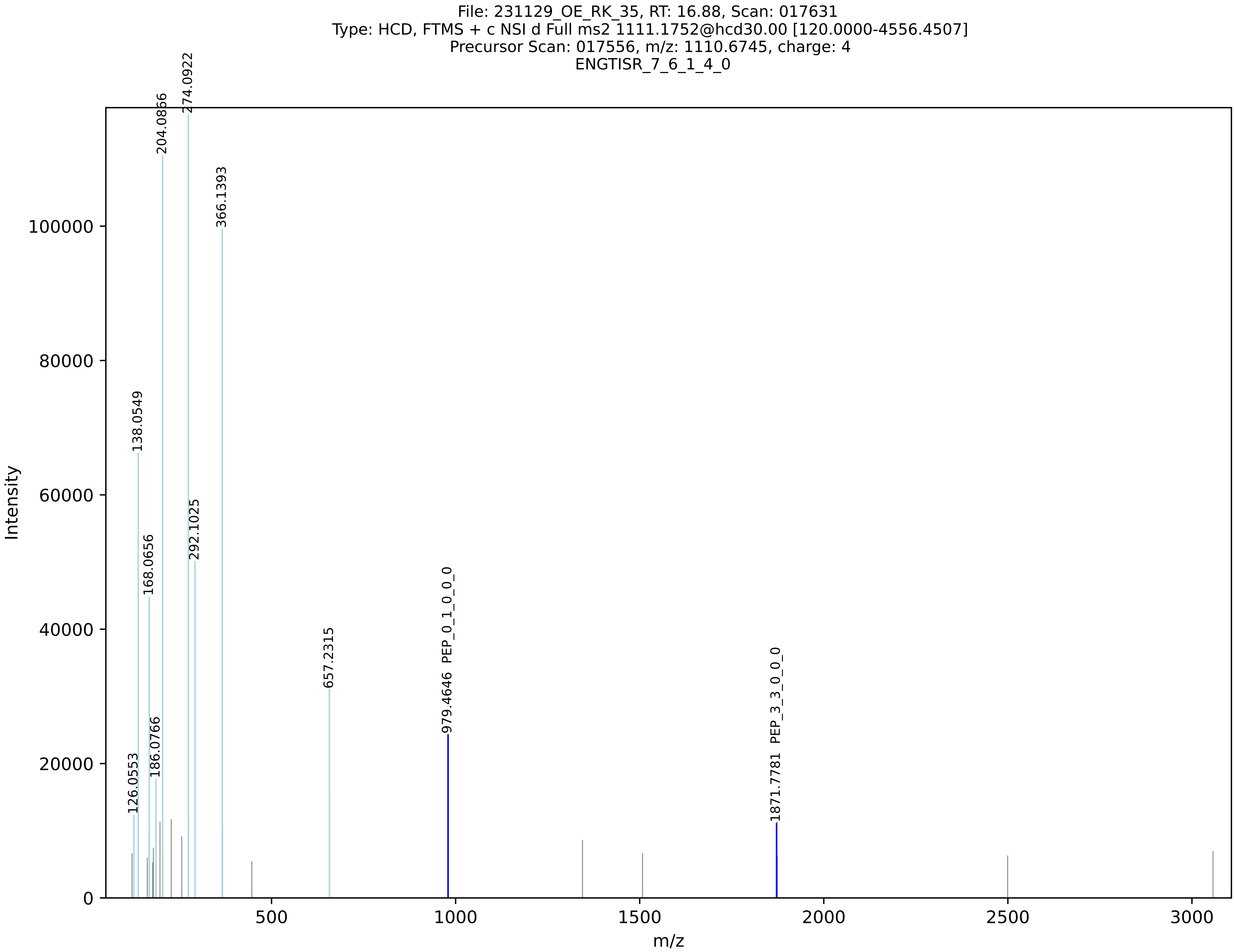

File: 231129_OE_RK_35, RT: 16.88, Scan: 017631
Type: HCD, FTMS + c NSI d Full ms2 1111.1752@hcd30.00 [120.0000-4556.4507]
Precursor Scan: 017556, m/z: 1110.6745, charge: 4
ENGTISR_7_6_1_4_0
Intensity
m/z
0
20000
40000
60000
80000
100000
500
1000
1500
2000
2500
3000
274.0922
204.0866
366.1393
138.0549
292.1025
168.0656
657.2315
979.4646 PEP_0_1_0_0_0
1871.7781 PEP_3_3_0_0_0
186.0766
126.0553

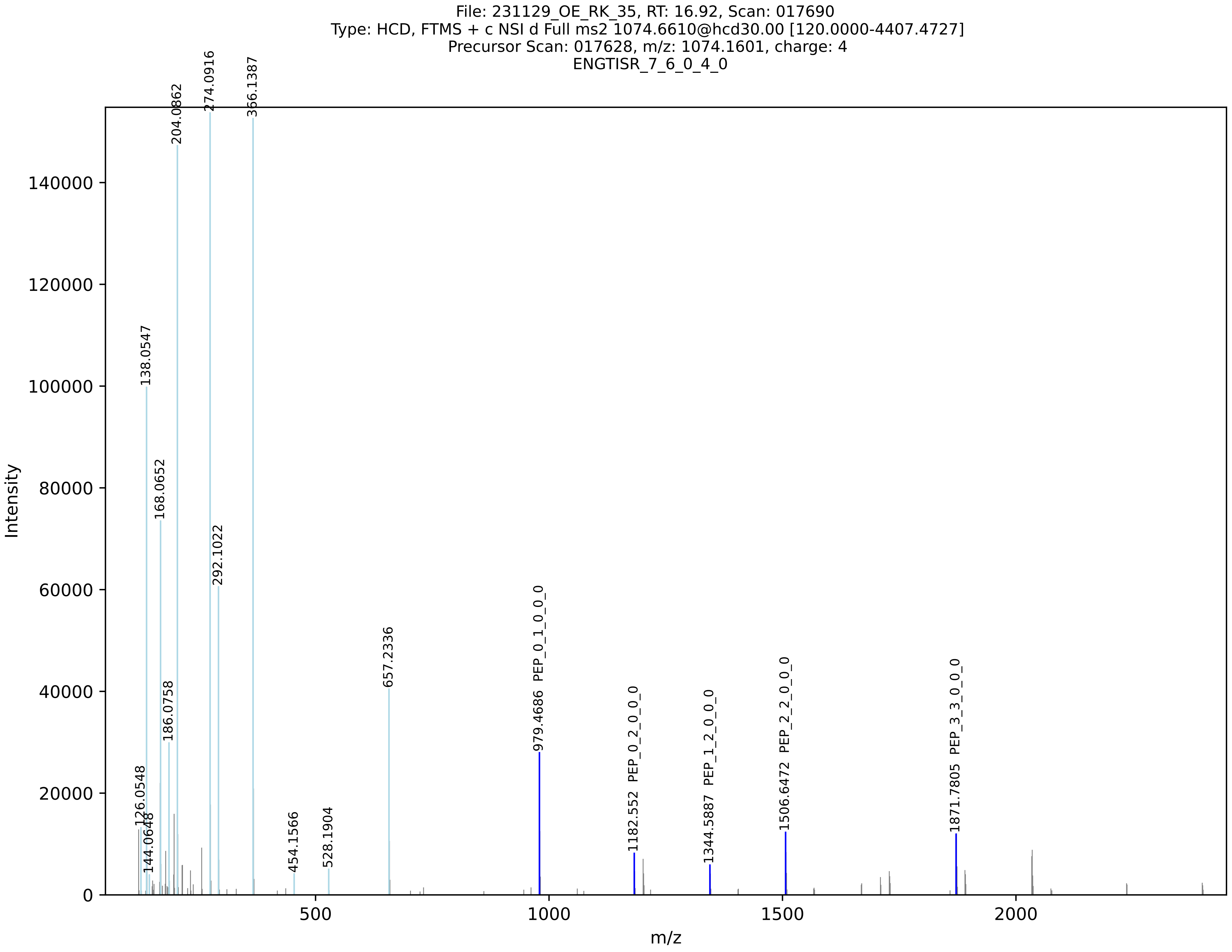

File: 231129_OE_RK_35, RT: 16.92, Scan: 017690
Type: HCD, FTMS + c NSI d Full ms2 1074.6610@hcd30.00 [120.0000-4407.4727]
Precursor Scan: 017628, m/z: 1074.1601, charge: 4
ENGTISR_7_6_0_4_0
Intensity
m/z
0
20000
40000
60000
80000
100000
120000
140000
500
1000
1500
2000
126.0548
138.0547
144.0648
168.0652
186.0758
204.0862
274.0916
292.1022
366.1387
454.1566
528.1904
657.2336
979.4686 PEP_0_1_0_0_0
1182.552 PEP_0_2_0_0_0
1344.5887 PEP_1_2_0_0_0
1506.6472 PEP_2_2_0_0_0
1871.7805 PEP_3_3_0_0_0

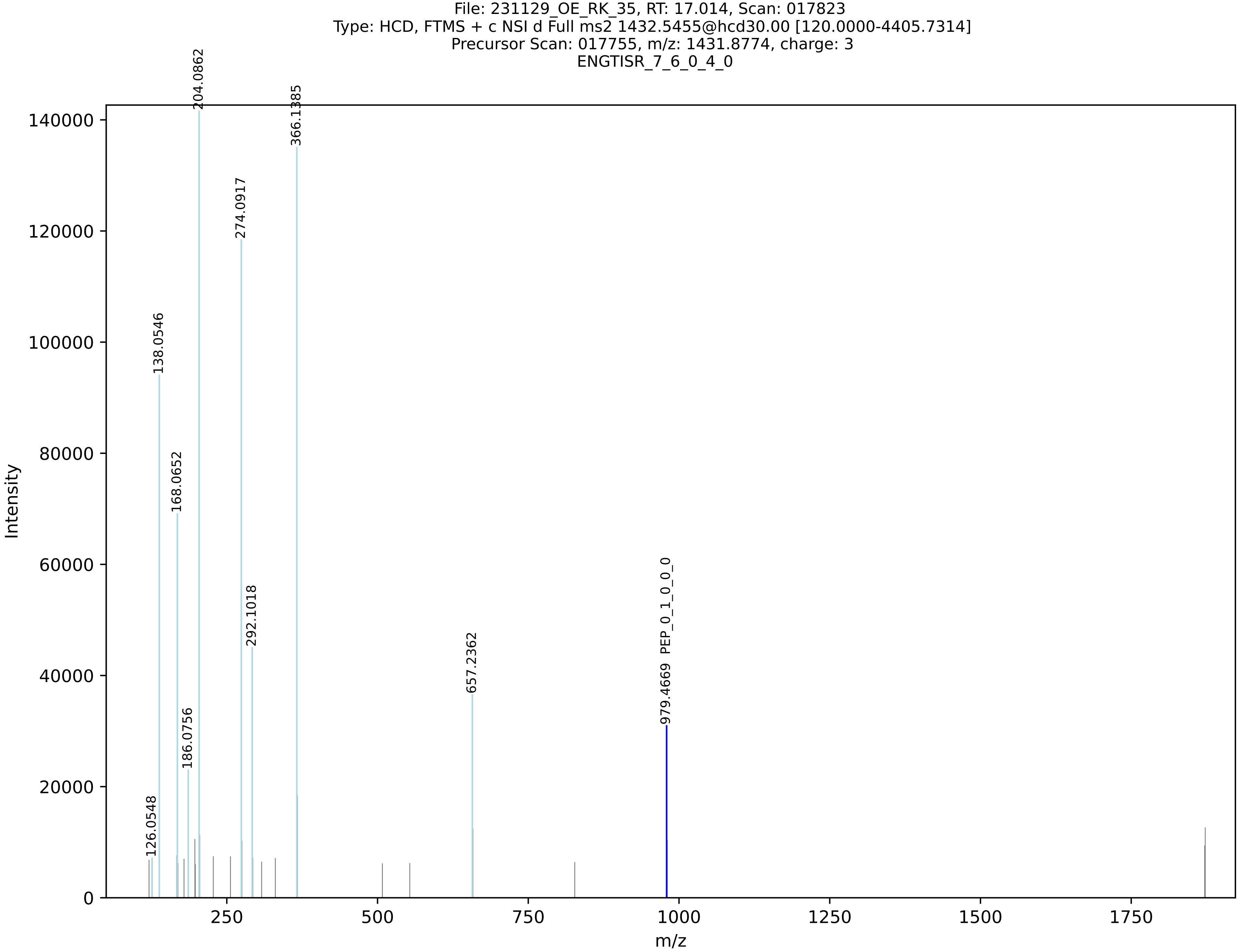

File: 231129_OE_RK_35, RT: 17.014, Scan: 017823
Type: HCD, FTMS + c NSI d Full ms2 1432.5455@hcd30.00 [120.0000-4405.7314]
Precursor Scan: 017755, m/z: 1431.8774, charge: 3
ENGTISR_7_6_0_4_0
Intensity
m/z
204.0862
366.1385
274.0917
138.0546
168.0652
292.1018
186.0756
126.0548
657.2362
979.4669 PEP_0_1_0_0_0

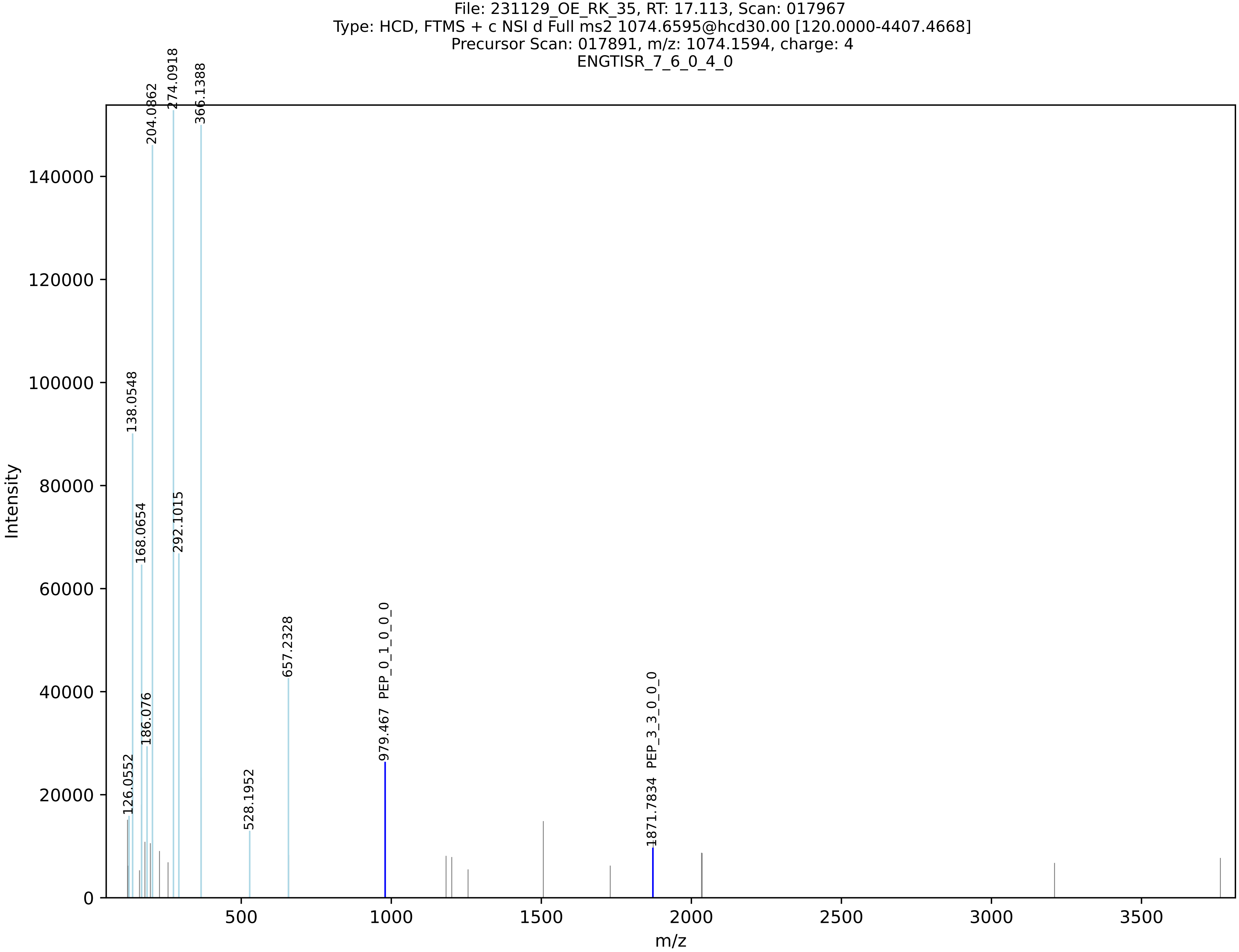

File: 231129_OE_RK_35, RT: 17.113, Scan: 017967
Type: HCD, FTMS + c NSI d Full ms2 1074.6595@hcd30.00 [120.0000-4407.4668]
Precursor Scan: 017891, m/z: 1074.1594, charge: 4
ENGTISR_7_6_0_4_0
Intensity
m/z
274.0918
366.1388
204.0862
138.0548
168.0654
292.1015
657.2328
979.467 PEP_0_1_0_0_0_
1871.7834 PEP_3_3_0_0_0_
186.076
126.0552
528.1952
0
20000
40000
60000
80000
100000
120000
140000
500
1000
1500
2000
2500
3000
3500

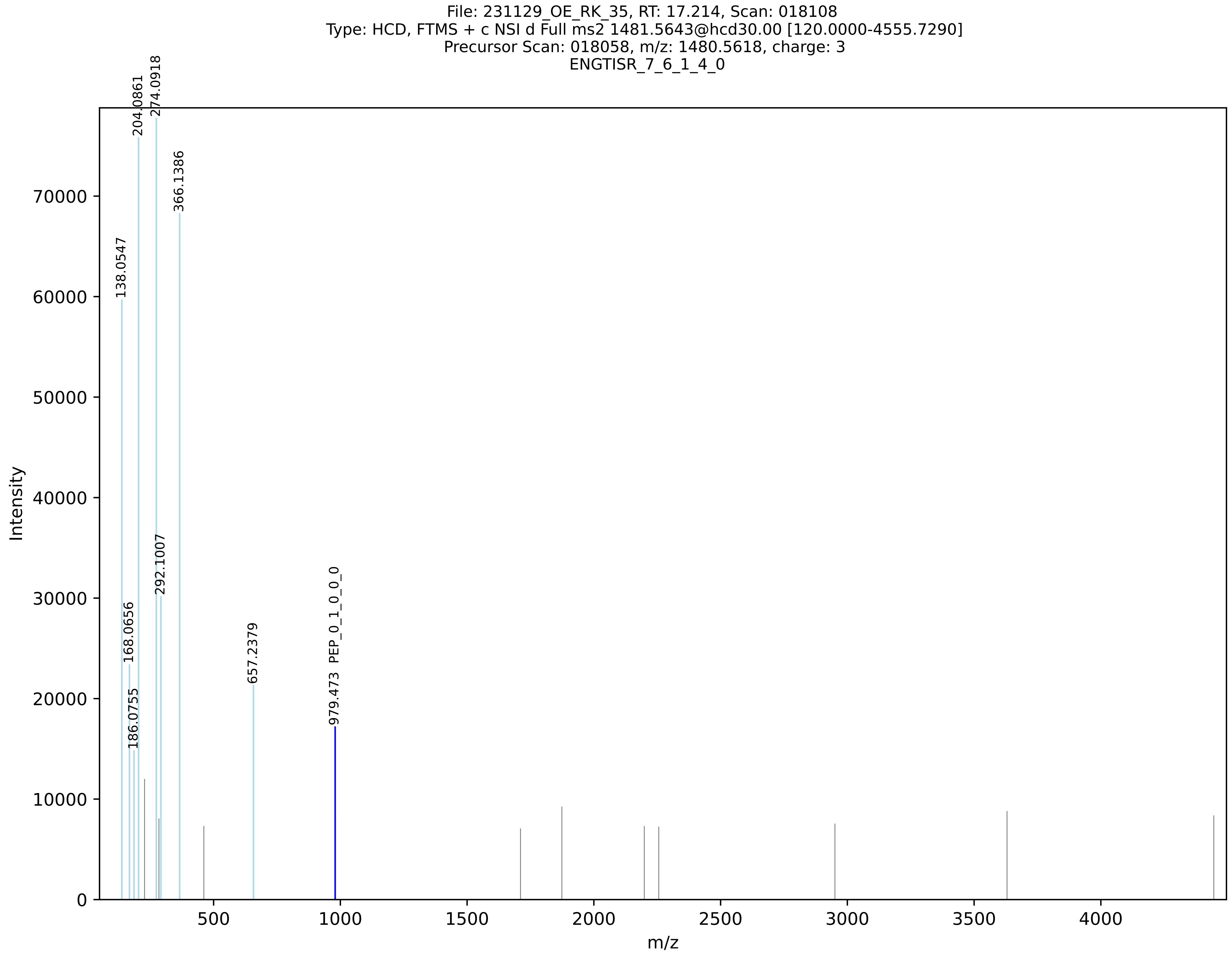
File: 231129_OE_RK_35, RT: 17.214, Scan: 018108
Type: HCD, FTMS + c NSI d Full ms2 1481.5643@hcd30.00 [120.0000-4555.7290]
Precursor Scan: 018058, m/z: 1480.5618, charge: 3
ENGTISR_7_6_1_4_0
204.0861
274.0918
366.1386
138.0547
292.1007
168.0656
186.0755
657.2379
979.473 PEP_0_1_0_0_0
Intensity
m/z
0
10000
20000
30000
40000
50000
60000
70000
500
1000
1500
2000
2500
3000
3500
4000

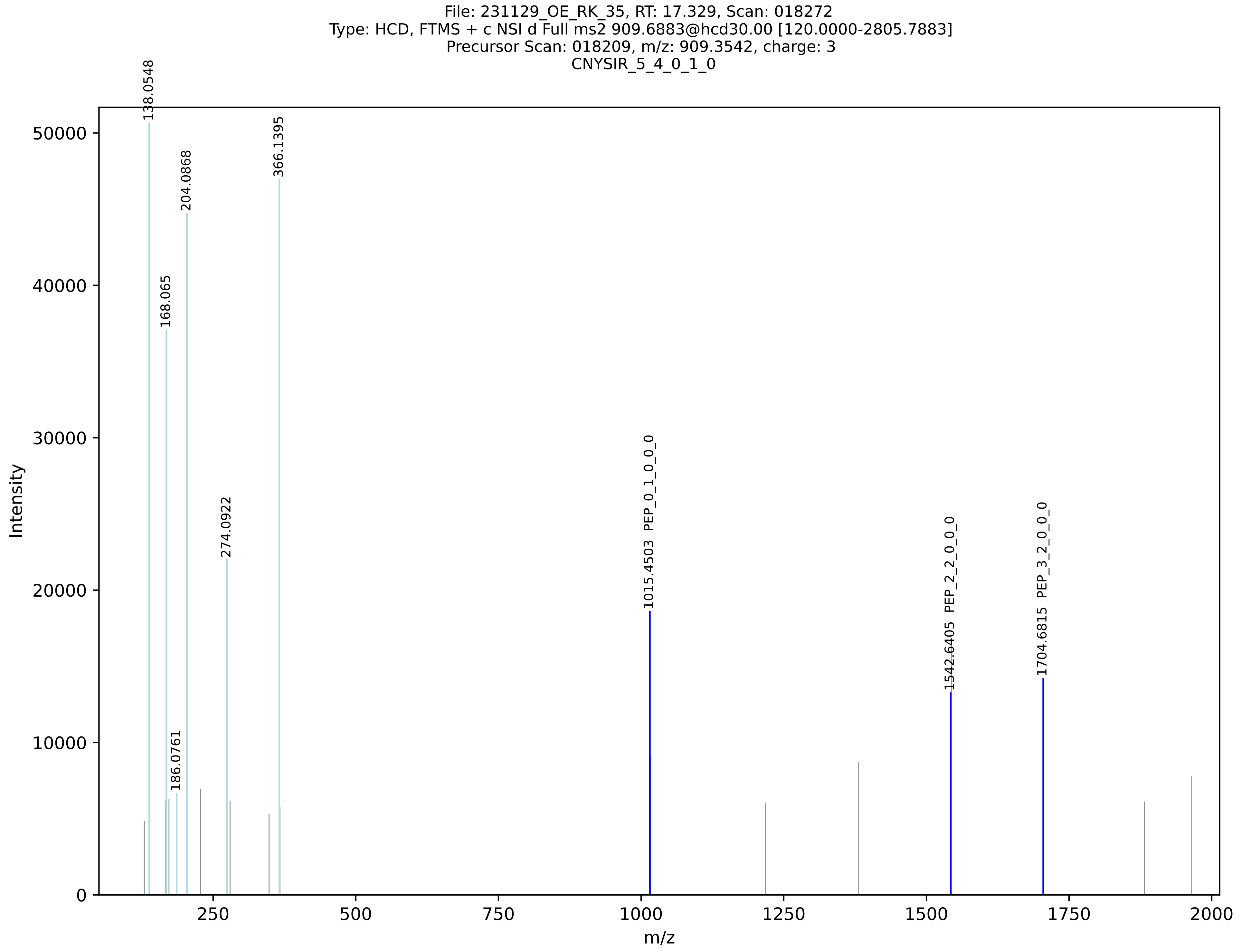
File: 231129_OE_RK_35, RT: 17.329, Scan: 018272
Type: HCD, FTMS + c NSI d Full ms2 909.6883@hcd30.00 [120.0000-2805.7883]
Precursor Scan: 018209, m/z: 909.3542, charge: 3
CNYSIR_5_4_0_1_0
138.0548
168.065
186.0761
204.0868
274.0922
366.1395
1015.4503 PEP_0_1_0_0_0
1542.6405 PEP_2_2_0_0_0
1704.6815 PEP_3_2_0_0_0
Intensity
m/z
0
10000
20000
30000
40000
50000
250
500
750
1000
1250
1500
1750
2000

File: 231129_OE_RK_35, RT: 17.375, Scan: 018338
Type: HCD, FTMS + c NSI d Full ms2 1149.4468@hcd30.00 [120.0000-3539.4490]
Precursor Scan: 018264, m/z: 1149.4468, charge: 3
DKMFSQNDTR_5_4_0_2_0

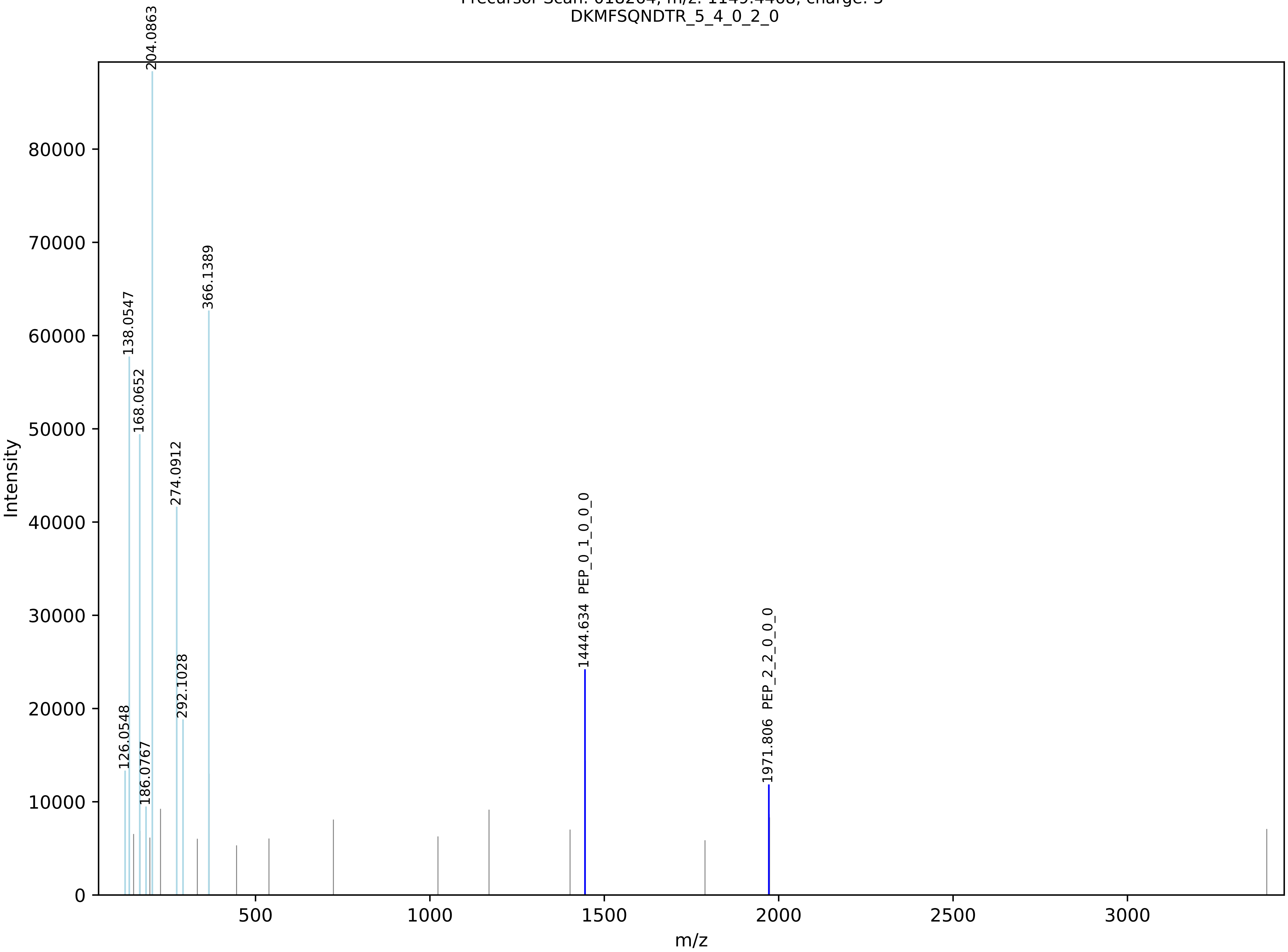

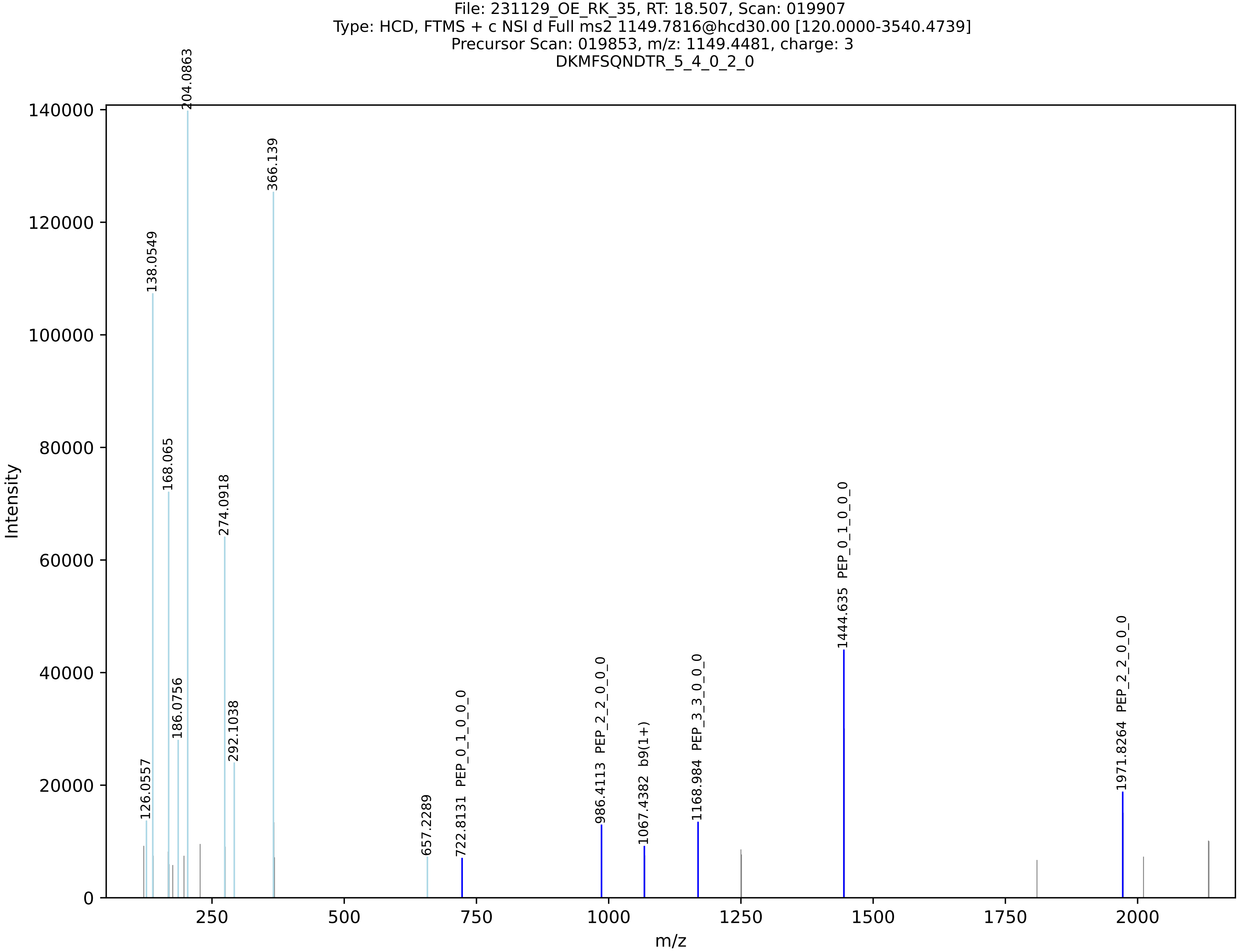

File: 231129_OE_RK_35, RT: 18.507, Scan: 019907
Type: HCD, FTMS + c NSI d Full ms2 1149.7816@hcd30.00 [120.0000-3540.4739]
Precursor Scan: 019853, m/z: 1149.4481, charge: 3
DKMFSQNDTR_5_4_0_2_0
Intensity
m/z
204.0863
366.139
138.0549
168.065
274.0918
186.0756
292.1038
126.0557
657.2289
722.8131 PEP_0_1_0_0_0
986.4113 PEP_2_2_0_0_0
1067.4382 b9(1+)
1168.984 PEP_3_3_0_0_0
1444.635 PEP_0_1_0_0_
1971.8264 PEP_2_2_0_0_

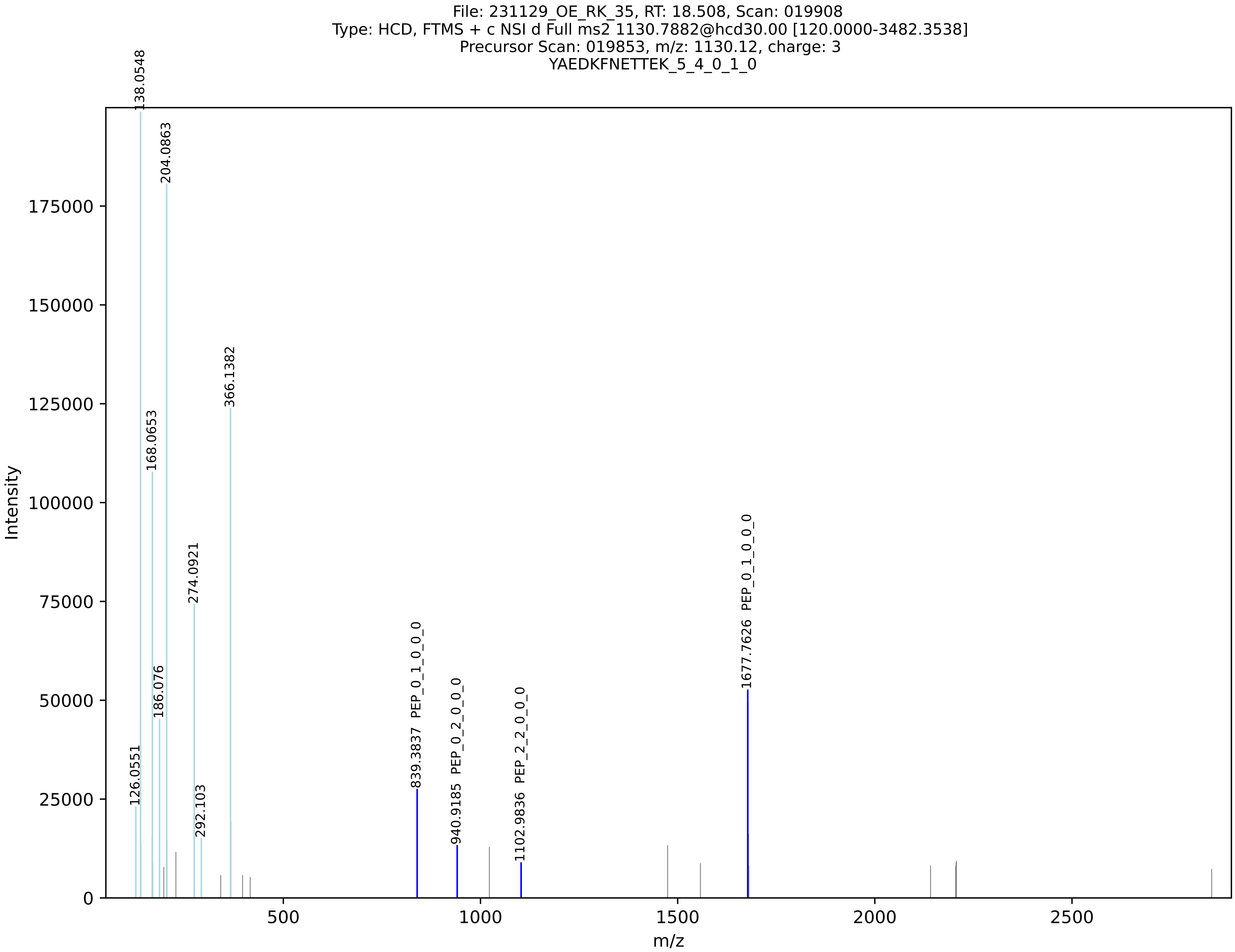

File: 231129_OE_RK_35, RT: 18.508, Scan: 019908
Type: HCD, FTMS + c NSI d Full ms2 1130.7882@hcd30.00 [120.0000-3482.3538]
Precursor Scan: 019853, m/z: 1130.12, charge: 3
YAEDKFNETTEK_5_4_0_1_0
138.0548
204.0863
366.1382
168.0653
274.0921
186.076
126.0551
292.103
839.3837 PEP_0_1_0_0_0
940.9185 PEP_0_2_0_0_0
1102.9836 PEP_2_2_0_0_0
1677.7626 PEP_0_1_0_0_0
Intensity
m/z
175000
150000
125000
100000
75000
50000
25000
0
500
1000
1500
2000
2500

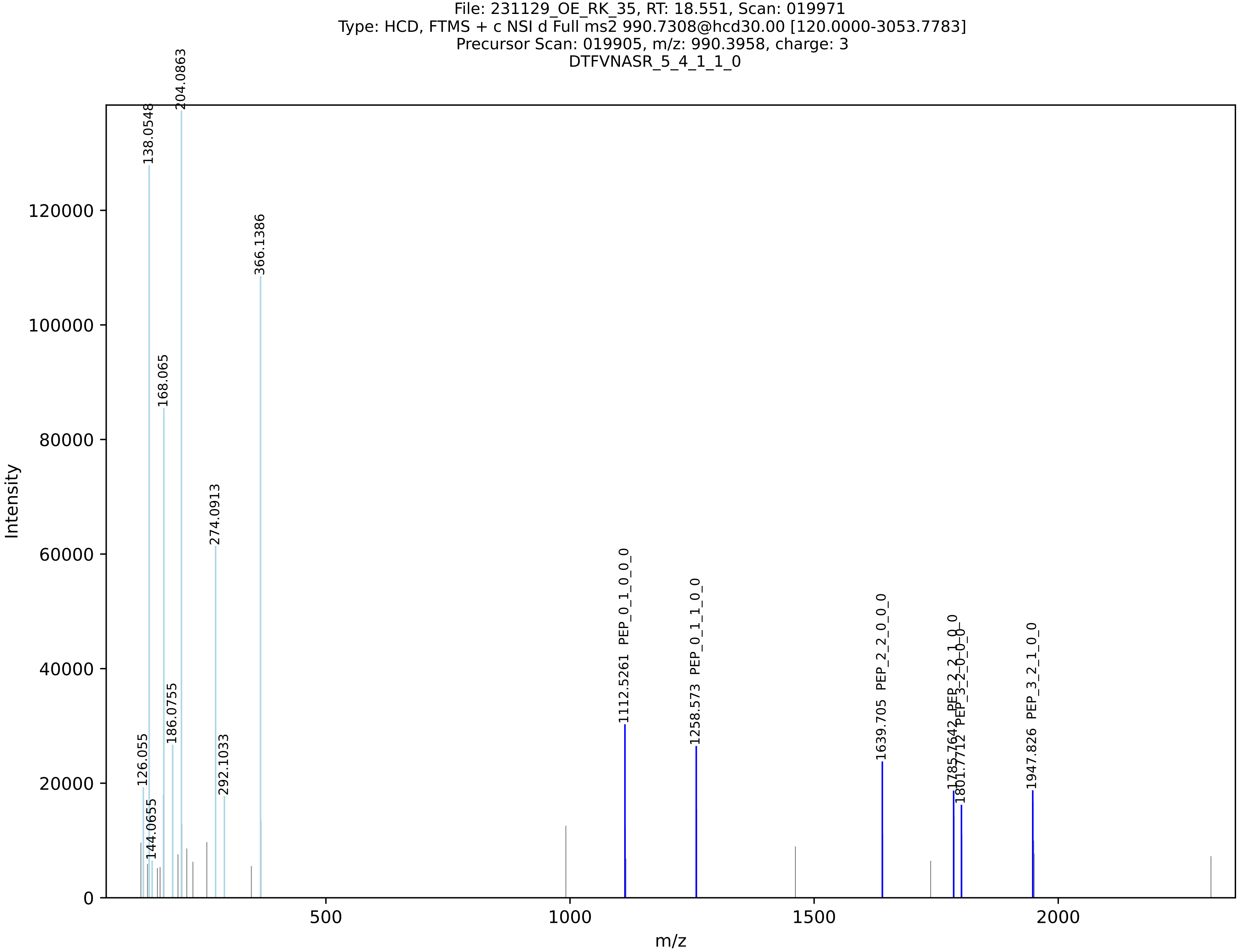

File: 231129_OE_RK_35, RT: 18.551, Scan: 019971
Type: HCD, FTMS + c NSI d Full ms2 990.7308@hcd30.00 [120.0000-3053.7783]
Precursor Scan: 019905, m/z: 990.3958, charge: 3
DTFVNASR_5_4_1_1_0
Intensity
m/z
204.0863
138.0548
366.1386
168.065
274.0913
186.0755
126.055
292.1033
144.0655
1112.5261 PEP_0_1_0_0_0
1258.573 PEP_0_1_1_0_0
1639.705 PEP_2_2_0_0_0
1785.7642 PEP_2_2_1_0_0
1801.7712 PEP_3_2_0_0_0
1947.826 PEP_3_2_1_0_0

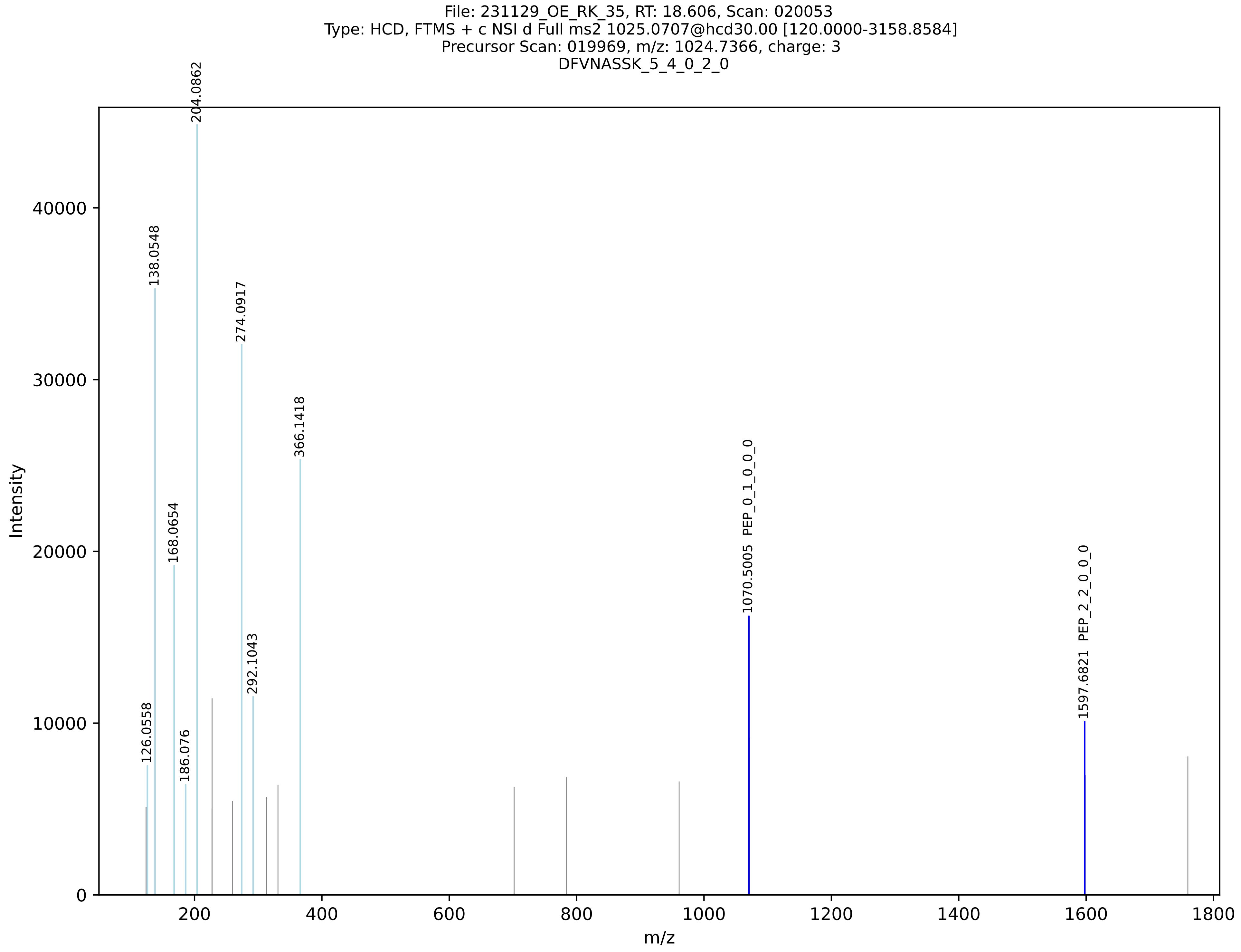
File: 231129_OE_RK_35, RT: 18.606, Scan: 020053
Type: HCD, FTMS + c NSI d Full ms2 1025.0707@hcd30.00 [120.0000-3158.8584]
Precursor Scan: 019969, m/z: 1024.7366, charge: 3
DFVNASSK_5_4_0_2_0
Intensity
m/z
204.0862
138.0548
274.0917
366.1418
168.0654
292.1043
126.0558
186.076
1070.5005 PEP_0_1_0_0_0
1597.6821 PEP_2_2_0_0_0
0
10000
20000
30000
40000
200
400
600
800
1000
1200
1400
1600
1800

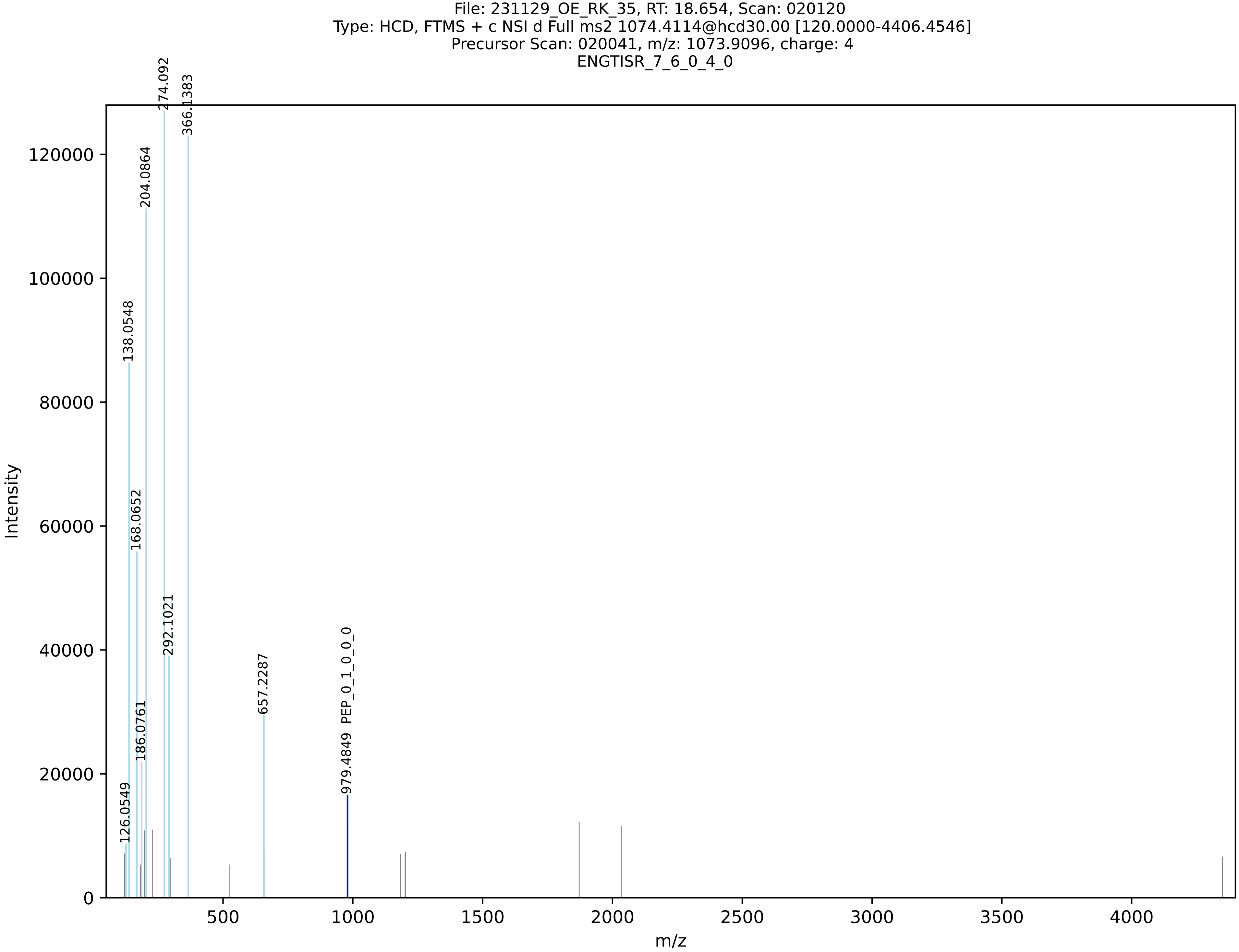

File: 231129_OE_RK_35, RT: 18.654, Scan: 020120
Type: HCD, FTMS + c NSI d Full ms2 1074.4114@hcd30.00 [120.0000-4406.4546]
Precursor Scan: 020041, m/z: 1073.9096, charge: 4
ENGTISR_7_6_0_4_0
274.092
366.1383
204.0864
138.0548
168.0652
292.1021
657.2287
PEP_0_1_0_0_0
186.0761
979.4849
126.0549
Intensity
m/z
0
20000
40000
60000
80000
100000
120000
500
1000
1500
2000
2500
3000
3500
4000

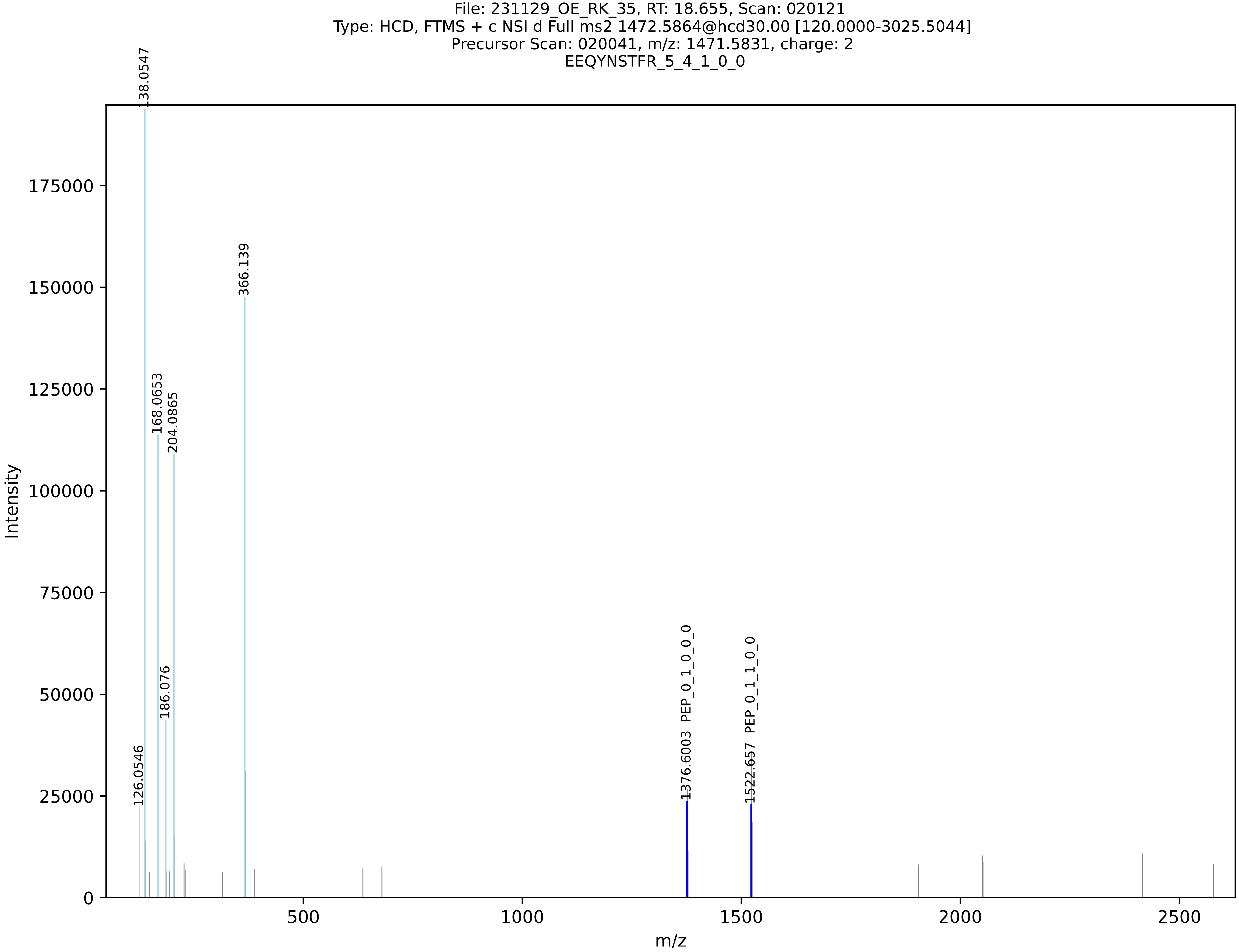

File: 231129_OE_RK_35, RT: 18.655, Scan: 020121
Type: HCD, FTMS + c NSI d Full ms2 1472.5864@hcd30.00 [120.0000-3025.5044]
Precursor Scan: 020041, m/z: 1471.5831, charge: 2
EEQYNSTFR_5_4_1_0_0
138.0547
366.139
168.0653
204.0865
186.076
126.0546
1376.6003 PEP_0_1_0_0_0
1522.657 PEP_0_1_1_0_0
Intensity
m/z

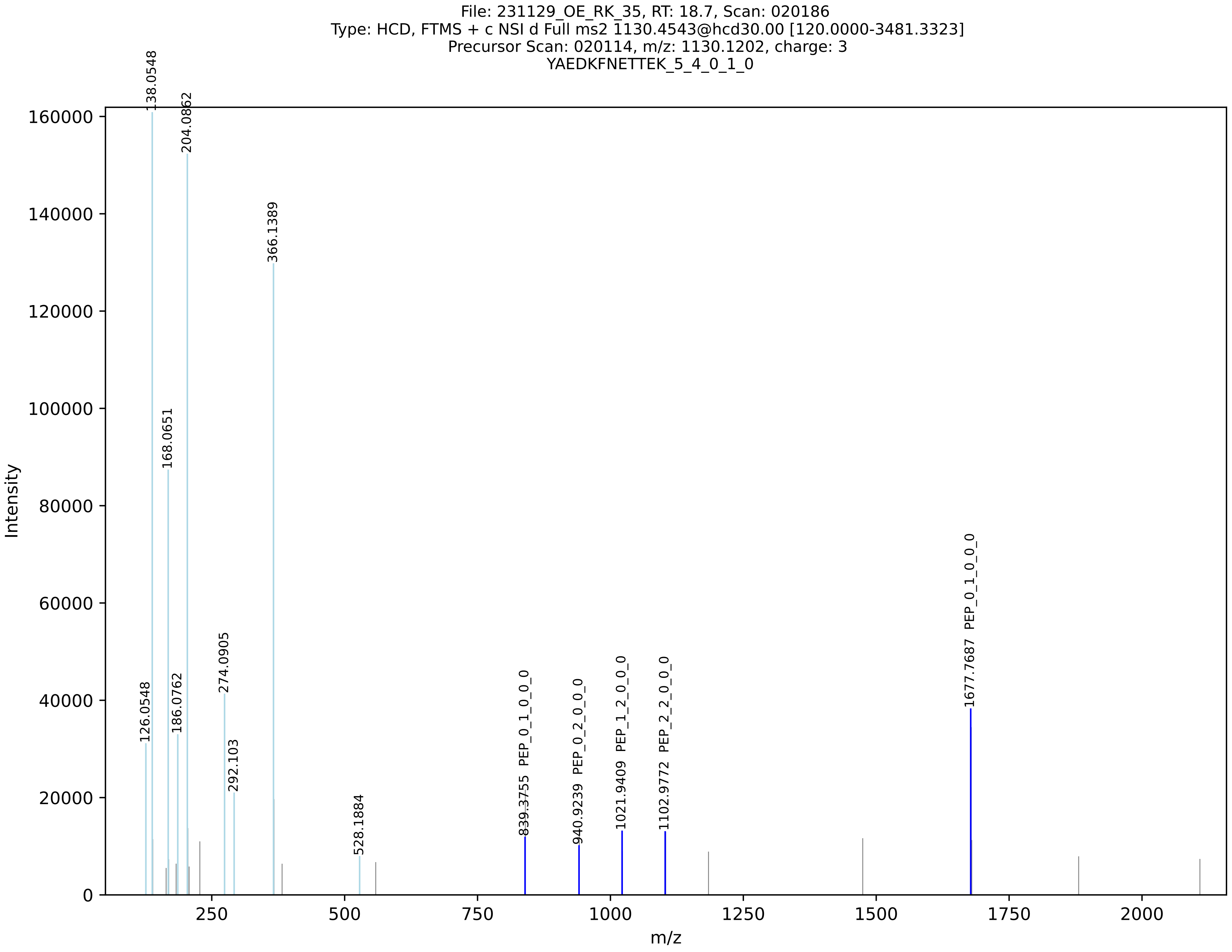
File: 231129_OE_RK_35, RT: 18.7, Scan: 020186
Type: HCD, FTMS + c NSI d Full ms2 1130.4543@hcd30.00 [120.0000-3481.3323]
Precursor Scan: 020114, m/z: 1130.1202, charge: 3
YAEDKFNETTEK_5_4_0_1_0
138.0548
204.0862
366.1389
168.0651
274.0905
126.0548
186.0762
292.103
528.1884
839.3755 PEP_0_1_0_0_0
940.9239 PEP_0_2_0_0_0
1021.9409 PEP_1_2_0_0_0
1102.9772 PEP_2_2_0_0_0
1677.7687 PEP_0_1_0_0_0
Intensity
m/z
0
20000
40000
60000
80000
100000
120000
140000
160000
250
500
750
1000
1250
1500
1750
2000

File: 231129_OE_RK_35, RT: 18.701, Scan: 020187
Type: HCD, FTMS + c NSI d Full ms2 1149.7822@hcd30.00 [120.0000-3540.4758]
Precursor Scan: 020114, m/z: 1149.4482, charge: 3
DKMFSQNDTR_5_4_0_2_0

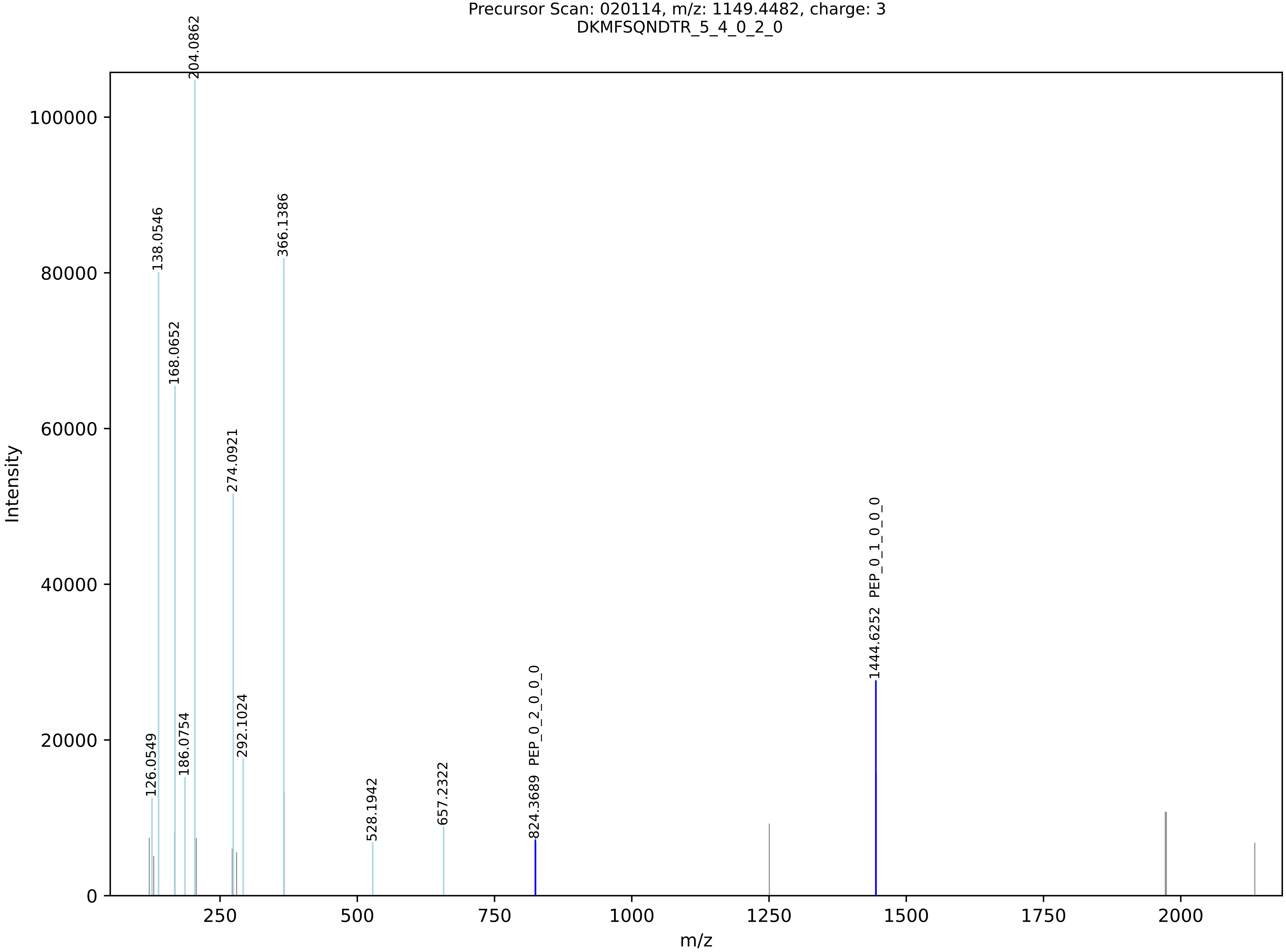

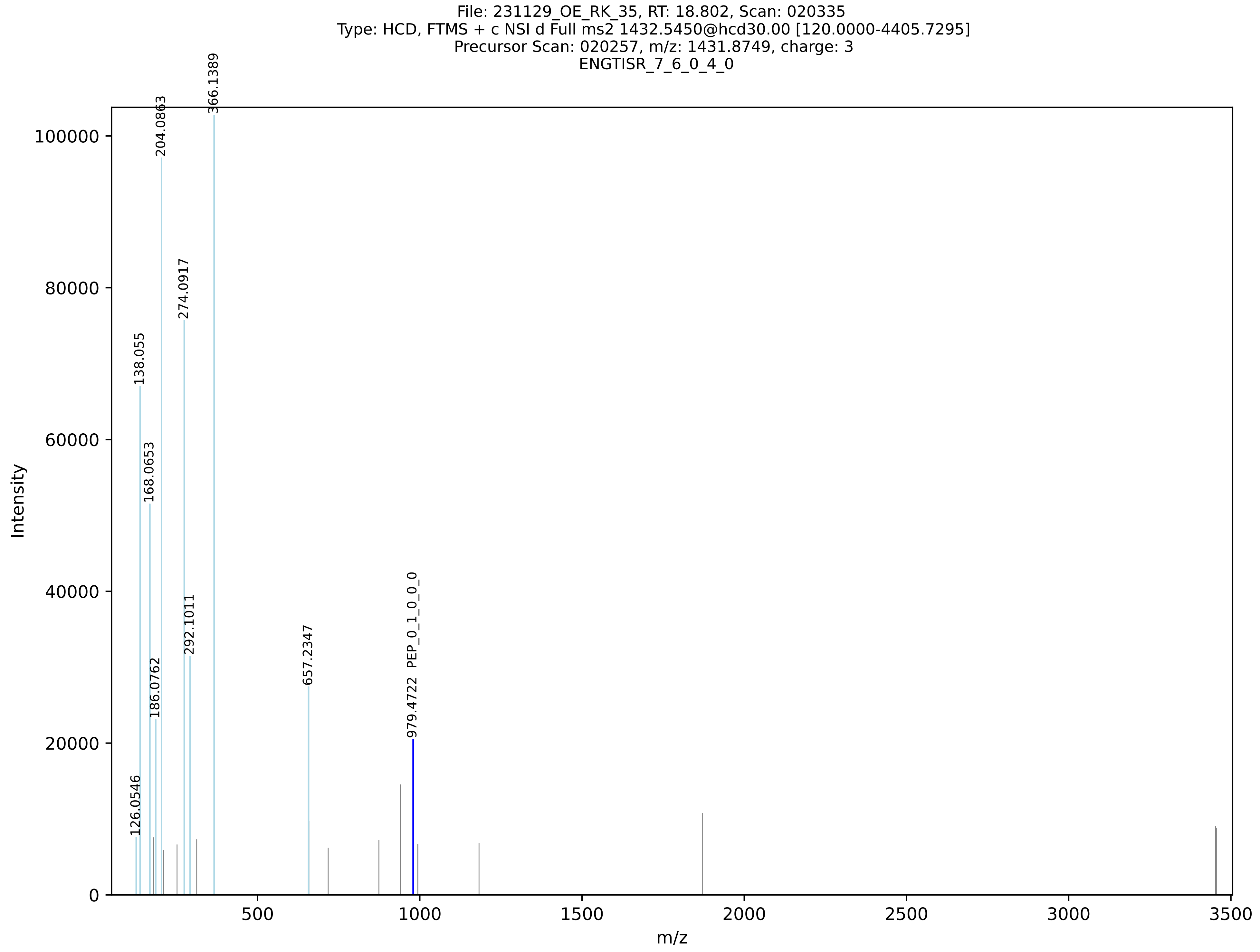

File: 231129_OE_RK_35, RT: 18.802, Scan: 020335
Type: HCD, FTMS + c NSI d Full ms2 1432.5450@hcd30.00 [120.0000-4405.7295]
Precursor Scan: 020257, m/z: 1431.8749, charge: 3
ENGTISR_7_6_0_4_0
366.1389
204.0863
274.0917
138.055
168.0653
292.1011
186.0762
126.0546
657.2347
979.4722 PEP_0_1_0_0_0
Intensity
m/z
0
20000
40000
60000
80000
100000
500
1000
1500
2000
2500
3000
3500

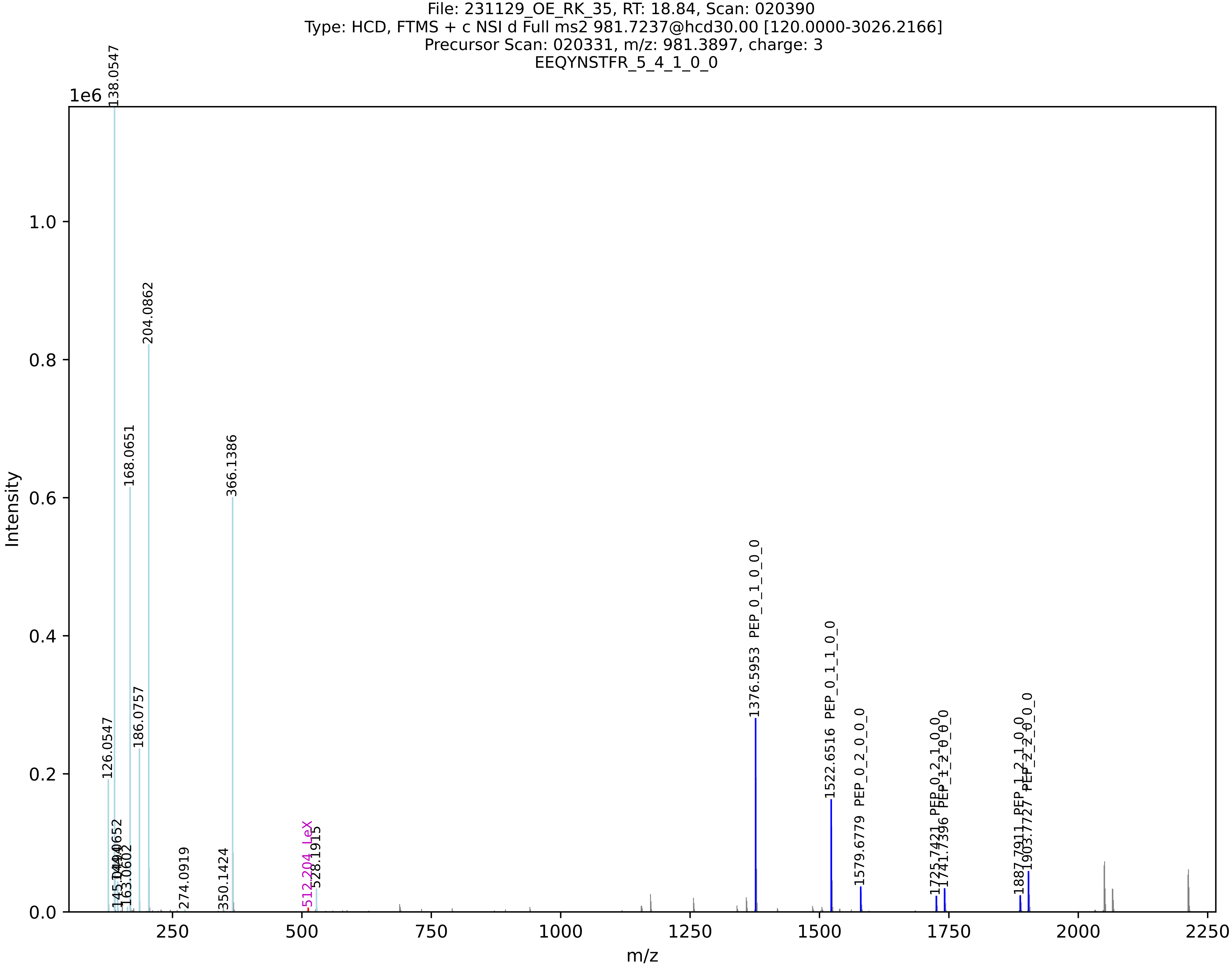
File: 231129_OE_RK_35, RT: 18.84, Scan: 020390
Type: HCD, FTMS + c NSI d Full ms2 981.7237@hcd30.00 [120.0000-3026.2166]
Precursor Scan: 020331, m/z: 981.3897, charge: 3
EEQYNSTFR_5_4_1_0_0
1e6
Intensity
m/z
138.0547
204.0862
168.0651
366.1386
126.0547
186.0757
274.0919
350.1424
512.204_LeX
528.1915
1376.5953 PEP_0_1_0_0_0
1522.6516 PEP_0_1_1_0_0
1579.6779 PEP_0_2_0_0_0
1887.7911 PEP_1_2_1_0_0
1903.7727 PEP_2_2_0_0_0

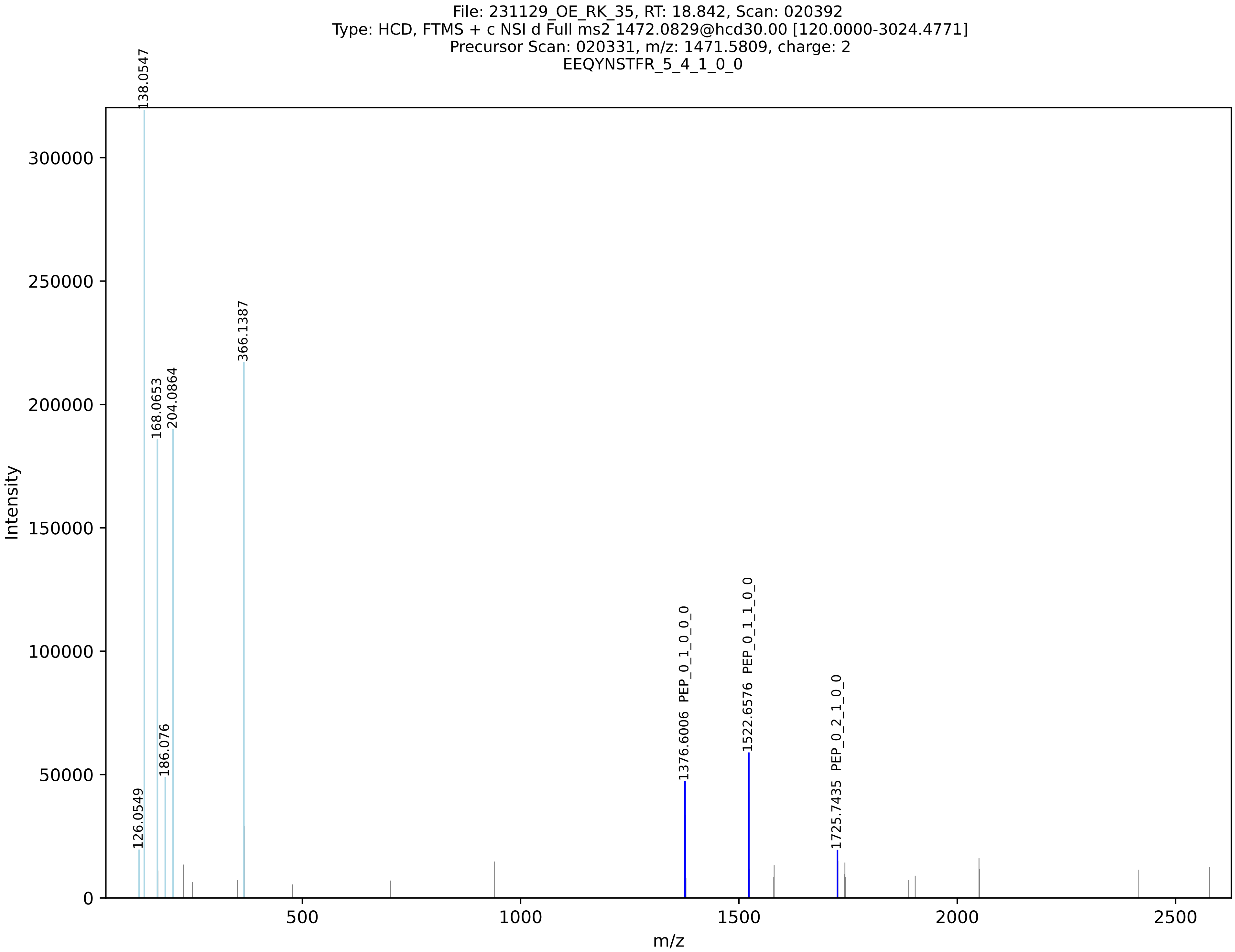
File: 231129_OE_RK_35, RT: 18.842, Scan: 020392
Type: HCD, FTMS + c NSI d Full ms2 1472.0829@hcd30.00 [120.0000-3024.4771]
Precursor Scan: 020331, m/z: 1471.5809, charge: 2
EEQYNSTFR_5_4_1_0_0
138.0547
366.1387
168.0653
204.0864
186.076
126.0549
1376.6006 PEP_0_1_0_0_0
1522.6576 PEP_0_1_1_0_0
1725.7435 PEP_0_2_1_0_0
Intensity
m/z
0
50000
100000
150000
200000
250000
300000
500
1000
1500
2000
2500

File: 231129_OE_RK_35, RT: 18.883, Scan: 020453
Type: HCD, FTMS + c NSI d Full ms2 1150.1165@hcd30.00 [120.0000-3541.4983]
Precursor Scan: 020389, m/z: 1149.4482, charge: 3
DKMFSQNDTR_5_4_0_2_0

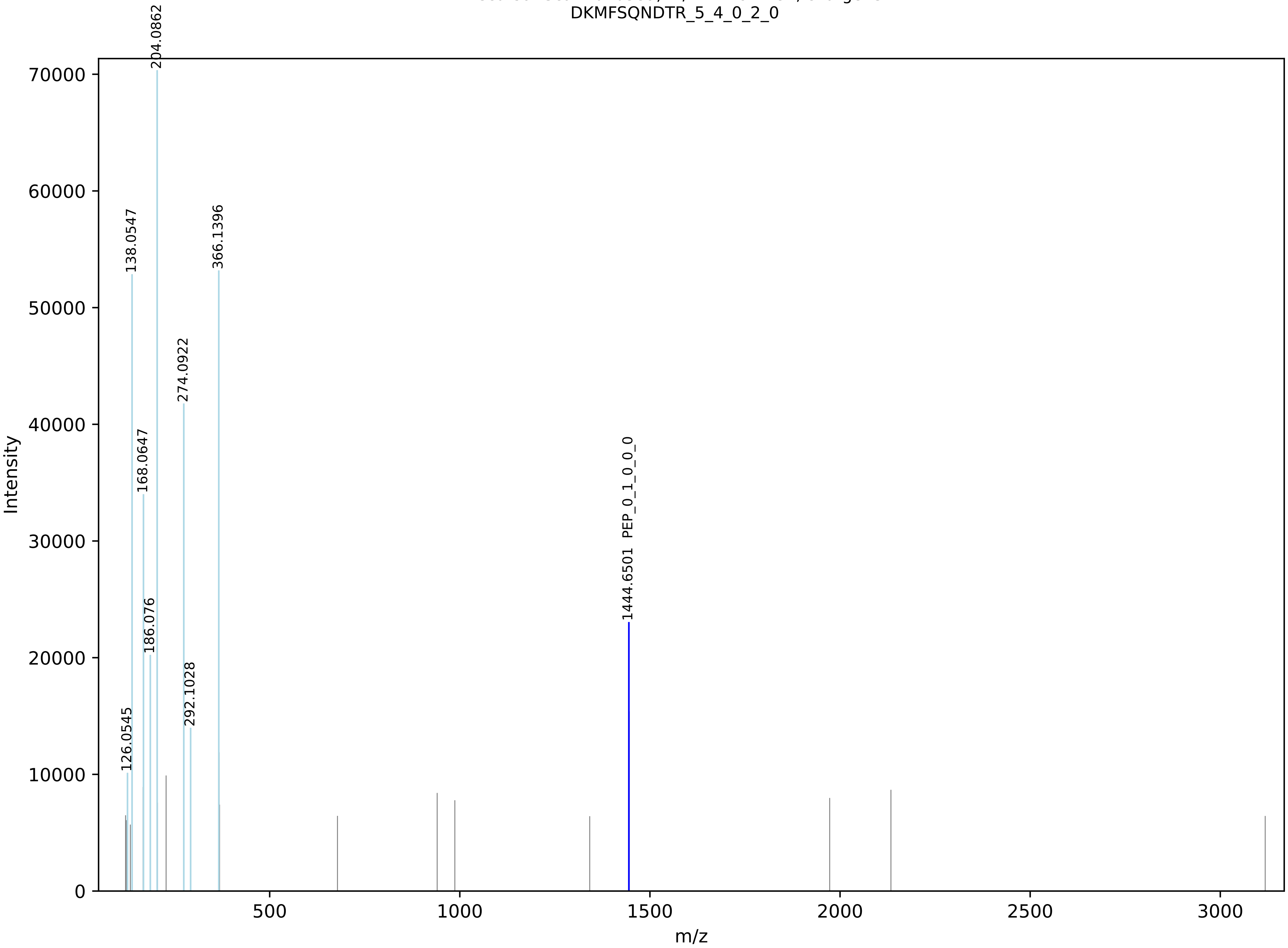

File: 231129_OE_RK_35, RT: 18.924, Scan: 020509
Type: HCD, FTMS + c NSI d Full ms2 1049.4169@hcd30.00 [120.0000-3233.3574]
Precursor Scan: 020445, m/z: 1049.0828, charge: 3
EEQYNSTFR_5_5_1_0_0

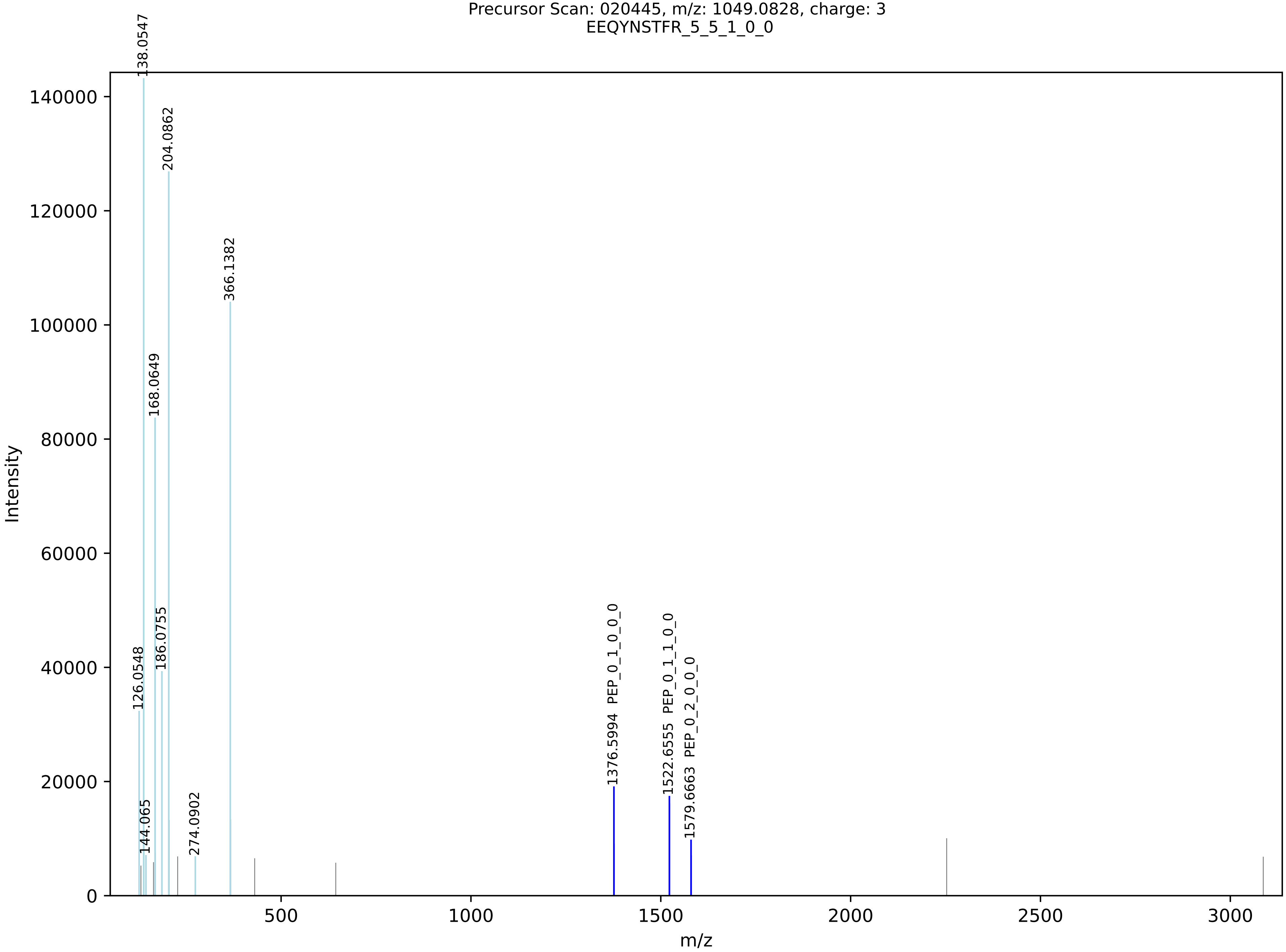

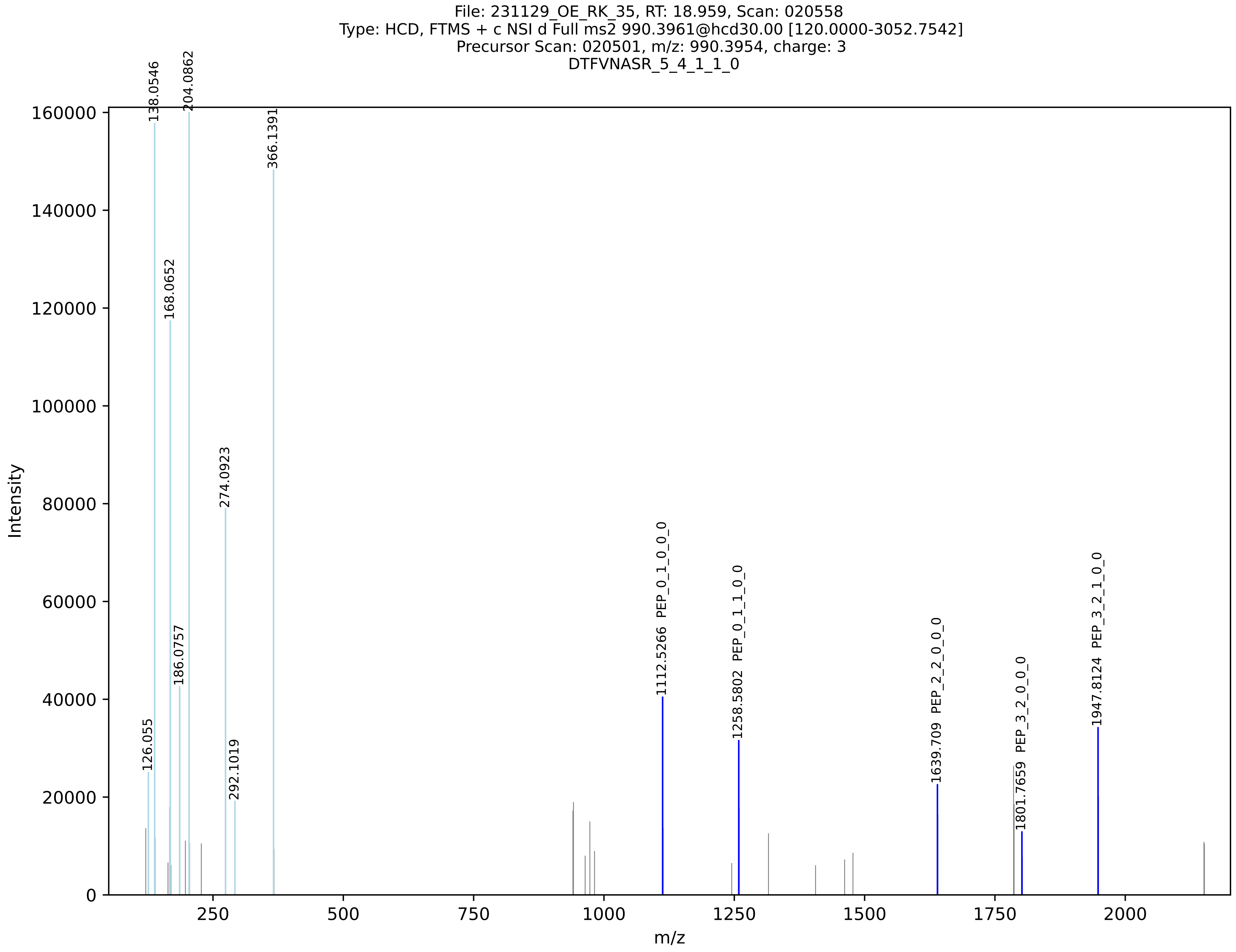
File: 231129_OE_RK_35, RT: 18.959, Scan: 020558
Type: HCD, FTMS + c NSI d Full ms2 990.3961@hcd30.00 [120.0000-3052.7542]
Precursor Scan: 020501, m/z: 990.3954, charge: 3
DTFVNASR_5_4_1_1_0
Intensity
m/z
0
20000
40000
60000
80000
100000
120000
140000
160000
250
500
750
1000
1250
1500
1750
2000
138.0546
204.0862
366.1391
168.0652
186.0757
274.0923
126.055
292.1019
1112.5266 PEP_0_1_0_0_0
1258.5802 PEP_0_1_1_0_0
1639.709 PEP_2_2_0_0_0
1801.7659 PEP_3_2_0_0_0
1947.8124 PEP_3_2_1_0_0

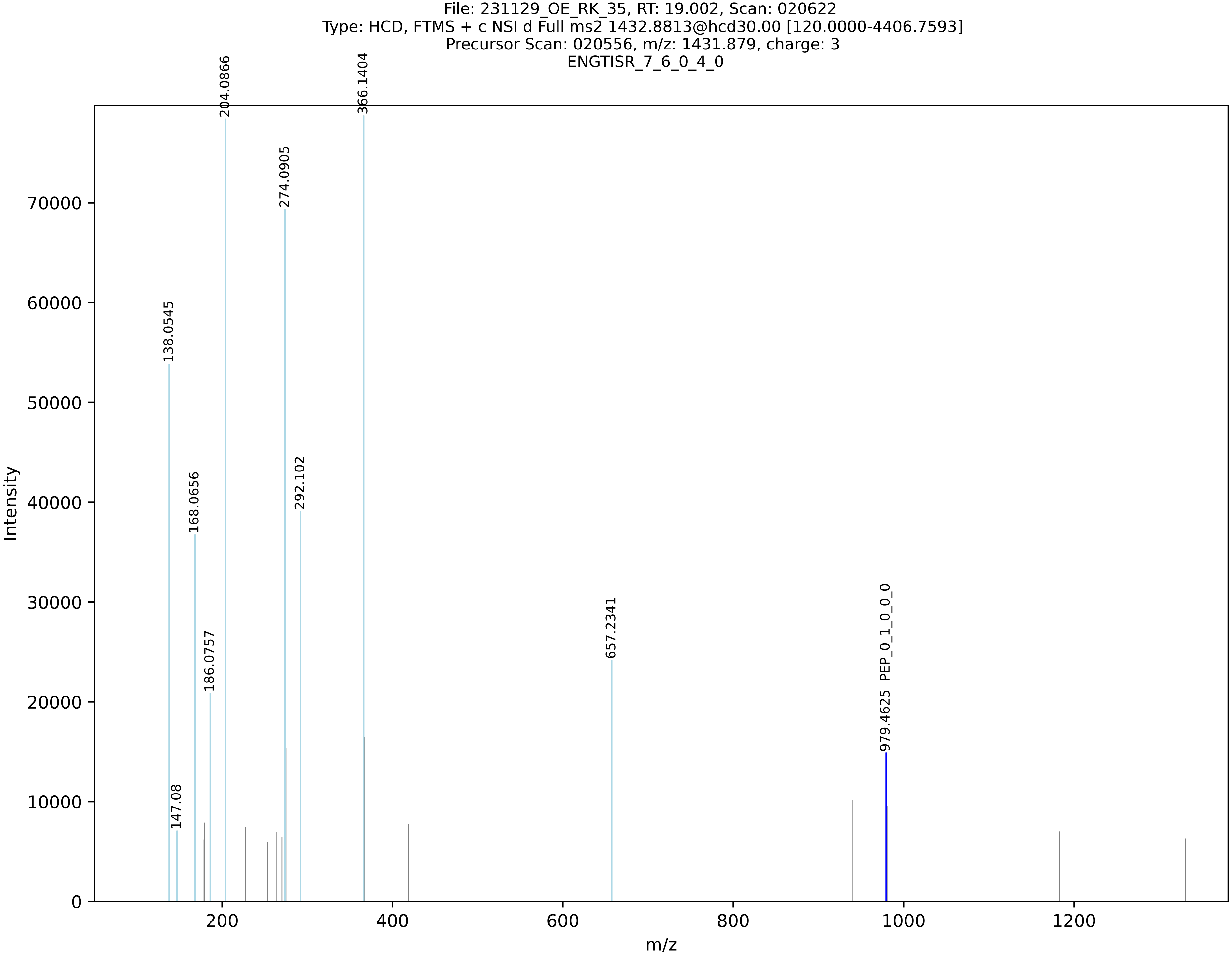
File: 231129_OE_RK_35, RT: 19.002, Scan: 020622
Type: HCD, FTMS + c NSI d Full ms2 1432.8813@hcd30.00 [120.0000-4406.7593]
Precursor Scan: 020556, m/z: 1431.879, charge: 3
ENGTISR_7_6_0_4_0
204.0866
366.1404
274.0905
138.0545
168.0656
292.102
186.0757
147.08
657.2341
979.4625 PEP_0_1_0_0_0
Intensity
m/z
0
10000
20000
30000
40000
50000
60000
70000
200
400
600
800
1000
1200

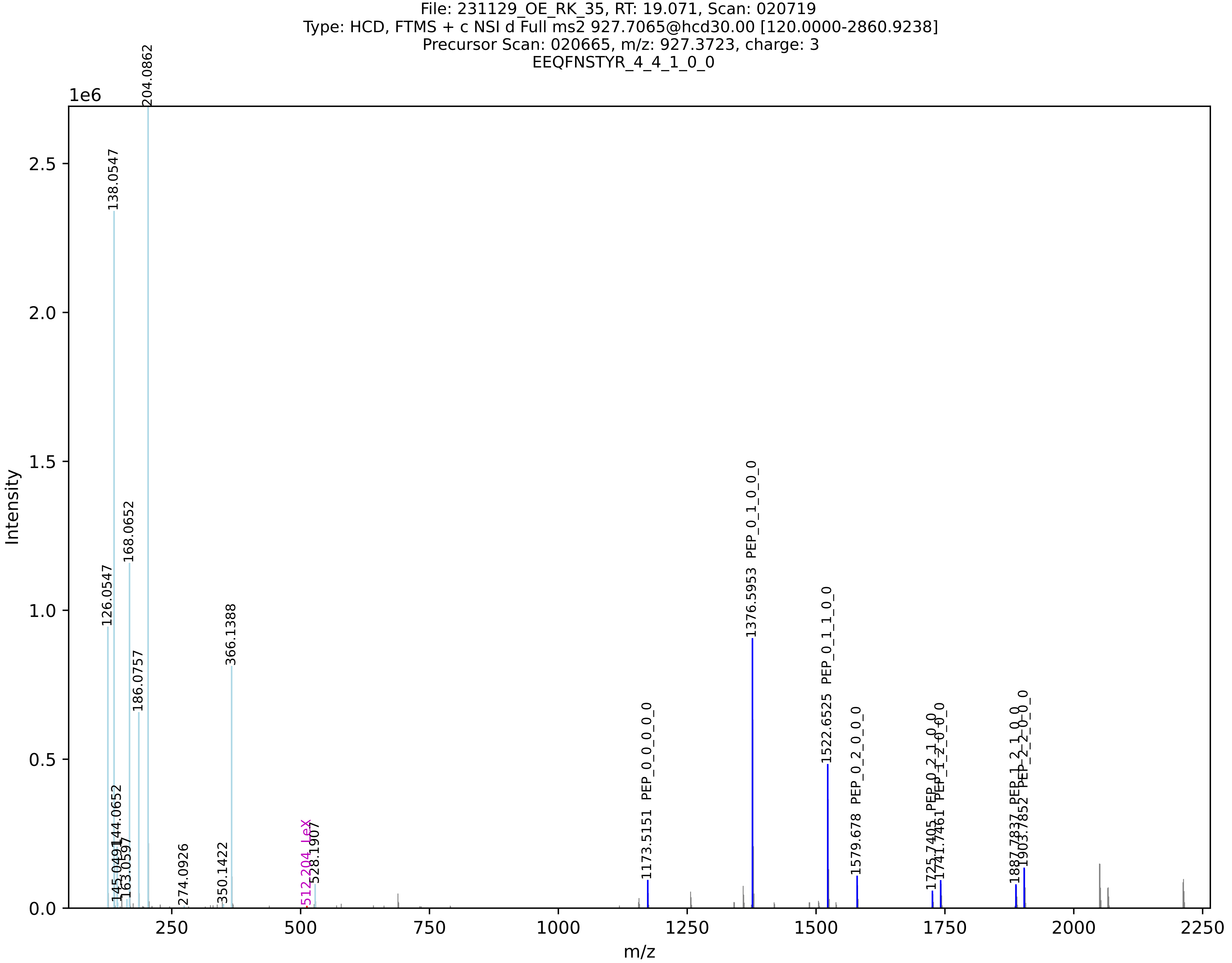
File: 231129_OE_RK_35, RT: 19.071, Scan: 020719
Type: HCD, FTMS + c NSI d Full ms2 927.7065@hcd30.00 [120.0000-2860.9238]
Precursor Scan: 020665, m/z: 927.3723, charge: 3
EEQFNSTYR_4_4_1_0_0
1e6
Intensity
m/z
204.0862
138.0547
168.0652
126.0547
186.0757
366.1388
144.0652
145.0491
163.0597
274.0926
350.1422
512.204_LeX
528.1907
1173.5151 PEP_0_0_0_0_0
1376.5953 PEP_0_1_0_0_0
1522.6525 PEP_0_1_1_0_0
1579.678 PEP_0_2_0_0_0
1725.7405 PEP_0_2_1_0_0
1741.7461 PEP_1_2_0_0
1887.7837 PEP_1_2_1_0_0
1903.7852 PEP_2_2_0_0_0

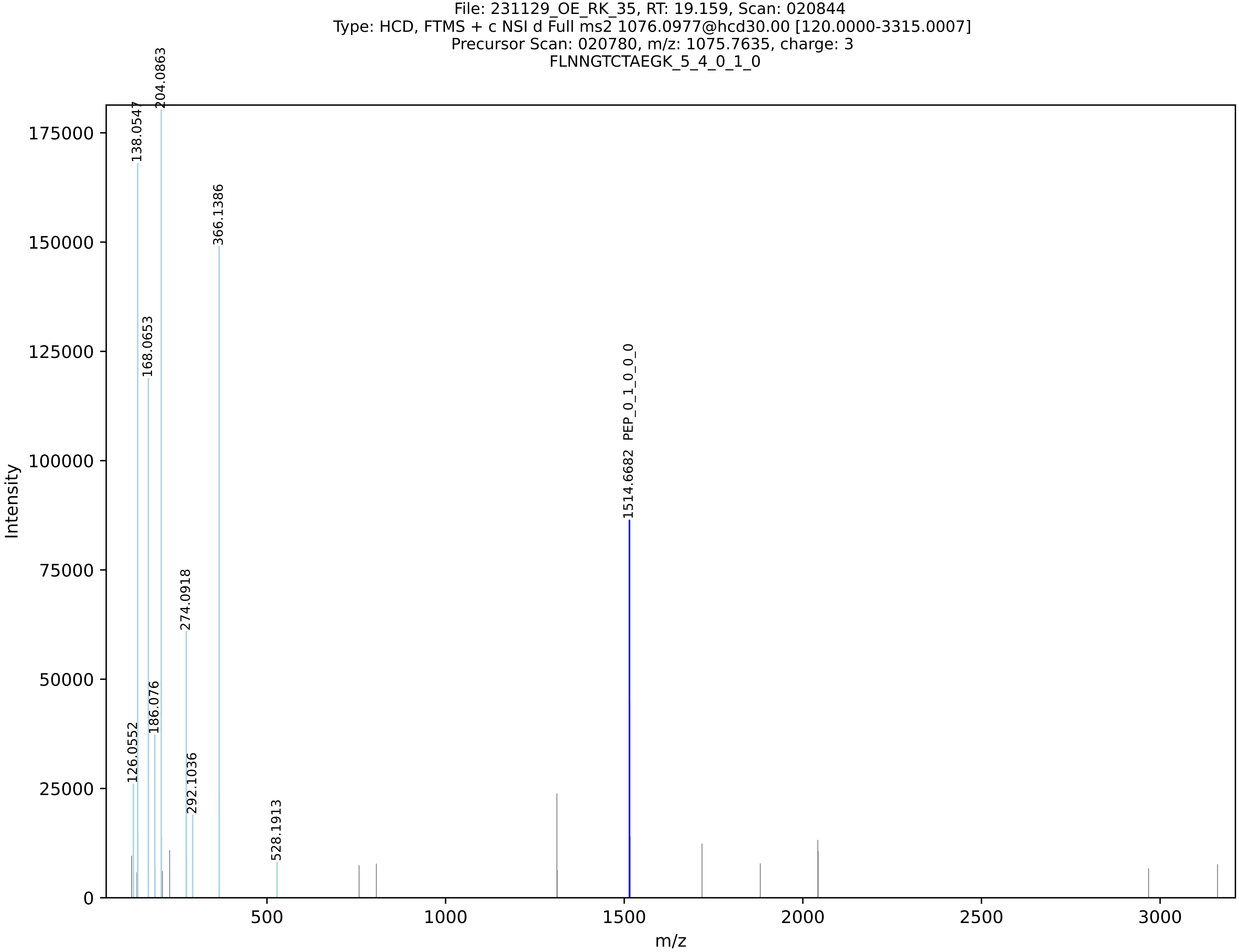
File: 231129_OE_RK_35, RT: 19.159, Scan: 020844
Type: HCD, FTMS + c NSI d Full ms2 1076.0977@hcd30.00 [120.0000-3315.0007]
Precursor Scan: 020780, m/z: 1075.7635, charge: 3
FLNNGTCTAEGK_5_4_0_1_0
204.0863
138.0547
366.1386
168.0653
1514.6682 PEP_0_1_0_0_0
274.0918
186.076
126.0552
292.1036
528.1913
Intensity
m/z
0
25000
50000
75000
100000
125000
150000
175000
500
1000
1500
2000
2500
3000

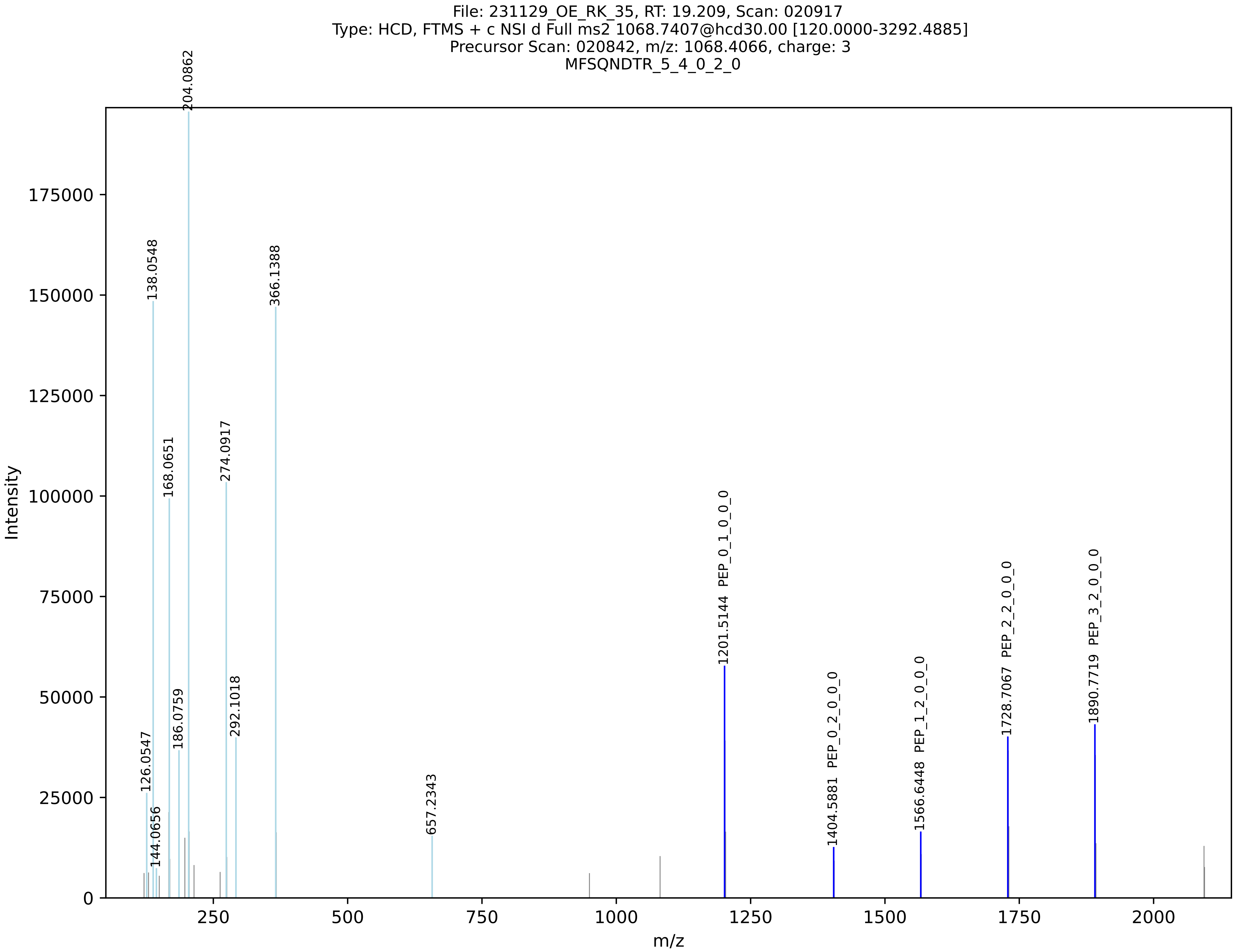
File: 231129_OE_RK_35, RT: 19.209, Scan: 020917
Type: HCD, FTMS + c NSI d Full ms2 1068.7407@hcd30.00 [120.0000-3292.4885]
Precursor Scan: 020842, m/z: 1068.4066, charge: 3
MFSQNDTR_5_4_0_2_0
Intensity
m/z
204.0862
138.0548
366.1388
168.0651
274.0917
186.0759
292.1018
126.0547
144.0656
657.2343
1201.5144 PEP_0_1_0_0_0
1404.5881 PEP_0_2_0_0_0
1566.6448 PEP_1_2_0_0_0
1728.7067 PEP_2_2_0_0_0
1890.7719 PEP_3_2_0_0_0
0
25000
50000
75000
100000
125000
150000
175000
250
500
750
1000
1250
1500
1750
2000

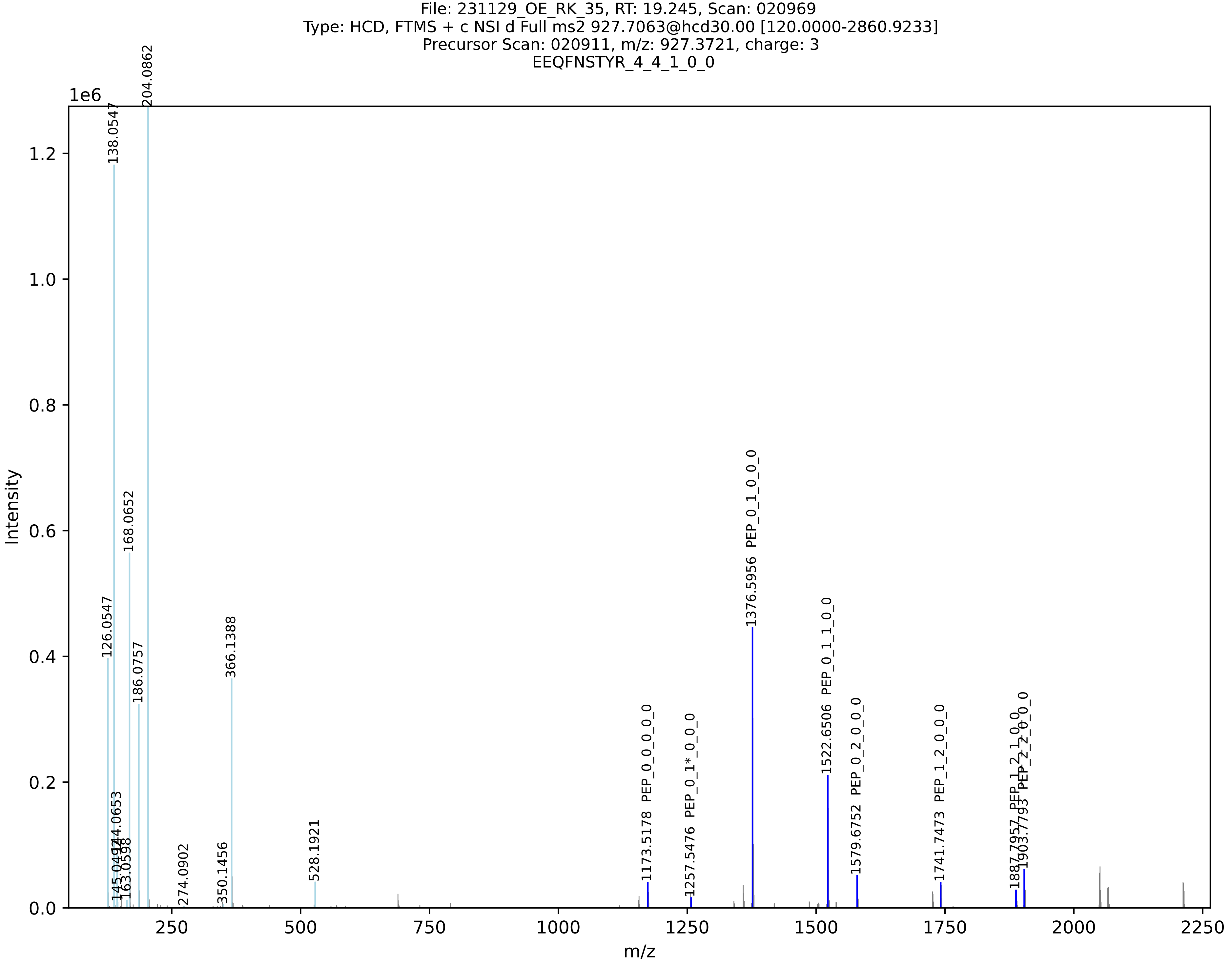

File: 231129_OE_RK_35, RT: 19.245, Scan: 020969
Type: HCD, FTMS + c NSI d Full ms2 927.7063@hcd30.00 [120.0000-2860.9233]
Precursor Scan: 020911, m/z: 927.3721, charge: 3
EEQFNSTYR_4_4_1_0_0
1e6
Intensity
m/z
204.0862
138.0547
168.0652
126.0547
186.0757
366.1388
144.0653
145.0492
163.0598
274.0902
350.1456
528.1921
1173.5178 PEP_0_0_0_0_0
1257.5476 PEP_0_1*_0_0_0
1376.5956 PEP_0_1_0_0_0
1522.6506 PEP_0_1_1_0_0
1579.6752 PEP_0_2_0_0_0
1741.7473 PEP_1_2_0_0_0
1887.7957 PEP_1_2_1_0_0
1903.7793 PEP_2_2_0_0_0

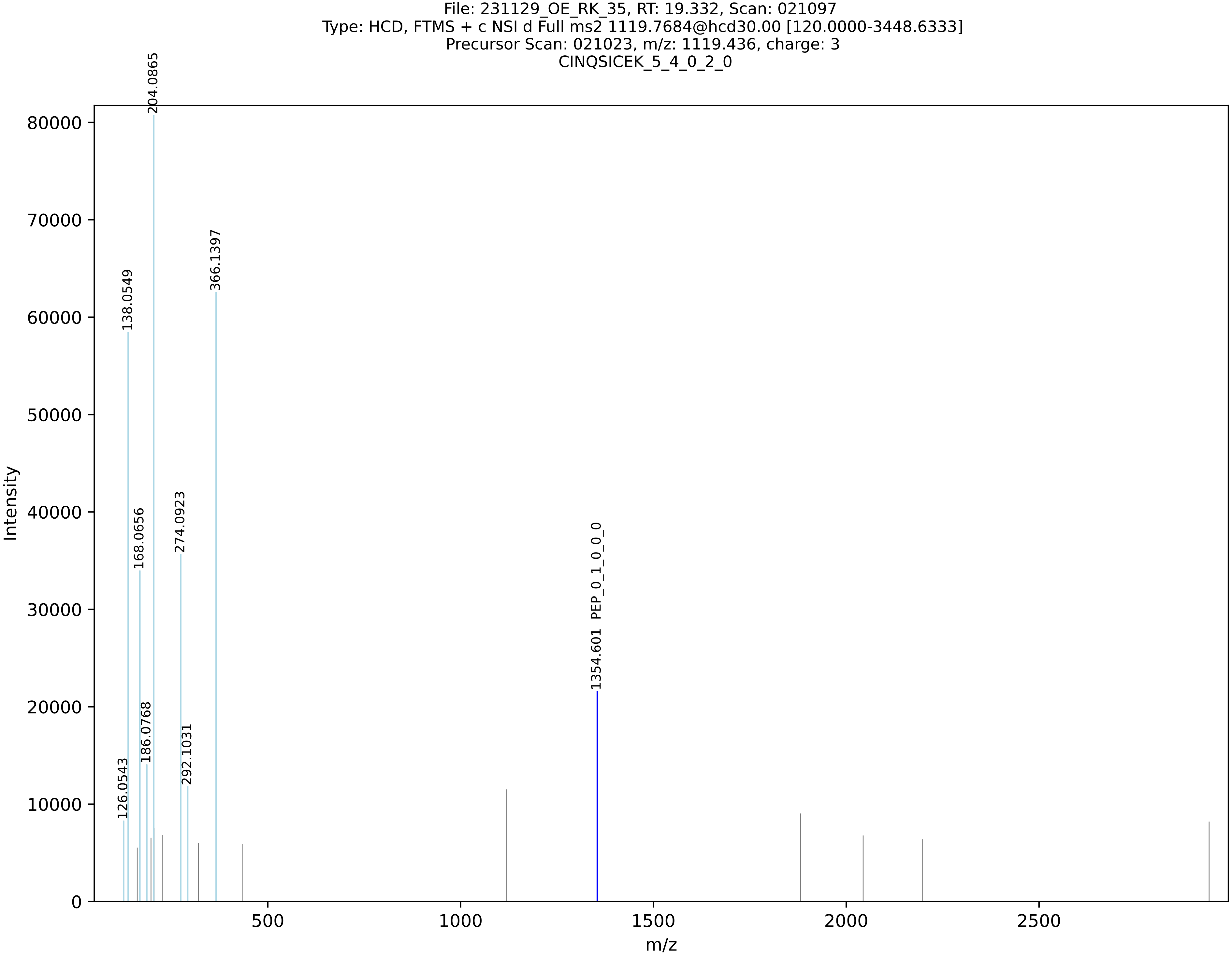
File: 231129_OE_RK_35, RT: 19.332, Scan: 021097
Type: HCD, FTMS + c NSI d Full ms2 1119.7684@hcd30.00 [120.0000-3448.6333]
Precursor Scan: 021023, m/z: 1119.436, charge: 3
CINQSICEK_5_4_0_2_0
Intensity
m/z
204.0865
366.1397
138.0549
168.0656
274.0923
186.0768
292.1031
126.0543
1354.601 PEP_0_1_0_0_0
0
10000
20000
30000
40000
50000
60000
70000
80000
500
1000
1500
2000
2500

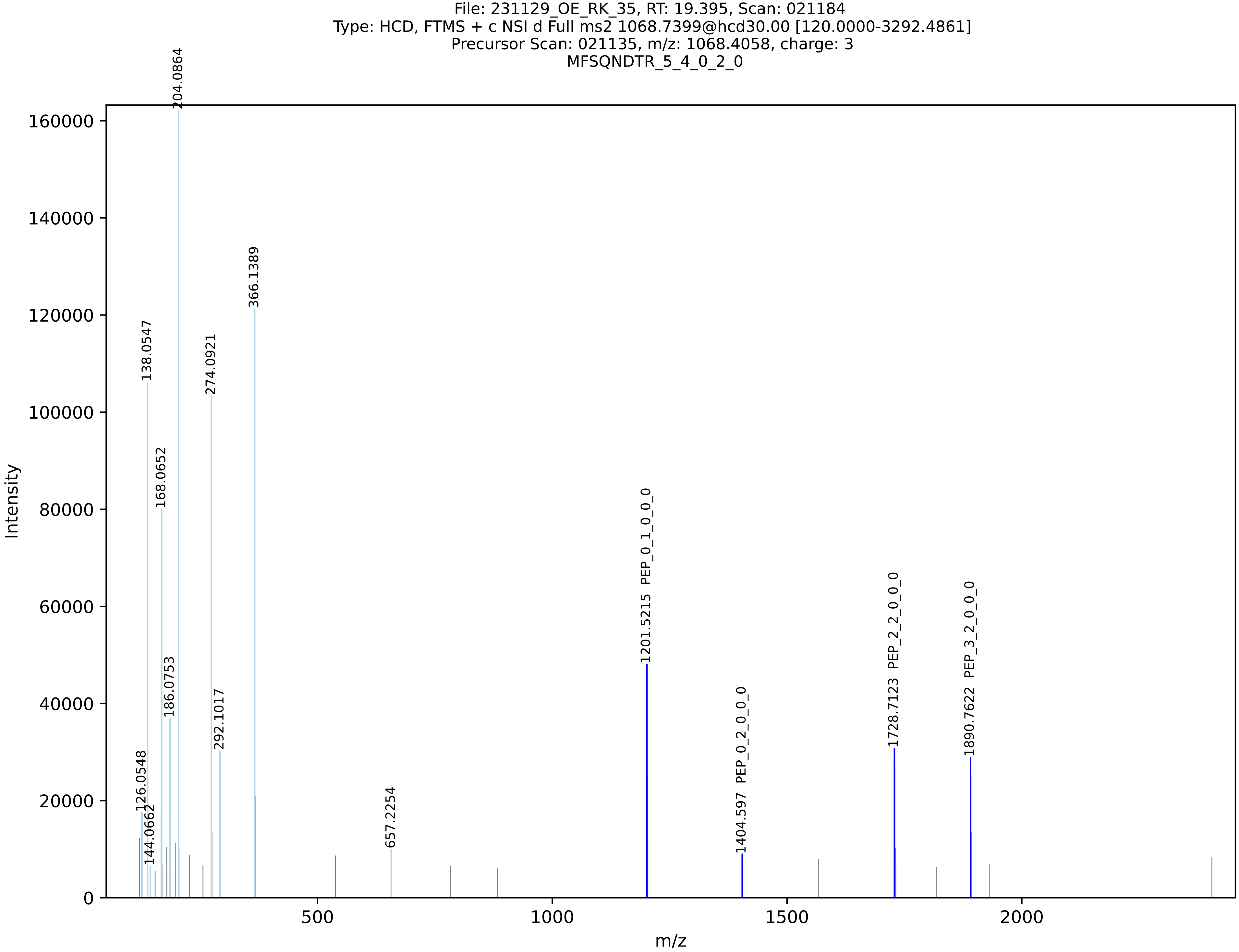
File: 231129_OE_RK_35, RT: 19.395, Scan: 021184
Type: HCD, FTMS + c NSI d Full ms2 1068.7399@hcd30.00 [120.0000-3292.4861]
Precursor Scan: 021135, m/z: 1068.4058, charge: 3
MFSQNDTR_5_4_0_2_0
Intensity
m/z
204.0864
366.1389
138.0547
274.0921
168.0652
186.0753
292.1017
126.0548
144.0662
657.2254
1201.5215 PEP_0_1_0_0_0
1404.597 PEP_0_2_0_0_0
1728.7123 PEP_2_2_0_0_0
1890.7622 PEP_3_2_0_0_0
0
20000
40000
60000
80000
100000
120000
140000
160000
500
1000
1500
2000

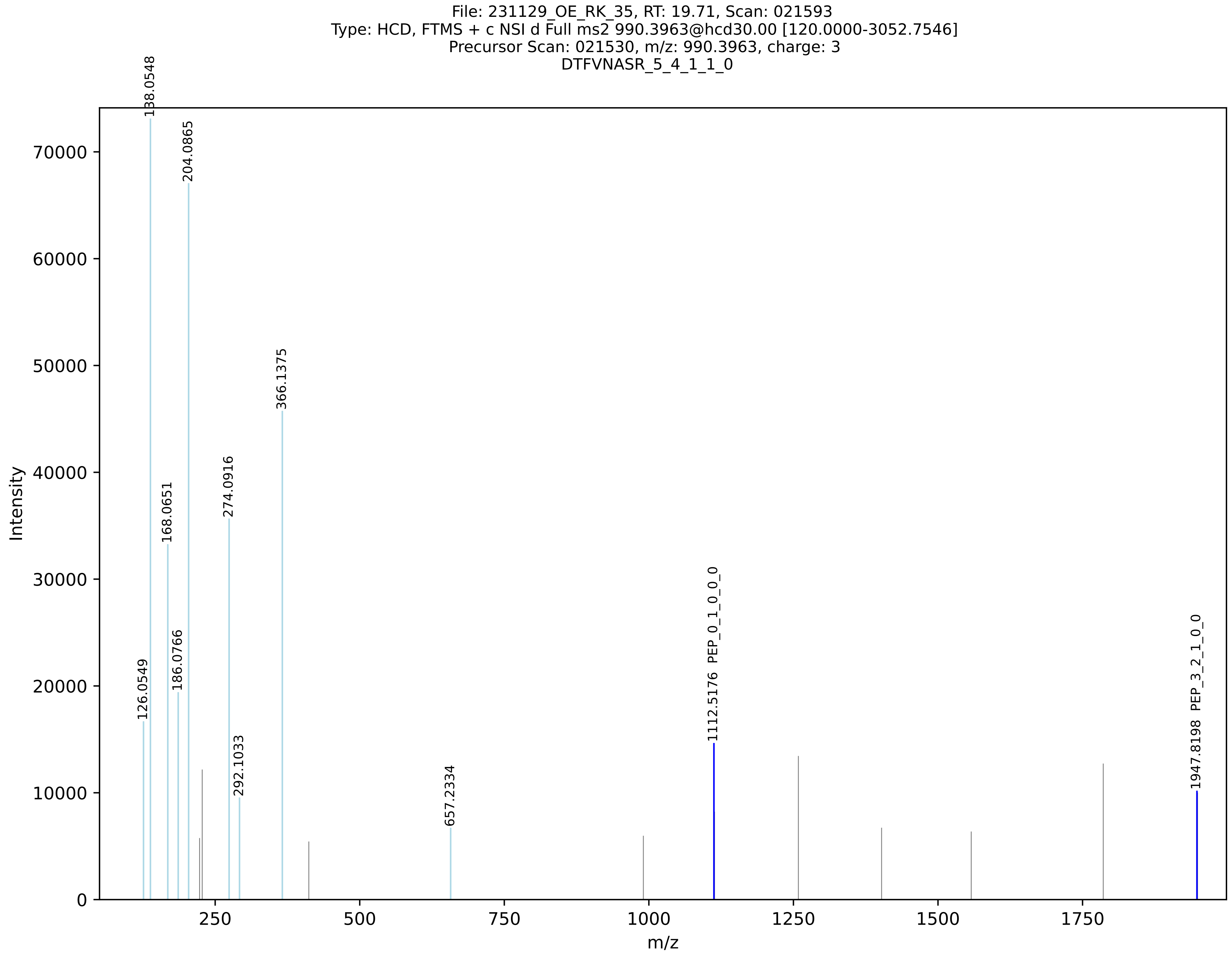

File: 231129_OE_RK_35, RT: 19.71, Scan: 021593
Type: HCD, FTMS + c NSI d Full ms2 990.3963@hcd30.00 [120.0000-3052.7546]
Precursor Scan: 021530, m/z: 990.3963, charge: 3
DTFVNASR_5_4_1_1_0
Intensity
m/z
138.0548
204.0865
366.1375
274.0916
168.0651
186.0766
126.0549
292.1033
657.2334
1112.5176 PEP_0_1_0_0_0
1947.8198 PEP_3_2_1_0_0
0
10000
20000
30000
40000
50000
60000
70000
250
500
750
1000
1250
1500
1750

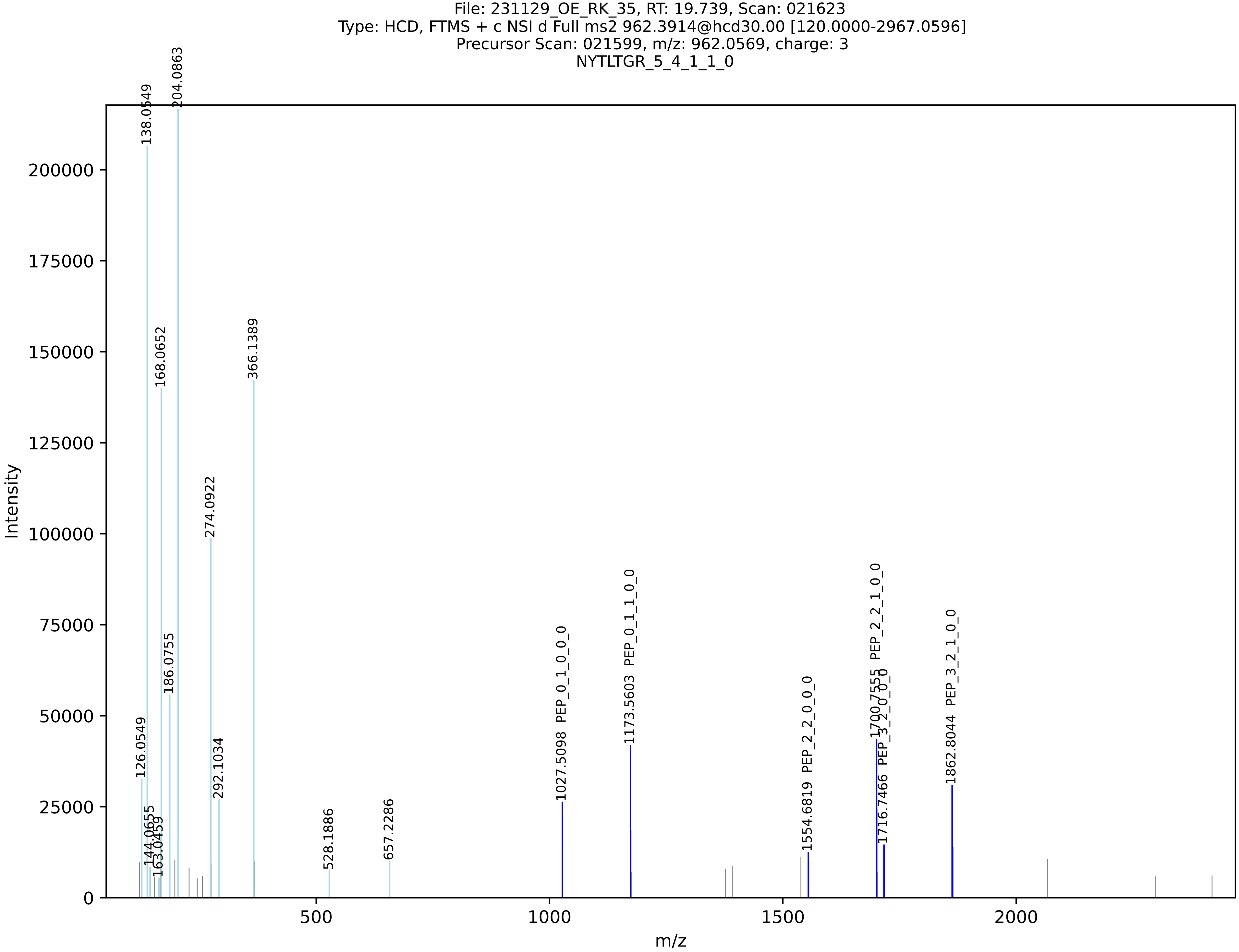
File: 231129_OE_RK_35, RT: 19.739, Scan: 021623
Type: HCD, FTMS + c NSI d Full ms2 962.3914@hcd30.00 [120.0000-2967.0596]
Precursor Scan: 021599, m/z: 962.0569, charge: 3
NYTLTGR_5_4_1_1_0
Intensity
m/z
204.0863
138.0549
168.0652
366.1389
274.0922
186.0755
126.0549
292.1034
144.0655
163.0459
528.1886
657.2286
1027.5098 PEP_0_1_0_0_0
1173.5603 PEP_0_1_1_0_0
1554.6819 PEP_2_2_0_0_0
1700.7555 PEP_2_2_1_0_0
1716.7466 PEP_3_2_0_0_0
1862.8044 PEP_3_2_1_0_0

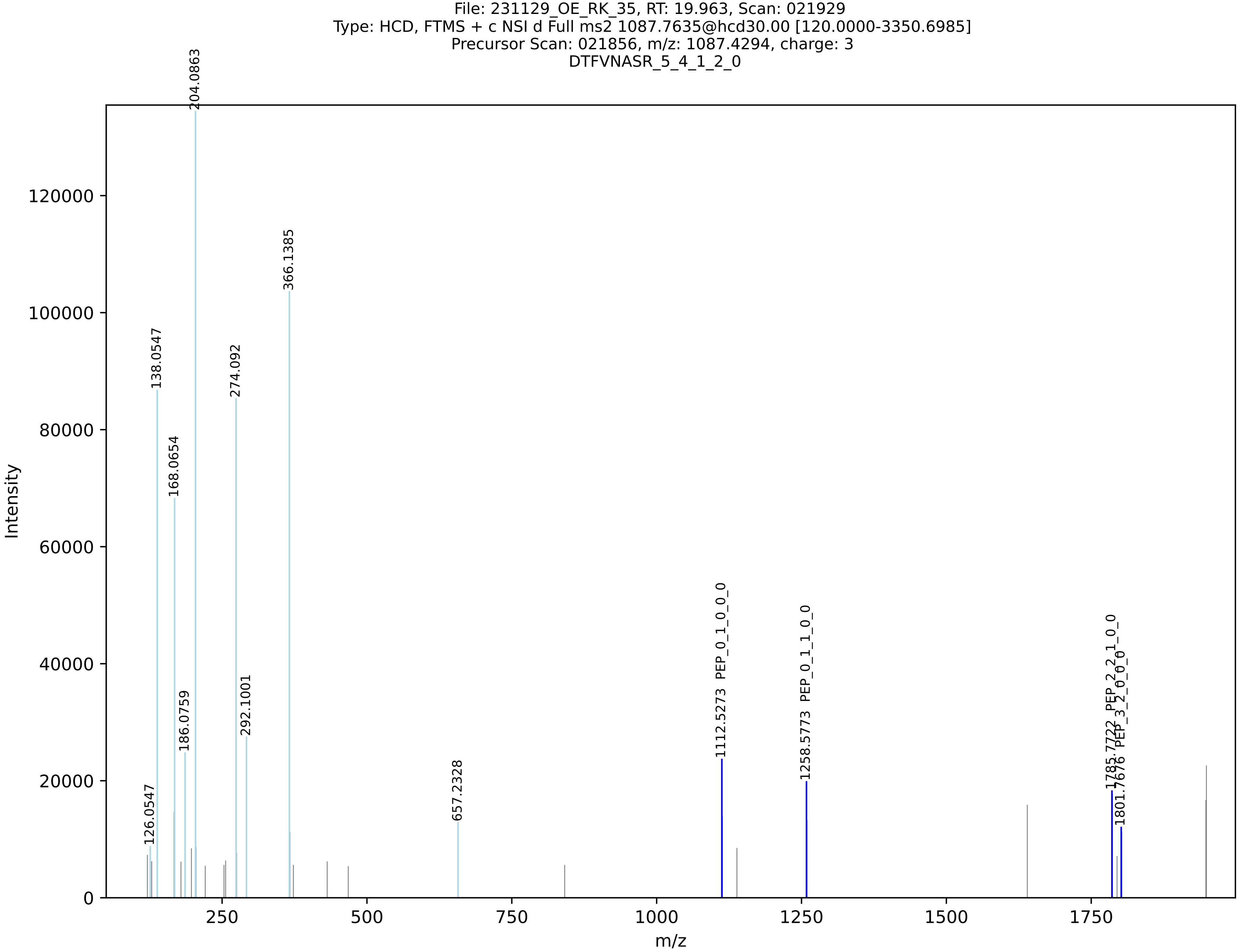
File: 231129_OE_RK_35, RT: 19.963, Scan: 021929
Type: HCD, FTMS + c NSI d Full ms2 1087.7635@hcd30.00 [120.0000-3350.6985]
Precursor Scan: 021856, m/z: 1087.4294, charge: 3
DTFVNASR_5_4_1_2_0
Intensity
m/z
204.0863
366.1385
138.0547
274.092
168.0654
186.0759
292.1001
126.0547
657.2328
1112.5273 PEP_0_1_0_0_0
1258.5773 PEP_0_1_1_0_0
1785.7722 PEP_2_2_1_0_0
1801.7676 PEP_3_2_0_0_0
0
20000
40000
60000
80000
100000
120000
250
500
750
1000
1250
1500
1750

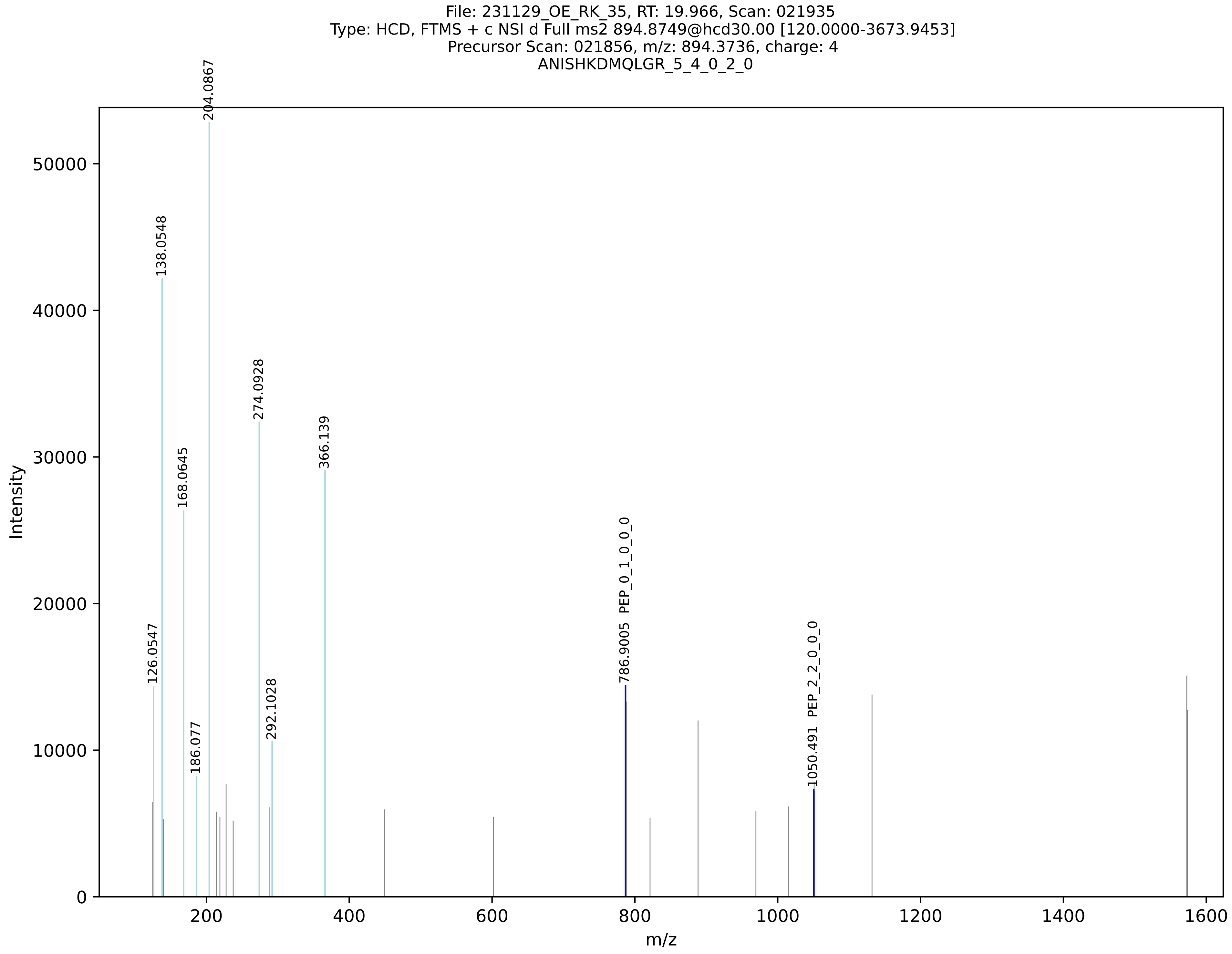
File: 231129_OE_RK_35, RT: 19.966, Scan: 021935
Type: HCD, FTMS + c NSI d Full ms2 894.8749@hcd30.00 [120.0000-3673.9453]
Precursor Scan: 021856, m/z: 894.3736, charge: 4
ANISHKDMQLGR_5_4_0_2_0
Intensity
m/z
204.0867
138.0548
168.0645
274.0928
366.139
126.0547
186.077
292.1028
786.9005 PEP_0_1_0_0_0
1050.491 PEP_2_2_0_0_0
0
10000
20000
30000
40000
50000
200
400
600
800
1000
1200
1400
1600

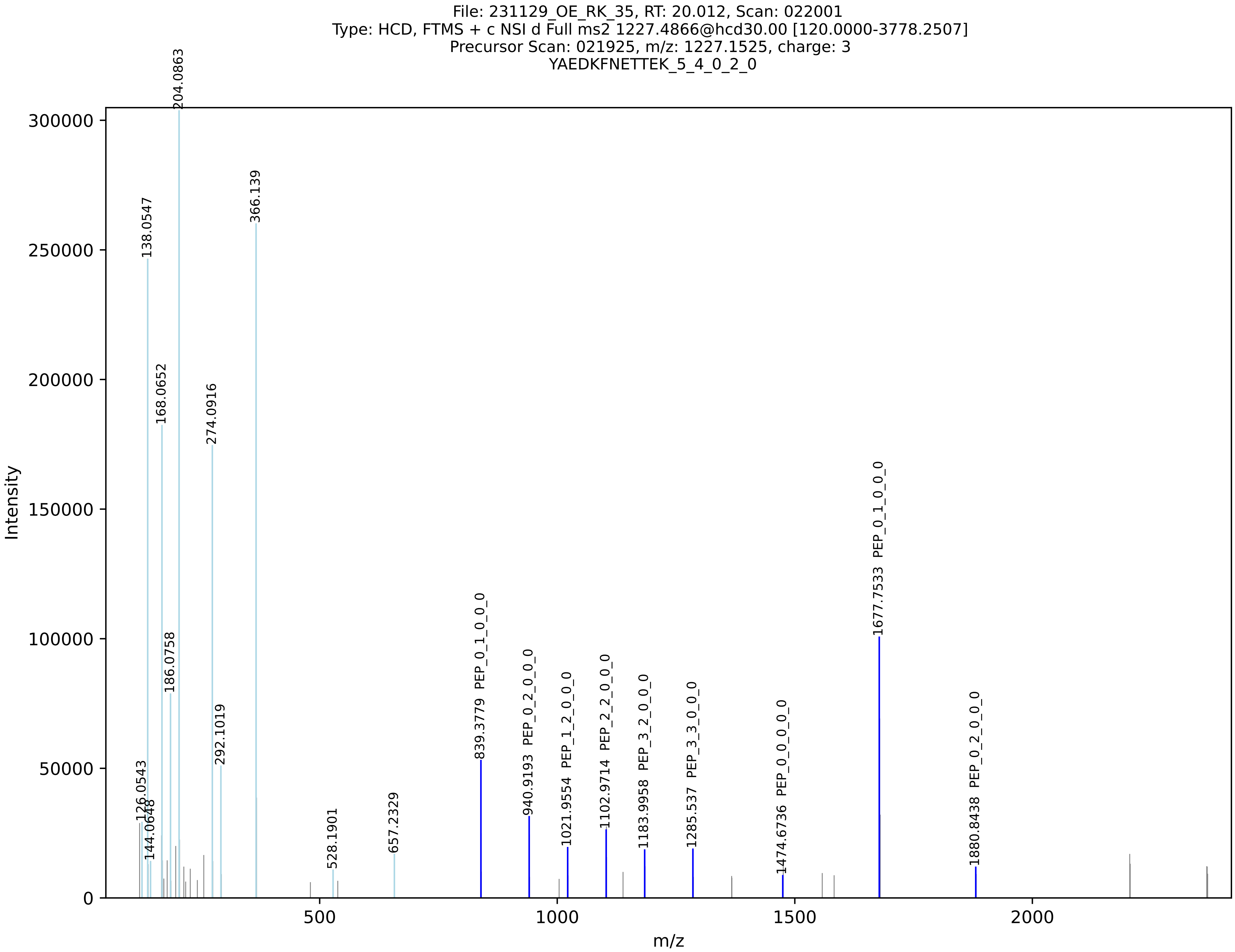
File: 231129_OE_RK_35, RT: 20.012, Scan: 022001
Type: HCD, FTMS + c NSI d Full ms2 1227.4866@hcd30.00 [120.0000-3778.2507]
Precursor Scan: 021925, m/z: 1227.1525, charge: 3
YAEDKFNETTEK_5_4_0_2_0
Intensity
m/z
204.0863
138.0547
366.139
168.0652
274.0916
186.0758
292.1019
126.0543
144.0648
528.1901
657.2329
839.3779 PEP_0_1_0_0_0
940.9193 PEP_0_2_0_0_0
1021.9554 PEP_1_2_0_0_0
1102.9714 PEP_2_2_0_0_0
1183.9958 PEP_3_2_0_0_0
1285.537 PEP_3_3_0_0_0
1474.6736 PEP_0_0_0_0_0
1677.7533 PEP_0_1_0_0_0
1880.8438 PEP_0_2_0_0_0

File: 231129_OE_RK_35, RT: 20.055, Scan: 022064
Type: HCD, FTMS + c NSI d Full ms2 920.8664@hcd30.00 [120.0000-3779.9910]
Precursor Scan: 021998, m/z: 920.6159, charge: 4
YAEDKFNETTEK_5_4_0_2_0

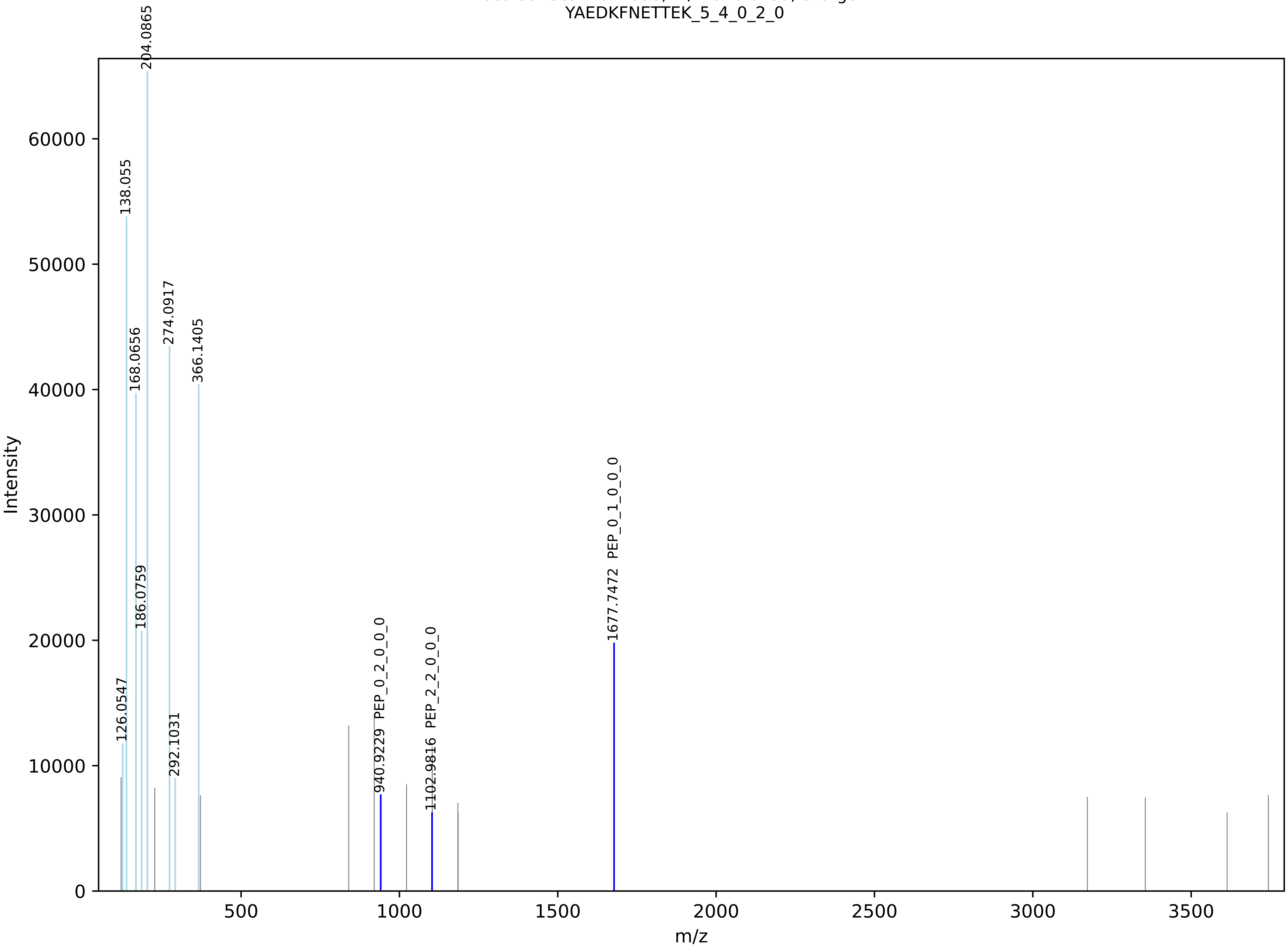

File: 231129_OE_RK_35, RT: 20.101, Scan: 022130
Type: HCD, FTMS + c NSI d Full ms2 962.3928@hcd30.00 [120.0000-2967.0642]
Precursor Scan: 022059, m/z: 962.0587, charge: 3
NYTLTGR_5_4_1_1_0

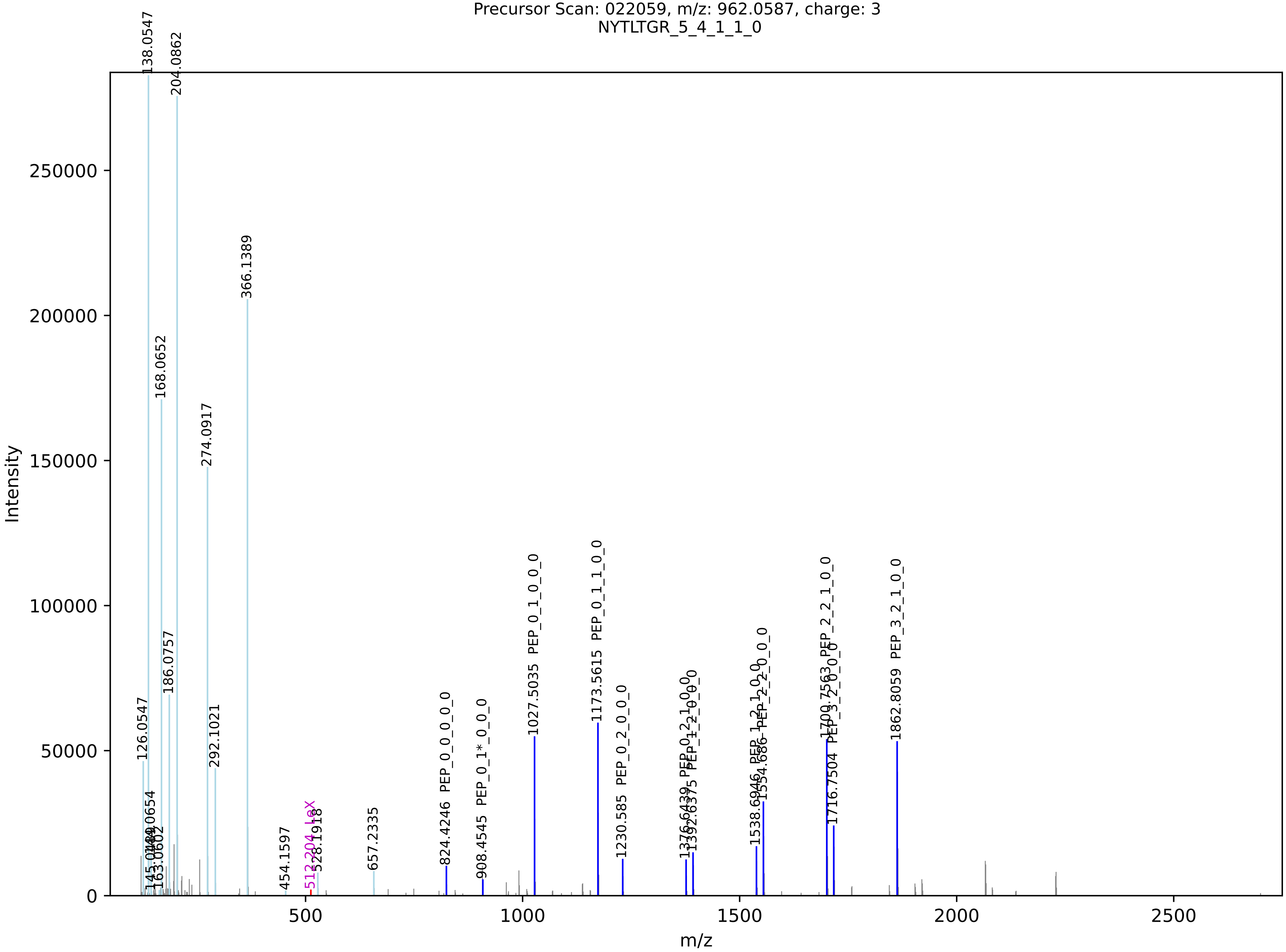

File: 231129_OE_RK_35, RT: 20.14, Scan: 022186
Type: HCD, FTMS + c NSI d Full ms2 1088.0979@hcd30.00 [120.0000-3351.7214]
Precursor Scan: 022129, m/z: 1087.4296, charge: 3
DTFVNASR_5_4_1_2_0

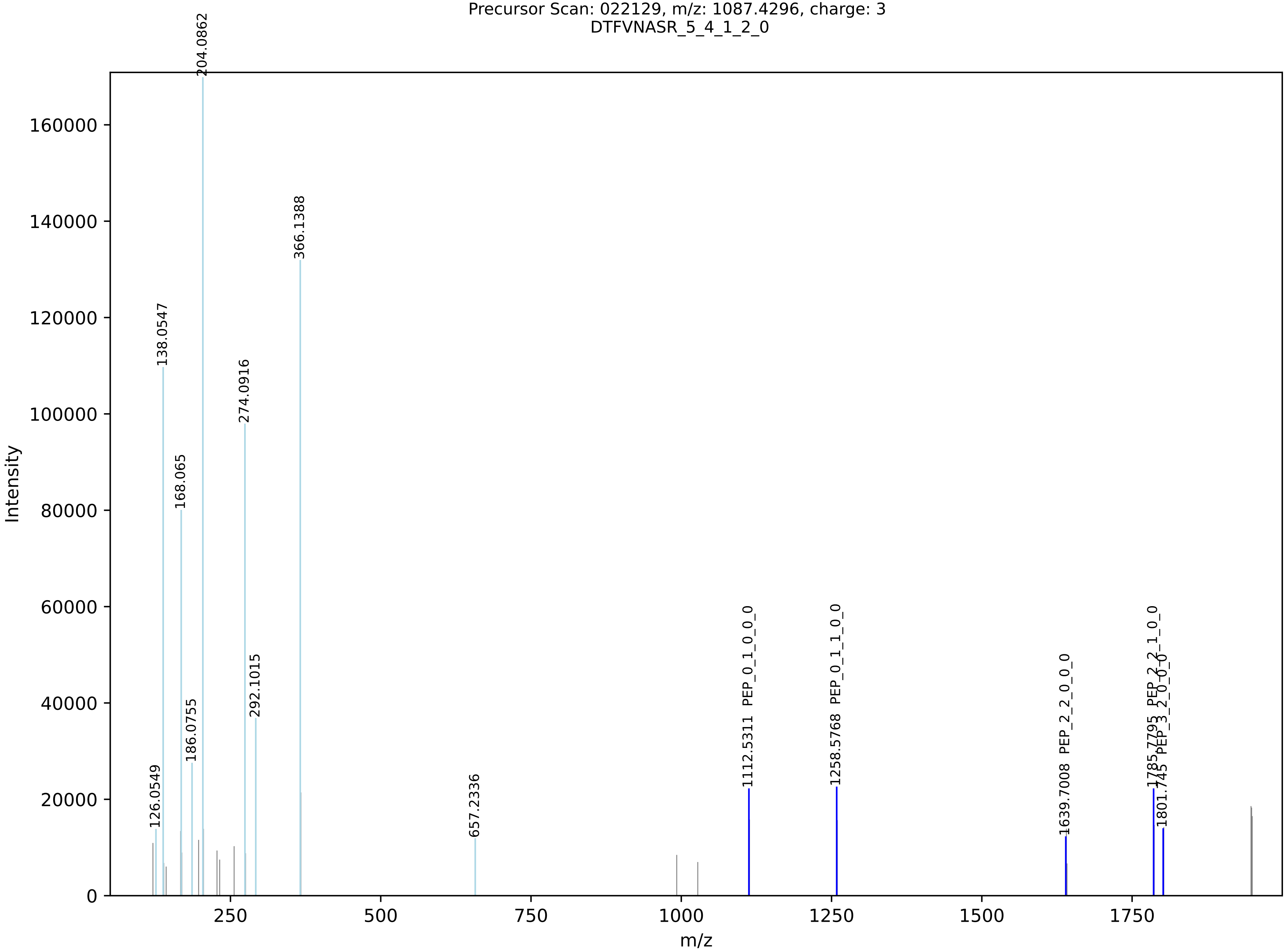

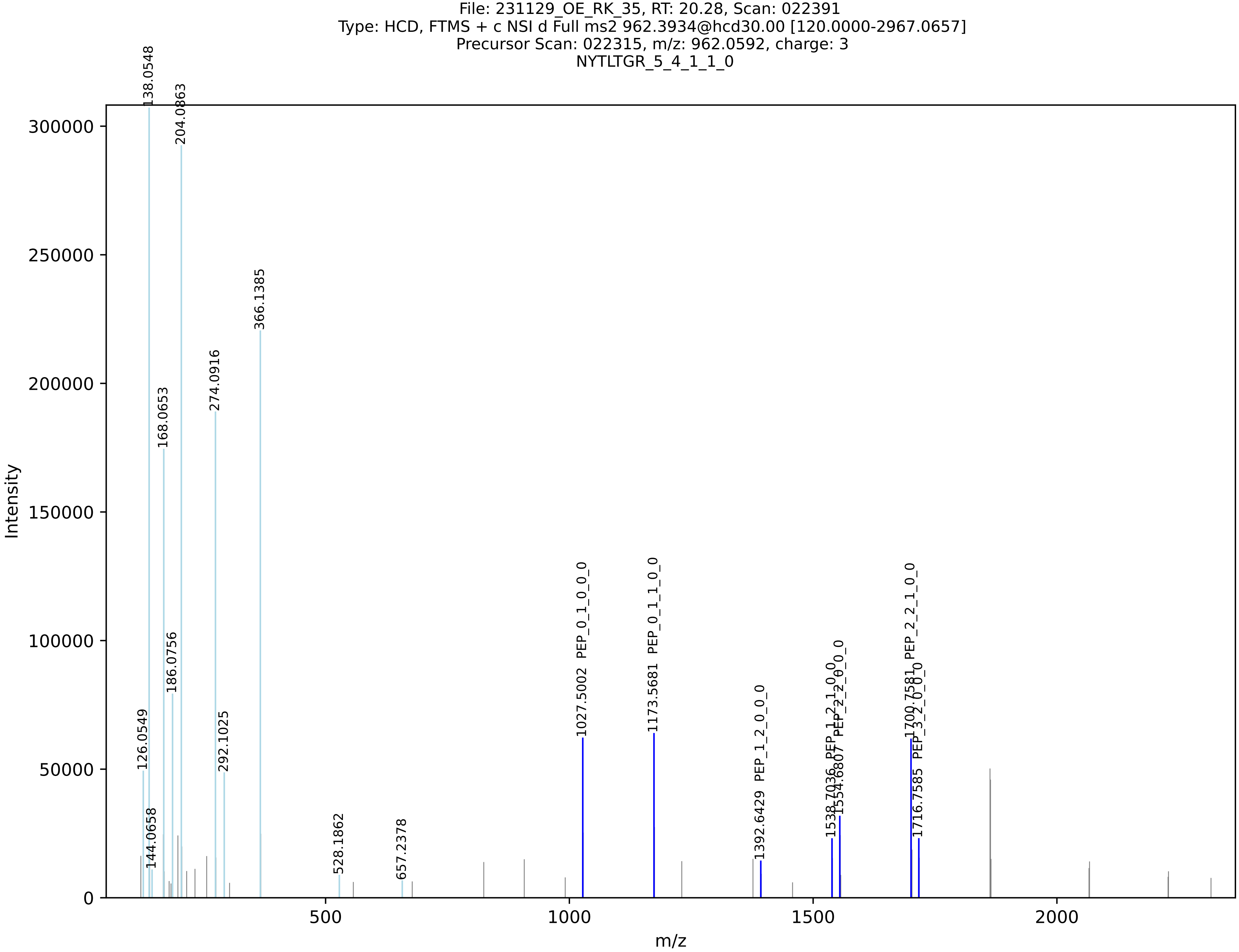
File: 231129_OE_RK_35, RT: 20.28, Scan: 022391
Type: HCD, FTMS + c NSI d Full ms2 962.3934@hcd30.00 [120.0000-2967.0657]
Precursor Scan: 022315, m/z: 962.0592, charge: 3
NYTLTGR_5_4_1_1_0
Intensity
m/z
138.0548
204.0863
366.1385
274.0916
168.0653
186.0756
126.0549
144.0658
292.1025
528.1862
657.2378
1027.5002 PEP_0_1_0_0_0
1173.5681 PEP_0_1_1_0_0
1392.6429 PEP_1_2_0_0_0
1700.7581 PEP_2_2_1_0_0
1716.7585 PEP_3_2_0_0_0

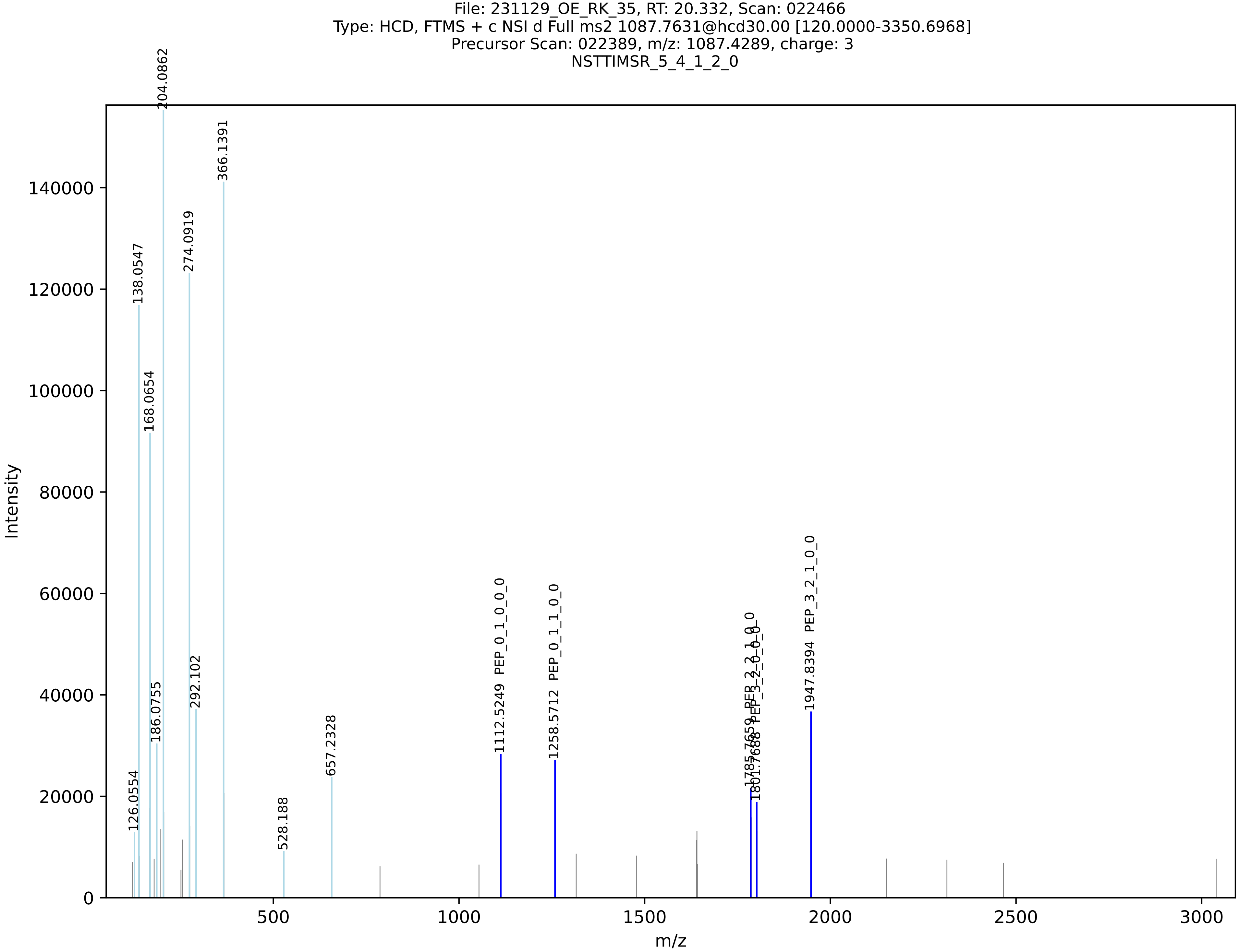

File: 231129_OE_RK_35, RT: 20.332, Scan: 022466
Type: HCD, FTMS + c NSI d Full ms2 1087.7631@hcd30.00 [120.0000-3350.6968]
Precursor Scan: 022389, m/z: 1087.4289, charge: 3
NSTTIMSR_5_4_1_2_0
Intensity
m/z
0
20000
40000
60000
80000
100000
120000
140000
500
1000
1500
2000
2500
3000
204.0862
366.1391
274.0919
138.0547
168.0654
186.0755
292.102
126.0554
528.188
657.2328
1112.5249 PEP_0_1_0_0_0
1258.5712 PEP_0_1_1_0_0
1785.7659 PEP_2_2_1_0_0
1801.7688 PEP_3_2_0_0_0
1947.8394 PEP_3_2_1_0_0

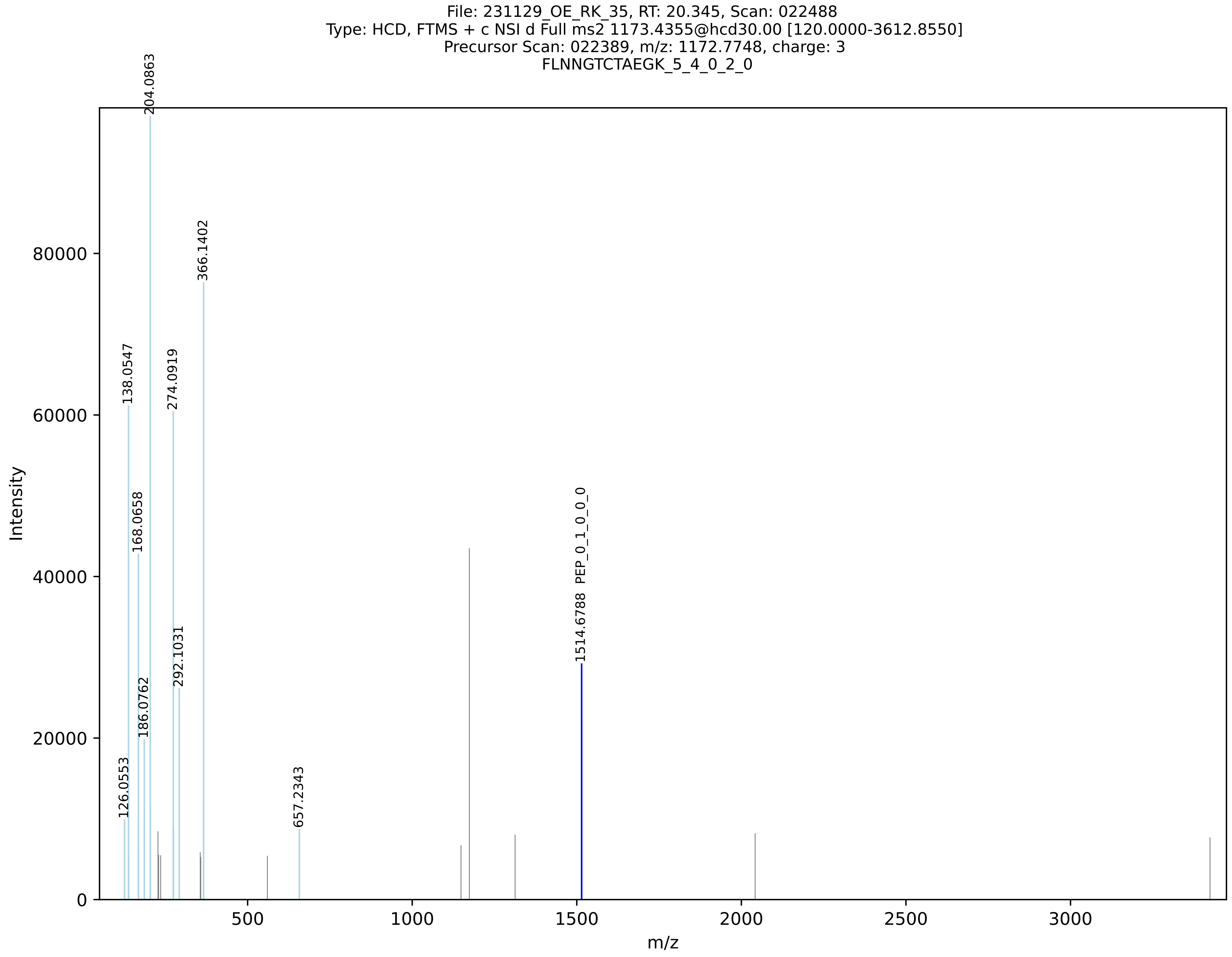
File: 231129_OE_RK_35, RT: 20.345, Scan: 022488
Type: HCD, FTMS + c NSI d Full ms2 1173.4355@hcd30.00 [120.0000-3612.8550]
Precursor Scan: 022389, m/z: 1172.7748, charge: 3
FLNNGTCTAEGK_5_4_0_2_0
204.0863
366.1402
138.0547
274.0919
168.0658
292.1031
186.0762
126.0553
657.2343
1514.6788 PEP_0_1_0_0_0
Intensity
m/z
0
20000
40000
60000
80000
500
1000
1500
2000
2500
3000

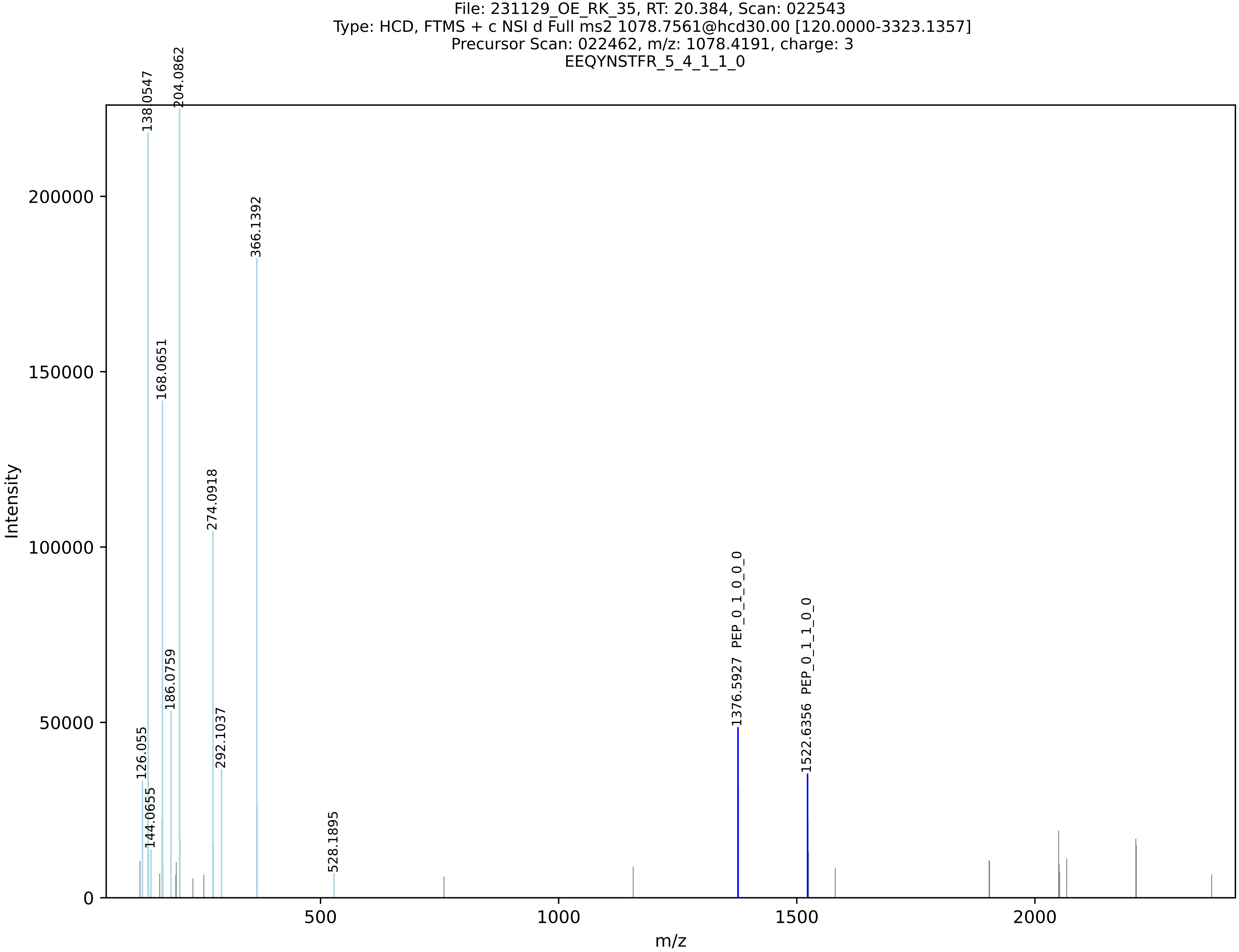
File: 231129_OE_RK_35, RT: 20.384, Scan: 022543
Type: HCD, FTMS + c NSI d Full ms2 1078.7561@hcd30.00 [120.0000-3323.1357]
Precursor Scan: 022462, m/z: 1078.4191, charge: 3
EEQYNSTFR_5_4_1_1_0
Intensity
m/z
0
50000
100000
150000
200000
500
1000
1500
2000
126.055
138.0547
144.0655
168.0651
186.0759
204.0862
274.0918
292.1037
366.1392
528.1895
1376.5927 PEP_0_1_0_0_0
1522.6356 PEP_0_1_1_0_0

File: 231129_OE_RK_35, RT: 20.431, Scan: 022611
Type: HCD, FTMS + c NSI d Full ms2 1227.8204@hcd30.00 [120.0000-3779.2727]
Precursor Scan: 022536, m/z: 1227.1524, charge: 3
YAEDKFNETTEK_5_4_0_2_0

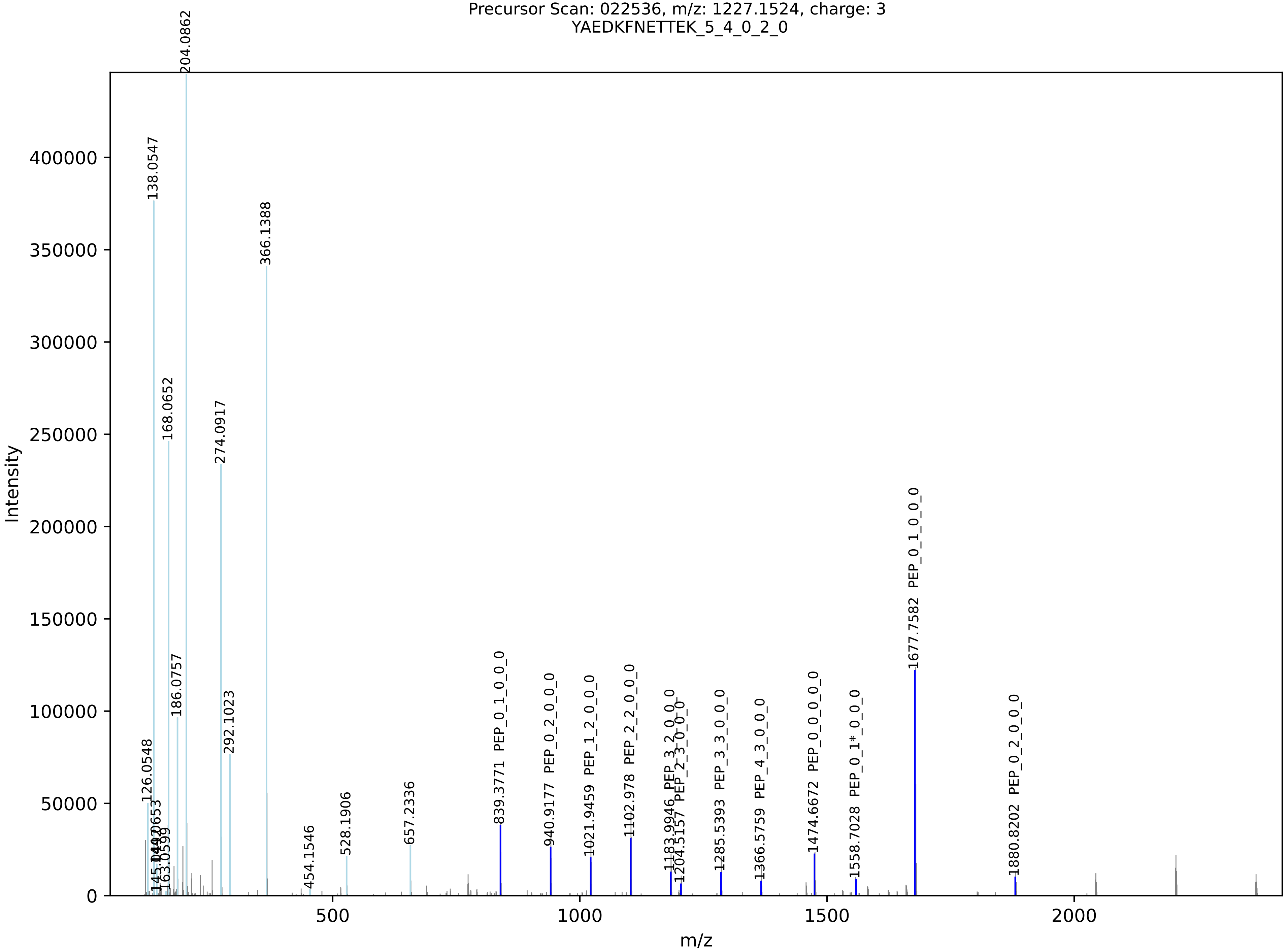

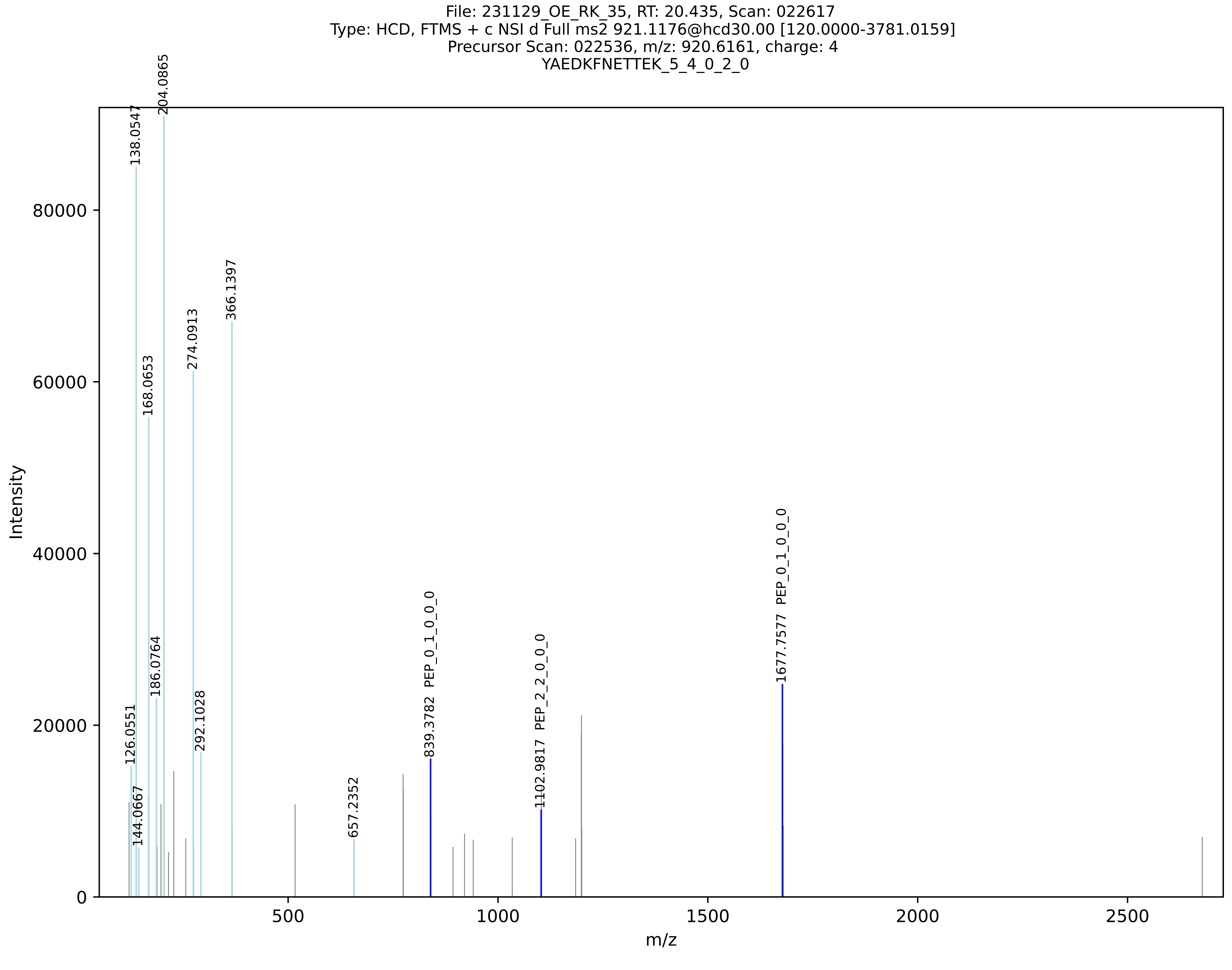
File: 231129_OE_RK_35, RT: 20.435, Scan: 022617
Type: HCD, FTMS + c NSI d Full ms2 921.1176@hcd30.00 [120.0000-3781.0159]
Precursor Scan: 022536, m/z: 920.6161, charge: 4
YAEDKFNETTEK_5_4_0_2_0
204.0865
138.0547
366.1397
274.0913
168.0653
186.0764
292.1028
126.0551
144.0667
657.2352
839.3782 PEP_0_1_0_0_0
1102.9817 PEP_2_2_0_0_0
1677.7577 PEP_0_1_0_0_0
Intensity
m/z
0
20000
40000
60000
80000
500
1000
1500
2000
2500

File: 231129_OE_RK_35, RT: 20.438, Scan: 022622
Type: HCD, FTMS + c NSI d Full ms2 1007.0540@hcd30.00 [120.0000-3103.7273]
Precursor Scan: 022536, m/z: 1006.3864, charge: 3
CNYSIR_5_4_0_2_0

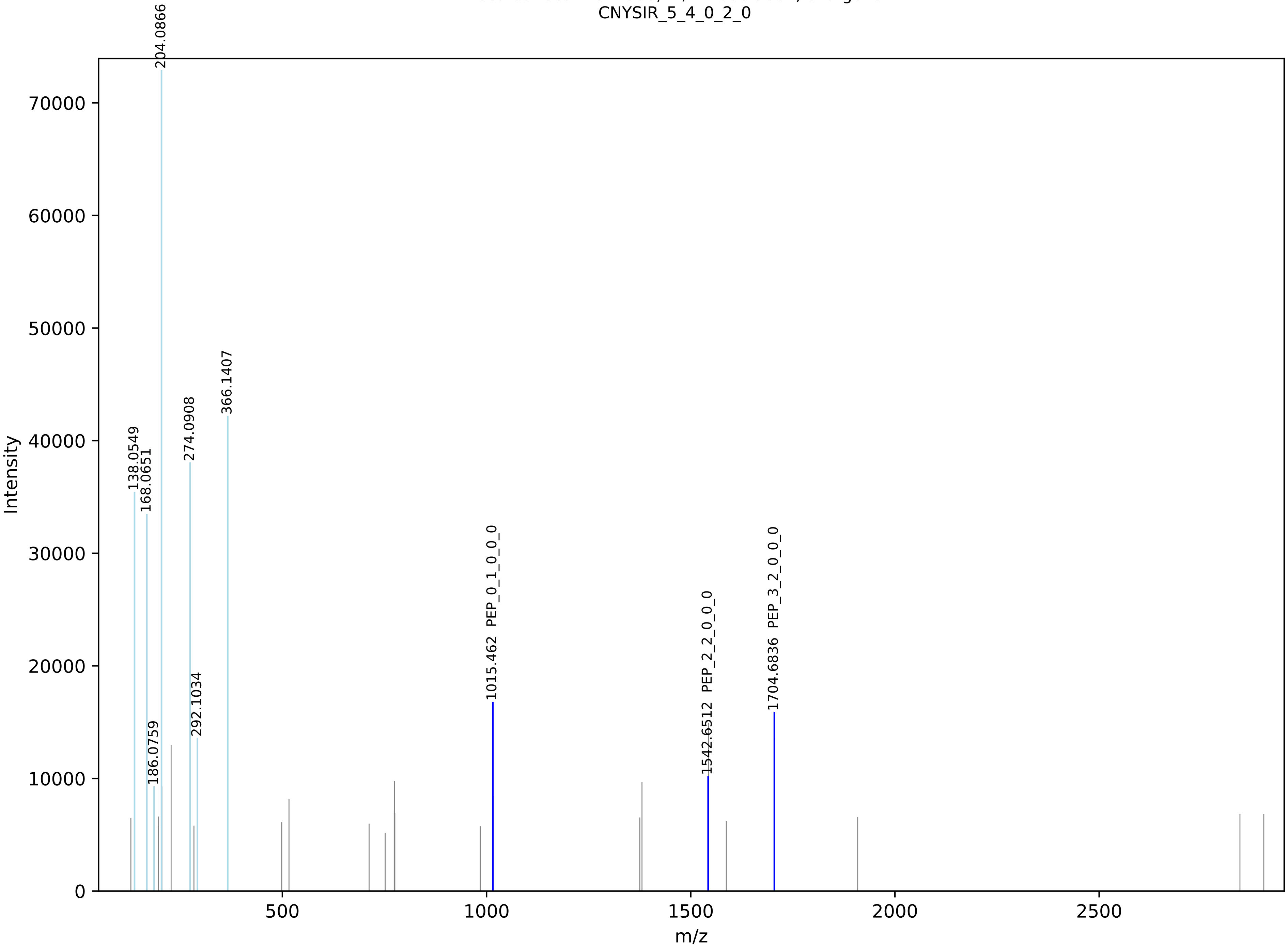

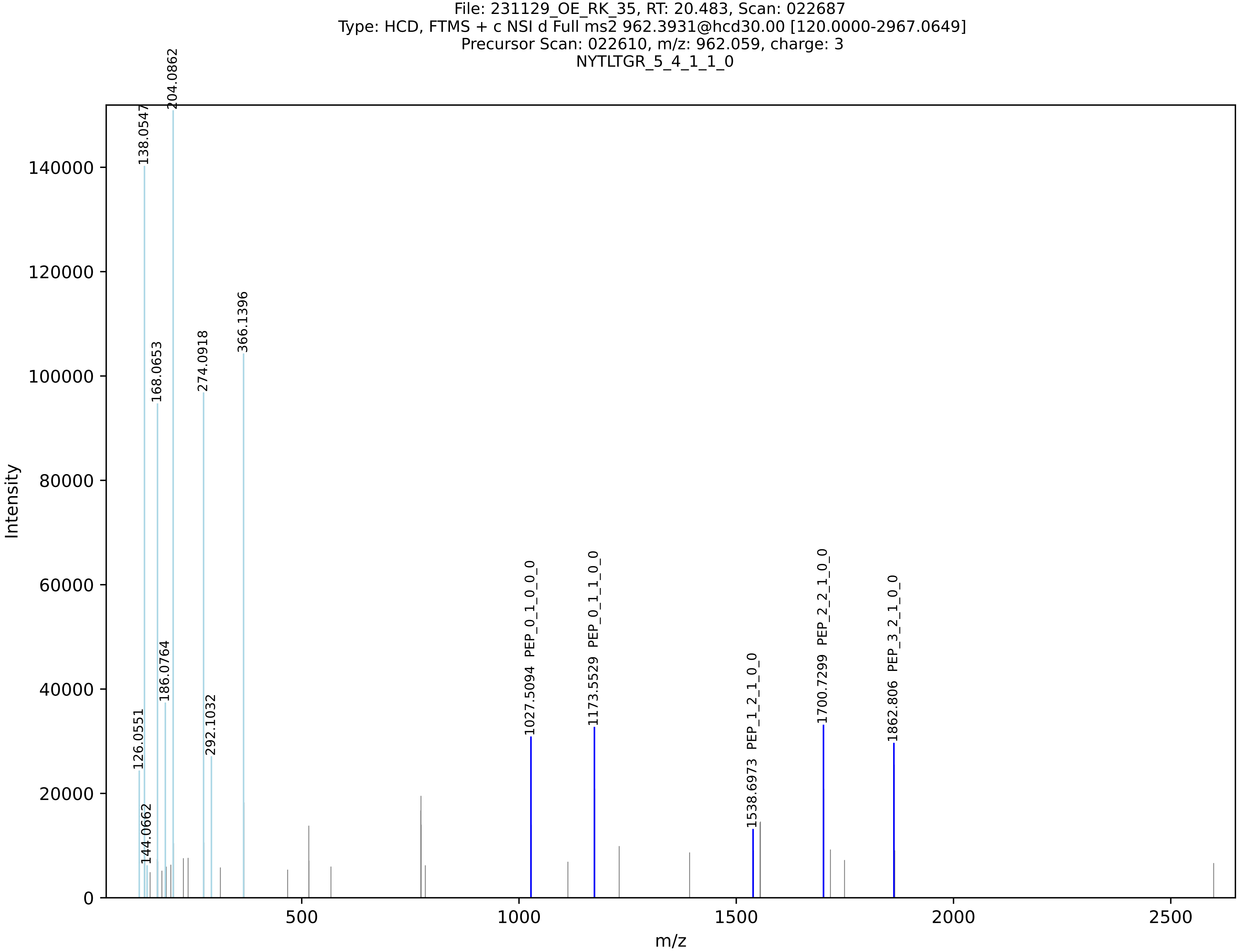
File: 231129_OE_RK_35, RT: 20.483, Scan: 022687
Type: HCD, FTMS + c NSI d Full ms2 962.3931@hcd30.00 [120.0000-2967.0649]
Precursor Scan: 022610, m/z: 962.059, charge: 3
NYTLTGR_5_4_1_1_0
Intensity
m/z
204.0862
138.0547
168.0653
274.0918
366.1396
186.0764
292.1032
126.0551
144.0662
1027.5094 PEP_0_1_0_0_0_
1173.5529 PEP_0_1_1_0_0
1538.6973 PEP_1_2_1_0_0
1700.7299 PEP_2_2_1_0_0
1862.806 PEP_3_2_1_0_0
0
20000
40000
60000
80000
100000
120000
140000
500
1000
1500
2000
2500

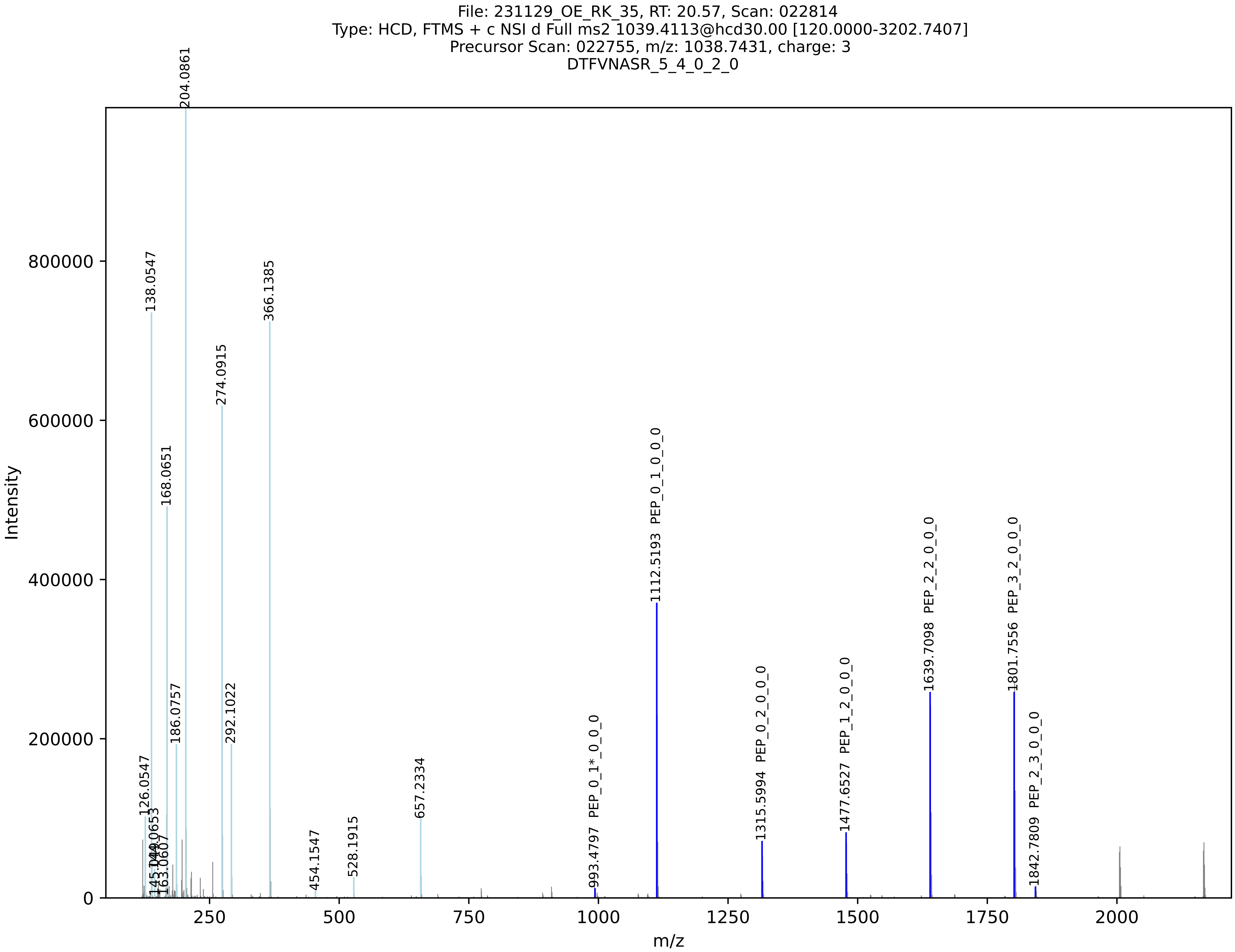

File: 231129_OE_RK_35, RT: 20.57, Scan: 022814
Type: HCD, FTMS + c NSI d Full ms2 1039.4113@hcd30.00 [120.0000-3202.7407]
Precursor Scan: 022755, m/z: 1038.7431, charge: 3
DTFVNASR_5_4_0_2_0
Intensity
m/z
0
200000
400000
600000
800000
250
500
750
1000
1250
1500
1750
2000
204.0861
138.0547
366.1385
274.0915
168.0651
186.0757
292.1022
126.0547
145.0490
163.0607
454.1547
528.1915
657.2334
993.4797 PEP_0_1*_0_0_0
1112.5193 PEP_0_1_0_0_0
1315.5994 PEP_0_2_0_0_0
1477.6527 PEP_1_2_0_0_0
1639.7098 PEP_2_2_0_0_0
1801.7556 PEP_3_2_0_0_0
1842.7809 PEP_2_3_0_0_0

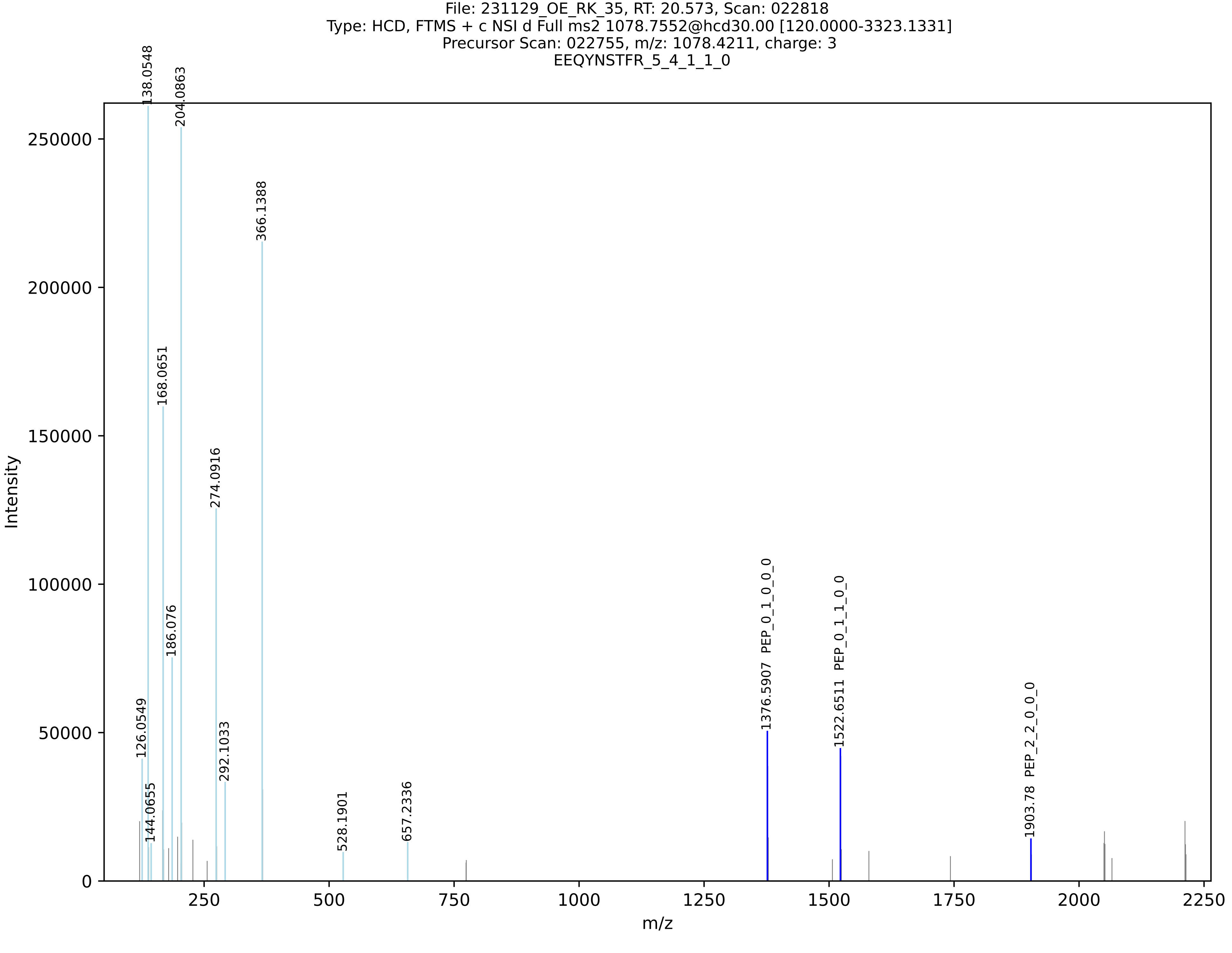

File: 231129_OE_RK_35, RT: 20.573, Scan: 022818
Type: HCD, FTMS + c NSI d Full ms2 1078.7552@hcd30.00 [120.0000-3323.1331]
Precursor Scan: 022755, m/z: 1078.4211, charge: 3
EEQYNSTFR_5_4_1_1_0
138.0548
204.0863
366.1388
168.0651
274.0916
186.076
126.0549
292.1033
144.0655
528.1901
657.2336
1376.5907 PEP_0_1_0_0_0
1522.6511 PEP_0_1_1_0_0
1903.78 PEP_2_2_0_0_0
Intensity
m/z
250000
200000
150000
100000
50000
0
250
500
750
1000
1250
1500
1750
2000
2250

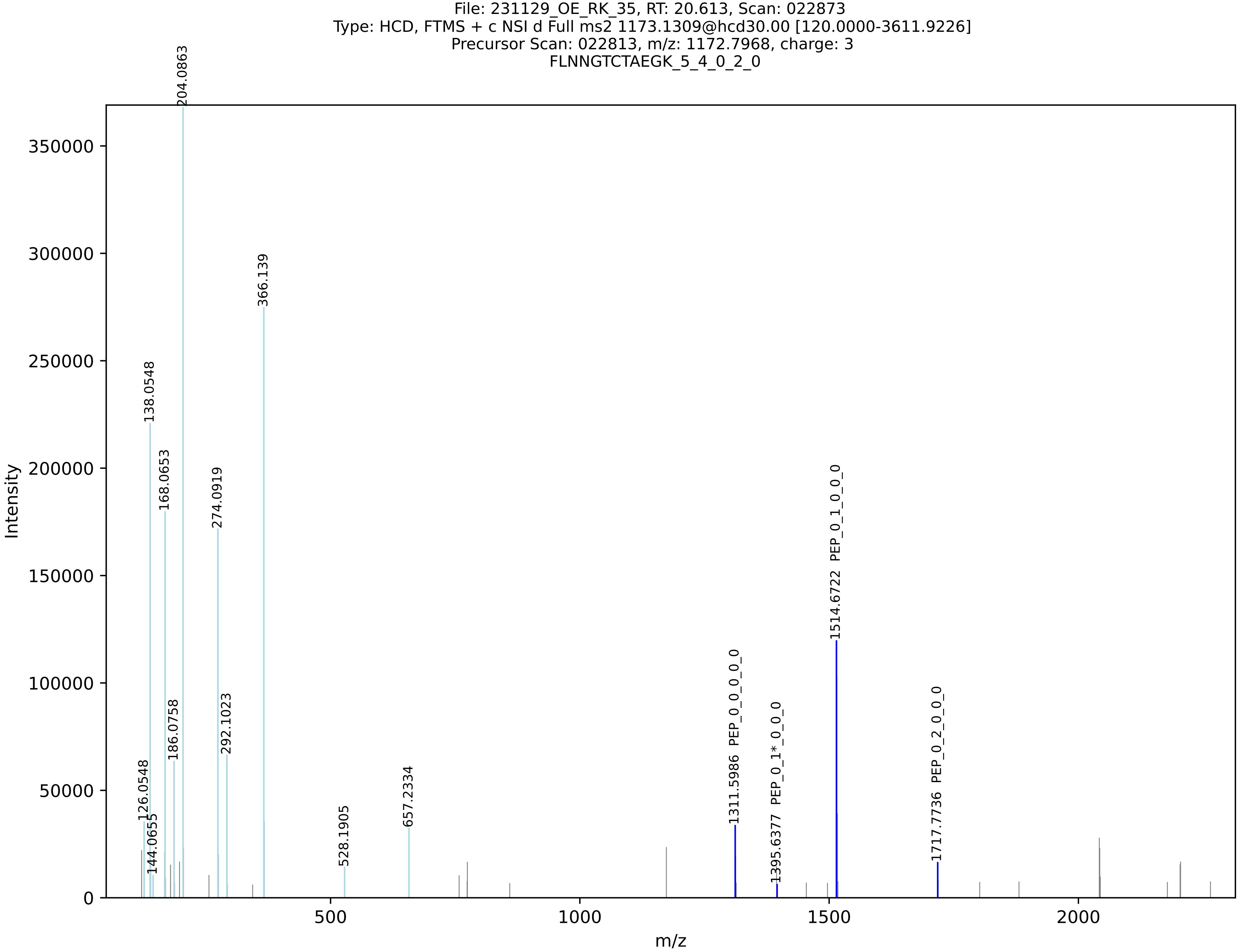
File: 231129_OE_RK_35, RT: 20.613, Scan: 022873
Type: HCD, FTMS + c NSI d Full ms2 1173.1309@hcd30.00 [120.0000-3611.9226]
Precursor Scan: 022813, m/z: 1172.7968, charge: 3
FLNNGTCTAEGK_5_4_0_2_0
204.0863
366.139
138.0548
168.0653
274.0919
186.0758
292.1023
126.0548
144.0655
528.1905
657.2334
1311.5986 PEP_0_0_0_0_0
1395.6377 PEP_0_1*_0_0_0
1514.6722 PEP_0_1_0_0_0
1717.7736 PEP_0_2_0_0_0
Intensity
m/z
0
50000
100000
150000
200000
250000
300000
350000
500
1000
1500
2000

File: 231129_OE_RK_35, RT: 20.626, Scan: 022895
Type: HCD, FTMS + c NSI d Full ms2 1172.2224@hcd30.00 [120.0000-3609.1426]
Precursor Scan: 022813, m/z: 1172.2224, charge: 3
FLNNGTCTAEGK_5_4_0_2_0

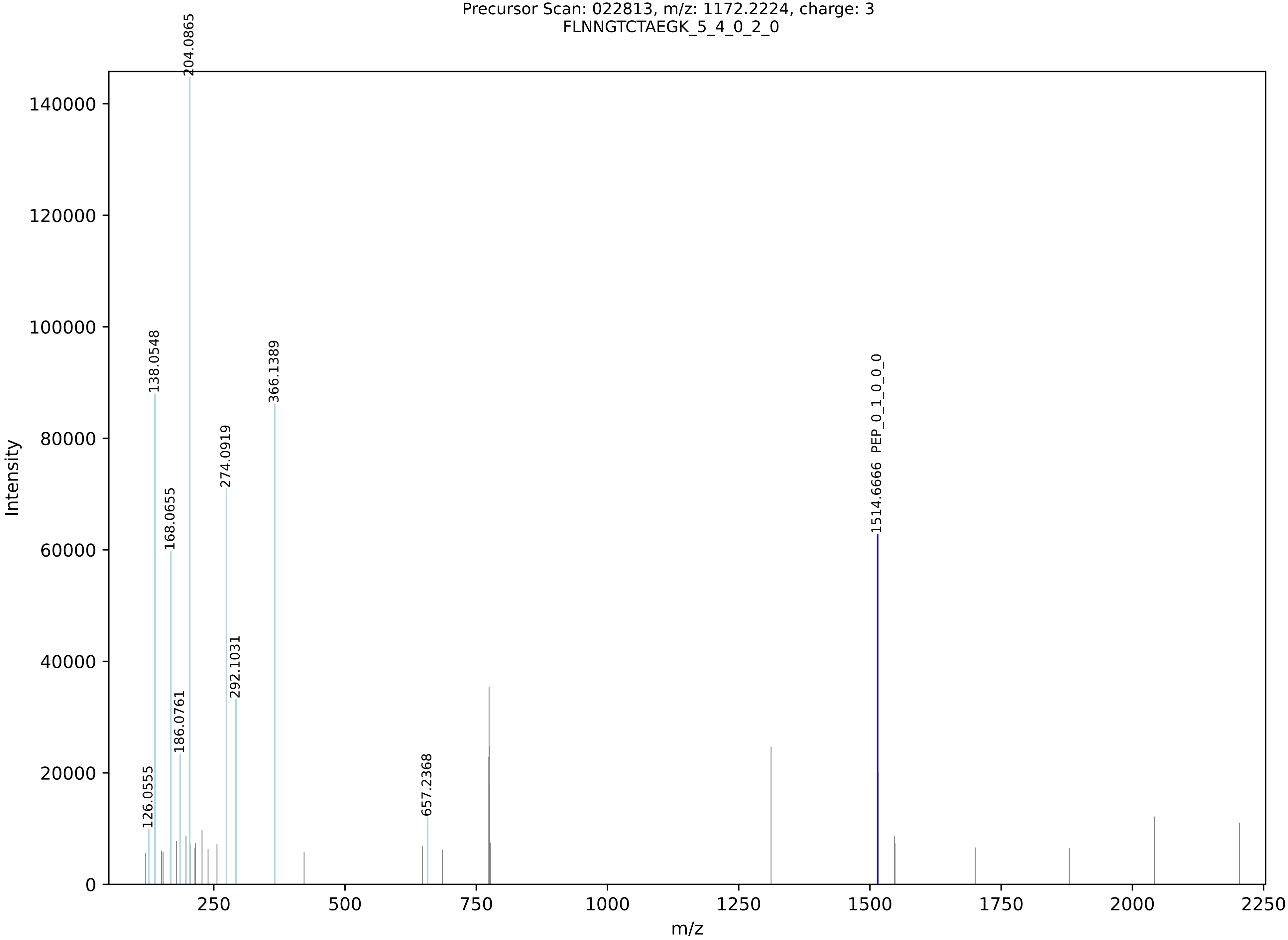

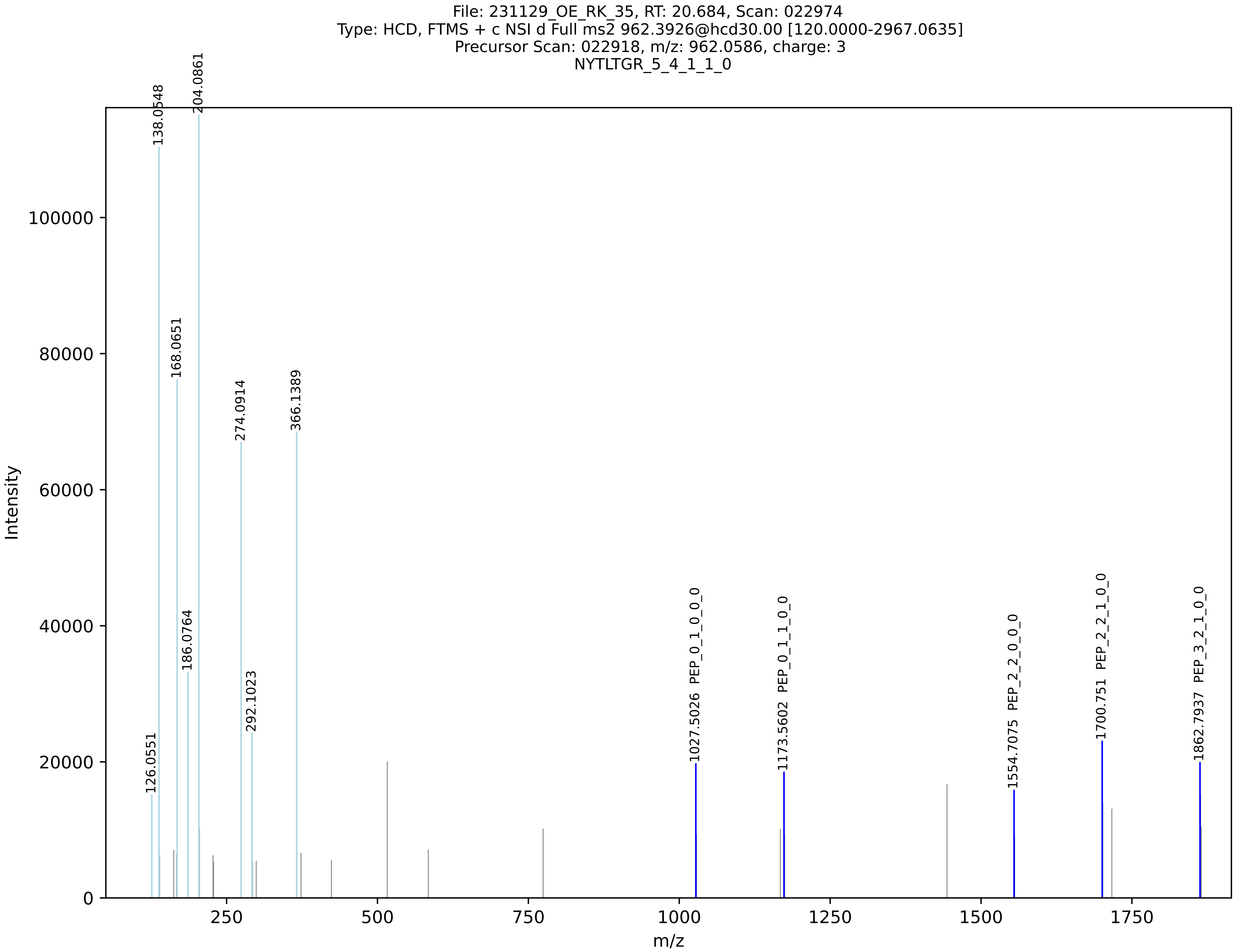
File: 231129_OE_RK_35, RT: 20.684, Scan: 022974
Type: HCD, FTMS + c NSI d Full ms2 962.3926@hcd30.00 [120.0000-2967.0635]
Precursor Scan: 022918, m/z: 962.0586, charge: 3
NYTLTGR_5_4_1_1_0
Intensity
m/z
126.0551
138.0548
168.0651
186.0764
204.0861
274.0914
292.1023
366.1389
1027.5026 PEP_0_1_0_0_0
1173.5602 PEP_0_1_1_0_0
1554.7075 PEP_2_2_0_0_0
1700.751 PEP_2_2_1_0_0
1862.7937 PEP_3_2_1_0_0
0
20000
40000
60000
80000
100000
250
500
750
1000
1250
1500
1750

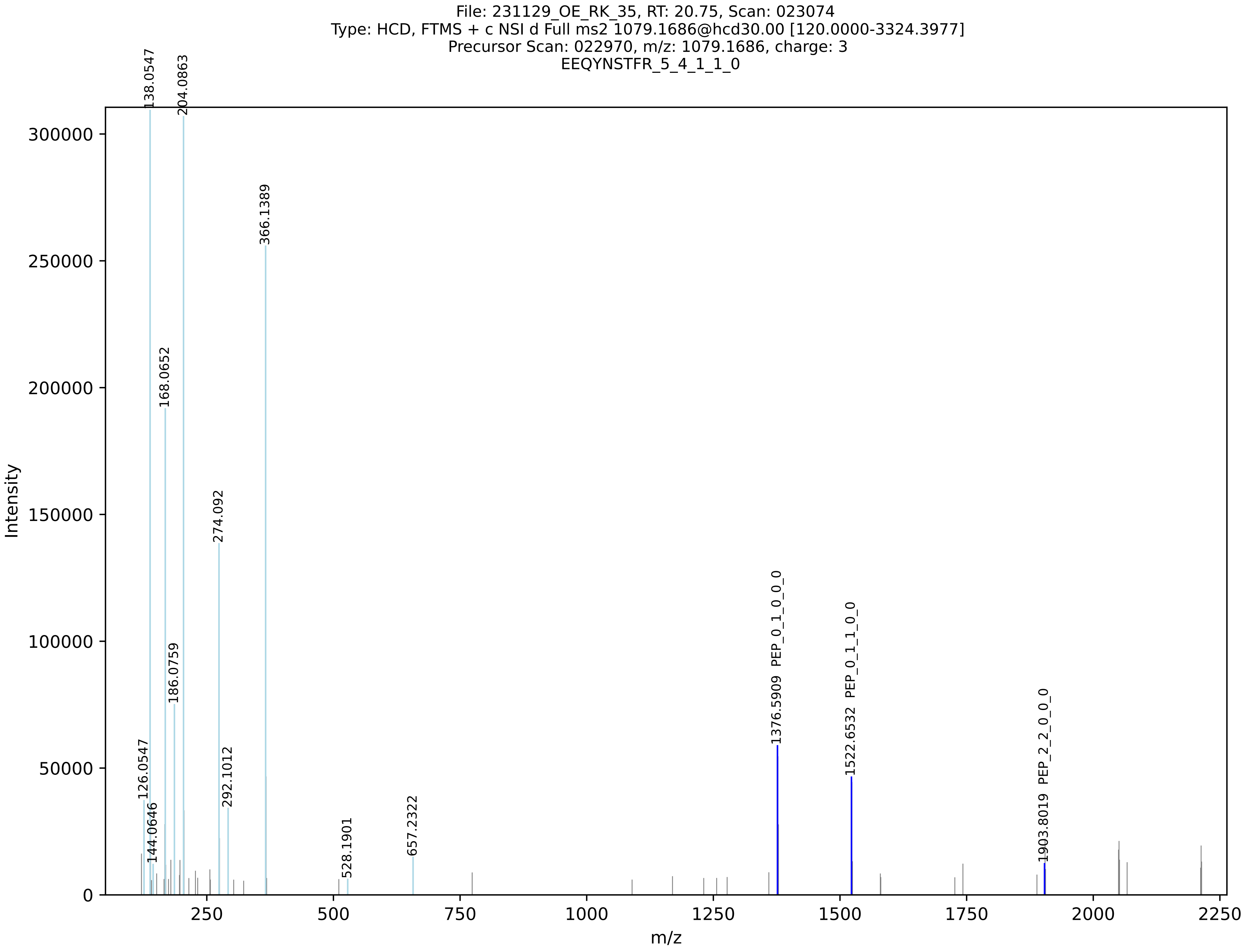
File: 231129_OE_RK_35, RT: 20.75, Scan: 023074
Type: HCD, FTMS + c NSI d Full ms2 1079.1686@hcd30.00 [120.0000-3324.3977]
Precursor Scan: 022970, m/z: 1079.1686, charge: 3
EEQYNSTFR_5_4_1_1_0
Intensity
m/z
138.0547
204.0863
366.1389
168.0652
274.092
186.0759
126.0547
144.0646
292.1012
528.1901
657.2322
1376.5909 PEP_0_1_0_0_0
1522.6532 PEP_0_1_1_0_0
1903.8019 PEP_2_2_0_0_0
0
50000
100000
150000
200000
250000
300000
250
500
750
1000
1250
1500
1750
2000
2250

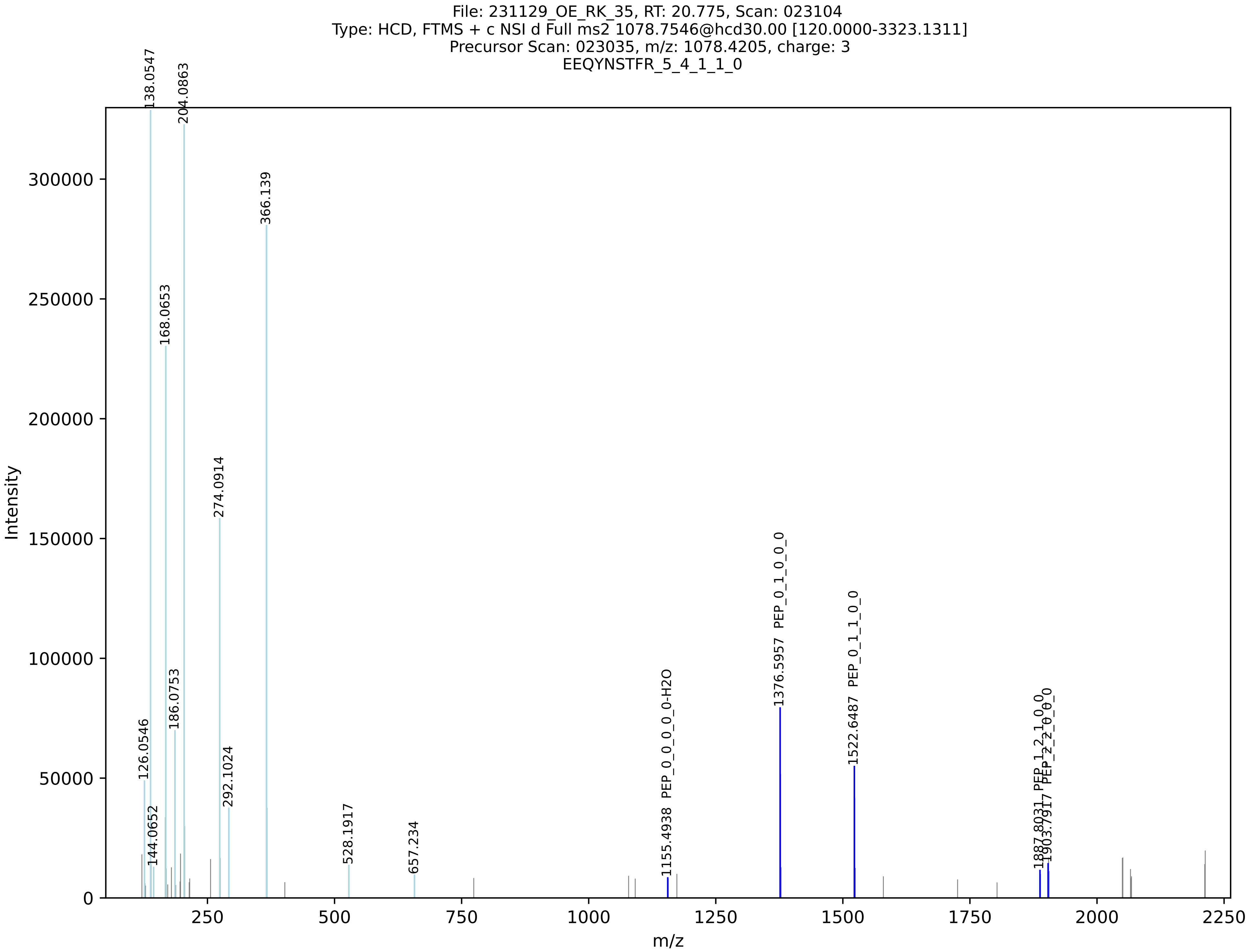
File: 231129_OE_RK_35, RT: 20.775, Scan: 023104
Type: HCD, FTMS + c NSI d Full ms2 1078.7546@hcd30.00 [120.0000-3323.1311]
Precursor Scan: 023035, m/z: 1078.4205, charge: 3
EEQYNSTFR_5_4_1_1_0
138.0547
204.0863
366.139
168.0653
274.0914
186.0753
126.0546
292.1024
144.0652
528.1917
657.234
1155.4938 PEP_0_0_0_0_0-H2O
1376.5957 PEP_0_1_0_0_0
1522.6487 PEP_0_1_1_0_0
1887.8031 PEP_1_2_1_0_0
1903.7917 PEP_2_2_0_0_0
Intensity
m/z
0
50000
100000
150000
200000
250000
300000
250
500
750
1000
1250
1500
1750
2000
2250

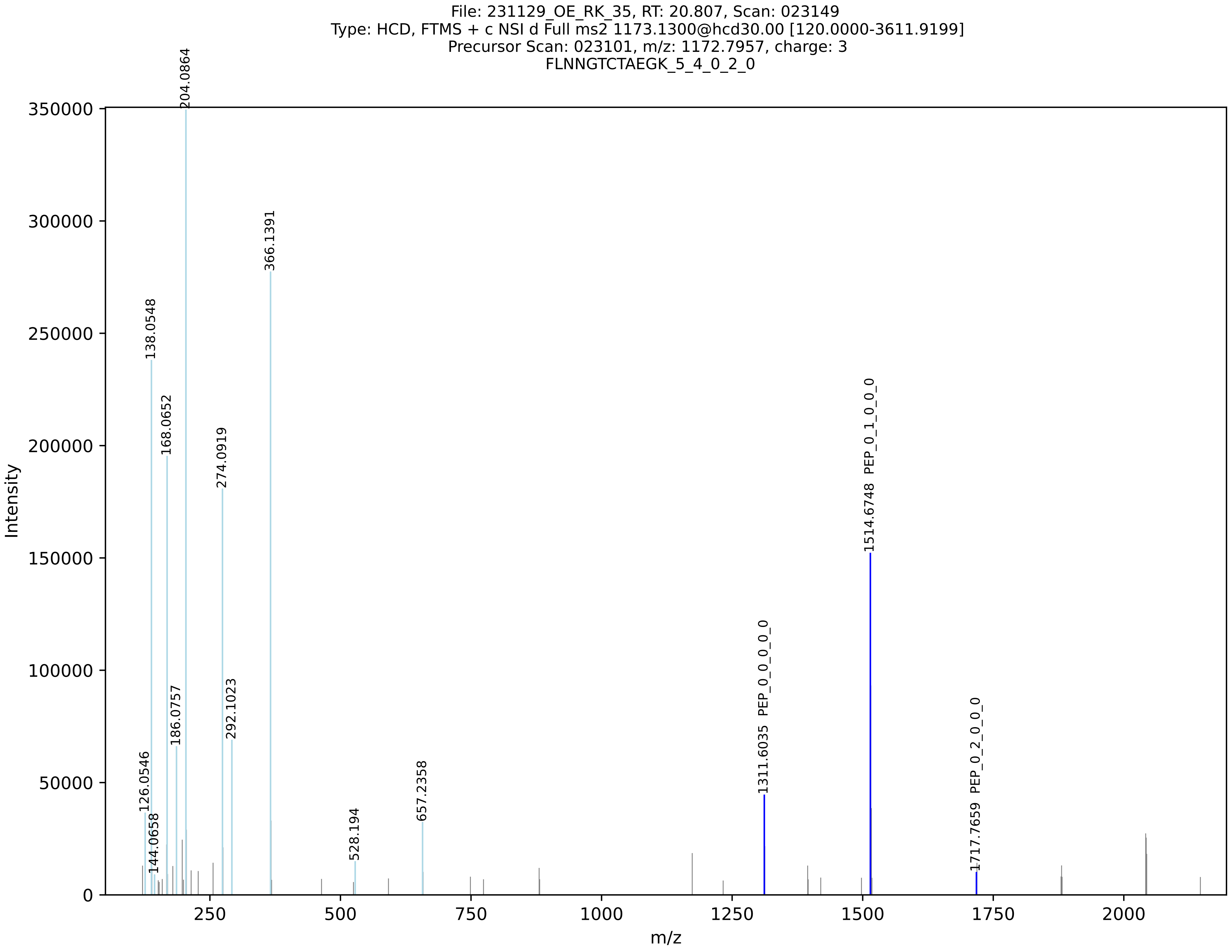
File: 231129_OE_RK_35, RT: 20.807, Scan: 023149
Type: HCD, FTMS + c NSI d Full ms2 1173.1300@hcd30.00 [120.0000-3611.9199]
Precursor Scan: 023101, m/z: 1172.7957, charge: 3
FLNNGTCTAEGK_5_4_0_2_0
Intensity
m/z
204.0864
366.1391
138.0548
168.0652
274.0919
186.0757
292.1023
126.0546
144.0658
528.194
657.2358
1311.6035 PEP_0_0_0_0_0
1514.6748 PEP_0_1_0_0_0
1717.7659 PEP_0_2_0_0_0

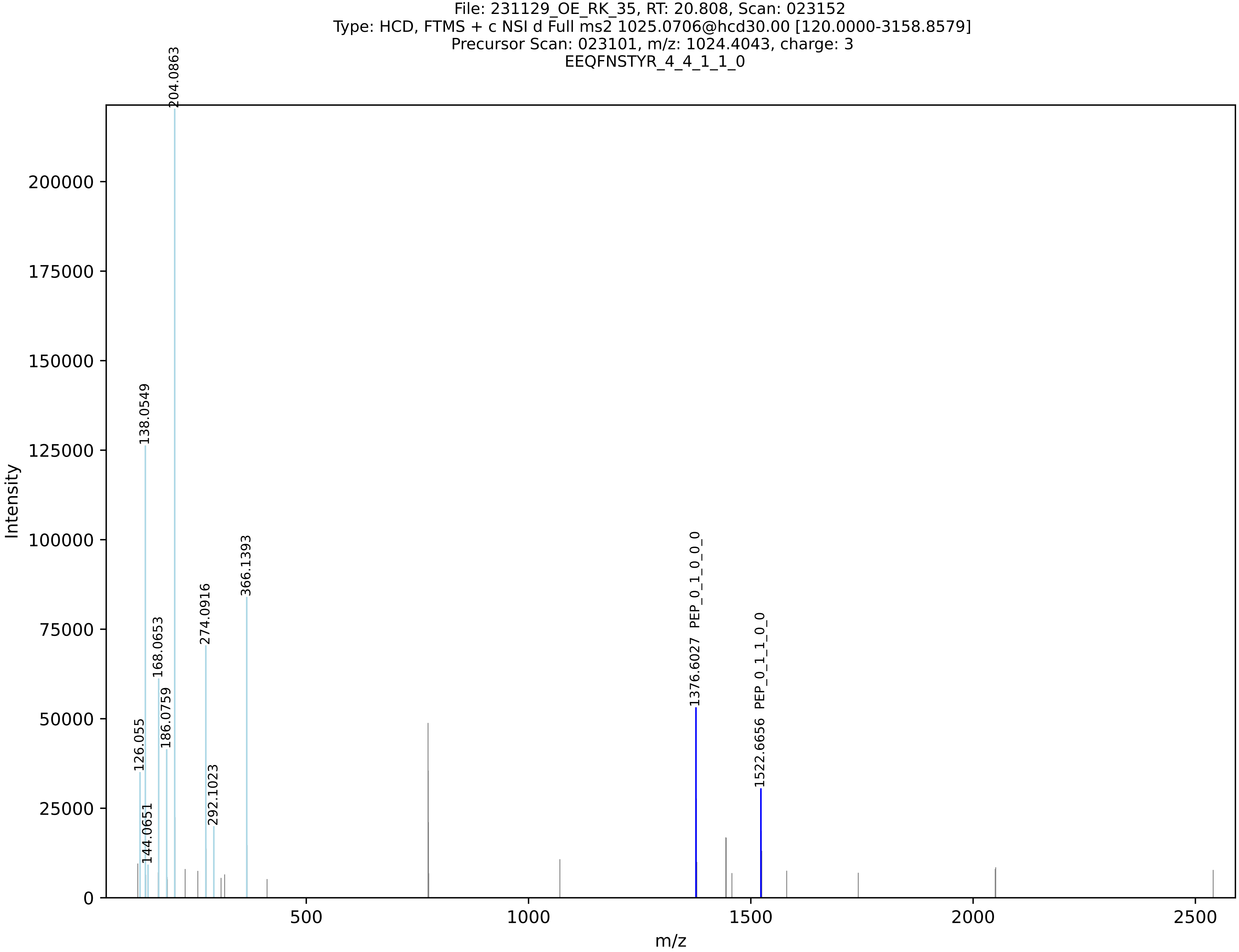

File: 231129_OE_RK_35, RT: 20.808, Scan: 023152
Type: HCD, FTMS + c NSI d Full ms2 1025.0706@hcd30.00 [120.0000-3158.8579]
Precursor Scan: 023101, m/z: 1024.4043, charge: 3
EEQFNSTYR_4_4_1_1_0
Intensity
m/z
204.0863
138.0549
366.1393
274.0916
168.0653
186.0759
126.055
292.1023
144.0651
1376.6027 PEP_0_1_0_0_0
1522.6656 PEP_0_1_1_0_0

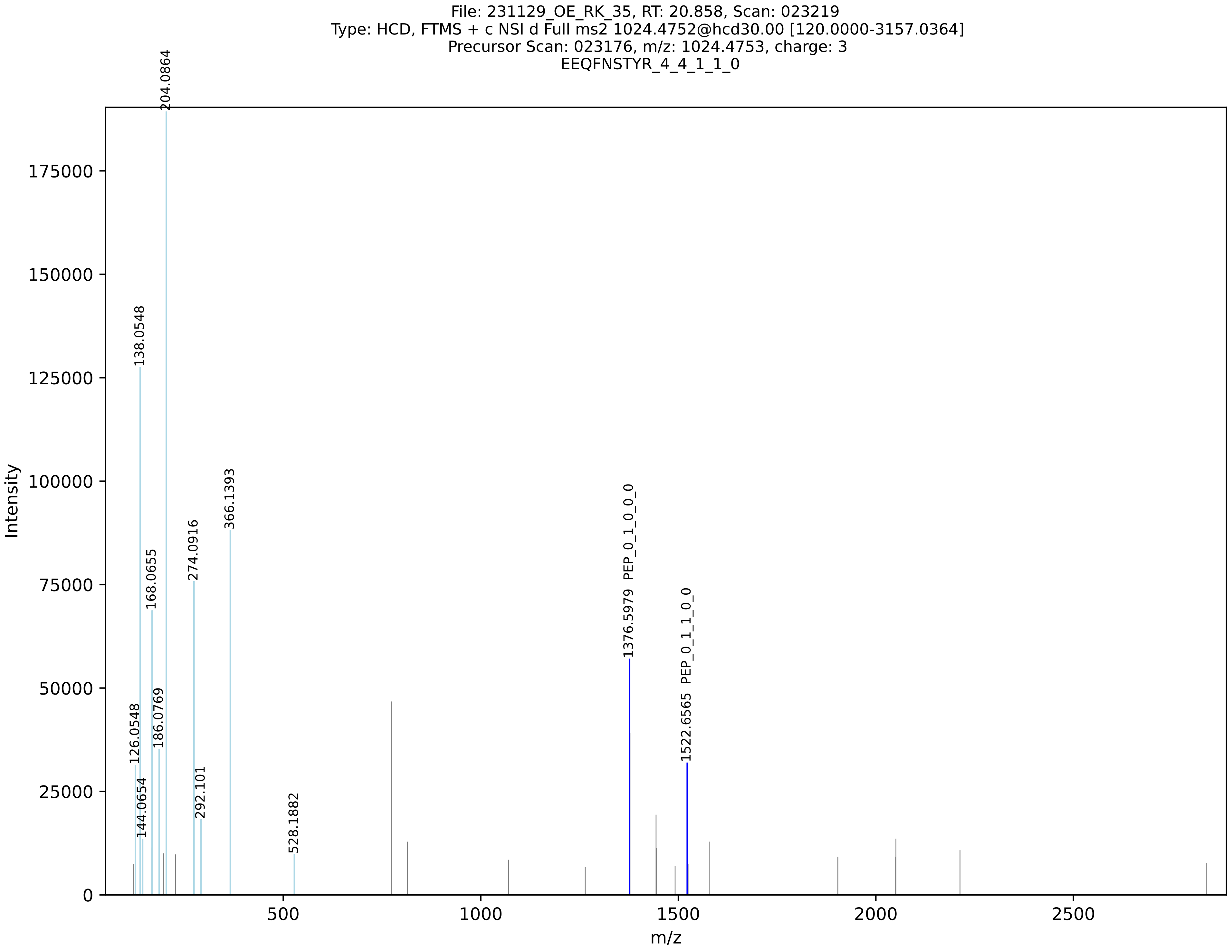

File: 231129_OE_RK_35, RT: 20.858, Scan: 023219
Type: HCD, FTMS + c NSI d Full ms2 1024.4752@hcd30.00 [120.0000-3157.0364]
Precursor Scan: 023176, m/z: 1024.4753, charge: 3
EEQFNSTYR_4_4_1_1_0
Intensity
m/z
204.0864
138.0548
168.0655
126.0548
186.0769
144.0654
274.0916
292.101
366.1393
528.1882
1376.5979 PEP_0_1_0_0_0
1522.6565 PEP_0_1_1_0_0
0
25000
50000
75000
100000
125000
150000
175000
500
1000
1500
2000
2500

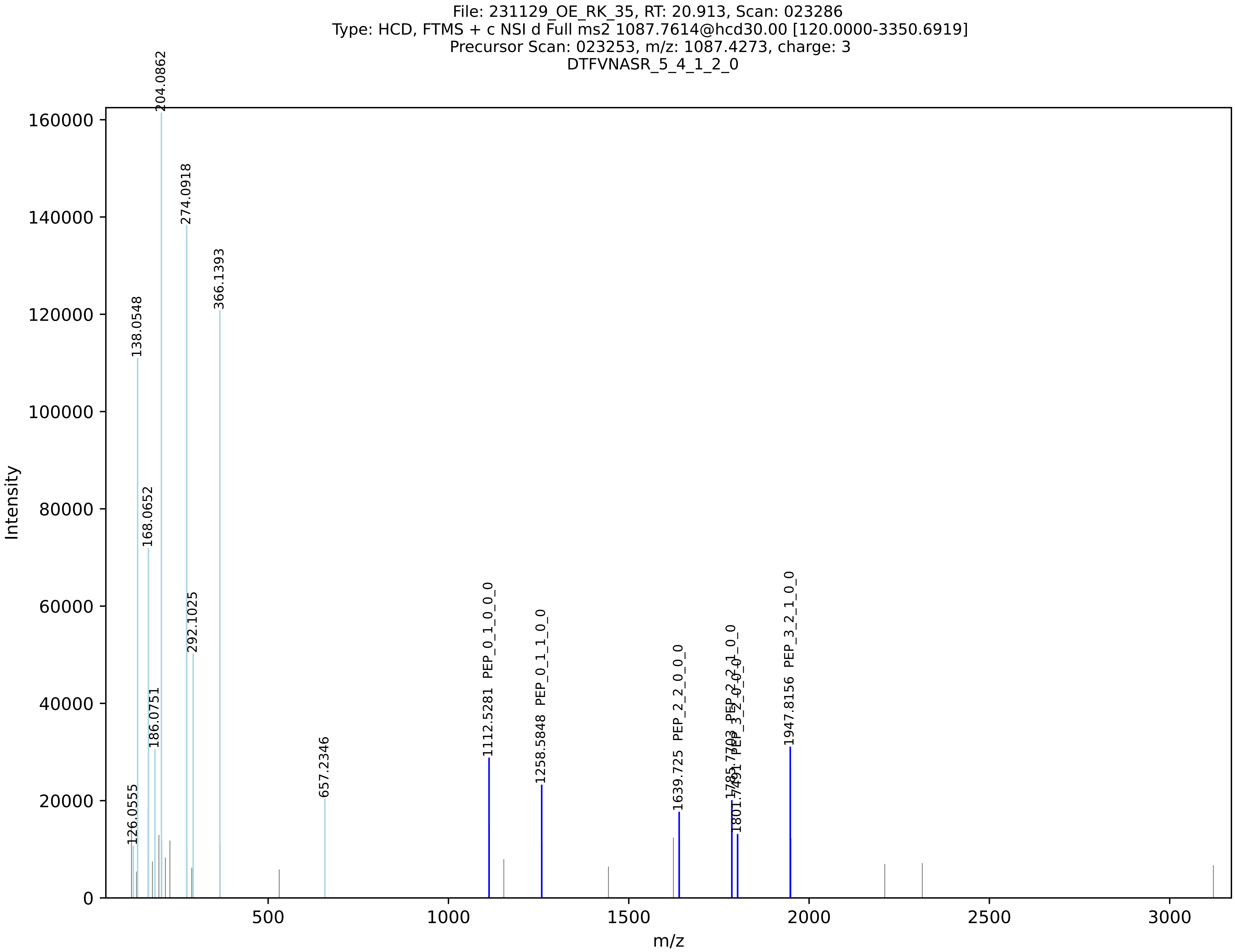
File: 231129_OE_RK_35, RT: 20.913, Scan: 023286
Type: HCD, FTMS + c NSI d Full ms2 1087.7614@hcd30.00 [120.0000-3350.6919]
Precursor Scan: 023253, m/z: 1087.4273, charge: 3
DTFVNASR_5_4_1_2_0
Intensity
m/z
204.0862
274.0918
366.1393
138.0548
168.0652
292.1025
186.0751
126.0555
657.2346
1112.5281 PEP_0_1_0_0_0
1258.5848 PEP_0_1_1_0_0
1639.725 PEP_2_2_0_0_0
1947.8156 PEP_3_2_1_0_0

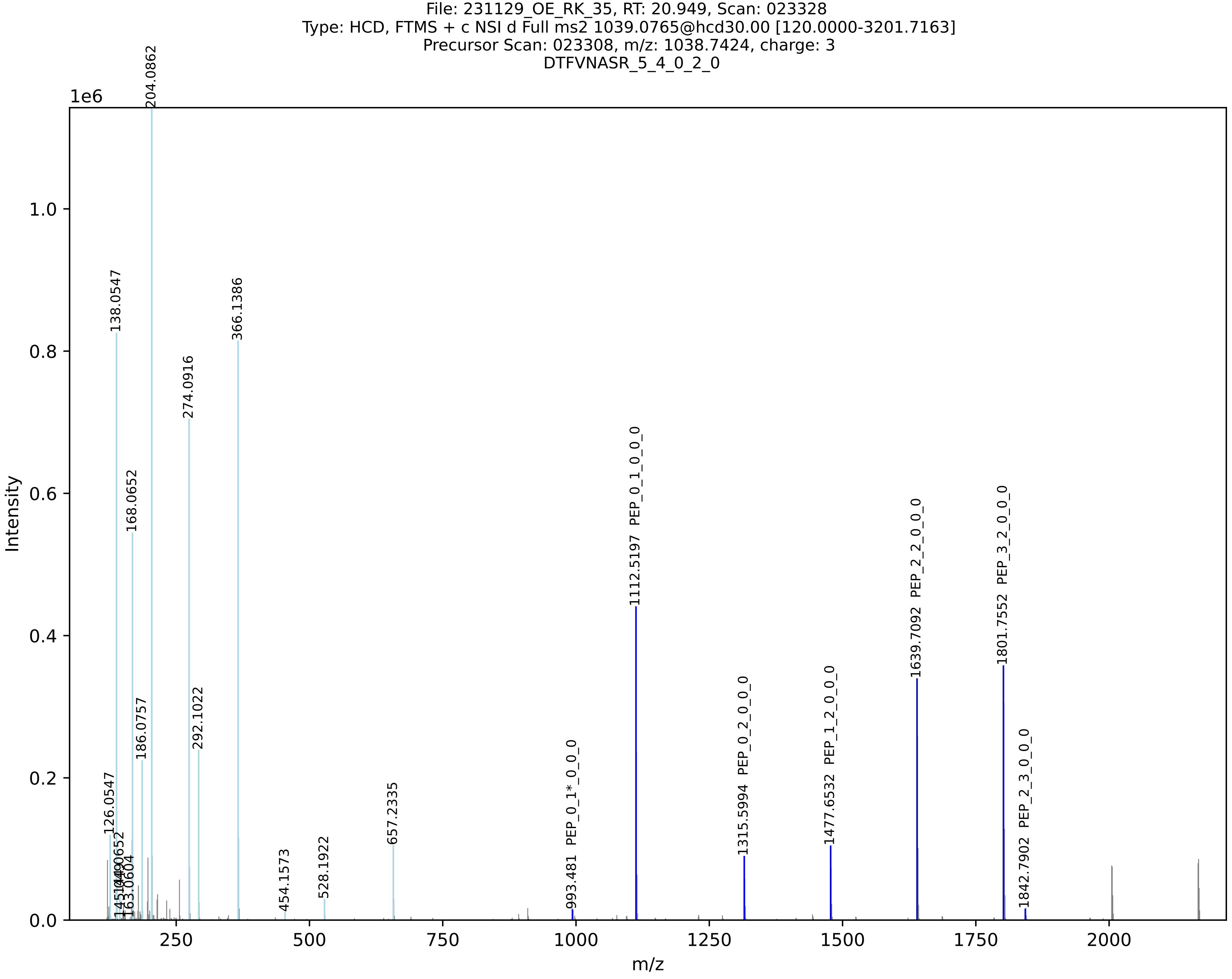
File: 231129_OE_RK_35, RT: 20.949, Scan: 023328
Type: HCD, FTMS + c NSI d Full ms2 1039.0765@hcd30.00 [120.0000-3201.7163]
Precursor Scan: 023308, m/z: 1038.7424, charge: 3
DTFVNASR_5_4_0_2_0
1e6
Intensity
m/z
204.0862
138.0547
366.1386
274.0916
168.0652
186.0757
292.1022
126.0547
163.0604
454.1573
528.1922
657.2335
993.481 PEP_0_1*_0_0_0
1112.5197 PEP_0_1_0_0_0
1315.5994 PEP_0_2_0_0_0
1477.6532 PEP_1_2_0_0_0
1639.7092 PEP_2_2_0_0_0
1801.7552 PEP_3_2_0_0_0
1842.7902 PEP_2_3_0_0_0

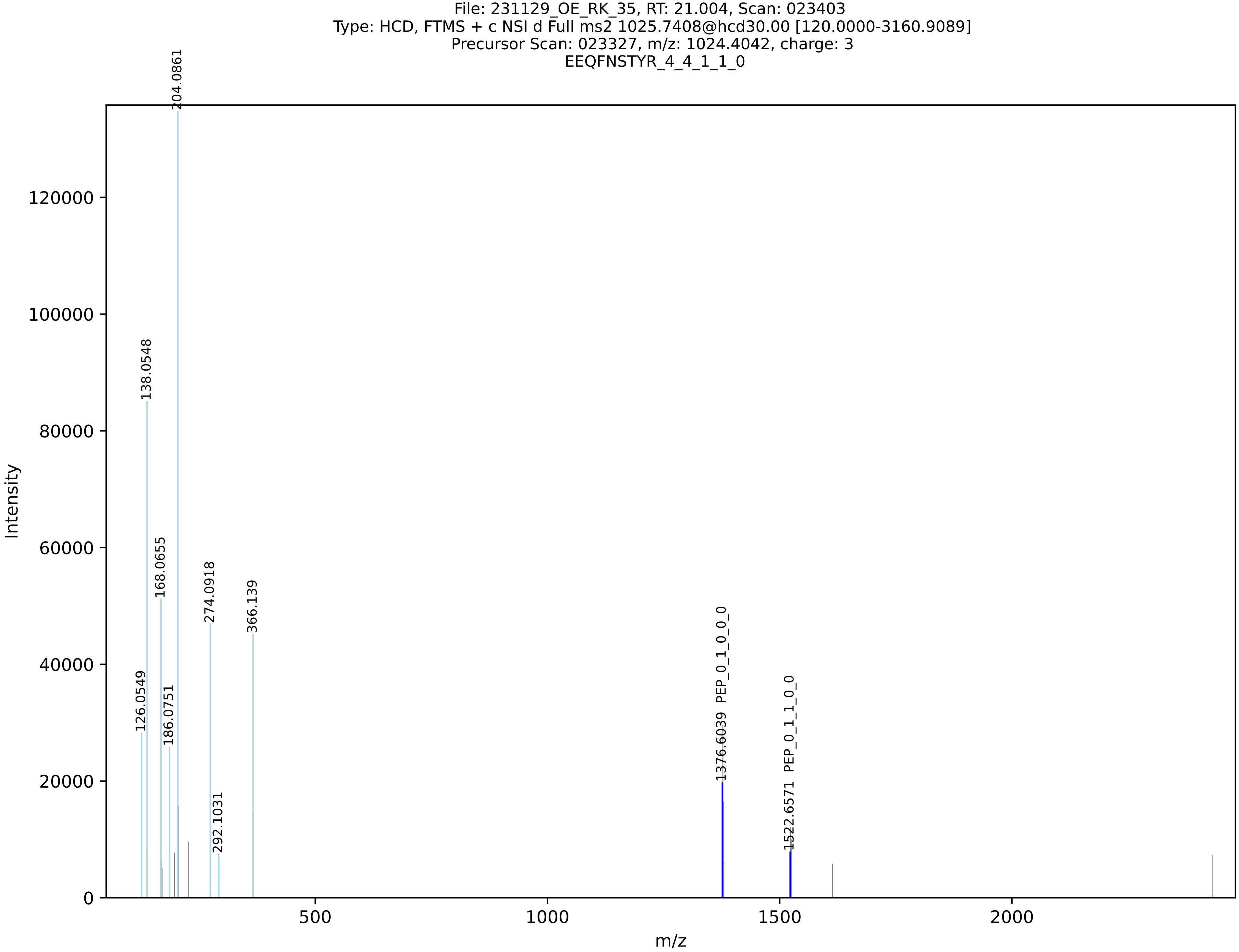
File: 231129_OE_RK_35, RT: 21.004, Scan: 023403
Type: HCD, FTMS + c NSI d Full ms2 1025.7408@hcd30.00 [120.0000-3160.9089]
Precursor Scan: 023327, m/z: 1024.4042, charge: 3
EEQFNSTYR_4_4_1_1_0
Intensity
m/z
0
20000
40000
60000
80000
100000
120000
500
1000
1500
2000
204.0861
138.0548
168.0655
274.0918
366.139
126.0549
186.0751
292.1031
1376.6039 PEP_0_1_0_0_0
1522.6571 PEP_0_1_1_0_0

File: 231129_OE_RK_35, RT: 21.019, Scan: 023420
Type: HCD, FTMS + c NSI d Full ms2 1311.8618@hcd30.00 [120.0000-4036.4392]
Precursor Scan: 023381, m/z: 1311.5269, charge: 3
RNHSCEPCQTLAVR_5_4_0_2_0

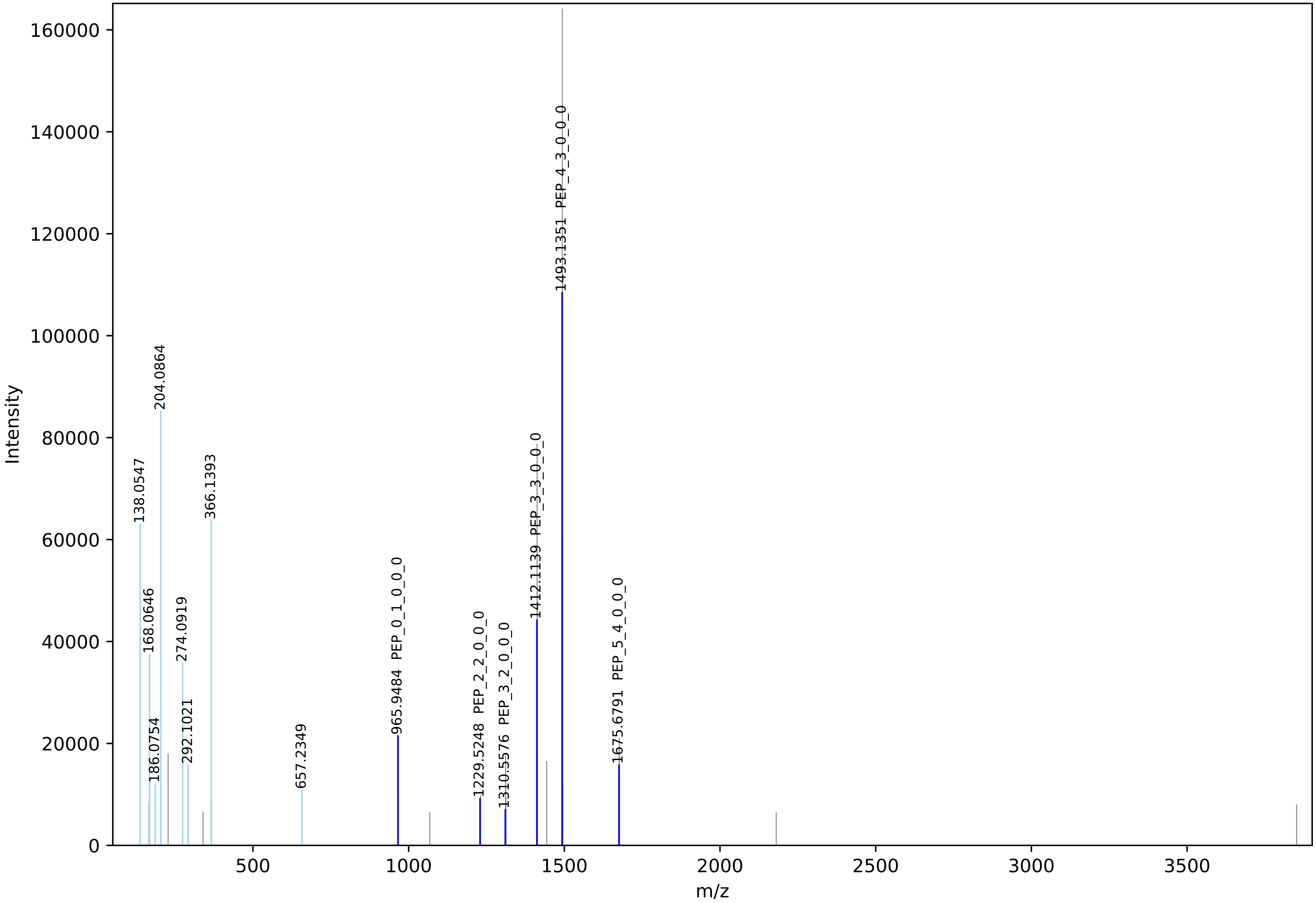

File: 231129_OE_RK_35, RT: 21.05, Scan: 023463
Type: HCD, FTMS + c NSI d Full ms2 1024.0720@hcd30.00 [120.0000-3155.8022]
Precursor Scan: 023381, m/z: 1024.4032, charge: 3
EEQFNSTYR_4_4_1_1_0

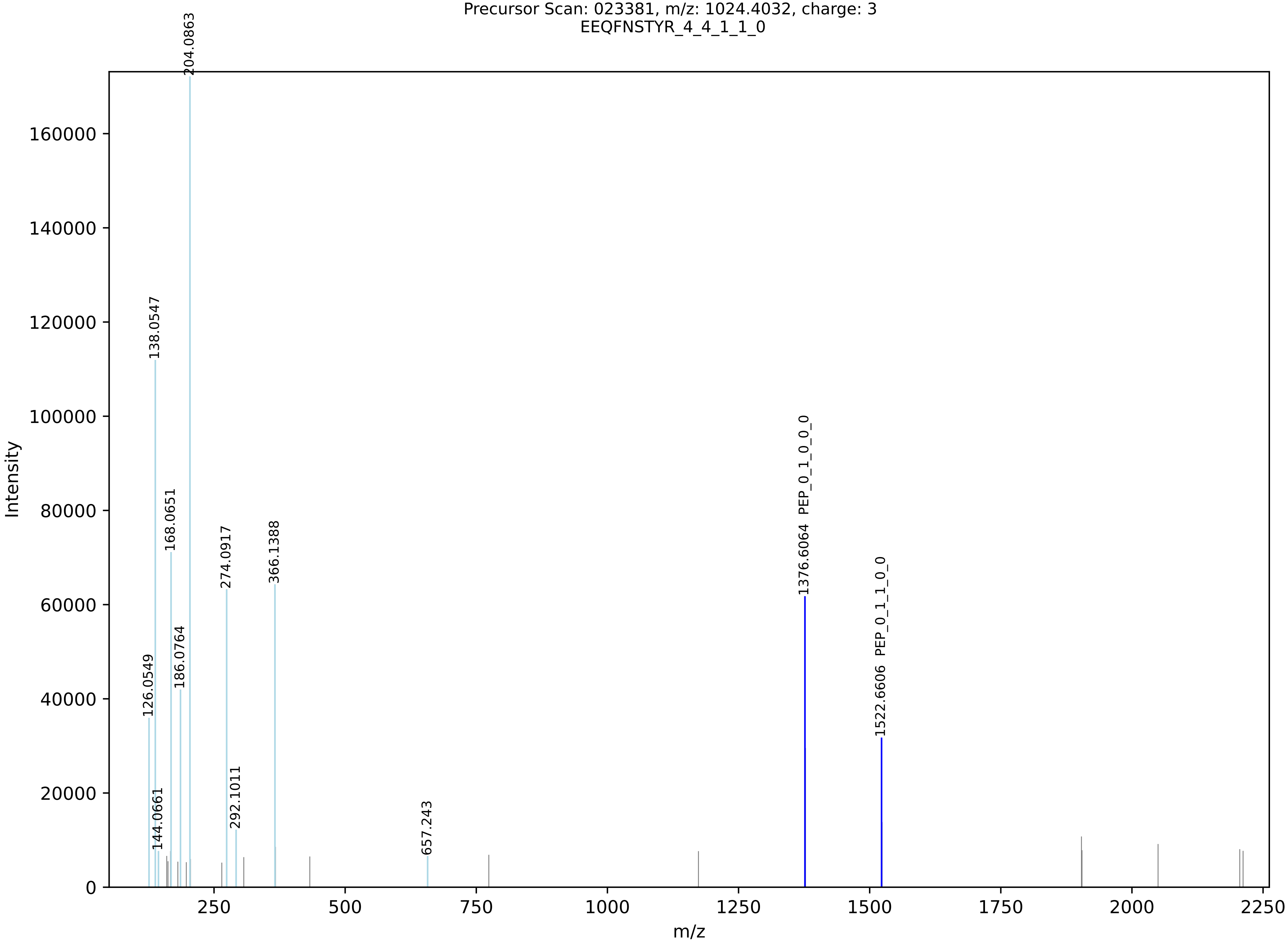

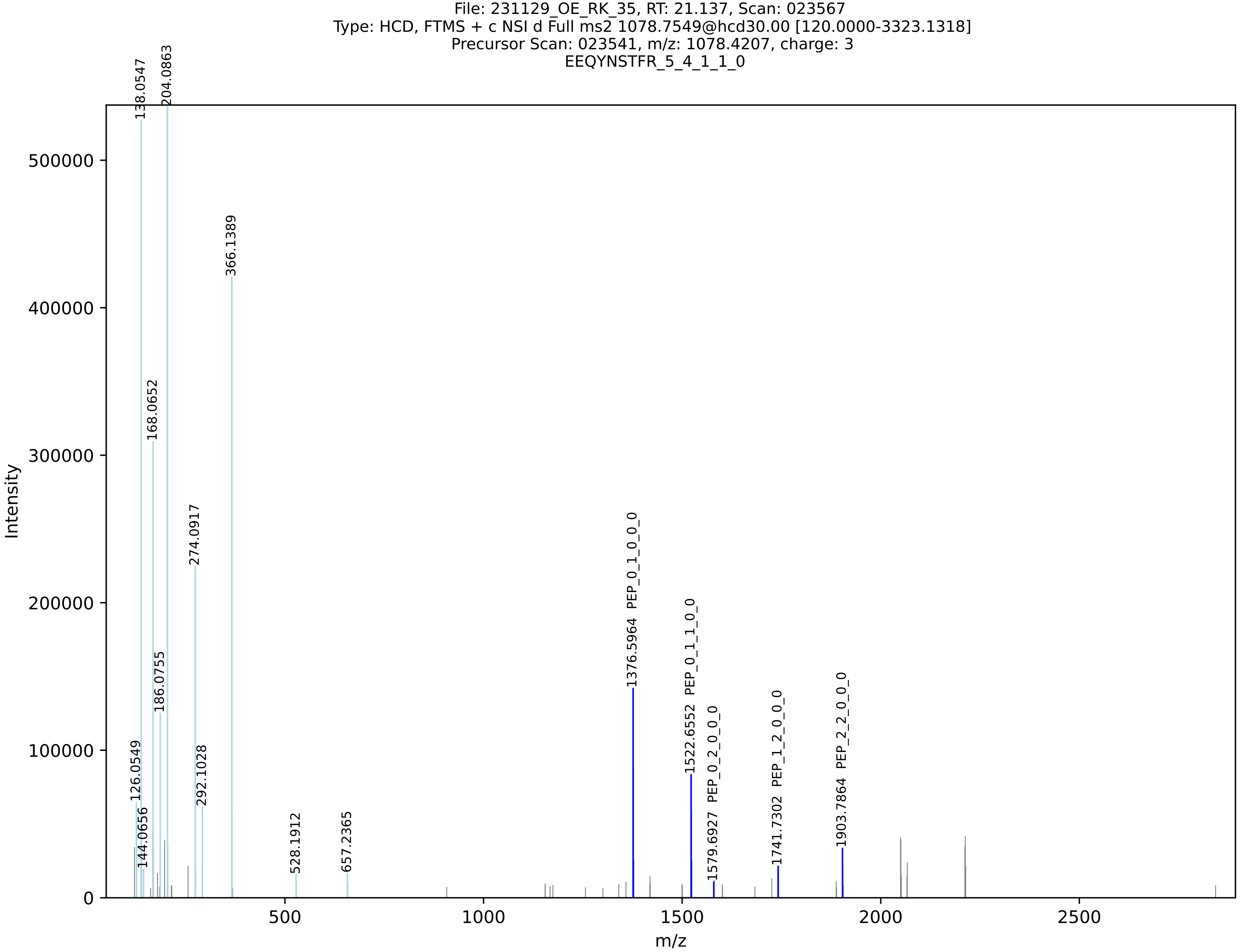

File: 231129_OE_RK_35, RT: 21.137, Scan: 023567
Type: HCD, FTMS + c NSI d Full ms2 1078.7549@hcd30.00 [120.0000-3323.1318]
Precursor Scan: 023541, m/z: 1078.4207, charge: 3
EEQYNSTFR_5_4_1_1_0
Intensity
m/z
138.0547
204.0863
366.1389
168.0652
274.0917
186.0755
126.0549
292.1028
144.0656
528.1912
657.2365
1376.5964 PEP_0_1_0_0_0
1522.6552 PEP_0_1_1_0_0
1579.6927 PEP_0_2_0_0_0
1741.7302 PEP_1_2_0_0_0
1903.7864 PEP_2_2_0_0_0

File: 231129_OE_RK_35, RT: 21.186, Scan: 023631
Type: HCD, FTMS + c NSI d Full ms2 1173.4644@hcd30.00 [120.0000-3612.9429]
Precursor Scan: 023588, m/z: 1172.7961, charge: 3
FLNNGTCTAEGK_5_4_0_2_0

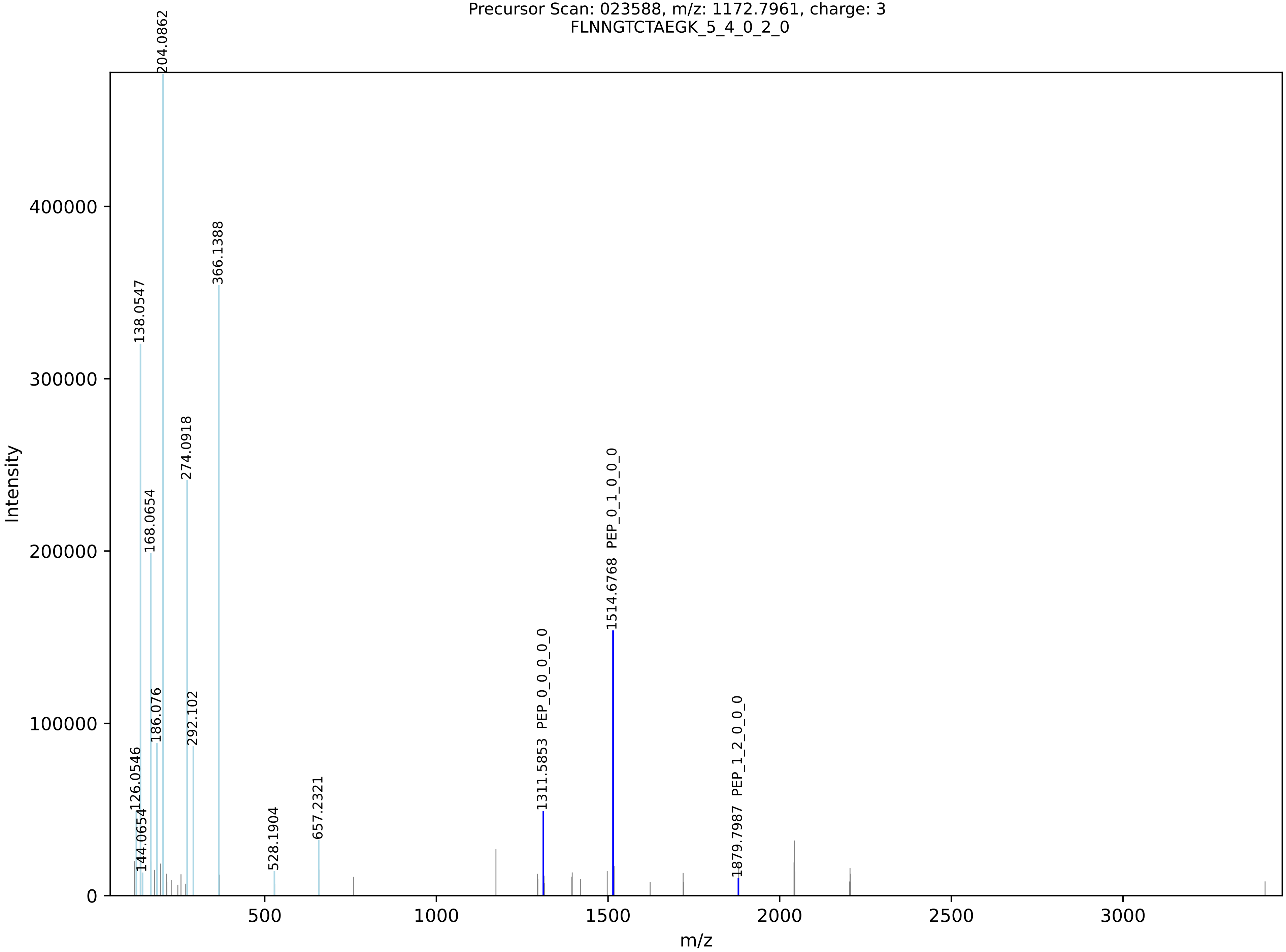

File: 231129_OE_RK_35, RT: 21.187, Scan: 023632
Type: HCD, FTMS + c NSI d Full ms2 1025.0714@hcd30.00 [120.0000-3158.8604]
Precursor Scan: 023588, m/z: 1024.4032, charge: 3
EEQFNSTYR_4_4_1_1_0

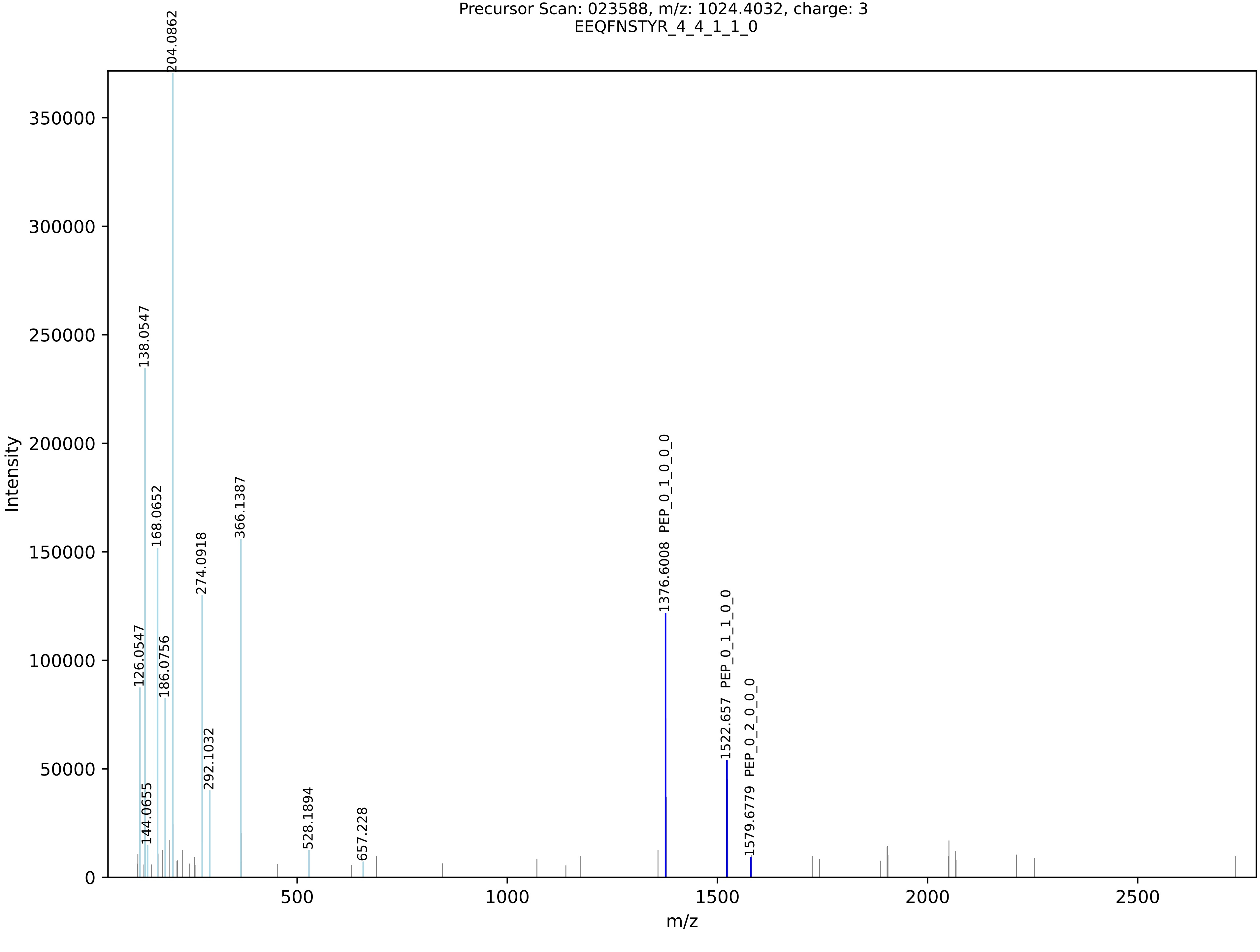

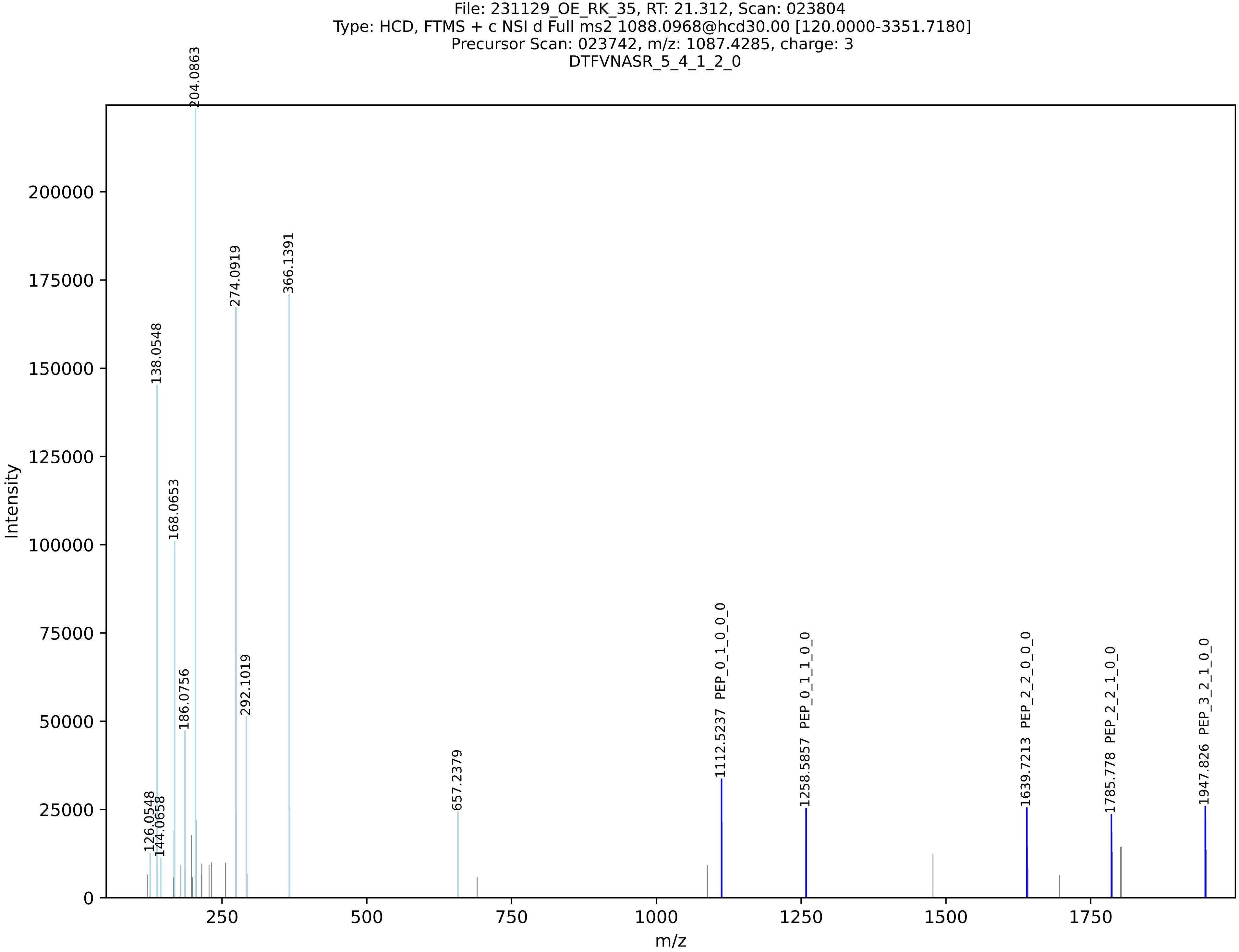

File: 231129_OE_RK_35, RT: 21.312, Scan: 023804
Type: HCD, FTMS + c NSI d Full ms2 1088.0968@hcd30.00 [120.0000-3351.7180]
Precursor Scan: 023742, m/z: 1087.4285, charge: 3
DTFVNASR_5_4_1_2_0
Intensity
m/z
0
25000
50000
75000
100000
125000
150000
175000
200000
250
500
750
1000
1250
1500
1750
204.0863
366.1391
274.0919
138.0548
168.0653
292.1019
186.0756
126.0548
144.0658
657.2379
1112.5237 PEP_0_1_0_0_0
1258.5857 PEP_0_1_1_0_0
1639.7213 PEP_2_2_0_0_0
1785.778 PEP_2_2_1_0_0
1947.826 PEP_3_2_1_0_0

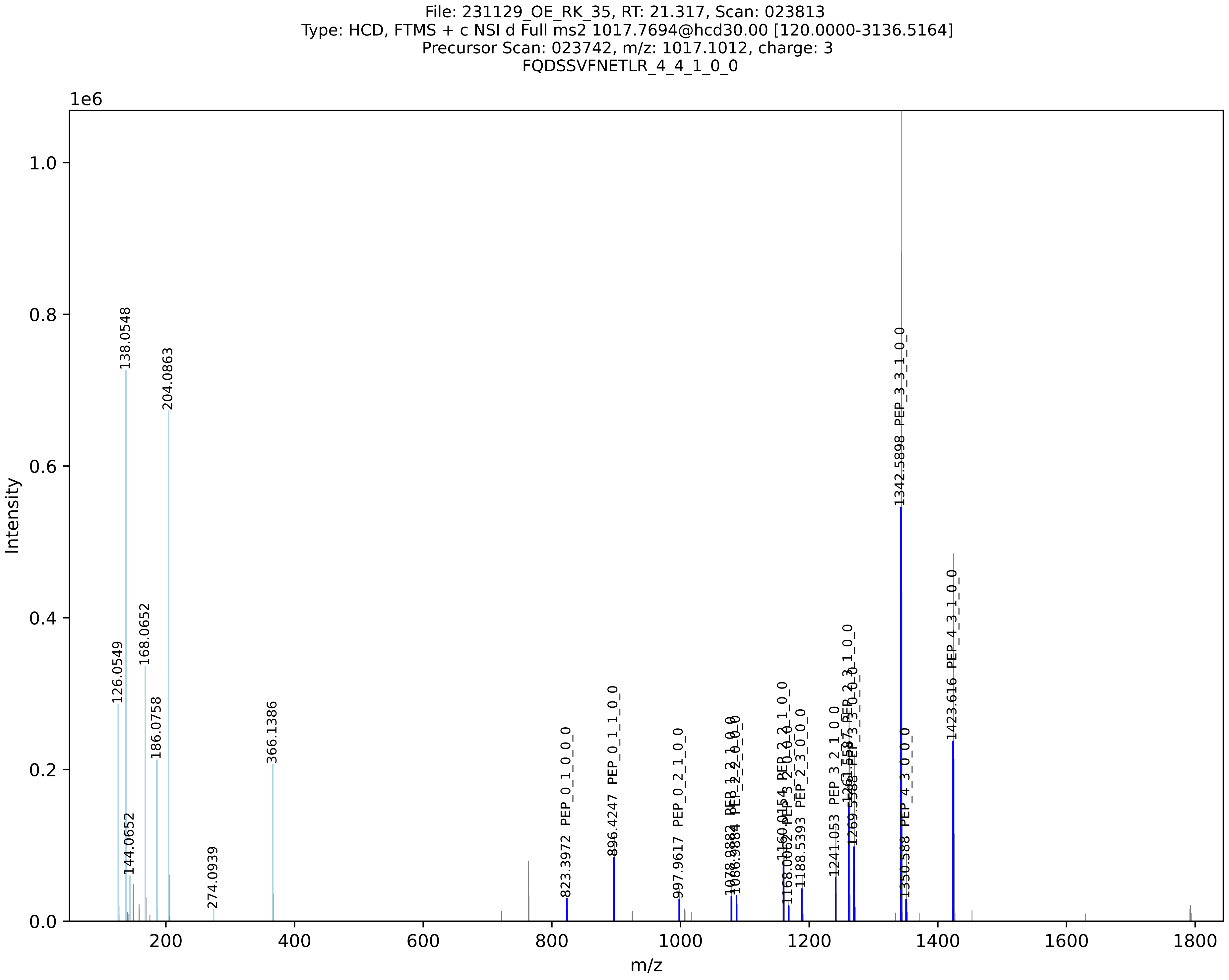
File: 231129_OE_RK_35, RT: 21.317, Scan: 023813
Type: HCD, FTMS + c NSI d Full ms2 1017.7694@hcd30.00 [120.0000-3136.5164]
Precursor Scan: 023742, m/z: 1017.1012, charge: 3
FQDSSVFNETLR_4_4_1_0_0
1e6
Intensity
m/z
138.0548
204.0863
168.0652
126.0549
186.0758
366.1386
144.0652
274.0939
823.3972 PEP_0_1_0_0_0
896.4247 PEP_0_1_1_0_0
997.9617 PEP_0_2_1_0_0
1188.5393 PEP_2_3_0_0_0
1241.053 PEP_3_2_1_0_0
1342.5898 PEP_3_3_1_0_0
1350.588 PEP_4_3_0_0_0
1423.616 PEP_4_3_1_0_0

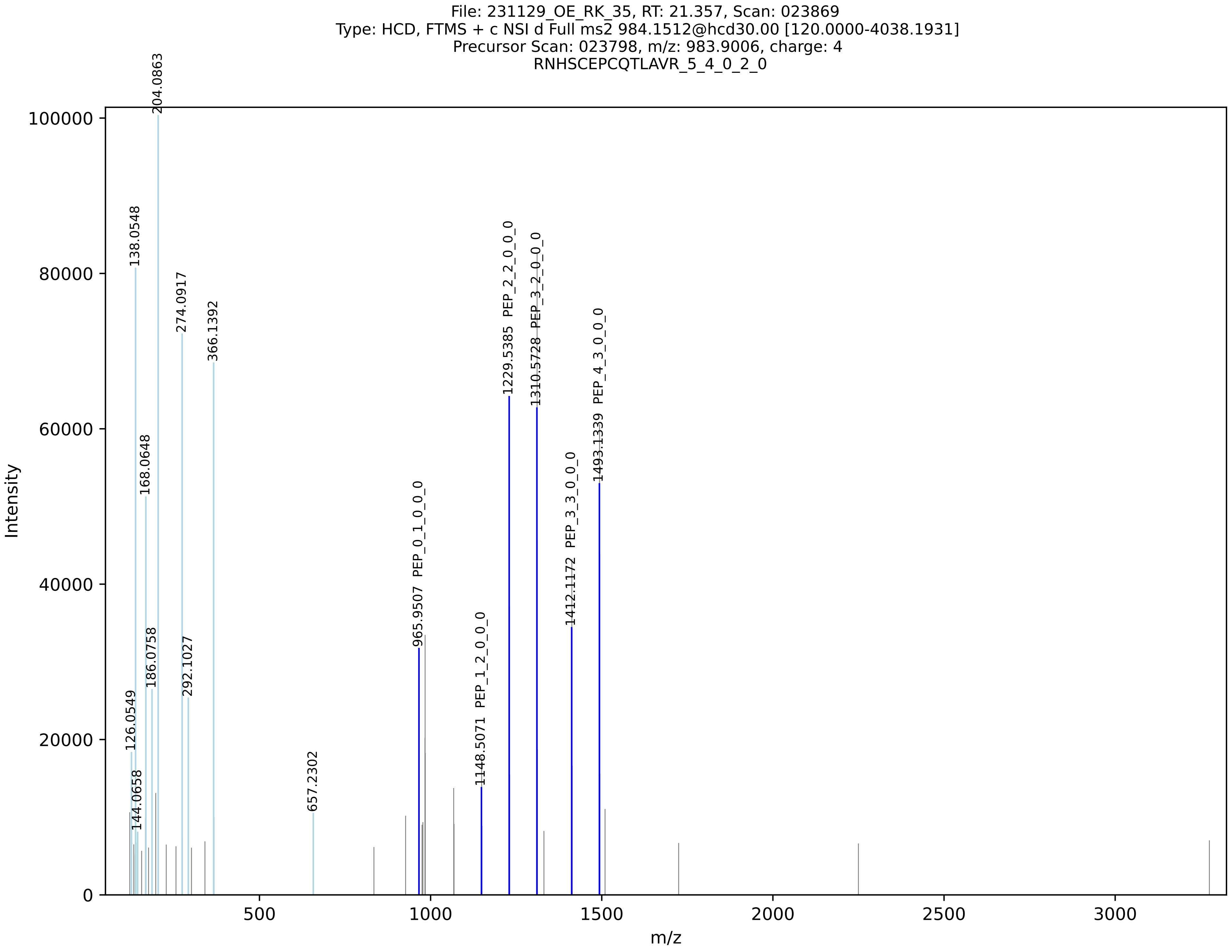
File: 231129_OE_RK_35, RT: 21.357, Scan: 023869
Type: HCD, FTMS + c NSI d Full ms2 984.1512@hcd30.00 [120.0000-4038.1931]
Precursor Scan: 023798, m/z: 983.9006, charge: 4
RNHSCEPCQTLAVR_5_4_0_2_0
Intensity
m/z
204.0863
138.0548
274.0917
366.1392
168.0648
186.0758
292.1027
126.0549
144.0658
657.2302
965.9507 PEP_0_1_0_0_0
1148.5071 PEP_1_2_0_0_0
1229.5385 PEP_2_2_0_0_0
1310.5728 PEP_3_2_0_0_0
1412.1172 PEP_3_3_0_0_0
1493.1339 PEP_4_3_0_0_0

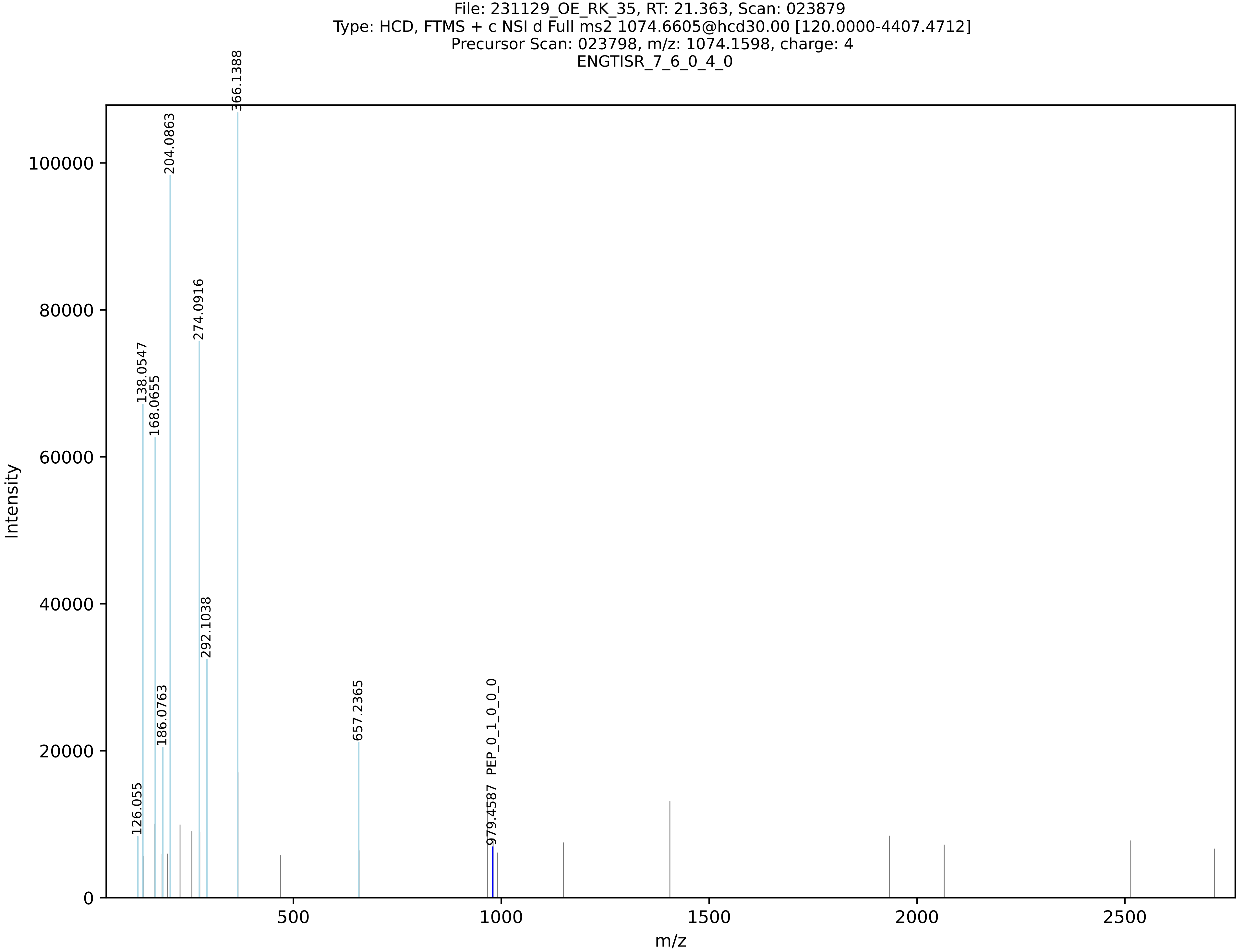

File: 231129_OE_RK_35, RT: 21.363, Scan: 023879
Type: HCD, FTMS + c NSI d Full ms2 1074.6605@hcd30.00 [120.0000-4407.4712]
Precursor Scan: 023798, m/z: 1074.1598, charge: 4
ENGTISR_7_6_0_4_0
366.1388
204.0863
274.0916
138.0547
168.0655
292.1038
186.0763
657.2365
PEP_0_1_0_0_0
979.4587
126.055
Intensity
m/z
0
20000
40000
60000
80000
100000
500
1000
1500
2000
2500

File: 231129_OE_RK_35, RT: 21.389, Scan: 023920
Type: HCD, FTMS + c NSI d Full ms2 1018.3998@hcd30.00 [120.0000-3138.4456]
Precursor Scan: 023798, m/z: 1018.3998, charge: 3
ELRYNESLTNFSCHGK_3_3_0_0_0

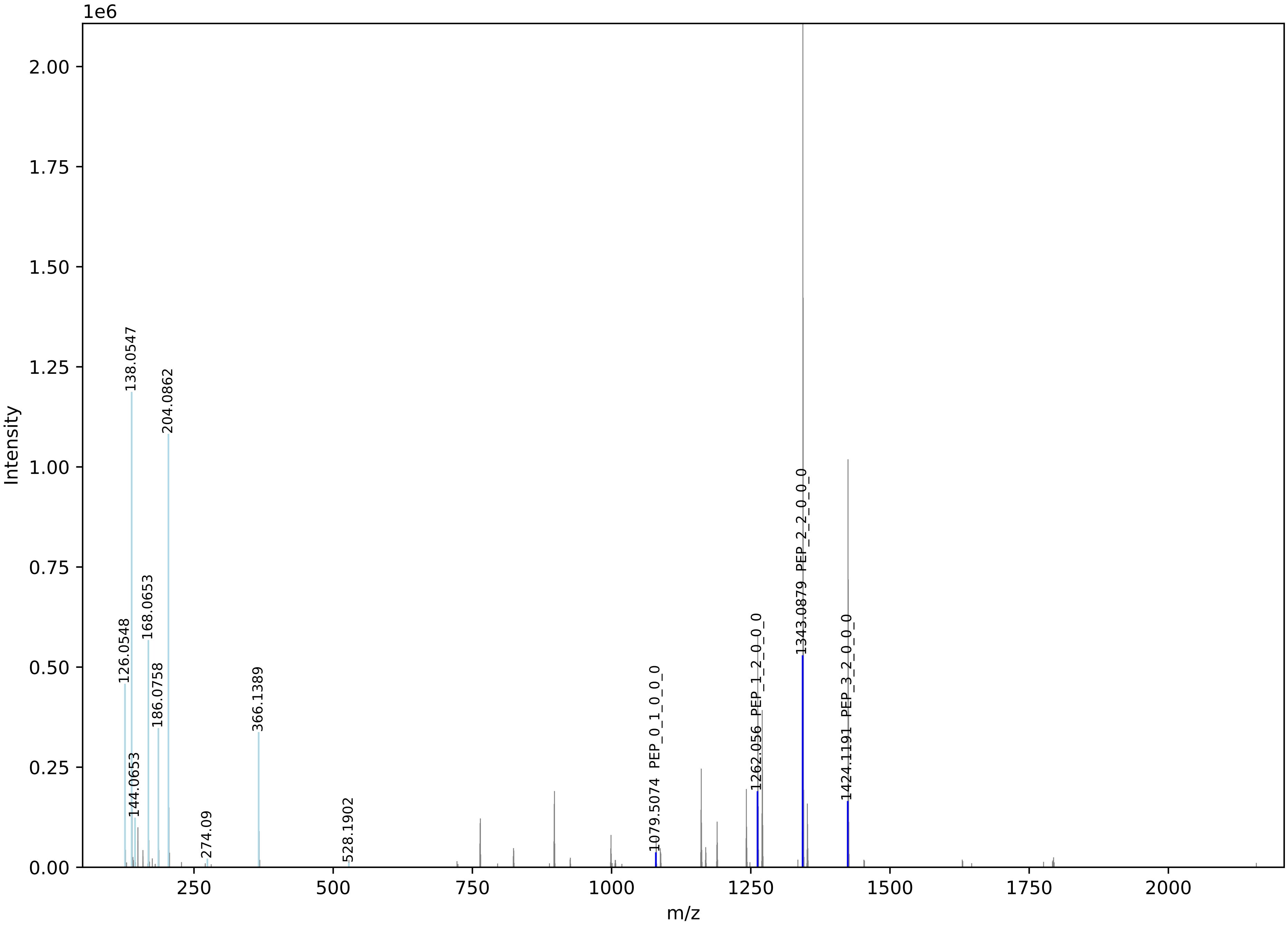

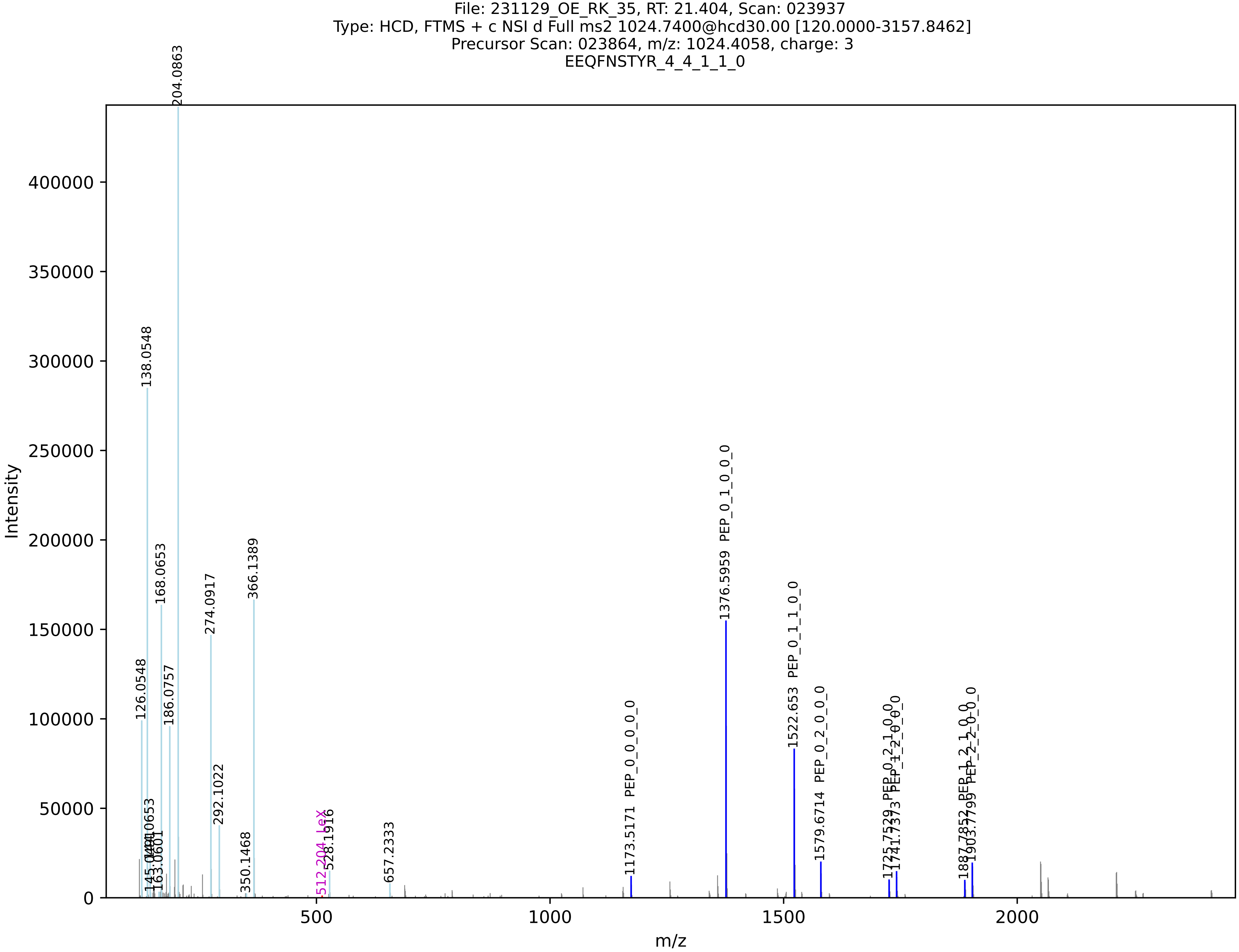

File: 231129_OE_RK_35, RT: 21.404, Scan: 023937
Type: HCD, FTMS + c NSI d Full ms2 1024.7400@hcd30.00 [120.0000-3157.8462]
Precursor Scan: 023864, m/z: 1024.4058, charge: 3
EEQFNSTYR_4_4_1_1_0
Intensity
m/z
204.0863
138.0548
168.0653
274.0917
366.1389
126.0548
186.0757
292.1022
350.1468
512.204_LeX
528.1916
657.2333
163.0601
1173.5171 PEP_0_0_0_0_0
1376.5959 PEP_0_1_0_0_0
1522.653 PEP_0_1_1_0_0
1579.6714 PEP_0_2_0_0_0
1725.7529 PEP_0_2_1_0_0
1741.7373 PEP_1_2_0_0_0
1887.7852 PEP_1_2_1_0_0
1903.7799 PEP_2_2_0_0_0

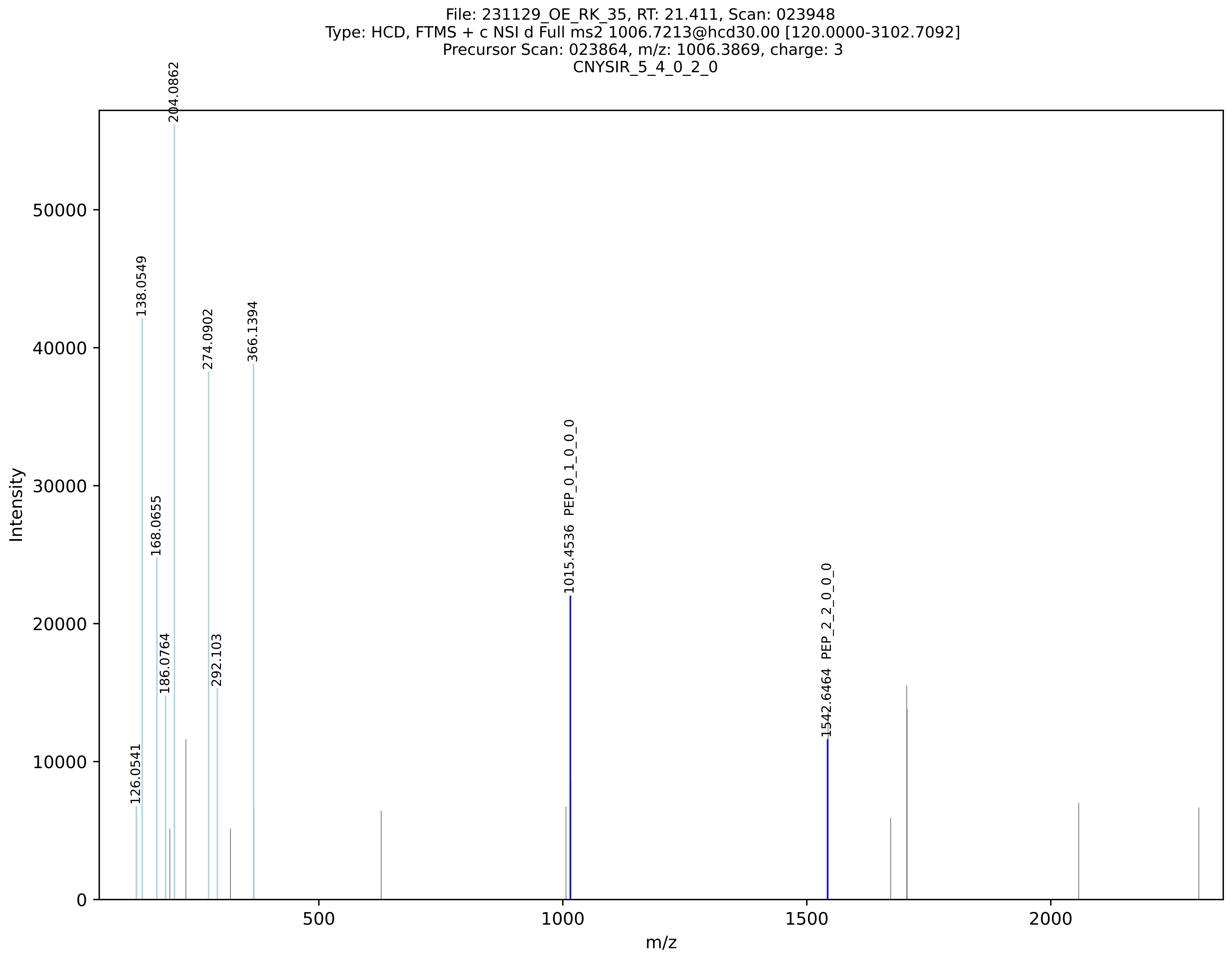
File: 231129_OE_RK_35, RT: 21.411, Scan: 023948
Type: HCD, FTMS + c NSI d Full ms2 1006.7213@hcd30.00 [120.0000-3102.7092]
Precursor Scan: 023864, m/z: 1006.3869, charge: 3
CNYSIR_5_4_0_2_0
Intensity
m/z
0
10000
20000
30000
40000
50000
500
1000
1500
2000
204.0862
138.0549
274.0902
366.1394
168.0655
186.0764
292.103
126.0541
1015.4536 PEP_0_1_0_0_0
1542.6464 PEP_2_2_0_0_0

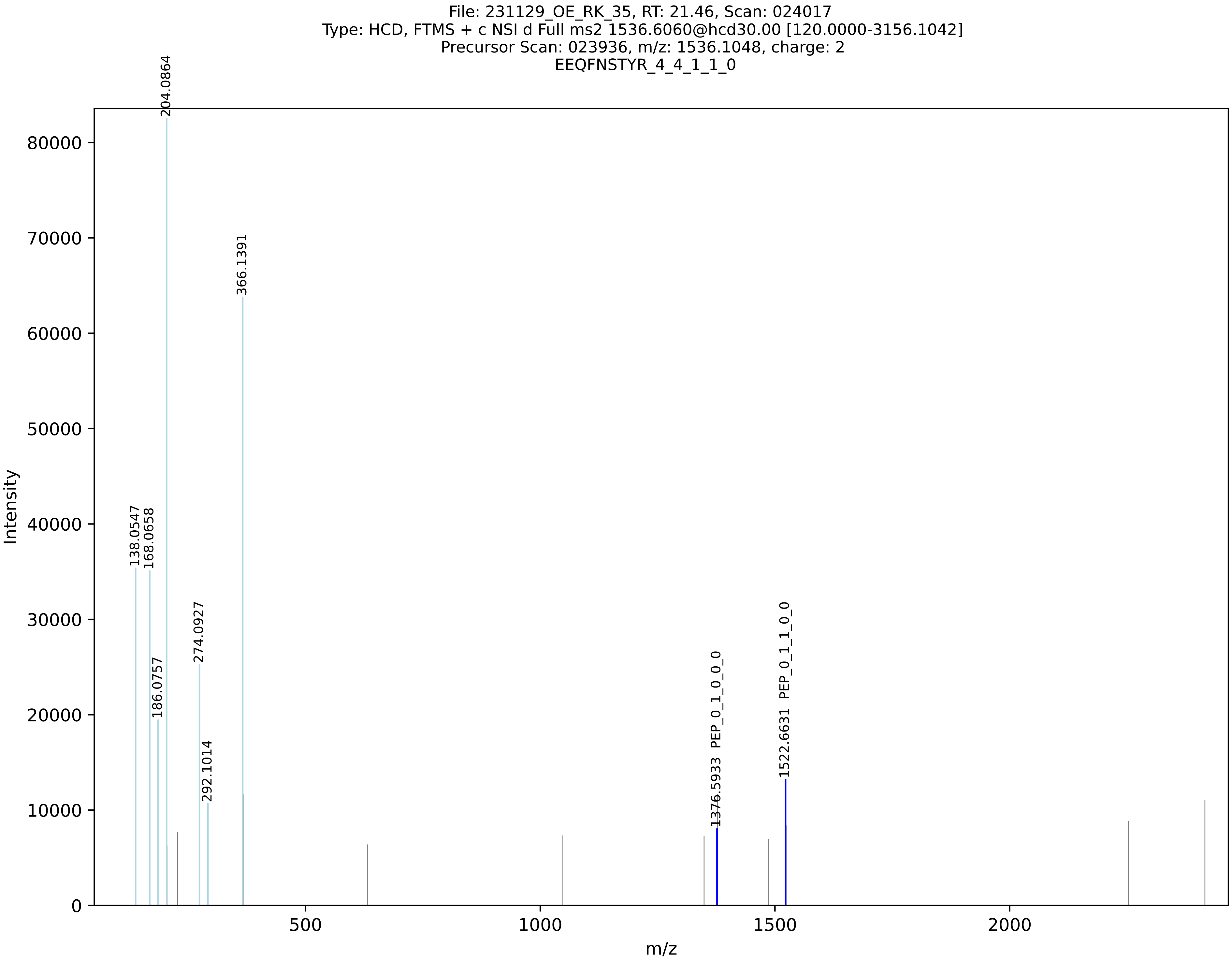

File: 231129_OE_RK_35, RT: 21.46, Scan: 024017
Type: HCD, FTMS + c NSI d Full ms2 1536.6060@hcd30.00 [120.0000-3156.1042]
Precursor Scan: 023936, m/z: 1536.1048, charge: 2
EEQFNSTYR_4_4_1_1_0
204.0864
366.1391
138.0547
168.0658
274.0927
186.0757
292.1014
1376.5933 PEP_0_1_0_0_0
1522.6631 PEP_0_1_1_0_0
Intensity
m/z
0
10000
20000
30000
40000
50000
60000
70000
80000
500
1000
1500
2000

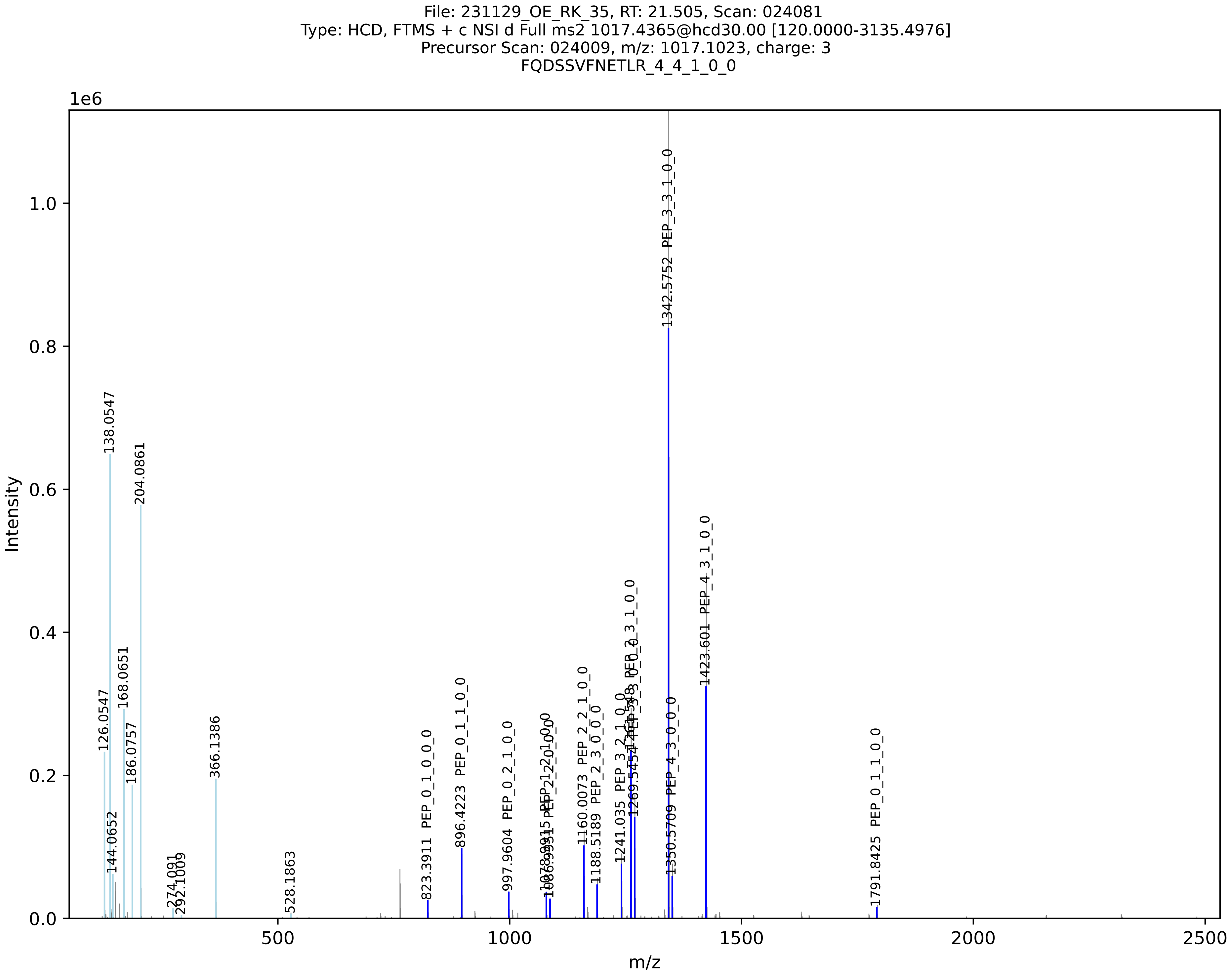
File: 231129_OE_RK_35, RT: 21.505, Scan: 024081
Type: HCD, FTMS + c NSI d Full ms2 1017.4365@hcd30.00 [120.0000-3135.4976]
Precursor Scan: 024009, m/z: 1017.1023, charge: 3
FQDSSVFNETLR_4_4_1_0_0
1e6
Intensity
m/z
138.0547
204.0861
168.0651
126.0547
186.0757
144.0652
366.1386
274.091
292.1009
528.1863
823.3911 PEP_0_1_0_0_0
896.4223 PEP_0_1_1_0_0
997.9604 PEP_0_2_1_0_0
1160.0073 PEP_2_2_1_0_0
1188.5189 PEP_2_3_0_0_0
1241.035 PEP_3_2_1_0_0
1342.5752 PEP_3_3_1_0_0
1350.5709 PEP_4_3_0_0_0
1423.601 PEP_4_3_1_0_0
1791.8425 PEP_0_1_1_0_0

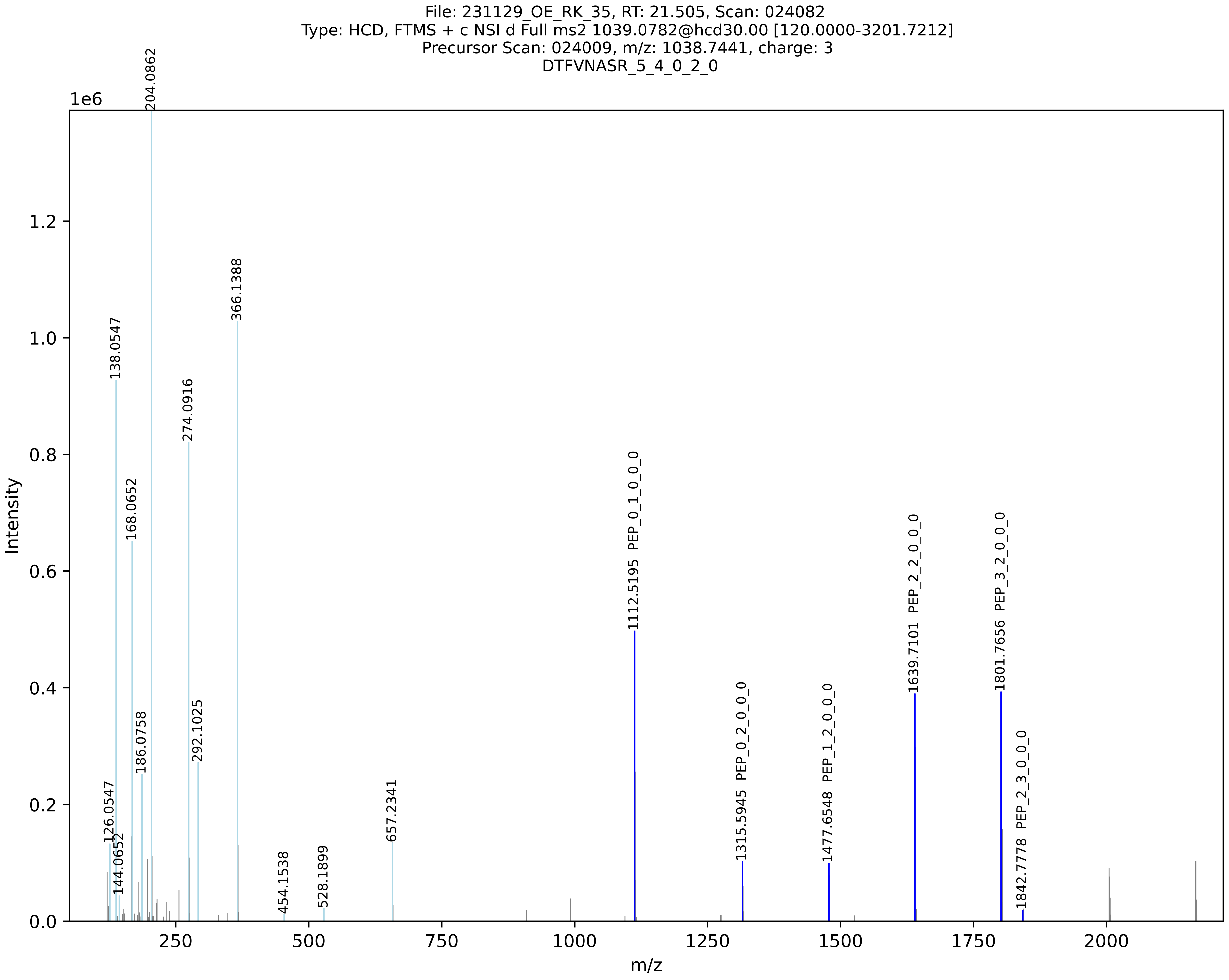
File: 231129_OE_RK_35, RT: 21.505, Scan: 024082
Type: HCD, FTMS + c NSI d Full ms2 1039.0782@hcd30.00 [120.0000-3201.7212]
Precursor Scan: 024009, m/z: 1038.7441, charge: 3
DTFVNASR_5_4_0_2_0
1e6
Intensity
m/z
204.0862
366.1388
138.0547
274.0916
168.0652
186.0758
292.1025
126.0547
144.0652
454.1538
528.1899
657.2341
1112.5195 PEP_0_1_0_0_0
1315.5945 PEP_0_2_0_0_0
1477.6548 PEP_1_2_0_0_0
1639.7101 PEP_2_2_0_0_0
1801.7656 PEP_3_2_0_0_0
1842.7778 PEP_2_3_0_0_0

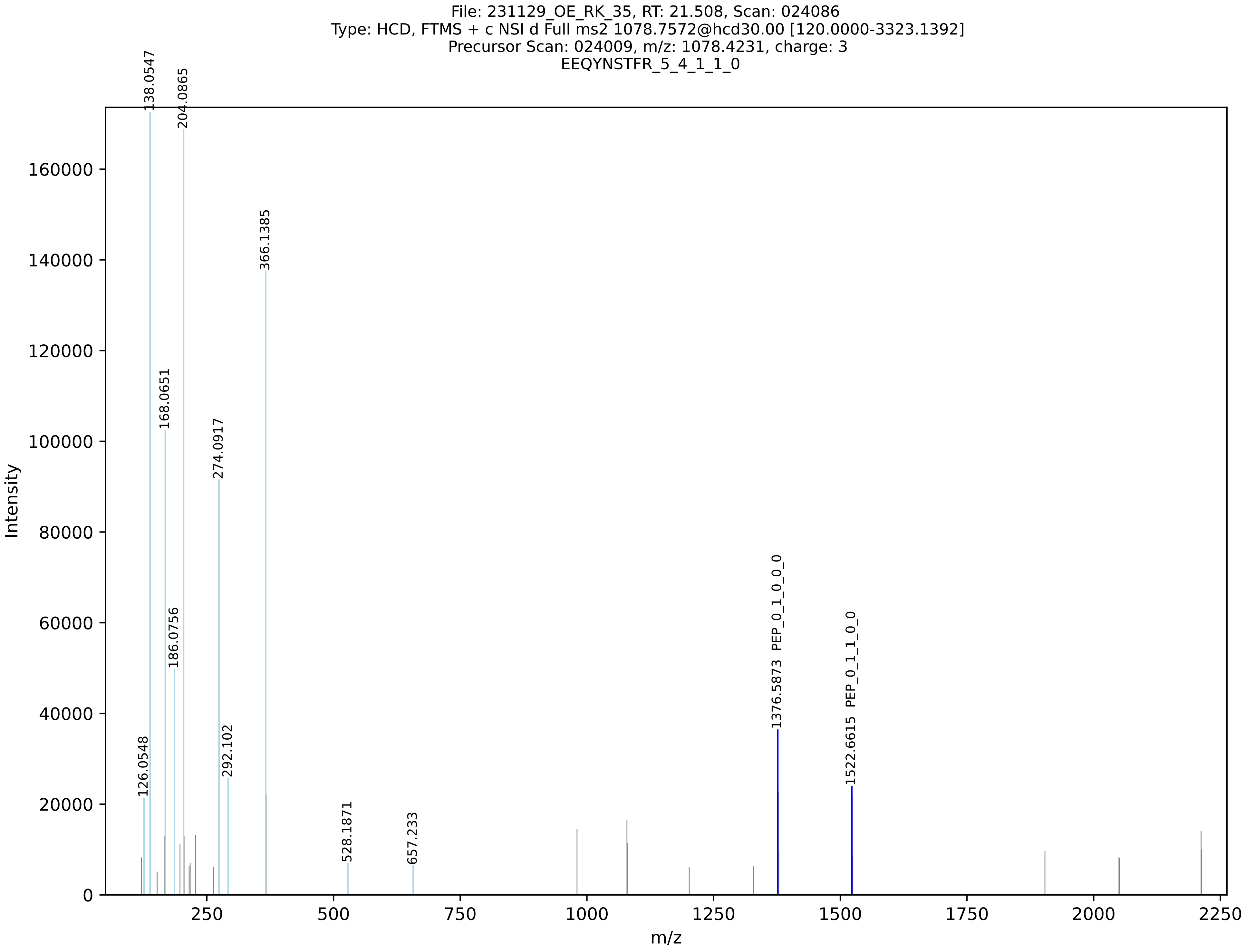
File: 231129_OE_RK_35, RT: 21.508, Scan: 024086
Type: HCD, FTMS + c NSI d Full ms2 1078.7572@hcd30.00 [120.0000-3323.1392]
Precursor Scan: 024009, m/z: 1078.4231, charge: 3
EEQYNSTFR_5_4_1_1_0
138.0547
204.0865
366.1385
168.0651
274.0917
186.0756
126.0548
292.102
528.1871
657.233
1376.5873 PEP_0_1_0_0_0
1522.6615 PEP_0_1_1_0_0
Intensity
m/z
0
20000
40000
60000
80000
100000
120000
140000
160000
250
500
750
1000
1250
1500
1750
2000
2250

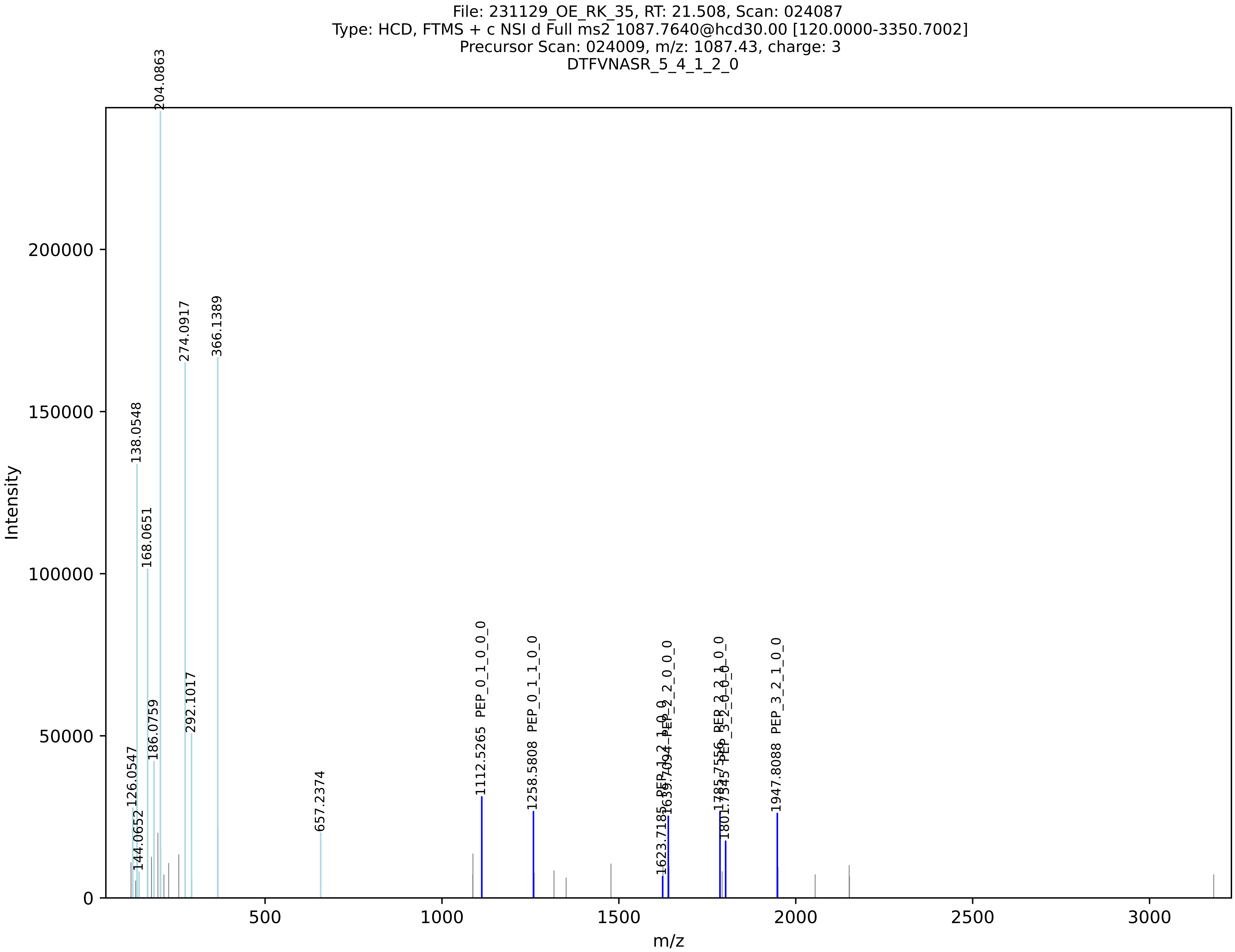
File: 231129_OE_RK_35, RT: 21.508, Scan: 024087
Type: HCD, FTMS + c NSI d Full ms2 1087.7640@hcd30.00 [120.0000-3350.7002]
Precursor Scan: 024009, m/z: 1087.43, charge: 3
DTFVNASR_5_4_1_2_0
204.0863
274.0917
366.1389
138.0548
168.0651
292.1017
186.0759
126.0547
144.0652
657.2374
1112.5265 PEP_0_1_0_0_0
1258.5808 PEP_0_1_1_0_0
1623.7185 PEP_1_2_1_0_0
1639.7094 PEP_2_2_0_0_0
1785.7556 PEP_2_2_1_0_0
1801.7545 PEP_3_2_0_0_0
1947.8088 PEP_3_2_1_0_0
Intensity
0
50000
100000
150000
200000
500
1000
1500
2000
2500
3000
m/z

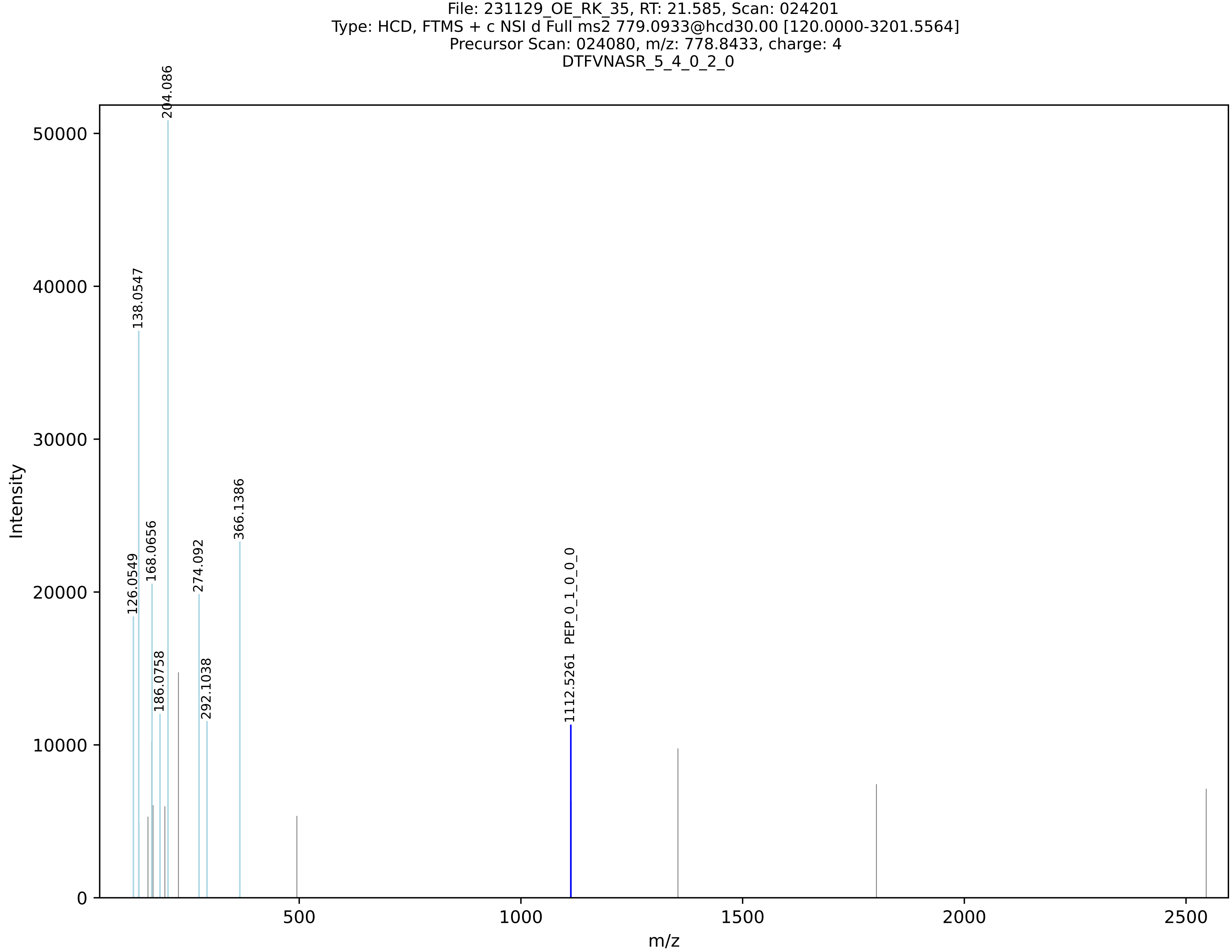
File: 231129_OE_RK_35, RT: 21.585, Scan: 024201
Type: HCD, FTMS + c NSI d Full ms2 779.0933@hcd30.00 [120.0000-3201.5564]
Precursor Scan: 024080, m/z: 778.8433, charge: 4
DTFVNASR_5_4_0_2_0
204.086
138.0547
366.1386
168.0656
274.092
126.0549
186.0758
292.1038
1112.5261 PEP_0_1_0_0_0
Intensity
m/z
0
10000
20000
30000
40000
50000
500
1000
1500
2000
2500

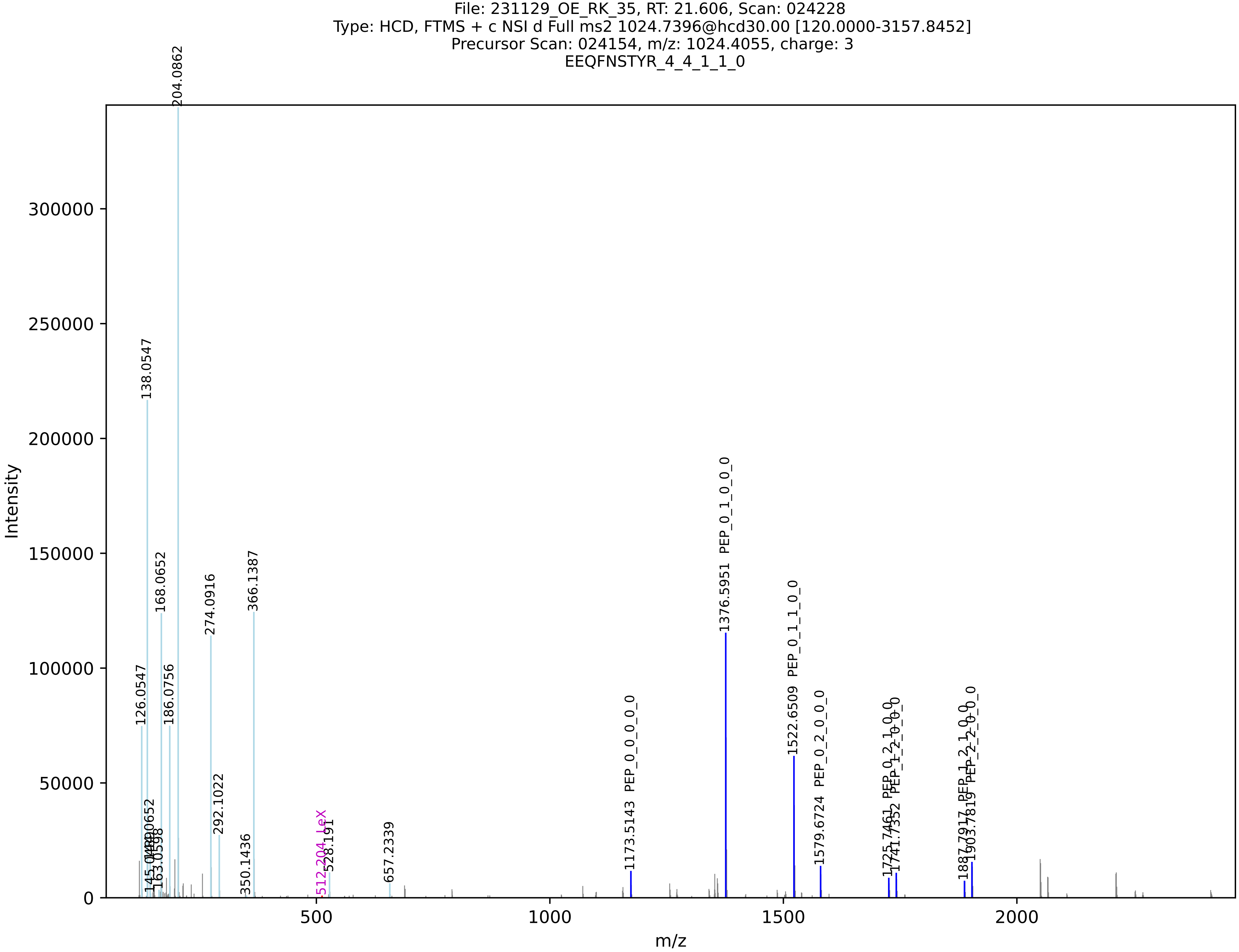

File: 231129_OE_RK_35, RT: 21.606, Scan: 024228
Type: HCD, FTMS + c NSI d Full ms2 1024.7396@hcd30.00 [120.0000-3157.8452]
Precursor Scan: 024154, m/z: 1024.4055, charge: 3
EEQFNSTYR_4_4_1_1_0
Intensity
m/z
204.0862
138.0547
168.0652
274.0916
366.1387
126.0547
186.0756
292.1022
350.1436
163.0598
512.204_LeX
528.191
657.2339
1173.5143 PEP_0_0_0_0_0
1376.5951 PEP_0_1_0_0_0
1522.6509 PEP_0_1_1_0_0
1579.6724 PEP_0_2_0_0_0
1741.7352 PEP_1_2_0_0_0
1903.7819 PEP_2_2_0_0_0

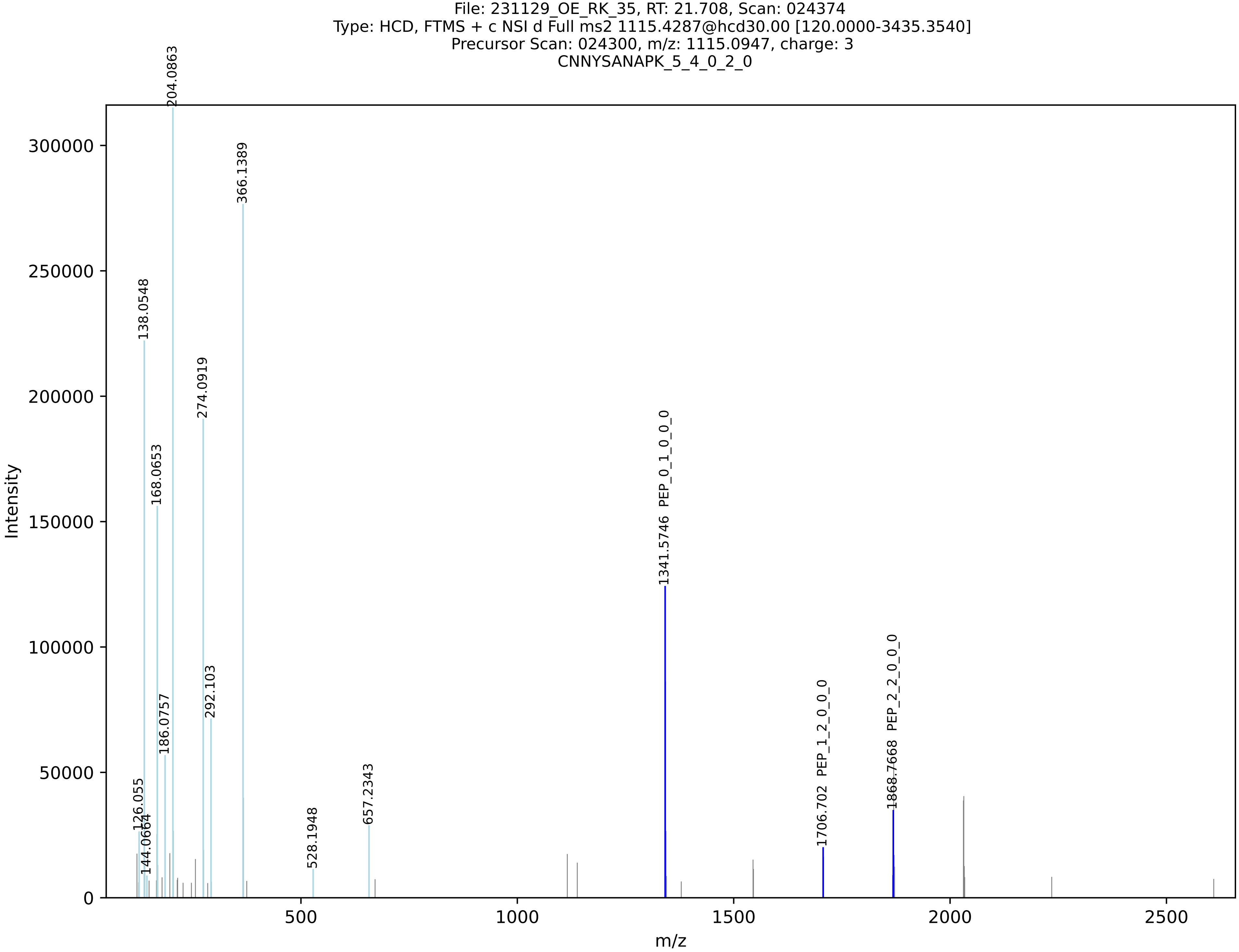

File: 231129_OE_RK_35, RT: 21.708, Scan: 024374
Type: HCD, FTMS + c NSI d Full ms2 1115.4287@hcd30.00 [120.0000-3435.3540]
Precursor Scan: 024300, m/z: 1115.0947, charge: 3
CNNYSANAPK_5_4_0_2_0
Intensity
m/z
204.0863
366.1389
138.0548
274.0919
168.0653
292.103
186.0757
126.055
144.0664
528.1948
657.2343
1341.5746 PEP_0_1_0_0_0
1706.702 PEP_1_2_0_0_0
1868.7668 PEP_2_2_0_0_0

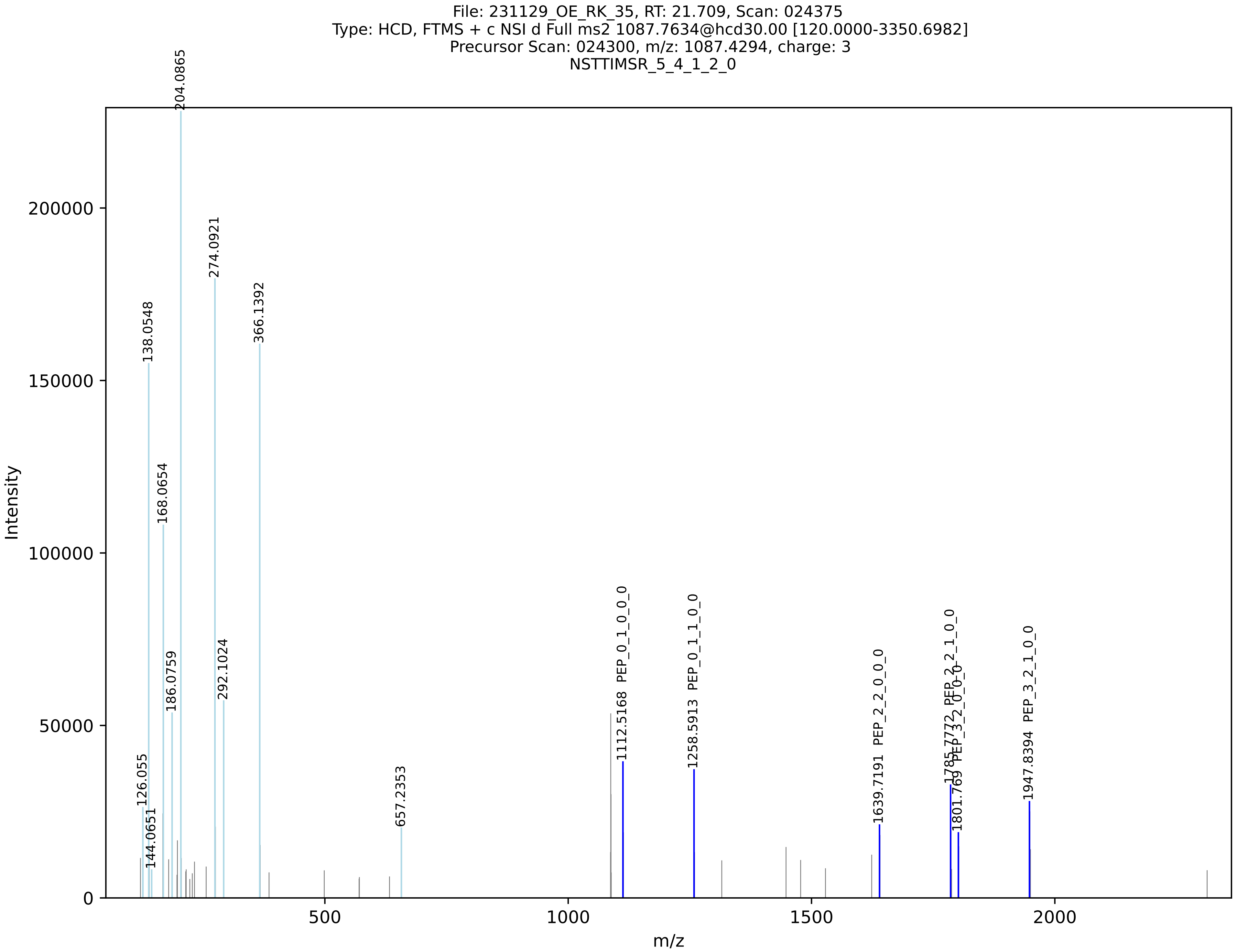
File: 231129_OE_RK_35, RT: 21.709, Scan: 024375
Type: HCD, FTMS + c NSI d Full ms2 1087.7634@hcd30.00 [120.0000-3350.6982]
Precursor Scan: 024300, m/z: 1087.4294, charge: 3
NSTTIMSR_5_4_1_2_0
204.0865
274.0921
366.1392
138.0548
168.0654
186.0759
292.1024
126.055
144.0651
657.2353
1112.5168 PEP_0_1_0_0_0
1258.5913 PEP_0_1_1_0_0
1639.7191 PEP_2_2_0_0_0
1785.7772 PEP_2_2_1_0_0
1801.769 PEP_3_2_0_0_0
1947.8394 PEP_3_2_1_0_0
Intensity
m/z
0
50000
100000
150000
200000
500
1000
1500
2000

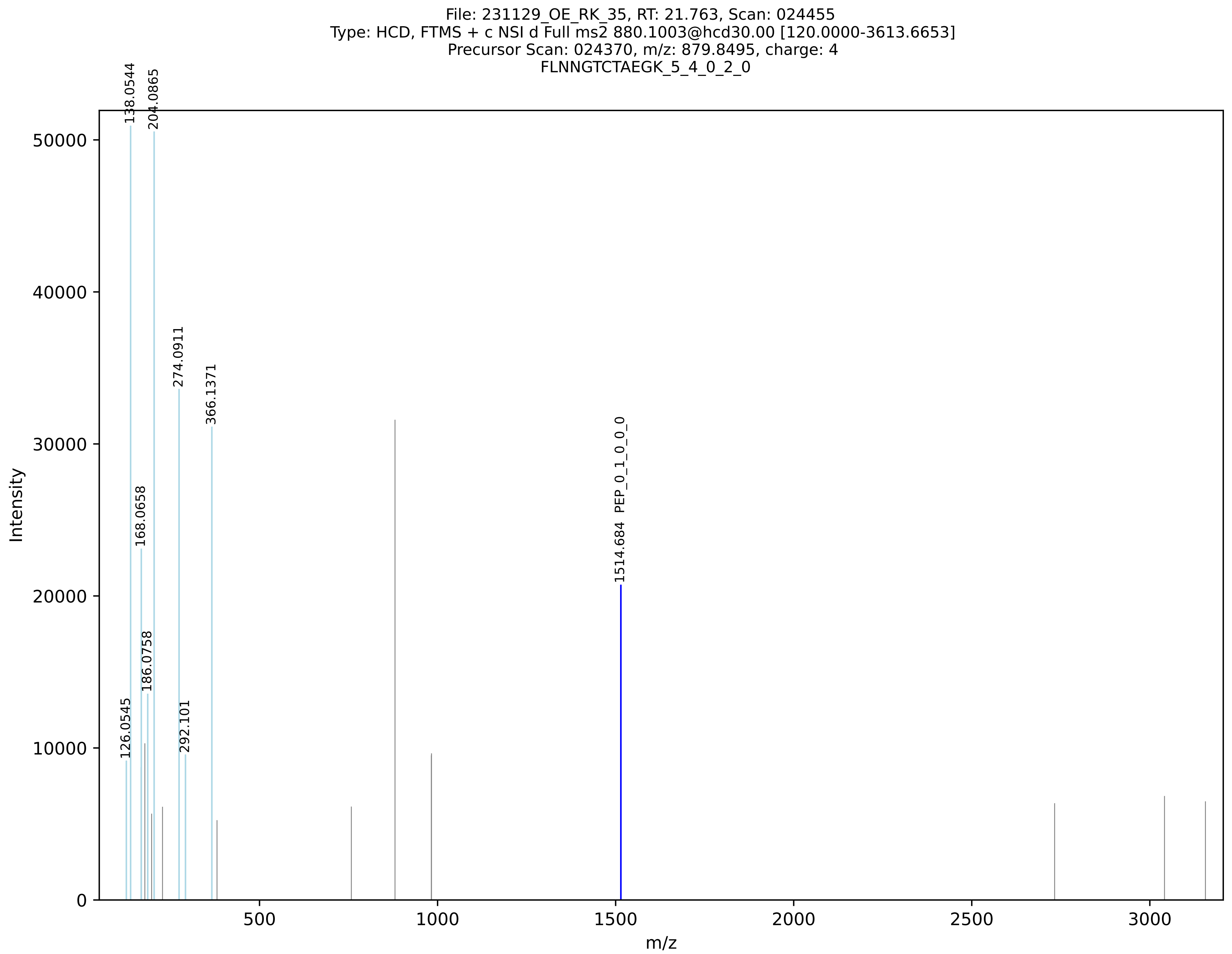

File: 231129_OE_RK_35, RT: 21.763, Scan: 024455
Type: HCD, FTMS + c NSI d Full ms2 880.1003@hcd30.00 [120.0000-3613.6653]
Precursor Scan: 024370, m/z: 879.8495, charge: 4
FLNNGTCTAEGK_5_4_0_2_0
138.0544
204.0865
274.0911
366.1371
168.0658
186.0758
126.0545
292.101
1514.684 PEP_0_1_0_0_0
Intensity
m/z
0
10000
20000
30000
40000
50000
500
1000
1500
2000
2500
3000

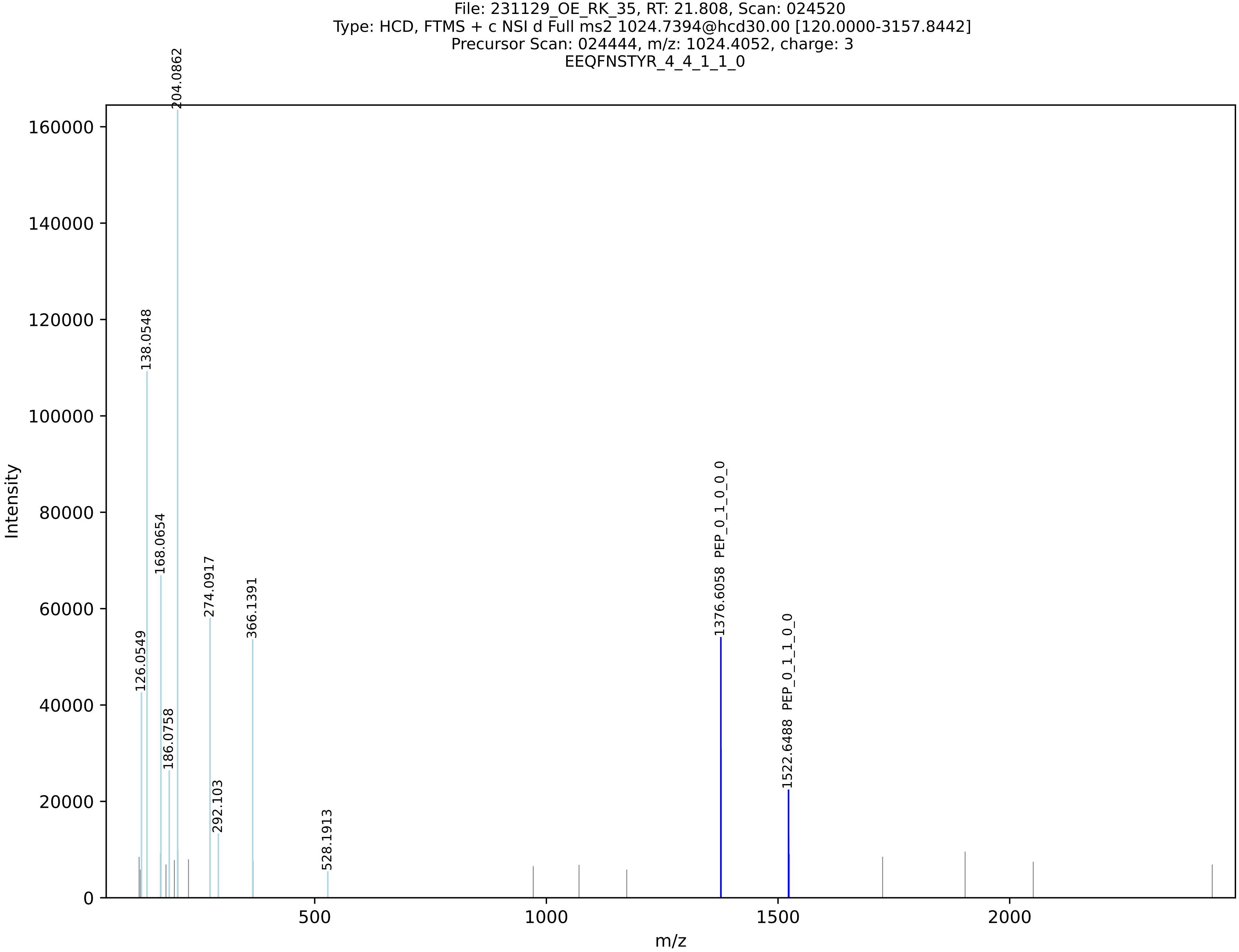
File: 231129_OE_RK_35, RT: 21.808, Scan: 024520
Type: HCD, FTMS + c NSI d Full ms2 1024.7394@hcd30.00 [120.0000-3157.8442]
Precursor Scan: 024444, m/z: 1024.4052, charge: 3
EEQFNSTYR_4_4_1_1_0
Intensity
m/z
204.0862
138.0548
168.0654
126.0549
186.0758
274.0917
292.103
366.1391
528.1913
1376.6058 PEP_0_1_0_0_0
1522.6488 PEP_0_1_1_0_0
0
20000
40000
60000
80000
100000
120000
140000
160000
500
1000
1500
2000

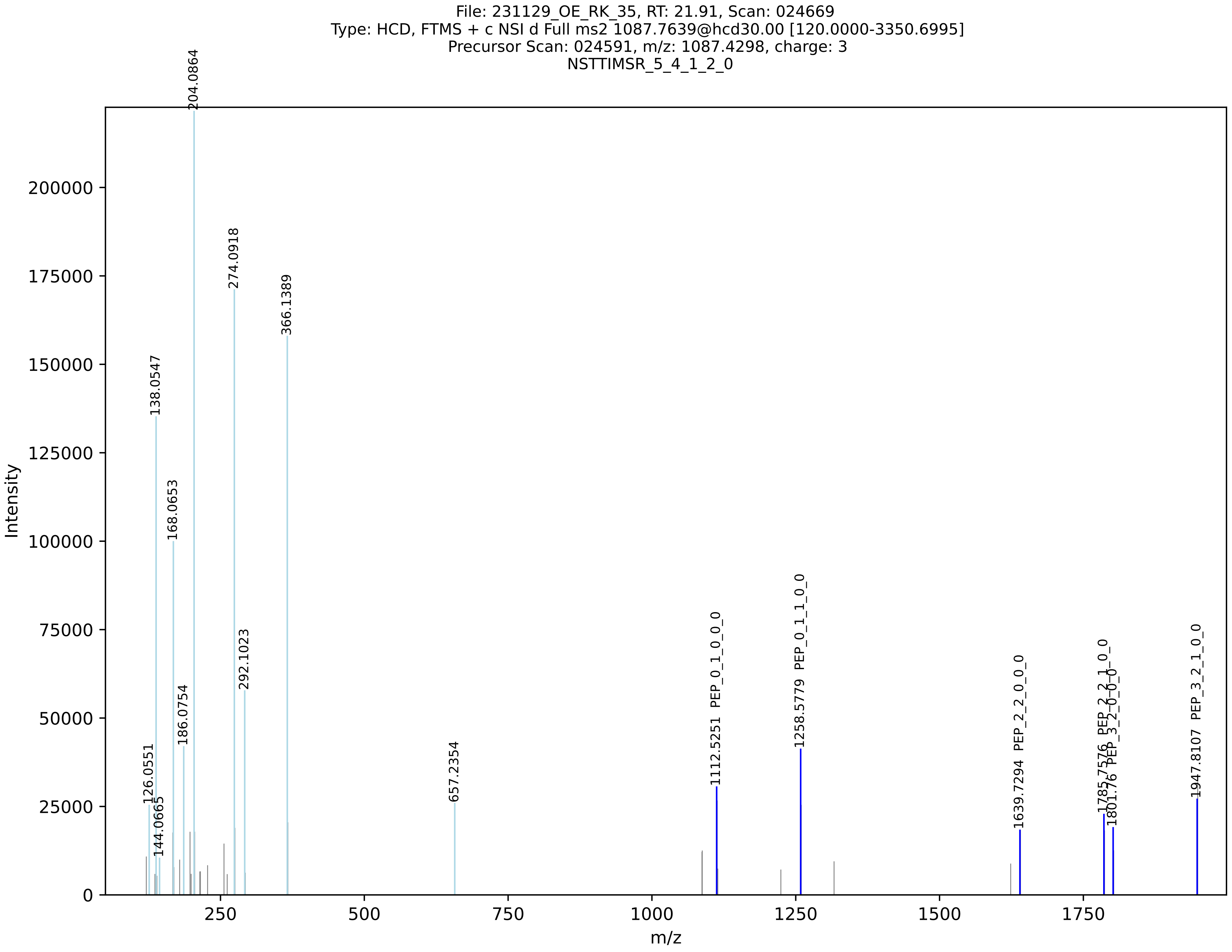

File: 231129_OE_RK_35, RT: 21.91, Scan: 024669
Type: HCD, FTMS + c NSI d Full ms2 1087.7639@hcd30.00 [120.0000-3350.6995]
Precursor Scan: 024591, m/z: 1087.4298, charge: 3
NSTTIMSR_5_4_1_2_0
Intensity
m/z
204.0864
274.0918
366.1389
138.0547
168.0653
292.1023
186.0754
126.0551
144.0665
657.2354
1112.5251 PEP_0_1_0_0_0
1258.5779 PEP_0_1_1_0_0
1639.7294 PEP_2_2_0_0_0
1785.7576 PEP_2_2_1_0_0
1801.76 PEP_3_2_0_0_0
1947.8107 PEP_3_2_1_0_0

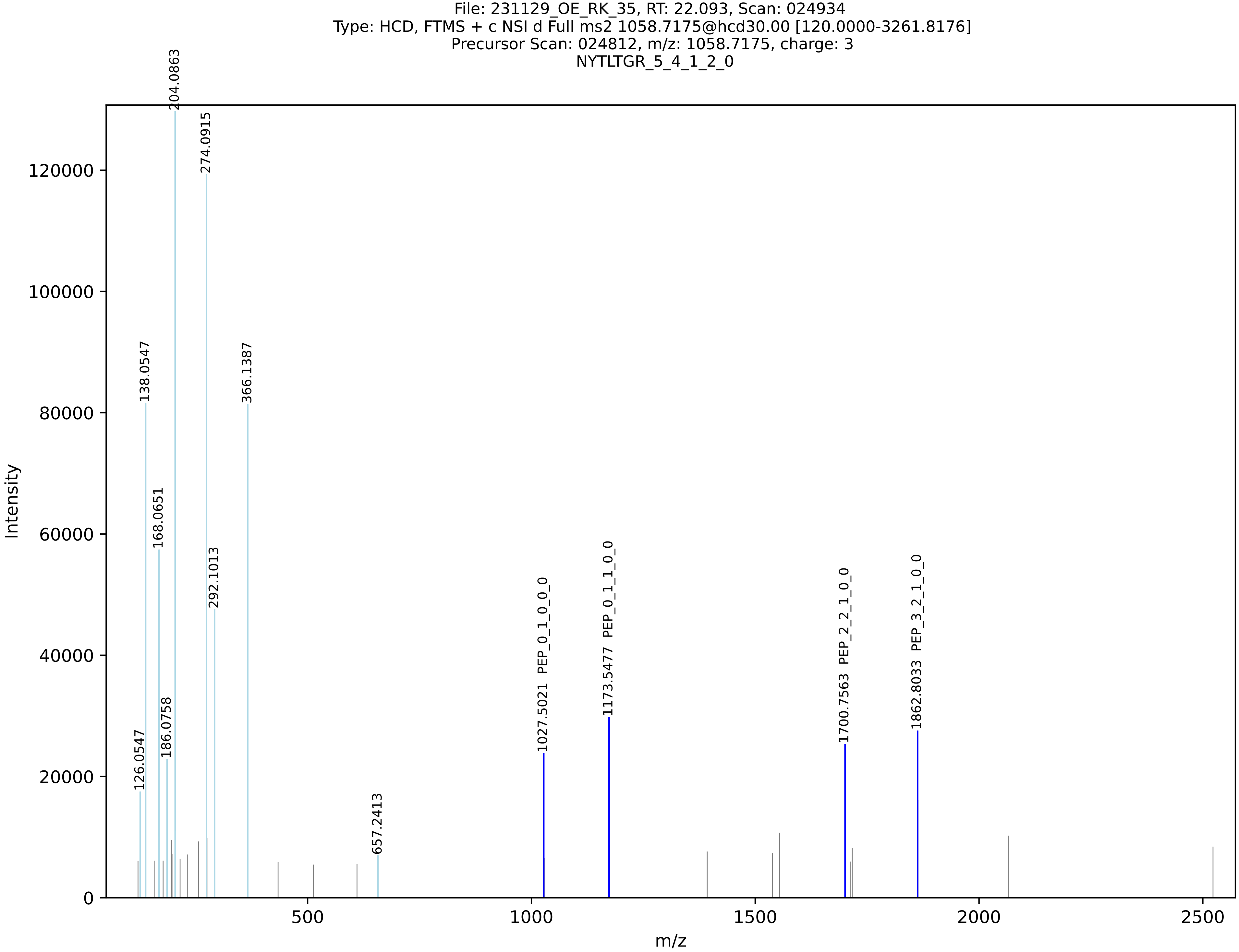
File: 231129_OE_RK_35, RT: 22.093, Scan: 024934
Type: HCD, FTMS + c NSI d Full ms2 1058.7175@hcd30.00 [120.0000-3261.8176]
Precursor Scan: 024812, m/z: 1058.7175, charge: 3
NYTLTGR_5_4_1_2_0
Intensity
m/z
204.0863
274.0915
138.0547
366.1387
168.0651
292.1013
186.0758
126.0547
657.2413
1027.5021 PEP_0_1_0_0_0
1173.5477 PEP_0_1_1_0_0
1700.7563 PEP_2_2_1_0_0
1862.8033 PEP_3_2_1_0_0
0
20000
40000
60000
80000
100000
120000
500
1000
1500
2000
2500

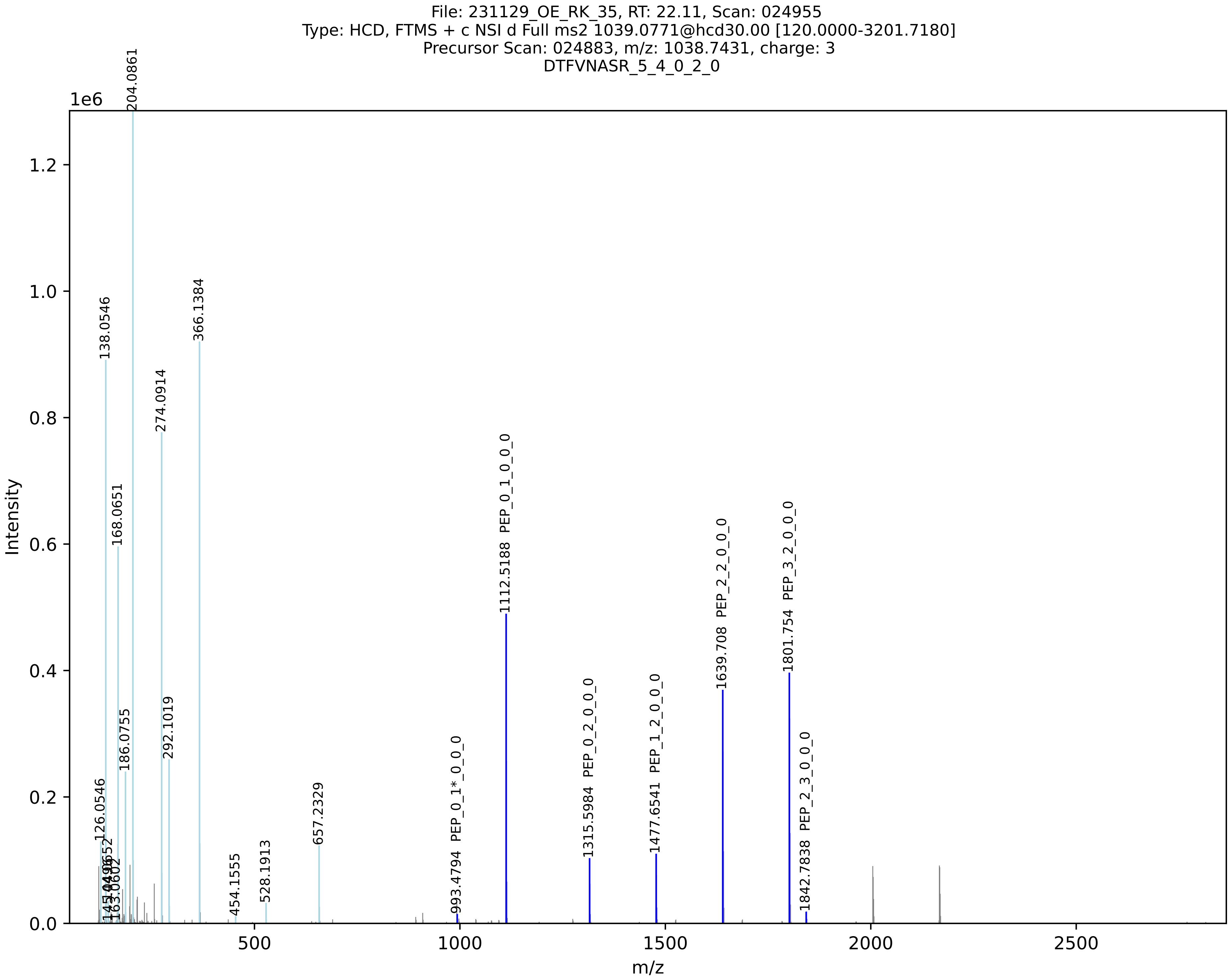
File: 231129_OE_RK_35, RT: 22.11, Scan: 024955
Type: HCD, FTMS + c NSI d Full ms2 1039.0771@hcd30.00 [120.0000-3201.7180]
Precursor Scan: 024883, m/z: 1038.7431, charge: 3
DTFVNASR_5_4_0_2_0
1e6
Intensity
m/z
204.0861
138.0546
366.1384
274.0914
168.0651
186.0755
292.1019
126.0546
163.0602
454.1555
528.1913
657.2329
993.4794 PEP_0_1*_0_0_0
1112.5188 PEP_0_1_0_0_0
1315.5984 PEP_0_2_0_0_0
1477.6541 PEP_1_2_0_0_0
1639.708 PEP_2_2_0_0_0
1801.754 PEP_3_2_0_0_0
1842.7838 PEP_2_3_0_0_0

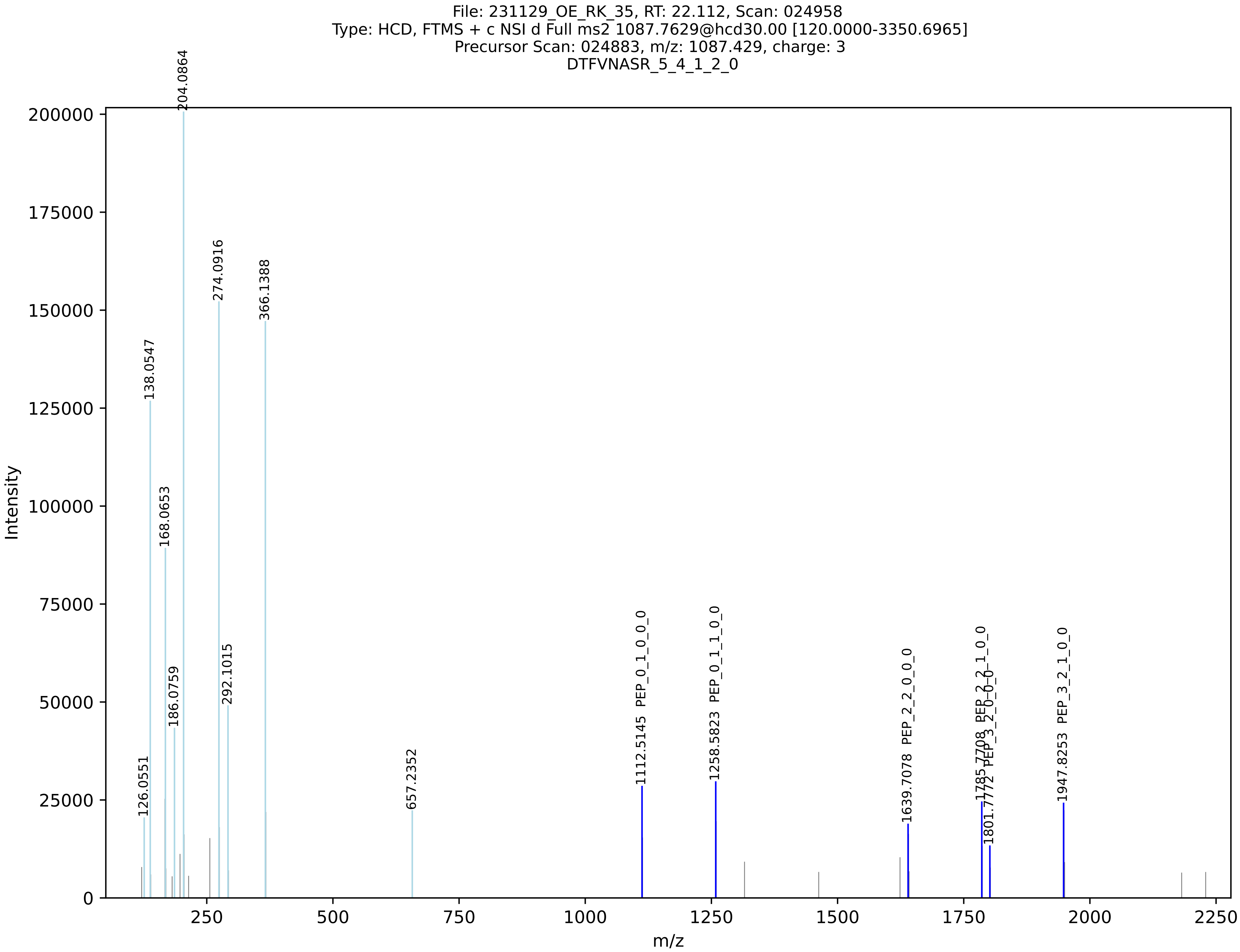
File: 231129_OE_RK_35, RT: 22.112, Scan: 024958
Type: HCD, FTMS + c NSI d Full ms2 1087.7629@hcd30.00 [120.0000-3350.6965]
Precursor Scan: 024883, m/z: 1087.429, charge: 3
DTFVNASR_5_4_1_2_0
Intensity
m/z
0
25000
50000
75000
100000
125000
150000
175000
200000
250
500
750
1000
1250
1500
1750
2000
2250
204.0864
274.0916
366.1388
138.0547
168.0653
186.0759
292.1015
126.0551
657.2352
1112.5145 PEP_0_1_0_0_0
1258.5823 PEP_0_1_1_0_0
1639.7078 PEP_2_2_0_0_0
1785.7708 PEP_2_2_1_0_0
1801.7772 PEP_3_2_0_0_0
1947.8253 PEP_3_2_1_0_0

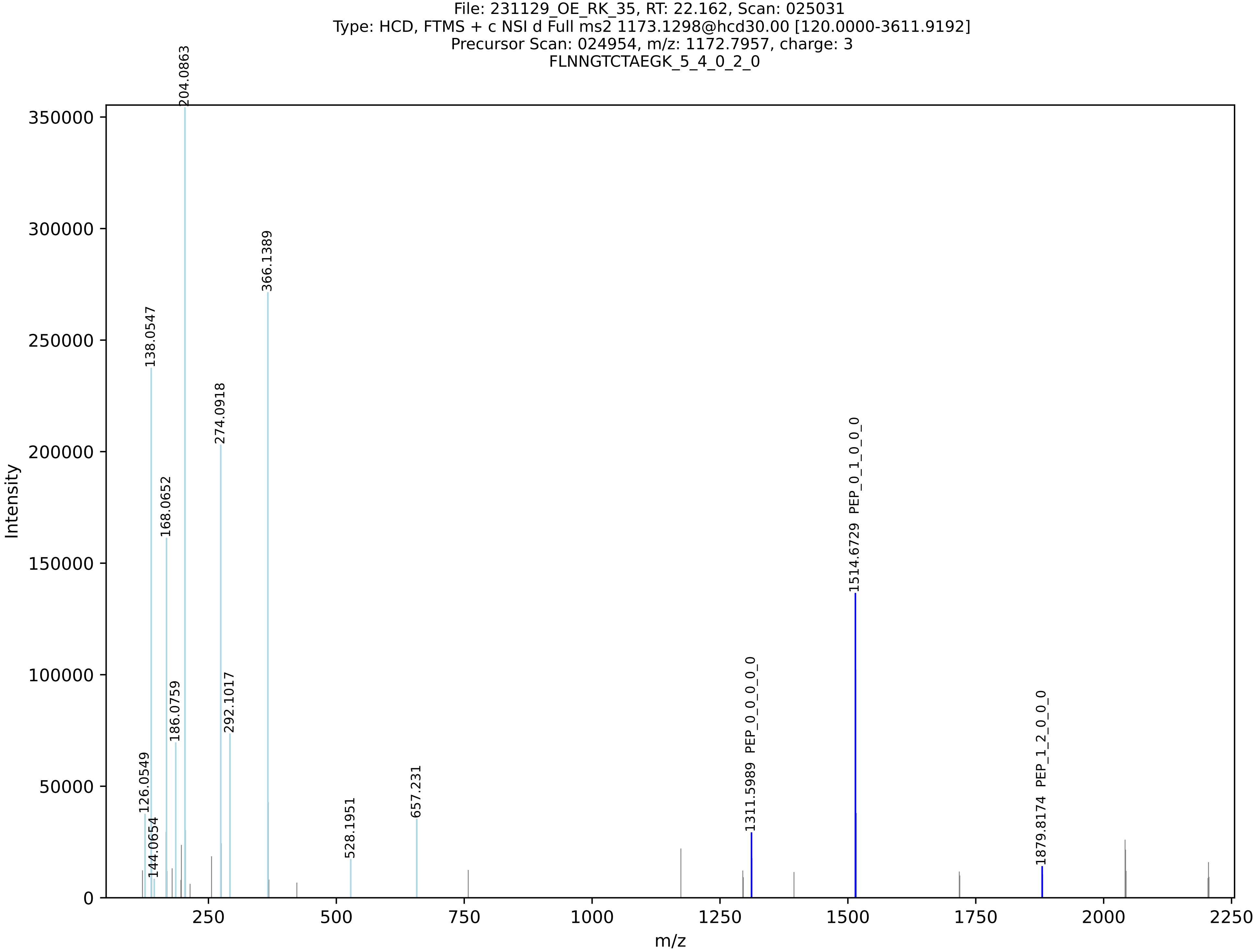

File: 231129_OE_RK_35, RT: 22.162, Scan: 025031
Type: HCD, FTMS + c NSI d Full ms2 1173.1298@hcd30.00 [120.0000-3611.9192]
Precursor Scan: 024954, m/z: 1172.7957, charge: 3
FLNNGTCTAEGK_5_4_0_2_0
Intensity
m/z
204.0863
366.1389
138.0547
274.0918
168.0652
186.0759
292.1017
126.0549
144.0654
528.1951
657.231
1311.5989 PEP_0_0_0_0_0
1514.6729 PEP_0_1_0_0_0
1879.8174 PEP_1_2_0_0_0

File: 231129_OE_RK_35, RT: 22.182, Scan: 025063
Type: HCD, FTMS + c NSI d Full ms2 1295.5305@hcd30.00 [120.0000-3986.4656]
Precursor Scan: 024954, m/z: 1295.1991, charge: 3
VVNCSDPGFVENAIR_5_4_0_2_0

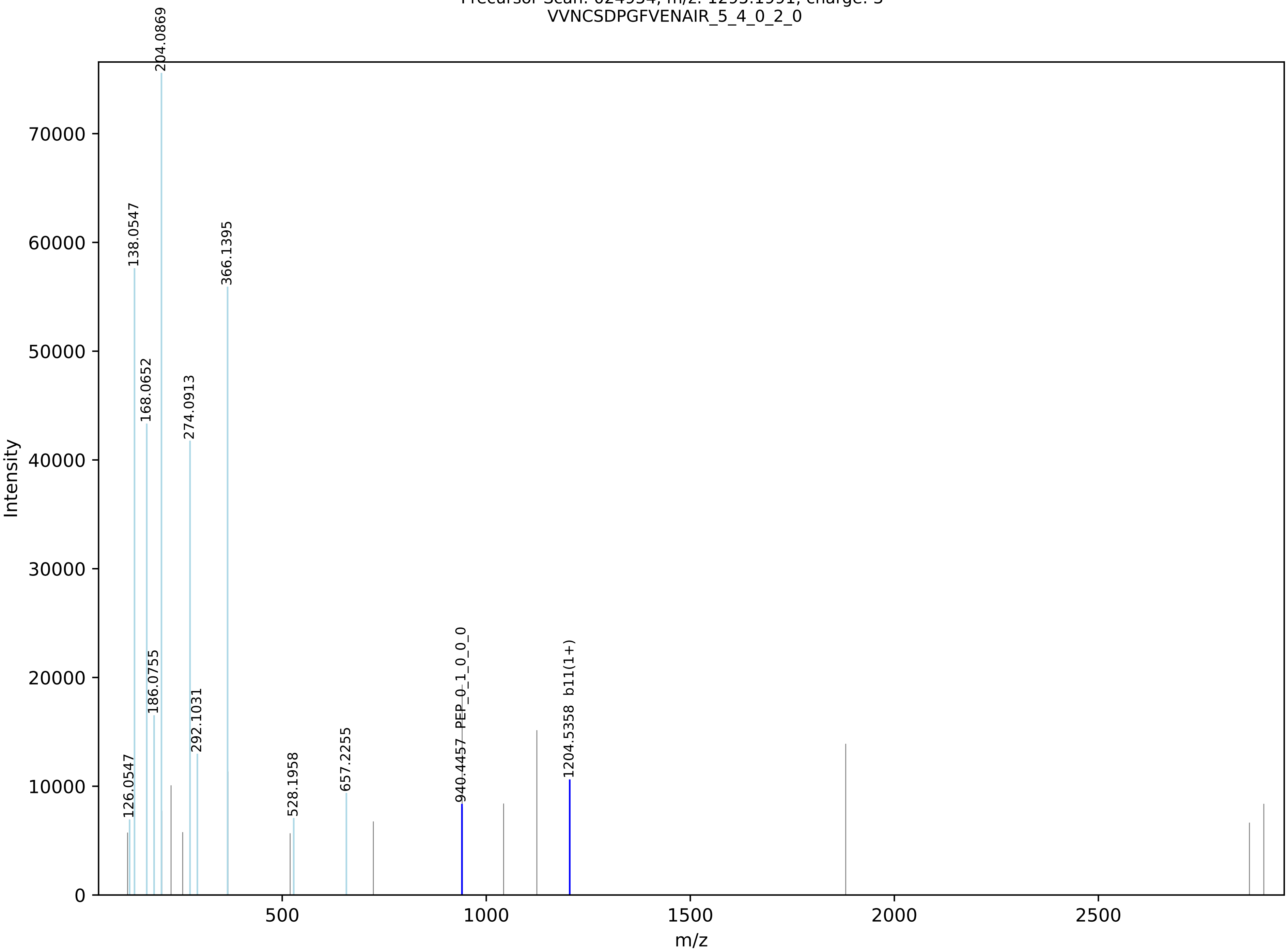

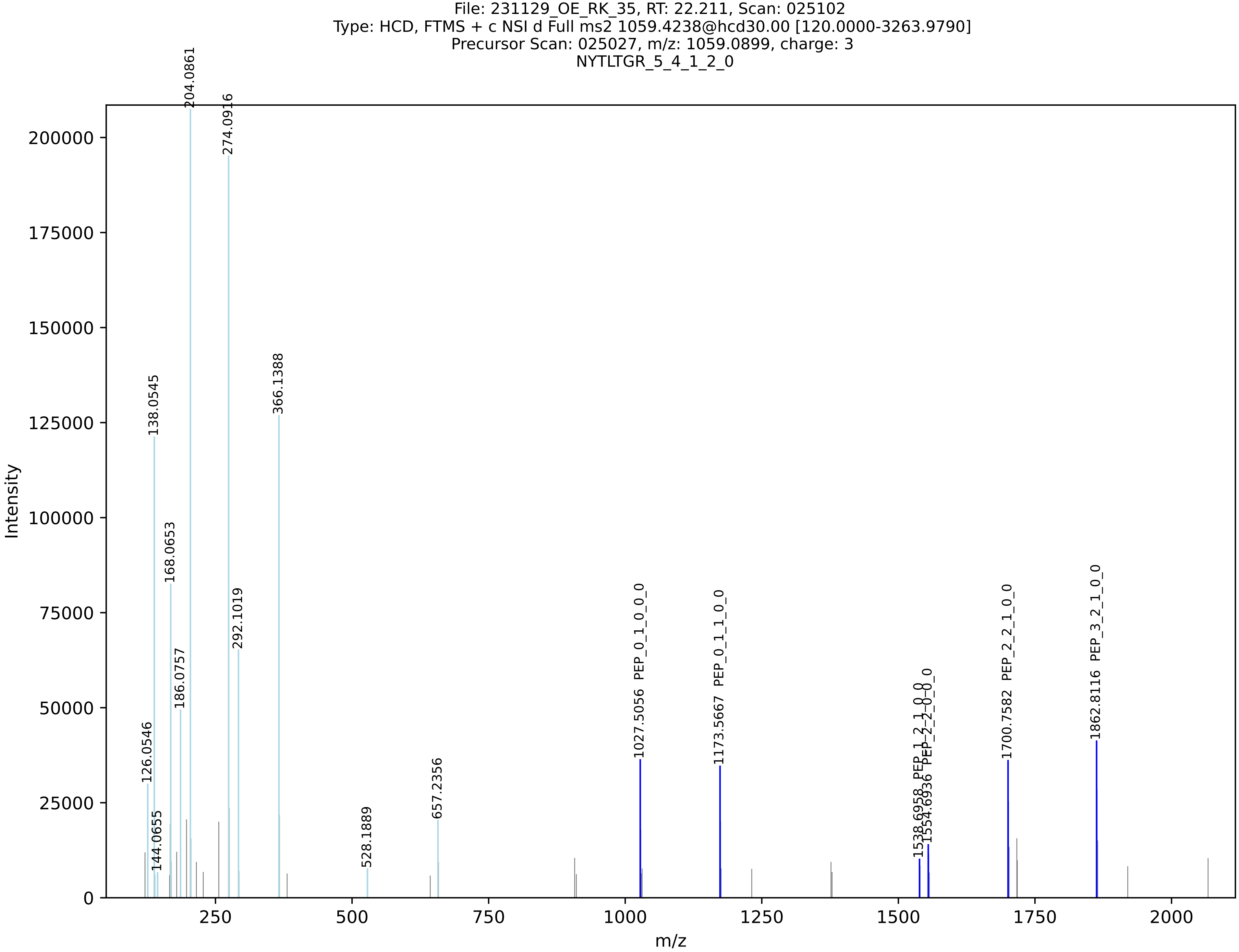

File: 231129_OE_RK_35, RT: 22.211, Scan: 025102
Type: HCD, FTMS + c NSI d Full ms2 1059.4238@hcd30.00 [120.0000-3263.9790]
Precursor Scan: 025027, m/z: 1059.0899, charge: 3
NYTLTGR_5_4_1_2_0
Intensity
m/z
204.0861
274.0916
366.1388
138.0545
168.0653
292.1019
186.0757
126.0546
144.0655
528.1889
657.2356
1027.5056 PEP_0_1_0_0_0
1173.5667 PEP_0_1_1_0_0
1538.6958 PEP_1_2_1_0_0
1554.6936 PEP_2_2_0_0_0
1700.7582 PEP_2_2_1_0_0
1862.8116 PEP_3_2_1_0_0

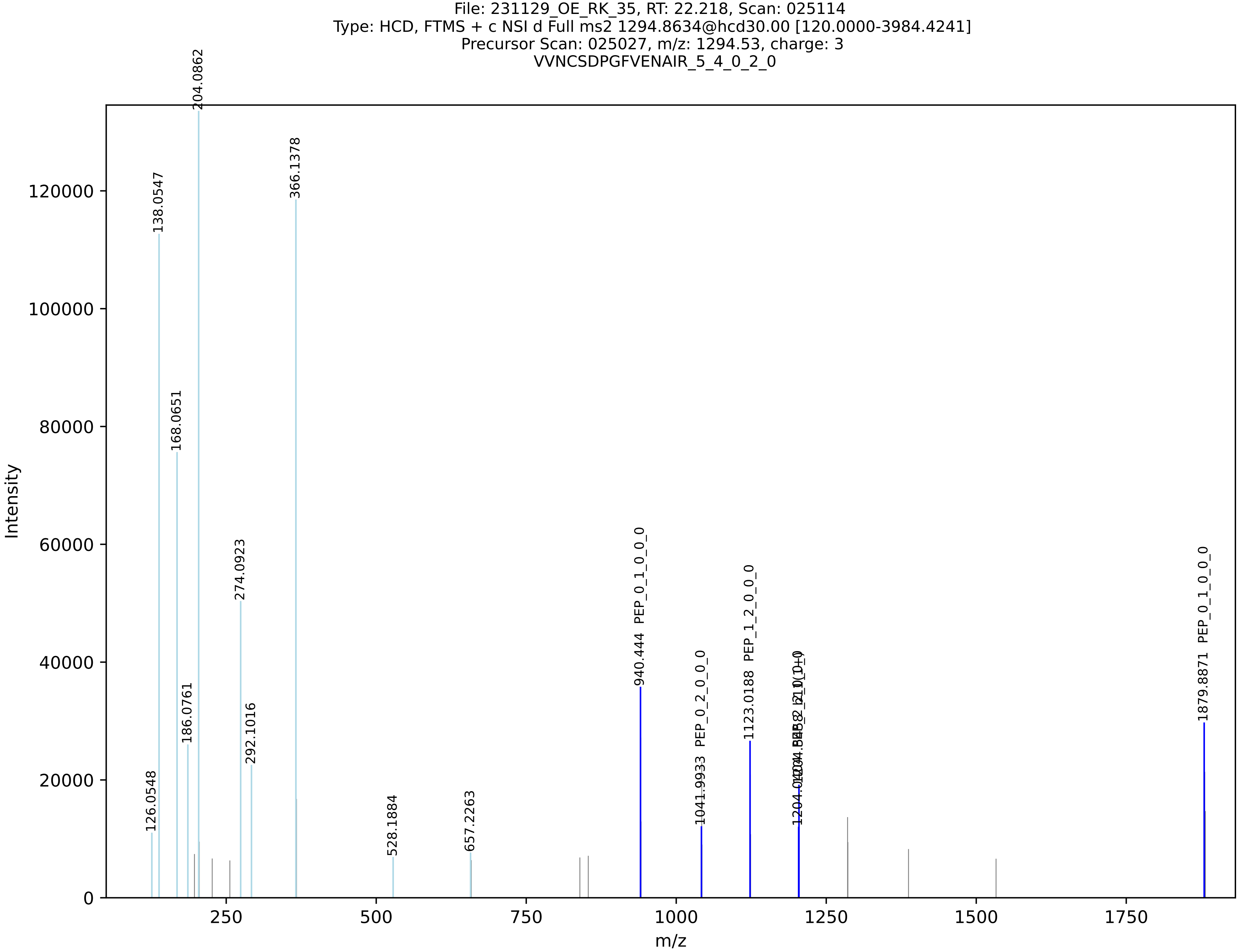

File: 231129_OE_RK_35, RT: 22.218, Scan: 025114
Type: HCD, FTMS + c NSI d Full ms2 1294.8634@hcd30.00 [120.0000-3984.4241]
Precursor Scan: 025027, m/z: 1294.53, charge: 3
VVNCSDPGFVENAIR_5_4_0_2_0
Intensity
m/z
0
20000
40000
60000
80000
100000
120000
250
500
750
1000
1250
1500
1750
126.0548
138.0547
168.0651
186.0761
204.0862
274.0923
292.1016
366.1378
528.1884
657.2263
940.444 PEP_0_1_0_0_0
1041.9933 PEP_0_2_0_0_0
1123.0188 PEP_1_2_0_0_0
1879.8871 PEP_0_1_0_0_0

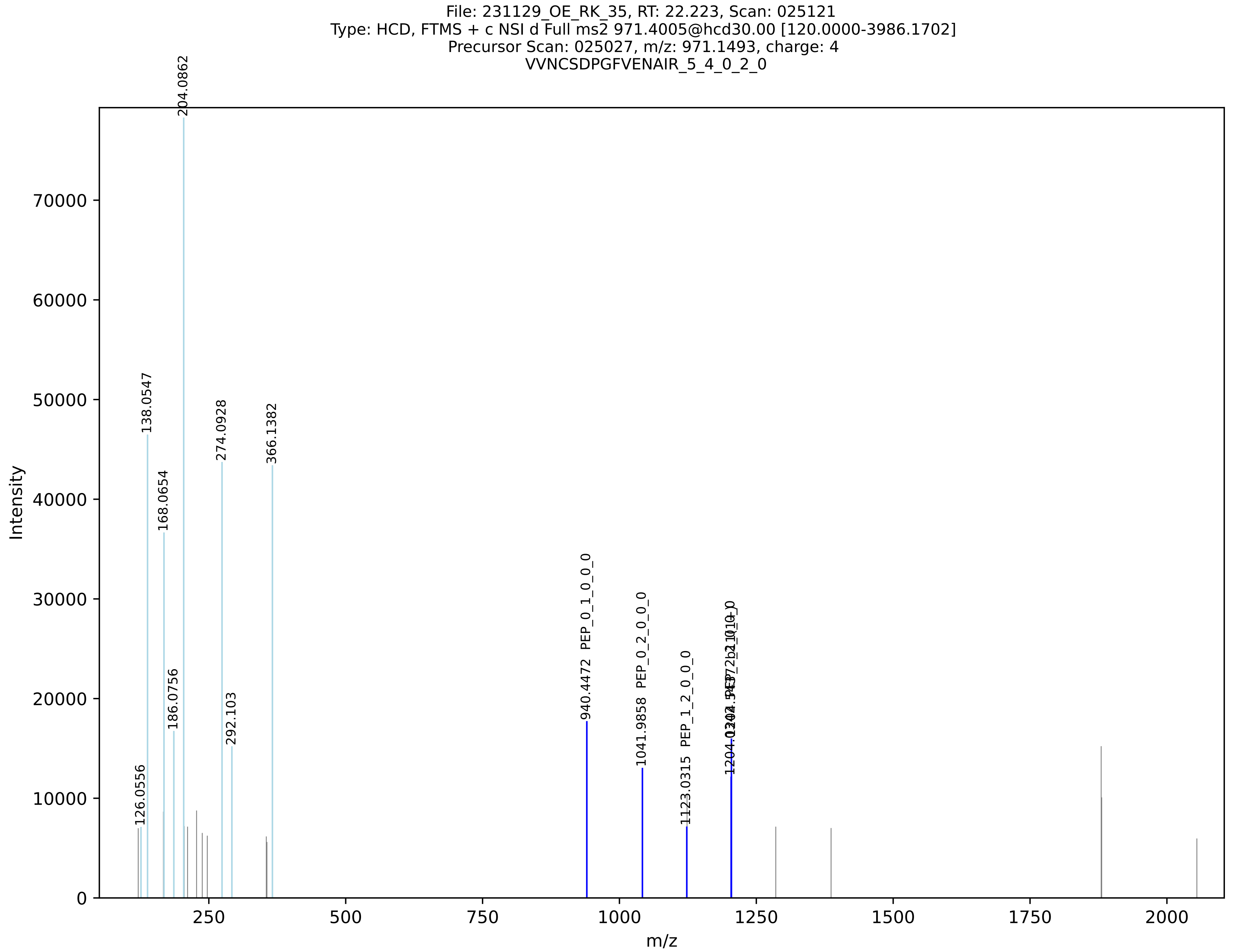
File: 231129_OE_RK_35, RT: 22.223, Scan: 025121
Type: HCD, FTMS + c NSI d Full ms2 971.4005@hcd30.00 [120.0000-3986.1702]
Precursor Scan: 025027, m/z: 971.1493, charge: 4
VVNCSDPGFVENAIR_5_4_0_2_0
204.0862
138.0547
168.0654
274.0928
366.1382
186.0756
292.103
126.0556
940.4472 PEP_0_1_0_0_0
1041.9858 PEP_0_2_0_0_0
1123.0315 PEP_1_2_0_0_0
Intensity
m/z
0
10000
20000
30000
40000
50000
60000
70000
250
500
750
1000
1250
1500
1750
2000

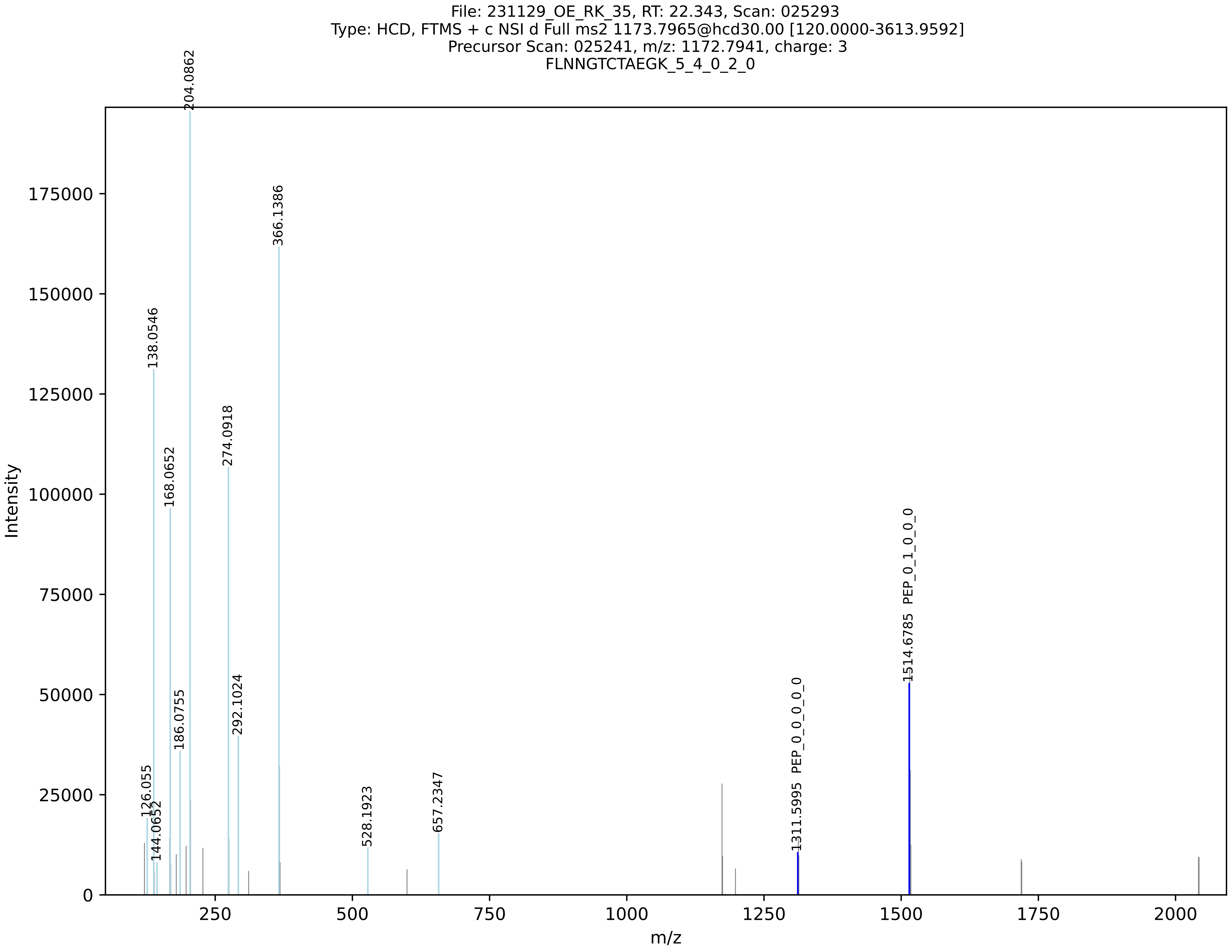

File: 231129_OE_RK_35, RT: 22.343, Scan: 025293
Type: HCD, FTMS + c NSI d Full ms2 1173.7965@hcd30.00 [120.0000-3613.9592]
Precursor Scan: 025241, m/z: 1172.7941, charge: 3
FLNNGTCTAEGK_5_4_0_2_0
Intensity
m/z
204.0862
366.1386
138.0546
274.0918
168.0652
186.0755
292.1024
126.055
144.0652
528.1923
657.2347
1311.5995 PEP_0_0_0_0_0
1514.6785 PEP_0_1_0_0_0

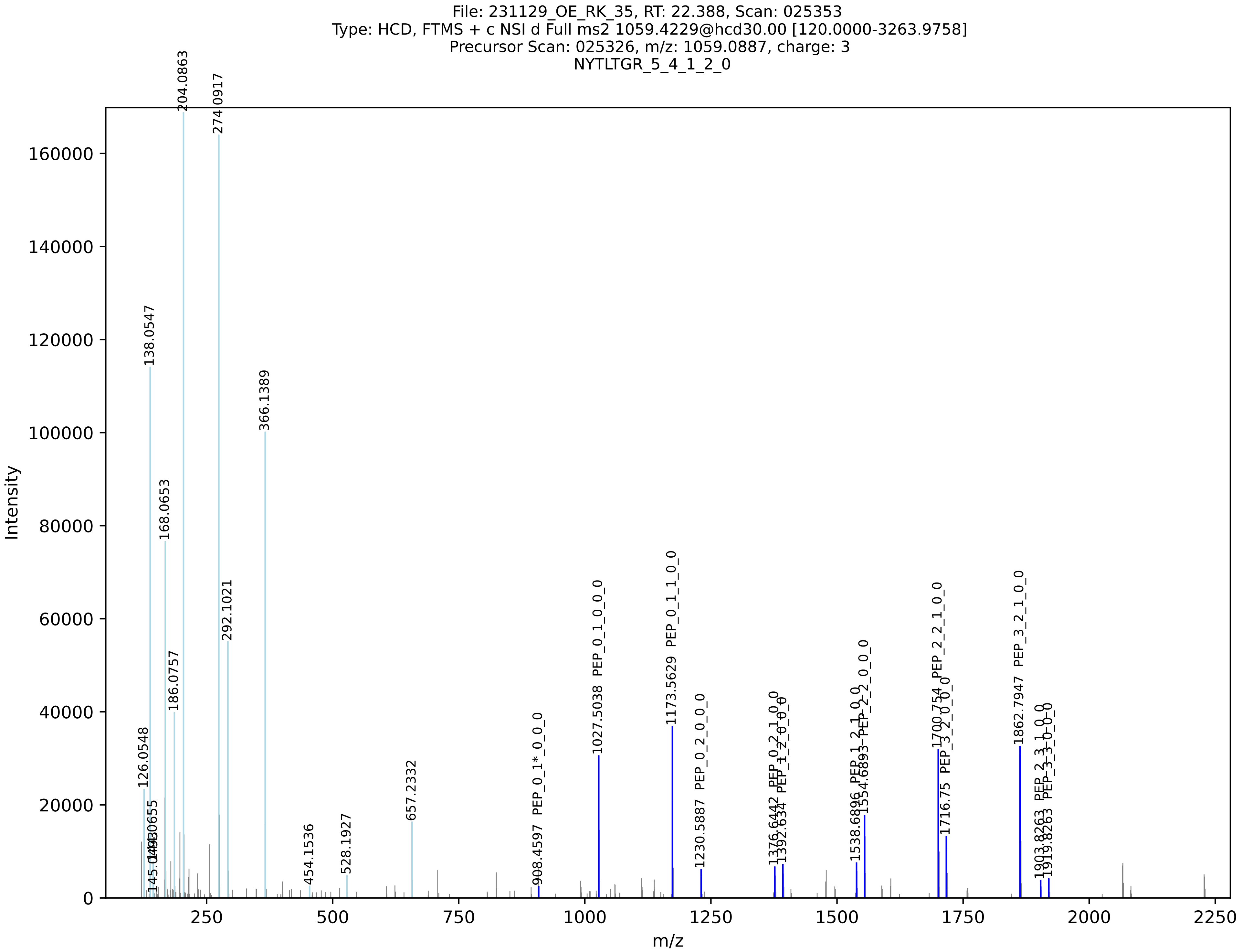
File: 231129_OE_RK_35, RT: 22.388, Scan: 025353
Type: HCD, FTMS + c NSI d Full ms2 1059.4229@hcd30.00 [120.0000-3263.9758]
Precursor Scan: 025326, m/z: 1059.0887, charge: 3
NYTLTGR_5_4_1_2_0
Intensity
m/z
0
20000
40000
60000
80000
100000
120000
140000
160000
250
500
750
1000
1250
1500
1750
2000
2250
204.0863
274.0917
138.0547
366.1389
168.0653
292.1021
186.0757
126.0548
657.2332
528.1927
454.1536
908.4597 PEP_0_1*_0_0_0
1027.5038 PEP_0_1_0_0_0
1173.5629 PEP_0_1_1_0_0
1230.5887 PEP_0_2_0_0_0
1376.6442 PEP_0_2_1_0_0
1392.634 PEP_1_2_0_0_0
1538.6896 PEP_1_2_1_0_0
1554.6893 PEP_2_2_0_0_0
1700.754 PEP_2_2_1_0_0
1716.75 PEP_3_2_0_0_0
1862.7947 PEP_3_2_1_0_0
1903.8263 PEP_2_3_1_0_0
1919.8263 PEP_3_3_0_0_0

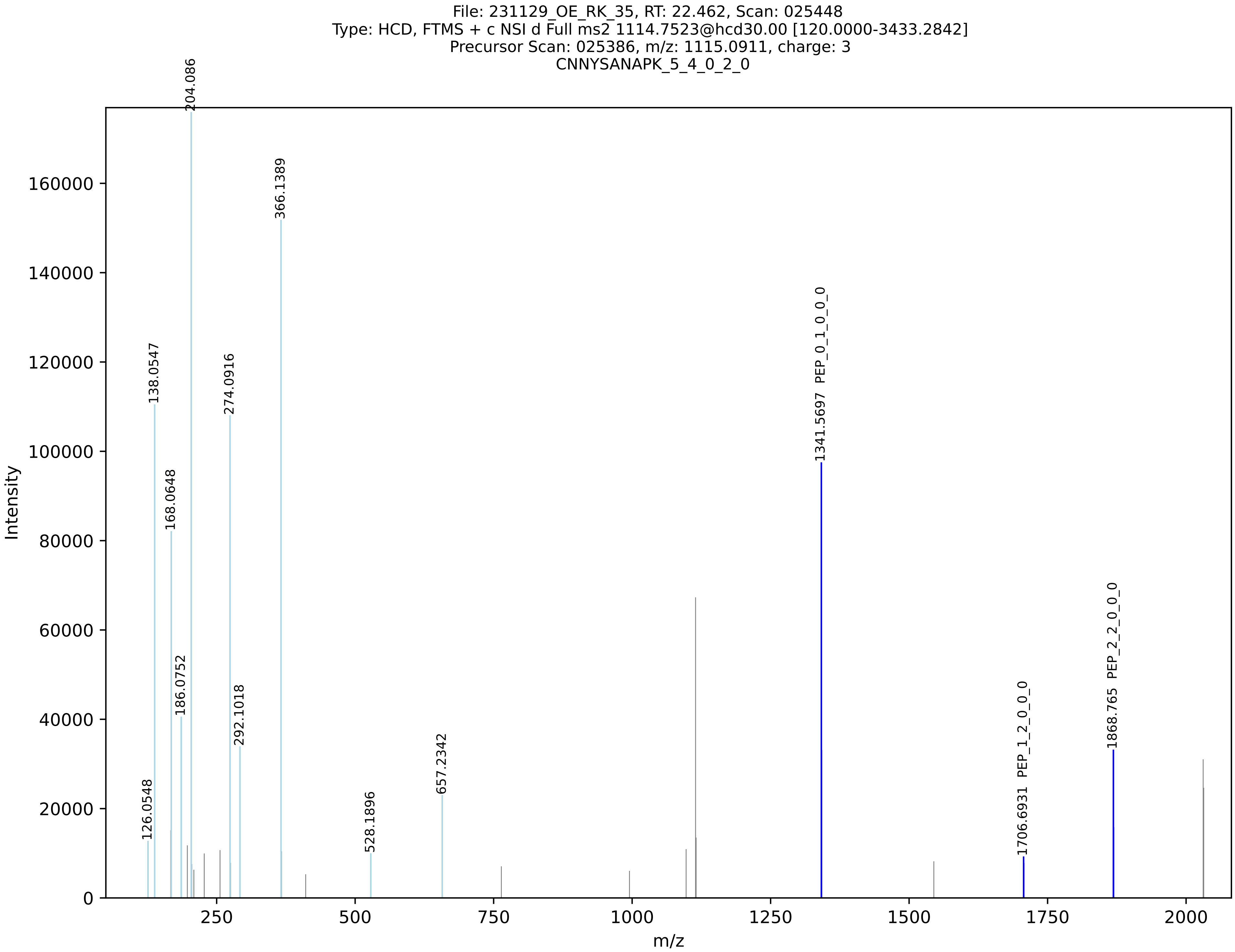

File: 231129_OE_RK_35, RT: 22.462, Scan: 025448
Type: HCD, FTMS + c NSI d Full ms2 1114.7523@hcd30.00 [120.0000-3433.2842]
Precursor Scan: 025386, m/z: 1115.0911, charge: 3
CNNYSANAPK_5_4_0_2_0
Intensity
m/z
204.086
366.1389
138.0547
274.0916
168.0648
186.0752
292.1018
126.0548
528.1896
657.2342
1341.5697 PEP_0_1_0_0_0
1706.6931 PEP_1_2_0_0_0
1868.765 PEP_2_2_0_0_0

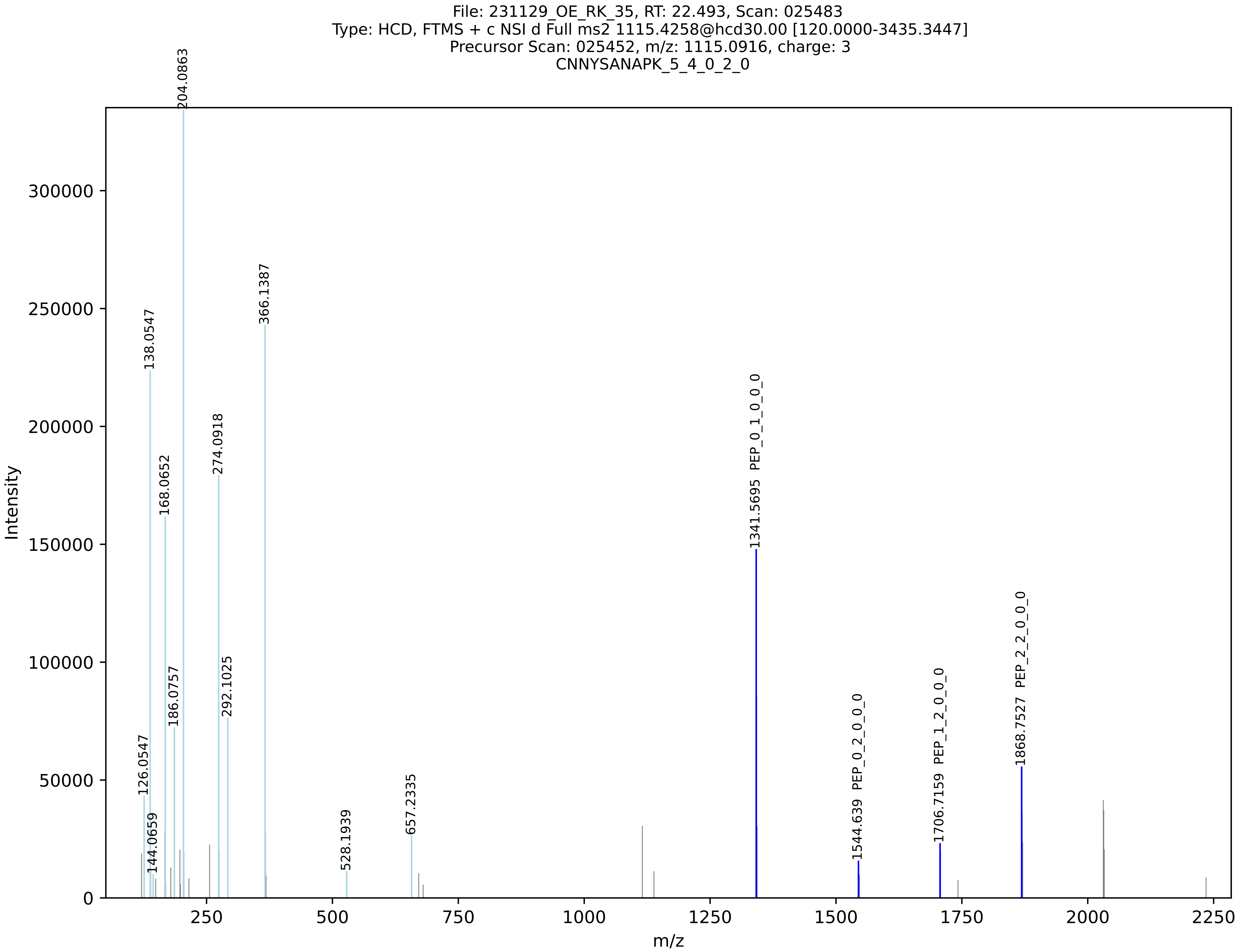

File: 231129_OE_RK_35, RT: 22.493, Scan: 025483
Type: HCD, FTMS + c NSI d Full ms2 1115.4258@hcd30.00 [120.0000-3435.3447]
Precursor Scan: 025452, m/z: 1115.0916, charge: 3
CNNYSANAPK_5_4_0_2_0
Intensity
m/z
204.0863
366.1387
138.0547
274.0918
168.0652
186.0757
292.1025
126.0547
144.0659
528.1939
657.2335
1341.5695 PEP_0_1_0_0_0
1544.639 PEP_0_2_0_0_0
1706.7159 PEP_1_2_0_0_0
1868.7527 PEP_2_2_0_0_0

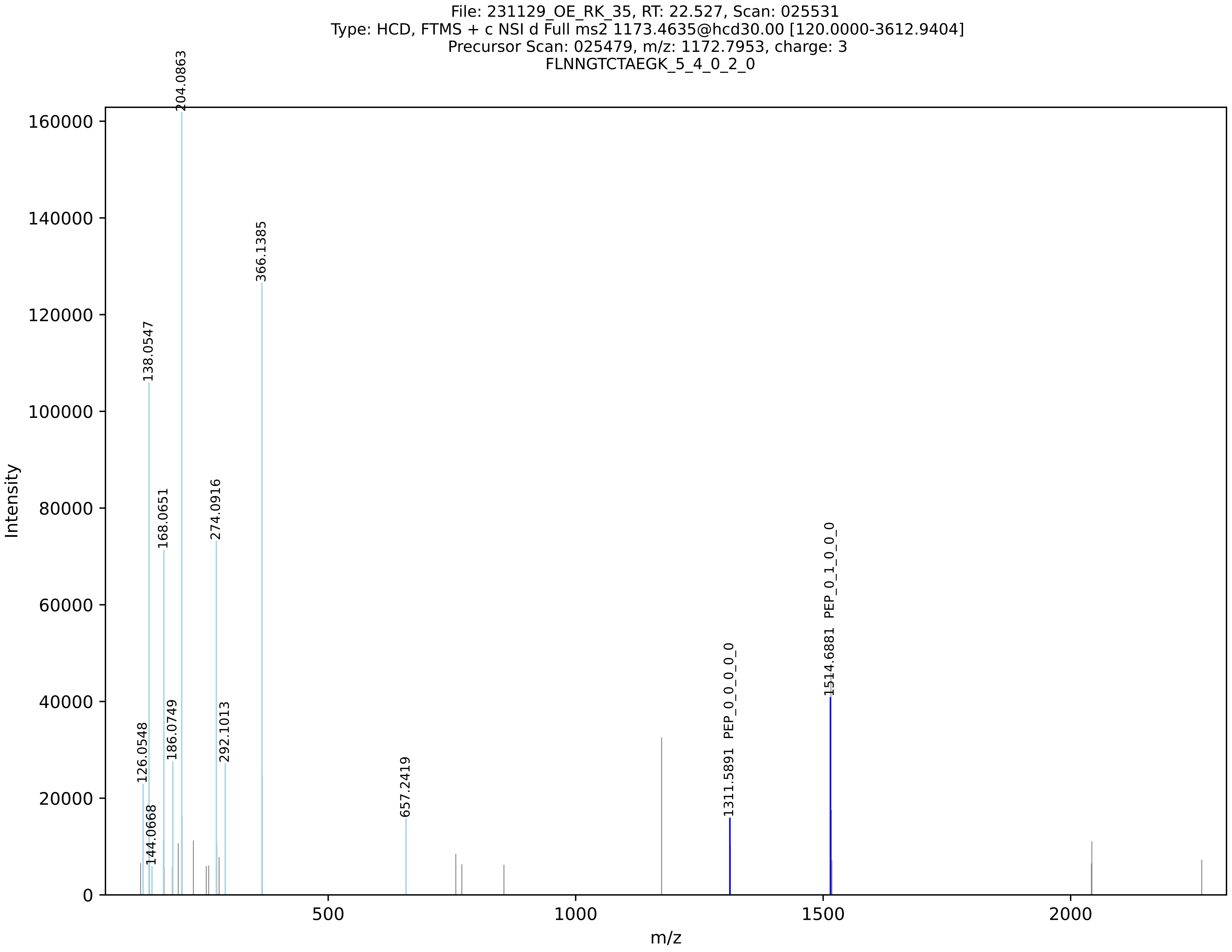
File: 231129_OE_RK_35, RT: 22.527, Scan: 025531
Type: HCD, FTMS + c NSI d Full ms2 1173.4635@hcd30.00 [120.0000-3612.9404]
Precursor Scan: 025479, m/z: 1172.7953, charge: 3
FLNNGTCTAEGK_5_4_0_2_0
Intensity
m/z
204.0863
366.1385
138.0547
168.0651
274.0916
126.0548
186.0749
292.1013
144.0668
657.2419
1311.5891 PEP_0_0_0_0_0
1514.6881 PEP_0_1_0_0_0
0
20000
40000
60000
80000
100000
120000
140000
160000
500
1000
1500
2000

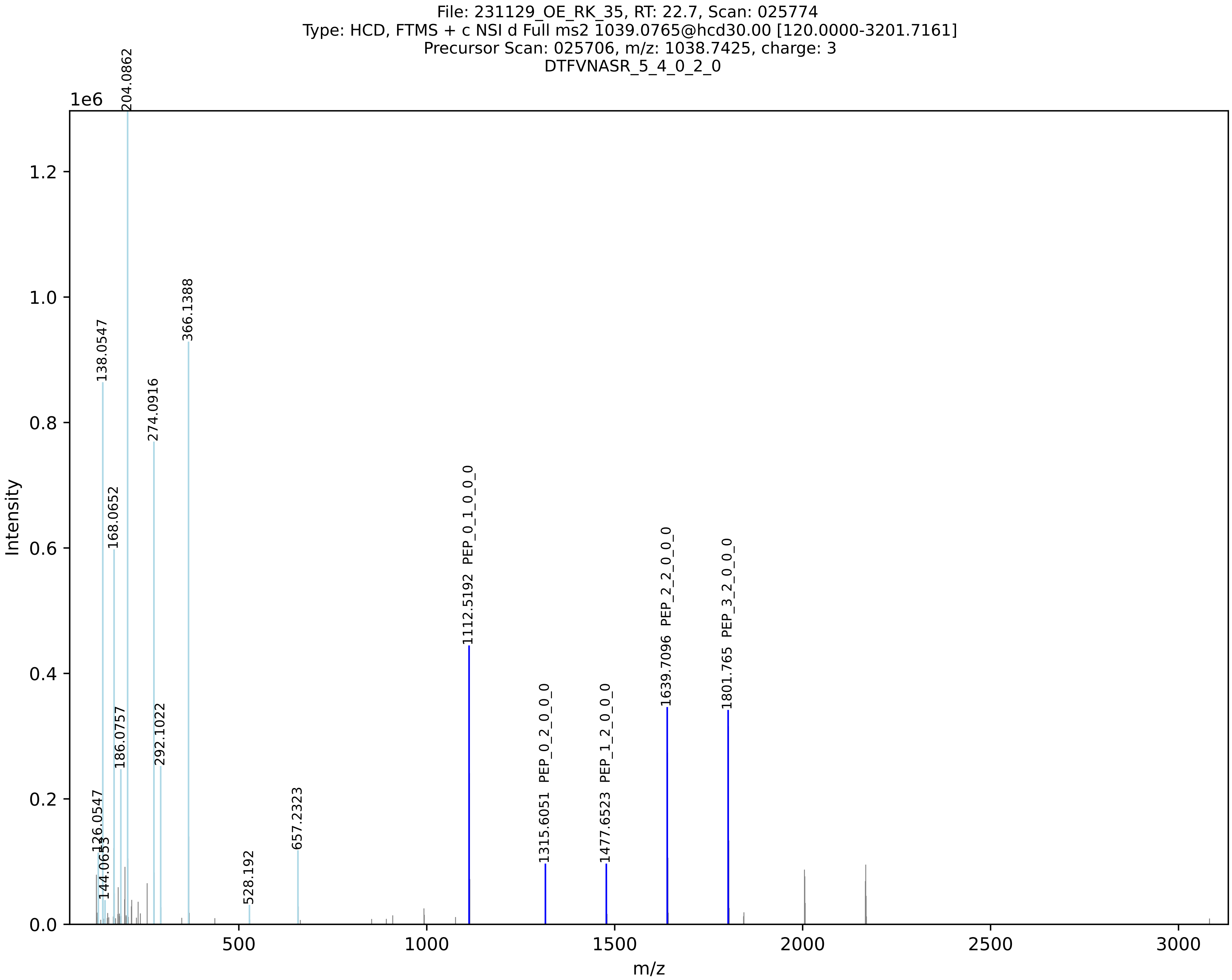
File: 231129_OE_RK_35, RT: 22.7, Scan: 025774
Type: HCD, FTMS + c NSI d Full ms2 1039.0765@hcd30.00 [120.0000-3201.7161]
Precursor Scan: 025706, m/z: 1038.7425, charge: 3
DTFVNASR_5_4_0_2_0
1e6
Intensity
m/z
0.0
0.2
0.4
0.6
0.8
1.0
1.2
500
1000
1500
2000
2500
3000
204.0862
138.0547
366.1388
274.0916
168.0652
186.0757
292.1022
126.0547
144.0653
528.192
657.2323
1112.5192 PEP_0_1_0_0_0
1315.6051 PEP_0_2_0_0_0
1477.6523 PEP_1_2_0_0_0
1639.7096 PEP_2_2_0_0_0
1801.765 PEP_3_2_0_0_0

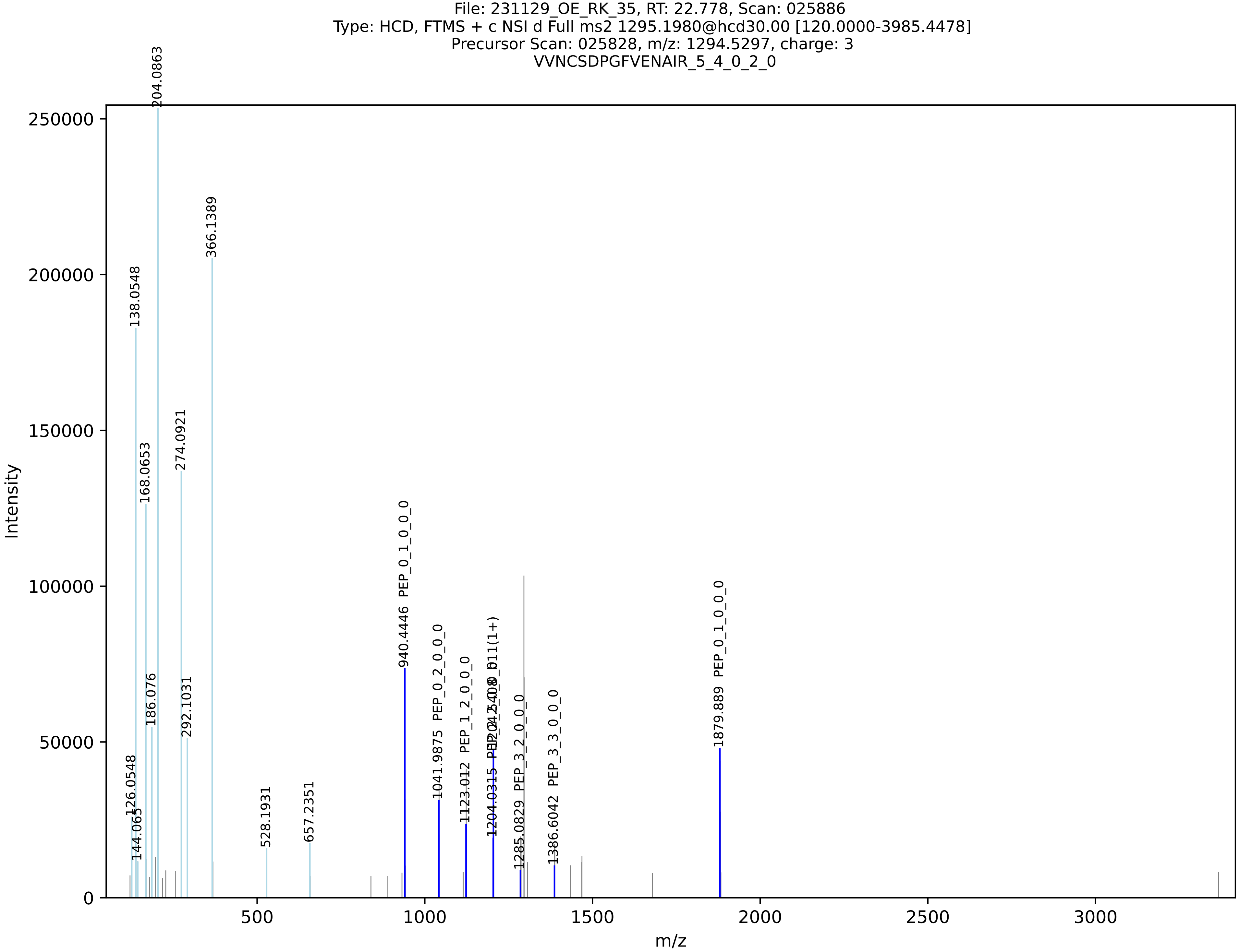
File: 231129_OE_RK_35, RT: 22.778, Scan: 025886
Type: HCD, FTMS + c NSI d Full ms2 1295.1980@hcd30.00 [120.0000-3985.4478]
Precursor Scan: 025828, m/z: 1294.5297, charge: 3
VVNCSDPGFVENAIR_5_4_0_2_0
Intensity
m/z
204.0863
366.1389
138.0548
274.0921
168.0653
186.076
292.1031
126.0548
144.065
528.1931
657.2351
940.4446 PEP_0_1_0_0_0
1041.9875 PEP_0_2_0_0_0
1123.012 PEP_1_2_0_0_0
1204.0315 PEP_2_2_0_0_0
1285.0829 PEP_3_2_0_0_0
1386.6042 PEP_3_3_0_0_0
1879.889 PEP_0_1_0_0_0

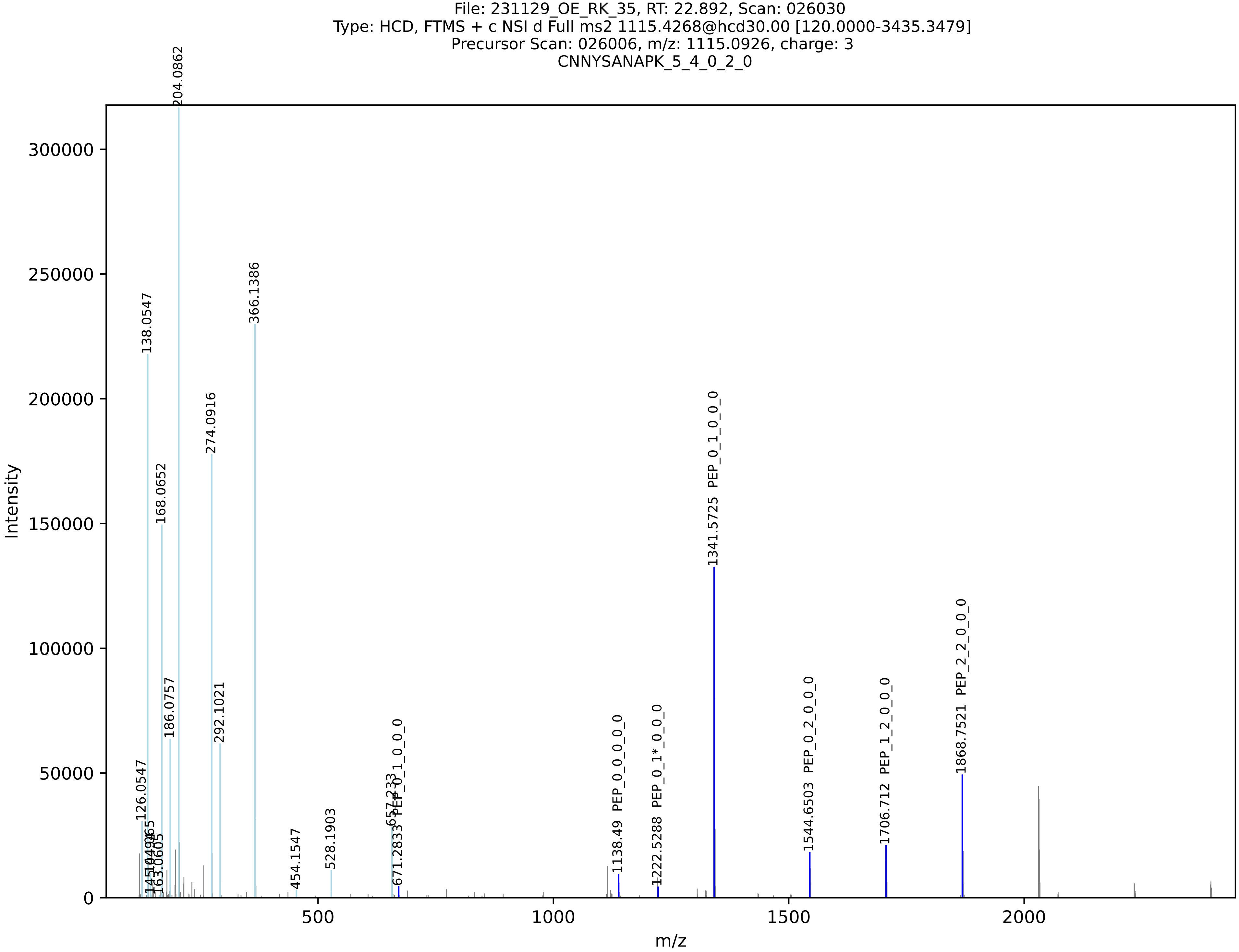
File: 231129_OE_RK_35, RT: 22.892, Scan: 026030
Type: HCD, FTMS + c NSI d Full ms2 1115.4268@hcd30.00 [120.0000-3435.3479]
Precursor Scan: 026006, m/z: 1115.0926, charge: 3
CNNYSANAPK_5_4_0_2_0
Intensity
m/z
0
50000
100000
150000
200000
250000
300000
500
1000
1500
2000
204.0862
138.0547
366.1386
274.0916
168.0652
186.0757
292.1021
126.0547
163.0605
454.1547
528.1903
657.233
671.2833 PEP_0_1_0_0_0
1138.49 PEP_0_0_0_0_0
1222.5288 PEP_0_1*_0_0_0
1341.5725 PEP_0_1_0_0_0
1544.6503 PEP_0_2_0_0_0
1706.712 PEP_1_2_0_0_0
1868.7521 PEP_2_2_0_0_0

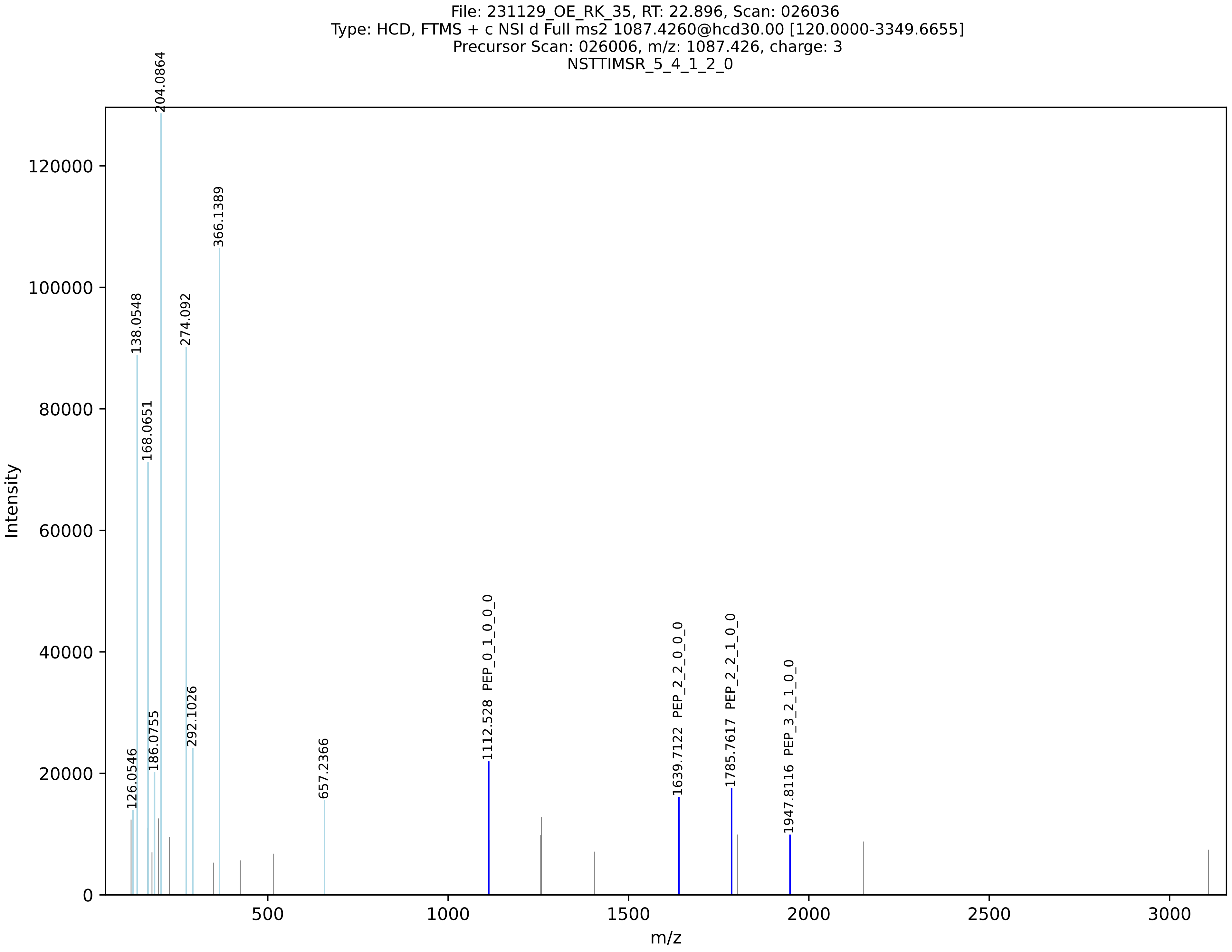

File: 231129_OE_RK_35, RT: 22.896, Scan: 026036
Type: HCD, FTMS + c NSI d Full ms2 1087.4260@hcd30.00 [120.0000-3349.6655]
Precursor Scan: 026006, m/z: 1087.426, charge: 3
NSTTIMSR_5_4_1_2_0
Intensity
m/z
0
20000
40000
60000
80000
100000
120000
500
1000
1500
2000
2500
3000
204.0864
366.1389
138.0548
274.092
168.0651
126.0546
186.0755
292.1026
657.2366
1112.528 PEP_0_1_0_0_0
1639.7122 PEP_2_2_0_0_0
1785.7617 PEP_2_2_1_0_0
1947.8116 PEP_3_2_1_0_0

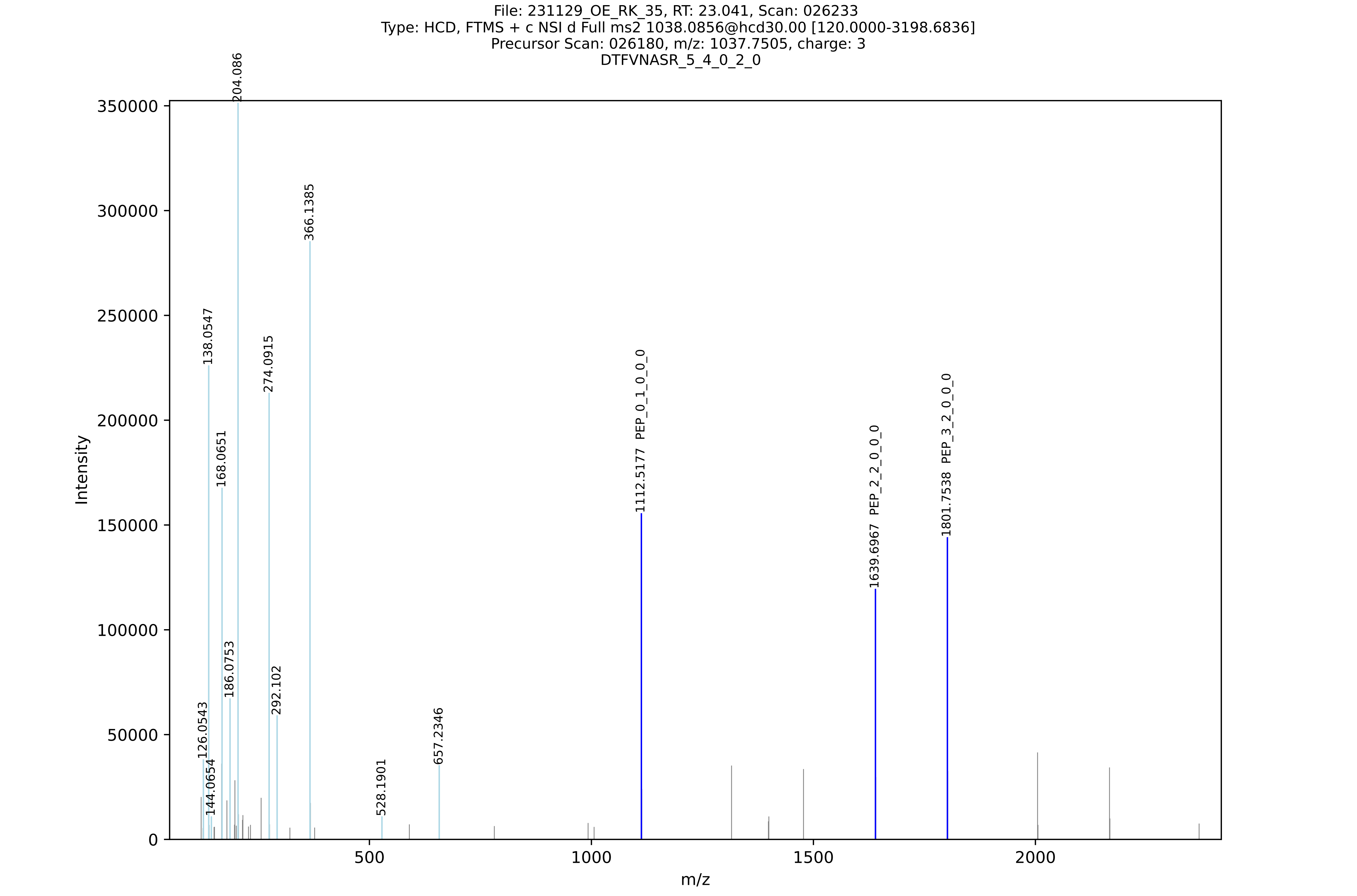
File: 231129_OE_RK_35, RT: 23.041, Scan: 026233
Type: HCD, FTMS + c NSI d Full ms2 1038.0856@hcd30.00 [120.0000-3198.6836]
Precursor Scan: 026180, m/z: 1037.7505, charge: 3
DTFVNASR_5_4_0_2_0
Intensity
m/z
204.086
366.1385
138.0547
274.0915
168.0651
186.0753
292.102
126.0543
144.0654
528.1901
657.2346
1112.5177 PEP_0_1_0_0_0
1639.6967 PEP_2_2_0_0_0
1801.7538 PEP_3_2_0_0_0

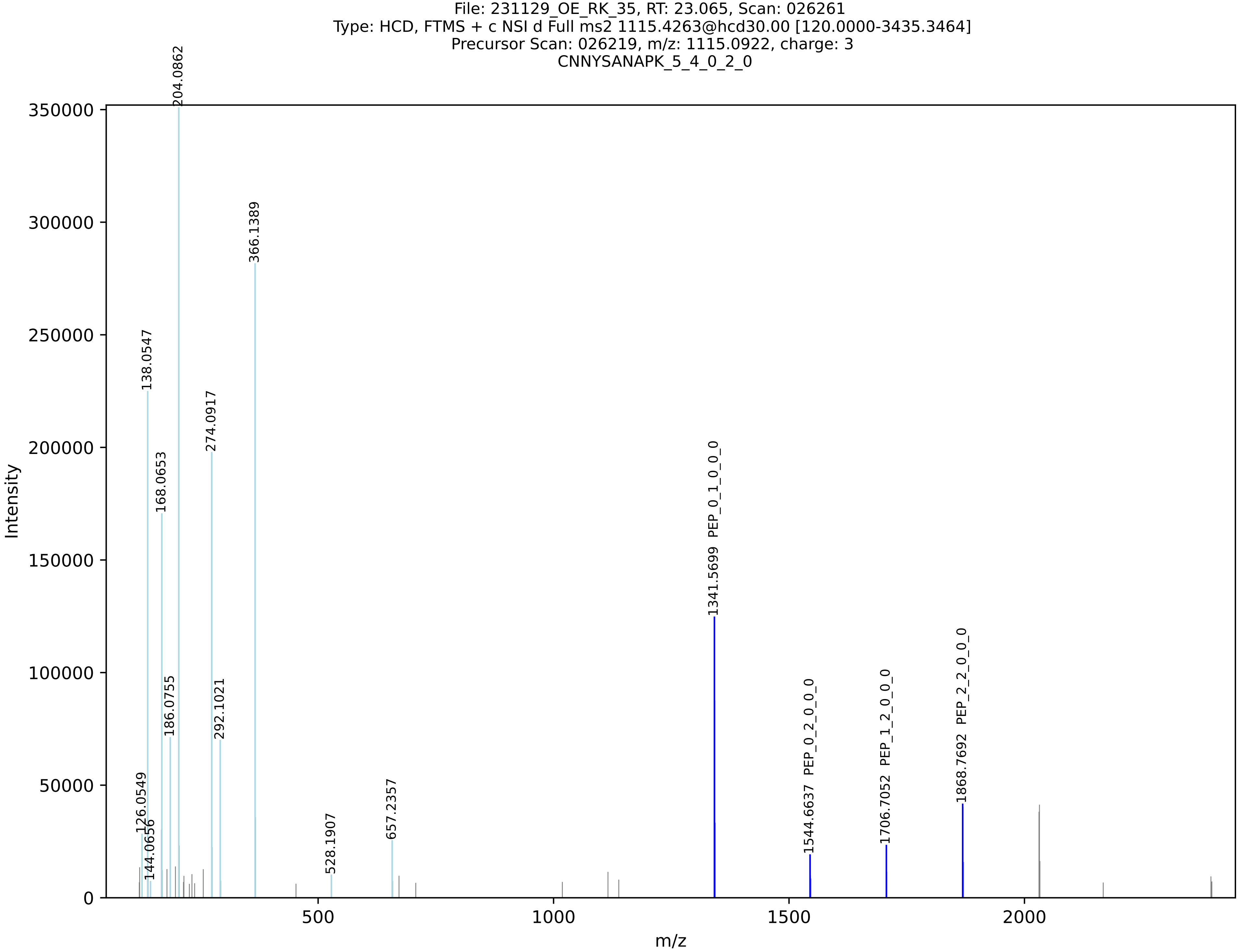
File: 231129_OE_RK_35, RT: 23.065, Scan: 026261
Type: HCD, FTMS + c NSI d Full ms2 1115.4263@hcd30.00 [120.0000-3435.3464]
Precursor Scan: 026219, m/z: 1115.0922, charge: 3
CNNYSANAPK_5_4_0_2_0
Intensity
m/z
204.0862
366.1389
138.0547
274.0917
168.0653
186.0755
292.1021
126.0549
144.0656
528.1907
657.2357
1341.5699 PEP_0_1_0_0_0
1544.6637 PEP_0_2_0_0_0
1706.7052 PEP_1_2_0_0_0
1868.7692 PEP_2_2_0_0_0
350000
300000
250000
200000
150000
100000
50000
0
500
1000
1500
2000

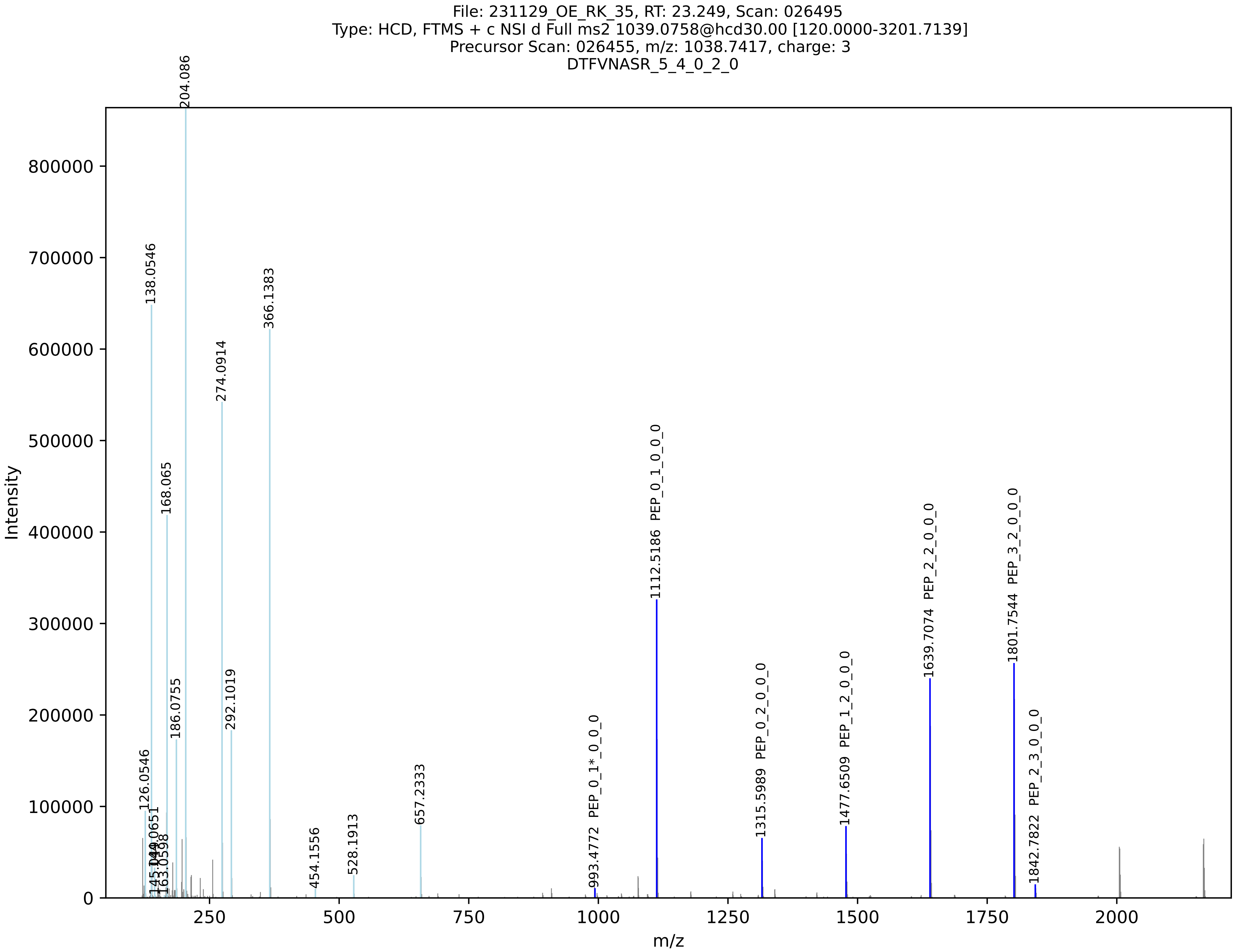
File: 231129_OE_RK_35, RT: 23.249, Scan: 026495
Type: HCD, FTMS + c NSI d Full ms2 1039.0758@hcd30.00 [120.0000-3201.7139]
Precursor Scan: 026455, m/z: 1038.7417, charge: 3
DTFVNASR_5_4_0_2_0
Intensity
m/z
204.086
138.0546
366.1383
274.0914
168.065
186.0755
292.1019
126.0546
657.2333
528.1913
454.1556
993.4772 PEP_0_1*_0_0_0
1112.5186 PEP_0_1_0_0_0
1315.5989 PEP_0_2_0_0_0
1477.6509 PEP_1_2_0_0_0
1639.7074 PEP_2_2_0_0_0
1801.7544 PEP_3_2_0_0_0
1842.7822 PEP_2_3_0_0_0

File: 231129_OE_RK_35, RT: 23.419, Scan: 026736
Type: HCD, FTMS + c NSI d Full ms2 1039.0765@hcd30.00 [120.0000-3201.7163]
Precursor Scan: 026680, m/z: 1038.7425, charge: 3
DTFVNASR_5_4_0_2_0

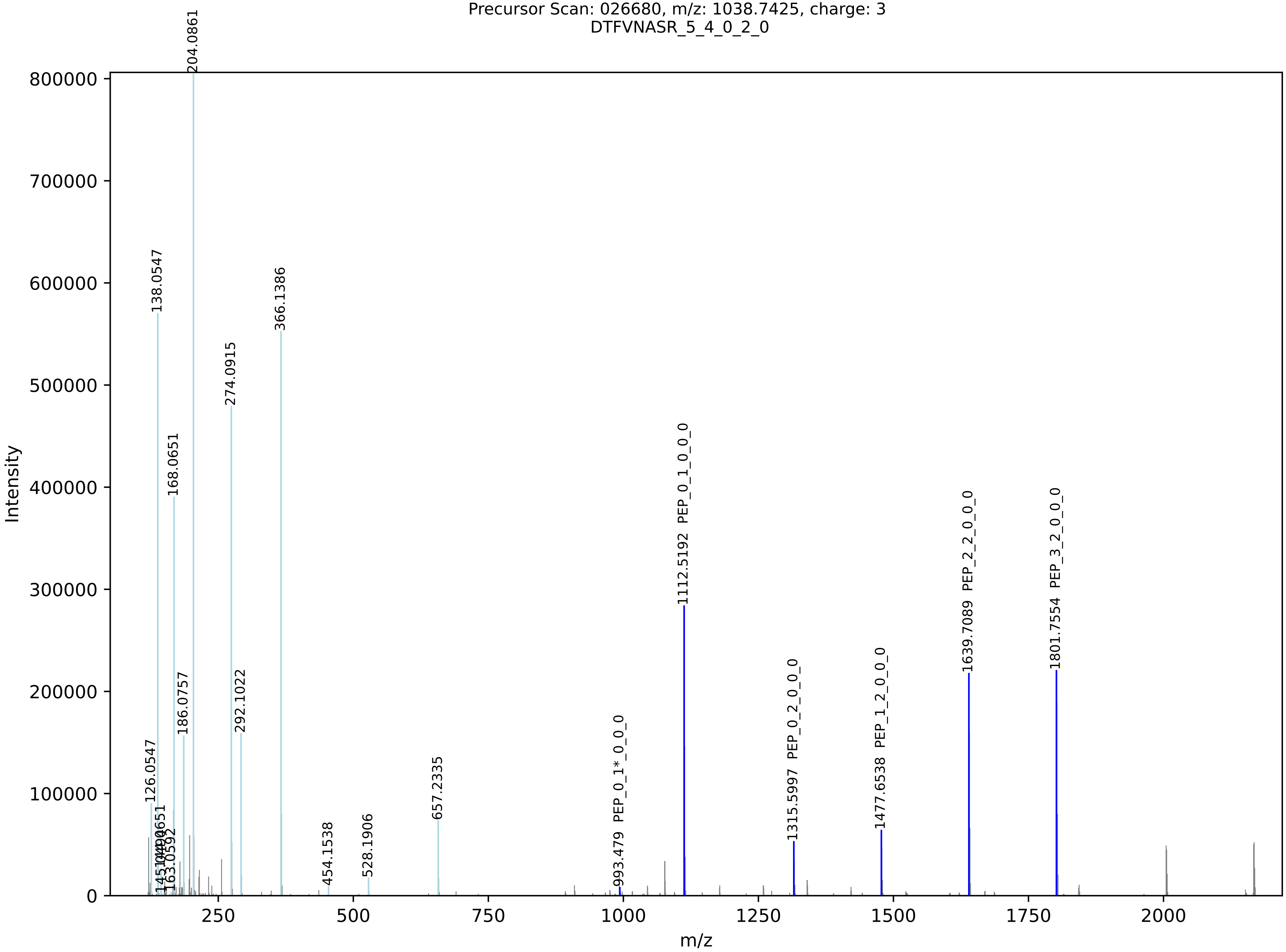

File: 231129_OE_RK_35, RT: 23.464, Scan: 026801
Type: HCD, FTMS + c NSI d Full ms2 1266.1736@hcd30.00 [120.0000-3896.6331]
Precursor Scan: 026735, m/z: 1265.5046, charge: 3
LQNNENNISCVER_5_4_0_2_0

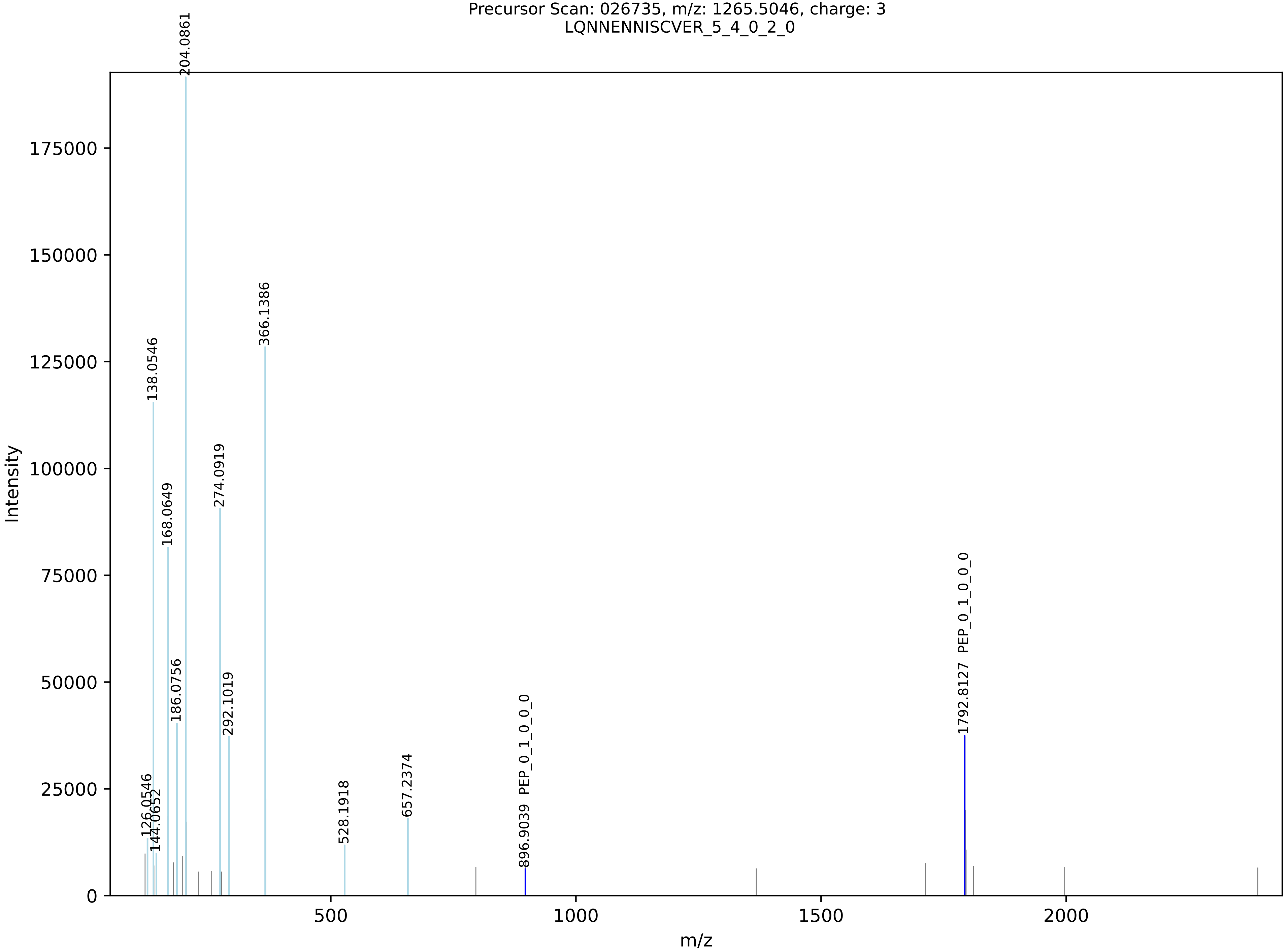

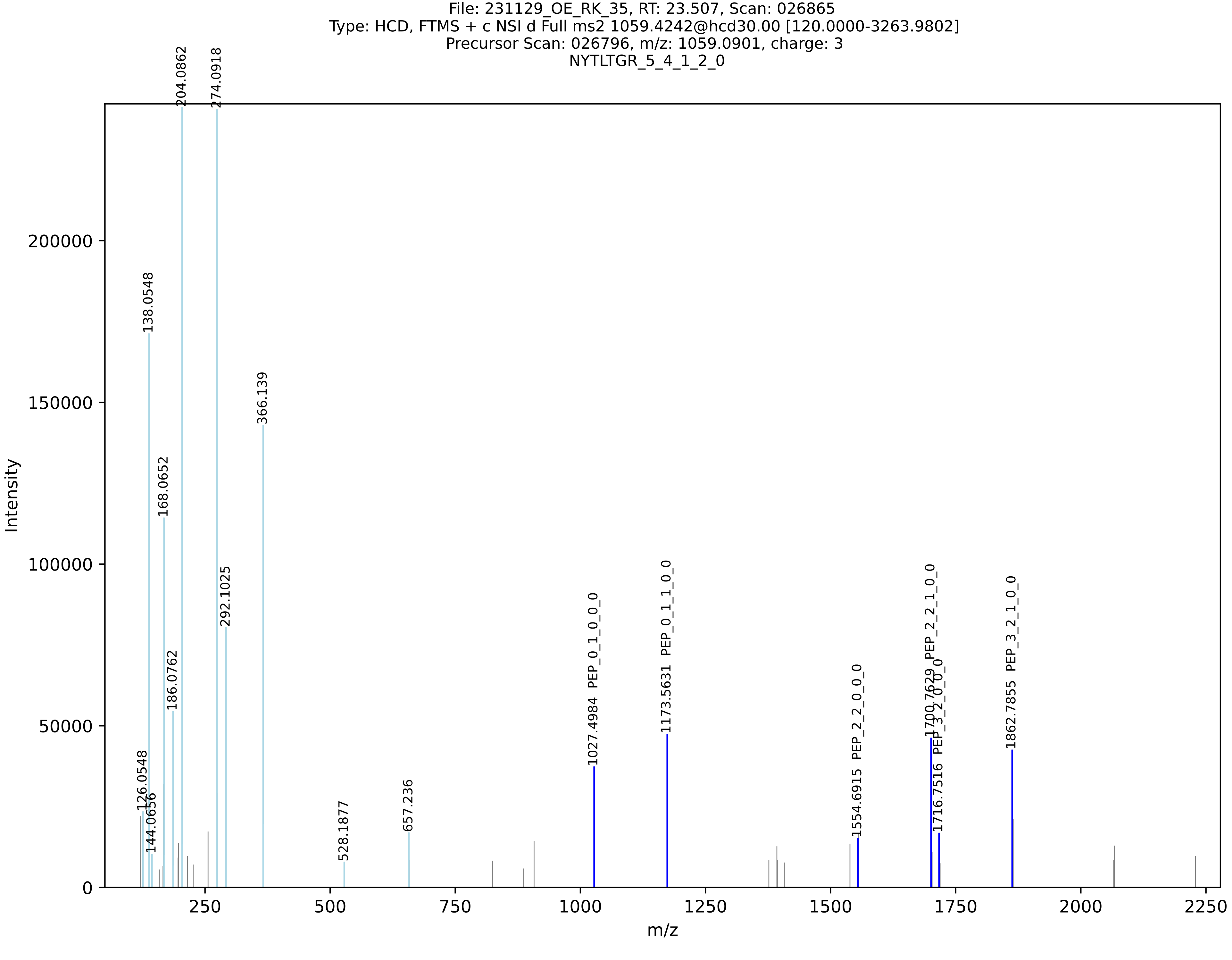

File: 231129_OE_RK_35, RT: 23.507, Scan: 026865
Type: HCD, FTMS + c NSI d Full ms2 1059.4242@hcd30.00 [120.0000-3263.9802]
Precursor Scan: 026796, m/z: 1059.0901, charge: 3
NYTLTGR_5_4_1_2_0
Intensity
m/z
204.0862
274.0918
138.0548
366.139
168.0652
292.1025
186.0762
126.0548
144.0656
528.1877
657.236
1027.4984 PEP_0_1_0_0_0
1173.5631 PEP_0_1_1_0_0
1554.6915 PEP_2_2_0_0_0
1700.7629 PEP_2_2_1_0_0
1716.7516 PEP_3_2_0_0_0
1862.7855 PEP_3_2_1_0_0

File: 231129_OE_RK_35, RT: 23.56, Scan: 026939
Type: HCD, FTMS + c NSI d Full ms2 1063.7928@hcd30.00 [120.0000-3277.3481]
Precursor Scan: 026862, m/z: 1063.4586, charge: 3
FVEGSHNSTVSLTTK_6_3_0_0_0

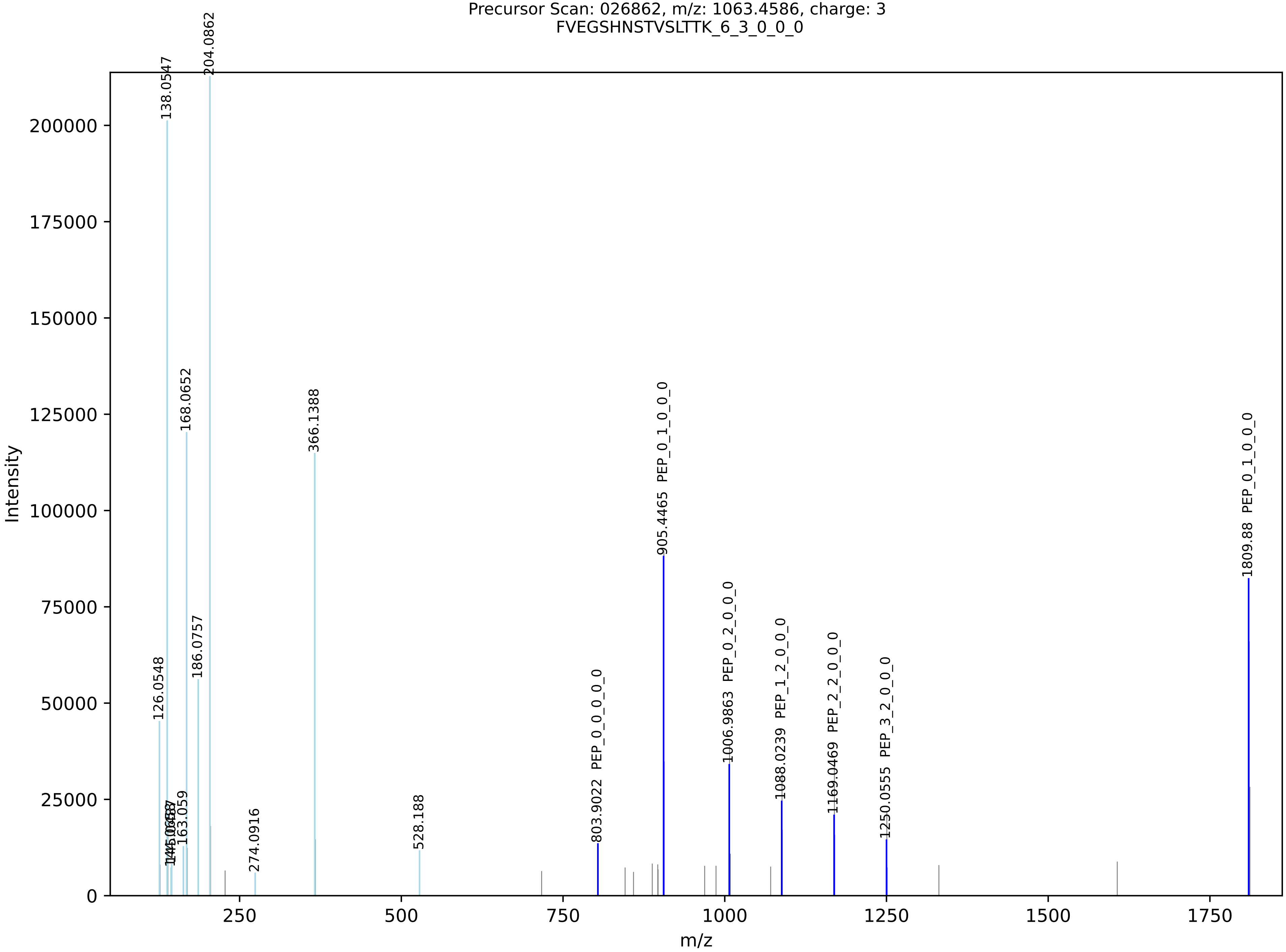

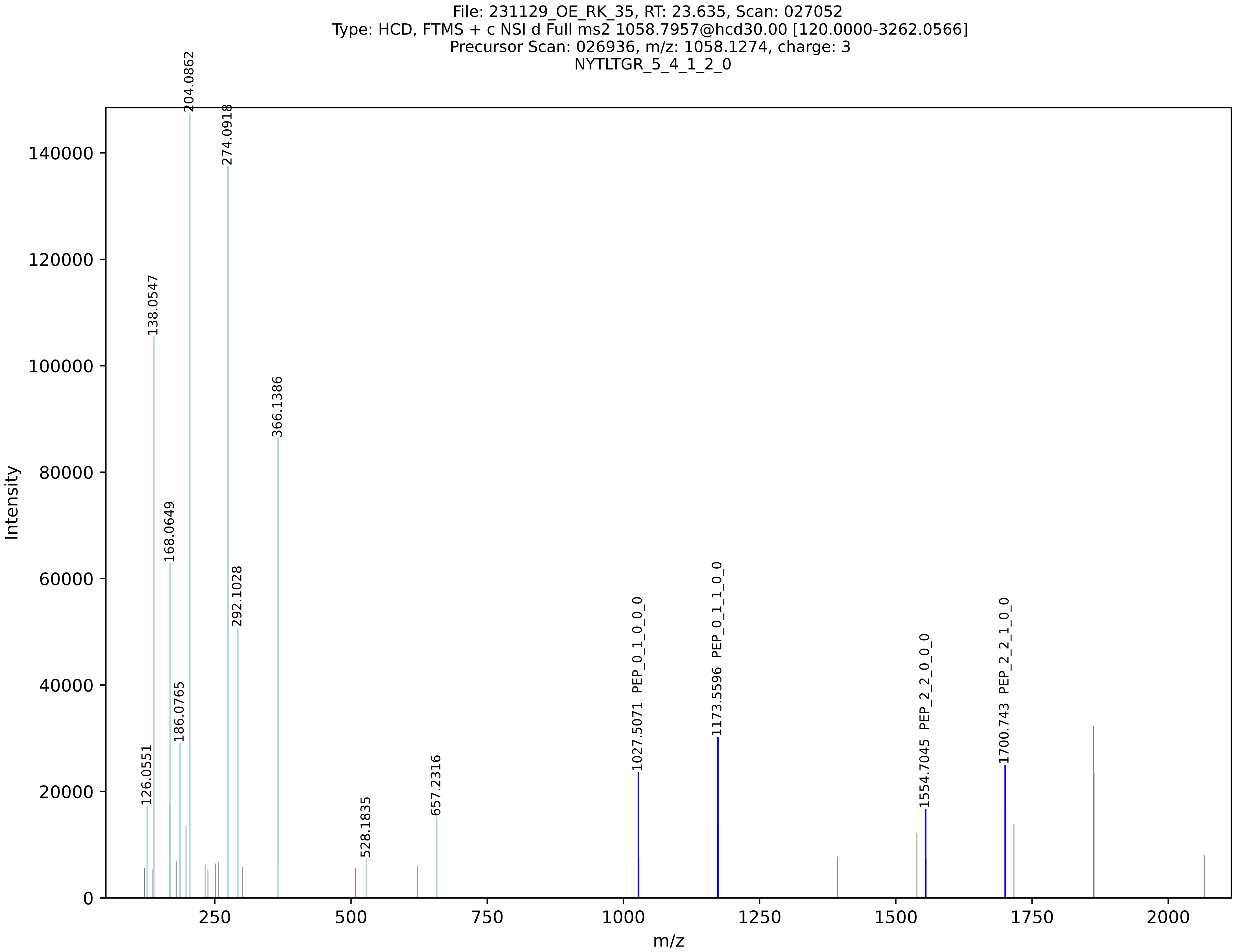

File: 231129_OE_RK_35, RT: 23.635, Scan: 027052
Type: HCD, FTMS + c NSI d Full ms2 1058.7957@hcd30.00 [120.0000-3262.0566]
Precursor Scan: 026936, m/z: 1058.1274, charge: 3
NYTLTGR_5_4_1_2_0
Intensity
m/z
204.0862
274.0918
138.0547
366.1386
168.0649
292.1028
186.0765
126.0551
528.1835
657.2316
1027.5071 PEP_0_1_0_0_0
1173.5596 PEP_0_1_1_0_0
1554.7045 PEP_2_2_0_0_0
1700.743 PEP_2_2_1_0_0

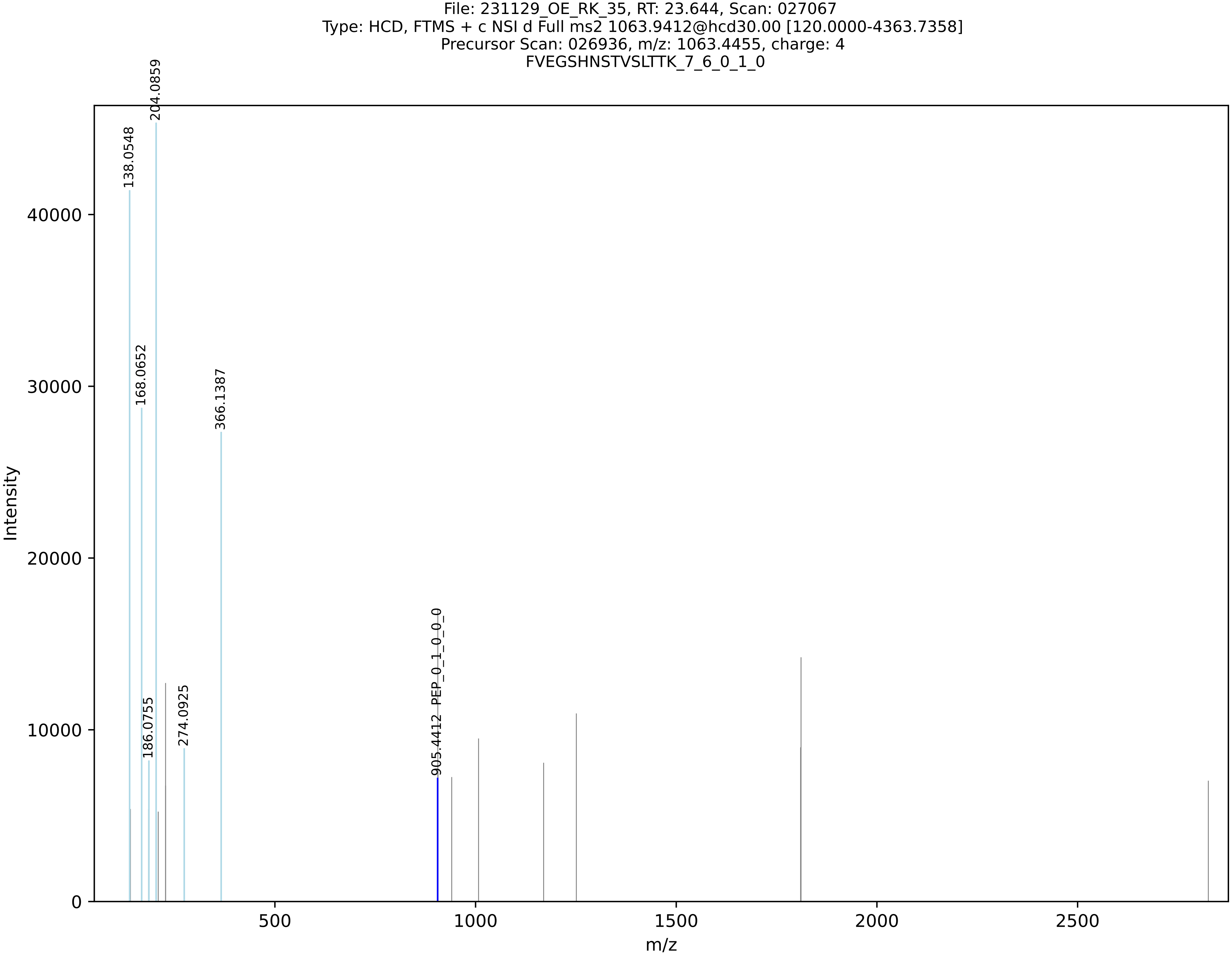

File: 231129_OE_RK_35, RT: 23.644, Scan: 027067
Type: HCD, FTMS + c NSI d Full ms2 1063.9412@hcd30.00 [120.0000-4363.7358]
Precursor Scan: 026936, m/z: 1063.4455, charge: 4
FVEGSHNSTVSLTTK_7_6_0_1_0
204.0859
138.0548
168.0652
366.1387
186.0755
274.0925
905.4412_PEP_0_1_0_0_0
Intensity
m/z
0
10000
20000
30000
40000
500
1000
1500
2000
2500

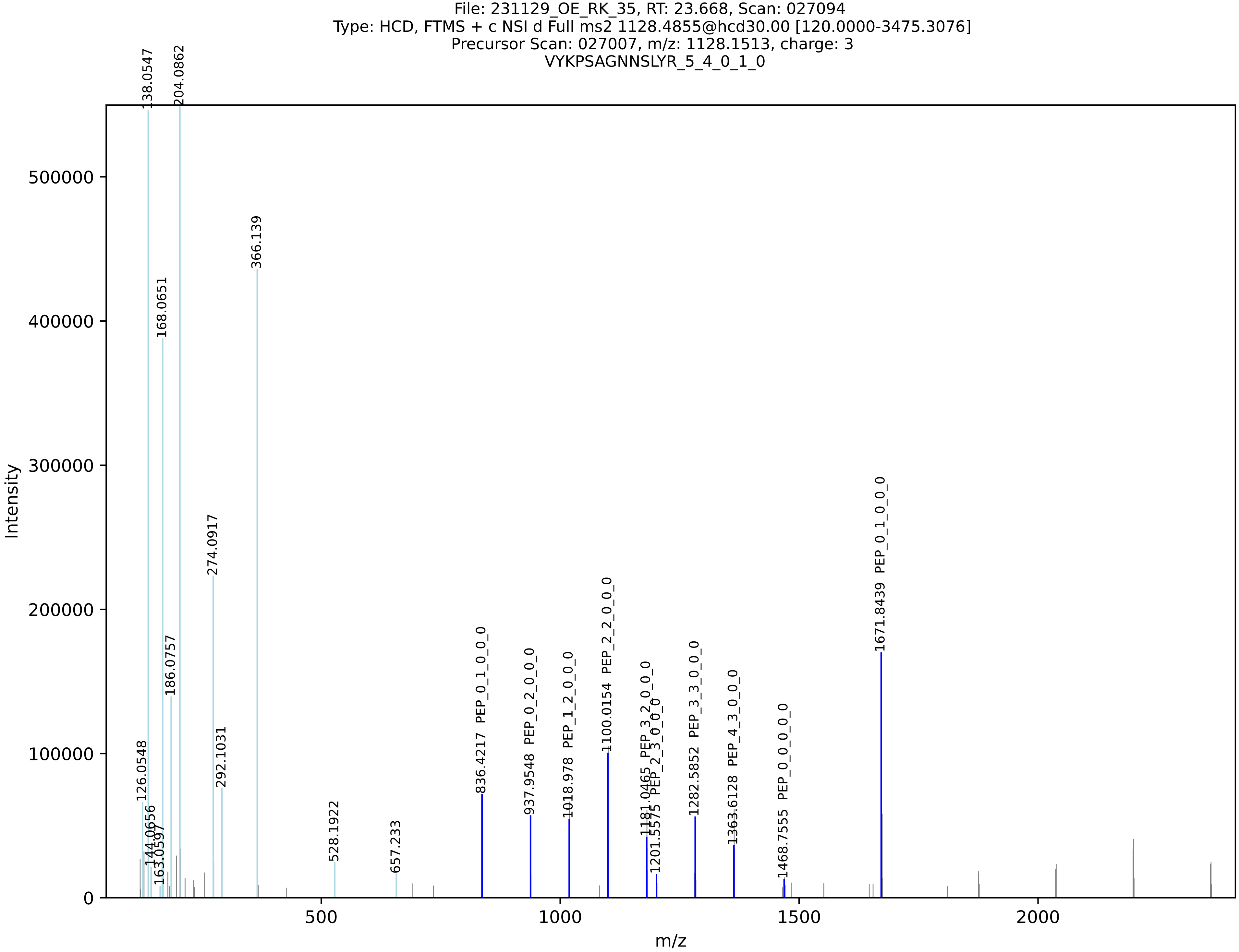
File: 231129_OE_RK_35, RT: 23.668, Scan: 027094
Type: HCD, FTMS + c NSI d Full ms2 1128.4855@hcd30.00 [120.0000-3475.3076]
Precursor Scan: 027007, m/z: 1128.1513, charge: 3
VYKPSAGNNSLYR_5_4_0_1_0
Intensity
m/z
138.0547
204.0862
366.139
168.0651
274.0917
186.0757
126.0548
292.1031
144.0656
163.0597
528.1922
657.233
836.4217 PEP_0_1_0_0_0
937.9548 PEP_0_2_0_0_0
1018.978 PEP_1_2_0_0_0
1100.0154 PEP_2_2_0_0_0
1181.0465 PEP_3_2_0_0_0
1201.5575 PEP_2_3_0_0_0
1282.5852 PEP_3_3_0_0_0
1363.6128 PEP_4_3_0_0_0
1468.7555 PEP_0_0_0_0_0
1671.8439 PEP_0_1_0_0_0

File: 231129_OE_RK_35, RT: 23.68, Scan: 027114
Type: HCD, FTMS + c NSI d Full ms2 1127.4519@hcd30.00 [120.0000-3472.1448]
Precursor Scan: 027007, m/z: 1127.1204, charge: 3
VYKPSAGNNSLYR_5_4_0_1_0

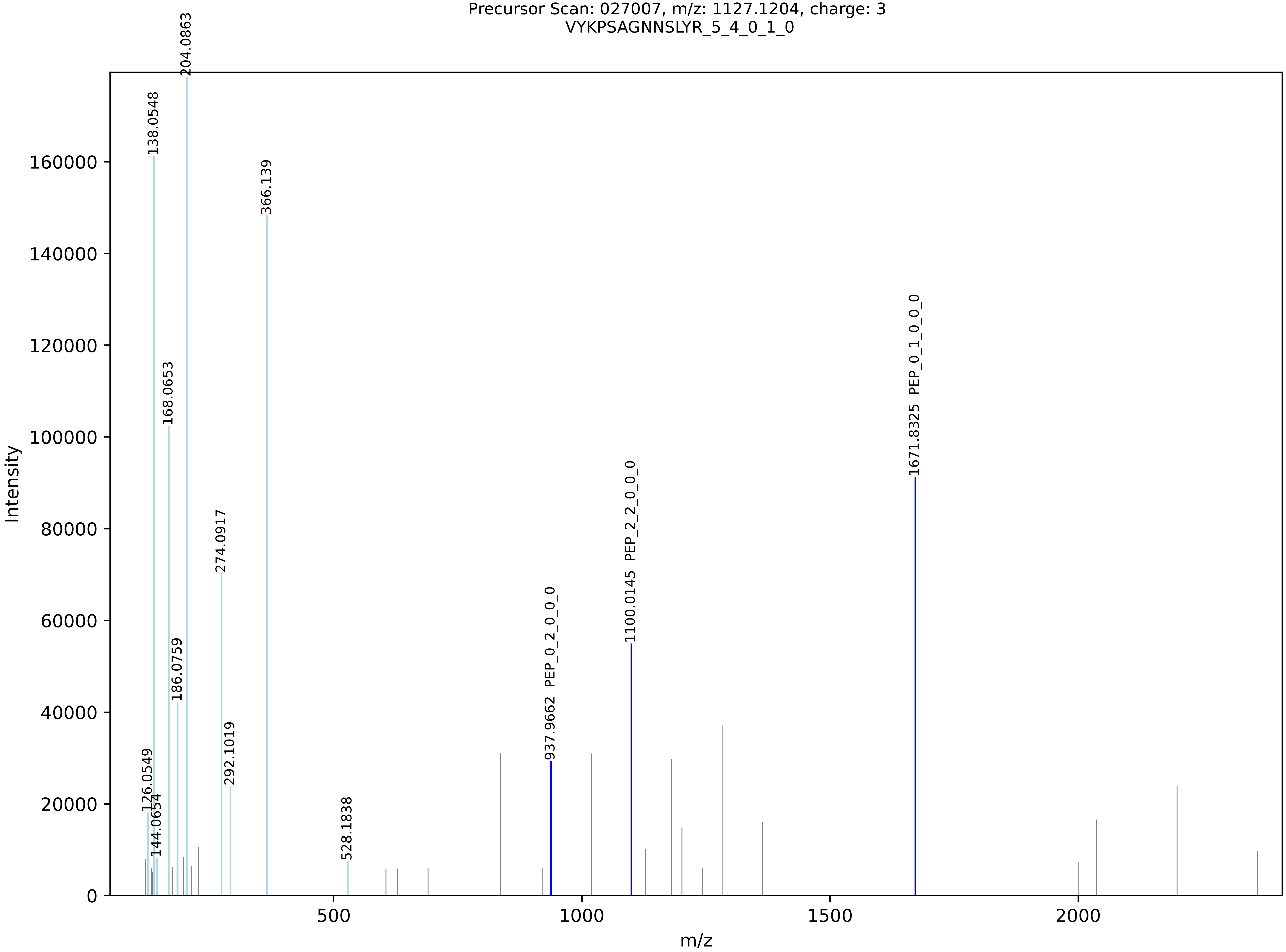

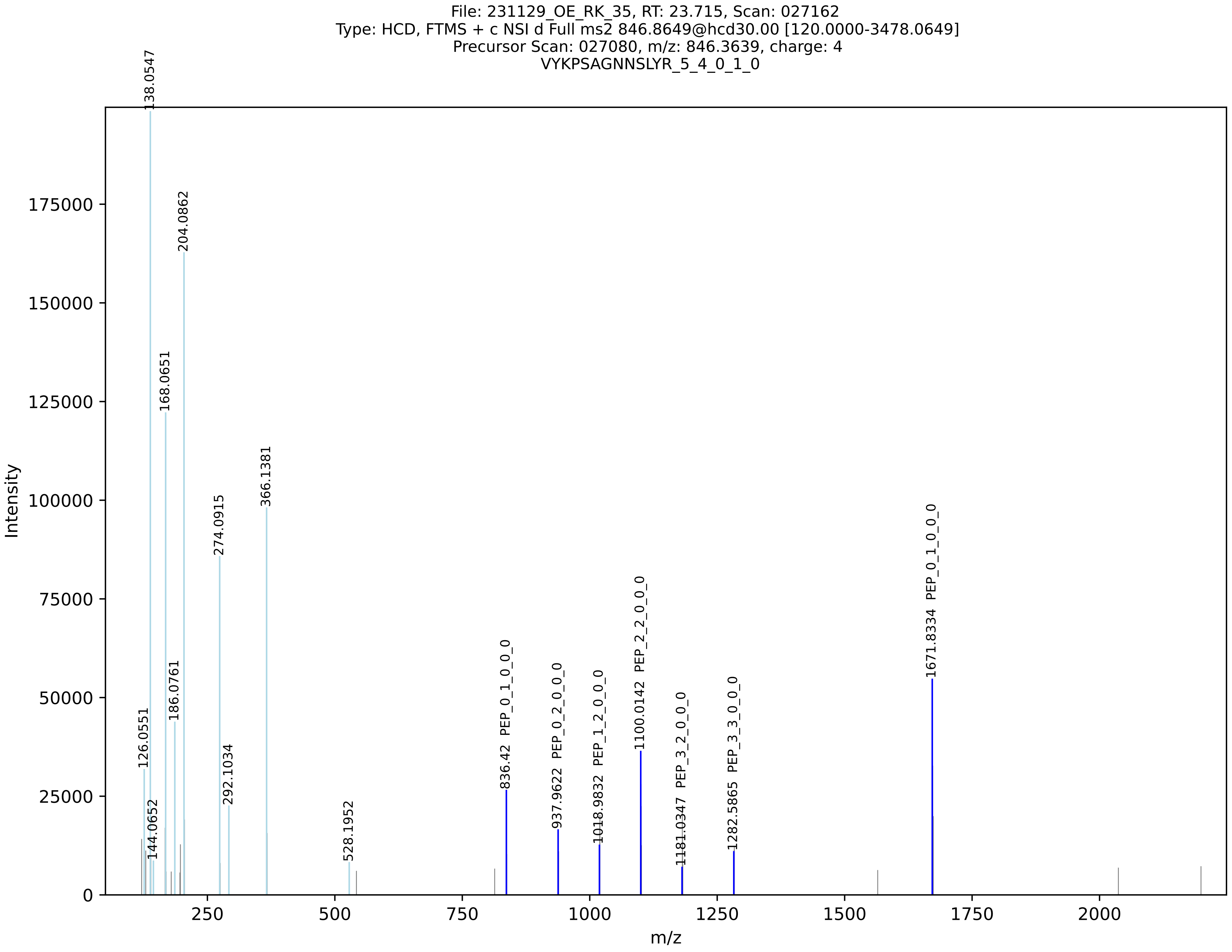

File: 231129_OE_RK_35, RT: 23.715, Scan: 027162
Type: HCD, FTMS + c NSI d Full ms2 846.8649@hcd30.00 [120.0000-3478.0649]
Precursor Scan: 027080, m/z: 846.3639, charge: 4
VYKPSAGNNSLYR_5_4_0_1_0
Intensity
m/z
138.0547
204.0862
168.0651
366.1381
274.0915
186.0761
126.0551
292.1034
144.0652
528.1952
836.42 PEP_0_1_0_0_0
937.9622 PEP_0_2_0_0_0
1018.9832 PEP_1_2_0_0_0
1100.0142 PEP_2_2_0_0_0
1181.0347 PEP_3_2_0_0_0
1282.5865 PEP_3_3_0_0_0
1671.8334 PEP_0_1_0_0_0

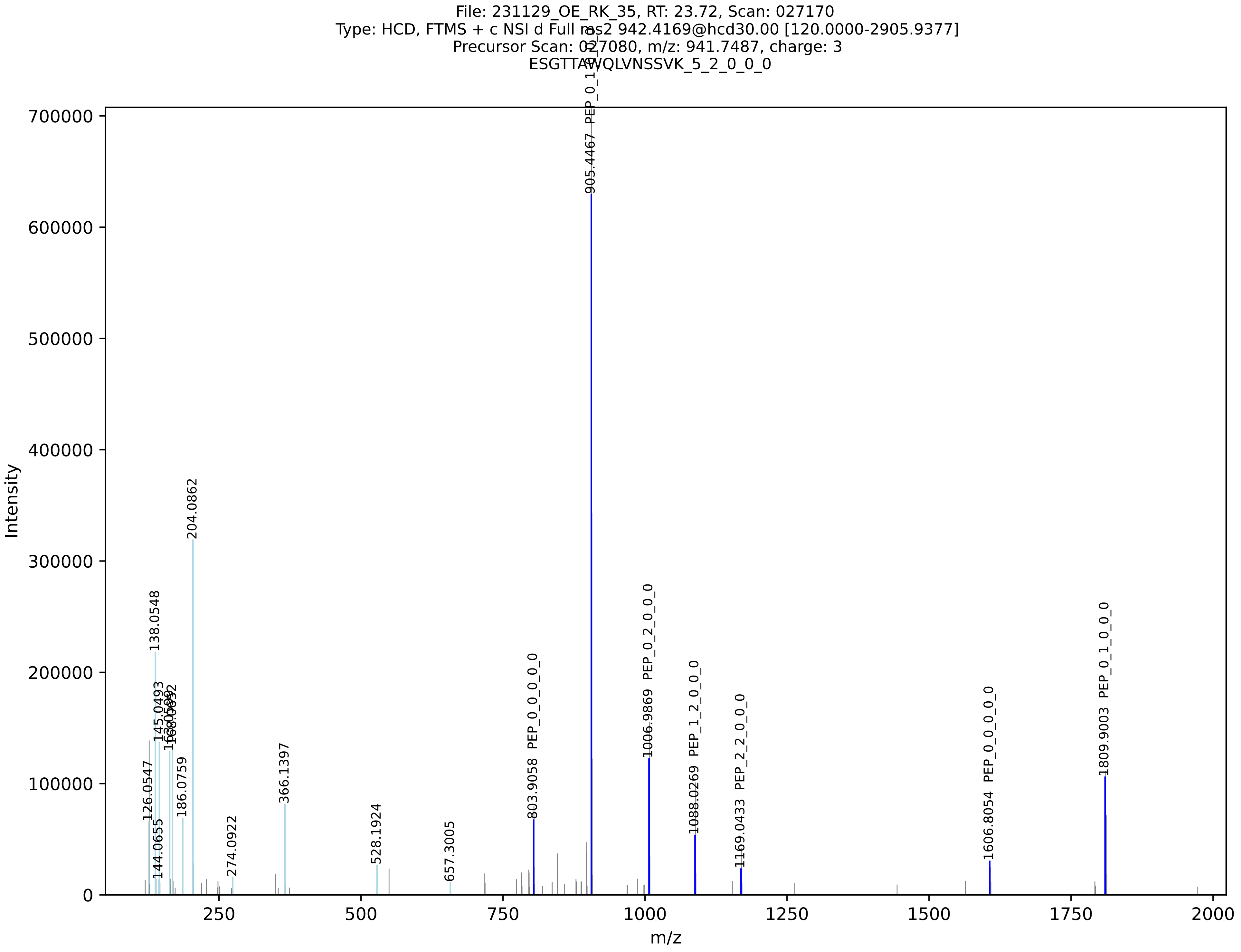
File: 231129_OE_RK_35, RT: 23.72, Scan: 027170
Type: HCD, FTMS + c NSI d Full ms2 942.4169@hcd30.00 [120.0000-2905.9377]
Precursor Scan: 027080, m/z: 941.7487, charge: 3
ESGTTAWQLVNSSVK_5_2_0_0_0
Intensity
m/z
905.4467 PEP_0_1
204.0862
138.0548
145.0493
126.0547
186.0759
144.0655
274.0922
366.1397
528.1924
657.3005
803.9058 PEP_0_0_0_0_0
1006.9869 PEP_0_2_0_0_0
1088.0269 PEP_1_2_0_0_0
1169.0433 PEP_2_2_0_0_0
1606.8054 PEP_0_0_0_0_0
1809.9003 PEP_0_1_0_0_0

File: 231129_OE_RK_35, RT: 23.741, Scan: 027204
Type: HCD, FTMS + c NSI d Full ms2 1010.1088@hcd30.00 [120.0000-3113.0747]
Precursor Scan: 027080, m/z: 1009.4421, charge: 3
FVEGSHNSTVSLTTK_5_3_0_0_0

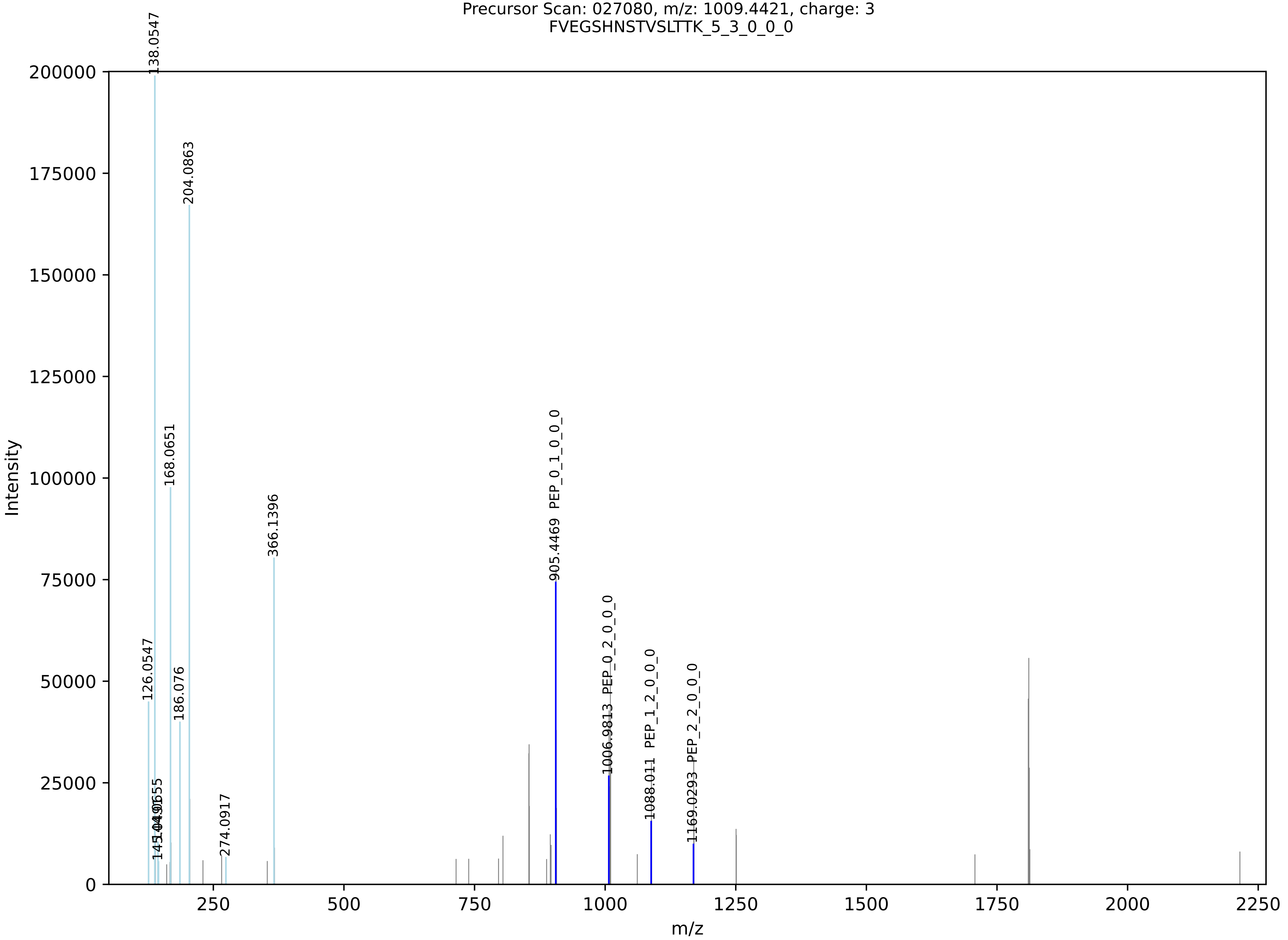

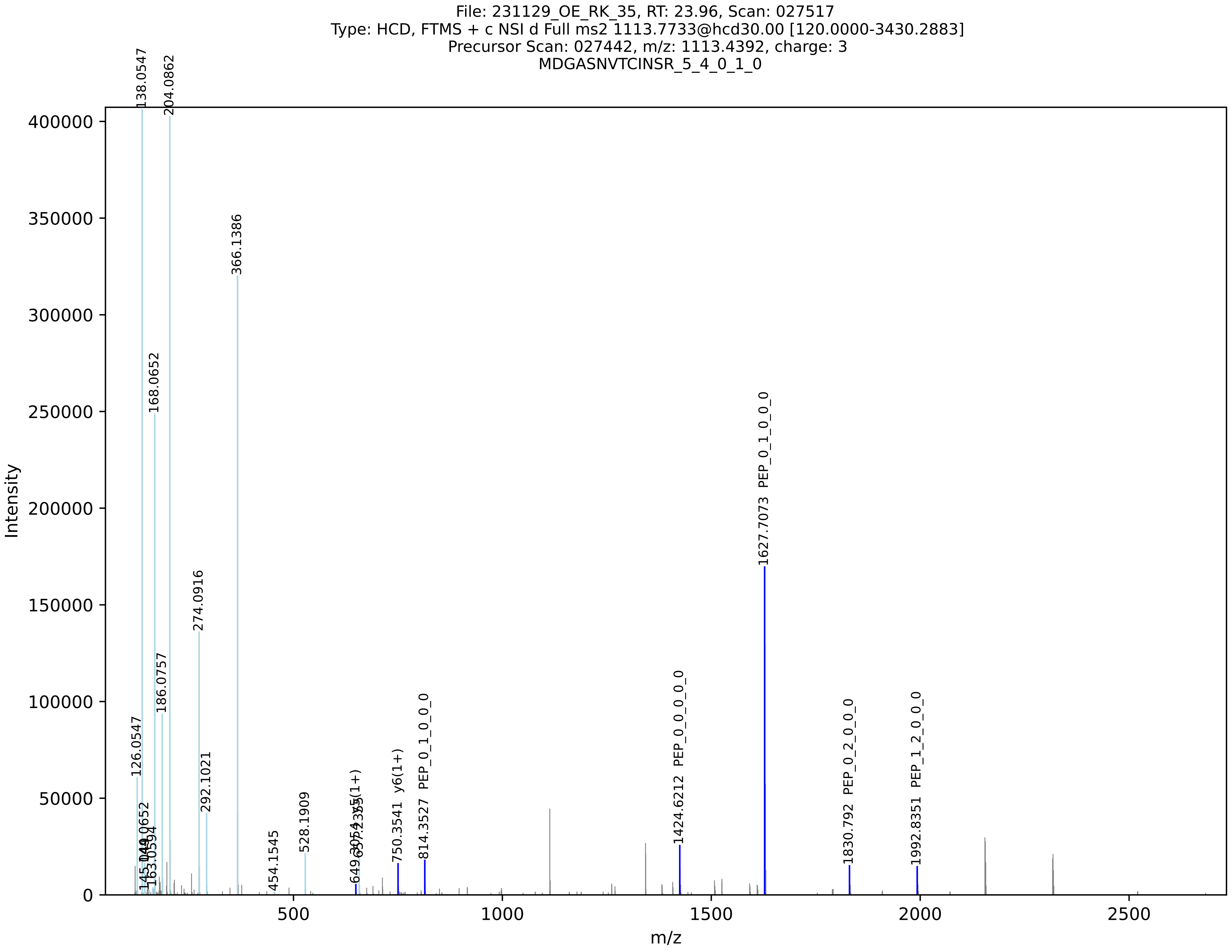
File: 231129_OE_RK_35, RT: 23.96, Scan: 027517
Type: HCD, FTMS + c NSI d Full ms2 1113.7733@hcd30.00 [120.0000-3430.2883]
Precursor Scan: 027442, m/z: 1113.4392, charge: 3
MDGASNVTCINSR_5_4_0_1_0
Intensity
m/z
138.0547
204.0862
366.1386
168.0652
274.0916
186.0757
126.0547
292.1021
163.0594
454.1545
528.1909
750.3541 y6(1+)
814.3527 PEP_0_1_0_0_0
1424.6212 PEP_0_0_0_0_0
1627.7073 PEP_0_1_0_0_0
1830.792 PEP_0_2_0_0_0
1992.8351 PEP_1_2_0_0_0

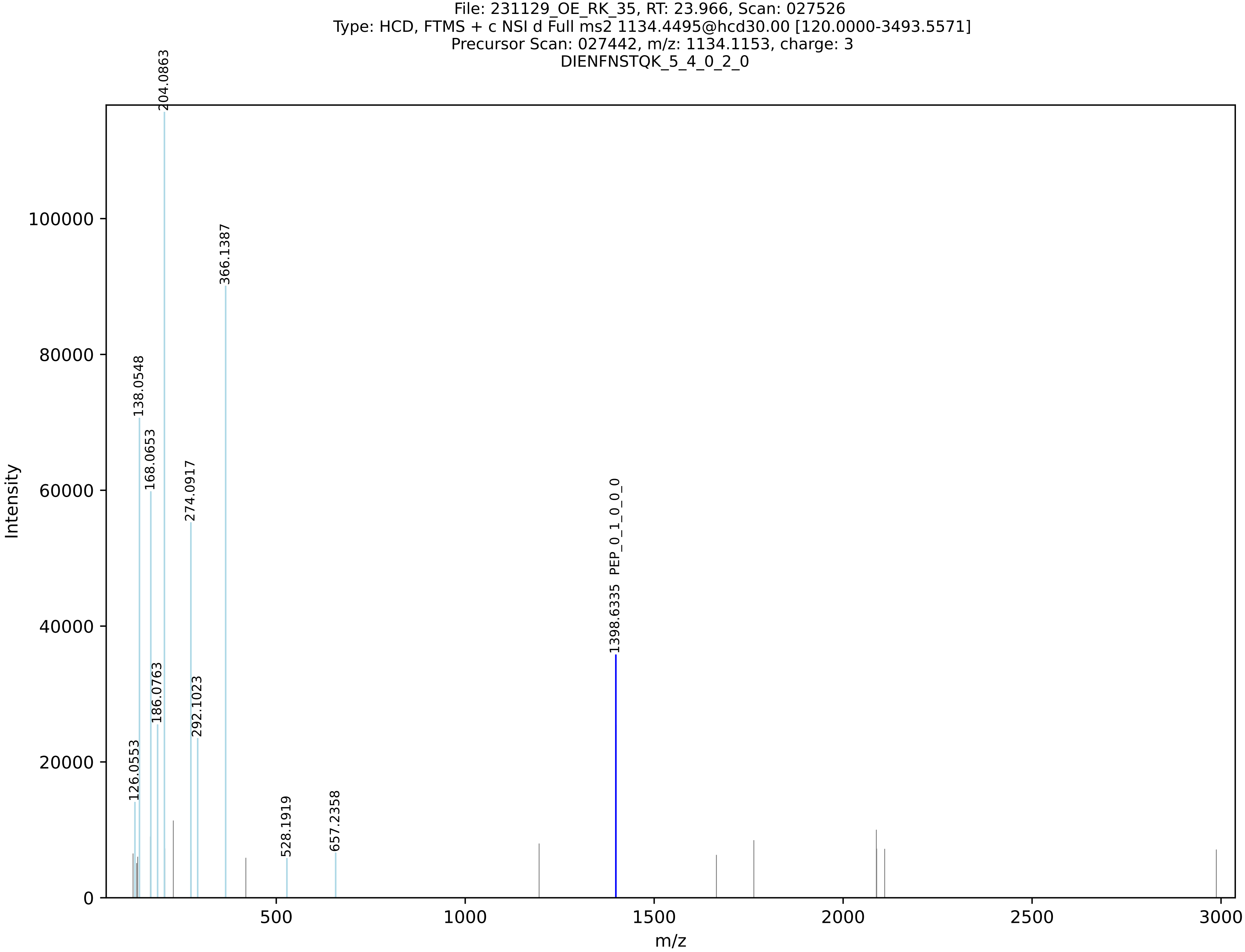
File: 231129_OE_RK_35, RT: 23.966, Scan: 027526
Type: HCD, FTMS + c NSI d Full ms2 1134.4495@hcd30.00 [120.0000-3493.5571]
Precursor Scan: 027442, m/z: 1134.1153, charge: 3
DIENFNSTQK_5_4_0_2_0
204.0863
366.1387
138.0548
168.0653
274.0917
1398.6335 PEP_0_1_0_0_0
186.0763
292.1023
126.0553
528.1919
657.2358
Intensity
m/z
0
20000
40000
60000
80000
100000
500
1000
1500
2000
2500
3000

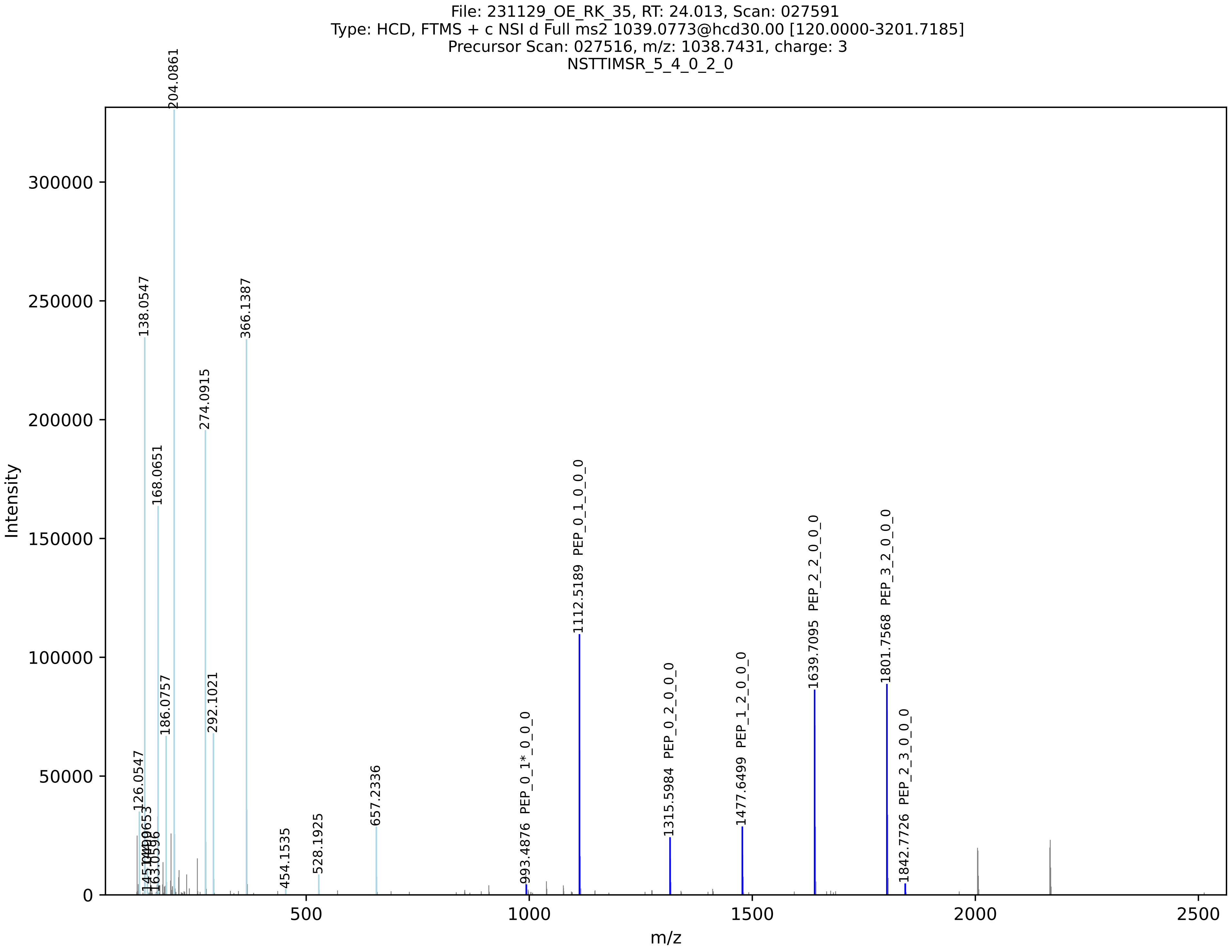

File: 231129_OE_RK_35, RT: 24.013, Scan: 027591
Type: HCD, FTMS + c NSI d Full ms2 1039.0773@hcd30.00 [120.0000-3201.7185]
Precursor Scan: 027516, m/z: 1038.7431, charge: 3
NSTTIMSR_5_4_0_2_0
204.0861
138.0547
366.1387
274.0915
168.0651
186.0757
292.1021
126.0547
163.0596
454.1535
528.1925
657.2336
993.4876 PEP_0_1*_0_0_0
1112.5189 PEP_0_1_0_0_0
1315.5984 PEP_0_2_0_0_0
1477.6499 PEP_1_2_0_0_0
1639.7095 PEP_2_2_0_0_0
1801.7568 PEP_3_2_0_0_0
1842.7726 PEP_2_3_0_0_0
Intensity
m/z
0
50000
100000
150000
200000
250000
300000
500
1000
1500
2000
2500

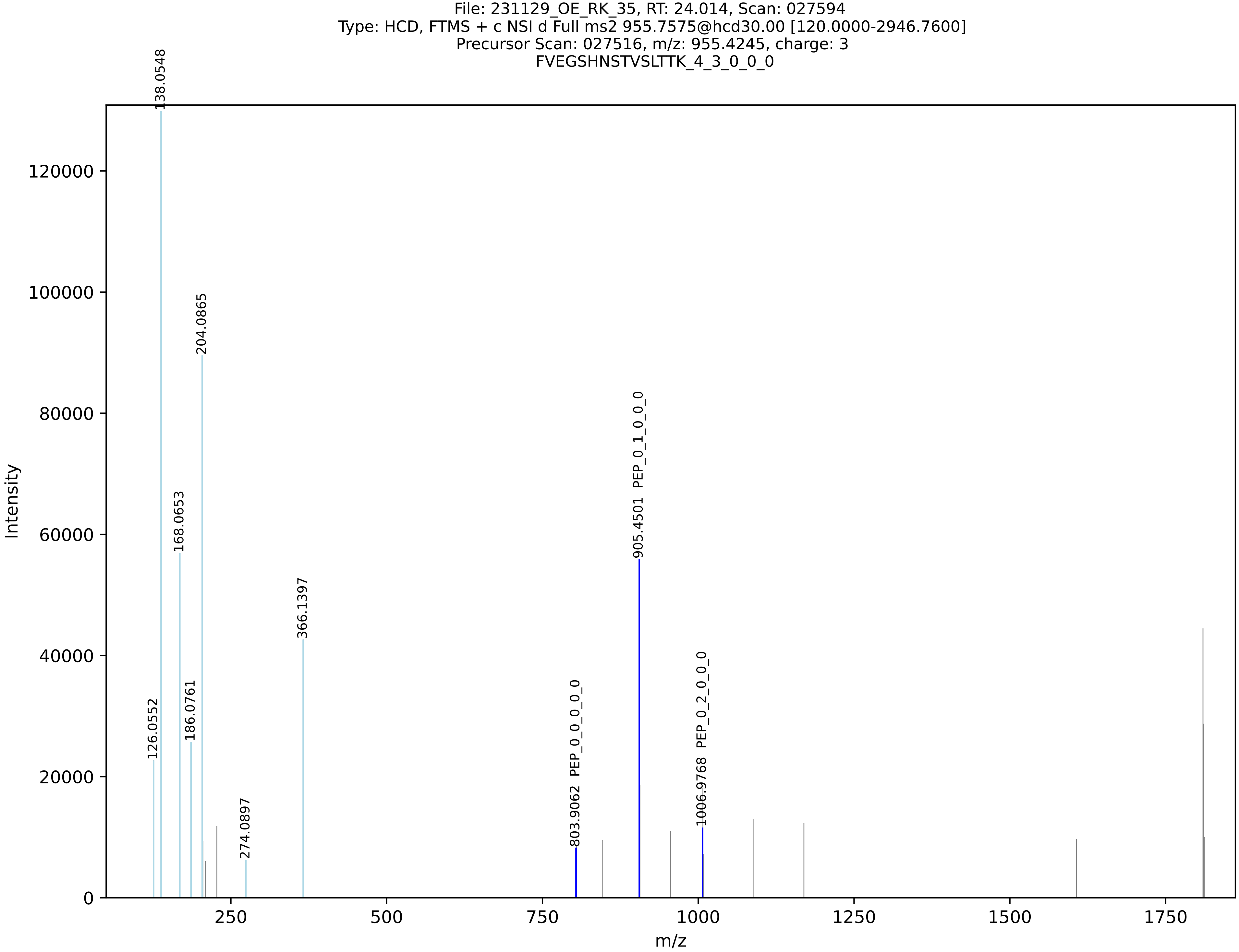
File: 231129_OE_RK_35, RT: 24.014, Scan: 027594
Type: HCD, FTMS + c NSI d Full ms2 955.7575@hcd30.00 [120.0000-2946.7600]
Precursor Scan: 027516, m/z: 955.4245, charge: 3
FVEGSHNSTVSLTTK_4_3_0_0_0
138.0548
204.0865
168.0653
366.1397
186.0761
126.0552
274.0897
803.9062 PEP_0_0_0_0_0
905.4501 PEP_0_1_0_0_0
1006.9768 PEP_0_2_0_0_0
Intensity
m/z
0
20000
40000
60000
80000
100000
120000
250
500
750
1000
1250
1500
1750

File: 231129_OE_RK_35, RT: 24.215, Scan: 027884
Type: HCD, FTMS + c NSI d Full ms2 949.8839@hcd30.00 [120.0000-3898.3821]
Precursor Scan: 027805, m/z: 949.3817, charge: 4
LQNNENNISCVER_5_4_0_2_0

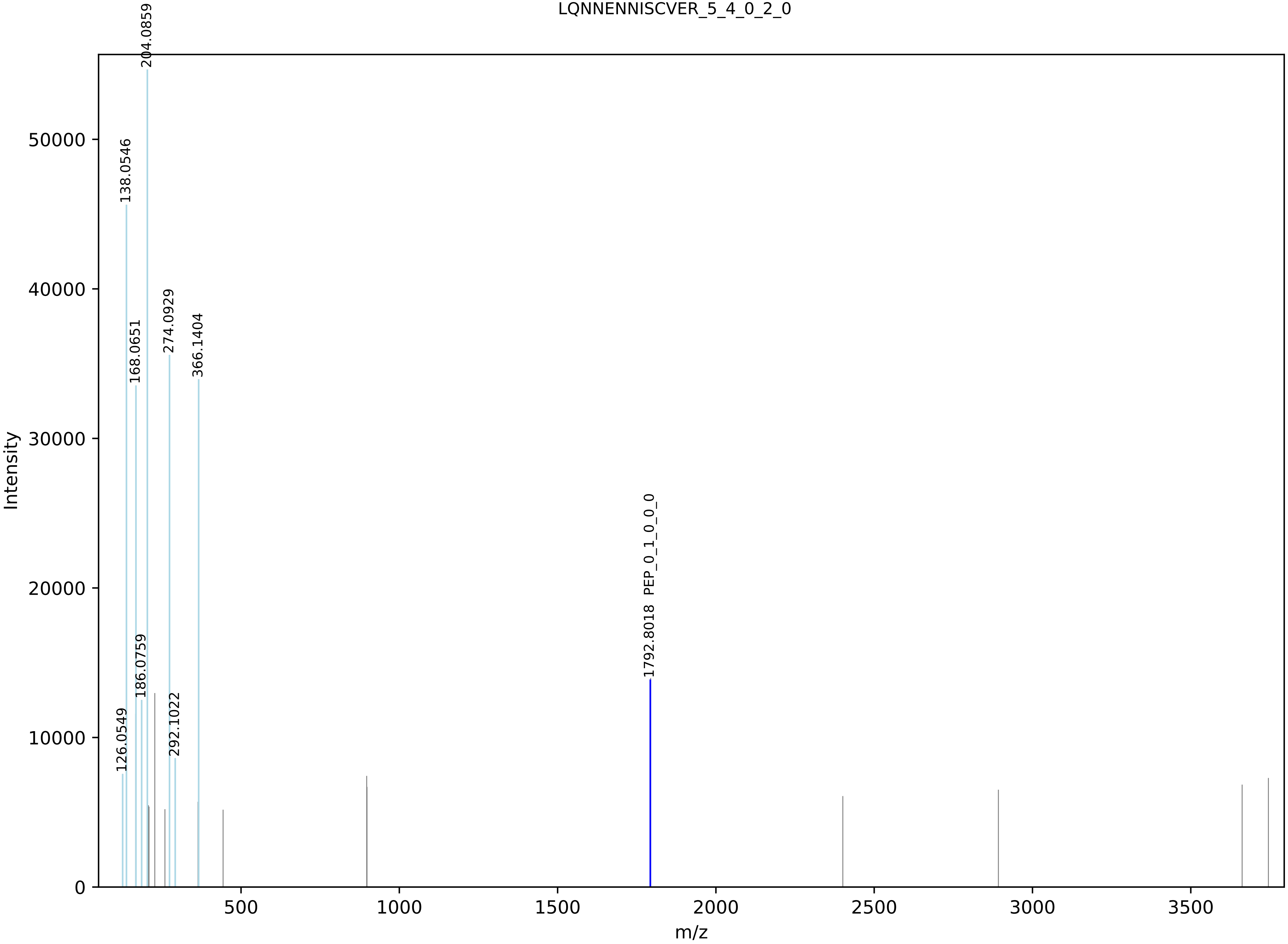

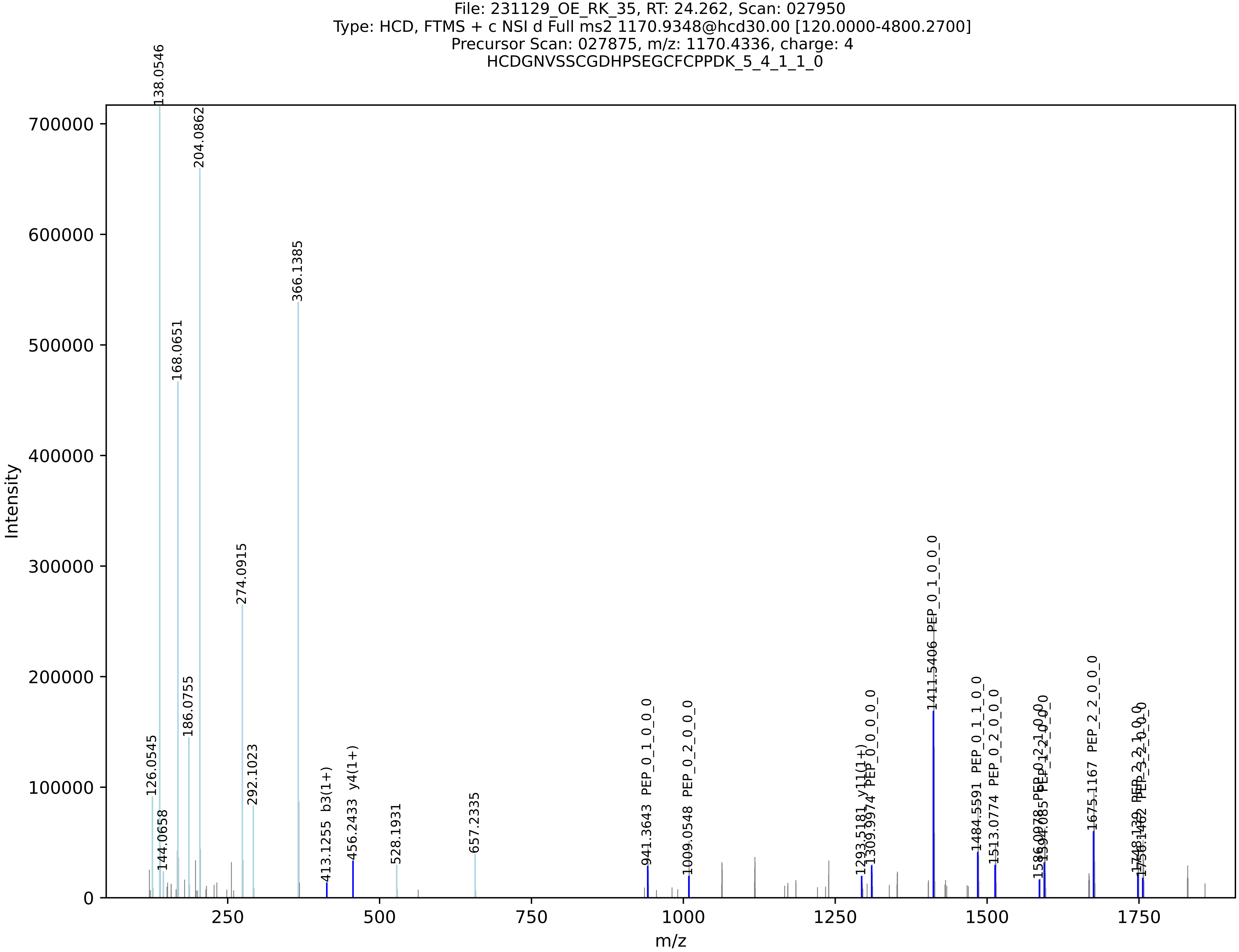
File: 231129_OE_RK_35, RT: 24.262, Scan: 027950
Type: HCD, FTMS + c NSI d Full ms2 1170.9348@hcd30.00 [120.0000-4800.2700]
Precursor Scan: 027875, m/z: 1170.4336, charge: 4
HCDGNVSSCGDHPSEGCFCPPDK_5_4_1_1_0
Intensity
m/z
138.0546
204.0862
366.1385
168.0651
274.0915
186.0755
126.0545
292.1023
144.0658
413.1255 b3(1+)
456.2433 y4(1+)
528.1931
657.2335
941.3643 PEP_0_1_0_0_0
1009.0548 PEP_0_2_0_0_0
1293.5181 y11(1+)
1309.9974 PEP_0_0_0_0_0
1411.5406 PEP_0_1_0_0_0
1484.5591 PEP_0_1_1_0_0
1513.0774 PEP_0_2_0_0_0
1675.1167 PEP_2_2_0_0_0

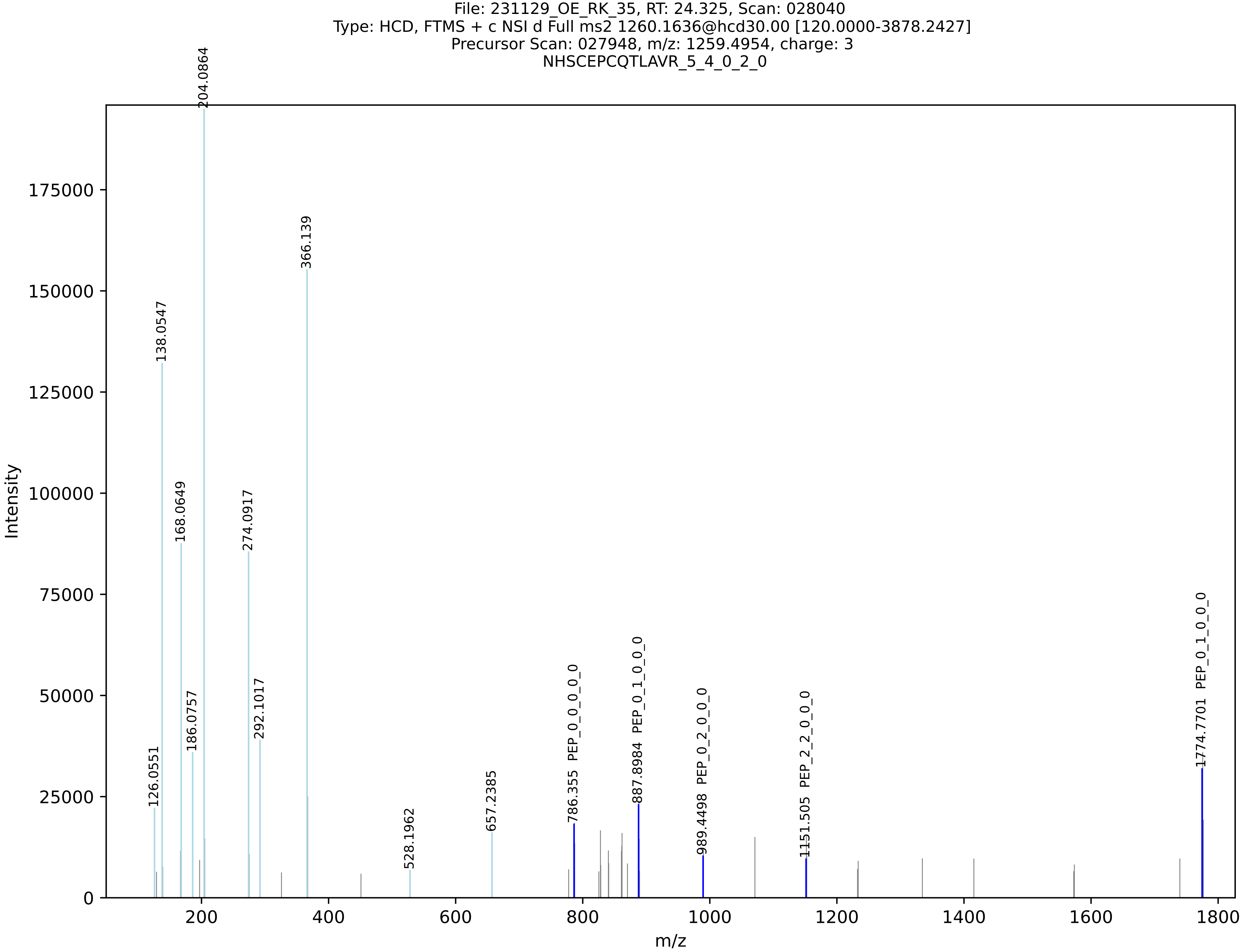
File: 231129_OE_RK_35, RT: 24.325, Scan: 028040
Type: HCD, FTMS + c NSI d Full ms2 1260.1636@hcd30.00 [120.0000-3878.2427]
Precursor Scan: 027948, m/z: 1259.4954, charge: 3
NHSCEPCQTLAVR_5_4_0_2_0
Intensity
m/z
204.0864
366.139
138.0547
168.0649
274.0917
186.0757
292.1017
126.0551
528.1962
657.2385
786.355 PEP_0_0_0_0_0
887.8984 PEP_0_1_0_0_0
989.4498 PEP_0_2_0_0_0
1151.505 PEP_2_2_0_0_0
1774.7701 PEP_0_1_0_0_0

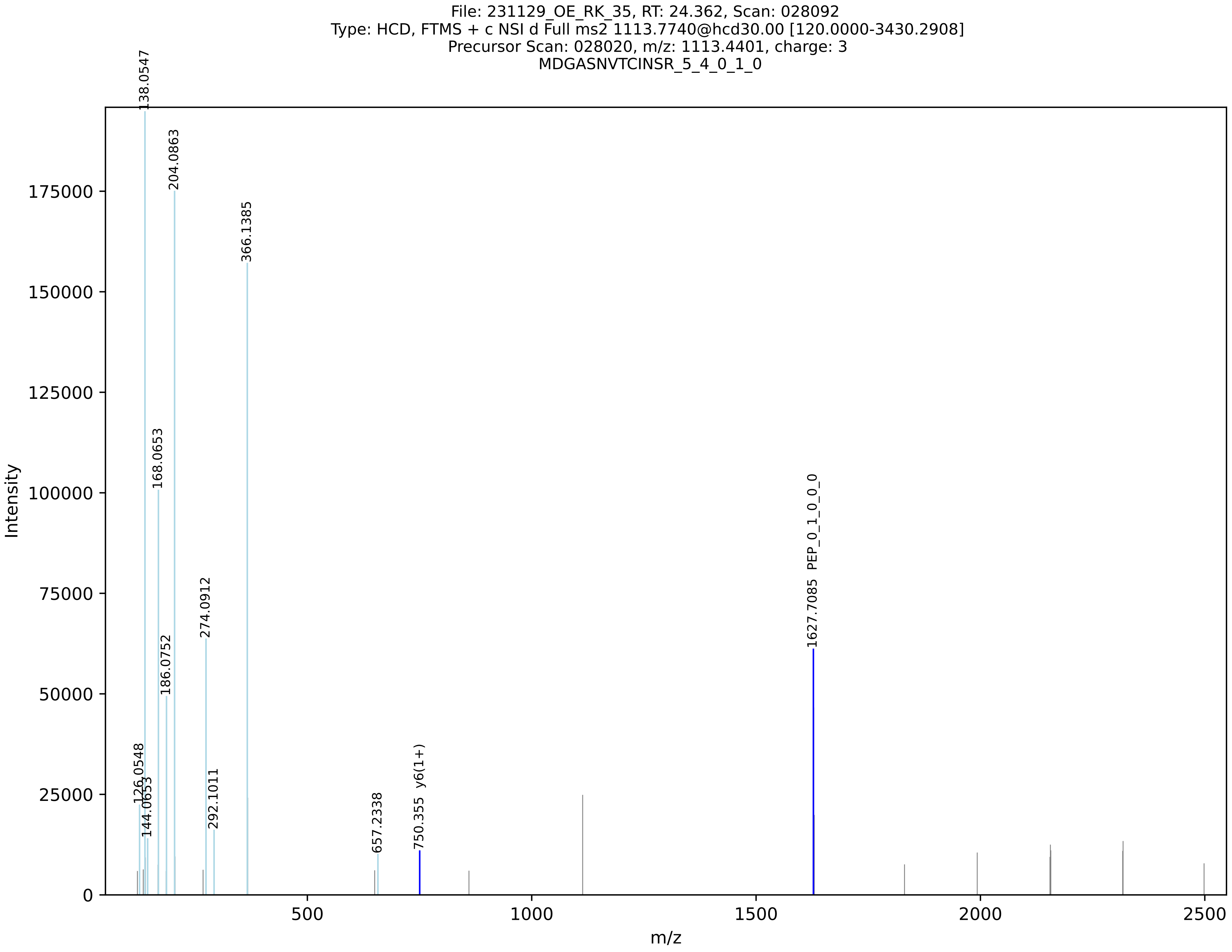
File: 231129_OE_RK_35, RT: 24.362, Scan: 028092
Type: HCD, FTMS + c NSI d Full ms2 1113.7740@hcd30.00 [120.0000-3430.2908]
Precursor Scan: 028020, m/z: 1113.4401, charge: 3
MDGASNVTCINSR_5_4_0_1_0
138.0547
204.0863
366.1385
168.0653
274.0912
186.0752
126.0548
144.0653
292.1011
657.2338
750.355 y6(1+)
1627.7085 PEP_0_1_0_0_0
Intensity
m/z

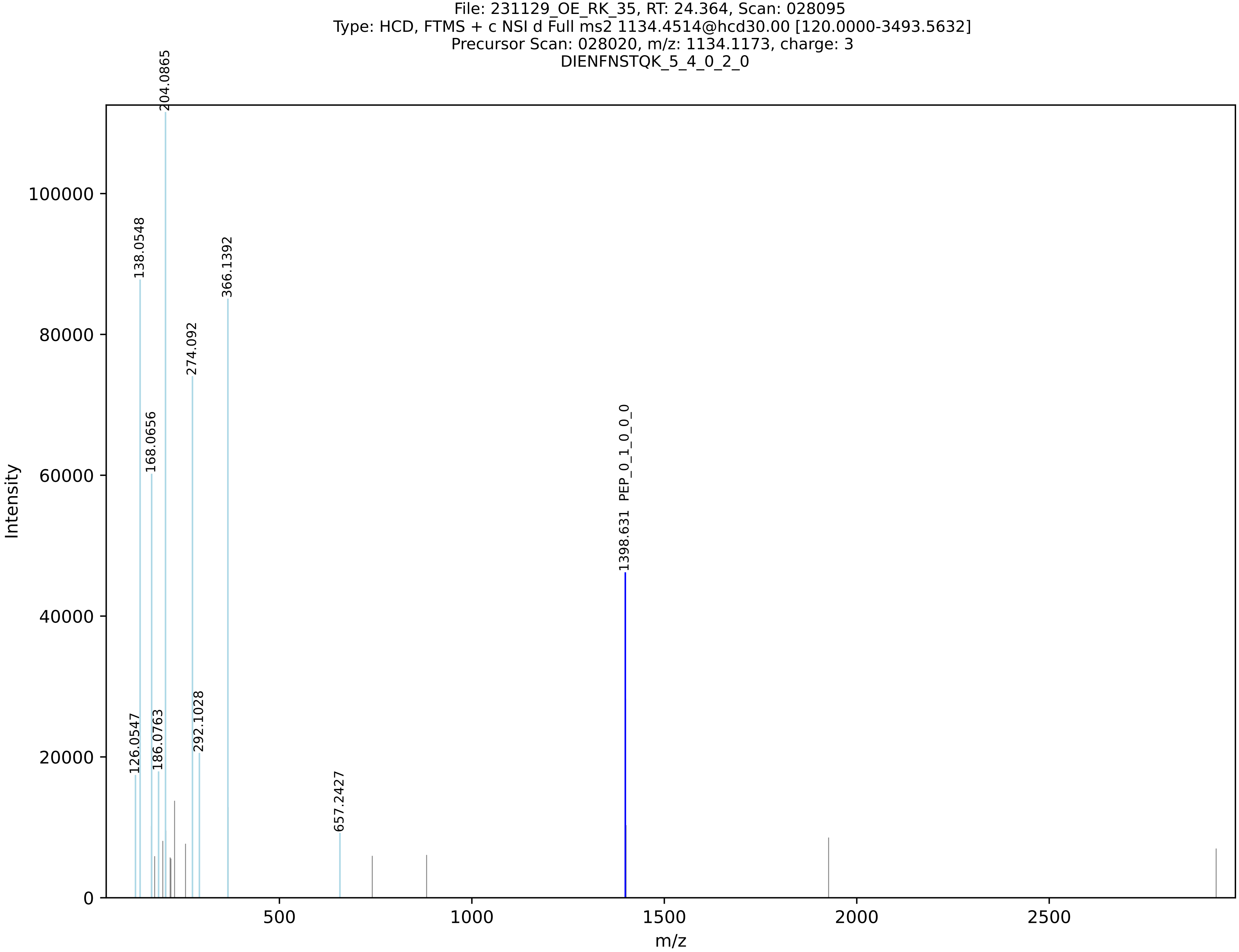
File: 231129_OE_RK_35, RT: 24.364, Scan: 028095
Type: HCD, FTMS + c NSI d Full ms2 1134.4514@hcd30.00 [120.0000-3493.5632]
Precursor Scan: 028020, m/z: 1134.1173, charge: 3
DIENFNSTQK_5_4_0_2_0
204.0865
138.0548
366.1392
274.092
168.0656
1398.631 PEP_0_1_0_0_0
126.0547
186.0763
292.1028
657.2427
Intensity
m/z
0
20000
40000
60000
80000
100000
500
1000
1500
2000
2500

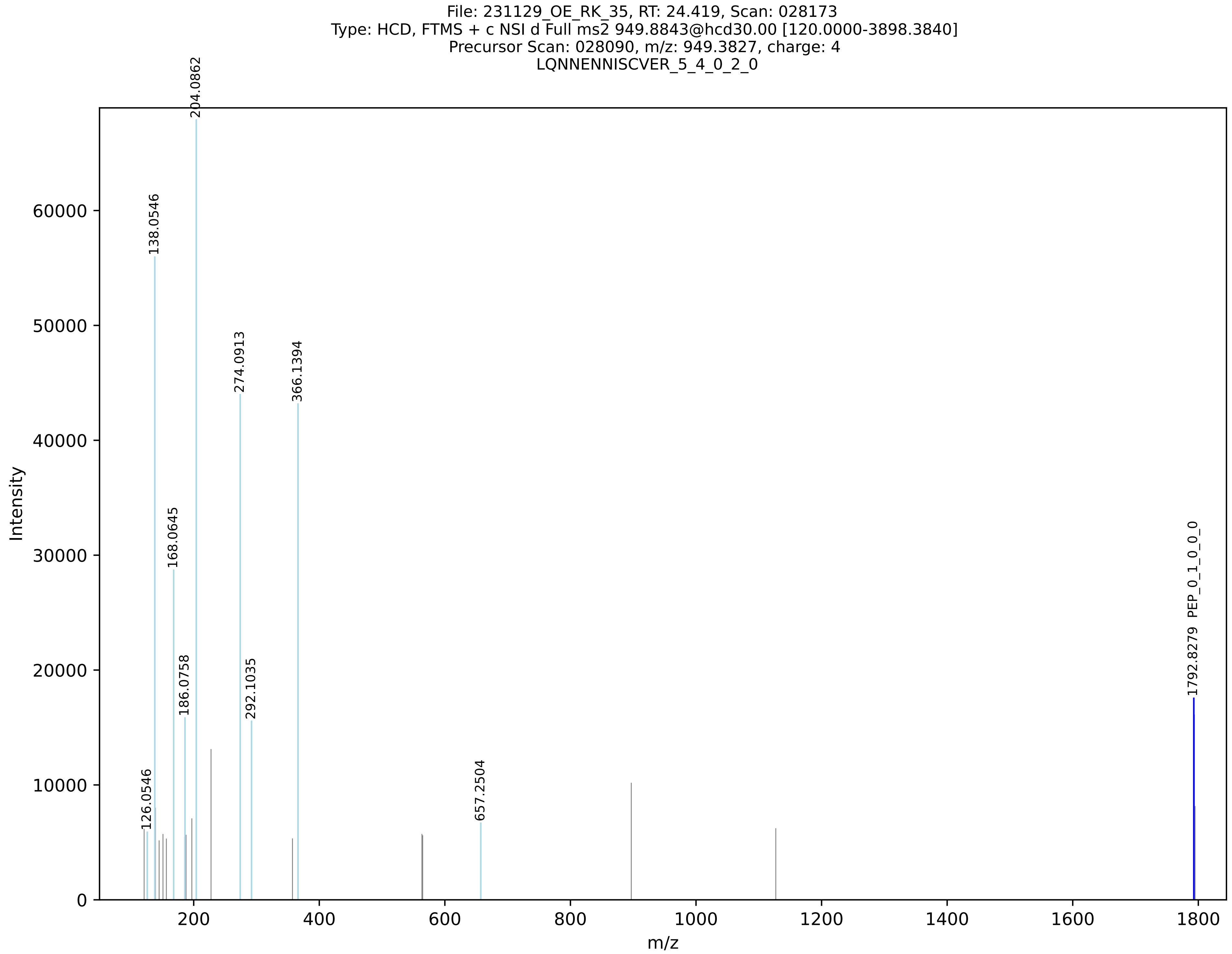
File: 231129_OE_RK_35, RT: 24.419, Scan: 028173
Type: HCD, FTMS + c NSI d Full ms2 949.8843@hcd30.00 [120.0000-3898.3840]
Precursor Scan: 028090, m/z: 949.3827, charge: 4
LQNNENNISCVER_5_4_0_2_0
204.0862
138.0546
274.0913
366.1394
168.0645
186.0758
292.1035
126.0546
657.2504
1792.8279 PEP_0_1_0_0_0
Intensity
m/z
0
10000
20000
30000
40000
50000
60000
200
400
600
800
1000
1200
1400
1600
1800

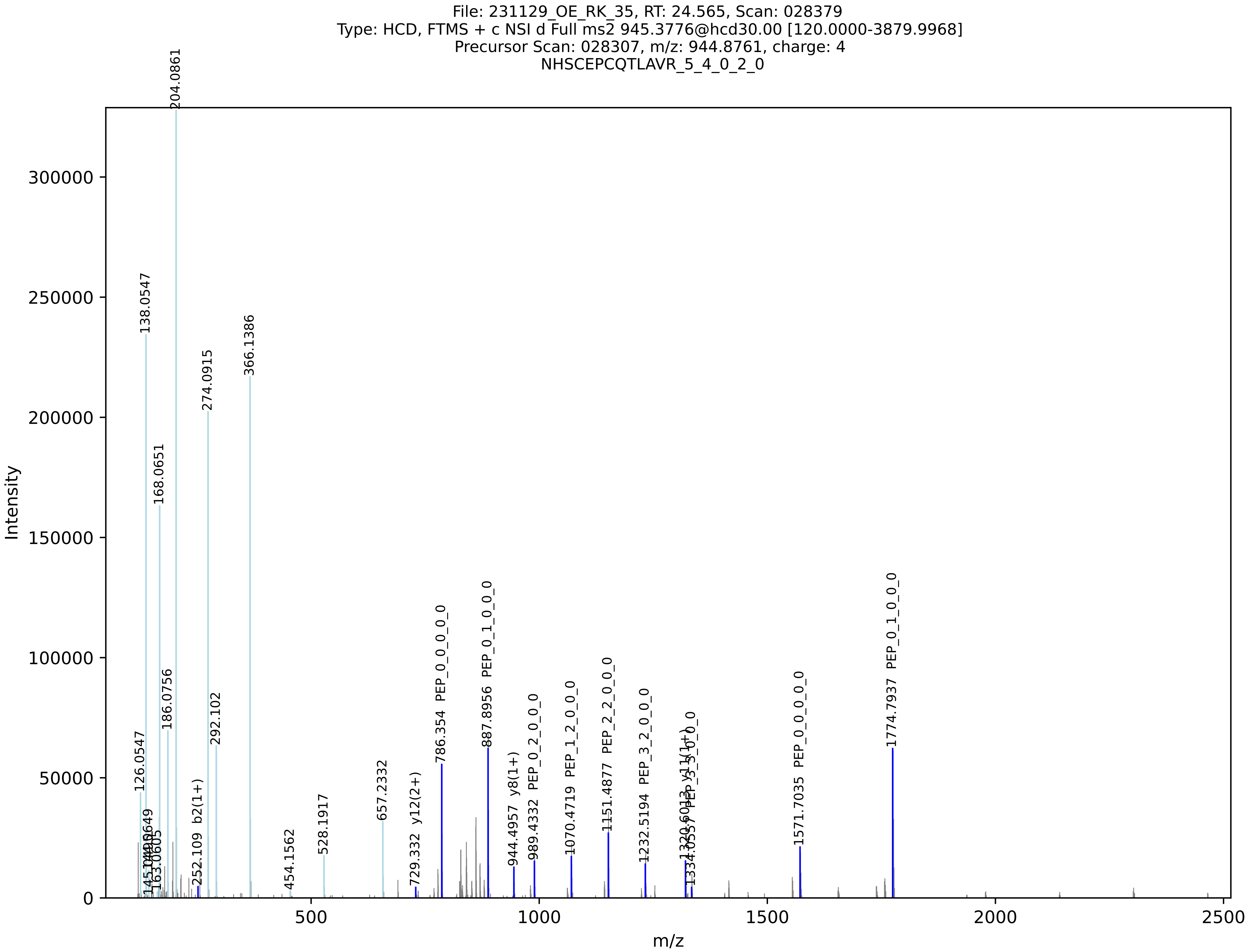
File: 231129_OE_RK_35, RT: 24.565, Scan: 028379
Type: HCD, FTMS + c NSI d Full ms2 945.3776@hcd30.00 [120.0000-3879.9968]
Precursor Scan: 028307, m/z: 944.8761, charge: 4
NHSCEPCQTLAVR_5_4_0_2_0
Intensity
m/z
0
50000
100000
150000
200000
250000
300000
500
1000
1500
2000
2500
204.0861
138.0547
366.1386
274.0915
168.0651
186.0756
292.102
126.0547
163.0605
252.109 b2(1+)
454.1562
528.1917
657.2332
729.332 y12(2+)
786.354 PEP_0_0_0_0_0_
887.8956 PEP_0_1_0_0_0_
944.4957 y8(1+)
989.4332 PEP_0_2_0_0_0
1070.4719 PEP_1_2_0_0_0
1151.4877 PEP_2_2_0_0_0
1232.5194 PEP_3_2_0_0_0
1571.7035 PEP_0_0_0_0_0
1774.7937 PEP_0_1_0_0_0_

File: 231129_OE_RK_35, RT: 24.616, Scan: 028454
Type: HCD, FTMS + c NSI d Full ms2 940.6031@hcd30.00 [120.0000-4820.6460]
Precursor Scan: 028378, m/z: 940.0017, charge: 5
GNVAVTVSGHTCQHWSAQTPHTHNR_5_4_0_1_0

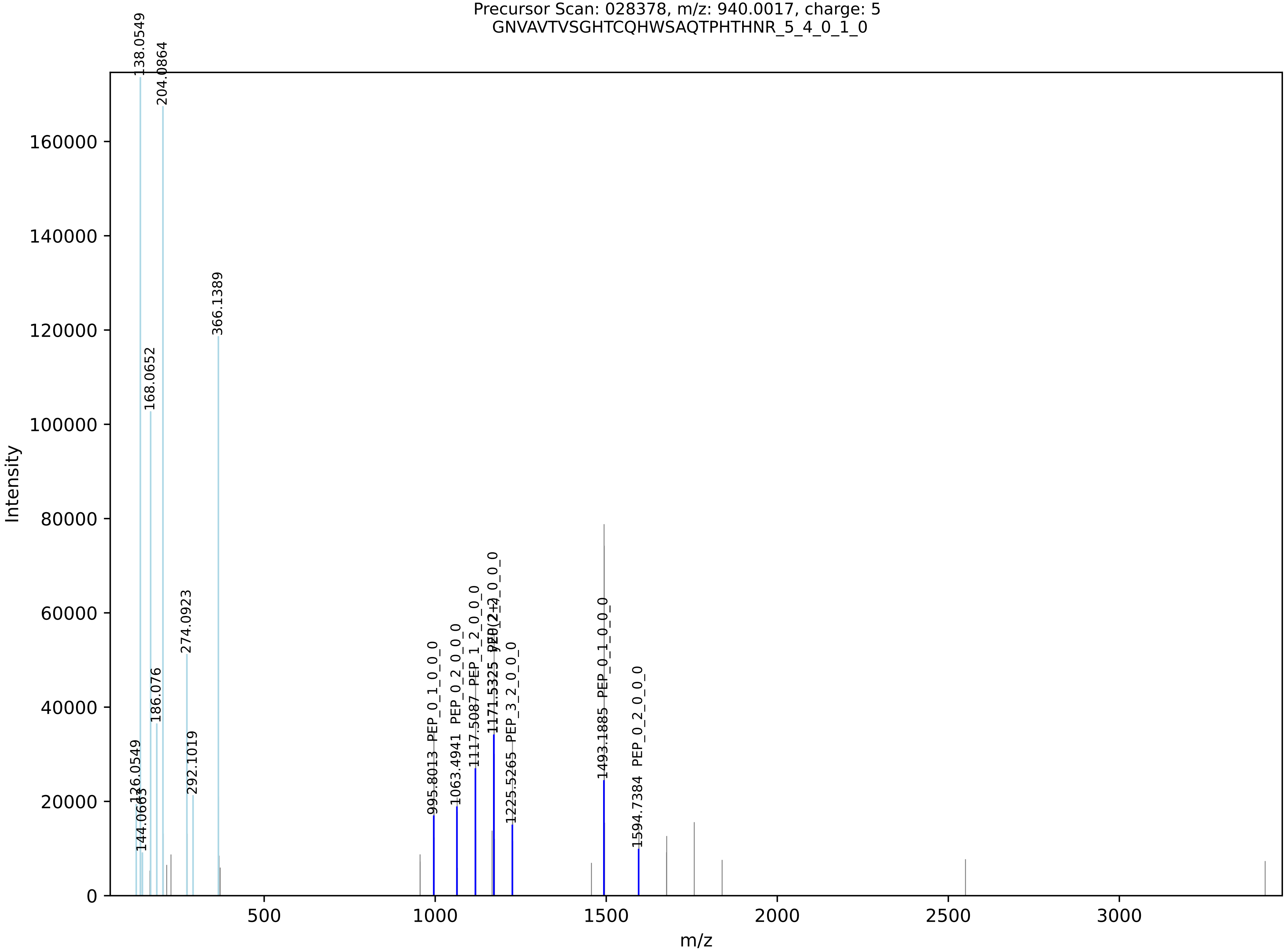

File: 231129_OE_RK_35, RT: 24.623, Scan: 028464
Type: HCD, FTMS + c NSI d Full ms2 1166.8320@hcd30.00 [120.0000-3592.6479]
Precursor Scan: 028378, m/z: 1166.4979, charge: 3
LINDYVKNGTR_5_4_0_2_0

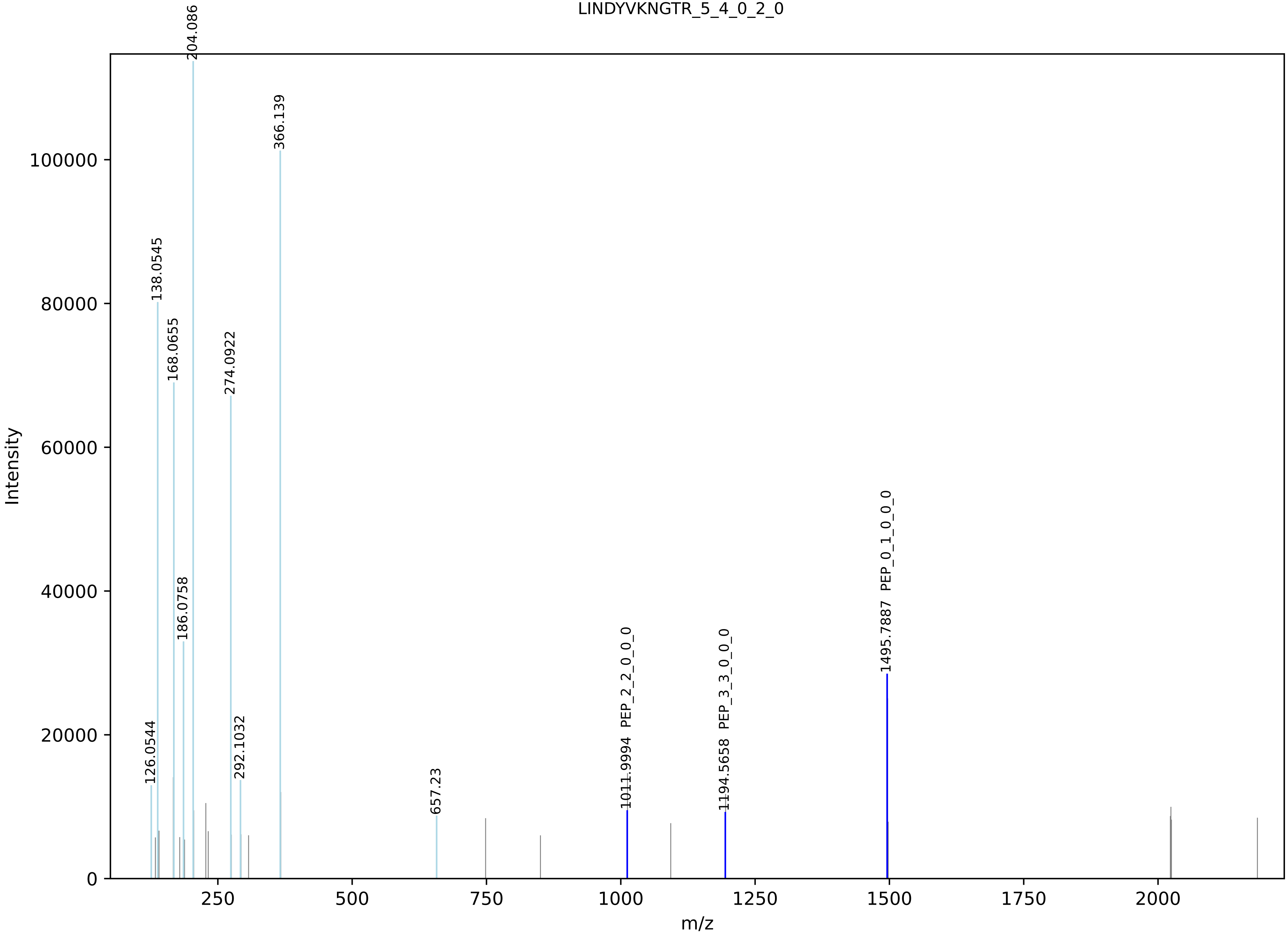

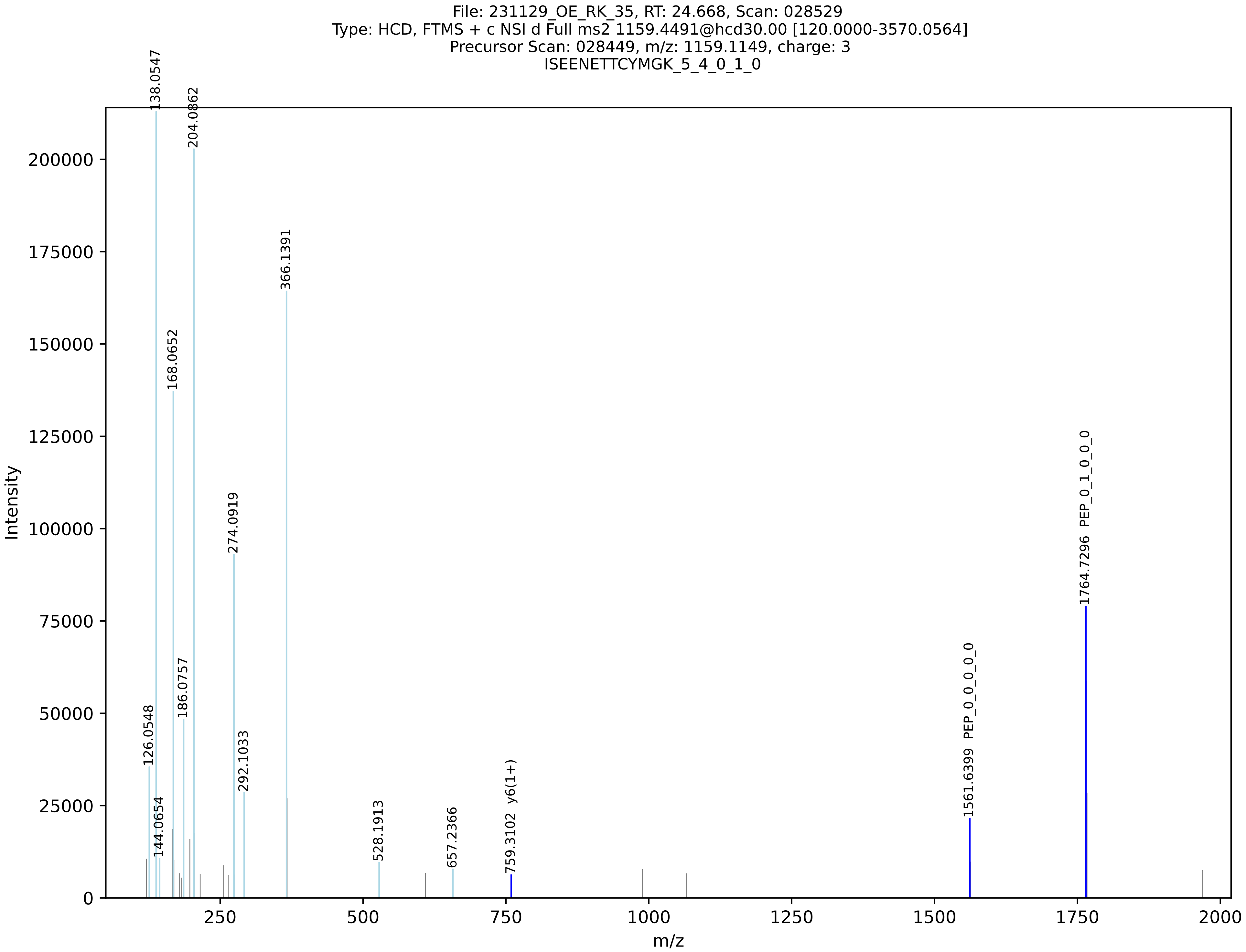
File: 231129_OE_RK_35, RT: 24.668, Scan: 028529
Type: HCD, FTMS + c NSI d Full ms2 1159.4491@hcd30.00 [120.0000-3570.0564]
Precursor Scan: 028449, m/z: 1159.1149, charge: 3
ISEENETTCYMGK_5_4_0_1_0
Intensity
m/z
138.0547
204.0862
366.1391
168.0652
274.0919
186.0757
126.0548
292.1033
144.0654
528.1913
657.2366
759.3102 y6(1+)
1561.6399 PEP_0_0_0_0_0
1764.7296 PEP_0_1_0_0_0
0
25000
50000
75000
100000
125000
150000
175000
200000
250
500
750
1000
1250
1500
1750
2000

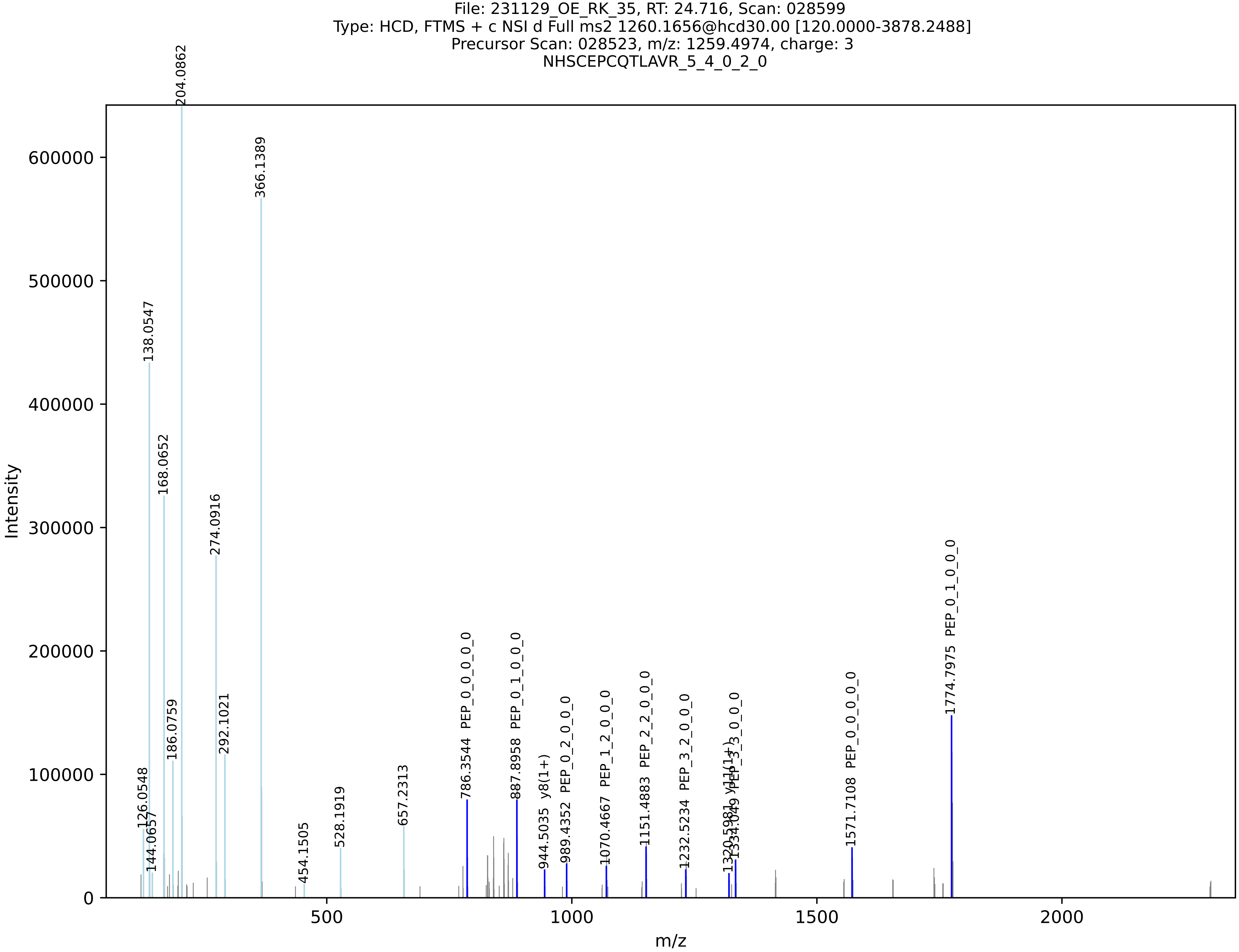

File: 231129_OE_RK_35, RT: 24.716, Scan: 028599
Type: HCD, FTMS + c NSI d Full ms2 1260.1656@hcd30.00 [120.0000-3878.2488]
Precursor Scan: 028523, m/z: 1259.4974, charge: 3
NHSCEPCQTLAVR_5_4_0_2_0
Intensity
m/z
0
100000
200000
300000
400000
500000
600000
500
1000
1500
2000
204.0862
366.1389
138.0547
168.0652
274.0916
186.0759
292.1021
126.0548
144.0657
454.1505
528.1919
657.2313
786.3544 PEP_0_0_0_0_0
887.8958 PEP_0_1_0_0_0
944.5035 y8(1+)
989.4352 PEP_0_2_0_0_0
1070.4667 PEP_1_2_0_0_0
1151.4883 PEP_2_2_0_0_0
1232.5234 PEP_3_2_0_0_0
1571.7108 PEP_0_0_0_0_0
1774.7975 PEP_0_1_0_0_0

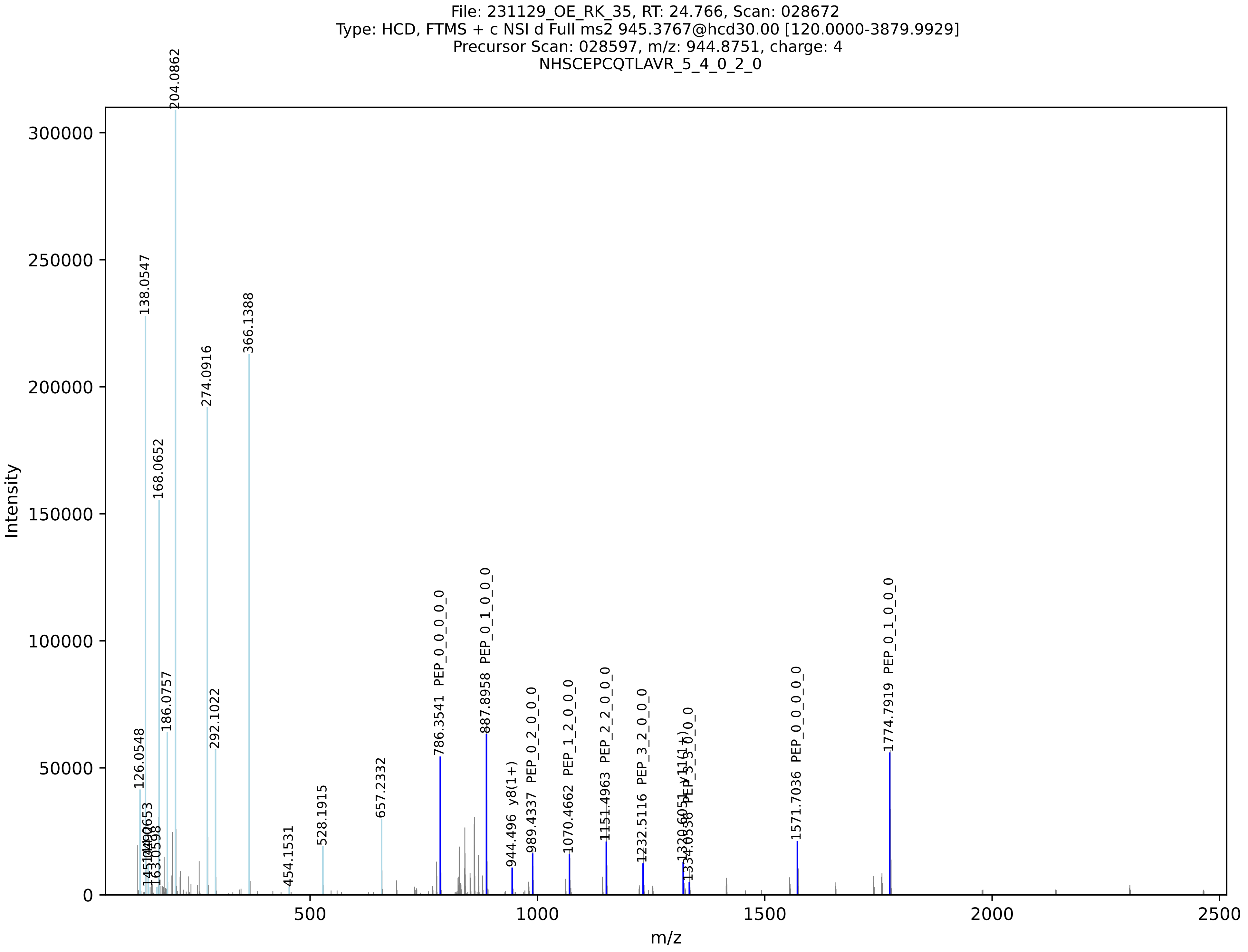
File: 231129_OE_RK_35, RT: 24.766, Scan: 028672
Type: HCD, FTMS + c NSI d Full ms2 945.3767@hcd30.00 [120.0000-3879.9929]
Precursor Scan: 028597, m/z: 944.8751, charge: 4
NHSCEPCQTLAVR_5_4_0_2_0
Intensity
m/z
204.0862
138.0547
366.1388
274.0916
168.0652
186.0757
292.1022
126.0548
163.0598
454.1531
528.1915
657.2332
786.3541 PEP_0_0_0_0_0_
887.8958 PEP_0_1_0_0_0_
944.496 y8(1+)
989.4337 PEP_0_2_0_0_0_
1070.4662 PEP_1_2_0_0_0_
1151.4963 PEP_2_2_0_0_0_
1232.5116 PEP_3_2_0_0_0_
1571.7036 PEP_0_0_0_0_0_
1774.7919 PEP_0_1_0_0_0_

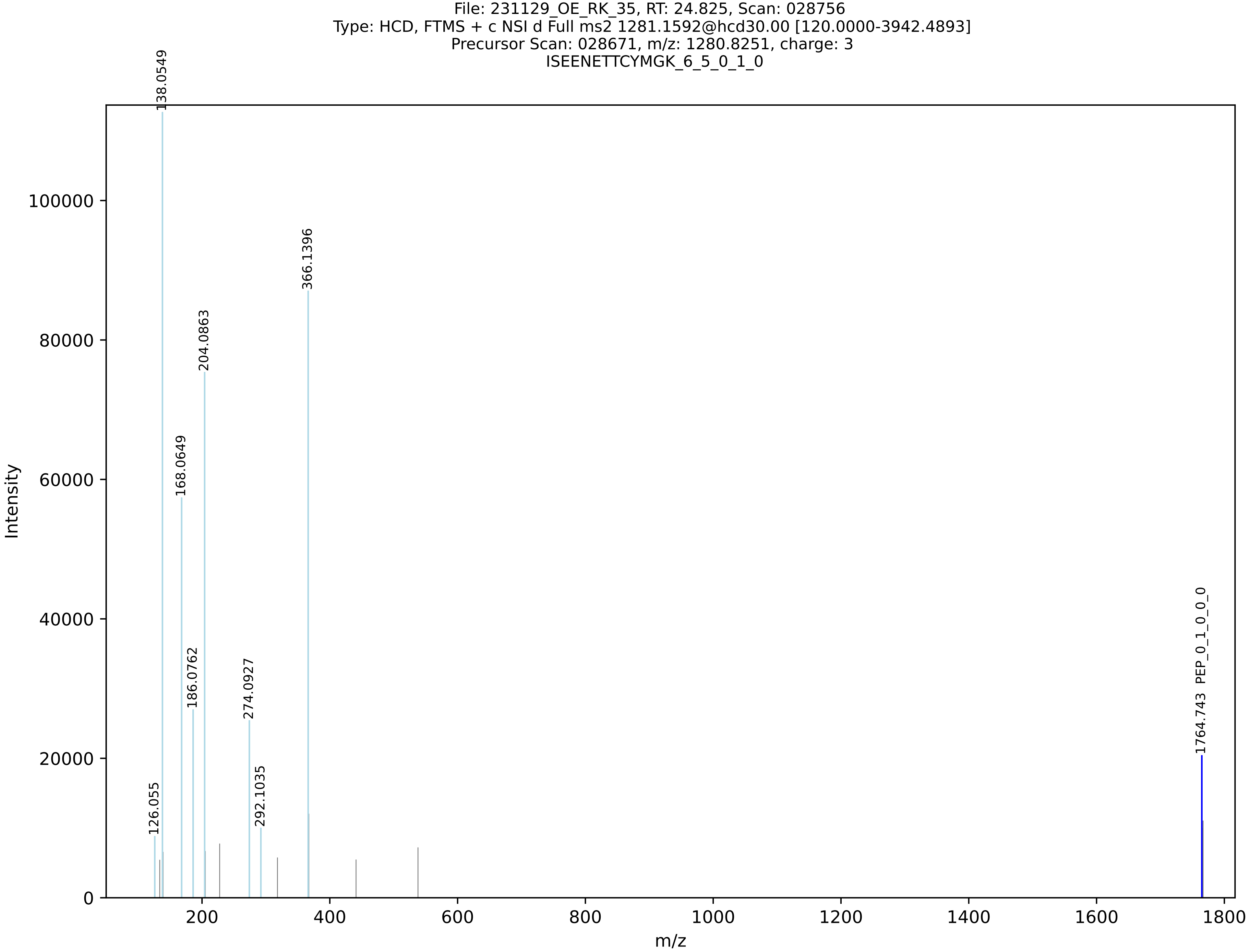
File: 231129_OE_RK_35, RT: 24.825, Scan: 028756
Type: HCD, FTMS + c NSI d Full ms2 1281.1592@hcd30.00 [120.0000-3942.4893]
Precursor Scan: 028671, m/z: 1280.8251, charge: 3
ISEENETTCYMGK_6_5_0_1_0
138.0549
366.1396
204.0863
168.0649
186.0762
274.0927
292.1035
126.055
1764.743 PEP_0_1_0_0_0
Intensity
m/z
0
20000
40000
60000
80000
100000
200
400
600
800
1000
1200
1400
1600
1800

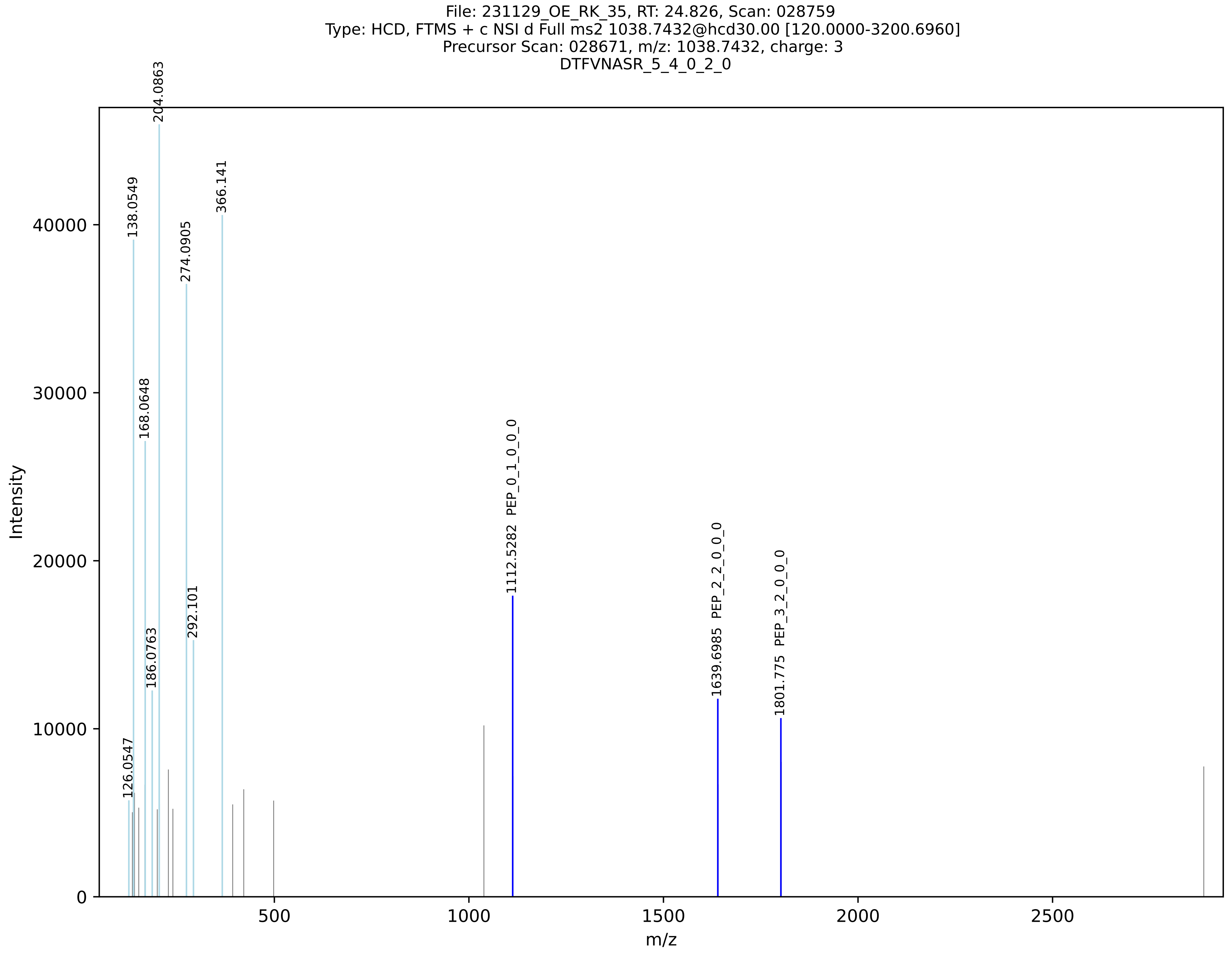

File: 231129_OE_RK_35, RT: 24.826, Scan: 028759
Type: HCD, FTMS + c NSI d Full ms2 1038.7432@hcd30.00 [120.0000-3200.6960]
Precursor Scan: 028671, m/z: 1038.7432, charge: 3
DTFVNASR_5_4_0_2_0
204.0863
138.0549
366.141
274.0905
168.0648
186.0763
292.101
126.0547
1112.5282 PEP_0_1_0_0_0
1639.6985 PEP_2_2_0_0_0
1801.775 PEP_3_2_0_0_0
Intensity
m/z
0
10000
20000
30000
40000
500
1000
1500
2000
2500

File: 231129_OE_RK_35, RT: 24.868, Scan: 028818
Type: HCD, FTMS + c NSI d Full ms2 977.7638@hcd30.00 [120.0000-3014.0994]
Precursor Scan: 028745, m/z: 977.4297, charge: 3
TPLTANITK_5_5_1_0_0

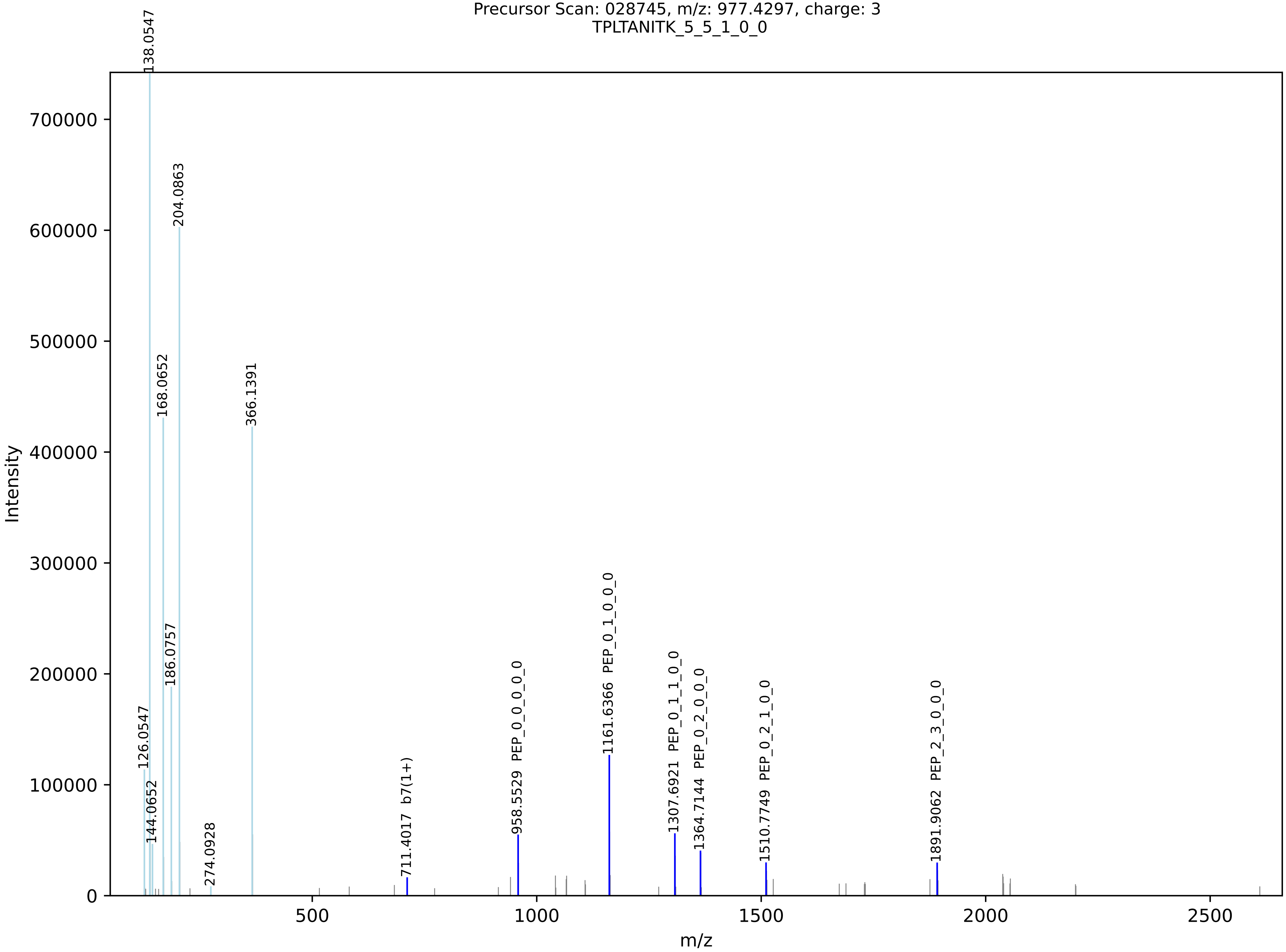

File: 231129_OE_RK_35, RT: 24.869, Scan: 028821
Type: HCD, FTMS + c NSI d Full ms2 1159.4481@hcd30.00 [120.0000-3570.0532]
Precursor Scan: 028745, m/z: 1159.114, charge: 3
ISEENETTCYMGK_5_4_0_1_0

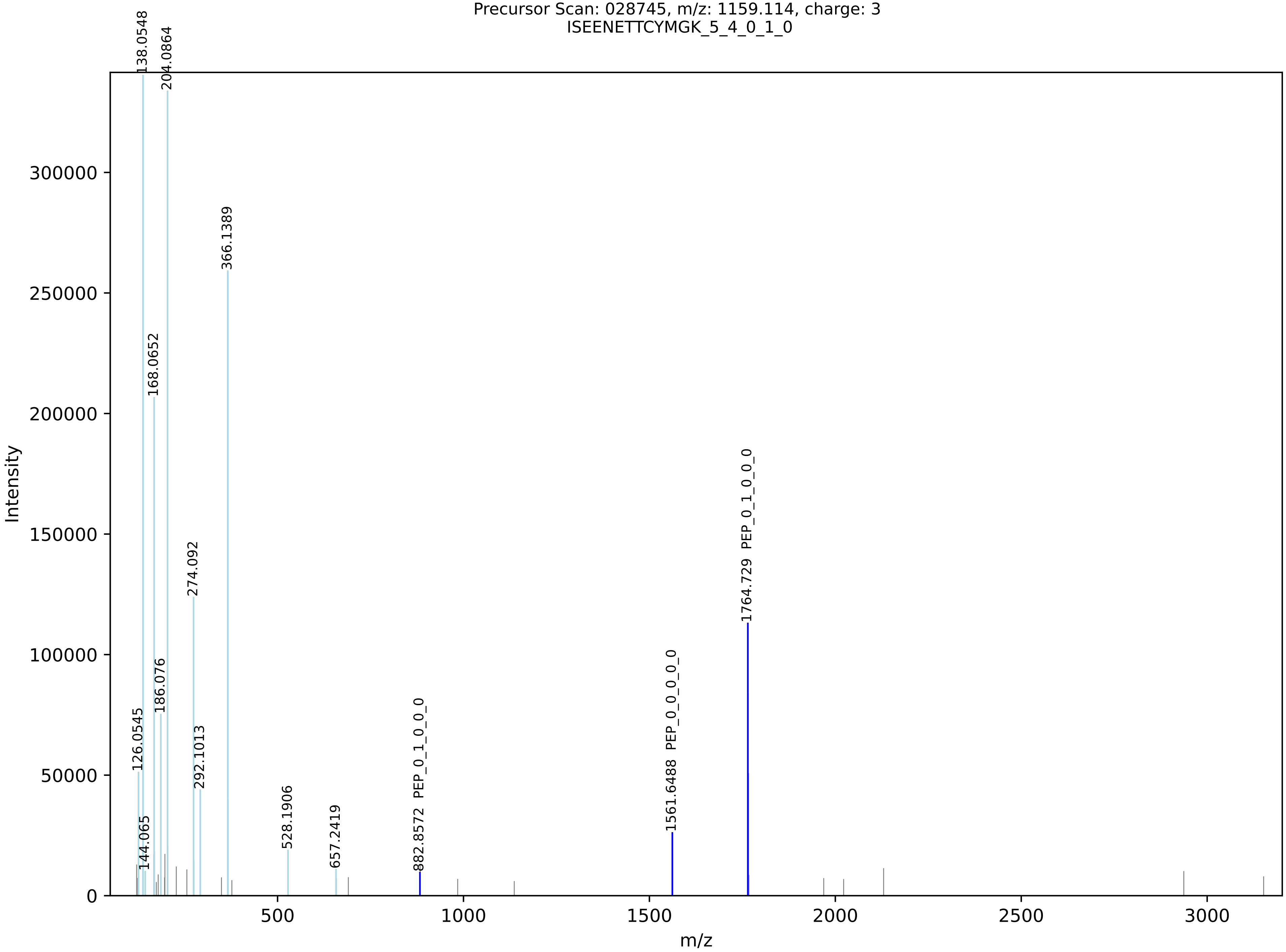

File: 231129_OE_RK_35, RT: 24.871, Scan: 028823
Type: HCD, FTMS + c NSI d Full ms2 1266.1740@hcd30.00 [120.0000-3896.6345]
Precursor Scan: 028745, m/z: 1265.5058, charge: 3
LQNNENNISCVER_5_4_0_2_0

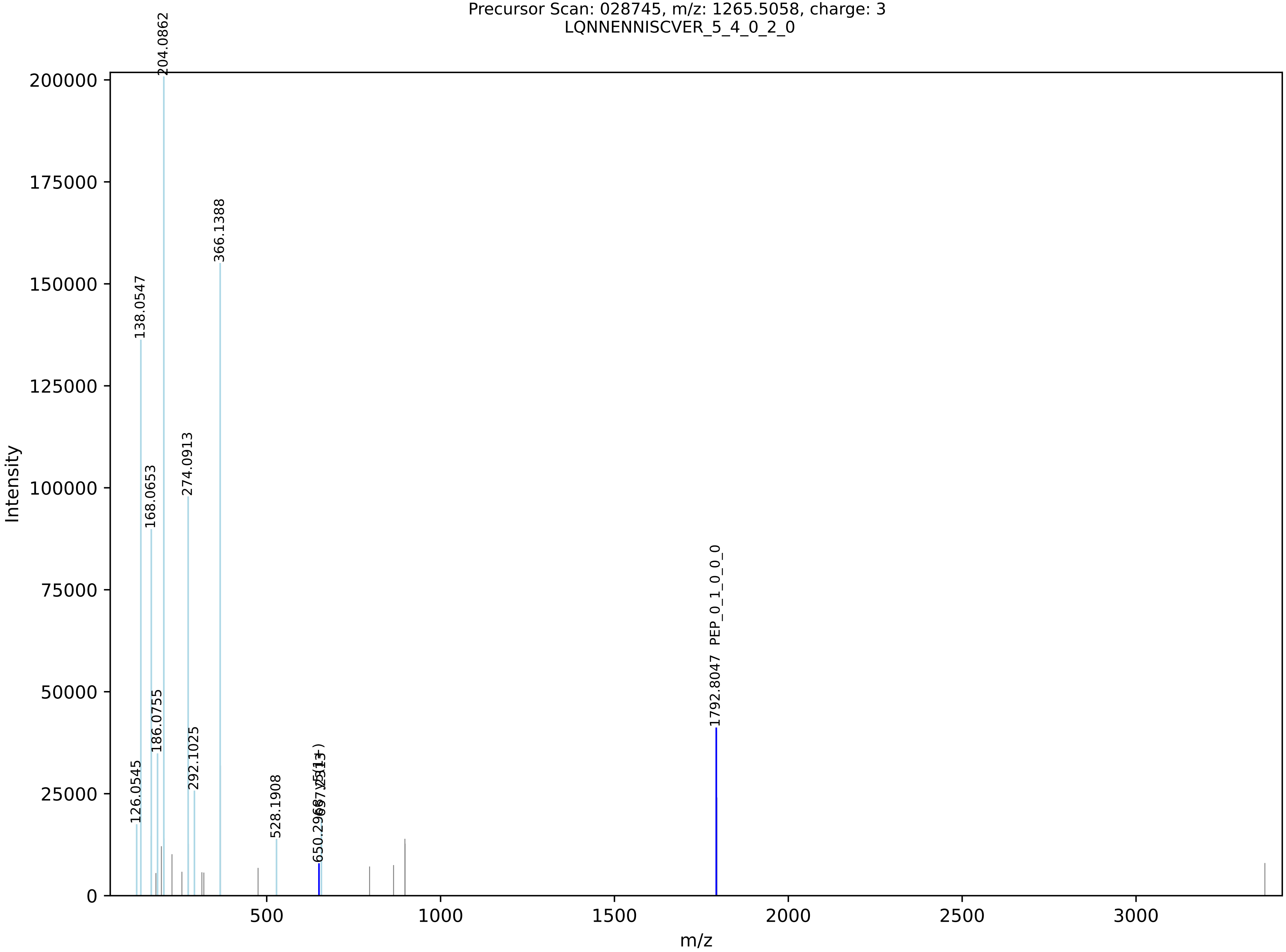

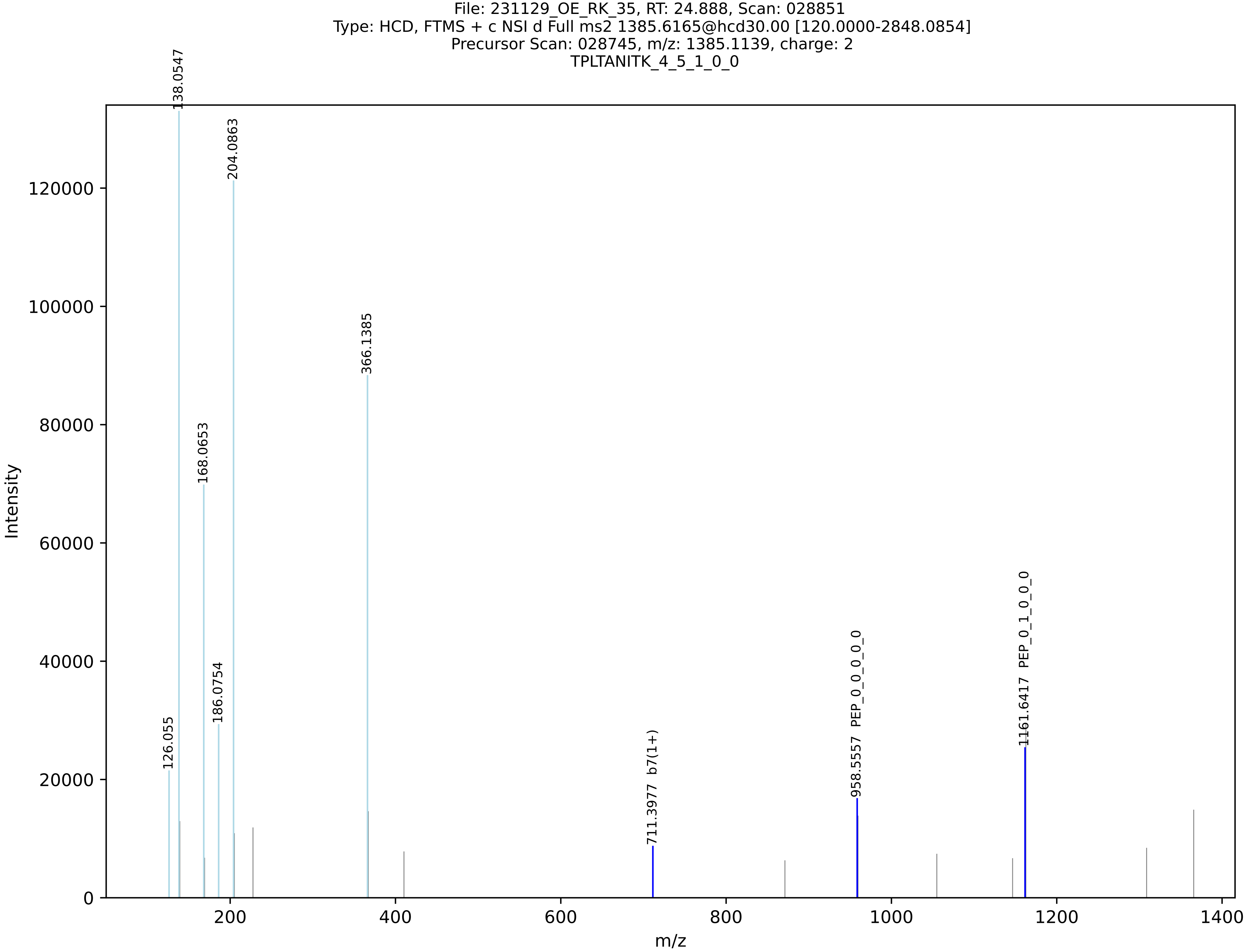
File: 231129_OE_RK_35, RT: 24.888, Scan: 028851
Type: HCD, FTMS + c NSI d Full ms2 1385.6165@hcd30.00 [120.0000-2848.0854]
Precursor Scan: 028745, m/z: 1385.1139, charge: 2
TPLTANITK_4_5_1_0_0
138.0547
204.0863
366.1385
168.0653
186.0754
126.055
711.3977 b7(1+)
958.5557 PEP_0_0_0_0_0
1161.6417 PEP_0_1_0_0_0
Intensity
0
20000
40000
60000
80000
100000
120000
200
400
600
800
1000
1200
1400
m/z

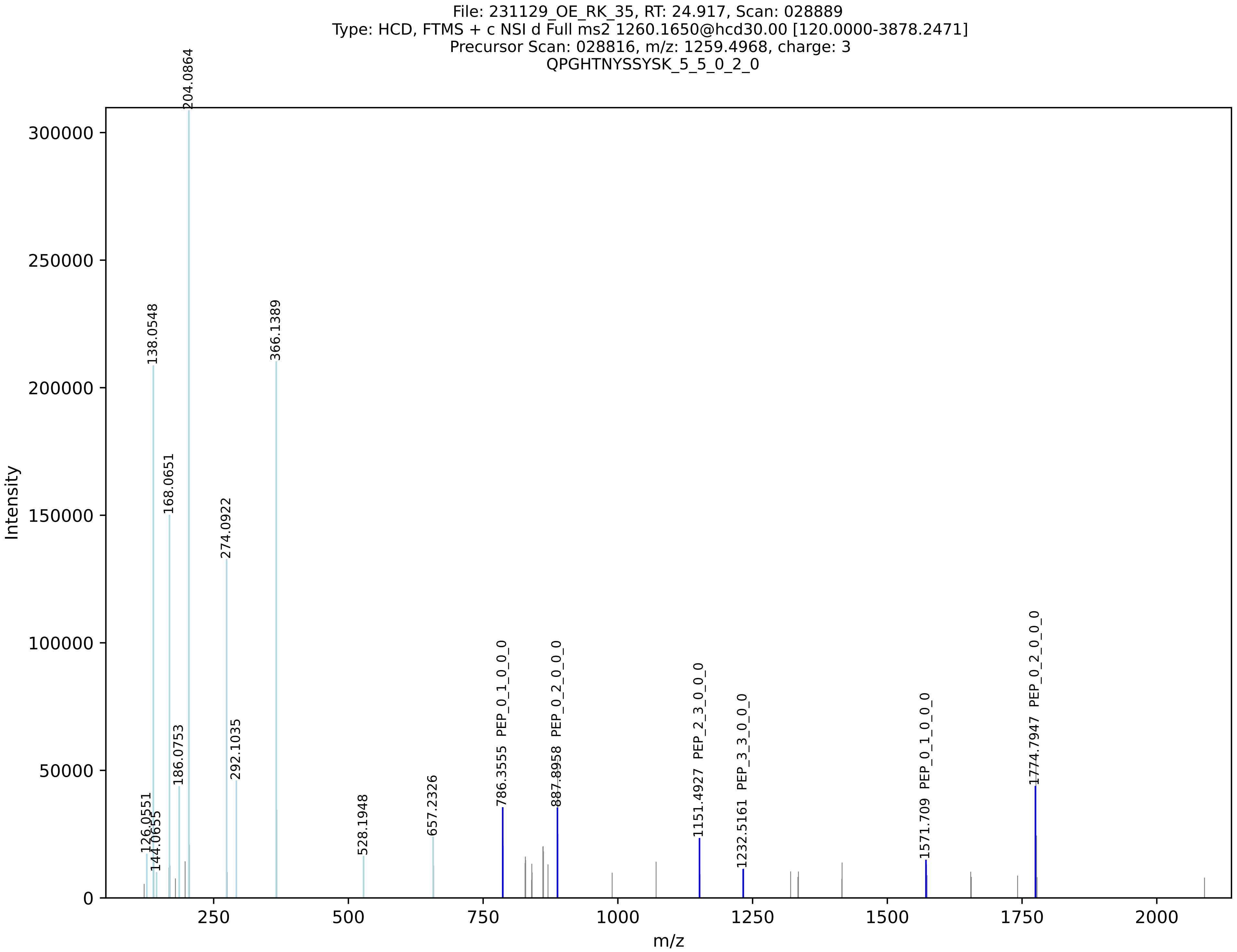
File: 231129_OE_RK_35, RT: 24.917, Scan: 028889
Type: HCD, FTMS + c NSI d Full ms2 1260.1650@hcd30.00 [120.0000-3878.2471]
Precursor Scan: 028816, m/z: 1259.4968, charge: 3
QPGHTNYSSYSK_5_5_0_2_0
Intensity
m/z
204.0864
138.0548
366.1389
168.0651
274.0922
186.0753
292.1035
126.0551
144.0655
528.1948
657.2326
786.3555 PEP_0_1_0_0_0
887.8958 PEP_0_2_0_0_0
1151.4927 PEP_2_3_0_0_0
1232.5161 PEP_3_3_0_0_0
1571.709 PEP_0_1_0_0_0
1774.7947 PEP_0_2_0_0_0

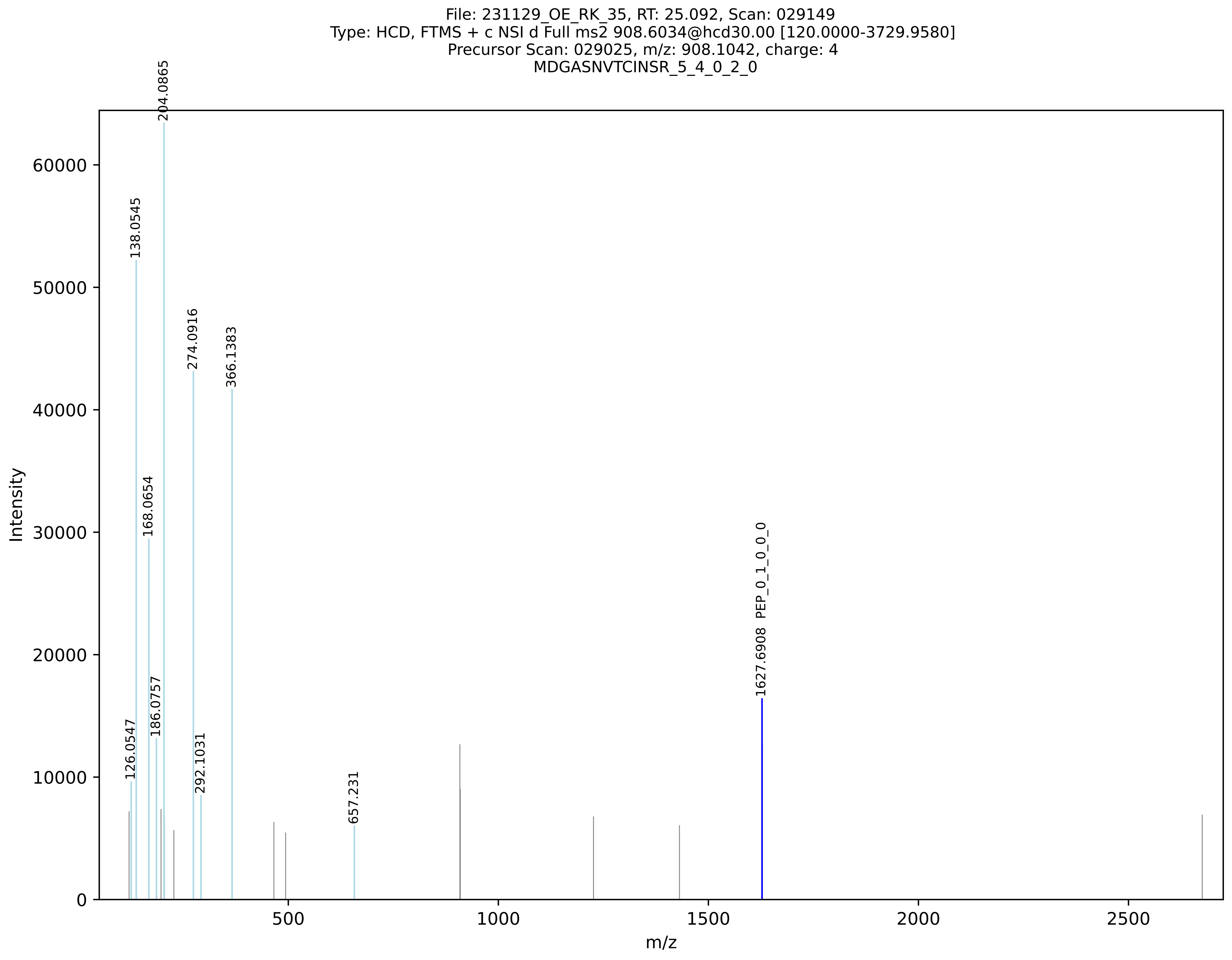
File: 231129_OE_RK_35, RT: 25.092, Scan: 029149
Type: HCD, FTMS + c NSI d Full ms2 908.6034@hcd30.00 [120.0000-3729.9580]
Precursor Scan: 029025, m/z: 908.1042, charge: 4
MDGASNVTCINSR_5_4_0_2_0
Intensity
m/z
204.0865
138.0545
274.0916
366.1383
168.0654
186.0757
126.0547
292.1031
657.231
1627.6908 PEP_0_1_0_0_0

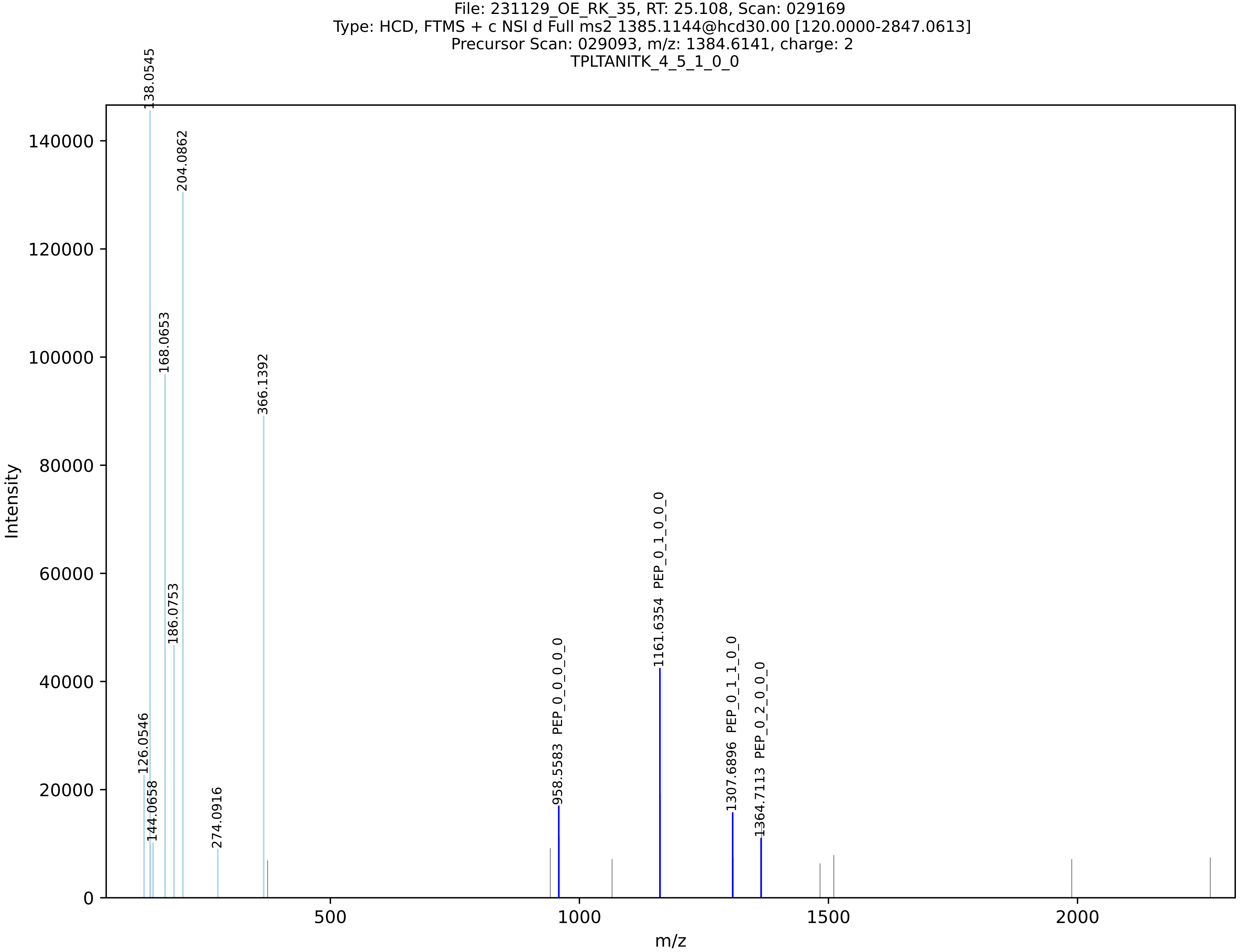
File: 231129_OE_RK_35, RT: 25.108, Scan: 029169
Type: HCD, FTMS + c NSI d Full ms2 1385.1144@hcd30.00 [120.0000-2847.0613]
Precursor Scan: 029093, m/z: 1384.6141, charge: 2
TPLTANITK_4_5_1_0_0
Intensity
m/z
138.0545
204.0862
168.0653
366.1392
186.0753
126.0546
144.0658
274.0916
958.5583 PEP_0_0_0_0_0
1161.6354 PEP_0_1_0_0_0
1307.6896 PEP_0_1_1_0_0
1364.7113 PEP_0_2_0_0_0

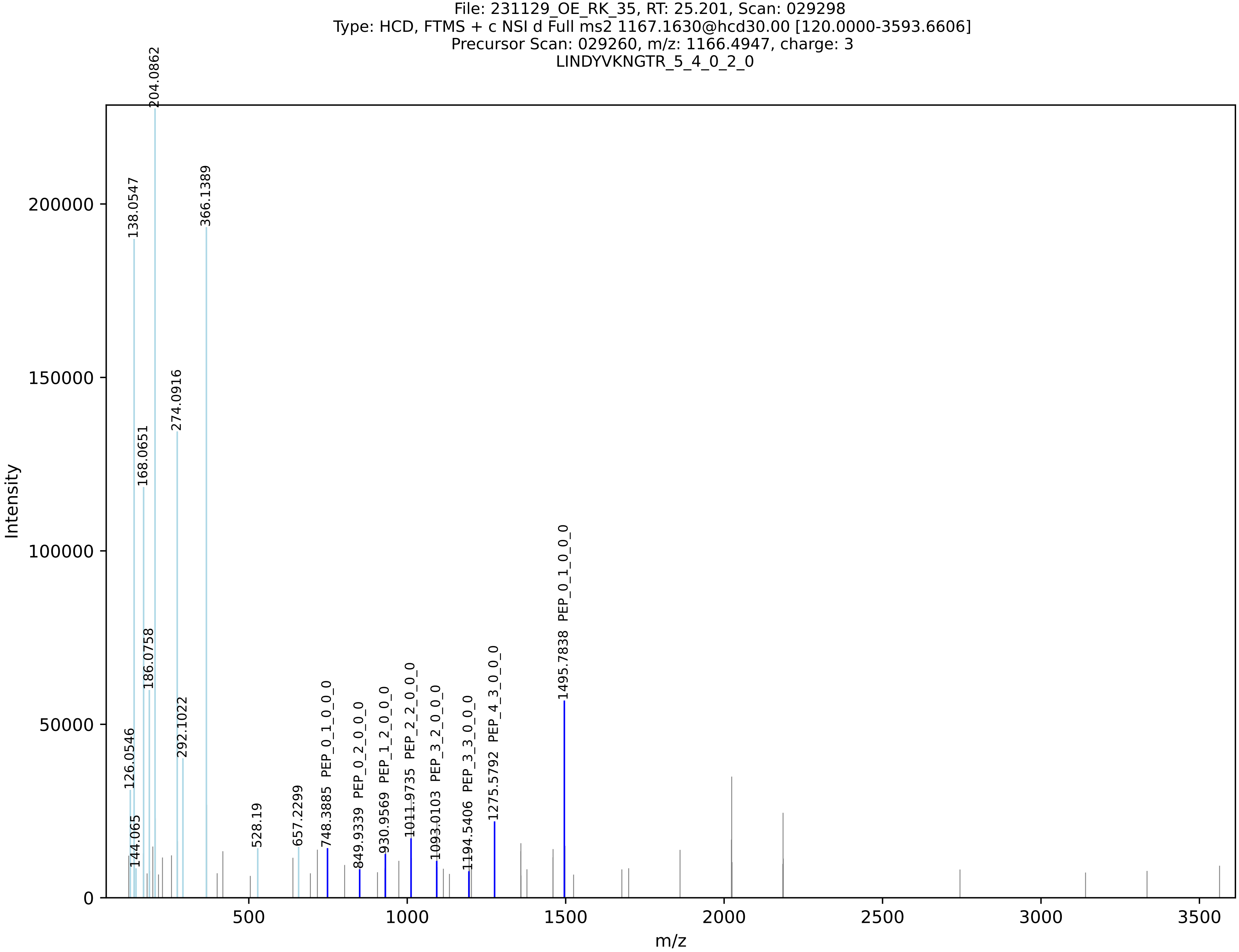

File: 231129_OE_RK_35, RT: 25.201, Scan: 029298
Type: HCD, FTMS + c NSI d Full ms2 1167.1630@hcd30.00 [120.0000-3593.6606]
Precursor Scan: 029260, m/z: 1166.4947, charge: 3
LINDYVKNGTR_5_4_0_2_0
204.0862
366.1389
138.0547
274.0916
168.0651
186.0758
1495.7838 PEP_0_1_0_0_0
1275.5792 PEP_4_3_0_0_0
1011.9735 PEP_2_2_0_0_0
1093.0103 PEP_3_2_0_0_0
930.9569 PEP_1_2_0_0_0
748.3885 PEP_0_1_0_0_0
1194.5406 PEP_3_3_0_0_0
849.9339 PEP_0_2_0_0_0
292.1022
126.0546
657.2299
528.19
144.065
Intensity
m/z

File: 231129_OE_RK_35, RT: 25.228, Scan: 029331
Type: HCD, FTMS + c NSI d Full ms2 1303.5850@hcd30.00 [120.0000-2680.7415]
Precursor Scan: 029295, m/z: 1303.5865, charge: 2
TPLTANITK_3_5_1_0_0

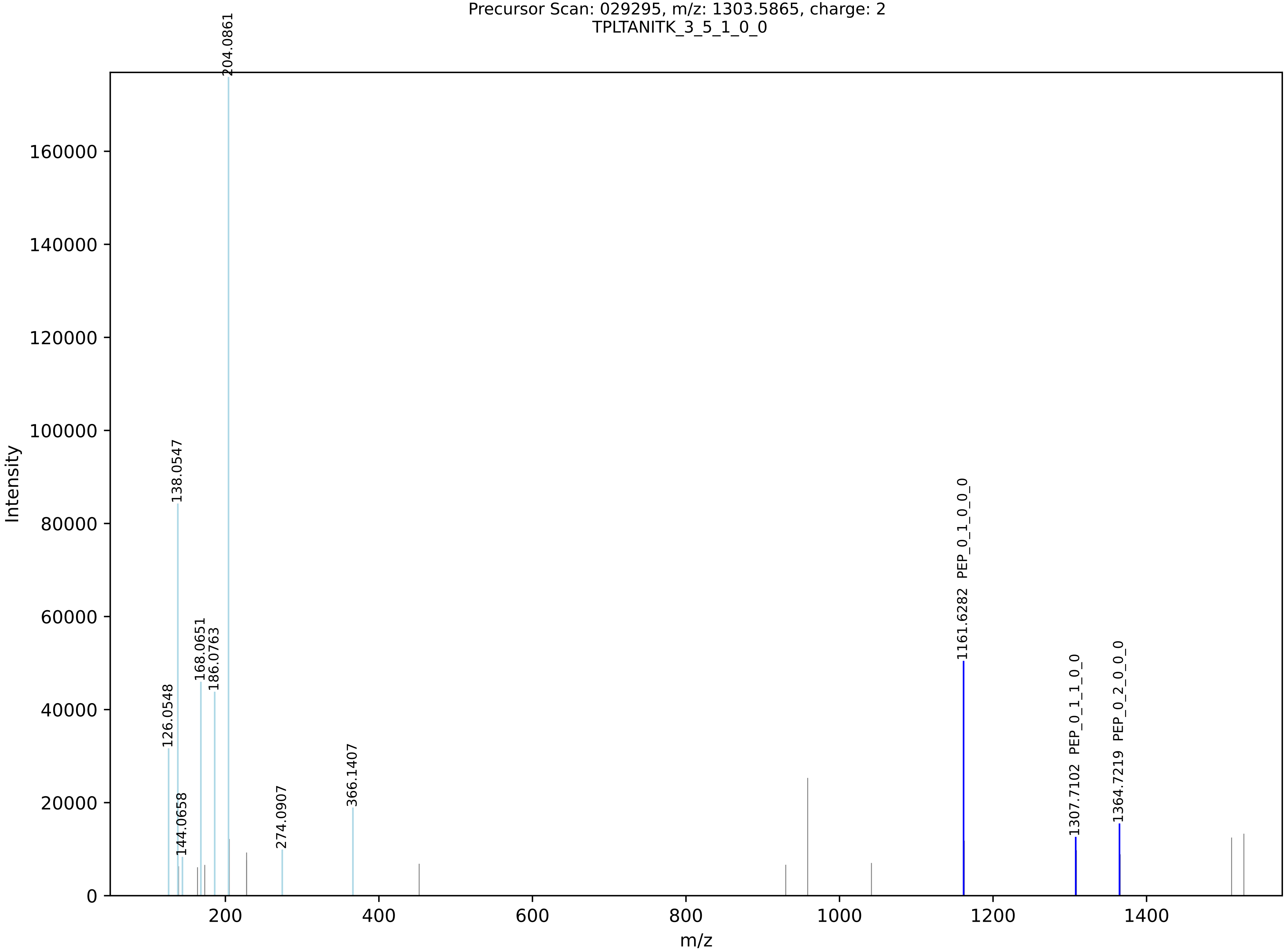

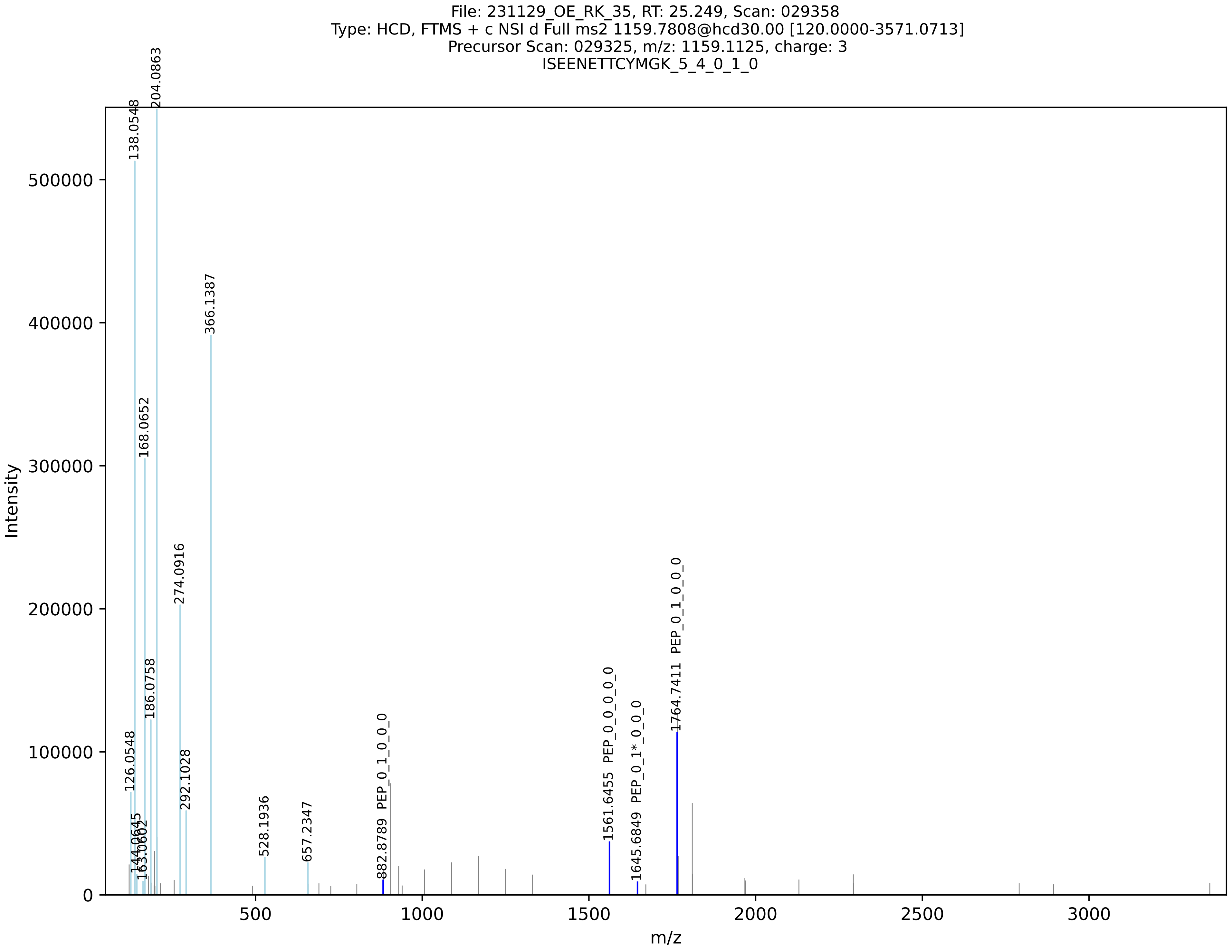

File: 231129_OE_RK_35, RT: 25.249, Scan: 029358
Type: HCD, FTMS + c NSI d Full ms2 1159.7808@hcd30.00 [120.0000-3571.0713]
Precursor Scan: 029325, m/z: 1159.1125, charge: 3
ISEENETTCYMGK_5_4_0_1_0
Intensity
m/z
204.0863
138.0548
366.1387
168.0652
274.0916
186.0758
126.0548
292.1028
144.0645
163.0602
528.1936
657.2347
882.8789 PEP_0_1_0_0_
1561.6455 PEP_0_0_0_0_0_
1645.6849 PEP_0_1*_0_0_
1764.7411 PEP_0_1_0_0_

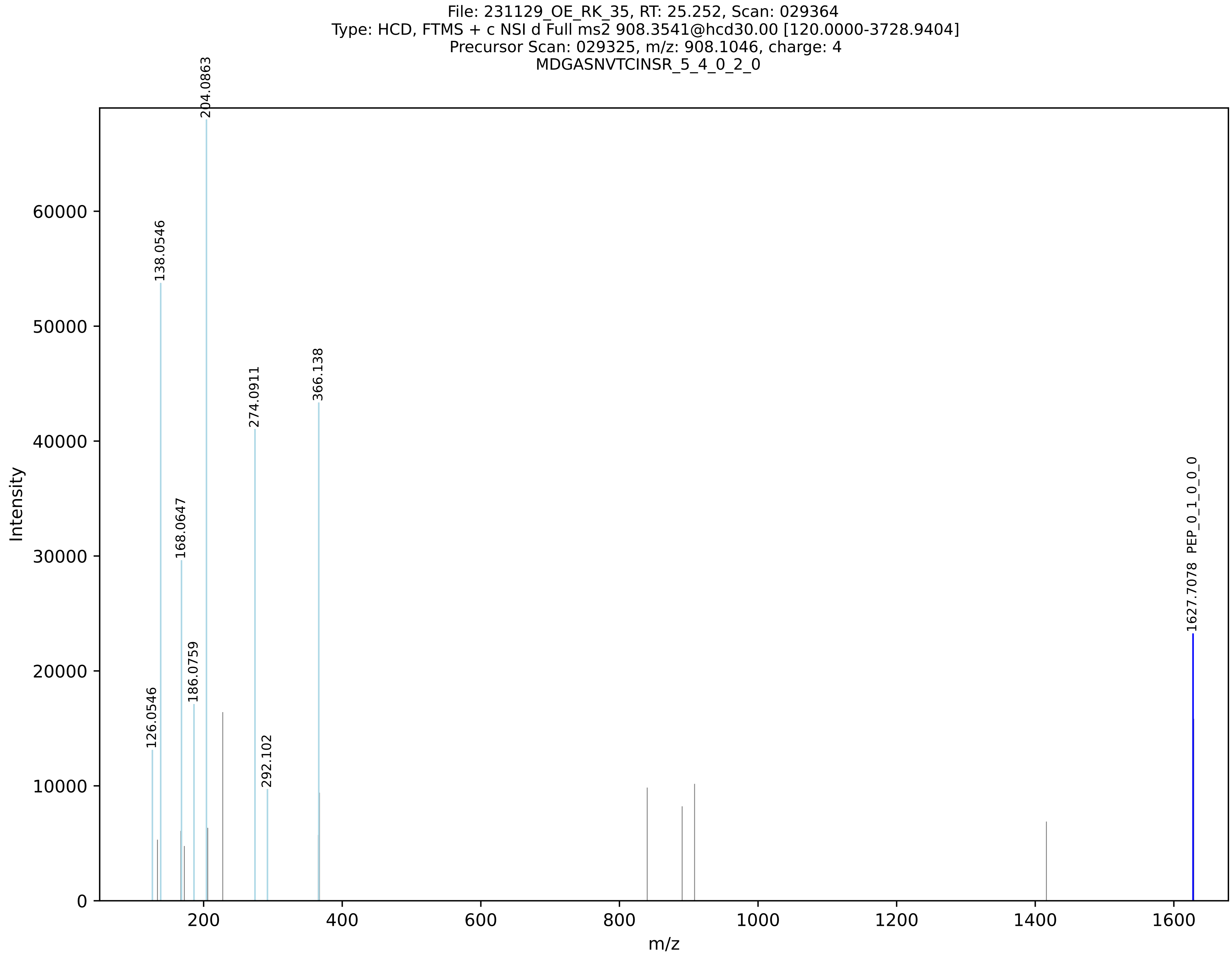
File: 231129_OE_RK_35, RT: 25.252, Scan: 029364
Type: HCD, FTMS + c NSI d Full ms2 908.3541@hcd30.00 [120.0000-3728.9404]
Precursor Scan: 029325, m/z: 908.1046, charge: 4
MDGASNVTCINSR_5_4_0_2_0
204.0863
138.0546
168.0647
186.0759
126.0546
274.0911
292.102
366.138
1627.7078 PEP_0_1_0_0_0
Intensity
m/z
0
10000
20000
30000
40000
50000
60000
200
400
600
800
1000
1200
1400
1600

File: 231129_OE_RK_35, RT: 25.363, Scan: 029514
Type: HCD, FTMS + c NSI d Full ms2 1134.7819@hcd30.00 [120.0000-3494.5747]
Precursor Scan: 029463, m/z: 1134.1157, charge: 3
DIENFNSTQK_5_4_0_2_0

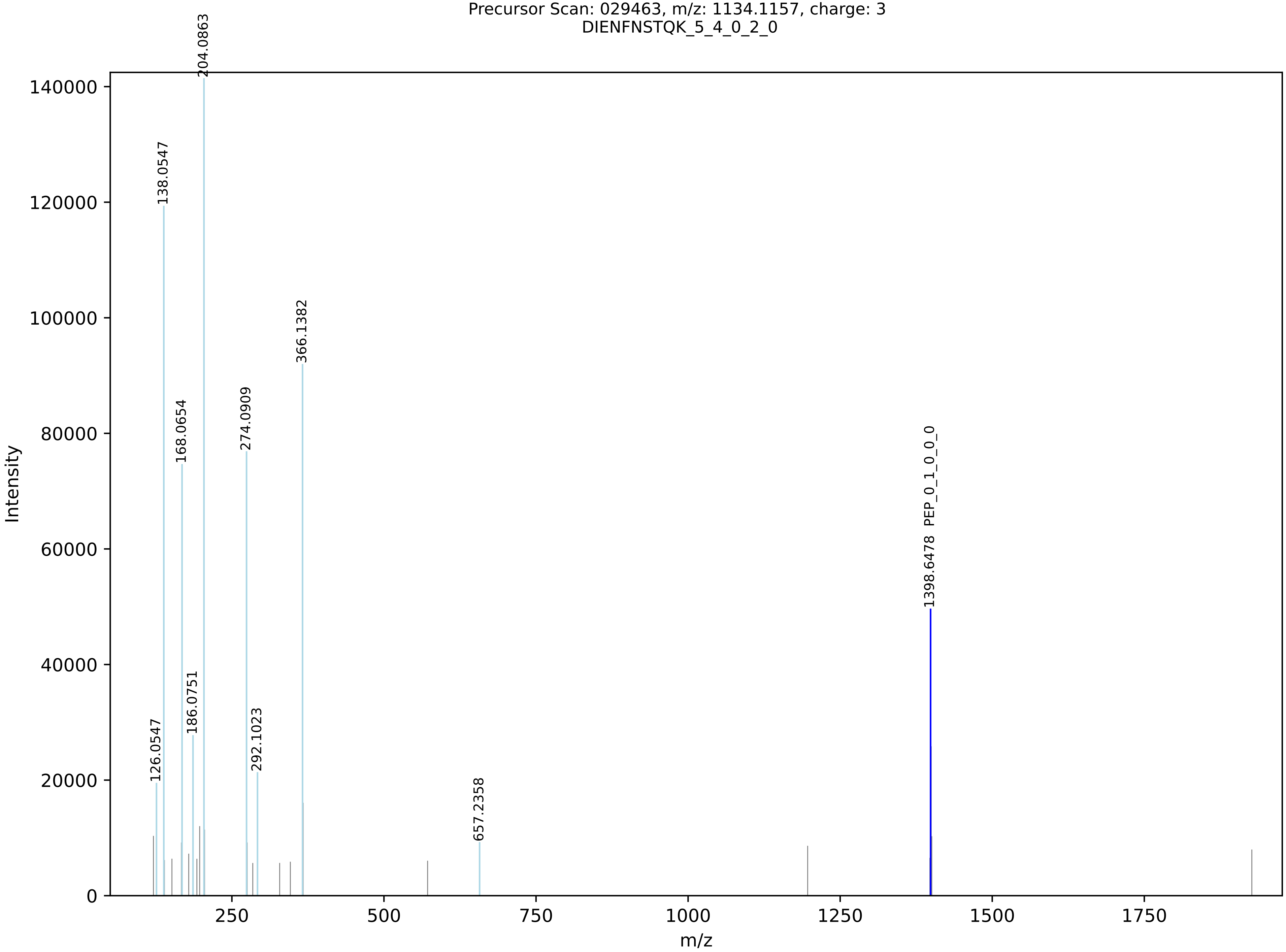

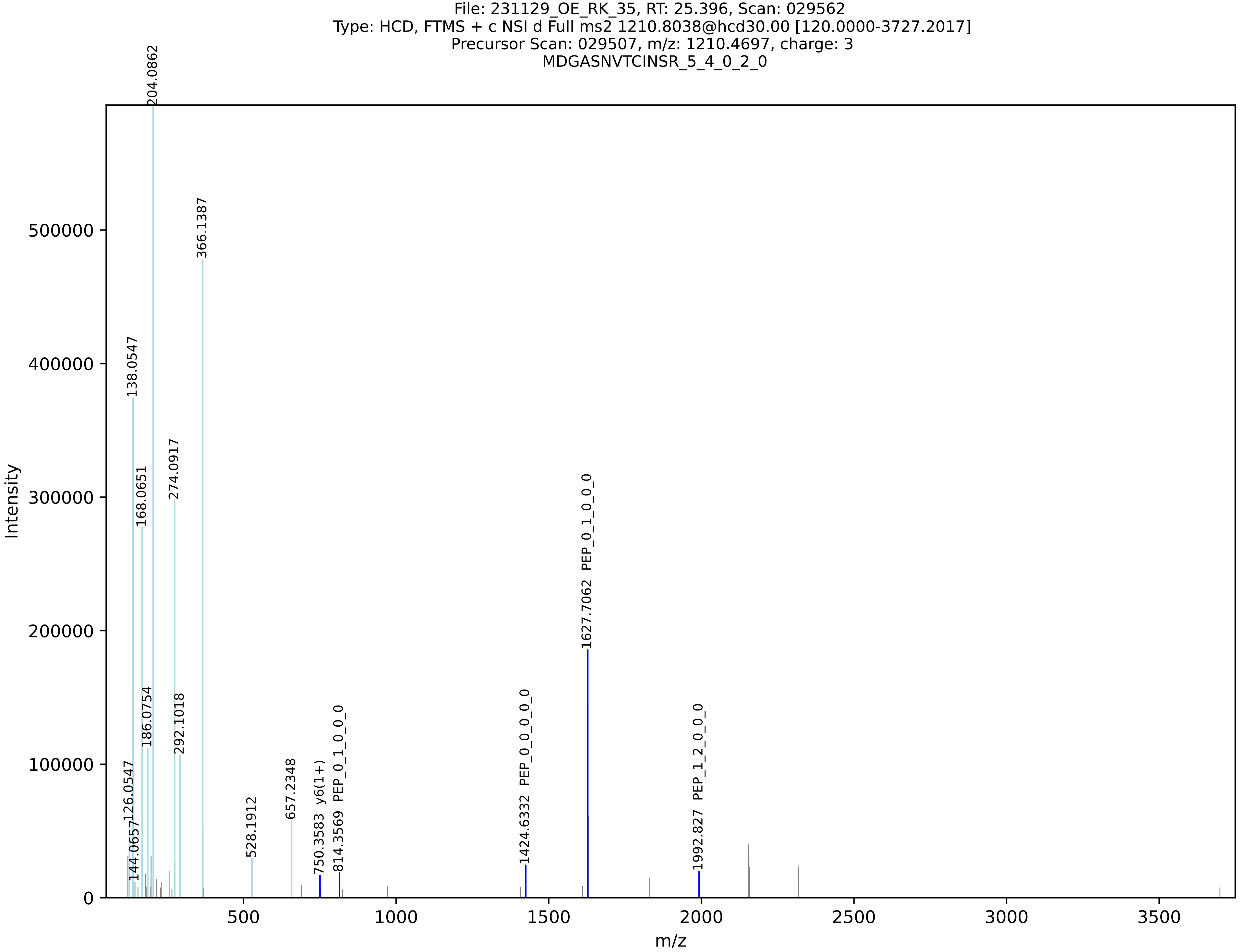
File: 231129_OE_RK_35, RT: 25.396, Scan: 029562
Type: HCD, FTMS + c NSI d Full ms2 1210.8038@hcd30.00 [120.0000-3727.2017]
Precursor Scan: 029507, m/z: 1210.4697, charge: 3
MDGASNVTCINSR_5_4_0_2_0
Intensity
m/z
204.0862
366.1387
138.0547
274.0917
168.0651
186.0754
292.1018
126.0547
144.0657
528.1912
657.2348
750.3583 y6(1+)
814.3569 PEP_0_1_0_0_0
1424.6332 PEP_0_0_0_0_0
1627.7062 PEP_0_1_0_0_
1992.827 PEP_1_2_0_0_0

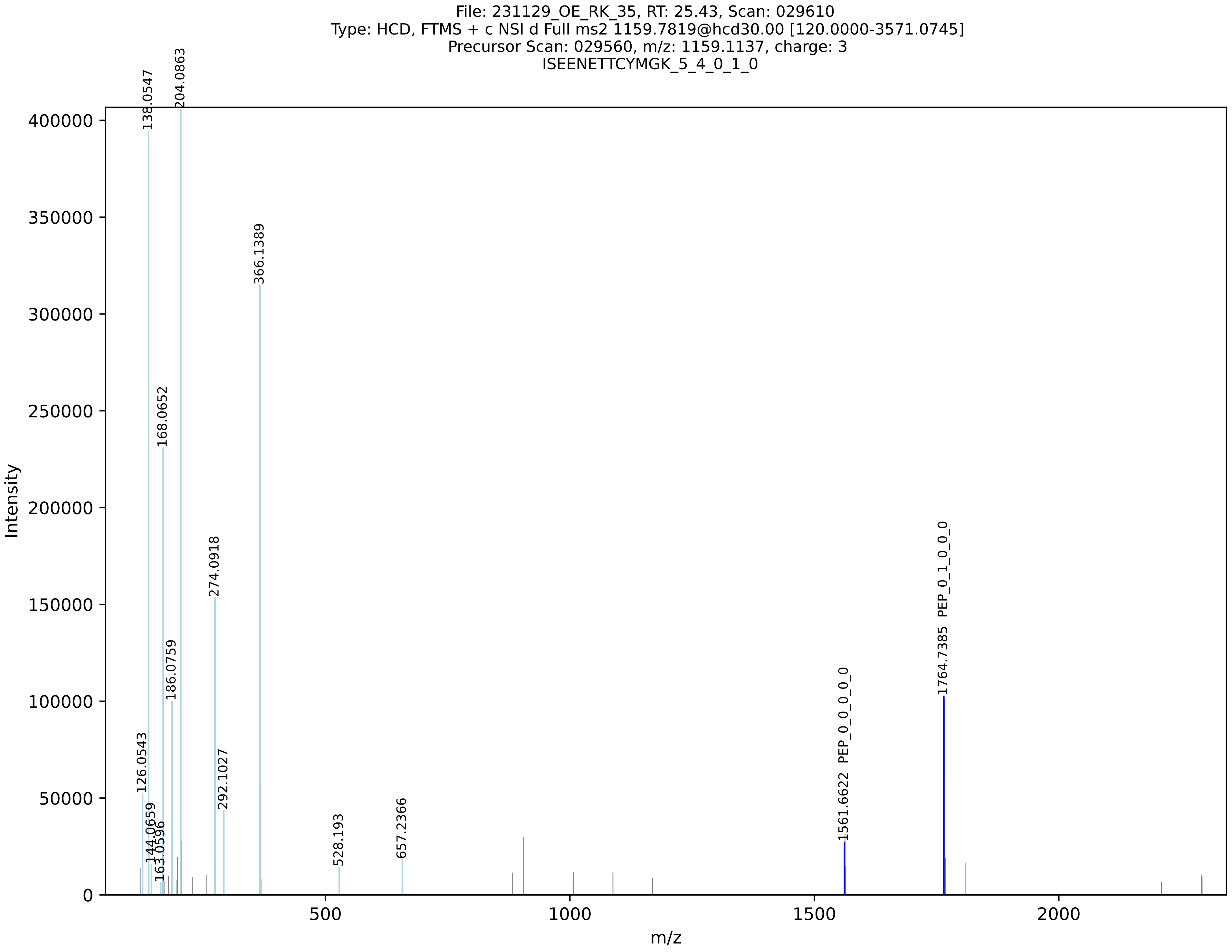
File: 231129_OE_RK_35, RT: 25.43, Scan: 029610
Type: HCD, FTMS + c NSI d Full ms2 1159.7819@hcd30.00 [120.0000-3571.0745]
Precursor Scan: 029560, m/z: 1159.1137, charge: 3
ISEENETTCYMGK_5_4_0_1_0
Intensity
m/z
204.0863
138.0547
366.1389
168.0652
274.0918
186.0759
126.0543
292.1027
144.0659
163.0596
528.193
657.2366
1561.6622 PEP_0_0_0_0_0
1764.7385 PEP_0_1_0_0_0

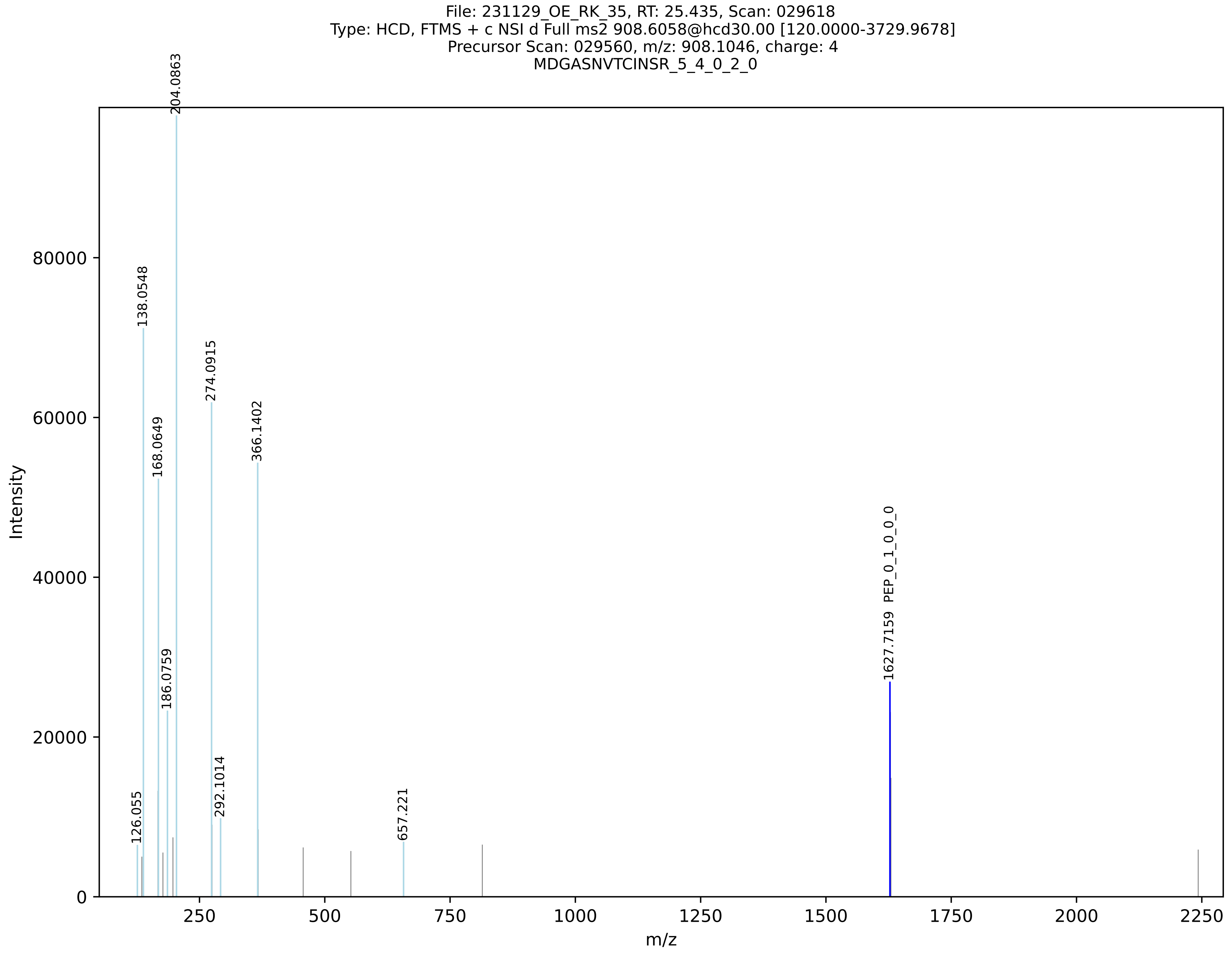

File: 231129_OE_RK_35, RT: 25.435, Scan: 029618
Type: HCD, FTMS + c NSI d Full ms2 908.6058@hcd30.00 [120.0000-3729.9678]
Precursor Scan: 029560, m/z: 908.1046, charge: 4
MDGASNVTCINSR_5_4_0_2_0
Intensity
m/z
0
20000
40000
60000
80000
250
500
750
1000
1250
1500
1750
2000
2250
204.0863
138.0548
168.0649
274.0915
366.1402
186.0759
126.055
292.1014
657.221
1627.7159 PEP_0_1_0_0_0_

File: 231129_OE_RK_35, RT: 25.574, Scan: 029816
Type: HCD, FTMS + c NSI d Full ms2 1211.1384@hcd30.00 [120.0000-3728.2256]
Precursor Scan: 029739, m/z: 1210.4702, charge: 3
MDGASNVTCINSR_5_4_0_2_0

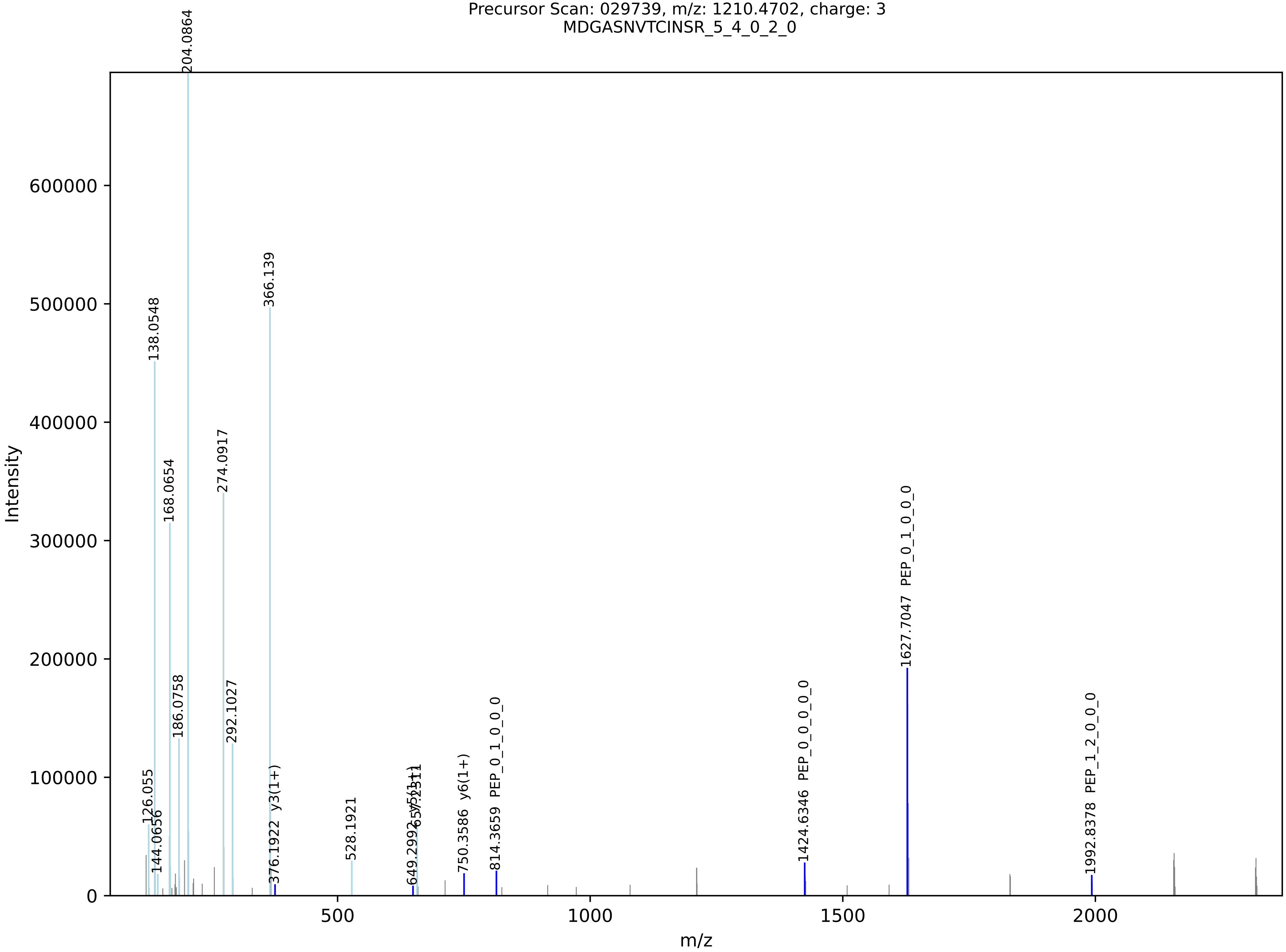

File: 231129_OE_RK_35, RT: 25.585, Scan: 029833
Type: HCD, FTMS + c NSI d Full ms2 1243.4542@hcd30.00 [120.0000-5096.1489]
Precursor Scan: 029739, m/z: 1243.2036, charge: 4
HCDGNVSSCGDHPSEGCFCPPDK_5_4_1_2_0

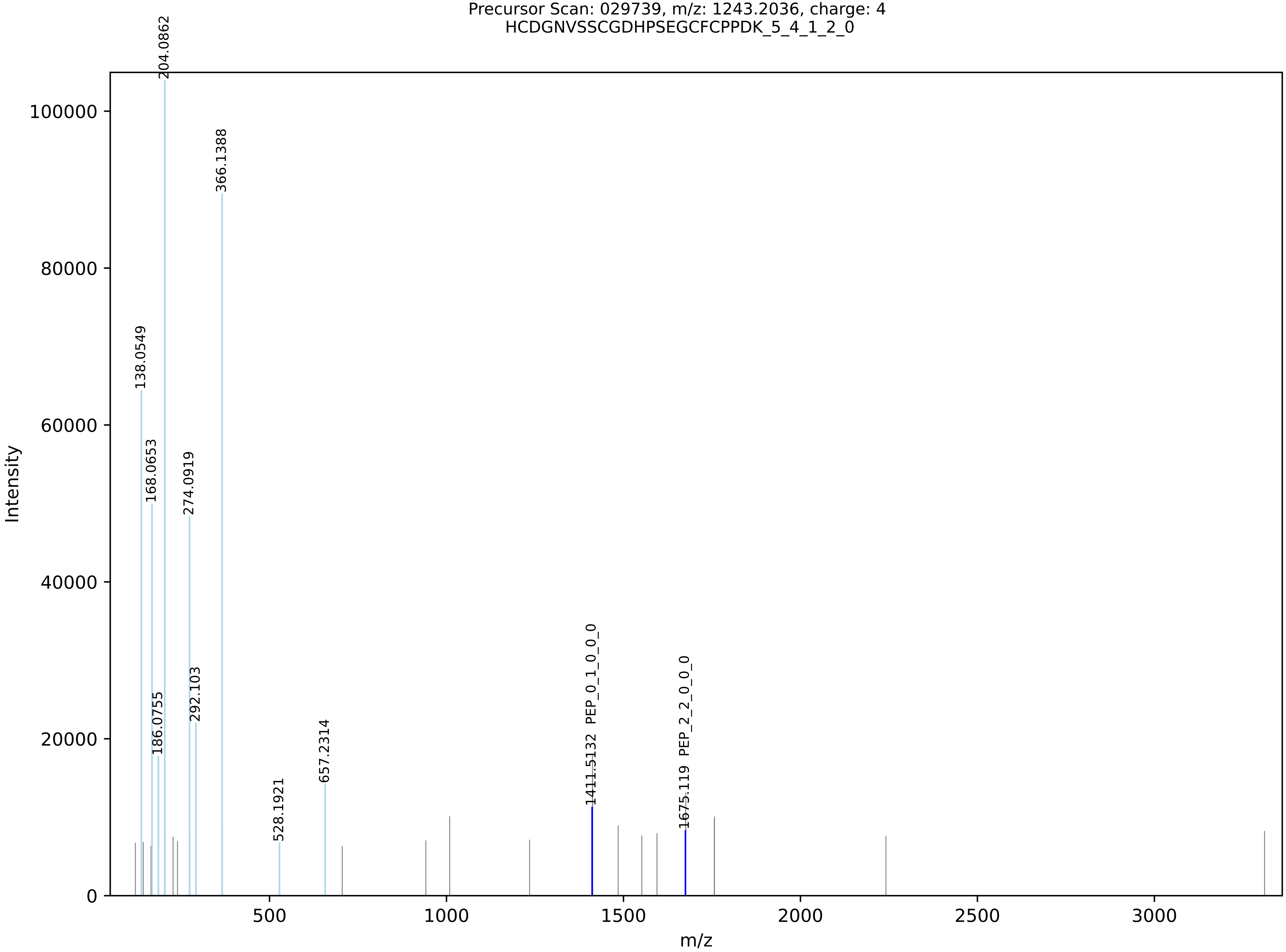

File: 231129_OE_RK_35, RT: 25.593, Scan: 029847
Type: HCD, FTMS + c NSI d Full ms2 1266.1904@hcd30.00 [120.0000-3896.6846]
Precursor Scan: 029739, m/z: 1265.1913, charge: 3
MDGASNVTCINSR_6_4_0_2_0

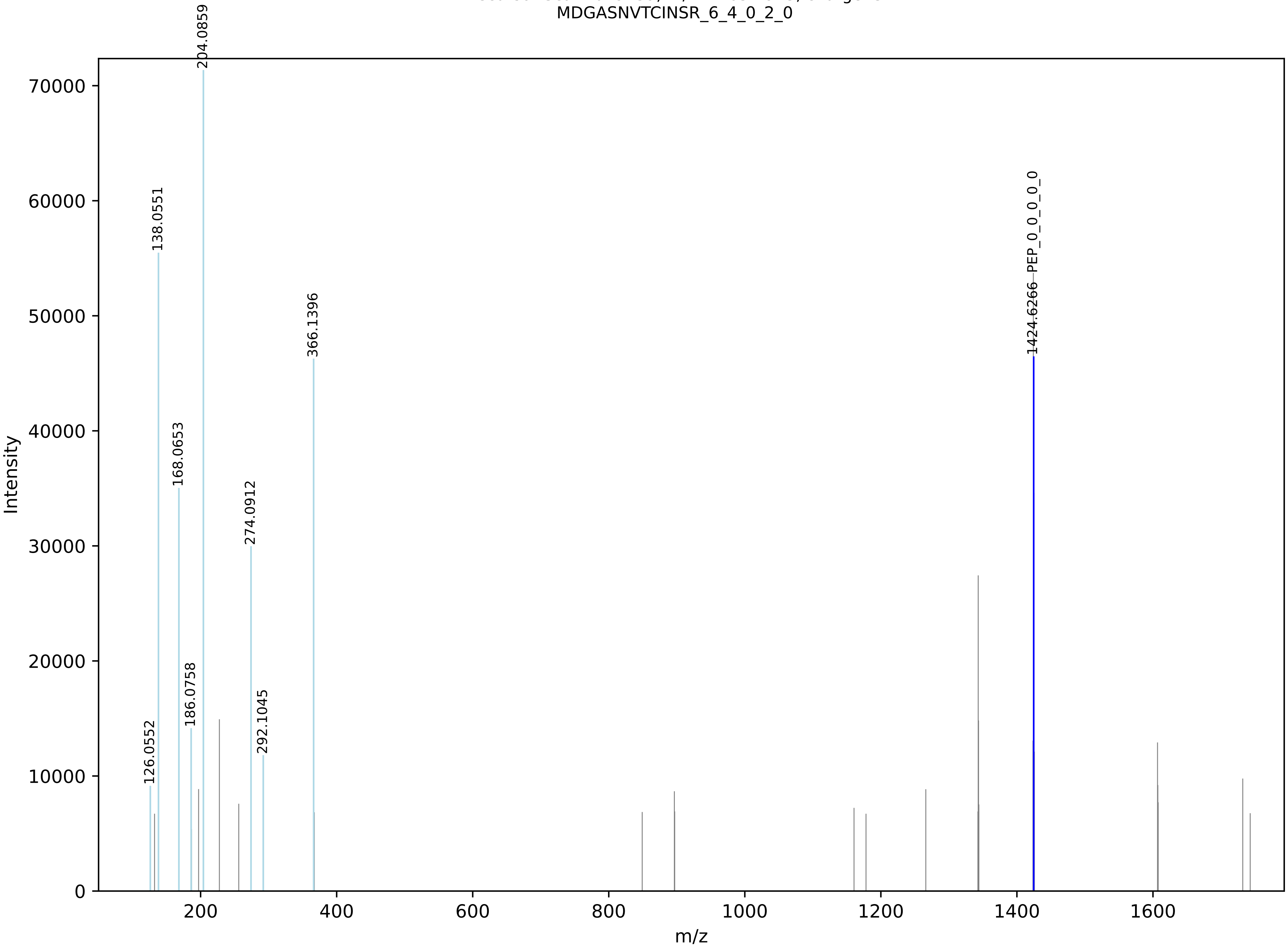

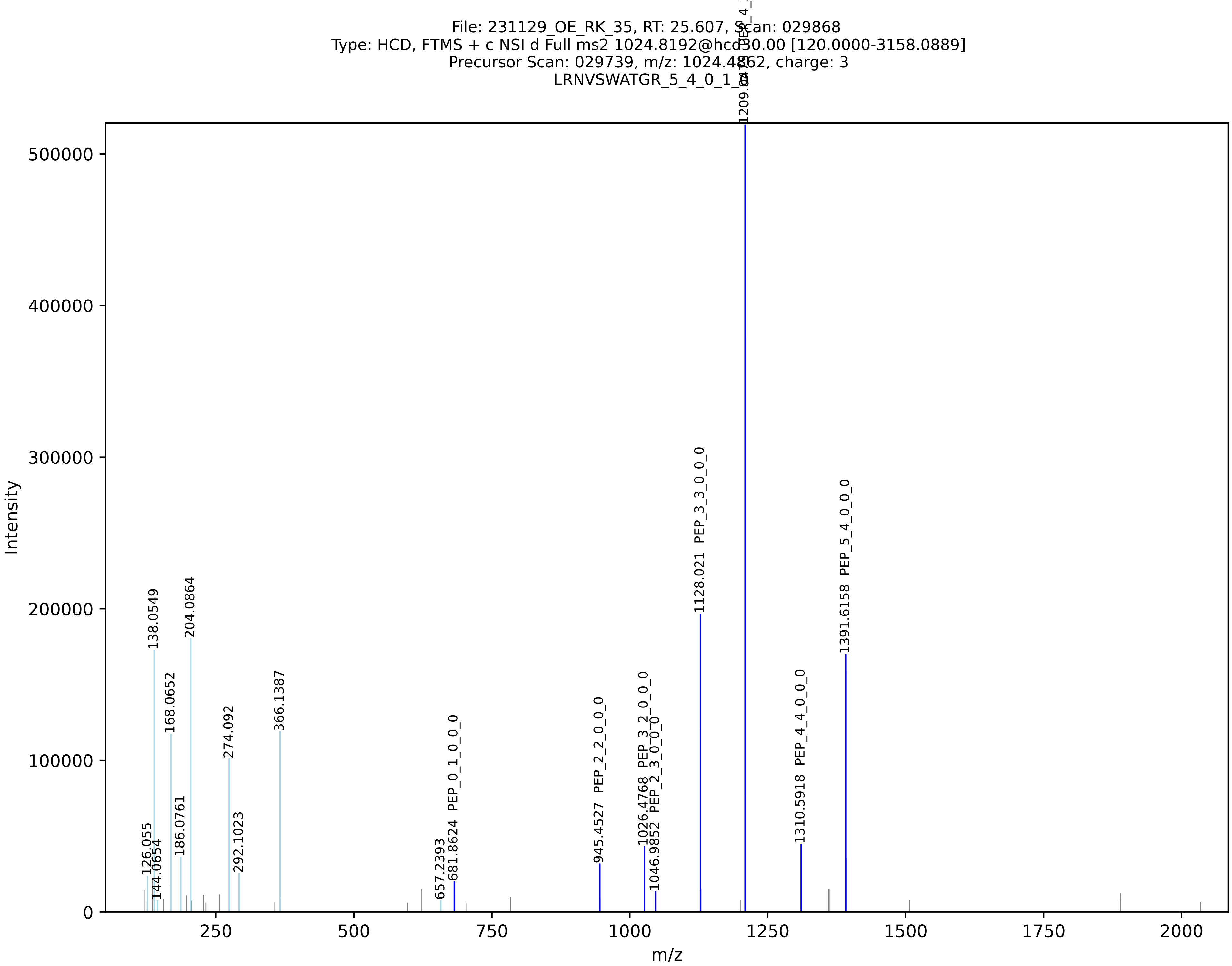
File: 231129_OE_RK_35, RT: 25.607, Scan: 029868
Type: HCD, FTMS + c NSI d Full ms2 1024.8192@hcd30.00 [120.0000-3158.0889]
Precursor Scan: 029739, m/z: 1024.4862, charge: 3
LRNVSWATGR_5_4_0_1_0
Intensity
m/z
126.055
144.0654
138.0549
168.0652
186.0761
204.0864
274.092
292.1023
366.1387
657.2393
681.8624 PEP_0_1_0_0_0
945.4527 PEP_2_2_0_0_0
1026.4768 PEP_3_2_0_0_0
1046.9852 PEP_2_3_0_0_0
1128.021 PEP_3_3_0_0_0
1310.5918 PEP_4_4_0_0_0
1391.6158 PEP_5_4_0_0_0
0
100000
200000
300000
400000
500000
250
500
750
1000
1250
1500
1750
2000

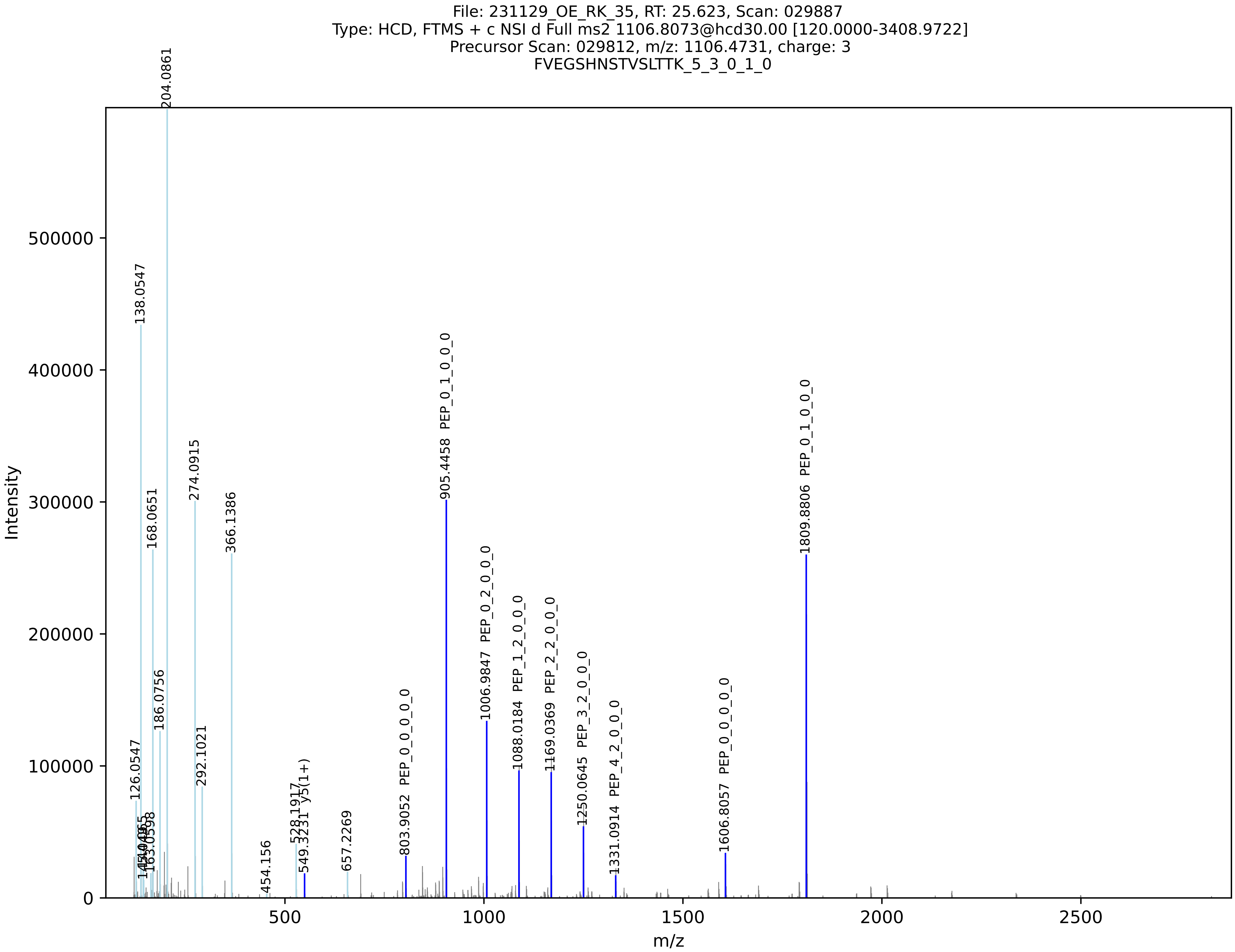
File: 231129_OE_RK_35, RT: 25.623, Scan: 029887
Type: HCD, FTMS + c NSI d Full ms2 1106.8073@hcd30.00 [120.0000-3408.9722]
Precursor Scan: 029812, m/z: 1106.4731, charge: 3
FVEGSHNSTVSLTTK_5_3_0_1_0
Intensity
m/z
204.0861
138.0547
168.0651
274.0915
366.1386
186.0756
292.1021
126.0547
163.0598
454.156
528.1917
549.3231 y5(1+)
657.2269
803.9052 PEP_0_0_0_0_0_
905.4458 PEP_0_1_0_0_0_
1006.9847 PEP_0_2_0_0_0_
1088.0184 PEP_1_2_0_0_0_
1169.0369 PEP_2_2_0_0_0_
1250.0645 PEP_3_2_0_0_0_
1331.0914 PEP_4_2_0_0_0_
1606.8057 PEP_0_0_0_0_0_
1809.8806 PEP_0_1_0_0_0_
0
100000
200000
300000
400000
500000
500
1000
1500
2000
2500

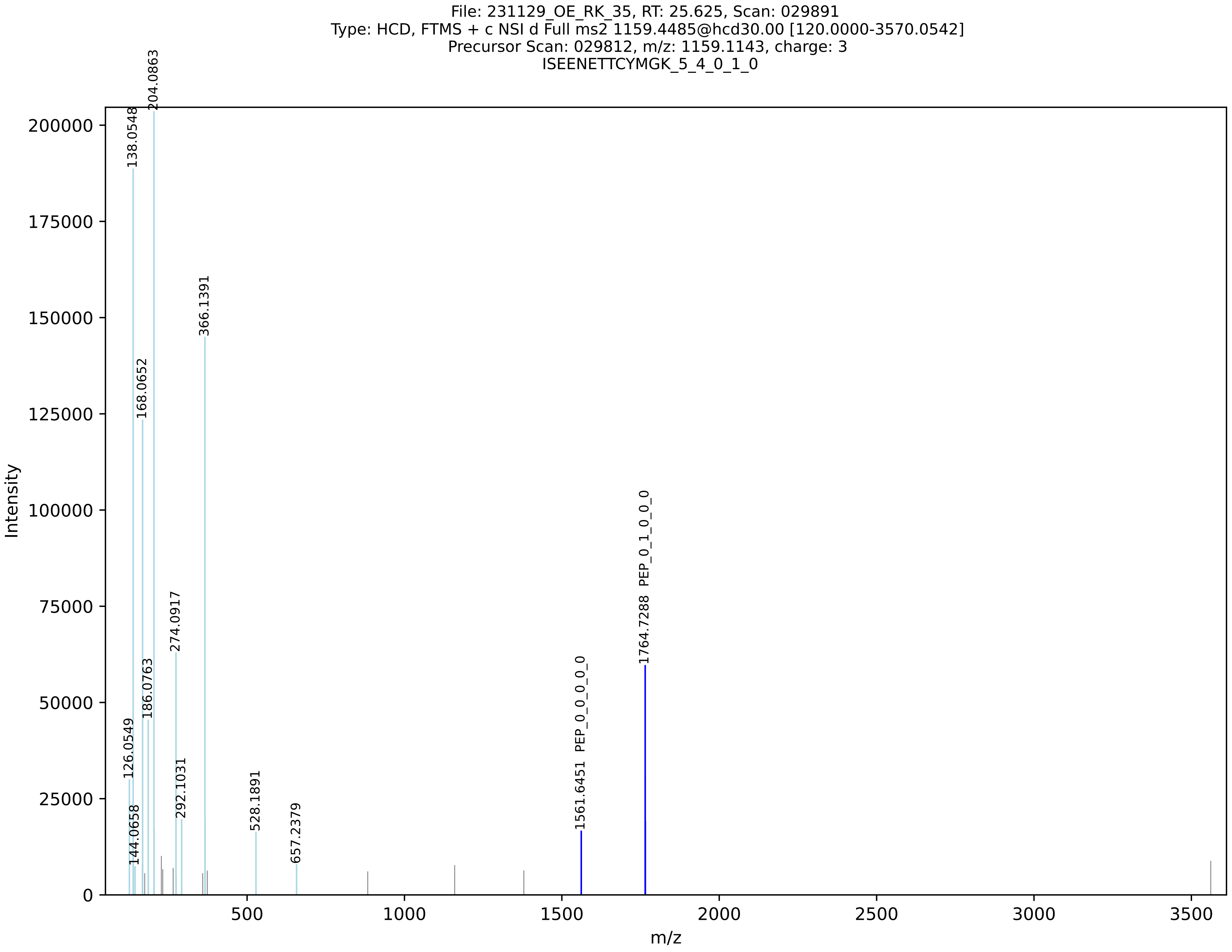

File: 231129_OE_RK_35, RT: 25.625, Scan: 029891
Type: HCD, FTMS + c NSI d Full ms2 1159.4485@hcd30.00 [120.0000-3570.0542]
Precursor Scan: 029812, m/z: 1159.1143, charge: 3
ISEENETTCYMGK_5_4_0_1_0
Intensity
m/z
204.0863
138.0548
366.1391
168.0652
274.0917
186.0763
126.0549
144.0658
292.1031
528.1891
657.2379
1561.6451 PEP_0_0_0_0_0
1764.7288 PEP_0_1_0_0_0

File: 231129_OE_RK_35, RT: 25.629, Scan: 029896
Type: HCD, FTMS + c NSI d Full ms2 1085.4452@hcd30.00 [120.0000-3343.6045]
Precursor Scan: 029812, m/z: 1085.1092, charge: 3
AFGSNPNLTK_5_4_0_2_0

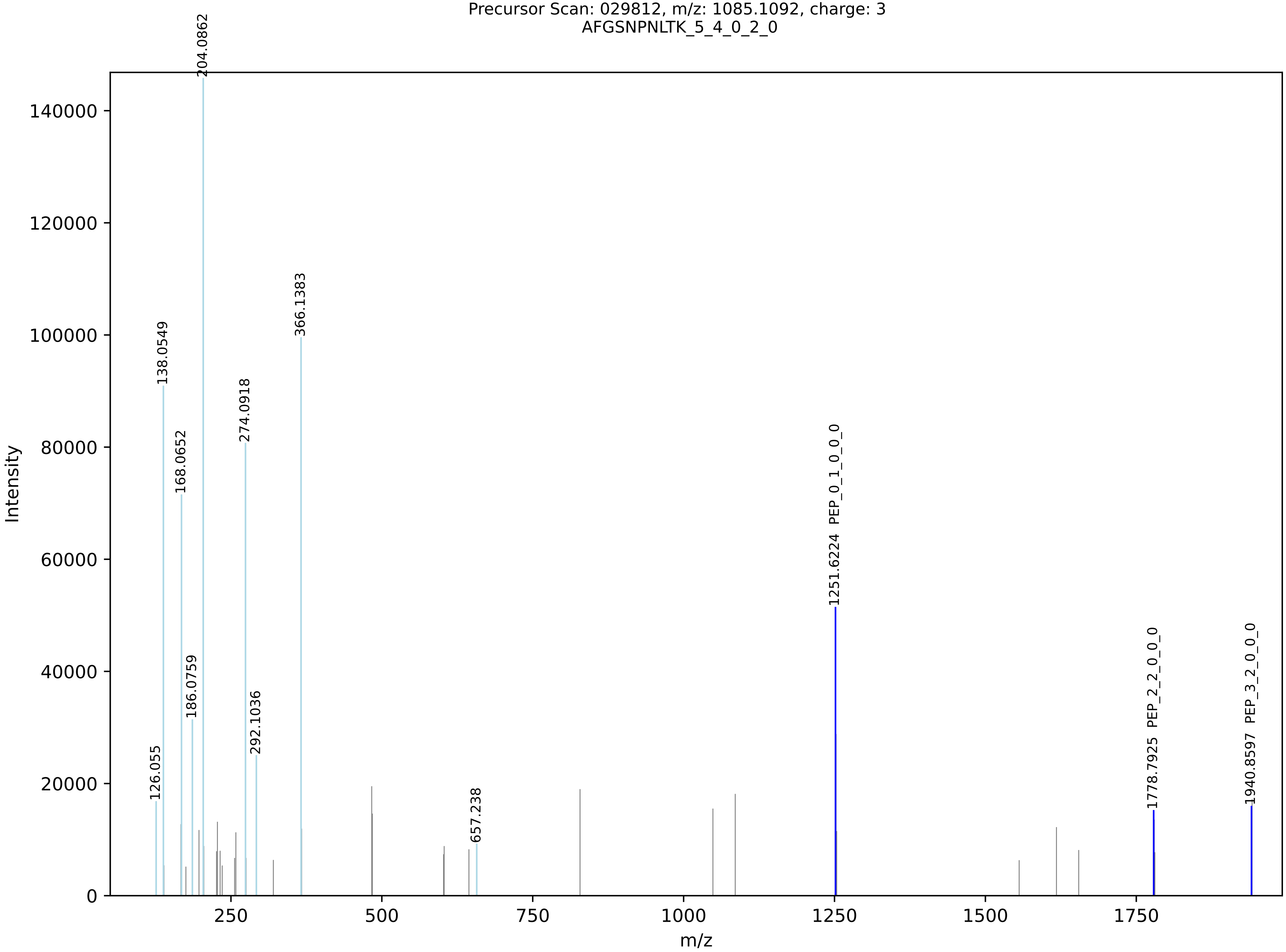

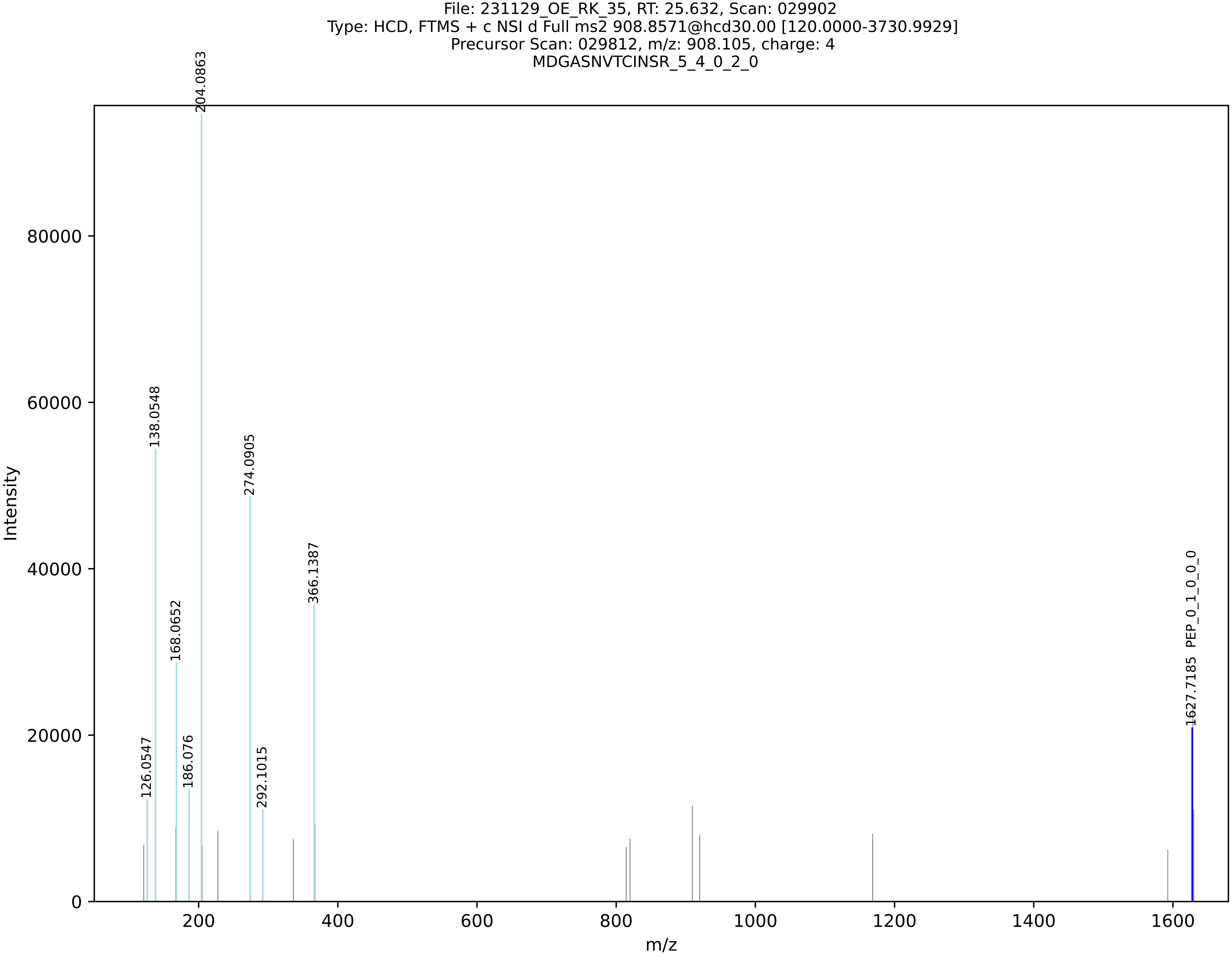
File: 231129_OE_RK_35, RT: 25.632, Scan: 029902
Type: HCD, FTMS + c NSI d Full ms2 908.8571@hcd30.00 [120.0000-3730.9929]
Precursor Scan: 029812, m/z: 908.105, charge: 4
MDGASNVTCINSR_5_4_0_2_0
204.0863
138.0548
274.0905
366.1387
168.0652
126.0547
186.076
292.1015
1627.7185 PEP_0_1_0_0_0
Intensity
m/z
0
20000
40000
60000
80000
200
400
600
800
1000
1200
1400
1600

File: 231129_OE_RK_35, RT: 25.677, Scan: 029963
Type: HCD, FTMS + c NSI d Full ms2 1052.7897@hcd30.00 [120.0000-3243.6785]
Precursor Scan: 029886, m/z: 1052.4555, charge: 3
FVEGSHNSTVSLTTK_4_3_0_1_0

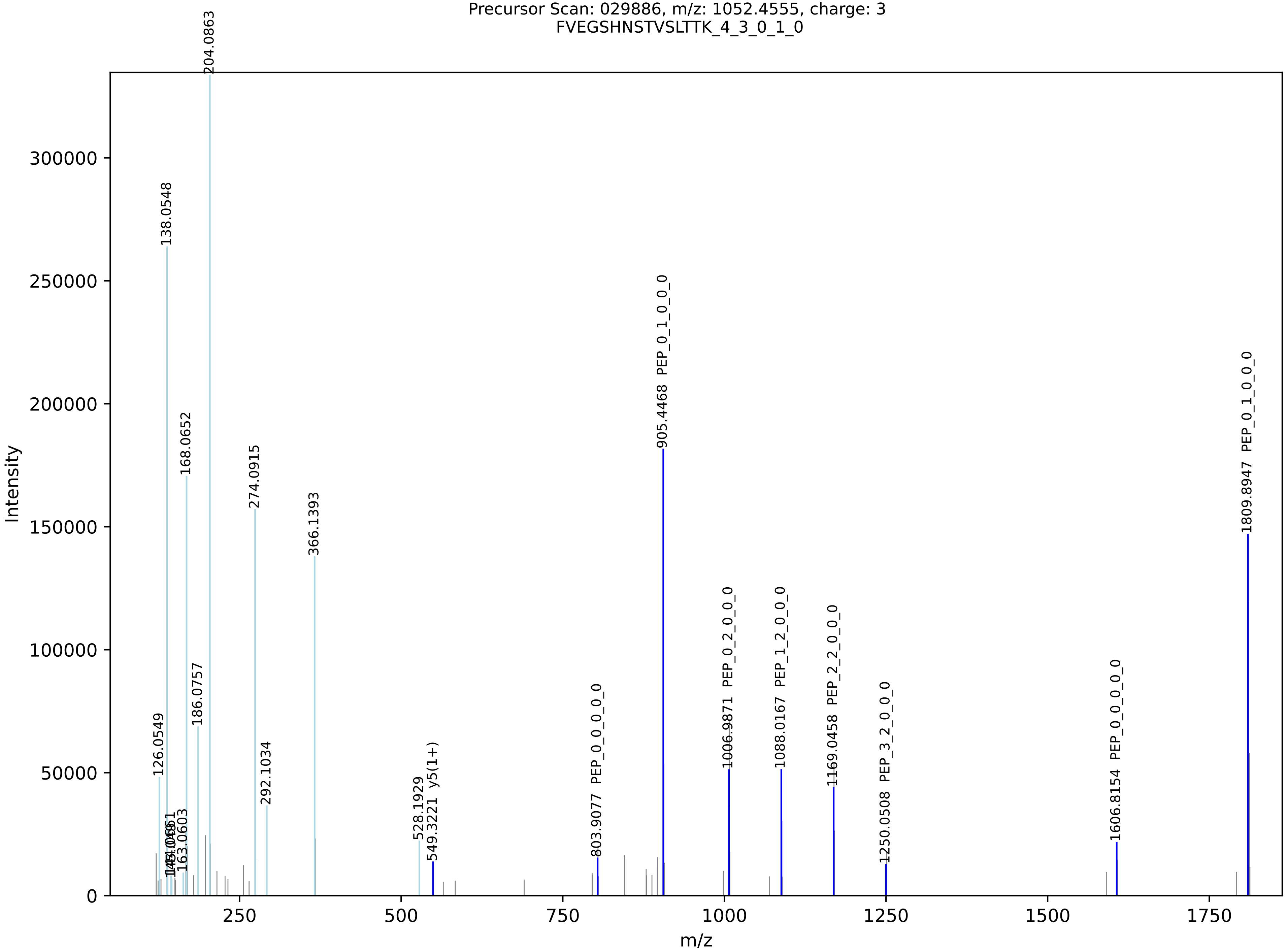

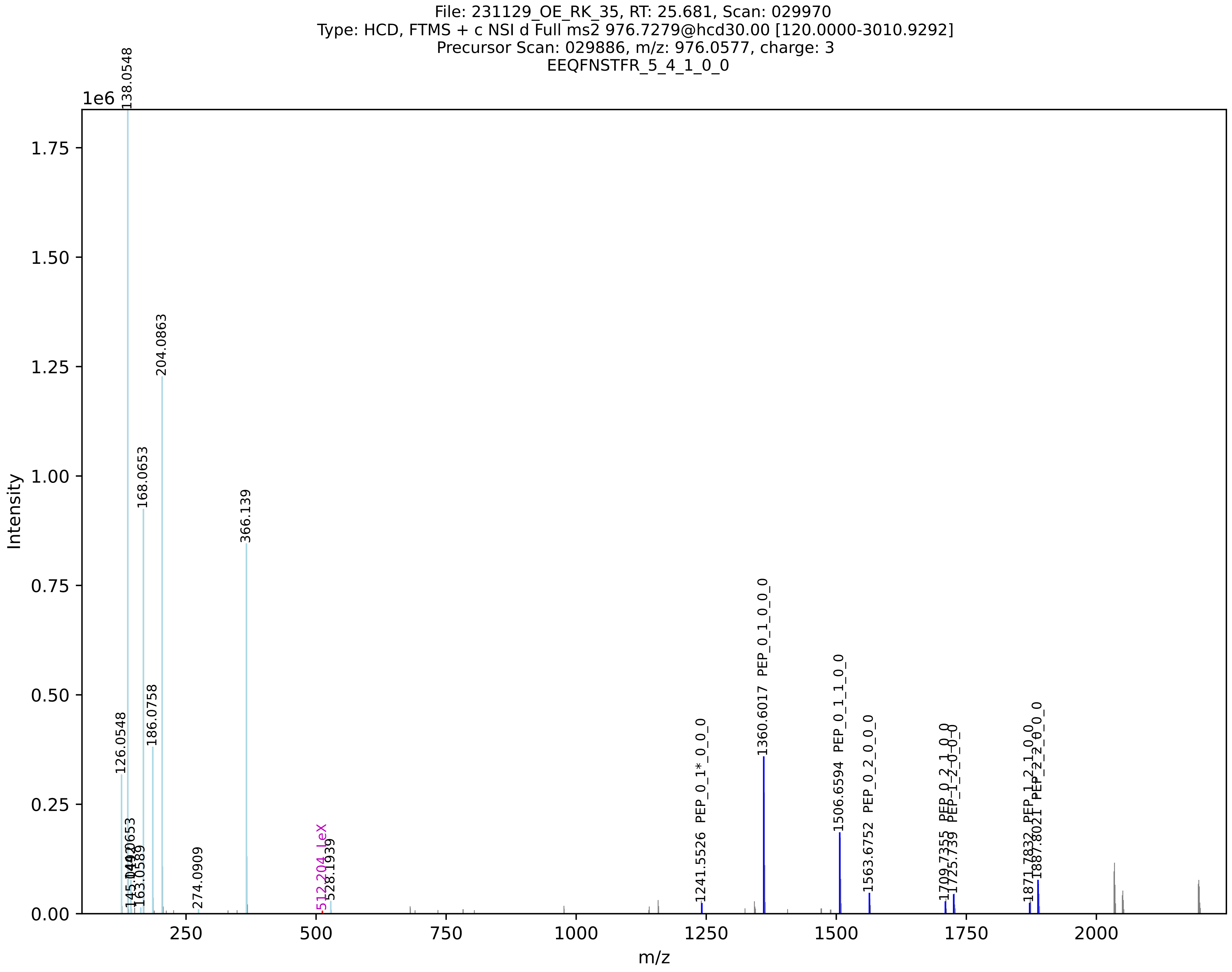
File: 231129_OE_RK_35, RT: 25.681, Scan: 029970
Type: HCD, FTMS + c NSI d Full ms2 976.7279@hcd30.00 [120.0000-3010.9292]
Precursor Scan: 029886, m/z: 976.0577, charge: 3
EEQFNSTFR_5_4_1_0_0
1e6
Intensity
m/z
138.0548
204.0863
168.0653
366.139
186.0758
126.0548
274.0909
512.204_LeX
528.1939
1241.5526 PEP_0_1*_0_0_0
1360.6017 PEP_0_1_0_0_0
1506.6594 PEP_0_1_1_0_0
1563.6752 PEP_0_2_0_0_0
1709.7355 PEP_0_2_1_0_0
1725.739 PEP_1_2_0_0_0
1871.7832 PEP_1_2_1_0_0
1887.8021 PEP_2_2_0_0_0

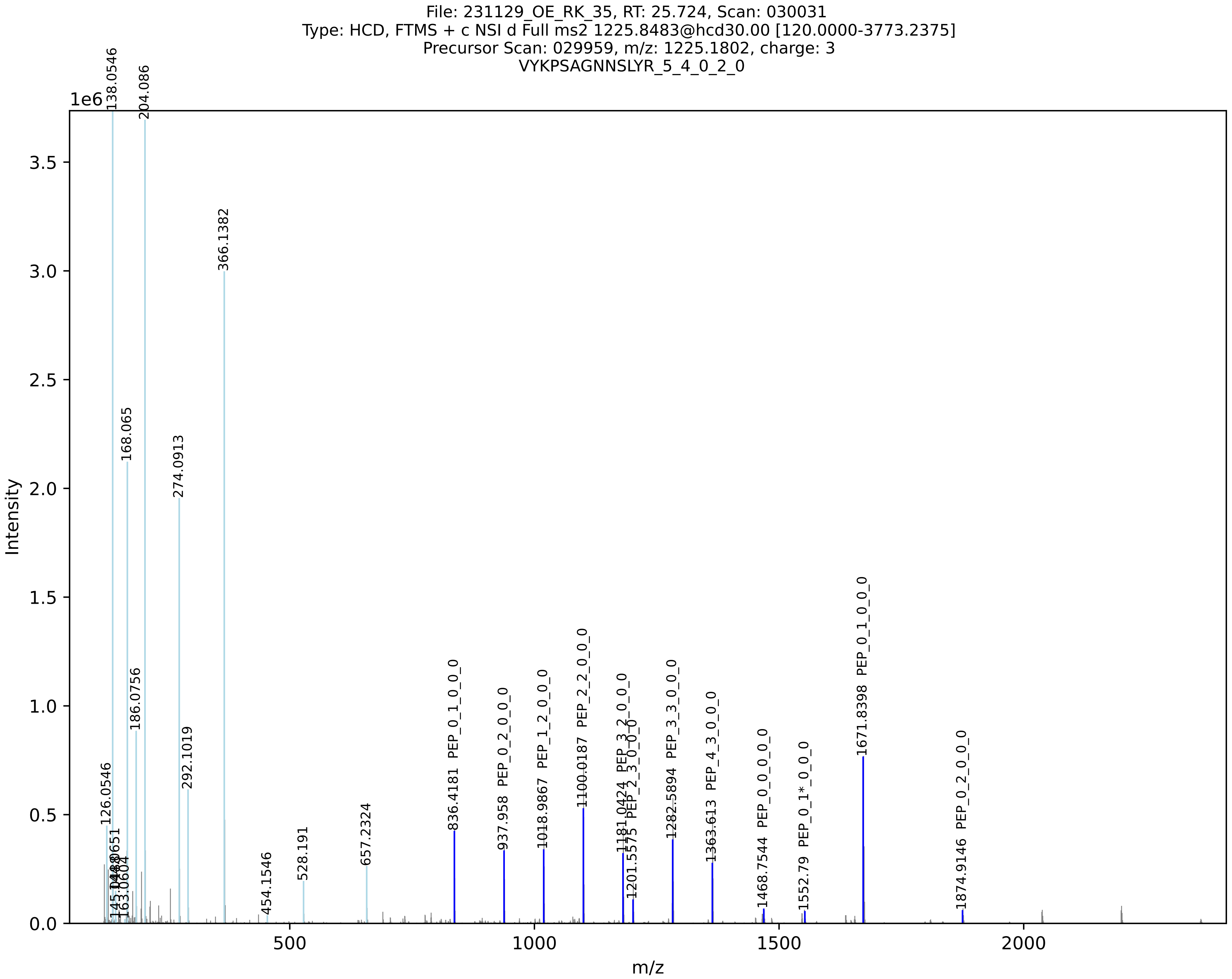
File: 231129_OE_RK_35, RT: 25.724, Scan: 030031
Type: HCD, FTMS + c NSI d Full ms2 1225.8483@hcd30.00 [120.0000-3773.2375]
Precursor Scan: 029959, m/z: 1225.1802, charge: 3
VYKPSAGNNSLYR_5_4_0_2_0
1e6
Intensity
m/z
0.0
0.5
1.0
1.5
2.0
2.5
3.0
3.5
500
1000
1500
2000
126.0546
138.0546
168.065
186.0756
204.086
274.0913
292.1019
366.1382
454.1546
528.191
657.2324
836.4181 PEP_0_1_0_0_0
937.958 PEP_0_2_0_0_0
1018.9867 PEP_1_2_0_0_0
1100.0187 PEP_2_2_0_0_0
1181.0424 PEP_3_2_0_0_0
1201.5575 PEP_2_3_0_0_0
1282.5894 PEP_3_3_0_0_0
1363.613 PEP_4_3_0_0_0
1468.7544 PEP_0_0_0_0_0
1552.79 PEP_0_1*_0_0_0
1671.8398 PEP_0_1_0_0_0
1874.9146 PEP_0_2_0_0_0

File: 231129_OE_RK_35, RT: 25.725, Scan: 030033
Type: HCD, FTMS + c NSI d Full ms2 919.6387@hcd30.00 [120.0000-3774.9817]
Precursor Scan: 029959, m/z: 919.137, charge: 4
VYKPSAGNNSLYR_5_4_0_2_0

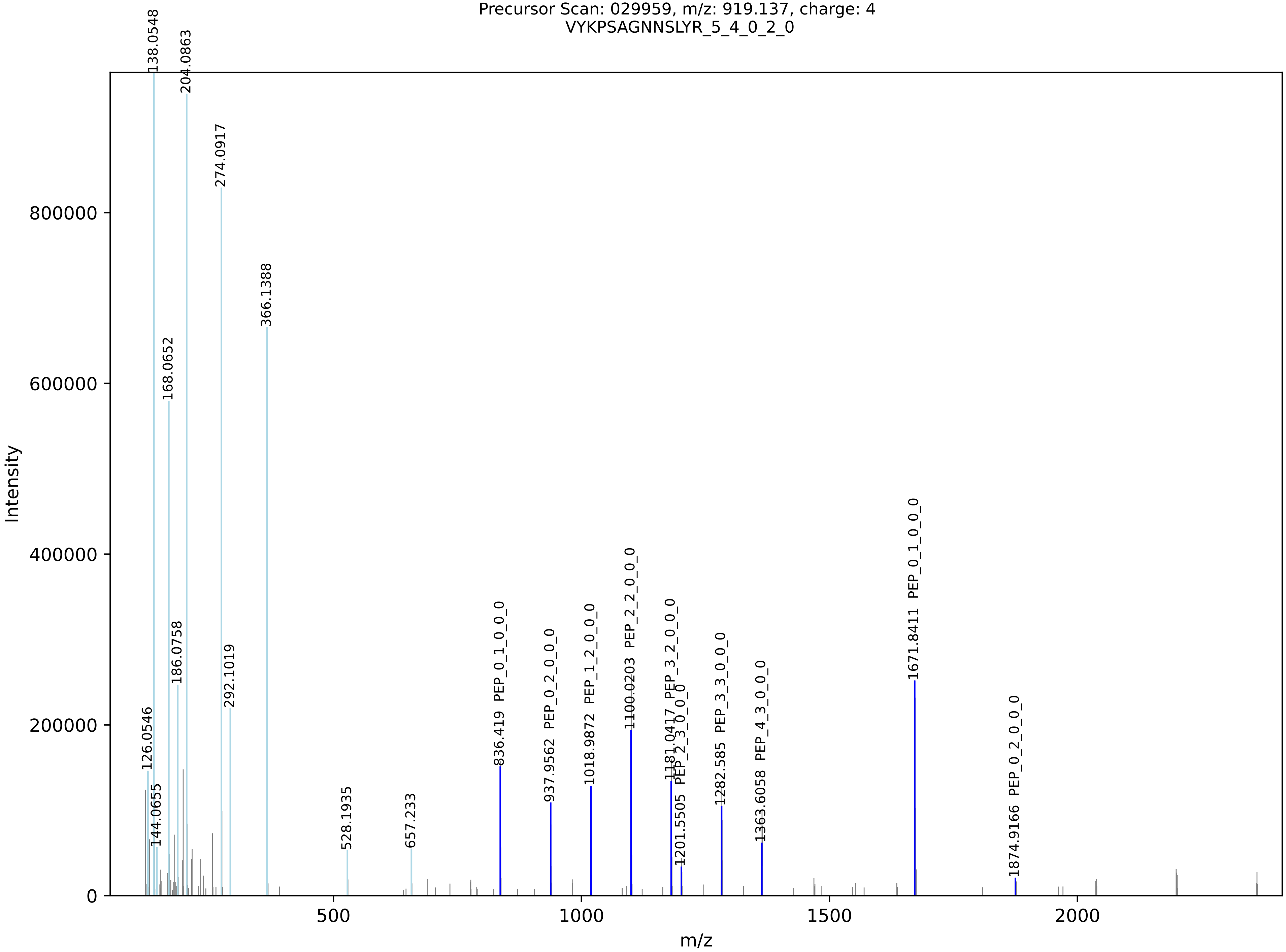

File: 231129_OE_RK_35, RT: 25.727, Scan: 030036
Type: HCD, FTMS + c NSI d Full ms2 1025.4420@hcd30.00 [120.0000-3159.9944]
Precursor Scan: 029959, m/z: 1025.1079, charge: 3
LRNVSWATGR_5_4_0_1_0

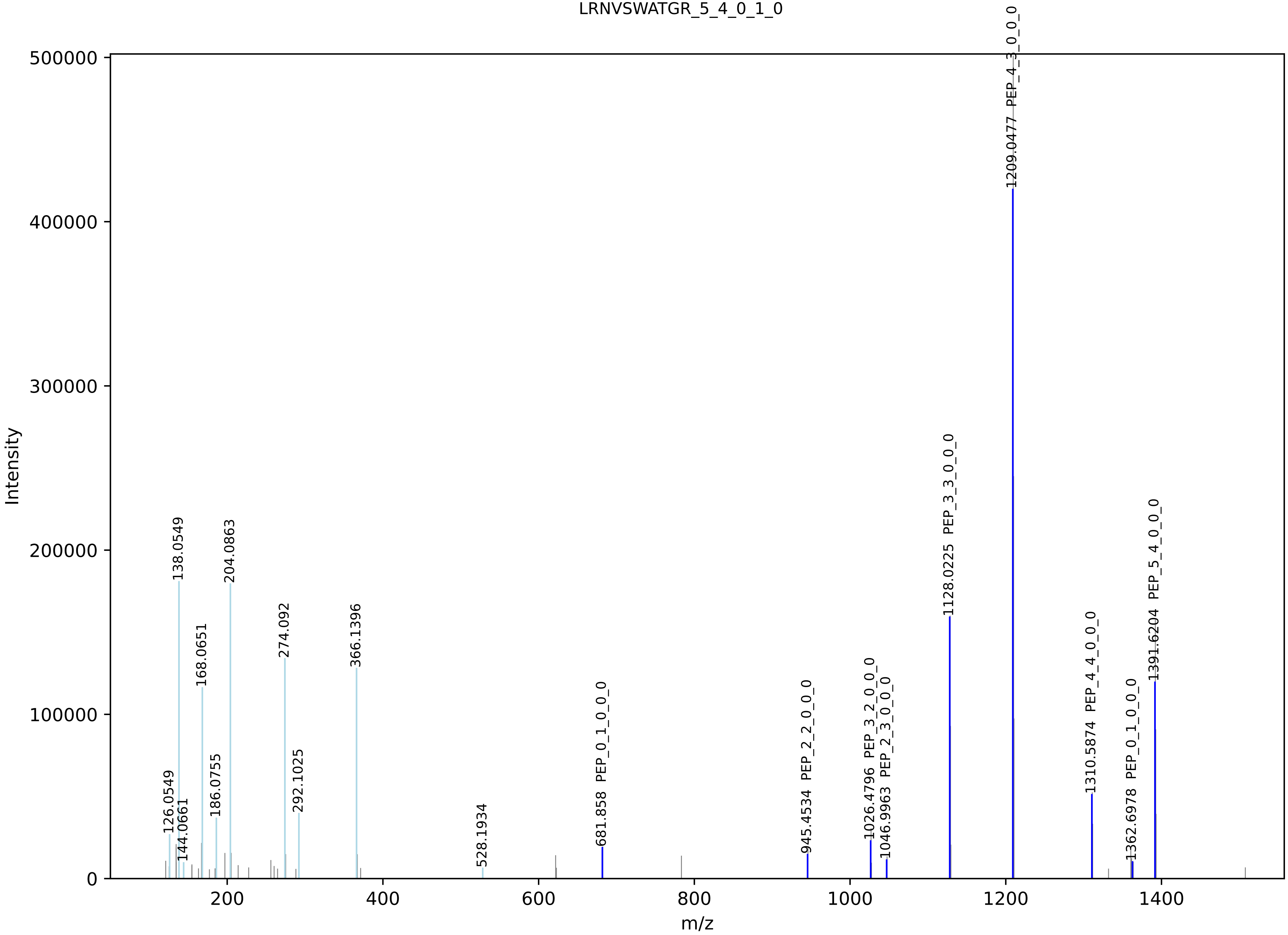

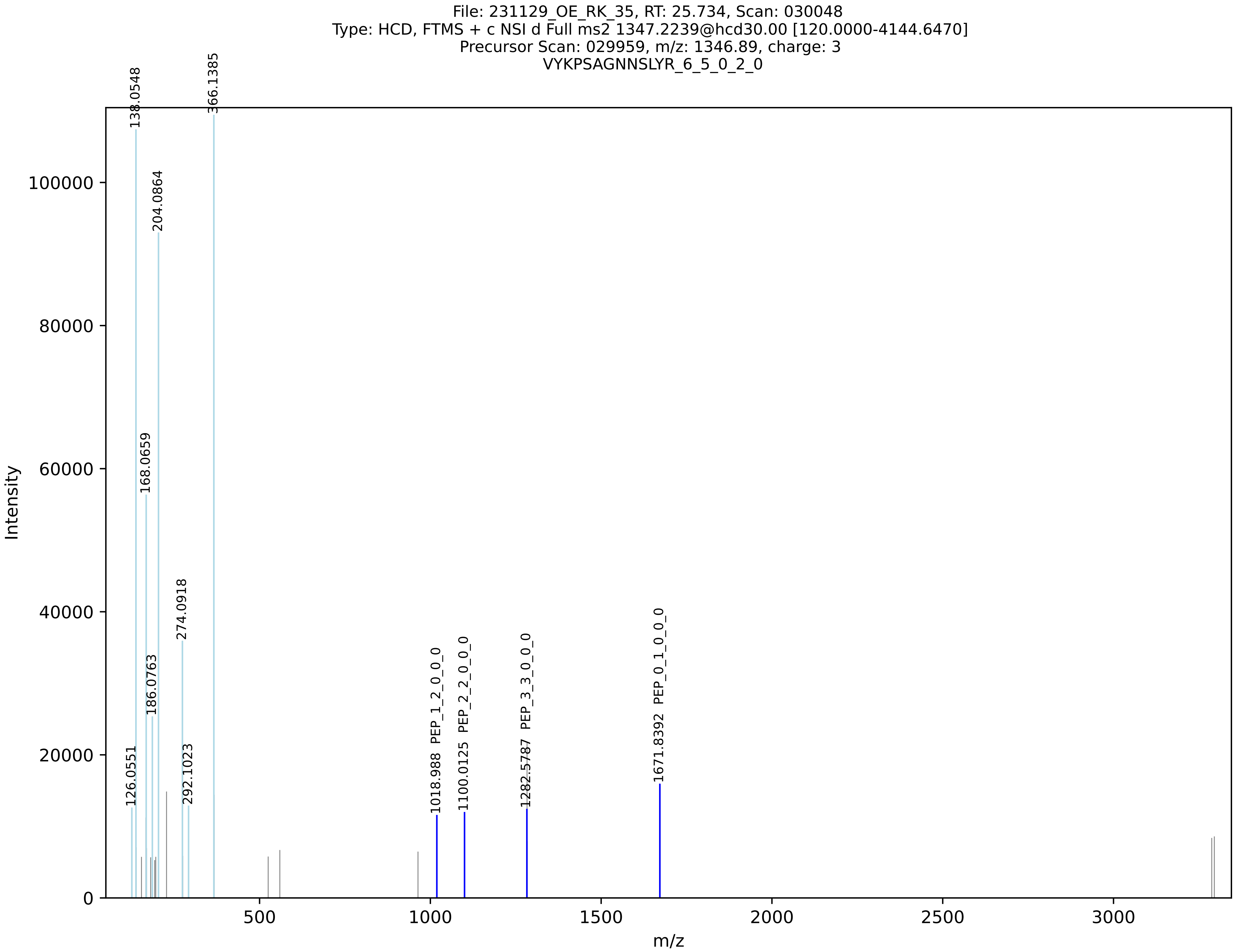

File: 231129_OE_RK_35, RT: 25.734, Scan: 030048
Type: HCD, FTMS + c NSI d Full ms2 1347.2239@hcd30.00 [120.0000-4144.6470]
Precursor Scan: 029959, m/z: 1346.89, charge: 3
VYKPSAGNNSLYR_6_5_0_2_0
138.0548
366.1385
204.0864
168.0659
274.0918
186.0763
126.0551
292.1023
1018.988 PEP_1_2_0_0_0
1100.0125 PEP_2_2_0_0_0
1282.5787 PEP_3_3_0_0_0
1671.8392 PEP_0_1_0_0_0
Intensity
m/z
0
20000
40000
60000
80000
100000
500
1000
1500
2000
2500
3000

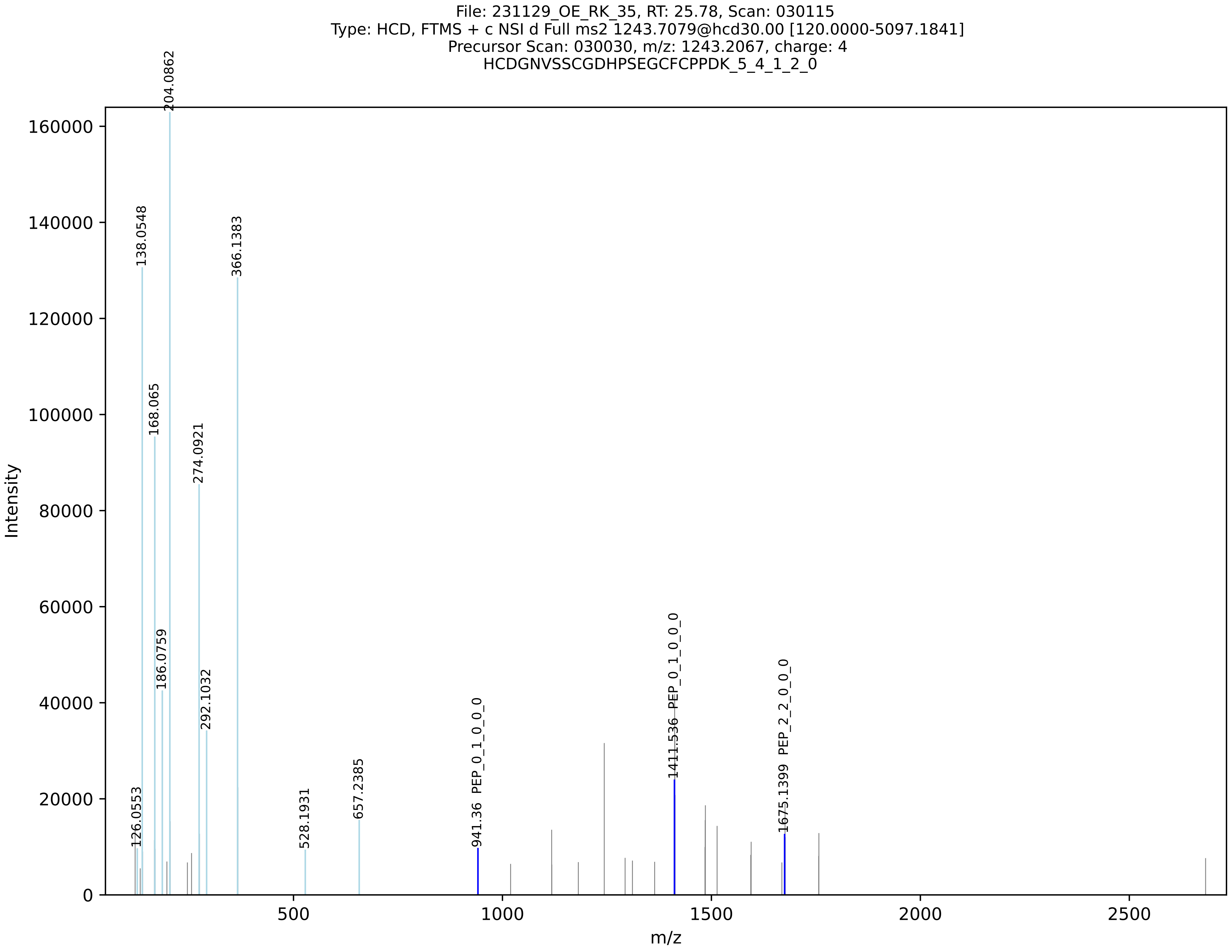
File: 231129_OE_RK_35, RT: 25.78, Scan: 030115
Type: HCD, FTMS + c NSI d Full ms2 1243.7079@hcd30.00 [120.0000-5097.1841]
Precursor Scan: 030030, m/z: 1243.2067, charge: 4
HCDGNVSSCGDHPSEGCFCPPDK_5_4_1_2_0
Intensity
m/z
204.0862
138.0548
366.1383
168.065
274.0921
186.0759
292.1032
126.0553
528.1931
657.2385
941.36 PEP_0_1_0_0_0
1411.536 PEP_0_1_0_0_0
1675.1399 PEP_2_2_0_0_0
0
20000
40000
60000
80000
100000
120000
140000
160000
500
1000
1500
2000
2500

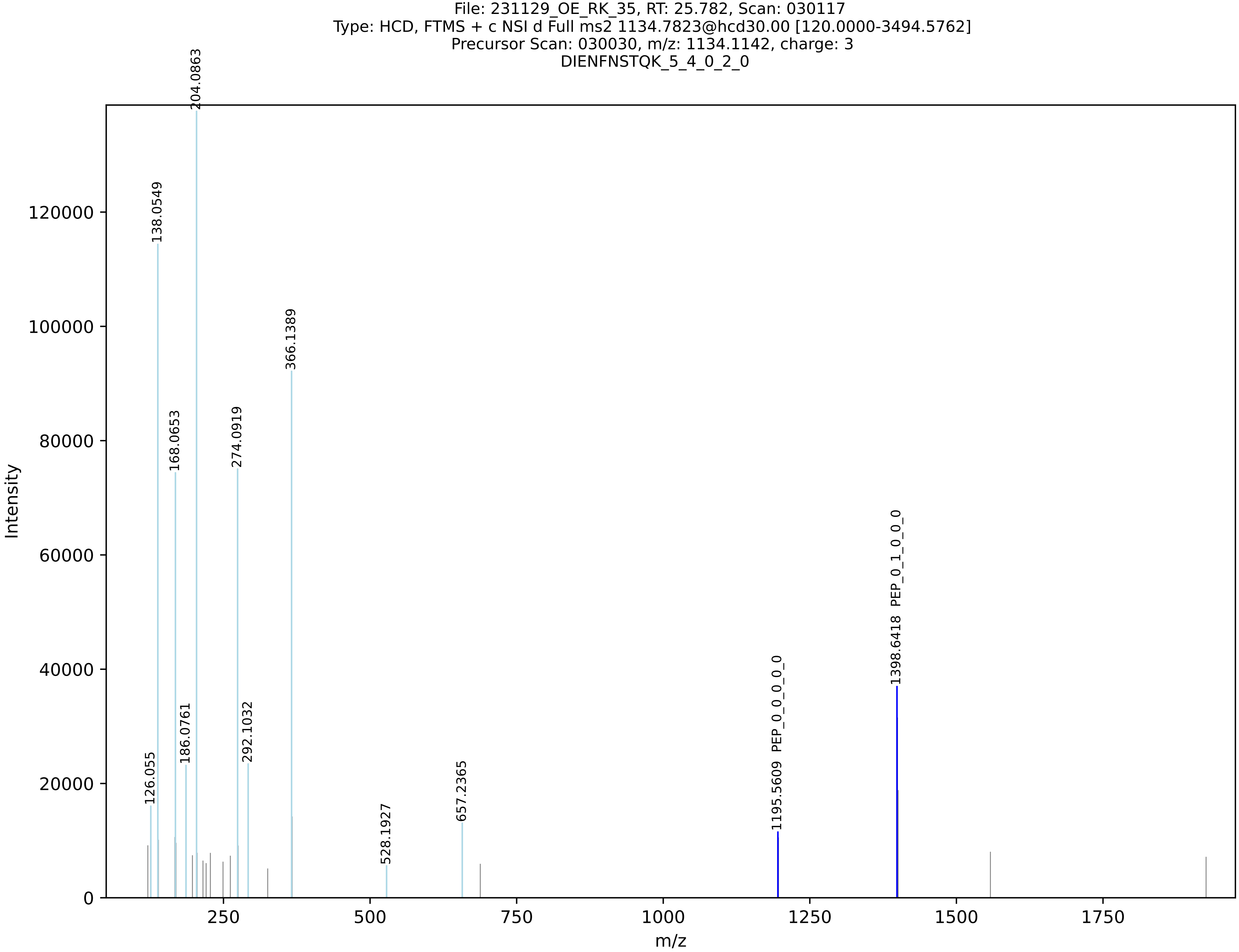

File: 231129_OE_RK_35, RT: 25.782, Scan: 030117
Type: HCD, FTMS + c NSI d Full ms2 1134.7823@hcd30.00 [120.0000-3494.5762]
Precursor Scan: 030030, m/z: 1134.1142, charge: 3
DIENFNSTQK_5_4_0_2_0
Intensity
m/z
204.0863
138.0549
366.1389
168.0653
274.0919
186.0761
292.1032
126.055
528.1927
657.2365
1195.5609 PEP_0_0_0_0_0
1398.6418 PEP_0_1_0_0_0
0
20000
40000
60000
80000
100000
120000
250
500
750
1000
1250
1500
1750

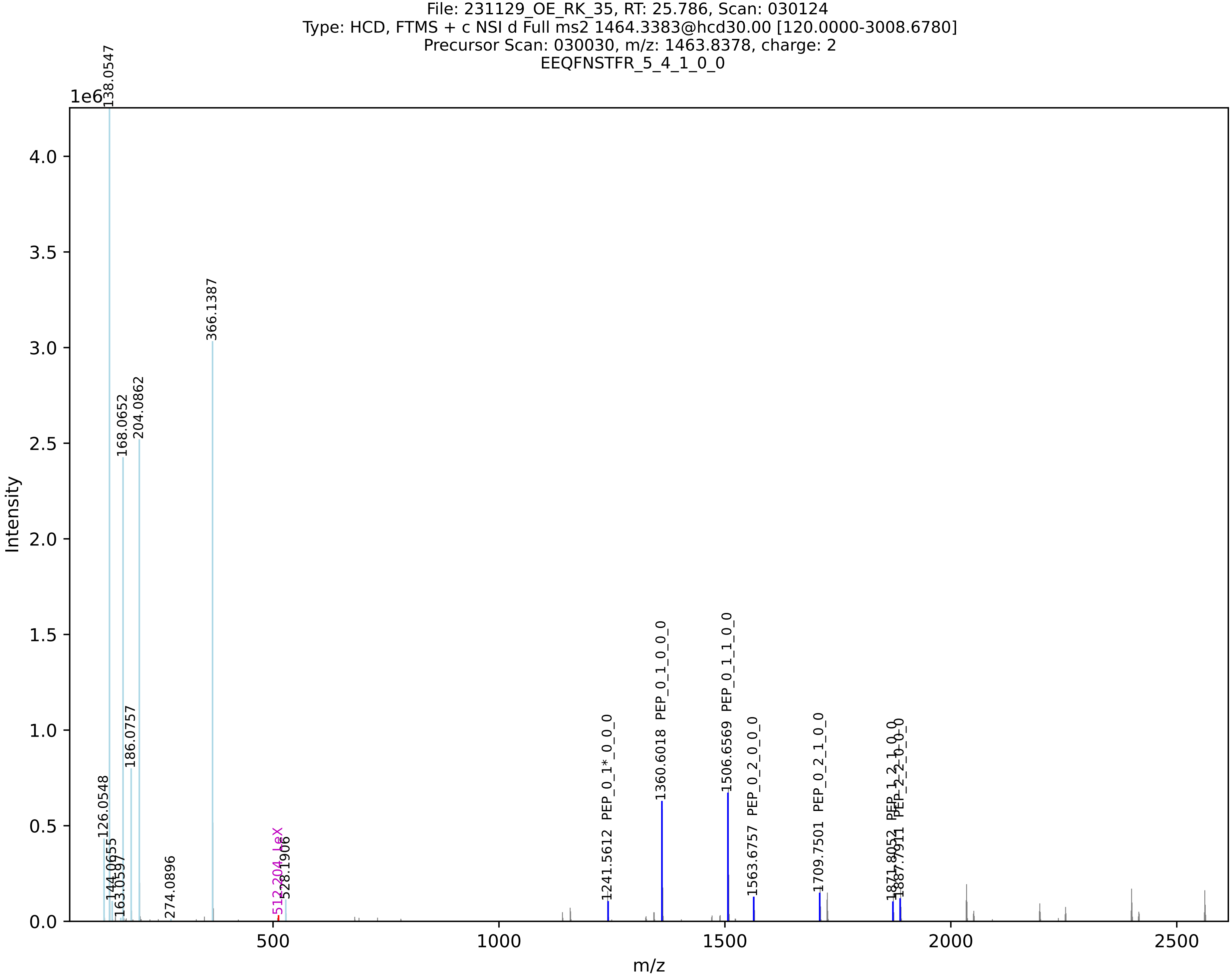
File: 231129_OE_RK_35, RT: 25.786, Scan: 030124
Type: HCD, FTMS + c NSI d Full ms2 1464.3383@hcd30.00 [120.0000-3008.6780]
Precursor Scan: 030030, m/z: 1463.8378, charge: 2
EEQFNSTFR_5_4_1_0_0
1e6
Intensity
m/z
138.0547
366.1387
168.0652
204.0862
186.0757
126.0548
144.0655
163.0597
274.0896
512.204 LeX
528.1906
1241.5612 PEP_0_1*_0_0_0
1360.6018 PEP_0_1_0_0_0
1506.6569 PEP_0_1_1_0_0
1563.6757 PEP_0_2_0_0_0
1709.7501 PEP_0_2_1_0_0
1871.8052 PEP_1_2_1_0_0
1887.7911 PEP_2_2_0_0_0

File: 231129_OE_RK_35, RT: 25.827, Scan: 030182
Type: HCD, FTMS + c NSI d Full ms2 1383.0582@hcd30.00 [120.0000-2842.8667]
Precursor Scan: 030103, m/z: 1382.5571, charge: 2
EEQFNSTFR_4_4_1_0_0

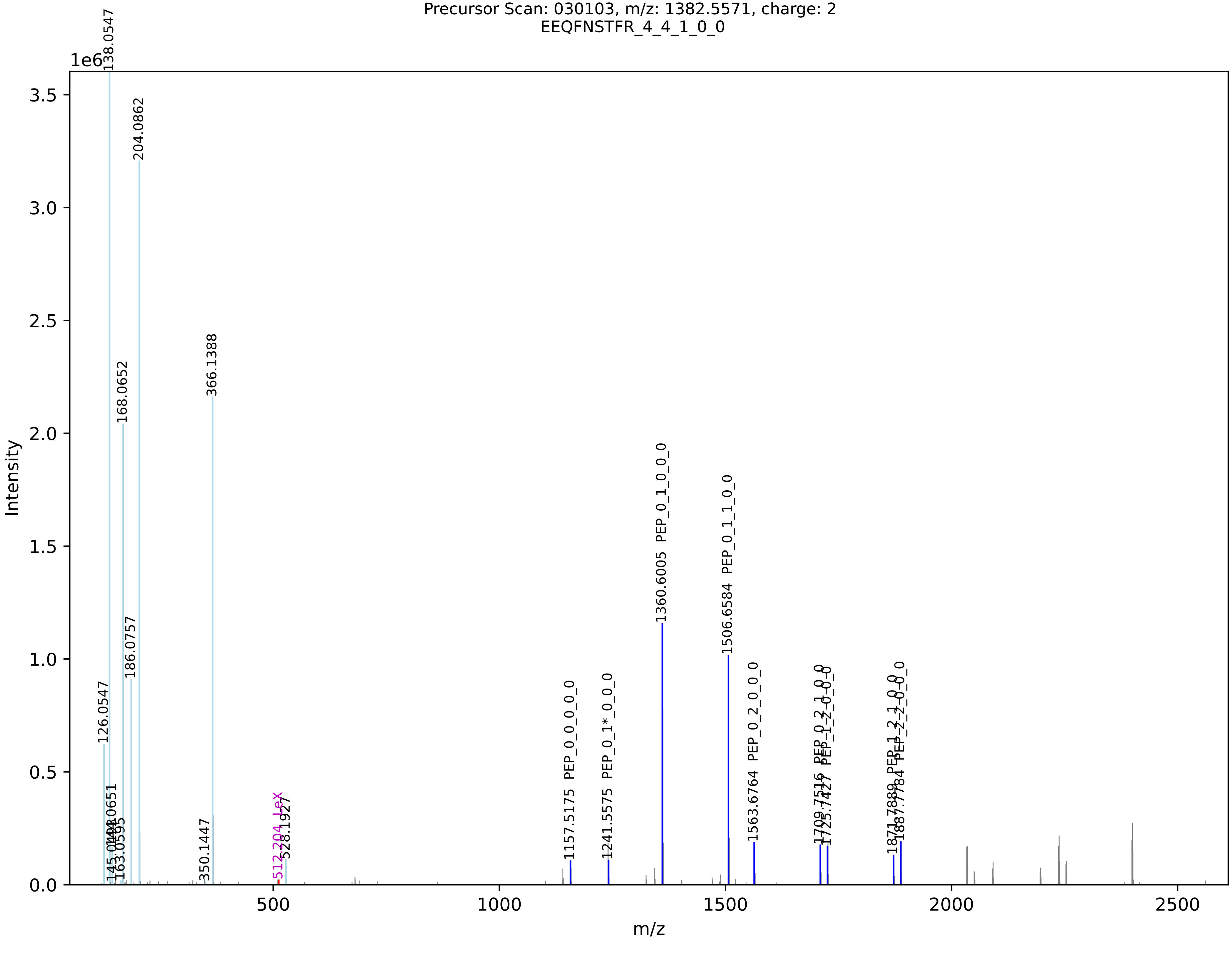

File: 231129_OE_RK_35, RT: 25.831, Scan: 030189
Type: HCD, FTMS + c NSI d Full ms2 998.6196@hcd30.00 [120.0000-5116.5303]
Precursor Scan: 030103, m/z: 998.2187, charge: 5
GNVAVTVSGHTCQHWSAQTPHTHNR_5_4_0_2_0

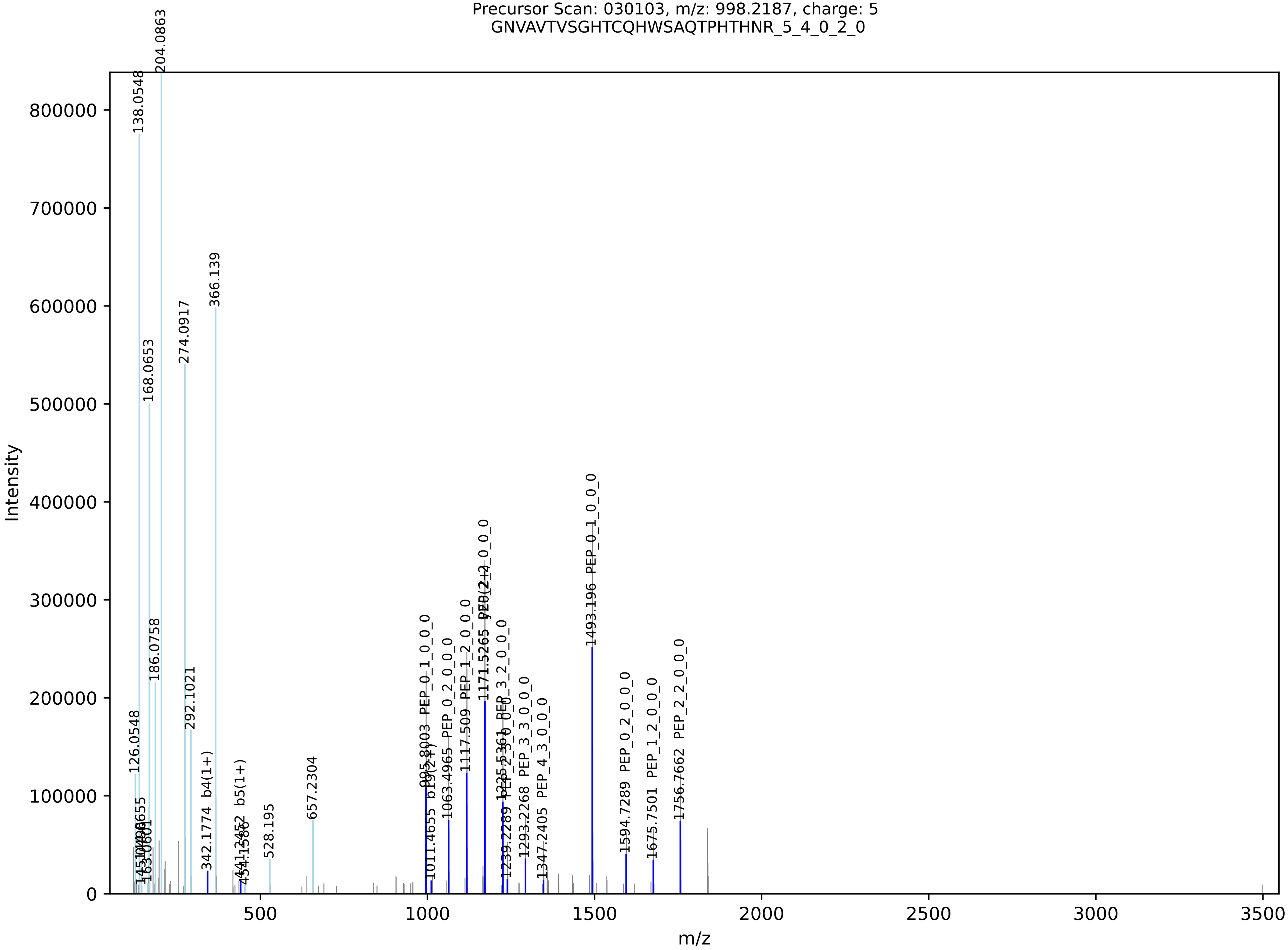

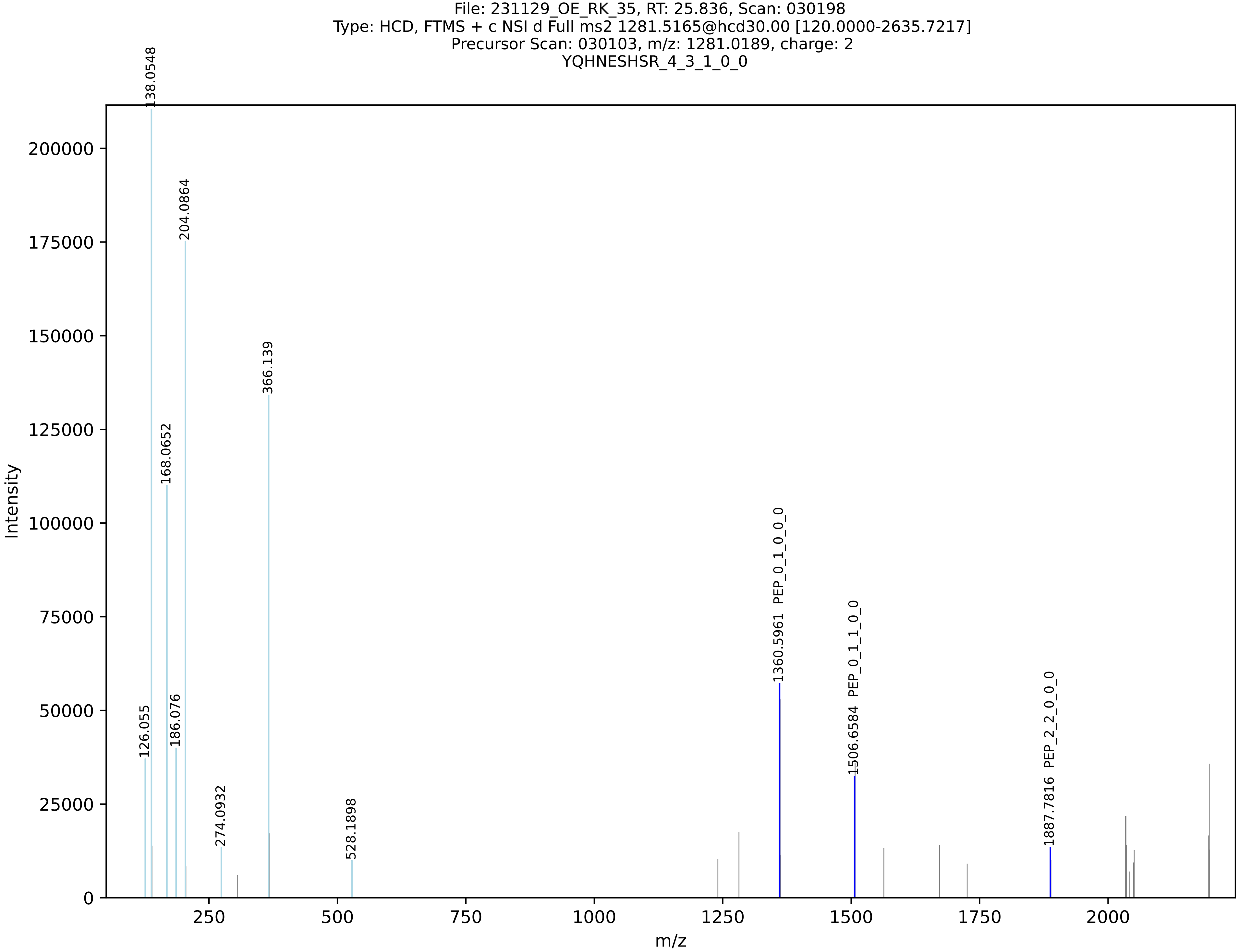
File: 231129_OE_RK_35, RT: 25.836, Scan: 030198
Type: HCD, FTMS + c NSI d Full ms2 1281.5165@hcd30.00 [120.0000-2635.7217]
Precursor Scan: 030103, m/z: 1281.0189, charge: 2
YQHNESHSR_4_3_1_0_0
Intensity
m/z
138.0548
204.0864
366.139
168.0652
126.055
186.076
274.0932
528.1898
1360.5961 PEP_0_1_0_0_0
1506.6584 PEP_0_1_1_0_0
1887.7816 PEP_2_2_0_0_0
0
25000
50000
75000
100000
125000
150000
175000
200000
250
500
750
1000
1250
1500
1750
2000

File: 231129_OE_RK_35, RT: 25.853, Scan: 030224
Type: HCD, FTMS + c NSI d Full ms2 832.3535@hcd30.00 [120.0000-5118.2871]
Precursor Scan: 030103, m/z: 832.0168, charge: 6
GNVAVTVSGHTCQHWSAQTPHTHNR_5_4_0_2_0

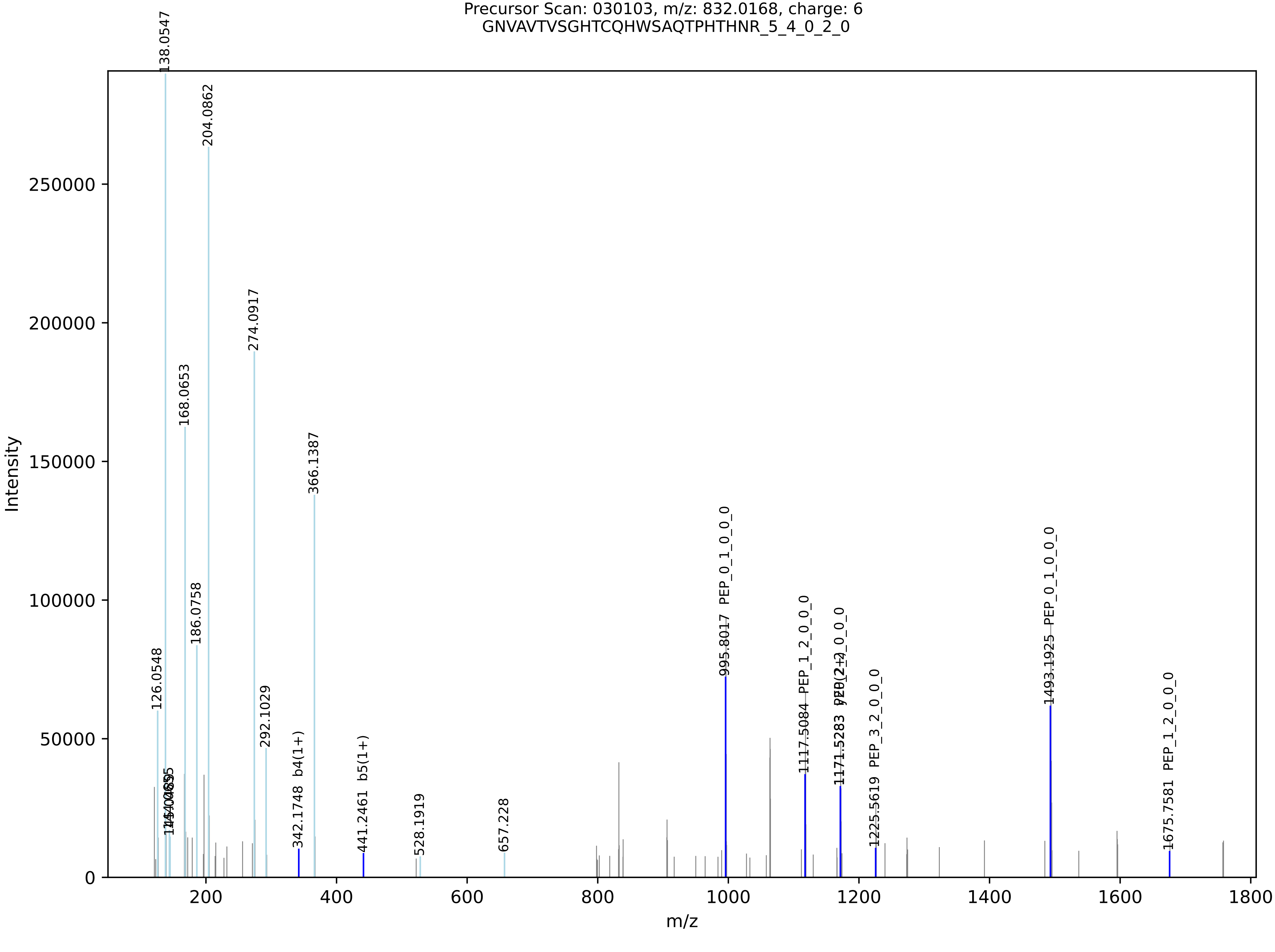

File: 231129_OE_RK_35, RT: 25.862, Scan: 030238
Type: HCD, FTMS + c NSI d Full ms2 1044.4180@hcd30.00 [120.0000-3218.0608]
Precursor Scan: 030103, m/z: 1044.0867, charge: 3
EEQFNSTFR_5_5_1_0_0

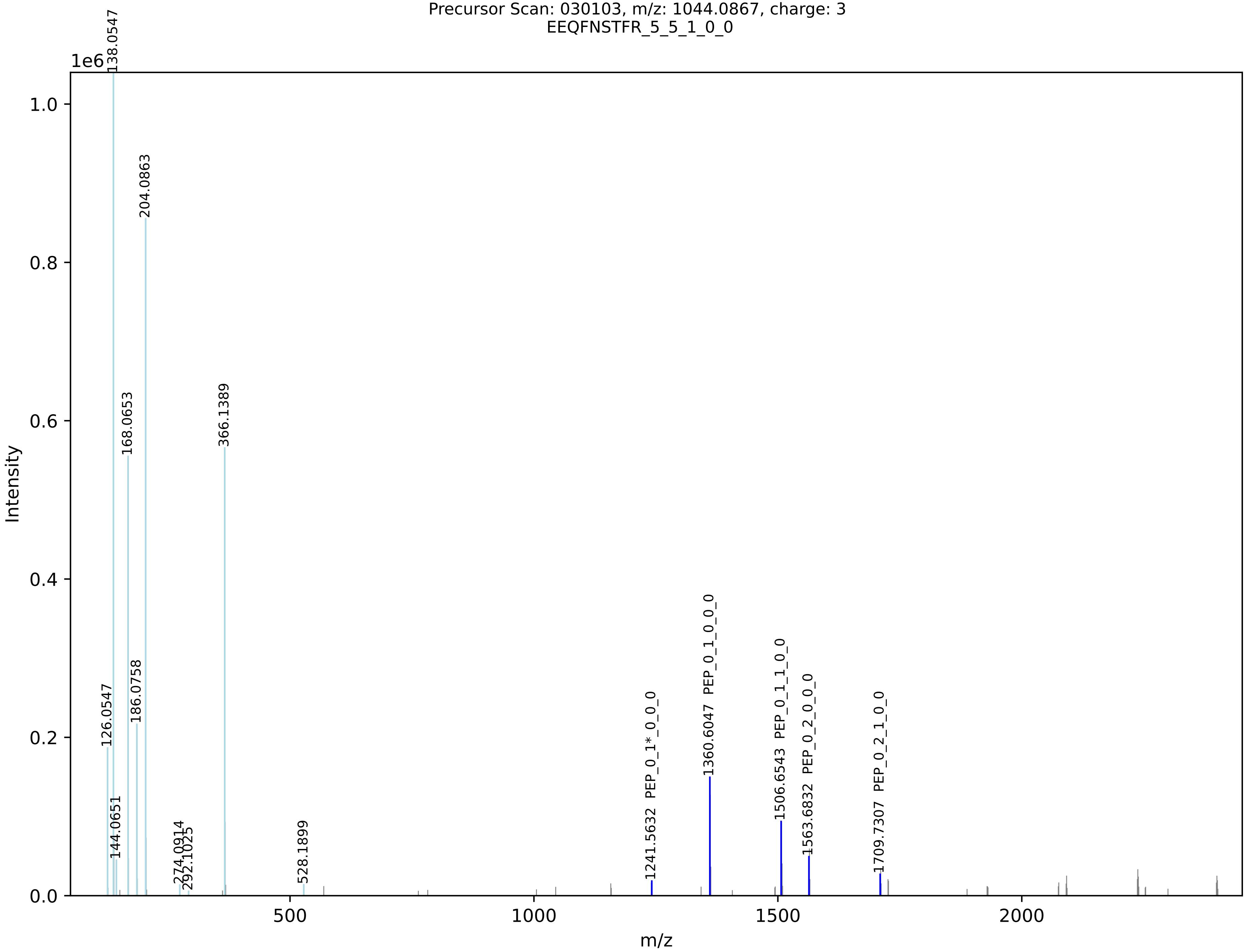

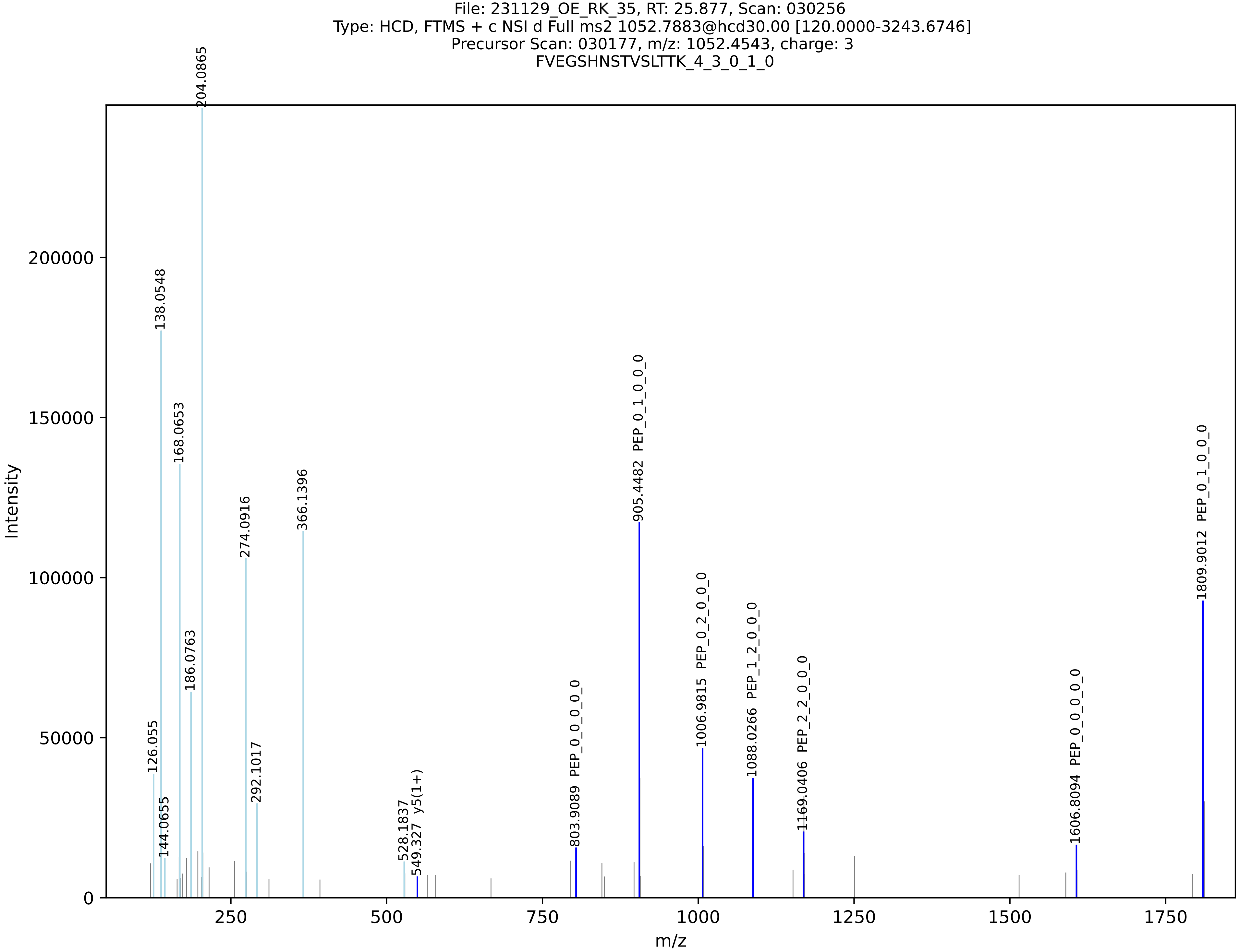

File: 231129_OE_RK_35, RT: 25.877, Scan: 030256
Type: HCD, FTMS + c NSI d Full ms2 1052.7883@hcd30.00 [120.0000-3243.6746]
Precursor Scan: 030177, m/z: 1052.4543, charge: 3
FVEGSHNSTVSLTTK_4_3_0_1_0
Intensity
m/z
204.0865
138.0548
168.0653
186.0763
126.055
144.0655
274.0916
292.1017
366.1396
528.1837
549.327 y5(1+)
803.9089 PEP_0_0_0_0_0
905.4482 PEP_0_1_0_0_0
1006.9815 PEP_0_2_0_0_0
1088.0266 PEP_1_2_0_0_0
1169.0406 PEP_2_2_0_0_0
1606.8094 PEP_0_0_0_0_0
1809.9012 PEP_0_1_0_0_0

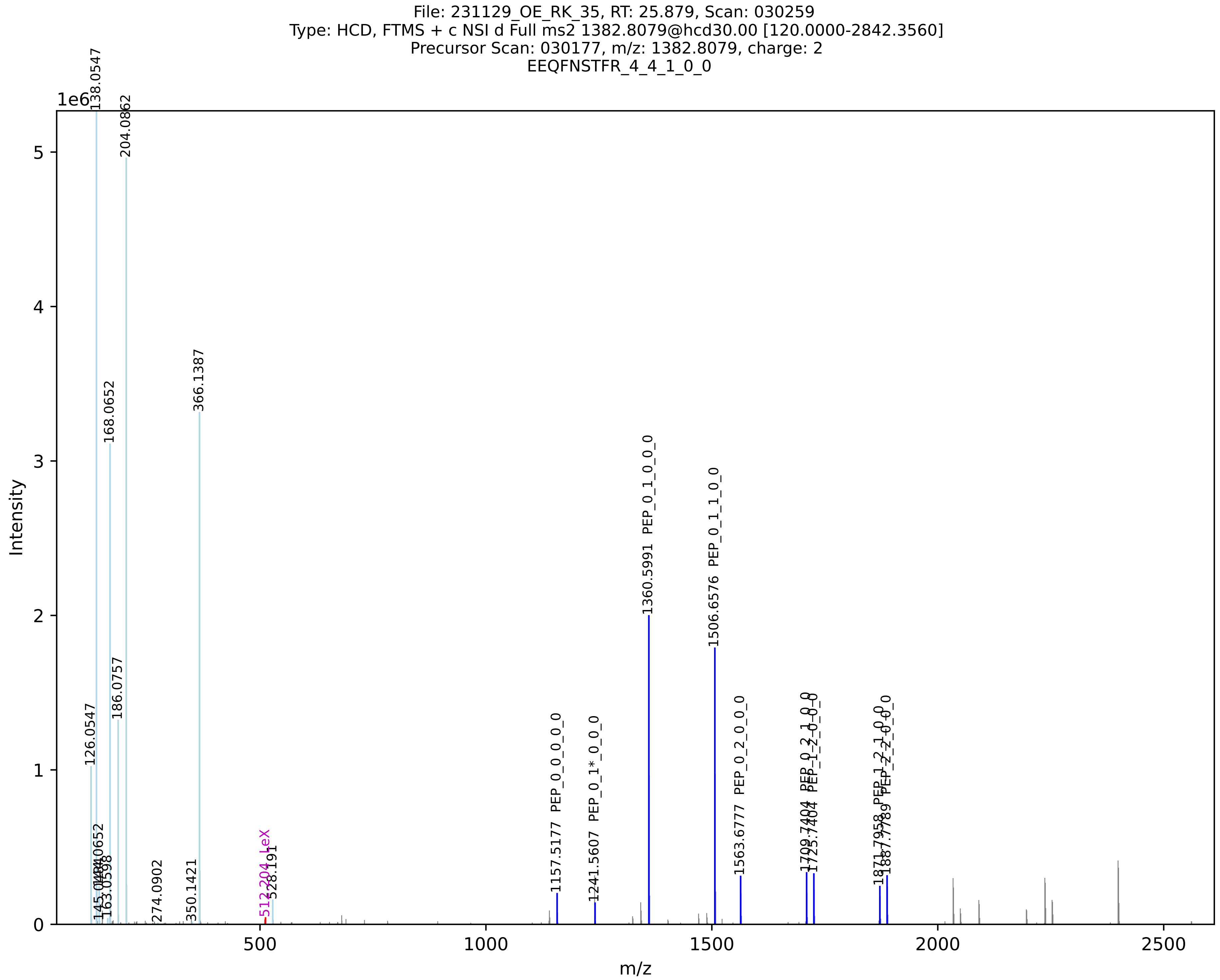

File: 231129_OE_RK_35, RT: 25.879, Scan: 030259
Type: HCD, FTMS + c NSI d Full ms2 1382.8079@hcd30.00 [120.0000-2842.3560]
Precursor Scan: 030177, m/z: 1382.8079, charge: 2
EEQFNSTFR_4_4_1_0_0
1e6
Intensity
m/z
138.0547
204.0862
366.1387
168.0652
186.0757
126.0547
163.0598
274.0902
350.1421
512.204_LeX
528.191
1157.5177 PEP_0_0_0_0_0
1241.5607 PEP_0_1*_0_0_0
1360.5991 PEP_0_1_0_0_0
1506.6576 PEP_0_1_1_0_0
1563.6777 PEP_0_2_0_0_0
1709.7404 PEP_0_2_1_0_0
1725.7404 PEP_1_2_0_0_0
1871.7958 PEP_1_2_1_0_0
1887.7789 PEP_2_2_0_0_0

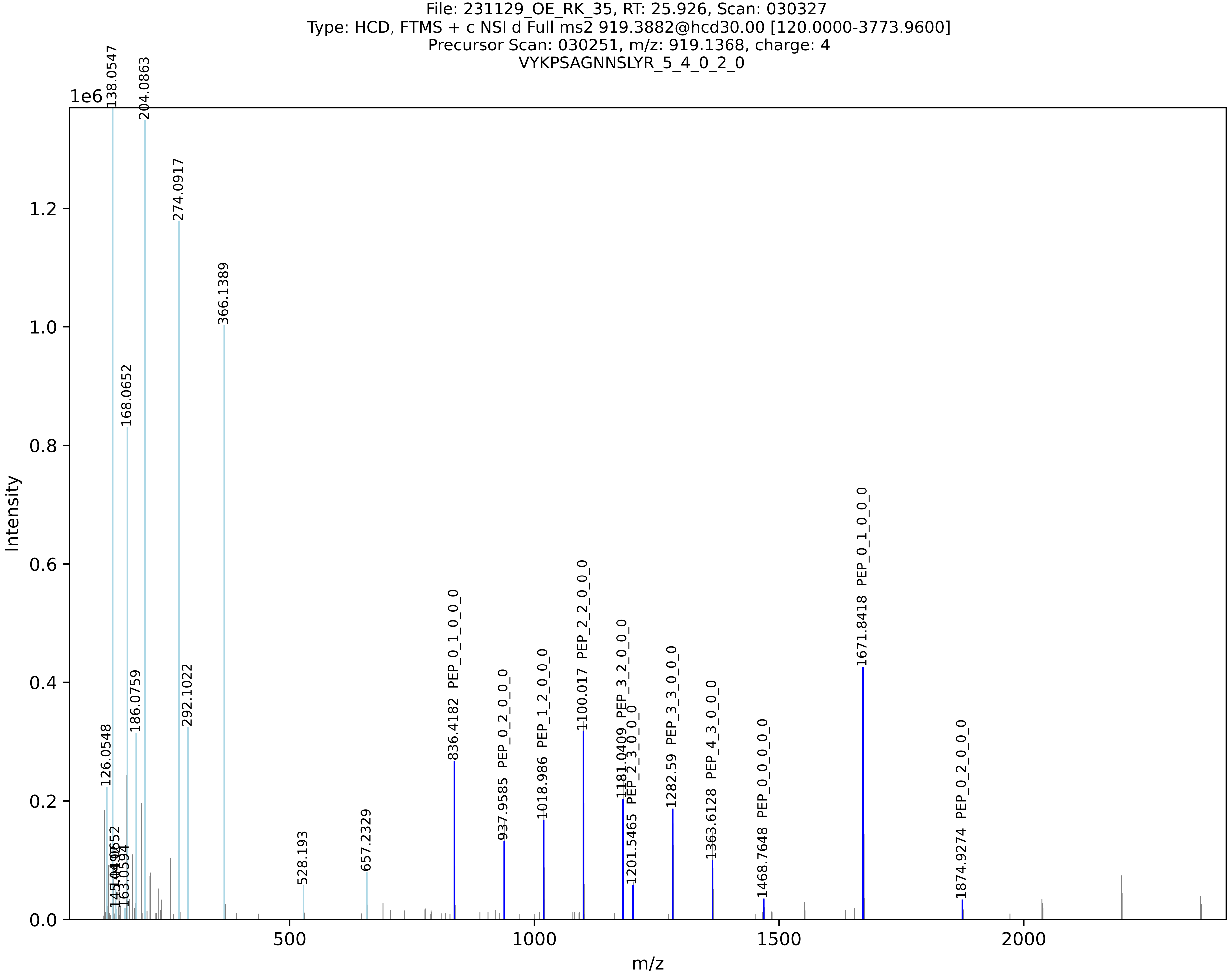
File: 231129_OE_RK_35, RT: 25.926, Scan: 030327
Type: HCD, FTMS + c NSI d Full ms2 919.3882@hcd30.00 [120.0000-3773.9600]
Precursor Scan: 030251, m/z: 919.1368, charge: 4
VYKPSAGNNSLYR_5_4_0_2_0
1e6
Intensity
m/z
0.0
0.2
0.4
0.6
0.8
1.0
1.2
500
1000
1500
2000
138.0547
204.0863
274.0917
366.1389
168.0652
186.0759
292.1022
126.0548
163.0594
528.193
657.2329
836.4182 PEP_0_1_0_0_0
937.9585 PEP_0_2_0_0_0
1018.986 PEP_1_2_0_0_0
1100.017 PEP_2_2_0_0_0
1181.0409 PEP_3_2_0_0_0
1201.5465 PEP_2_3_0_0_0
1282.59 PEP_3_3_0_0_0
1363.6128 PEP_4_3_0_0_0
1468.7648 PEP_0_0_0_0_0
1671.8418 PEP_0_1_0_0_0
1874.9274 PEP_0_2_0_0_0

File: 231129_OE_RK_35, RT: 25.929, Scan: 030333
Type: HCD, FTMS + c NSI d Full ms2 956.1523@hcd30.00 [120.0000-3923.9578]
Precursor Scan: 030251, m/z: 955.6508, charge: 4
VYKPSAGNNSLYR_5_4_1_2_0

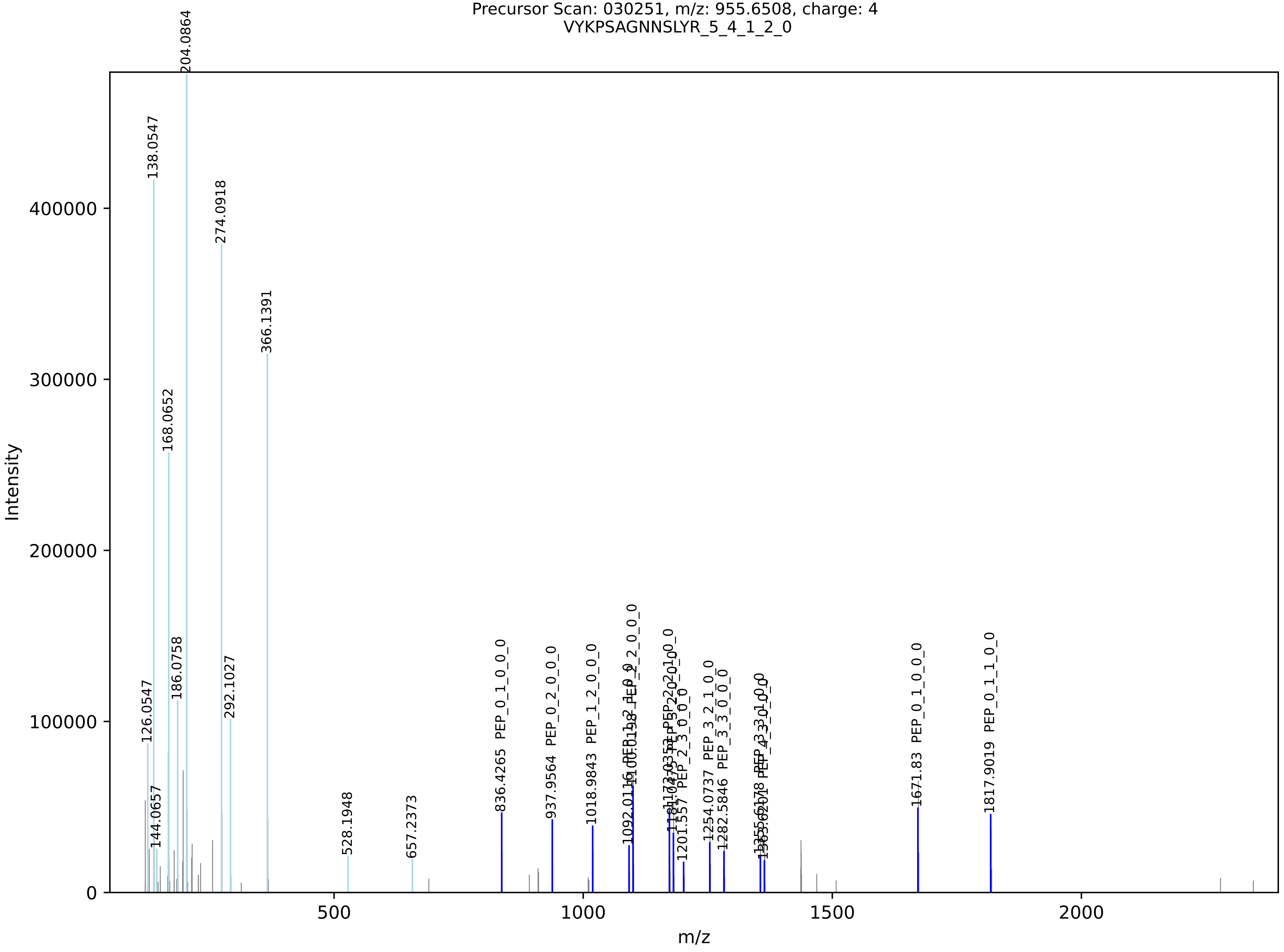

File: 231129_OE_RK_35, RT: 25.977, Scan: 030402
Type: HCD, FTMS + c NSI d Full ms2 1210.8048@hcd30.00 [120.0000-3727.2046]
Precursor Scan: 030324, m/z: 1210.4707, charge: 3
MDGASNVTCINSR_5_4_0_2_0

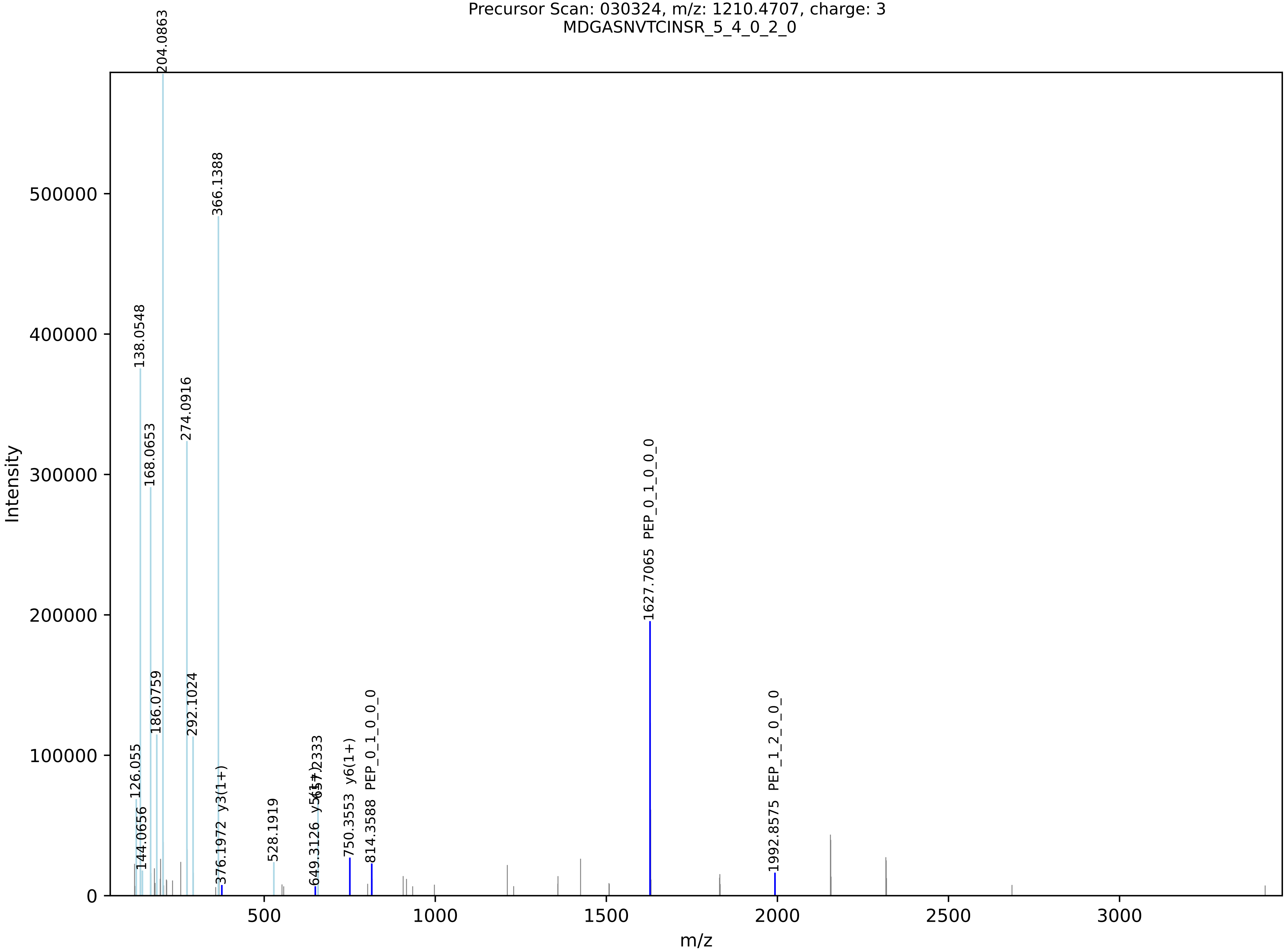

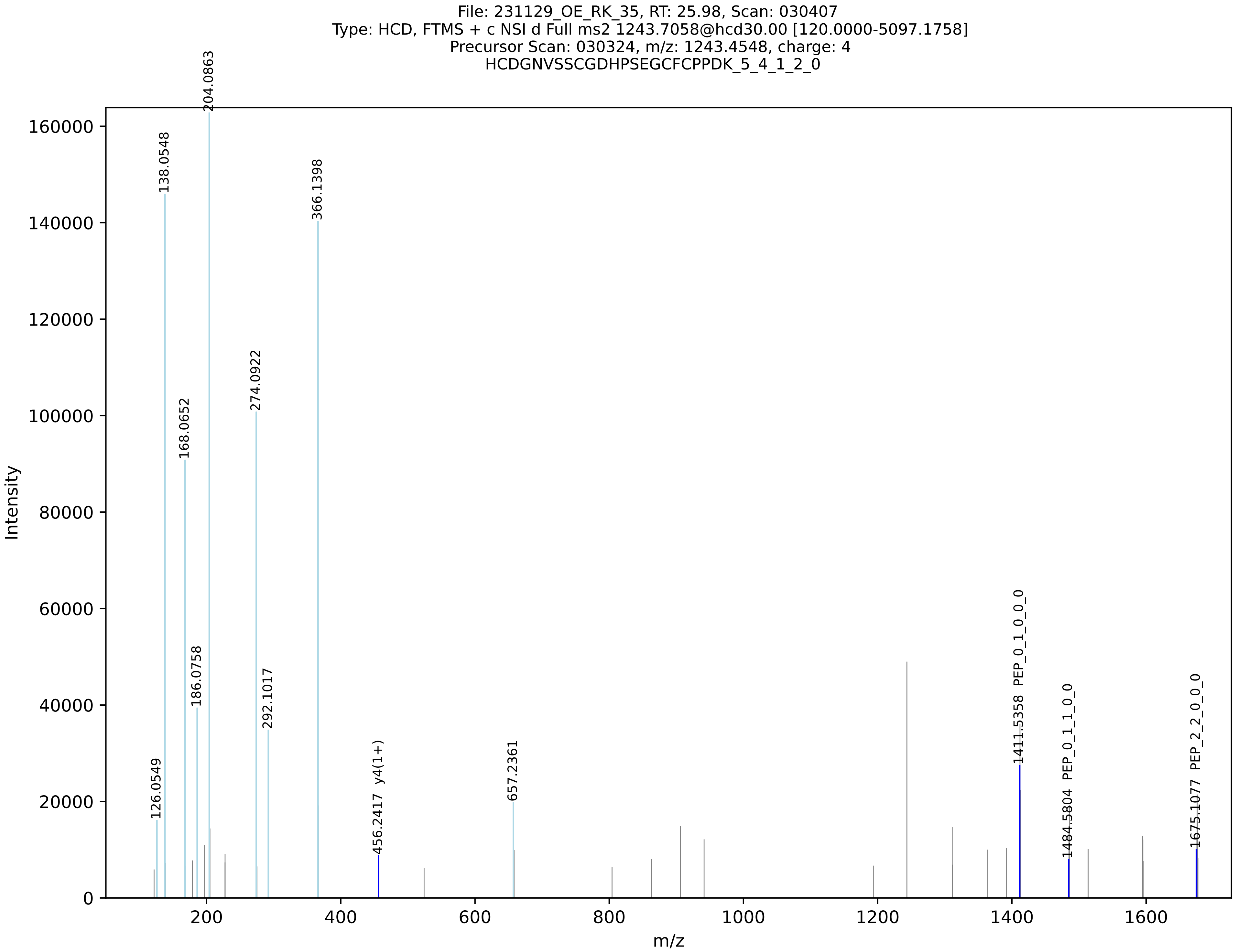
File: 231129_OE_RK_35, RT: 25.98, Scan: 030407
Type: HCD, FTMS + c NSI d Full ms2 1243.7058@hcd30.00 [120.0000-5097.1758]
Precursor Scan: 030324, m/z: 1243.4548, charge: 4
HCDGNVSSCGDHPSEGCFCPPDK_5_4_1_2_0
Intensity
m/z
204.0863
138.0548
366.1398
274.0922
168.0652
186.0758
292.1017
126.0549
456.2417 y4(1+)
657.2361
1411.5358 PEP_0_1_0_0_0
1484.5804 PEP_0_1_1_0_0
1675.1077 PEP_2_2_0_0_0

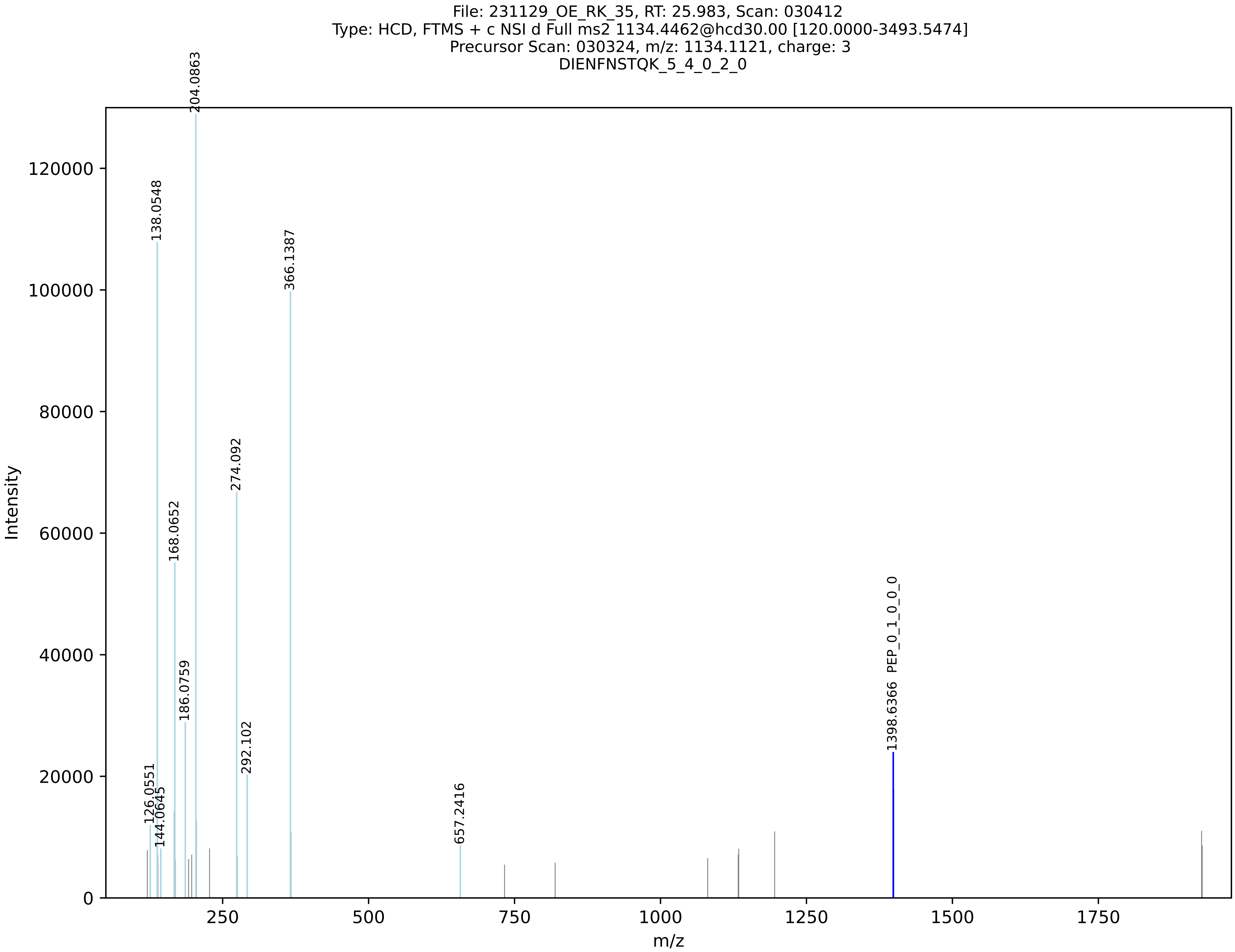
File: 231129_OE_RK_35, RT: 25.983, Scan: 030412
Type: HCD, FTMS + c NSI d Full ms2 1134.4462@hcd30.00 [120.0000-3493.5474]
Precursor Scan: 030324, m/z: 1134.1121, charge: 3
DIENFNSTQK_5_4_0_2_0
Intensity
m/z
204.0863
138.0548
366.1387
274.092
168.0652
186.0759
292.102
126.0551
144.0645
657.2416
1398.6366 PEP_0_1_0_0_0_
0
20000
40000
60000
80000
100000
120000
250
500
750
1000
1250
1500
1750

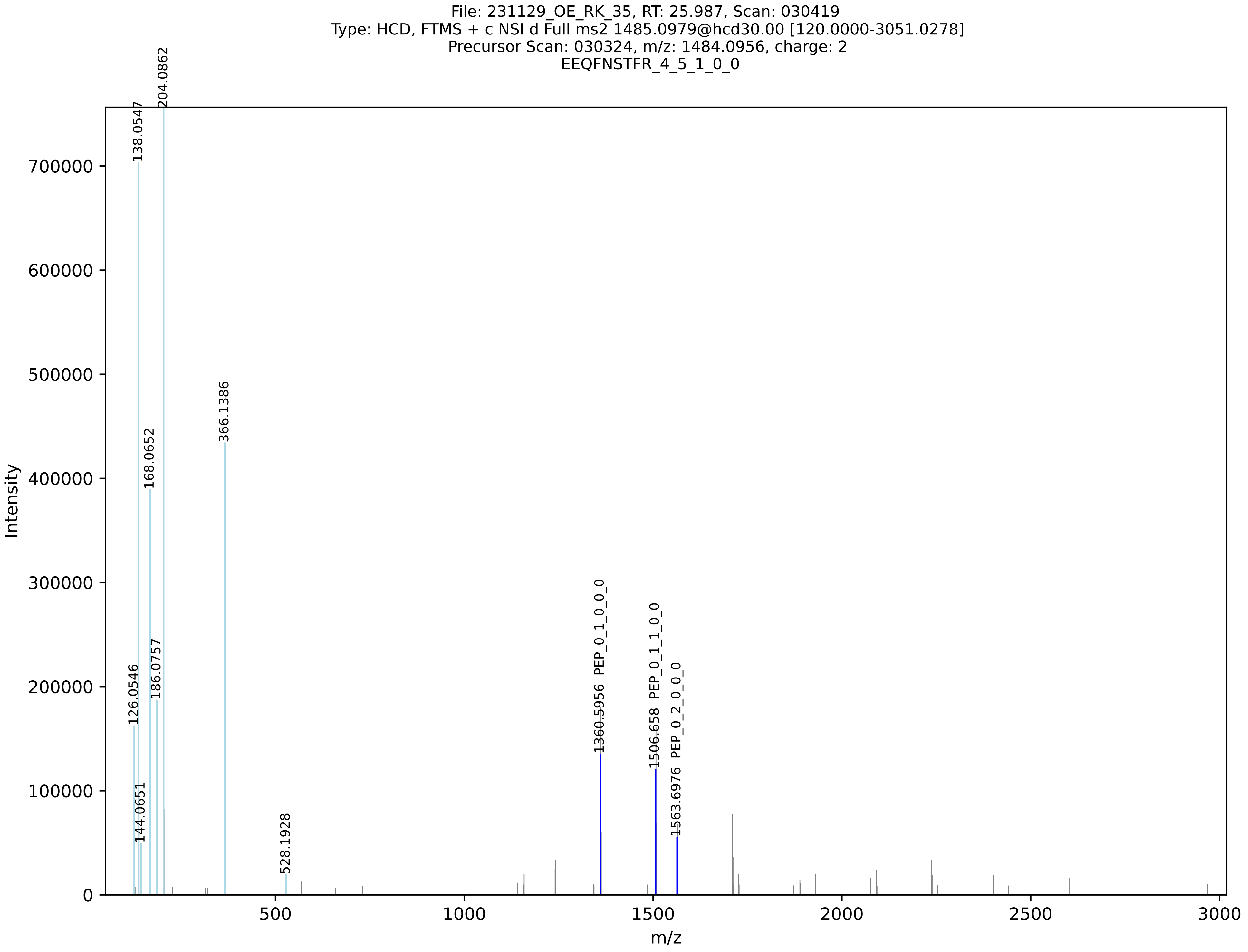
File: 231129_OE_RK_35, RT: 25.987, Scan: 030419
Type: HCD, FTMS + c NSI d Full ms2 1485.0979@hcd30.00 [120.0000-3051.0278]
Precursor Scan: 030324, m/z: 1484.0956, charge: 2
EEQFNSTFR_4_5_1_0_0
204.0862
138.0547
366.1386
168.0652
186.0757
126.0546
144.0651
528.1928
1360.5956 PEP_0_1_0_0_0
1506.658 PEP_0_1_1_0_0
1563.6976 PEP_0_2_0_0_0
Intensity
m/z
0
100000
200000
300000
400000
500000
600000
700000
500
1000
1500
2000
2500
3000

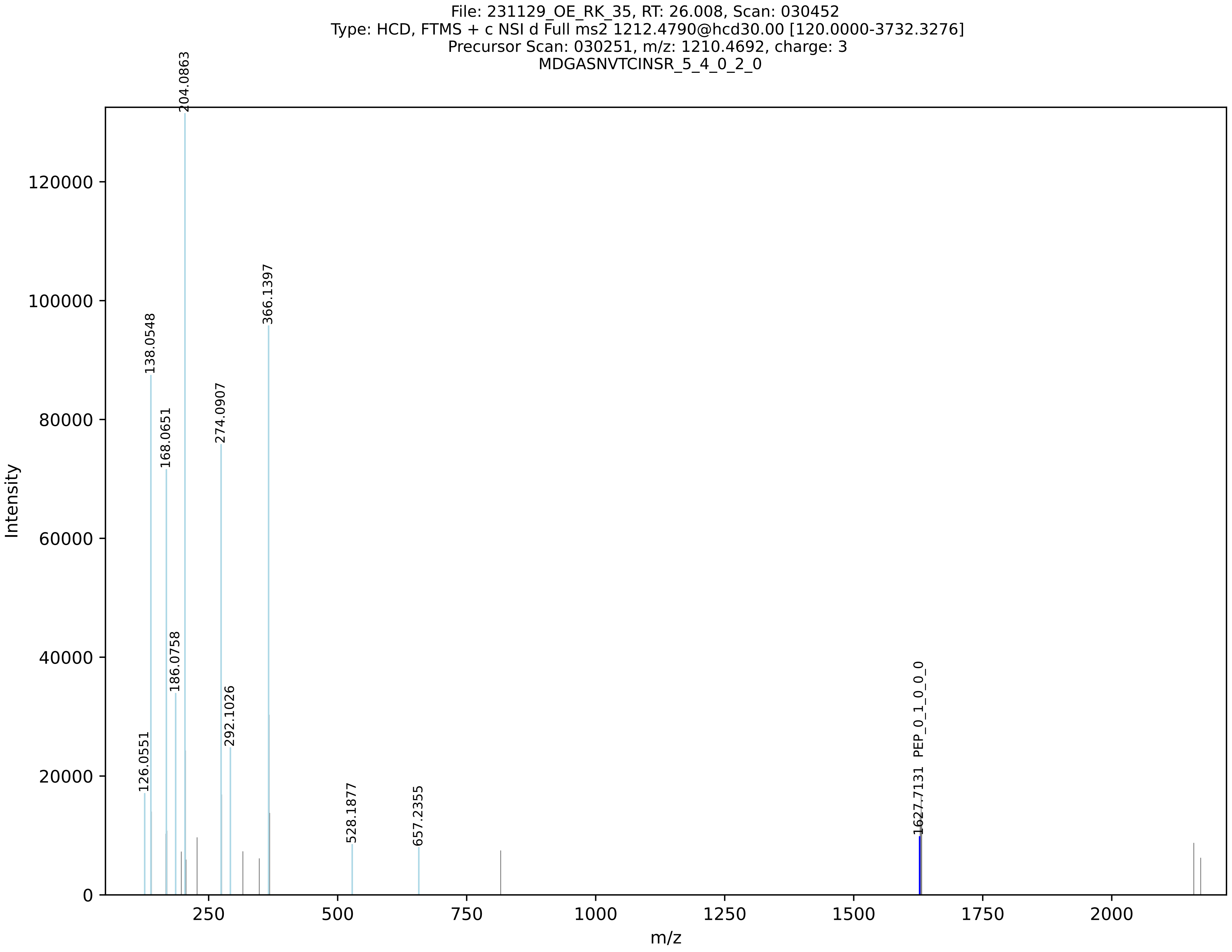
File: 231129_OE_RK_35, RT: 26.008, Scan: 030452
Type: HCD, FTMS + c NSI d Full ms2 1212.4790@hcd30.00 [120.0000-3732.3276]
Precursor Scan: 030251, m/z: 1210.4692, charge: 3
MDGASNVTCINSR_5_4_0_2_0
204.0863
366.1397
138.0548
168.0651
274.0907
186.0758
292.1026
126.0551
528.1877
657.2355
1627.7131 PEP_0_1_0_0_0
Intensity
m/z
0
20000
40000
60000
80000
100000
120000
250
500
750
1000
1250
1500
1750
2000

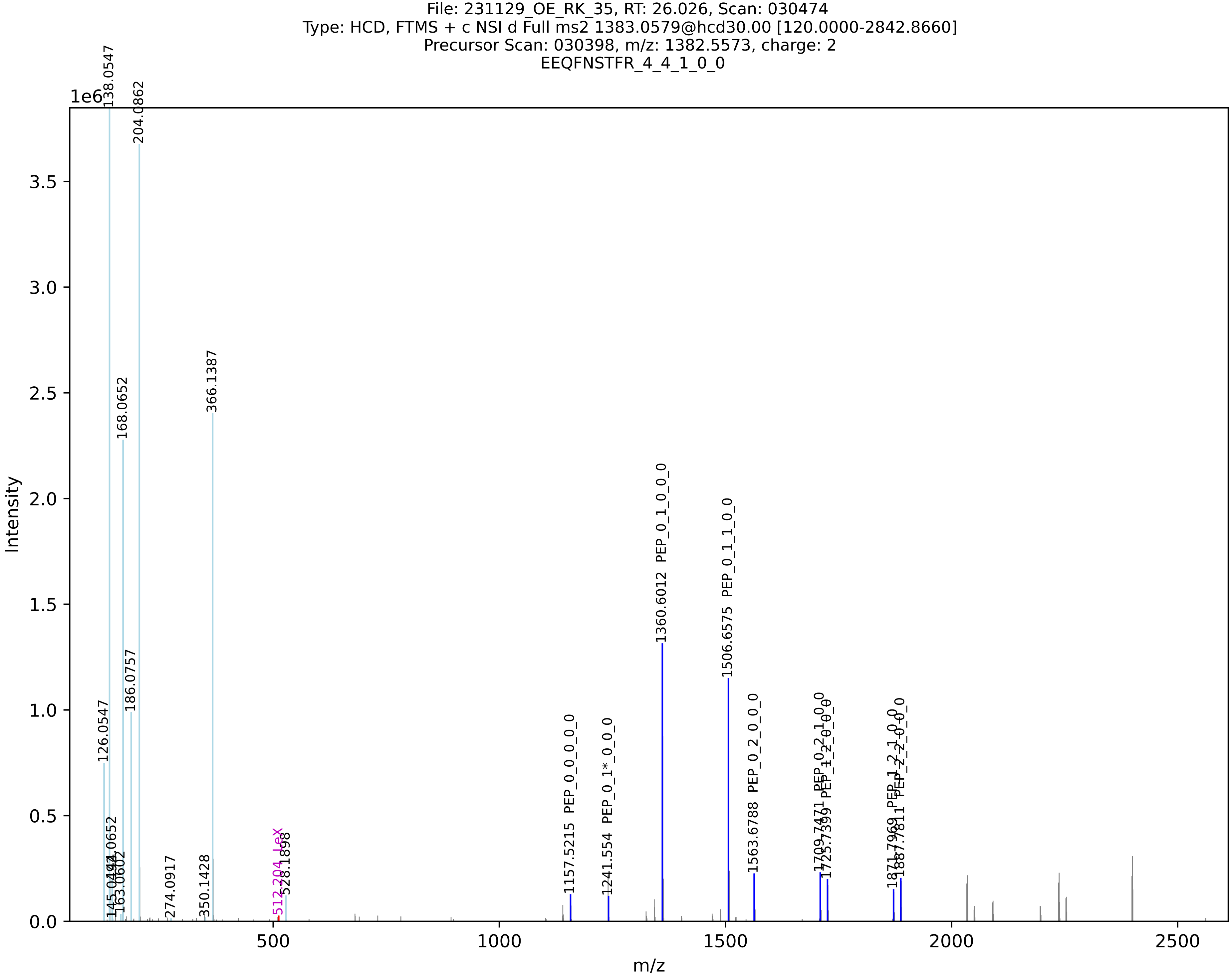
File: 231129_OE_RK_35, RT: 26.026, Scan: 030474
Type: HCD, FTMS + c NSI d Full ms2 1383.0579@hcd30.00 [120.0000-2842.8660]
Precursor Scan: 030398, m/z: 1382.5573, charge: 2
EEQFNSTFR_4_4_1_0_0
1e6
Intensity
m/z
138.0547
204.0862
366.1387
168.0652
186.0757
126.0547
274.0917
350.1428
512.204_LeX
528.1898
1157.5215 PEP_0_0_0_0_0
1241.554 PEP_0_1*_0_0_0
1360.6012 PEP_0_1_0_0_0
1506.6575 PEP_0_1_1_0_0
1563.6788 PEP_0_2_0_0_0
1709.7471 PEP_0_2_1_0_0
1725.7399 PEP_1_2_0_0_0
1871.7969 PEP_1_2_1_0_0
1887.7811 PEP_2_2_0_0_0

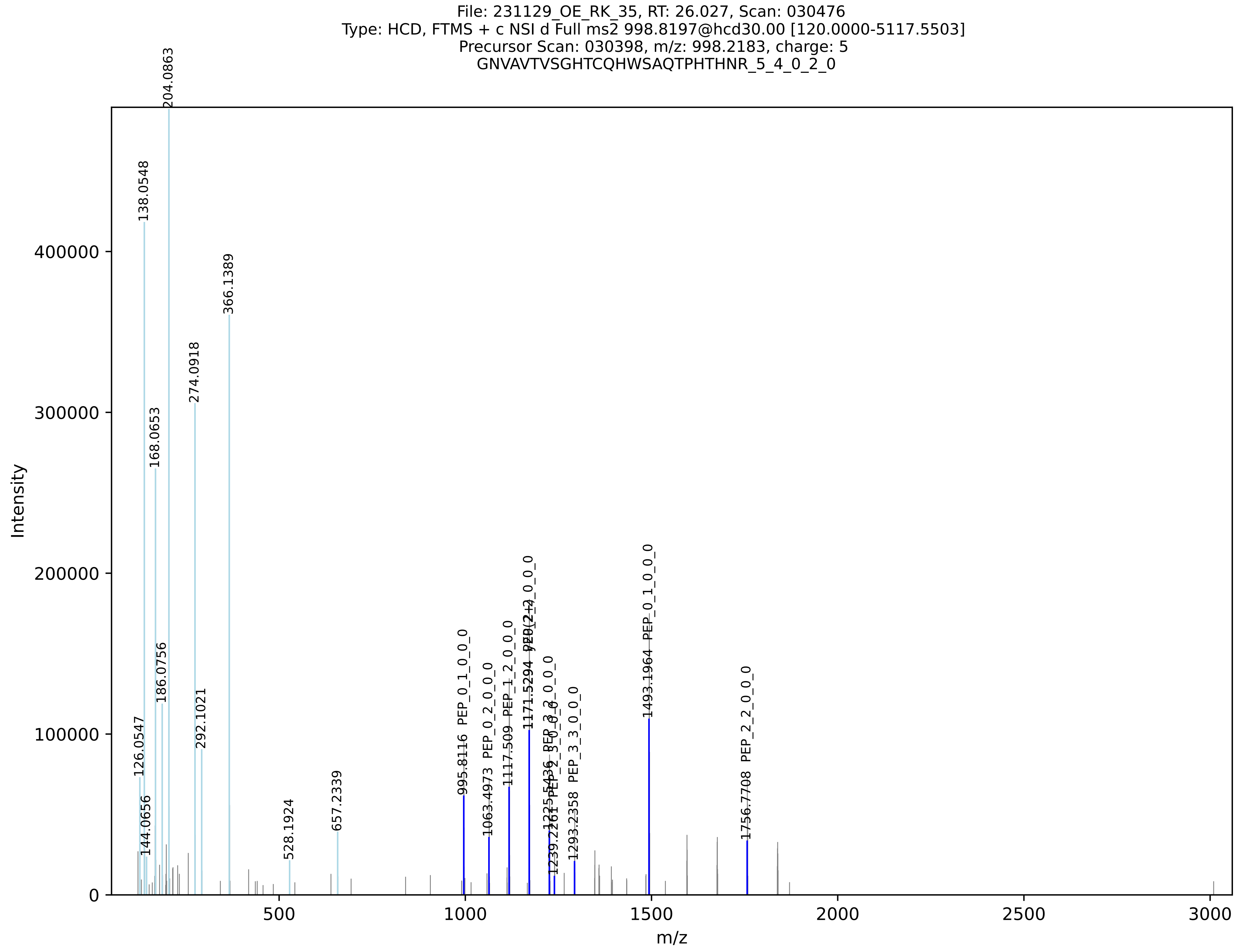

File: 231129_OE_RK_35, RT: 26.027, Scan: 030476
Type: HCD, FTMS + c NSI d Full ms2 998.8197@hcd30.00 [120.0000-5117.5503]
Precursor Scan: 030398, m/z: 998.2183, charge: 5
GNVAVTVSGHTCQHWSAQTPHTHNR_5_4_0_2_0
Intensity
m/z
204.0863
138.0548
168.0653
186.0756
126.0547
144.0656
274.0918
292.1021
366.1389
528.1924
657.2339
995.8116 PEP_0_1_0_0_0
1063.4973 PEP_0_2_0_0_0
1117.509 PEP_1_2_0_0_0
1493.1964 PEP_0_1_0_0_0
1756.7708 PEP_2_2_0_0_0
1293.2358 PEP_3_3_0_0_0
1225.5436 PEP_3_2_0_0_0
0
100000
200000
300000
400000
500
1000
1500
2000
2500
3000

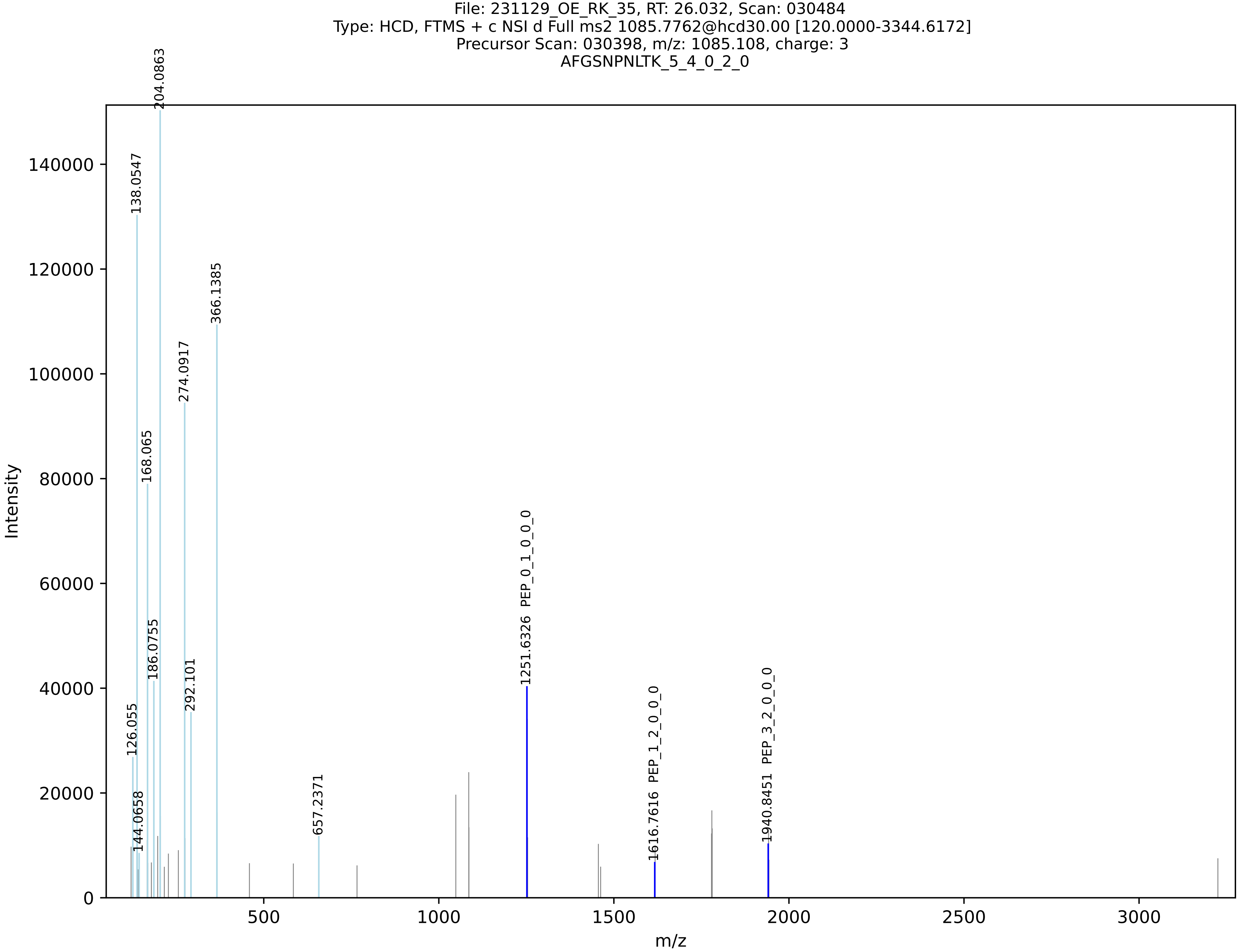

File: 231129_OE_RK_35, RT: 26.032, Scan: 030484
Type: HCD, FTMS + c NSI d Full ms2 1085.7762@hcd30.00 [120.0000-3344.6172]
Precursor Scan: 030398, m/z: 1085.108, charge: 3
AFGSNPNLTK_5_4_0_2_0
Intensity
m/z
204.0863
138.0547
366.1385
274.0917
168.065
186.0755
292.101
126.055
144.0658
657.2371
1251.6326 PEP_0_1_0_0_0
1616.7616 PEP_1_2_0_0_0
1940.8451 PEP_3_2_0_0_0

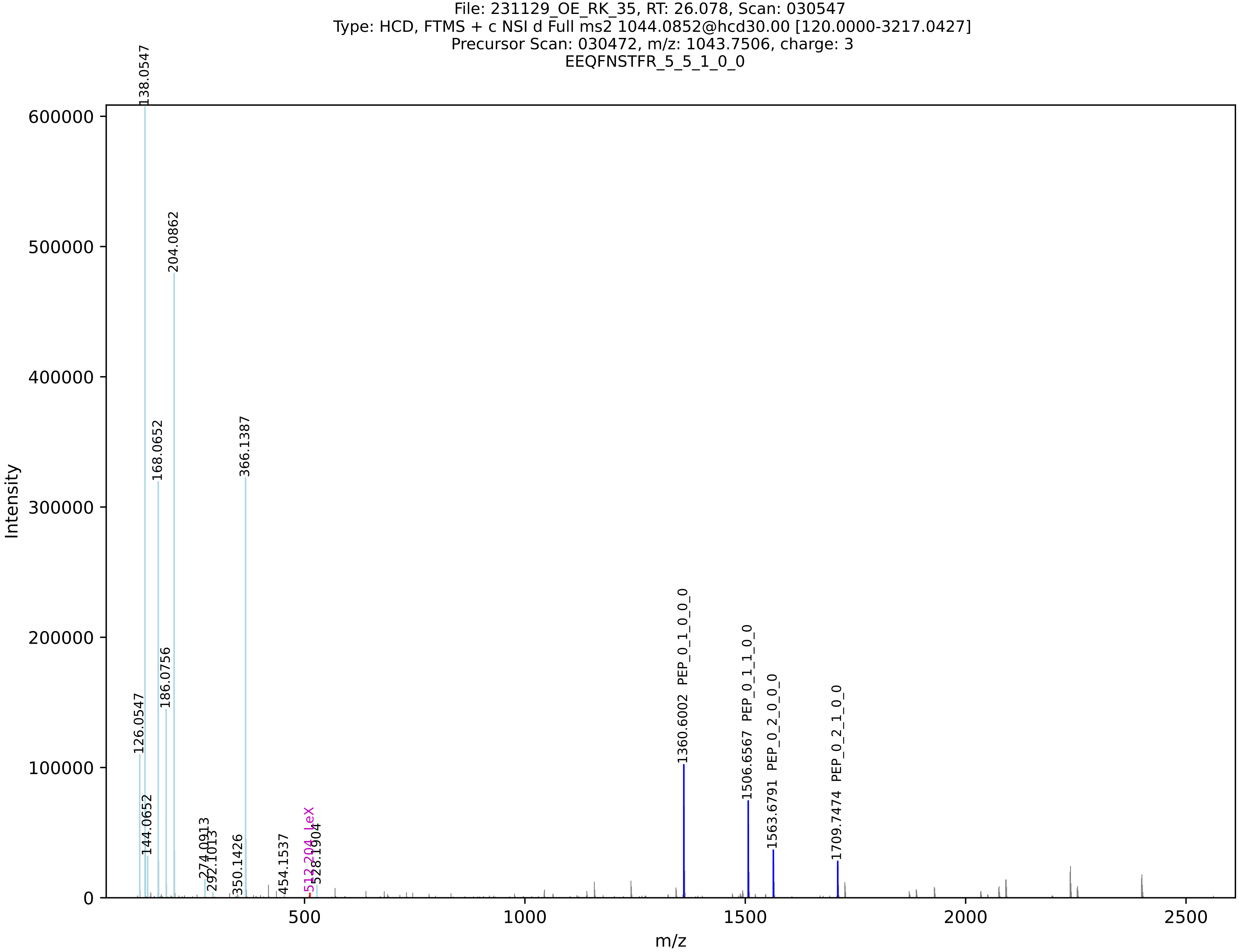
File: 231129_OE_RK_35, RT: 26.078, Scan: 030547
Type: HCD, FTMS + c NSI d Full ms2 1044.0852@hcd30.00 [120.0000-3217.0427]
Precursor Scan: 030472, m/z: 1043.7506, charge: 3
EEQFNSTFR_5_5_1_0_0
Intensity
m/z
138.0547
204.0862
168.0652
366.1387
186.0756
126.0547
144.0652
274.0913
292.1013
350.1426
454.1537
512.204_LeX
528.1904
1360.6002 PEP_0_1_0_0_0
1506.6567 PEP_0_1_1_0_0
1563.6791 PEP_0_2_0_0_0
1709.7474 PEP_0_2_1_0_0

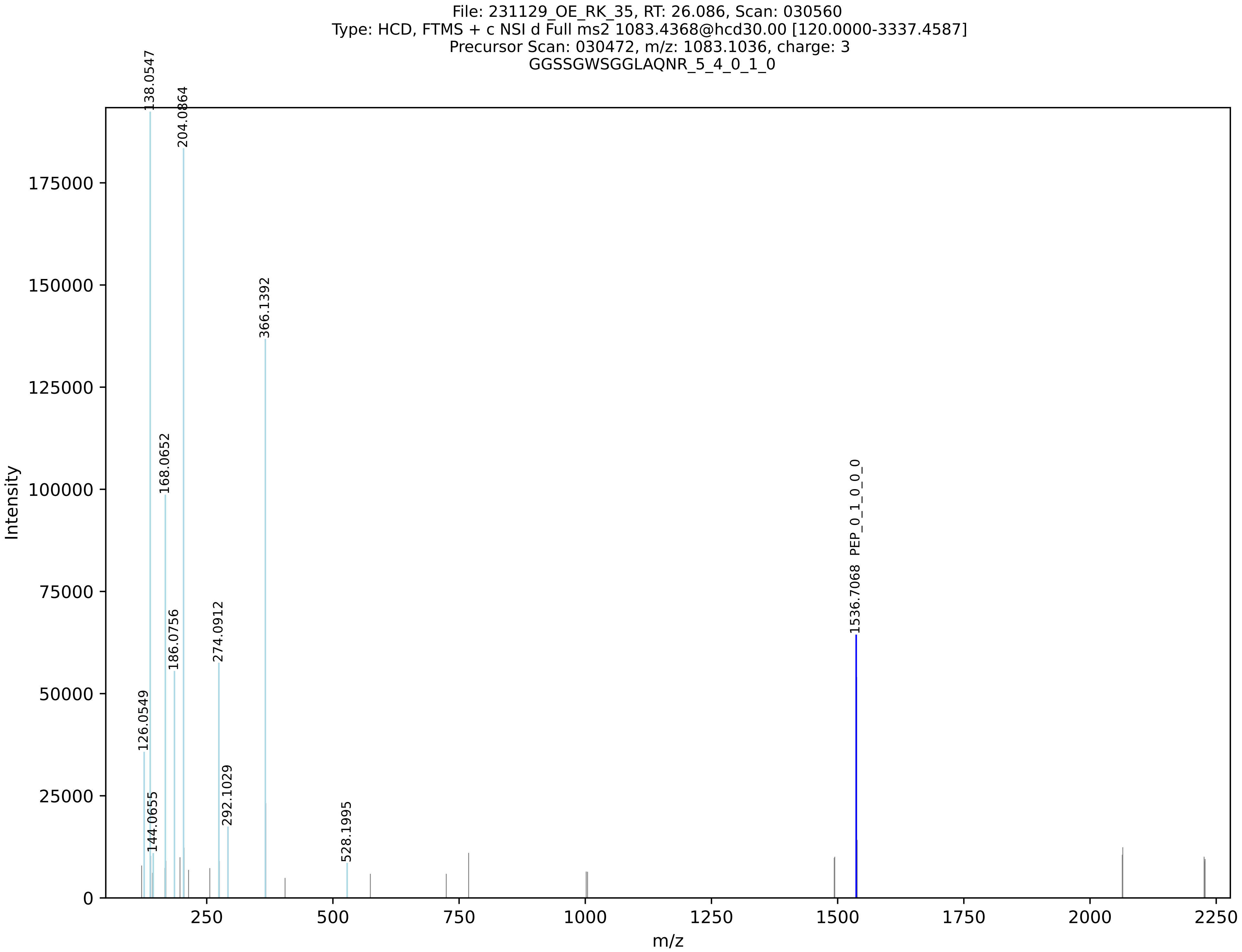
File: 231129_OE_RK_35, RT: 26.086, Scan: 030560
Type: HCD, FTMS + c NSI d Full ms2 1083.4368@hcd30.00 [120.0000-3337.4587]
Precursor Scan: 030472, m/z: 1083.1036, charge: 3
GGSSGWSGGLAQNR_5_4_0_1_0
Intensity
m/z
138.0547
204.0864
366.1392
168.0652
186.0756
274.0912
126.0549
292.1029
144.0655
528.1995
1536.7068 PEP_0_1_0_0_0
0
25000
50000
75000
100000
125000
150000
175000
250
500
750
1000
1250
1500
1750
2000
2250

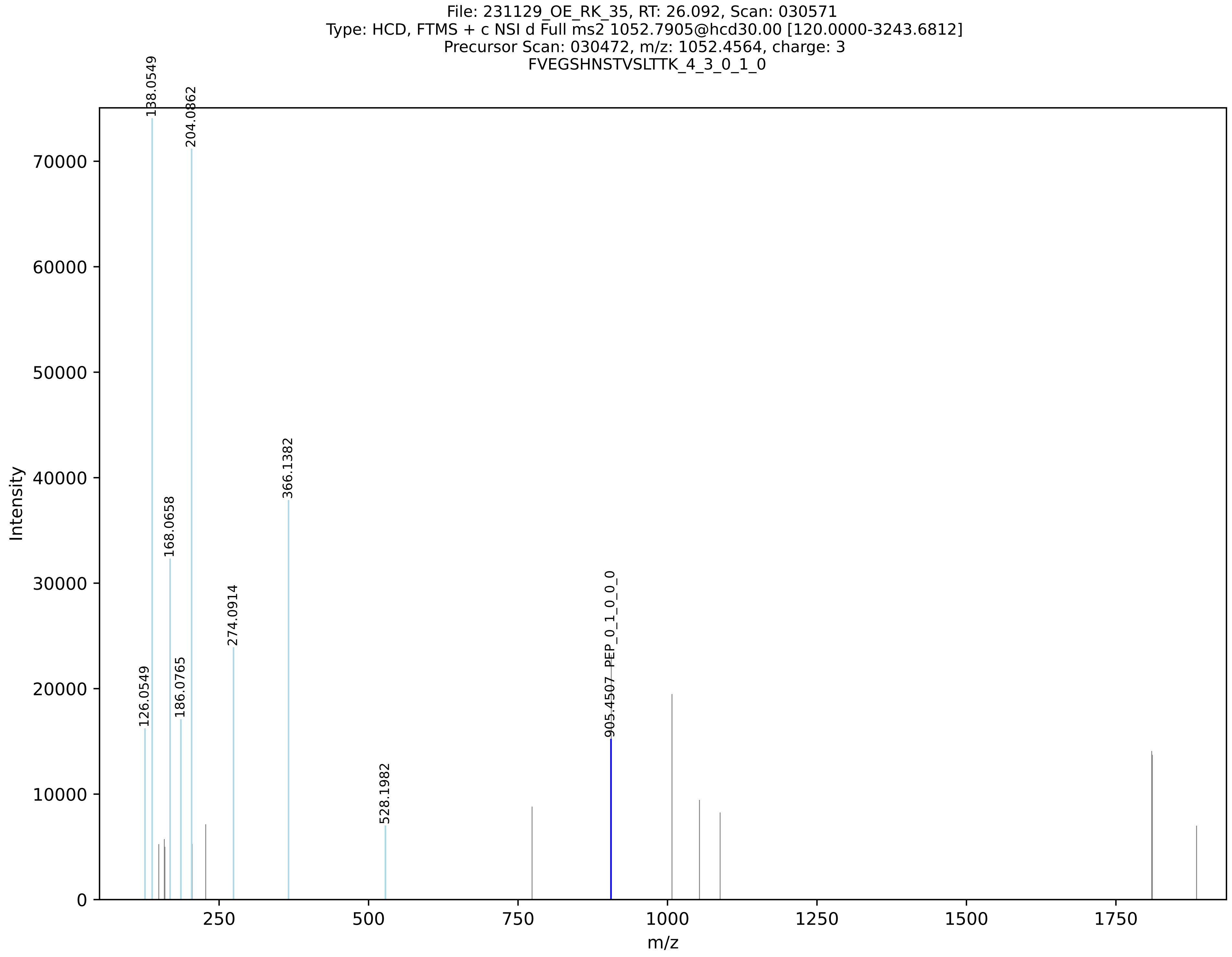
File: 231129_OE_RK_35, RT: 26.092, Scan: 030571
Type: HCD, FTMS + c NSI d Full ms2 1052.7905@hcd30.00 [120.0000-3243.6812]
Precursor Scan: 030472, m/z: 1052.4564, charge: 3
FVEGSHNSTVSLTTK_4_3_0_1_0
Intensity
m/z
138.0549
204.0862
366.1382
168.0658
274.0914
126.0549
186.0765
528.1982
905.4507_PEP_0_1_0_0_0
0
10000
20000
30000
40000
50000
60000
70000
250
500
750
1000
1250
1500
1750

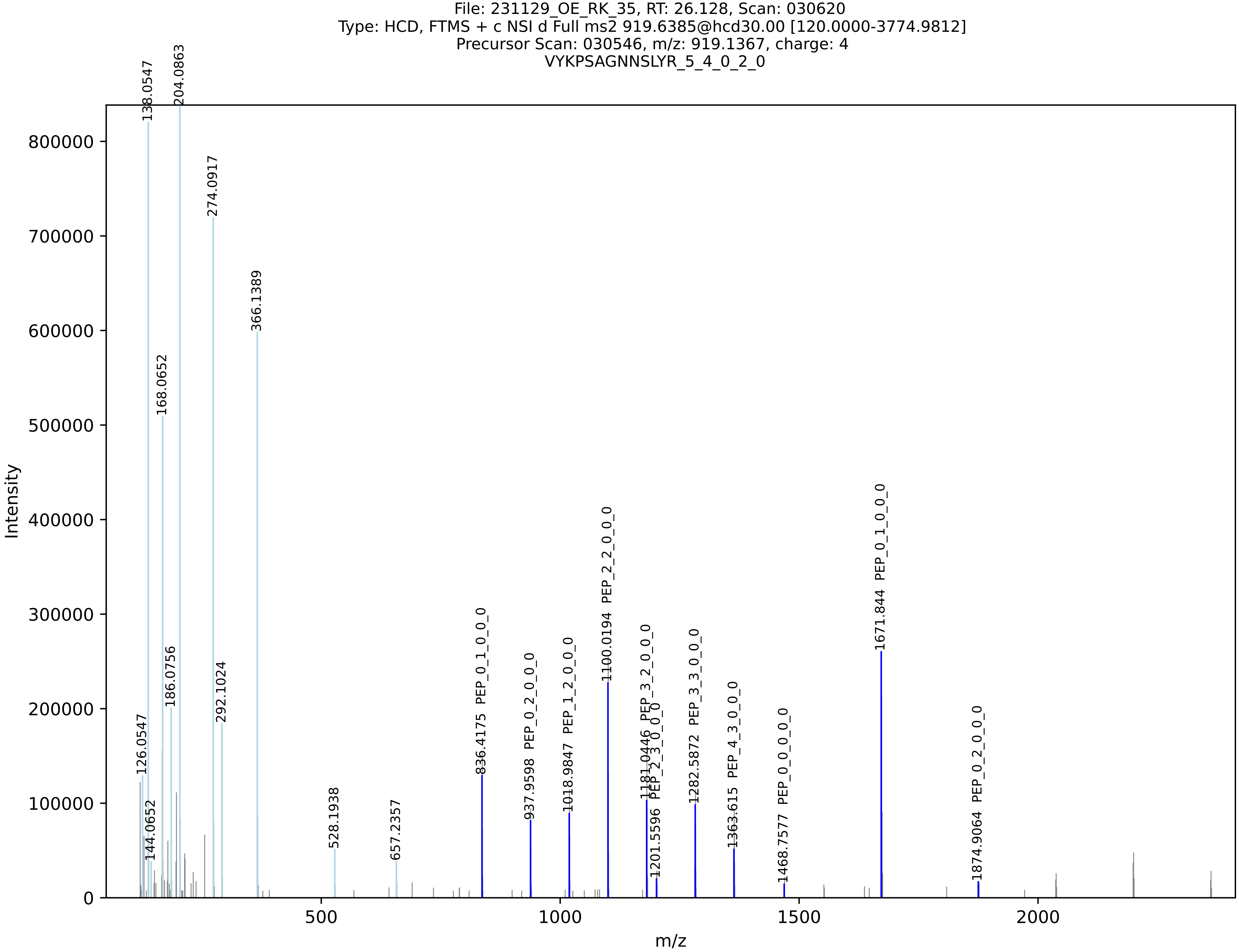
File: 231129_OE_RK_35, RT: 26.128, Scan: 030620
Type: HCD, FTMS + c NSI d Full ms2 919.6385@hcd30.00 [120.0000-3774.9812]
Precursor Scan: 030546, m/z: 919.1367, charge: 4
VYKPSAGNNSLYR_5_4_0_2_0
Intensity
m/z
138.0547
204.0863
274.0917
366.1389
168.0652
186.0756
292.1024
126.0547
144.0652
528.1938
657.2357
836.4175 PEP_0_1_0_0_0
937.9598 PEP_0_2_0_0_0
1018.9847 PEP_1_2_0_0_0
1100.0194 PEP_2_2_0_0_0
1181.0446 PEP_3_2_0_0_0
1201.5596 PEP_2_3_0_0_0
1282.5872 PEP_3_3_0_0_0
1363.615 PEP_4_3_0_0_0
1468.7577 PEP_0_0_0_0_0
1671.844 PEP_0_1_0_0_0
1874.9064 PEP_0_2_0_0_0

File: 231129_OE_RK_35, RT: 26.179, Scan: 030696
Type: HCD, FTMS + c NSI d Full ms2 1211.1388@hcd30.00 [120.0000-3728.2266]
Precursor Scan: 030617, m/z: 1210.4709, charge: 3
MDGASNVTCINSR_5_4_0_2_0

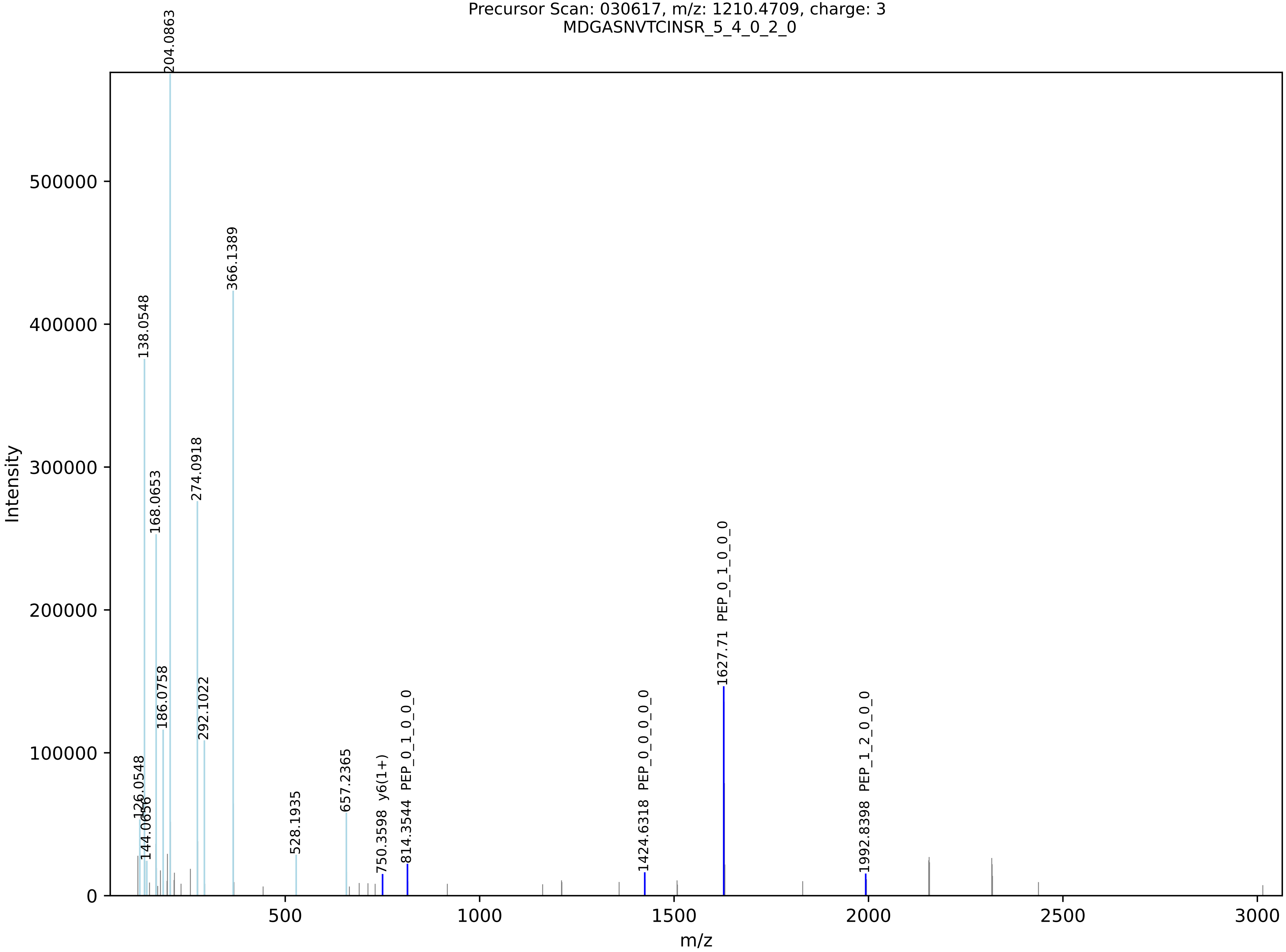

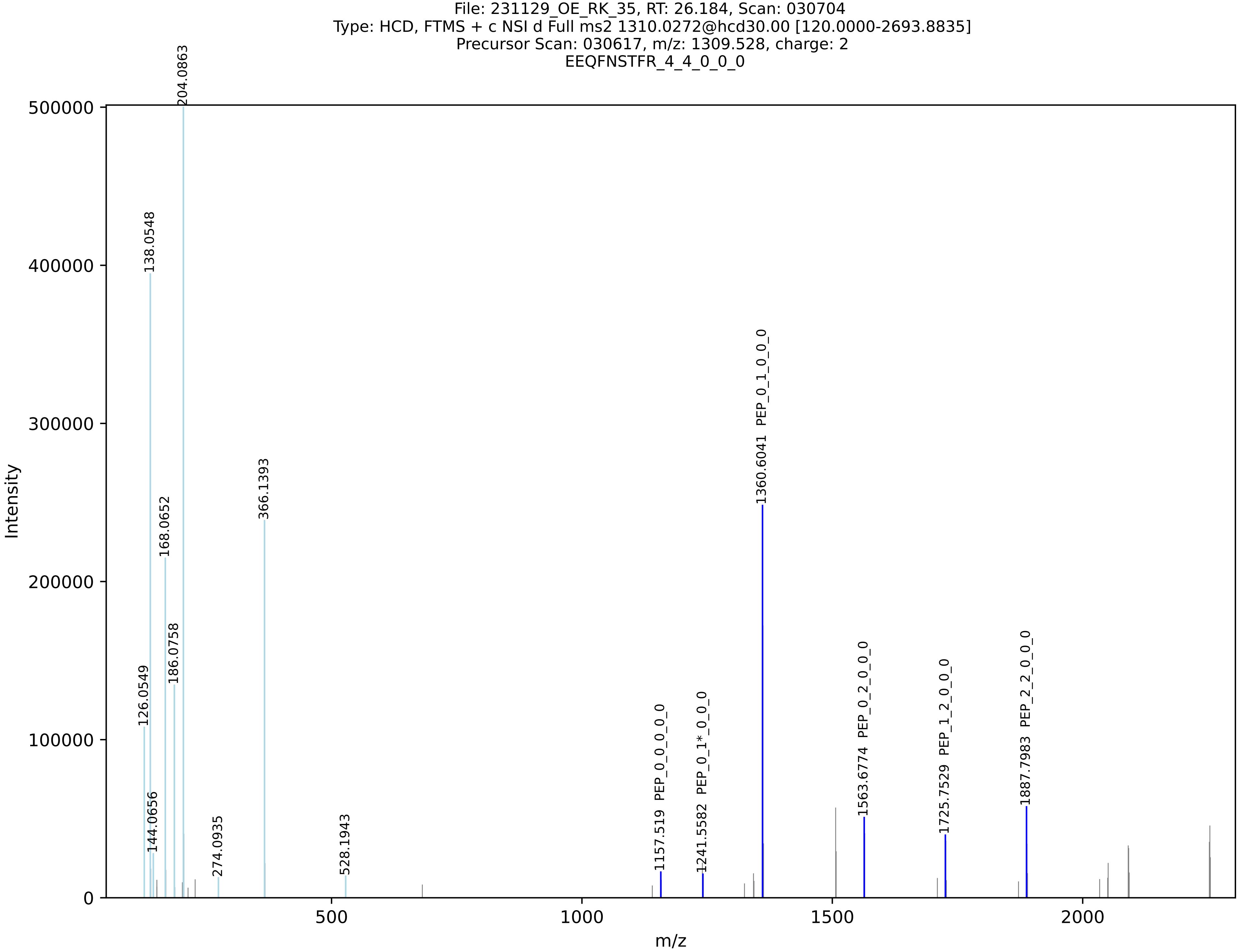
File: 231129_OE_RK_35, RT: 26.184, Scan: 030704
Type: HCD, FTMS + c NSI d Full ms2 1310.0272@hcd30.00 [120.0000-2693.8835]
Precursor Scan: 030617, m/z: 1309.528, charge: 2
EEQFNSTFR_4_4_0_0_0
Intensity
m/z
204.0863
138.0548
168.0652
186.0758
126.0549
144.0656
274.0935
366.1393
528.1943
1157.519 PEP_0_0_0_0_0
1241.5582 PEP_0_1*_0_0_0
1360.6041 PEP_0_1_0_0_0
1563.6774 PEP_0_2_0_0_0
1725.7529 PEP_1_2_0_0_0
1887.7983 PEP_2_2_0_0_0

File: 231129_OE_RK_35, RT: 26.228, Scan: 030767
Type: HCD, FTMS + c NSI d Full ms2 1383.0580@hcd30.00 [120.0000-2842.8662]
Precursor Scan: 030691, m/z: 1382.5572, charge: 2
EEQFNSTFR_4_4_1_0_0

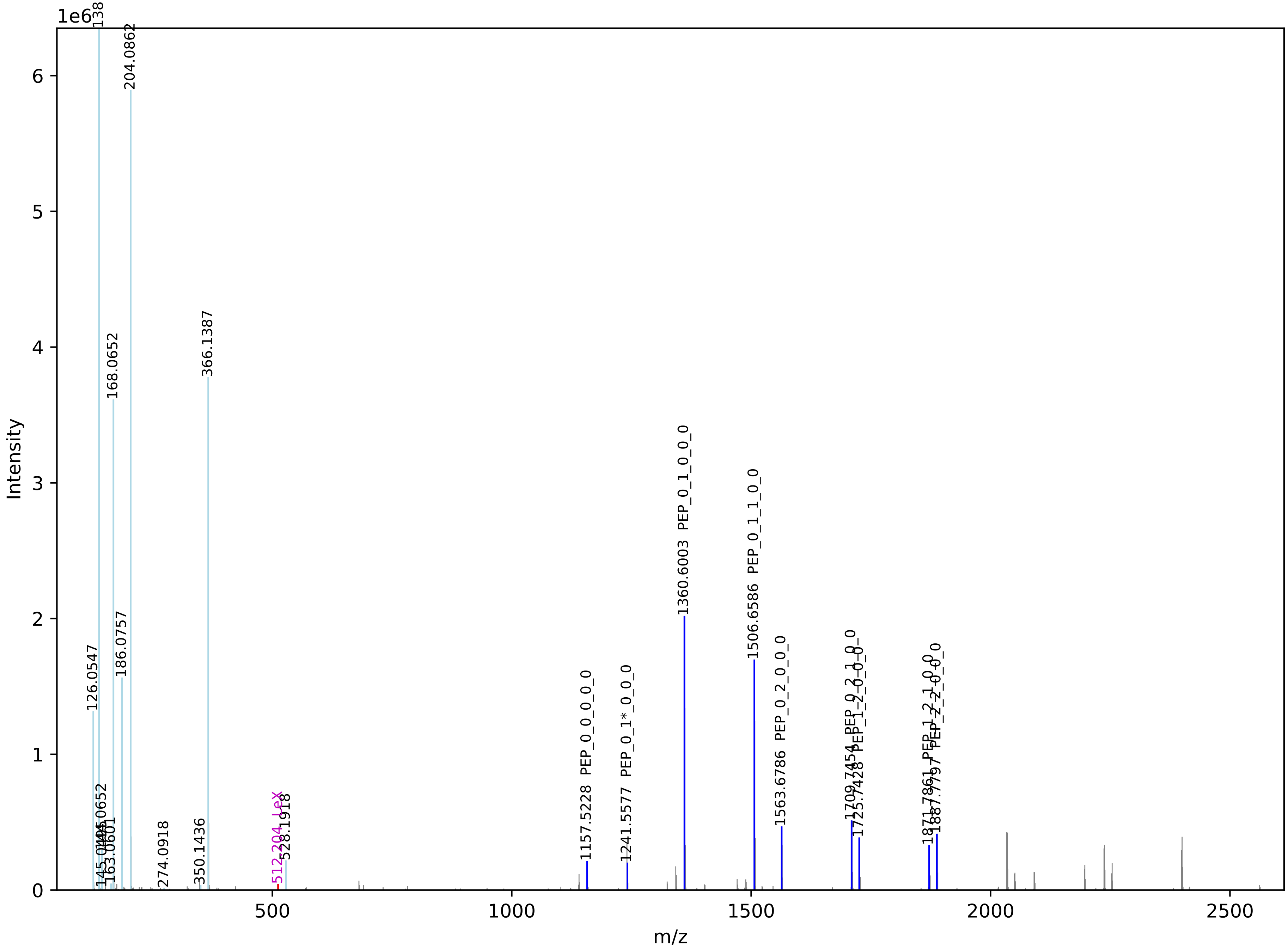

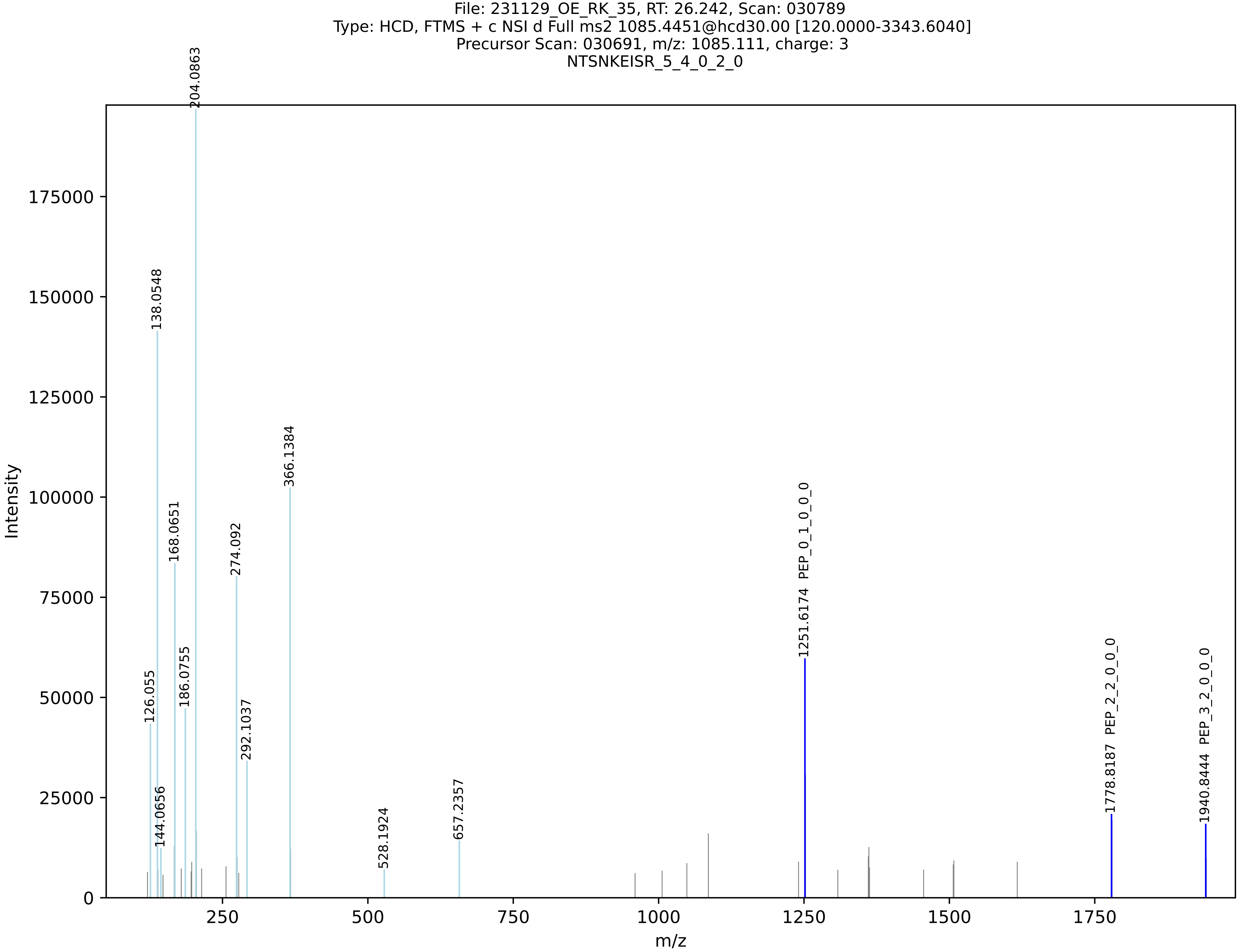

File: 231129_OE_RK_35, RT: 26.242, Scan: 030789
Type: HCD, FTMS + c NSI d Full ms2 1085.4451@hcd30.00 [120.0000-3343.6040]
Precursor Scan: 030691, m/z: 1085.111, charge: 3
NTSNKEISR_5_4_0_2_0
Intensity
m/z
204.0863
138.0548
366.1384
168.0651
274.092
186.0755
126.055
292.1037
144.0656
528.1924
657.2357
1251.6174 PEP_0_1_0_0_0
1778.8187 PEP_2_2_0_0_0
1940.8444 PEP_3_2_0_0_0

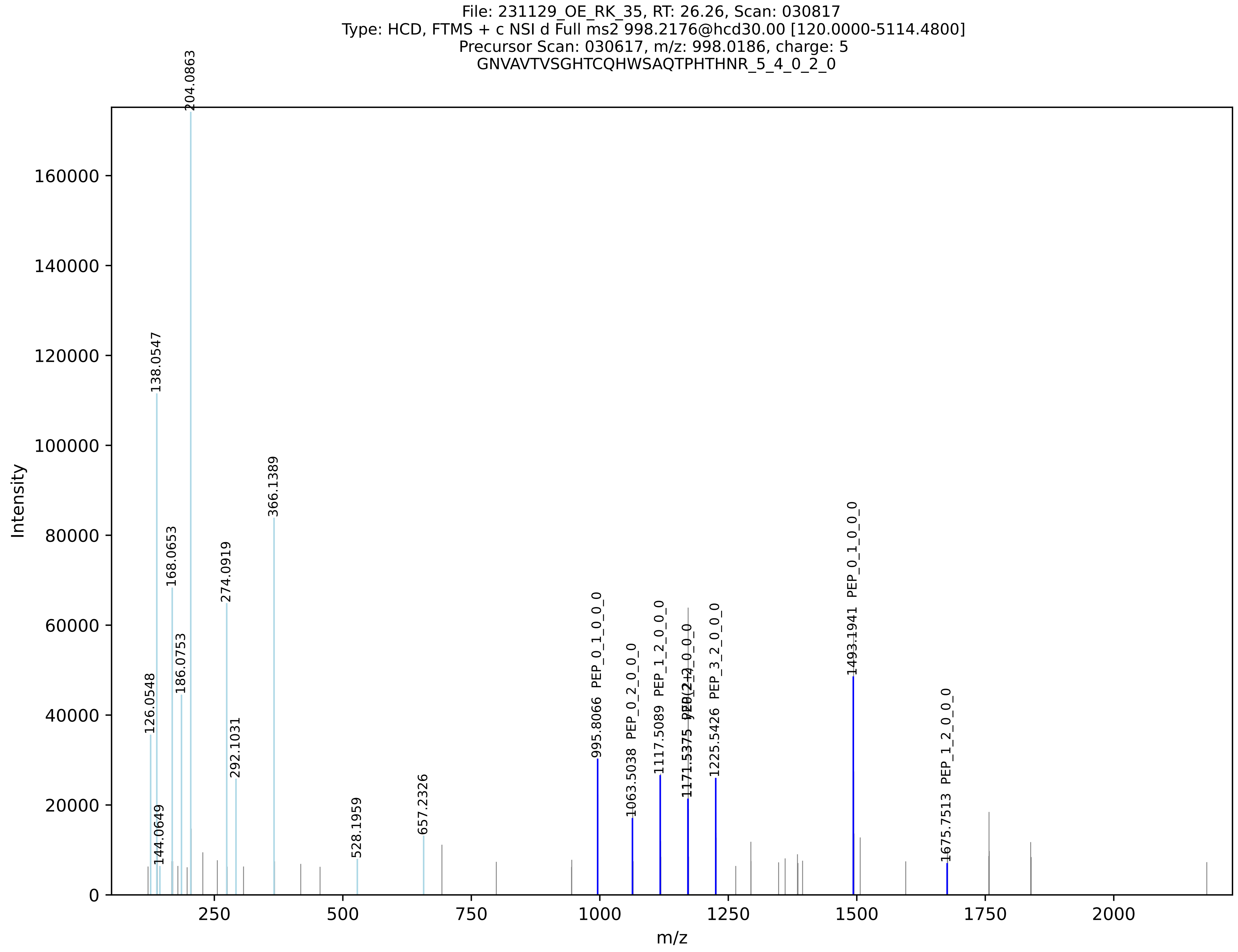
File: 231129_OE_RK_35, RT: 26.26, Scan: 030817
Type: HCD, FTMS + c NSI d Full ms2 998.2176@hcd30.00 [120.0000-5114.4800]
Precursor Scan: 030617, m/z: 998.0186, charge: 5
GNVAVTVSGHTCQHWSAQTPHTHNR_5_4_0_2_0
Intensity
m/z
0
20000
40000
60000
80000
100000
120000
140000
160000
250
500
750
1000
1250
1500
1750
2000
204.0863
138.0547
126.0548
144.0649
168.0653
186.0753
274.0919
292.1031
366.1389
528.1959
657.2326
995.8066 PEP_0_1_0_0_0
1063.5038 PEP_0_2_0_0_0
1117.5089 PEP_1_2_0_0_0
1171.5375
1225.5426 PEP_3_2_0_0_0
1493.1941 PEP_0_1_0_0_0
1675.7513 PEP_1_2_0_0_0

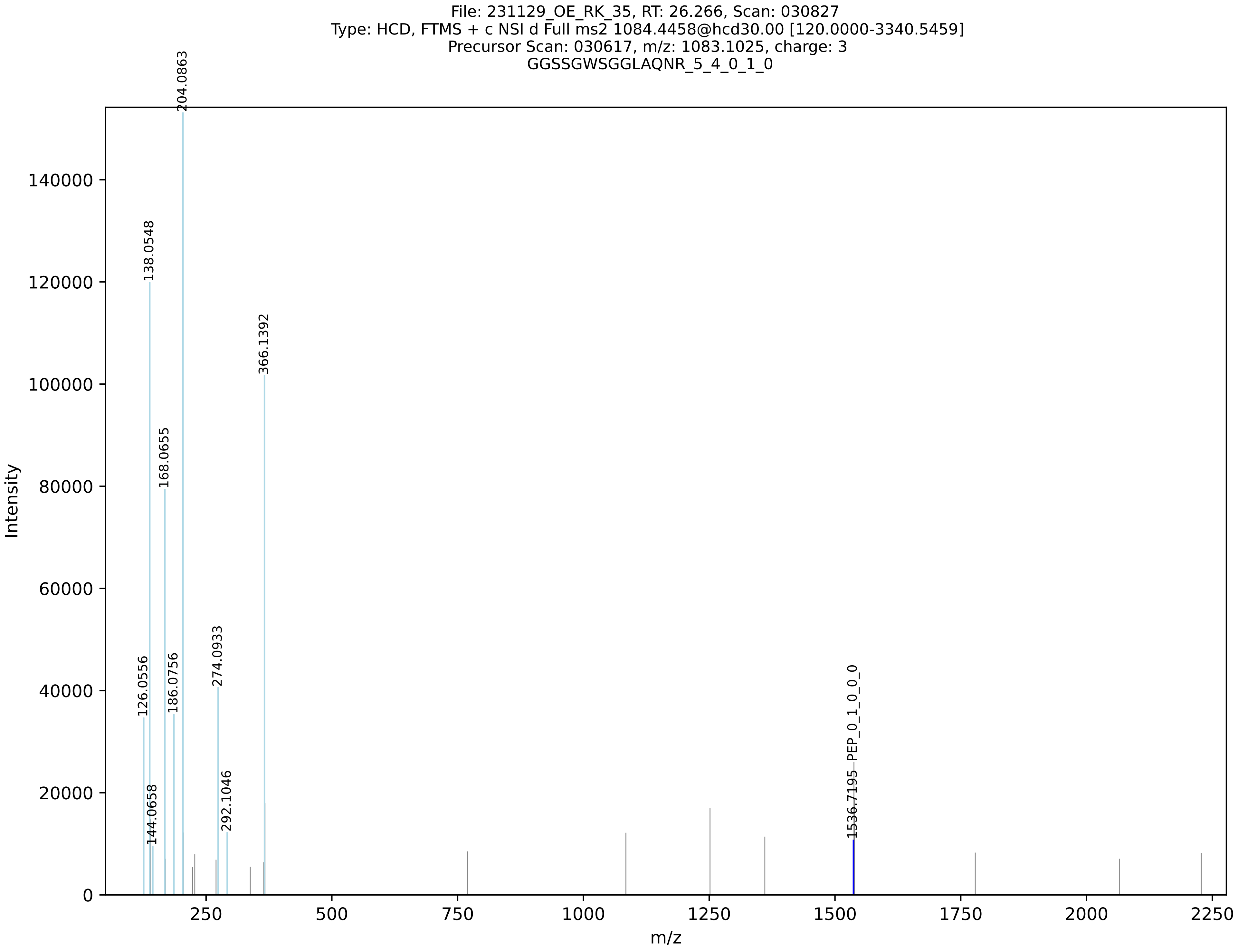
File: 231129_OE_RK_35, RT: 26.266, Scan: 030827
Type: HCD, FTMS + c NSI d Full ms2 1084.4458@hcd30.00 [120.0000-3340.5459]
Precursor Scan: 030617, m/z: 1083.1025, charge: 3
GGSSGWSGGLAQNR_5_4_0_1_0
204.0863
138.0548
366.1392
168.0655
126.0556
186.0756
274.0933
144.0658
292.1046
1536.7195_PEP_0_1_0_0_0
Intensity
m/z
0
20000
40000
60000
80000
100000
120000
140000
250
500
750
1000
1250
1500
1750
2000
2250

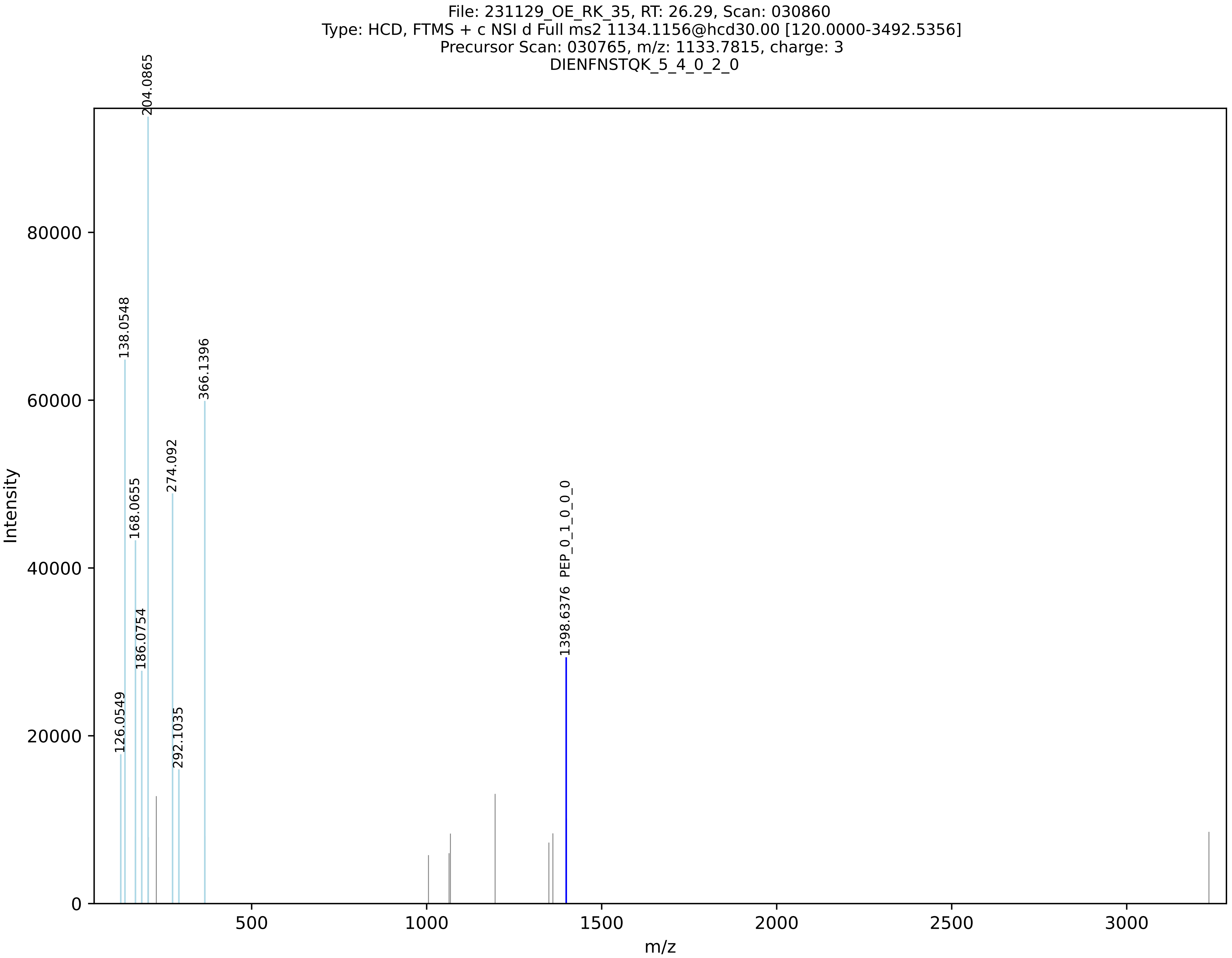

File: 231129_OE_RK_35, RT: 26.29, Scan: 030860
Type: HCD, FTMS + c NSI d Full ms2 1134.1156@hcd30.00 [120.0000-3492.5356]
Precursor Scan: 030765, m/z: 1133.7815, charge: 3
DIENFNSTQK_5_4_0_2_0
204.0865
138.0548
366.1396
274.092
168.0655
186.0754
126.0549
292.1035
1398.6376 PEP_0_1_0_0_0
Intensity
m/z
0
20000
40000
60000
80000
500
1000
1500
2000
2500
3000

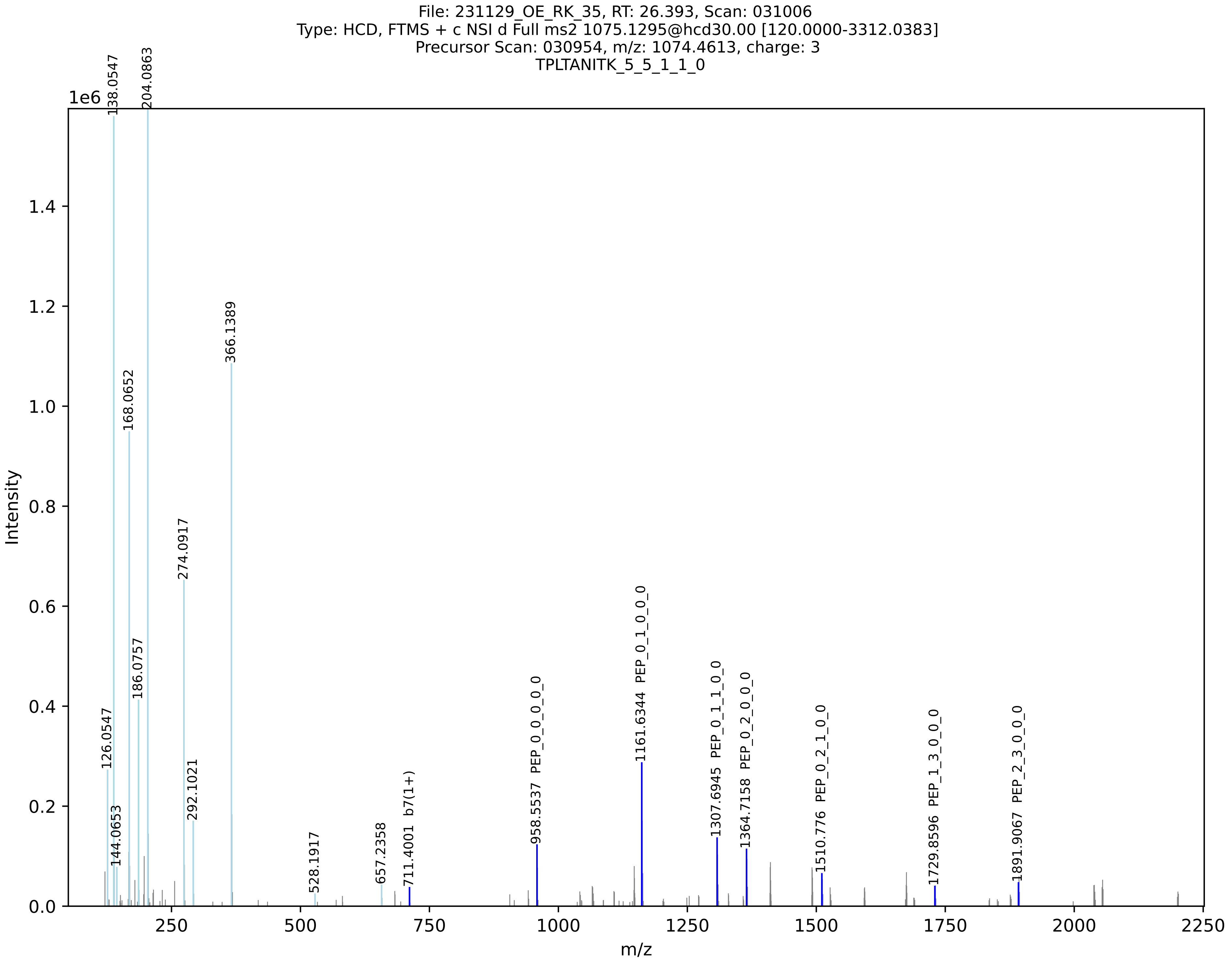

File: 231129_OE_RK_35, RT: 26.393, Scan: 031006
Type: HCD, FTMS + c NSI d Full ms2 1075.1295@hcd30.00 [120.0000-3312.0383]
Precursor Scan: 030954, m/z: 1074.4613, charge: 3
TPLTANITK_5_5_1_1_0
1e6
Intensity
m/z
138.0547
204.0863
366.1389
168.0652
274.0917
186.0757
126.0547
292.1021
144.0653
528.1917
657.2358
711.4001 b7(1+)
958.5537 PEP_0_0_0_0_0
1161.6344 PEP_0_1_0_0_0
1307.6945 PEP_0_1_1_0_0
1364.7158 PEP_0_2_0_0_0
1510.776 PEP_0_2_1_0_0
1729.8596 PEP_1_3_0_0_0
1891.9067 PEP_2_3_0_0_0

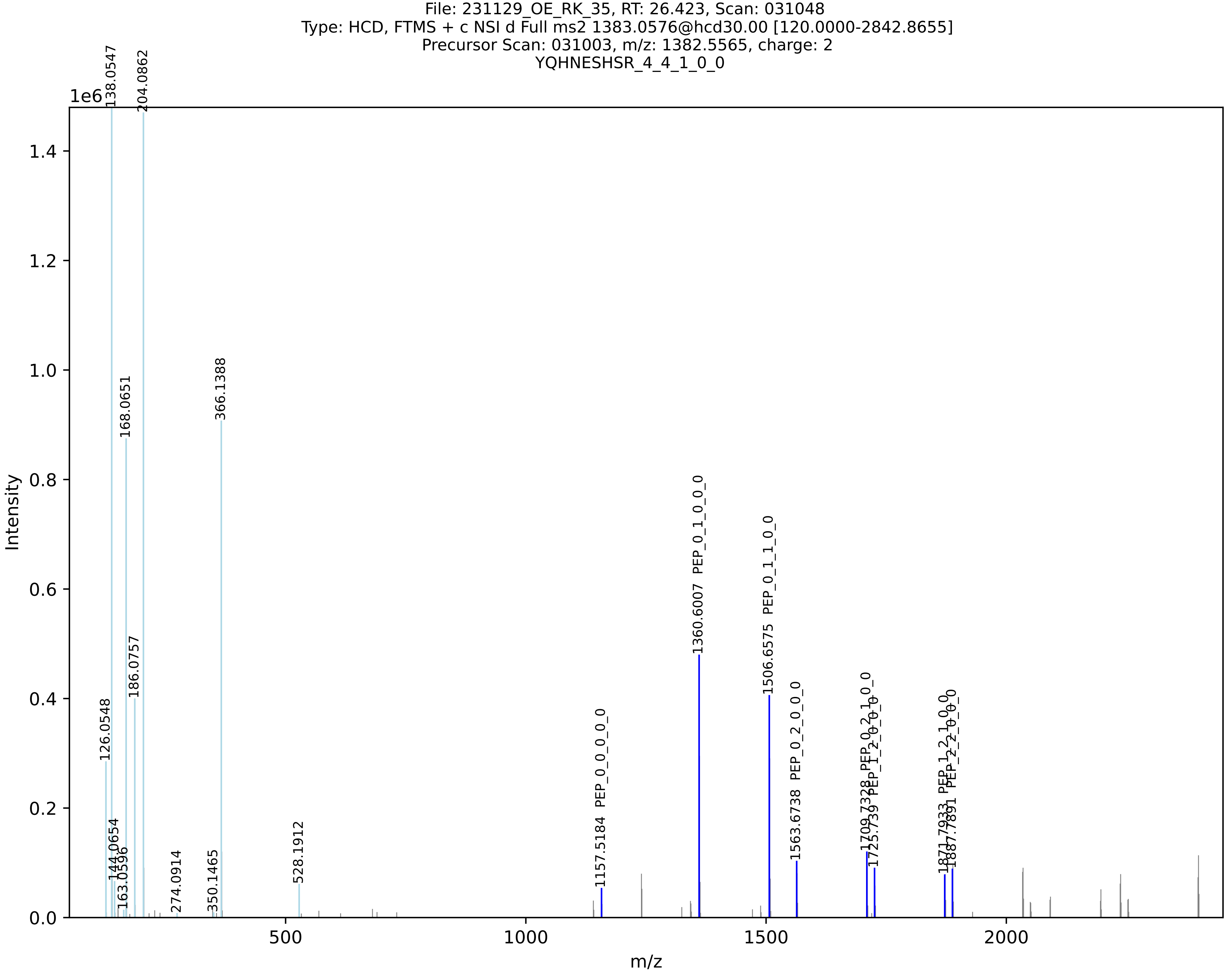
File: 231129_OE_RK_35, RT: 26.423, Scan: 031048
Type: HCD, FTMS + c NSI d Full ms2 1383.0576@hcd30.00 [120.0000-2842.8655]
Precursor Scan: 031003, m/z: 1382.5565, charge: 2
YQHNESHSR_4_4_1_0_0
1e6
Intensity
m/z
138.0547
204.0862
168.0651
366.1388
186.0757
126.0548
144.0654
163.0596
274.0914
350.1465
528.1912
1157.5184 PEP_0_0_0_0_0
1360.6007 PEP_0_1_0_0_0
1506.6575 PEP_0_1_1_0_0
1563.6738 PEP_0_2_0_0_0
1709.7328 PEP_0_2_1_0_0
1725.739 PEP_1_2_0_0_0
1871.7933 PEP_1_2_1_0_0
1887.7891 PEP_2_2_0_0_0

File: 231129_OE_RK_35, RT: 26.476, Scan: 031126
Type: HCD, FTMS + c NSI d Full ms2 1612.1888@hcd30.00 [120.0000-3310.2932]
Precursor Scan: 031046, m/z: 1611.1868, charge: 2
TPLTANITK_5_5_1_1_0

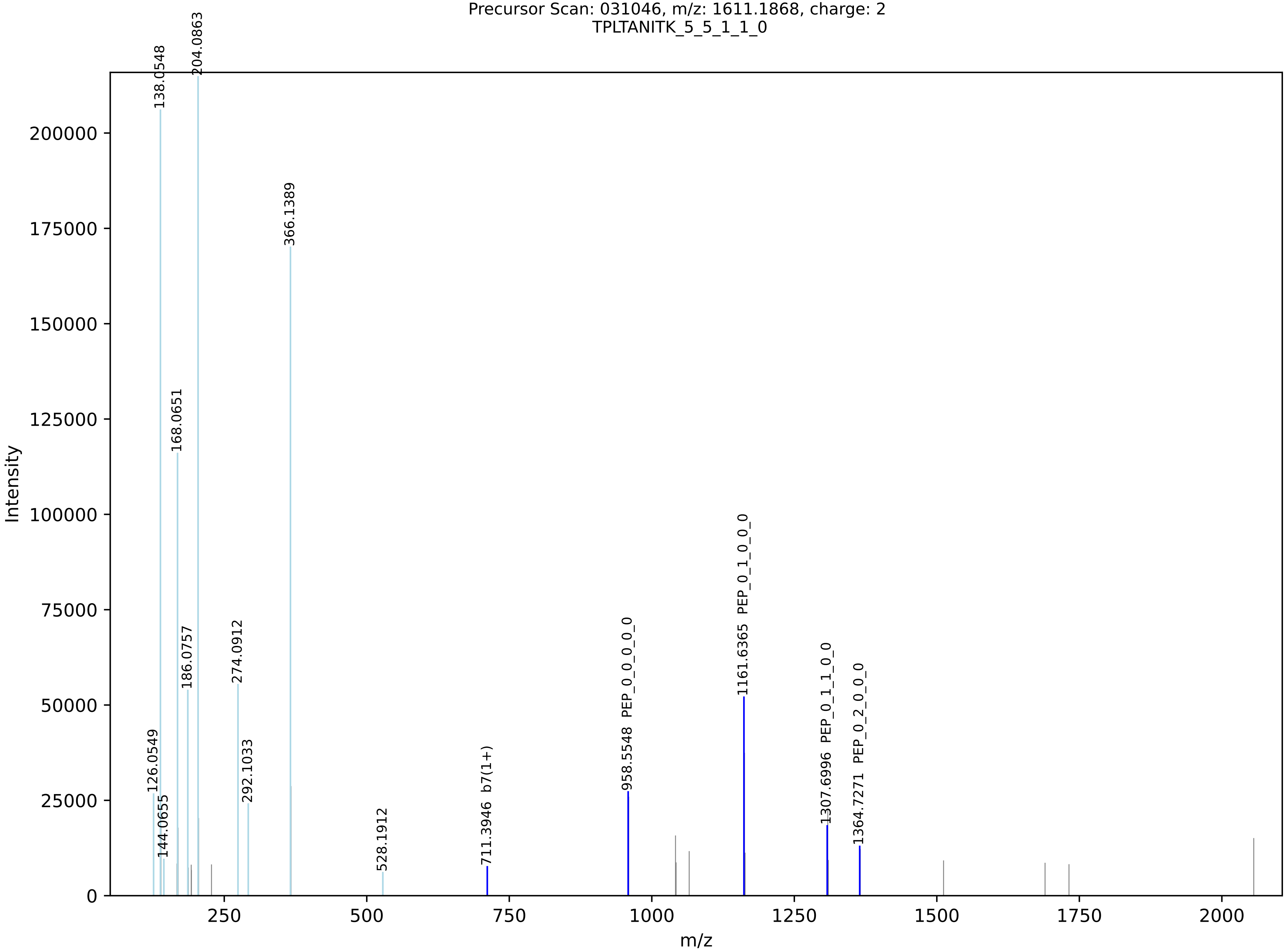

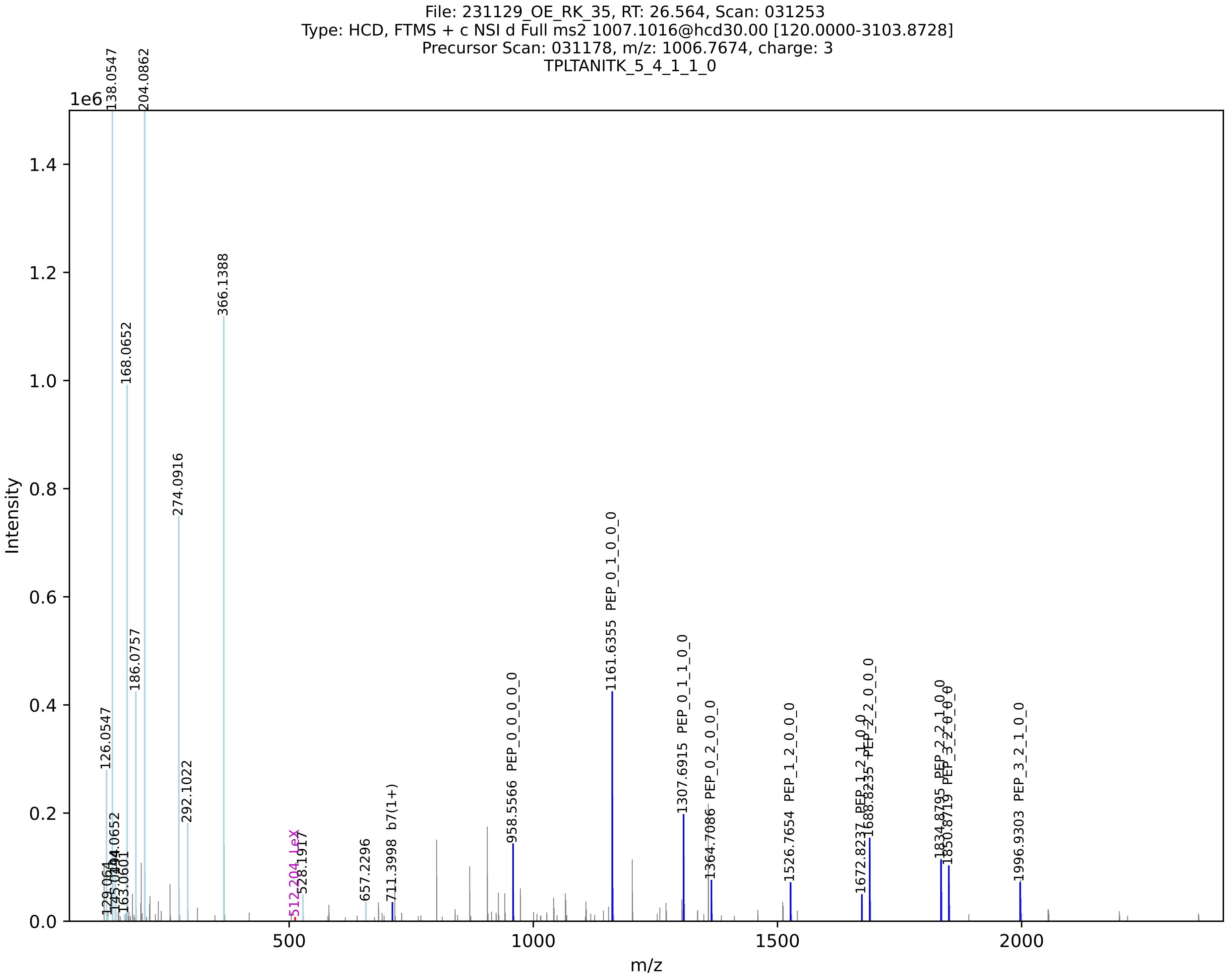

File: 231129_OE_RK_35, RT: 26.564, Scan: 031253
Type: HCD, FTMS + c NSI d Full ms2 1007.1016@hcd30.00 [120.0000-3103.8728]
Precursor Scan: 031178, m/z: 1006.7674, charge: 3
TPLTANITK_5_4_1_1_0
1e6
Intensity
m/z
138.0547
204.0862
366.1388
168.0652
274.0916
186.0757
126.0547
292.1022
512.204 LeX
528.1917
657.2296
711.3998 b7(1+)
958.5566 PEP_0_0_0_0_0
1161.6355 PEP_0_1_0_0_0
1307.6915 PEP_0_1_1_0_0
1364.7086 PEP_0_2_0_0_0
1526.7654 PEP_1_2_0_0_0
1672.8237 PEP_1_2_1_0_0
1688.8235 PEP_2_2_0_0_0
1834.8795 PEP_2_2_1_0_0
1850.8719 PEP_3_2_0_0_0
1996.9303 PEP_3_2_1_0_0

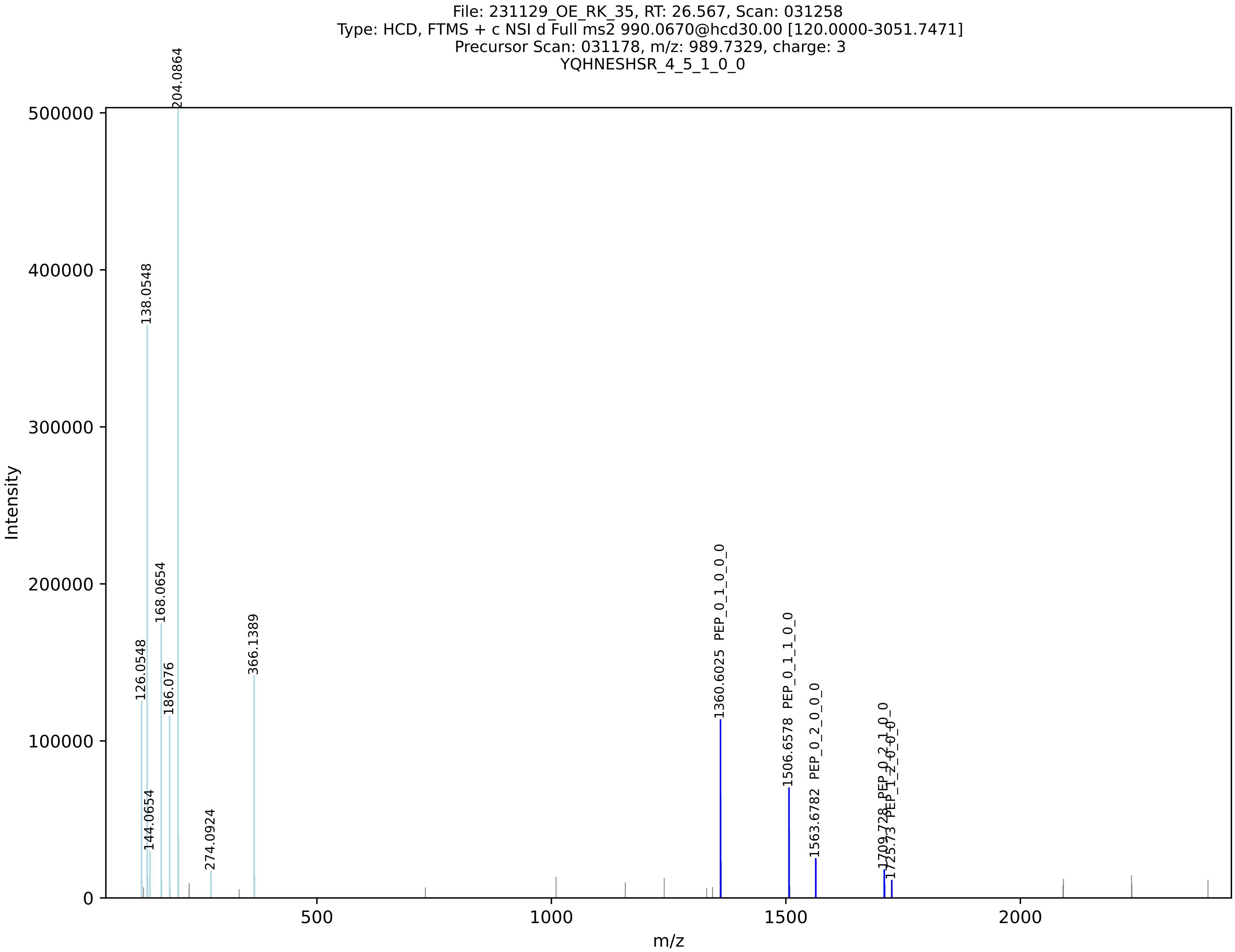
File: 231129_OE_RK_35, RT: 26.567, Scan: 031258
Type: HCD, FTMS + c NSI d Full ms2 990.0670@hcd30.00 [120.0000-3051.7471]
Precursor Scan: 031178, m/z: 989.7329, charge: 3
YQHNESHSR_4_5_1_0_0
Intensity
m/z
204.0864
138.0548
168.0654
126.0548
186.076
366.1389
144.0654
274.0924
1360.6025 PEP_0_1_0_0_0
1506.6578 PEP_0_1_1_0_0
1563.6782 PEP_0_2_0_0_0
1709.728 PEP_0_2_1_0_0
1725.73 PEP_1_2_0_0_0
0
100000
200000
300000
400000
500000
500
1000
1500
2000

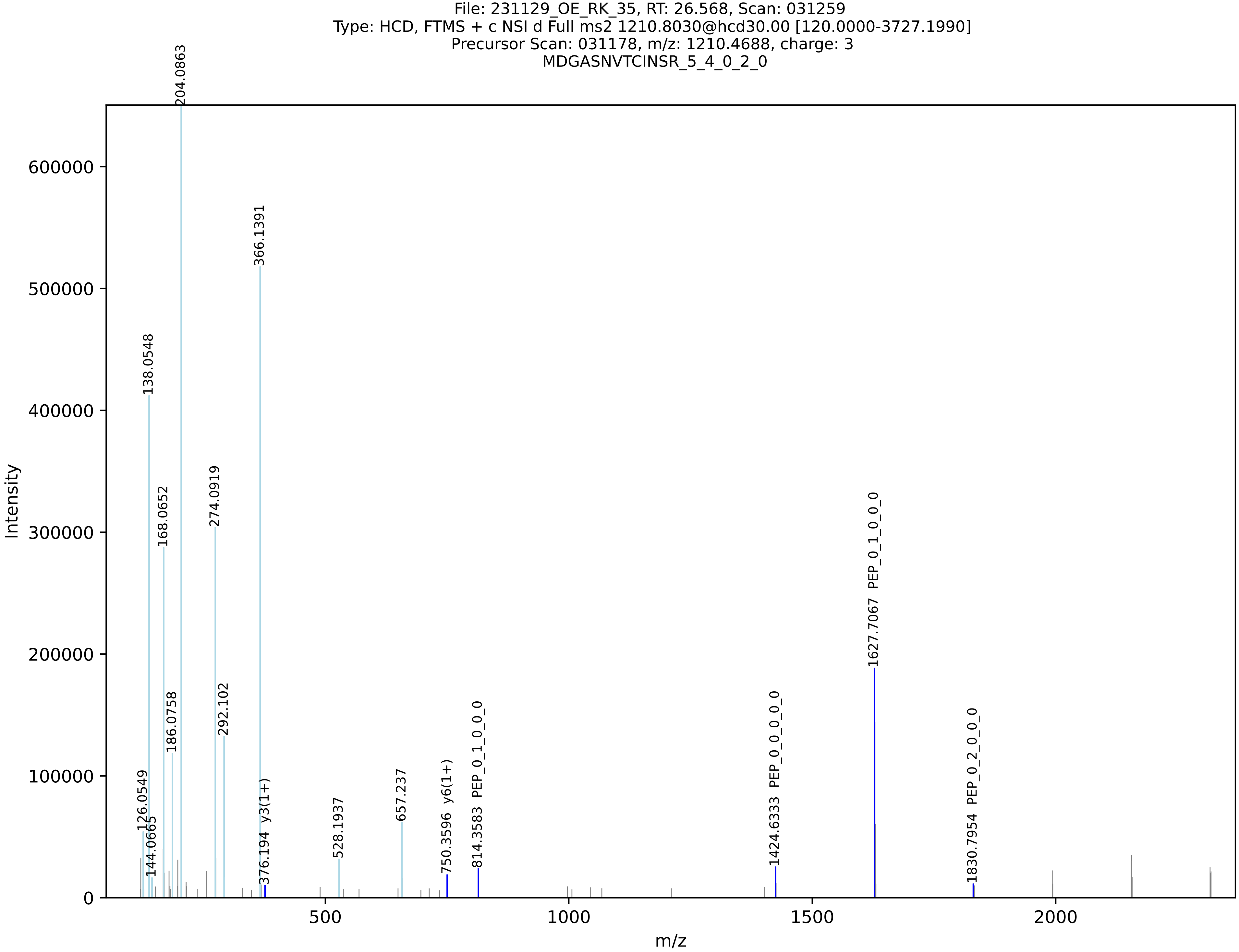
File: 231129_OE_RK_35, RT: 26.568, Scan: 031259
Type: HCD, FTMS + c NSI d Full ms2 1210.8030@hcd30.00 [120.0000-3727.1990]
Precursor Scan: 031178, m/z: 1210.4688, charge: 3
MDGASNVTCINSR_5_4_0_2_0
204.0863
366.1391
138.0548
274.0919
168.0652
292.102
186.0758
126.0549
144.0665
376.194 y3(1+)
528.1937
657.237
750.3596 y6(1+)
814.3583 PEP_0_1_0_0_0
1424.6333 PEP_0_0_0_0_0
1627.7067 PEP_0_1_0_0_0
1830.7954 PEP_0_2_0_0_0
Intensity
m/z
0
100000
200000
300000
400000
500000
600000
500
1000
1500
2000

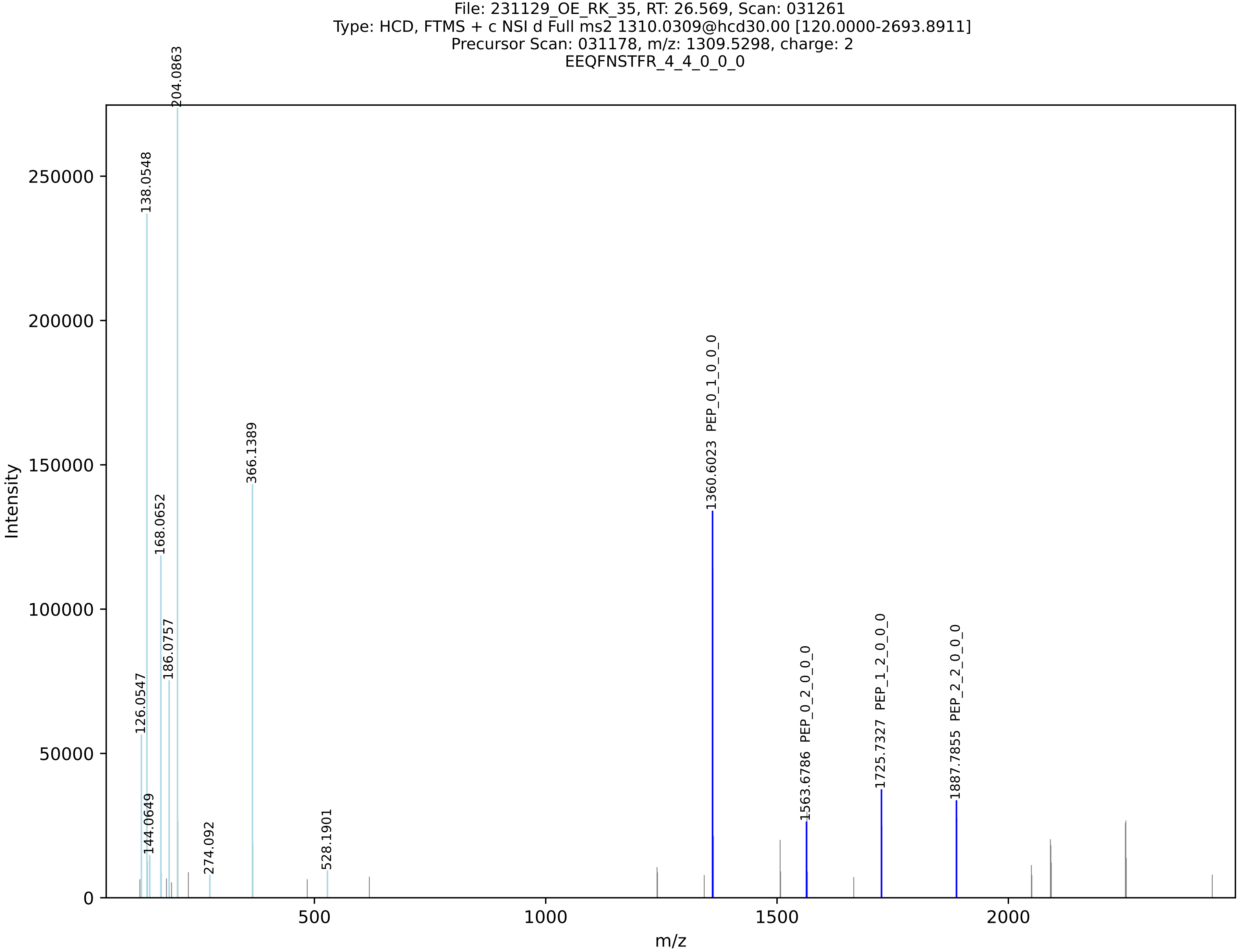
File: 231129_OE_RK_35, RT: 26.569, Scan: 031261
Type: HCD, FTMS + c NSI d Full ms2 1310.0309@hcd30.00 [120.0000-2693.8911]
Precursor Scan: 031178, m/z: 1309.5298, charge: 2
EEQFNSTFR_4_4_0_0_0
Intensity
m/z
204.0863
138.0548
168.0652
186.0757
126.0547
144.0649
274.092
366.1389
528.1901
1360.6023 PEP_0_1_0_0_0
1563.6786 PEP_0_2_0_0_0
1725.7327 PEP_1_2_0_0_0
1887.7855 PEP_2_2_0_0_0

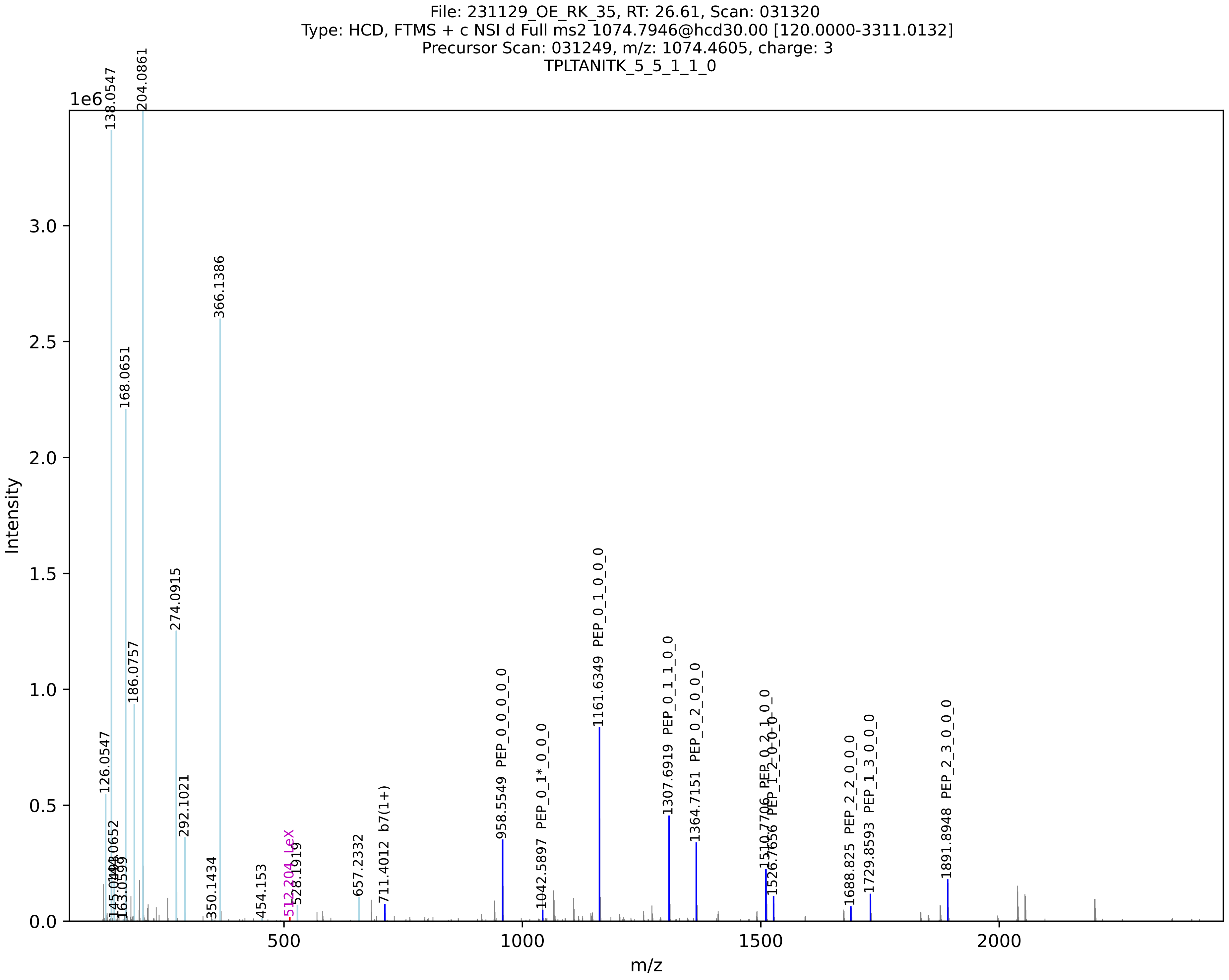
File: 231129_OE_RK_35, RT: 26.61, Scan: 031320
Type: HCD, FTMS + c NSI d Full ms2 1074.7946@hcd30.00 [120.0000-3311.0132]
Precursor Scan: 031249, m/z: 1074.4605, charge: 3
TPLTANITK_5_5_1_1_0
1e6
Intensity
m/z
138.0547
204.0861
168.0651
366.1386
274.0915
186.0757
126.0547
292.1021
350.1434
454.153
512.204_LeX
528.1919
657.2332
711.4012 b7(1+)
958.5549 PEP_0_0_0_0_0
1042.5897 PEP_0_1*_0_0_0
1161.6349 PEP_0_1_0_0_0
1307.6919 PEP_0_1_1_0_0
1364.7151 PEP_0_2_0_0_0
1688.825 PEP_2_2_0_0_0
1729.8593 PEP_1_3_0_0_0
1891.8948 PEP_2_3_0_0_0

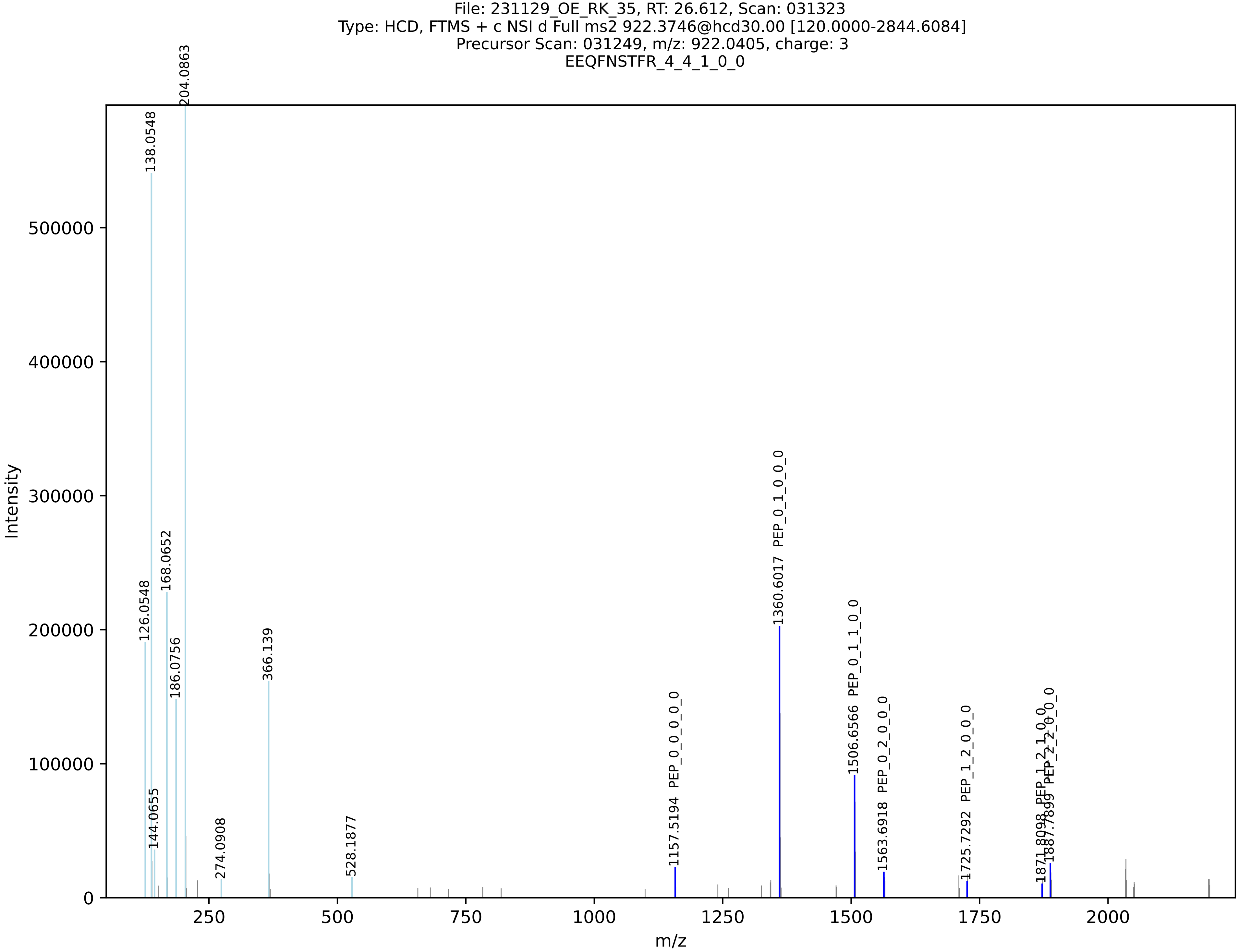

File: 231129_OE_RK_35, RT: 26.612, Scan: 031323
Type: HCD, FTMS + c NSI d Full ms2 922.3746@hcd30.00 [120.0000-2844.6084]
Precursor Scan: 031249, m/z: 922.0405, charge: 3
EEQFNSTFR_4_4_1_0_0
Intensity
m/z
204.0863
138.0548
168.0652
126.0548
186.0756
366.139
144.0655
274.0908
528.1877
1157.5194 PEP_0_0_0_0_0
1360.6017 PEP_0_1_0_0_0
1506.6566 PEP_0_1_1_0_0
1563.6918 PEP_0_2_0_0_0
1725.7292 PEP_1_2_0_0_0
1871.8098 PEP_1_2_1_0_0
1887.7899 PEP_2_2_0_0_0

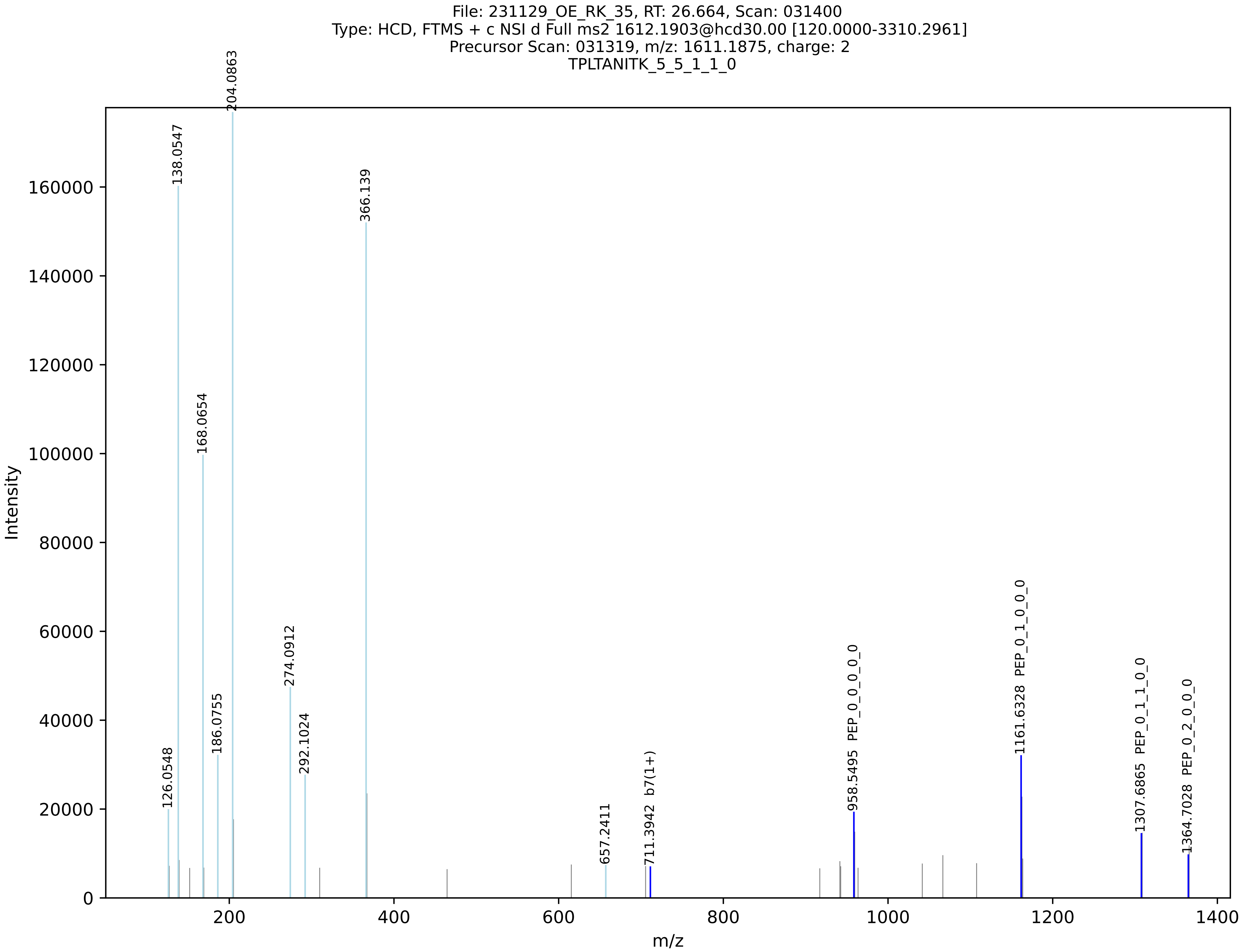

File: 231129_OE_RK_35, RT: 26.664, Scan: 031400
Type: HCD, FTMS + c NSI d Full ms2 1612.1903@hcd30.00 [120.0000-3310.2961]
Precursor Scan: 031319, m/z: 1611.1875, charge: 2
TPLTANITK_5_5_1_1_0
Intensity
m/z
204.0863
138.0547
366.139
168.0654
274.0912
292.1024
186.0755
126.0548
657.2411
711.3942 b7(1+)
958.5495 PEP_0_0_0_0_0
1161.6328 PEP_0_1_0_0_0
1307.6865 PEP_0_1_1_0_0
1364.7028 PEP_0_2_0_0_0

File: 231129_OE_RK_35, RT: 26.665, Scan: 031401
Type: HCD, FTMS + c NSI d Full ms2 806.5986@hcd30.00 [120.0000-3313.7783]
Precursor Scan: 031319, m/z: 806.0974, charge: 4
TPLTANITK_5_5_1_1_0

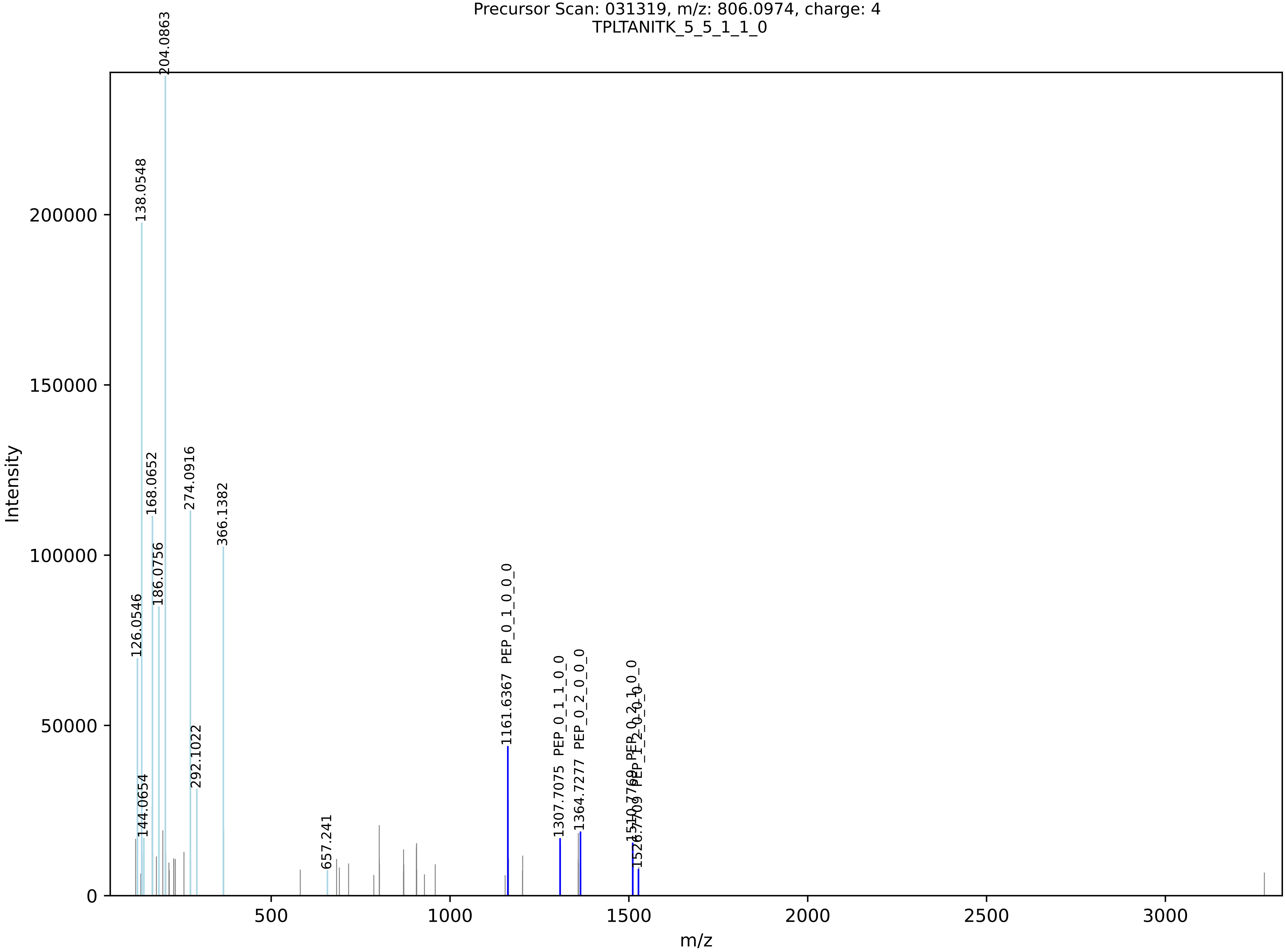

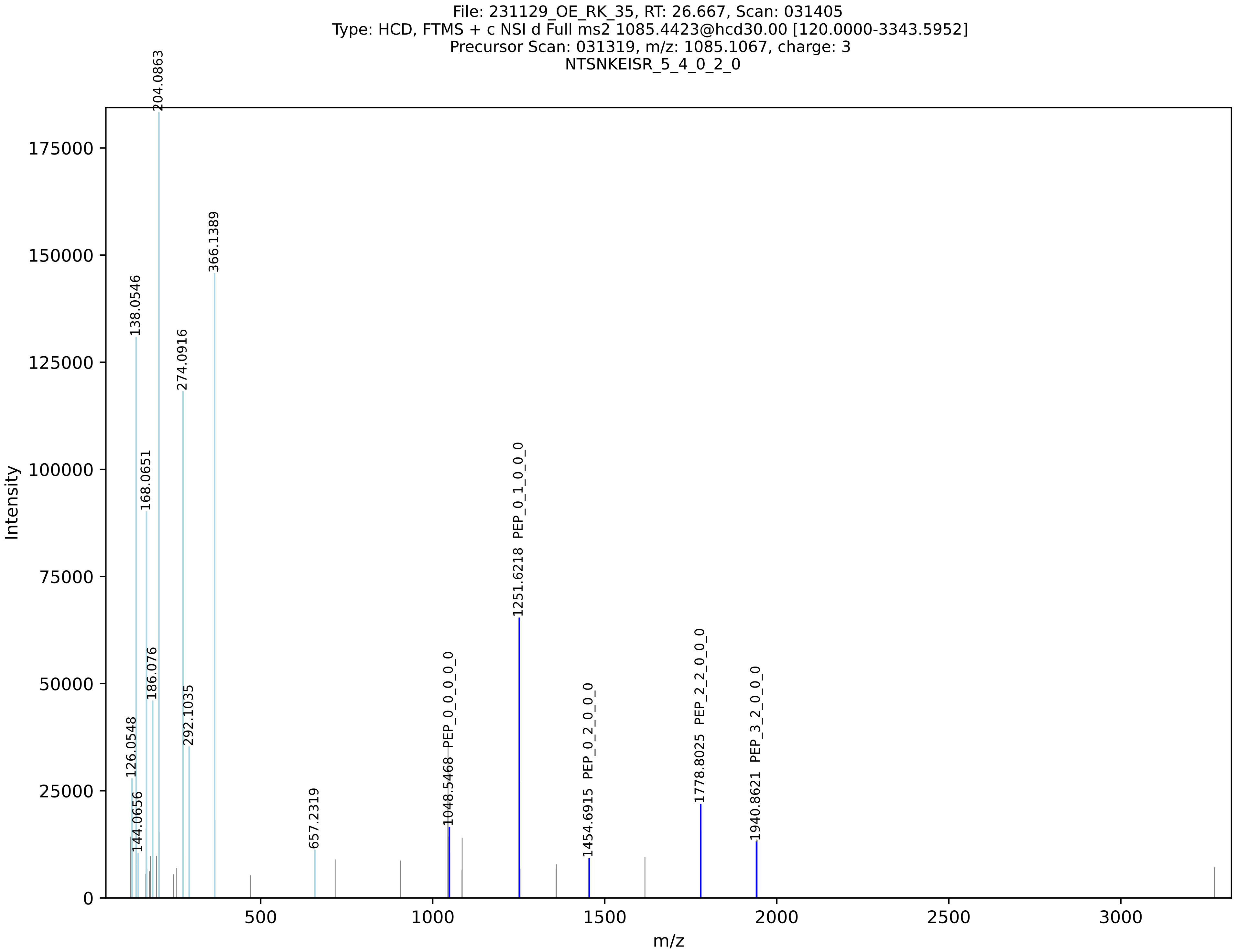
File: 231129_OE_RK_35, RT: 26.667, Scan: 031405
Type: HCD, FTMS + c NSI d Full ms2 1085.4423@hcd30.00 [120.0000-3343.5952]
Precursor Scan: 031319, m/z: 1085.1067, charge: 3
NTSNKEISR_5_4_0_2_0
Intensity
m/z
204.0863
366.1389
138.0546
274.0916
168.0651
186.076
292.1035
126.0548
144.0656
657.2319
1048.5468 PEP_0_0_0_0_0
1251.6218 PEP_0_1_0_0_0
1454.6915 PEP_0_2_0_0_0
1778.8025 PEP_2_2_0_0_0
1940.8621 PEP_3_2_0_0_0
0
25000
50000
75000
100000
125000
150000
175000
500
1000
1500
2000
2500
3000

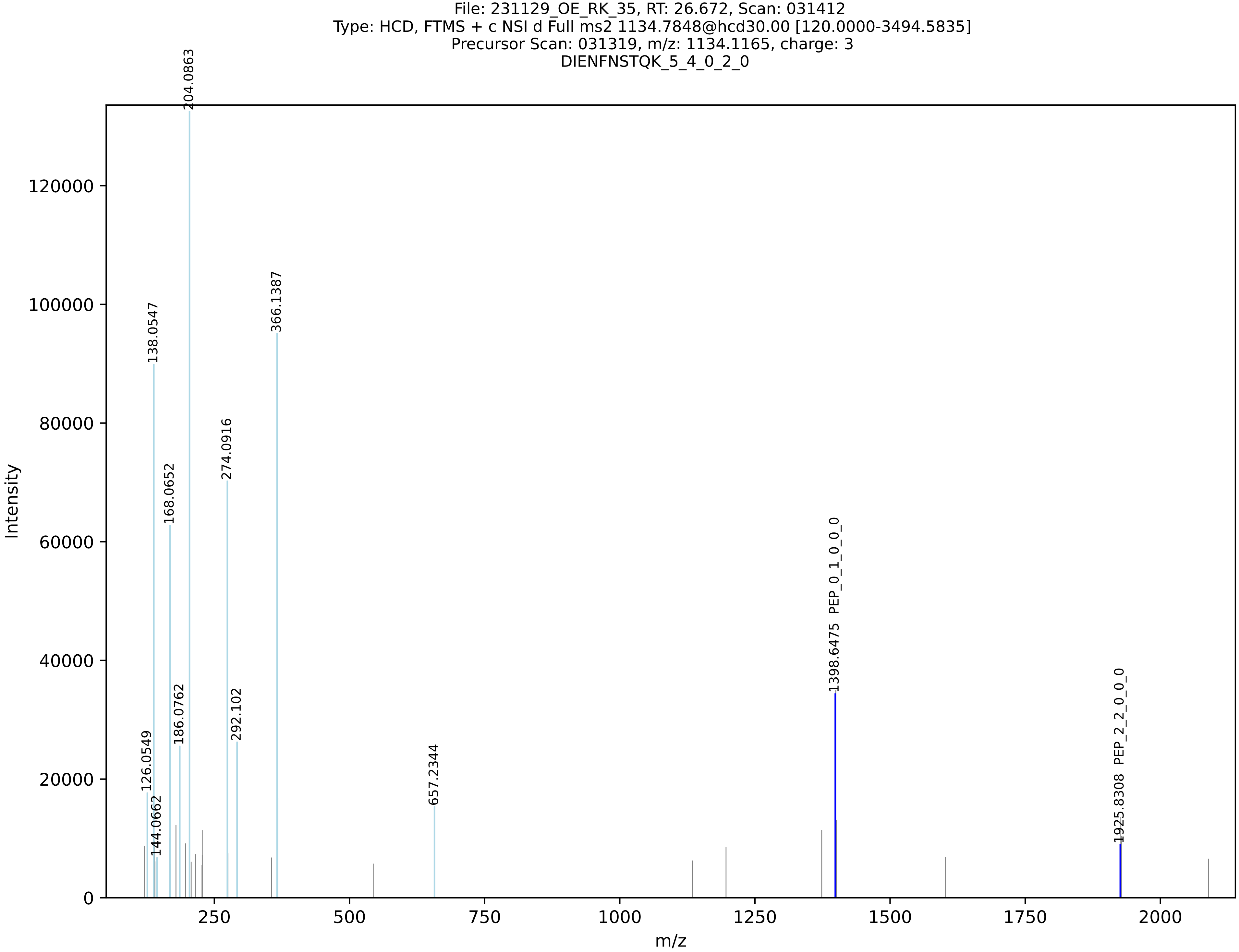
File: 231129_OE_RK_35, RT: 26.672, Scan: 031412
Type: HCD, FTMS + c NSI d Full ms2 1134.7848@hcd30.00 [120.0000-3494.5835]
Precursor Scan: 031319, m/z: 1134.1165, charge: 3
DIENFNSTQK_5_4_0_2_0
Intensity
m/z
204.0863
138.0547
366.1387
274.0916
168.0652
186.0762
292.102
126.0549
144.0662
657.2344
1398.6475 PEP_0_1_0_0_0
1925.8308 PEP_2_2_0_0_0

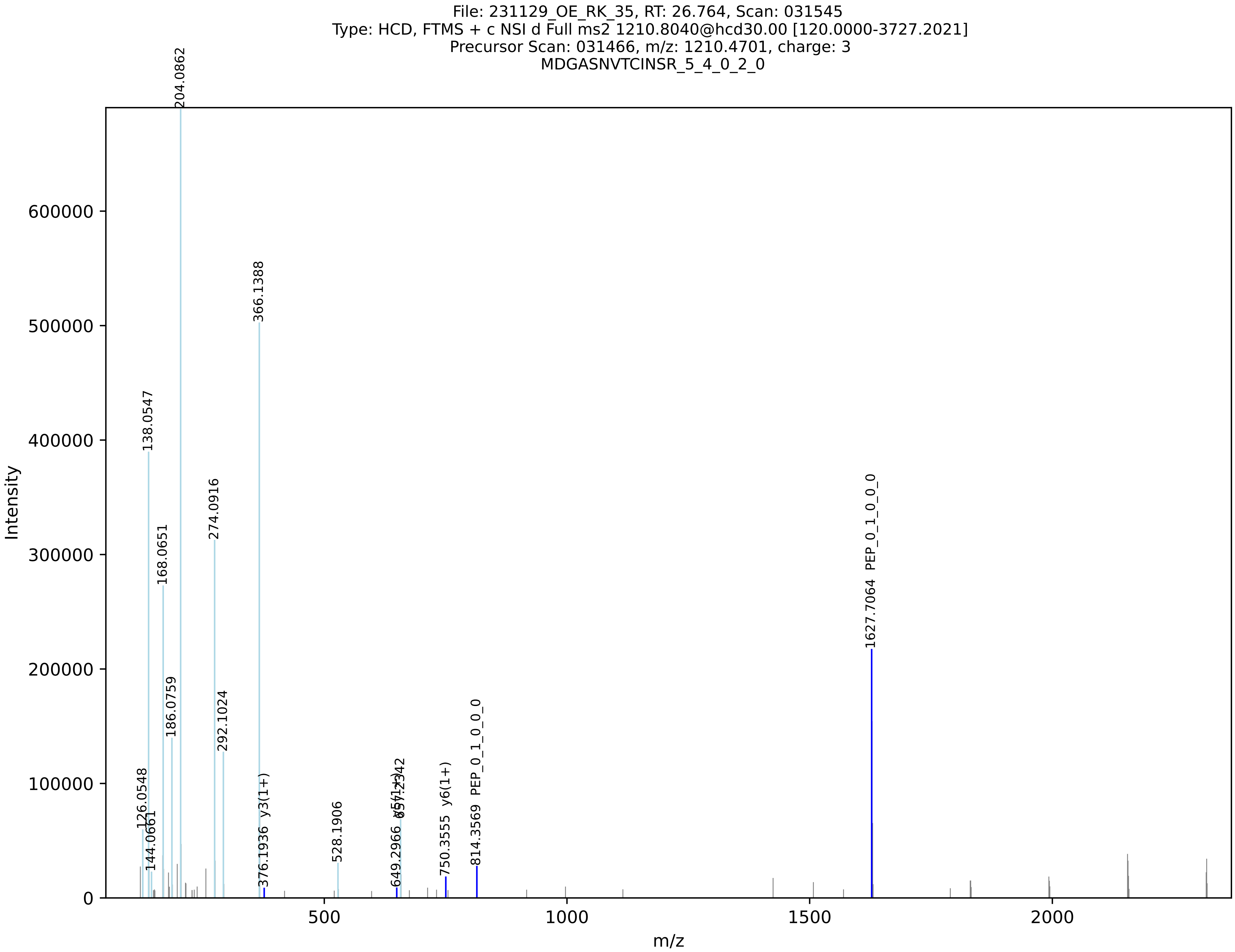

File: 231129_OE_RK_35, RT: 26.764, Scan: 031545
Type: HCD, FTMS + c NSI d Full ms2 1210.8040@hcd30.00 [120.0000-3727.2021]
Precursor Scan: 031466, m/z: 1210.4701, charge: 3
MDGASNVTCINSR_5_4_0_2_0
Intensity
m/z
204.0862
366.1388
138.0547
274.0916
168.0651
186.0759
292.1024
126.0548
144.0661
376.1936 y3(1+)
528.1906
649.2966 y5(1+)
657.2342
750.3555 y6(1+)
814.3569 PEP_0_1_0_0_0
1627.7064 PEP_0_1_0_0_0

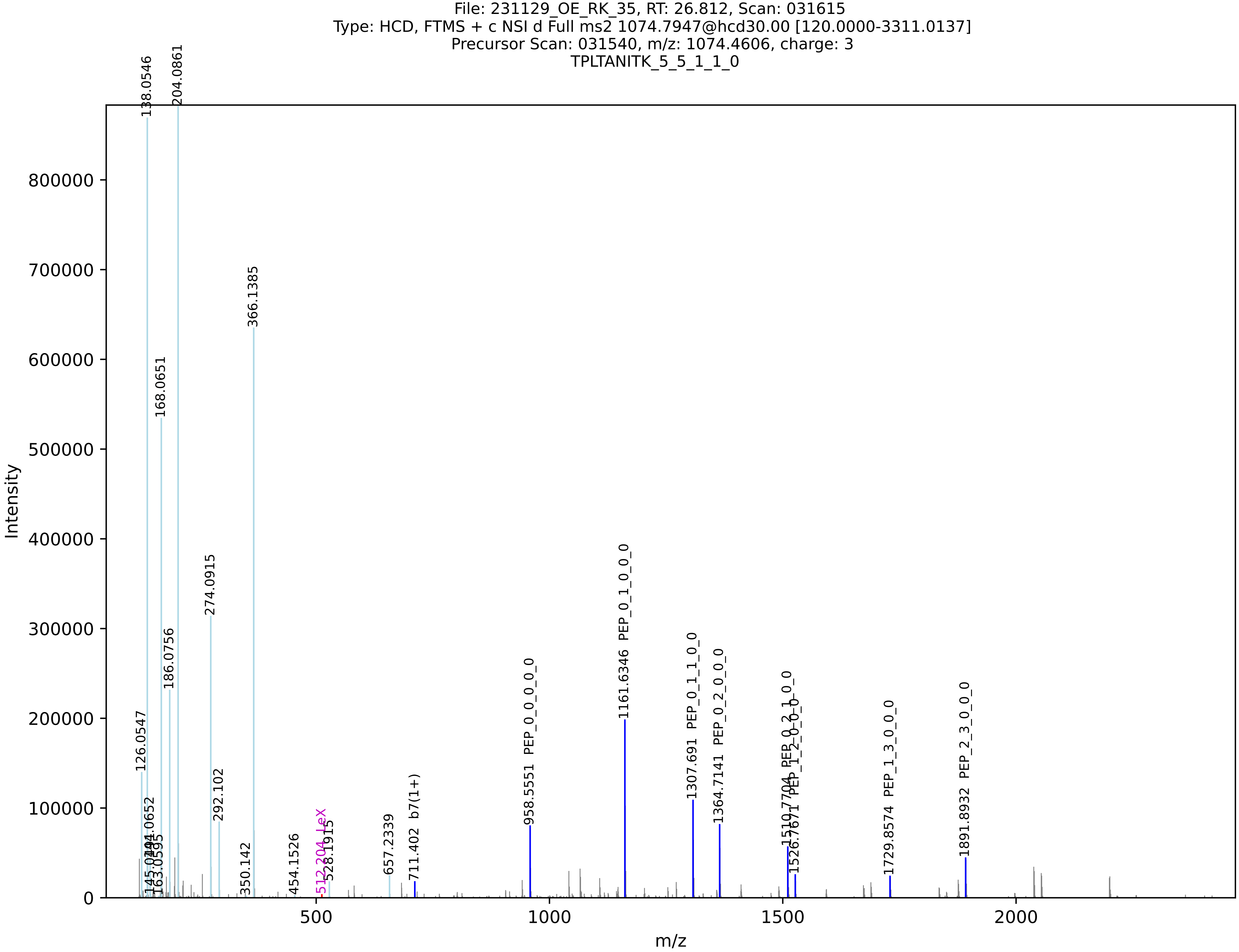

File: 231129_OE_RK_35, RT: 26.812, Scan: 031615
Type: HCD, FTMS + c NSI d Full ms2 1074.7947@hcd30.00 [120.0000-3311.0137]
Precursor Scan: 031540, m/z: 1074.4606, charge: 3
TPLTANITK_5_5_1_1_0
Intensity
m/z
138.0546
204.0861
366.1385
168.0651
186.0756
274.0915
126.0547
292.102
350.142
454.1526
512.204_LeX
528.1915
657.2339
711.402 b7(1+)
958.5551 PEP_0_0_0_0_0_
1161.6346 PEP_0_1_0_0_0_
1307.691 PEP_0_1_1_0_0_
1364.7141 PEP_0_2_0_0_0_
1510.7704 PEP_0_2_1_0_0_
1526.7671 PEP_1_2_0_0_0
1729.8574 PEP_1_3_0_0_0
1891.8932 PEP_2_3_0_0_0_

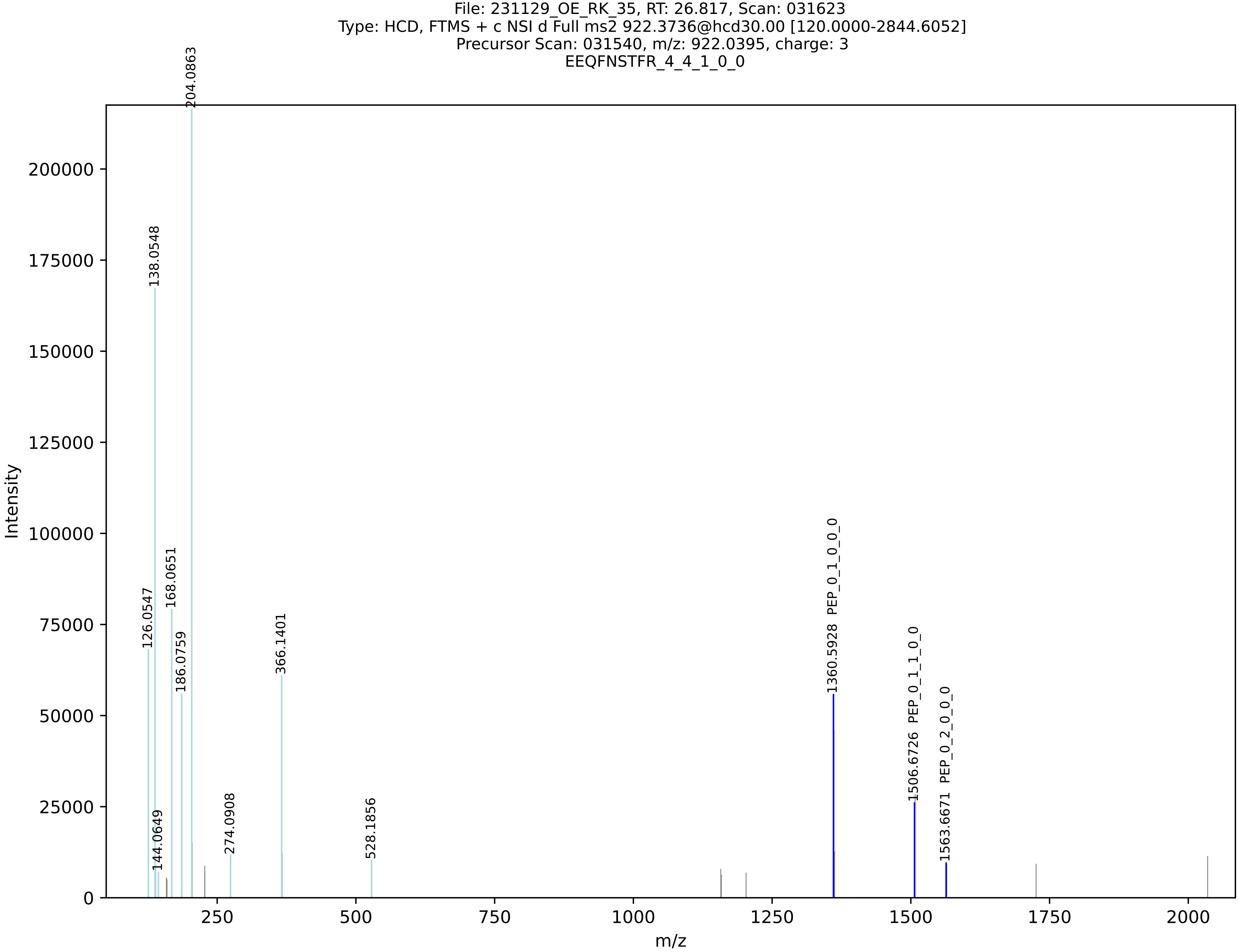
File: 231129_OE_RK_35, RT: 26.817, Scan: 031623
Type: HCD, FTMS + c NSI d Full ms2 922.3736@hcd30.00 [120.0000-2844.6052]
Precursor Scan: 031540, m/z: 922.0395, charge: 3
EEQFNSTFR_4_4_1_0_0
Intensity
m/z
204.0863
138.0548
168.0651
126.0547
186.0759
366.1401
144.0649
274.0908
528.1856
1360.5928 PEP_0_1_0_0_
1506.6726 PEP_0_1_1_0_0
1563.6671 PEP_0_2_0_0_0

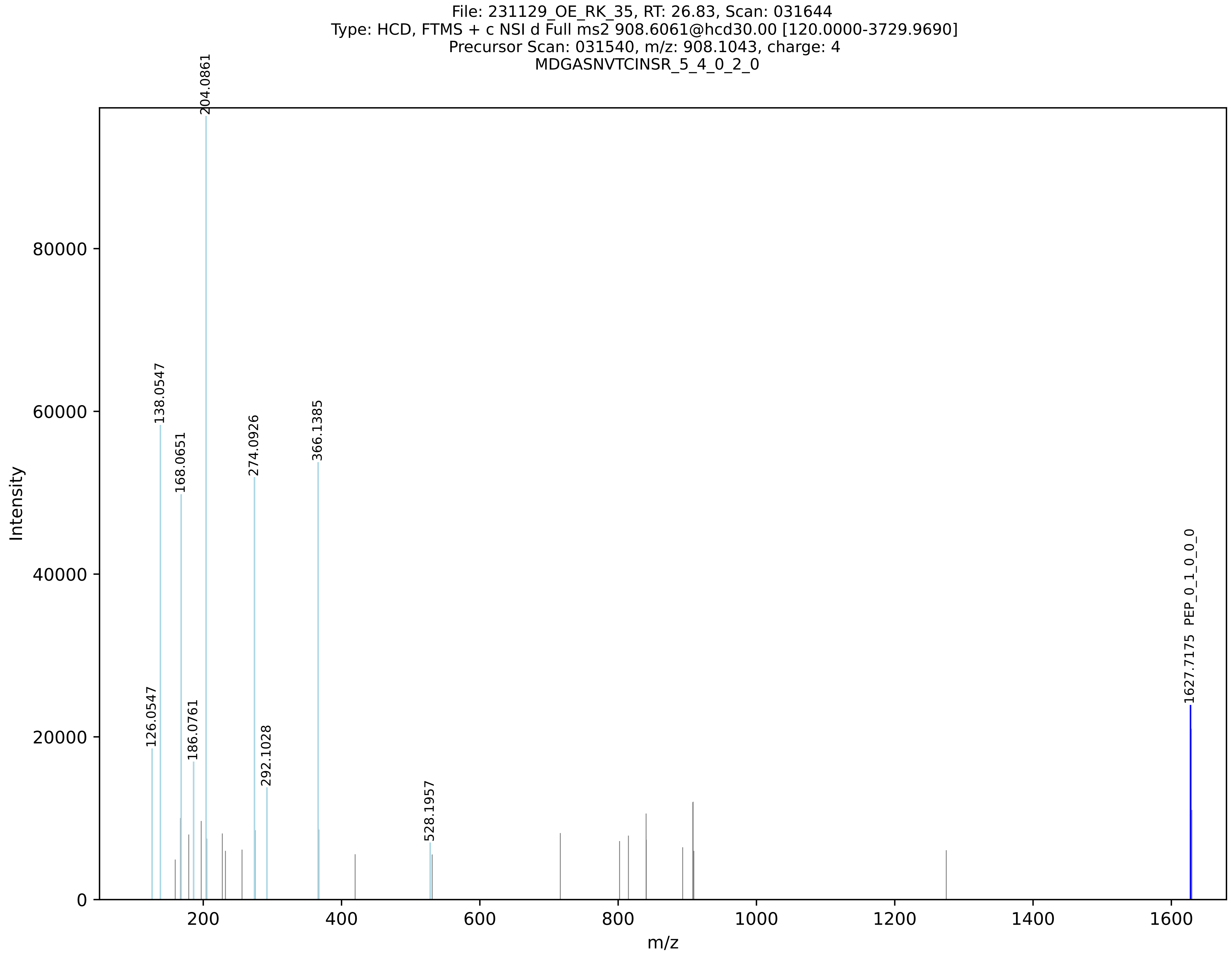
File: 231129_OE_RK_35, RT: 26.83, Scan: 031644
Type: HCD, FTMS + c NSI d Full ms2 908.6061@hcd30.00 [120.0000-3729.9690]
Precursor Scan: 031540, m/z: 908.1043, charge: 4
MDGASNVTCINSR_5_4_0_2_0
204.0861
138.0547
168.0651
274.0926
366.1385
126.0547
186.0761
292.1028
528.1957
1627.7175 PEP_0_1_0_0_0
Intensity
m/z
0
20000
40000
60000
80000
200
400
600
800
1000
1200
1400
1600

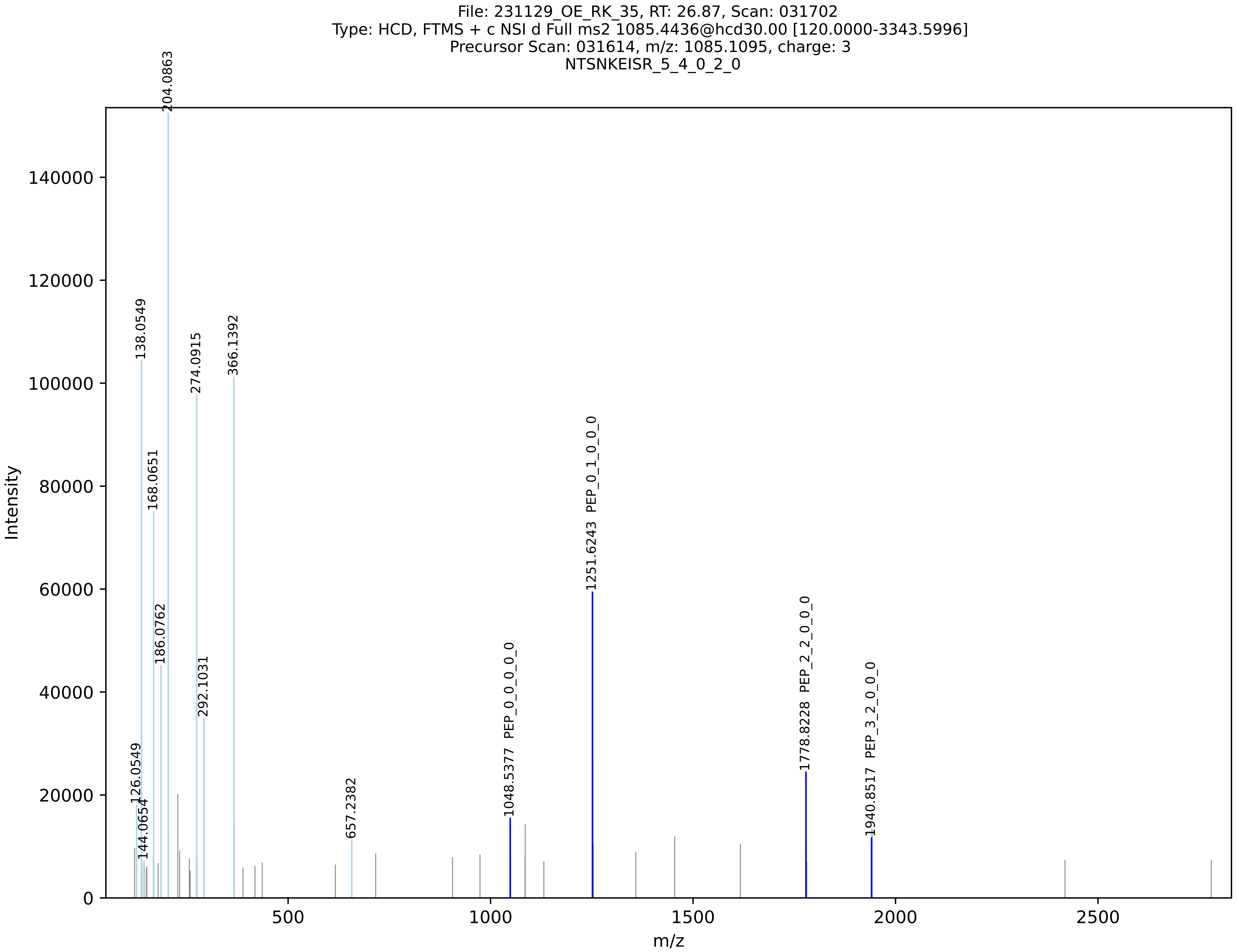
File: 231129_OE_RK_35, RT: 26.87, Scan: 031702
Type: HCD, FTMS + c NSI d Full ms2 1085.4436@hcd30.00 [120.0000-3343.5996]
Precursor Scan: 031614, m/z: 1085.1095, charge: 3
NTSNKEISR_5_4_0_2_0
Intensity
m/z
204.0863
138.0549
366.1392
274.0915
168.0651
186.0762
292.1031
126.0549
144.0654
657.2382
1048.5377 PEP_0_0_0_0_0
1251.6243 PEP_0_1_0_0_0
1778.8228 PEP_2_2_0_0_0
1940.8517 PEP_3_2_0_0_0

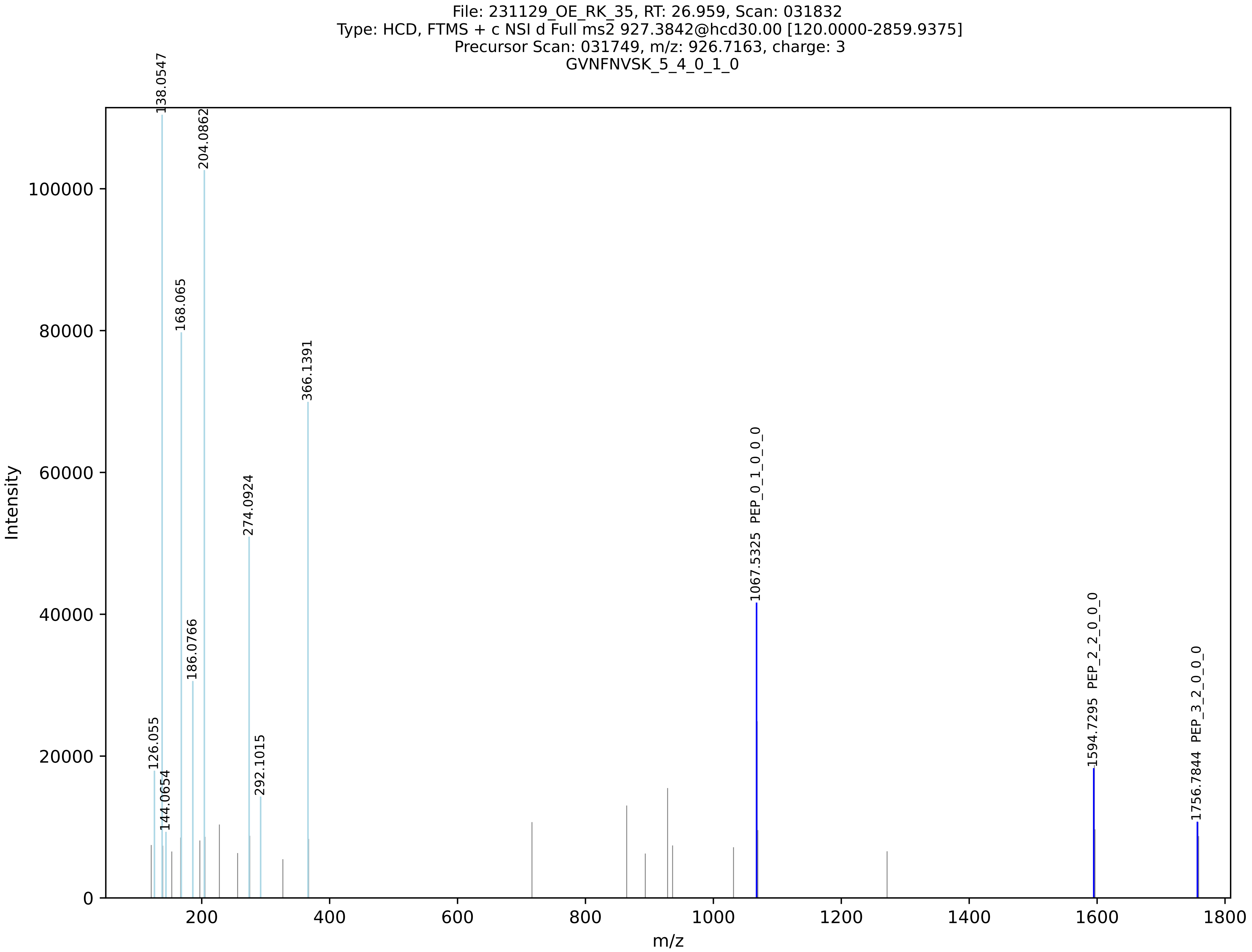

File: 231129_OE_RK_35, RT: 26.959, Scan: 031832
Type: HCD, FTMS + c NSI d Full ms2 927.3842@hcd30.00 [120.0000-2859.9375]
Precursor Scan: 031749, m/z: 926.7163, charge: 3
GVNFNVSK_5_4_0_1_0
Intensity
m/z
138.0547
204.0862
168.065
366.1391
274.0924
186.0766
126.055
144.0654
292.1015
1067.5325 PEP_0_1_0_0_0
1594.7295 PEP_2_2_0_0_0
1756.7844 PEP_3_2_0_0_0

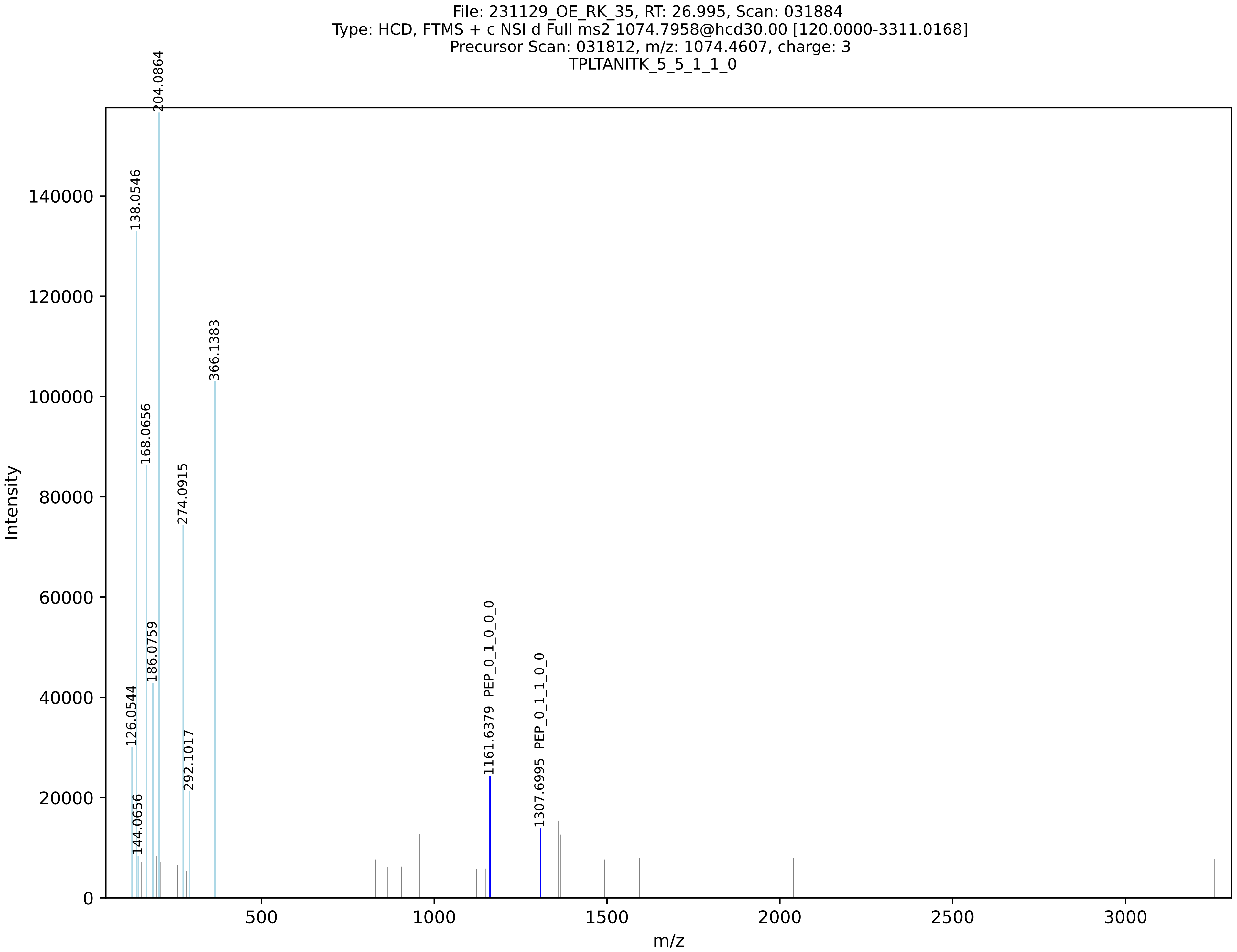

File: 231129_OE_RK_35, RT: 26.995, Scan: 031884
Type: HCD, FTMS + c NSI d Full ms2 1074.7958@hcd30.00 [120.0000-3311.0168]
Precursor Scan: 031812, m/z: 1074.4607, charge: 3
TPLTANITK_5_5_1_1_0
Intensity
m/z
204.0864
138.0546
366.1383
168.0656
274.0915
186.0759
126.0544
292.1017
144.0656
1161.6379 PEP_0_1_0_0_0
1307.6995 PEP_0_1_1_0_0
0
20000
40000
60000
80000
100000
120000
140000
500
1000
1500
2000
2500
3000

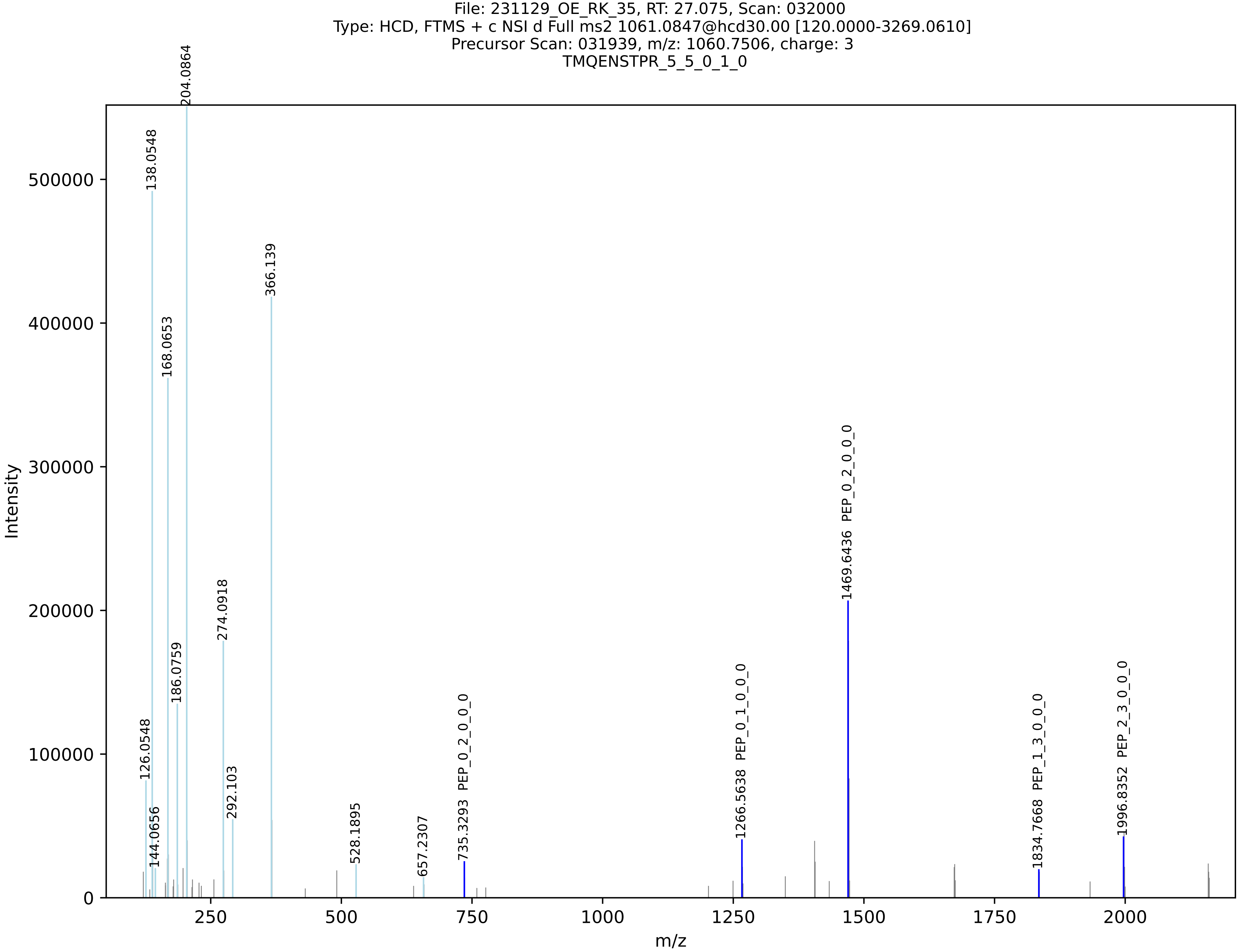
File: 231129_OE_RK_35, RT: 27.075, Scan: 032000
Type: HCD, FTMS + c NSI d Full ms2 1061.0847@hcd30.00 [120.0000-3269.0610]
Precursor Scan: 031939, m/z: 1060.7506, charge: 3
TMQENSTPR_5_5_0_1_0
Intensity
m/z
204.0864
138.0548
366.139
168.0653
274.0918
186.0759
126.0548
292.103
144.0656
528.1895
657.2307
735.3293 PEP_0_2_0_0_0
1266.5638 PEP_0_1_0_0_0
1469.6436 PEP_0_2_0_0_0
1834.7668 PEP_1_3_0_0_0
1996.8352 PEP_2_3_0_0_0
0
100000
200000
300000
400000
500000
250
500
750
1000
1250
1500
1750
2000

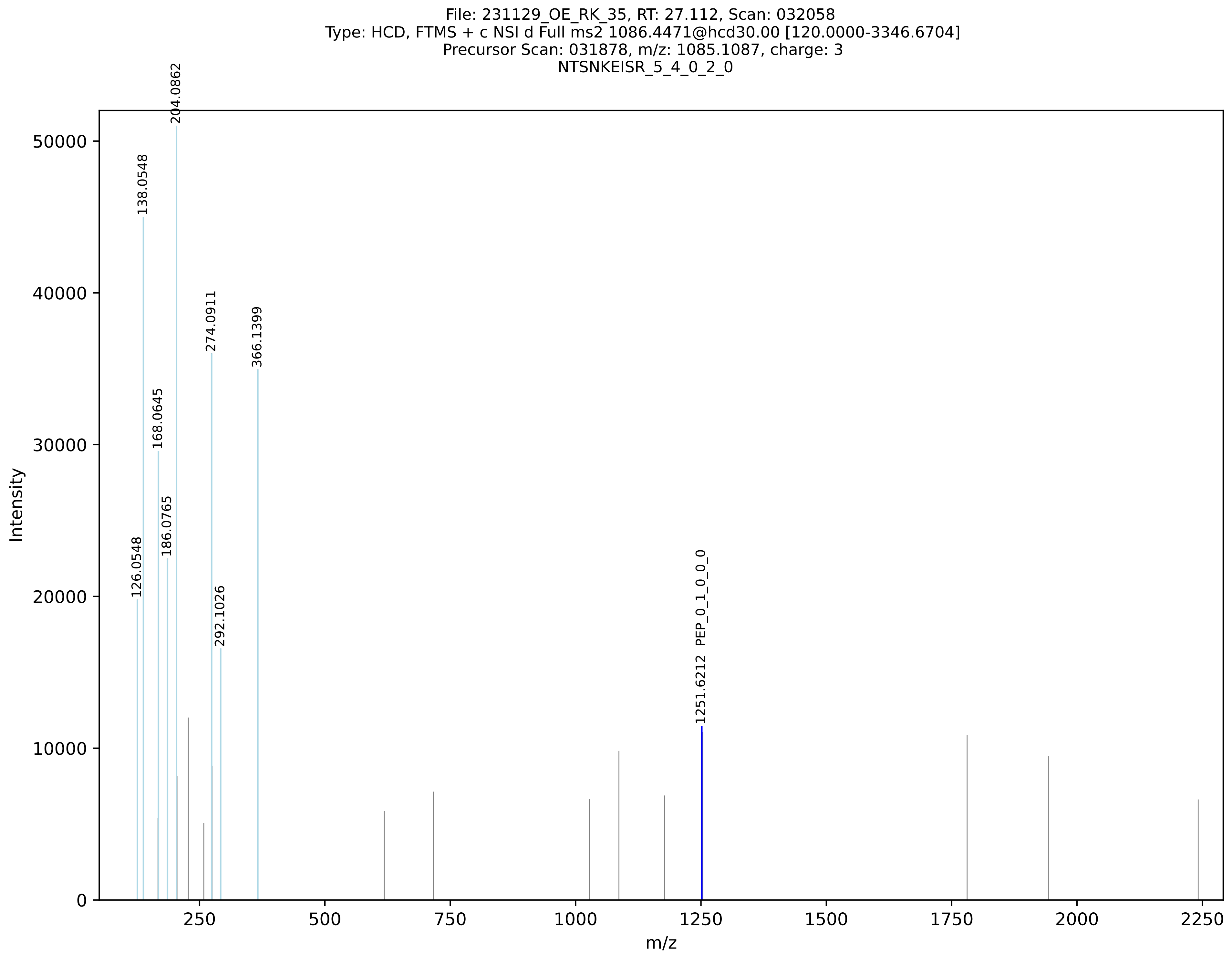
File: 231129_OE_RK_35, RT: 27.112, Scan: 032058
Type: HCD, FTMS + c NSI d Full ms2 1086.4471@hcd30.00 [120.0000-3346.6704]
Precursor Scan: 031878, m/z: 1085.1087, charge: 3
NTSNKEISR_5_4_0_2_0
204.0862
138.0548
274.0911
366.1399
168.0645
186.0765
126.0548
292.1026
1251.6212 PEP_0_1_0_0_0
Intensity
m/z
0
10000
20000
30000
40000
50000
250
500
750
1000
1250
1500
1750
2000
2250

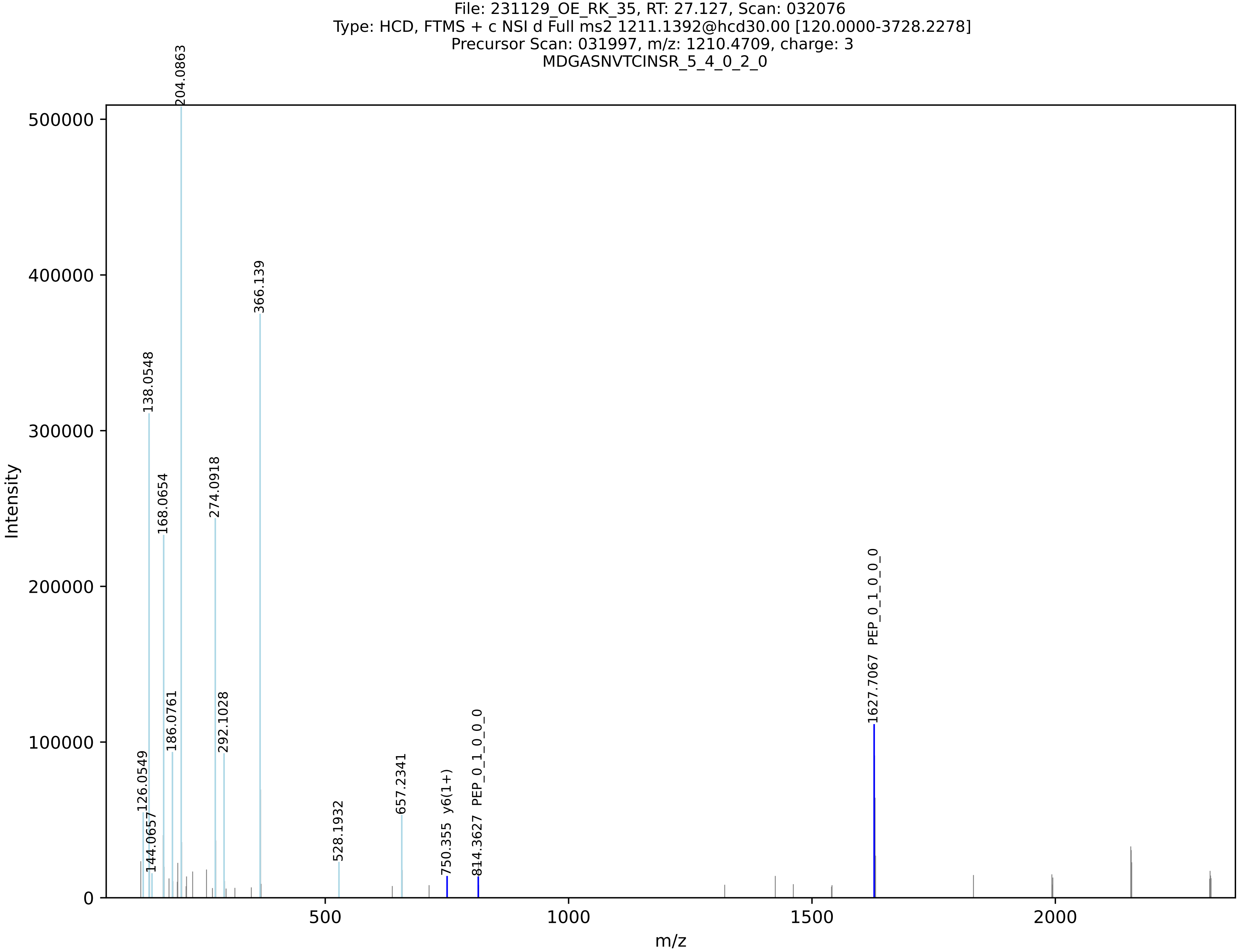
File: 231129_OE_RK_35, RT: 27.127, Scan: 032076
Type: HCD, FTMS + c NSI d Full ms2 1211.1392@hcd30.00 [120.0000-3728.2278]
Precursor Scan: 031997, m/z: 1210.4709, charge: 3
MDGASNVTCINSR_5_4_0_2_0
Intensity
m/z
204.0863
366.139
138.0548
168.0654
274.0918
186.0761
292.1028
126.0549
144.0657
528.1932
657.2341
750.355 y6(1+)
814.3627 PEP_0_1_0_0_0
1627.7067 PEP_0_1_0_0_0
0
100000
200000
300000
400000
500000
500
1000
1500
2000

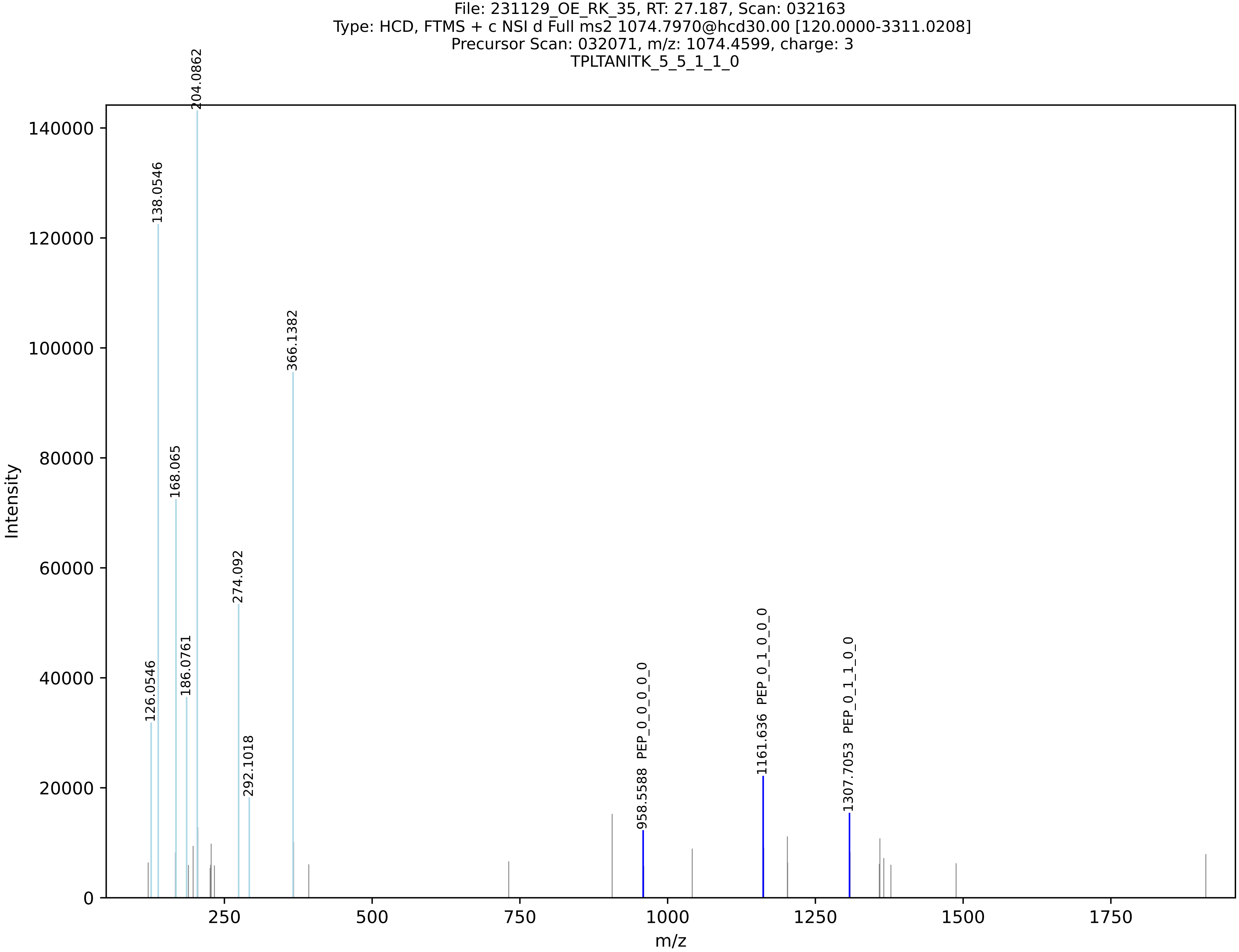
File: 231129_OE_RK_35, RT: 27.187, Scan: 032163
Type: HCD, FTMS + c NSI d Full ms2 1074.7970@hcd30.00 [120.0000-3311.0208]
Precursor Scan: 032071, m/z: 1074.4599, charge: 3
TPLTANITK_5_5_1_1_0
Intensity
m/z
204.0862
138.0546
366.1382
168.065
274.092
126.0546
186.0761
292.1018
958.5588 PEP_0_0_0_0_0
1161.636 PEP_0_1_0_0_0
1307.7053 PEP_0_1_1_0_0
0
20000
40000
60000
80000
100000
120000
140000
250
500
750
1000
1250
1500
1750

File: 231129_OE_RK_35, RT: 27.243, Scan: 032245
Type: HCD, FTMS + c NSI d Full ms2 1271.5313@hcd30.00 [120.0000-3913.0276]
Precursor Scan: 032145, m/z: 1271.1971, charge: 3
ESGTTAWQLVNSSVK_5_4_0_2_0

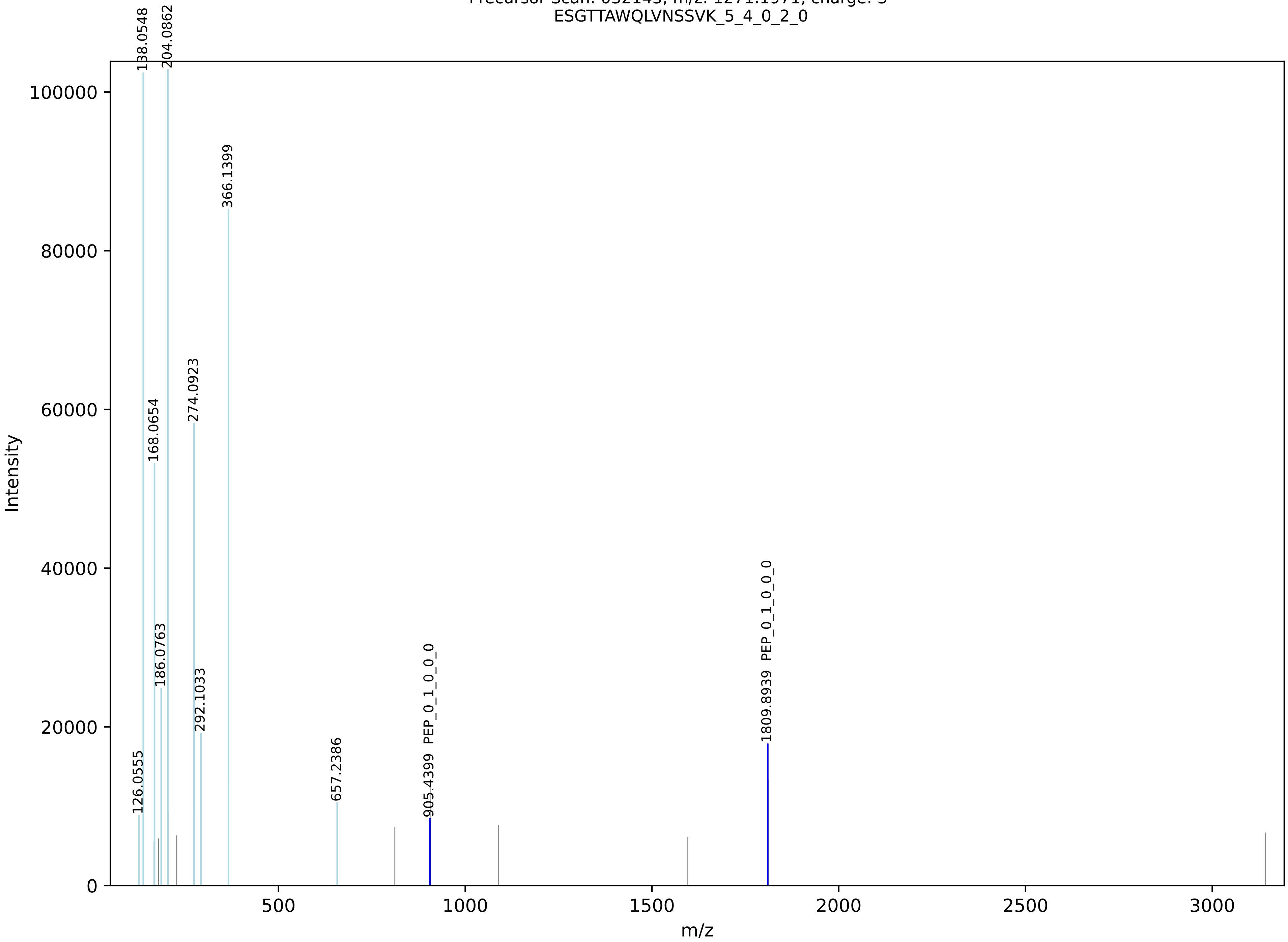

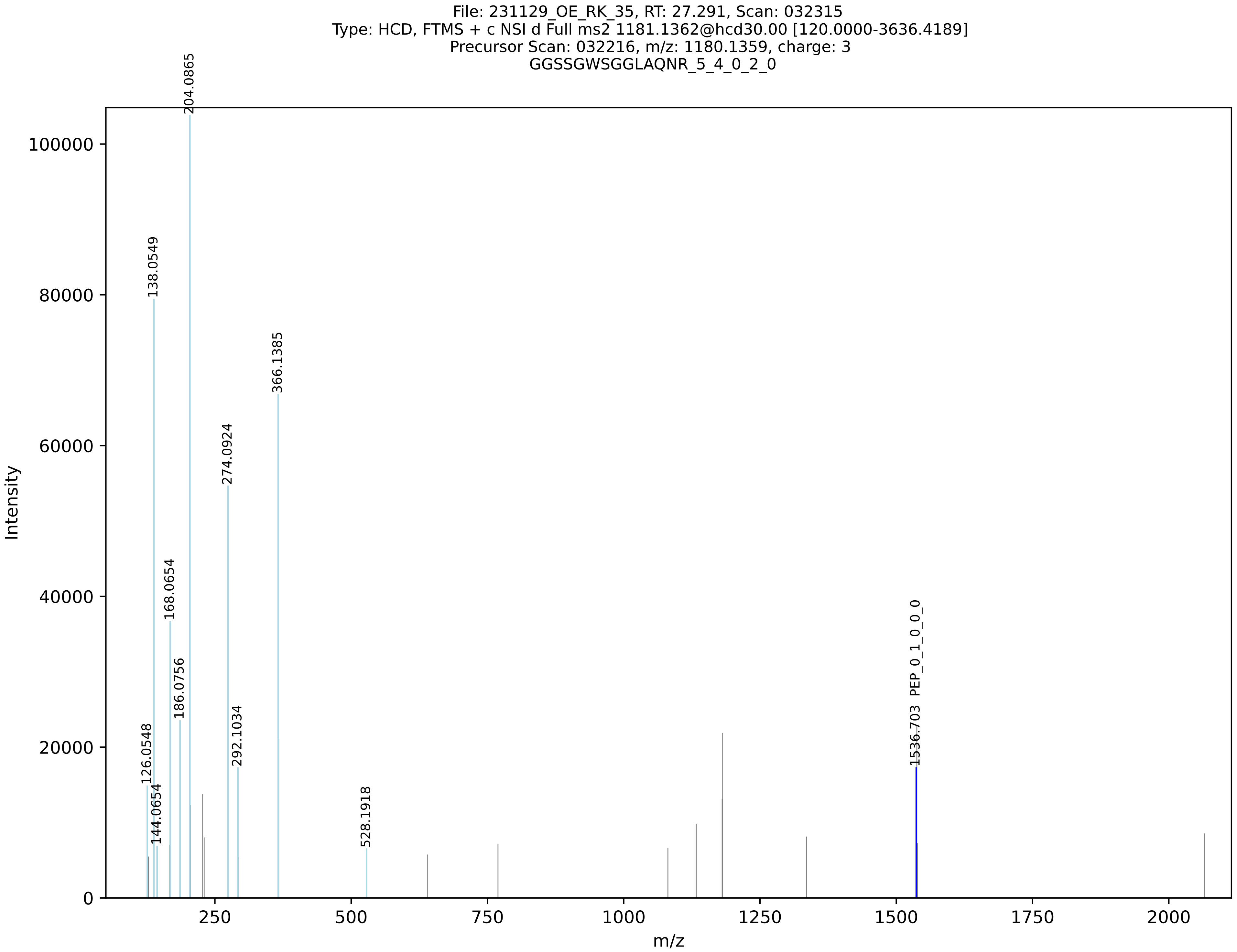
File: 231129_OE_RK_35, RT: 27.291, Scan: 032315
Type: HCD, FTMS + c NSI d Full ms2 1181.1362@hcd30.00 [120.0000-3636.4189]
Precursor Scan: 032216, m/z: 1180.1359, charge: 3
GGSSGWSGGLAQNR_5_4_0_2_0
Intensity
m/z
204.0865
138.0549
366.1385
274.0924
168.0654
186.0756
292.1034
126.0548
144.0654
528.1918
1536.703 PEP_0_1_0_0_0
0
20000
40000
60000
80000
100000
250
500
750
1000
1250
1500
1750
2000

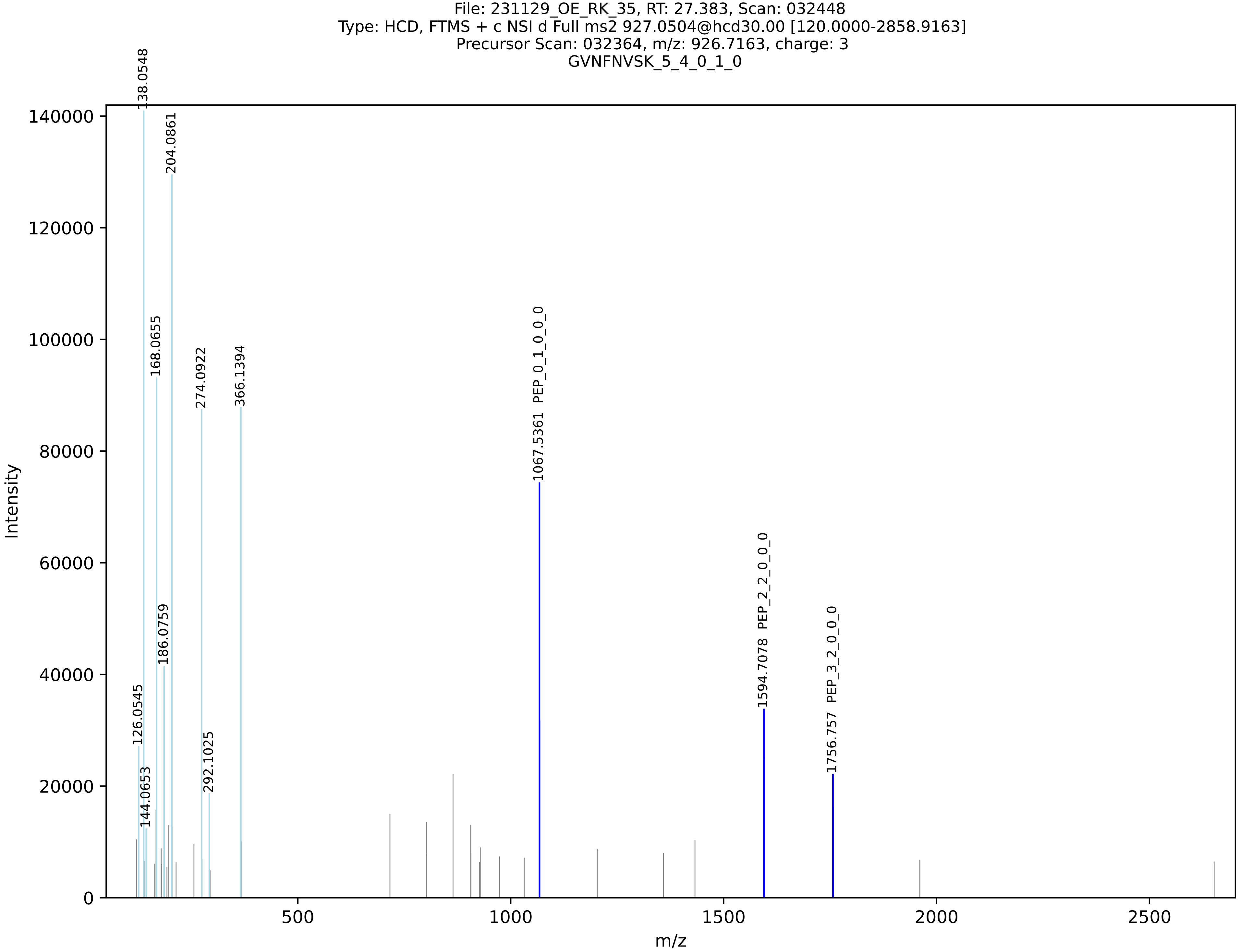
File: 231129_OE_RK_35, RT: 27.383, Scan: 032448
Type: HCD, FTMS + c NSI d Full ms2 927.0504@hcd30.00 [120.0000-2858.9163]
Precursor Scan: 032364, m/z: 926.7163, charge: 3
GVNFNVSK_5_4_0_1_0
Intensity
m/z
138.0548
204.0861
168.0655
274.0922
366.1394
186.0759
126.0545
144.0653
292.1025
1067.5361 PEP_0_1_0_0_0
1594.7078 PEP_2_2_0_0_0
1756.757 PEP_3_2_0_0_0
140000
120000
100000
80000
60000
40000
20000
0
500
1000
1500
2000
2500

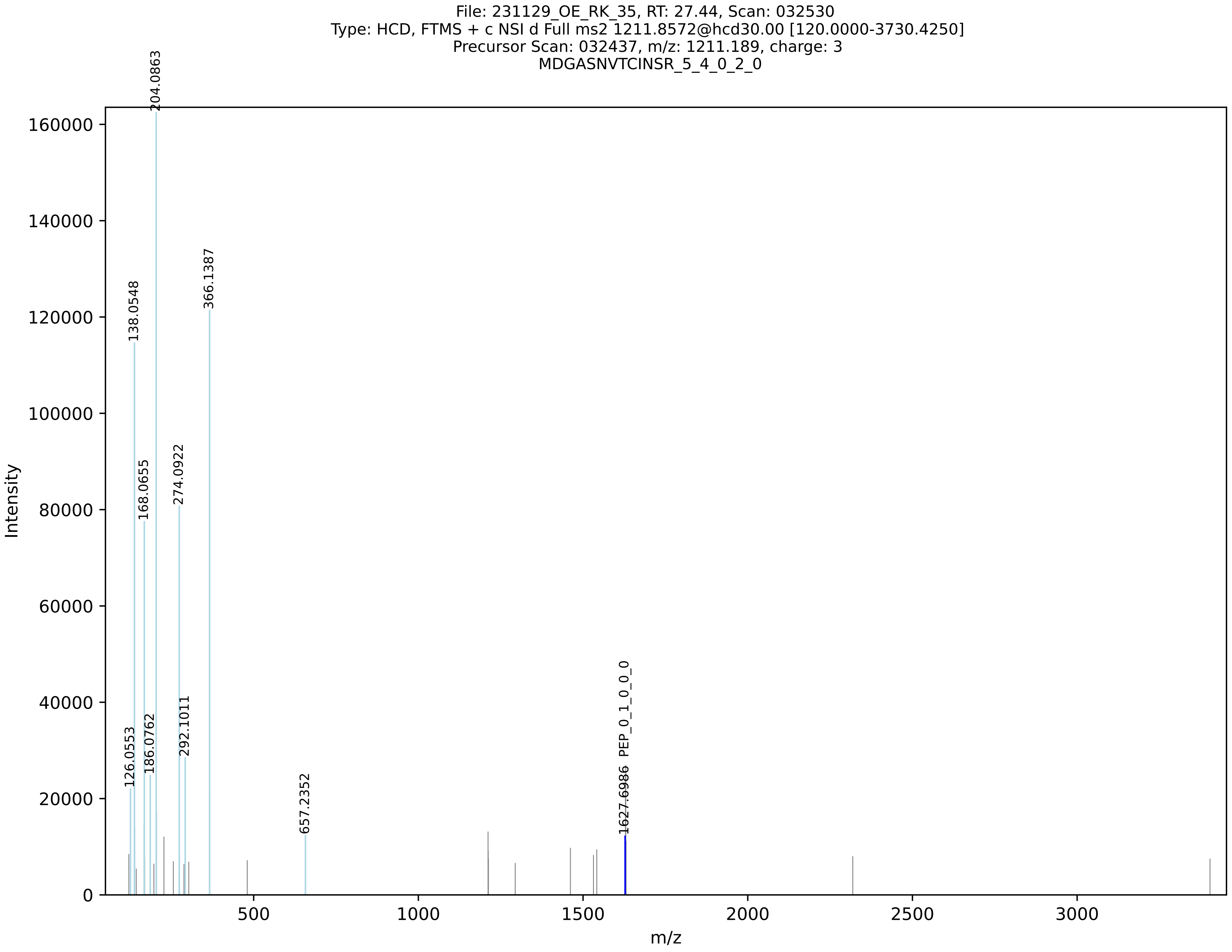
File: 231129_OE_RK_35, RT: 27.44, Scan: 032530
Type: HCD, FTMS + c NSI d Full ms2 1211.8572@hcd30.00 [120.0000-3730.4250]
Precursor Scan: 032437, m/z: 1211.189, charge: 3
MDGASNVTCINSR_5_4_0_2_0
Intensity
m/z
204.0863
366.1387
138.0548
168.0655
274.0922
292.1011
186.0762
126.0553
657.2352
1627.6986 PEP_0_1_0_0_0
0
20000
40000
60000
80000
100000
120000
140000
160000
500
1000
1500
2000
2500
3000

File: 231129_OE_RK_35, RT: 27.478, Scan: 032584
Type: HCD, FTMS + c NSI d Full ms2 1125.1416@hcd30.00 [120.0000-3465.0752]
Precursor Scan: 032510, m/z: 1124.8075, charge: 3
NLFLNHSENATAK_5_4_0_1_0

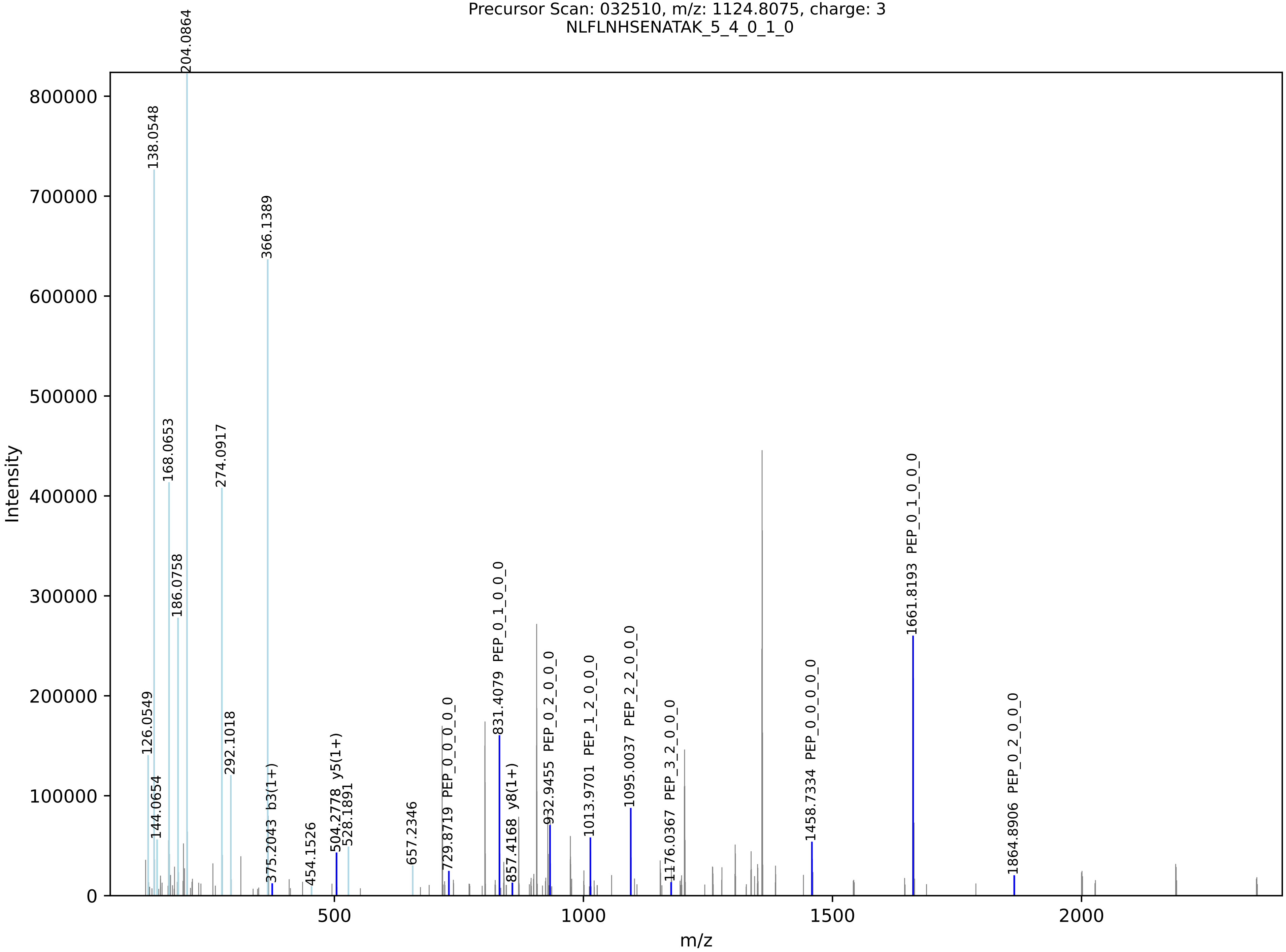

File: 231129_OE_RK_35, RT: 27.529, Scan: 032659
Type: HCD, FTMS + c NSI d Full ms2 1211.1389@hcd30.00 [120.0000-3728.2271]
Precursor Scan: 032581, m/z: 1210.4707, charge: 3
MDGASNVTCINSR_5_4_0_2_0

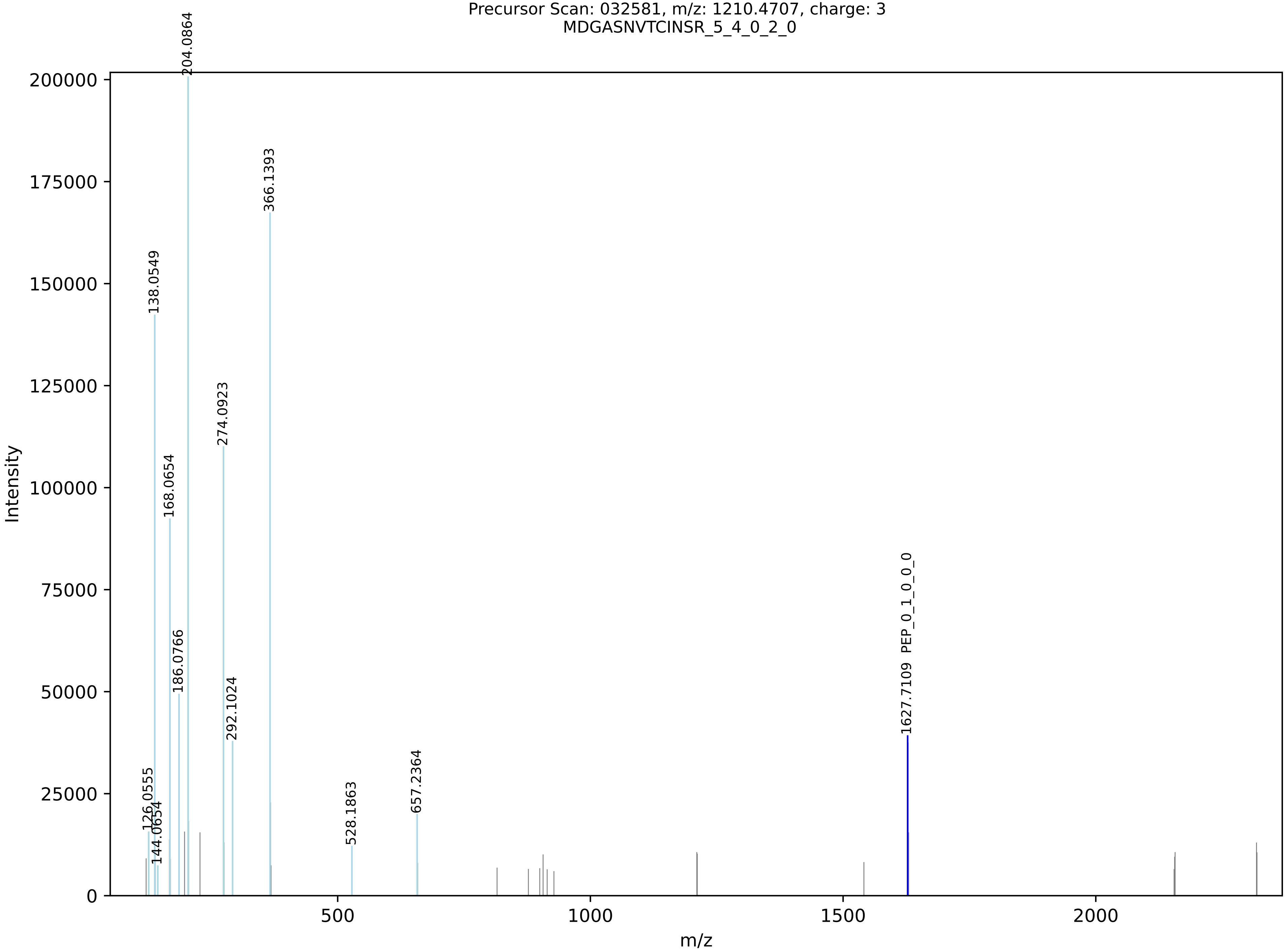

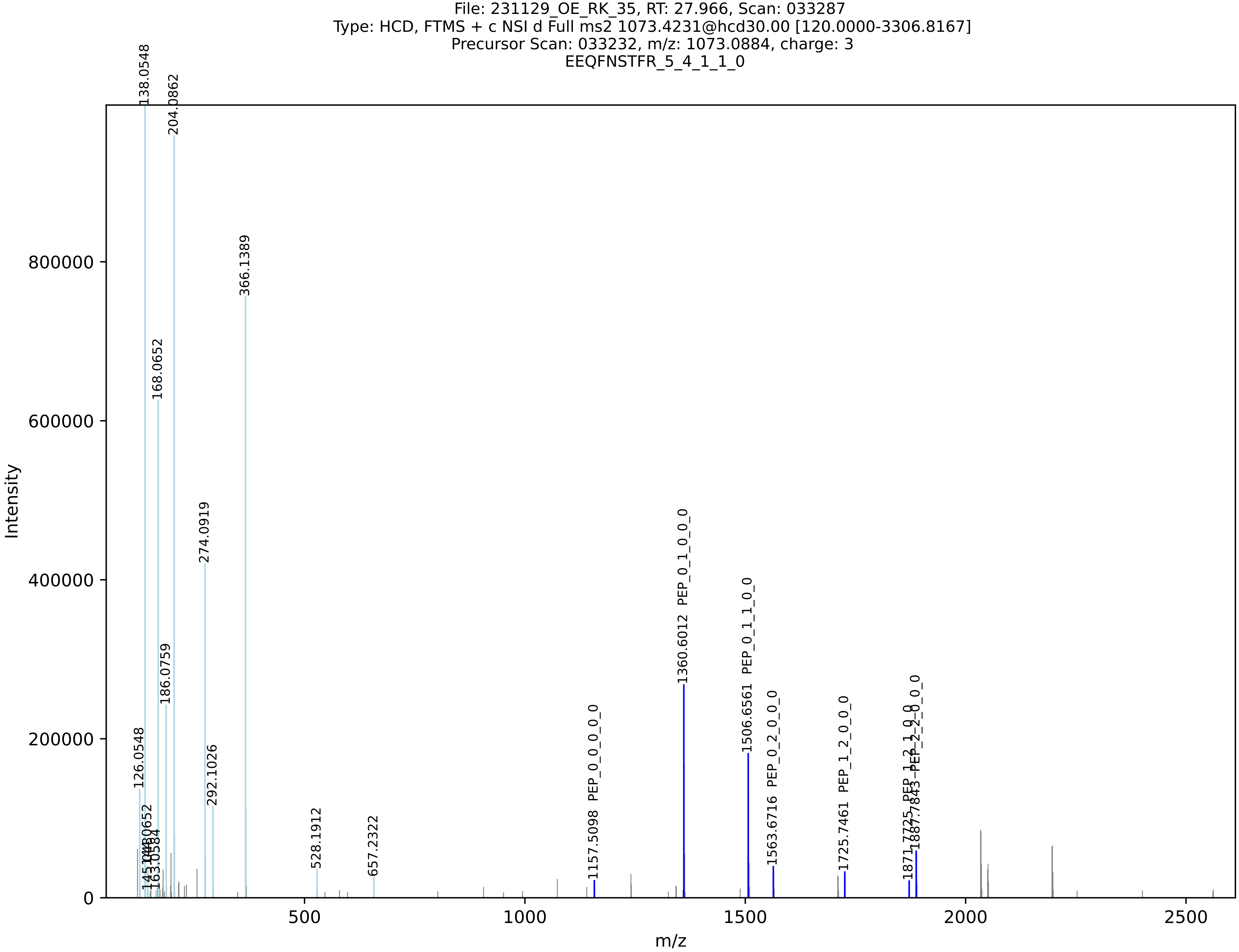

File: 231129_OE_RK_35, RT: 27.966, Scan: 033287
Type: HCD, FTMS + c NSI d Full ms2 1073.4231@hcd30.00 [120.0000-3306.8167]
Precursor Scan: 033232, m/z: 1073.0884, charge: 3
EEQFNSTFR_5_4_1_1_0
Intensity
m/z
138.0548
204.0862
366.1389
168.0652
274.0919
186.0759
126.0548
292.1026
163.0584
528.1912
657.2322
1157.5098 PEP_0_0_0_0_0
1360.6012 PEP_0_1_0_0_0
1506.6561 PEP_0_1_1_0_0
1563.6716 PEP_0_2_0_0_0
1725.7461 PEP_1_2_0_0_0
1871.7725 PEP_1_2_1_0_0
1887.7843 PEP_2_2_0_0_0

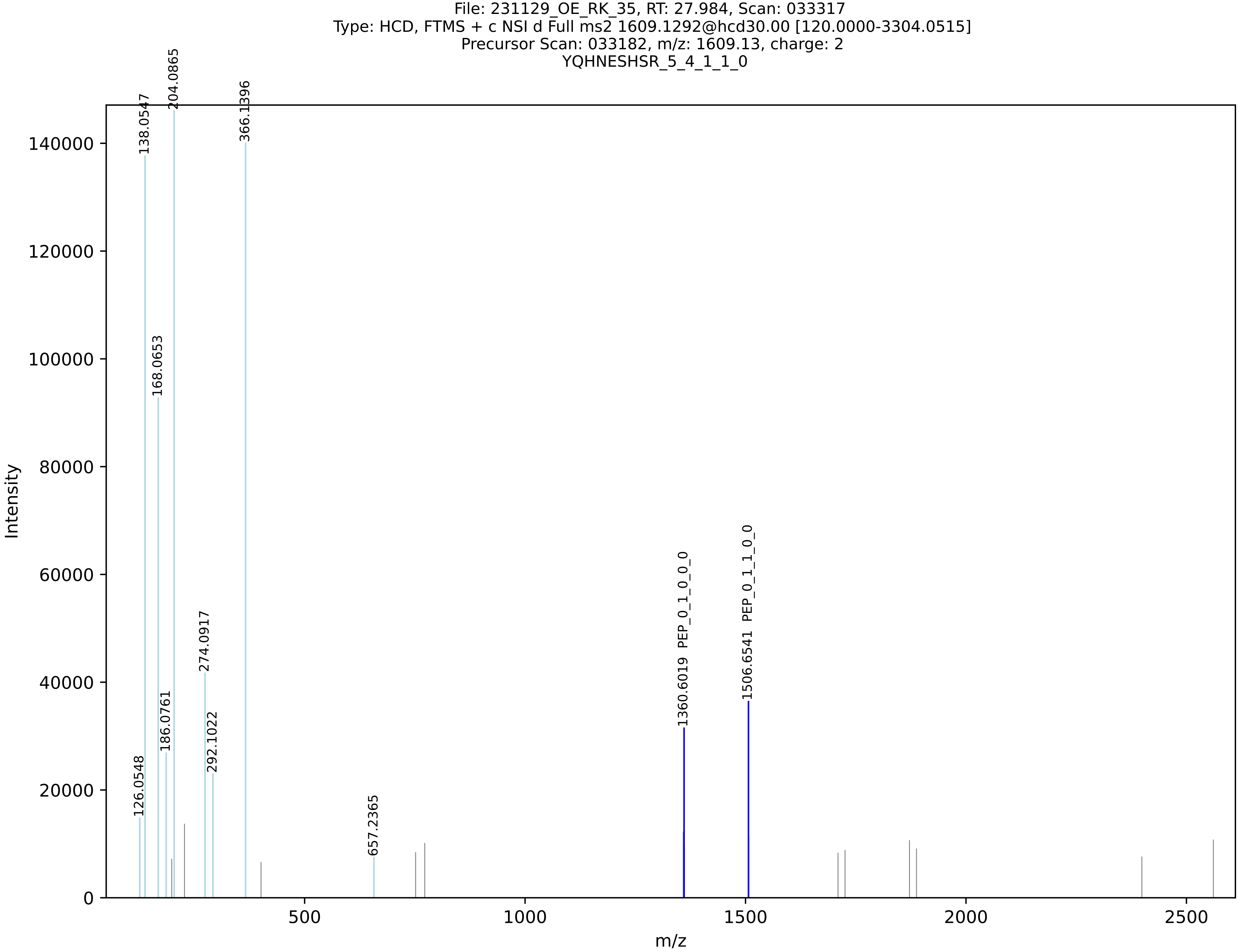
File: 231129_OE_RK_35, RT: 27.984, Scan: 033317
Type: HCD, FTMS + c NSI d Full ms2 1609.1292@hcd30.00 [120.0000-3304.0515]
Precursor Scan: 033182, m/z: 1609.13, charge: 2
YQHNESHSR_5_4_1_1_0
Intensity
m/z
138.0547
204.0865
366.1396
168.0653
274.0917
186.0761
292.1022
126.0548
657.2365
1360.6019 PEP_0_1_0_0_0
1506.6541 PEP_0_1_1_0_0
0
20000
40000
60000
80000
100000
120000
140000
500
1000
1500
2000
2500

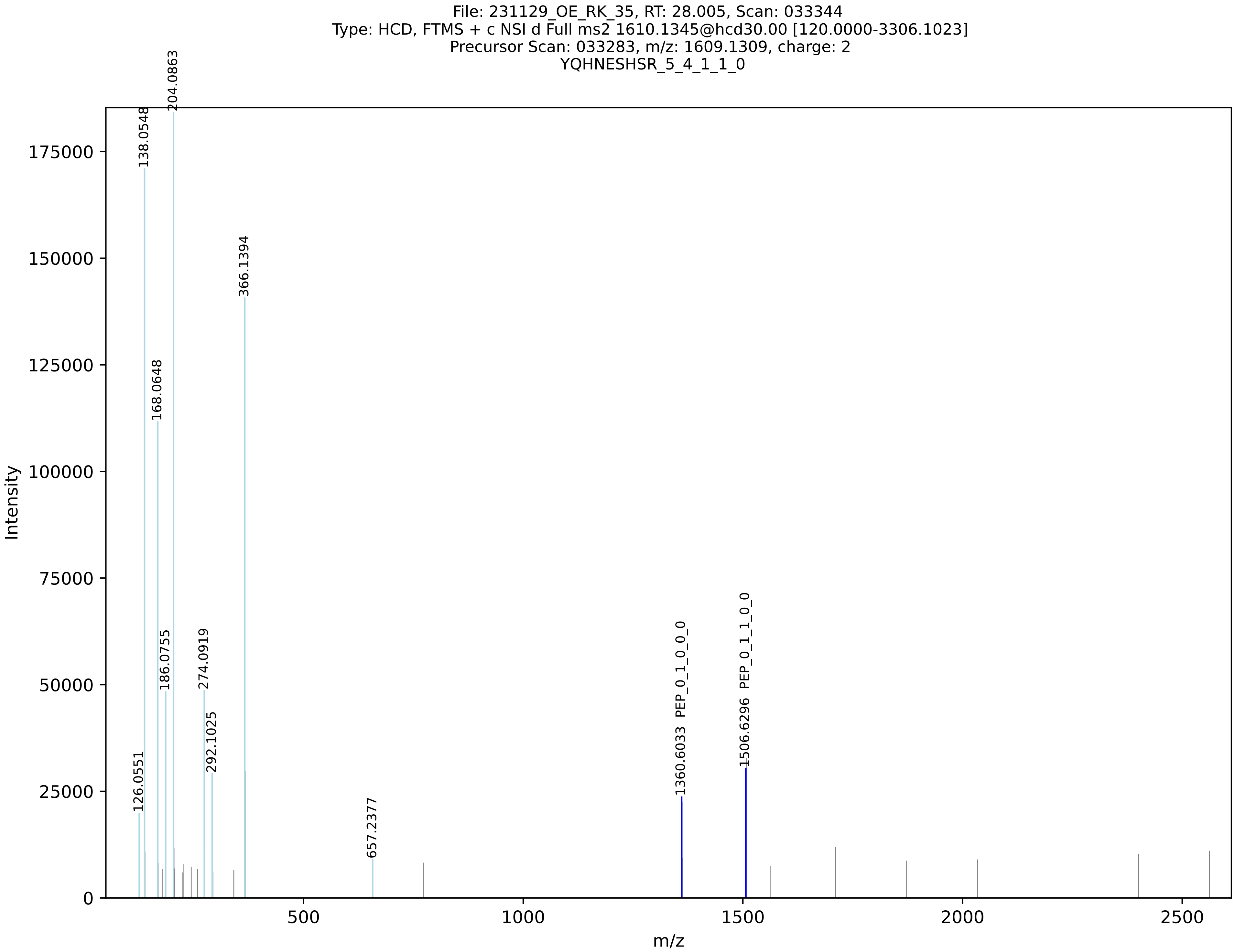
File: 231129_OE_RK_35, RT: 28.005, Scan: 033344
Type: HCD, FTMS + c NSI d Full ms2 1610.1345@hcd30.00 [120.0000-3306.1023]
Precursor Scan: 033283, m/z: 1609.1309, charge: 2
YQHNESHSR_5_4_1_1_0
Intensity
m/z
0
25000
50000
75000
100000
125000
150000
175000
500
1000
1500
2000
2500
126.0551
138.0548
168.0648
186.0755
204.0863
274.0919
292.1025
366.1394
657.2377
1360.6033 PEP_0_1_0_0_0
1506.6296 PEP_0_1_1_0_0

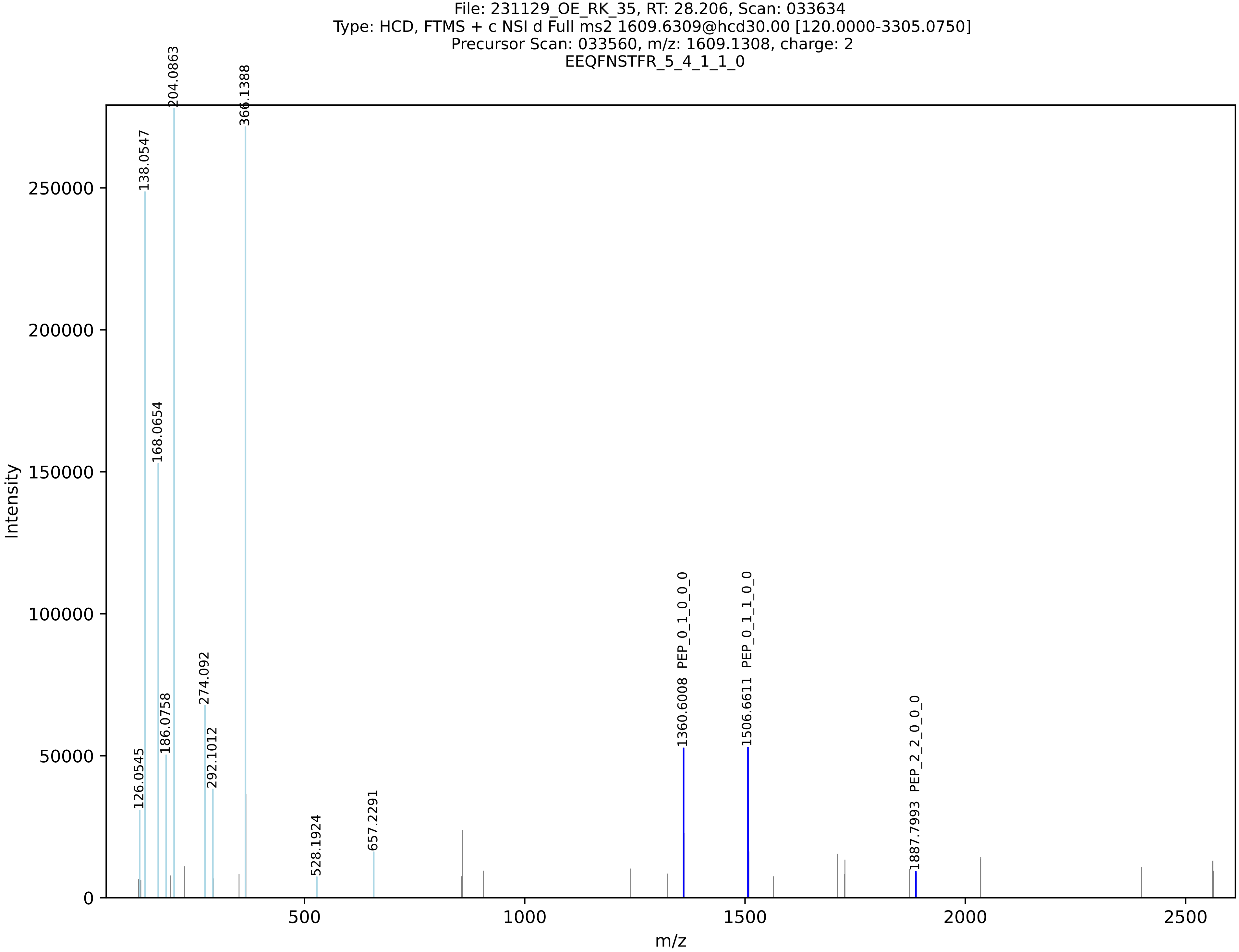

File: 231129_OE_RK_35, RT: 28.206, Scan: 033634
Type: HCD, FTMS + c NSI d Full ms2 1609.6309@hcd30.00 [120.0000-3305.0750]
Precursor Scan: 033560, m/z: 1609.1308, charge: 2
EEQFNSTFR_5_4_1_1_0
204.0863
366.1388
138.0547
168.0654
274.092
186.0758
292.1012
126.0545
528.1924
657.2291
1360.6008 PEP_0_1_0_0_0
1506.6611 PEP_0_1_1_0_0
1887.7993 PEP_2_2_0_0_0
Intensity
m/z
0
50000
100000
150000
200000
250000
500
1000
1500
2000
2500

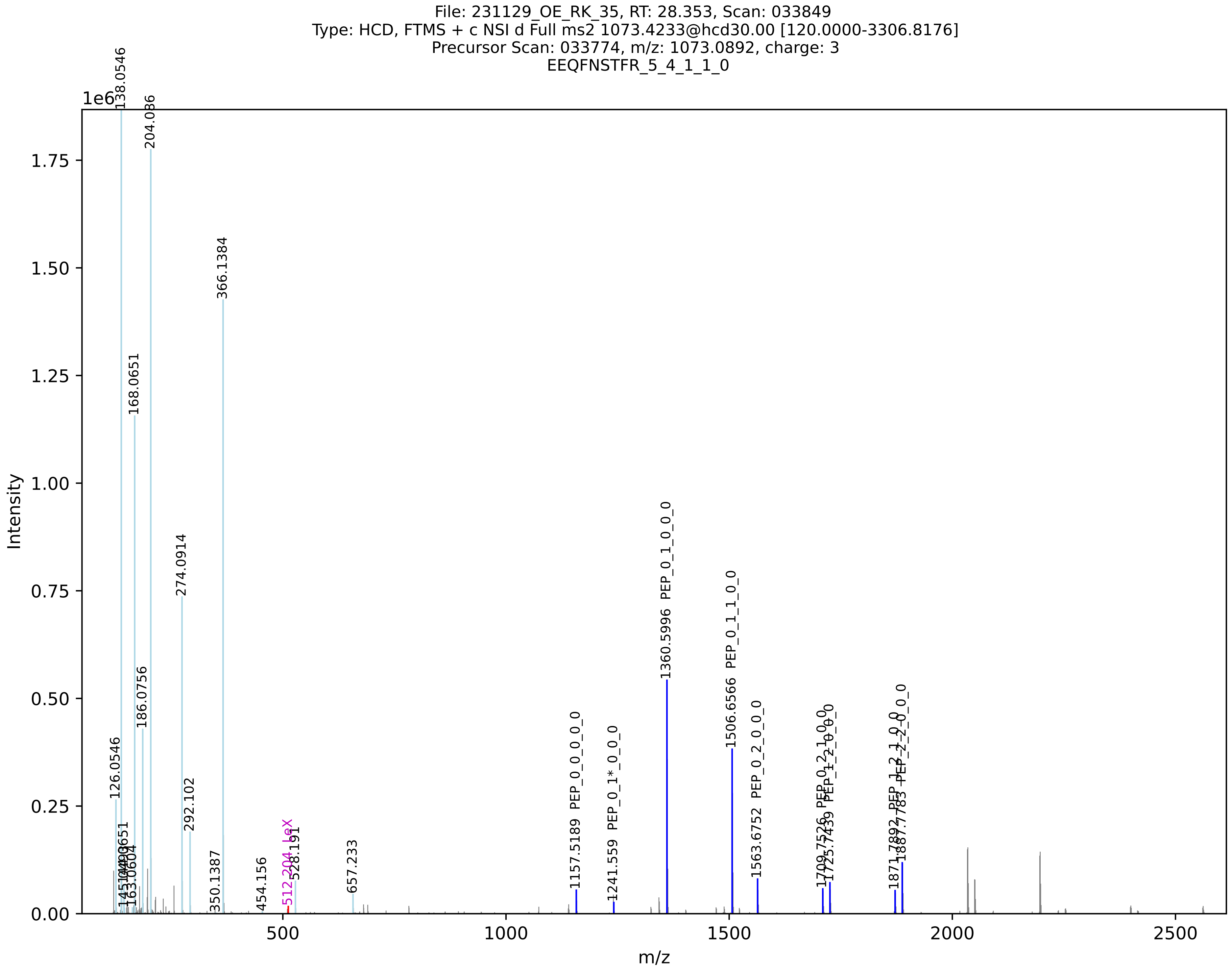
File: 231129_OE_RK_35, RT: 28.353, Scan: 033849
Type: HCD, FTMS + c NSI d Full ms2 1073.4233@hcd30.00 [120.0000-3306.8176]
Precursor Scan: 033774, m/z: 1073.0892, charge: 3
EEQFNSTFR_5_4_1_1_0
1e6
Intensity
m/z
138.0546
204.086
366.1384
168.0651
274.0914
186.0756
126.0546
292.102
163.0604
350.1387
454.156
512.204 LeX
528.191
657.233
1157.5189 PEP_0_0_0_0_0
1241.559 PEP_0_1*_0_0_0
1360.5996 PEP_0_1_0_0_0
1506.6566 PEP_0_1_1_0_0
1563.6752 PEP_0_2_0_0_0
1725.7439
1871.7892
1887.7783

File: 231129_OE_RK_35, RT: 28.405, Scan: 033925
Type: HCD, FTMS + c NSI d Full ms2 1609.6320@hcd30.00 [120.0000-3305.0771]
Precursor Scan: 033848, m/z: 1609.1309, charge: 2
EEQFNSTFR_5_4_1_1_0

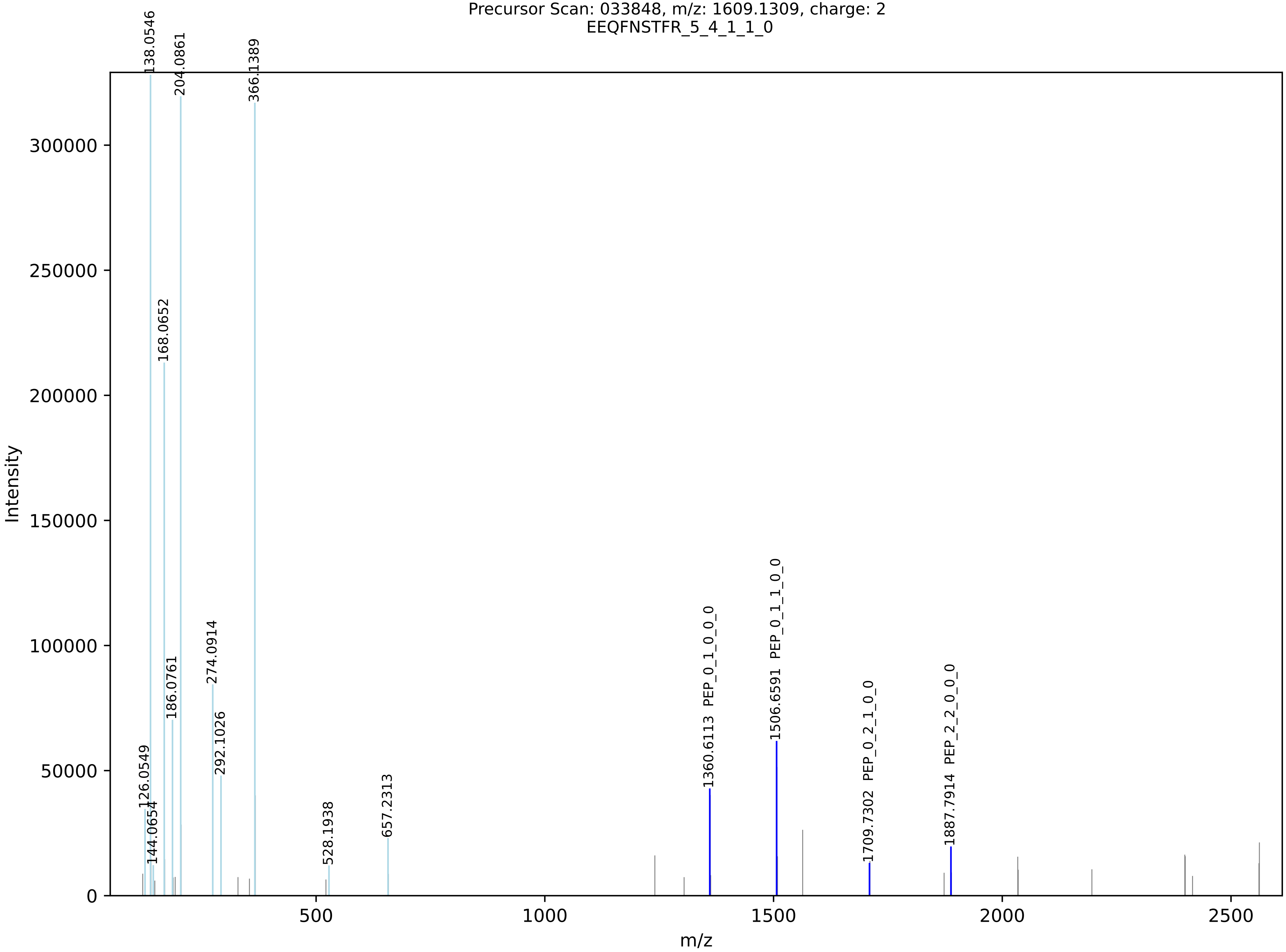

File: 231129_OE_RK_35, RT: 28.417, Scan: 033945
Type: HCD, FTMS + c NSI d Full ms2 1104.1316@hcd30.00 [120.0000-3400.7847]
Precursor Scan: 033848, m/z: 1103.7973, charge: 3
TPLTANITK_5_4_1_2_0

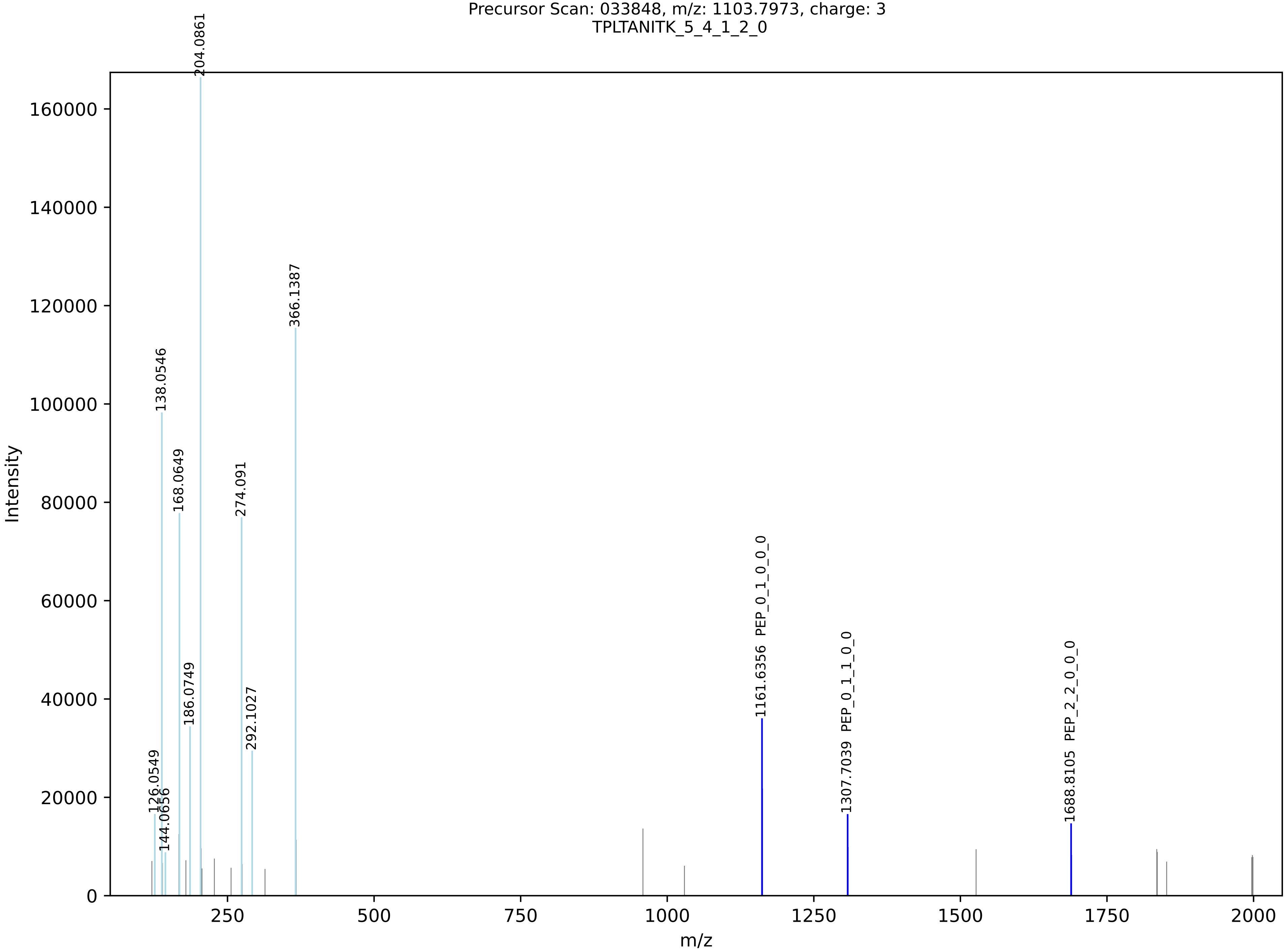

File: 231129_OE_RK_35, RT: 28.443, Scan: 033986
Type: HCD, FTMS + c NSI d Full ms2 1444.2603@hcd30.00 [120.0000-4441.5781]
Precursor Scan: 033774, m/z: 1443.9235, charge: 3
VYKPSAGNNSLYR_6_5_0_3_0

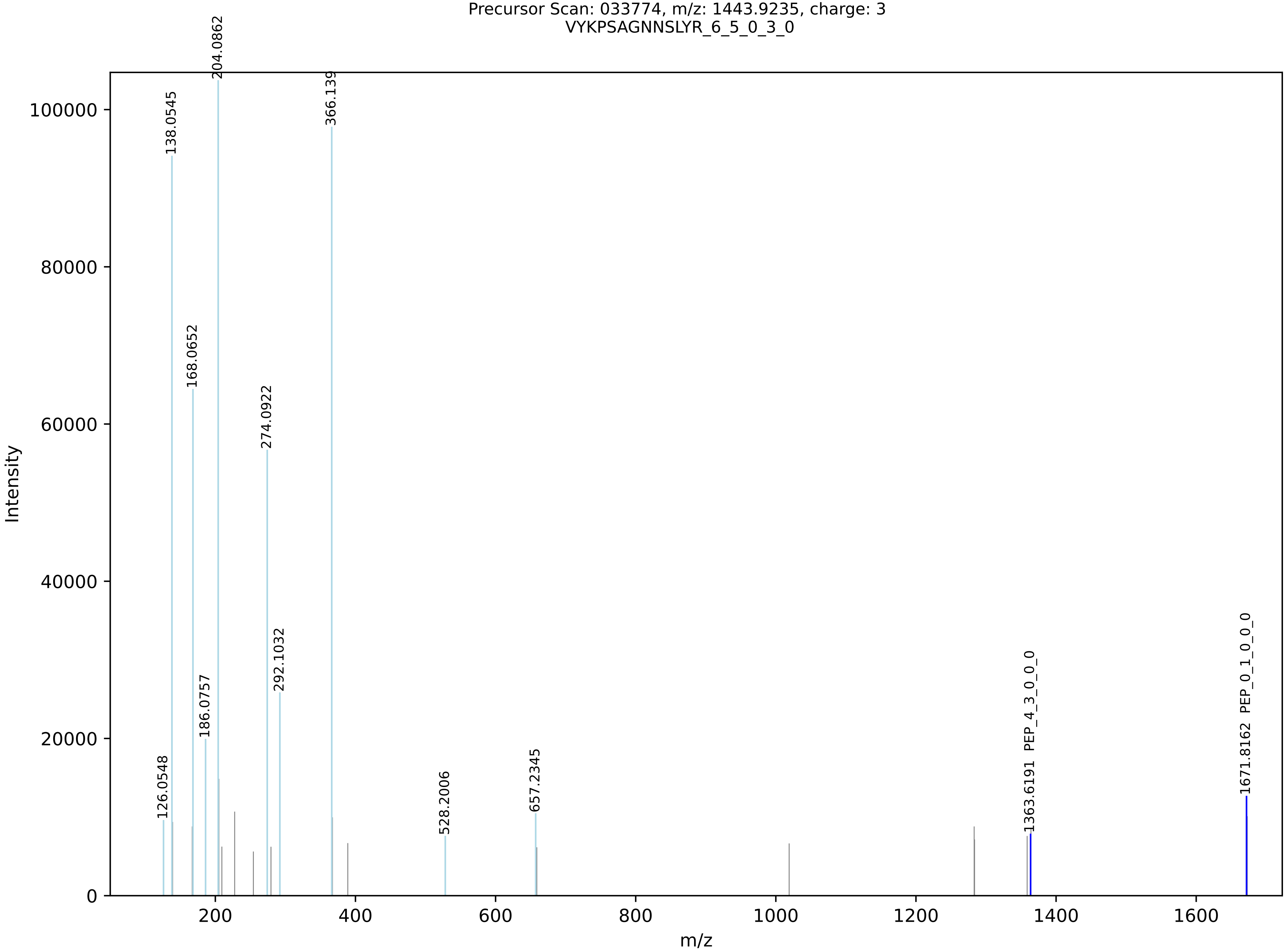

File: 231129_OE_RK_35, RT: 28.523, Scan: 034100
Type: HCD, FTMS + c NSI d Full ms2 1444.5947@hcd30.00 [120.0000-4442.6021]
Precursor Scan: 033995, m/z: 1443.9265, charge: 3
VYKPSAGNNSLYR_6_5_0_3_0

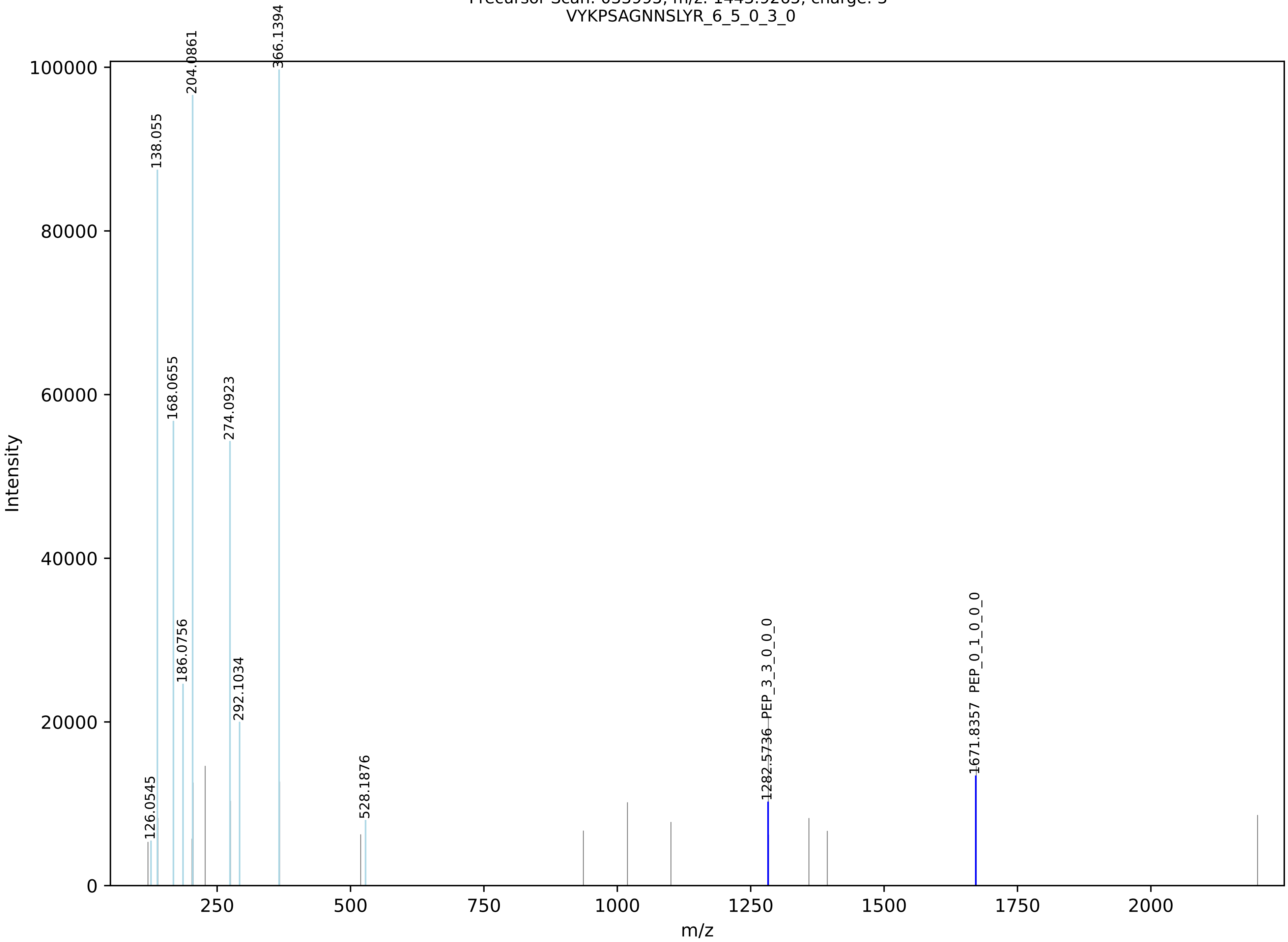

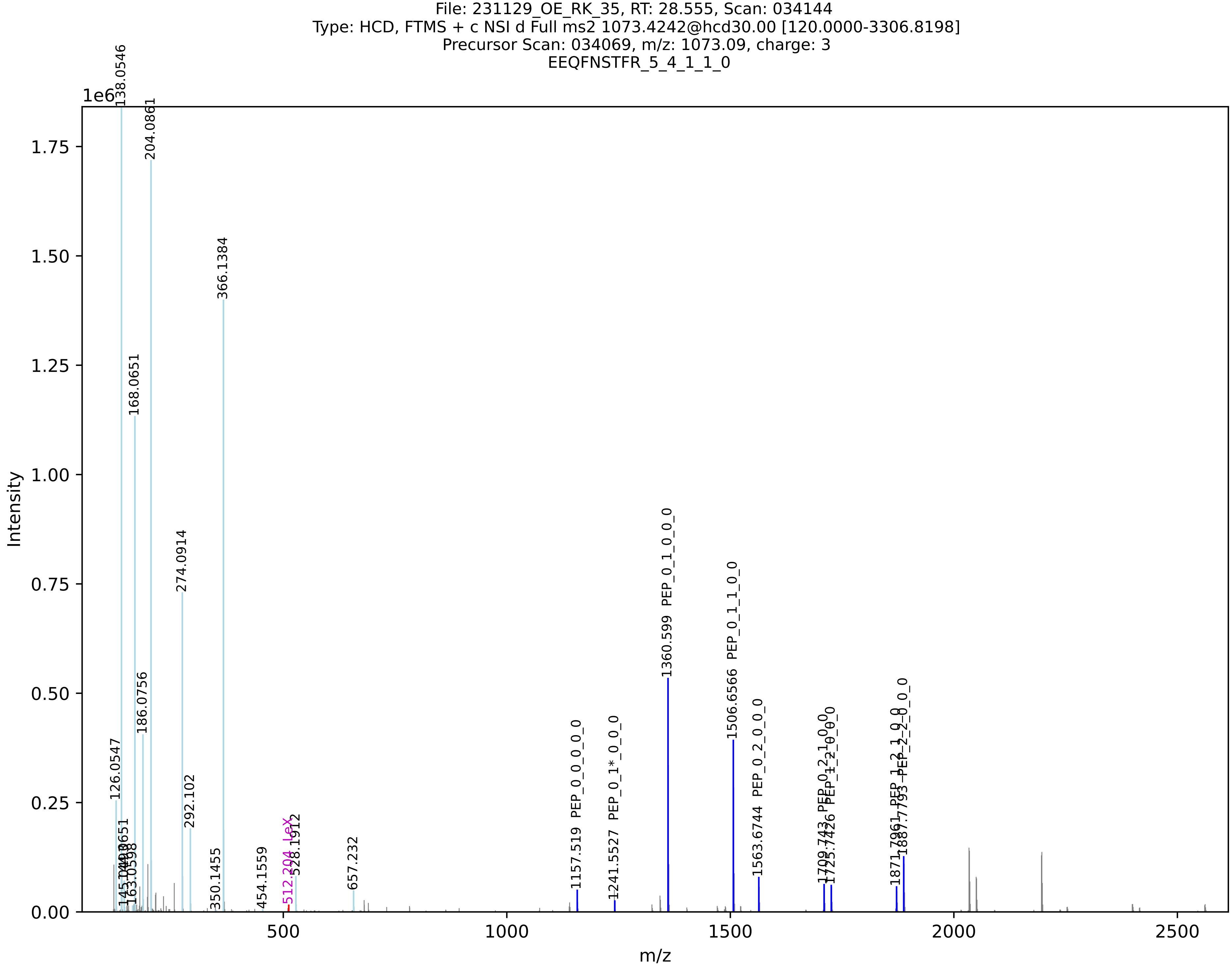
File: 231129_OE_RK_35, RT: 28.555, Scan: 034144
Type: HCD, FTMS + c NSI d Full ms2 1073.4242@hcd30.00 [120.0000-3306.8198]
Precursor Scan: 034069, m/z: 1073.09, charge: 3
EEQFNSTFR_5_4_1_1_0
1e6
Intensity
m/z
138.0546
204.0861
366.1384
168.0651
274.0914
186.0756
126.0547
292.102
350.1455
454.1559
512.204_LeX
528.1912
657.232
1157.519 PEP_0_0_0_0_0
1241.5527 PEP_0_1*_0_0_0
1360.599 PEP_0_1_0_0_0
1506.6566 PEP_0_1_1_0_0
1563.6744 PEP_0_2_0_0_0

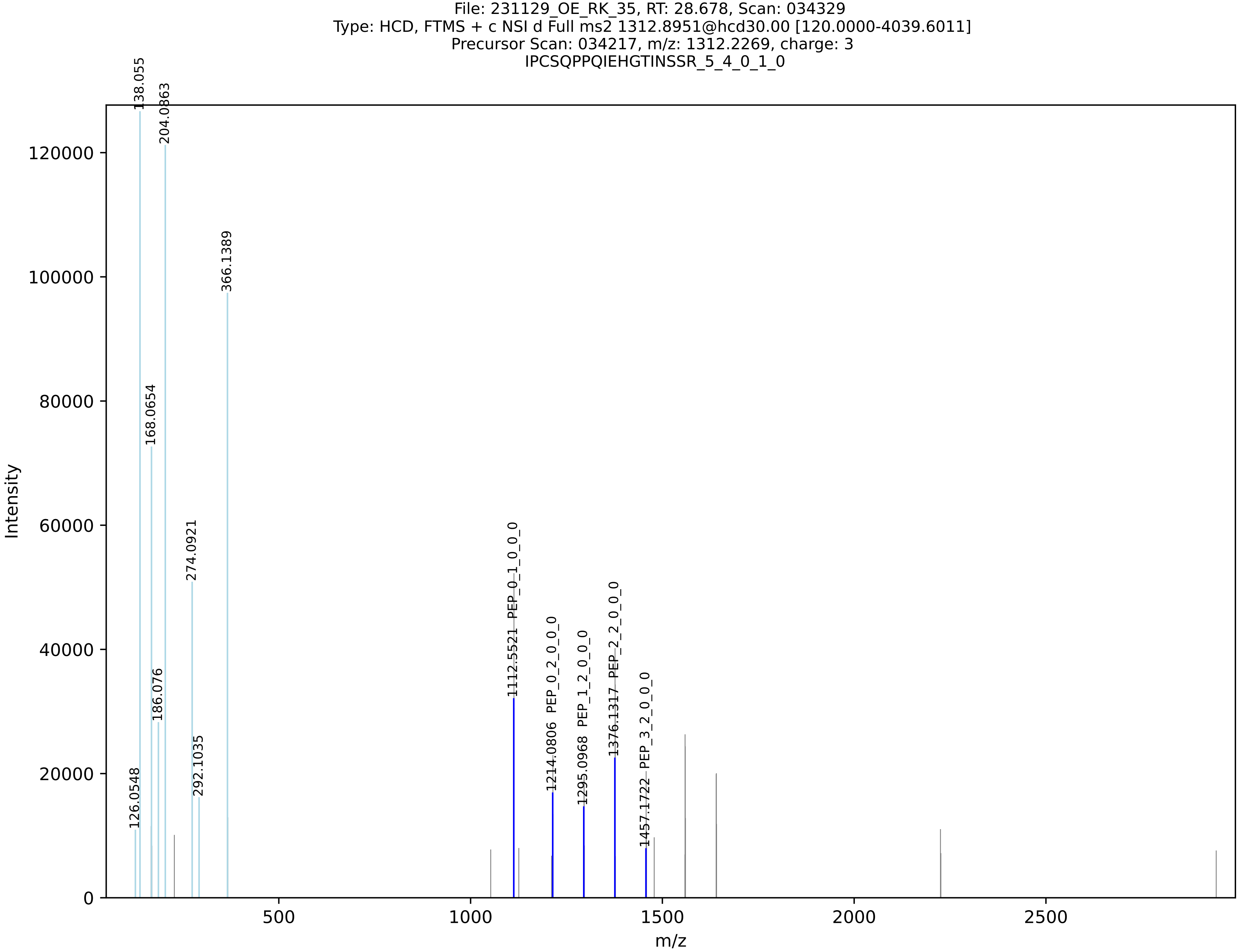

File: 231129_OE_RK_35, RT: 28.678, Scan: 034329
Type: HCD, FTMS + c NSI d Full ms2 1312.8951@hcd30.00 [120.0000-4039.6011]
Precursor Scan: 034217, m/z: 1312.2269, charge: 3
IPCSQPPQIEHGTINSSR_5_4_0_1_0
138.055
204.0863
366.1389
168.0654
274.0921
186.076
292.1035
126.0548
1112.5521_PEP_0_1_0_0_0
1214.0806_PEP_0_2_0_0_0
1295.0968_PEP_1_2_0_0_0
1376.1317_PEP_2_2_0_0_0
1457.1722_PEP_3_2_0_0_0
Intensity
m/z

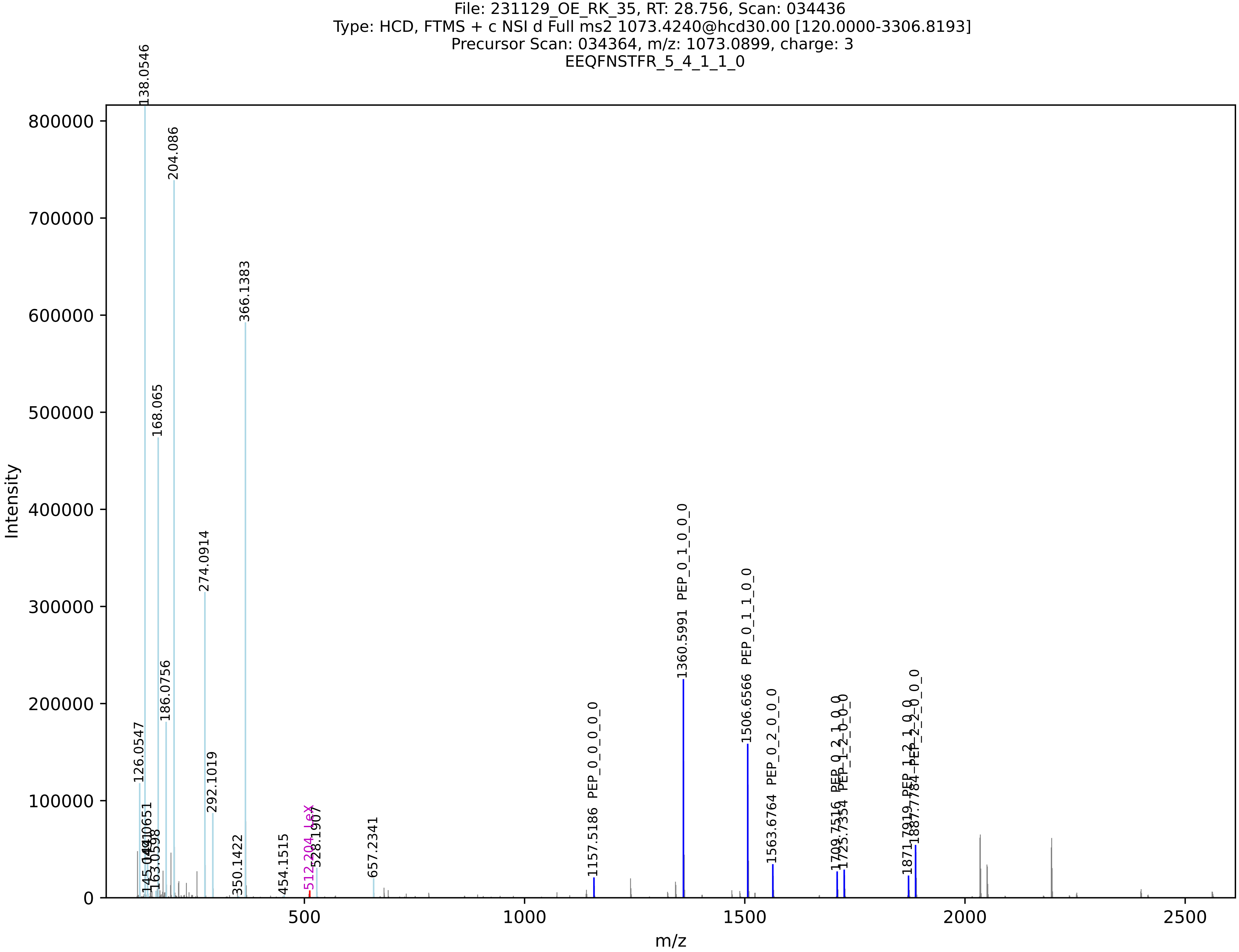
File: 231129_OE_RK_35, RT: 28.756, Scan: 034436
Type: HCD, FTMS + c NSI d Full ms2 1073.4240@hcd30.00 [120.0000-3306.8193]
Precursor Scan: 034364, m/z: 1073.0899, charge: 3
EEQFNSTFR_5_4_1_1_0
Intensity
m/z
138.0546
204.086
366.1383
168.065
274.0914
186.0756
126.0547
292.1019
350.1422
454.1515
512.204_LeX
528.1907
657.2341
1157.5186 PEP_0_0_0_0_0
1360.5991 PEP_0_1_0_0_0
1506.6566 PEP_0_1_1_0_0
1563.6764 PEP_0_2_0_0_0
1887.7784 PEP_2_2_0_0_0

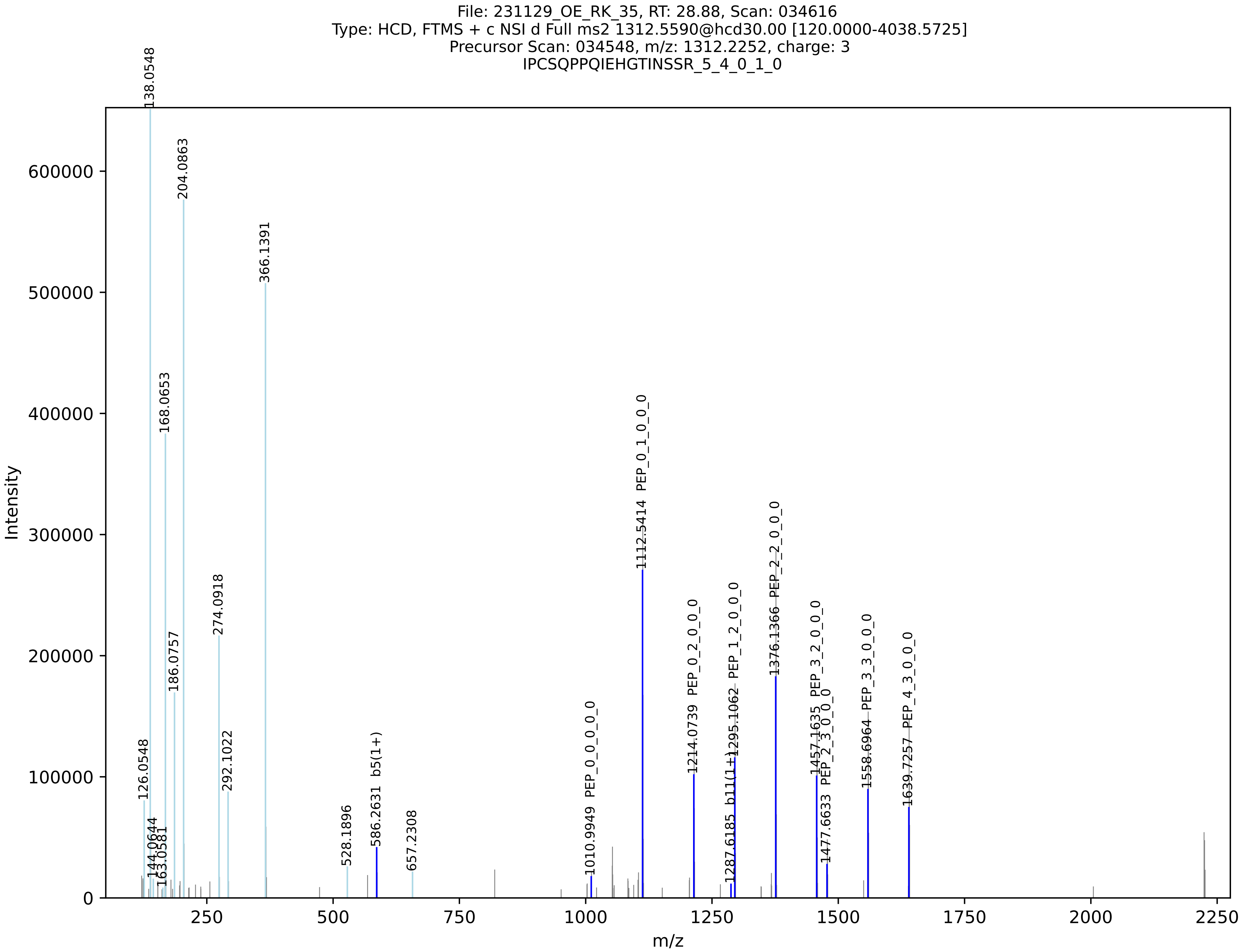

File: 231129_OE_RK_35, RT: 28.88, Scan: 034616
Type: HCD, FTMS + c NSI d Full ms2 1312.5590@hcd30.00 [120.0000-4038.5725]
Precursor Scan: 034548, m/z: 1312.2252, charge: 3
IPCSQPPQIEHGTINSSR_5_4_0_1_0
Intensity
m/z
138.0548
204.0863
366.1391
168.0653
186.0757
274.0918
292.1022
126.0548
144.0644
163.0581
528.1896
586.2631 b5(1+)
657.2308
1010.9949 PEP_0_0_0_0_0
1112.5414 PEP_0_1_0_0_0
1214.0739 PEP_0_2_0_0_0
1287.6185 b11(1+)
1295.1062 PEP_1_2_0_0_0
1376.1366 PEP_2_2_0_0_0
1457.1635 PEP_3_2_0_0_0
1477.6633 PEP_2_3_0_0_0
1558.6964 PEP_3_3_0_0_0
1639.7257 PEP_4_3_0_0_0

File: 231129_OE_RK_35, RT: 28.902, Scan: 034652
Type: HCD, FTMS + c NSI d Full ms2 1059.7490@hcd30.00 [120.0000-3264.9741]
Precursor Scan: 034548, m/z: 1059.749, charge: 3
EEQFNSTFR_6_3_1_1_0

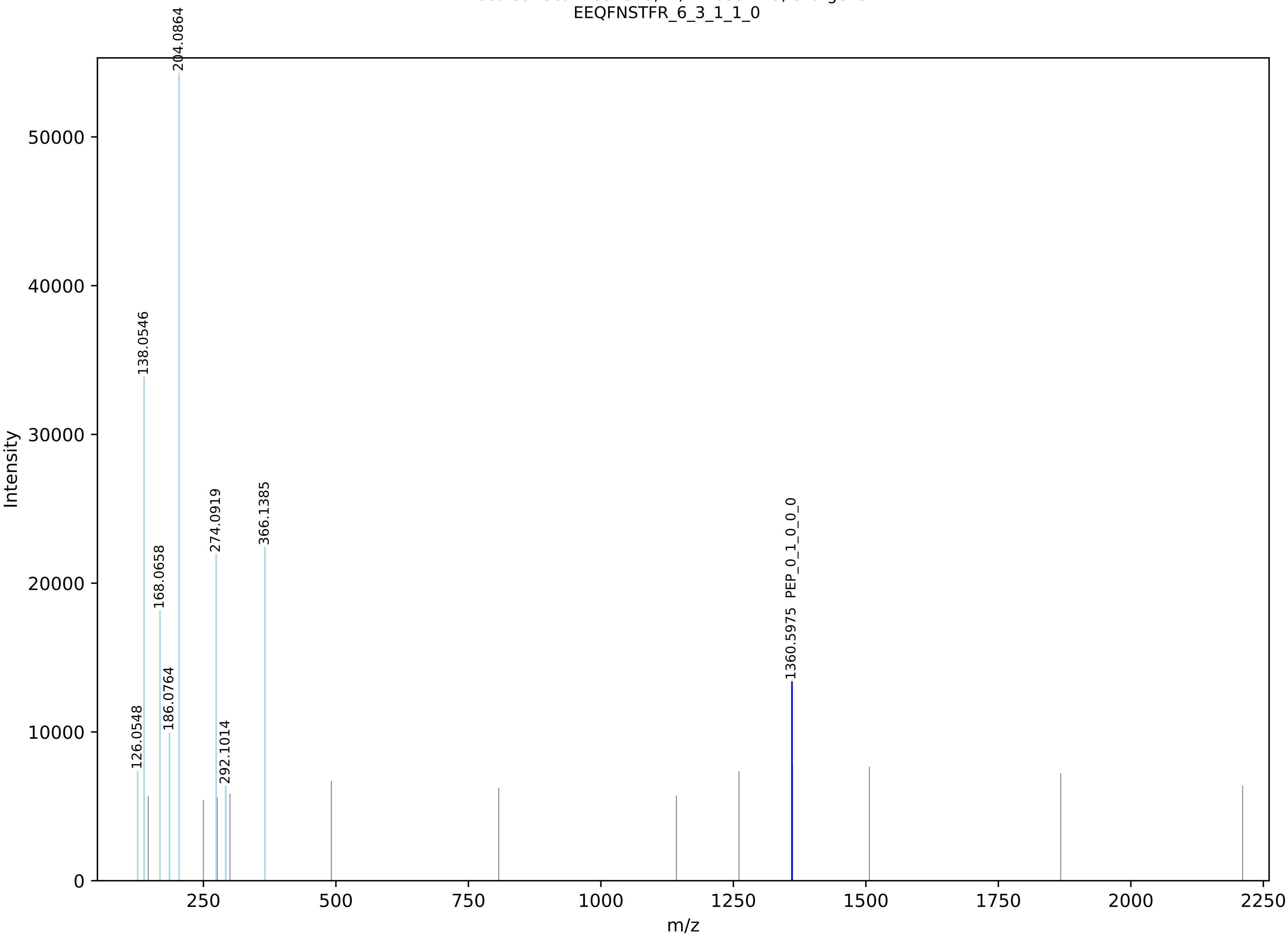

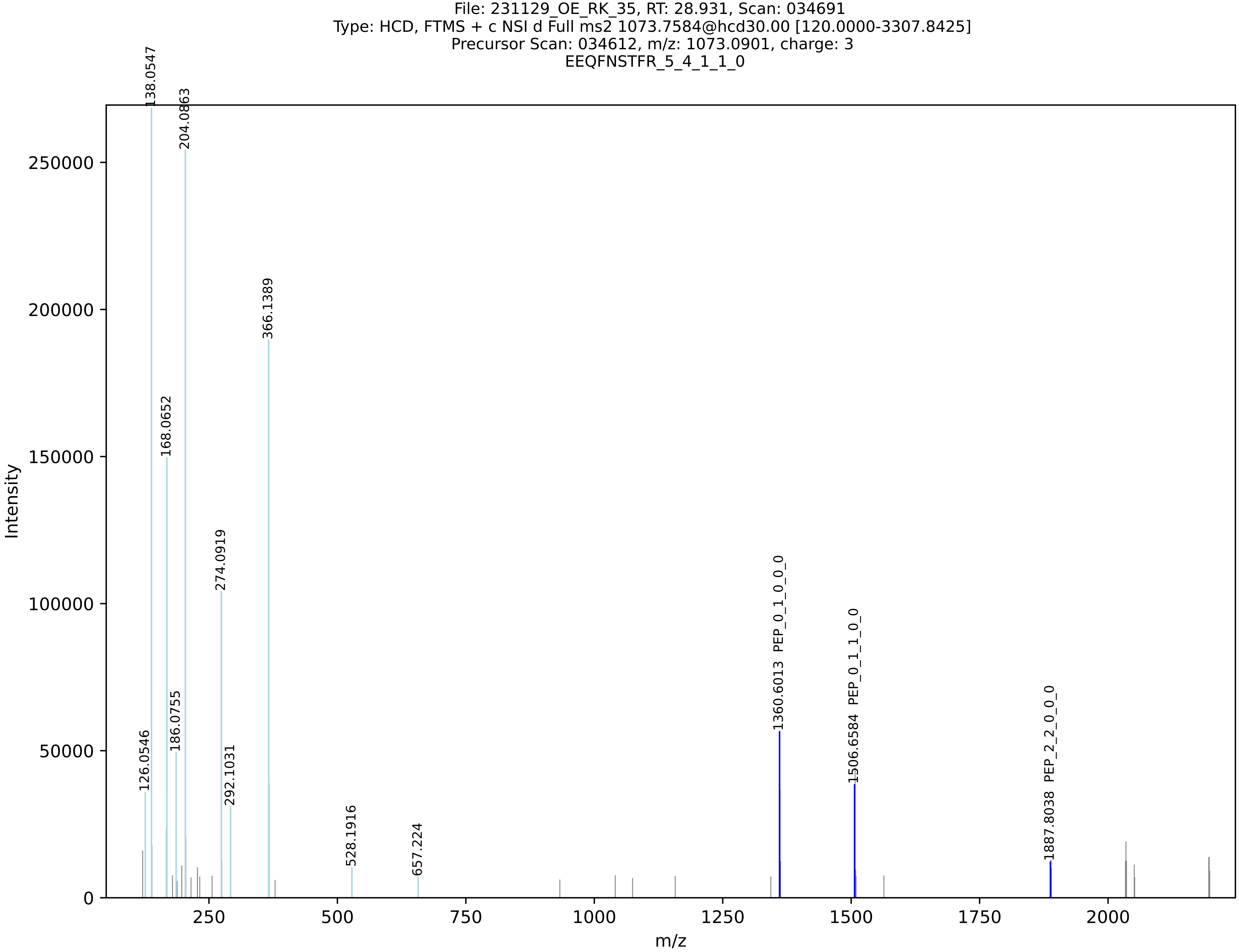
File: 231129_OE_RK_35, RT: 28.931, Scan: 034691
Type: HCD, FTMS + c NSI d Full ms2 1073.7584@hcd30.00 [120.0000-3307.8425]
Precursor Scan: 034612, m/z: 1073.0901, charge: 3
EEQFNSTFR_5_4_1_1_0
Intensity
m/z
138.0547
204.0863
366.1389
168.0652
274.0919
186.0755
126.0546
292.1031
528.1916
657.224
1360.6013 PEP_0_1_0_0_0
1506.6584 PEP_0_1_1_0_0
1887.8038 PEP_2_2_0_0_0
0
50000
100000
150000
200000
250000
250
500
750
1000
1250
1500
1750
2000

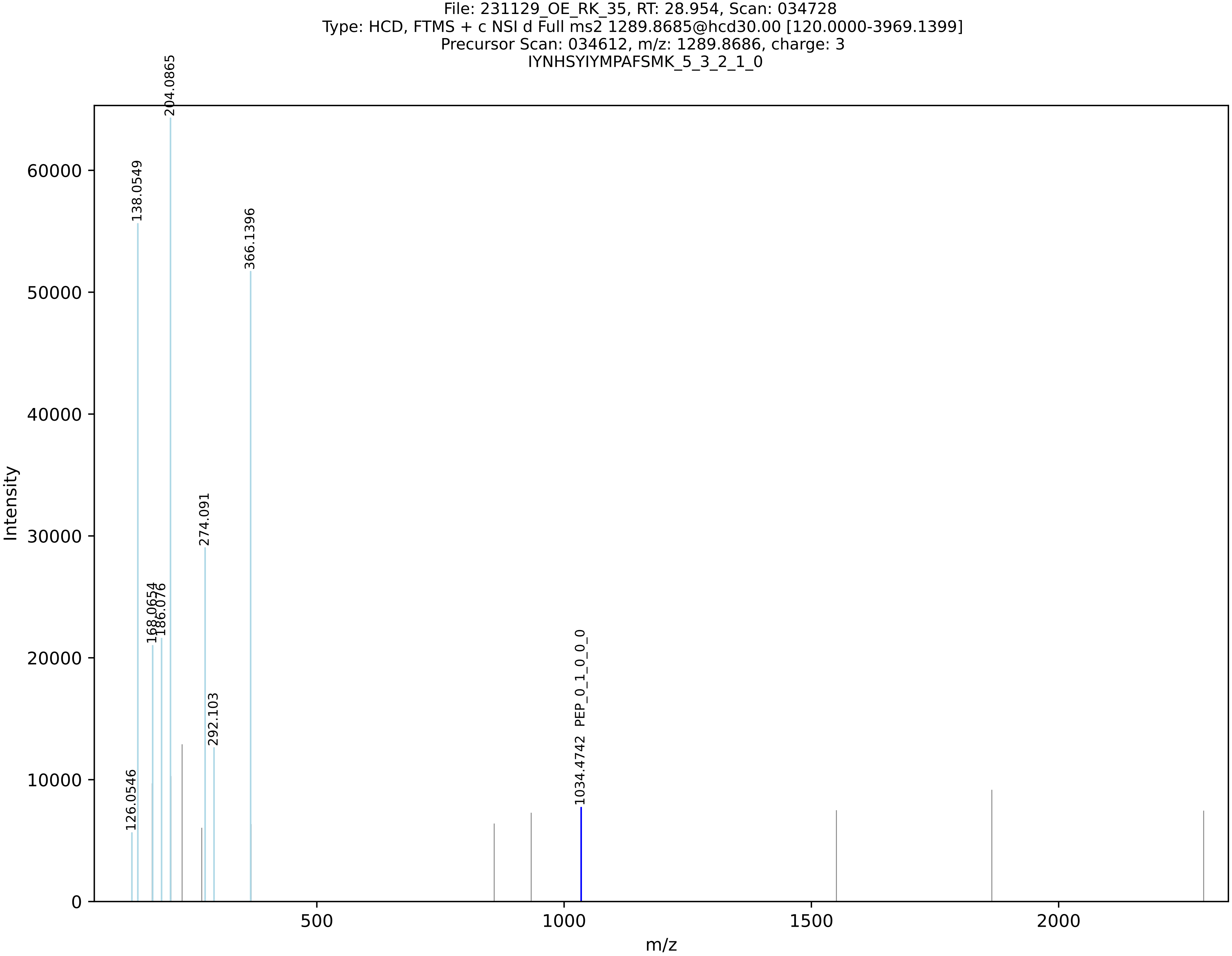
File: 231129_OE_RK_35, RT: 28.954, Scan: 034728
Type: HCD, FTMS + c NSI d Full ms2 1289.8685@hcd30.00 [120.0000-3969.1399]
Precursor Scan: 034612, m/z: 1289.8686, charge: 3
IYNHSYIYMPAFSMK_5_3_2_1_0
204.0865
138.0549
366.1396
274.091
168.0654
186.076
292.103
126.0546
1034.4742 PEP_0_1_0_0_0
Intensity
m/z
0
10000
20000
30000
40000
50000
60000
500
1000
1500
2000

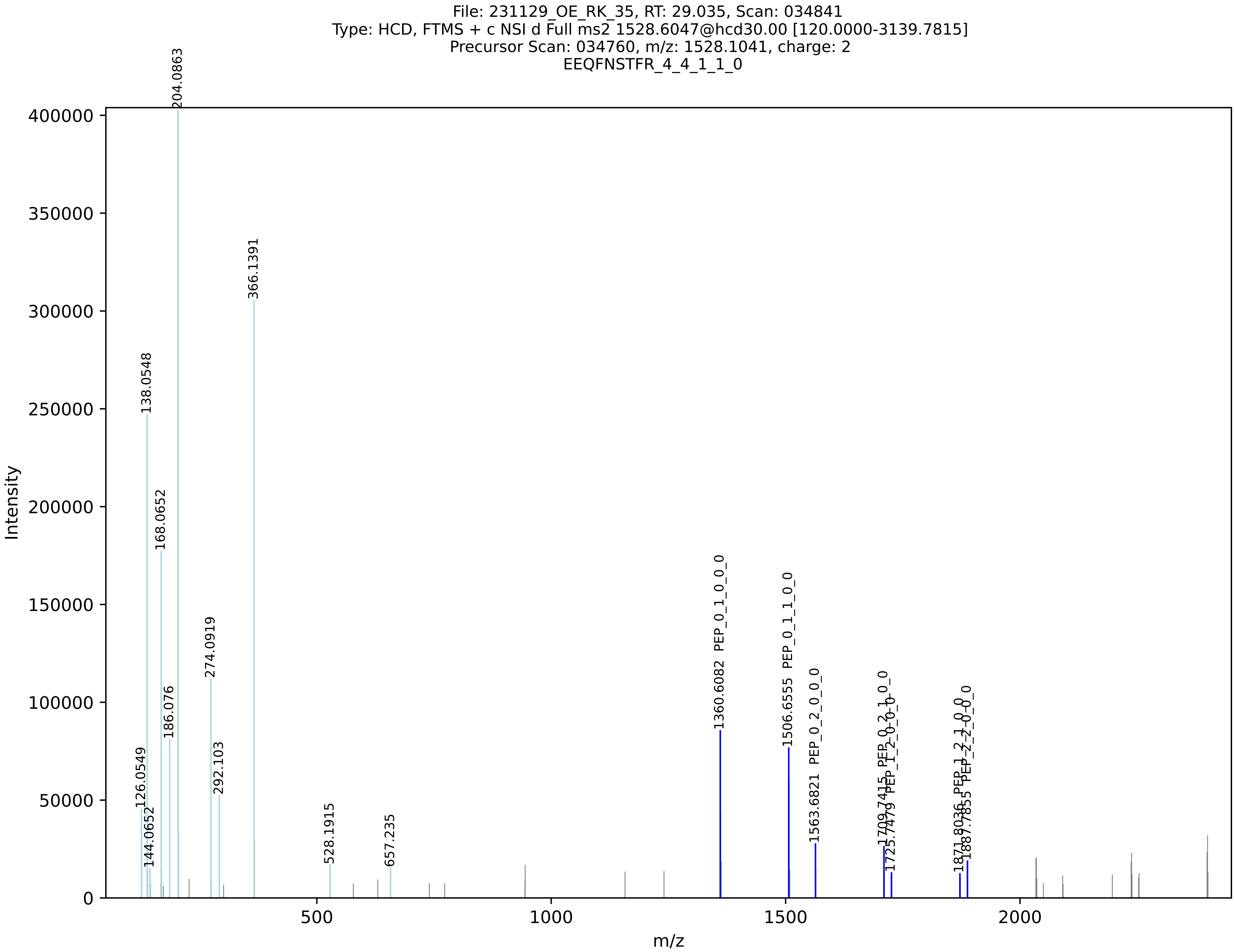
File: 231129_OE_RK_35, RT: 29.035, Scan: 034841
Type: HCD, FTMS + c NSI d Full ms2 1528.6047@hcd30.00 [120.0000-3139.7815]
Precursor Scan: 034760, m/z: 1528.1041, charge: 2
EEQFNSTFR_4_4_1_1_0
Intensity
m/z
0
50000
100000
150000
200000
250000
300000
350000
400000
500
1000
1500
2000
204.0863
366.1391
138.0548
168.0652
274.0919
186.076
292.103
126.0549
144.0652
528.1915
657.235
1360.6082 PEP_0_1_0_0_0
1506.6555 PEP_0_1_1_0_0
1563.6821 PEP_0_2_0_0_0
1709.7415 PEP_0_2_1_0_0
1725.7479 PEP_1_2_0_0_0
1871.8036 PEP_1_2_1_0_0
1887.7855 PEP_2_2_0_0_0

File: 231129_OE_RK_35, RT: 29.044, Scan: 034855
Type: HCD, FTMS + c NSI d Full ms2 1357.2225@hcd30.00 [120.0000-4175.2432]
Precursor Scan: 034760, m/z: 1357.2226, charge: 3
FNSSYLQGTNQITGR_7_4_1_1_0

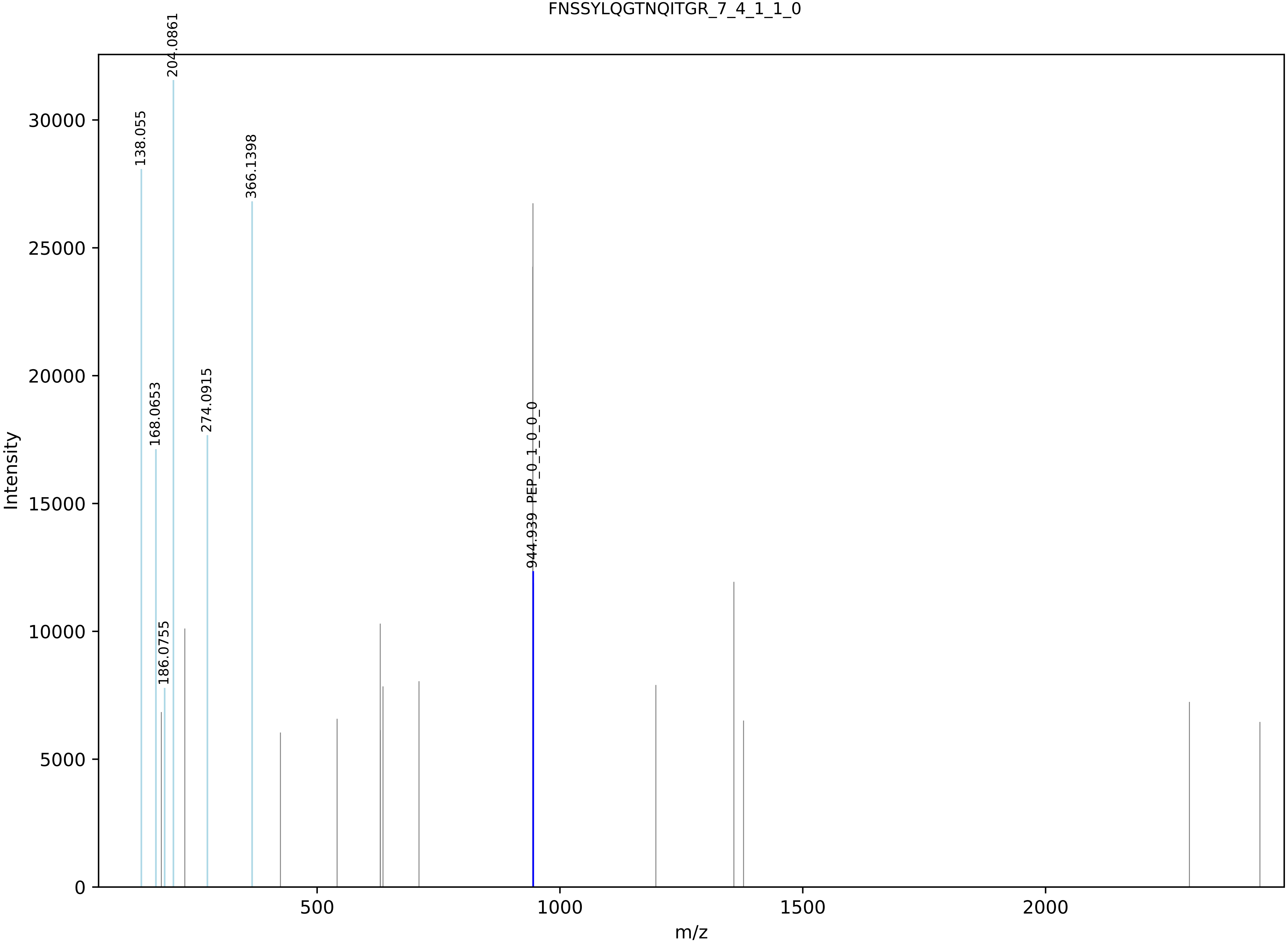

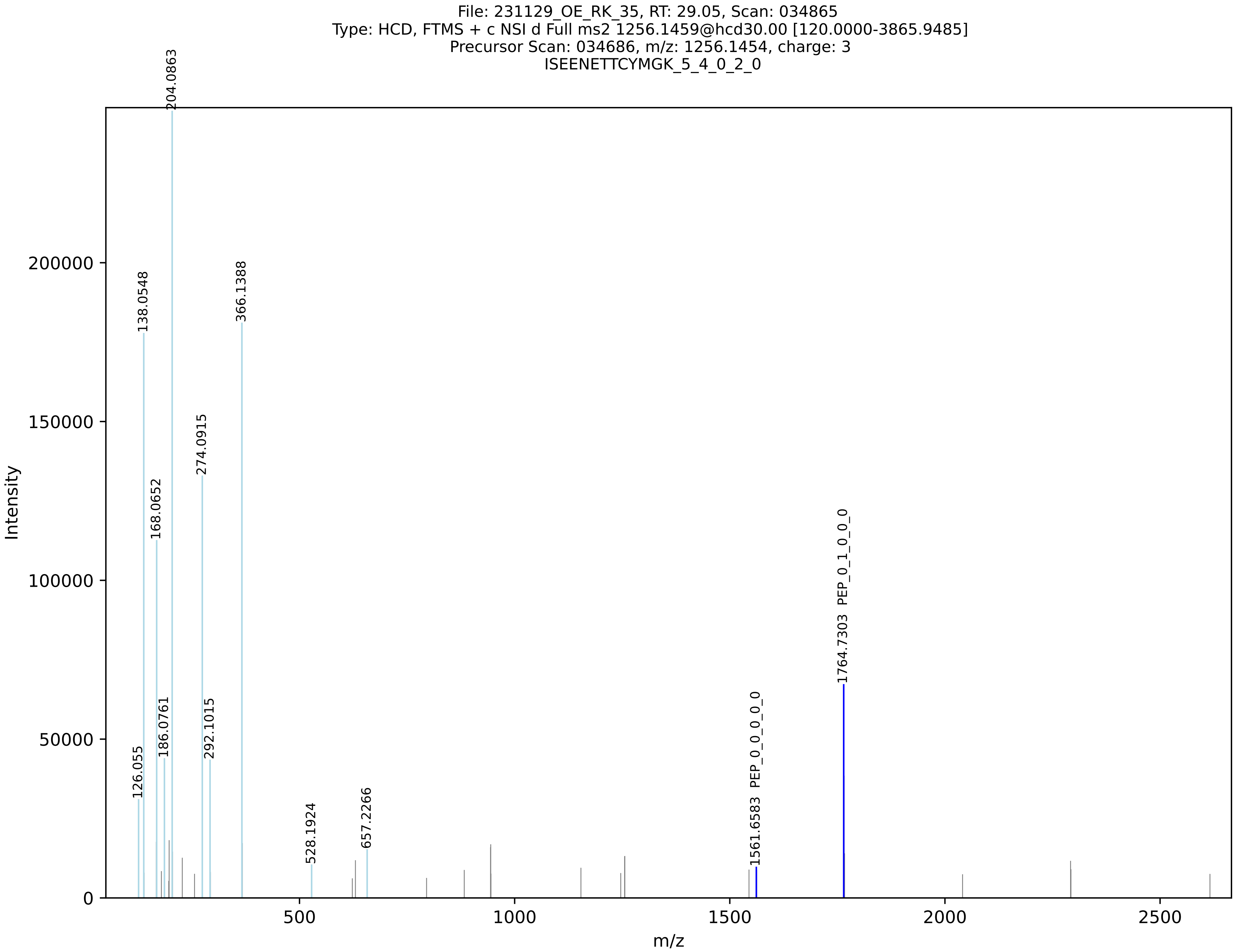
File: 231129_OE_RK_35, RT: 29.05, Scan: 034865
Type: HCD, FTMS + c NSI d Full ms2 1256.1459@hcd30.00 [120.0000-3865.9485]
Precursor Scan: 034686, m/z: 1256.1454, charge: 3
ISEENETTCYMGK_5_4_0_2_0
204.0863
138.0548
366.1388
274.0915
168.0652
186.0761
292.1015
126.055
528.1924
657.2266
1561.6583 PEP_0_0_0_0_0
1764.7303 PEP_0_1_0_0_0
Intensity
m/z
0
50000
100000
150000
200000
500
1000
1500
2000
2500

File: 231129_OE_RK_35, RT: 29.079, Scan: 034904
Type: HCD, FTMS + c NSI d Full ms2 1312.5593@hcd30.00 [120.0000-4038.5735]
Precursor Scan: 034830, m/z: 1312.2252, charge: 3
IPCSQPPQIEHGTINSSR_5_4_0_1_0

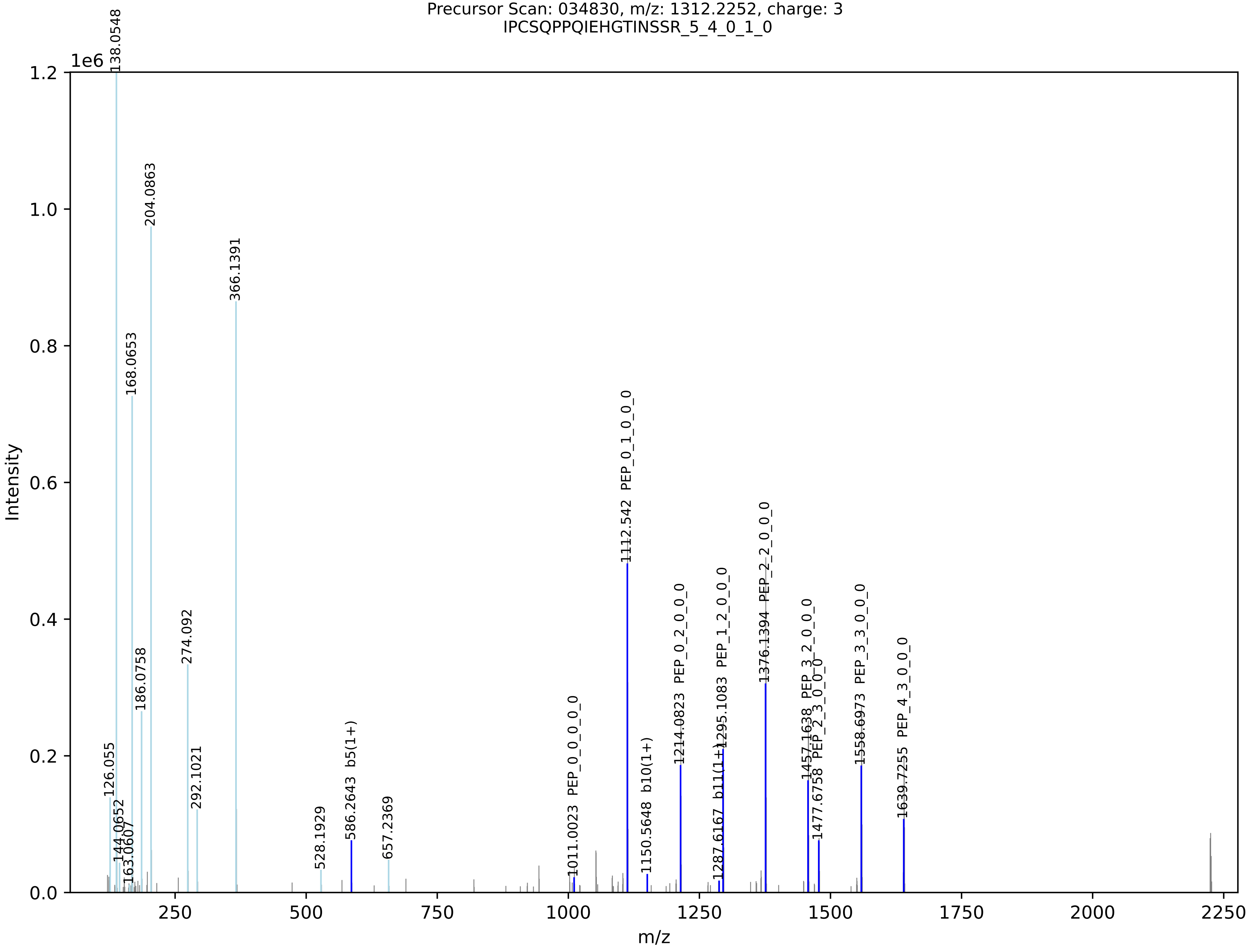

File: 231129_OE_RK_35, RT: 29.084, Scan: 034913
Type: HCD, FTMS + c NSI d Full ms2 1256.8123@hcd30.00 [120.0000-3867.9873]
Precursor Scan: 034830, m/z: 1256.1471, charge: 3
ISEENETTCYMGK_5_4_0_2_0

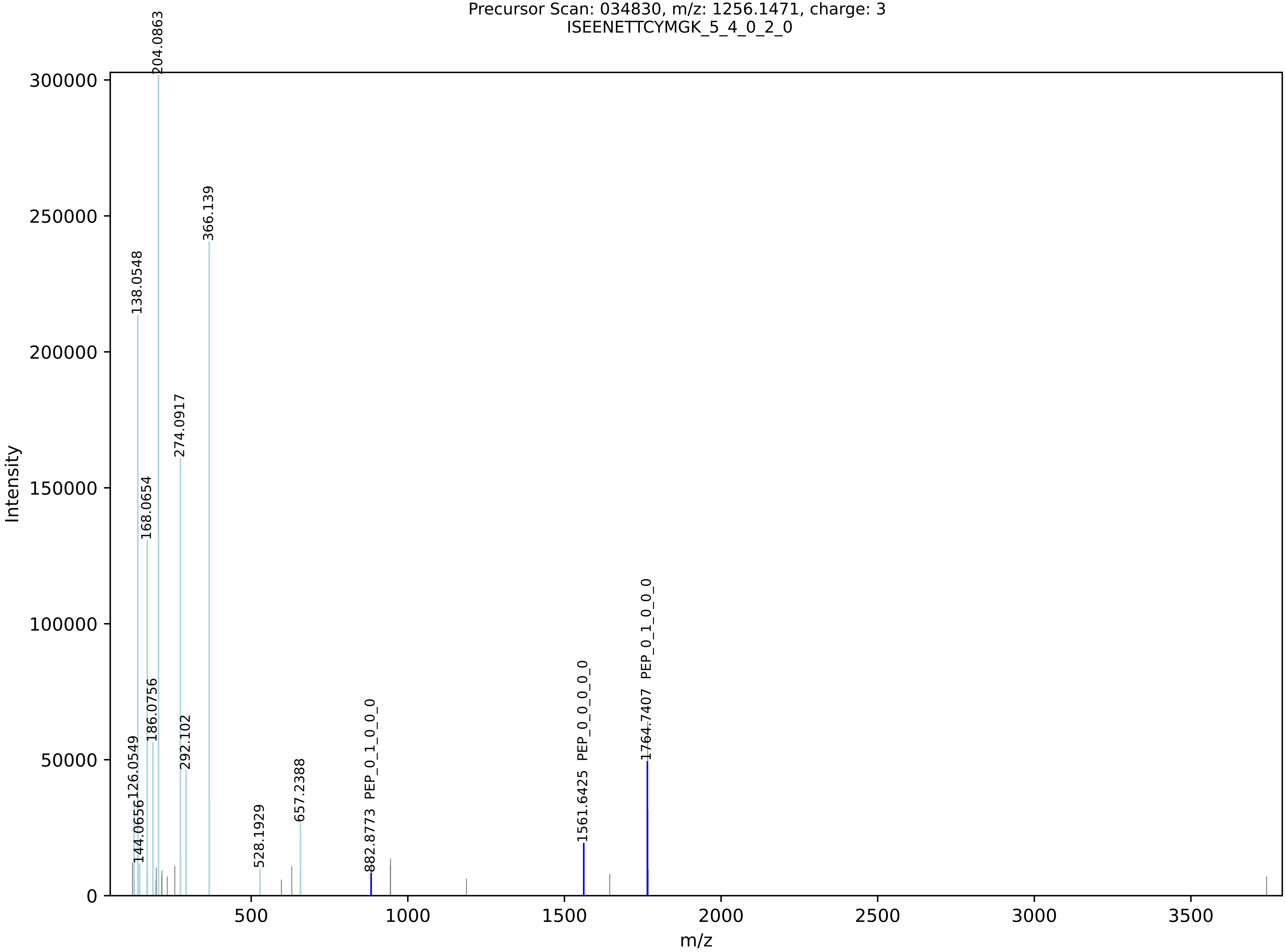

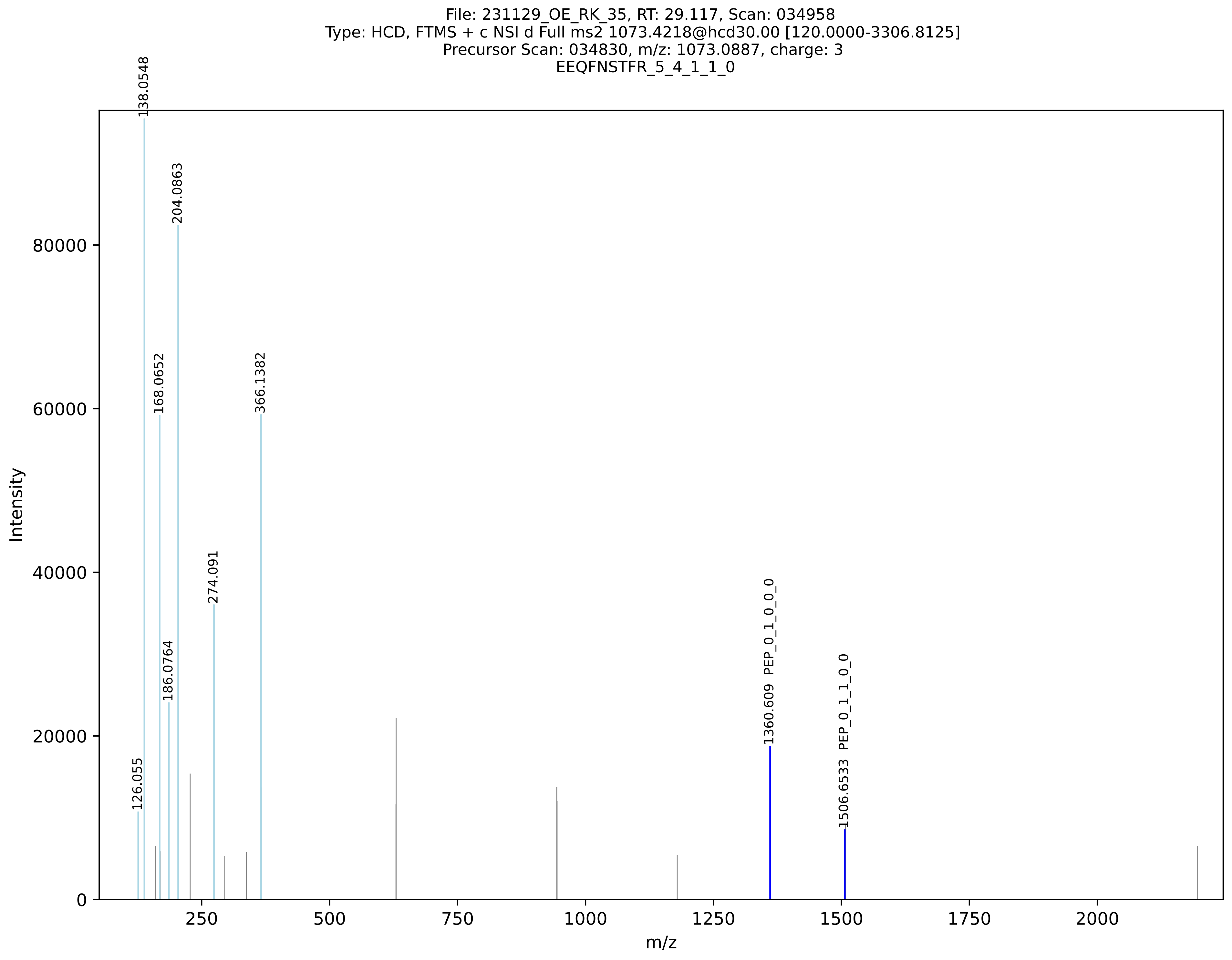

File: 231129_OE_RK_35, RT: 29.117, Scan: 034958
Type: HCD, FTMS + c NSI d Full ms2 1073.4218@hcd30.00 [120.0000-3306.8125]
Precursor Scan: 034830, m/z: 1073.0887, charge: 3
EEQFNSTFR_5_4_1_1_0
138.0548
204.0863
168.0652
366.1382
274.091
186.0764
126.055
1360.609 PEP_0_1_0_0_0
1506.6533 PEP_0_1_1_0_0
Intensity
m/z
0
20000
40000
60000
80000
250
500
750
1000
1250
1500
1750
2000

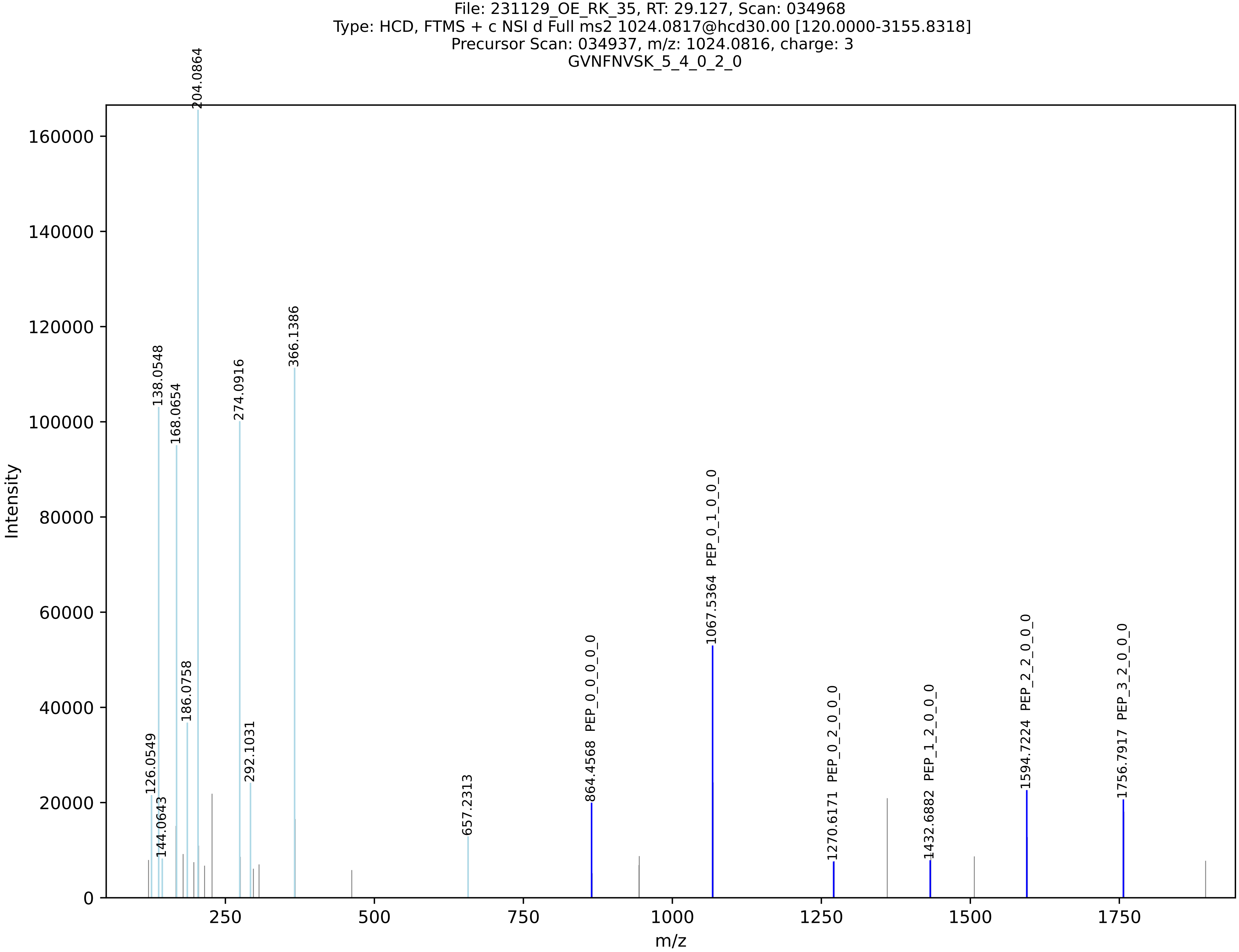

File: 231129_OE_RK_35, RT: 29.127, Scan: 034968
Type: HCD, FTMS + c NSI d Full ms2 1024.0817@hcd30.00 [120.0000-3155.8318]
Precursor Scan: 034937, m/z: 1024.0816, charge: 3
GVNFNVSK_5_4_0_2_0
Intensity
m/z
0
20000
40000
60000
80000
100000
120000
140000
160000
250
500
750
1000
1250
1500
1750
204.0864
138.0548
168.0654
274.0916
366.1386
186.0758
126.0549
144.0643
292.1031
657.2313
864.4568 PEP_0_0_0_0_0
1067.5364 PEP_0_1_0_0_0
1270.6171 PEP_0_2_0_0_0
1432.6882 PEP_1_2_0_0_0
1594.7224 PEP_2_2_0_0_0
1756.7917 PEP_3_2_0_0_0

File: 231129_OE_RK_35, RT: 29.218, Scan: 035087
Type: HCD, FTMS + c NSI d Full ms2 1018.7639@hcd30.00 [120.0000-3139.5593]
Precursor Scan: 035049, m/z: 1018.7638, charge: 3
EEQFNSTFR_4_4_1_1_0

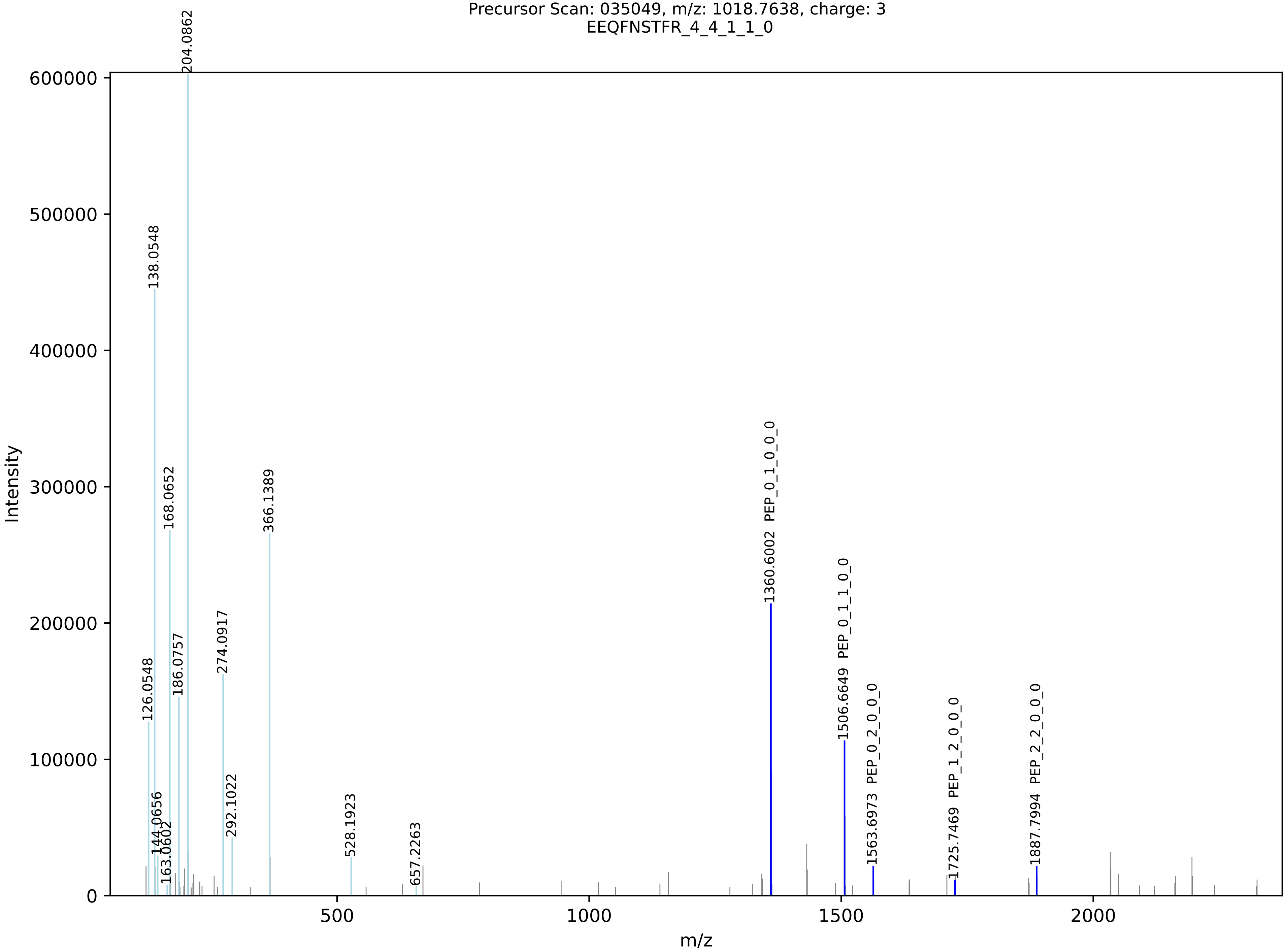

File: 231129_OE_RK_35, RT: 29.264, Scan: 035147
Type: HCD, FTMS + c NSI d Full ms2 1312.5586@hcd30.00 [120.0000-4038.5713]
Precursor Scan: 035109, m/z: 1312.225, charge: 3
IPCSQPPQIEHGTINSSR_5_4_0_1_0

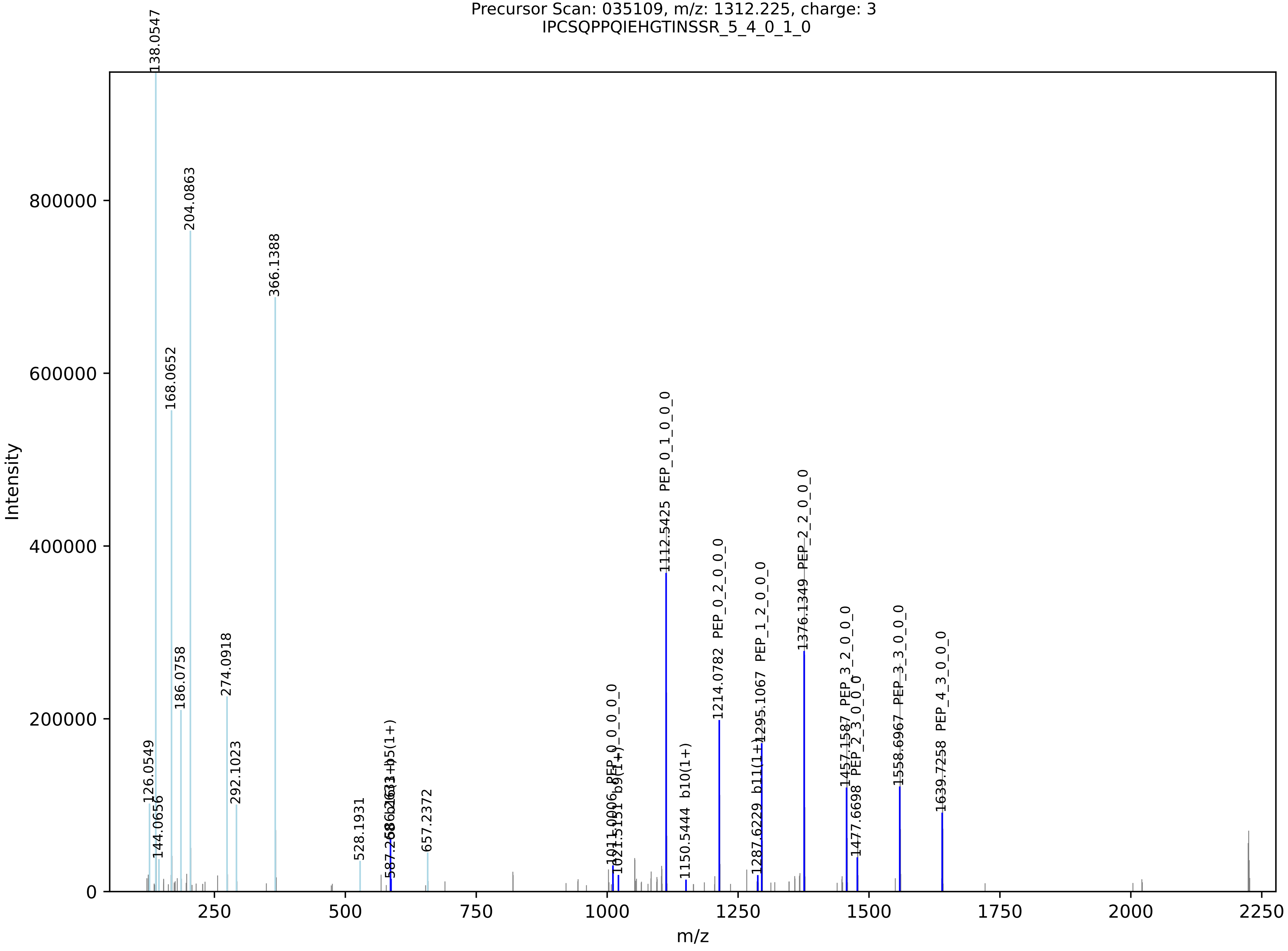

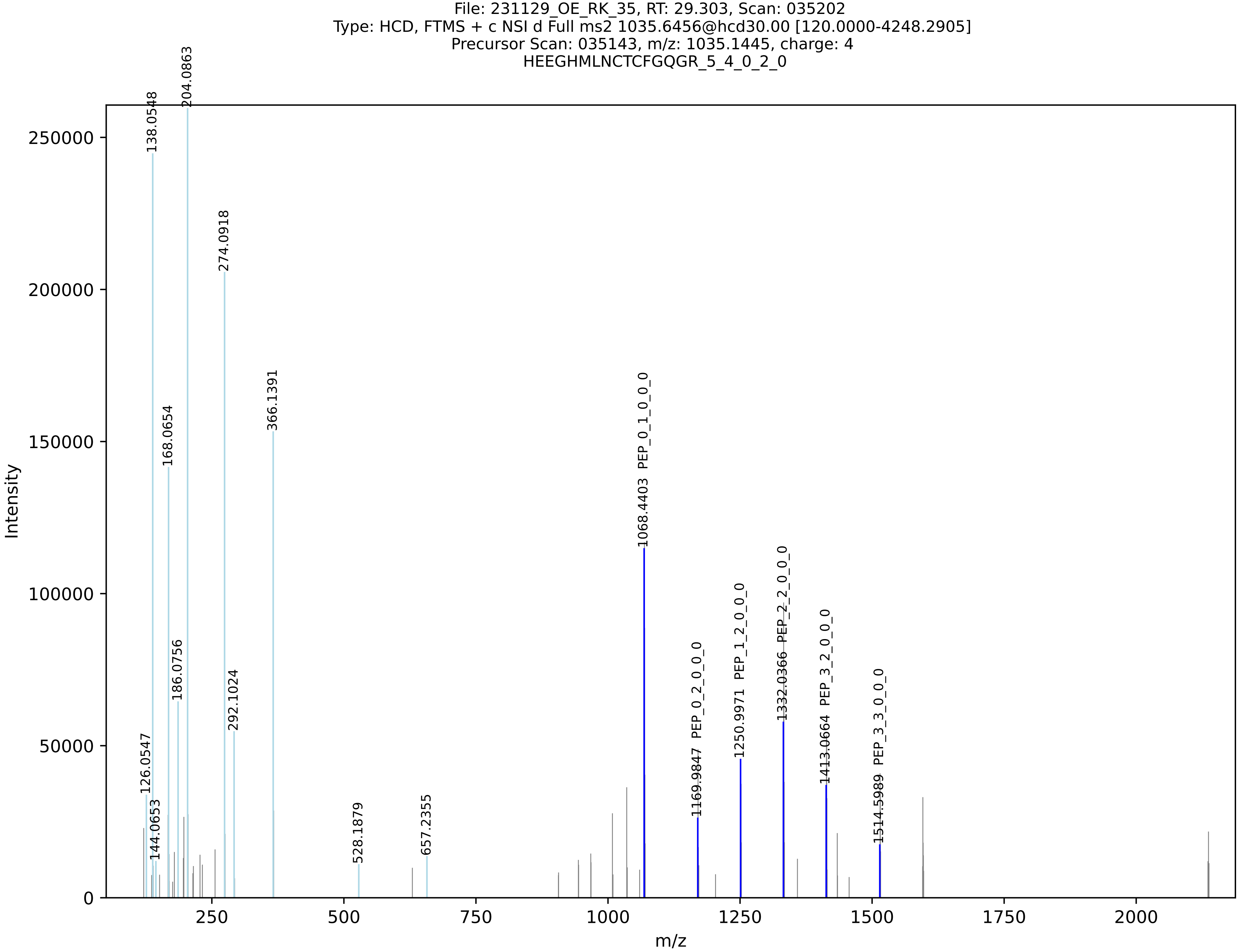

File: 231129_OE_RK_35, RT: 29.303, Scan: 035202
Type: HCD, FTMS + c NSI d Full ms2 1035.6456@hcd30.00 [120.0000-4248.2905]
Precursor Scan: 035143, m/z: 1035.1445, charge: 4
HEEGHMLNCTCFGQGR_5_4_0_2_0
Intensity
m/z
138.0548
204.0863
274.0918
168.0654
366.1391
186.0756
292.1024
126.0547
144.0653
528.1879
657.2355
1068.4403 PEP_0_1_0_0_0
1169.9847 PEP_0_2_0_0_0
1250.9971 PEP_1_2_0_0_0
1332.0366 PEP_2_2_0_0_0
1413.0664 PEP_3_2_0_0_0
1514.5989 PEP_3_3_0_0_0

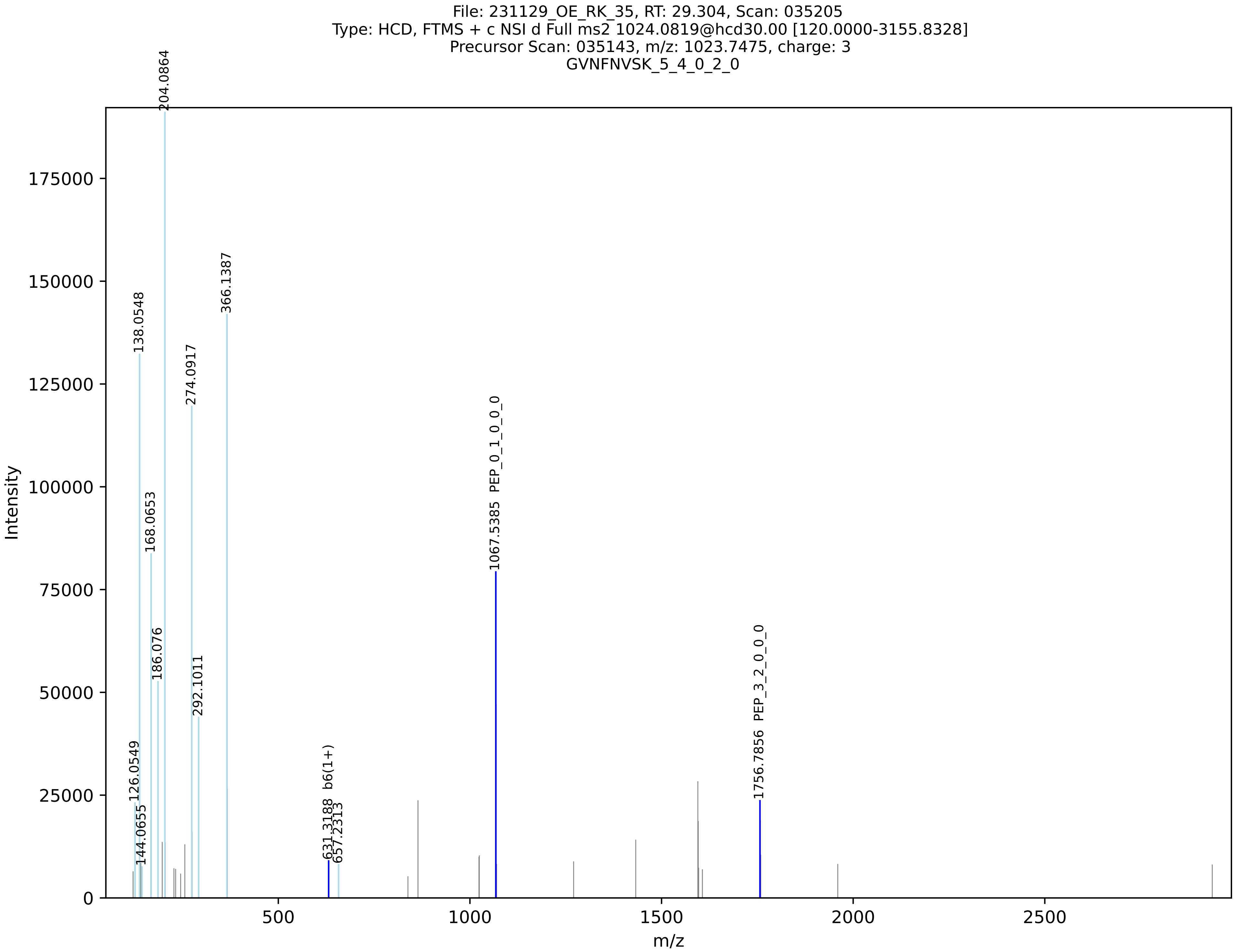
File: 231129_OE_RK_35, RT: 29.304, Scan: 035205
Type: HCD, FTMS + c NSI d Full ms2 1024.0819@hcd30.00 [120.0000-3155.8328]
Precursor Scan: 035143, m/z: 1023.7475, charge: 3
GVNFNVSK_5_4_0_2_0
Intensity
m/z
204.0864
366.1387
138.0548
274.0917
168.0653
186.076
292.1011
126.0549
144.0655
631.3188 b6(1+)
657.2313
1067.5385 PEP_0_1_0_0_
1756.7856 PEP_3_2_0_0_
0
25000
50000
75000
100000
125000
150000
175000
500
1000
1500
2000
2500

File: 231129_OE_RK_35, RT: 29.361, Scan: 035290
Type: HCD, FTMS + c NSI d Full ms2 1020.1027@hcd30.00 [120.0000-3143.6565]
Precursor Scan: 035143, m/z: 1018.7631, charge: 3
YQHNESHSR_4_4_1_1_0

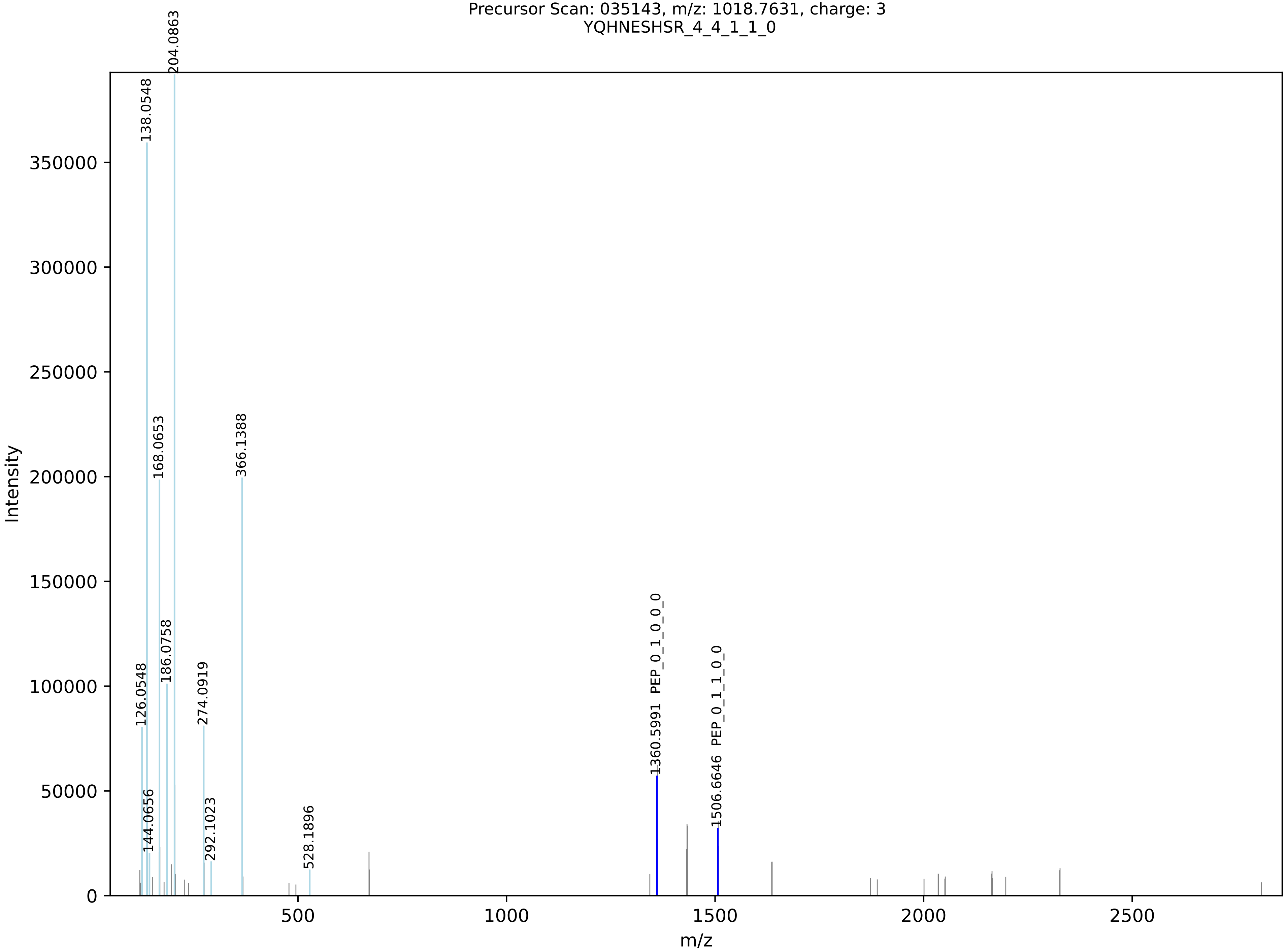

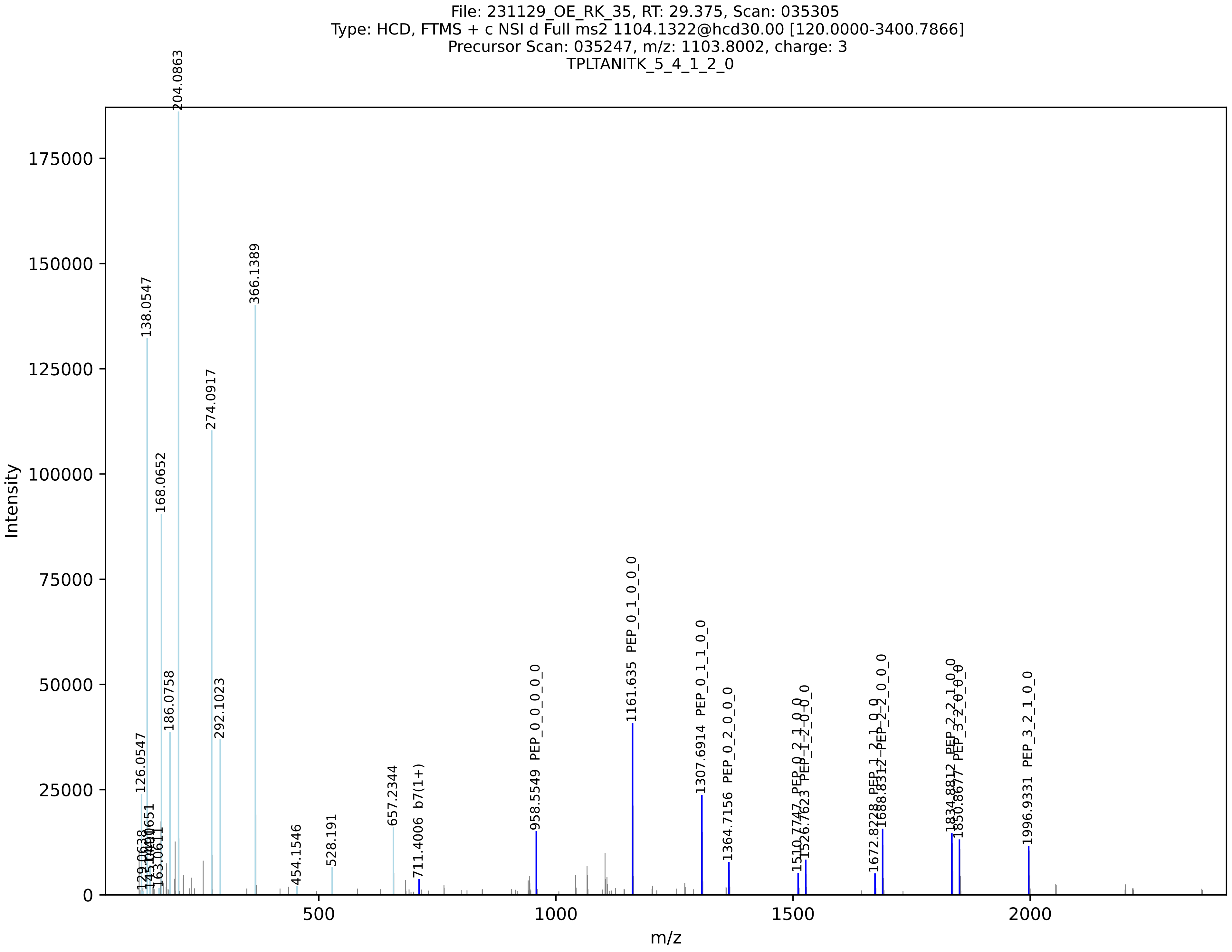

File: 231129_OE_RK_35, RT: 29.375, Scan: 035305
Type: HCD, FTMS + c NSI d Full ms2 1104.1322@hcd30.00 [120.0000-3400.7866]
Precursor Scan: 035247, m/z: 1103.8002, charge: 3
TPLTANITK_5_4_1_2_0
Intensity
m/z
204.0863
366.1389
138.0547
274.0917
168.0652
186.0758
292.1023
126.0547
454.1546
528.191
657.2344
711.4006 b7(1+)
958.5549 PEP_0_0_0_0_0
1161.635 PEP_0_1_0_0_0
1307.6914 PEP_0_1_1_0_0
1364.7156 PEP_0_2_0_0_0
1996.9331 PEP_3_2_1_0_0

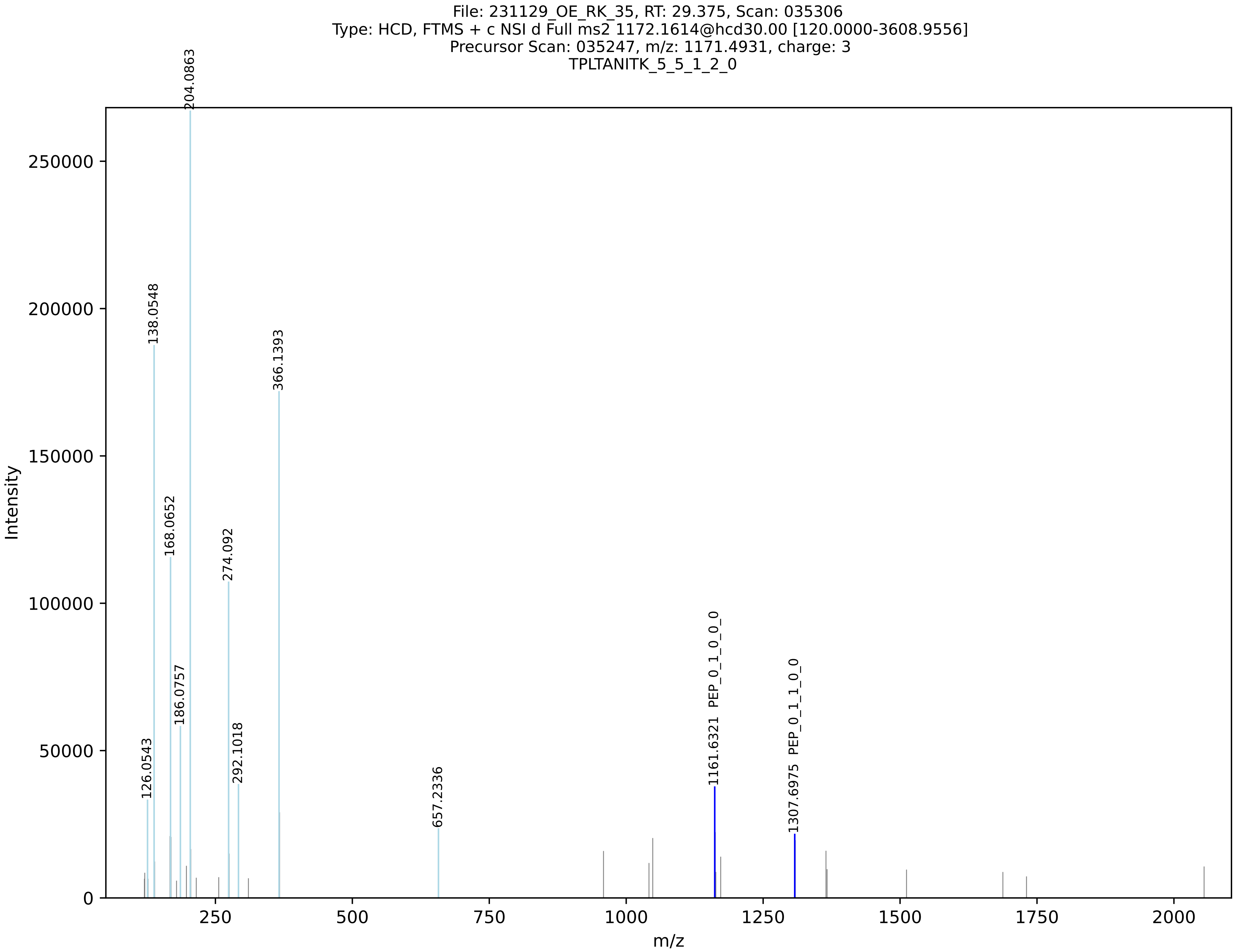
File: 231129_OE_RK_35, RT: 29.375, Scan: 035306
Type: HCD, FTMS + c NSI d Full ms2 1172.1614@hcd30.00 [120.0000-3608.9556]
Precursor Scan: 035247, m/z: 1171.4931, charge: 3
TPLTANITK_5_5_1_2_0
204.0863
138.0548
366.1393
168.0652
274.092
186.0757
126.0543
292.1018
657.2336
1161.6321 PEP_0_1_0_0_0
1307.6975 PEP_0_1_1_0_0
Intensity
m/z
0
50000
100000
150000
200000
250000
250
500
750
1000
1250
1500
1750
2000

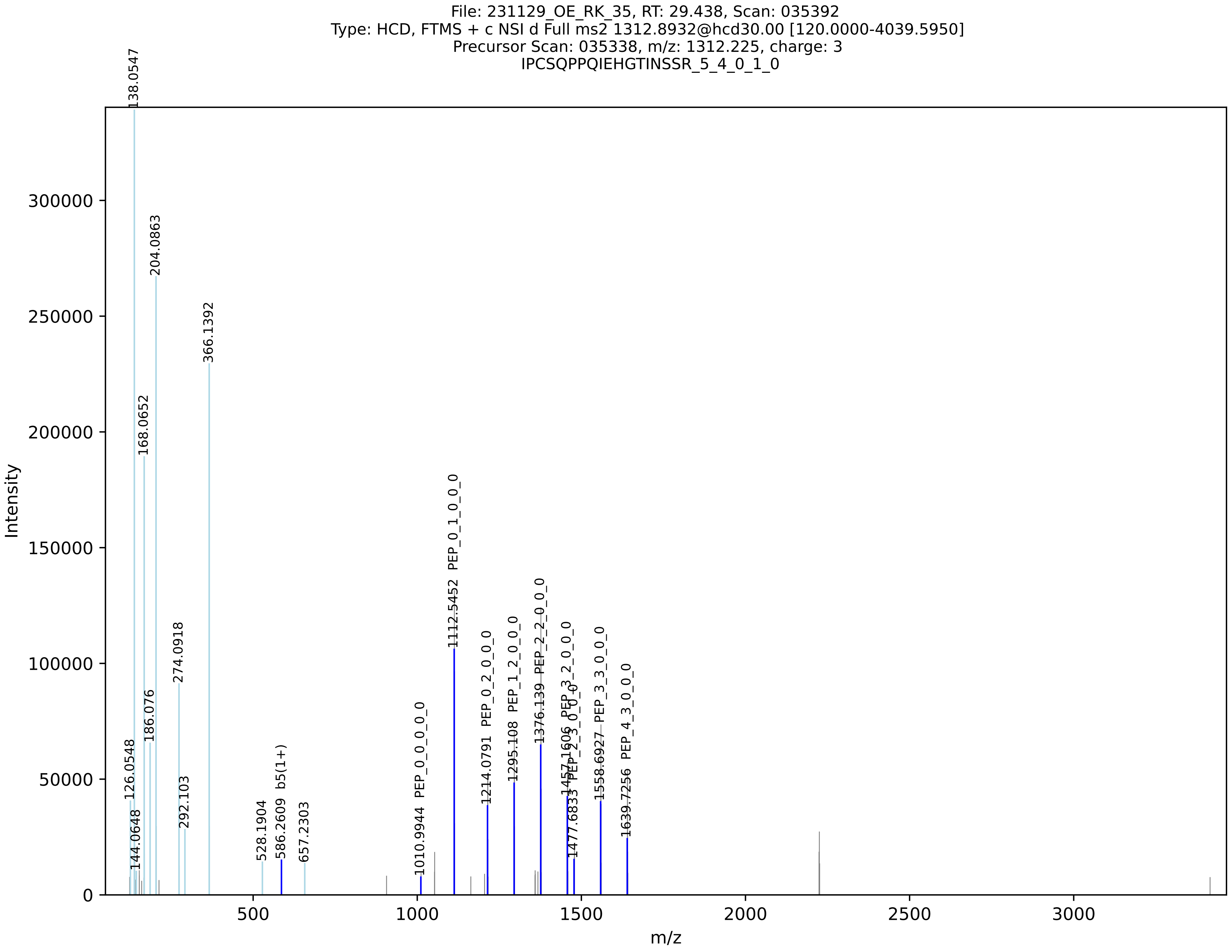

File: 231129_OE_RK_35, RT: 29.438, Scan: 035392
Type: HCD, FTMS + c NSI d Full ms2 1312.8932@hcd30.00 [120.0000-4039.5950]
Precursor Scan: 035338, m/z: 1312.225, charge: 3
IPCSQPPQIEHGTINSSR_5_4_0_1_0
Intensity
m/z
138.0547
204.0863
366.1392
168.0652
274.0918
186.076
126.0548
292.103
144.0648
528.1904
586.2609 b5(1+)
657.2303
1010.9944 PEP_0_0_0_0_0
1112.5452 PEP_0_1_0_0_0
1214.0791 PEP_0_2_0_0_0
1295.108 PEP_1_2_0_0_0
1376.139 PEP_2_2_0_0_0
1457.1606 PEP_3_2_0_0_0
1477.6833 PEP_2_3_0_0_0
1558.6927 PEP_3_3_0_0_0
1639.7256 PEP_4_3_0_0_0

File: 231129_OE_RK_35, RT: 29.447, Scan: 035407
Type: HCD, FTMS + c NSI d Full ms2 1380.5243@hcd30.00 [120.0000-4246.5464]
Precursor Scan: 035338, m/z: 1379.8577, charge: 3
HEEGHMLNCTCFGQGR_5_4_0_2_0

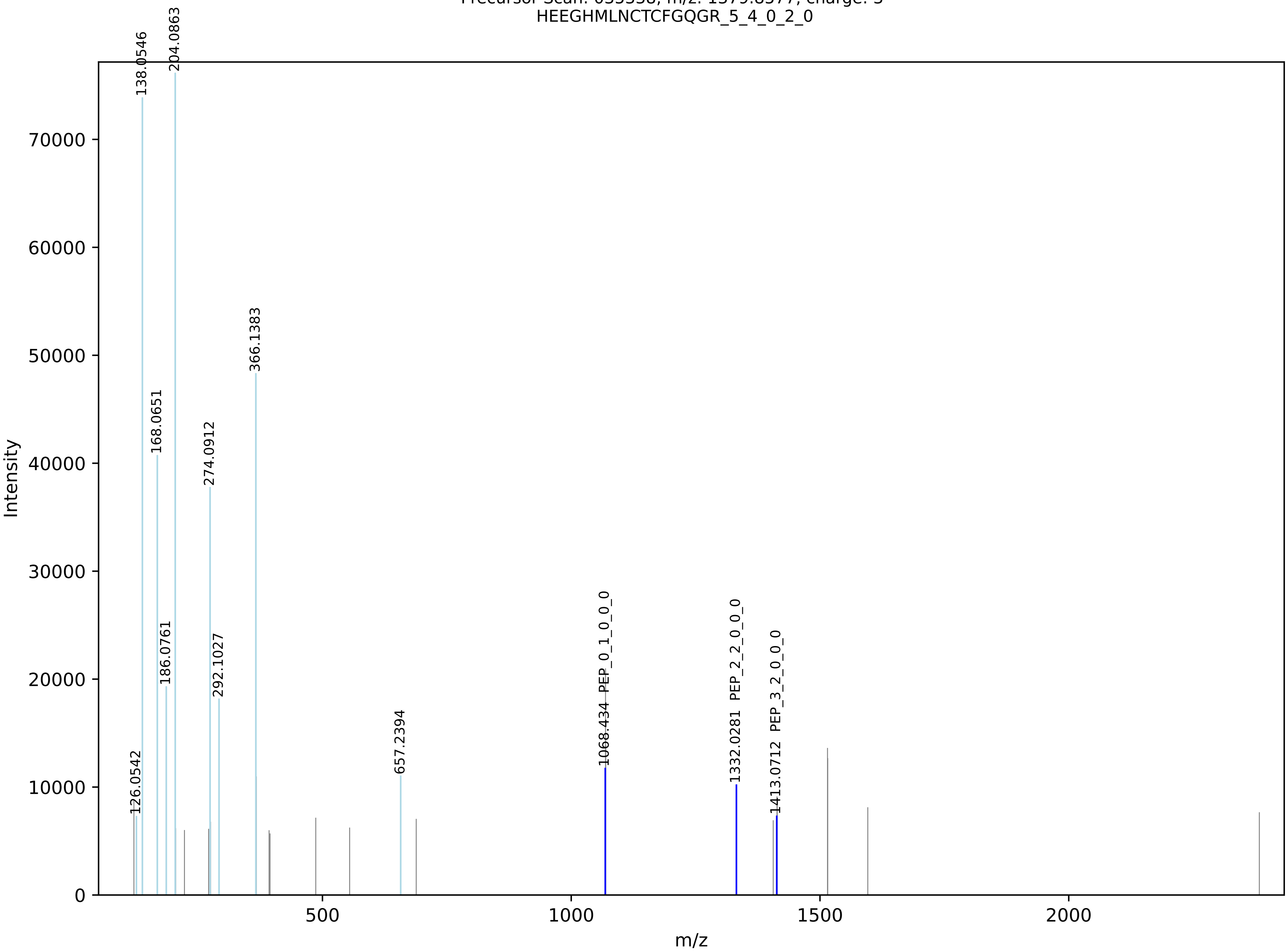

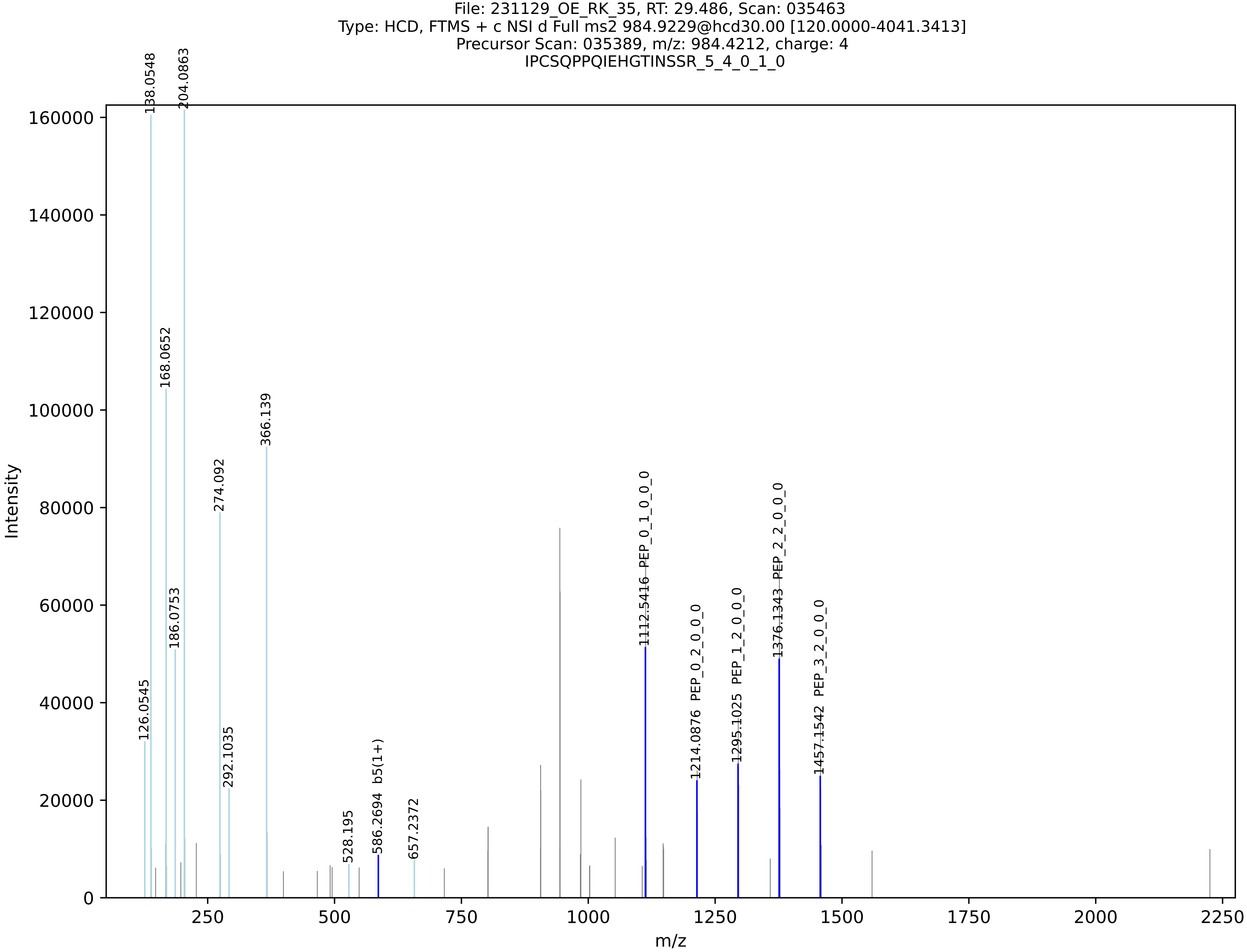
File: 231129_OE_RK_35, RT: 29.486, Scan: 035463
Type: HCD, FTMS + c NSI d Full ms2 984.9229@hcd30.00 [120.0000-4041.3413]
Precursor Scan: 035389, m/z: 984.4212, charge: 4
IPCSQPPQIEHGTINSSR_5_4_0_1_0
Intensity
m/z
138.0548
204.0863
168.0652
366.139
274.092
186.0753
126.0545
292.1035
528.195
586.2694 b5(1+)
657.2372
1112.5416 PEP_0_1_0_0_0
1214.0876 PEP_0_2_0_0_0
1295.1025 PEP_1_2_0_0_0
1376.1343 PEP_2_2_0_0_0
1457.1542 PEP_3_2_0_0_0

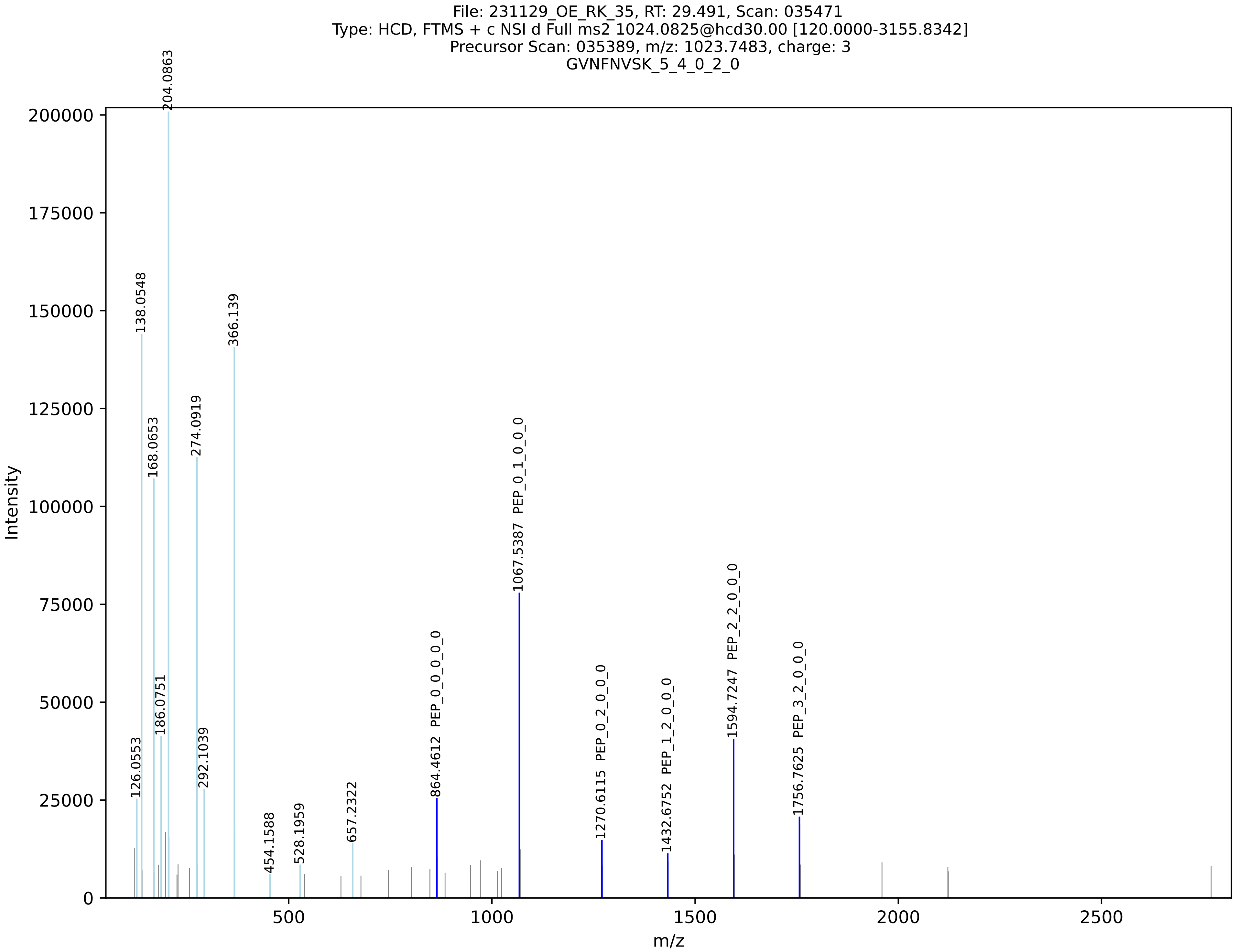

File: 231129_OE_RK_35, RT: 29.491, Scan: 035471
Type: HCD, FTMS + c NSI d Full ms2 1024.0825@hcd30.00 [120.0000-3155.8342]
Precursor Scan: 035389, m/z: 1023.7483, charge: 3
GVNFNVSK_5_4_0_2_0
Intensity
m/z
0
25000
50000
75000
100000
125000
150000
175000
200000
500
1000
1500
2000
2500
204.0863
138.0548
366.139
168.0653
274.0919
186.0751
126.0553
292.1039
454.1588
528.1959
657.2322
864.4612 PEP_0_0_0_0_0_
1067.5387 PEP_0_1_0_0_0_
1270.6115 PEP_0_2_0_0_0_
1432.6752 PEP_1_2_0_0_0_
1594.7247 PEP_2_2_0_0_0_
1756.7625 PEP_3_2_0_0_0_

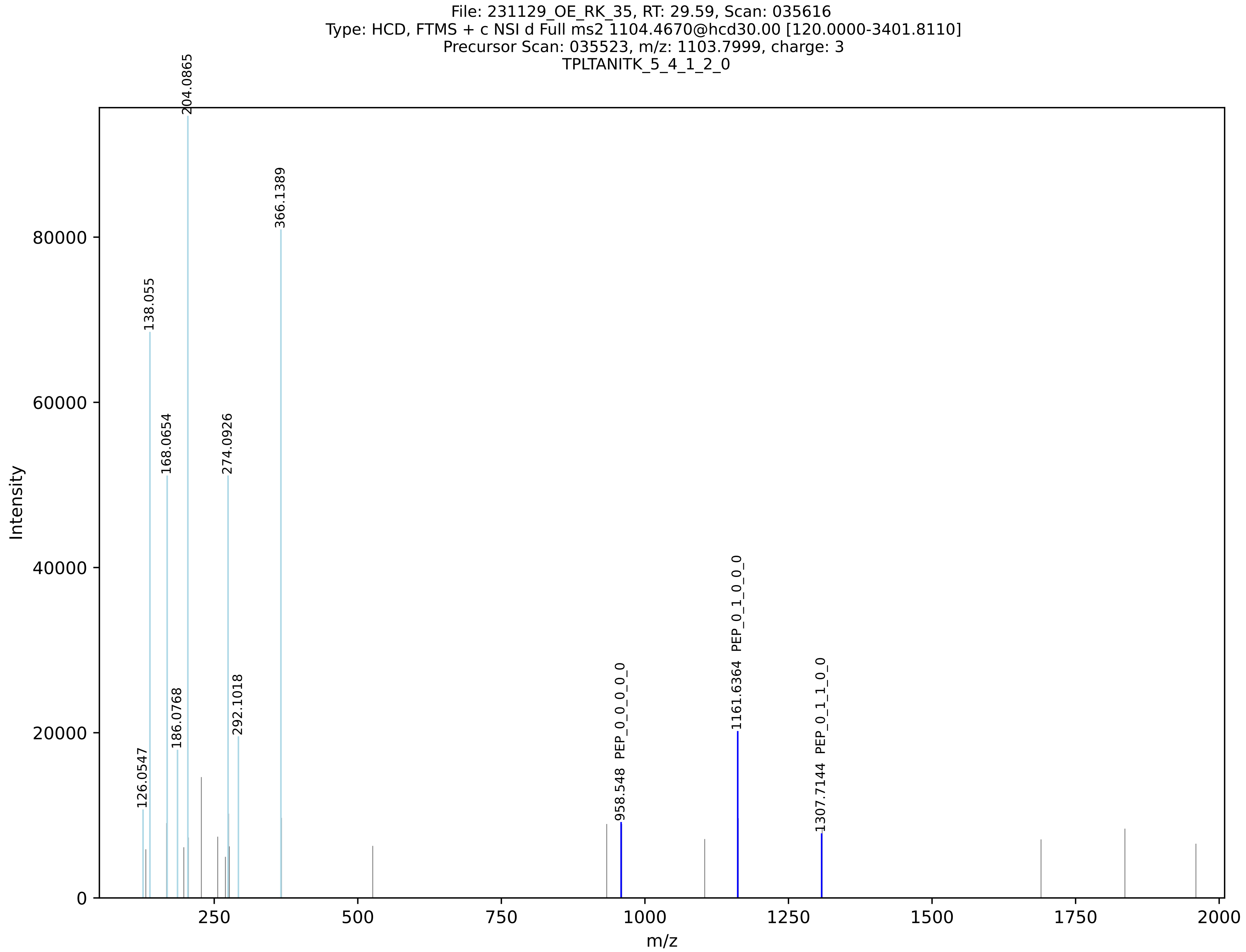
File: 231129_OE_RK_35, RT: 29.59, Scan: 035616
Type: HCD, FTMS + c NSI d Full ms2 1104.4670@hcd30.00 [120.0000-3401.8110]
Precursor Scan: 035523, m/z: 1103.7999, charge: 3
TPLTANITK_5_4_1_2_0
Intensity
m/z
0
20000
40000
60000
80000
250
500
750
1000
1250
1500
1750
2000
204.0865
366.1389
138.055
168.0654
274.0926
186.0768
292.1018
126.0547
958.548 PEP_0_0_0_0_0_
1161.6364 PEP_0_1_0_0_0_
1307.7144 PEP_0_1_1_0_0_

File: 231129_OE_RK_35, RT: 29.628, Scan: 035671
Type: HCD, FTMS + c NSI d Full ms2 1312.5577@hcd30.00 [120.0000-4038.5686]
Precursor Scan: 035597, m/z: 1312.5577, charge: 3
IPCSQPPQIEHGTINSSR_5_4_0_1_0

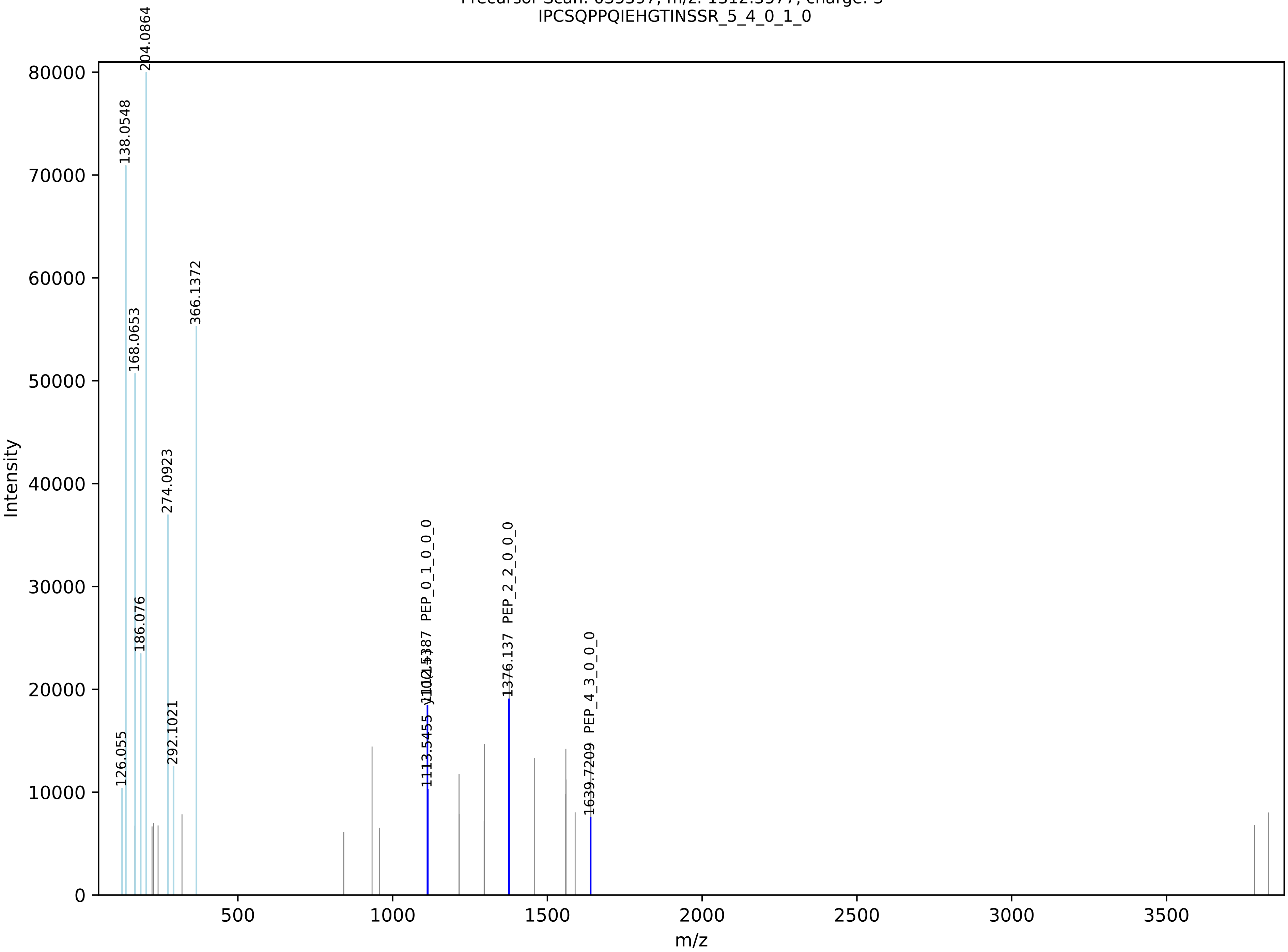

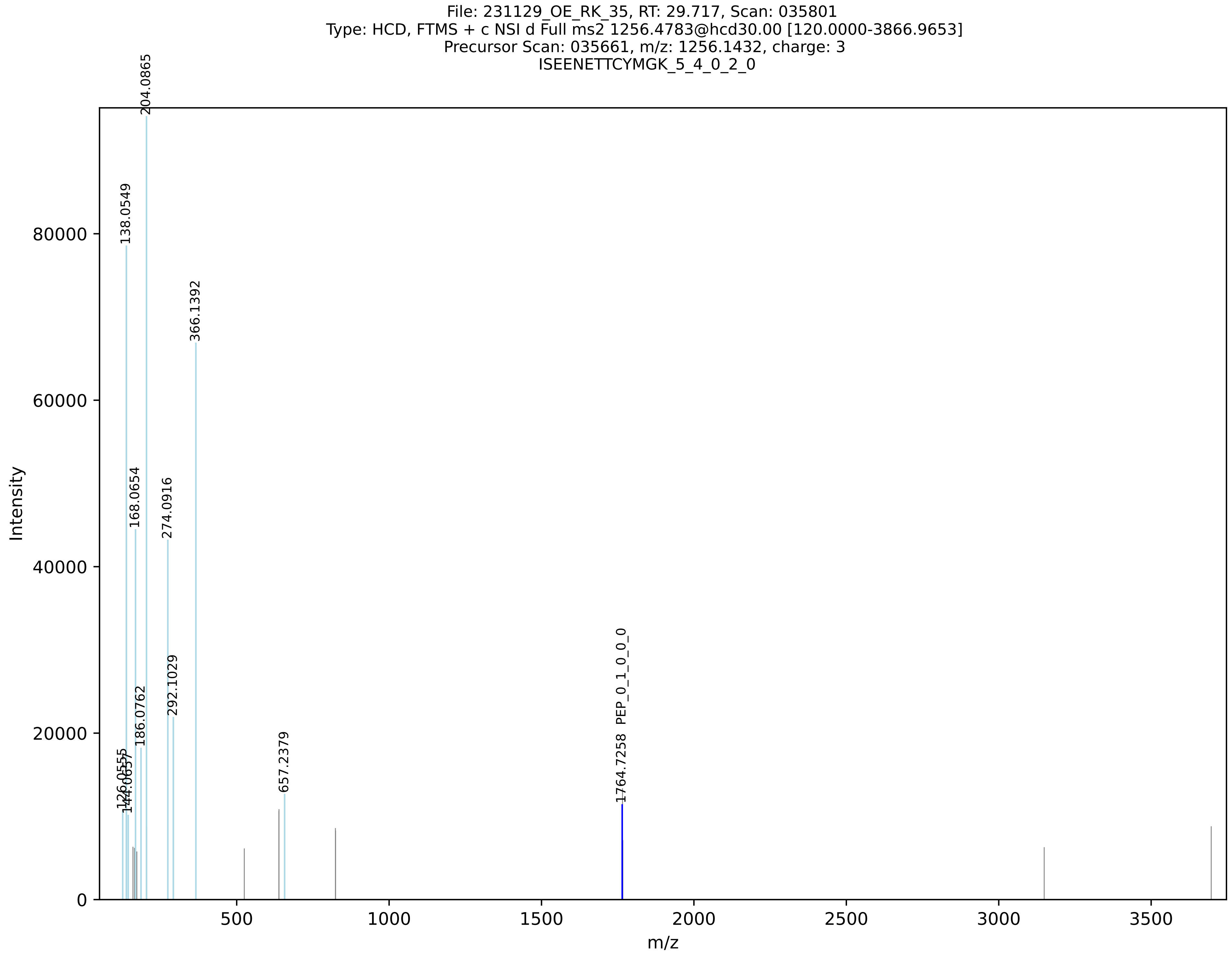
File: 231129_OE_RK_35, RT: 29.717, Scan: 035801
Type: HCD, FTMS + c NSI d Full ms2 1256.4783@hcd30.00 [120.0000-3866.9653]
Precursor Scan: 035661, m/z: 1256.1432, charge: 3
ISEENETTCYMGK_5_4_0_2_0
204.0865
138.0549
366.1392
168.0654
274.0916
292.1029
186.0762
126.0555
144.0657
657.2379
1764.7258 PEP_0_1_0_0_0
Intensity
m/z
0
20000
40000
60000
80000
500
1000
1500
2000
2500
3000
3500

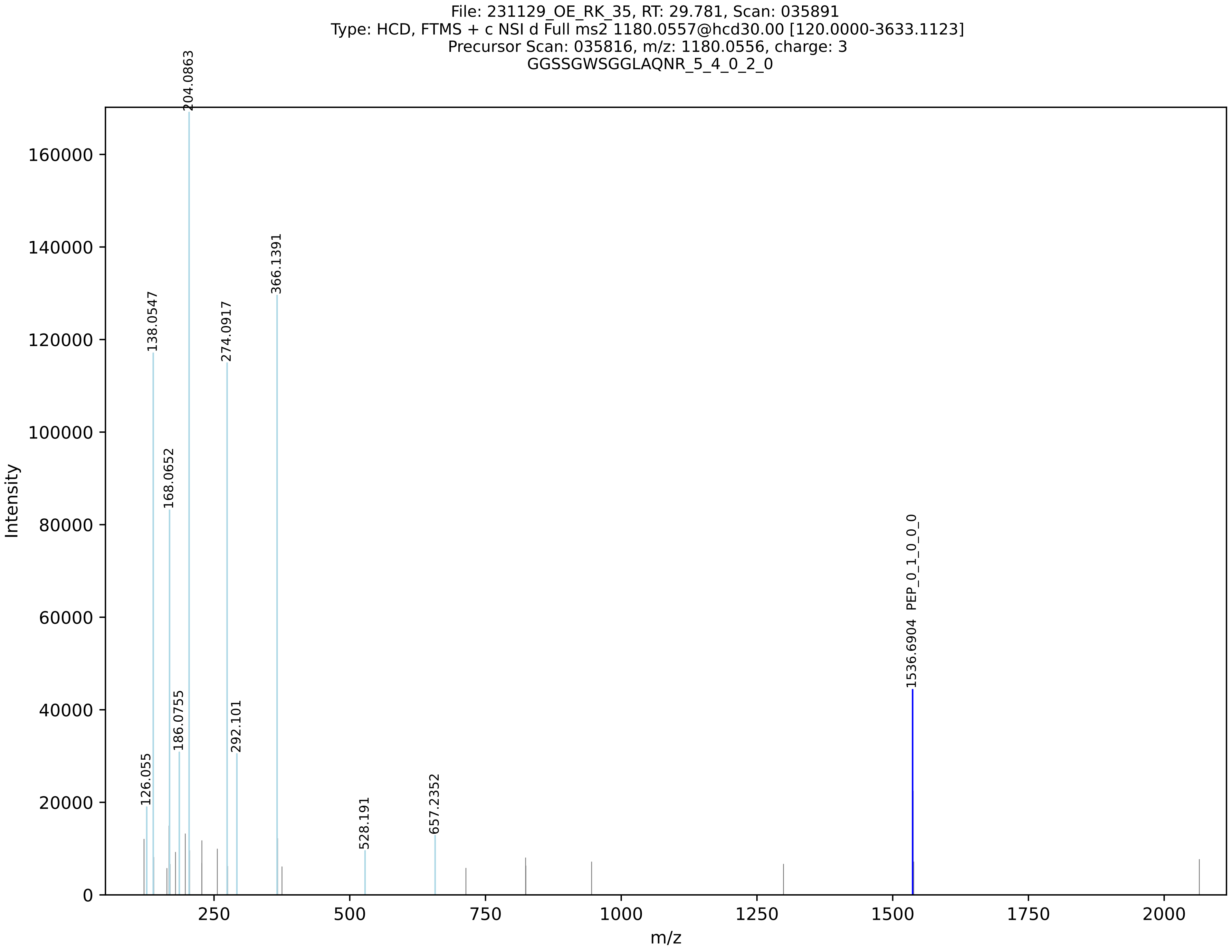

File: 231129_OE_RK_35, RT: 29.781, Scan: 035891
Type: HCD, FTMS + c NSI d Full ms2 1180.0557@hcd30.00 [120.0000-3633.1123]
Precursor Scan: 035816, m/z: 1180.0556, charge: 3
GGSSGWSGGLAQNR_5_4_0_2_0
Intensity
m/z
204.0863
366.1391
138.0547
274.0917
168.0652
186.0755
292.101
126.055
528.191
657.2352
1536.6904 PEP_0_1_0_0_0

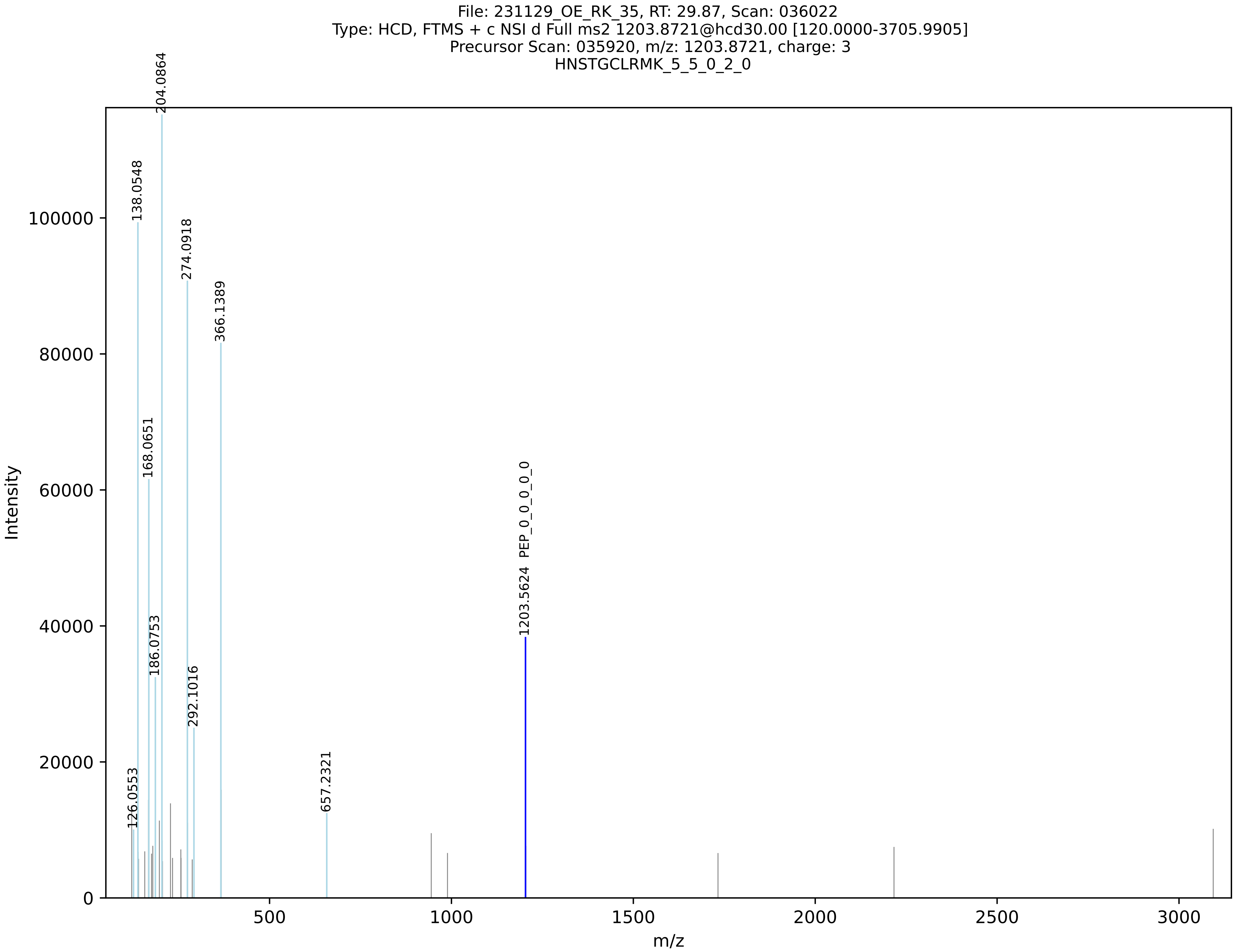

File: 231129_OE_RK_35, RT: 29.87, Scan: 036022
Type: HCD, FTMS + c NSI d Full ms2 1203.8721@hcd30.00 [120.0000-3705.9905]
Precursor Scan: 035920, m/z: 1203.8721, charge: 3
HNSTGCLRMK_5_5_0_2_0
Intensity
m/z
204.0864
138.0548
274.0918
366.1389
168.0651
186.0753
292.1016
126.0553
657.2321
1203.5624 PEP_0_0_0_0_0_
0
20000
40000
60000
80000
100000
500
1000
1500
2000
2500
3000

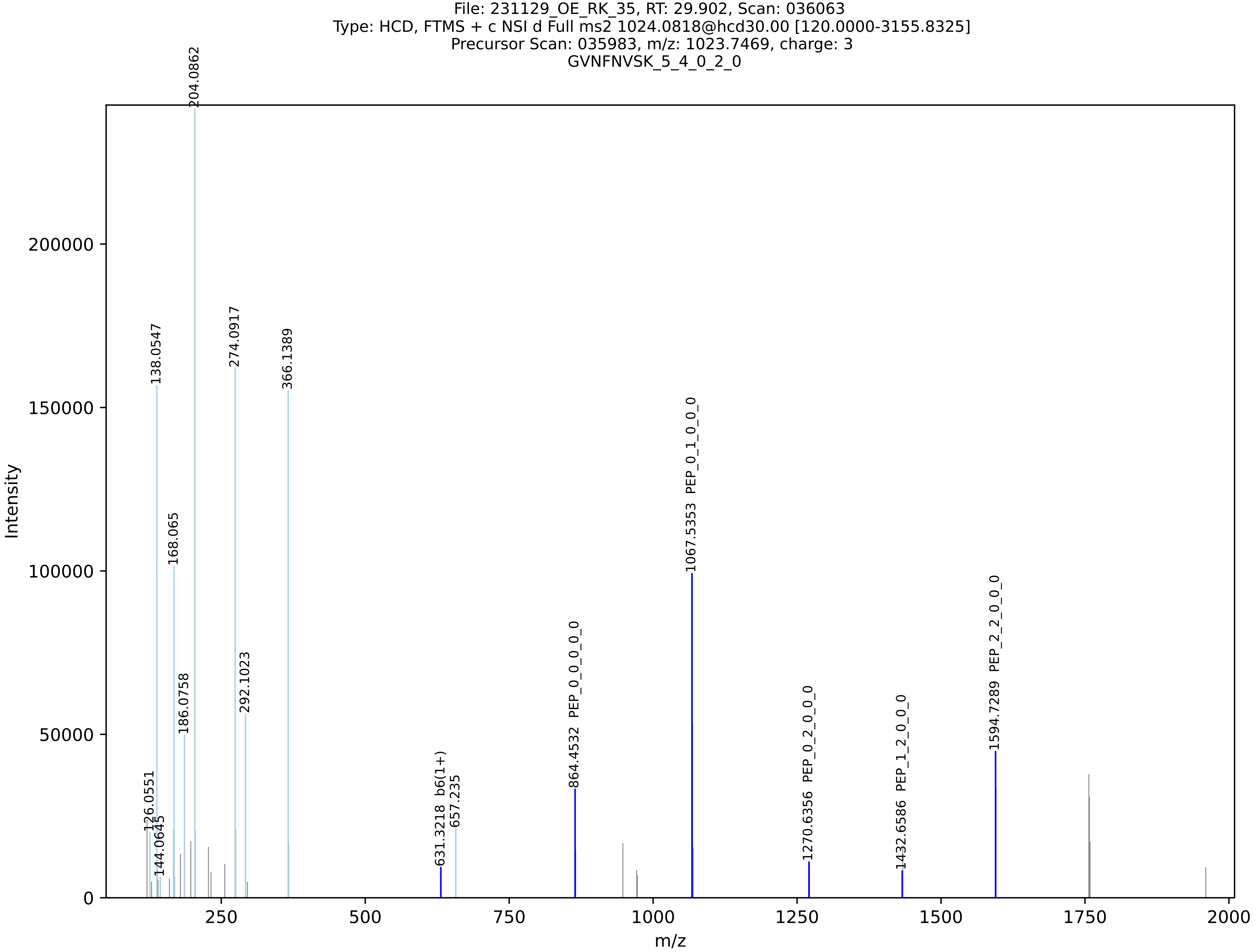
File: 231129_OE_RK_35, RT: 29.902, Scan: 036063
Type: HCD, FTMS + c NSI d Full ms2 1024.0818@hcd30.00 [120.0000-3155.8325]
Precursor Scan: 035983, m/z: 1023.7469, charge: 3
GVNFNVSK_5_4_0_2_0
Intensity
m/z
204.0862
138.0547
274.0917
366.1389
168.065
186.0758
292.1023
126.0551
144.0645
631.3218 b6(1+)
657.235
864.4532 PEP_0_0_0_0_0_
1067.5353 PEP_0_1_0_0_0_
1270.6356 PEP_0_2_0_0_0_
1432.6586 PEP_1_2_0_0_0_
1594.7289 PEP_2_2_0_0_0_

File: 231129_OE_RK_35, RT: 30.021, Scan: 036239
Type: HCD, FTMS + c NSI d Full ms2 1158.1508@hcd30.00 [120.0000-3566.0835]
Precursor Scan: 036122, m/z: 1157.4834, charge: 3
TMQENSTPR_5_5_0_2_0

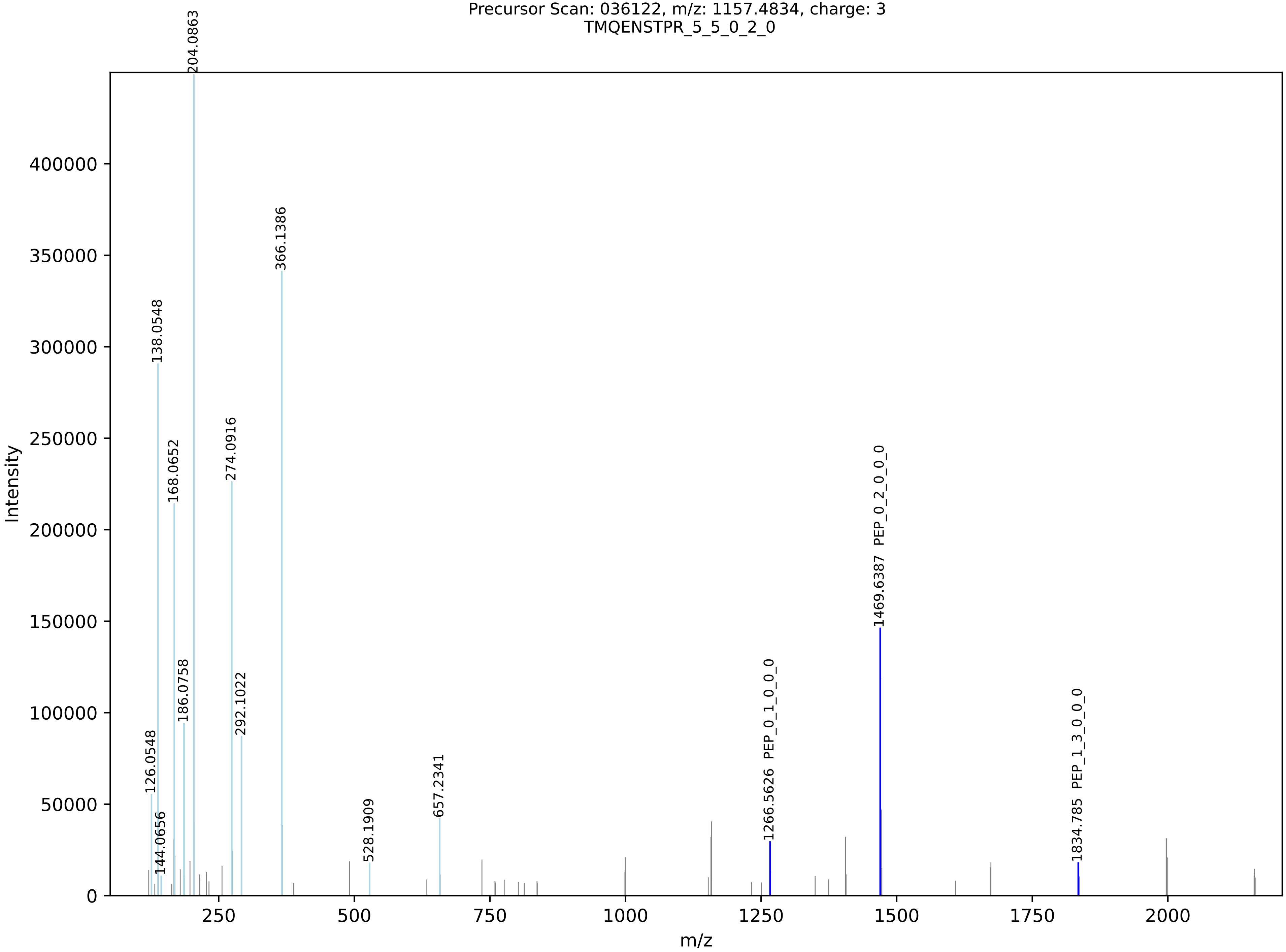

File: 231129_OE_RK_35, RT: 30.095, Scan: 036345
Type: HCD, FTMS + c NSI d Full ms2 1024.0808@hcd30.00 [120.0000-3155.8293]
Precursor Scan: 036269, m/z: 1023.747, charge: 3
GVNFNVSK_5_4_0_2_0

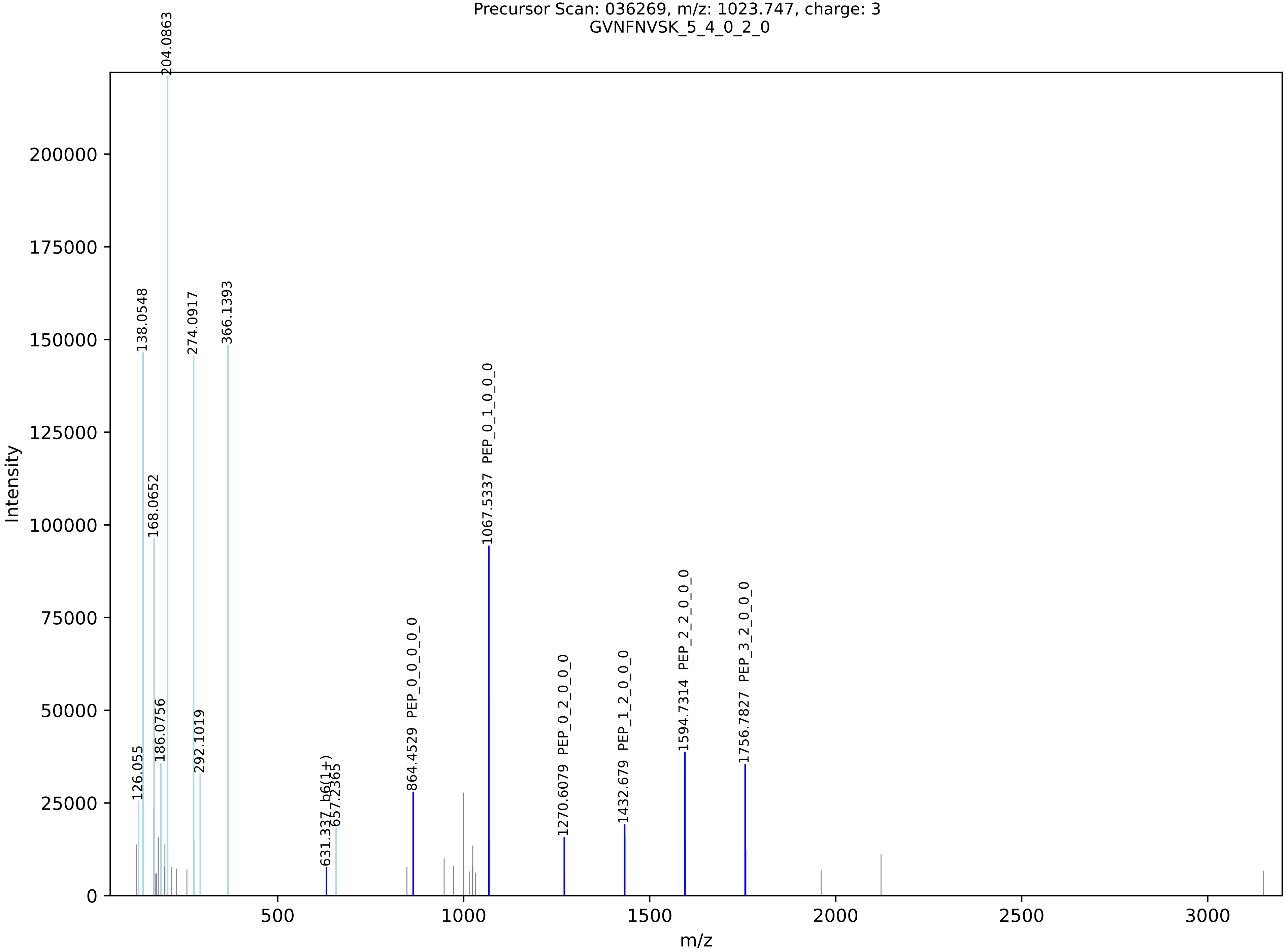

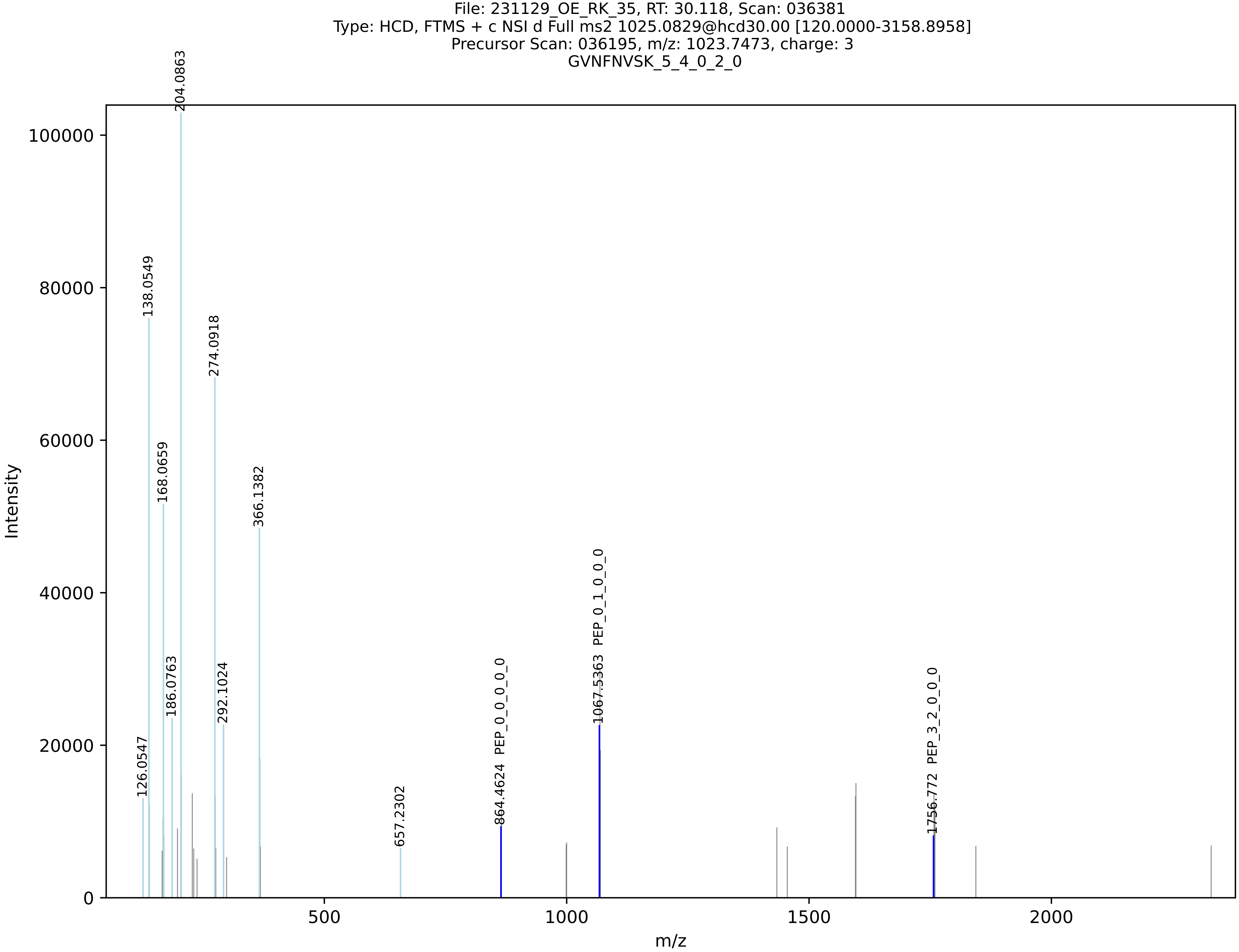

File: 231129_OE_RK_35, RT: 30.118, Scan: 036381
Type: HCD, FTMS + c NSI d Full ms2 1025.0829@hcd30.00 [120.0000-3158.8958]
Precursor Scan: 036195, m/z: 1023.7473, charge: 3
GVNFNVSK_5_4_0_2_0
Intensity
m/z
0
20000
40000
60000
80000
100000
500
1000
1500
2000
204.0863
138.0549
274.0918
168.0659
366.1382
186.0763
292.1024
126.0547
657.2302
864.4624 PEP_0_0_0_0_0
1067.5363 PEP_0_1_0_0_0
1756.772 PEP_3_2_0_0_0

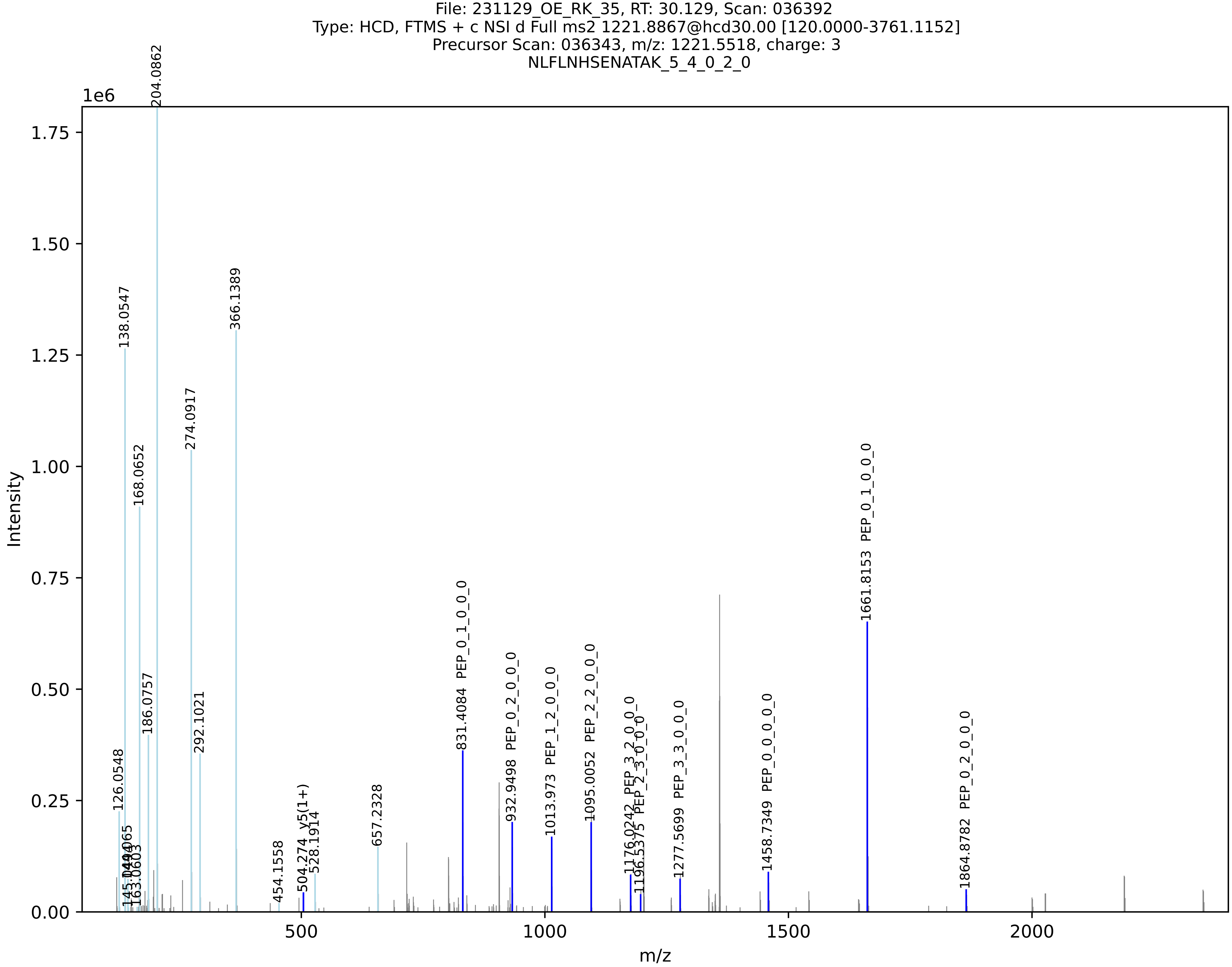

File: 231129_OE_RK_35, RT: 30.129, Scan: 036392
Type: HCD, FTMS + c NSI d Full ms2 1221.8867@hcd30.00 [120.0000-3761.1152]
Precursor Scan: 036343, m/z: 1221.5518, charge: 3
NLFLNHSENATAK_5_4_0_2_0
1e6
Intensity
m/z
204.0862
138.0547
366.1389
274.0917
168.0652
186.0757
292.1021
126.0548
163.0603
454.1558
504.274 y5(1+)
528.1914
657.2328
831.4084 PEP_0_1_0_0_0
932.9498 PEP_0_2_0_0_0
1013.973 PEP_1_2_0_0_0
1095.0052 PEP_2_2_0_0_0
1176.0242 PEP_3_2_0_0_0
1196.5375 PEP_2_3_0_0_0
1277.5699 PEP_3_3_0_0_0
1458.7349 PEP_0_0_0_0_0
1661.8153 PEP_0_1_0_0_0
1864.8782 PEP_0_2_0_0_0

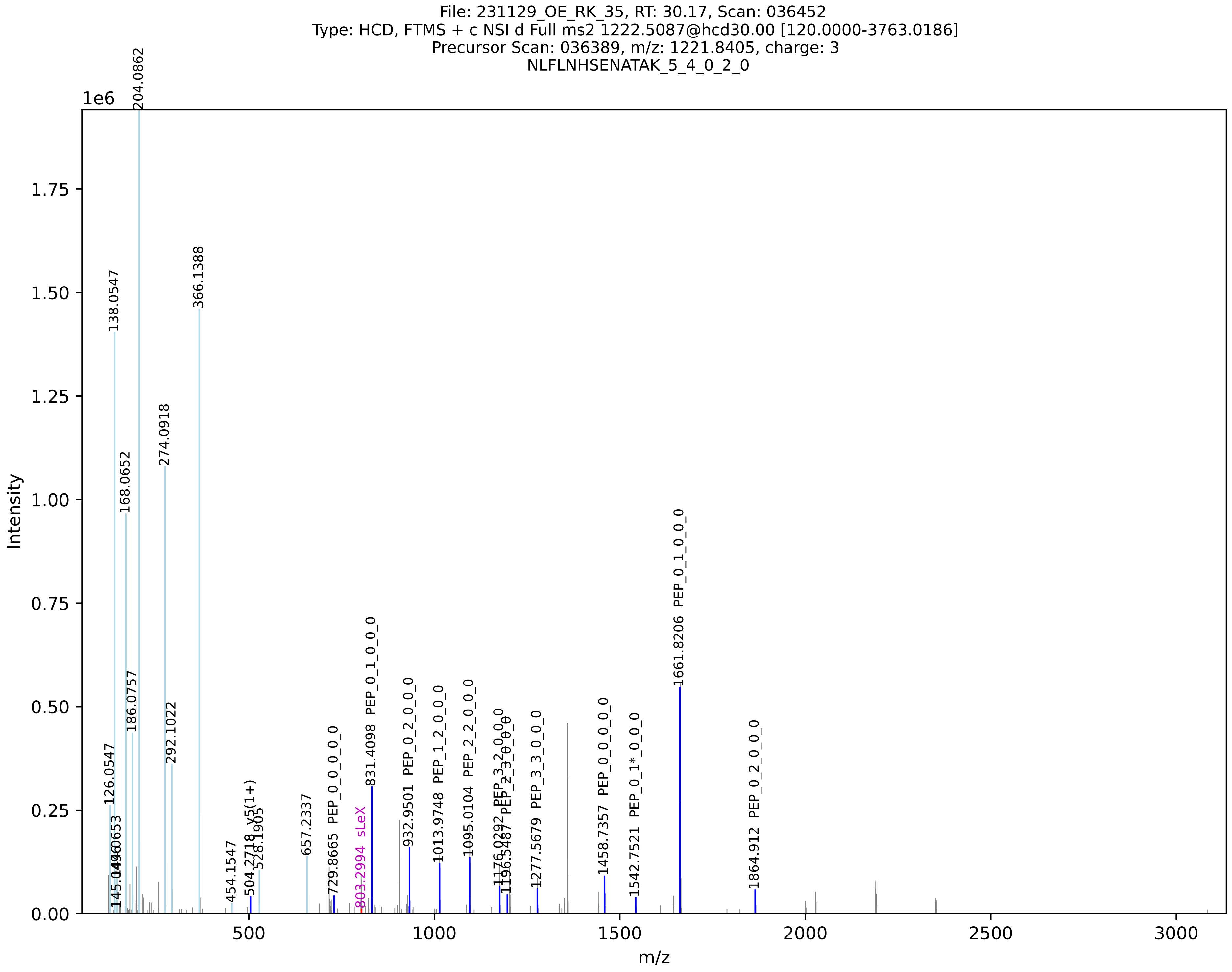
File: 231129_OE_RK_35, RT: 30.17, Scan: 036452
Type: HCD, FTMS + c NSI d Full ms2 1222.5087@hcd30.00 [120.0000-3763.0186]
Precursor Scan: 036389, m/z: 1221.8405, charge: 3
NLFLNHSENATAK_5_4_0_2_0
1e6
Intensity
m/z
204.0862
138.0547
366.1388
274.0918
168.0652
186.0757
292.1022
126.0547
454.1547
504.2718 y5(1+)
528.1905
657.2337
729.8665 PEP_0_0_0_0_0
803.2994 sLeX
831.4098 PEP_0_1_0_0_0
932.9501 PEP_0_2_0_0_0
1013.9748 PEP_1_2_0_0_0
1095.0104 PEP_2_2_0_0_0
1176.0292 PEP_3_2_0_0_0
1196.5487 PEP_2_3_0_0_0
1277.5679 PEP_3_3_0_0_0
1458.7357 PEP_0_0_0_0_0
1542.7521 PEP_0_1*_0_0_0
1661.8206 PEP_0_1_0_0_0
1864.912 PEP_0_2_0_0_0
0.00
0.25
0.50
0.75
1.00
1.25
1.50
1.75
500
1000
1500
2000
2500
3000

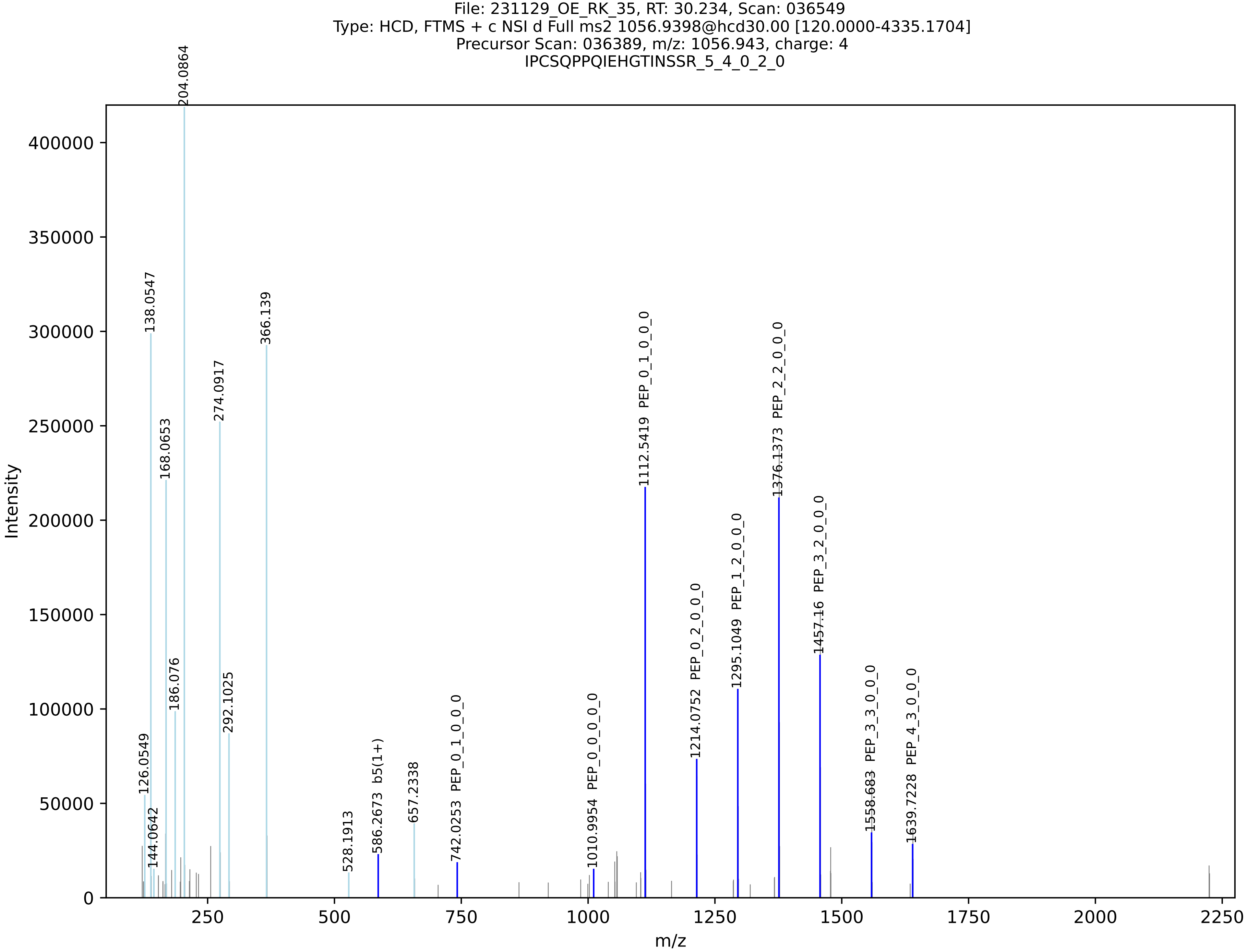

File: 231129_OE_RK_35, RT: 30.234, Scan: 036549
Type: HCD, FTMS + c NSI d Full ms2 1056.9398@hcd30.00 [120.0000-4335.1704]
Precursor Scan: 036389, m/z: 1056.943, charge: 4
IPCSQPPQIEHGTINSSR_5_4_0_2_0
Intensity
m/z
204.0864
138.0547
366.139
274.0917
168.0653
186.076
292.1025
126.0549
144.0642
528.1913
586.2673 b5(1+)
657.2338
742.0253 PEP_0_1_0_0_0
1010.9954 PEP_0_0_0_0_0
1112.5419 PEP_0_1_0_0_
1214.0752 PEP_0_2_0_0_0
1295.1049 PEP_1_2_0_0_0
1376.1373 PEP_2_2_0_0_0
1457.16 PEP_3_2_0_0_0
1558.683 PEP_3_3_0_0_0
1639.7228 PEP_4_3_0_0_0

File: 231129_OE_RK_35, RT: 30.238, Scan: 036555
Type: HCD, FTMS + c NSI d Full ms2 1157.8168@hcd30.00 [120.0000-3565.0613]
Precursor Scan: 036389, m/z: 1157.7817, charge: 3
TMQENSTPR_5_5_0_2_0

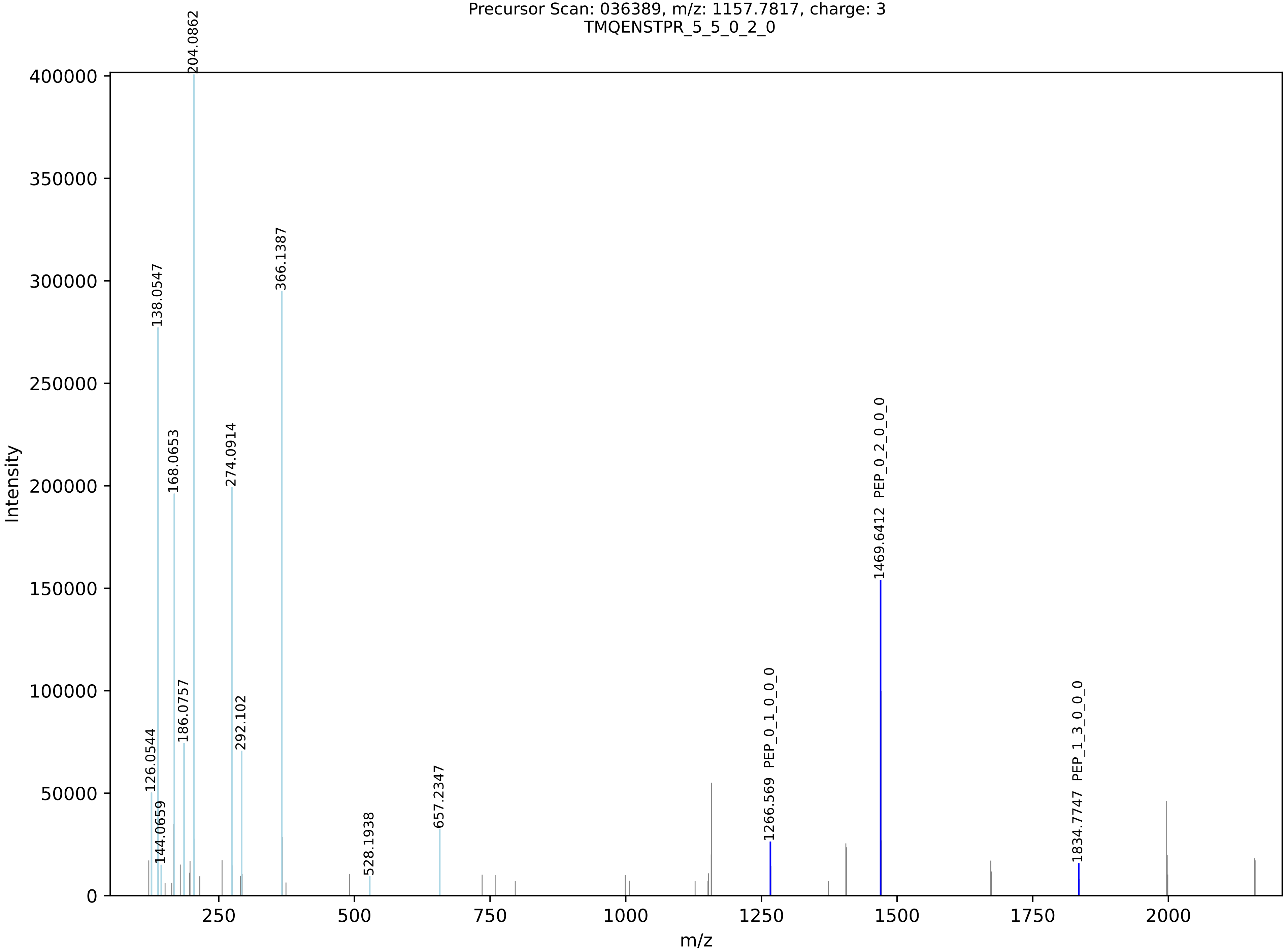

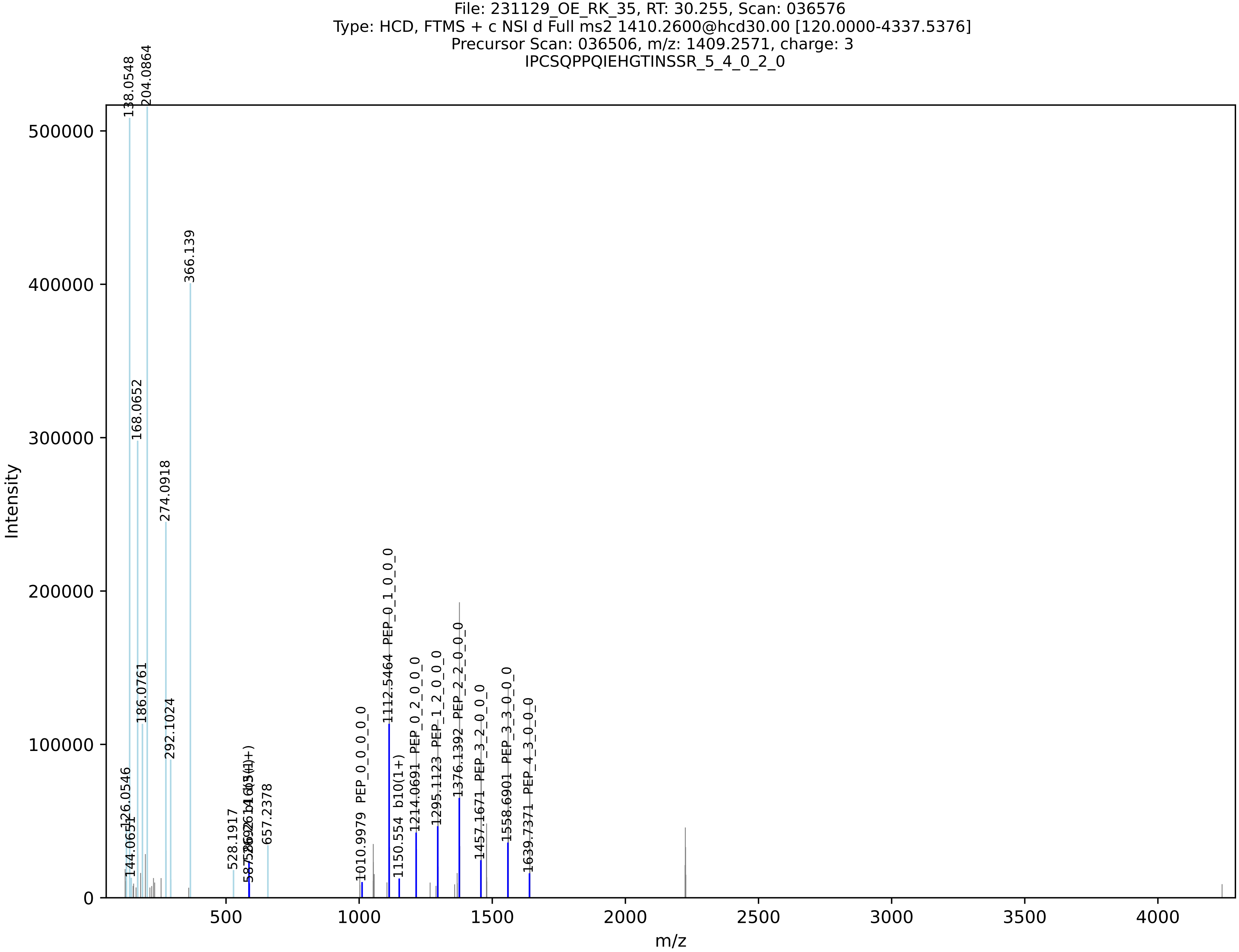
File: 231129_OE_RK_35, RT: 30.255, Scan: 036576
Type: HCD, FTMS + c NSI d Full ms2 1410.2600@hcd30.00 [120.0000-4337.5376]
Precursor Scan: 036506, m/z: 1409.2571, charge: 3
IPCSQPPQIEHGTINSSR_5_4_0_2_0
Intensity
m/z
138.0548
204.0864
366.139
168.0652
274.0918
186.0761
292.1024
126.0546
144.0651
528.1917
657.2378
1010.9979 PEP_0_0_0_0_0
1112.5464 PEP_0_1_0_0_0
1150.554 b10(1+)
1214.0691 PEP_0_2_0_0_0
1295.1123 PEP_1_2_0_0_0
1376.1392 PEP_2_2_0_0_0
1457.1671 PEP_3_2_0_0_0
1558.6901 PEP_3_3_0_0_0
1639.7371 PEP_4_3_0_0_0

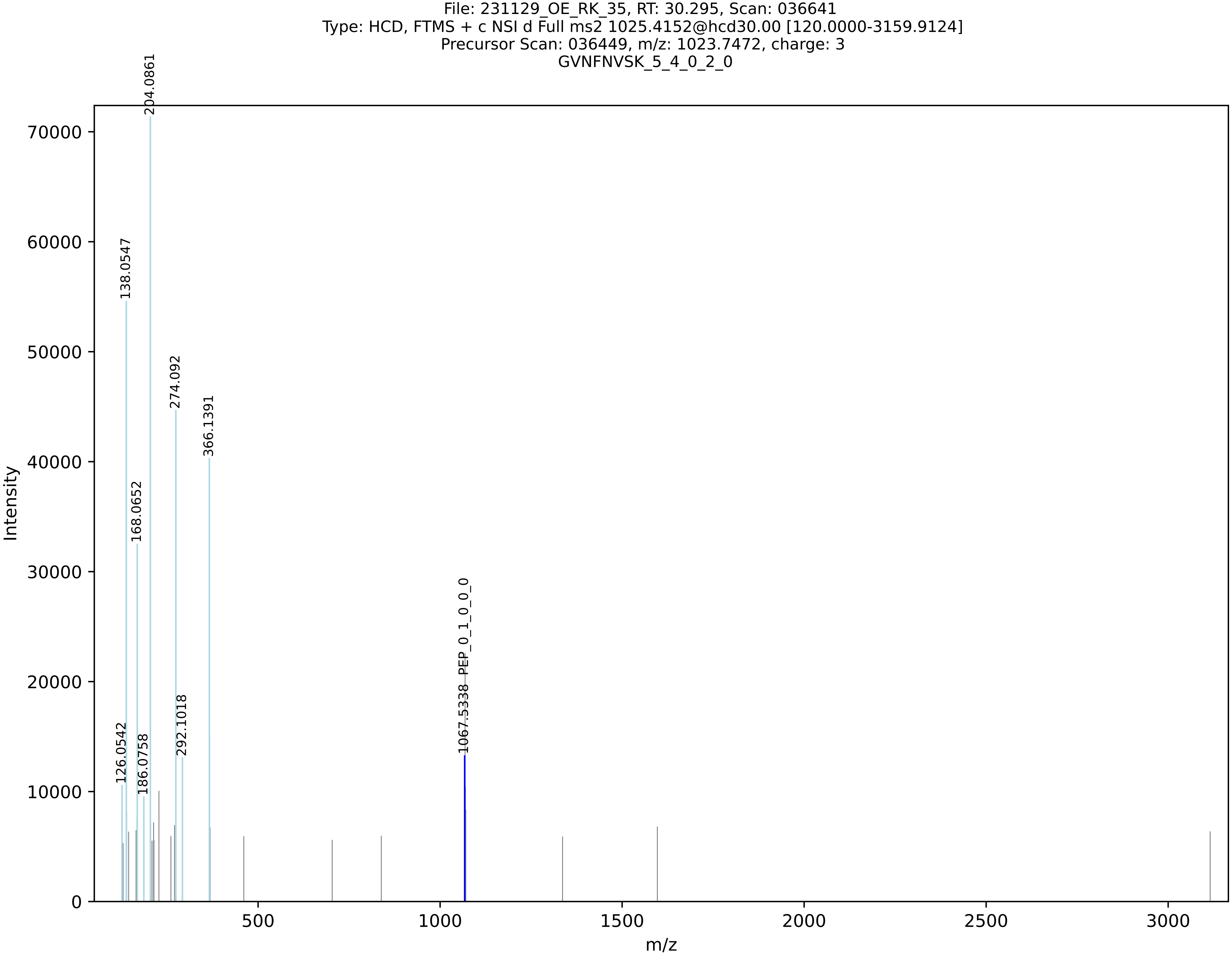
File: 231129_OE_RK_35, RT: 30.295, Scan: 036641
Type: HCD, FTMS + c NSI d Full ms2 1025.4152@hcd30.00 [120.0000-3159.9124]
Precursor Scan: 036449, m/z: 1023.7472, charge: 3
GVNFNVSK_5_4_0_2_0
204.0861
138.0547
168.0652
274.092
366.1391
1067.5338_PEP_0_1_0_0_0
126.0542
186.0758
292.1018
Intensity
m/z
0
10000
20000
30000
40000
50000
60000
70000
500
1000
1500
2000
2500
3000

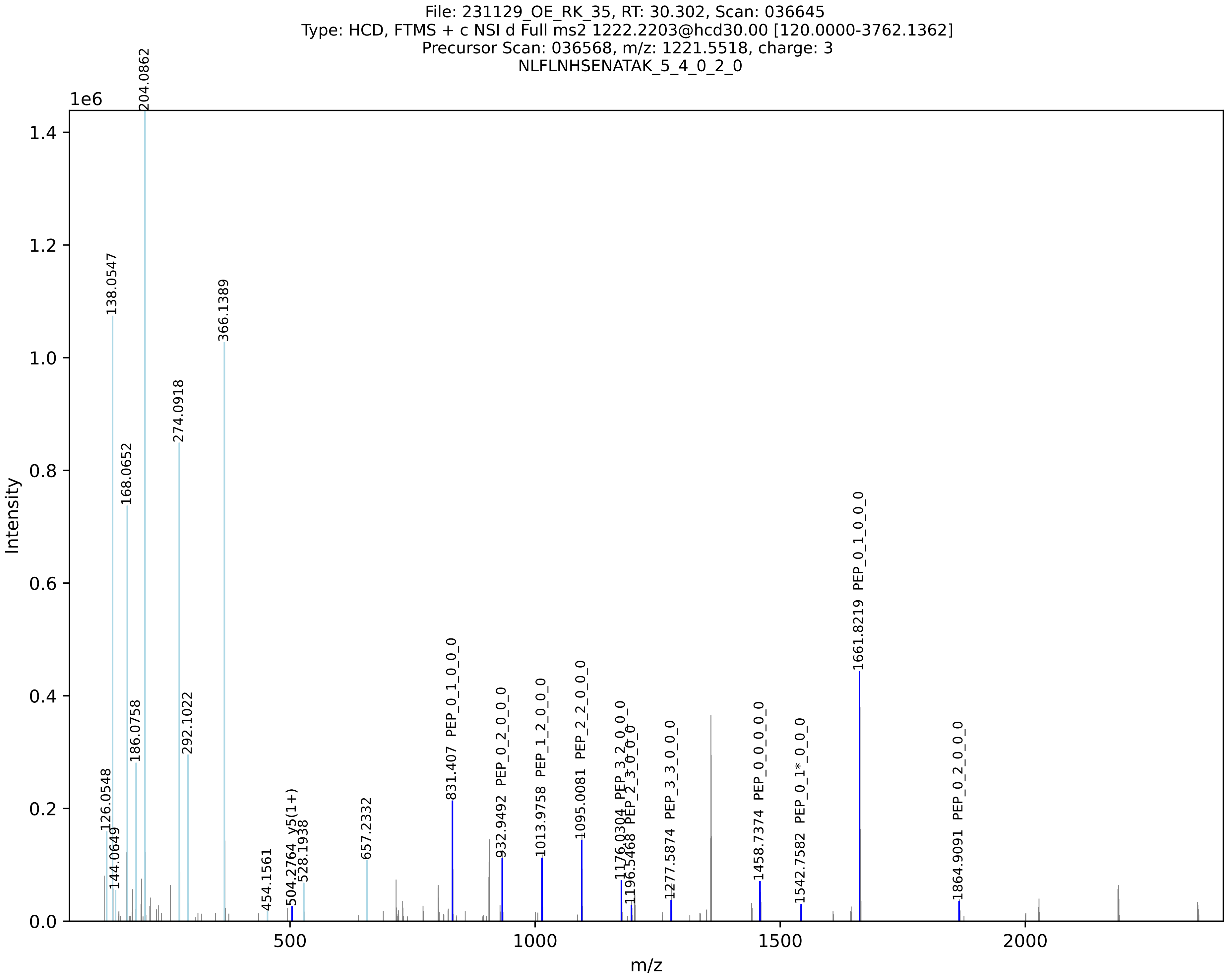
File: 231129_OE_RK_35, RT: 30.302, Scan: 036645
Type: HCD, FTMS + c NSI d Full ms2 1222.2203@hcd30.00 [120.0000-3762.1362]
Precursor Scan: 036568, m/z: 1221.5518, charge: 3
NLFLNHSENATAK_5_4_0_2_0
1e6
Intensity
m/z
204.0862
138.0547
366.1389
274.0918
168.0652
186.0758
292.1022
126.0548
144.0649
454.1561
504.2764 y5(1+)
528.1938
657.2332
831.407 PEP_0_1_0_0_0_
932.9492 PEP_0_2_0_0_0_
1013.9758 PEP_1_2_0_0_0_
1095.0081 PEP_2_2_0_0_0_
1176.0304 PEP_3_2_0_0_0_
1196.5468 PEP_2_3_0_0_0_
1277.5874 PEP_3_3_0_0_0_
1458.7374 PEP_0_0_0_0_0_
1542.7582 PEP_0_1*_0_0_0_
1661.8219 PEP_0_1_0_0_0_
1864.9091 PEP_0_2_0_0_0_

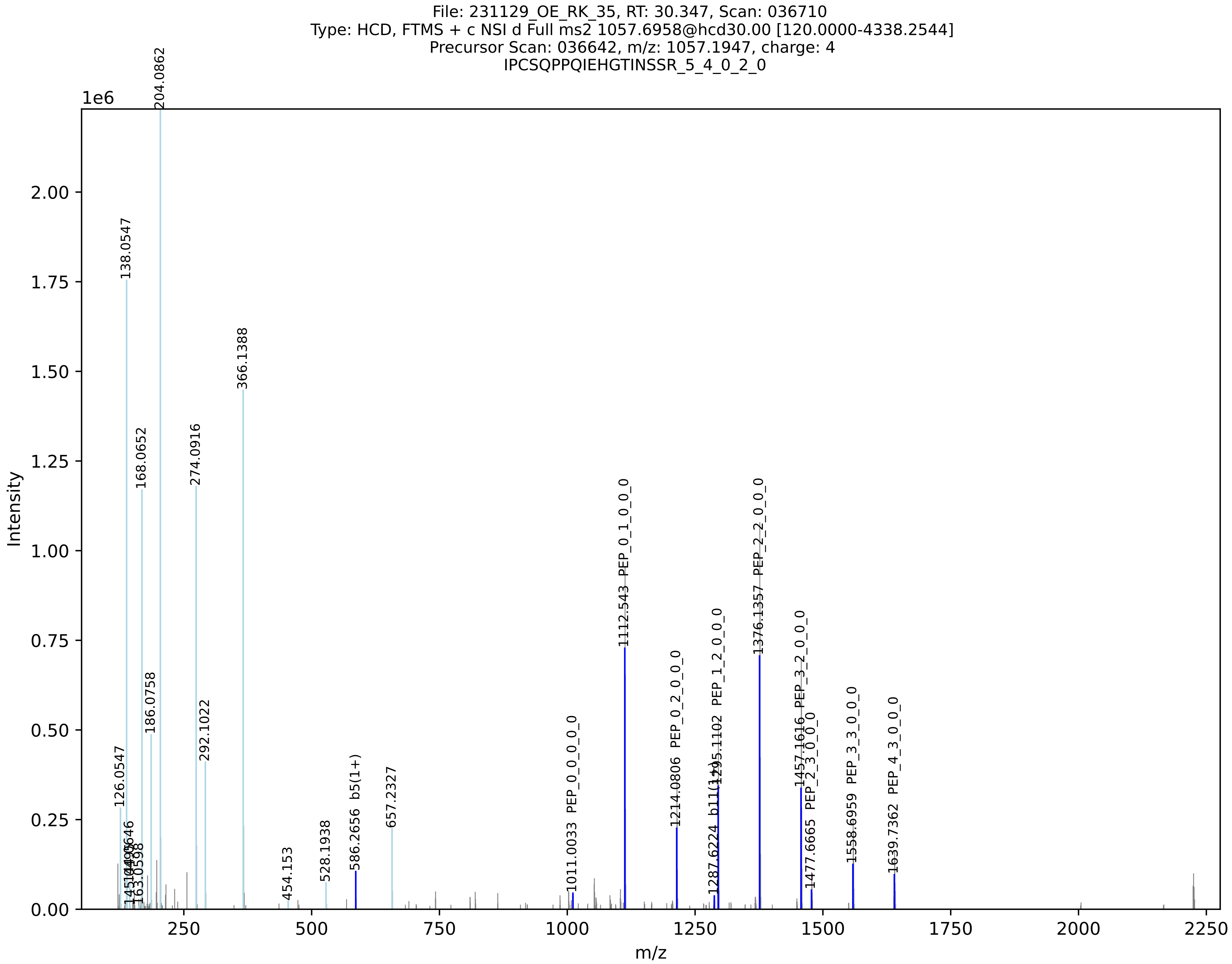
File: 231129_OE_RK_35, RT: 30.347, Scan: 036710
Type: HCD, FTMS + c NSI d Full ms2 1057.6958@hcd30.00 [120.0000-4338.2544]
Precursor Scan: 036642, m/z: 1057.1947, charge: 4
IPCSQPPQIEHGTINSSR_5_4_0_2_0
1e6
Intensity
m/z
204.0862
138.0547
366.1388
168.0652
274.0916
186.0758
292.1022
126.0547
163.0598
454.153
528.1938
586.2656 b5(1+)
657.2327
1011.0033 PEP_0_0_0_0_0
1112.543 PEP_0_1_0_0_0
1214.0806 PEP_0_2_0_0_0
1287.6224 b11(1+)
1295.1102 PEP_1_2_0_0_0
1376.1357 PEP_2_2_0_0_0
1457.1616 PEP_3_2_0_0_0
1477.6665 PEP_2_3_0_0_0
1558.6959 PEP_3_3_0_0_0
1639.7362 PEP_4_3_0_0_0

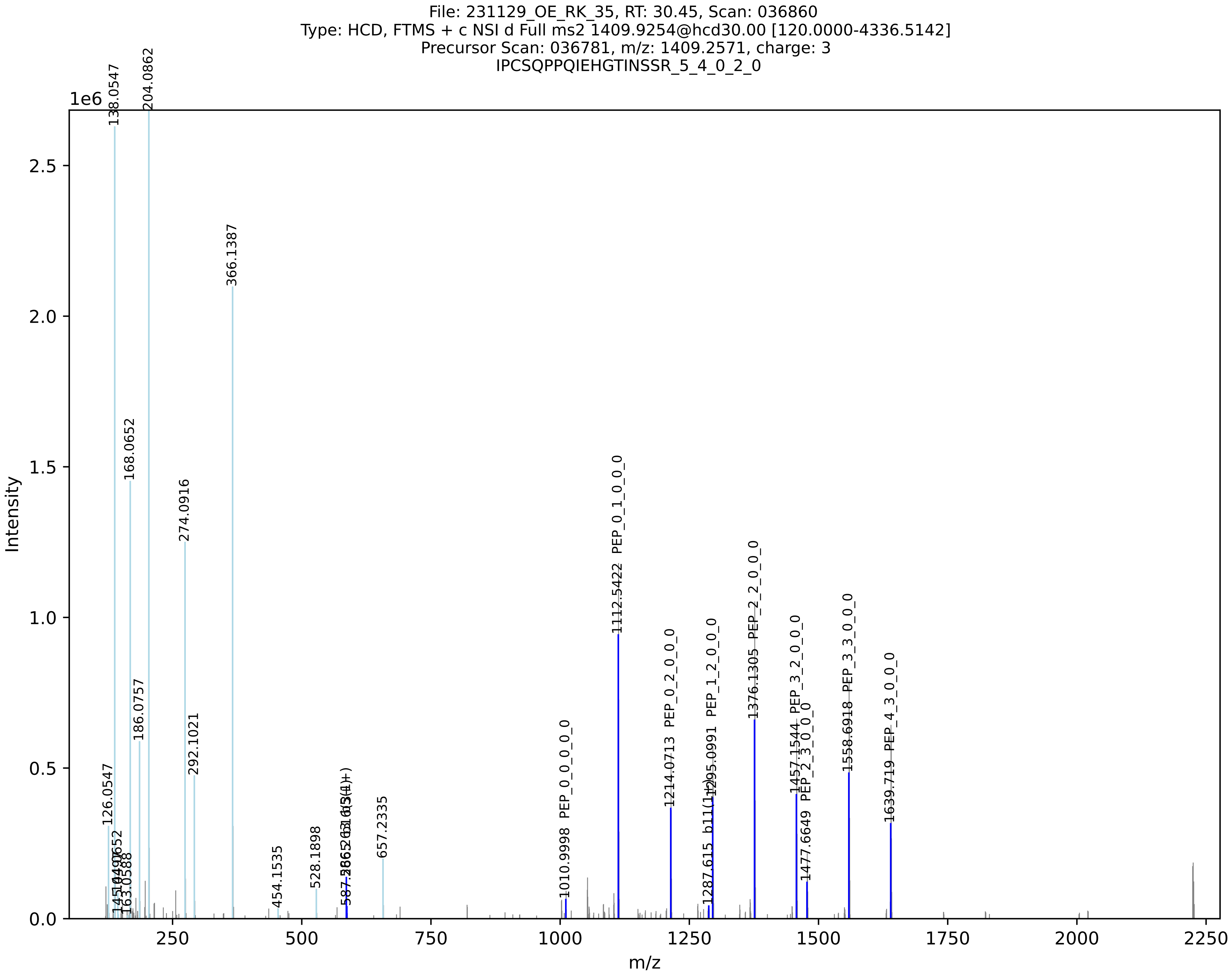
File: 231129_OE_RK_35, RT: 30.45, Scan: 036860
Type: HCD, FTMS + c NSI d Full ms2 1409.9254@hcd30.00 [120.0000-4336.5142]
Precursor Scan: 036781, m/z: 1409.2571, charge: 3
IPCSQPPQIEHGTINSSR_5_4_0_2_0
1e6
Intensity
m/z
138.0547
204.0862
366.1387
168.0652
274.0916
186.0757
292.1021
126.0547
163.0588
454.1535
528.1898
657.2335
1010.9998 PEP_0_0_0_0_0
1112.5422 PEP_0_1_0_0_0
1214.0713 PEP_0_2_0_0_0
1287.615 b11(1+)
1295.0991 PEP_1_2_0_0_0
1376.1305 PEP_2_2_0_0_0
1457.1544 PEP_3_2_0_0_0
1477.6649 PEP_2_3_0_0_0
1558.6918 PEP_3_3_0_0_0
1639.719 PEP_4_3_0_0_0

File: 231129_OE_RK_35, RT: 32.346, Scan: 039607
Type: HCD, FTMS + c NSI d Full ms2 1110.1329@hcd30.00 [120.0000-3419.1489]
Precursor Scan: 039543, m/z: 1109.798, charge: 3
ITYSIVQTNCSK_5_4_0_1_0

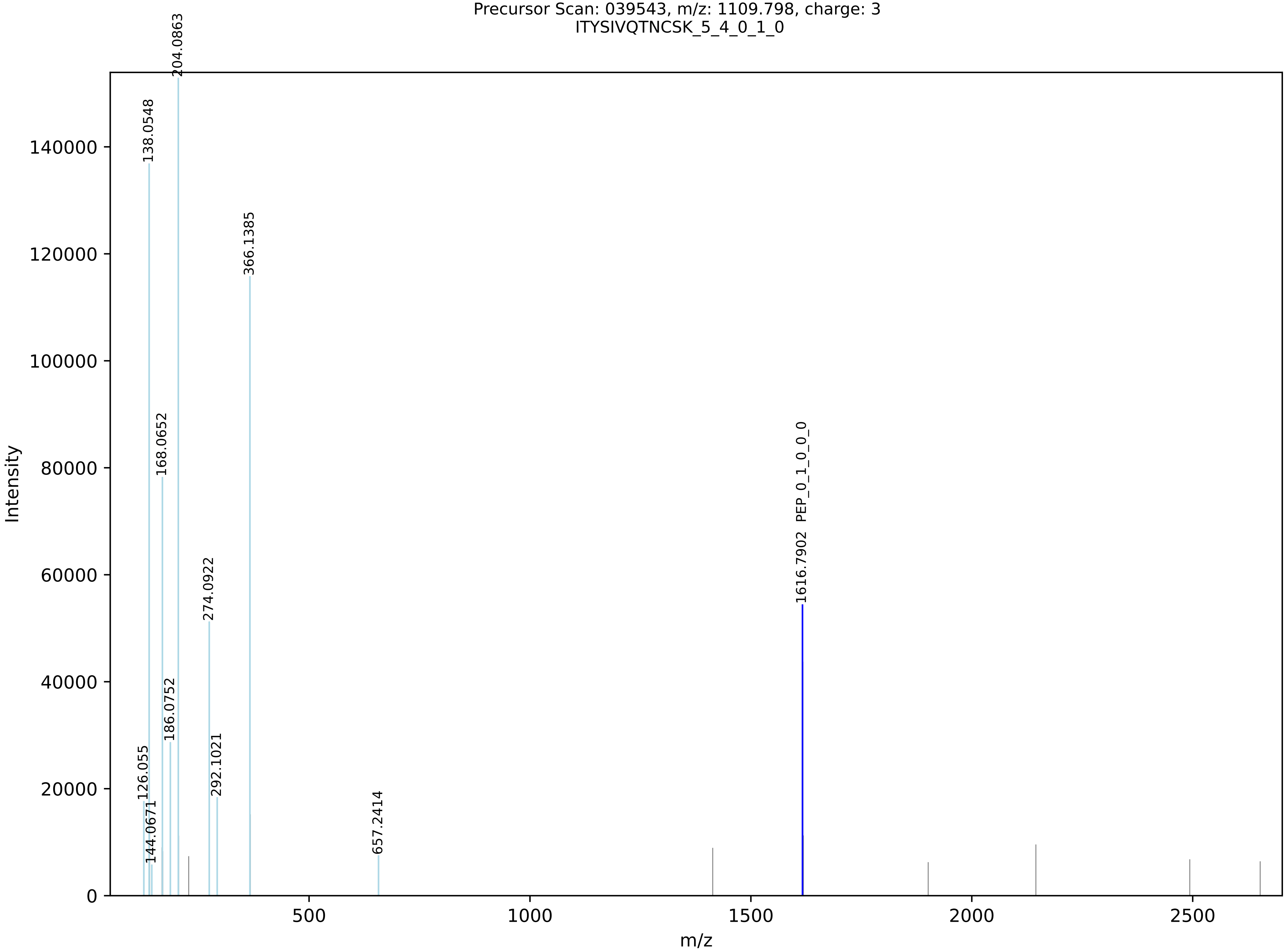

File: 231129_OE_RK_35, RT: 32.383, Scan: 039660
Type: HCD, FTMS + c NSI d Full ms2 1145.8032@hcd30.00 [120.0000-3528.3000]
Precursor Scan: 039599, m/z: 1145.135, charge: 3
ENISDPTSPLR_5_4_0_2_0

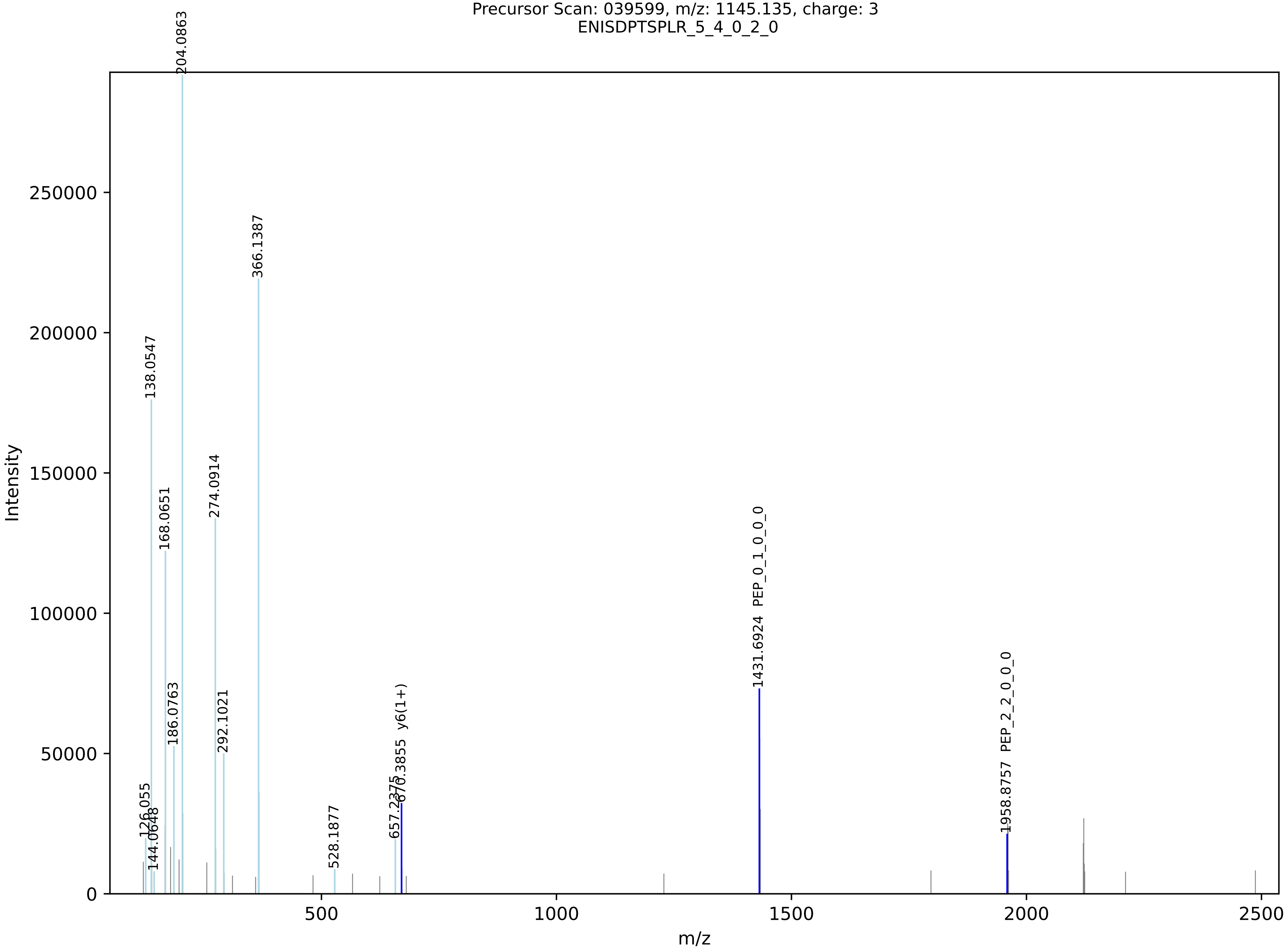

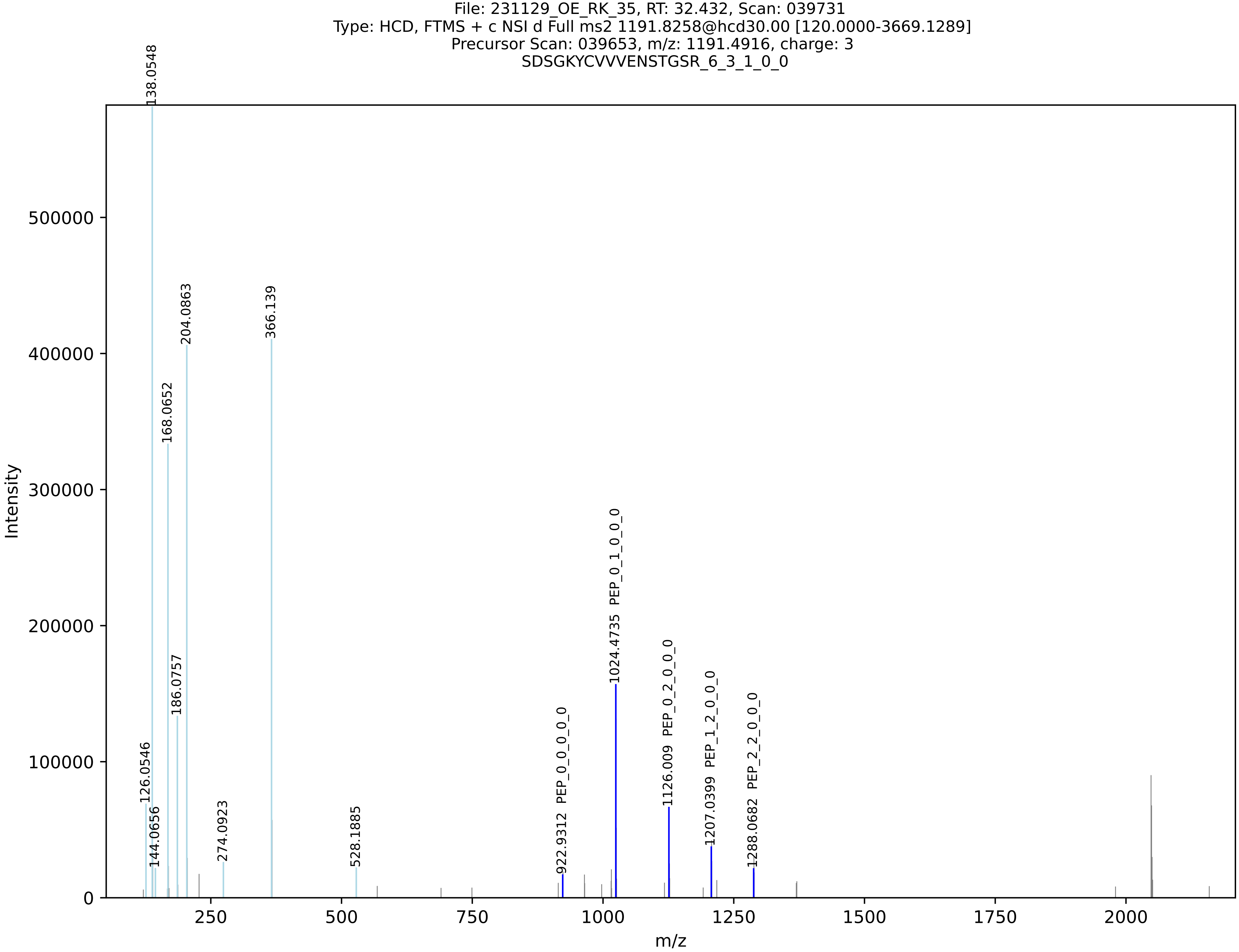
File: 231129_OE_RK_35, RT: 32.432, Scan: 039731
Type: HCD, FTMS + c NSI d Full ms2 1191.8258@hcd30.00 [120.0000-3669.1289]
Precursor Scan: 039653, m/z: 1191.4916, charge: 3
SDSGKYCVVVENSTGSR_6_3_1_0_0
138.0548
204.0863
366.139
168.0652
186.0757
126.0546
144.0656
274.0923
528.1885
922.9312 PEP_0_0_0_0_0
1024.4735 PEP_0_1_0_0_0
1126.009 PEP_0_2_0_0_0
1207.0399 PEP_1_2_0_0_0
1288.0682 PEP_2_2_0_0_0
Intensity
m/z
0
100000
200000
300000
400000
500000
250
500
750
1000
1250
1500
1750
2000

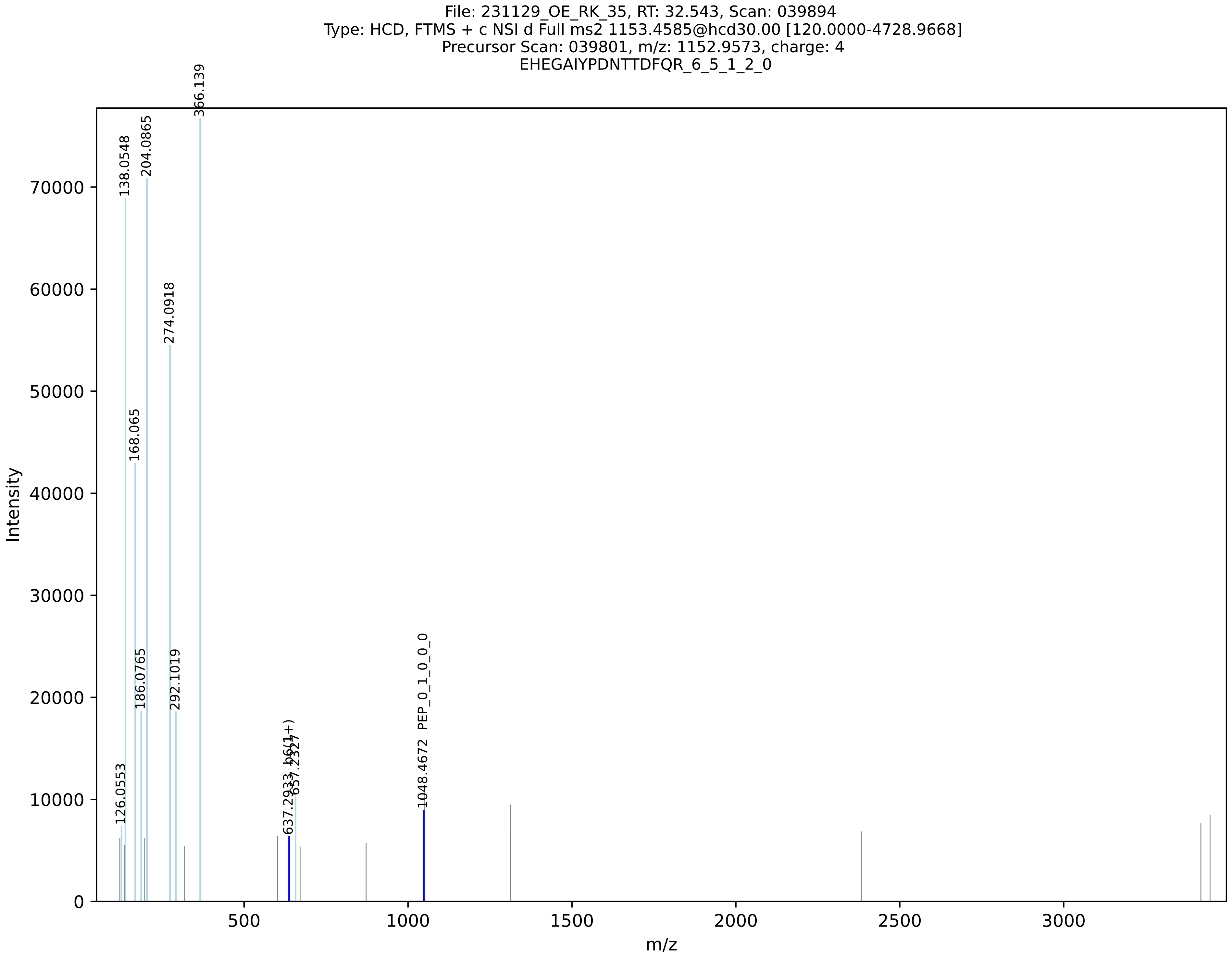

File: 231129_OE_RK_35, RT: 32.543, Scan: 039894
Type: HCD, FTMS + c NSI d Full ms2 1153.4585@hcd30.00 [120.0000-4728.9668]
Precursor Scan: 039801, m/z: 1152.9573, charge: 4
EHEGAIYPDNTTDFQR_6_5_1_2_0
366.139
138.0548
204.0865
274.0918
168.065
186.0765
292.1019
126.0553
637.2933_b6(1+)
657.2327
1048.4672_PEP_0_1_0_0_0
Intensity
0
10000
20000
30000
40000
50000
60000
70000
500
1000
1500
2000
2500
3000
m/z

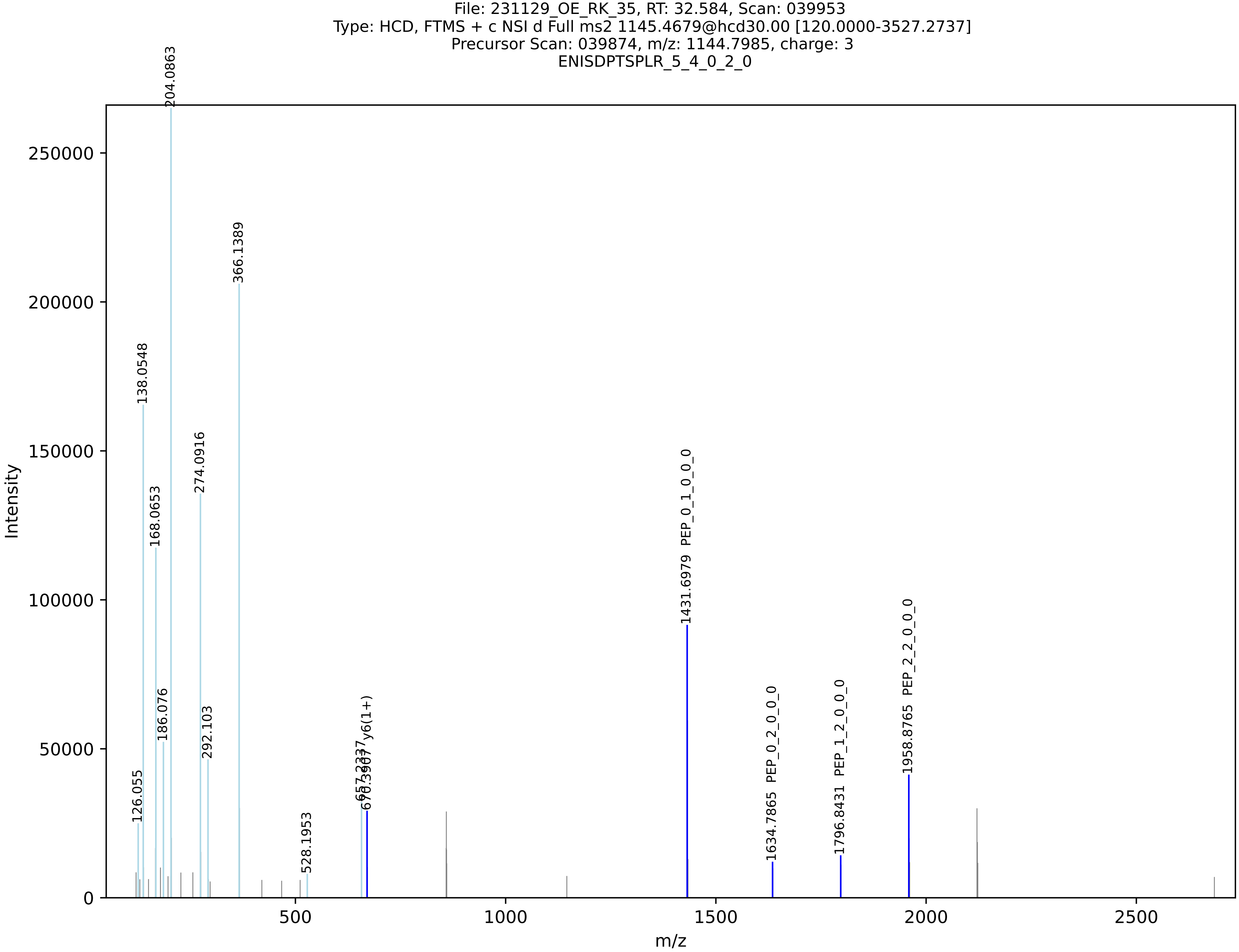

File: 231129_OE_RK_35, RT: 32.584, Scan: 039953
Type: HCD, FTMS + c NSI d Full ms2 1145.4679@hcd30.00 [120.0000-3527.2737]
Precursor Scan: 039874, m/z: 1144.7985, charge: 3
ENISDPTSPLR_5_4_0_2_0
Intensity
m/z
204.0863
366.1389
138.0548
274.0916
168.0653
186.076
292.103
126.055
528.1953
657.2237
670.3907 y6(1+)
1431.6979 PEP_0_1_0_0_0
1634.7865 PEP_0_2_0_0_0
1796.8431 PEP_1_2_0_0_0
1958.8765 PEP_2_2_0_0_0

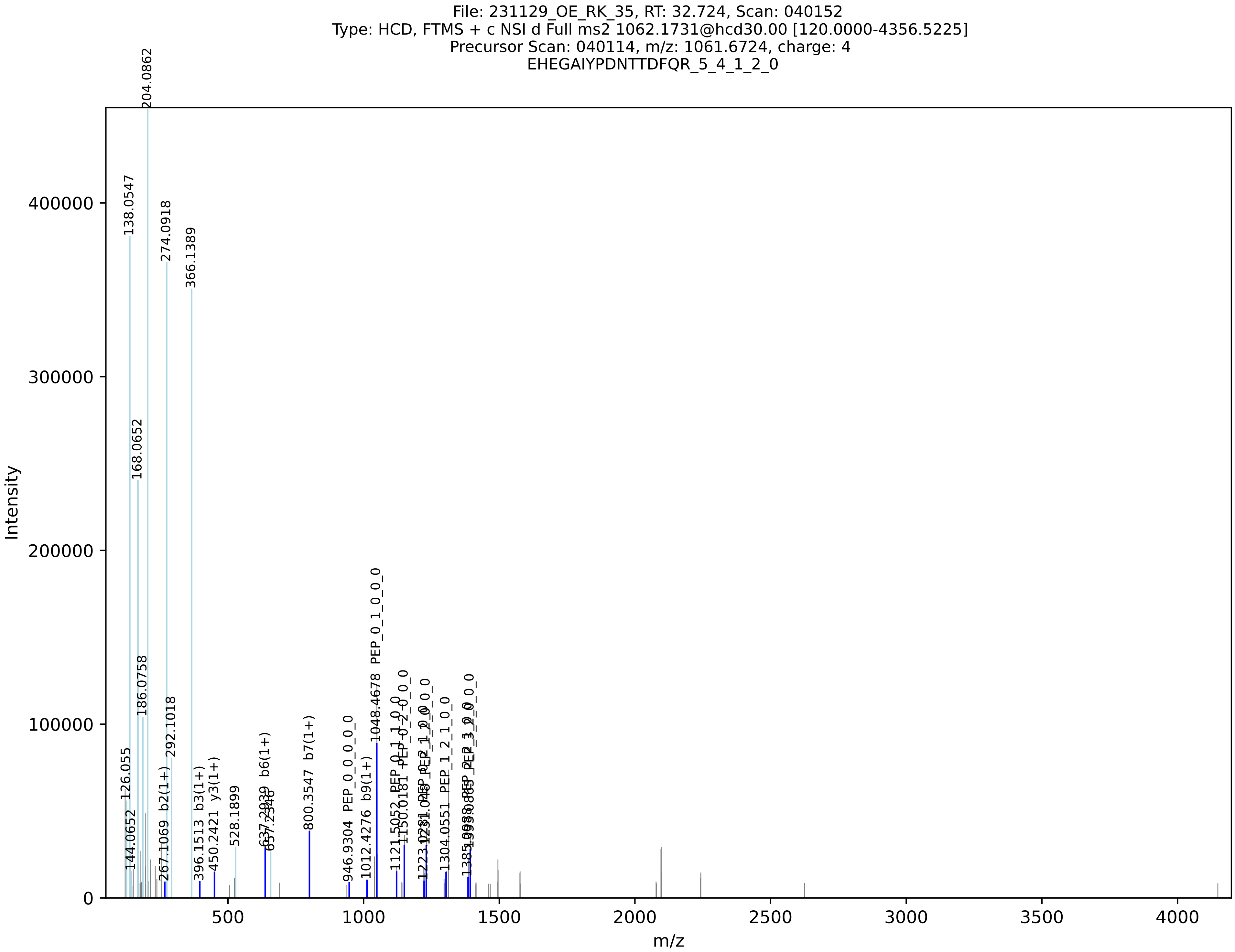
File: 231129_OE_RK_35, RT: 32.724, Scan: 040152
Type: HCD, FTMS + c NSI d Full ms2 1062.1731@hcd30.00 [120.0000-4356.5225]
Precursor Scan: 040114, m/z: 1061.6724, charge: 4
EHEGAIYPDNTTDFQR_5_4_1_2_0
Intensity
m/z
204.0862
138.0547
274.0918
366.1389
168.0652
186.0758
292.1018
126.055
144.0652
267.1069 b2(1+)
396.1513 b3(1+)
450.2421 y3(1+)
528.1899
637.2939 b6(1+)
800.3547 b7(1+)
946.9304 PEP_0_0_0_0_0
1012.4276 b9(1+)
1048.4678 PEP_0_1_0_0_0
1304.0551 PEP_1_2_1_0_0

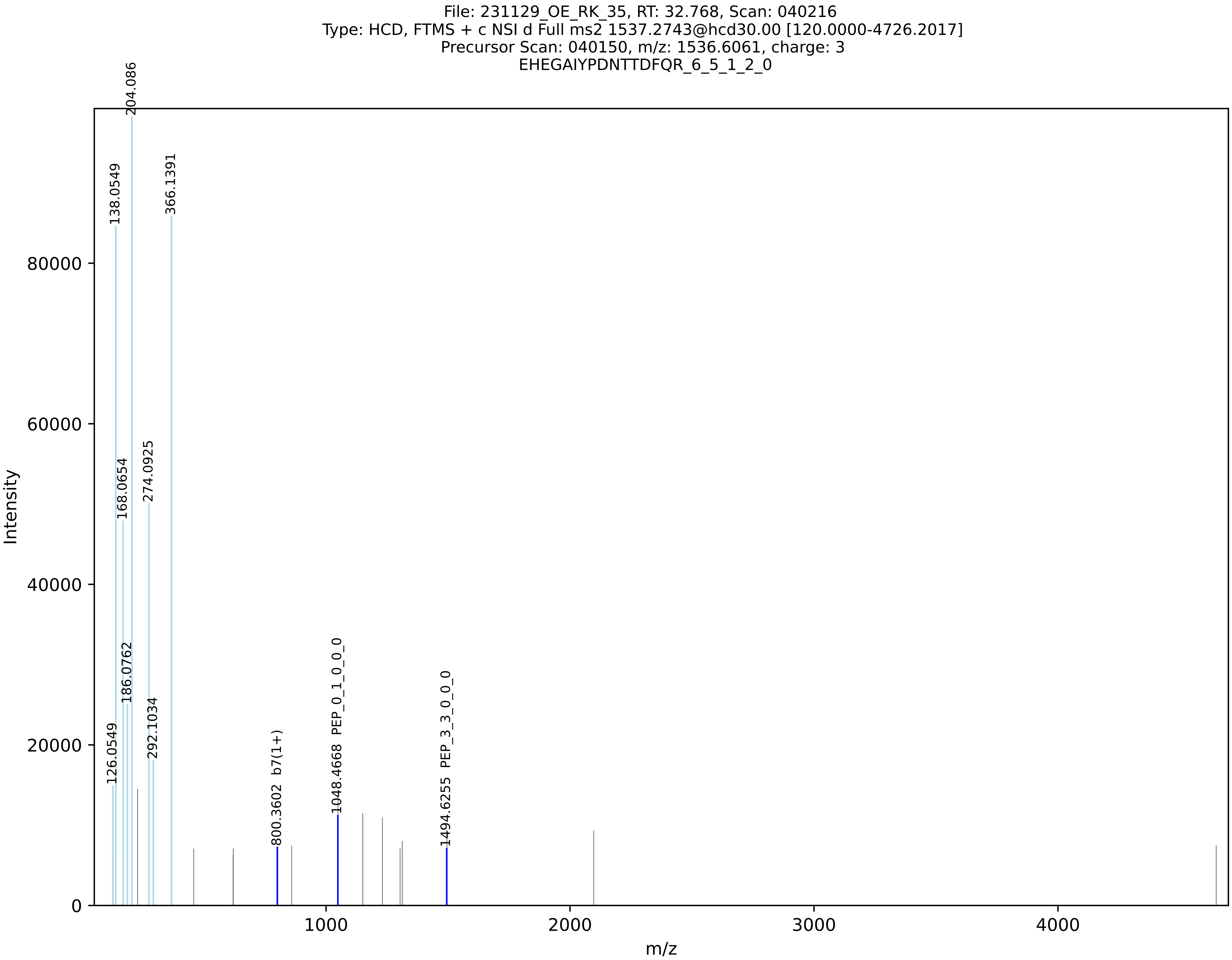
File: 231129_OE_RK_35, RT: 32.768, Scan: 040216
Type: HCD, FTMS + c NSI d Full ms2 1537.2743@hcd30.00 [120.0000-4726.2017]
Precursor Scan: 040150, m/z: 1536.6061, charge: 3
EHEGAIYPDNTTDFQR_6_5_1_2_0
204.086
138.0549
366.1391
168.0654
274.0925
186.0762
292.1034
126.0549
800.3602 b7(1+)
1048.4668 PEP_0_1_0_0_0
1494.6255 PEP_3_3_0_0_0
Intensity
80000
60000
40000
20000
0
1000
2000
3000
4000
m/z

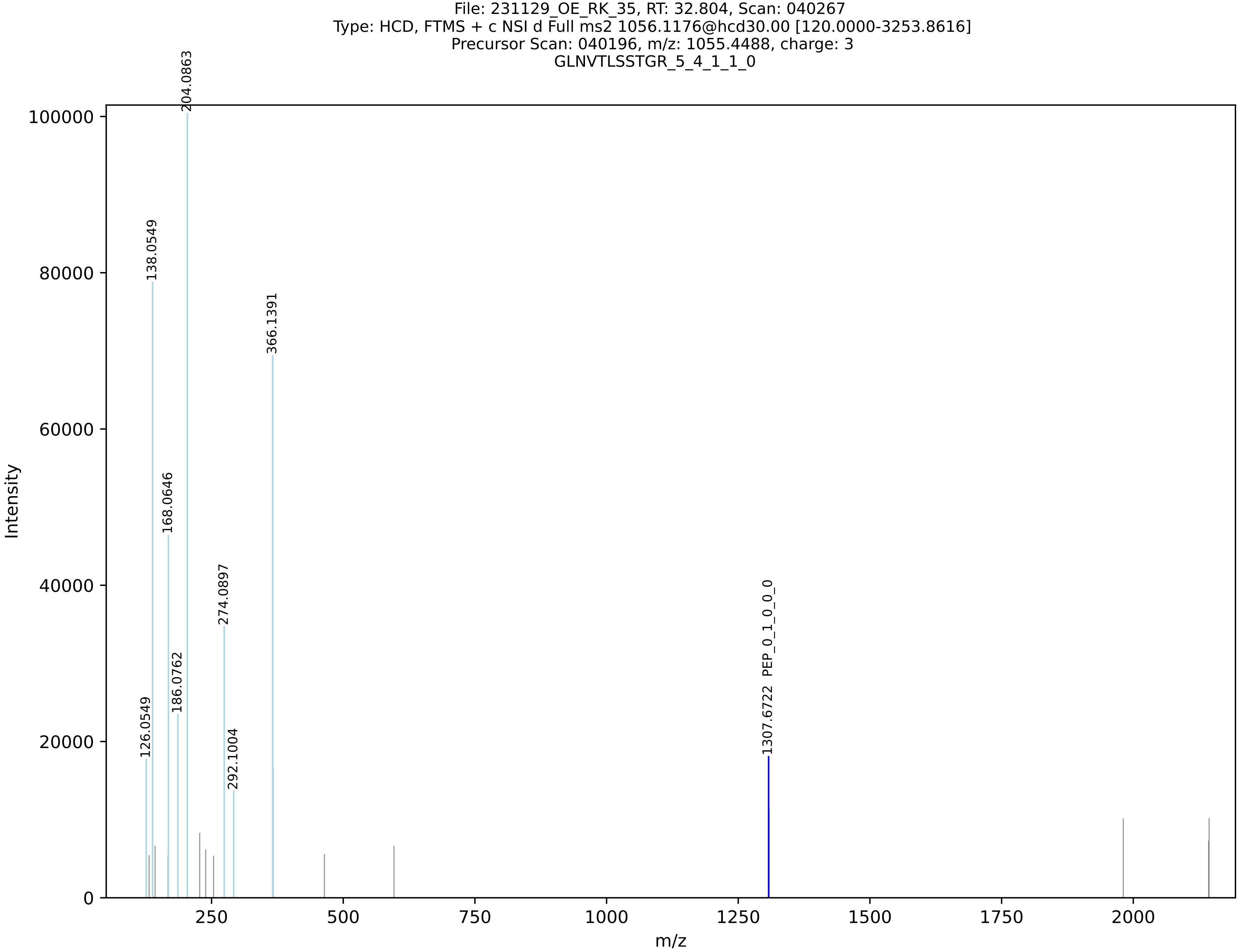

File: 231129_OE_RK_35, RT: 32.804, Scan: 040267
Type: HCD, FTMS + c NSI d Full ms2 1056.1176@hcd30.00 [120.0000-3253.8616]
Precursor Scan: 040196, m/z: 1055.4488, charge: 3
GLNVTLSSTGR_5_4_1_1_0
Intensity
m/z
204.0863
138.0549
366.1391
168.0646
274.0897
186.0762
126.0549
292.1004
1307.6722 PEP_0_1_0_0_0
0
20000
40000
60000
80000
100000
250
500
750
1000
1250
1500
1750
2000

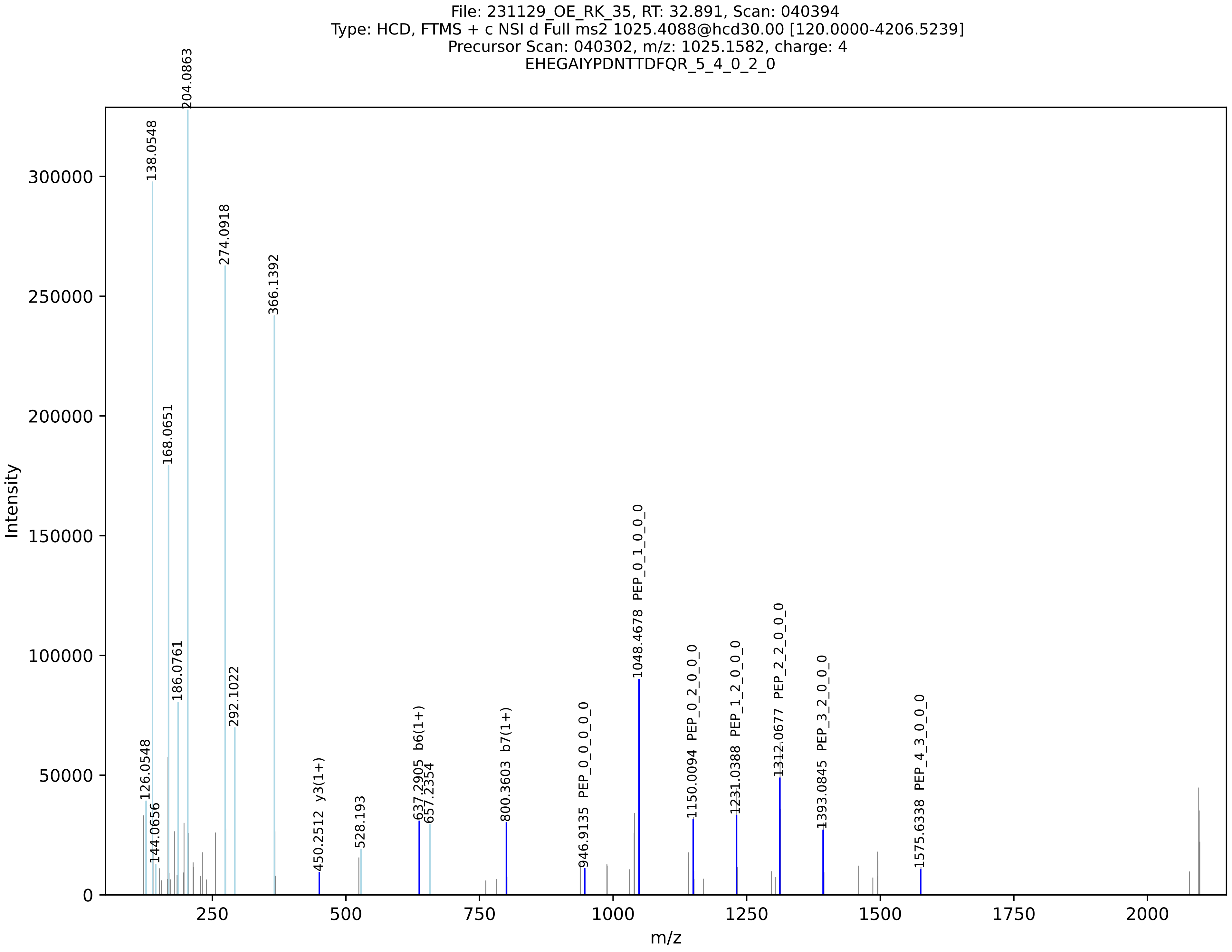
File: 231129_OE_RK_35, RT: 32.891, Scan: 040394
Type: HCD, FTMS + c NSI d Full ms2 1025.4088@hcd30.00 [120.0000-4206.5239]
Precursor Scan: 040302, m/z: 1025.1582, charge: 4
EHEGAIYPDNTTDFQR_5_4_0_2_0
Intensity
m/z
204.0863
138.0548
274.0918
366.1392
168.0651
186.0761
292.1022
126.0548
144.0656
450.2512 y3(1+)
528.193
637.2905 b6(1+)
657.2354
800.3603 b7(1+)
946.9135 PEP_0_0_0_0_0
1048.4678 PEP_0_1_0_0_0
1150.0094 PEP_0_2_0_0_0
1231.0388 PEP_1_2_0_0_0
1312.0677 PEP_2_2_0_0_0
1393.0845 PEP_3_2_0_0_0
1575.6338 PEP_4_3_0_0_0

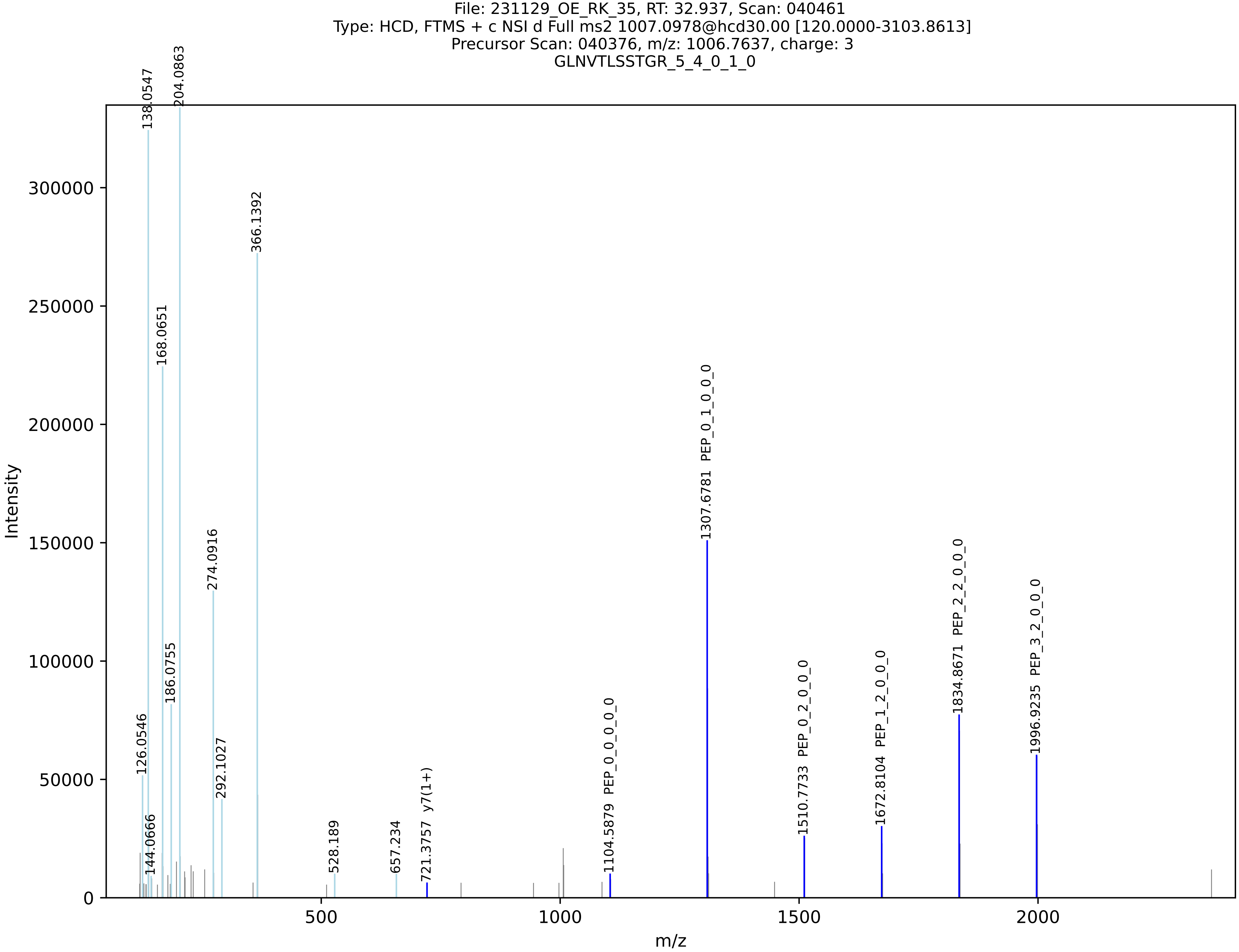

File: 231129_OE_RK_35, RT: 32.937, Scan: 040461
Type: HCD, FTMS + c NSI d Full ms2 1007.0978@hcd30.00 [120.0000-3103.8613]
Precursor Scan: 040376, m/z: 1006.7637, charge: 3
GLNVTLSSTGR_5_4_0_1_0
Intensity
m/z
0
50000
100000
150000
200000
250000
300000
500
1000
1500
2000
138.0547
204.0863
366.1392
168.0651
274.0916
186.0755
126.0546
292.1027
144.0666
528.189
657.234
721.3757 y7(1+)
1104.5879 PEP_0_0_0_0_0
1307.6781 PEP_0_1_0_0_0
1510.7733 PEP_0_2_0_0_0
1672.8104 PEP_1_2_0_0_0
1834.8671 PEP_2_2_0_0_0
1996.9235 PEP_3_2_0_0_0

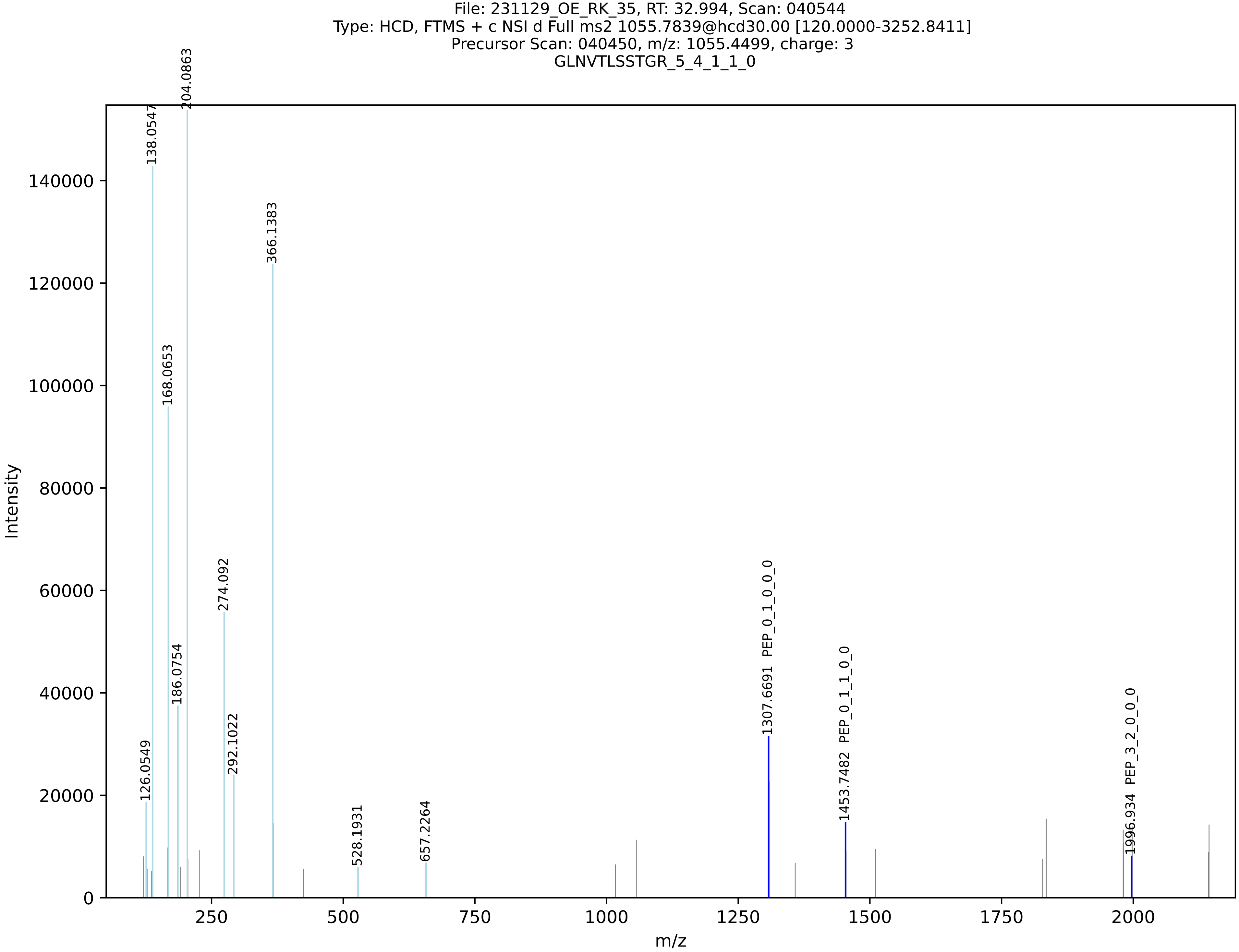
File: 231129_OE_RK_35, RT: 32.994, Scan: 040544
Type: HCD, FTMS + c NSI d Full ms2 1055.7839@hcd30.00 [120.0000-3252.8411]
Precursor Scan: 040450, m/z: 1055.4499, charge: 3
GLNVTLSSTGR_5_4_1_1_0
Intensity
m/z
204.0863
138.0547
366.1383
168.0653
274.092
186.0754
292.1022
126.0549
528.1931
657.2264
1307.6691 PEP_0_1_0_0_0
1453.7482 PEP_0_1_1_0_0
1996.934 PEP_3_2_0_0_0
0
20000
40000
60000
80000
100000
120000
140000
250
500
750
1000
1250
1500
1750
2000

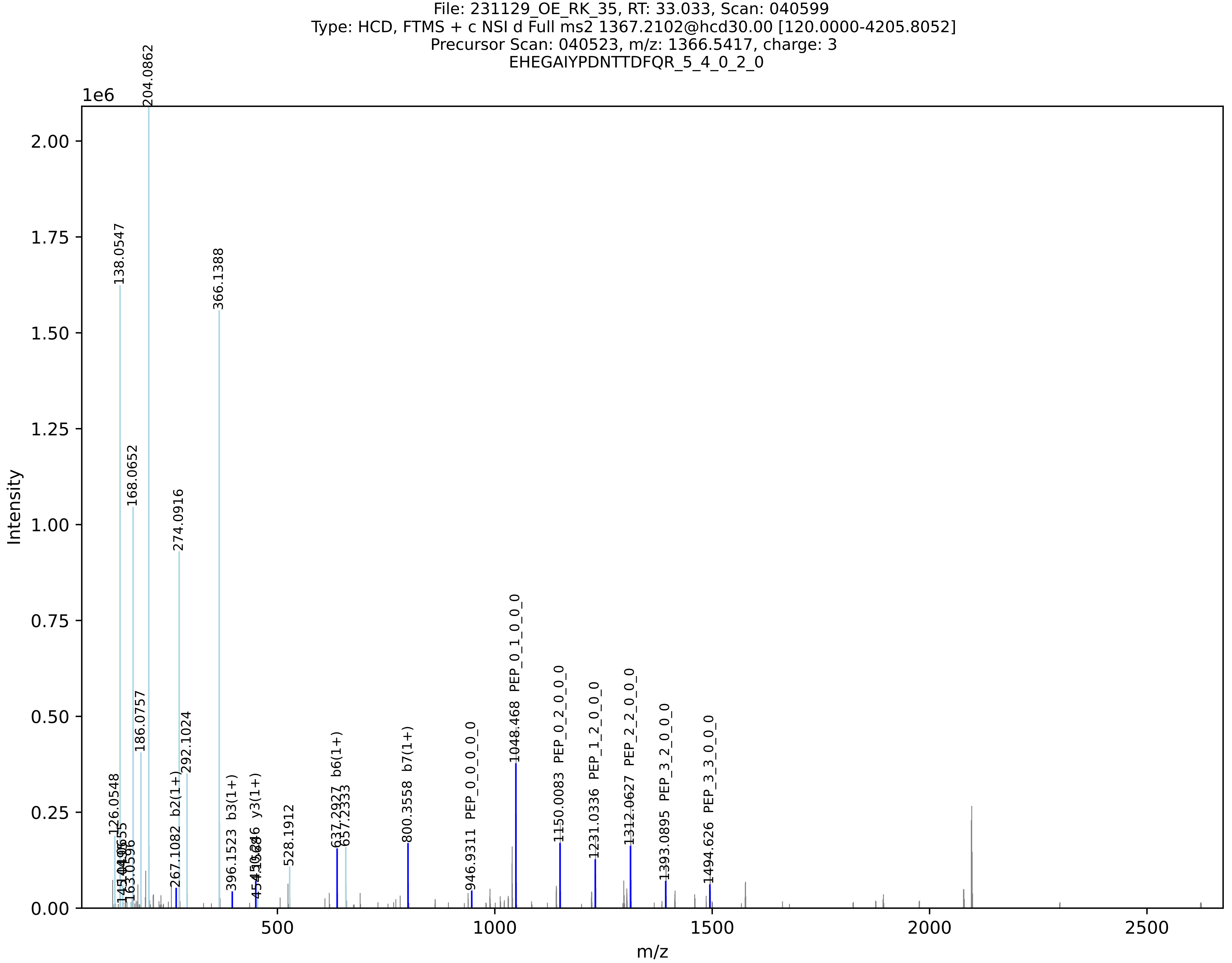

File: 231129_OE_RK_35, RT: 33.033, Scan: 040599
Type: HCD, FTMS + c NSI d Full ms2 1367.2102@hcd30.00 [120.0000-4205.8052]
Precursor Scan: 040523, m/z: 1366.5417, charge: 3
EHEGAIYPDNTTDFQR_5_4_0_2_0
1e6
Intensity
m/z
204.0862
138.0547
366.1388
168.0652
274.0916
186.0757
292.1024
126.0548
267.1082 b2(1+)
396.1523 b3(1+)
y3(1+)
528.1912
637.2927 b6(1+)
657.2333
800.3558 b7(1+)
946.9311 PEP_0_0_0_0_0
1048.468 PEP_0_1_0_0_0
1150.0083 PEP_0_2_0_0_0
1231.0336 PEP_1_2_0_0_0
1312.0627 PEP_2_2_0_0_0
1393.0895 PEP_3_2_0_0_0
1494.626 PEP_3_3_0_0_0

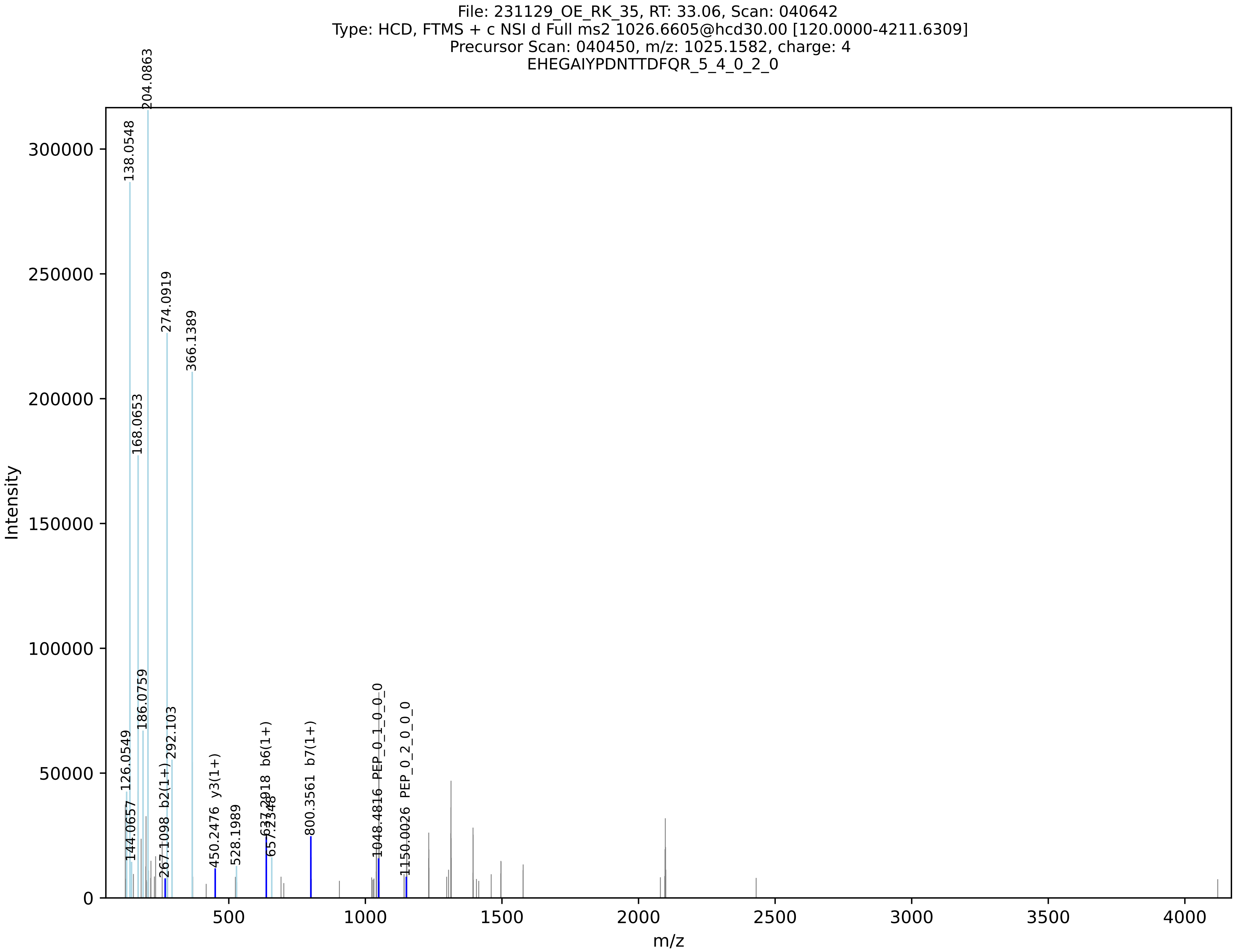
File: 231129_OE_RK_35, RT: 33.06, Scan: 040642
Type: HCD, FTMS + c NSI d Full ms2 1026.6605@hcd30.00 [120.0000-4211.6309]
Precursor Scan: 040450, m/z: 1025.1582, charge: 4
EHEGAIYPDNTTDFQR_5_4_0_2_0
Intensity
m/z
204.0863
138.0548
274.0919
366.1389
168.0653
186.0759
292.103
126.0549
144.0657
267.1098 b2(1+)
450.2476 y3(1+)
528.1989
637.2918 b6(1+)
657.2348
800.3561 b7(1+)
1048.4816 PEP_0_1_0_0_0
1150.0026 PEP_0_2_0_0_0
0
50000
100000
150000
200000
250000
300000
500
1000
1500
2000
2500
3000
3500
4000

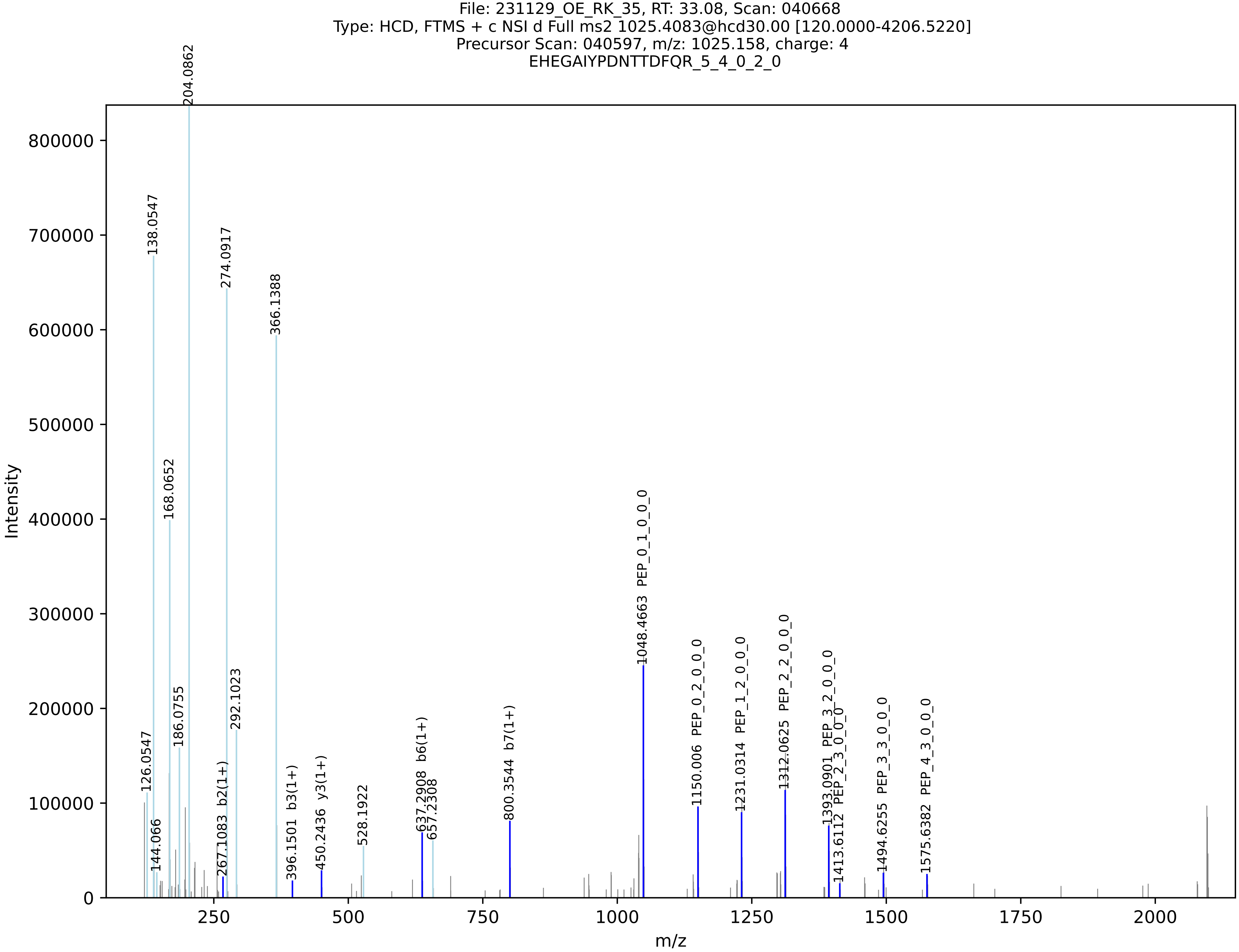

File: 231129_OE_RK_35, RT: 33.08, Scan: 040668
Type: HCD, FTMS + c NSI d Full ms2 1025.4083@hcd30.00 [120.0000-4206.5220]
Precursor Scan: 040597, m/z: 1025.158, charge: 4
EHEGAIYPDNTTDFQR_5_4_0_2_0
Intensity
m/z
204.0862
138.0547
274.0917
366.1388
168.0652
186.0755
292.1023
126.0547
144.066
267.1083 b2(1+)
396.1501 b3(1+)
450.2436 y3(1+)
528.1922
637.2908 b6(1+)
657.2308
800.3544 b7(1+)
1048.4663 PEP_0_1_0_0_0
1150.006 PEP_0_2_0_0_0
1231.0314 PEP_1_2_0_0_0
1312.0625 PEP_2_2_0_0_0
1393.0901 PEP_3_2_0_0_0
1413.6112 PEP_2_3_0_0_0
1494.6255 PEP_3_3_0_0_0
1575.6382 PEP_4_3_0_0_0

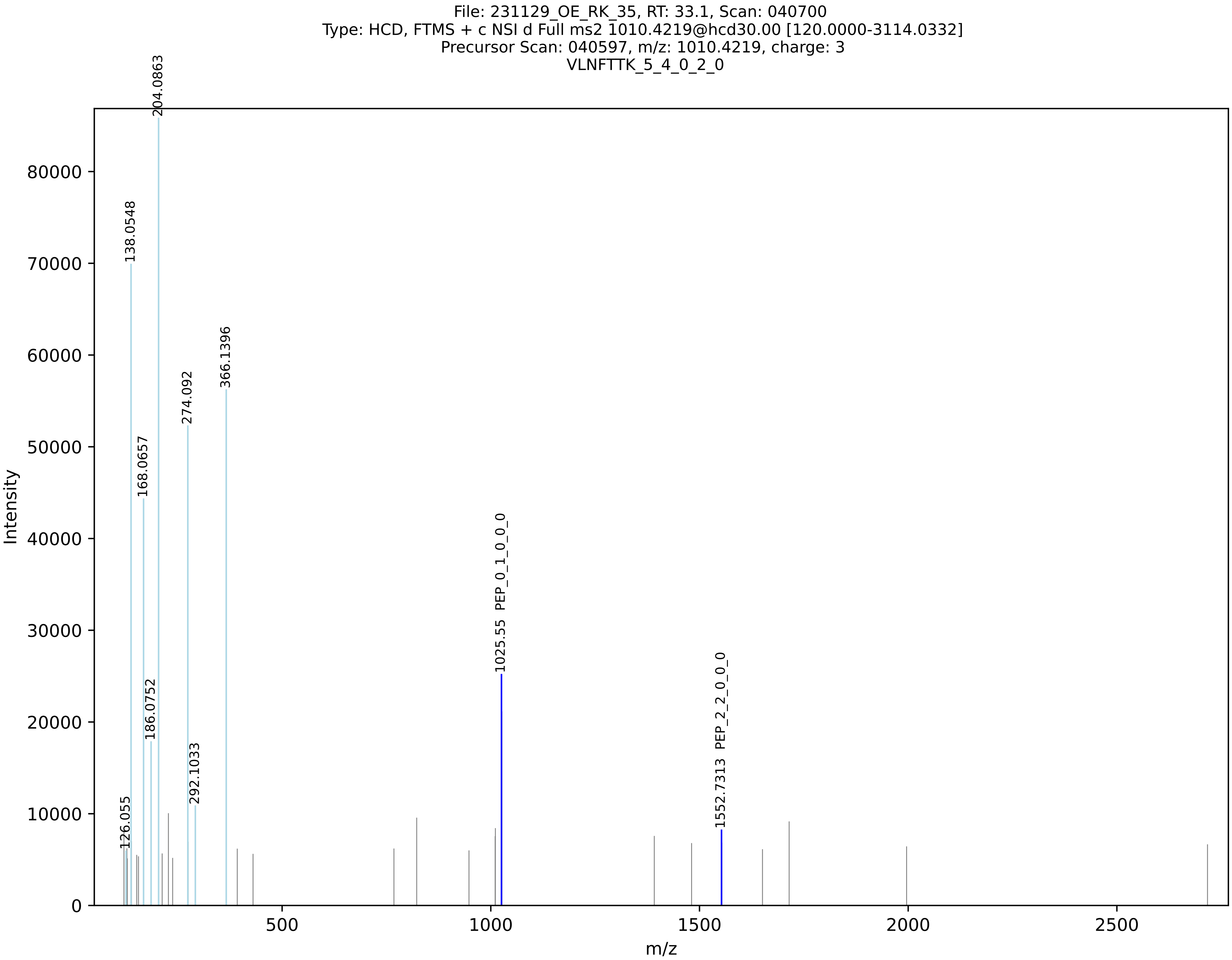

File: 231129_OE_RK_35, RT: 33.1, Scan: 040700
Type: HCD, FTMS + c NSI d Full ms2 1010.4219@hcd30.00 [120.0000-3114.0332]
Precursor Scan: 040597, m/z: 1010.4219, charge: 3
VLNFTTK_5_4_0_2_0
204.0863
138.0548
366.1396
274.092
168.0657
1025.55 PEP_0_1_0_0_0
1552.7313 PEP_2_2_0_0_0
186.0752
292.1033
126.055
Intensity
m/z
0
10000
20000
30000
40000
50000
60000
70000
80000
500
1000
1500
2000
2500

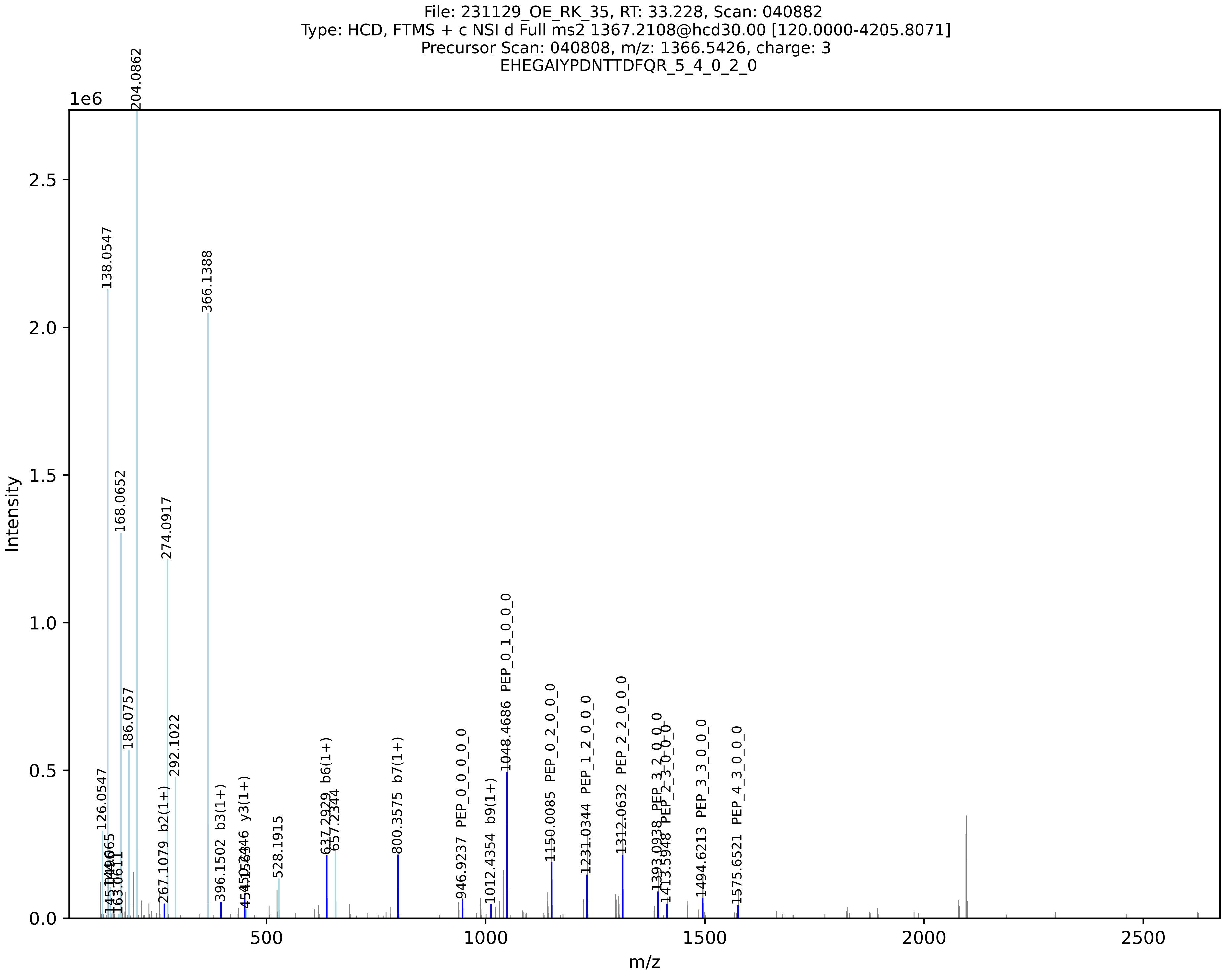
File: 231129_OE_RK_35, RT: 33.228, Scan: 040882
Type: HCD, FTMS + c NSI d Full ms2 1367.2108@hcd30.00 [120.0000-4205.8071]
Precursor Scan: 040808, m/z: 1366.5426, charge: 3
EHEGAIYPDNTTDFQR_5_4_0_2_0
1e6
Intensity
m/z
204.0862
138.0547
366.1388
168.0652
274.0917
186.0757
292.1022
126.0547
267.1079 b2(1+)
396.1502 b3(1+)
y3(1+)
528.1915
637.2929 b6(1+)
657.2344
800.3575 b7(1+)
946.9237 PEP_0_0_0_0_0
1012.4354 b9(1+)
1048.4686 PEP_0_1_0_0_0
1150.0085 PEP_0_2_0_0_0
1231.0344 PEP_1_2_0_0_0
1312.0632 PEP_2_2_0_0_0
1393.0938 PEP_3_2_0_0_0
1413.5948 PEP_2_3_0_0_0
1494.6213 PEP_3_3_0_0_0
1575.6521 PEP_4_3_0_0_0

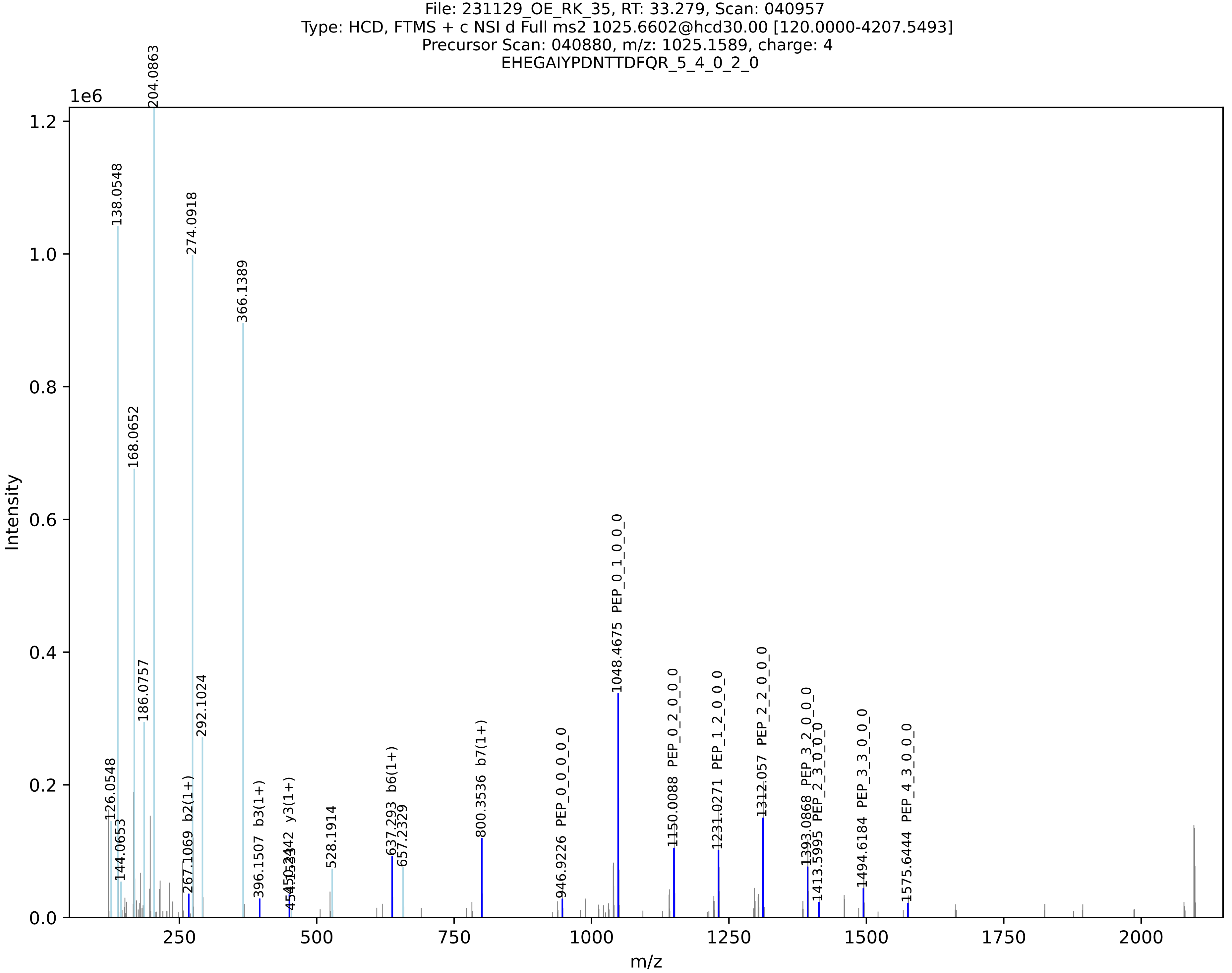

File: 231129_OE_RK_35, RT: 33.279, Scan: 040957
Type: HCD, FTMS + c NSI d Full ms2 1025.6602@hcd30.00 [120.0000-4207.5493]
Precursor Scan: 040880, m/z: 1025.1589, charge: 4
EHEGAIYPDNTTDFQR_5_4_0_2_0
1e6
Intensity
m/z
204.0863
138.0548
274.0918
366.1389
168.0652
186.0757
292.1024
126.0548
144.0653
267.1069 b2(1+)
396.1507 b3(1+)
454.2442 y3(1+)
528.1914
637.293 b6(1+)
657.2329
800.3536 b7(1+)
946.9226 PEP_0_0_0_0_0_
1048.4675 PEP_0_1_0_0_0_
1150.0088 PEP_0_2_0_0_0_
1231.0271 PEP_1_2_0_0_0_
1312.057 PEP_2_2_0_0_0_
1393.0868 PEP_3_2_0_0_0_
1413.5995 PEP_2_3_0_0_0_
1494.6184 PEP_3_3_0_0_0_
1575.6444 PEP_4_3_0_0_0_

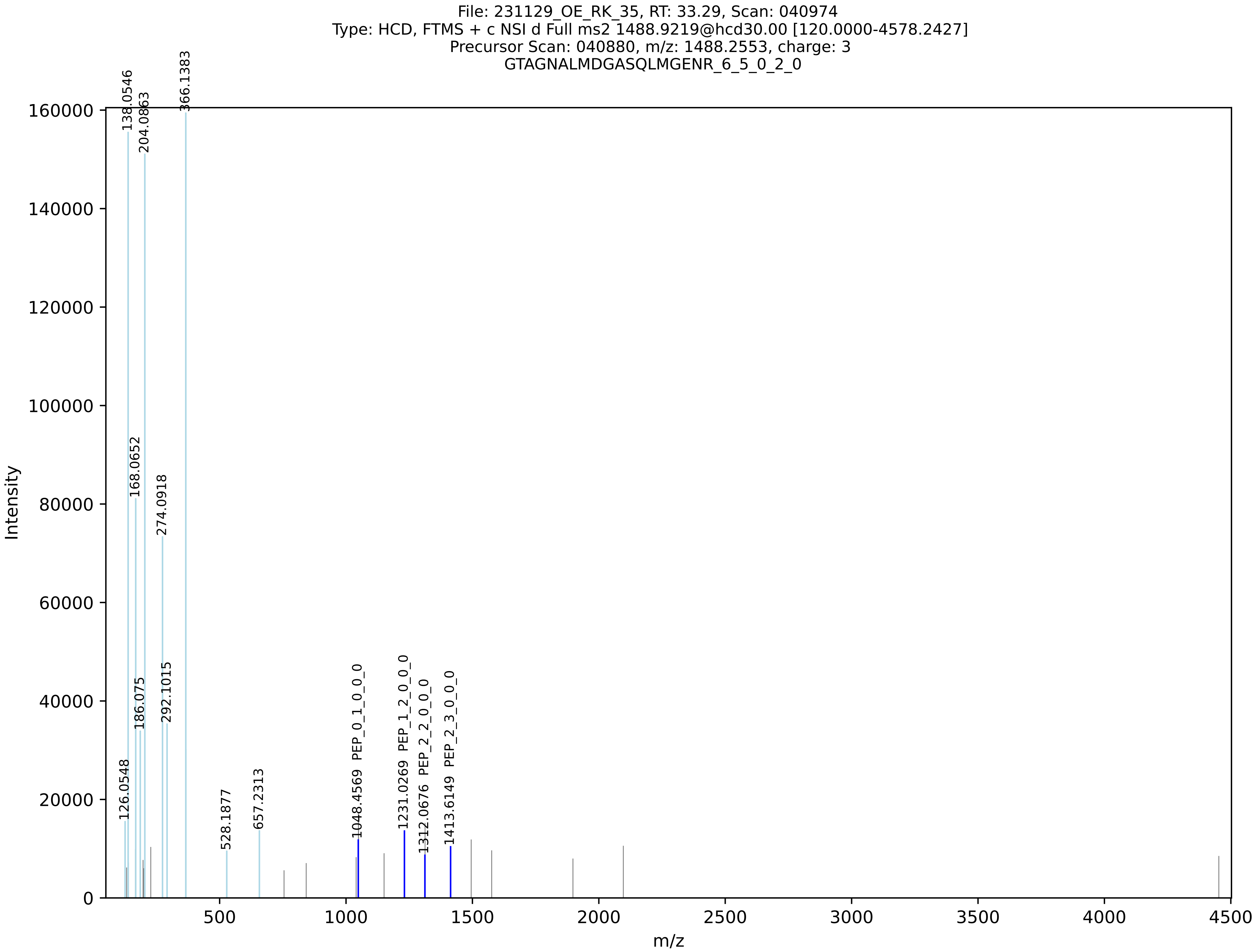

File: 231129_OE_RK_35, RT: 33.29, Scan: 040974
Type: HCD, FTMS + c NSI d Full ms2 1488.9219@hcd30.00 [120.0000-4578.2427]
Precursor Scan: 040880, m/z: 1488.2553, charge: 3
GTAGNALMDGASQLMGENR_6_5_0_2_0
Intensity
m/z
138.0546
204.0863
366.1383
168.0652
274.0918
186.075
292.1015
126.0548
528.1877
657.2313
1048.4569 PEP_0_1_0_0_0
1231.0269 PEP_1_2_0_0_0
1312.0676 PEP_2_2_0_0_0
1413.6149 PEP_2_3_0_0_0

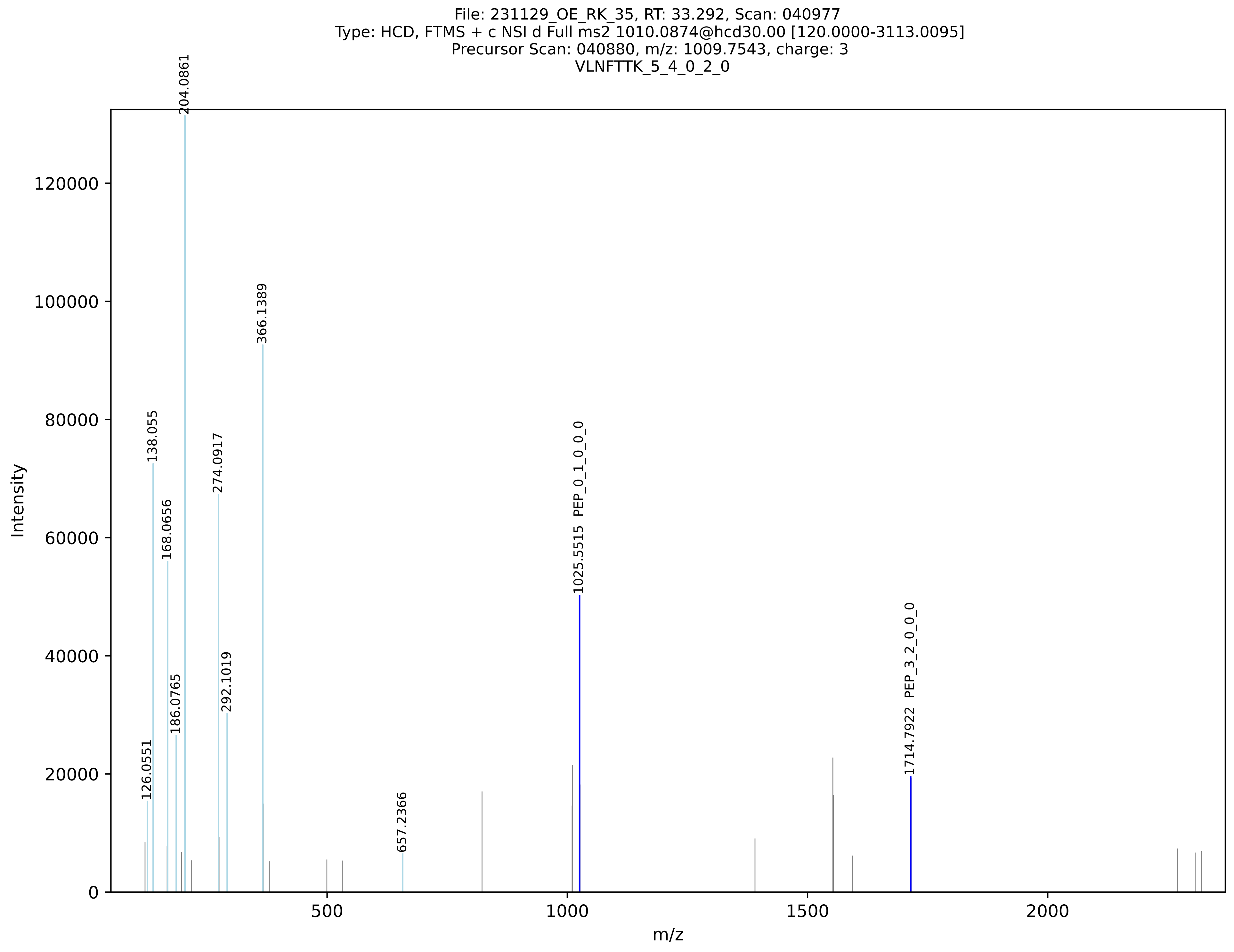
File: 231129_OE_RK_35, RT: 33.292, Scan: 040977
Type: HCD, FTMS + c NSI d Full ms2 1010.0874@hcd30.00 [120.0000-3113.0095]
Precursor Scan: 040880, m/z: 1009.7543, charge: 3
VLNFTTK_5_4_0_2_0
204.0861
366.1389
138.055
274.0917
168.0656
1025.5515 PEP_0_1_0_0_0
1714.7922 PEP_3_2_0_0_0
292.1019
186.0765
126.0551
657.2366
Intensity
m/z
0
20000
40000
60000
80000
100000
120000
500
1000
1500
2000

File: 231129_OE_RK_35, RT: 33.379, Scan: 041102
Type: HCD, FTMS + c NSI d Full ms2 1007.0984@hcd30.00 [120.0000-3103.8630]
Precursor Scan: 041028, m/z: 1006.7643, charge: 3
GLNVTLSSTGR_5_4_0_1_0

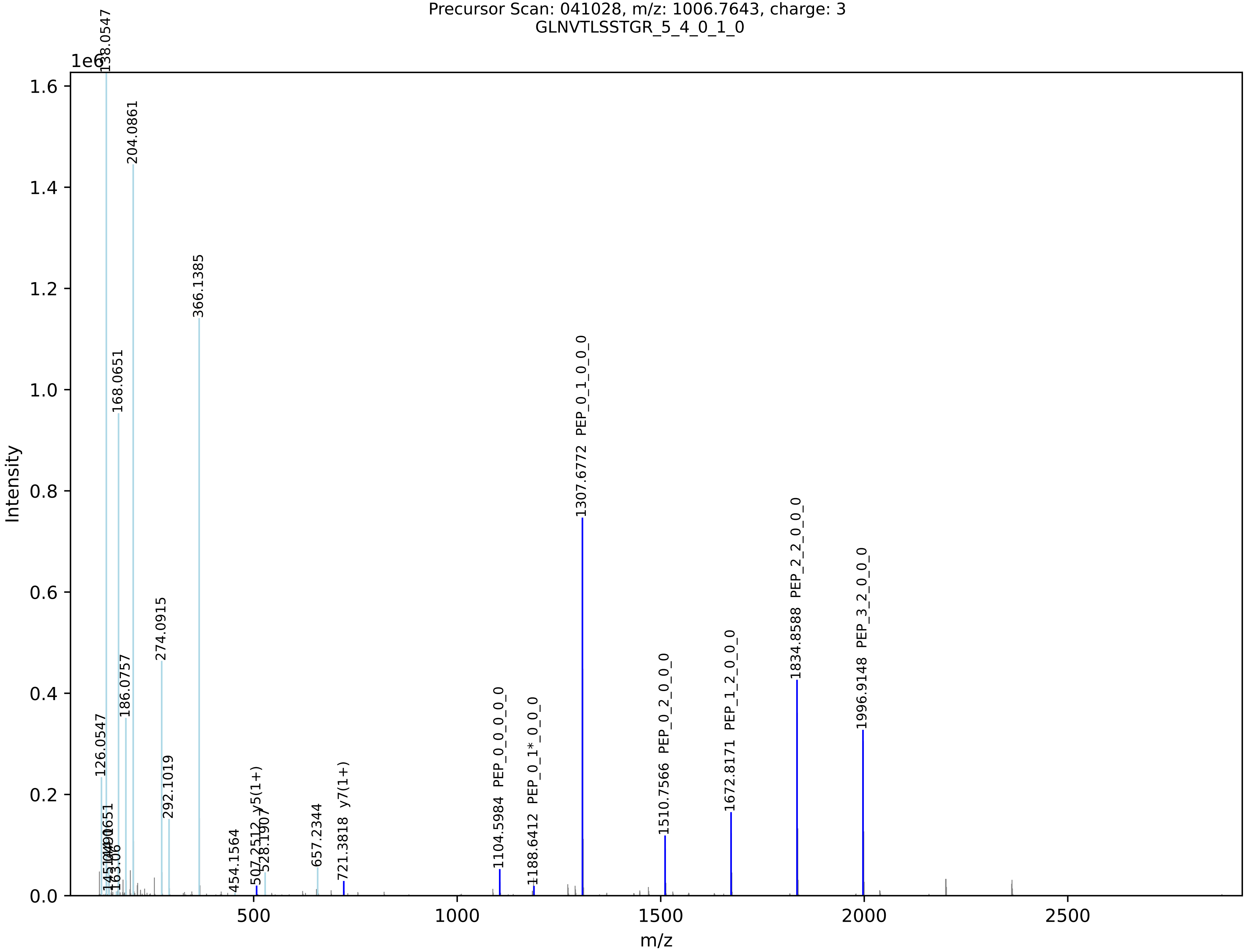

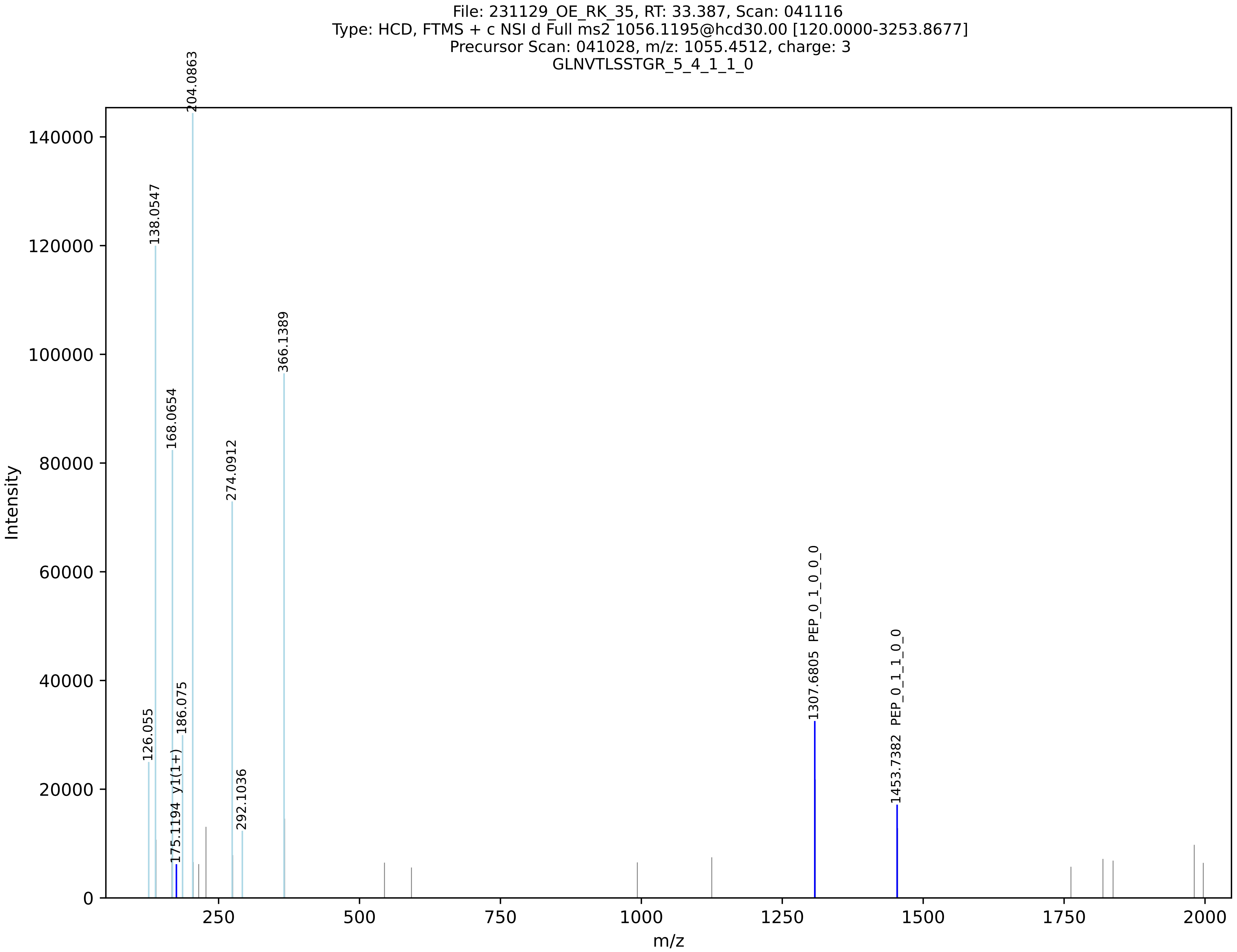
File: 231129_OE_RK_35, RT: 33.387, Scan: 041116
Type: HCD, FTMS + c NSI d Full ms2 1056.1195@hcd30.00 [120.0000-3253.8677]
Precursor Scan: 041028, m/z: 1055.4512, charge: 3
GLNVTLSSTGR_5_4_1_1_0
Intensity
m/z
0
20000
40000
60000
80000
100000
120000
140000
250
500
750
1000
1250
1500
1750
2000
204.0863
138.0547
366.1389
168.0654
274.0912
126.055
186.075
175.1194 y1(1+)
292.1036
1307.6805 PEP_0_1_0_0_0
1453.7382 PEP_0_1_1_0_0

File: 231129_OE_RK_35, RT: 33.429, Scan: 041175
Type: HCD, FTMS + c NSI d Full ms2 1367.2109@hcd30.00 [120.0000-4205.8076]
Precursor Scan: 041101, m/z: 1366.5429, charge: 3
EHEGAIYPDNTTDFQR_5_4_0_2_0

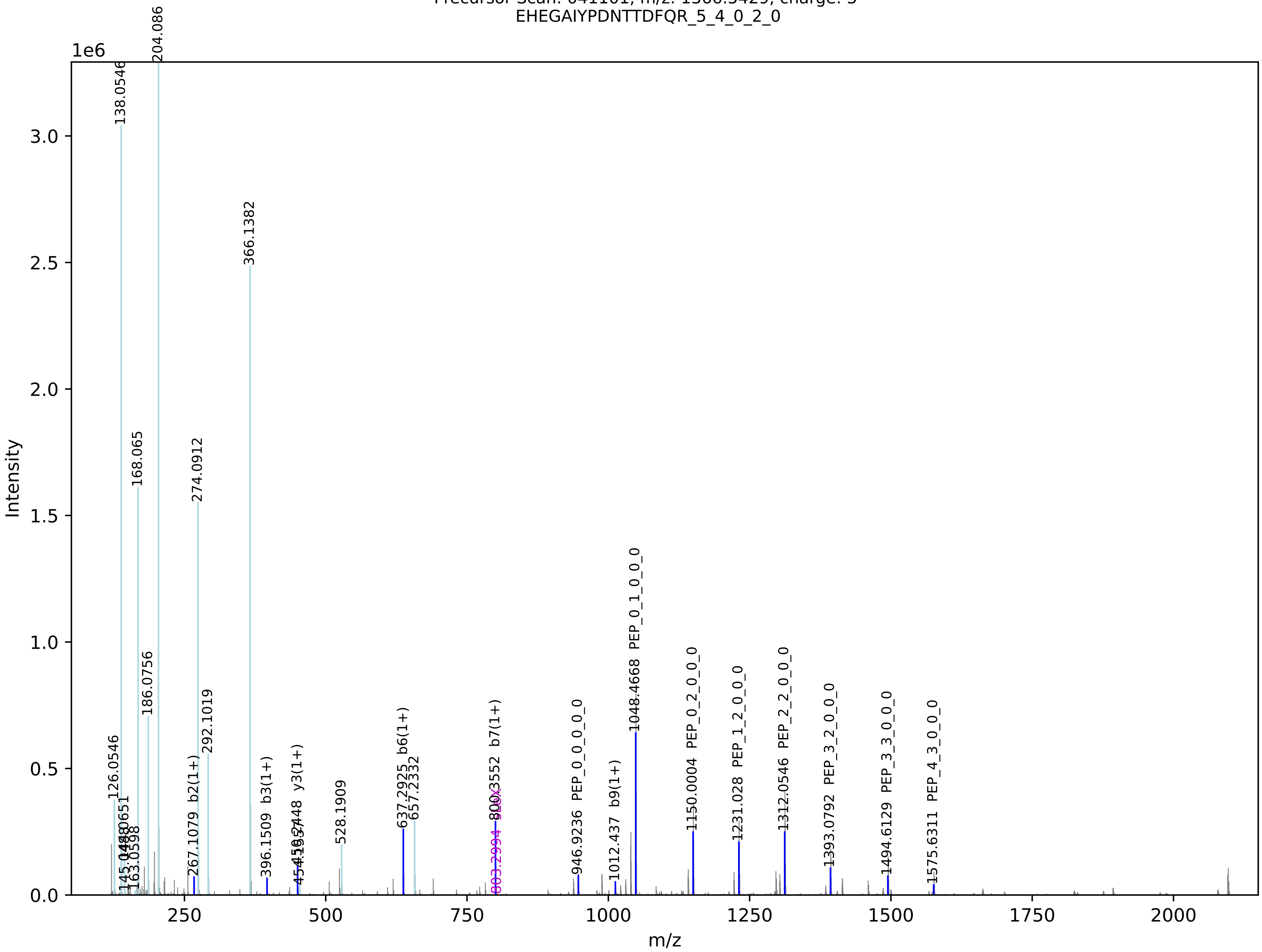

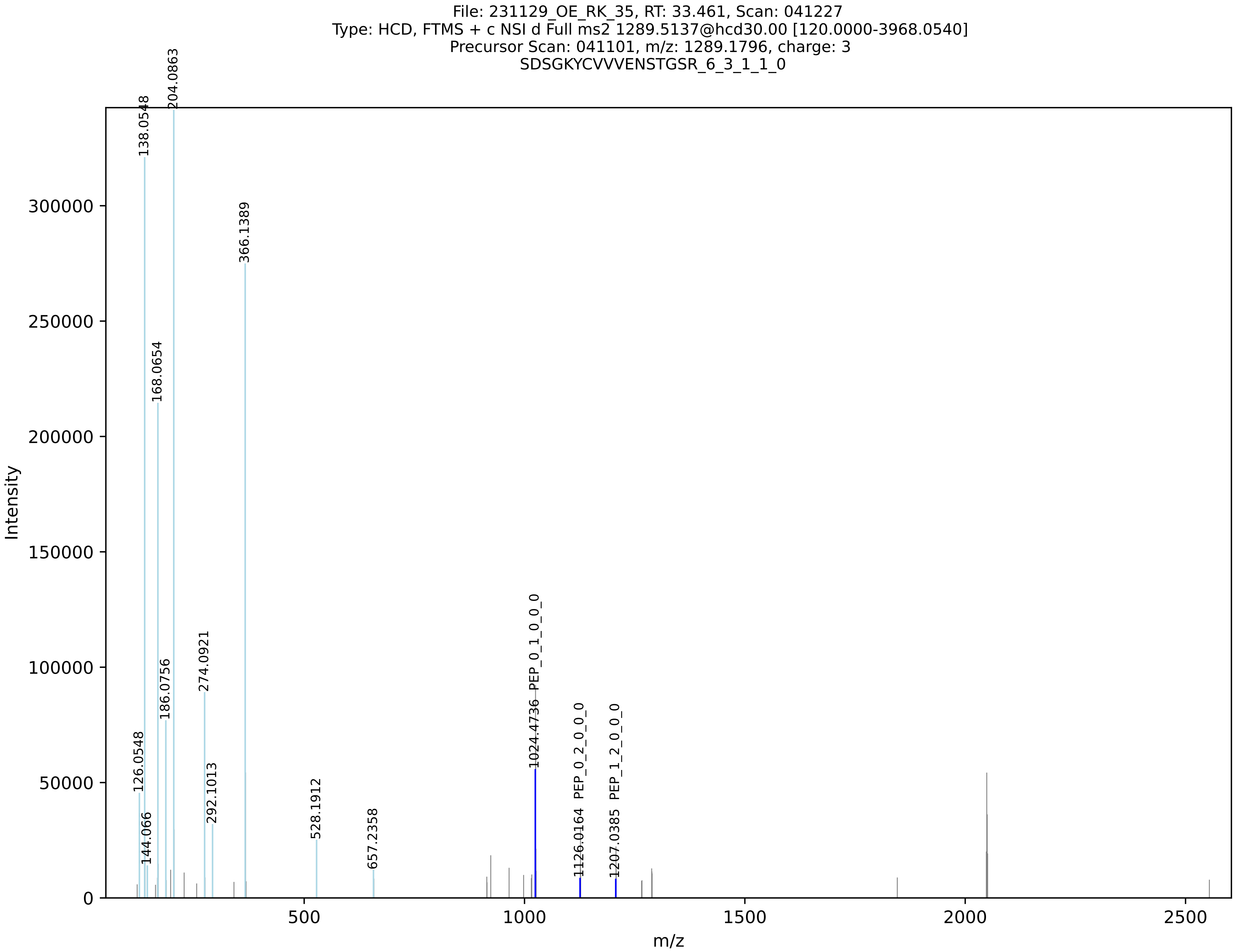

File: 231129_OE_RK_35, RT: 33.461, Scan: 041227
Type: HCD, FTMS + c NSI d Full ms2 1289.5137@hcd30.00 [120.0000-3968.0540]
Precursor Scan: 041101, m/z: 1289.1796, charge: 3
SDSGKYCVVVENSTGSR_6_3_1_1_0
Intensity
m/z
126.0548
138.0548
144.066
168.0654
186.0756
204.0863
274.0921
292.1013
366.1389
528.1912
657.2358
1024.4736 PEP_0_1_0_0_0
1126.0164 PEP_0_2_0_0_0
1207.0385 PEP_1_2_0_0_0

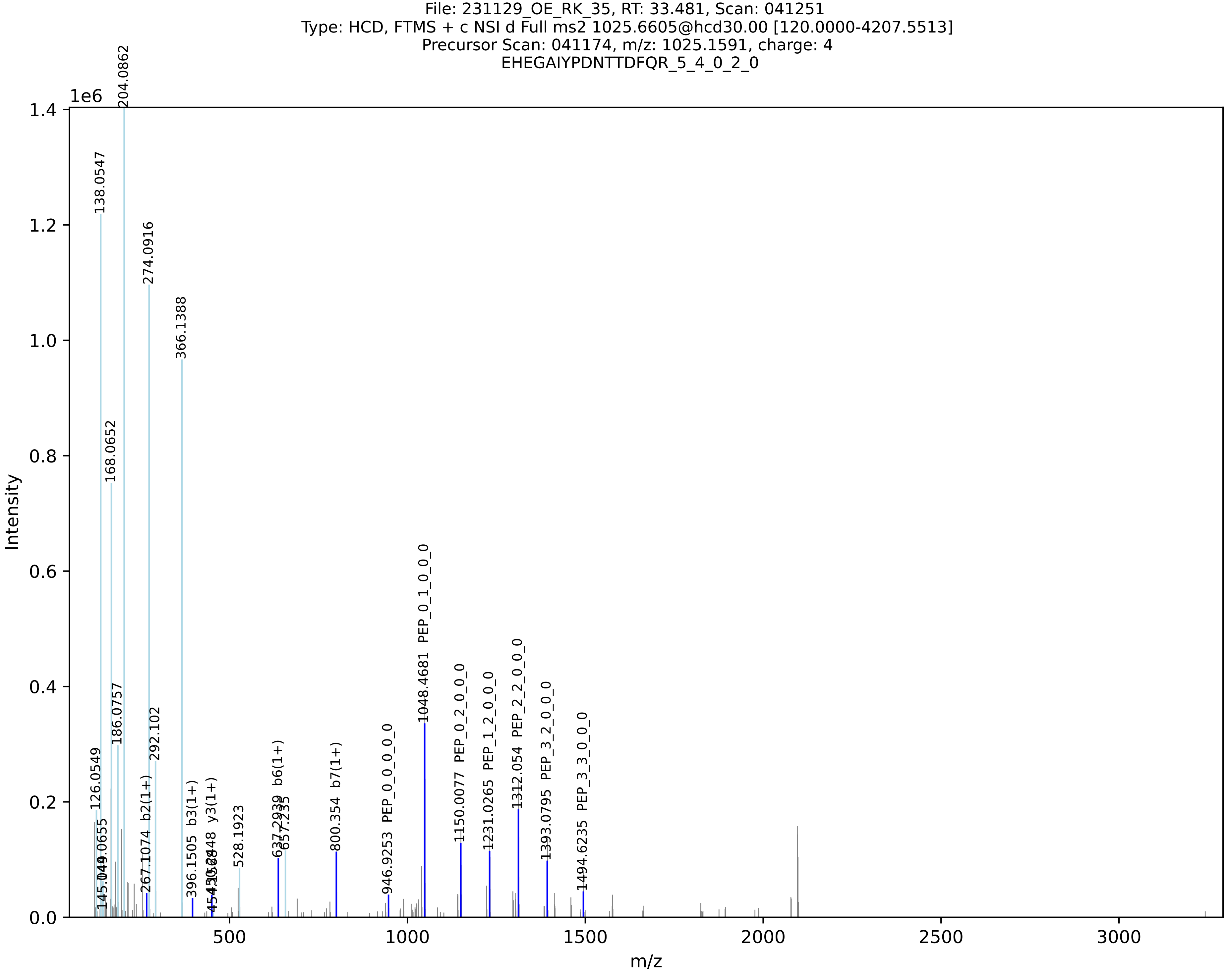

File: 231129_OE_RK_35, RT: 33.481, Scan: 041251
Type: HCD, FTMS + c NSI d Full ms2 1025.6605@hcd30.00 [120.0000-4207.5513]
Precursor Scan: 041174, m/z: 1025.1591, charge: 4
EHEGAIYPDNTTDFQR_5_4_0_2_0
1e6
Intensity
m/z
204.0862
138.0547
274.0916
366.1388
168.0652
186.0757
292.102
126.0549
267.1074 b2(1+)
396.1505 b3(1+)
528.1923
637.2939 b6(1+)
657.235
800.354 b7(1+)
946.9253 PEP_0_0_0_0_0
1048.4681 PEP_0_1_0_0_0
1150.0077 PEP_0_2_0_0_0
1231.0265 PEP_1_2_0_0_0
1312.054 PEP_2_2_0_0_0
1393.0795 PEP_3_2_0_0_0
1494.6235 PEP_3_3_0_0_0

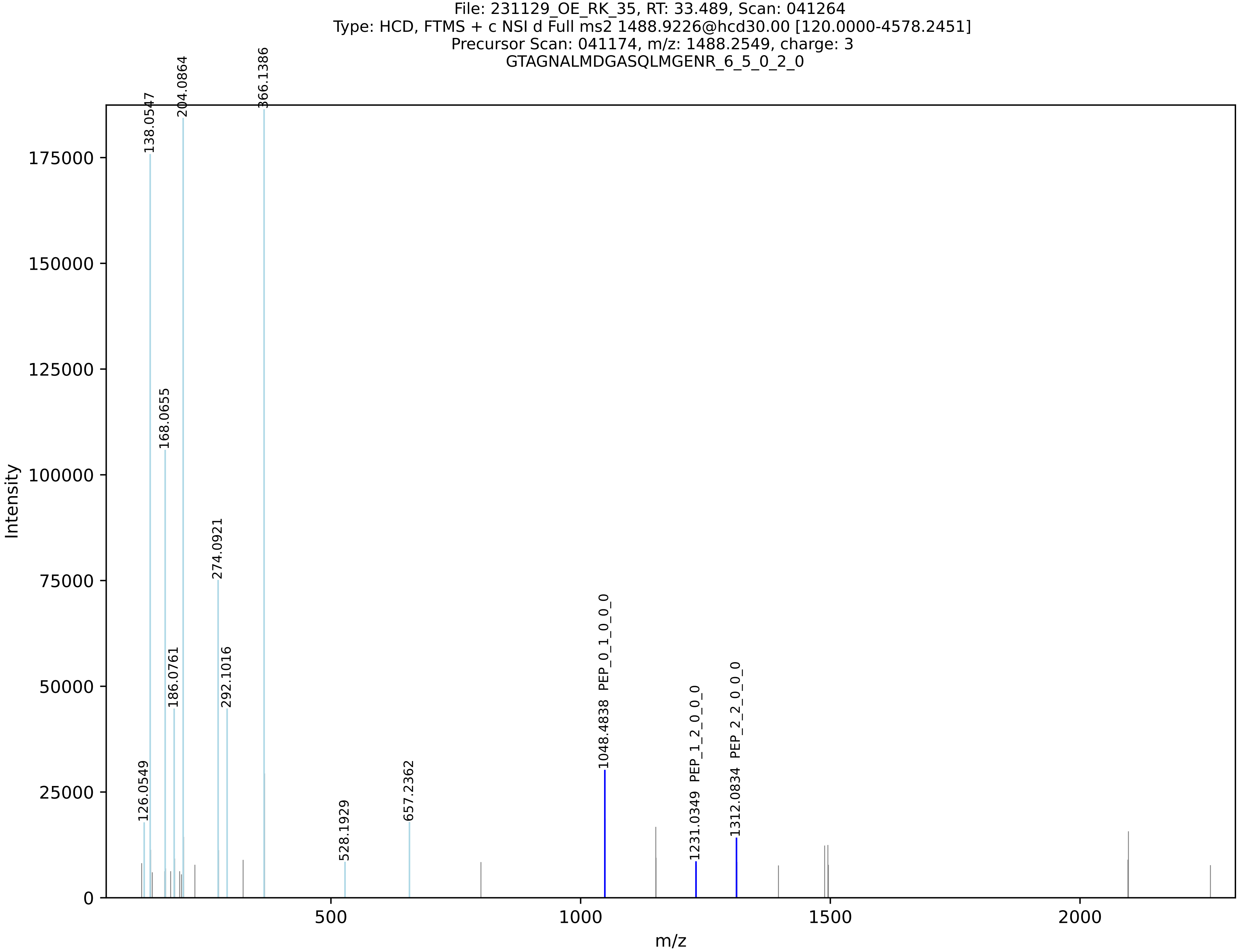

File: 231129_OE_RK_35, RT: 33.489, Scan: 041264
Type: HCD, FTMS + c NSI d Full ms2 1488.9226@hcd30.00 [120.0000-4578.2451]
Precursor Scan: 041174, m/z: 1488.2549, charge: 3
GTAGNALMDGASQLMGENR_6_5_0_2_0
Intensity
m/z
126.0549
138.0547
168.0655
186.0761
204.0864
274.0921
292.1016
366.1386
528.1929
657.2362
1048.4838 PEP_0_1_0_0_0
1231.0349 PEP_1_2_0_0_0
1312.0834 PEP_2_2_0_0_0

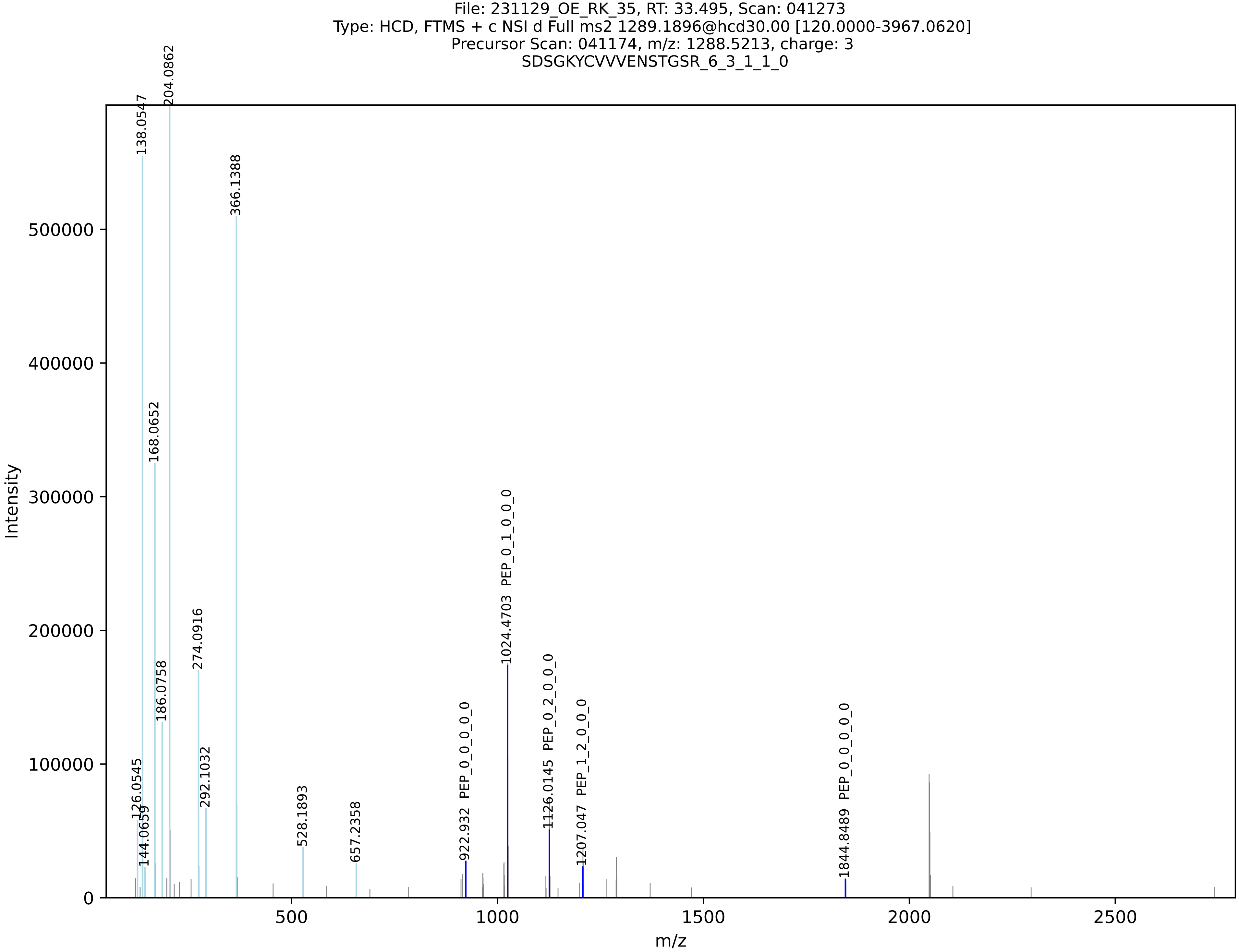

File: 231129_OE_RK_35, RT: 33.495, Scan: 041273
Type: HCD, FTMS + c NSI d Full ms2 1289.1896@hcd30.00 [120.0000-3967.0620]
Precursor Scan: 041174, m/z: 1288.5213, charge: 3
SDSGKYCVVVENSTGSR_6_3_1_1_0
Intensity
m/z
204.0862
138.0547
366.1388
168.0652
274.0916
186.0758
126.0545
144.0659
292.1032
528.1893
657.2358
922.932 PEP_0_0_0_0_0_
1024.4703 PEP_0_1_0_0_0_
1126.0145 PEP_0_2_0_0_0_
1207.047 PEP_1_2_0_0_0_
1844.8489 PEP_0_0_0_0_0_

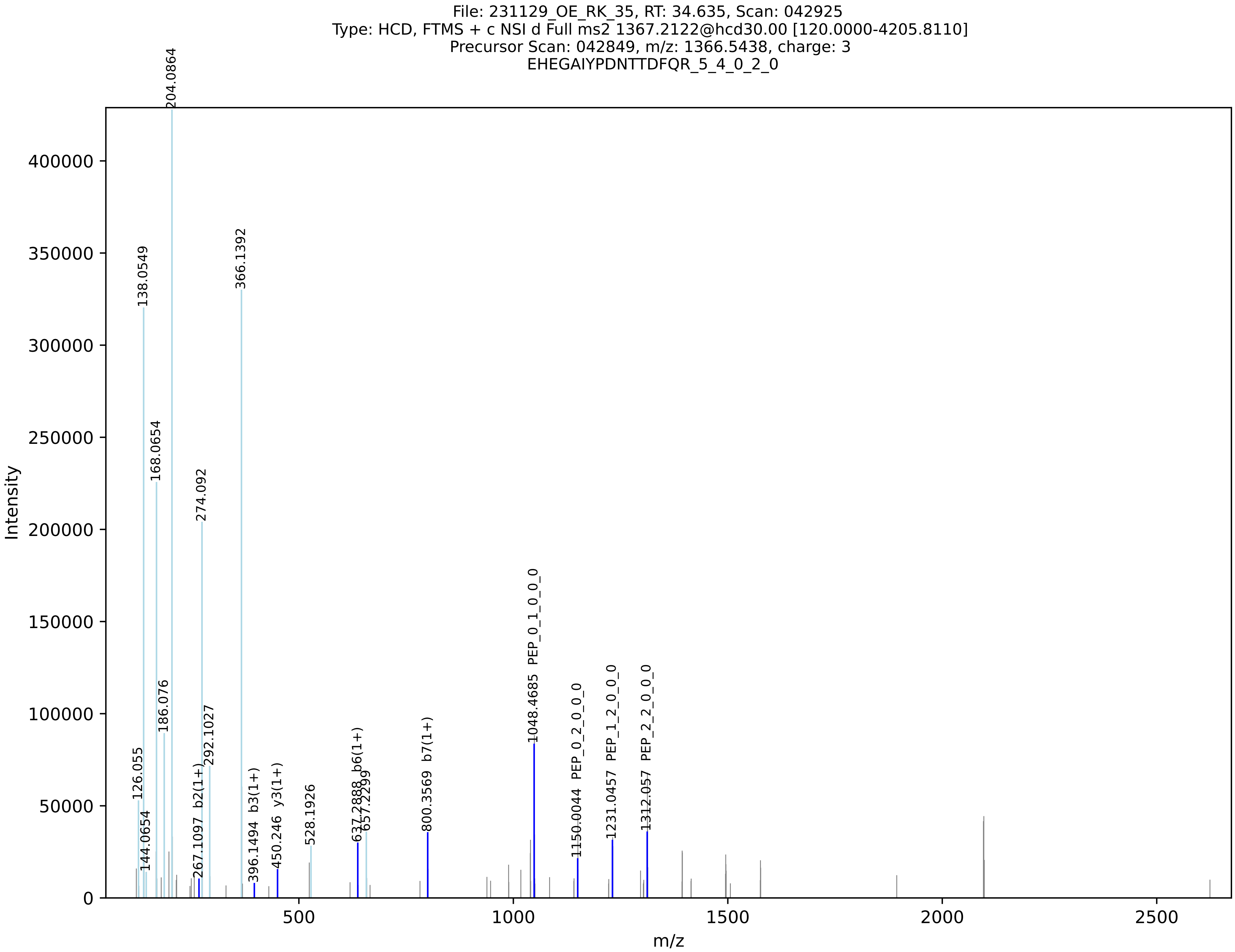

File: 231129_OE_RK_35, RT: 34.635, Scan: 042925
Type: HCD, FTMS + c NSI d Full ms2 1367.2122@hcd30.00 [120.0000-4205.8110]
Precursor Scan: 042849, m/z: 1366.5438, charge: 3
EHEGAIYPDNTTDFQR_5_4_0_2_0
Intensity
m/z
204.0864
138.0549
366.1392
168.0654
274.092
186.076
292.1027
126.055
144.0654
267.1097 b2(1+)
396.1494 b3(1+)
450.246 y3(1+)
528.1926
637.2888 b6(1+)
657.2299
800.3569 b7(1+)
1048.4685 PEP_0_1_0_0_0
1150.0044 PEP_0_2_0_0_0
1231.0457 PEP_1_2_0_0_0
1312.057 PEP_2_2_0_0_0

File: 231129_OE_RK_35, RT: 34.638, Scan: 042929
Type: HCD, FTMS + c NSI d Full ms2 1074.9691@hcd30.00 [120.0000-4408.7300]
Precursor Scan: 042849, m/z: 1074.4678, charge: 4
NNATVHEQVGGPSLTSDLQAQSK_5_4_0_1_0

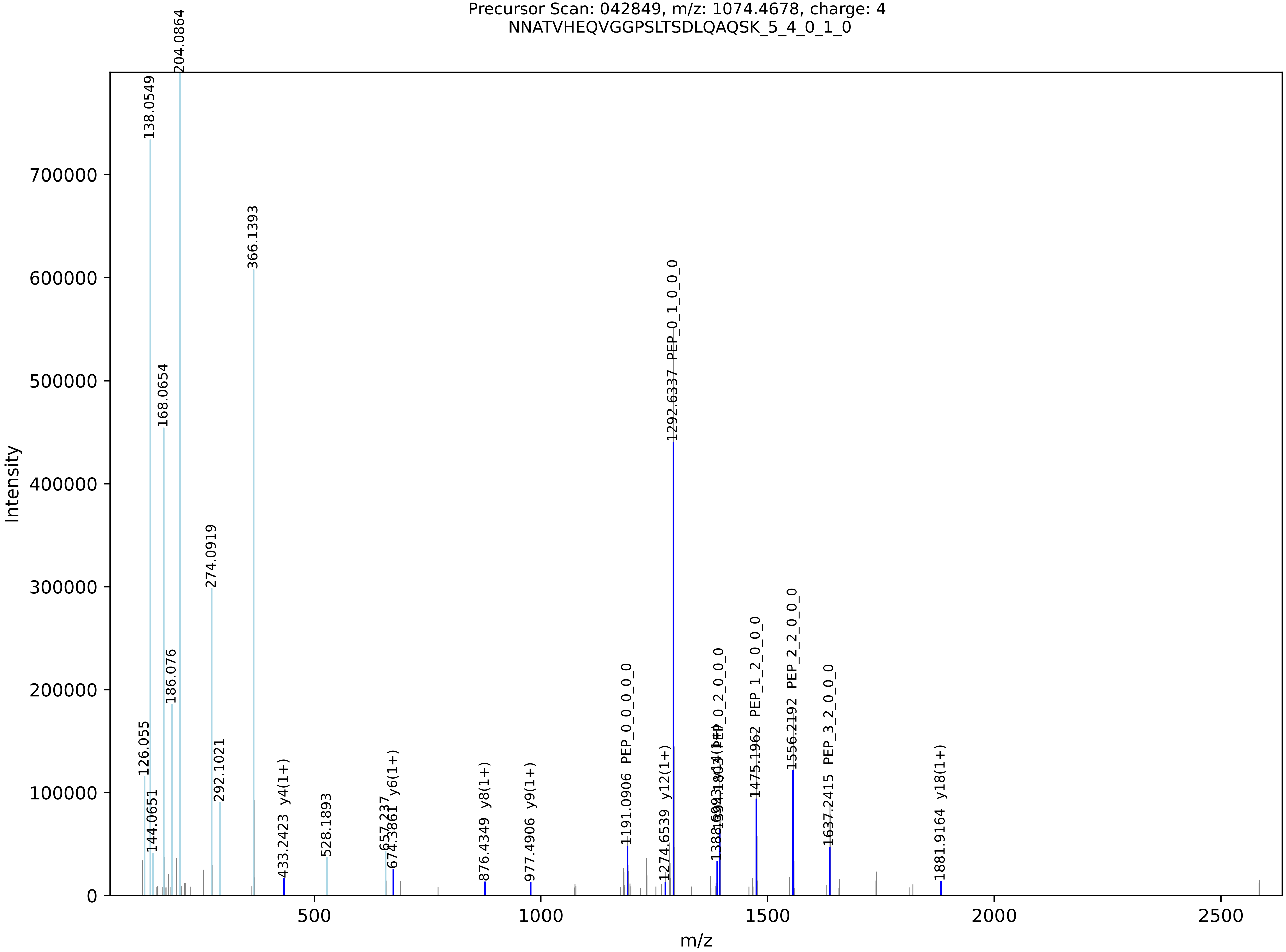

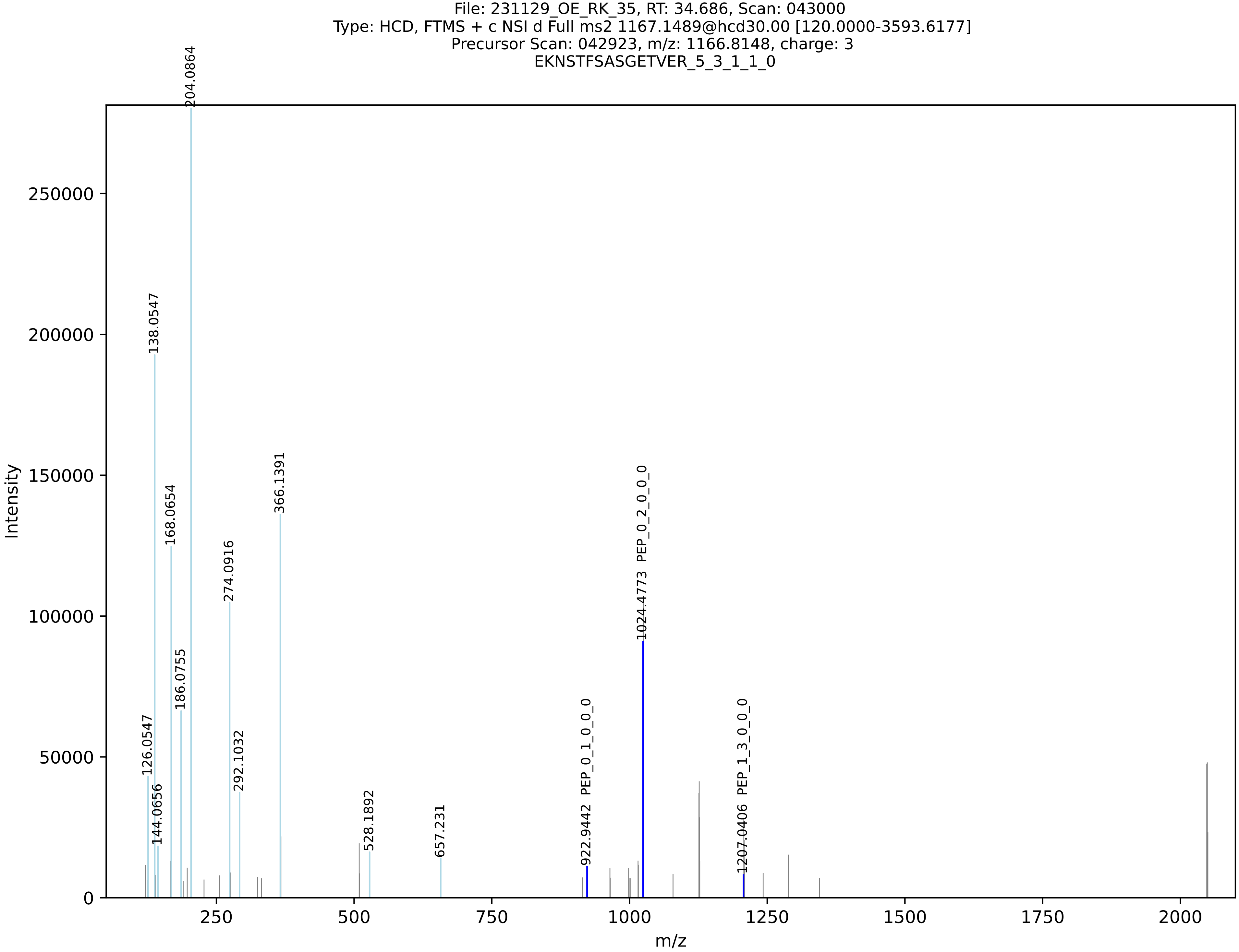
File: 231129_OE_RK_35, RT: 34.686, Scan: 043000
Type: HCD, FTMS + c NSI d Full ms2 1167.1489@hcd30.00 [120.0000-3593.6177]
Precursor Scan: 042923, m/z: 1166.8148, charge: 3
EKNSTFSASGETVER_5_3_1_1_0
Intensity
m/z
204.0864
138.0547
168.0654
186.0755
126.0547
144.0656
274.0916
292.1032
366.1391
528.1892
657.231
922.9442 PEP_0_1_0_0_0
1024.4773 PEP_0_2_0_0_0
1207.0406 PEP_1_3_0_0_0
0
50000
100000
150000
200000
250000
250
500
750
1000
1250
1500
1750
2000

File: 231129_OE_RK_35, RT: 34.687, Scan: 043001
Type: HCD, FTMS + c NSI d Full ms2 1207.5009@hcd30.00 [120.0000-3717.0945]
Precursor Scan: 042923, m/z: 1206.8326, charge: 3
ITYSIVQTNCSK_5_4_0_2_0

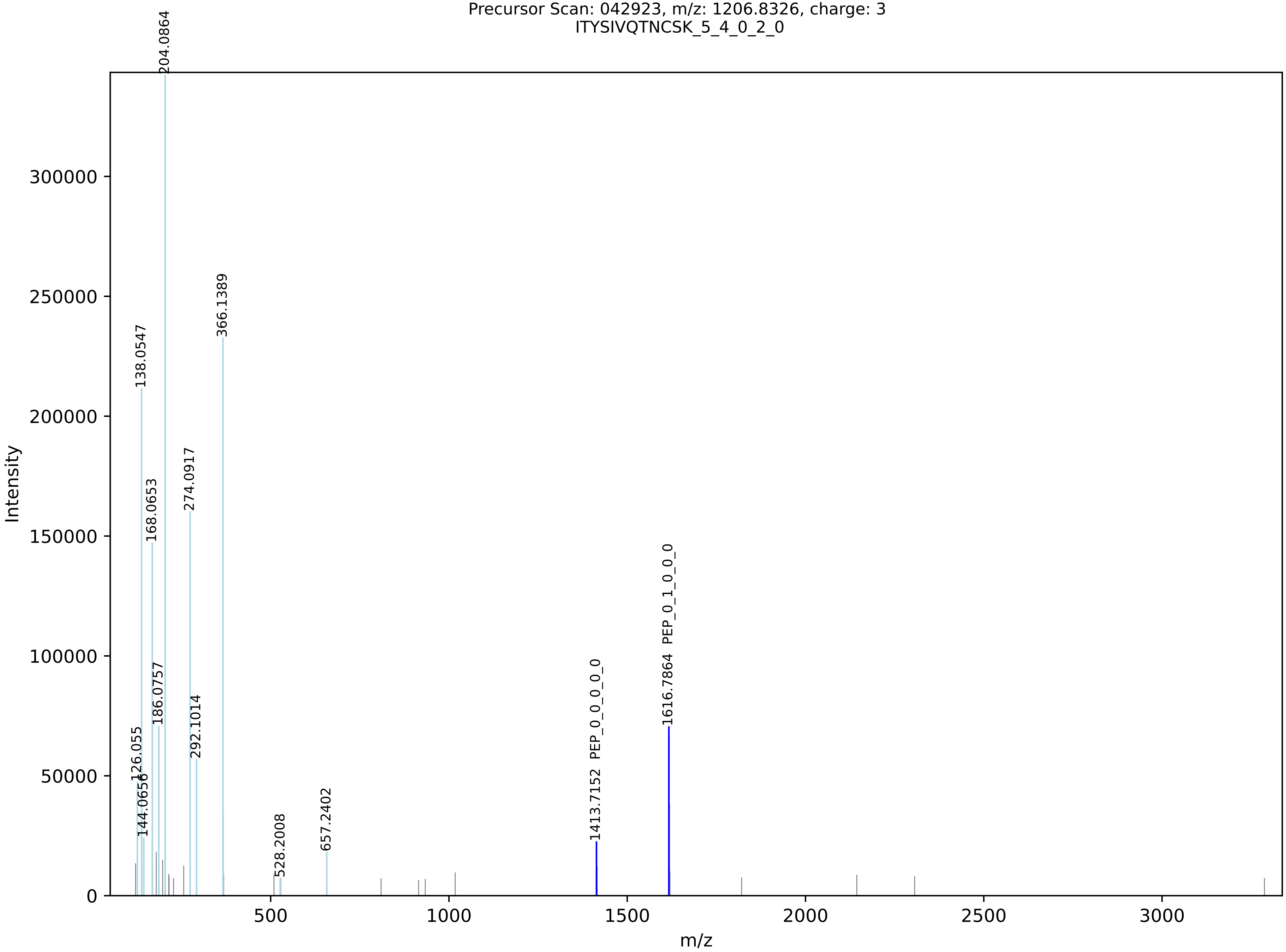

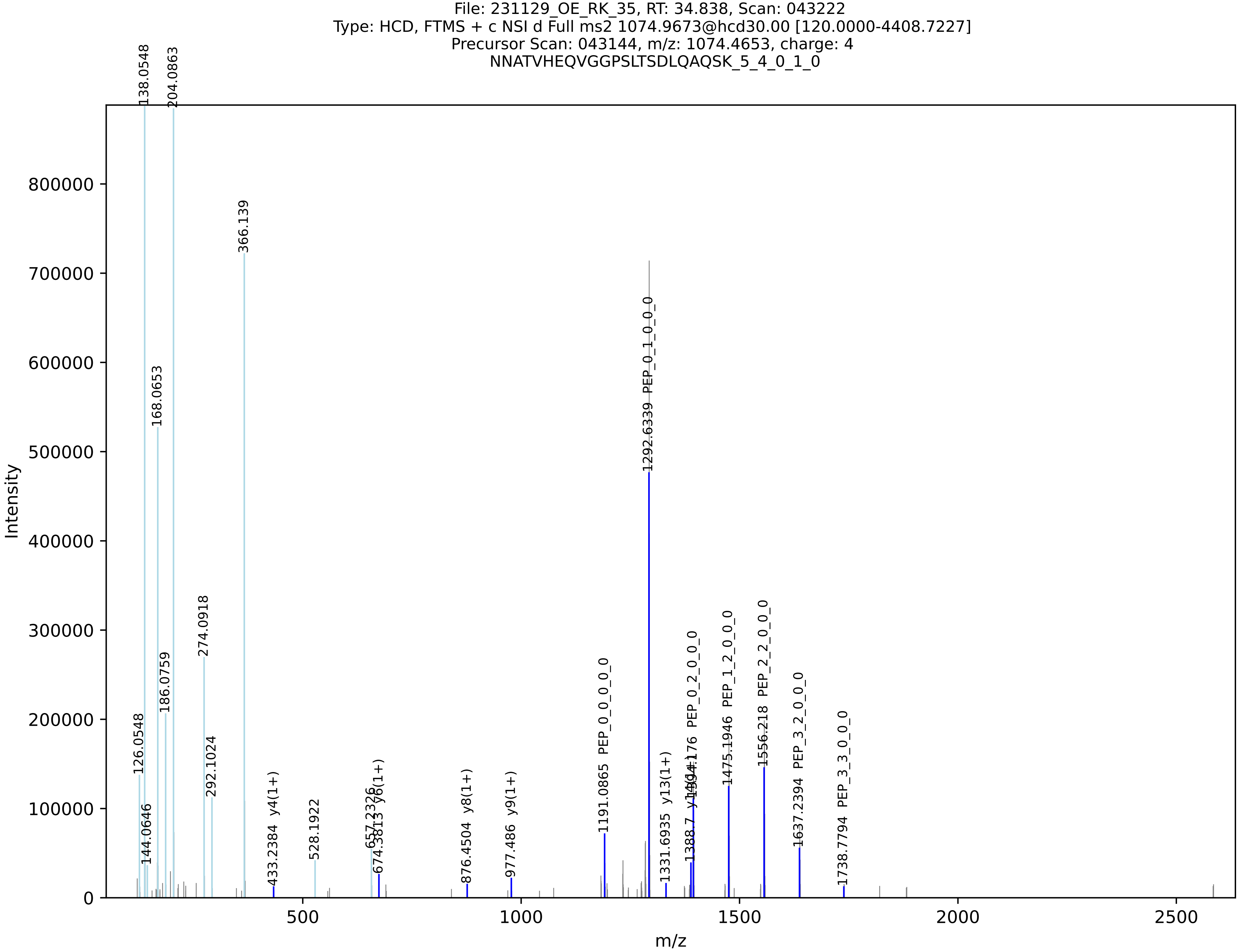
File: 231129_OE_RK_35, RT: 34.838, Scan: 043222
Type: HCD, FTMS + c NSI d Full ms2 1074.9673@hcd30.00 [120.0000-4408.7227]
Precursor Scan: 043144, m/z: 1074.4653, charge: 4
NNATVHEQVGGPSLTSDLQAQSK_5_4_0_1_0
Intensity
m/z
138.0548
204.0863
366.139
168.0653
186.0759
274.0918
126.0548
292.1024
144.0646
433.2384 y4(1+)
528.1922
657.2326
674.3813 y6(1+)
876.4504 y8(1+)
977.486 y9(1+)
1191.0865 PEP_0_0_0_0_0
1292.6339 PEP_0_1_0_0_0
1331.6935 y13(1+)
1388.7 y14(1+)
1394.176 PEP_0_2_0_0_0
1475.1946 PEP_1_2_0_0_0
1556.218 PEP_2_2_0_0_0
1637.2394 PEP_3_2_0_0_0
1738.7794 PEP_3_3_0_0_0

File: 231129_OE_RK_35, RT: 34.844, Scan: 043232
Type: HCD, FTMS + c NSI d Full ms2 1145.8031@hcd30.00 [120.0000-3528.2996]
Precursor Scan: 043144, m/z: 1145.1359, charge: 3
ENISDPTSPLR_5_4_0_2_0

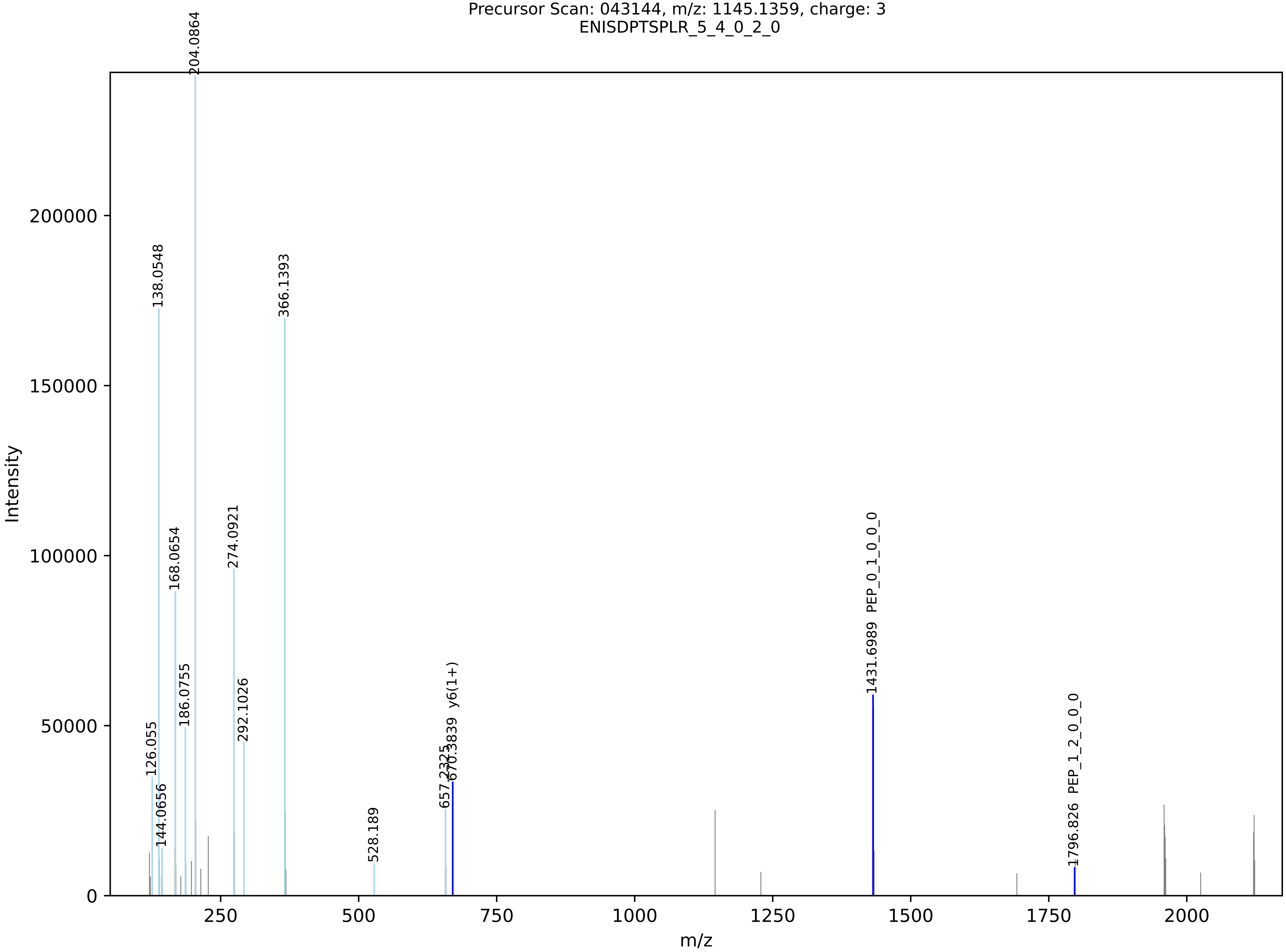

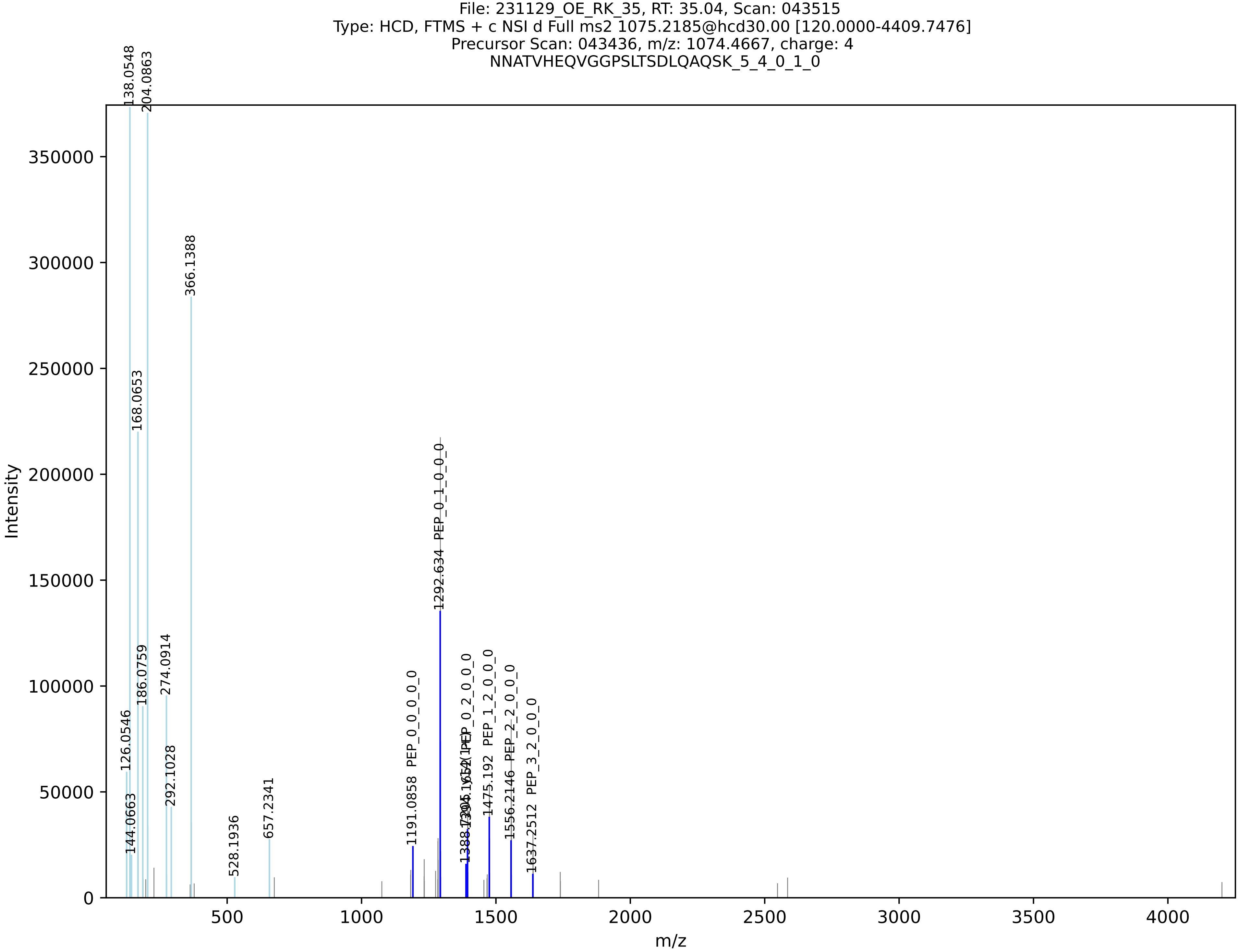
File: 231129_OE_RK_35, RT: 35.04, Scan: 043515
Type: HCD, FTMS + c NSI d Full ms2 1075.2185@hcd30.00 [120.0000-4409.7476]
Precursor Scan: 043436, m/z: 1074.4667, charge: 4
NNATVHEQVGGPSLTSDLQAQSK_5_4_0_1_0
138.0548
204.0863
366.1388
168.0653
186.0759
274.0914
126.0546
292.1028
144.0663
528.1936
657.2341
1191.0858 PEP_0_0_0_0_0_
1292.634 PEP_0_1_0_0_0_
1475.192 PEP_1_2_0_0_0_
1556.2146 PEP_2_2_0_0_0_
1637.2512 PEP_3_2_0_0_0_
Intensity
m/z
0
50000
100000
150000
200000
250000
300000
350000
500
1000
1500
2000
2500
3000
3500
4000

File: 231129_OE_RK_35, RT: 35.102, Scan: 043608
Type: HCD, FTMS + c NSI d Full ms2 1585.6223@hcd30.00 [120.0000-4874.1460]
Precursor Scan: 043510, m/z: 1585.2867, charge: 3
EHEGAIYPDNTTDFQR_6_5_0_3_0

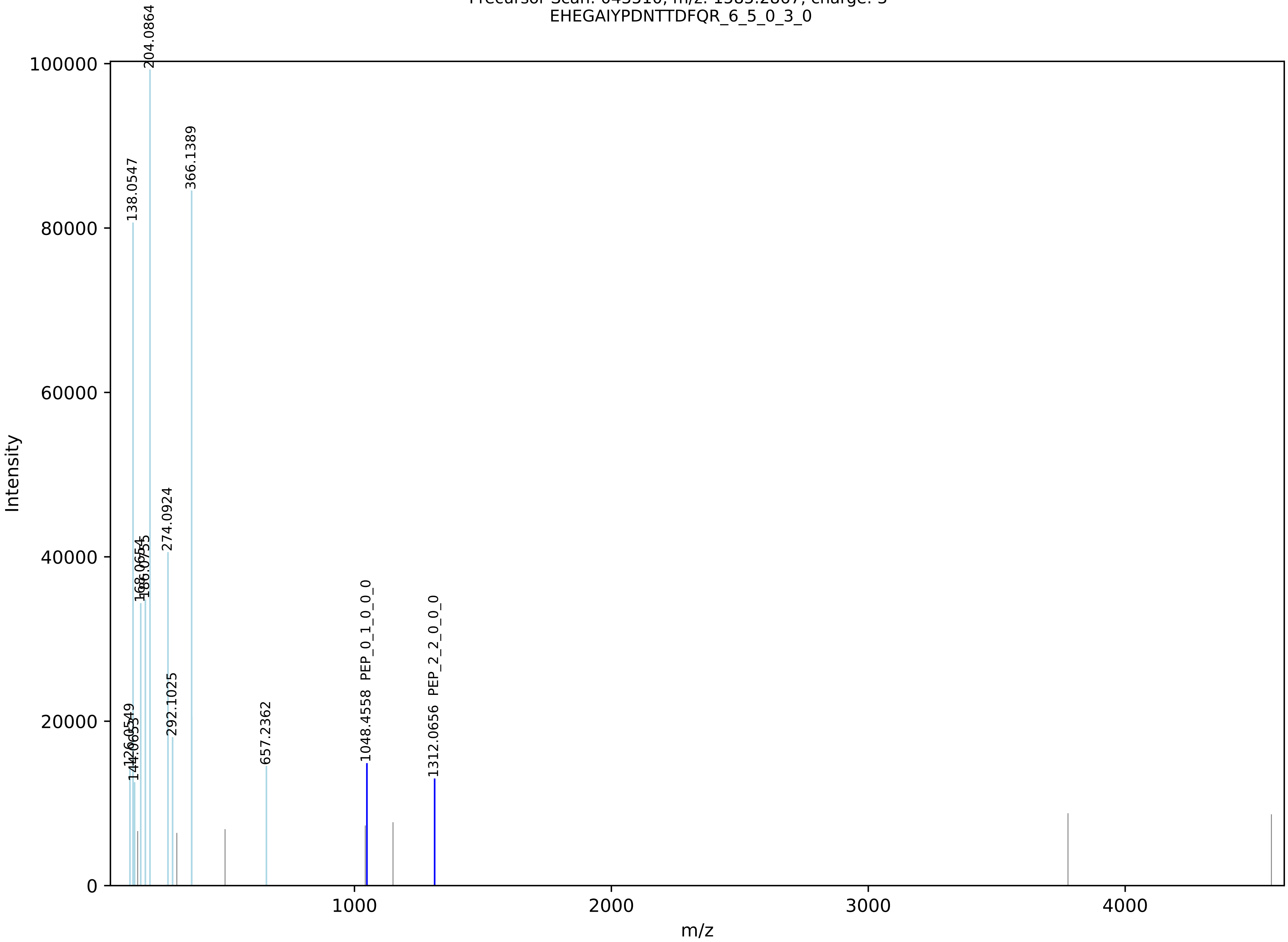

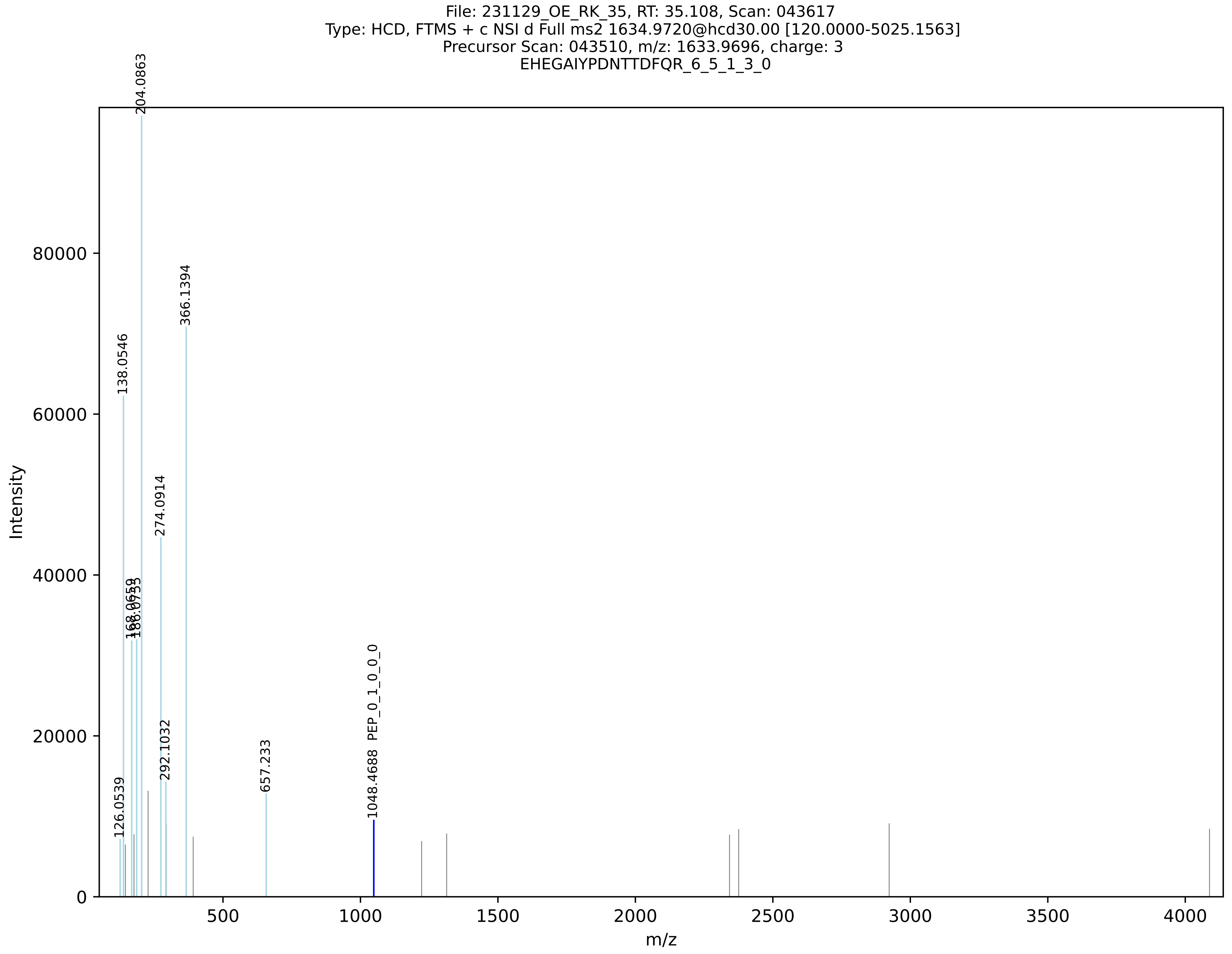
File: 231129_OE_RK_35, RT: 35.108, Scan: 043617
Type: HCD, FTMS + c NSI d Full ms2 1634.9720@hcd30.00 [120.0000-5025.1563]
Precursor Scan: 043510, m/z: 1633.9696, charge: 3
EHEGAIYPDNTTDFQR_6_5_1_3_0
204.0863
366.1394
138.0546
274.0914
168.0659
186.0753
292.1032
657.233
126.0539
1048.4688 PEP_0_1_0_0_0
Intensity
m/z
0
20000
40000
60000
80000
500
1000
1500
2000
2500
3000
3500
4000

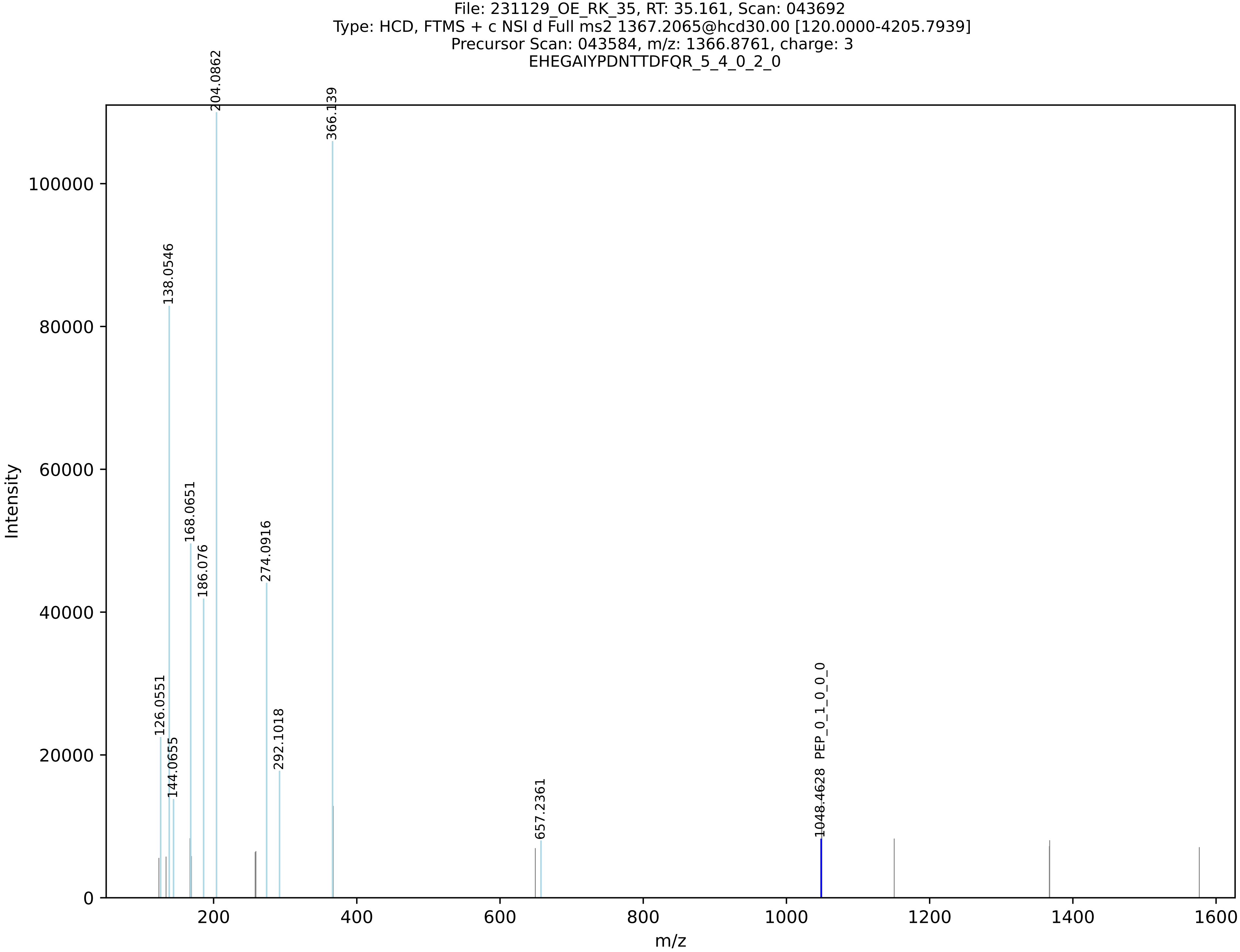

File: 231129_OE_RK_35, RT: 35.161, Scan: 043692
Type: HCD, FTMS + c NSI d Full ms2 1367.2065@hcd30.00 [120.0000-4205.7939]
Precursor Scan: 043584, m/z: 1366.8761, charge: 3
EHEGAIYPDNTTDFQR_5_4_0_2_0
204.0862
366.139
138.0546
168.0651
186.076
274.0916
126.0551
144.0655
292.1018
657.2361
1048.4628
PEP_0_1_0_0_0
Intensity
m/z
0
20000
40000
60000
80000
100000
200
400
600
800
1000
1200
1400
1600

File: 231129_OE_RK_35, RT: 35.24, Scan: 043805
Type: HCD, FTMS + c NSI d Full ms2 1104.1304@hcd30.00 [120.0000-3400.7810]
Precursor Scan: 043729, m/z: 1103.7963, charge: 3
GLNVTLSSTGR_5_4_0_2_0

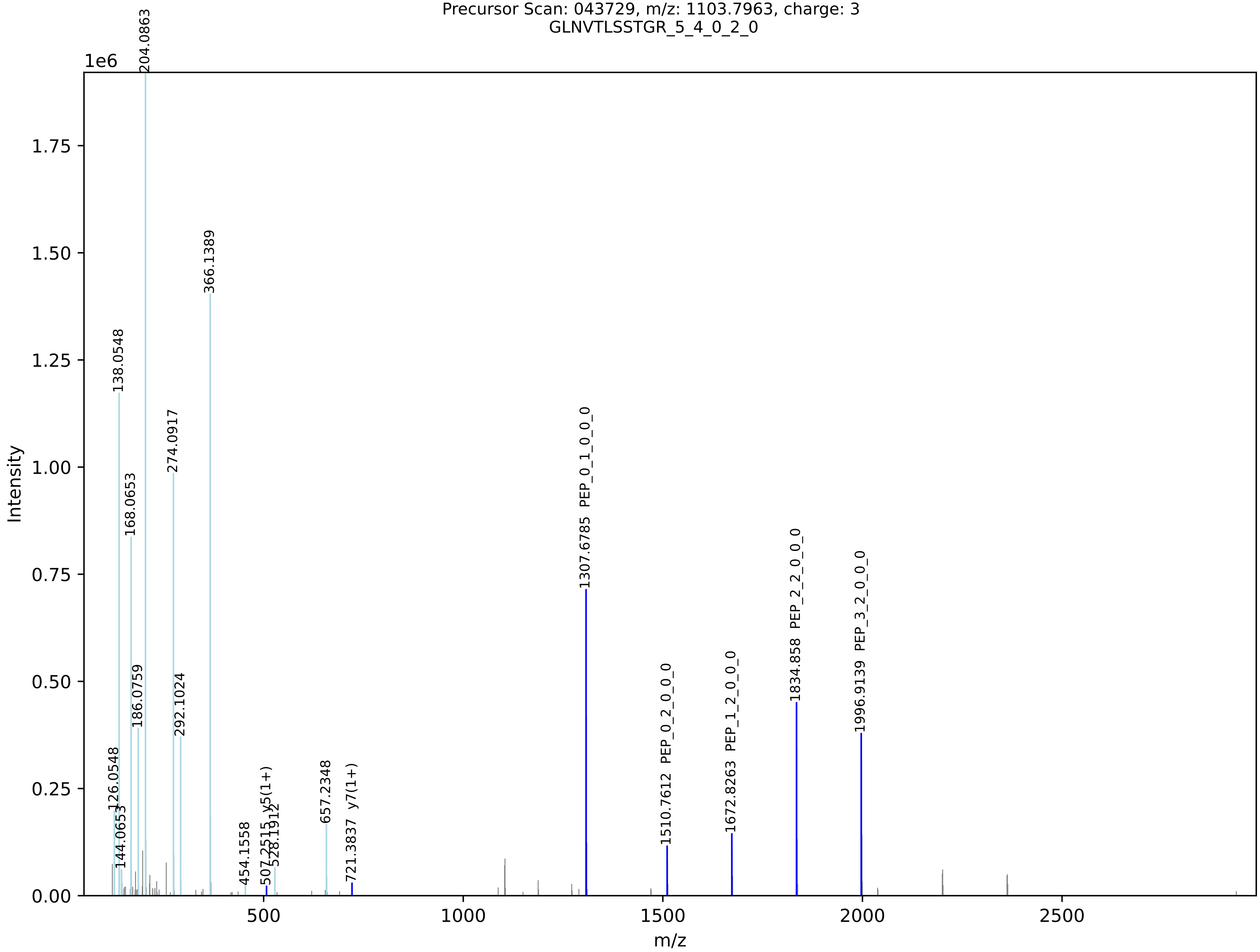

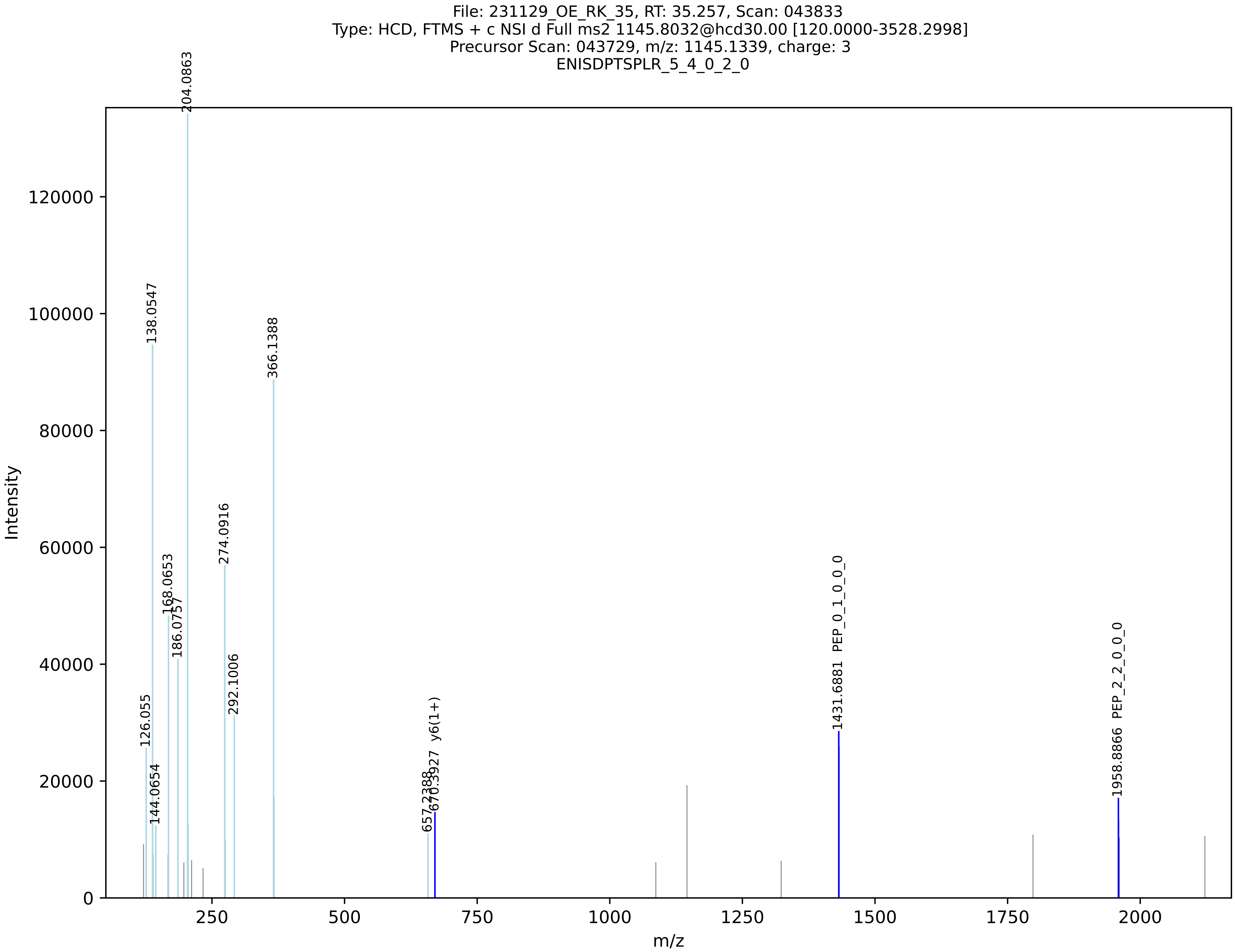

File: 231129_OE_RK_35, RT: 35.257, Scan: 043833
Type: HCD, FTMS + c NSI d Full ms2 1145.8032@hcd30.00 [120.0000-3528.2998]
Precursor Scan: 043729, m/z: 1145.1339, charge: 3
ENISDPTSPLR_5_4_0_2_0
Intensity
m/z
204.0863
138.0547
366.1388
274.0916
168.0653
186.0757
292.1006
126.055
144.0654
657.2388
670.3927 y6(1+)
1431.6881 PEP_0_1_0_0_0
1958.8866 PEP_2_2_0_0_0

File: 231129_OE_RK_35, RT: 35.346, Scan: 043957
Type: HCD, FTMS + c NSI d Full ms2 828.3499@hcd30.00 [120.0000-3402.5234]
Precursor Scan: 043877, m/z: 828.0987, charge: 4
GLNVTLSSTGR_5_4_0_2_0

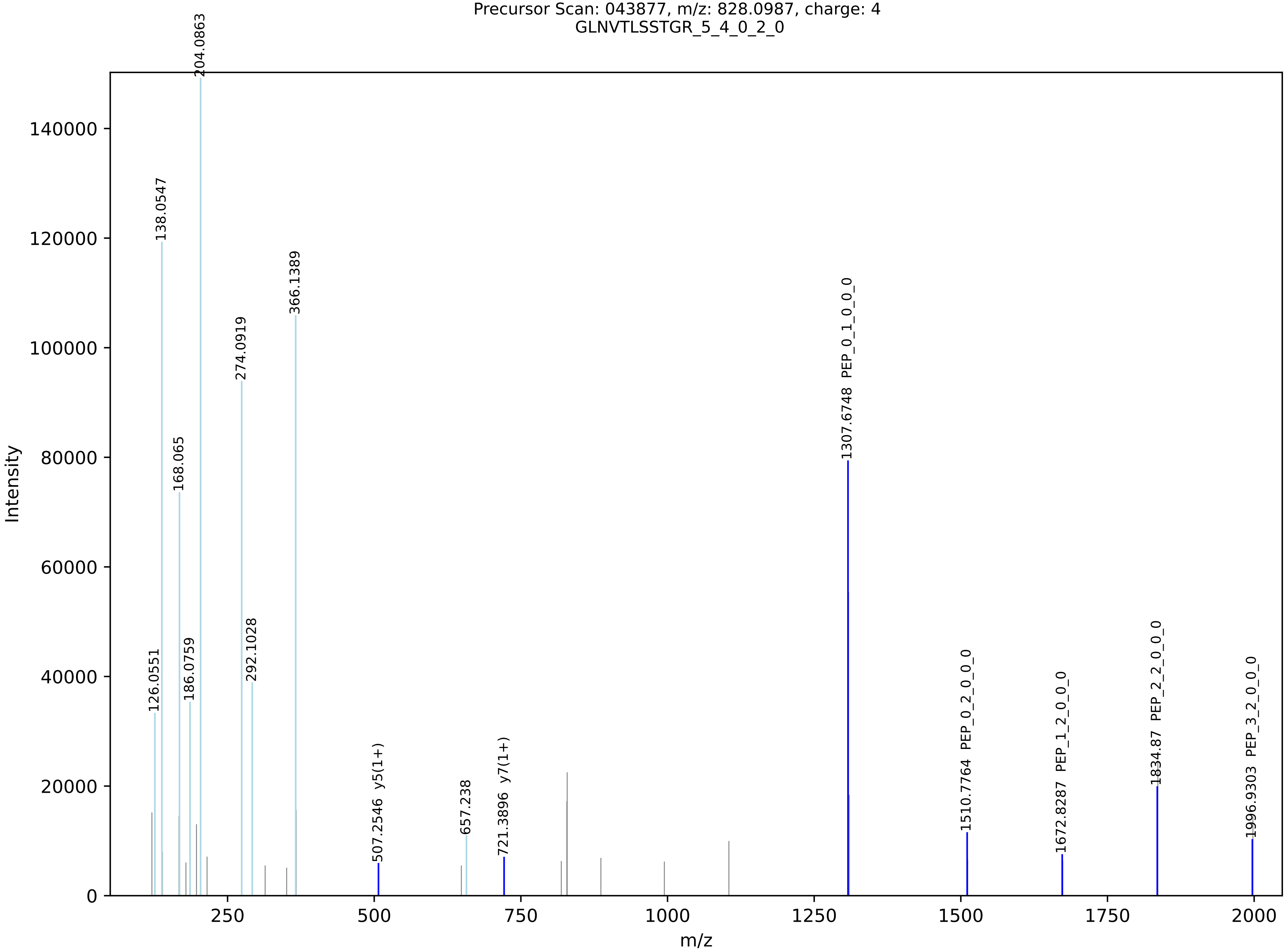

File: 231129_OE_RK_35, RT: 35.352, Scan: 043967
Type: HCD, FTMS + c NSI d Full ms2 1270.1742@hcd30.00 [120.0000-3908.8750]
Precursor Scan: 043877, m/z: 1269.506, charge: 3
CIQANYSLMENGK_6_5_0_1_0

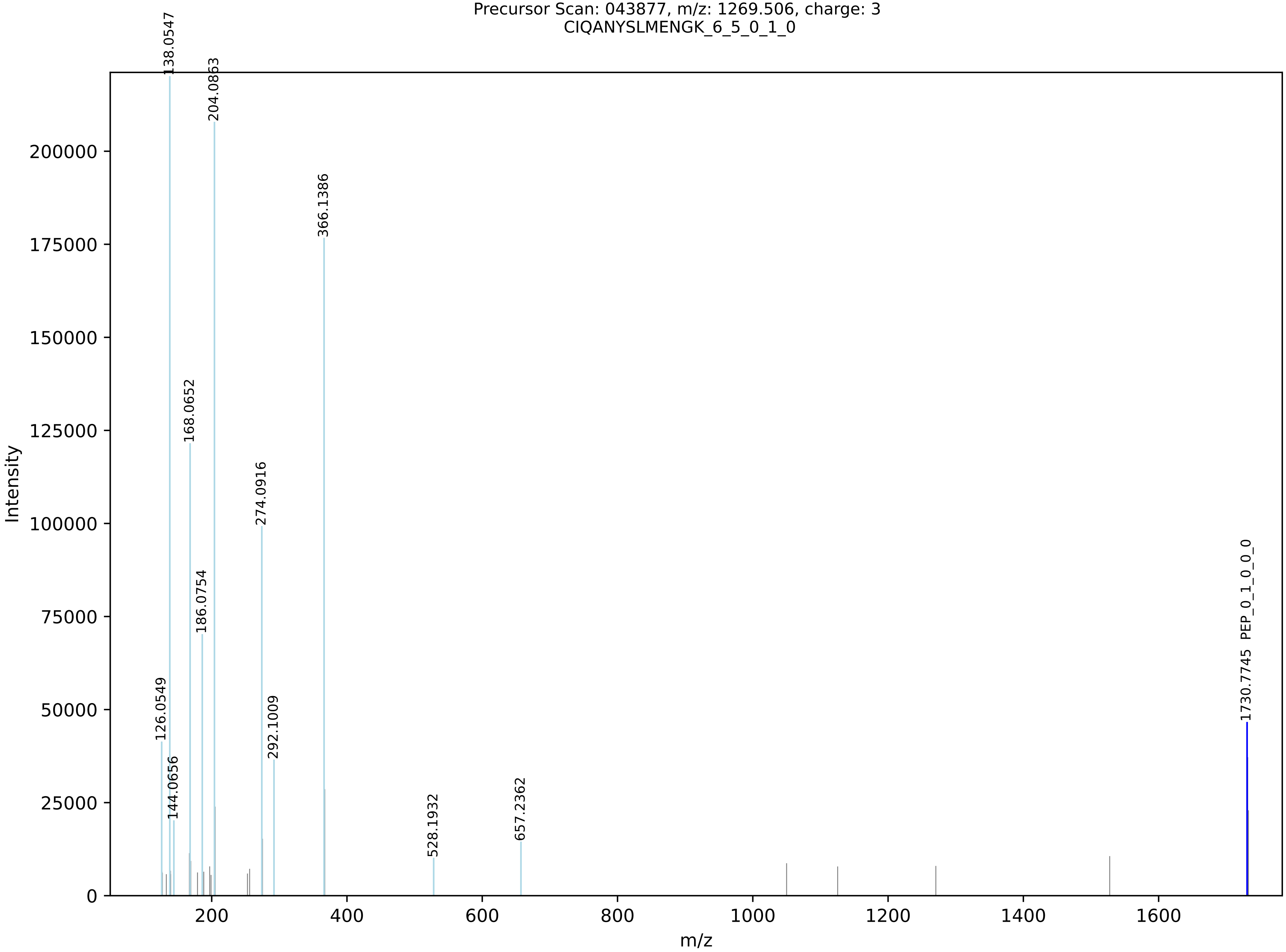

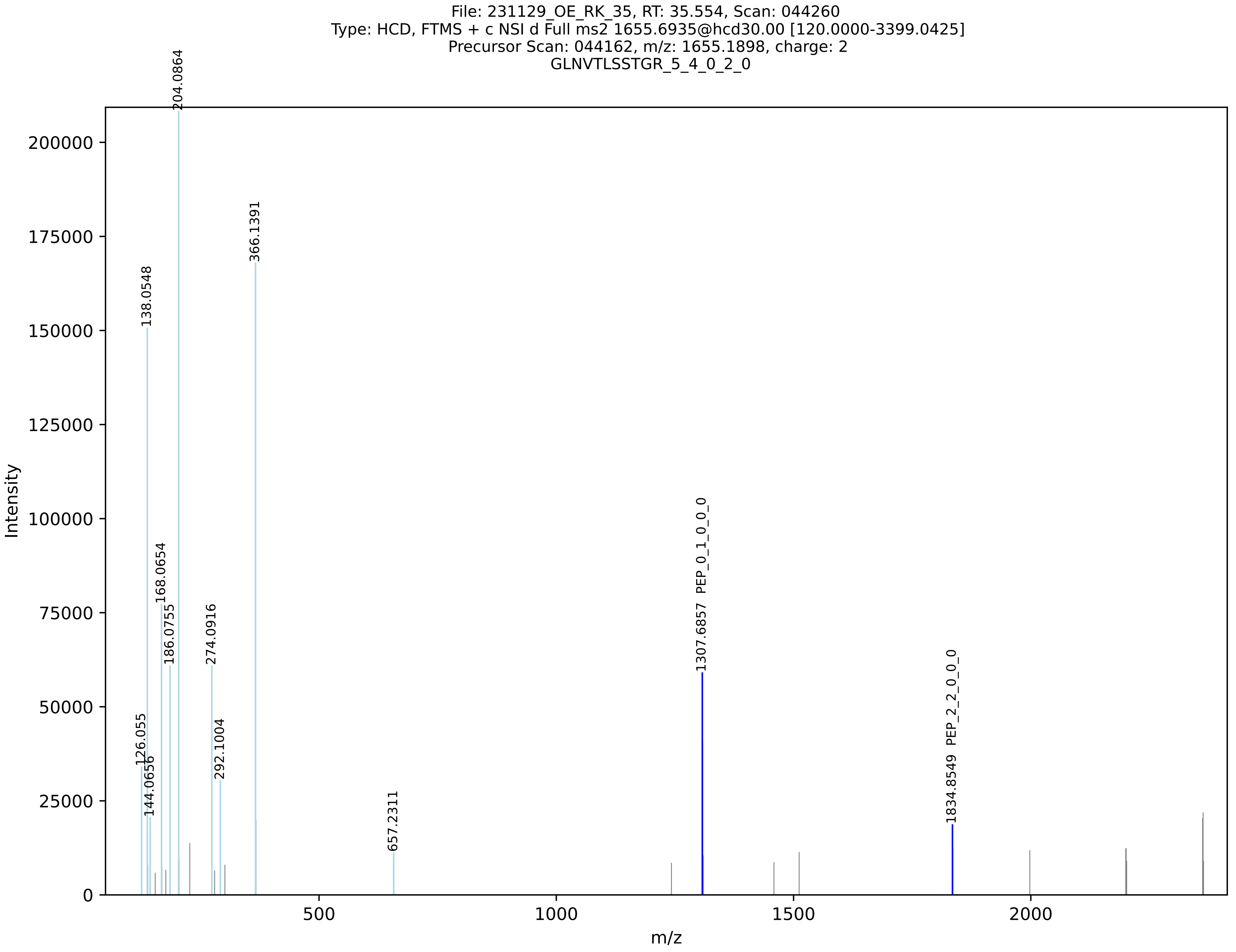

File: 231129_OE_RK_35, RT: 35.554, Scan: 044260
Type: HCD, FTMS + c NSI d Full ms2 1655.6935@hcd30.00 [120.0000-3399.0425]
Precursor Scan: 044162, m/z: 1655.1898, charge: 2
GLNVTLSSTGR_5_4_0_2_0
Intensity
m/z
204.0864
366.1391
138.0548
168.0654
186.0755
274.0916
292.1004
126.055
144.0656
657.2311
1307.6857 PEP_0_1_0_0_0
1834.8549 PEP_2_2_0_0_0

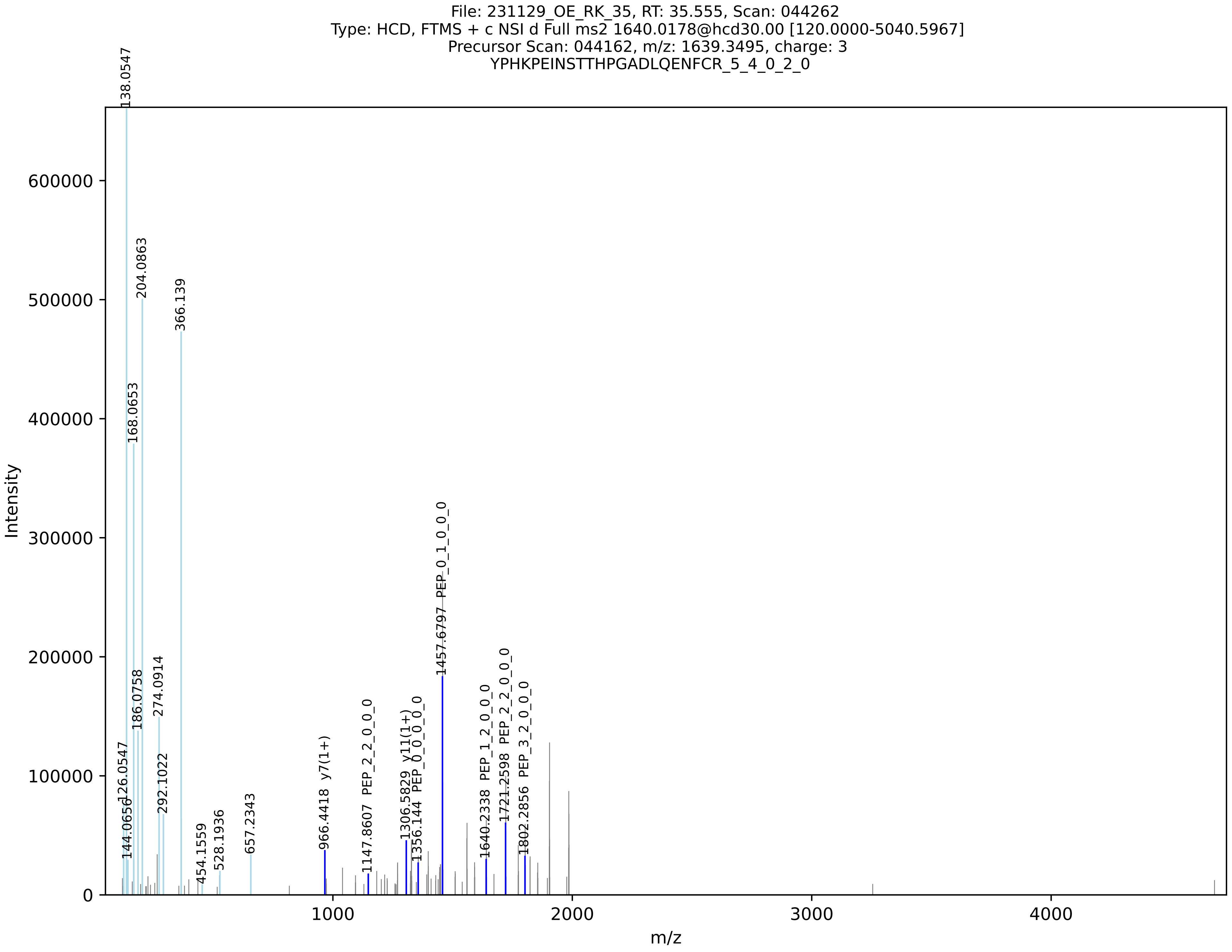
File: 231129_OE_RK_35, RT: 35.555, Scan: 044262
Type: HCD, FTMS + c NSI d Full ms2 1640.0178@hcd30.00 [120.0000-5040.5967]
Precursor Scan: 044162, m/z: 1639.3495, charge: 3
YPHKPEINSTTHPGADLQENFCR_5_4_0_2_0
Intensity
m/z
138.0547
204.0863
366.139
168.0653
186.0758
274.0914
126.0547
144.0656
292.1022
454.1559
528.1936
657.2343
966.4418 y7(1+)
1147.8607 PEP_2_2_0_0_0
1306.5829 y11(1+)
1356.144 PEP_0_0_0_0_0
1457.6797 PEP_0_1_0_0_0
1640.2338 PEP_1_2_0_0_0
1721.2598 PEP_2_2_0_0_0
1802.2856 PEP_3_2_0_0_0

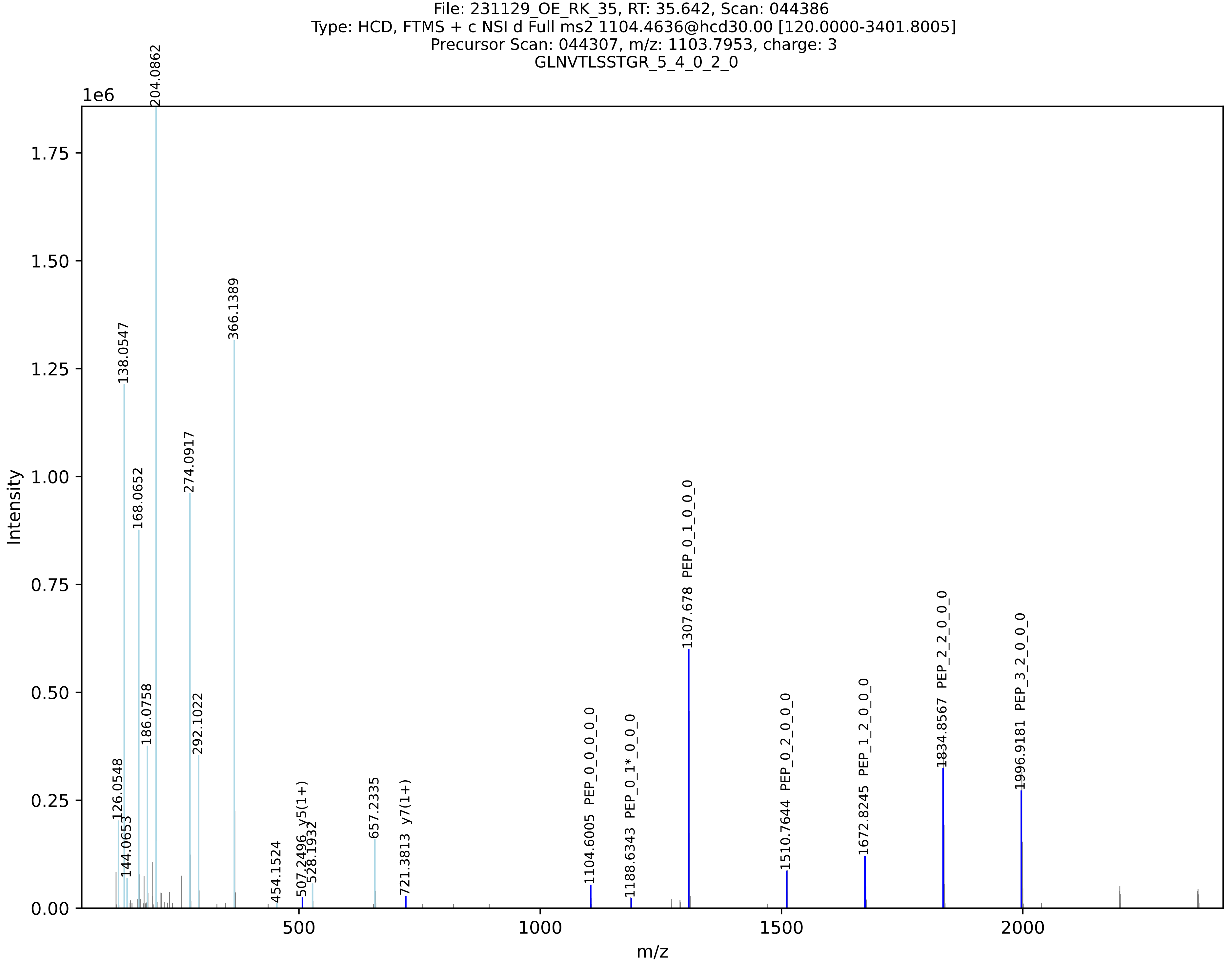
File: 231129_OE_RK_35, RT: 35.642, Scan: 044386
Type: HCD, FTMS + c NSI d Full ms2 1104.4636@hcd30.00 [120.0000-3401.8005]
Precursor Scan: 044307, m/z: 1103.7953, charge: 3
GLNVTLSSTGR_5_4_0_2_0
1e6
Intensity
m/z
204.0862
366.1389
138.0547
274.0917
168.0652
186.0758
292.1022
126.0548
144.0653
454.1524
507.2496 y5(1+)
528.1932
657.2335
721.3813 y7(1+)
1104.6005 PEP_0_0_0_0_0
1188.6343 PEP_0_1*_0_0_0
1307.678 PEP_0_1_0_0_0
1510.7644 PEP_0_2_0_0_0
1672.8245 PEP_1_2_0_0_0
1834.8567 PEP_2_2_0_0_0
1996.9181 PEP_3_2_0_0_0

File: 231129_OE_RK_35, RT: 35.756, Scan: 044555
Type: HCD, FTMS + c NSI d Full ms2 1270.1733@hcd30.00 [120.0000-3908.8723]
Precursor Scan: 044455, m/z: 1269.8374, charge: 3
CIQANYSLMENGK_6_5_0_1_0

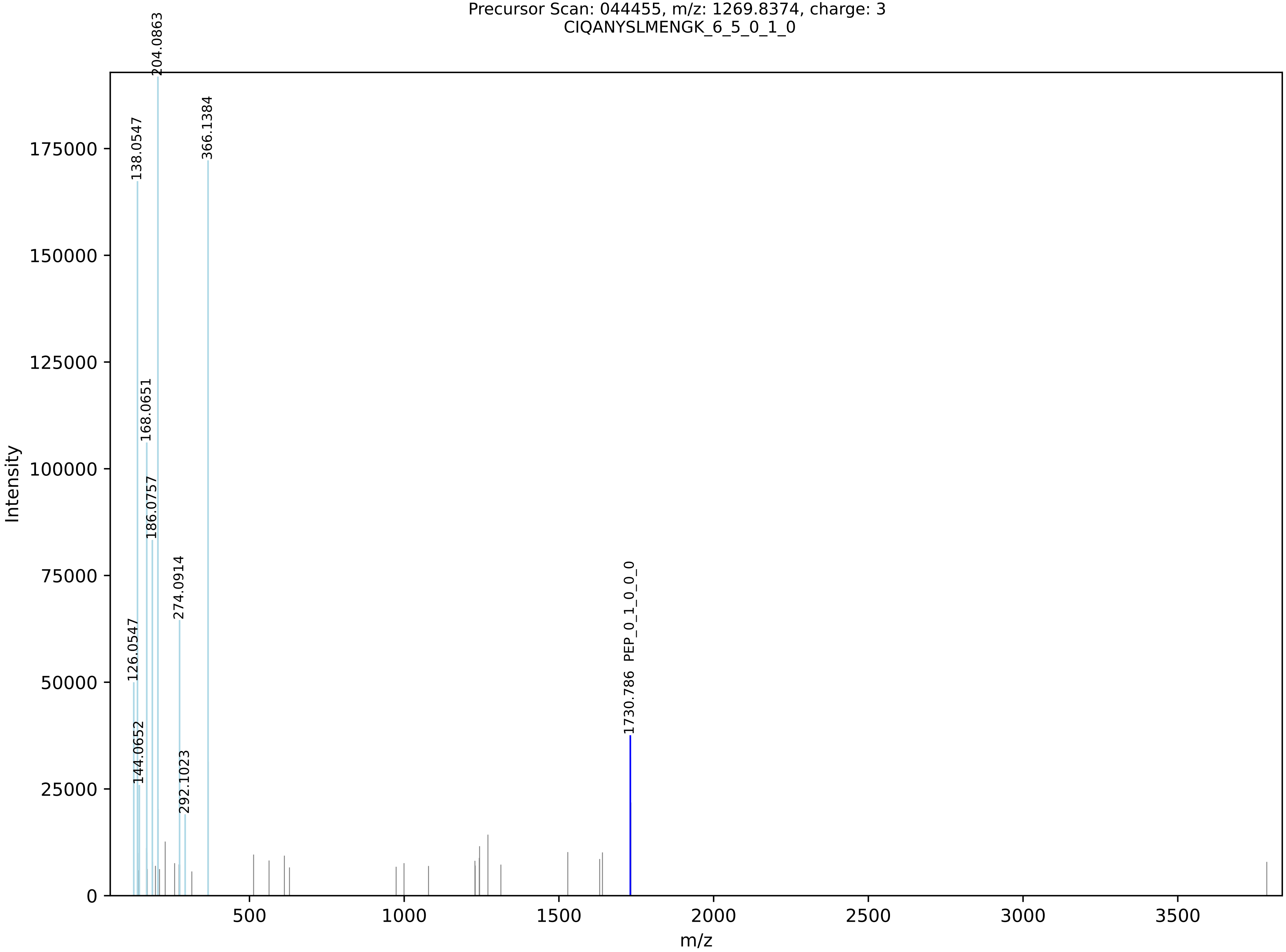

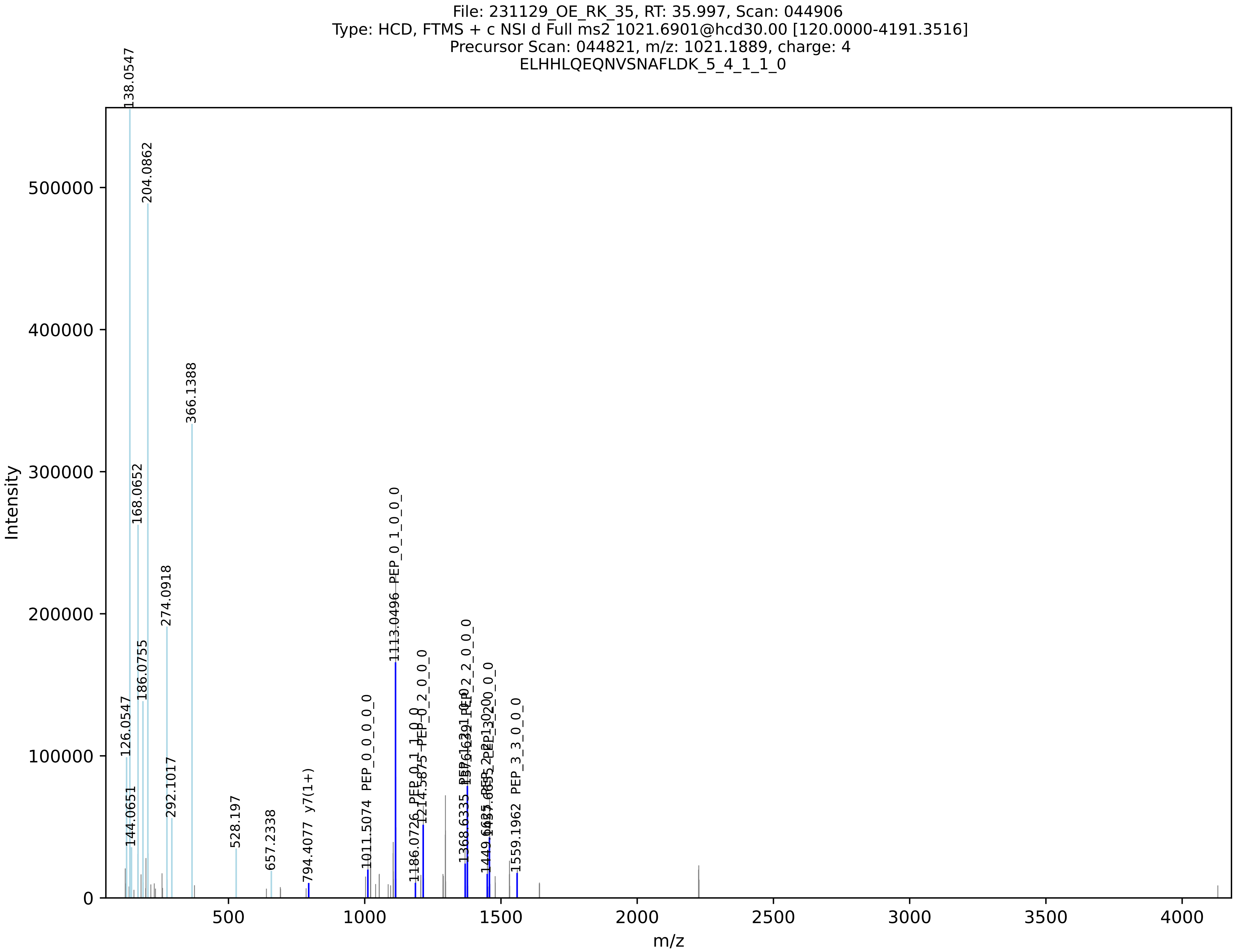

File: 231129_OE_RK_35, RT: 35.997, Scan: 044906
Type: HCD, FTMS + c NSI d Full ms2 1021.6901@hcd30.00 [120.0000-4191.3516]
Precursor Scan: 044821, m/z: 1021.1889, charge: 4
ELHHLQEQNVSNAFLDK_5_4_1_1_0
Intensity
m/z
138.0547
204.0862
366.1388
168.0652
274.0918
186.0755
126.0547
144.0651
292.1017
528.197
657.2338
794.4077 y7(1+)
1011.5074 PEP_0_0_0_0_0
1113.0496 PEP_0_1_0_0_0
1186.0726 PEP_0_1_1_0_0
1214.5875 PEP_0_2_0_0_0
1368.6335 PEP_1_2_0_0_0
1559.1962 PEP_3_3_0_0_0

File: 231129_OE_RK_35, RT: 36.119, Scan: 045086
Type: HCD, FTMS + c NSI d Full ms2 1104.7646@hcd30.00 [120.0000-3402.7217]
Precursor Scan: 044966, m/z: 1104.7646, charge: 3
GLNVTLSSTGR_5_4_0_2_0

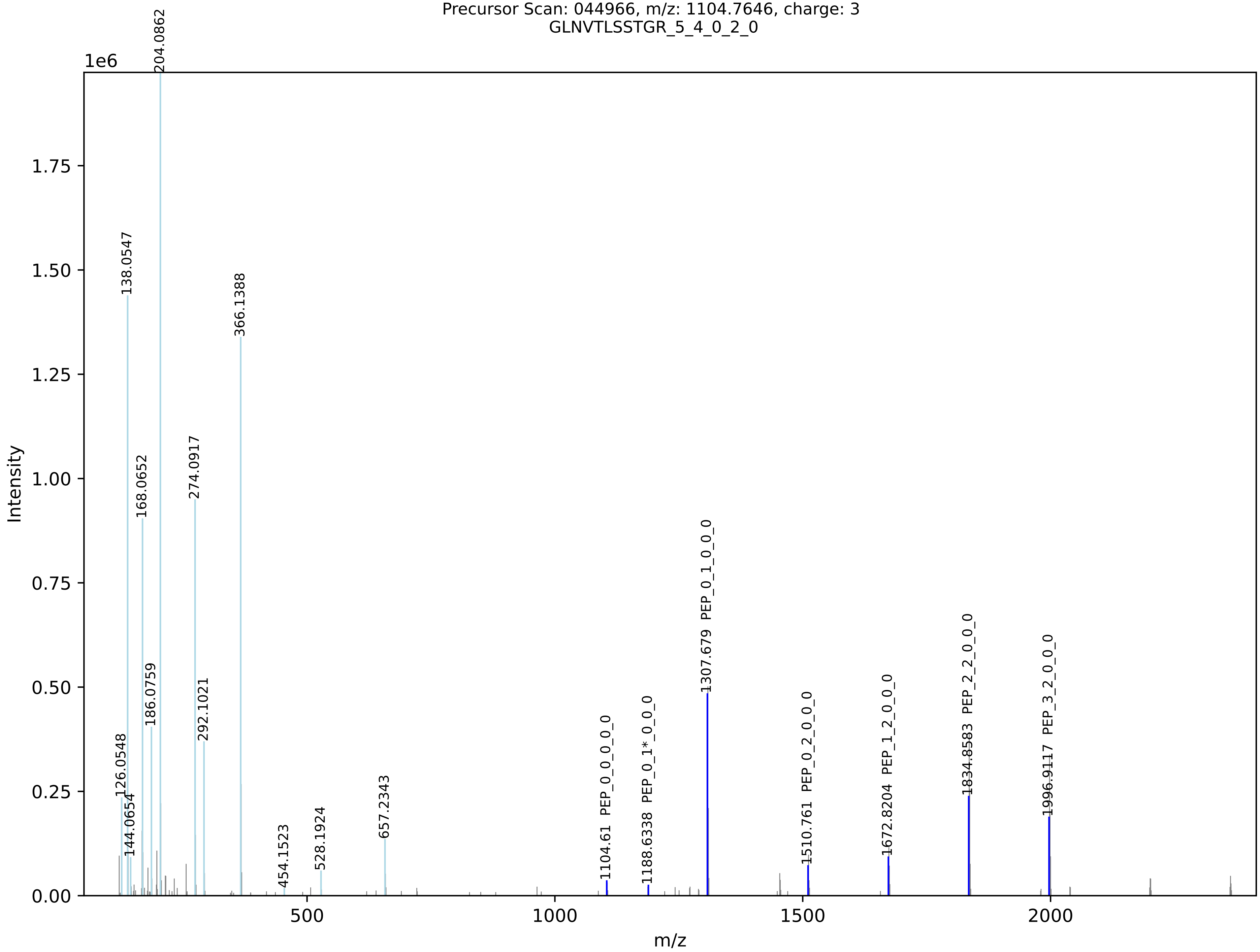

File: 231129_OE_RK_35, RT: 36.191, Scan: 045186
Type: HCD, FTMS + c NSI d Full ms2 1039.9191@hcd30.00 [120.0000-4265.7256]
Precursor Scan: 045112, m/z: 1039.4182, charge: 4
EKNSTFSASGETVER_6_4_1_2_0

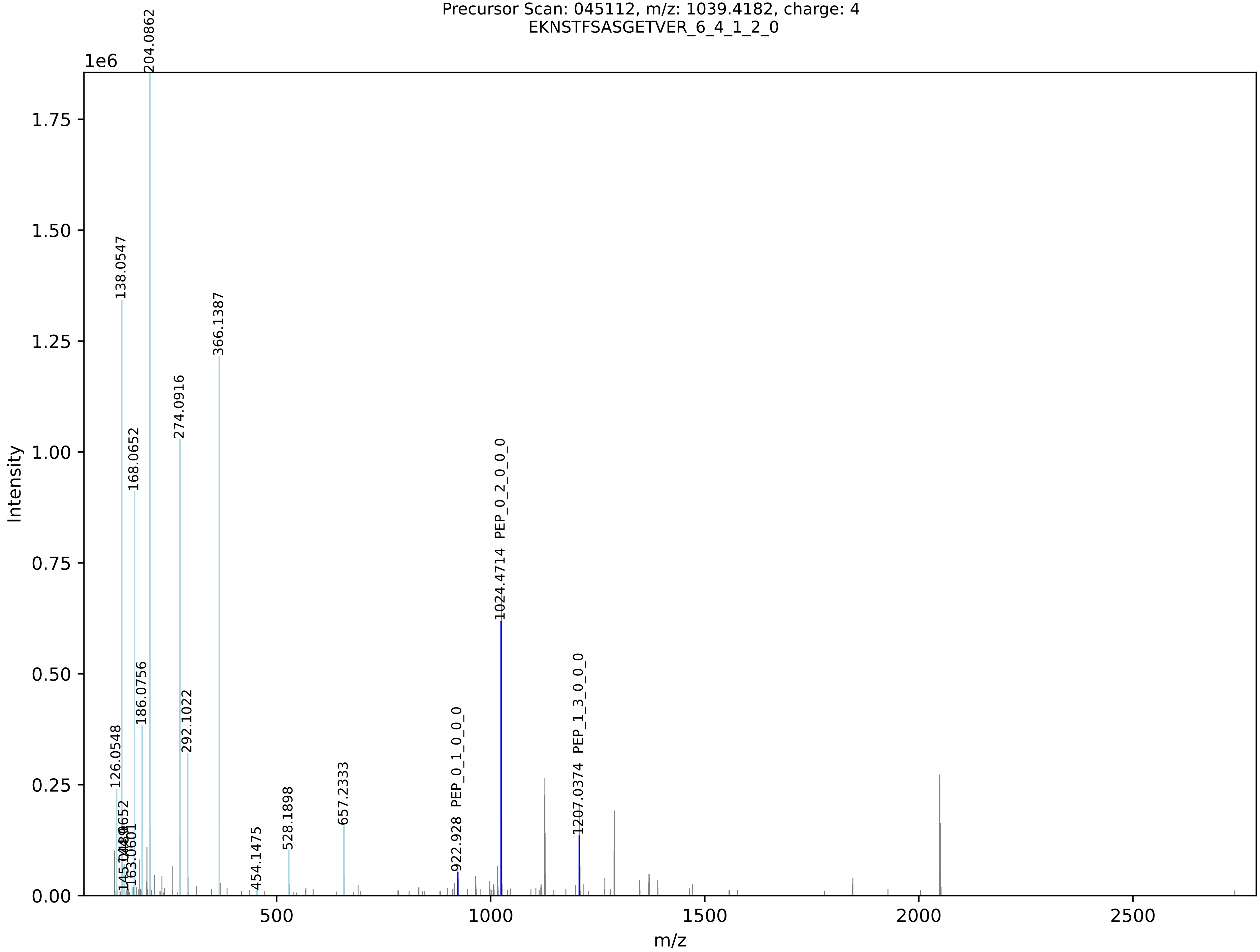

File: 231129_OE_RK_35, RT: 36.242, Scan: 045260
Type: HCD, FTMS + c NSI d Full ms2 1104.1301@hcd30.00 [120.0000-3400.7800]
Precursor Scan: 045183, m/z: 1103.7963, charge: 3
GLNVTLSSTGR_5_4_0_2_0

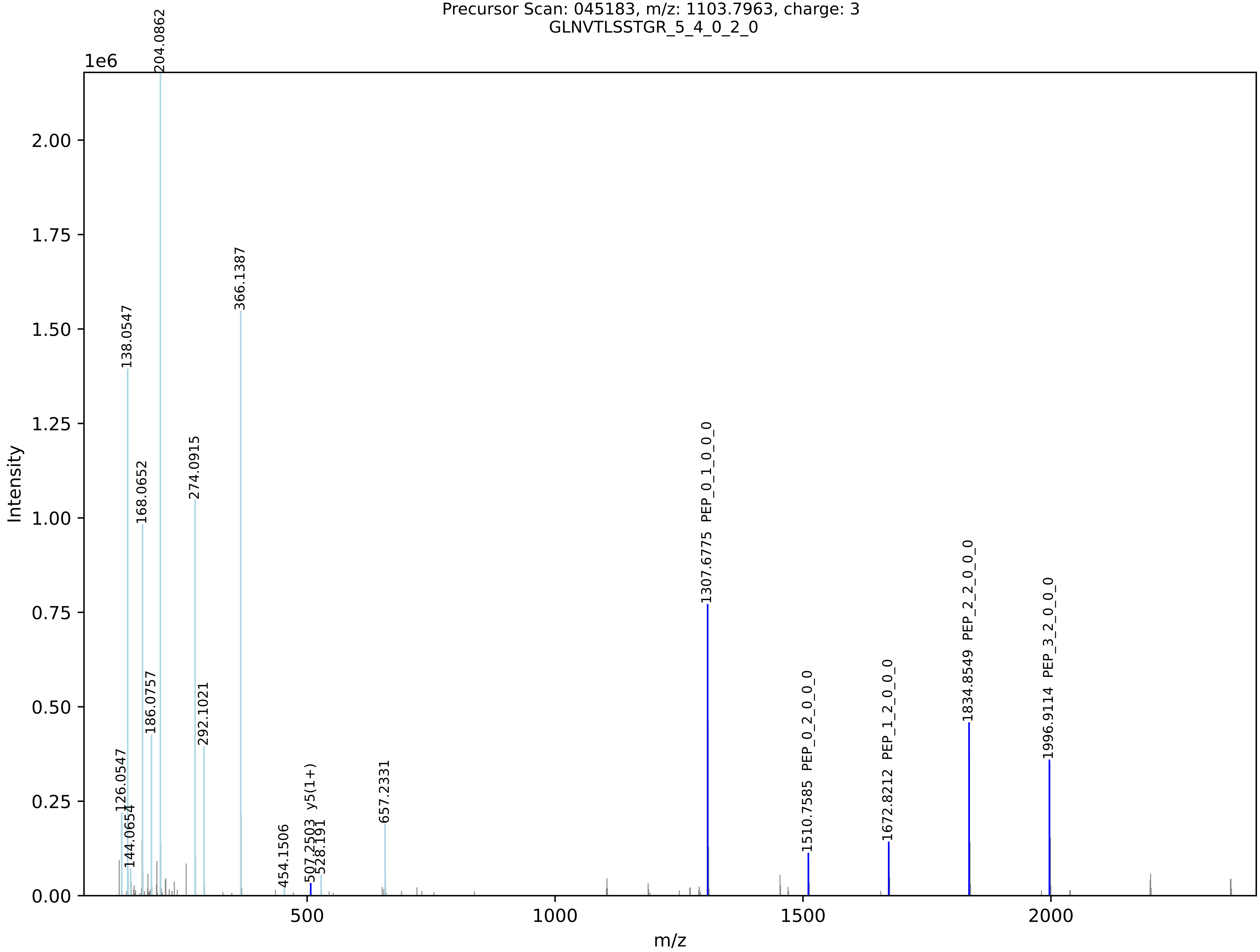

File: 231129_OE_RK_35, RT: 36.386, Scan: 045470
Type: HCD, FTMS + c NSI d Full ms2 1529.9855@hcd30.00 [120.0000-4703.8975]
Precursor Scan: 045404, m/z: 1529.3175, charge: 3
NNATVHEQVGGPSLTSDLQAQSK_5_4_0_2_0

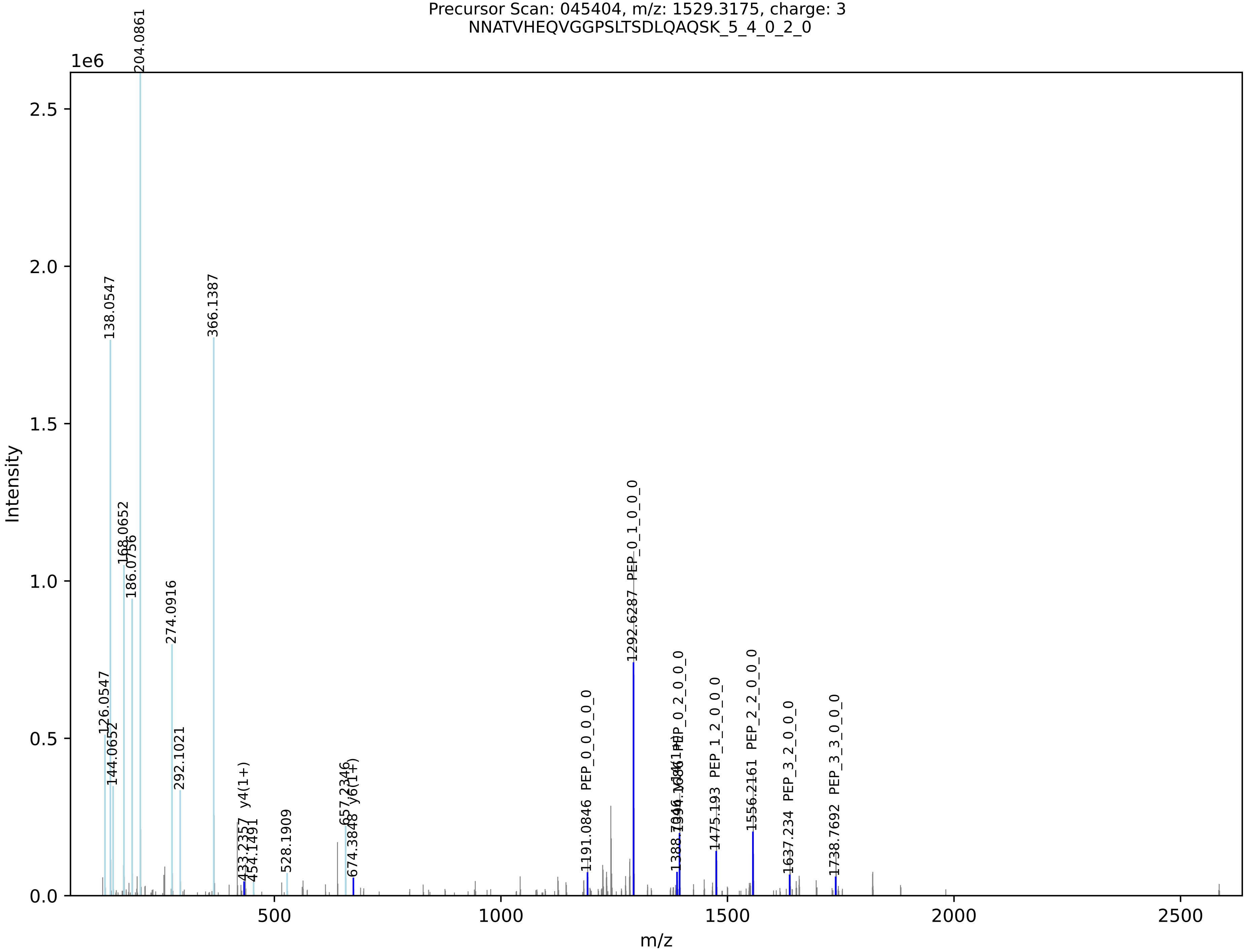

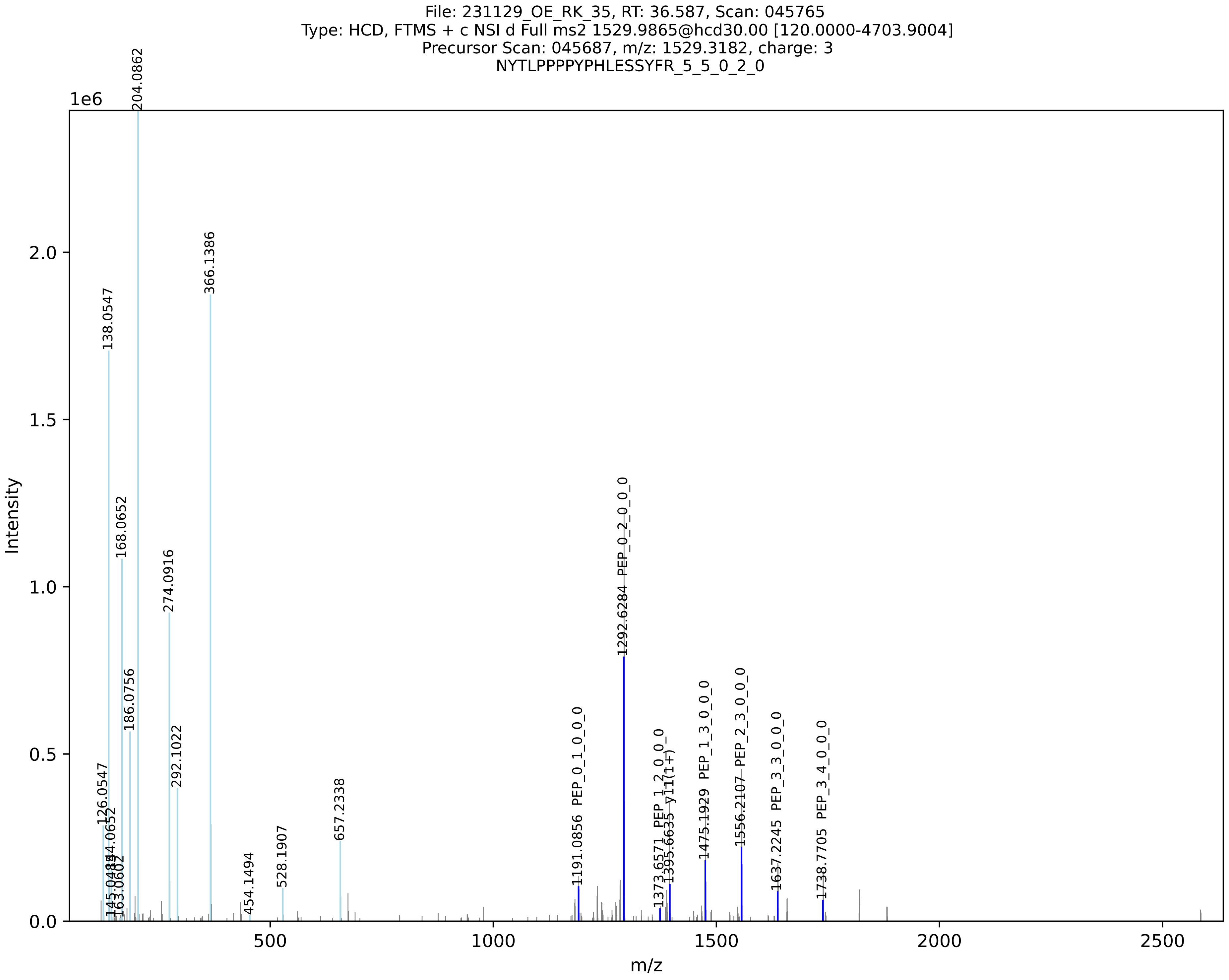
File: 231129_OE_RK_35, RT: 36.587, Scan: 045765
Type: HCD, FTMS + c NSI d Full ms2 1529.9865@hcd30.00 [120.0000-4703.9004]
Precursor Scan: 045687, m/z: 1529.3182, charge: 3
NYTLPPPPYPHLESSYFR_5_5_0_2_0
1e6
Intensity
m/z
204.0862
366.1386
138.0547
168.0652
274.0916
186.0756
292.1022
126.0547
657.2338
528.1907
454.1494
1292.6284 PEP_0_2_0_0_0
1191.0856 PEP_0_1_0_0_0
1373.6571 PEP_1_2_0_0_0
1395.6635 y11(1+)
1475.1929 PEP_1_3_0_0_0
1556.2107 PEP_2_3_0_0_0
1637.2245 PEP_3_3_0_0_0
1738.7705 PEP_3_4_0_0_0
0.0
0.5
1.0
1.5
2.0
500
1000
1500
2000
2500

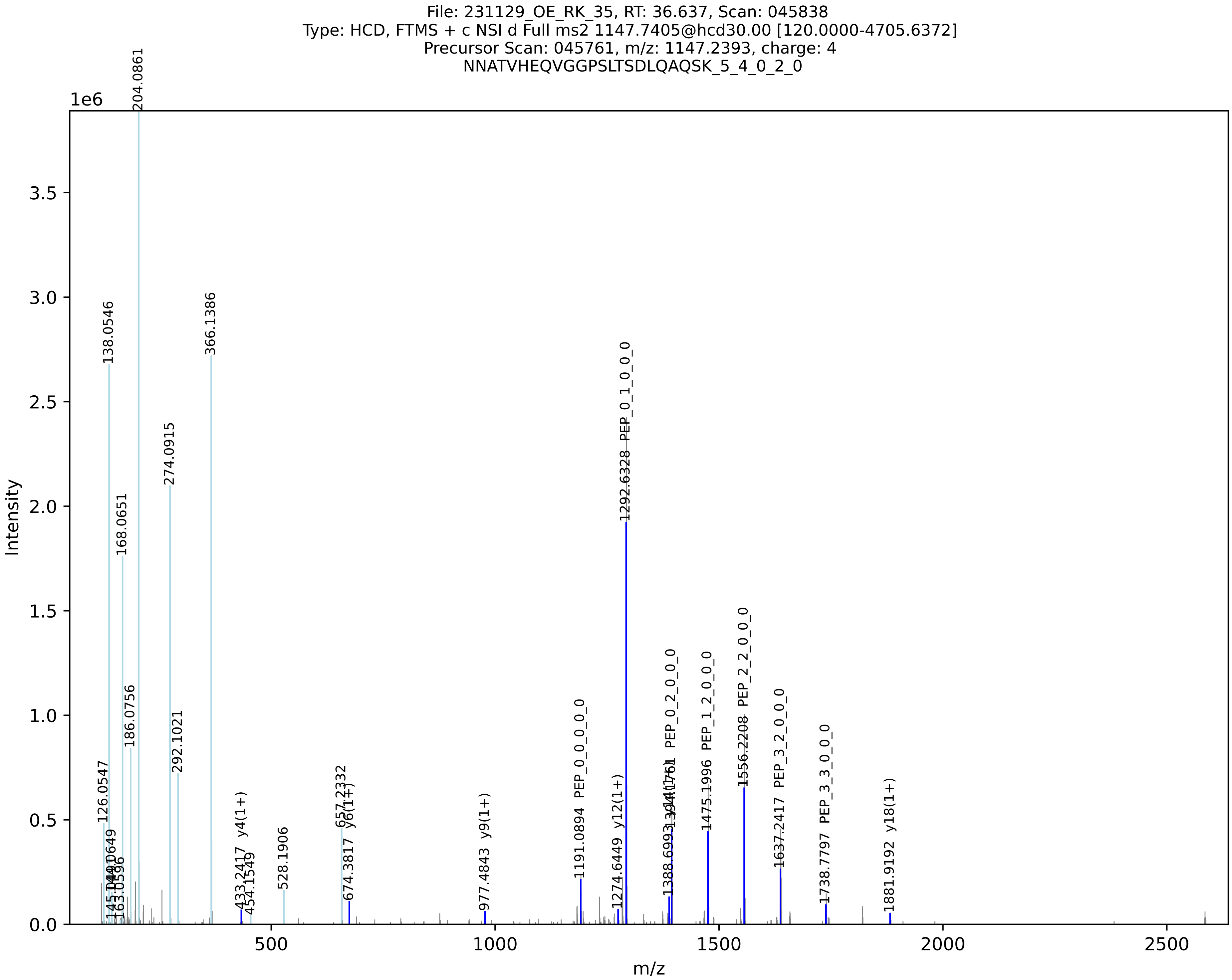

File: 231129_OE_RK_35, RT: 36.637, Scan: 045838
Type: HCD, FTMS + c NSI d Full ms2 1147.7405@hcd30.00 [120.0000-4705.6372]
Precursor Scan: 045761, m/z: 1147.2393, charge: 4
NNATVHEQVGGPSLTSDLQAQSK_5_4_0_2_0
1e6
Intensity
m/z
204.0861
138.0546
366.1386
274.0915
168.0651
186.0756
292.1021
126.0547
433.2417 y4(1+)
454.1549
528.1906
657.2332
674.3817 y6(1+)
977.4843 y9(1+)
1191.0894 PEP_0_0_0_0_0
1274.6449 y12(1+)
1292.6328 PEP_0_1_0_0_0
PEP_0_2_0_0_0
1475.1996 PEP_1_2_0_0_0
1556.2208 PEP_2_2_0_0_0
1637.2417 PEP_3_2_0_0_0
1738.7797 PEP_3_3_0_0_0
1881.9192 y18(1+)

File: 231129_OE_RK_35, RT: 36.771, Scan: 046038
Type: HCD, FTMS + c NSI d Full ms2 918.3942@hcd30.00 [120.0000-4707.3804]
Precursor Scan: 045835, m/z: 917.9934, charge: 5
NNATVHEQVGGPSLTSDLQAQSK_5_4_0_2_0

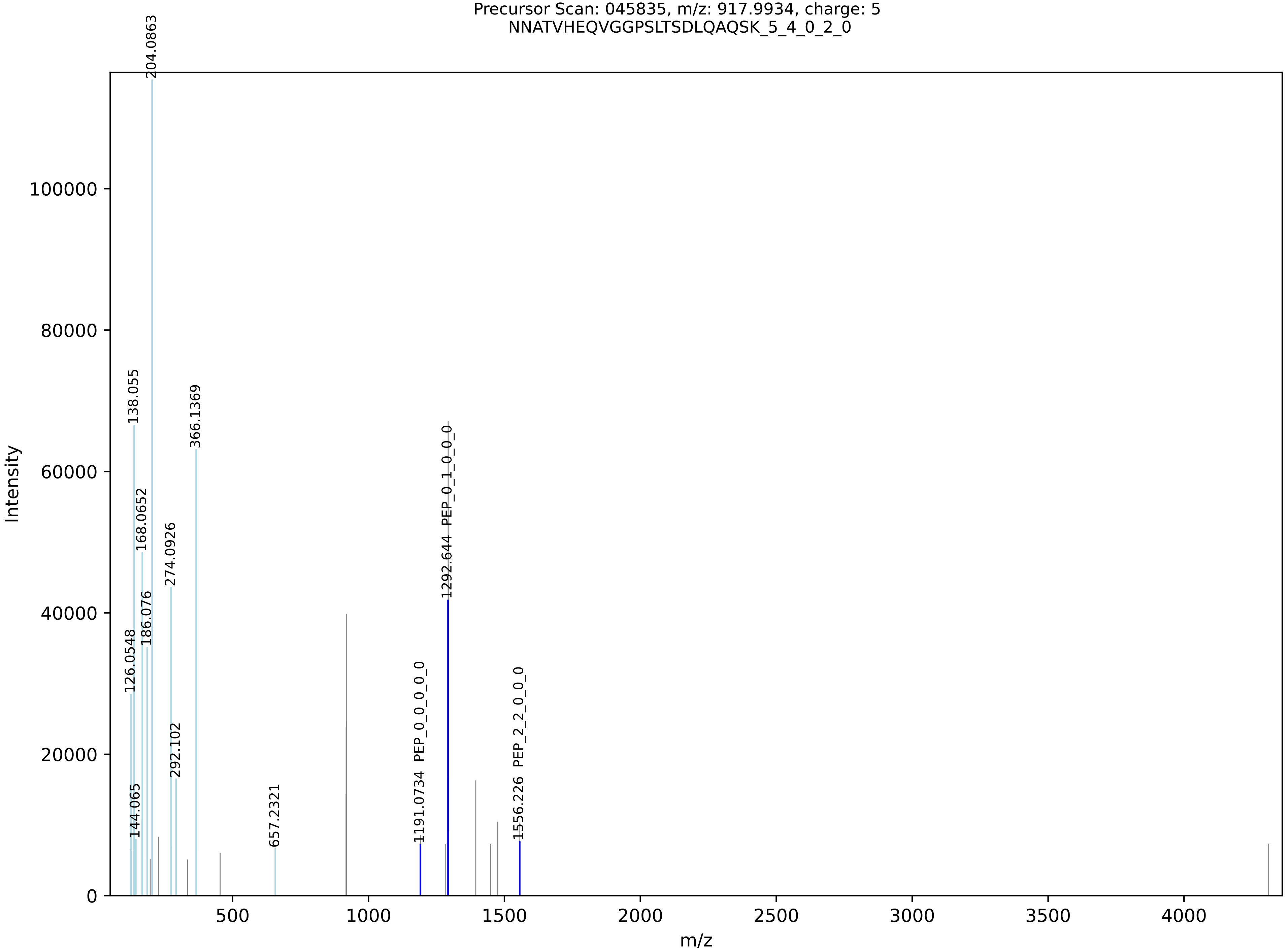

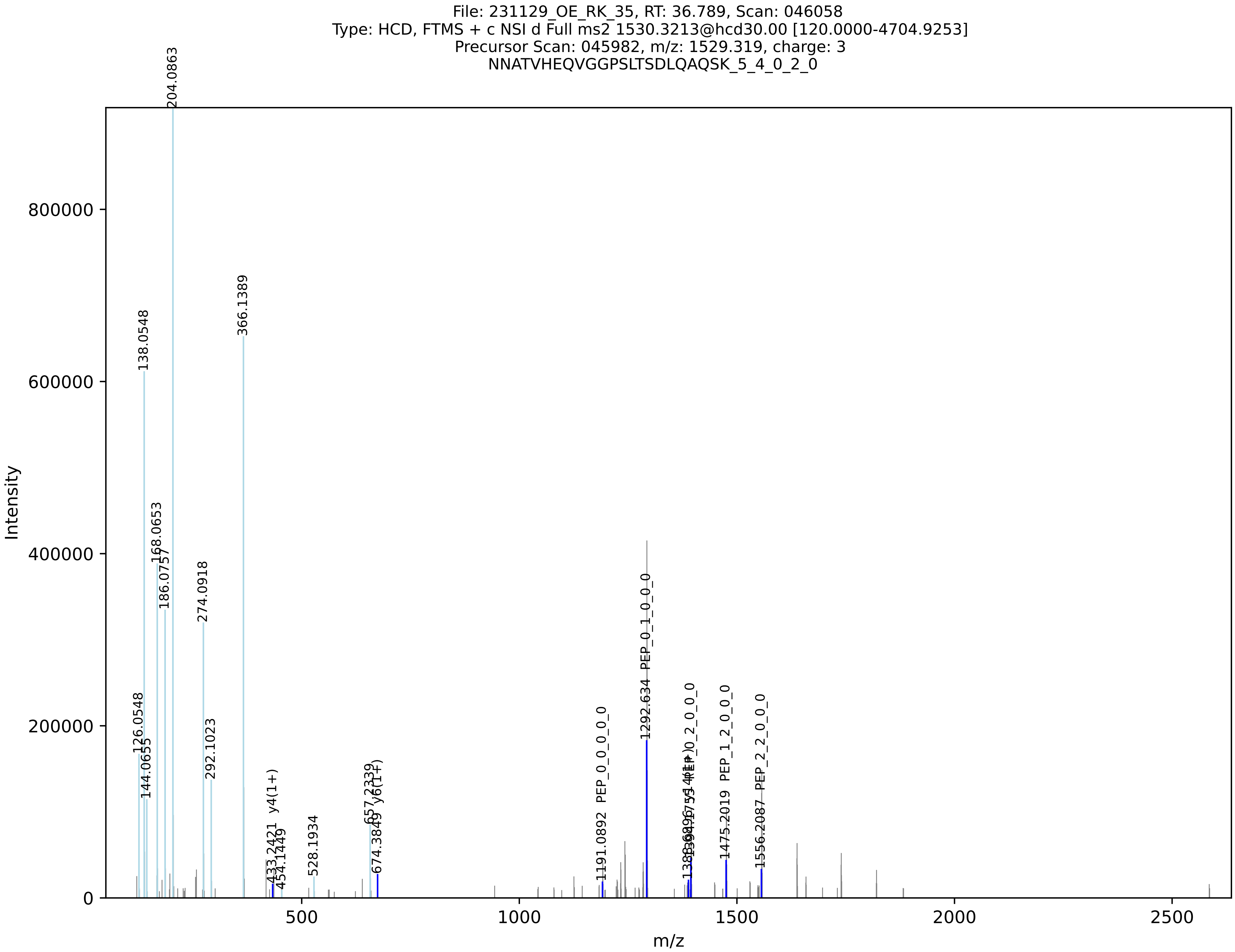

File: 231129_OE_RK_35, RT: 36.789, Scan: 046058
Type: HCD, FTMS + c NSI d Full ms2 1530.3213@hcd30.00 [120.0000-4704.9253]
Precursor Scan: 045982, m/z: 1529.319, charge: 3
NNATVHEQVGGPSLTSDLQAQSK_5_4_0_2_0
204.0863
138.0548
366.1389
168.0653
186.0757
274.0918
126.0548
144.0655
292.1023
433.2421 y4(1+)
454.1449
528.1934
657.2339
674.3849 y6(1+)
1191.0892 PEP_0_0_0_0_0
1292.634 PEP_0_1_0_0_0
1475.2019 PEP_1_2_0_0_0
1556.2087 PEP_2_2_0_0_0
Intensity
m/z

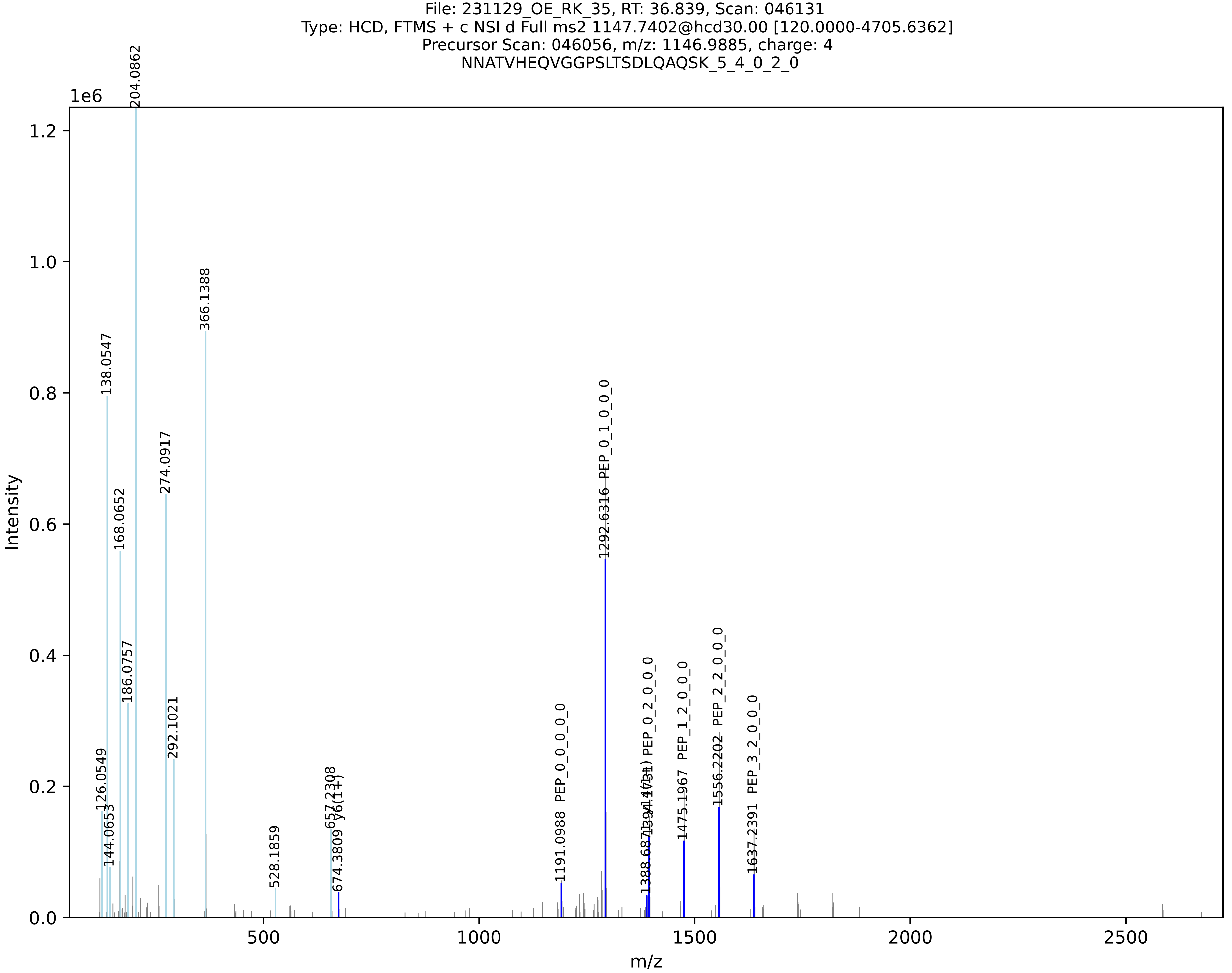

File: 231129_OE_RK_35, RT: 36.839, Scan: 046131
Type: HCD, FTMS + c NSI d Full ms2 1147.7402@hcd30.00 [120.0000-4705.6362]
Precursor Scan: 046056, m/z: 1146.9885, charge: 4
NNATVHEQVGGPSLTSDLQAQSK_5_4_0_2_0
1e6
Intensity
m/z
204.0862
366.1388
138.0547
274.0917
168.0652
186.0757
292.1021
126.0549
144.0653
528.1859
657.2308
674.3809 y6(1+)
1191.0988 PEP_0_0_0_0_0_
1292.6316 PEP_0_1_0_0_0_
PEP_0_2_0_0_0_
1475.1967 PEP_1_2_0_0_0_
1556.2202 PEP_2_2_0_0_0_
1637.2391 PEP_3_2_0_0_0_

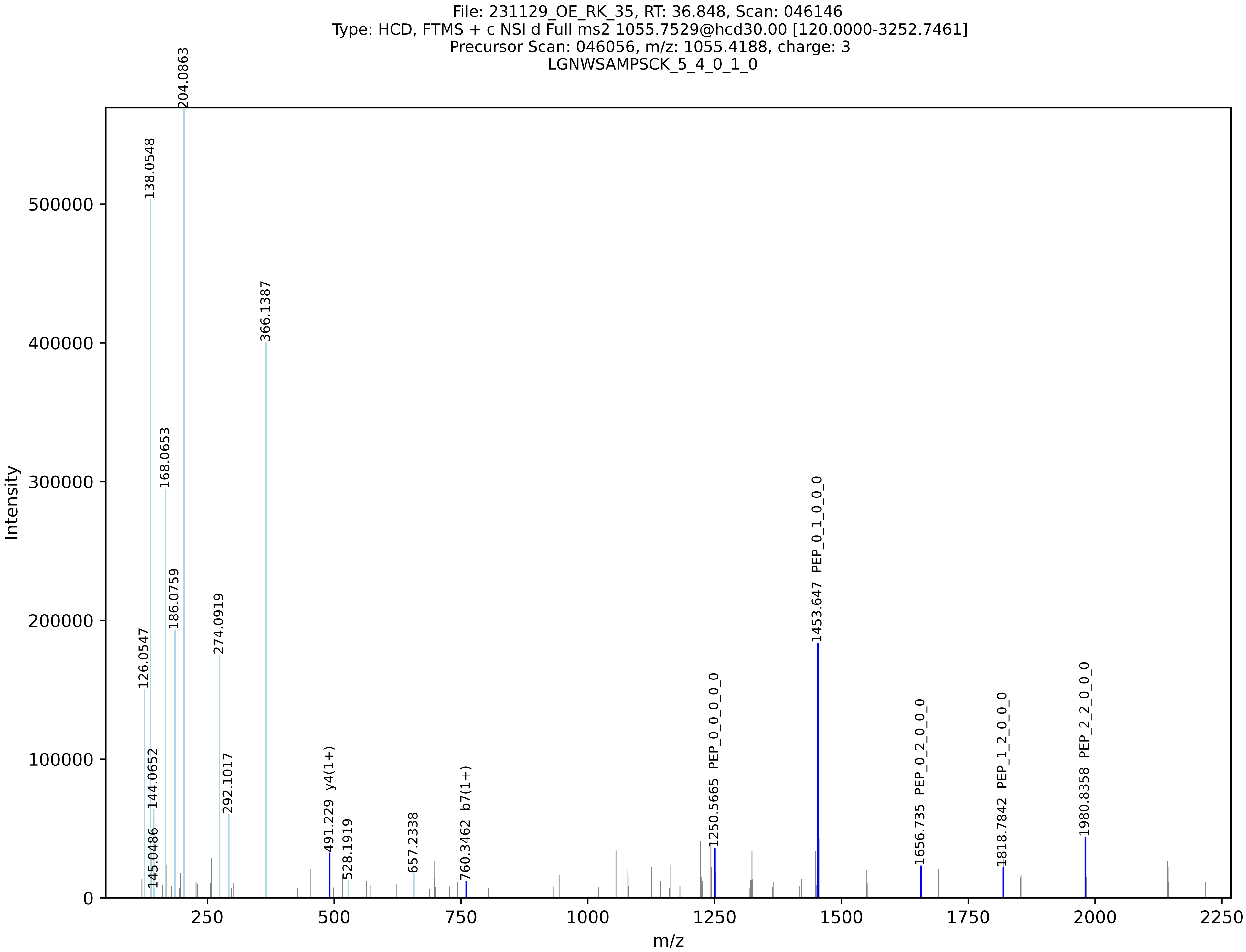
File: 231129_OE_RK_35, RT: 36.848, Scan: 046146
Type: HCD, FTMS + c NSI d Full ms2 1055.7529@hcd30.00 [120.0000-3252.7461]
Precursor Scan: 046056, m/z: 1055.4188, charge: 3
LGNWSAMPSCK_5_4_0_1_0
Intensity
m/z
204.0863
138.0548
366.1387
168.0653
186.0759
274.0919
126.0547
144.0652
292.1017
145.0486
491.229 y4(1+)
528.1919
657.2338
760.3462 b7(1+)
1250.5665 PEP_0_0_0_0_0
1453.647 PEP_0_1_0_0_0
1656.735 PEP_0_2_0_0_0
1818.7842 PEP_1_2_0_0_0
1980.8358 PEP_2_2_0_0_0

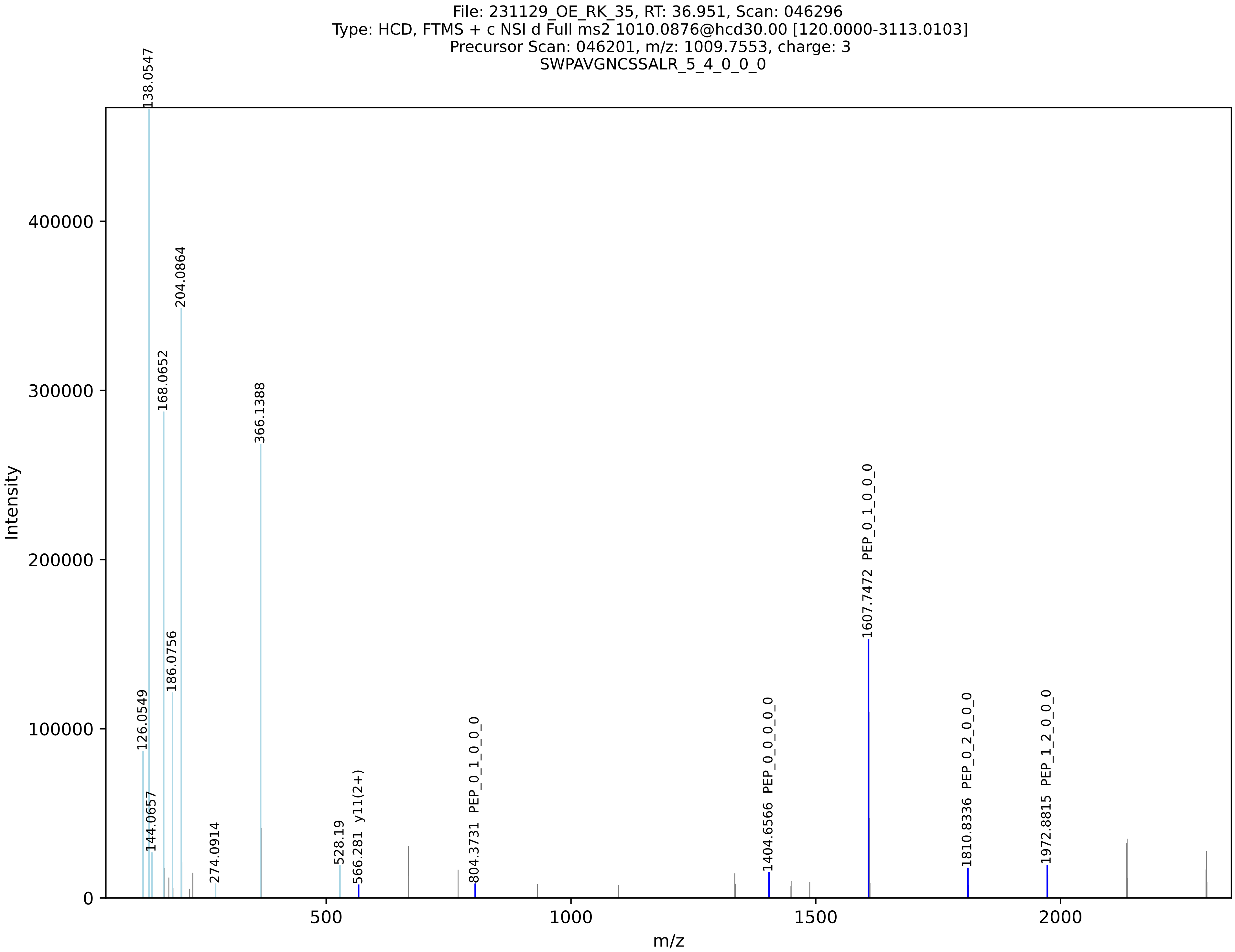
File: 231129_OE_RK_35, RT: 36.951, Scan: 046296
Type: HCD, FTMS + c NSI d Full ms2 1010.0876@hcd30.00 [120.0000-3113.0103]
Precursor Scan: 046201, m/z: 1009.7553, charge: 3
SWPAVGNCSSALR_5_4_0_0_0
Intensity
m/z
0
100000
200000
300000
400000
500
1000
1500
2000
138.0547
204.0864
168.0652
366.1388
186.0756
126.0549
144.0657
274.0914
528.19
566.281 y11(2+)
804.3731 PEP_0_1_0_0_0
1404.6566 PEP_0_0_0_0_0
1607.7472 PEP_0_1_0_0_0
1810.8336 PEP_0_2_0_0_0
1972.8815 PEP_1_2_0_0_0

File: 231129_OE_RK_35, RT: 37.044, Scan: 046431
Type: HCD, FTMS + c NSI d Full ms2 1147.9918@hcd30.00 [120.0000-4706.6626]
Precursor Scan: 046349, m/z: 1147.24, charge: 4
NNATVHEQVGGPSLTSDLQAQSK_5_4_0_2_0

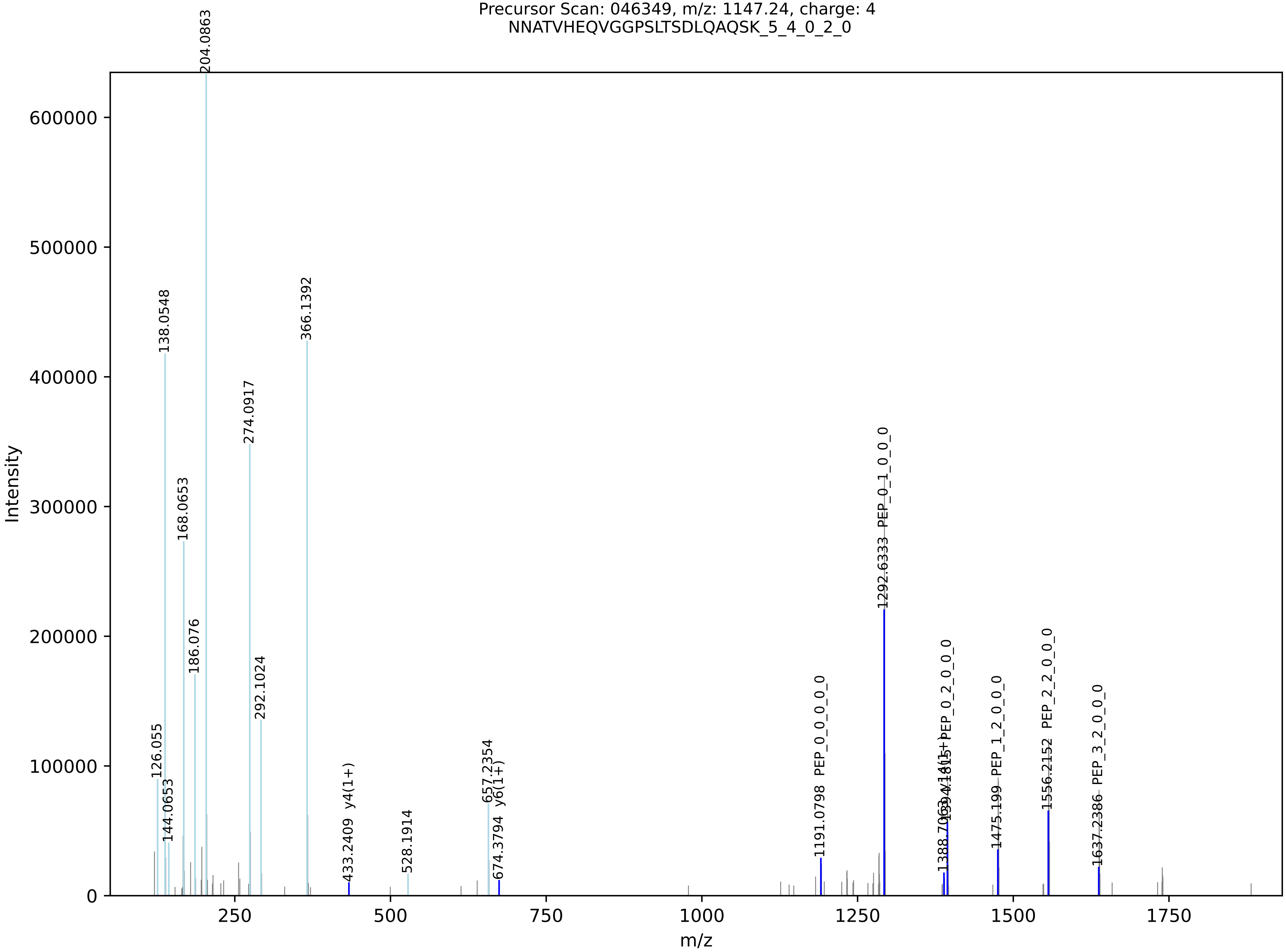

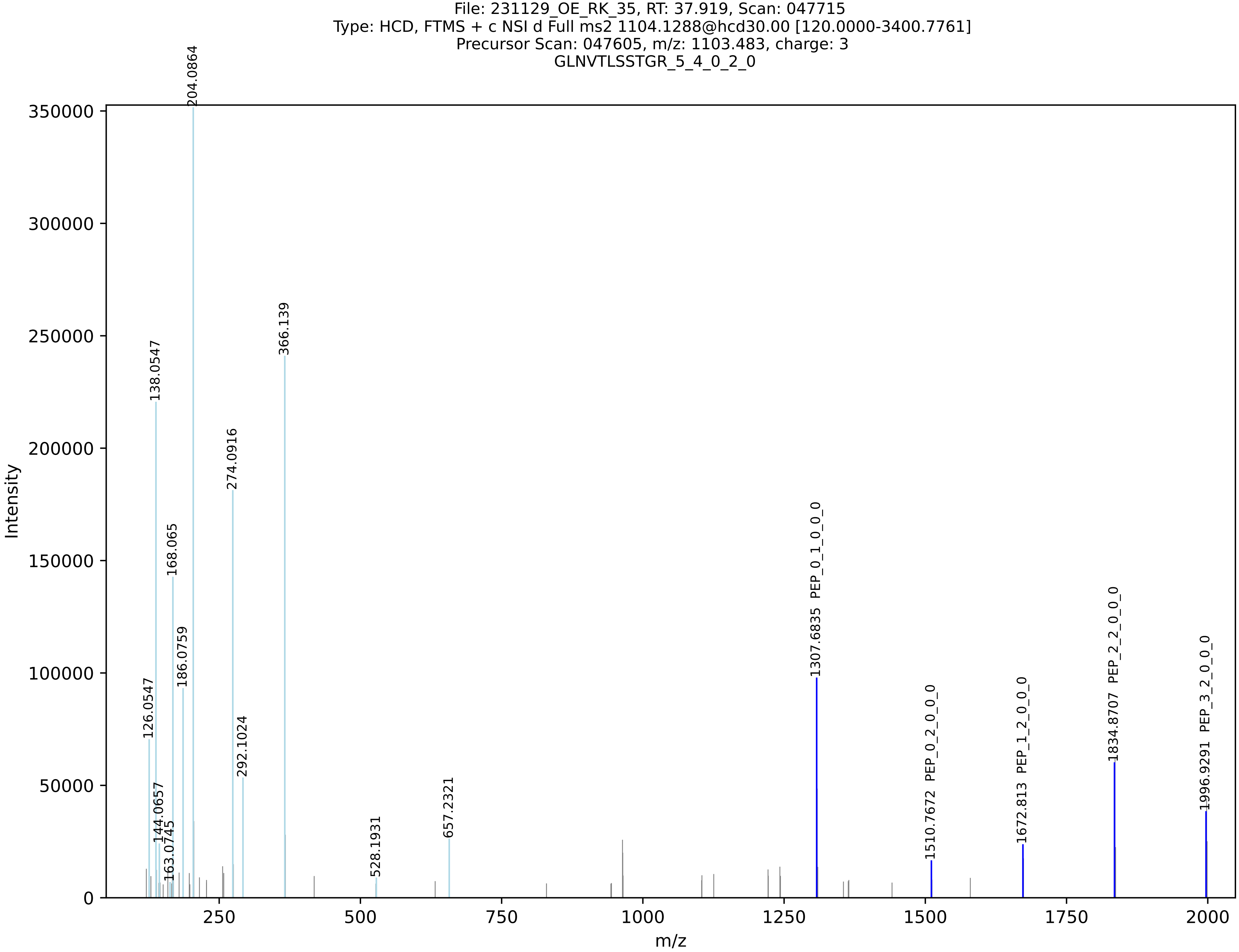
File: 231129_OE_RK_35, RT: 37.919, Scan: 047715
Type: HCD, FTMS + c NSI d Full ms2 1104.1288@hcd30.00 [120.0000-3400.7761]
Precursor Scan: 047605, m/z: 1103.483, charge: 3
GLNVTLSSTGR_5_4_0_2_0
Intensity
m/z
204.0864
366.139
138.0547
274.0916
168.065
186.0759
126.0547
292.1024
144.0657
163.0745
528.1931
657.2321
1307.6835 PEP_0_1_0_0_0
1510.7672 PEP_0_2_0_0_0
1672.813 PEP_1_2_0_0_0
1834.8707 PEP_2_2_0_0_0
1996.9291 PEP_3_2_0_0_0

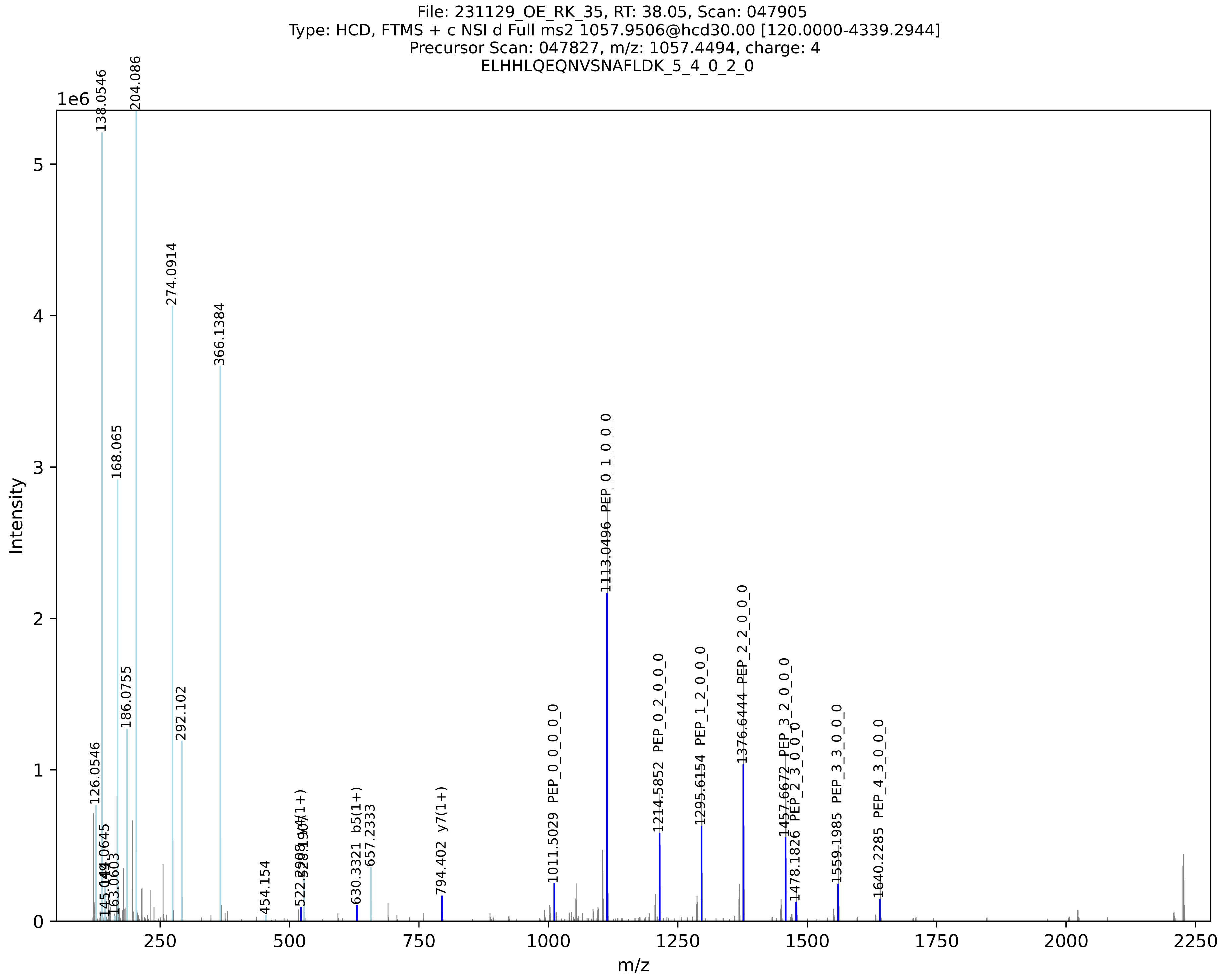
File: 231129_OE_RK_35, RT: 38.05, Scan: 047905
Type: HCD, FTMS + c NSI d Full ms2 1057.9506@hcd30.00 [120.0000-4339.2944]
Precursor Scan: 047827, m/z: 1057.4494, charge: 4
ELHHLQEQNVSNAFLDK_5_4_0_2_0
Intensity
m/z
1e6
138.0546
204.086
274.0914
366.1384
168.065
186.0755
292.102
126.0546
1113.0496 PEP_0_1_0_0_0
1214.5852 PEP_0_2_0_0_0
1295.6154 PEP_1_2_0_0_0
1376.6444 PEP_2_2_0_0_0
1457.6672 PEP_3_2_0_0_0
1478.1826 PEP_2_3_0_0_0
1559.1985 PEP_3_3_0_0_0
1640.2285 PEP_4_3_0_0_0
1011.5029 PEP_0_0_0_0_0
794.402 y7(1+)
630.3321 b5(1+)
657.2333
454.154
163.0603

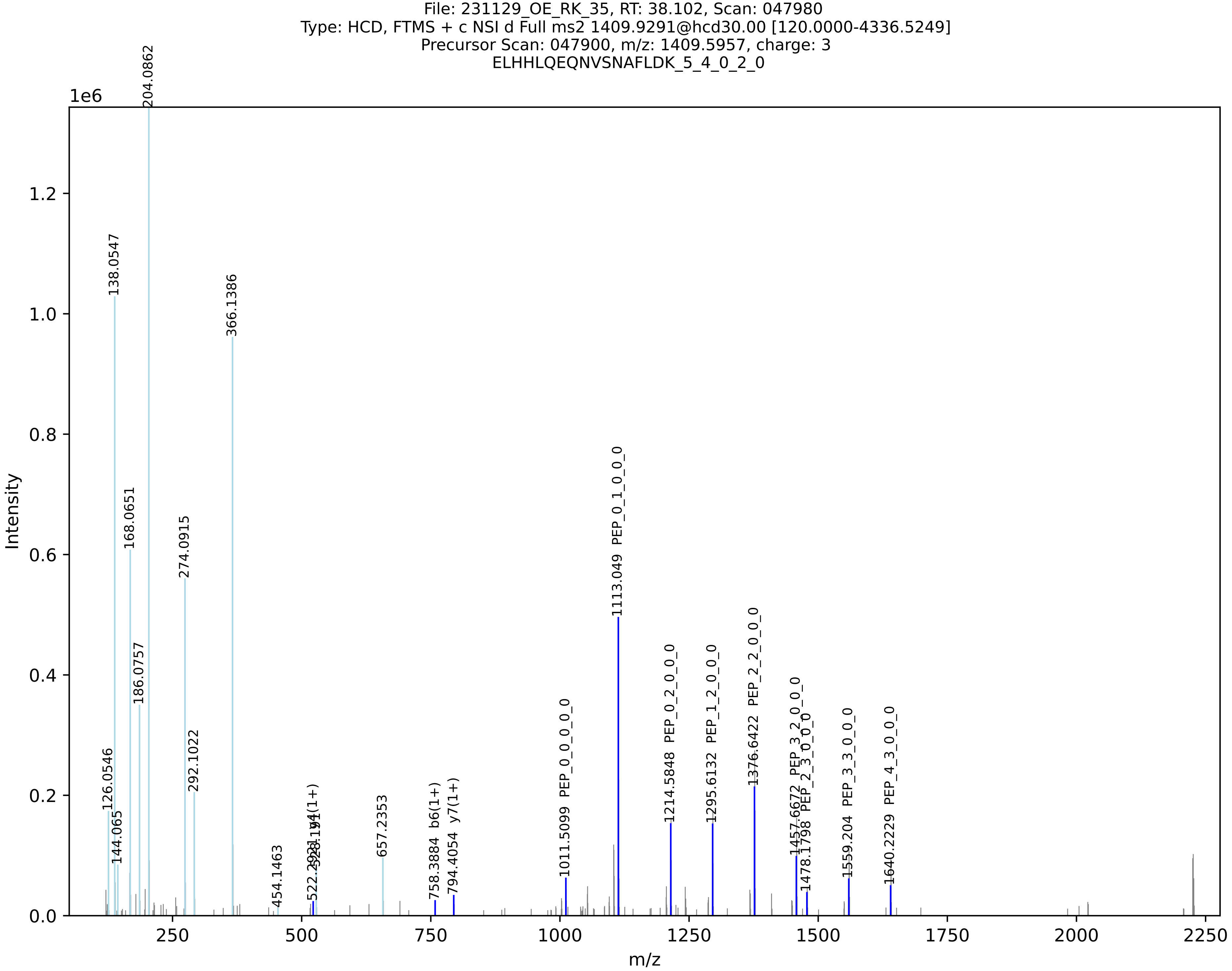
File: 231129_OE_RK_35, RT: 38.102, Scan: 047980
Type: HCD, FTMS + c NSI d Full ms2 1409.9291@hcd30.00 [120.0000-4336.5249]
Precursor Scan: 047900, m/z: 1409.5957, charge: 3
ELHHLQEQNVSNAFLDK_5_4_0_2_0
1e6
Intensity
m/z
0.0
0.2
0.4
0.6
0.8
1.0
1.2
250
500
750
1000
1250
1500
1750
2000
2250
204.0862
138.0547
366.1386
168.0651
274.0915
186.0757
292.1022
126.0546
144.065
454.1463
522.2921 y4(1+)
528.191
657.2353
758.3884 b6(1+)
794.4054 y7(1+)
1011.5099 PEP_0_0_0_0_0
1113.049 PEP_0_1_0_0_0
1214.5848 PEP_0_2_0_0_0
1295.6132 PEP_1_2_0_0_0
1376.6422 PEP_2_2_0_0_0
1457.6672 PEP_3_2_0_0_0
1478.1798 PEP_2_3_0_0_0
1559.204 PEP_3_3_0_0_0
1640.2229 PEP_4_3_0_0_0

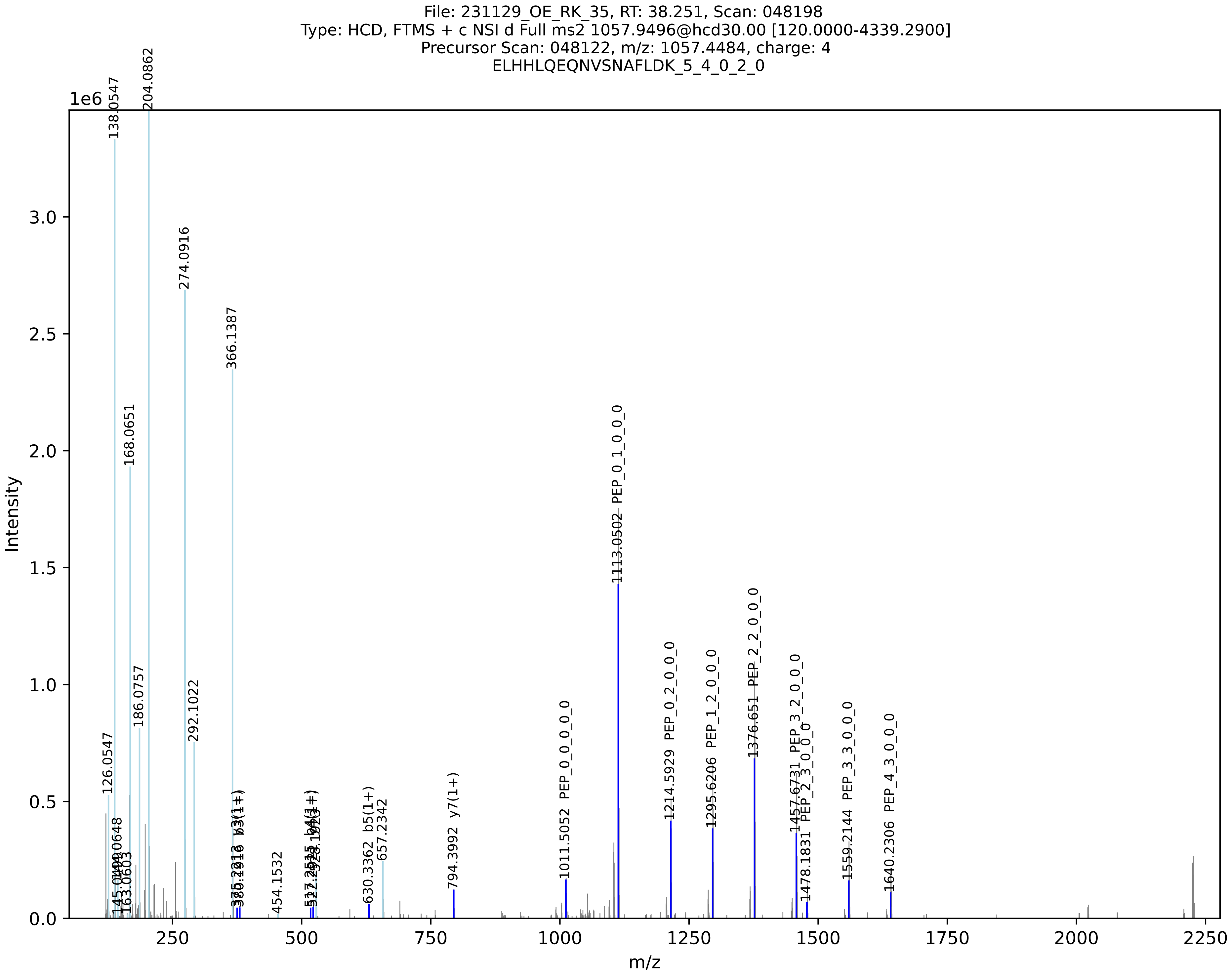

File: 231129_OE_RK_35, RT: 38.251, Scan: 048198
Type: HCD, FTMS + c NSI d Full ms2 1057.9496@hcd30.00 [120.0000-4339.2900]
Precursor Scan: 048122, m/z: 1057.4484, charge: 4
ELHHLQEQNVSNAFLDK_5_4_0_2_0
1e6
Intensity
m/z
138.0547
204.0862
274.0916
366.1387
168.0651
186.0757
292.1022
126.0547
1113.0502 PEP_0_1_0_0_0
1214.5929 PEP_0_2_0_0_0
1295.6206 PEP_1_2_0_0_0
1376.651 PEP_2_2_0_0_0
1457.6731 PEP_3_2_0_0_0
1478.1831 PEP_2_3_0_0_0
1559.2144 PEP_3_3_0_0_0
1640.2306 PEP_4_3_0_0_0
1011.5052 PEP_0_0_0_0_0
794.3992 y7(1+)
630.3362 b5(1+)
657.2342
454.1532

File: 231129_OE_RK_35, RT: 38.305, Scan: 048278
Type: HCD, FTMS + c NSI d Full ms2 1153.1200@hcd30.00 [120.0000-3550.6892]
Precursor Scan: 048196, m/z: 1152.4518, charge: 3
LGNWSAMPSCK_5_4_0_2_0

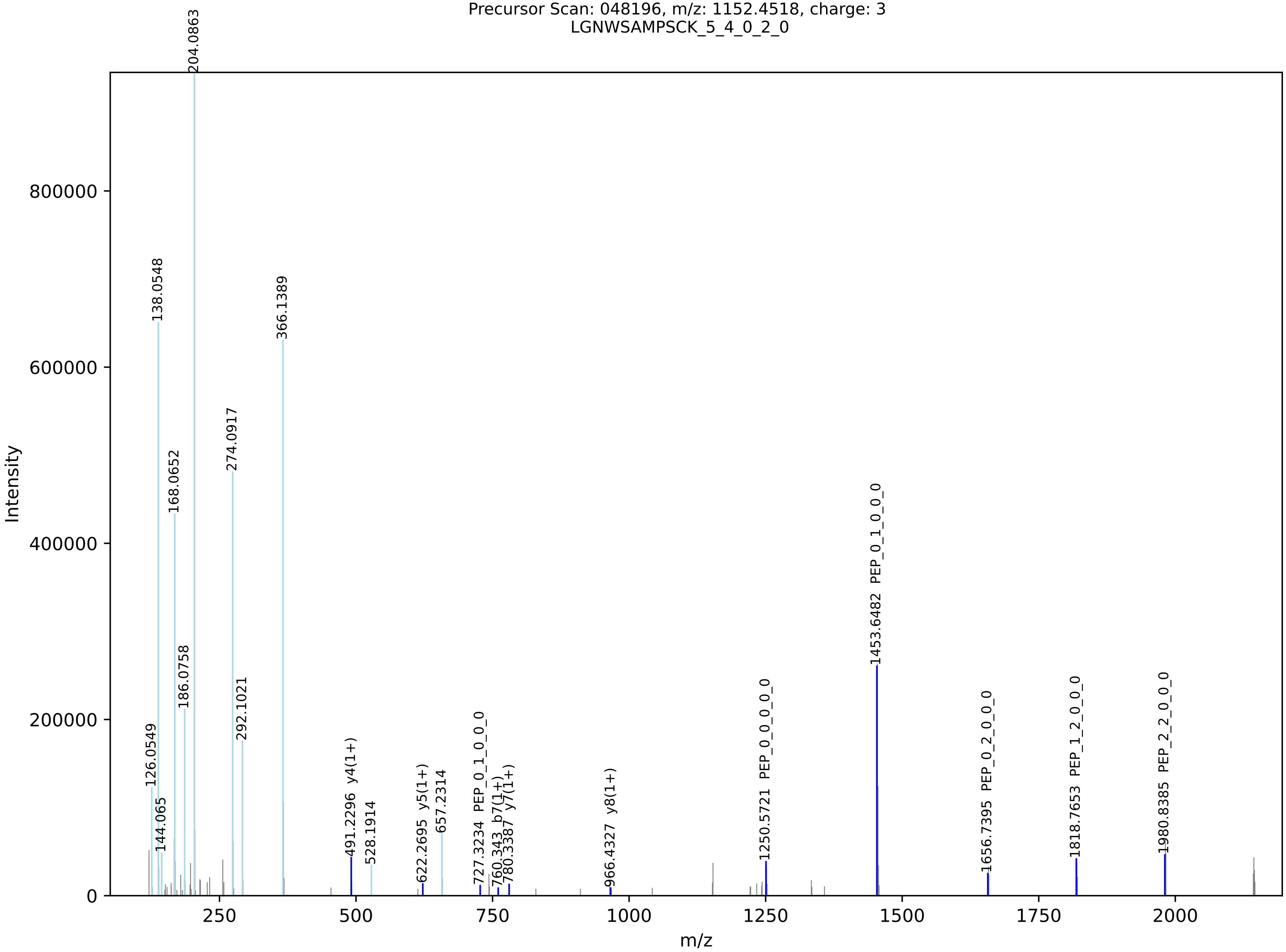

File: 231129_OE_RK_35, RT: 38.305, Scan: 048279
Type: HCD, FTMS + c NSI d Full ms2 1410.5977@hcd30.00 [120.0000-4338.5708]
Precursor Scan: 048196, m/z: 1409.5959, charge: 3
ELHHLQEQNVSNAFLDK_5_4_0_2_0

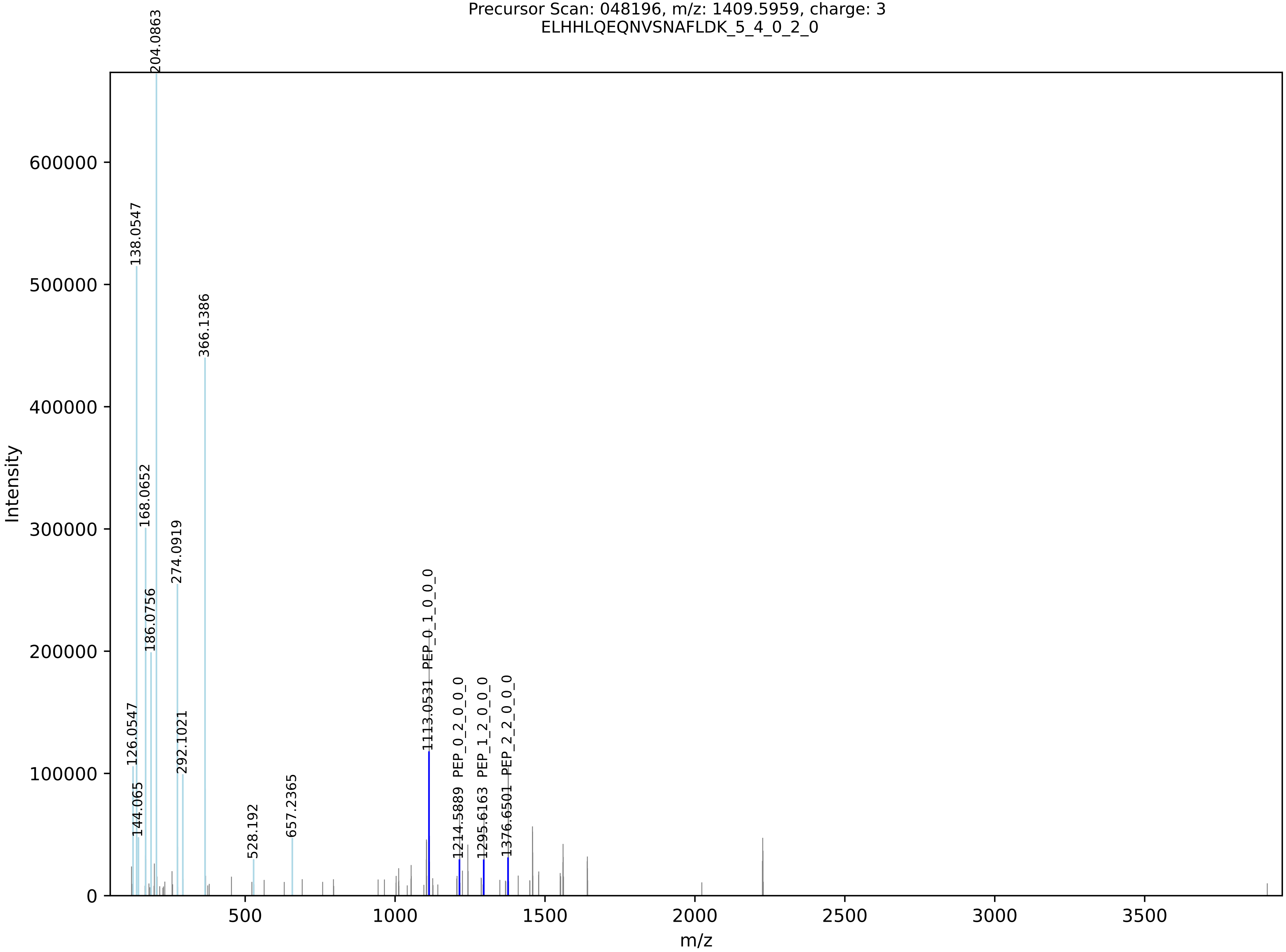

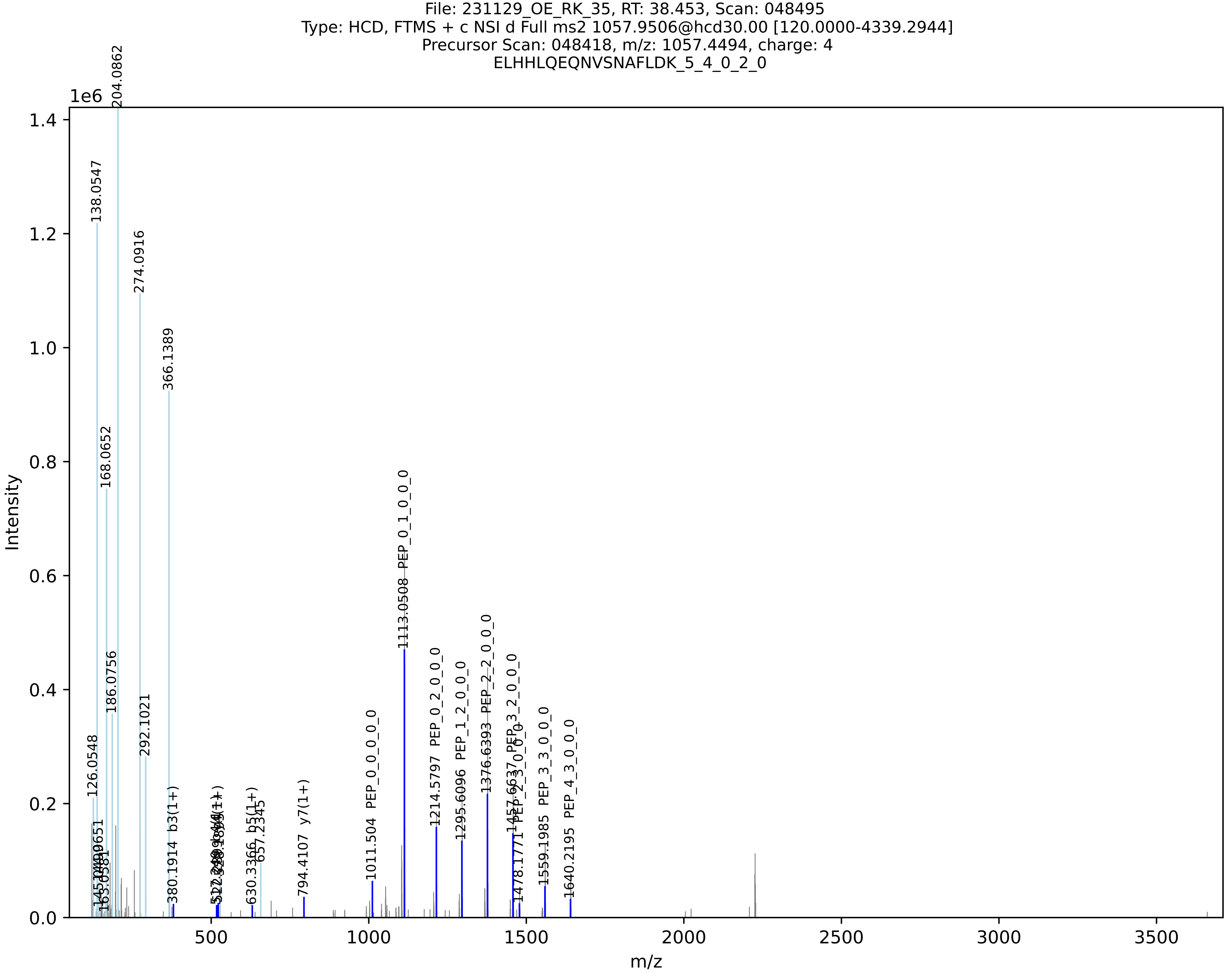
File: 231129_OE_RK_35, RT: 38.453, Scan: 048495
Type: HCD, FTMS + c NSI d Full ms2 1057.9506@hcd30.00 [120.0000-4339.2944]
Precursor Scan: 048418, m/z: 1057.4494, charge: 4
ELHHLQEQNVSNAFLDK_5_4_0_2_0
1e6
Intensity
m/z
204.0862
138.0547
274.0916
366.1389
168.0652
186.0756
292.1021
126.0548
380.1914 b3(1+)
630.3366 b5(1+)
657.2345
794.4107 y7(1+)
1011.504 PEP_0_0_0_0_0
1113.0508 PEP_0_1_0_0_0
1214.5797 PEP_0_2_0_0_0
1295.6096 PEP_1_2_0_0_0
1376.6393 PEP_2_2_0_0_0
1457.6637 PEP_3_2_0_0_0
1478.1771 PEP_2_3_0_0_0
1559.1985 PEP_3_3_0_0_0
1640.2195 PEP_4_3_0_0_0

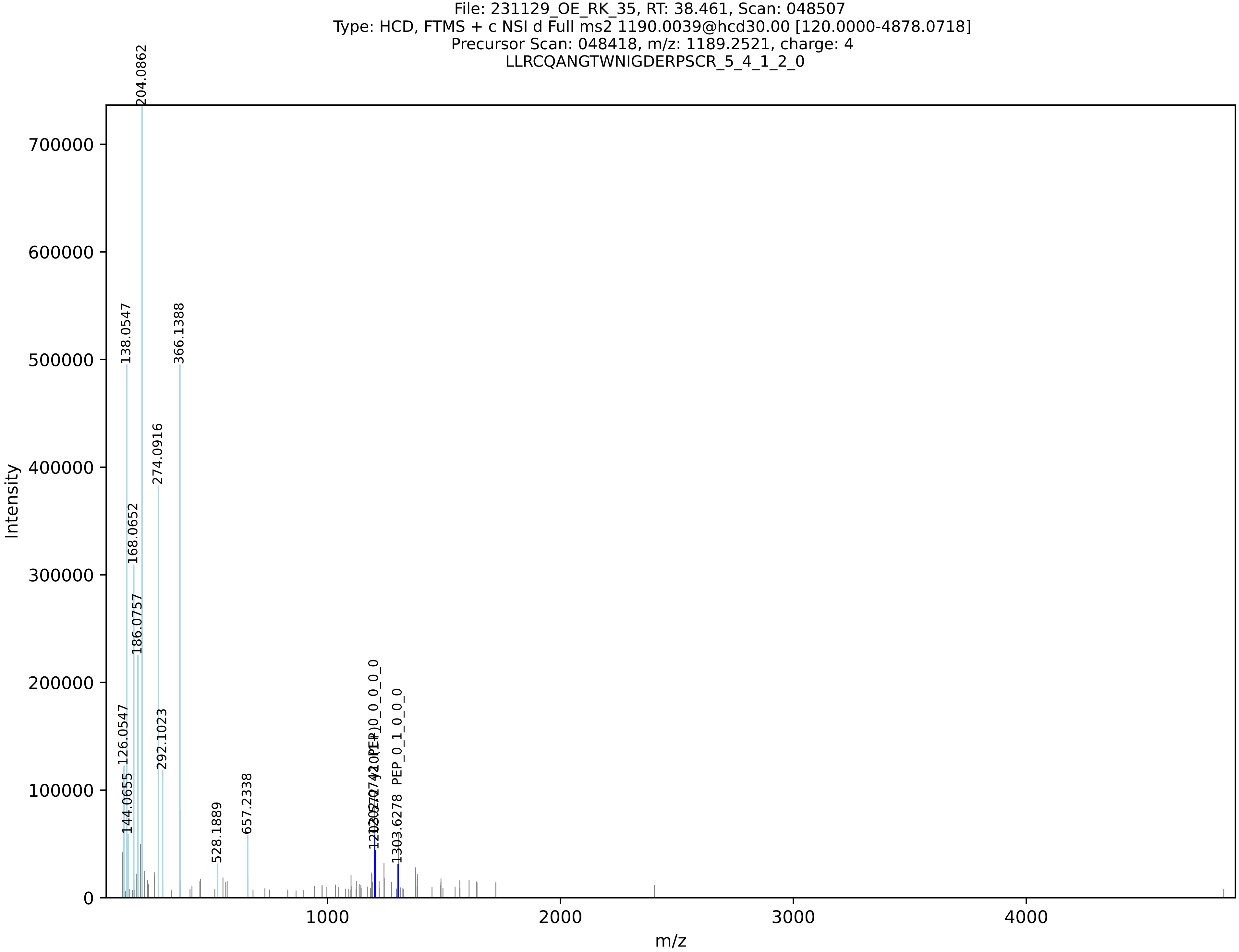

File: 231129_OE_RK_35, RT: 38.461, Scan: 048507
Type: HCD, FTMS + c NSI d Full ms2 1190.0039@hcd30.00 [120.0000-4878.0718]
Precursor Scan: 048418, m/z: 1189.2521, charge: 4
LLRCQANGTWNIGDERPSCR_5_4_1_2_0
204.0862
138.0547
366.1388
274.0916
168.0652
186.0757
126.0547
292.1023
144.0655
528.1889
657.2338
PEP_0_1_0_0_0
1303.6278
Intensity
m/z
0
100000
200000
300000
400000
500000
600000
700000
1000
2000
3000
4000

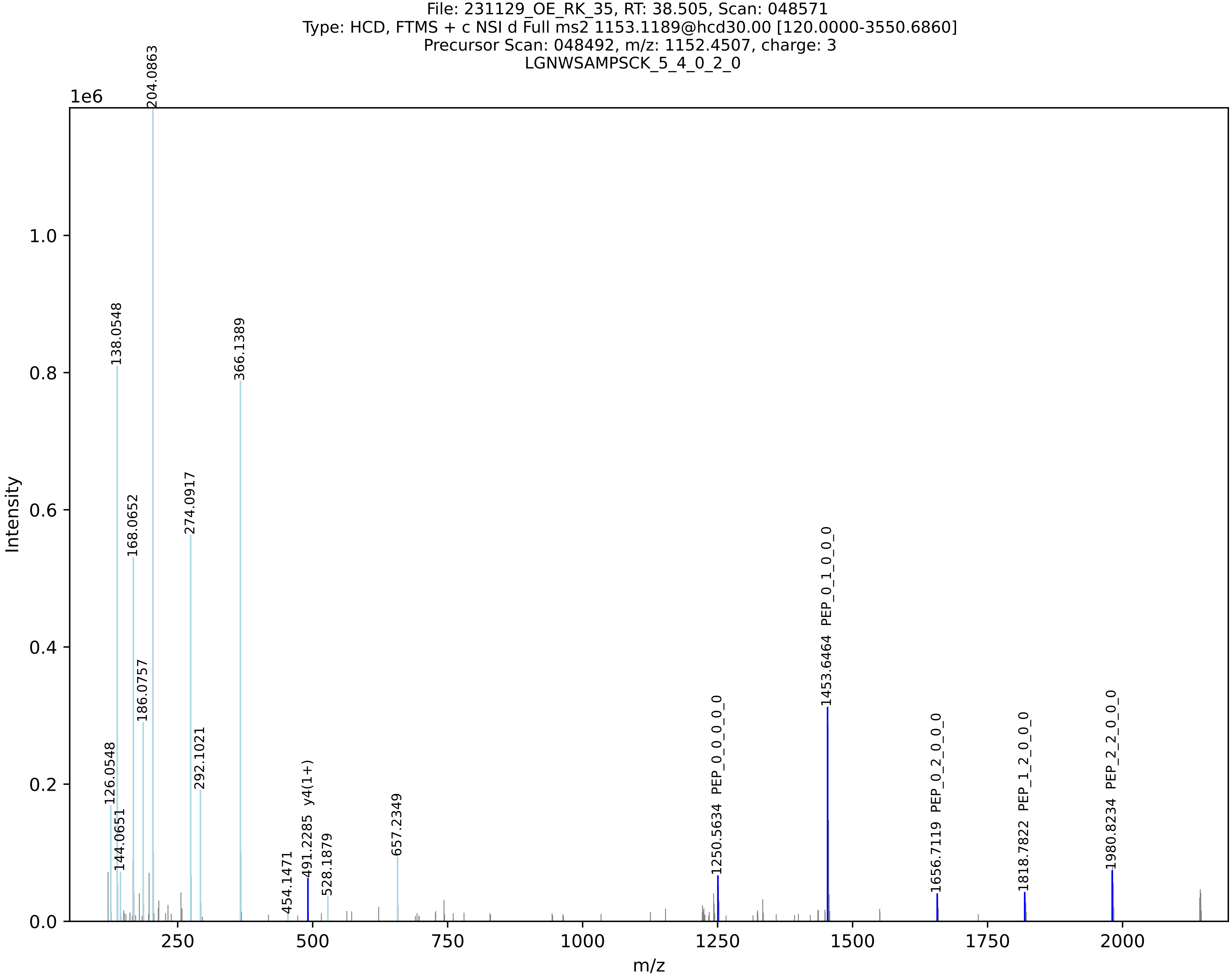

File: 231129_OE_RK_35, RT: 38.505, Scan: 048571
Type: HCD, FTMS + c NSI d Full ms2 1153.1189@hcd30.00 [120.0000-3550.6860]
Precursor Scan: 048492, m/z: 1152.4507, charge: 3
LGNWSAMPSCK_5_4_0_2_0
1e6
Intensity
m/z
204.0863
138.0548
366.1389
168.0652
274.0917
186.0757
292.1021
126.0548
144.0651
454.1471
491.2285 y4(1+)
528.1879
657.2349
1250.5634 PEP_0_0_0_0_0
1453.6464 PEP_0_1_0_0_0
1656.7119 PEP_0_2_0_0_0
1818.7822 PEP_1_2_0_0_0
1980.8234 PEP_2_2_0_0_0

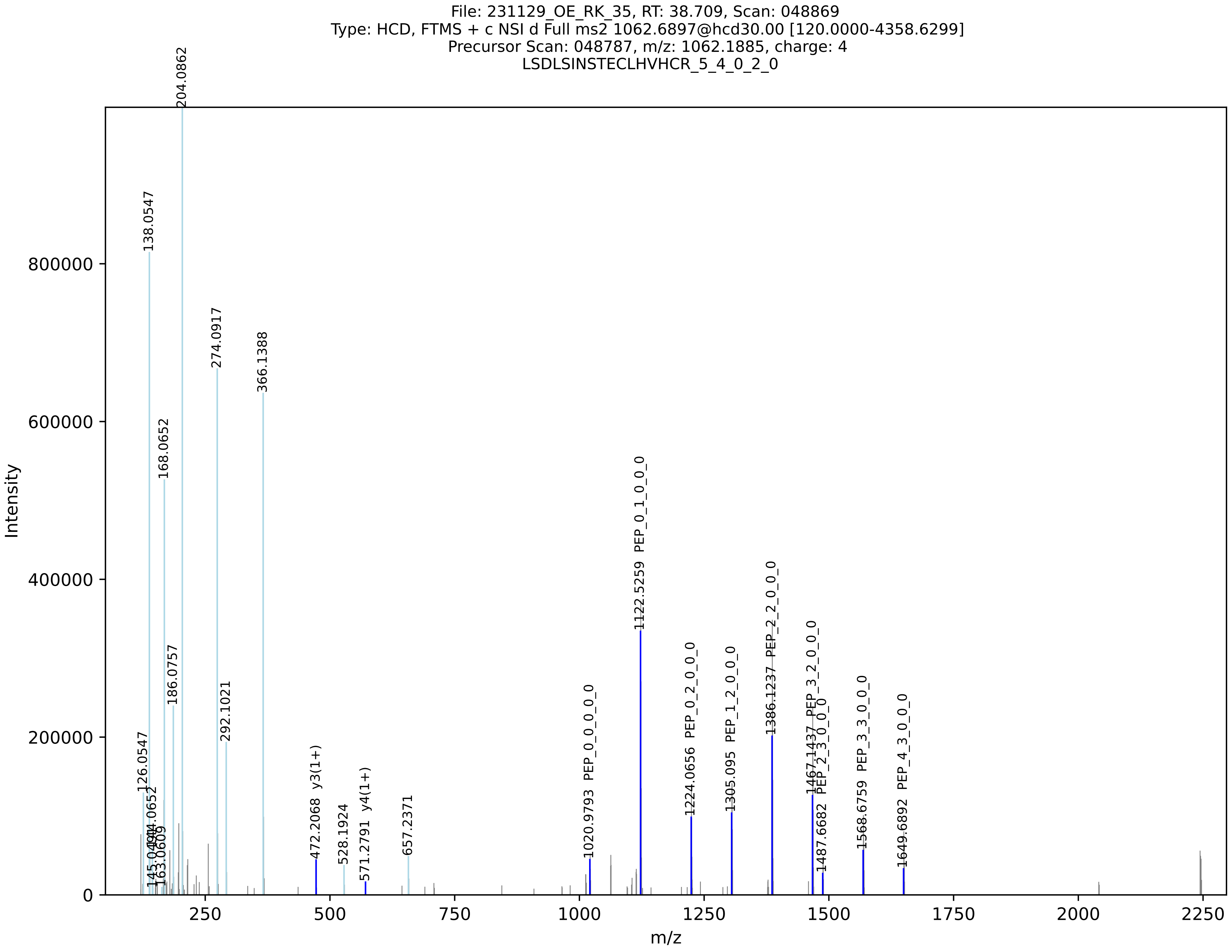
File: 231129_OE_RK_35, RT: 38.709, Scan: 048869
Type: HCD, FTMS + c NSI d Full ms2 1062.6897@hcd30.00 [120.0000-4358.6299]
Precursor Scan: 048787, m/z: 1062.1885, charge: 4
LSDLSINSTECLHVHCR_5_4_0_2_0
Intensity
m/z
204.0862
138.0547
274.0917
366.1388
168.0652
186.0757
292.1021
126.0547
163.0609
472.2068 y3(1+)
528.1924
571.2791 y4(1+)
657.2371
1020.9793 PEP_0_0_0_0_0
1122.5259 PEP_0_1_0_0_0
1224.0656 PEP_0_2_0_0_0
1305.095 PEP_1_2_0_0_0
1386.1237 PEP_2_2_0_0_0
1467.1437 PEP_3_2_0_0_0
1487.6682 PEP_2_3_0_0_0
1568.6759 PEP_3_3_0_0_0
1649.6892 PEP_4_3_0_0_0
0
200000
400000
600000
800000
250
500
750
1000
1250
1500
1750
2000
2250

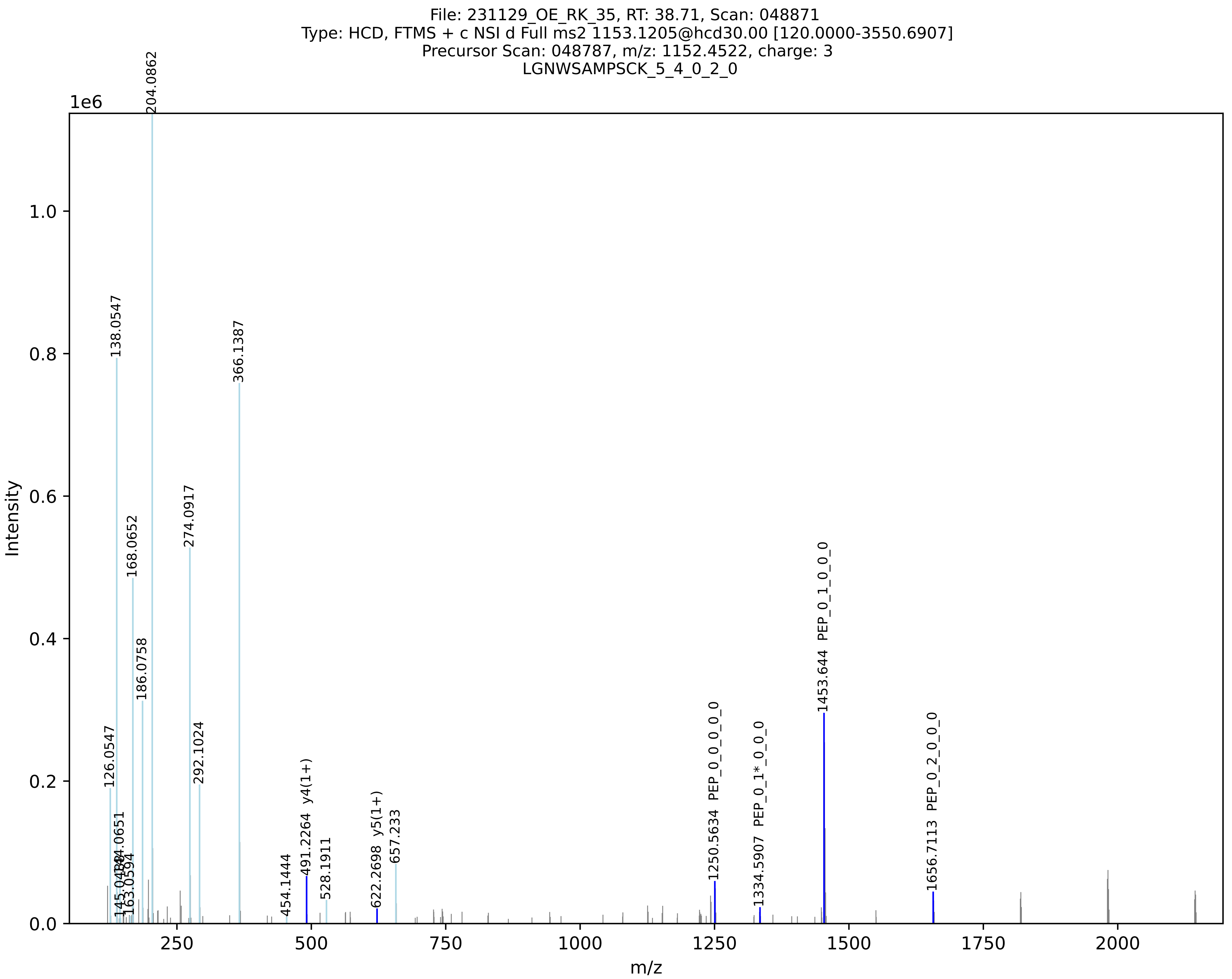
File: 231129_OE_RK_35, RT: 38.71, Scan: 048871
Type: HCD, FTMS + c NSI d Full ms2 1153.1205@hcd30.00 [120.0000-3550.6907]
Precursor Scan: 048787, m/z: 1152.4522, charge: 3
LGNWSAMPSCK_5_4_0_2_0
1e6
Intensity
m/z
204.0862
138.0547
366.1387
274.0917
168.0652
186.0758
126.0547
292.1024
163.0594
454.1444
491.2264 y4(1+)
528.1911
622.2698 y5(1+)
657.233
1250.5634 PEP_0_0_0_0_0
1334.5907 PEP_0_1*_0_0_0
1453.644 PEP_0_1_0_0_0
1656.7113 PEP_0_2_0_0_0

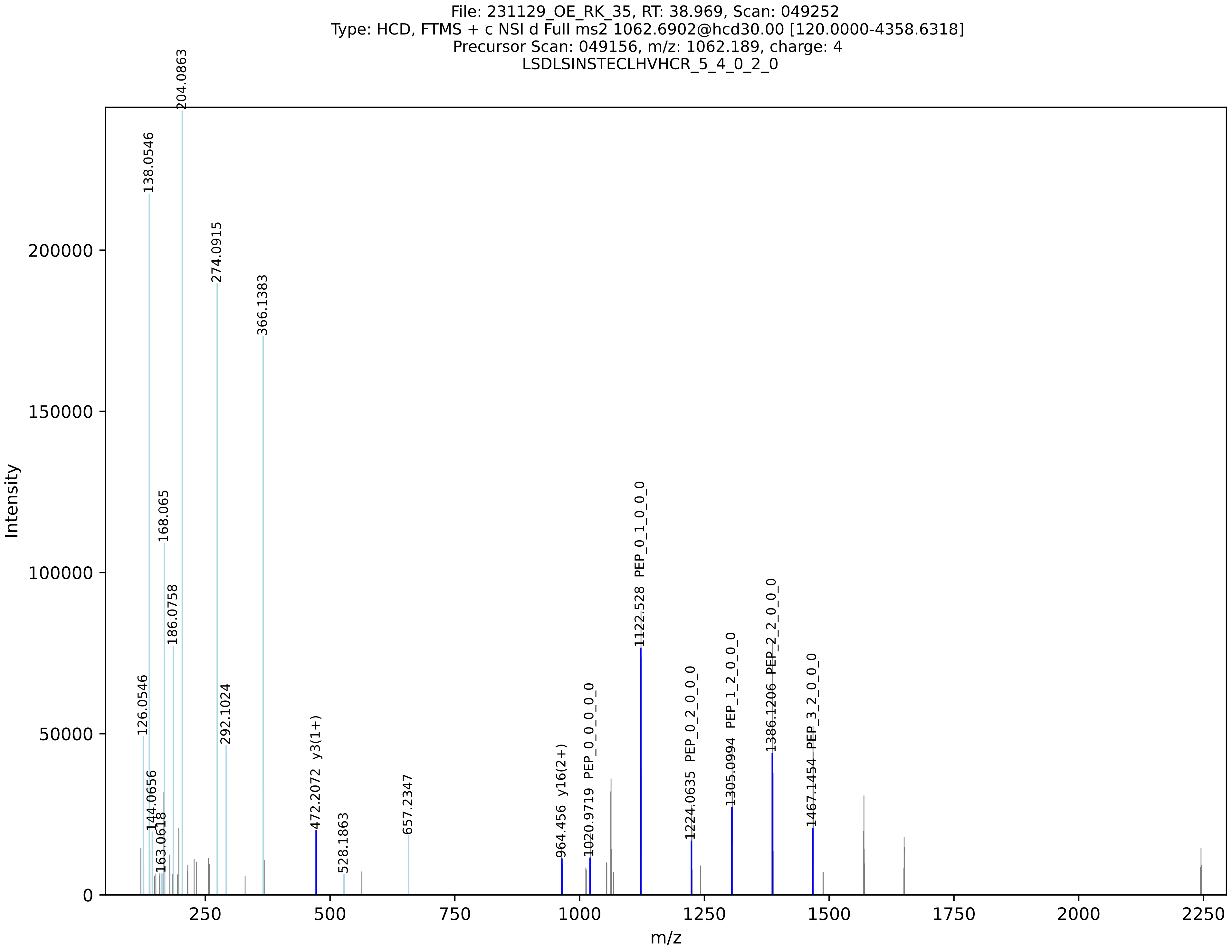

File: 231129_OE_RK_35, RT: 38.969, Scan: 049252
Type: HCD, FTMS + c NSI d Full ms2 1062.6902@hcd30.00 [120.0000-4358.6318]
Precursor Scan: 049156, m/z: 1062.189, charge: 4
LSDLSINSTECLHVHCR_5_4_0_2_0
Intensity
m/z
204.0863
138.0546
274.0915
366.1383
168.065
186.0758
126.0546
292.1024
144.0656
163.0618
472.2072 y3(1+)
528.1863
657.2347
964.456 y16(2+)
1020.9719 PEP_0_0_0_0_0_
1122.528 PEP_0_1_0_0_0_
1224.0635 PEP_0_2_0_0_0_
1305.0994 PEP_1_2_0_0_0_
1386.1206 PEP_2_2_0_0_0_
1467.1454 PEP_3_2_0_0_0_

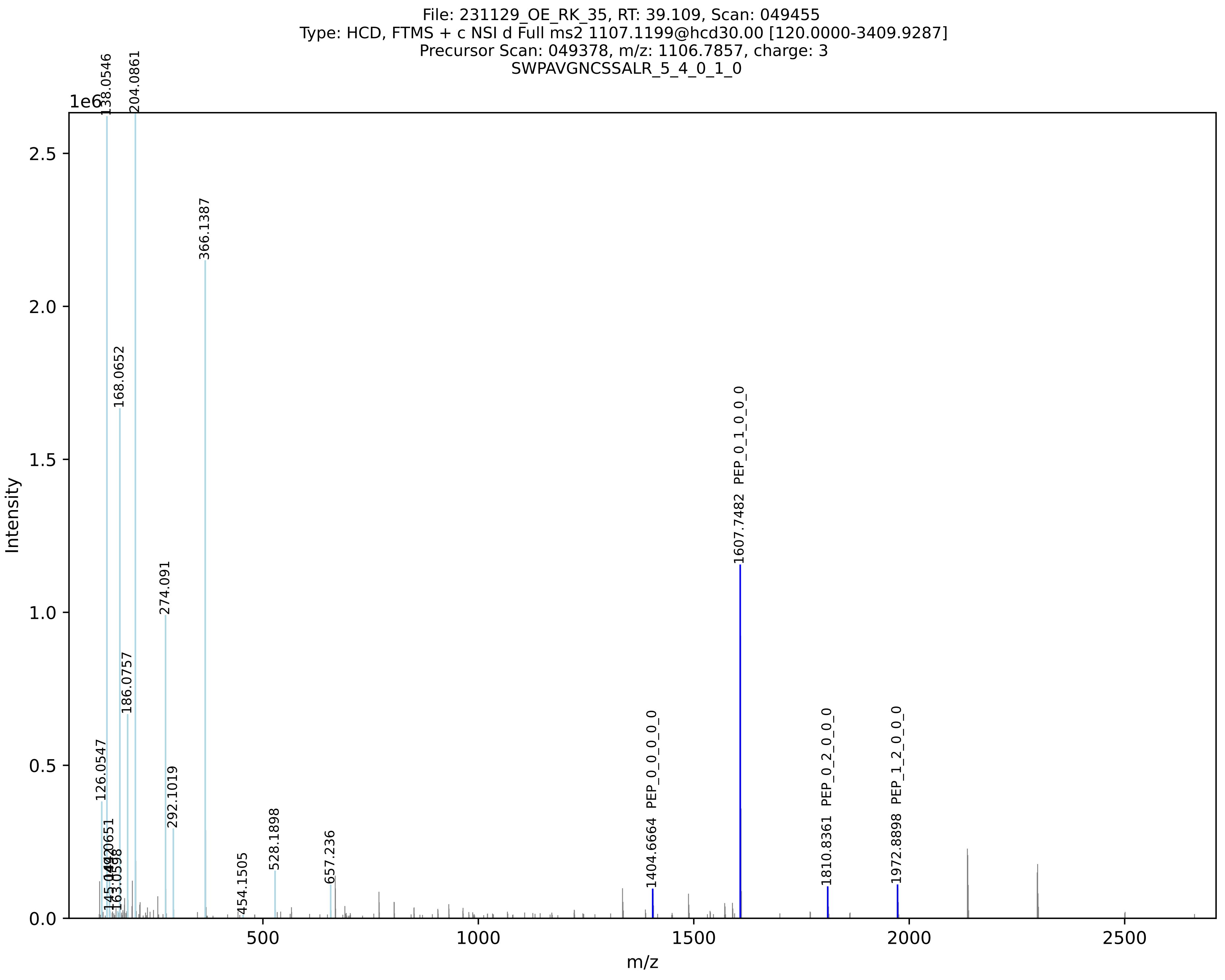
File: 231129_OE_RK_35, RT: 39.109, Scan: 049455
Type: HCD, FTMS + c NSI d Full ms2 1107.1199@hcd30.00 [120.0000-3409.9287]
Precursor Scan: 049378, m/z: 1106.7857, charge: 3
SWPAVGNCSSALR_5_4_0_1_0
1e6
138.0546
204.0861
366.1387
168.0652
274.091
186.0757
126.0547
292.1019
528.1898
657.236
454.1505
163.0598
1404.6664 PEP_0_0_0_0_0
1607.7482 PEP_0_1_0_0_0
1810.8361 PEP_0_2_0_0_0
1972.8898 PEP_1_2_0_0_0
Intensity
m/z
0.0
0.5
1.0
1.5
2.0
2.5
500
1000
1500
2000
2500

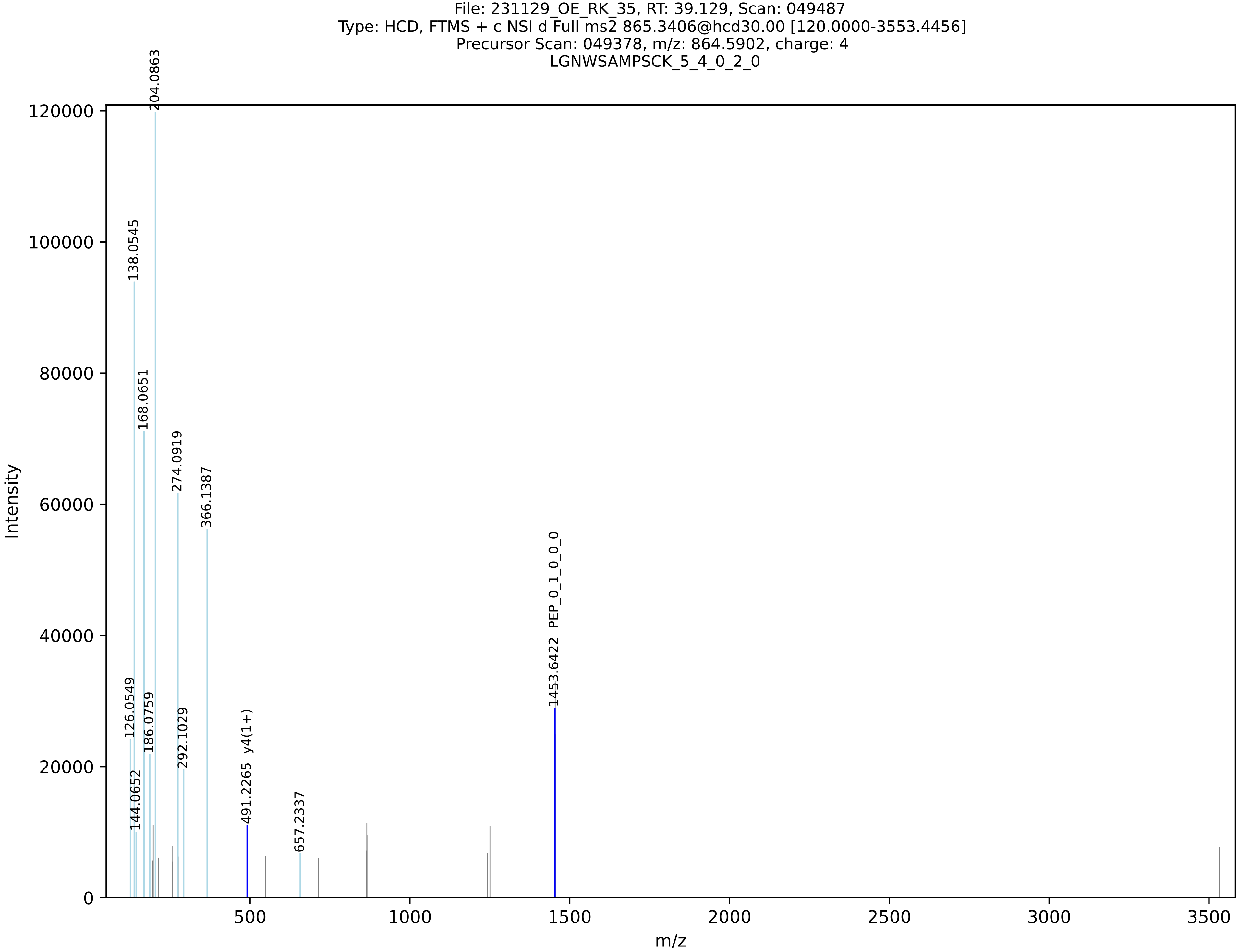
File: 231129_OE_RK_35, RT: 39.129, Scan: 049487
Type: HCD, FTMS + c NSI d Full ms2 865.3406@hcd30.00 [120.0000-3553.4456]
Precursor Scan: 049378, m/z: 864.5902, charge: 4
LGNWSAMPSCK_5_4_0_2_0
Intensity
m/z
204.0863
138.0545
168.0651
274.0919
366.1387
1453.6422 PEP_0_1_0_0_0_
126.0549
186.0759
292.1029
491.2265 y4(1+)
144.0652
657.2337
0
20000
40000
60000
80000
100000
120000
500
1000
1500
2000
2500
3000
3500

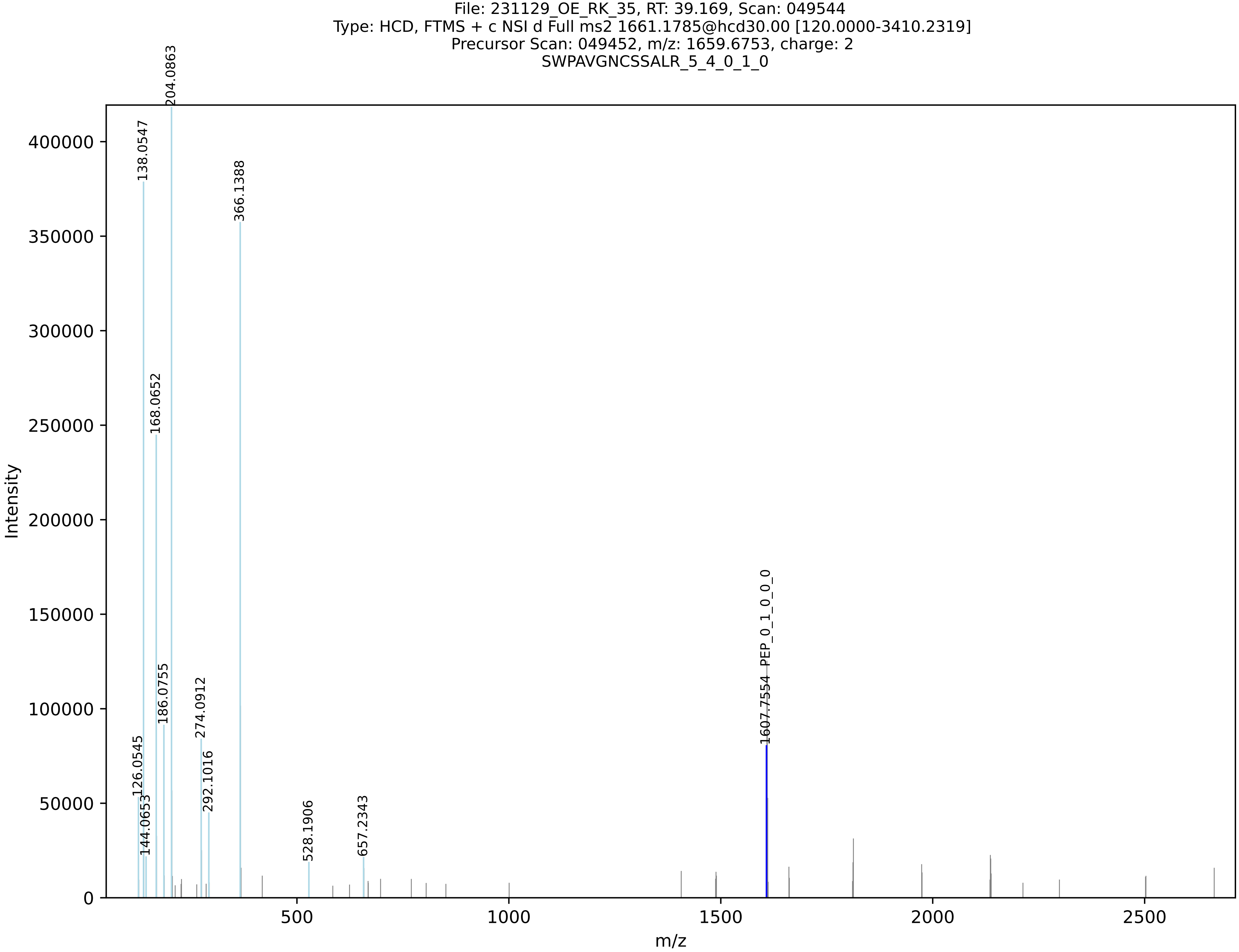

File: 231129_OE_RK_35, RT: 39.169, Scan: 049544
Type: HCD, FTMS + c NSI d Full ms2 1661.1785@hcd30.00 [120.0000-3410.2319]
Precursor Scan: 049452, m/z: 1659.6753, charge: 2
SWPAVGNCSSALR_5_4_0_1_0
Intensity
m/z
204.0863
138.0547
366.1388
168.0652
186.0755
274.0912
292.1016
126.0545
144.0653
528.1906
657.2343
1607.7554_PEP_0_1_0_0_0

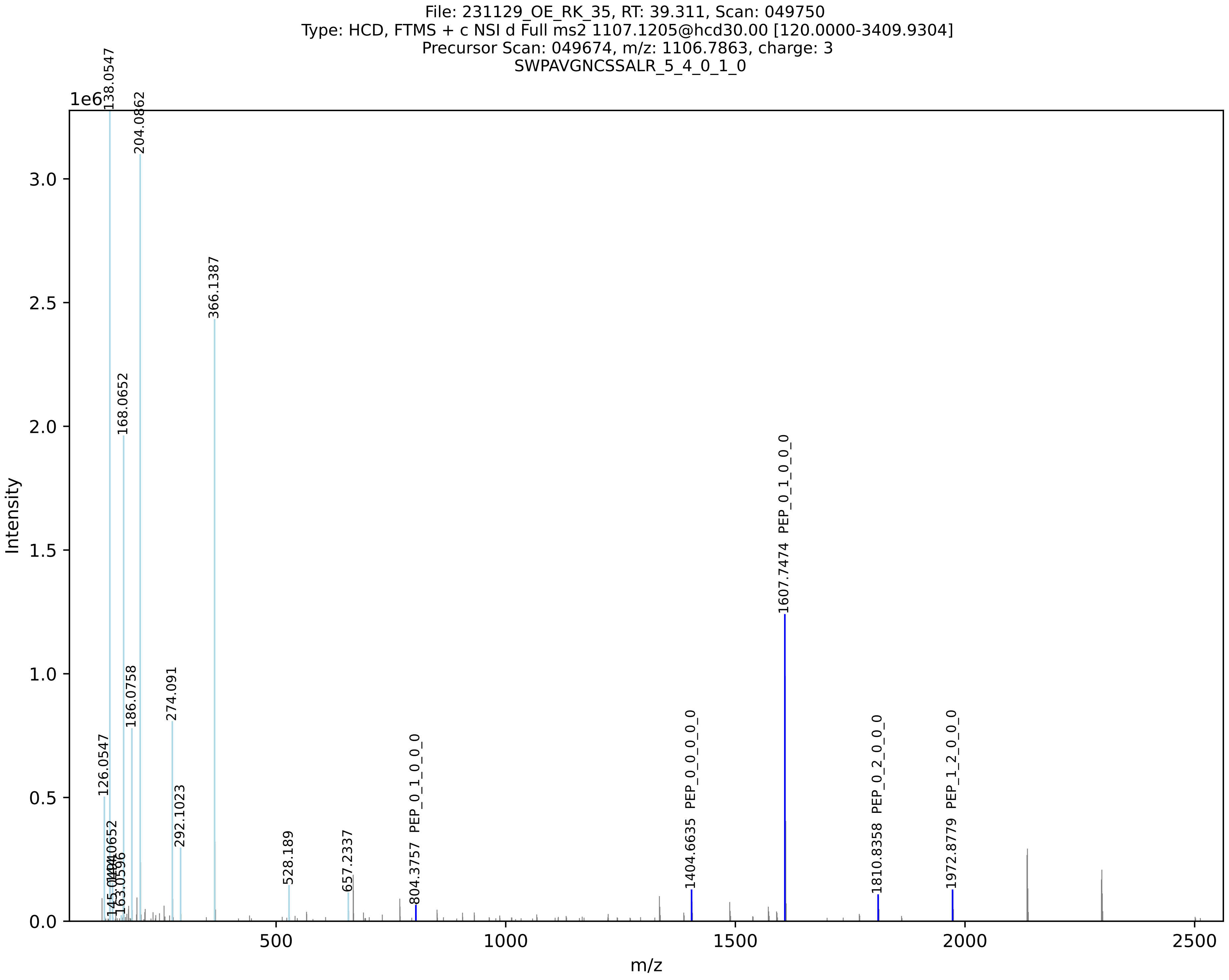
File: 231129_OE_RK_35, RT: 39.311, Scan: 049750
Type: HCD, FTMS + c NSI d Full ms2 1107.1205@hcd30.00 [120.0000-3409.9304]
Precursor Scan: 049674, m/z: 1106.7863, charge: 3
SWPAVGNCSSALR_5_4_0_1_0
1e6
Intensity
m/z
138.0547
204.0862
366.1387
168.0652
186.0758
274.091
126.0547
292.1023
528.189
657.2337
804.3757 PEP_0_1_0_0_0
1404.6635 PEP_0_0_0_0_0
1607.7474 PEP_0_1_0_0_0
1810.8358 PEP_0_2_0_0_0
1972.8779 PEP_1_2_0_0_0

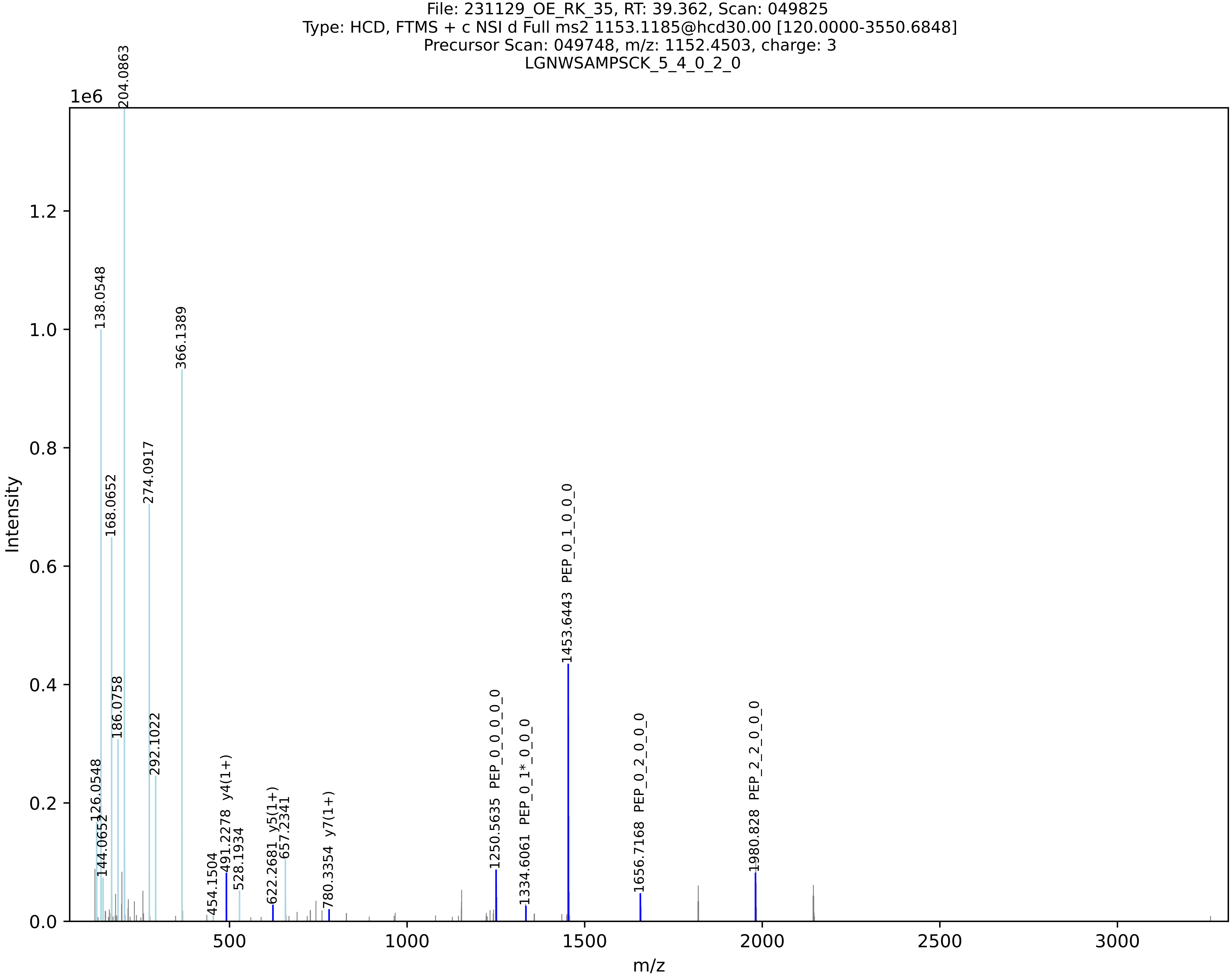
File: 231129_OE_RK_35, RT: 39.362, Scan: 049825
Type: HCD, FTMS + c NSI d Full ms2 1153.1185@hcd30.00 [120.0000-3550.6848]
Precursor Scan: 049748, m/z: 1152.4503, charge: 3
LGNWSAMPSCK_5_4_0_2_0
1e6
Intensity
m/z
204.0863
138.0548
366.1389
274.0917
168.0652
186.0758
292.1022
126.0548
144.0652
454.1504
491.2278 y4(1+)
528.1934
622.2681 y5(1+)
657.2341
780.3354 y7(1+)
1250.5635 PEP_0_0_0_0_0
1334.6061 PEP_0_1*_0_0_0
1453.6443 PEP_0_1_0_0_0
1656.7168 PEP_0_2_0_0_0
1980.828 PEP_2_2_0_0_0
0.0
0.2
0.4
0.6
0.8
1.0
1.2
500
1000
1500
2000
2500
3000

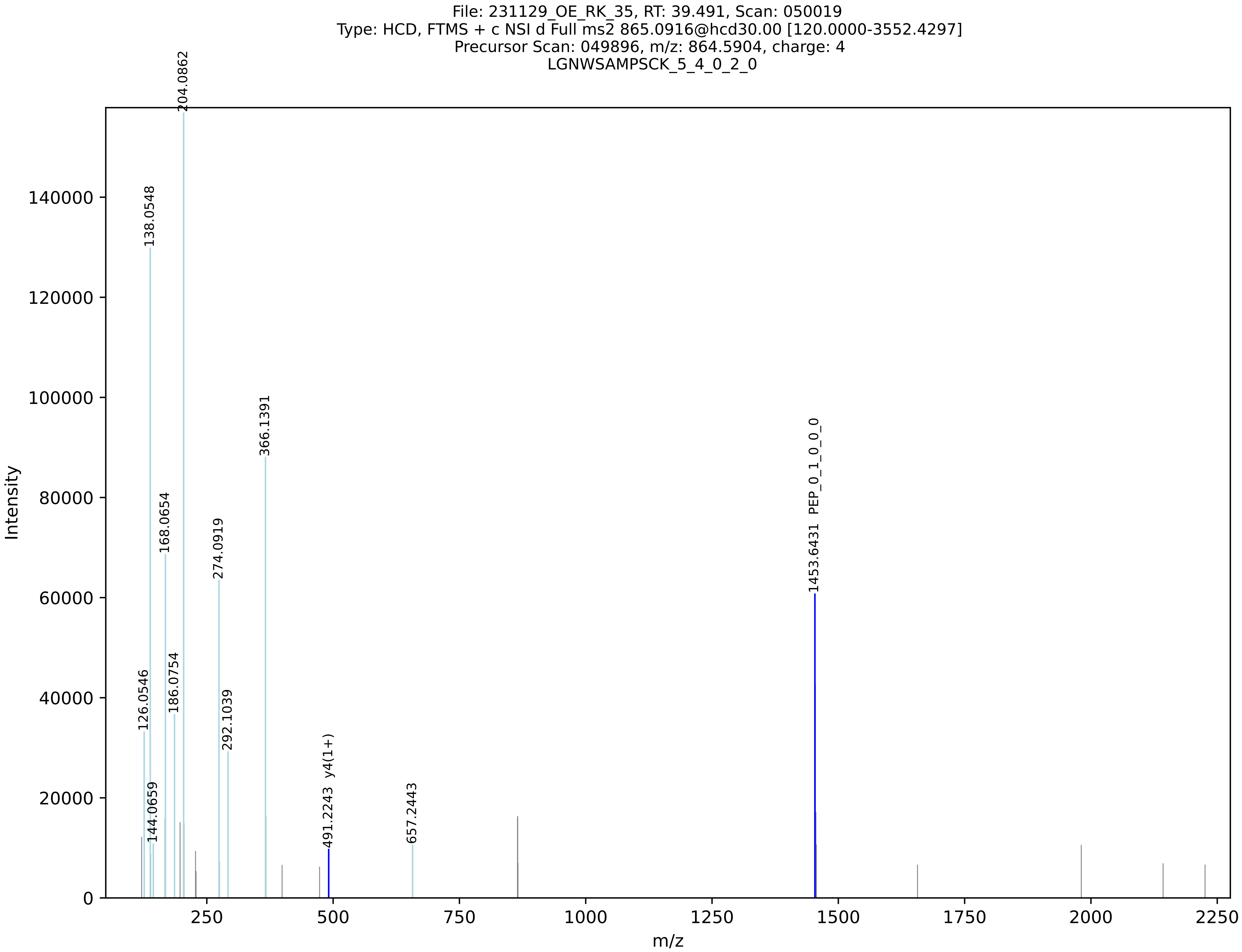
File: 231129_OE_RK_35, RT: 39.491, Scan: 050019
Type: HCD, FTMS + c NSI d Full ms2 865.0916@hcd30.00 [120.0000-3552.4297]
Precursor Scan: 049896, m/z: 864.5904, charge: 4
LGNWSAMPSCK_5_4_0_2_0
Intensity
m/z
204.0862
138.0548
168.0654
126.0546
186.0754
144.0659
274.0919
292.1039
366.1391
491.2243 y4(1+)
657.2443
1453.6431 PEP_0_1_0_0_0_
0
20000
40000
60000
80000
100000
120000
140000
250
500
750
1000
1250
1500
1750
2000
2250

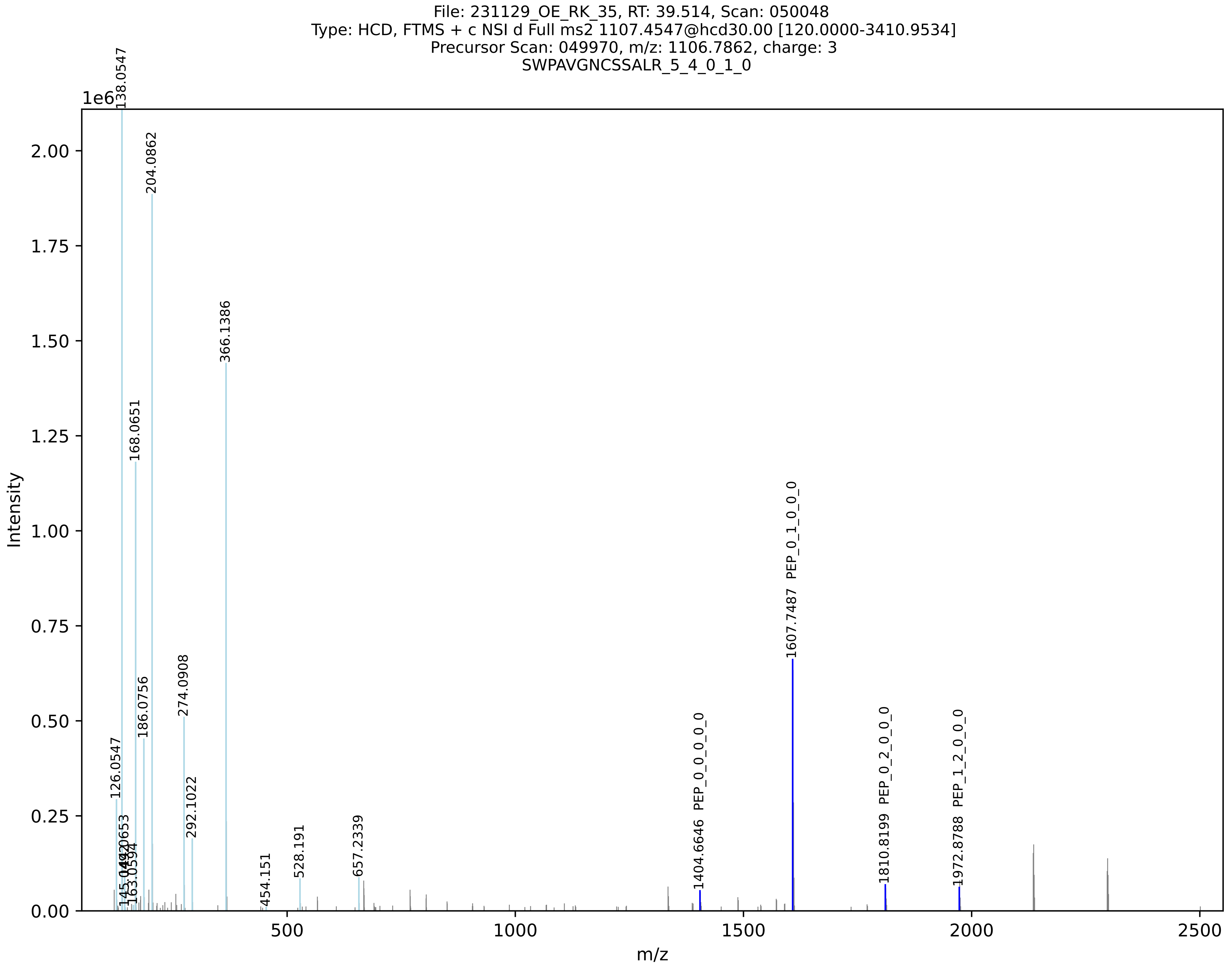
File: 231129_OE_RK_35, RT: 39.514, Scan: 050048
Type: HCD, FTMS + c NSI d Full ms2 1107.4547@hcd30.00 [120.0000-3410.9534]
[truncated: 402,213 more chars]
